# Surface Proteomics of Cancer-Derived Extracellular Vesicles Reveals Universal Heat Shock Proteins and Cancer Signatures

**DOI:** 10.1016/j.mcpro.2026.101649

**Published:** 2026-08-22

**Authors:** Leyla Ayse Erozenci, Fenna Feenstra, Sander R. Piersma, Thang V. Pham, Jianing Liu, Joke Wortel, Jan R.T. van Weering, Natasja FJ. Dits, Guido Jenster, R. Jeroen A. van Moorselaar, Irene V. Bijnsdorp, Connie R. Jimenez

**Affiliations:** 1 Amsterdam UMC location Vrije Universiteit Amsterdam, OncoProteomics Laboratory, Department of Medical Oncolgy, Amsterdam, The Netherlands; 2 Cancer Center Amsterdam, Research Program, Amsterdam, The Netherlands; 3 Amsterdam UMC location Vrije Universiteit Amsterdam, Department of Urology, Amsterdam, The Netherlands; 4 Amsterdam UMC location Vrije Universiteit Amsterdam, Department of Human Genetics, Center for Neurogenomics and Cognitive Research, Amsterdam Neuroscience, Amsterdam, The Netherlands; 5 Erasmus MC, Department of Urology, Rotterdam, The Netherlands

**Keywords:** cancer, extracellular vesicle, LC-MS/MS proteomics, pan-cancer, surface proteins

## Abstract

Extracellular vesicles (EVs) secreted by cancer cells are a valuable source of noninvasive biomarkers, as they are released into biofluids and reflect the molecular makeup of their cells of origin. To enable direct capture and detection of cancer-derived EVs, detailed knowledge of their surface proteome is essential. Here, we combined mild enzymatic treatment with mass spectrometry to analyze outward-facing proteins of EVs across a panel of 18 cell lines. This approach identified 1094 EV-associated surface proteins, including 144 transmembrane proteins involved in cellular communication and transport. Peptide-level analyses revealed that nearly 50% of multi-pass transmembrane proteins, such as ATP1A1, displayed their intracellular domains on the external EV surface. Out of a core set of 112 EV surface proteins shared across all cancer types, we report a top list of 15 highly abundant EV-surface proteins including ATP1A1, heat shock proteins (HSP)A8 and HSP90AB1 and nuclear proteins linked to EV biogenesis and cancer progression. This comprehensive profiling of EV surface proteomes from diverse cancer types reveals a robust set of pan-cancer EV surface markers with potential for diagnostic analysis.

## Highlights

• Identification of 1094 EV surface proteins across 18 cell lines.
• ∼50% of multi-pass membrane proteins show reversed orientation.
• Diverse biology is enriched in a core set of 112 pan-cancer EV proteins.
• Top 15 pan-cancer EV surface proteins are associated with cancer progression.
• EV surfaceome reveals HSPs as markers for diagnostic EV capture.

## Graphical Abstract

**Figure:**
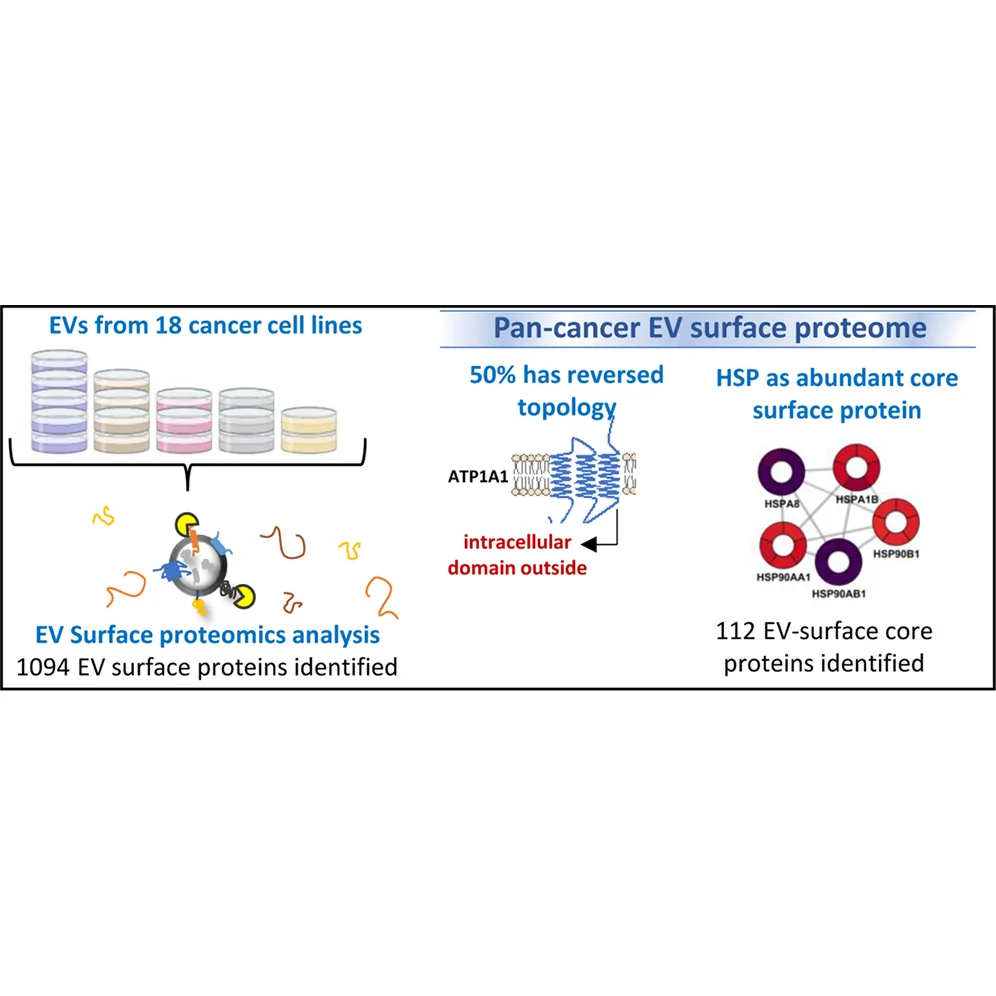

## In Brief

EV surface proteins are promising tools for diagnostic EV capture, yet knowledge of outward-facing EV proteins across cancers is limited. We identified a core set of 112 pan-cancer EV surface proteins across 18 cell lines, implicated in a wide range of biological processes. Multiple transmembrane proteins displayed reversed topology. The top 15 EV surface proteins were involved in cancer progression and included HSPs. This outward-facing EV surfaceome across multiple cancer types may serve as promising biomarkers.

Extracellular vesicles (EVs) are small lipid bilayer-enclosed vesicles secreted by cells and can be found in various biofluids such as blood, urine, saliva, and cerebrospinal fluid (1). EVs are broadly categorized based on their biogenesis into exosomes, originating from the endosomal system, and microvesicles, which are shed directly from the plasma membrane (2). These vesicles carry a complex cargo of nucleic acids, proteins, lipids, and metabolites, some of which are selectively sorted during EV formation and can reflect the physiological or pathological state of their cell of origin (3). As such, EVs have emerged as a promising, noninvasive source of biomarkers for liquid biopsy-based diagnostics in a variety of diseases, including cancer (4), neurodegenerative disorders (5), cardiovascular disease, and inflammatory conditions.

In addition to their diagnostic potential, EVs play crucial roles in intercellular communication, influencing recipient cell behavior by transferring bioactive molecules or through interactions between EV surface proteins and cell surface receptors (6, 7). This mode of action is particularly relevant in cancer, where EVs are known to modulate the tumor microenvironment, promote immune evasion, stimulate angiogenesis, and contribute to metastasis. A growing body of evidence suggests that the EV surface proteome, or "surfaceome", is functionally involved in these processes and may serve as a critical interface for EV-cell interactions.

Because EVs are readily accessible in body fluids and their surface proteins are directly exposed to the extracellular environment, the EV surfaceome represents an attractive target for the development of diagnostic, prognostic, and therapeutic tools. For example, surface proteins may serve as markers for EV capture and enrichment from complex biological samples, improving the sensitivity and specificity of downstream analyses. They may also be exploited for targeted drug delivery applications or as targets for immune recognition. However, despite increasing interest, the molecular composition and topology of proteins at the EV surface remain incompletely characterized, particularly in cancer-derived EVs.

Previous studies have employed strategies such as protease shaving, surface biotinylation, and click chemistry to enrich and identify surface-exposed EV proteins by mass spectrometry (8, 9, 10, 11, 12, 13, 14, 15, 16, 17, 18). These studies revealed not only expected transmembrane proteins but also cytosolic and even nuclear proteins associated with the EV surface, challenging classical models of protein topology and EV biogenesis. While these findings hint at the complexity and heterogeneity of EV subtypes and their cargo, systematic and comparative analyses across a panel of cell lines representing multiple cancer types remain limited.

To address this gap, we performed a comprehensive, mass spectrometry-based surface proteomic analysis of EVs secreted by 18 cell lines, representing prostate, colorectal, lung, cervical, and bladder cancers.

Our findings expand our understanding of the EV surfaceome and provide a valuable resource for the identification of cancer-derived EVs. The data support the feasibility of using abundant pan-cancer EV surface proteins, including heat shock proteins, as universal markers for EV-based diagnostics, while also identifying tumor type-specific proteins that may inform future biomarker development and functional studies.

## Experimental procedures

### Experimental Design and Statistical Rationale

#### Cell Culture

A total of 18 immortalized human cell lines representative of prostate (DU145, PC3, 22RV1, LAPC4, LNCaP, RWPE1), colorectal (HCT116, Lovo92, HT29, CaCo2), cervical (SiHa, HeLa, Caski), lung (H522, H1703, H1650) and bladder (RT112, T24) cancer were used in this study. Cell lines were cultured with Dulbecco’s modified Eagle’s medium (DMEM) or Roswell Park Memorial Institute (RPMI) 1640 Medium following manufacture recommendations, supplemented with 10% heat-inactivated fetal bovine serum (Biowest) and 1% antibiotic-antimycotic (Penicillin-Streptomycin), at 37 ^°^C in 5% CO2 atmosphere. Cells were expanded in 20 × T145 flasks, and grown overnight in serum-free media for EV isolation. The cell lines were regularly (1x per 3 months) confirmed to be mycoplasma-free (e-MycoTM *Mycoplasma* PCR Detection Kit, iNtRON Bio).

#### Isolation of EVs and Preparation EV Fractions

Isolation of EVs with ultracentrifugation was performed as follows: 400 ml conditioned medium from each cell line grown in 20 × T145 flasks overnight in serum-free media was collected and centrifuged at 500*g* for 10 min and subsequently at 2000*g* for 20 min. The supernatant was ultracentrifuged at 20,000*g* for 30 min to clear larger vesicles, and subsequently at 100,000*g* for 1.5 h to pellet the EVs in Optima LE-80 K ultracentrifuge with SW 32 Ti rotor (Beckman Coulter, Brea, CA). All the centrifugation and ultracentrifugation steps were performed at 4 ^°^C. The EV pellet was washed once at 100000*g* for 1.5 h with 45 ml PBS, and subsequently resuspended in 270ul PBS and immediately processed for the EV surface-protein isolation protocol (See Fig. 1 for a schematic overview of the protocol).

**Fig. 1:**
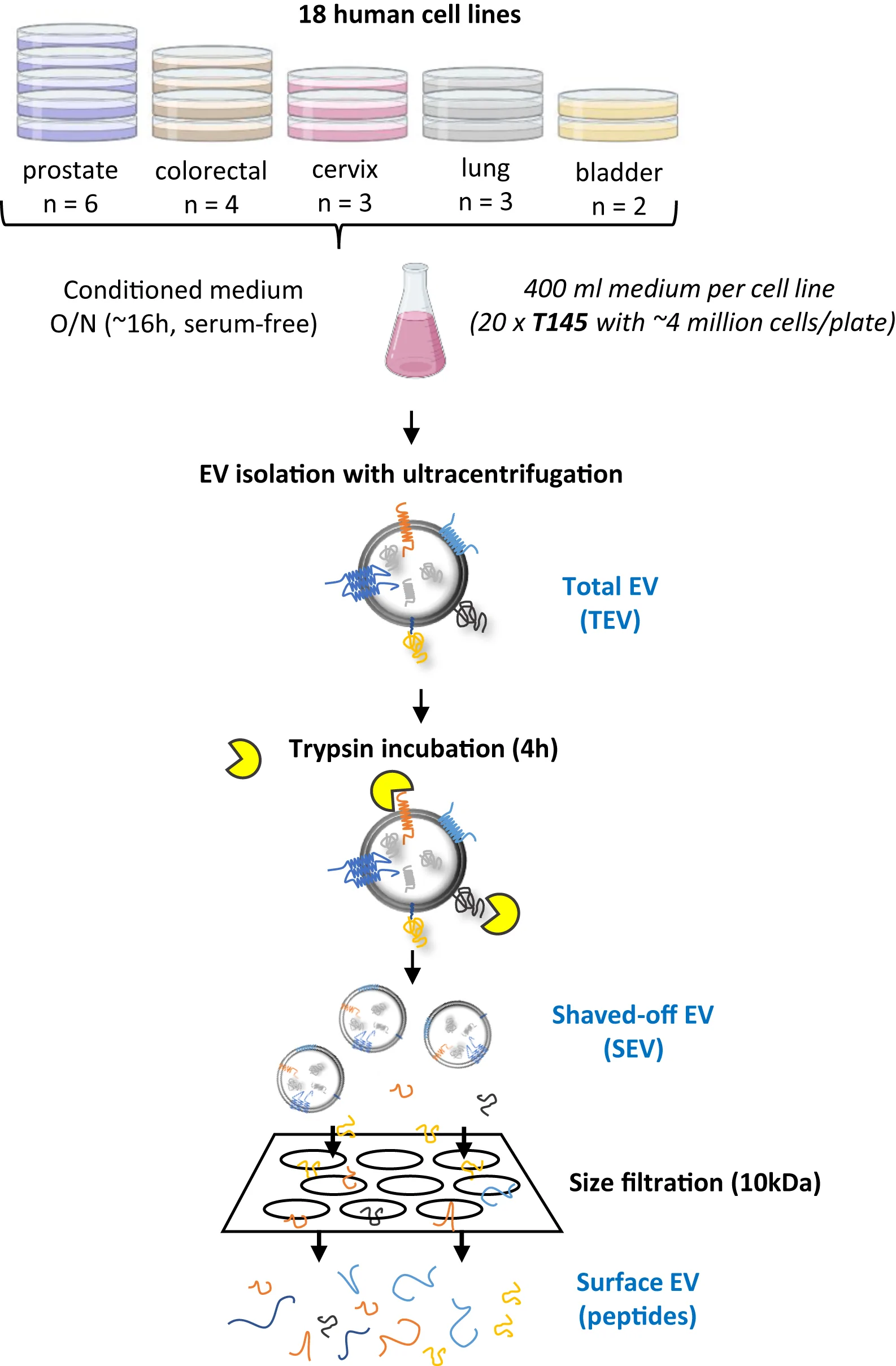
**Schematic illustration of the study 18 cell lines were expanded to ∼4 million cells per plate in 20 x T145 plates, and were grown overnight (O/N; ∼16 h) in serum-free media.** EVs were isolated with ultracentrifugation from 400 ml of conditioned medium for each cell line, and were subsequently treated with trypsin in duplicate to shave-off the parts of the proteins facing outside of the EV membrane (*i*.*e*., surface peptides). Surface peptides were separated from the intact EVs (TEV) by size filtration to obtain (SEV) fraction. All three fractions (surface, TEV and SEV) were analyzed with label-free mass spectrometry. SEV, shaved off EV; TEV, total EV.

Each cell line was processed as biological duplicate. For the total EV (TEV) fraction, EVs resuspended in PBS were aliquoted (35 μl), which was further dissolved in in LDS sample buffer containing 10% Dithiothreitol (Sigma-Aldrich chemicals, Zwijndrecht, The Netherlands) and stored at −80 ^°^C until processing for proteomics. The EV surface-associated proteome (100 μl) was collected after incubation for 4 h at RT with 6.25 ng/ml trypsin (Promega, sequence grade V5111) in 50 mM ammonium bicarbonate, to generate tryptic fragments of the proteins facing outside on the EV membrane (surface peptides). The tryptic surface peptides were collected with filtration *via* the Amicon Ultra 10 K filter (Millipore), subsequently desalted with 10 mg OASIS HLB column (Waters, Milford) and stored at −80 ^°^C until processing for proteomics as the surface fraction. Lastly, the undigested proteins and remaining intact EVs in the filter were resuspended in LDS sample buffer (containing 10% Dithiothreitol, Sigma-Aldrich chemicals, Zwijndrecht, The Netherlands) and stored at −80 ^°^C until processing for proteomics as the shaved-off EV (SEV) fraction. In addition to aliquots for proteomics, TEV and SEV fractions were also stored in PBS fixed with 4% paraformaldehyde (PFA) for further analysis by electron microscopy (EM) analysis.

#### Mass Spectrometry Measurement of TEV, SEV and Surface EV Fractions

The TEV and SEV samples were applied on a home-made 12% acrylamide SDS page gel (BioRad, Hercules, CA). The gels were stained with Coomassie brilliant blue G-250 (Pierce, Rockford, IL) and in-gel digested as previously described (19). Briefly, gels were washed twice in 50 mM ammonium bicarbonate (ABC) and dehydrated twice in 50 mM ABC/50% acetonitrile (ACN). Cysteine bonds were reduced by incubation with 10 mM DTT/50 mM ABC at 56 °C for 1 h and alkylated with 50 mM iodoacetamide/50 mM ABC at RT for 45 min. After washing sequentially with ABC and ABC/50% ACN, the whole gel was sliced in 1 band for each lane. Gel bands were sliced into cubes of 1mm3, which were incubated overnight with 6.25 ng/ml trypsin (Promega, sequence grade V5111). Peptides were extracted once in 1% formic acid and twice in 5% formic acid/50% ACN.

Extracted peptides of TEV and SEV EV fractions and filter-collected tryptic surface peptides were desalted using a 10 mg OASIS HLB column (Waters, Milford) after washing in 0.1% TFA. Samples were eluted in 80% ACN/0.1%TFA and were dried in a vacuum centrifuge. Peptides were redissolved in 20 μl loading solvent (4% ACN in 0.5% TFA) for LC-MS analysis. Extracted peptides were separated on a 75 μm × 42 cm custom packed Reprosil C18 aqua column (1.9 μm, 120 Å) in a 90 min gradient (2–32% Acetonitrile+0.5% Acetic acid at 300 nl/min) using a U3000 RSLC high pressure nanoLC (Dionex). Eluting peptides were measured on-line by a Q Exactive mass spectrometer (Thermo Fisher Scientific, Bremen, Germany) operating in data-dependent acquisition mode. Intact peptide ions were detected at a resolution of 70,000 (at m/z 200) and fragment ions at a resolution of 17,500 (at m/z 200); the MS mass range was 350–1400 Da. AGC Target settings for MS were 3E6 charges and for MS/MS 1E6 charges. Peptides were selected for Higher-energy C-trap dissociation fragmentation at an underfill ratio of 1% and a quadrupole isolation window of 1.6 Da, peptides were fragmented at a normalized collision energy of 28.

#### Identification and Label-free Quantification of Proteins

DDA Raw Files Were Processed Using MaxQuant 1.6.4.0. MS/MS Spectra Were Searched against a Swissprot FASTA file downloaded Feb 2019 (42,417 entries). The precursor and fragment mass tolerance were set to 4.5 and 20 ppm, respectively. Peptides with minimum of seven amino-acid length were considered with both the peptides and proteins filtered to a false discovery rate of 1%. Enzyme specificity was set to trypsin and up to two missed cleavages were allowed. Cysteine carbamidomethylation was searched as a fixed modification, whereas protein N-terminal acetylation and methionine oxidation were searched for as variable modifications. Searches were performed separately for each fraction with the label-free quantification option selected. 5 out of 108 samples were excluded from further analysis due to low protein identification supplmental Fig. S1*A*. These samples were only excluded from the surface fraction, for the TEV and SEV no samples were excluded in the analyses.

#### Data Analysis

Quantification of proteins was done separately for each fraction (*i*.*e*., surface EV, TEV and SEV fractions) using spectral counting. For each sample, the number of spectral counts identified per protein was normalized on the total number of spectral counts of all identified proteins in the sample. Averaged values of the biological duplicates were used for the following descriptive and comparative analyses. All analysis and plotting was done in R (https://www.Rproject.org/). Protein expression differences between the different cancer cell lines were calculated using the beta-binominal test for unpaired data. The *p*-values <0.05 were considered statistically significant. The clustering analyses were performed using R package ComplexHeatmaps (20). For protein expression heatmaps, the protein abundances were normalized to zero mean and unit variance for each individual protein. Subsequently, the Euclidean distance measure was used for protein clustering. R package ggplot2 (https://ggplot2.tidyverse.org) was used for all plots other than heatmaps and networks. Gene Ontology (GO) term analyses of the core urinary EV proteome were performed using DAVID (21, 22). Redundant GO terms were manually collapsed into one parent term and were presented in the GO barplots. Protein networks were created using STRING (23) and visualization was further modified using Cytoscape (24) and OmicsVis (25).

Membrane-associated EV surface proteins were annotated in detail based on multiple sources (1): subcellular localization according to the UniProt database (26) using the keywords Transmembrane (KW-0812), Multi-pass (SL-9909), Single-pass (SL-9904), Peripheral Membrane (SL-9903), Lipid-anchor (SL-9901) and GPI-anchor (KW-0336/SL-9902) (2); transmembrane domain, signal peptide and surface localization prediction using web-based tools TOPCONS (27), TMHMM (28) and SurfaceGenie (29); and (3) 2-dimensional topology provided in Protter (30). The membrane localization and functional classes of single-pass transmembrane proteins were obtained from the Membranome database: the whole human single-pass transmembrane proteome of >2000 proteins (31, 32) and the associated comprehensive study by Pogozheva *et al*. (33). These categories were used to annotate multi-pass and single-pass transmembrane proteins.

Two-dimensional topology illustrations of 51 multi-pass transmembrane proteins were obtained from Protter (30) and identified peptides in the mass spectrometry were mapped onto topology illustrations. We selected to annotate only the multi-pass proteins, since they span the membrane multiple times with multiple domains on either side of the membrane, and therefore would provide more reliable results. The multi-pass proteins were then annotated for their orientation on EV membrane into three categories based on the localization of identified peptides in the surface fraction. Proteins that have surface peptides identified on their cytoplasmic (on vesicle membrane) or extracellular (on plasma membrane) parts as annotated by Protter (30) were categorized as having “predicted orientation”; whereas proteins with surface peptides identified on their luminal (on vesicle membrane) or cytoplasmic (on plasma membrane) domains were classified as having “reverse orientation”. Proteins which have surface peptides mapped to their domains on both sides of the membrane were annotated as “both”. Statistical testing to investigate cancer-enriched EV surface proteomes was done in R using beta-binominal test (34). Gene Set Enrichment analysis was performed using BROAD tool (35, 36) with default settings and KEGG pathways (37).

#### Electron Microscopy

EVs isolated by ultracentrifugation from HCT116, HT29 and DU145 cell lines were resuspended in a solution of 4% PFA in PBS. The EVs were spotted on carbon/formvar-coated EM grids and air-dried. Samples were fixed to the EM grid in 1% PFA, washed and double-labelled/labelled for CD63 (BD Bioscience, NJ, Cat No 556019), ATP1A1 (Sigma-Aldrich, Cat No SAB4501974) with an anti-mouse IgG (Rockland Inc, Cat No 610-4102). The grids were contrasted by 2% uranyl acetate and analyzed in a Tecnai12 (Thermo Fisher Scientific) TEM electron microscope at 100 kV.

## Results

### Isolation and Characterization of EV Fractions from a Multi-Cancer Cell Line Panel

To investigate the multi-cancer EV surface proteome, we isolated EVs using ultracentrifugation from conditioned medium of a panel of 18 cancer cell lines consisting of 5 cancer types, including prostate (PCa; 5 cell lines), colorectal (CRC; 4 cell lines), cervical (CC; 3 cell lines), lung (LC; 3 cell lines) and bladder (BC; 2 cell lines) cancers, and healthy prostate (RWPE1) (Fig. 1). To identify the proteins at the outward EV membrane, we applied mild tryptic cleavage (Fig. 1). The EV-integrity was not affected by this short enzymatic treatment, as demonstrated by the similar morphology and size of the trypsinized-EVs (SEV) to that of the untreated samples (TEV) (Fig. 2, *A* and *B*).

**Fig. 2:**
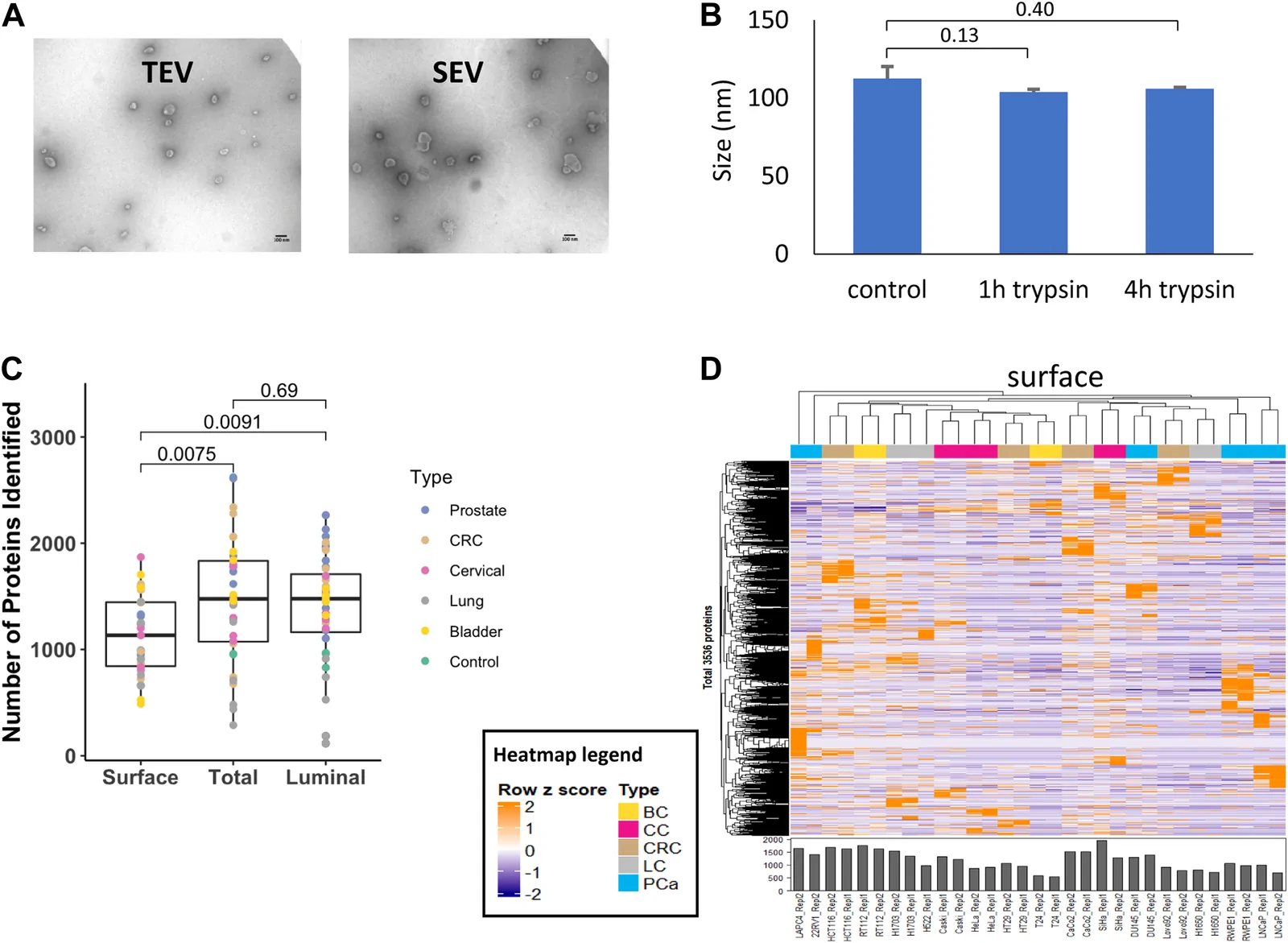
**Characterization of EV fractions and EV surface proteome dataset overview.***A*, representative EM images of TEV and SEV fractions, showing no change in EV morphology after 4 h of trypsinization. *B*, EM quantifications of average EV sizes of the SEV fraction, showing no clear difference in size after 1 or 4 h of trypsinization. About 950 vesicles were quantified per condition, over a total of 182 EM images. *C*, number of proteins identified by mass spectrometry in surface EV, TEV and SEV fractions, showing a lower number in the surface fraction compared to the other fractions. *D*, unsupervised hierarchical clustering of EV surface proteome, showing no clear clustering between cancer types. Color intensity represents the Row Z-score, which scales protein abundance by subtracting the row mean and dividing by the row standard deviation. This normalization allows for the visualization of relative protein expression levels across samples, independent of absolute intensity scales. EM, electron microscopy; EV, extracellular vesicle; SEV, shaved off EV; TEV, total EV.

The surface peptides (EV surface fraction) and shaved-off remnant EV fraction (SEV), together with (TEV, were analyzed by mass spectrometry-based proteomics (Fig. 1 and supplmental Table S1, *A–D*). The measured EV surface peptides were derived from a total of 3536 proteins, while TEV and the SEV fractions included 4161 and 3621 proteins, with an average of around 1100 proteins per measured sample (Fig. 2*C*). A comparable number of proteins was identified for biological replicates of all EV fractions (supplemental Fig. S1*B*), showing the overall reproducibility of the EV-surface isolations and proteomics workflow. The surface fraction had a significant lower number of identified peptides compared to TEV and SEV, as expected (supplemental Fig. S1*B*).

Unsupervised clustering of the EV surface proteome revealed cell line-specific protein expression patterns with clustering of biological replicates, while clustering by cancer type was not clearly observed (Fig. 2*D*). Comparable findings were obtained with the TEV and SEV clustering (supplemental Fig. S2). Together, these findings indicate that the trypsin-treated EVs keep their membrane integrity, and have cell type enriched proteins present at the outer surface.

### Highly Connected EV Surface Transmembrane Proteome is Involved in the Regulation of Communication and Transport

To investigate the associated biology of the proteins that are present at the EV surface, we performed functional data mining. To this end, we filtered for robustly identified EV surface proteins with a data presence of 50% across all samples. This threshold was chosen to ensure that subsequent analyses were grounded in robustly detected signals, minimizing the impact of stochastic missing values. Analysis of this set of 1084 EV surface proteins showed that extracellular exosome was the most enriched term in the EV surface proteome (Fig. 3*A*). In addition, as can be expected from surface proteins, a clear enrichment of membrane-related cellular component terms was observed (Fig. 3*A*). The enrichment of intracellular terms such as cytosol, ribosome and nucleoplasm on EV surface (Fig. 3*A*) is in agreement to previous observations that demonstrate that the protein corona of the EV surface could be more complex than initially thought (38, 39).

**Fig. 3:**
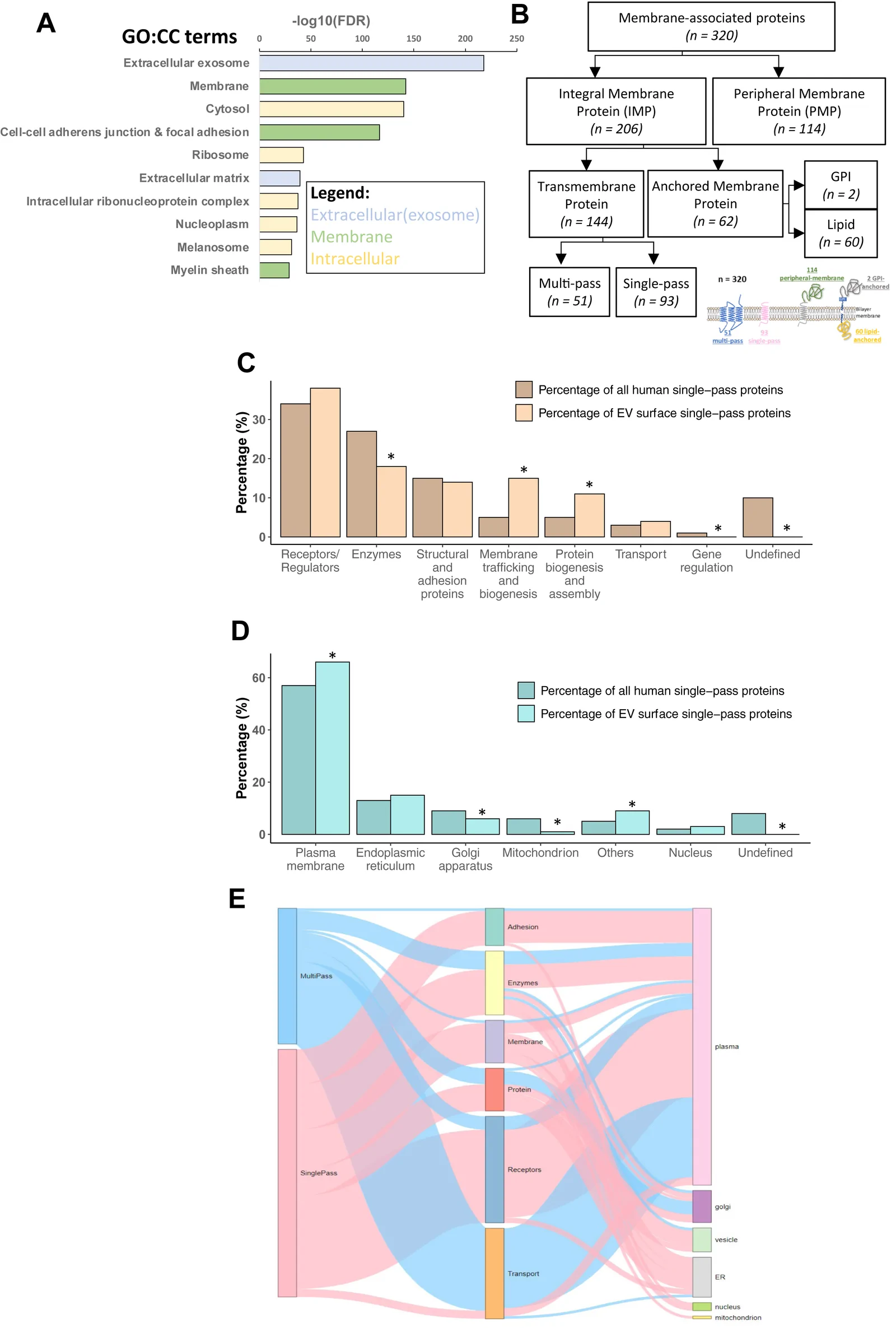
**Cellular components and membrane-associated proteins of EV surface.***A*, top10 CC gene ontology terms enriched in EV surface proteome. gene ontology:cellular component analysis is performed using DAVID (21, 22) and terms are ranked based on –log10(false discovery rate) values. Bars are colored based on the general location of enriched terms; as in *blue* for extracellular (exosome), *green* for membrane, and *yellow* for intracellular-related terms. *B*, schematic representation of membrane-associated proteins of the EV surface. Total of 320 proteins that were found to be membrane-associated are shown in the figure for each category. *C*, comparison of functional classes and *D*. membrane locations of the identified single-pass transmembrane proteins on EV surface with the whole *homo sapiens* single-pass transmembrane proteome. Barplots show the distribution of proteins (in percentage) identified in each category in the all human and in the identified EV surface single-pass transmembrane proteome, respectively. *Bars* marked with *∗* indicate *p* < 0.05 as measured using the *Fisher’s* Exact test. *E*, functional classes and locations of multi-pass (*blue*) and single-pass (*pink*) transmembrane EV surface proteins. The categories provided in the Membranome database (31, 32, 33) were used to annotate transmembrane EV surface proteins into 6 functional classes. Membrane = Membrane trafficking and biogenesis, Protein = protein biogenesis, assembly and transport, adhesion = structural and adhesion proteins. EV, extracellular vesicle.

To determine what type of membrane proteins are associated with the EV surface, we annotated the classified 320 membrane proteins (Fig. 3*B*, supplemental Fig. S3 and supplemental Table S2). The majority was classified as transmembrane integral membrane proteins (IMPs) (144 proteins), and peripheral membrane proteins (114 proteins) (Fig. 3*B*). Both multi-pass and single-pass transmembrane-IMPs are embedded in the EV membrane, they are expected to contribute to the EV surface proteome. See supplemental Table S3 for the list of 144 multi-pass and single-pass transmembrane-IMPs. The membrane localization and functional classes of the predicted 93 single-pass EV surface transmembrane proteins at the surface of the EVs showed a distribution largely comparable to the single-pass transmembrane proteins as encoded by the whole human proteome. Single-pass EV surface proteins were mostly receptors implicated in signaling, as well as membrane/protein biogenesis and adhesion; further emphasizing the role of EVs in intercellular communication (Fig. 3*C*). EVs showed a significant enrichment compared to total proteome on membrane trafficking and protein biogenesis and assembly, and significant decrease in enzymes, and gene regulation (Fig. 3*C*). Single-pass transmembrane EV surface proteins were mostly derived from plasma or vesicular organelle membrane (Fig. 3*D*), as expected due to the process of EV biogenesis; while almost no proteins were found to be associated with the mitochondrial-membrane (Fig. 3*D*). Compared to the total proteome, EVs were enriched for plasma membrane, while golgi and mitochondrial proteins were significantly reduced (Fig. 3*D*).

In contrast to the observed diversity of single-pass proteins, ∼84% of the identified multi-pass EV surface proteins function in regulation Fig. 3*E*) and are plasma membrane-proteins (Fig. 3*E*). These multi-pass transporters consisted of members of two main transporter superfamilies: ATPases and solute carrier (SLC) transporters (supplemental Fig. S3). Importantly, network-based analysis showed that both multi-and single-pass EV surface transmembrane proteins were highly inter-connected (supplemental Fig. S3), suggesting their association in functional complexes that could mediate EV-related signaling, regulation and transport events. Together, these results demonstrate the connectivity and multi-functionality of the EV surface transmembrane proteome.

### Reverse Topology of Almost Half the Multi-Pass Transmembrane Proteins Identified on the EV Surface

The topology of EV membrane-bound proteins is important, because their direction determines their signaling status, as well as their recognition by target proteins/molecules. To investigate topology in our dataset, we mapped the mass spectrometry-identified peptides of the multi-pass transmembrane EV surface proteins on their topological illustrations using Protter (30), to determine which parts are facing outside on EV membrane. Almost half (45%) of the multi-pass transmembrane proteins on EV surface showed topologically reverse orientation compared to their predicted orientation, while 31% had surface peptides identified on both sides of the EV membrane (Fig. 4*A*, supplemental Table S3). Importantly, no peptides belonging to transmembrane domains were identified in the surface fraction.

**Fig. 4:**
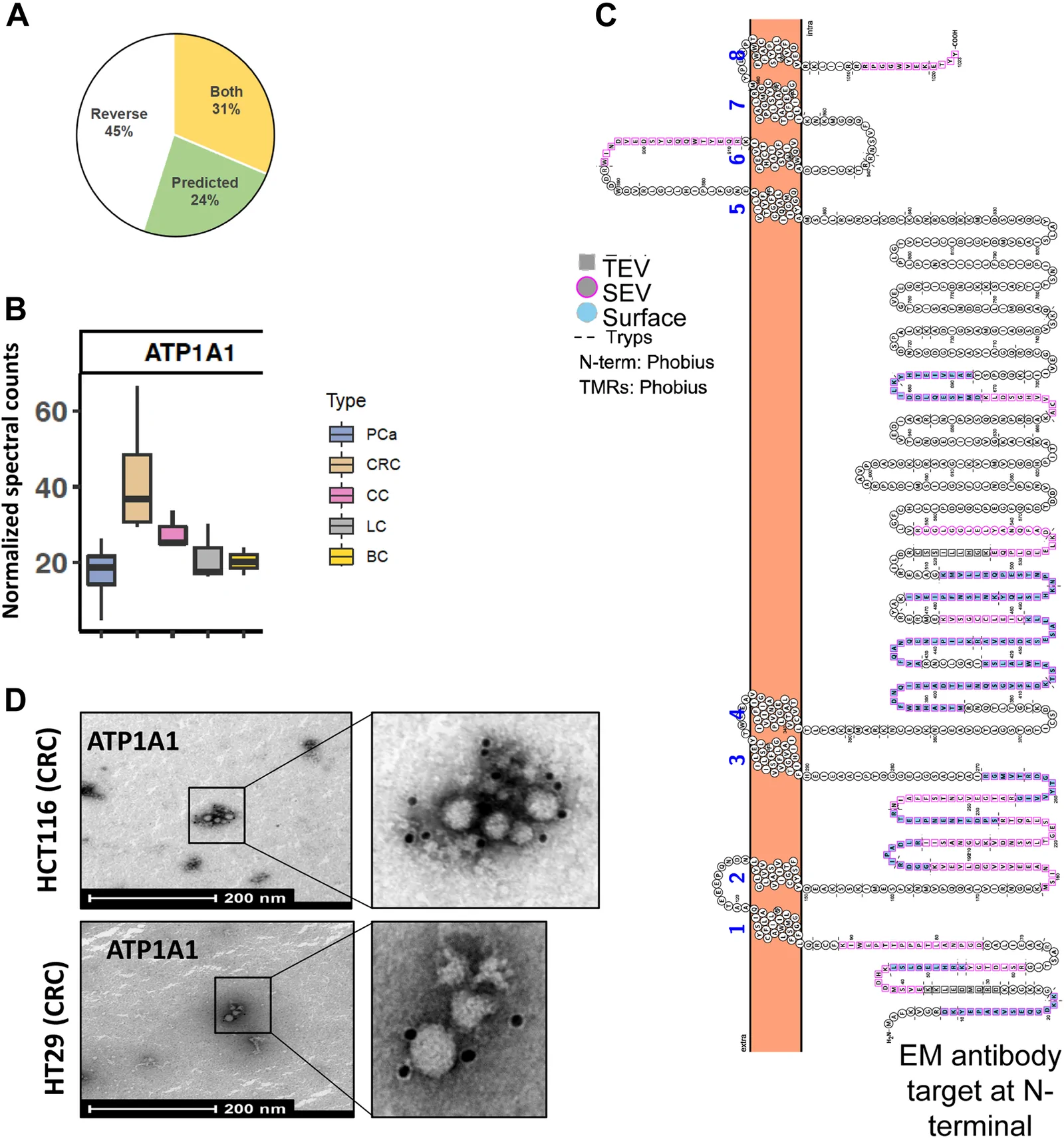
**Orientation of EV surface multi-pass transmembembrane proteins.***A*, distribution of orientation of multi-pass transmembrane EV surface proteins, showing that most of the multi-pass transmembrane proteins found on EV surface have reverse orientation. Predicted: EV surface proteins that have surface peptides identified on their cytoplasmic (on vesicle membrane) or extracellular (on plasma membrane) parts; Reverse: EV surface proteins that have surface peptides identified on their luminal (on vesicle membrane) or cytoplasmic (on plasma membrane) domains; Both: EV surface proteins which have surface peptides mapped to their domains on both sides of the membrane. *B*, ATP1A1 expression in surface EV fraction detected by mass spectrometry. *C*, ATP1A1 Protter (30) mapping of surface EV, TEV and SEV peptides. *D*, ATP1A1 immuno-EM in TEV fraction showing EV surface localization of ATP1A1 in HCT116 and HT29 CRC cell lines. This scale bar represents 200 nm. EM, electron microscopy; EV, extracellular vesicle.

Our mass spectrometry-based findings show that the orientation of many proteins on the surface of EVs are not in the predicted orientation on the cell membrane. We selected the multi-pass transmembrane protein ATP1A1, that is abundantly expressed on the EV surface of all cancer cell lines for measurement on TEM (Fig. 4*B*). This protein has a large intracellular domain that exhibited a reverse orientation on the EV surface membrane as deduced from the fact that almost all intracellular domain peptides were identified in the surface fraction (Fig. 4*C*, in blue). Immuno-staining with an antibody targeting the N-terminal intracellular tail of the protein coupled to EM of indicated indeed a reverse orientation of ATP1A1 on EV membrane, with its N-terminal facing outwards (Fig. 4*D*). In addition, several other proteins that have larger domains on both sides of the membrane (intracellular and extracellular), such as ANO6 and SLC12A4 were also detected in reversed order (supplemental Fig. S3*B*). Taken together, the mapping of transmembrane EV surface proteins show that many membrane-associated proteins on EV surface can have a reversed topology.

### EV Surface Proteome Harbors Cancer Type-Enriched Signatures

To investigate whether cancer type-specific protein expression patterns can be detected on EV surface, we compared the EV surface proteome of each cancer type to the other cancer types in multiple two-group comparisons, resulting in 4 comparisons per cancer type. Gene set enrichment analysis using KEGG pathways showed enrichment of several different biological pathways per cancer type (Fig. 5*A*). Functional data mining of cancer type-enriched EV surface proteins revealed RNA-related terms such as splicing to be enriched in PCa, and epithelial-mesenchymal transition (EMT)-related processes such as extracellular matrix-receptor interaction and cell adhesion molecules in CRC, CC, LC and BC (Fig. 5*A*). Interestingly, PCa exhibits a distinct biological profile, characterized by a strong involvement of the spliceosome, which appears to be specific to PCa. In contrast, the altered pathways in other cancers are more broadly shared of which CRC shows enrichment of pathways related to infectious and adhesion signaling, while cervical cancer is characterized by enrichment in extracellular matrix–receptor interaction pathways.

**Fig. 5:**
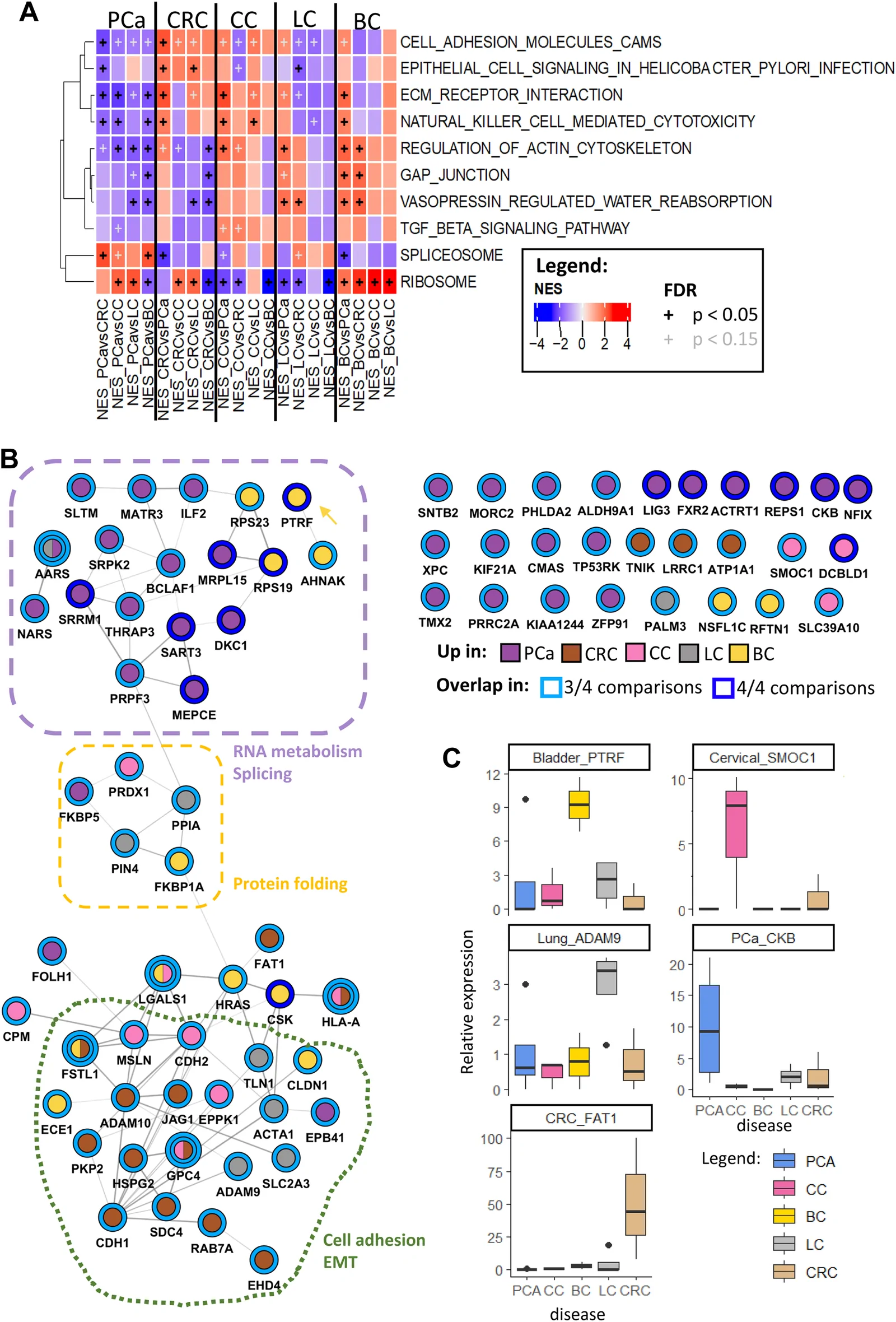
**Cancer type-enriched EV surface proteome.***A*, gene set enrichment analysis using KEGG pathways shows cancer type-enriched processes on EV surface. Pathways that have a normalized enrichment score > 0 in at least 2/4 comparisons and false discovery rate <0.15 in at least 2/4 comparisons are shown. *B*, protein-protein interaction network of EV surface proteins enriched per cancer type. *C*, *top* cancer type-enriched EV surface proteins detected by mass spectrometry. EV, extracellular vesicle.

To identify the most discriminatory cancer type-enriched EV surface proteins, we selected the significantly (*p* < 0.05) upregulated EV surface proteins per cancer type that overlap in 3 out of the 4 comparisons (supplemental Fig. S4). This resulted in 35 PCa-, 15 CRC-, 11 CC-, 8 LC- and 13 BC-enriched proteins (supplemental Fig. S4 and supplemental Table S4). Protein-protein interaction network of the cancer-specific EV surface proteome of the combined 77 proteins showed a clear cluster of proteins involved in RNA metabolism and splicing to be specifically upregulated on PCa-derived EV surface (Fig. 5*B*). The other large protein cluster was inter-mixed with EV surface proteins specific to several cancer types, with EMT and cell adhesion as the main deregulated pathways (Fig. 5*B*). Of each cancer type, the top proteins are plotted that was cancer-type enriched (Fig. 5*C*). Together, these analyses demonstrate that cancer type-enriched biological pathways and associated proteins are present on the EV surface and could possibly be employed for cancer-specific EV capture and detection.

### Pan-Cancer Core EV Surface Proteome is Enriched for Cytosolic and Nuclear Proteins

Proteins that are present on the EV surface independent of cancer type would be of high interest to develop an EV-capture assay. To investigate the pan-cancer EV surface proteome in our dataset, we selected the proteins detected with 100% presence at the EV surface of the 17 cancer cell lines. We excluded the ‘normal’ cell line from subsequent analysis, because this cell line had a faster growth rate than most used other cell lines. The universal EV surface proteome included a total of 112 proteins facing outward on the EV membrane (supplemental Table S5), which we refer to as the pan-cancer core EV surface proteome. Highly inter-connected protein-protein interaction network of the pan-cancer core membrane-bound EV surface proteins contained only 8 transmembrane proteins, including ATP1A1, and was remarkably enriched for cytosolic proteins (Fig. 6*A*). Importantly, 5 members of the heat-shock protein (HSP) family showed very high abundance on the EV surface independent of cancer type (Fig. 6*A*). Further analysis of molecular functions revealed that ∼80% of the core EV surface proteins are significantly enriched for nucleic acid binding proteins (Fig. 6*B*). Other enriched functions include regulation of gene expression such as transcription, translation and post-translational protein modification (Fig. 6*B*). Interestingly, 23 structural constituents of the ribosome were identified as the core EV surface proteome, though with not as high abundant compared to other components (Fig. 6, *A* and *B*). The gene expression regulatory protein cluster was inter-connected with other cytosolic proteins on the EV surface, including proteins related to EV structure and biogenesis such as cytoskeletal and vesicle-associated proteins; as well as proteins involved in intra-cellular/intra-vesicular signaling, protein trafficking and folding such as 14-3-3 and RAB proteins (Fig. 6*A*), further emphasizing the role of EVs in communication. Of note, a subset of these pan-cancer abundant EV surface proteins was also abundant on the EV surface of the “normal’ cell line (supplemental Fig. S2*C*).

**Fig. 6:**
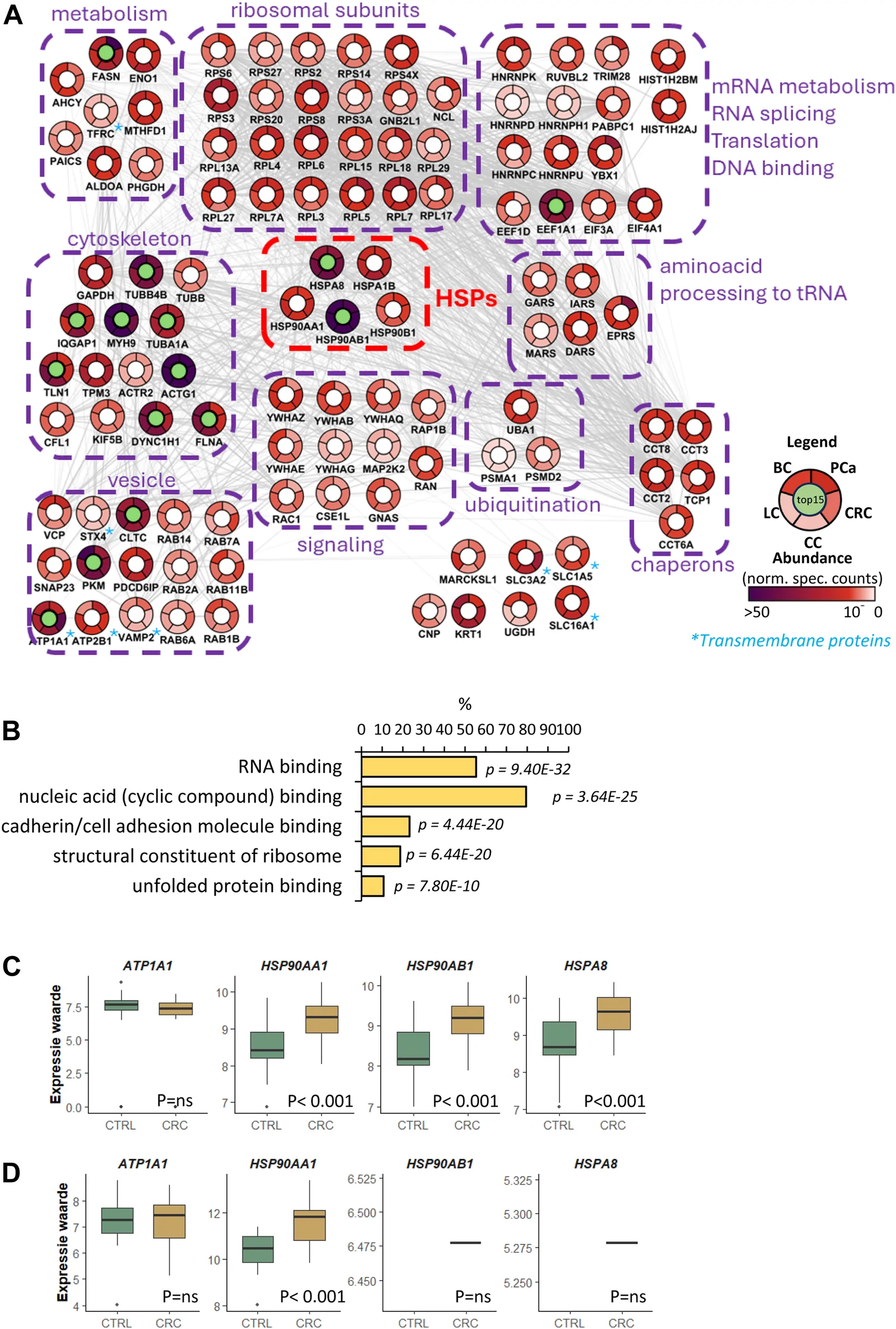
**Pan-cancer core EV surface proteome.***A*, core EV surface protein-protein network annotated with biological process and abundance on surface EV fraction. *B*, molecular function bargraph of the core EV surface proteins. *C*, expression of key identified proteins on plasma EVs of control versus colorectal cancer patients. *D*, expression of key identified proteins on plasma EVs of control *versus* colorectal cancer patients of an external dataset of Wang *et al*. (41). Signed informed consent was obtained from all patients. EV, extracellular vesicle.

The top 15 most abundant EV surface proteins comprise cytoskeletal, chaperone, and metabolic components linked to cancer and vesicle biogenesis (supplemental Table S6). Actin- and tubulin-associated proteins (ACTG1, MYH9, TUBB4B, TUBA1A, FLNA, TLN1, IQGAP1) reflect the cytoskeletal origin of EVs and their role in budding, motility, and cargo sorting. Metabolic enzymes PKM, FASN, EEF1A1 and ATP1A1 indicate that EVs mirror the metabolic state of tumor cells. Cadherin-binding proteins (FLNA, TLN1, IQGAP1) suggest roles in adhesion and intercellular signaling, while transport-related proteins DYNC1H1 and CLTC highlight active cytoskeletal dynamics in EV biogenesis. HSP90AB1 and HSPA8, are consistent with functions in EV-mediated tumor progression and immune modulation. To explore whether the top 15 most abundant EV surface proteins identified in our cancer cell line panel showed altered levels in the plasma EV proteome of patients with cancer, we analyzed their expression in an in-house plasma EV proteomics dataset of controls and patients with CRC (n = 59) (Fig. 6, *C–D* and supplemental Fig. S5). In the in-house dataset of plasma EVs isolated with magnetic beads (40), the heat shock proteins HSP90AB1, HSP90AA1 and HSPA8 were significantly enriched in CRC patients relative to controls, whereas ATP1A1 was unchanged (Fig. 6*C*). This enrichment was not limited to the heat shock proteins: of the remaining top-15 proteins detectable in this dataset, ACTG1, CLTC, EEF1A1, MYH9, PKM, TLN1 and TUBB4B were also significantly elevated in CRC (*p* < 0.001; PKM *p* < 0.01), while DYNC1H1, FASN, FLNA and IQGAP1 were not detected (supplemental Fig. S5). The heat shock protein signal was largely reproduced in the external CRC dataset (n = 60) of plasma EVs isolated with the exoEasy Maxi Kit (41), in which HSP90AA1 was again significantly enriched in CRC (Fig. 6*D*), although HSP90AB1 and HSPA8 were detected in only a single CRC patient and could therefore not be statistically tested. In this cohort FLNA and MYH9 were also significantly higher in CRC plasma EVs, whereas the remaining proteins showed no significant difference or were not detected (supplemental Fig. S5). Although these proteins were enriched in cancer patient EVs, further investigation is needed to confirm their surface localization and regulation in EVs isolated from biofluids of patients with other cancer types. Due to the availability of a commercial capture assay (42), HSPs are especially promising EV surface proteins for EV-based cancer diagnostics. Together, these findings underscore the multifunctional EV surface landscape contributing to tumor progression and communication.

## Discussion

Characterizing proteins accessible on the outer surface of EVs is essential both for understanding EV biology and for developing effective (cancer)-EV capture and detection assays. In this study, we employed mass spectrometry-based proteomics to comprehensively profile the outward-facing proteins of EVs derived from a panel of 18 cell lines. Our analysis revealed that the EV surface-accessible proteome is highly diverse, encompassing transmembrane, cytosolic, and nuclear proteins, with almost half of the transmembrane proteins having a reversed orientation. We identified both cancer type-enriched EV surface proteins and a top set of 15 core EV surface proteins that were consistently and abundantly present across all cancer cell lines. These EV surface proteins and HSP90AB1 and HSPA8 in particular, represent promising capture protein candidates for cancer EV isolation and detection in biofluids.

As expected, proteins with a transmembrane domain, that is EV membrane-embedded single-pass and multi-pass proteins were found to be enriched on EV surface based on the cellular component analysis. The identified single-pass transmembrane surface proteins were mostly receptors known to contribute to EV-associated tumorigenesis *via* mediating intercellular communication, such as integrins in cancer metastasis (43, 44), ephrins in tumor angiogenesis (6) and NOTCH2 in cell differentiation (45). In addition, EV surface enriched multi-pass membrane transporters belonging to ABC, SLC, and P-type pump families are well-known for their role in EV-directed chemoresistance (46), and in controlling cancer cell metabolism, and thereby growth and survival (47, 48).

A surprising finding of the detailed peptide mapping of the multi-pass transmembrane EV surface proteins was the reverse’ topology of 45% of the proteins, including ATP1A1 that exhibited its cytoplasmic terminal facing outward at the EV membrane. Our observation that a subpopulation of EV surface proteins display inside-out topology is not the only one. For example, Cvjetkovic *et al*. (2016) used surface-accessible protease digestion and biotin-labelling of EVs, and identified numerous transmembrane proteins which cytosolic loops or termini accessible on the EV surface (“inside-out”) (10). Hallal *et al*. (2022) similarly summarized that a comprehensive understanding of EV surface topology is necessary for interpreting EV-mediated signalling and biomarker studies (49).

Cancer type-specific proteins on the surface of EVs could eventually serve as markers to enrich cancer EVs from biofluids. In pancreatic cancer (pancreatic ductal adenocarcinoma), exosomes were captured from blood using a panel of EV surface proteins identified in pancreatic ductal adenocarcinoma cell lines, and showed successful enrichment of tumor-specific material (50). In our study, we identified several cancer type-enriched deregulated biological pathways and proteins in the EV surface fraction. PCa cells showed a clear enrichment in splicing, a known hallmark of PCa aggressiveness (41). Furthermore, the clinical PCa biomarker FOLH1 (prostate surface membrane antigen) protein (11) was identified exclusively on the surface of PCa EVs, suggesting the suitability of EV surface proteins for cancer-specific detection or isolation of prostate cancer specific EVs. Proteins involved in EMT, cell adhesion and natural killer cytotoxicity were enriched in all cancer types that in our body are in contact with the outside world (CRC, CC, LC, BC), with CRC displaying enriched abundance of FAT1, a known protein to be involved and mutated in CRC progression and a potential target for immunotherapy (51, 52). SMOC1 was identified as a possible CC-specific protein, which was reported as enriched in CC cell lines (HPA), and involved in cancer progression in various cancer types, though the relation between cervical cancer progression remains unclear (53, 54). Finally, we identified LC-enriched ADAM9, an important protein in malignant transformation and metastasis in several types of cancer including lung (55, 56); and cancer drug resistance-associated PTRF (57) to be upregulated in BC. While our study has its limitations such as the sample size for certain cancer types, *e.g*. 4 cell lines for CRC which has 4 subtypes, we nevertheless show several known pathways and proteins specific to cancer type on the EV surface.

A striking finding of our pan-cancer analysis of abundant EV surface proteins was the high enrichment of cytosolic (HSPs among others) and nuclear DNA/RNA binding proteins, as part of the core EV surface proteome. There has been growing research on the EV-associated DNA and RNA in recent years, showing their implication in cancer progression and their potential as biomarkers and therapeutic targets (58, 59). Enrichment of DNA and RNA binding proteins on EV surface was also observed in other studies (60, 61), and fragments of DNA and RNA have been reported to be associated with the EV surface (9, 61). While it remains possible that EVs are co-isolated with nonvesicular complexes associated with extracellular DNA and RNA (13, 62, 63, 64), the robust detection of these proteins across the whole set of our diverse cell lines may suggest a stable, structural association rather than random contamination (13, 64, 65, 66). Alternatively, the nucleic acid binding proteins could be contributing to EV ‘coronal’ surface proteome as interacting partners (65) to promote alterations in the receptor cell and/or reflecting the tumorigenic state of the cancer cell, such as ribosomal proteins (66, 67). This oncogenic stress signature was also underscored by the high abundance of HSPs on the EV surface. High-resolution surfaceomics studies have repeatedly demonstrated that the aberrant subcellular localization of intracellular proteins is a distinct biological feature of tumorigenesis and vascular remodeling (68, 69). Ultimately, while the functional relevance of every identified EV surface-associated protein remains to be fully explored in future studies, the consistent identification of these proteins across our cell lines, points toward a legitimate and accessible surface architecture on cancer-derived EVs.

The top 15 most abundant pan-cancer EV-surface proteins are all implicated in cancer progression and 7 of these proteins are involved in EV biogenesis. Out of this EV surface top list, PKM, besides its metabolic function may be involved in tumor EV release by the phosphorylation of SNAP23 (70). Importantly, SNAP23 was also observed in our 112-protein list of pan-cancer surface proteins. The cytoskeletal proteins ACTG1, MYH9, FLNA, TLN1, TUBA1A, and TUBB4B play key roles in actin and microtubule dynamics, which are essential for vesicle formation, and transport. Importantly, these proteins are also implicated in tumor invasion, migration, and angiogenesis, suggesting that their presence on EV surface may be associated to malignant progression by cytoskeletal remodeling and angiogenesis stimulation (71, 72, 73, 74, 75). CLTC, DYNC1H1, and IQGAP1 are involved in vesicle trafficking and membrane dynamics further support a link between EV surface composition and mechanisms that drive EV formation and secretion (76, 77, 78)). The transporter ATP1A1 regulates Na^+^/K^+^ gradients and has been linked to tumor growth and therapy resistance, may influence EV cargo selection and membrane homeostasis during vesicle release (79). The reversed position of this protein is further interesting to investigate in respect of cancer progression. Finally, HSP90AB1 and HSPA8 are known EV-proteins that are involved in signaling pathways involved in tumor growth and survival (80, 81). EV-associated HSPs are frequently dysregulated in cancer and contribute to tumor progression, where they are anchored to the vesicle surface through lipid interactions (82, 83). Pan-cancer EV surface proteins HSPA8 and HSP90AB1 are very abundant and therefore promising candidates for cancer EV capture. Importantly, there is a commercial kit that precipitates EVs using the Vn96 peptide that binds HSPs on the EV surface, as was shown in the original research study (42). We previously benchmarked this HSP-based EV capture method against ultracentrifugation using cancer cell secretome and urine, underscoring that the method enriches for exosome related EVs (84, 85). Furthermore, multiple studies have used the HSP-based EV capture approach successfully for molecular profiling of EVs in clinical samples including blood and urine (86, 87, 88, 89, 90), underscoring the potential use of EV surface proteome data to develop EV capture assays.

### Limitations

While our study provides a comprehensive overview across 18 cell lines, we acknowledge that certain cancer types, such as bladder and cervical cancer, are represented by only a small number of lines. Consequently, these results may not fully account for the extensive intra-tumor heterogeneity characteristic of these diseases, and further validation in broader cohorts is warranted. Furthermore, while our proteomics data suggests the surface localization of typically cytosolic or nuclear proteins, subsequent biochemical validation, such as proteinase protection assays or surface-specific labeling, will be necessary to definitively confirm their topological orientation and explore functional consequences. Protein quantification was performed using spectral counting and statistical tests were not adjusted for multiple testing. Newer mass spectrometry workflows such as data independent acquisition with a more stringent statistical control could further refine the between-cell-line comparisons. Several proteins were identified based on single peptides (corresponding spectra provided in supplemental Figs. S6–S8). Proteins of the cancer surface EV core proteome set are abundant on EVs, yet may not be unique for cancer EVs as the “normal” cell line that exhibited very fast proliferation also displayed high surface EV abundance. Future studies, ideally using EVs isolated from patient material and controls are needed to establish enrichment on the surface of (cancer) EVs.

In conclusion, this study provides a comprehensive characterization of EV surface proteins across multiple cancer types, revealing the presence of membrane proteins with reverse topology as well as the unexpected enrichment of cytosolic and nuclear proteins on the EV surface. The highly abundant surface proteins consistently detected across all cancer cell lines in our multi-cancer panel represent promising targets for the development of capture reagents to enable high-throughput profiling of biofluid-derived EVs in cancer.

Underscoring potential clinical relevance, a subset of the abundant top 15 pan-cancer core set, including HSPA8 and HSP90AB1, was also enriched in plasma EVs from colorectal cancer patient relative to controls in two clinical cohorts. Further studies are warranted to profile biofluid EVs isolated in different cancer types to determine which of these proteins are truly cancer-enriched *versus* generally abundant on EVs. Altogether, our data supports the feasibility of using abundant EV surface proteins, including heat shock proteins, as universal EV capture proteins for EV-based diagnostics, while also identifying tumor type-specific proteins that may inform future biomarker development and functional studies.

## Data Availability

The mass spectrometry proteomics data have been deposited to the ProteomeXchange Consortium *via* the PRIDE (91, 92) partner repository with the dataset identifier PXD075520.

## Supplemental Data

This article contains supplemental data.

## Conflict of Interest

The authors declare no competing interests.

## Supplemental Data

Supplemental TablesSupplementary Table 1A: Overview of the 108 samples used in the project. Supplementary Table 1B: protein Report of the identified surface proteins. Supplementary Table 1C: protein Report of the identified TEV proteins. Supplementary Table 1D: protein Report of the identified SEV proteins. Supplementary Table 2: Annotated membrane protein to Multi Pass, Single Pass, GPI_anchored, Lipid_anchored and Peripheral Membrane. Supplementary Table 3. Annotated single and multipass proteins to functional classes and membrane location. Supplementary Table 4. List of cancer type enriched proteins. Supplementary Table 5. List of the core cancer surface proteins. Supplementary Table 6. Top 15 core EV surface proteins.

Supplemental Figs. S1–S5 Supplementary Figure 1: Dataset overviewA. Number of proteins identified by mass spectrometry in surface EV, TEV and SEV fractions in all samples per cancer type/cell line/replicates. The 5 excluded samples, because of too low protein identification number, are shown with a black line.B. Number of peptides identified between surface EV, TEV and SEV, showing that surface fraction contains less peptide as expected compared to TEV and SEV. Supplementary Figure 2: heatmaps of TEV and SEV fractions Unsupervised hierarchical clustering of EV TEV (A) and SEV (B) proteomes, showing no clear clustering between cancer types. Color intensity represents the Row Z-score, which scales protein abundance by subtracting the row mean and dividing by the row standard deviation. This normalization allows for the visualization of relative protein expression levels across samples, independent of absolute intensity scales. C, heatmap showing the average expression (Z-score) of the control cell line versus the cancer cell lines of the top 112 proteins detected. Supplementary Figure 3: Multi- and Single-pass transmembrane EV surface protein network A. Protein-protein interaction network of multi-and single-pass EV surface transmembrane proteins was created using STRING (23), and visualization was further modifed using Cytoscape (24) and OmicsVis (25). Fill shows if the protein is multi- (blue) or single-pass (pink); inner ring shows the membrane locations; and outer ring shows the functional classes. B. Examples of 2 proteins with reversed detection using Protter mapping. Supplementary Figure 4: Identificaton of surface-enriched EV proteins per cancer type EV surface proteome of each cancer type was compared to the other cancer types in multiple two-group comparisons, resulting in 4 comparisons per cancer type. Significantly (p < 0.05) upregulated EV surface proteins per cancer type that overlap in 3 out of the 4 comparisons were selected as cancer type-enriched EV surface proteins. Intersecting proteins that are shown in Fig. 5C are highlighted. Supplementary Figure 5: Identification of surface enriched proteins on plasma EVs of colorectal cancer patients. Plasma EV protein expression of both our internal data, and external dataset (41), showing that several of the top15 surface proteins are enriched on plasma EVs of CRC patients compared to controls.

Supplemental Fig. S6 Supplementary Figure 6: single peptide report Total EV fraction.

Supplemental Fig. S7 Supplementary Figure 7: single peptide report SEV fraction.

Supplemental Fig. S8 Supplementary Figure 8: single peptide report surface fraction
