# Supplementary material for: Surface Proteomics of Cancer-Derived Extracellular Vesicles Reveals Universal Heat Shock Proteins and Cancer Signatures
Source: Mol Cell Proteomics. 2026 Aug 22;25(10):101649. doi: 10.1016/j.mcpro.2026.101649 (PMC13634791; doi:10.1016/j.mcpro.2026.101649)

Suppl. Figure 1:

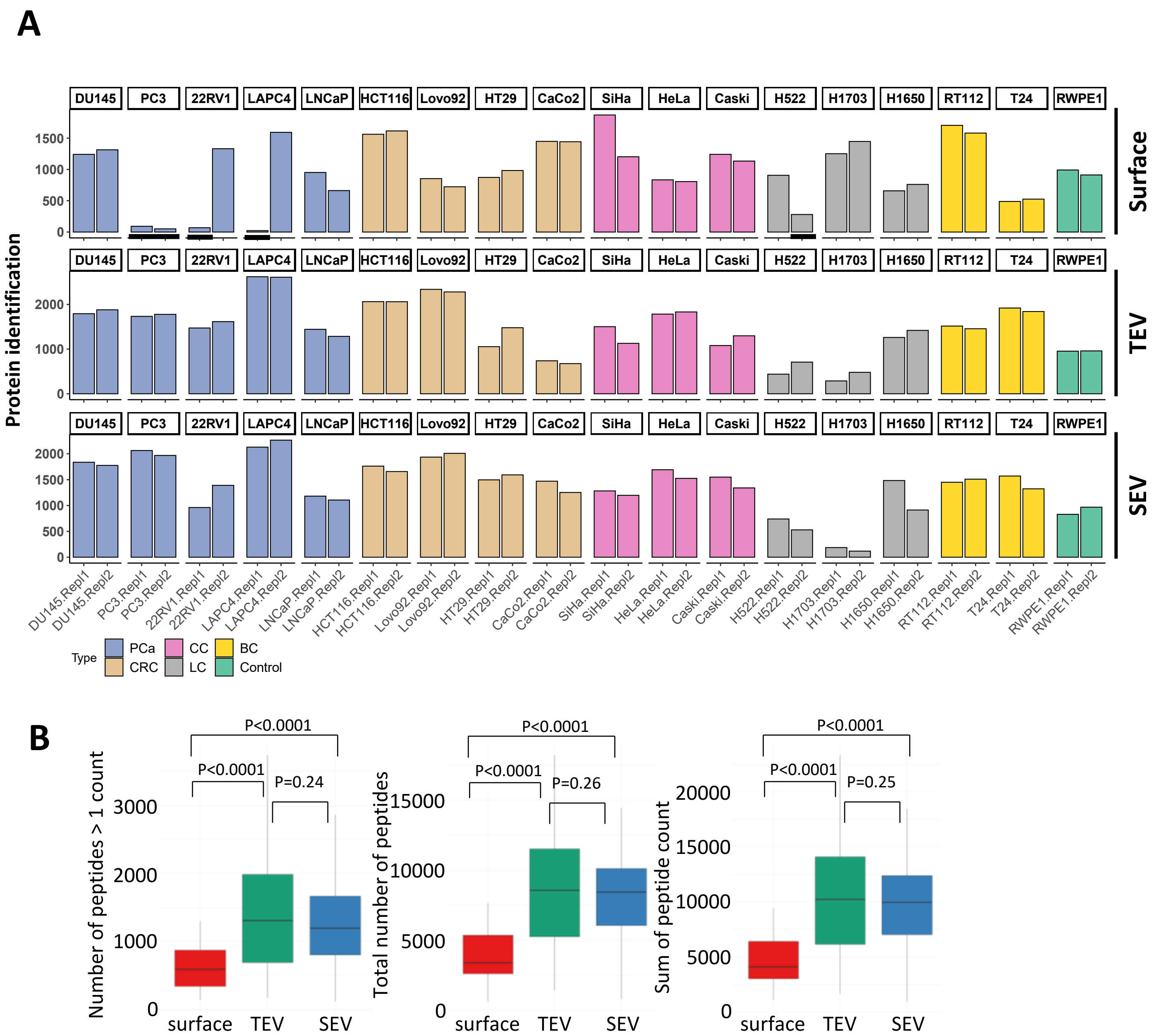

Suppl. Figure 2:

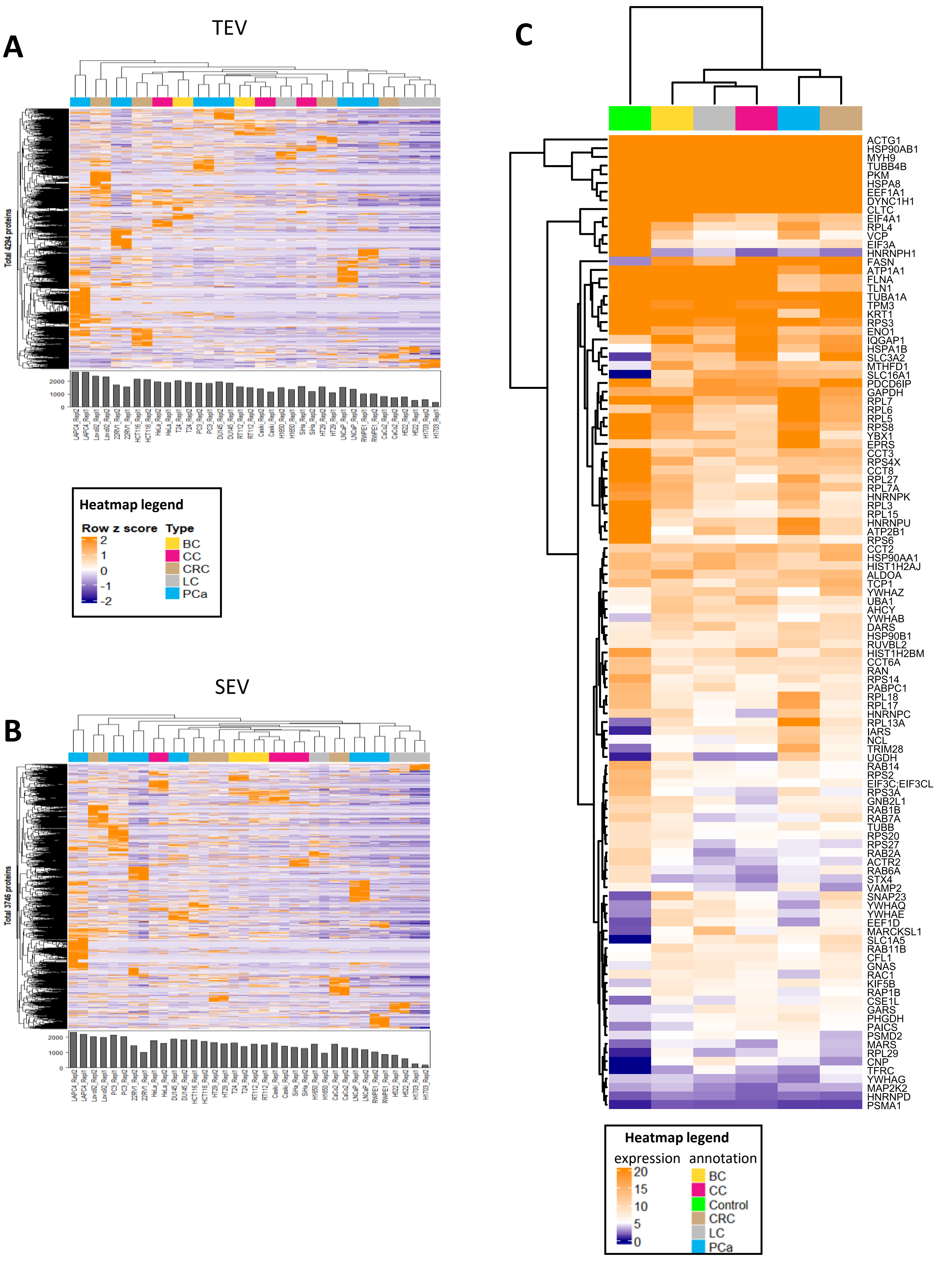

Suppl. Figure 3: Multi- and Single-pass transmembrane EV surface protein network

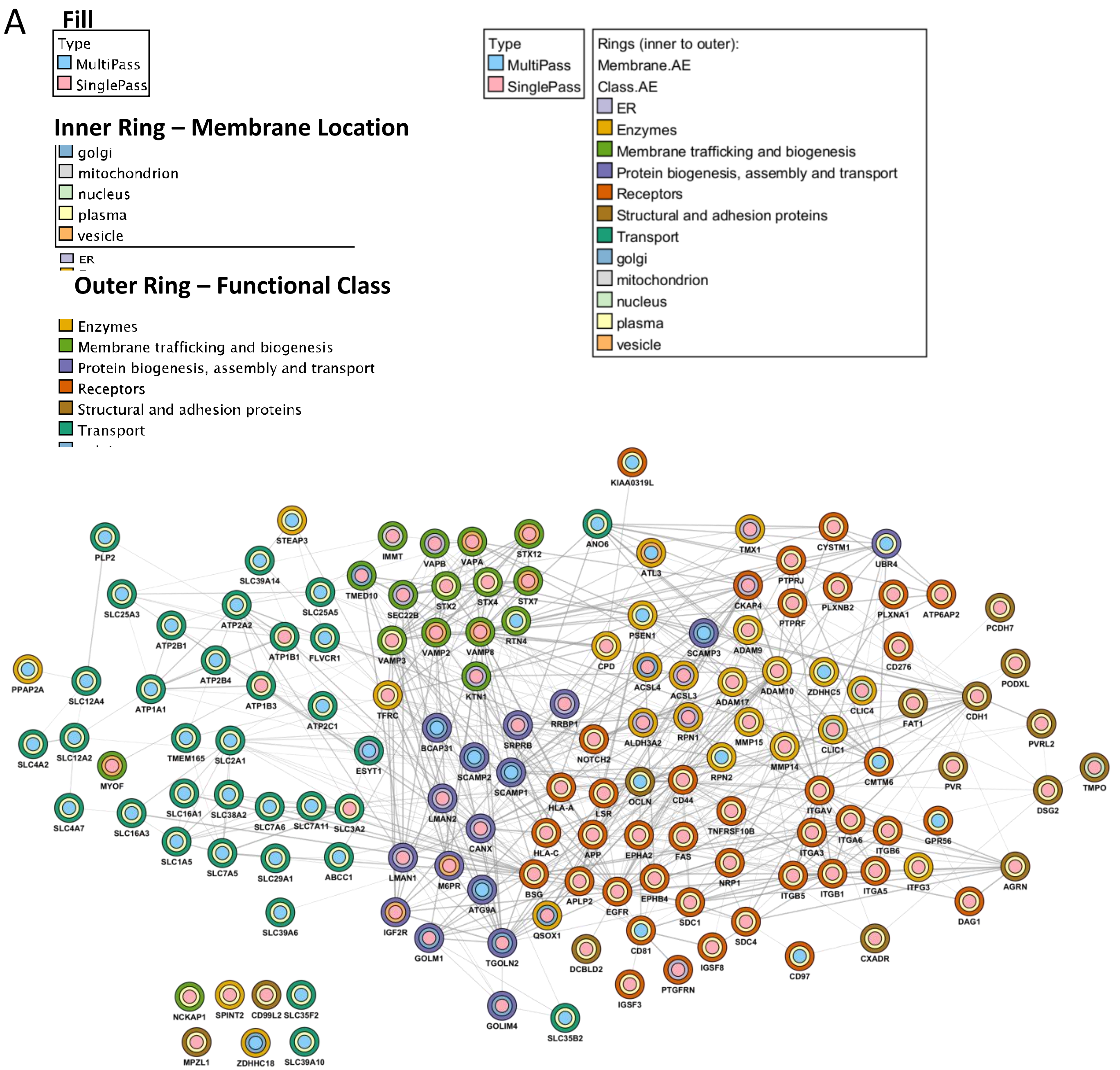

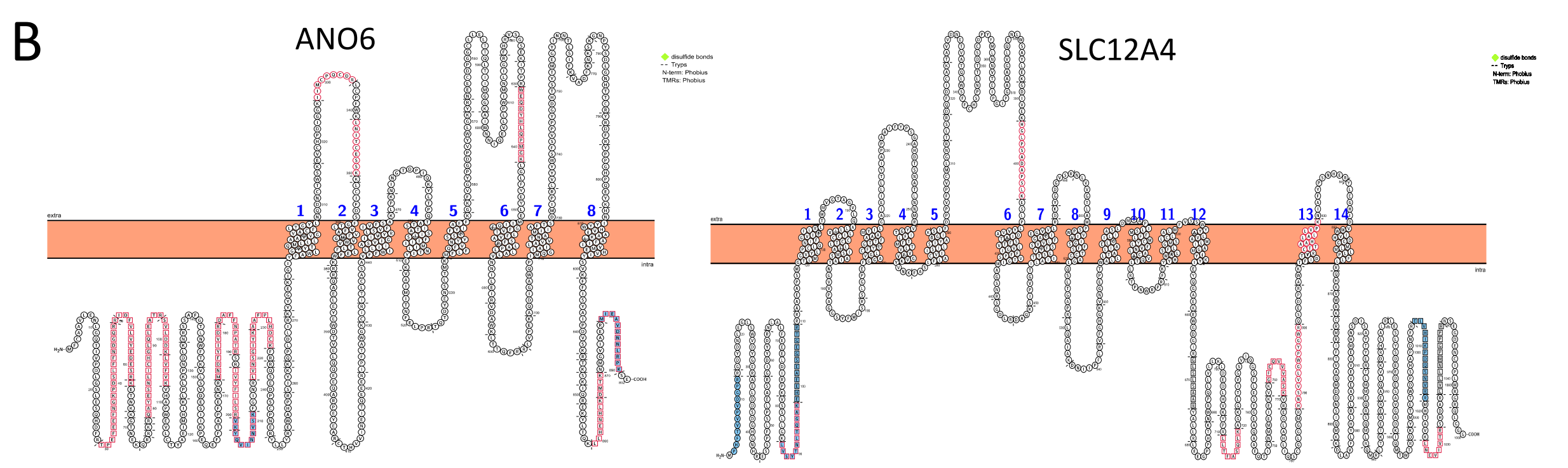

LEGEND (Protter)

TEV: Square

SEV: red outline

Surface: Blue fill

Default: Round

Suppl. Figure 4: Identificaton of surface-enriched EV proteins per cancer type

Comparisons per cancer type

PCa-vs.-	SURFACE EV FRACTION		
	# Samples PCa, n = 4	# Total Protein	UP in PCa $p < 0.05$
CRC	4	3067	87
CC	3	3037	69
LC	3	2906	68
BC	2	2906	60

CRC-vs.-	SURFACE EV FRACTION		
	# Samples CRC, n = 4	# Total Protein	UP in CRC $p < 0.05$
PCa	4	3127	101
CC	3	2948	28
LC	3	2840	49
BC	2	2795	29

CC-vs.-	SURFACE EV FRACTION		
	# Samples CC, n = 3	# Total Protein	UP in CC $p < 0.05$
PCa	4	3037	110
CRC	4	2948	51
LC	3	2681	46
BC	2	2596	25

LC-vs.-	SURFACE EV FRACTION		
	# Samples LC, n = 3	# Total Protein	UP in LC $p < 0.05$
PCa	4	2906	75
CRC	4	2840	47
CC	3	2681	21
BC	2	2449	23

BC-vs.-	SURFACE EV FRACTION		
	# Samples BC, n = 2	# Total Protein	UP in BC $p < 0.05$
PCa	4	2906	75
CRC	4	2795	27
CC	3	2596	26
LC	3	2449	43

Overlap min 3 out of 4 comparisons

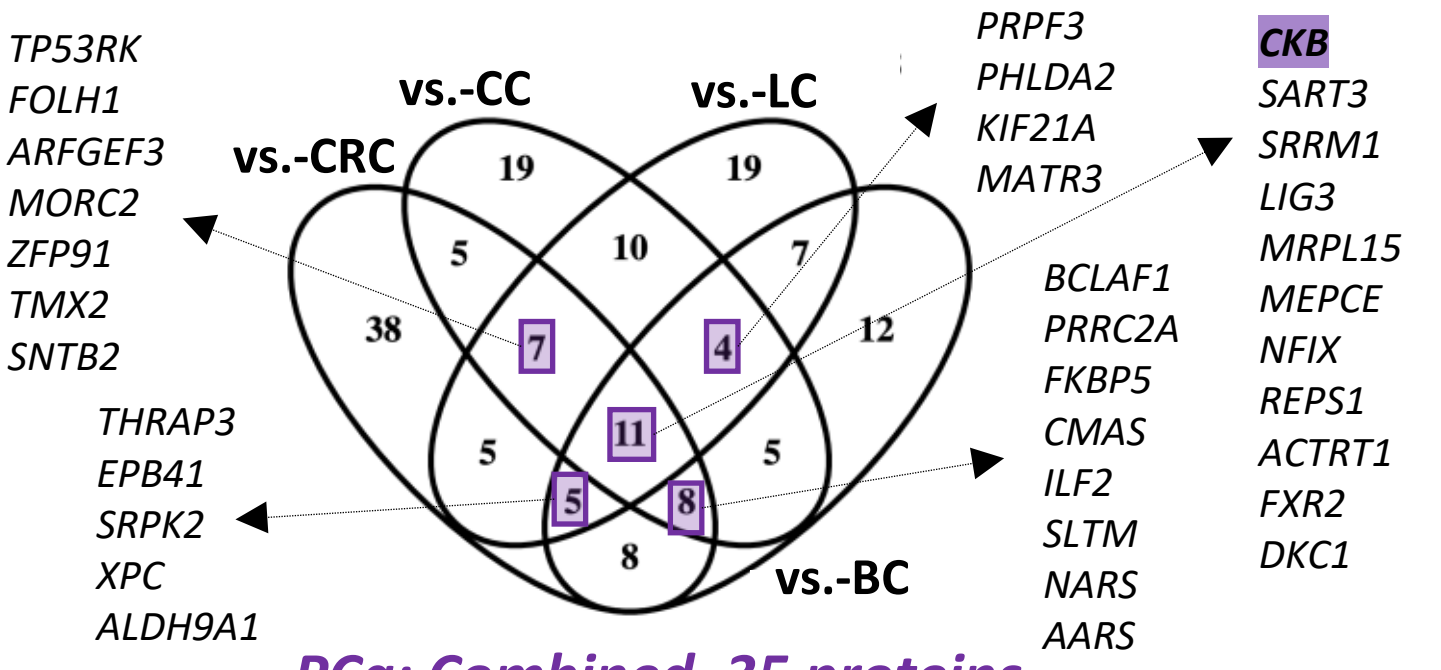

PCa: Combined 35 proteins

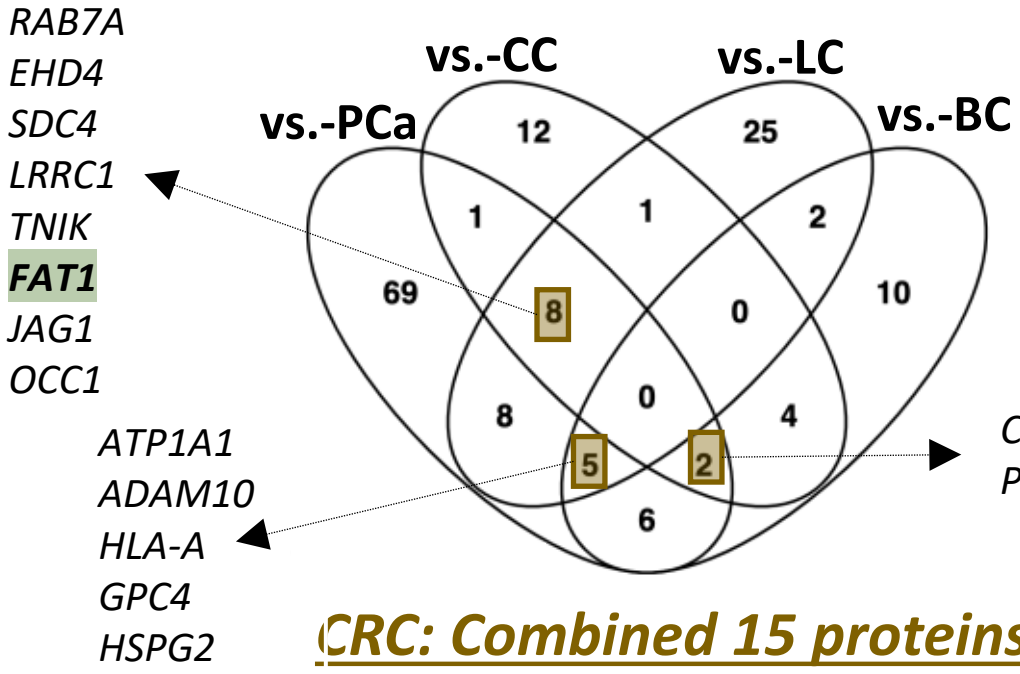

CRC: Combined 15 proteins

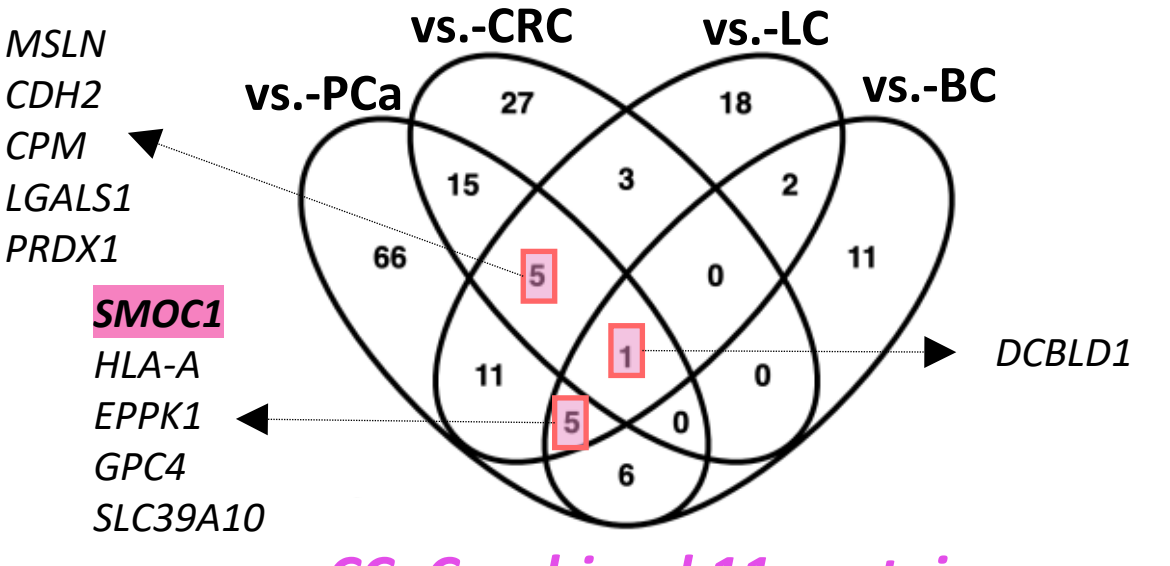

CC: Combined 11 proteins

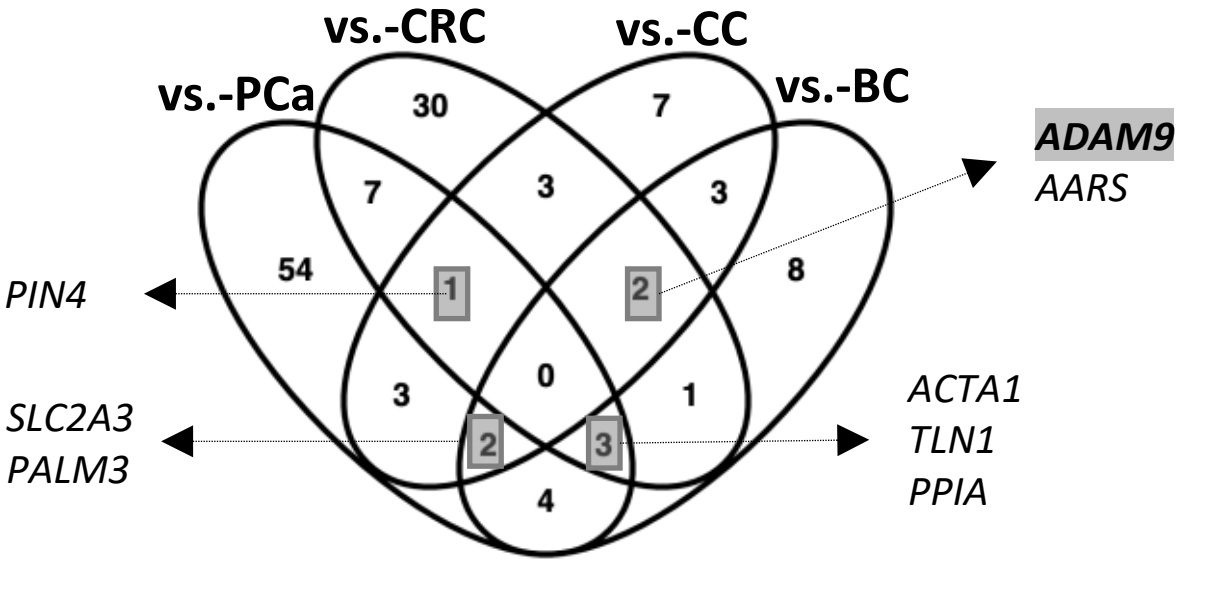

LC: Combined 8 proteins

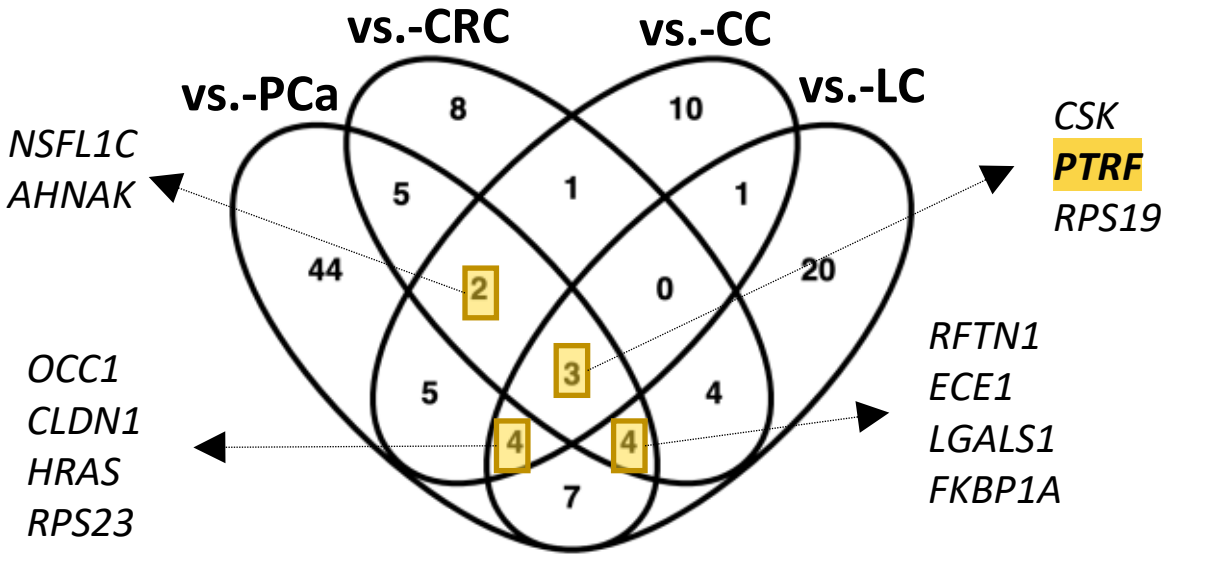

BC: Combined 13 proteins

Suppl. Figure 5: Identificaton of surface-enriched EV proteins per cancer type

Plasma EV protein expression – internal data

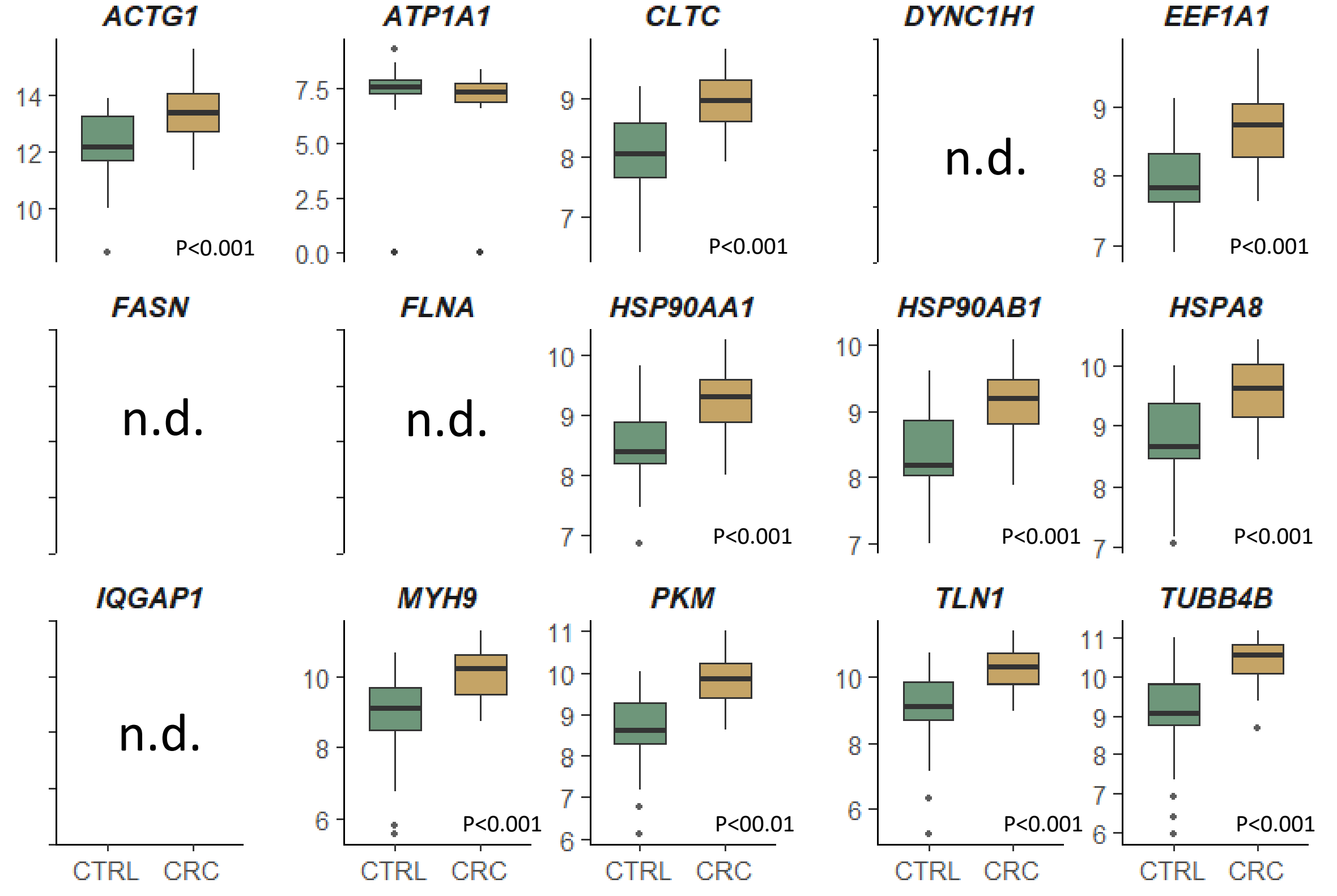

Plasma EV protein expression – external data

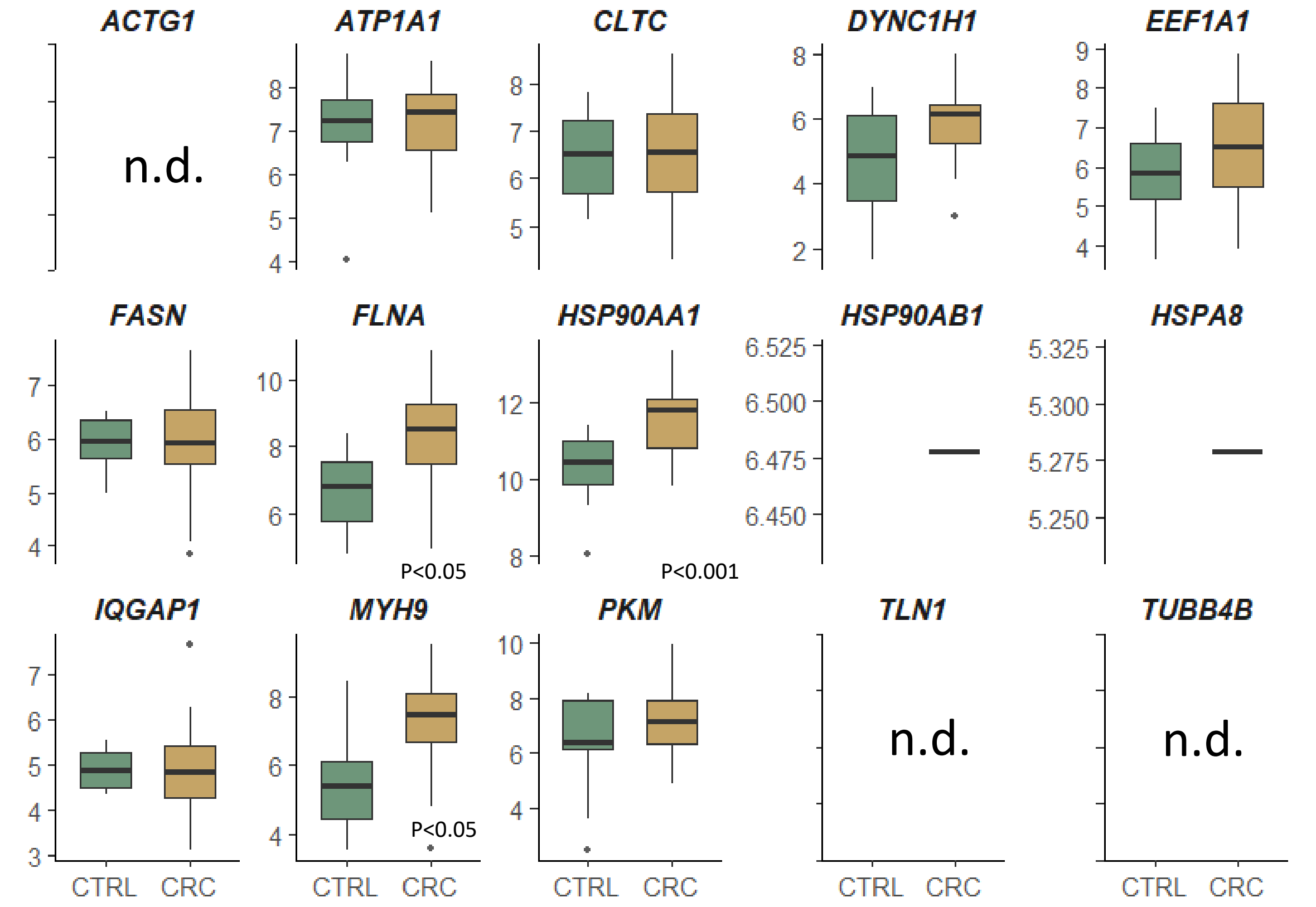

Supplement: Supplementary file 1 [file mmc2.pdf]
