# Supplementary material for: Surface Proteomics of Cancer-Derived Extracellular Vesicles Reveals Universal Heat Shock Proteins and Cancer Signatures
Source: Mol Cell Proteomics. 2026 Aug 22;25(10):101649. doi: 10.1016/j.mcpro.2026.101649 (PMC13634791; doi:10.1016/j.mcpro.2026.101649)
Supplement: Supplementary file 2 [file mmc3.pdf]

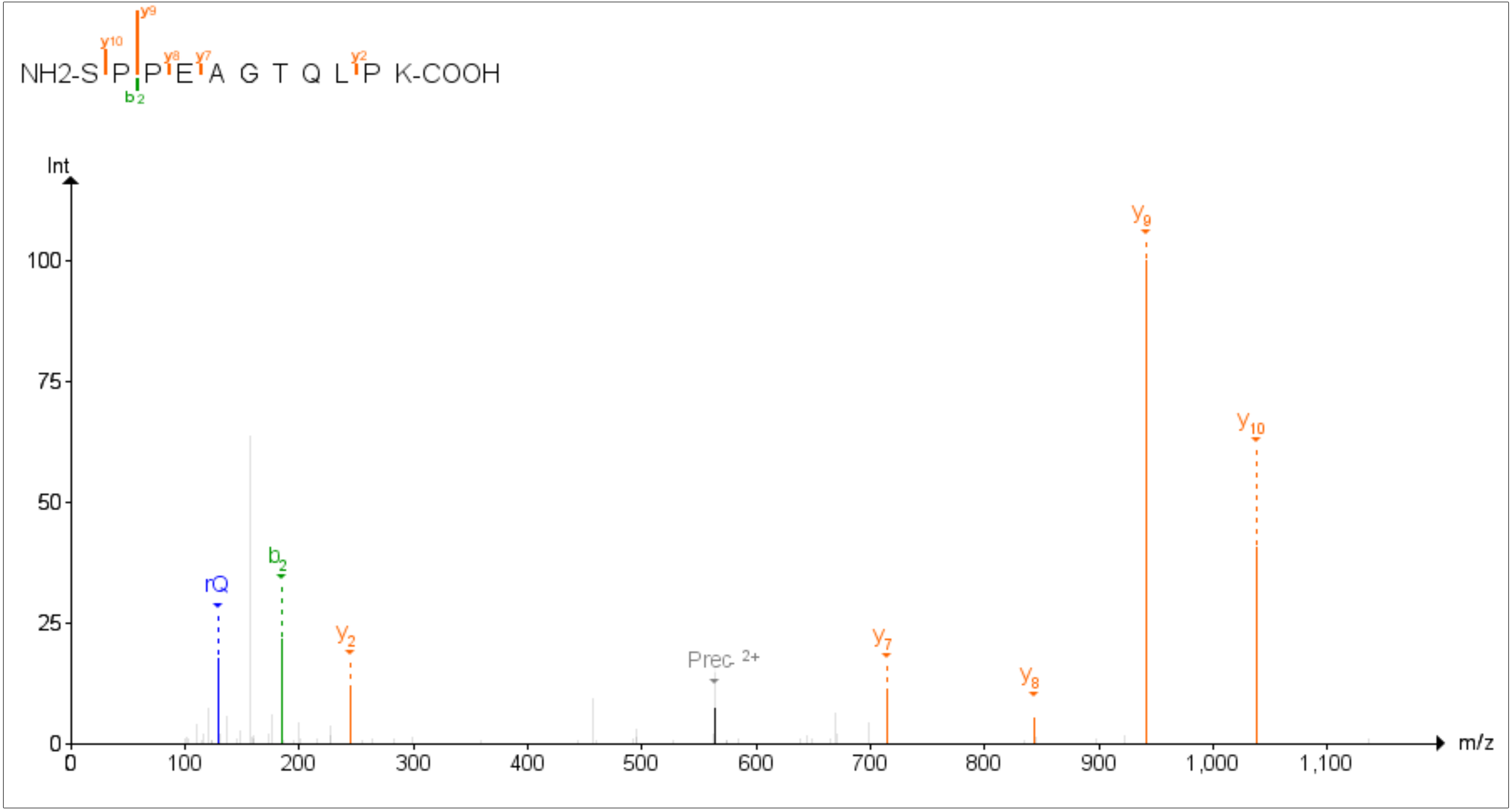
NH2-S P P E A G T Q L P K-COOH
y10
y9
y8
y7
y2
b2
Int
100
75
50
25
0
rQ
b2
y2
Prec 2+
y7
y8
y9
y10
0
100
200
300
400
500
600
700
800
900
1,000
1,100
m/z

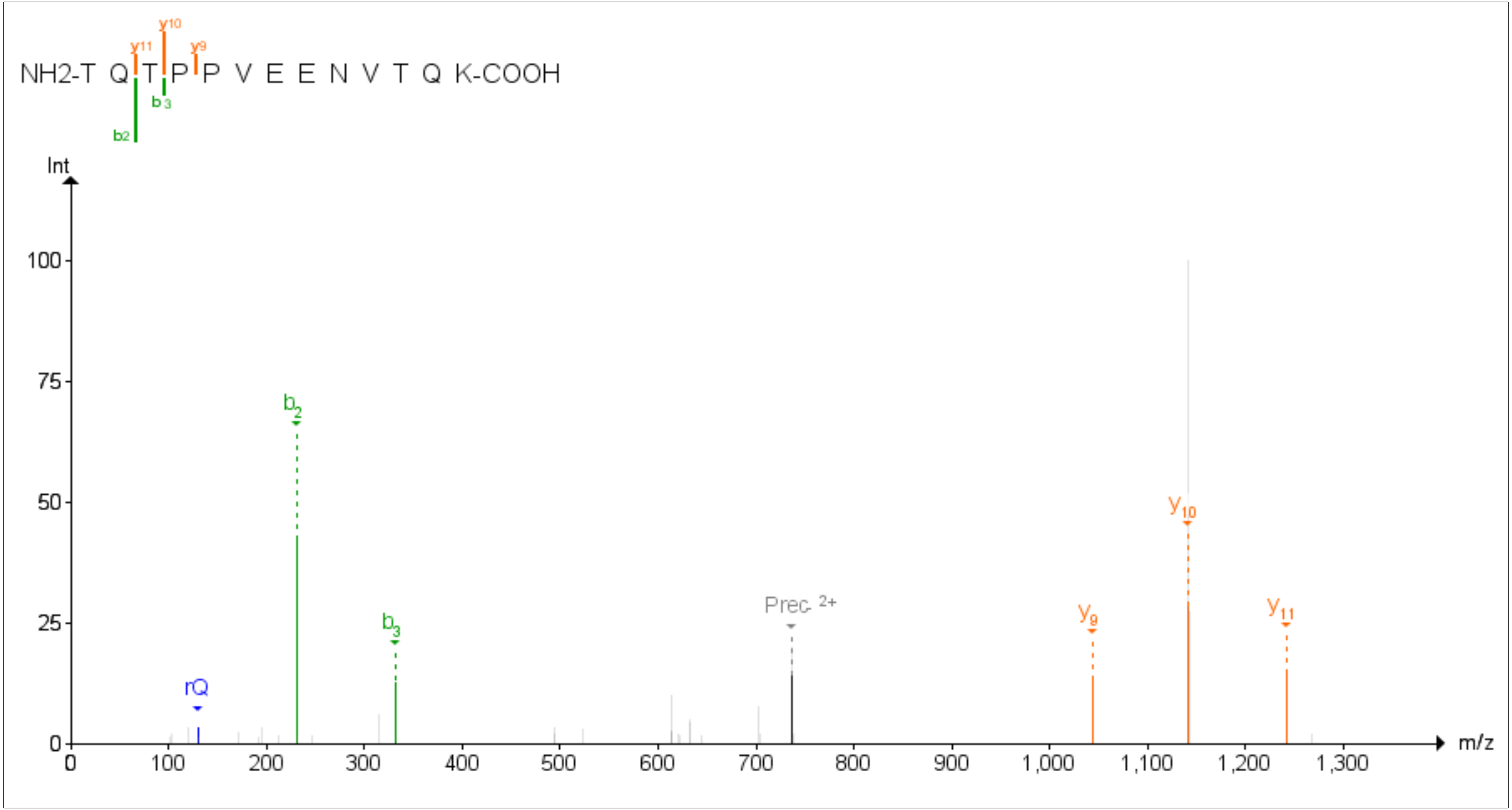

NH2-T Q T P P V E E N V T Q K-COOH
y10
y11
y9
b3
b2
Int
100
75
50
25
0
b2
b3
rQ
Prec 2+
y9
y10
y11
0
100
200
300
400
500
600
700
800
900
1,000
1,100
1,200
1,300
m/z

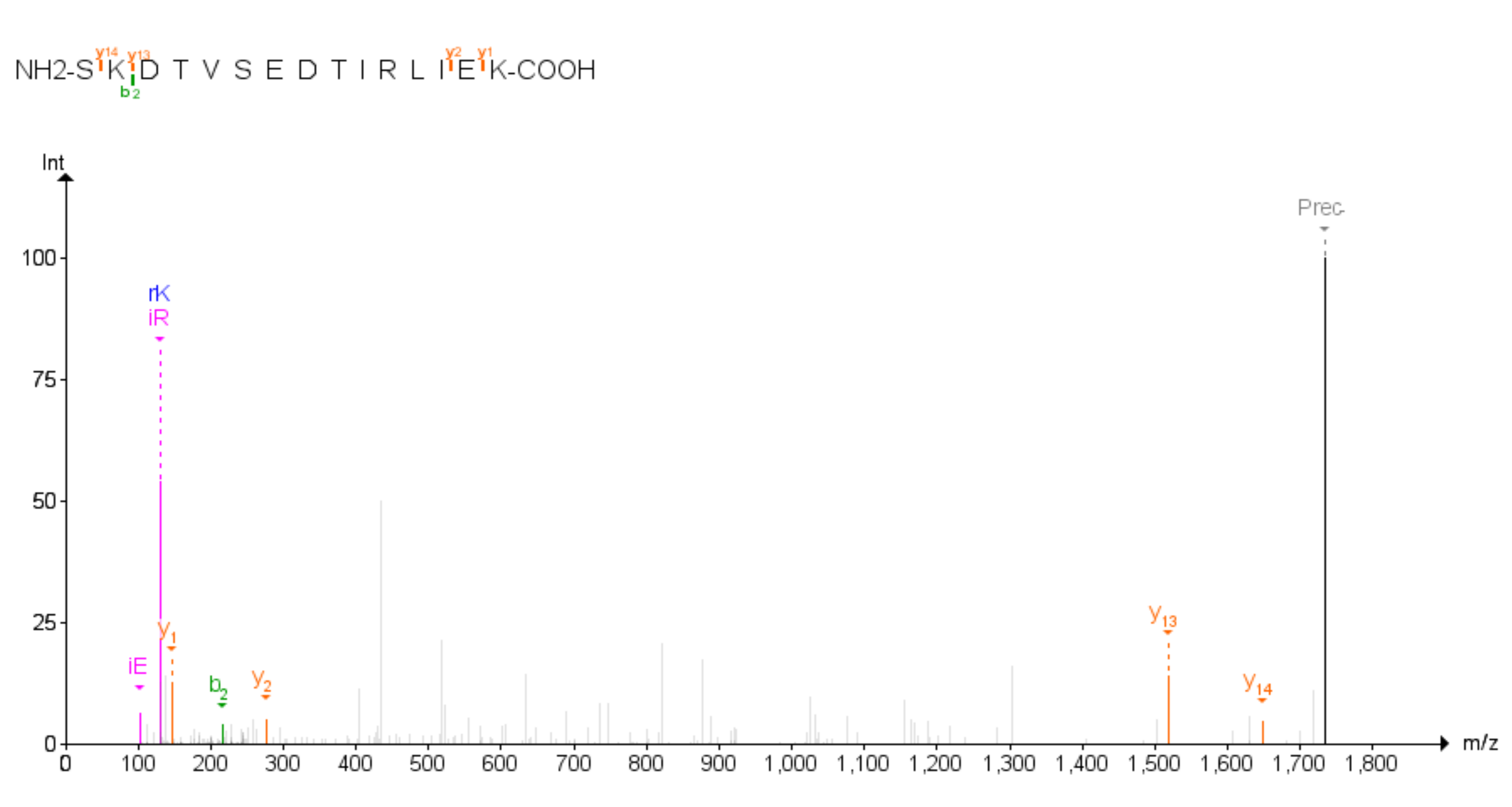
NH2-S K D T V S E D T I R L I E K-COOH
y14
y13
b2
y2
y1
Int
m/z
Prec.
rK
iR
iE
y1
b2
y2
y13
y14
0
25
50
75
100
0
100
200
300
400
500
600
700
800
900
1,000
1,100
1,200
1,300
1,400
1,500
1,600
1,700
1,800

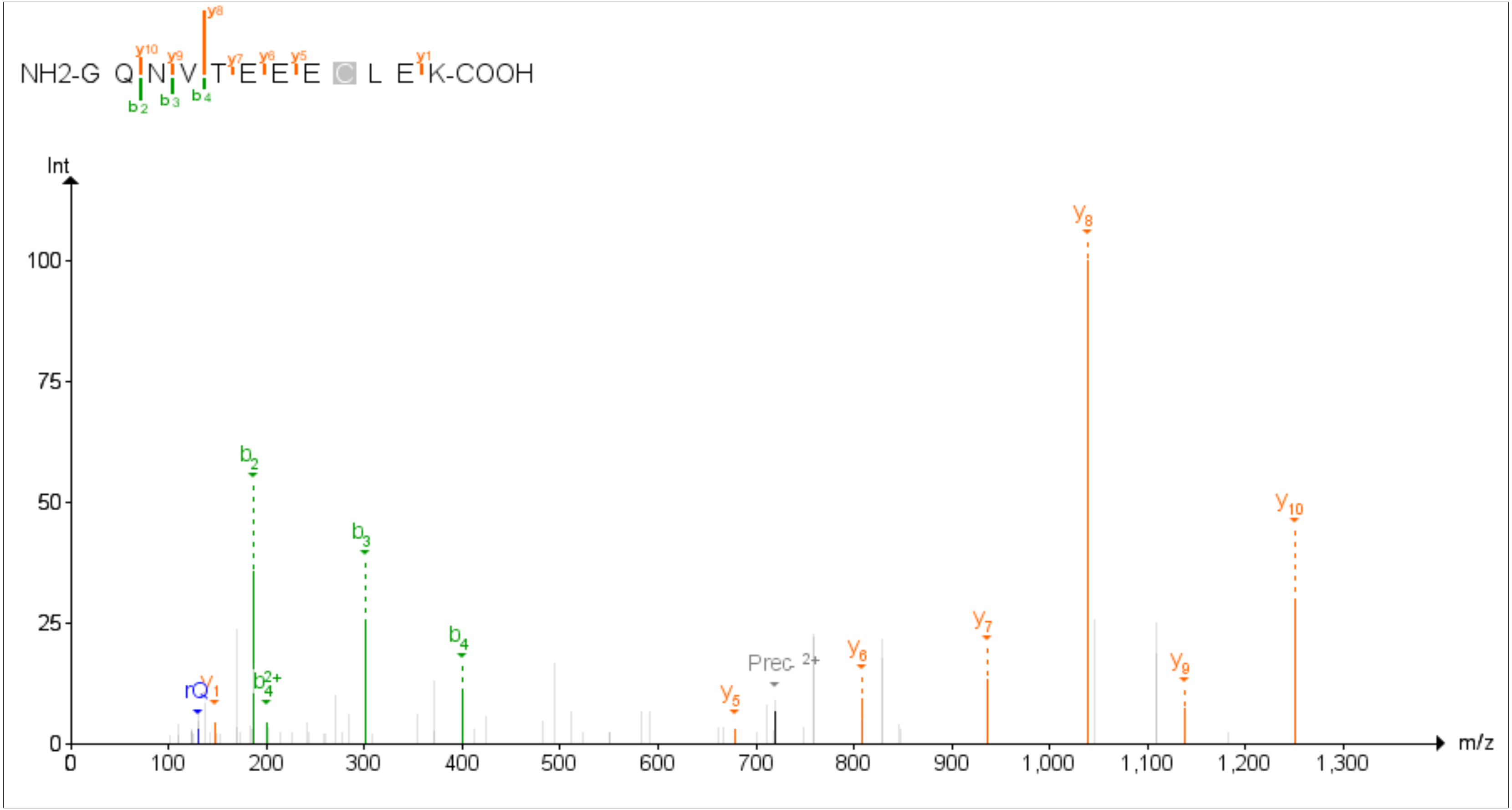

NH2-G Q N V T E E E C L E K-COOH
y10 y9 y8 y7 y6 y5 y1
b2 b3 b4
Int
100
75
50
25
0
m/z
0 100 200 300 400 500 600 700 800 900 1,000 1,100 1,200 1,300
rQ
y1
b2
b4 2+
b3
b4
Prec 2+
y5
y6
y7
y8
y9
y10

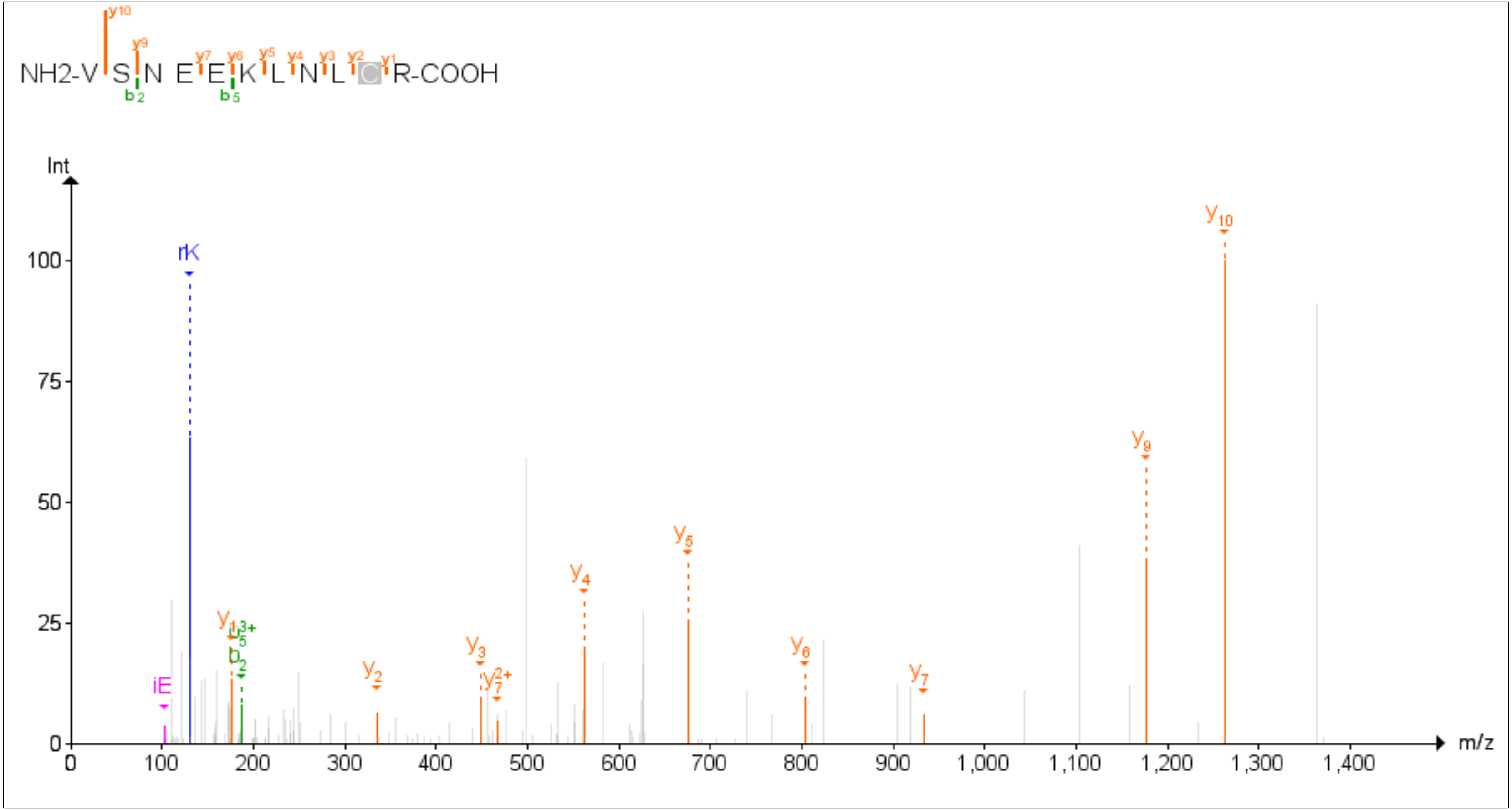

NH2-V S N E E K L N L C R-COOH
Int
m/z
rK
iE
y10
y9
y5
y4
y6
y7
y3
y2
y1
b2
b5
y7 2+
b5 3+
0
25
50
75
100
0
100
200
300
400
500
600
700
800
900
1,000
1,100
1,200
1,300
1,400

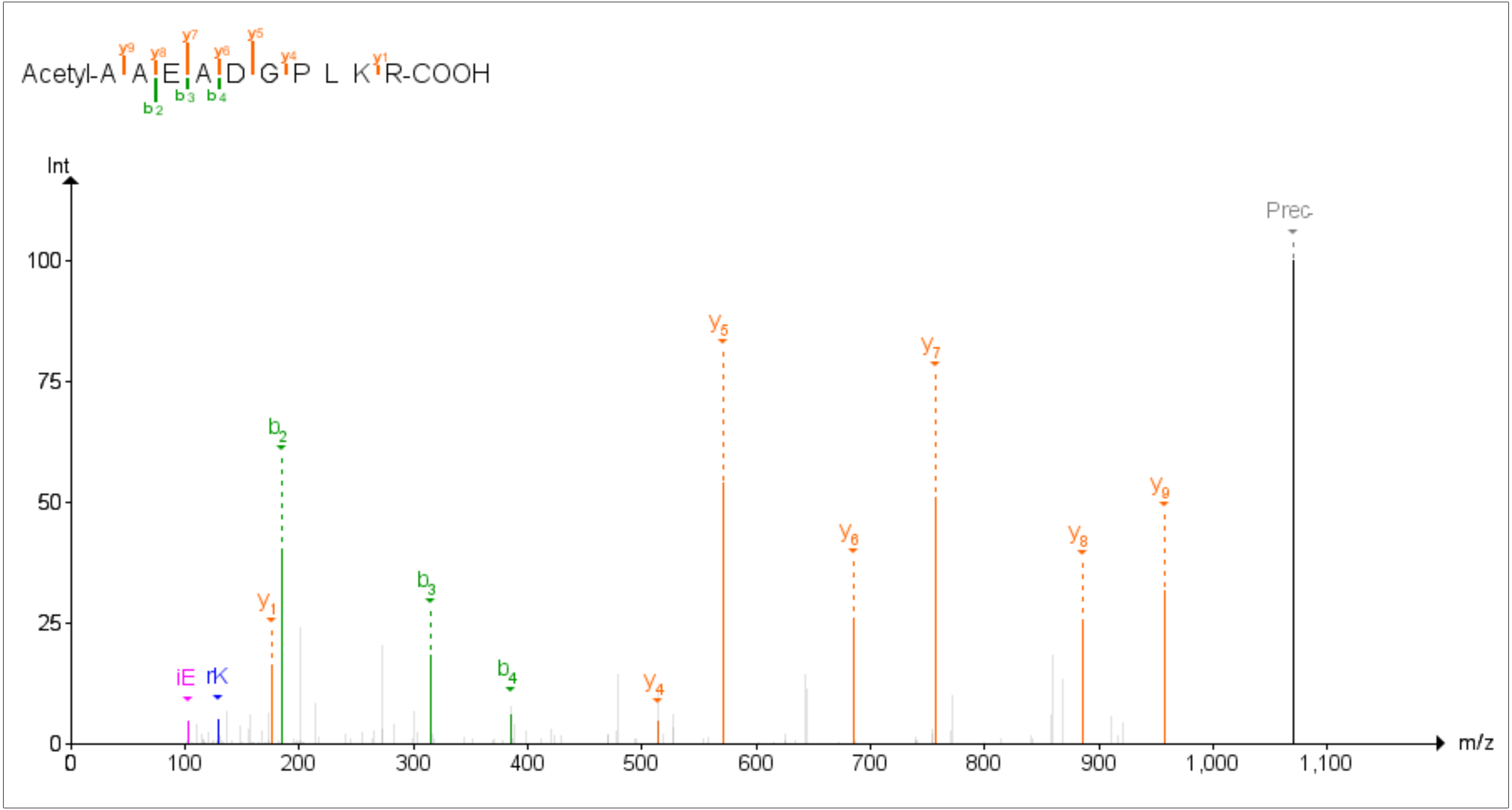

Acetyl-A A E A D G P L K R-COOH
y9 y8 y7 y6 y5 y4 y1
b2 b3 b4
Int
100
75
50
25
0
Prec.
y5
y7
b2
y9
y6
y8
b3
y1
b4
y4
iE
rK
0
100
200
300
400
500
600
700
800
900
1,000
1,100
m/z

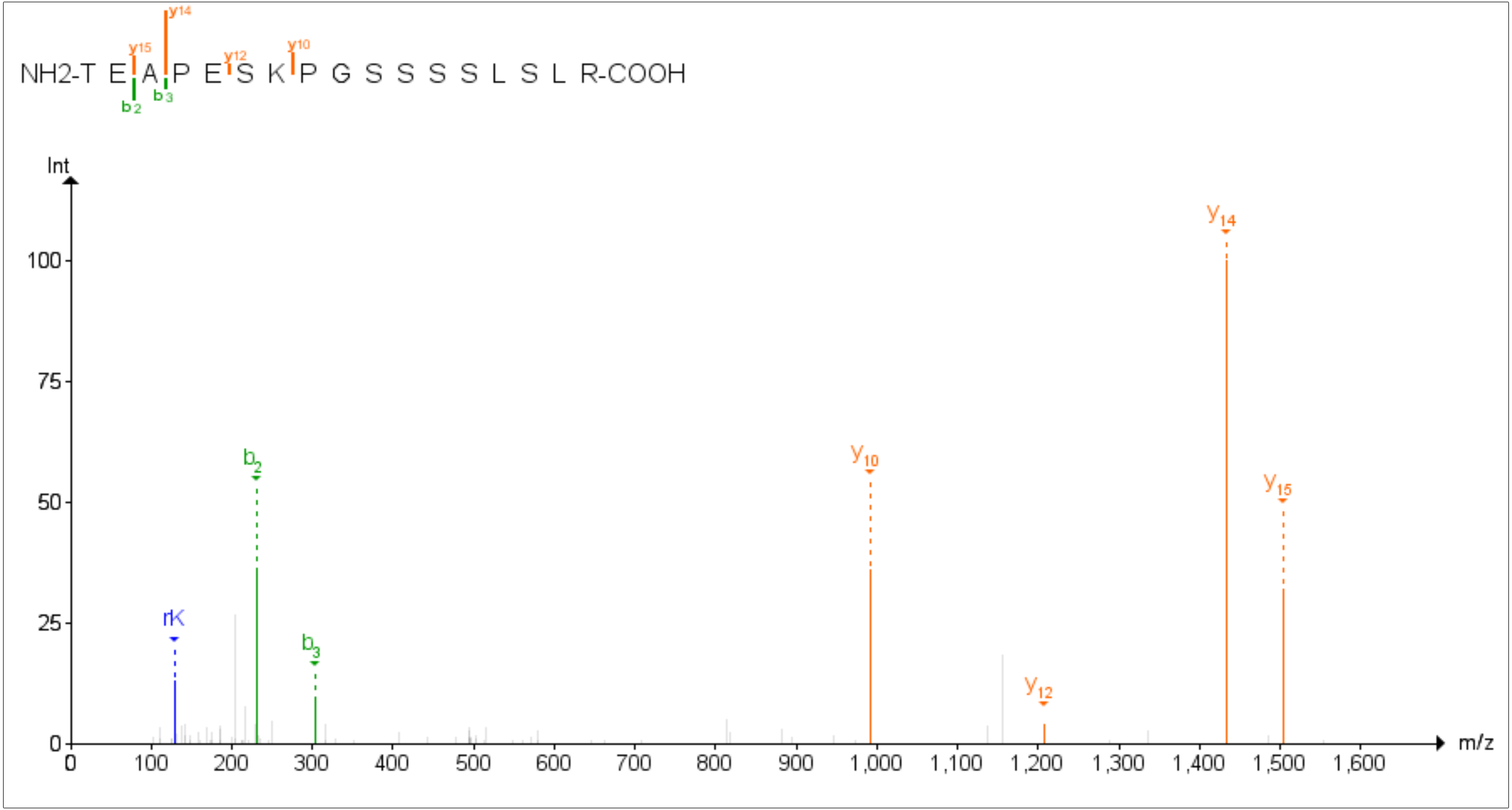

NH2-T E A P E S K P G S S S S L S L R-COOH
y14
y15
y12
y10
b2
b3
Int
100
75
50
25
0
rK
b2
b3
y10
y12
y14
y15
0
100
200
300
400
500
600
700
800
900
1,000
1,100
1,200
1,300
1,400
1,500
1,600
m/z

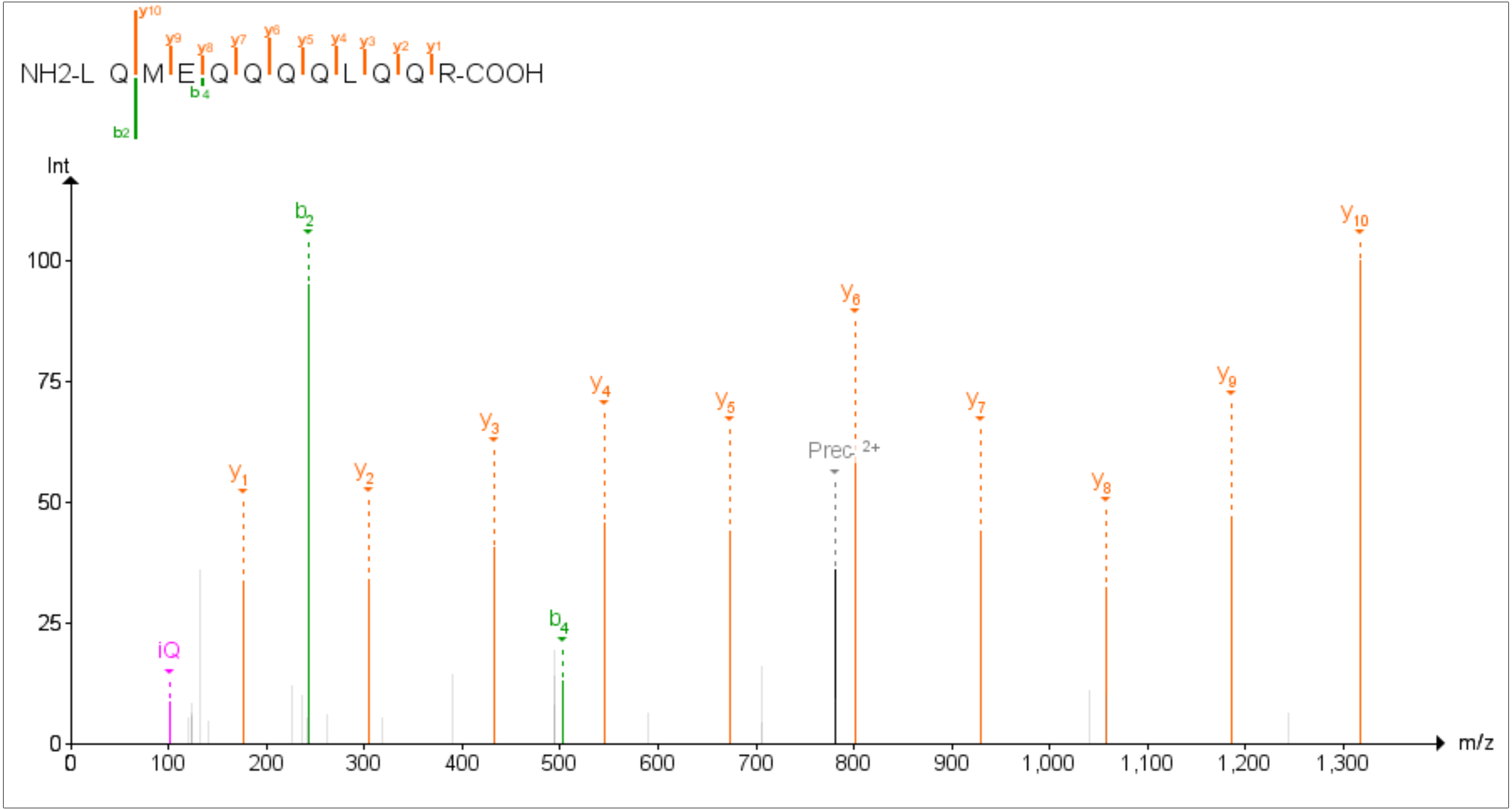

NH2-L Q M E Q Q Q Q L Q Q R-COOH
y10 y9 y8 y7 y6 y5 y4 y3 y2 y1
b2 b4
Int
m/z
iQ
y1 y2 y3 y4 y5 y6 y7 y8 y9 y10
b2 b4
Prec 2+
0 25 50 75 100
0 100 200 300 400 500 600 700 800 900 1,000 1,100 1,200 1,300

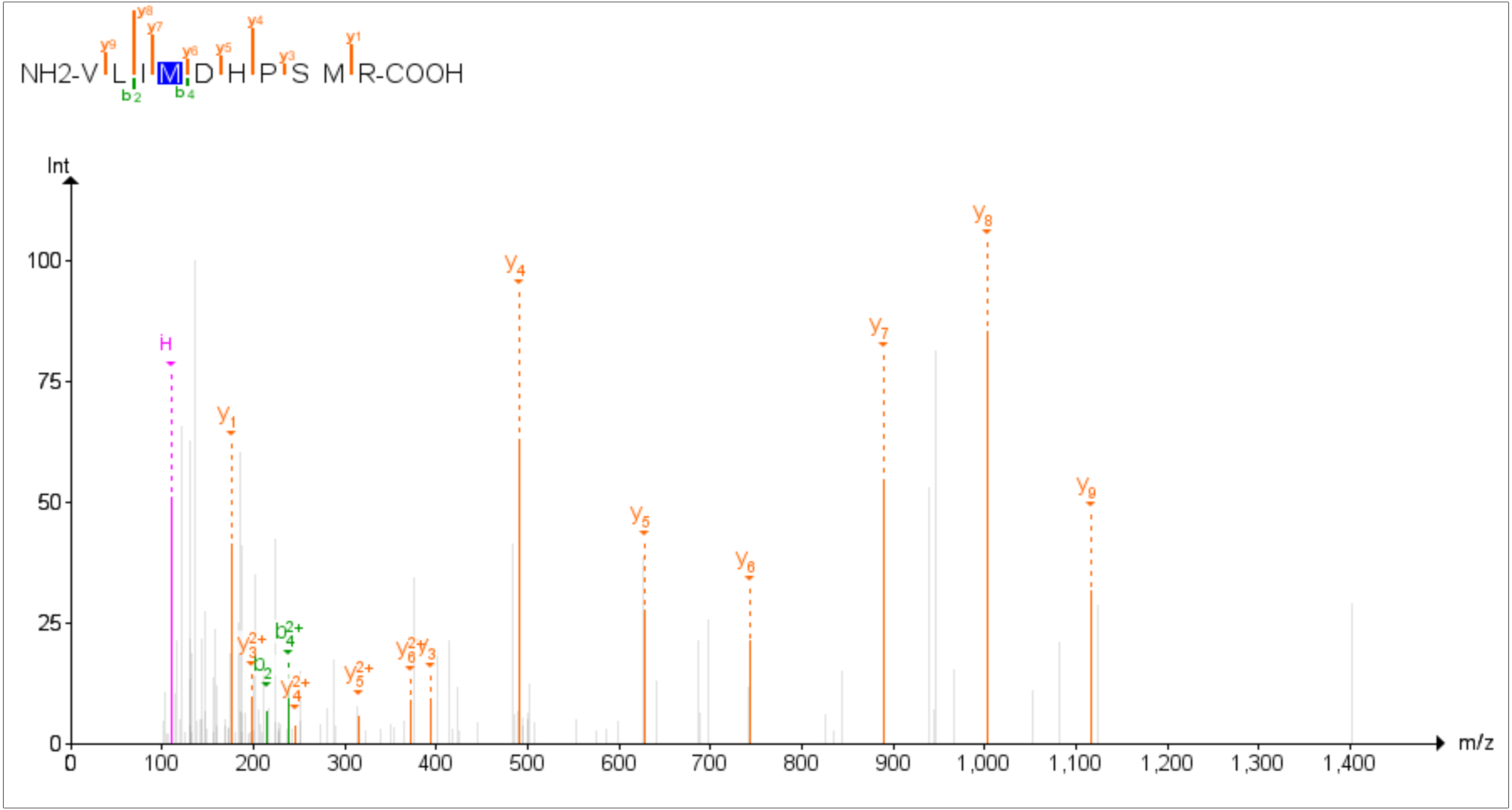
NH2-V L I M D H P S M R-COOH
y9 y8 y7 y6 y5 y4 y3 y1
b2 b4
Int
100
75
50
25
0
H
y1
y4
y5
y6
y7
y8
y9
y3
b2
b4
m/z
0
100
200
300
400
500
600
700
800
900
1,000
1,100
1,200
1,300
1,400

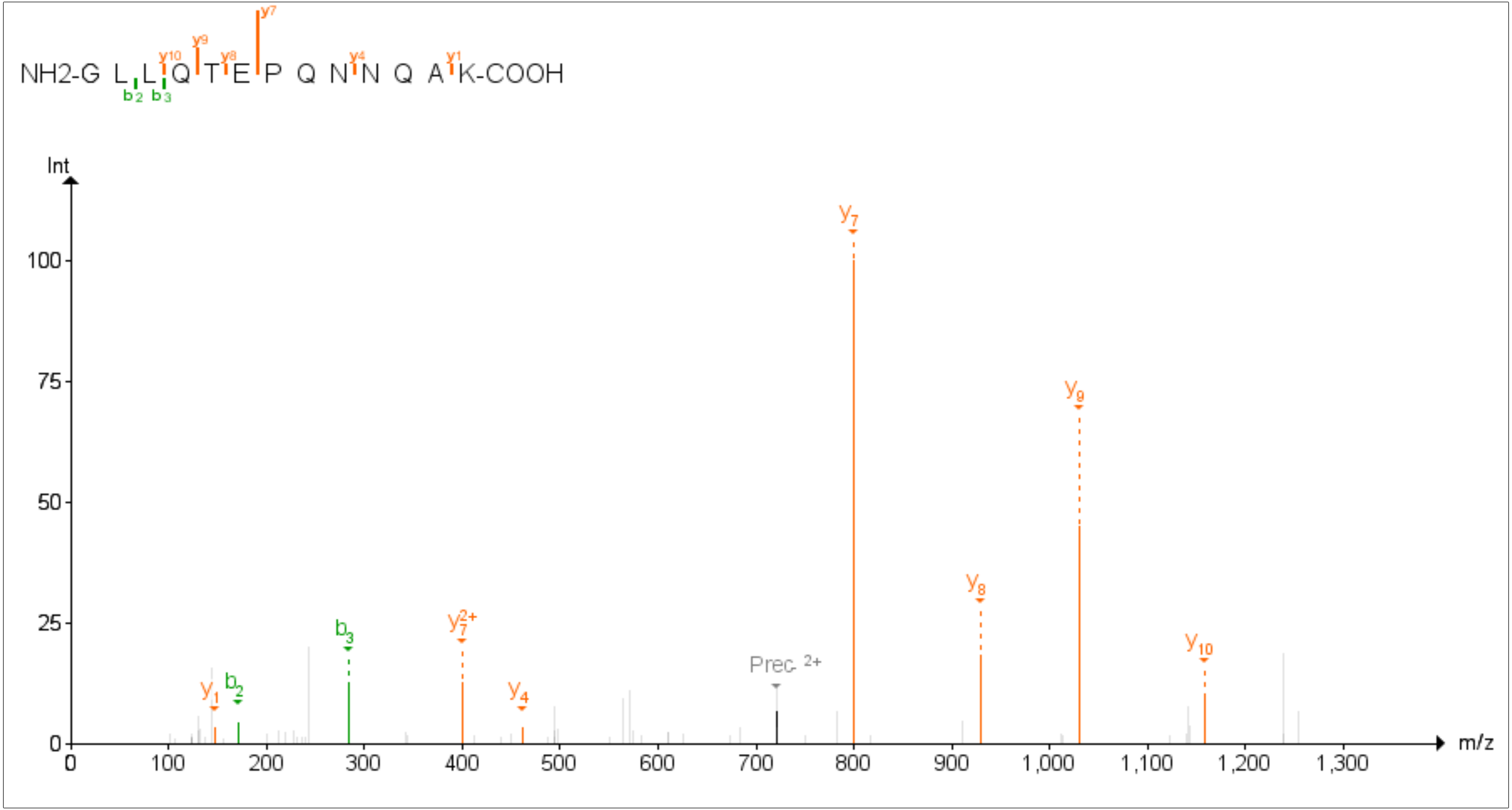
NH2-G L L Q T E P Q N N Q A K-COOH
y10 y9 y8 y7 y4 y1
b2 b3
Int
100
75
50
25
0
y7
y9
y8
y10
b3
y7 2+
y1
b2
y4
Prec- 2+
0 100 200 300 400 500 600 700 800 900 1,000 1,100 1,200 1,300
m/z

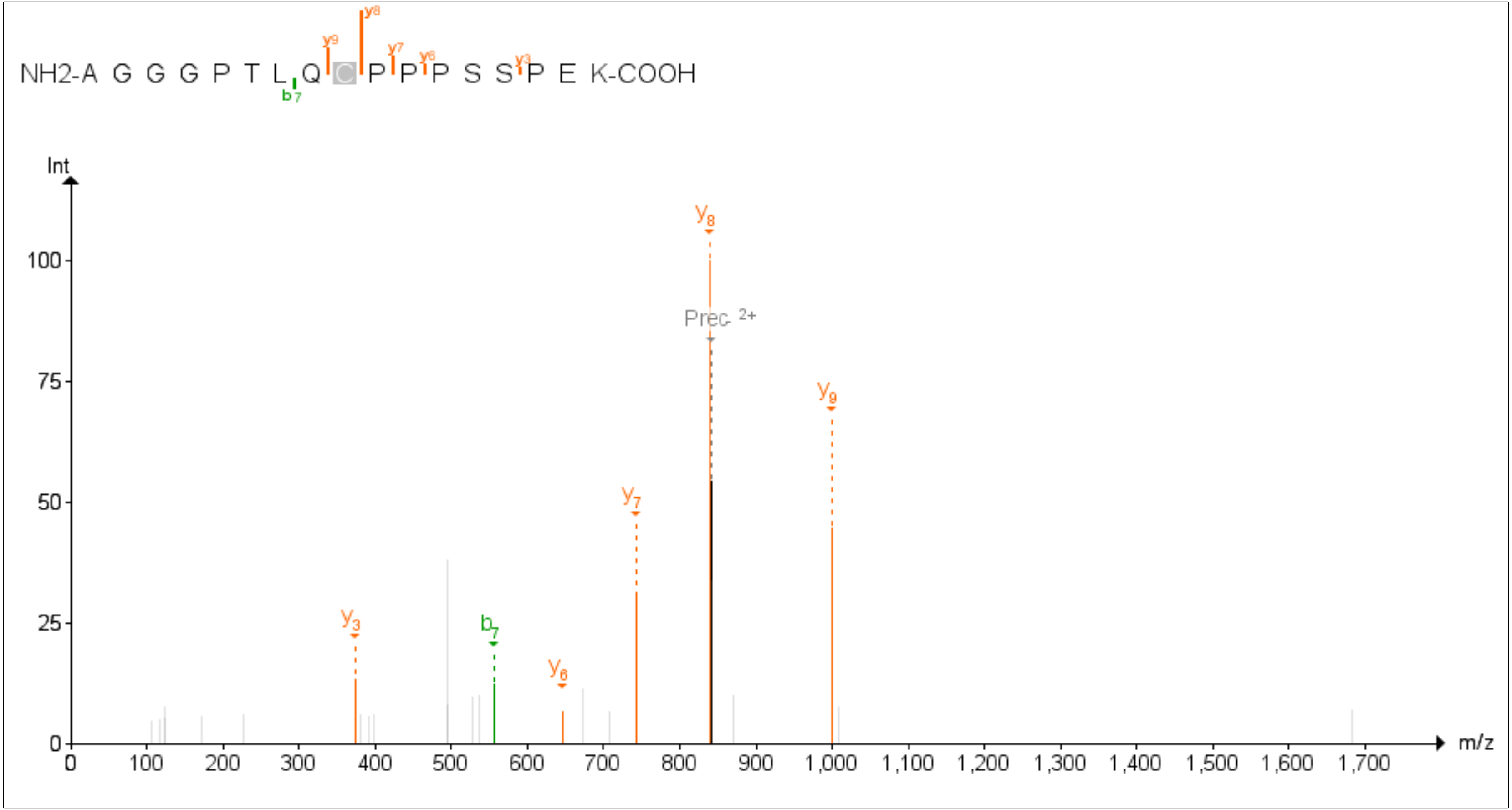

NH2-A G G G P T L Q C P P P S S P E K-COOH
y9
y8
y7
y6
y3
b7
Int
100
75
50
25
0
Prec 2+
0
100
200
300
400
500
600
700
800
900
1,000
1,100
1,200
1,300
1,400
1,500
1,600
1,700
m/z

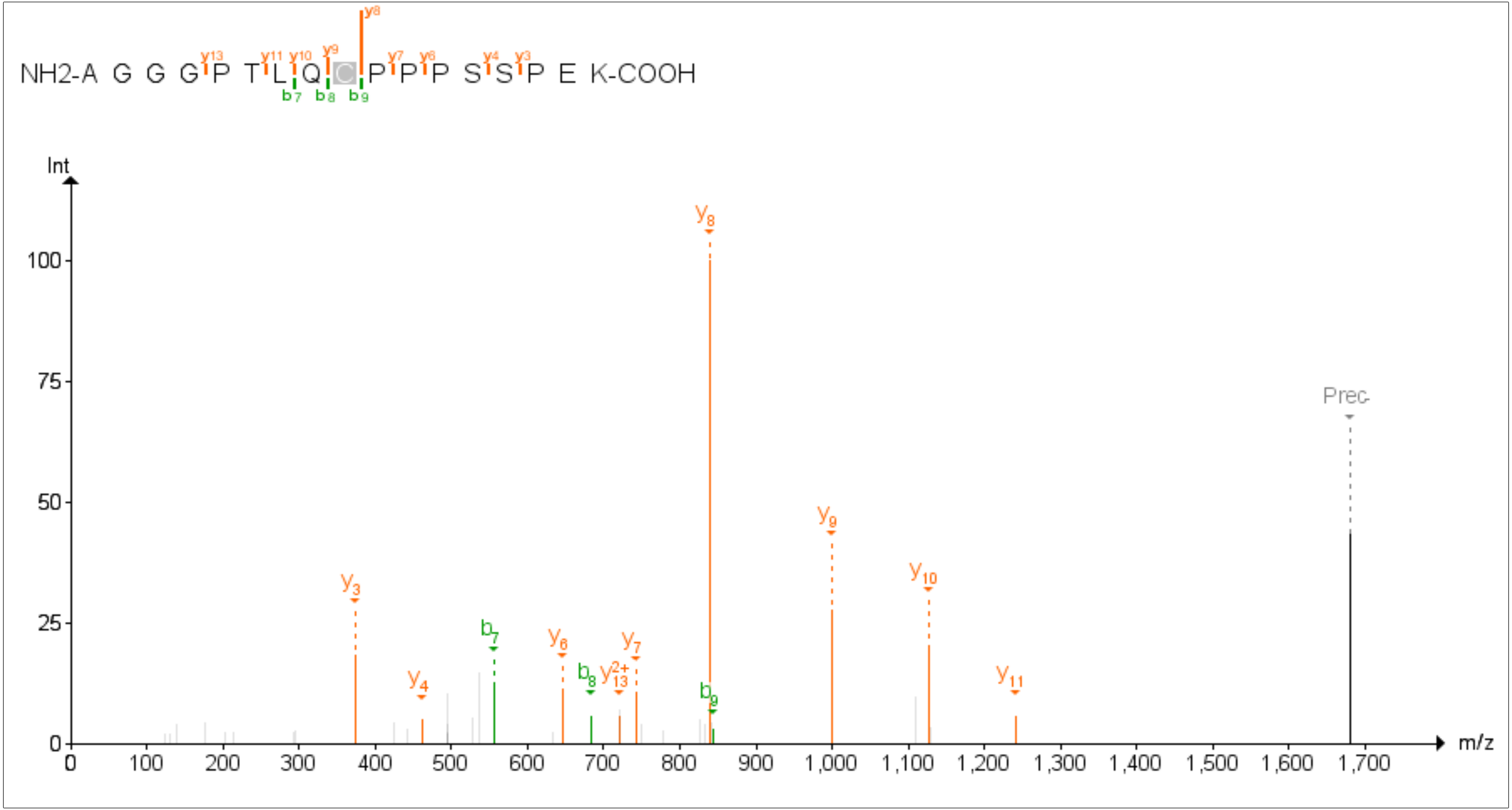
NH2-A G G G P T L Q C P P P S S P E K-COOH
y13 y11 y10 y9 y8 y7 y6 y4 y3
b7 b8 b9
Int
100
75
50
25
0
y8
Prec
y9
y10
y3
y4
b7
y6
b8
y2+13
y7
b9
y11
0 100 200 300 400 500 600 700 800 900 1,000 1,100 1,200 1,300 1,400 1,500 1,600 1,700
m/z

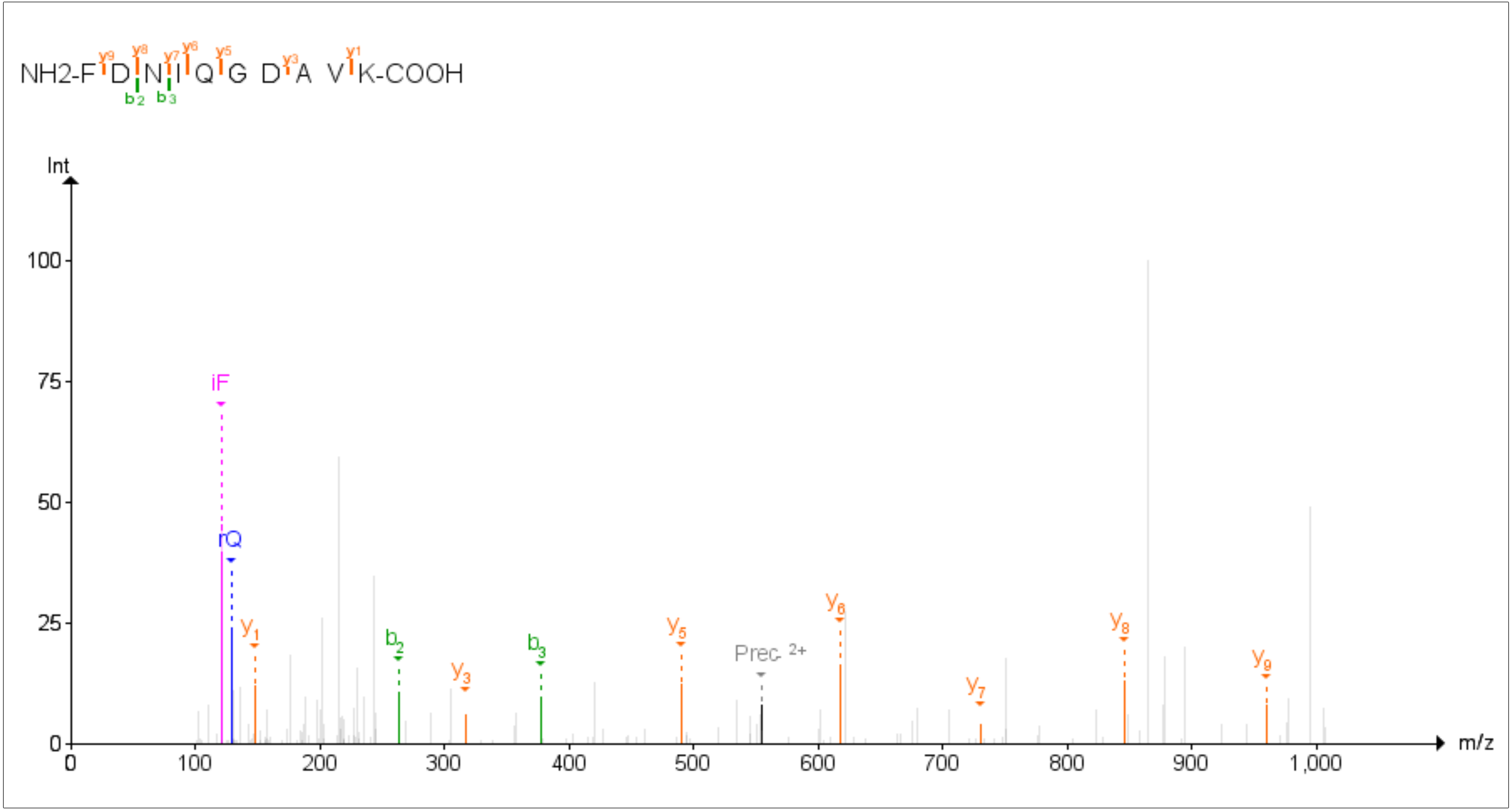
NH2-F D N I Q G D A V K-COOH
y9 y8 y7 y6 y5 y3 y1
b2 b3
Int
100
75
50
25
0
iF
rQ
y1
b2
y3
b3
y5
Prec 2+
y6
y7
y8
y9
0
100
200
300
400
500
600
700
800
900
1,000
m/z

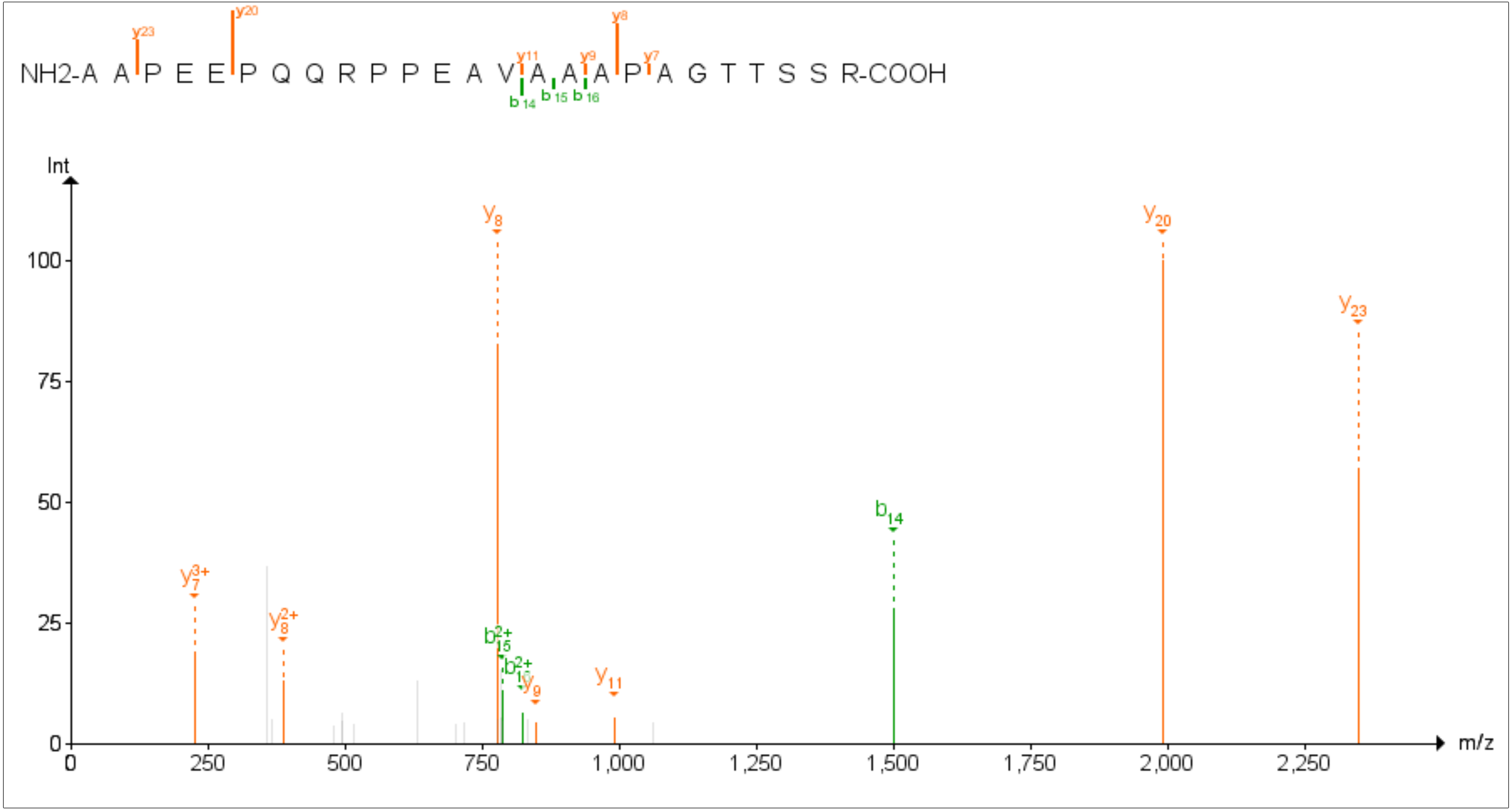

NH2-A A P E E P Q Q R P P E A V A A A P A G T T S S R-COOH
y23
y20
y11
y9
y8
y7
b 14
b 15
b 16
Int
m/z
100
75
50
25
0
250
500
750
1,000
1,250
1,500
1,750
2,000
2,250
y8
y20
y23
b14
y7 3+
y8 2+
b15 2+
b16 2+
y9
y11

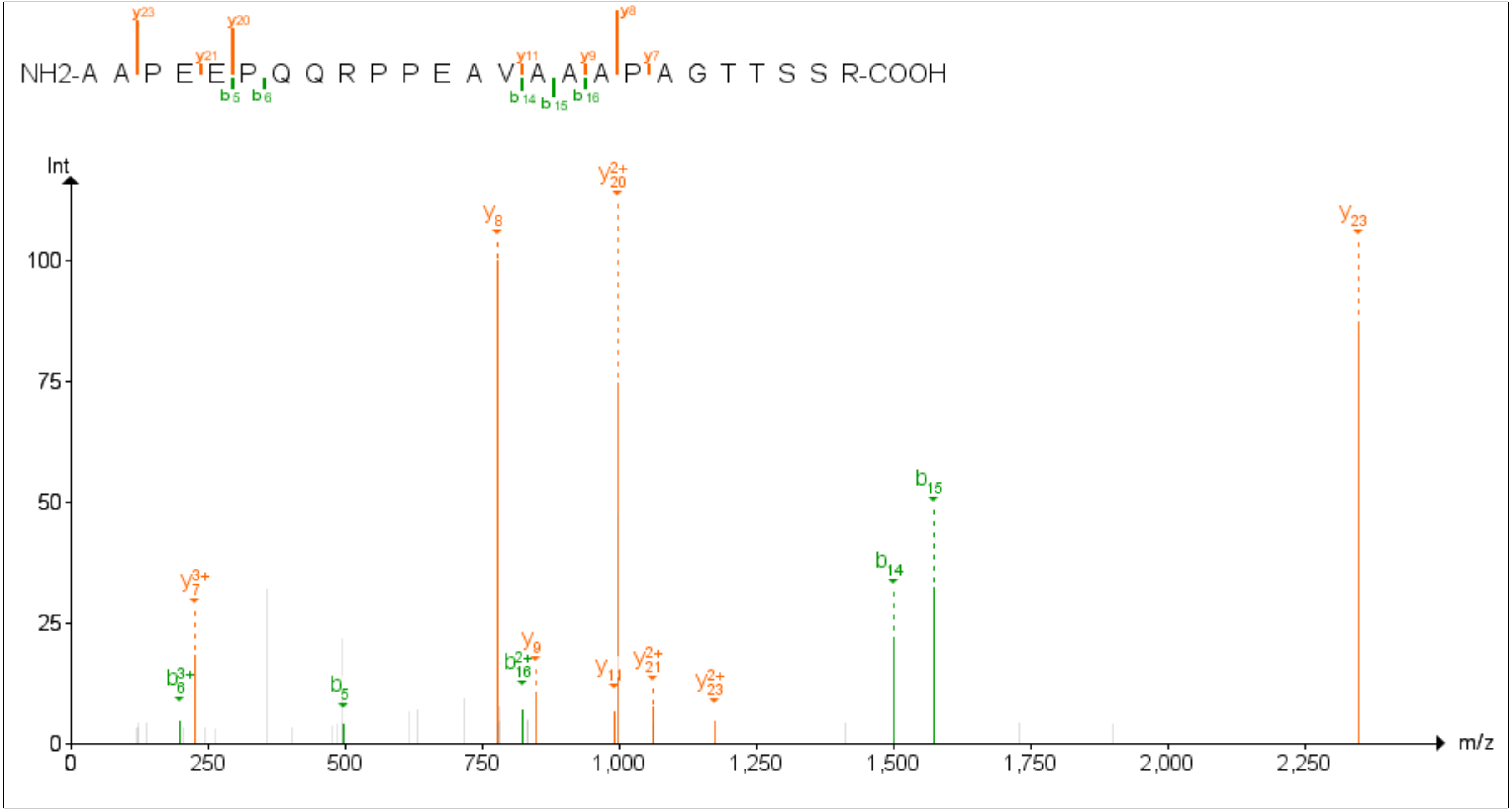

NH2-A A P E E P Q Q R P P E A V A A A P A G T T S S R-COOH
y23 y21 y20 y11 y9 y8 y7
b5 b6 b14 b15 b16
Int
100
75
50
25
0
m/z
0 250 500 750 1,000 1,250 1,500 1,750 2,000 2,250
y_8 y_{20}^{2+} y_{23} b_{15} b_{14} y_7^{3+} b_6^{3+} b_5 b_{16}^{2+} y_9 y_{11} y_{21}^{2+} y_{23}^{2+}

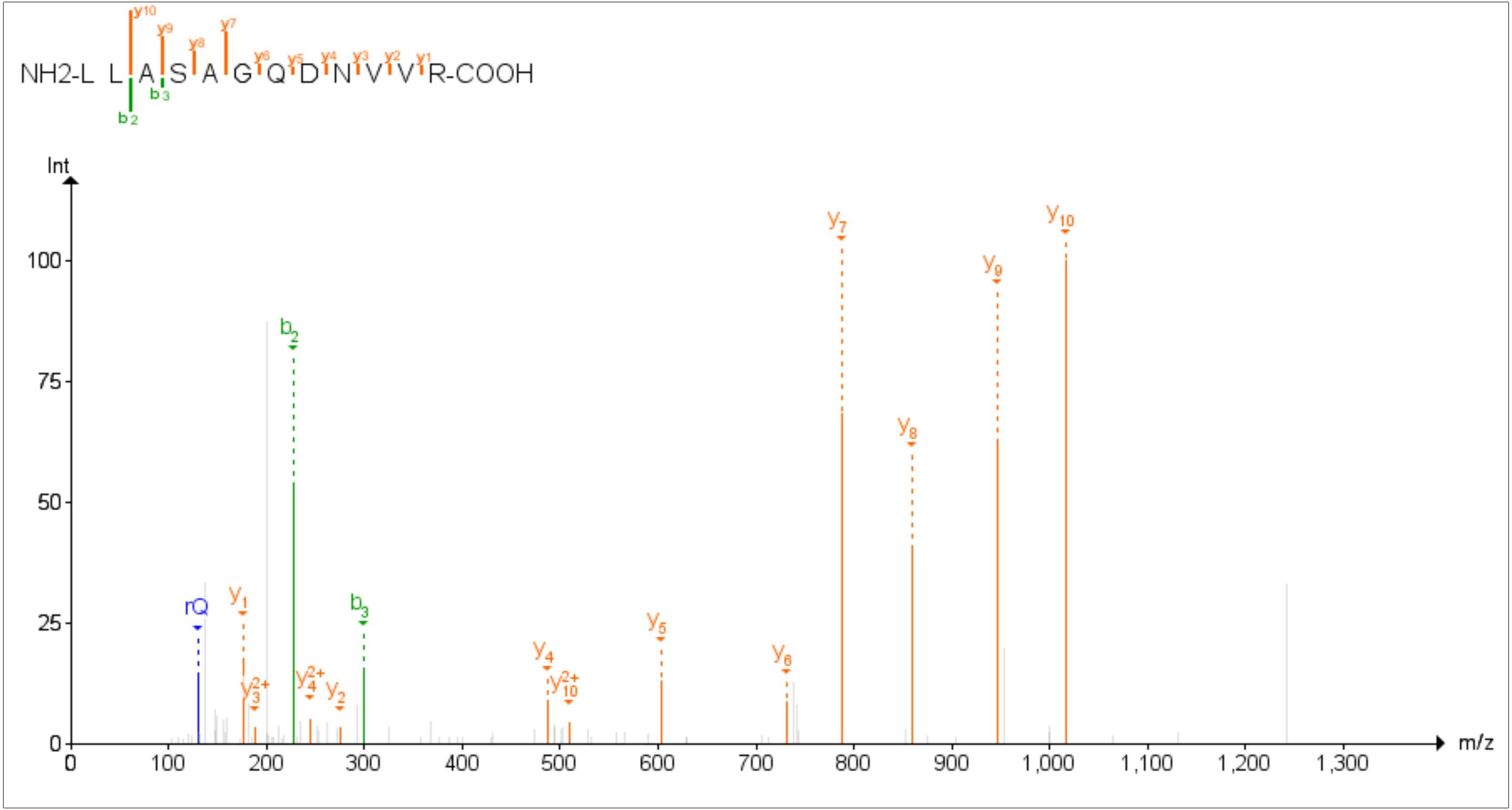

NH2-L L A S A G Q D N V V R-COOH
y10 y9 y8 y7 y6 y5 y4 y3 y2 y1
b2 b3
Int
m/z
100
75
50
25
0
0 100 200 300 400 500 600 700 800 900 1,000 1,100 1,200 1,300
rQ
y1
y3 2+
b2
y4 2+
y2
b3
y4
y10 2+
y5
y6
y7
y8
y9
y10

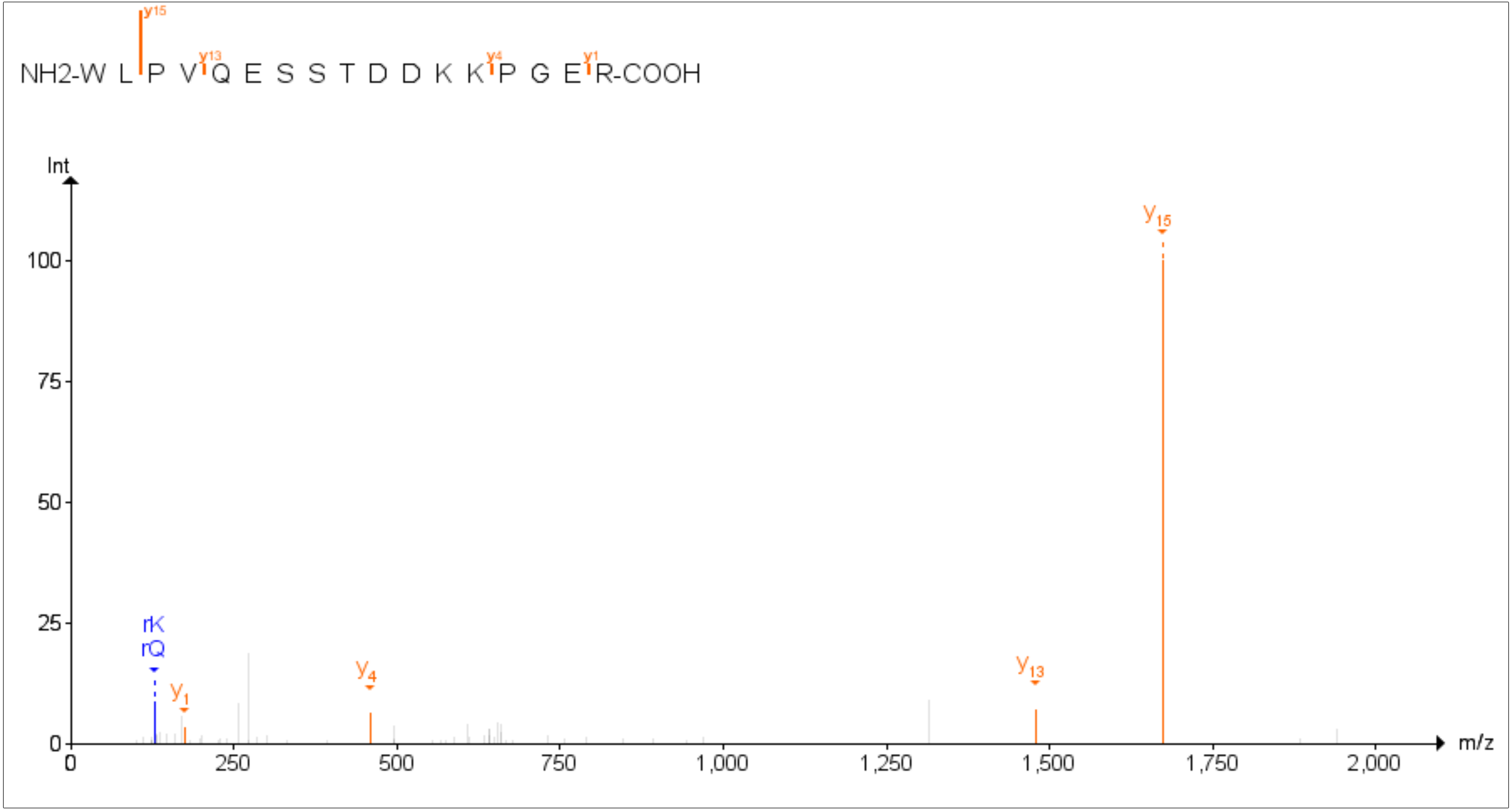

NH2-W L P V Q E S S T D D K K P G E R-COOH
y15
y13
y4
y1
Int
100
75
50
25
0
rK
rQ
y_{1}
y_{4}
y_{13}
y_{15}
0
250
500
750
1,000
1,250
1,500
1,750
2,000
m/z

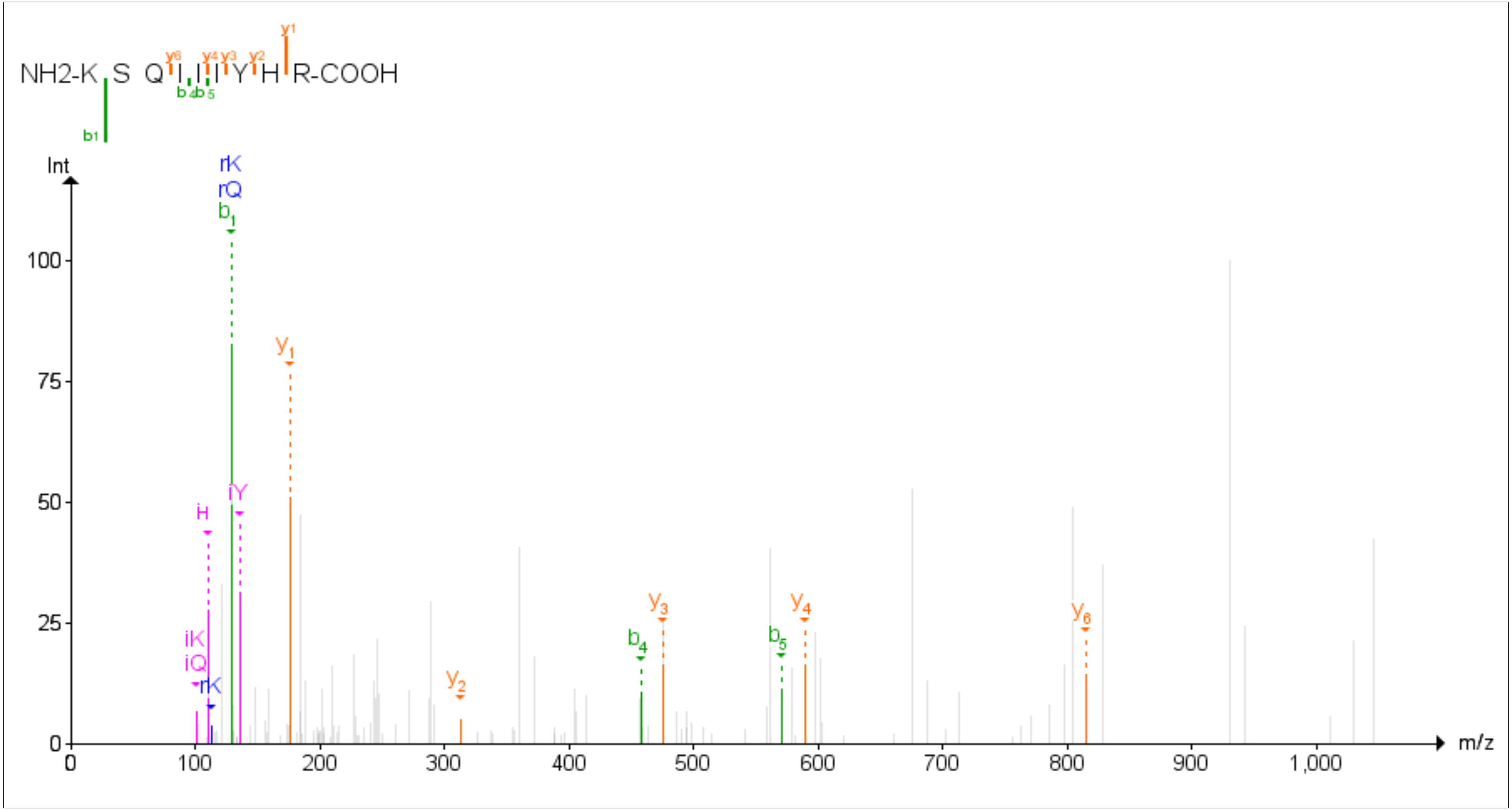

NH2-K S Q I I I Y H R-COOH
y1 y2 y3 y4 y6
b1 b4 b5
Int
m/z
rK
rQ
b1
y1
iH
iY
iK
iQ
rK
y2
b4
y3
b5
y4
y6

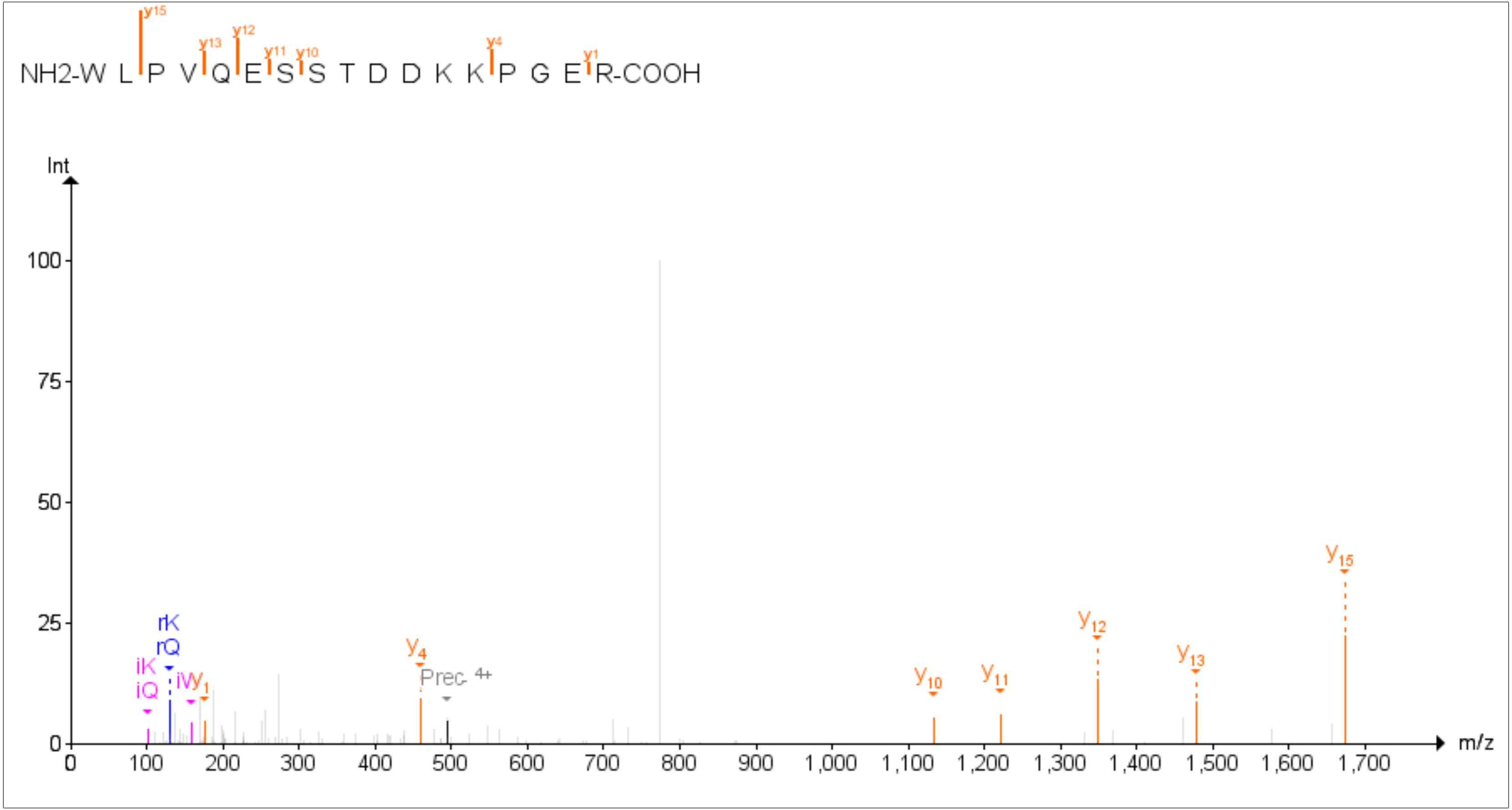

NH2-W L P V Q E S S T D D K K P G E R-COOH
y15
y13
y12
y11
y10
y4
y1
Int
100
75
50
25
0
rK
rQ
iK
iQ
iW
y1
y4
Prec 4+
y10
y11
y12
y13
y15
0
100
200
300
400
500
600
700
800
900
1,000
1,100
1,200
1,300
1,400
1,500
1,600
1,700
m/z

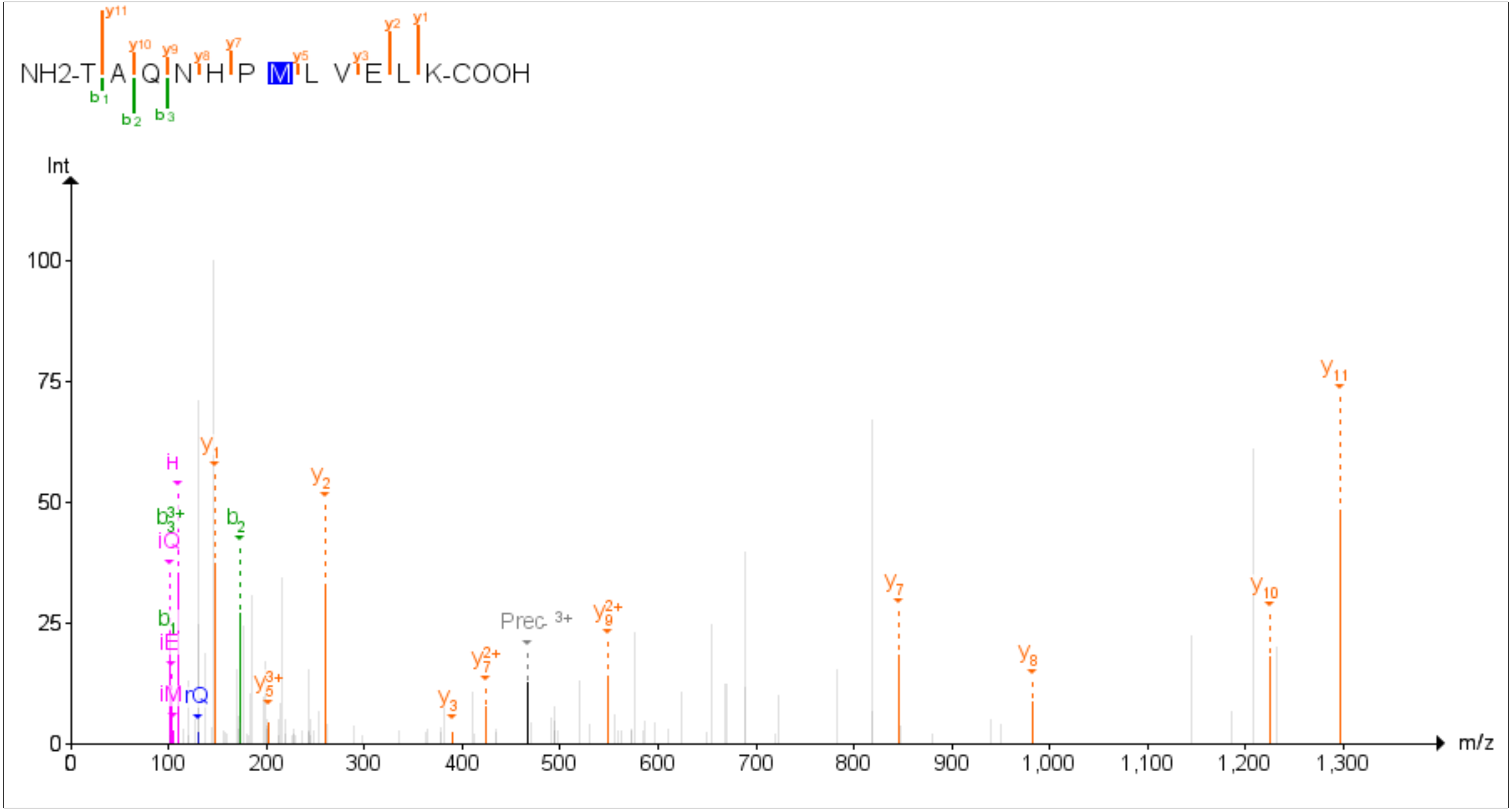

NH2-T A Q N H P M L V E L K-COOH
y11 y10 y9 y8 y7 y5 y3 y2 y1
b1 b2 b3
Int
m/z
0
25
50
75
100
0
100
200
300
400
500
600
700
800
900
1,000
1,100
1,200
1,300
y1
y2
y3
y7
y8
y10
y11
b2
b1
iH
iQ
iE
iM
rQ
Prec. 3+

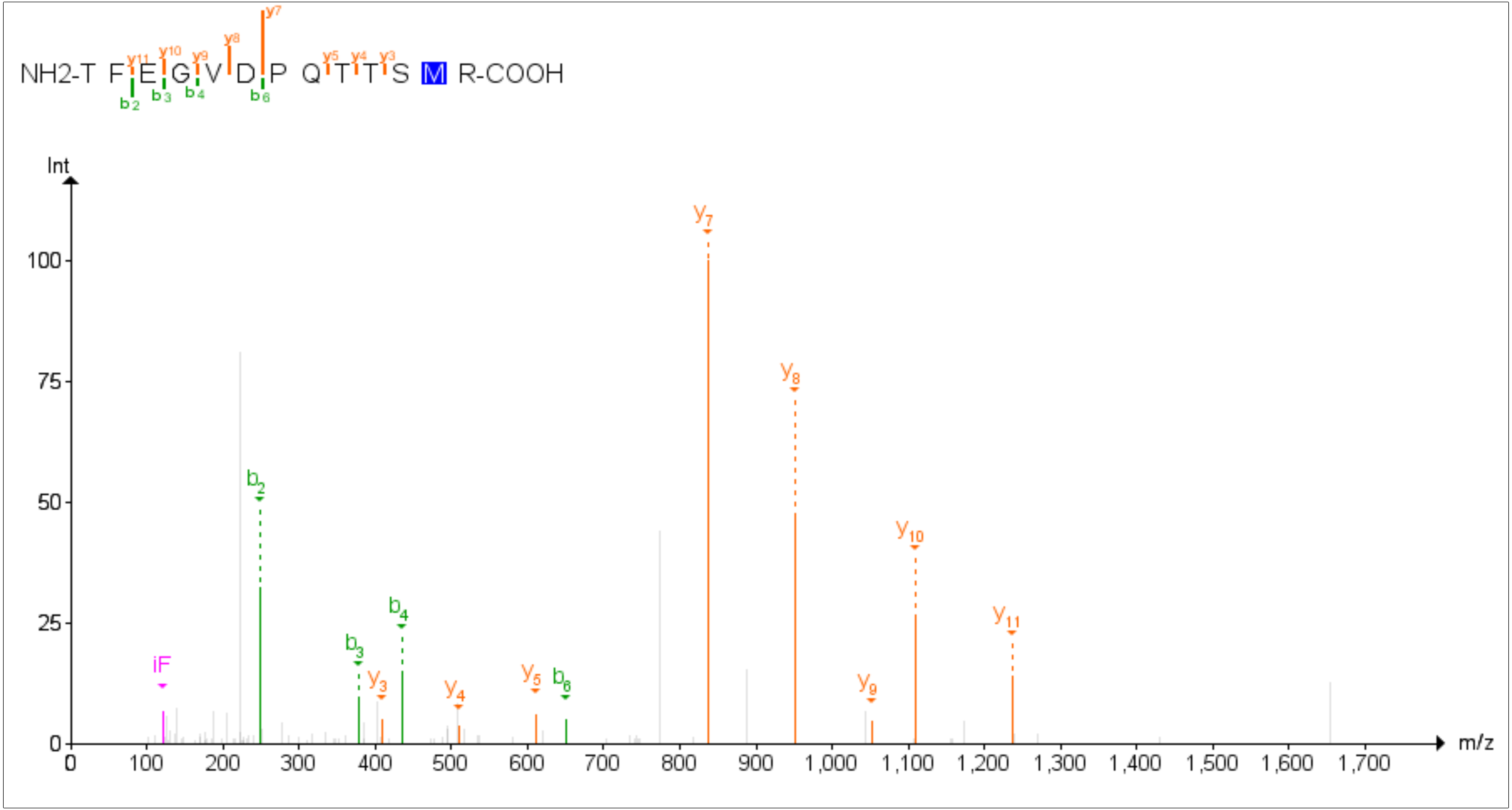

NH2-T F E G V D P Q T T S M R-COOH
y11 y10 y9 y8 y7 y5 y4 y3
b2 b3 b4 b6
Int
100
75
50
25
0
iF
b2
b3
y3
b4
y4
y5
b6
y7
y8
y9
y10
y11
0 100 200 300 400 500 600 700 800 900 1,000 1,100 1,200 1,300 1,400 1,500 1,600 1,700
m/z

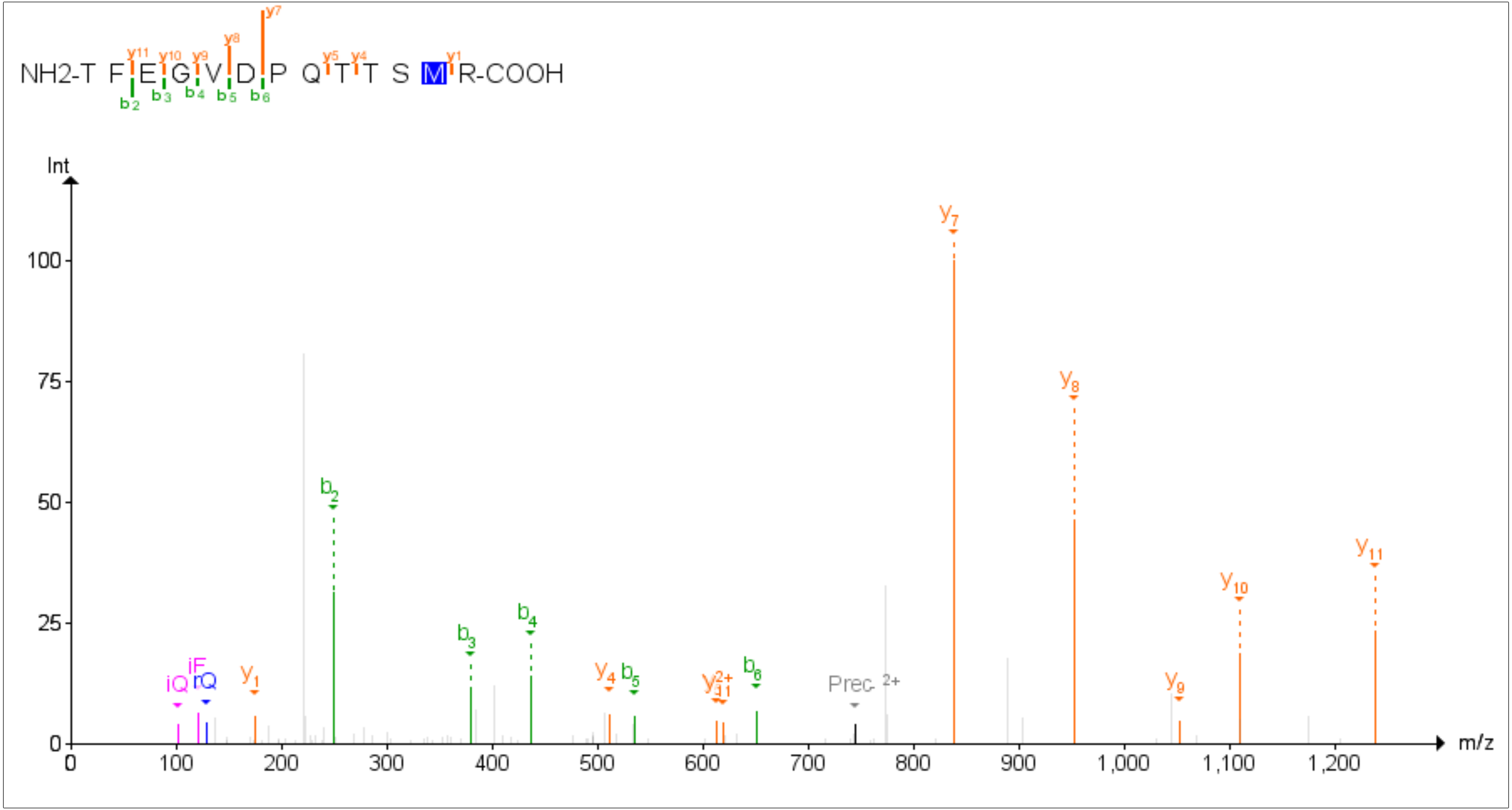
NH2-T F E G V D P Q T T S M R-COOH
y11 y10 y9 y8 y7 y5 y4 y1
b2 b3 b4 b5 b6
Int
100
75
50
25
0
iQ
iF
rQ
y1
b2
b3
b4
y4
b5
b6
Prec 2+
y7
y8
y9
y10
y11
0
100
200
300
400
500
600
700
800
900
1,000
1,100
1,200
m/z

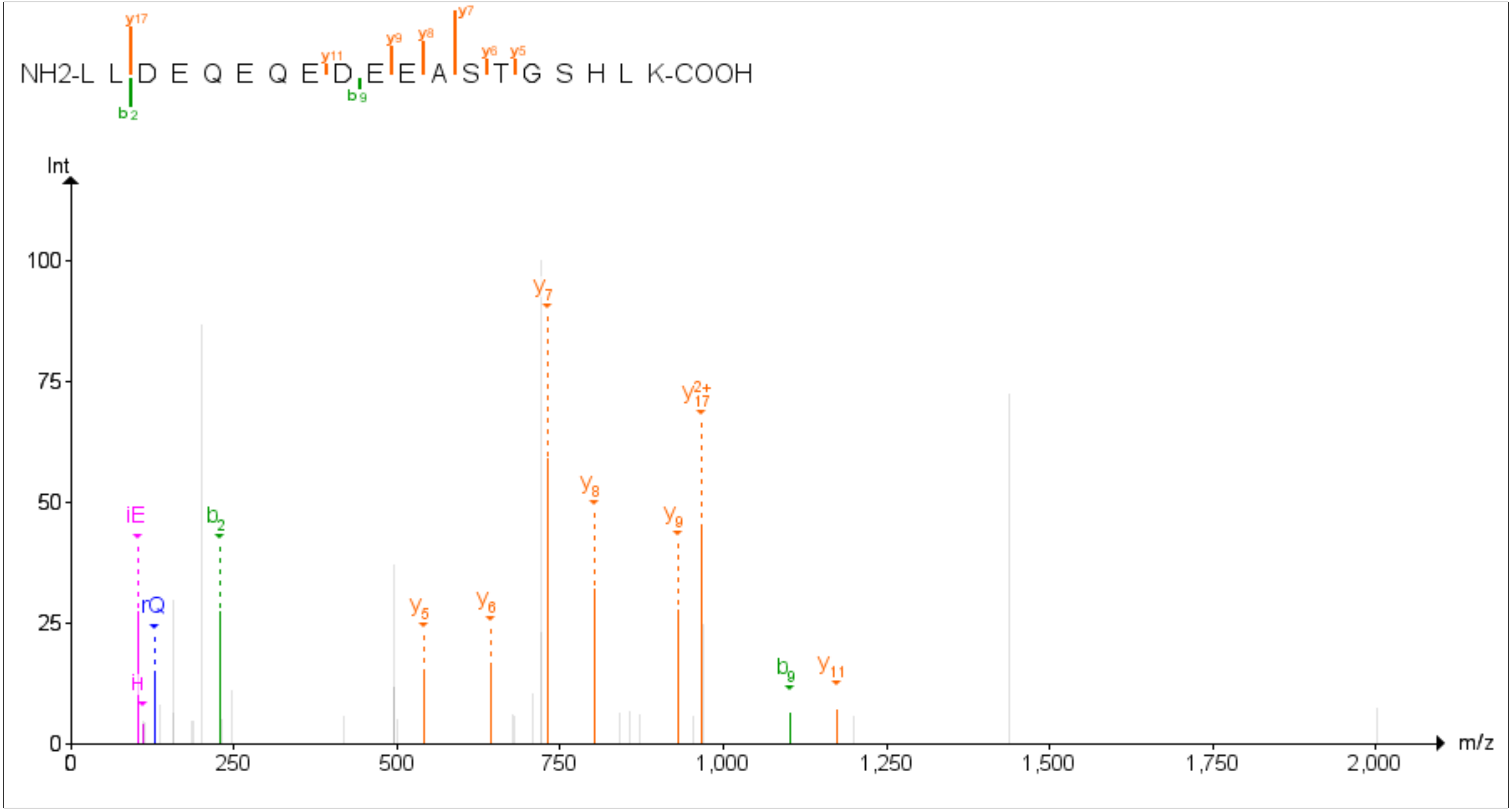
NH2-L L D E Q E Q E D E E A S T G S H L K-COOH
y17
y11
y9
y8
y7
y6
y5
b 2
b 9
Int
100
75
50
25
0
iE
b2
rQ
iH
y7
y8
y9
y_{17}^{2+}
y5
y6
b9
y11
0
250
500
750
1,000
1,250
1,500
1,750
2,000
m/z

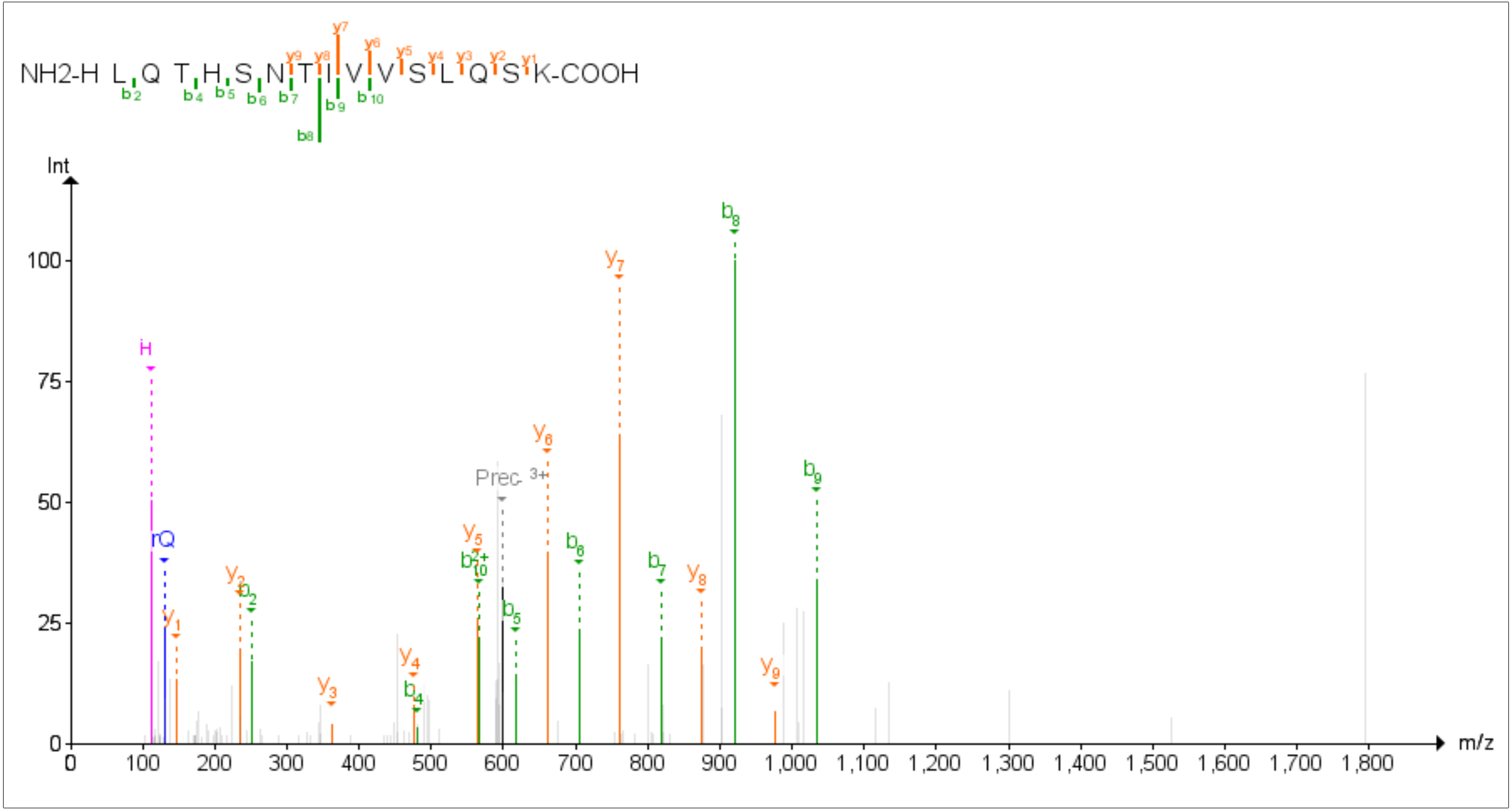

NH2-H L Q T H S N T I V V S L Q S K-COOH
b2 b4 b5 b6 b7 b8 b9 b10
y9 y8 y7 y6 y5 y4 y3 y2 y1
Int
m/z
Prec 3+
rQ

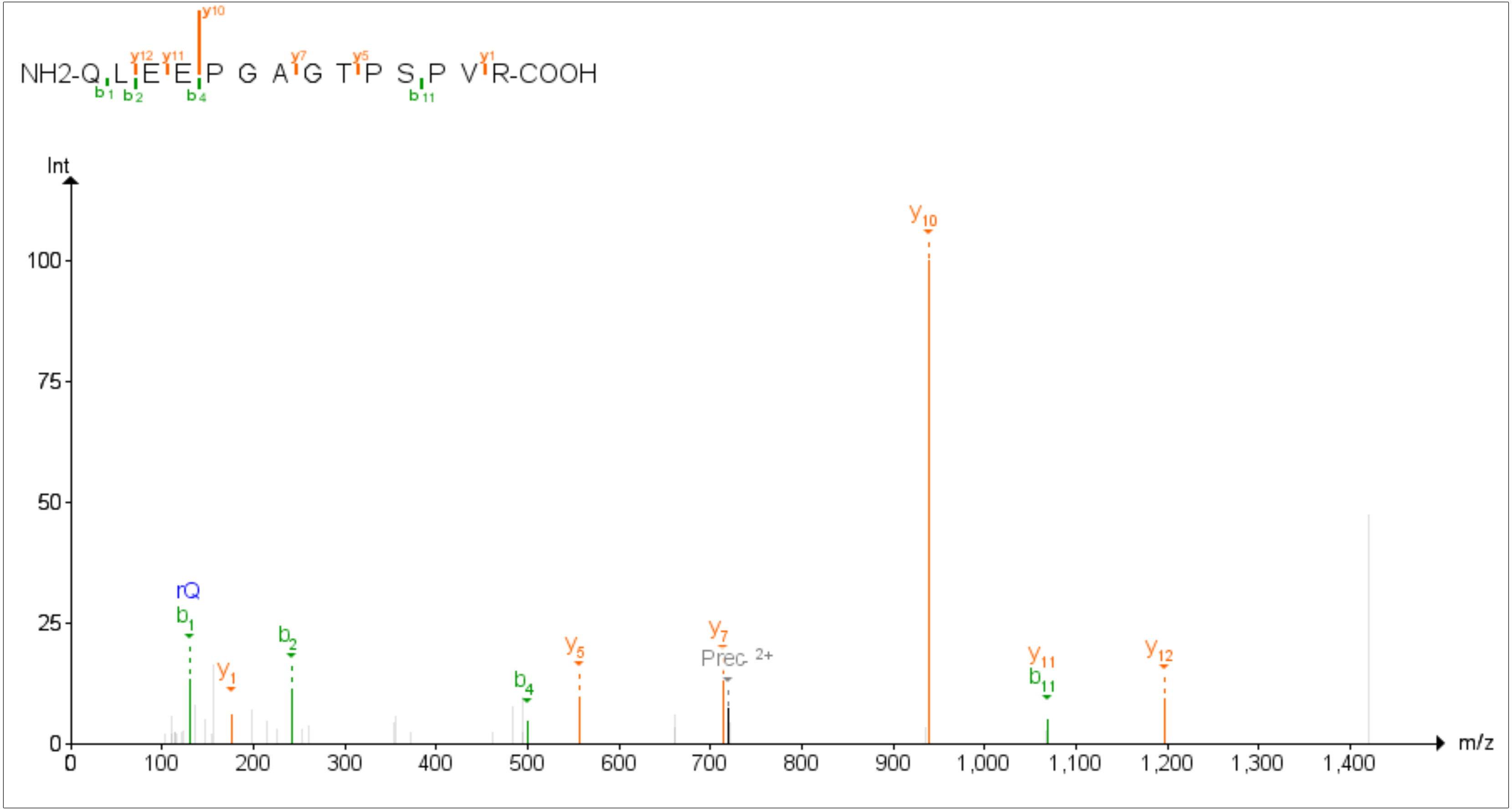

NH2-QLEEPGAGTPSPVR-COOH
y10
y12
y11
y7
y5
y1
b1
b2
b4
b11
Int
100
75
50
25
0
rQ
Prec 2+
m/z
0
100
200
300
400
500
600
700
800
900
1,000
1,100
1,200
1,300
1,400

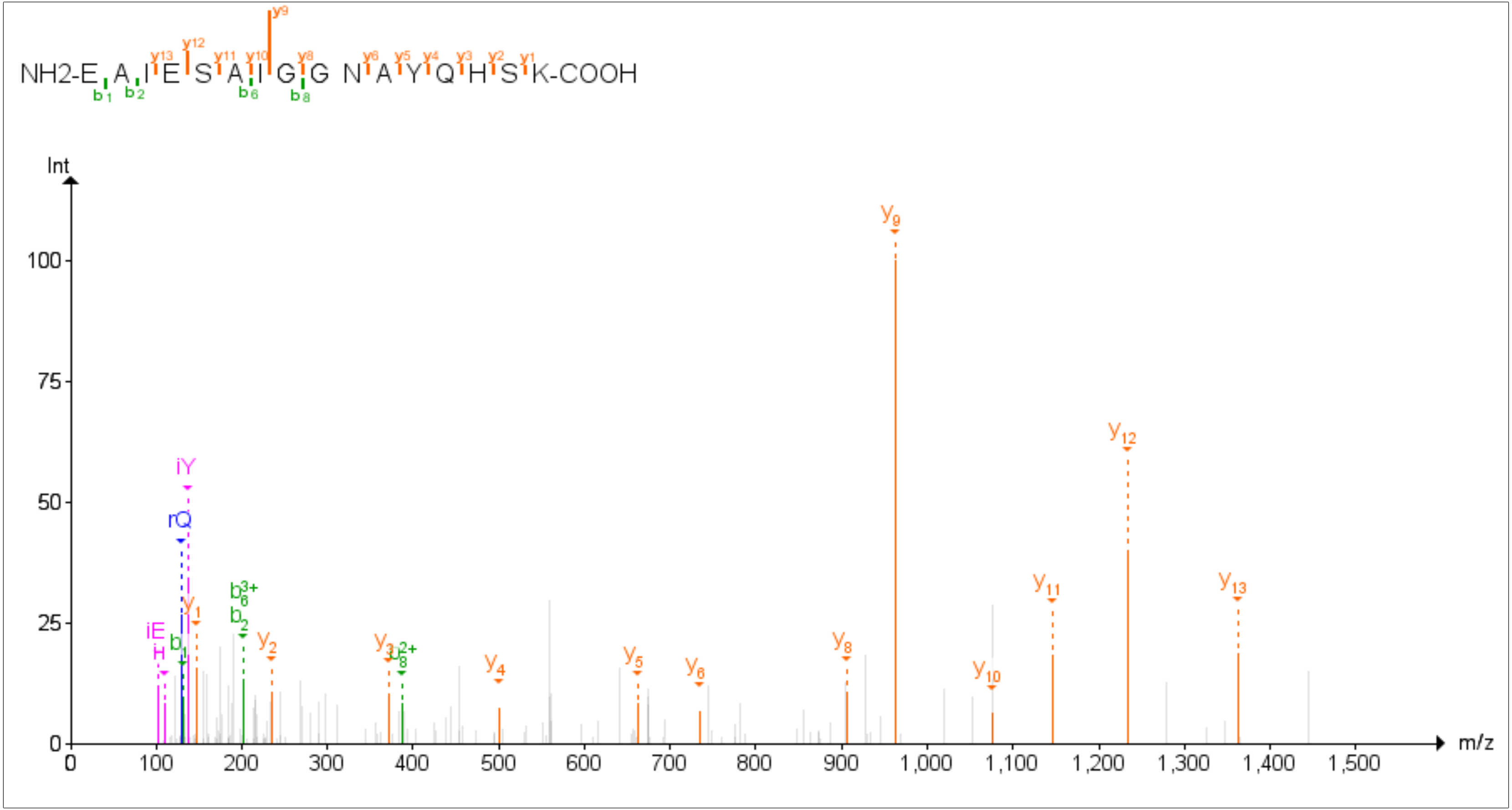

NH2-E A I E S A I G G N A Y Q H S K-COOH
Int
m/z
y9
y12
y11
y13
y10
y8
y4
y5
y6
y3
y2
y1
b2
b1
iY
rQ
iE
H
0
25
50
75
100
0
100
200
300
400
500
600
700
800
900
1,000
1,100
1,200
1,300
1,400
1,500

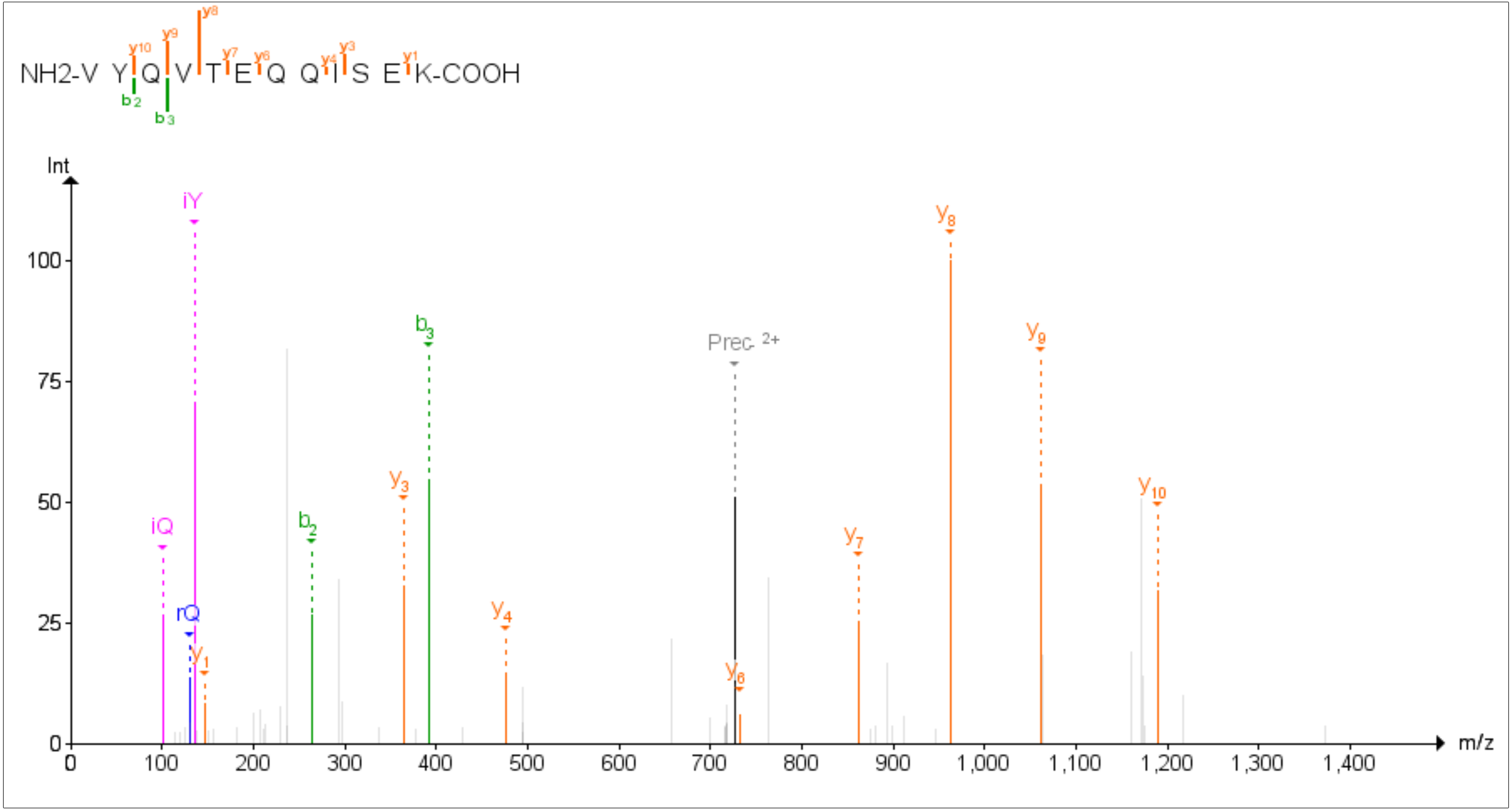

NH2-V Y Q V T E Q Q I S E K-COOH
y10 y9 y8 y7 y6 y4 y3 y1
b2 b3
Int
m/z
iY
iQ
rQ
y1
b2
b3
y3
y4
Prec 2+
y6
y7
y8
y9
y10
0
25
50
75
100
0
100
200
300
400
500
600
700
800
900
1,000
1,100
1,200
1,300
1,400

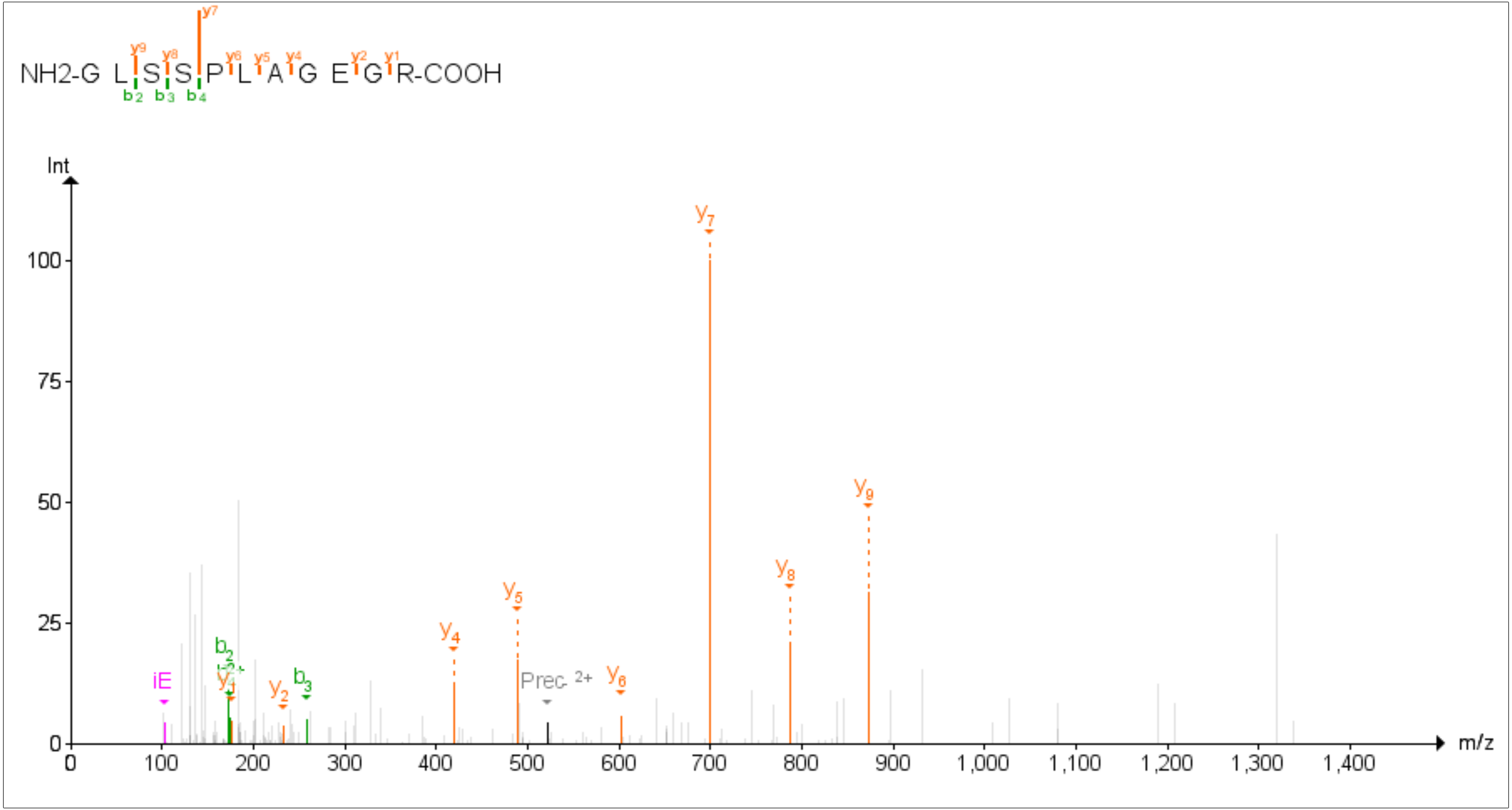

NH2-G L S S P L A G E G R-COOH
y9 y8 y7 y6 y5 y4 y2 y1
b2 b3 b4
Int
100
75
50
25
0
y7
y9
y8
y5
y4
b2
iE
y2
b3
Prec 2+
y6
0 100 200 300 400 500 600 700 800 900 1,000 1,100 1,200 1,300 1,400
m/z

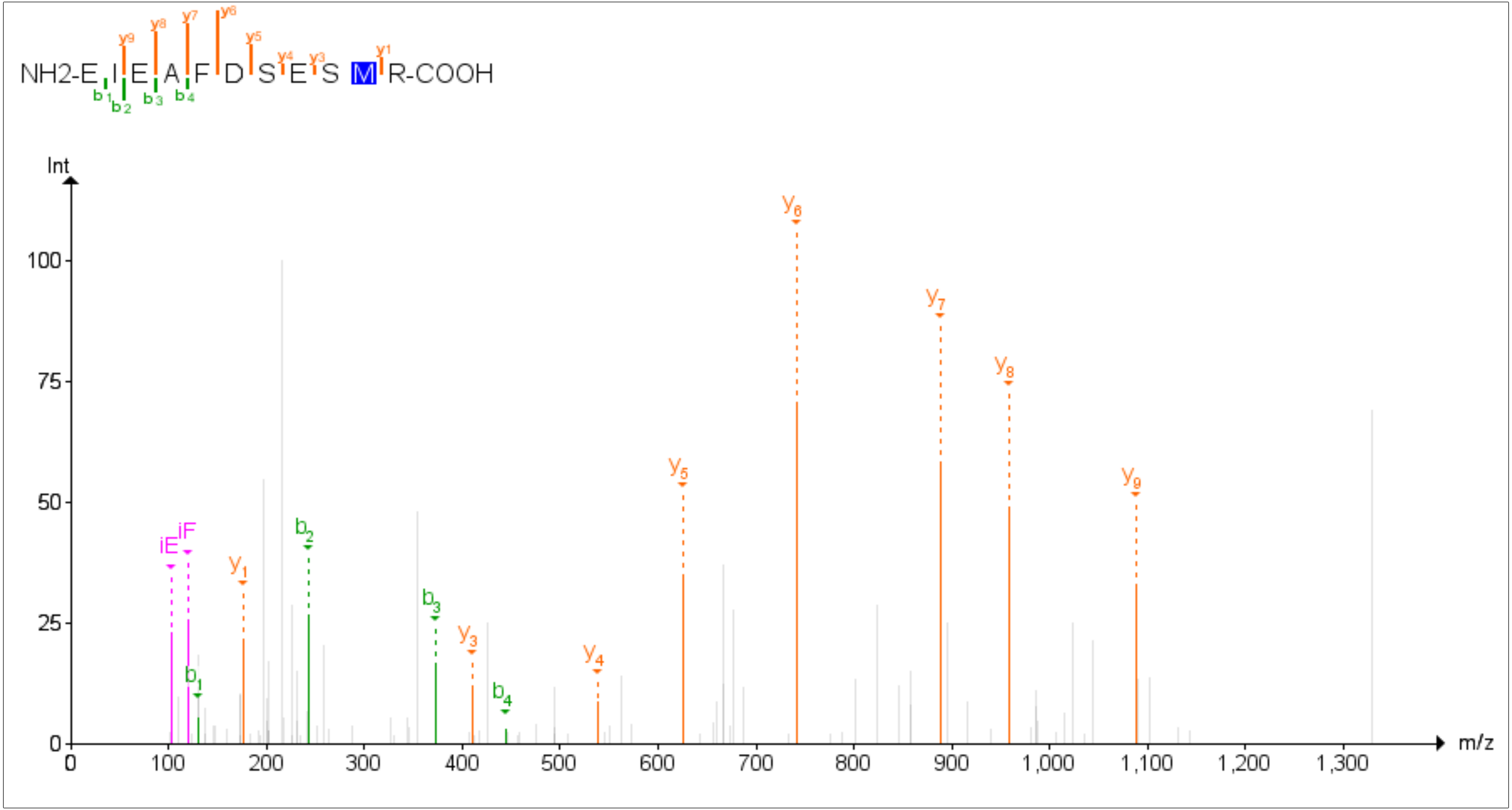
NH2-E I E A F D S E S M R-COOH
Int
m/z
y9 y8 y7 y6 y5 y4 y3 y1
b1 b2 b3 b4
iE iF
0 25 50 75 100
0 100 200 300 400 500 600 700 800 900 1,000 1,100 1,200 1,300

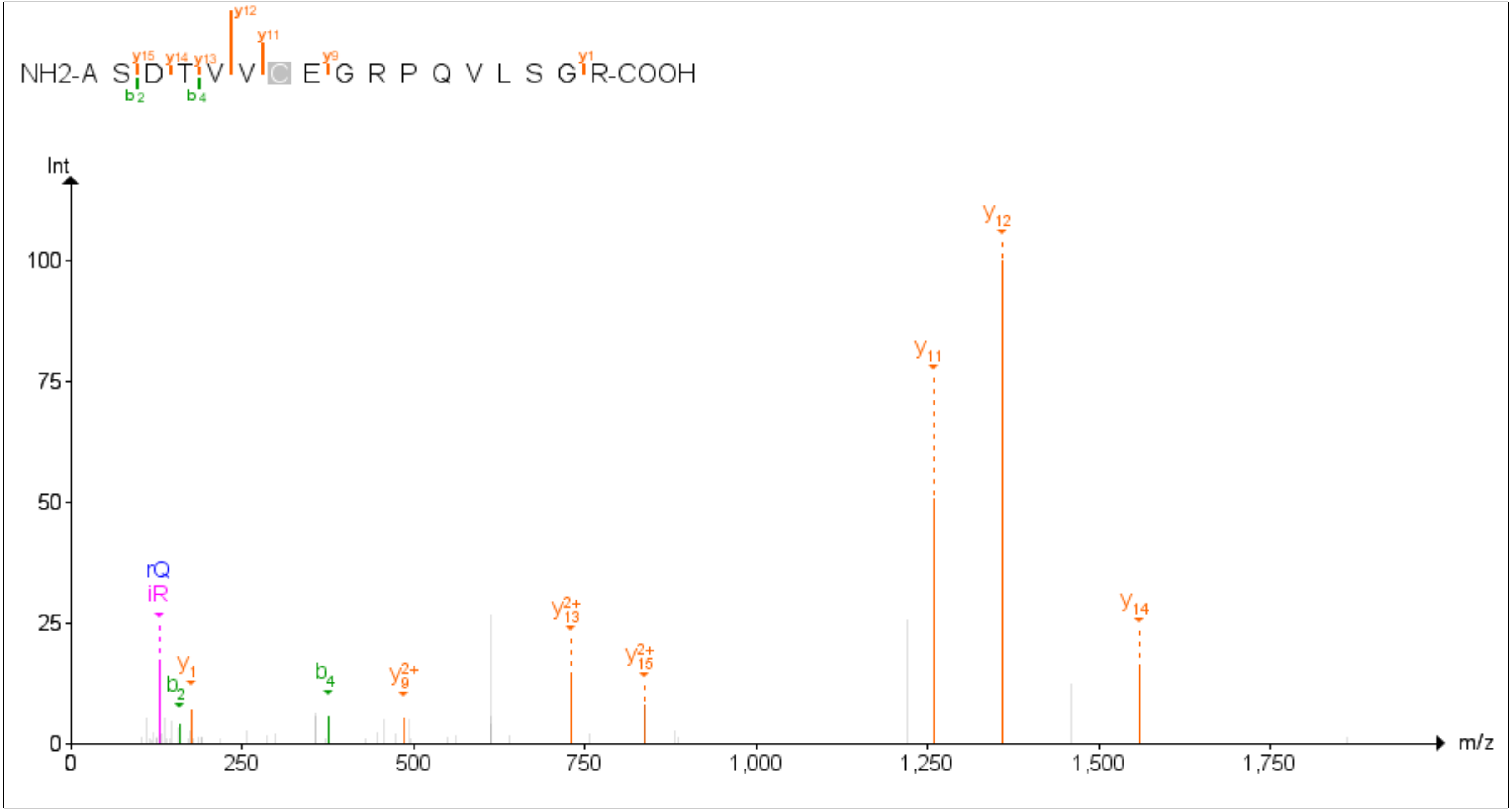

NH2-A S D T V V C E G R P Q V L S G R-COOH
y12
y11
y15
y14
y13
y9
y1
b2
b4
Int
100
75
50
25
0
m/z
0
250
500
750
1,000
1,250
1,500
1,750
rQ
iR
y1
b2
b4
y9 2+
y13 2+
y15 2+
y11
y12
y14

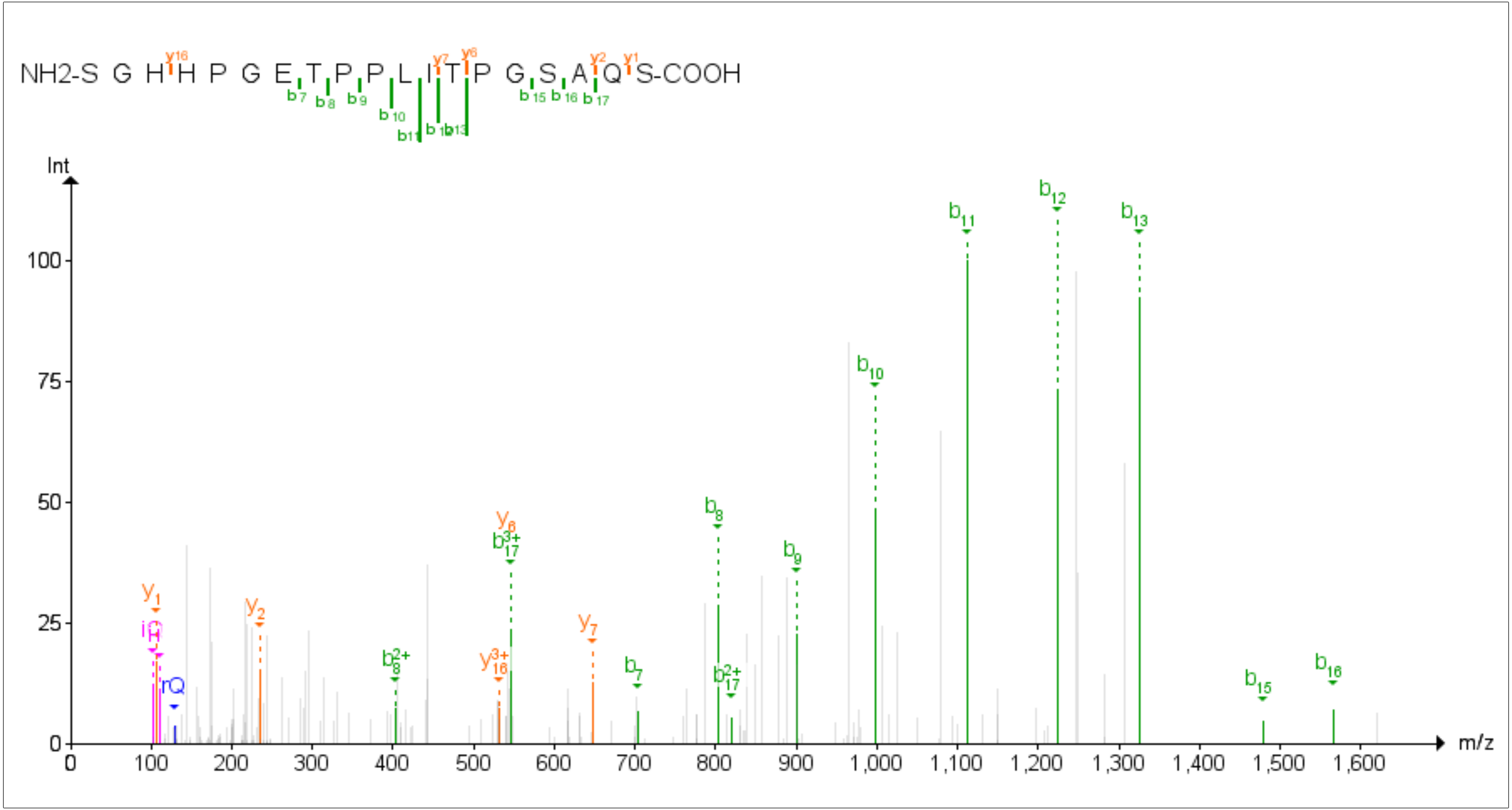

NH2-S G H H P G E T P P L I T P G S A Q S-COOH
Int
m/z

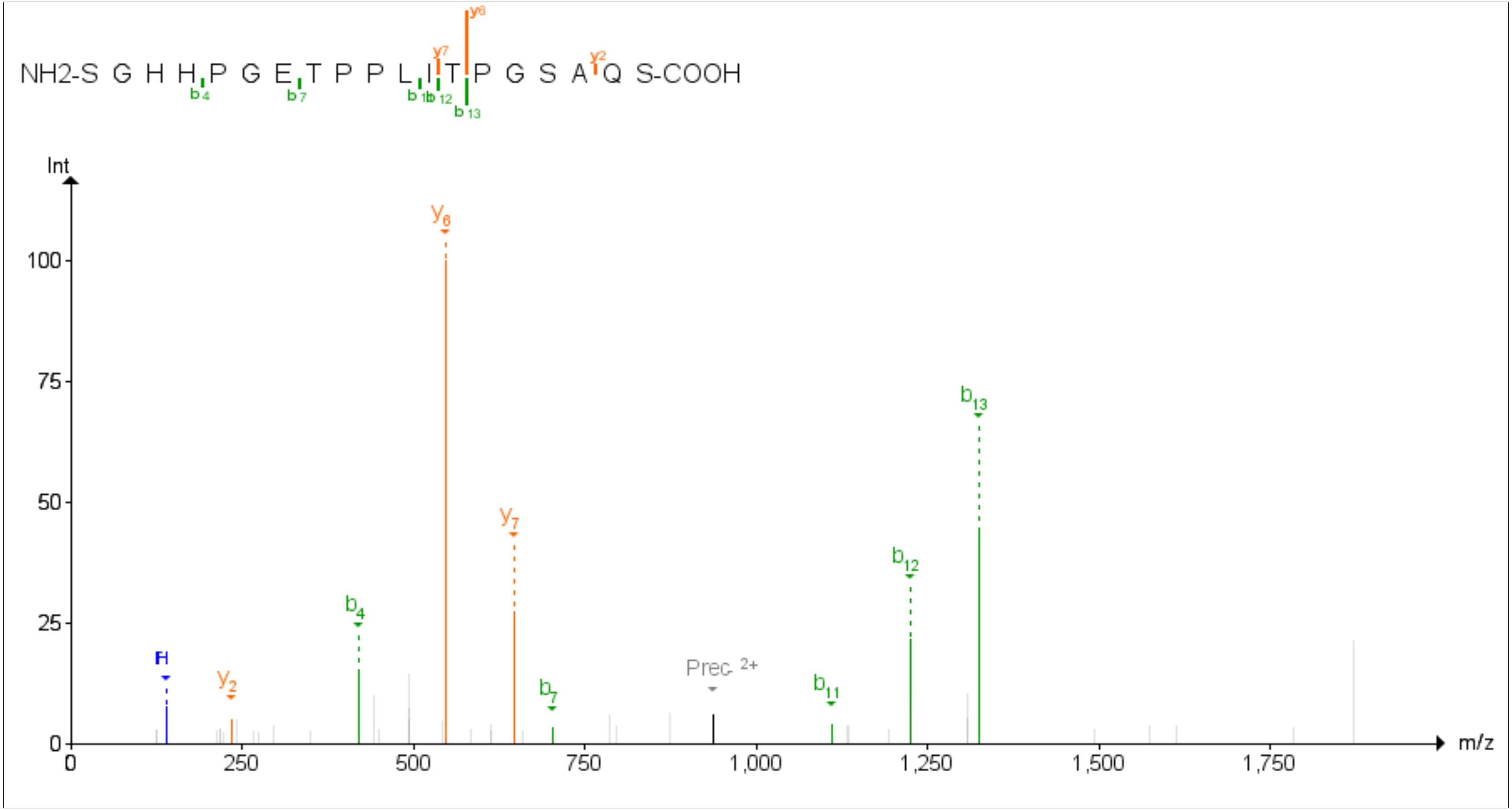
NH2-S G H H P G E T P P L I T P G S A Q S-COOH
b4
b7
b11
b12
b13
y7
y6
y2
Int
100
75
50
25
0
m/z
250
500
750
1,000
1,250
1,500
1,750
y6
y7
b4
b7
y2
b11
b12
b13
Prec 2+

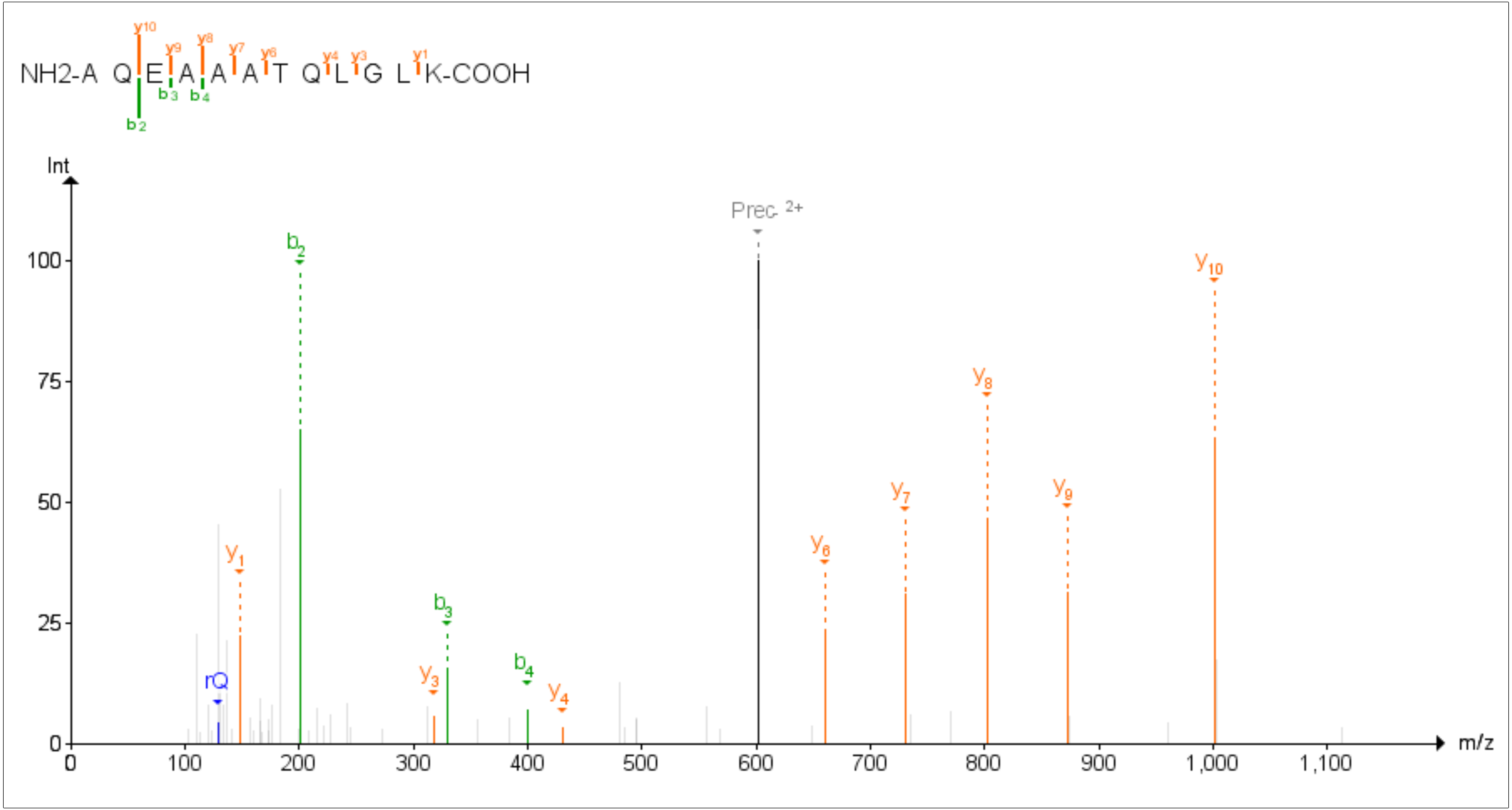
NH2-A Q E A A A T Q L G L K-COOH
y10 y9 y8 y7 y6 y4 y3 y1
b2 b3 b4
Int
Prec 2+
b2
y10
y8
y7
y9
y6
y1
b3
rQ
y3
b4
y4
100
75
50
25
0
0
100
200
300
400
500
600
700
800
900
1,000
1,100
m/z

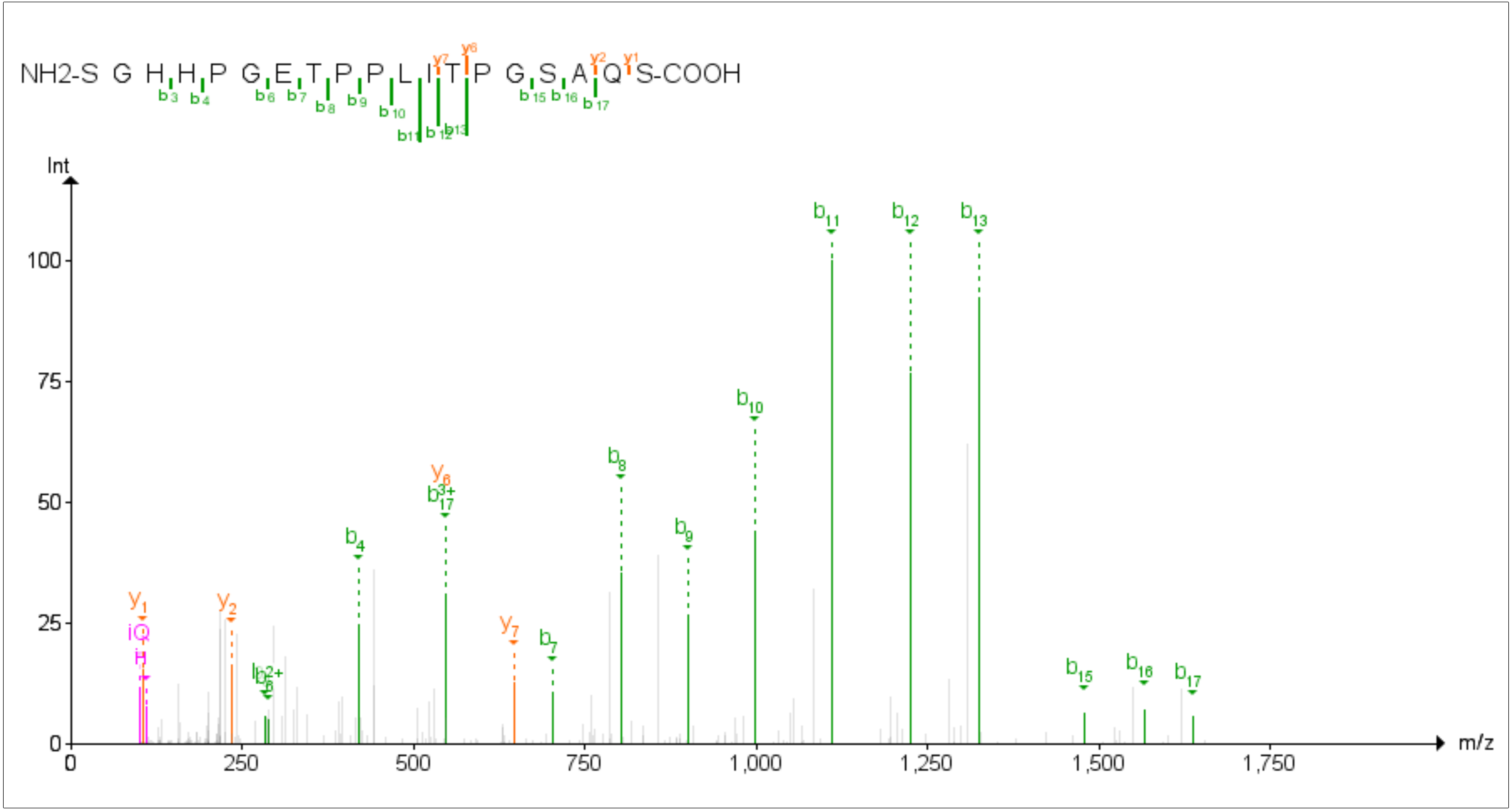

NH2-S G H H P G E T P P L I T P G S A Q S-COOH
Int
m/z
100
75
50
25
0
250
500
750
1,000
1,250
1,500
1,750

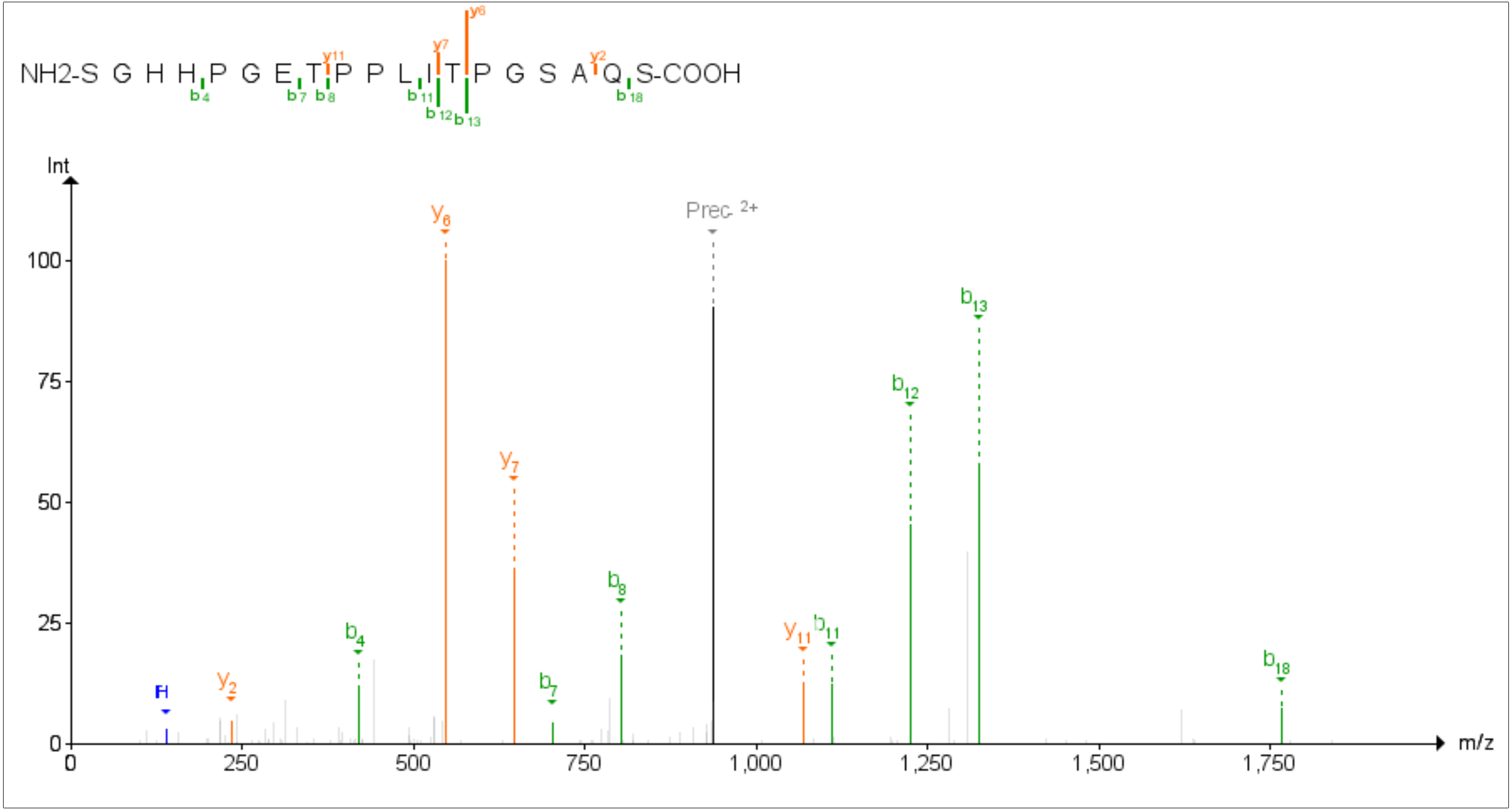

NH2-S G H H P G E T P P L I T P G S A Q S-COOH
y11 y7 y6 y2
b4 b7 b8 b11 b12 b13 b18
Int
100
75
50
25
0
y6
Prec. 2+
b13
b12
y7
b8
y11 b11
b4
b7
y2
H
b18
0
250
500
750
1,000
1,250
1,500
1,750
m/z

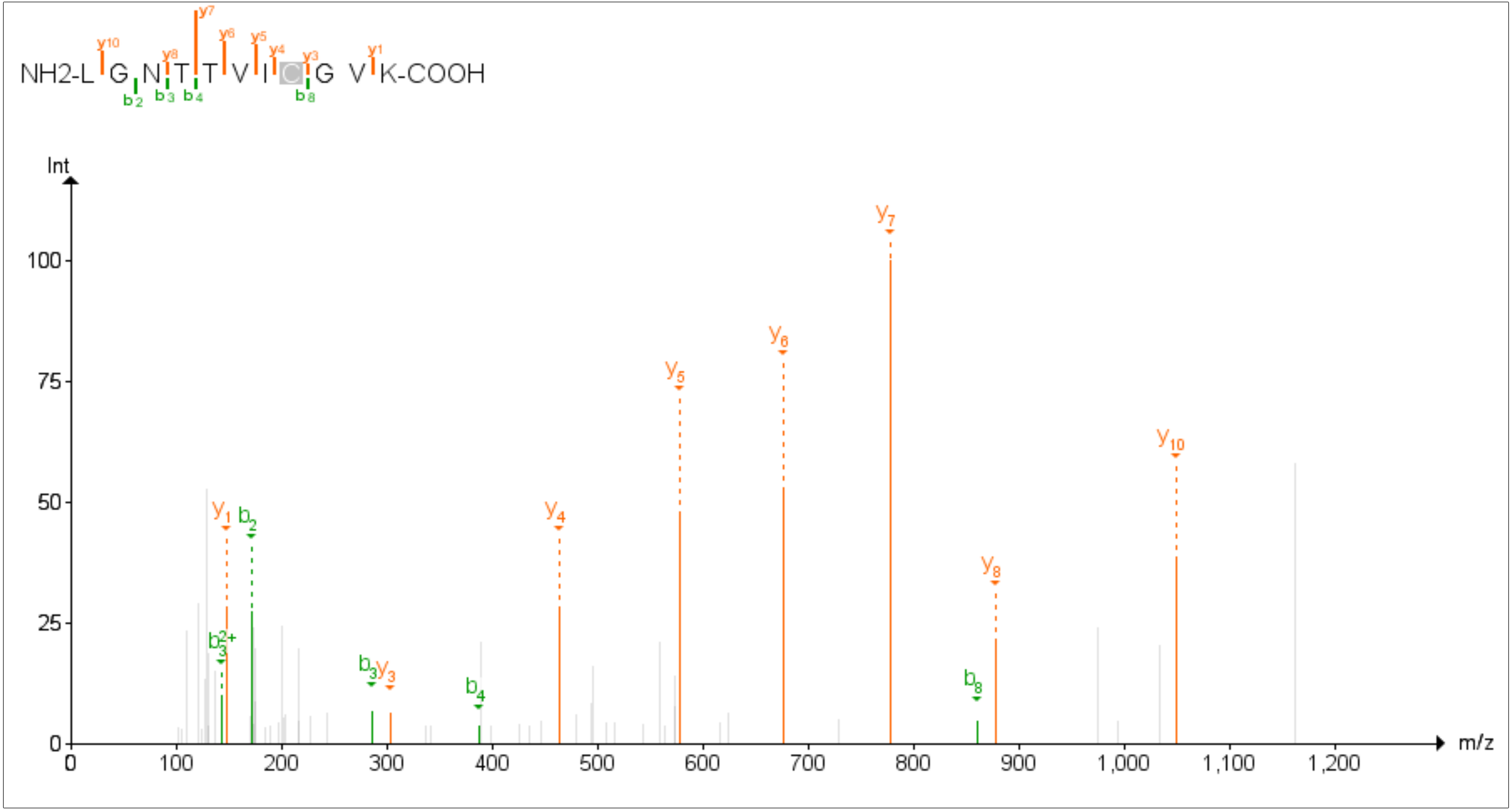

NH2-L G N T T V I C G V K-COOH
y10 y8 y7 y6 y5 y4 y3 y1
b2 b3 b4 b8
Int
100
75
50
25
0
y7
y6
y5
y10
y1
b2
y4
y8
b3 2+
b3 y3
b4
b8
0 100 200 300 400 500 600 700 800 900 1,000 1,100 1,200
m/z

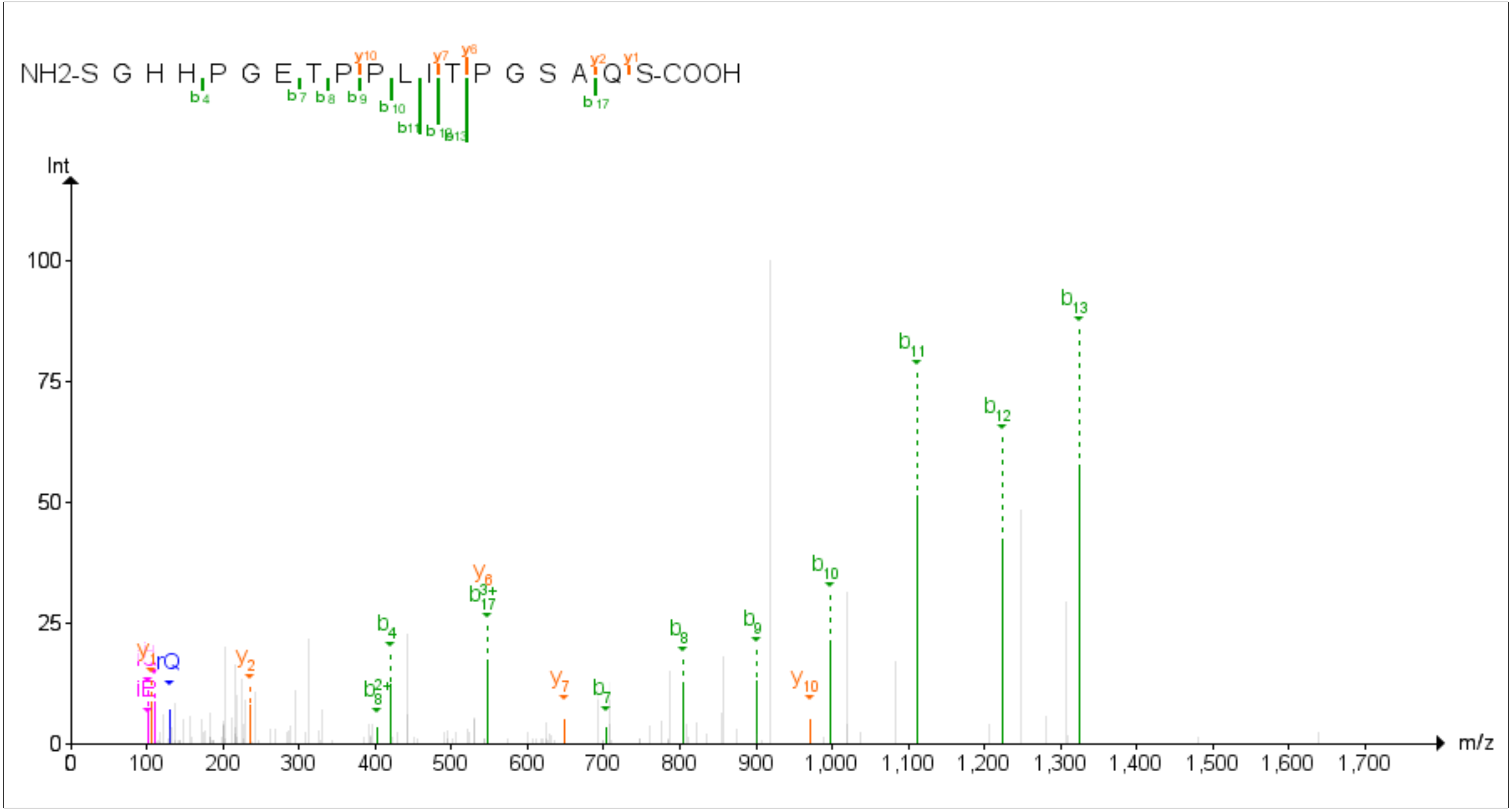

NH2-S G H H P G E T P P L I T P G S A Q S-COOH
Int
m/z
100
75
50
25
0
b13
b11
b12
b10
y6
b17 3+
b4
b8
b9
y2
rQ
y7
b7
y10
b8 2+

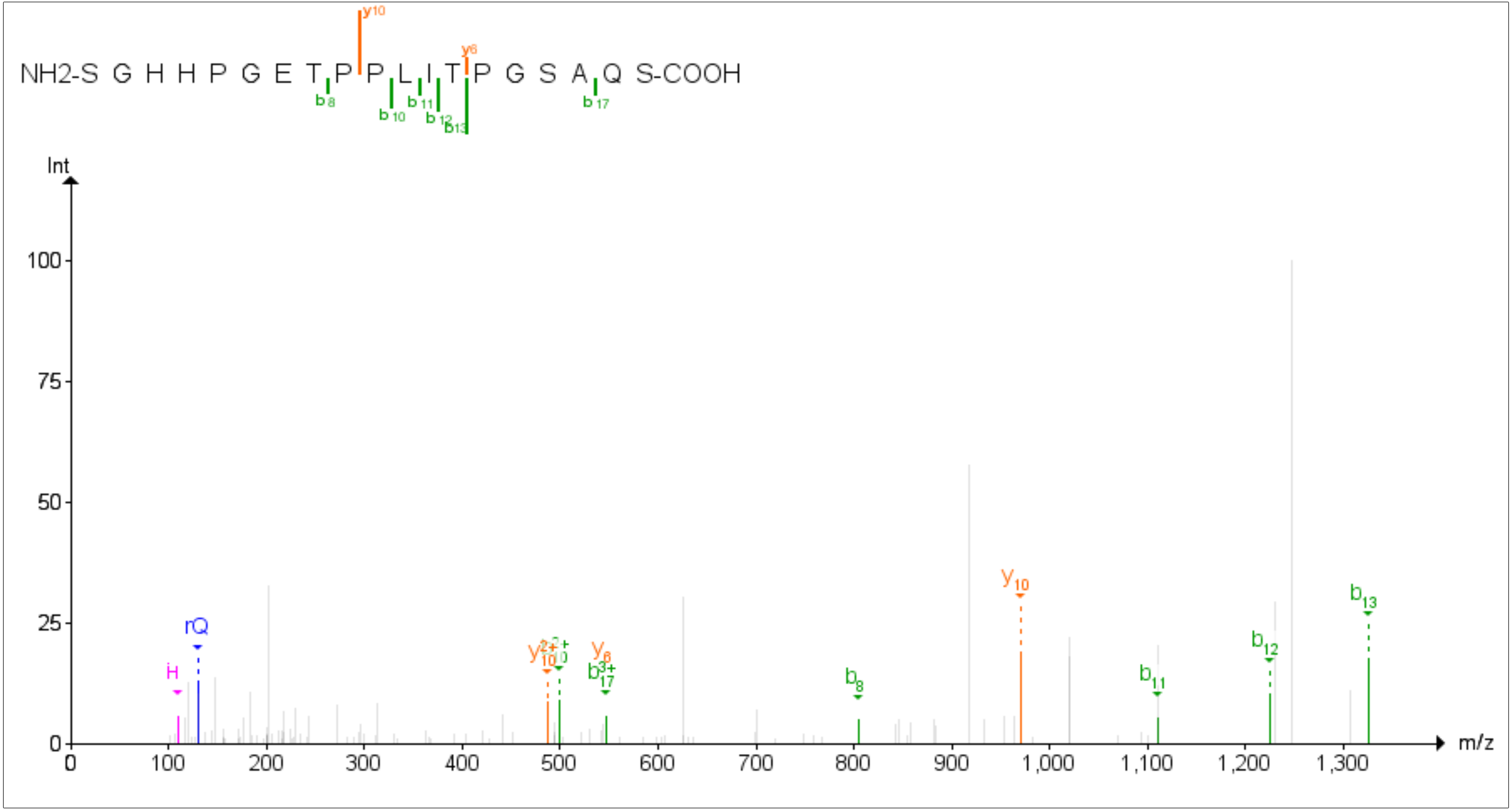

NH2-S G H H P G E T P P L I T P G S A Q S-COOH
y10
y6
b 8
b 10
b 11
b 12
b13
b 17
Int
100
75
50
25
0
rQ
H
y10
y6
b8
b11
b12
b13
0
100
200
300
400
500
600
700
800
900
1,000
1,100
1,200
1,300
m/z

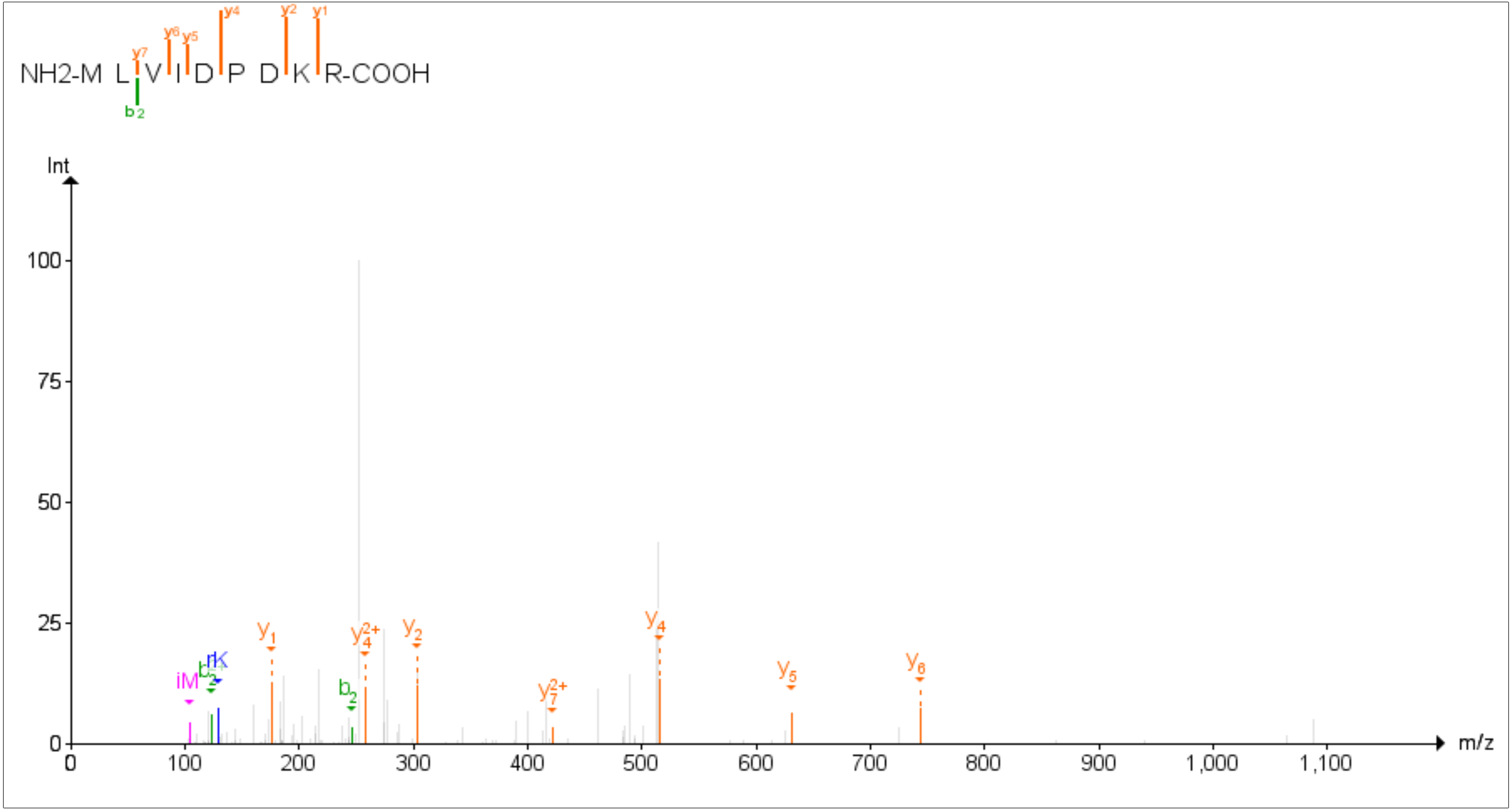

NH2-M L V I D P D K R-COOH
y7
y6
y5
y4
y2
y1
b2
Int
100
75
50
25
0
iM
b2
rK
y1
y4 2+
y2
b2
y7 2+
y4
y5
y6
0
100
200
300
400
500
600
700
800
900
1,000
1,100
m/z

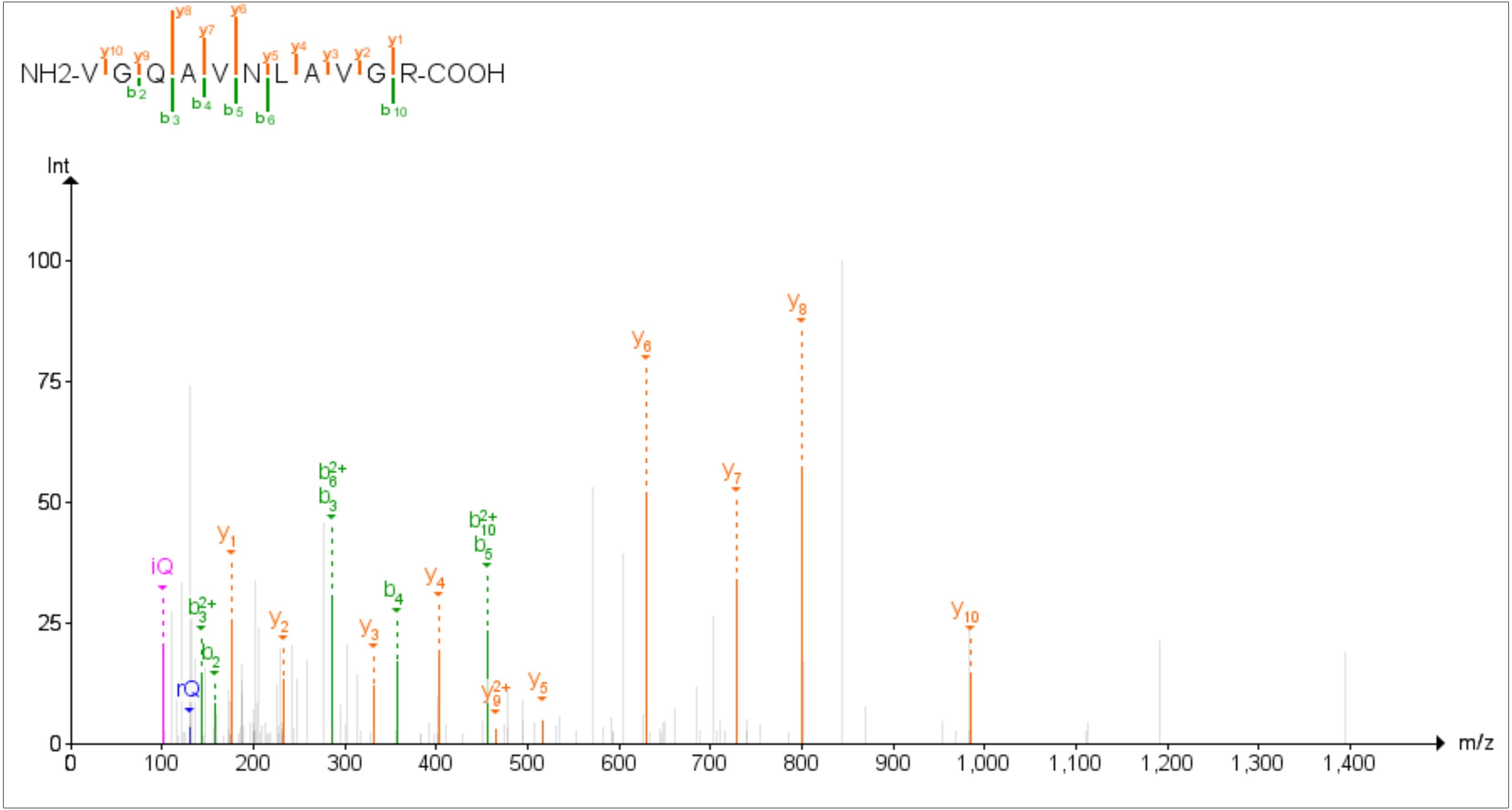

NH2-V G Q A V N L A V G R-COOH
y10 y9 y8 y7 y6 y5 y4 y3 y2 y1
b2 b3 b4 b5 b6 b10
Int
100
75
50
25
0
iQ
rQ
y1
y2
y3
y4
y5
y6
y7
y8
y10
b4
m/z
0 100 200 300 400 500 600 700 800 900 1,000 1,100 1,200 1,300 1,400

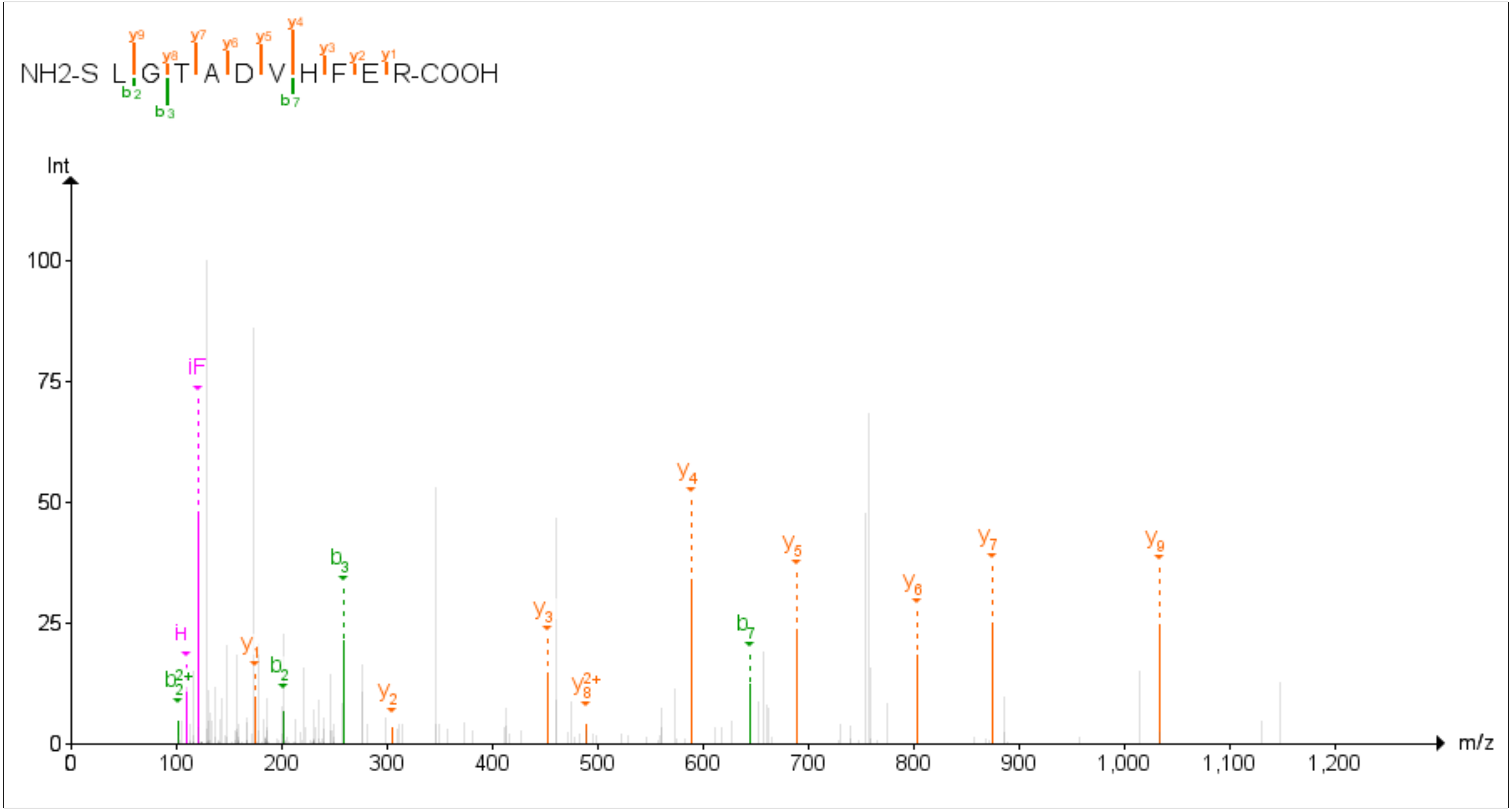
NH2-S L G T A D V H F E R-COOH
y9 y8 y7 y6 y5 y4 y3 y2 y1
b2 b3 b7
Int
m/z
iF
H
y1 y2 y3 y4 y5 y6 y7 y9
b2 b3 b7
b2 2+
y8 2+
0
25
50
75
100
0
100
200
300
400
500
600
700
800
900
1,000
1,100
1,200

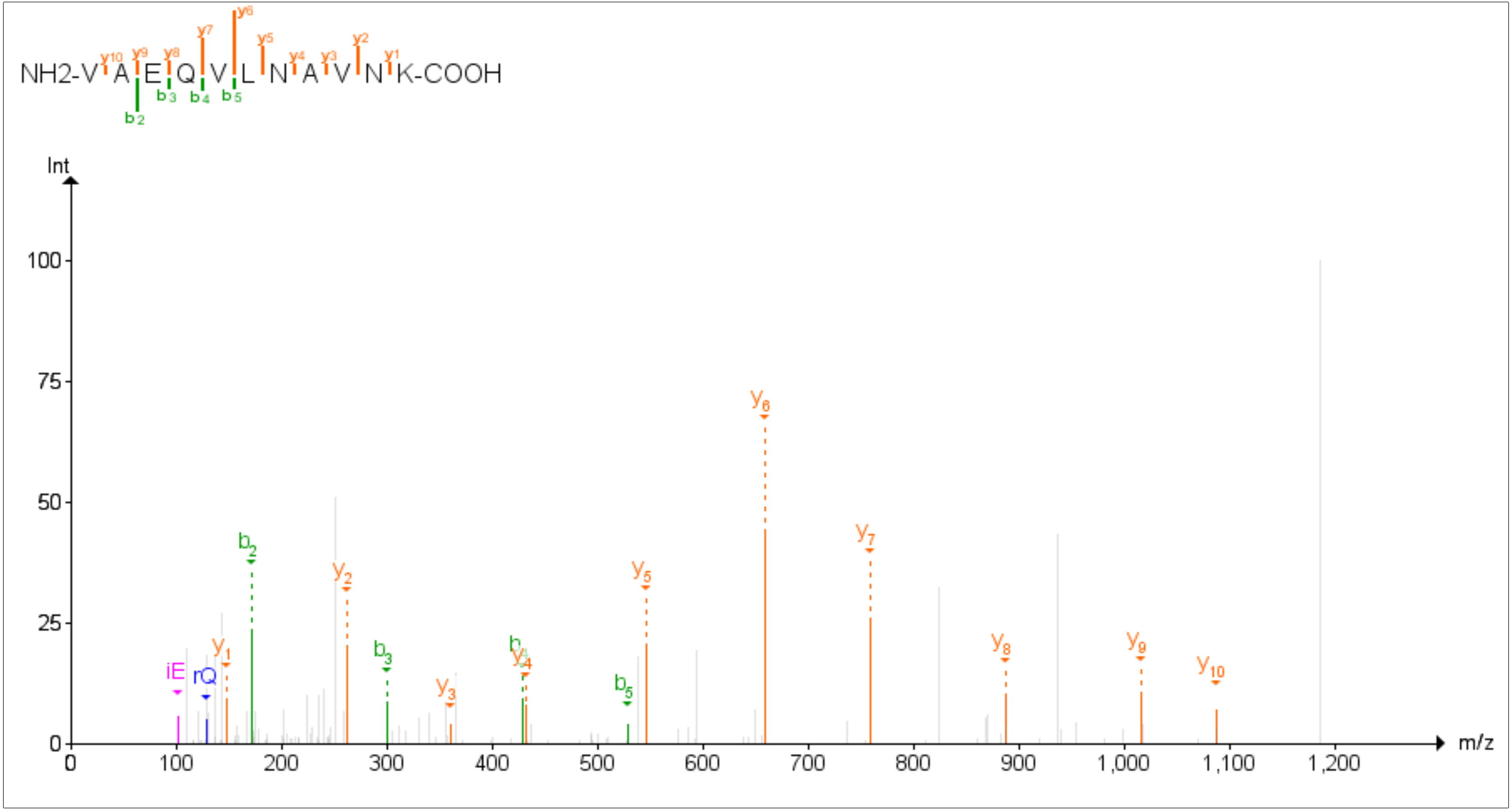

NH2-VAEQVLNAVNK-COOH
y10 y9 y8 y7 y6 y5 y4 y3 y2 y1
b2 b3 b4 b5
Int
100
75
50
25
0
m/z
0 100 200 300 400 500 600 700 800 900 1,000 1,100 1,200
iE
rQ
y1 y2 y3 y4 y5 y6 y7 y8 y9 y10
b2 b3 b4 b5

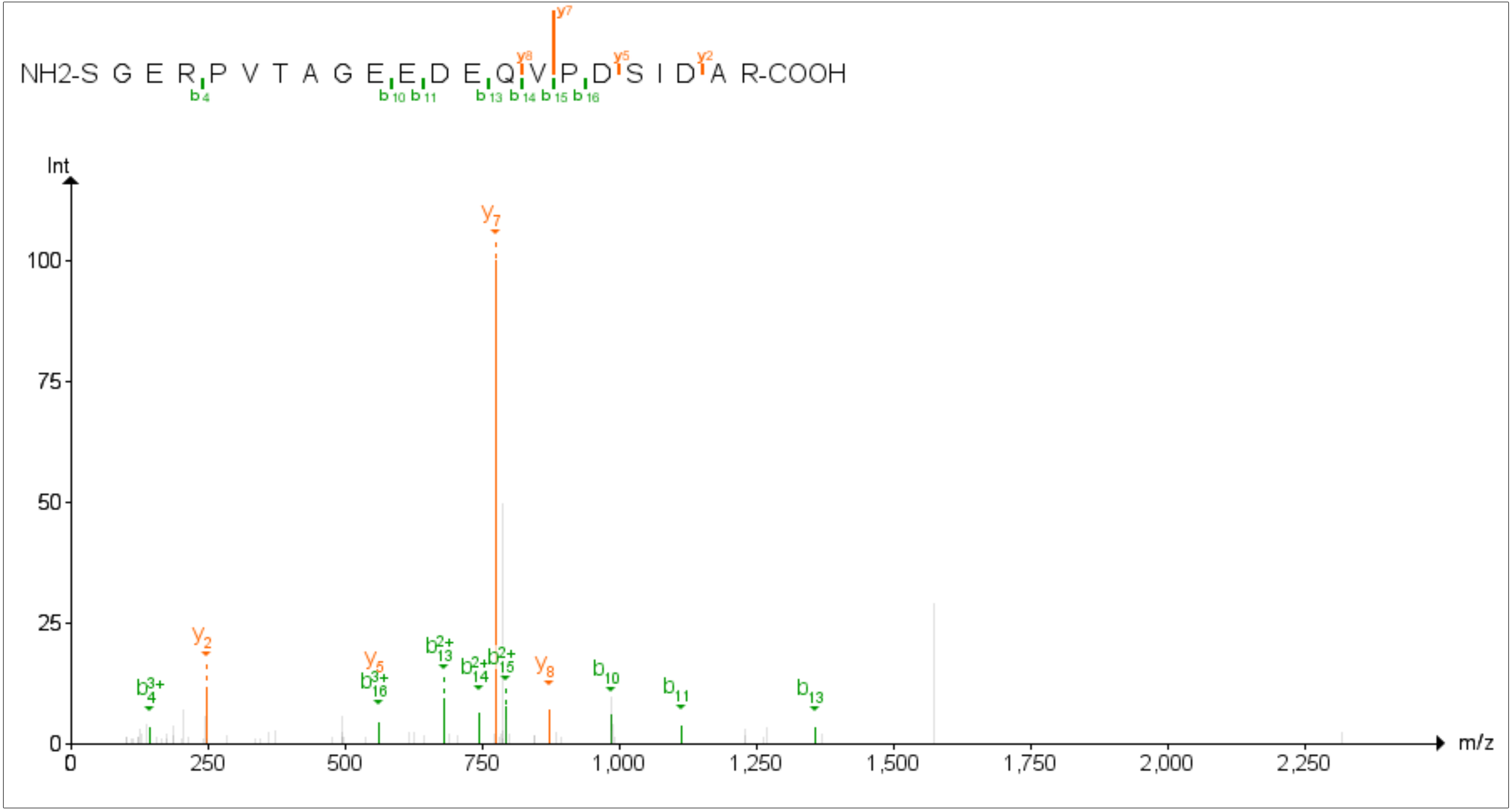
NH2-S G E R P V T A G E E D E Q V P D S I D A R-COOH
y7 y8 y5 y2
b4 b10 b11 b13 b14 b15 b16
Int
100
75
50
25
0
y7
y2
y5
y8
b10
b11
b13
m/z
0 250 500 750 1,000 1,250 1,500 1,750 2,000 2,250

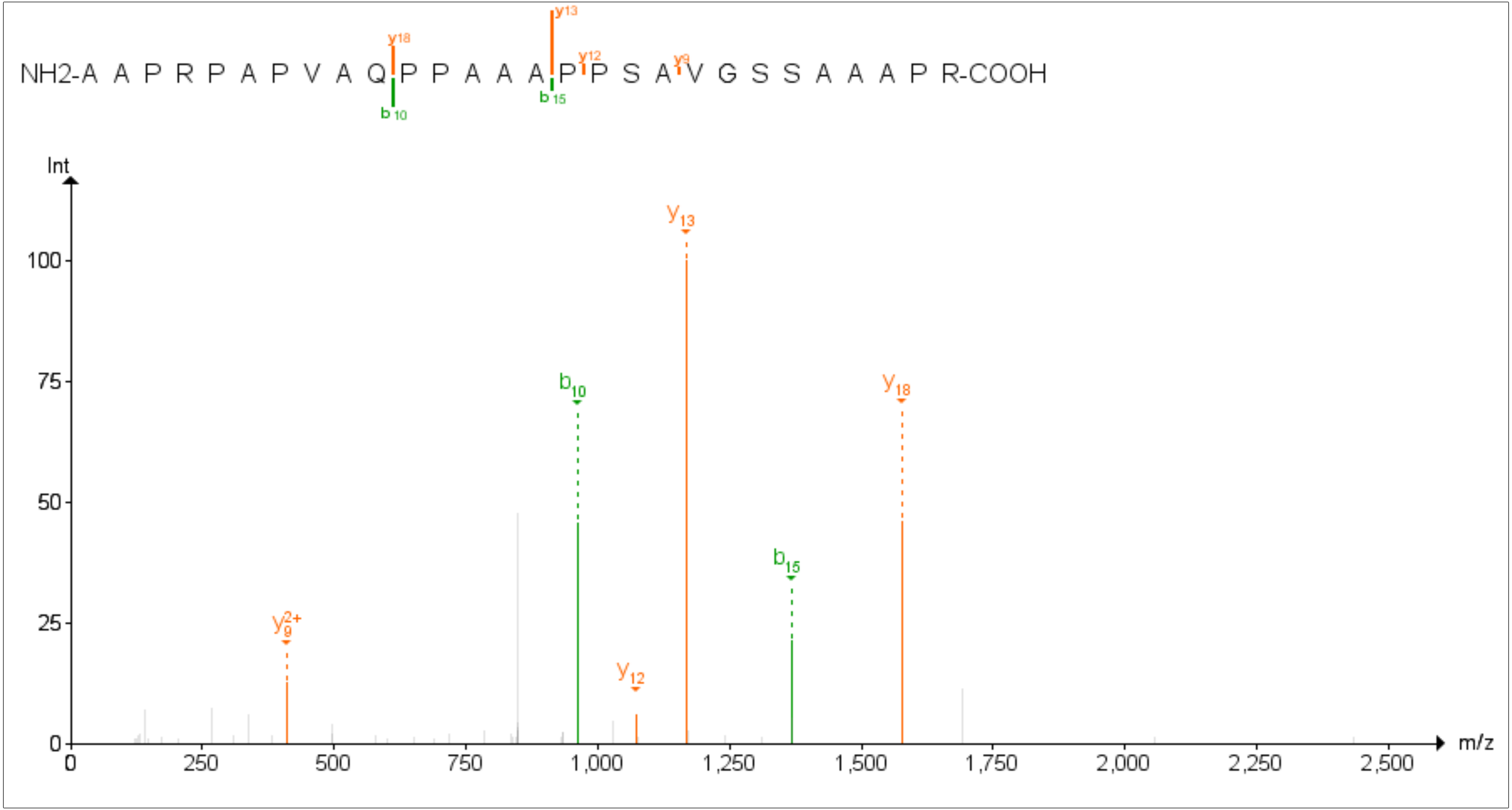

NH2-A A P R P A P V A Q P P A A A P P S A V G S S A A A P R-COOH
y18
y13
y12
y9
b 10
b 15
Int
100
75
50
25
0
y13
b10
y18
b15
y9 2+
y12
0
250
500
750
1,000
1,250
1,500
1,750
2,000
2,250
2,500
m/z

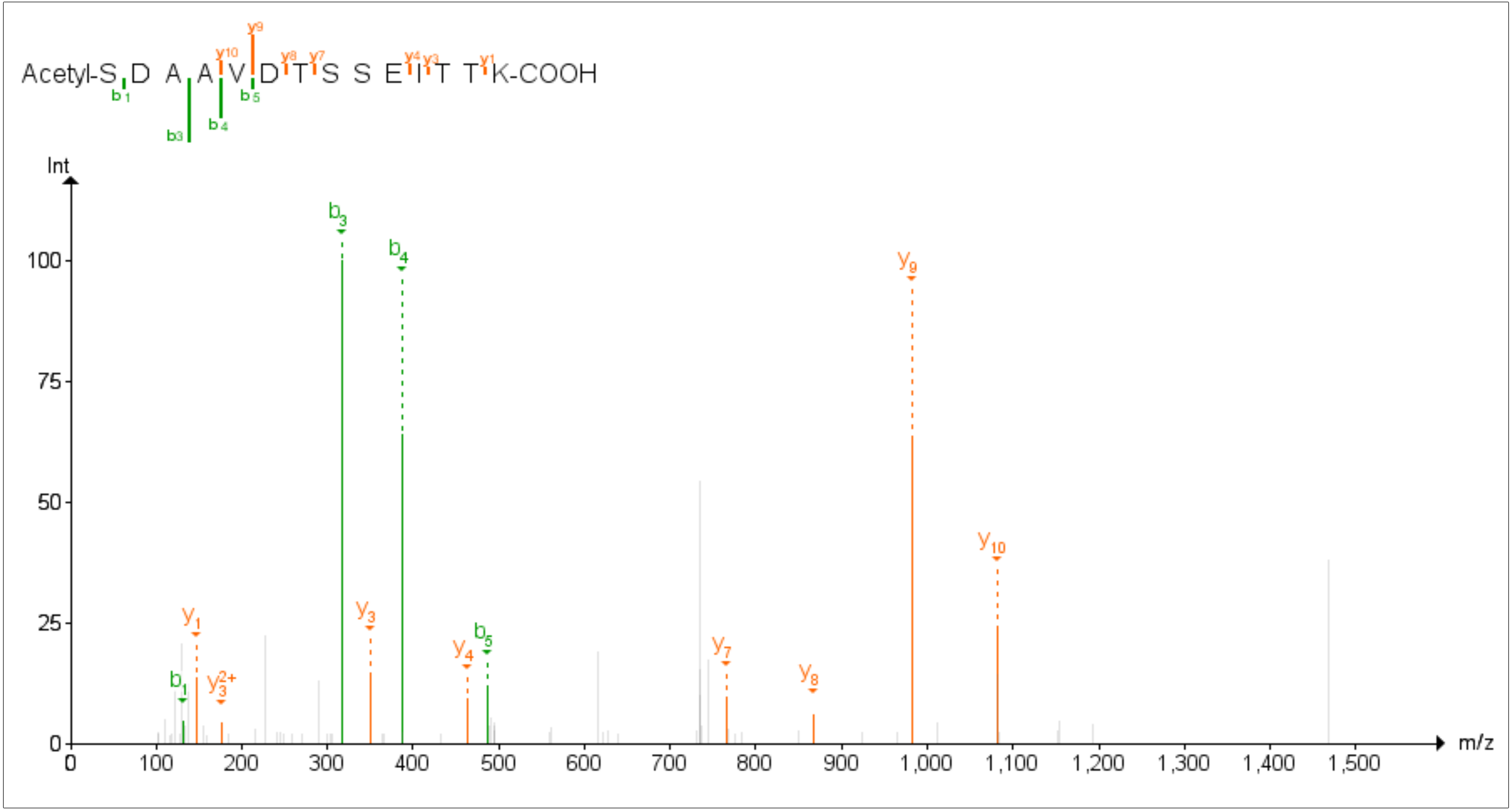

Acetyl-S D A A V D T S S E I T T K-COOH
b1 b3 b4 b5
y10 y9 y8 y7 y4 y3 y1
Int
m/z
b3
b4
y9
y10
y1
b1
y3 2+
y3
y4
b5
y7
y8
0
25
50
75
100
0
100
200
300
400
500
600
700
800
900
1,000
1,100
1,200
1,300
1,400
1,500

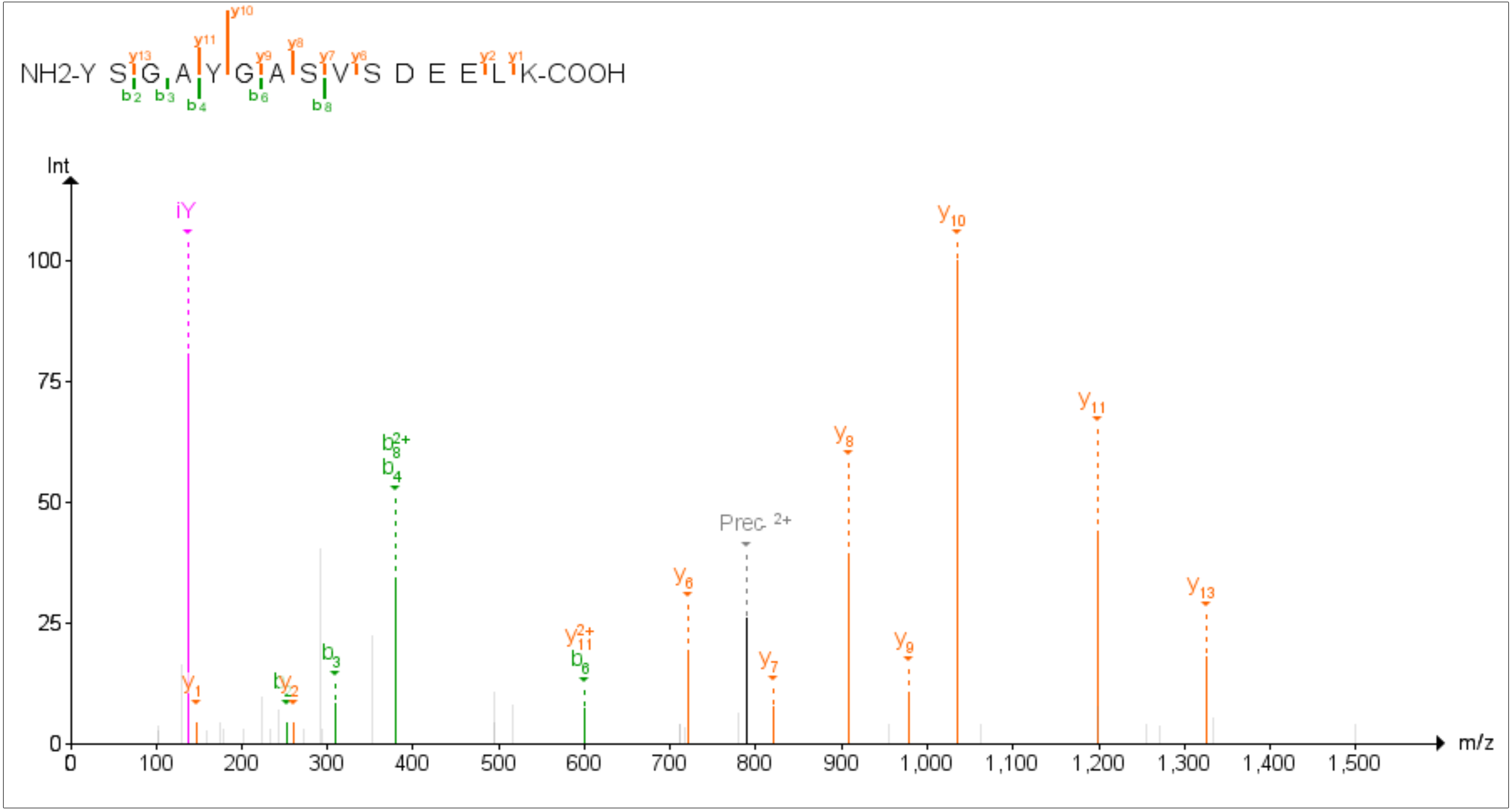

NH2-Y S G A Y G A S V S D E E L K-COOH
y13 y11 y10 y9 y8 y7 y6 y2 y1
b2 b3 b4 b6 b8
Int
iY
y10
y11
y8
b8 2+
b4
Prec 2+
y6
y13
y9
y7
y11 2+
b6
b3
y1
b y2
0
25
50
75
100
0 100 200 300 400 500 600 700 800 900 1,000 1,100 1,200 1,300 1,400 1,500
m/z

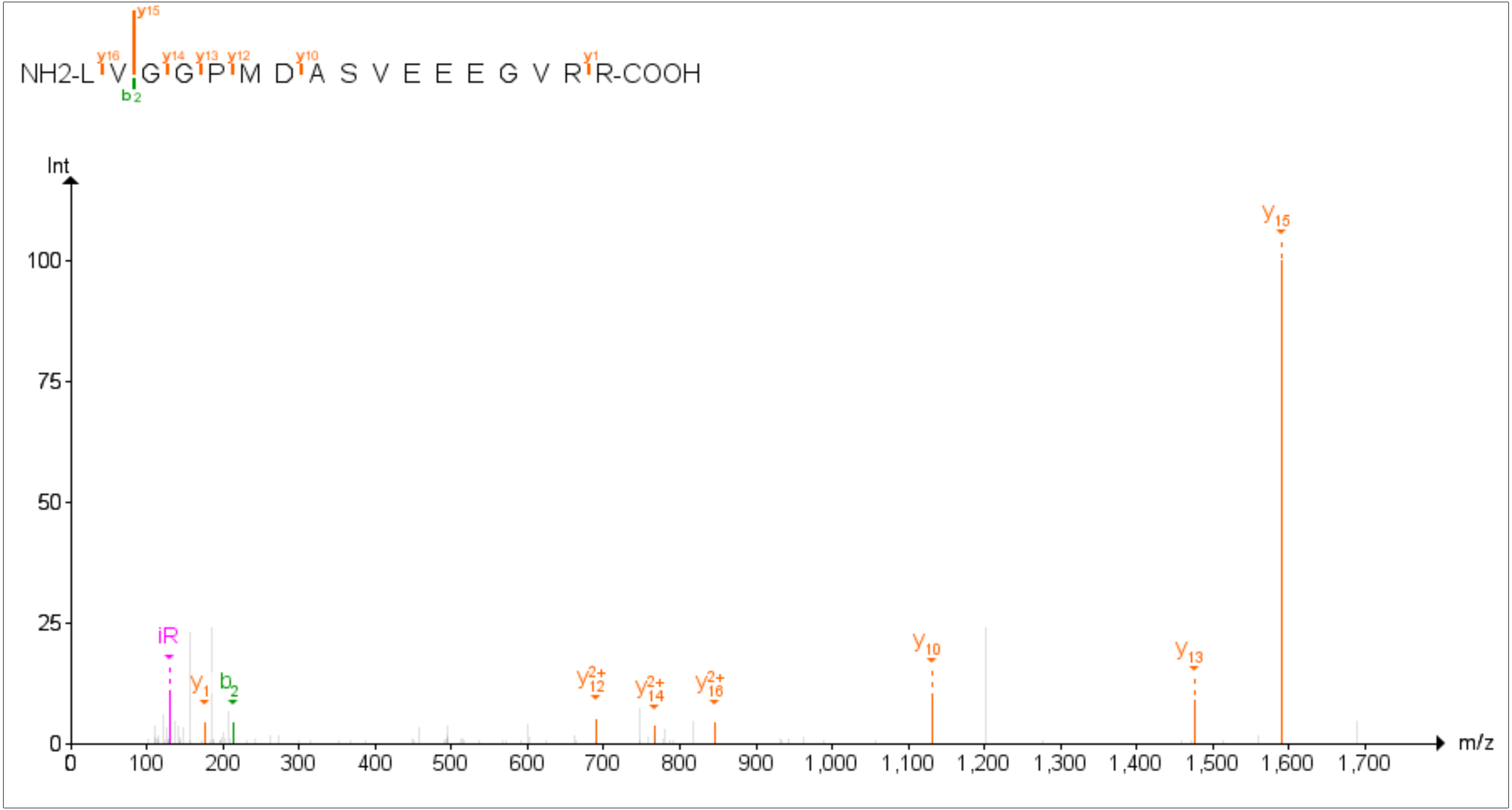

NH2-L V G G P M D A S V E E E G V R R-COOH
y15
y16
y14
y13
y12
y10
y1
b2
Int
100
75
50
25
0
iR
y1
b2
y_{12}^{2+}
y_{14}^{2+}
y_{16}^{2+}
y10
y13
y15
0
100
200
300
400
500
600
700
800
900
1,000
1,100
1,200
1,300
1,400
1,500
1,600
1,700
m/z

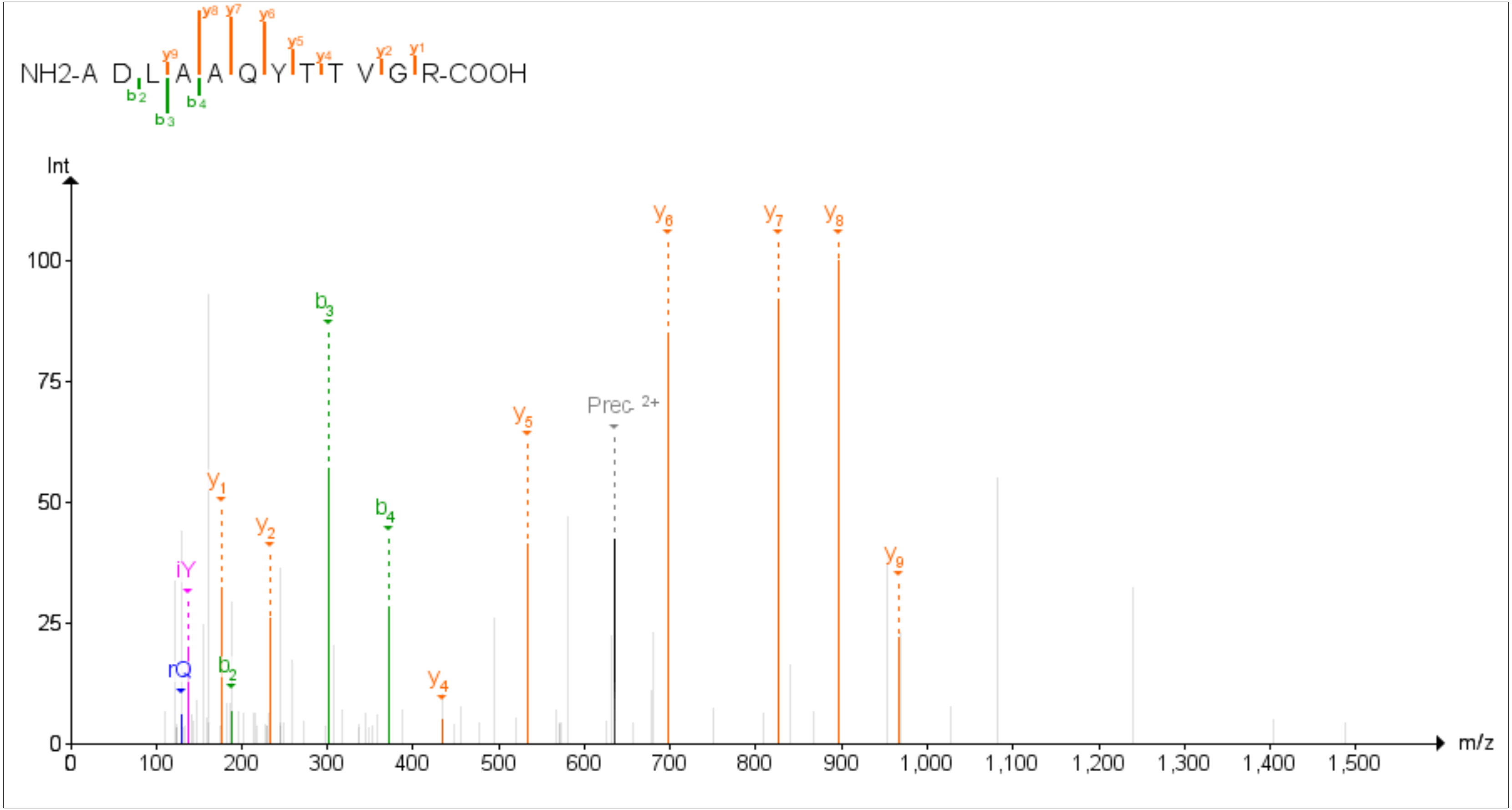

NH2-A D L A A Q Y T T V G R-COOH
y9 y8 y7 y6 y5 y4 y2 y1
b2 b3 b4
Int
m/z
y6 y7 y8
b3
Prec 2+
y5
y1
y2
b4
iY
y9
rQ
b2
y4
0 25 50 75 100
0 100 200 300 400 500 600 700 800 900 1,000 1,100 1,200 1,300 1,400 1,500

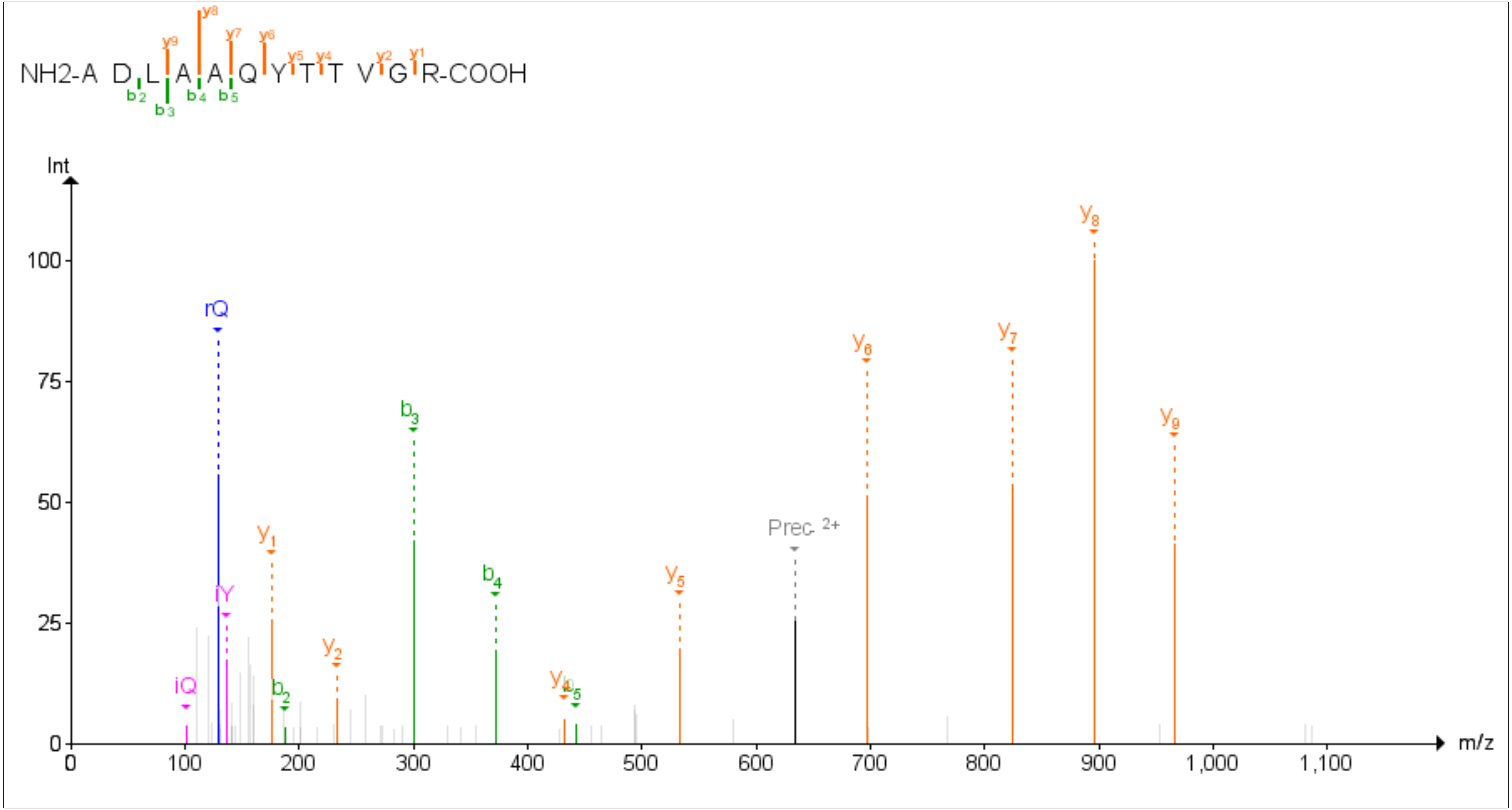

NH2-A D L A A Q Y T T V G R-COOH
y9 y8 y7 y6 y5 y4 y2 y1
b2 b3 b4 b5
Int
100
75
50
25
0
rQ
iY
iQ
y1
y2
b2
b3
b4
y4
b5
y5
Prec 2+
y6
y7
y8
y9
0 100 200 300 400 500 600 700 800 900 1,000 1,100
m/z

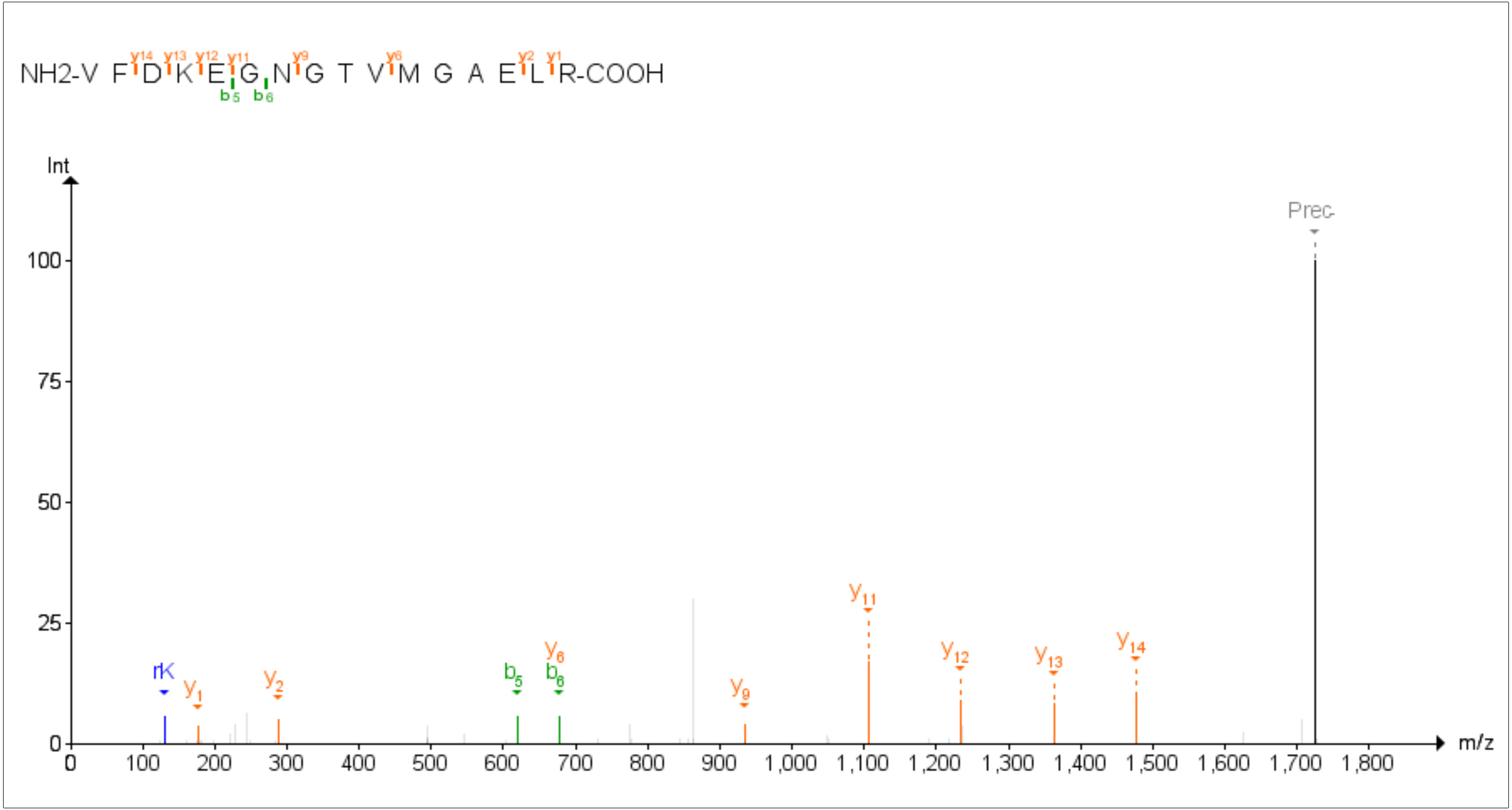
NH2-V F D K E G N G T V M G A E L R-COOH
y14 y13 y12 y11 y9 y6 y2 y1
b5 b6
Int
100
75
50
25
0
Prec
rK
y1
y2
b5
b6
y6
y9
y11
y12
y13
y14
0 100 200 300 400 500 600 700 800 900 1,000 1,100 1,200 1,300 1,400 1,500 1,600 1,700 1,800
m/z

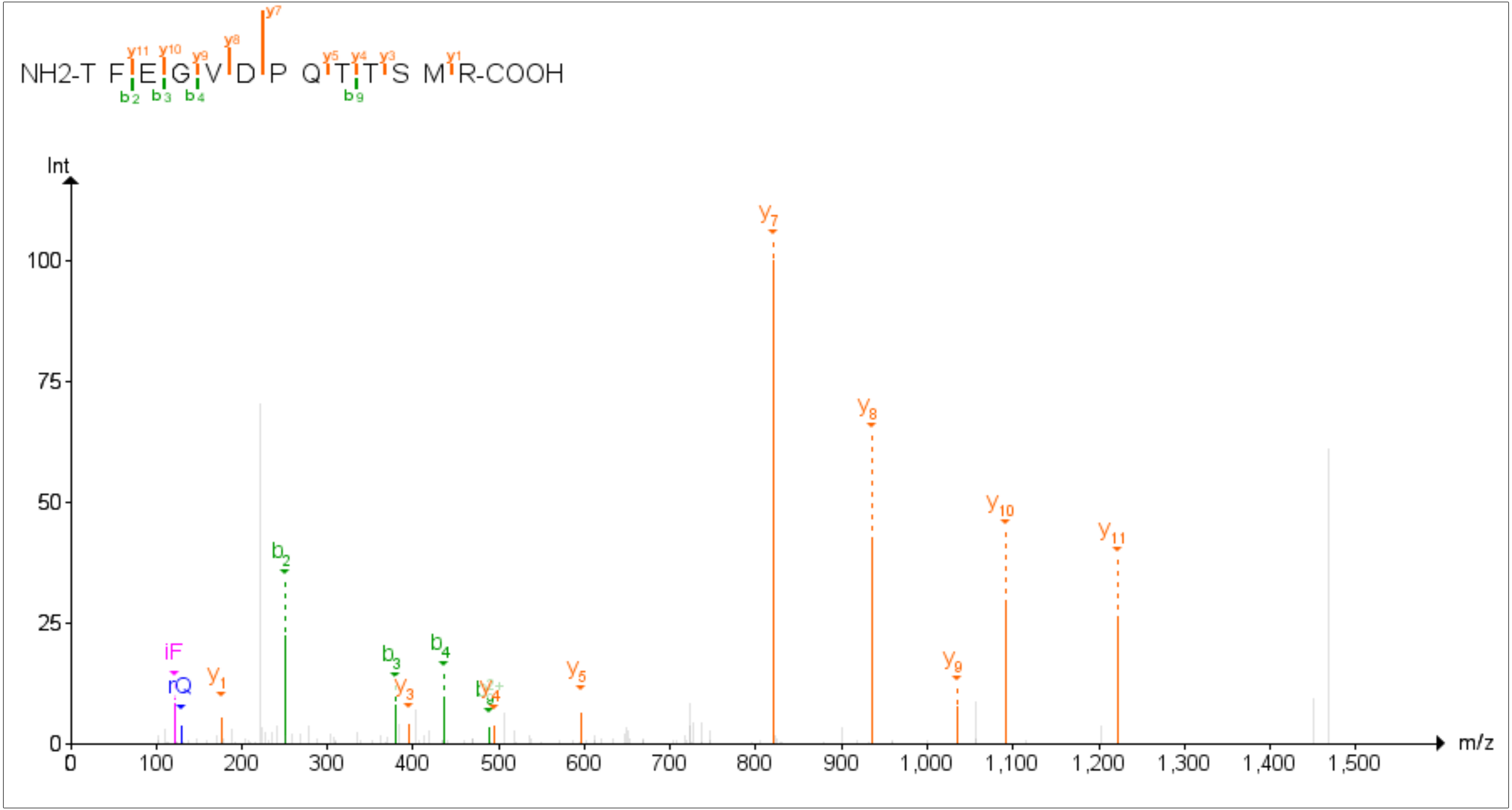

NH2-T F E G V D P Q T T S M R-COOH
y11 y10 y9 y8 y7 y5 y4 y3 y1
b2 b3 b4 b9
Int
100
75
50
25
0
y7
y8
y10
y11
b2
b3
b4
y9
y5
y3
y1
iF
rQ
m/z
0 100 200 300 400 500 600 700 800 900 1,000 1,100 1,200 1,300 1,400 1,500

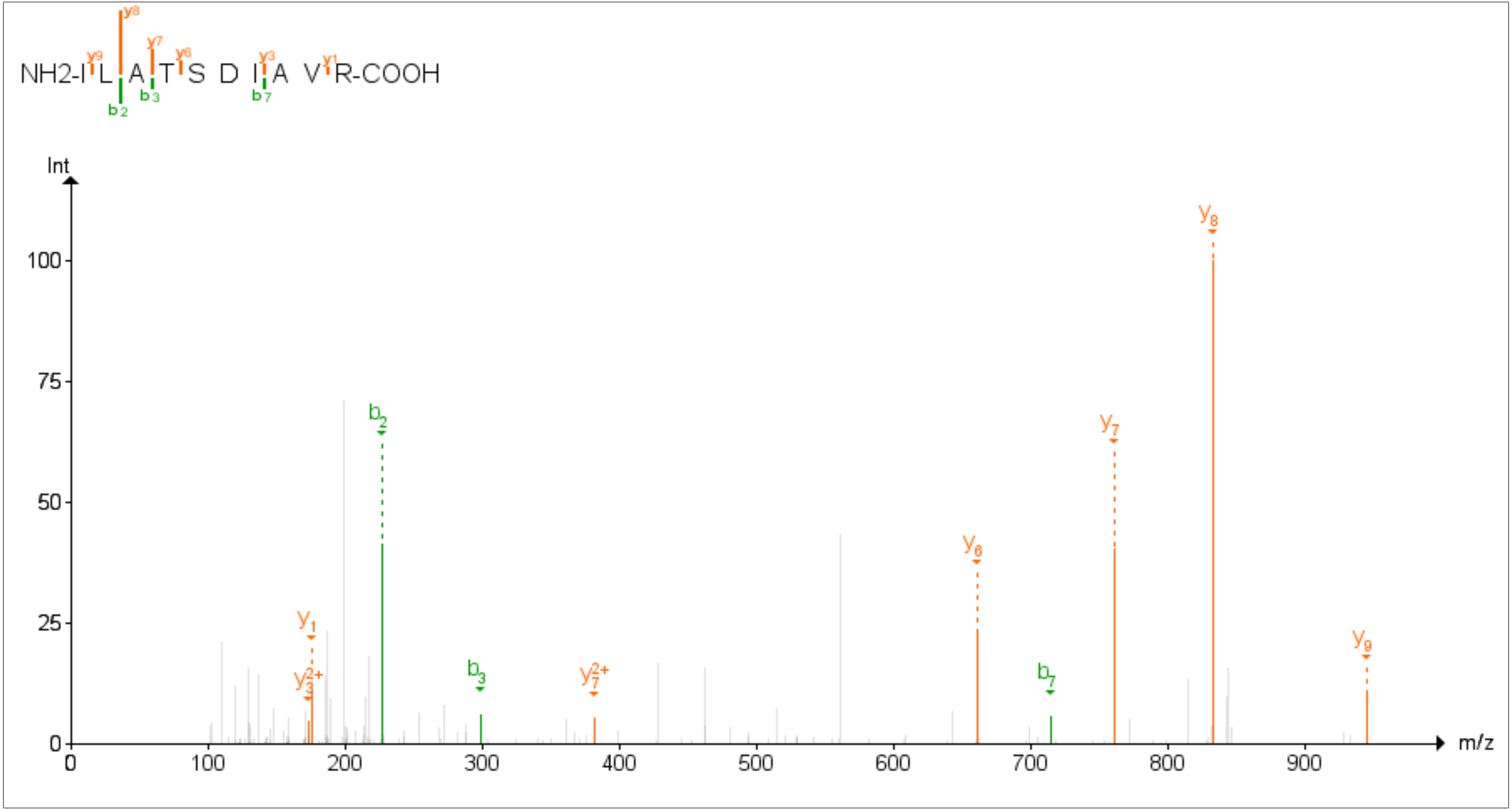
NH2-I L A T S D I A V R-COOH
y9 y8 y7 y6 y3 y1
b2 b3 b7
Int
100
75
50
25
0
m/z
0 100 200 300 400 500 600 700 800 900
y8
y7
b2
y6
y1
y_3^{2+}
b3
y_7^{2+}
b7
y9

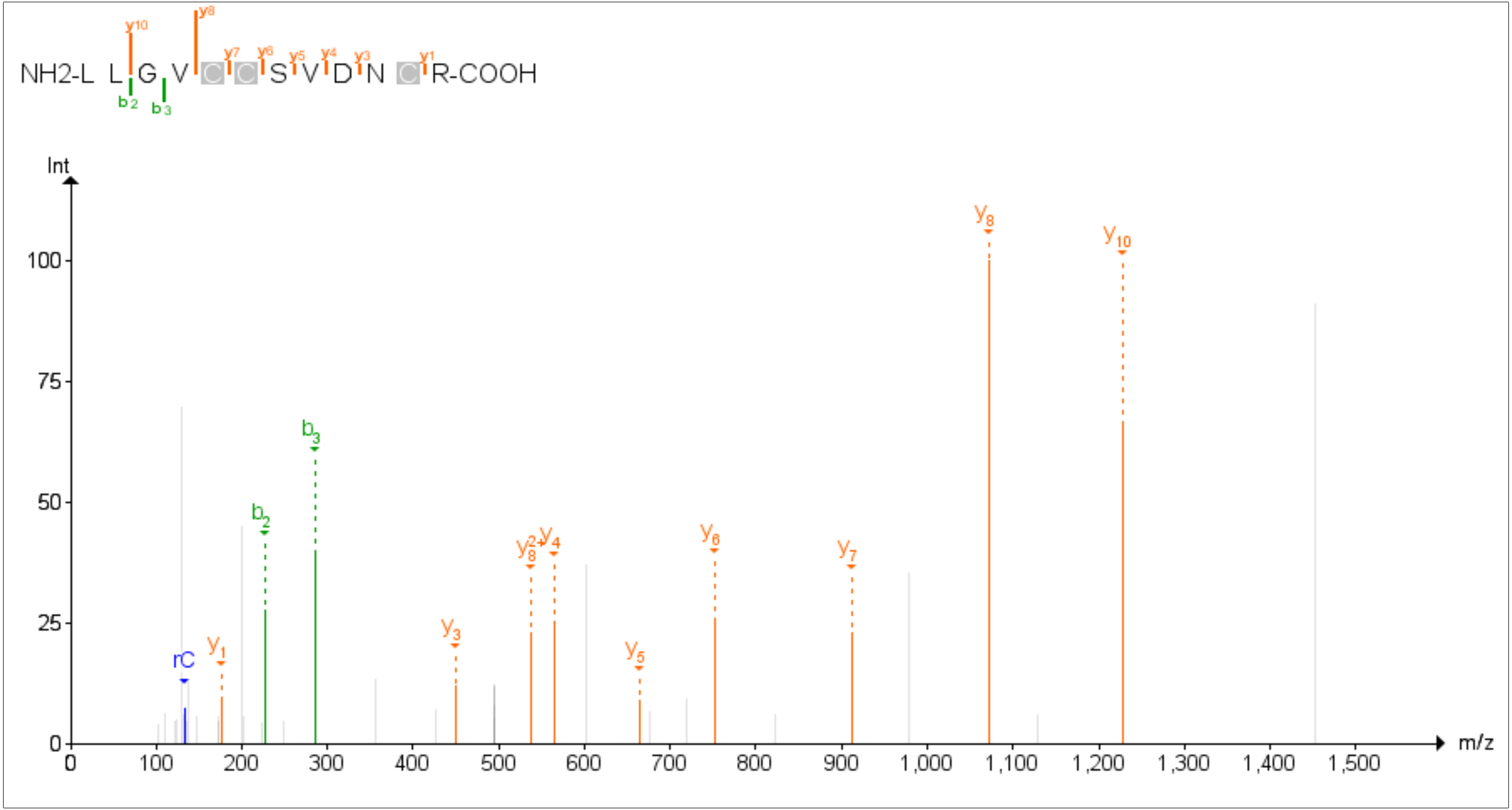
NH2-L L G V C C S V D N C R-COOH
y10 y8 y7 y6 y5 y4 y3 y1
b2 b3
Int
100
75
50
25
0
rC
y1
b2
b3
y3
y8 2+ y4
y5
y6
y7
y8
y10
0 100 200 300 400 500 600 700 800 900 1,000 1,100 1,200 1,300 1,400 1,500
m/z

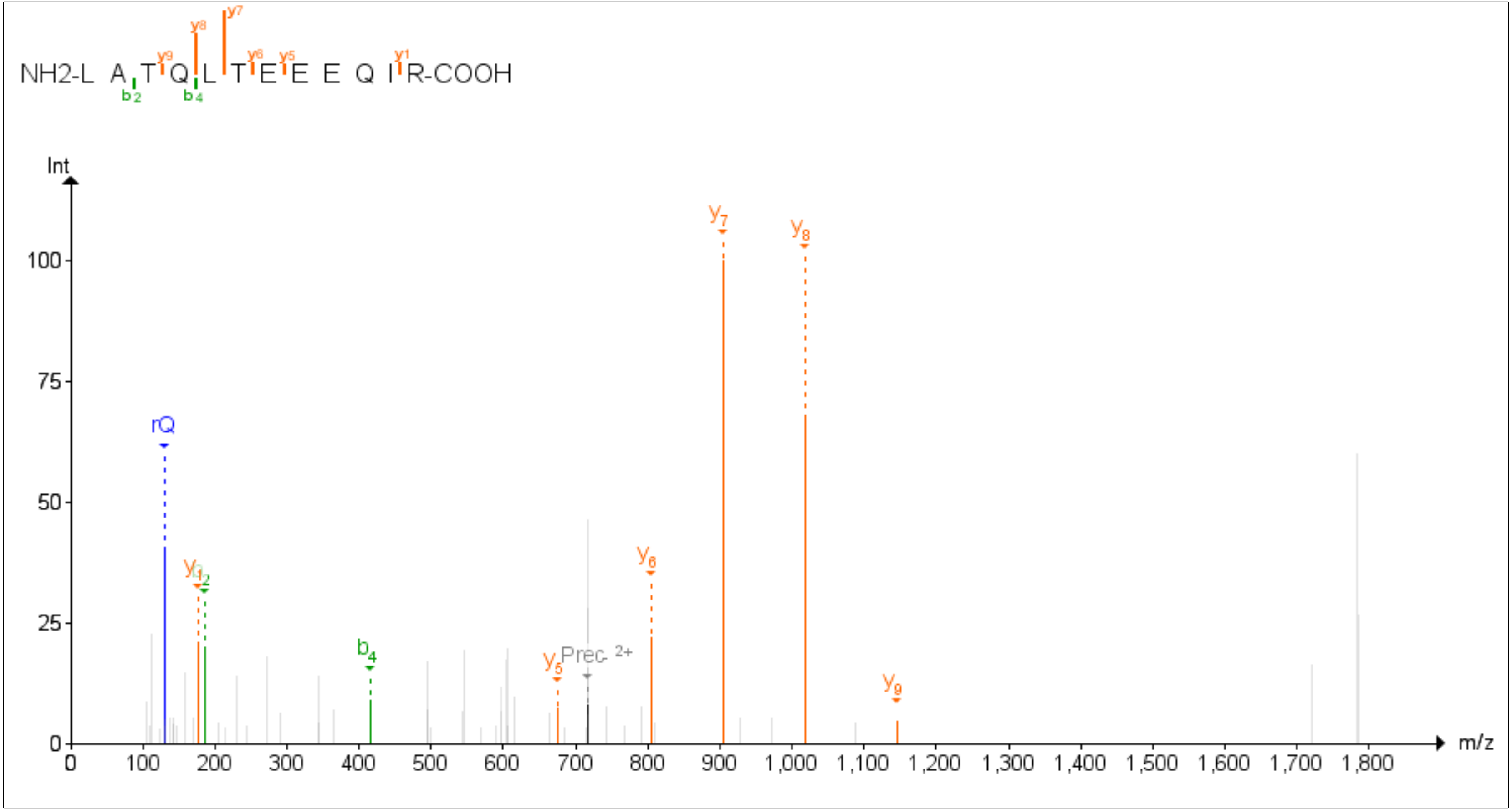

NH2-L A T Q L T E E E Q I R-COOH
y7
y8
y9
y6
y5
y1
b2
b4
Int
100
75
50
25
0
rQ
y1
y7
y8
y6
b4
y5
Prec 2+
y9
0
100
200
300
400
500
600
700
800
900
1,000
1,100
1,200
1,300
1,400
1,500
1,600
1,700
1,800
m/z

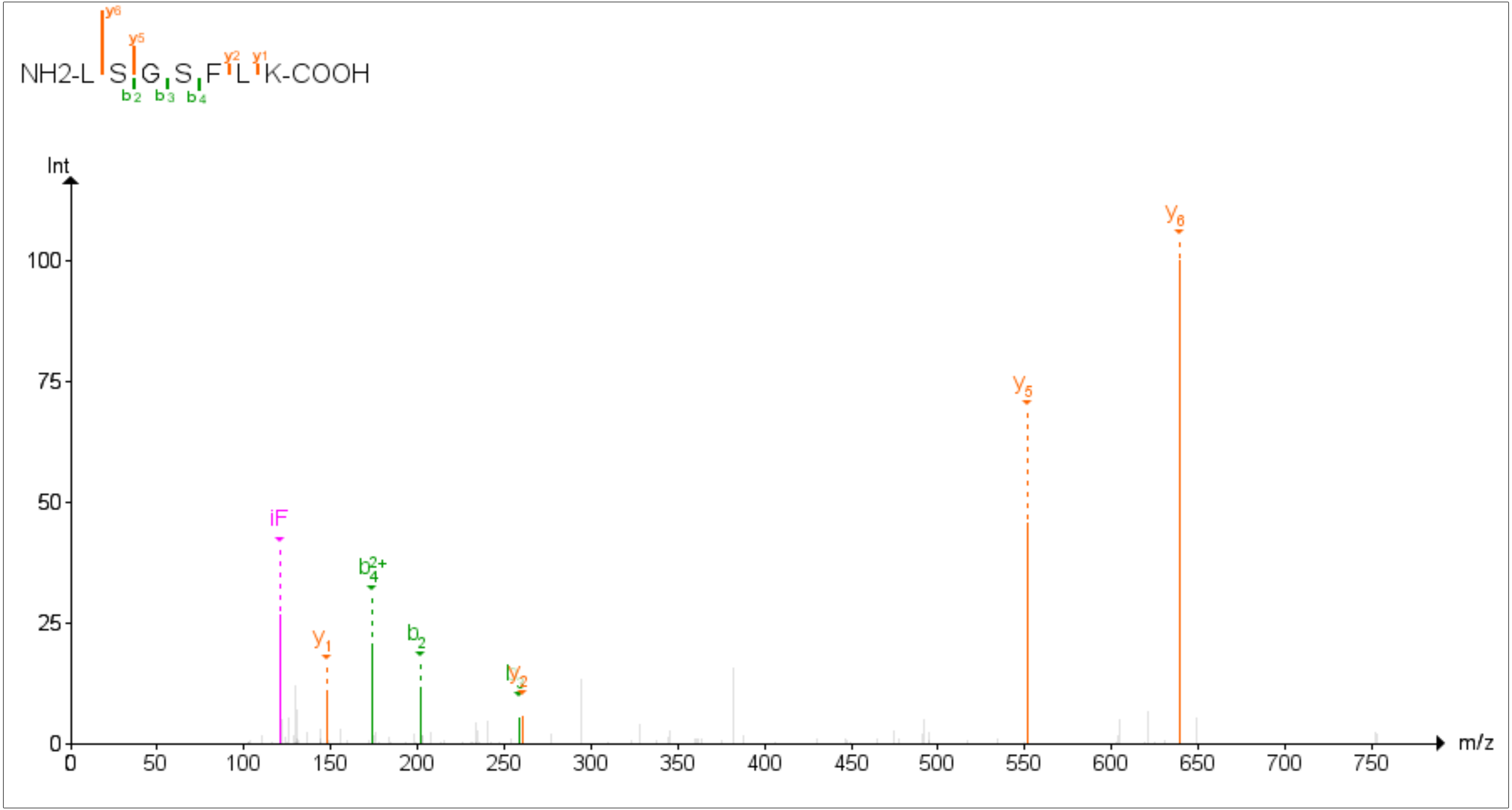

NH2-L S G S F L K-COOH
y6
y5
y2
y1
b2
b3
b4
Int
100
75
50
25
0
iF
y1
b4 2+
b2
y2
y5
y6
0
50
100
150
200
250
300
350
400
450
500
550
600
650
700
750
m/z

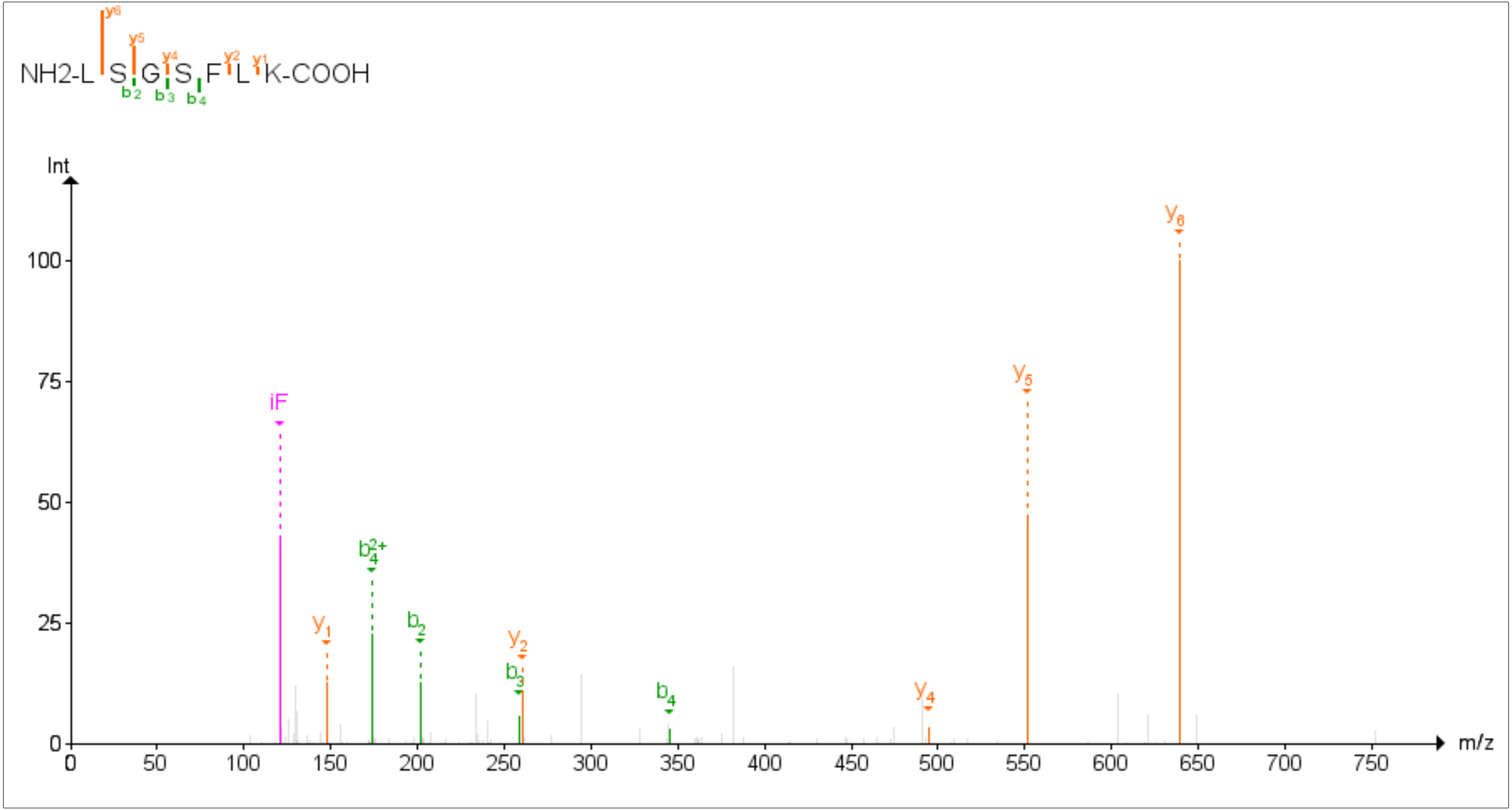

NH2-L S G S F L K-COOH
y6
y5
y4
y2
y1
b2
b3
b4
Int
m/z
iF
y1
b4 2+
b2
y2
b3
b4
y4
y5
y6
0
25
50
75
100
0
50
100
150
200
250
300
350
400
450
500
550
600
650
700
750

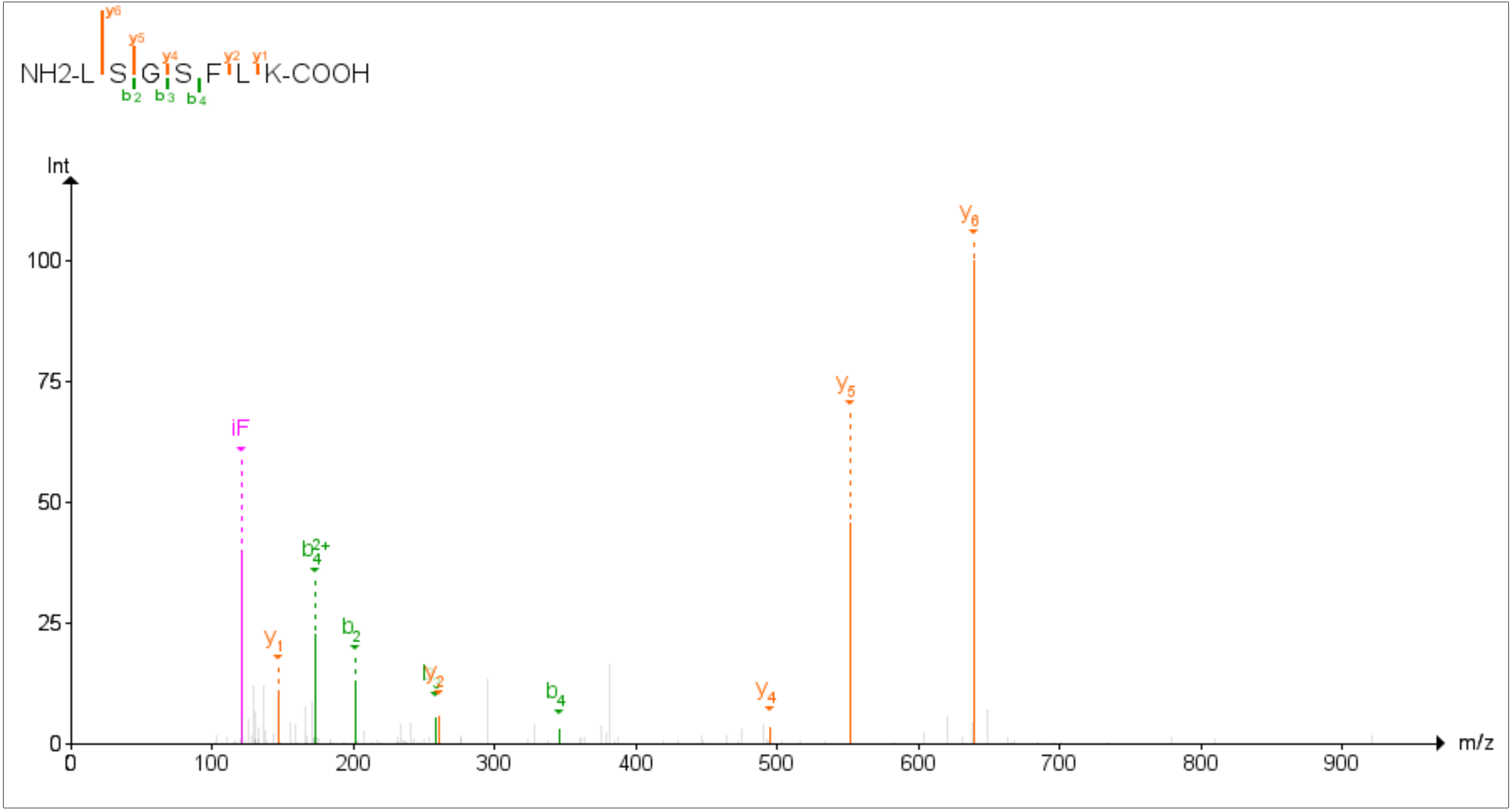

NH2-L S G S F L K-COOH
y6
y5
y4
y2
y1
b2
b3
b4
Int
100
75
50
25
0
m/z
0
100
200
300
400
500
600
700
800
900
iF
b4 2+
y1
b2
y2
b4
y4
y5
y6

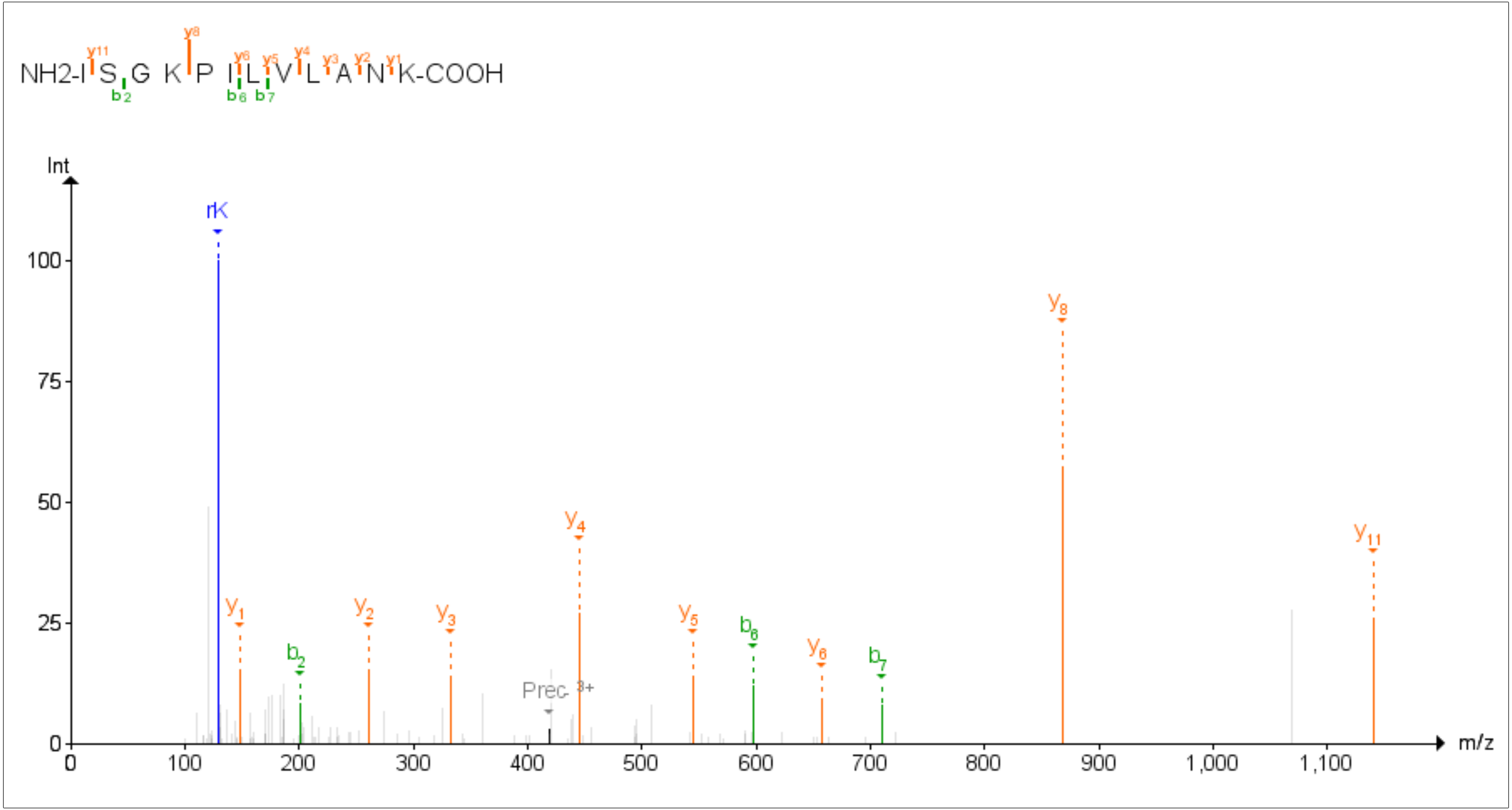

NH2-I S G K P I L V L A N K-COOH
y11 y8 y6 y5 y4 y3 y2 y1
b2 b6 b7
Int
m/z
rK
y1 y2 y3 y4 y5 y6 y8 y11
b2 b6 b7
Prec 3+
0
25
50
75
100
0
100
200
300
400
500
600
700
800
900
1,000
1,100

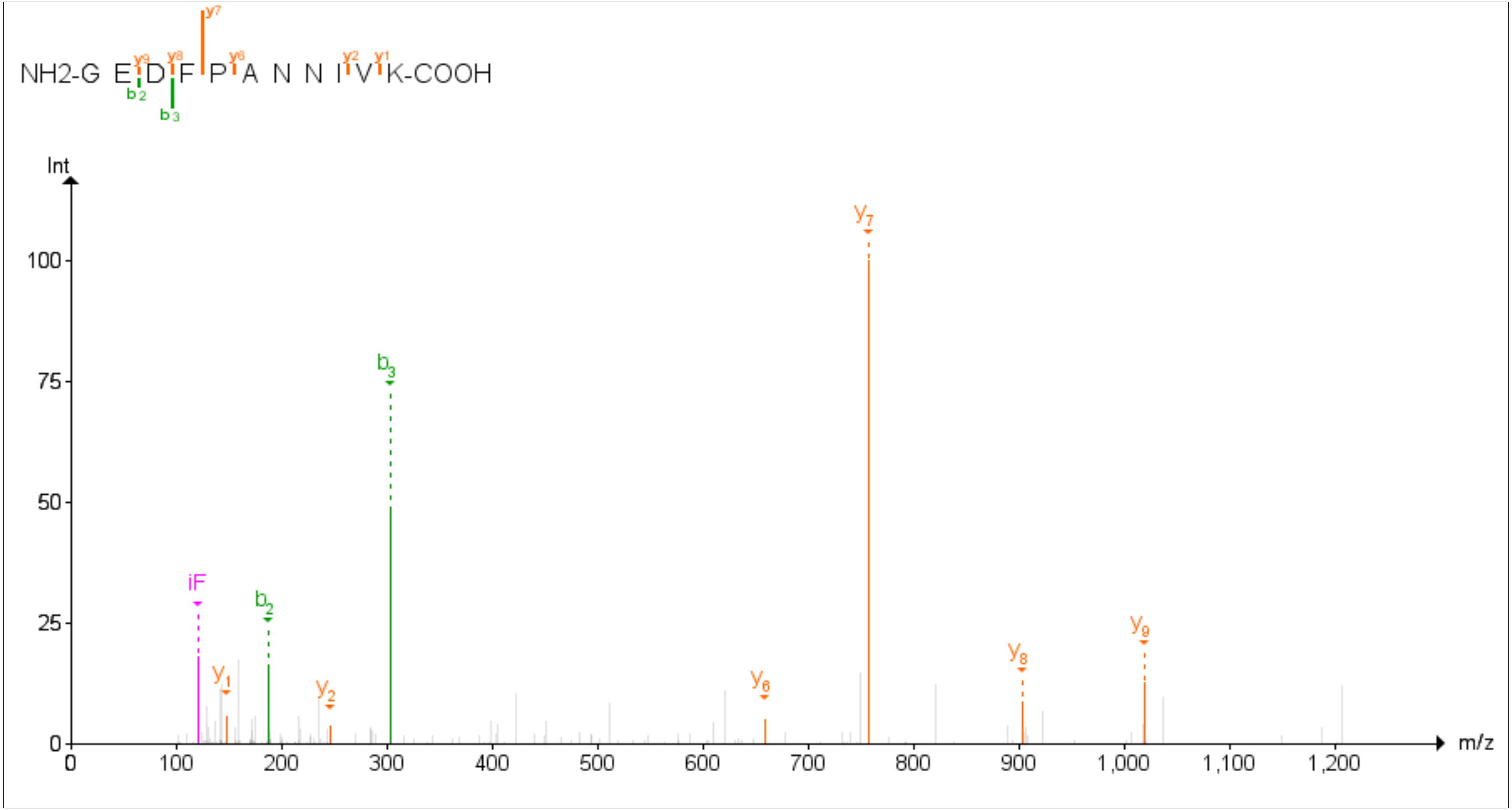

NH2-G E D F P A N N I V K-COOH
y9
y8
y7
y6
y2
y1
b2
b3
Int
100
75
50
25
0
y7
b3
iF
b2
y1
y2
y6
y8
y9
0
100
200
300
400
500
600
700
800
900
1,000
1,100
1,200
m/z

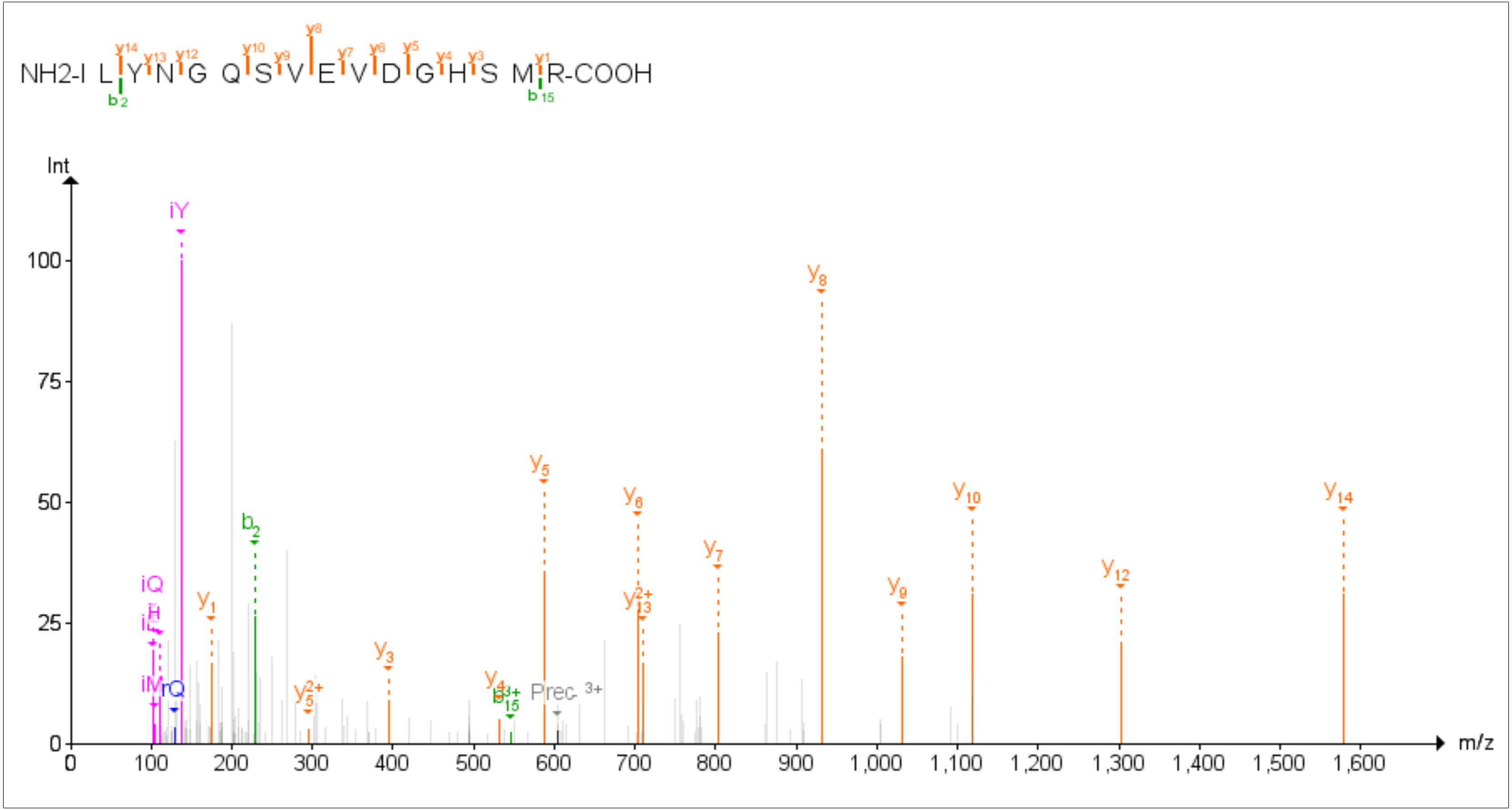

NH2-I L Y N G Q S V E V D G H S M R-COOH
y14 y13 y12 y10 y9 y8 y7 y6 y5 y4 y3 y1
b2 b15
Int
m/z
iY
y8
y5
y6
y10
y14
b2
y7
iQ
y1
y13 2+
y9
y12
y3
iM
rQ
y5 2+
y4
b15 3+
Prec 3+

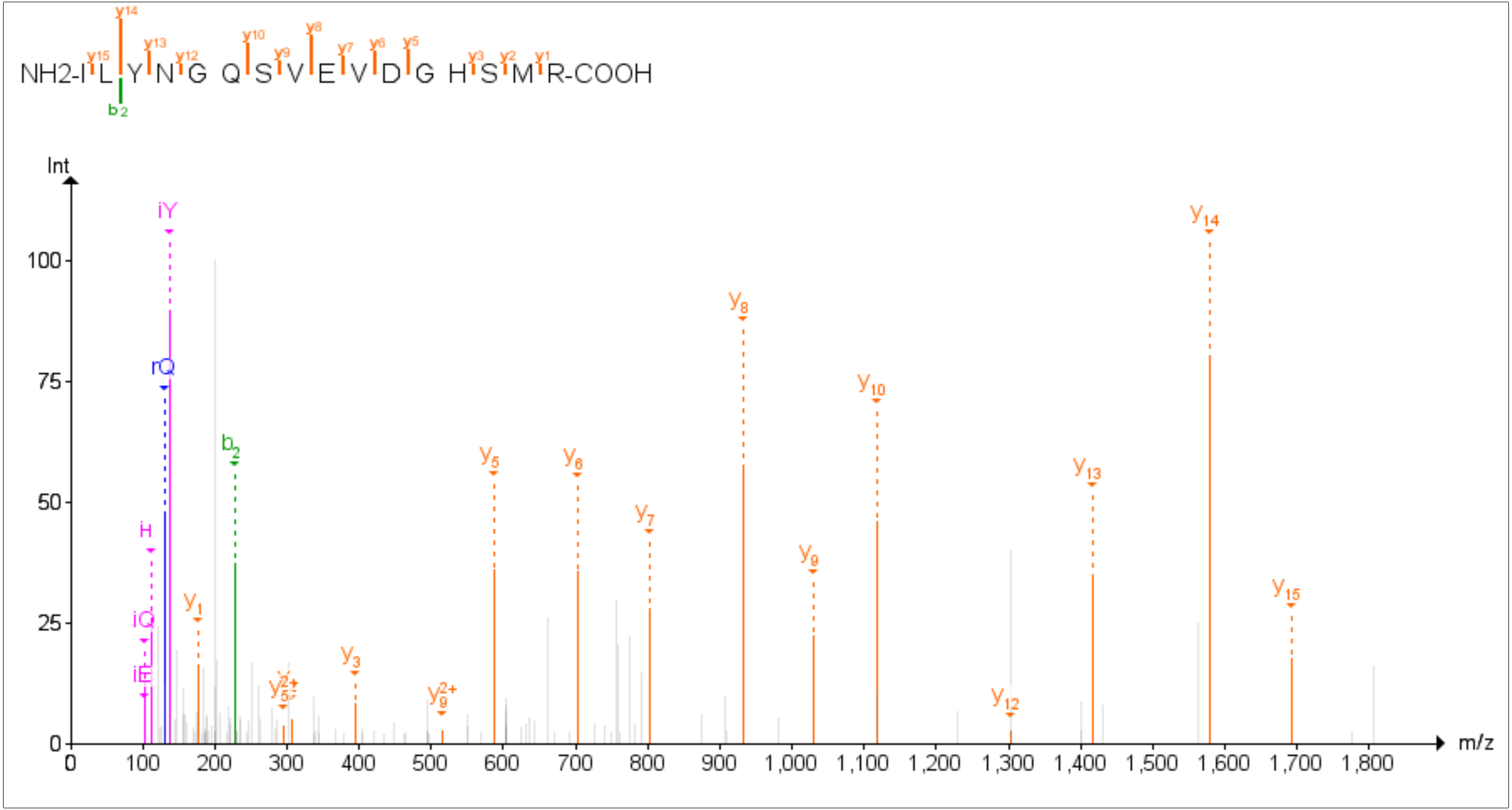

NH2-ILYNGQSVEVDGHSMR-COOH
Int
m/z
iY
rQ
b2
iH
iQ
iE
y1
y2+5
y3
y2+9
y5
y6
y7
y8
y9
y10
y12
y13
y14
y15

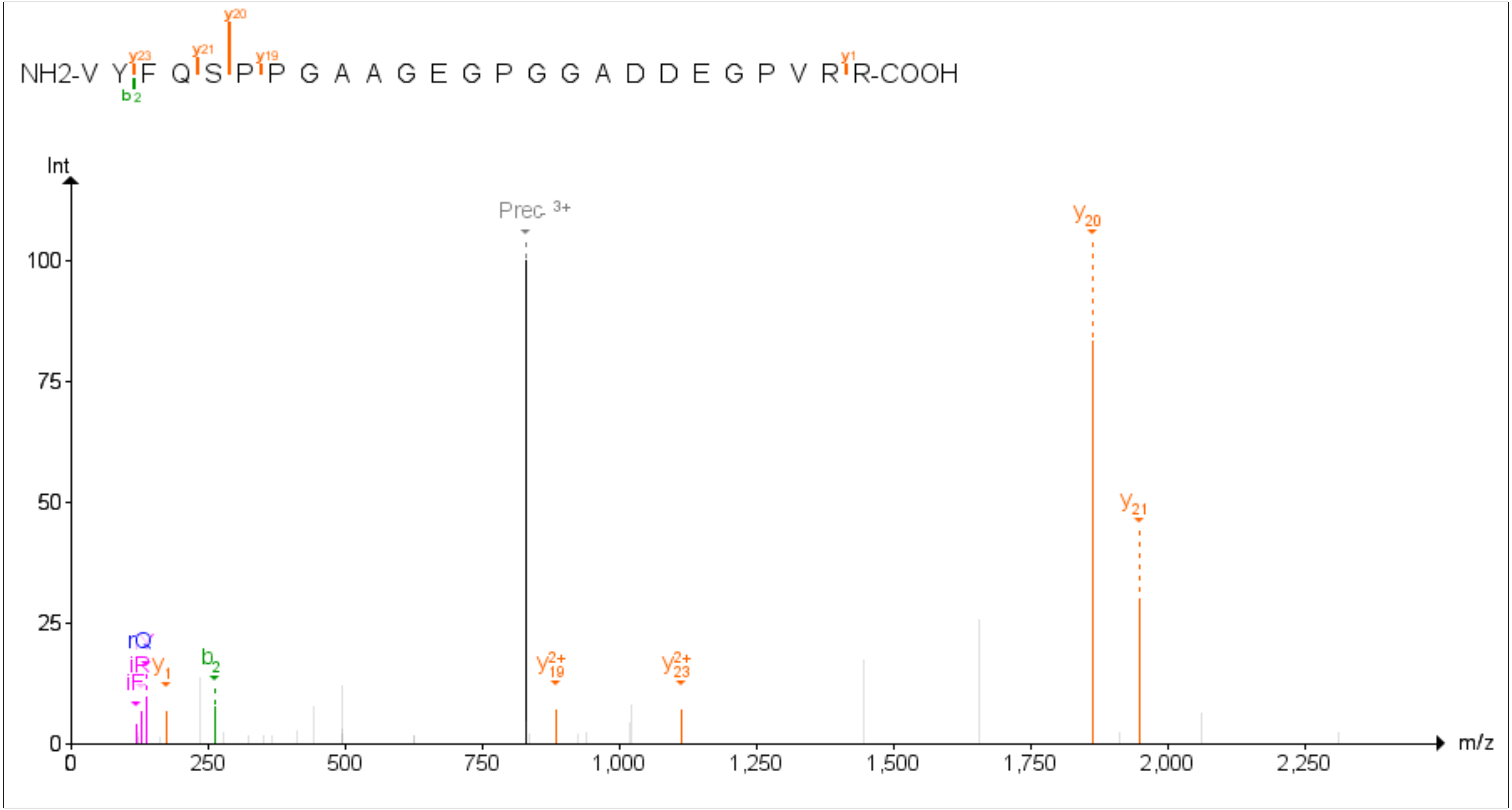

NH2-V Y F Q S P P G A A G E G P G G A D D E G P V R R-COOH
y23
b2
y21
y20
y19
y1
Int
m/z
Prec 3+
y20
y21
rQ
iR
iF
y1
b2
y19 2+
y23 2+
100
75
50
25
0
0
250
500
750
1,000
1,250
1,500
1,750
2,000
2,250

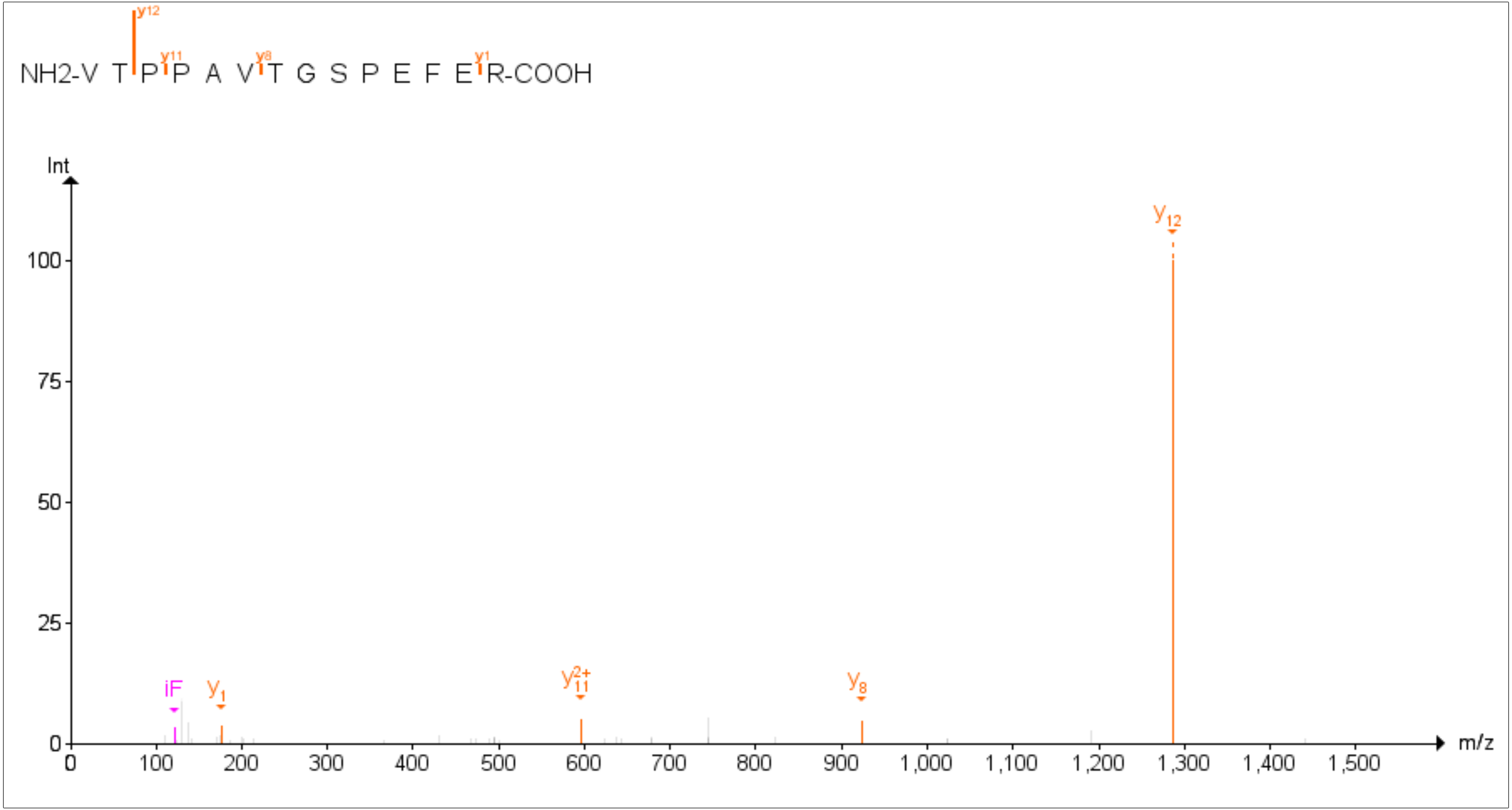

NH2-V T P P A V T G S P E F E R-COOH
y12
y11
y8
y1
Int
100
75
50
25
0
iF
y1
y11 2+
y8
y12
0
100
200
300
400
500
600
700
800
900
1,000
1,100
1,200
1,300
1,400
1,500
m/z

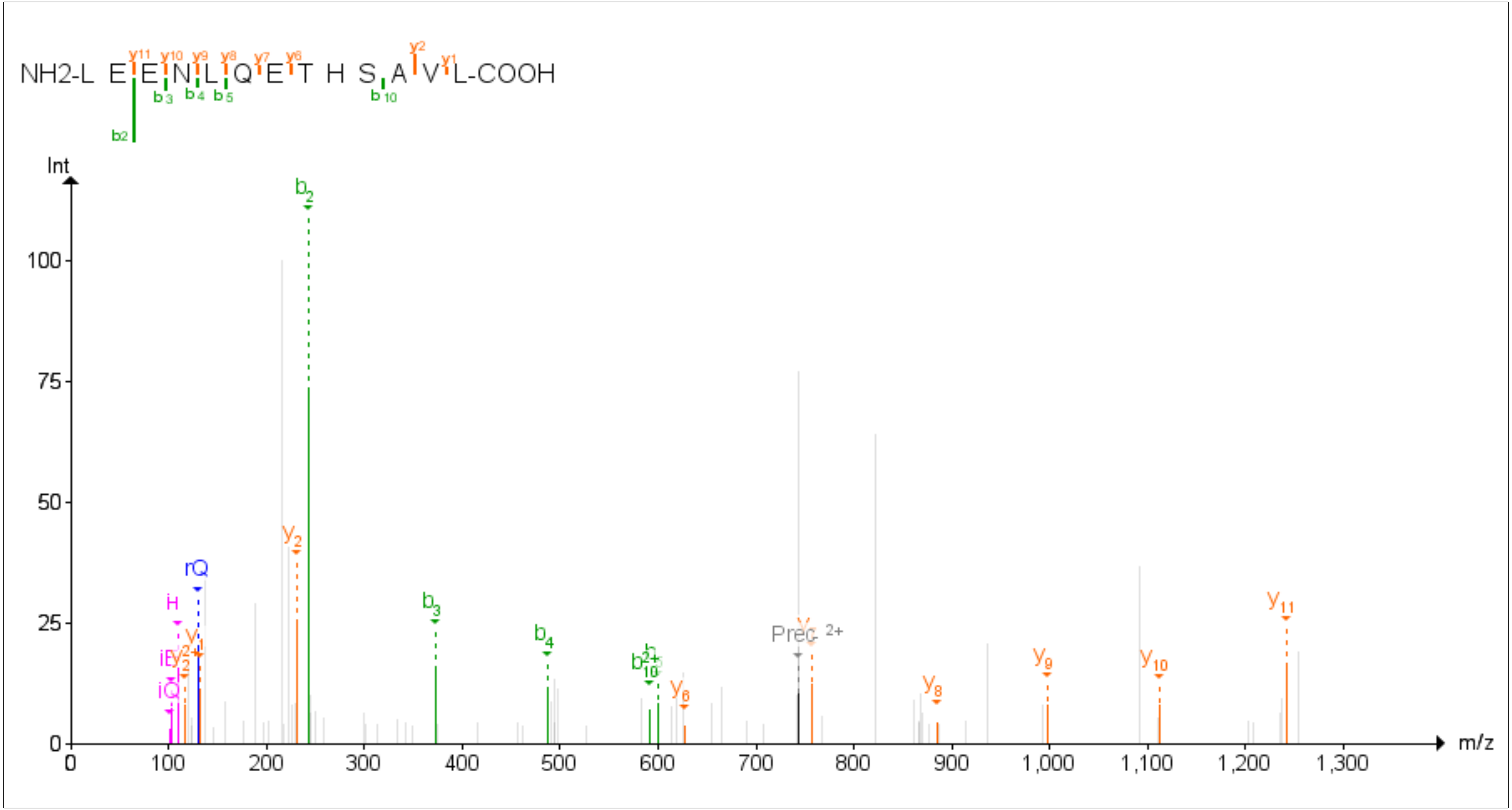
NH2-L E E N L Q E T H S A V L-COOH
y11 y10 y9 y8 y7 y6 y2 y1
b2 b3 b4 b5 b10
Int
m/z
100
75
50
25
0
0 100 200 300 400 500 600 700 800 900 1,000 1,100 1,200 1,300
b2
y2
rQ
iH
y1
iE
iQ
b3
b4
b10 2+
y6
Prec 2+
y8
y9
y10
y11

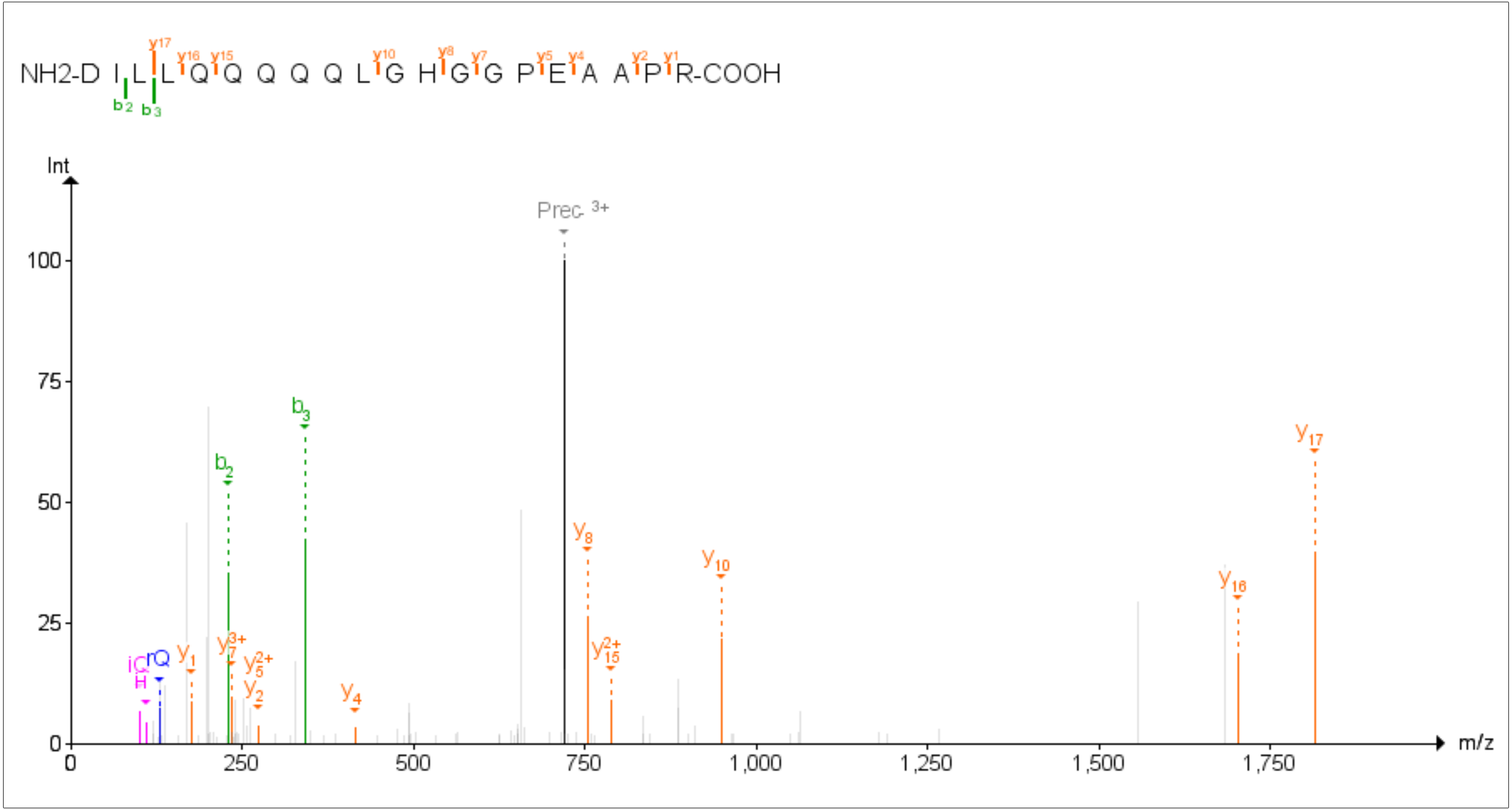

NH2-D I L L Q Q Q Q Q L G H G G P E A A P R-COOH
y17 y16 y15 y10 y8 y7 y5 y4 y2 y1
b2 b3
Int
Prec- 3+
100
75
50
25
0
b3
b2
y17
y8
y10
y16
y1
y7 3+
y5 2+
y2
y4
y15 2+
iC
rQ
H
0
250
500
750
1,000
1,250
1,500
1,750
m/z

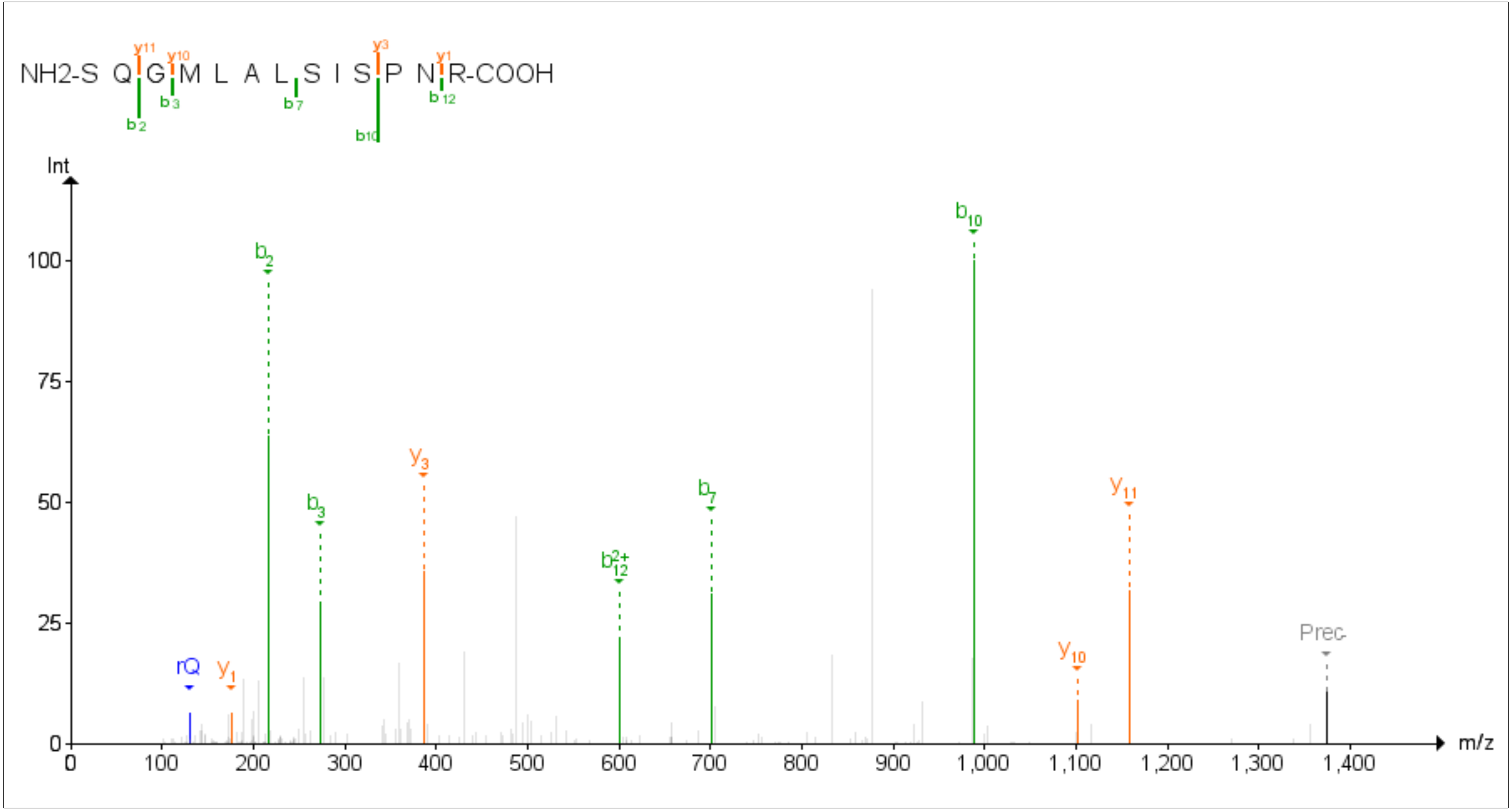
NH2-S Q G M L A L S I S P N R-COOH
y11 y10 y3 y1
b2 b3 b7 b10 b12
Int
m/z
b2
b3
y3
b7
b10
y11
y10
rQ
y1
Prec
0 25 50 75 100
0 100 200 300 400 500 600 700 800 900 1,000 1,100 1,200 1,300 1,400

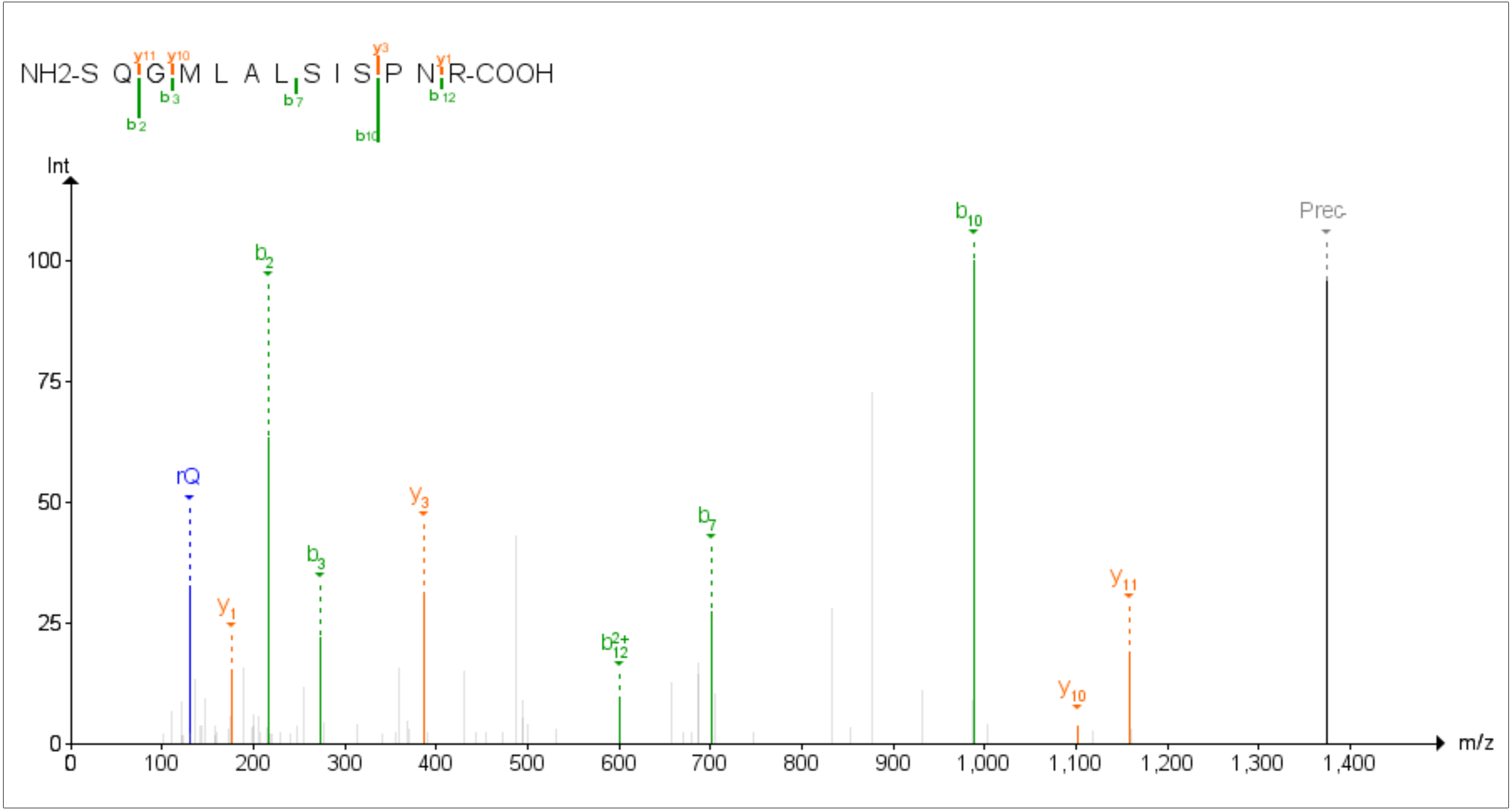

NH2-S Q G M L A L S I S P N R-COOH
y11 y10 y3 y1
b2 b3 b7 b10 b12
Int
100
75
50
25
0
rQ
y1
b2
b3
y3
b7
b12 2+
b10
y10
y11
Prec
0 100 200 300 400 500 600 700 800 900 1,000 1,100 1,200 1,300 1,400
m/z

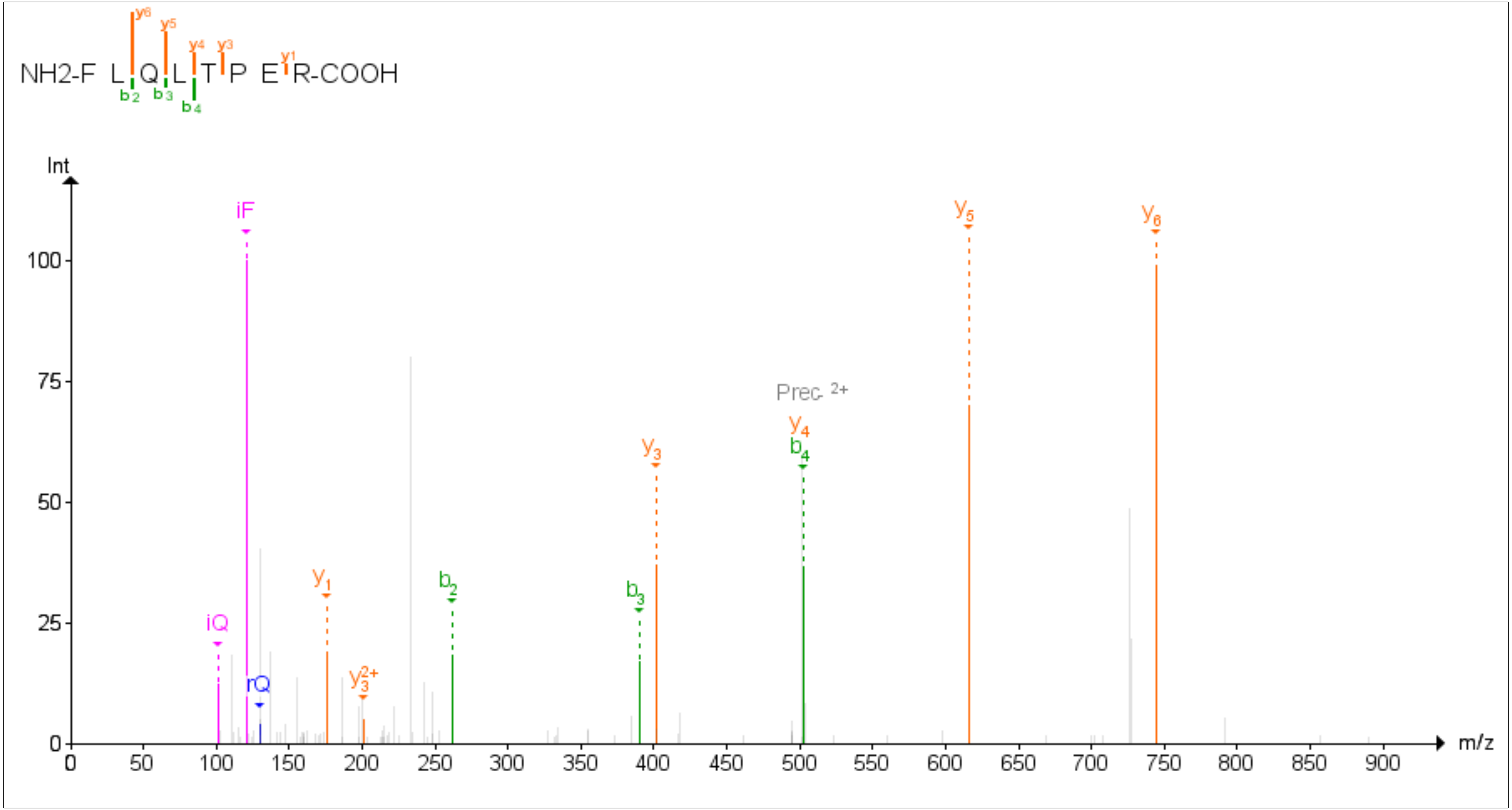

NH2-F L Q L T P E R-COOH
y6
y5
y4
y3
y1
b2
b3
b4
Int
100
75
50
25
0
iF
iQ
rQ
y1
y_3^{2+}
b2
b3
y3
y4
b4
Prec 2+
y5
y6
0 50 100 150 200 250 300 350 400 450 500 550 600 650 700 750 800 850 900
m/z

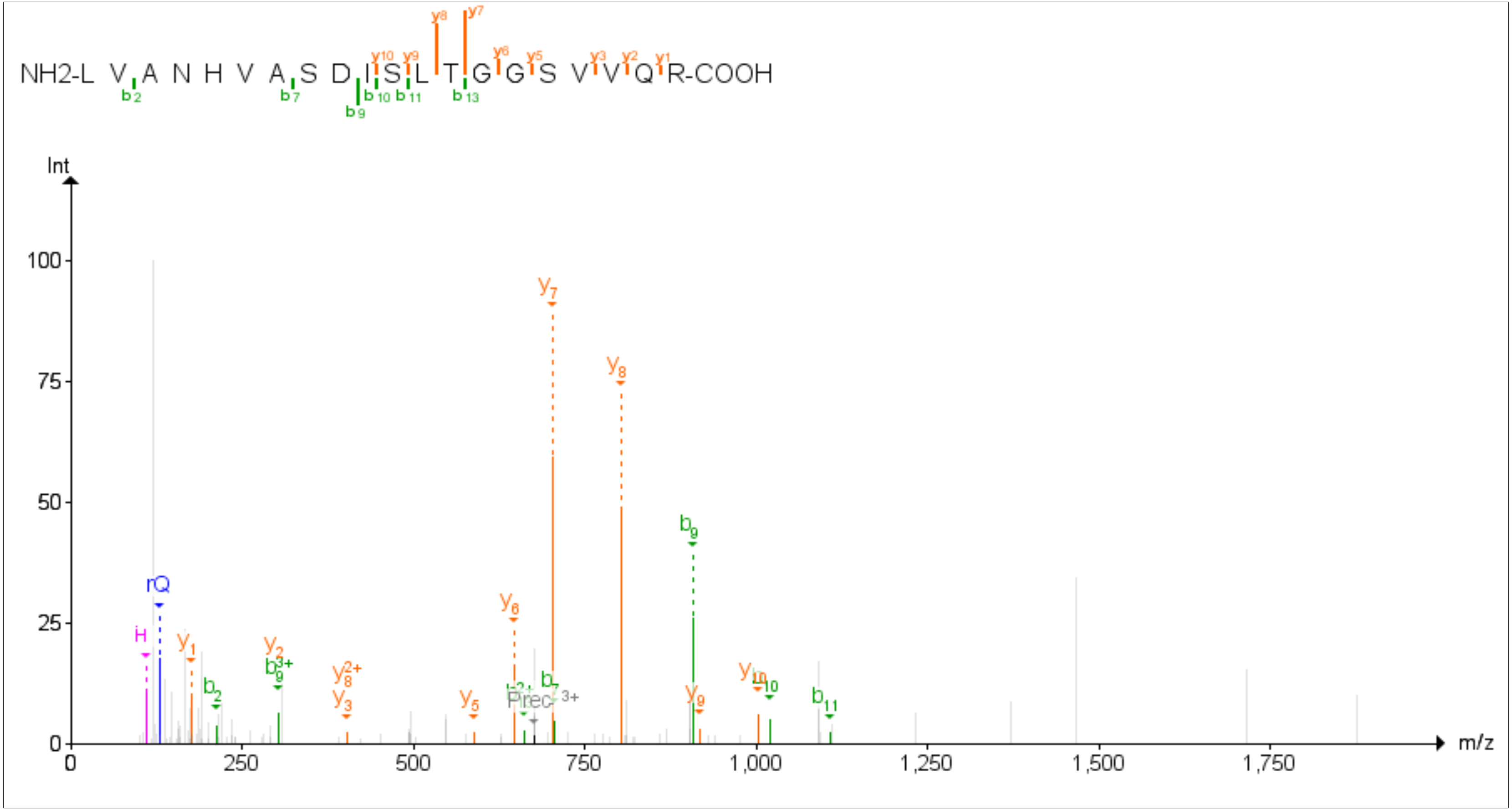
NH2-L V A N H V A S D I S L T G G S V V Q R-COOH
Int
m/z
0
25
50
75
100
250
500
750
1,000
1,250
1,500
1,750
y7
y8
b9
rQ
y6
y1
y2
b2
y5
y9
b11
Prec

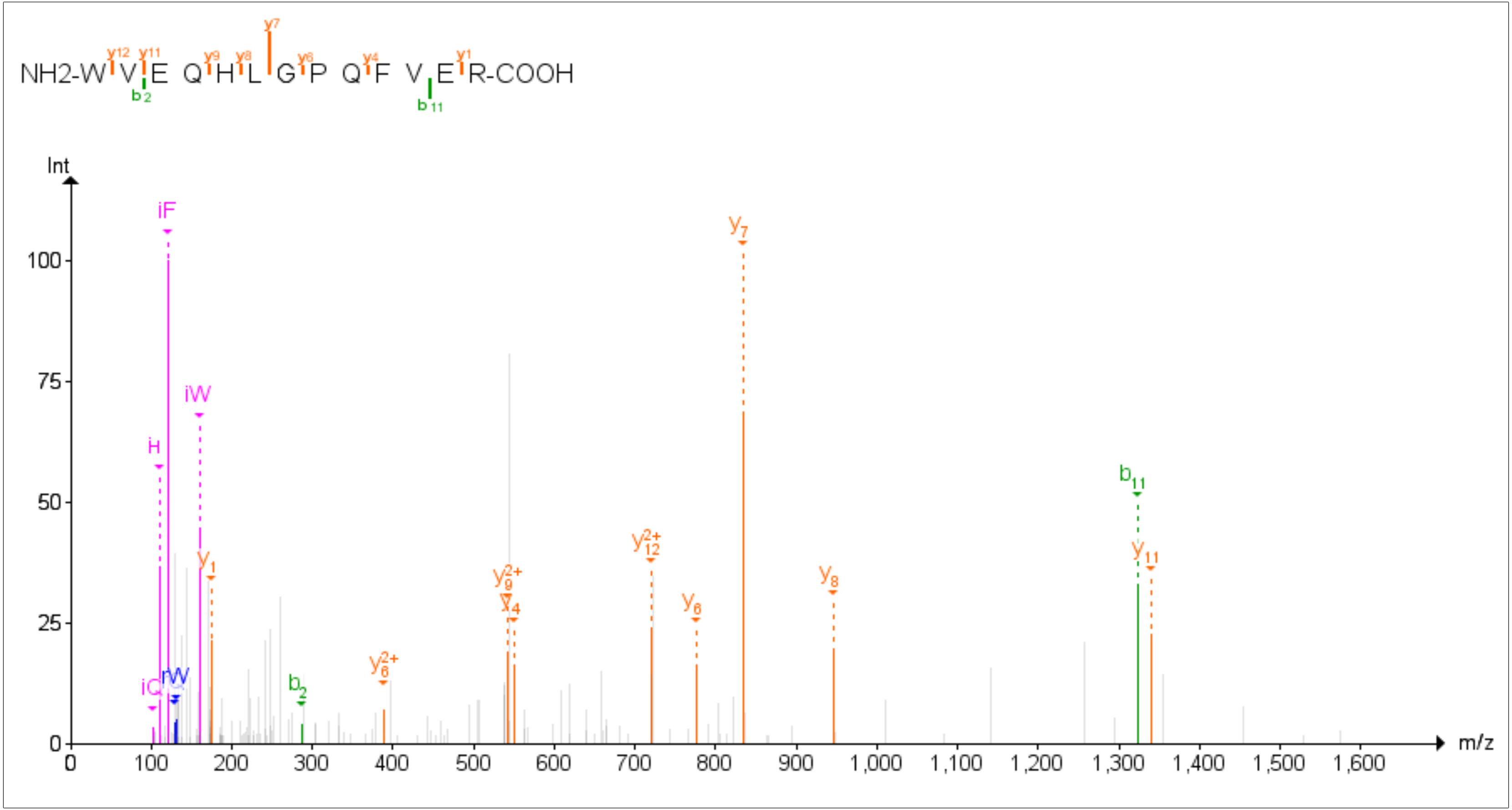

NH2-W V E Q H L G P Q F V E R-COOH
y12 y11 y9 y8 y7 y6 y4 y1
b2 b11
Int
100
75
50
25
0
m/z
0 100 200 300 400 500 600 700 800 900 1,000 1,100 1,200 1,300 1,400 1,500 1,600
iF
iW
iH
iQ
rW
y1
b2
y6 2+
y9 2+
y4
y12 2+
y6
y7
y8
b11
y11

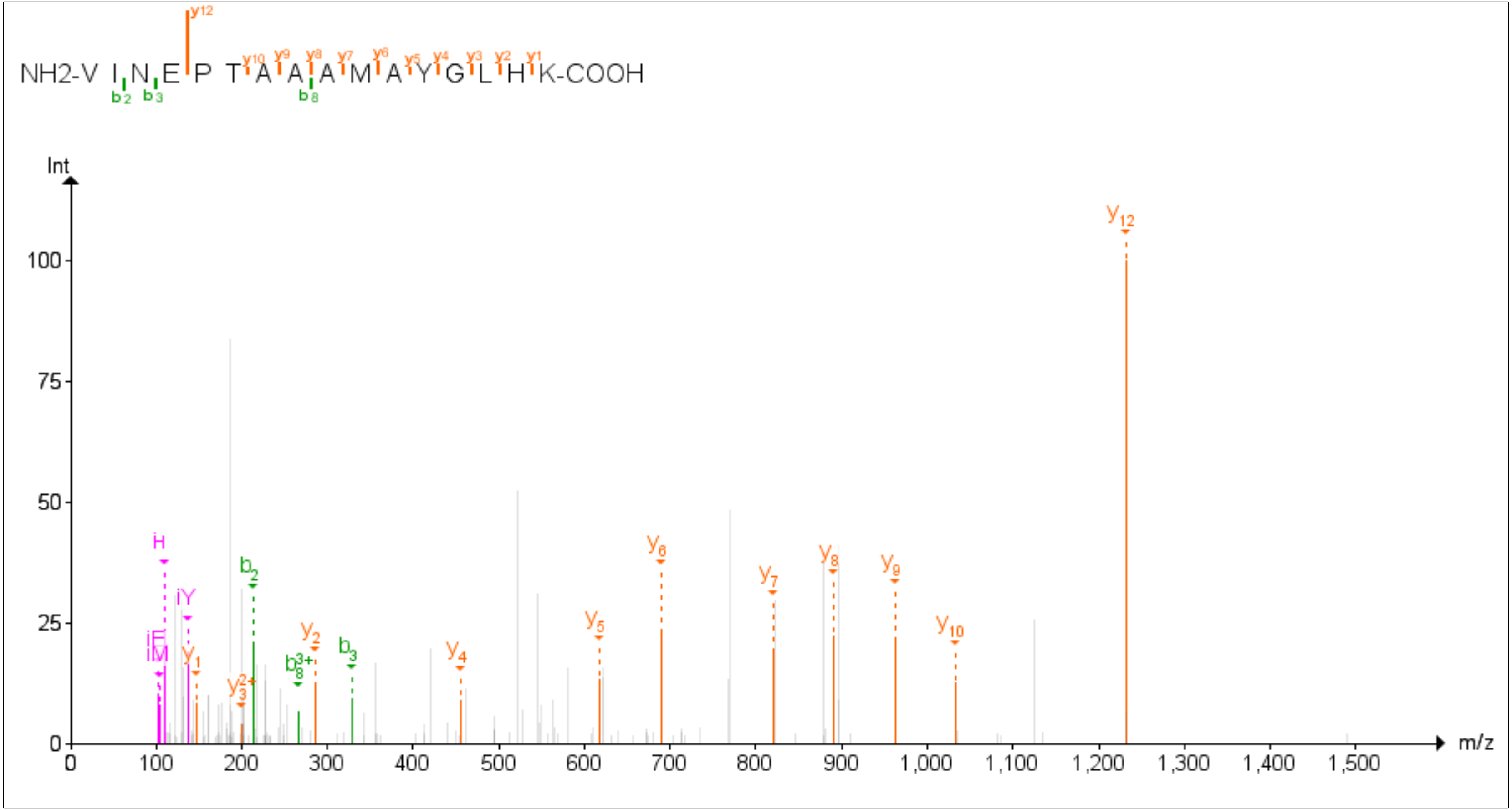

NH2-V I N E P T A A A M A Y G L H K-COOH
y12 y10 y9 y8 y7 y6 y5 y4 y3 y2 y1
b2 b3 b8
Int
100
75
50
25
0
y12
y6
y8
y7
y9
y5
y10
y4
iH
iY
iF
iM
y1
b2
y2
b3
m/z
0 100 200 300 400 500 600 700 800 900 1,000 1,100 1,200 1,300 1,400 1,500

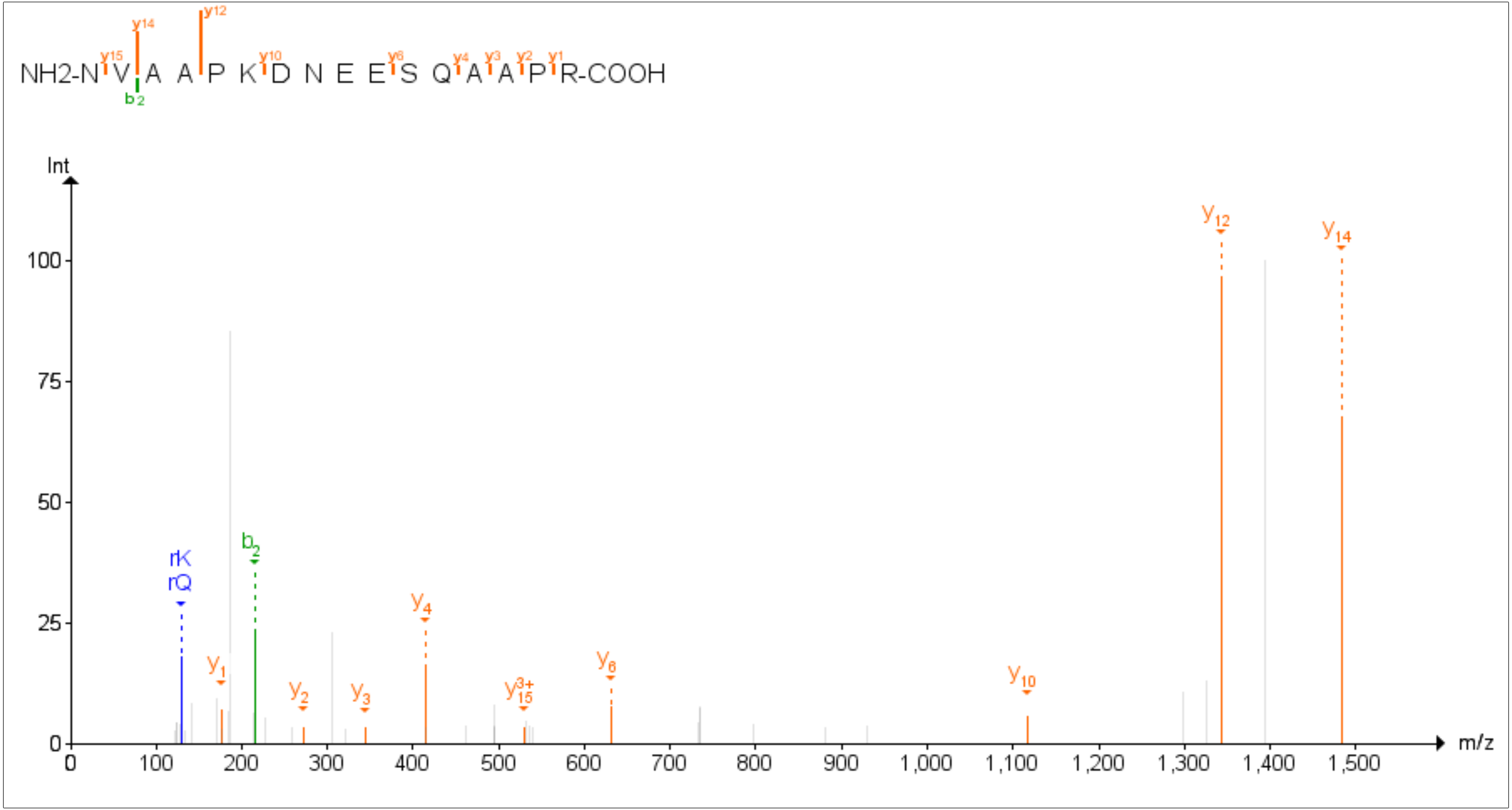

NH2-N V A A P K D N E E S Q A A P R-COOH
y15
y14
y12
y10
y6
y4
y3
y2
y1
b2
Int
100
75
50
25
0
rK
rQ
b2
y1
y2
y3
y4
y15 3+
y6
y10
y12
y14
0
100
200
300
400
500
600
700
800
900
1,000
1,100
1,200
1,300
1,400
1,500
m/z

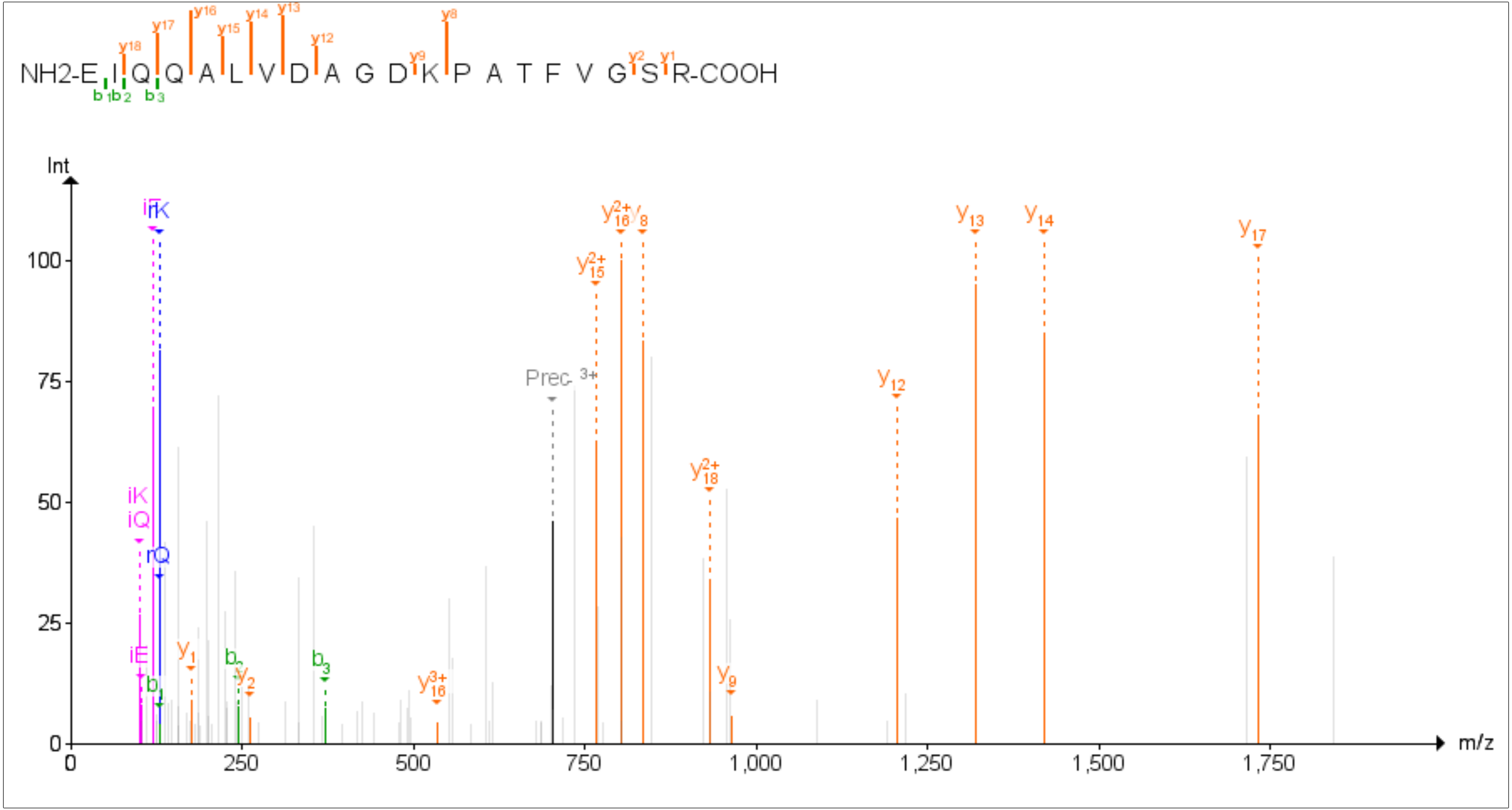
NH2-E I Q Q A L V D A G D K P A T F V G S R-COOH
Int
m/z
Prec 3+
0
250
500
750
1,000
1,250
1,500
1,750
25
50
75
100

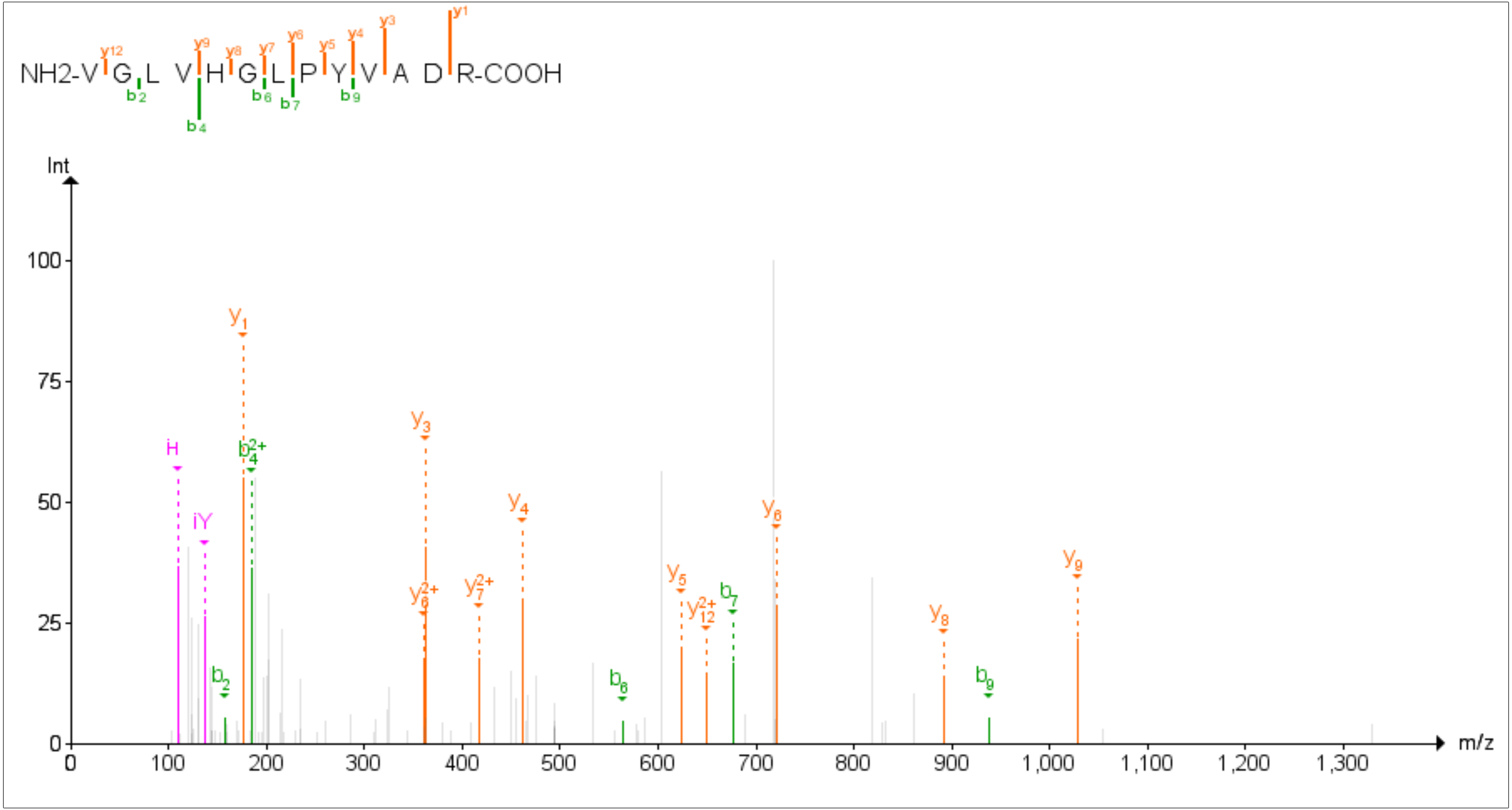

NH2-V G L V H G L P Y V A D R-COOH
y12 y9 y8 y7 y6 y5 y4 y3 y1
b2 b4 b6 b7 b9
Int
100
75
50
25
0
iH
iY
b2
y1
b4 2+
y3
y6 2+
y7 2+
y4
b6
y5
y12 2+
b7
y6
y8
b9
y9
0
100
200
300
400
500
600
700
800
900
1,000
1,100
1,200
1,300
m/z

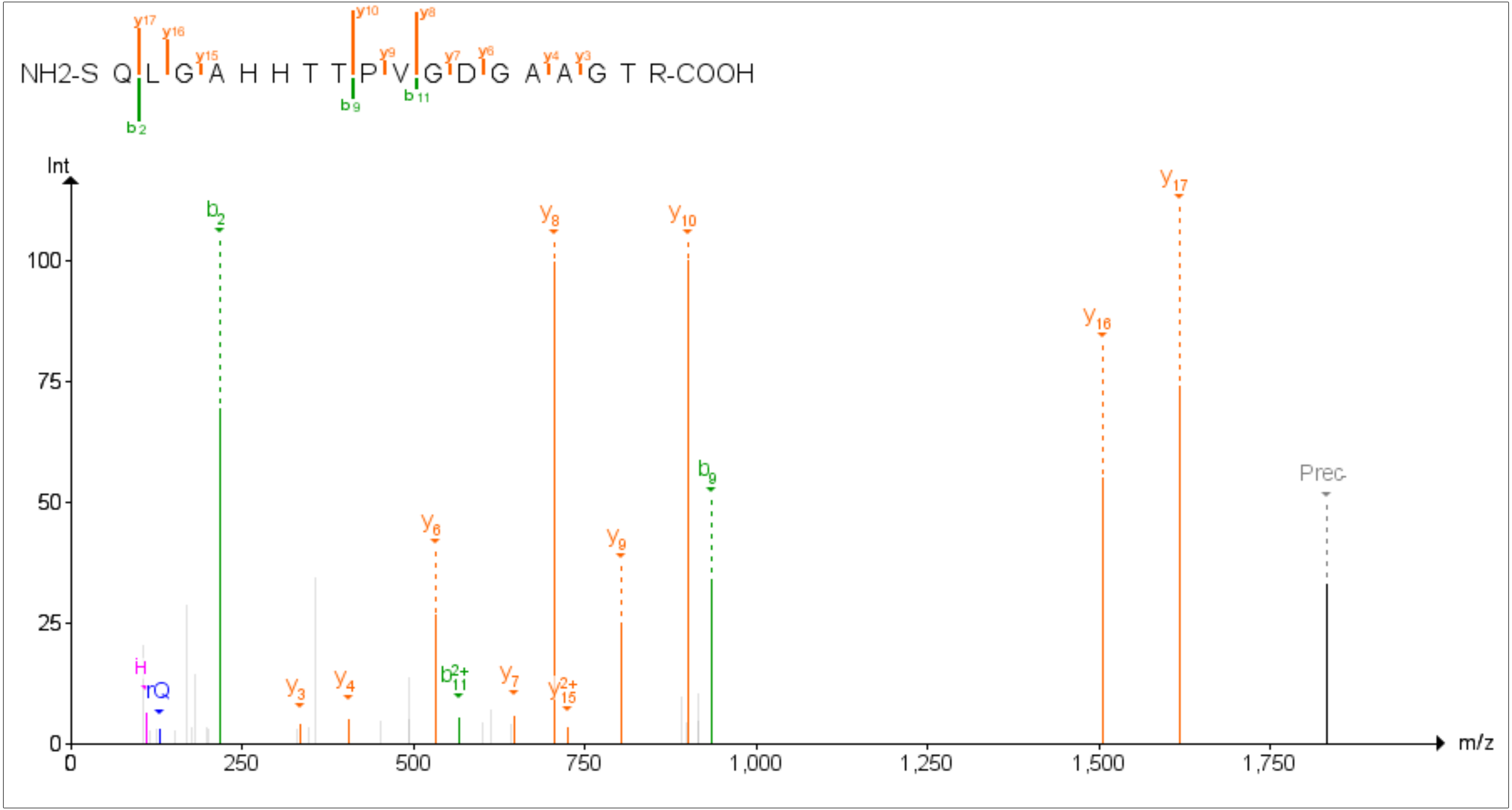

NH2-S Q L G A H H T T P V G D G A A G T R-COOH
y17 y16 y15 y10 y9 y8 y7 y6 y4 y3
b2 b9 b11
Int
m/z
0
25
50
75
100
250
500
750
1,000
1,250
1,500
1,750
b2
y8
y10
y17
y16
b9
Prec
y6
y9
y3
y4
b11 2+
y7
y15 2+
H
rQ

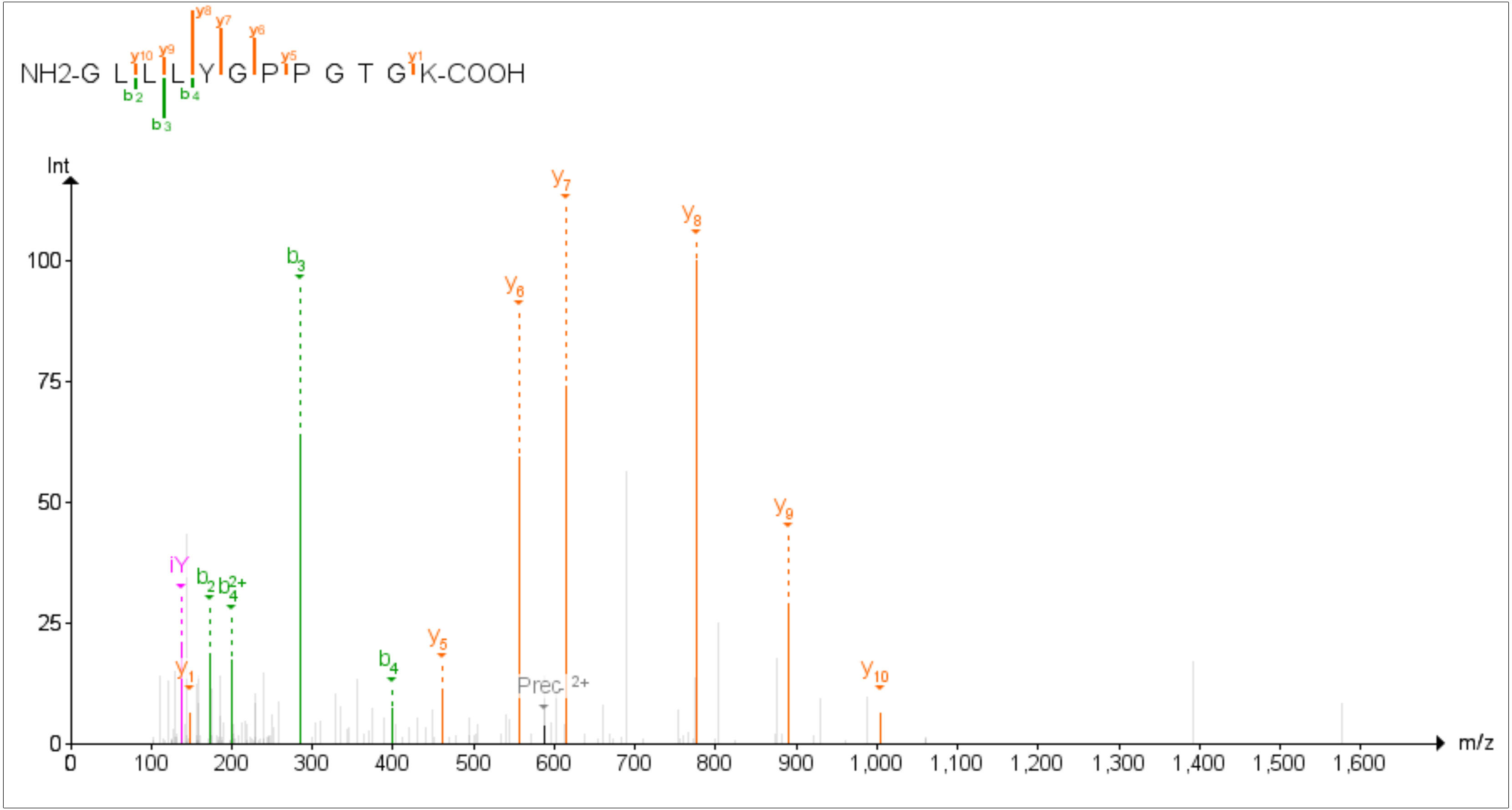

NH2-G L L L Y G P P G T G K-COOH
y10 y9 y8 y7 y6 y5 y1
b2 b3 b4
Int
100
75
50
25
0
b3
y6
y7
y8
y9
y10
iY
b2
b4 2+
y1
b4
y5
Prec 2+
0 100 200 300 400 500 600 700 800 900 1,000 1,100 1,200 1,300 1,400 1,500 1,600
m/z

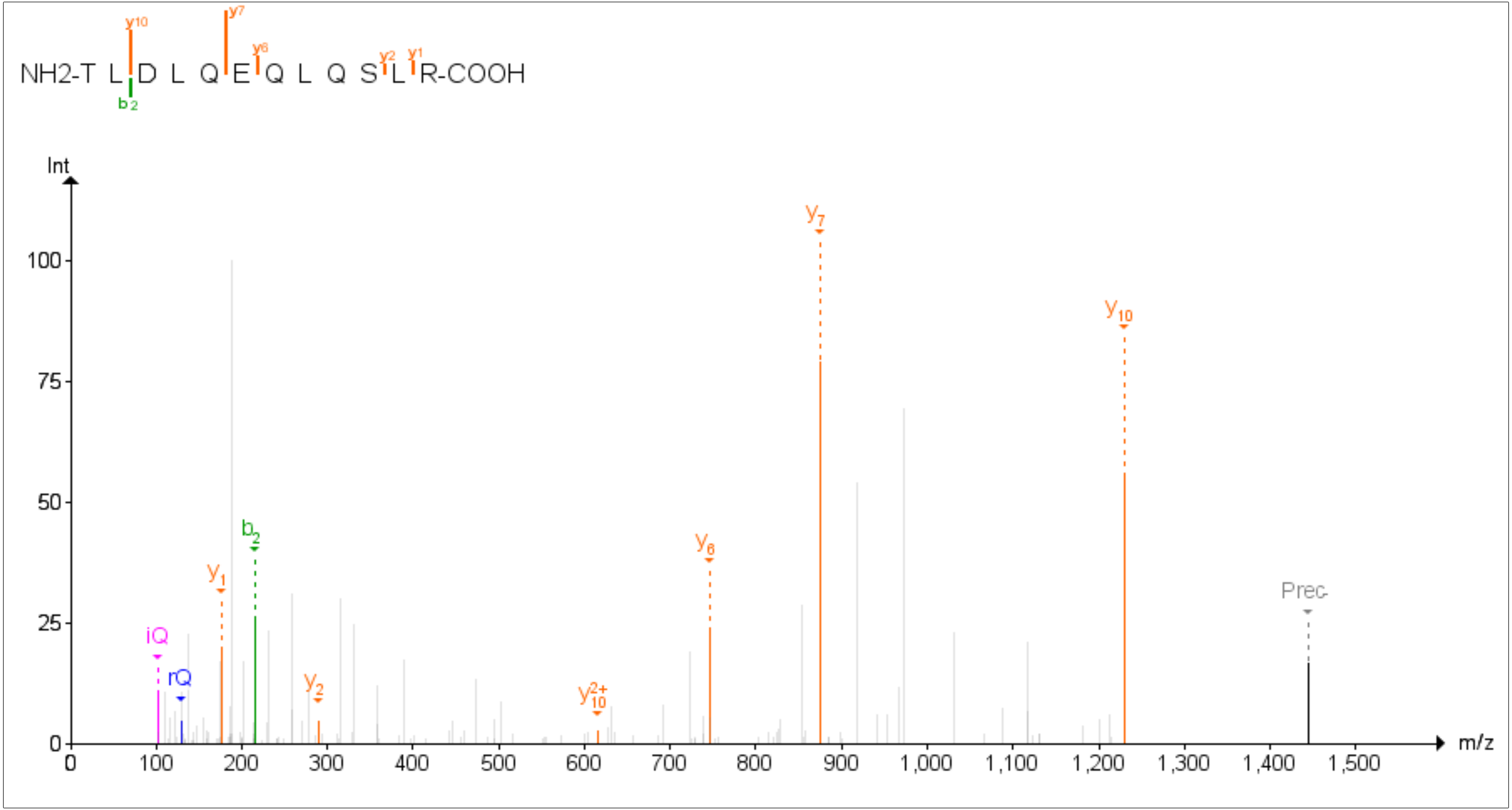
NH2-T L D L Q E Q L Q S L R-COOH
y10
y7
y6
y2
y1
b2
Int
100
75
50
25
0
y7
y10
b2
y1
y6
iQ
rQ
y2
y10 2+
Prec
0
100
200
300
400
500
600
700
800
900
1,000
1,100
1,200
1,300
1,400
1,500
m/z

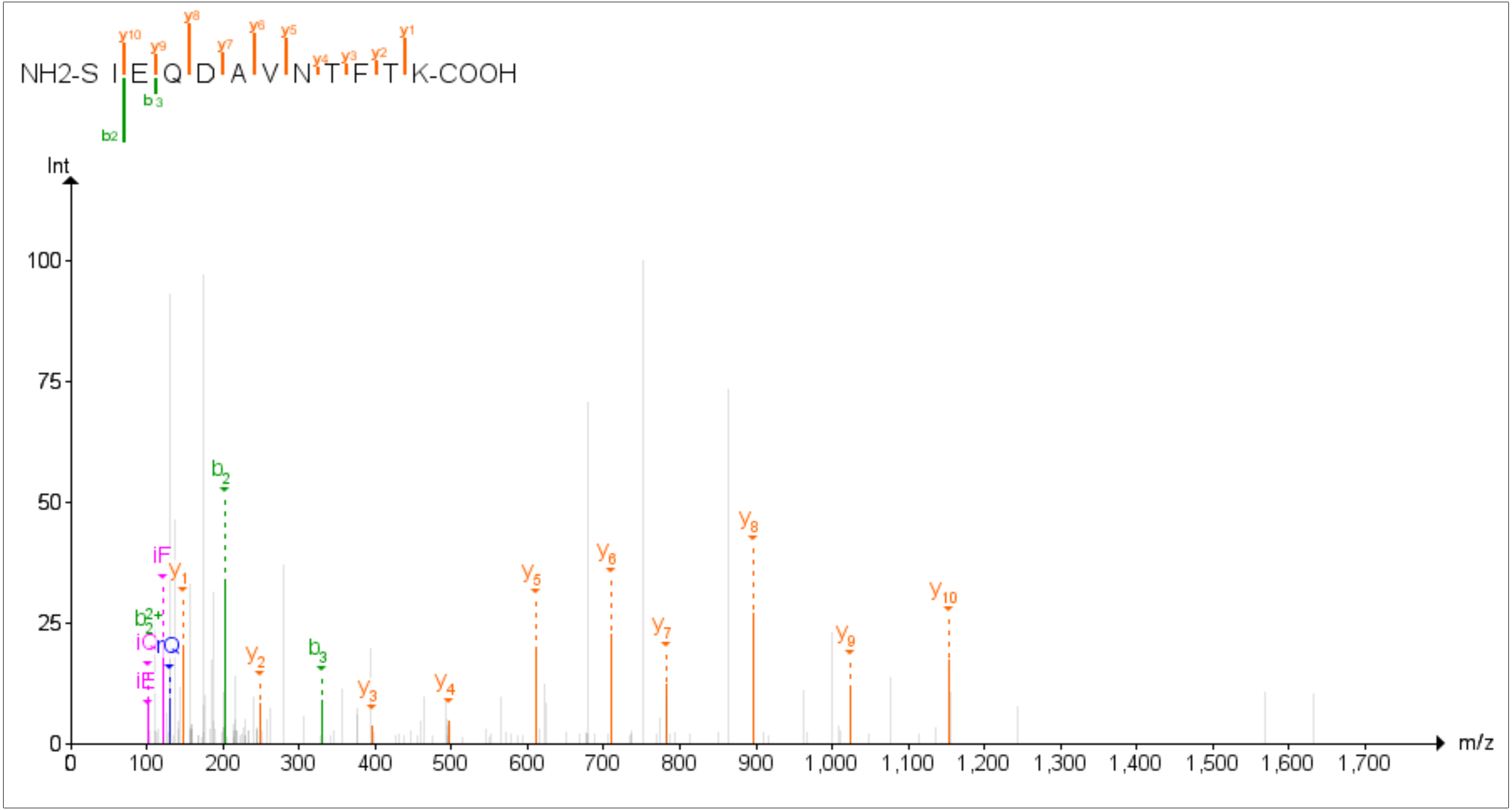

NH2-S I E Q D A V N T F T K-COOH
y10 y9 y8 y7 y6 y5 y4 y3 y2 y1
b2 b3
Int
m/z
100
75
50
25
0
0 100 200 300 400 500 600 700 800 900 1,000 1,100 1,200 1,300 1,400 1,500 1,600 1,700
b2
iF
y1
b2 2+
iQ
rQ
iE
y2
b3
y3
y4
y5
y6
y7
y8
y9
y10

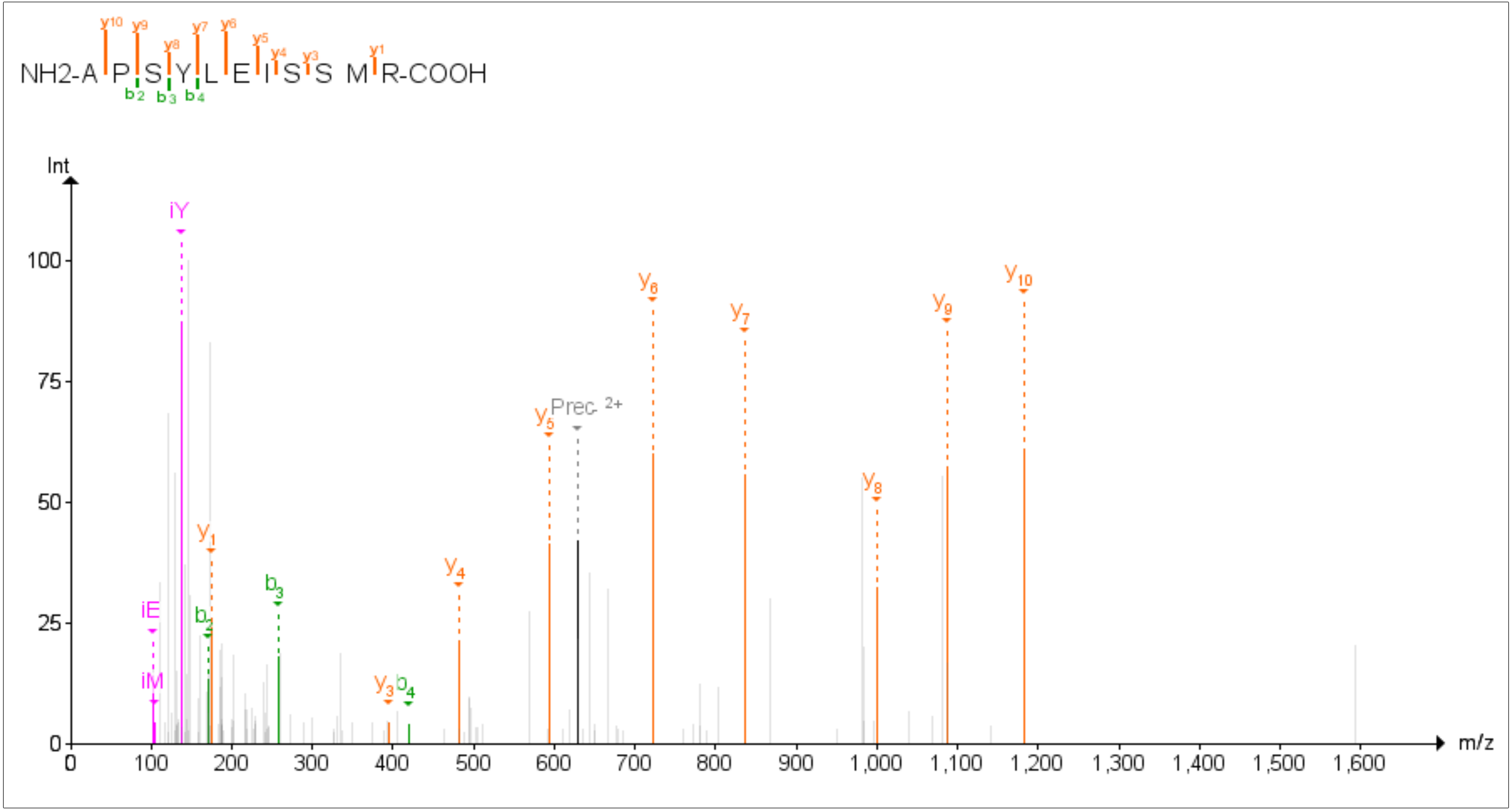

NH2-A P S Y L E I S S M R-COOH
y10 y9 y8 y7 y6 y5 y4 y3 y1
b2 b3 b4
Int
iY
y6
y10
y9
y7
y5
Prec. 2+
y8
y1
y4
b3
iE
b2
iM
y3
b4
m/z
0
25
50
75
100
0 100 200 300 400 500 600 700 800 900 1,000 1,100 1,200 1,300 1,400 1,500 1,600

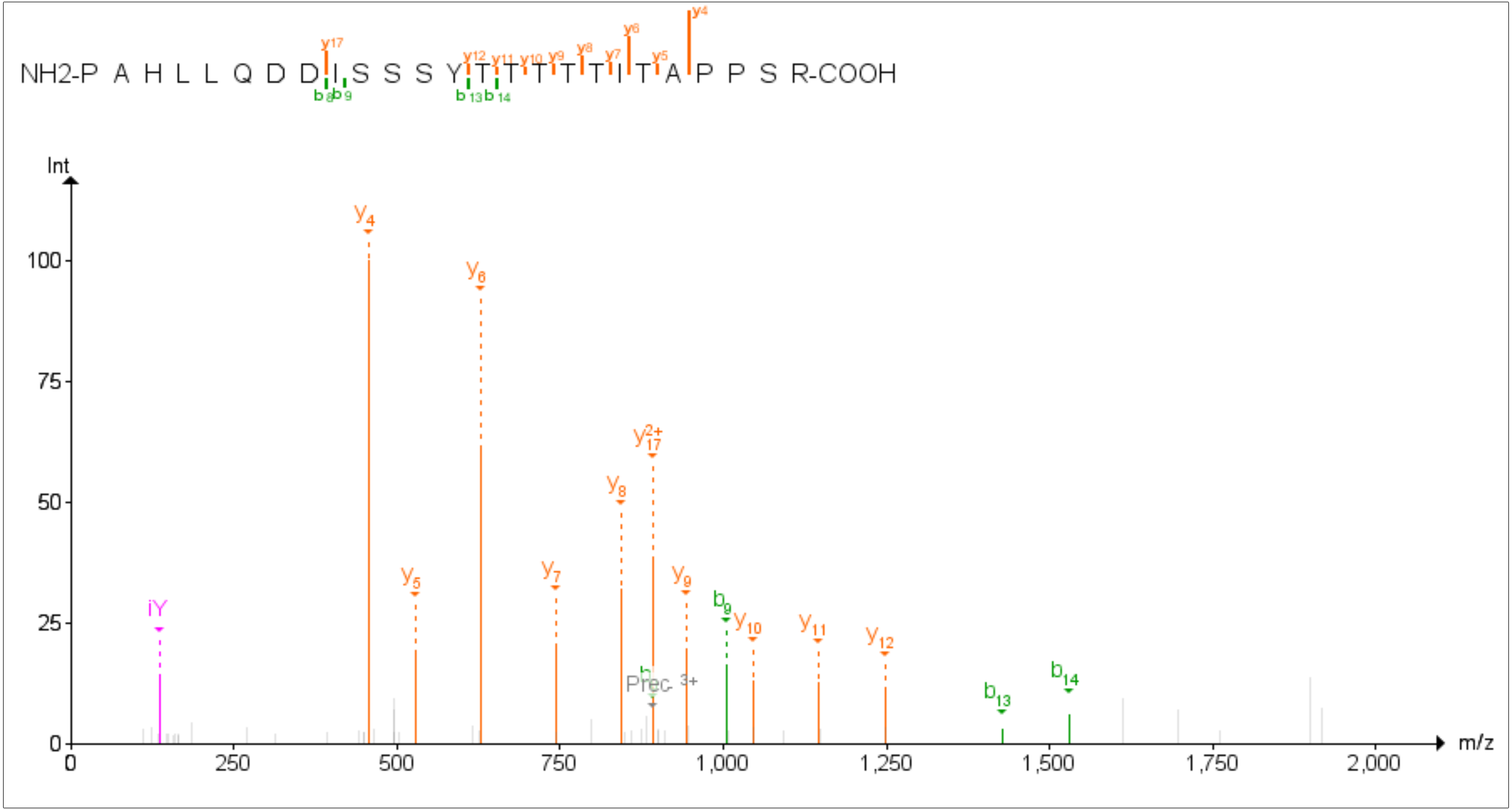

NH2-P A H L L Q D D I S S S Y T T T T T T I T A P P S R-COOH
y17 y12 y11 y10 y9 y8 y7 y6 y5 y4
b8 b9 b13 b14
Int
100
75
50
25
0
iY
y4
y5
y6
y7
y8
y_{17}^{2+}
y9
b9
y10
y11
y12
Prec 3+
b13
b14
0
250
500
750
1,000
1,250
1,500
1,750
2,000
m/z

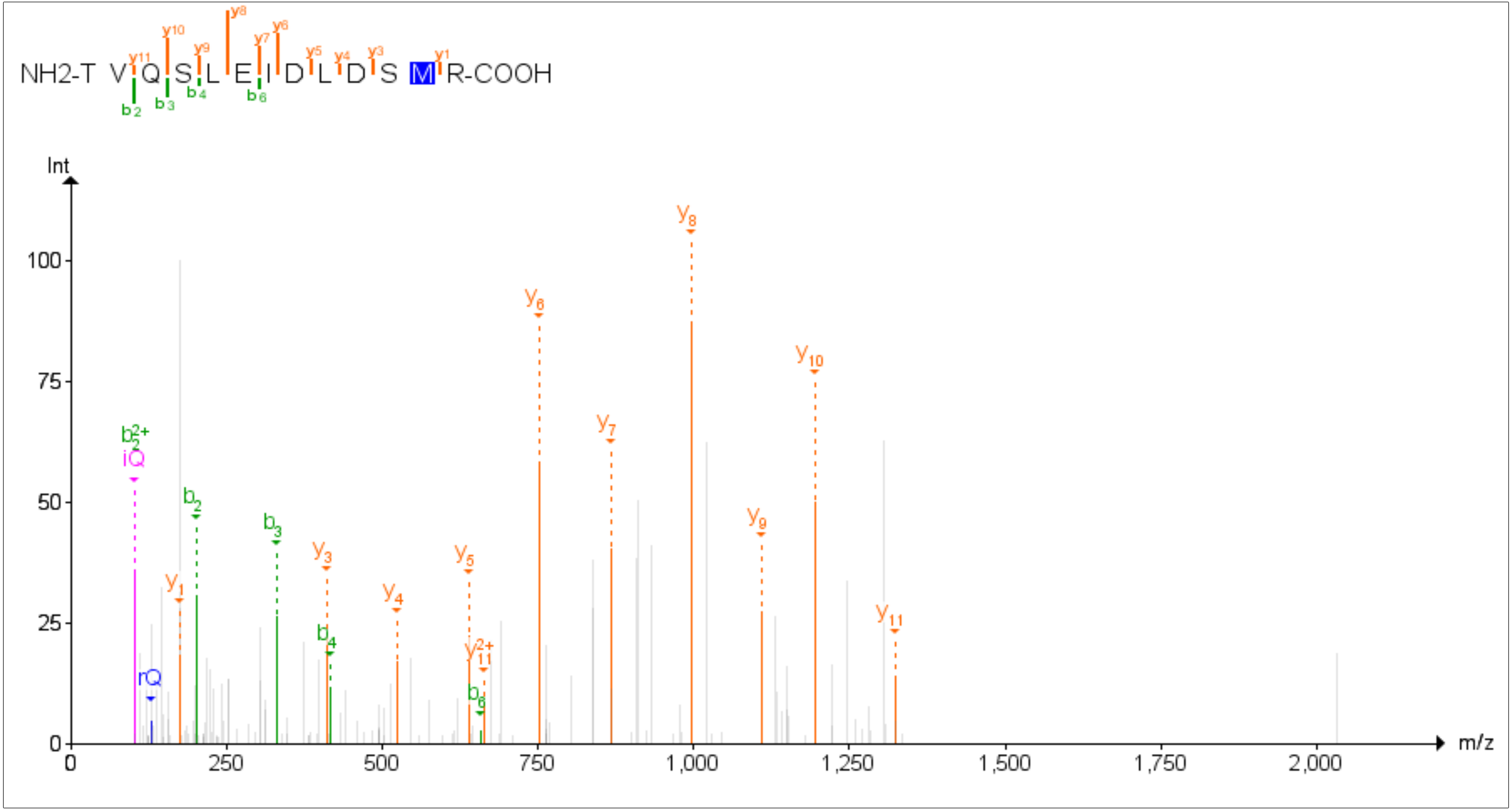

NH2-T V Q S L E I D L D S M R-COOH
y11 y10 y9 y8 y7 y6 y5 y4 y3 y1
b2 b3 b4 b6
Int
m/z
0
25
50
75
100
250
500
750
1,000
1,250
1,500
1,750
2,000
iQ
rQ

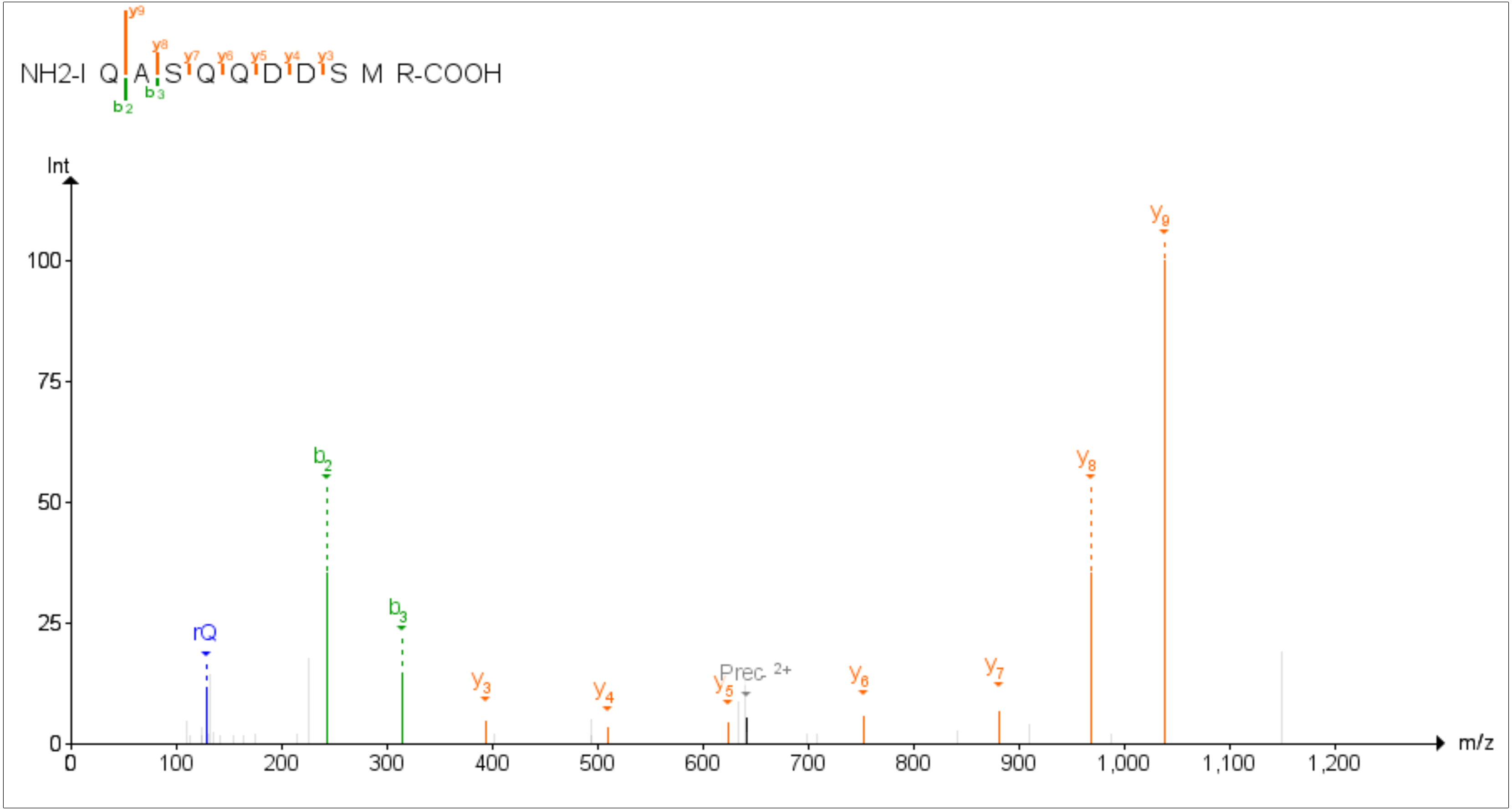

NH2-I Q A S Q Q D D S M R-COOH
y9 y8 y7 y6 y5 y4 y3
b2 b3
Int
100
75
50
25
0
rQ
b2
b3
y3
y4
y5
Prec- 2+
y6
y7
y8
y9
0 100 200 300 400 500 600 700 800 900 1,000 1,100 1,200
m/z

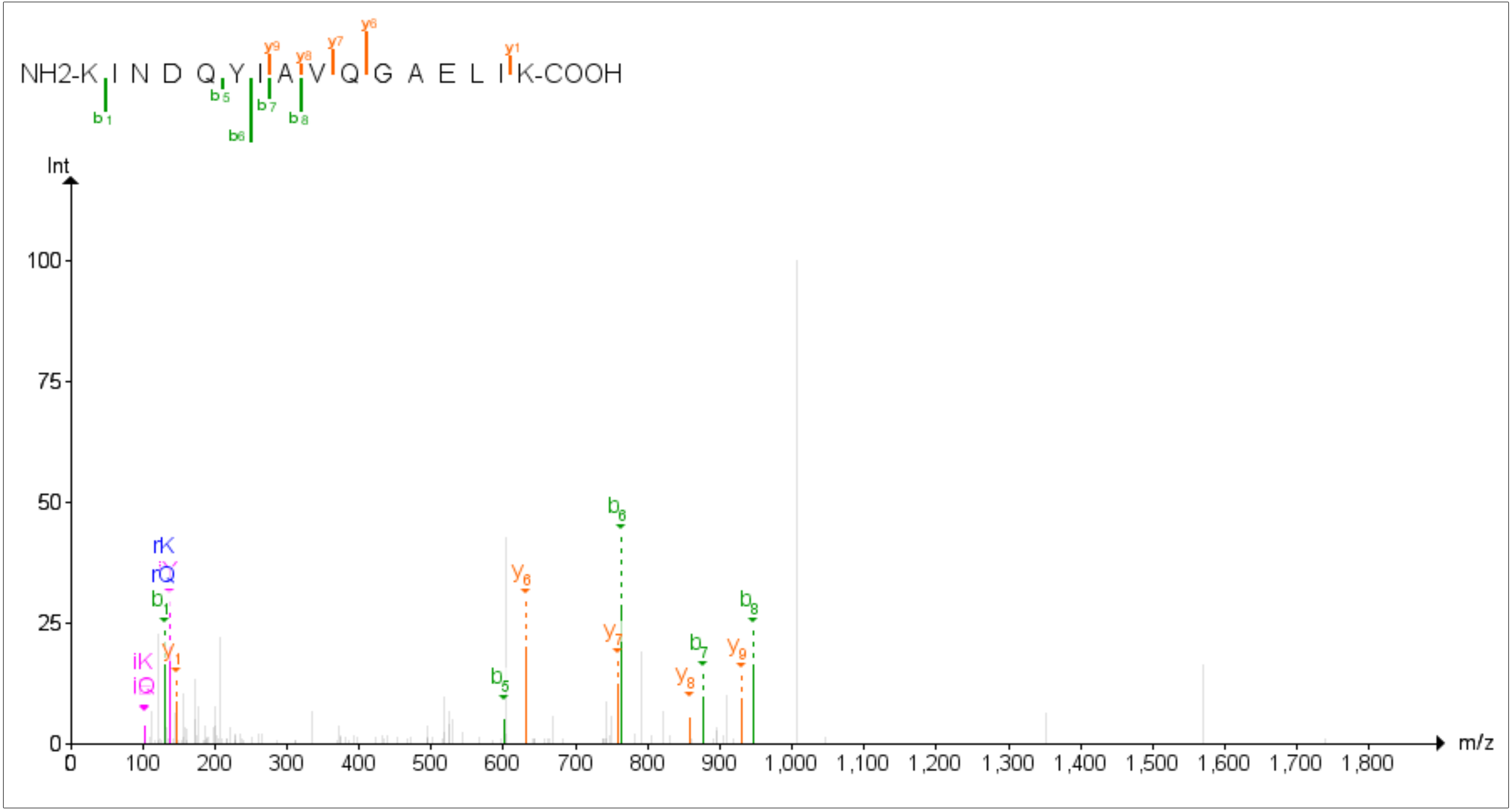

NH2-K I N D Q Y I A V Q G A E L I K-COOH
b1
b5
b6
b7
b8
y9
y8
y7
y6
y1
Int
100
75
50
25
0
m/z
0
100
200
300
400
500
600
700
800
900
1,000
1,100
1,200
1,300
1,400
1,500
1,600
1,700
1,800
rK
rQ
iK
iQ
b1
y1
b5
y6
b6
y7
y8
b7
y9
b8

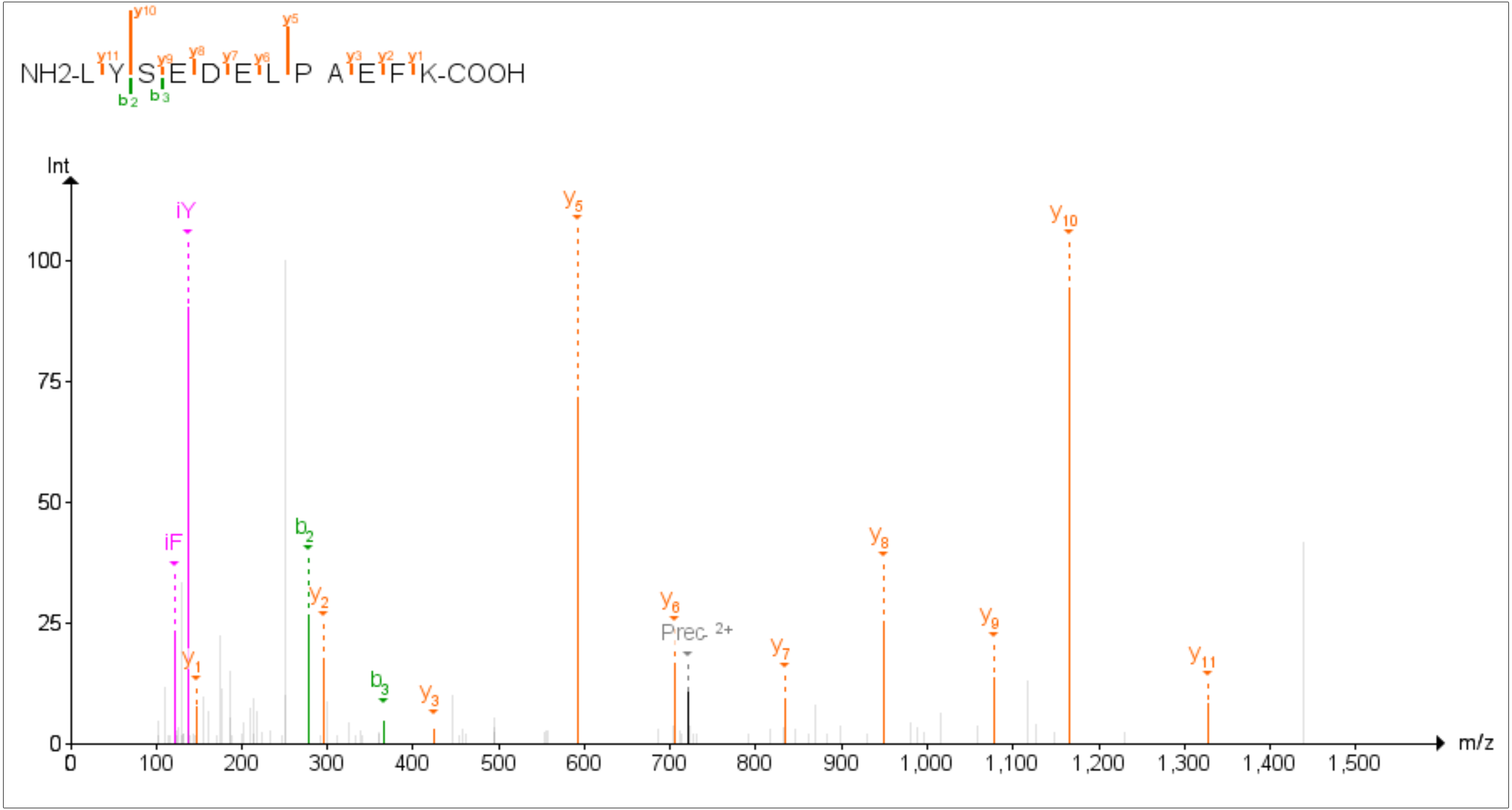

NH2-LYSEDELPAEFK-COOH
y11 y10 y9 y8 y7 y6 y5 y3 y2 y1
b2 b3
Int
m/z
100
75
50
25
0
100
200
300
400
500
600
700
800
900
1,000
1,100
1,200
1,300
1,400
1,500
iY
iF
y5
y10
b2
y2
y1
b3
y3
y6
Prec- 2+
y7
y8
y9
y11

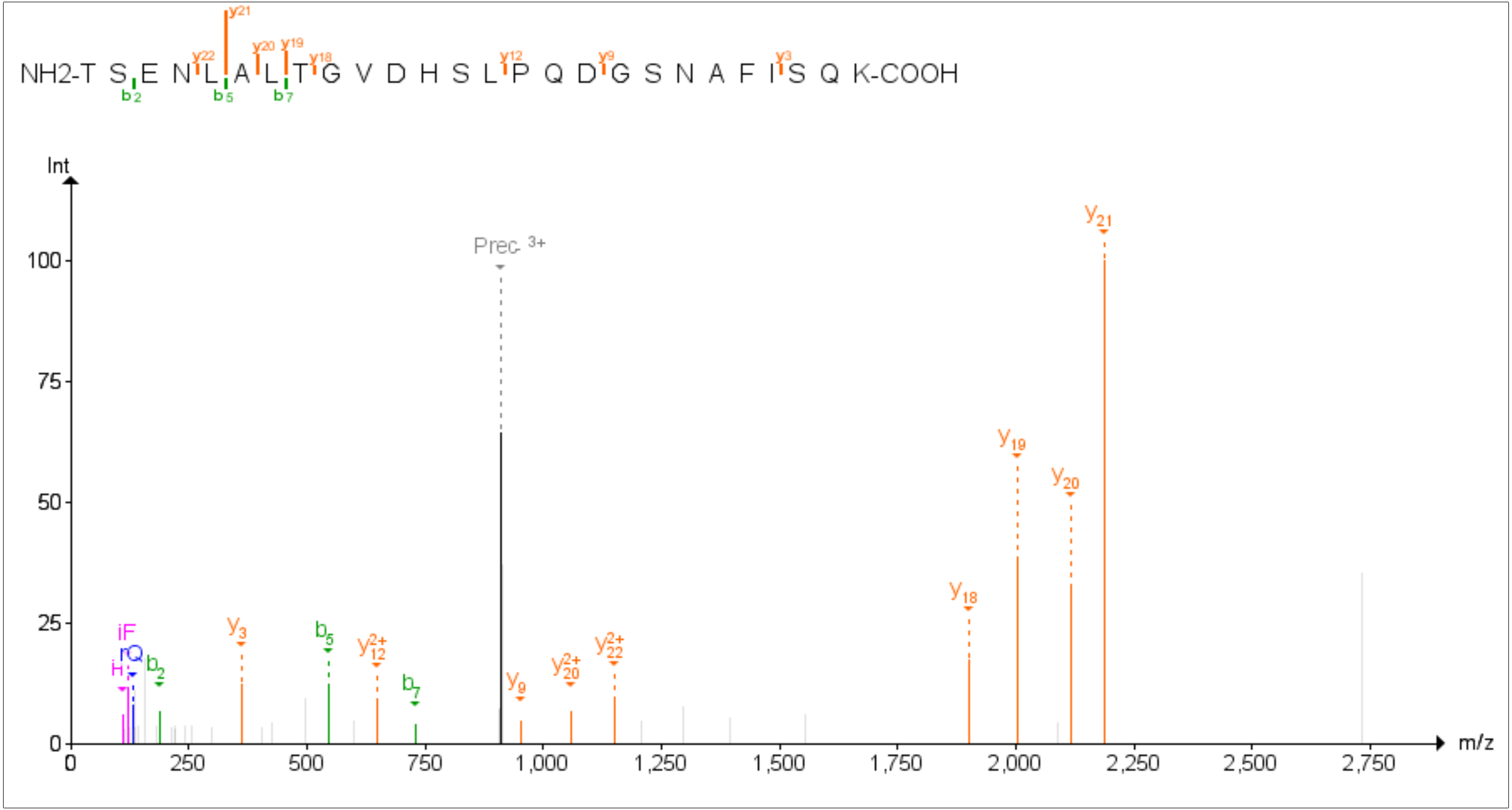

NH2-T S E N L A L T G V D H S L P Q D G S N A F I S Q K-COOH
y21
y22
y20
y19
y18
y12
y9
y3
b2
b5
b7
Int
Prec 3+
y21
y19
y20
y18
iF
rQ
H
b2
y3
b5
y12 2+
b7
y9
y20 2+
y22 2+
100
75
50
25
0
0
250
500
750
1,000
1,250
1,500
1,750
2,000
2,250
2,500
2,750
m/z

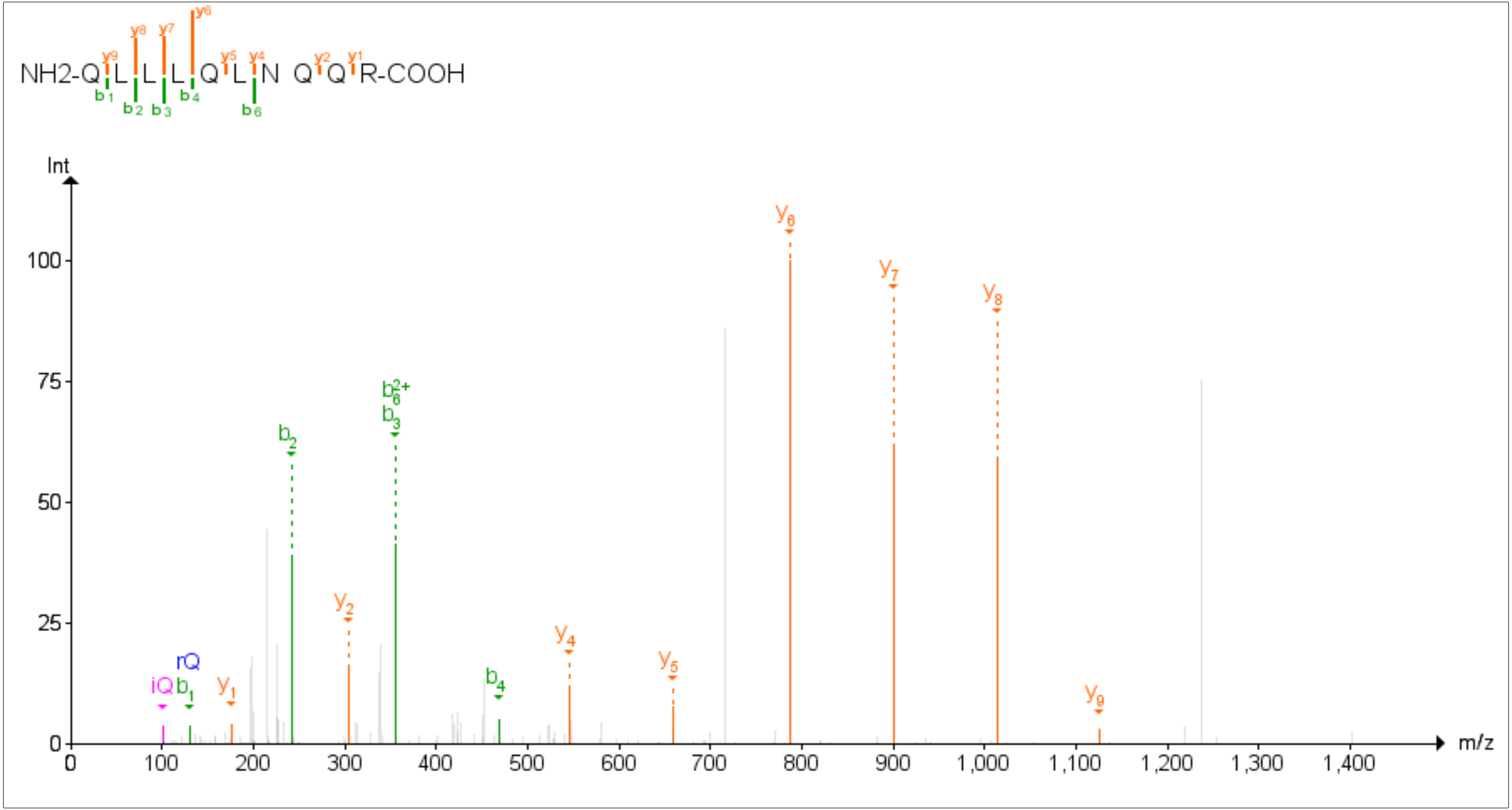
NH2-Q L L L Q L N Q Q R-COOH
y9 y8 y7 y6 y5 y4 y2 y1
b1 b2 b3 b4 b6
Int
m/z
y6 y7 y8 y9 y5 y4 y2 y1
b2 b3 b6 2+ b4 b1
rQ iQ
0 25 50 75 100
0 100 200 300 400 500 600 700 800 900 1,000 1,100 1,200 1,300 1,400

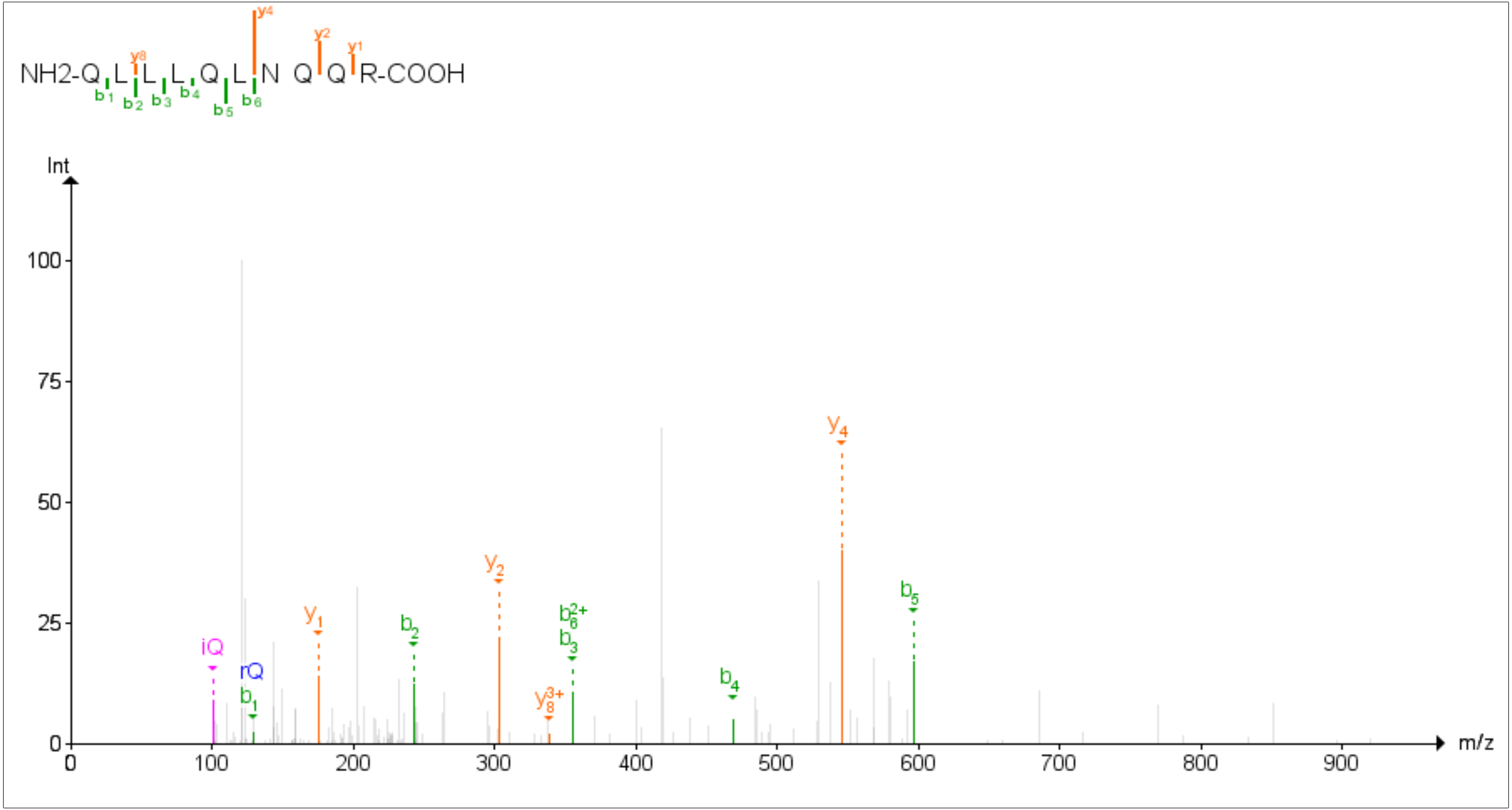

NH2-Q L L L Q L N Q Q R-COOH
y8
y4
y2
y1
b1
b2
b3
b4
b5
b6
Int
100
75
50
25
0
iQ
rQ
b1
y1
b2
y2
y8 3+
b6 2+
b3
b4
y4
b5
0
100
200
300
400
500
600
700
800
900
m/z

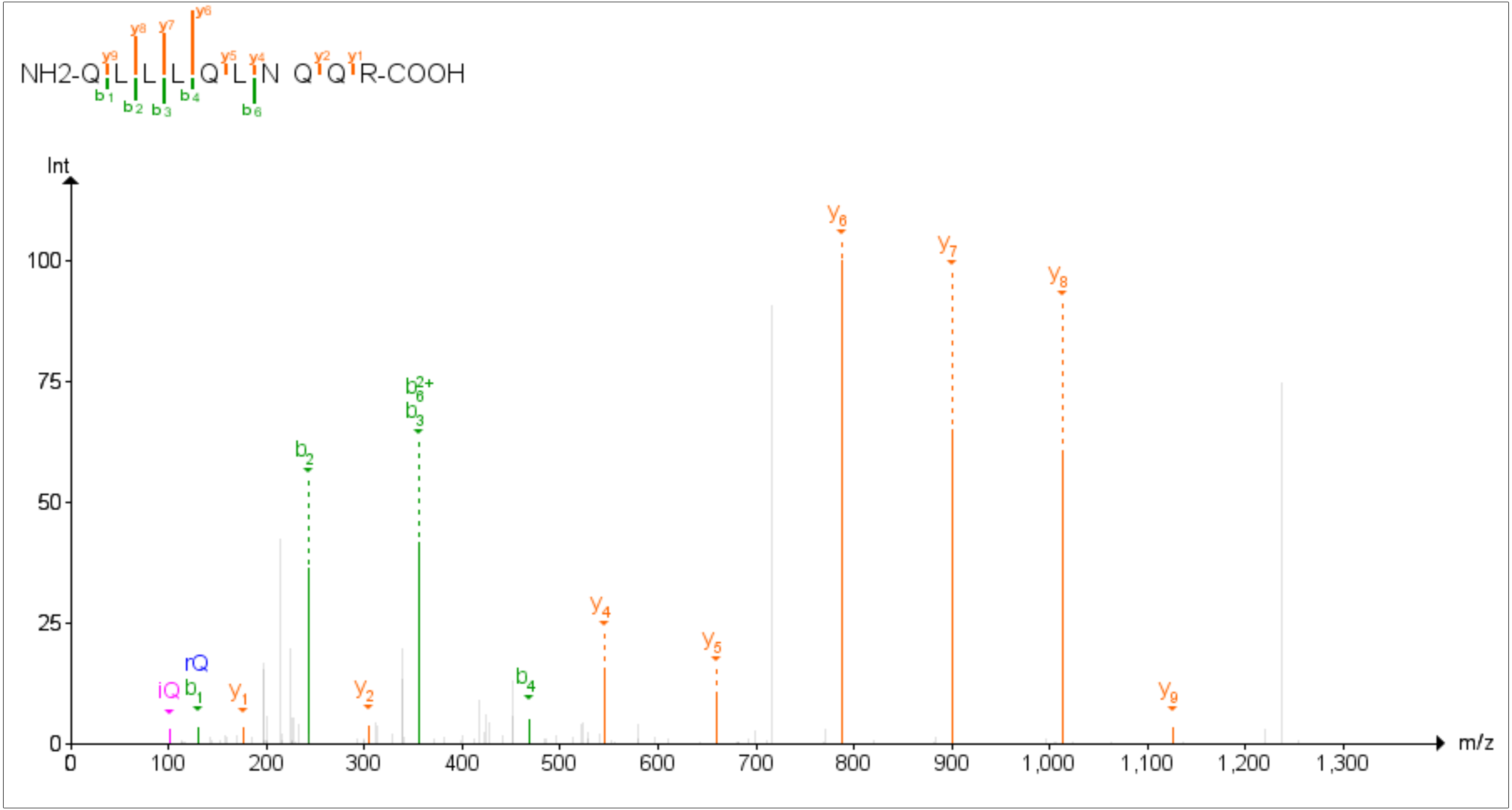

NH2-Q L L L Q L N Q Q R-COOH
y9 y8 y7 y6 y5 y4 y2 y1
b1 b2 b3 b4 b6
Int
m/z
y6
y7
y8
b6 2+
b3
b2
y4
y5
rQ
iQ
b1
y1
y2
b4
y9
0
25
50
75
100
0
100
200
300
400
500
600
700
800
900
1,000
1,100
1,200
1,300

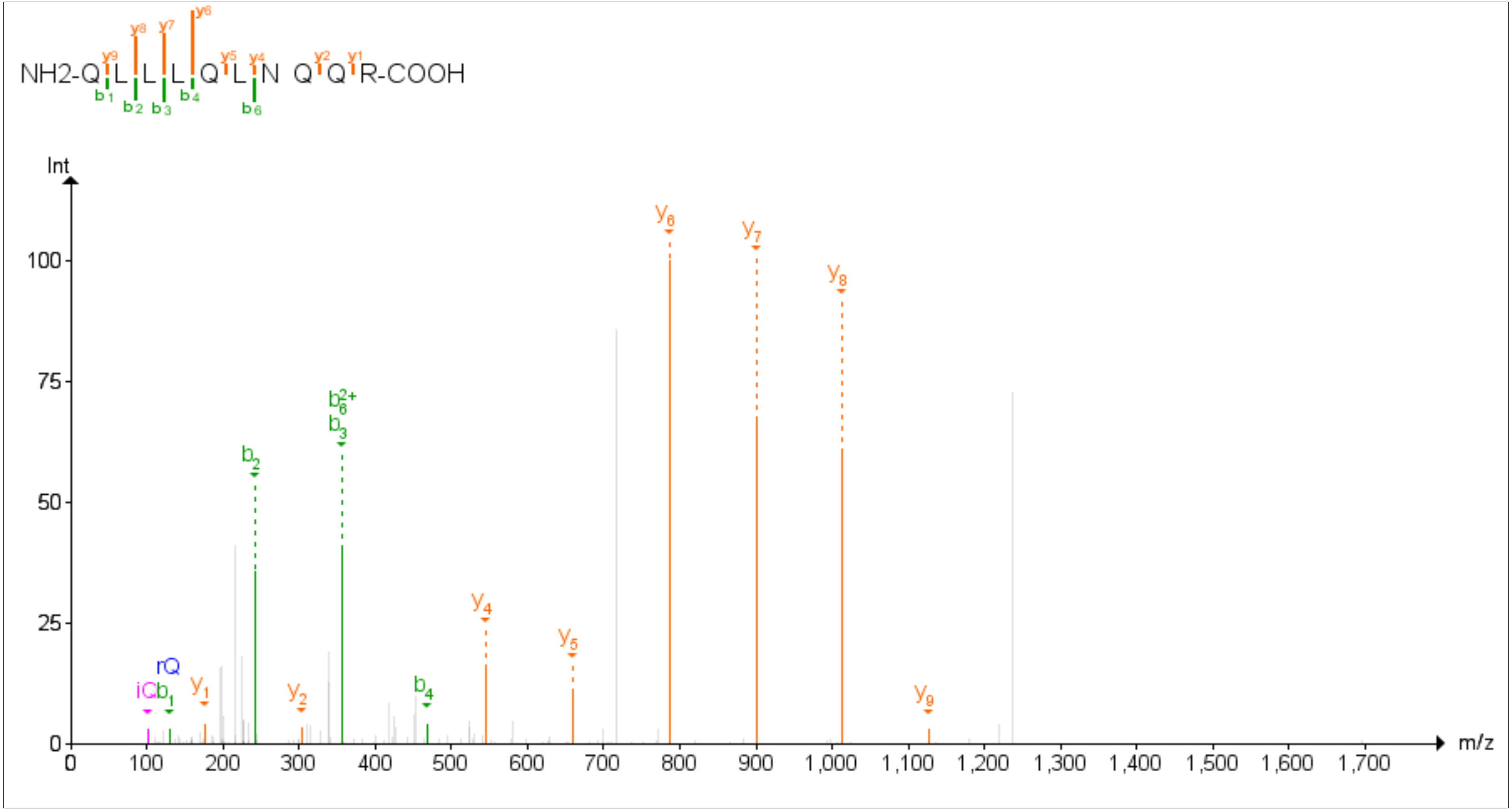

NH2-Q L L L Q L N Q Q R-COOH
y9 y8 y7 y6 y5 y4 y2 y1
b1 b2 b3 b4 b6
Int
100
75
50
25
0
y6
y7
y8
b2
b_6^{2+}
b3
y4
y5
rQ
iQ
b1
y1
y2
b4
y9
m/z
0 100 200 300 400 500 600 700 800 900 1,000 1,100 1,200 1,300 1,400 1,500 1,600 1,700

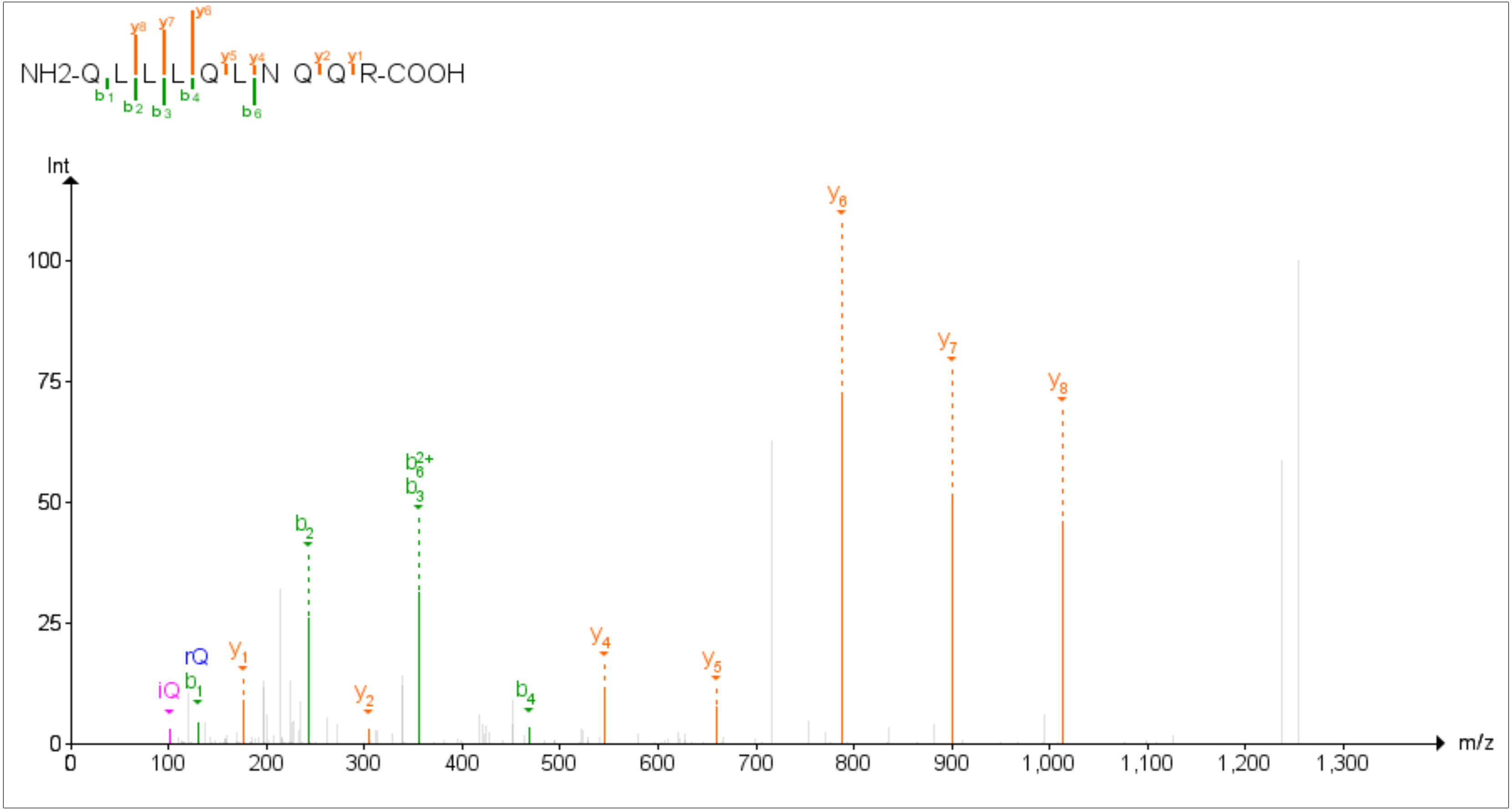

NH2-Q L L L L Q L N Q Q R-COOH
y8 y7 y6 y5 y4 y2 y1
b1 b2 b3 b4 b6
Int
100
75
50
25
0
y6
y7
y8
b6 2+
b3
b2
rQ
y1
iQ
b1
y2
b4
y4
y5
0
100
200
300
400
500
600
700
800
900
1,000
1,100
1,200
1,300
m/z

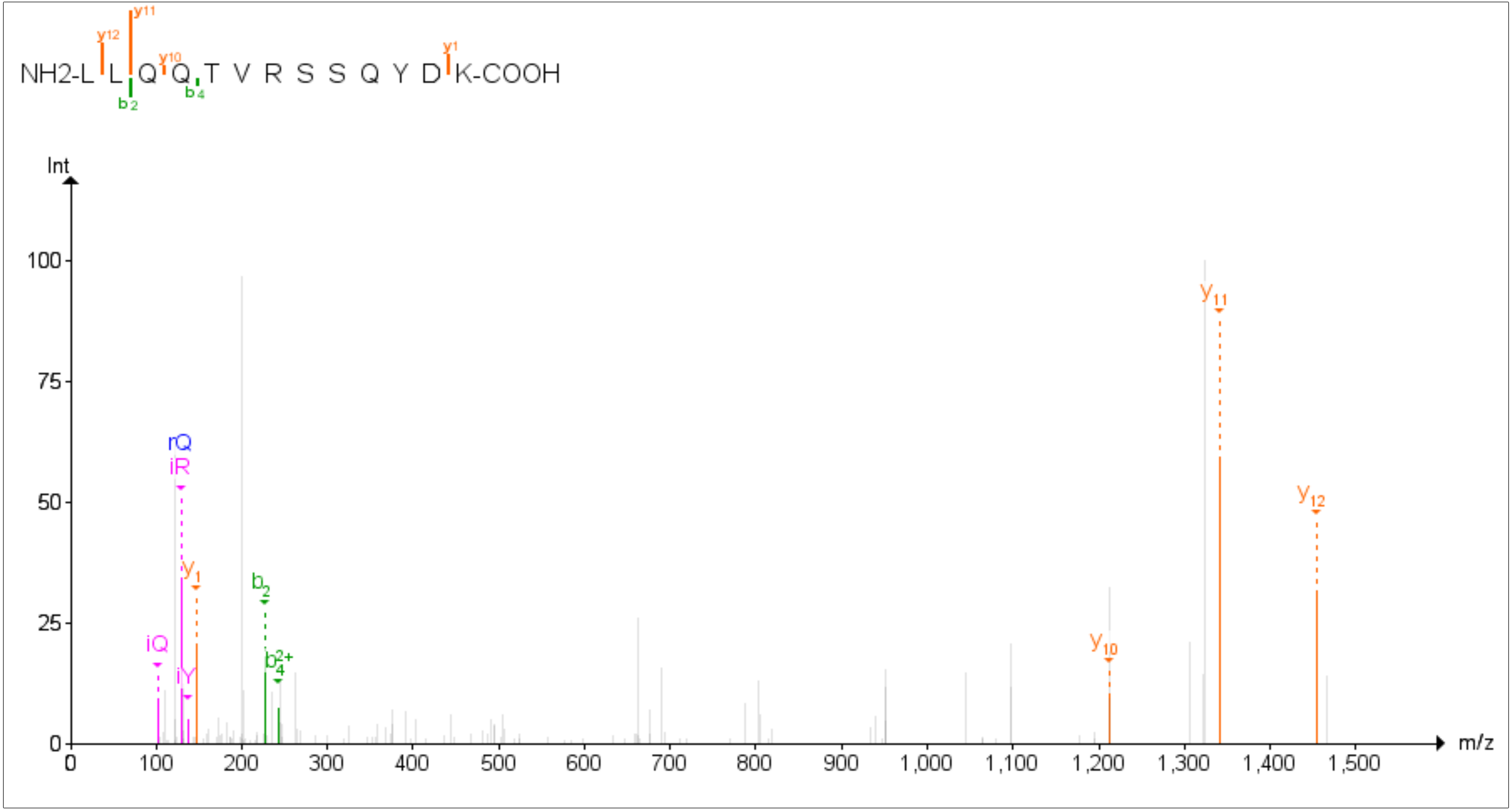

NH2-L L Q Q T V R S S Q Y D K-COOH
y12
y11
y10
y1
b2
b4
Int
100
75
50
25
0
rQ
iR
iQ
iY
y1
b2
b4 2+
y10
y11
y12
0
100
200
300
400
500
600
700
800
900
1,000
1,100
1,200
1,300
1,400
1,500
m/z

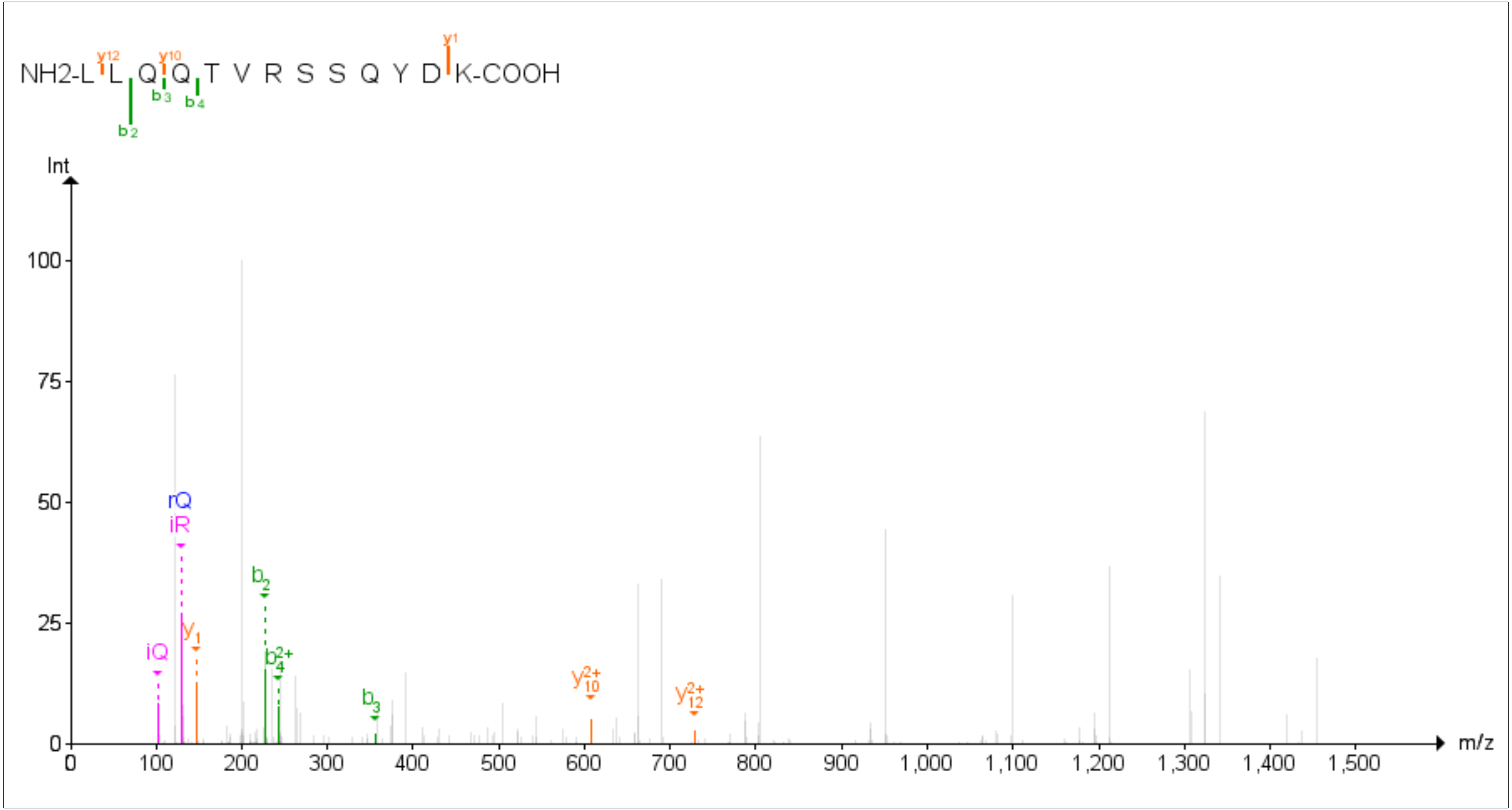

NH2-L L Q Q T V R S S Q Y D K-COOH
y12
y10
y1
b2
b3
b4
Int
100
75
50
25
0
rQ
iR
iQ
y1
b2
b4 2+
b3
y10 2+
y12 2+
0
100
200
300
400
500
600
700
800
900
1,000
1,100
1,200
1,300
1,400
1,500
m/z

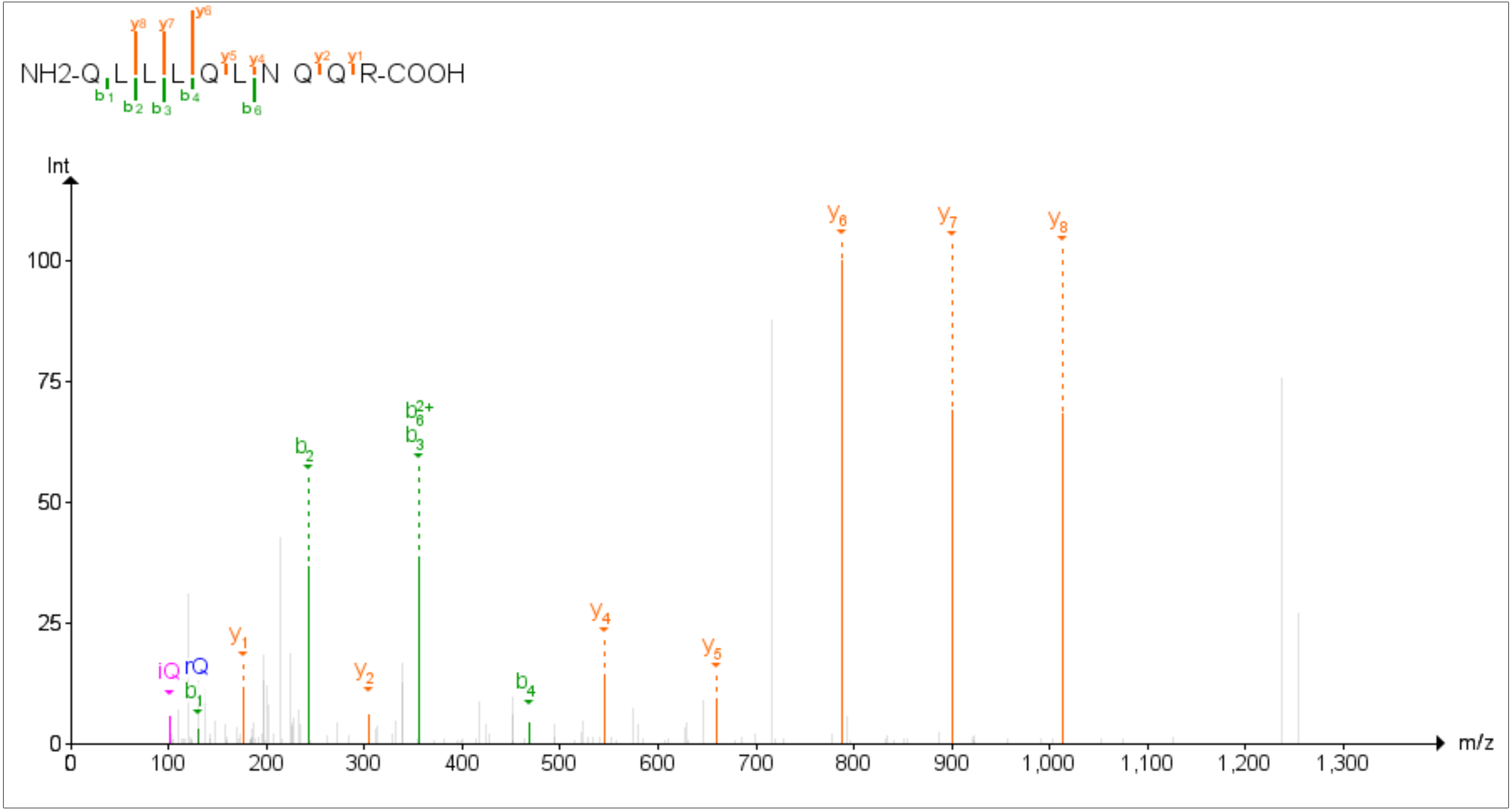
NH2-Q L L L Q L N Q Q R-COOH
y8 y7 y6 y5 y4 y2 y1
b1 b2 b3 b4 b6
Int
m/z
y6
y7
y8
b2
b3
b6 2+
y1
y2
y4
y5
b1
b4
iQ
rQ
0
25
50
75
100
0
100
200
300
400
500
600
700
800
900
1,000
1,100
1,200
1,300

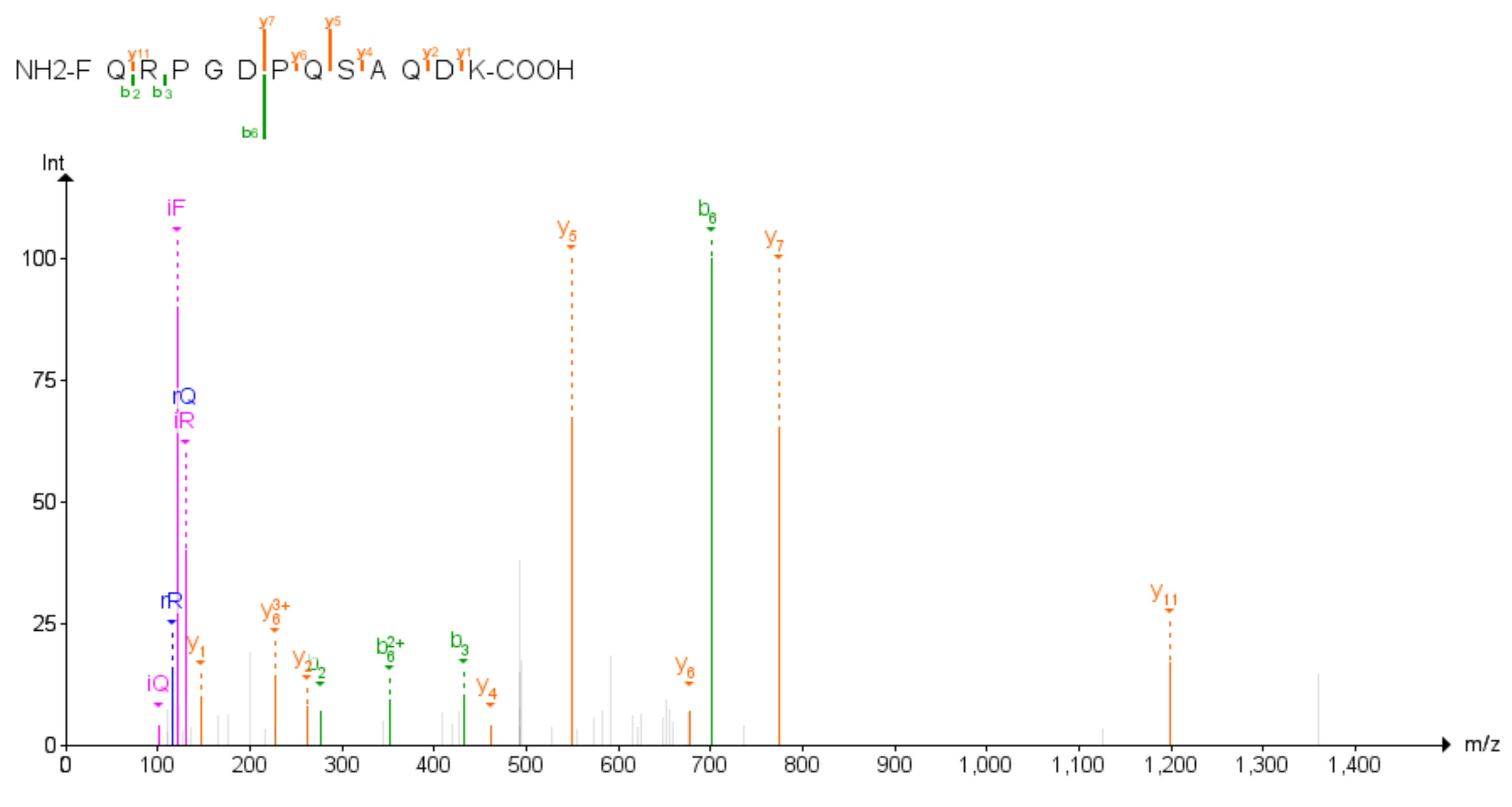

NH2-F Q R P G D P Q S A Q D K-COOH
y11 y7 y6 y5 y4 y2 y1
b2 b3 b6
Int
iF
rQ
iR
rR
iQ
y1
y6 3+
y2
b2
b6 2+
b3
y4
y5
b6
y7
y6
y11
0 25 50 75 100
0 100 200 300 400 500 600 700 800 900 1,000 1,100 1,200 1,300 1,400
m/z

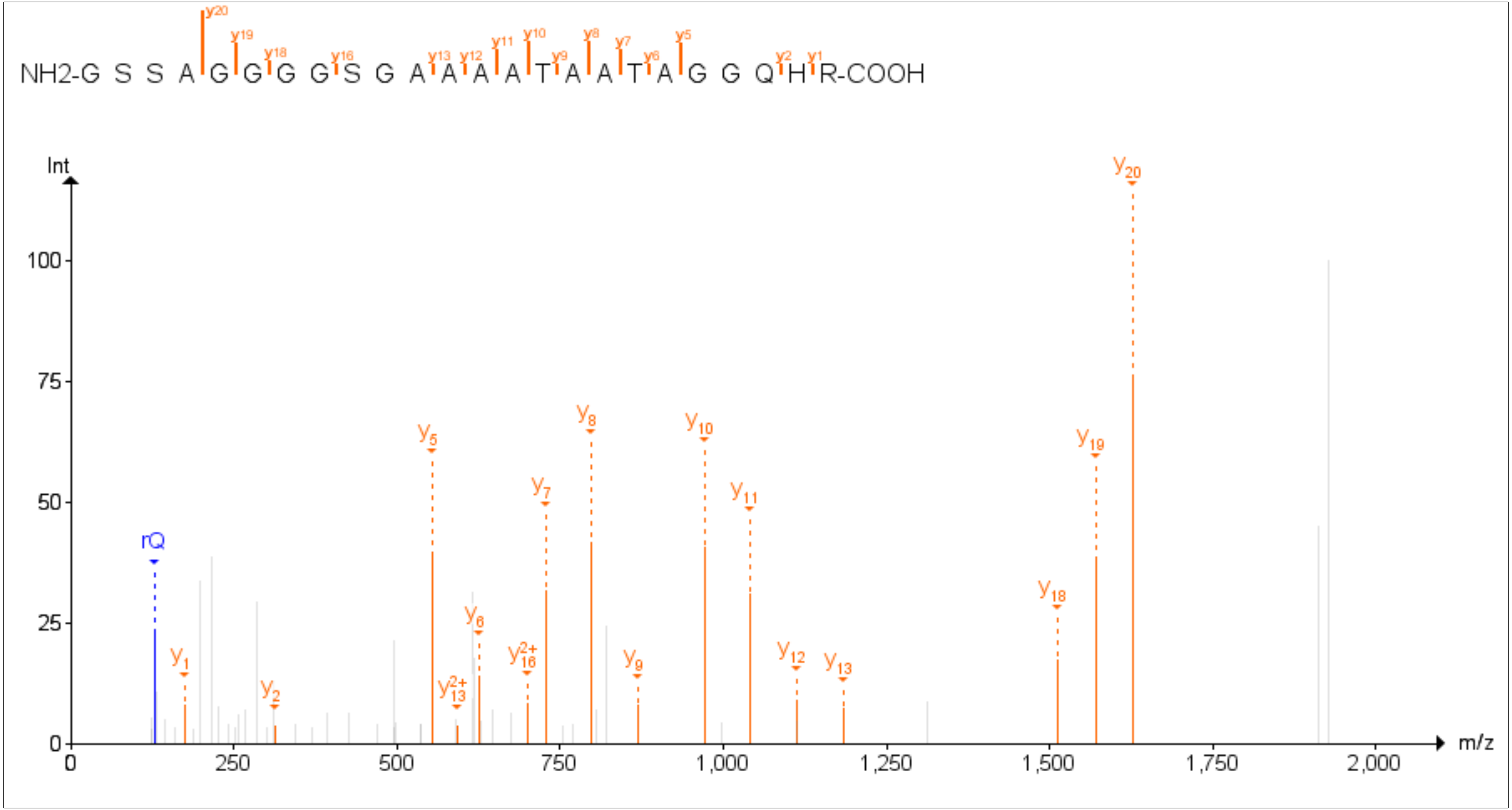
NH2-G S S A G G G G S G A A A A T A A T A G G Q H R-COOH
y20 y19 y18 y16 y13 y12 y11 y10 y9 y8 y7 y6 y5 y2 y1
Int
100
75
50
25
0
rQ
y1 y2 y5 y2+ 13 y6 y2+ 16 y7 y8 y9 y10 y11 y12 y13 y18 y19 y20
0 250 500 750 1,000 1,250 1,500 1,750 2,000
m/z

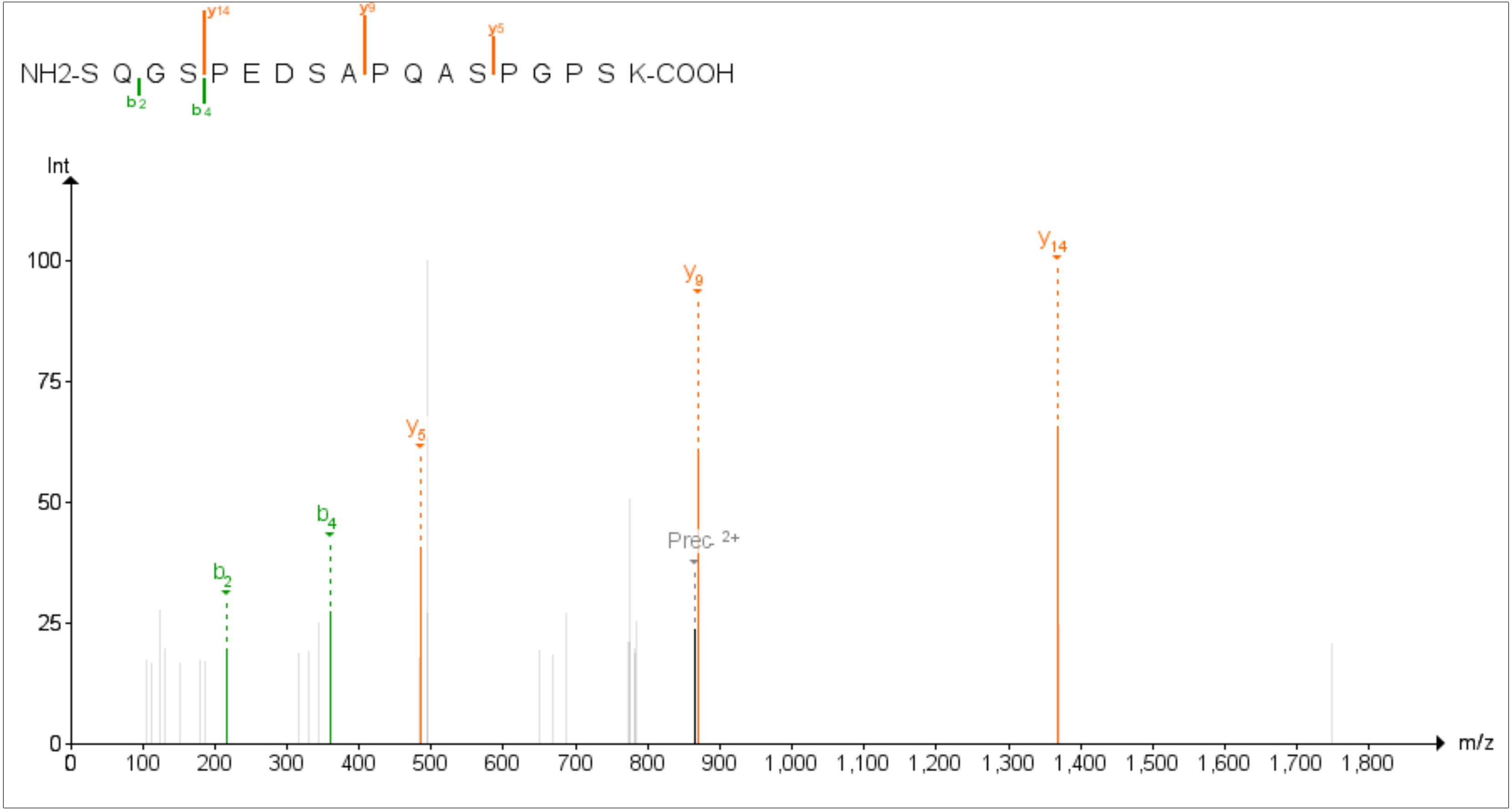

NH2-S Q G S P E D S A P Q A S P G P S K-COOH
y14
y9
y5
b2
b4
Int
100
75
50
25
0
y14
y9
y5
b4
b2
Prec. 2+
m/z
0
100
200
300
400
500
600
700
800
900
1,000
1,100
1,200
1,300
1,400
1,500
1,600
1,700
1,800

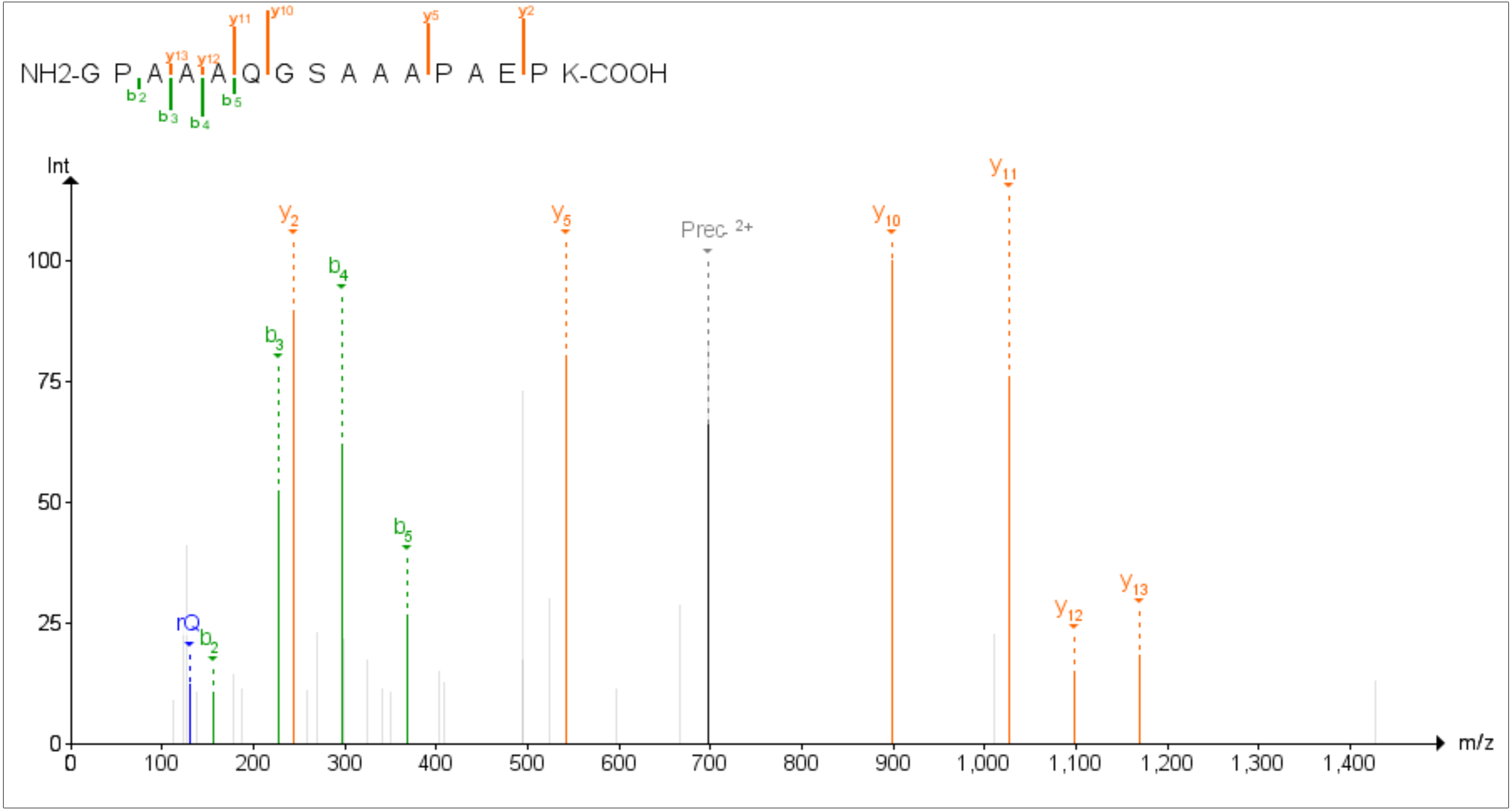

NH2-G P A A A Q G S A A A P A E P K-COOH
y13 y12 y11 y10 y5 y2
b2 b3 b4 b5
Int
m/z
y2
y5
Prec. 2+
y10
y11
b4
b3
b5
rQ
b2
y12
y13
0
25
50
75
100
0 100 200 300 400 500 600 700 800 900 1,000 1,100 1,200 1,300 1,400

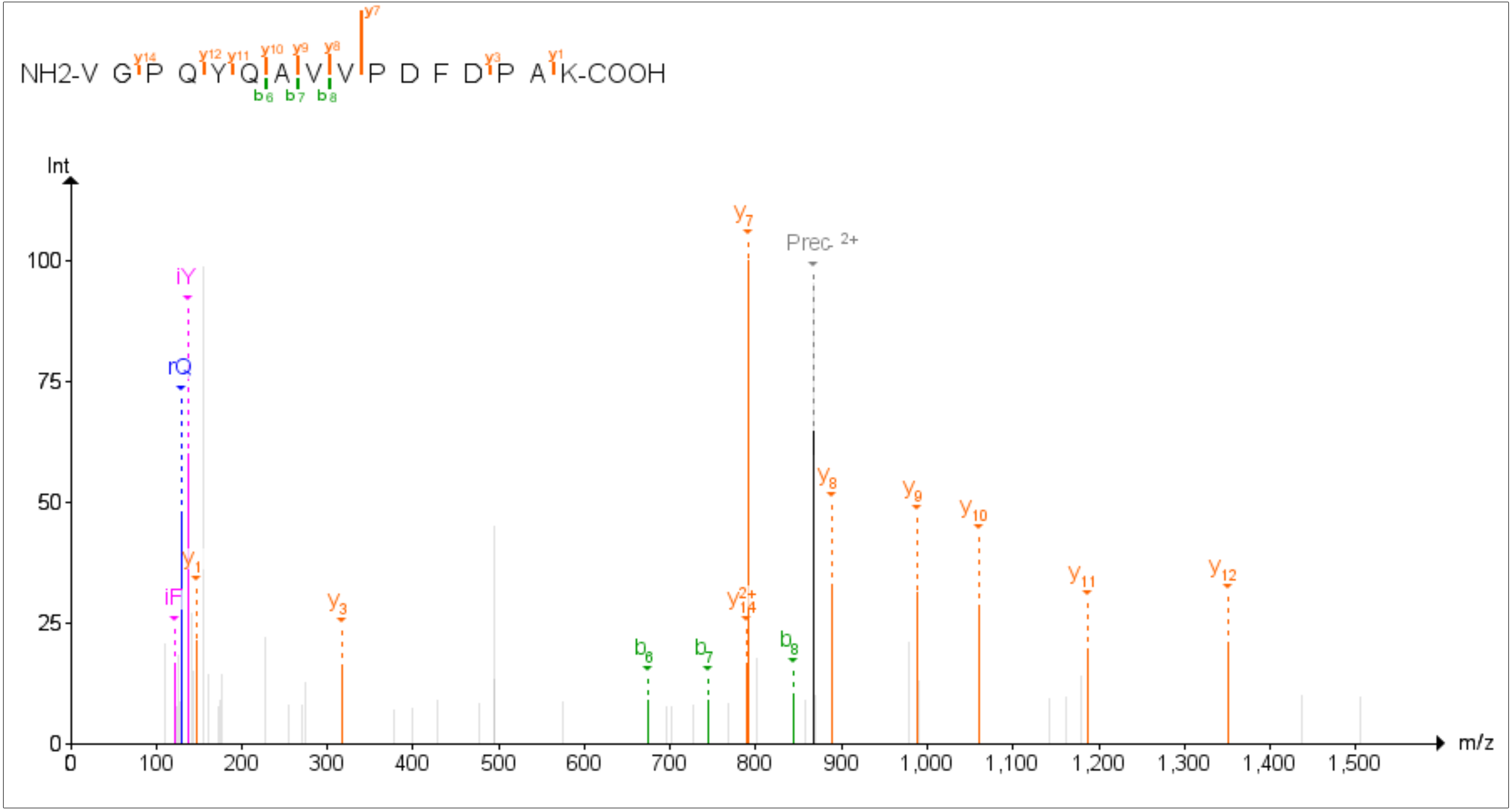

NH2-V G P Q Y Q A V V P D F D P A K-COOH
y14 y12 y11 y10 y9 y8 y7 y3 y1
b6 b7 b8
Int
100
75
50
25
0
iY
rQ
iF
y1
y3
b6
b7
y14 2+
b8
y7
Prec 2+
y8
y9
y10
y11
y12
0 100 200 300 400 500 600 700 800 900 1,000 1,100 1,200 1,300 1,400 1,500
m/z

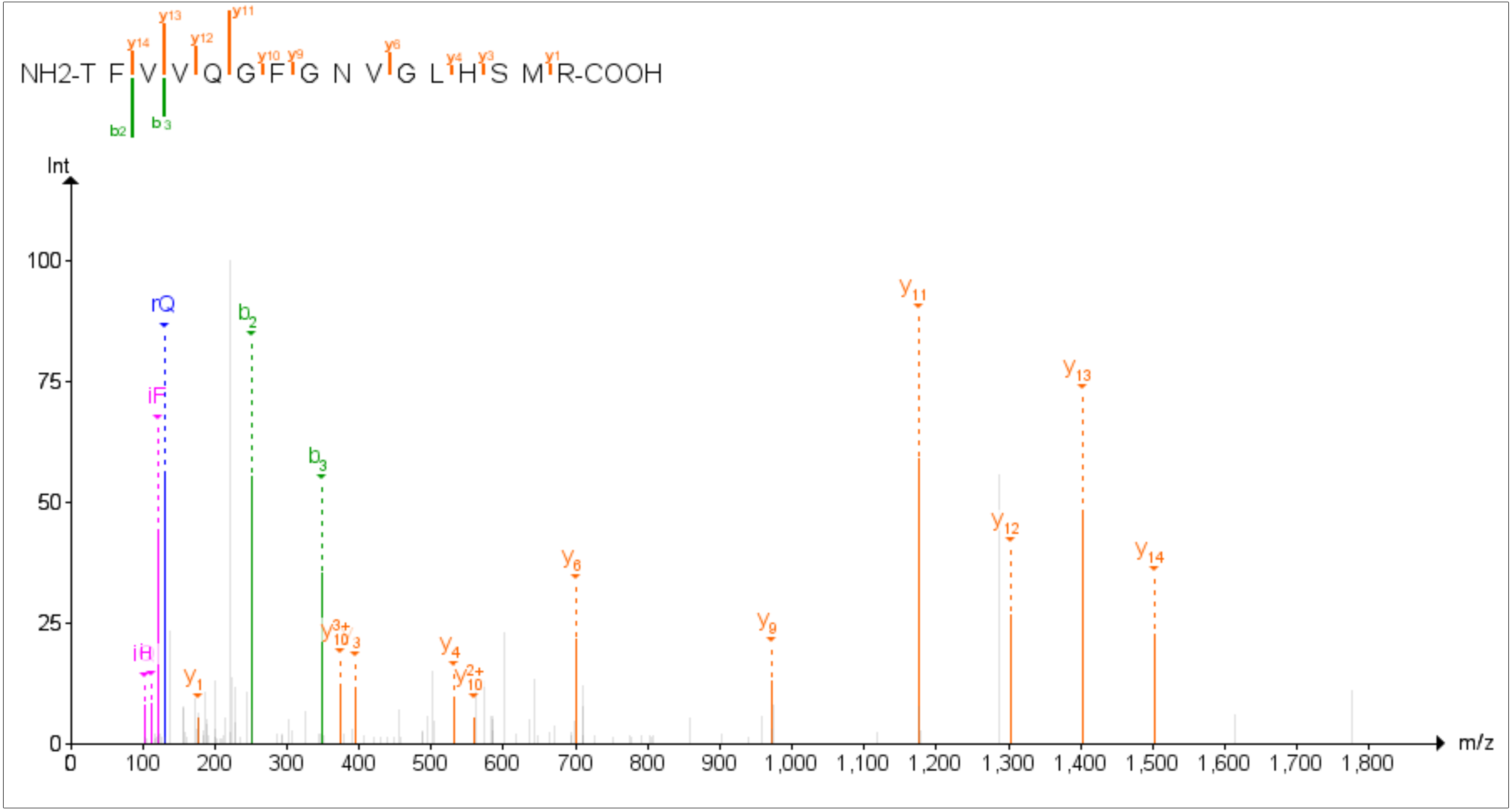

NH2-T F V V Q G F G N V G L H S M R-COOH
y14
y13
y12
y11
y10
y9
y6
y4
y3
y1
b2
b3
Int
100
75
50
25
0
rQ
iF
iH
b2
b3
y1
y10 3+
y3
y4
y10 2+
y6
y9
y11
y12
y13
y14
m/z
0
100
200
300
400
500
600
700
800
900
1,000
1,100
1,200
1,300
1,400
1,500
1,600
1,700
1,800

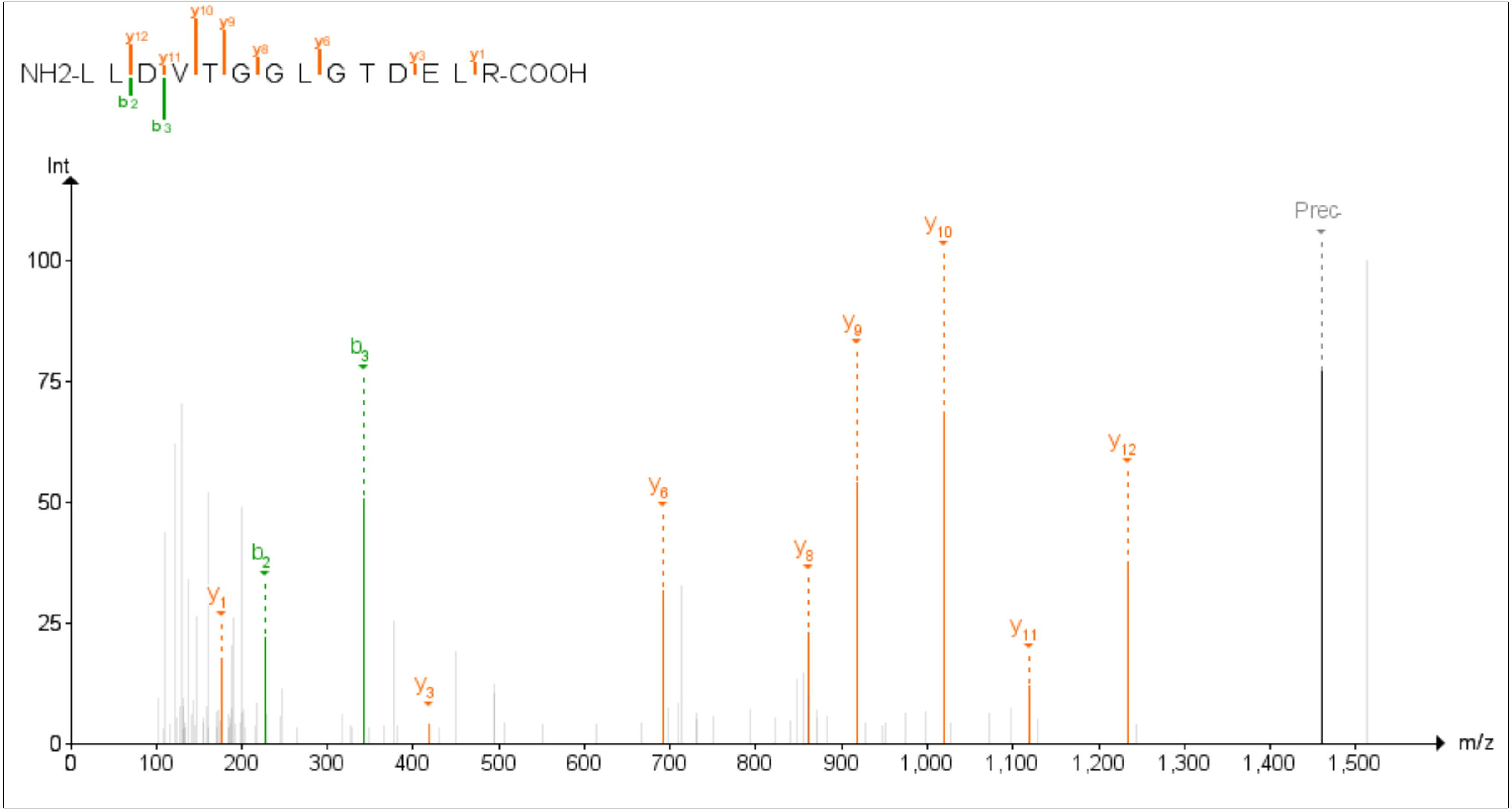

NH2-L L D V T G G L G T D E L R-COOH
y12 y11 y10 y9 y8 y6 y3 y1
b2 b3
Int
m/z
Prec.

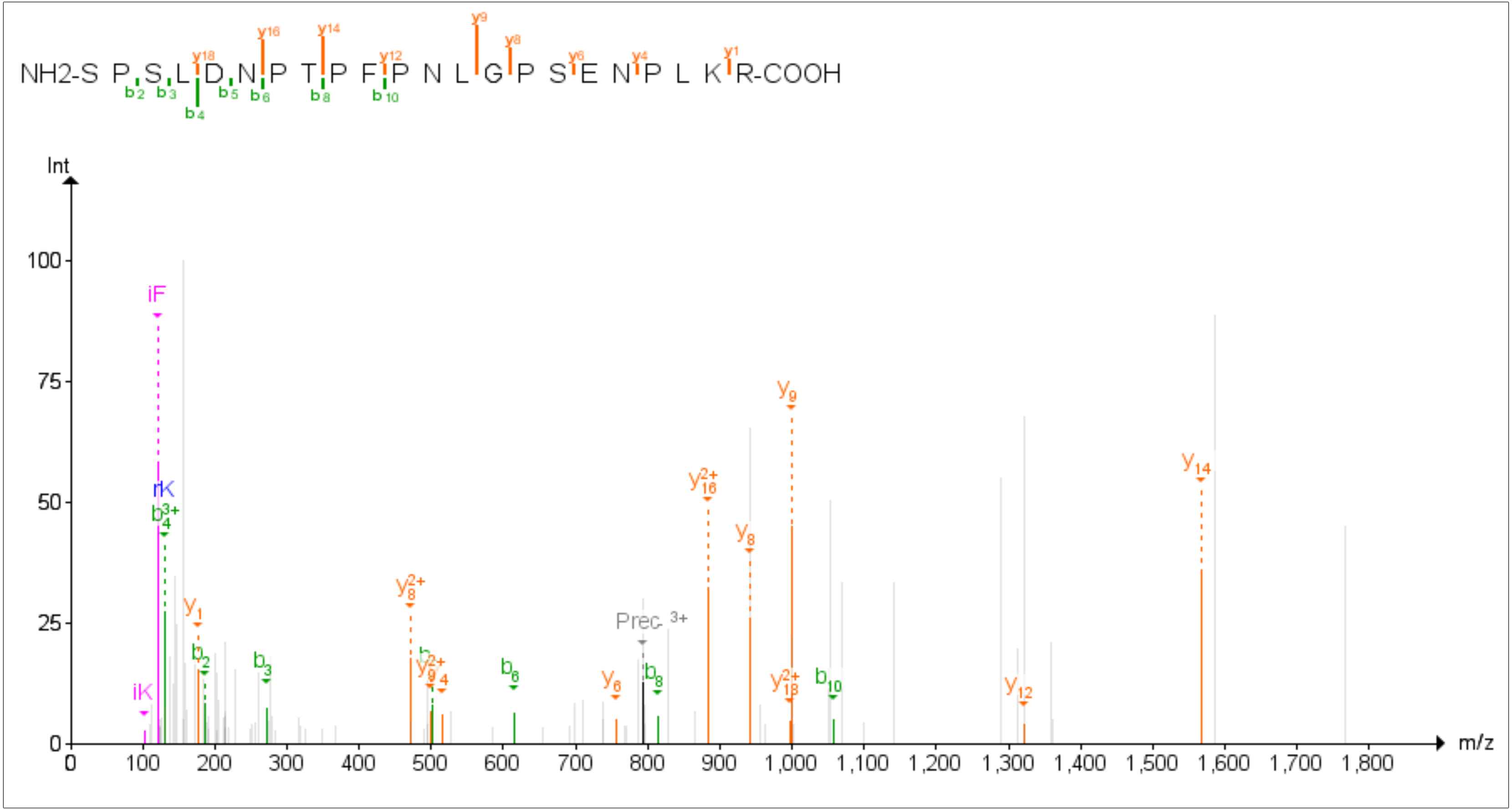
NH2-S P S L D N P T P F P N L G P S E N P L K R-COOH
y18 y16 y14 y12 y9 y8 y6 y4 y1
b2 b3 b4 b5 b6 b8 b10
Int
100
75
50
25
0
iF
rK
iK
y1
b2
b3
y8 2+
y9 2+
y4
b6
y6
Prec 3+
b8
y16 2+
y8
y9
y18 2+
b10
y12
y14
0 100 200 300 400 500 600 700 800 900 1,000 1,100 1,200 1,300 1,400 1,500 1,600 1,700 1,800
m/z

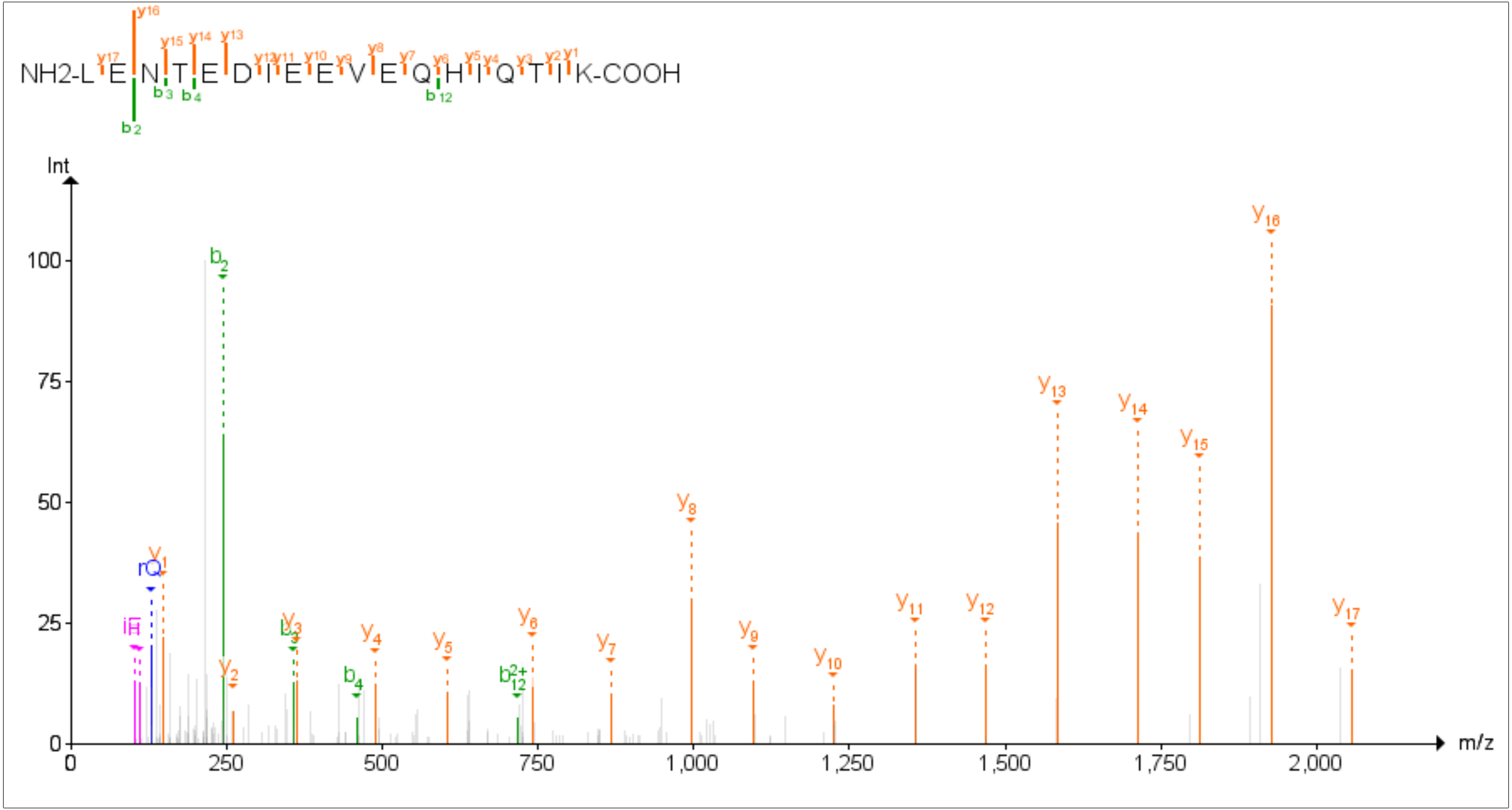

NH2-L E N T E D I E E V E Q H I Q T I K-COOH
Int
m/z
b2
y1
rQ
iH
y2
b3
y3
b4
y4
y5
b12 2+
y6
y7
y8
y9
y10
y11
y12
y13
y14
y15
y16
y17

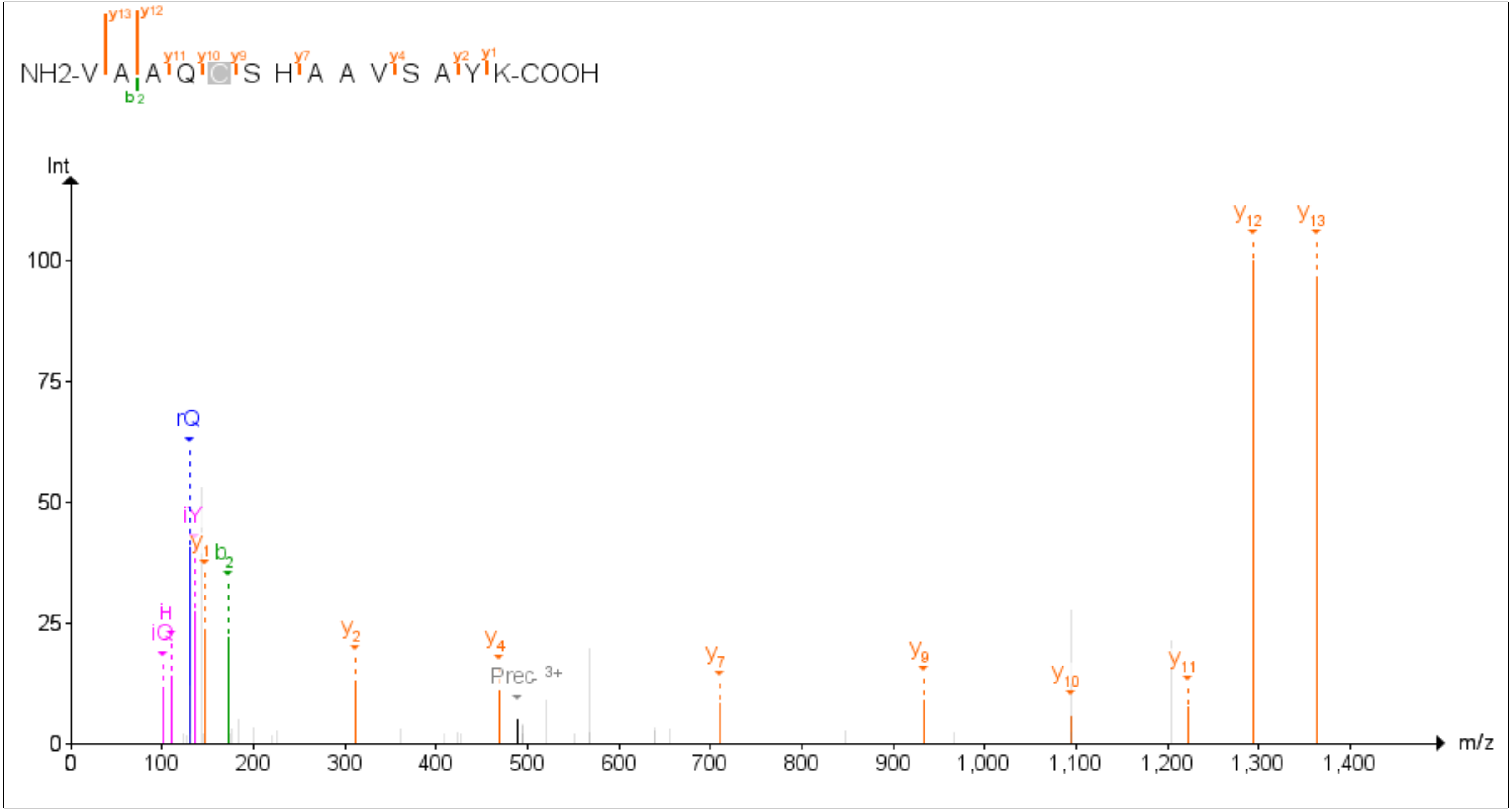

NH2-V A A Q C S H A A V S A Y K-COOH
y13 y12 y11 y10 y9 y7 y4 y2 y1
b2
Int
100
75
50
25
0
rQ
iY
y1
b2
iH
iQ
y2
y4
Prec- 3+
y7
y9
y10
y11
y12
y13
0 100 200 300 400 500 600 700 800 900 1,000 1,100 1,200 1,300 1,400
m/z

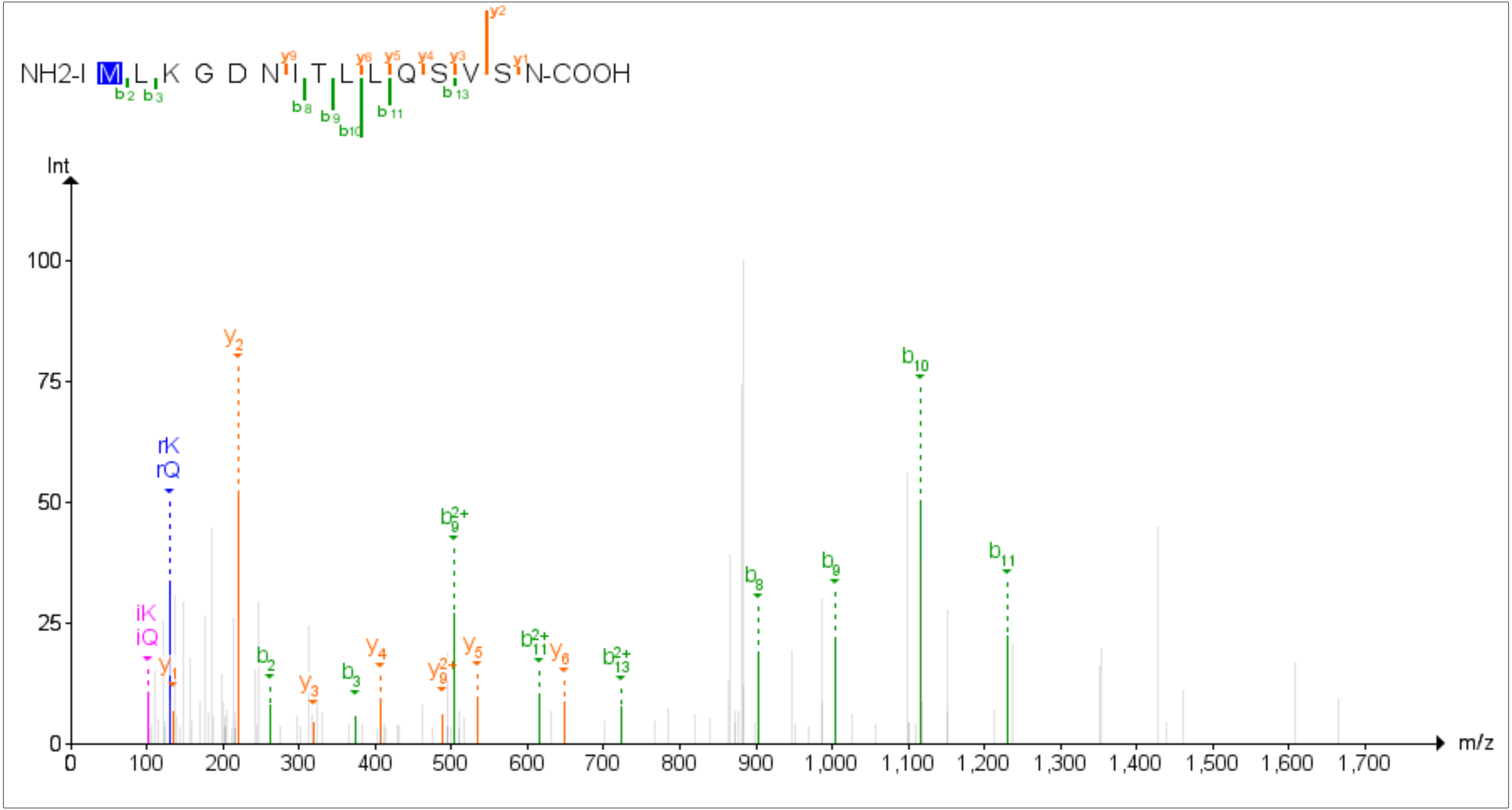

NH2-I M L K G D N I T L L Q S V S N-COOH
Int
m/z
0
25
50
75
100
100
200
300
400
500
600
700
800
900
1,000
1,100
1,200
1,300
1,400
1,500
1,600
1,700

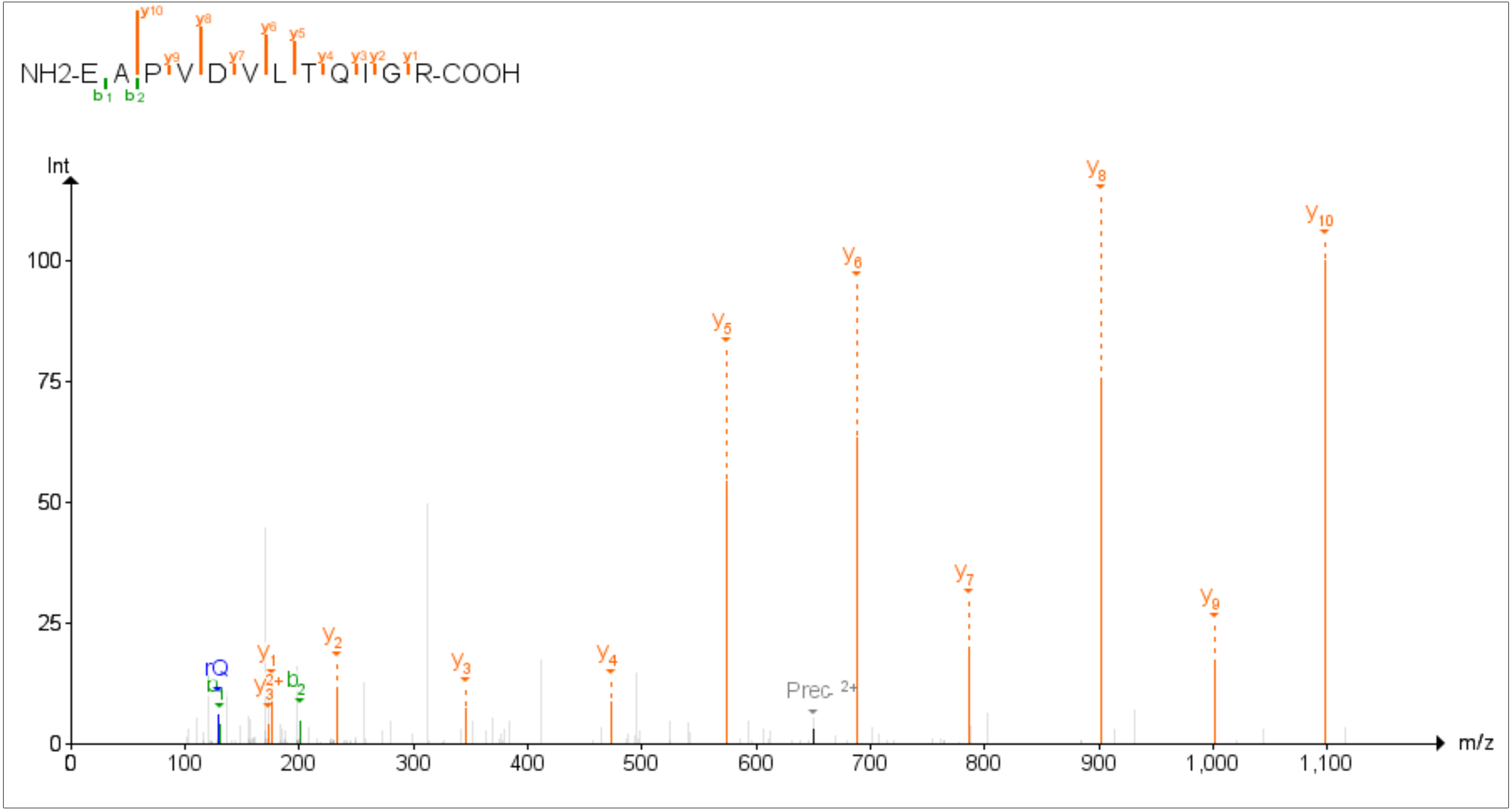

NH2-E A P V D V L T Q I G R-COOH
b1 b2
y10 y9 y8 y7 y6 y5 y4 y3 y2 y1
Int
m/z
0
25
50
75
100
0
100
200
300
400
500
600
700
800
900
1,000
1,100
rQ
b1
y1
y3 2+
b2
y2
y3
y4
y5
y6
y7
y8
y9
y10
Prec 2+

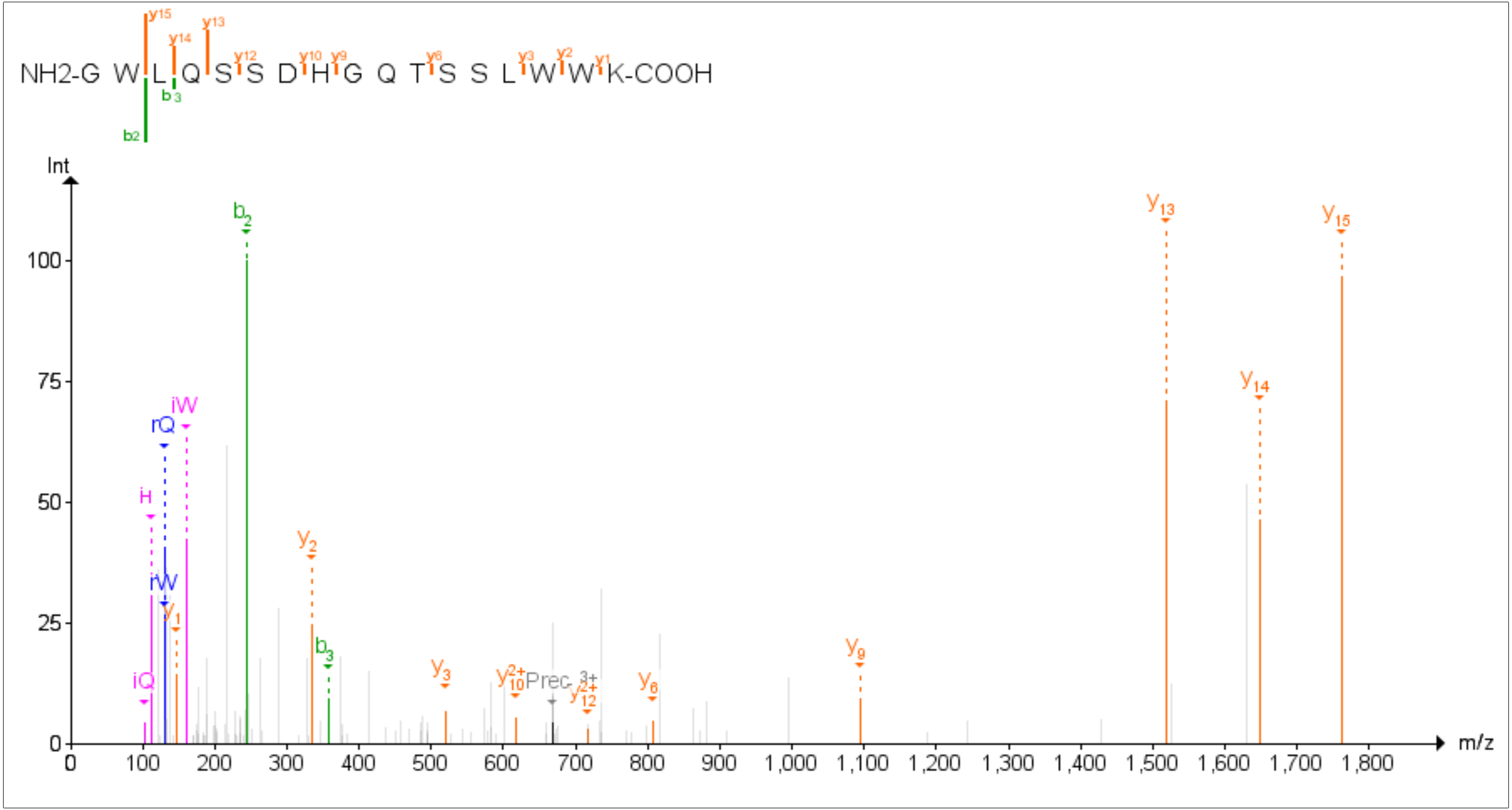

NH2-G W L Q S S D H G Q T S S L W W K-COOH
y15
y14
y13
y12
y10
y9
y6
y3
y2
y1
b2
b3
Int
100
75
50
25
0
m/z
0
100
200
300
400
500
600
700
800
900
1,000
1,100
1,200
1,300
1,400
1,500
1,600
1,700
1,800
b2
b3
y1
y2
y3
y6
y9
y13
y14
y15
iW
rQ
iH
rW
iQ
Prec 3+

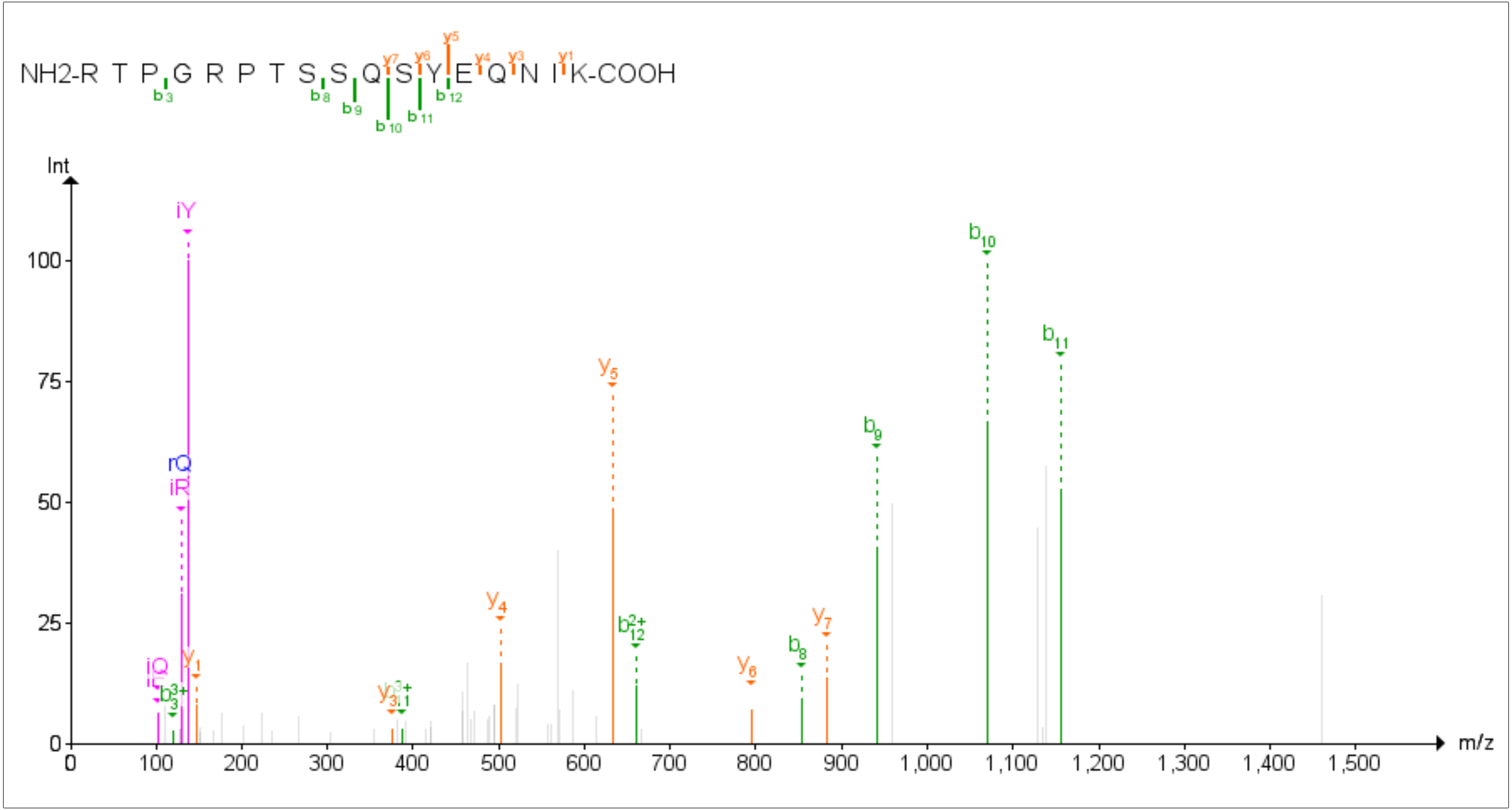

NH2-R T P G R P T S S Q S Y E Q N I K-COOH
b3
b8
b9
b10
b11
b12
y7
y6
y5
y4
y3
y1
Int
iY
rQ
iR
iQ
y1
y5
y4
b10
b11
b9
y7
y6
b8
0
25
50
75
100
0
100
200
300
400
500
600
700
800
900
1,000
1,100
1,200
1,300
1,400
1,500
m/z

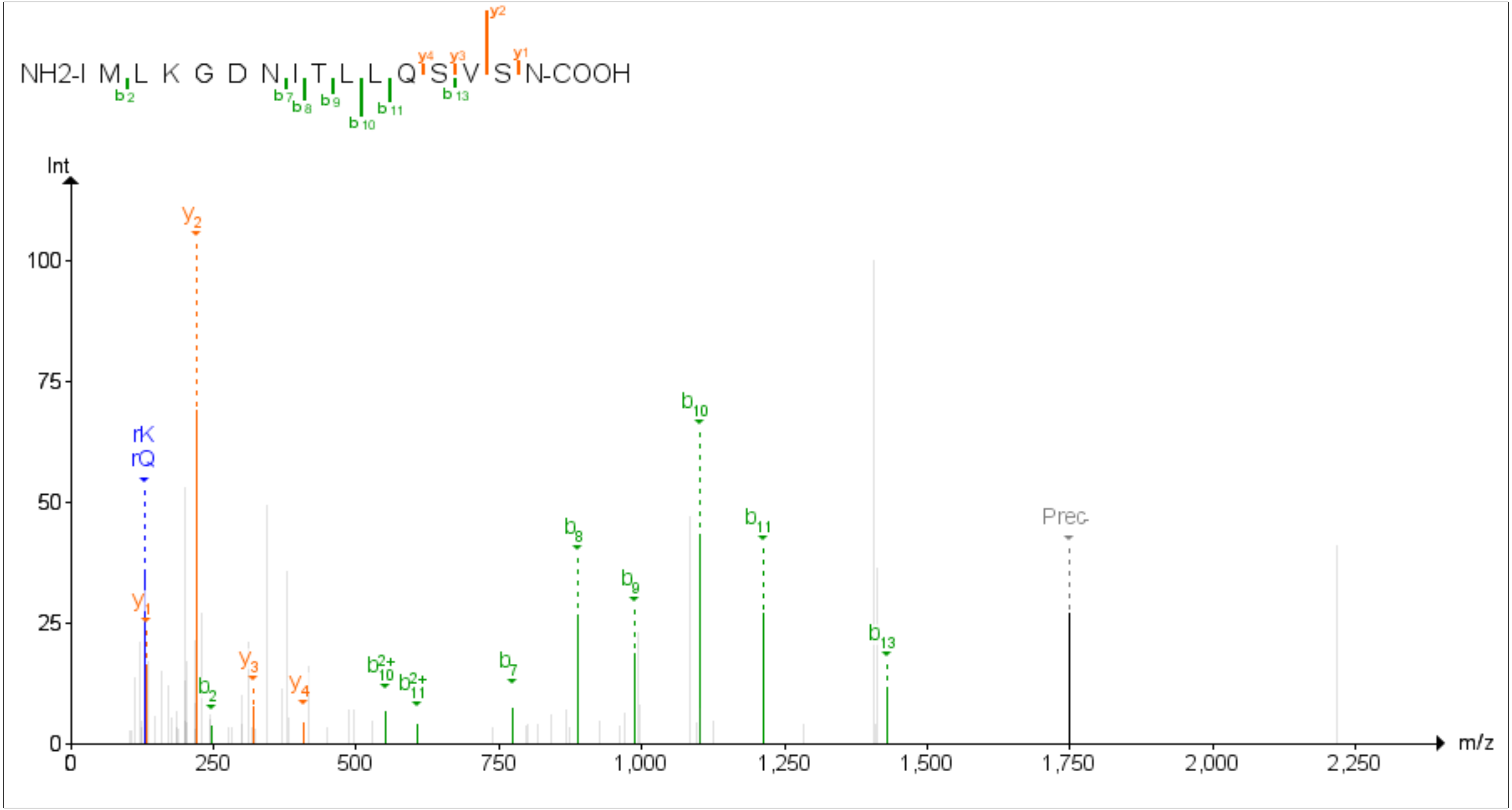

NH2-I M L K G D N I T L L Q S V S N-COOH
b2 b7 b8 b9 b10 b11 b13
y4 y3 y2 y1
Int
100
75
50
25
0
y2
rK
rQ
y1
y3
y4
b2
b10 2+
b11 2+
b7
b8
b9
b10
b11
b13
Prec
0
250
500
750
1,000
1,250
1,500
1,750
2,000
2,250
m/z

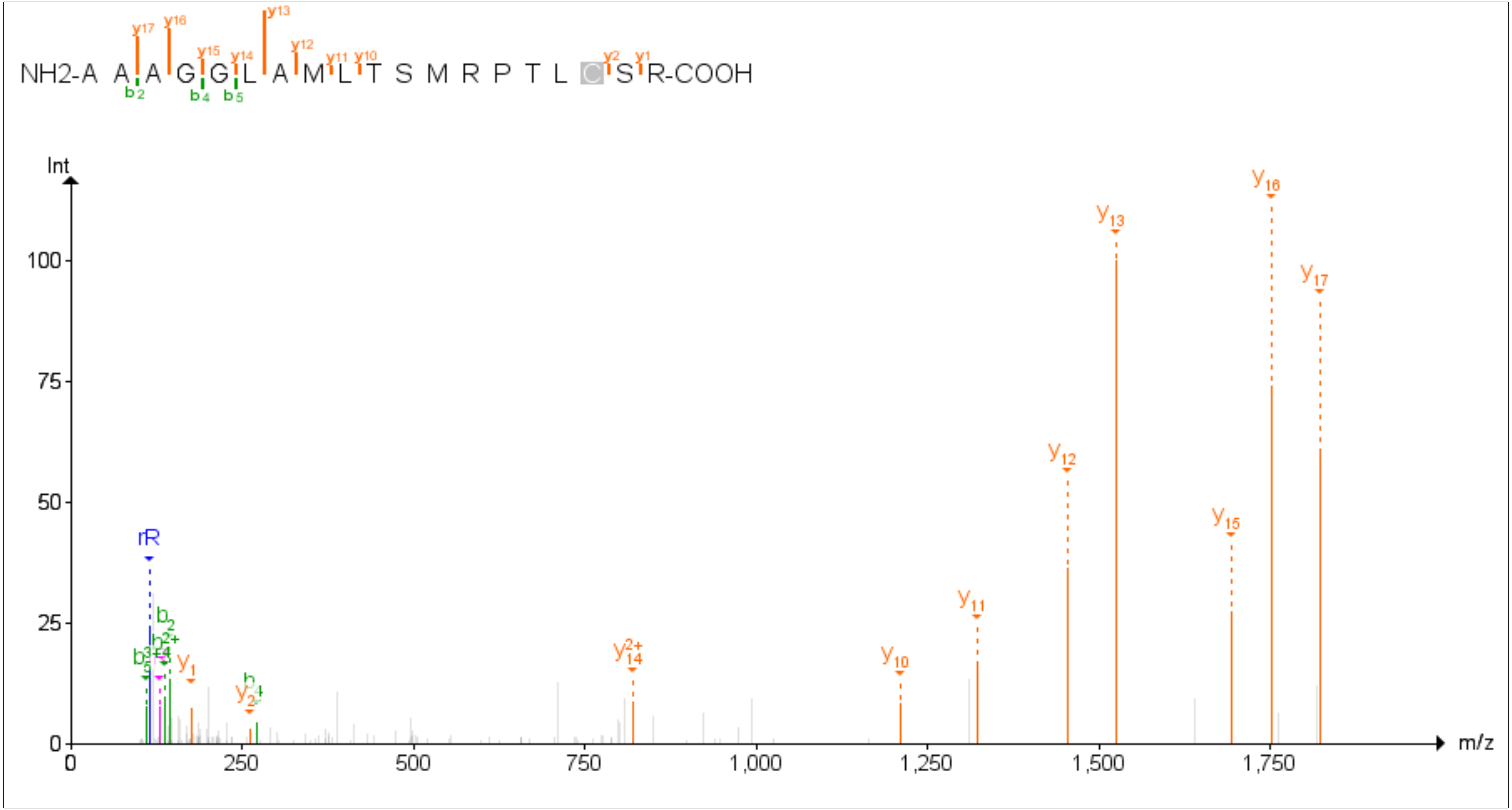

NH2-A A A G G L A M L T S M R P T L C S R-COOH
Int
m/z
y17
y16
y15
y14
y13
y12
y11
y10
y2
y1
b2
b4
b5
rR
0
25
50
75
100
250
500
750
1,000
1,250
1,500
1,750

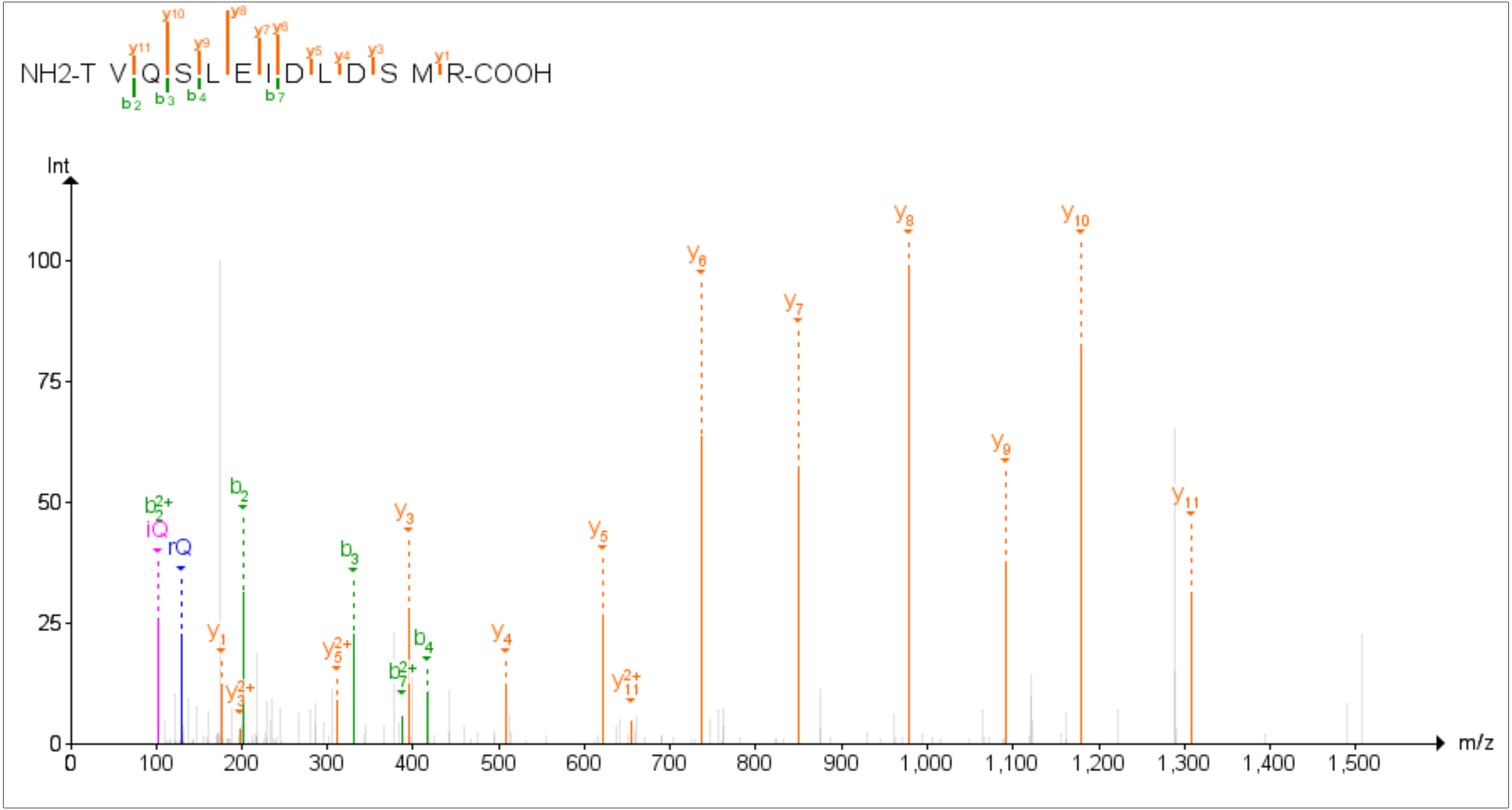

NH2-T V Q S L E I D L D S M R-COOH
y11 y10 y9 y8 y7 y6 y5 y4 y3 y1
b2 b3 b4 b7
Int
100
75
50
25
0
m/z
0 100 200 300 400 500 600 700 800 900 1,000 1,100 1,200 1,300 1,400 1,500
y8 y10 y6 y7 y9 y11 y5 y3 y4 y1
b2 b3 b4
iQ rQ

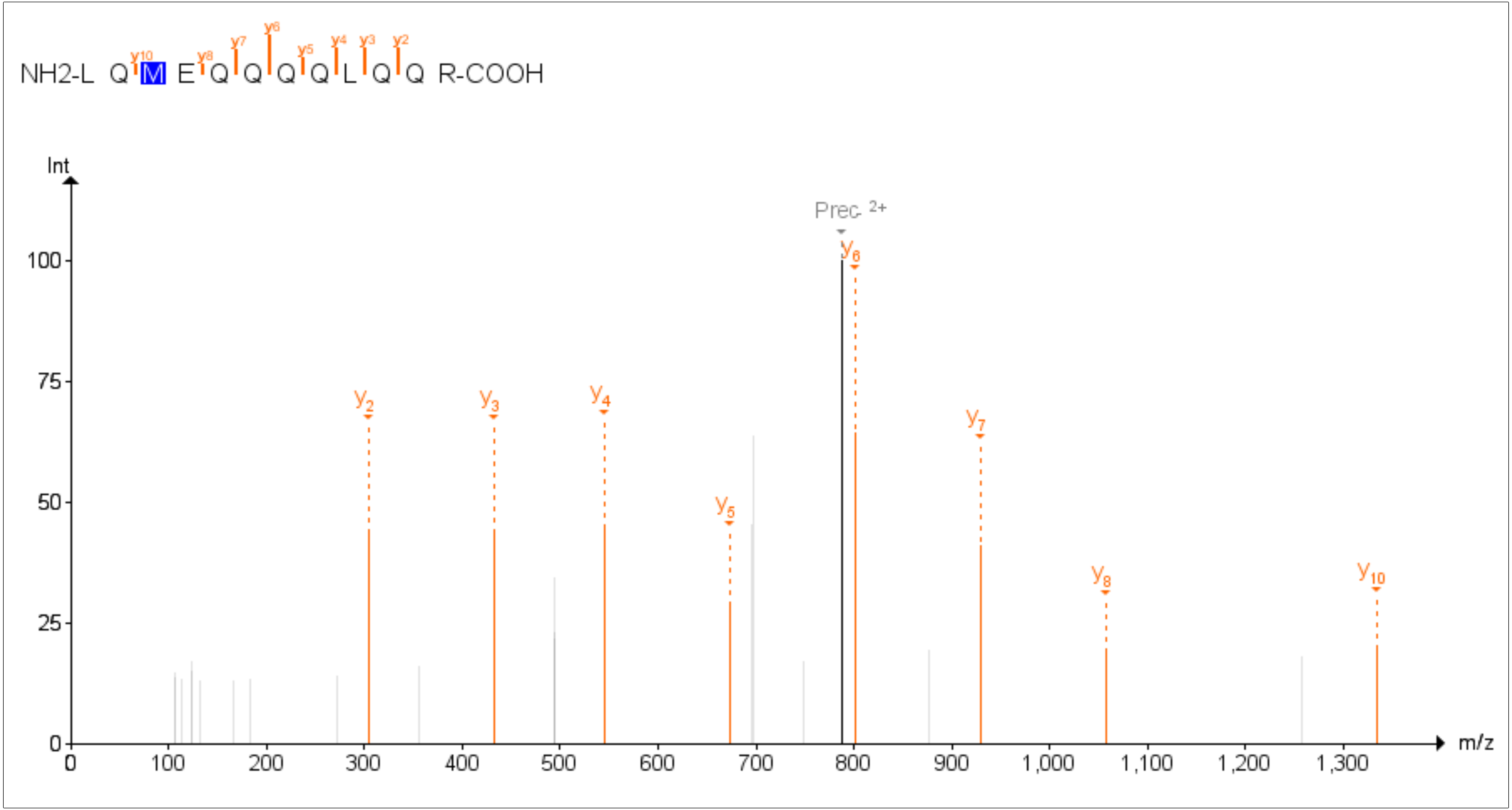

NH2-L Q M E Q Q Q Q L Q Q R-COOH
y10 y8 y7 y6 y5 y4 y3 y2
Int
100
75
50
25
0
Prec 2+
y2
y3
y4
y5
y6
y7
y8
y10
0 100 200 300 400 500 600 700 800 900 1,000 1,100 1,200 1,300
m/z

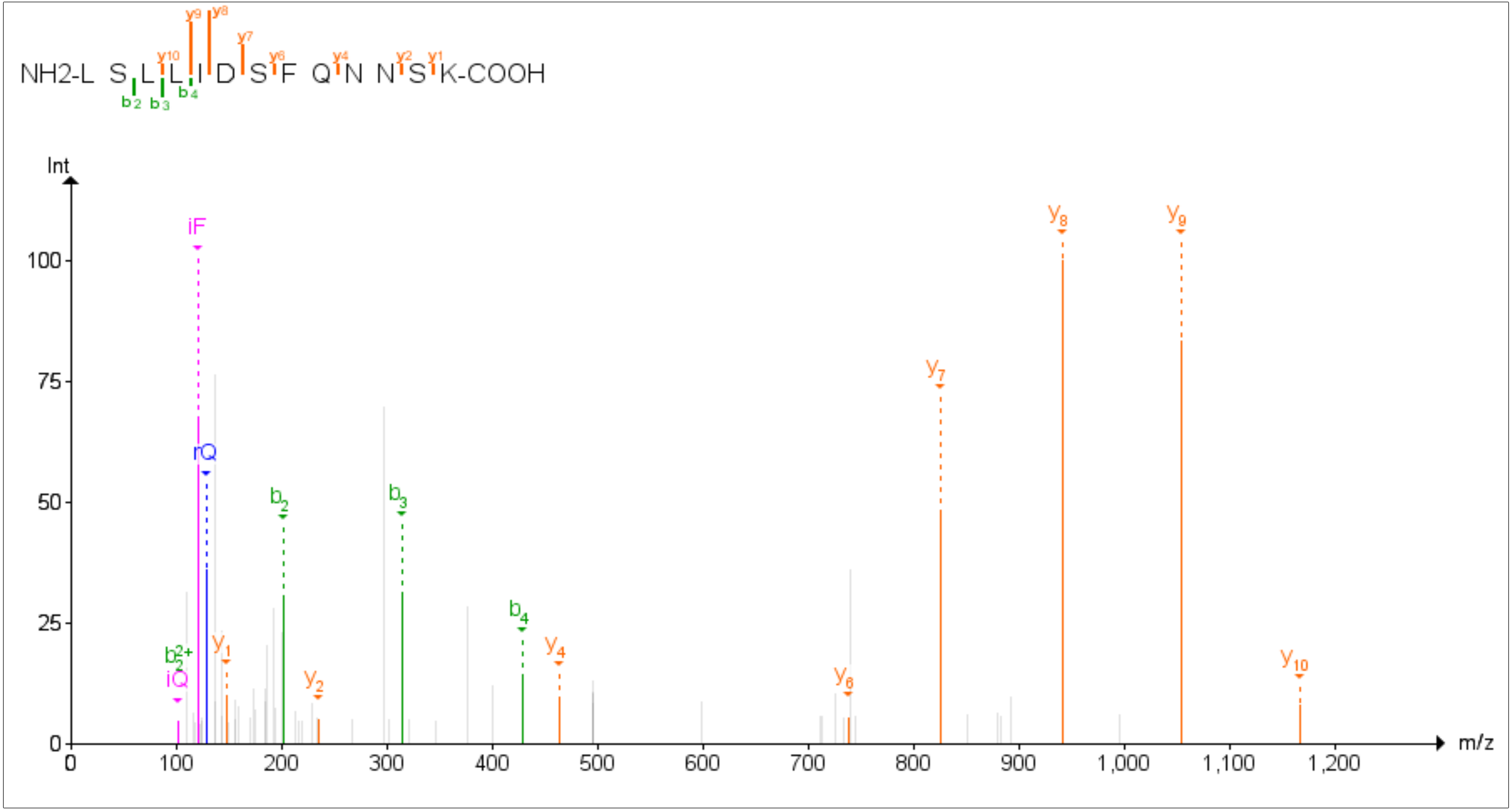

NH2-L S L L I D S F Q N N S K-COOH
y9 y8 y10 y7 y6 y4 y2 y1
b2 b3 b4
Int
m/z
iF
rQ
iQ
b2 b3 b4
y1 y2 y4 y6 y7 y8 y9 y10
0
25
50
75
100
0
100
200
300
400
500
600
700
800
900
1,000
1,100
1,200

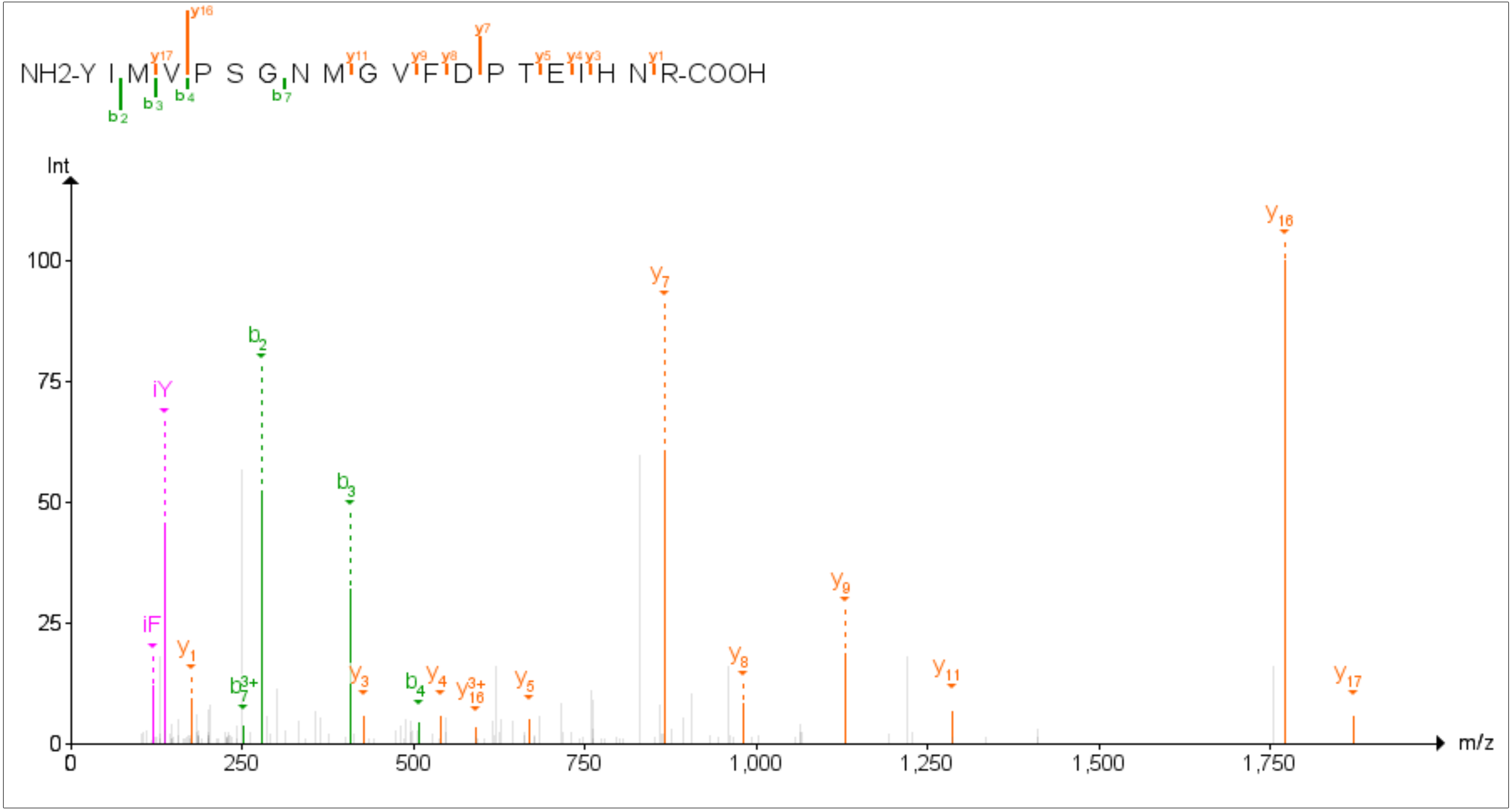

NH2-Y I M V P S G N M G V F D P T E I H N R-COOH
y16 y17 y11 y9 y8 y7 y5 y4 y3 y1
b2 b3 b4 b7
Int
m/z
0
25
50
75
100
250
500
750
1,000
1,250
1,500
1,750
iY
iF
y1
b7 3+
b2
b3
y3
b4
y4
y16 3+
y5
y7
y8
y9
y11
y16
y17

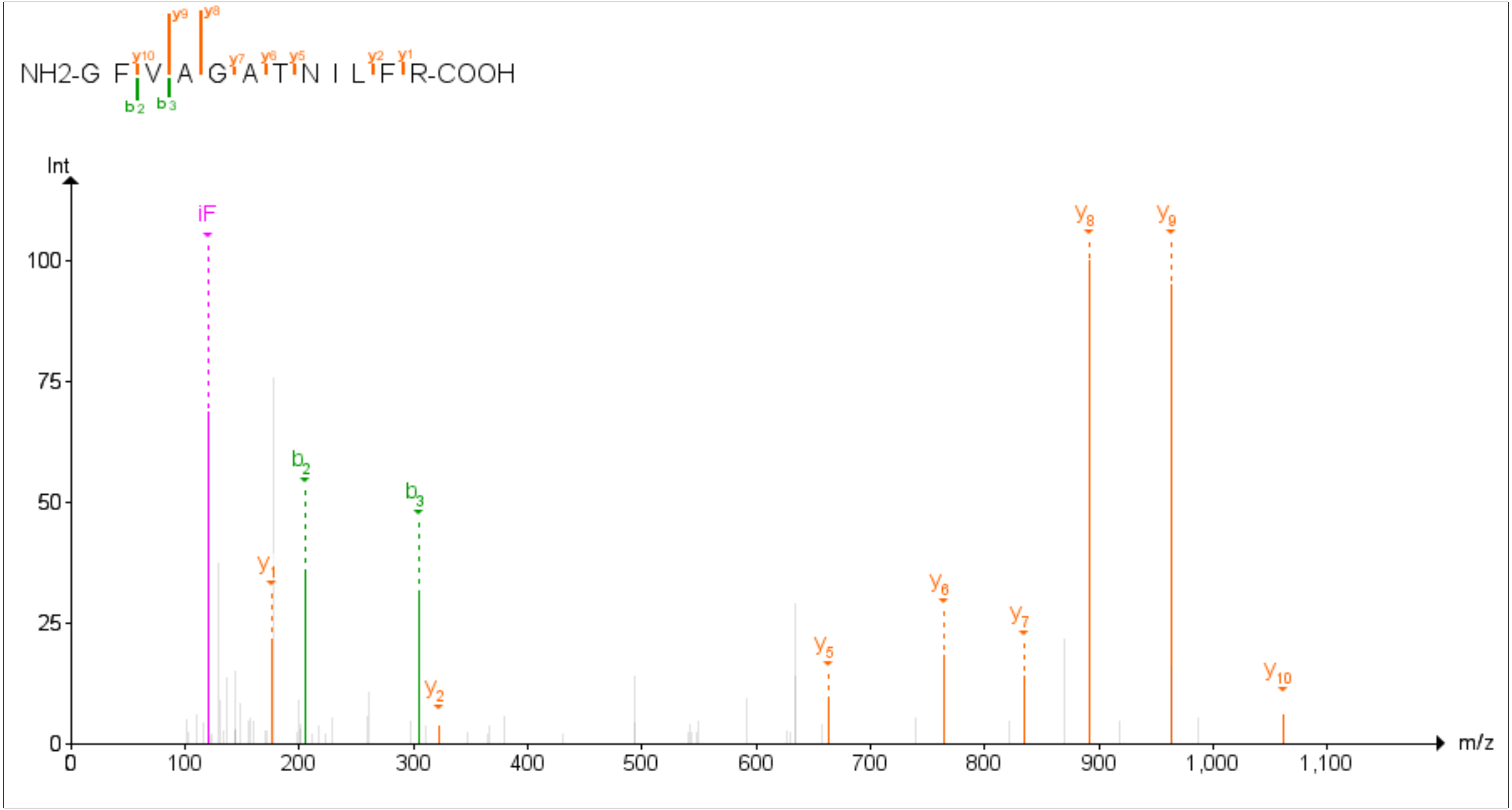
NH2-G F V A G A T N I L F R-COOH
y10 y9 y8 y7 y6 y5 y2 y1
b2 b3
Int
iF
y8
y9
b2
b3
y1
y2
y5
y6
y7
y10
0
25
50
75
100
0 100 200 300 400 500 600 700 800 900 1,000 1,100
m/z

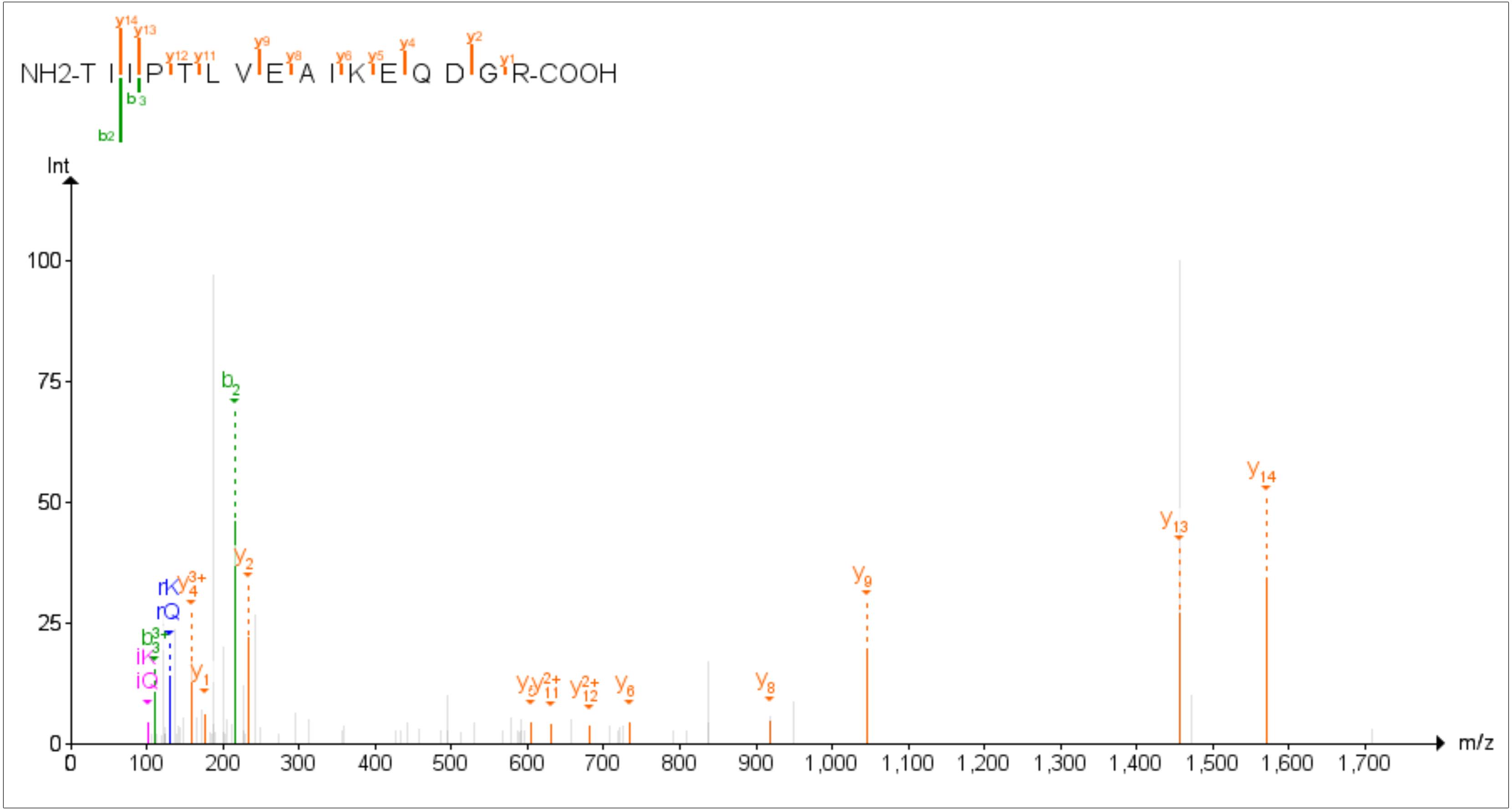
NH2-T I I P T L V E A I K E Q D G R-COOH
y14
y13
y12
y11
y9
y8
y6
y5
y4
y2
y1
b2
b3
Int
100
75
50
25
0
b2
y2
rK
y4 3+
rQ
b3 3+
iK
iQ
y1
y9
y13
y14
y8
y6
y 2+ 11
y 2+ 12
m/z
0
100
200
300
400
500
600
700
800
900
1,000
1,100
1,200
1,300
1,400
1,500
1,600
1,700

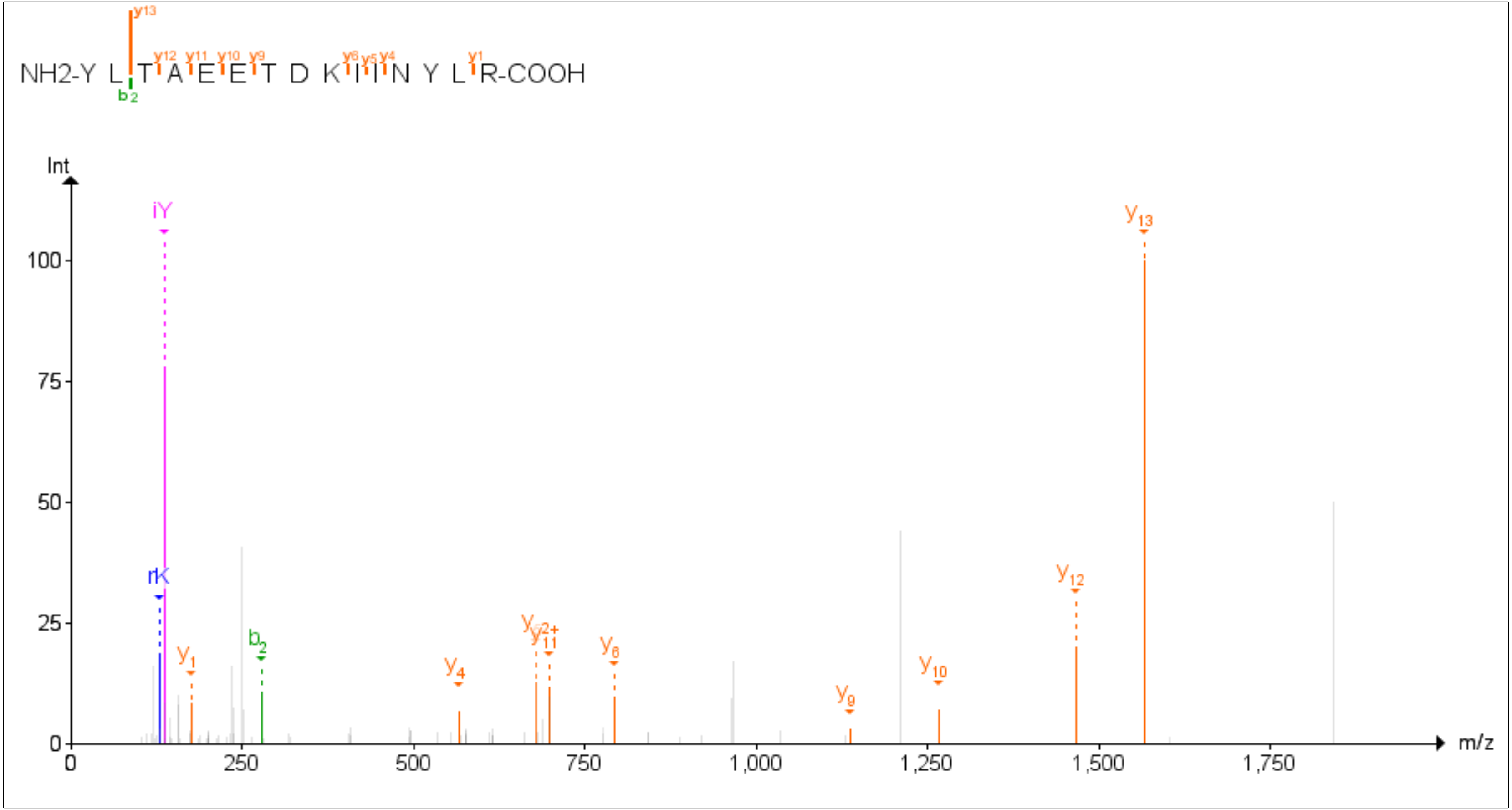

NH2-Y L T A E E T D K I I N Y L R-COOH
y13 y12 y11 y10 y9 y6 y5 y4 y1
b2
Int
iY
rK
y1
b2
y4
y11 2+
y11
y6
y9
y10
y12
y13
100
75
50
25
0
0
250
500
750
1,000
1,250
1,500
1,750
m/z

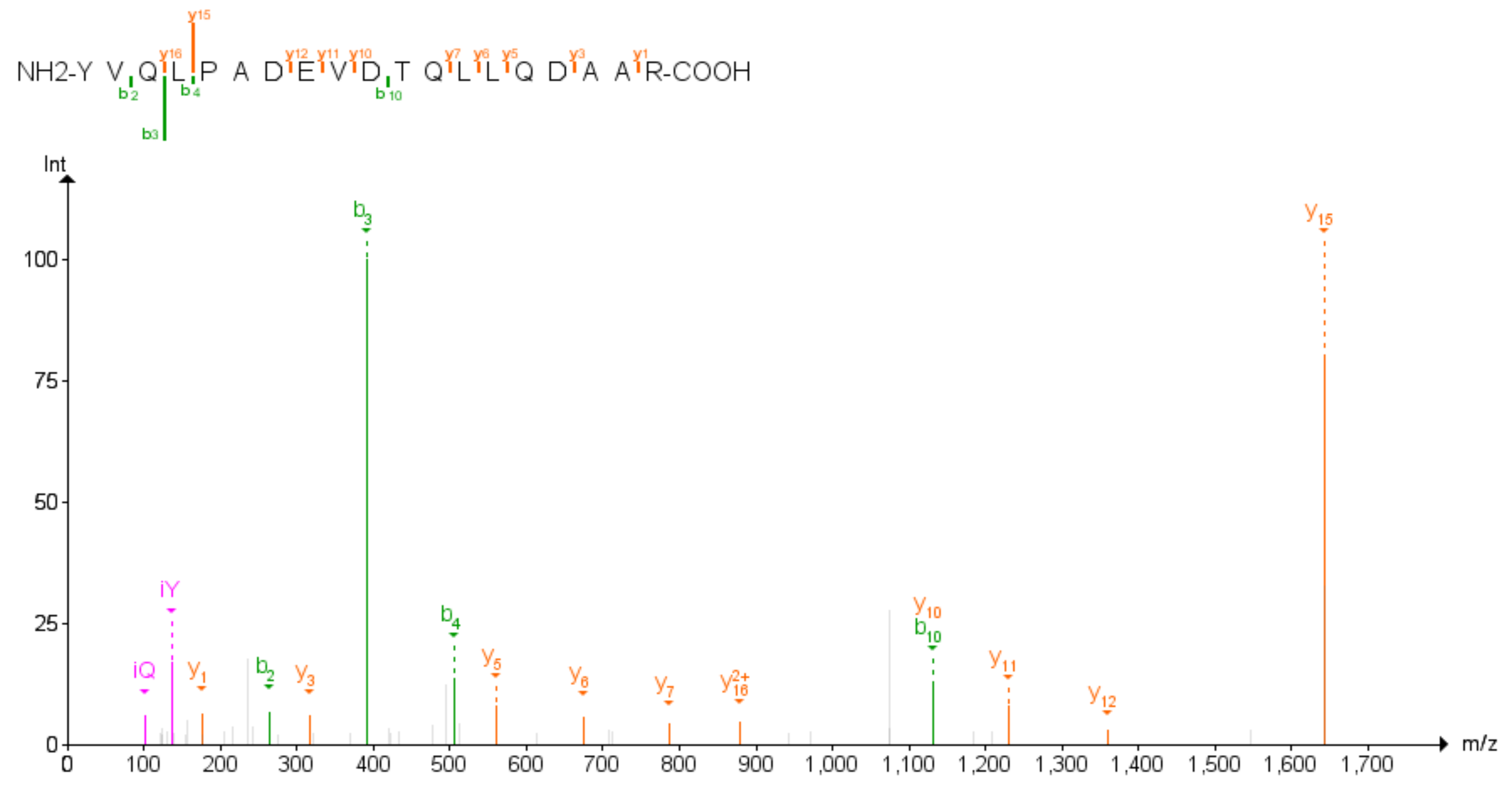

NH2-Y V Q L P A D E V D T Q L L Q D A A R-COOH
y15
y16
y12
y11
y10
y7
y6
y5
y3
y1
b2
b4
b10
b3
Int
100
75
50
25
0
b3
y15
iY
iQ
y1
b2
y3
b4
y5
y6
y7
y16 2+
y10
b10
y11
y12
m/z
0
100
200
300
400
500
600
700
800
900
1,000
1,100
1,200
1,300
1,400
1,500
1,600
1,700

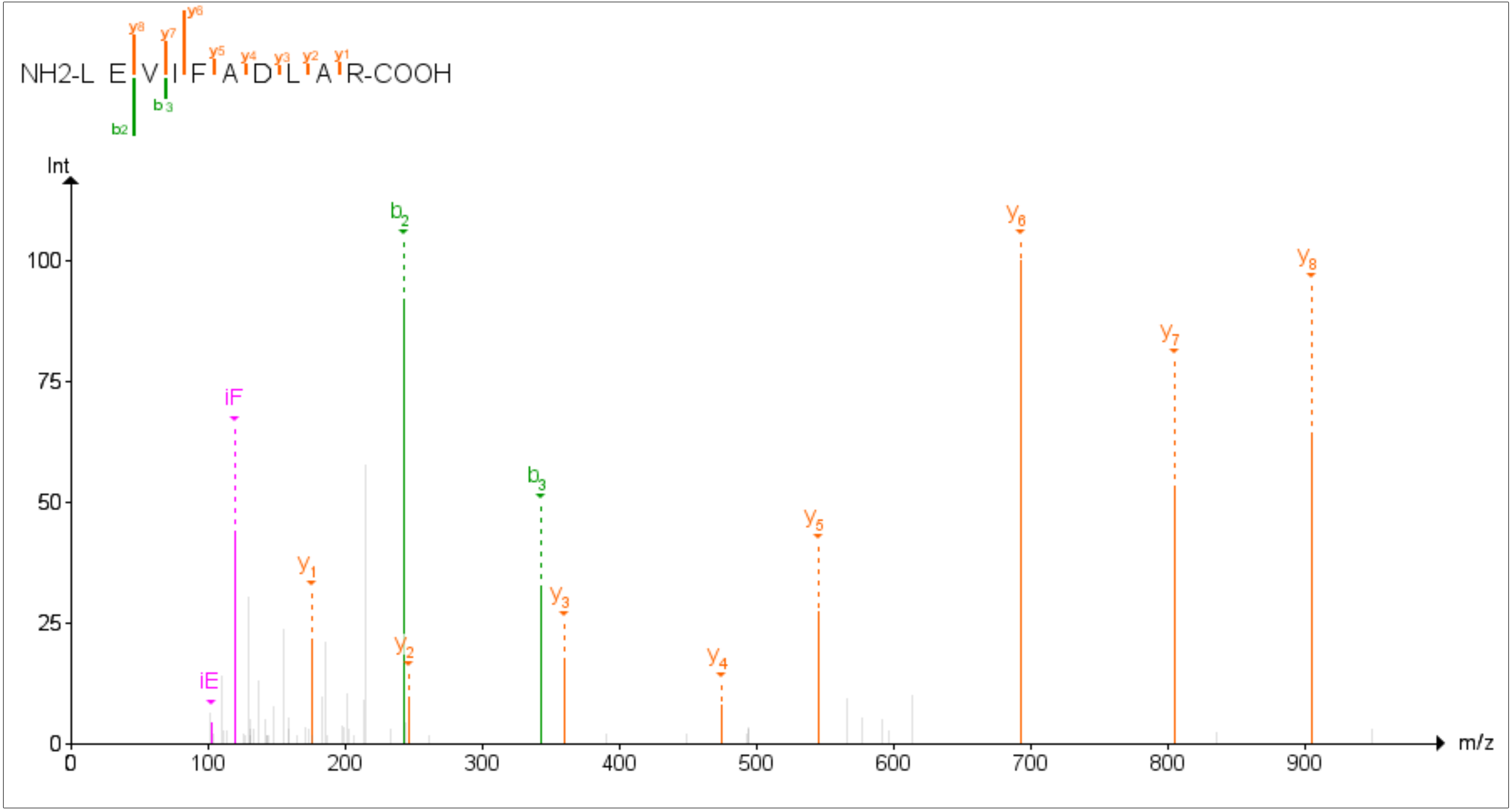
NH2-L E V I F A D L A R-COOH
y8 y7 y6 y5 y4 y3 y2 y1
b2 b3
Int
m/z
b2 y6 y8 y7 iF b3 y5 y1 y3 y2 y4 iE
0 25 50 75 100
0 100 200 300 400 500 600 700 800 900

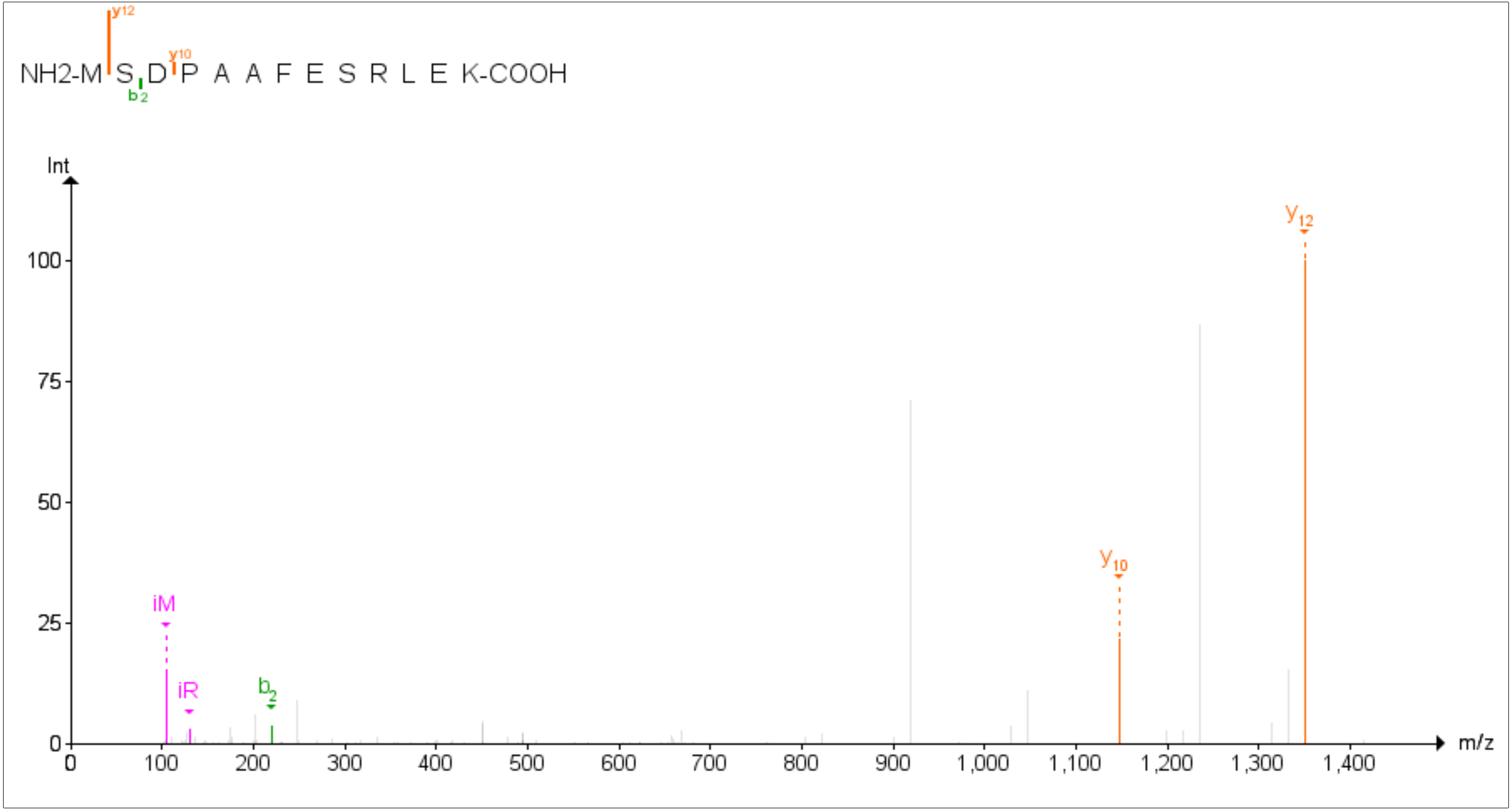

NH2-M S D P A A F E S R L E K-COOH
y12
y10
b2
Int
m/z
iM
iR
b2
y10
y12
0
25
50
75
100
0
100
200
300
400
500
600
700
800
900
1,000
1,100
1,200
1,300
1,400

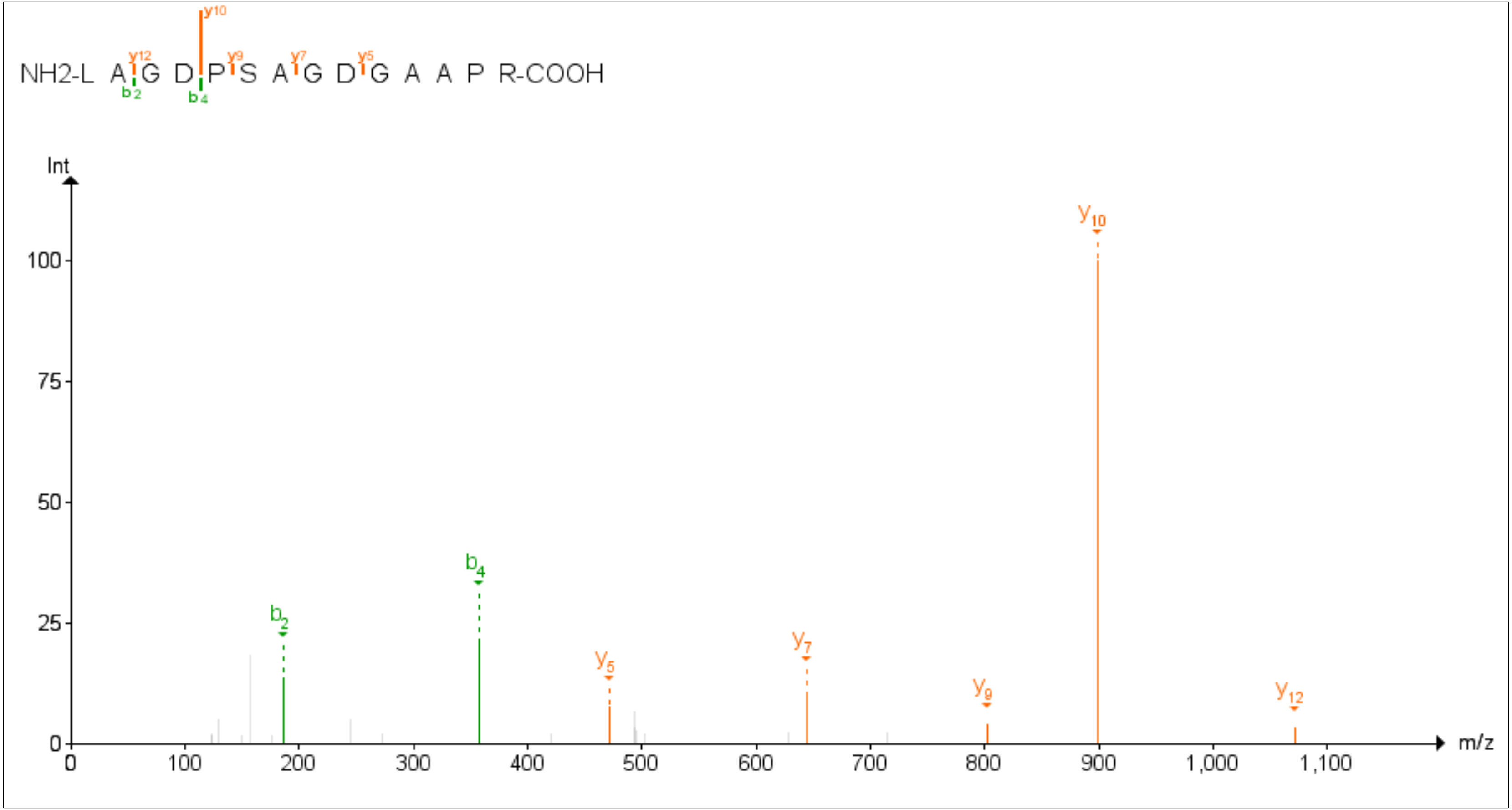

NH2-L A G D P S A G D G A A P R-COOH
y10
y12
y9
y7
y5
b2
b4
Int
100
75
50
25
0
m/z
0
100
200
300
400
500
600
700
800
900
1,000
1,100
y10
b4
b2
y5
y7
y9
y12

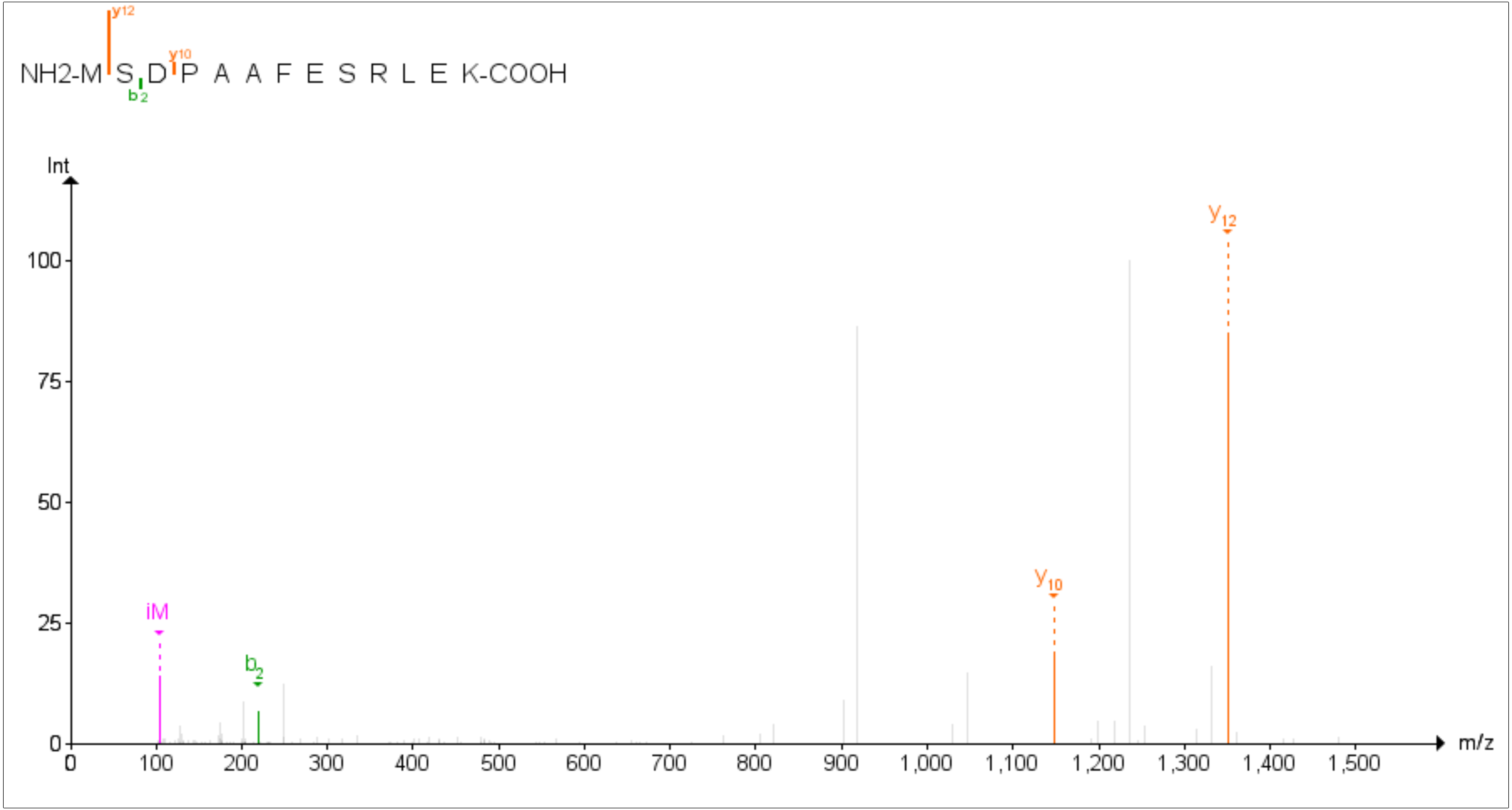

NH2-M S D P A A F E S R L E K-COOH
y12
y10
b2
Int
m/z
iM
0
25
50
75
100
100
200
300
400
500
600
700
800
900
1,000
1,100
1,200
1,300
1,400
1,500

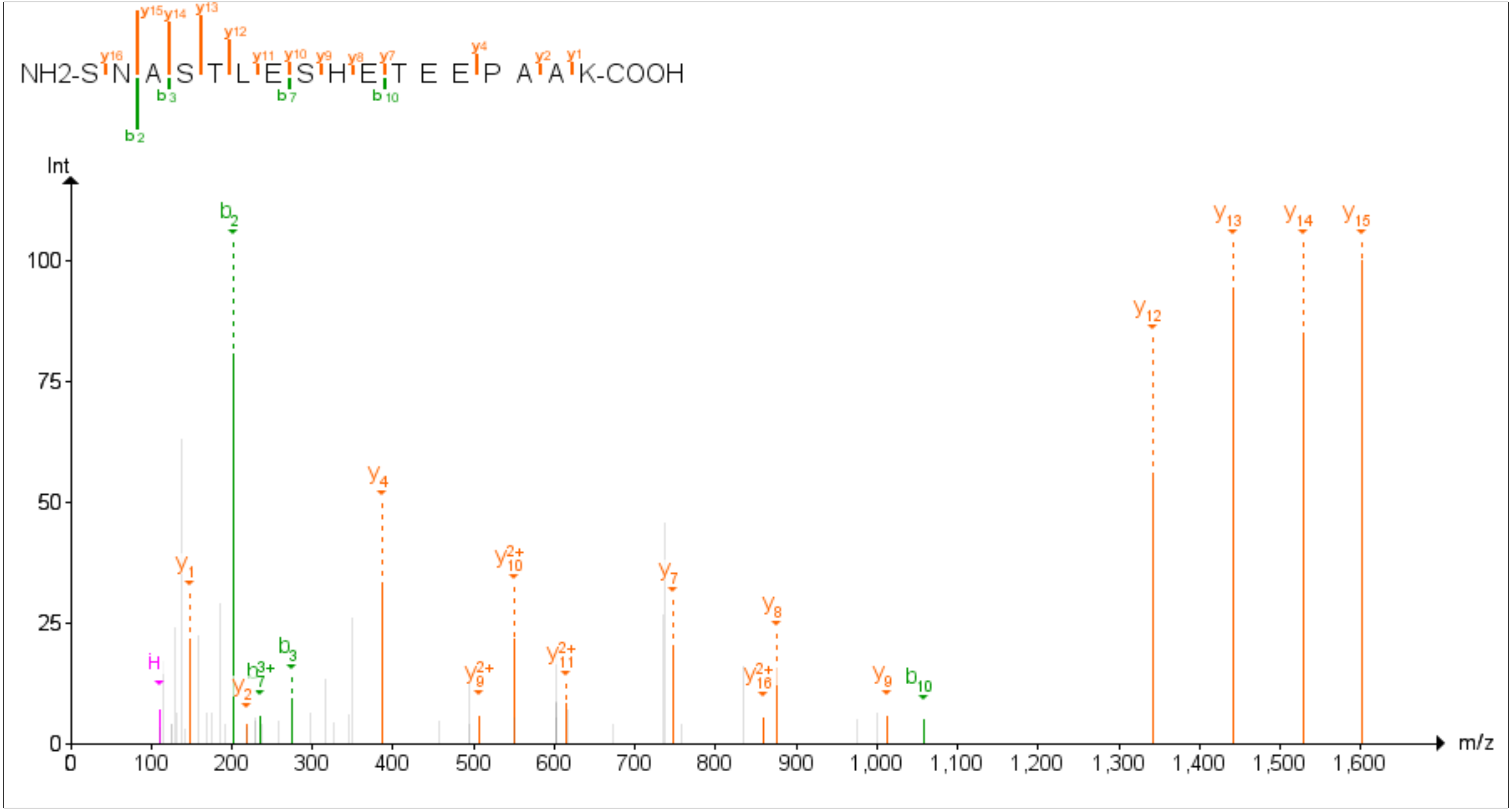

NH2-S N A S T L E S H E T E E P A A K-COOH
y16 y15 y14 y13 y12 y11 y10 y9 y8 y7 y4 y2 y1
b2 b3 b7 b10
Int
m/z
0
25
50
75
100
0 100 200 300 400 500 600 700 800 900 1,000 1,100 1,200 1,300 1,400 1,500 1,600
b2
y13 y14 y15
y12
y4
y1
y10 2+
y7
y8
H
y2
b7 3+
b3
y9 2+
y11 2+
y16 2+
y9
b10

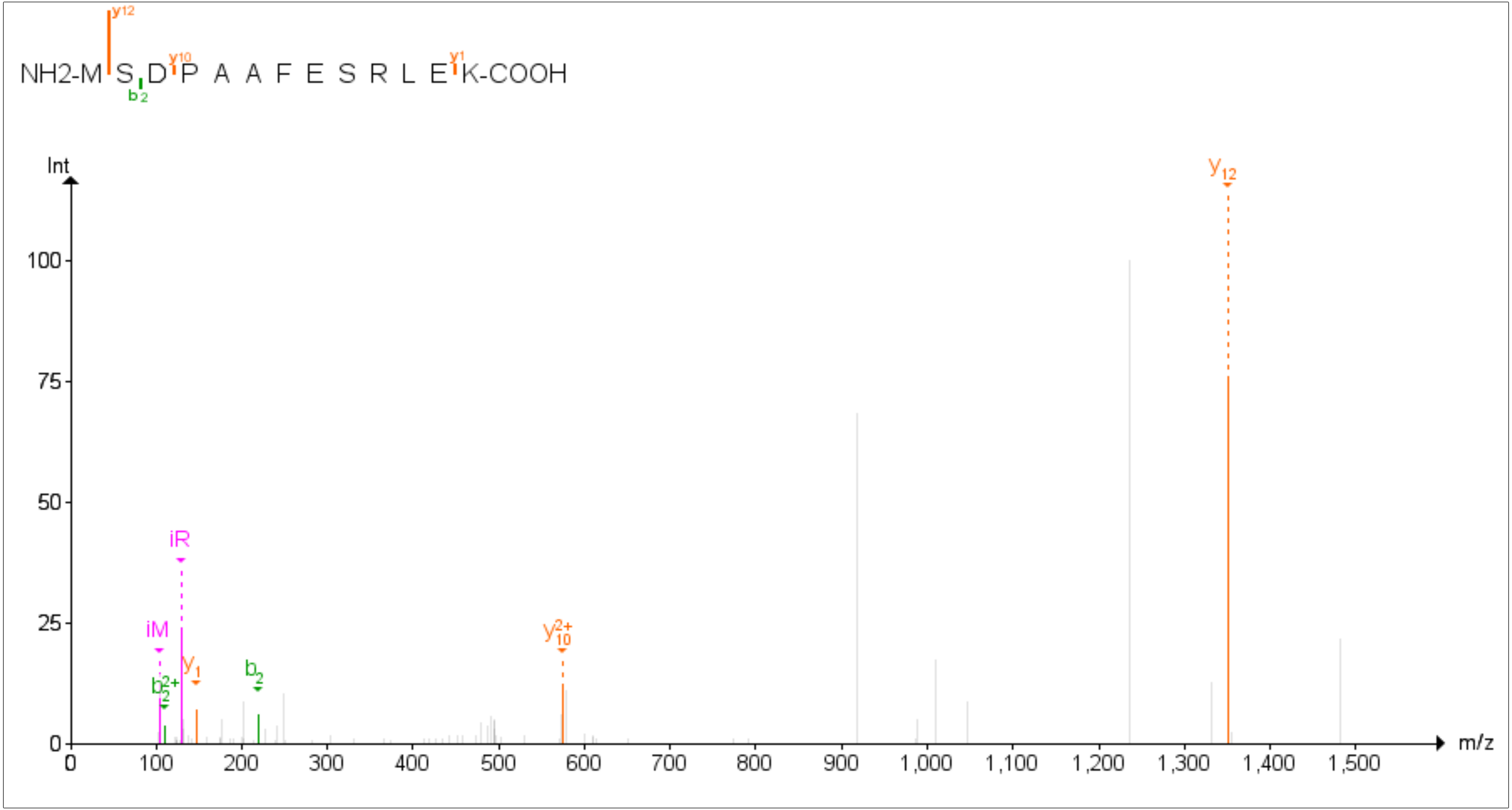

NH2-M S D P A A F E S R L E K-COOH
y12
y10
y1
b2
Int
100
75
50
25
0
y12
iR
iM
y1
b2
b_2^{2+}
y_{10}^{2+}
0
100
200
300
400
500
600
700
800
900
1,000
1,100
1,200
1,300
1,400
1,500
m/z

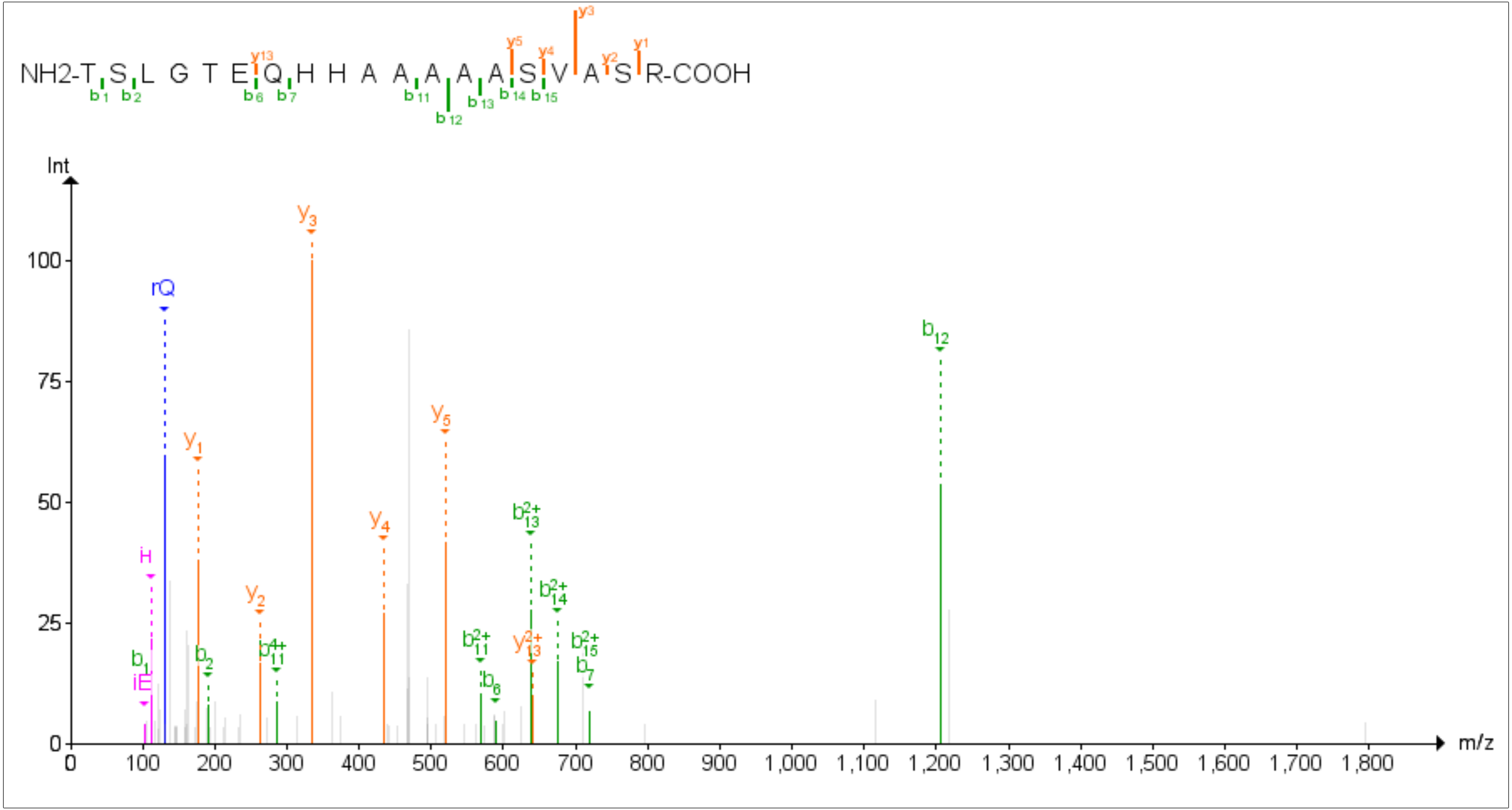

NH2-T S L G T E Q H H A A A A A S V A S R-COOH
b1 b2 b6 b7 b11 b12 b13 b14 b15
y13 y5 y4 y3 y2 y1
Int
rQ
y3
y1
y5
y4
y2
iH
b1
iE
b2
b11 4+
b11 2+
b13 2+
b14 2+
y13 2+
b15 2+
b6
b7
b12
m/z
0 25 50 75 100
0 100 200 300 400 500 600 700 800 900 1,000 1,100 1,200 1,300 1,400 1,500 1,600 1,700 1,800

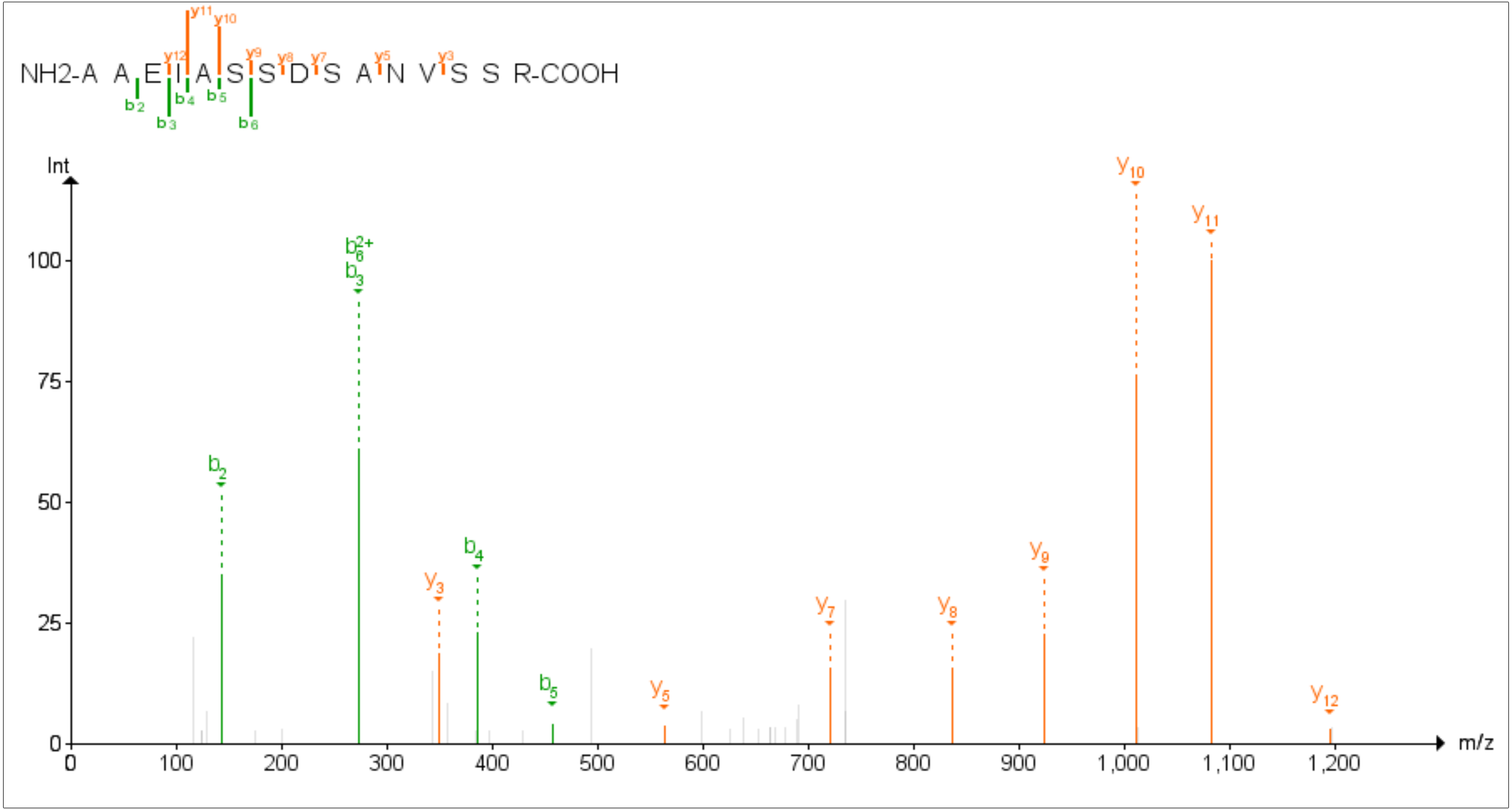

NH2-A A E I I A S S D S A N V S S R-COOH
Int
m/z

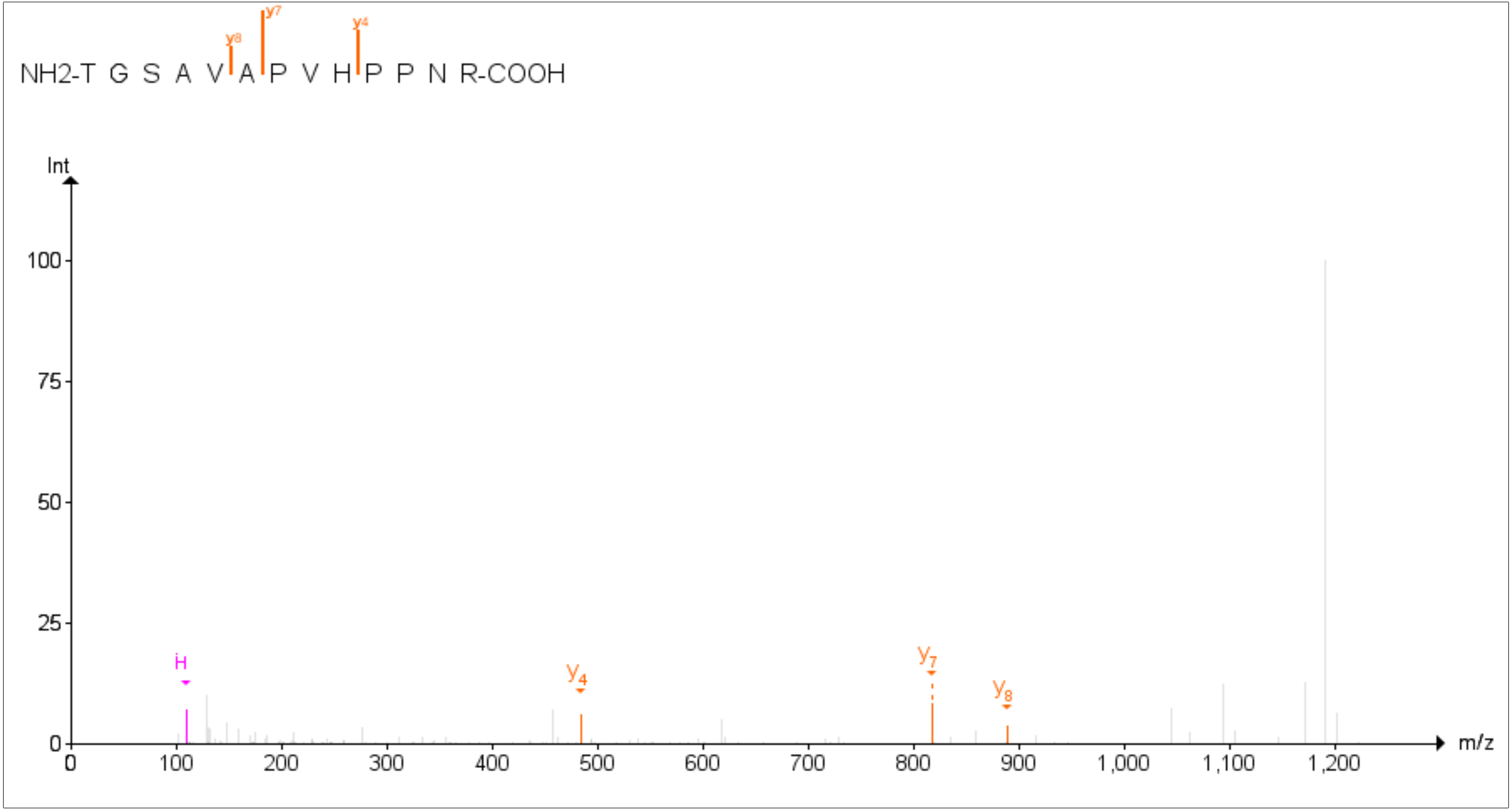
NH2-T G S A V A P V H P P N R-COOH
y8
y7
y4
Int
100
75
50
25
0
H
y4
y7
y8
0
100
200
300
400
500
600
700
800
900
1,000
1,100
1,200
m/z

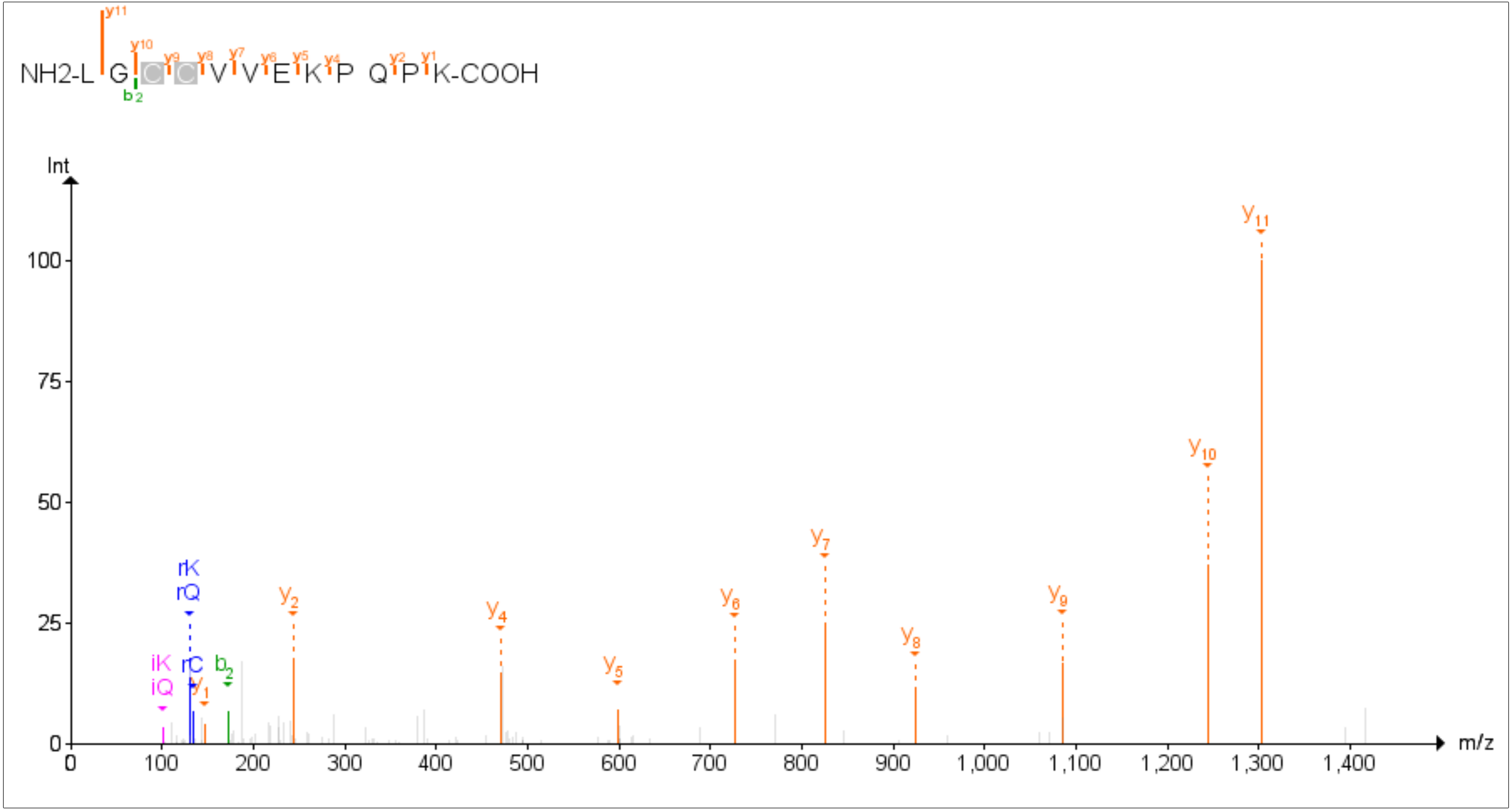

NH2-L G C C V V E K P Q P K-COOH
y11
y10
y9
y8
y7
y6
y5
y4
y2
y1
b2
Int
m/z
rK
rQ
rC
iK
iQ

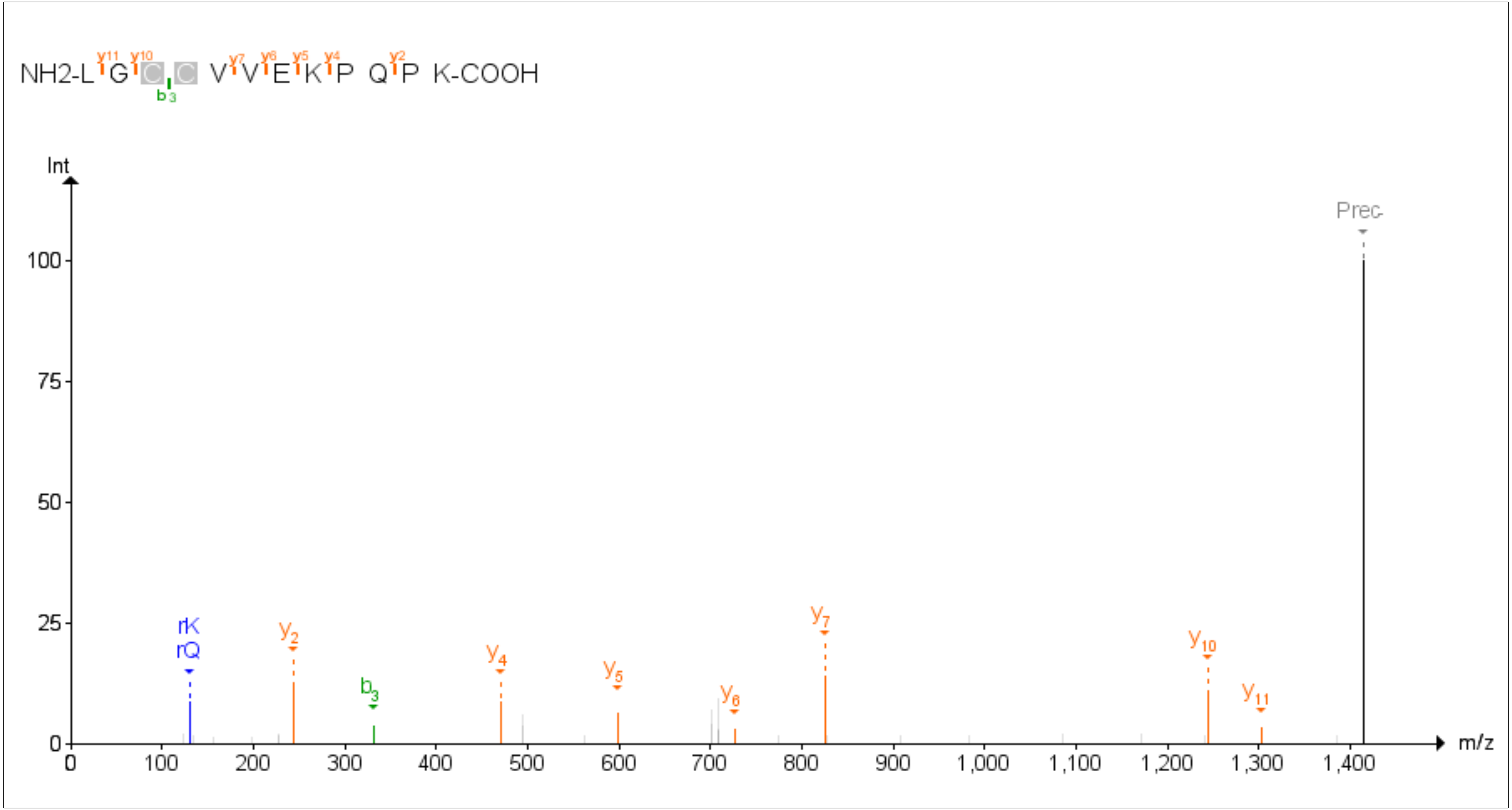
NH2-L G C C V V E K P Q P K-COOH
y11 y10 y7 y6 y5 y4 y2
b3
Int
100
75
50
25
0
Prec
rK
rQ
y2
b3
y4
y5
y6
y7
y10
y11
0 100 200 300 400 500 600 700 800 900 1,000 1,100 1,200 1,300 1,400
m/z

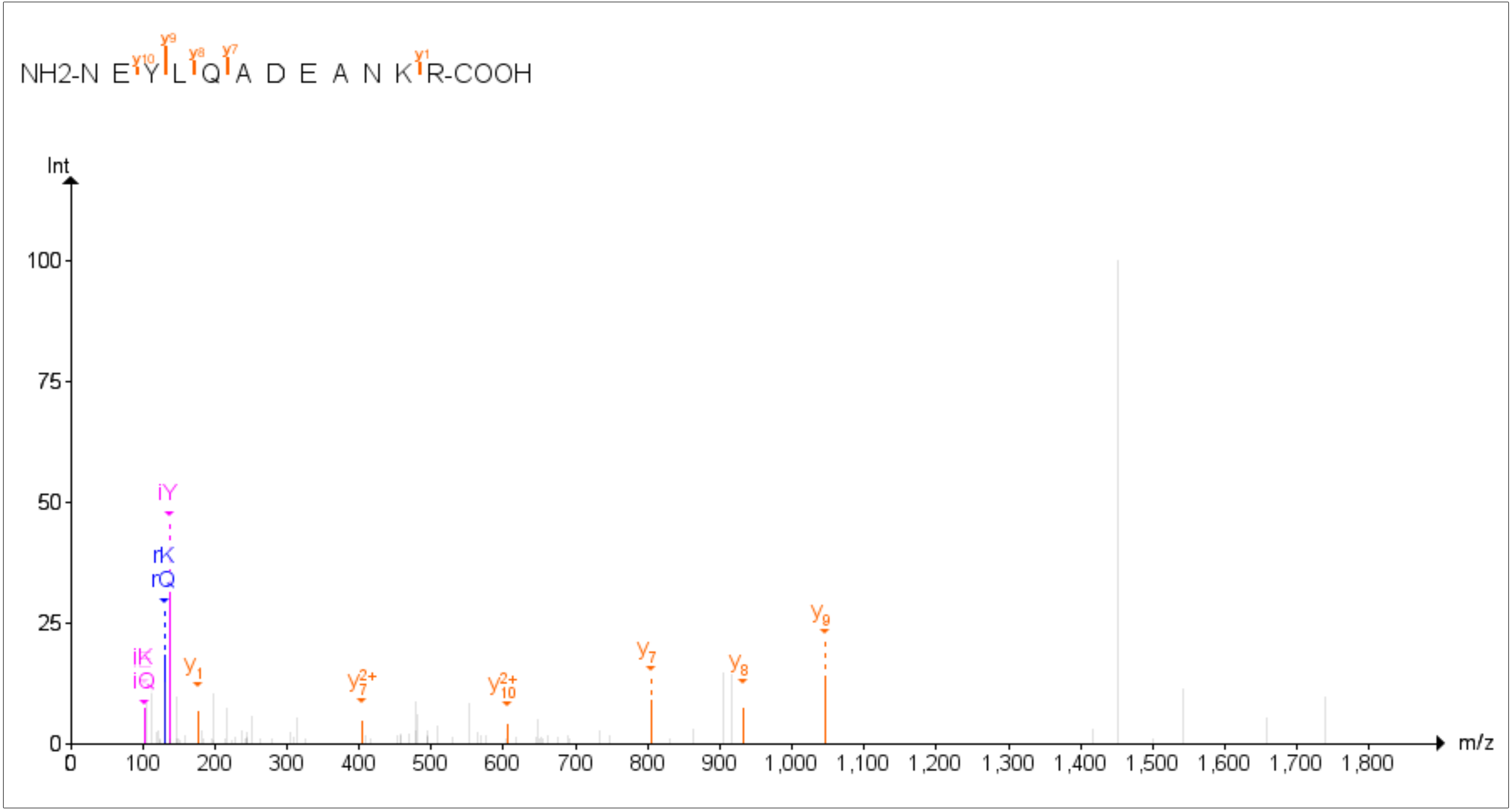

NH2-N E Y L Q A D E A N K R-COOH
y10 y9 y8 y7 y1
Int
100
75
50
25
0
iY
rK
rQ
iK
iQ
y1
y7 2+
y10 2+
y7
y8
y9
0 100 200 300 400 500 600 700 800 900 1,000 1,100 1,200 1,300 1,400 1,500 1,600 1,700 1,800
m/z

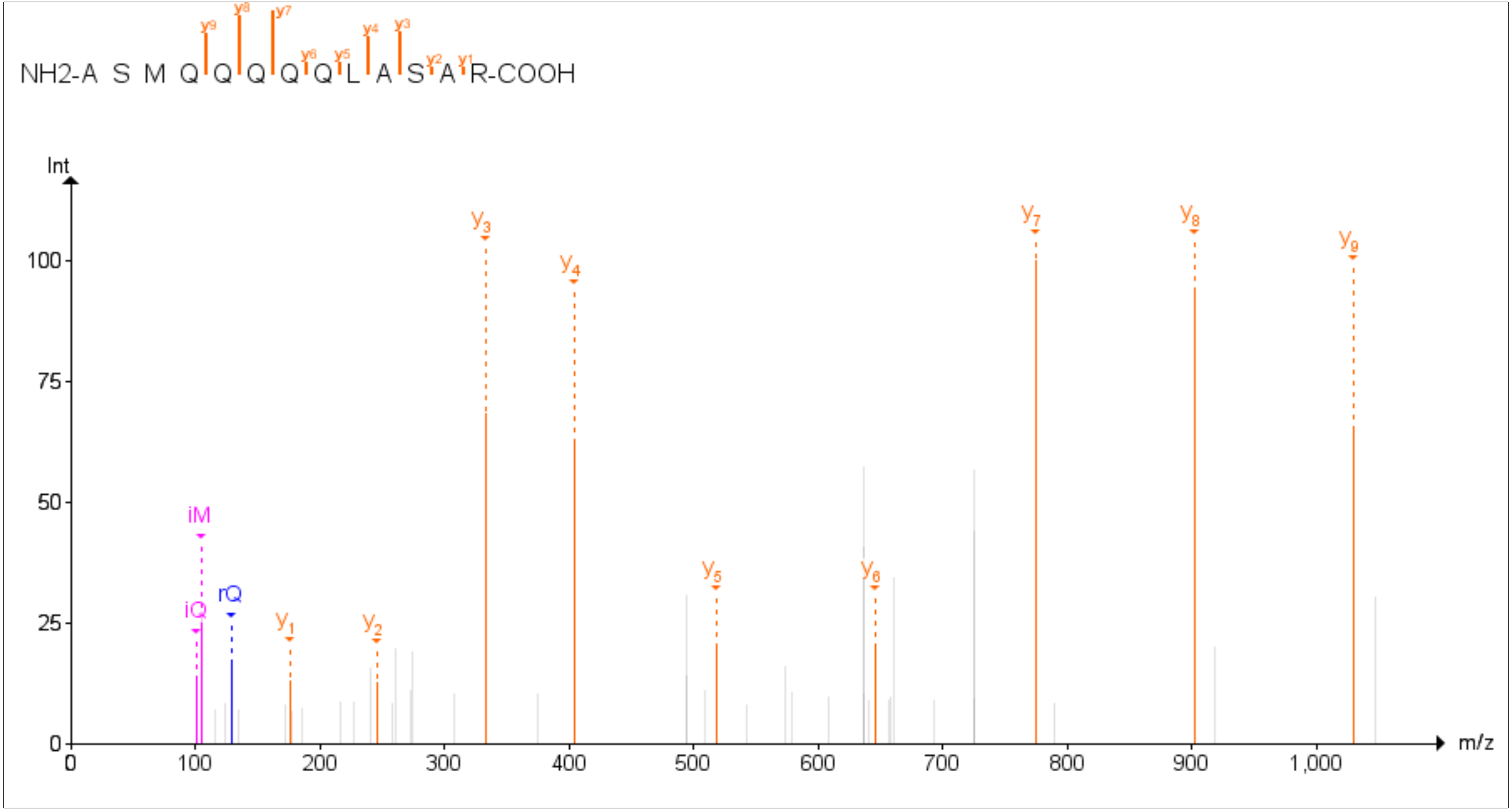
NH2-A S M Q Q Q Q Q L A S A R-COOH
y9 y8 y7 y6 y5 y4 y3 y2 y1
Int
100
75
50
25
0
iM
iQ
rQ
y1 y2 y3 y4 y5 y6 y7 y8 y9
0 100 200 300 400 500 600 700 800 900 1,000
m/z

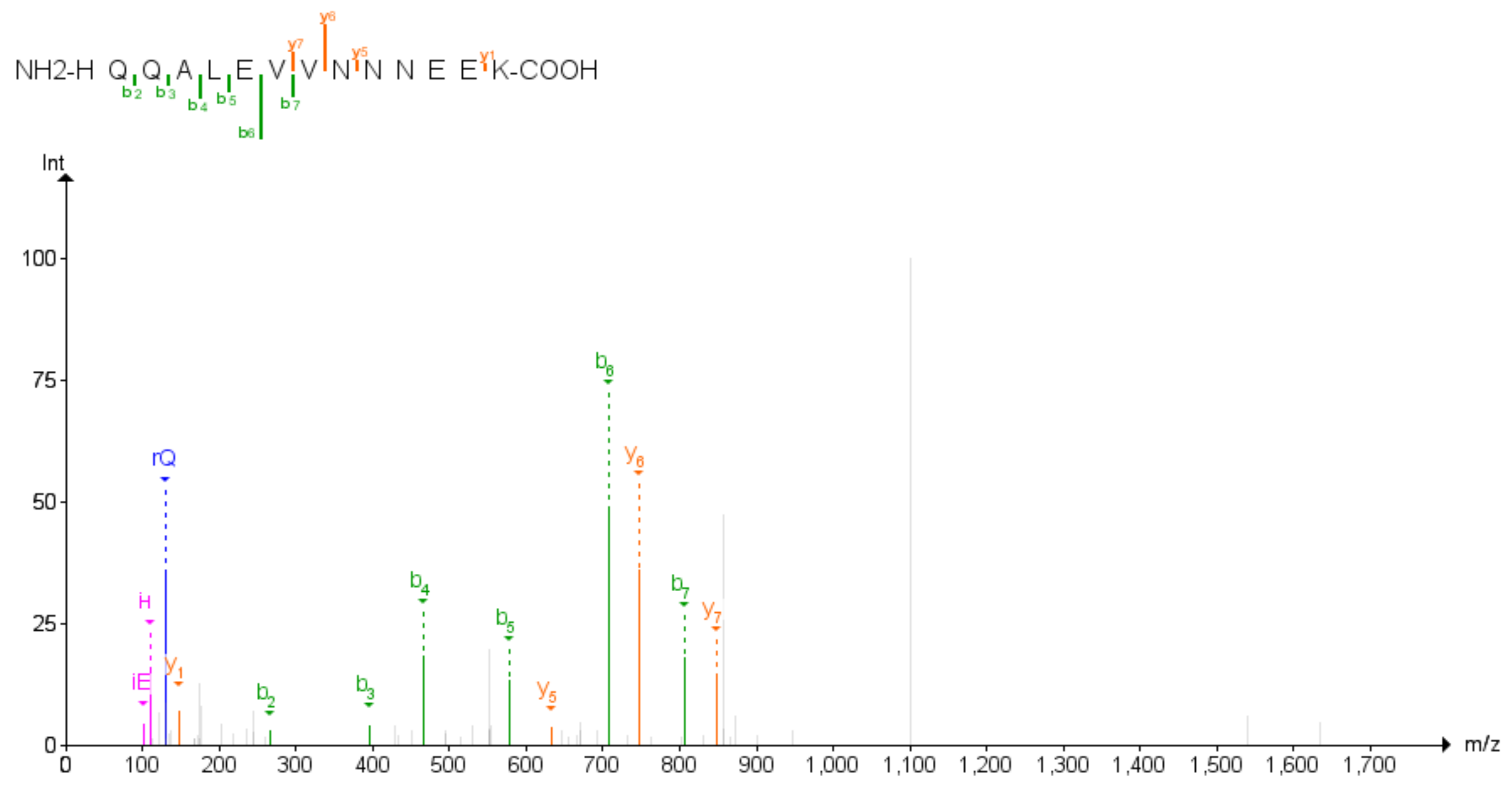

NH2-H Q Q A L E V V N N N E E K-COOH
b2 b3 b4 b5 b6 b7
y7 y6 y5 y1
Int
m/z
rQ
iH
iE
y1
b2
b3
b4
b5
y5
b6
y6
b7
y7

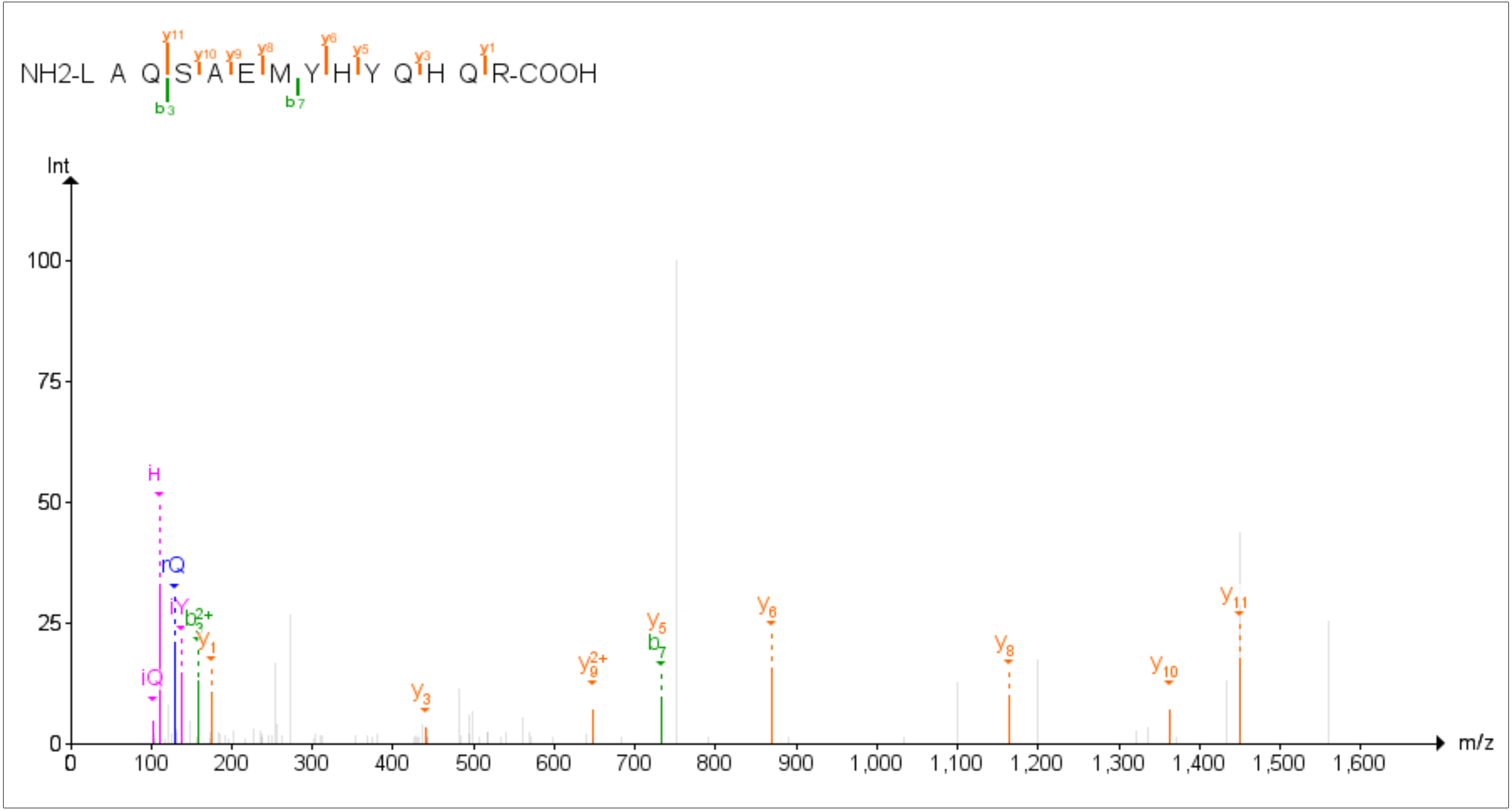
NH2-L A Q S A E M Y H Y Q H Q R-COOH
y11 y10 y9 y8 y6 y5 y3 y1
b3 b7
Int
m/z
0
25
50
75
100
100
200
300
400
500
600
700
800
900
1,000
1,100
1,200
1,300
1,400
1,500
1,600
iH
rQ
iY
iQ
y1
y3
y5
b7
y6
y8
y10
y11

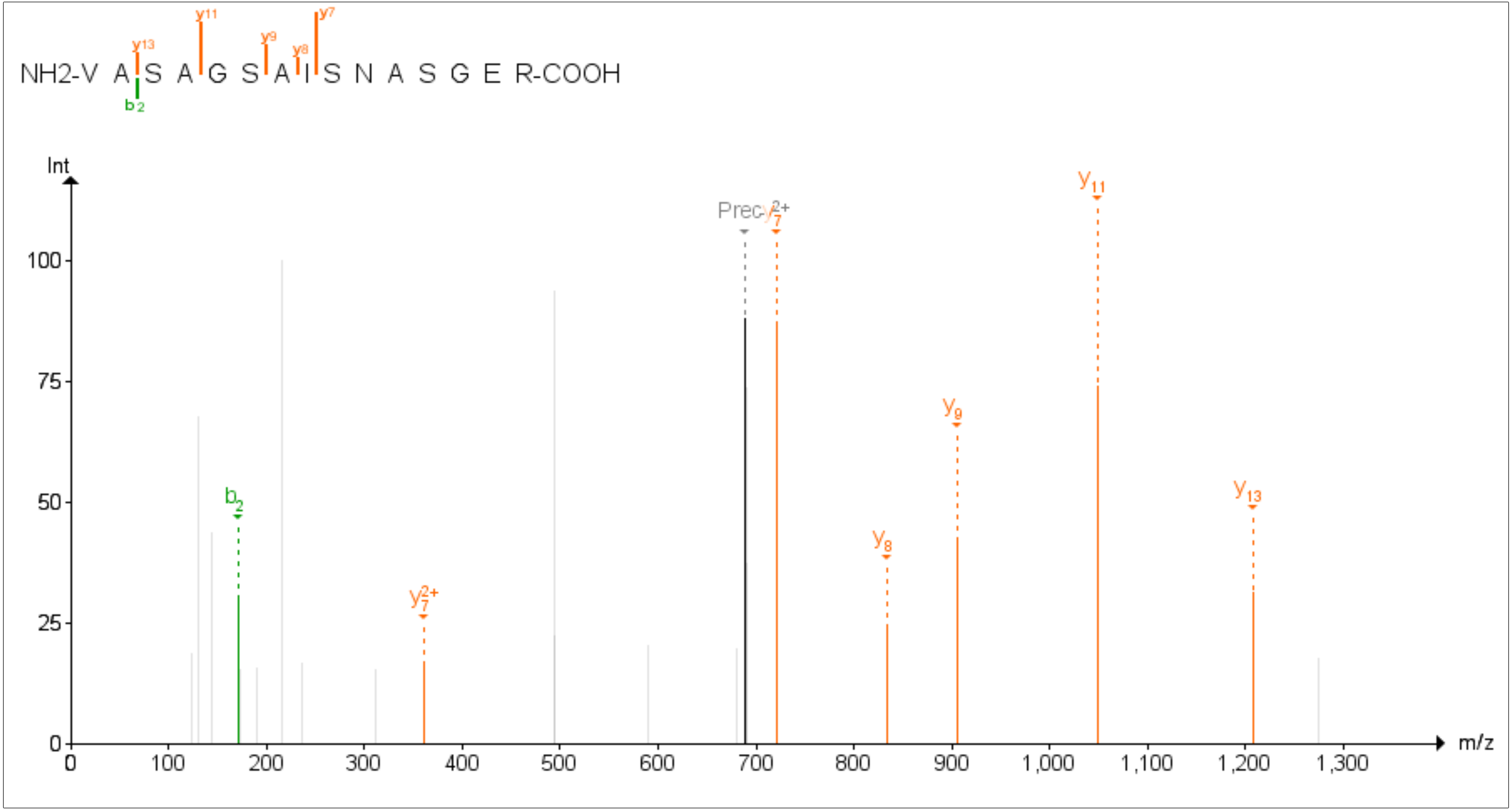

NH2-V A S A G S A I S N A S G E R-COOH
y13
y11
y9
y8
y7
b2
Int
100
75
50
25
0
b2
y7 2+
Prec
y7 2+
y11
y9
y8
y13
m/z
0
100
200
300
400
500
600
700
800
900
1,000
1,100
1,200
1,300

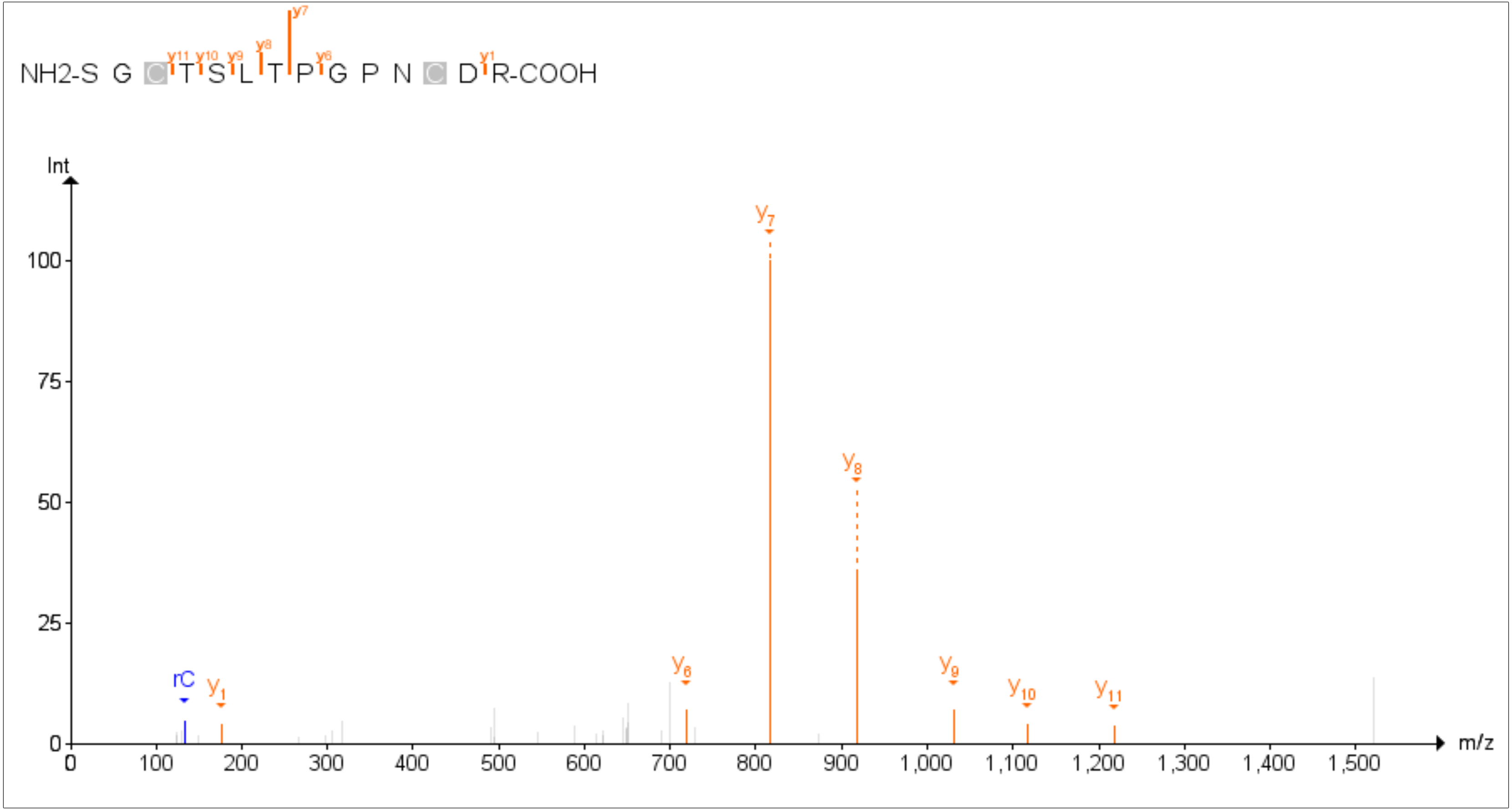

NH2-S G C T S L T P G P N C D R-COOH
y11 y10 y9 y8 y7 y6 y1
Int
100
75
50
25
0
rC
y1
y6
y7
y8
y9
y10
y11
0 100 200 300 400 500 600 700 800 900 1,000 1,100 1,200 1,300 1,400 1,500
m/z

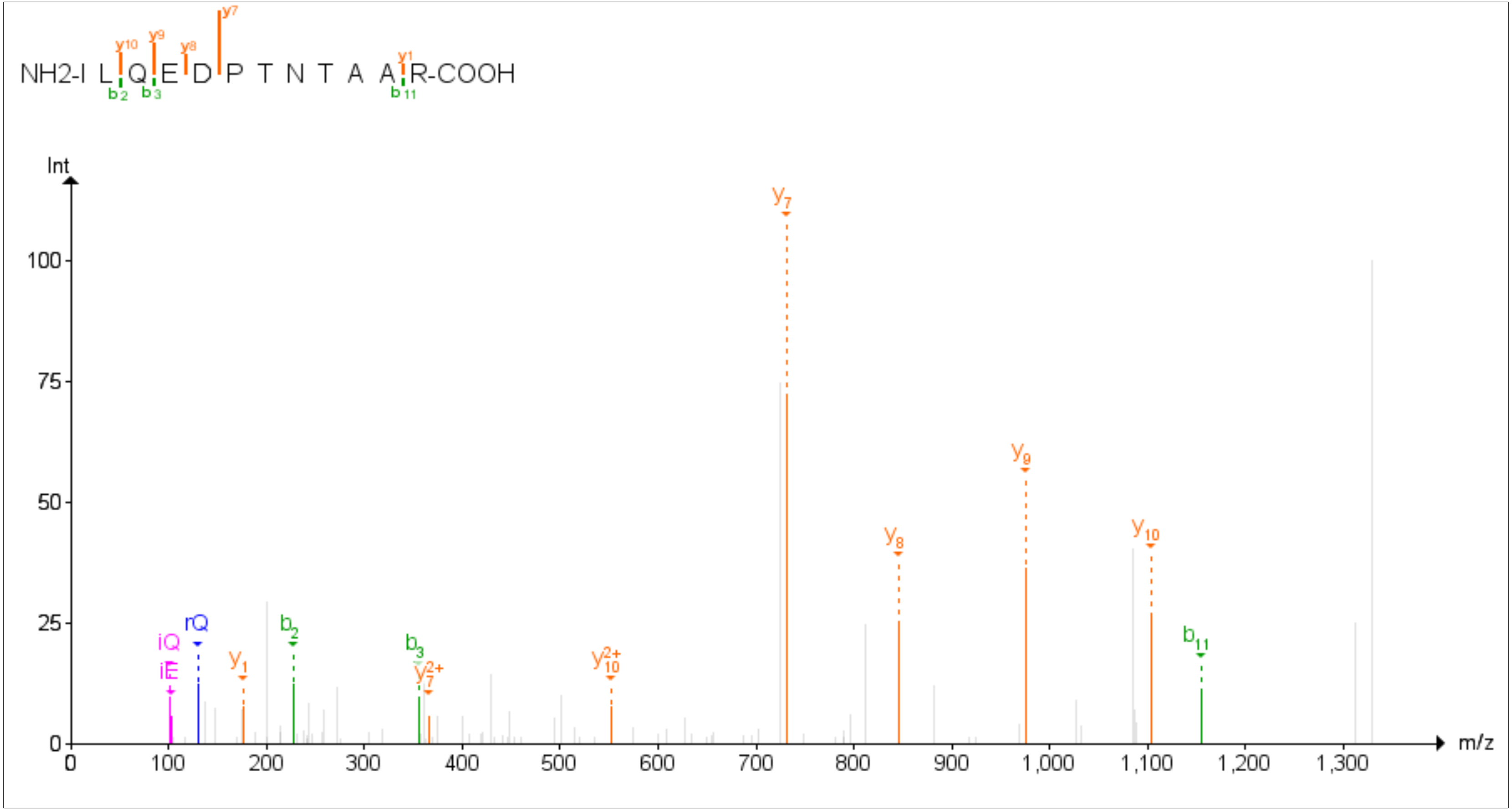

NH2-I L Q E D P T N T A A R-COOH
y10
y9
y8
y7
y1
b2
b3
b11
Int
m/z
y7
y8
y9
y10
b11
b2
b3
y1
rQ
iQ
iE
y7 2+
y10 2+
0
25
50
75
100
0
100
200
300
400
500
600
700
800
900
1,000
1,100
1,200
1,300

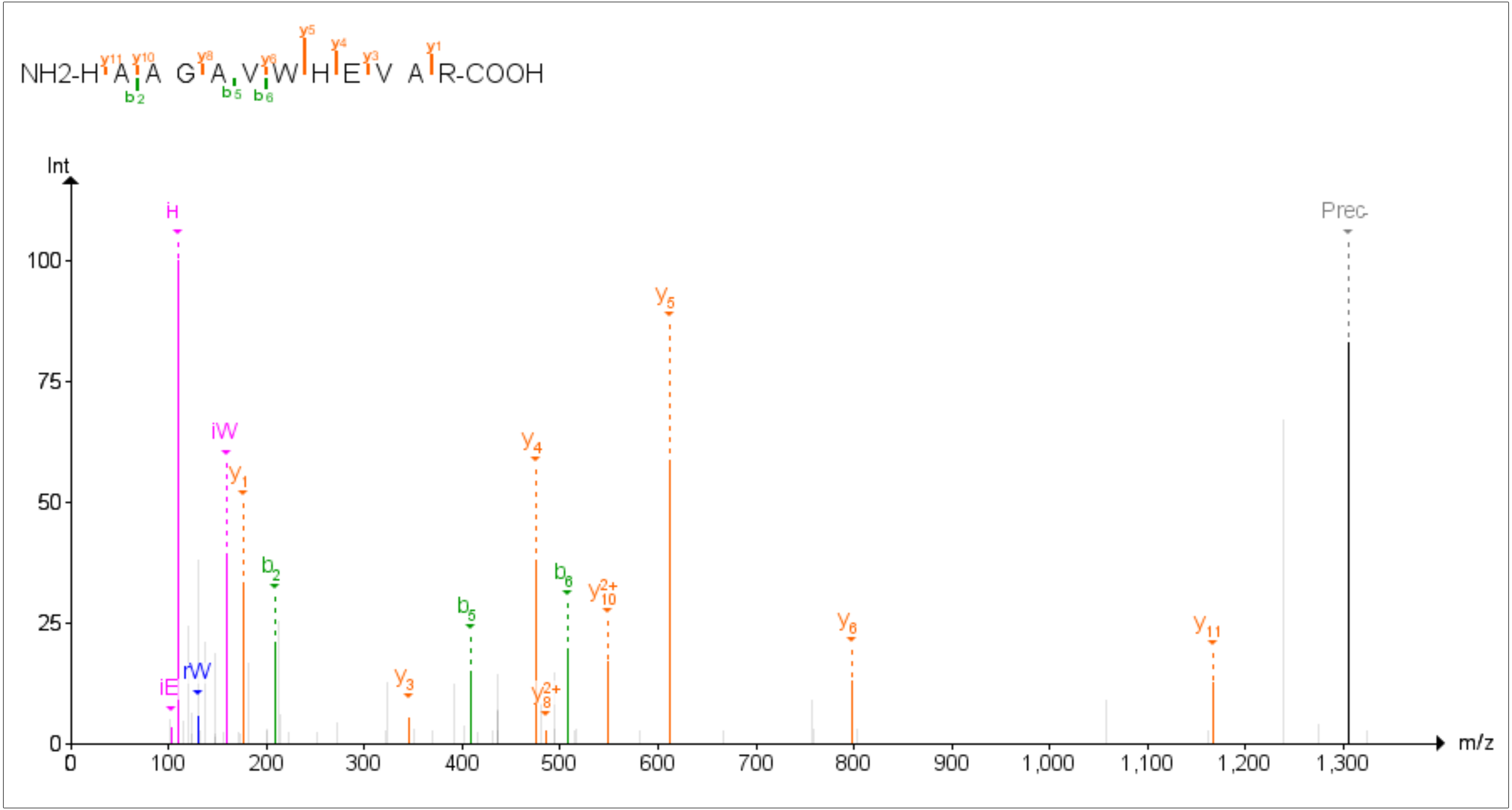
NH2-H A A G A V W H E V A R-COOH
y11 y10 y8 y6 y5 y4 y3 y1
b2 b5 b6
Int
m/z
iH
iW
y1
b2
y3
b5
y4
y8 2+
b6
y10 2+
y5
y6
y11
Prec.
iE
rW
0
25
50
75
100
0 100 200 300 400 500 600 700 800 900 1,000 1,100 1,200 1,300

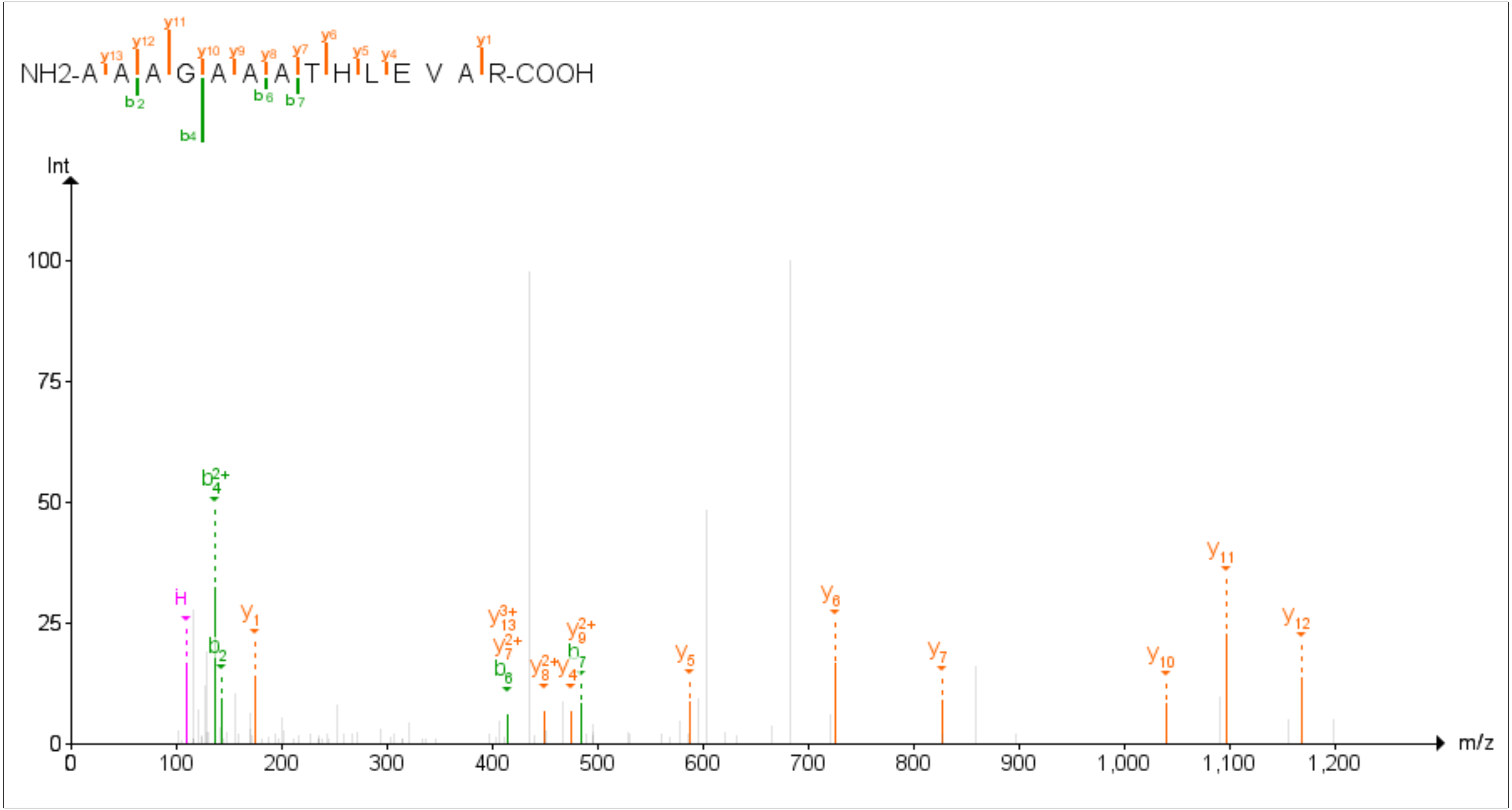

NH2-A A A G A A A A T H L E V A R-COOH
y13 y12 y11 y10 y9 y8 y7 y6 y5 y4 y1
b2 b4 b6 b7
Int
m/z
b4 2+
H
y1
b2
y13 3+
y7 2+
b6
y8 2+
y4
y9 2+
b7
y5
y6
y7
y10
y11
y12

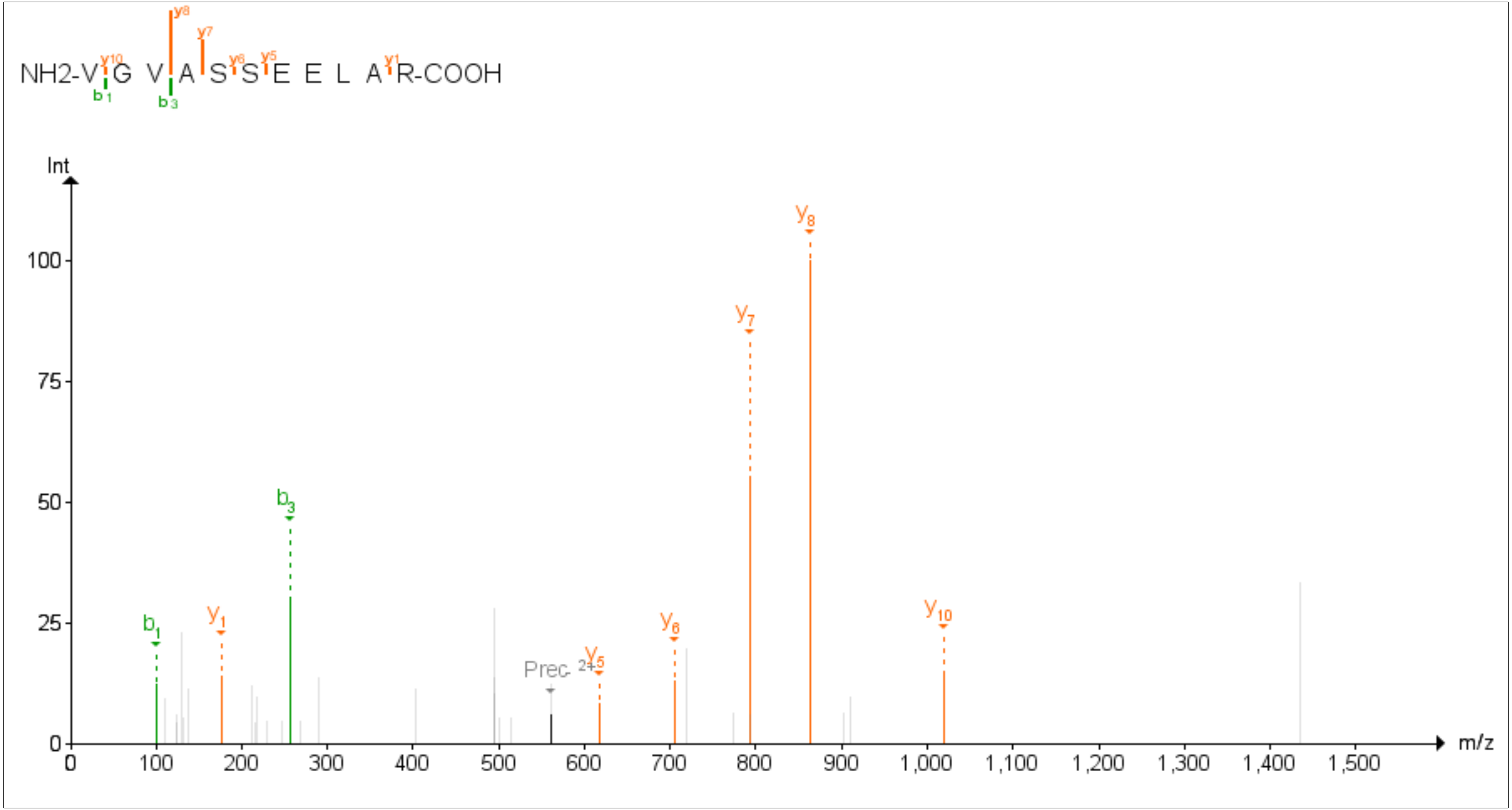
NH2-V G V A S S E E L A R-COOH
y10 y8 y7 y6 y5 y1
b1 b3
Int
100
75
50
25
0
b1
y1
b3
Prec- 2+
y5
y6
y7
y8
y10
0 100 200 300 400 500 600 700 800 900 1,000 1,100 1,200 1,300 1,400 1,500
m/z

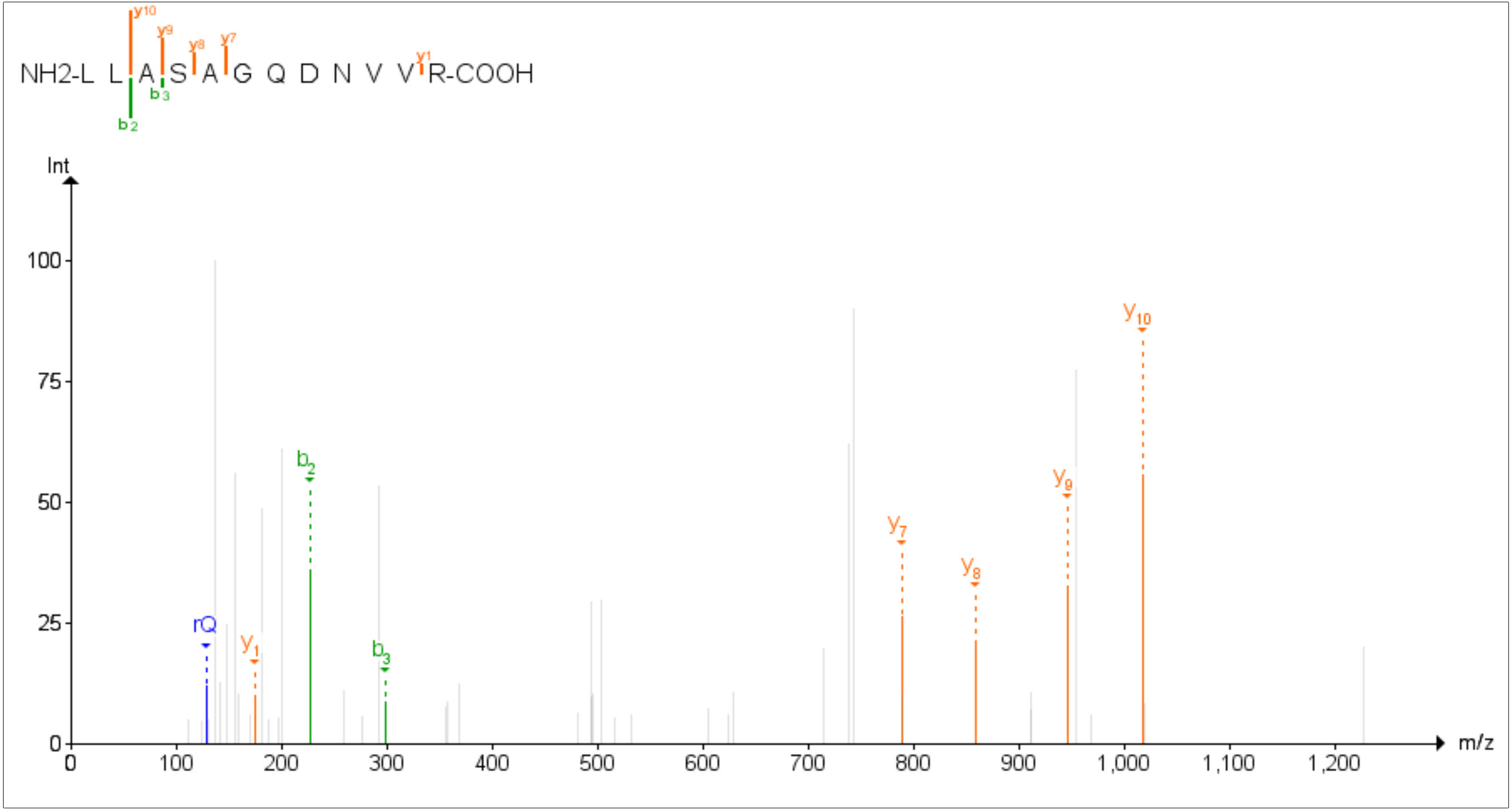

NH2-L L A S A G Q D N V V R-COOH
y10
y9
y8
y7
y1
b2
b3
Int
100
75
50
25
0
rQ
y1
b2
b3
y7
y8
y9
y10
0
100
200
300
400
500
600
700
800
900
1,000
1,100
1,200
m/z

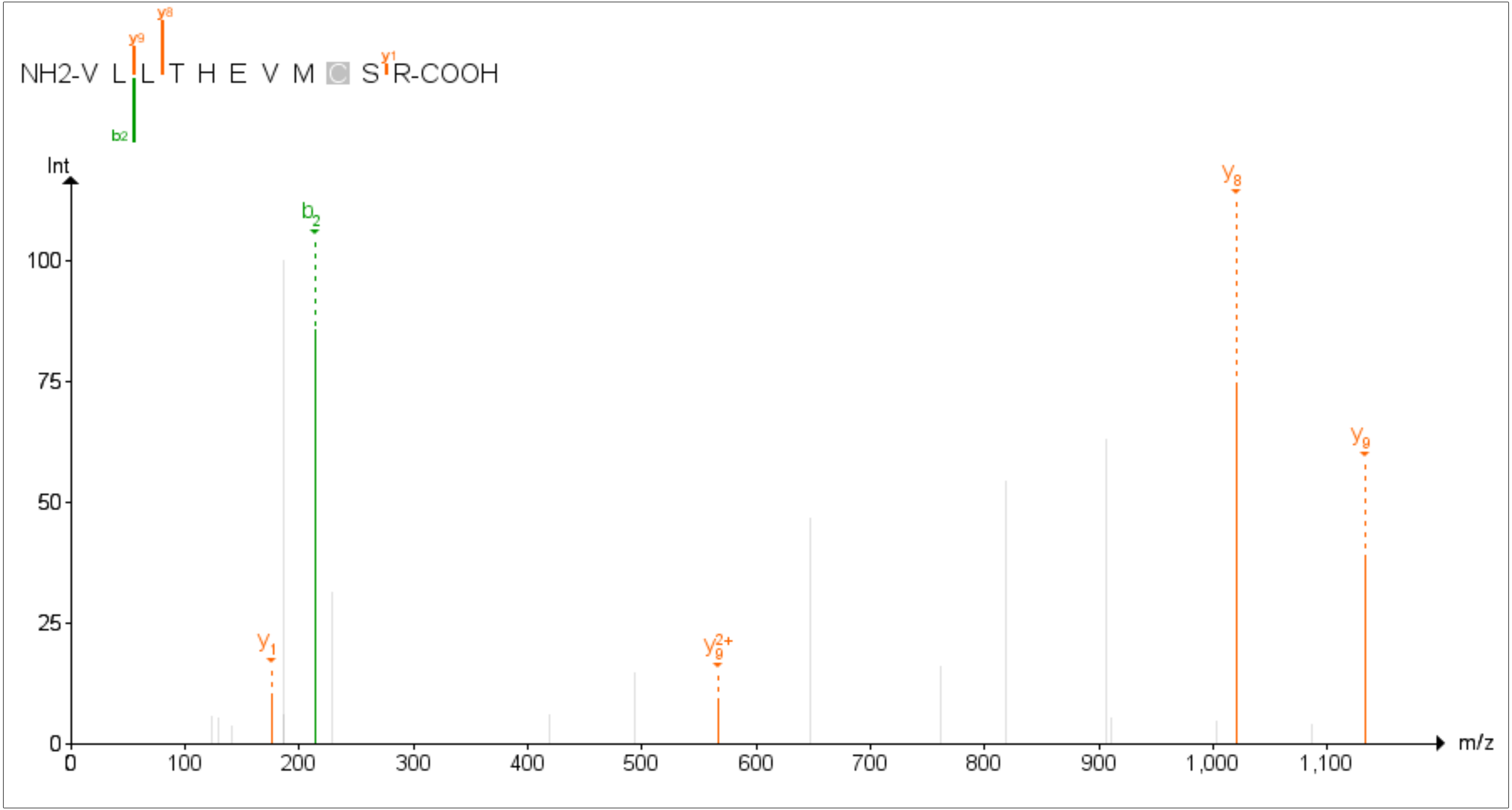
NH2-V L L T H E V M C S R-COOH
y8
y9
y1
b2
Int
100
75
50
25
0
b2
y1
y9 2+
y8
y9
m/z
0
100
200
300
400
500
600
700
800
900
1,000
1,100

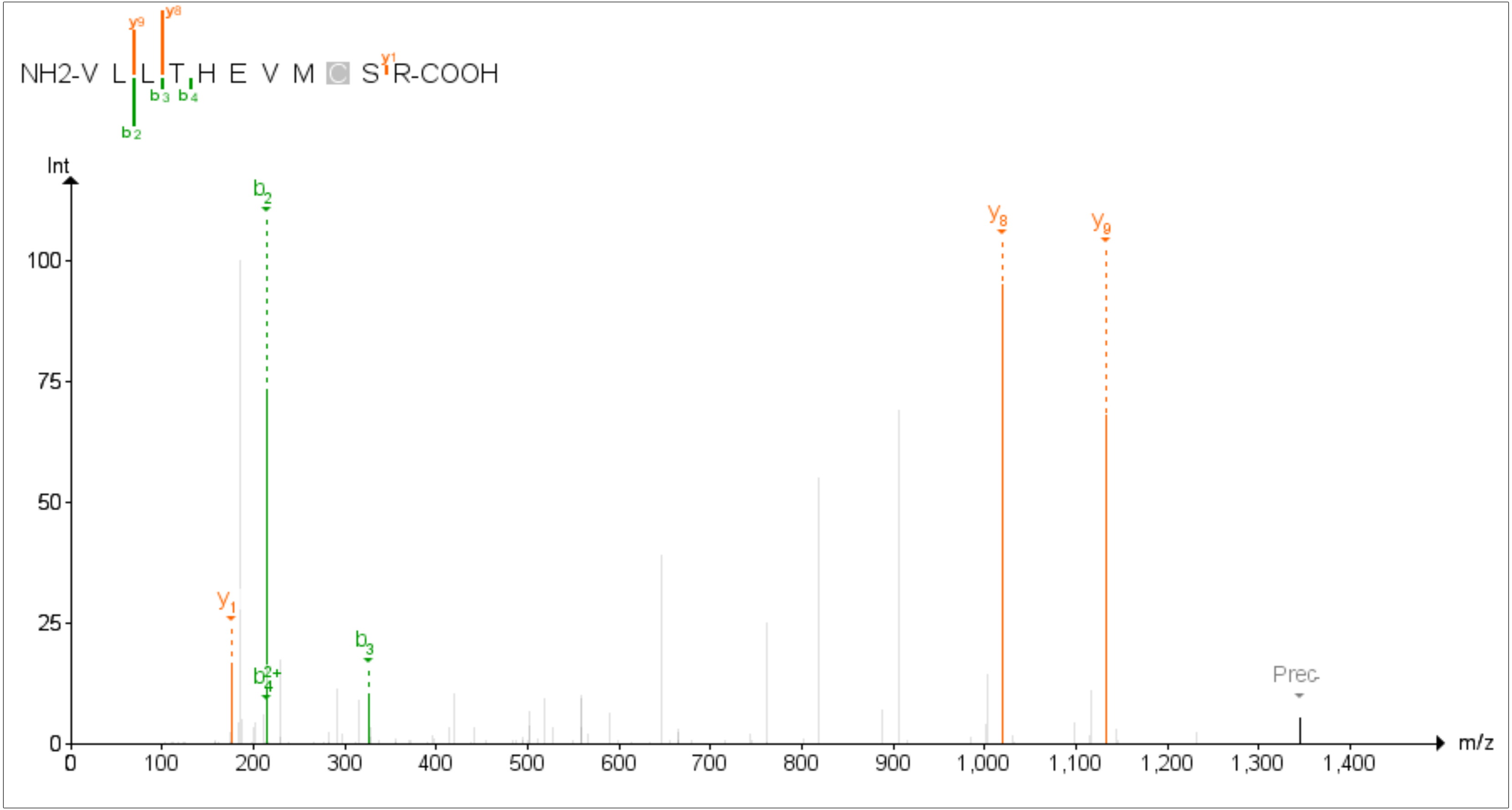

NH2-V L L T H E V M C S R-COOH
y9
y8
y1
b2
b3
b4
Int
100
75
50
25
0
b2
y8
y9
y1
b3
b4 2+
Prec.
0
100
200
300
400
500
600
700
800
900
1,000
1,100
1,200
1,300
1,400
m/z

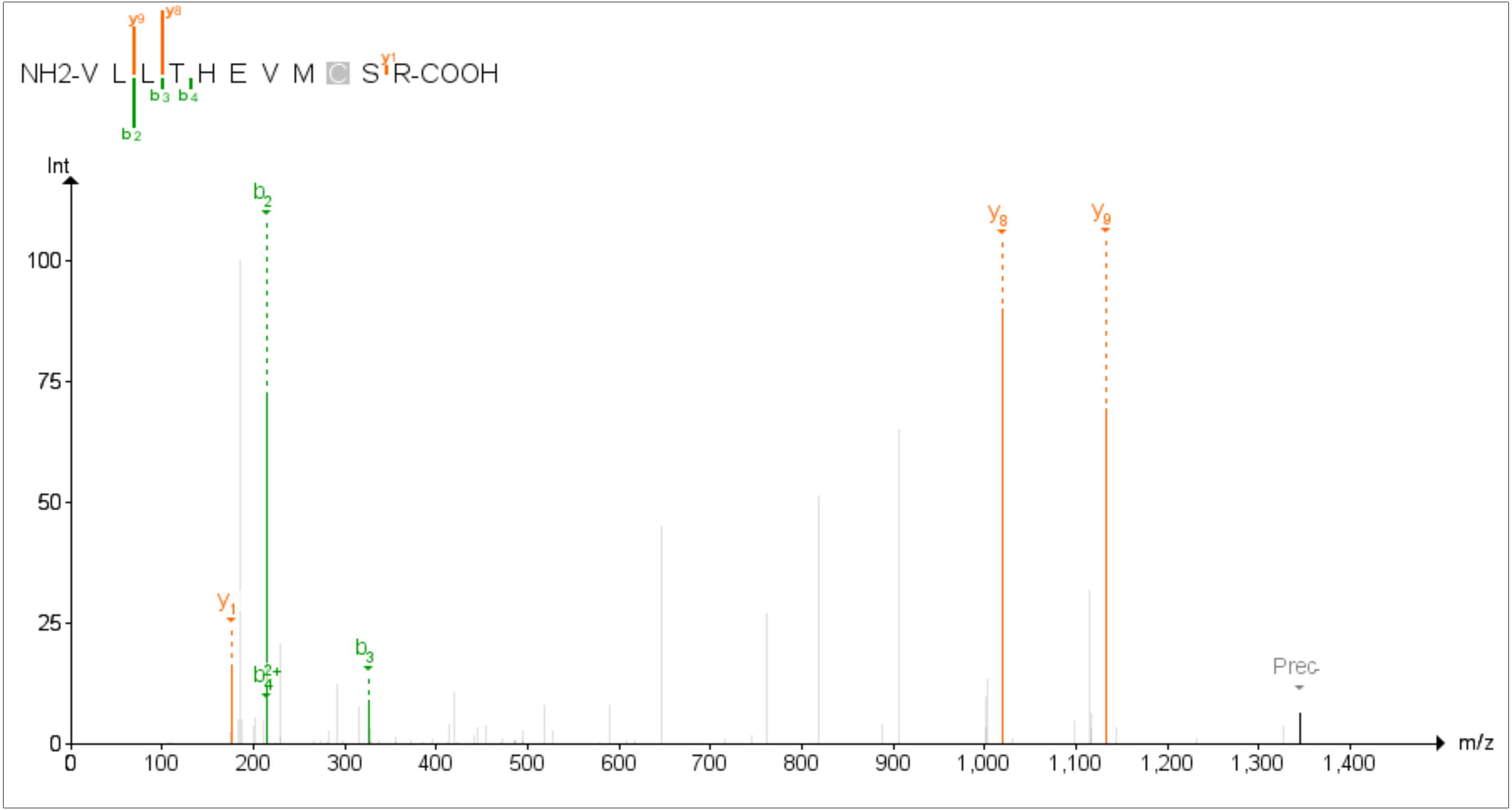

NH2-V L L T H E V M C S R-COOH
y9
y8
y1
b2
b3
b4
Int
100
75
50
25
0
b2
y8
y9
y1
b4 2+
b3
Prec
0
100
200
300
400
500
600
700
800
900
1,000
1,100
1,200
1,300
1,400
m/z

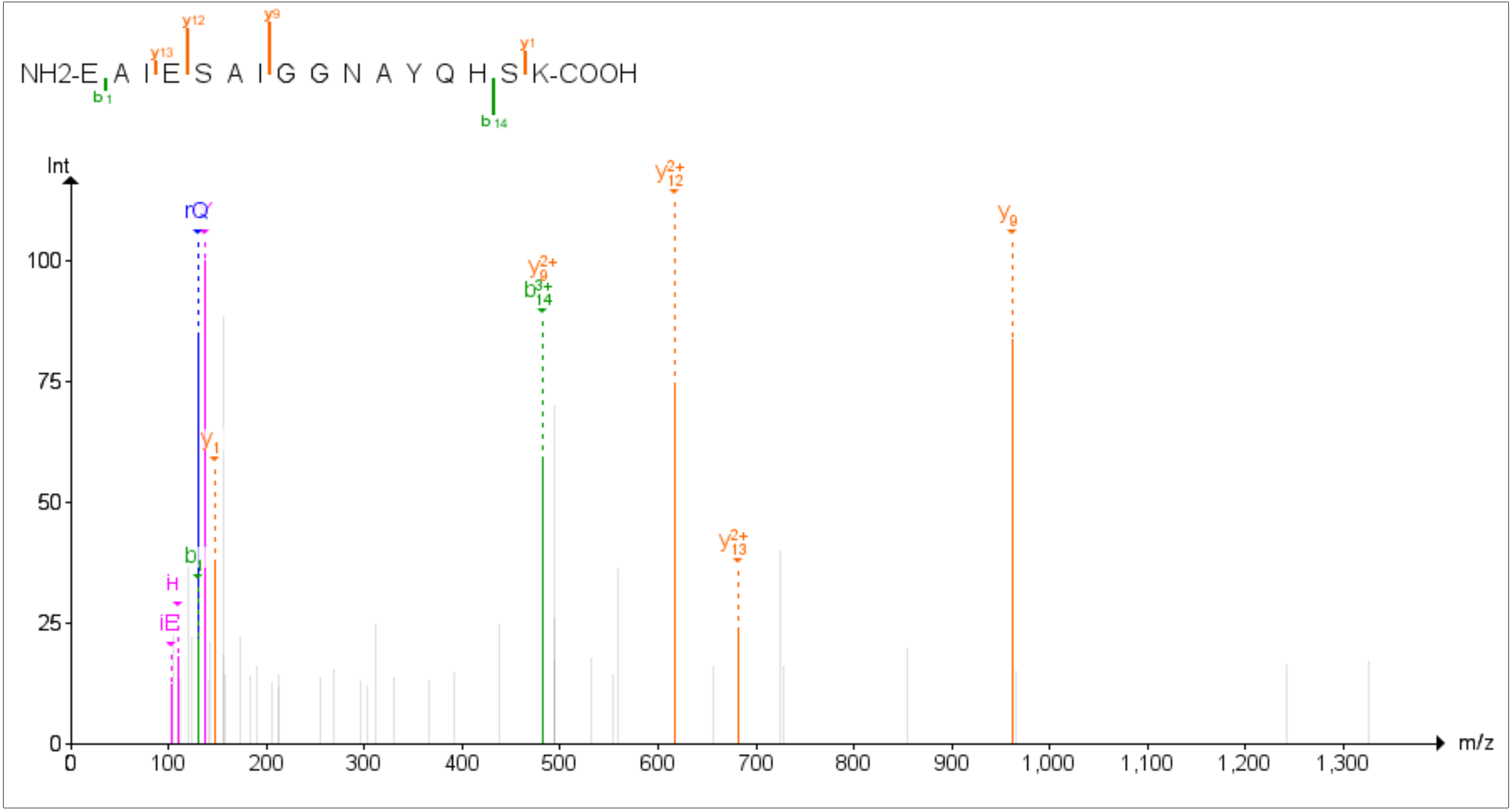

NH2-E A I E S A I G G N A Y Q H S K-COOH
b 1
b 14
y13
y12
y9
y1
Int
rQ
y12 2+
y9
y9 2+
b14 3+
y1
b1
H
iE
y13 2+
0
25
50
75
100
0
100
200
300
400
500
600
700
800
900
1,000
1,100
1,200
1,300
m/z

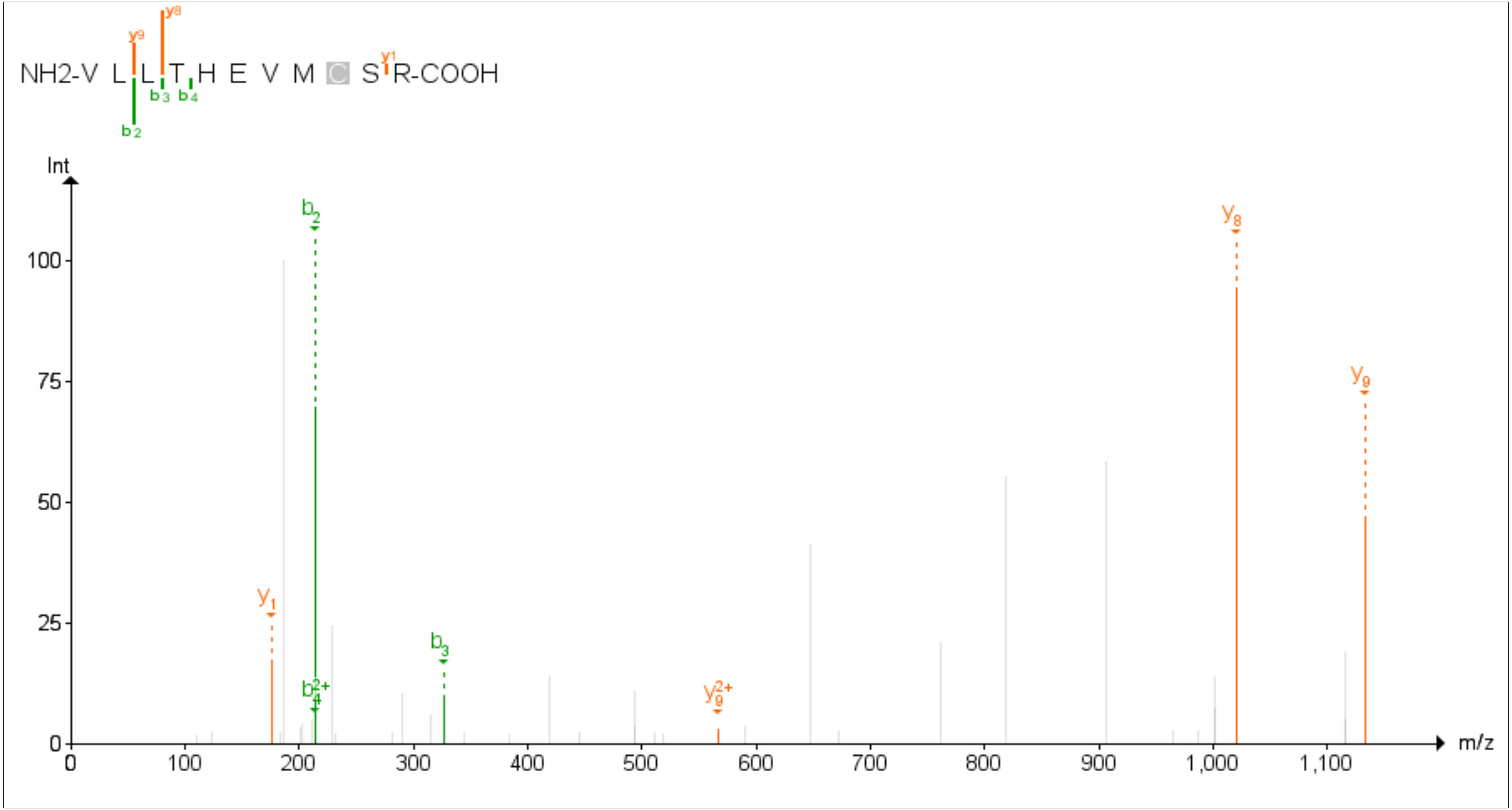

NH2-V L L T H E V M C S R-COOH
y9
y8
y1
b2
b3
b4
Int
100
75
50
25
0
b2
y8
y9
y1
b3
b4 2+
y9 2+
0
100
200
300
400
500
600
700
800
900
1,000
1,100
m/z

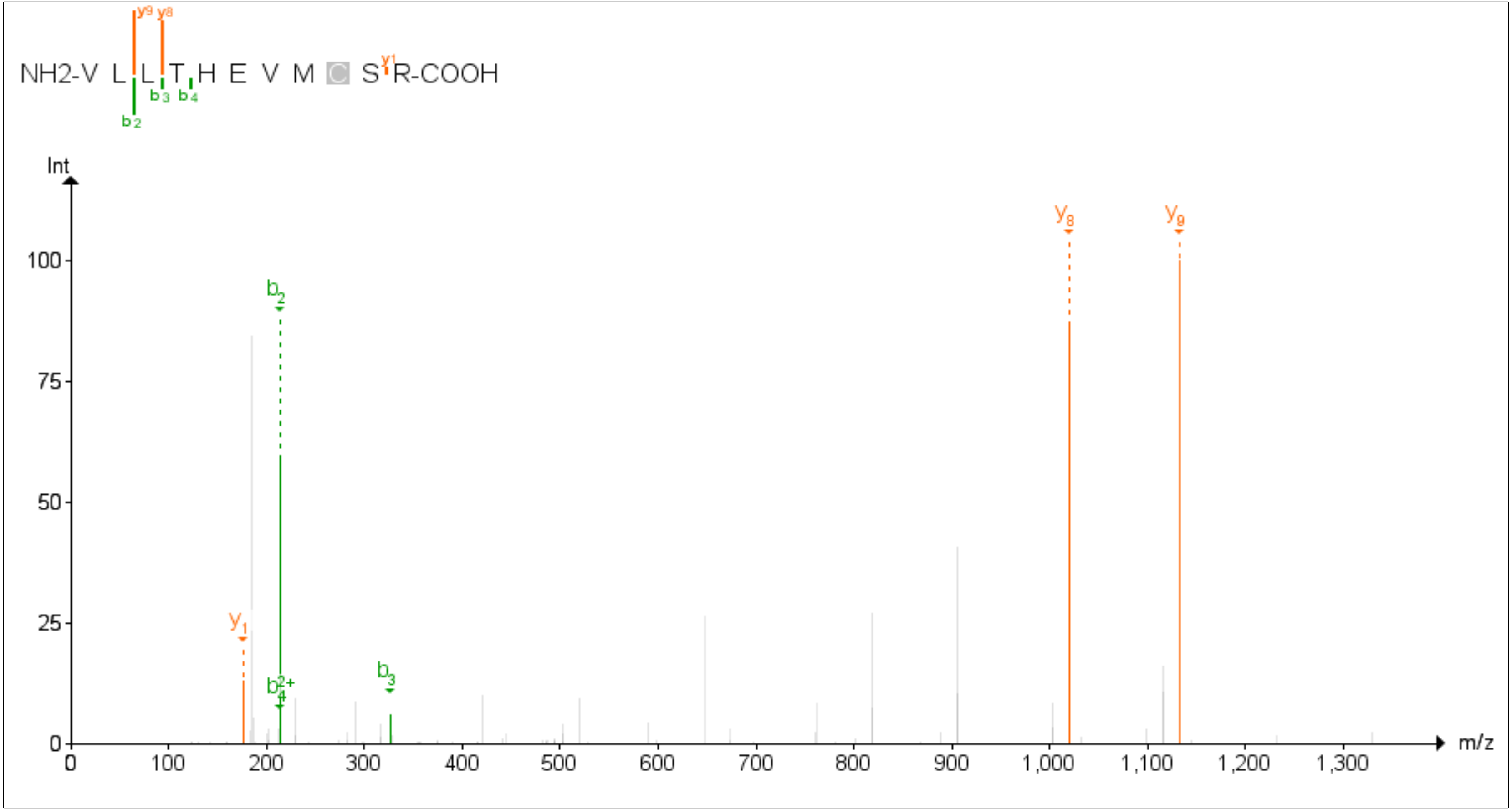

NH2-V L L T H E V M C S R-COOH
y9 y8 y1
b2 b3 b4
Int
100
75
50
25
0
y8
y9
b2
y1
b4 2+
b3
0 100 200 300 400 500 600 700 800 900 1,000 1,100 1,200 1,300
m/z

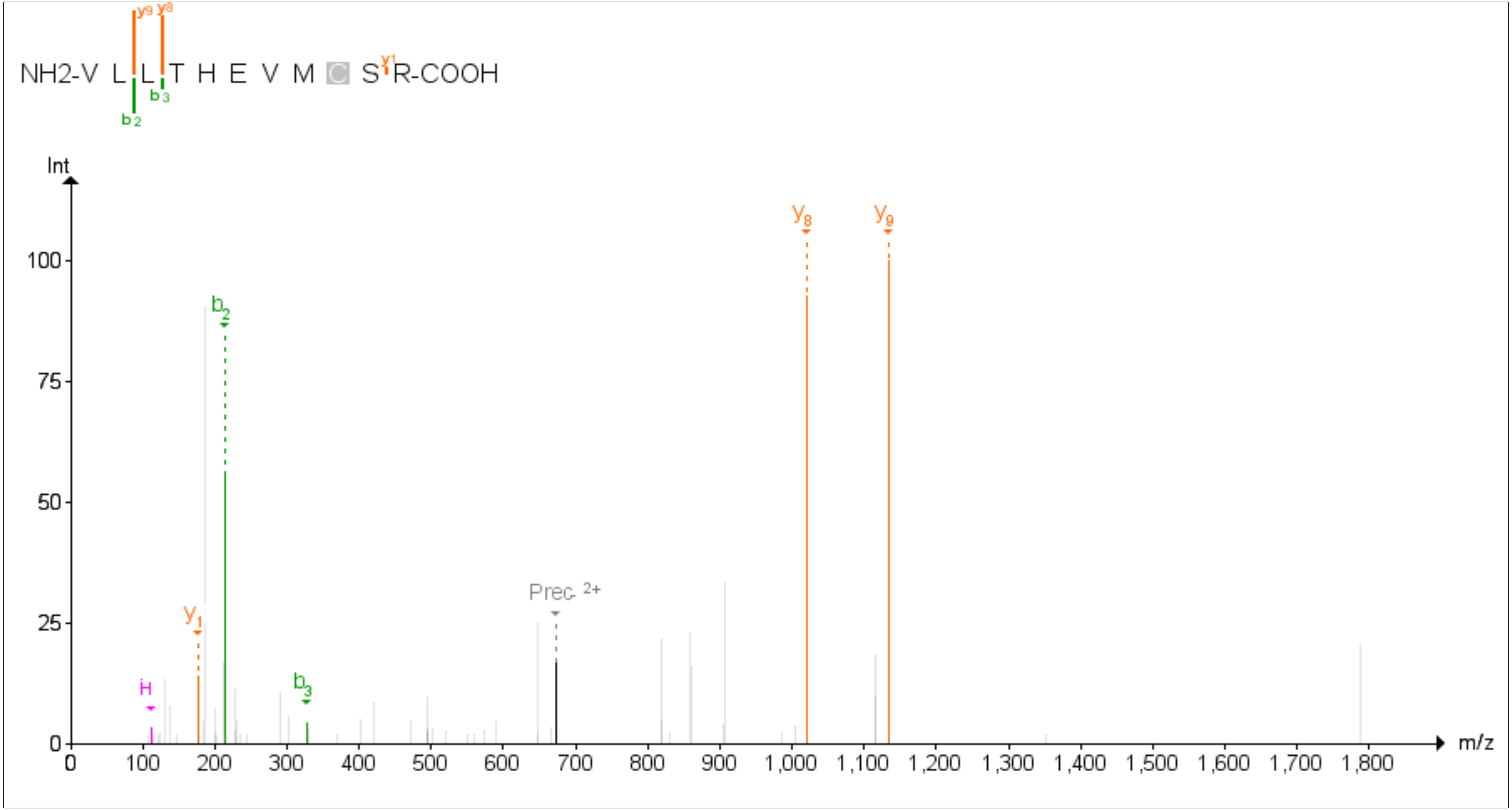
NH2-V L L T H E V M C S R-COOH
y9 y8
b2 b3
y1
Int
100
75
50
25
0
m/z
0 100 200 300 400 500 600 700 800 900 1,000 1,100 1,200 1,300 1,400 1,500 1,600 1,700 1,800
y8
y9
b2
y1
b3
H
Prec 2+

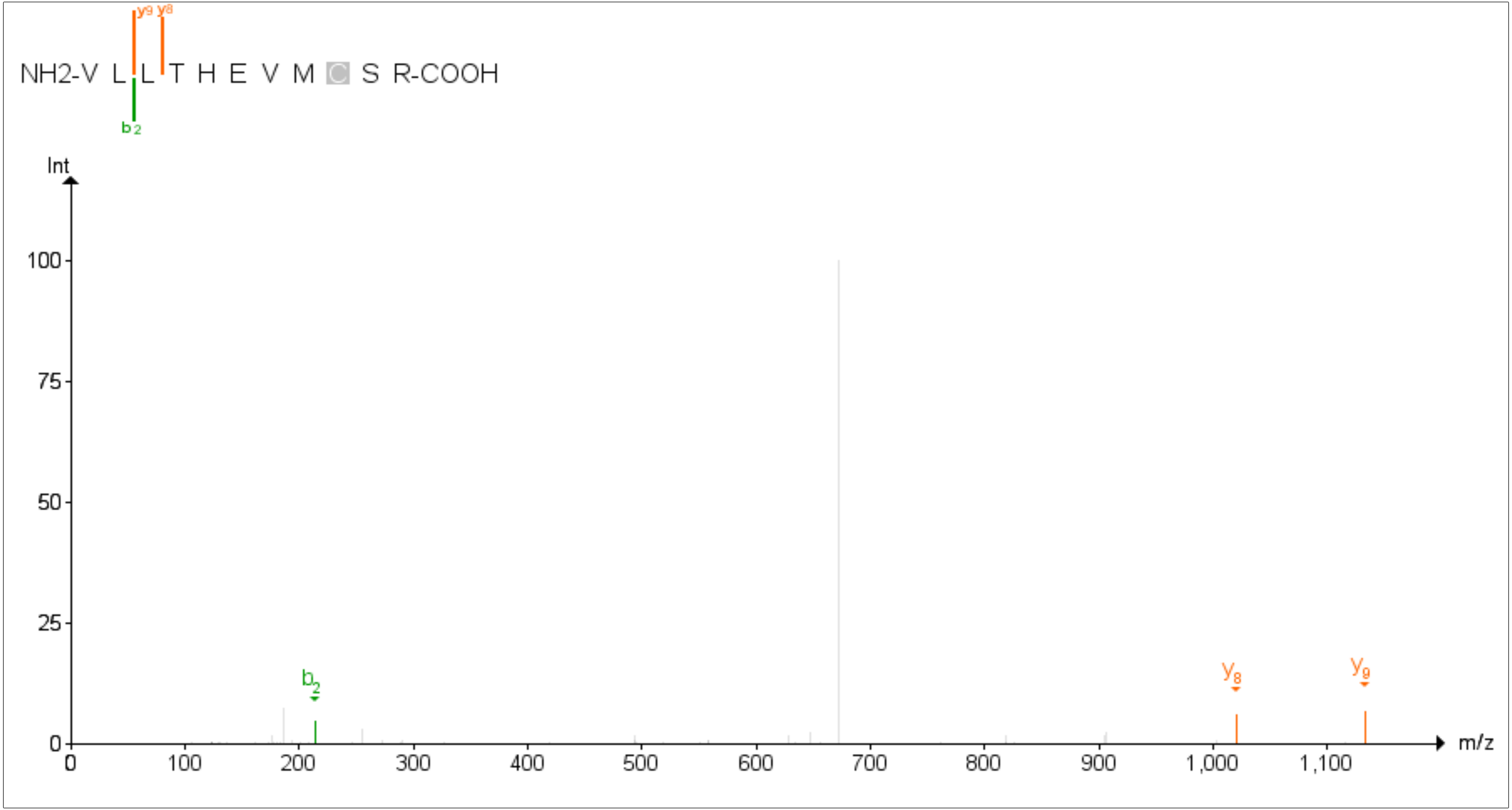

y9 y8
NH2-V L L T H E V M C S R-COOH
b2
Int
100
75
50
25
0
b2
y8
y9
0
100
200
300
400
500
600
700
800
900
1,000
1,100
m/z

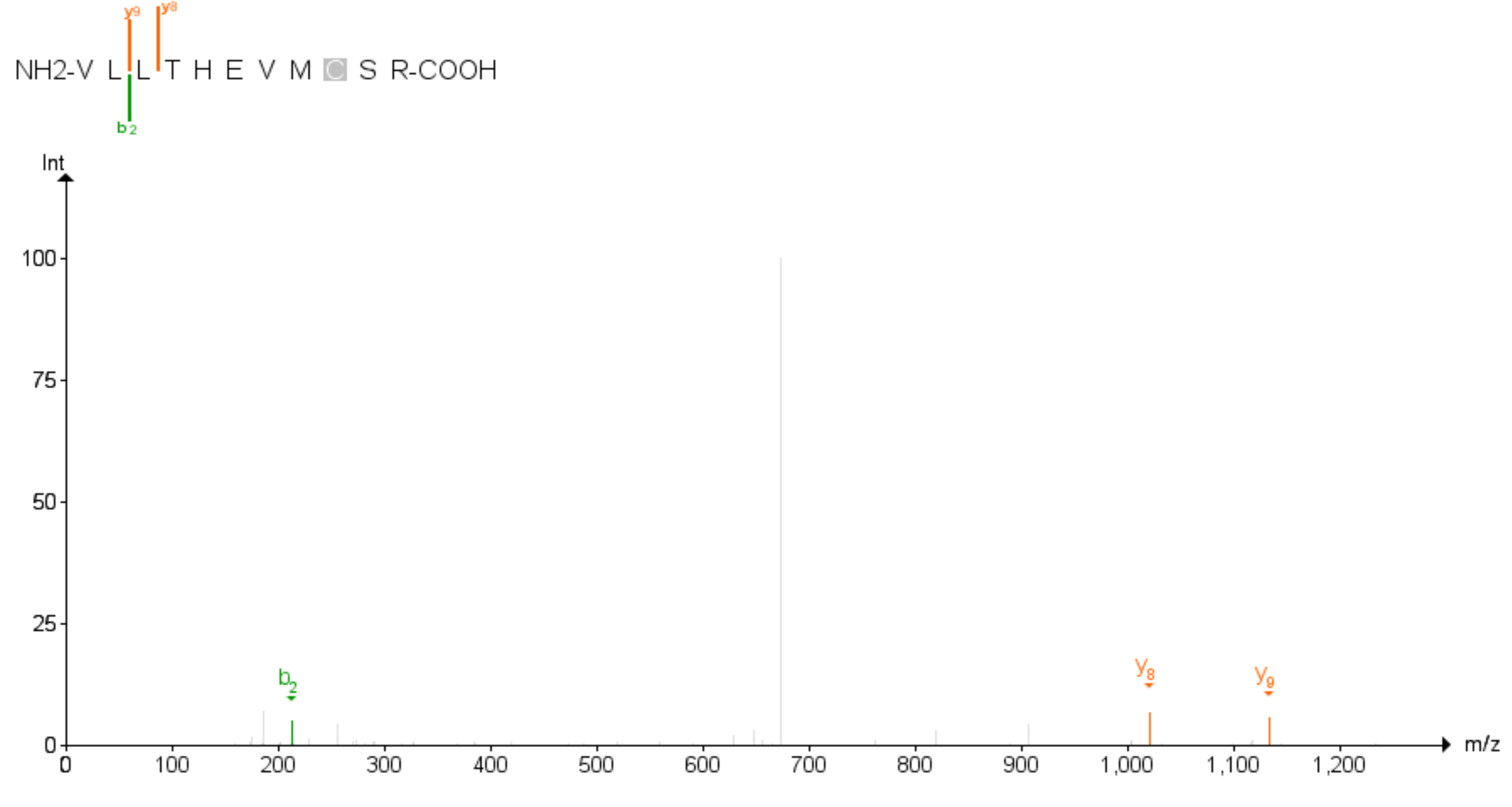

NH2-V L L T H E V M C S R-COOH
y9
y8
b2
Int
100
75
50
25
0
m/z
0
100
200
300
400
500
600
700
800
900
1,000
1,100
1,200
b2
y8
y9

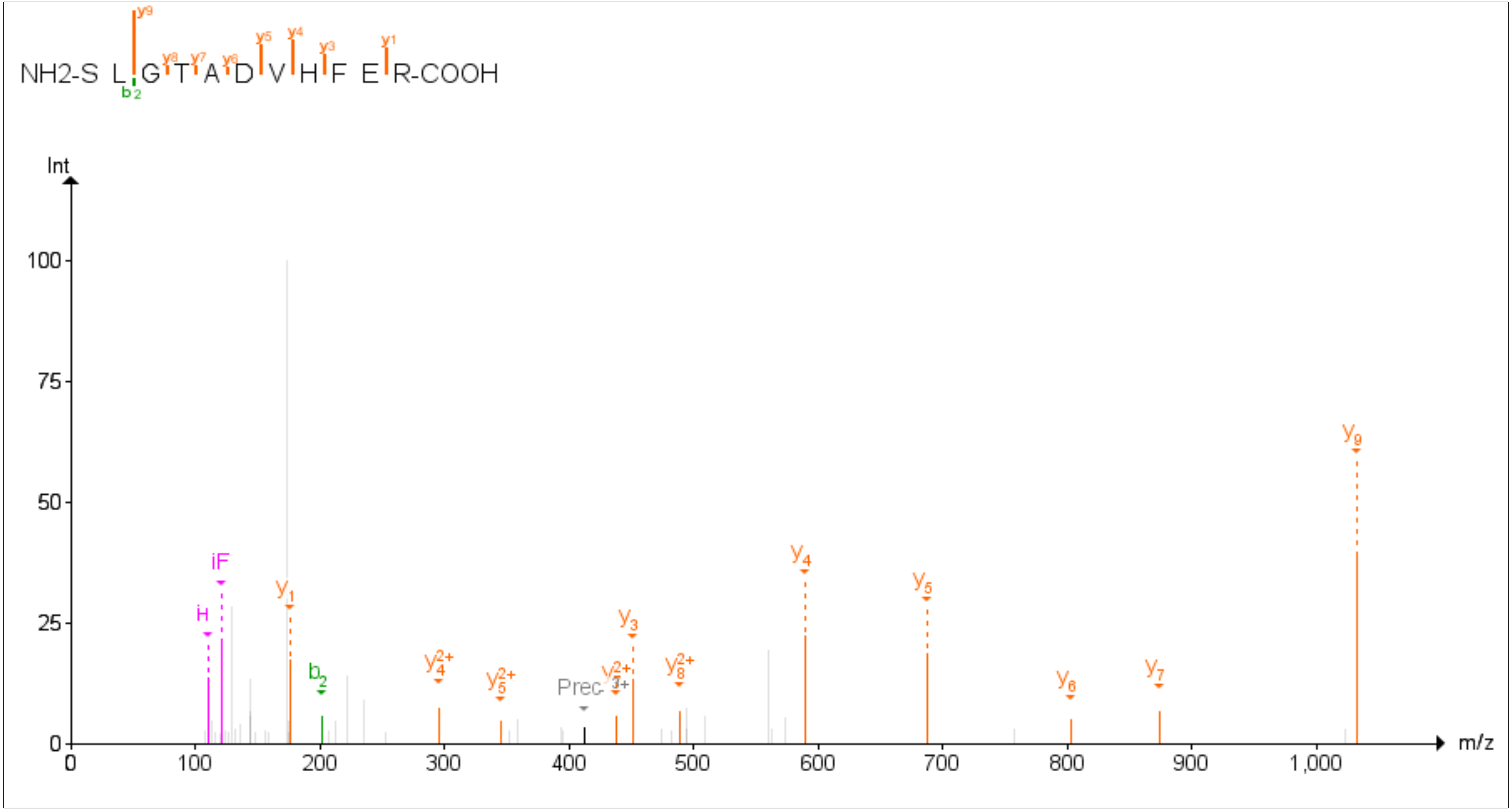

NH2-S L G T A D V H F E R-COOH
y9 y8 y7 y6 y5 y4 y3 y1
b2
Int
100
75
50
25
0
m/z
0 100 200 300 400 500 600 700 800 900 1,000
iF
iH
y1
b2
y4 2+
y5 2+
Prec 3+
y7 2+
y3
y8 2+
y4
y5
y6
y7
y9

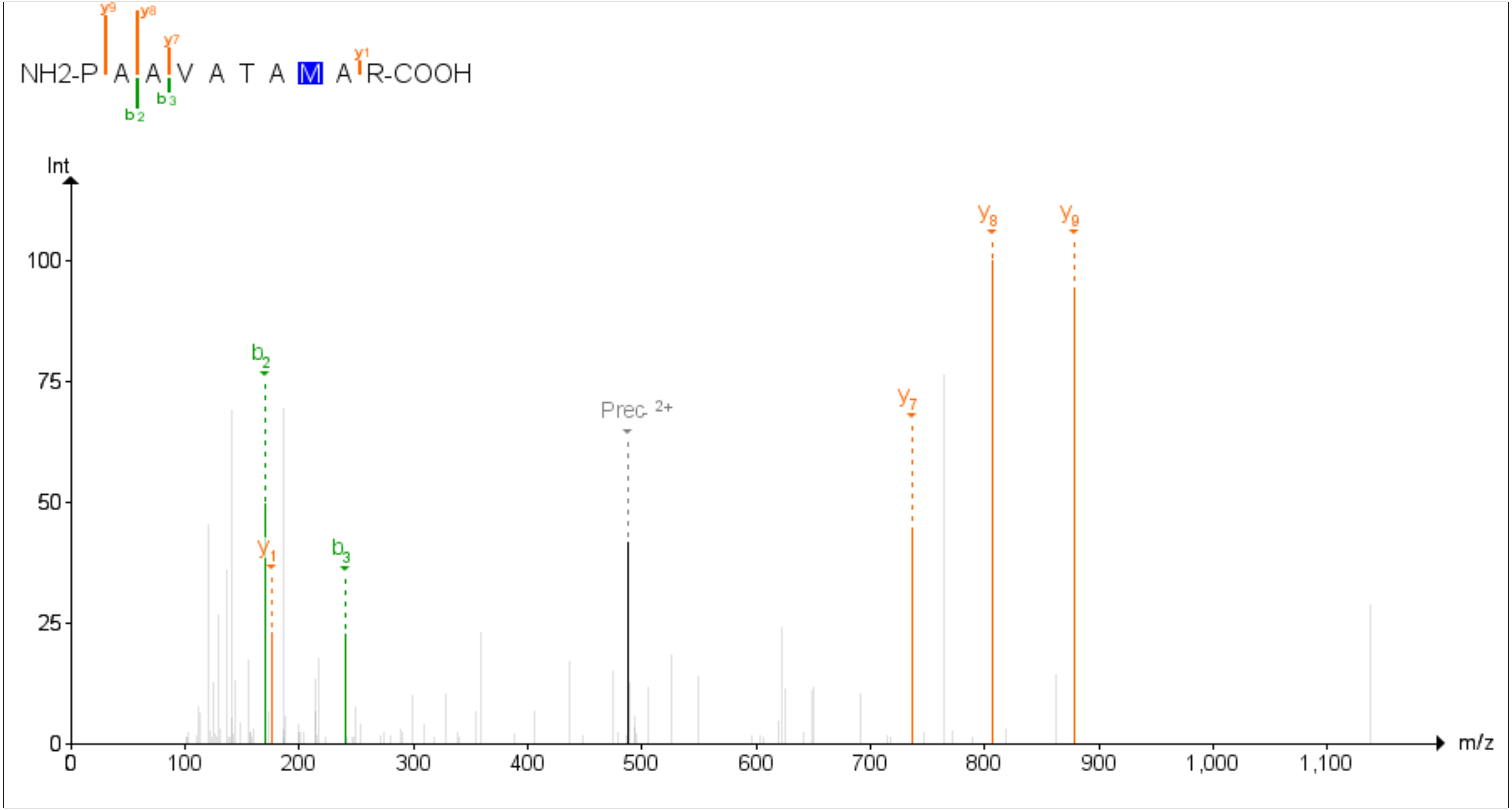

y9
y8
y7
y1
NH2-P A A V A T A M A R-COOH
b2
b3
Int
100
75
50
25
0
b2
y1
b3
Prec 2+
y7
y8
y9
0
100
200
300
400
500
600
700
800
900
1,000
1,100
m/z

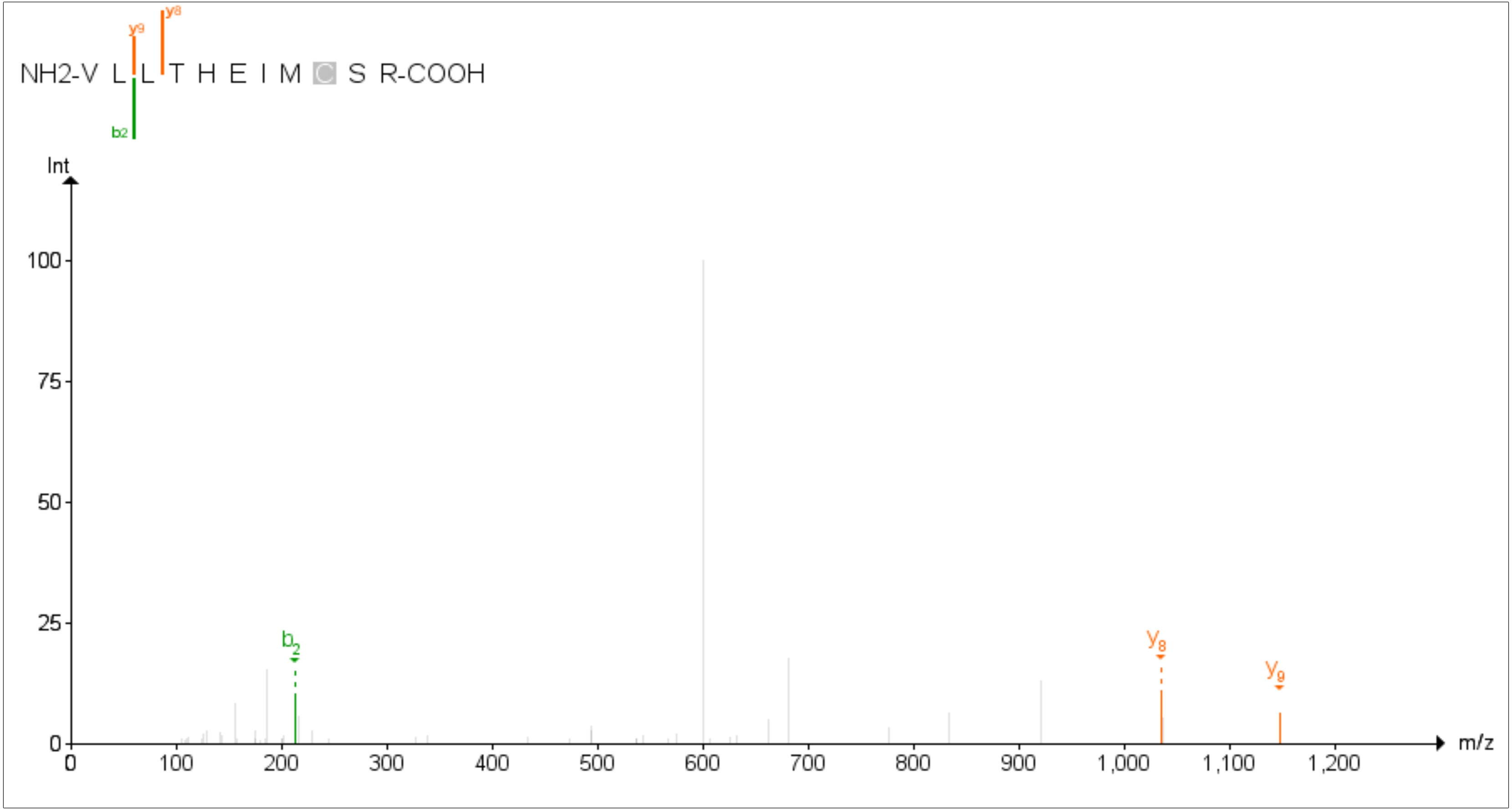
NH2-V L L T H E I M C S R-COOH
y9
y8
b2
Int
100
75
50
25
0
b2
y8
y9
0
100
200
300
400
500
600
700
800
900
1,000
1,100
1,200
m/z

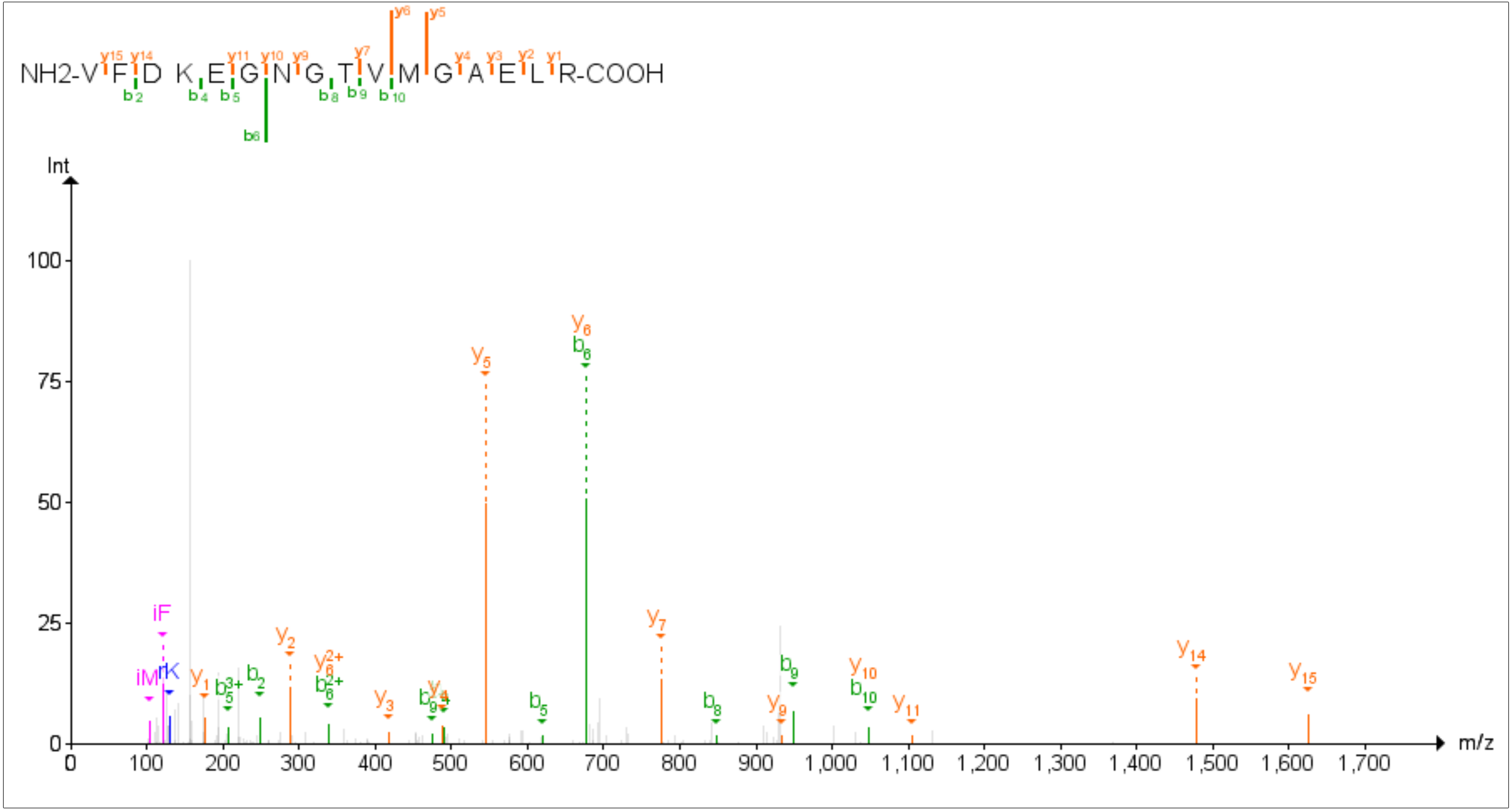
NH2-VFDKEGNGTVMGAELR-COOH
Int
m/z
y5
y6
b6
y7
y14
y15
y10
b10
y11
b9
b8
y9
b5
y4
b9
y3
y2
y1
b2
iF
iM
rK

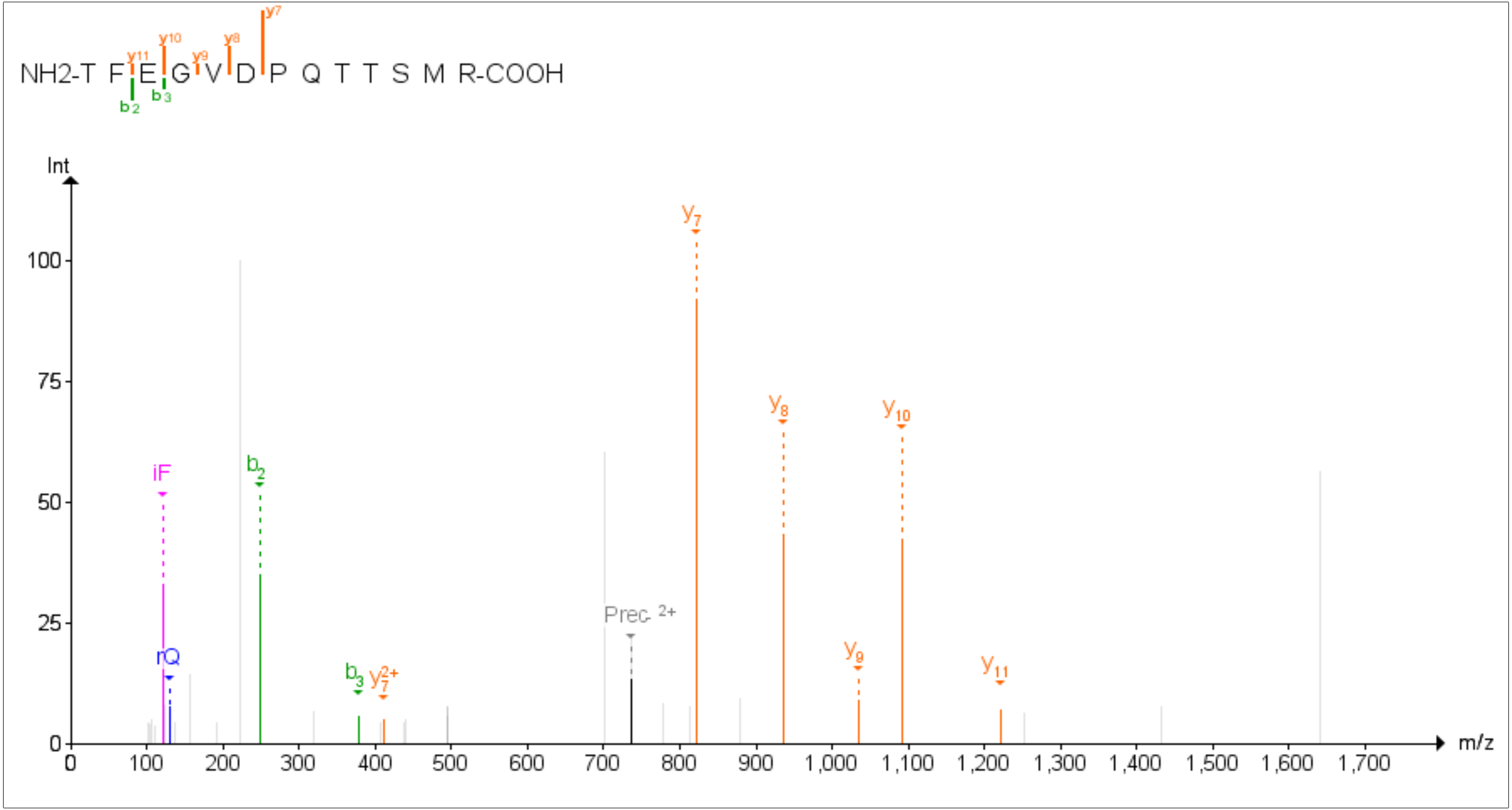

NH2-T F E G V D P Q T T S M R-COOH
y11 y10 y9 y8 y7
b2 b3
Int
100
75
50
25
0
iF
b2
rQ
b3
y7 2+
Prec 2+
y7
y8
y9
y10
y11
0 100 200 300 400 500 600 700 800 900 1,000 1,100 1,200 1,300 1,400 1,500 1,600 1,700
m/z

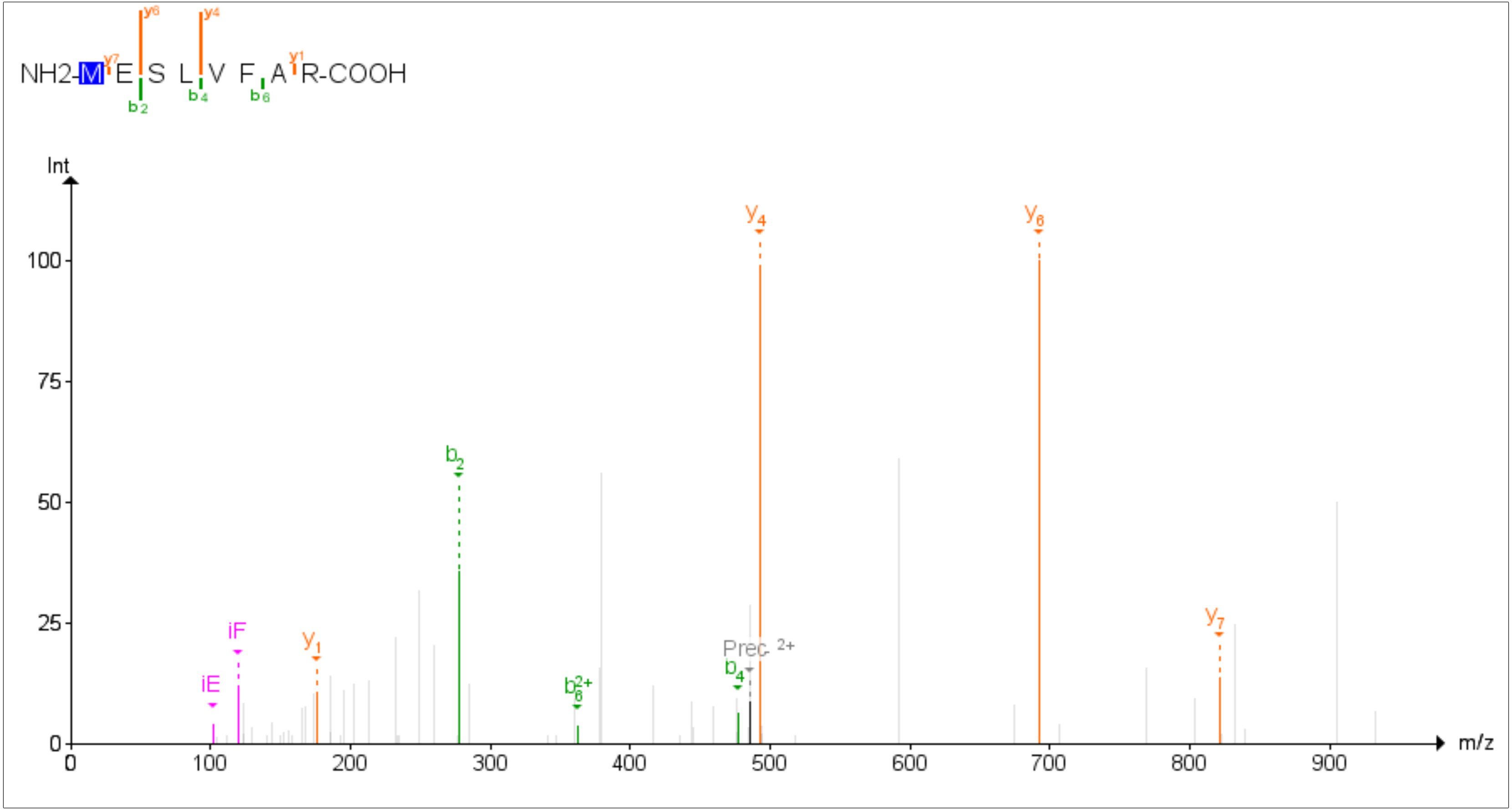

NH2-M E S L V F A R-COOH
y7
y6
y4
y1
b2
b4
b6
Int
100
75
50
25
0
y4
y6
b2
y7
iF
y1
iE
b6 2+
b4
Prec 2+
0
100
200
300
400
500
600
700
800
900
m/z

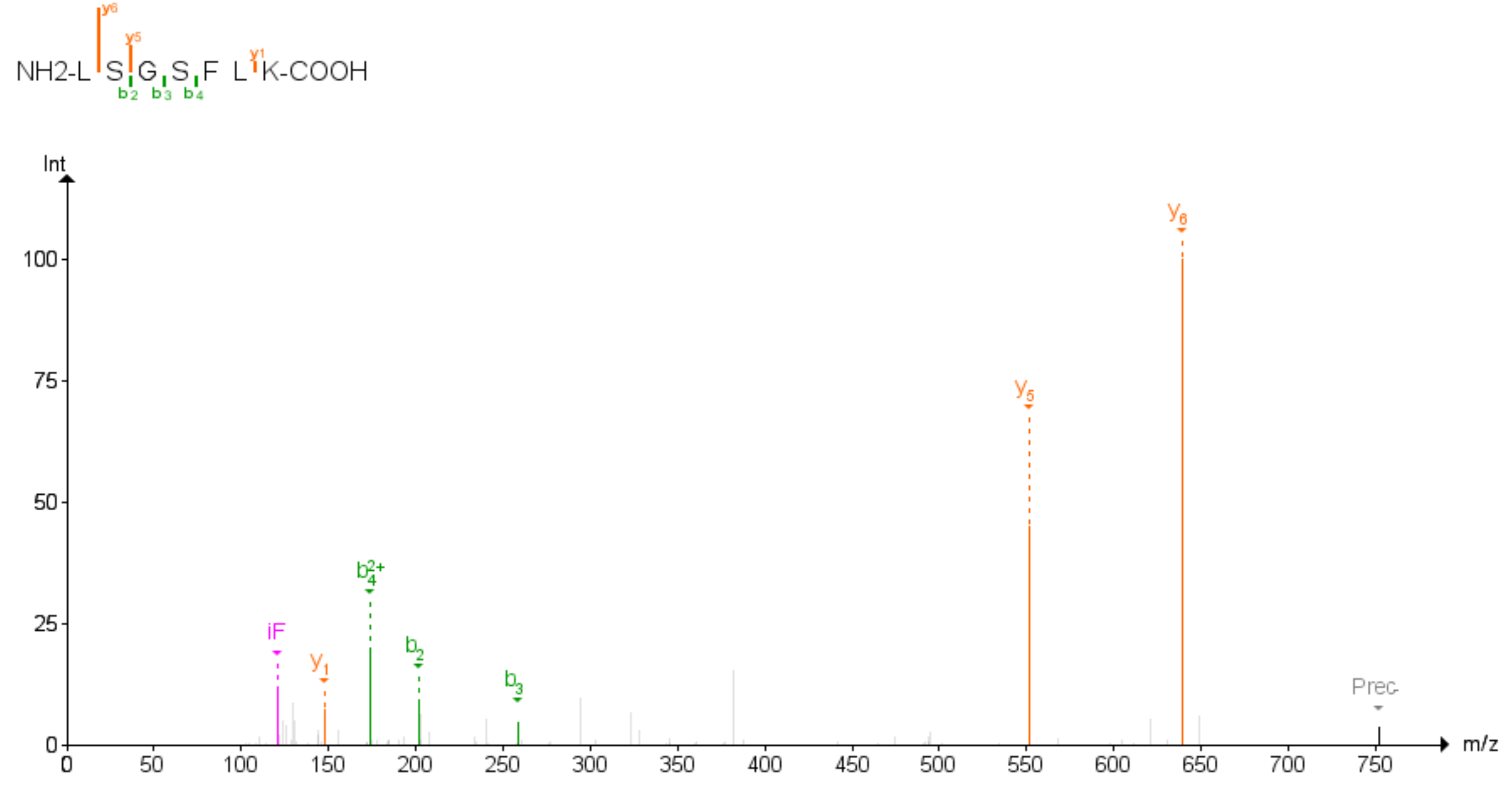

NH2-L S G S F L K-COOH
y6
y5
y1
b2
b3
b4
Int
100
75
50
25
0
y6
y5
b4 2+
iF
y1
b2
b3
Prec
0
50
100
150
200
250
300
350
400
450
500
550
600
650
700
750
m/z

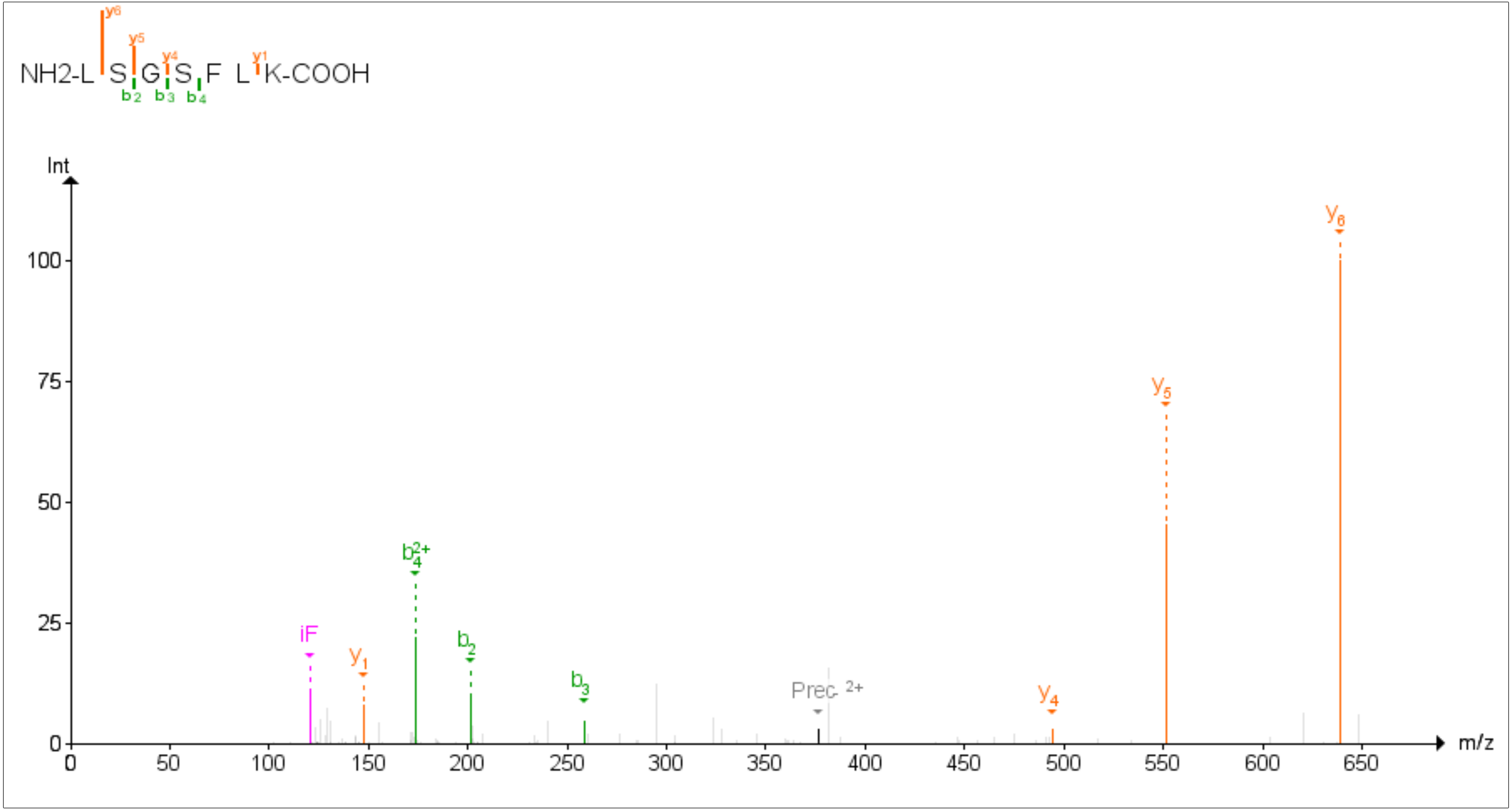
NH2-L S G S F L K-COOH
y6
y5
y4
y1
b2
b3
b4
Int
100
75
50
25
0
iF
y1
b4 2+
b2
b3
Prec 2+
y4
y5
y6
0
50
100
150
200
250
300
350
400
450
500
550
600
650
m/z

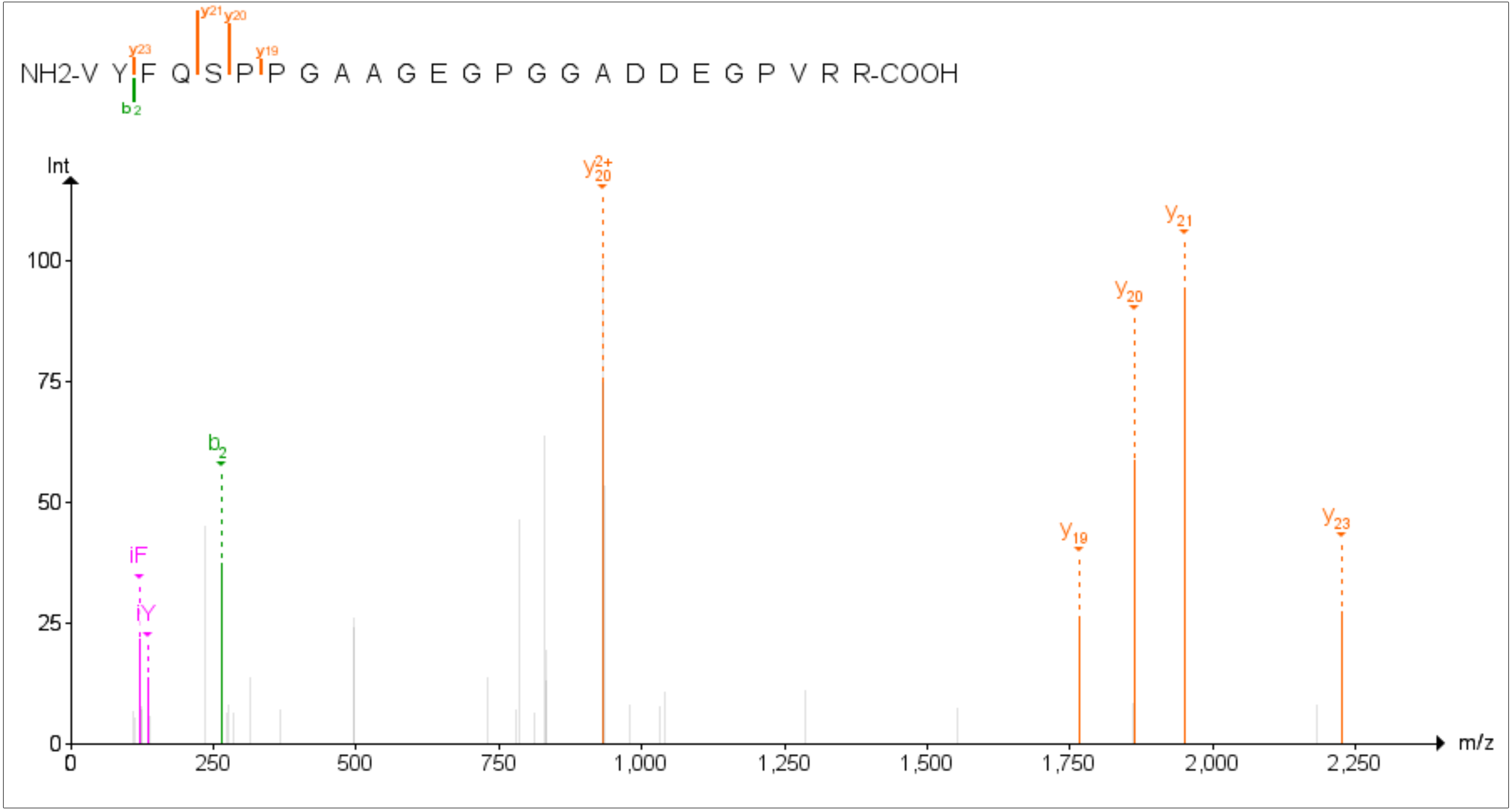

NH2-V Y F Q S P P G A A G E G P G G A D D E G P V R R-COOH
y23
y21
y20
y19
b2
Int
100
75
50
25
0
m/z
0
250
500
750
1,000
1,250
1,500
1,750
2,000
2,250
y_{20}^{2+}
y_{21}
y_{20}
b_2
iF
iY
y_{19}
y_{23}

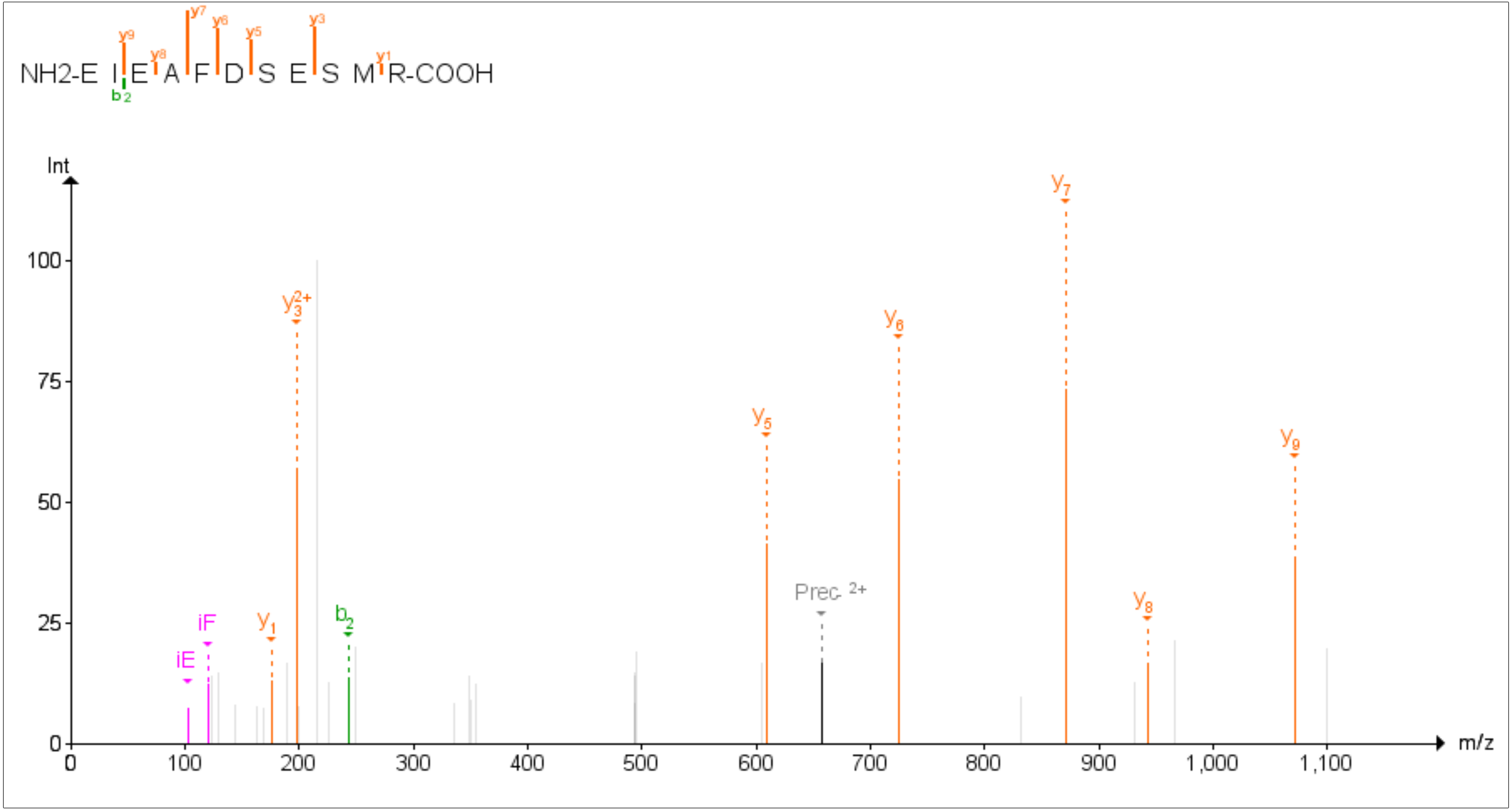

NH2-E I E A F D S E S M R-COOH
y9
y8
y7
y6
y5
y3
y1
b2
Int
100
75
50
25
0
y3 2+
y7
y6
y5
y9
y8
Prec 2+
iF
iE
y1
b2
0
100
200
300
400
500
600
700
800
900
1,000
1,100
m/z

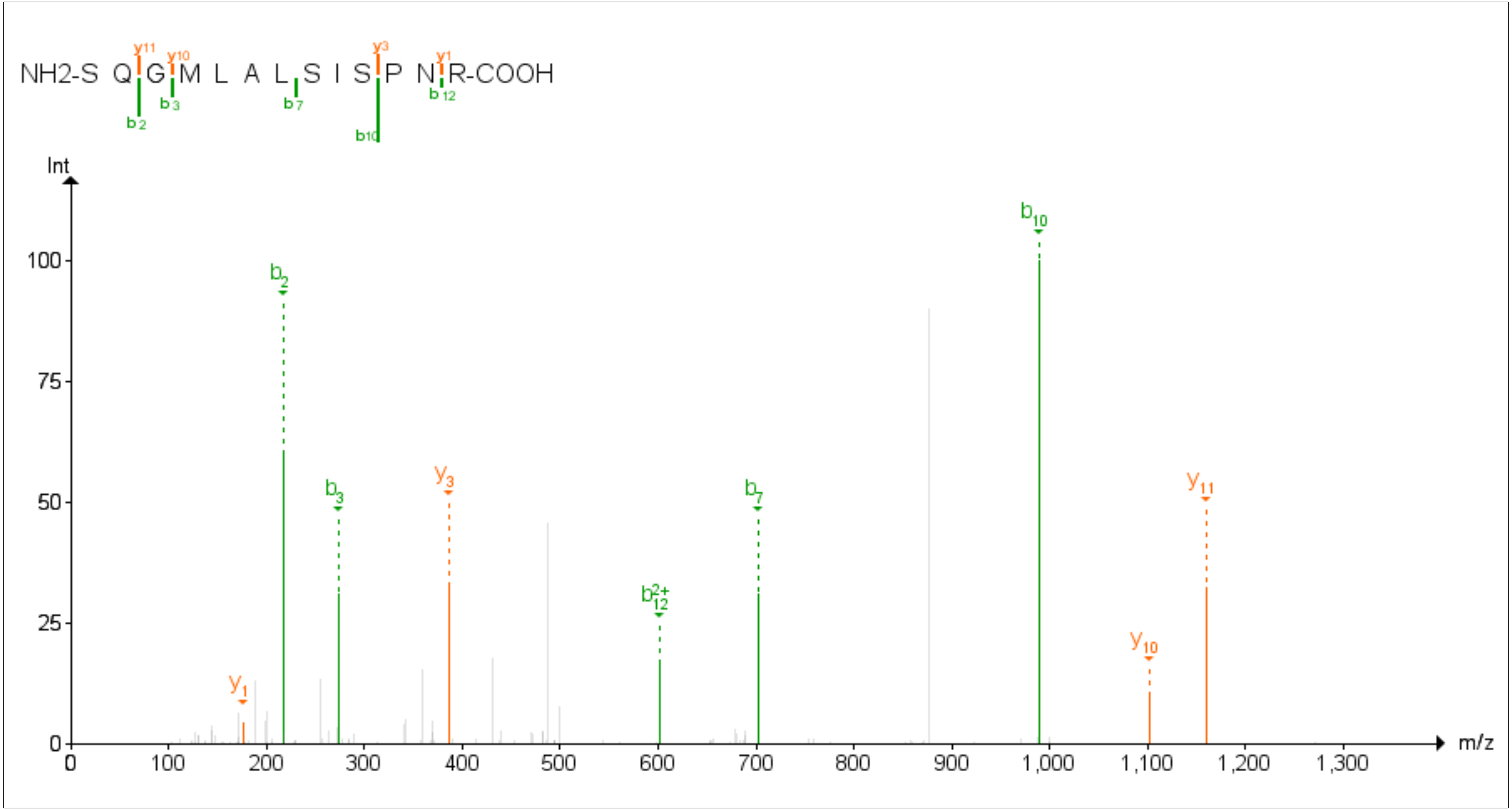
NH2-S Q G M L A L S I S P N R-COOH
y11
y10
y3
y1
b2
b3
b7
b10
b12
Int
100
75
50
25
0
b2
b3
y3
y1
b12 2+
b7
b10
y10
y11
0
100
200
300
400
500
600
700
800
900
1,000
1,100
1,200
1,300
m/z

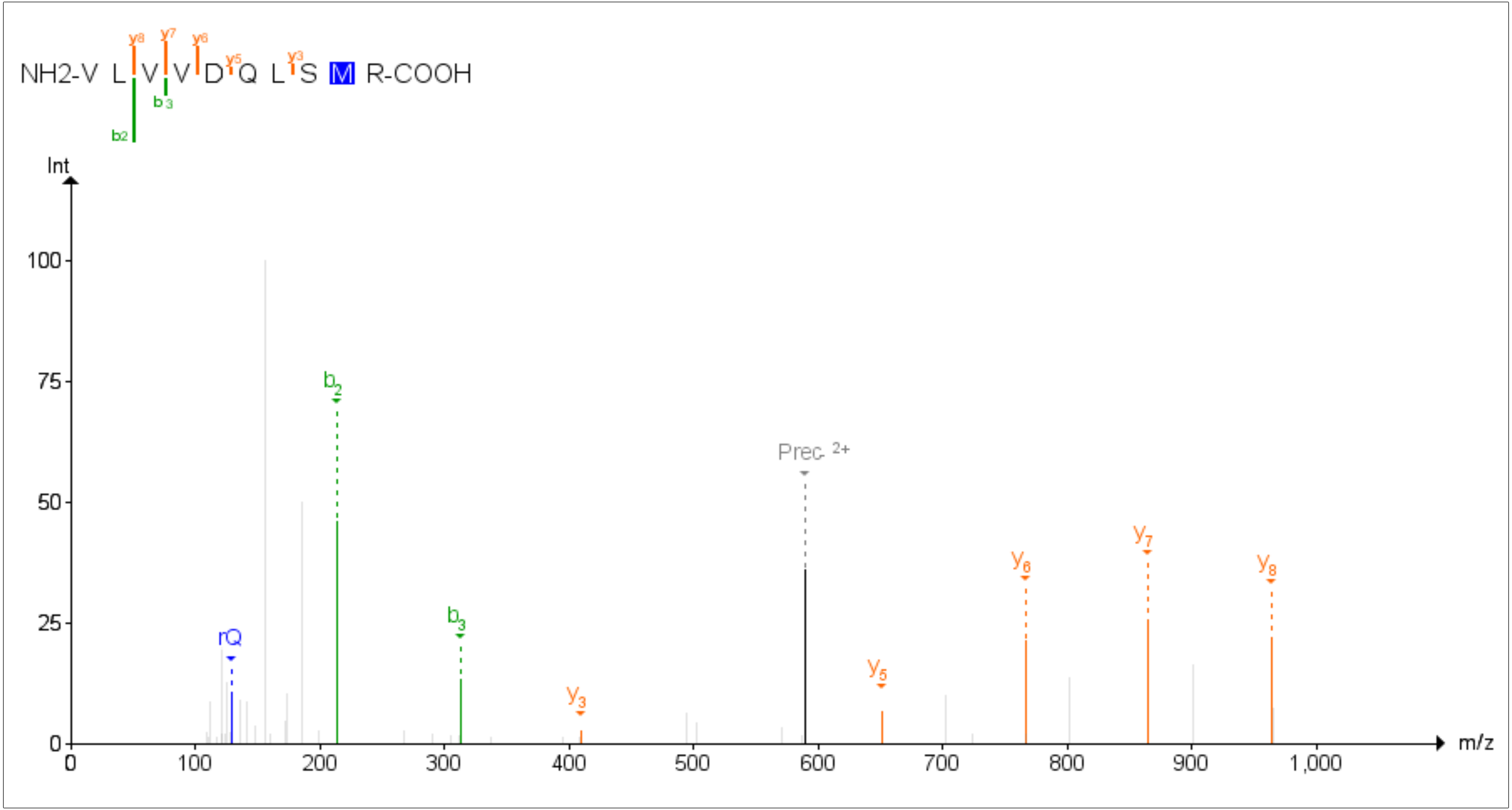

NH2-V L V V D Q L S M R-COOH
y8 y7 y6 y5 y3
b2 b3
Int
100
75
50
25
0
b2
b3
rQ
y3
Prec 2+
y5
y6
y7
y8
0 100 200 300 400 500 600 700 800 900 1,000
m/z

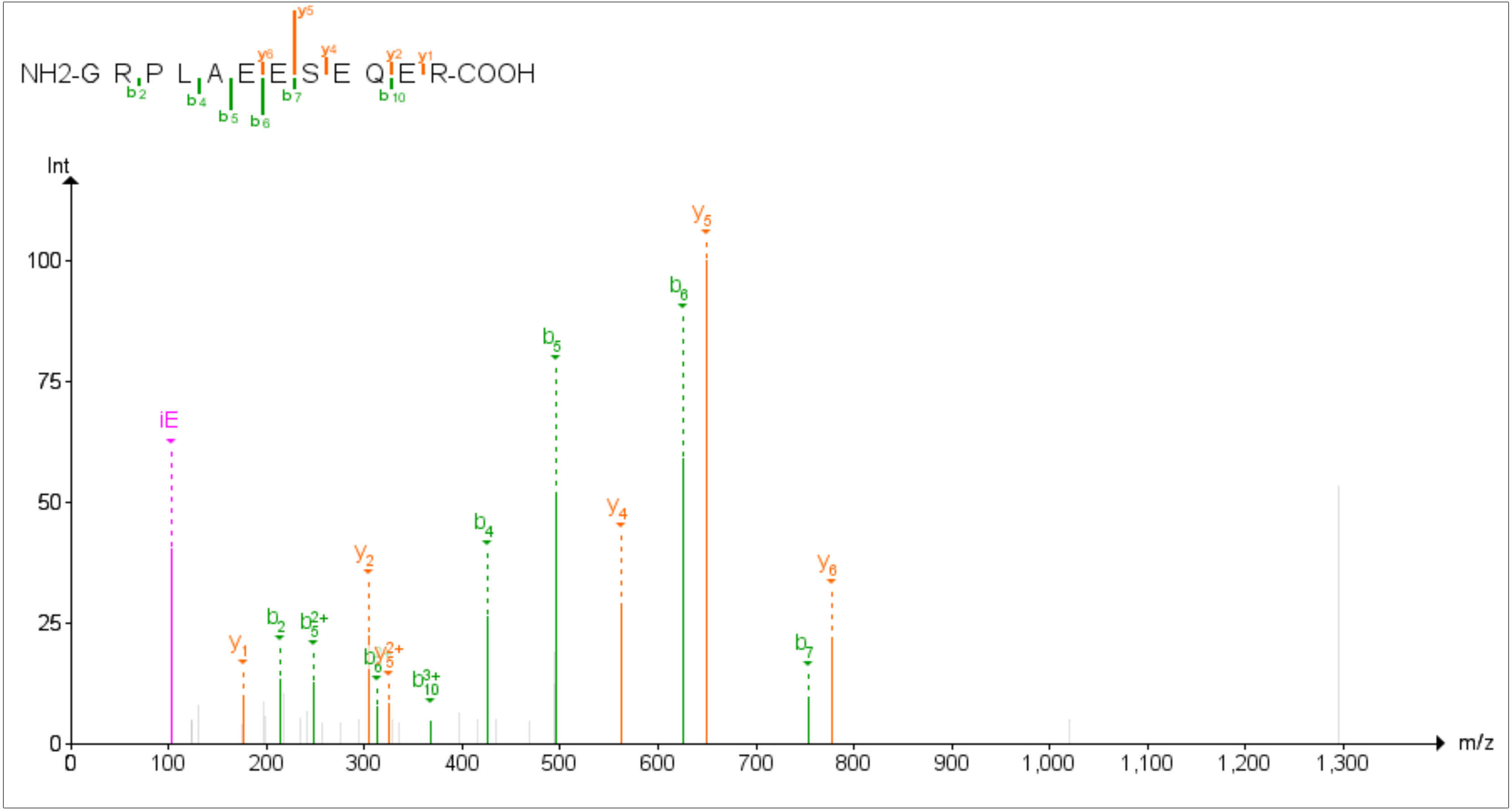

NH2-G R P L A E E S E Q E R-COOH
b2 b4 b5 b6 b7 b10
y6 y5 y4 y2 y1
Int
100
75
50
25
0
iE
y1
b2
b5 2+
y2
b6
y5 2+
b10 3+
b4
b5
y4
b6
y5
b7
y6
0 100 200 300 400 500 600 700 800 900 1,000 1,100 1,200 1,300
m/z

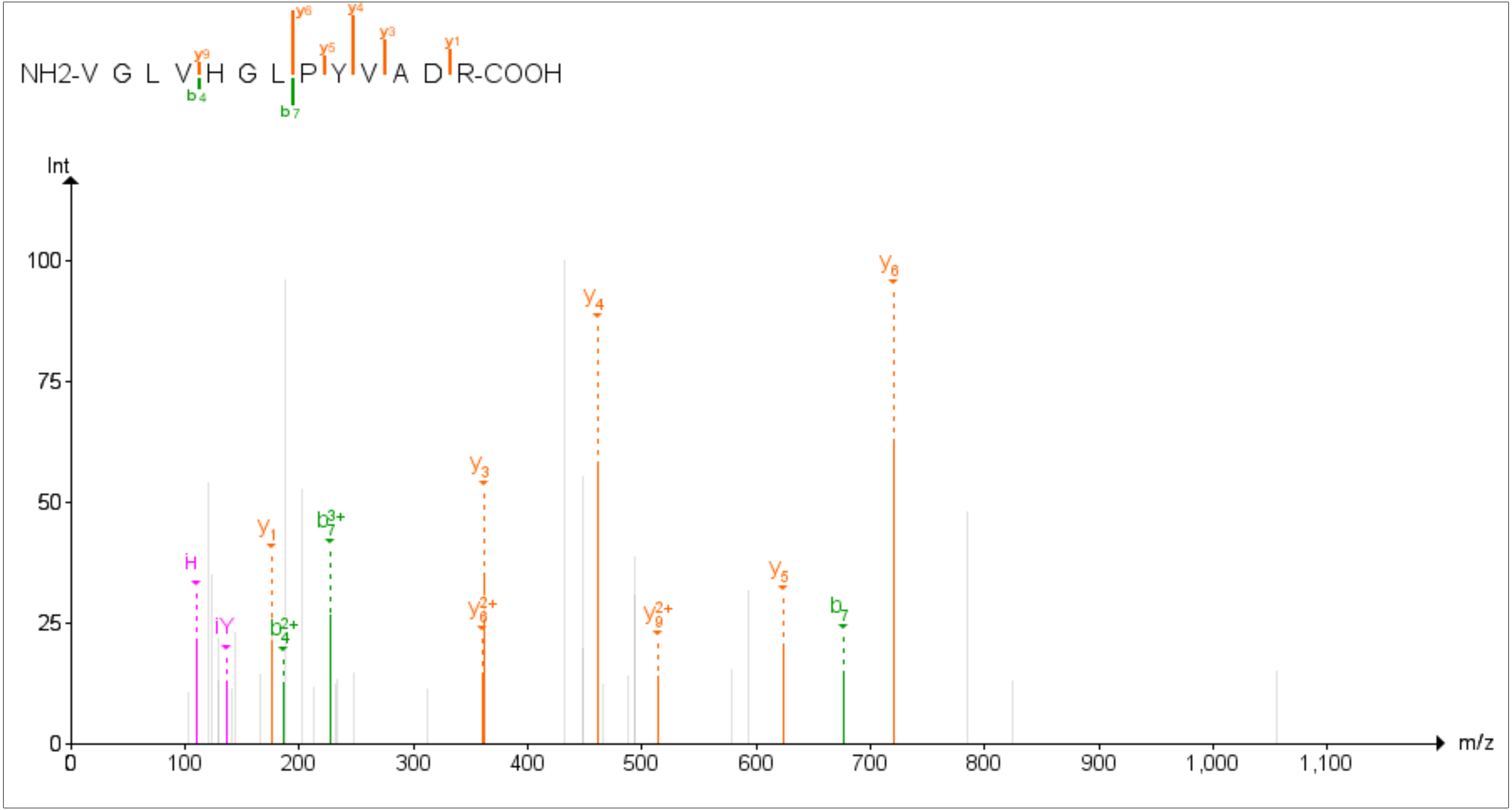
NH2-V G L V H G L P Y V A D R-COOH
y9
y6
y5
y4
y3
y1
b4
b7
Int
100
75
50
25
0
y6
y4
y3
y1
b7 3+
iH
iY
b4 2+
y6 2+
y9 2+
y5
b7
0
100
200
300
400
500
600
700
800
900
1,000
1,100
m/z

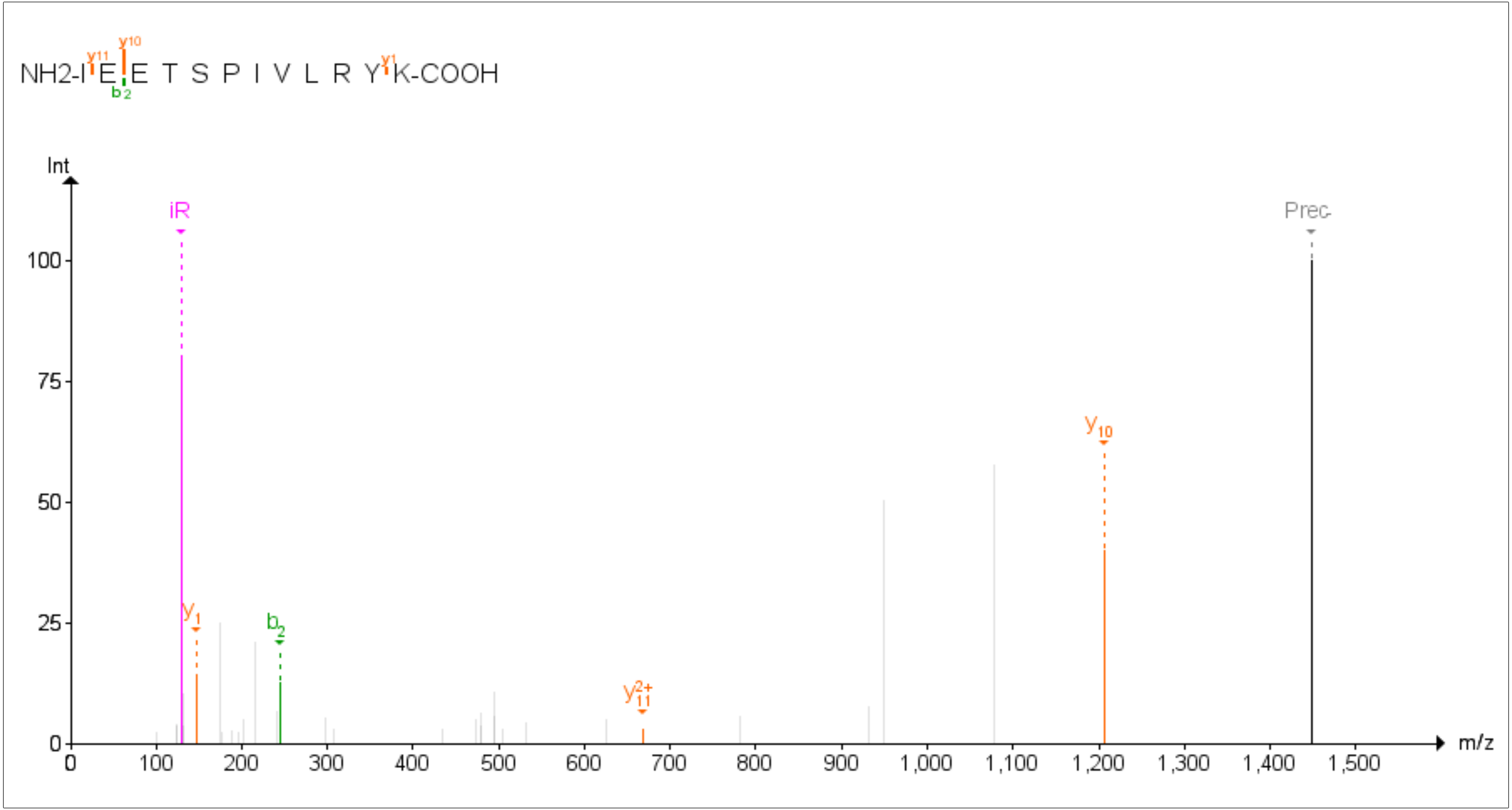

NH2-I E E T S P I V L R Y K-COOH
y11
y10
b2
y1
Int
iR
Prec.
y10
y1
b2
y11 2+
m/z
0
25
50
75
100
0
100
200
300
400
500
600
700
800
900
1,000
1,100
1,200
1,300
1,400
1,500

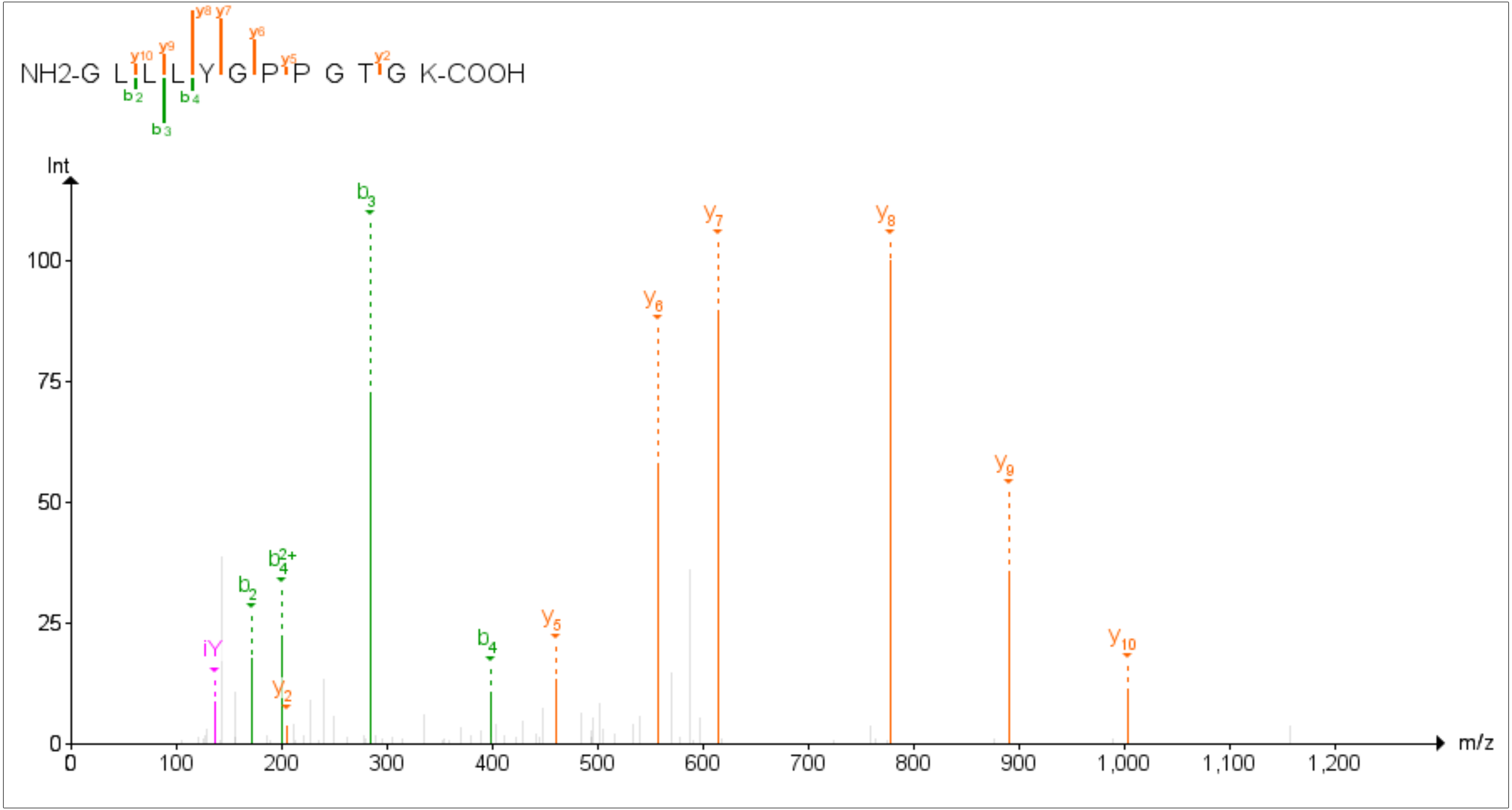

NH2-G L L L Y G P P G T G K-COOH
y10 y9 y8 y7 y6 y5 y2
b2 b3 b4
Int
100
75
50
25
0
b3
y7
y8
y6
y9
b2
b4 2+
iY
y2
b4
y5
y10
0
100
200
300
400
500
600
700
800
900
1,000
1,100
1,200
m/z

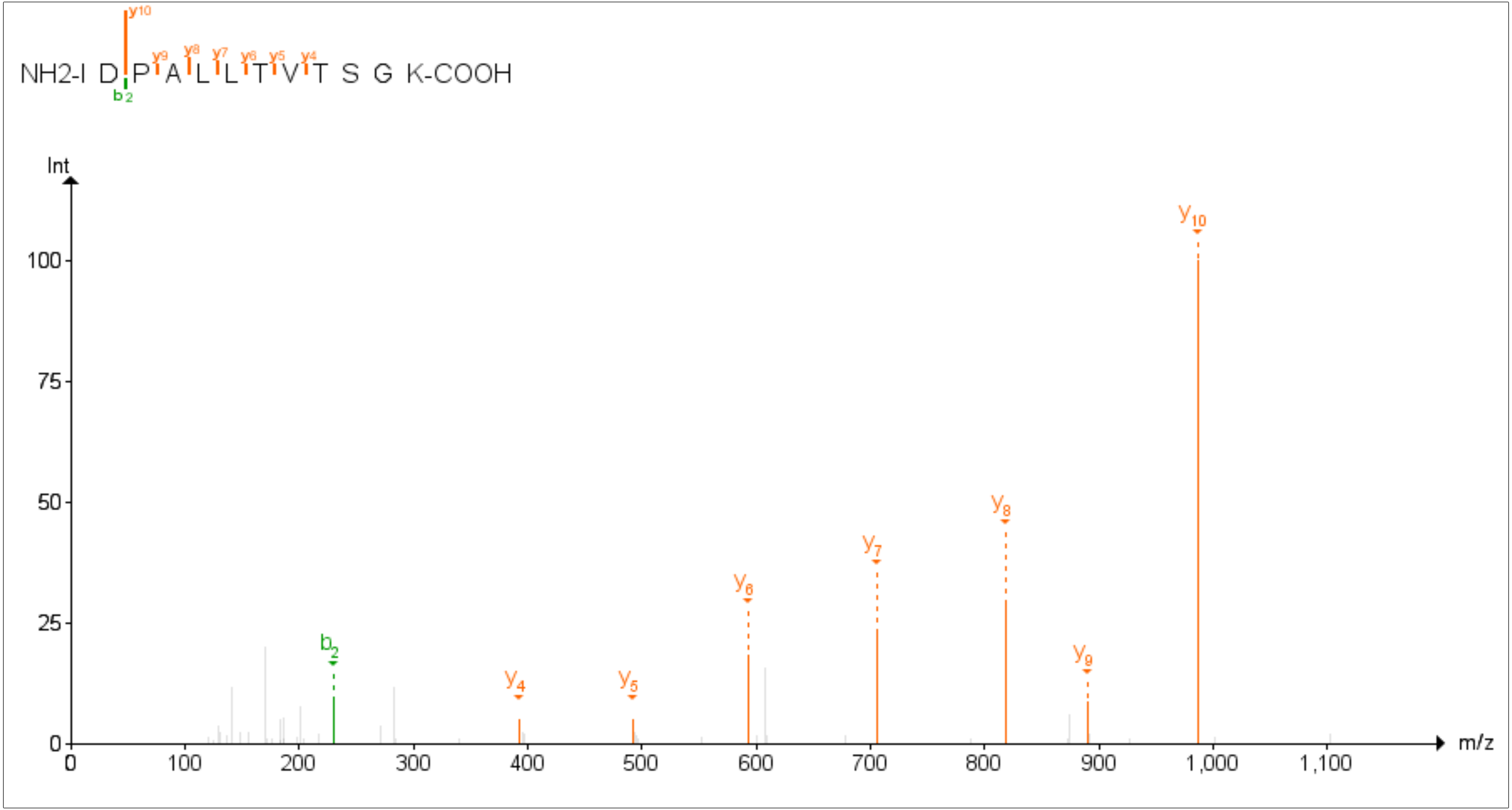

NH2-I D P A L L T V T S G K-COOH
y10 y9 y8 y7 y6 y5 y4
b2
Int
100
75
50
25
0
m/z
0 100 200 300 400 500 600 700 800 900 1,000 1,100
y10 y8 y7 y6 y9 b2 y4 y5

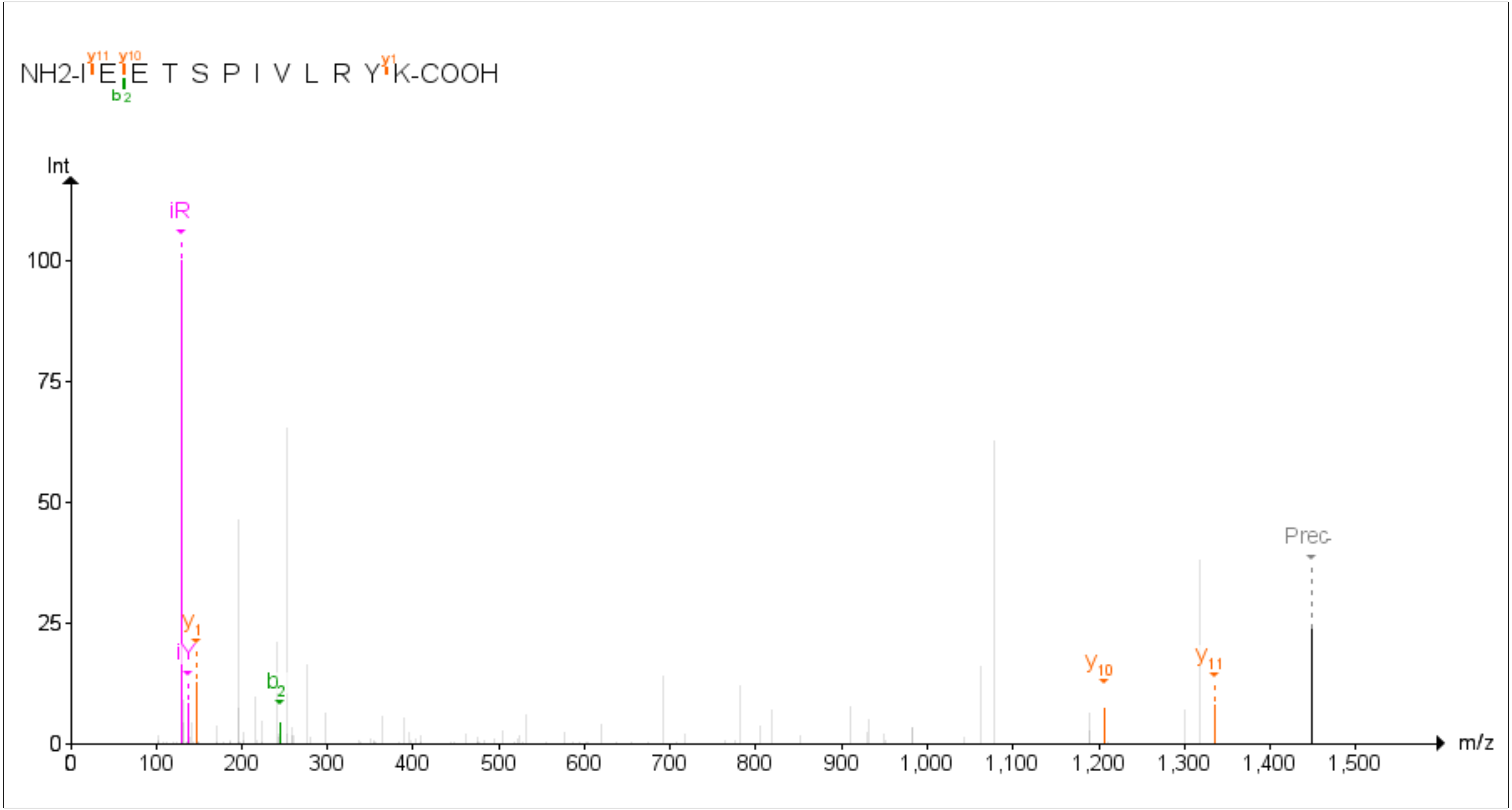

NH2-I E E T S P I V L R Y K-COOH
y11
y10
y1
b2
Int
iR
100
75
50
25
0
y1
iY
b2
y10
y11
Prec
0
100
200
300
400
500
600
700
800
900
1,000
1,100
1,200
1,300
1,400
1,500
m/z

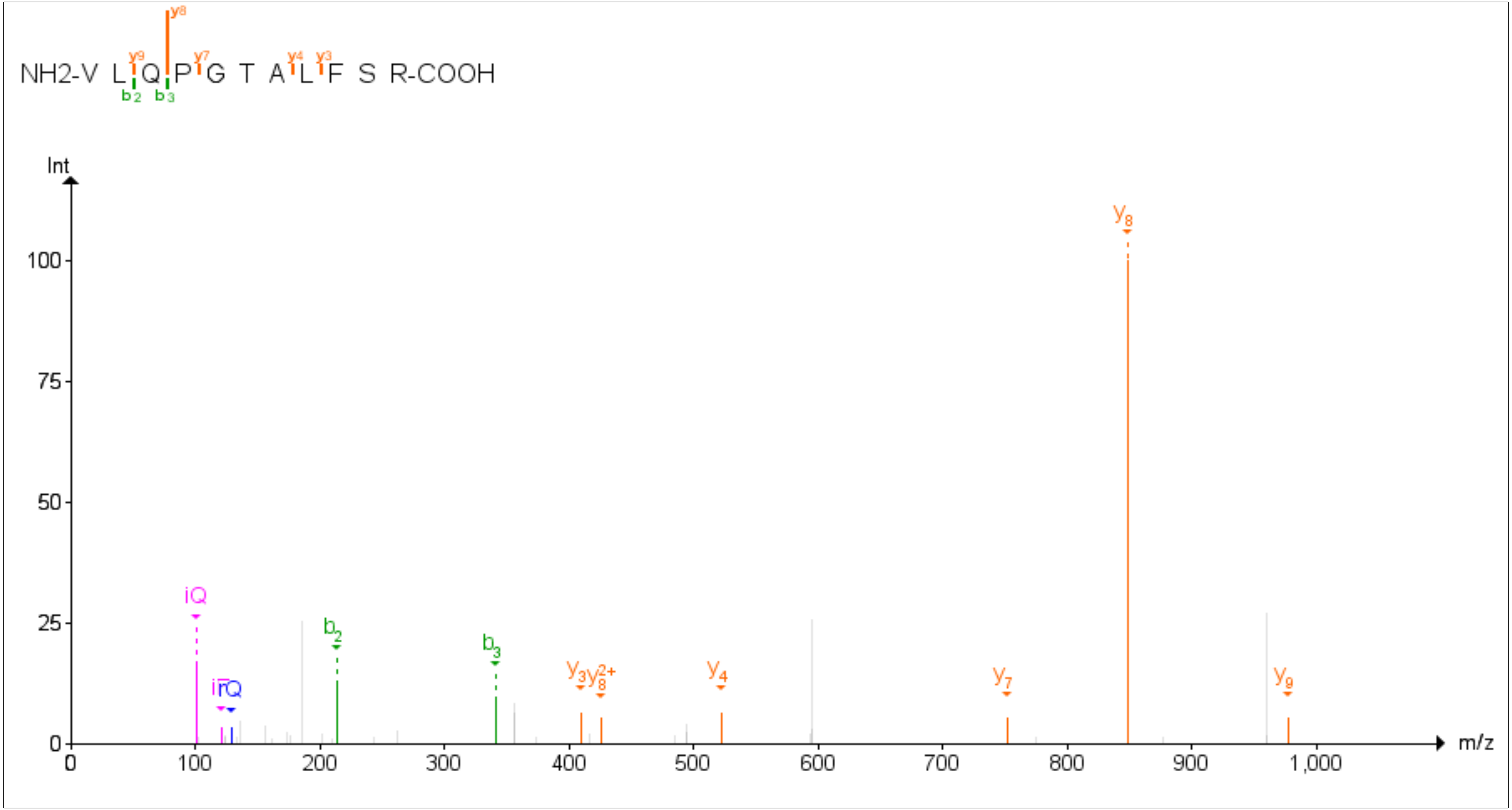

NH2-V L Q P G T A L F S R-COOH
y8
y9
y7
y4
y3
b2
b3
Int
100
75
50
25
0
iQ
iQ
rQ
b2
b3
y3
y8 2+
y4
y7
y8
y9
0
100
200
300
400
500
600
700
800
900
1,000
m/z

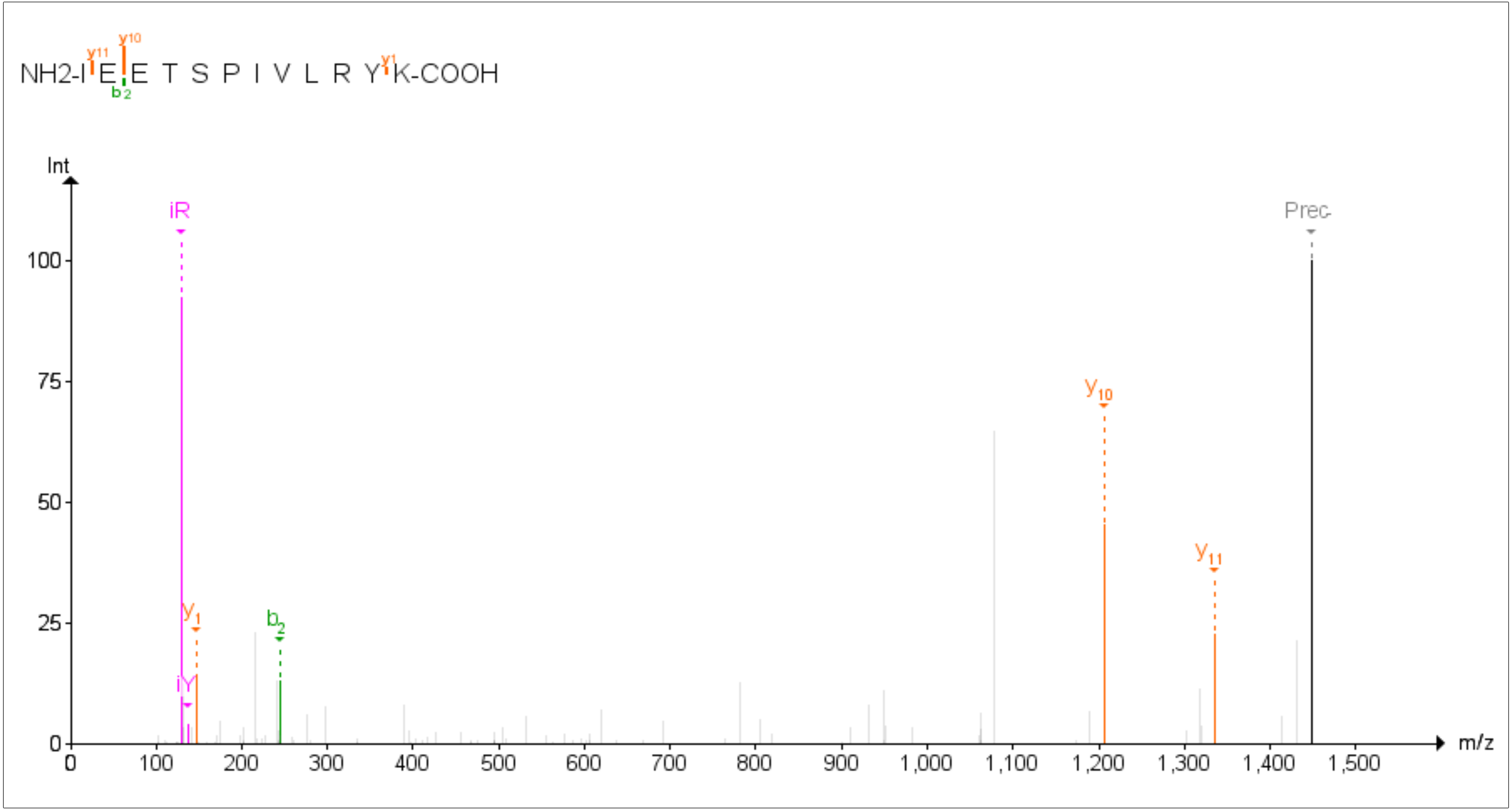

NH2-I E E T S P I V L R Y K-COOH
y11
y10
y1
b2
Int
iR
Prec
100
75
50
25
0
y10
y11
y1
b2
iY
0
100
200
300
400
500
600
700
800
900
1,000
1,100
1,200
1,300
1,400
1,500
m/z

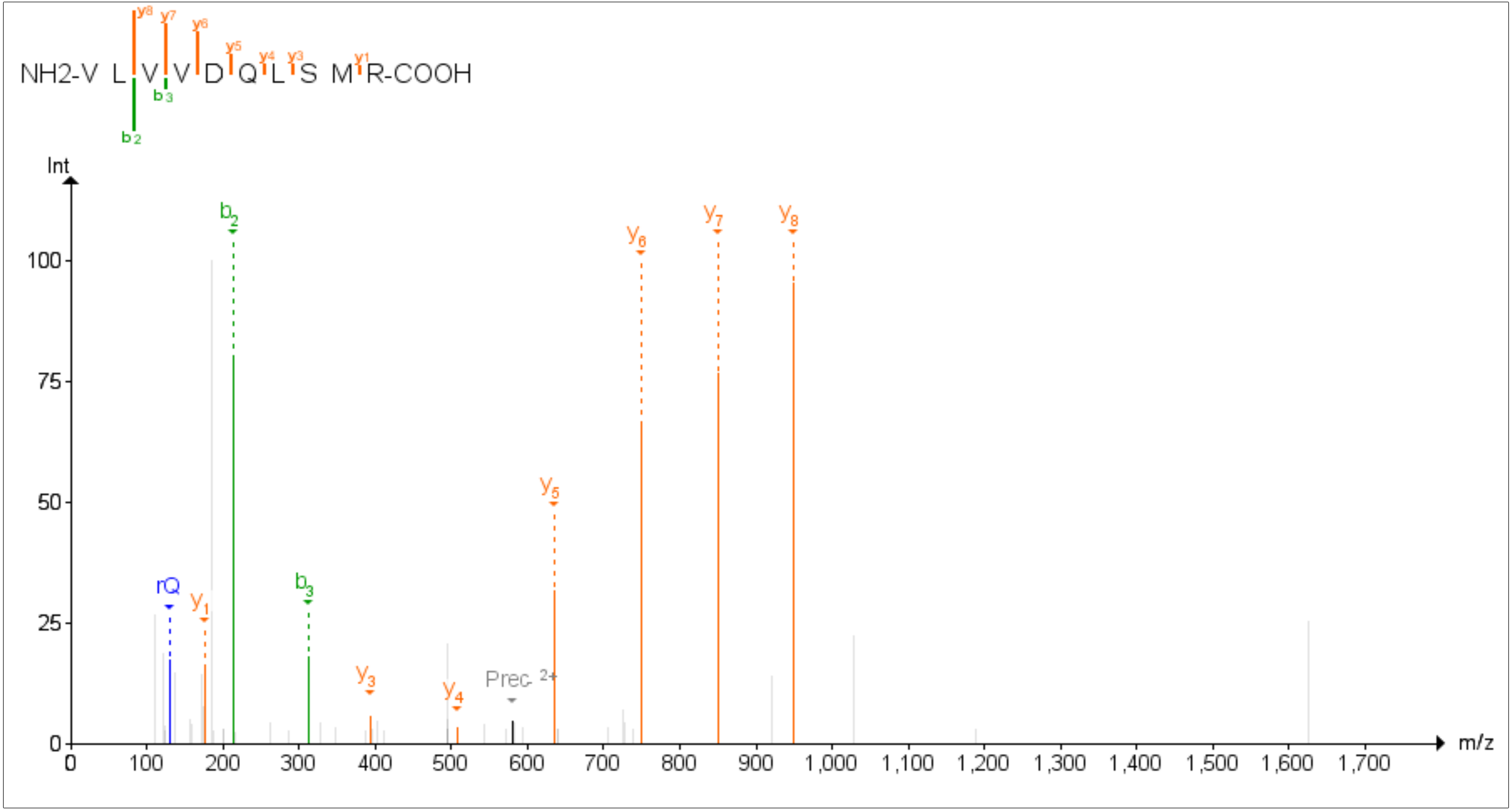

NH2-V L V V D Q L S M R-COOH
y8
y7
y6
y5
y4
y3
y1
b2
b3
Int
100
75
50
25
0
b2
y6
y7
y8
y5
rQ
y1
b3
y3
y4
Prec 2+
0
100
200
300
400
500
600
700
800
900
1,000
1,100
1,200
1,300
1,400
1,500
1,600
1,700
m/z

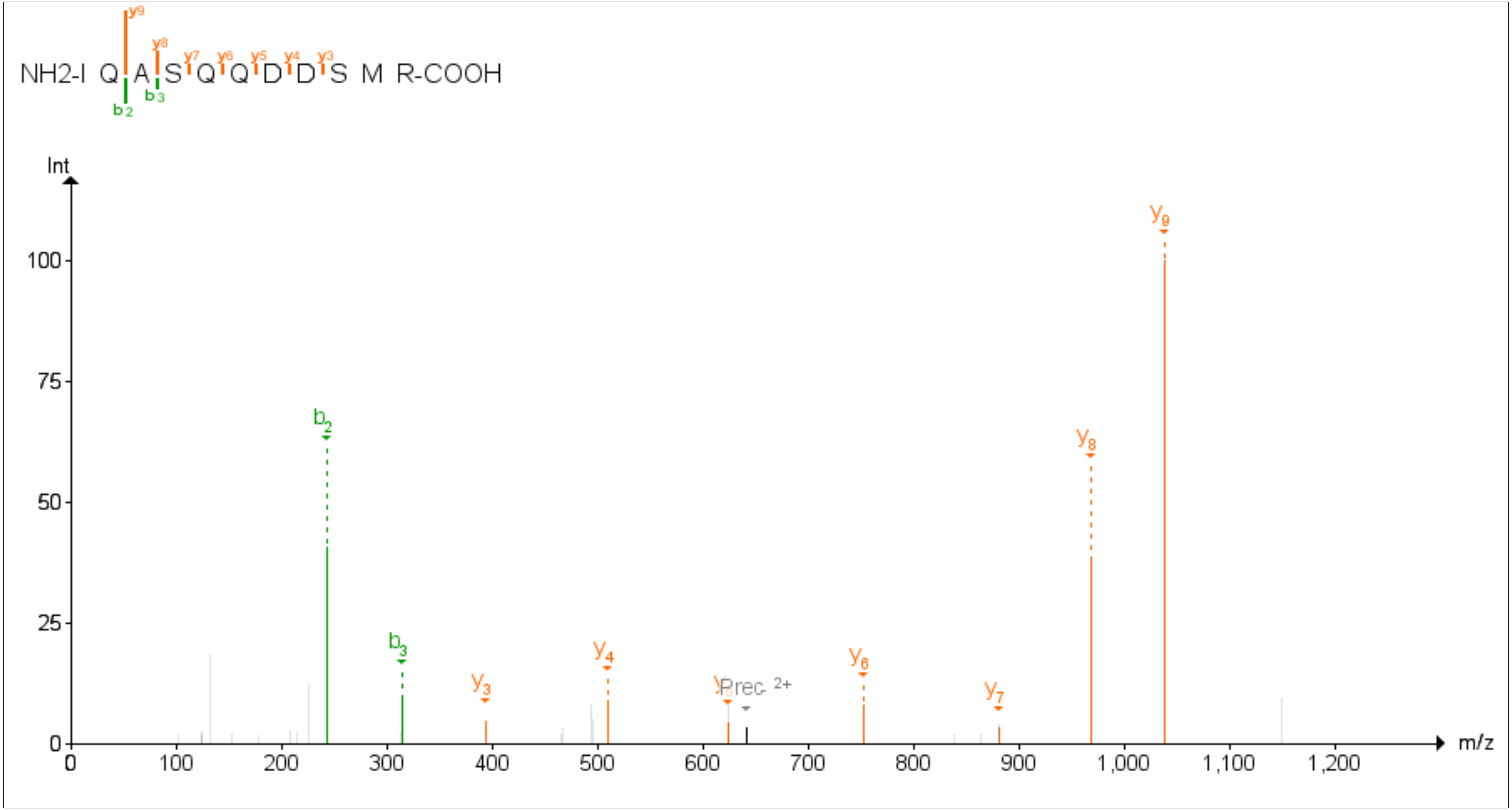

NH2-I Q A S Q Q D D S M R-COOH
y9
y8
y7
y6
y5
y4
y3
b 2
b 3
Int
100
75
50
25
0
b2
b3
y3
y4
Prec. 2+
y6
y7
y8
y9
0
100
200
300
400
500
600
700
800
900
1,000
1,100
1,200
m/z

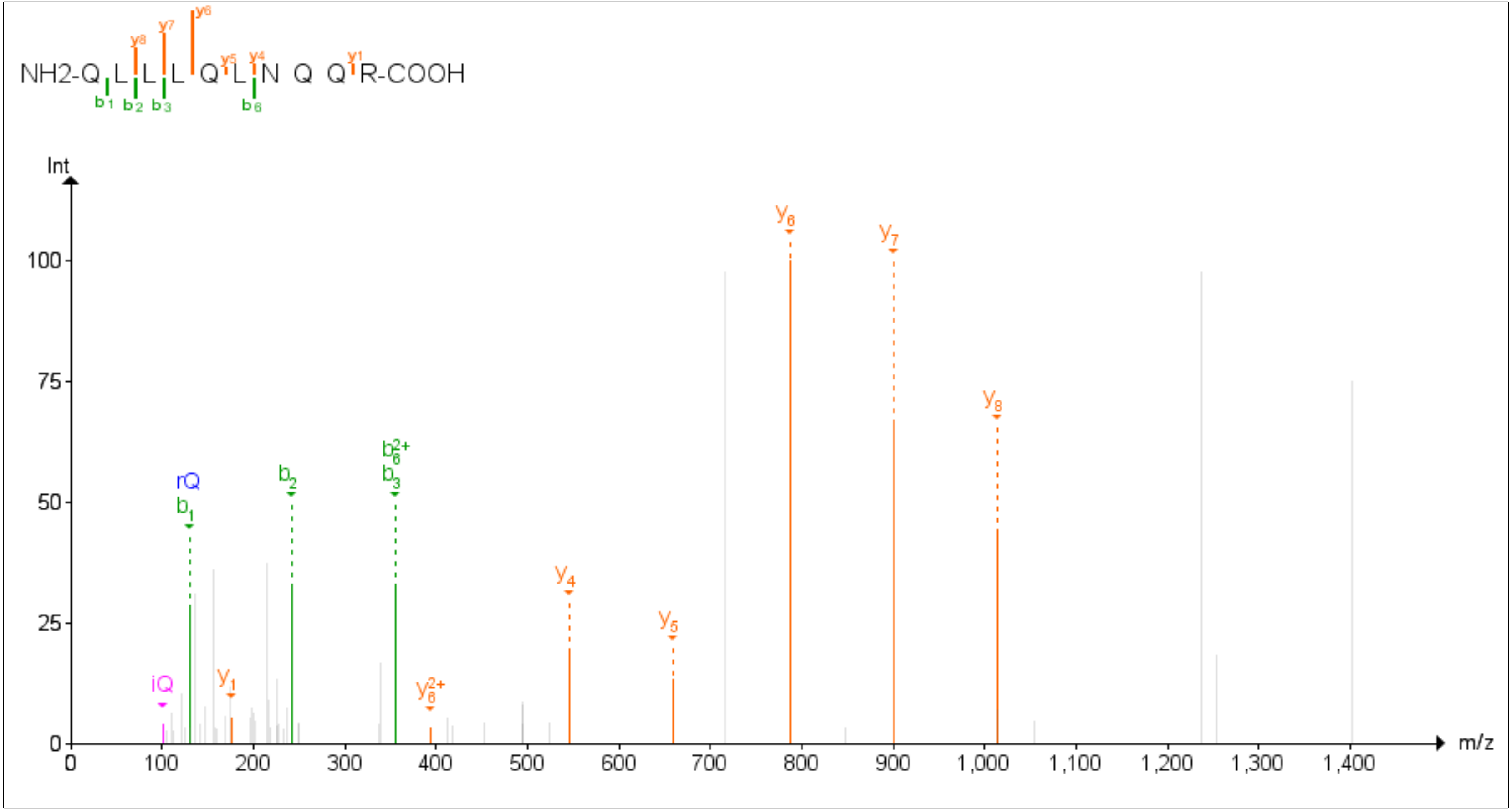
NH2-Q L L L Q L N Q Q R-COOH
y8 y7 y6 y5 y4 y1
b1 b2 b3 b6
Int
100
75
50
25
0
m/z
0 100 200 300 400 500 600 700 800 900 1,000 1,100 1,200 1,300 1,400
iQ
rQ
b_1
y_1
b_2
b_3
b_6^{2+}
y_6^{2+}
y_4
y_5
y_6
y_7
y_8

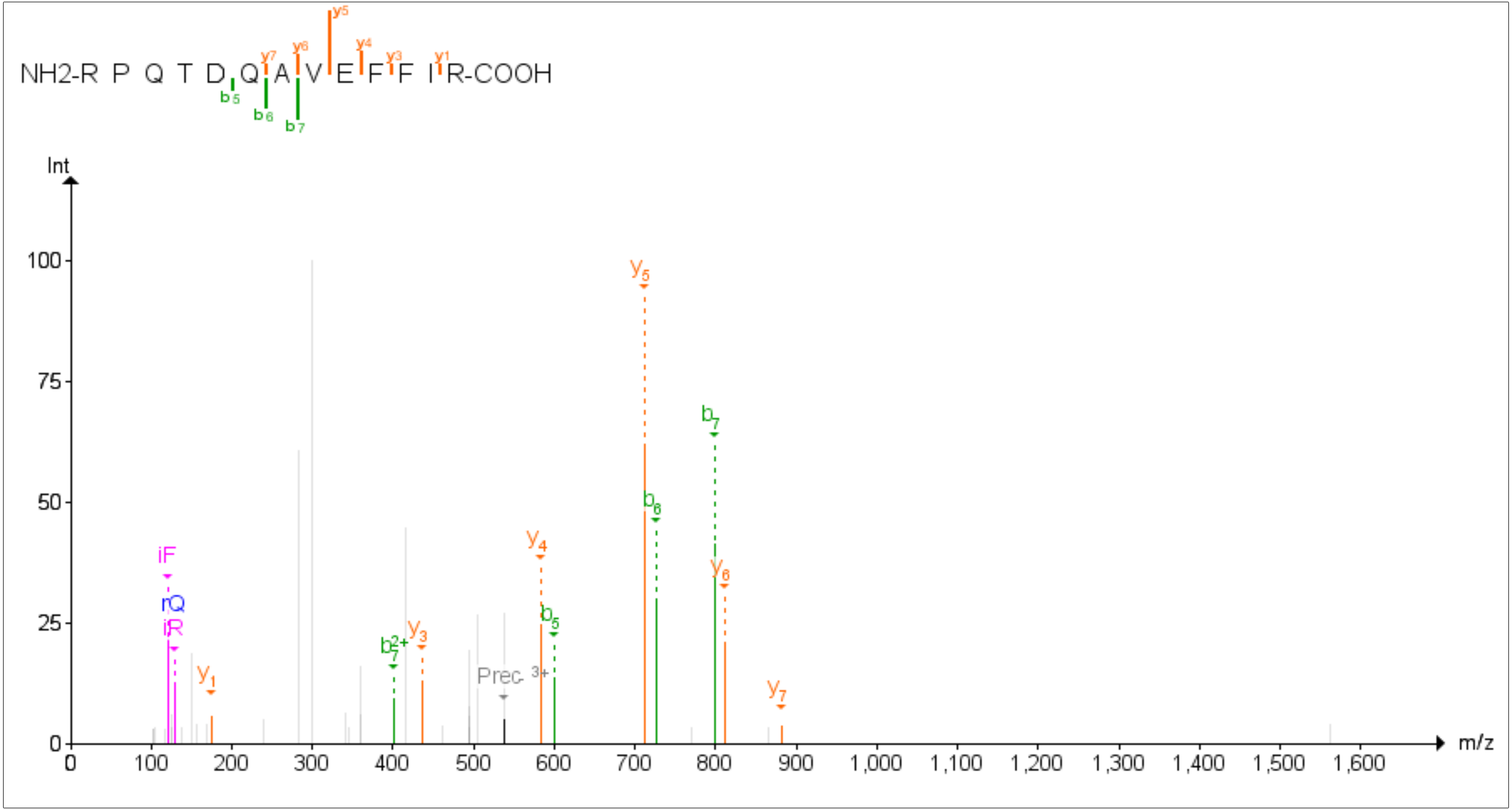

NH2-R P Q T D Q A V E F F I R-COOH
y5 y7 y6 y4 y3 y1
b5 b6 b7
Int
m/z
y5
b7
b6
y4
y6
b5
y3
b7 2+
y1
y7
iF
rQ
iR
Prec 3+

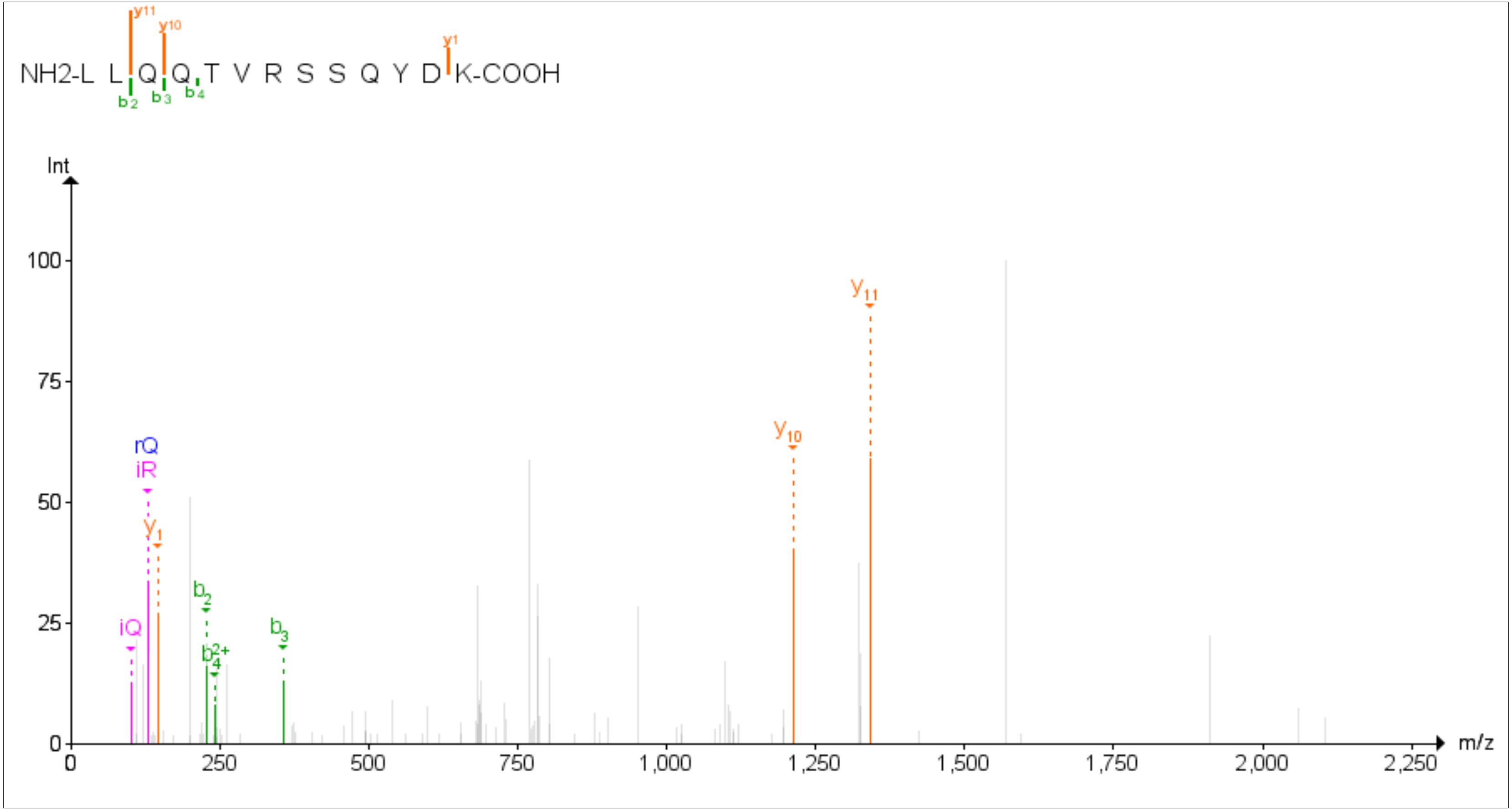

NH2-L L Q Q T V R S S Q Y D K-COOH
y11
y10
y1
b2
b3
b4
Int
100
75
50
25
0
rQ
iR
y1
iQ
b2
b4 2+
b3
y10
y11
0
250
500
750
1,000
1,250
1,500
1,750
2,000
2,250
m/z

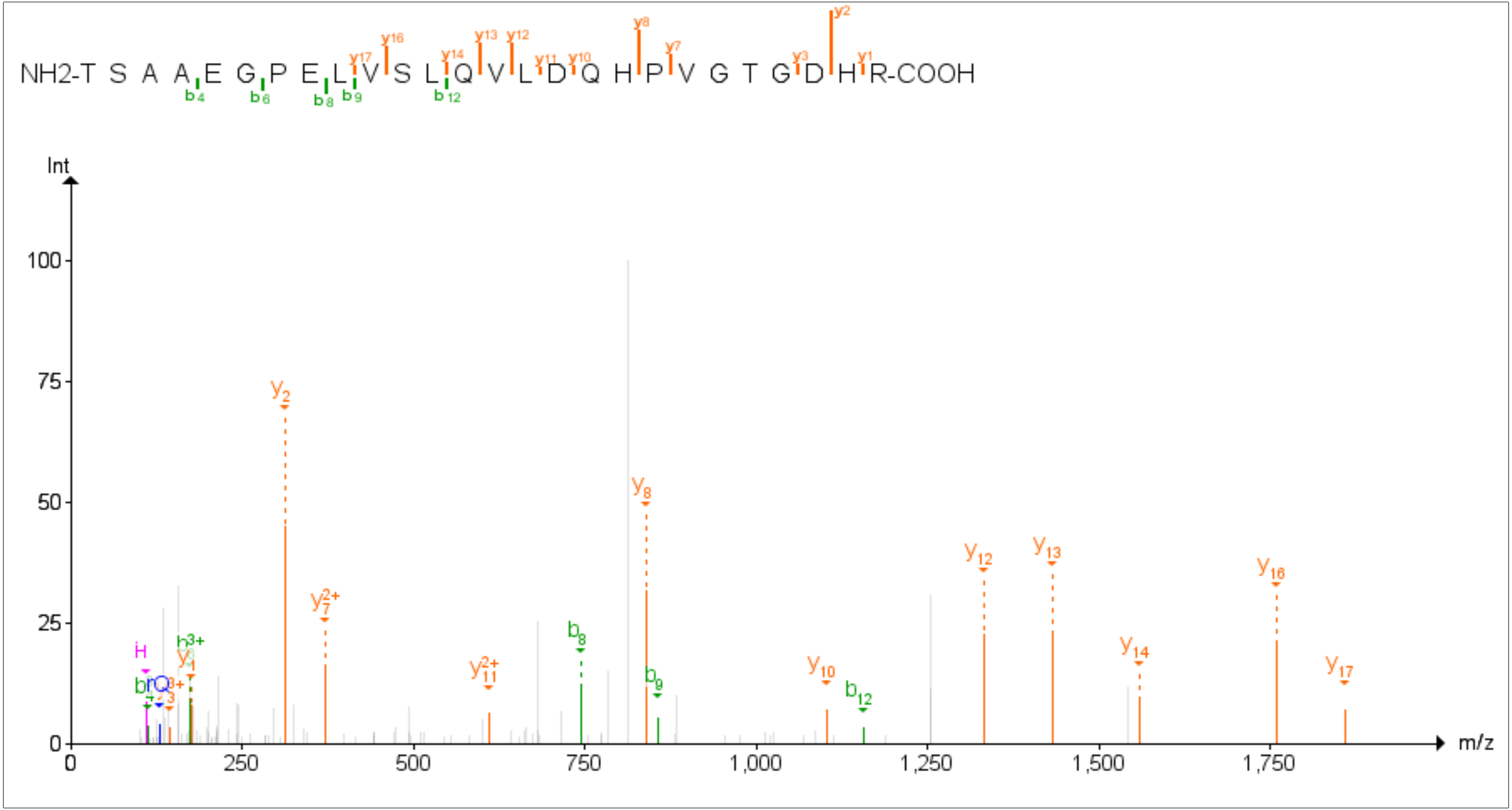

NH2-T S A A E G P E L V S L Q V L D Q H P V G T G D H R-COOH
b4 b6 b8 b9 b12
y17 y16 y14 y13 y12 y11 y10 y8 y7 y3 y2 y1
Int
100
75
50
25
0
y2
y8
y12
y13
y16
y14
y17
y10
b12
b9
b8
y7 2+
y11 2+
m/z
0
250
500
750
1,000
1,250
1,500
1,750

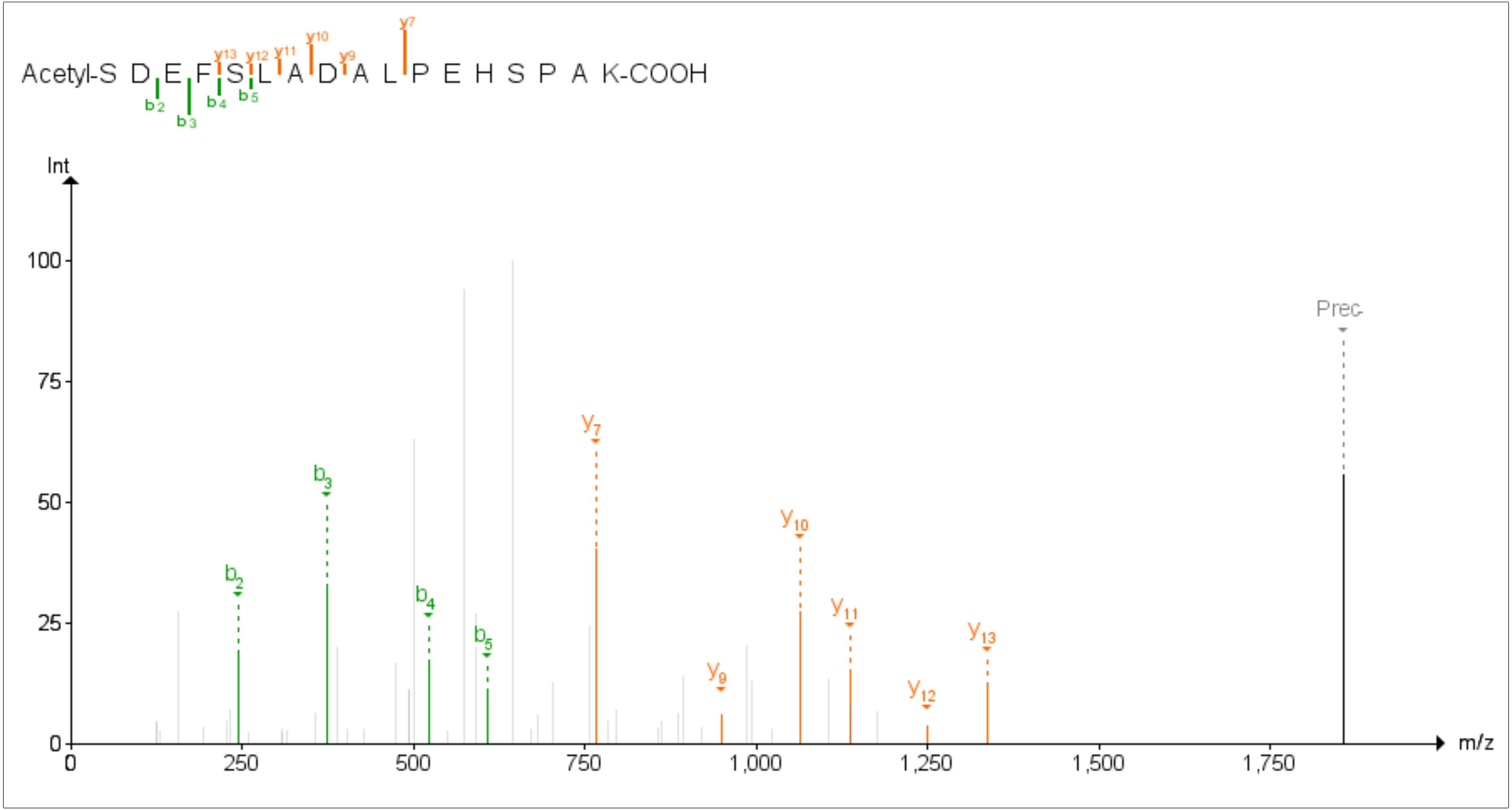

Acetyl-S D E F S L A D A L P E H S P A K-COOH
b2
b3
b4
b5
y13
y12
y11
y10
y9
y7
Int
100
75
50
25
0
Prec.
m/z
0
250
500
750
1,000
1,250
1,500
1,750

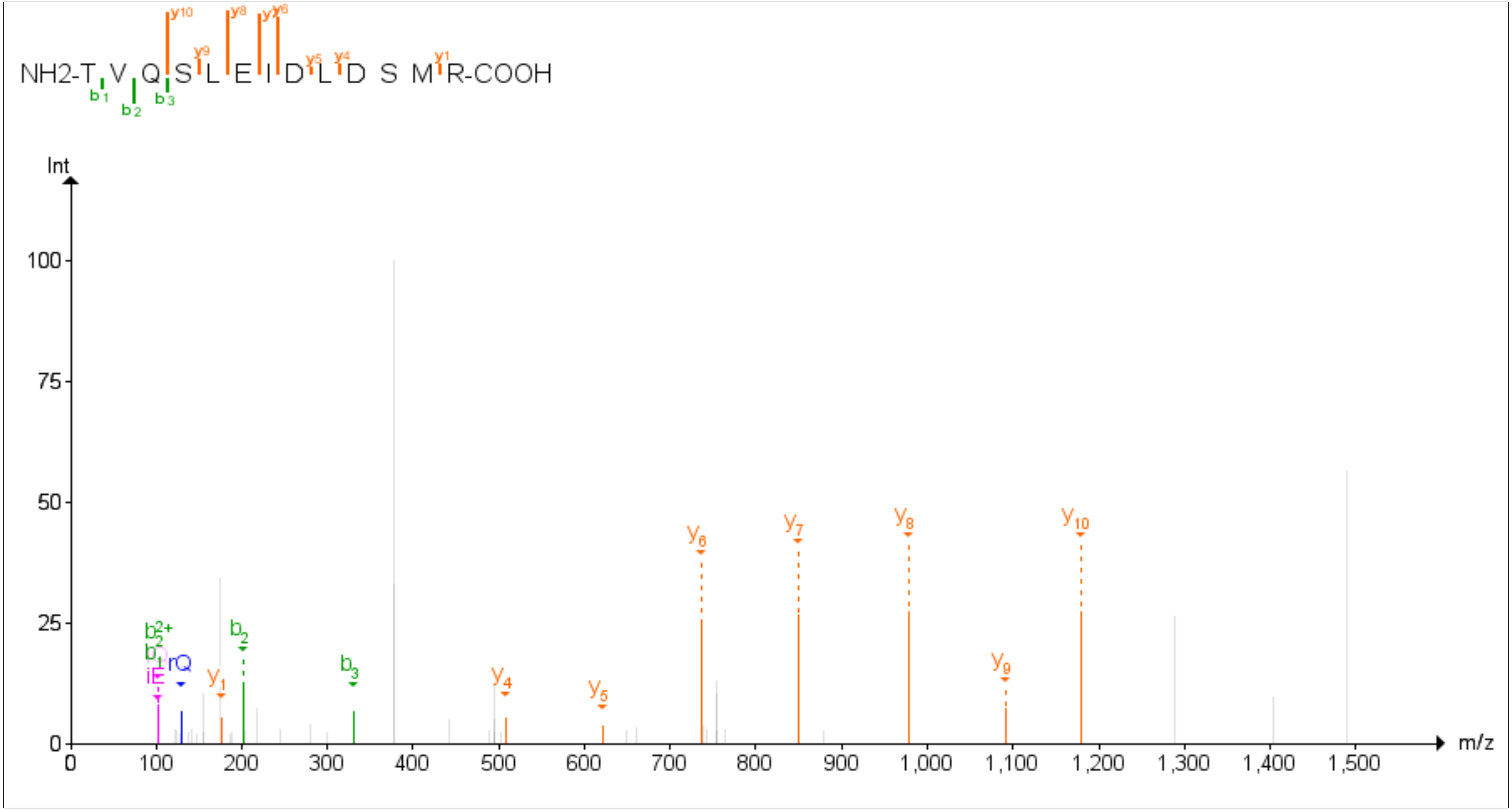

NH2-T V Q S L E I D L D S M R-COOH
y10 y9 y8 y7 y6 y5 y4 y1
b1 b2 b3
Int
100
75
50
25
0
m/z
0 100 200 300 400 500 600 700 800 900 1,000 1,100 1,200 1,300 1,400 1,500
y6 y7 y8 y10 y9 y4 y5 y1
b2 b3 rQ iE

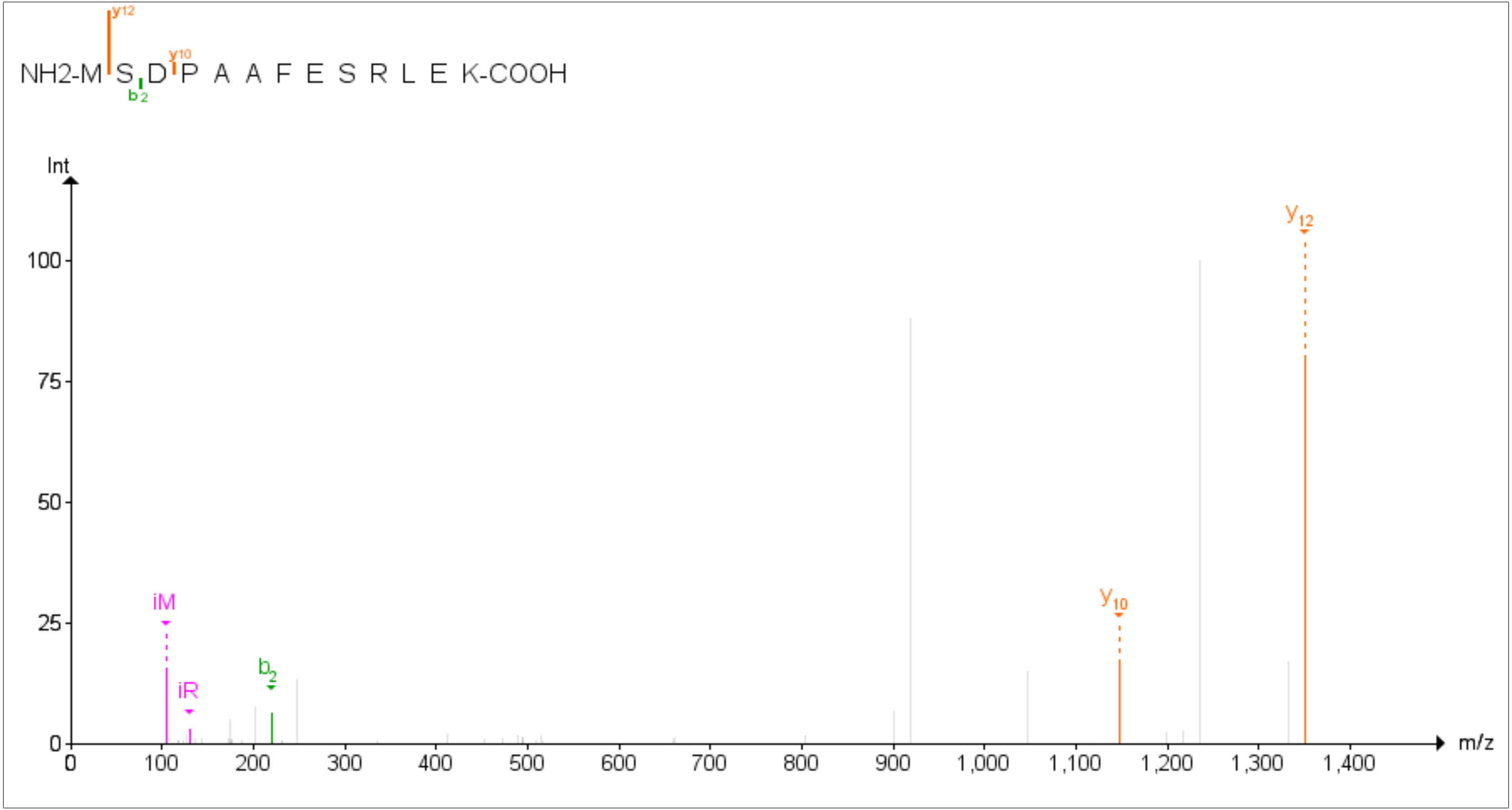

NH2-M S D P A A F E S R L E K-COOH
y12
y10
b2
Int
100
75
50
25
0
iM
iR
b2
y10
y12
m/z
0
100
200
300
400
500
600
700
800
900
1,000
1,100
1,200
1,300
1,400

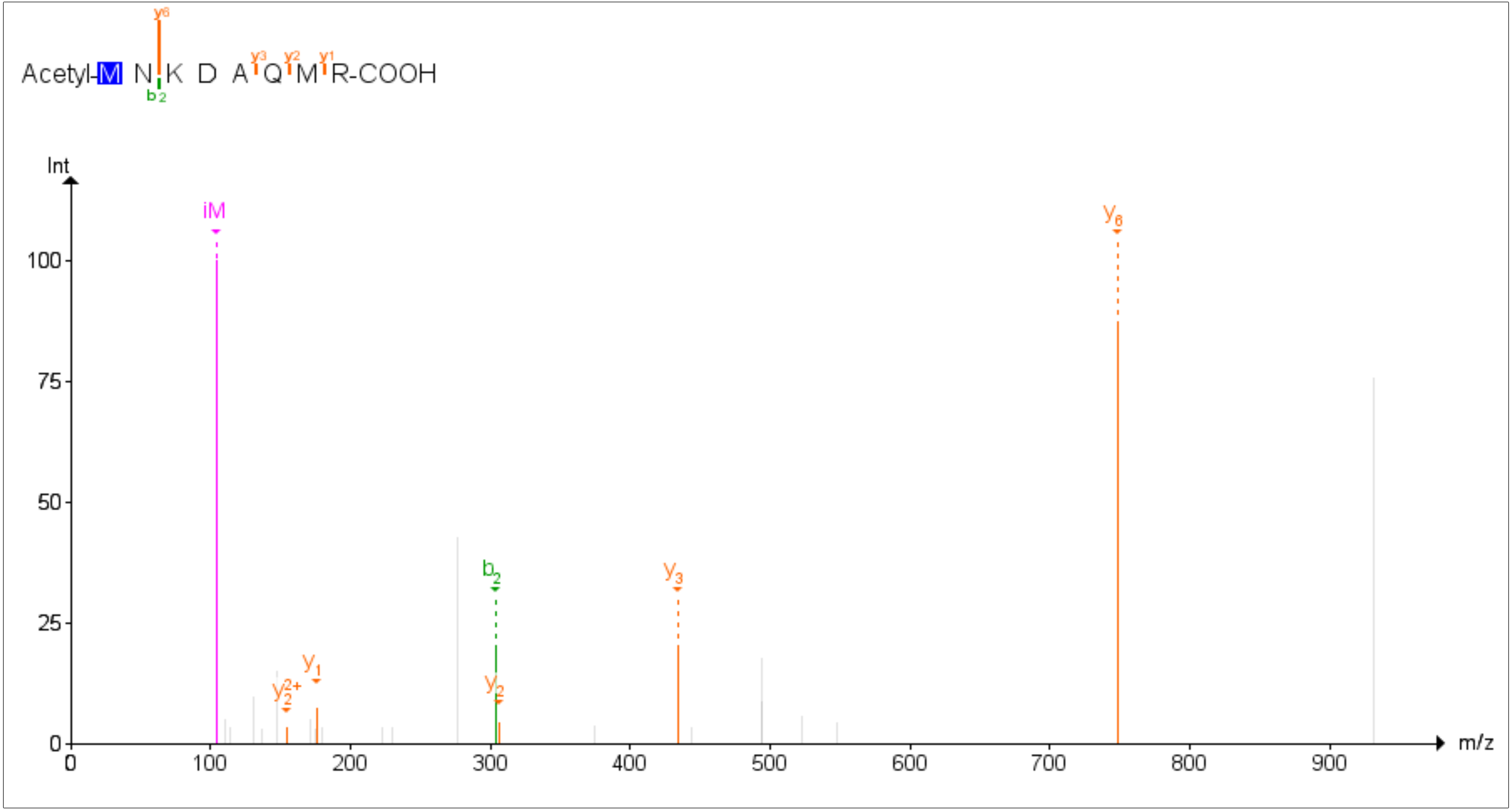

Acetyl-M N K D A Q M R-COOH
y6
y3
y2
y1
b2
Int
iM
y6
b2
y3
y1
y2
y2 2+
100
75
50
25
0
0
100
200
300
400
500
600
700
800
900
m/z

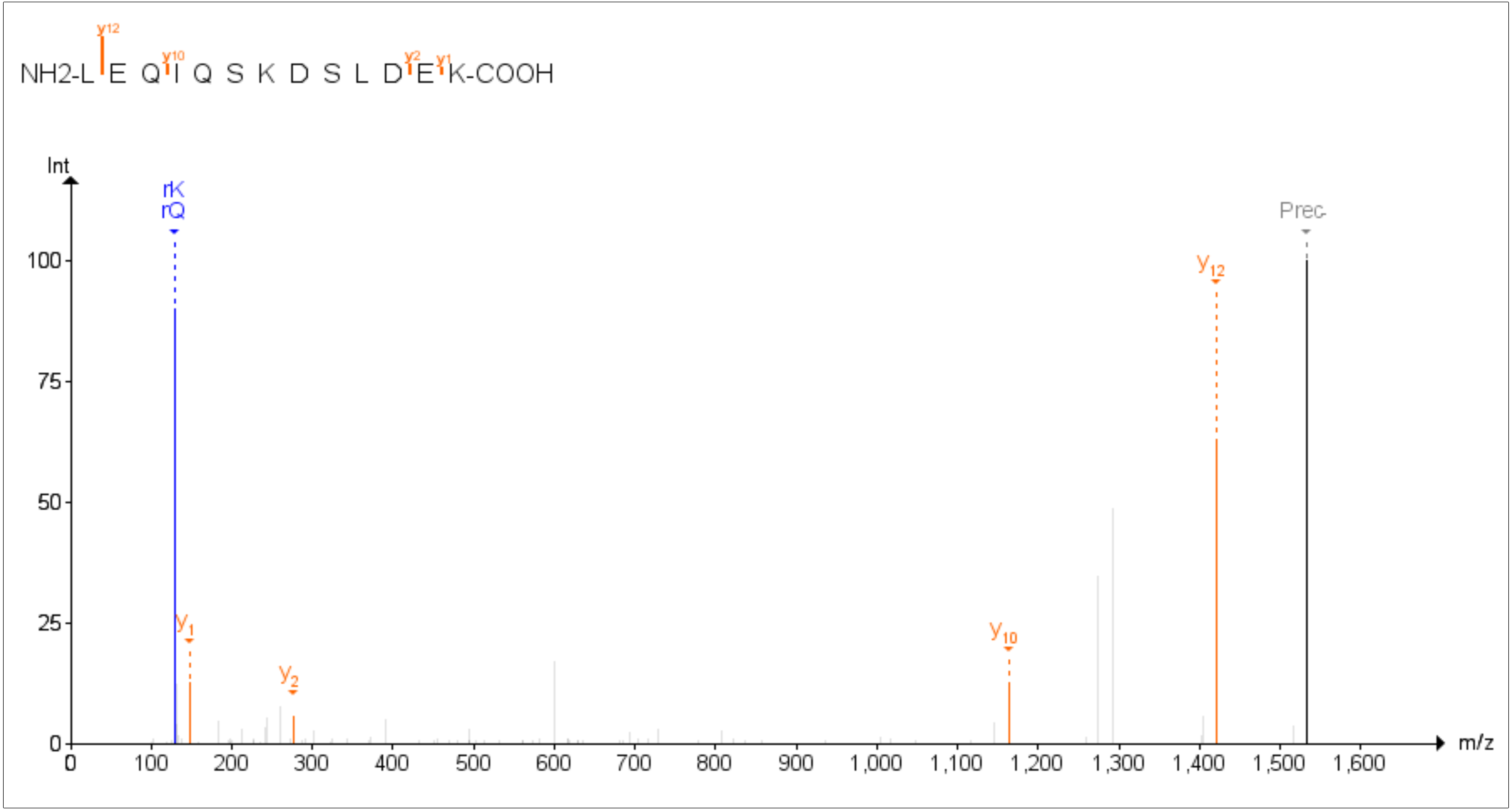

NH2-L E Q I Q S K D S L D E K-COOH
y12
y10
y2
y1
Int
rK
rQ
Prec
y_{12}
y_1
y_2
y_{10}
100
75
50
25
0
0
100
200
300
400
500
600
700
800
900
1,000
1,100
1,200
1,300
1,400
1,500
1,600
m/z

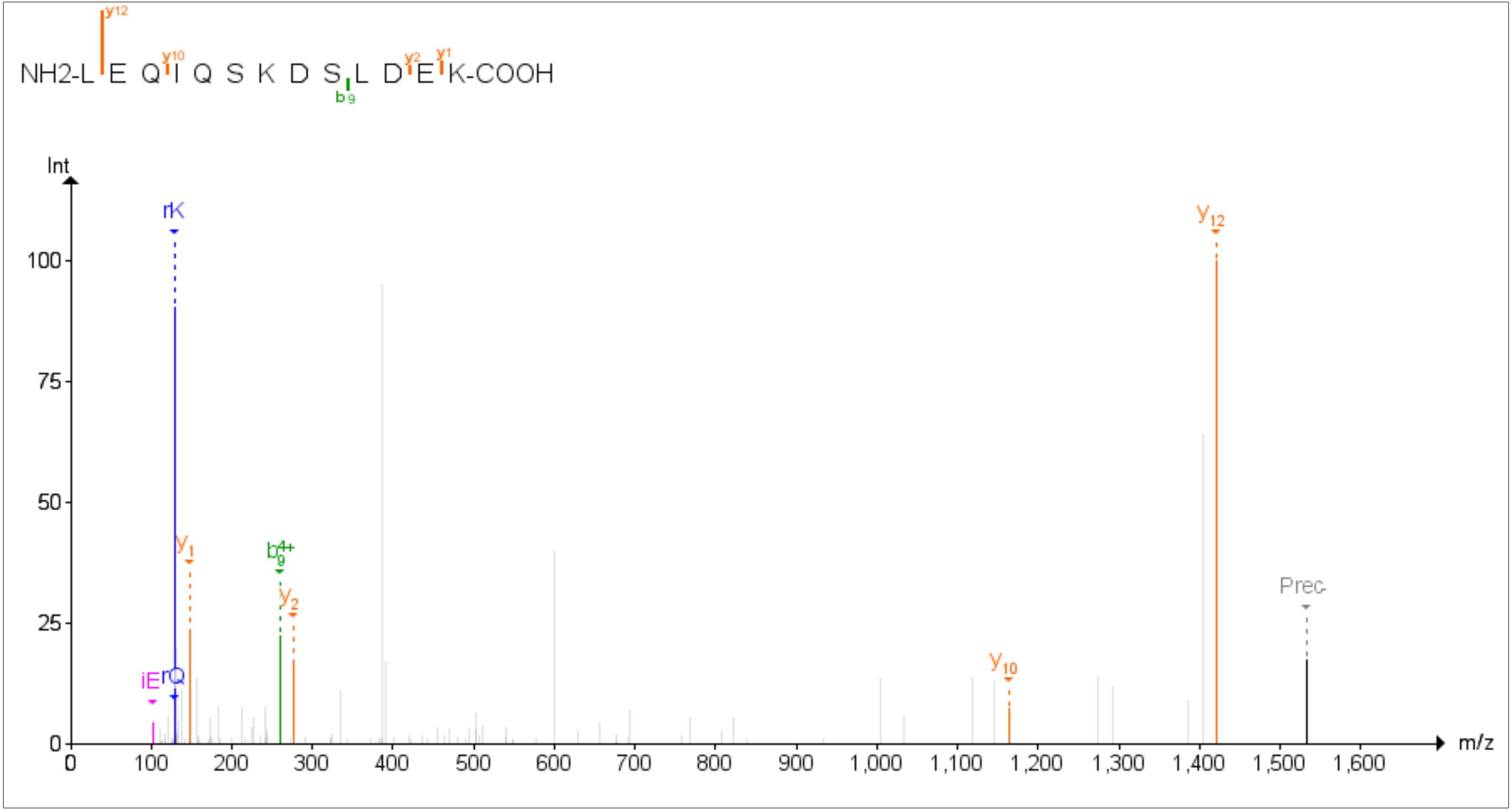
NH2-L E Q I Q S K D S L D E K-COOH
y12
y10
y2
y1
b9
Int
100
75
50
25
0
rK
y12
y1
b9 4+
y2
iE
rQ
y10
Prec
0
100
200
300
400
500
600
700
800
900
1,000
1,100
1,200
1,300
1,400
1,500
1,600
m/z

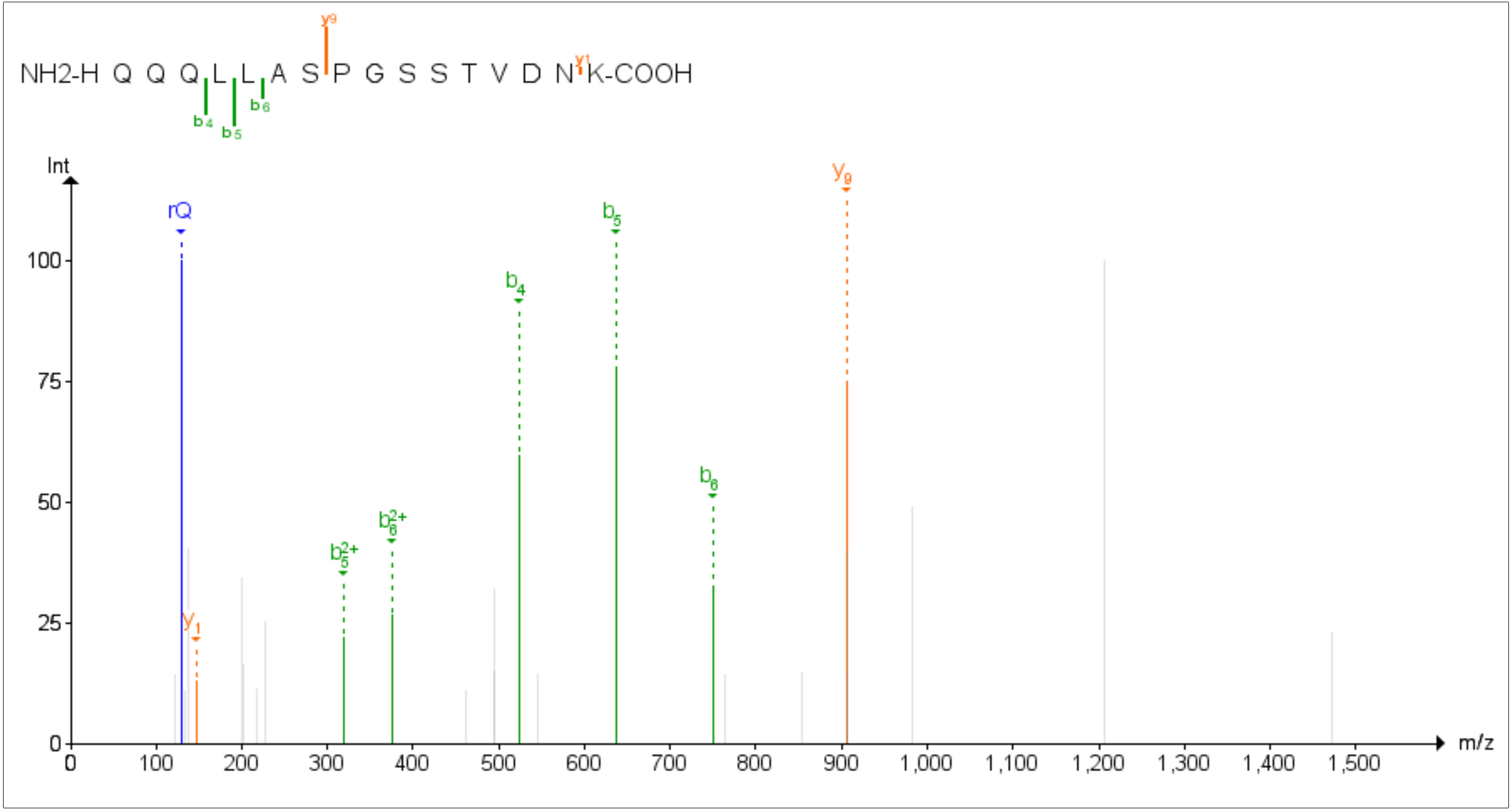

NH2-H Q Q Q L L A S P G S S T V D N K-COOH
y9
y1
b4
b5
b6
Int
m/z
rQ
y1
b5 2+
b6 2+
b4
b5
b6
y9
0
25
50
75
100
0
100
200
300
400
500
600
700
800
900
1,000
1,100
1,200
1,300
1,400
1,500

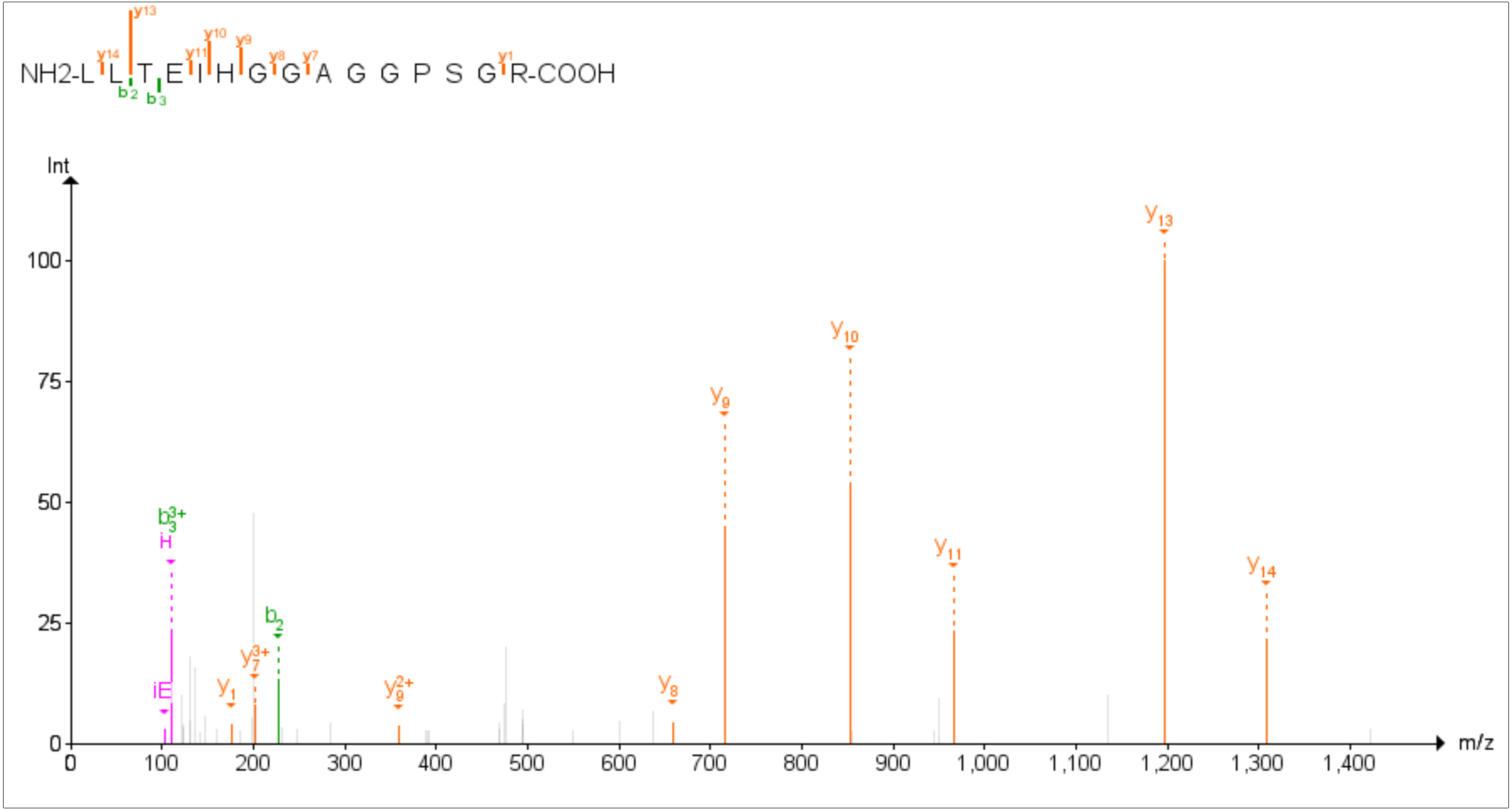

NH2-L L T E I H G G A G G P S G R-COOH
y14
y13
y11
y10
y9
y8
y7
y1
b2
b3
Int
100
75
50
25
0
m/z
0
100
200
300
400
500
600
700
800
900
1,000
1,100
1,200
1,300
1,400
y13
y10
y9
y11
y14
b3 3+
iH
b2
y7 3+
y1
iE
y9 2+
y8

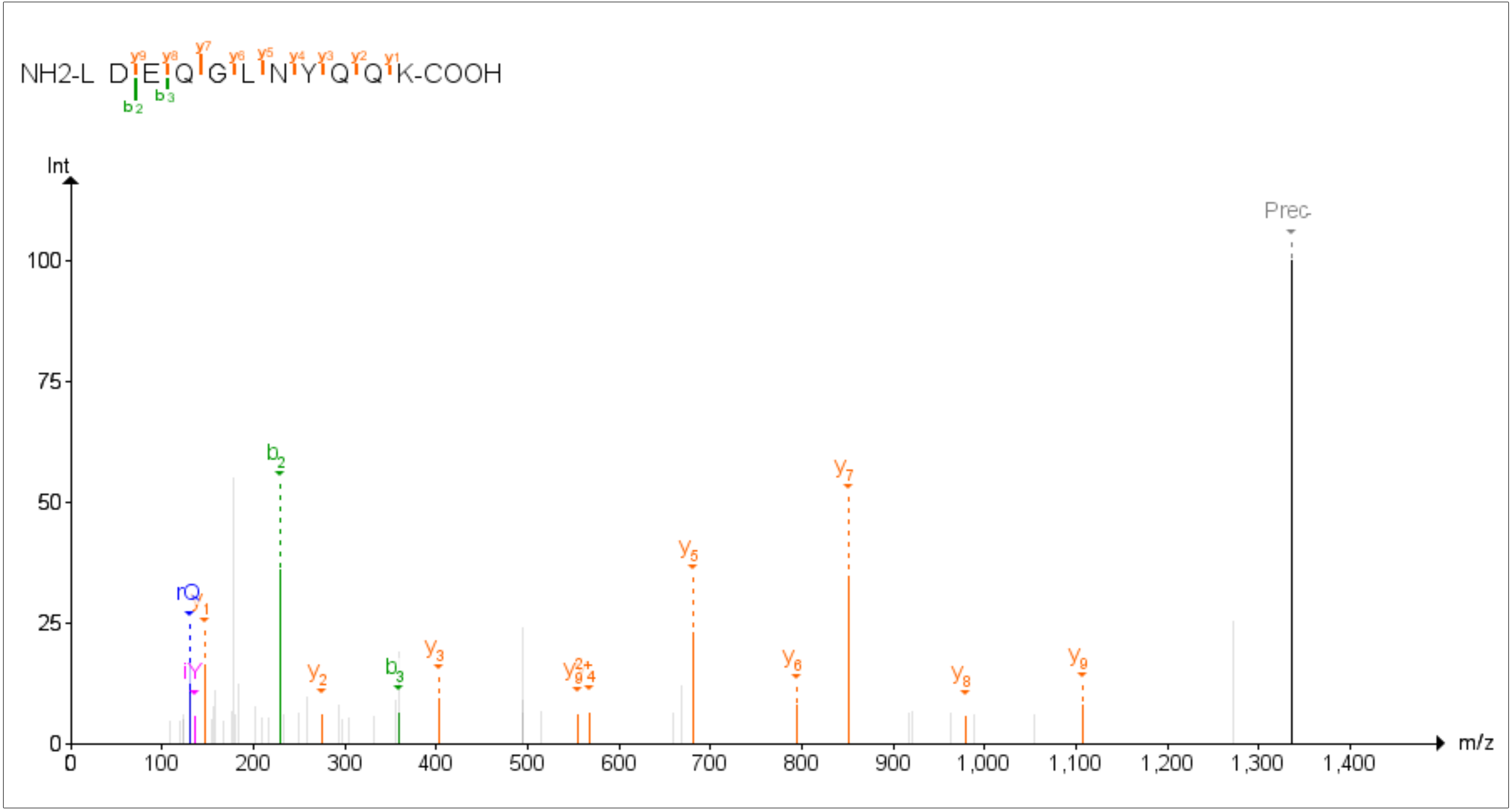
NH2-L D E Q G L N Y Q Q K-COOH
y9 y8 y7 y6 y5 y4 y3 y2 y1
b2 b3
Int
100
75
50
25
0
Prec
b2
y7
y5
rQ
y1
iY
y2
b3
y3
y9 2+
y4
y6
y8
y9
0 100 200 300 400 500 600 700 800 900 1,000 1,100 1,200 1,300 1,400
m/z

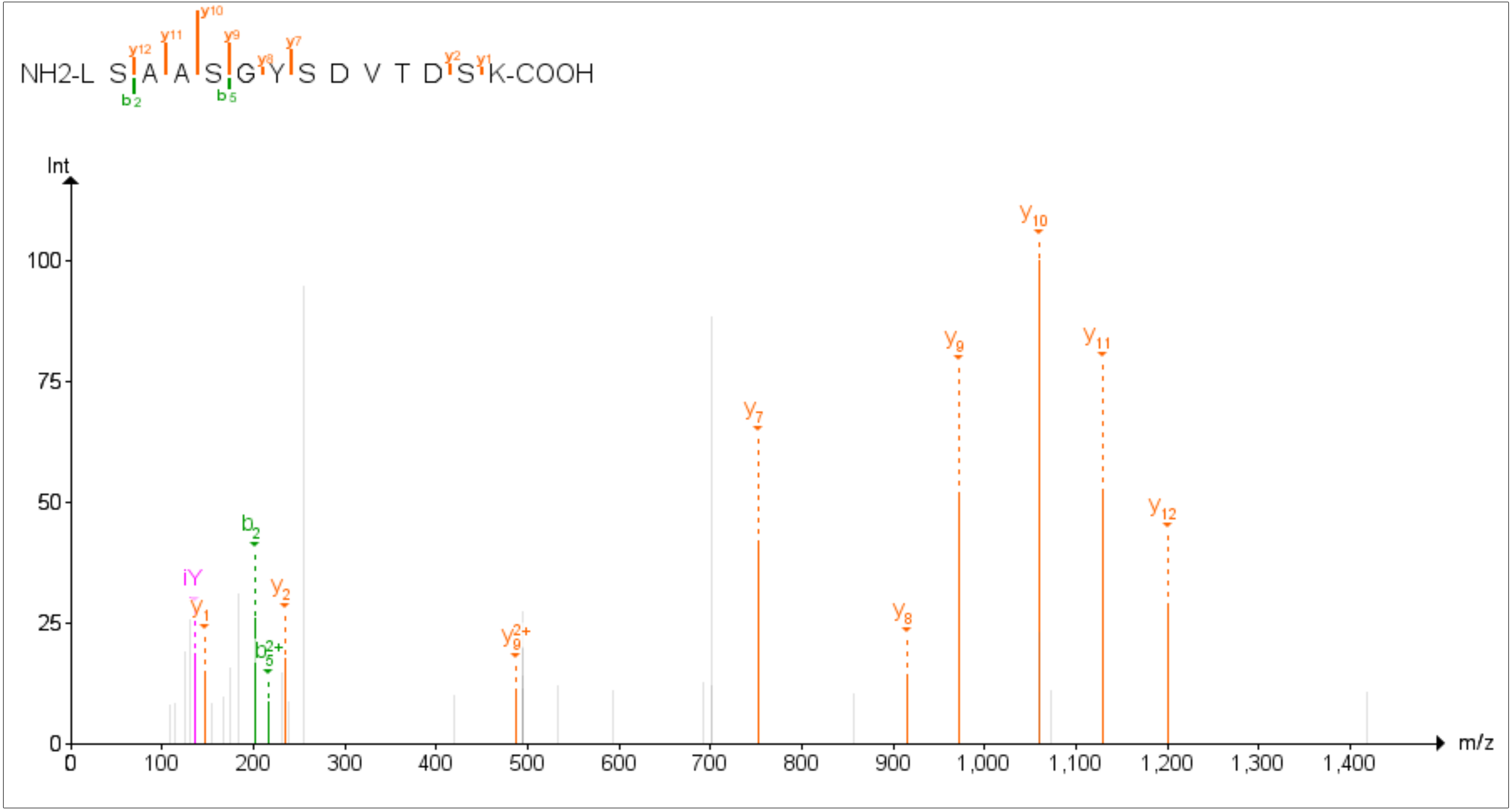

NH2-L S A A S G Y S D V T D S K-COOH
y12 y11 y10 y9 y8 y7 y2 y1
b2 b5
Int
100
75
50
25
0
m/z
0 100 200 300 400 500 600 700 800 900 1,000 1,100 1,200 1,300 1,400
y10
y9
y11
y7
y12
b2
iY
y1
y2
y9 2+
b5 2+
y8

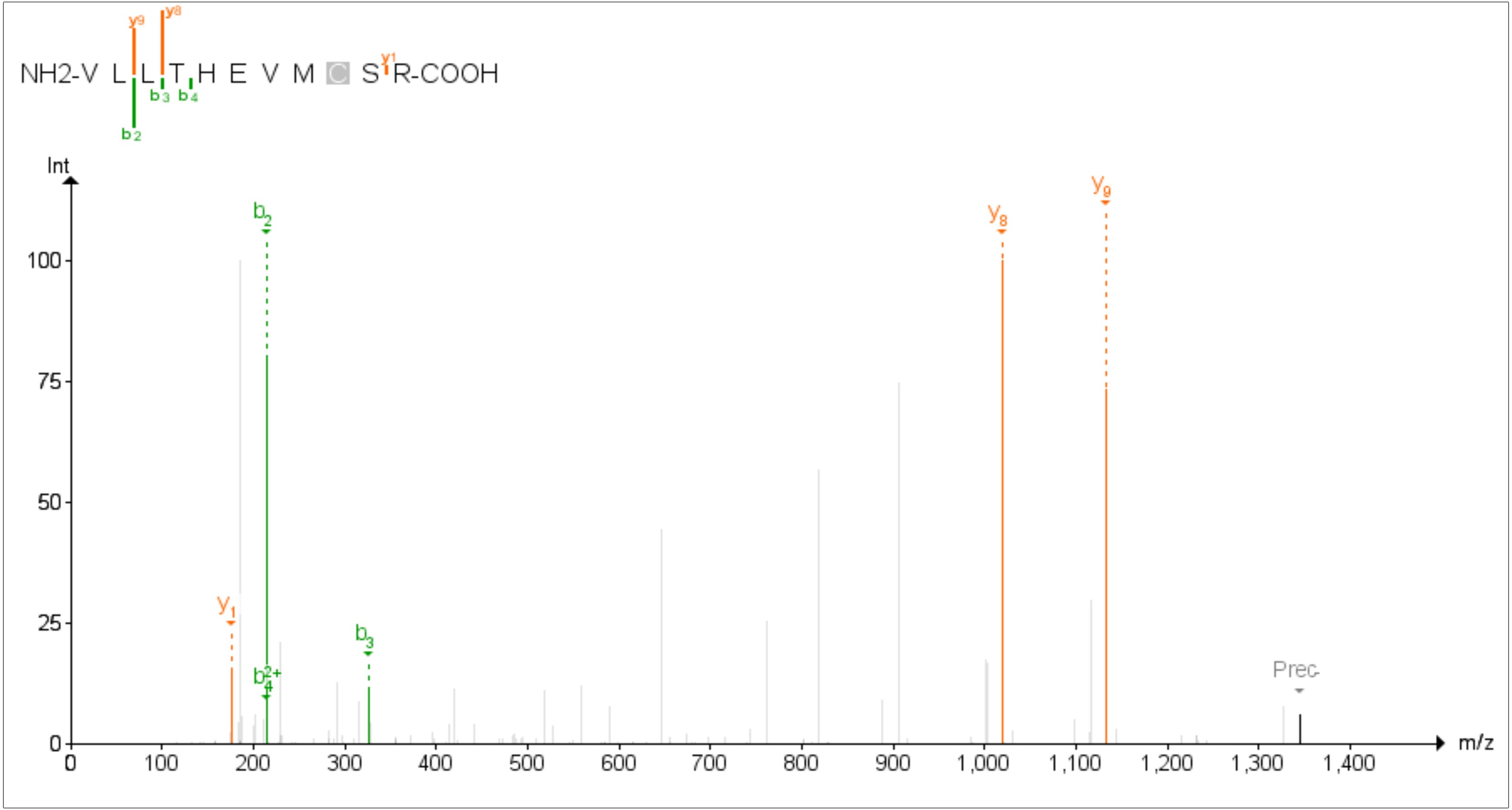

NH2-V L L T H E V M C S R-COOH
y9
y8
y1
b2
b3
b4
Int
100
75
50
25
0
b2
y8
y9
y1
b3
b4 2+
Prec.
0
100
200
300
400
500
600
700
800
900
1,000
1,100
1,200
1,300
1,400
m/z

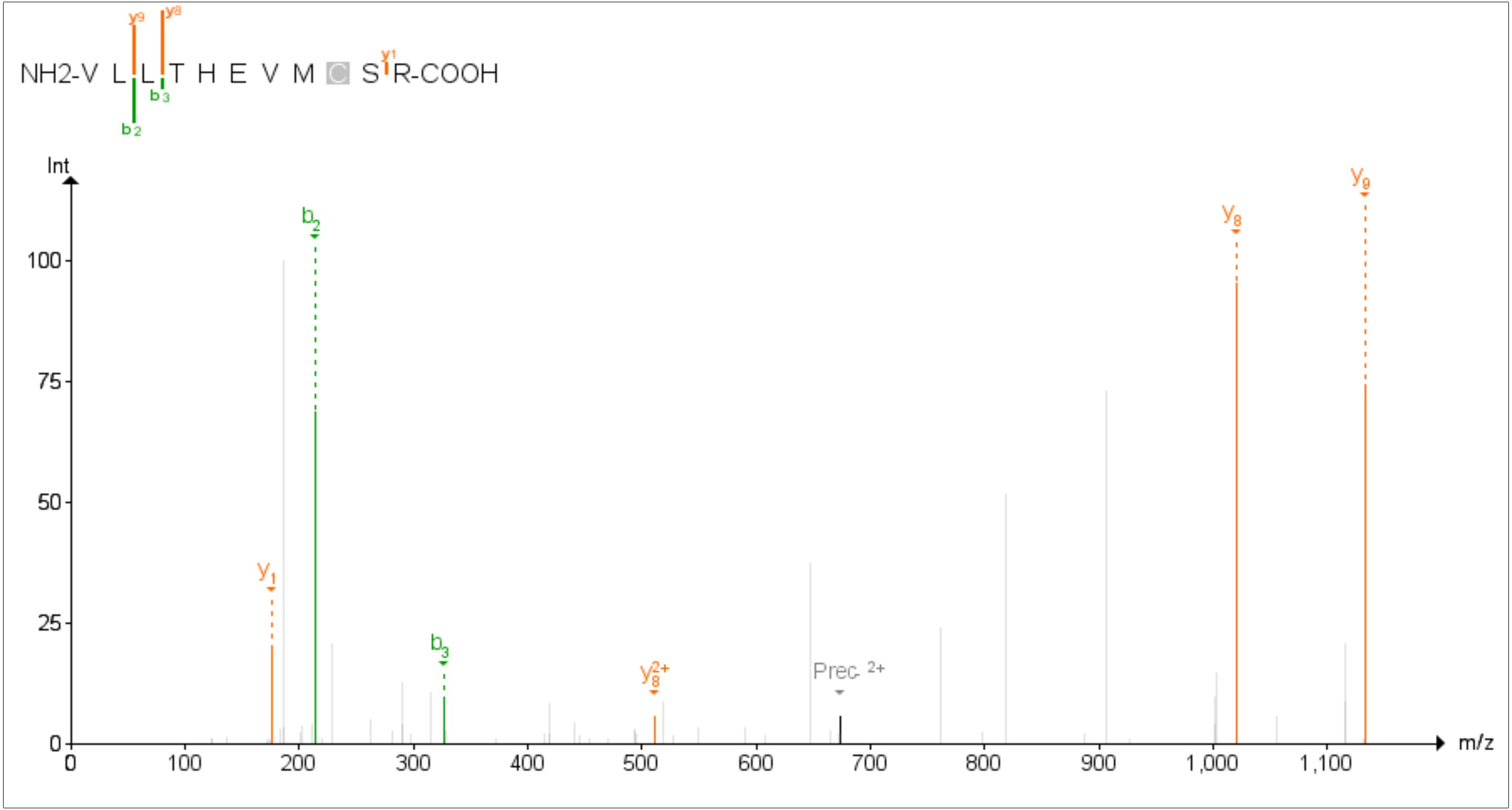

NH2-V L L T H E V M C S R-COOH
y9
y8
y1
b2
b3
Int
m/z
b2
y8
y9
y1
b3
y8 2+
Prec. 2+
0
25
50
75
100
0
100
200
300
400
500
600
700
800
900
1,000
1,100

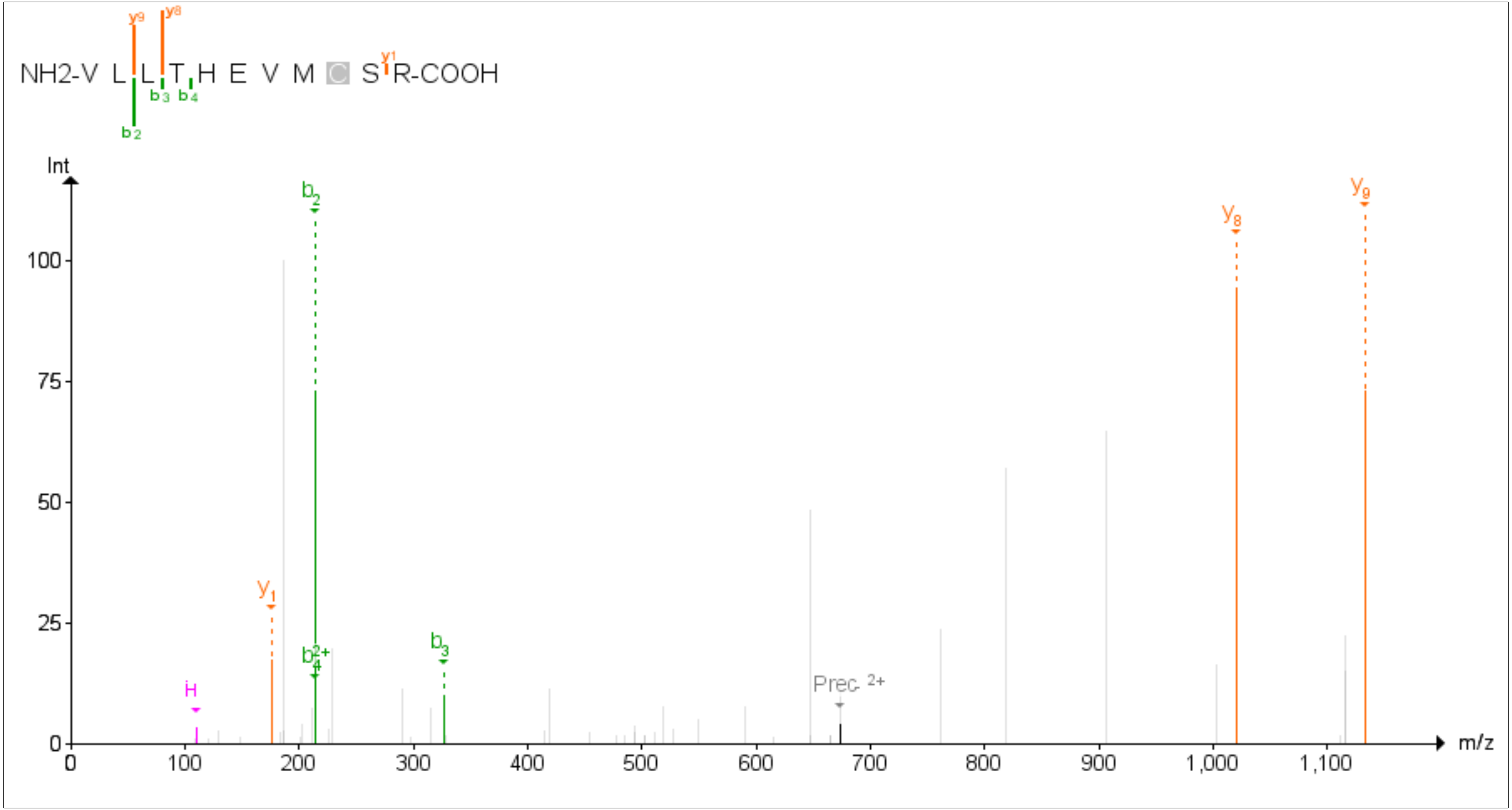
NH2-V L L T H E V M C S R-COOH
y9 y8 y1
b2 b3 b4
Int
100
75
50
25
0
b2
y8
y9
y1
b4 2+
b3
H
Prec. 2+
0 100 200 300 400 500 600 700 800 900 1,000 1,100
m/z

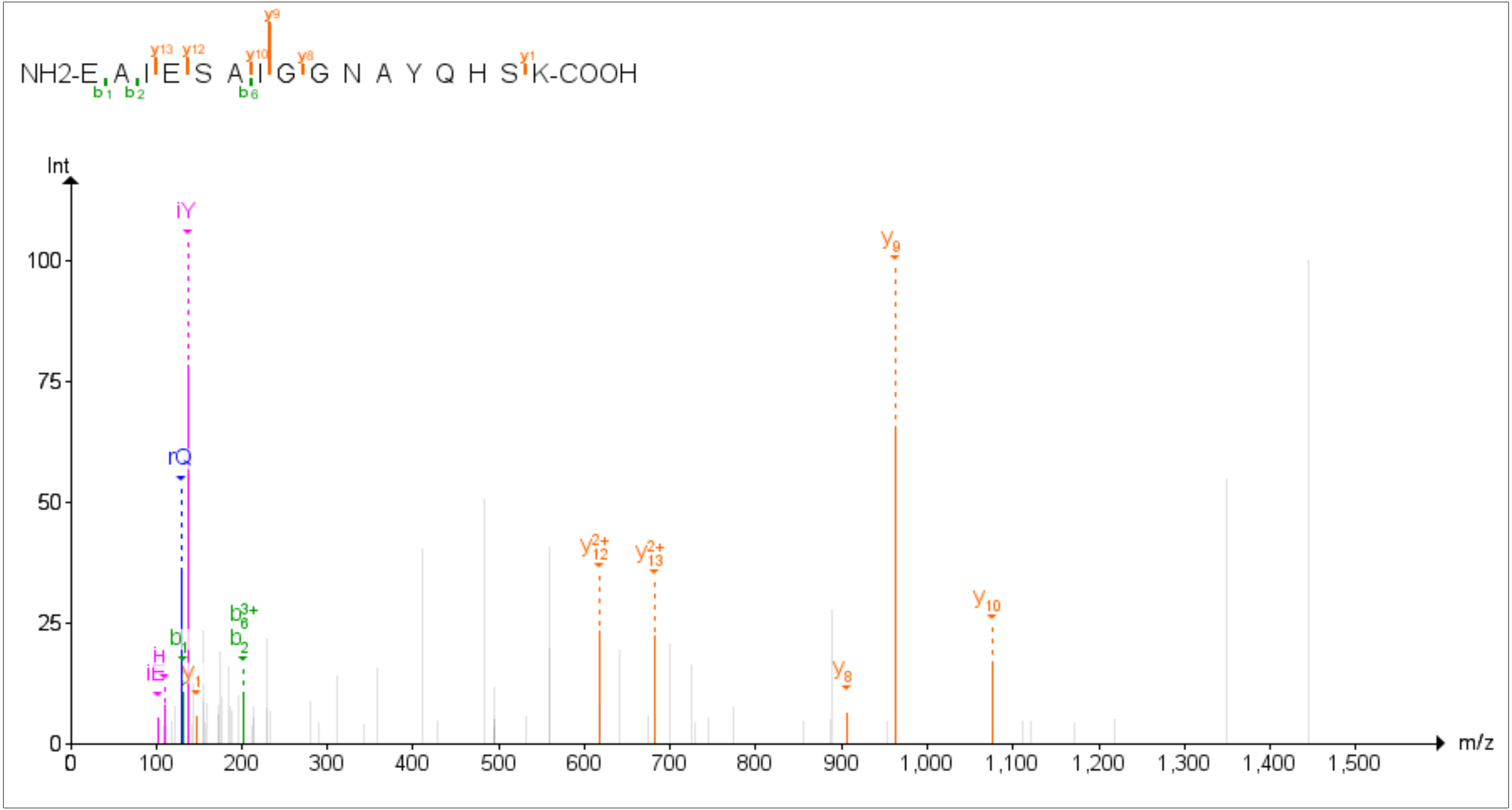

NH2-E A I E S A I G G N A Y Q H S K-COOH
y13 y12 y10 y9 y8 y1
b1 b2 b6
Int
iY
rQ
y9
y10
y8
y1
b1
b2
iH
iE
100
75
50
25
0
0 100 200 300 400 500 600 700 800 900 1,000 1,100 1,200 1,300 1,400 1,500
m/z

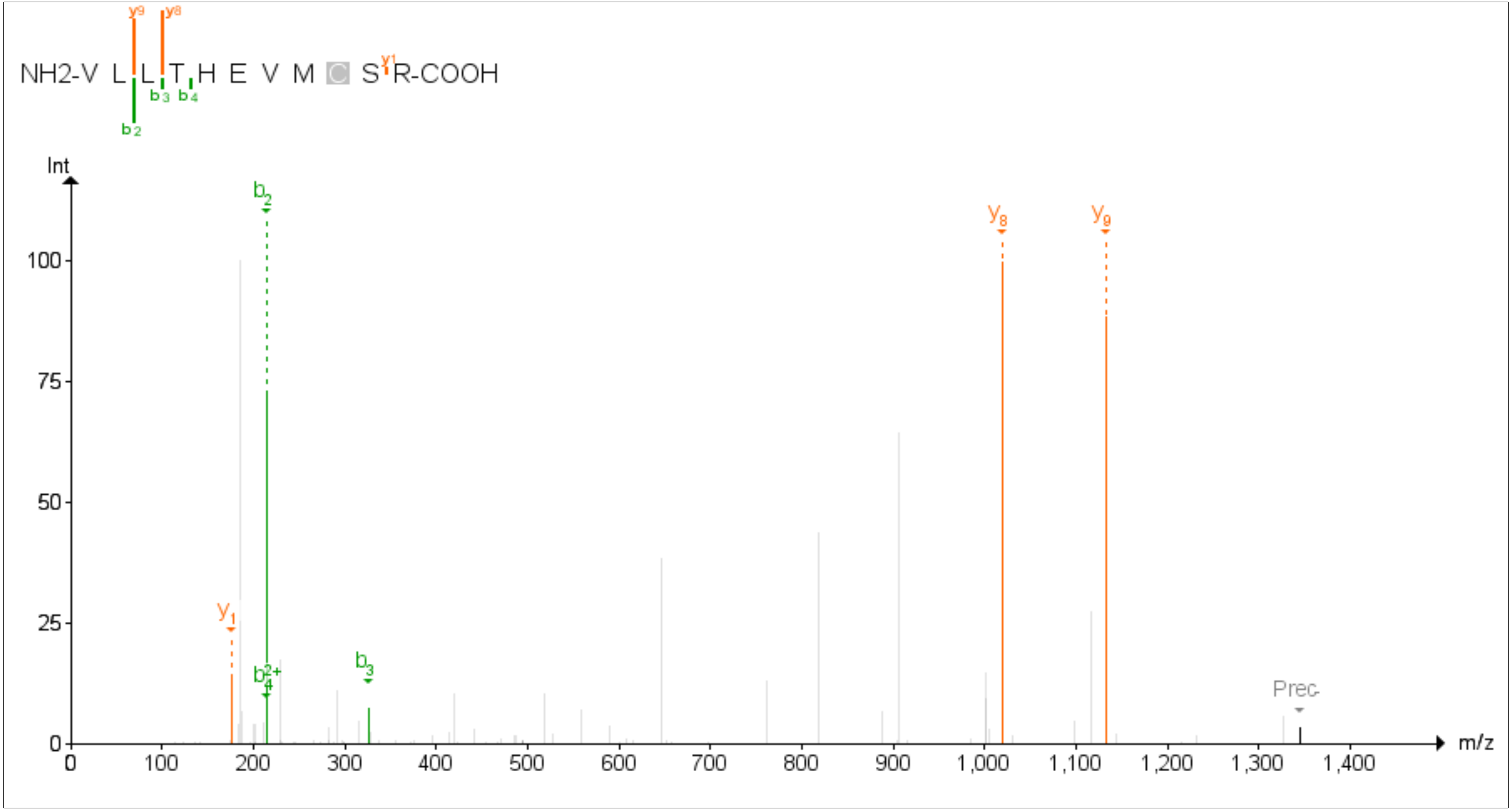

NH2-V L L T H E V M C S R-COOH
y9
y8
y1
b2
b3
b4
Int
100
75
50
25
0
b2
y8
y9
y1
b4 2+
b3
Prec
0
100
200
300
400
500
600
700
800
900
1,000
1,100
1,200
1,300
1,400
m/z

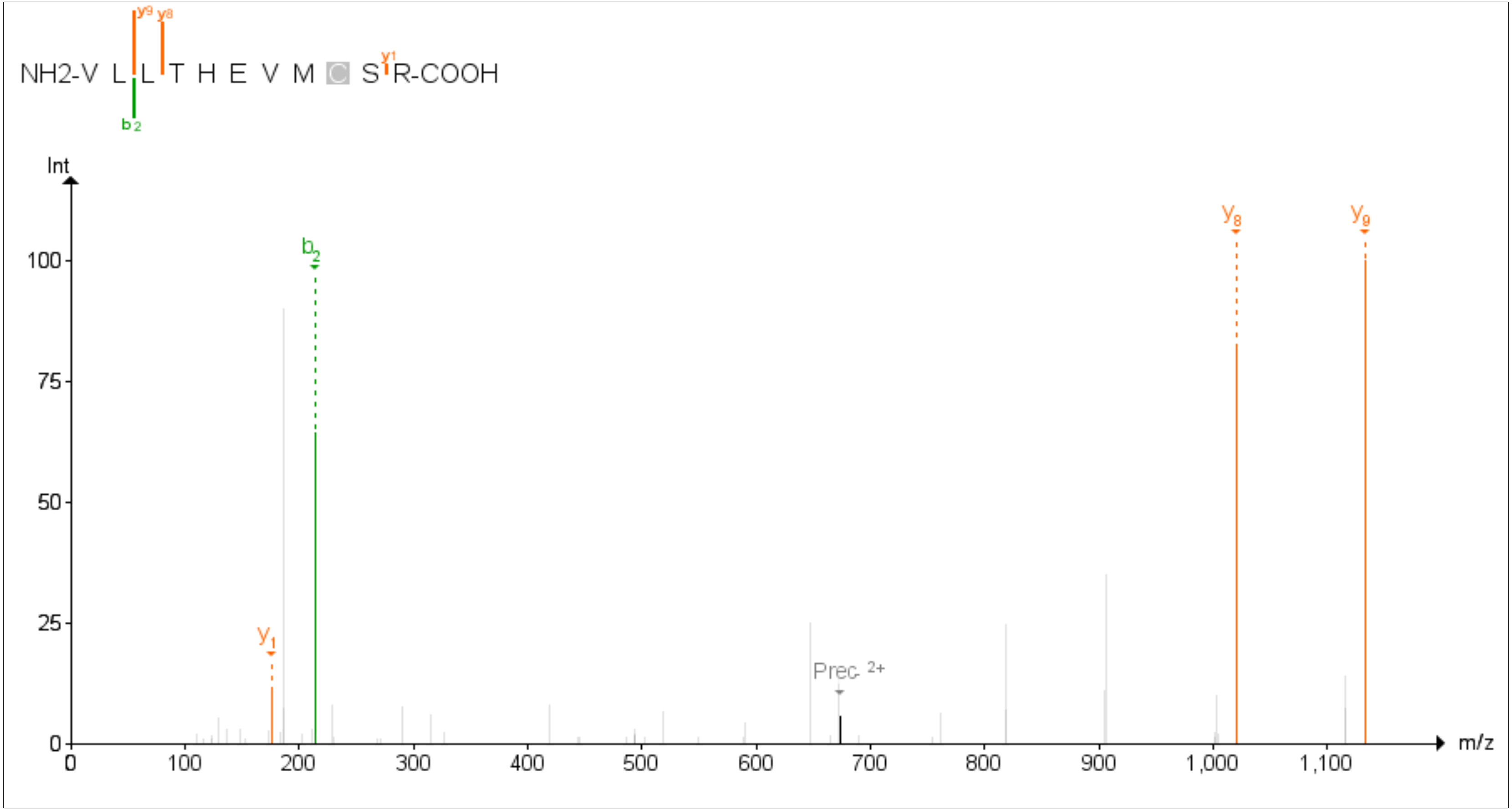

NH2-V L L T H E V M C S R-COOH
y9 y8 y1 b2
Int
100
75
50
25
0
y8
y9
b2
y1
Prec. 2+
0 100 200 300 400 500 600 700 800 900 1,000 1,100
m/z

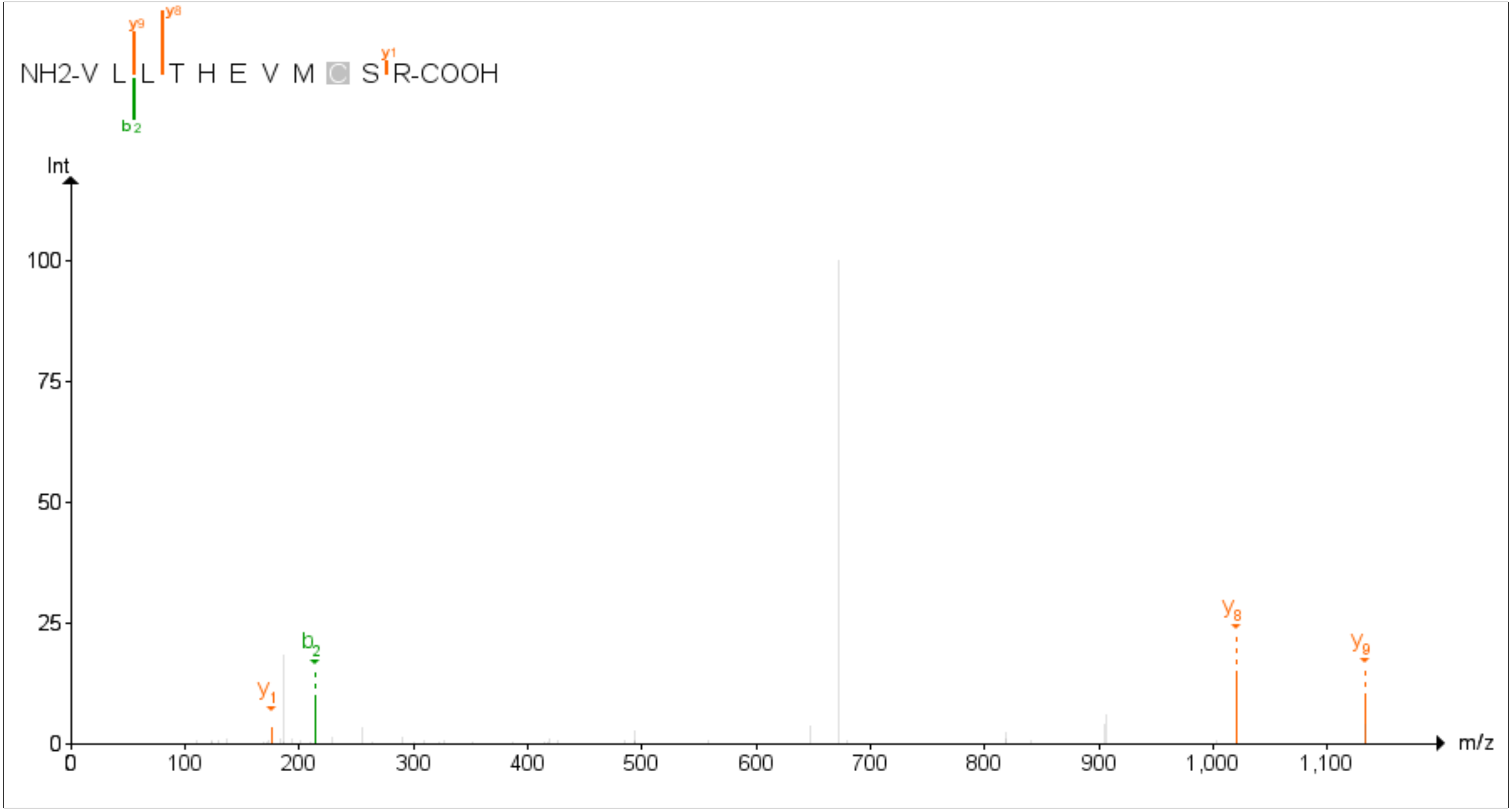

NH2-V L L T H E V M C S R-COOH
y9
y8
y1
b2
Int
100
75
50
25
0
m/z
0
100
200
300
400
500
600
700
800
900
1,000
1,100
y1
b2
y8
y9

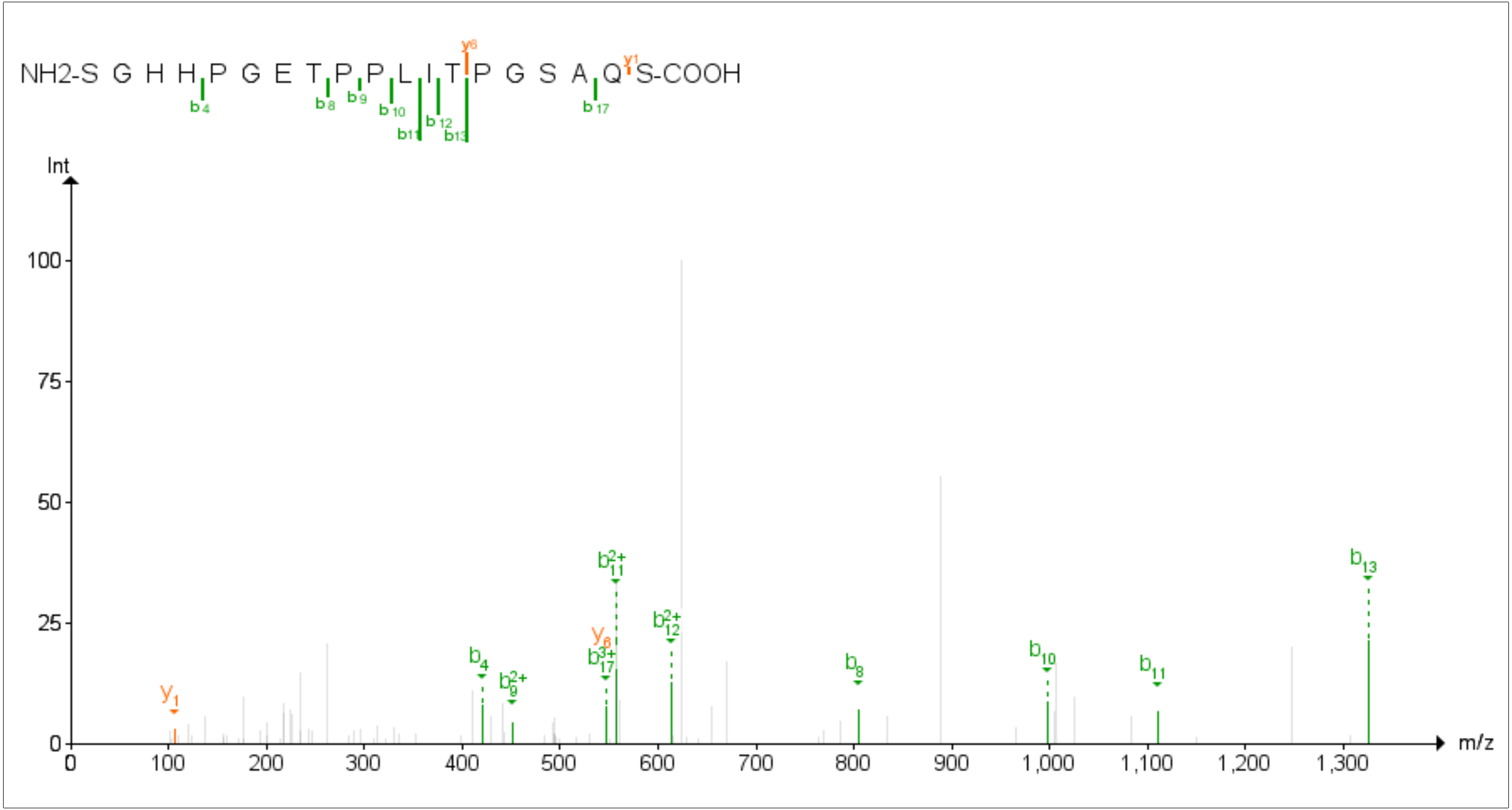

NH2-S G H H P G E T P P L I T P G S A Q S-COOH
y6
y1
b4
b8
b9
b10
b11
b12
b13
b17
Int
100
75
50
25
0
m/z
0
100
200
300
400
500
600
700
800
900
1,000
1,100
1,200
1,300
y1
b4
b9 2+
y6
b17 3+
b11 2+
b12 2+
b8
b10
b11
b13

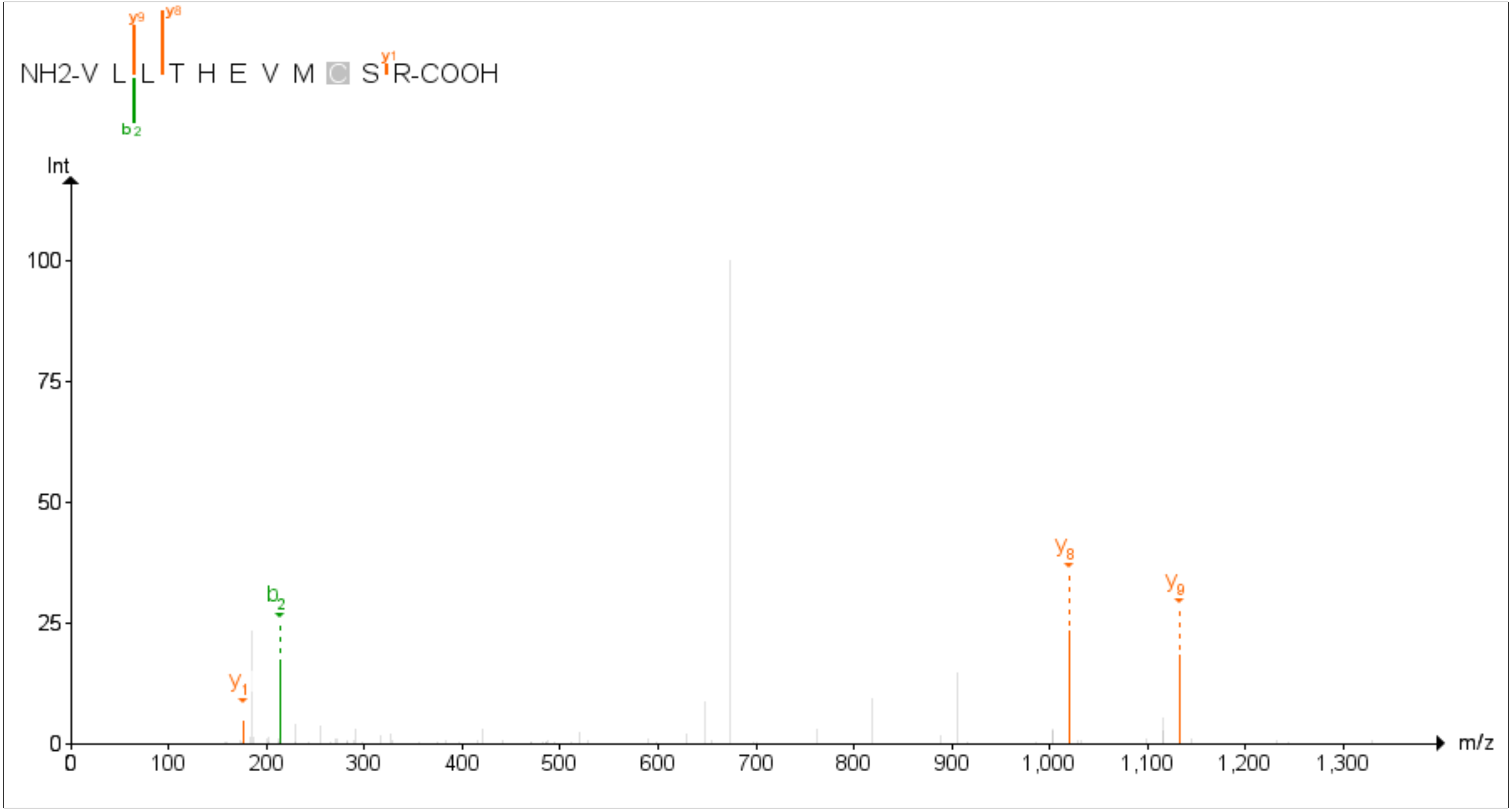

NH2-V L L T H E V M C S R-COOH
y9
y8
y1
b2
Int
100
75
50
25
0
m/z
0
100
200
300
400
500
600
700
800
900
1,000
1,100
1,200
1,300
y1
b2
y8
y9

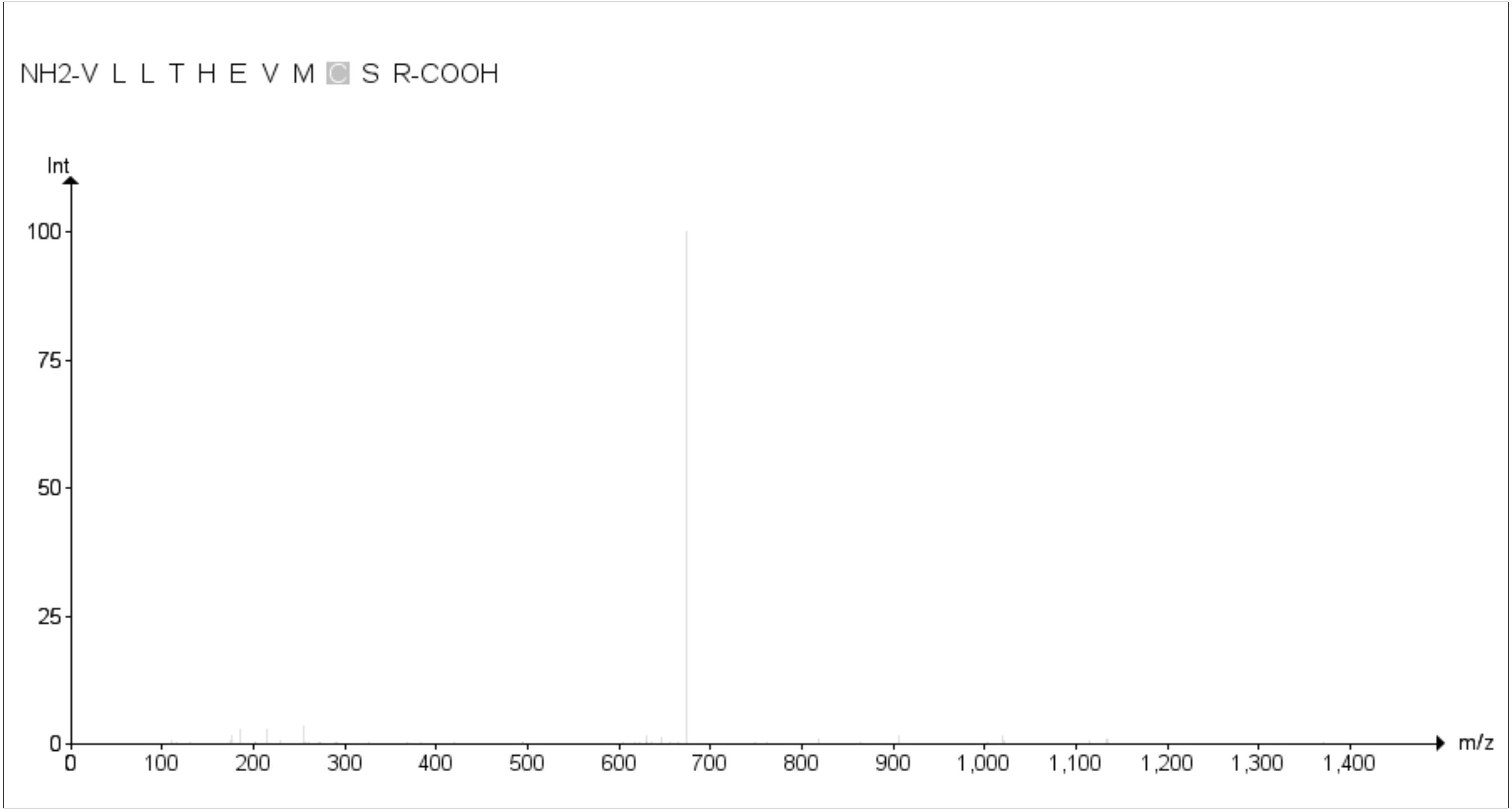
NH2-V L L T H E V M C S R-COOH
Int
100
75
50
25
0
m/z
0
100
200
300
400
500
600
700
800
900
1,000
1,100
1,200
1,300
1,400

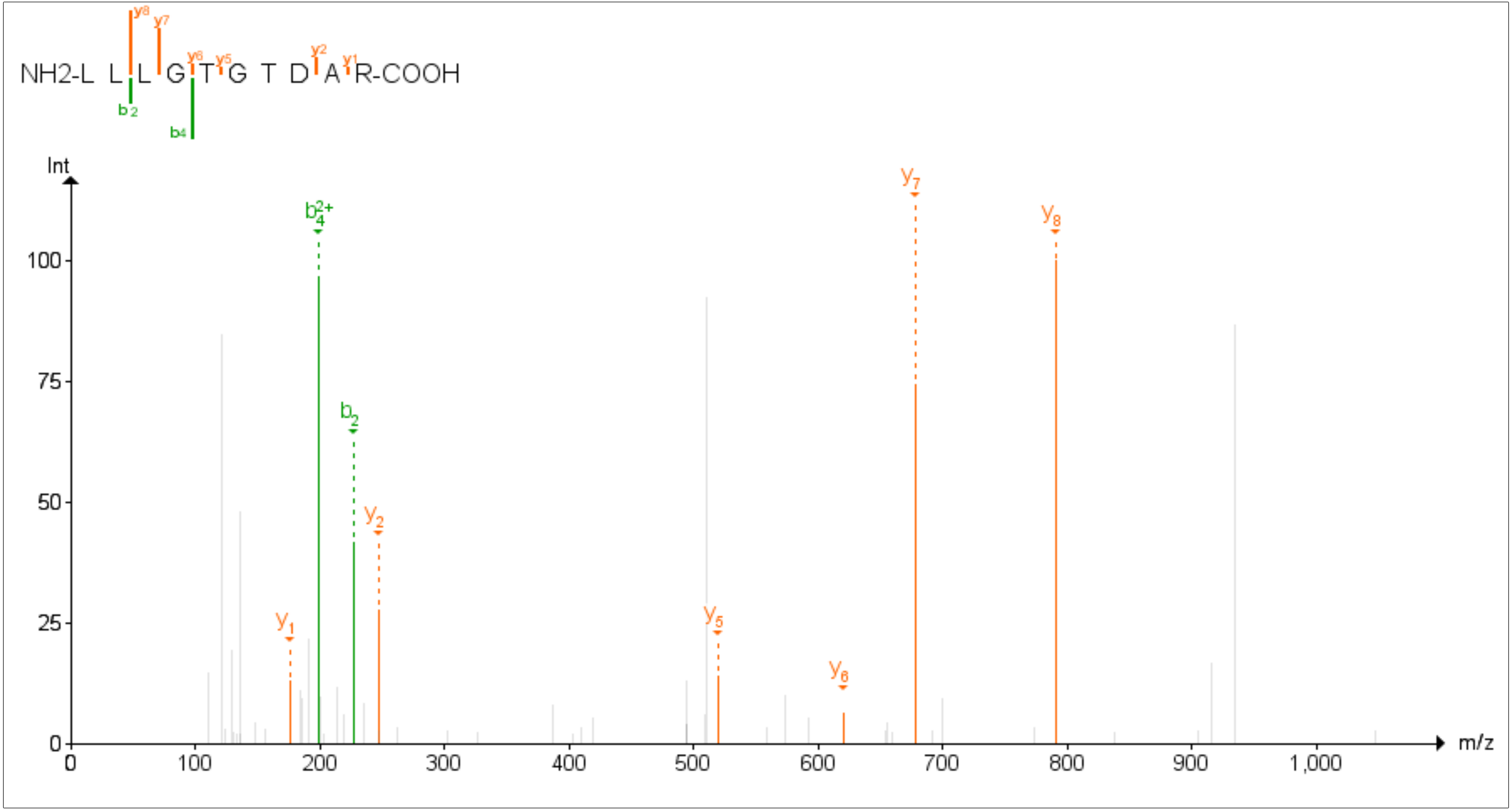

NH2-L L L G T G T D A R-COOH
y8
y7
y6
y5
y2
y1
b2
b4
Int
100
75
50
25
0
0
100
200
300
400
500
600
700
800
900
1,000
m/z
y1
b4 2+
b2
y2
y5
y6
y7
y8

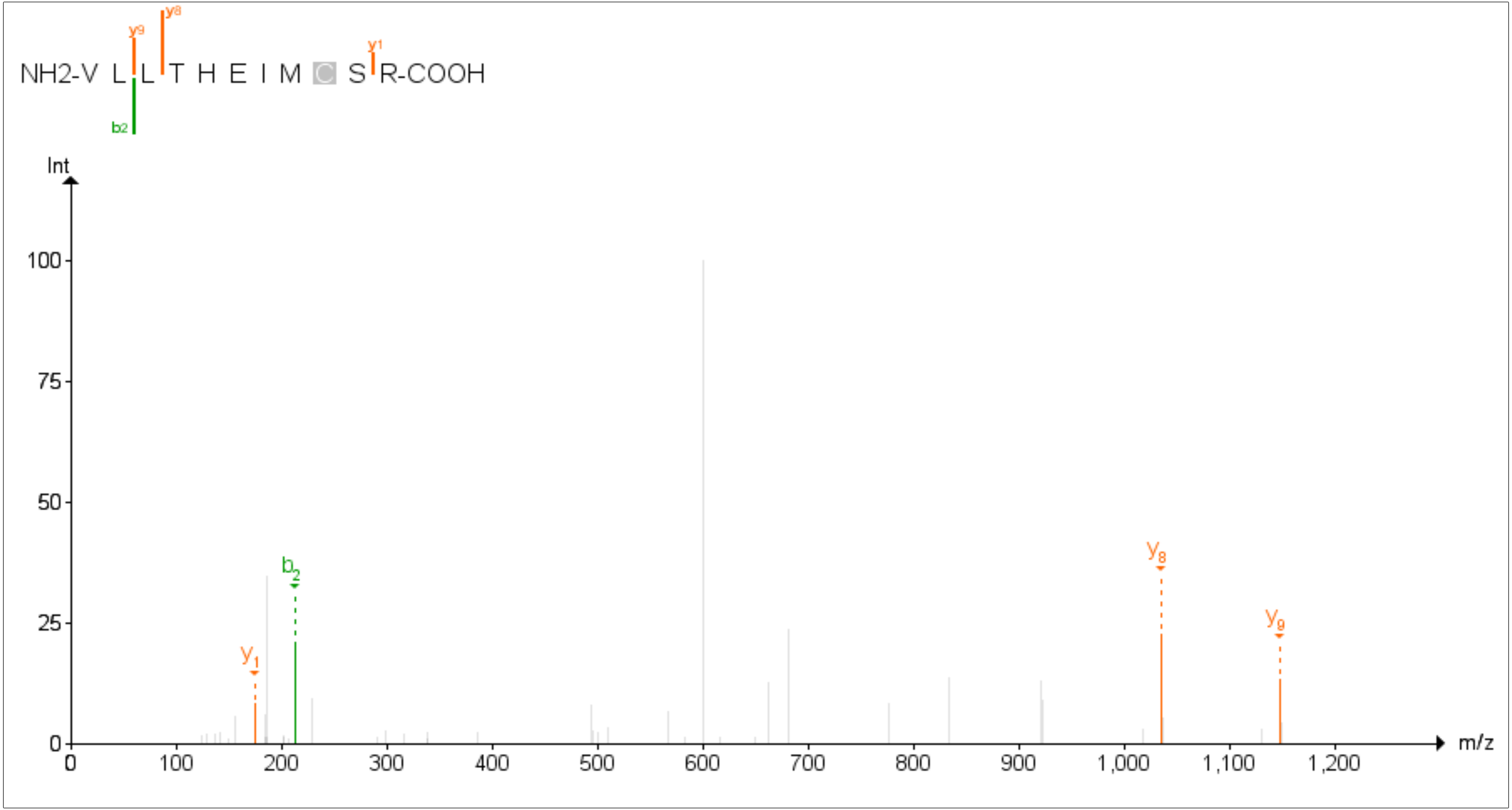

NH2-V L L T H E I M C S R-COOH
y9
y8
y1
b2
Int
100
75
50
25
0
0
100
200
300
400
500
600
700
800
900
1,000
1,100
1,200
m/z
y_1
b_2
y_8
y_9

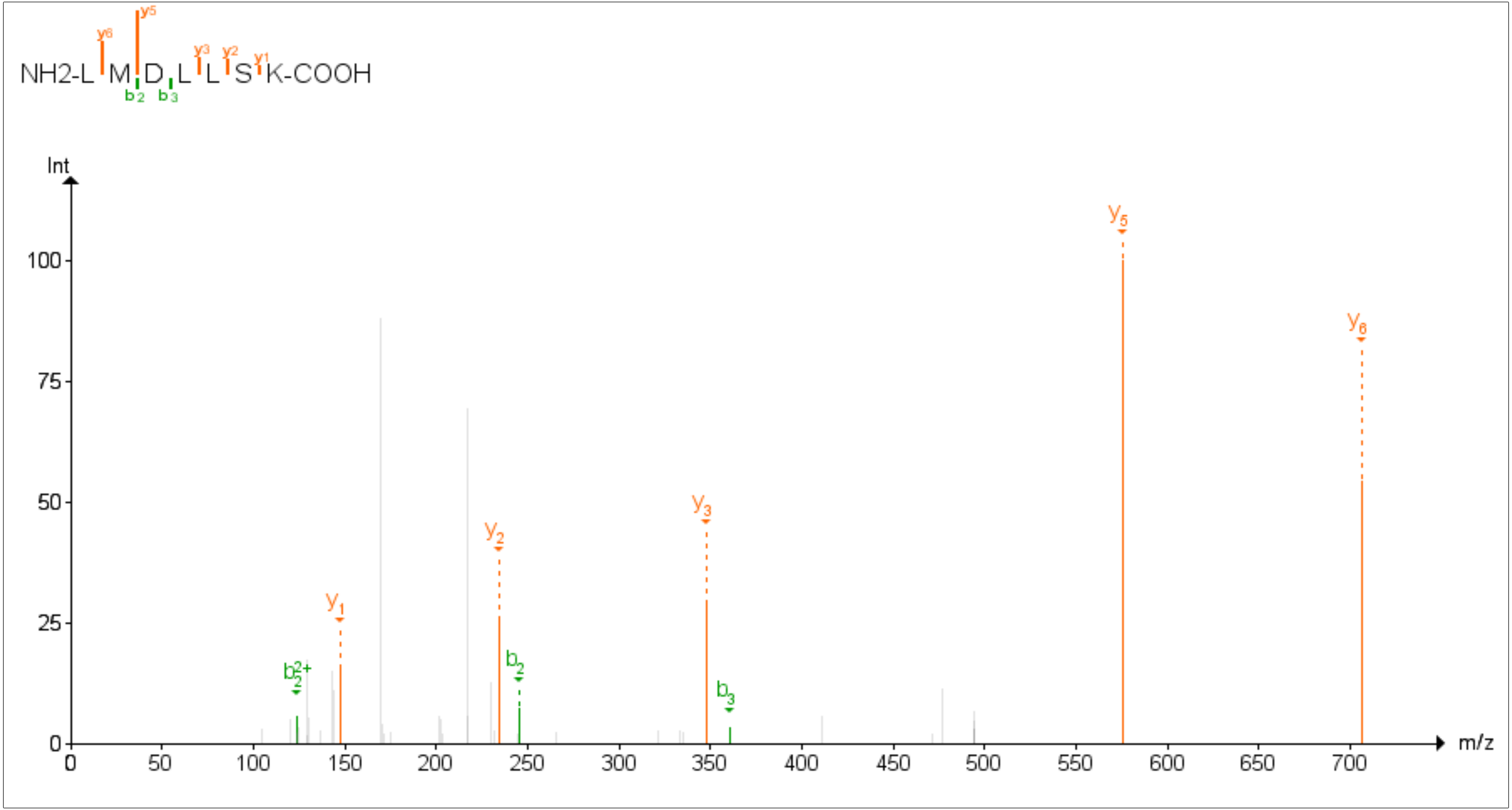

NH2-L M D L L S K-COOH
y6
y5
y3
y2
y1
b2
b3
Int
100
75
50
25
0
m/z
0
50
100
150
200
250
300
350
400
450
500
550
600
650
700
y5
y6
y3
y2
y1
b2
b3
b2 2+

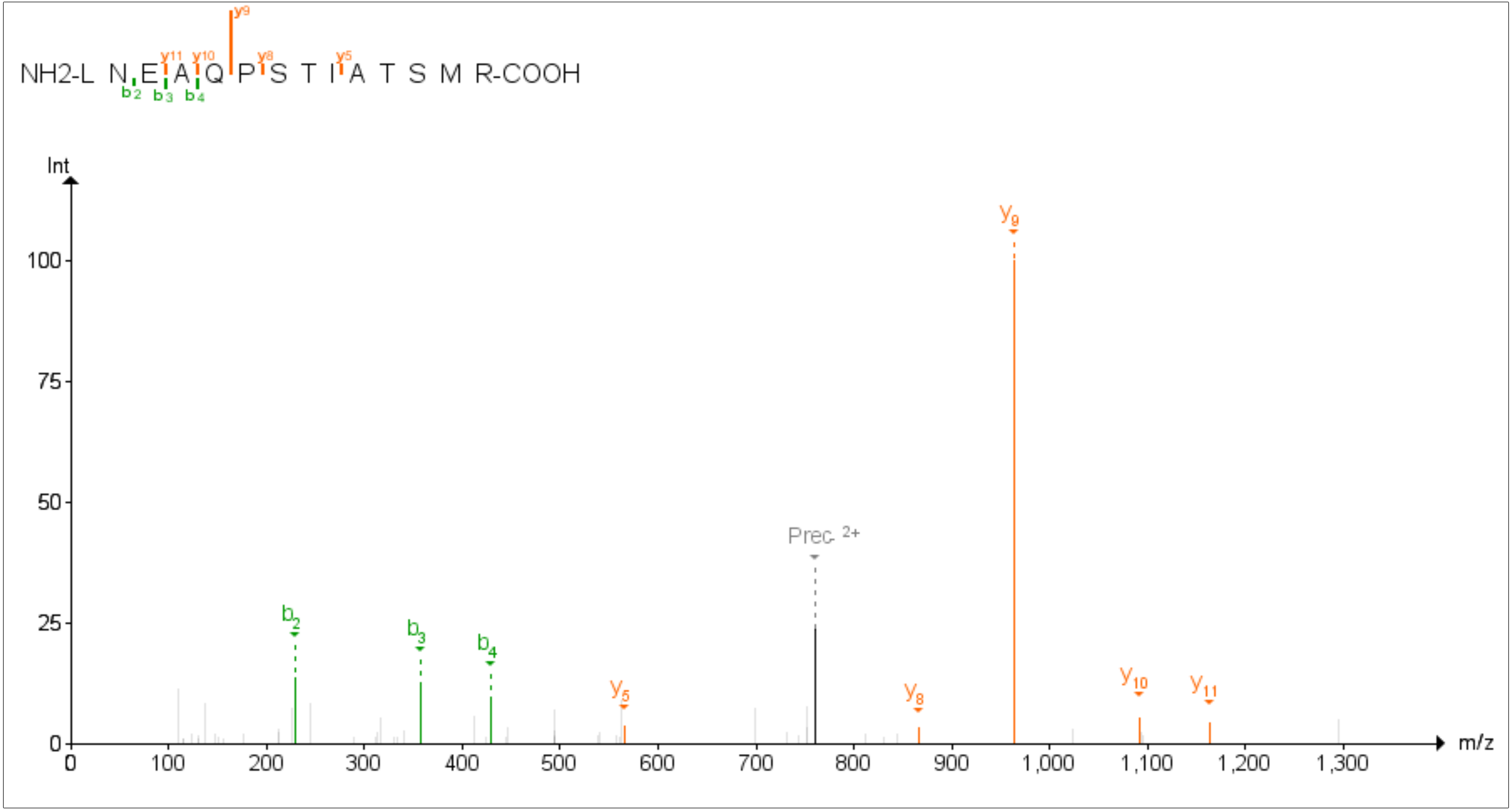

NH2-L N E A Q P S T I A T S M R-COOH
y9
y11
y10
y8
y5
b2
b3
b4
Int
100
75
50
25
0
Prec- 2+
m/z
0
100
200
300
400
500
600
700
800
900
1,000
1,100
1,200
1,300

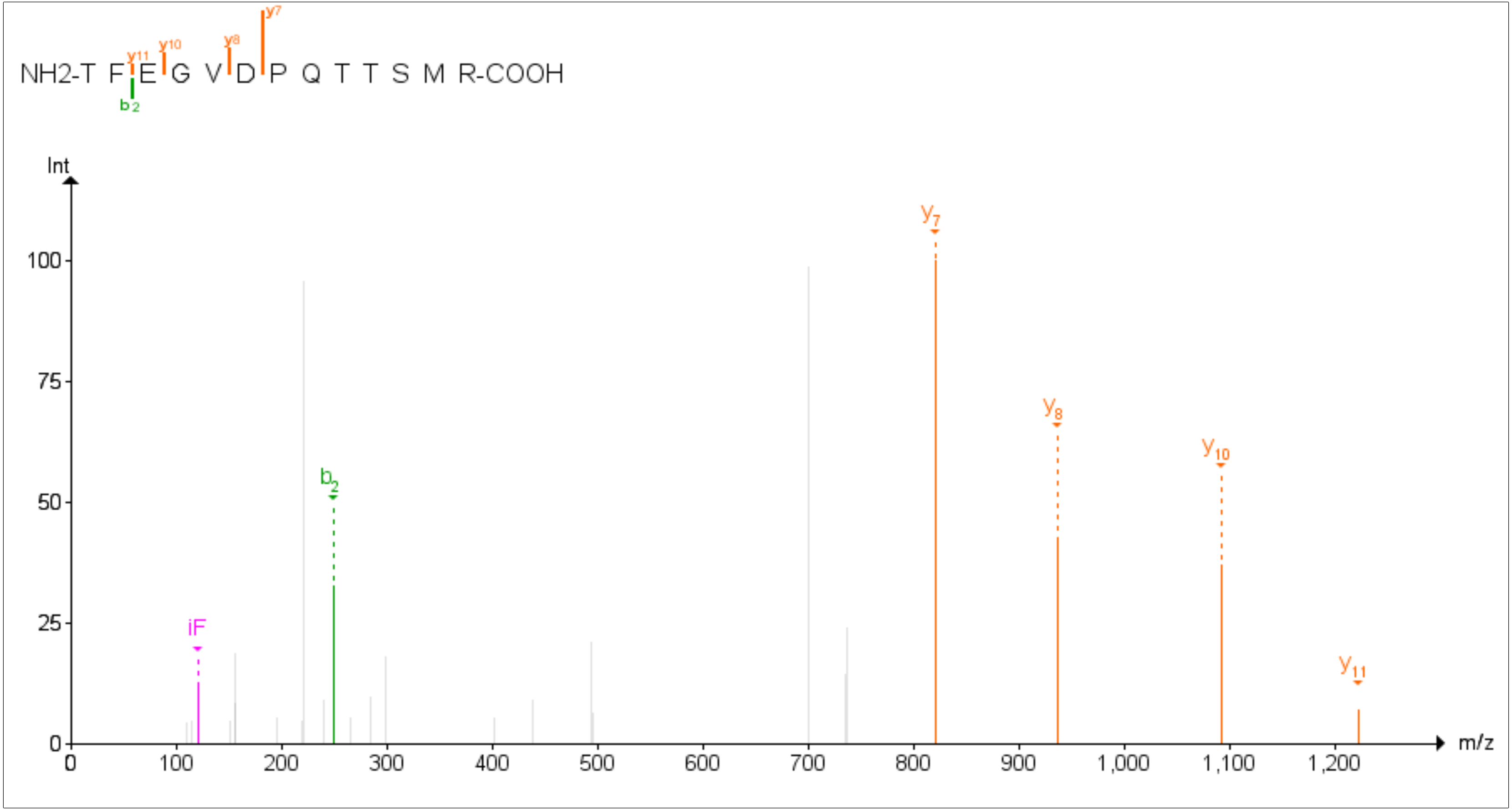

NH2-T F E G V D P Q T T S M R-COOH
y7
y8
y10
y11
b2
Int
100
75
50
25
0
iF
b2
y7
y8
y10
y11
m/z
0
100
200
300
400
500
600
700
800
900
1,000
1,100
1,200

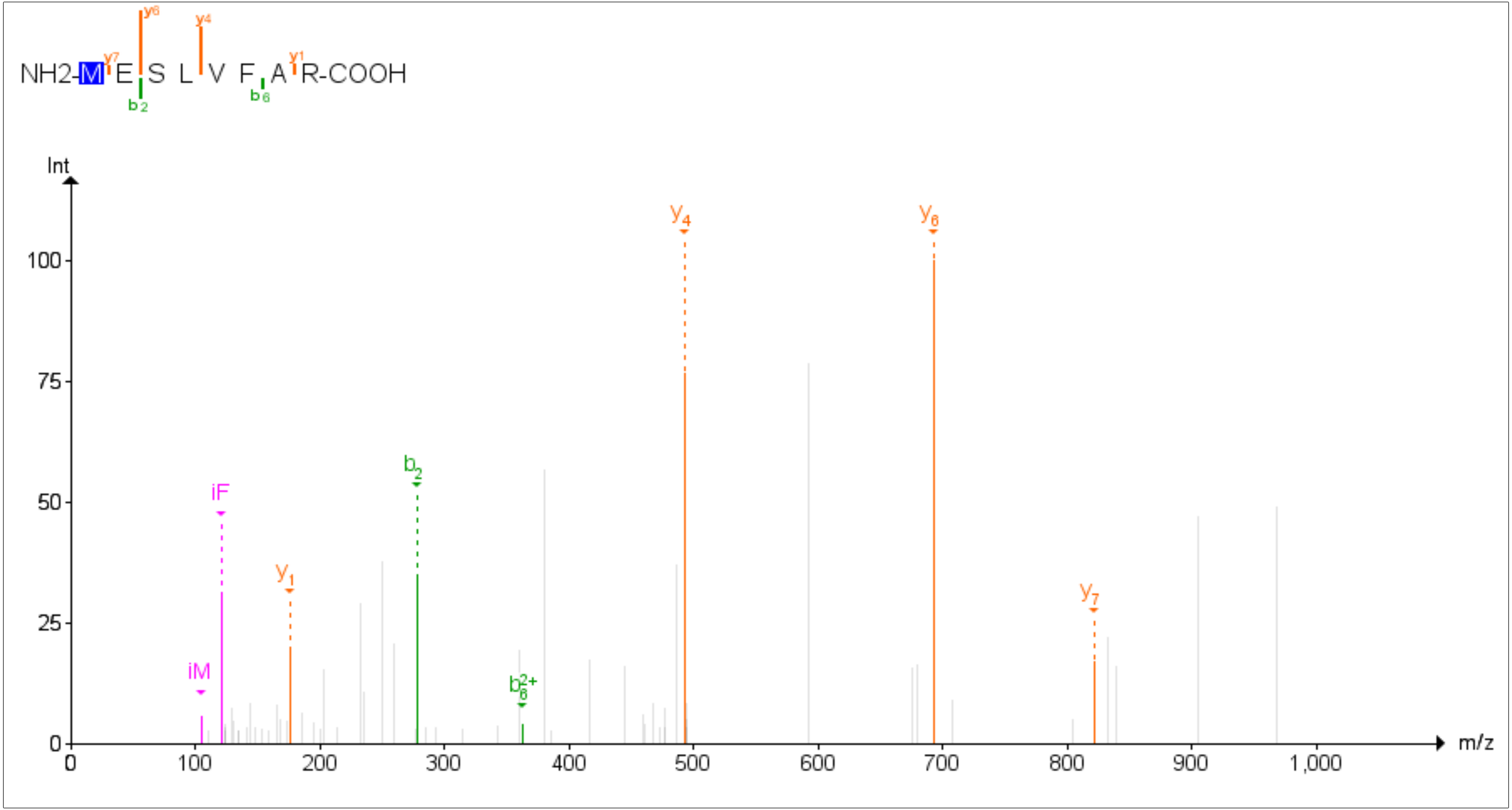

NH2-M E S L V F A R-COOH
y7
y6
y4
y1
b2
b6
Int
100
75
50
25
0
iM
iF
y1
b2
b6 2+
y4
y6
y7
0
100
200
300
400
500
600
700
800
900
1,000
m/z

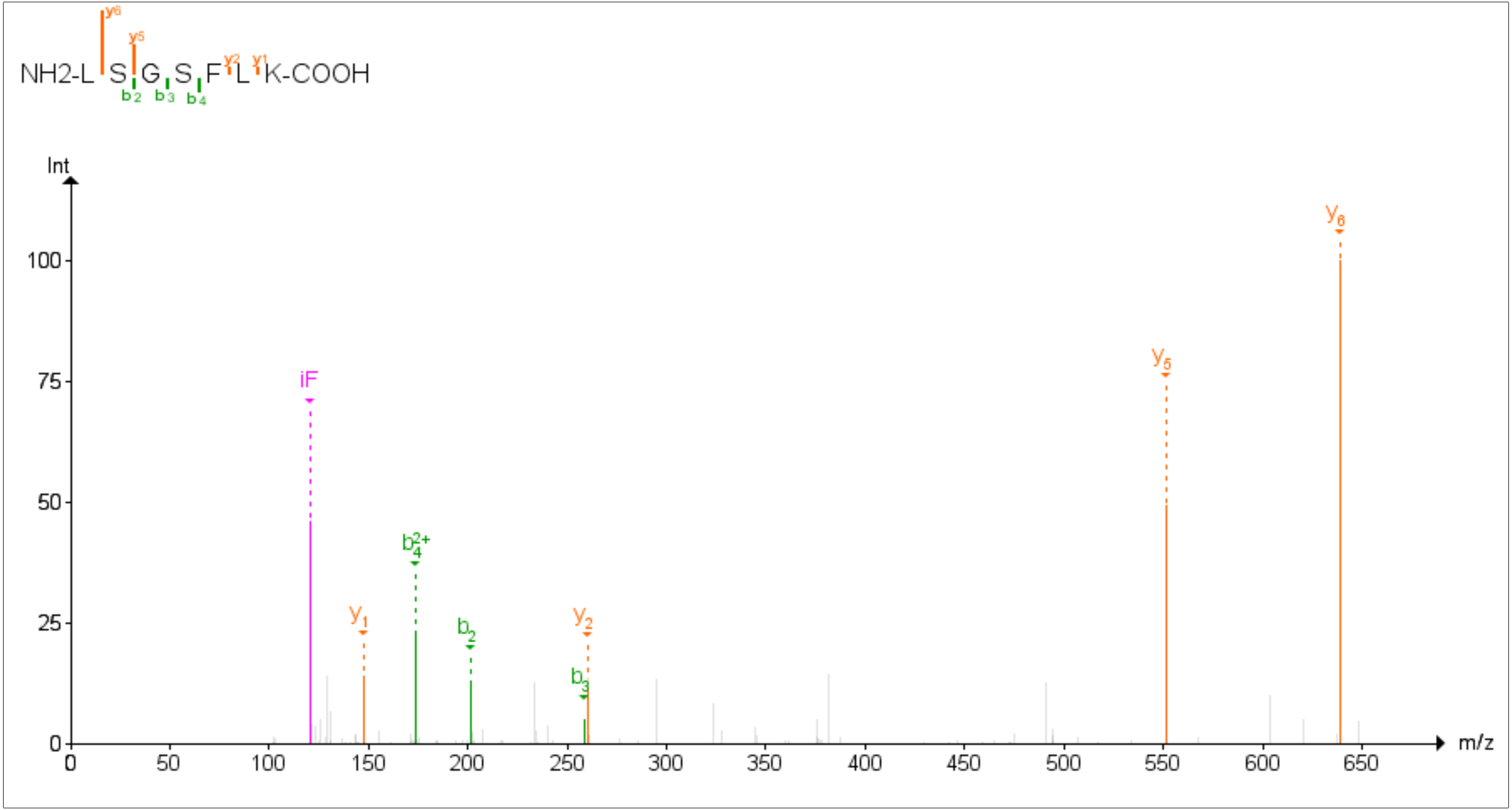

NH2-L S G S F L K-COOH
y6
y5
y2
y1
b2
b3
b4
Int
100
75
50
25
0
iF
y1
b4 2+
b2
y2
b3
y5
y6
0
50
100
150
200
250
300
350
400
450
500
550
600
650
m/z

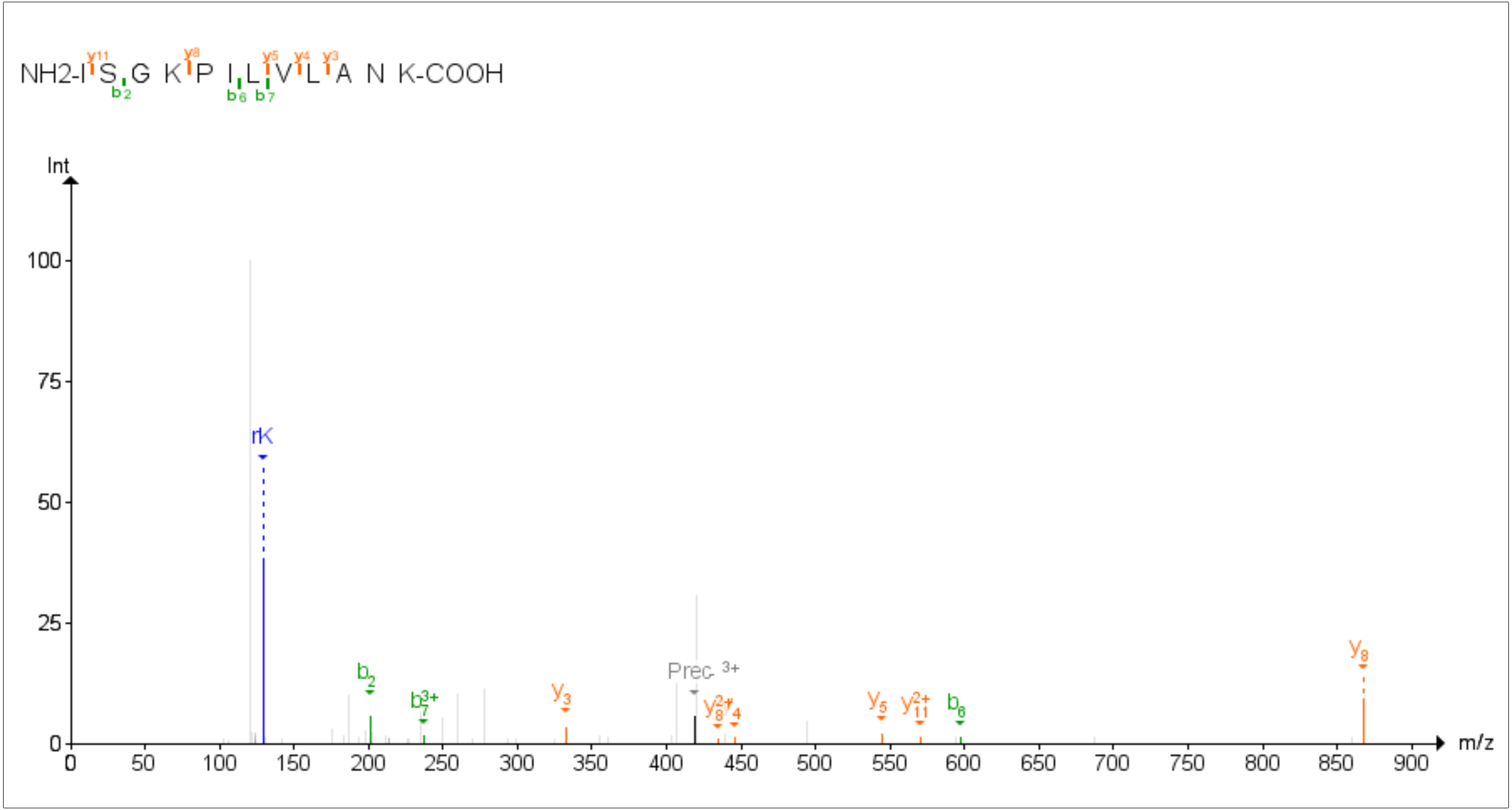
NH2-I S G K P I L V L A N K-COOH
y11 y8 y5 y4 y3
b2 b6 b7
Int
100
75
50
25
0
rK
b2
b7 3+
y3
Prec 3+
y8 2+
y4
y5
y11 2+
b6
y8
0 50 100 150 200 250 300 350 400 450 500 550 600 650 700 750 800 850 900
m/z

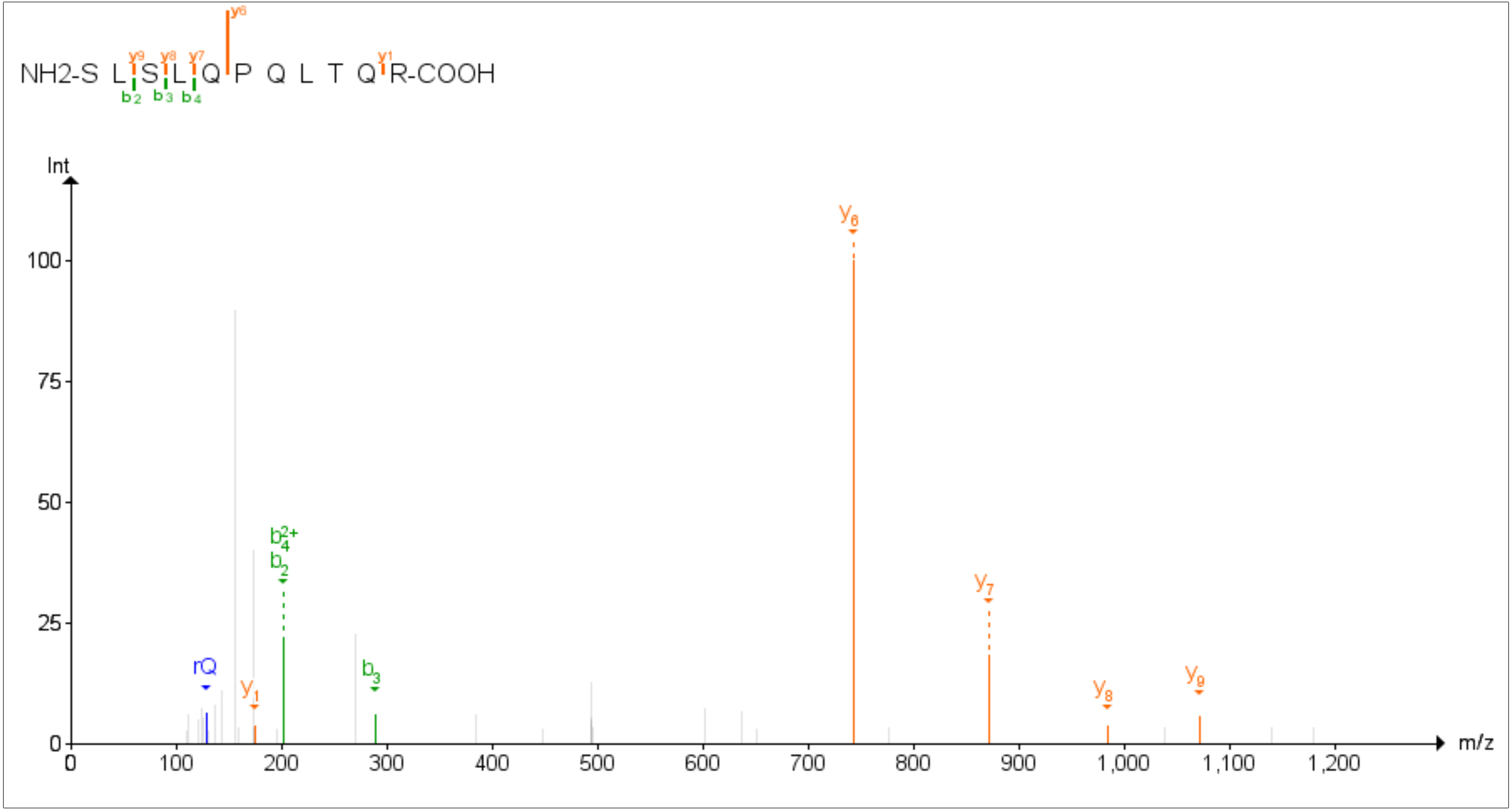

NH2-S L S L Q P Q L T Q R-COOH
y9 y8 y7 y6 y1
b2 b3 b4
Int
100
75
50
25
0
rQ
y1
b4 2+
b2
b3
y6
y7
y8
y9
0 100 200 300 400 500 600 700 800 900 1,000 1,100 1,200
m/z

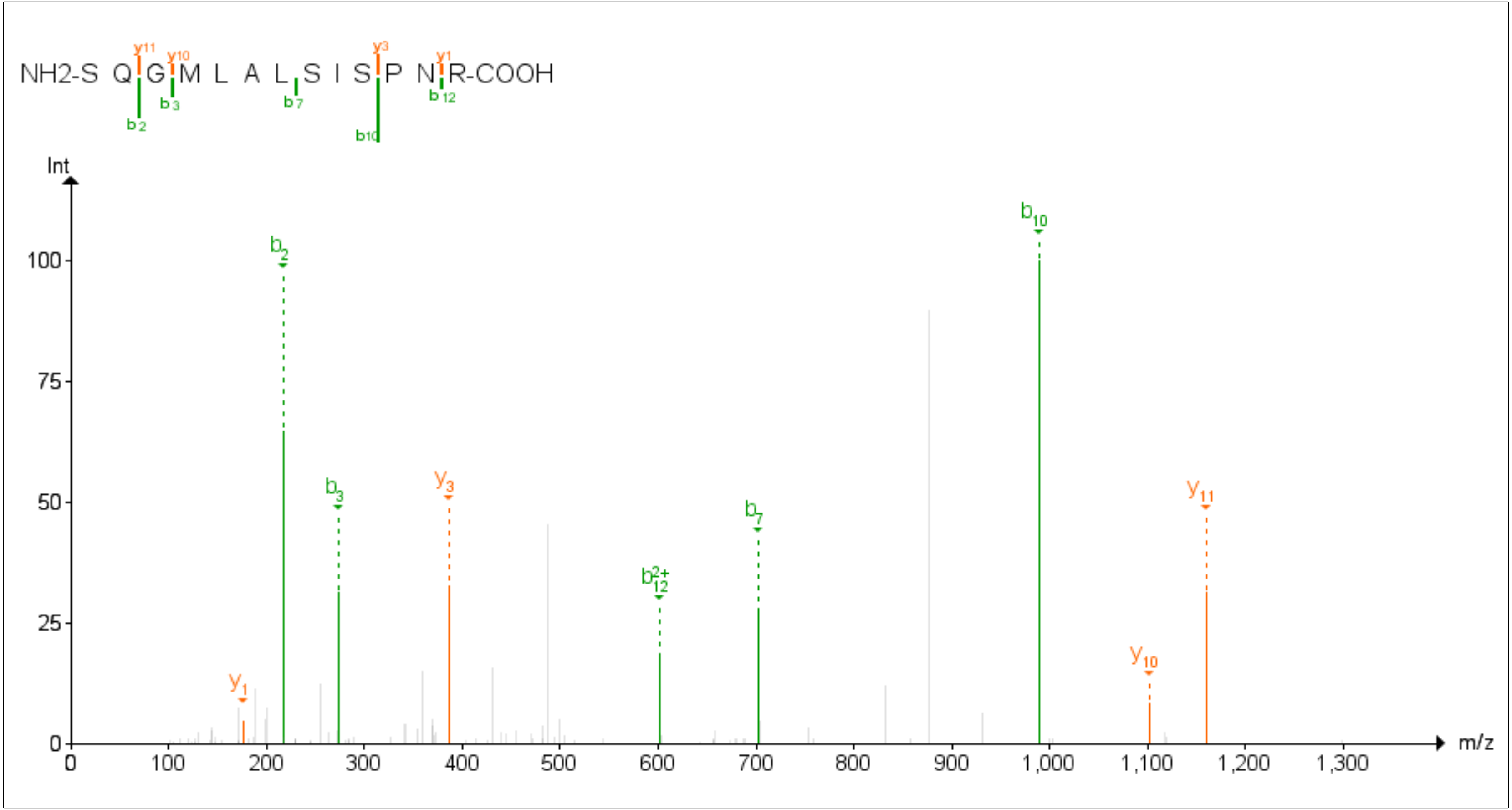

NH2-S Q G M L A L S I S P N R-COOH
y11
y10
y3
y1
b 2
b 3
b 7
b10
b 12
Int
100
75
50
25
0
b2
b3
y3
y1
b12 2+
b7
b10
y10
y11
0
100
200
300
400
500
600
700
800
900
1,000
1,100
1,200
1,300
m/z

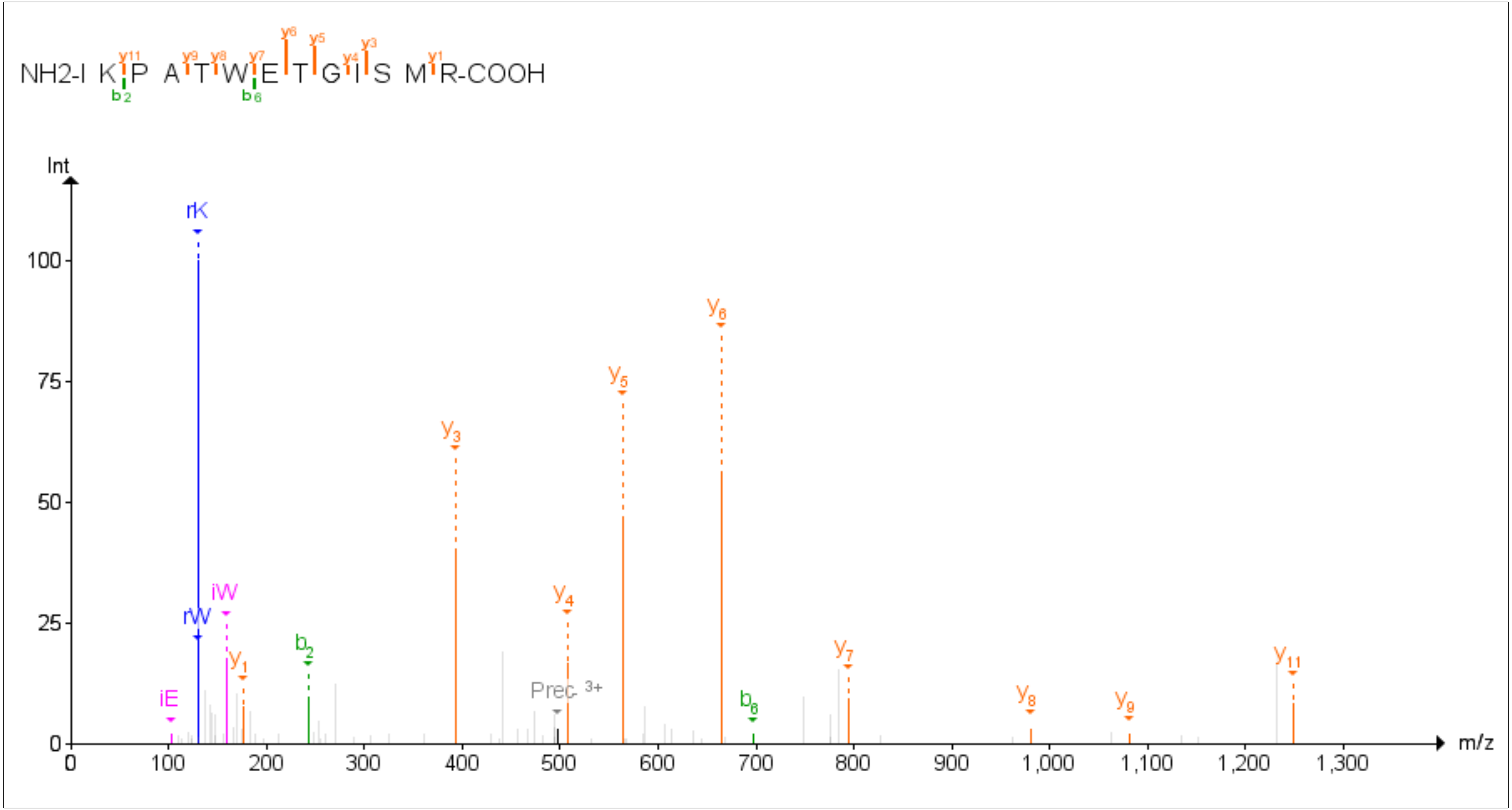

NH2-I K P A T W E T G I S M R-COOH
Int
m/z
rK
rW
iW
iE
y1
b2
y3
Prec 3+
y4
y5
y6
b6
y7
y8
y9
y11

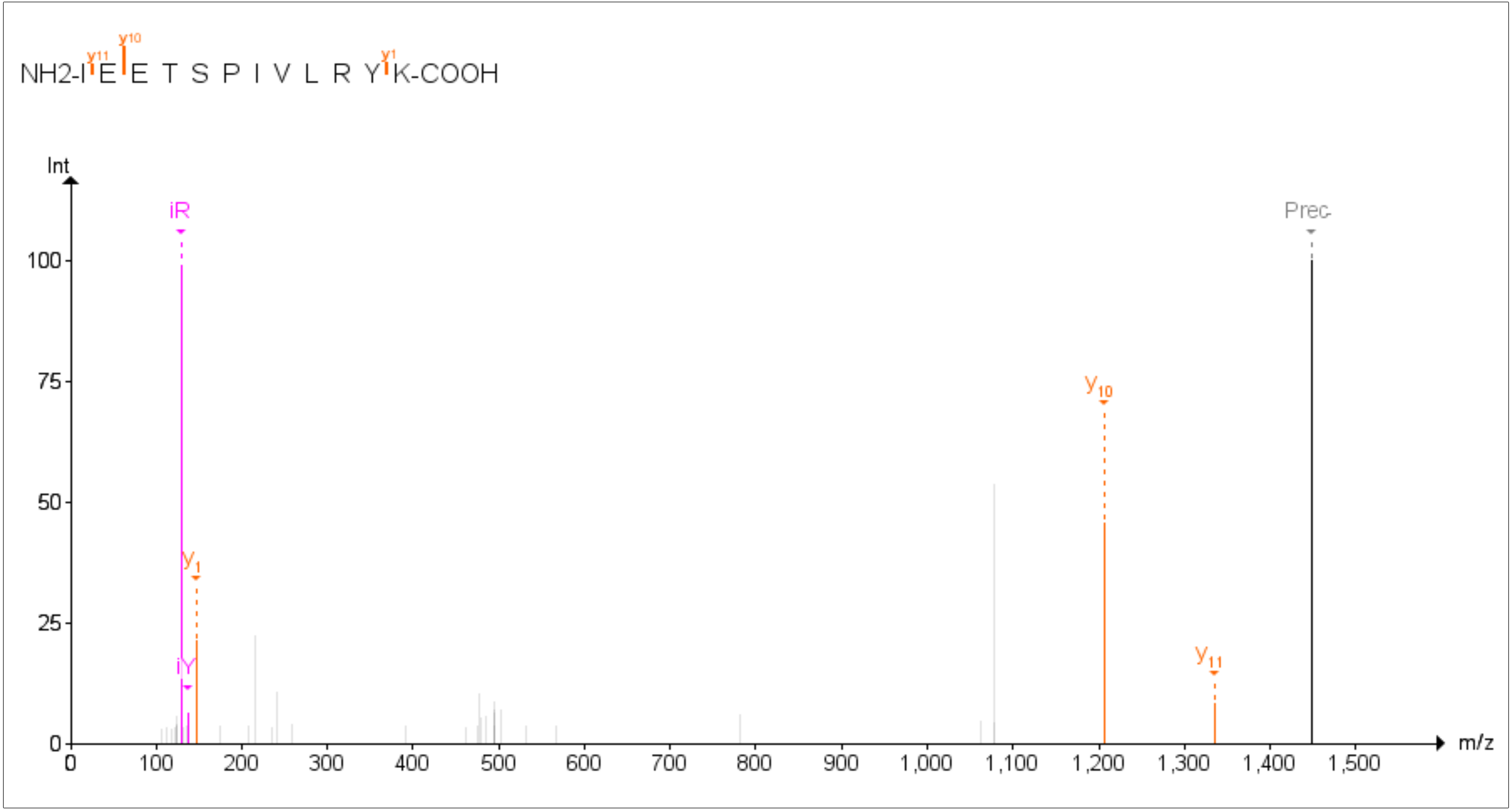

NH2-I E E T S P I V L R Y K-COOH
y11
y10
y1
Int
iR
Prec
100
75
50
25
0
y1
iY
y10
y11
0
100
200
300
400
500
600
700
800
900
1,000
1,100
1,200
1,300
1,400
1,500
m/z

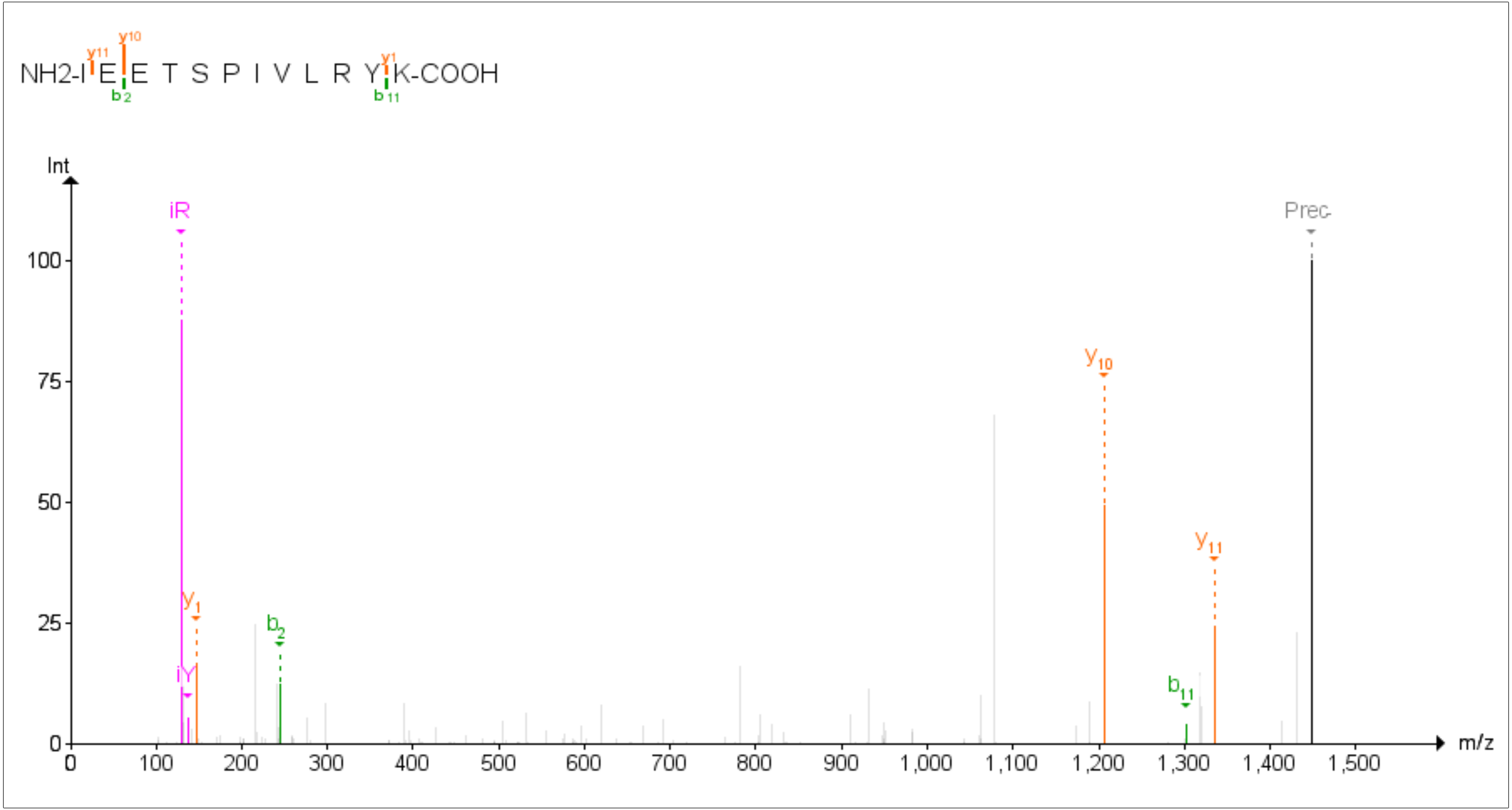

NH2-I E E T S P I V L R Y K-COOH
y11
y10
y1
b2
b11
Int
m/z
iR
Prec
y10
y11
y1
iY
b2
b11
0
25
50
75
100
0
100
200
300
400
500
600
700
800
900
1,000
1,100
1,200
1,300
1,400
1,500

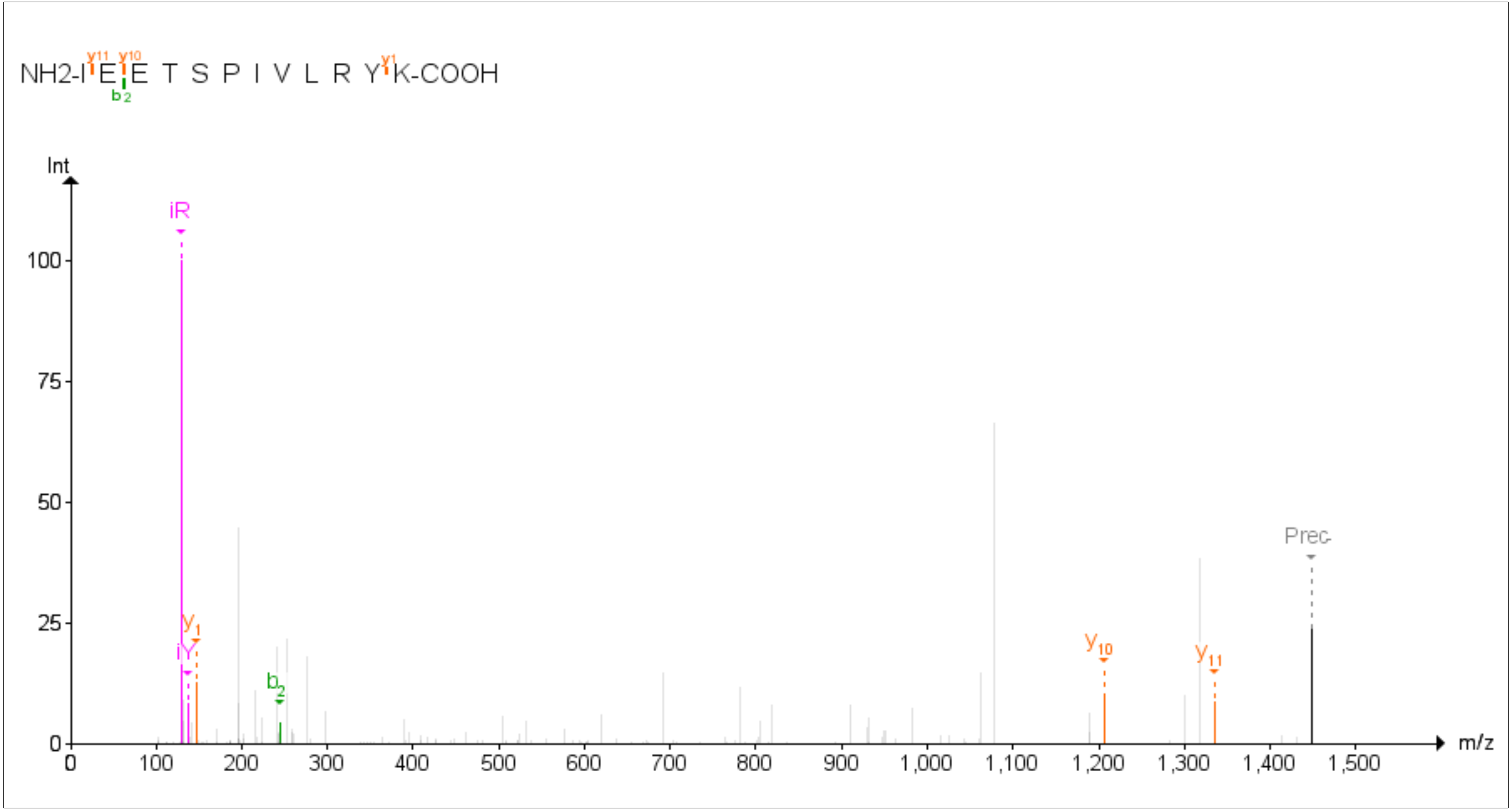

NH2-I E E T S P I V L R Y K-COOH
y11 y10 y1
b2
Int
iR
y1
iY
b2
y10
y11
Prec
100
75
50
25
0
0 100 200 300 400 500 600 700 800 900 1,000 1,100 1,200 1,300 1,400 1,500
m/z

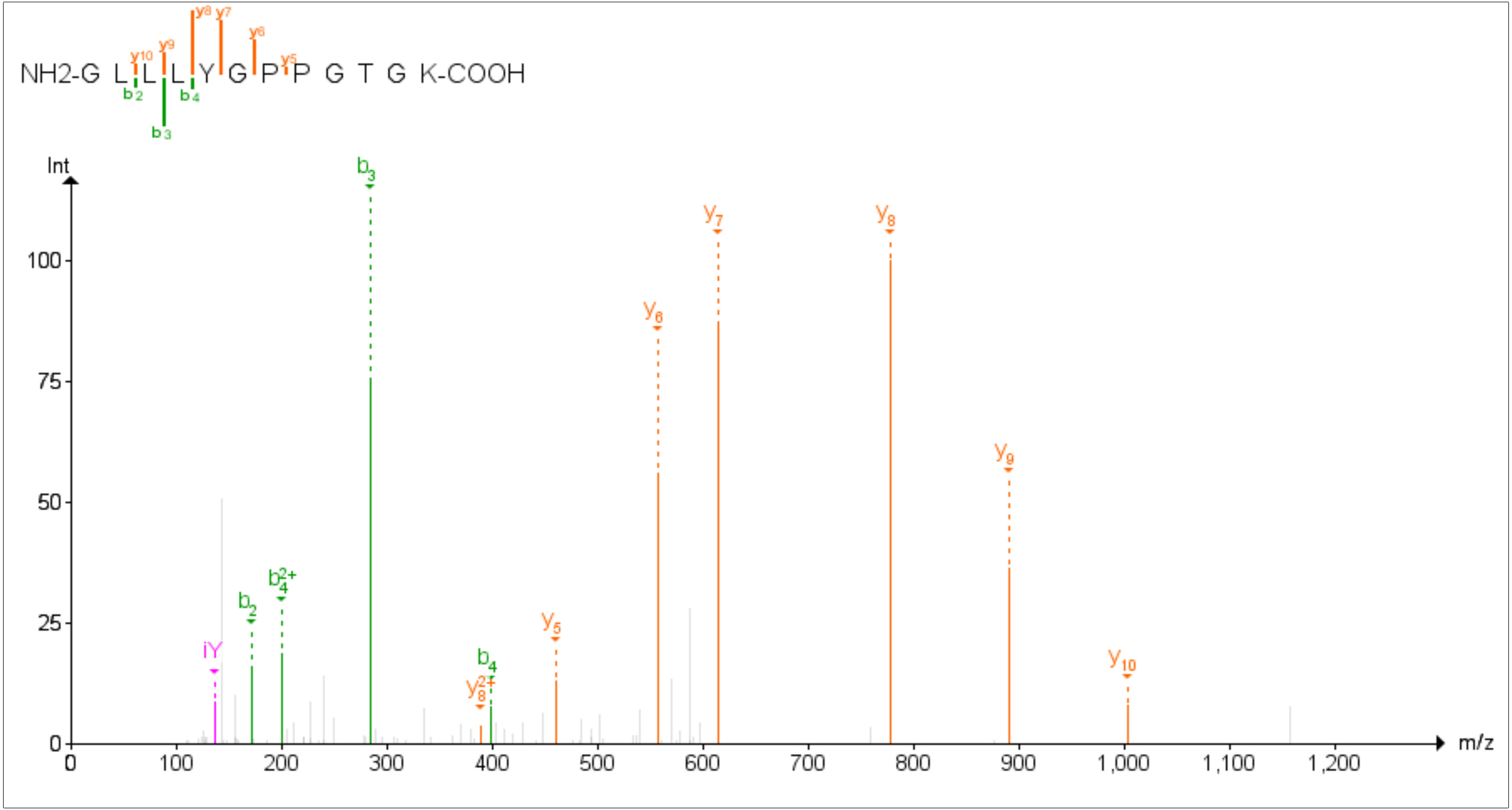

NH2-G L L L Y G P P G T G K-COOH
y10 y9 y8 y7 y6 y5
b2 b3 b4
Int
m/z
0
25
50
75
100
0
100
200
300
400
500
600
700
800
900
1,000
1,100
1,200
iY
b_2
b_4^{2+}
b_3
y_8^{2+}
b_4
y_5
y_6
y_7
y_8
y_9
y_{10}

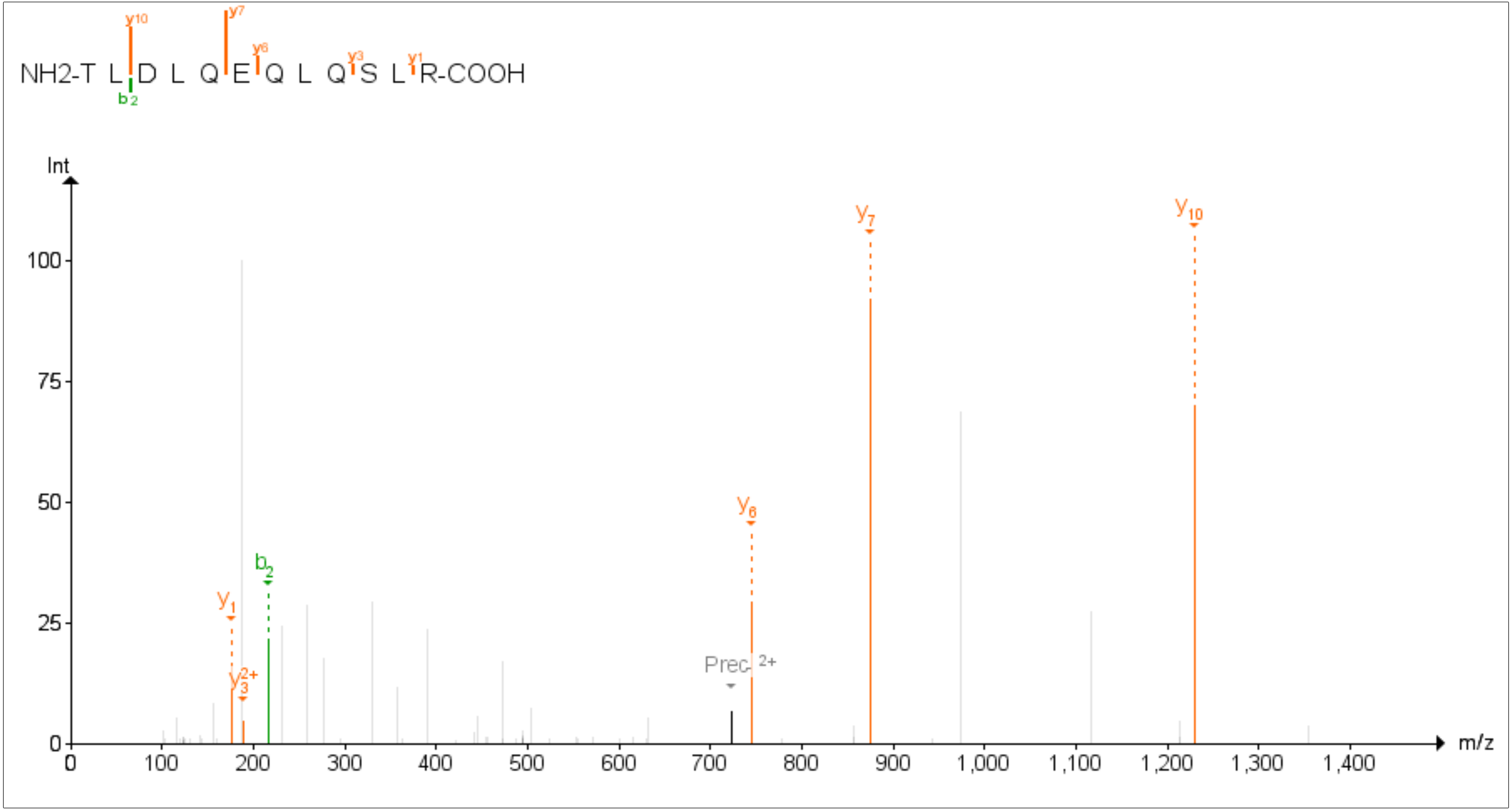

NH2-T L D L Q E Q L Q S L R-COOH
y10 y7 y6 y3 y1
b2
Int
100
75
50
25
0
y7
y10
y6
b2
y1
y3 2+
Prec 2+
0 100 200 300 400 500 600 700 800 900 1,000 1,100 1,200 1,300 1,400
m/z

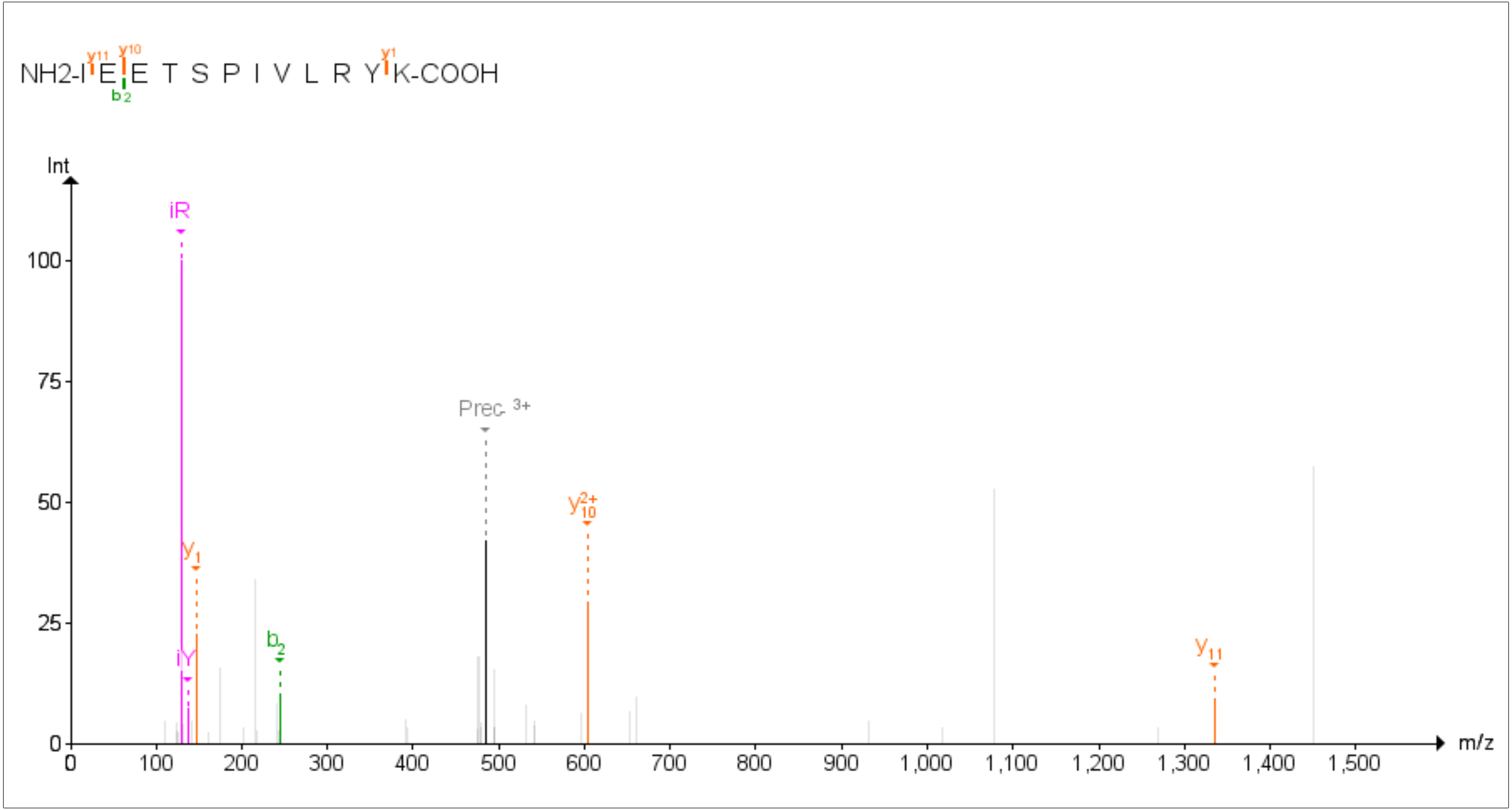

NH2-I E E T S P I V L R Y K-COOH
y11
y10
y1
b2
Int
iR
Prec 3+
y10 2+
y1
iY
b2
y11
m/z
0
25
50
75
100
0
100
200
300
400
500
600
700
800
900
1,000
1,100
1,200
1,300
1,400
1,500

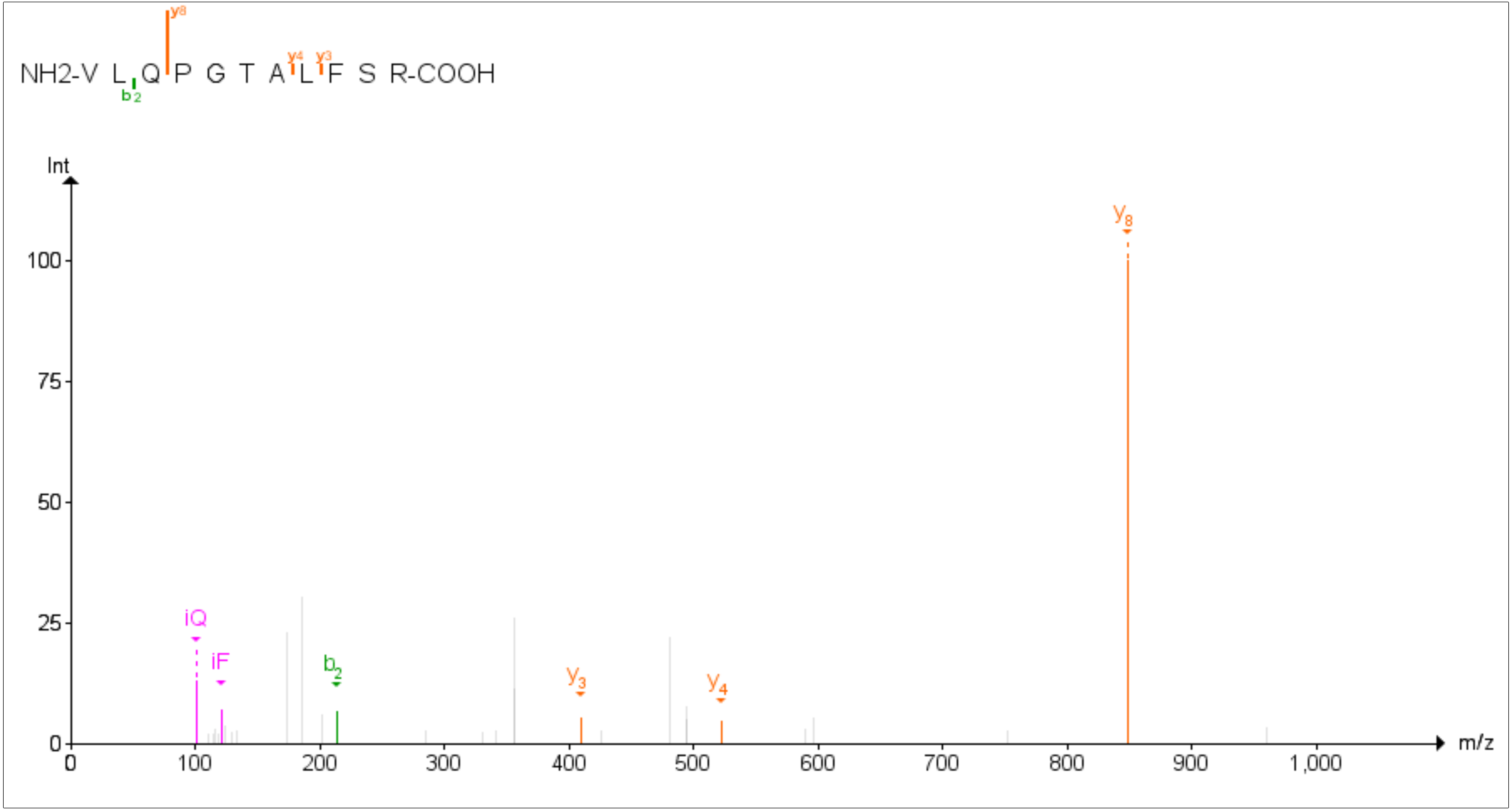
NH2-V L Q P G T A L F S R-COOH
y8
y4
y3
b2
Int
100
75
50
25
0
iQ
iF
b2
y3
y4
y8
m/z
0
100
200
300
400
500
600
700
800
900
1,000

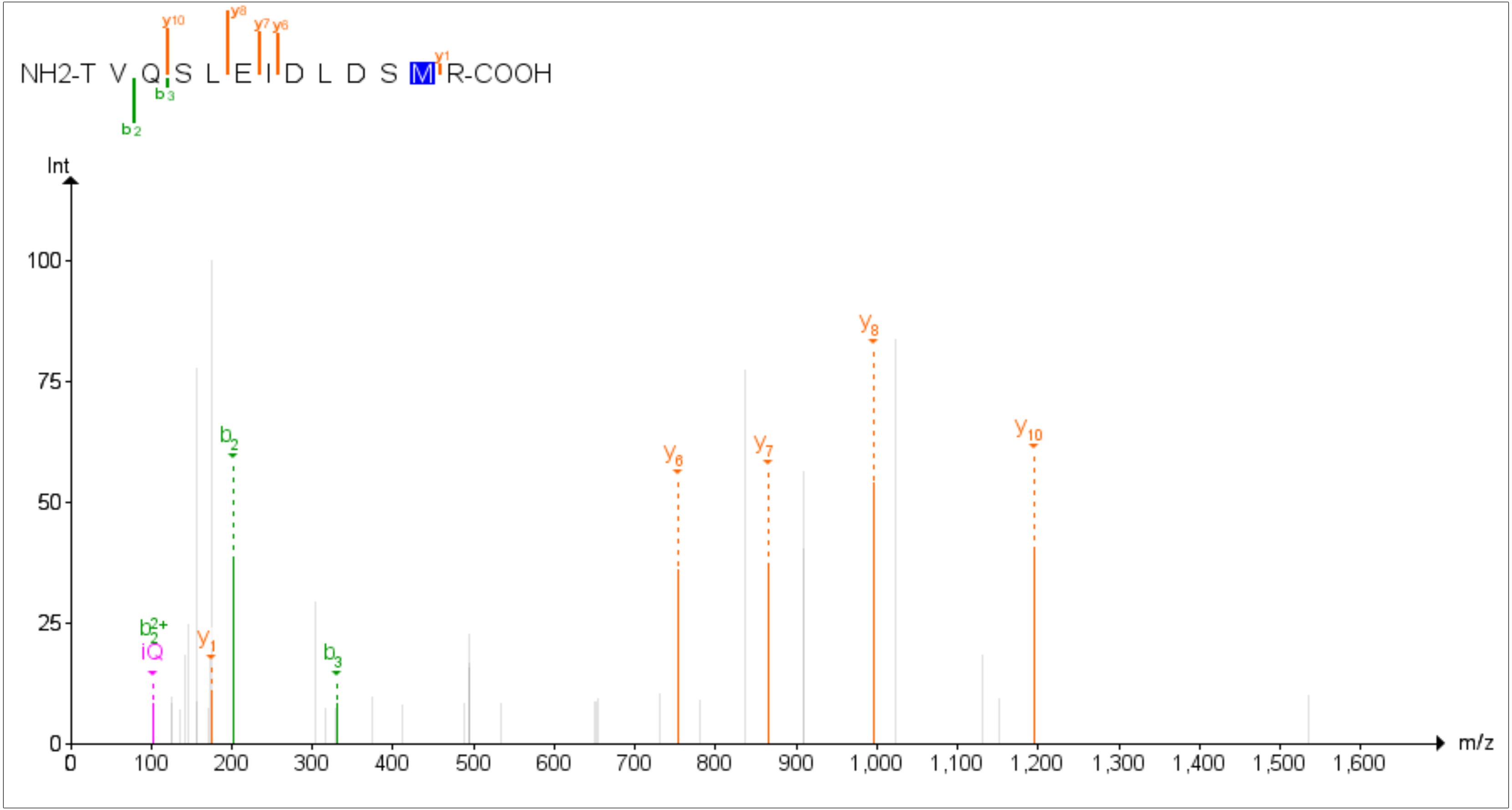

NH2-T V Q S L E I D L D S M R-COOH
y10
y8
y7
y6
y1
b2
b3
Int
m/z
100
75
50
25
0
0
100
200
300
400
500
600
700
800
900
1,000
1,100
1,200
1,300
1,400
1,500
1,600
b2
y6
y7
y8
y10
b2 2+
iQ
y1
b3

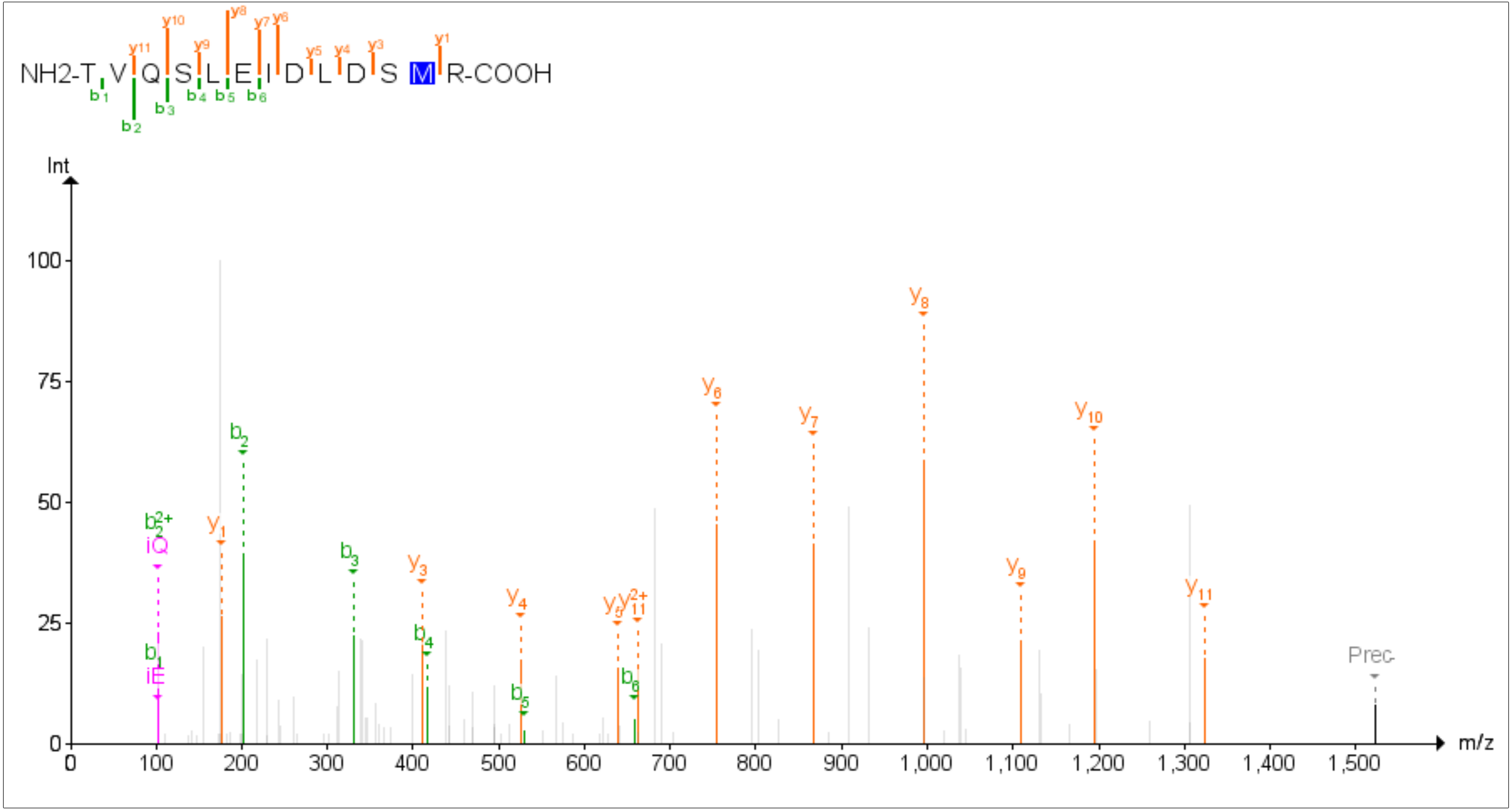

NH2-T V Q S L E I D L D S M R-COOH
b1 b2 b3 b4 b5 b6
y11 y10 y9 y8 y7 y6 y5 y4 y3 y1
Int
m/z
0
25
50
75
100
0
100
200
300
400
500
600
700
800
900
1,000
1,100
1,200
1,300
1,400
1,500
b2 2+
iQ
b1
iE
y1
b2
b3
y3
b4
y4
b5
y5
y11 2+
b6
y6
y7
y8
y9
y10
y11
Prec

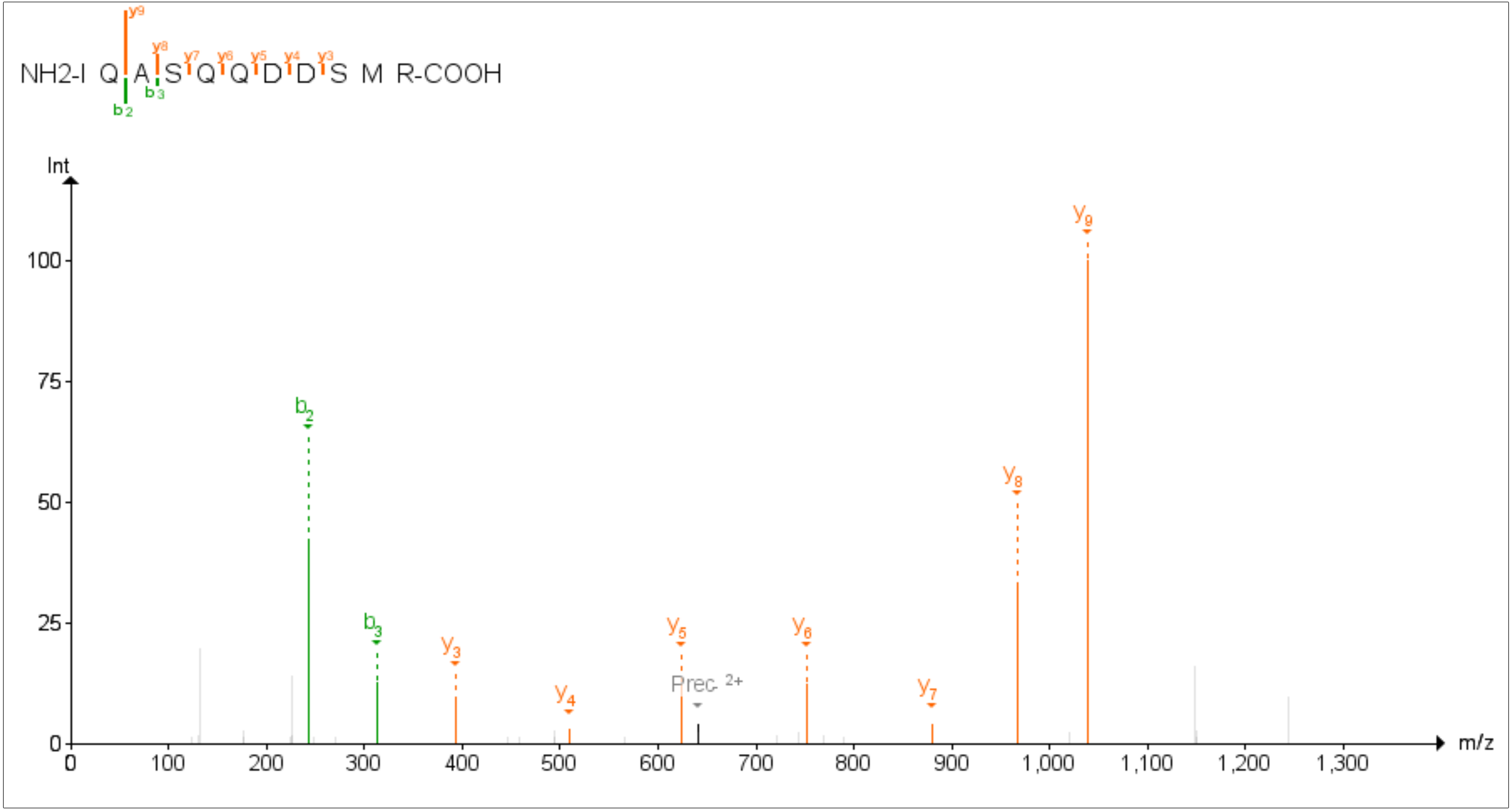

NH2-I Q A S Q Q D D S M R-COOH
y9
y8
y7
y6
y5
y4
y3
b2
b3
Int
m/z
0
25
50
75
100
0
100
200
300
400
500
600
700
800
900
1,000
1,100
1,200
1,300
b_2
b_3
y_3
y_4
y_5
Prec. 2+
y_6
y_7
y_8
y_9

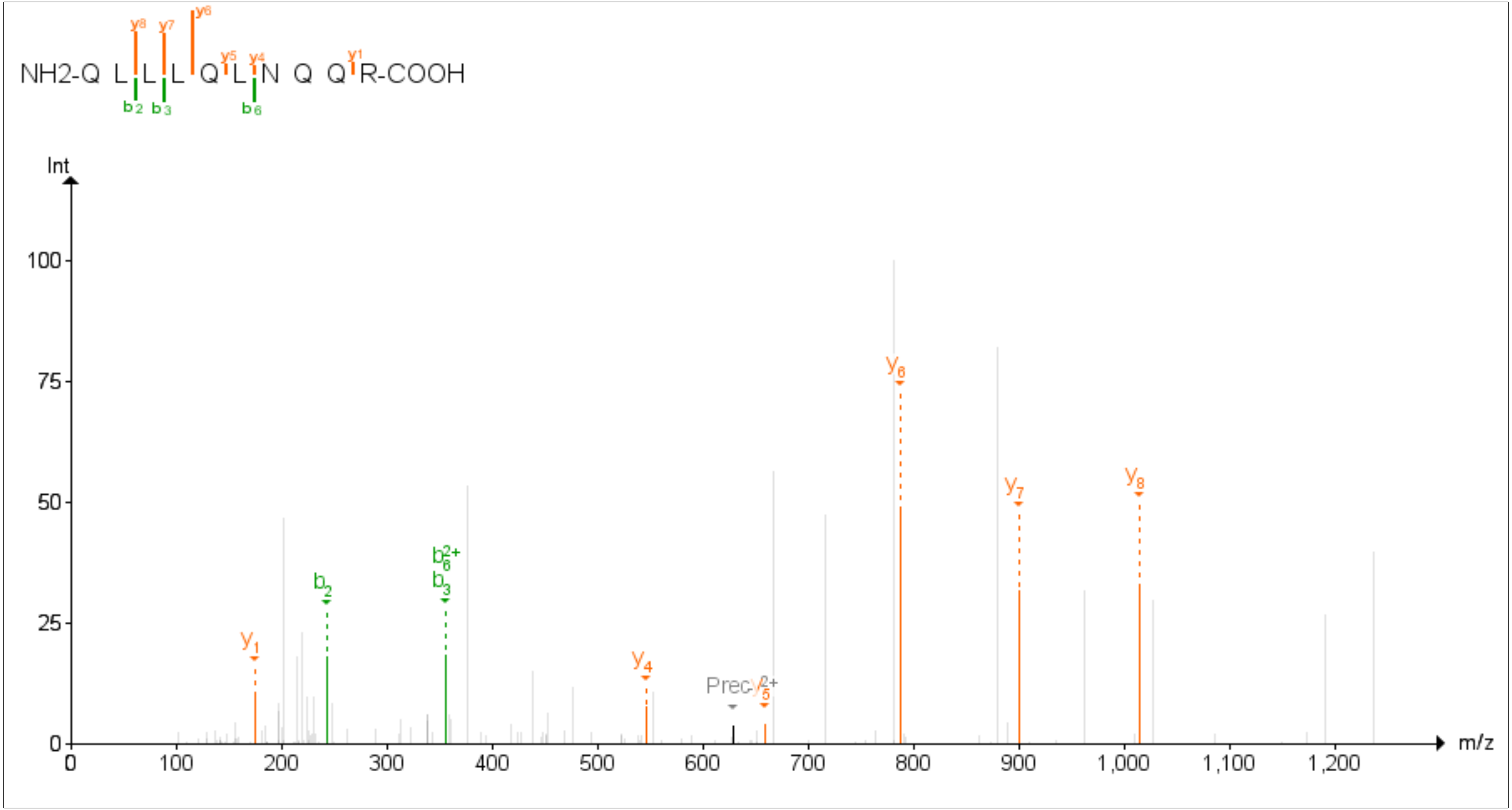

NH2-Q L L L Q L N Q Q R-COOH
y8 y7 y6 y5 y4 y1
b2 b3 b6
Int
100
75
50
25
0
y6
y7
y8
b2
b6 2+
b3
y1
y4
Prec 2+
y5
0 100 200 300 400 500 600 700 800 900 1,000 1,100 1,200
m/z

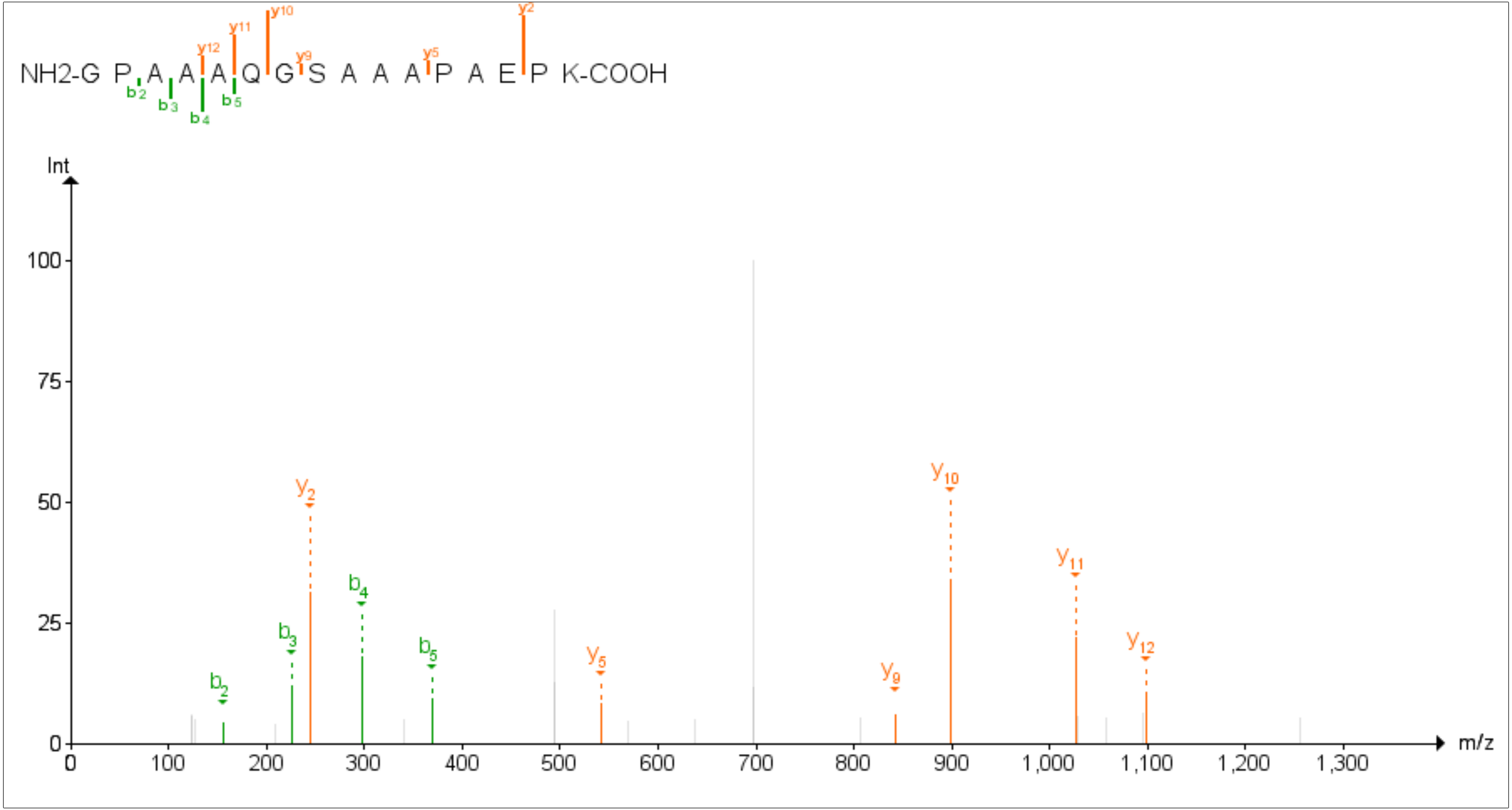
NH2-G P A A A Q G S A A A P A E P K-COOH
b2 b3 b4 b5
y12 y11 y10 y9 y5 y2
Int
100
75
50
25
0
m/z
0 100 200 300 400 500 600 700 800 900 1,000 1,100 1,200 1,300

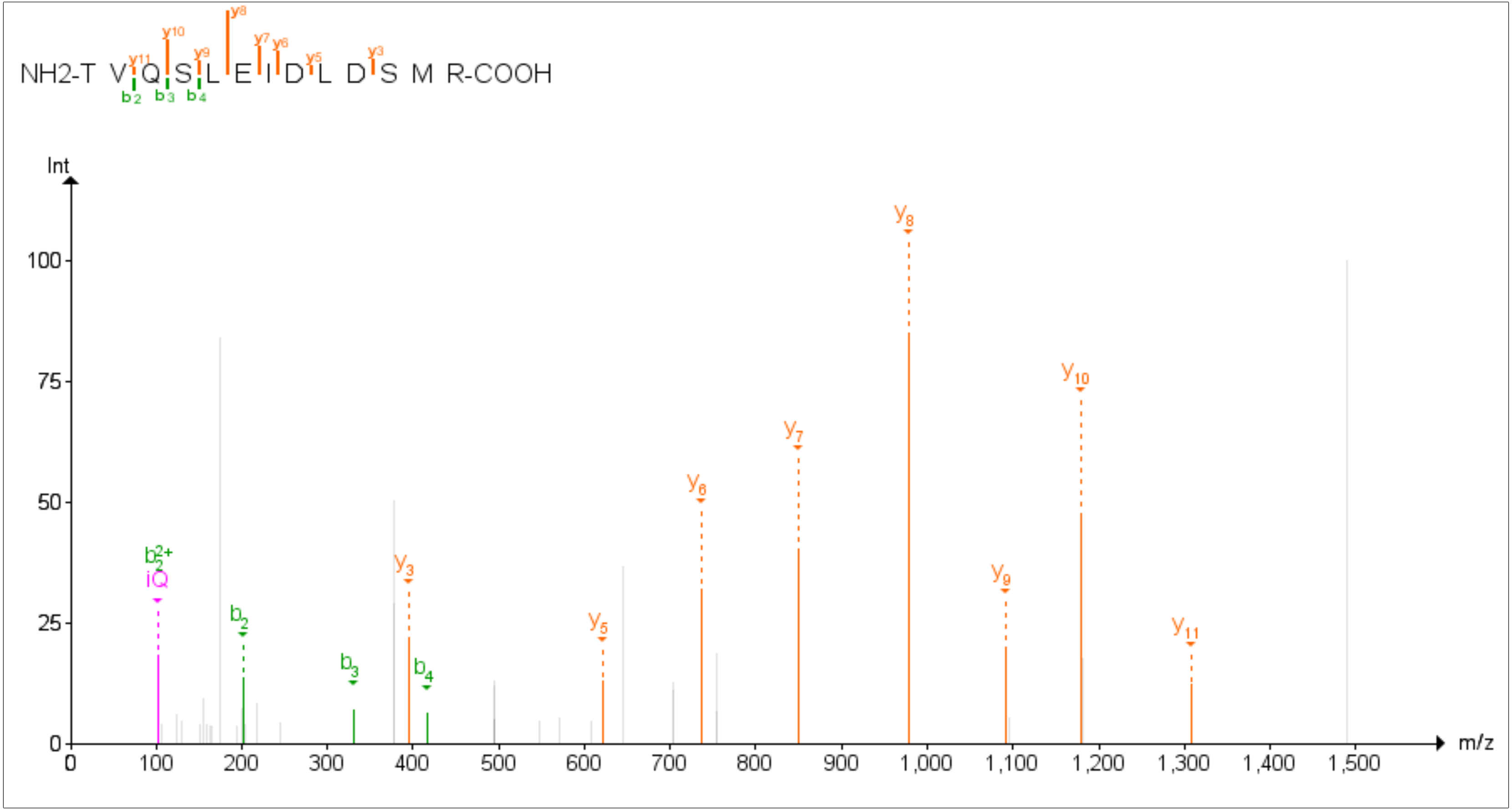
NH2-T V Q S L E I D L D S M R-COOH
y11 y10 y9 y8 y7 y6 y5 y3
b2 b3 b4
Int
100
75
50
25
0
m/z
0 100 200 300 400 500 600 700 800 900 1,000 1,100 1,200 1,300 1,400 1,500
y8 y10 y7 y6 y3 y9 y5 y11
b2 b3 b4
iQ

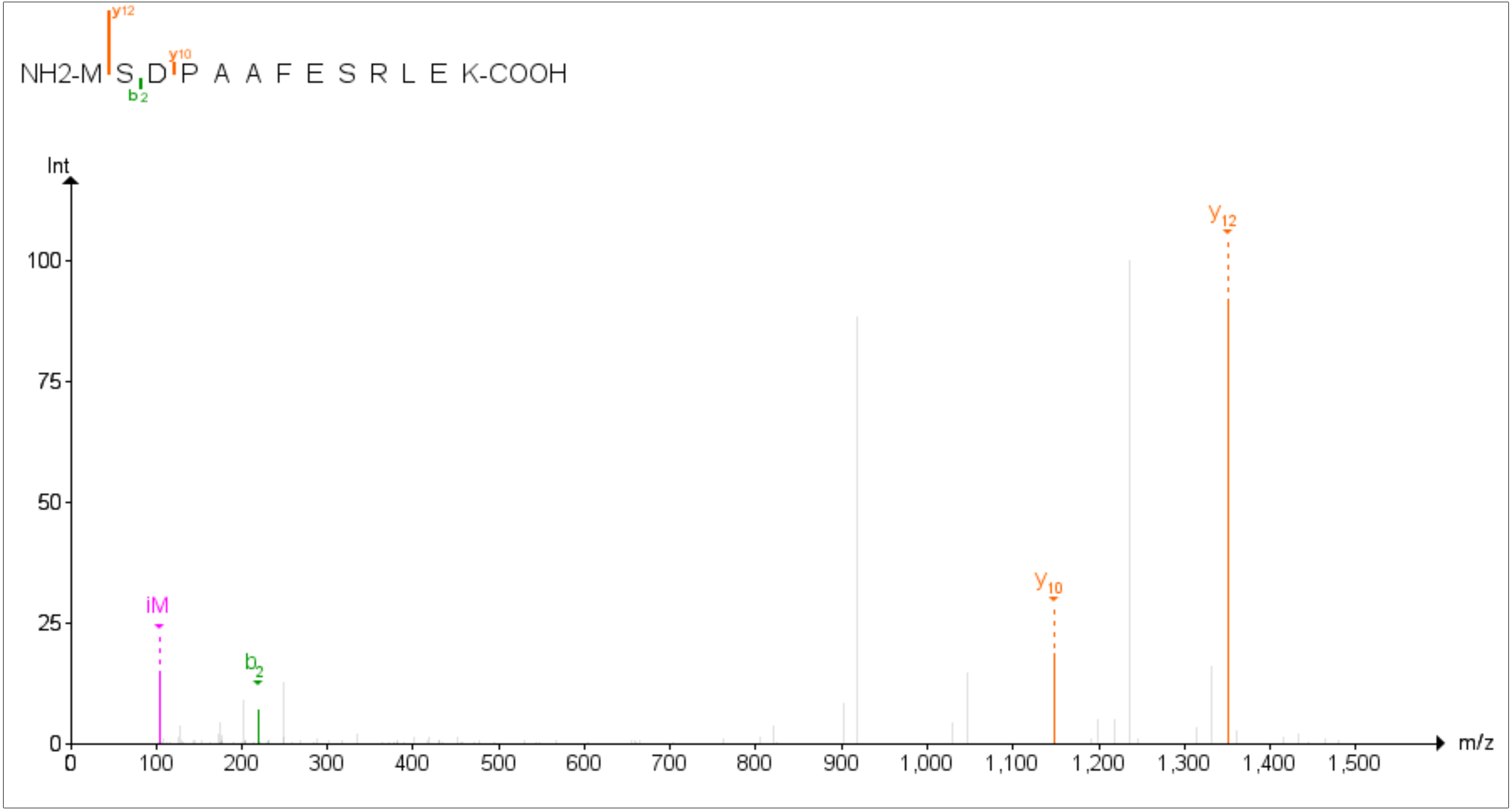

NH2-M S D P A A F E S R L E K-COOH
y12
y10
b2
Int
100
75
50
25
0
iM
b2
y10
y12
0
100
200
300
400
500
600
700
800
900
1,000
1,100
1,200
1,300
1,400
1,500
m/z

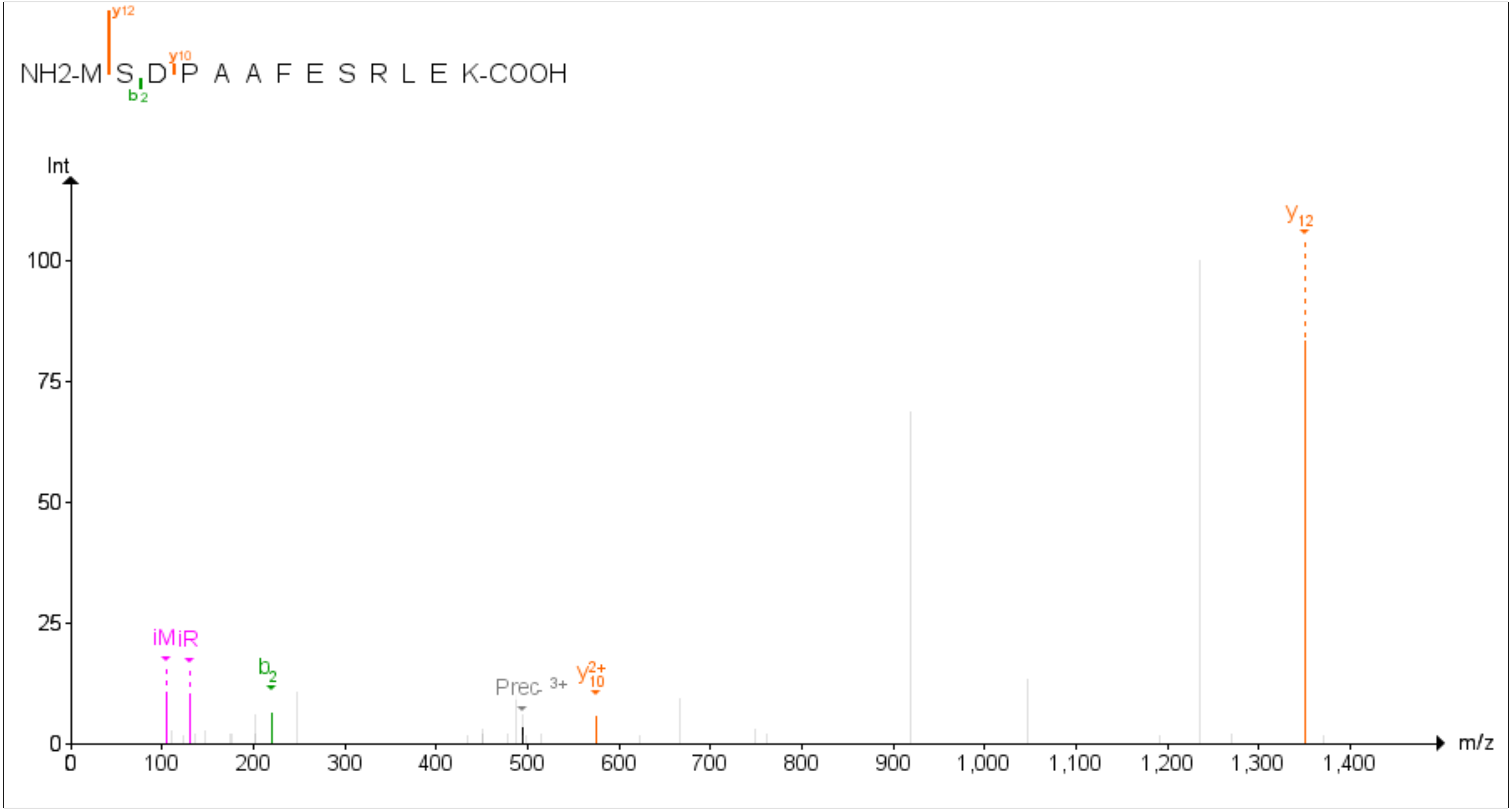
NH2-M S D P A A F E S R L E K-COOH
y12
y10
b2
Int
100
75
50
25
0
iM
iR
b2
Prec 3+
y10 2+
y12
m/z
0
100
200
300
400
500
600
700
800
900
1,000
1,100
1,200
1,300
1,400

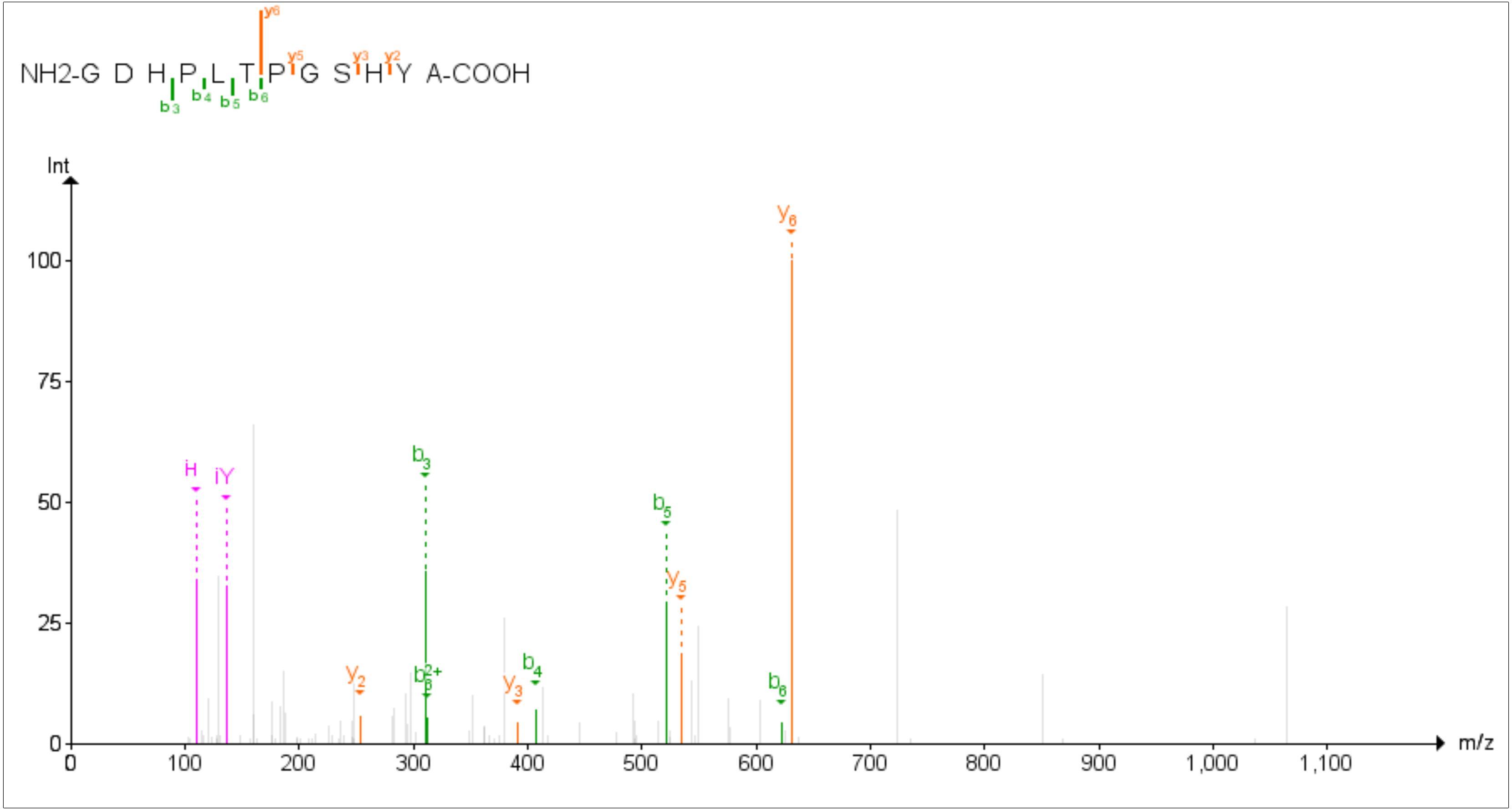

NH2-G D H P L T P G S H Y A-COOH
Int
m/z

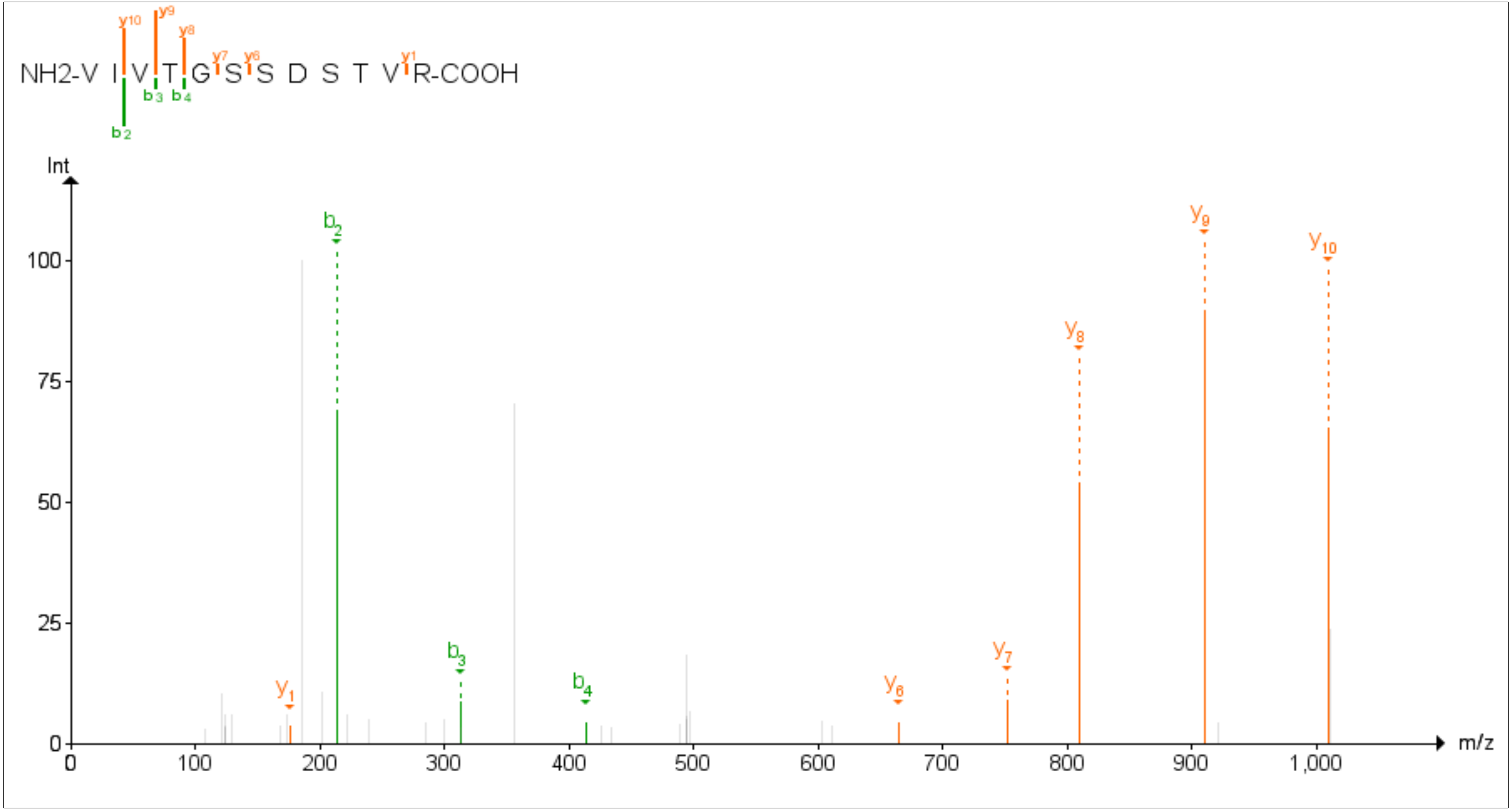
NH2-V I V T G S S D S T V R-COOH
y10
y9
y8
y7
y6
y1
b2
b3
b4
Int
m/z
0
25
50
75
100
0
100
200
300
400
500
600
700
800
900
1,000

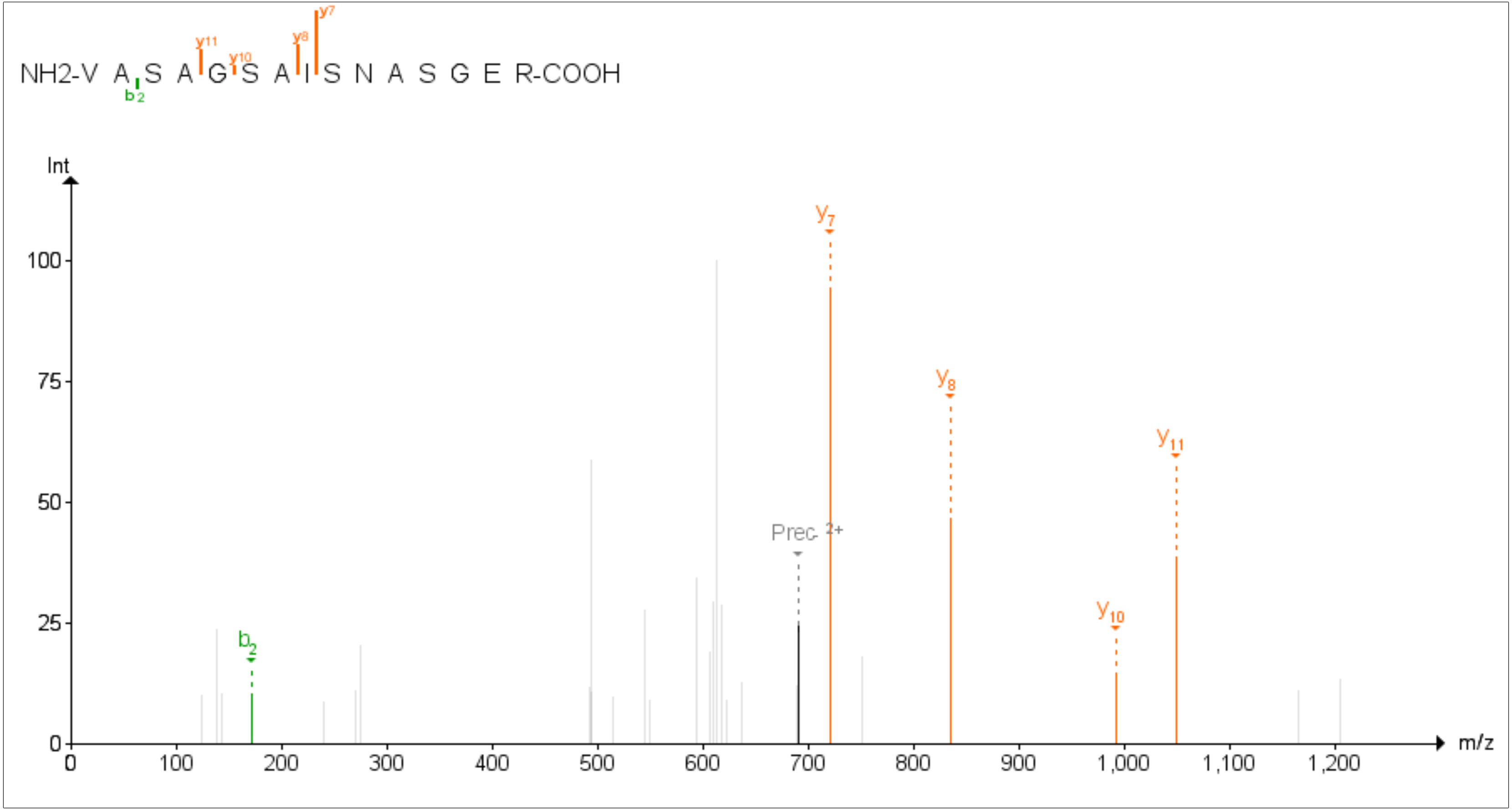
NH2-V A S A G S A I S N A S G E R-COOH
b2
y11
y10
y8
y7
Int
100
75
50
25
0
Prec 2+
m/z
0
100
200
300
400
500
600
700
800
900
1,000
1,100
1,200

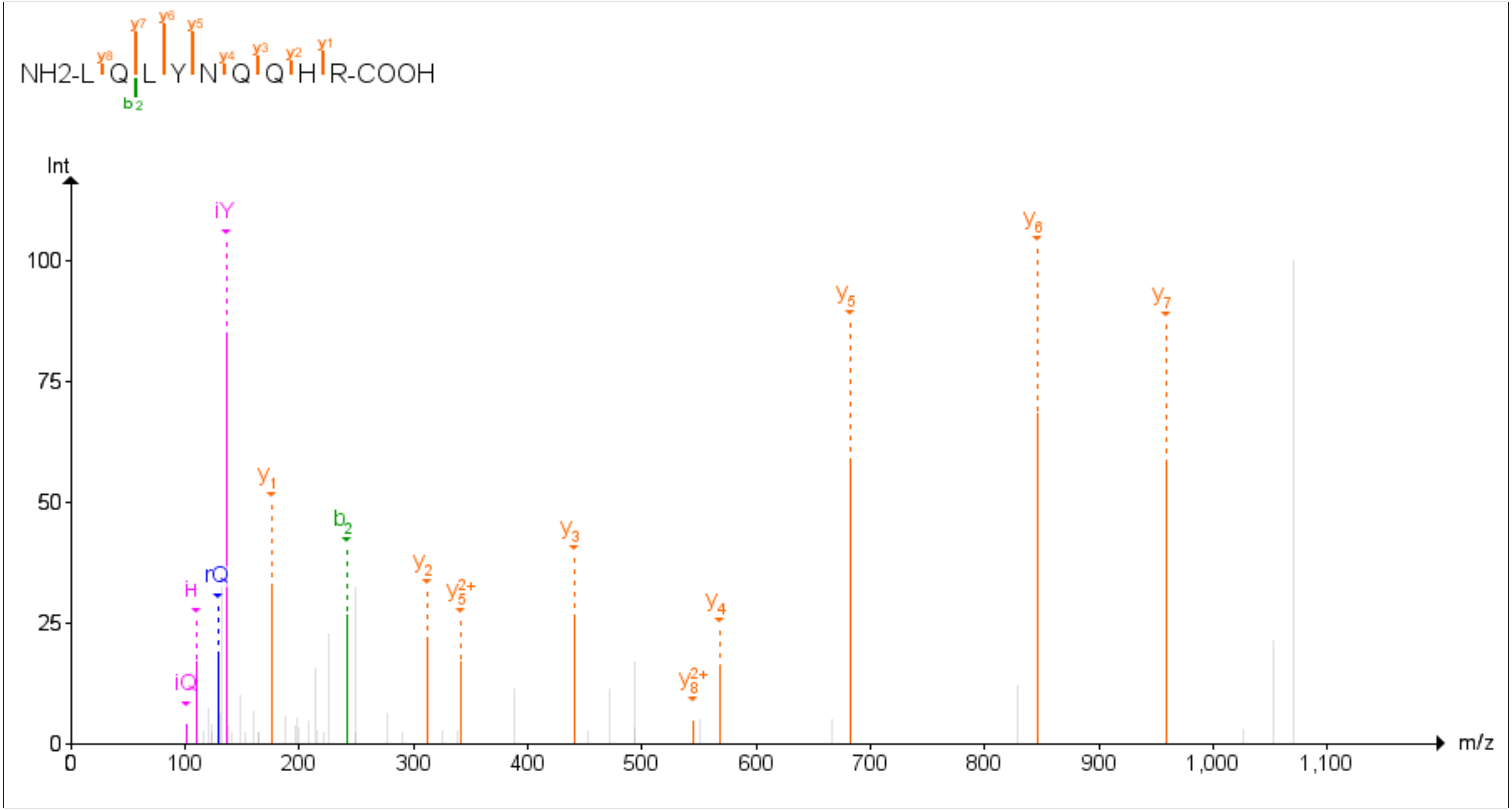

NH2-L Q L Y N Q Q H R-COOH
y8
y7
y6
y5
y4
y3
y2
y1
b2
Int
m/z
iY
y6
y5
y7
y1
b2
y3
y2
y5 2+
rQ
iH
y4
iQ
y8 2+
0
25
50
75
100
0
100
200
300
400
500
600
700
800
900
1,000
1,100

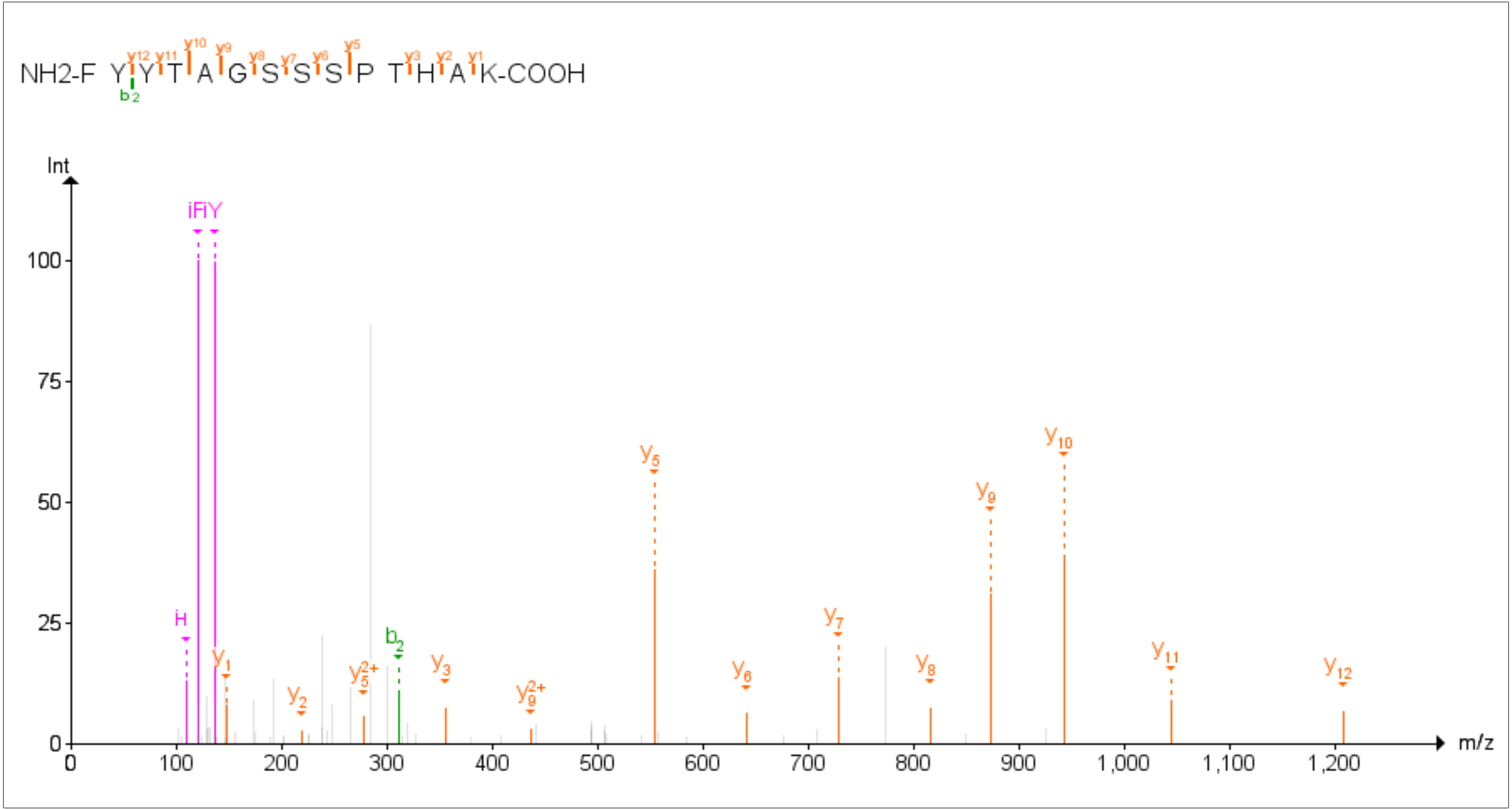

NH2-F Y Y T A G S S S P T H A K-COOH
y12 y11 y10 y9 y8 y7 y6 y5 y3 y2 y1
b2
Int
m/z
iFiY
iH
y1
y2
y5 2+
b2
y3
y9 2+
y5
y6
y7
y8
y9
y10
y11
y12
0
25
50
75
100
0
100
200
300
400
500
600
700
800
900
1,000
1,100
1,200

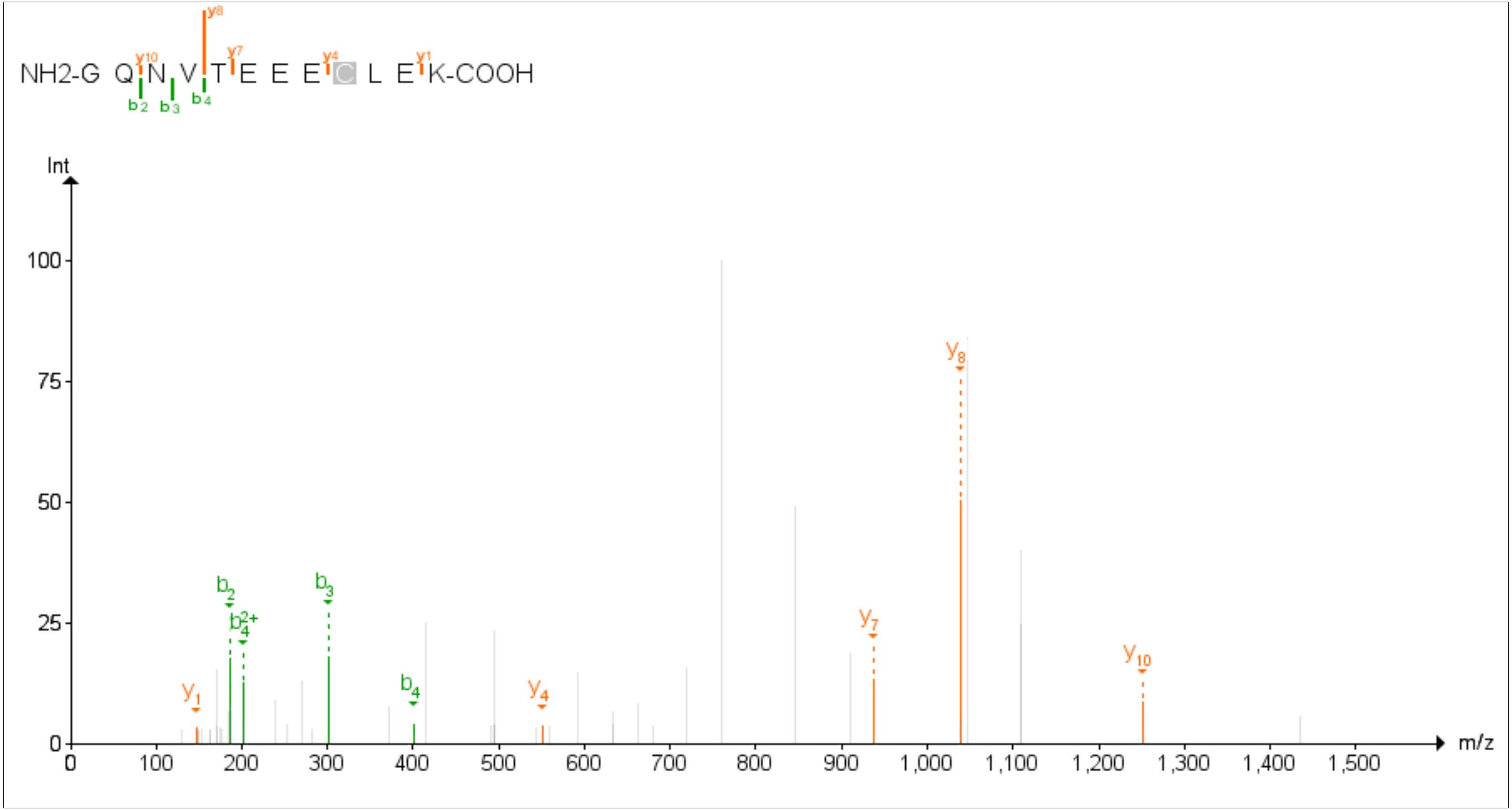

NH2-G Q N V T E E E C L E K-COOH
y10 y8 y7 y4 y1
b2 b3 b4
Int
100
75
50
25
0
m/z
0 100 200 300 400 500 600 700 800 900 1,000 1,100 1,200 1,300 1,400 1,500
y1 b2 b4 2+ b3 b4 y4 y7 y8 y10

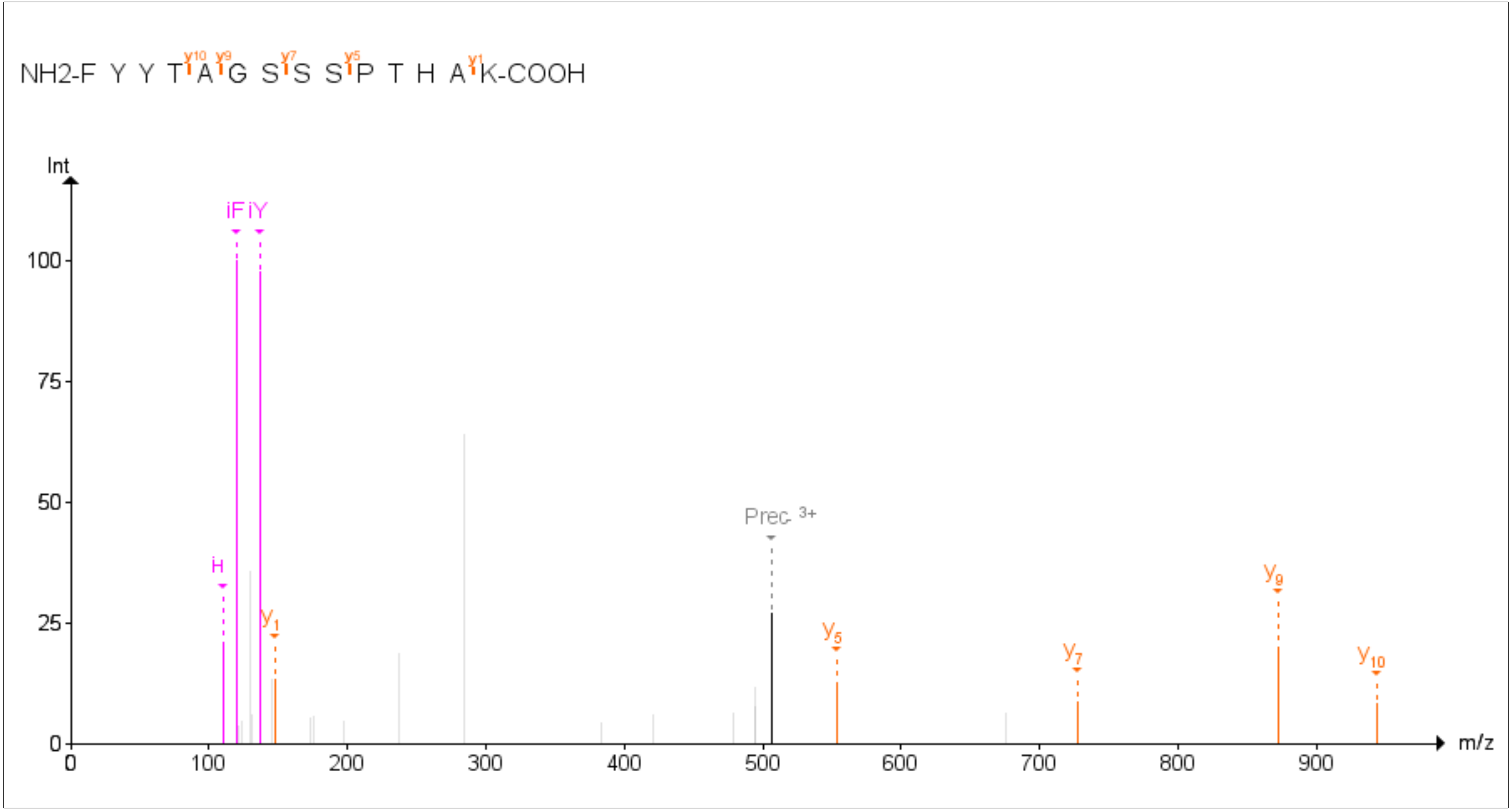

NH2-F Y Y T A G S S S P T H A K-COOH
y10 y9 y7 y5 y1
Int
100
75
50
25
0
iF iY
iH
y1
Prec 3+
y5
y7
y9
y10
0 100 200 300 400 500 600 700 800 900
m/z

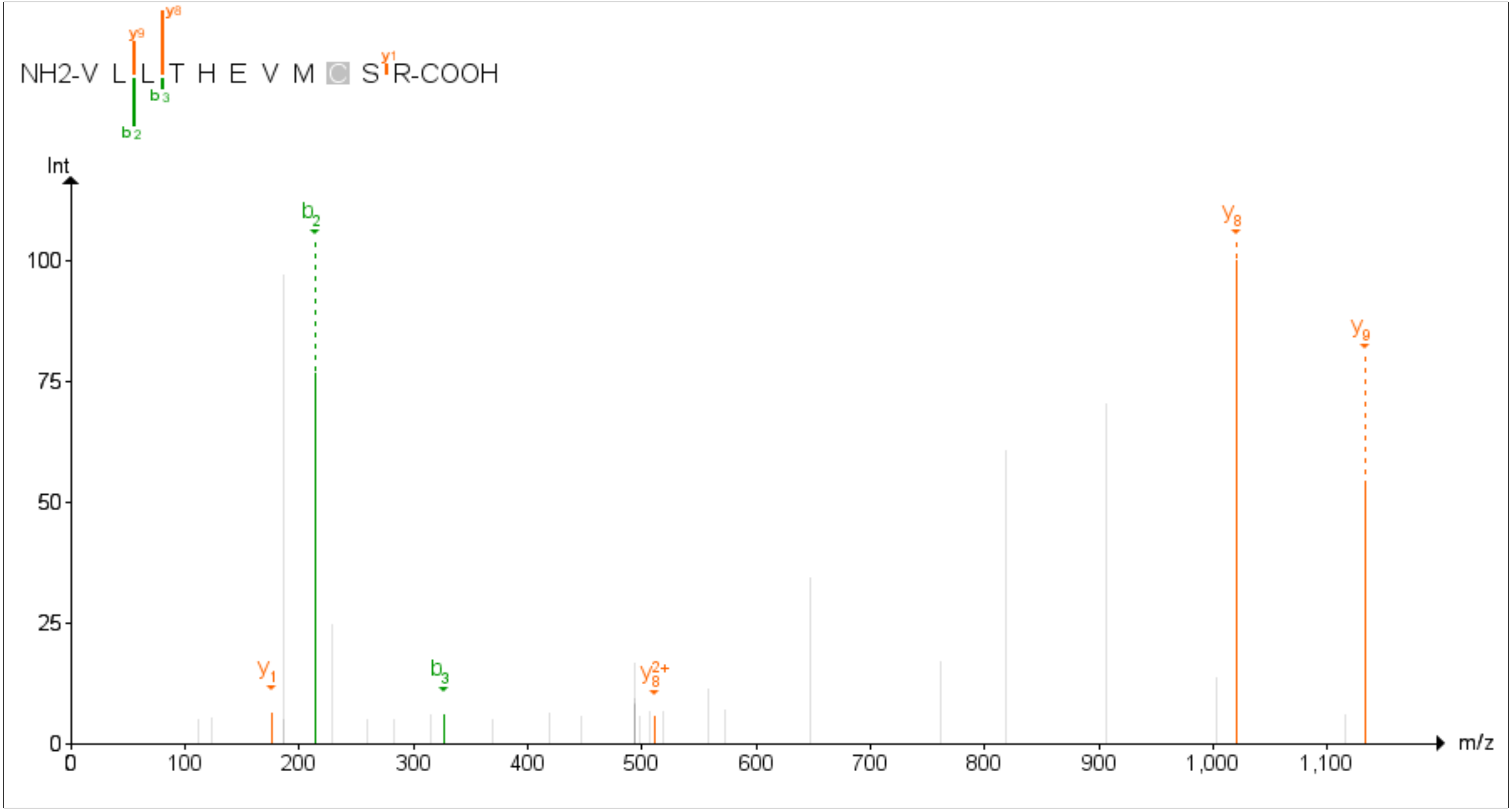

NH2-V L L T H E V M C S R-COOH
y9
y8
y1
b2
b3
Int
100
75
50
25
0
b2
y8
y9
y1
b3
y8 2+
0
100
200
300
400
500
600
700
800
900
1,000
1,100
m/z

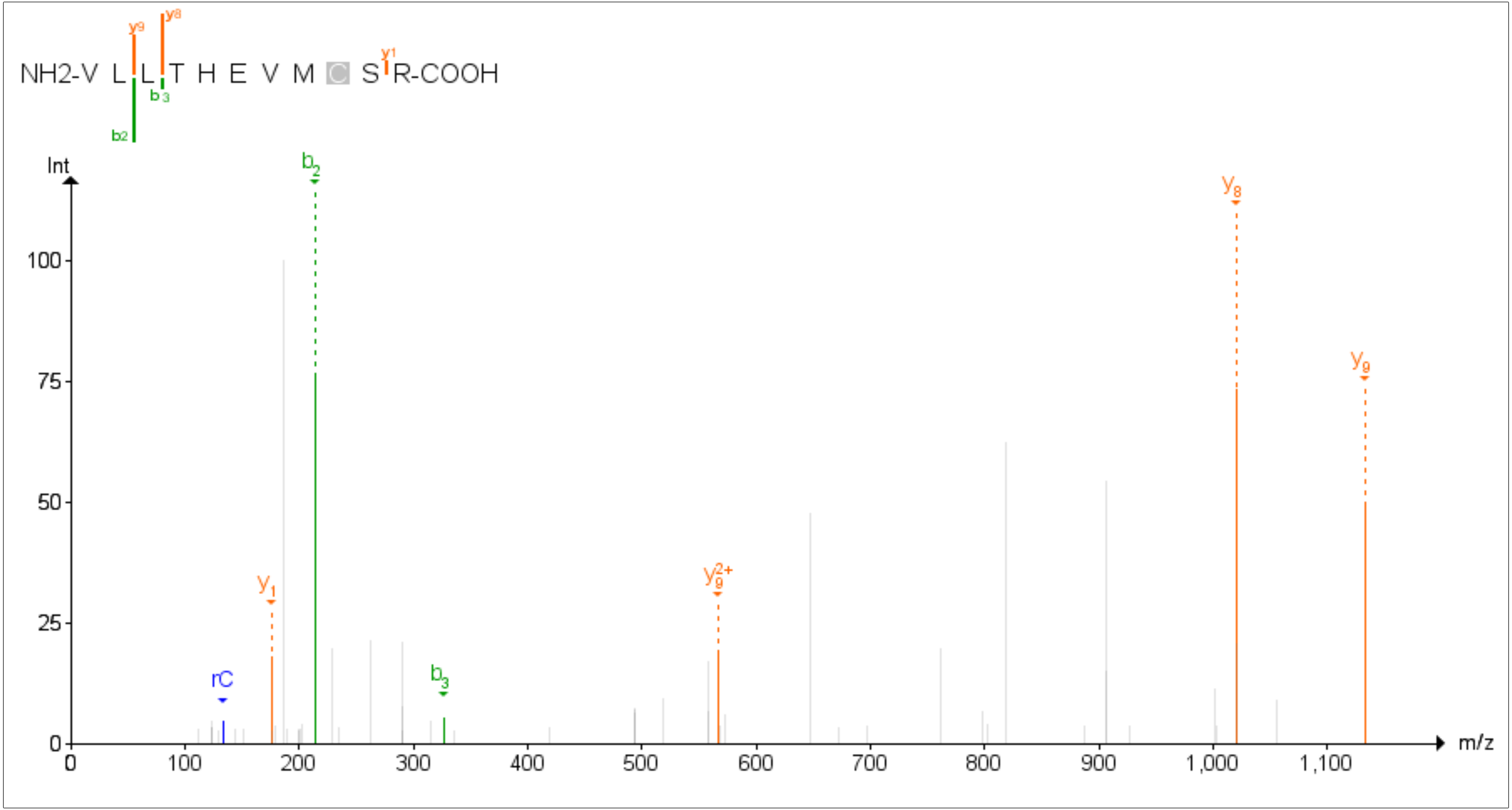

NH2-V L L T H E V M C S R-COOH
y9
y8
y1
b2
b3
Int
100
75
50
25
0
m/z
0
100
200
300
400
500
600
700
800
900
1,000
1,100
rC
y1
b2
b3
y9 2+
y8
y9

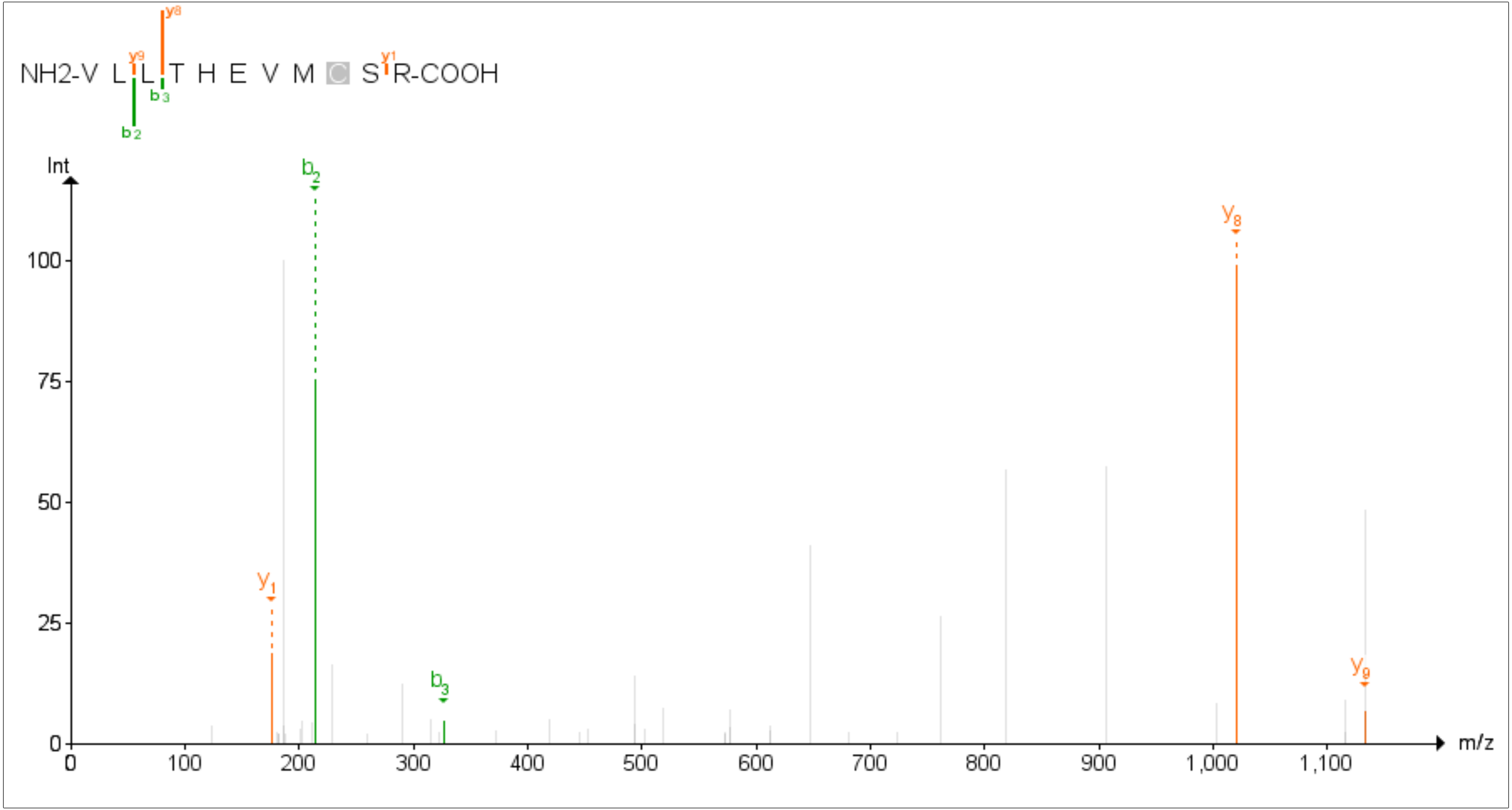

NH2-V L L T H E V M C S R-COOH
y8
y9
y1
b2
b3
Int
m/z
100
75
50
25
0
100
200
300
400
500
600
700
800
900
1,000
1,100

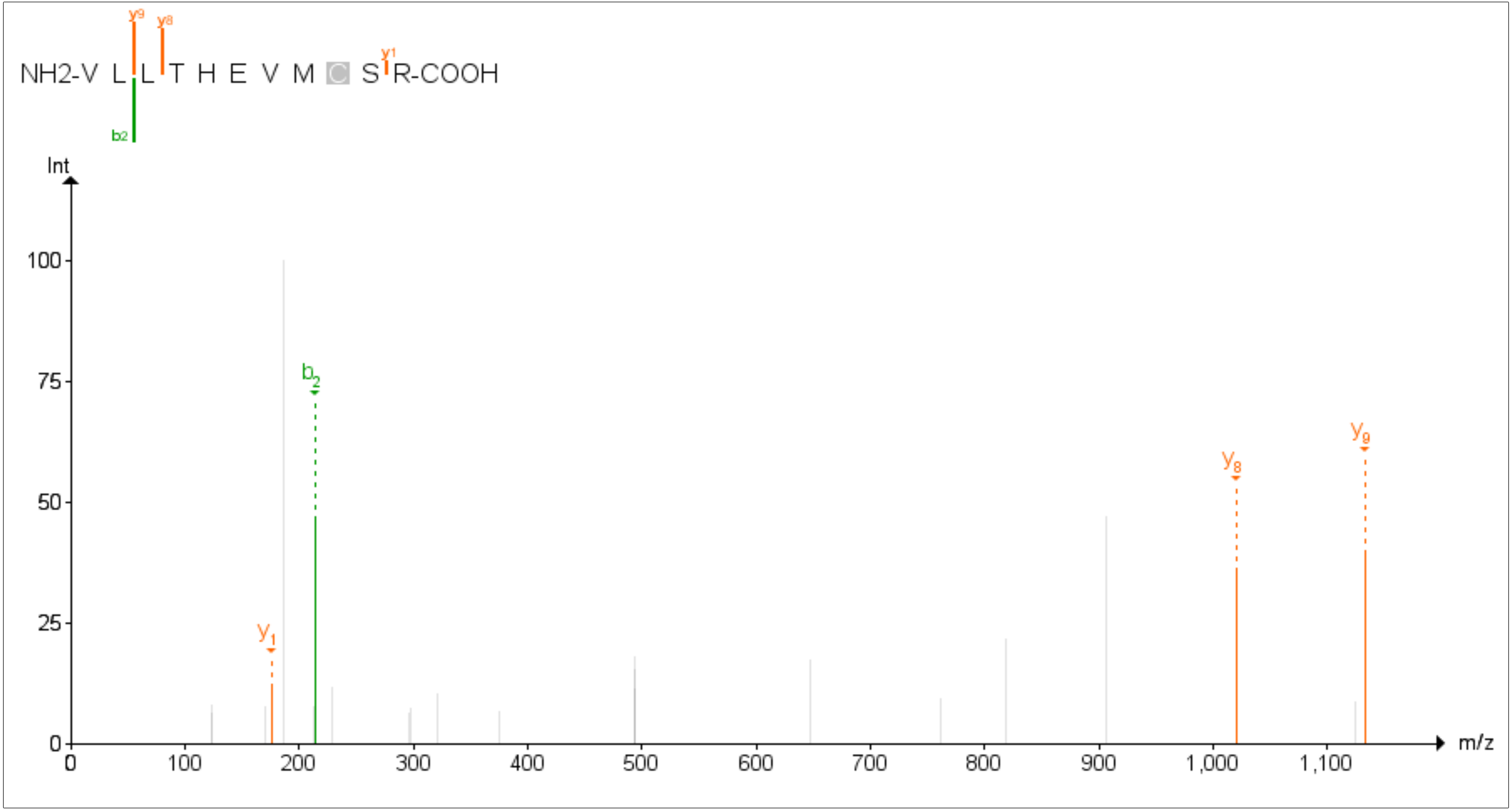

NH2-V L L T H E V M C S R-COOH
y9
y8
y1
b2
Int
100
75
50
25
0
b2
y1
y8
y9
0
100
200
300
400
500
600
700
800
900
1,000
1,100
m/z

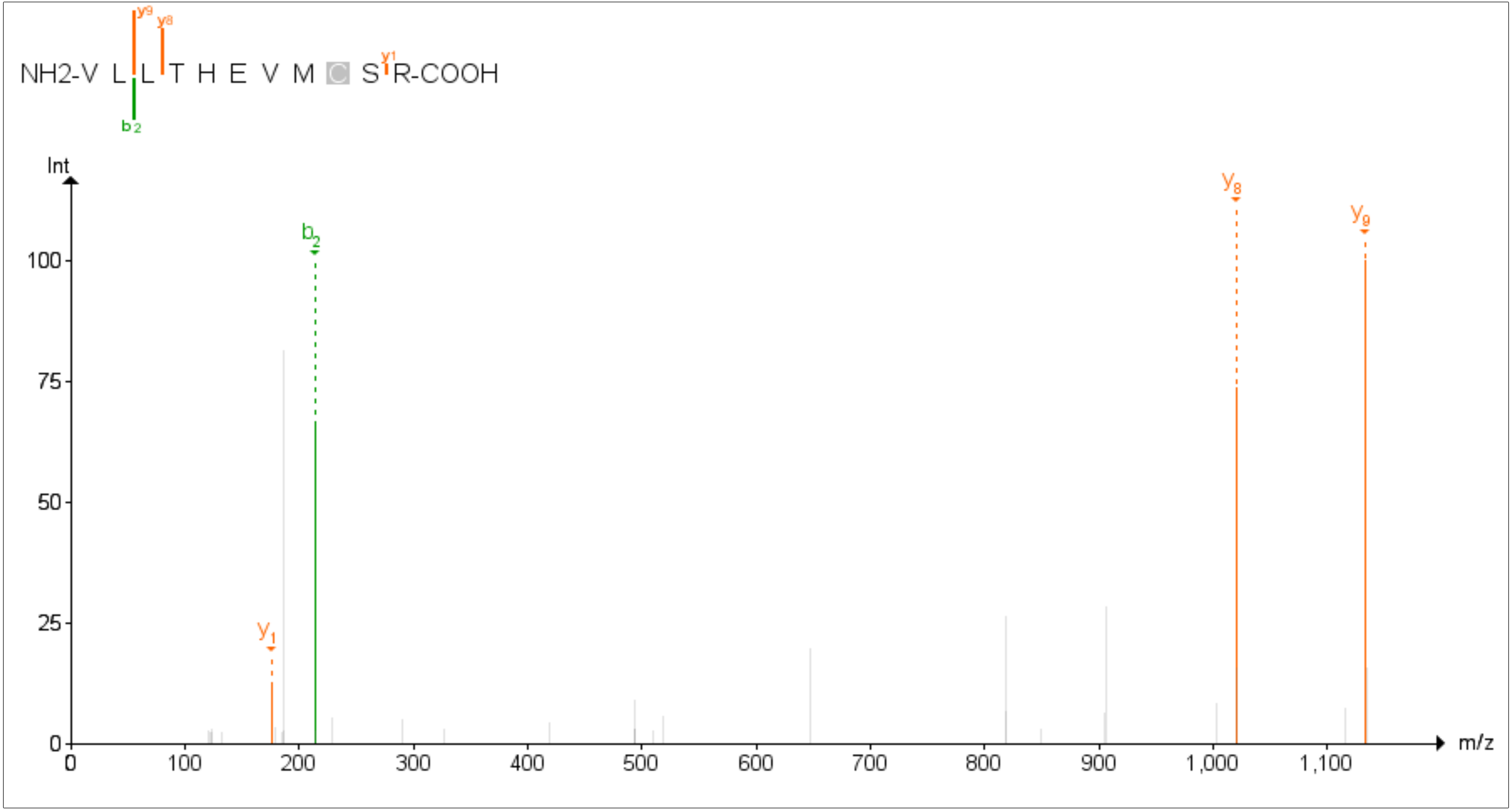

NH2-V L L T H E V M C S R-COOH
y9
y8
y1
b2
Int
100
75
50
25
0
0
100
200
300
400
500
600
700
800
900
1,000
1,100
m/z
y_8
y_9
b_2
y_1

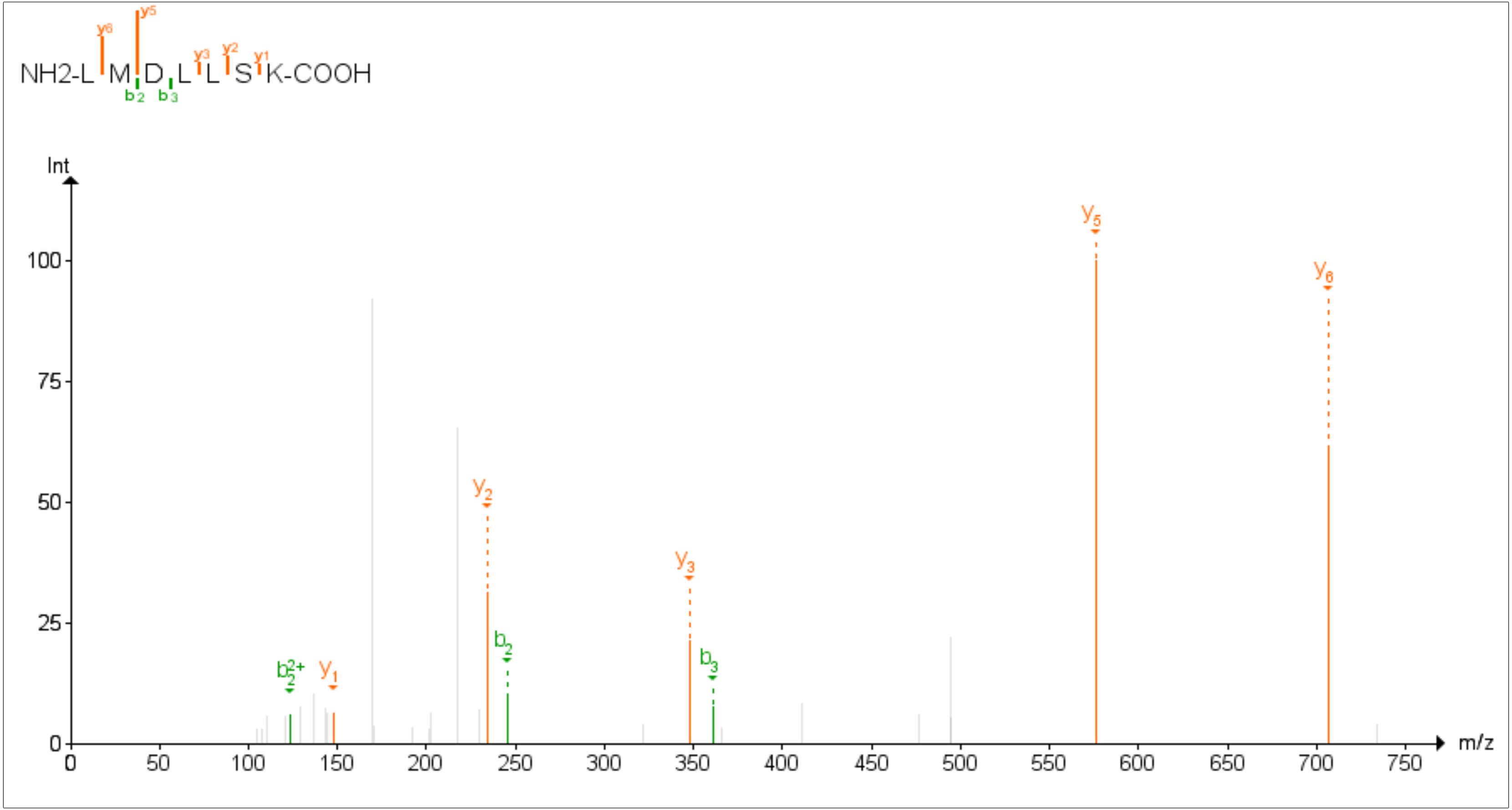
NH2-L M D L L S K-COOH
y6 y5 y3 y2 y1
b2 b3
Int
100
75
50
25
0
m/z
0 50 100 150 200 250 300 350 400 450 500 550 600 650 700 750
y5
y6
y2
y3
b2
b3
b2 2+
y1

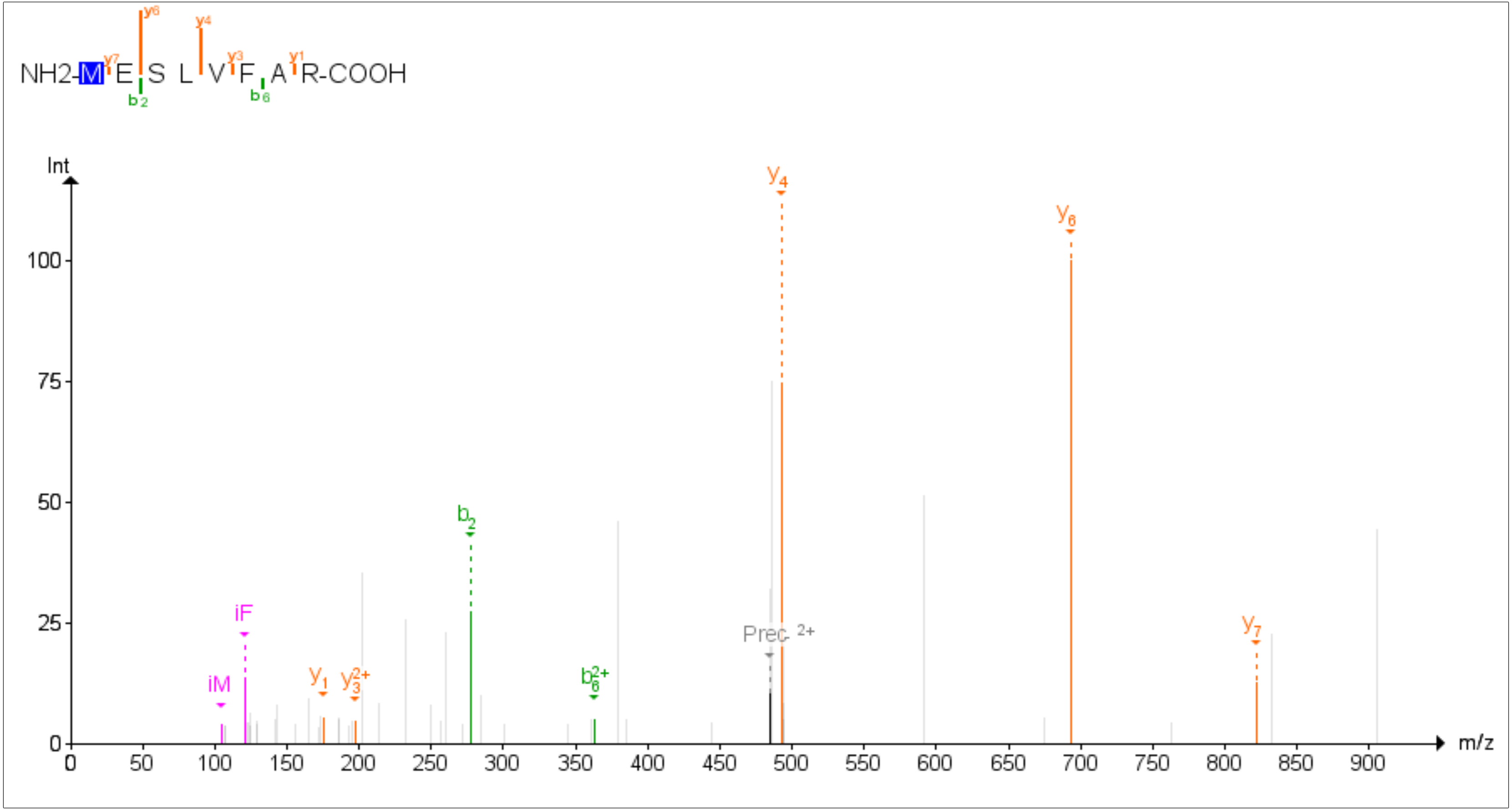
NH2-M E S L V F A R-COOH
y7 y6 y4 y3 y1
b2 b6
Int
m/z
0 25 50 75 100
0 50 100 150 200 250 300 350 400 450 500 550 600 650 700 750 800 850 900
y4
y6
b2
iF
iM
y1
y3 2+
b6 2+
Prec 2+
y7

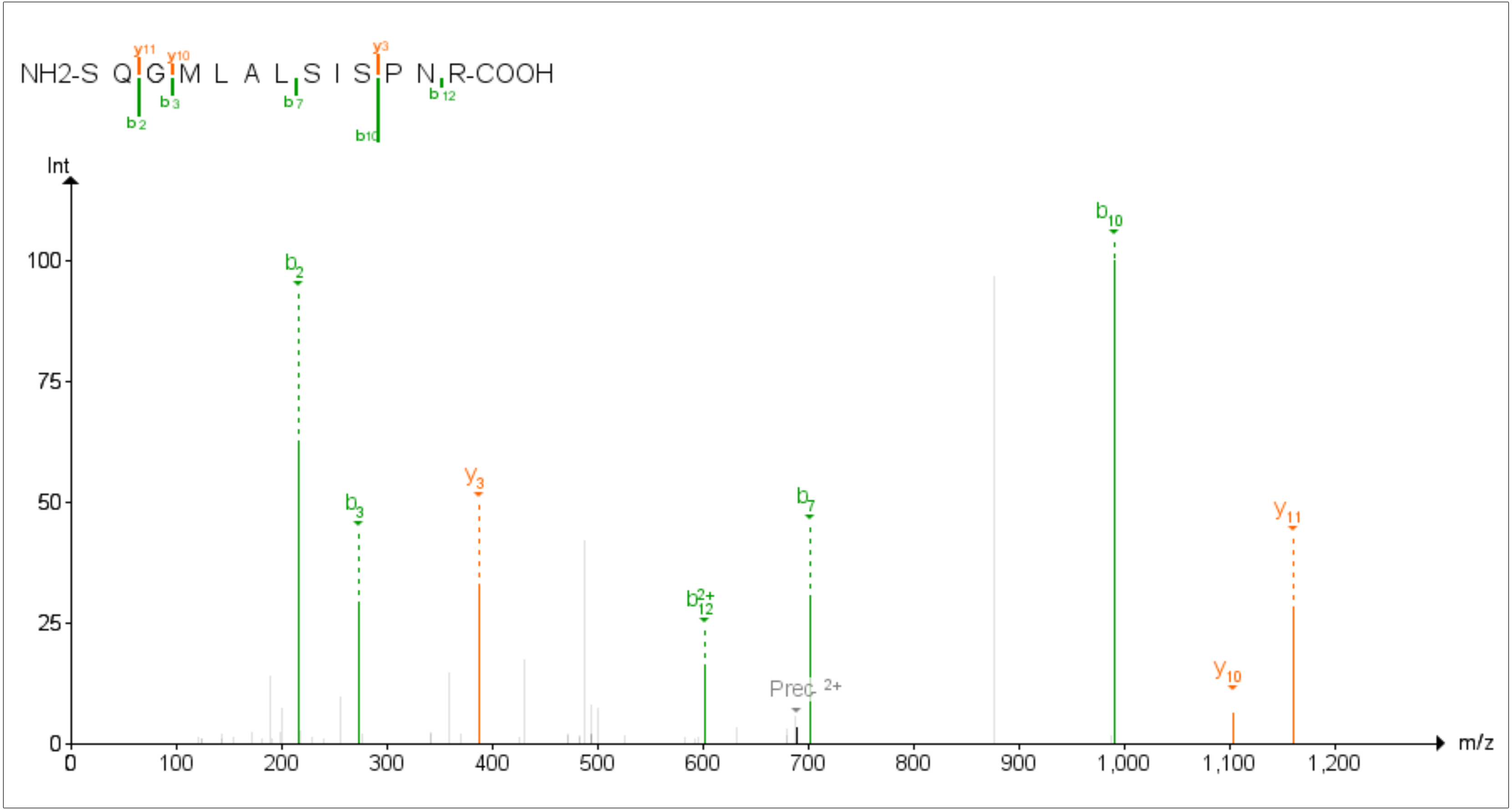

NH2-S Q G M L A L S I S P N R-COOH
y11
y10
y3
b2
b3
b7
b10
b12
Int
100
75
50
25
0
b2
b3
y3
b12 2+
Prec 2+
b7
b10
y10
y11
0
100
200
300
400
500
600
700
800
900
1,000
1,100
1,200
m/z

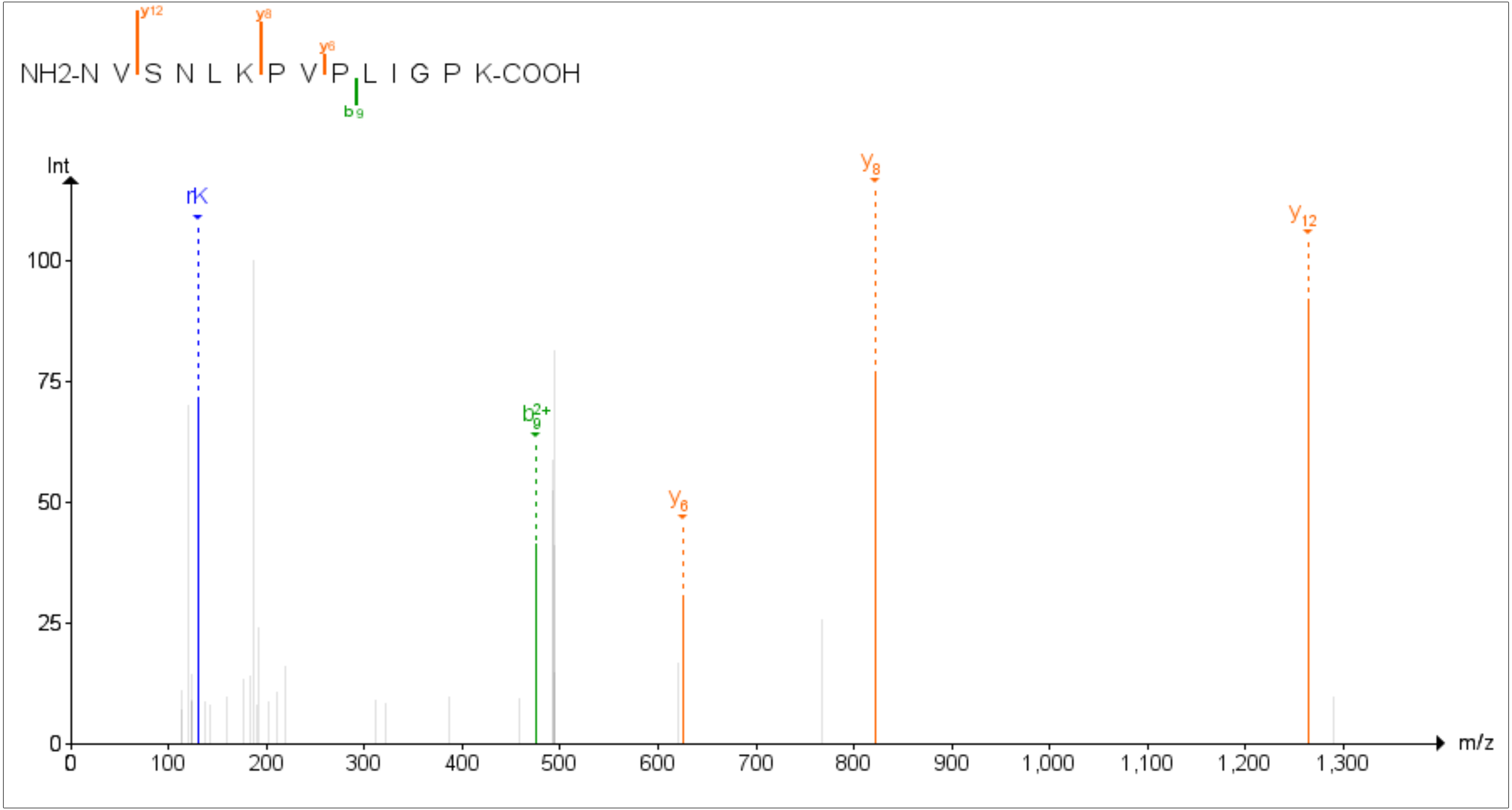
NH2-N V S N L K P V P L I G P K-COOH
y12
y8
y6
b9
Int
rK
b_9^{2+}
y_6
y_8
y_{12}
100
75
50
25
0
0 100 200 300 400 500 600 700 800 900 1,000 1,100 1,200 1,300
m/z

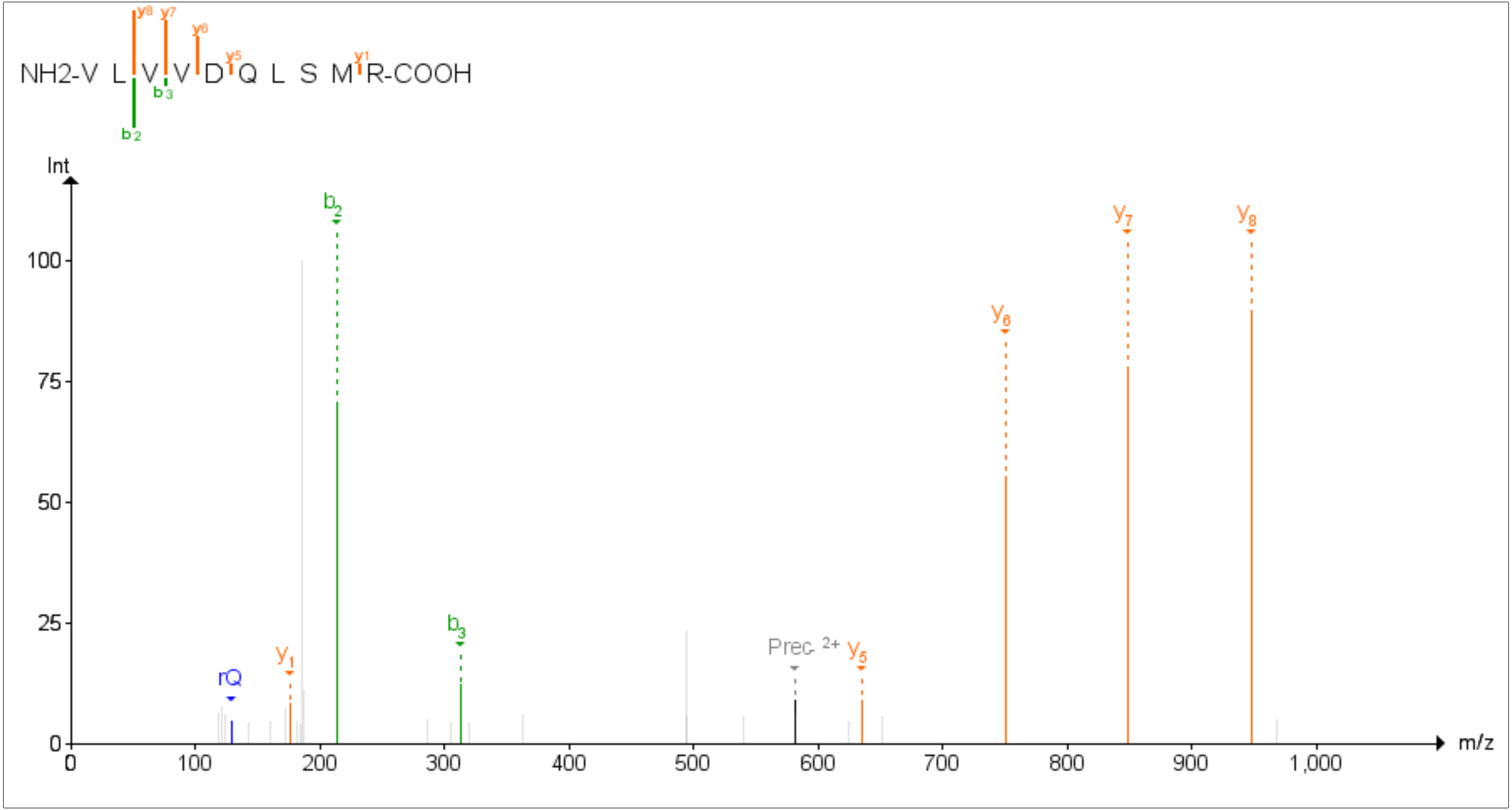

NH2-V L V V D Q L S M R-COOH
y8
y7
y6
y5
y1
b2
b3
Int
b2
y7
y8
y6
b3
rQ
y1
Prec- 2+
y5
100
75
50
25
0
0
100
200
300
400
500
600
700
800
900
1,000
m/z

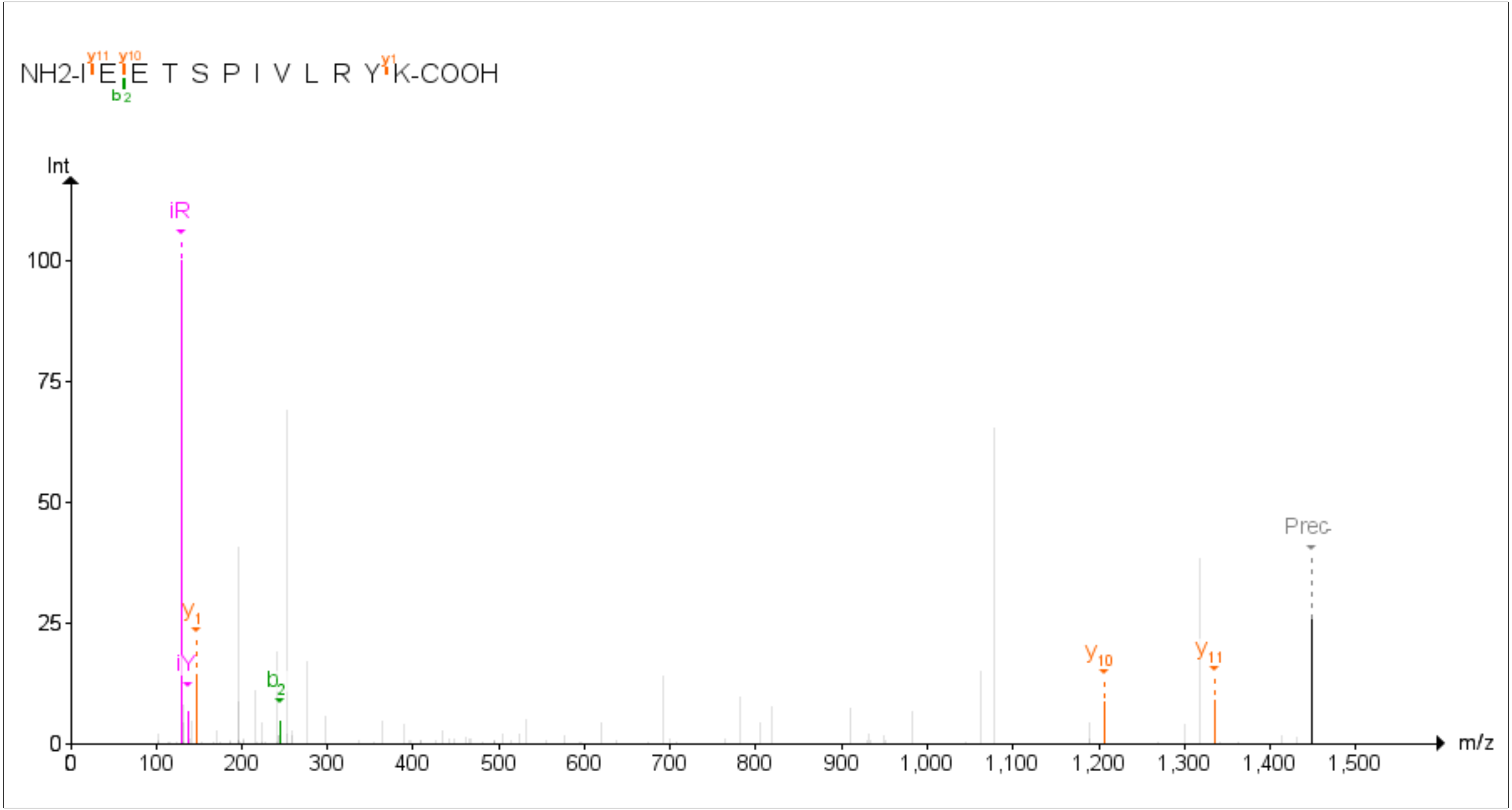
NH2-I E E T S P I V L R Y K-COOH
y11
y10
y1
b2
Int
iR
100
75
50
25
0
y1
iY
b2
y10
y11
Prec-
0
100
200
300
400
500
600
700
800
900
1,000
1,100
1,200
1,300
1,400
1,500
m/z

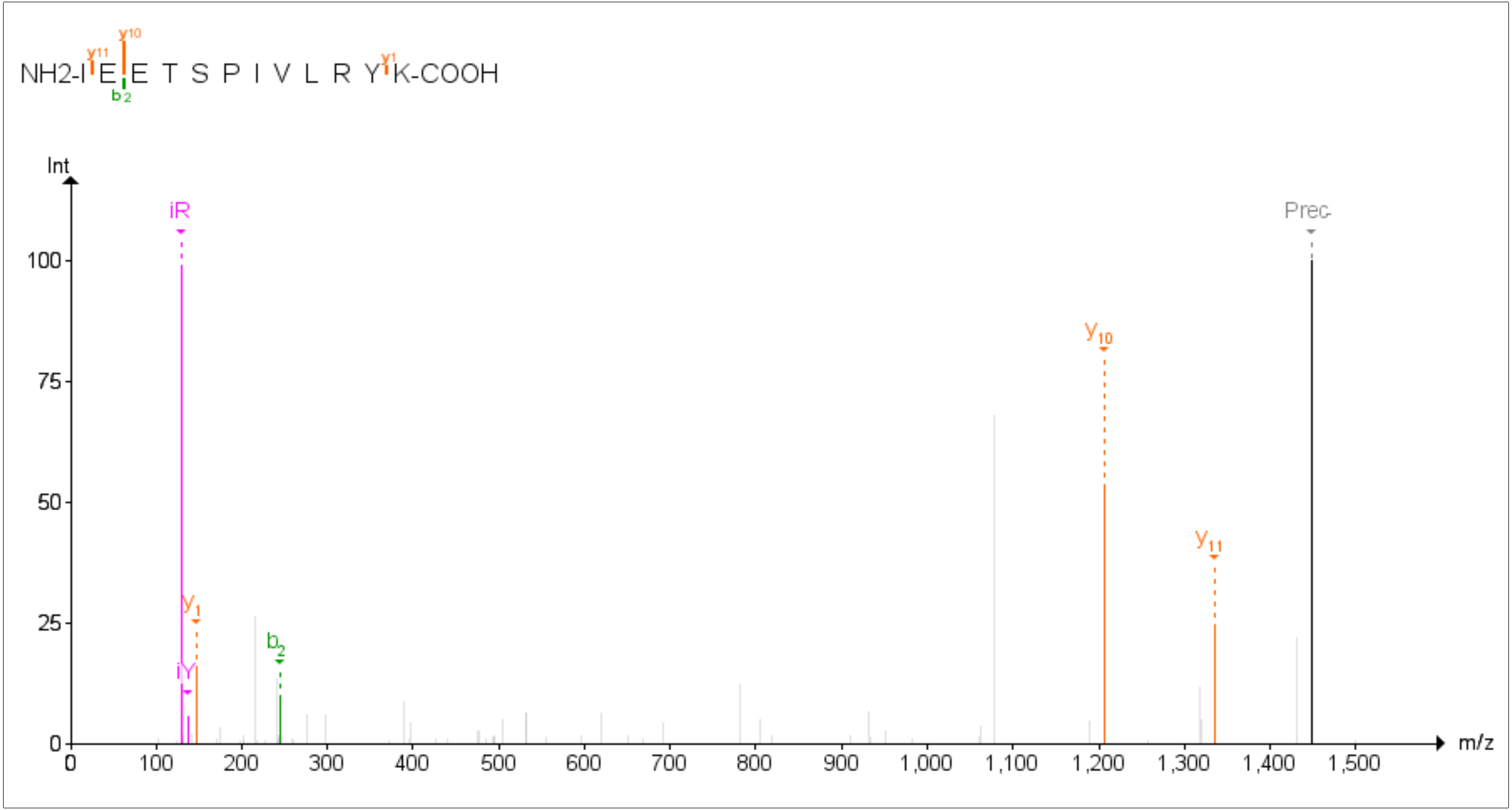

NH2-I E E T S P I V L R Y K-COOH
y11
y10
y1
b2
Int
iR
Prec
y10
y11
y1
iY
b2
100
75
50
25
0
0
100
200
300
400
500
600
700
800
900
1,000
1,100
1,200
1,300
1,400
1,500
m/z

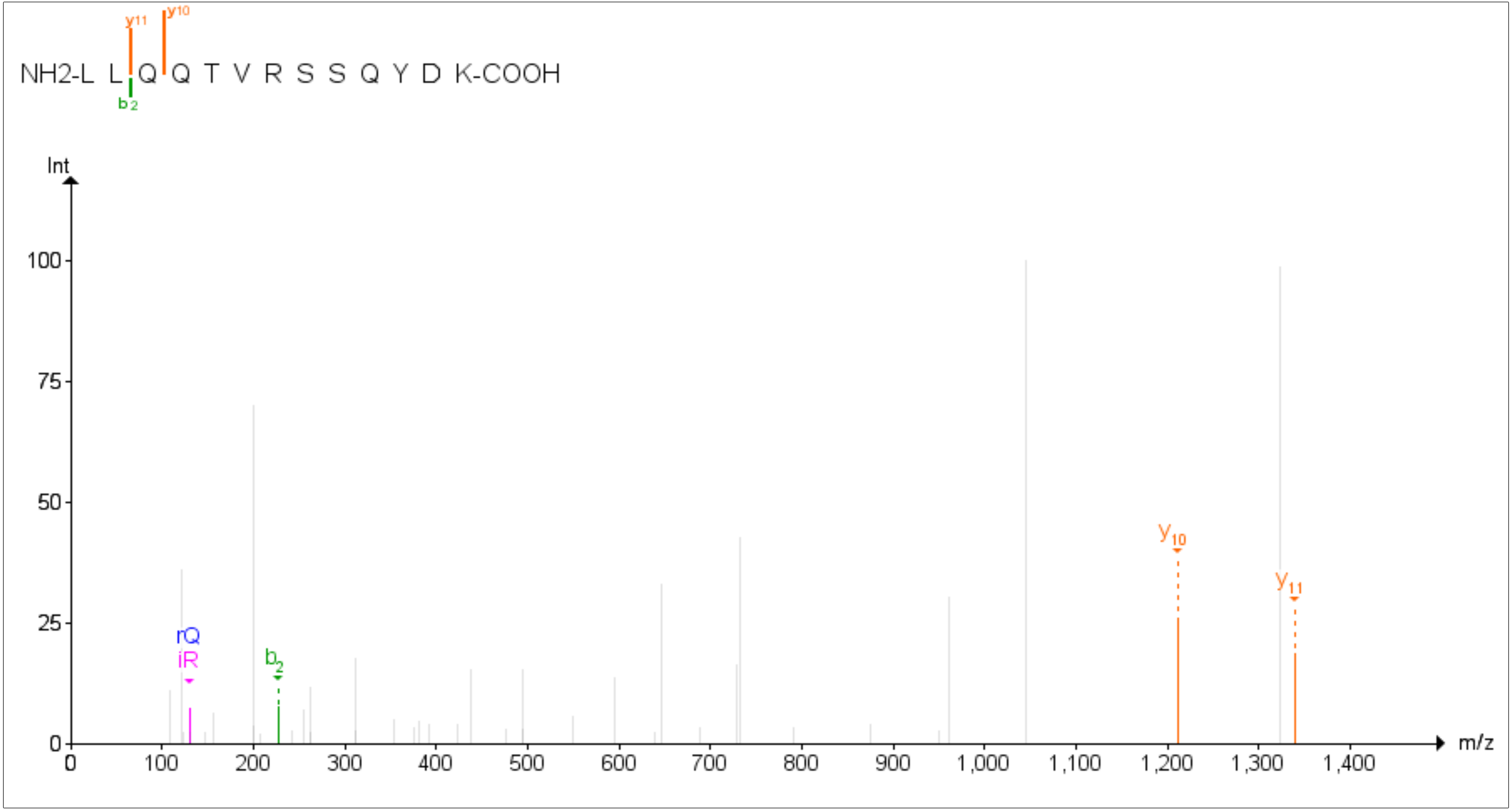

y11
y10
NH2-L L Q Q T V R S S Q Y D K-COOH
b2
Int
100
75
50
25
0
rQ
iR
b2
y10
y11
m/z
0
100
200
300
400
500
600
700
800
900
1,000
1,100
1,200
1,300
1,400

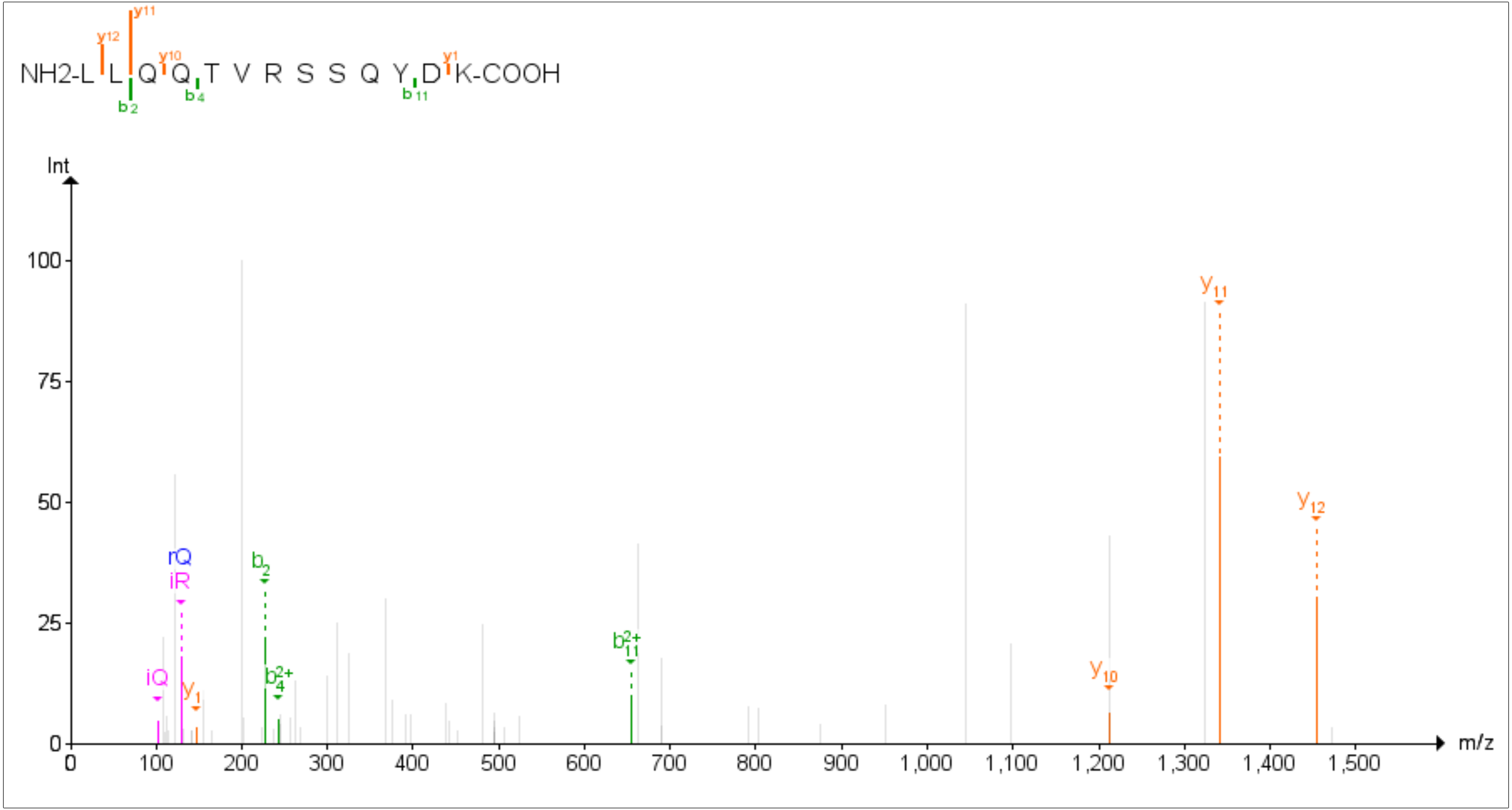

NH2-L L Q Q T V R S S Q Y D K-COOH
y12
y11
y10
y1
b2
b4
b11
Int
100
75
50
25
0
m/z
0
100
200
300
400
500
600
700
800
900
1,000
1,100
1,200
1,300
1,400
1,500
rQ
iR
iQ
y1
b2
b4 2+
b11 2+
y10
y11
y12

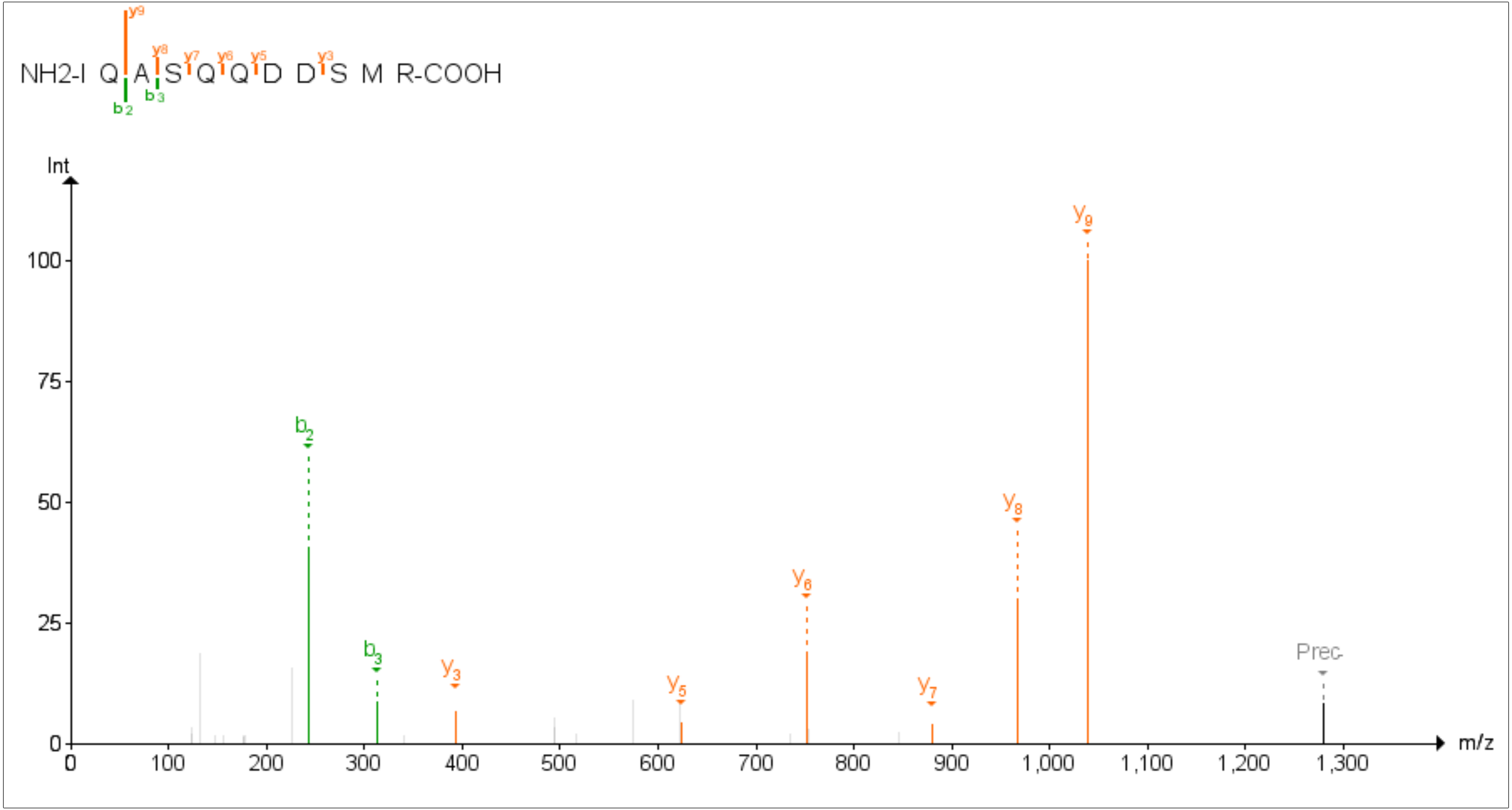
NH2-I Q A S Q Q D D S M R-COOH
y9
y8
y7
y6
y5
y3
b2
b3
Int
100
75
50
25
0
b2
b3
y3
y5
y6
y7
y8
y9
Prec
0
100
200
300
400
500
600
700
800
900
1,000
1,100
1,200
1,300
m/z

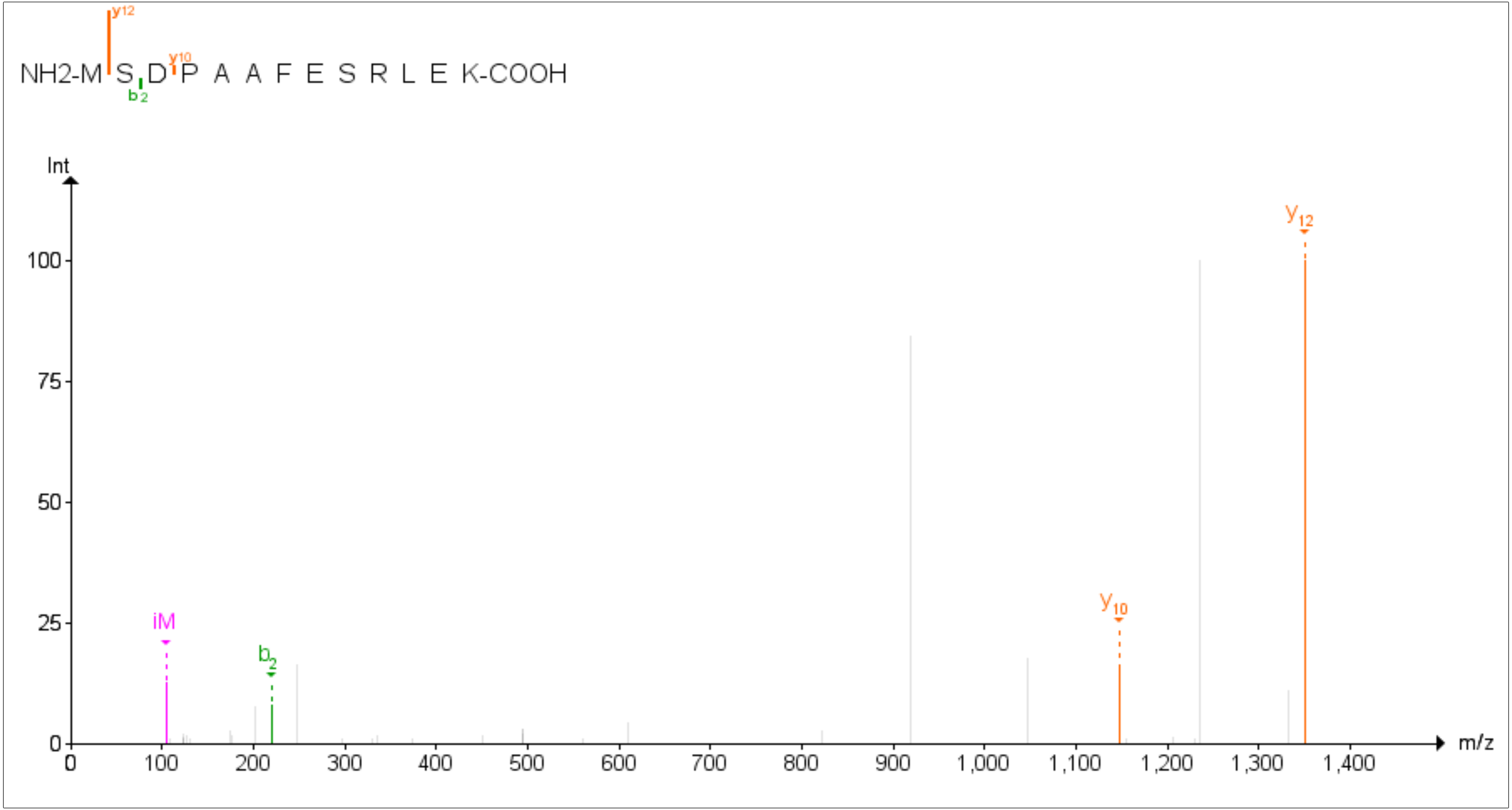

NH2-M S D P A A F E S R L E K-COOH
y12
y10
b2
Int
100
75
50
25
0
iM
b2
y10
y12
m/z
0
100
200
300
400
500
600
700
800
900
1,000
1,100
1,200
1,300
1,400

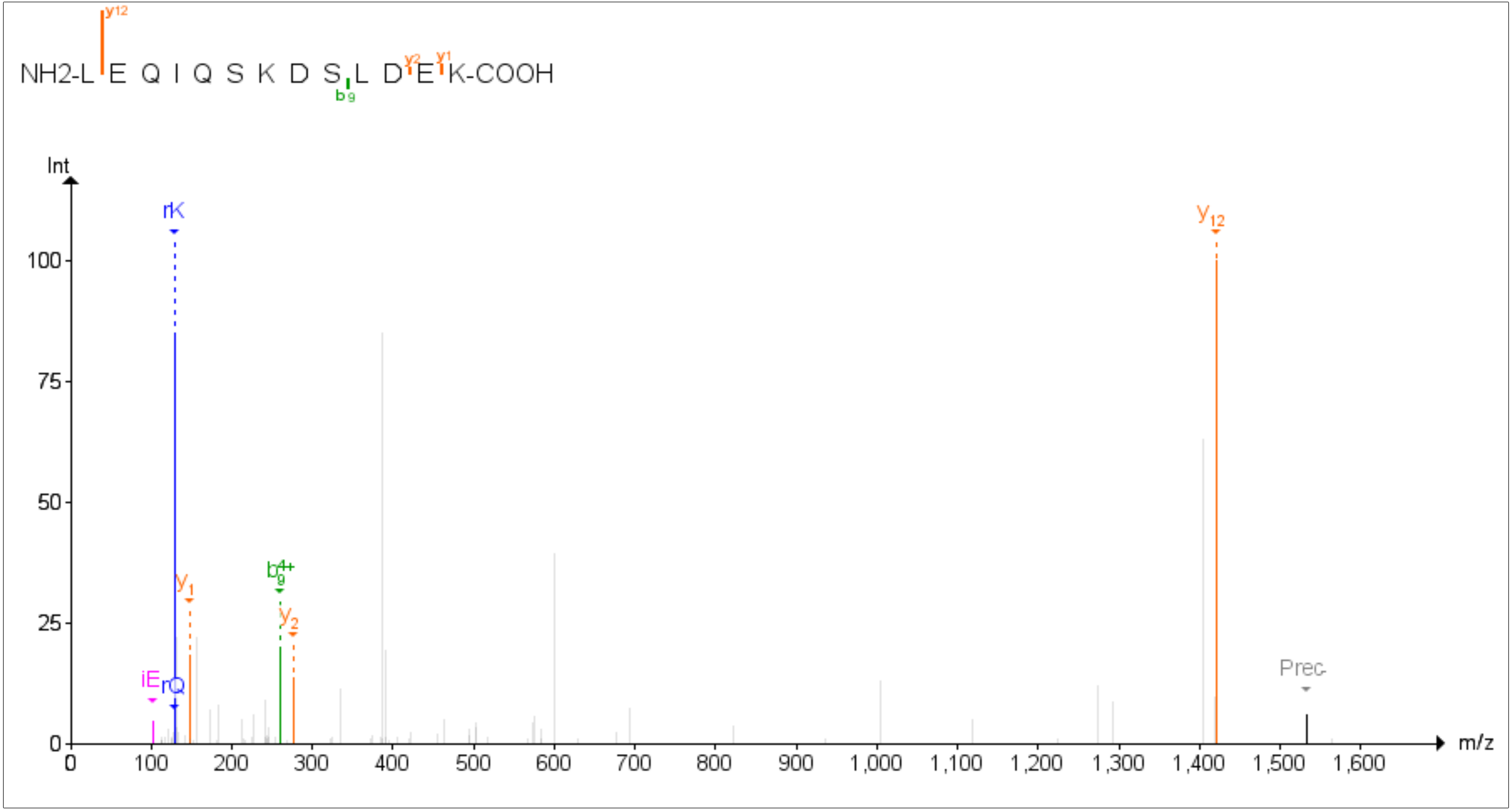

NH2-L E Q I Q S K D S L D E K-COOH
y12
y2
y1
b9
Int
100
75
50
25
0
rK
y12
y1
b9 4+
y2
iE
rQ
Prec
0
100
200
300
400
500
600
700
800
900
1,000
1,100
1,200
1,300
1,400
1,500
1,600
m/z

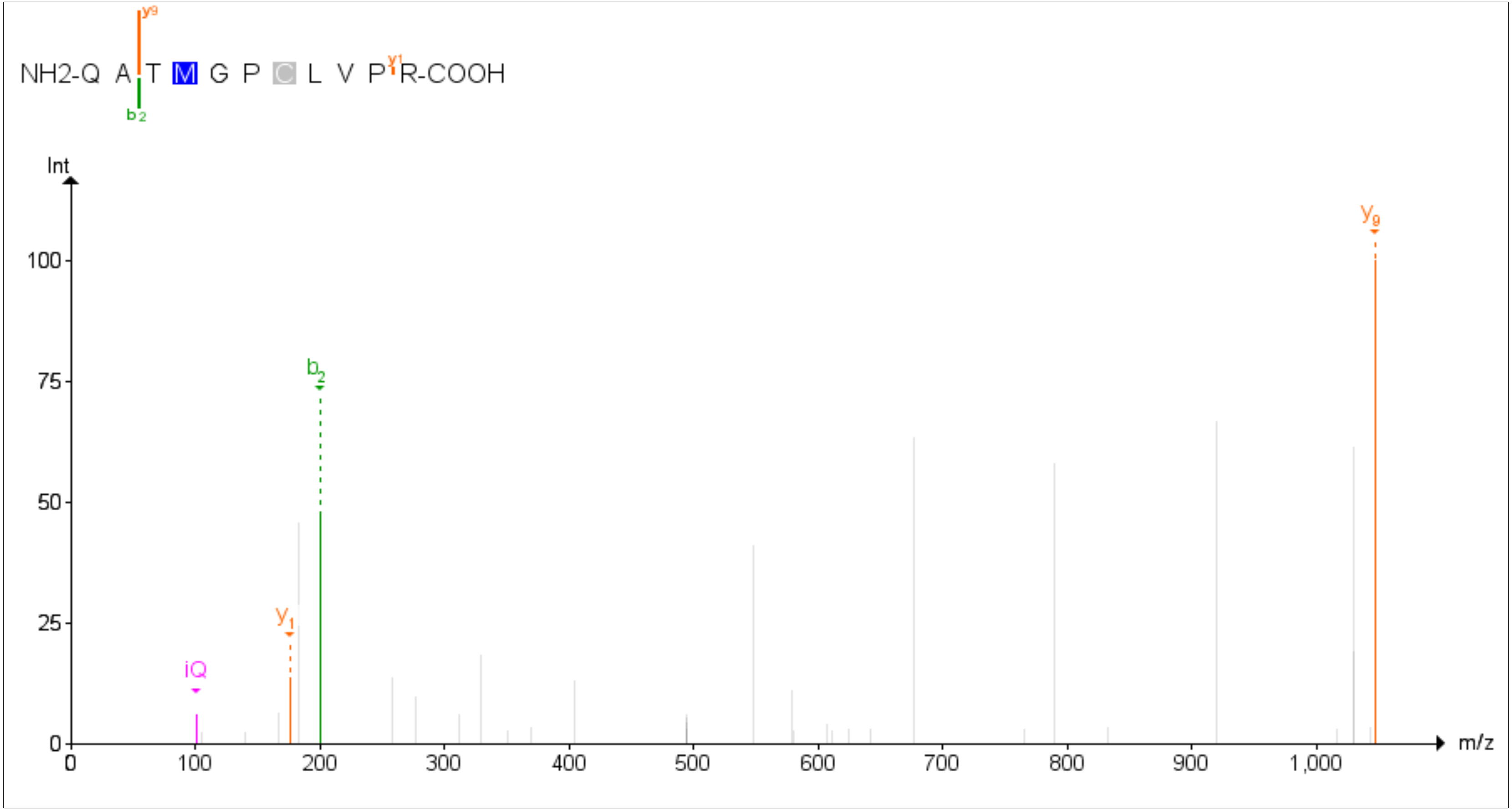

NH2-Q A T M G P C L V P R-COOH
y9
b2
y1
Int
100
75
50
25
0
iQ
y1
b2
y9
0
100
200
300
400
500
600
700
800
900
1,000
m/z

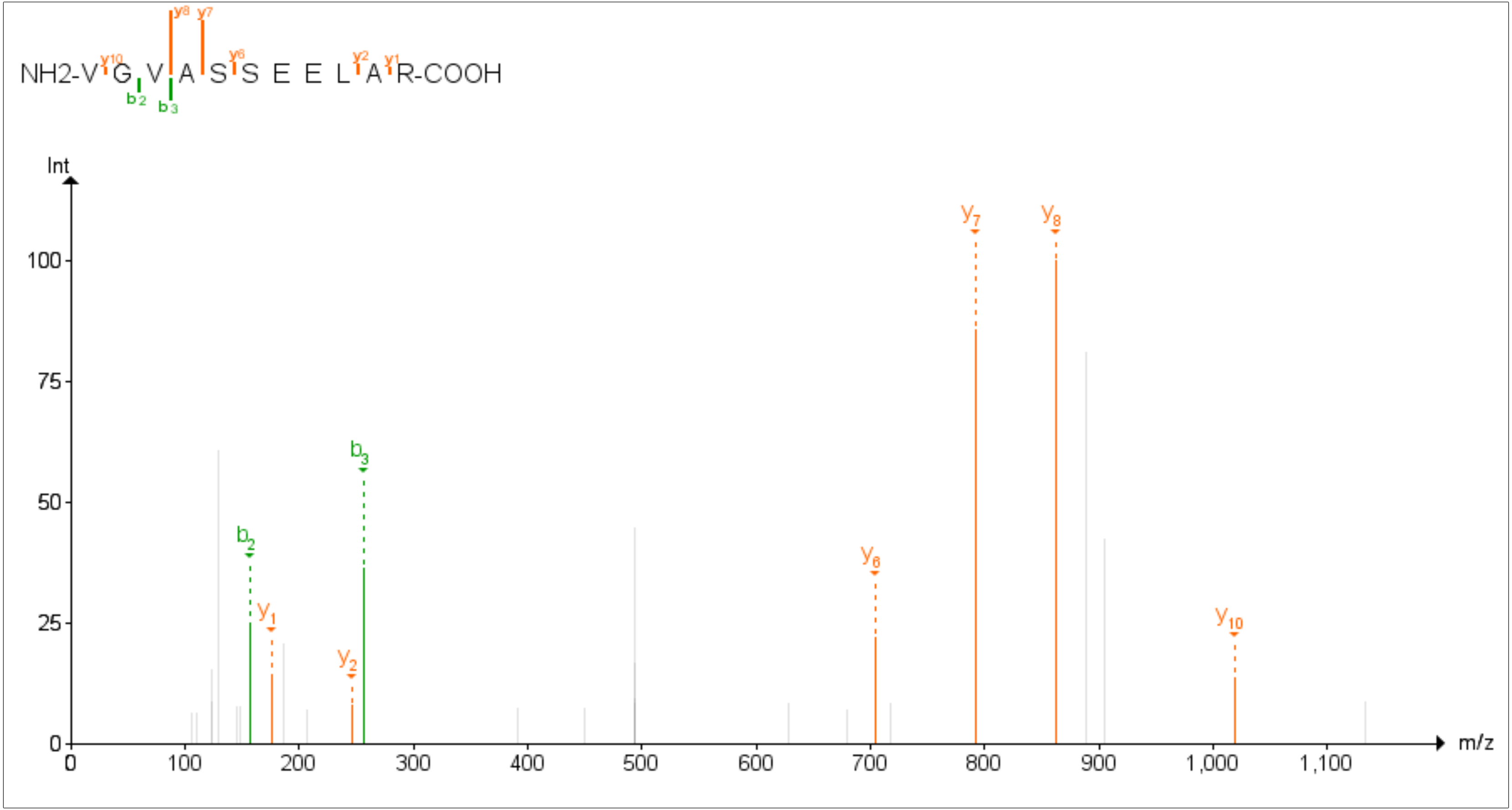

NH2-V G V A S S E E L A R-COOH
y10
y8
y7
y6
y2
y1
b2
b3
Int
100
75
50
25
0
m/z
0
100
200
300
400
500
600
700
800
900
1,000
1,100
b2
b3
y1
y2
y6
y7
y8
y10

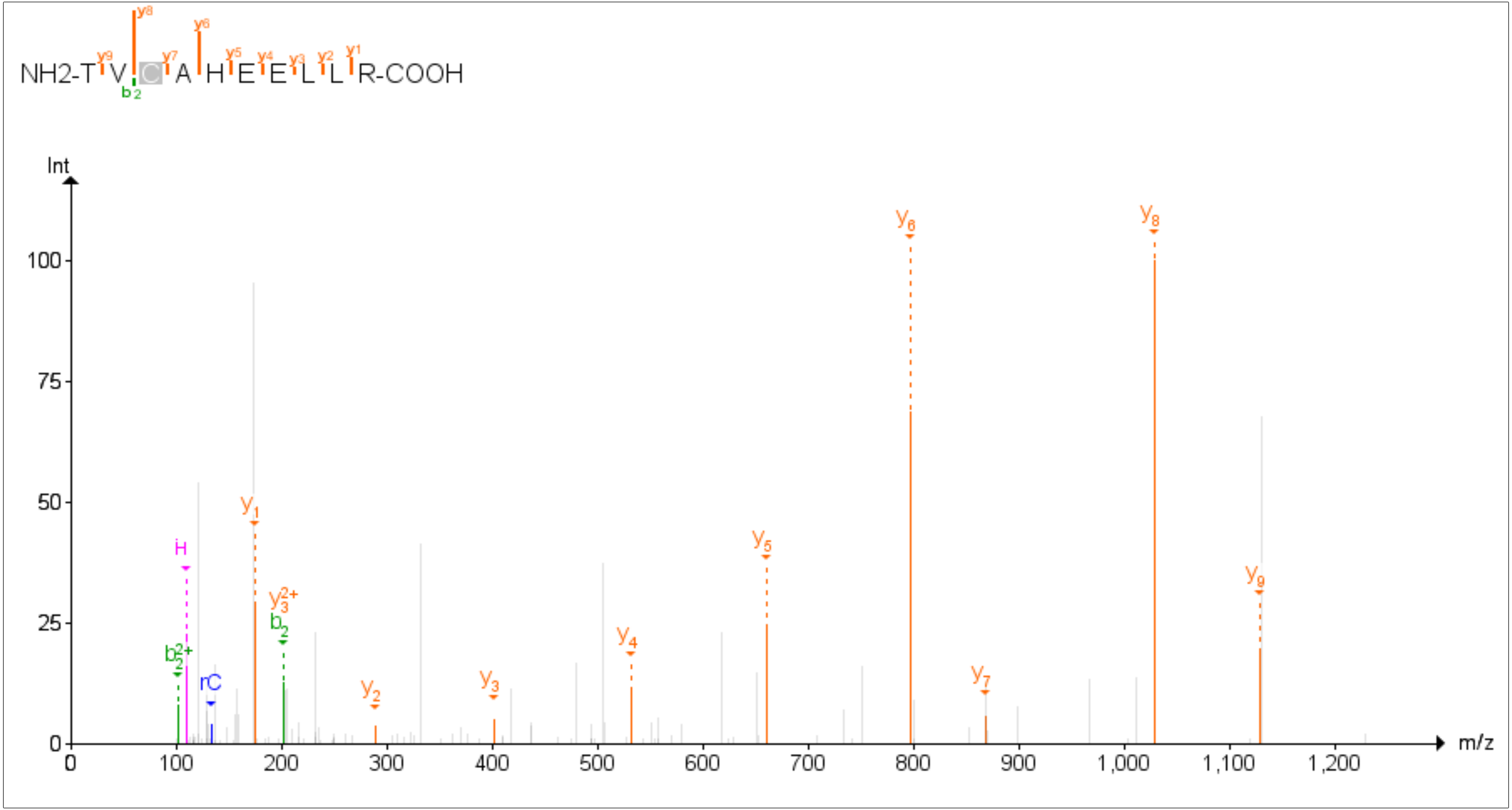
NH2-T V C A H E E L L R-COOH
y9 y8 y7 y6 y5 y4 y3 y2 y1
b2
Int
100
75
50
25
0
m/z
0 100 200 300 400 500 600 700 800 900 1,000 1,100 1,200
y6
y8
y1
H
y3 2+
b2
b2 2+
rC
y5
y4
y2
y3
y7
y9

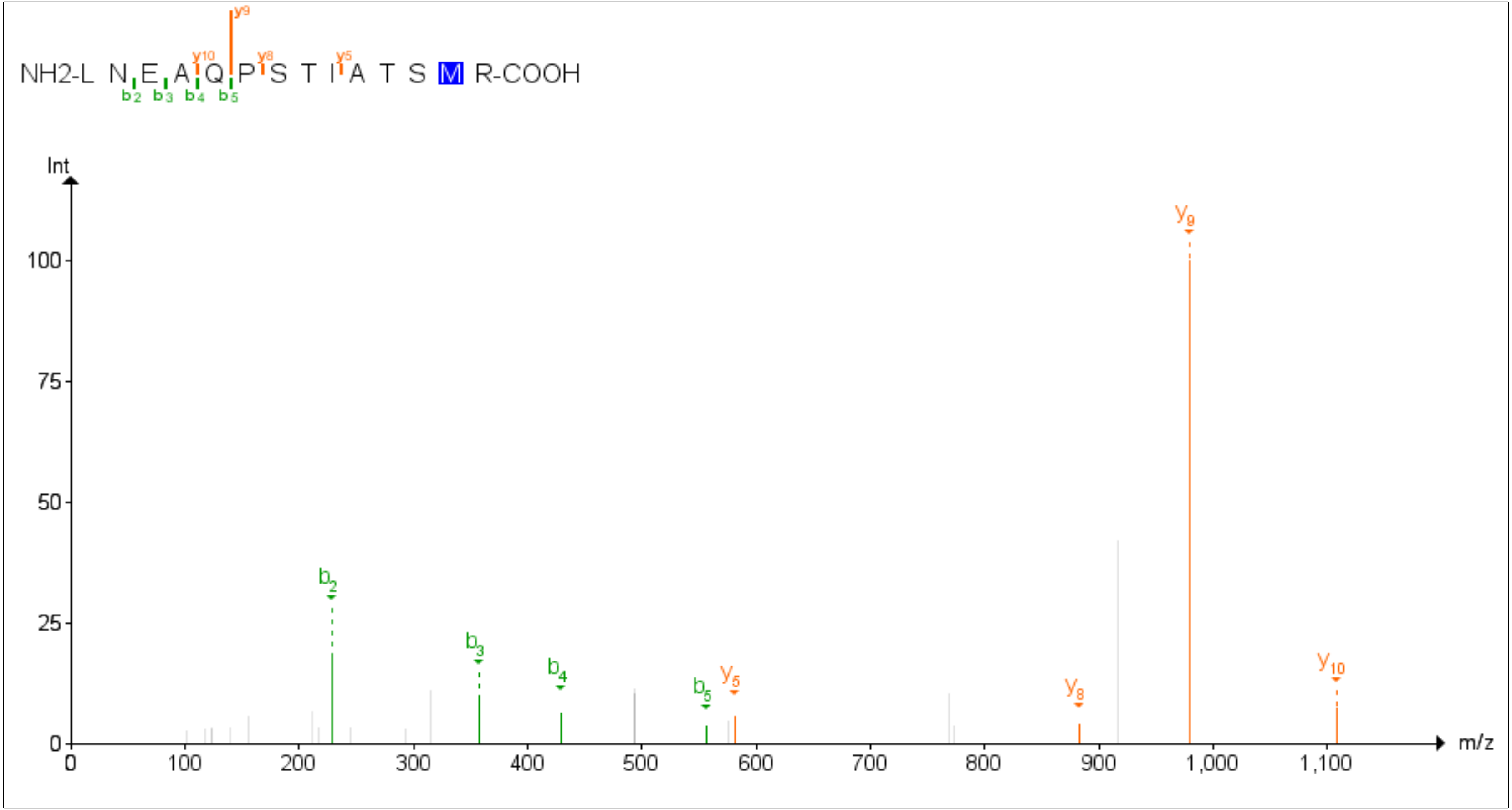

NH2-L N E A Q P S T I A T S M R-COOH
b2 b3 b4 b5
y10 y9 y8 y5
Int
0
25
50
75
100
m/z
0
100
200
300
400
500
600
700
800
900
1,000
1,100
b2
b3
b4
b5
y5
y8
y9
y10

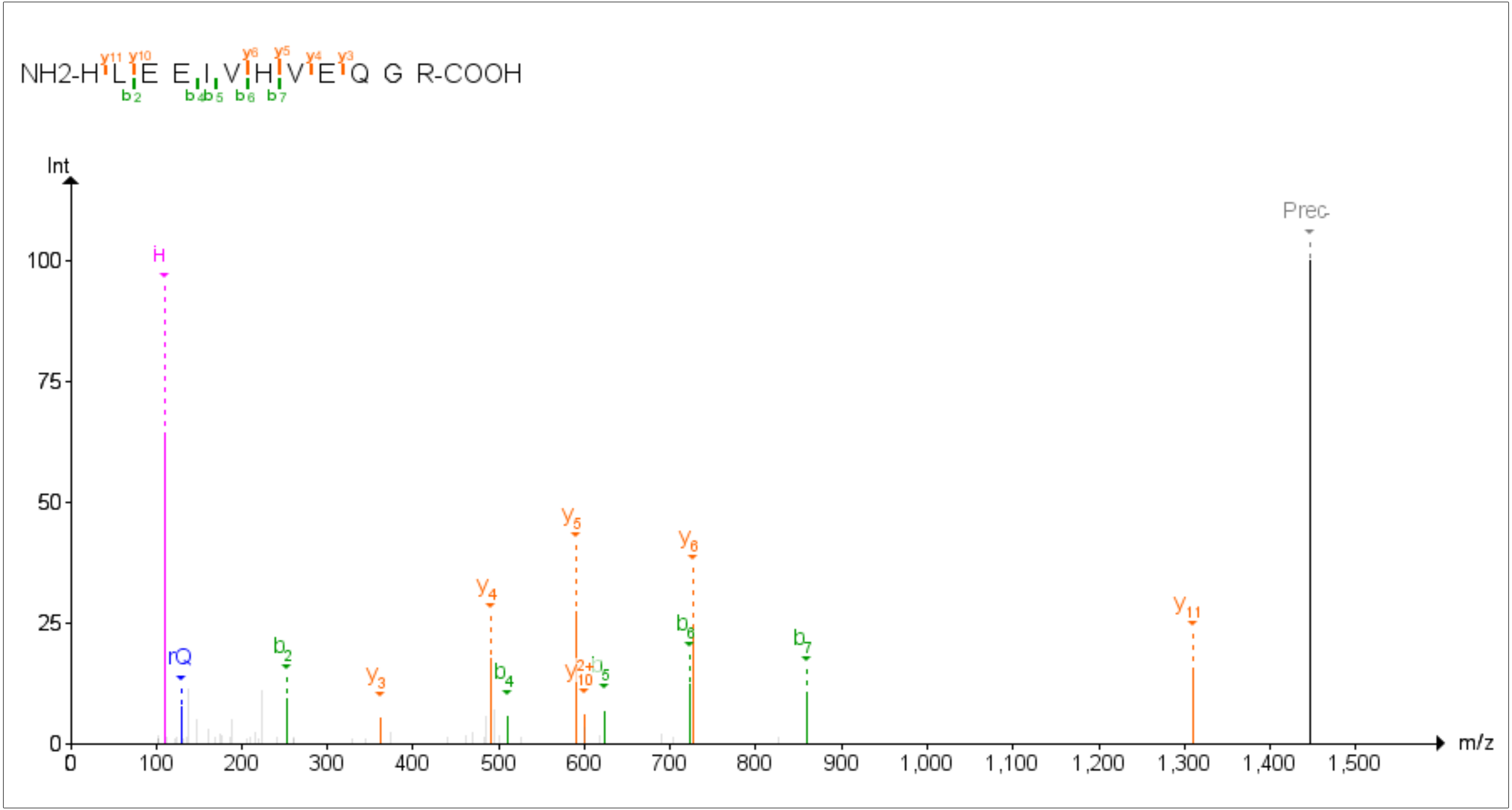

NH2-H L E E I V H V E Q G R-COOH
y11 y10 y6 y5 y4 y3
b2 b4 b5 b6 b7
Int
Prec
H
100
75
50
25
0
rQ
b2
y3
y4
b4
y5
y10 2+
b5
y6
b6
b7
y11
0 100 200 300 400 500 600 700 800 900 1,000 1,100 1,200 1,300 1,400 1,500
m/z

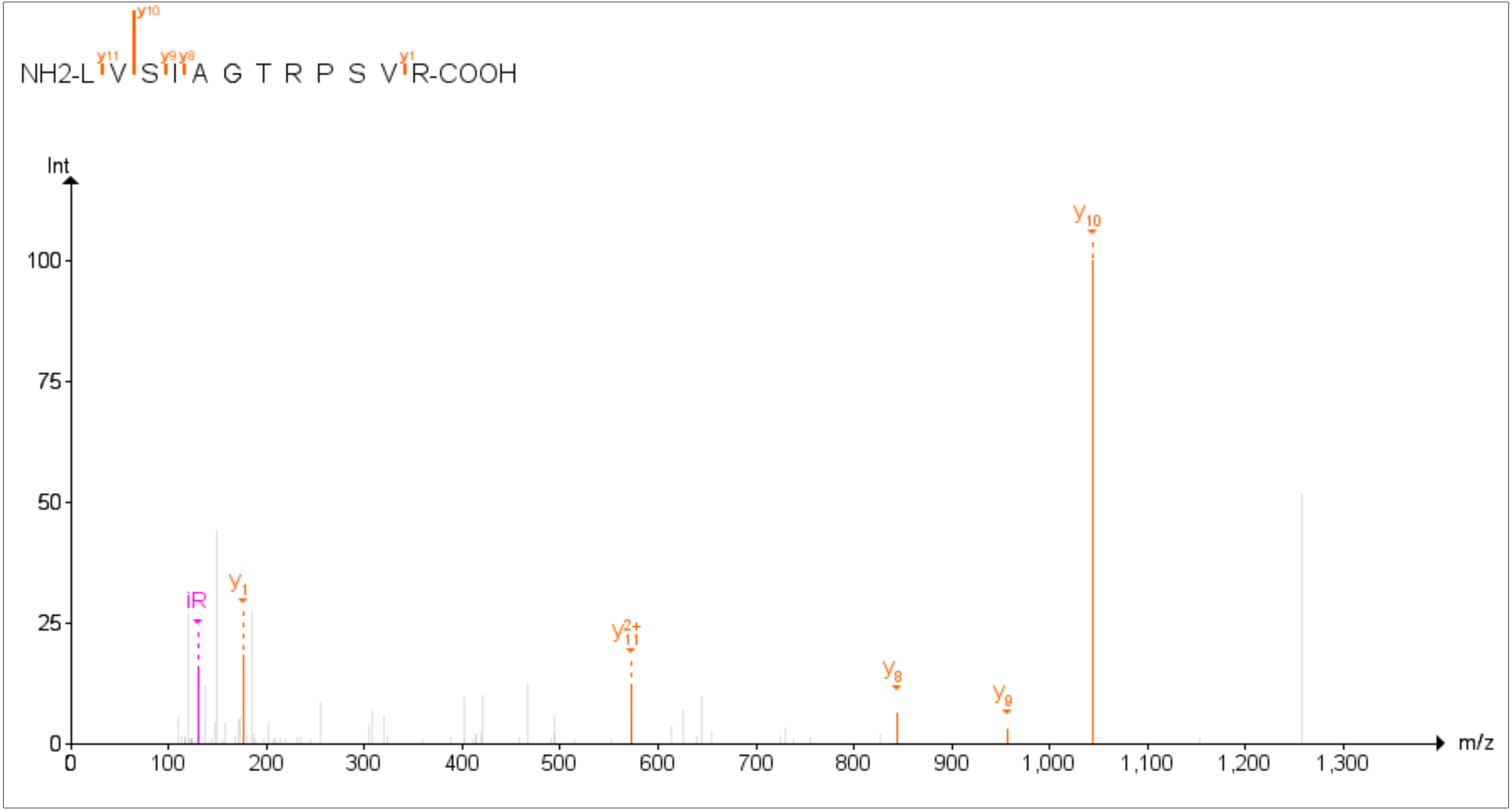

NH2-L V S I A G T R P S V R-COOH
y10
y11
y9
y8
y1
Int
100
75
50
25
0
iR
y1
y11 2+
y8
y9
y10
0
100
200
300
400
500
600
700
800
900
1,000
1,100
1,200
1,300
m/z

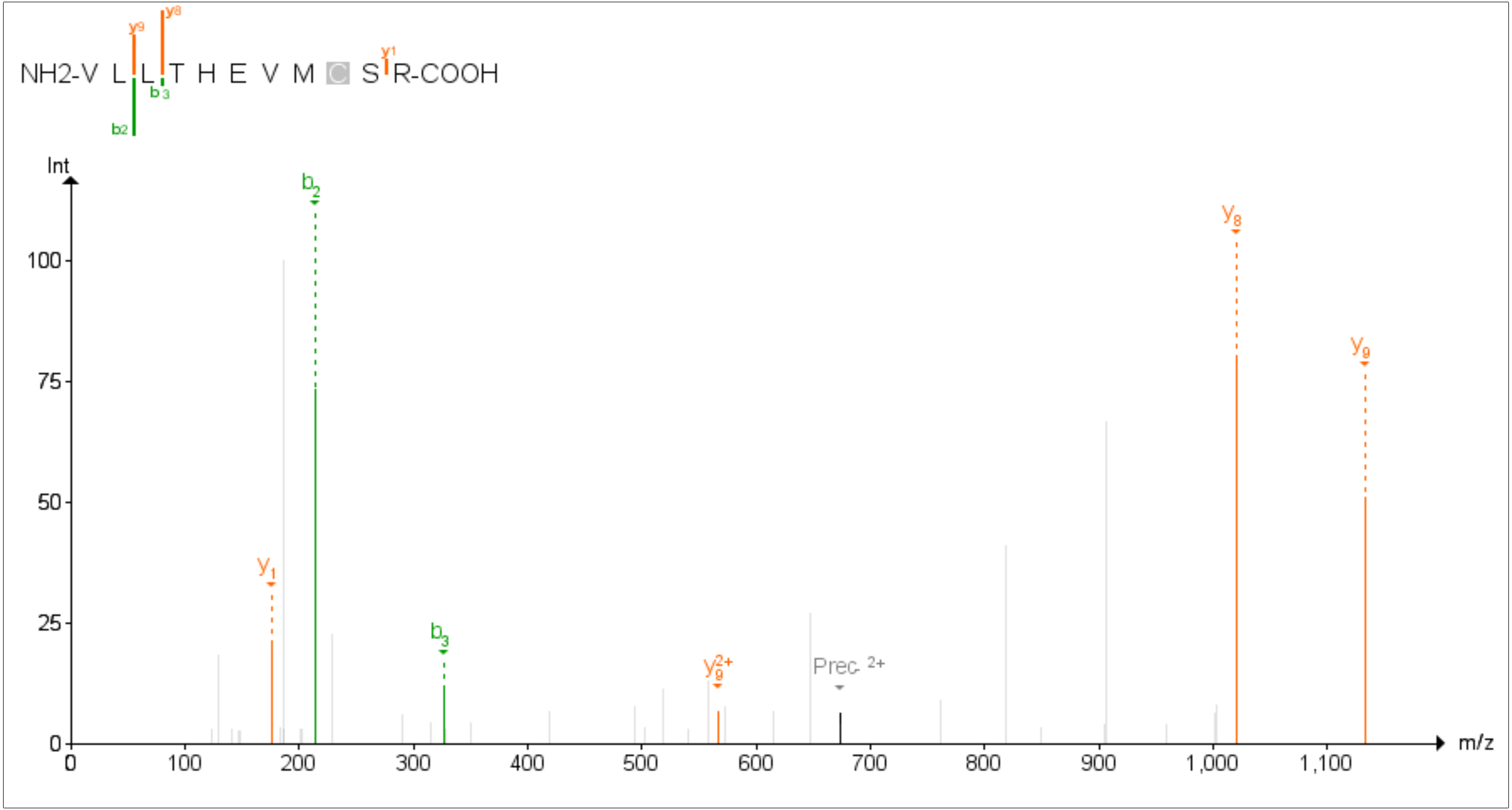

NH2-V L L T H E V M C S R-COOH
y9
y8
y1
b2
b3
Int
m/z
0
25
50
75
100
100
200
300
400
500
600
700
800
900
1,000
1,100
b2
b3
y1
y8
y9
y9 2+
Prec. 2+

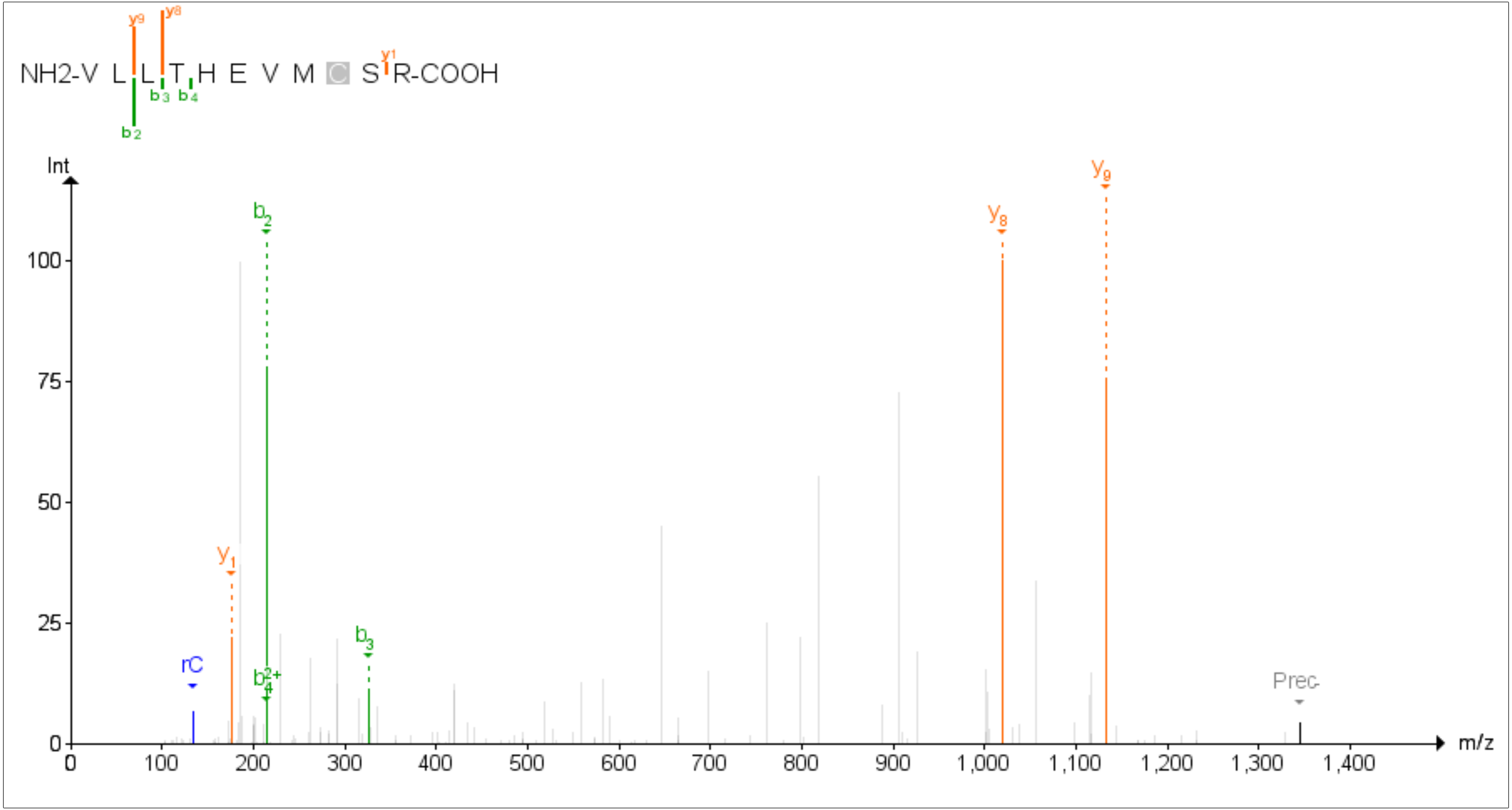

NH2-V L L T H E V M C S R-COOH
y9
y8
y1
b2
b3
b4
Int
100
75
50
25
0
b2
y8
y9
y1
rC
b4 2+
b3
Prec
m/z
0
100
200
300
400
500
600
700
800
900
1,000
1,100
1,200
1,300
1,400

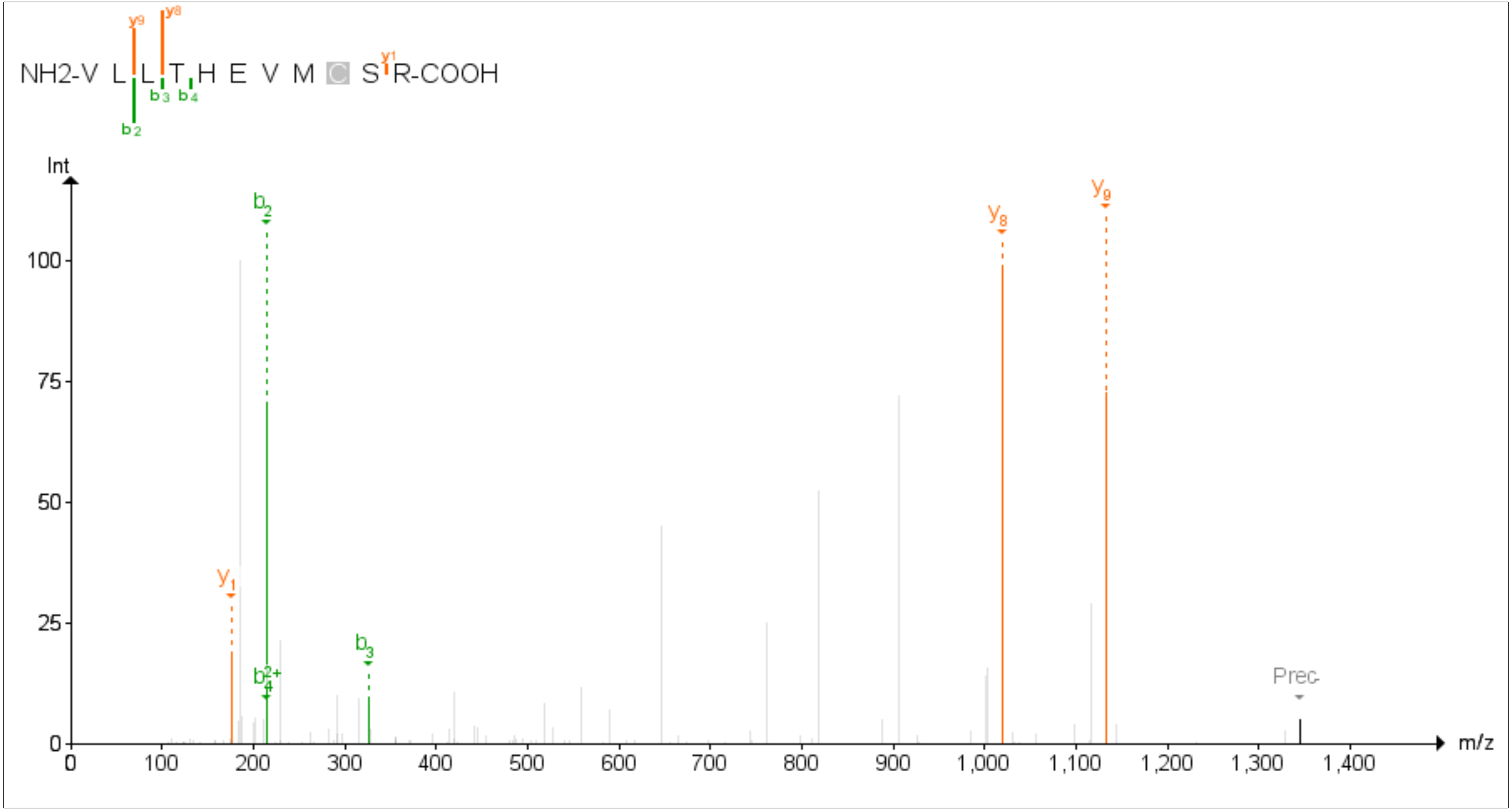

NH2-V L L T H E V M C S R-COOH
y9
y8
y1
b2
b3
b4
Int
100
75
50
25
0
m/z
0
100
200
300
400
500
600
700
800
900
1,000
1,100
1,200
1,300
1,400
b2
y8
y9
y1
b3
b4 2+
Prec.

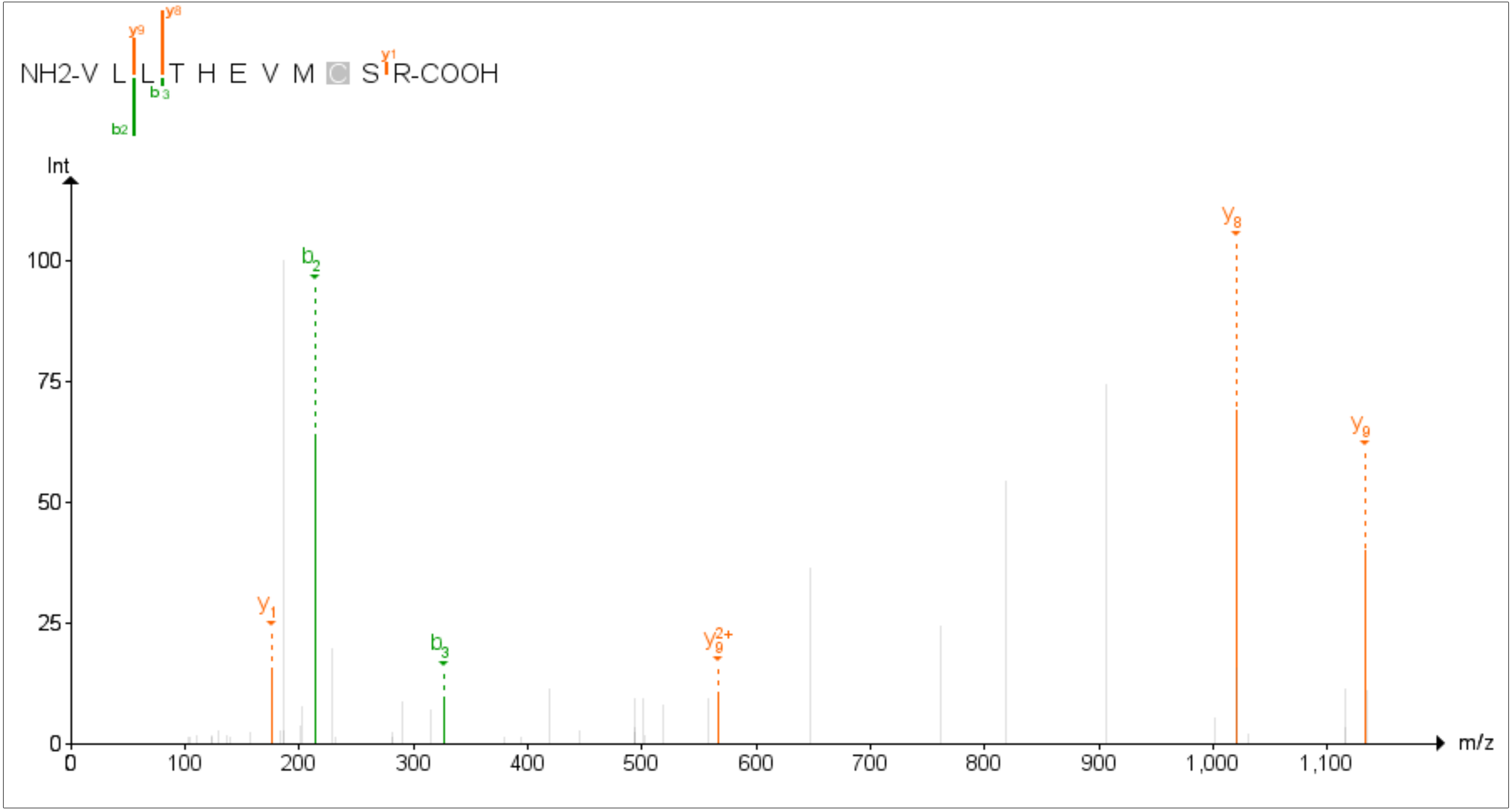

NH2-V L L T H E V M C S R-COOH
y9
y8
y1
b2
b3
Int
100
75
50
25
0
b2
b3
y1
y8
y9
y9 2+
0
100
200
300
400
500
600
700
800
900
1,000
1,100
m/z

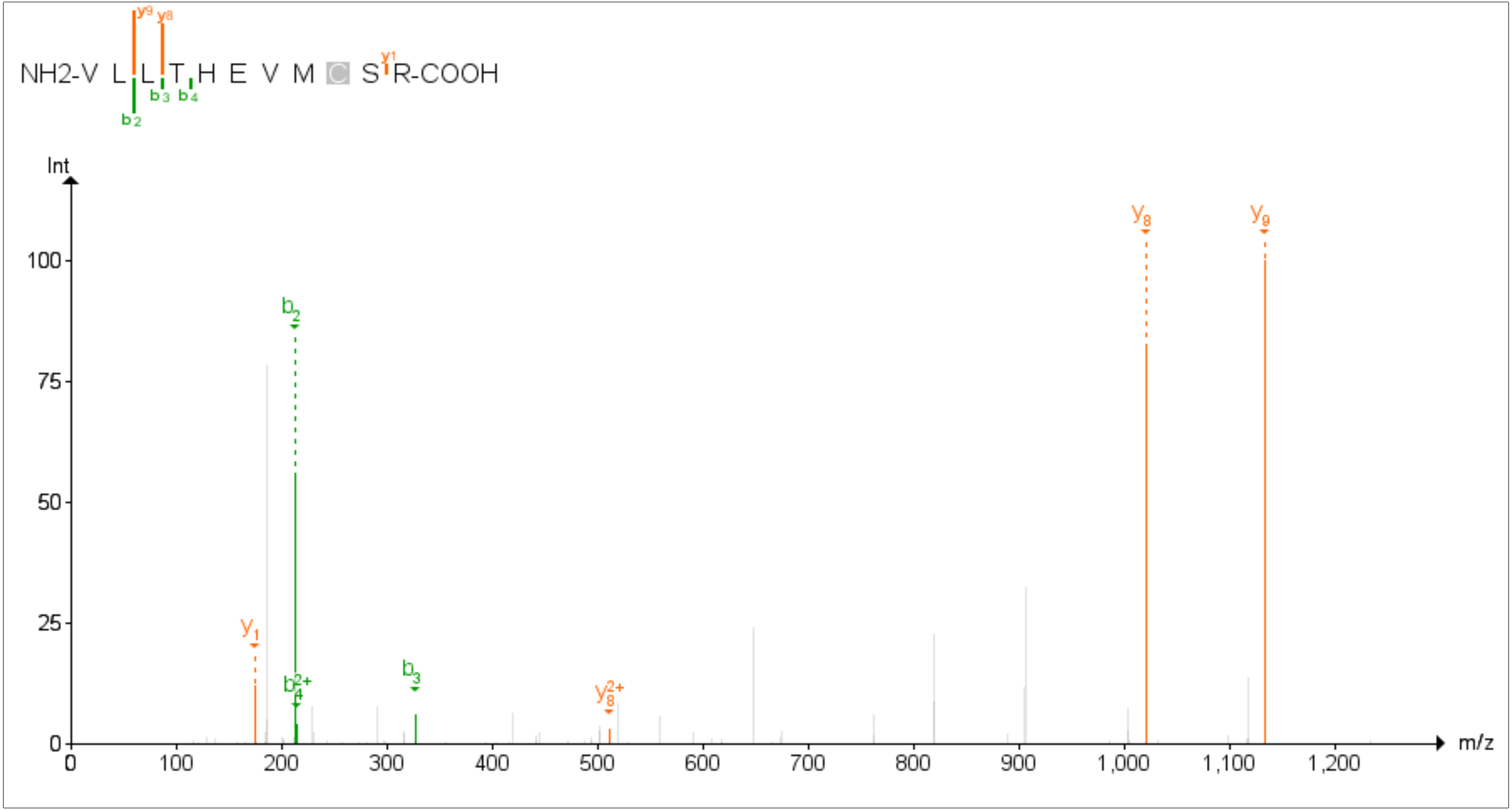

NH2-V L L T H E V M C S R-COOH
y9
y8
y1
b2
b3
b4
Int
m/z
y8
y9
b2
y1
b4 2+
b3
y8 2+
0
25
50
75
100
0
100
200
300
400
500
600
700
800
900
1,000
1,100
1,200

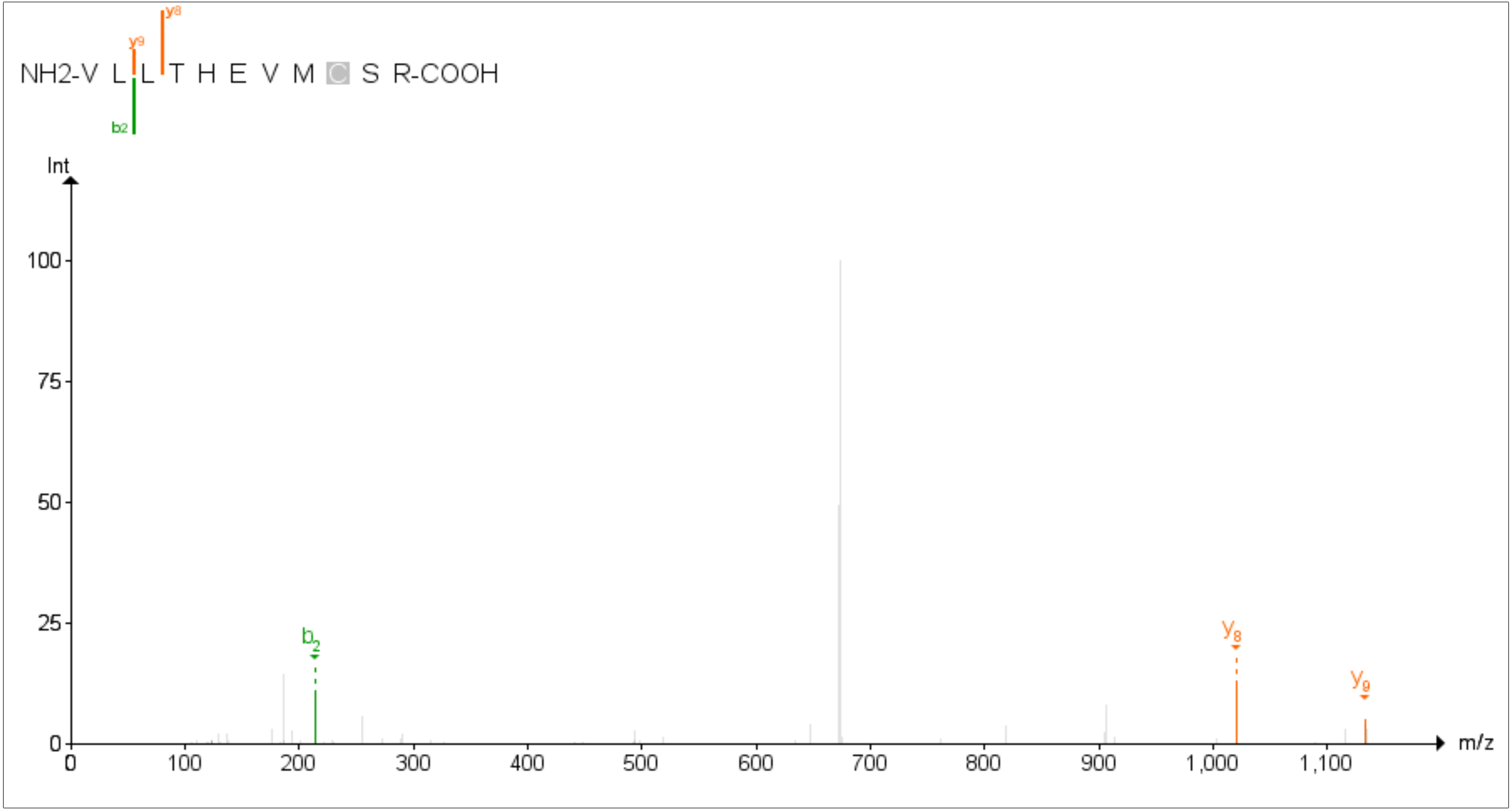

NH2-V L L T H E V M C S R-COOH
y8
y9
b2
Int
100
75
50
25
0
b2
y8
y9
0
100
200
300
400
500
600
700
800
900
1,000
1,100
m/z

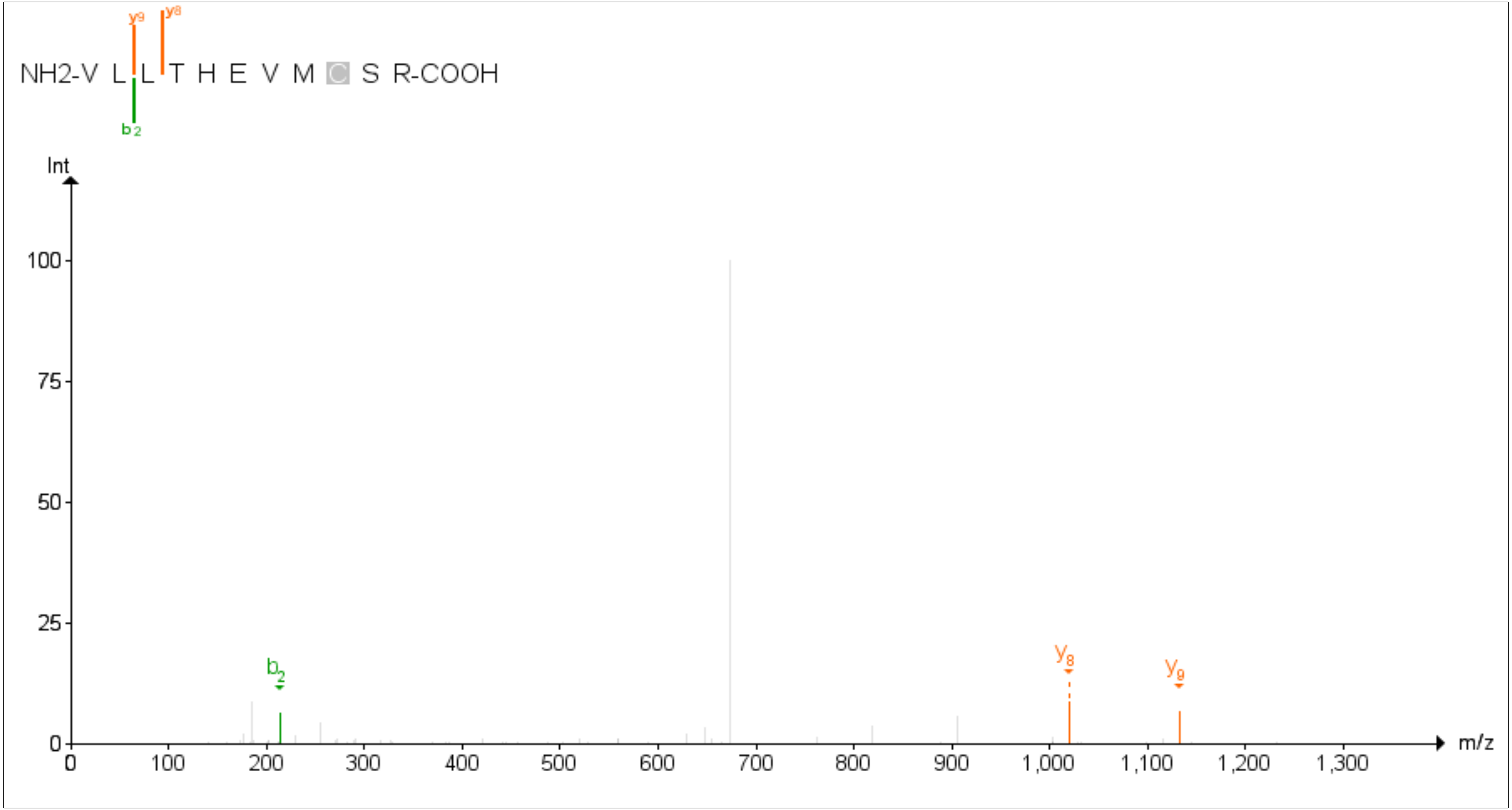

NH2-V L L T H E V M C S R-COOH
y9
y8
b2
Int
100
75
50
25
0
b2
y8
y9
0
100
200
300
400
500
600
700
800
900
1,000
1,100
1,200
1,300
m/z

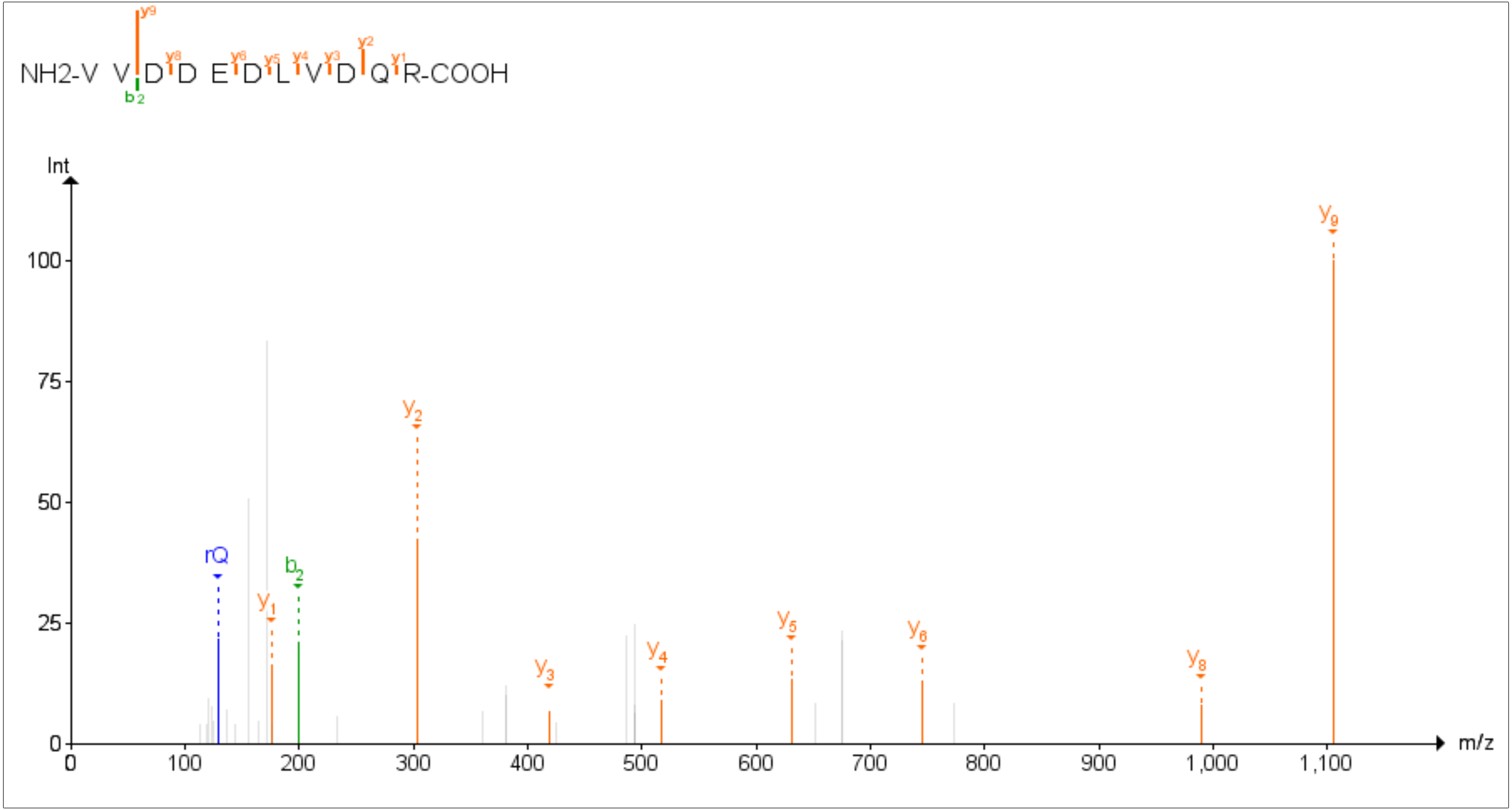

NH2-V V D D E D L V D Q R-COOH
y9
y8
y6
y5
y4
y3
y2
y1
b2
Int
100
75
50
25
0
rQ
b2
y1
y2
y3
y4
y5
y6
y8
y9
0
100
200
300
400
500
600
700
800
900
1,000
1,100
m/z

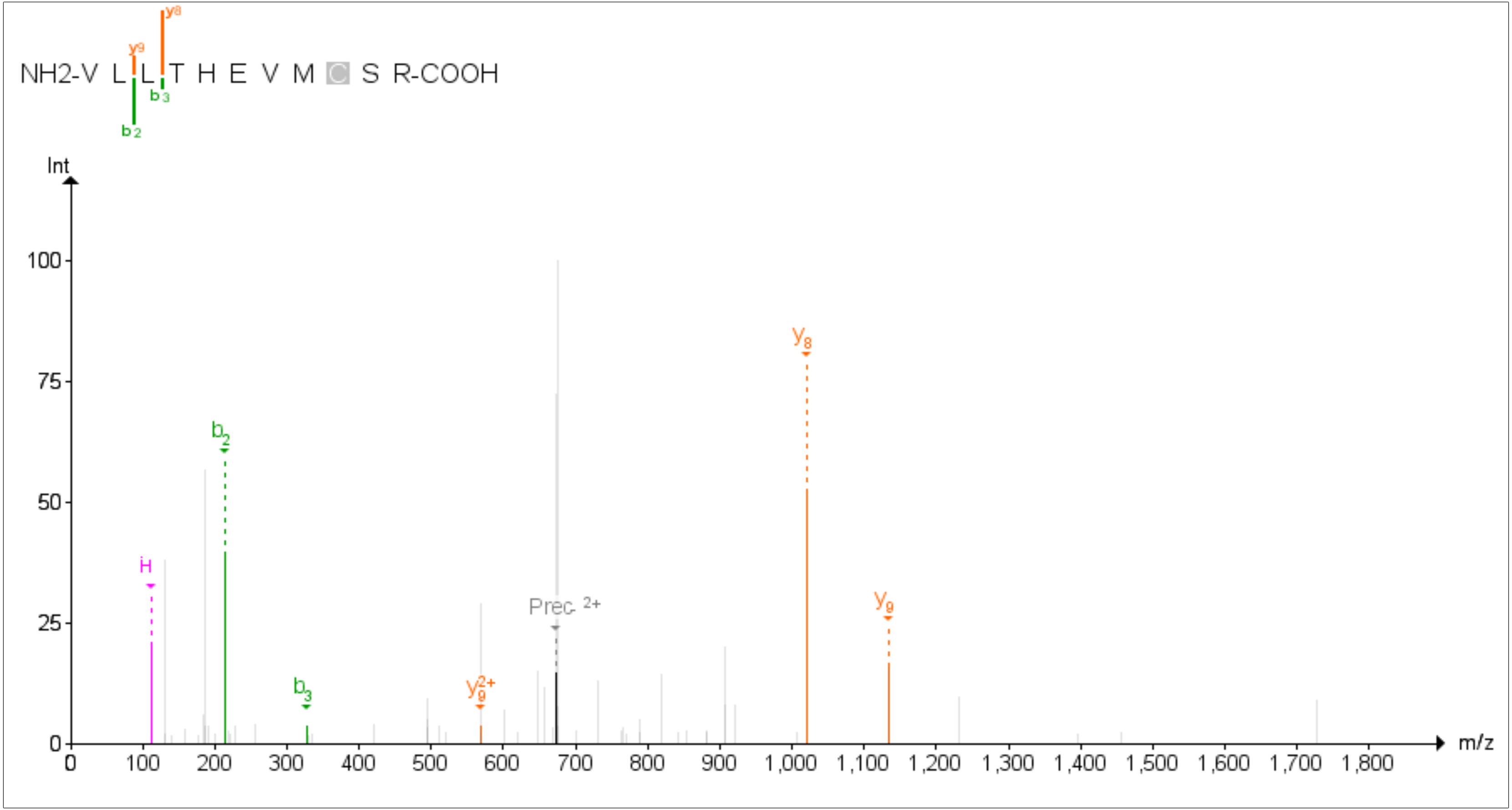

NH2-V L L T H E V M C S R-COOH
y9
y8
b2
b3
Int
100
75
50
25
0
H
b2
b3
y9 2+
Prec 2+
y8
y9
0
100
200
300
400
500
600
700
800
900
1,000
1,100
1,200
1,300
1,400
1,500
1,600
1,700
1,800
m/z

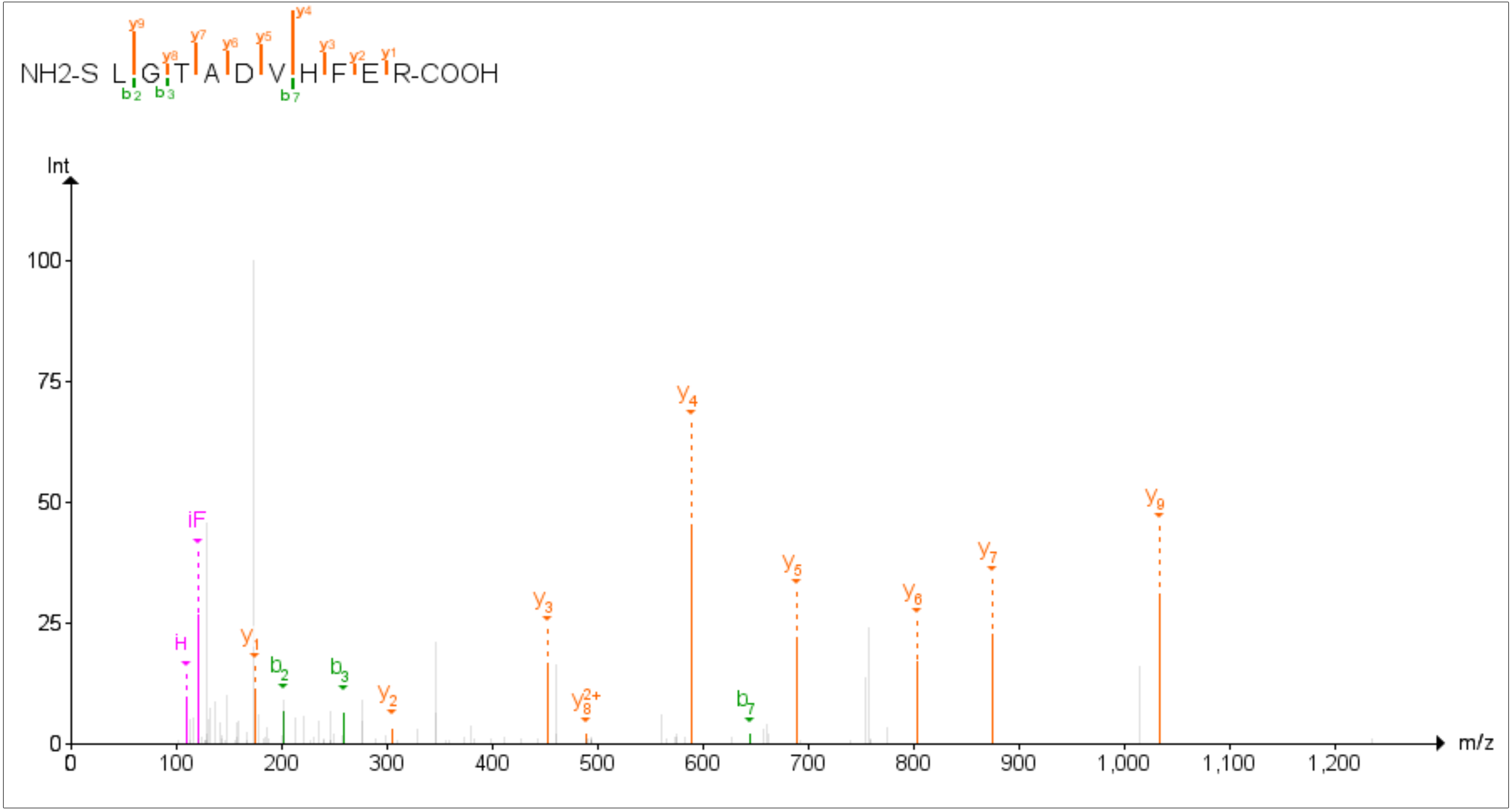

NH2-S L G T A D V H F E R-COOH
y9 y8 y7 y6 y5 y4 y3 y2 y1
b2 b3 b7
Int
100
75
50
25
0
iF
H
y1
b2
b3
y2
y3
y8 2+
y4
b7
y5
y6
y7
y9
0 100 200 300 400 500 600 700 800 900 1,000 1,100 1,200
m/z

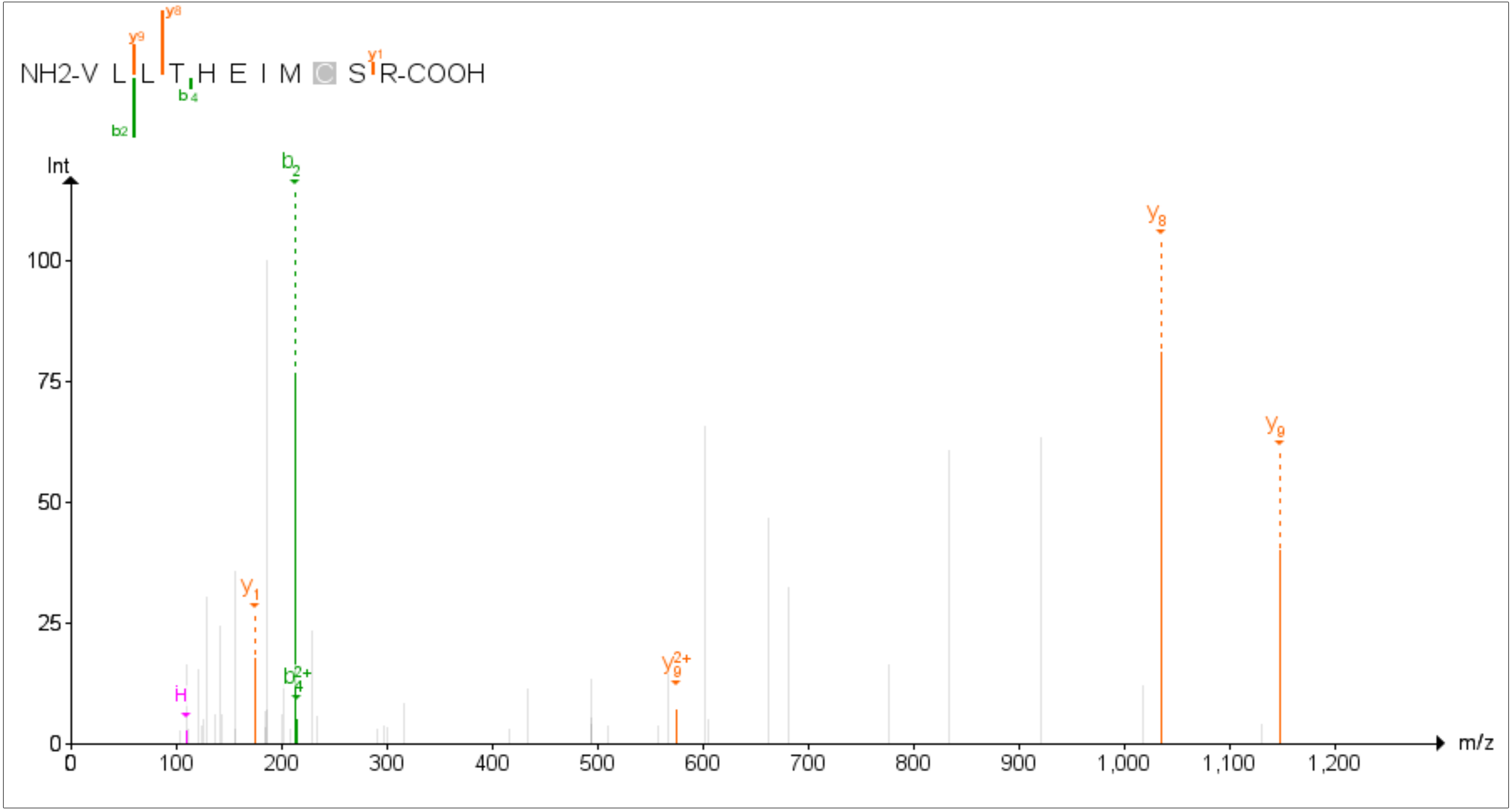

NH2-V L L T H E I M C S R-COOH
y9
y8
y1
b2
b4
Int
m/z
b2
y8
y9
y1
y9 2+
b4 2+
H
0
25
50
75
100
0
100
200
300
400
500
600
700
800
900
1,000
1,100
1,200

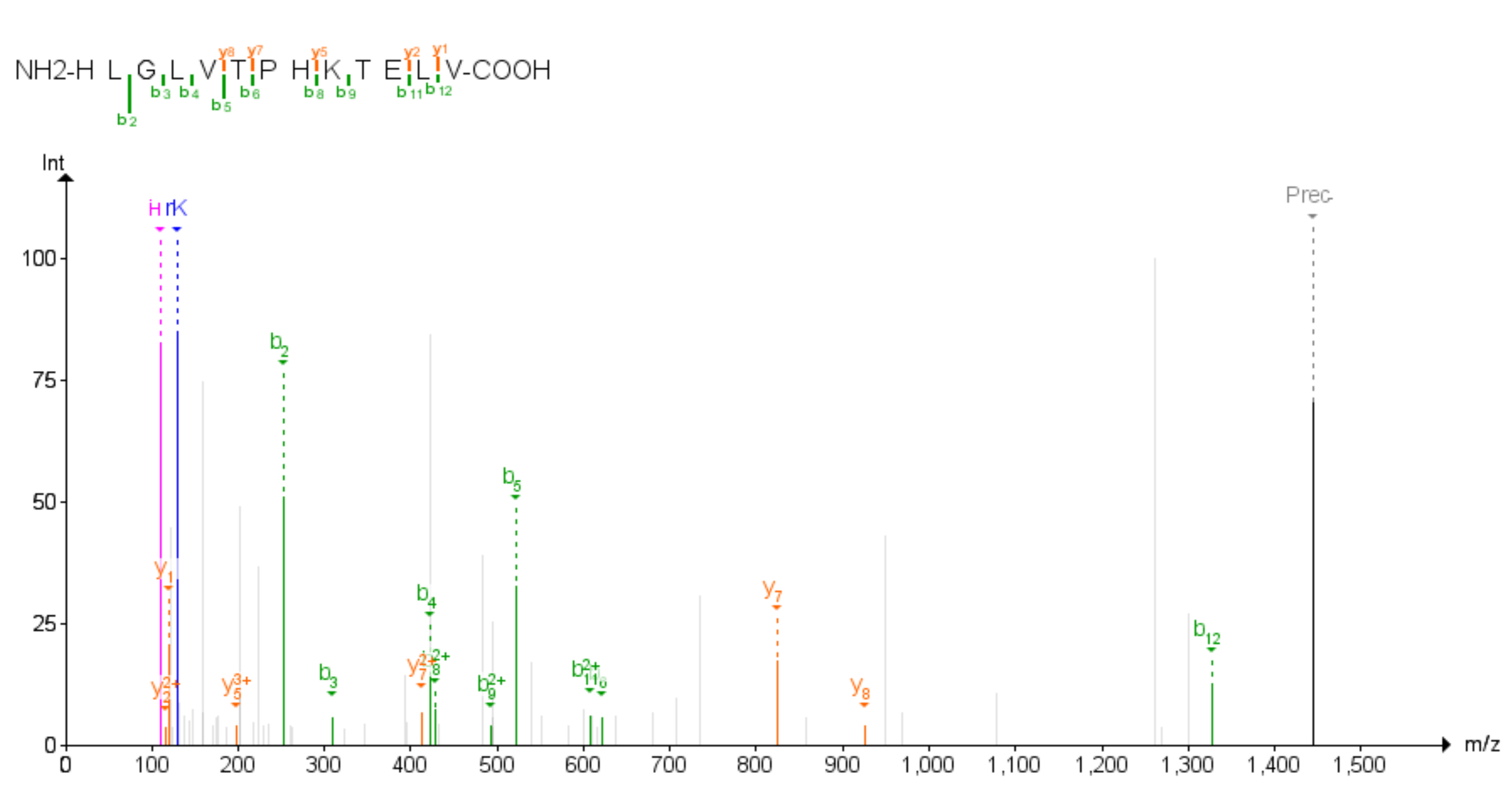

NH2-H L G L V T P H K T E L V-COOH
y8 y7 y5 y2 y1
b2 b3 b4 b5 b6 b8 b9 b11 b12
Int
iH
rK
Prec
b2
b5
b4
b3
y1
y7
y8
b12
m/z
0
25
50
75
100
0
100
200
300
400
500
600
700
800
900
1,000
1,100
1,200
1,300
1,400
1,500

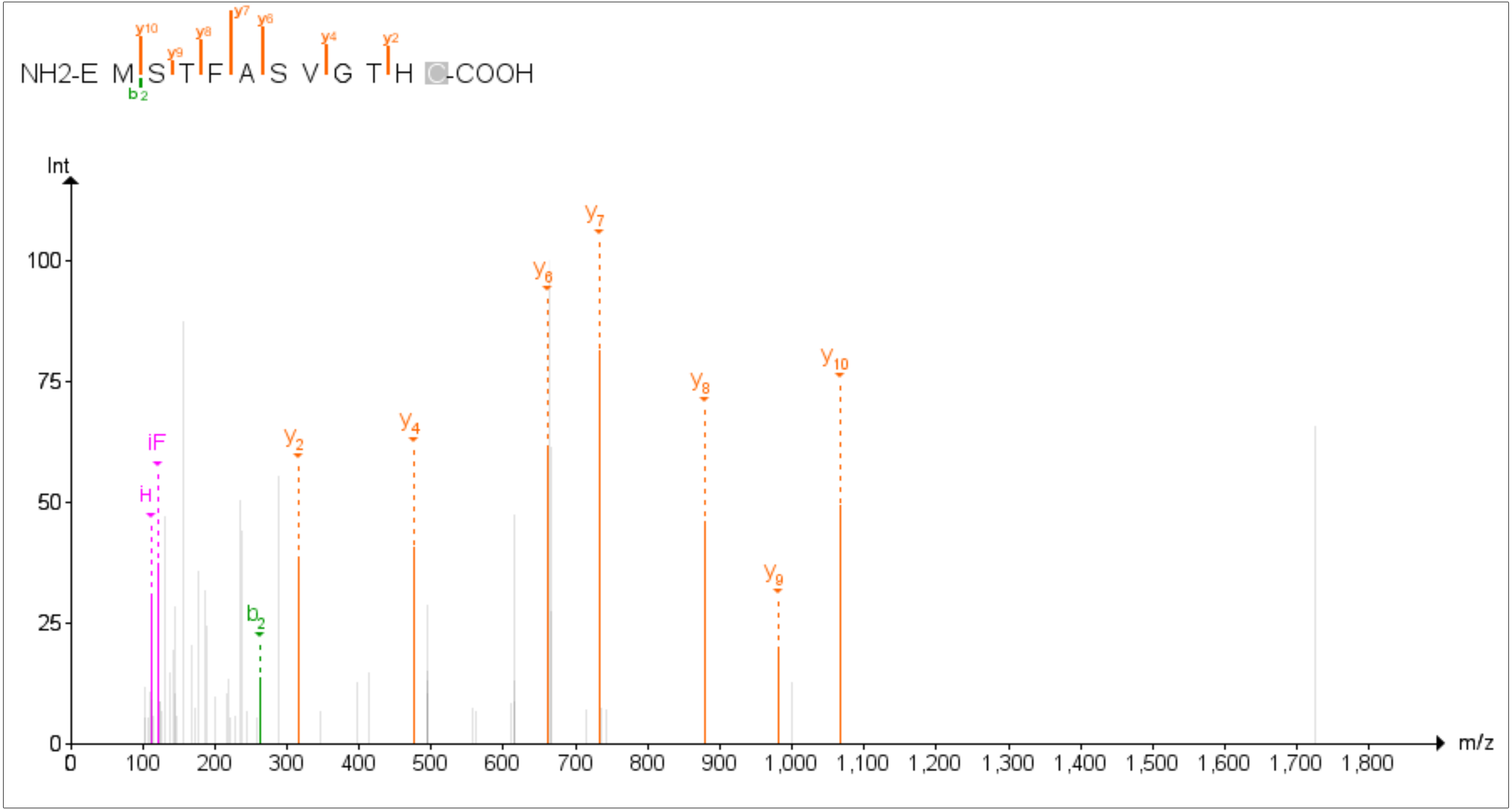

NH2-E M S T F A S V G T H C-COOH
y10 y9 y8 y7 y6 y4 y2
b2
Int
100
75
50
25
0
m/z
0 100 200 300 400 500 600 700 800 900 1,000 1,100 1,200 1,300 1,400 1,500 1,600 1,700 1,800
iF
iH
b2
y2
y4
y6
y7
y8
y9
y10

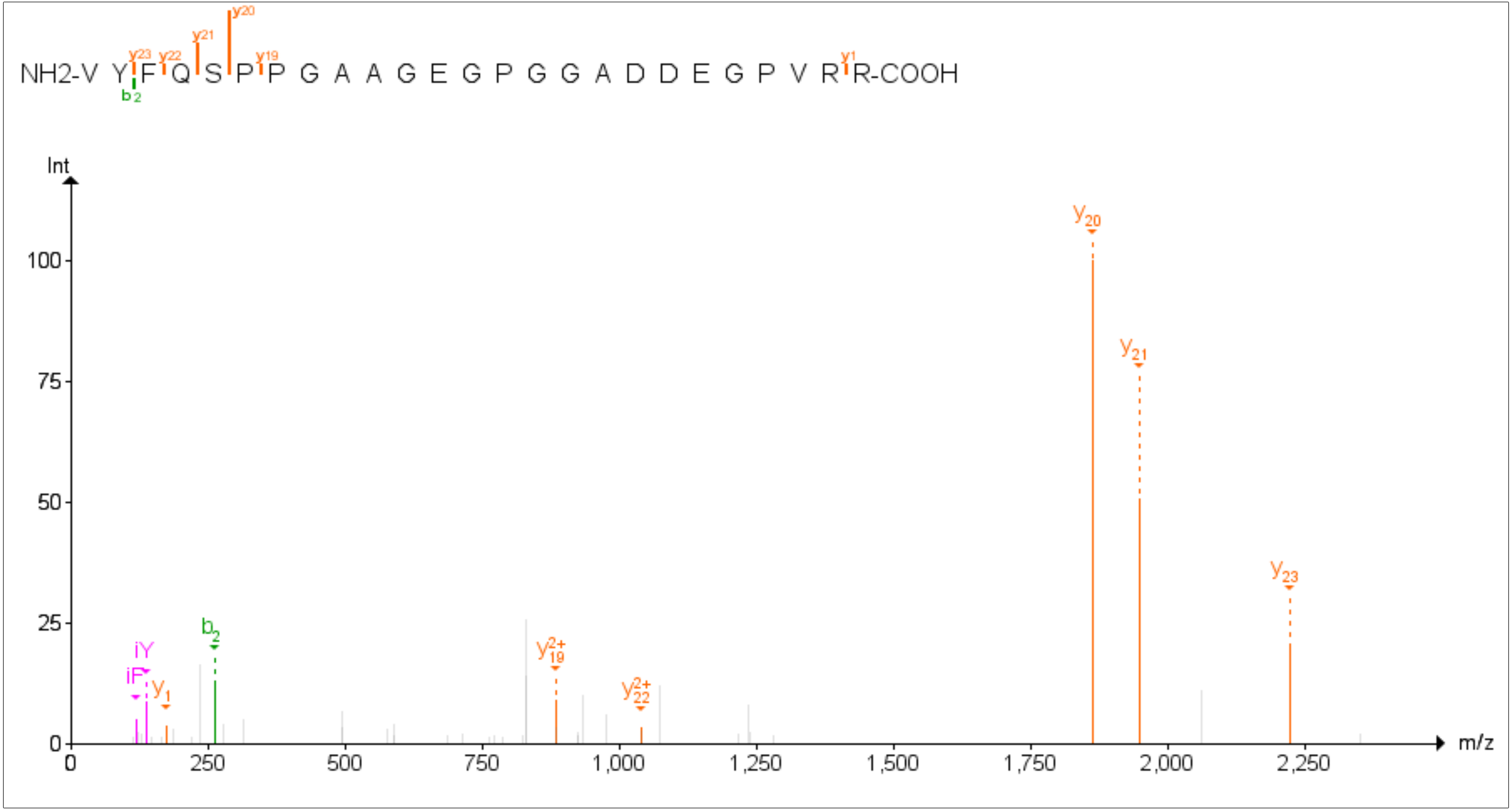

NH2-V Y F Q S P P G A A G E G P G G A D D E G P V R R-COOH
y23
y22
y21
y20
y19
y1
b2
Int
100
75
50
25
0
m/z
250
500
750
1,000
1,250
1,500
1,750
2,000
2,250
iY
iF
y1
b2
y19 2+
y22 2+
y20
y21
y23

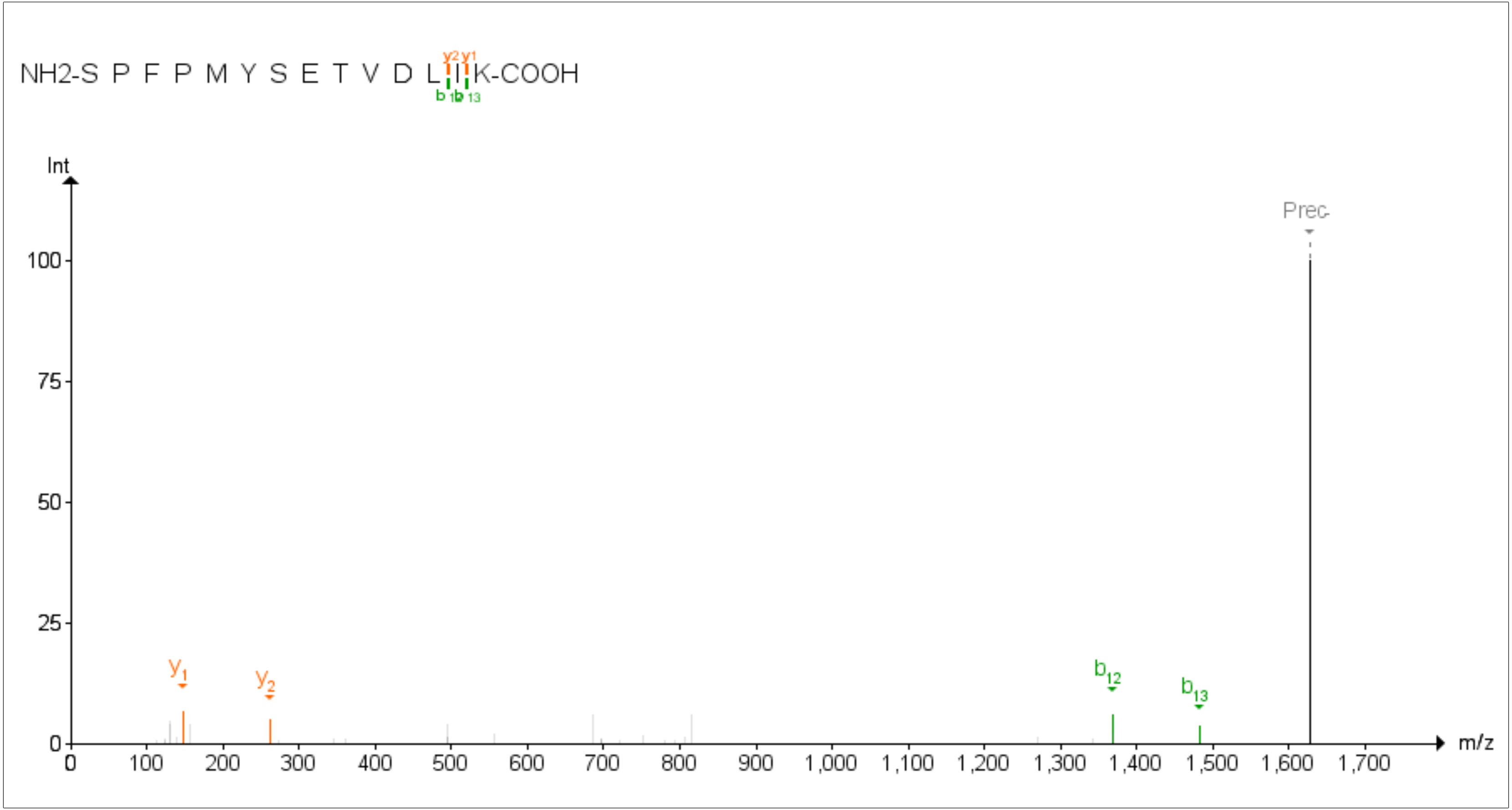

NH2-S P F P M Y S E T V D L I I K-COOH
y2 y1
b 12 b 13
Int
Prec
100
75
50
25
0
y1
y2
b12
b13
0
100
200
300
400
500
600
700
800
900
1,000
1,100
1,200
1,300
1,400
1,500
1,600
1,700
m/z

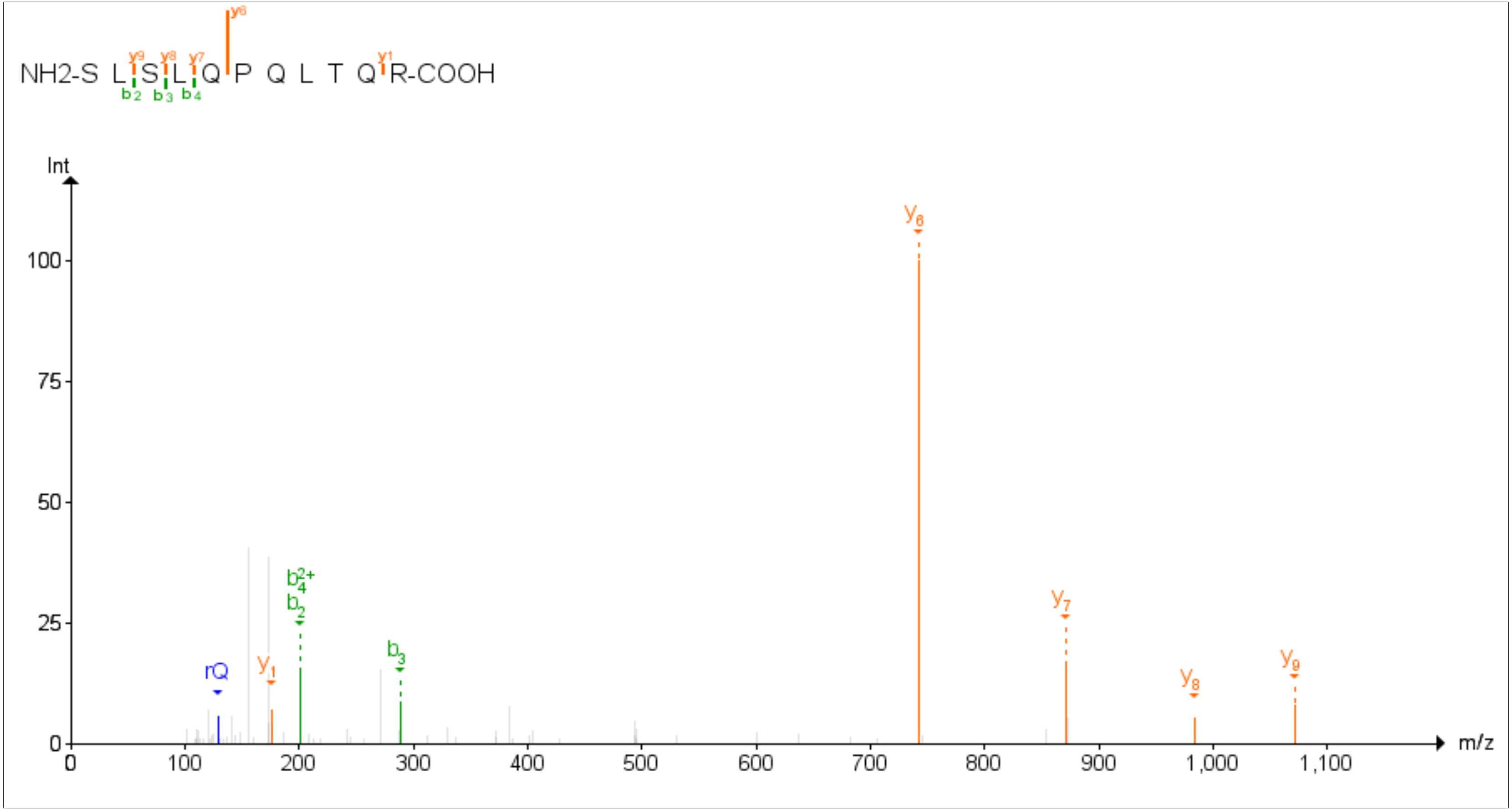

NH2-S L S L Q P Q L T Q R-COOH
y9 y8 y7 y6 y1
b2 b3 b4
Int
100
75
50
25
0
y6
y7
y8
y9
rQ
y1
b4 2+
b2
b3
0 100 200 300 400 500 600 700 800 900 1,000 1,100
m/z

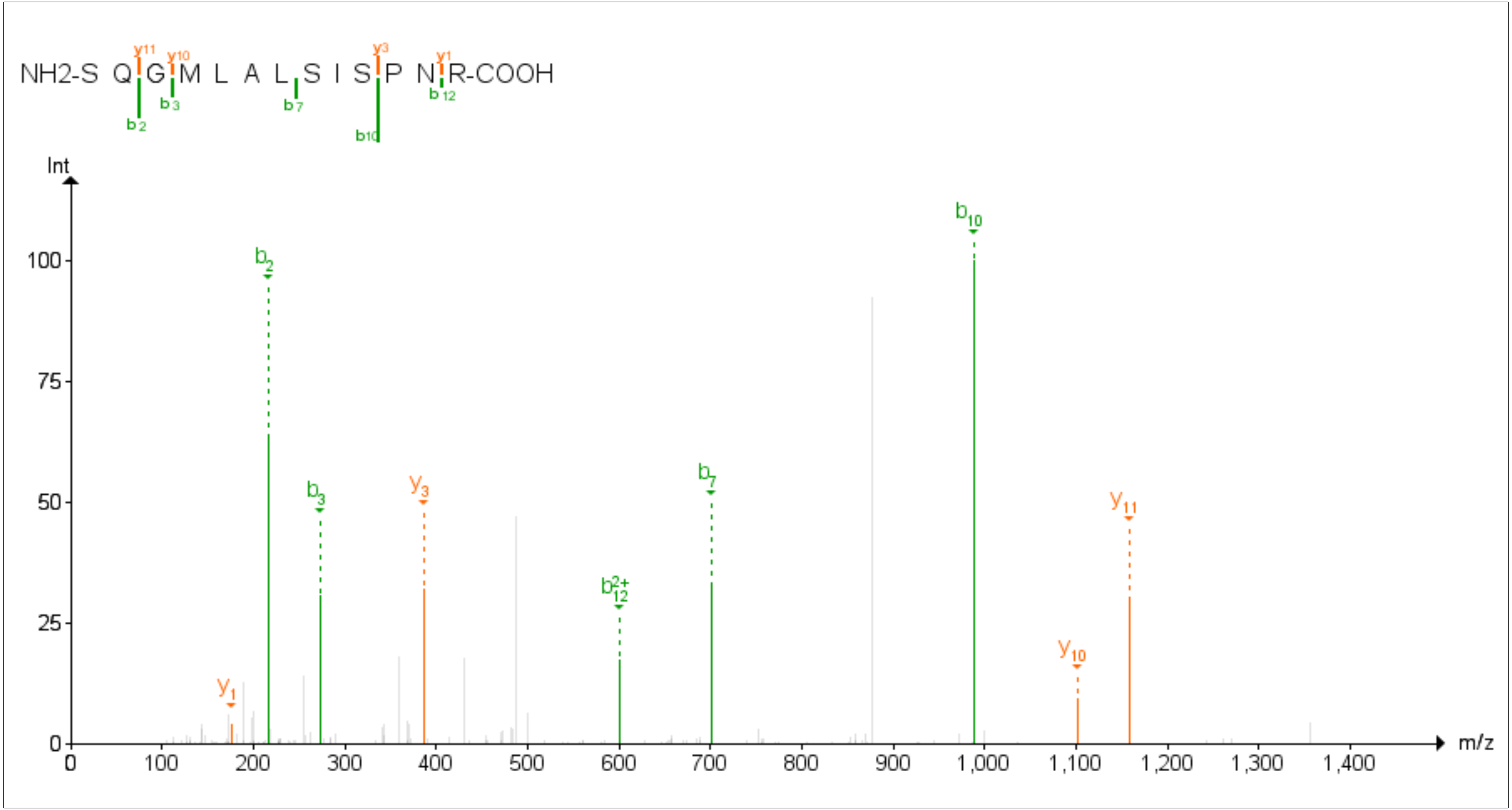

NH2-S Q G M L A L S I S P N R-COOH
y11
y10
y3
y1
b2
b3
b7
b10
b12
Int
100
75
50
25
0
b2
b3
y3
b7
b10
y11
y10
y1
b_{12}^{2+}
0
100
200
300
400
500
600
700
800
900
1,000
1,100
1,200
1,300
1,400
m/z

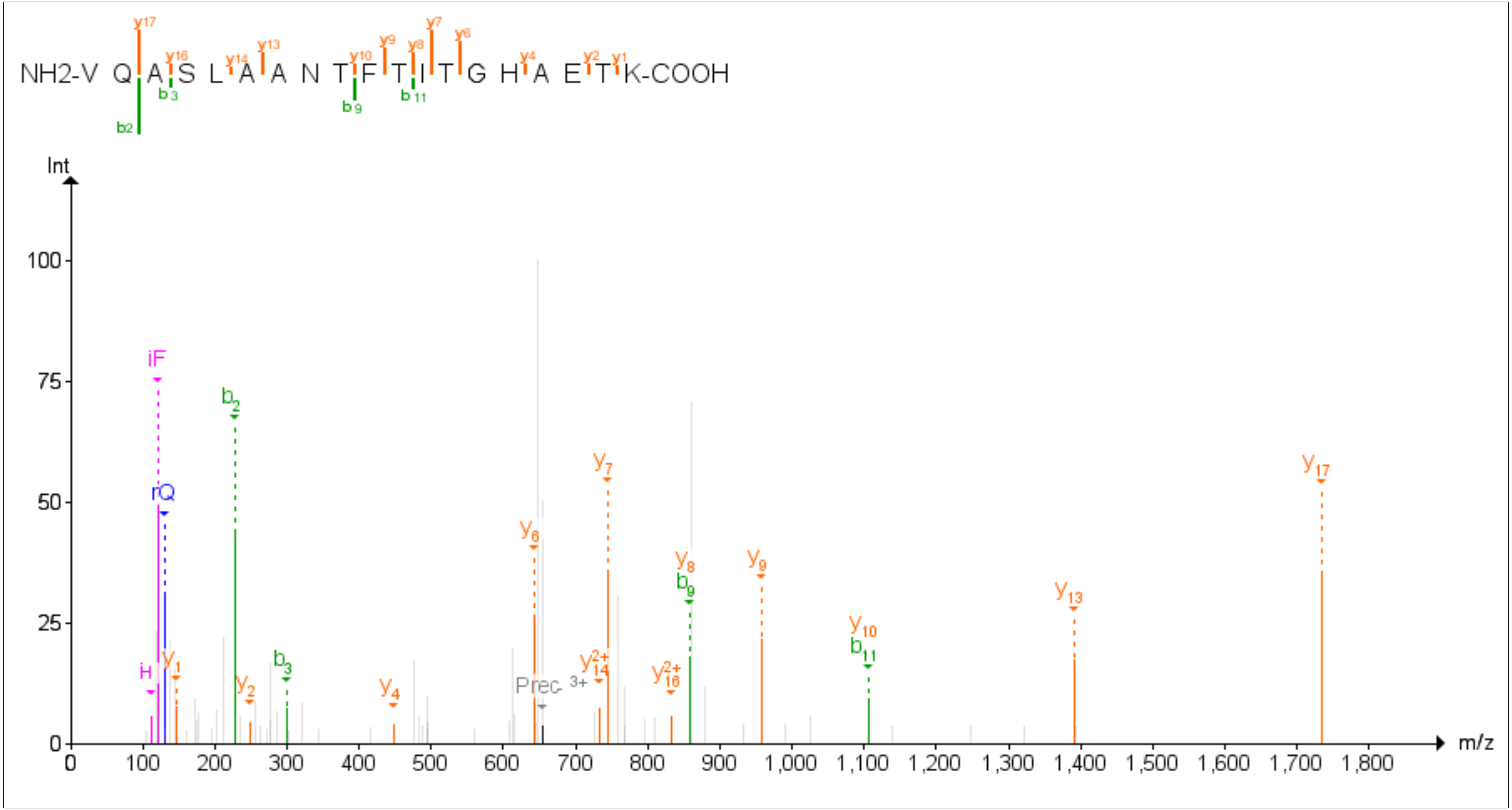

NH2-V Q A S L A A N T F T I T G H A E T K-COOH
y17 y16 y14 y13 y10 y9 y8 y7 y6 y4 y2 y1
b2 b3 b9 b11
Int
m/z
iF
rQ
iH
b2
y1
y2
b3
y4
y6
y7
Prec- 3+
y14 2+
y16 2+
y8
b9
y9
y10
b11
y13
y17

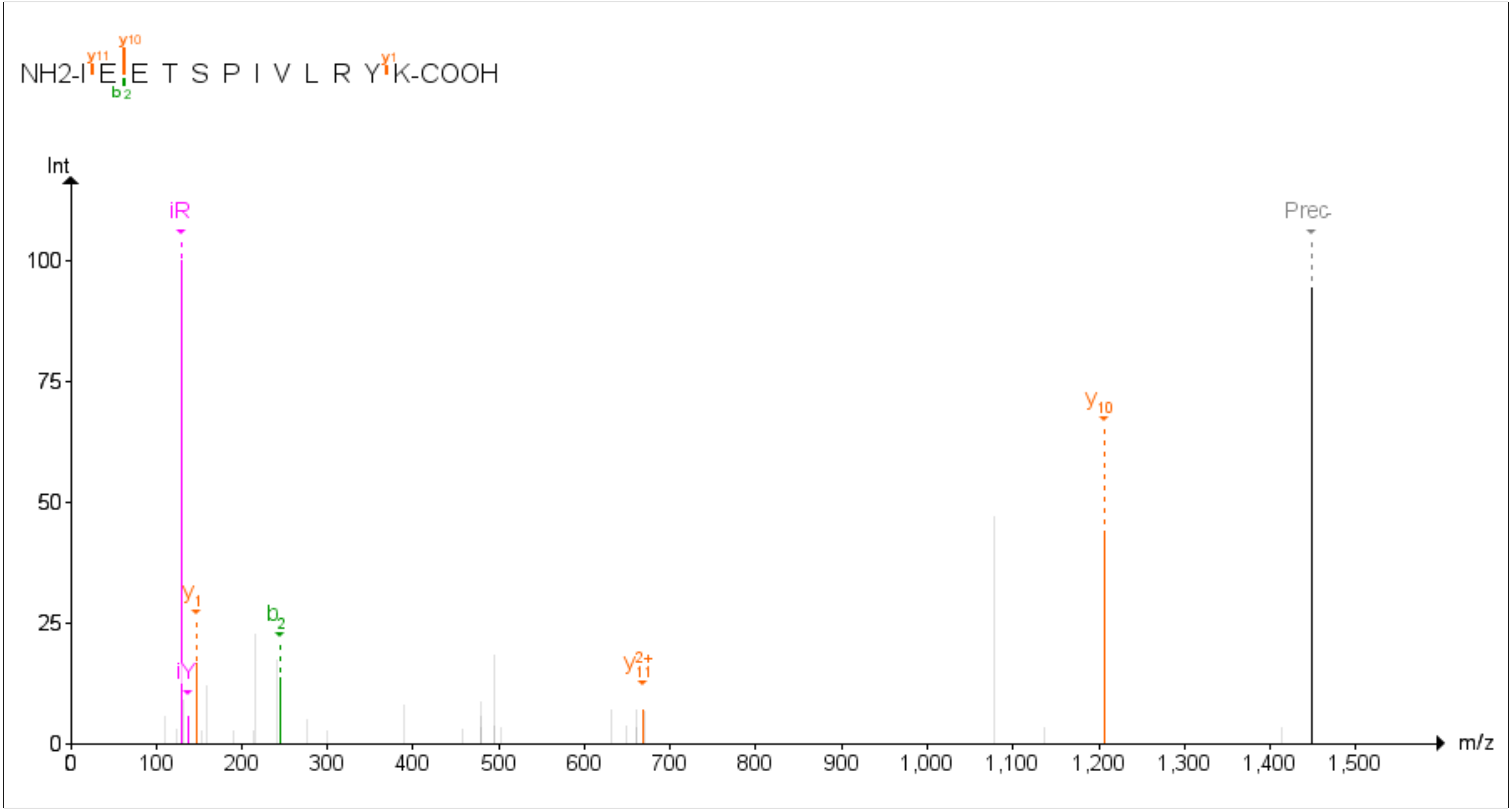

NH2-I E E T S P I V L R Y K-COOH
y11
y10
b2
y1
Int
iR
Prec
100
75
50
25
0
y10
y1
b2
iY
y11 2+
m/z
0
100
200
300
400
500
600
700
800
900
1,000
1,100
1,200
1,300
1,400
1,500

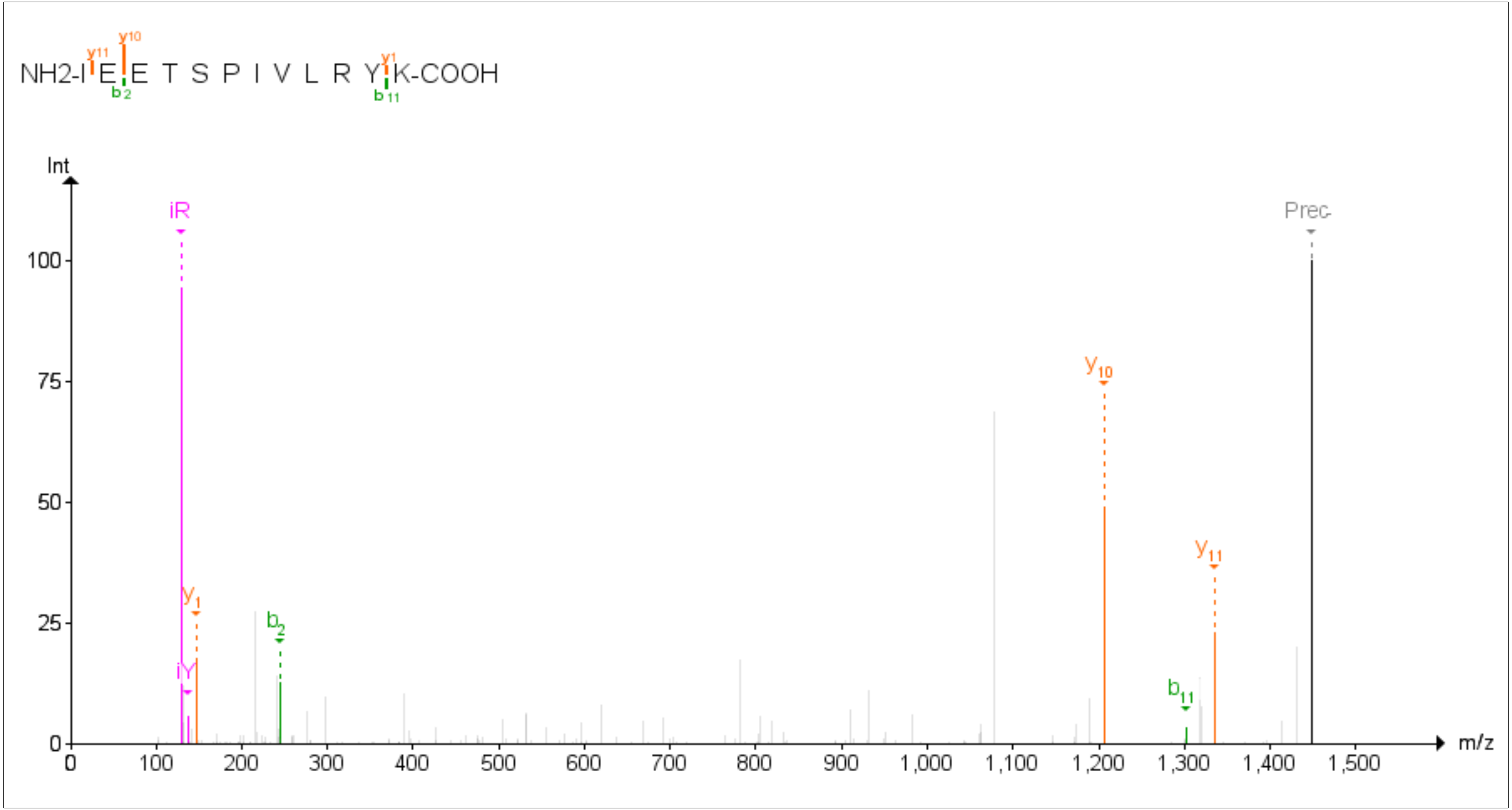

NH2-I E E T S P I V L R Y K-COOH
y11
y10
y1
b2
b11
Int
iR
Prec
100
75
50
25
0
y10
y11
y1
iY
b2
b11
0
100
200
300
400
500
600
700
800
900
1,000
1,100
1,200
1,300
1,400
1,500
m/z

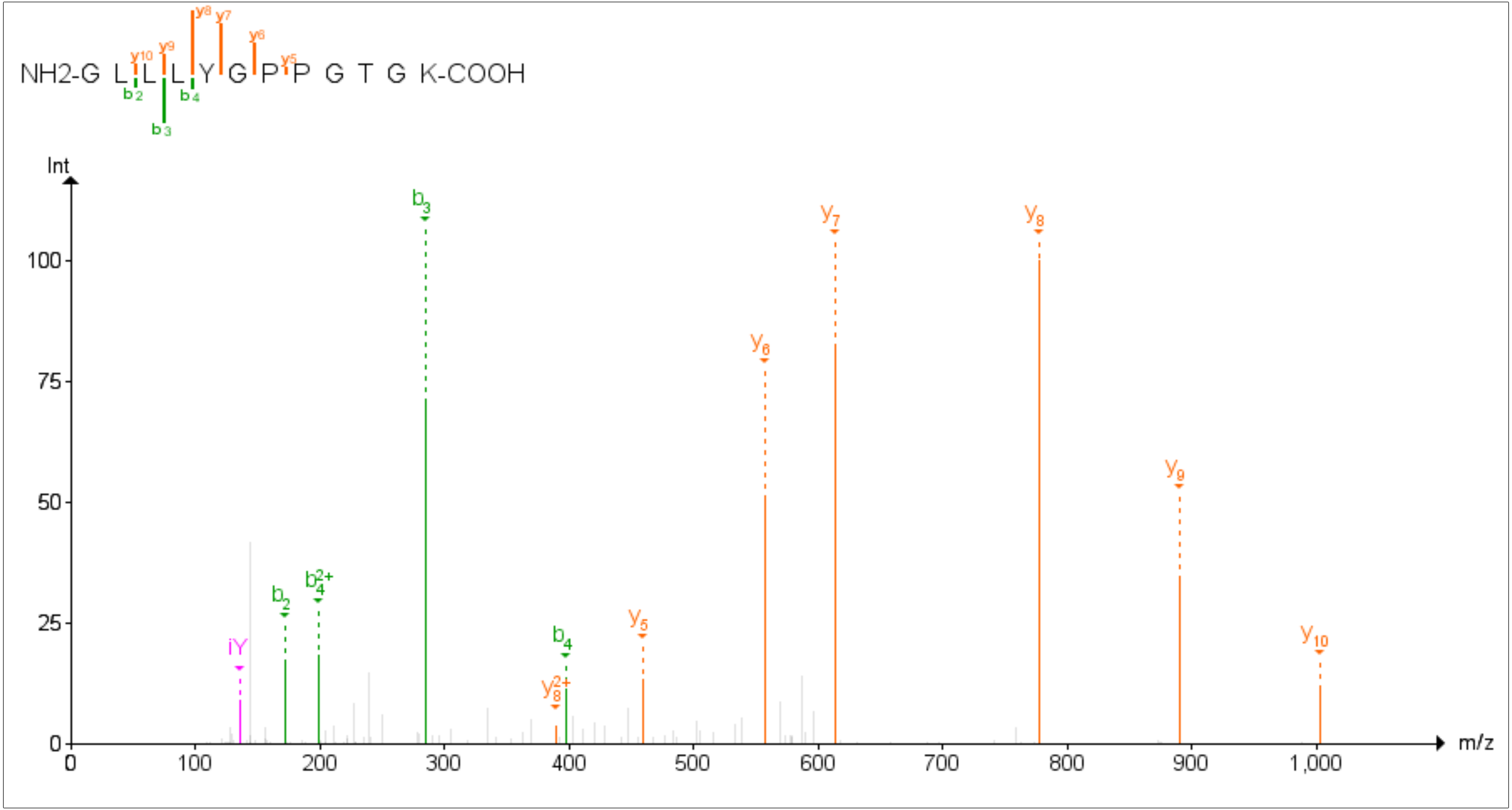
NH2-G L L L Y G P P G T G K-COOH
y10 y9 y8 y7 y6 y5
b2 b3 b4
Int
m/z
b3
y7
y8
y6
y9
b4 2+
b2
iY
b4
y5
y8 2+
y10
0
25
50
75
100
0
100
200
300
400
500
600
700
800
900
1,000

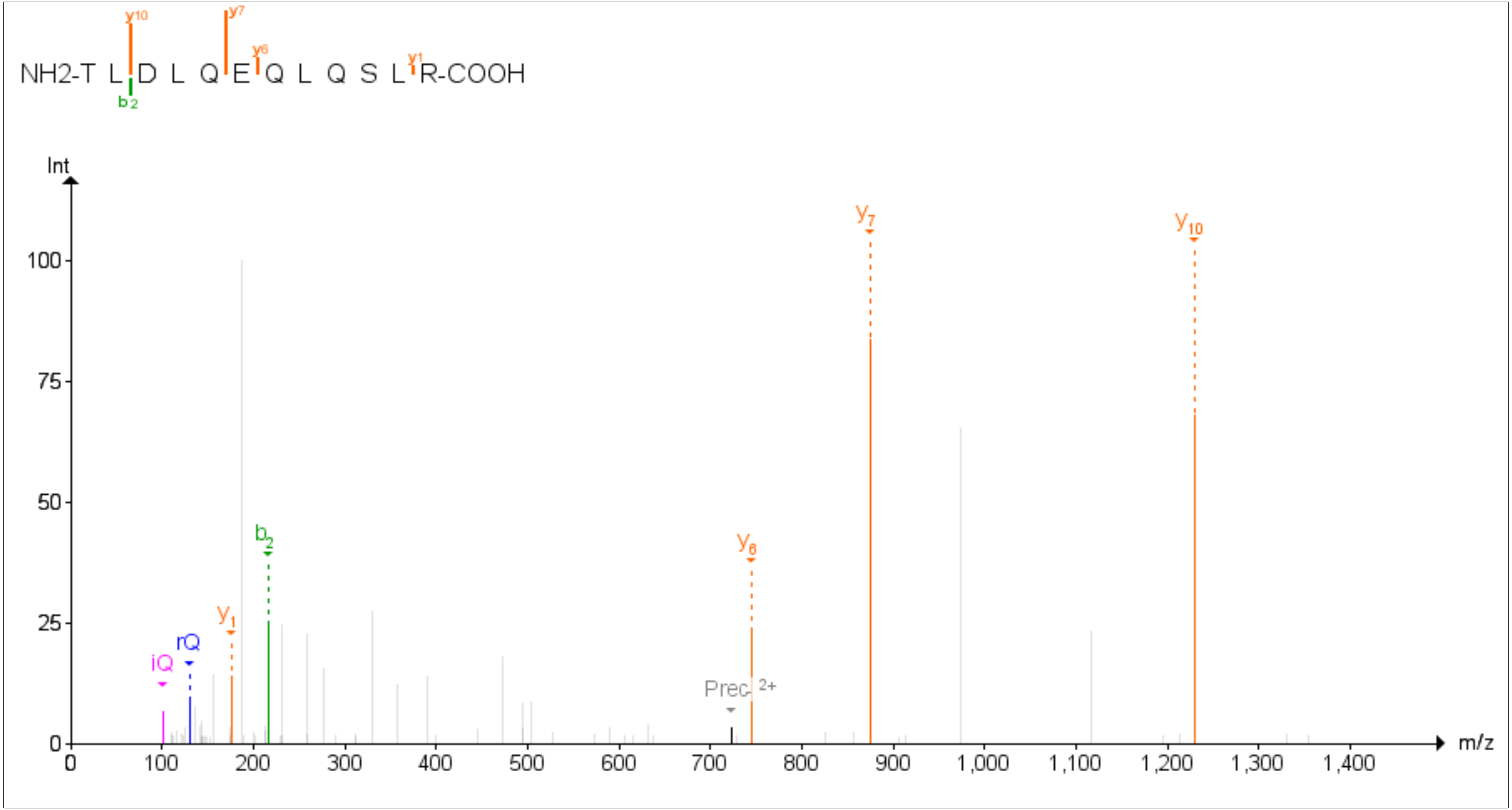

NH2-T L D L Q E Q L Q S L R-COOH
y10
y7
y6
y1
b2
Int
100
75
50
25
0
y7
y10
b2
y6
y1
rQ
iQ
Prec 2+
0
100
200
300
400
500
600
700
800
900
1,000
1,100
1,200
1,300
1,400
m/z

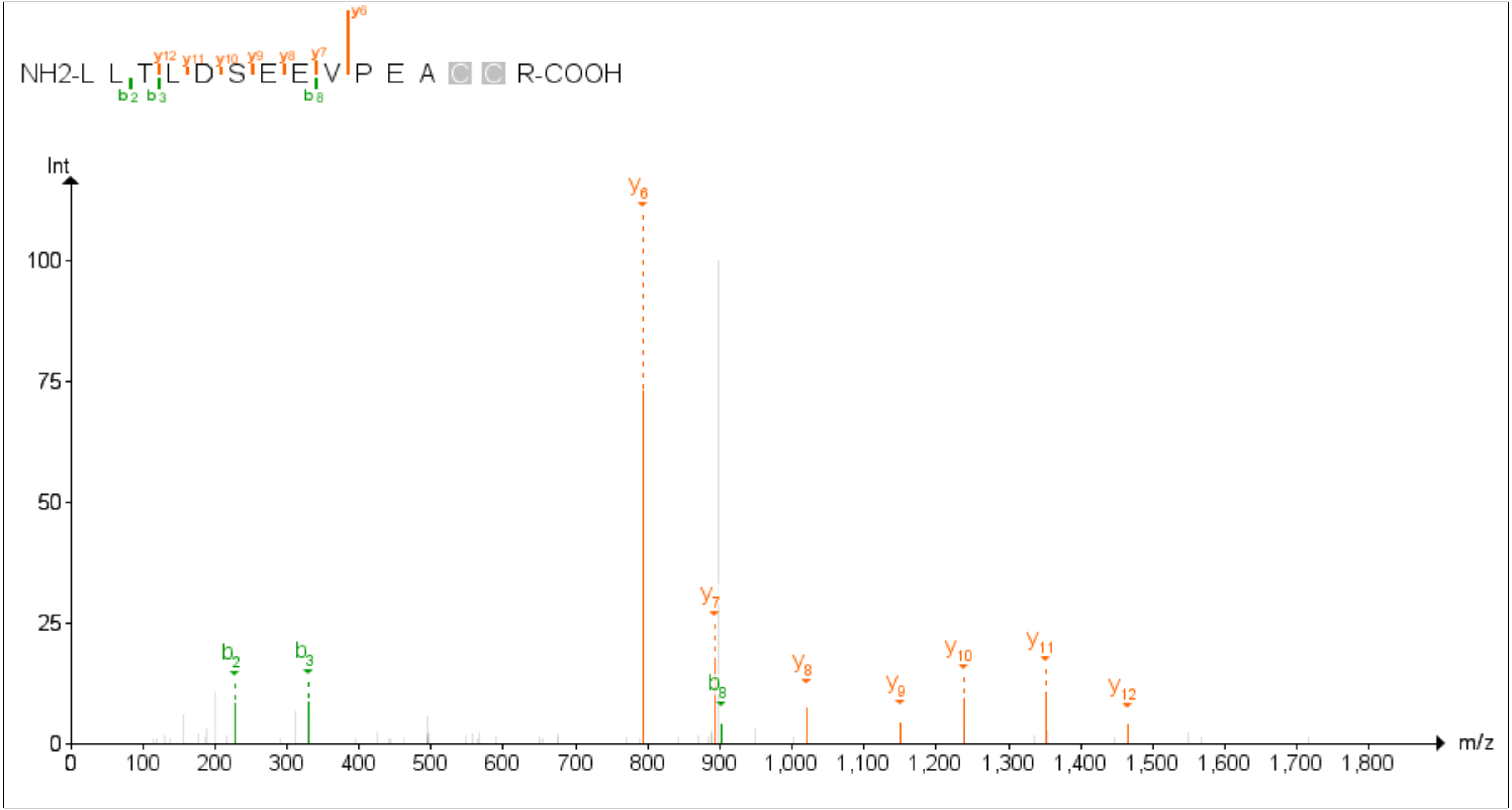

NH2-L L T I L D S E E V P E A C C R-COOH
y12 y11 y10 y9 y8 y7 y6
b2 b3 b8
Int
100
75
50
25
0
y6
y7
b2
b3
b8
y8
y9
y10
y11
y12
0 100 200 300 400 500 600 700 800 900 1,000 1,100 1,200 1,300 1,400 1,500 1,600 1,700 1,800
m/z

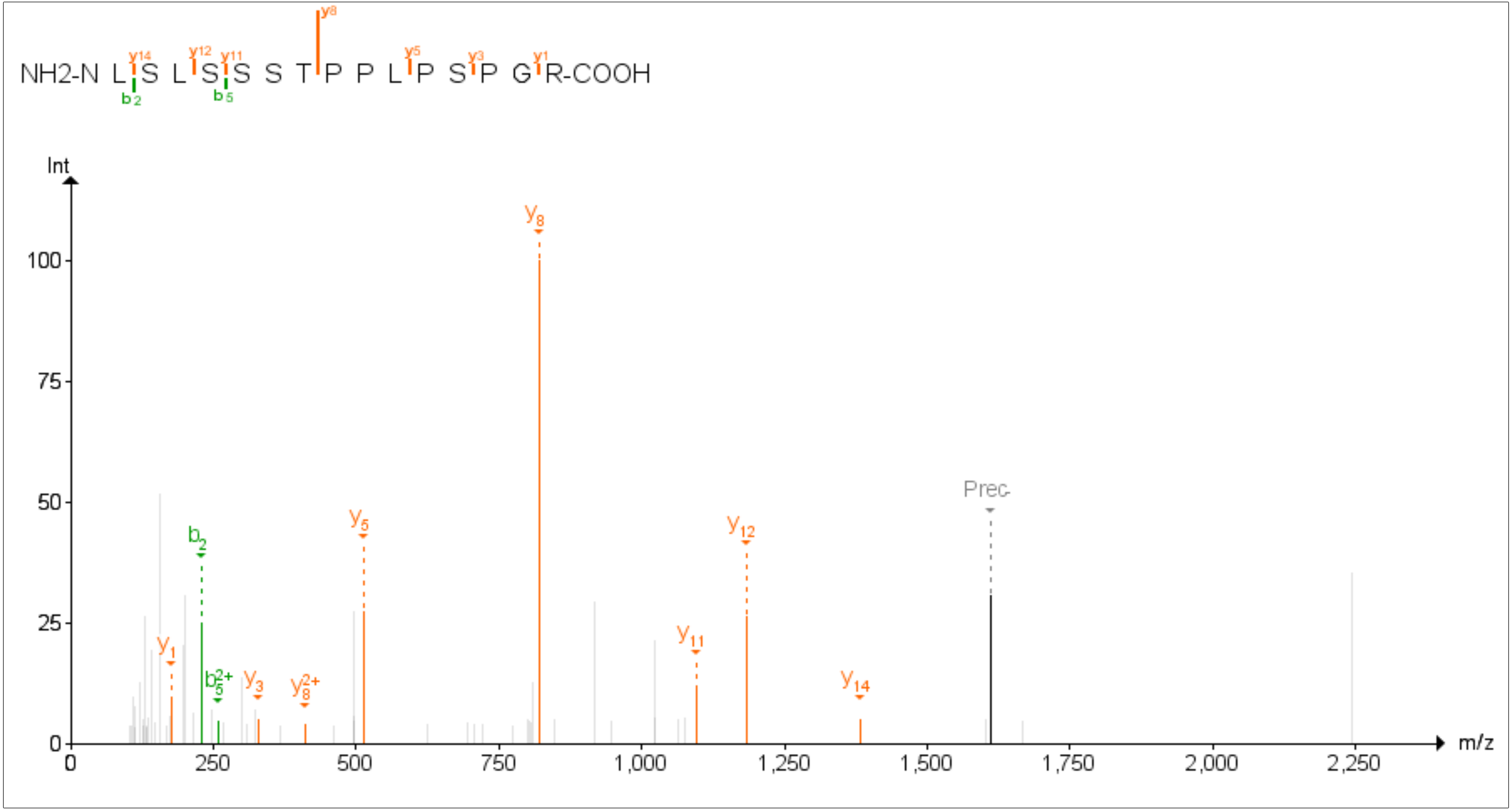
NH2-N L S L S S S T P P L P S P G R-COOH
y14 y12 y11 y8 y5 y3 y1
b2 b5
Int
m/z
y8
y5
y12
b2
y1
b5 2+
y3
y8 2+
y11
y14
Prec
0
25
50
75
100
0
250
500
750
1,000
1,250
1,500
1,750
2,000
2,250

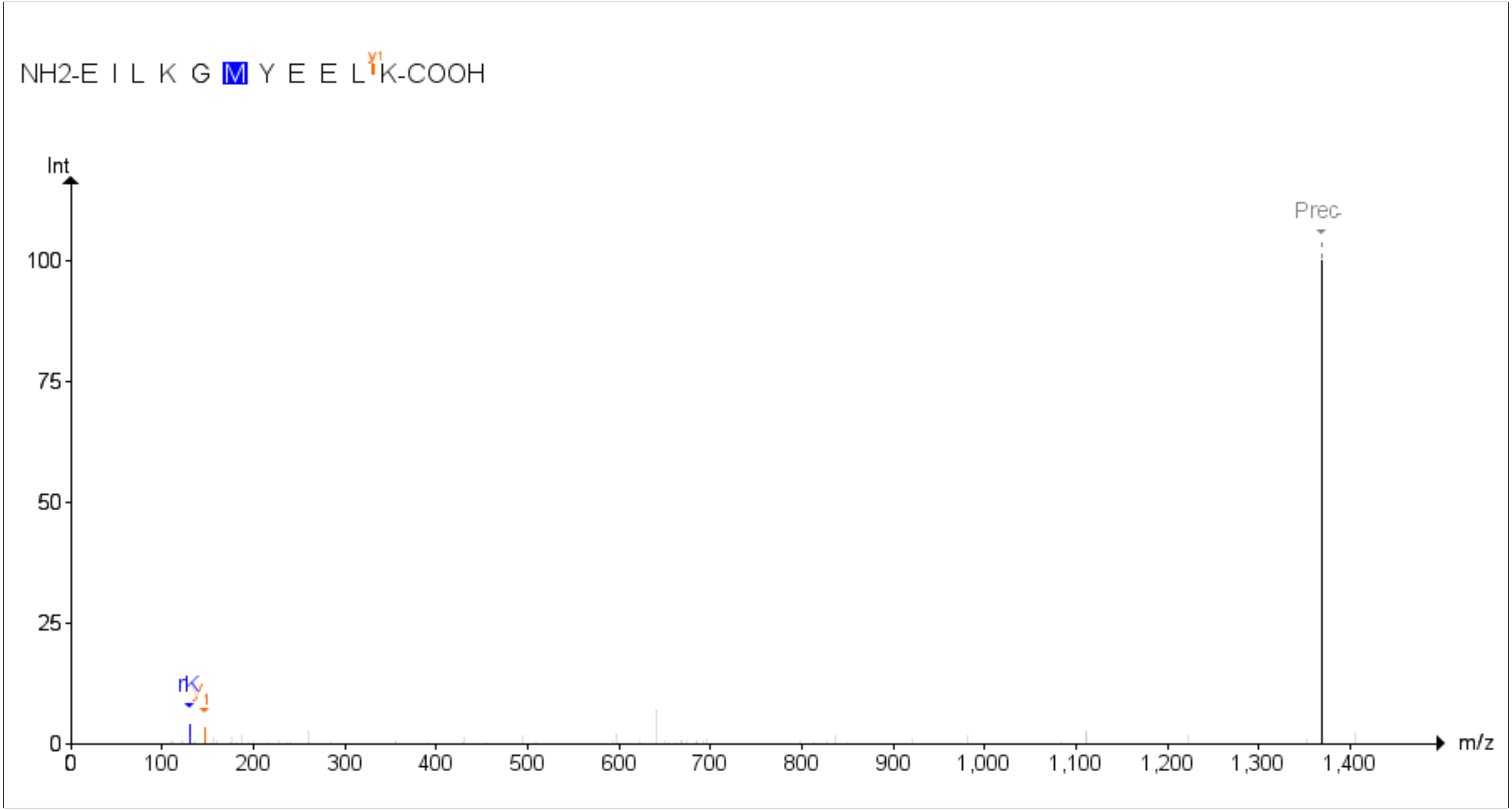
NH2-E I L K G M Y E E L K-COOH
y1
Int
Prec
100
75
50
25
0
rK
y1
m/z
0
100
200
300
400
500
600
700
800
900
1,000
1,100
1,200
1,300
1,400

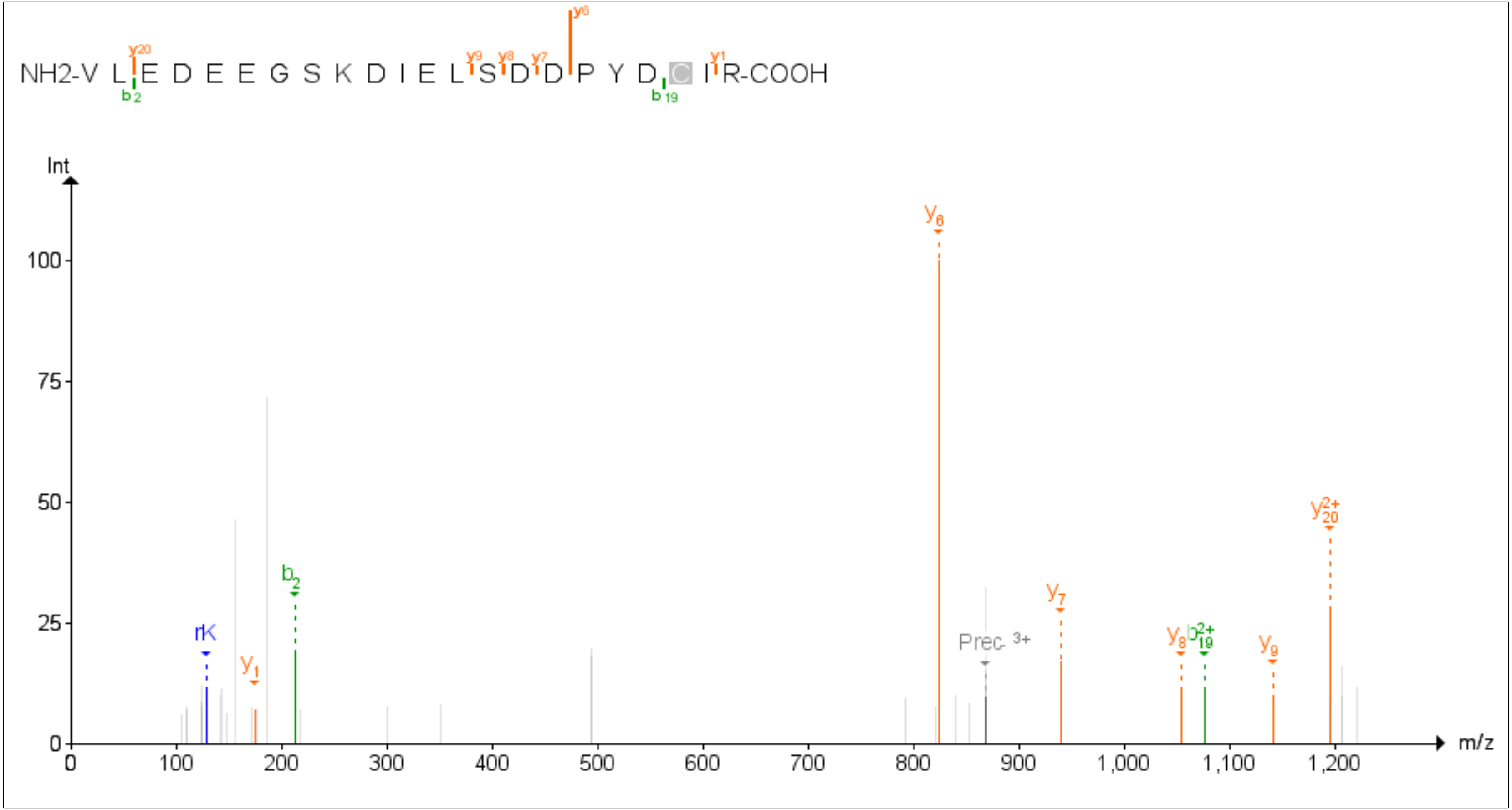
NH2-V L E D E E G S K D I E L S D D P Y D C I R-COOH
y20 y9 y8 y7 y6 y1
b2 b19
Int
100
75
50
25
0
m/z
0 100 200 300 400 500 600 700 800 900 1,000 1,100 1,200
rK
y1
b2
y6
Prec 3+
y7
y8
b19 2+
y9
y20 2+

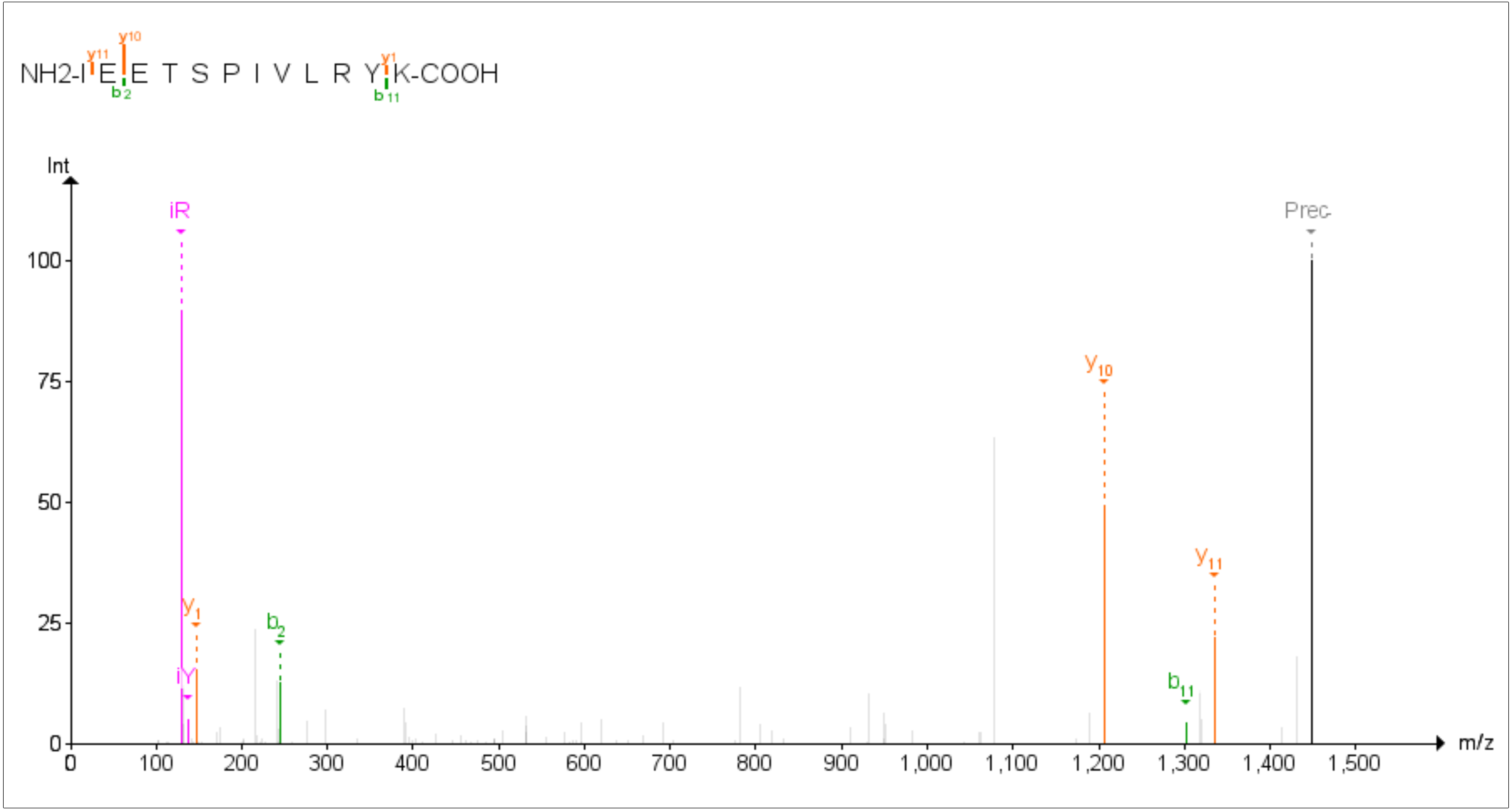

NH2-I E E T S P I V L R Y K-COOH
y11
y10
b2
y1
b11
Int
iR
Prec.
y10
y11
y1
b2
iY
b11
100
75
50
25
0
0
100
200
300
400
500
600
700
800
900
1,000
1,100
1,200
1,300
1,400
1,500
m/z

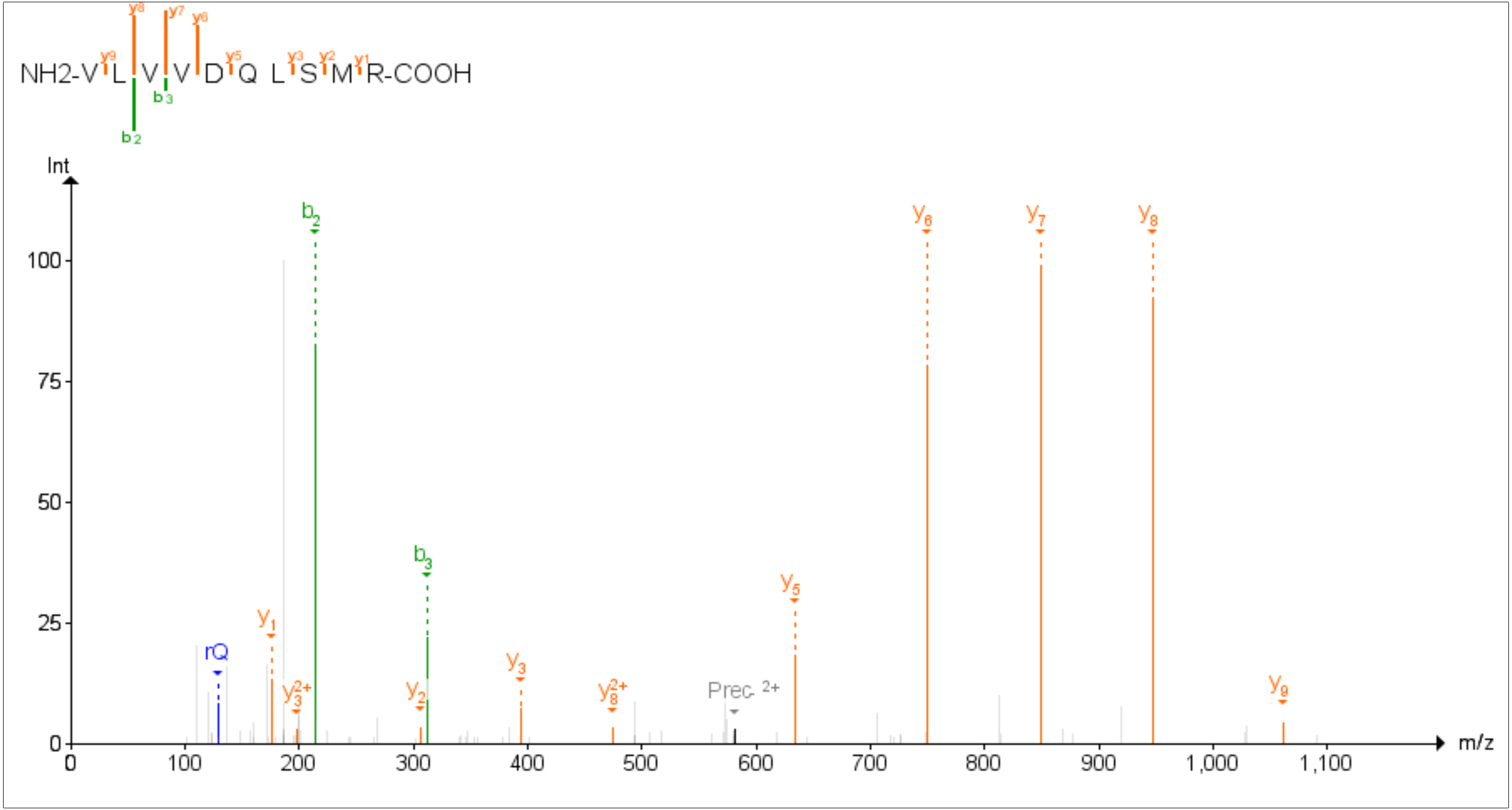

NH2-V L V V D Q L S M R-COOH
y9 y8 y7 y6 y5 y3 y2 y1
b2 b3
Int
m/z
b2
b3
y1
y2
y3
y5
y6
y7
y8
y9
rQ
y3 2+
y8 2+
Prec 2+
0
25
50
75
100
0
100
200
300
400
500
600
700
800
900
1,000
1,100

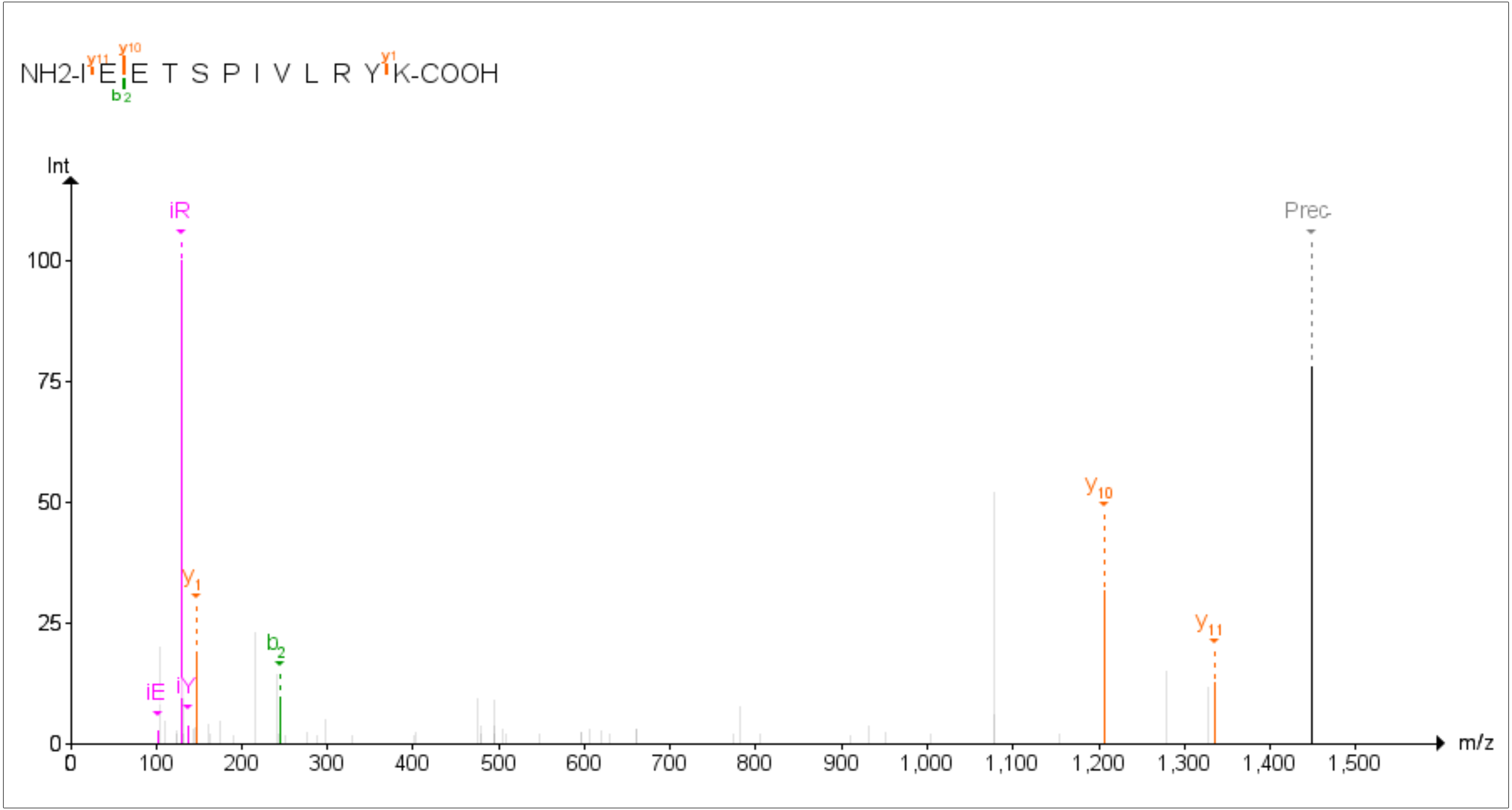

NH2-I E E T S P I V L R Y K-COOH
y11
y10
b2
y1
Int
iR
Prec
y10
y1
b2
y11
iE
iY
100
75
50
25
0
m/z
0
100
200
300
400
500
600
700
800
900
1,000
1,100
1,200
1,300
1,400
1,500

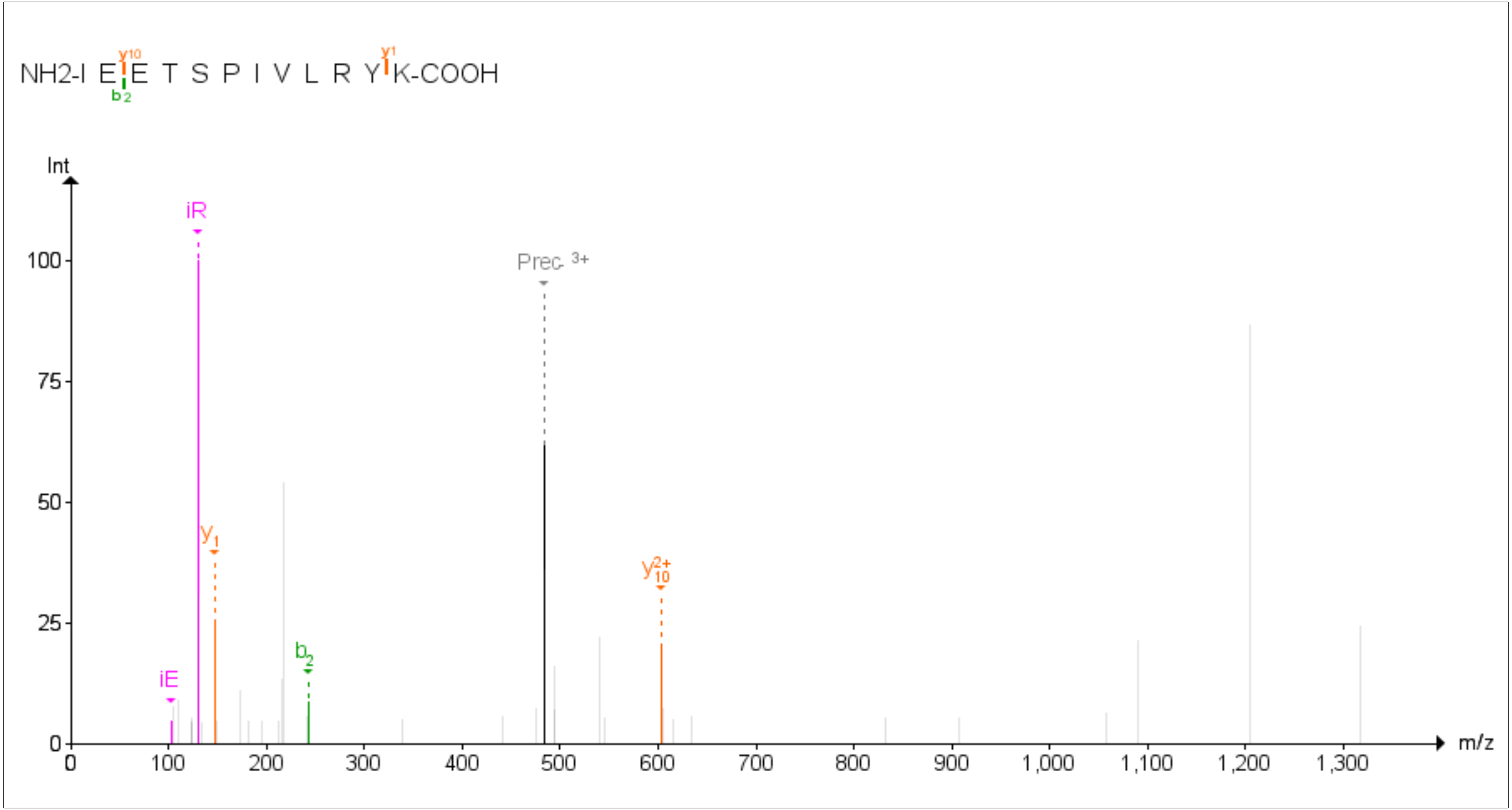

NH2-I E E T S P I V L R Y K-COOH
y10
b2
y1
Int
iR
Prec 3+
y1
y2+ 10
b2
iE
100
75
50
25
0
0
100
200
300
400
500
600
700
800
900
1,000
1,100
1,200
1,300
m/z

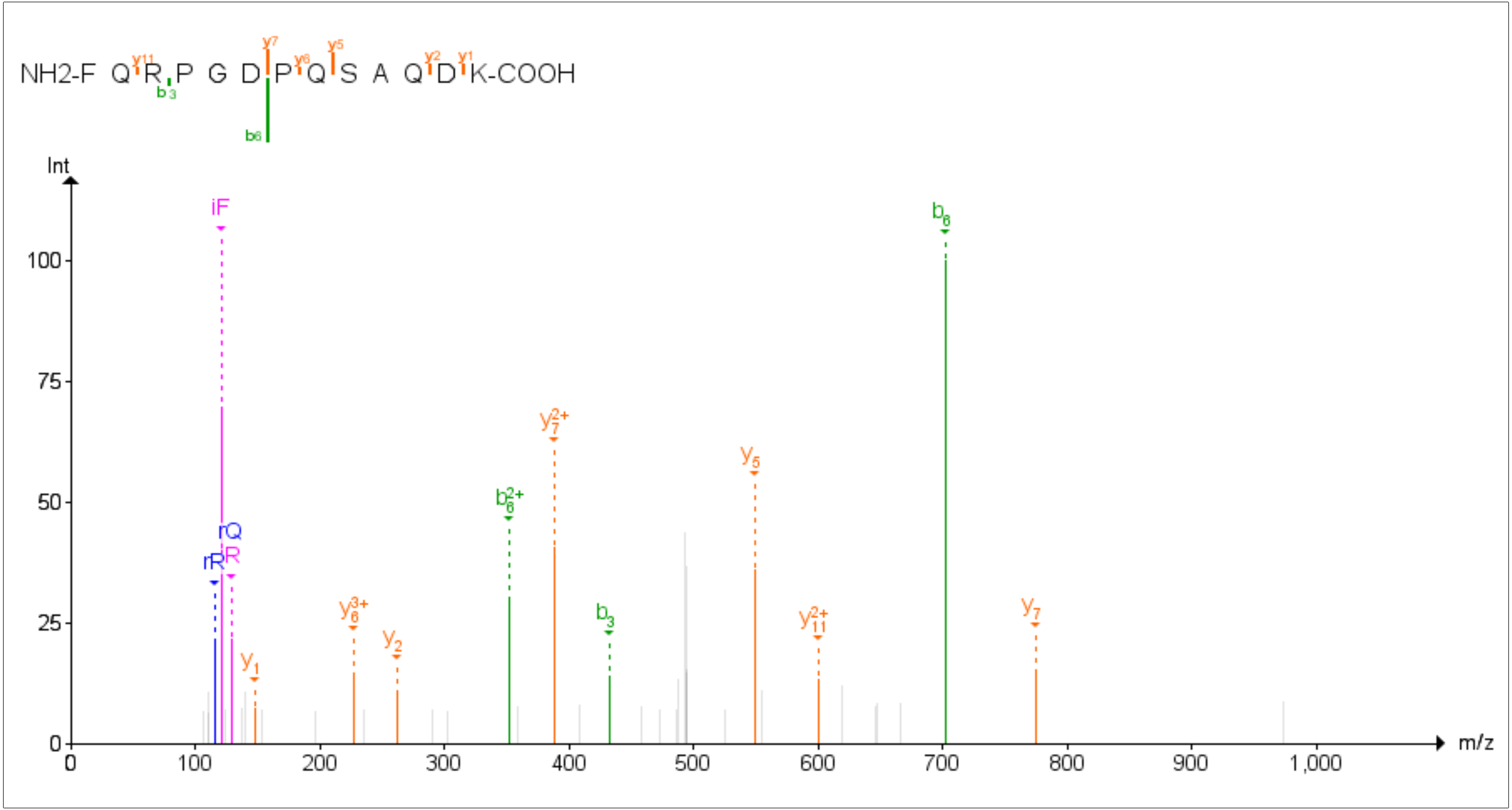

NH2-F Q R P G D P Q S A Q D K-COOH
y11
b3
y7
b6
y6
y5
y2
y1
Int
100
75
50
25
0
iF
rQ
rR
R
y1
y6 3+
y2
b6 2+
y7 2+
b3
y5
y11 2+
b6
y7
0
100
200
300
400
500
600
700
800
900
1,000
m/z

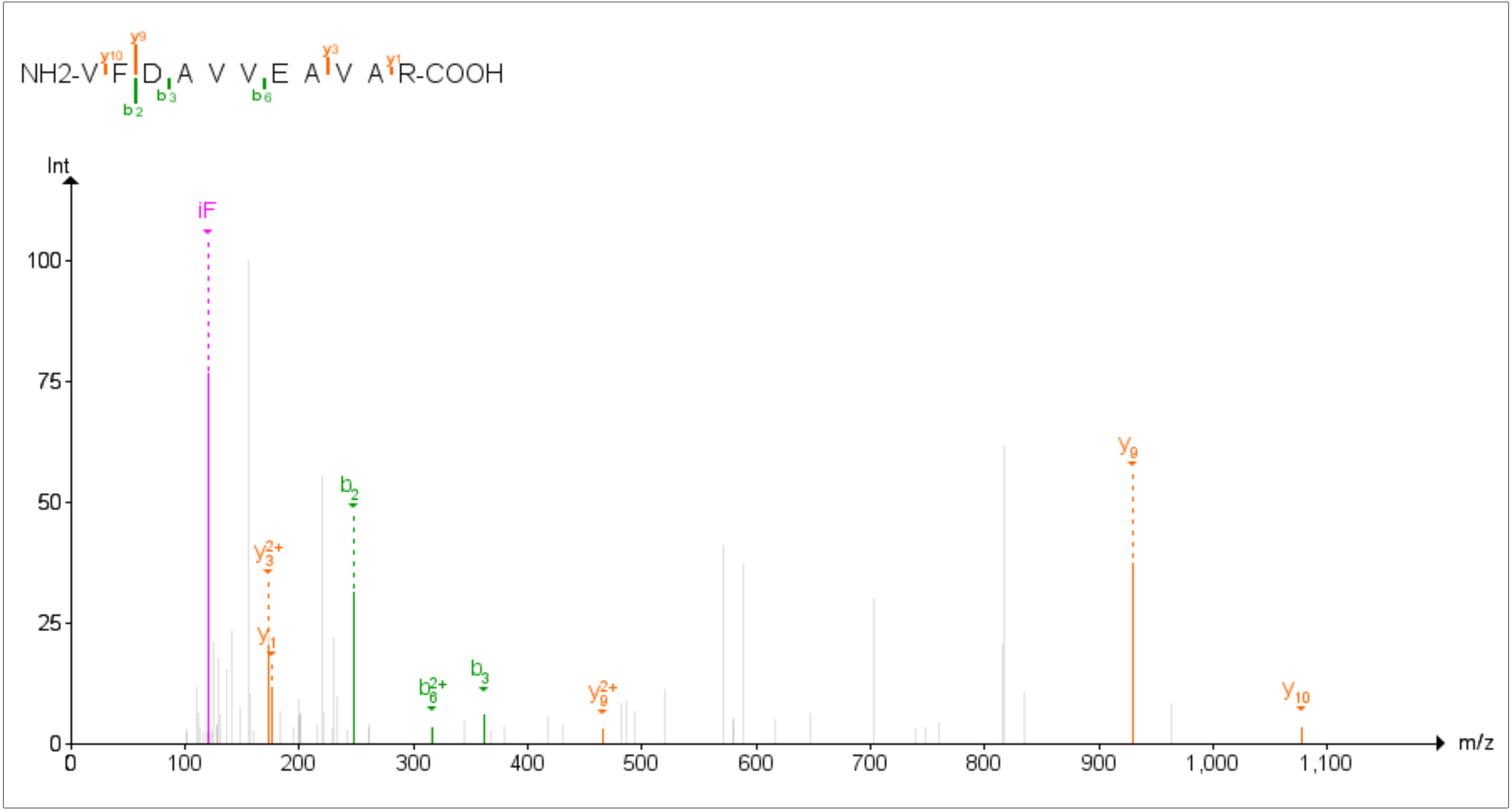

NH2-V F D A V V E A V A R-COOH
y10 y9 y3 y1
b2 b3 b6
Int
m/z
iF
b2
y9
y3 2+
y1
b6 2+
b3
y9 2+
y10
0
25
50
75
100
0
100
200
300
400
500
600
700
800
900
1,000
1,100

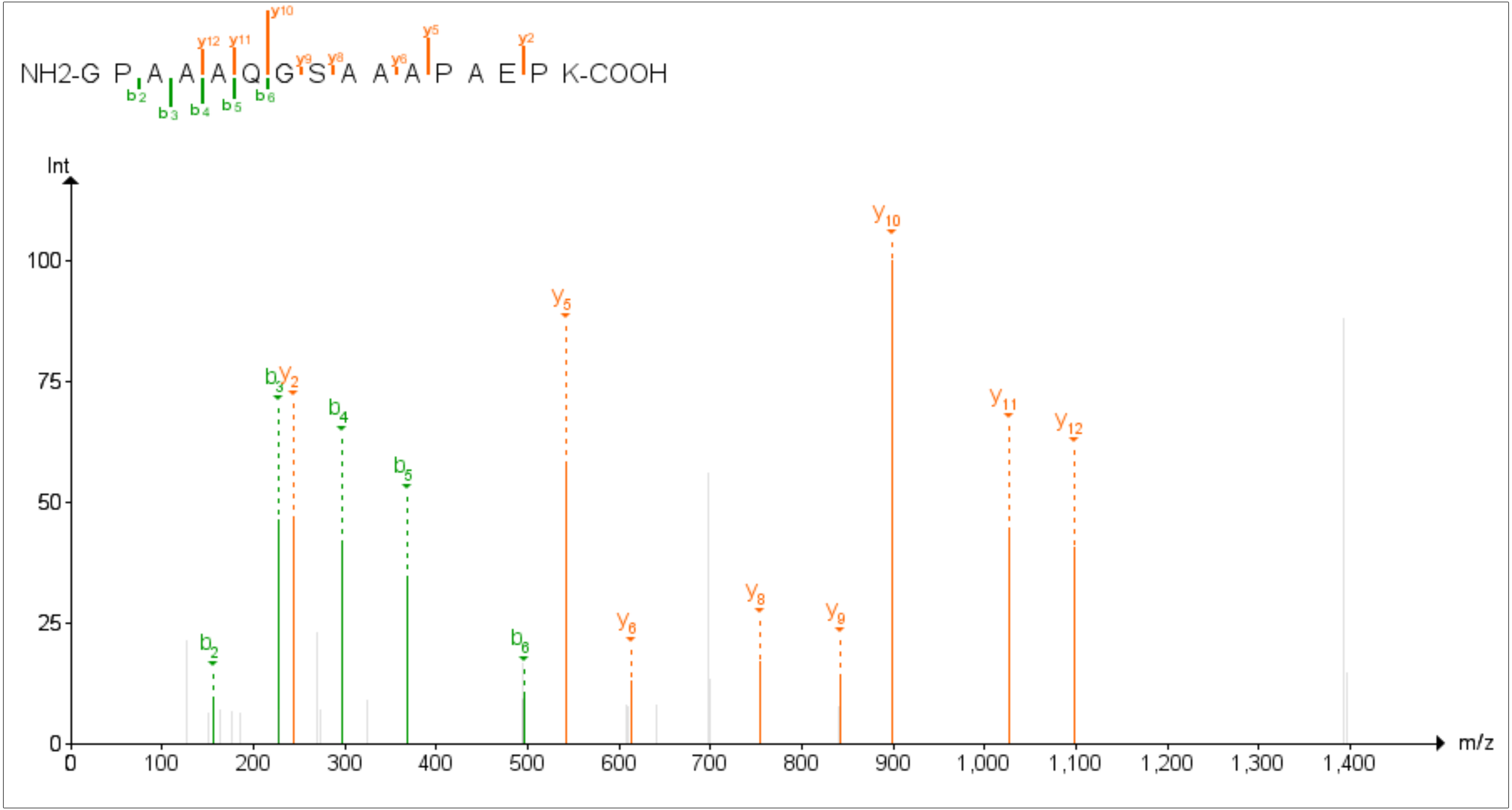

NH2-G P A A A Q G S A A A P A E P K-COOH
y10
y12
y11
y9
y8
y6
y5
y2
b2
b3
b4
b5
b6
Int
m/z
y10
y5
b3
y2
b4
b5
y11
y12
y8
y9
y6
b2
b6

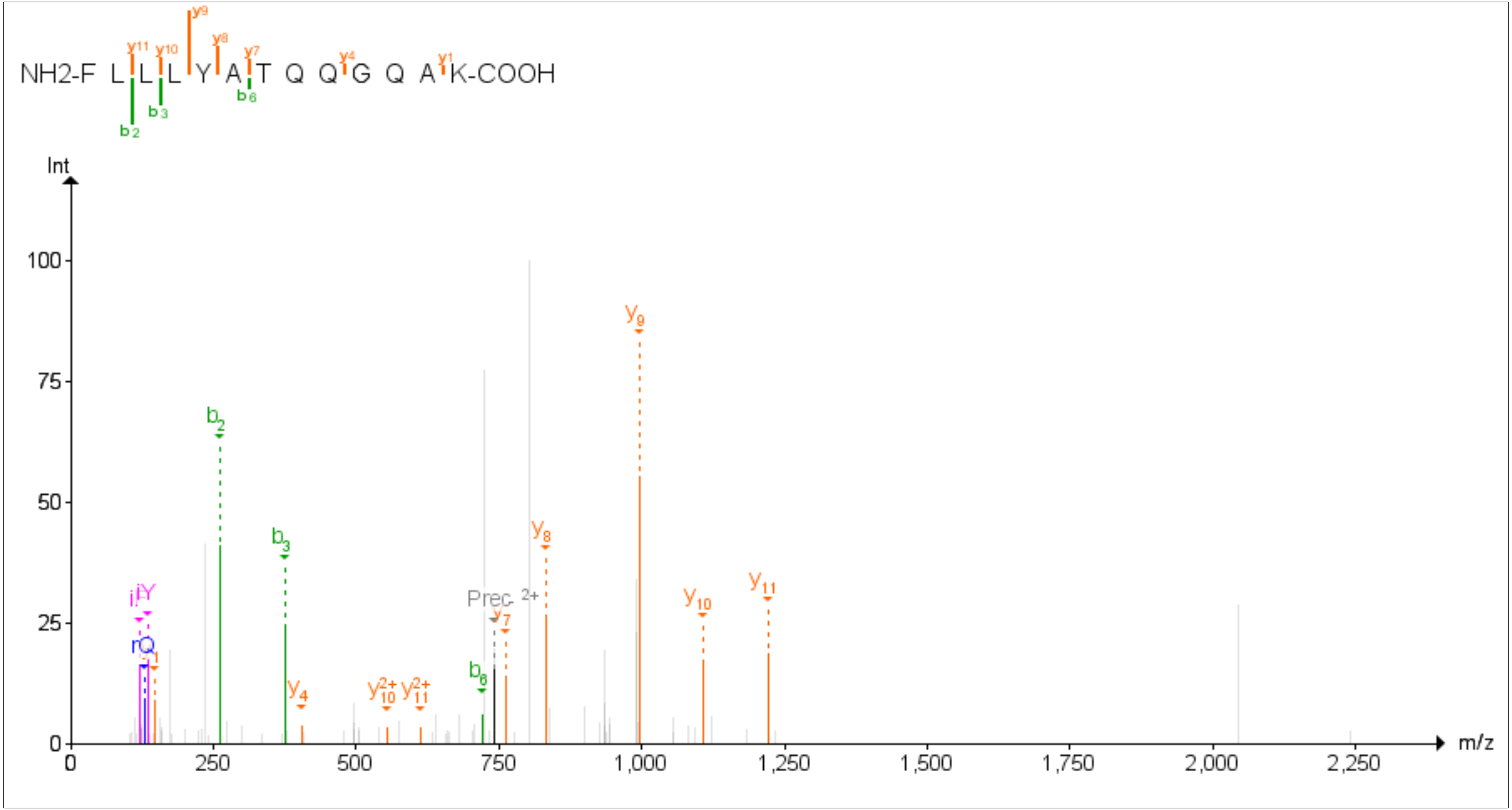

NH2-F L L L Y A T Q Q G Q A K-COOH
y11 y10 y9 y8 y7 y4 y1
b2 b3 b6
Int
100
75
50
25
0
b2
b3
y9
y8
Prec 2+
y7
y10
y11
iY
rQ
y4
y_{10}^{2+} y_{11}^{2+}
b6
0
250
500
750
1,000
1,250
1,500
1,750
2,000
2,250
m/z

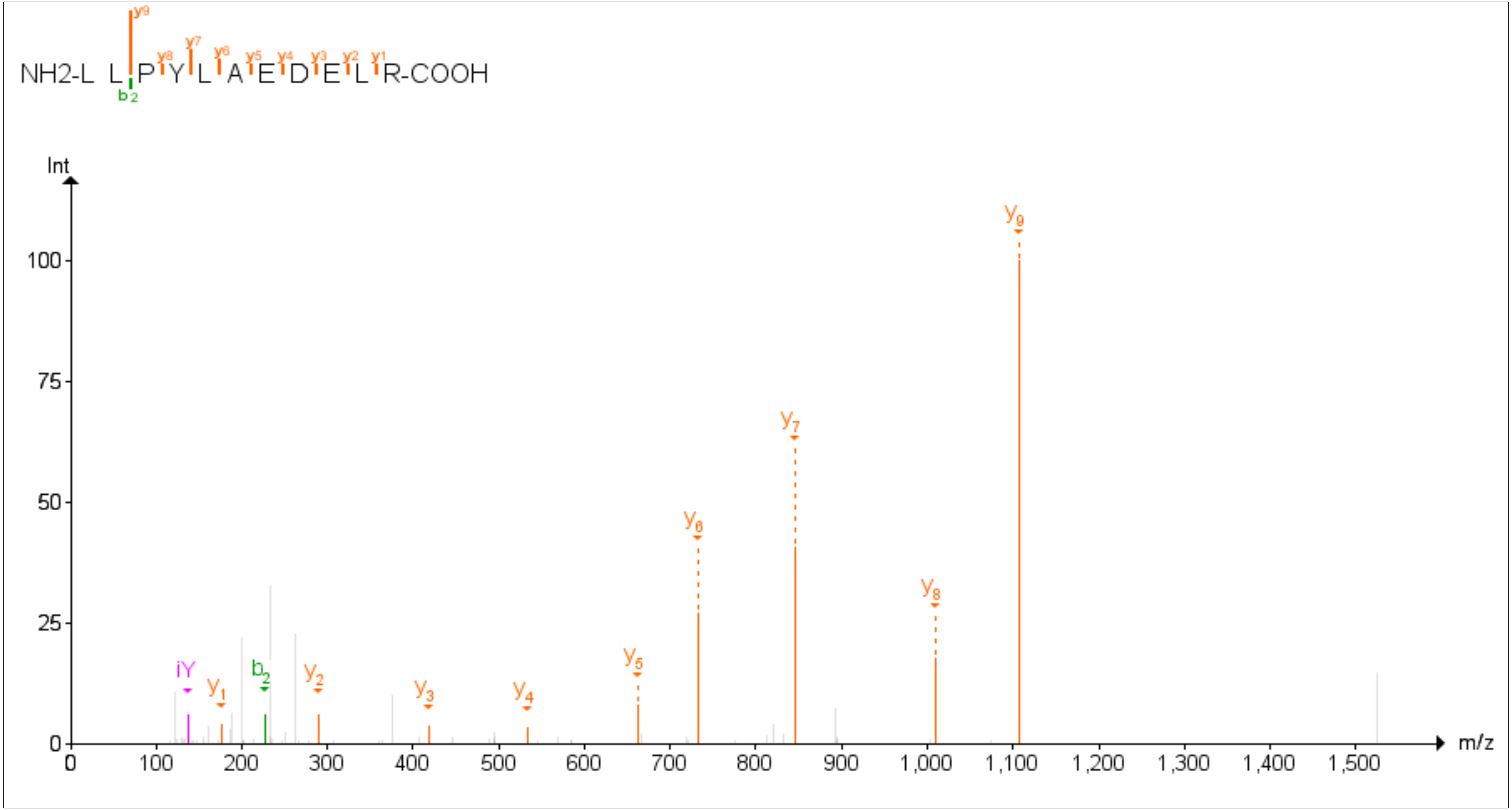

NH2-L L P Y L A E D E L R-COOH
y9 y8 y7 y6 y5 y4 y3 y2 y1
b2
Int
100
75
50
25
0
m/z
0 100 200 300 400 500 600 700 800 900 1,000 1,100 1,200 1,300 1,400 1,500
iY
y1
b2
y2
y3
y4
y5
y6
y7
y8
y9

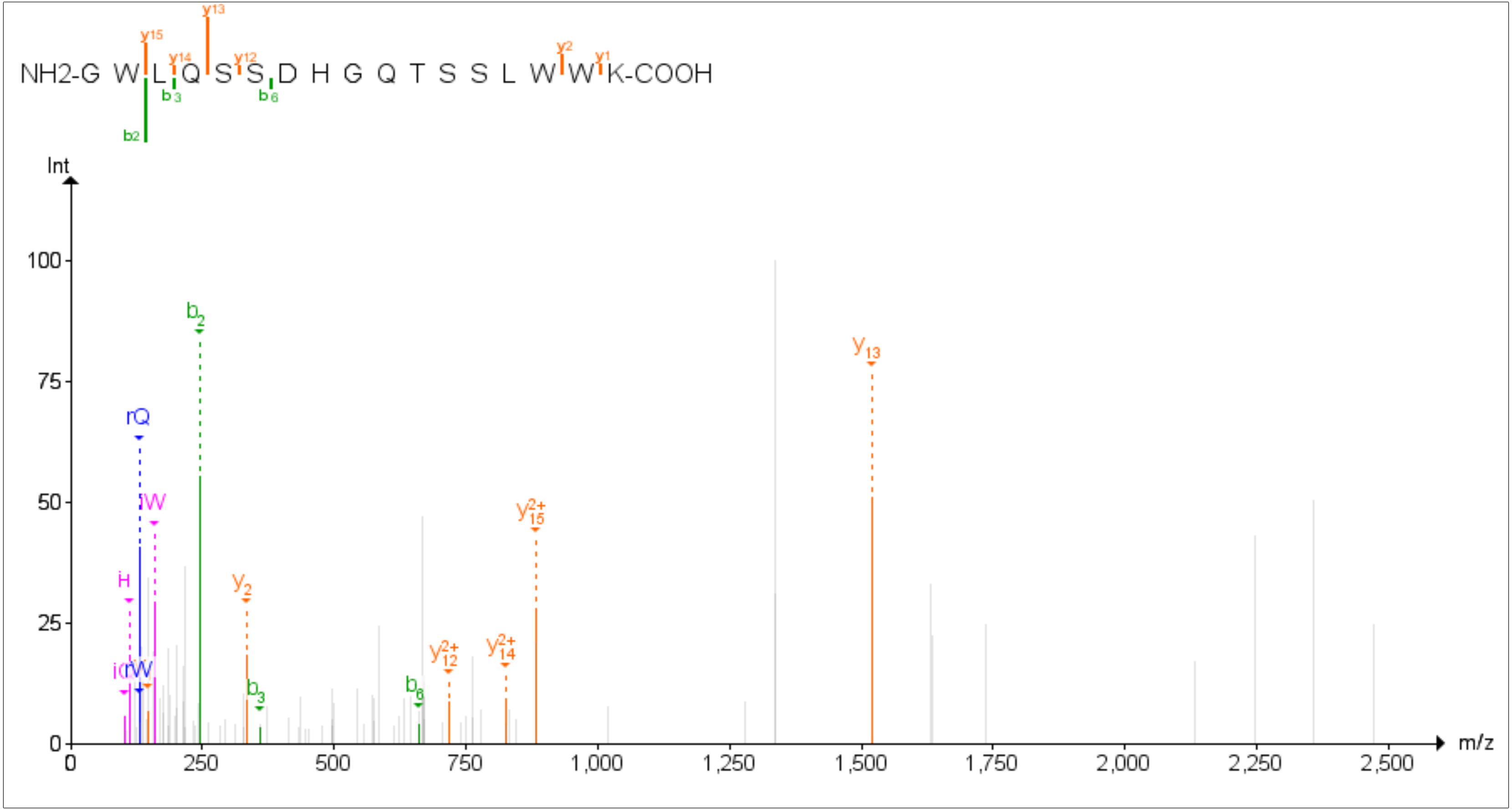

NH2-G W L Q S S D H G Q T S S L W W K-COOH
y15
y14
y13
y12
y2
y1
b2
b3
b6
Int
100
75
50
25
0
b2
rQ
iW
iH
icW
y2
b3
b6
y13
y2+ 15
y2+ 12
y2+ 14
0
250
500
750
1,000
1,250
1,500
1,750
2,000
2,250
2,500
m/z

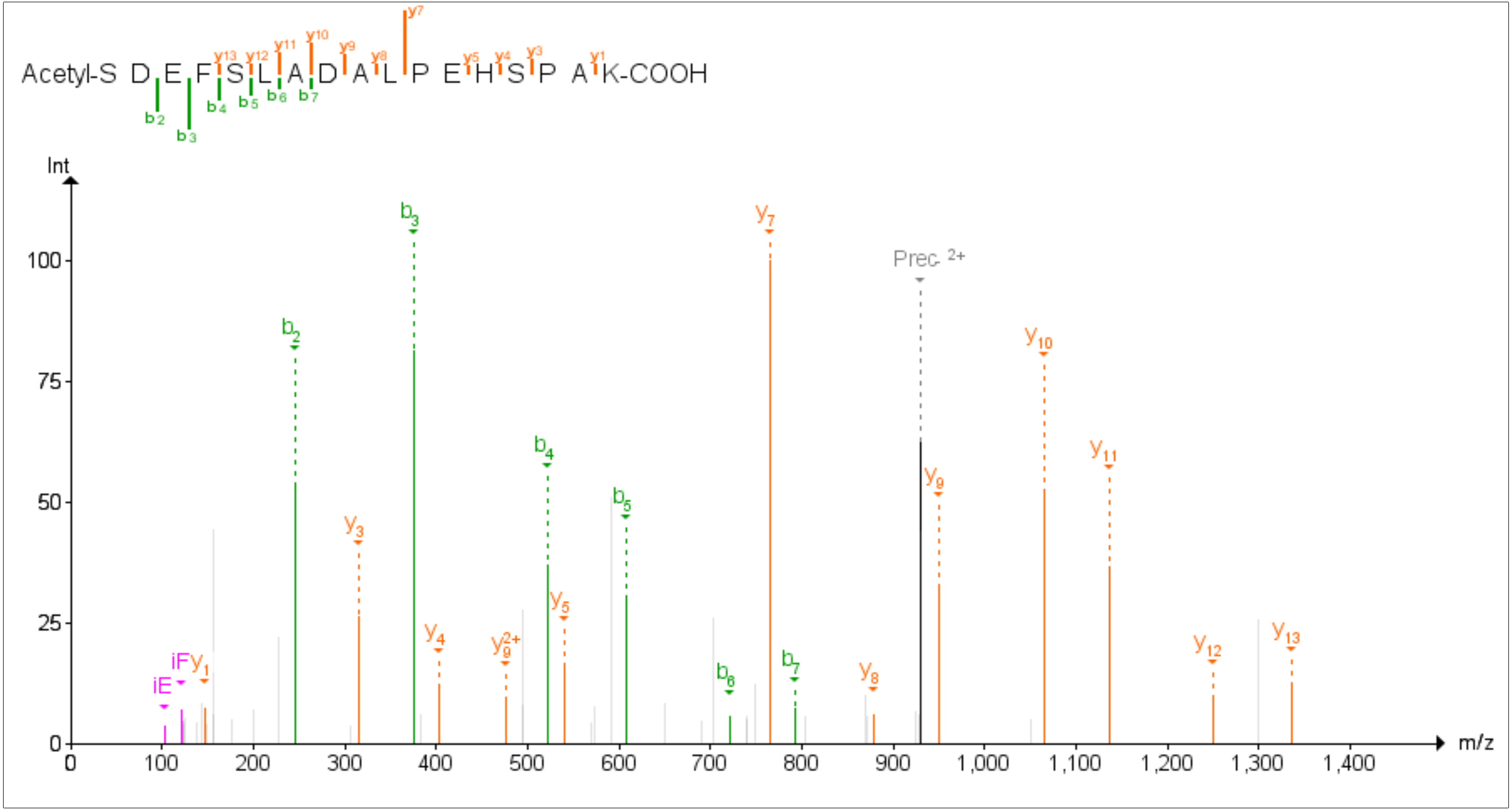

Acetyl-S D E F S L A D A L P E H S P A K-COOH
b2 b3 b4 b5 b6 b7
y13 y12 y11 y10 y9 y8 y7 y5 y4 y3 y1
Int
m/z
Prec. 2+
iE
iF
y9 2+
0
25
50
75
100
0
100
200
300
400
500
600
700
800
900
1,000
1,100
1,200
1,300
1,400

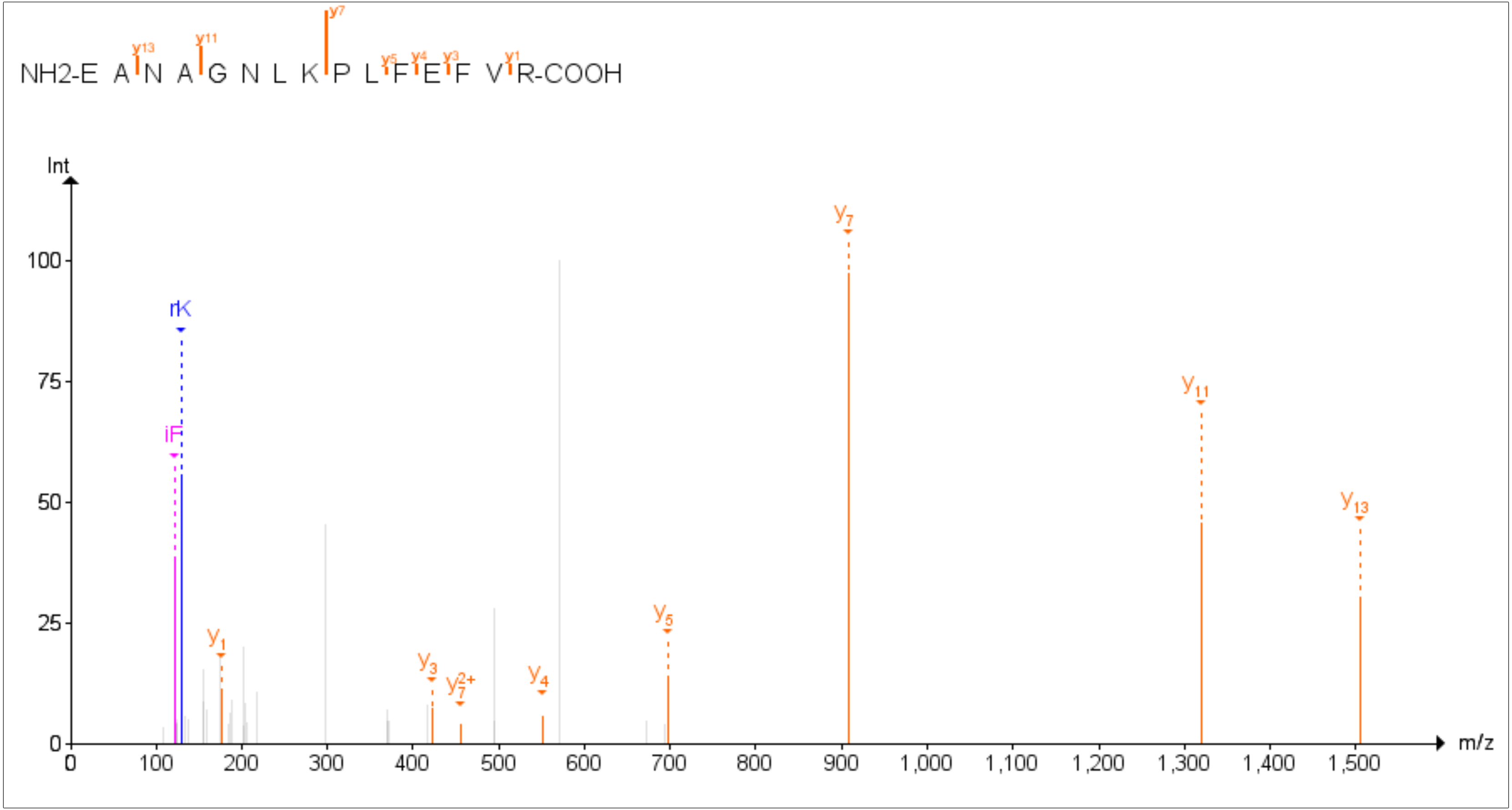

NH2-E A N A G N L K P L F E F V R-COOH
y13
y11
y7
y5
y4
y3
y1
Int
m/z
rK
iF
y1
y3
y7 2+
y4
y5
y7
y11
y13
0
25
50
75
100
0
100
200
300
400
500
600
700
800
900
1,000
1,100
1,200
1,300
1,400
1,500

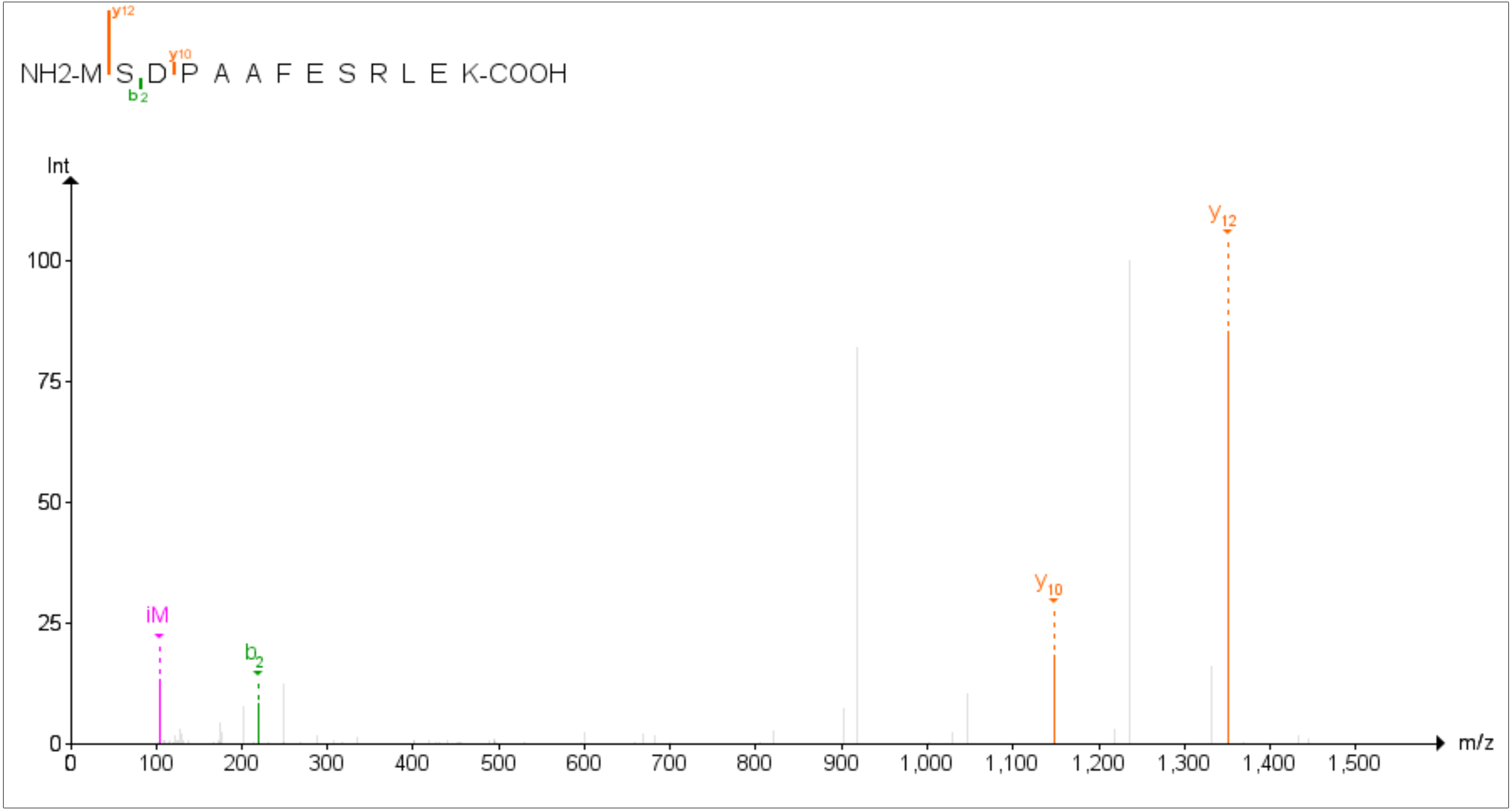

NH2-M S D P A A F E S R L E K-COOH
y12
y10
b2
Int
100
75
50
25
0
iM
b2
y10
y12
0
100
200
300
400
500
600
700
800
900
1,000
1,100
1,200
1,300
1,400
1,500
m/z

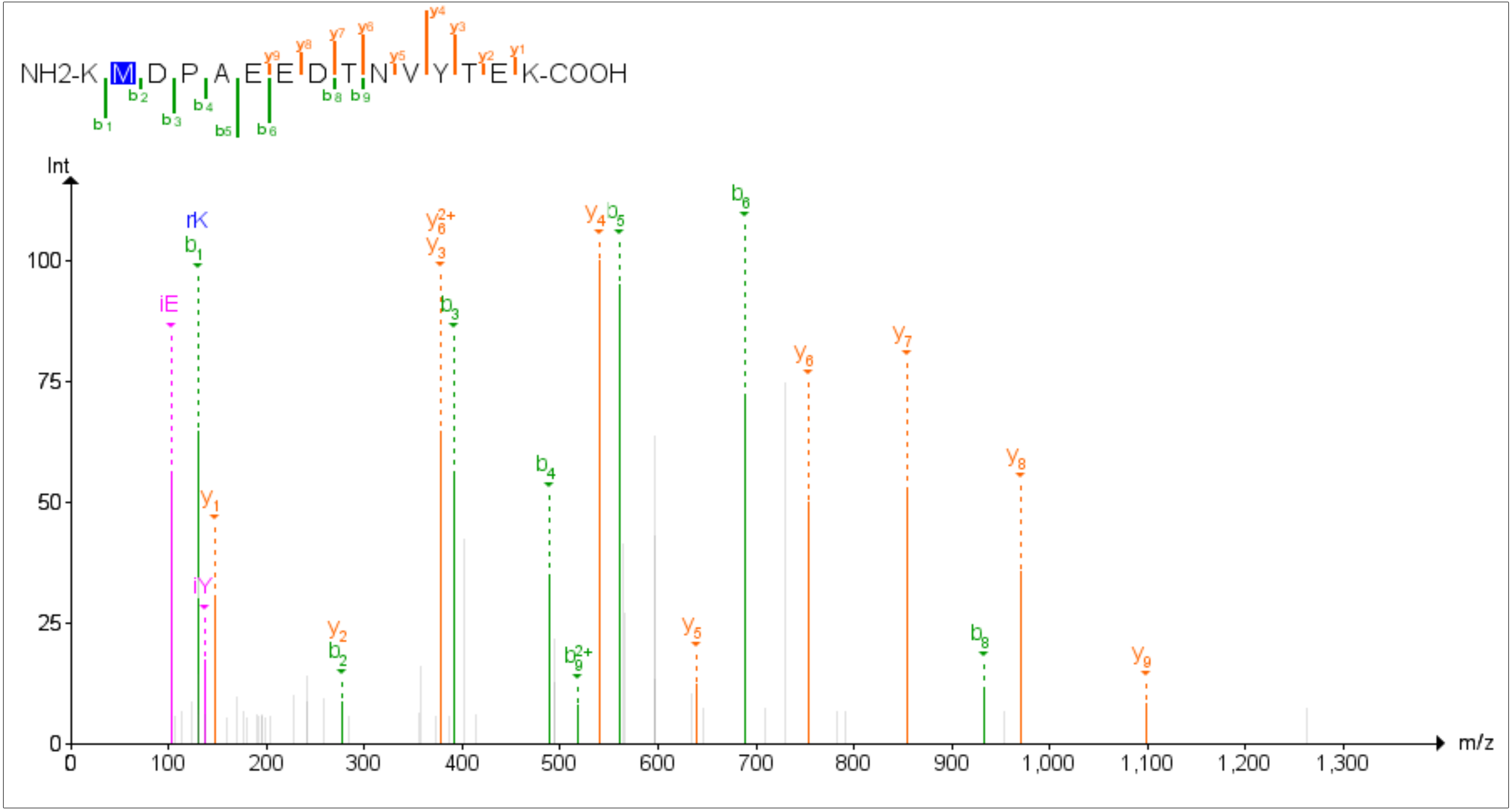
NH2-K M D P A E E D T N V Y T E K-COOH
b1 b2 b3 b4 b5 b6 b8 b9
y9 y8 y7 y6 y5 y4 y3 y2 y1
Int
100
75
50
25
0
rK
b1
iE
y1
iY
y2
b2
y6 2+
y3
b3
b4
b9 2+
y4
b5
y5
b6
y6
y7
b8
y8
y9
0 100 200 300 400 500 600 700 800 900 1,000 1,100 1,200 1,300
m/z

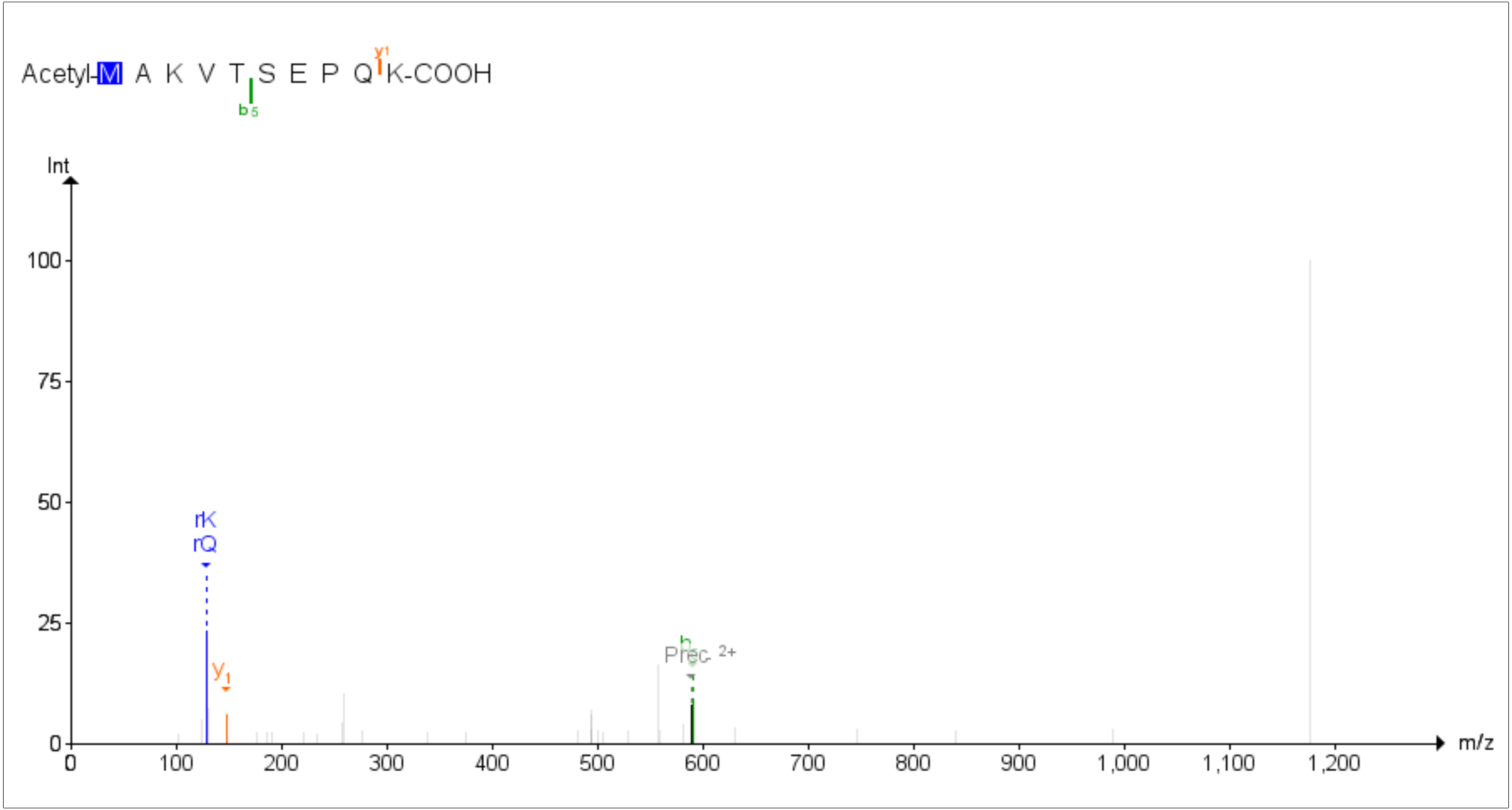
Acetyl-M A K V T S E P Q K-COOH
y1
b5
Int
100
75
50
25
0
rK
rQ
y1
Prec 2+
0
100
200
300
400
500
600
700
800
900
1,000
1,100
1,200
m/z

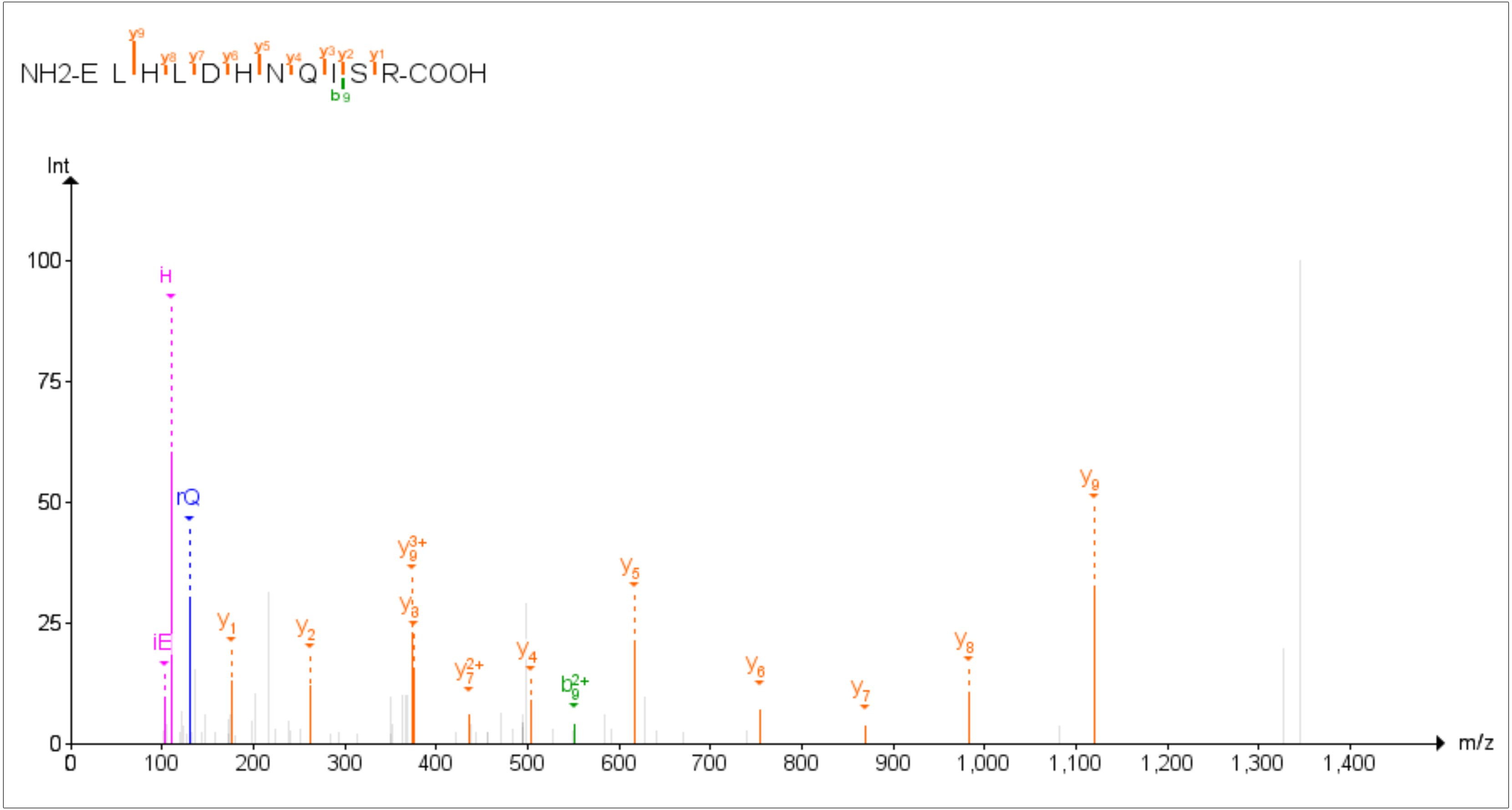

NH2-E L H L D H N Q I S R-COOH
y9 y8 y7 y6 y5 y4 y3 y2 y1
b9
Int
m/z
iH
rQ
iE
y1
y2
y3
y9 3+
y7 2+
y4
b9 2+
y5
y6
y7
y8
y9
0
25
50
75
100
0
100
200
300
400
500
600
700
800
900
1,000
1,100
1,200
1,300
1,400

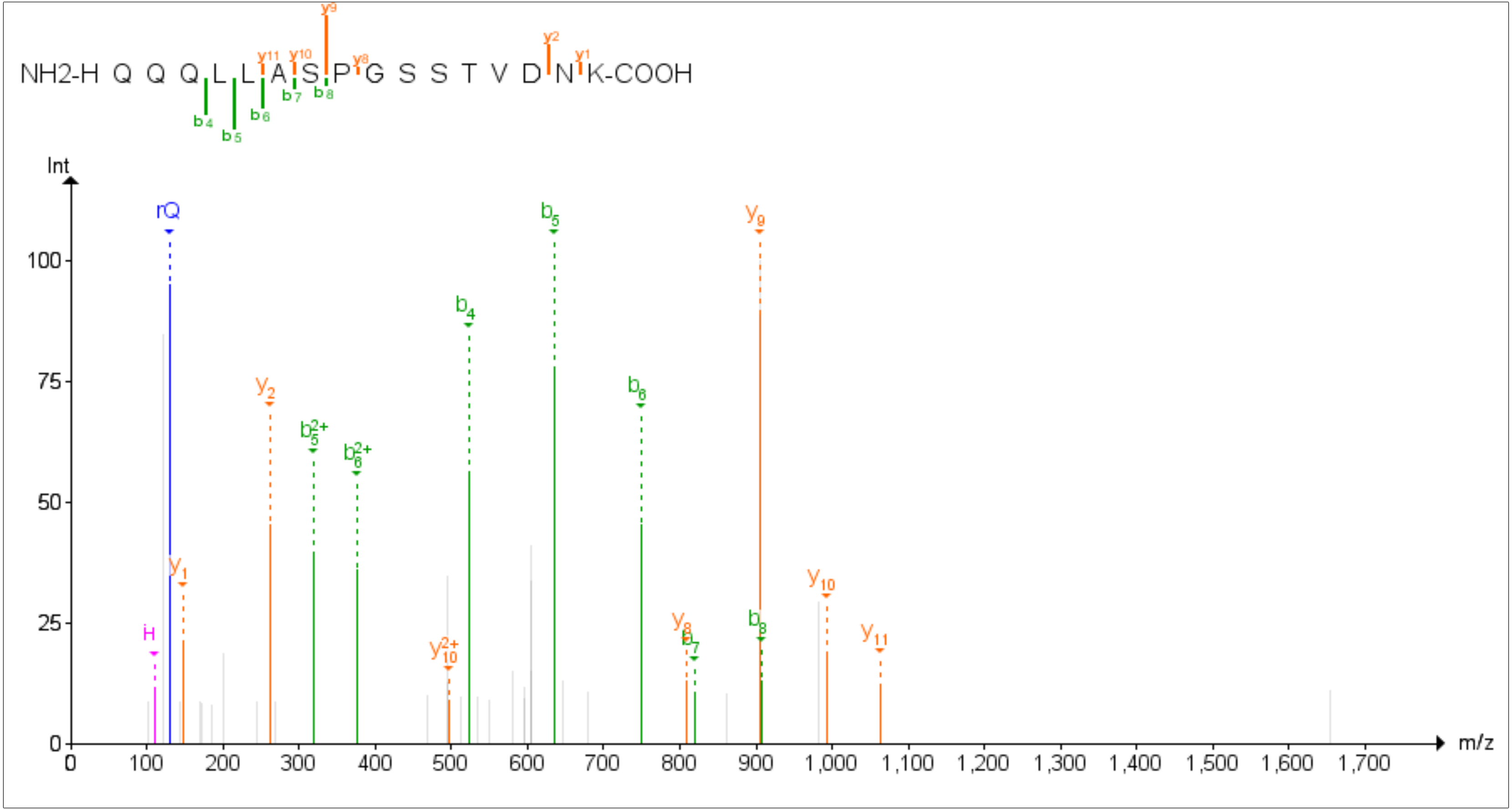
NH2-H Q Q Q L L A S P G S S T V D N K-COOH
y11 y10 y9 y8 y2 y1
b4 b5 b6 b7 b8
Int
rQ
b5
y9
b4
b6
y2
b5 2+
b6 2+
y1
H
y10 2+
y8
b7
b8
y10
y11
0
25
50
75
100
0 100 200 300 400 500 600 700 800 900 1,000 1,100 1,200 1,300 1,400 1,500 1,600 1,700
m/z

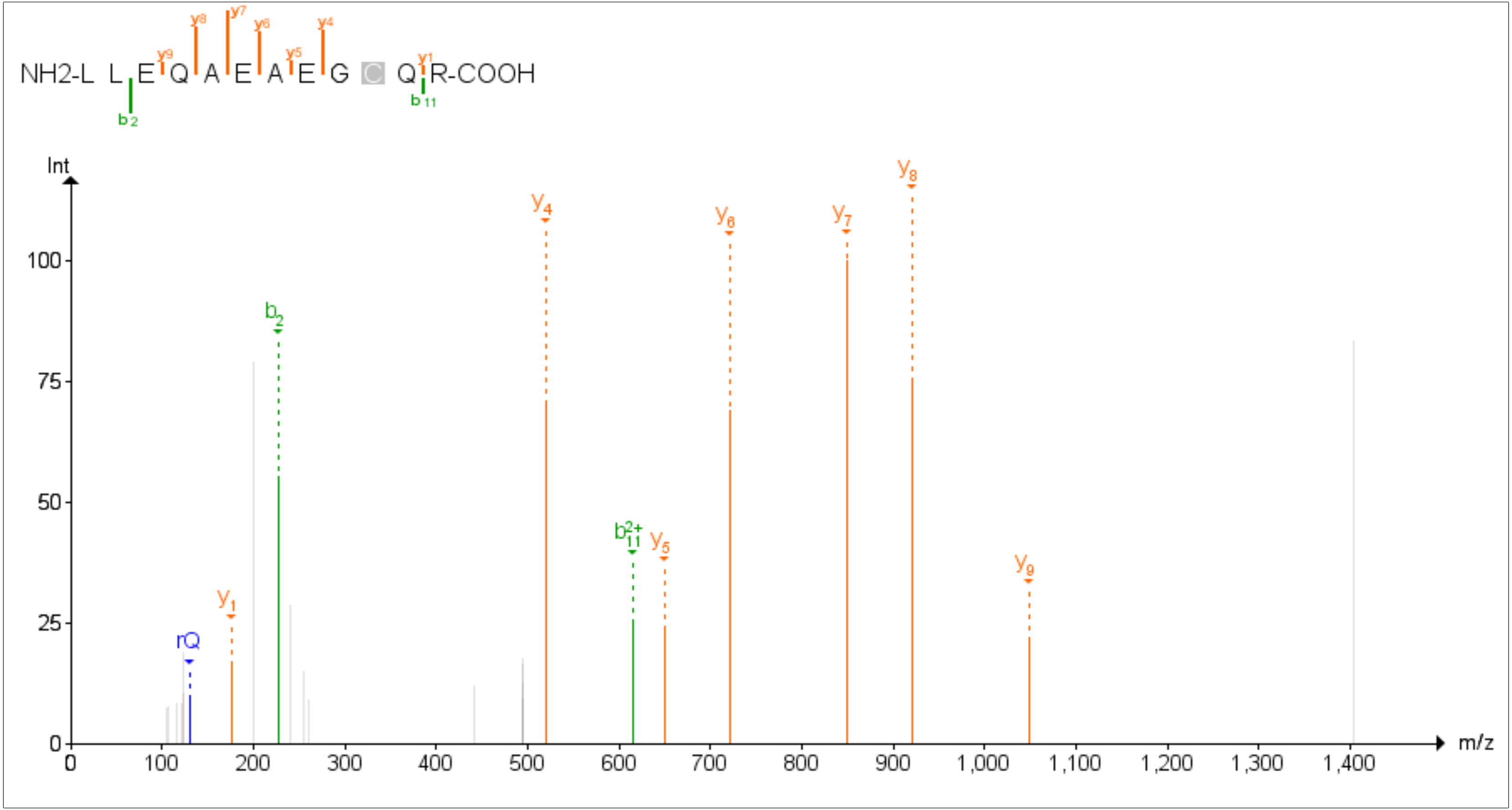

NH2-L L E Q A E A E G C Q R-COOH
b2
b11
y1
y4
y5
y6
y7
y8
y9
Int
100
75
50
25
0
rQ
y_1
b_2
y_4
b_{11}^{2+}
y_5
y_6
y_7
y_8
y_9
0
100
200
300
400
500
600
700
800
900
1,000
1,100
1,200
1,300
1,400
m/z

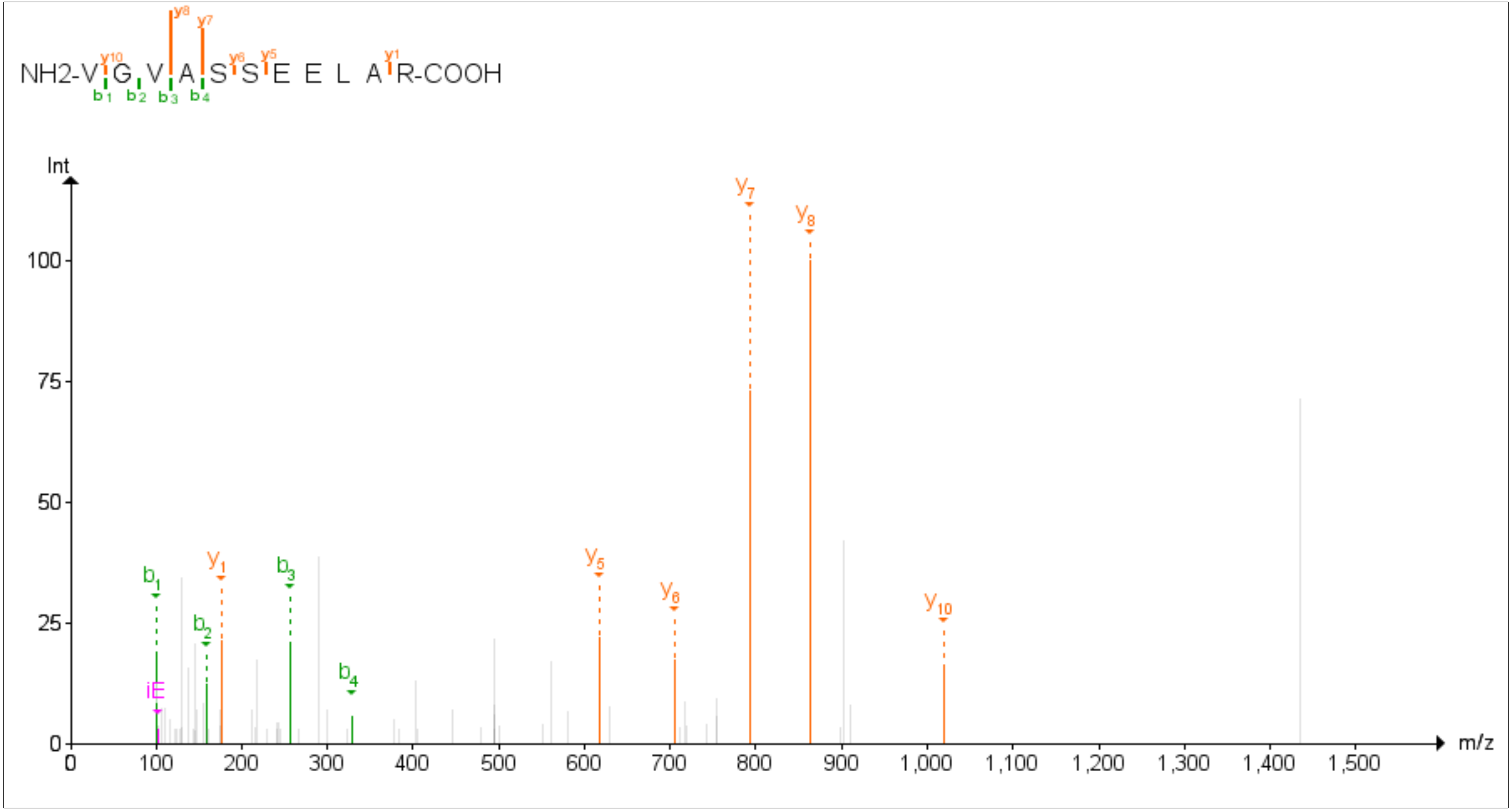
NH2-V G V A S S E E L A R-COOH
y10 y8 y7 y6 y5 y1
b1 b2 b3 b4
Int
0
25
50
75
100
b1 b2 b3 b4 iE
y1 y5 y6 y7 y8 y10
0 100 200 300 400 500 600 700 800 900 1,000 1,100 1,200 1,300 1,400 1,500
m/z

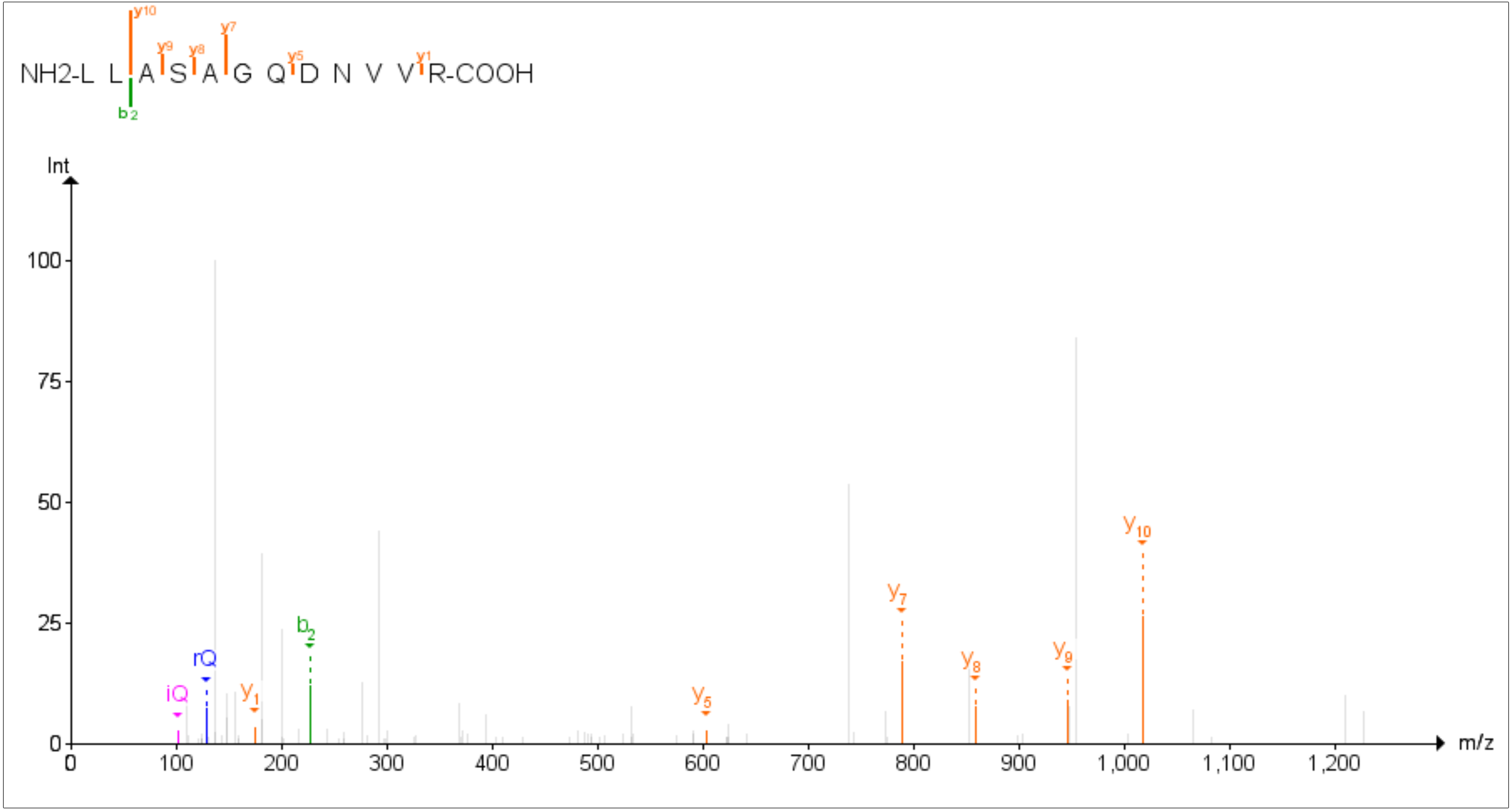
NH2-L L A S A G Q D N V V R-COOH
y10
y9
y8
y7
y5
y1
b2
Int
100
75
50
25
0
iQ
rQ
y1
b2
y5
y7
y8
y9
y10
0
100
200
300
400
500
600
700
800
900
1,000
1,100
1,200
m/z

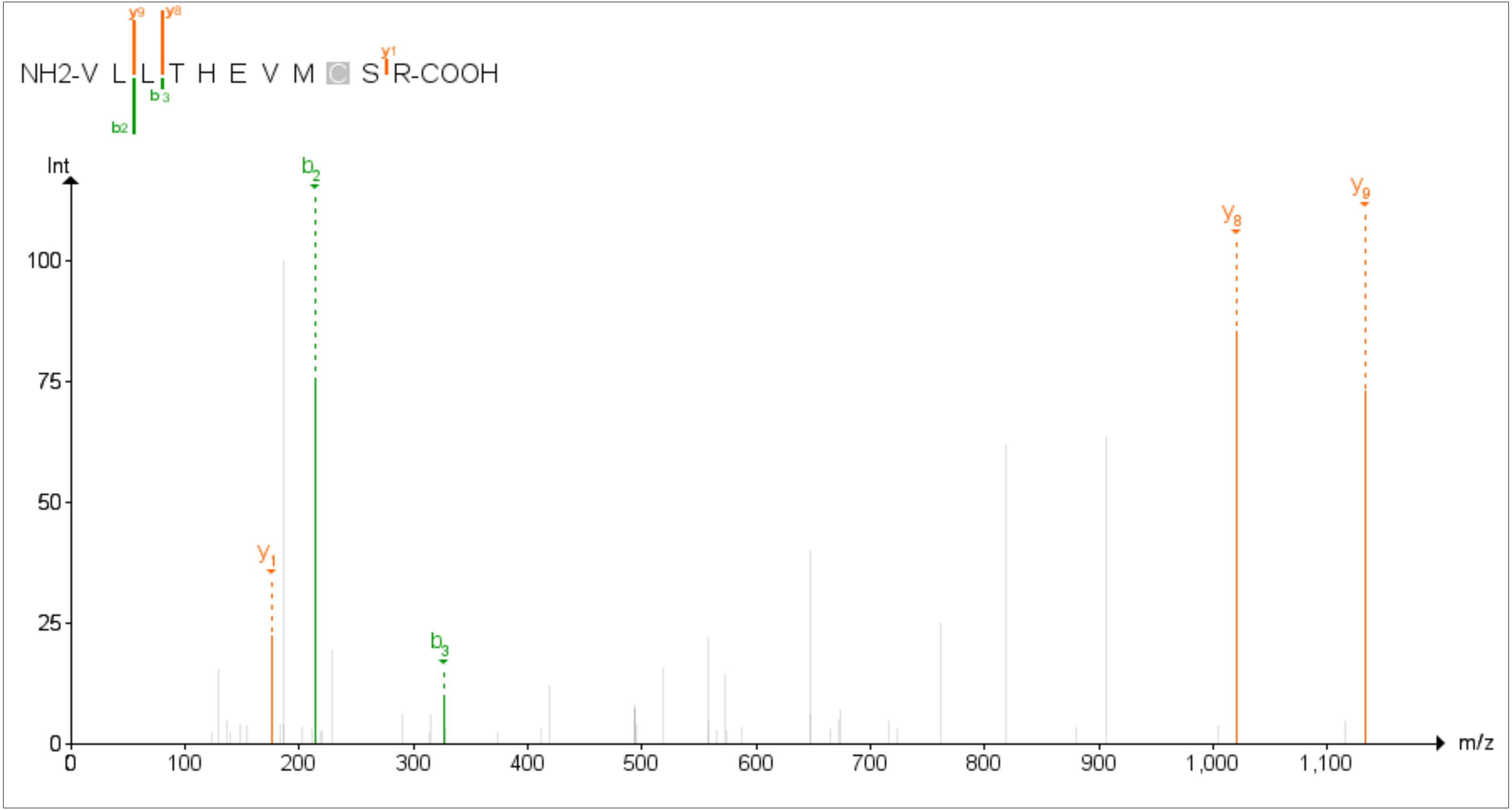

NH2-V L L T H E V M C S R-COOH
y9
y8
y1
b2
b3
Int
m/z
0
25
50
75
100
0
100
200
300
400
500
600
700
800
900
1,000
1,100

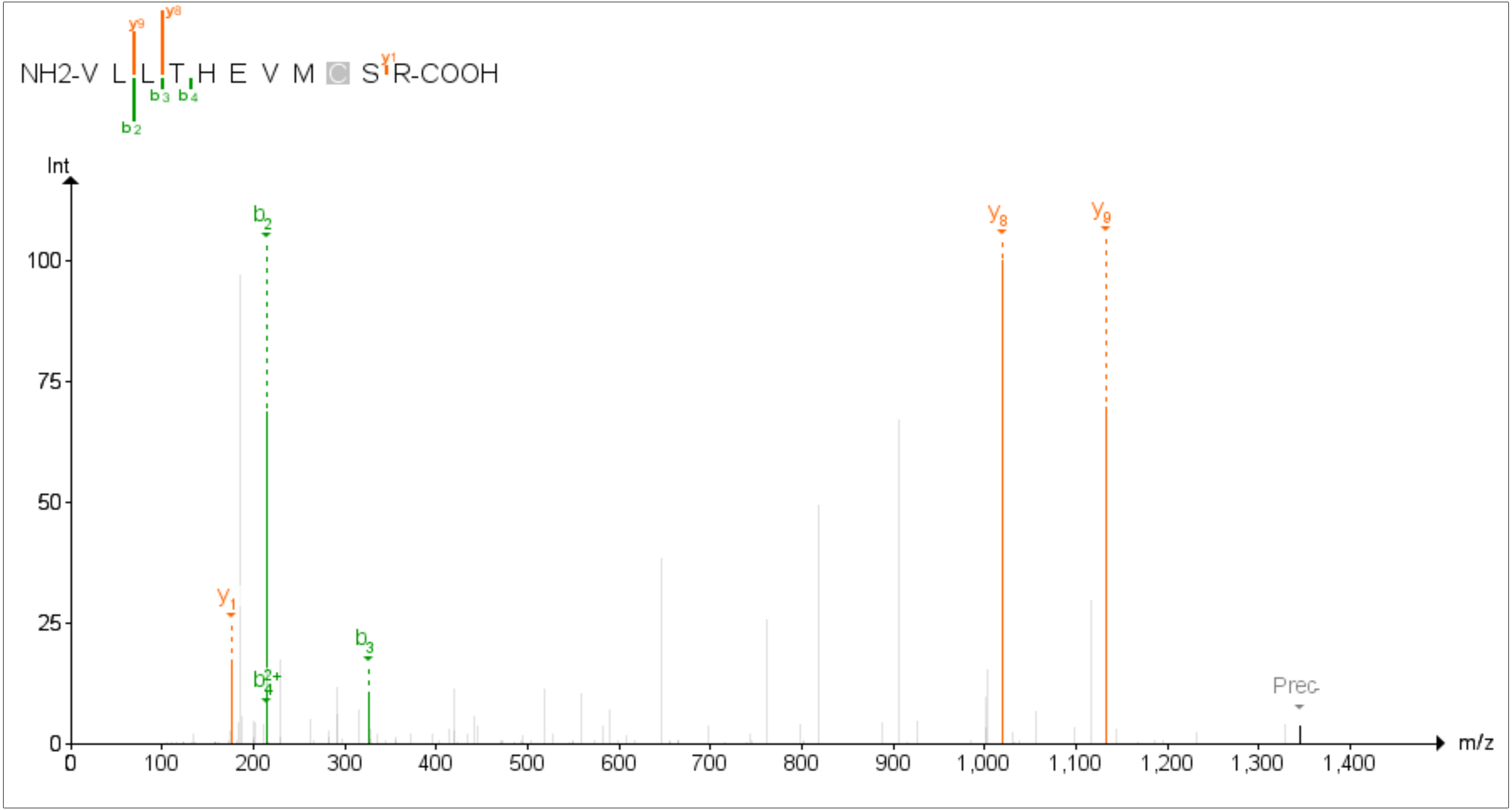
NH2-V L L T H E V M C S R-COOH
y9
y8
y1
b2
b3
b4
Int
100
75
50
25
0
b2
y8
y9
y1
b3
b4 2+
Prec.
m/z
0
100
200
300
400
500
600
700
800
900
1,000
1,100
1,200
1,300
1,400

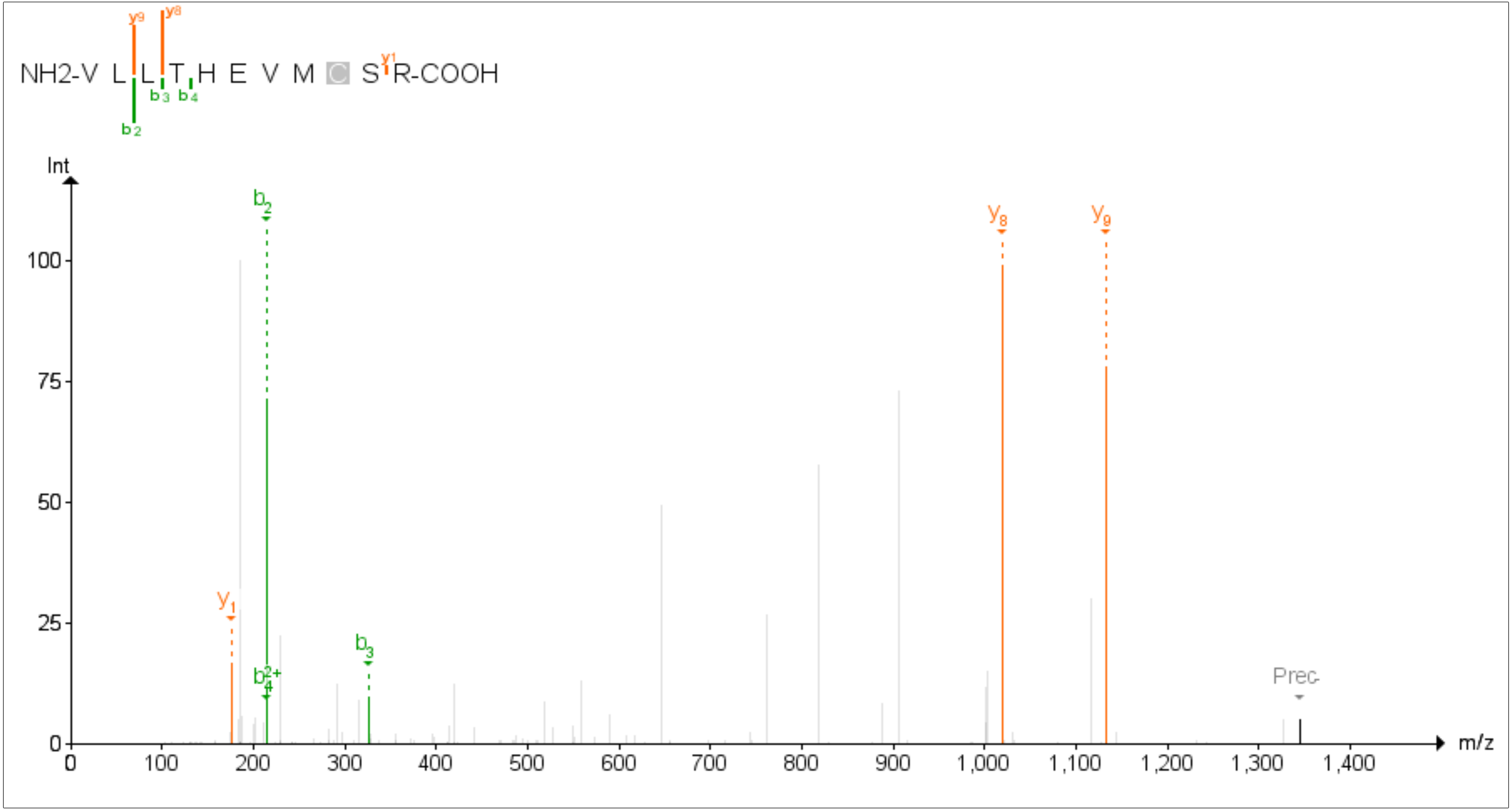

NH2-V L L T H E V M C S R-COOH
y9 y8 y1
b2 b3 b4
Int
m/z
0
25
50
75
100
0 100 200 300 400 500 600 700 800 900 1,000 1,100 1,200 1,300 1,400
b2
y8
y9
y1
b3
b4 2+
Prec

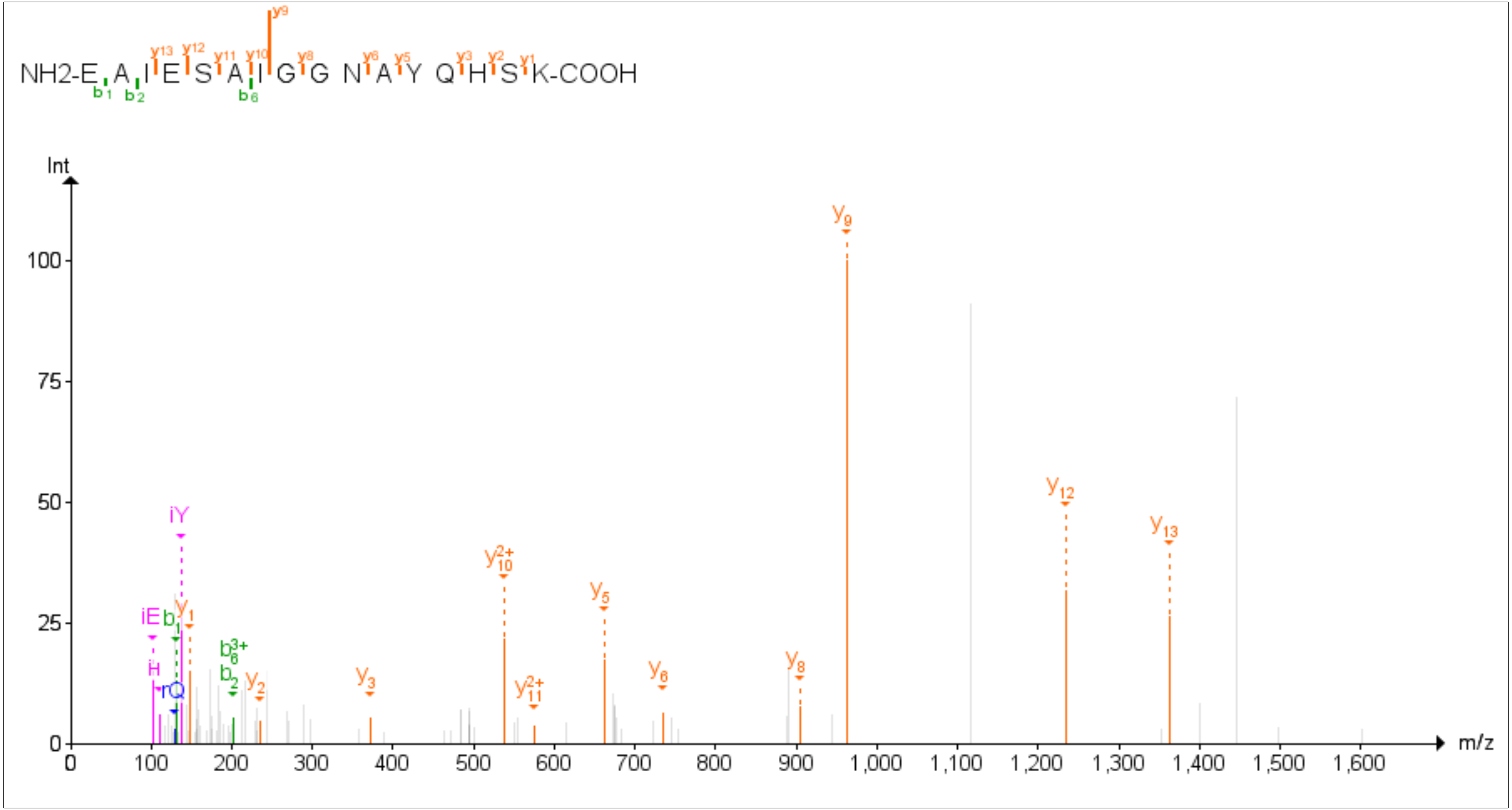

NH2-E A I E S A I G G N A Y Q H S K-COOH
b1 b2 b6
y13 y12 y11 y10 y9 y8 y6 y5 y3 y2 y1
Int
m/z
0
25
50
75
100
100
200
300
400
500
600
700
800
900
1,000
1,100
1,200
1,300
1,400
1,500
1,600
iY
iE
iH
rQ
b1
b2
b6 3+
y1
y2
y3
y10 2+
y11 2+
y5
y6
y8
y9
y12
y13

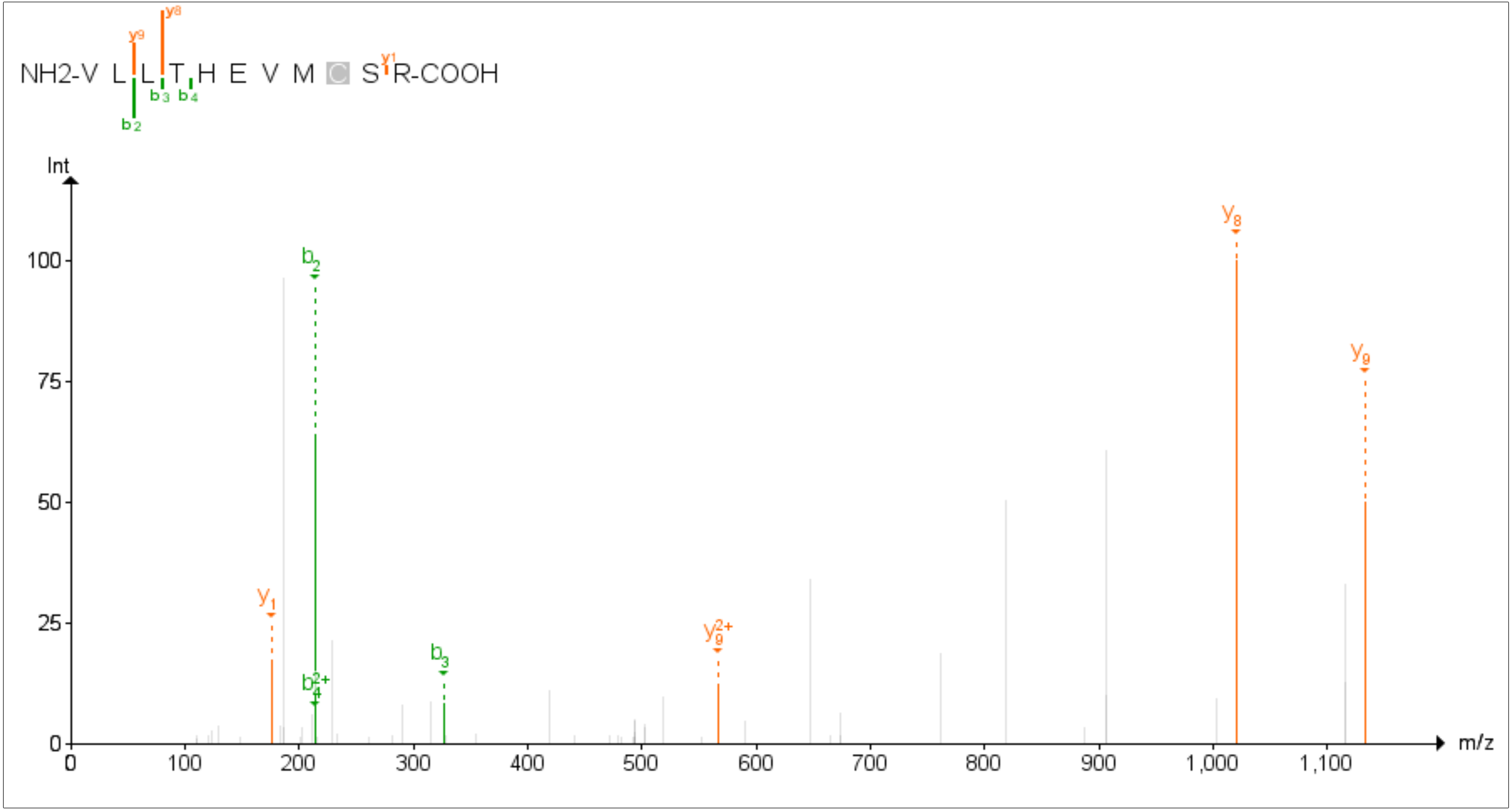

NH2-V L L T H E V M C S R-COOH
y9
y8
y1
b2
b3
b4
Int
100
75
50
25
0
m/z
0
100
200
300
400
500
600
700
800
900
1,000
1,100
y8
y9
b2
y1
b4 2+
b3
y9 2+

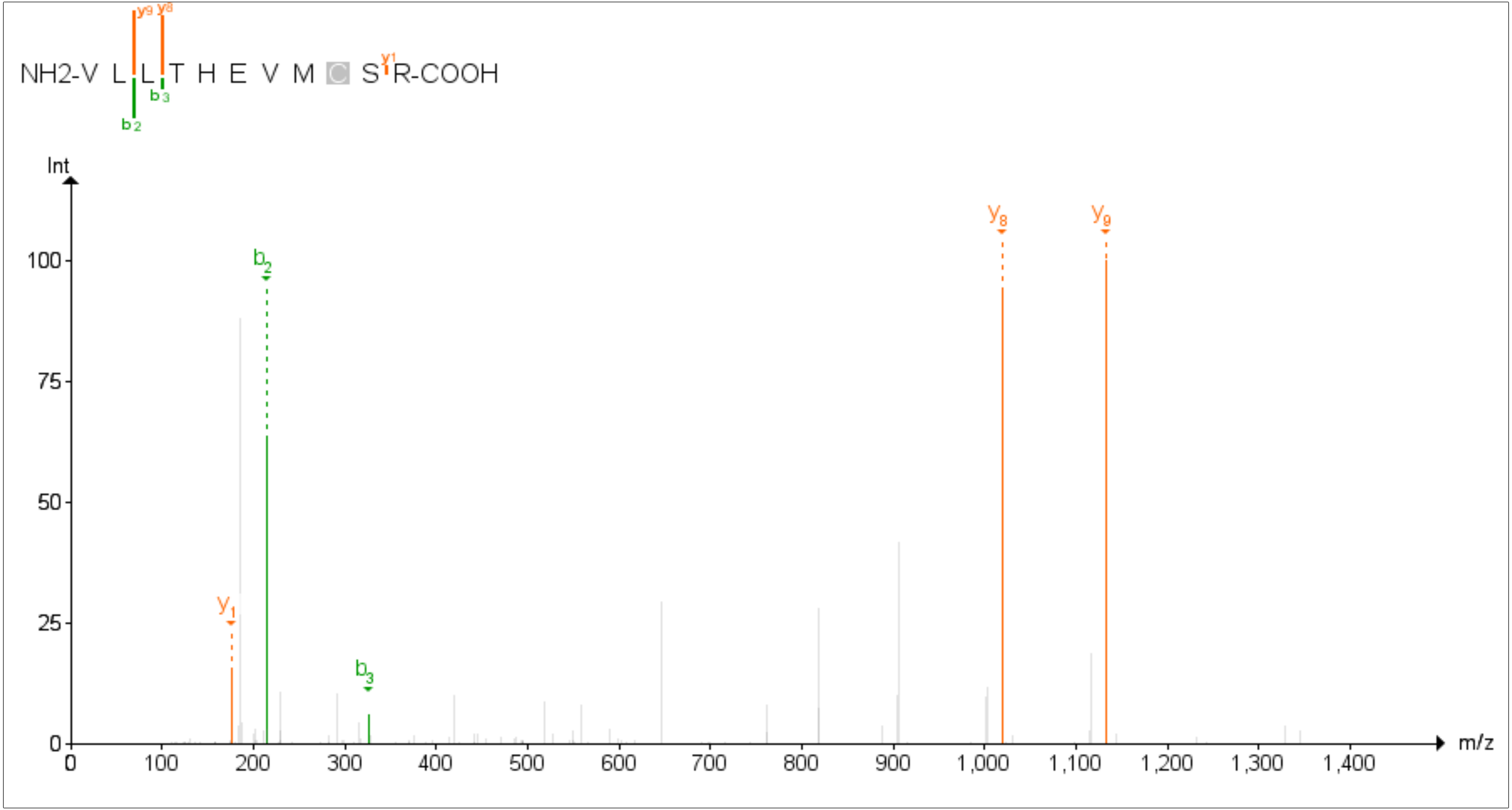

NH2-V L L T H E V M C S R-COOH
y9
y8
b2
b3
y1
Int
100
75
50
25
0
b2
y1
b3
y8
y9
0
100
200
300
400
500
600
700
800
900
1,000
1,100
1,200
1,300
1,400
m/z

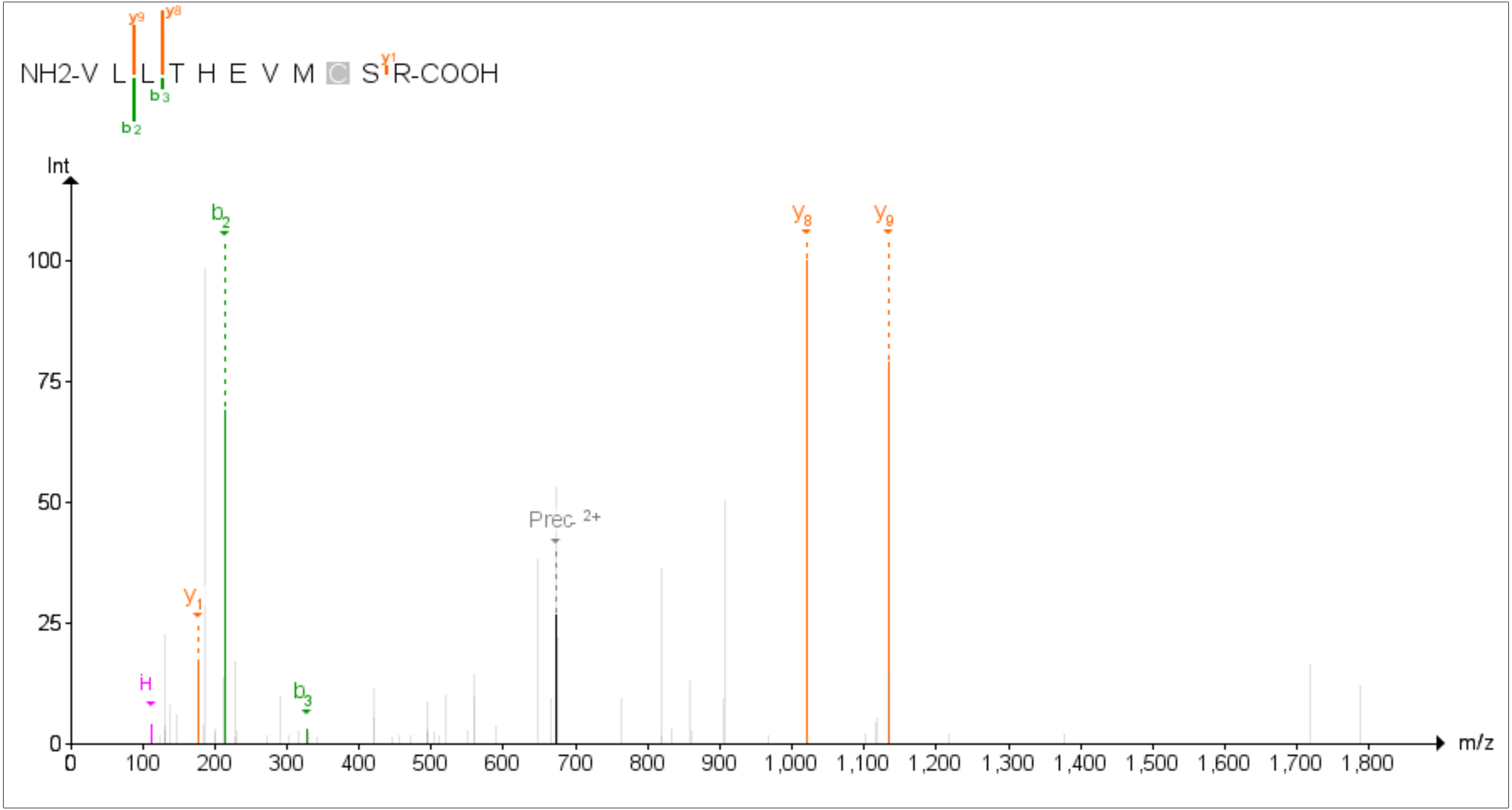

NH2-V L L T H E V M C S R-COOH
y9
y8
b2
b3
y1
Int
b2
y8
y9
Prec. 2+
y1
H
b3
100
75
50
25
0
0
100
200
300
400
500
600
700
800
900
1,000
1,100
1,200
1,300
1,400
1,500
1,600
1,700
1,800
m/z

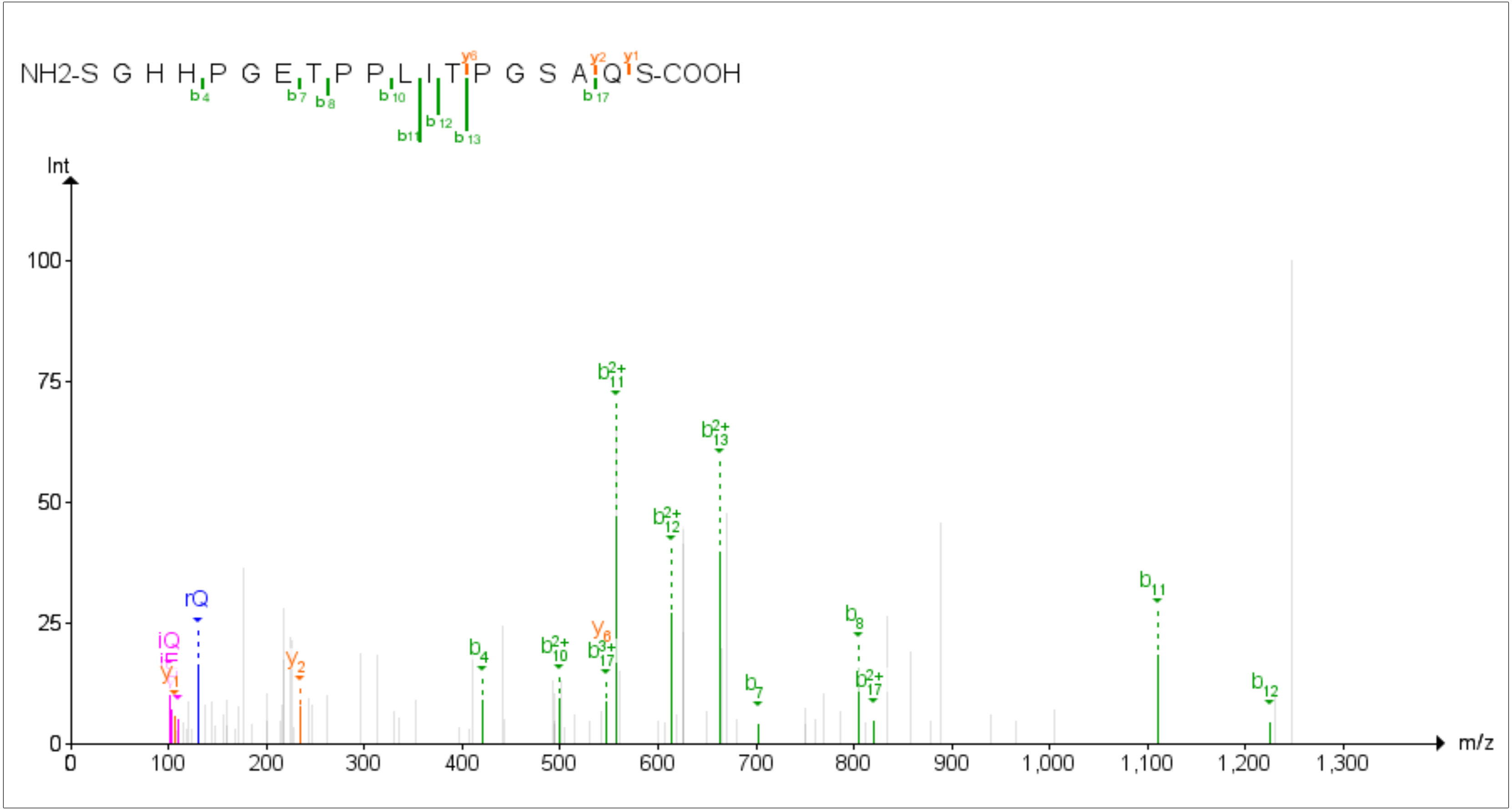
NH2-S G H H P G E T P P L I T P G S A Q S-COOH
b4 b7 b8 b10 b11 b12 b13 b17
y6 y2 y1
Int
100
75
50
25
0
m/z
0
100
200
300
400
500
600
700
800
900
1,000
1,100
1,200
1,300
rQ
iQ
y1
y2
b4
b10 2+
y6
b17 3+
b11 2+
b12 2+
b13 2+
b7
b8
b17 2+
b11
b12

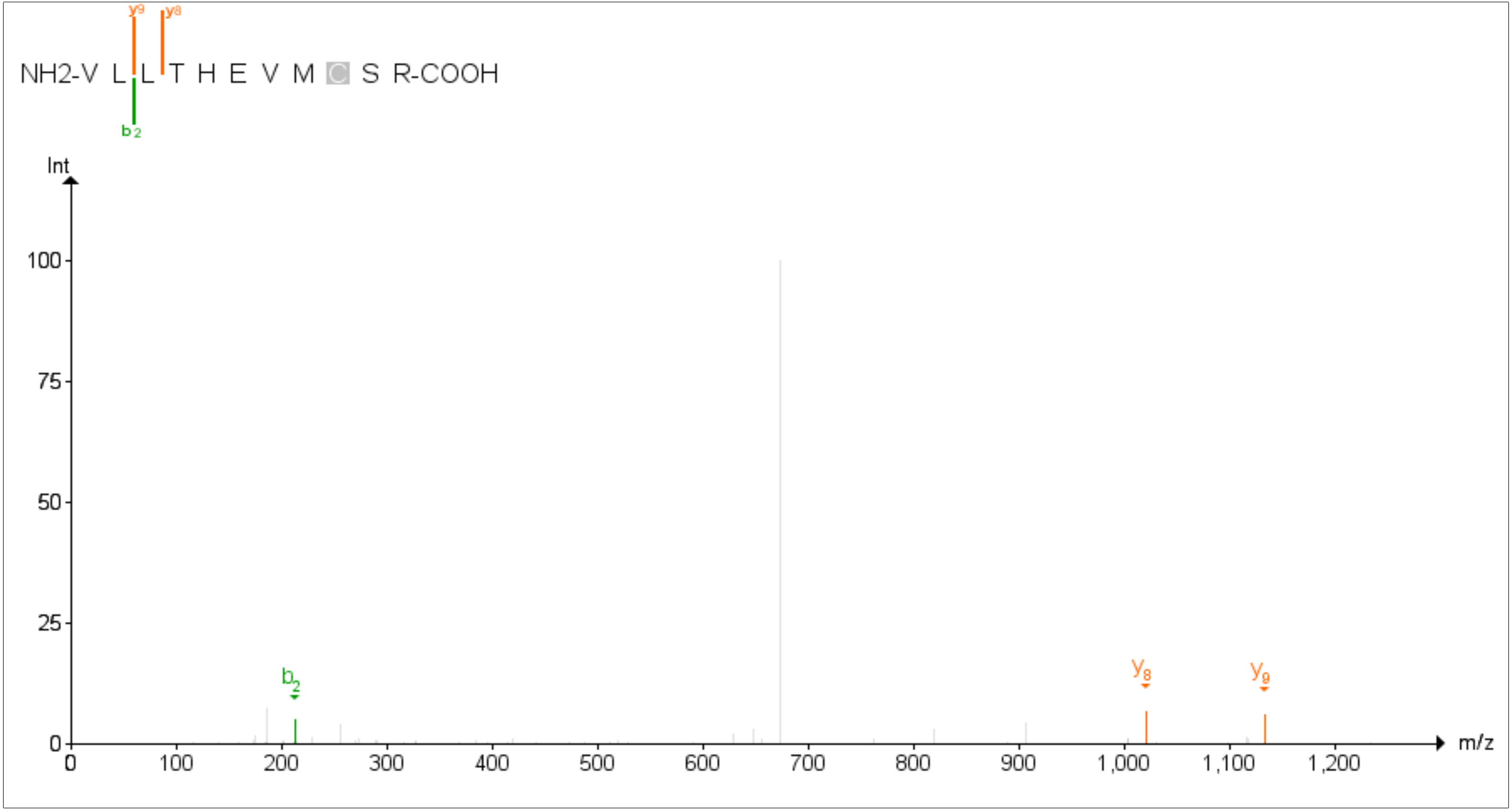

NH2-V L L T H E V M C S R-COOH
y9
y8
b2
Int
100
75
50
25
0
b2
y8
y9
0
100
200
300
400
500
600
700
800
900
1,000
1,100
1,200
m/z

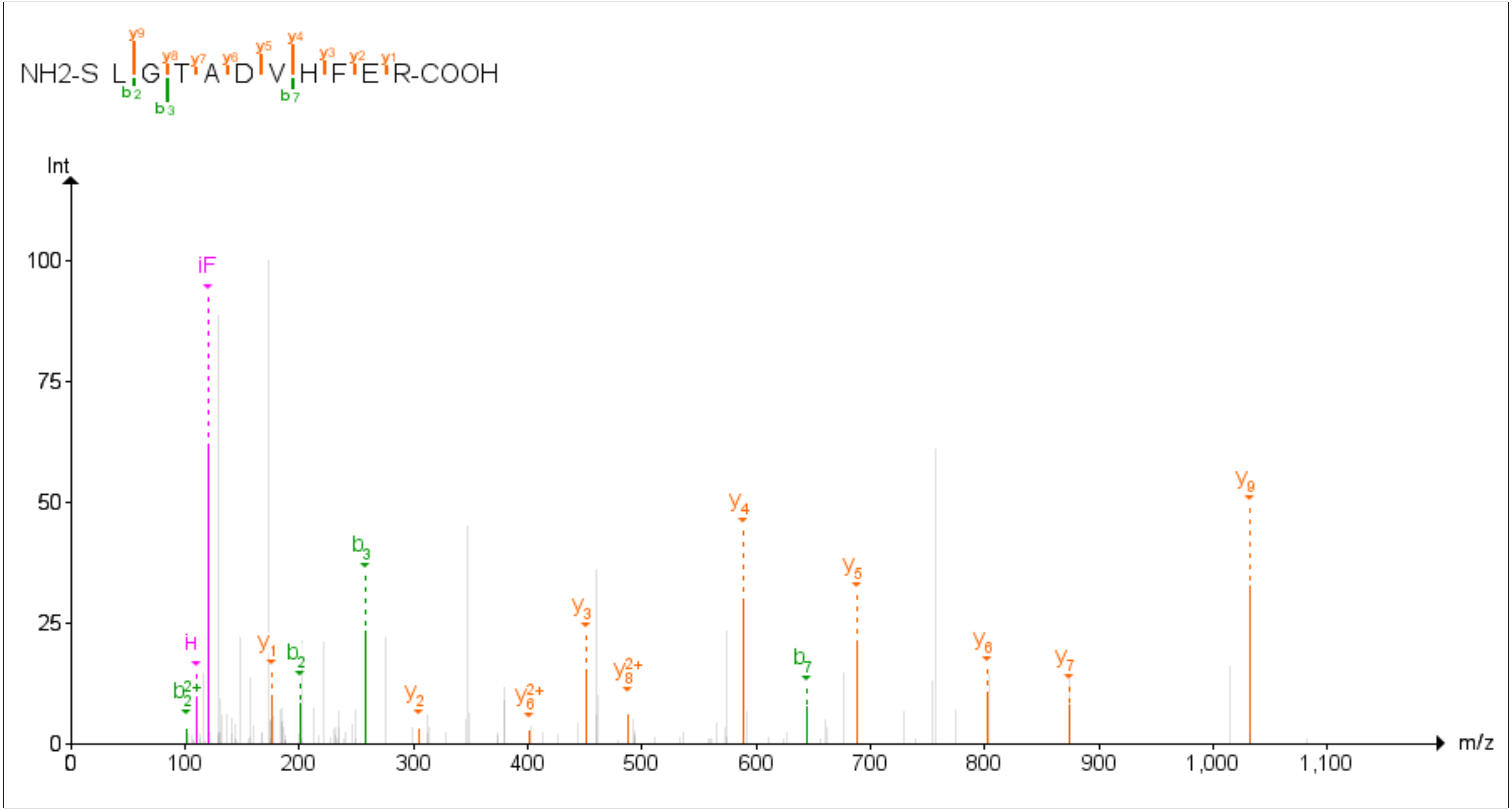

NH2-S L G T A D V H F E R-COOH
y9 y8 y7 y6 y5 y4 y3 y2 y1
b2 b3 b7
Int
100
75
50
25
0
iF
iH
b2 2+
y1
b2
b3
y2
y6 2+
y3
y8 2+
y4
b7
y5
y6
y7
y9
0 100 200 300 400 500 600 700 800 900 1,000 1,100
m/z

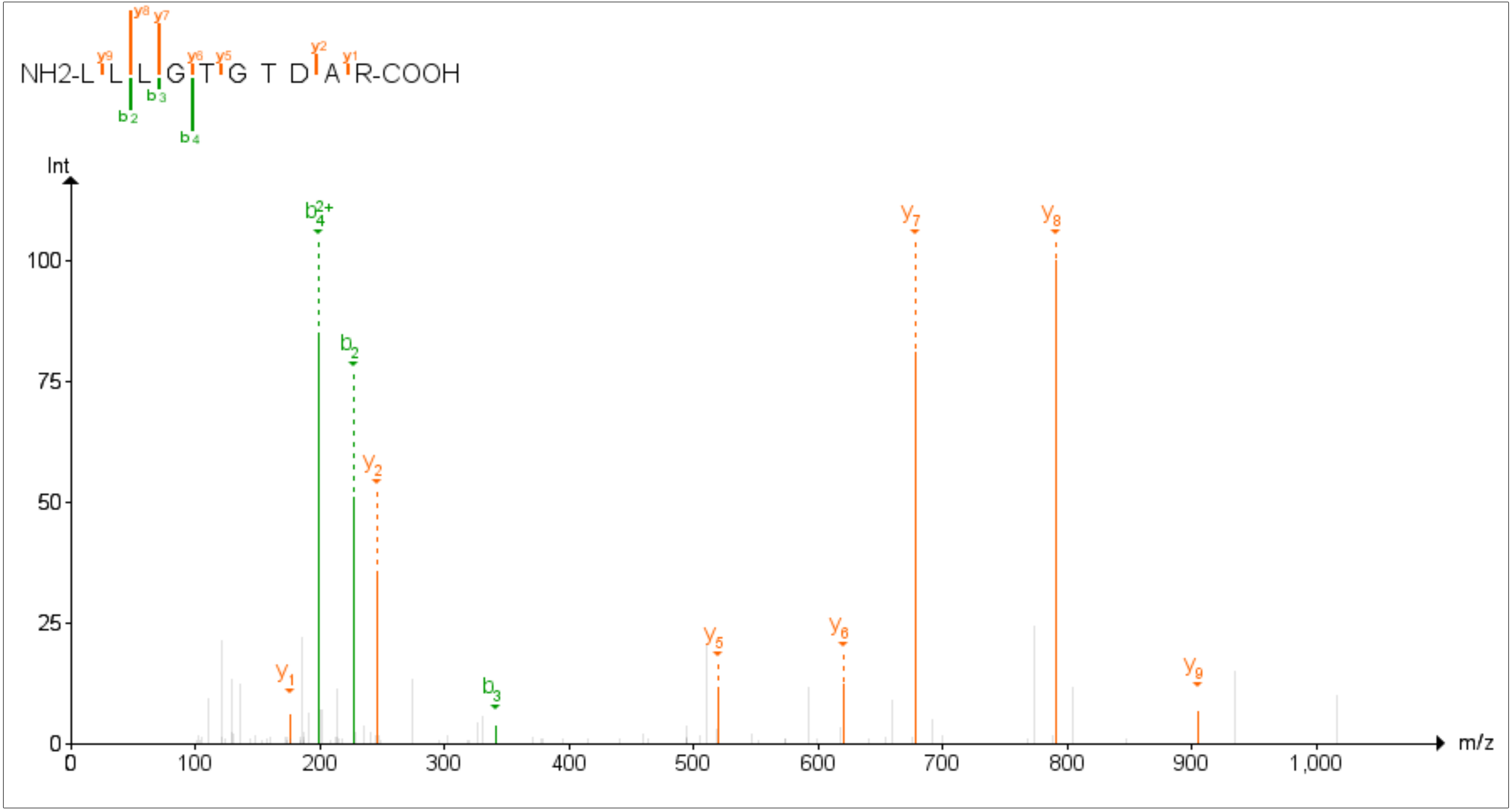

NH2-L L L G T G T D A R-COOH
y9 y8 y7 y6 y5 y2 y1
b2 b3 b4
Int
m/z
b4 2+
b2
y2
y1
b3
y5
y6
y7
y8
y9
0
25
50
75
100
0
100
200
300
400
500
600
700
800
900
1,000

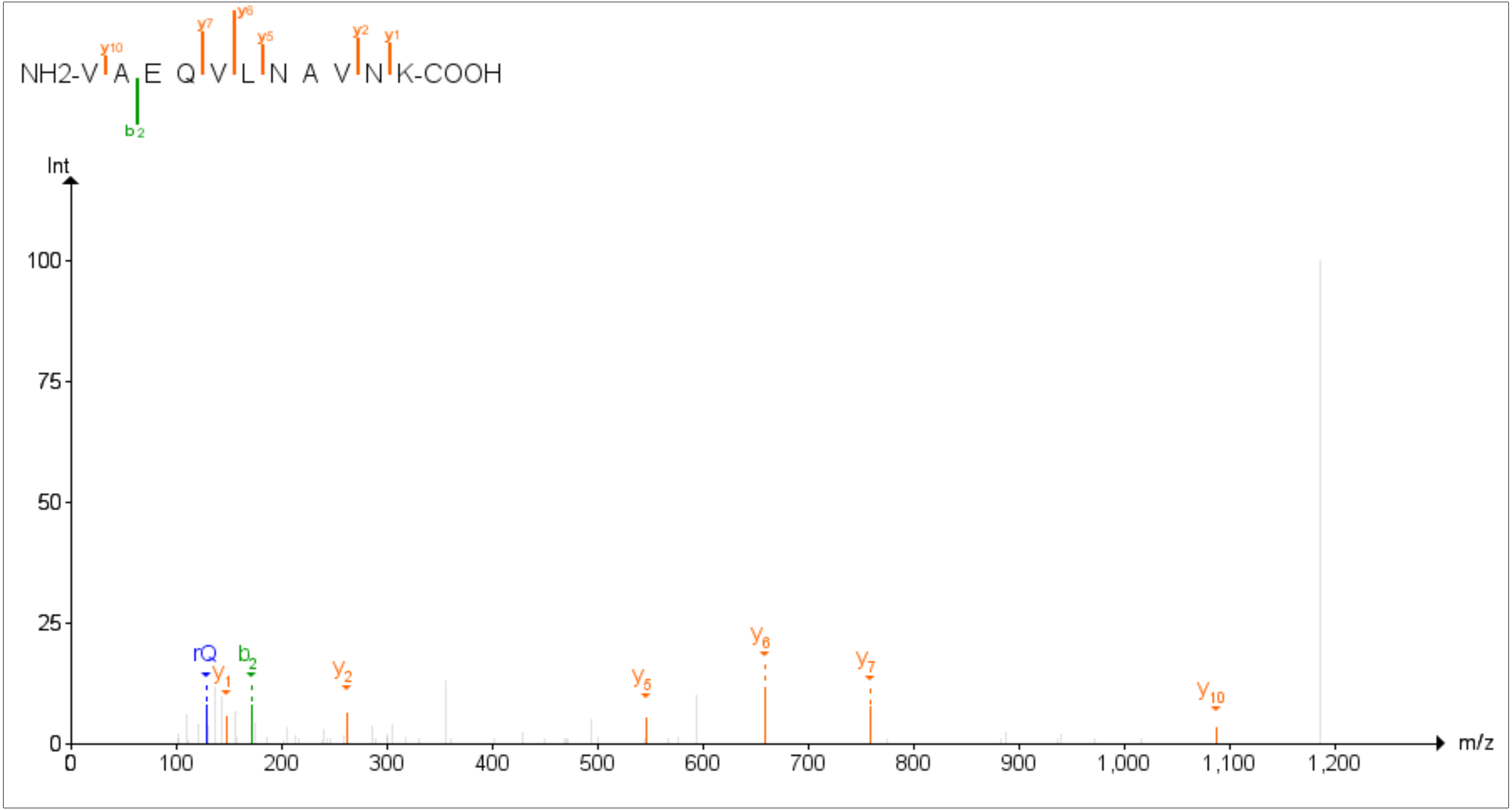

NH2-V A E Q V L N A V N K-COOH
y10
y7
y6
y5
y2
y1
b2
Int
100
75
50
25
0
rQ
y_1
b_2
y_2
y_5
y_6
y_7
y_{10}
0
100
200
300
400
500
600
700
800
900
1,000
1,100
1,200
m/z

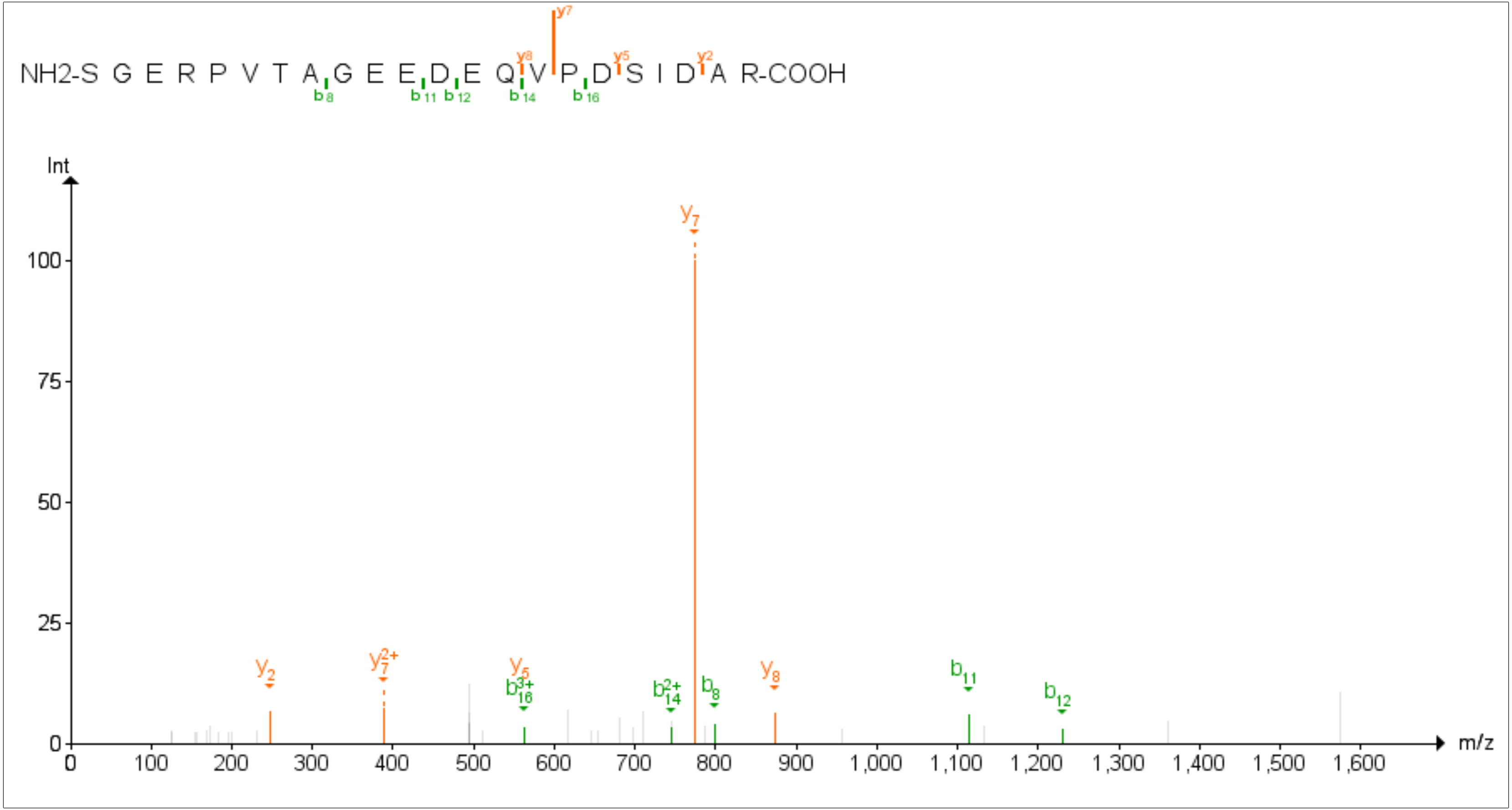

NH2-S G E R P V T A G E E D E Q V P D S I D A R-COOH
y7
y8
y5
y2
b8
b11
b12
b14
b16
Int
100
75
50
25
0
y7
y2
y7 2+
y5
b16 3+
b14 2+
b8
y8
b11
b12
m/z
0
100
200
300
400
500
600
700
800
900
1,000
1,100
1,200
1,300
1,400
1,500
1,600

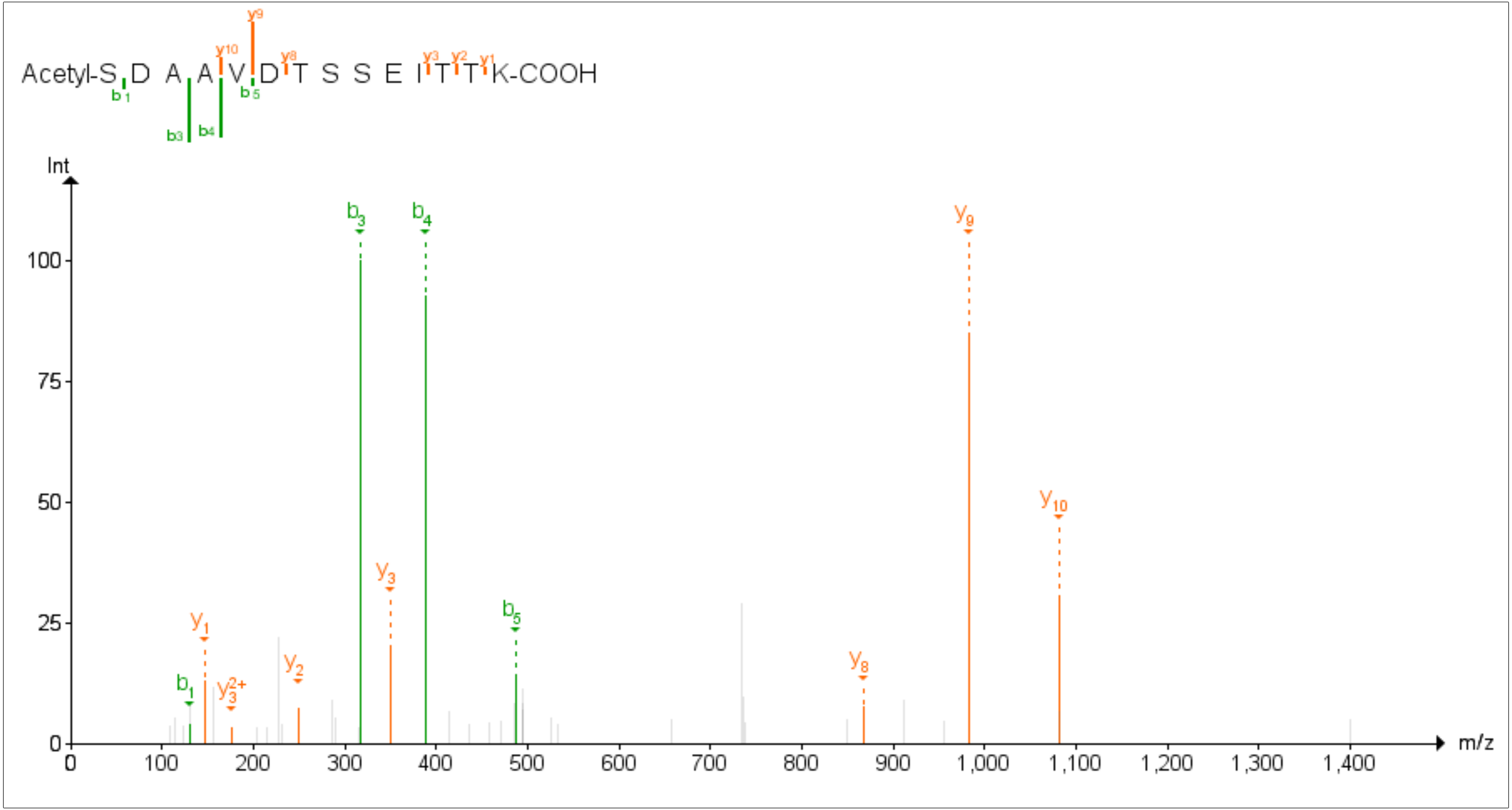
Acetyl-S D A A V D T S S E I T T K-COOH
b1
b3
b4
b5
y10
y9
y8
y3
y2
y1
Int
100
75
50
25
0
b3
b4
y9
y10
y3
b5
y1
b1
y3 2+
y2
y8
m/z
0
100
200
300
400
500
600
700
800
900
1,000
1,100
1,200
1,300
1,400

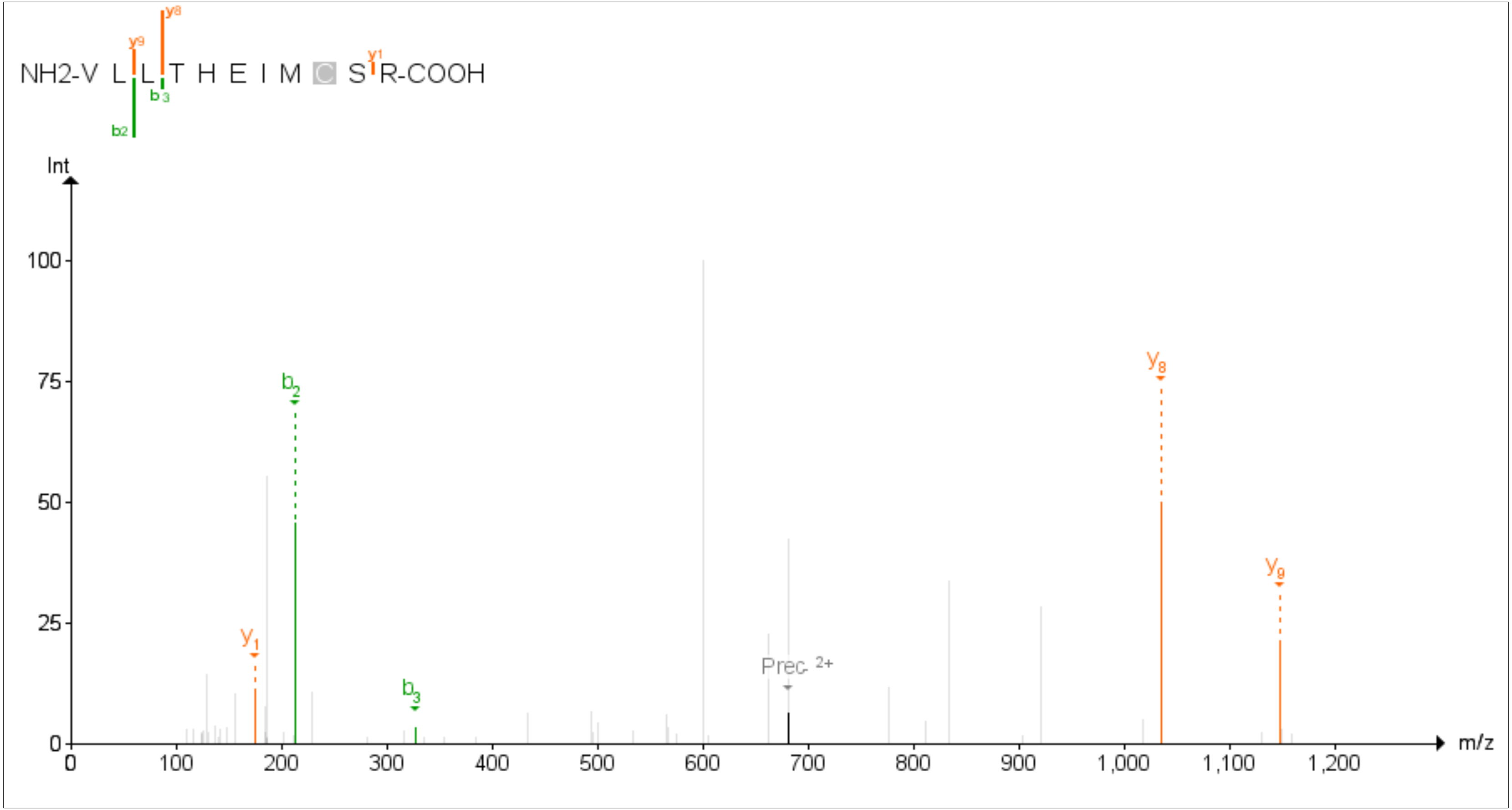

NH2-V L L T H E I M C S R-COOH
y9
y8
y1
b2
b3
Int
100
75
50
25
0
b2
y8
y9
y1
b3
Prec 2+
0
100
200
300
400
500
600
700
800
900
1,000
1,100
1,200
m/z

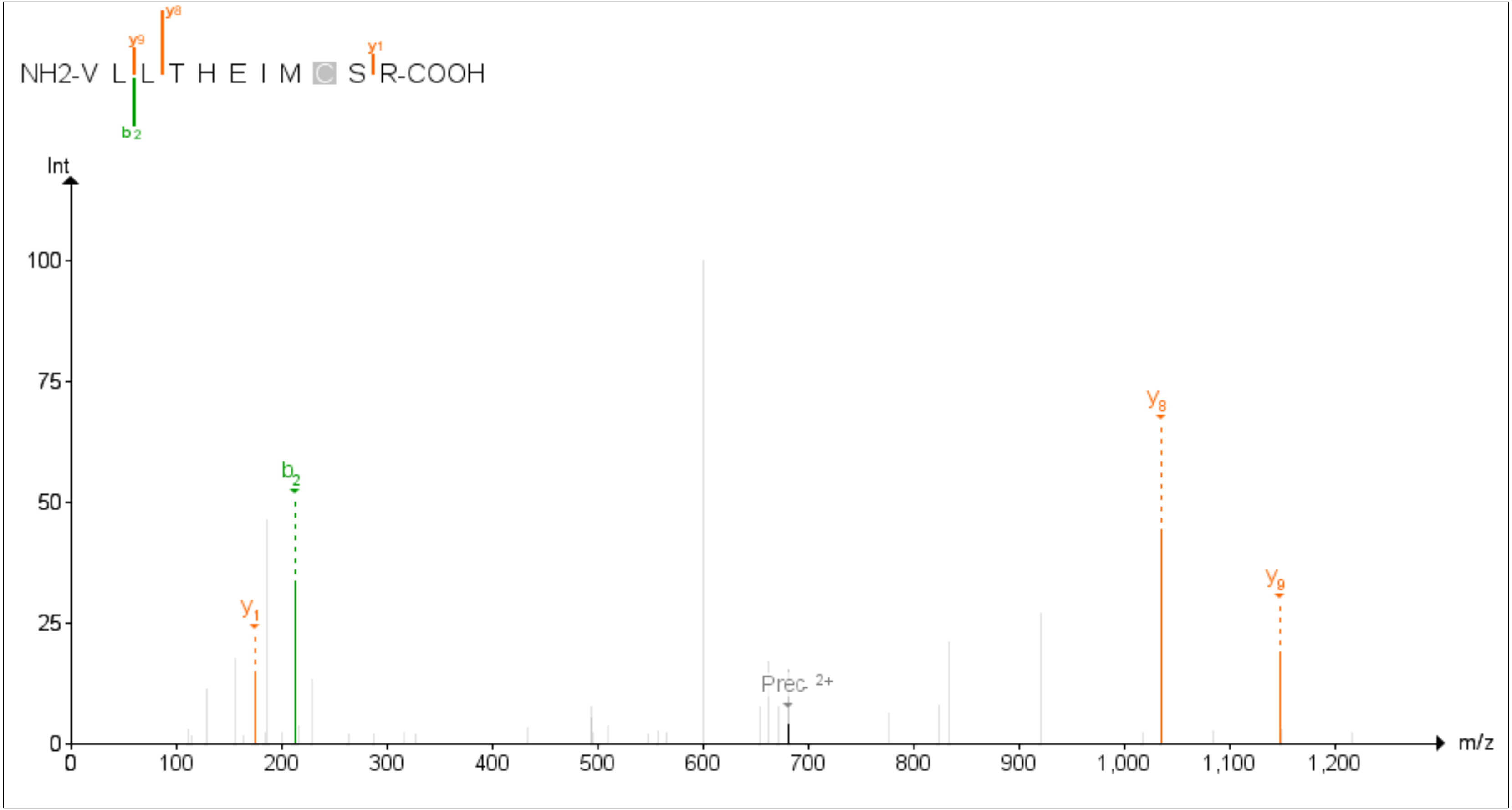

NH2-V L L T H E I M C S R-COOH
y9
y8
y1
b2
Int
100
75
50
25
0
0
100
200
300
400
500
600
700
800
900
1,000
1,100
1,200
m/z
y1
b2
Prec 2+
y8
y9

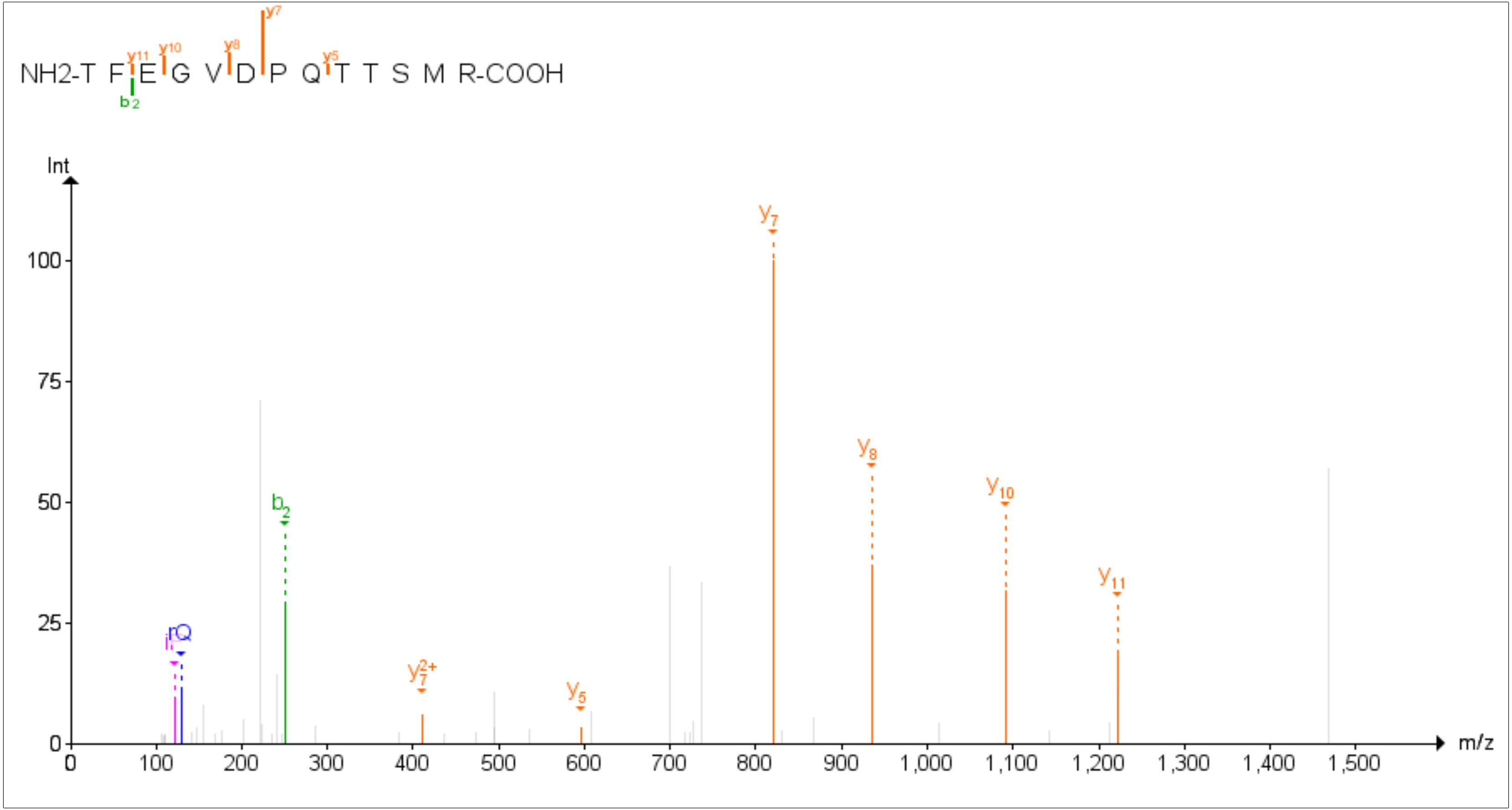

NH2-T F E G V D P Q T T S M R-COOH
y11
y10
y8
y7
y5
b2
Int
m/z
y7
y8
y10
y11
b2
y7 2+
y5
rQ
i
100
75
50
25
0
0
100
200
300
400
500
600
700
800
900
1,000
1,100
1,200
1,300
1,400
1,500

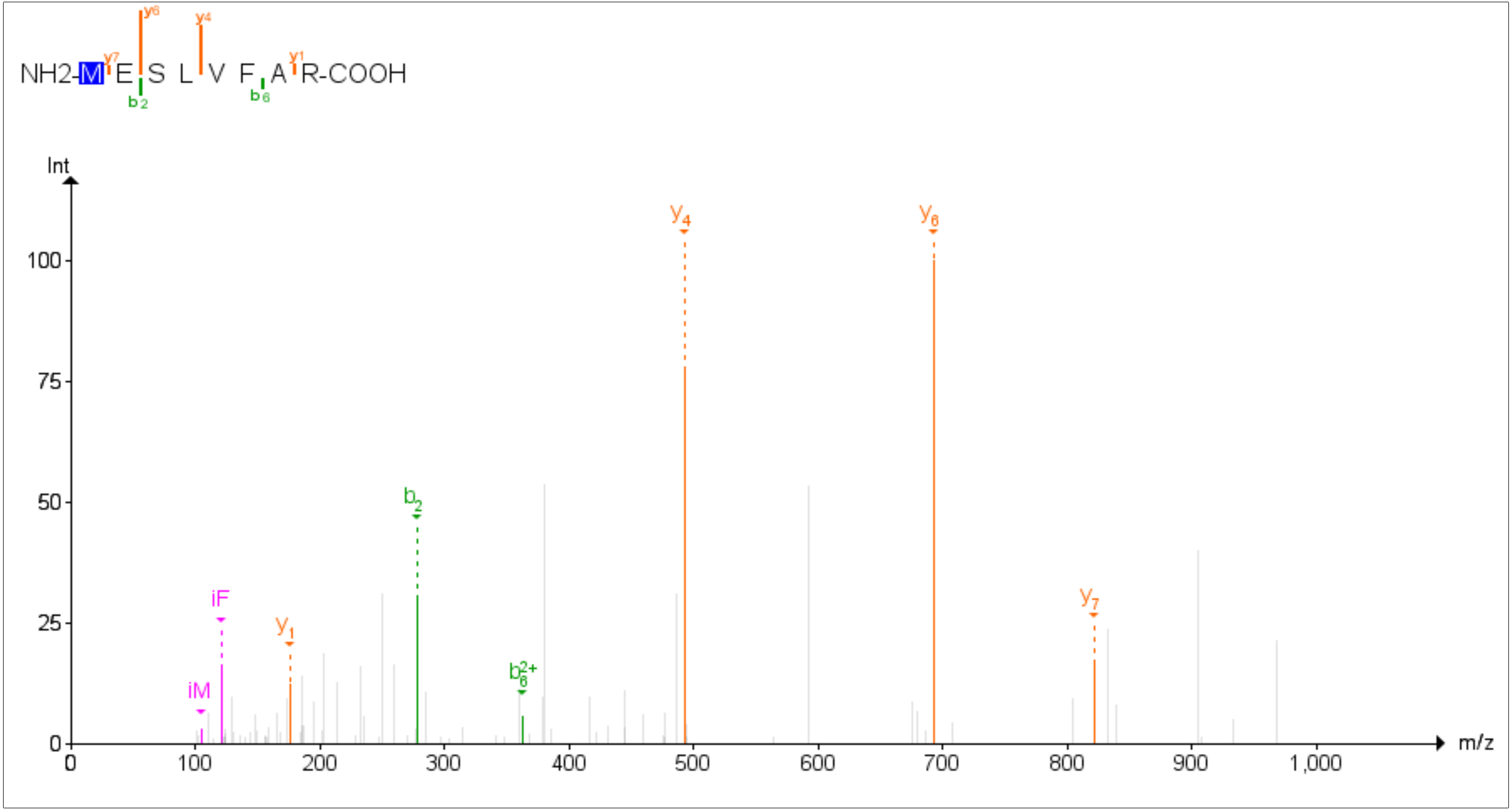

NH2-M E S L V F A R-COOH
y7 y6 y4 y1
b2 b6
Int
100
75
50
25
0
m/z
0 100 200 300 400 500 600 700 800 900 1,000
y4
y6
y7
y1
b2
b6 2+
iF
iM

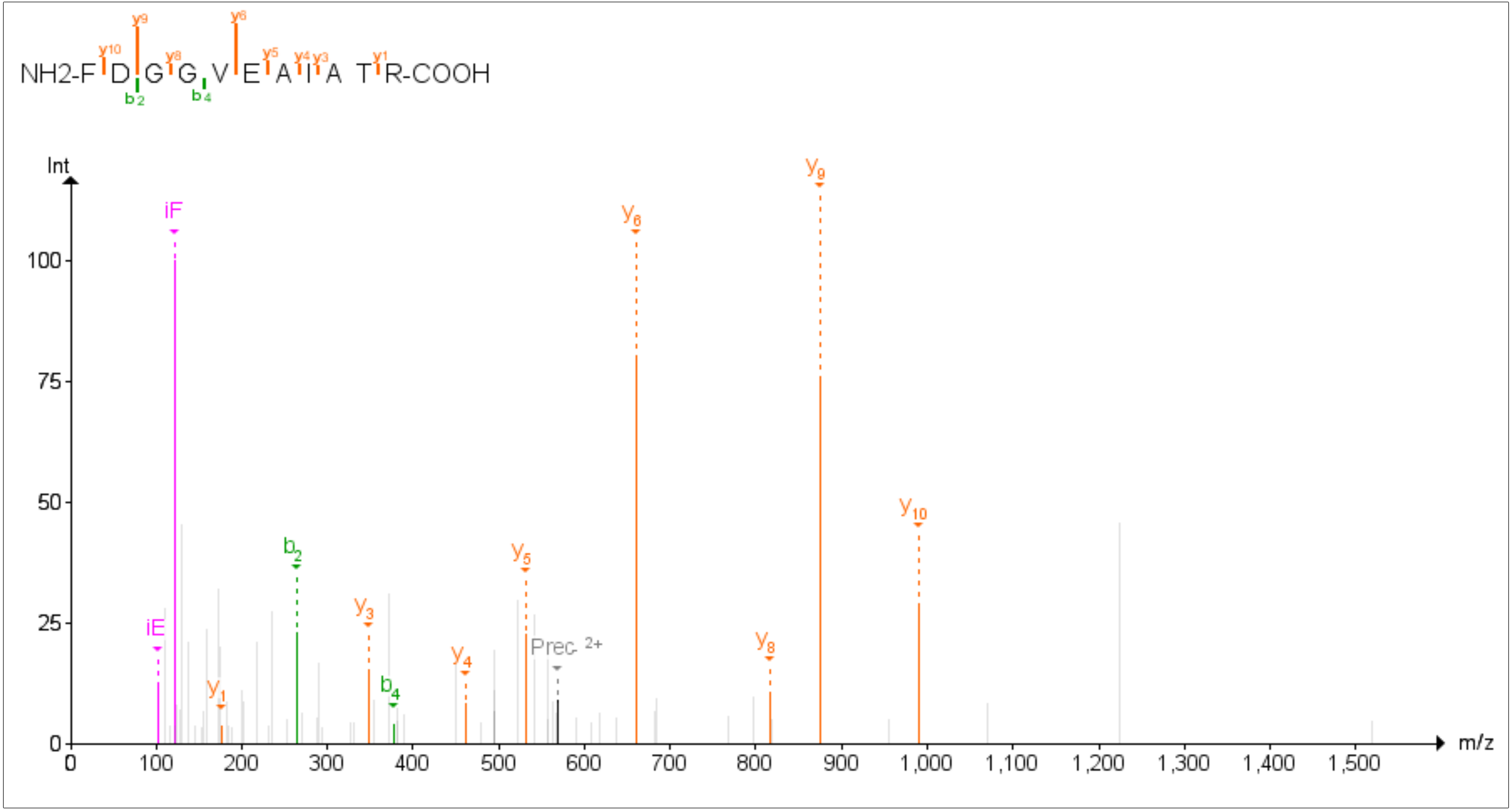

NH2-F D G G V E A I A T R-COOH
y10 y9 y8 y6 y5 y4 y3 y1
b2 b4
Int
100
75
50
25
0
iF
iE
y1
b2
y3
b4
y4
y5
Prec- 2+
y6
y8
y9
y10
0
100
200
300
400
500
600
700
800
900
1,000
1,100
1,200
1,300
1,400
1,500
m/z

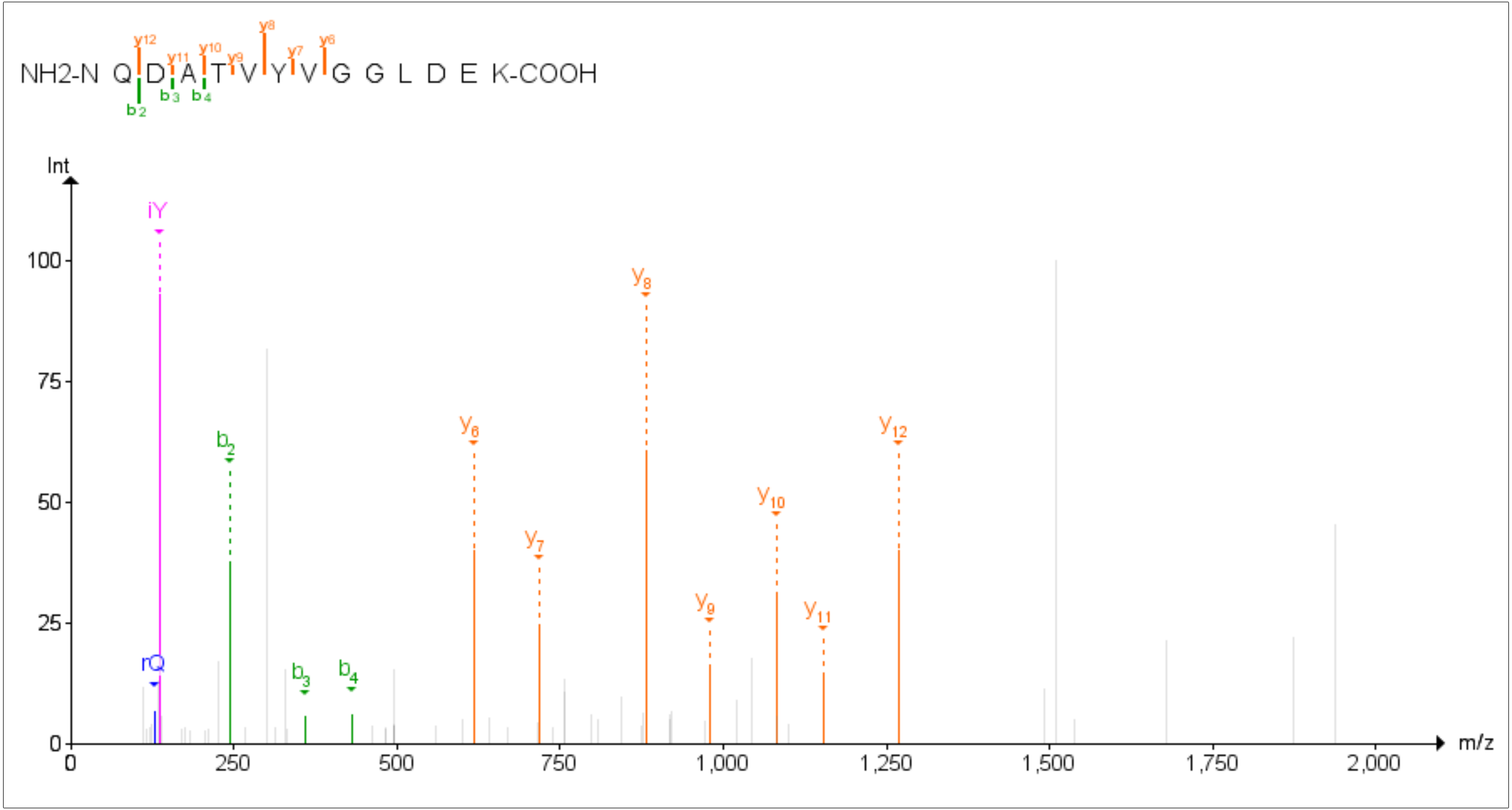

NH2-N Q D A T V Y V G G L D E K-COOH
y12 y11 y10 y9 y8 y7 y6
b2 b3 b4
Int
iY
rQ
b2
b3
b4
y6
y7
y8
y9
y10
y11
y12
0
25
50
75
100
0
250
500
750
1,000
1,250
1,500
1,750
2,000
m/z

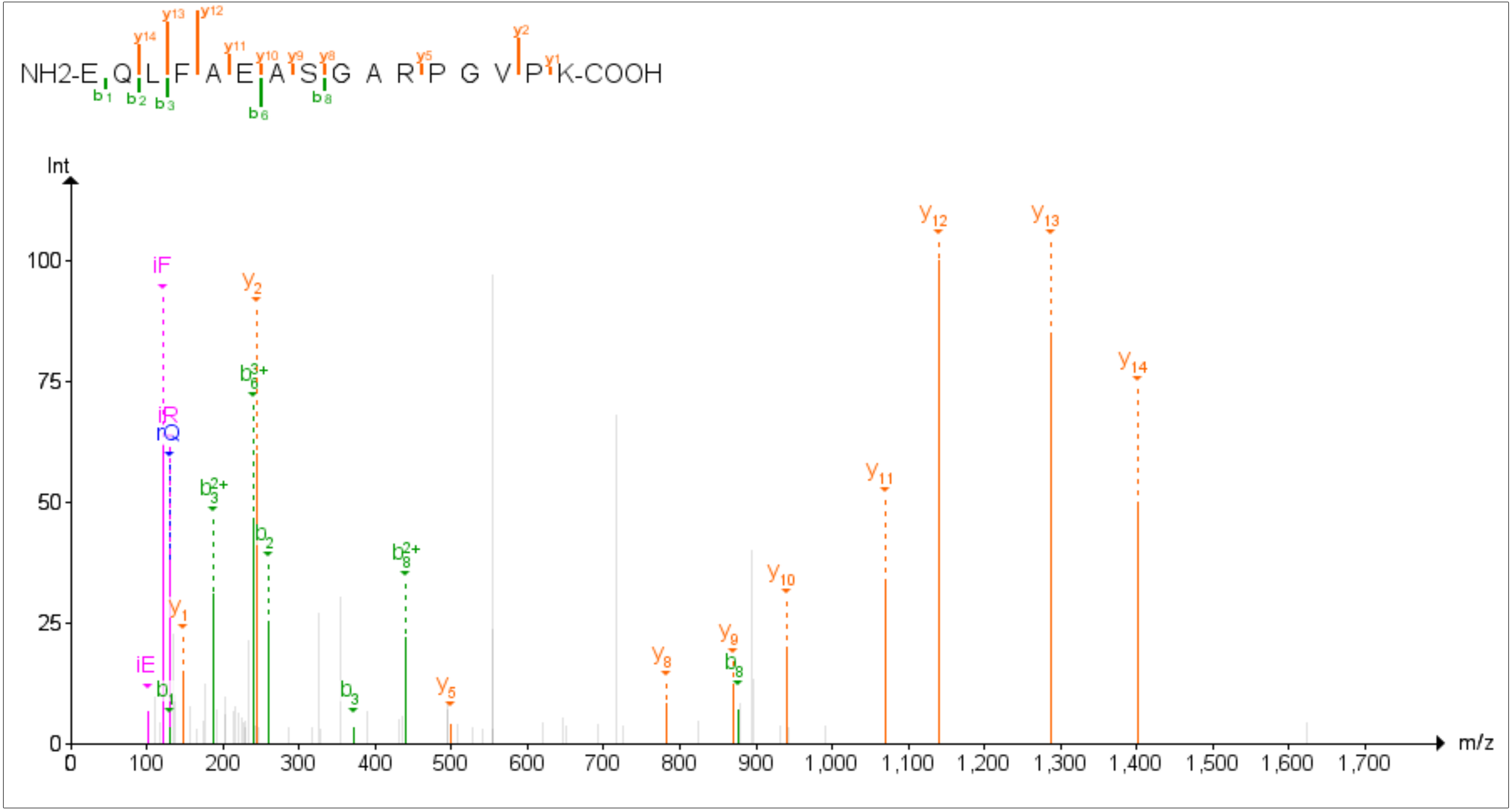

NH2-E Q L F A E A S G A R P G V P K-COOH
Int
m/z
y12
y13
y14
y11
y10
y9
y8
y5
y2
y1
b1
b2
b3
b6
b8
iF
iR
rQ
iE
0
25
50
75
100
0
100
200
300
400
500
600
700
800
900
1,000
1,100
1,200
1,300
1,400
1,500
1,600
1,700

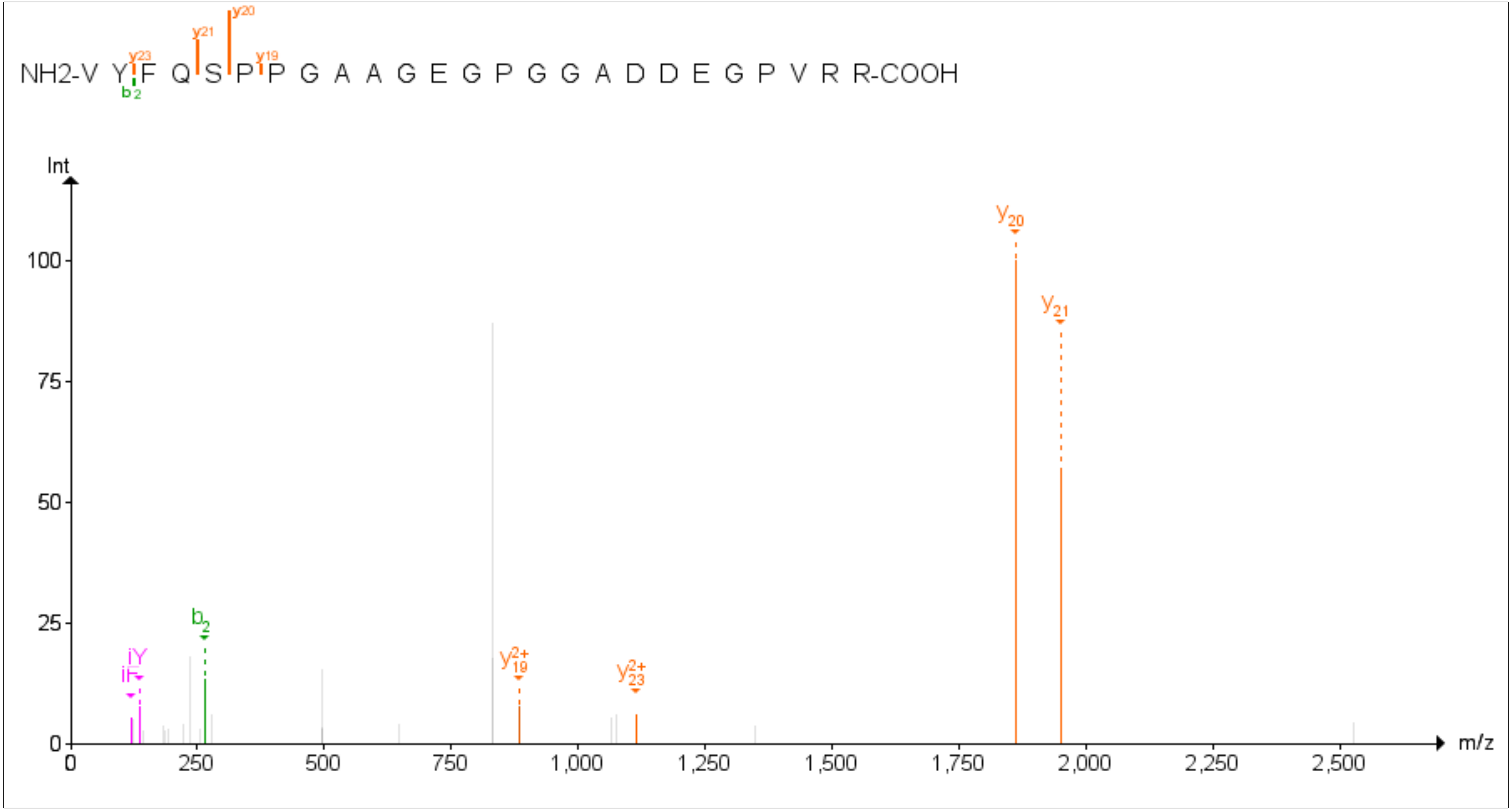

NH2-V Y F Q S P P G A A G E G P G G A D D E G P V R R-COOH
y23
y21
y20
y19
b2
Int
100
75
50
25
0
iY
iF
b2
y19 2+
y23 2+
y20
y21
0
250
500
750
1,000
1,250
1,500
1,750
2,000
2,250
2,500
m/z

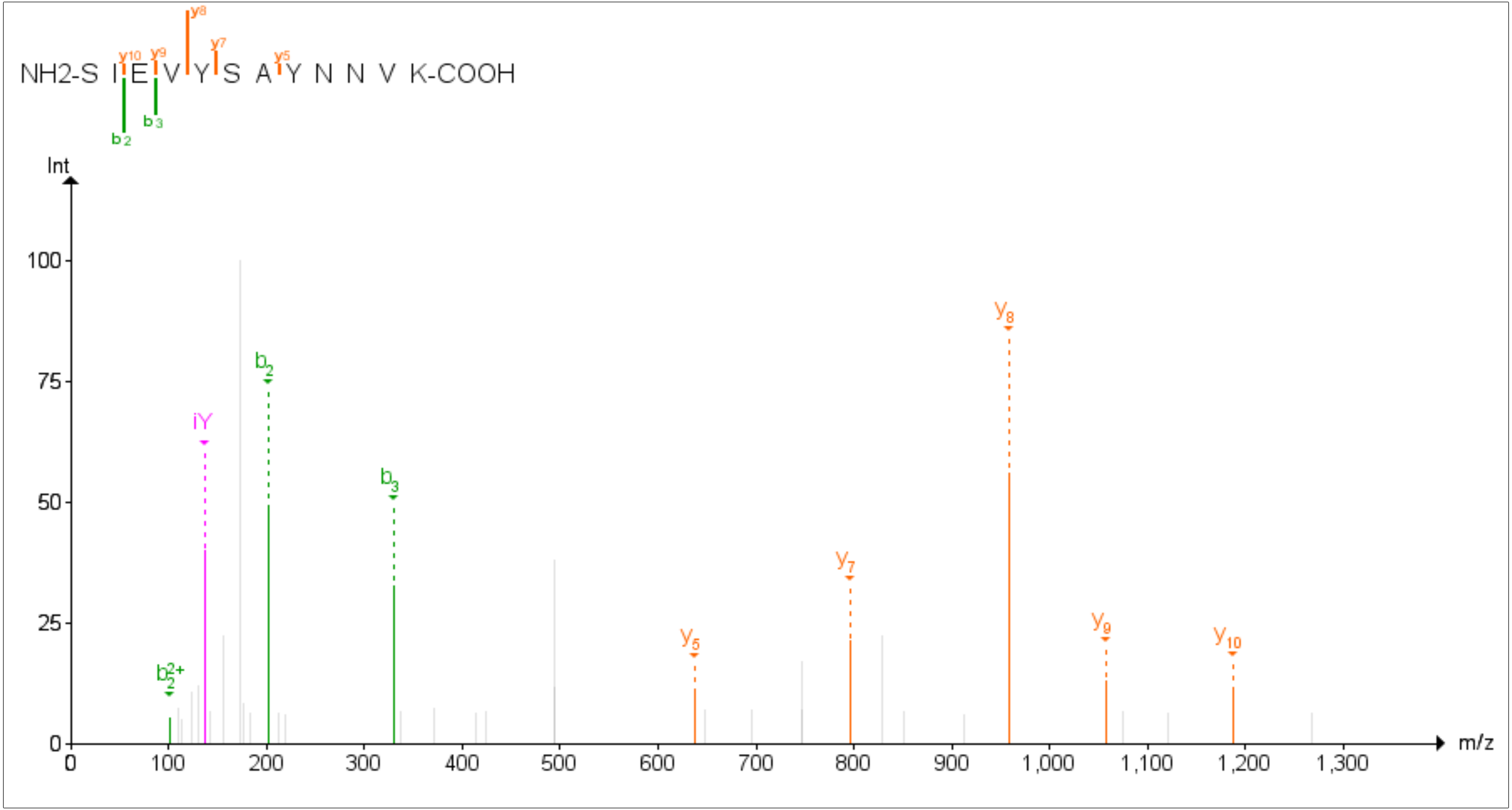

NH2-S I E V Y S A Y N N V K-COOH
y10 y9 y8 y7 y5
b2 b3
Int
100
75
50
25
0
iY
b2
b3
b2 2+
y5
y7
y8
y9
y10
0 100 200 300 400 500 600 700 800 900 1,000 1,100 1,200 1,300
m/z

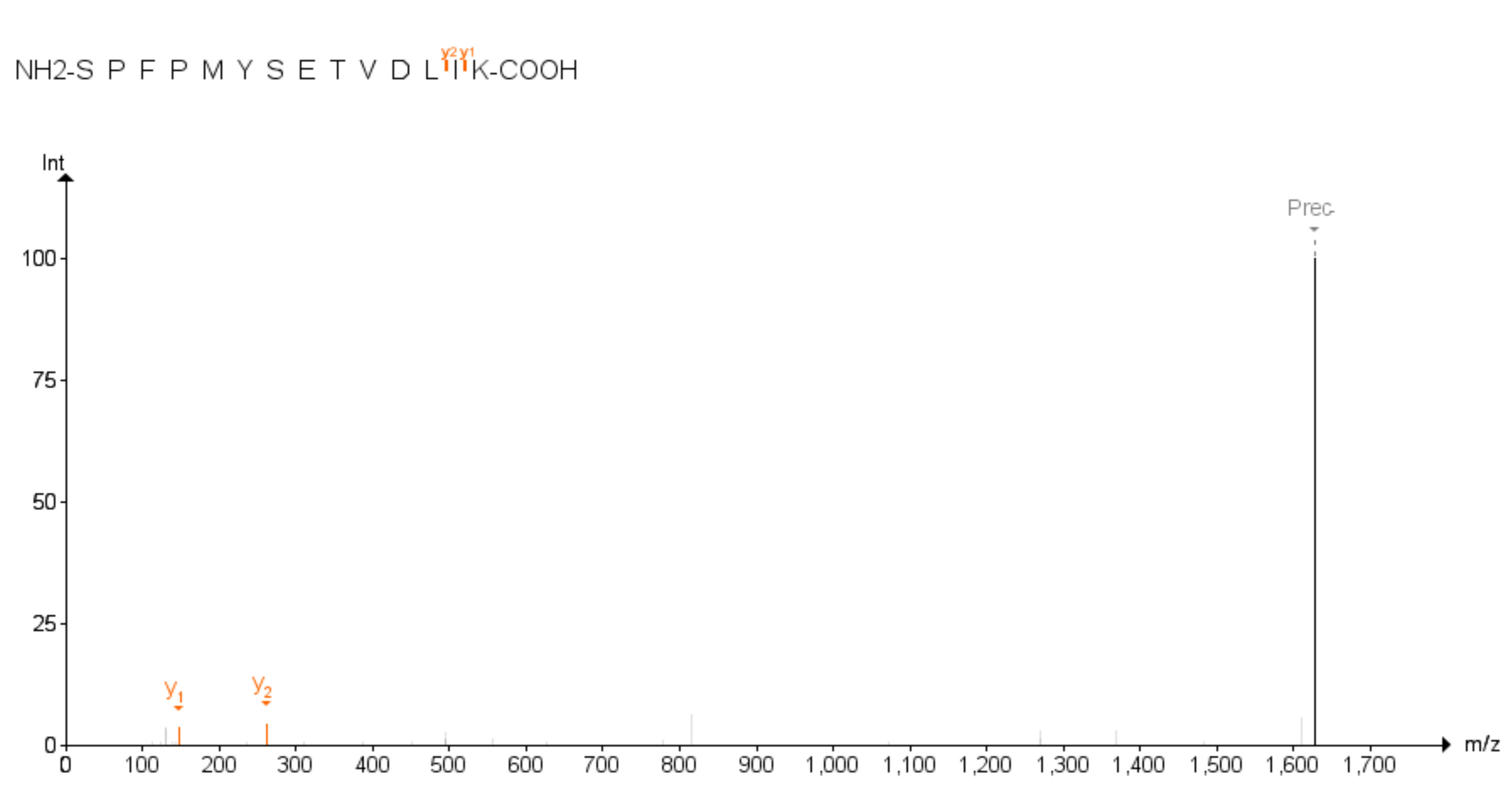

NH2-S P F P M Y S E T V D L I K-COOH
y2 y1
Int
Prec-
100
75
50
25
0
y1
y2
0 100 200 300 400 500 600 700 800 900 1,000 1,100 1,200 1,300 1,400 1,500 1,600 1,700
m/z

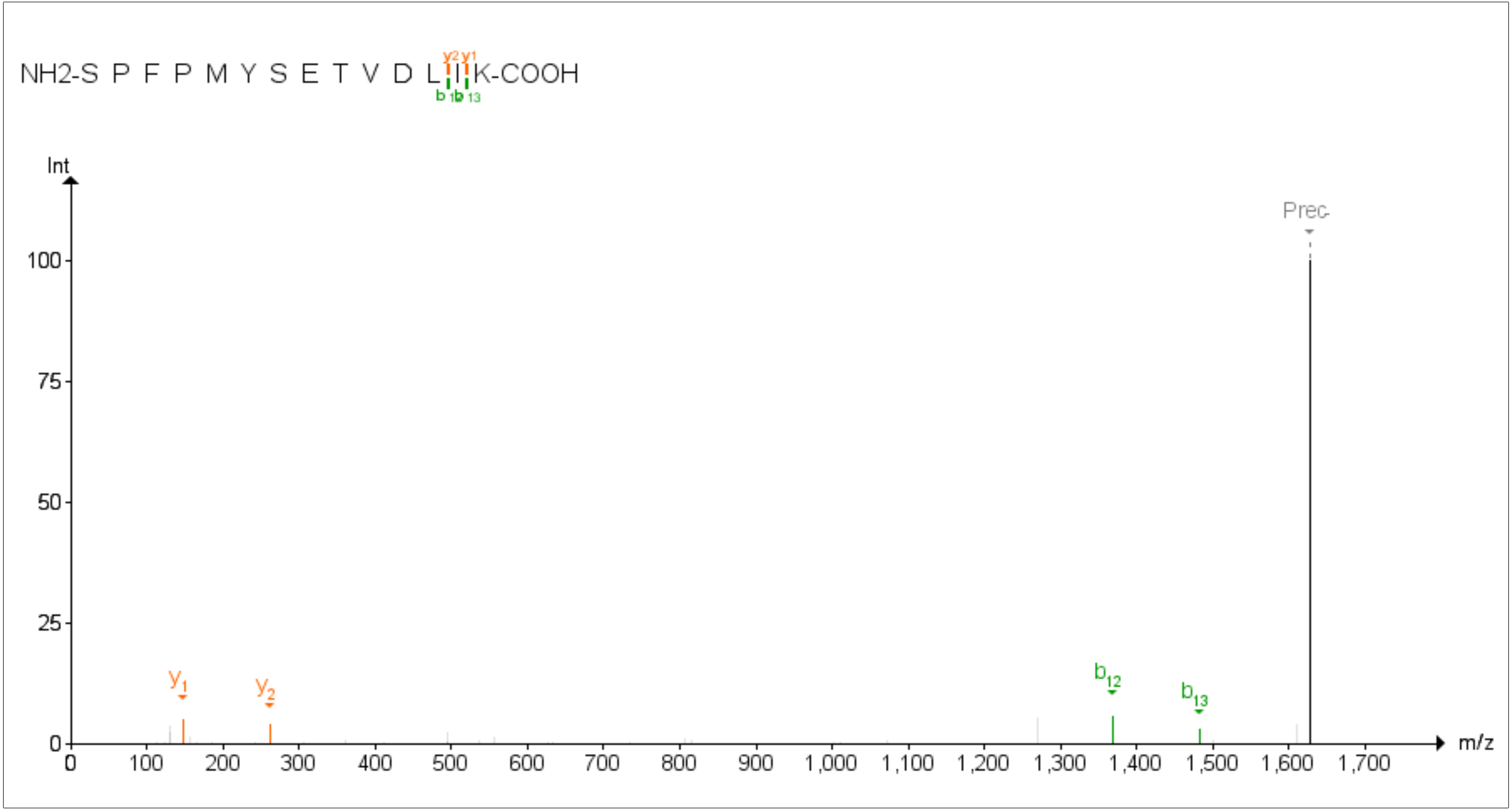
NH2-S P F P M Y S E T V D L I I K-COOH
y2 y1
b12 b13
Int
100
75
50
25
0
Prec
y1
y2
b12
b13
0 100 200 300 400 500 600 700 800 900 1,000 1,100 1,200 1,300 1,400 1,500 1,600 1,700
m/z

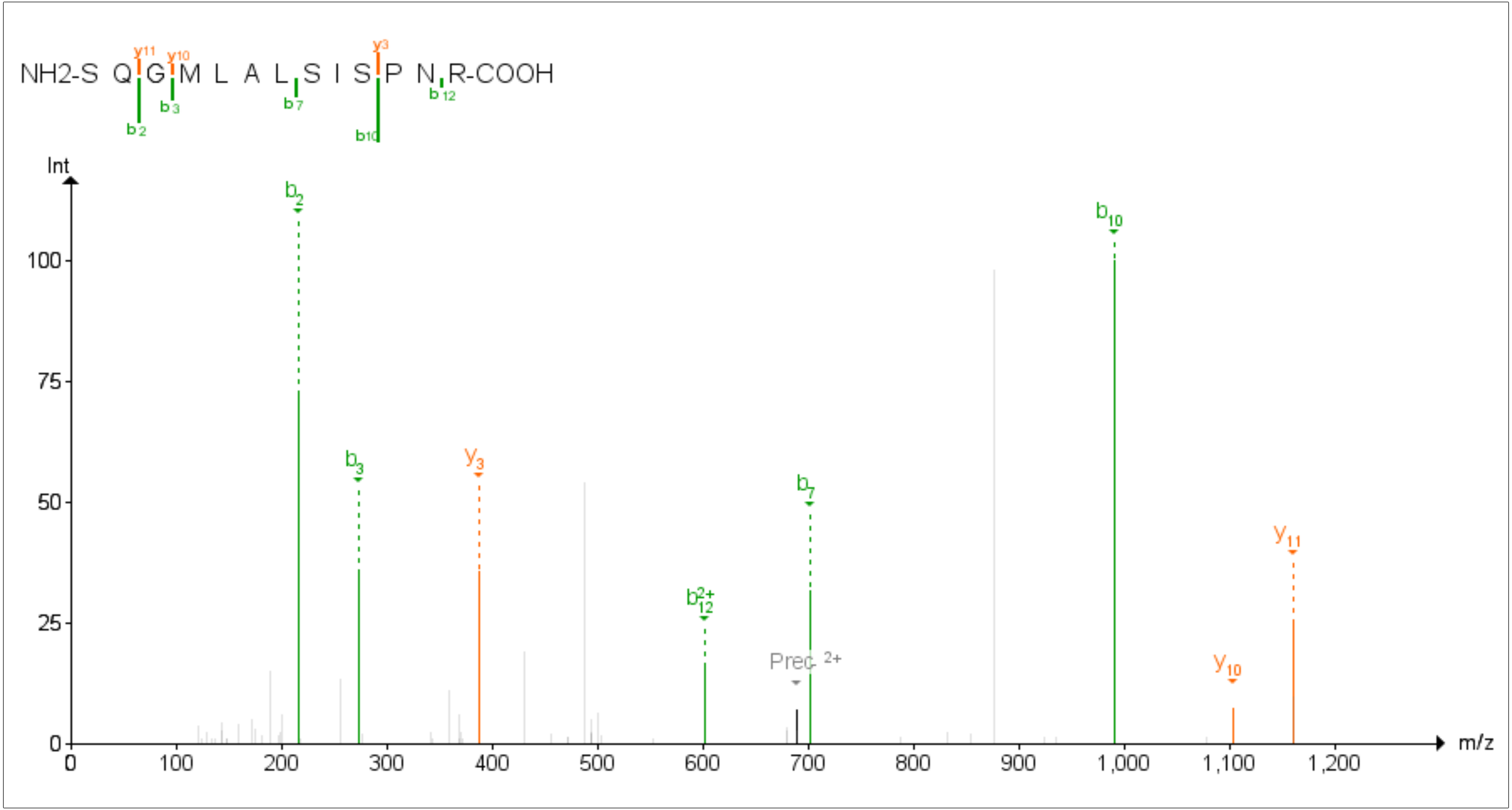
NH2-S Q G M L A L S I S P N R-COOH
y11
y10
y3
b2
b3
b7
b10
b12
Int
100
75
50
25
0
m/z
0
100
200
300
400
500
600
700
800
900
1,000
1,100
1,200
b2
b3
y3
b12 2+
Prec 2+
b7
b10
y10
y11

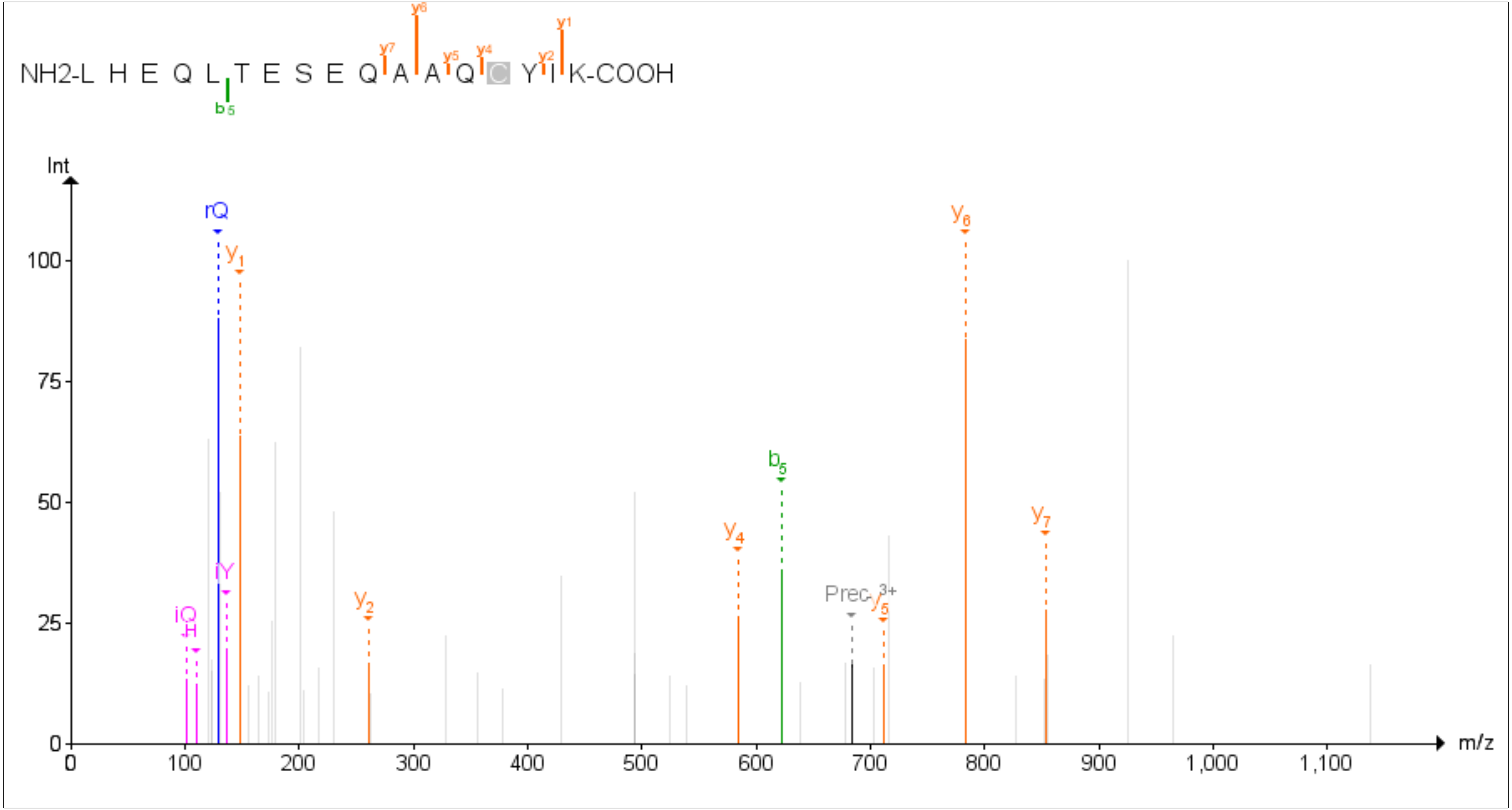

NH2-L H E Q L T E S E Q A A Q C Y I K-COOH
y8
y7
y5
y4
y2
y1
b5
Int
rQ
y1
y6
b5
y4
y7
iY
iQ
iH
y2
Prec 3+
y5
100
75
50
25
0
0
100
200
300
400
500
600
700
800
900
1,000
1,100
m/z

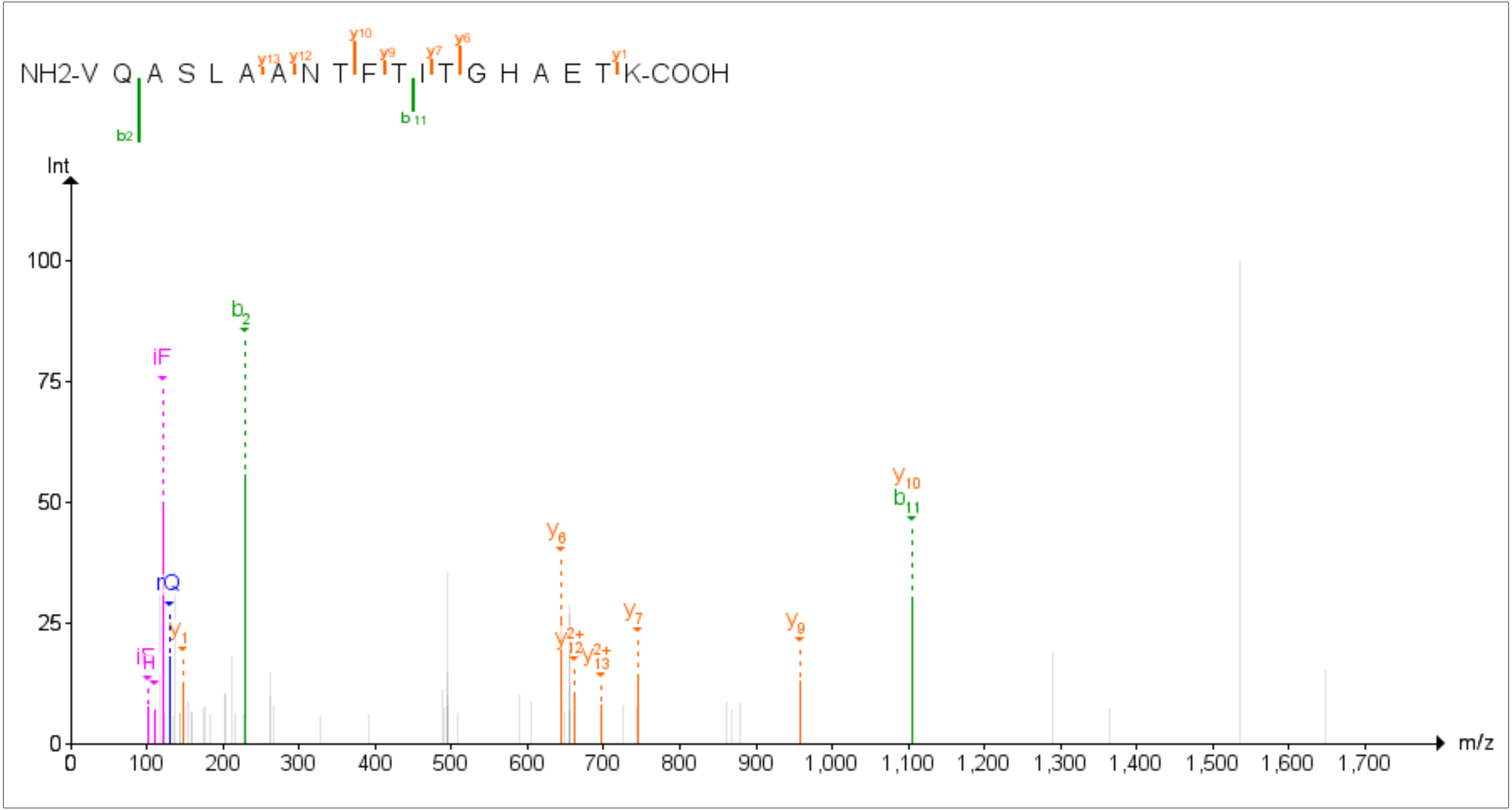

NH2-V Q A S L A A N T F T I T G H A E T K-COOH
b2
b 11
y13
y12
y10
y9
y7
y6
y1
Int
m/z
iF
iH
rQ
b2
y1
y6
y7
y9
y10
b11
100
75
50
25
0
100
200
300
400
500
600
700
800
900
1,000
1,100
1,200
1,300
1,400
1,500
1,600
1,700

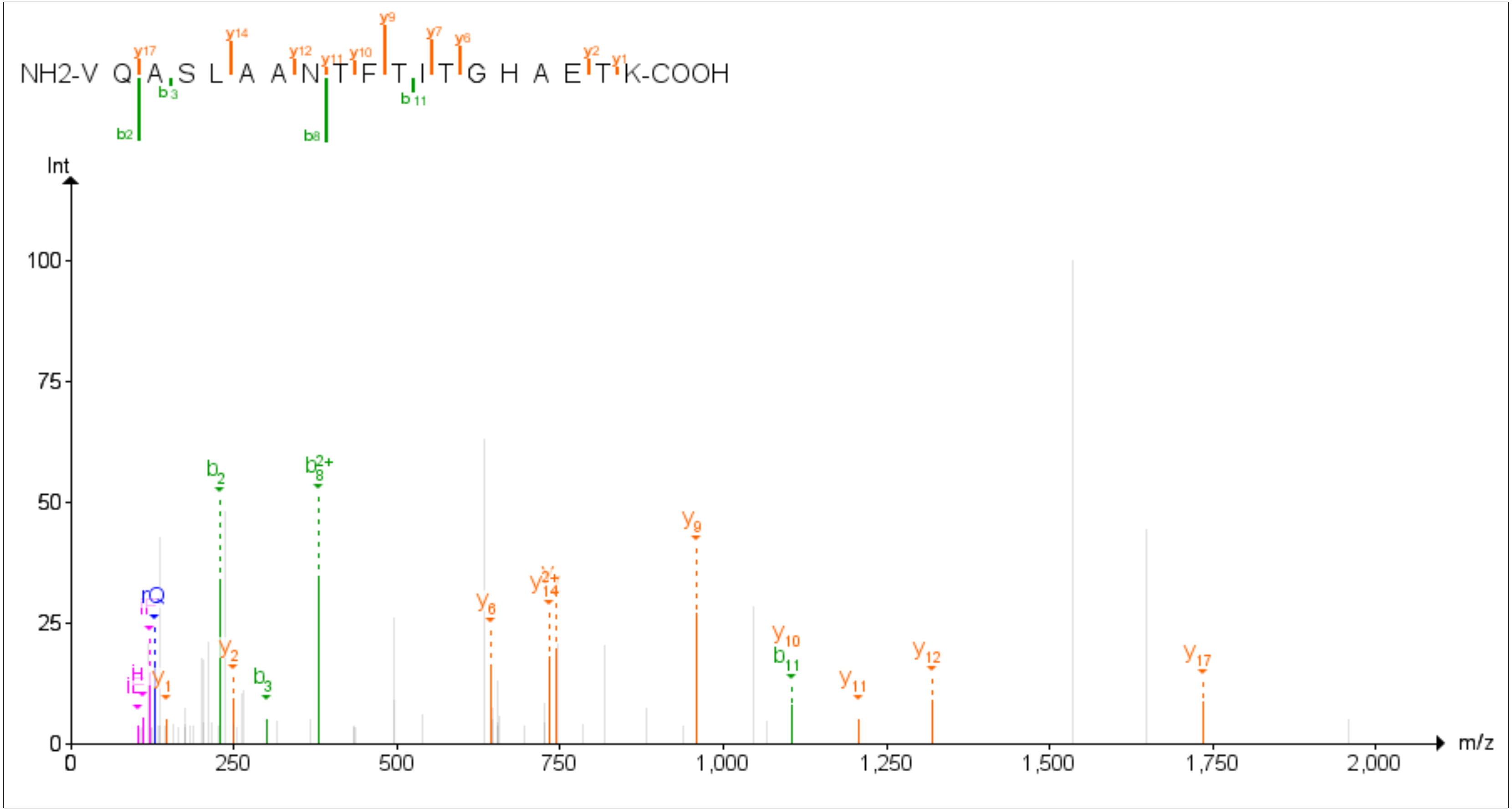
NH2-V Q A S L A A N T F T I T G H A E T K-COOH
Int
m/z
100
75
50
25
0
250
500
750
1,000
1,250
1,500
1,750
2,000
b2
b3
b8
b11
y17
y14
y12
y11
y10
y9
y7
y6
y2
y1
b8 2+
y14 2+
rQ
y10
b11
y11
y12
y17

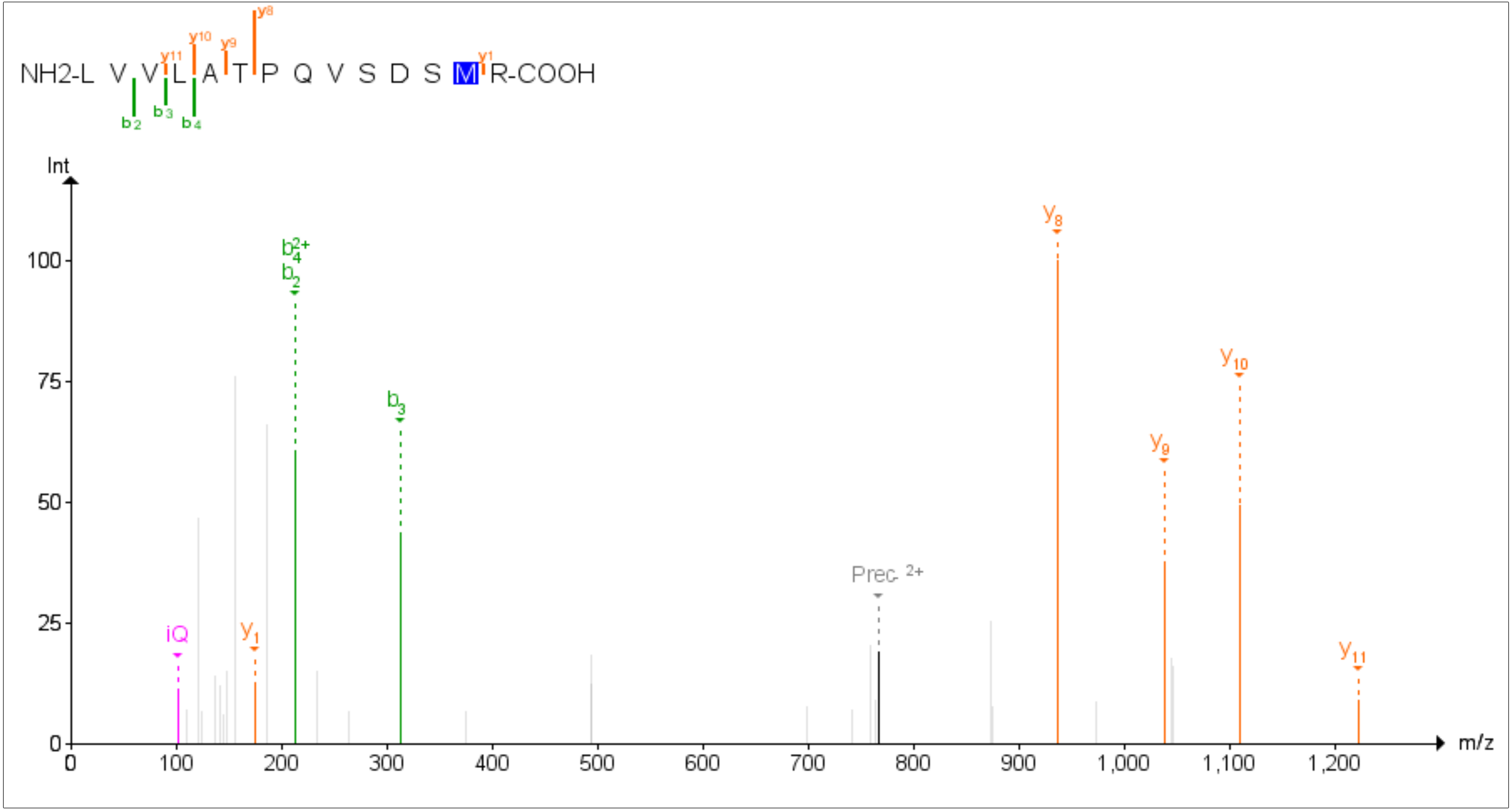

NH2-L V V L A T P Q V S D S M R-COOH
y8
y10
y9
y11
y1
b2
b3
b4
Int
100
75
50
25
0
b4 2+
b2
b3
y8
y10
y9
y11
y1
iQ
Prec 2+
0
100
200
300
400
500
600
700
800
900
1,000
1,100
1,200
m/z

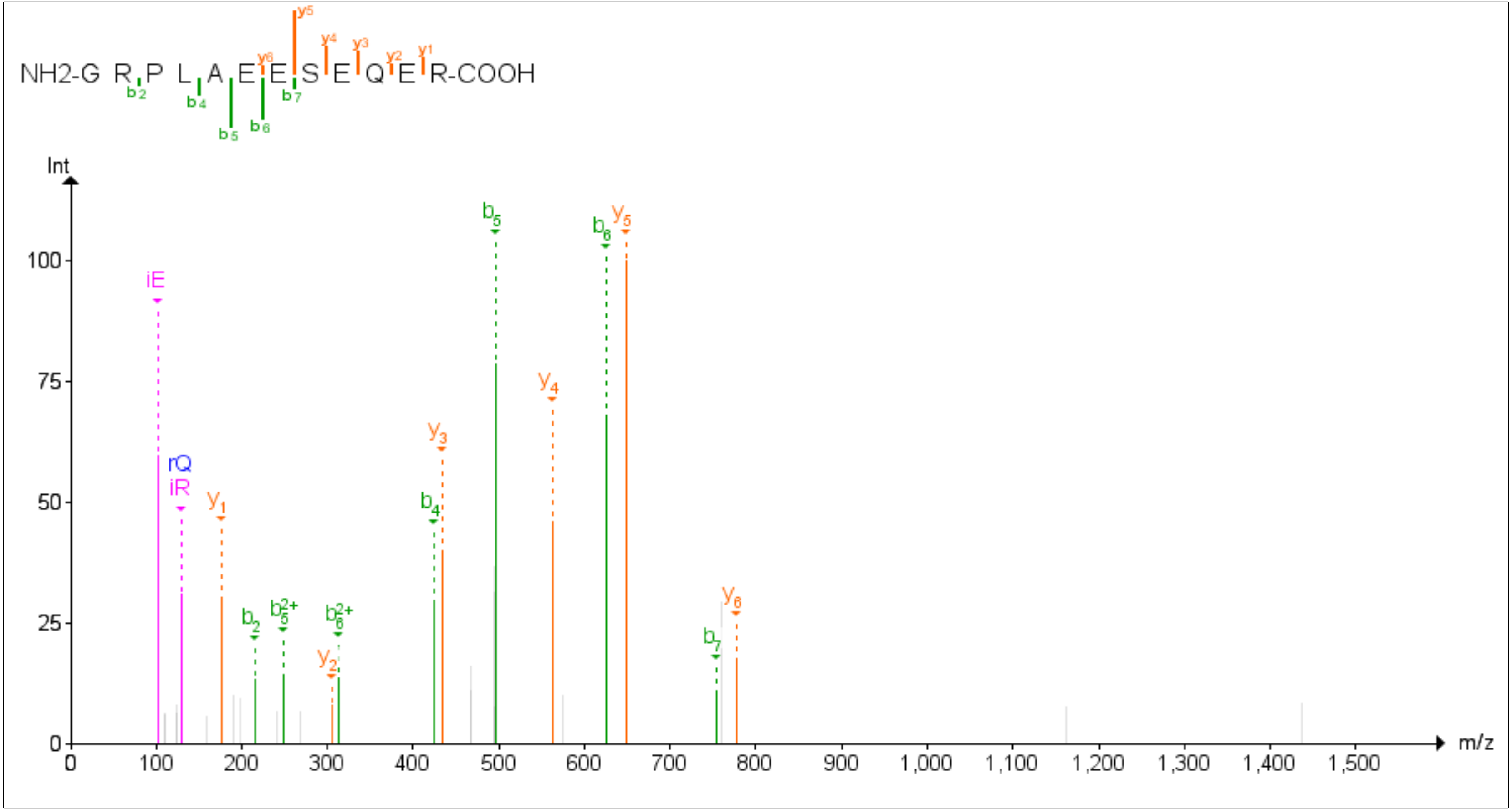
NH2-G R P L A E E S E Q E R-COOH
b2
b4
b5
b6
b7
y6
y5
y4
y3
y2
y1
Int
iE
rQ
iR
y1
b2
b5 2+
y2
b6 2+
b4
y3
b5
y4
b6
y5
b7
y6
100
75
50
25
0
100
200
300
400
500
600
700
800
900
1,000
1,100
1,200
1,300
1,400
1,500
m/z

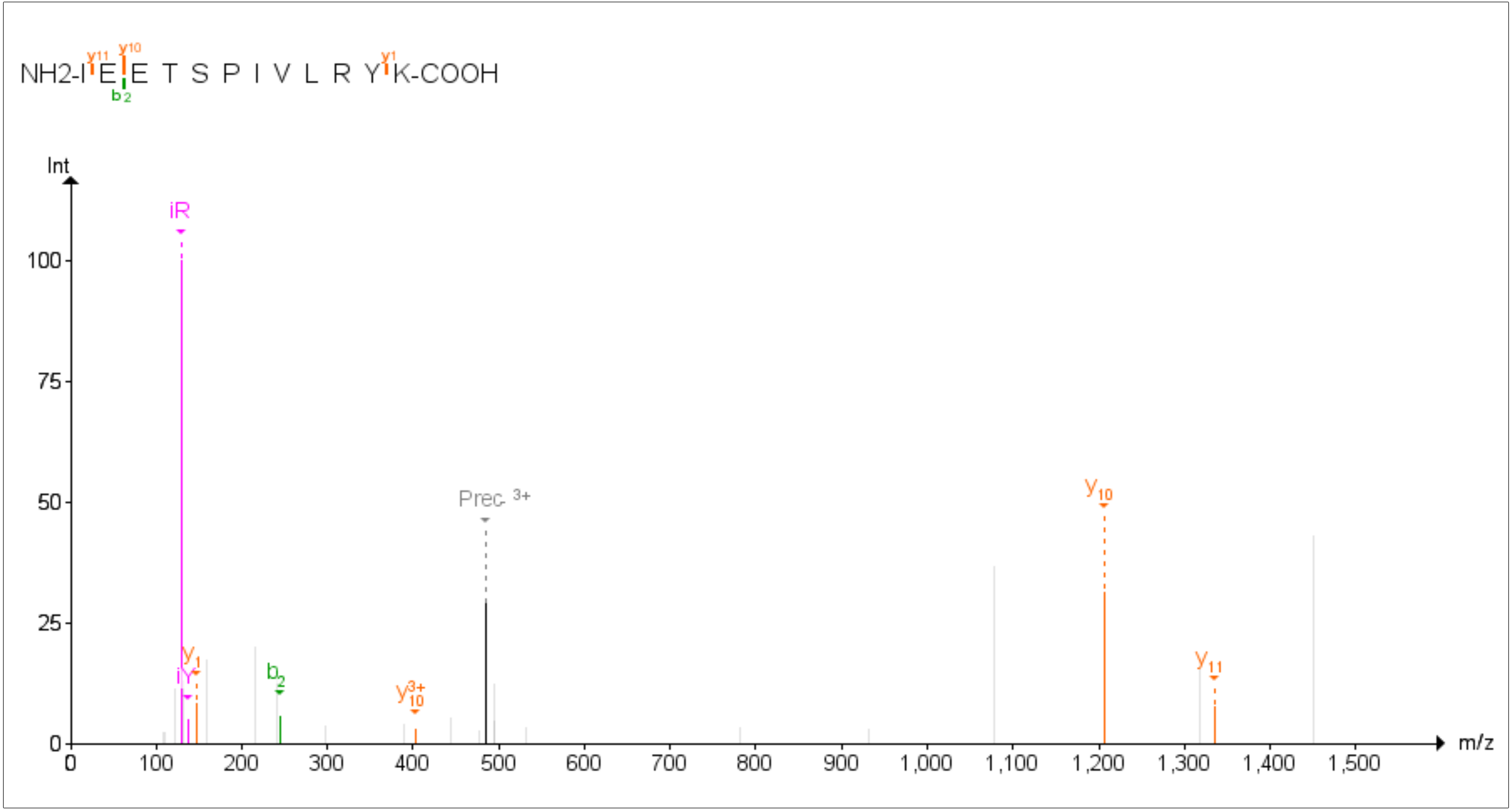

NH2-I E E T S P I V L R Y K-COOH
y11
y10
y1
b2
Int
iR
Prec 3+
y10
y11
y1
iY
b2
y10 3+
m/z
0
25
50
75
100
0
100
200
300
400
500
600
700
800
900
1,000
1,100
1,200
1,300
1,400
1,500

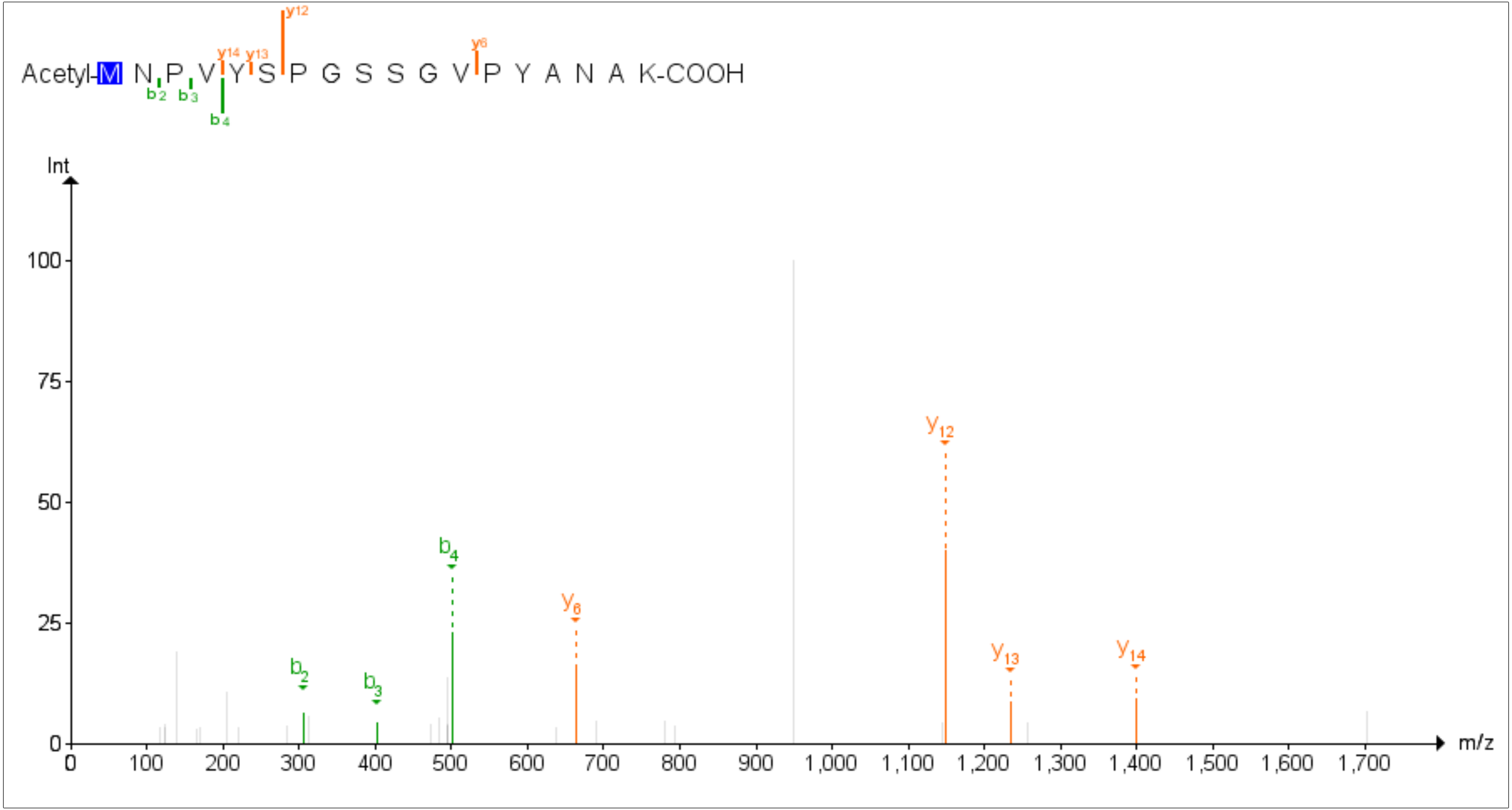

Acetyl-M N P V Y S P G S S G V P Y A N A K-COOH
b2
b3
b4
y14
y13
y12
y6
Int
100
75
50
25
0
m/z
0
100
200
300
400
500
600
700
800
900
1,000
1,100
1,200
1,300
1,400
1,500
1,600
1,700

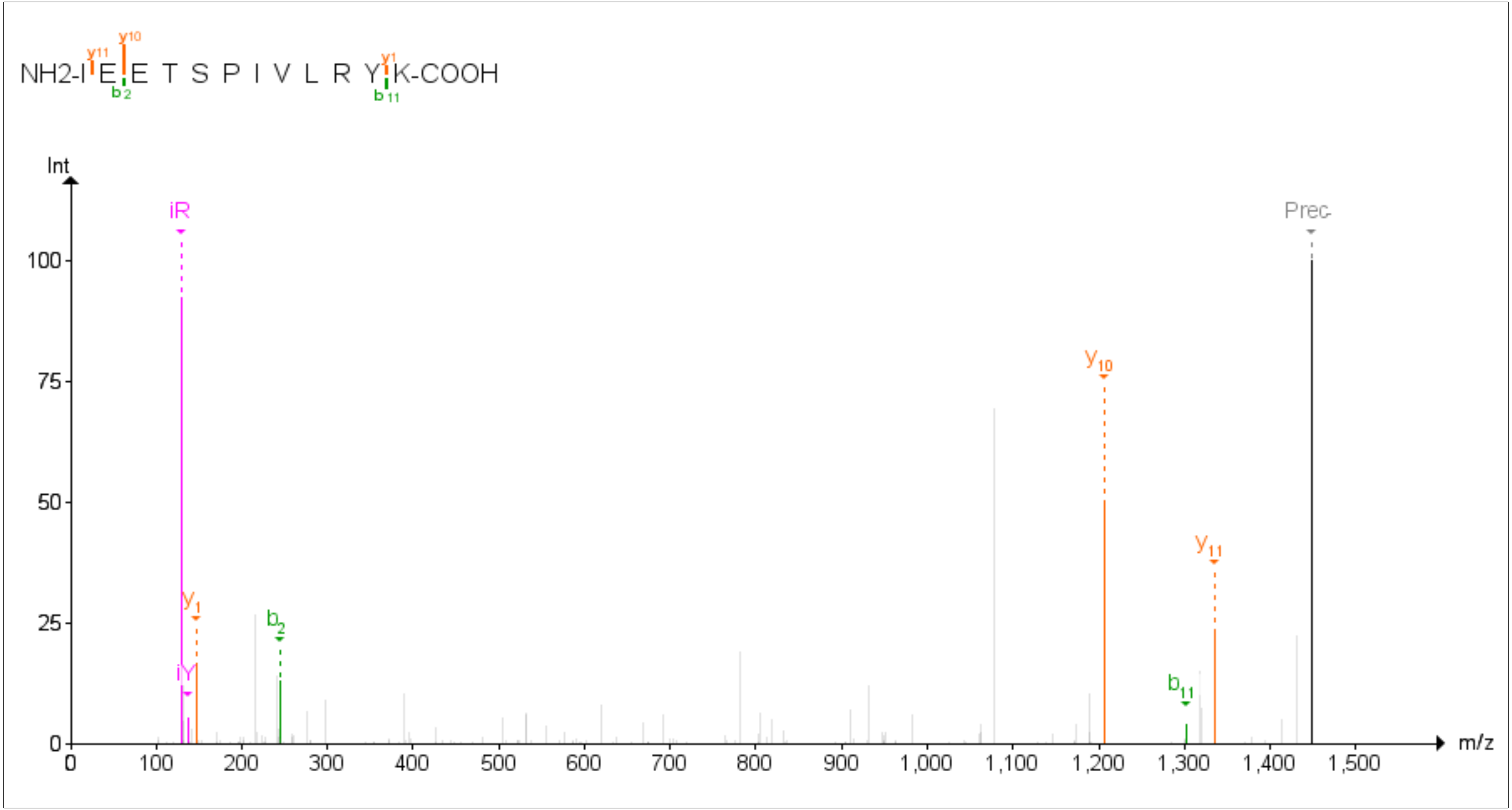

NH2-I E E T S P I V L R Y K-COOH
y11
y10
y1
b2
b11
Int
100
75
50
25
0
iR
iY
y1
b2
y10
b11
y11
Prec-
0
100
200
300
400
500
600
700
800
900
1,000
1,100
1,200
1,300
1,400
1,500
m/z

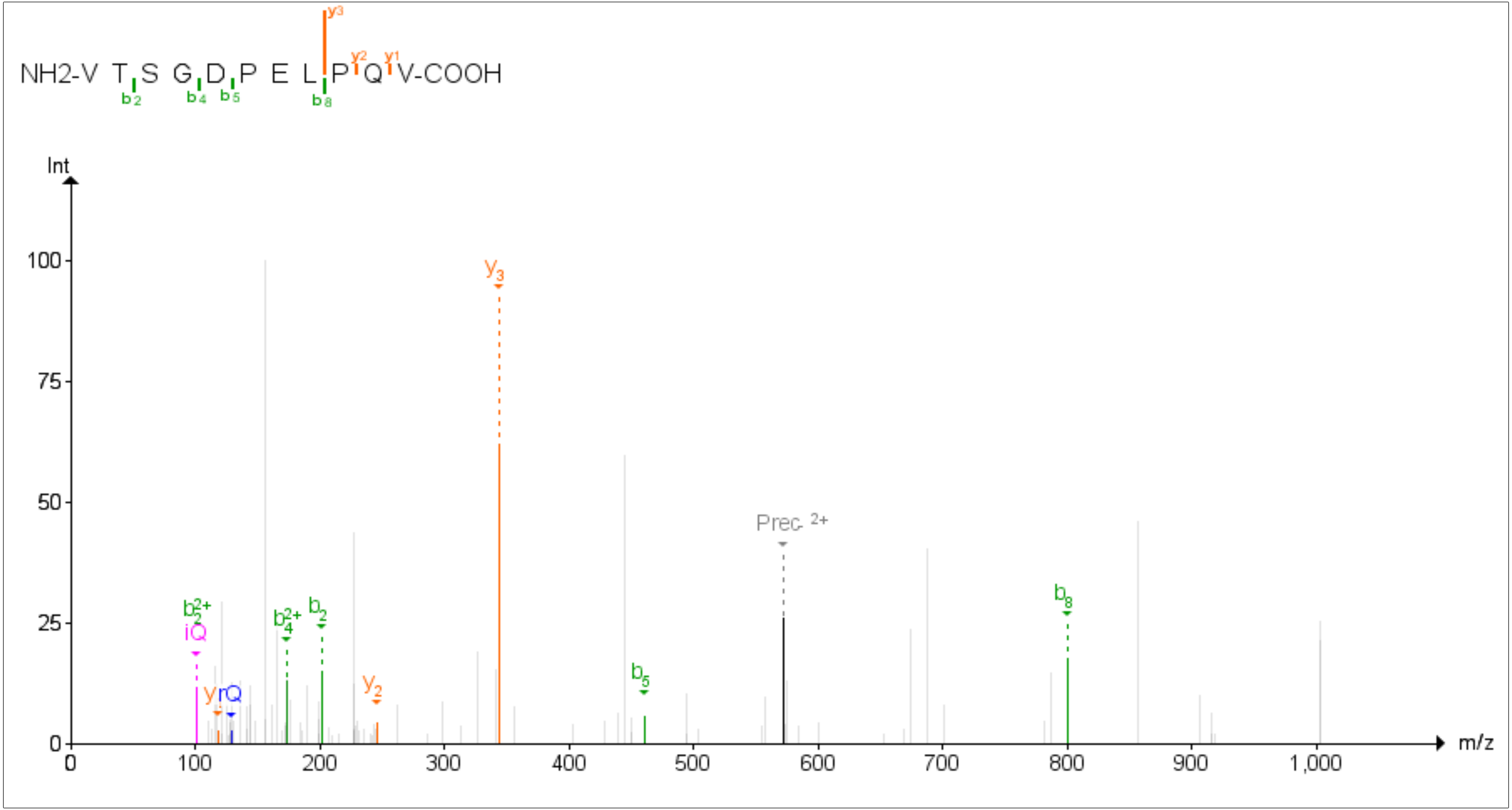

NH2-V T S G D P E L P Q V-COOH
b2 b4 b5 b8
y3 y2 y1
Int
m/z
y3
Prec 2+
b8
b5
y2
b2
b4 2+
b2 2+
iQ
y1
rQ
0
25
50
75
100
0
100
200
300
400
500
600
700
800
900
1,000

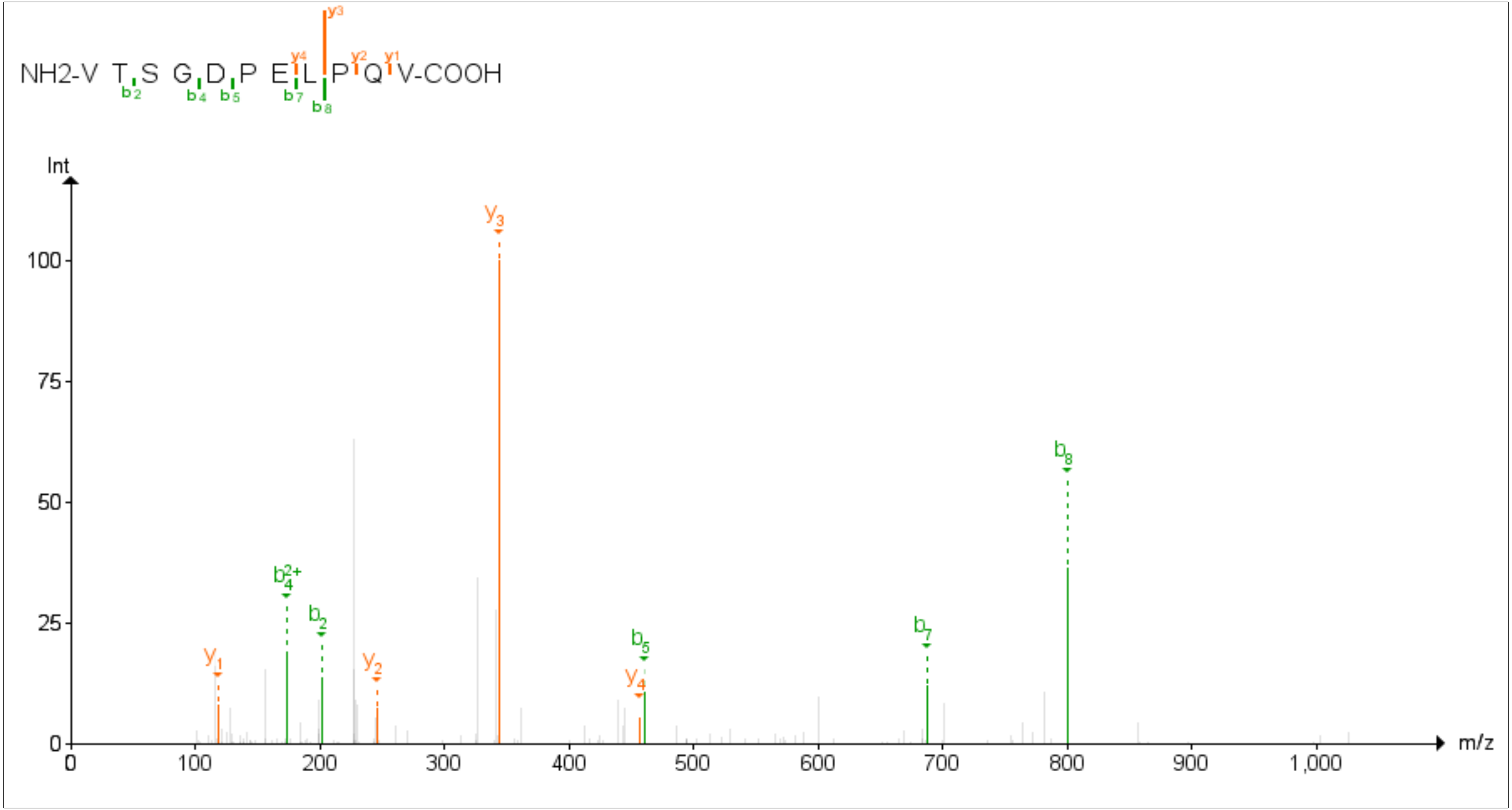
NH2-V T S G D P E L P Q V-COOH
b2 b4 b5 b7 b8
y4 y3 y2 y1
Int
100
75
50
25
0
y3
b8
b_4^{2+}
b2
y1
y2
b5
y4
b7
0 100 200 300 400 500 600 700 800 900 1,000
m/z

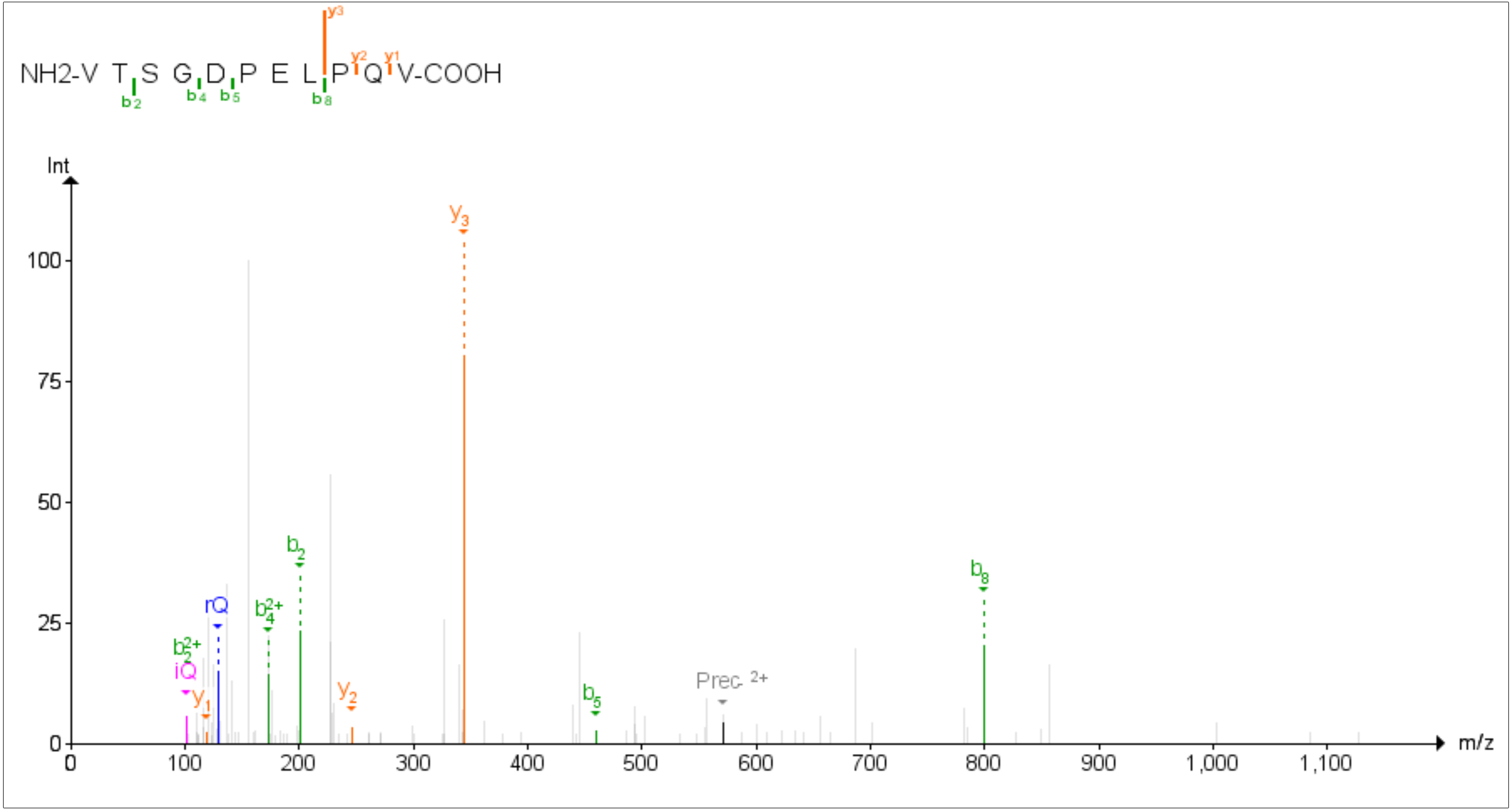

NH2-V T S G D P E L P Q V-COOH
b2 b4 b5 b8
y3 y2 y1
Int
m/z
y3
b2
b8
rQ
b4 2+
b2 2+
iQ
y1
y2
b5
Prec 2+

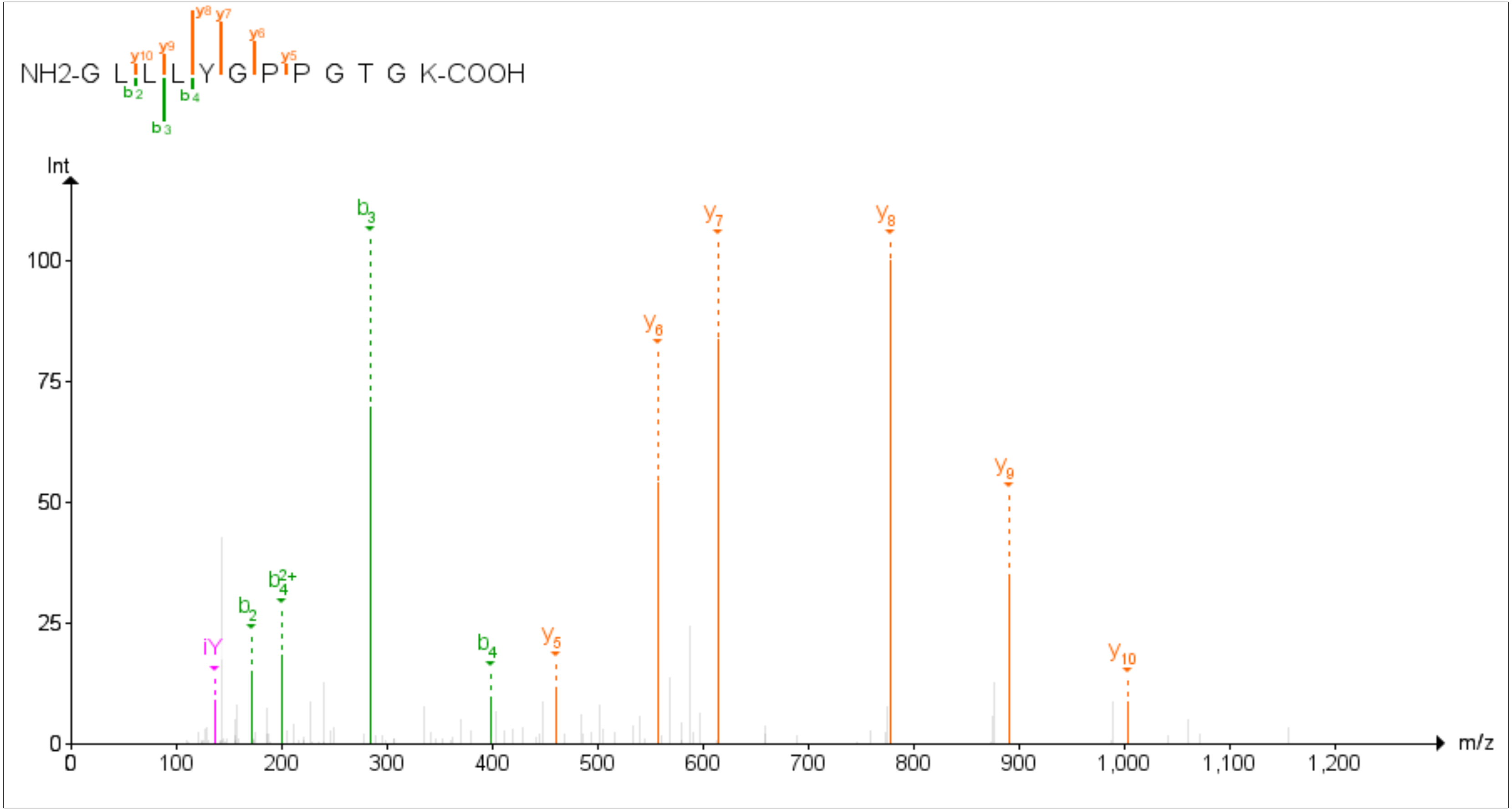

NH2-G L L L Y G P P G T G K-COOH
y10 y9 y8 y7 y6 y5
b2 b3 b4
Int
100
75
50
25
0
b3
y7
y8
y6
y9
b4 2+
b2
iY
b4
y5
y10
0 100 200 300 400 500 600 700 800 900 1,000 1,100 1,200
m/z

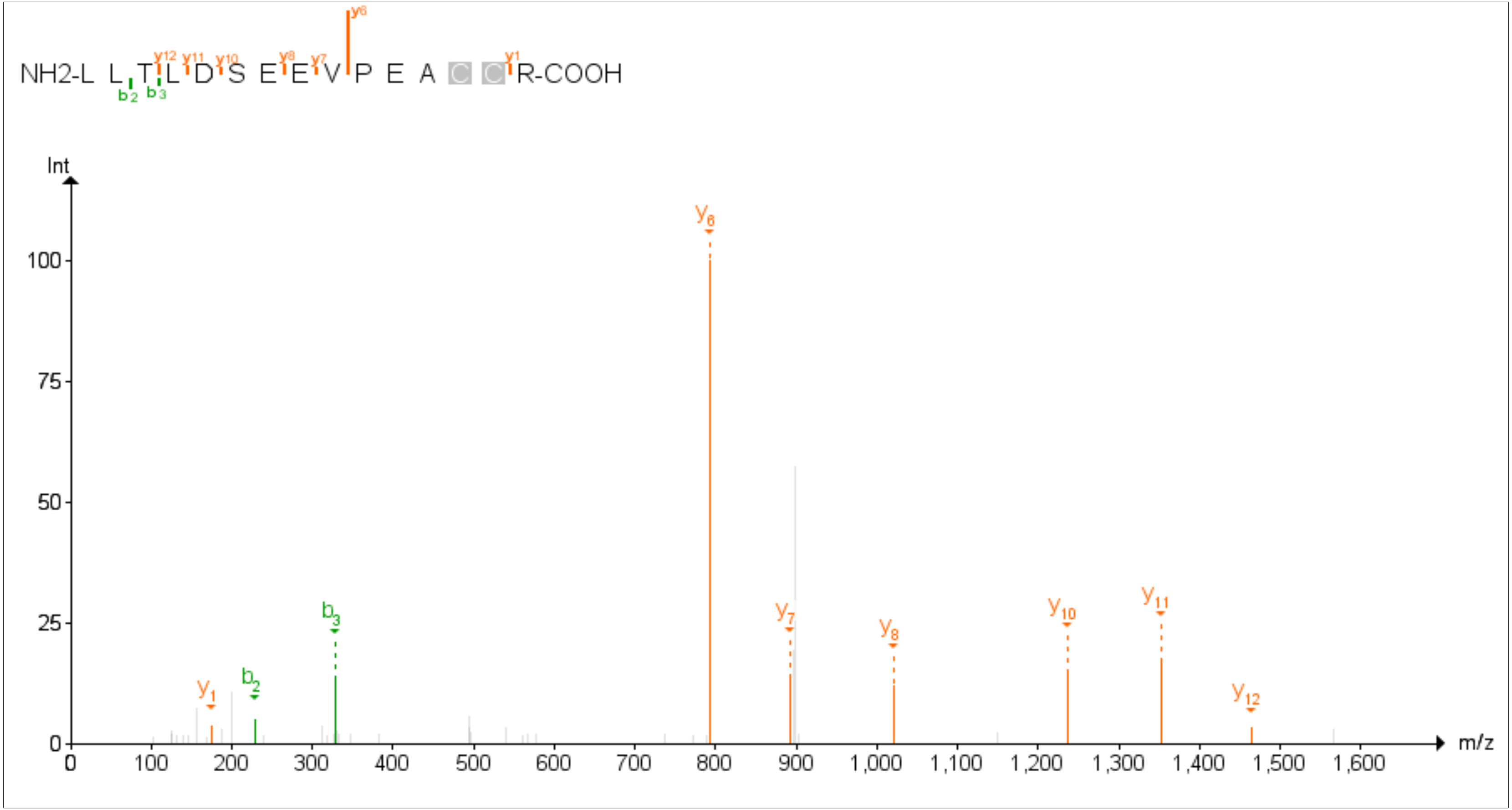
NH2-L L T L D S E E V P E A C C R-COOH
y12 y11 y10 y8 y7 y6 y1
b2 b3
Int
100
75
50
25
0
y6
y7
y8
y10
y11
y12
y1
b2
b3
0 100 200 300 400 500 600 700 800 900 1,000 1,100 1,200 1,300 1,400 1,500 1,600
m/z

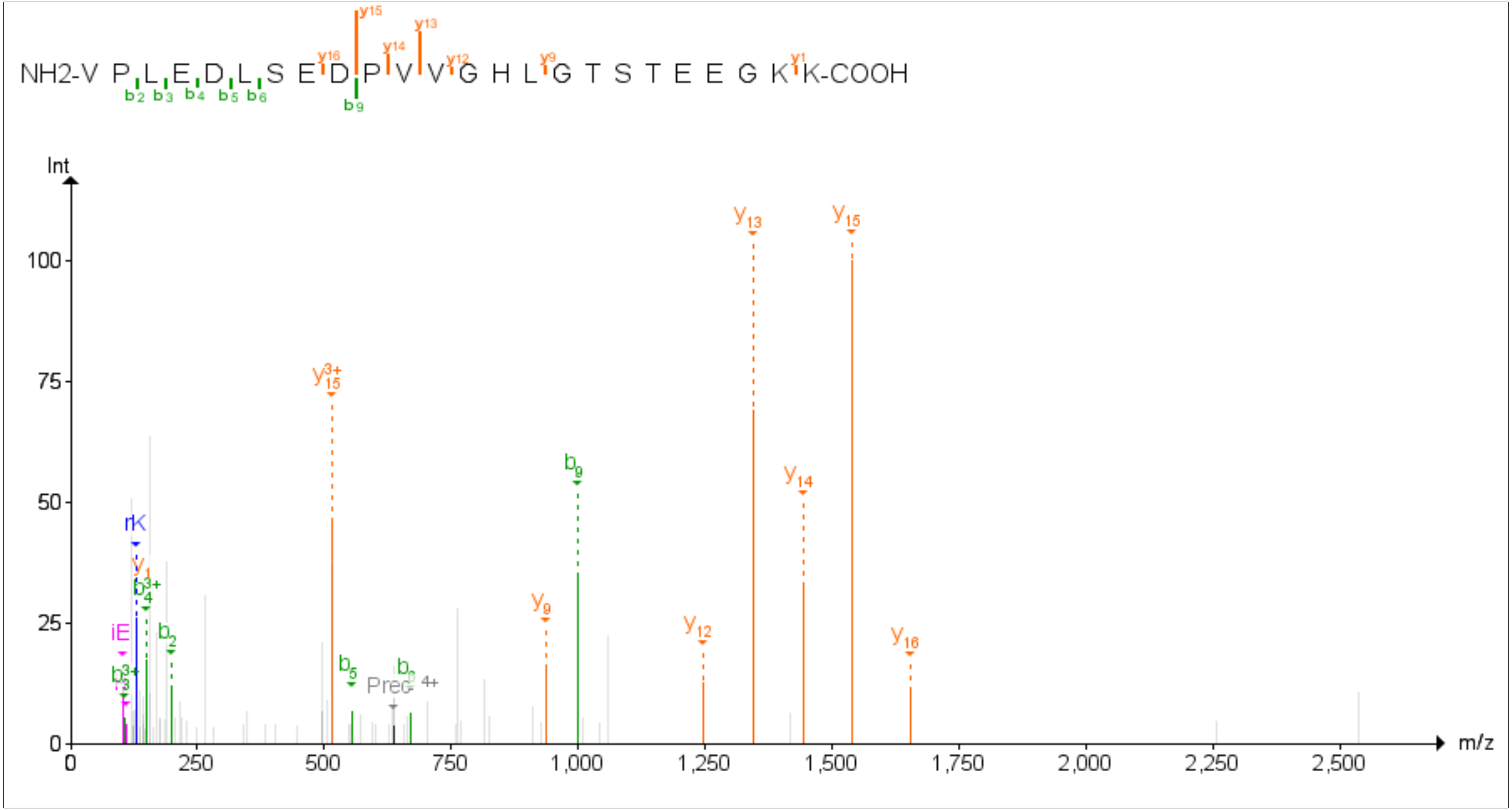

NH2-V P L E D L S E D P V V G H L G T S T E E G K K-COOH
b2 b3 b4 b5 b6 b9
y16 y15 y14 y13 y12 y9 y1
Int
m/z
0
25
50
75
100
250
500
750
1,000
1,250
1,500
1,750
2,000
2,250
2,500
y13
y15
y15 3+
b9
y14
y9
y12
y16
b5
b6
Prec 4+
rK
y1
b4 3+
iE
b2
b3 3+

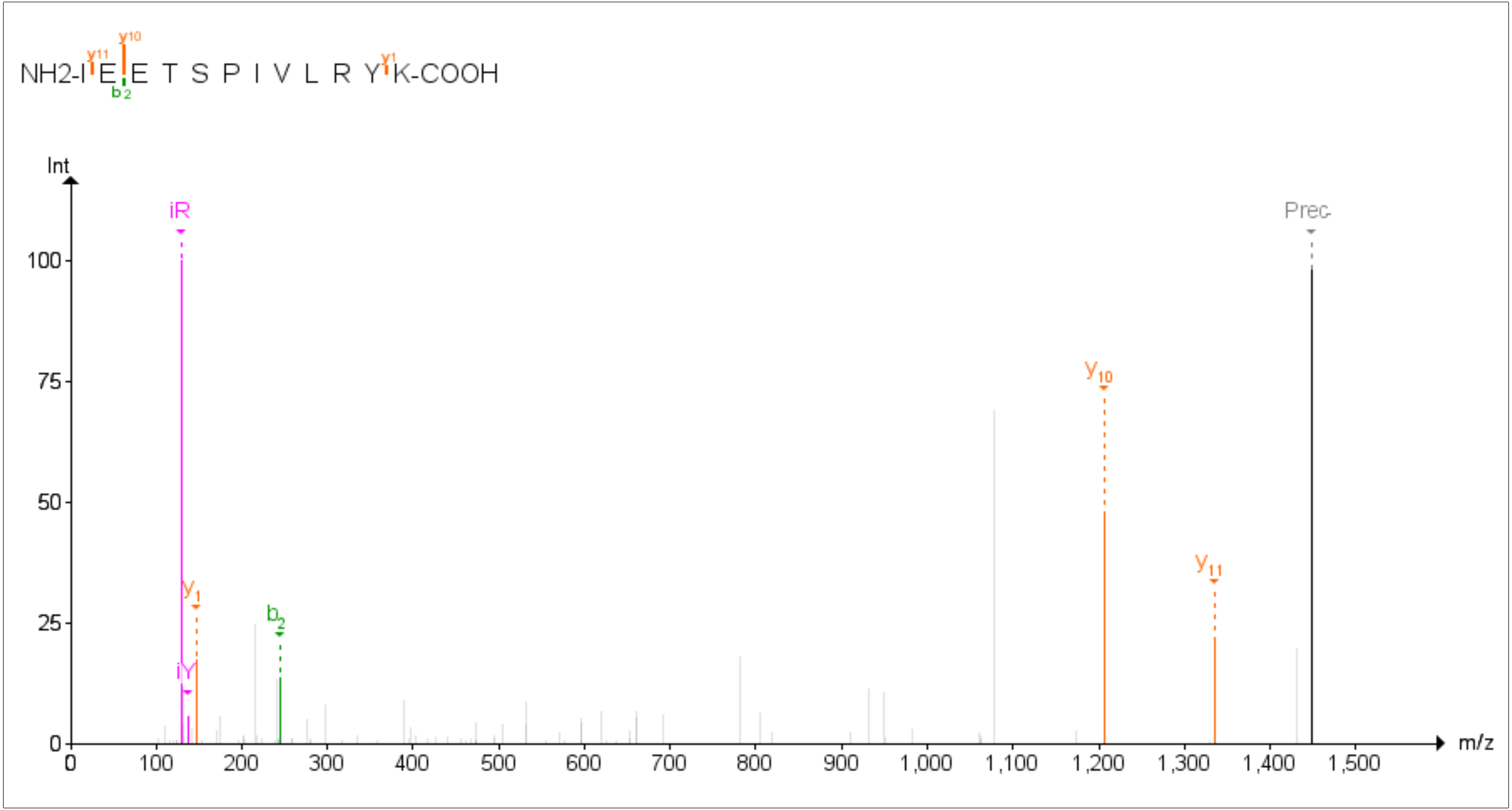
NH2-I E E T S P I V L R Y K-COOH
y11
y10
b2
y1
Int
iR
Prec
y10
y11
y1
b2
iY
100
75
50
25
0
0
100
200
300
400
500
600
700
800
900
1,000
1,100
1,200
1,300
1,400
1,500
m/z

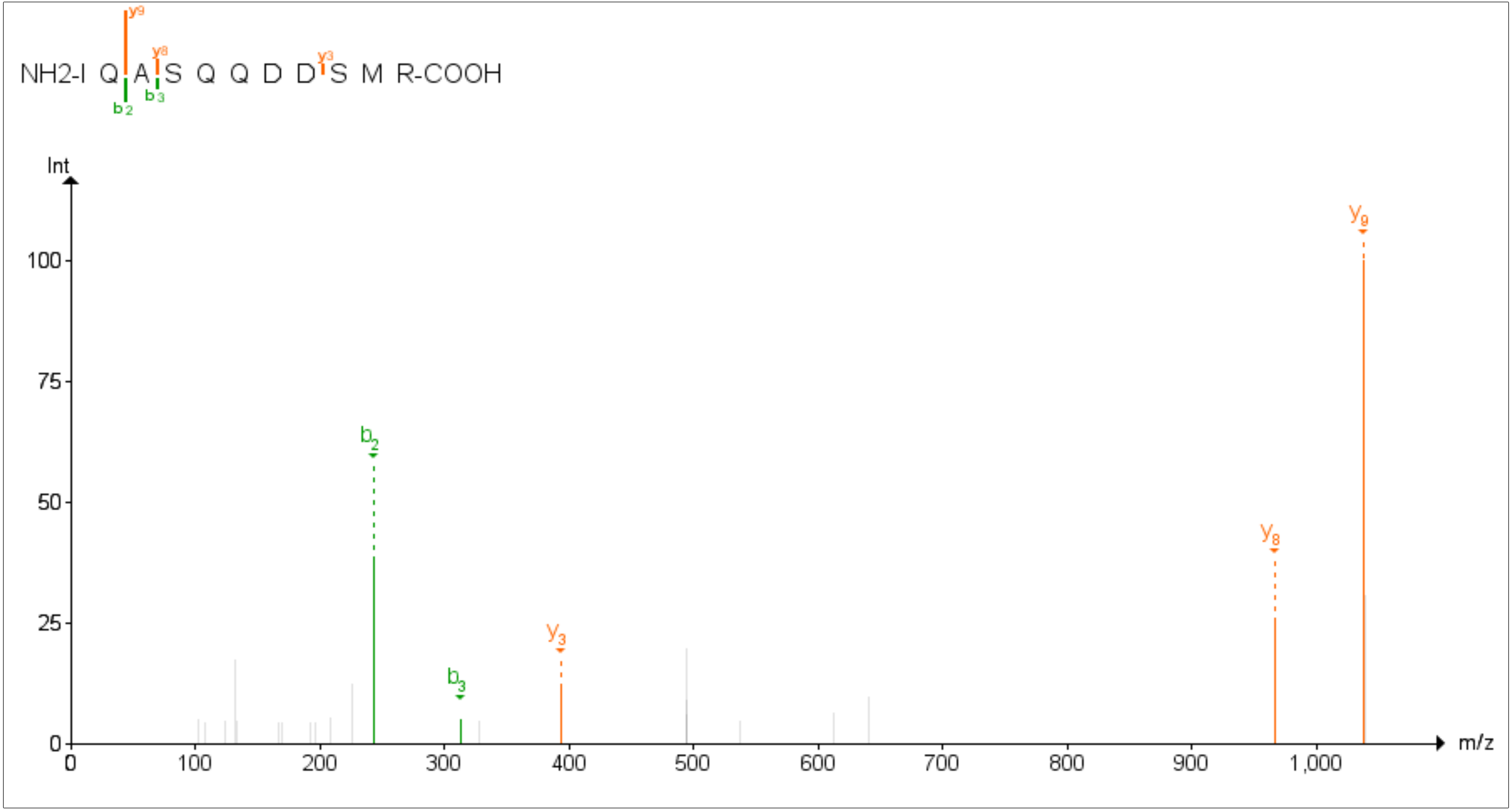

NH2-I Q A S Q Q D D S M R-COOH
y9
y8
y3
b2
b3
Int
100
75
50
25
0
m/z
0
100
200
300
400
500
600
700
800
900
1,000

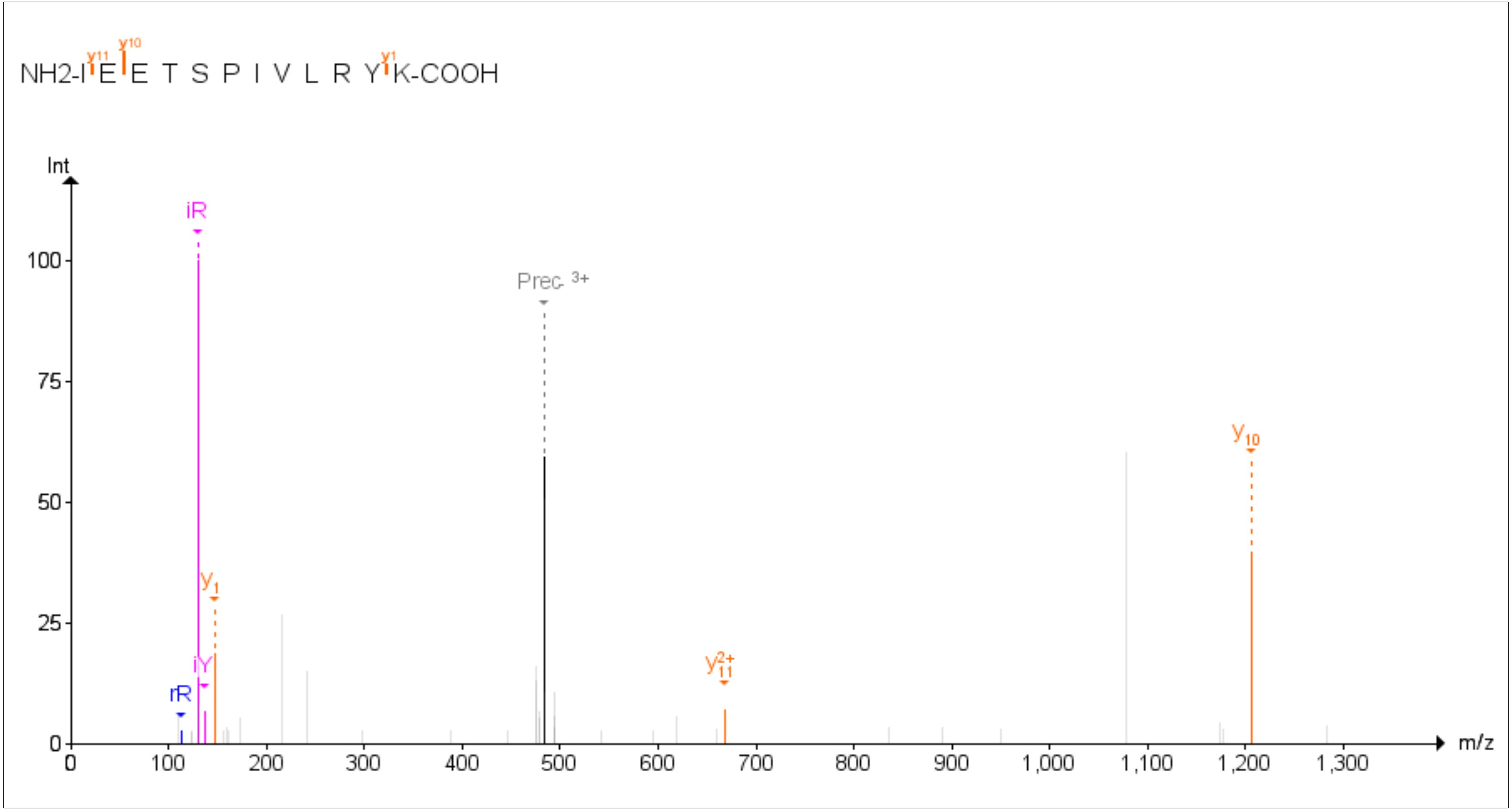
NH2-I E E T S P I V L R Y K-COOH
y11
y10
y1
Int
iR
Prec 3+
y_{10}
y_1
iY
rR
y_{11}^{2+}
100
75
50
25
0
0
100
200
300
400
500
600
700
800
900
1,000
1,100
1,200
1,300
m/z

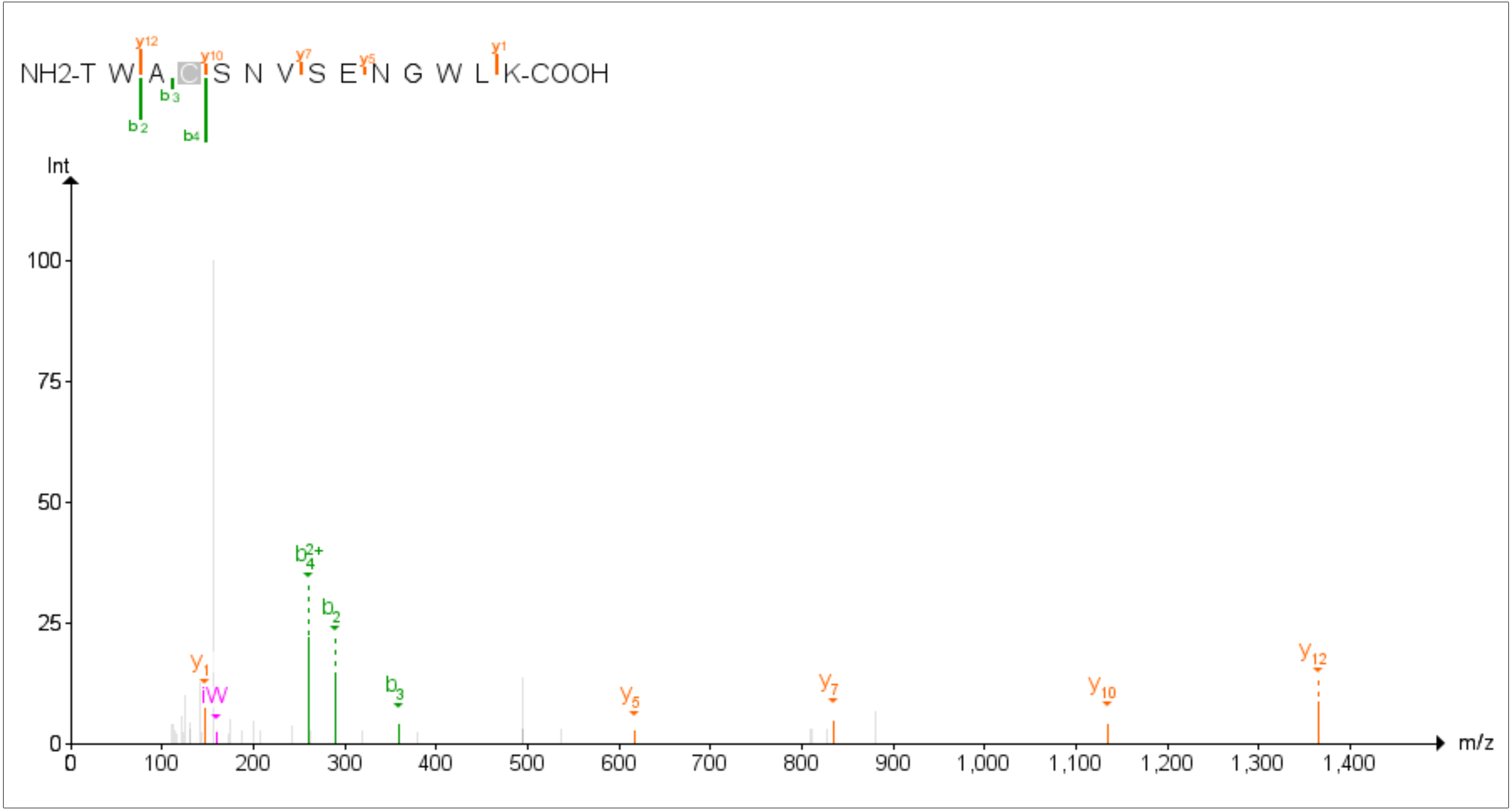

NH2-T W A C S N V S E N G W L K-COOH
y12
y10
y7
y5
y1
b2
b3
b4
Int
100
75
50
25
0
b4 2+
b2
b3
y1
iW
y5
y7
y10
y12
0
100
200
300
400
500
600
700
800
900
1,000
1,100
1,200
1,300
1,400
m/z

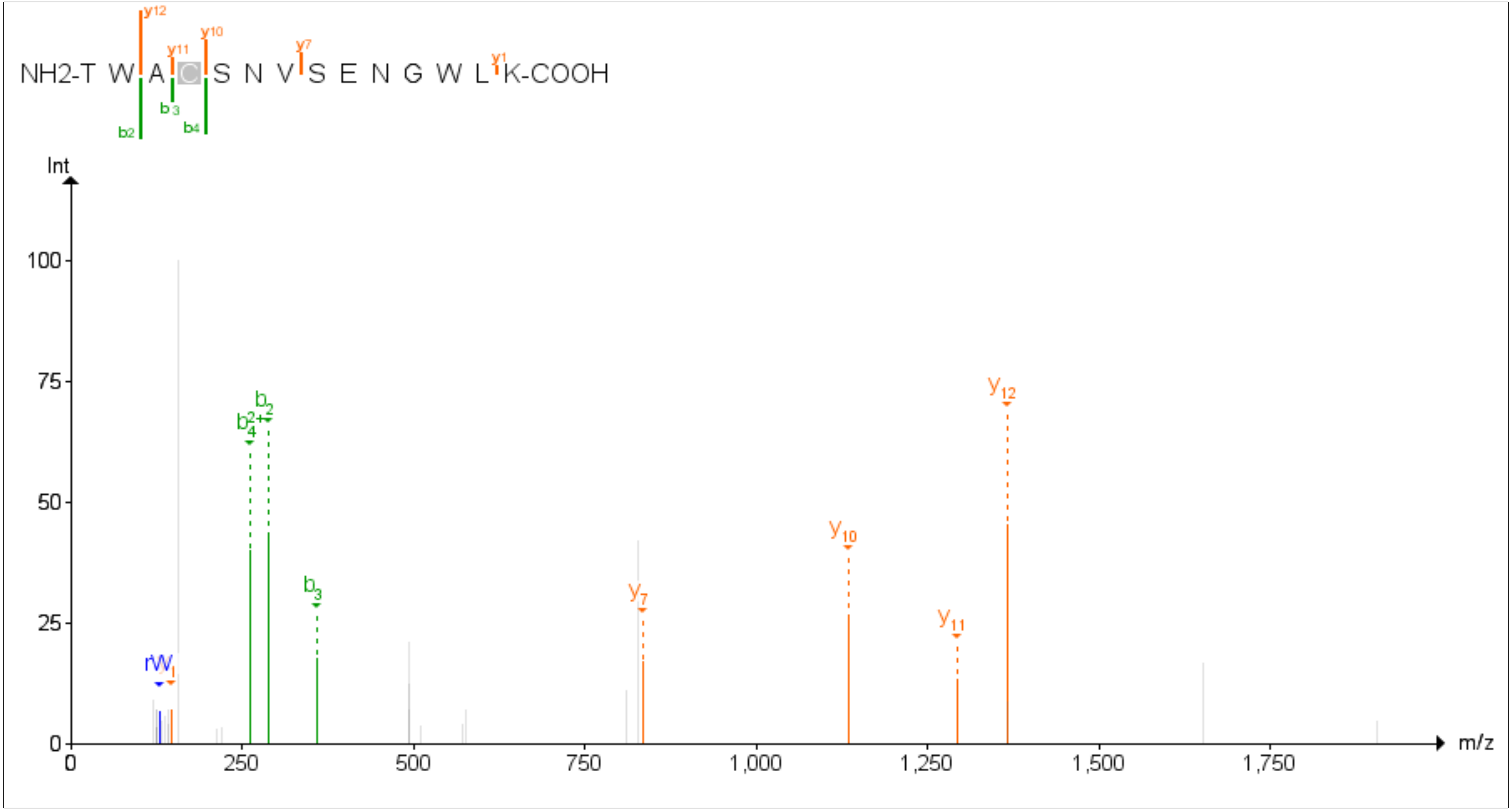
NH2-T W A C S N V S E N G W L K-COOH
y12
y11
y10
y7
y1
b2
b3
b4
Int
100
75
50
25
0
m/z
0
250
500
750
1,000
1,250
1,500
1,750
b2
b4 2+
b3
y7
y10
y11
y12
rW

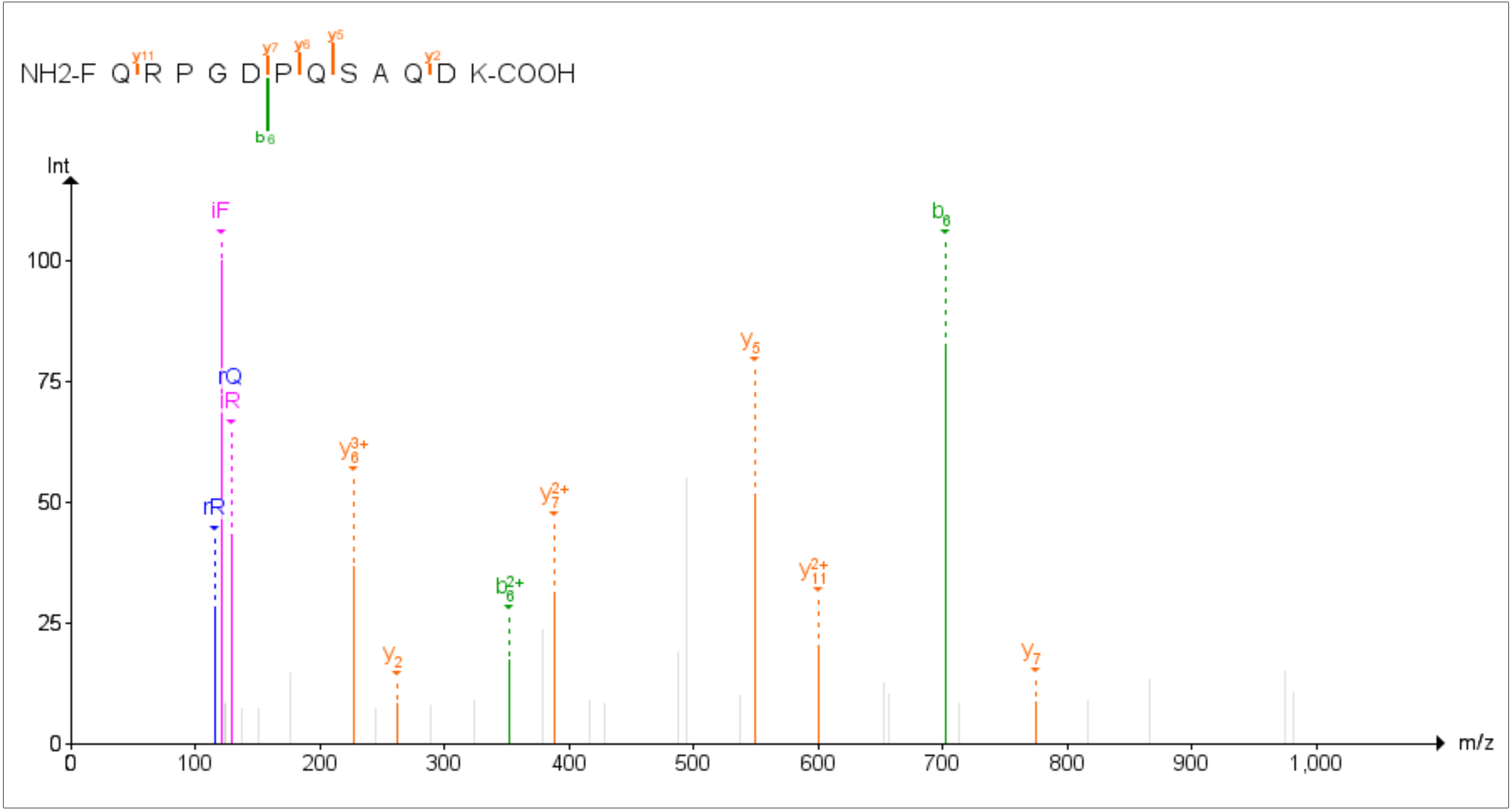

NH2-F Q R P G D P Q S A Q D K-COOH
y11
y7
y6
y5
y2
b6
Int
m/z
iF
rQ
iR
rR
y_6^{3+}
y_2
b_6^{2+}
y_7^{2+}
y_5
y_{11}^{2+}
b_6
y_7
0
25
50
75
100
0
100
200
300
400
500
600
700
800
900
1,000

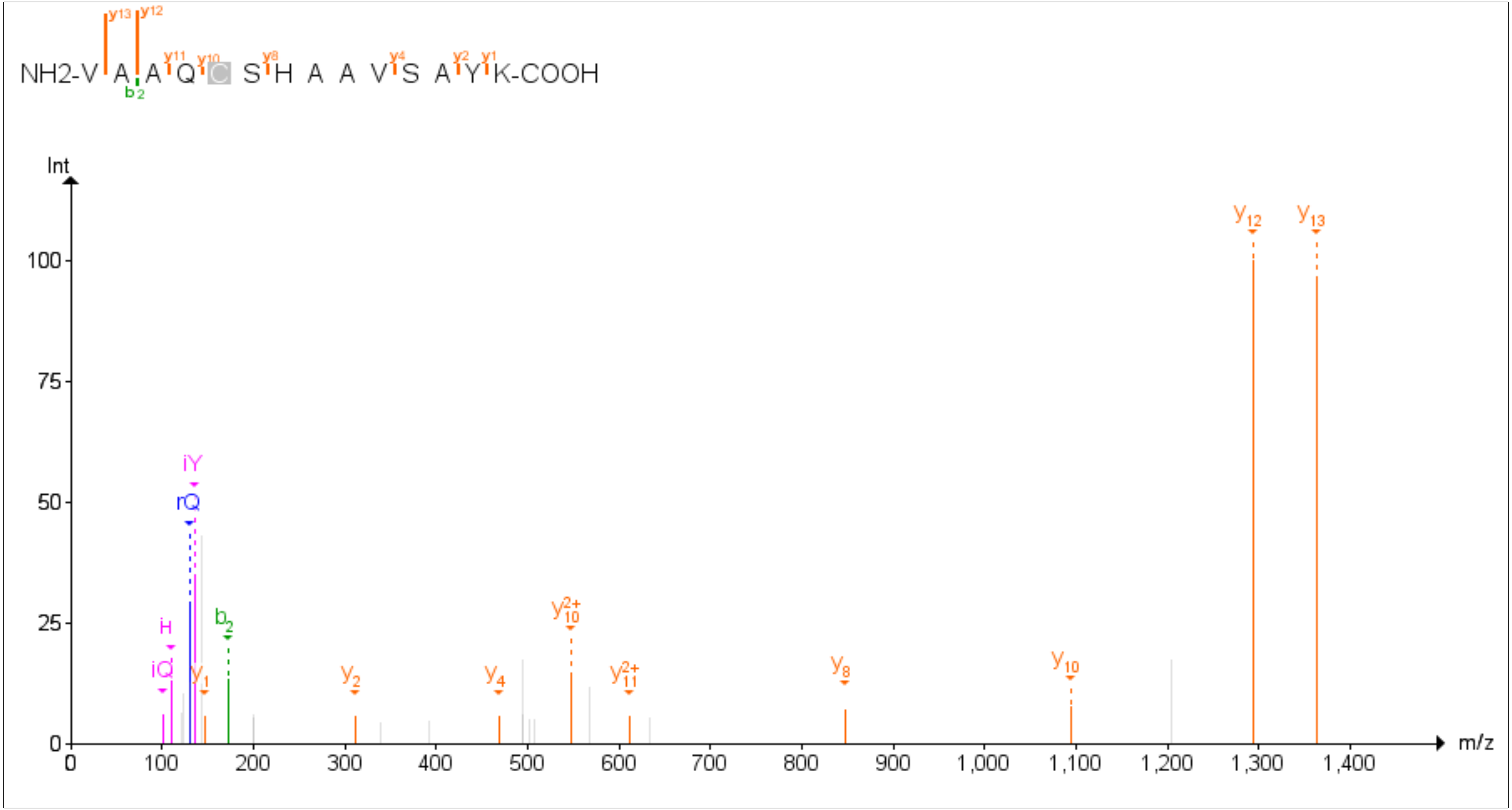
NH2-V A A Q C S H A A V S A Y K-COOH
y13 y12 y11 y10 y8 y4 y2 y1
b2
Int
100
75
50
25
0
iY
rQ
iH
iQ
y1
b2
y2
y4
y10 2+
y11 2+
y8
y10
y12
y13
0
100
200
300
400
500
600
700
800
900
1,000
1,100
1,200
1,300
1,400
m/z

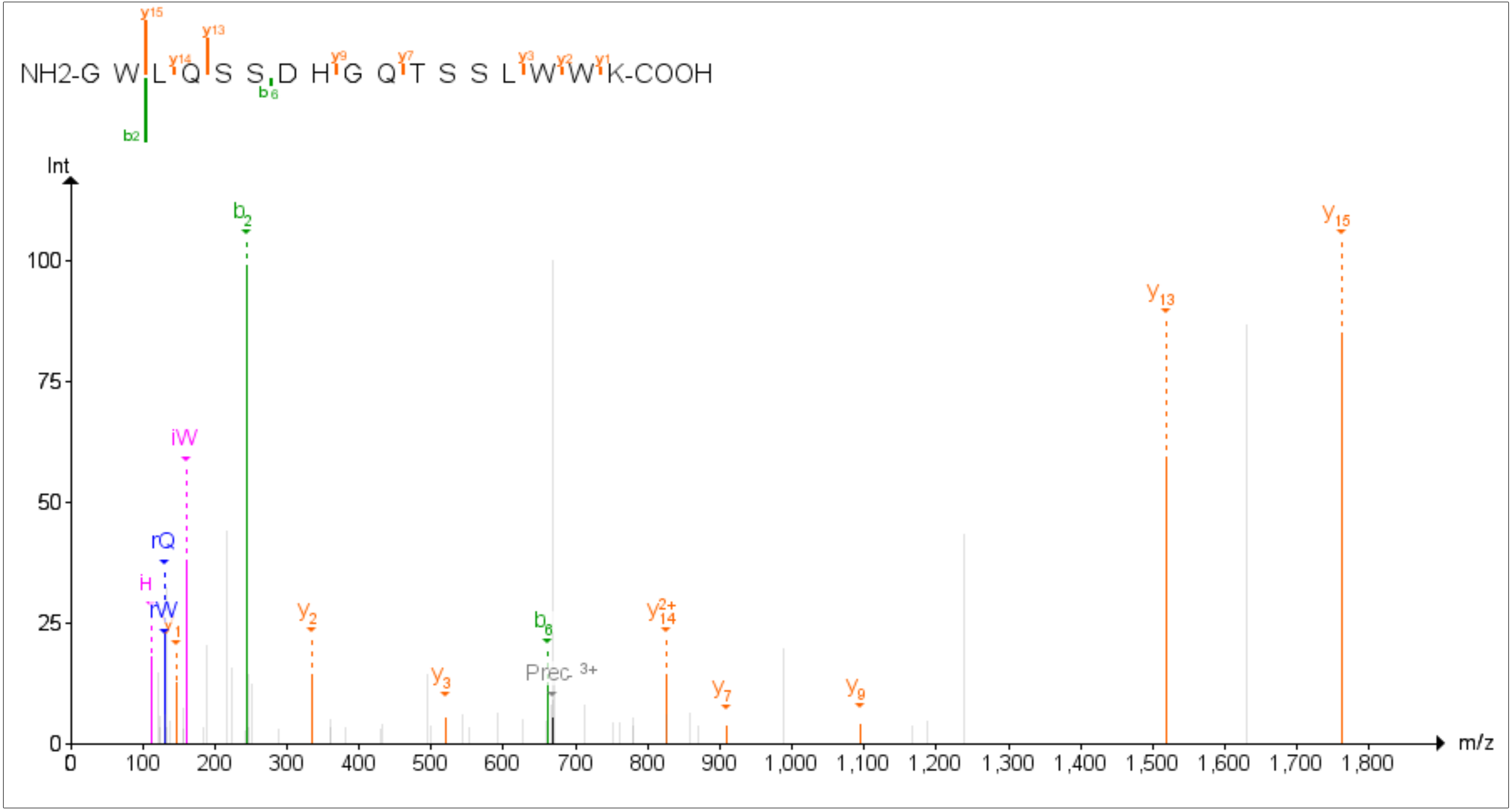

NH2-G W L Q S S D H G Q T S S L W W K-COOH
y15
y14
y13
y9
y7
y3
y2
y1
b2
b6
Int
100
75
50
25
0
b2
y15
y13
iW
rQ
iH
rW
y1
y2
y3
b6
Prec 3+
y14 2+
y7
y9
0
100
200
300
400
500
600
700
800
900
1,000
1,100
1,200
1,300
1,400
1,500
1,600
1,700
1,800
m/z

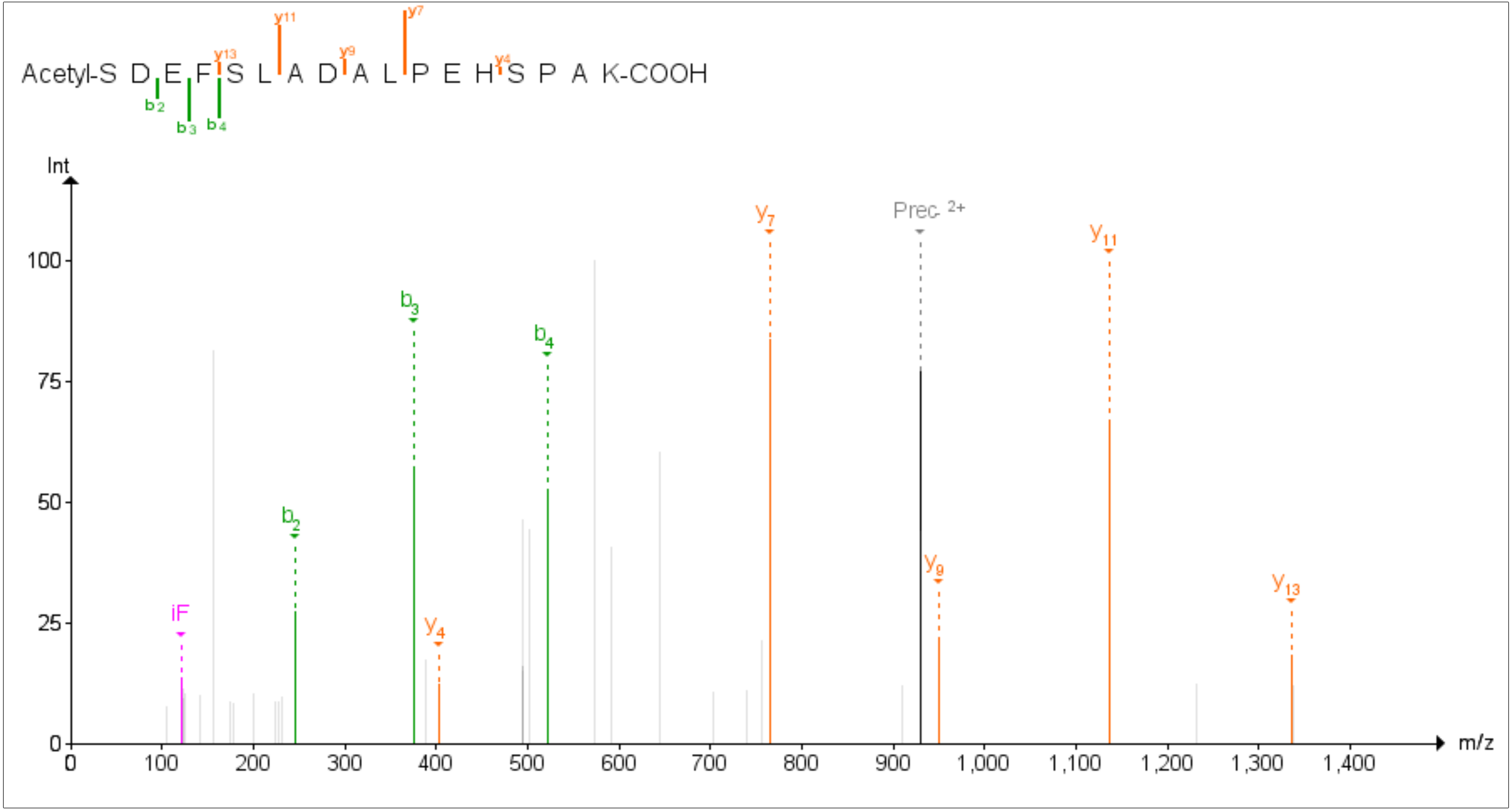

Acetyl-S D E F S L A D A L P E H S P A K-COOH
b2 b3 b4
y13 y11 y9 y7 y4
Int
m/z
Prec. 2+
iF

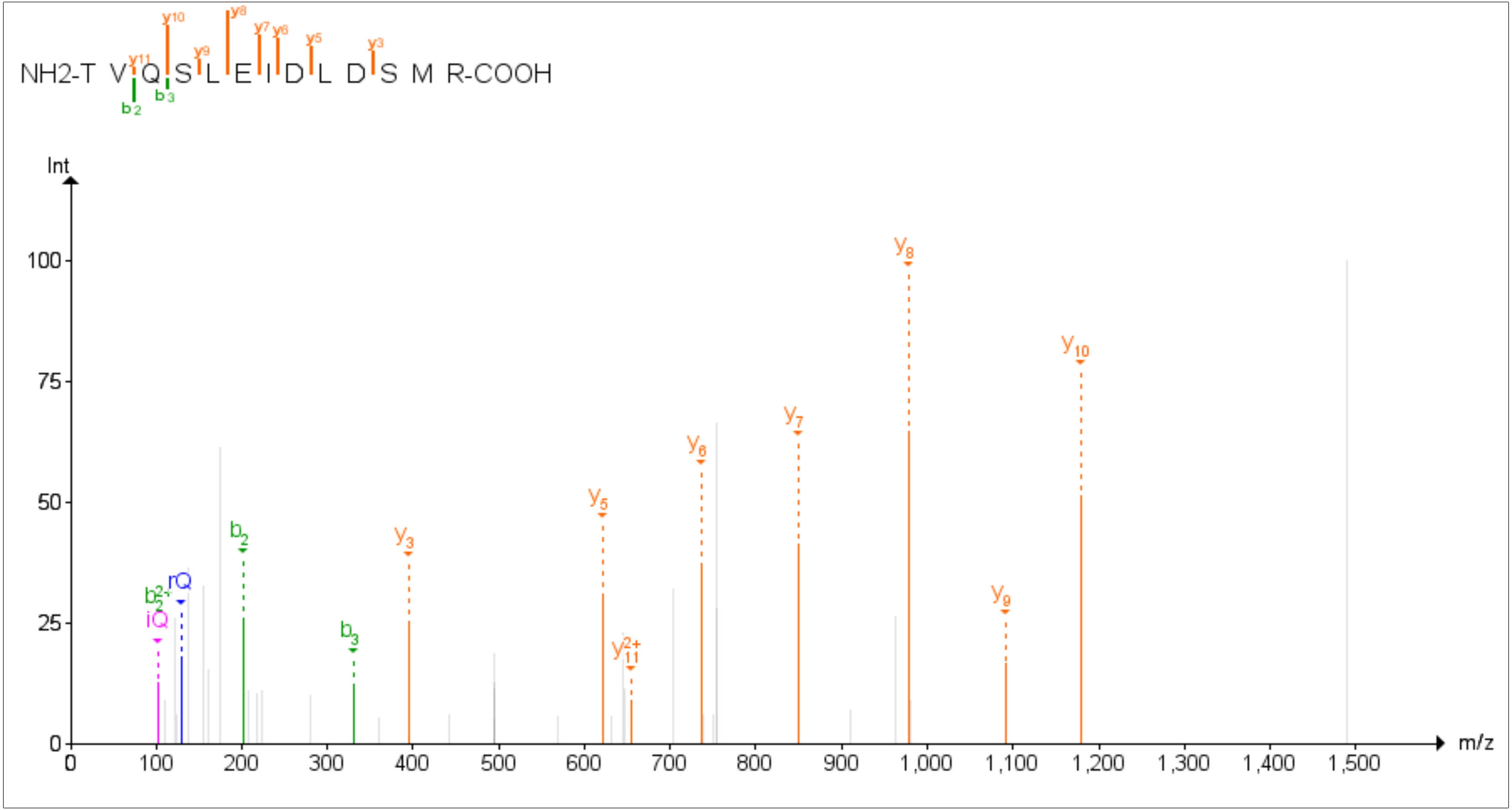

NH2-T V Q S L E I D L D S M R-COOH
y11 y10 y9 y8 y7 y6 y5 y3
b2 b3
Int
100
75
50
25
0
y8
y10
y7
y6
y5
b2
y3
rQ
b2 2+
iQ
b3
y11 2+
y9
0 100 200 300 400 500 600 700 800 900 1,000 1,100 1,200 1,300 1,400 1,500
m/z

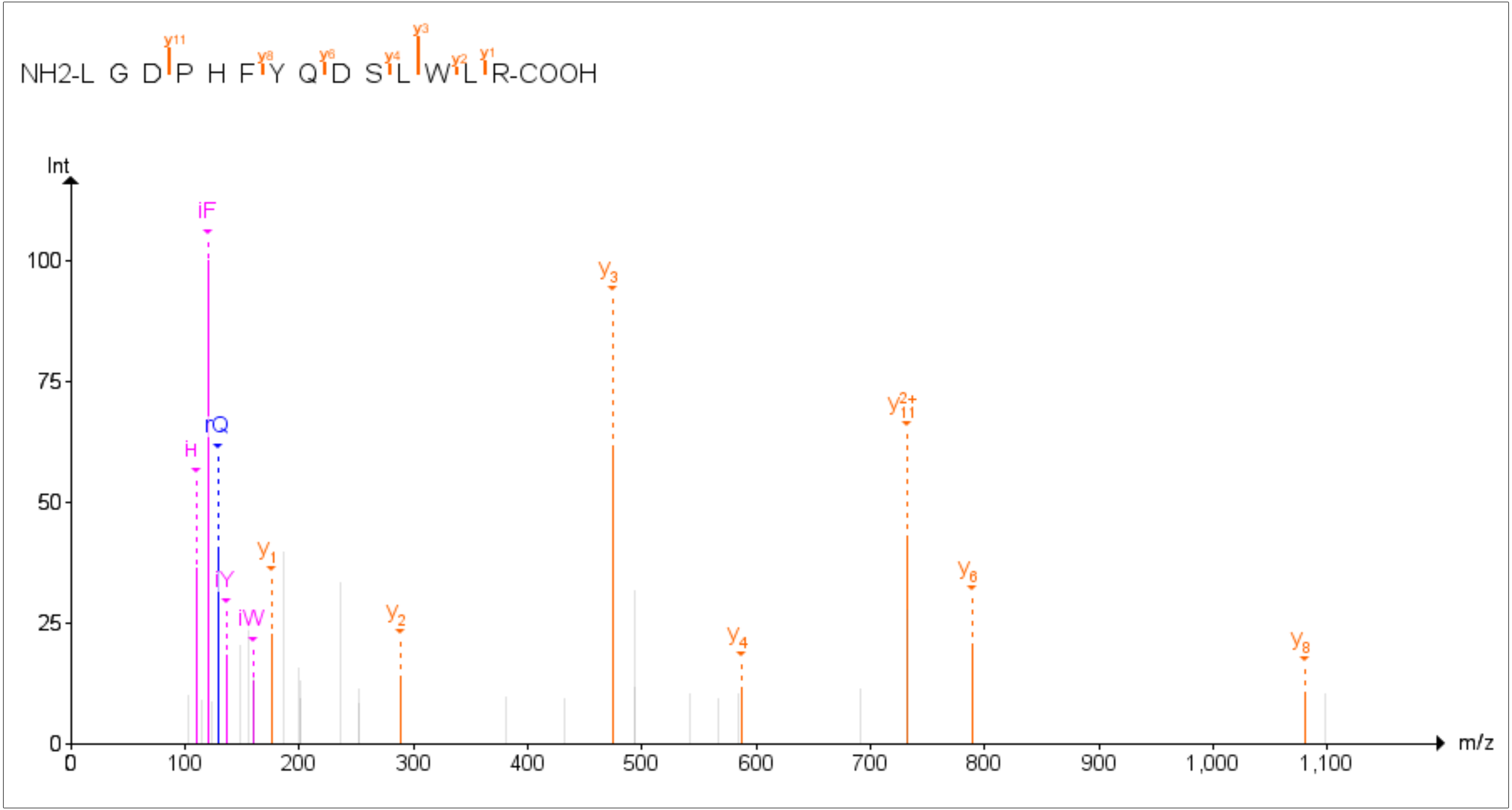

NH2-L G D P H F Y Q D S L W L R-COOH
y11
y8
y6
y4
y3
y2
y1
Int
iF
rQ
iH
iY
iW
y1
y2
y3
y4
y11 2+
y6
y8
m/z
0
25
50
75
100
0
100
200
300
400
500
600
700
800
900
1,000
1,100

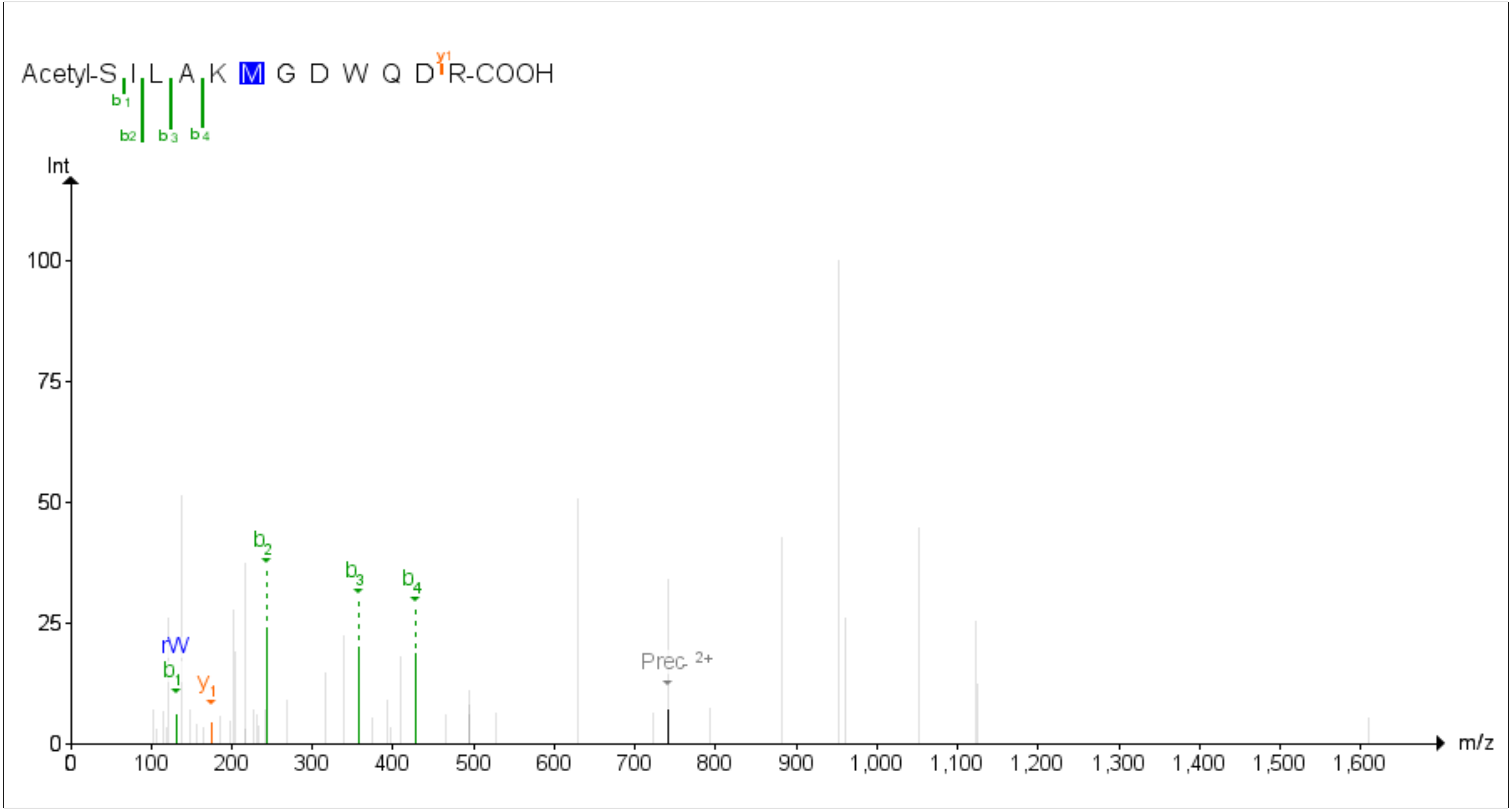

Acetyl-S I L A K M G D W Q D R-COOH
b1
b2
b3
b4
y1
Int
100
75
50
25
0
rW
b1
y1
b2
b3
b4
Prec- 2+
0
100
200
300
400
500
600
700
800
900
1,000
1,100
1,200
1,300
1,400
1,500
1,600
m/z

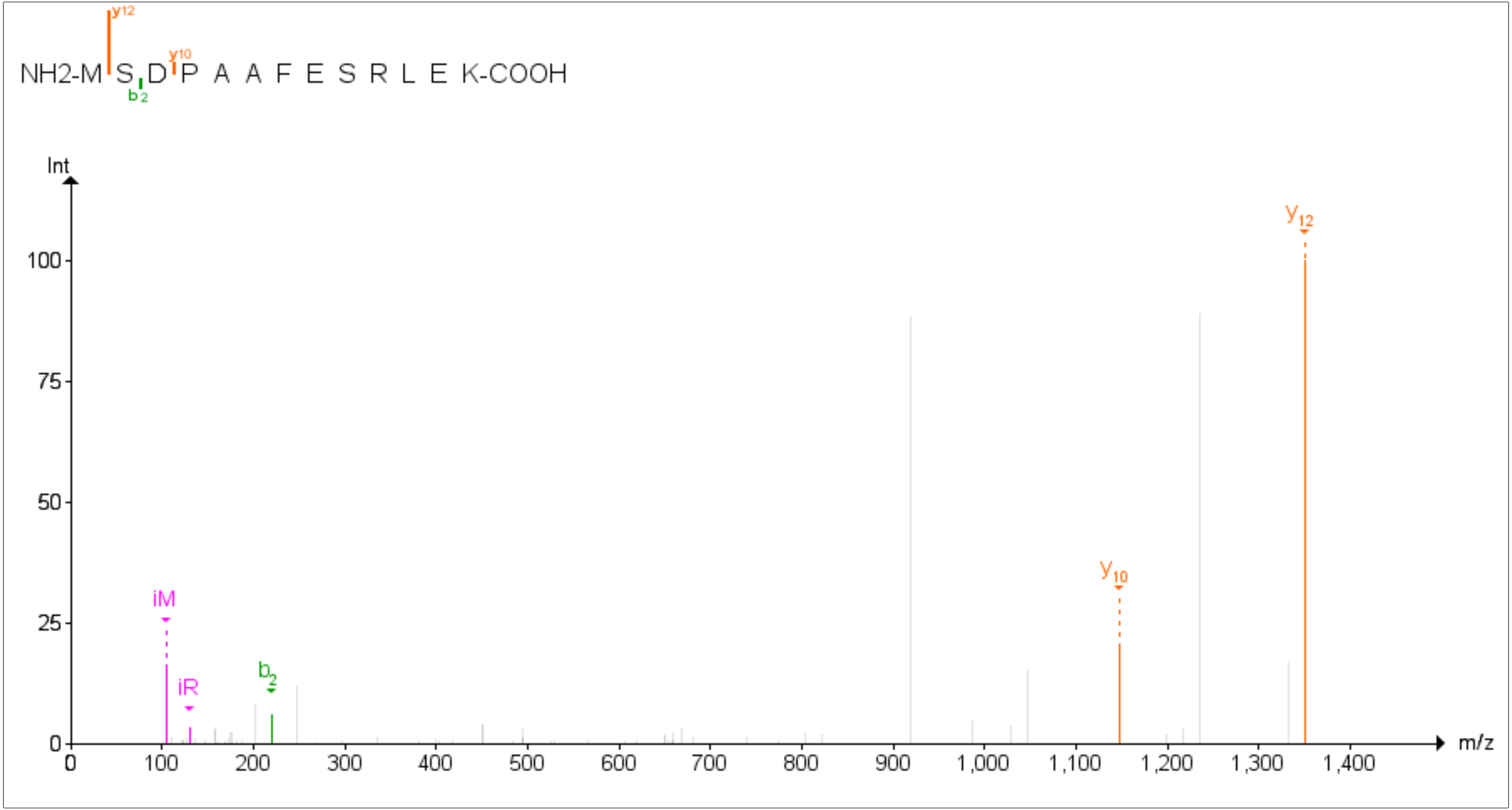

NH2-M S D P A A F E S R L E K-COOH
y12
y10
b2
Int
100
75
50
25
0
iM
iR
b2
y10
y12
0
100
200
300
400
500
600
700
800
900
1,000
1,100
1,200
1,300
1,400
m/z

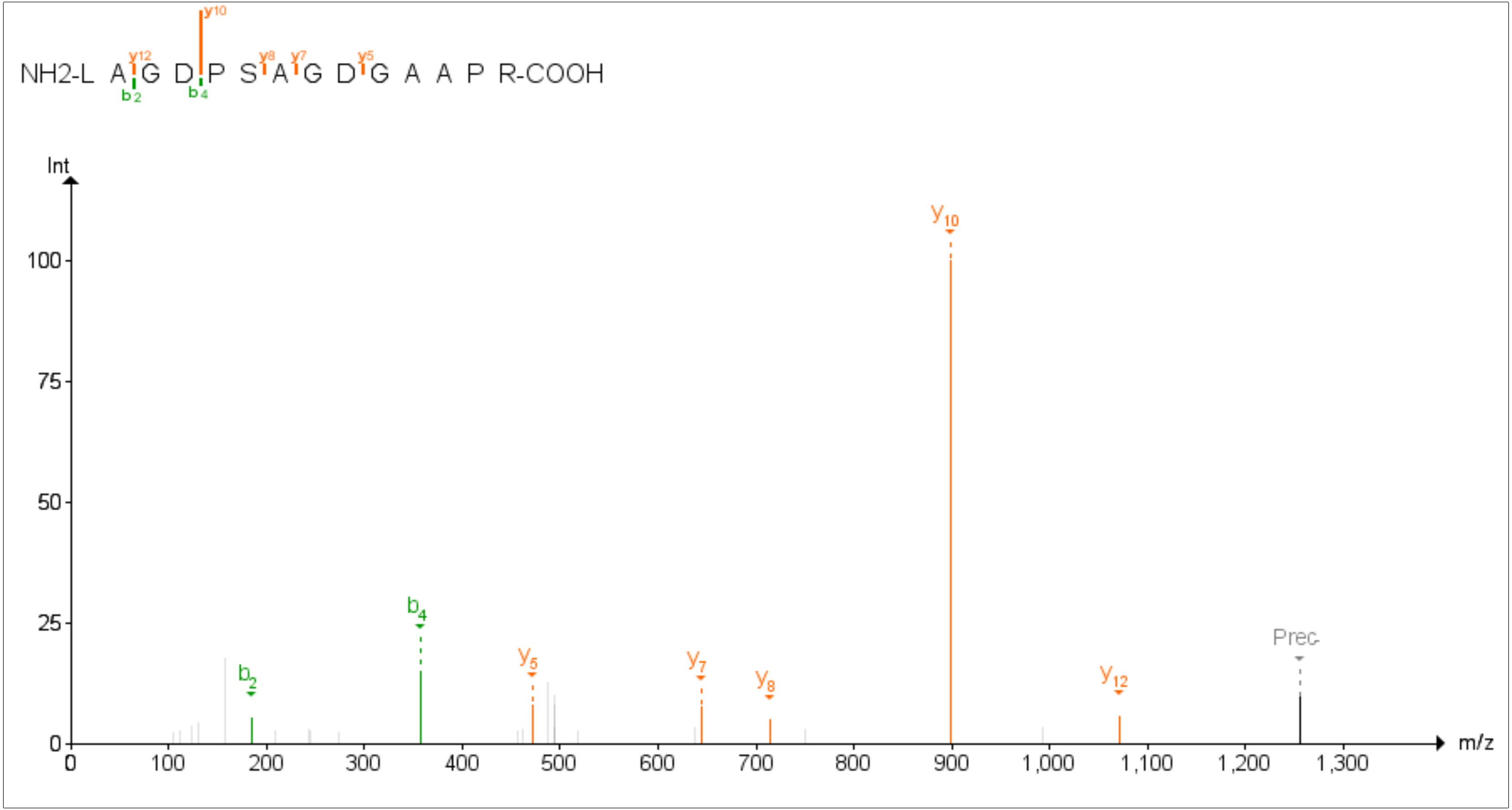

NH2-L A G D P S A G D G A A P R-COOH
y10
y12
y8
y7
y5
b2
b4
Int
100
75
50
25
0
y10
b4
y5
y7
y8
y12
b2
Prec
0
100
200
300
400
500
600
700
800
900
1,000
1,100
1,200
1,300
m/z

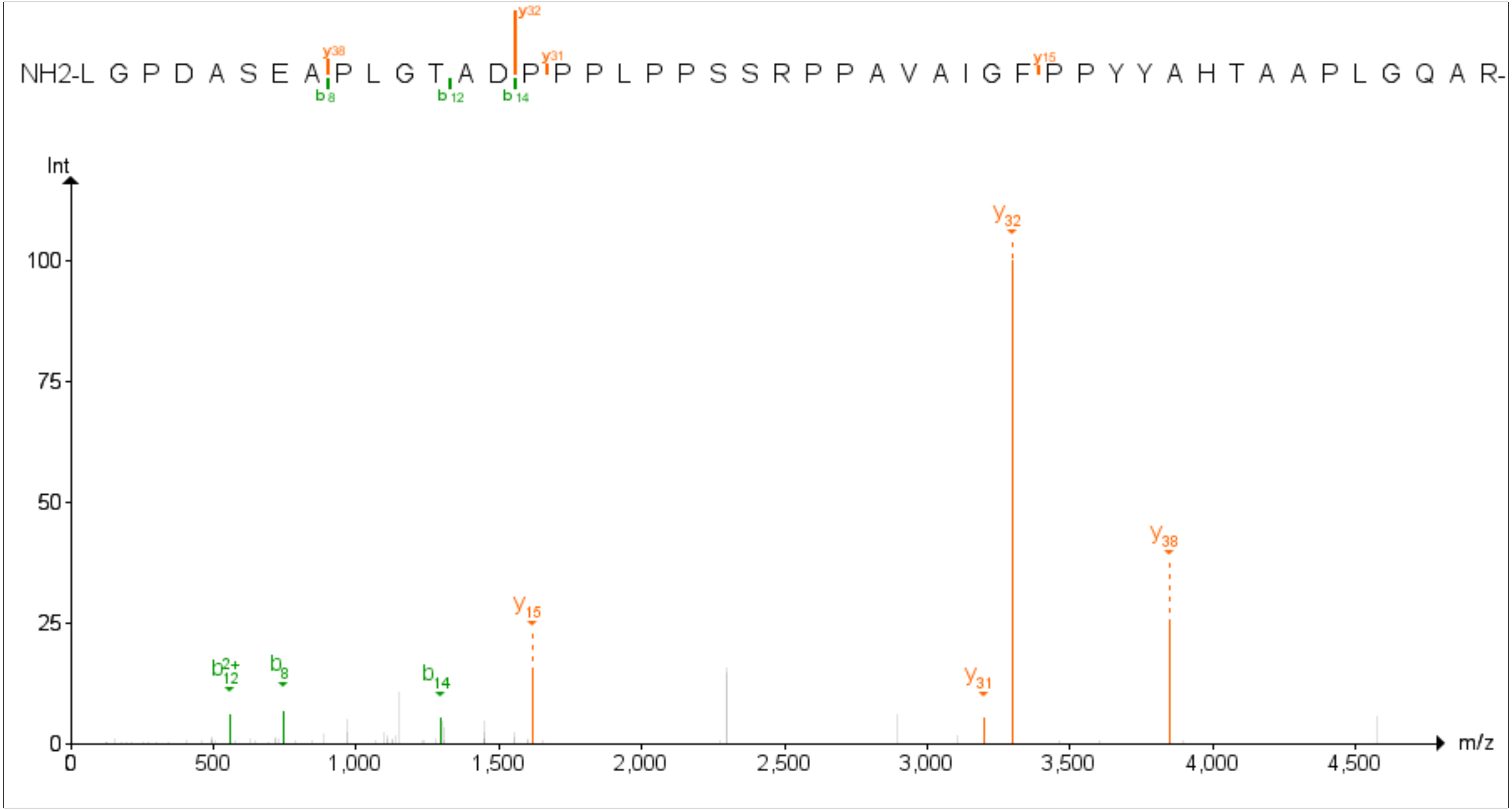

NH2-L G P D A S E A P L G T A D P P P L P P S S R P P A V A I G F P P Y Y A H T A A P L G Q A R-
y38
y32
y31
y15
b 8
b 12
b 14
Int
100
75
50
25
0
y32
y38
y15
b2+ 12
b8
b14
y31
0
500
1,000
1,500
2,000
2,500
3,000
3,500
4,000
4,500
m/z

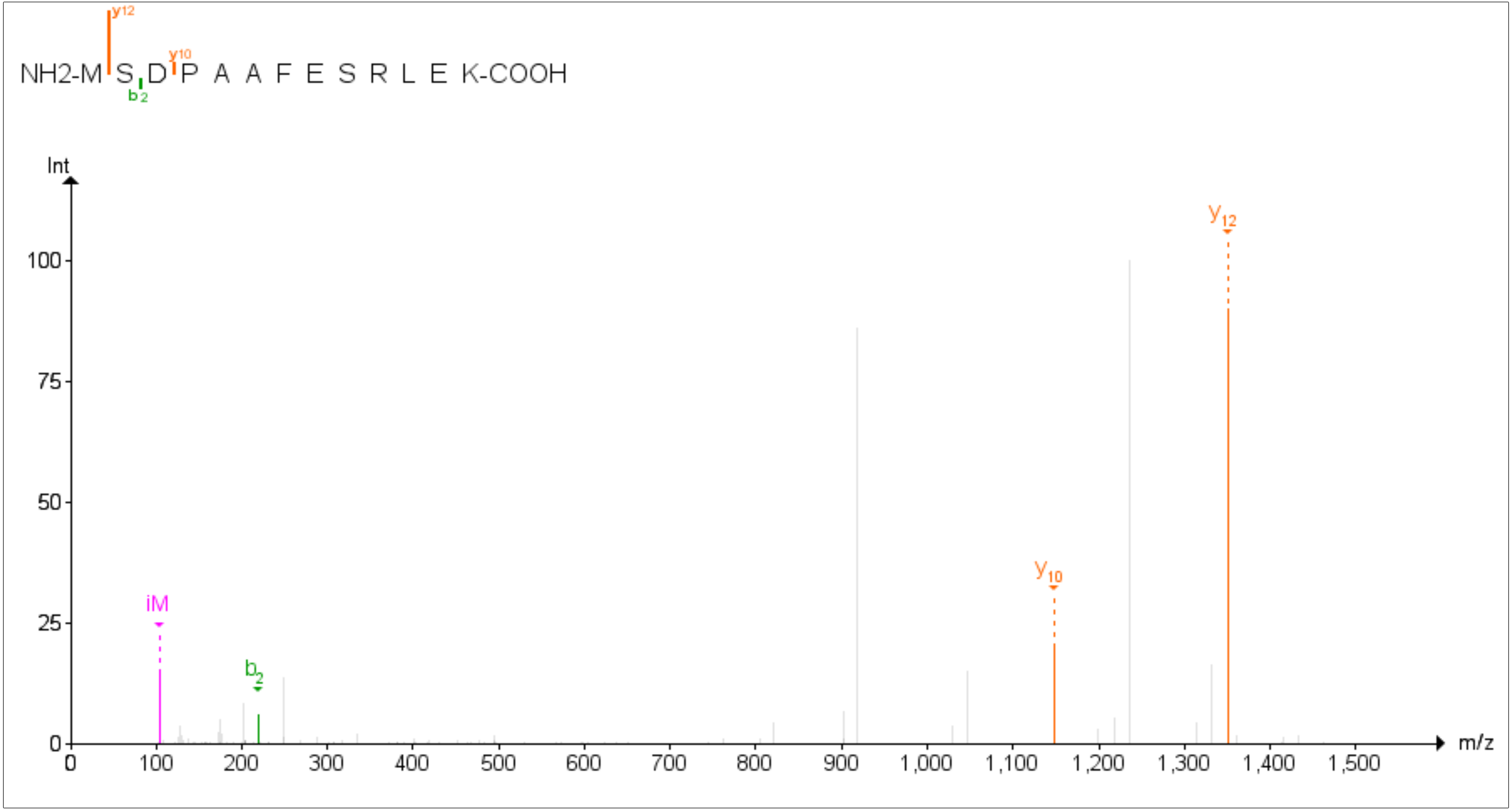

NH2-M S D P A A F E S R L E K-COOH
y12
y10
b2
Int
100
75
50
25
0
iM
b2
y10
y12
m/z
0
100
200
300
400
500
600
700
800
900
1,000
1,100
1,200
1,300
1,400
1,500

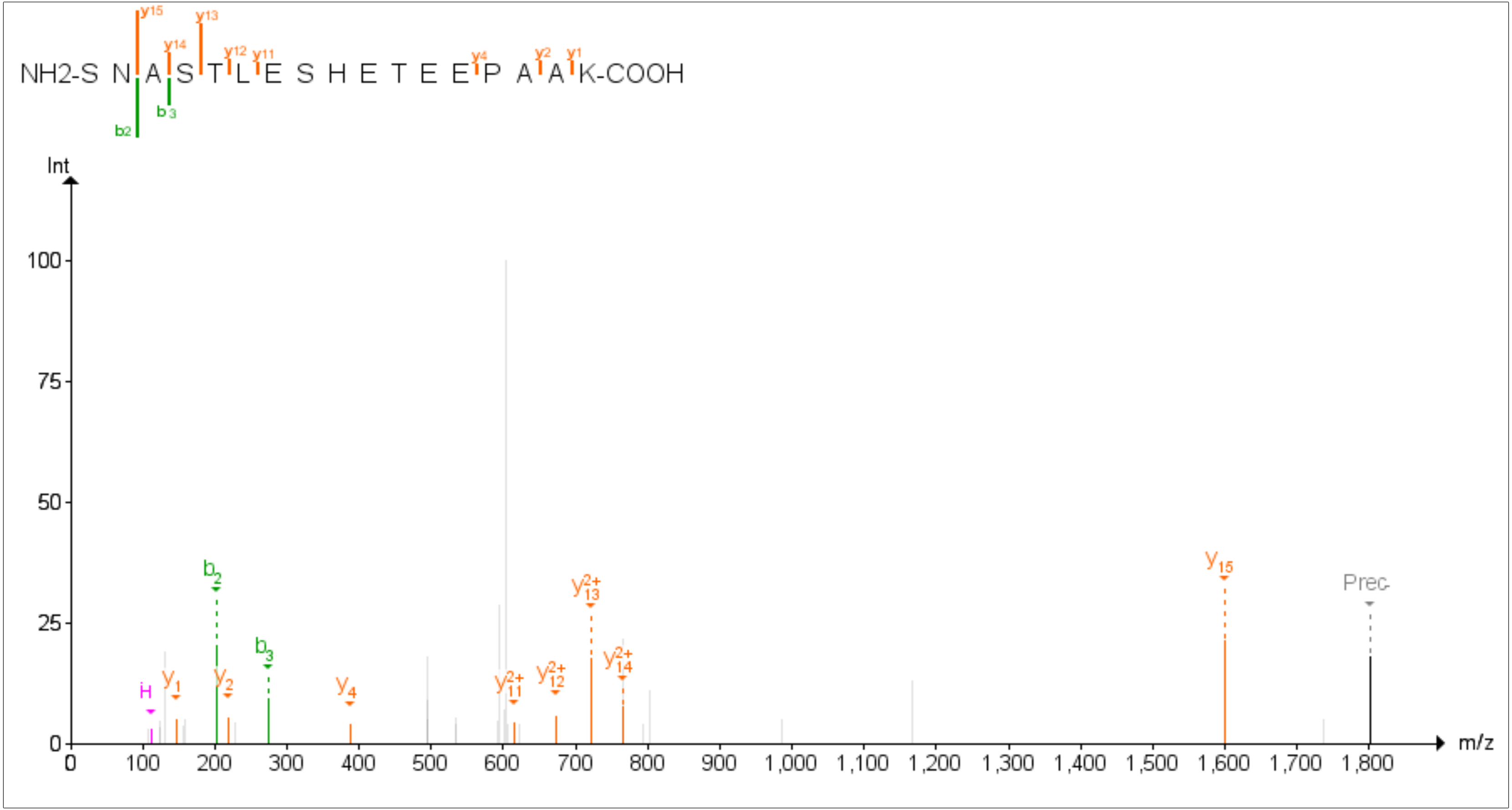

NH2-S N A S T L E S H E T E E P A A K-COOH
y15
y14
y13
y12
y11
y4
y2
y1
b2
b3
Int
100
75
50
25
0
H
y1
b2
y2
b3
y4
y11 2+
y12 2+
y13 2+
y14 2+
y15
Prec.
0
100
200
300
400
500
600
700
800
900
1,000
1,100
1,200
1,300
1,400
1,500
1,600
1,700
1,800
m/z

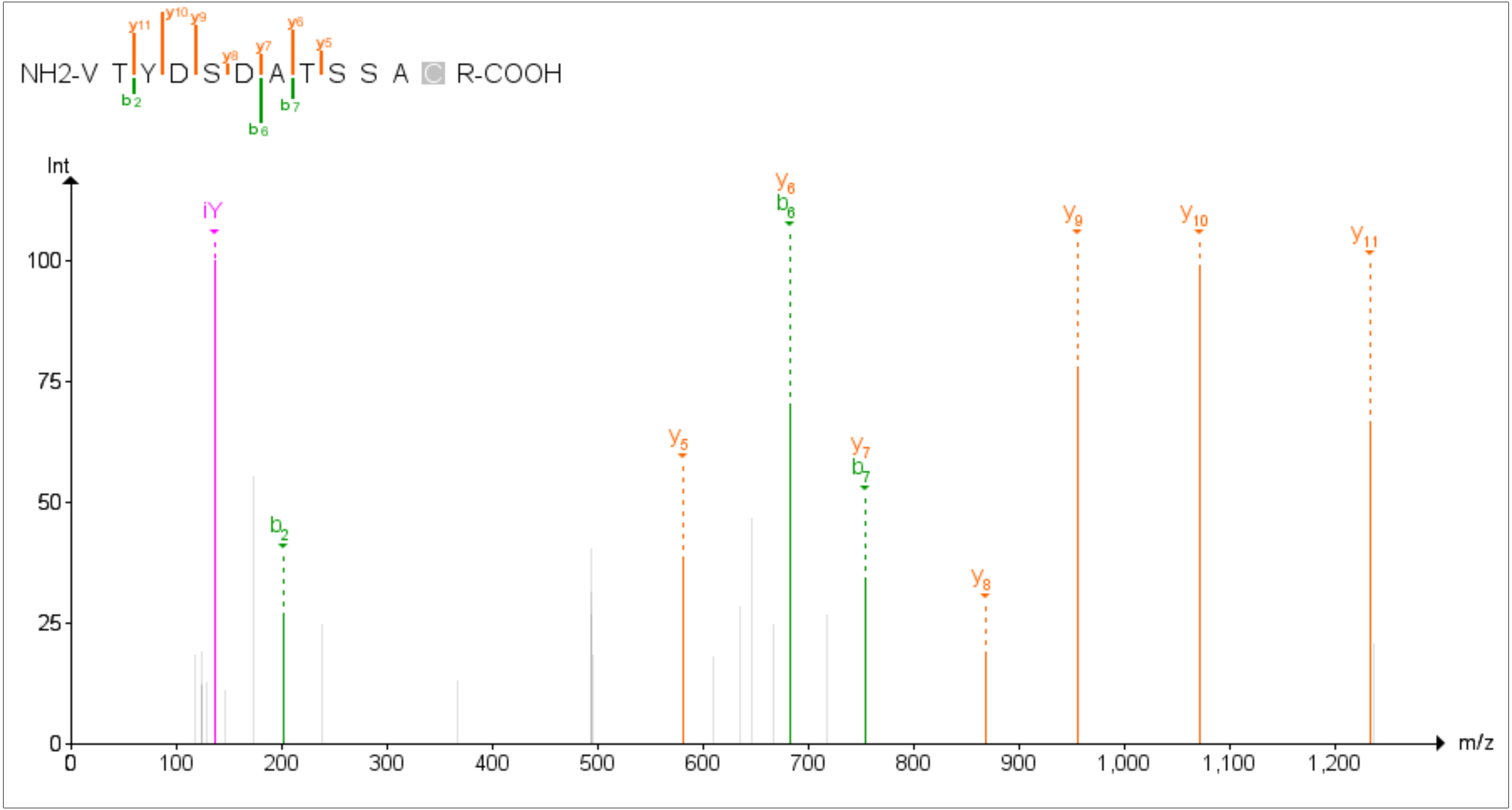

NH2-V T Y D S D A T S S A C R-COOH
y11 y10 y9 y8 y7 y6 y5
b2 b6 b7
Int
m/z
iY
b2
y5
y6
b6
y7
b7
y8
y9
y10
y11
0
25
50
75
100
0
100
200
300
400
500
600
700
800
900
1,000
1,100
1,200

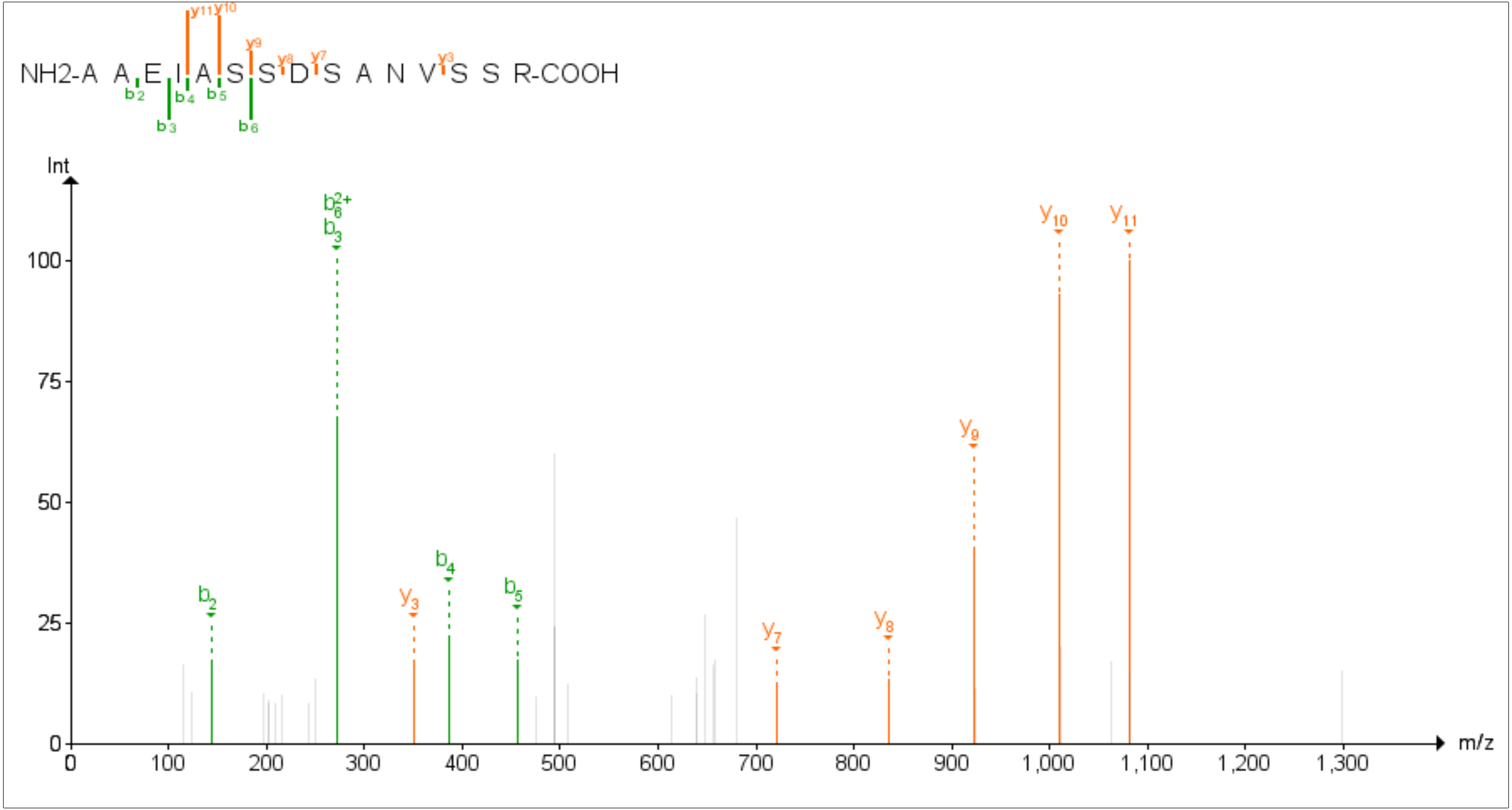

NH2-A A E I A S S D S A N V S S R-COOH
y11 y10 y9 y8 y7 y3
b2 b3 b4 b5 b6
Int
100
75
50
25
0
b6 2+
b3
b2
y3
b4
b5
y7
y8
y9
y10
y11
0
100
200
300
400
500
600
700
800
900
1,000
1,100
1,200
1,300
m/z

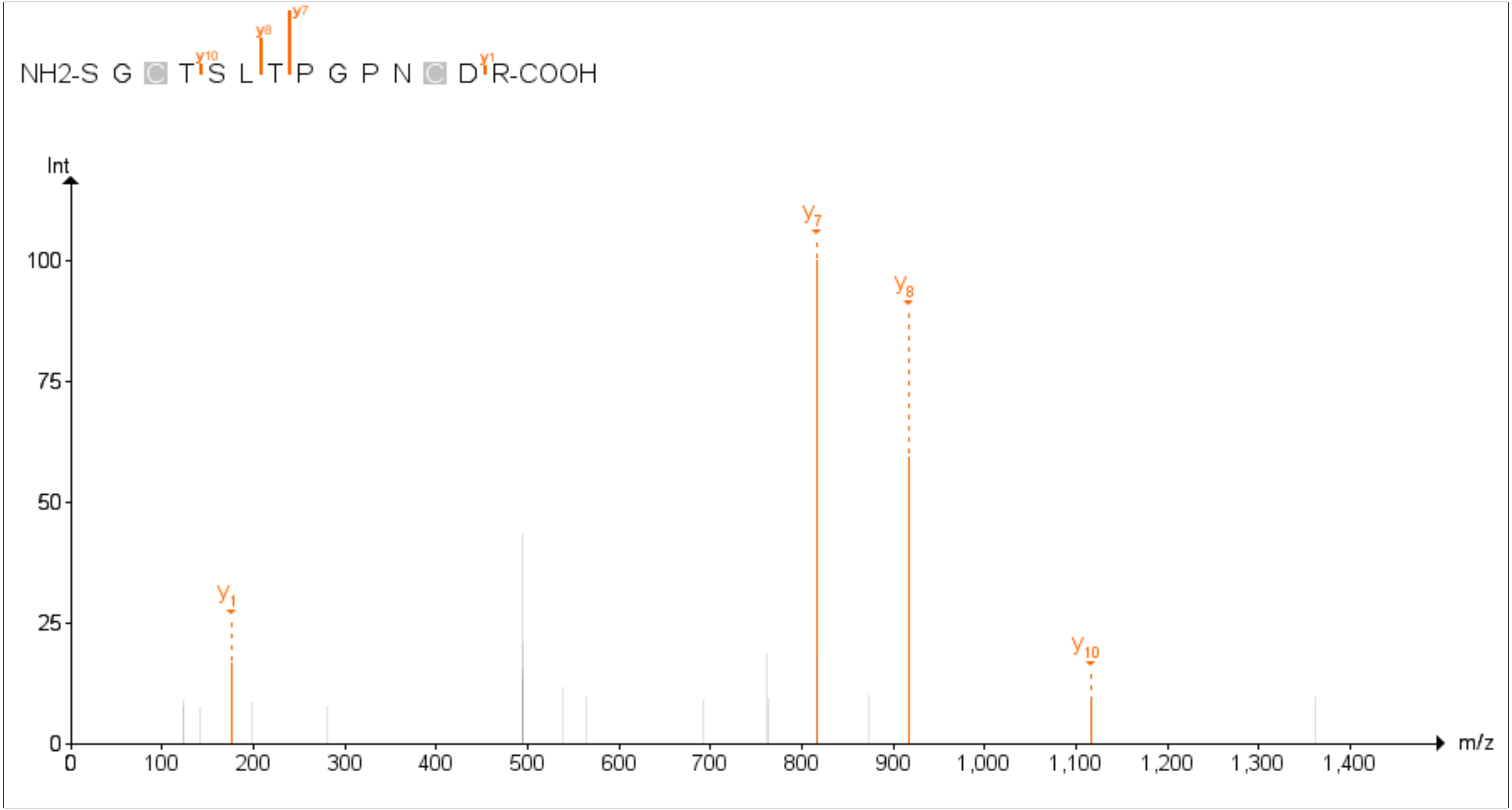

NH2-S G C T S L T P G P N C D R-COOH
y10
y8
y7
y1
Int
100
75
50
25
0
y7
y8
y1
y10
0
100
200
300
400
500
600
700
800
900
1,000
1,100
1,200
1,300
1,400
m/z

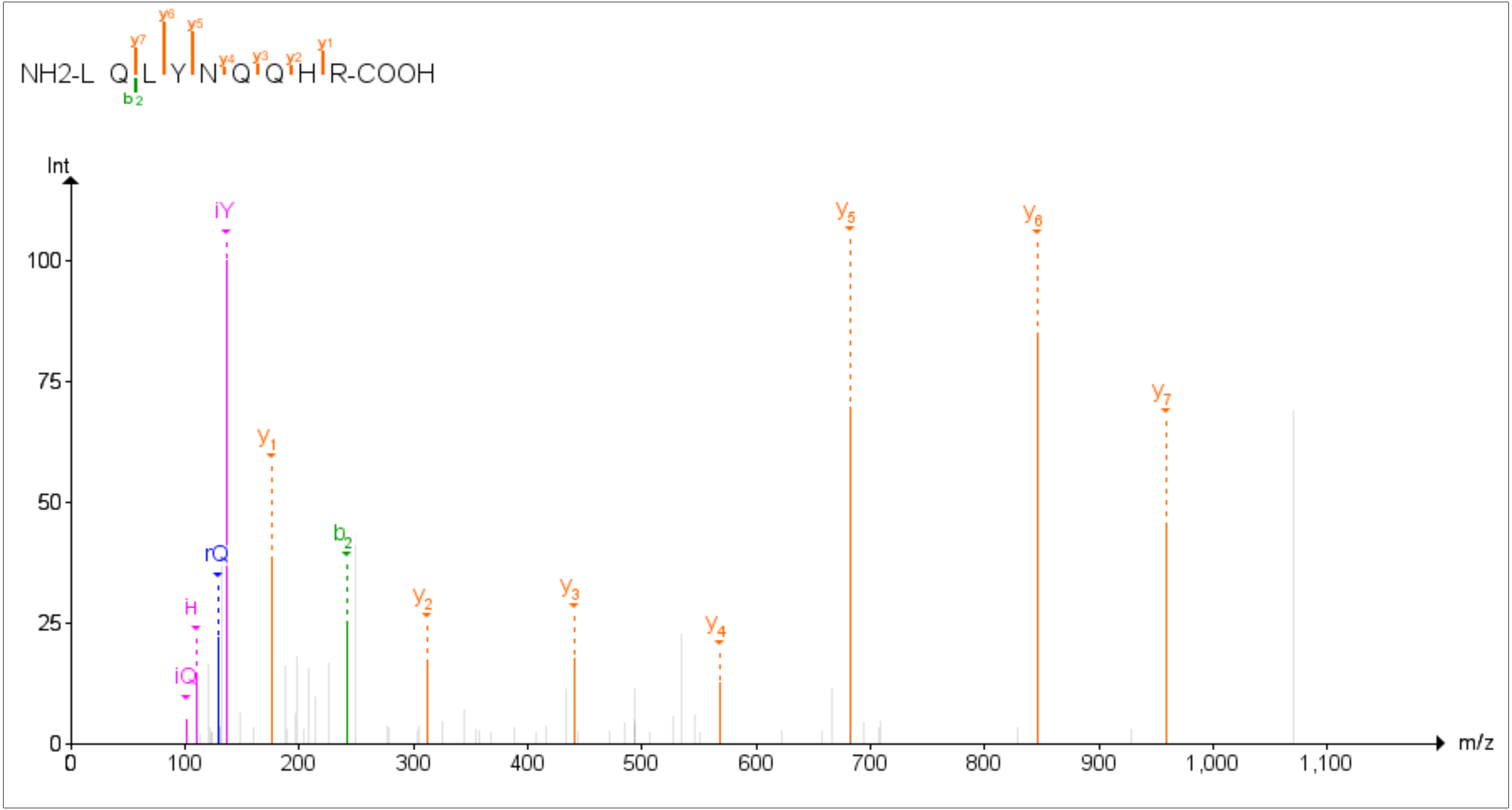

NH2-L Q L Y N Q Q H R-COOH
y7 y6 y5 y4 y3 y2 y1
b2
Int
100
75
50
25
0
iY
y5
y6
y7
y1
b2
rQ
iH
iQ
y2
y3
y4
0 100 200 300 400 500 600 700 800 900 1,000 1,100
m/z

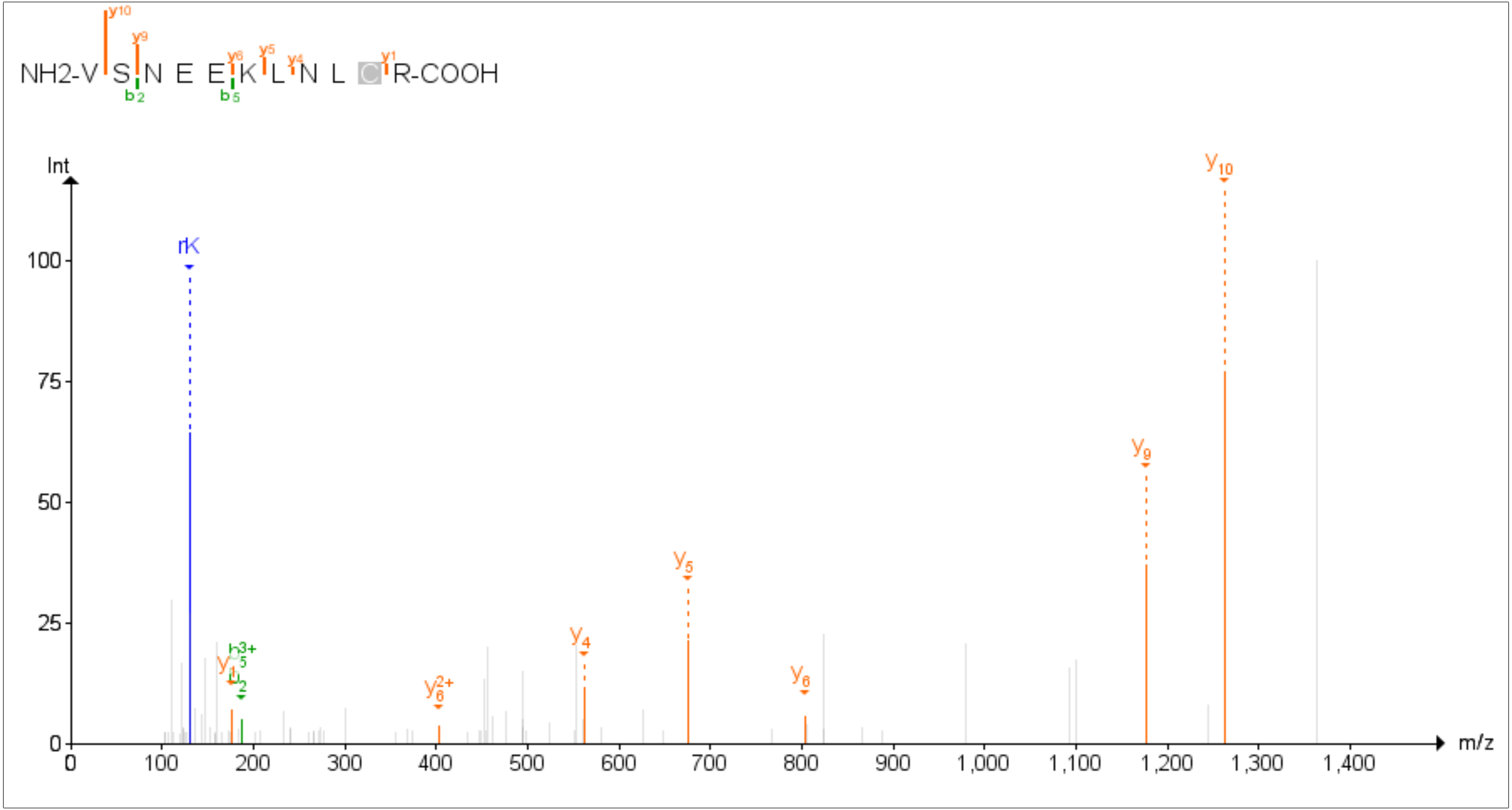
NH2-V S N E E K L N L C R-COOH
y10
y9
y6
y5
y4
y1
b2
b5
Int
rK
y10
y9
y5
y4
y6
y_6^{2+}
y1
b2
b_5^{3+}
m/z
0
25
50
75
100
0
100
200
300
400
500
600
700
800
900
1,000
1,100
1,200
1,300
1,400

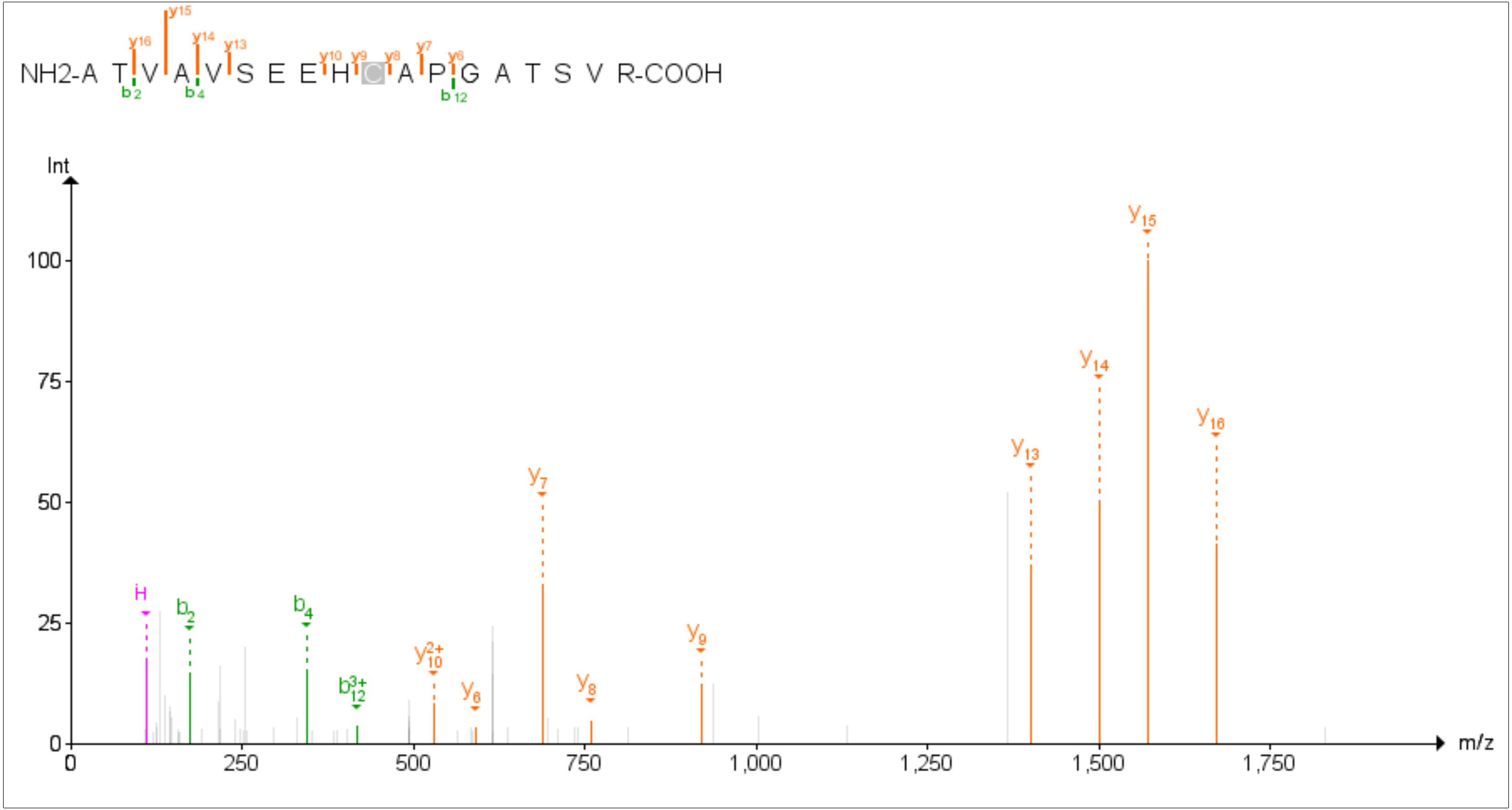

NH2-A T V A V S E E H C A P G A T S V R-COOH
y15
y16
y14
y13
y10
y9
y8
y7
y6
b 2
b 4
b 12
Int
100
75
50
25
0
iH
b2
b4
b12 3+
y10 2+
y6
y7
y8
y9
y13
y14
y15
y16
0
250
500
750
1,000
1,250
1,500
1,750
m/z

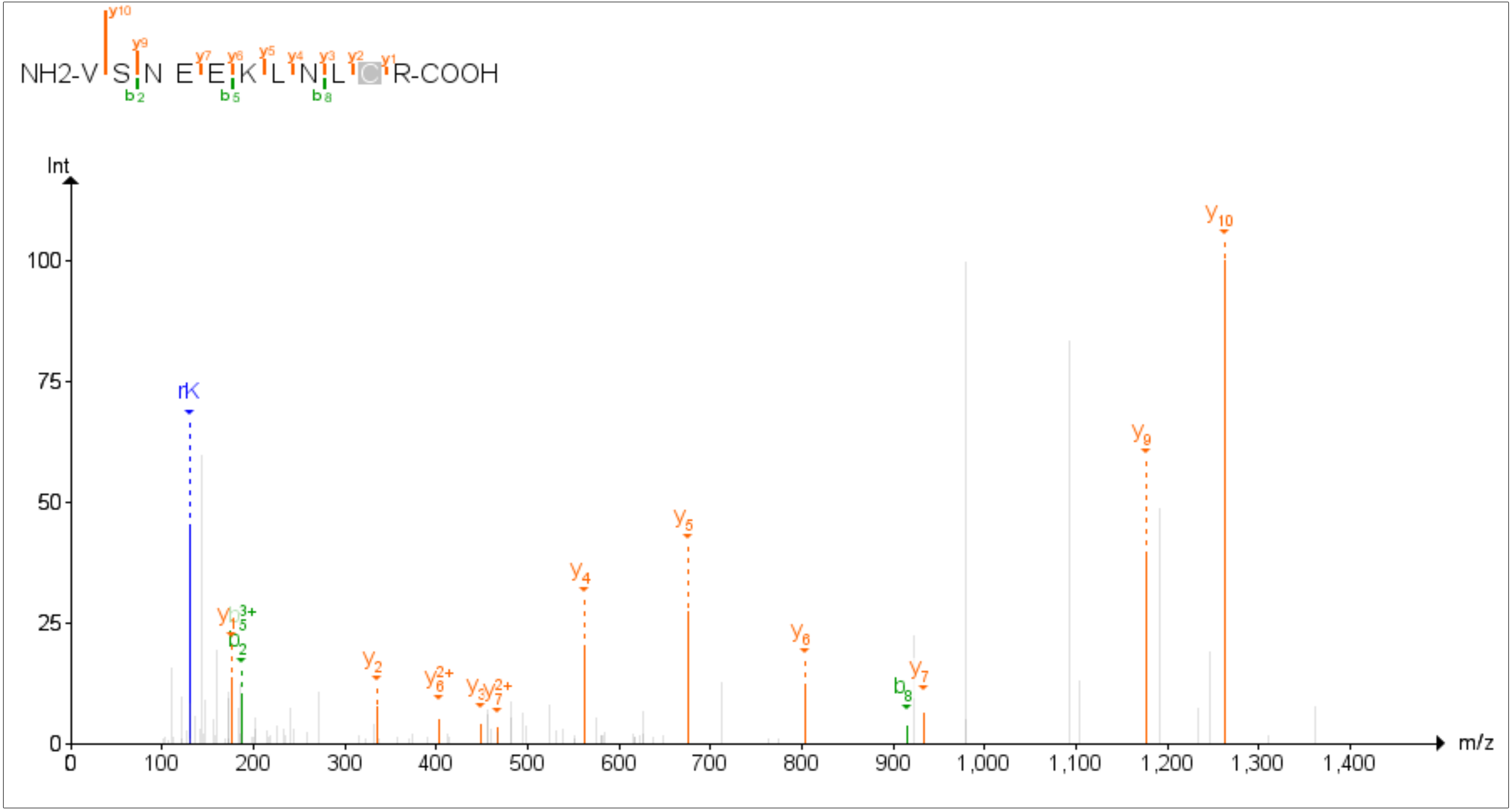

NH2-V S N E E K L N L C R-COOH
y10
y9
y7
y6
y5
y4
y3
y2
y1
b2
b5
b8
Int
m/z
rK
y10
y9
y5
y4
y6
y7
b8
y2
y3
b2
0
25
50
75
100
0
100
200
300
400
500
600
700
800
900
1,000
1,100
1,200
1,300
1,400

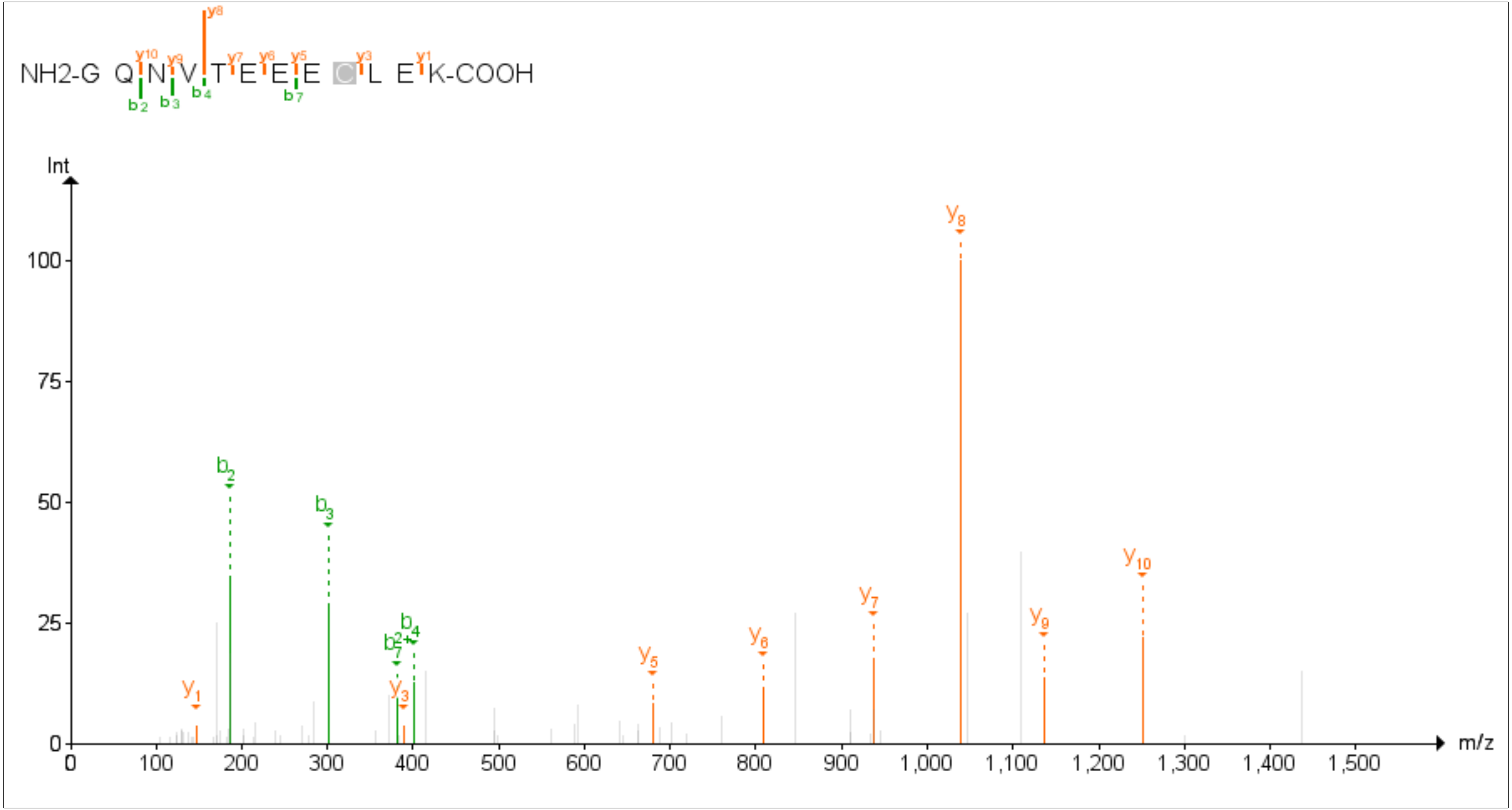

NH2-G Q N V T E E E C L E K-COOH
y10 y9 y8 y7 y6 y5 y3 y1
b2 b3 b4 b7
Int
100
75
50
25
0
b2
b3
b4
b_7^{2+}
y1
y3
y5
y6
y7
y8
y9
y10
0 100 200 300 400 500 600 700 800 900 1,000 1,100 1,200 1,300 1,400 1,500
m/z

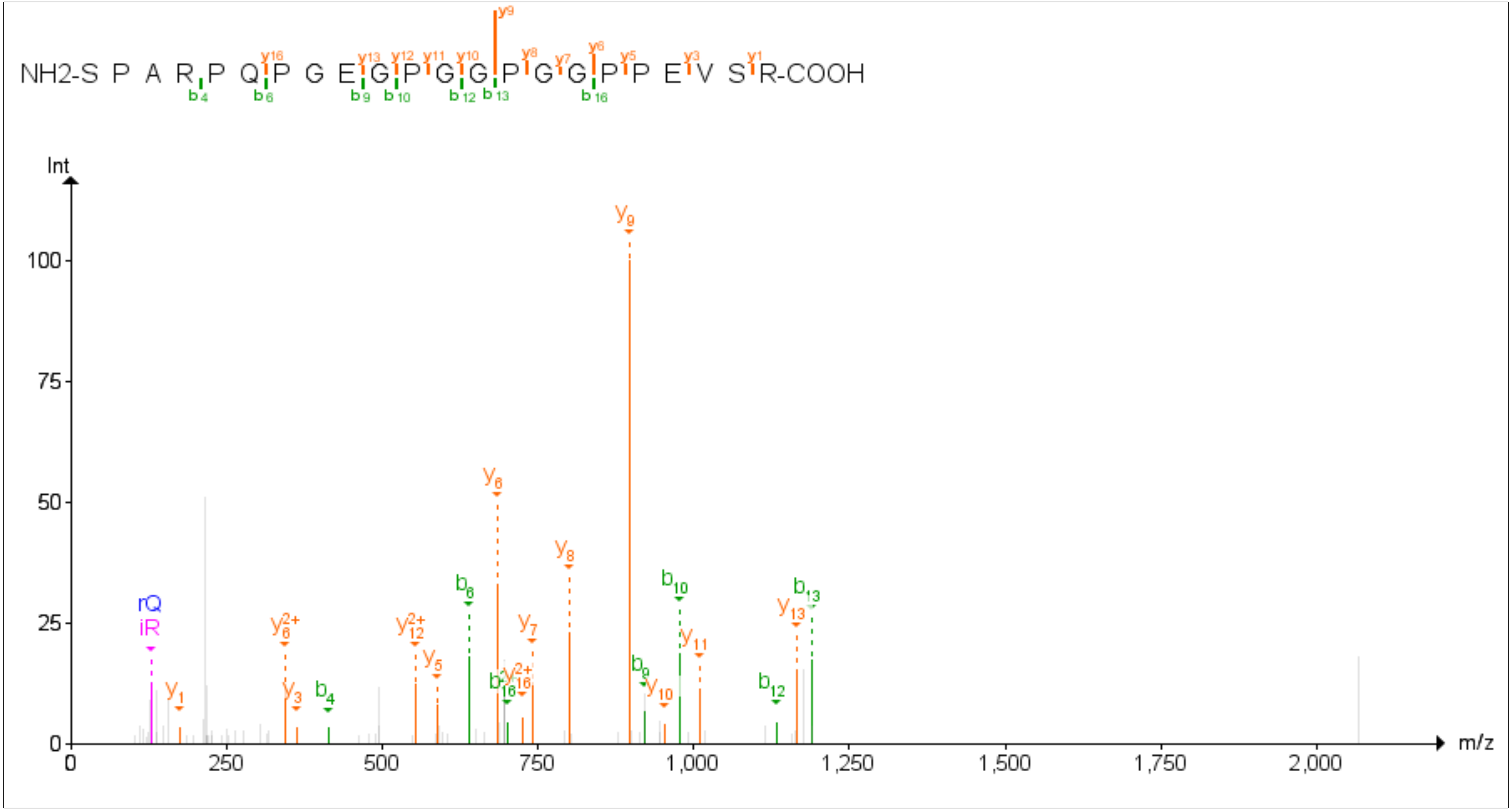

NH2-S P A R P Q P G E G P G G P G G P P E V S R-COOH
y16 y13 y12 y11 y10 y9 y8 y7 y6 y5 y3 y1
b4 b6 b9 b10 b12 b13 b16
Int
100
75
50
25
0
0
250
500
750
1,000
1,250
1,500
1,750
2,000
m/z
rQ
iR
y1
y3
y6 2+
b4
y12 2+
y5
b6
y6
b16
y16 2+
y7
y8
y9
b9
y10
b10
y11
b12
y13
b13

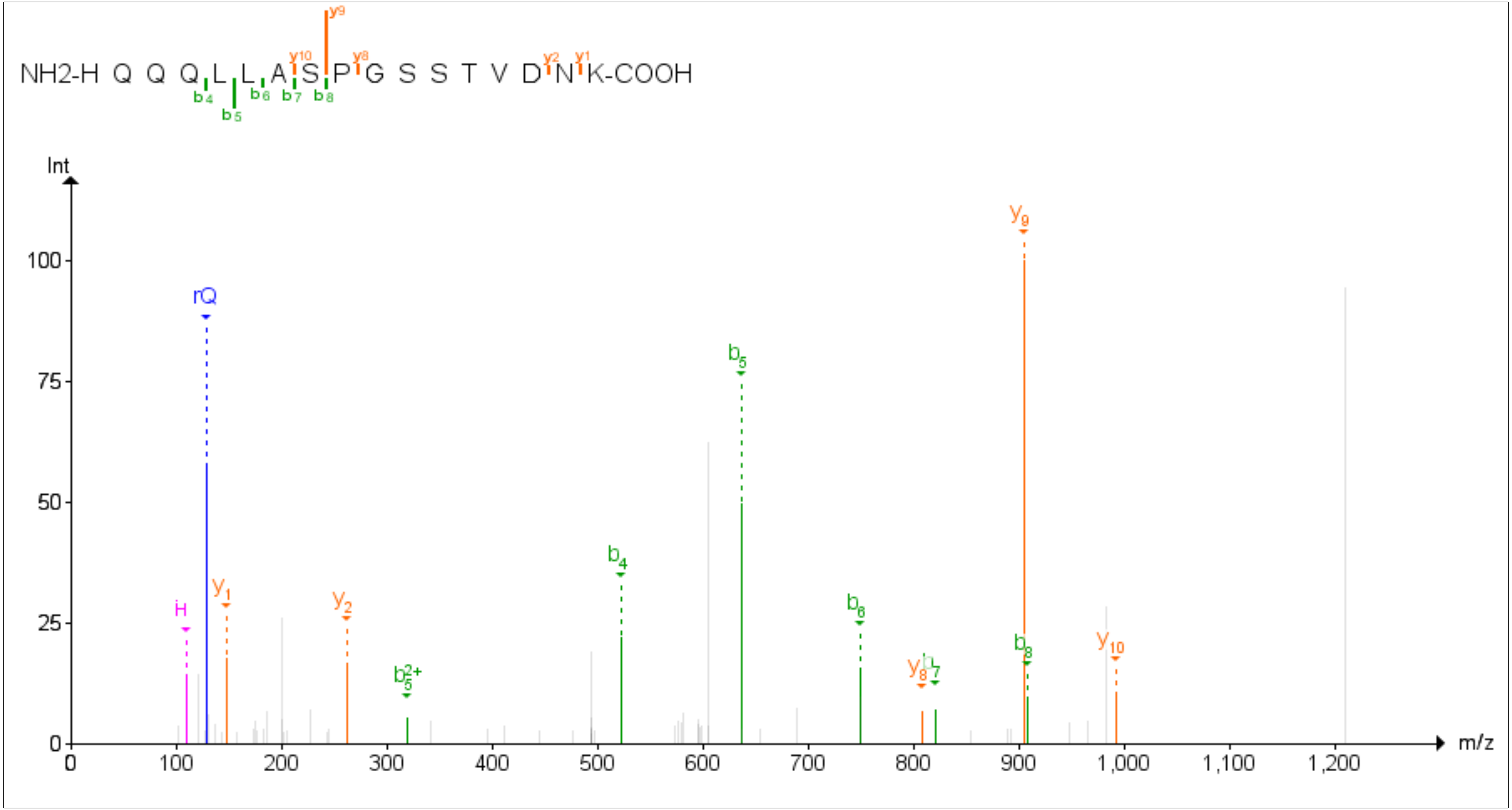

NH2-H Q Q Q L L A S P G S S T V D N K-COOH
y9 y10 y8 y2 y1
b4 b5 b6 b7 b8
Int
m/z
rQ
H
y1
y2
b5 2+
b4
b5
b6
y8
b7
b8
y9
y10
0
25
50
75
100
0
100
200
300
400
500
600
700
800
900
1,000
1,100
1,200

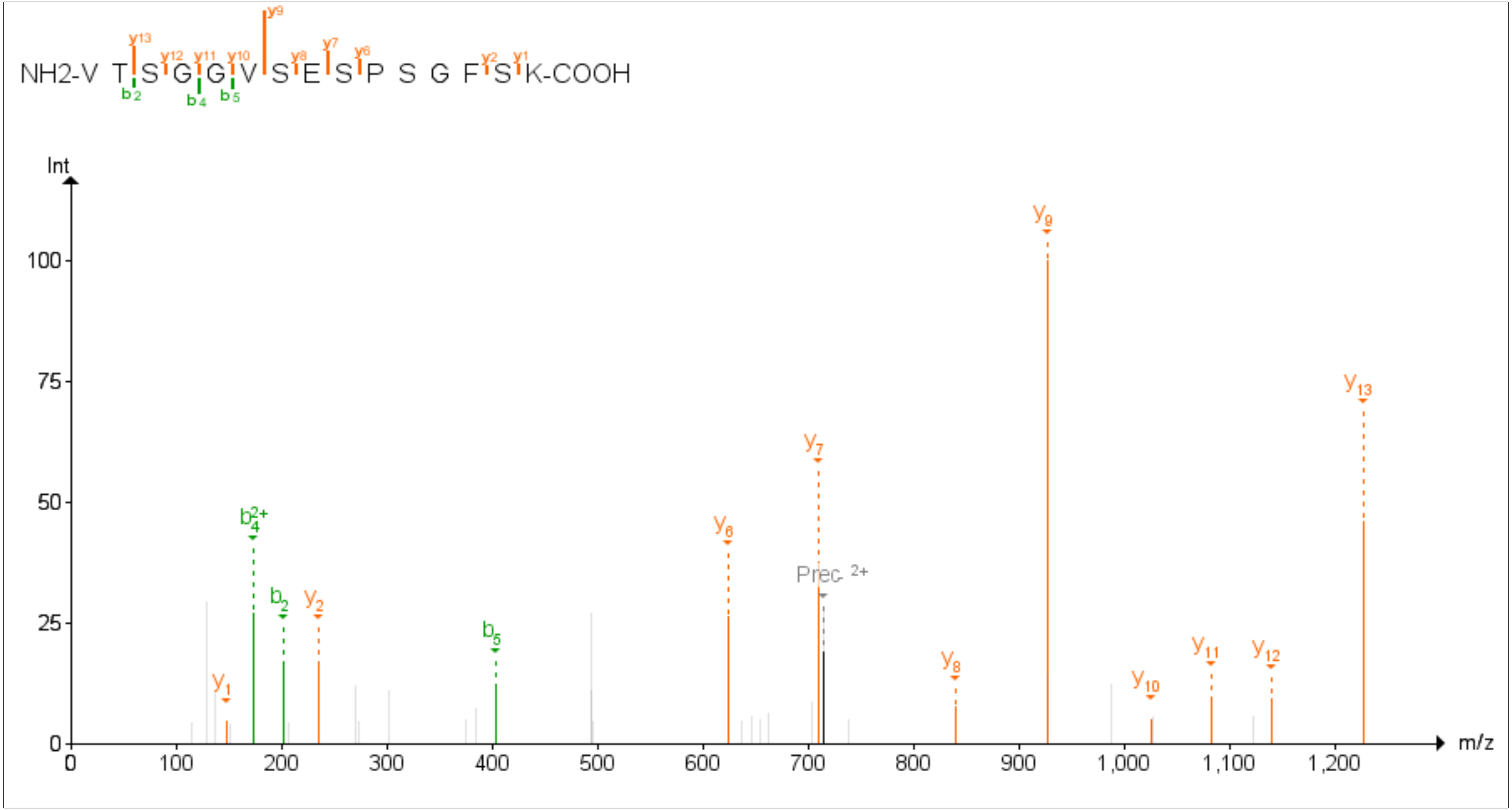

NH2-V T S G G V S E S P S G F S K-COOH
y13 y12 y11 y10 y9 y8 y7 y6 y2 y1
b2 b4 b5
Int
m/z
y9
y13
y7
y6
Prec- 2+
b4 2+
b2
y2
b5
y1
y8
y10
y11
y12
0
25
50
75
100
0
100
200
300
400
500
600
700
800
900
1,000
1,100
1,200

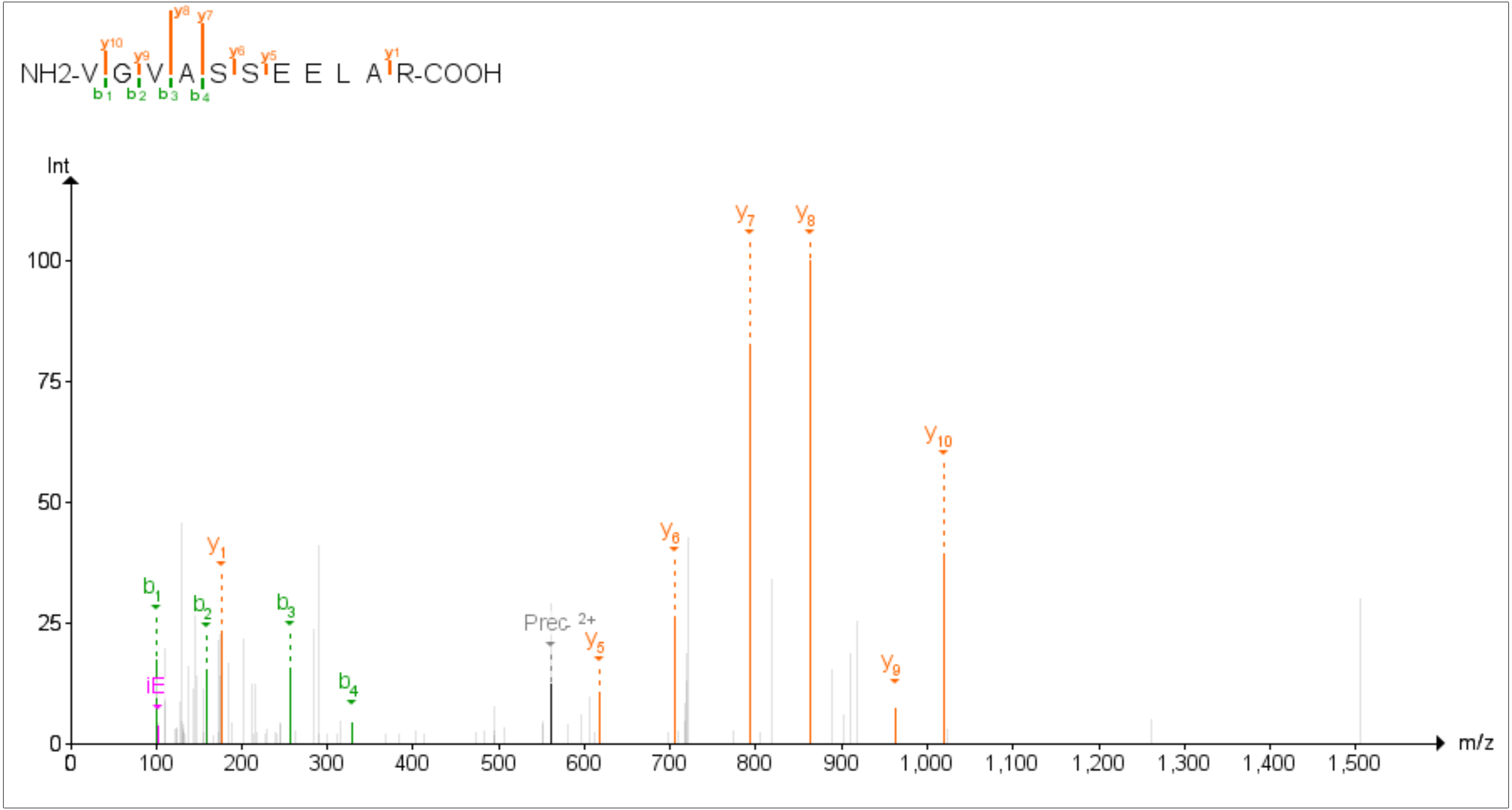

NH2-V G V A S S E E L A R-COOH
y10 y9 y8 y7 y6 y5 y1
b1 b2 b3 b4
Int
100
75
50
25
0
y7
y8
y10
y6
y1
b1
b2
b3
b4
iE
Prec- 2+
y5
y9
0 100 200 300 400 500 600 700 800 900 1,000 1,100 1,200 1,300 1,400 1,500
m/z

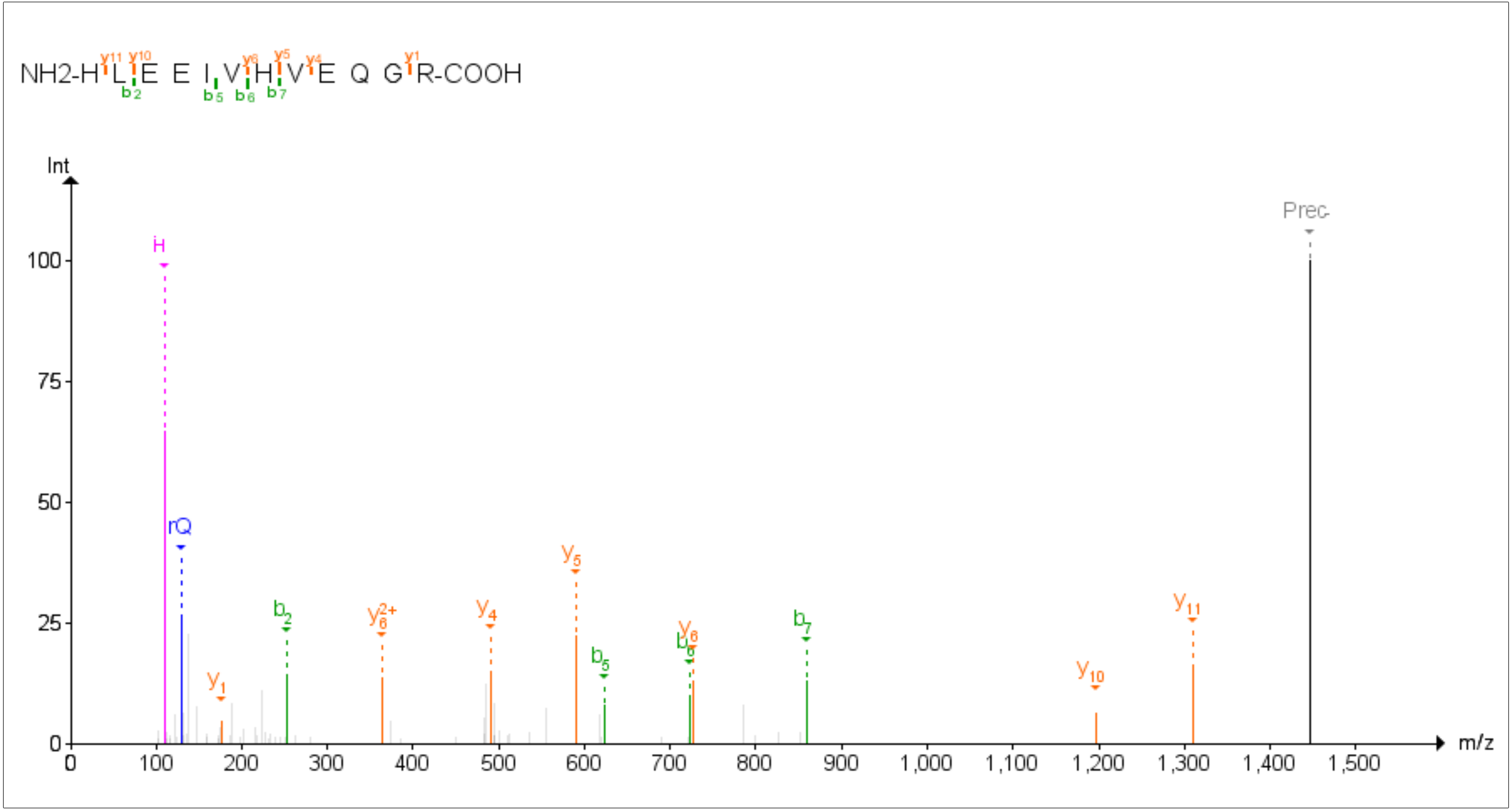

NH2-H L E E I V H V E Q G R-COOH
y11 y10 y6 y5 y4 y1
b2 b5 b6 b7
Int
m/z
H
rQ
Prec.
y1
b2
y6 2+
y4
y5
b5
b6
y6
b7
y10
y11

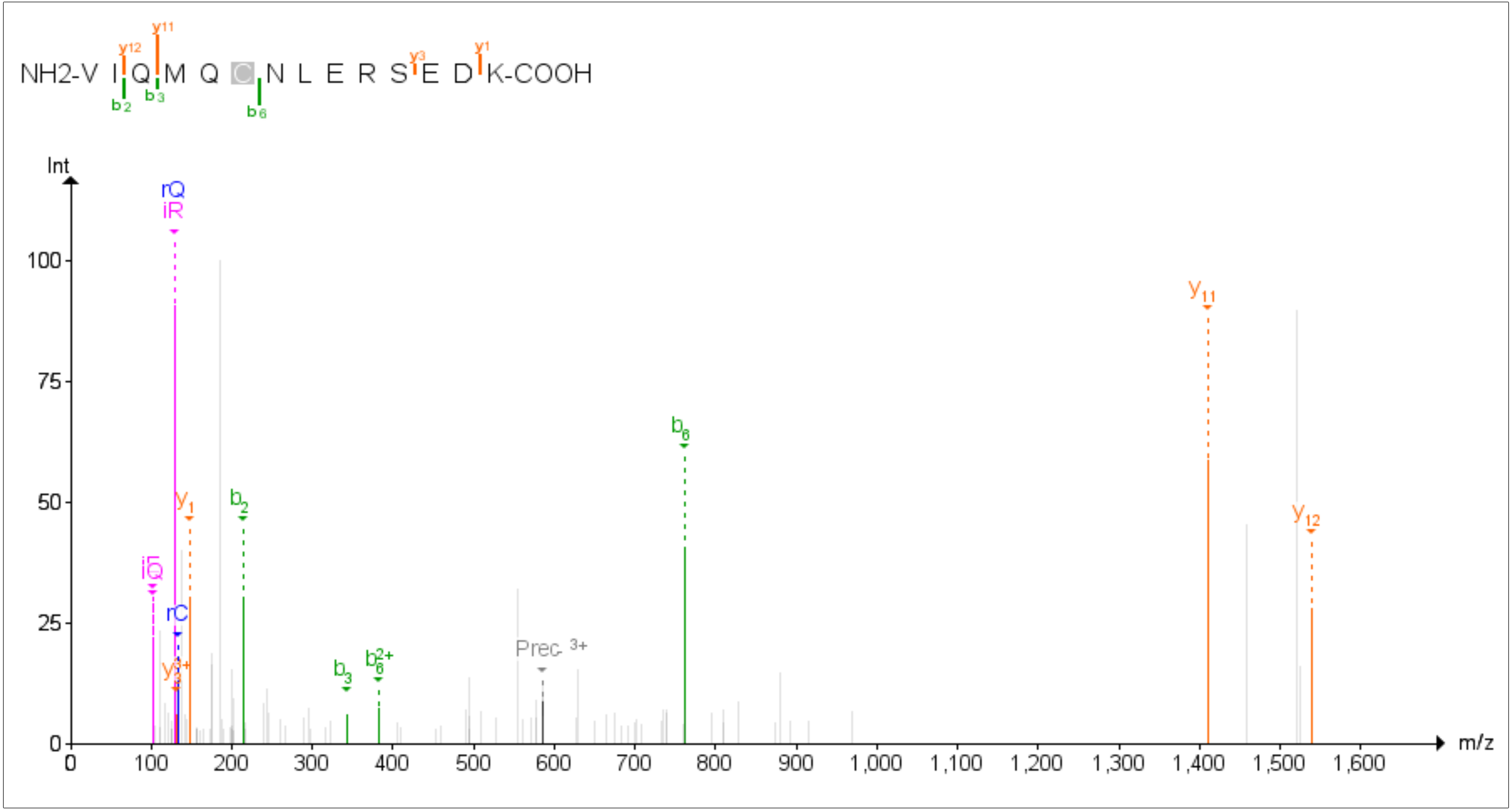

NH2-V I Q M Q C N L E R S E D K-COOH
y12
y11
y3
y1
b2
b3
b6
Int
rQ
iR
iQ
rC
y1
b2
b6
y11
y12
b3
b6 2+
y3 3+
Prec 3+
100
75
50
25
0
m/z
0
100
200
300
400
500
600
700
800
900
1,000
1,100
1,200
1,300
1,400
1,500
1,600

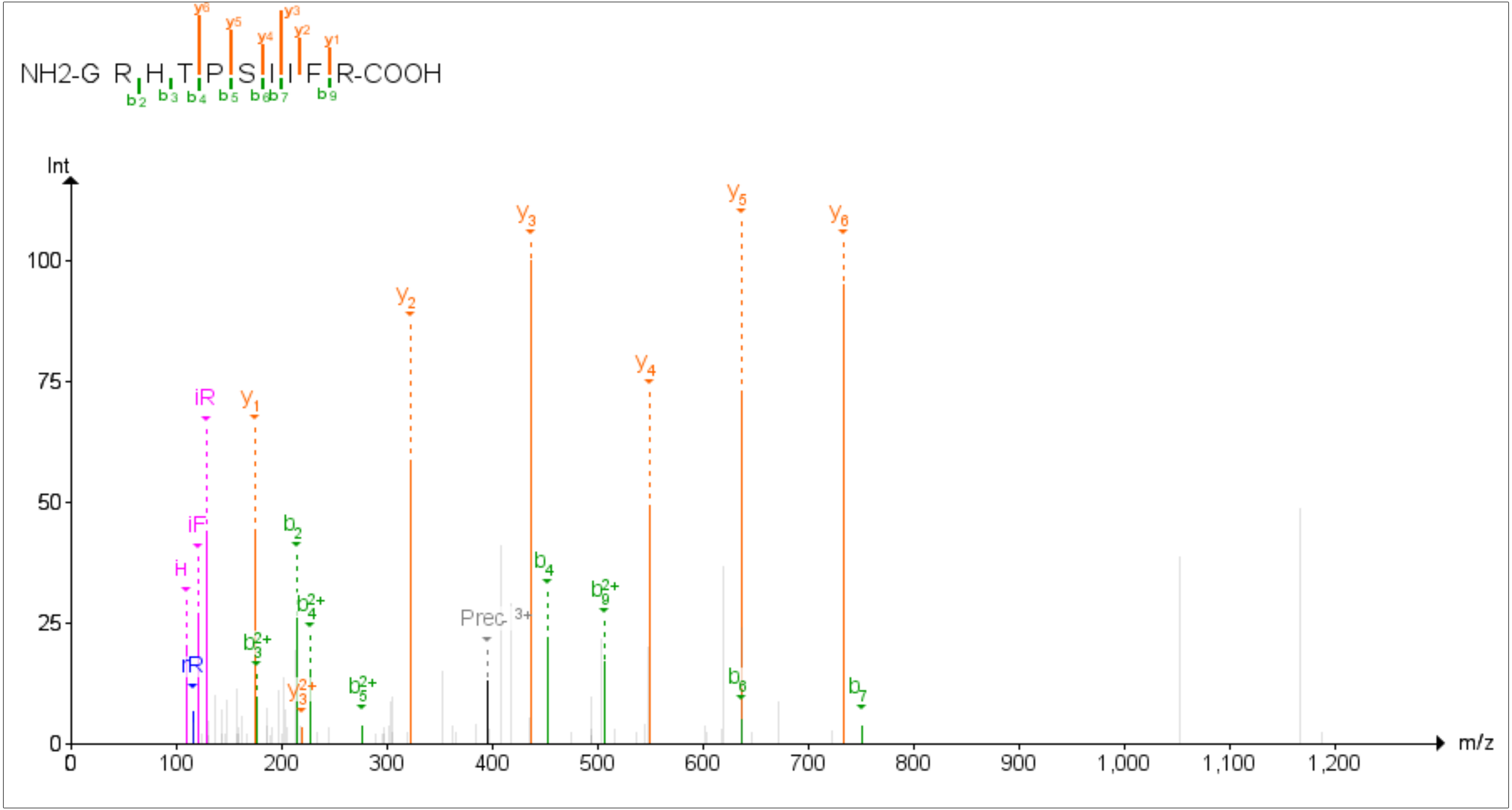

NH2-G R H T P S I I F R-COOH
y6 y5 y4 y3 y2 y1
b2 b3 b4 b5 b6 b7 b9
Int
100
75
50
25
0
iR
y1
iF
iH
b2
b4
b9 2+
y2
y3
y5
y6
y4
b4 2+
b3 2+
rR
y3 2+
b5 2+
Prec 3+
b6
b7
0
100
200
300
400
500
600
700
800
900
1,000
1,100
1,200
m/z

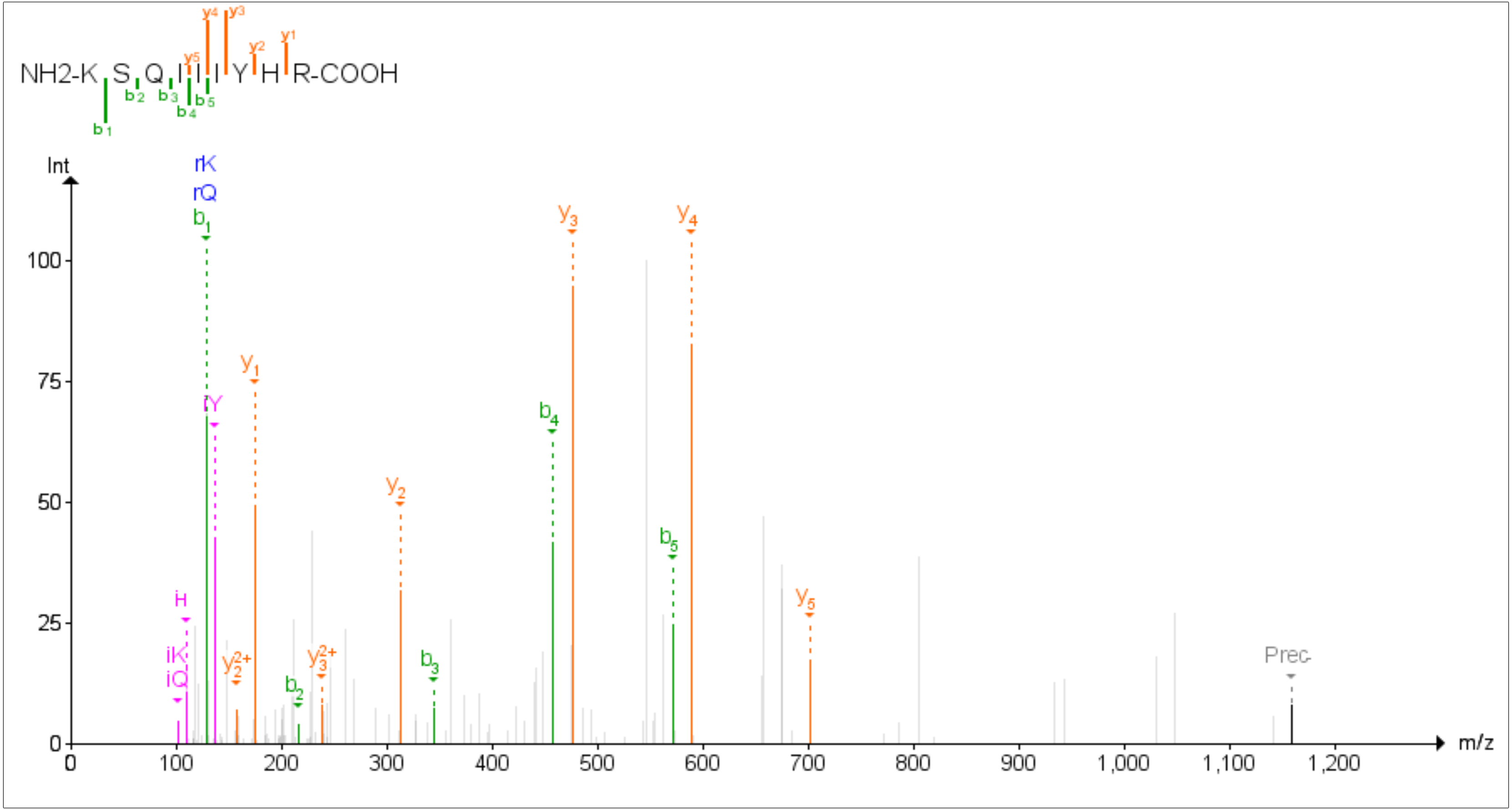

NH2-K S Q I I I Y H R-COOH
Int
m/z
Prec

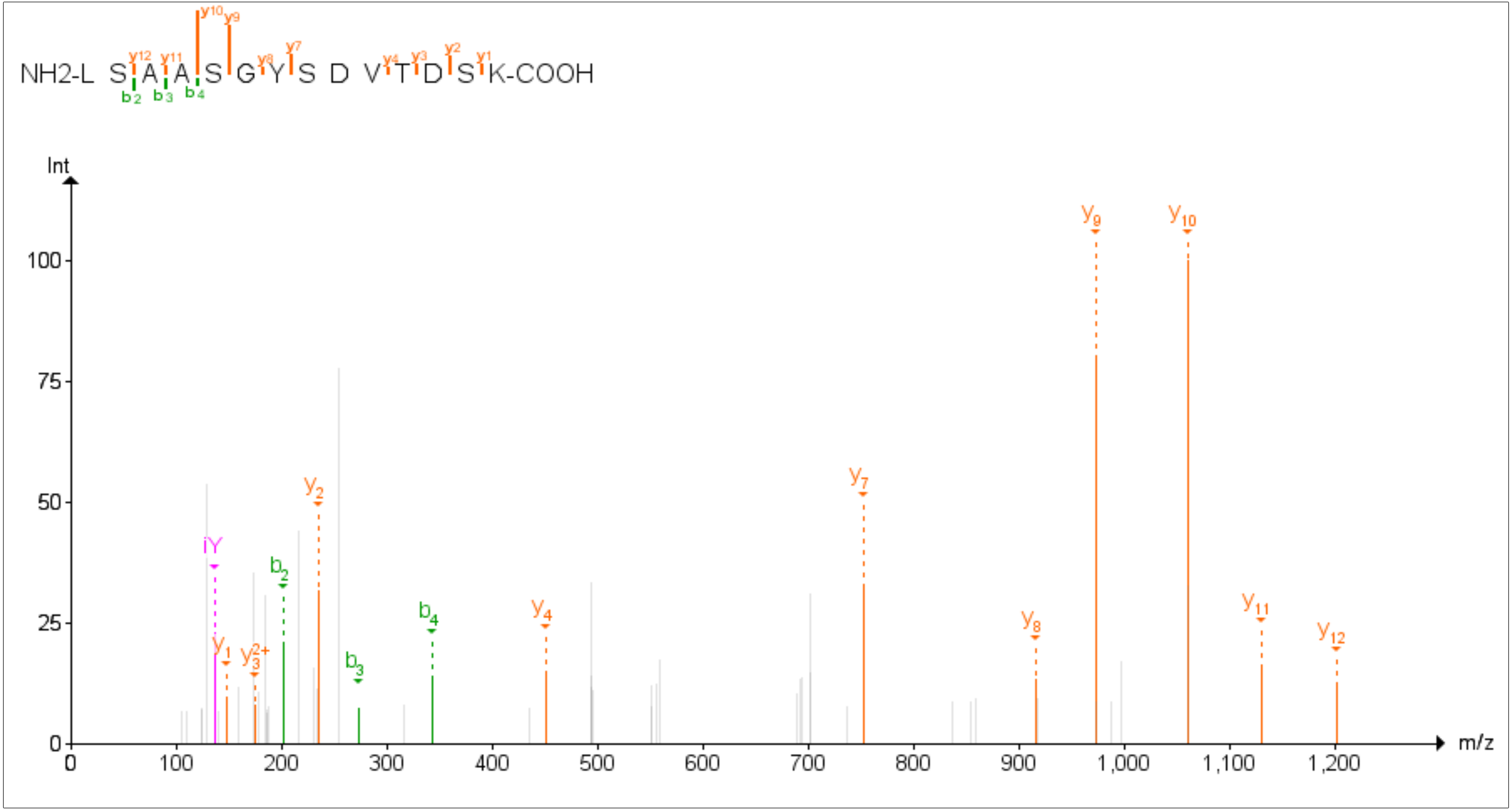

NH2-L S A A S G Y S D V T D S K-COOH
y12 y11 y10 y9 y8 y7 y4 y3 y2 y1
b2 b3 b4
Int
100
75
50
25
0
iY
y1
y3 2+
b2
y2
b3
b4
y4
y7
y8
y9
y10
y11
y12
0
100
200
300
400
500
600
700
800
900
1,000
1,100
1,200
m/z

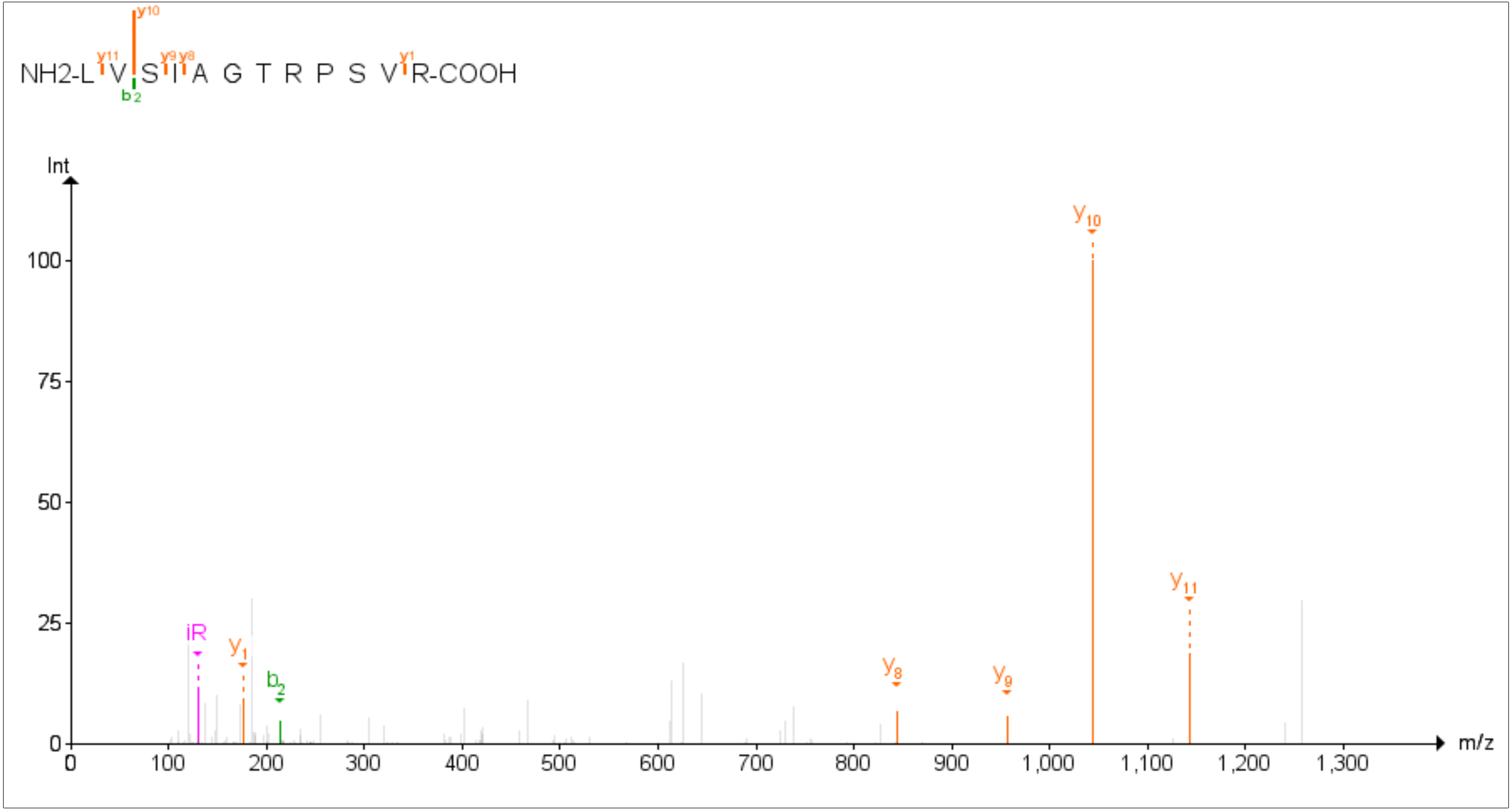

NH2-L V S I A G T R P S V R-COOH
y10
y11
y9
y8
y1
b2
Int
100
75
50
25
0
iR
y1
b2
y8
y9
y10
y11
m/z
0
100
200
300
400
500
600
700
800
900
1,000
1,100
1,200
1,300

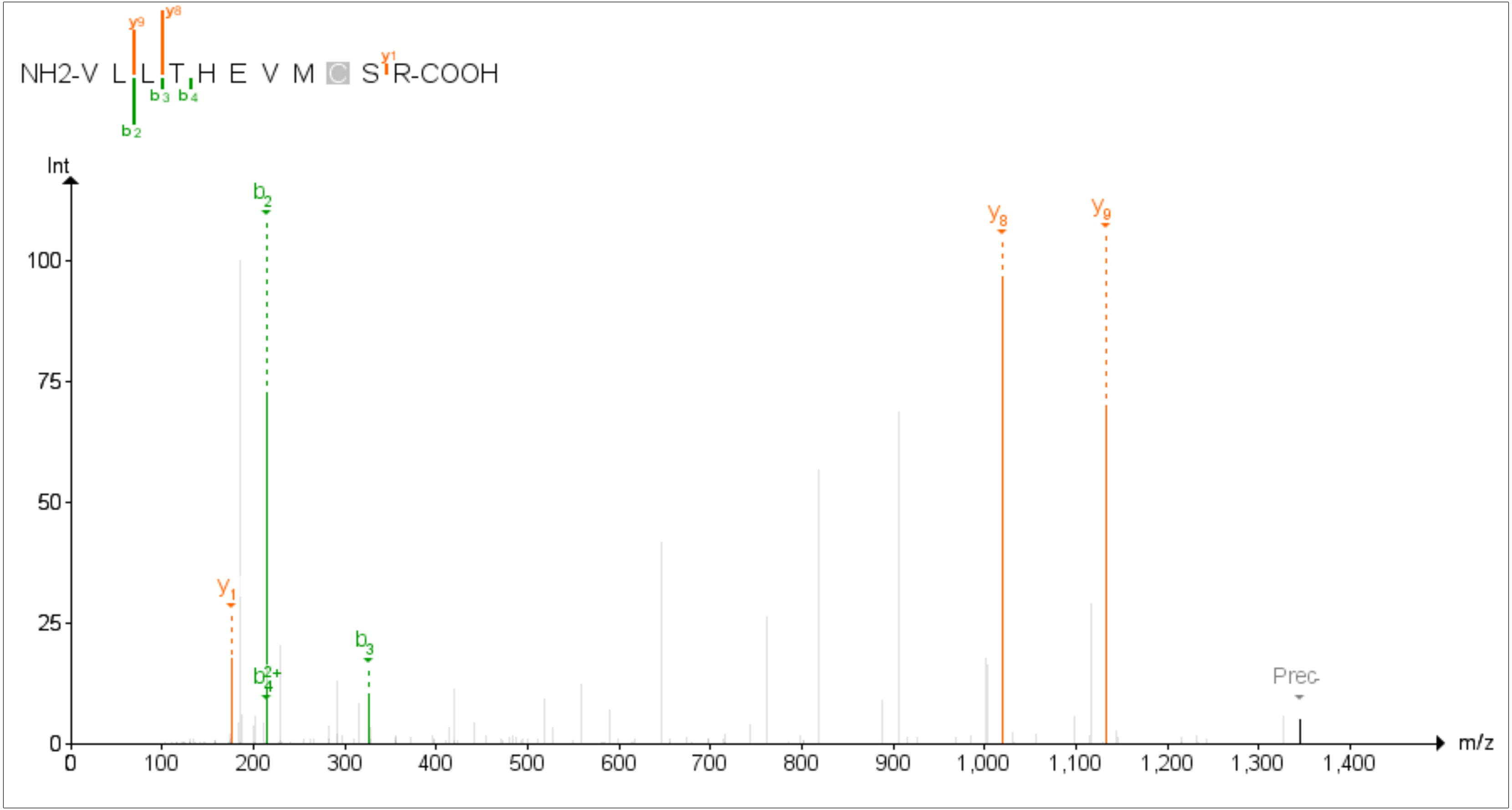

NH2-V L L T H E V M C S R-COOH
y9
y8
y1
b2
b3
b4
Int
100
75
50
25
0
b2
y8
y9
y1
b3
b4 2+
Prec.
m/z
0
100
200
300
400
500
600
700
800
900
1,000
1,100
1,200
1,300
1,400

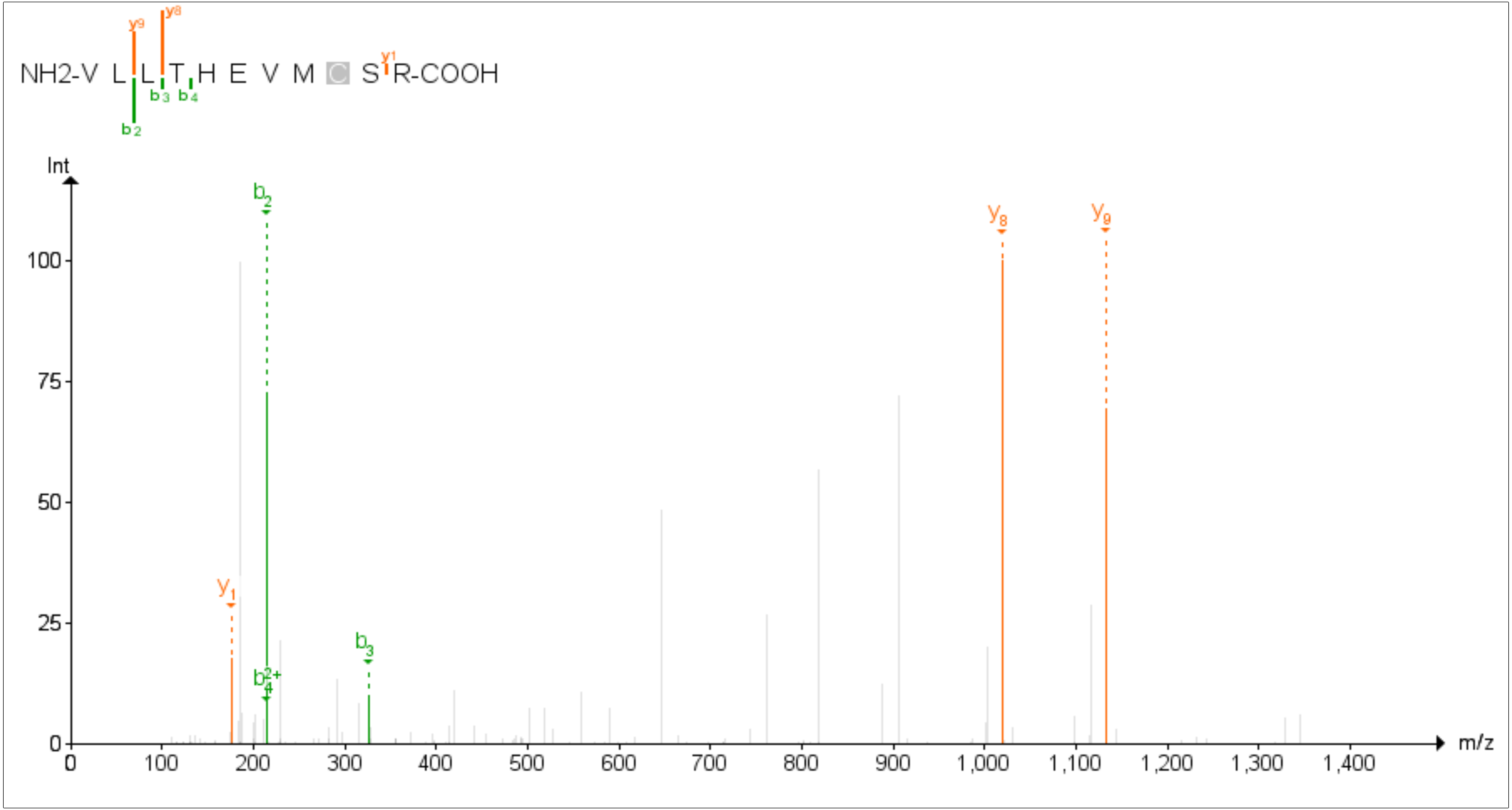

NH2-V L L T H E V M C S R-COOH
y9
y8
y1
b2
b3
b4
Int
100
75
50
25
0
m/z
0
100
200
300
400
500
600
700
800
900
1,000
1,100
1,200
1,300
1,400

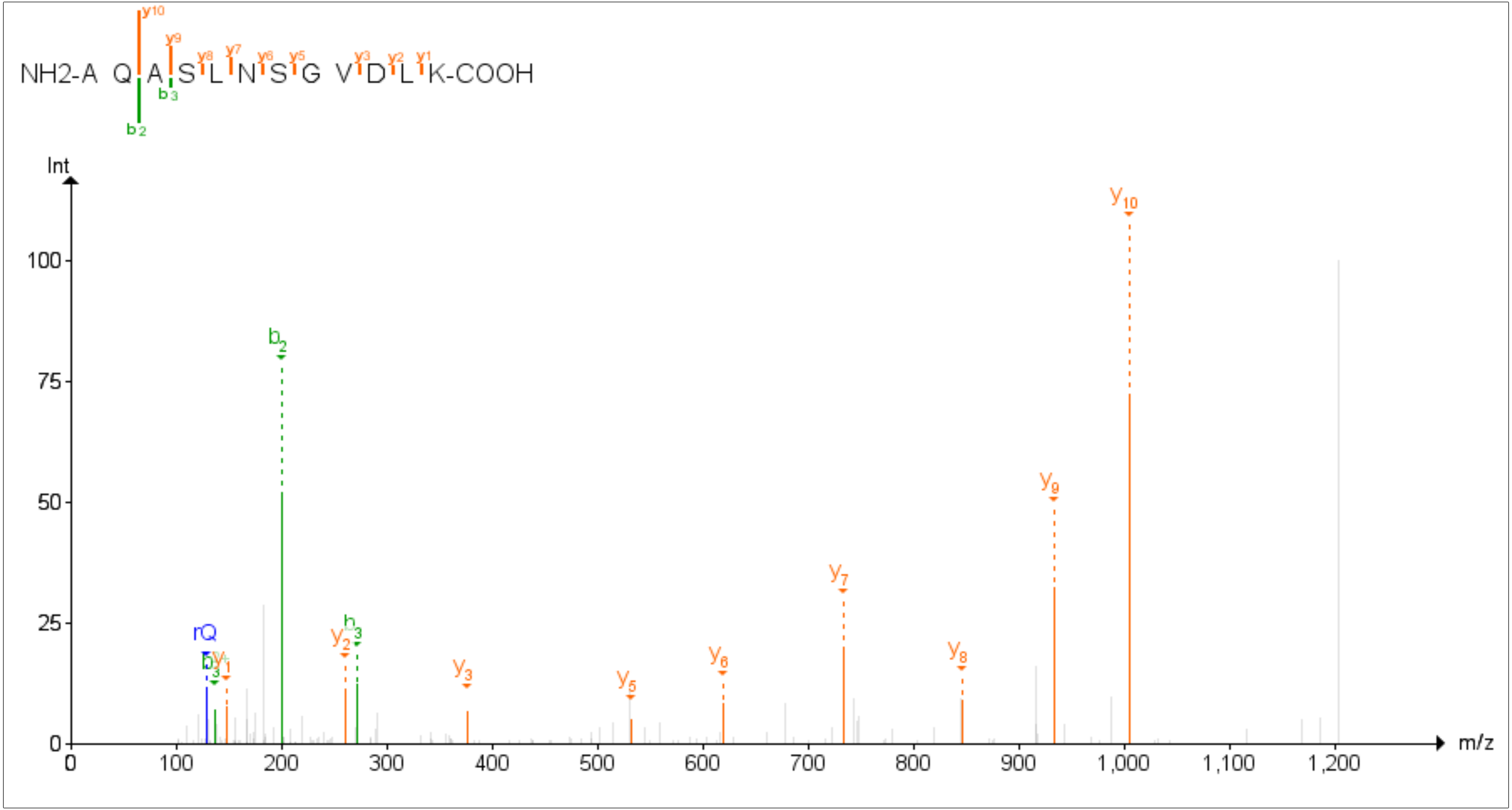
NH2-A Q A S L N S G V D L K-COOH
y10 y9 y8 y7 y6 y5 y3 y2 y1
b2 b3
Int
100
75
50
25
0
rQ
b2 b3 y1 y2 y3 y5 y6 y7 y8 y9 y10
0 100 200 300 400 500 600 700 800 900 1,000 1,100 1,200
m/z

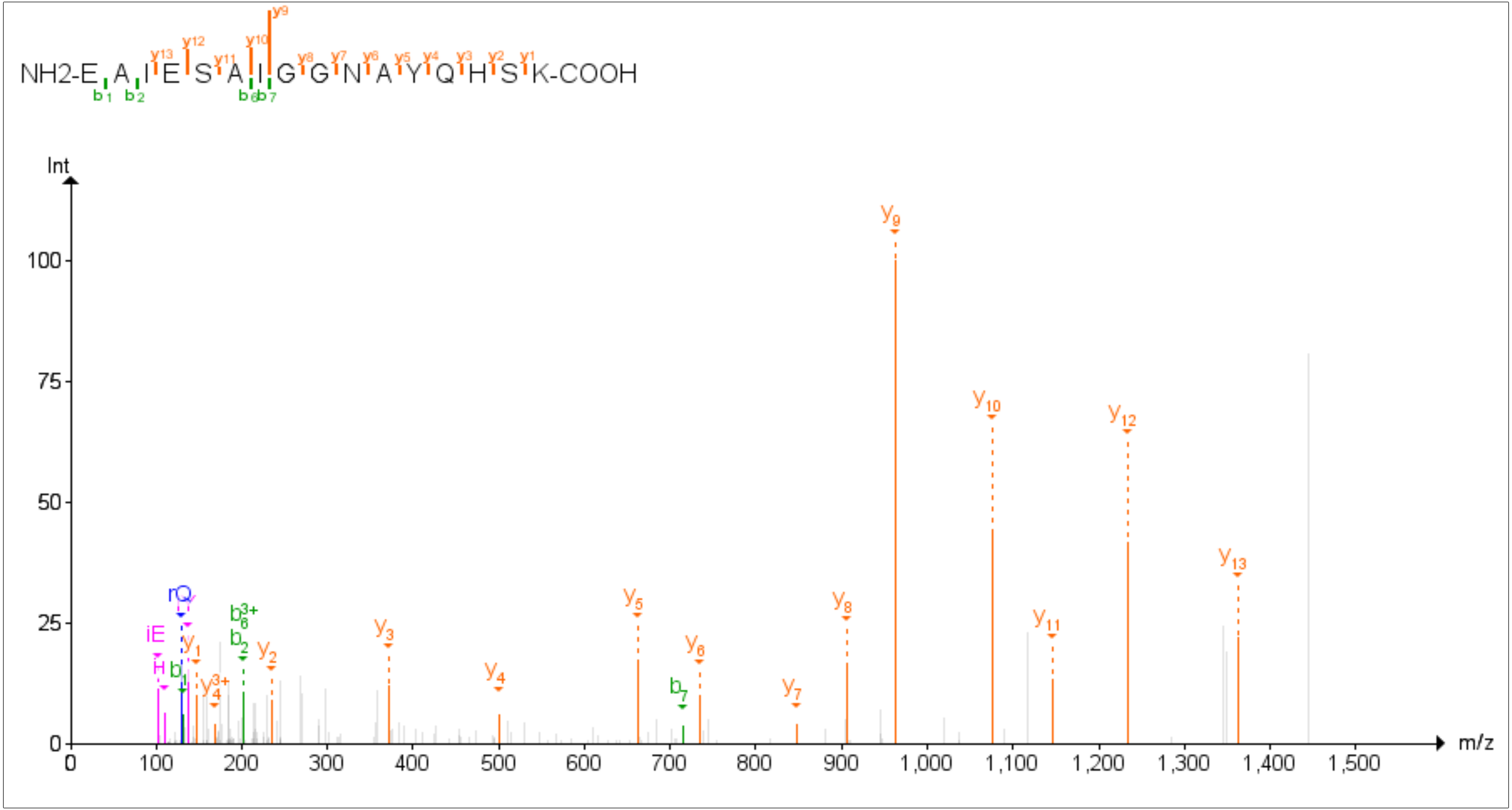

NH2-E A I E S A I I G G N A Y Q H S K-COOH
Int
m/z
y9
y10
y12
y13
y11
y5
y8
y3
y1
y2
y6
y4
y7
b7
rQ
iE
H
b1
b2
b6 3+
y4 3+

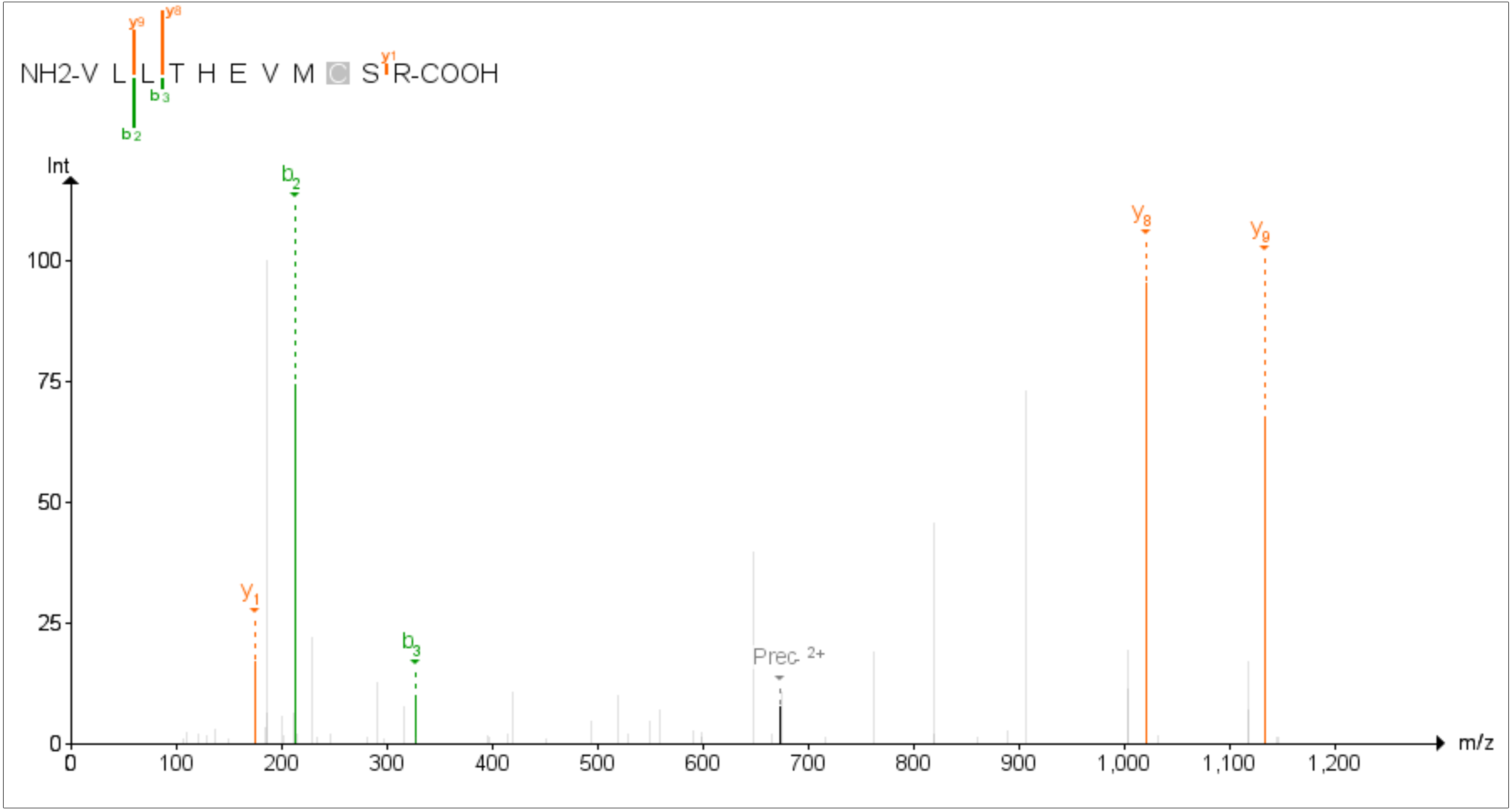
NH2-V L L T H E V M C S R-COOH
y9
y8
y1
b2
b3
Int
100
75
50
25
0
b2
y8
y9
y1
b3
Prec 2+
0
100
200
300
400
500
600
700
800
900
1,000
1,100
1,200
m/z

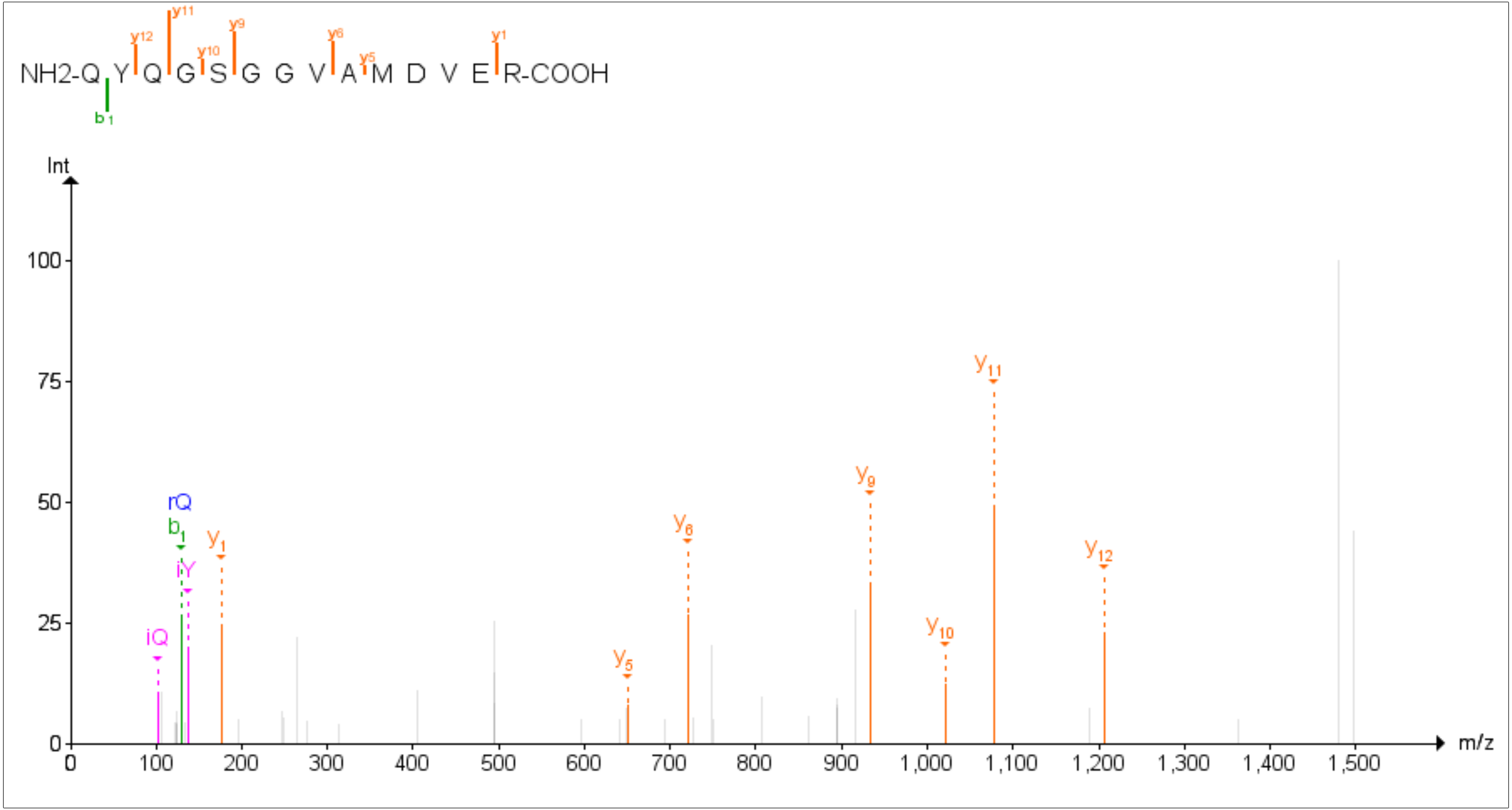
NH2-Q Y Q G S G G V A M D V E R-COOH
y12
y11
y10
y9
y6
y5
y1
b1
Int
100
75
50
25
0
rQ
b1
y1
iY
iQ
y5
y6
y9
y10
y11
y12
m/z
0
100
200
300
400
500
600
700
800
900
1,000
1,100
1,200
1,300
1,400
1,500

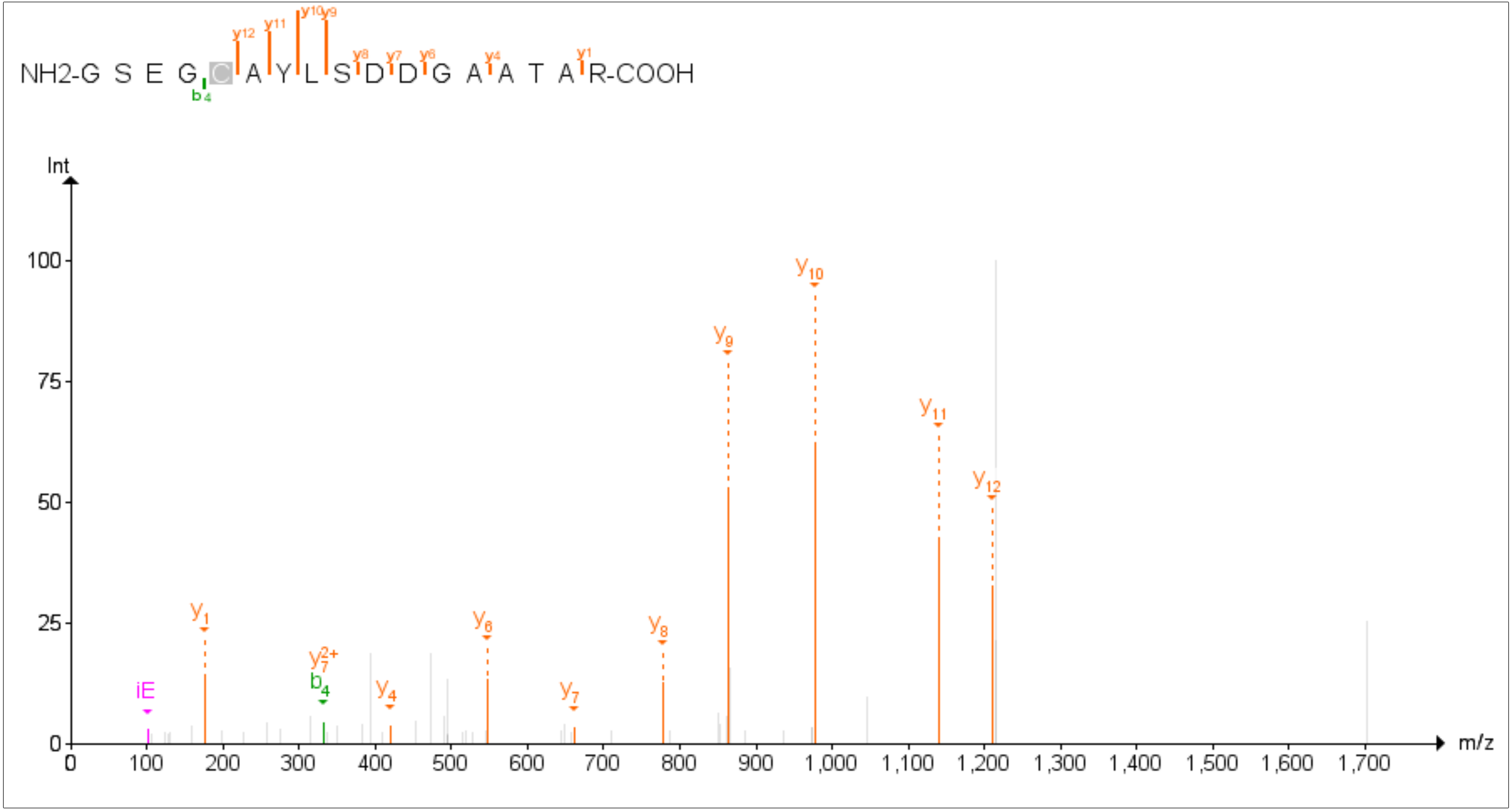

NH2-G S E G C A Y L S D D G A A T A R-COOH
b4
y12 y11 y10 y9 y8 y7 y6 y4 y1
Int
m/z
iE
y1
y_7^{2+}
b4
y4
y6
y7
y8
y9
y10
y11
y12
0
25
50
75
100
0
100
200
300
400
500
600
700
800
900
1,000
1,100
1,200
1,300
1,400
1,500
1,600
1,700

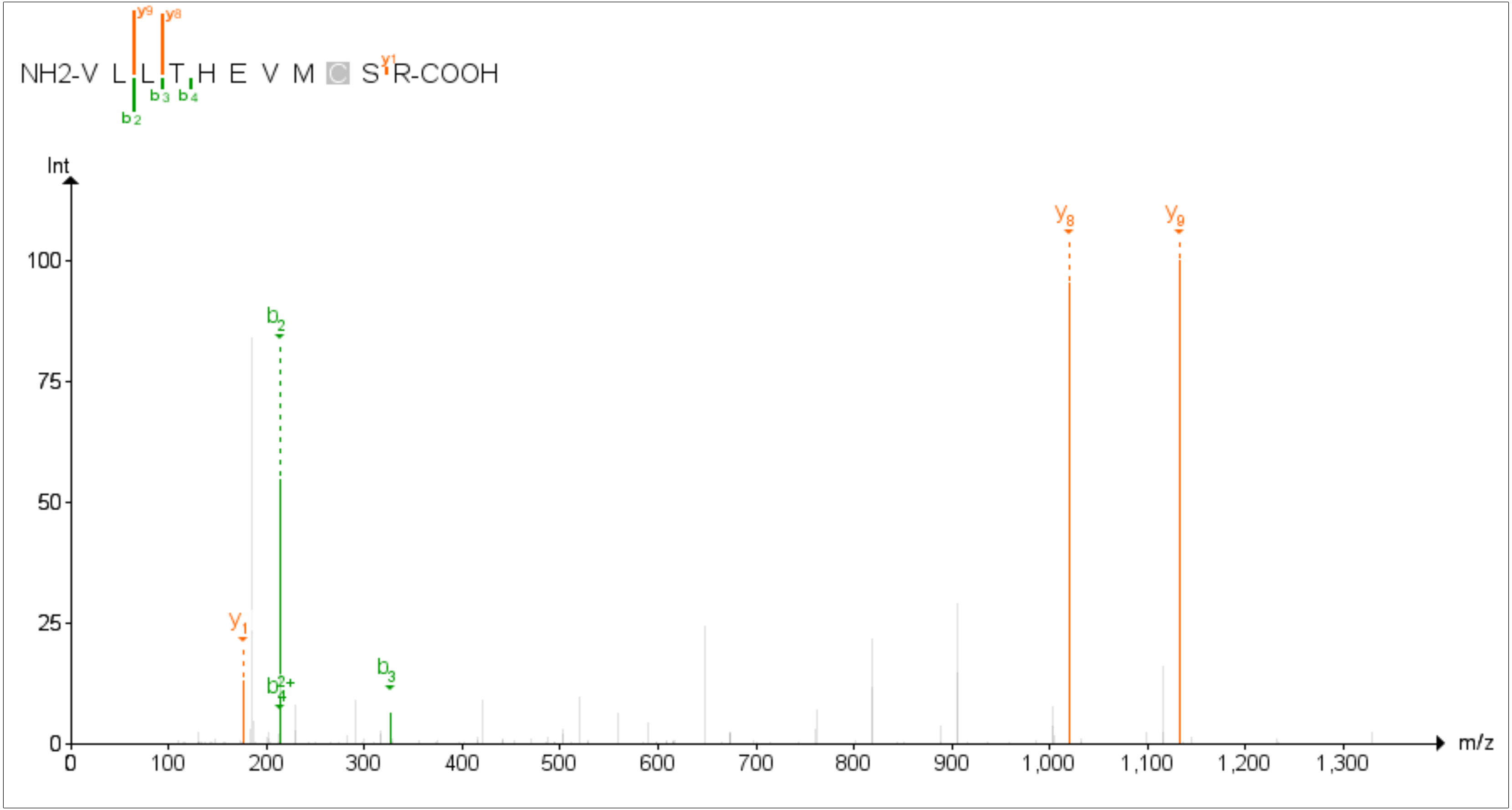

NH2-V L L T H E V M C S R-COOH
y9
y8
y1
b2
b3
b4
Int
100
75
50
25
0
y8
y9
b2
y1
b4 2+
b3
0
100
200
300
400
500
600
700
800
900
1,000
1,100
1,200
1,300
m/z

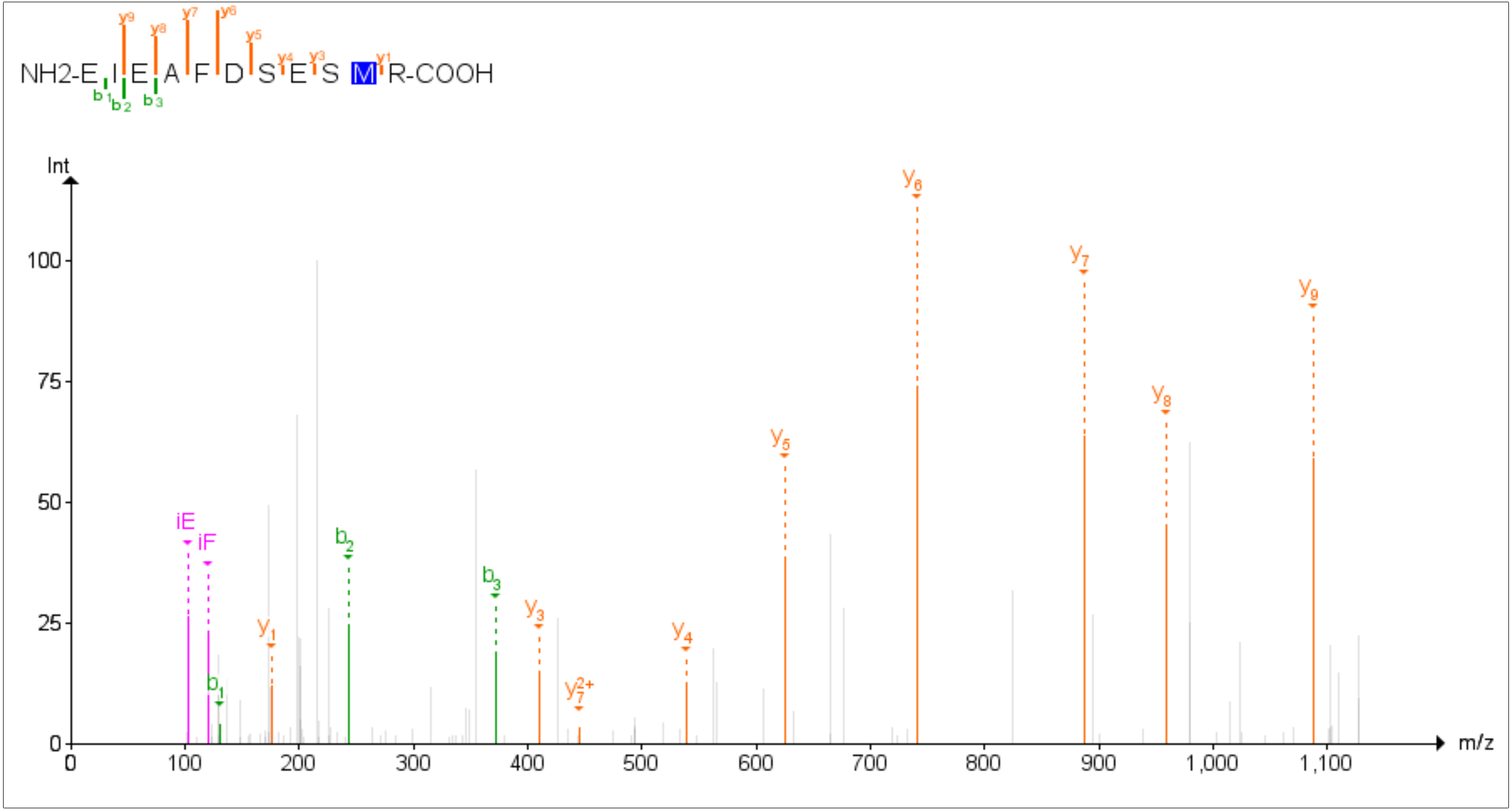

NH2-E I E A F D S E S M R-COOH
y9 y8 y7 y6 y5 y4 y3 y1
b1 b2 b3
Int
100
75
50
25
0
iE
iF
b1
y1
b2
b3
y3
y7 2+
y4
y5
y6
y7
y8
y9
0
100
200
300
400
500
600
700
800
900
1,000
1,100
m/z

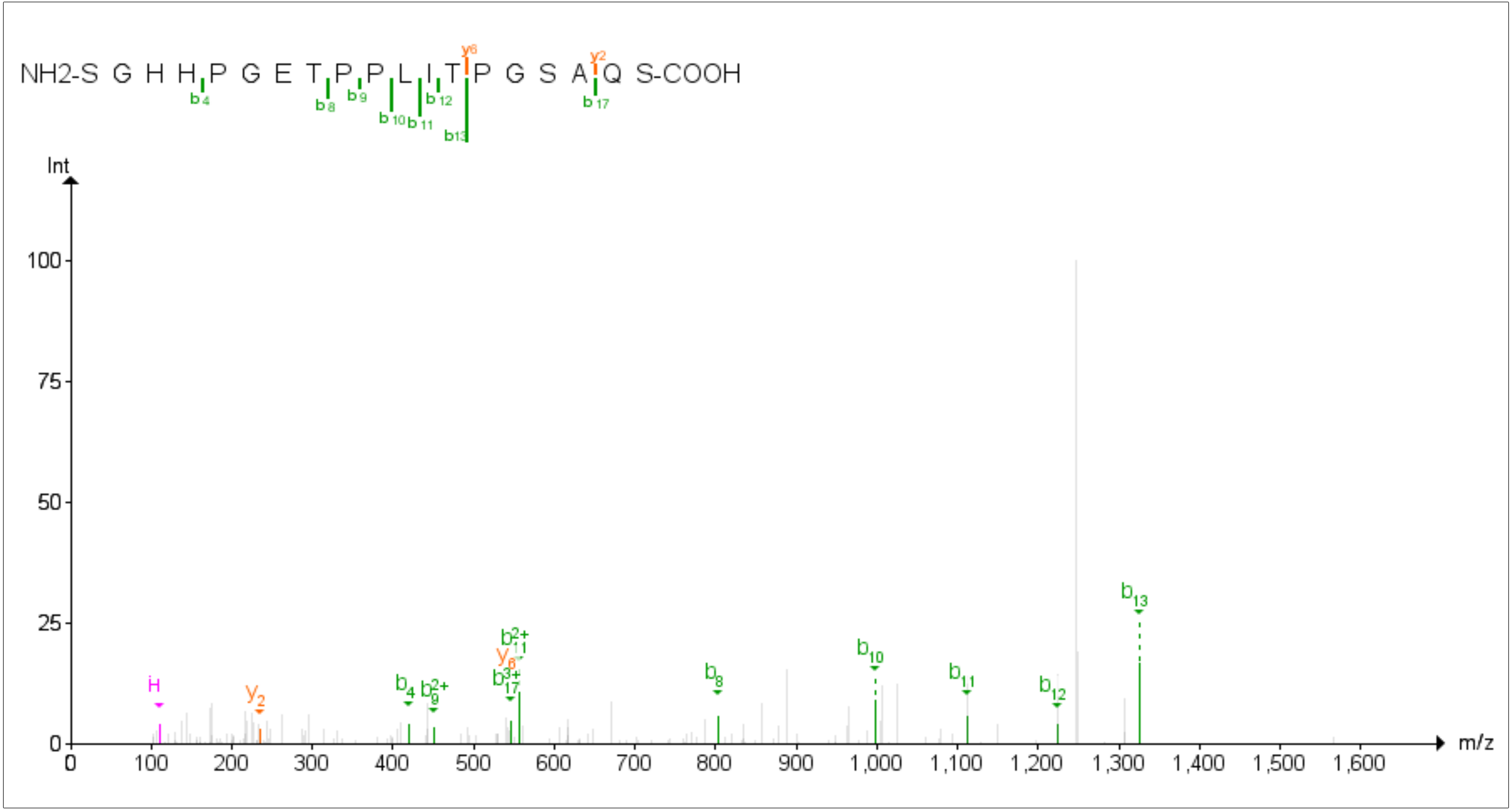

NH2-S G H H P G E T P P L I T P G S A Q S-COOH
y6
y2
b4
b8
b9
b10
b11
b12
b13
b17
Int
100
75
50
25
0
H
y2
b4
b9 2+
b17 3+
y6
b11 2+
b8
b10
b11
b12
b13
0
100
200
300
400
500
600
700
800
900
1,000
1,100
1,200
1,300
1,400
1,500
1,600
m/z

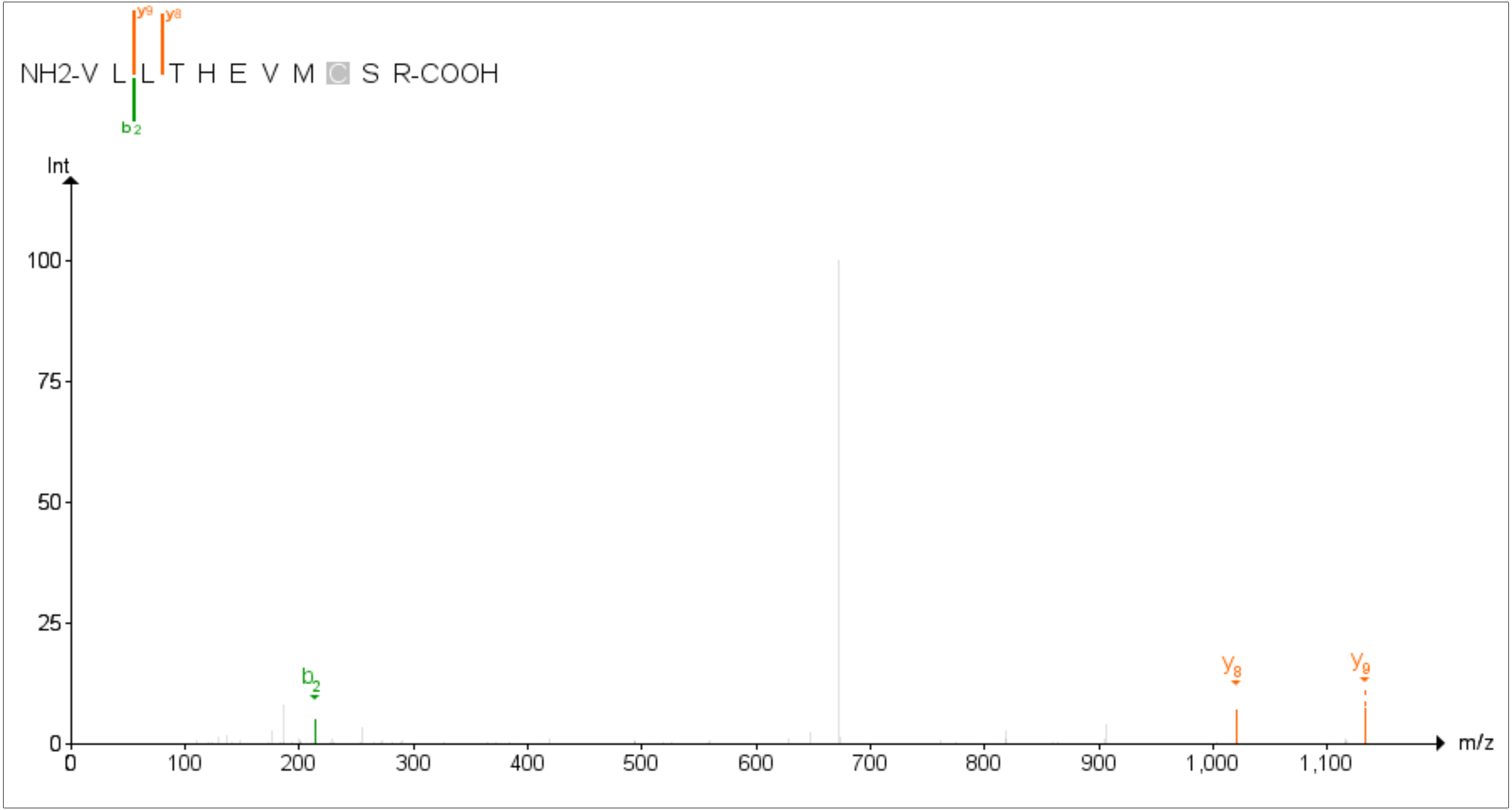

y9
y8
NH2-V L L T H E V M C S R-COOH
b2
Int
100
75
50
25
0
b2
y8
y9
0
100
200
300
400
500
600
700
800
900
1,000
1,100
m/z

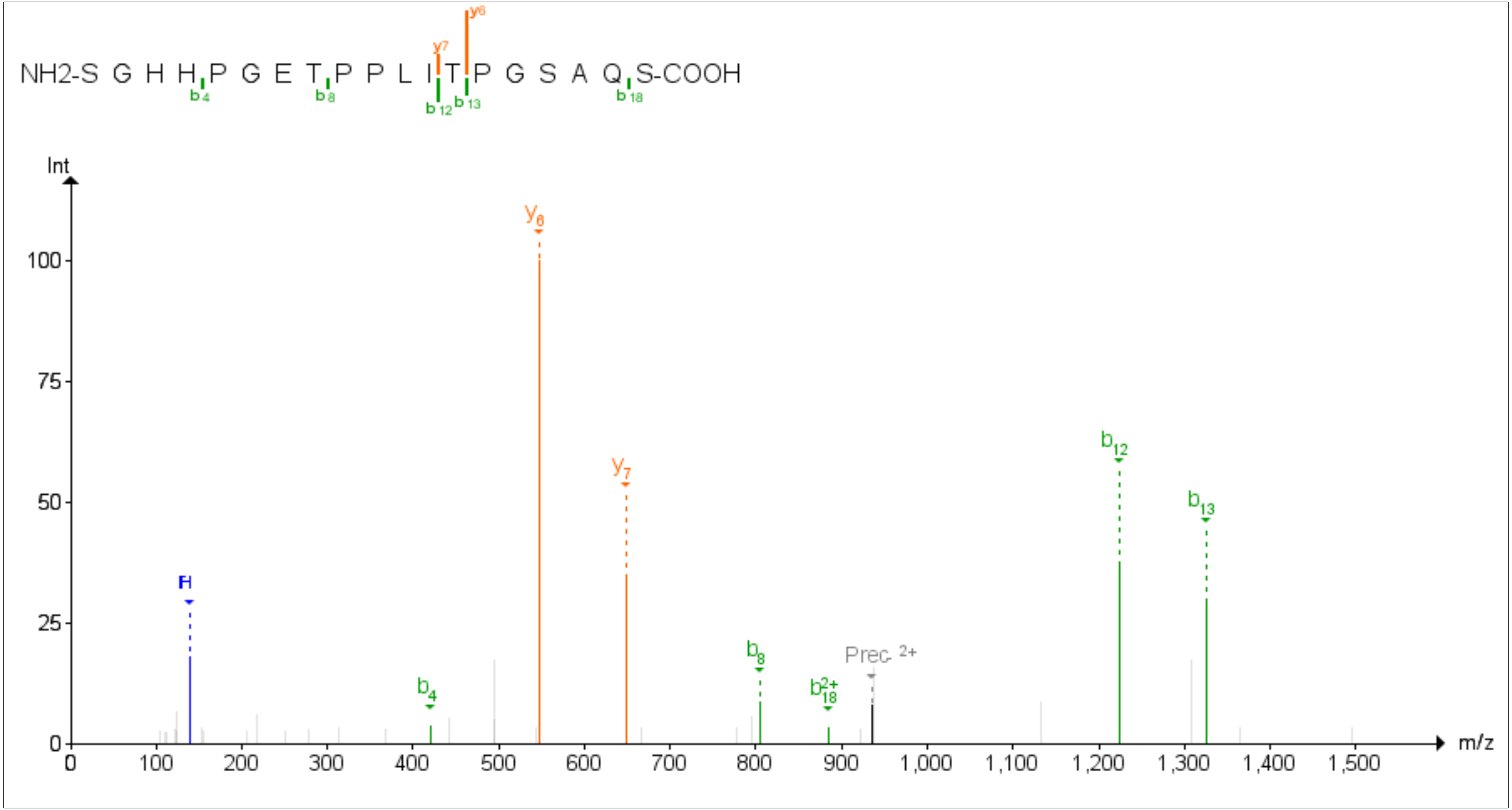

NH2-S G H H P G E T P P L I T P G S A Q S-COOH
y6
y7
b4
b8
b12
b13
b18
Int
100
75
50
25
0
H
b4
y6
y7
b8
b18 2+
Prec 2+
b12
b13
0
100
200
300
400
500
600
700
800
900
1,000
1,100
1,200
1,300
1,400
1,500
m/z

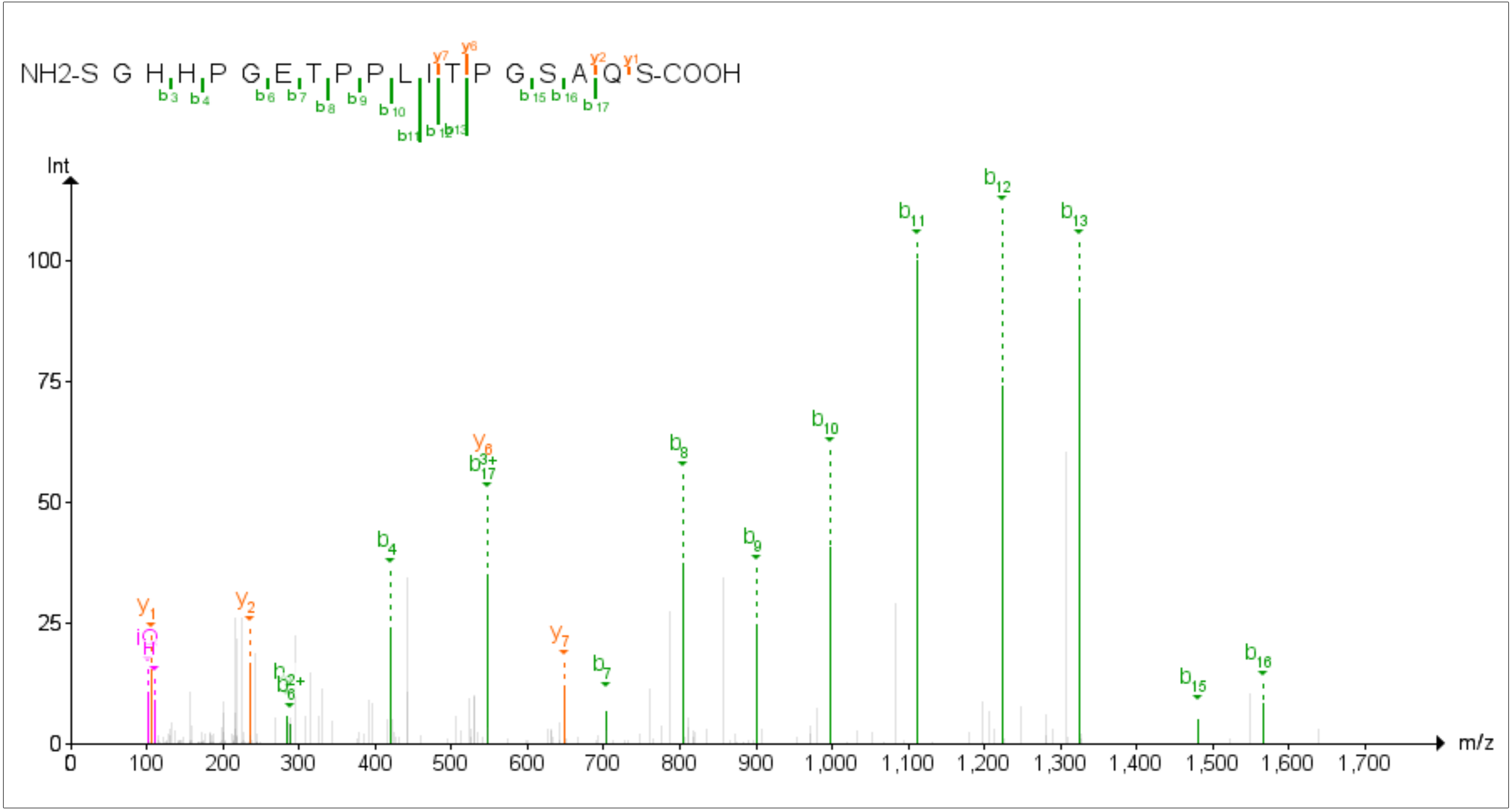
NH2-S G H H P G E T P P L I T P G S A Q S-COOH
Int
m/z
b11
b12
b13
b10
b8
b9
b4
y6
b_{17}^{3+}
y7
b7
y1
y2
b_{2}^{2+}
b6
b15
b16
iC
H

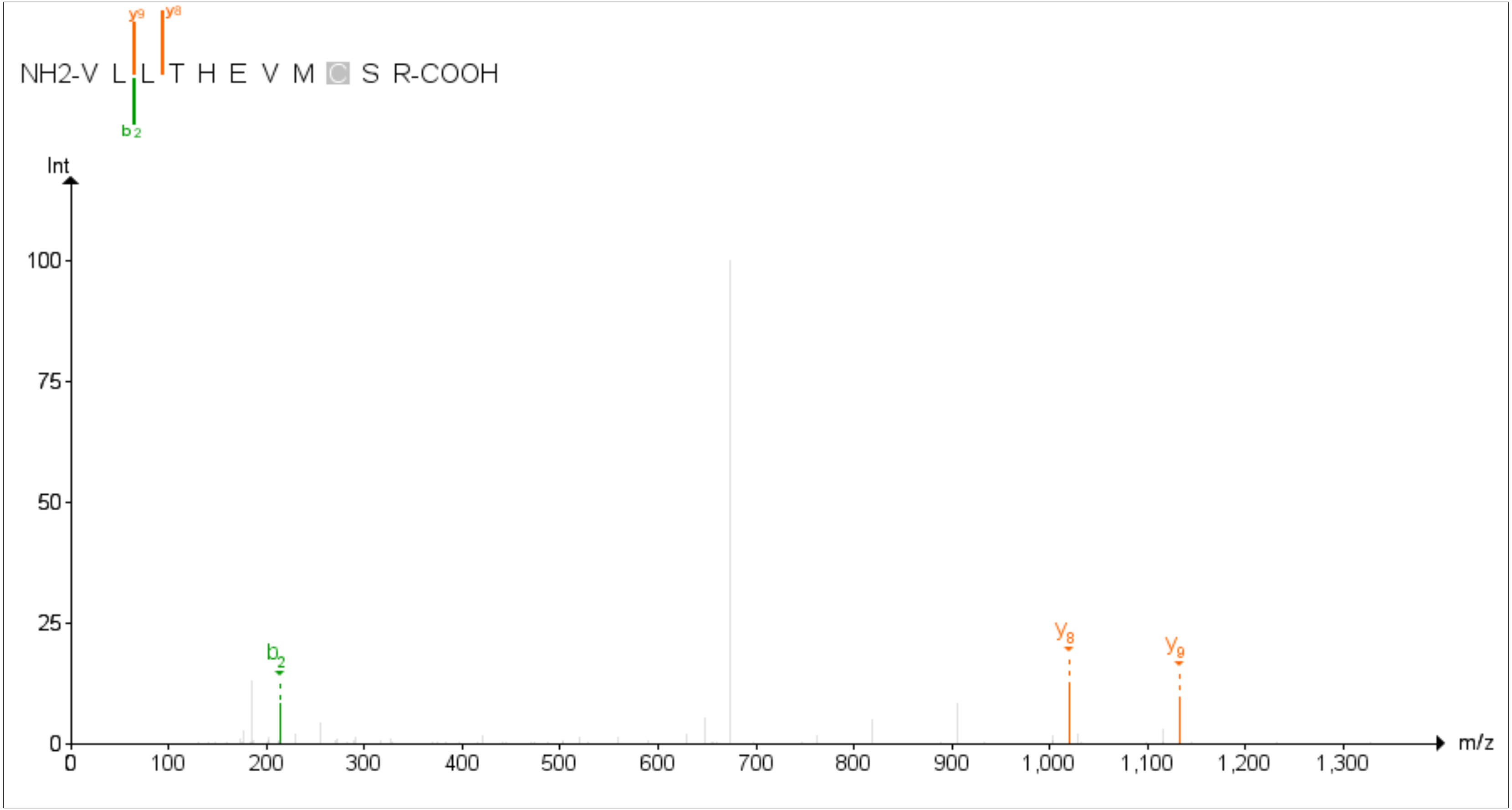

NH2-V L L T H E V M C S R-COOH
y9
y8
b2
Int
100
75
50
25
0
m/z
0
100
200
300
400
500
600
700
800
900
1,000
1,100
1,200
1,300
b2
y8
y9

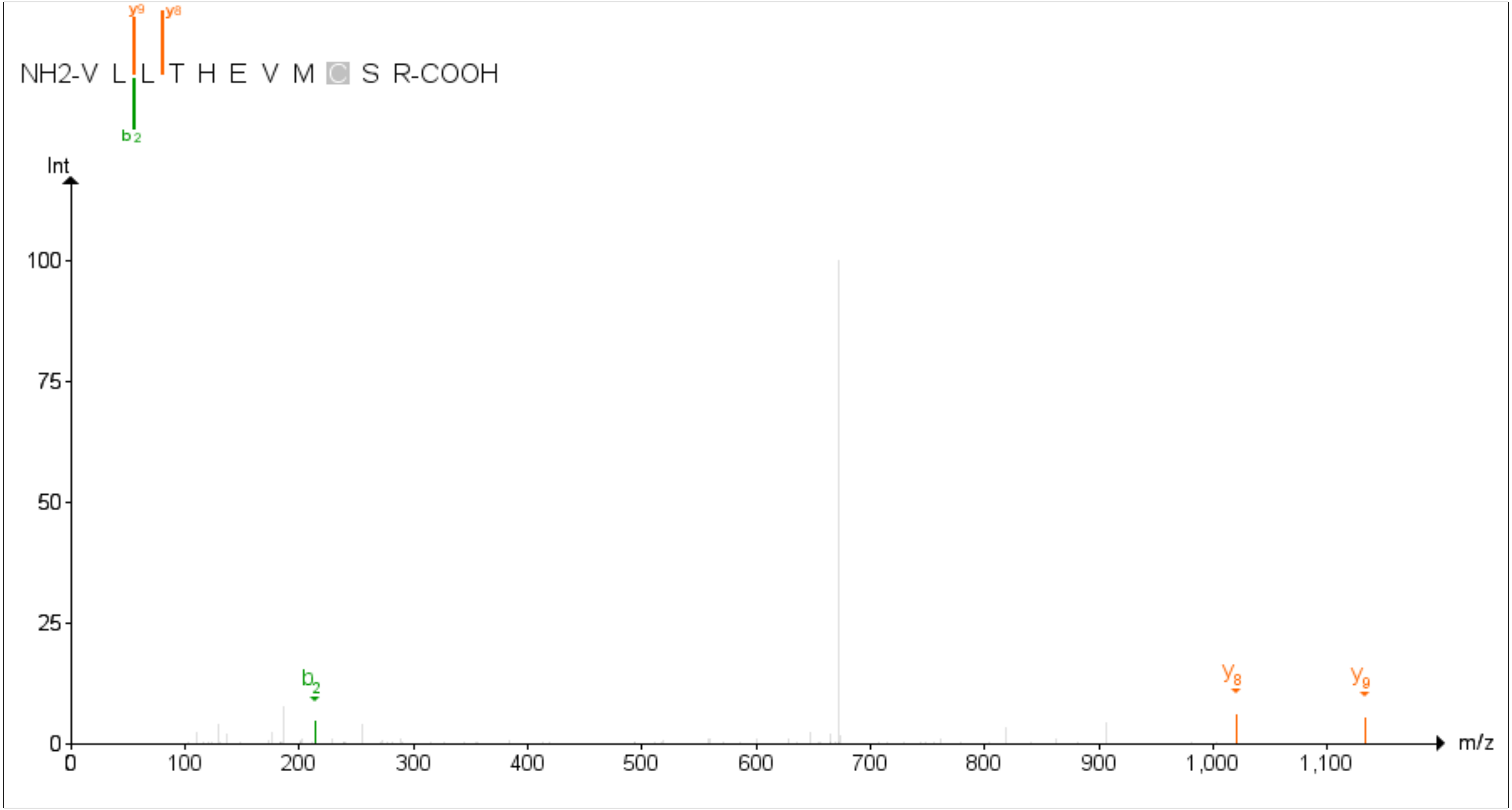

y9
y8
NH2-V L L T H E V M C S R-COOH
b2
Int
100
75
50
25
0
b2
y8
y9
0
100
200
300
400
500
600
700
800
900
1,000
1,100
m/z

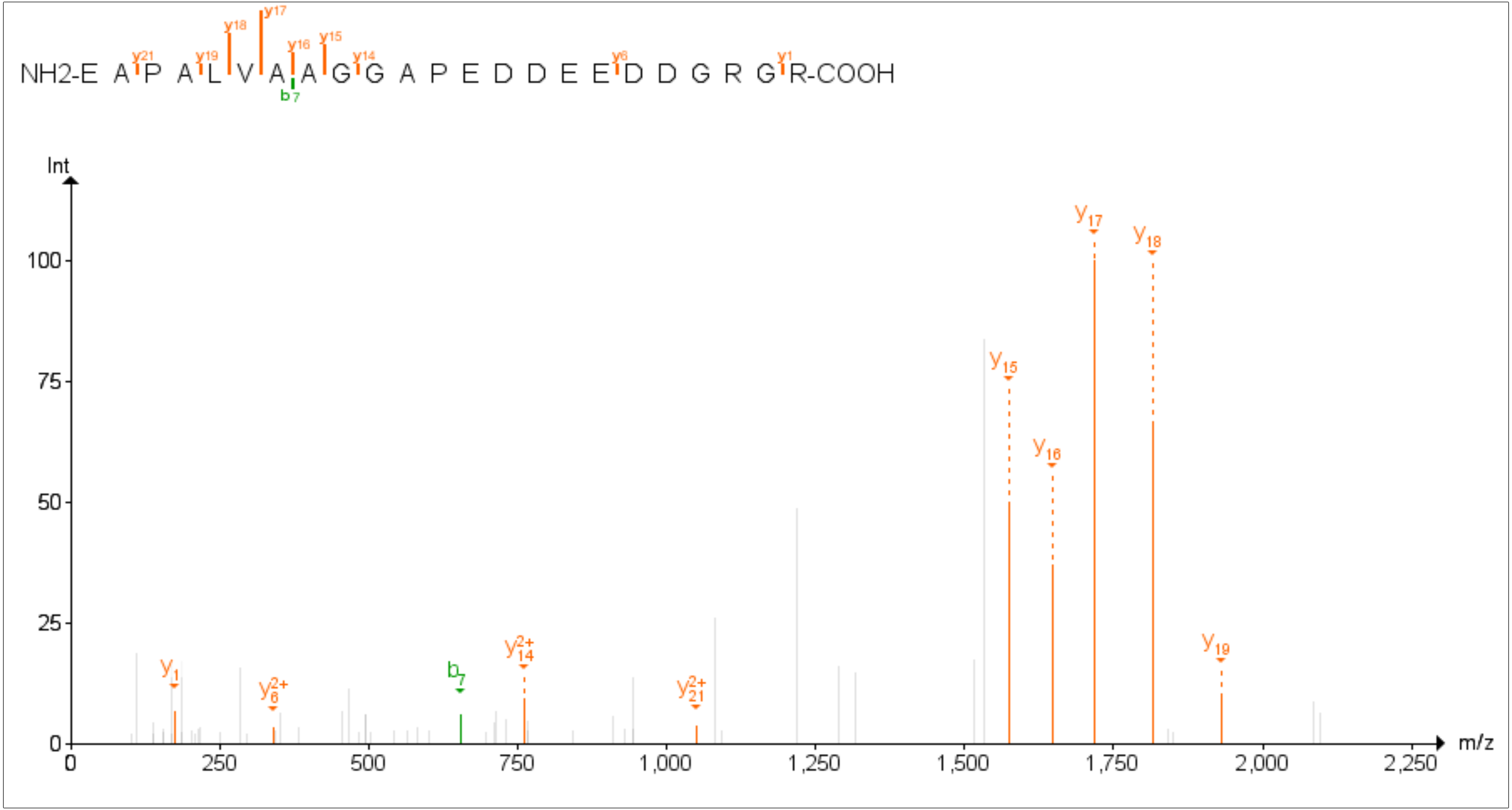

NH2-E A P A L V A A G G A P E D D E E D D G R G R-COOH
y21
y19
y18
y17
y16
y15
y14
y6
y1
b7
Int
100
75
50
25
0
m/z
0
250
500
750
1,000
1,250
1,500
1,750
2,000
2,250
y1
y6 2+
b7
y14 2+
y21 2+
y15
y16
y17
y18
y19

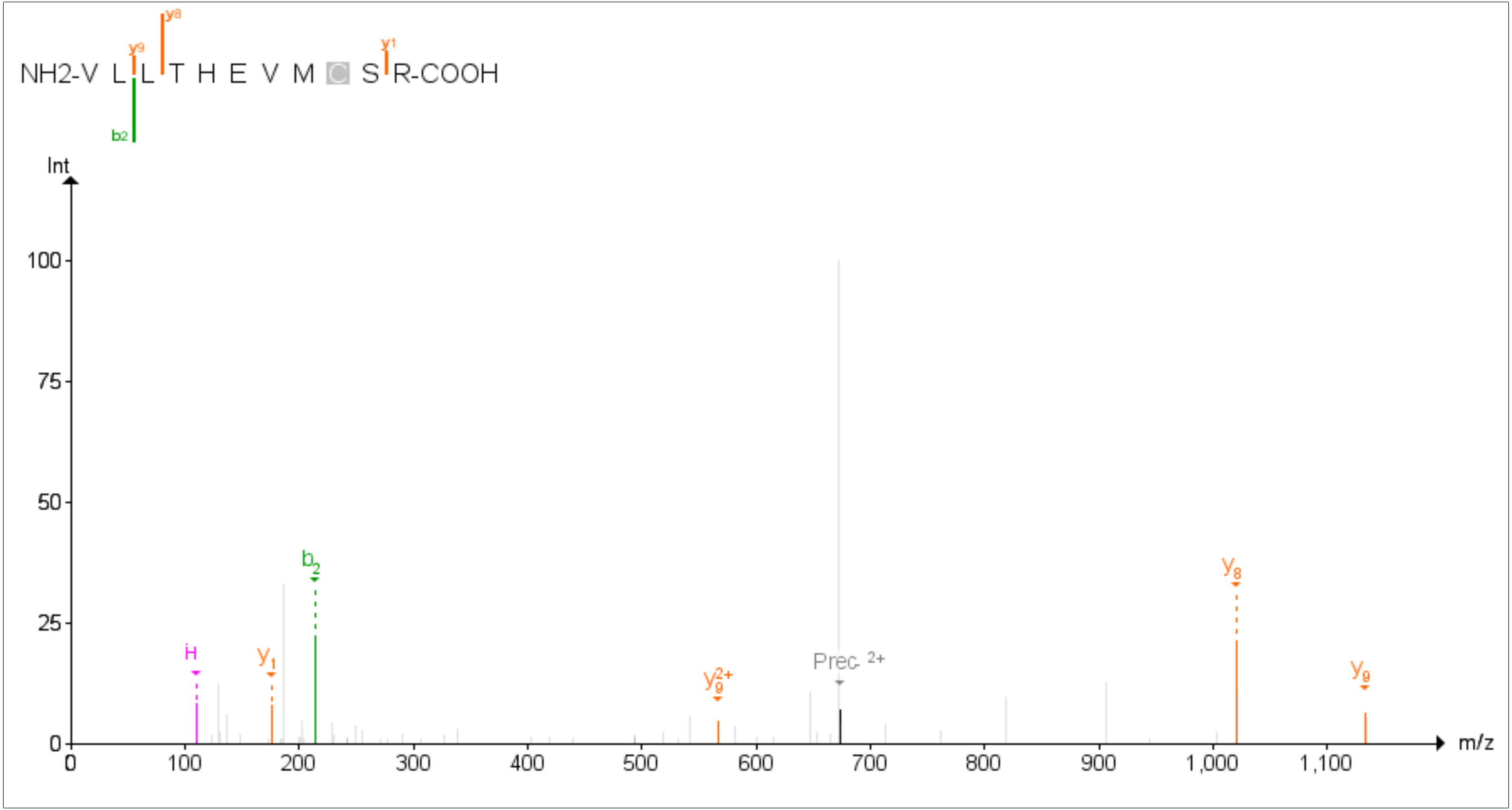

NH2-V L L T H E V M C S R-COOH
y9
y8
y1
b2
Int
100
75
50
25
0
b2
y8
y1
H
y9
y9 2+
Prec 2+
0
100
200
300
400
500
600
700
800
900
1,000
1,100
m/z

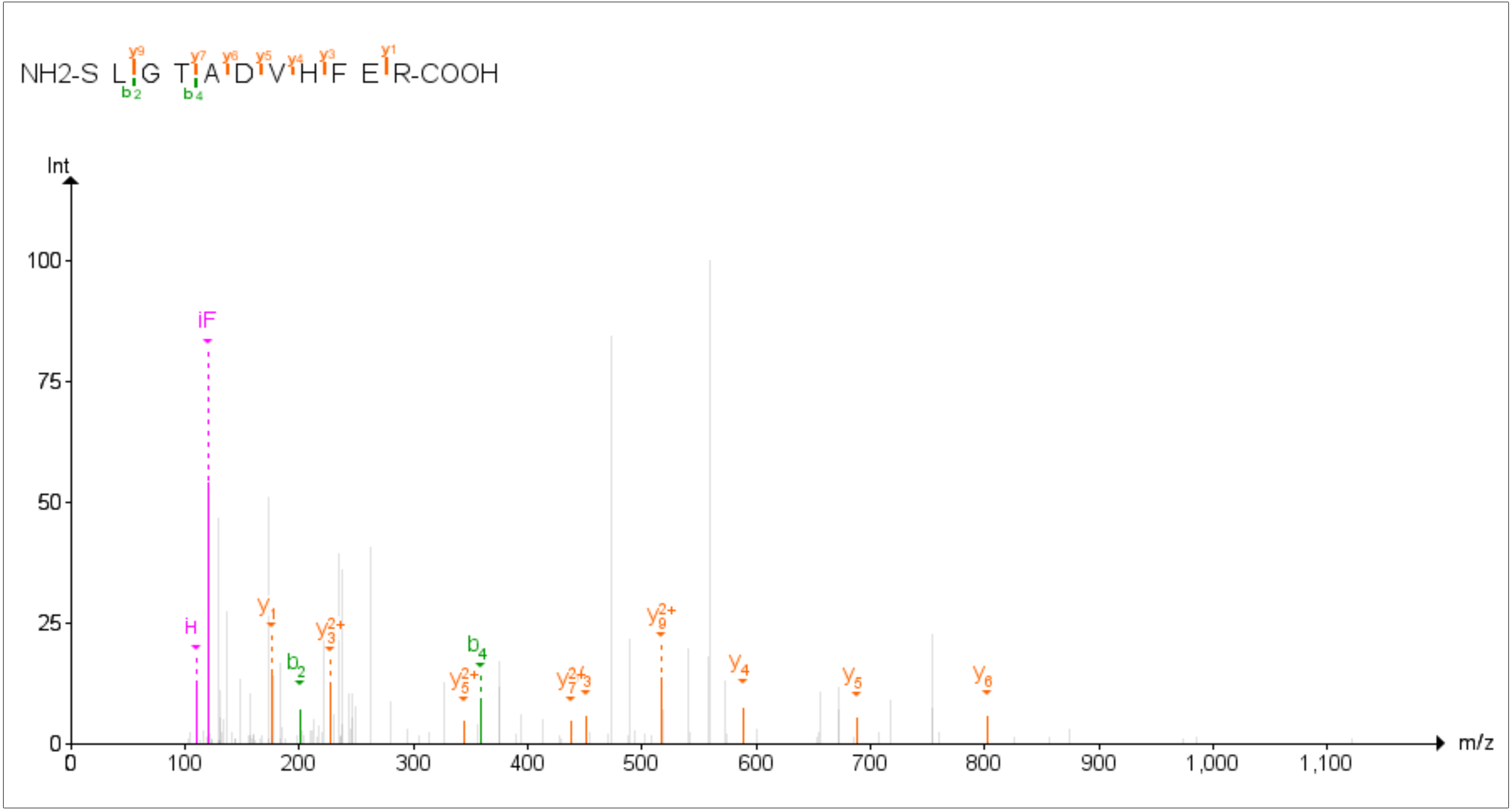

NH2-S L G T A D V H F E R-COOH
y9 y7 y6 y5 y4 y3 y1
b2 b4
Int
100
75
50
25
0
iF
iH
y1
b2
y3 2+
y5 2+
b4
y7 2+
y3
y9 2+
y4
y5
y6
m/z
0
100
200
300
400
500
600
700
800
900
1,000
1,100

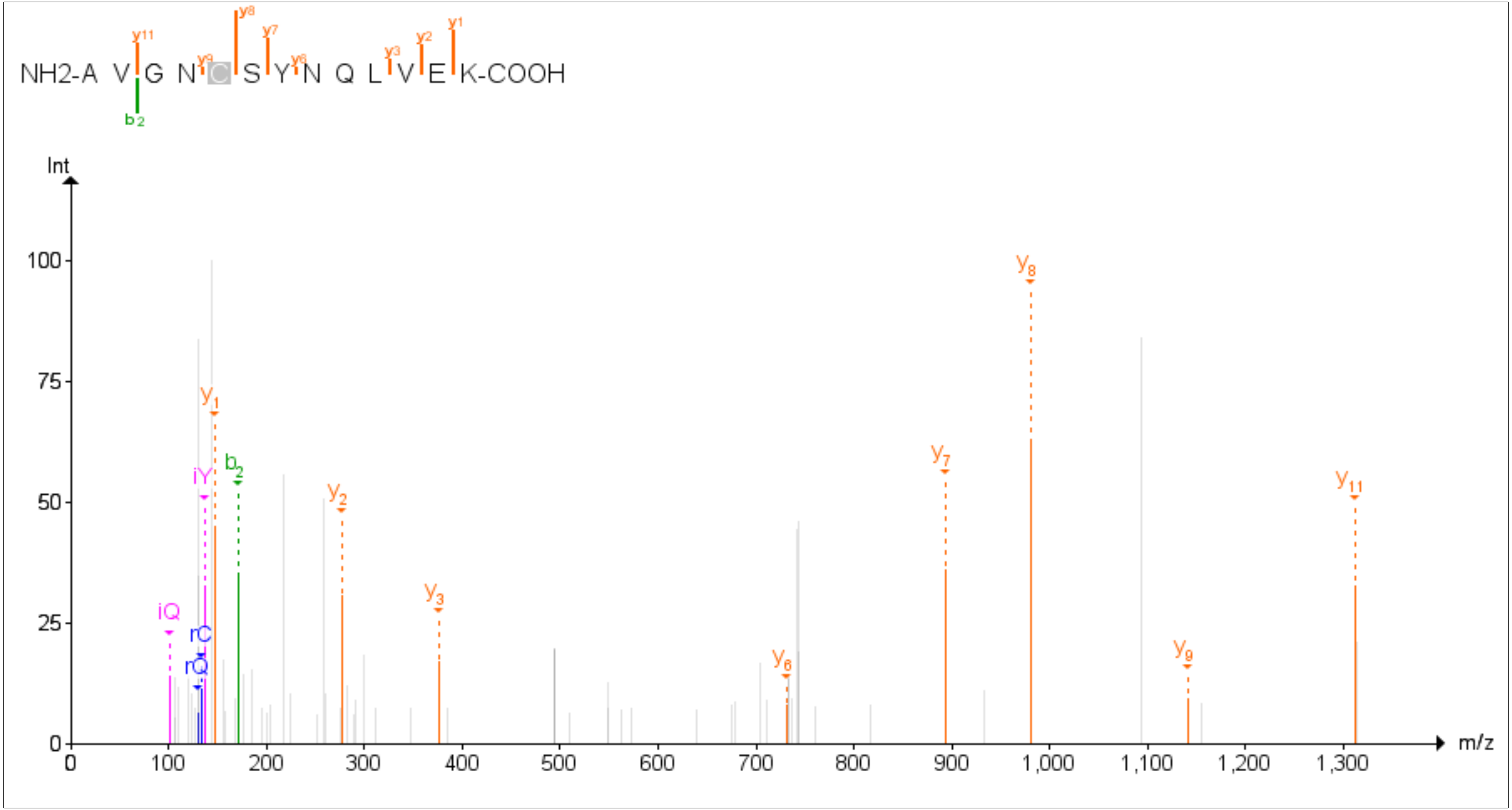

NH2-A V G N C S Y N Q L V E K-COOH
y11
y9
y8
y7
y6
y3
y2
y1
b2
Int
100
75
50
25
0
y8
y1
y7
b2
iY
y2
y11
y3
iQ
rC
rQ
y6
y9
0
100
200
300
400
500
600
700
800
900
1,000
1,100
1,200
1,300
m/z

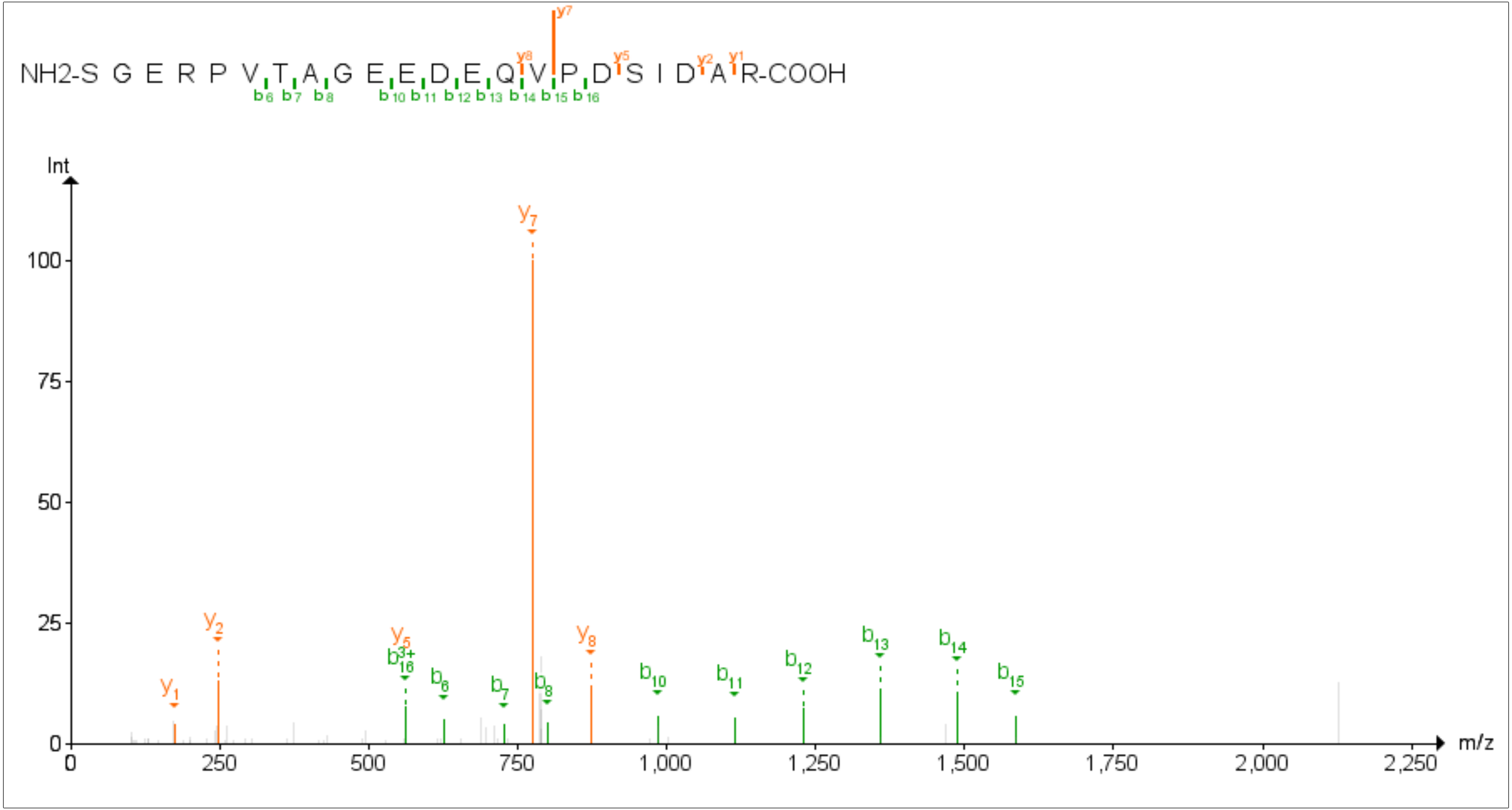

NH2-S G E R P V T A G E E D E Q V P D S I D A R-COOH
y7
y8
y5
y2
y1
b6 b7 b8 b10 b11 b12 b13 b14 b15 b16
Int
m/z
0
25
50
75
100
0
250
500
750
1,000
1,250
1,500
1,750
2,000
2,250
y7
y1
y2
y5
b3+ 16
b6
b7
b8
y8
b10
b11
b12
b13
b14
b15

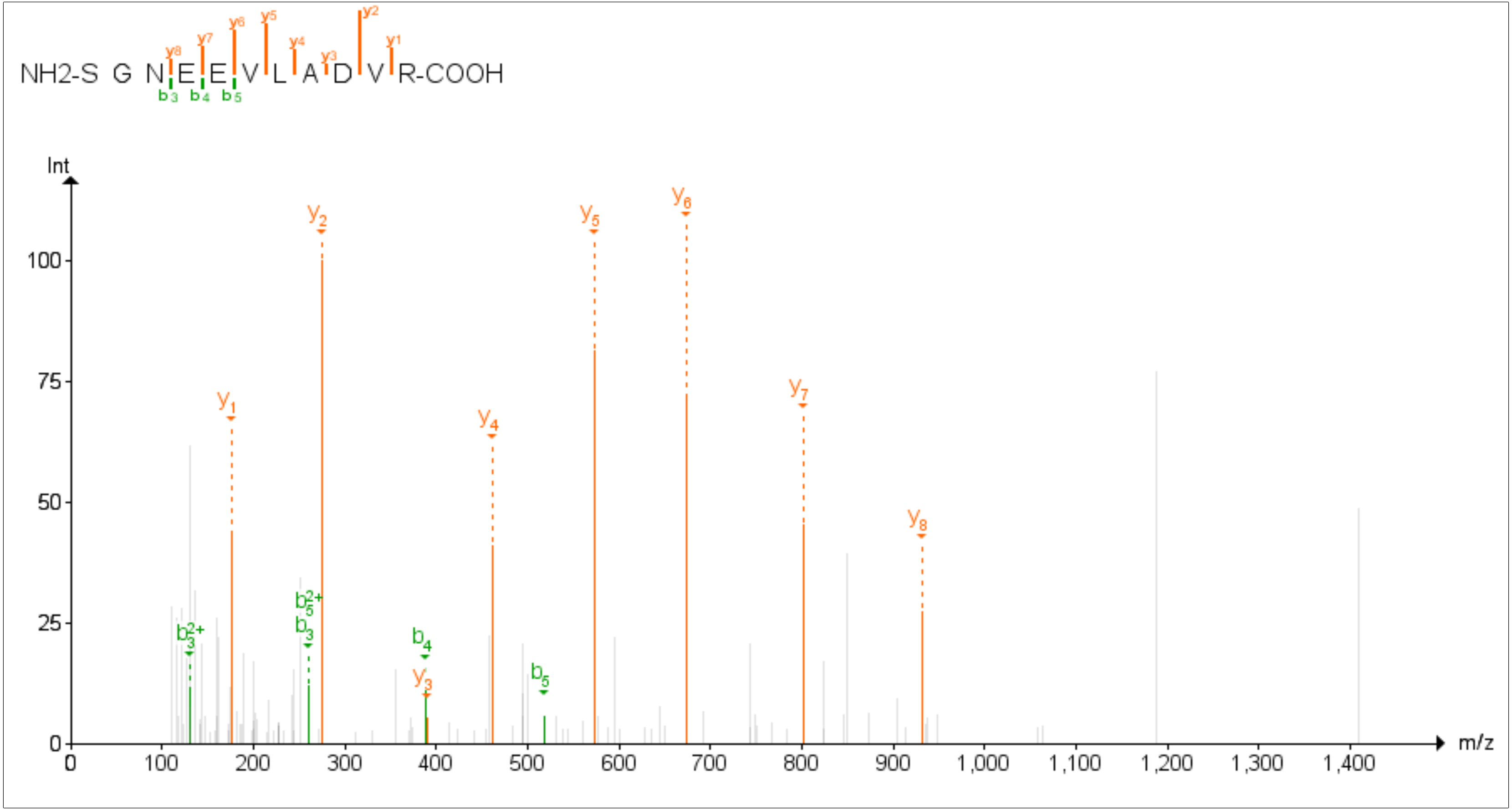

NH2-S G N E E V L A D V R-COOH
y8 y7 y6 y5 y4 y3 y2 y1
b3 b4 b5
Int
m/z
y1 y2 y3 y4 y5 y6 y7 y8
b3 b4 b5
b_3^{2+} b_5^{2+}
0 25 50 75 100
0 100 200 300 400 500 600 700 800 900 1,000 1,100 1,200 1,300 1,400

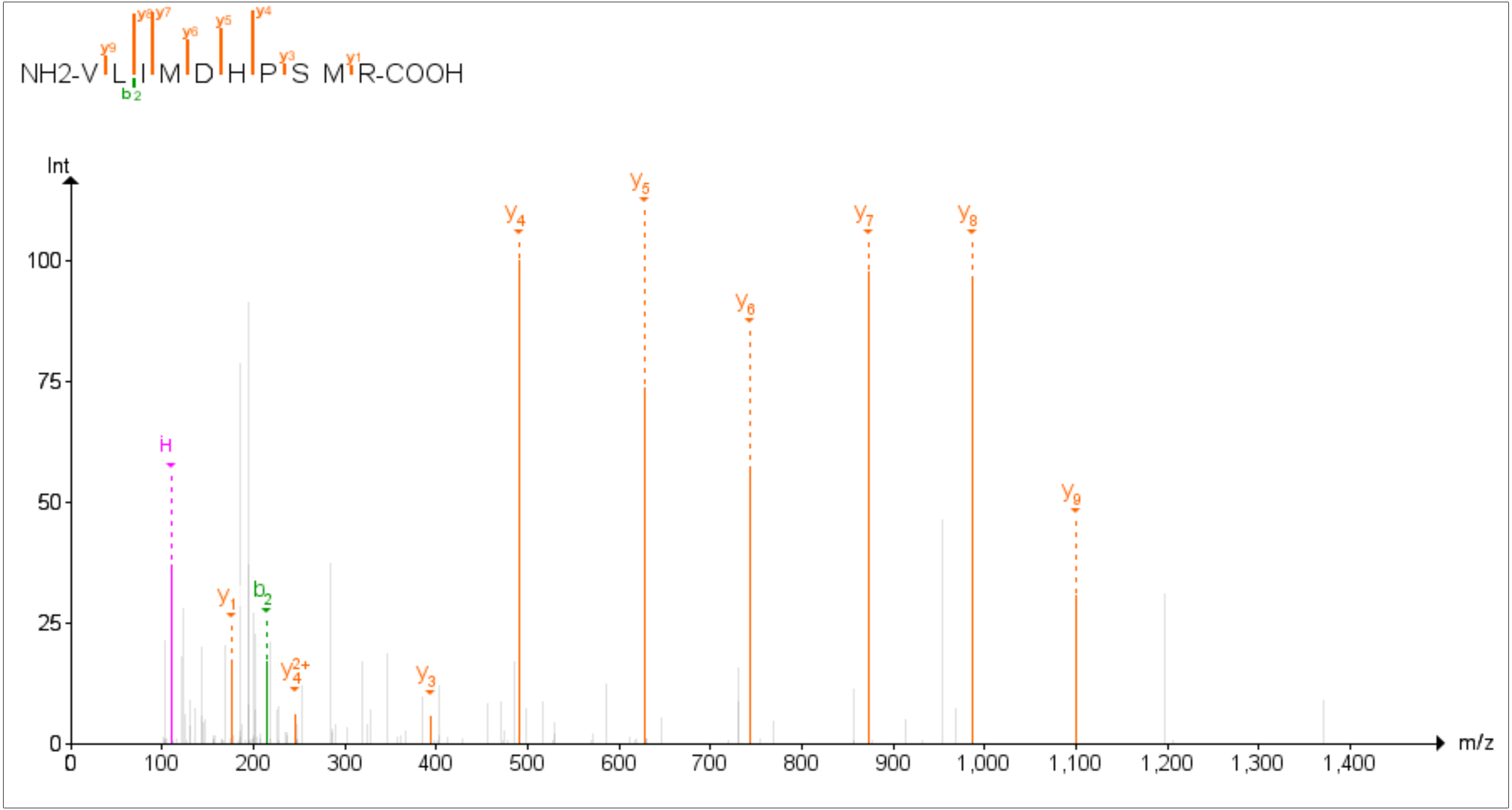

NH2-V L I M D H P S M R-COOH
y9 y8 y7 y6 y5 y4 y3 y1
b2
Int
100
75
50
25
0
m/z
0 100 200 300 400 500 600 700 800 900 1,000 1,100 1,200 1,300 1,400
H
y1
b2
y4 2+
y3
y4
y5
y6
y7
y8
y9

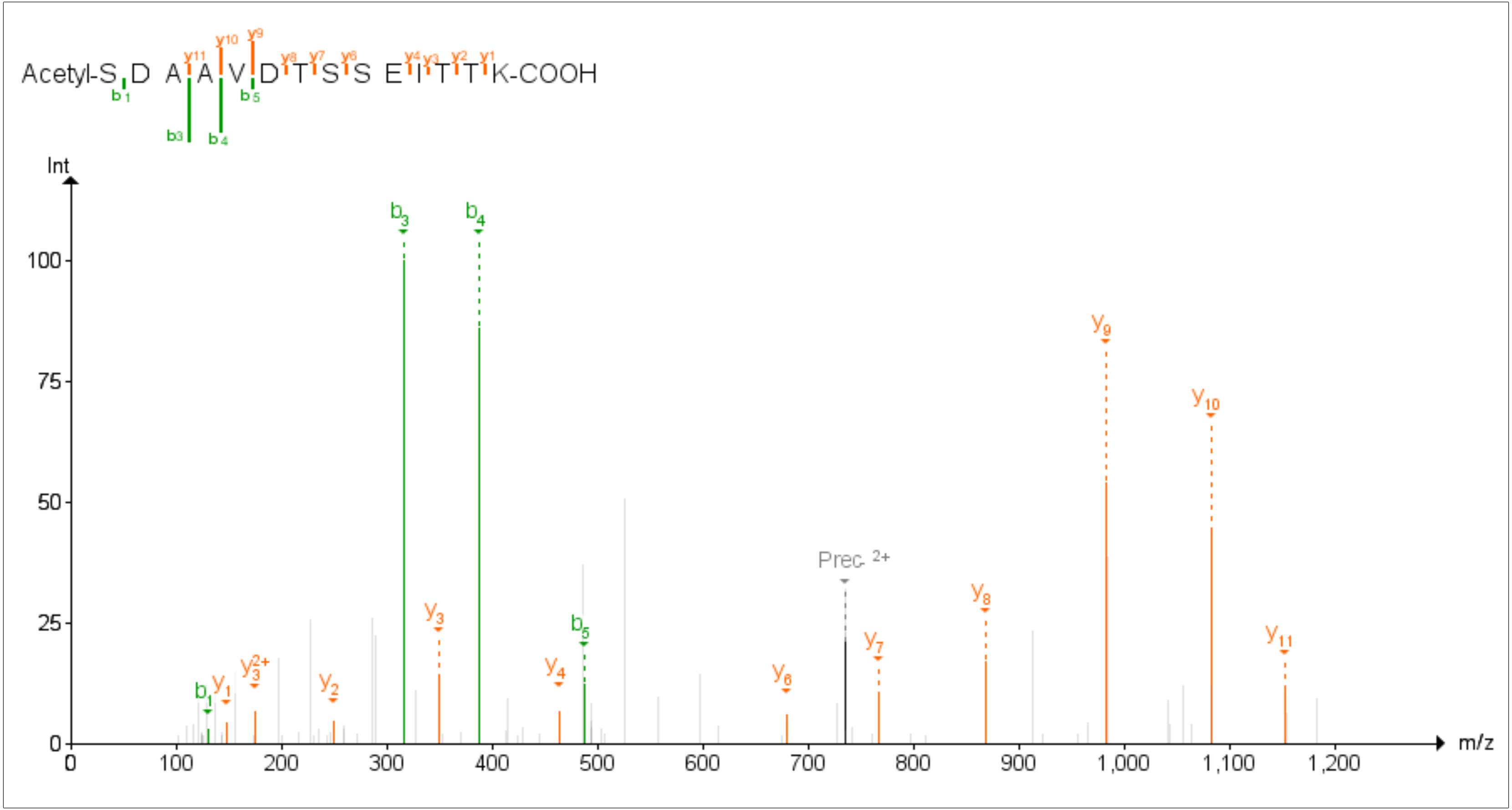
Acetyl-S D A A V D T S S E I T T K-COOH
Int
100
75
50
25
0
Prec 2+
m/z
0
100
200
300
400
500
600
700
800
900
1,000
1,100
1,200

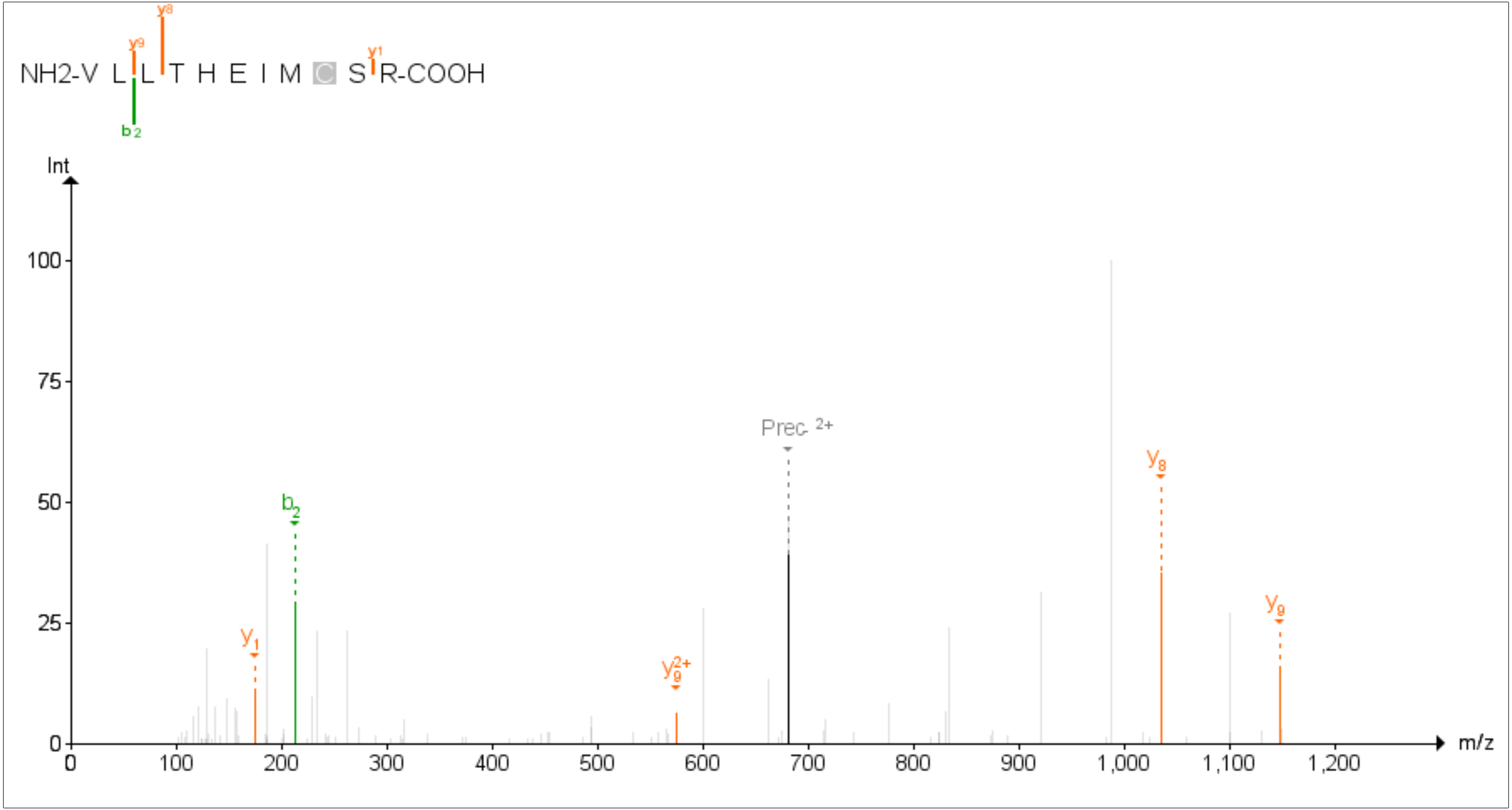

NH2-V L L T H E I M C S R-COOH
y8
y9
y1
b2
Int
100
75
50
25
0
Prec 2+
y8
y9
y1
b2
y9 2+
0
100
200
300
400
500
600
700
800
900
1,000
1,100
1,200
m/z

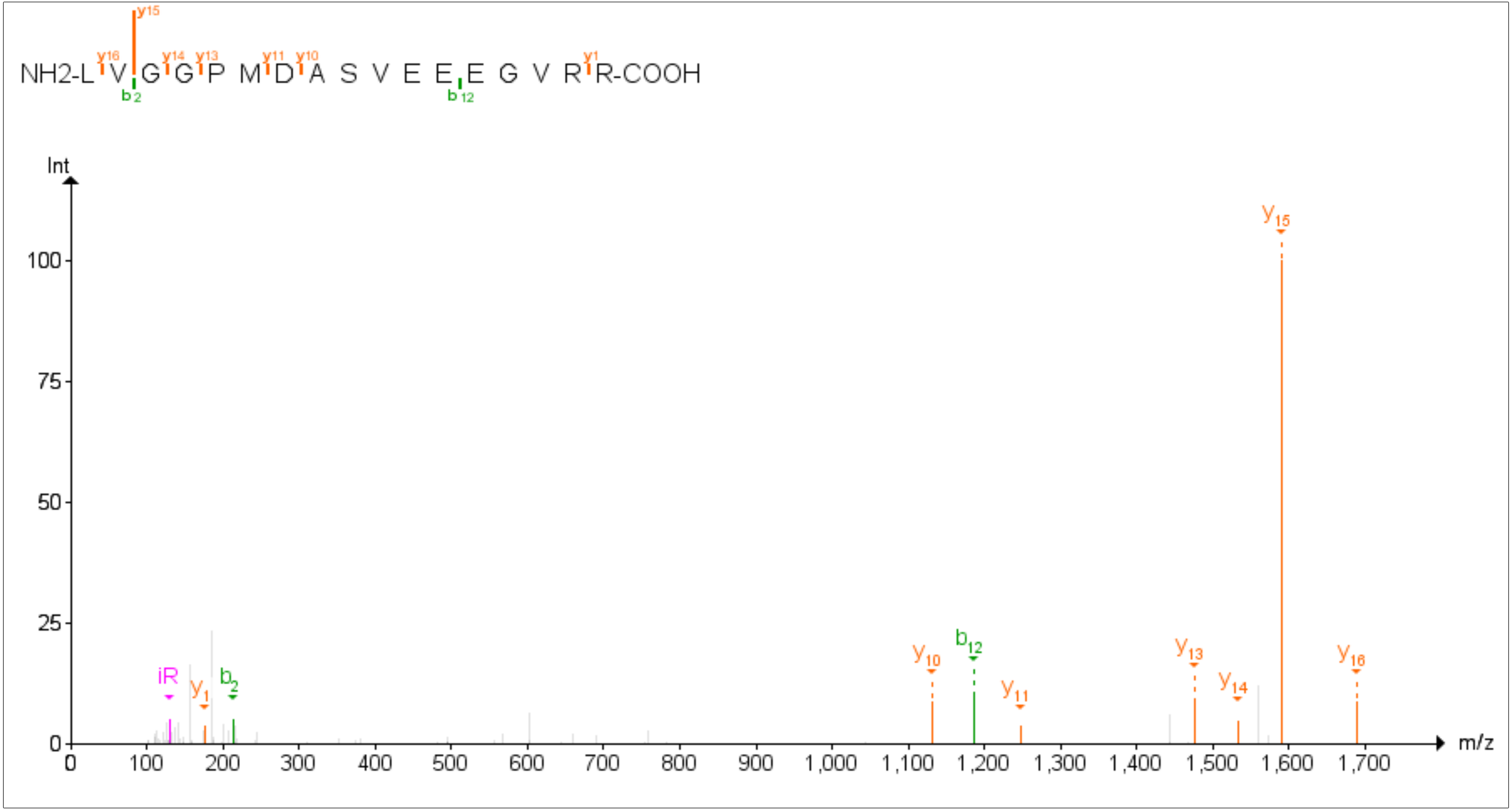

NH2-L V G G P M D A S V E E E G V R R-COOH
y16 y15 y14 y13 y11 y10 y1
b2 b12
Int
100
75
50
25
0
iR
y1
b2
y10
b12
y11
y13
y14
y15
y16
0 100 200 300 400 500 600 700 800 900 1,000 1,100 1,200 1,300 1,400 1,500 1,600 1,700
m/z

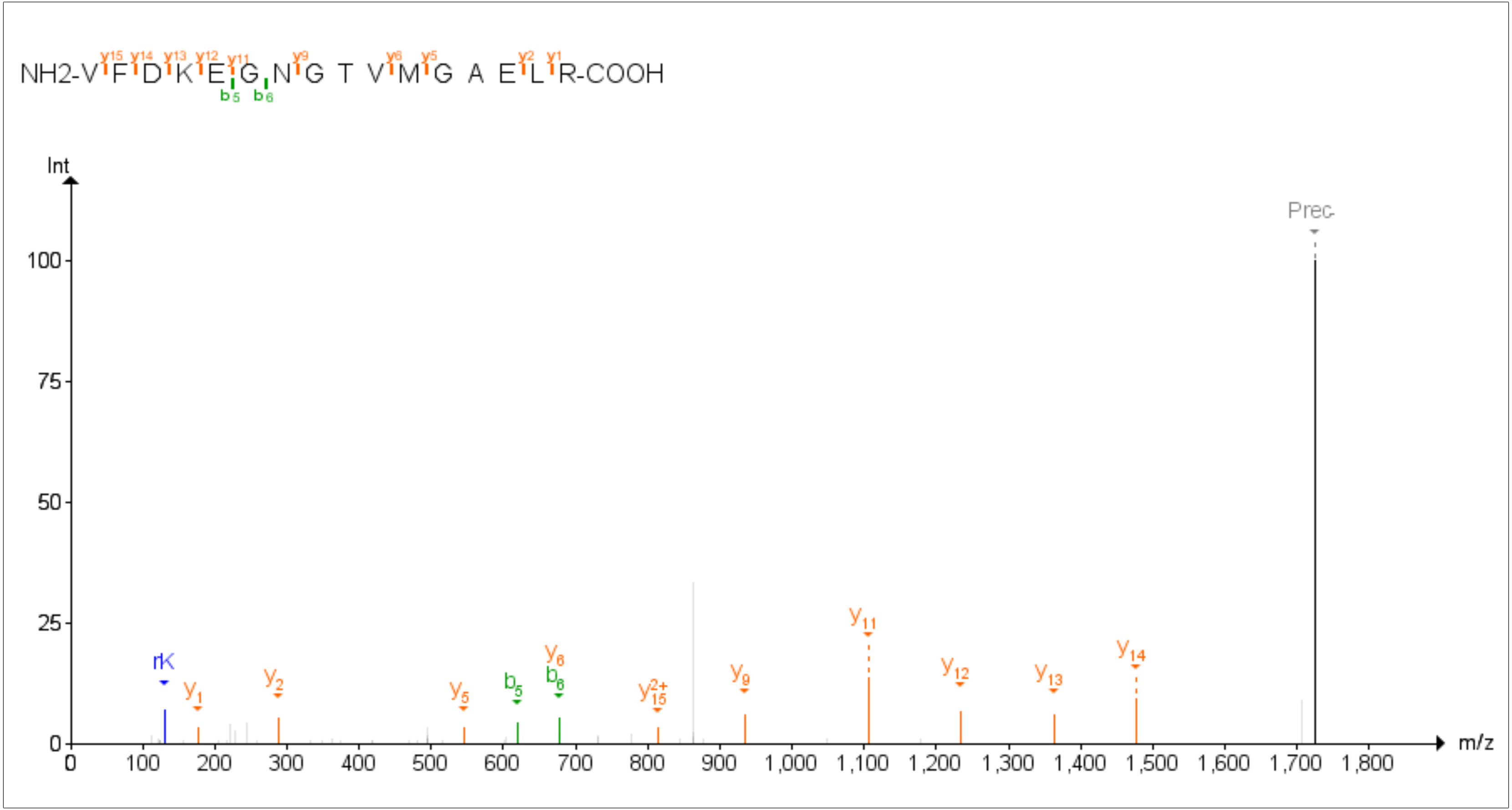
NH2-V F D K E G N G T V M G A E L R-COOH
y15 y14 y13 y12 y11 y9 y6 y5 y2 y1
b5 b6
Int
100
75
50
25
0
Prec
rK
y1
y2
y5
b5
y6
b6
y15 2+
y9
y11
y12
y13
y14
0 100 200 300 400 500 600 700 800 900 1,000 1,100 1,200 1,300 1,400 1,500 1,600 1,700 1,800
m/z

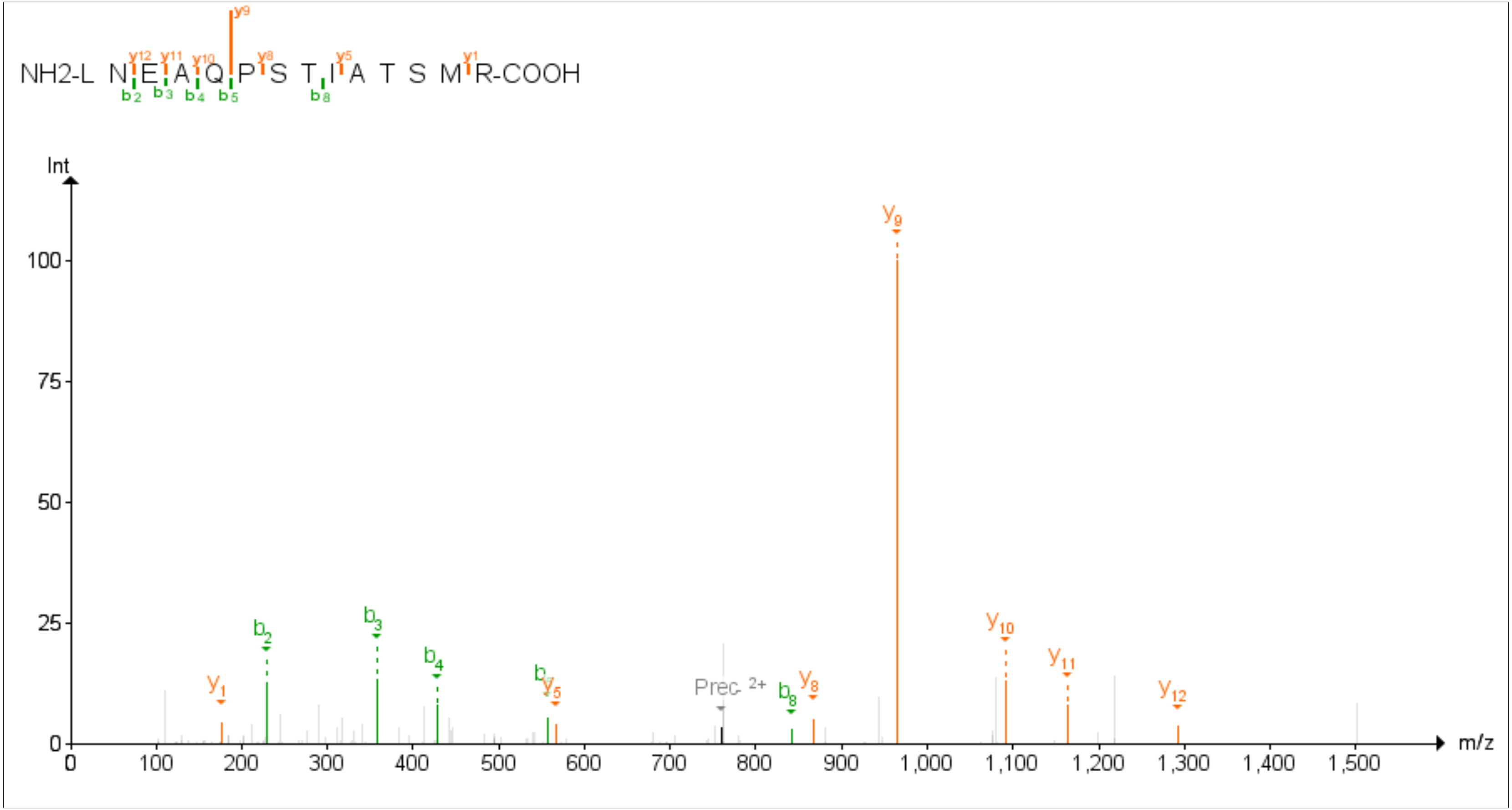

NH2-L N E A Q P S T I A T S M R-COOH
y12 y11 y10 y9 y8 y5 y1
b2 b3 b4 b5 b8
Int
100
75
50
25
0
y9
y10
y11
y12
y1
b2
b3
b4
b7
y5
Prec 2+
b8
y8
0 100 200 300 400 500 600 700 800 900 1,000 1,100 1,200 1,300 1,400 1,500
m/z

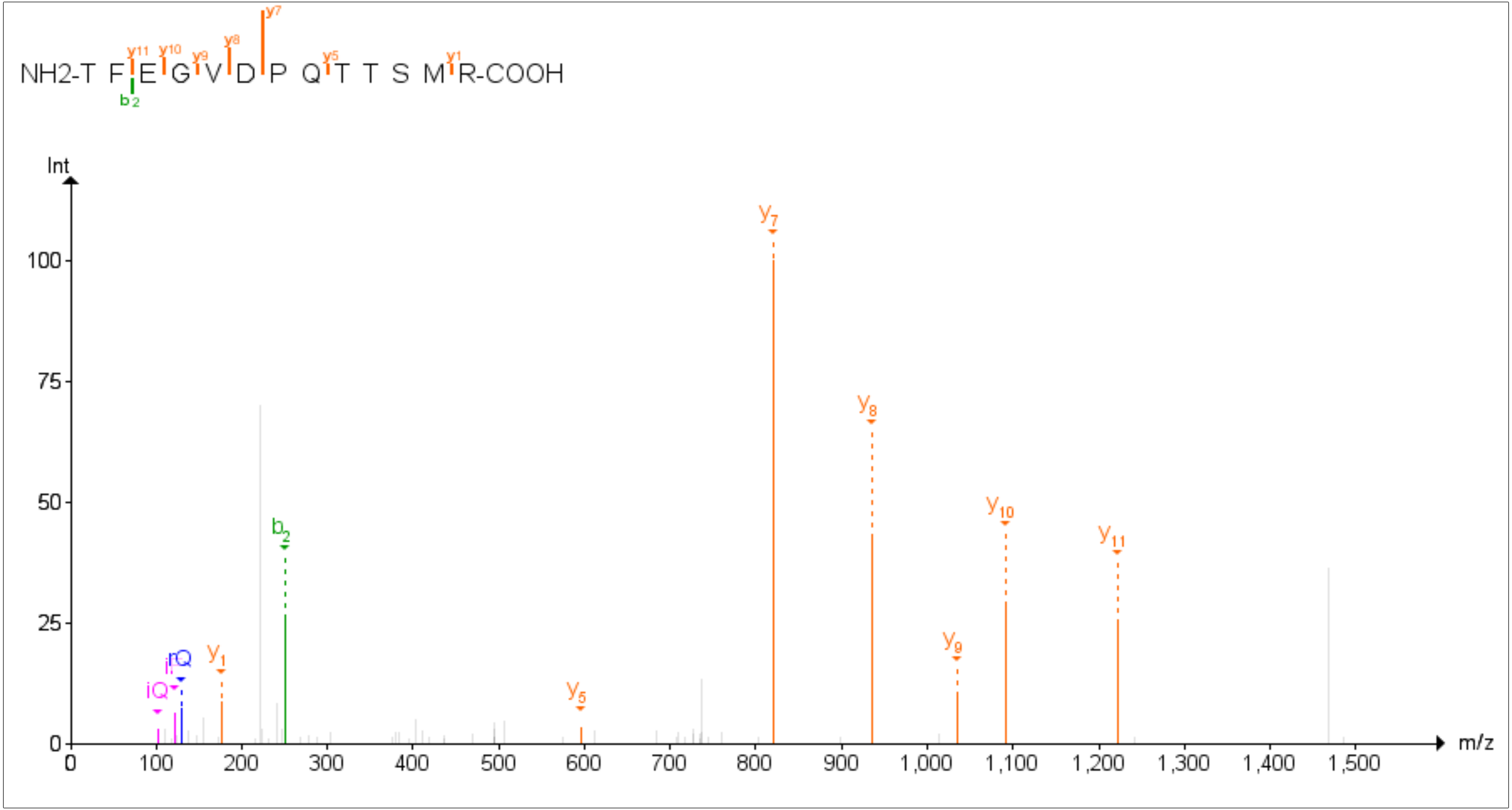

NH2-T F E G V D P Q T T S M R-COOH
y11
y10
y9
y8
y7
y5
y1
b2
Int
m/z
100
75
50
25
0
y7
y8
y10
y11
y9
b2
y1
y5
iQ
rQ
0
100
200
300
400
500
600
700
800
900
1,000
1,100
1,200
1,300
1,400
1,500

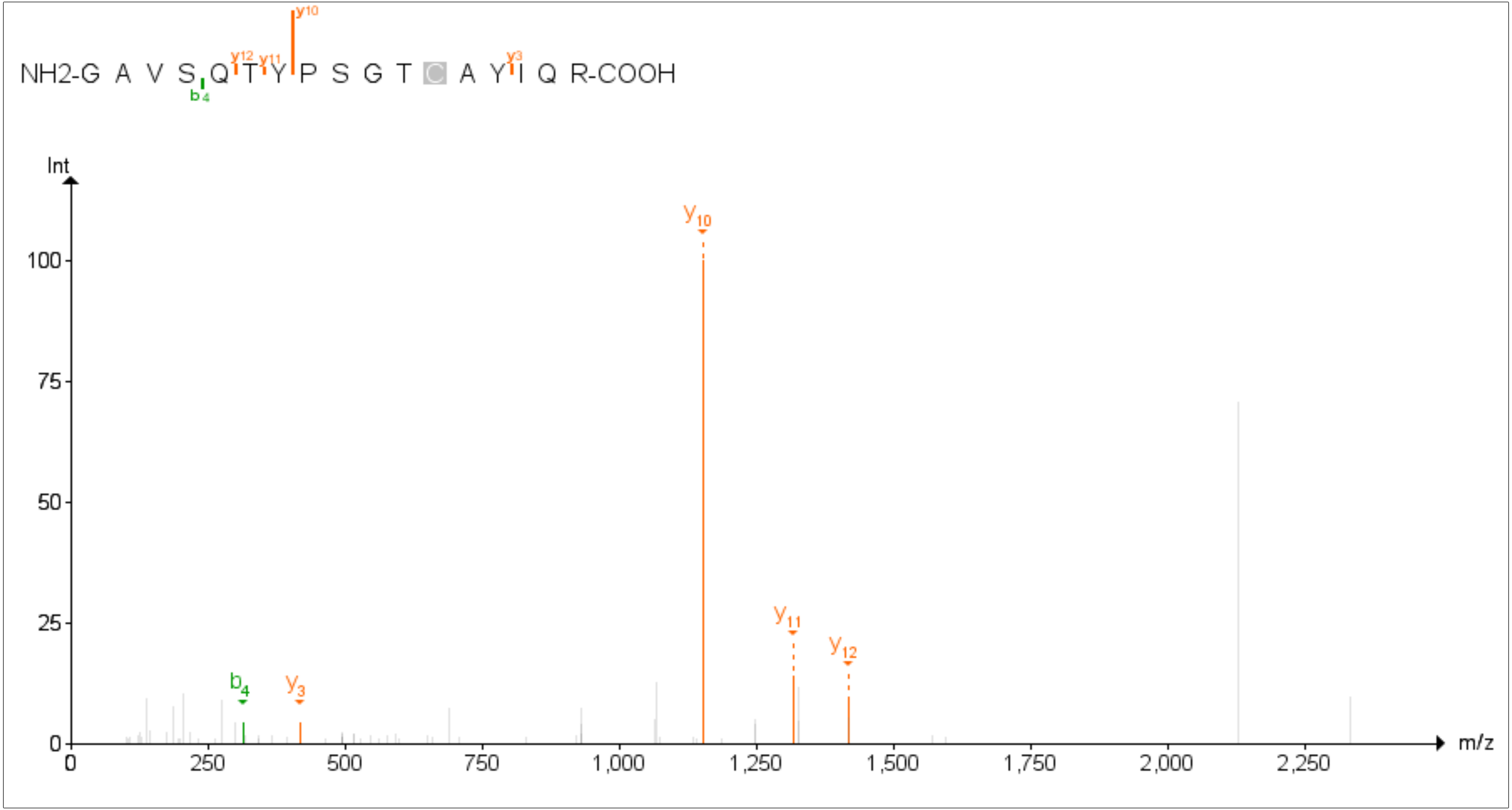

NH2-G A V S Q T Y P S G T C A Y I Q R-COOH
b4
y12
y11
y10
y3
Int
100
75
50
25
0
y10
y11
y12
b4
y3
0
250
500
750
1,000
1,250
1,500
1,750
2,000
2,250
m/z

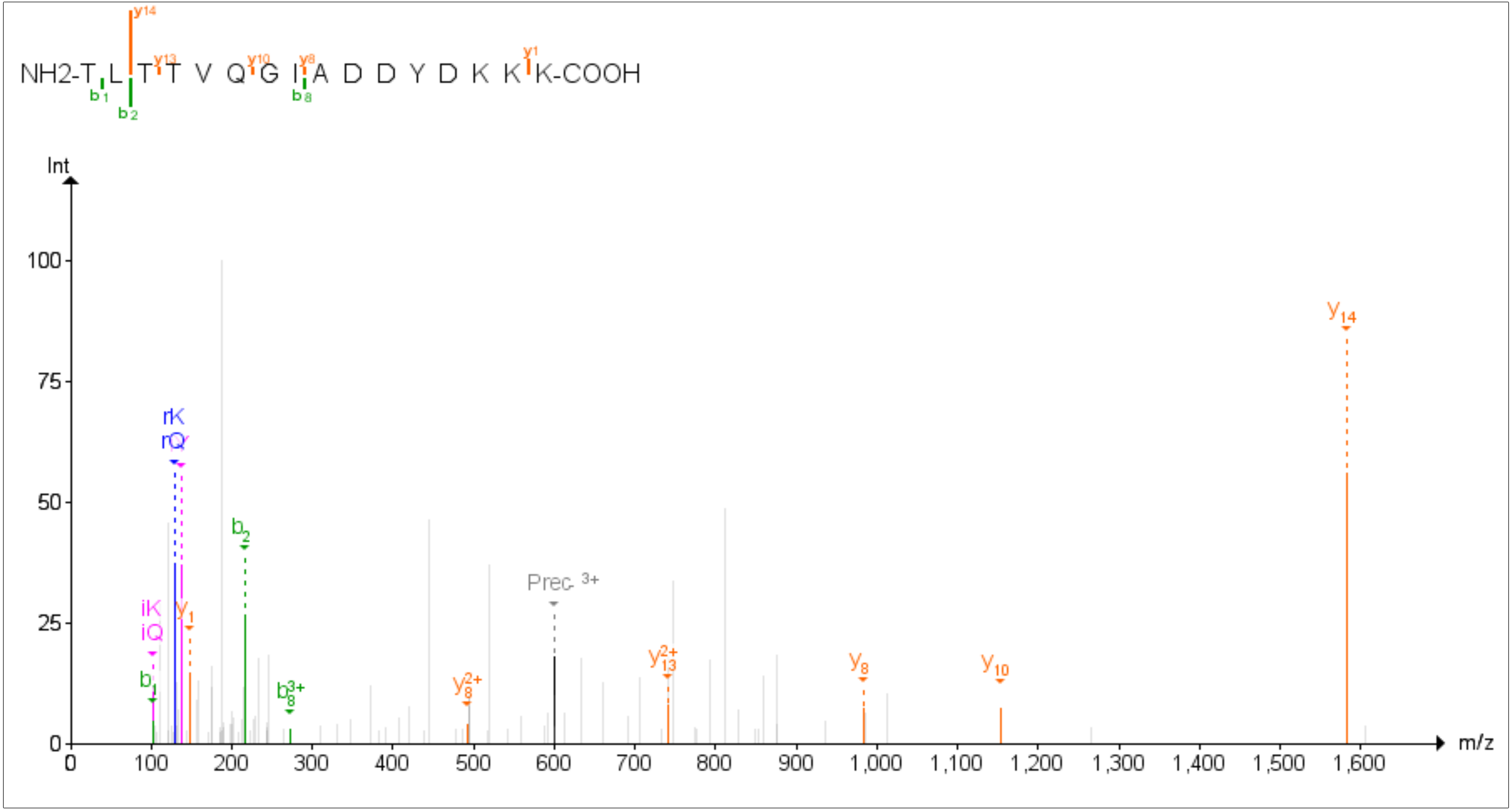

NH2-T L T T V Q G I A D D Y D K K K-COOH
y14
y13
y10
y8
y1
b1
b2
b8
Int
100
75
50
25
0
rK
rQ
iK
iQ
b1
b2
y1
b8 3+
y8 2+
Prec 3+
y13 2+
y8
y10
y14
0
100
200
300
400
500
600
700
800
900
1,000
1,100
1,200
1,300
1,400
1,500
1,600
m/z

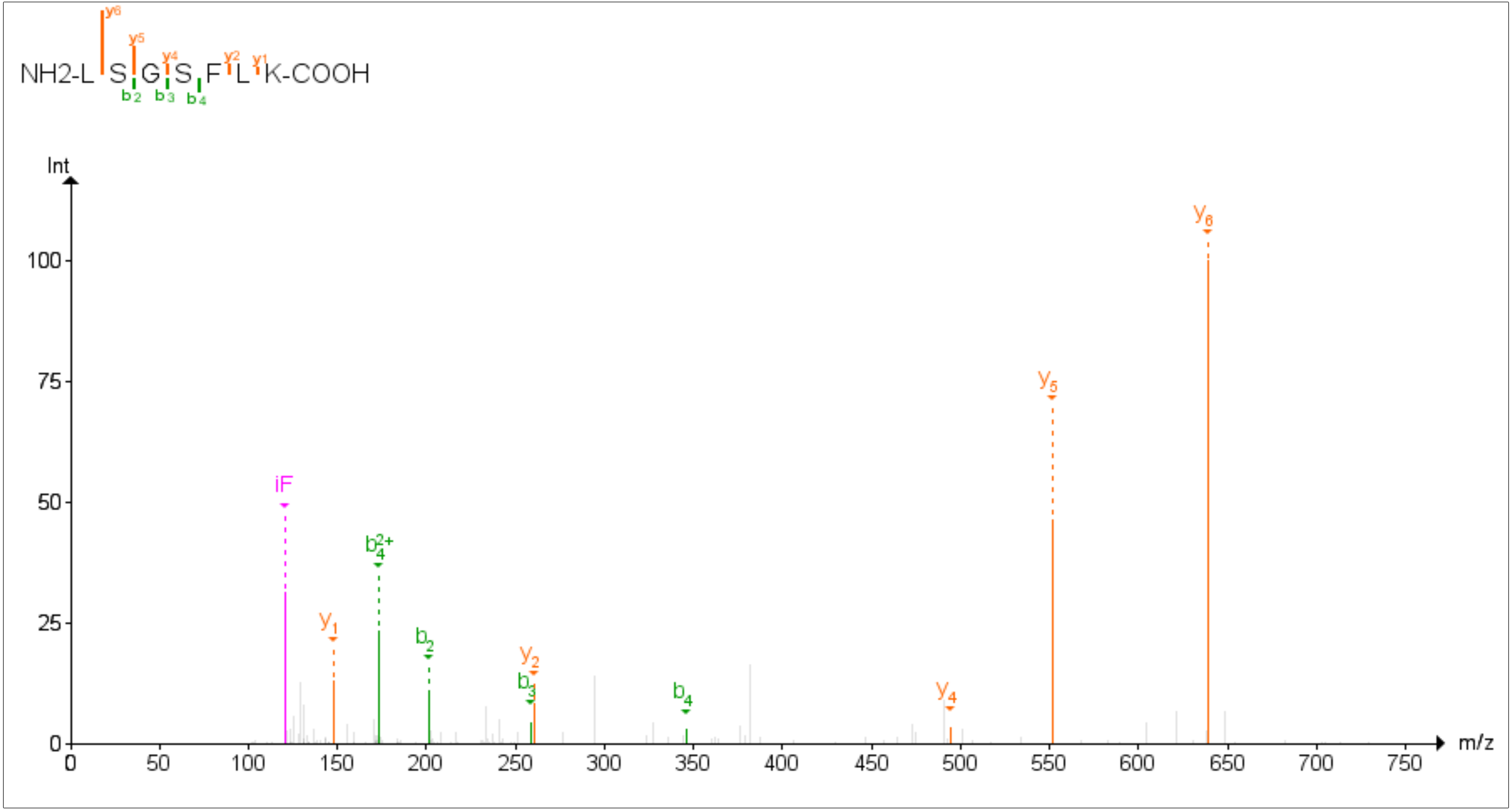

NH2-L S G S F L K-COOH
y6 y5 y4 y2 y1
b2 b3 b4
Int
m/z
0 25 50 75 100
0 50 100 150 200 250 300 350 400 450 500 550 600 650 700 750
iF
y1
b4 2+
b2
y2
b3
b4
y4
y5
y6

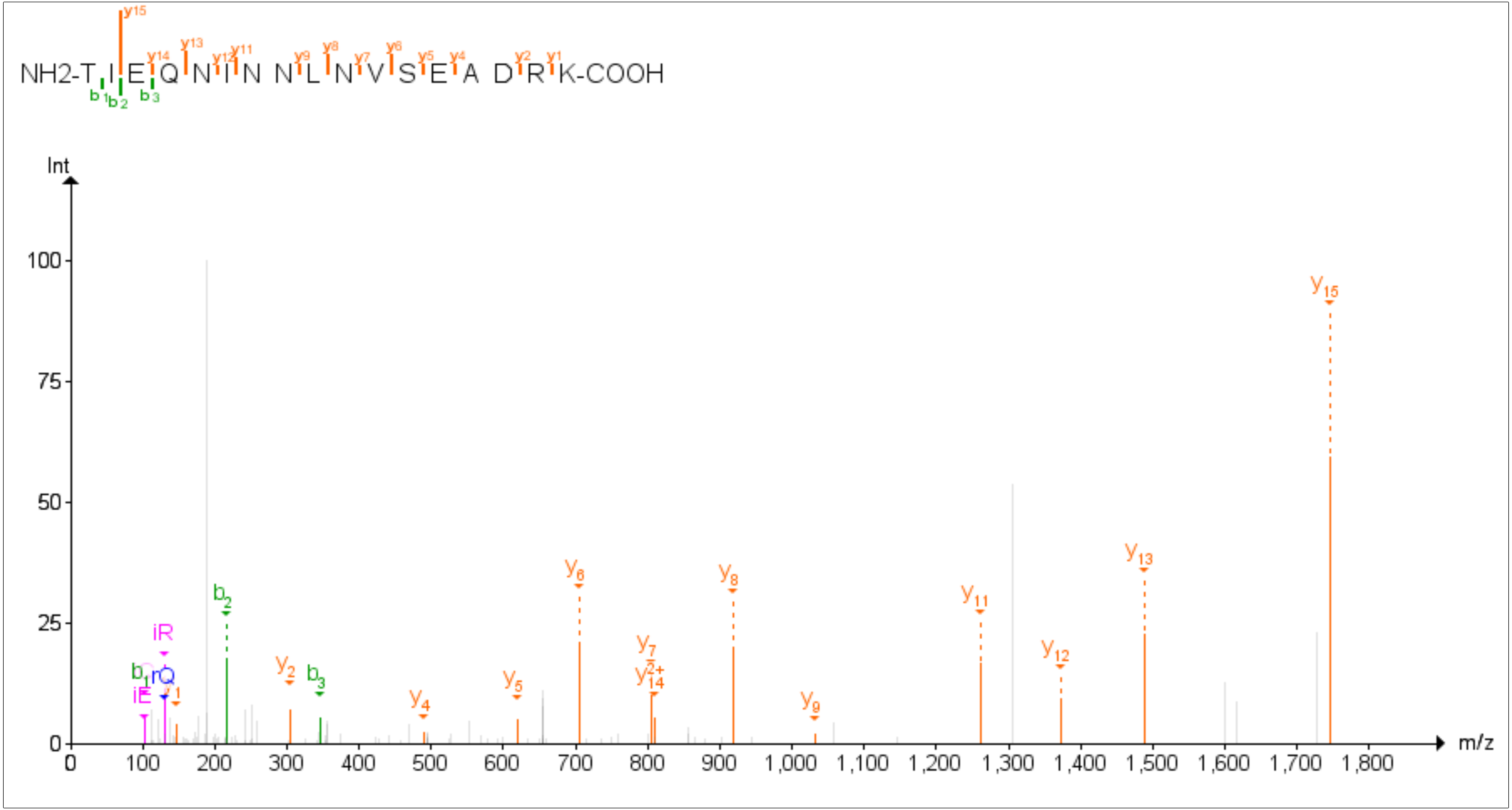

NH2-T I E Q N I N N L N V S E A D R K-COOH
Int
m/z
100
75
50
25
0
0
100
200
300
400
500
600
700
800
900
1,000
1,100
1,200
1,300
1,400
1,500
1,600
1,700
1,800

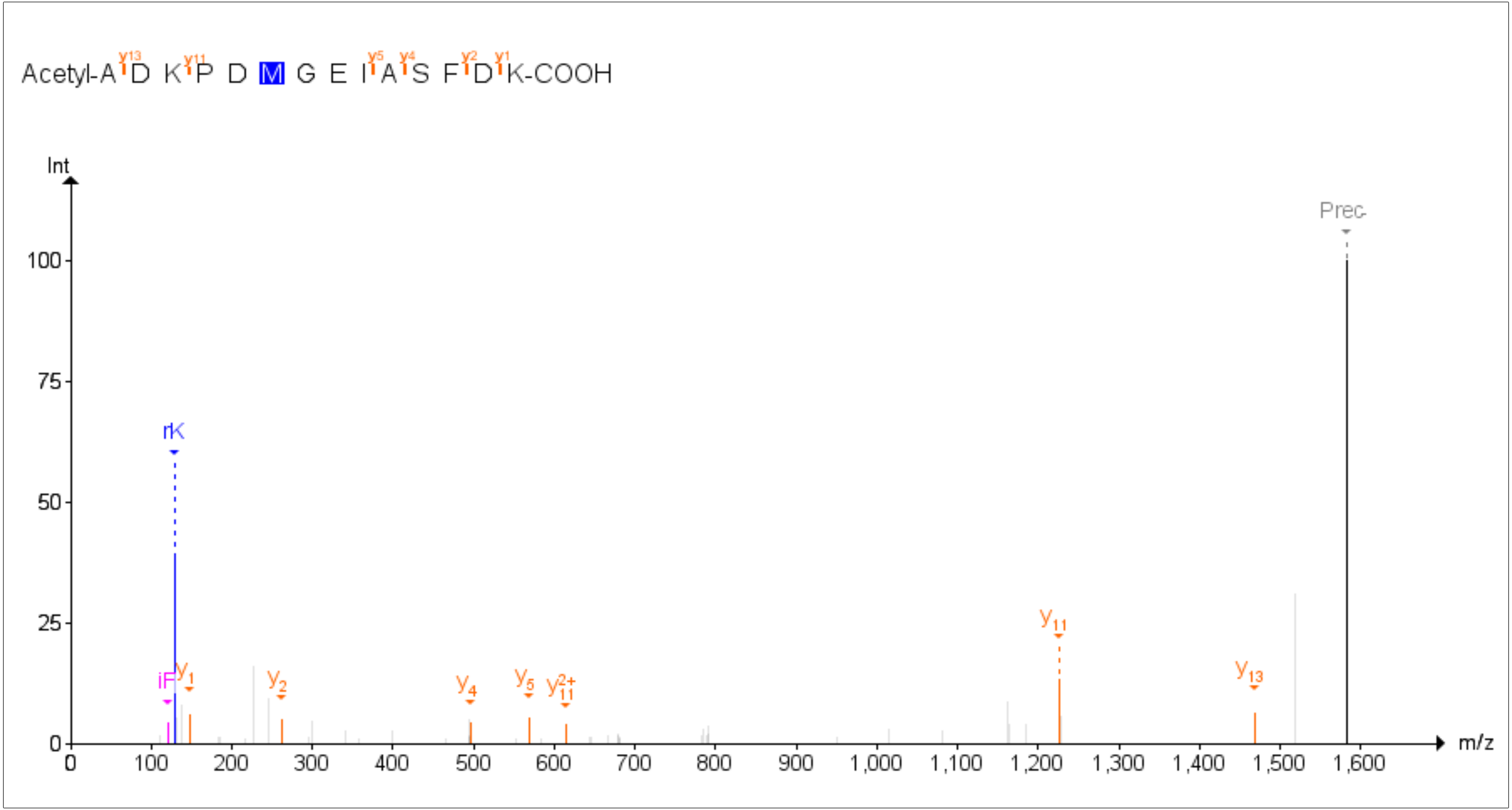

Acetyl-A D K P D M G E I A S F D K-COOH
y13
y11
y5
y4
y2
y1
Int
100
75
50
25
0
rK
iF
y1
y2
y4
y5
y11 2+
y11
y13
Prec
0
100
200
300
400
500
600
700
800
900
1,000
1,100
1,200
1,300
1,400
1,500
1,600
m/z

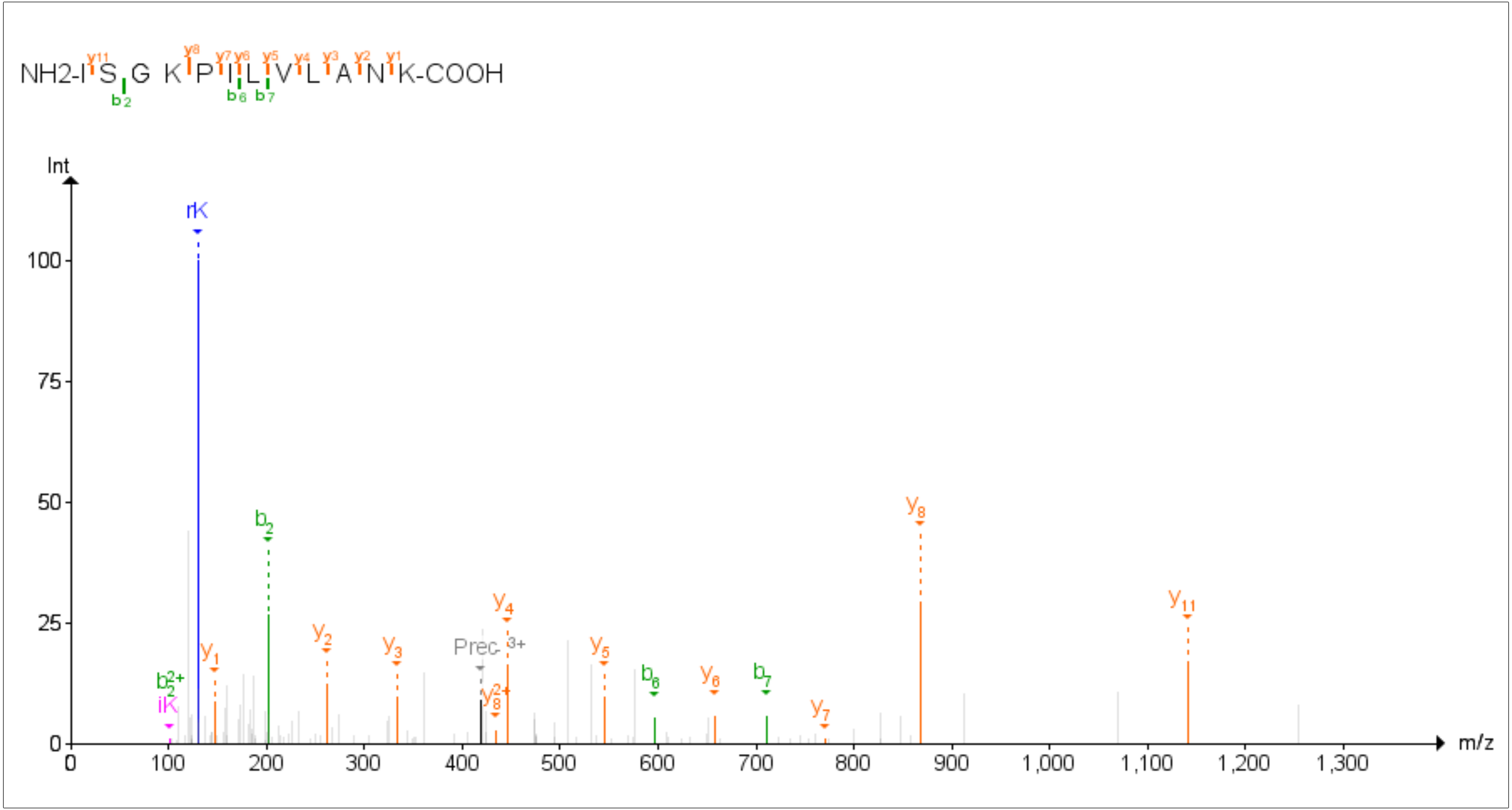

NH2-I S G K P I L V L A N K-COOH
y11 y8 y7 y6 y5 y4 y3 y2 y1
b2 b6 b7
Int
rK
b2
y8
y11
y4
y2
y3
y1
Prec- 3+
y5
b6
y6
b7
y7
b2 2+
y8 2+
iK
100
75
50
25
0
0 100 200 300 400 500 600 700 800 900 1,000 1,100 1,200 1,300
m/z

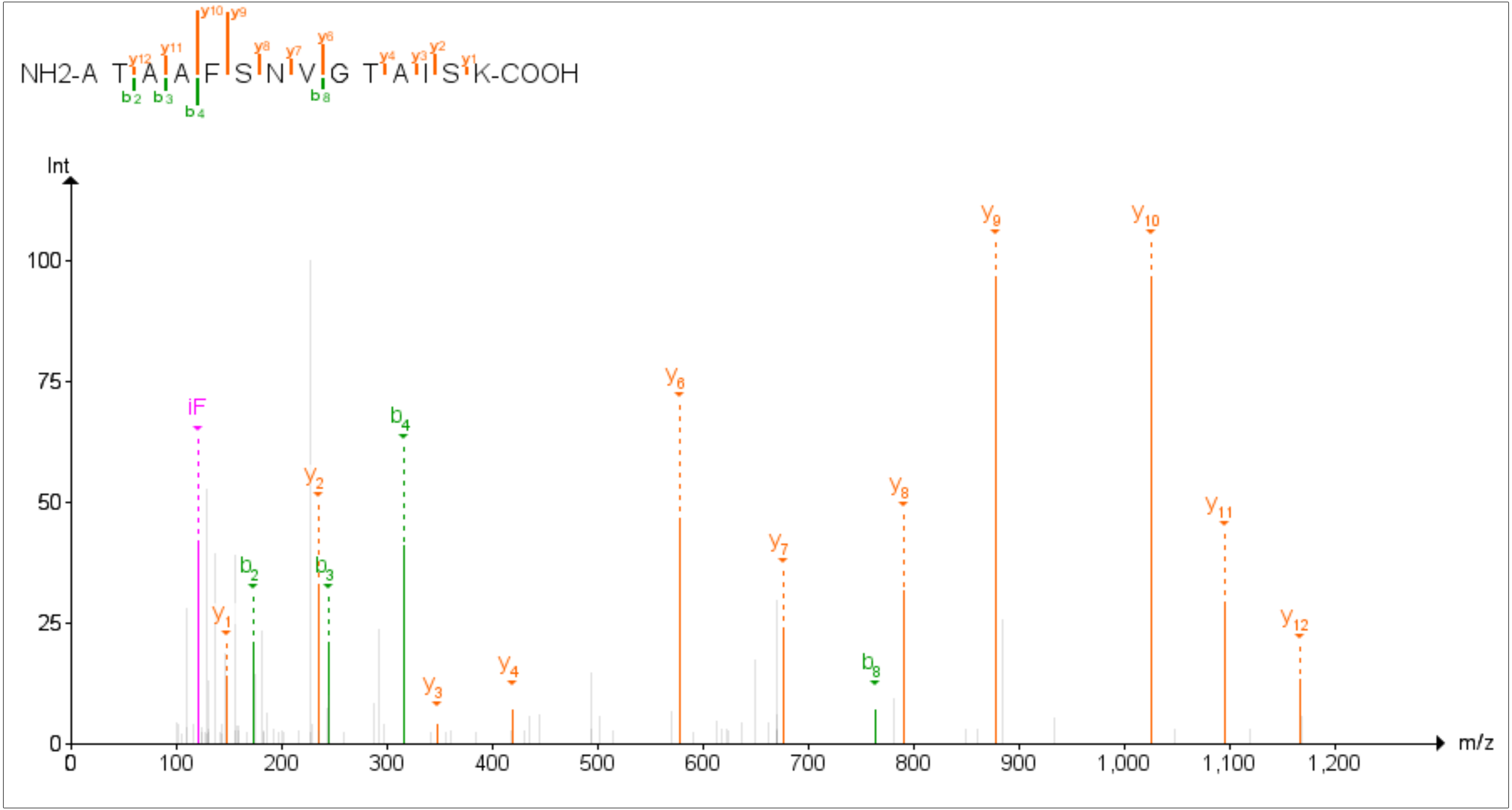

NH2-A T A A F S N V G T A I S K-COOH
y12 y11 y10 y9 y8 y7 y6 y4 y3 y2 y1
b2 b3 b4 b8
Int
100
75
50
25
0
iF
y1 b2 y2 b3 b4 y3 y4 y6 y7 b8 y8 y9 y10 y11 y12
0 100 200 300 400 500 600 700 800 900 1,000 1,100 1,200
m/z

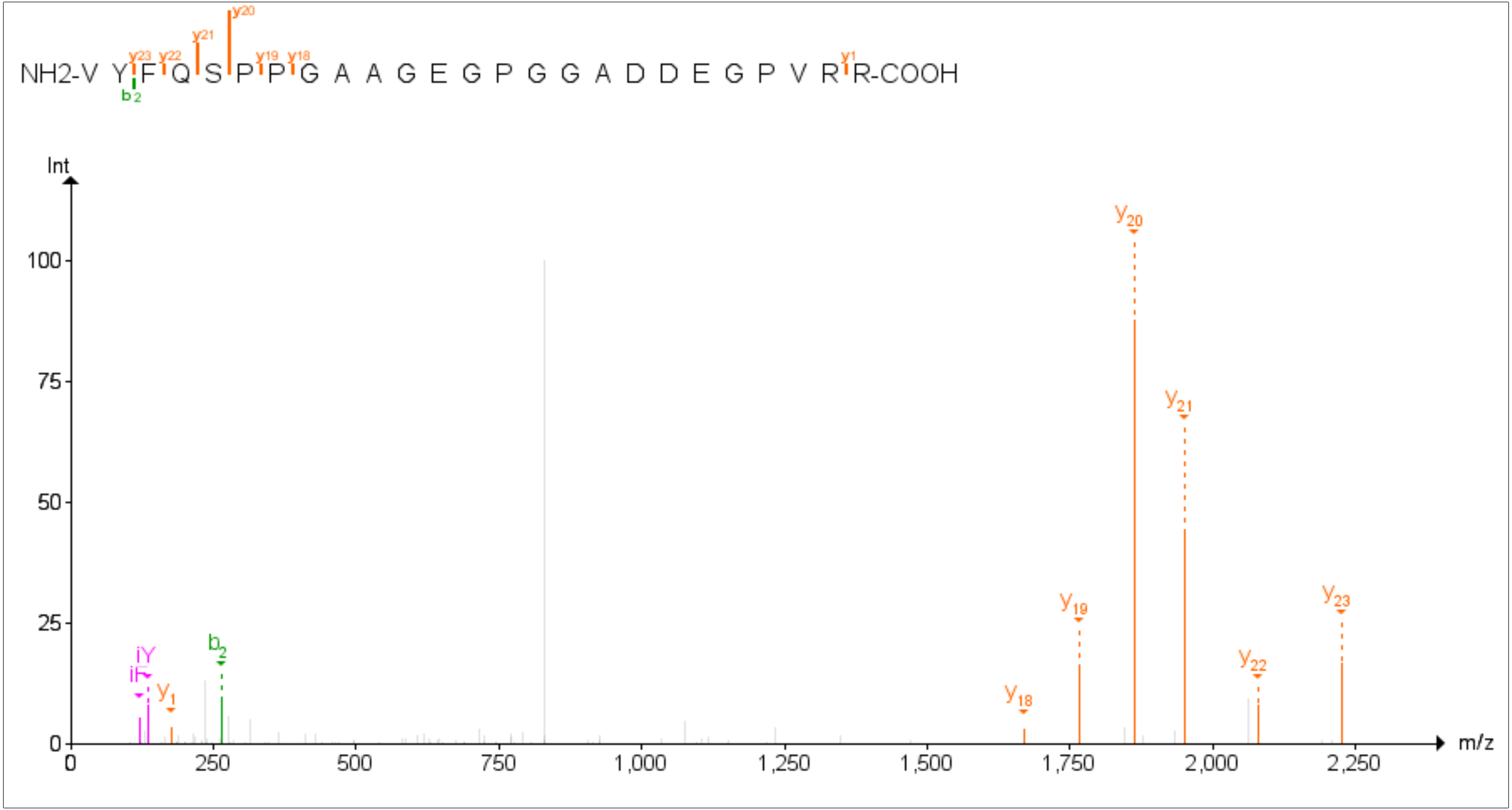

NH2-V Y F Q S P P G A A G E G P G G A D D E G P V R R-COOH
y23 y22 y21 y20 y19 y18 y1 b2
Int
100
75
50
25
0
iY iF y1 b2 y18 y19 y20 y21 y22 y23
0 250 500 750 1,000 1,250 1,500 1,750 2,000 2,250
m/z

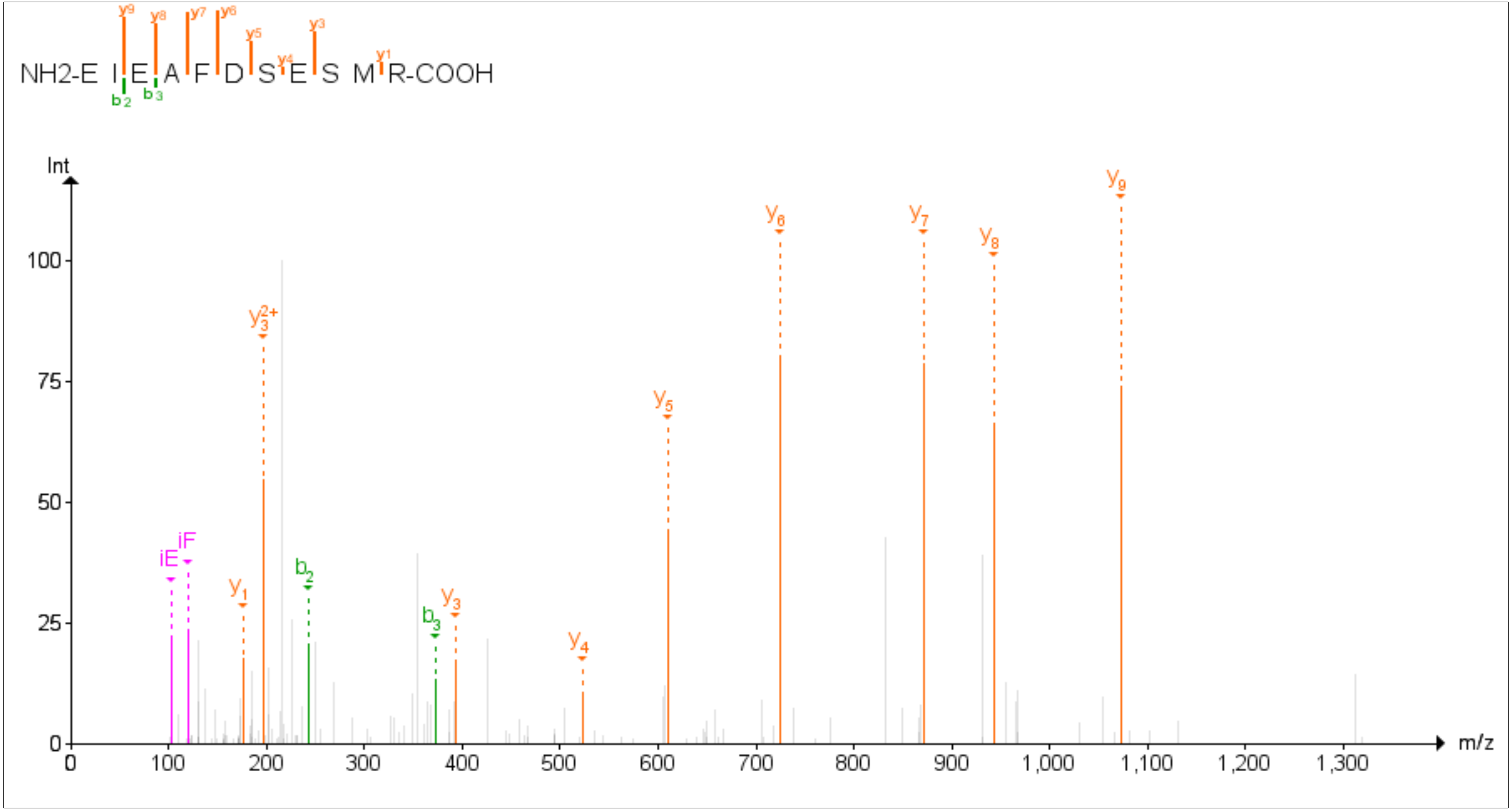
NH2-E I E A F D S E S M R-COOH
y9
y8
y7
y6
y5
y4
y3
y1
b2
b3
Int
100
75
50
25
0
m/z
0
100
200
300
400
500
600
700
800
900
1,000
1,100
1,200
1,300
iE
iF
y1
y3 2+
b2
b3
y3
y4
y5
y6
y7
y8
y9

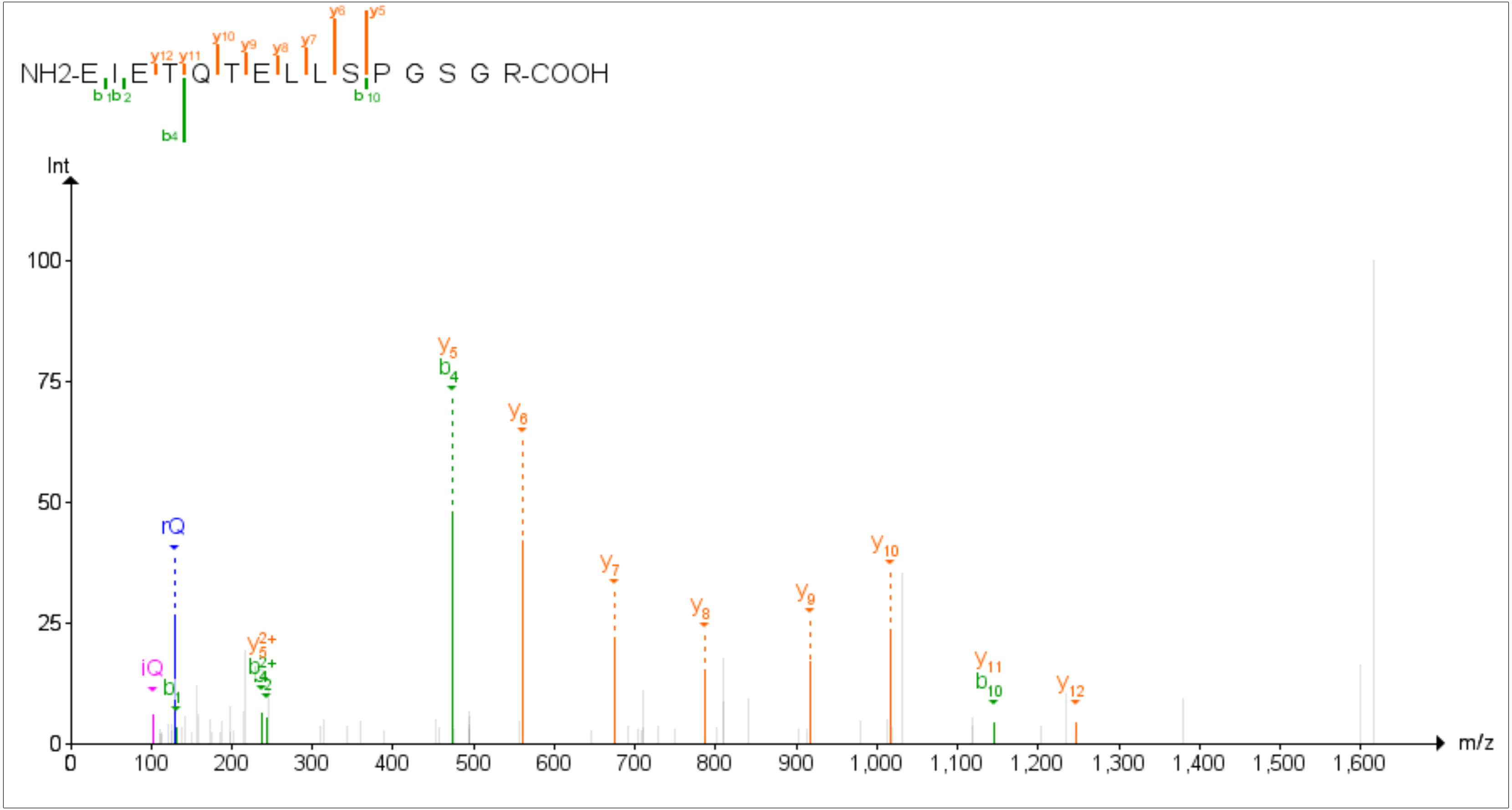

NH2-E I E T Q T E L L S P G S G R-COOH
Int
m/z
rQ
iQ

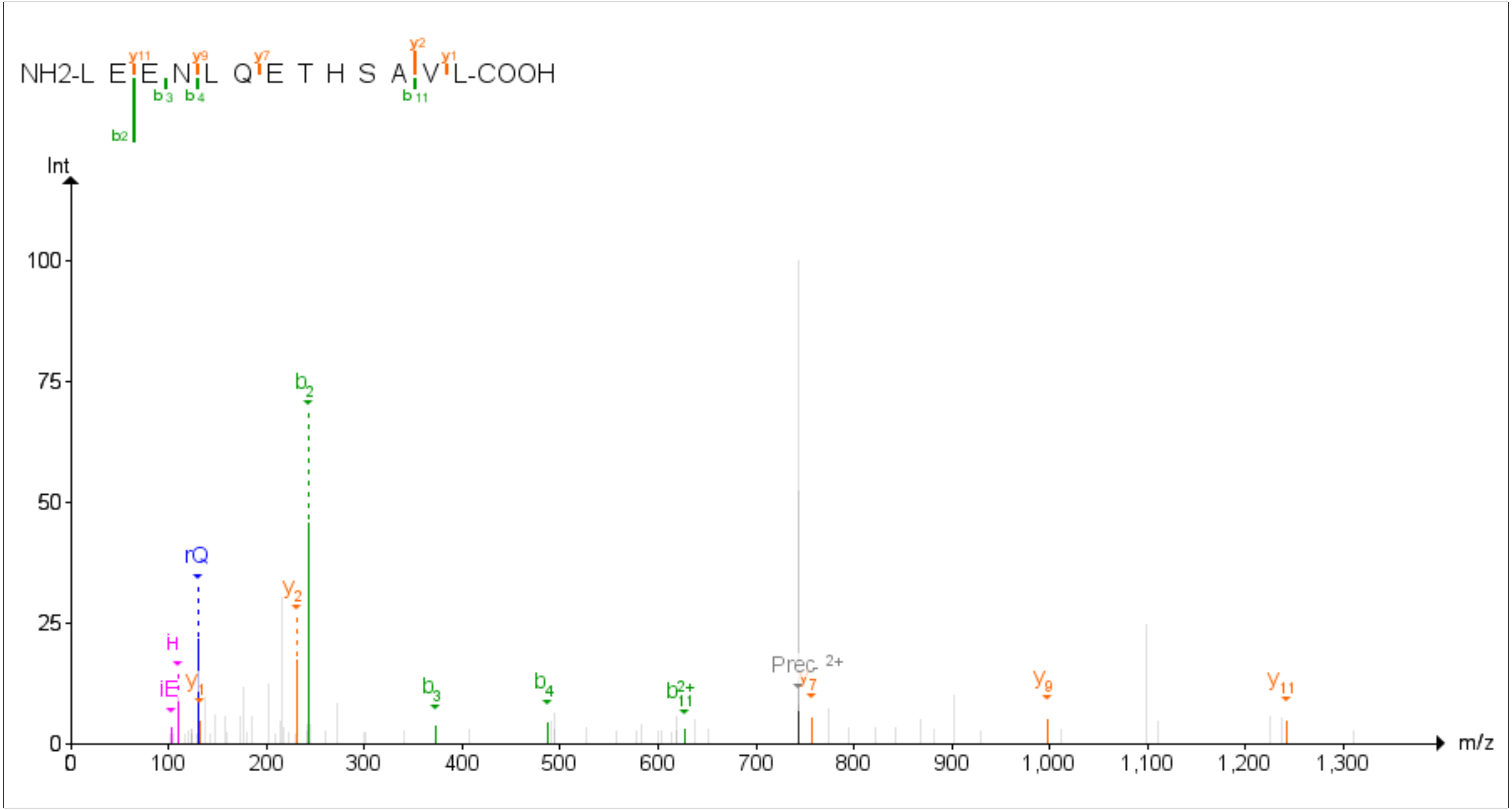

NH2-L E E N L Q E T H S A V L-COOH
y11 y9 y7 y2 y1
b2 b3 b4 b11
Int
100
75
50
25
0
b2
rQ
y2
H
iE
y1
b3
b4
b11 2+
Prec 2+
y7
y9
y11
0 100 200 300 400 500 600 700 800 900 1,000 1,100 1,200 1,300
m/z

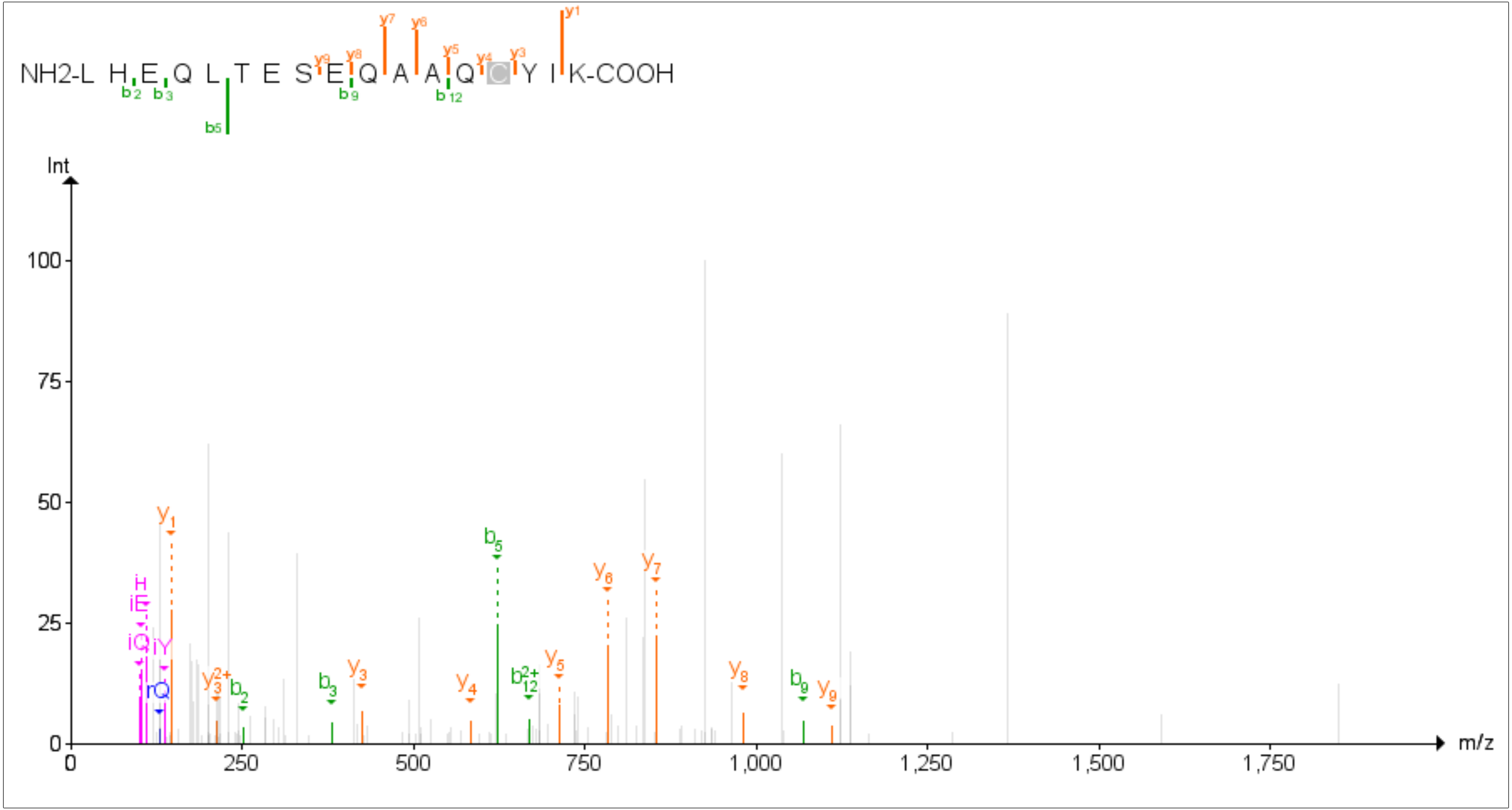

NH2-L H E Q L T E S E Q A A Q C Y I K-COOH
y7 y6 y5 y4 y3 y1 y9 y8
b2 b3 b5 b9 b12
Int
100
75
50
25
0
0
250
500
750
1,000
1,250
1,500
1,750
m/z
y1
b5
y6
y7
iH
iE
iQ
iY
rQ
y3 2+
b2
b3
y3
y4
b12 2+
y5
y8
b9
y9

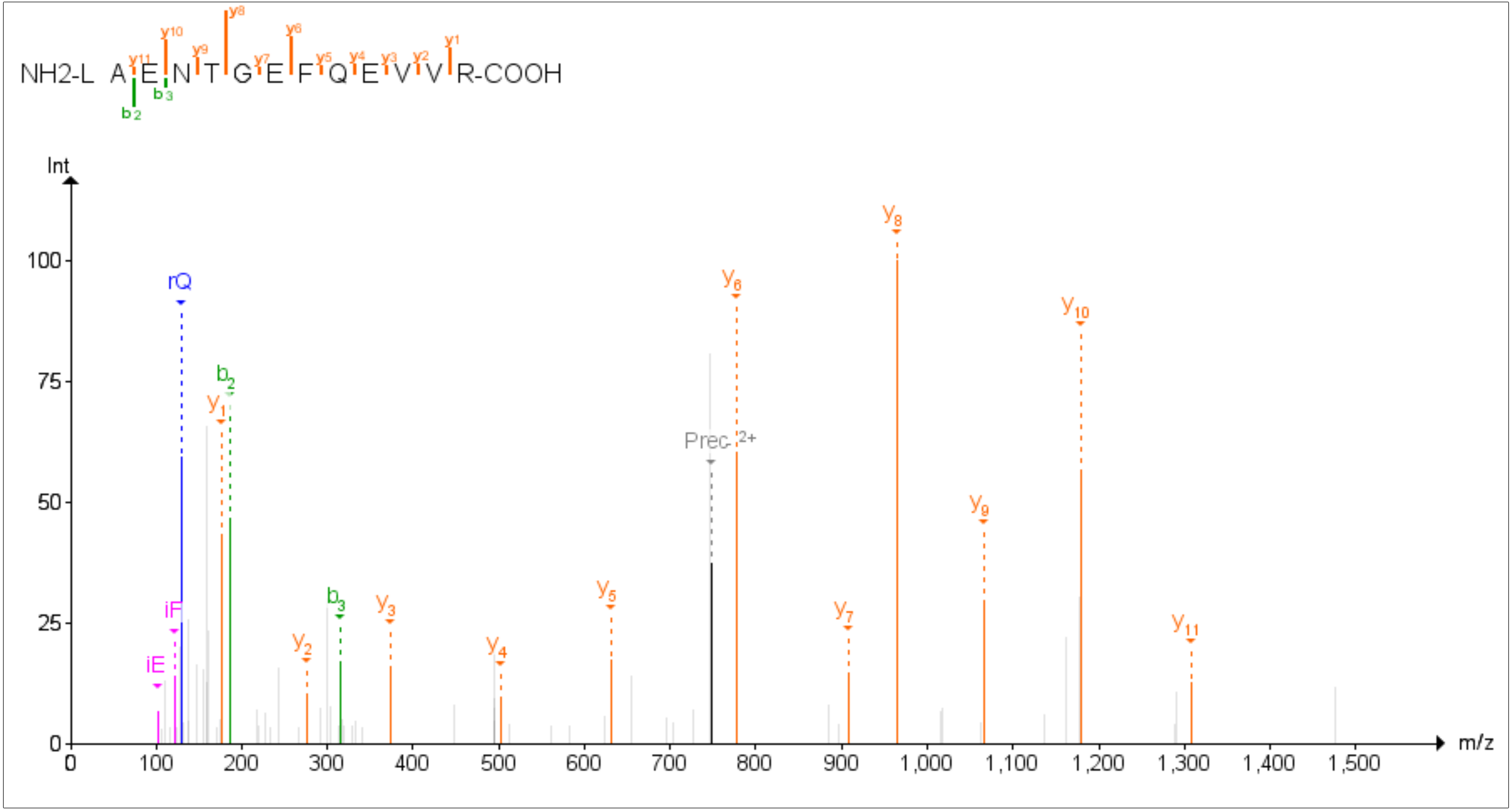

NH2-L A E N T G E F Q E V V R-COOH
y11 y10 y9 y8 y7 y6 y5 y4 y3 y2 y1
b2 b3
Int
100
75
50
25
0
rQ
b2
y1
iF
iE
y2
b3
y3
y4
y5
Prec 2+
y6
y7
y8
y9
y10
y11
0
100
200
300
400
500
600
700
800
900
1,000
1,100
1,200
1,300
1,400
1,500
m/z

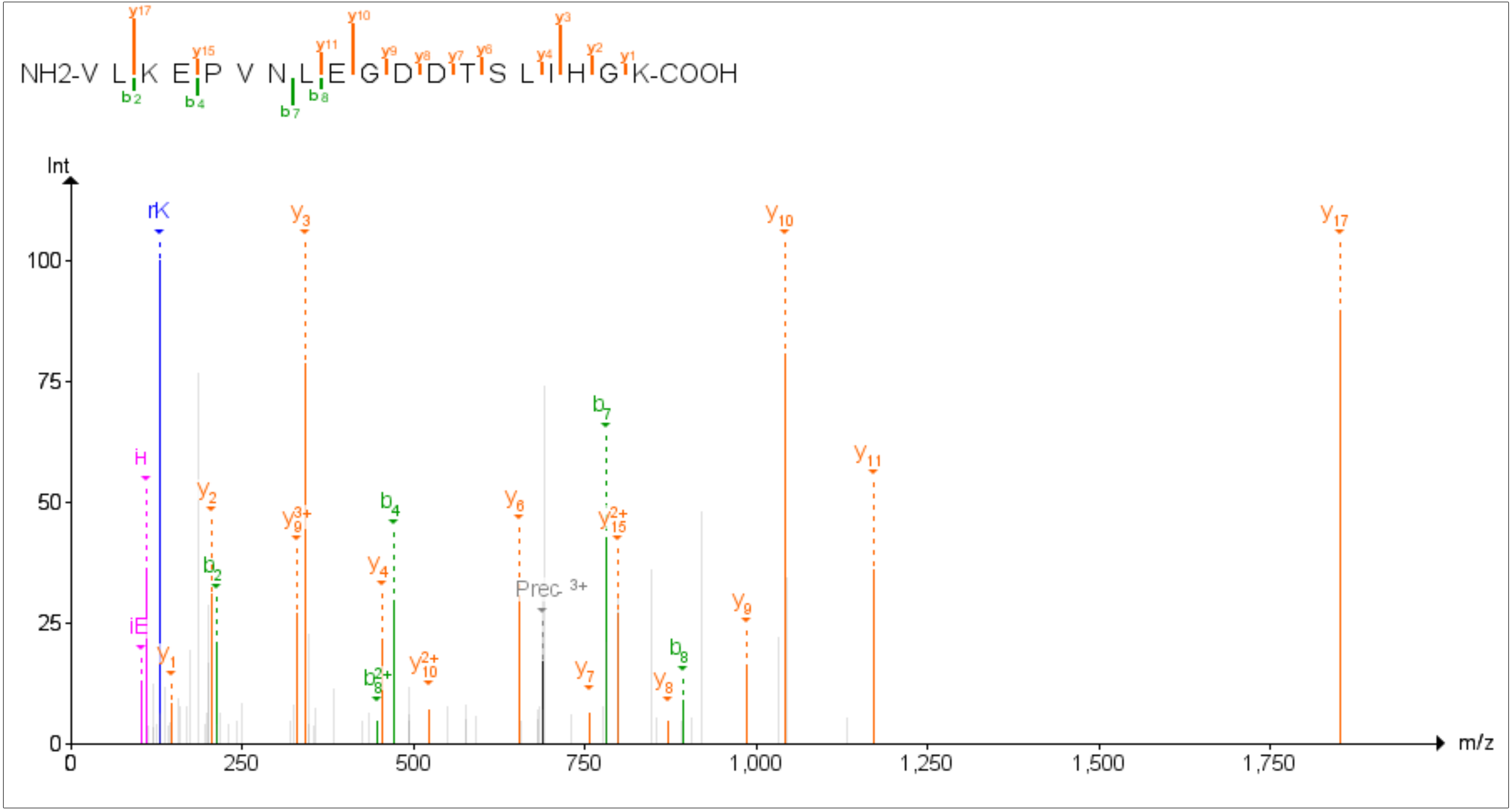

NH2-V L K E P V N L E G D D T S L I H G K-COOH
Int
m/z
rK
iH
iE
Prec 3+

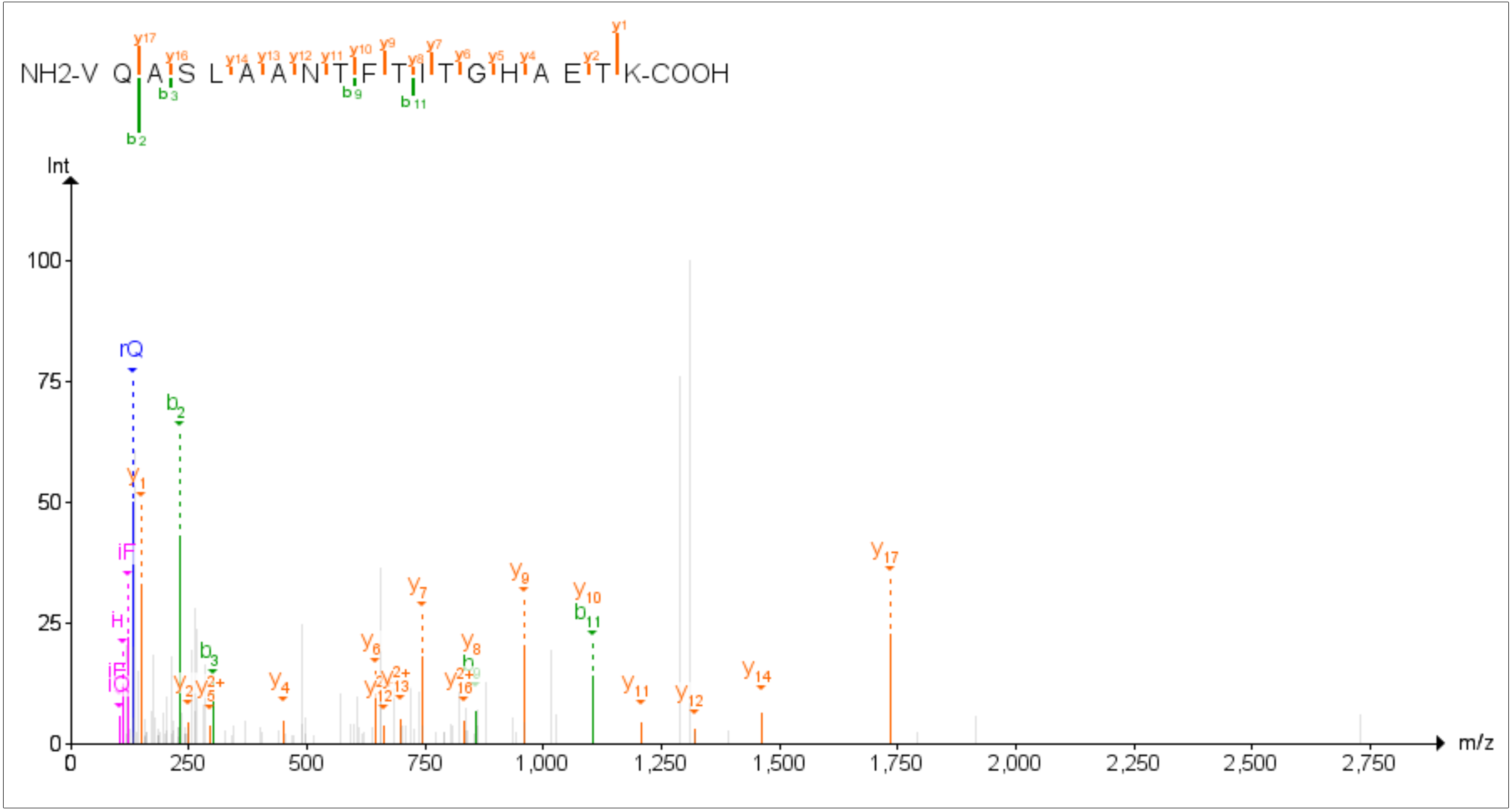

NH2-V Q A S L A A N T F T I T G H A E T K-COOH
Int
m/z
rQ
b2
b3
b11
y1
y7
y9
y10
y17
y14
y11
y12
y4
y6
y8

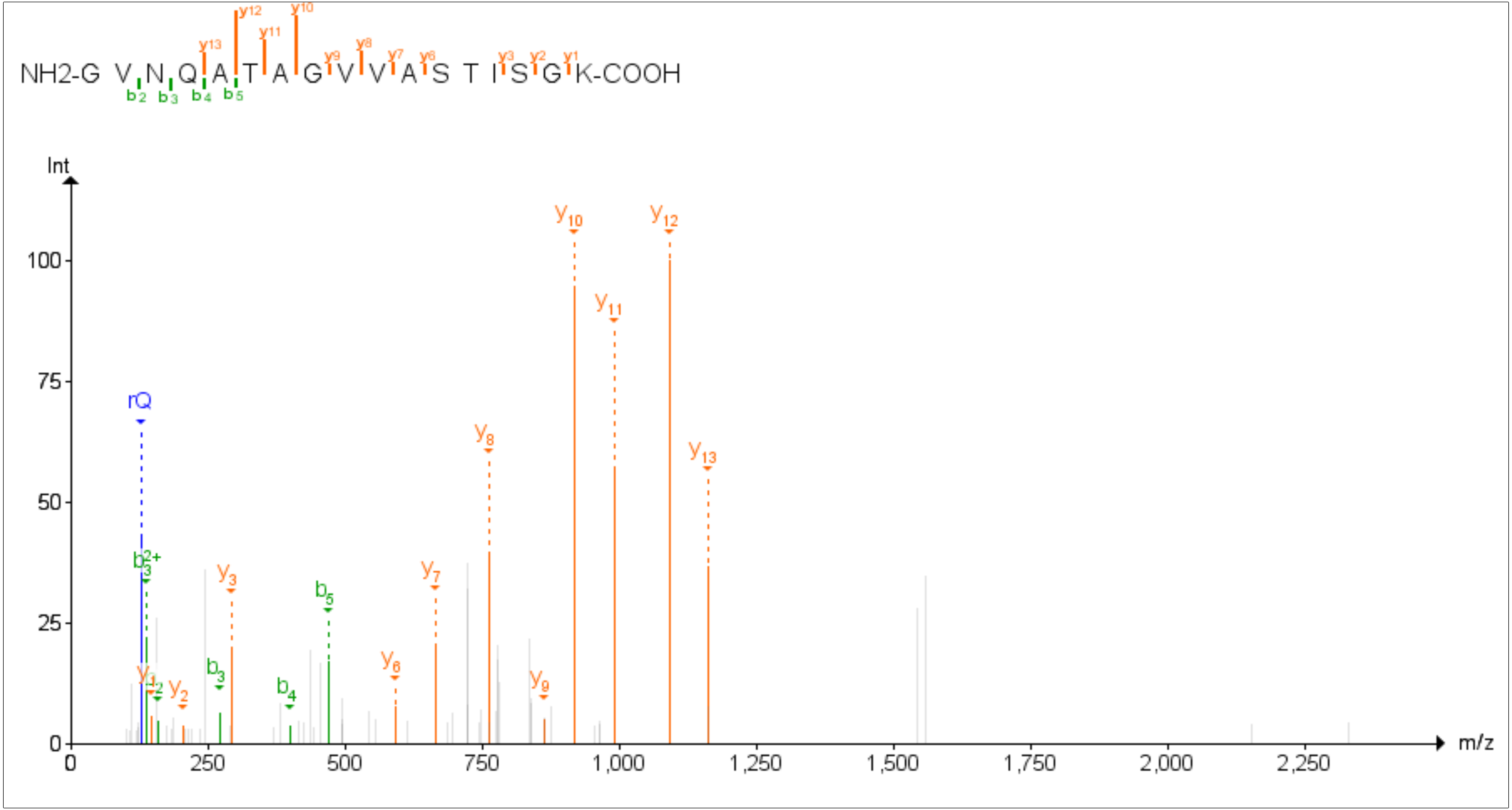

NH2-G V N Q A T A G V V A S T I S G K-COOH
y13 y12 y11 y10 y9 y8 y7 y6 y3 y2 y1
b2 b3 b4 b5
Int
m/z
rQ
y10 y12 y11 y8 y13 y3 y7 b5 y6 b3 b4 y2 y9
0 25 50 75 100
0 250 500 750 1,000 1,250 1,500 1,750 2,000 2,250

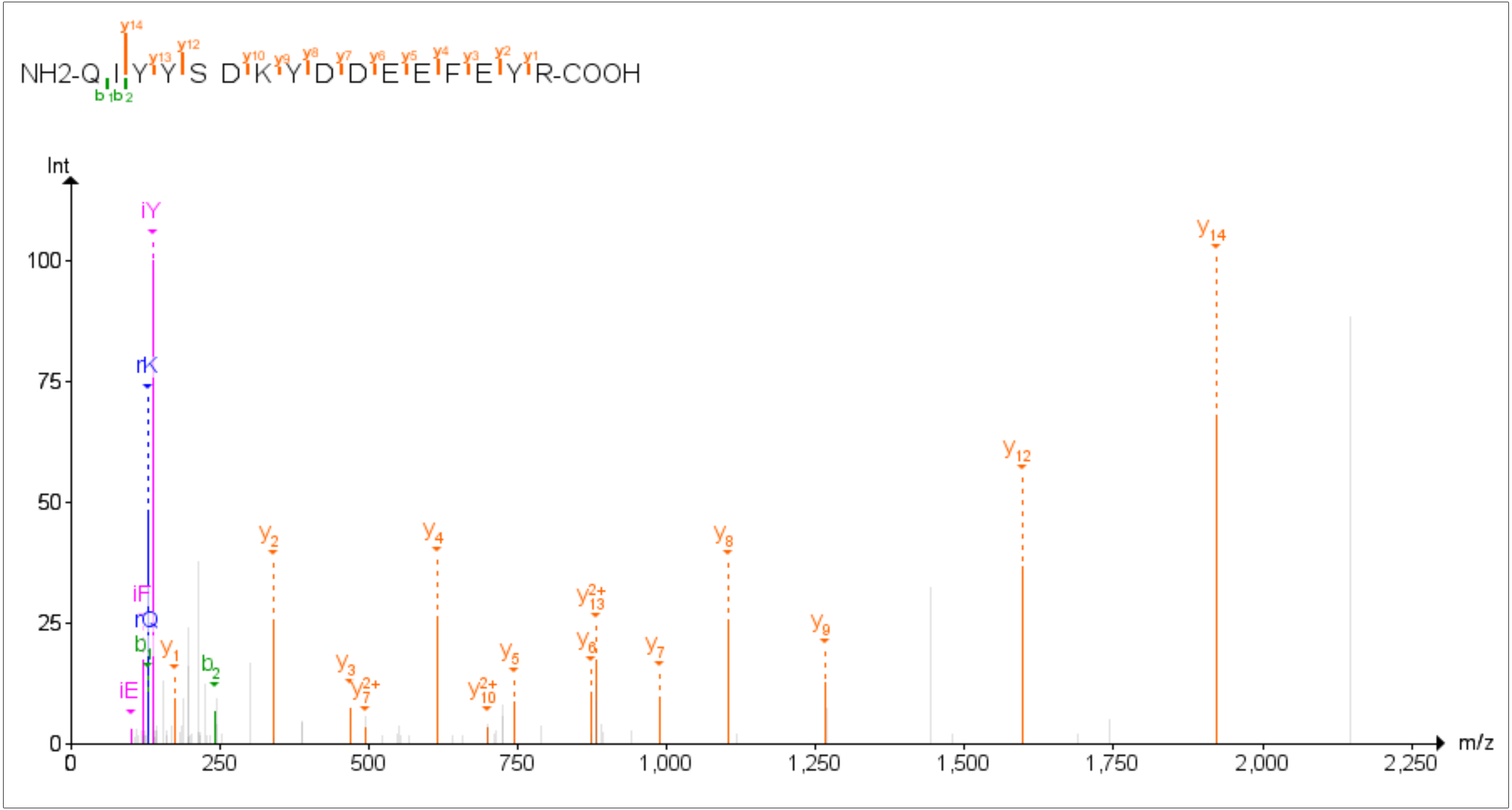

NH2-Q I Y Y S D K Y D D E E F E Y R-COOH
b1 b2
y14 y13 y12 y10 y9 y8 y7 y6 y5 y4 y3 y2 y1
Int
100
75
50
25
0
iY
rK
iF
rQ
b1
iE
y1
b2
y2
y3
y7 2+
y4
y10 2+
y5
y13 2+
y6
y7
y8
y9
y12
y14
0
250
500
750
1,000
1,250
1,500
1,750
2,000
2,250
m/z

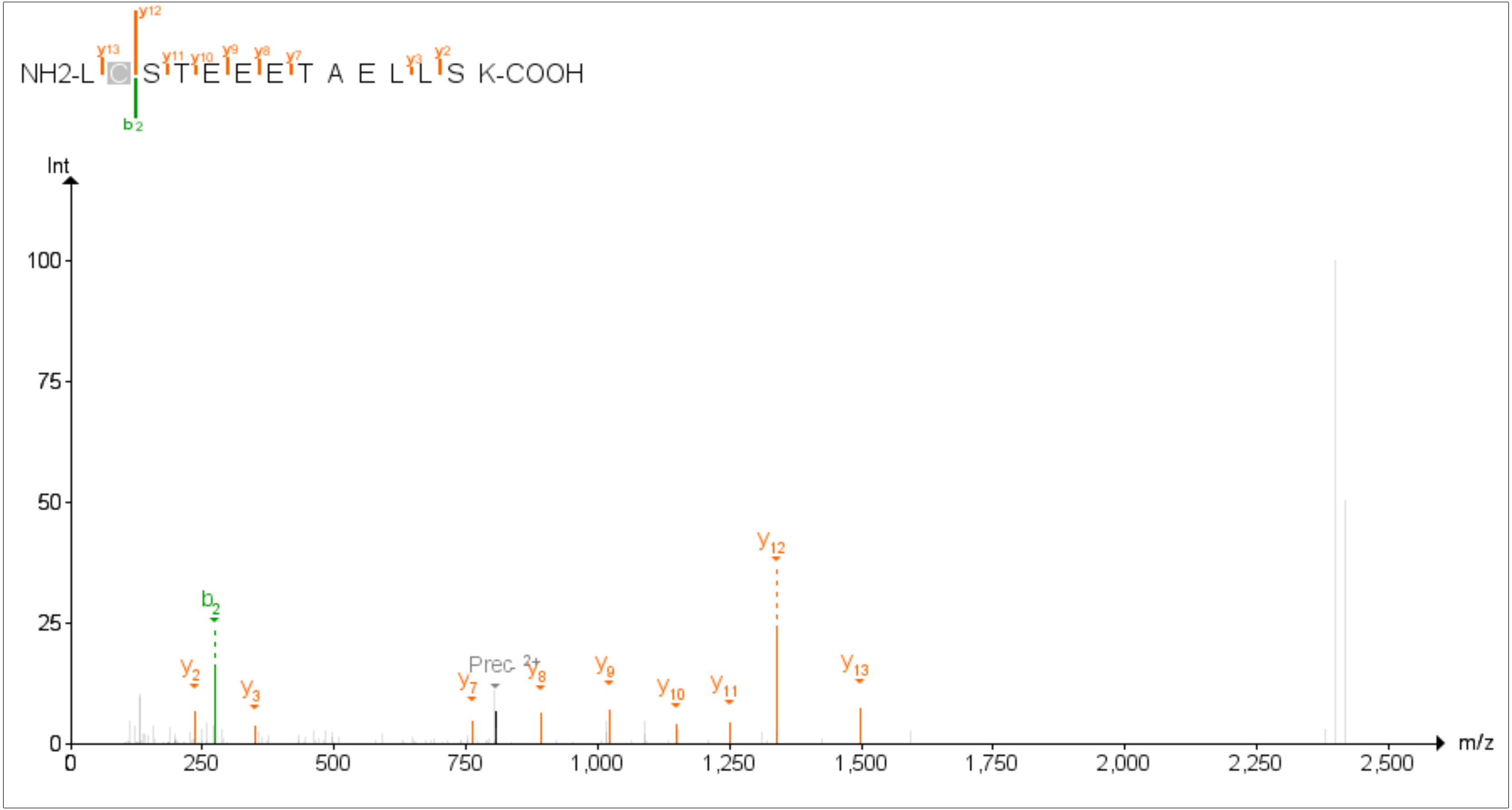

NH2-L C S T E E E T A E L L S K-COOH
y13
y12
y11
y10
y9
y8
y7
y3
y2
b2
Int
100
75
50
25
0
b2
y2
y3
Prec 2+
y7
y8
y9
y10
y11
y12
y13
0
250
500
750
1,000
1,250
1,500
1,750
2,000
2,250
2,500
m/z

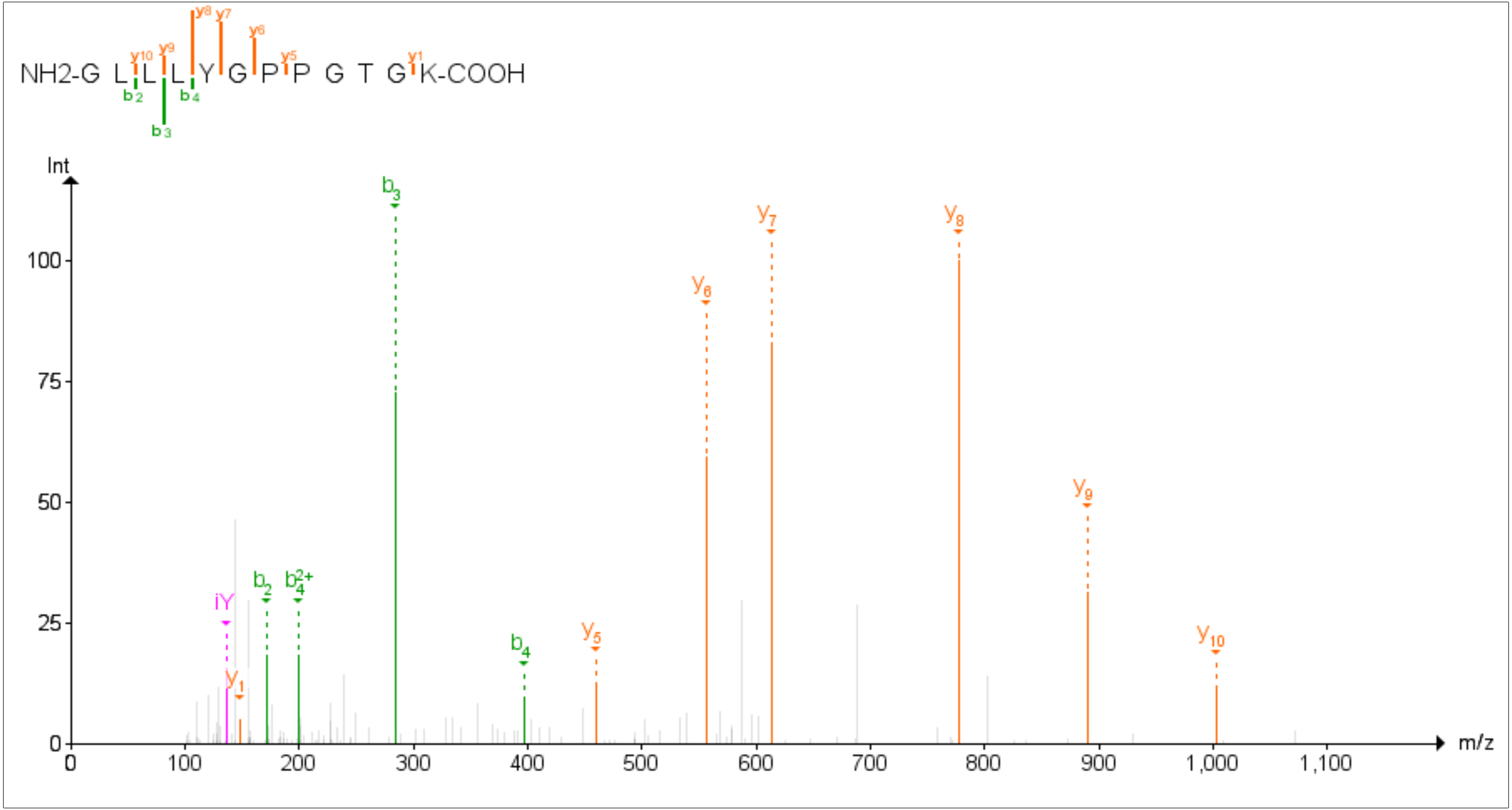

NH2-G L L L Y G P P G T G K-COOH
y10 y9 y8 y7 y6 y5 y1
b2 b3 b4
Int
m/z
b3
y7
y8
y6
y9
y10
y5
b4
iY
b2
b_4^{2+}
y1
0
25
50
75
100
0
100
200
300
400
500
600
700
800
900
1,000
1,100

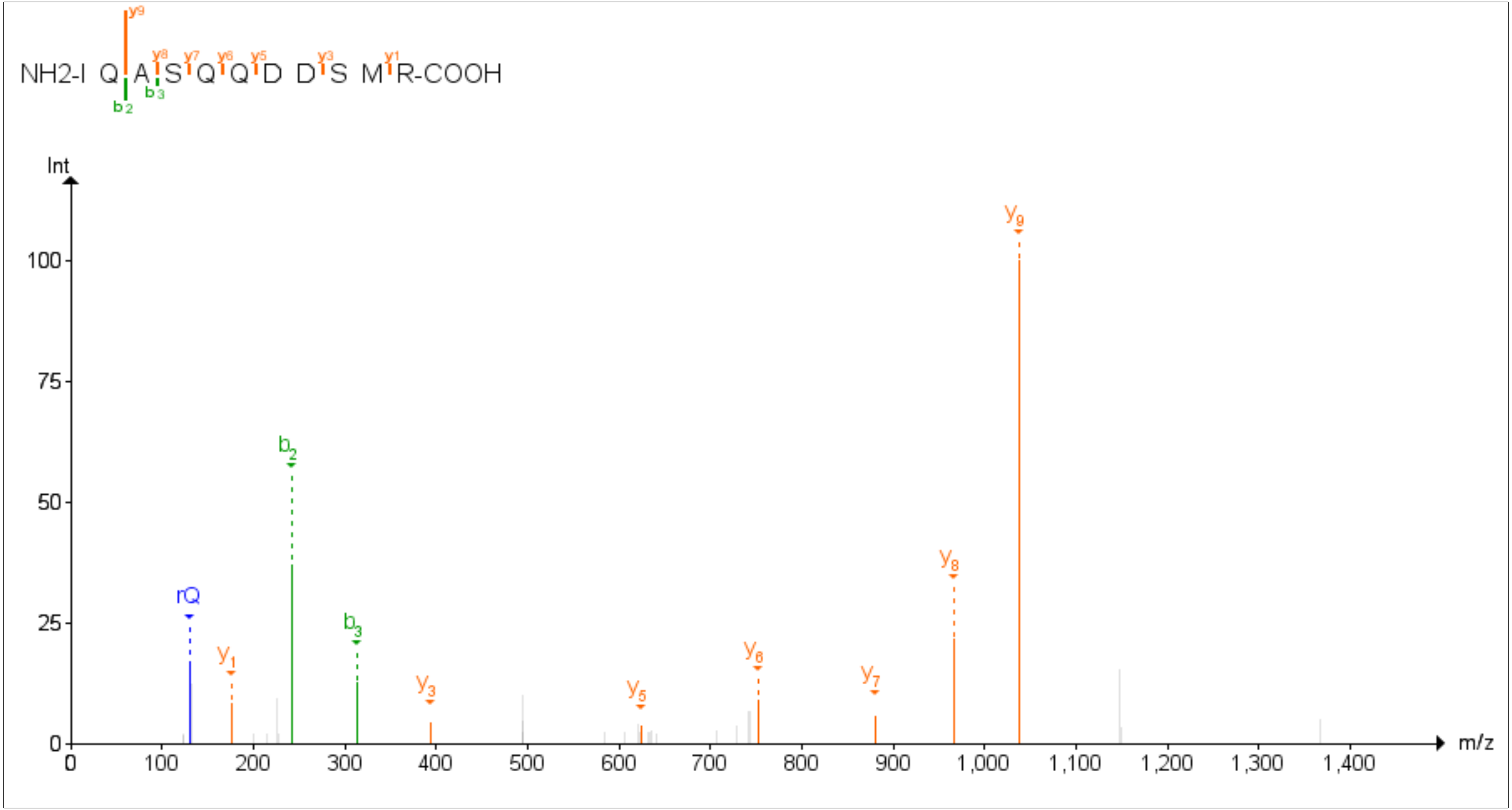

NH2-I Q A S Q Q D D S M R-COOH
y9
y8
y7
y6
y5
y3
y1
b2
b3
Int
100
75
50
25
0
rQ
y1
b2
b3
y3
y5
y6
y7
y8
y9
0
100
200
300
400
500
600
700
800
900
1,000
1,100
1,200
1,300
1,400
m/z

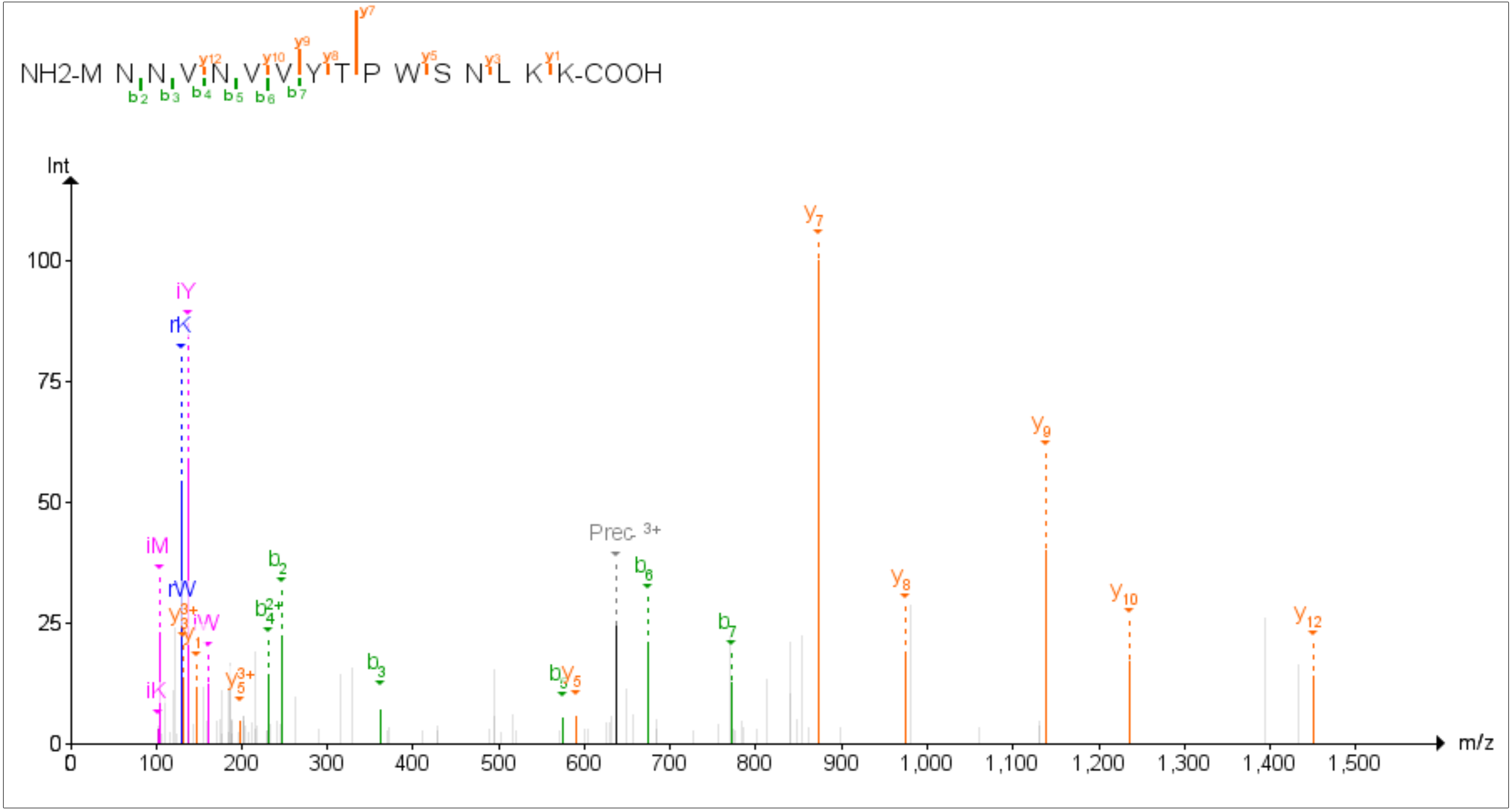

NH2-M N N V N V V Y T P W S N L K K-COOH
b2 b3 b4 b5 b6 b7
y12 y10 y9 y8 y7 y5 y3 y1
Int
m/z
Prec 3+

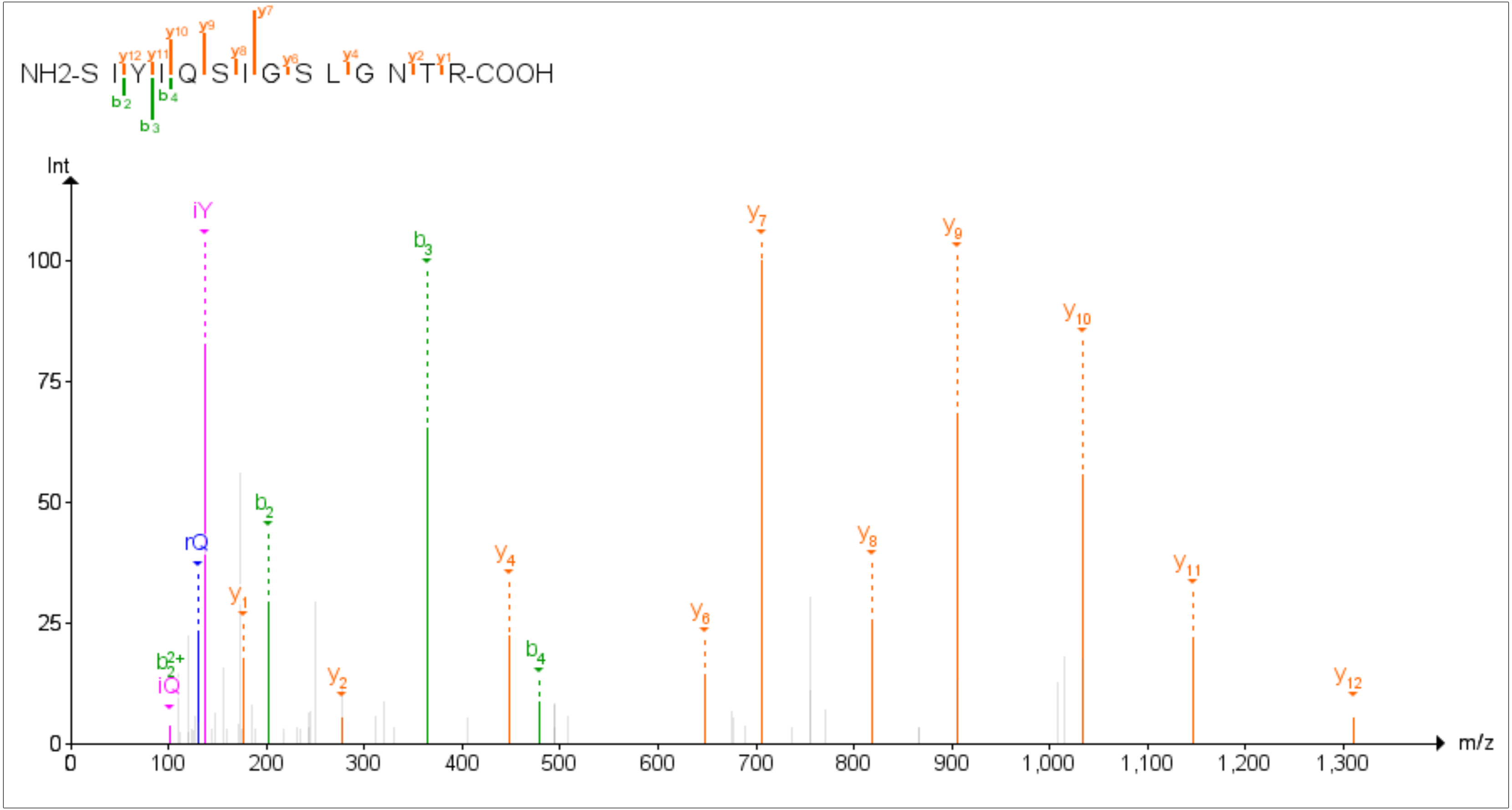

NH2-S I Y I Q S I G S L G N T R-COOH
y12 y11 y10 y9 y8 y7 y6 y4 y2 y1
b2 b3 b4
Int
m/z
iY
b3
y7
y9
y10
b2
rQ
y1
y4
y8
y11
y6
b4
b2 2+
iQ
y2
y12
0
25
50
75
100
0
100
200
300
400
500
600
700
800
900
1,000
1,100
1,200
1,300

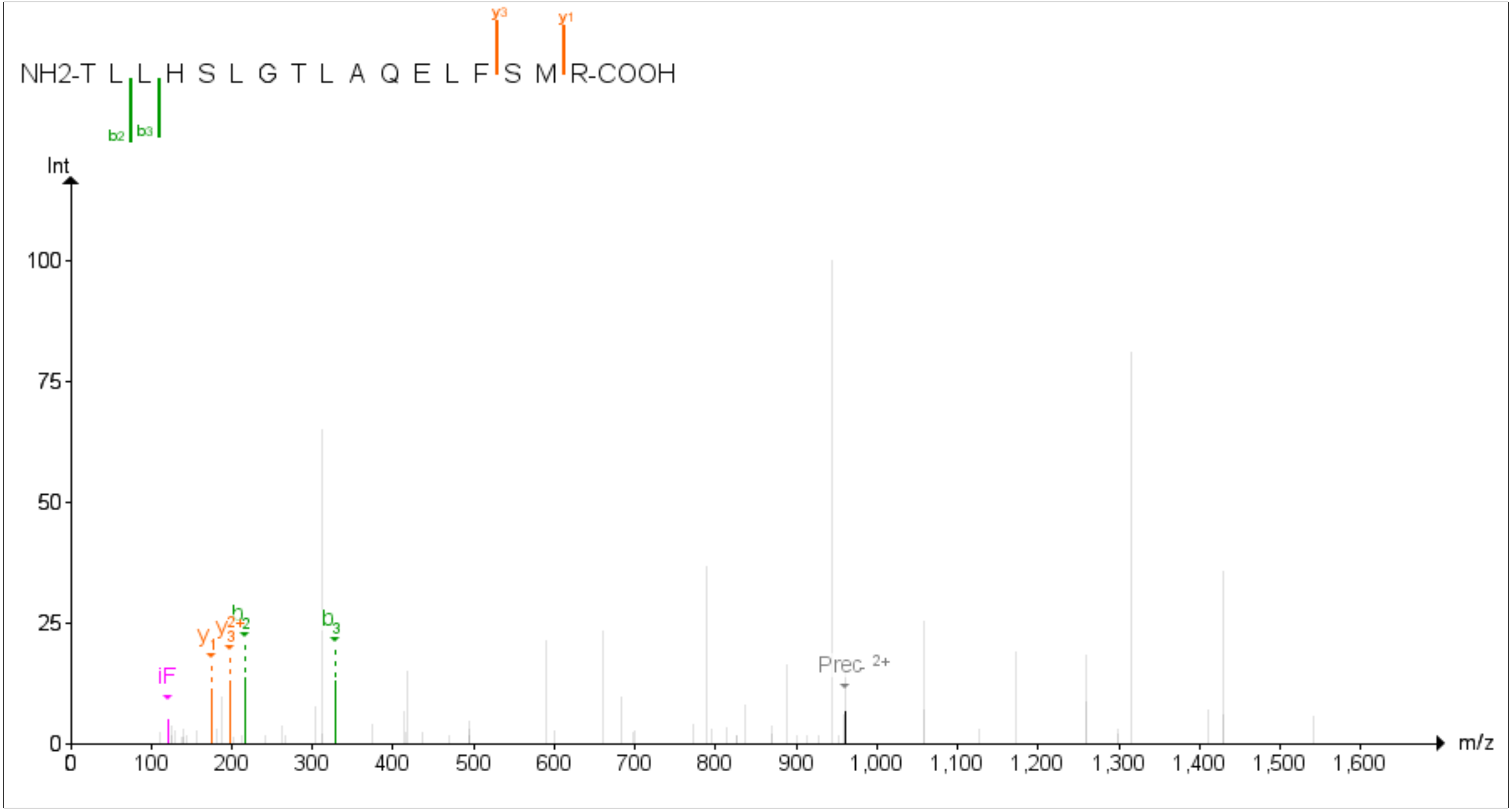
NH2-T L L H S L G T L A Q E L F S M R-COOH
y3
y1
b2
b3
Int
100
75
50
25
0
iF
y1
y3 2+
b2
b3
Prec 2+
0
100
200
300
400
500
600
700
800
900
1,000
1,100
1,200
1,300
1,400
1,500
1,600
m/z

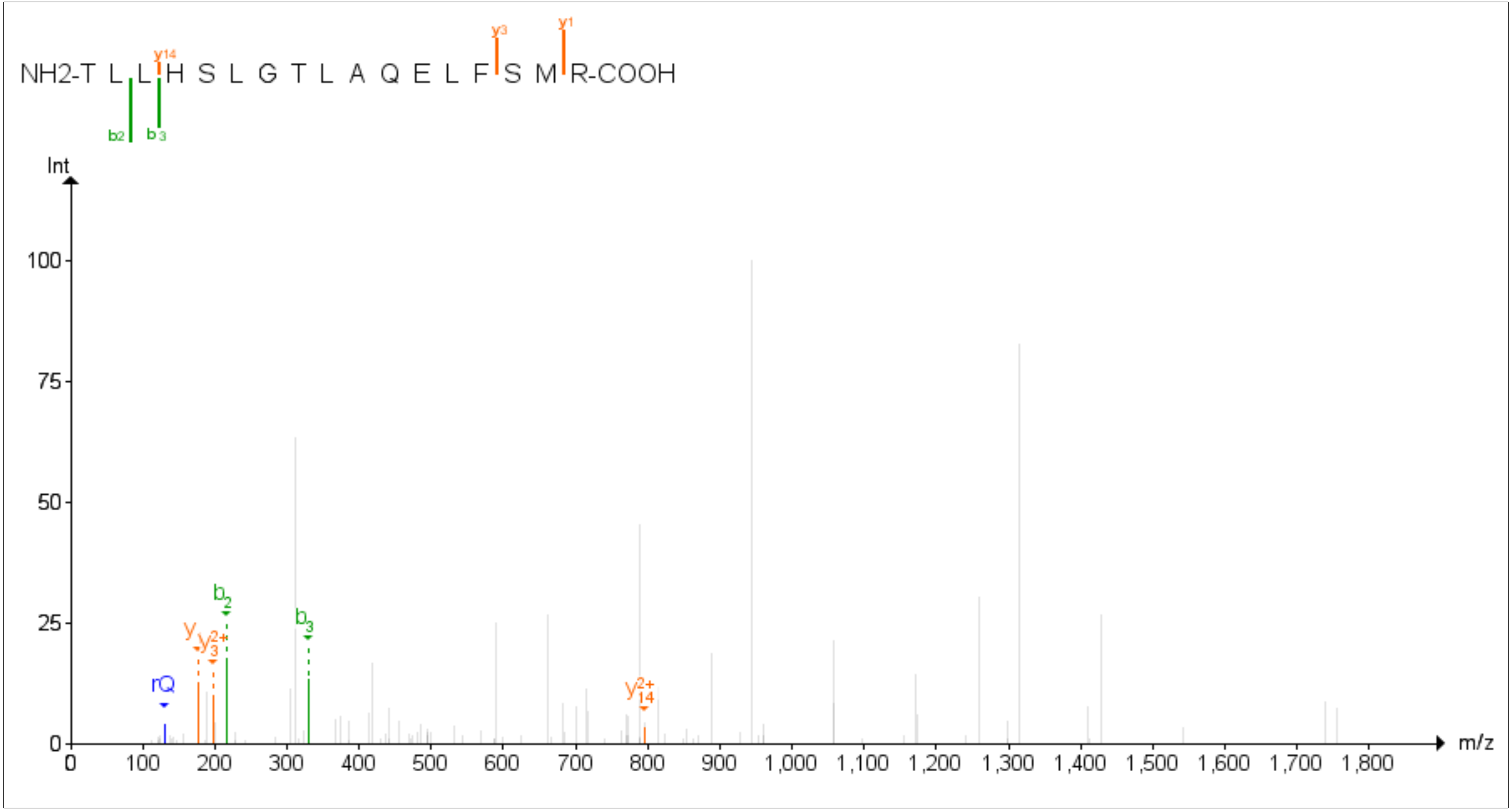

NH2-T L L H S L G T L A Q E L F S M R-COOH
y14
y3
y1
b2
b3
Int
100
75
50
25
0
rQ
y
y3 2+
b2
b3
y14 2+
0
100
200
300
400
500
600
700
800
900
1,000
1,100
1,200
1,300
1,400
1,500
1,600
1,700
1,800
m/z

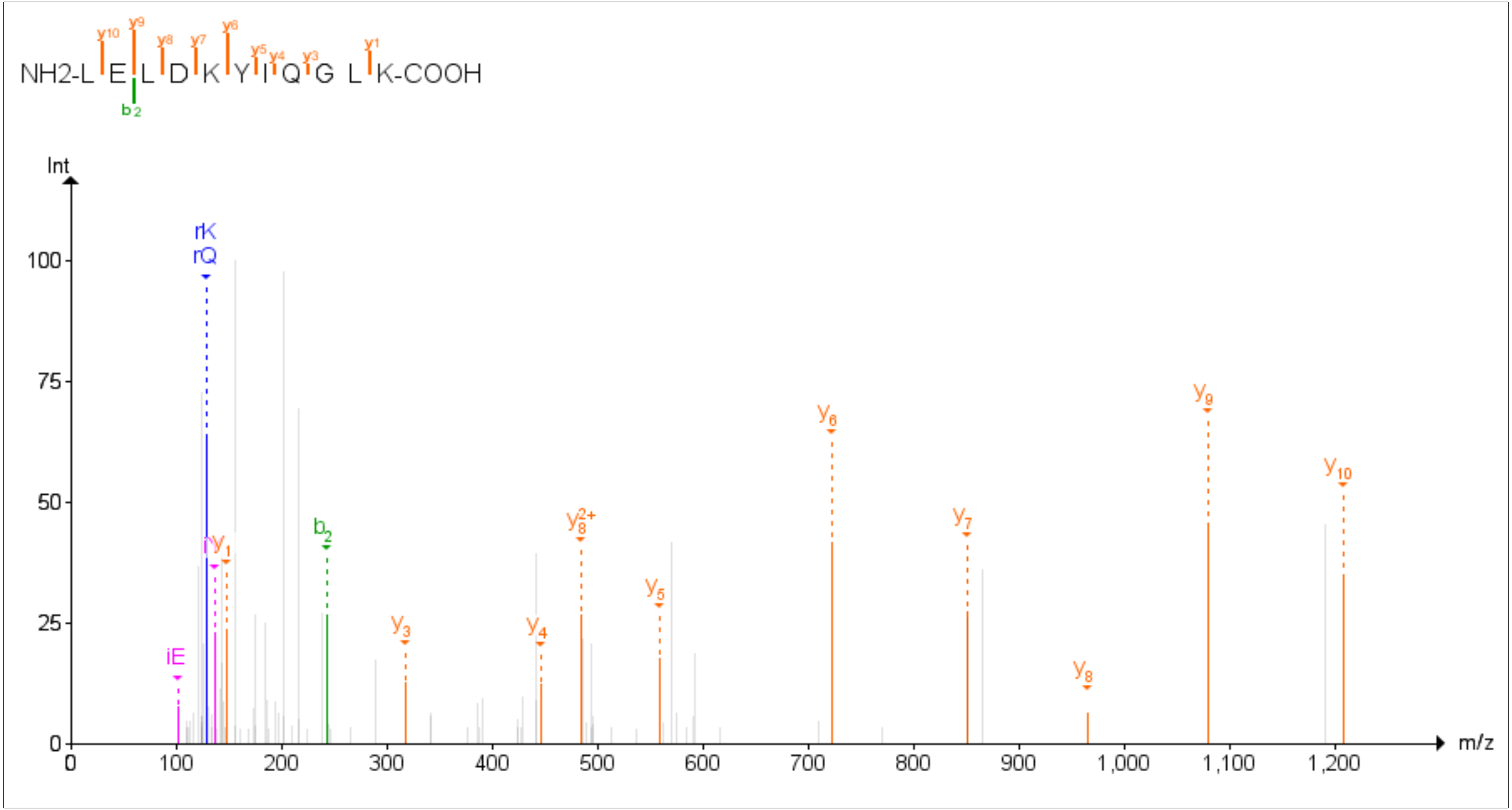

NH2-L E L D K Y I Q G L K-COOH
y10 y9 y8 y7 y6 y5 y4 y3 y1
b2
Int
100
75
50
25
0
rK
rQ
iE
y1
b2
y3
y4
y8 2+
y5
y6
y7
y8
y9
y10
0 100 200 300 400 500 600 700 800 900 1,000 1,100 1,200
m/z

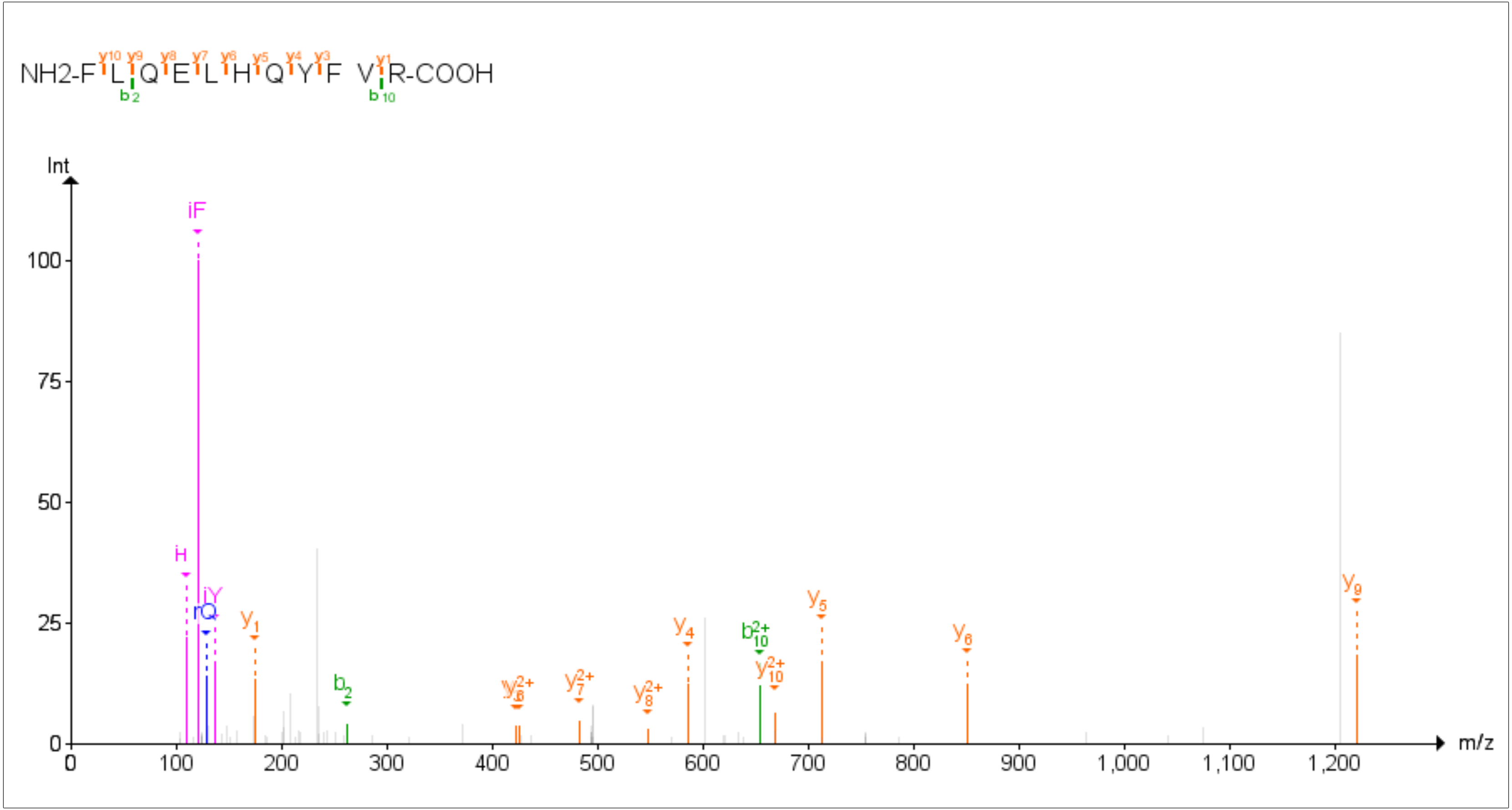

NH2-F L Q E L H Q Y F V R-COOH
y10 y9 y8 y7 y6 y5 y4 y3 y1
b2 b10
Int
100
75
50
25
0
iF
iH
iY
rQ
y1
b2
y6 2+
y7 2+
y8 2+
y4
b10 2+
y10 2+
y5
y6
y9
0
100
200
300
400
500
600
700
800
900
1,000
1,100
1,200
m/z

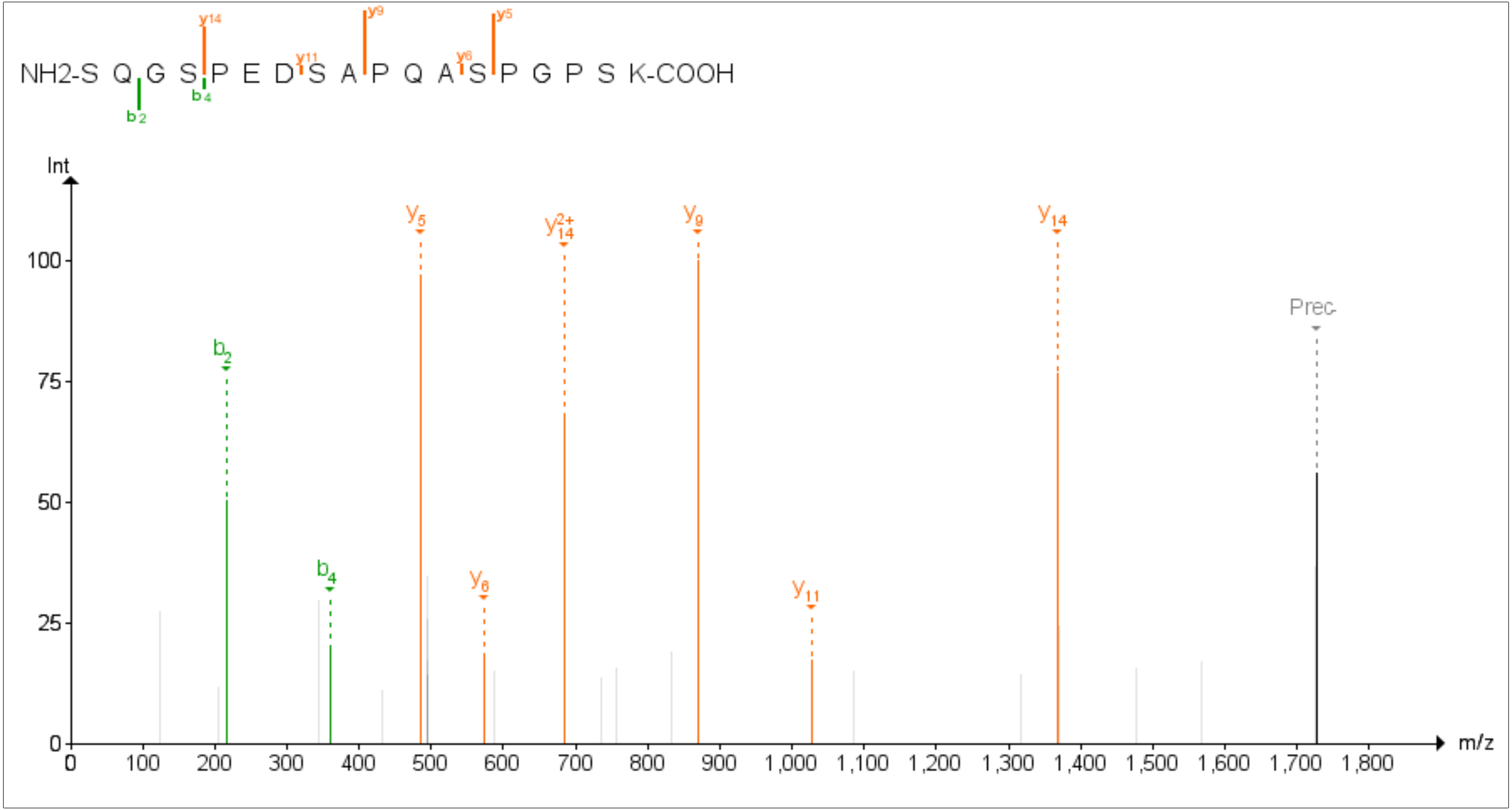

NH2-S Q G S P E D S A P Q A S P G P S K-COOH
y14
y9
y5
y11
y6
b2
b4
Int
100
75
50
25
0
y5
y14 2+
y9
y14
Prec
b2
b4
y6
y11
0 100 200 300 400 500 600 700 800 900 1,000 1,100 1,200 1,300 1,400 1,500 1,600 1,700 1,800
m/z

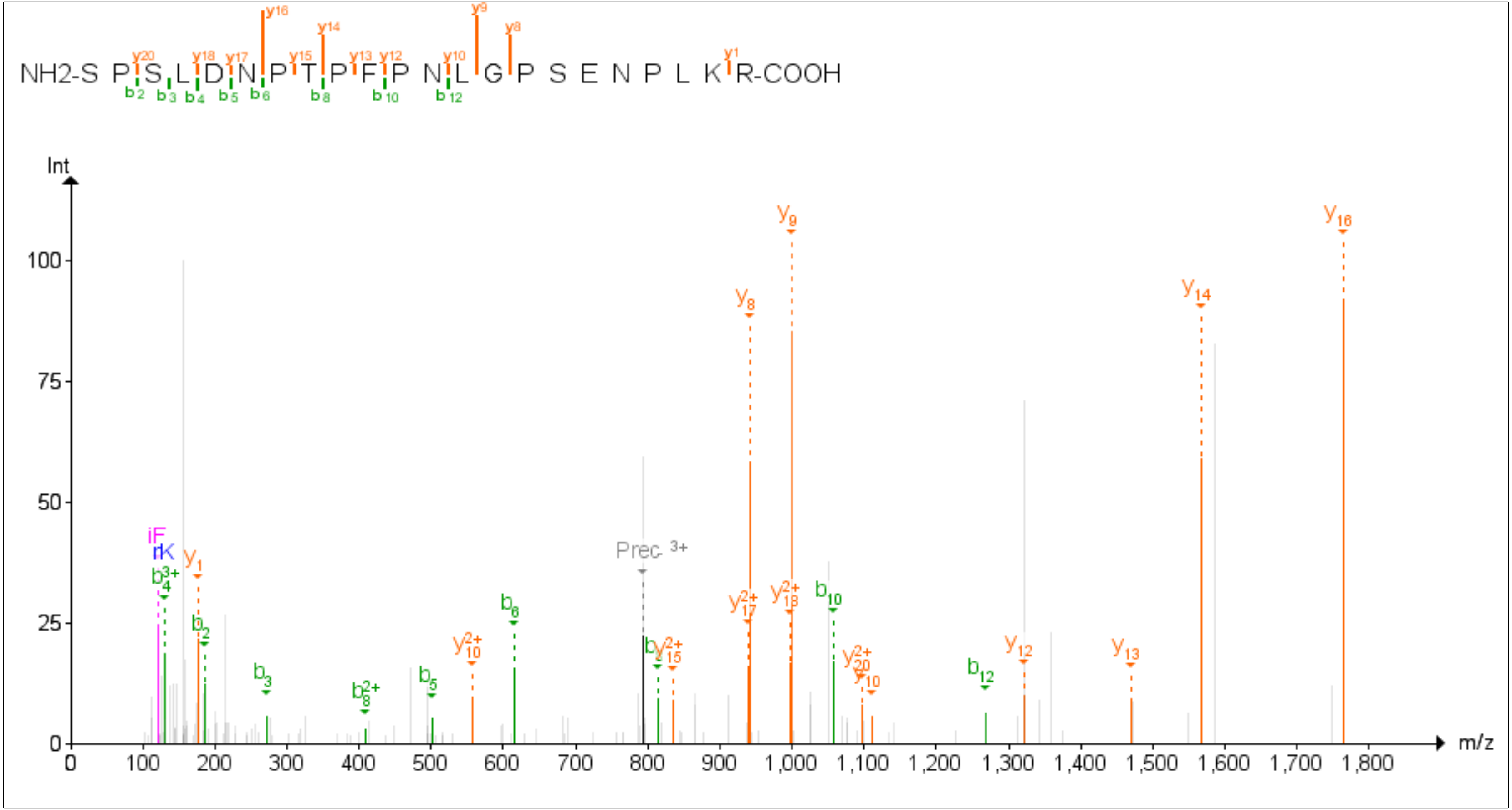

NH2-S P S L D N P T P F P N L G P S E N P L K R-COOH
y20 y18 y17 y16 y15 y14 y13 y12 y10 y9 y8 y1
b2 b3 b4 b5 b6 b8 b10 b12
Int
m/z
Prec 3+
iF
rK

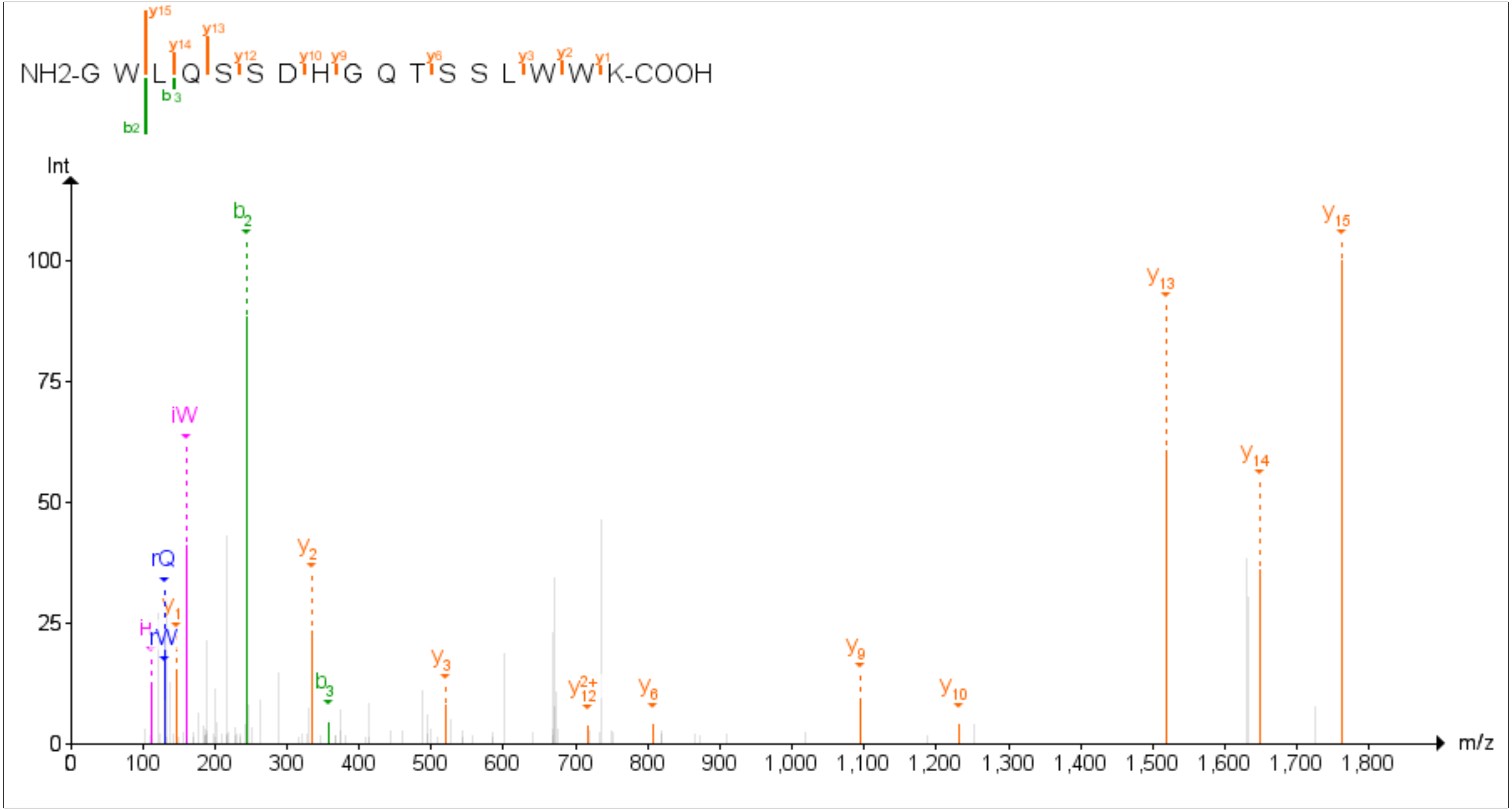

NH2-G W L Q S S D H G Q T S S L W W K-COOH
y15
y14
y13
y12
y10
y9
y6
y3
y2
y1
b2
b3
Int
100
75
50
25
0
b2
y15
y13
y14
iW
rQ
y1
iH
rW
y2
b3
y3
y2+12
y6
y9
y10
m/z
0
100
200
300
400
500
600
700
800
900
1,000
1,100
1,200
1,300
1,400
1,500
1,600
1,700
1,800

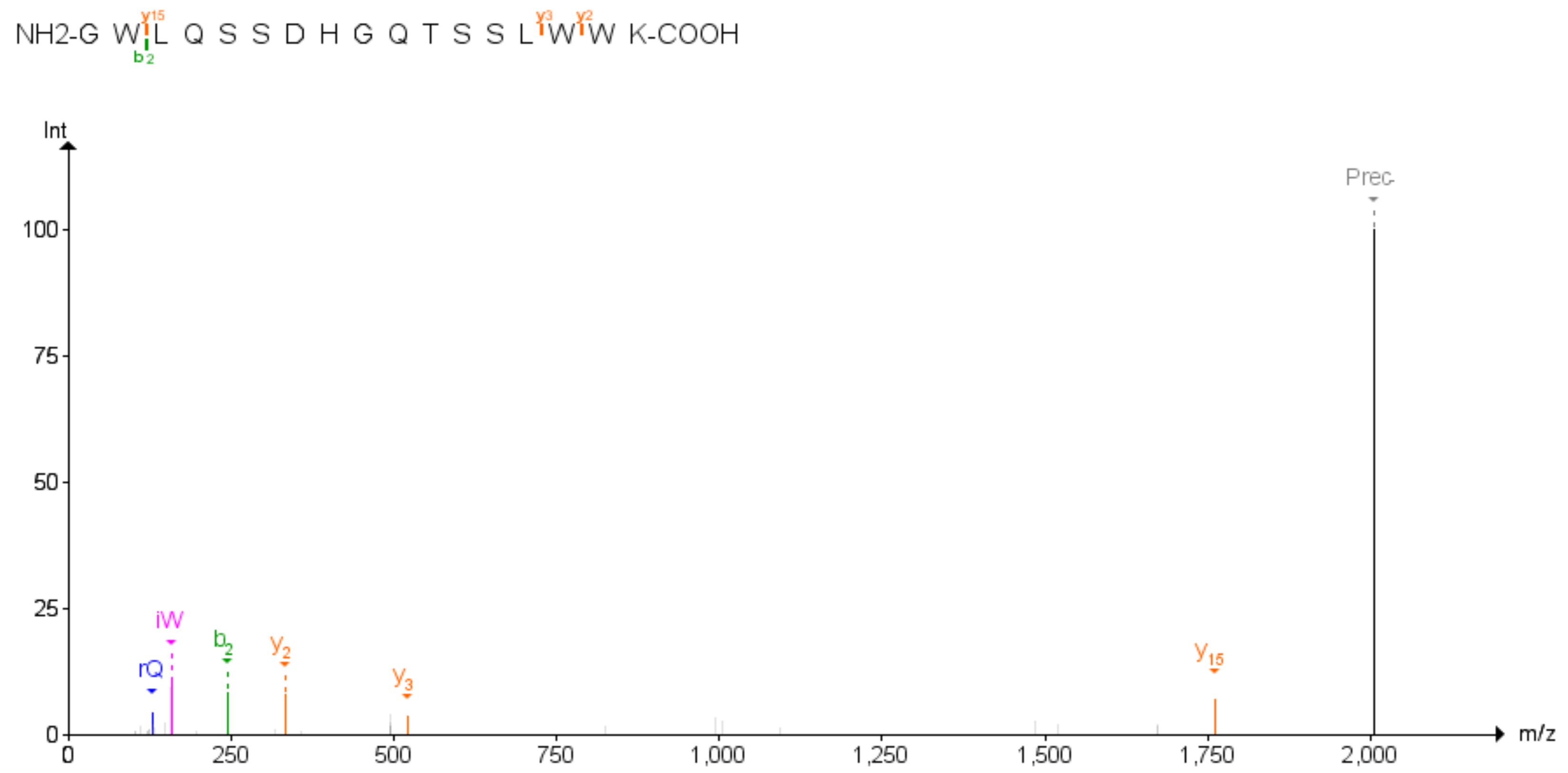

NH2-G W L Q S S D H G Q T S S L W W K-COOH
y15
b2
y3
y2
Int
Prec
100
75
50
25
0
iW
rQ
b2
y2
y3
y15
0
250
500
750
1,000
1,250
1,500
1,750
2,000
m/z

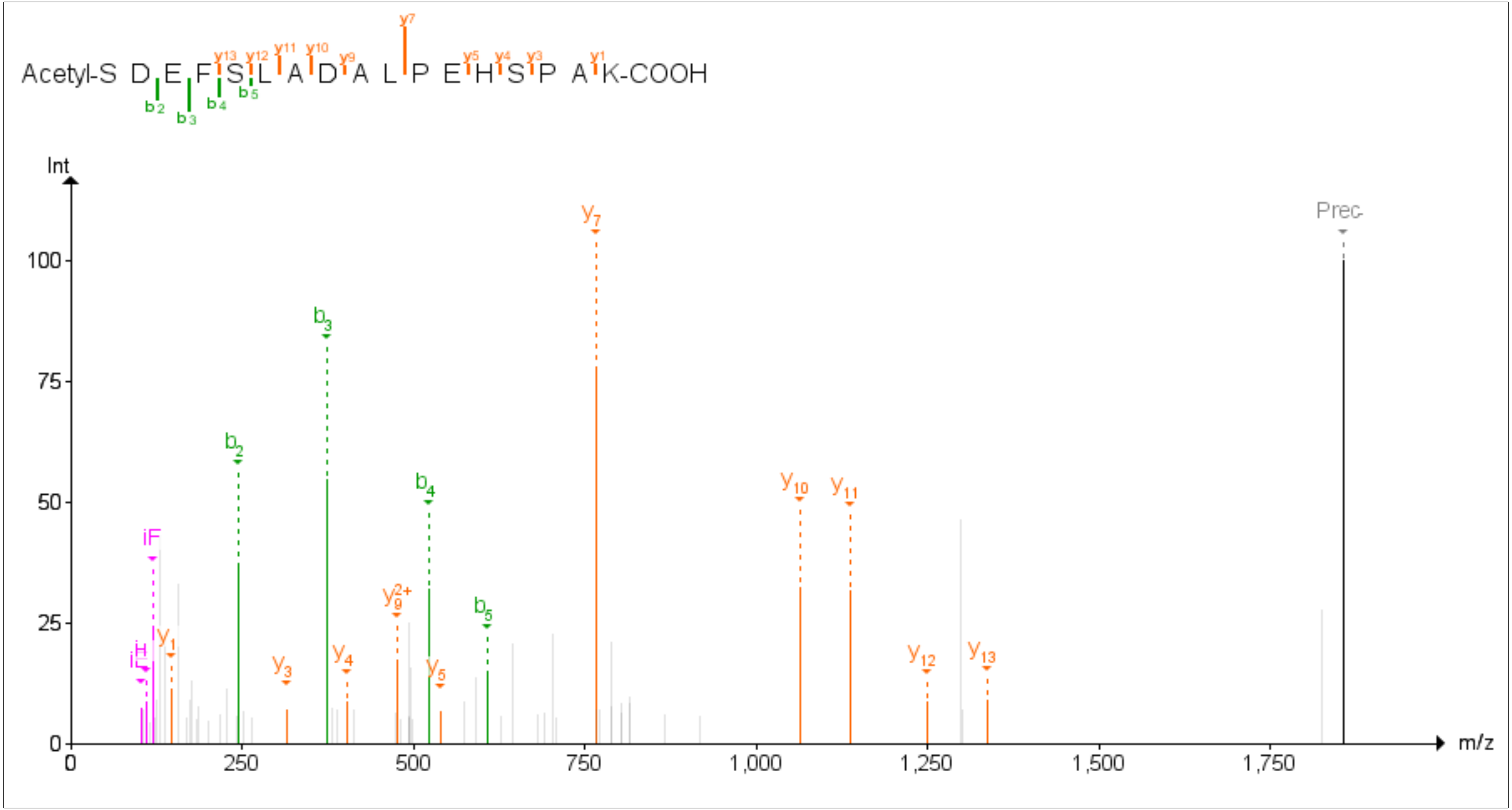

Acetyl-S D E F S L A D A L P E H S P A K-COOH
y13 y12 y11 y10 y9 y7 y5 y4 y3 y1
b2 b3 b4 b5
Int
m/z
Prec
y7
b3
b2
b4
y10
y11
iF
y9 2+
b5
y1
iH
iL
y3
y4
y5
y12
y13
0
25
50
75
100
0
250
500
750
1,000
1,250
1,500
1,750

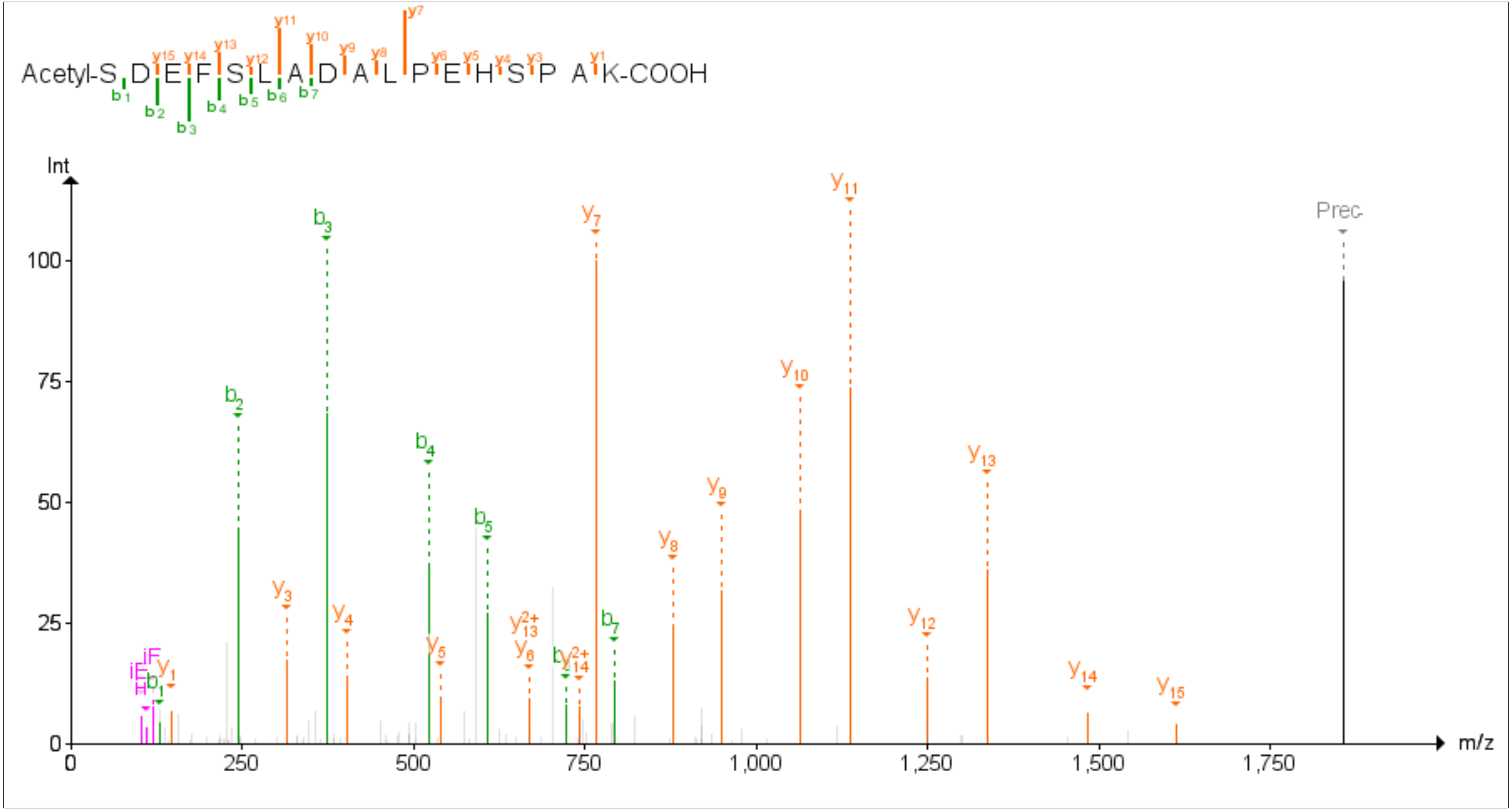

Acetyl-S D E F S L A D A L P E H S P A K-COOH
Int
m/z
Prec

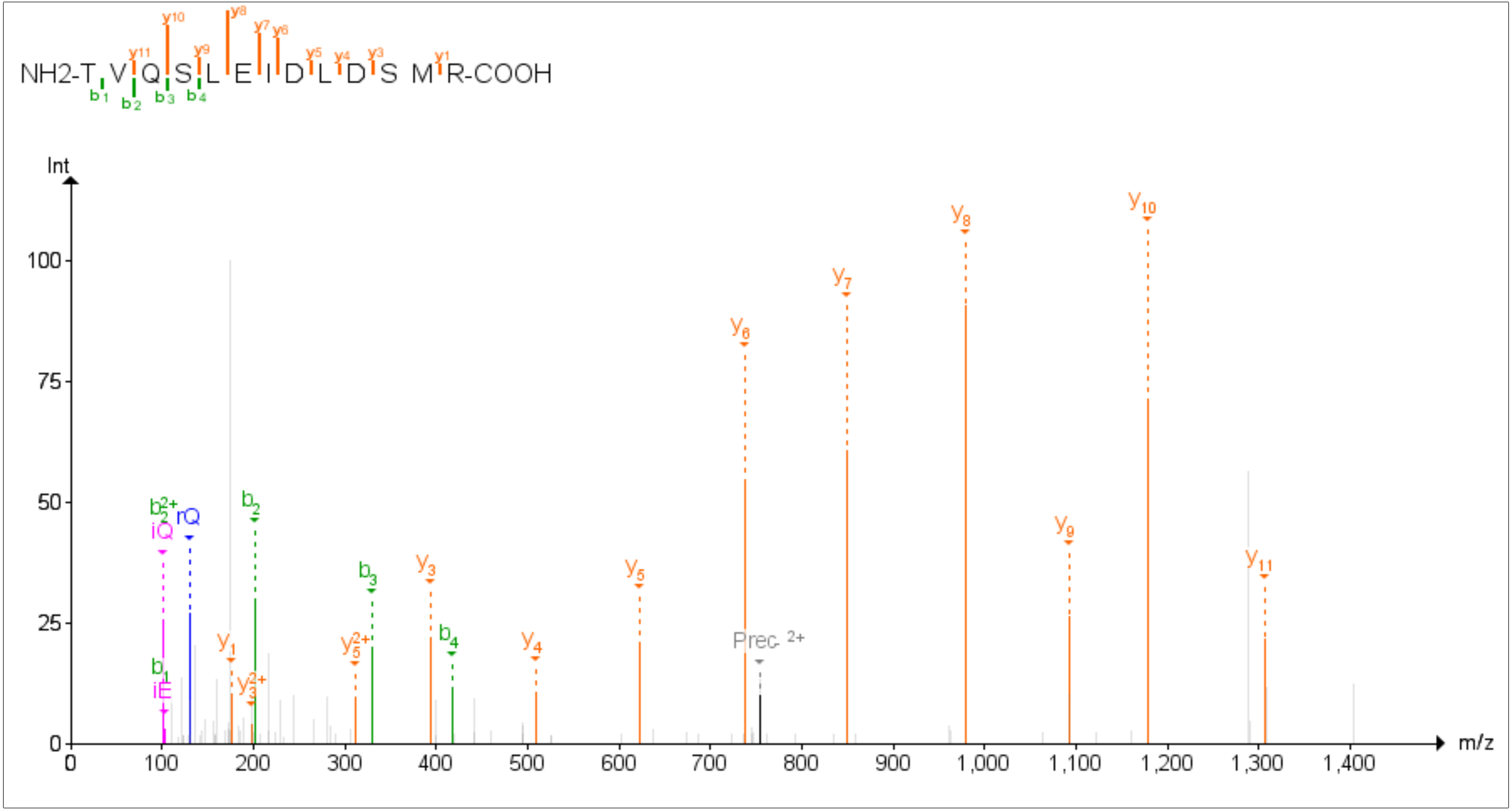

NH2-T V Q S L E I D L D S M R-COOH
b1 b2 b3 b4
y11 y10 y9 y8 y7 y6 y5 y4 y3 y1
Int
100
75
50
25
0
m/z
0 100 200 300 400 500 600 700 800 900 1,000 1,100 1,200 1,300 1,400
iQ
rQ
b2
b3
y3
b4
y4
y5
y6
y7
y8
y9
y10
y11
y1
b1
iE
Prec. 2+

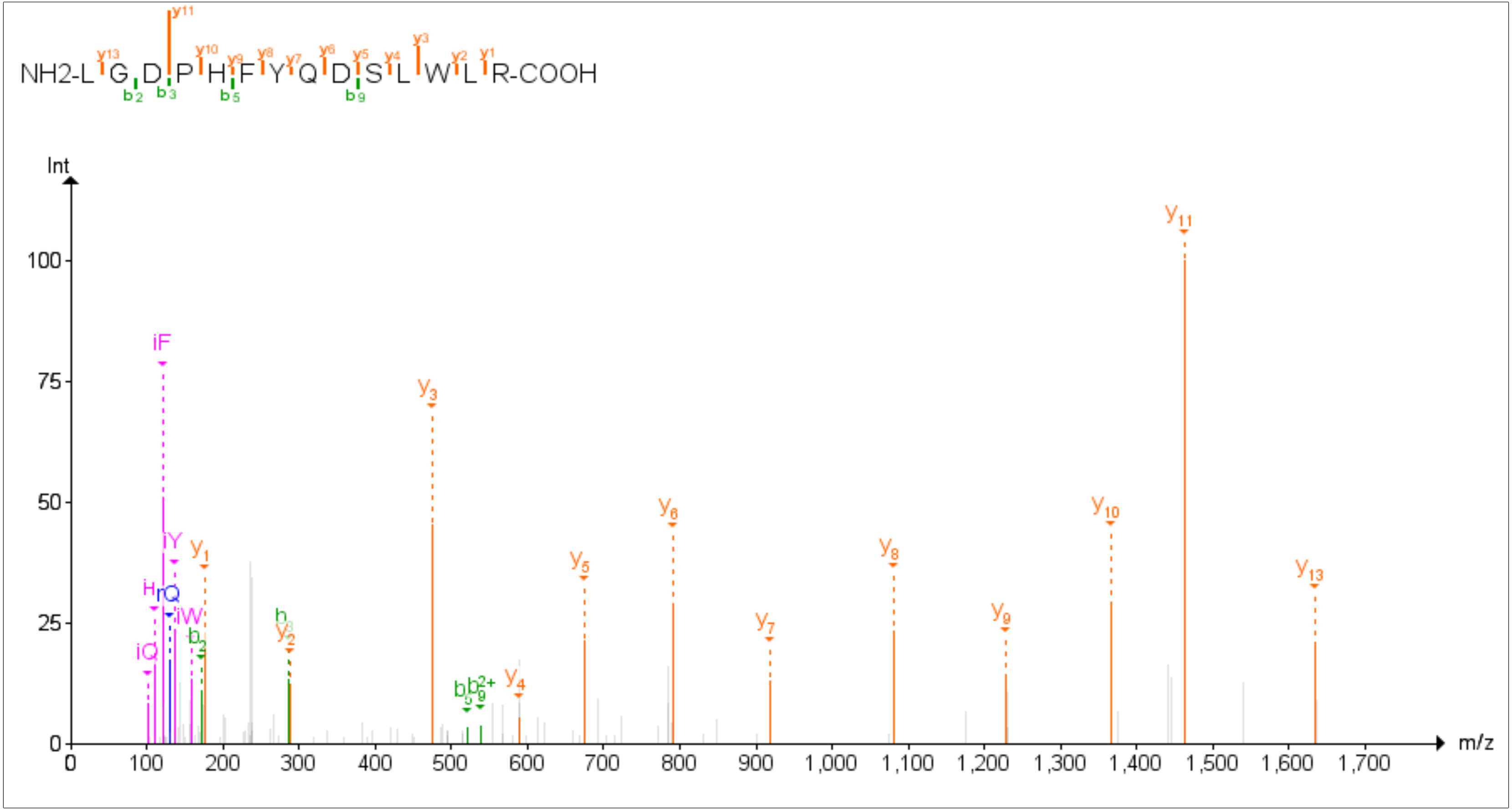

NH2-L G D P H F Y Q D S L W L R-COOH
y13 y11 y10 y9 y8 y7 y6 y5 y4 y3 y2 y1
b2 b3 b5 b9
Int
100
75
50
25
0
iF
iY
y1
iH
rQ
iW
iQ
b2
b3
y2
y3
b5
b9 2+
y4
y5
y6
y7
y8
y9
y10
y11
y13
0 100 200 300 400 500 600 700 800 900 1,000 1,100 1,200 1,300 1,400 1,500 1,600 1,700
m/z

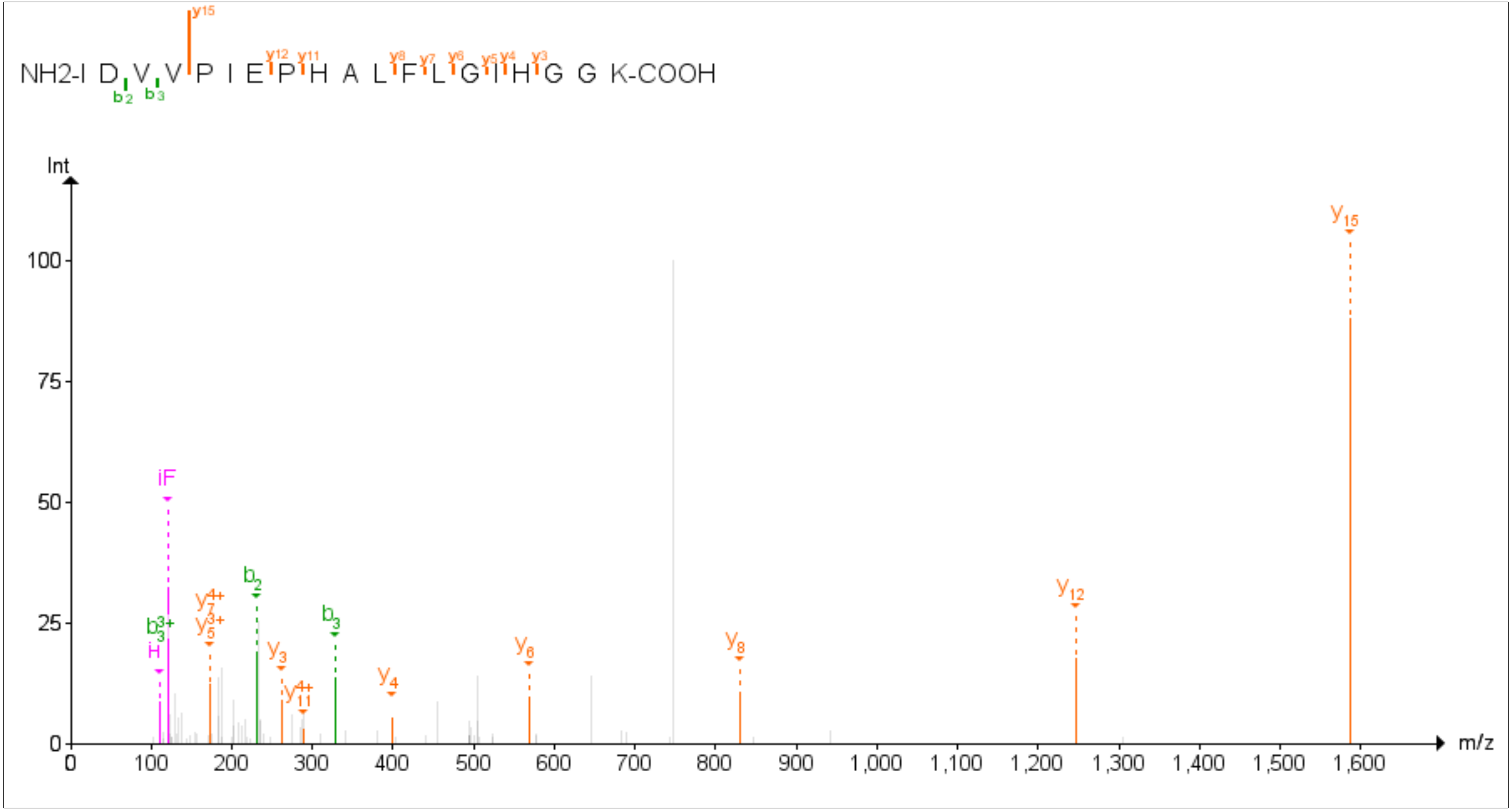

NH2-I D V V P I E P H A L F L G I H G G K-COOH
y15
y12
y11
y8
y7
y6
y5
y4
y3
b2
b3
Int
m/z
y15
y12
y8
y6
y4
y3
b2
b3
iF
H

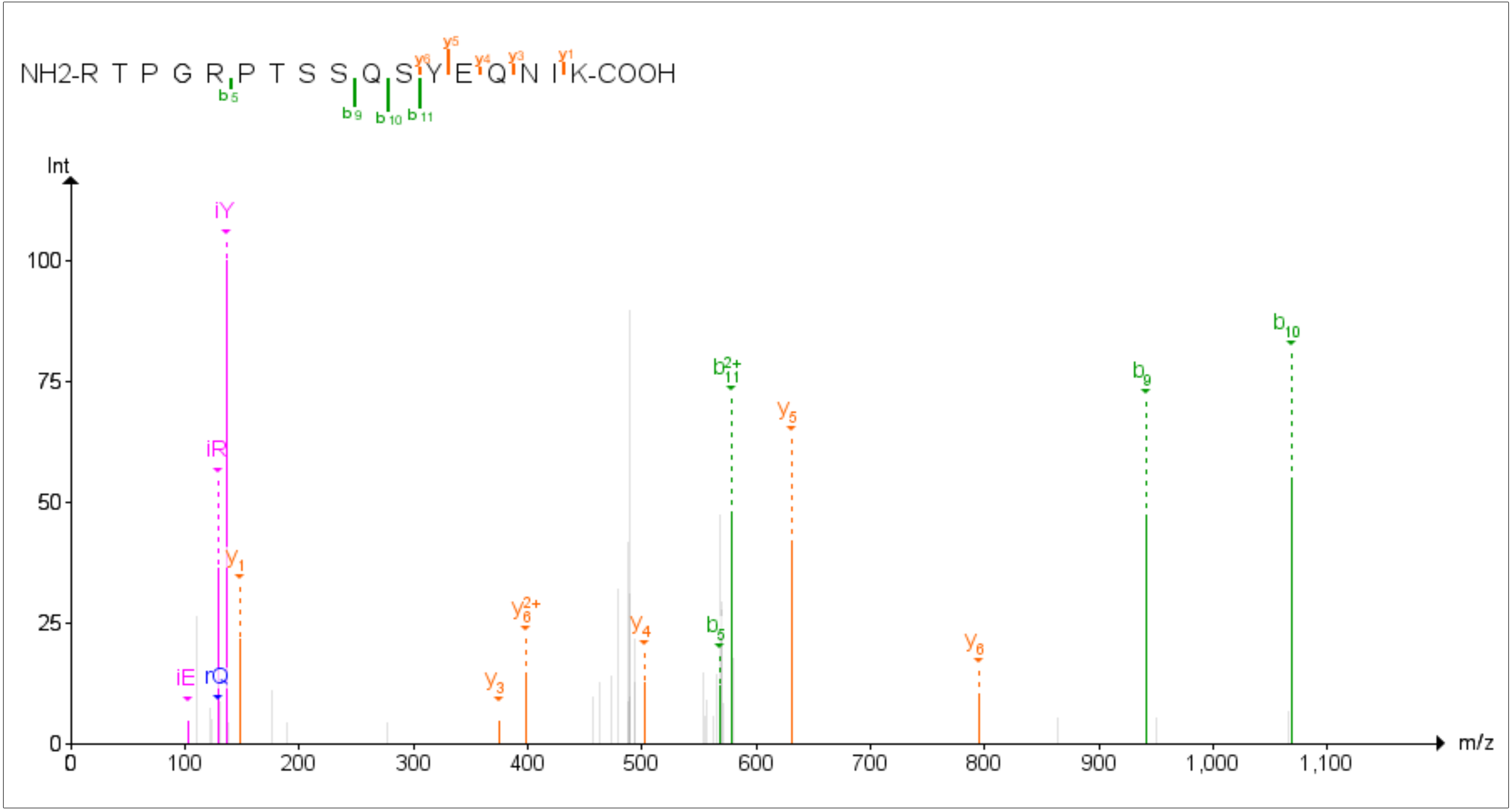

NH2-R T P G R P T S S Q S Y E Q N I K-COOH
b5 b9 b10 b11
y6 y5 y4 y3 y1
Int
m/z
iY
iR
iE
rQ
y1
y3
y6 2+
y4
b5
b11 2+
y5
y6
b9
b10

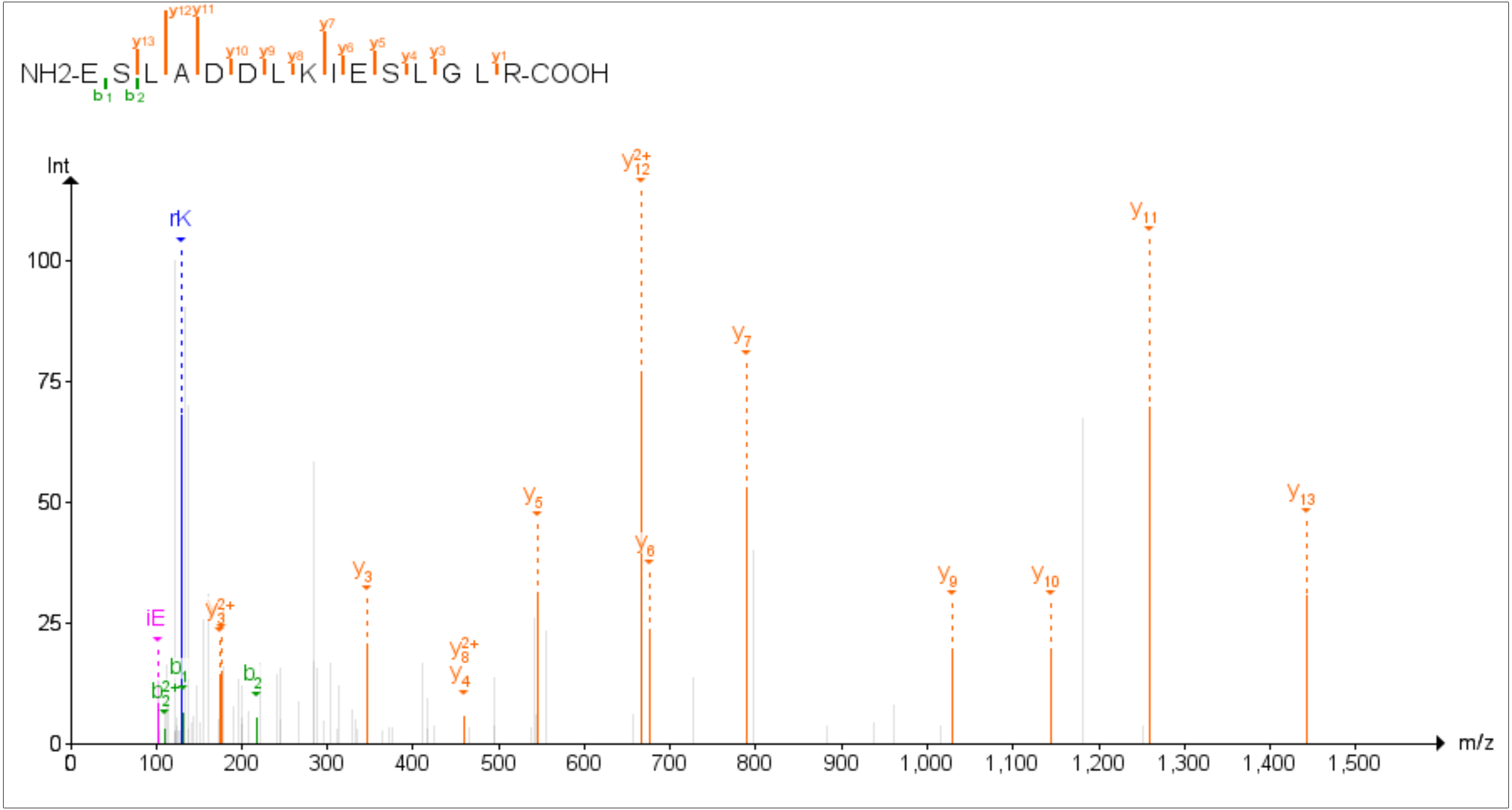
NH2-E S L A D D L K I E S L G L R-COOH
y13 y12 y11 y10 y9 y8 y7 y6 y5 y4 y3 y1
b1 b2
Int
m/z
rK
iE
y12 2+
y11
y7
y5
y6
y13
y3
y3 2+
y8 2+
y4
y9
y10
b1
b2
b2 2+
0
25
50
75
100
0 100 200 300 400 500 600 700 800 900 1,000 1,100 1,200 1,300 1,400 1,500

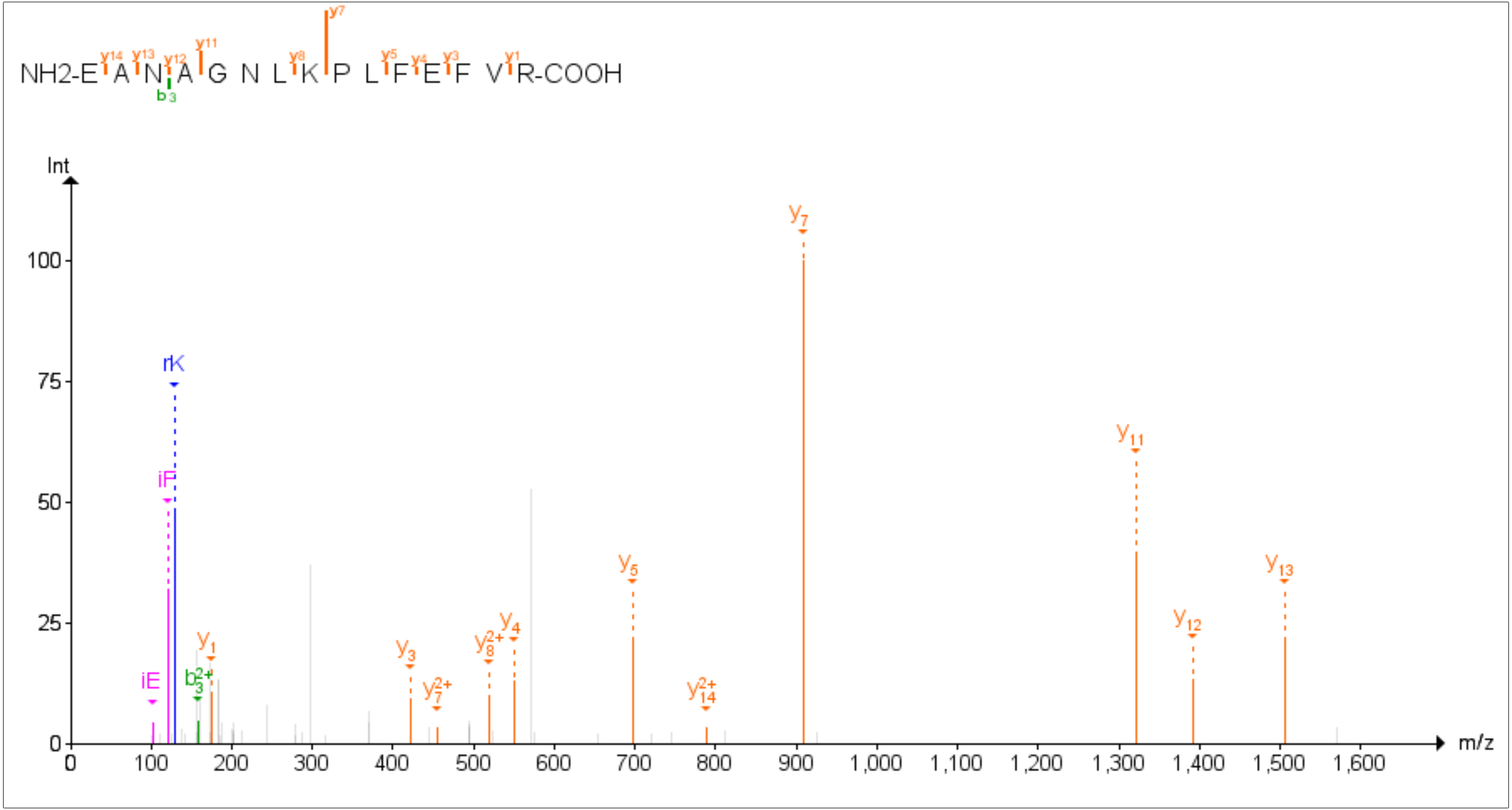
NH2-E A N A G N L K P L F E F V R-COOH
y14 y13 y12 y11 y8 y7 y5 y4 y3 y1
b3
Int
100
75
50
25
0
rK
iF
iE
b3 2+
y1
y3
y7 2+
y8 2+
y4
y5
y7
y14 2+
y11
y12
y13
0 100 200 300 400 500 600 700 800 900 1,000 1,100 1,200 1,300 1,400 1,500 1,600
m/z

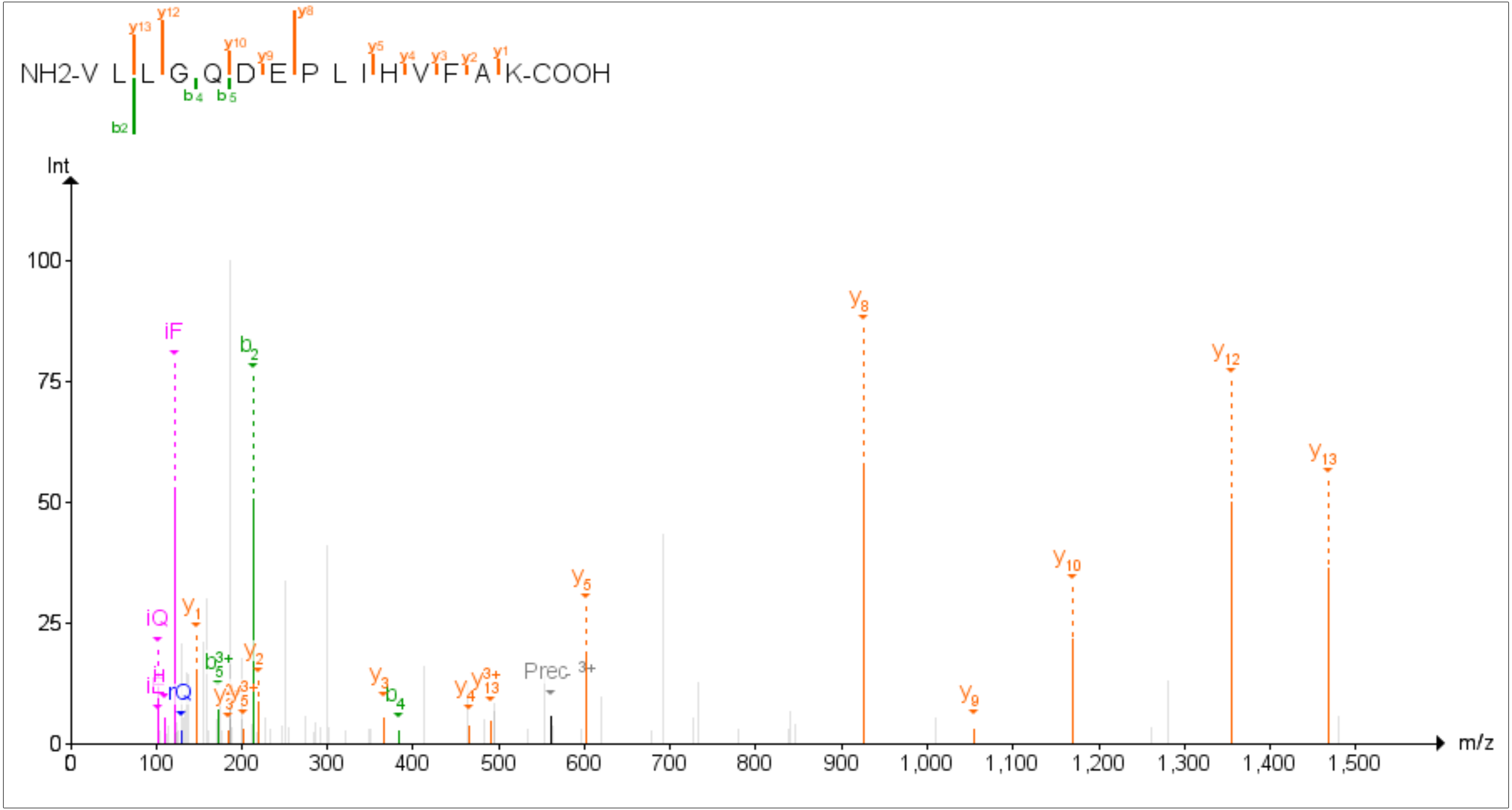

NH2-V L L G Q D E P L I H V F A K-COOH
y13 y12 y10 y9 y8 y5 y4 y3 y2 y1
b2 b4 b5
Int
m/z
y8
y12
y13
y10
y5
y9
iF
b2
iQ
y1
Prec 3+

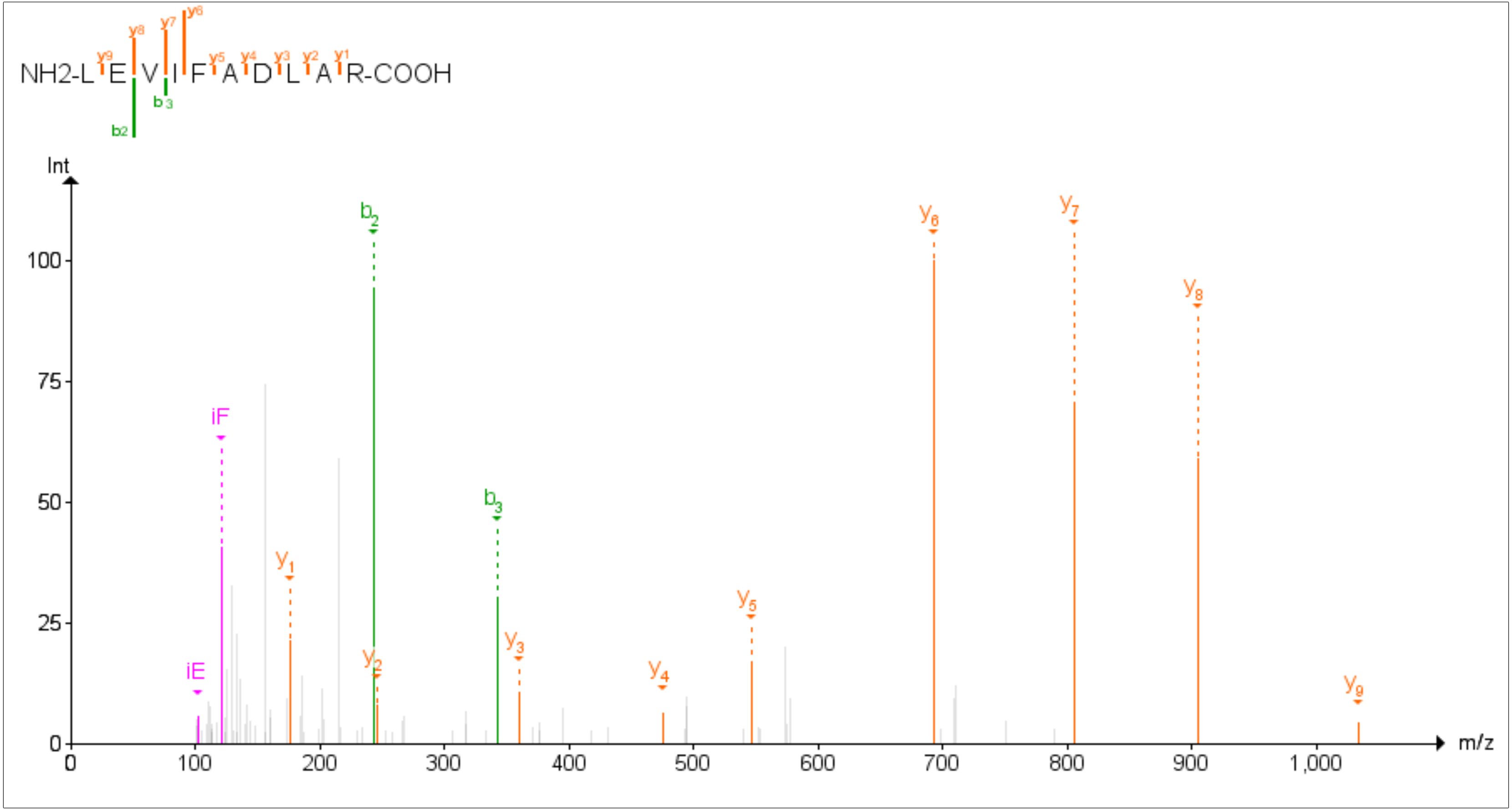
NH2-L E V I F A D L A R-COOH
y9 y8 y7 y6 y5 y4 y3 y2 y1
b2 b3
Int
m/z
b2 y6 y7 y8 iF b3 y1 y5 y3 y2 y4 iE y9
0 25 50 75 100
0 100 200 300 400 500 600 700 800 900 1,000

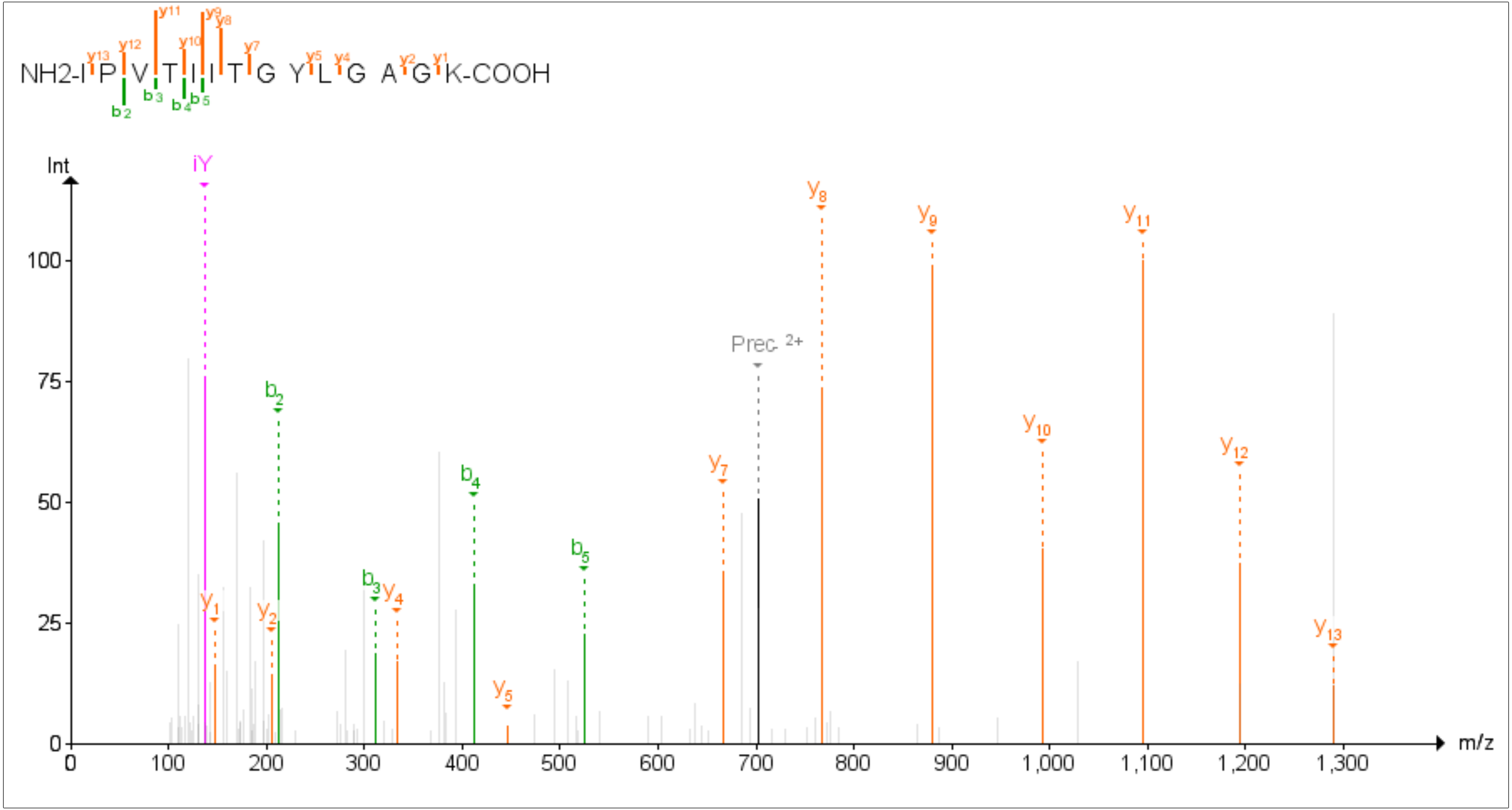

NH2-I P V T I I T G Y L G A G K-COOH
y13 y12 y11 y10 y9 y8 y7 y5 y4 y2 y1
b2 b3 b4 b5
Int
iY
Prec 2+
y8 y9 y11 y10 y12 y7 y13 b2 b4 b5 b3 y4 y1 y2 y5
0 25 50 75 100
0 100 200 300 400 500 600 700 800 900 1,000 1,100 1,200 1,300
m/z

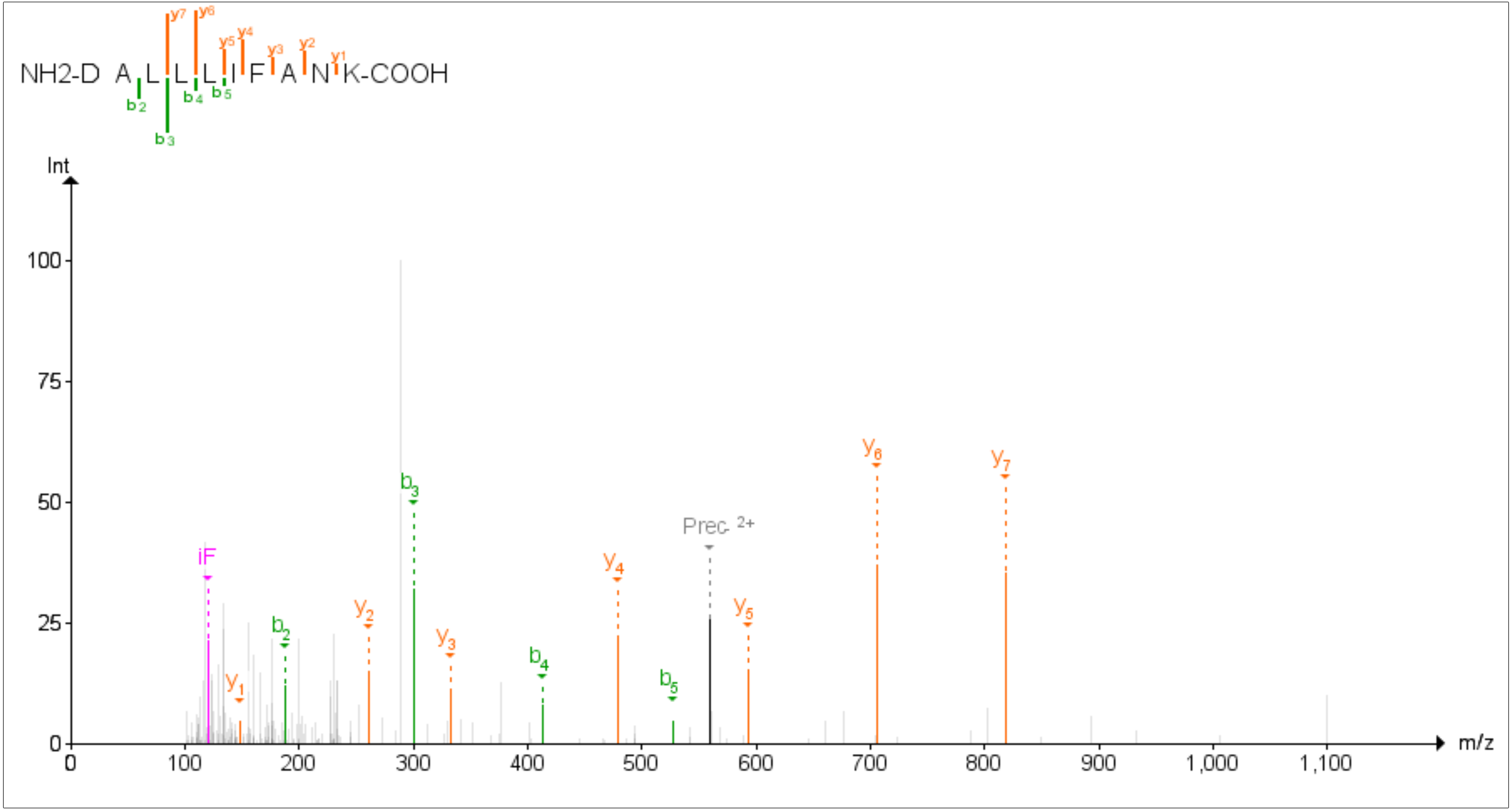

NH2-D A L L L I F A N K-COOH
y7 y6 y5 y4 y3 y2 y1
b2 b3 b4 b5
Int
100
75
50
25
0
iF
y1
b2
y2
b3
y3
b4
y4
b5
Prec 2+
y5
y6
y7
0
100
200
300
400
500
600
700
800
900
1,000
1,100
m/z

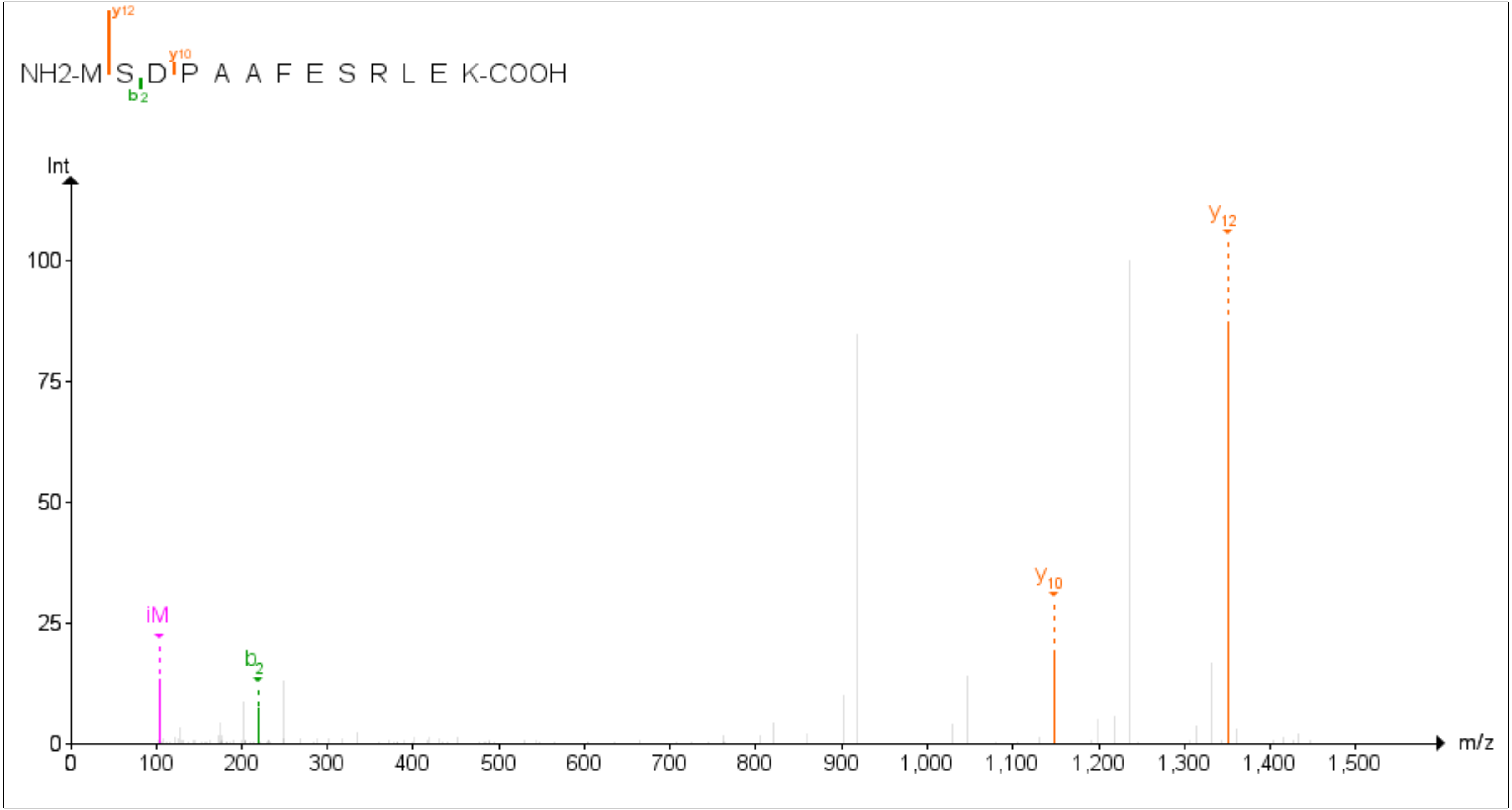

NH2-M S D P A A F E S R L E K-COOH
y12
y10
b2
Int
100
75
50
25
0
iM
b2
y10
y12
0
100
200
300
400
500
600
700
800
900
1,000
1,100
1,200
1,300
1,400
1,500
m/z

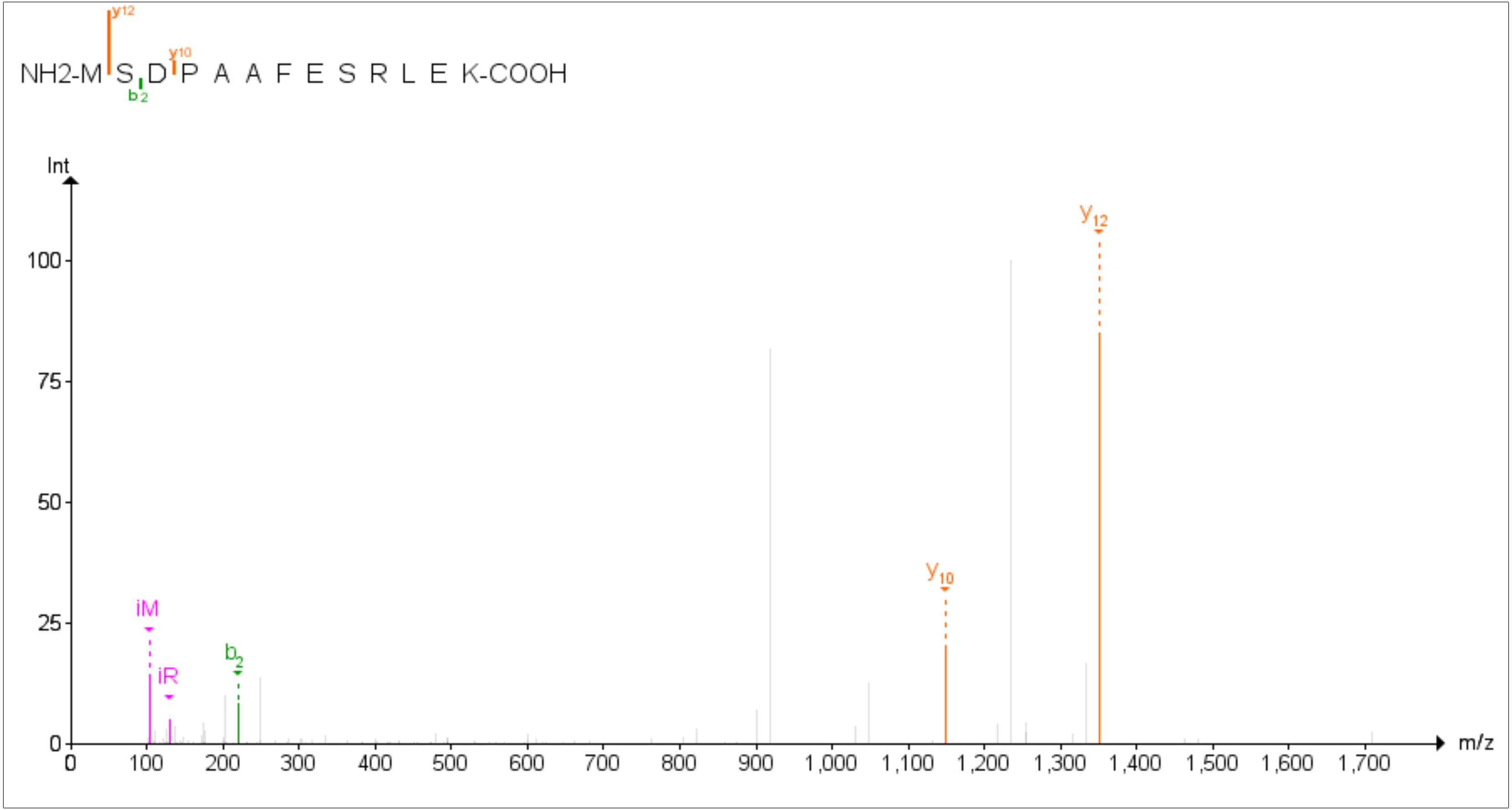

NH2-M S D P A A F E S R L E K-COOH
y12
y10
b2
Int
100
75
50
25
0
iM
iR
b2
y10
y12
0
100
200
300
400
500
600
700
800
900
1,000
1,100
1,200
1,300
1,400
1,500
1,600
1,700
m/z

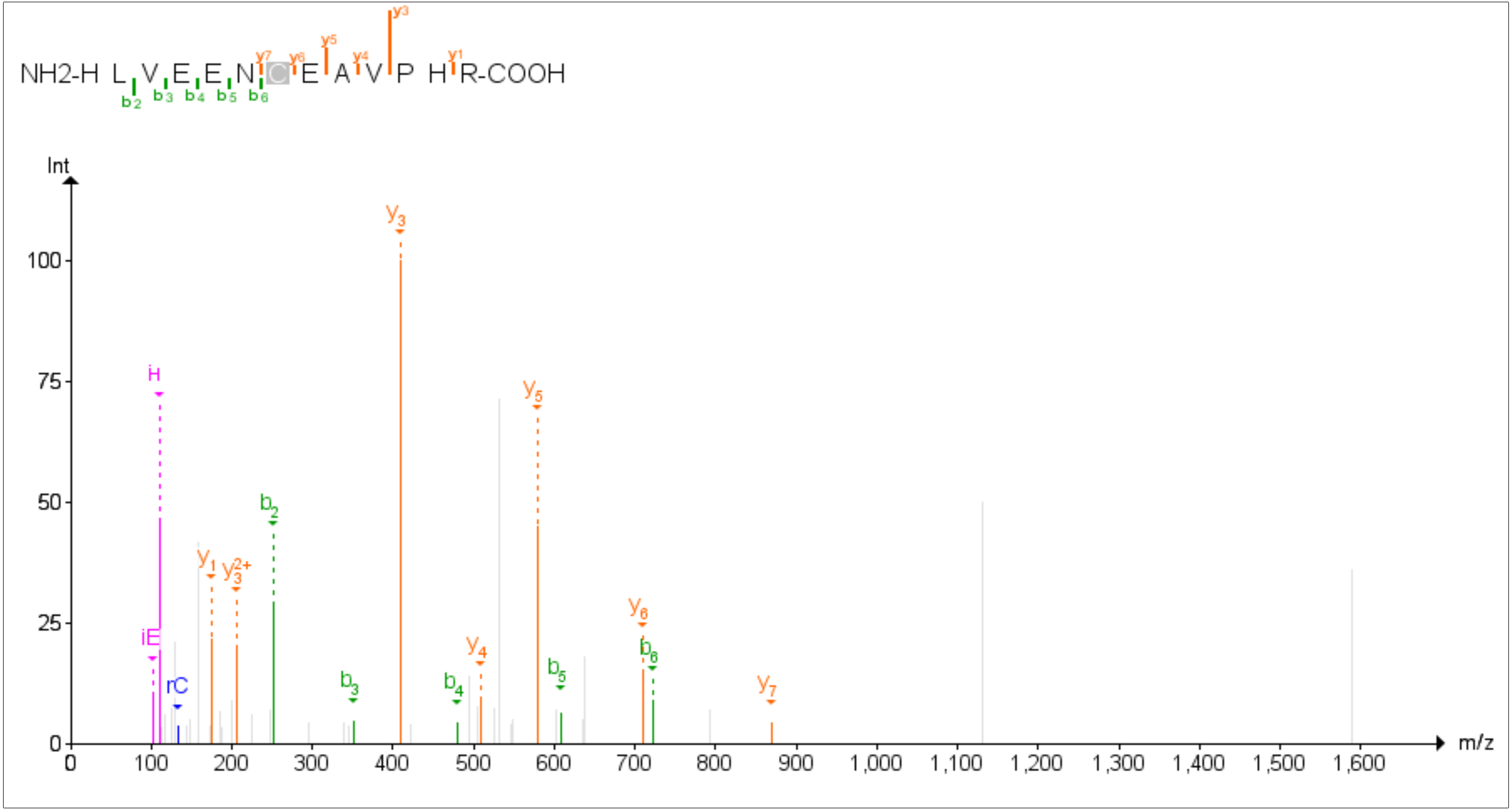

NH2-H L V E E N C E A V P H R-COOH
y7 y6 y5 y4 y3 y1
b2 b3 b4 b5 b6
Int
100
75
50
25
0
y3
iH
y5
b2
y1
y3 2+
iE
rC
y6
b6
y4
b5
b3
b4
y7
0 100 200 300 400 500 600 700 800 900 1,000 1,100 1,200 1,300 1,400 1,500 1,600
m/z

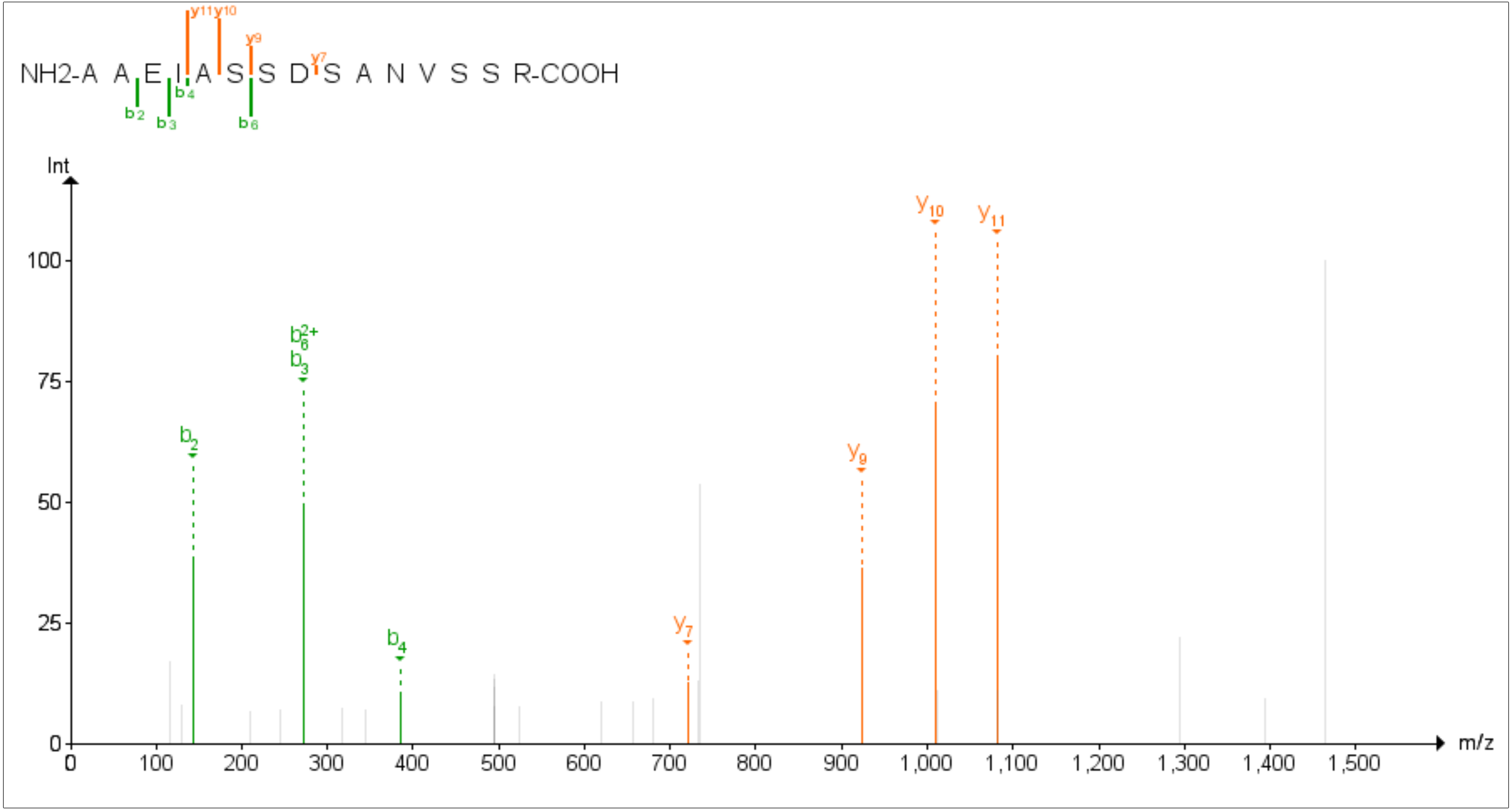
NH2-A A E I A S S D S A N V S S R-COOH
y11 y10
y9
y7
b2
b3
b4
b6
Int
100
75
50
25
0
b2
b6 2+
b3
b4
y7
y9
y10
y11
0
100
200
300
400
500
600
700
800
900
1,000
1,100
1,200
1,300
1,400
1,500
m/z

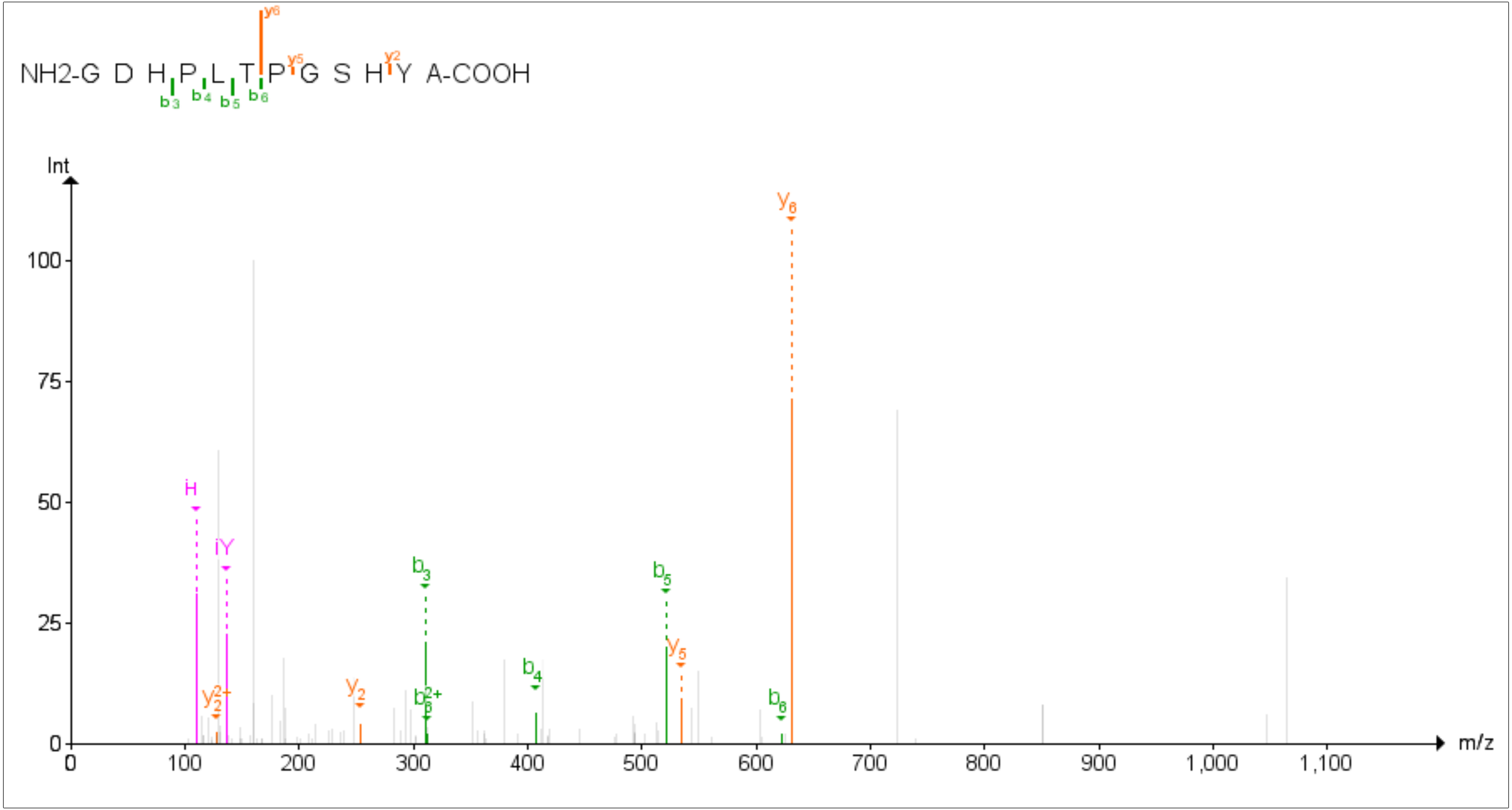
NH2-G D H P L T P G S H Y A-COOH
b3 b4 b5 b6
y6 y5 y2
Int
m/z
0
25
50
75
100
0
100
200
300
400
500
600
700
800
900
1,000
1,100
iH
iY
y6
b3
b5
y5
b4
y2
b6
y2 2+
b3 2+

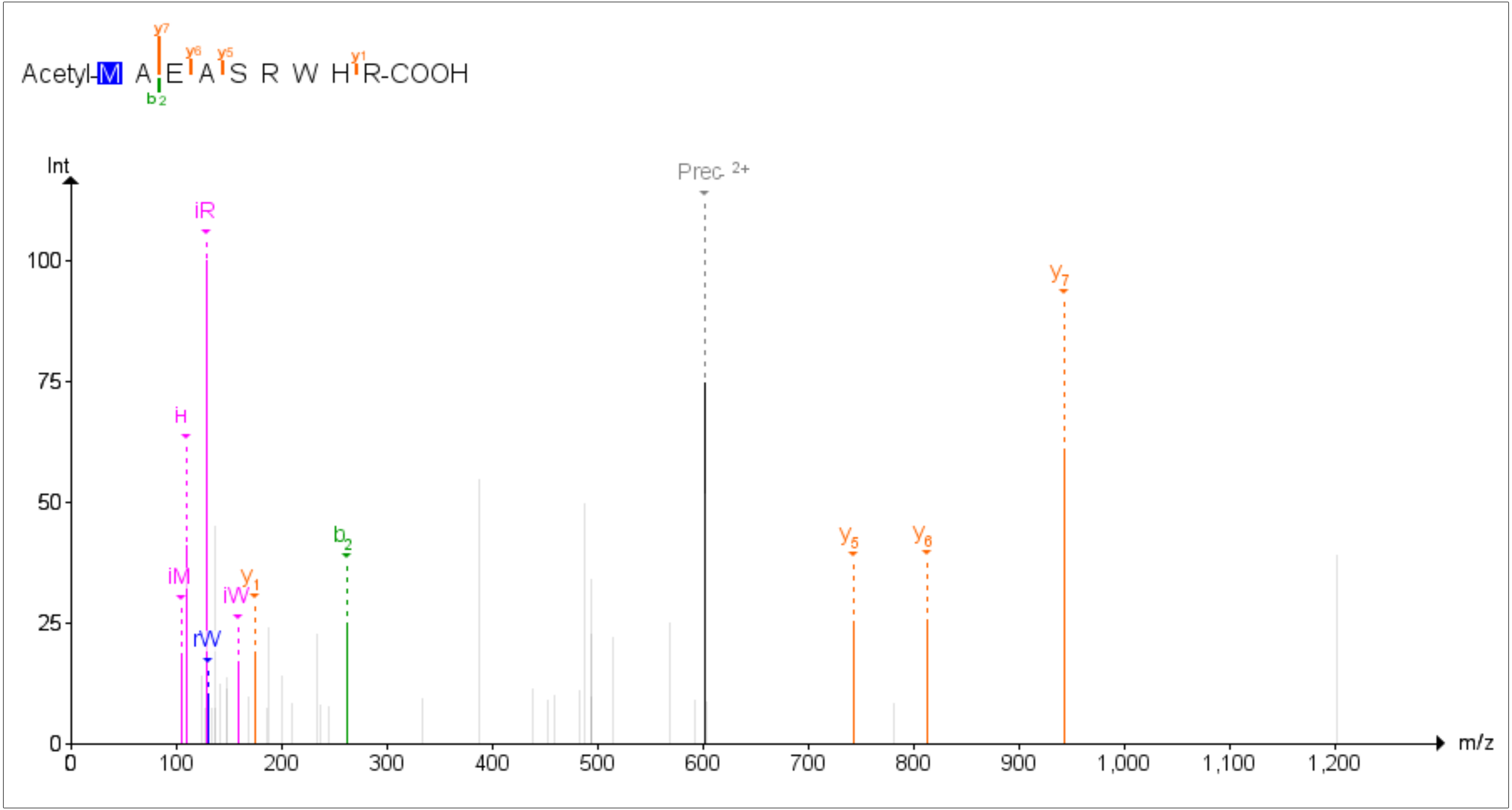

Acetyl-M A E A S R W H R-COOH
y7
y6
y5
y1
b2
Int
Prec 2+
iR
iH
iM
iW
rW
y1
b2
y5
y6
y7
100
75
50
25
0
0
100
200
300
400
500
600
700
800
900
1,000
1,100
1,200
m/z

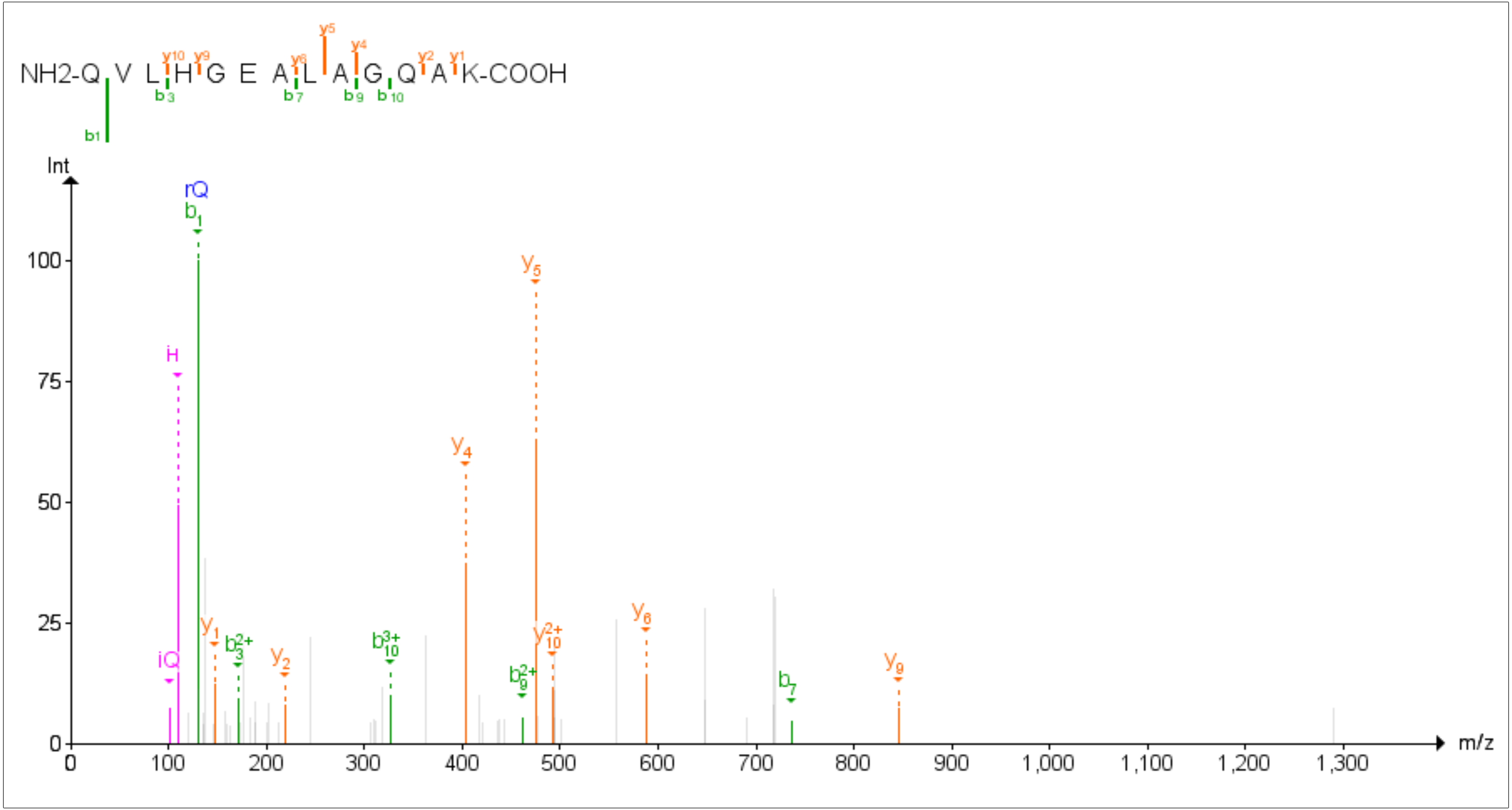

NH2-Q V L H G E A L A G Q A K-COOH
b1 b3 b7 b9 b10
y10 y9 y8 y5 y4 y2 y1
Int
rQ
b1
iH
iQ
y1
b3 2+
y2
b10 3+
y4
y5
b9 2+
y10 2+
y8
b7
y9
100
75
50
25
0
m/z
0 100 200 300 400 500 600 700 800 900 1,000 1,100 1,200 1,300

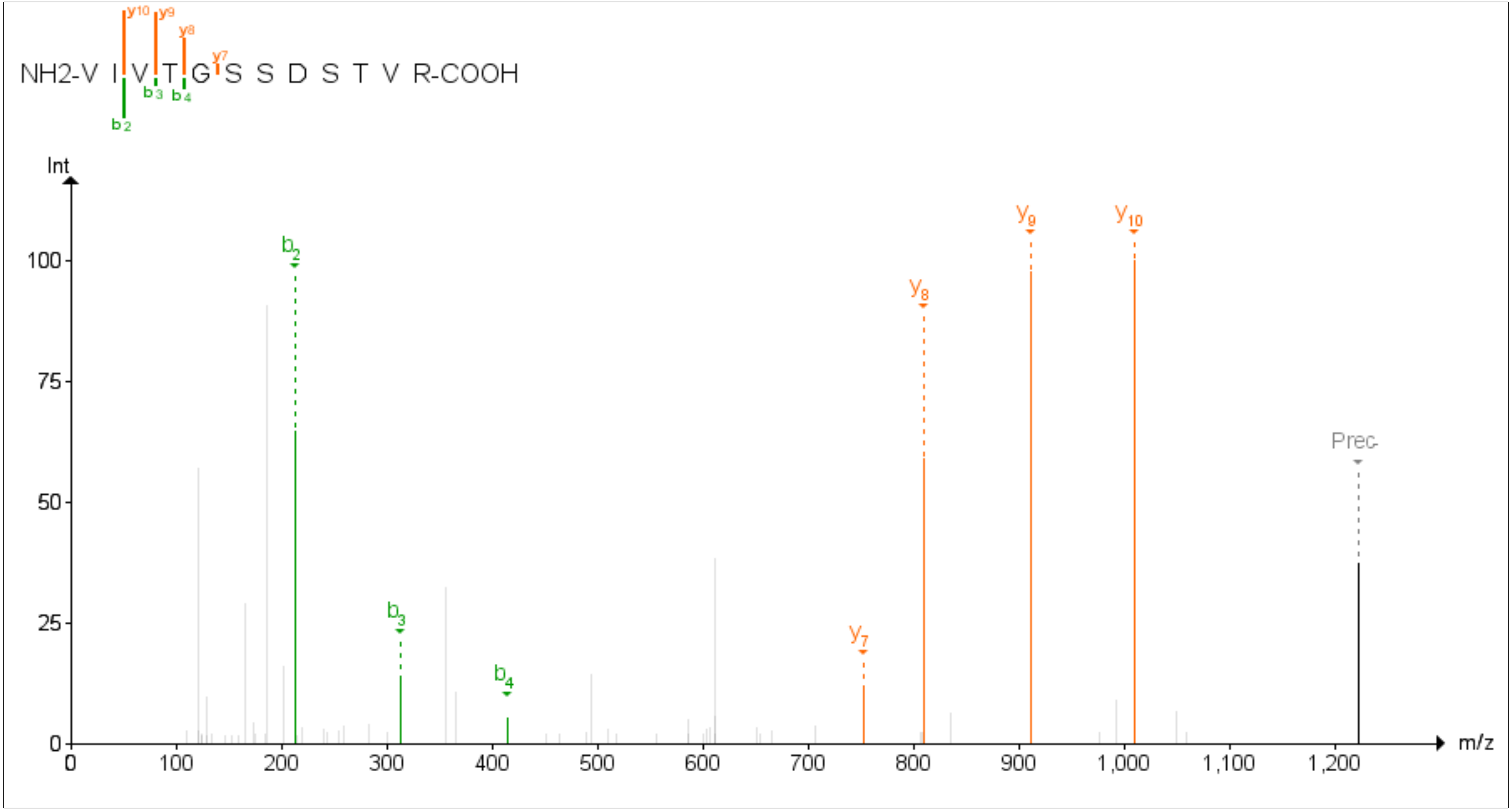

NH2-V I V T G S S D S T V R-COOH
y10 y9 y8 y7
b2 b3 b4
Int
m/z
Prec.
0 25 50 75 100
0 100 200 300 400 500 600 700 800 900 1,000 1,100 1,200

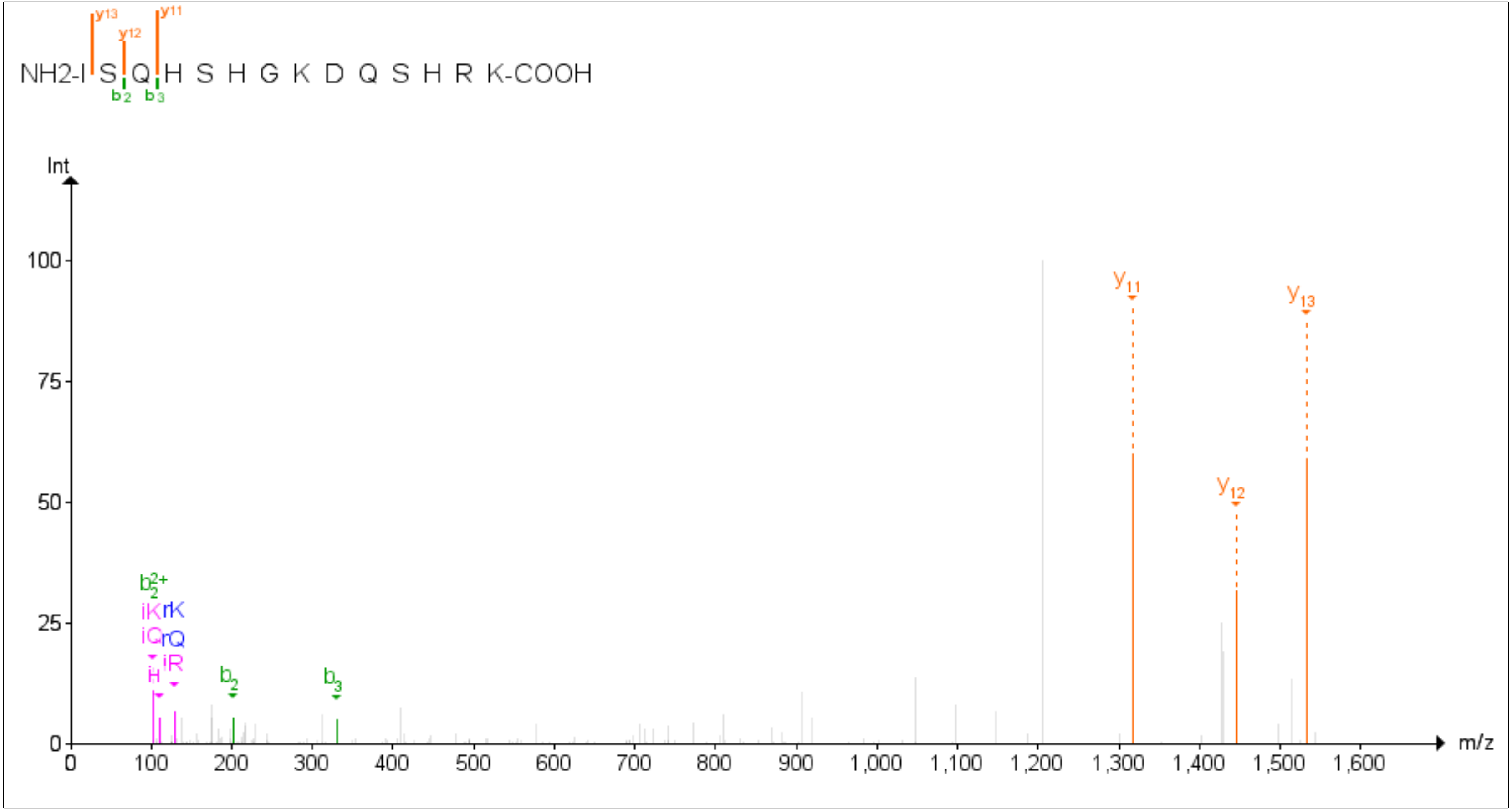
NH2-I S Q H S H G K D Q S H R K-COOH
y13
y12
y11
b2
b3
Int
100
75
50
25
0
m/z
0
100
200
300
400
500
600
700
800
900
1,000
1,100
1,200
1,300
1,400
1,500
1,600
y_{11}
y_{13}
y_{12}
b_2^{2+}
iK rK
iQ rQ
iR
iH
b_2
b_3

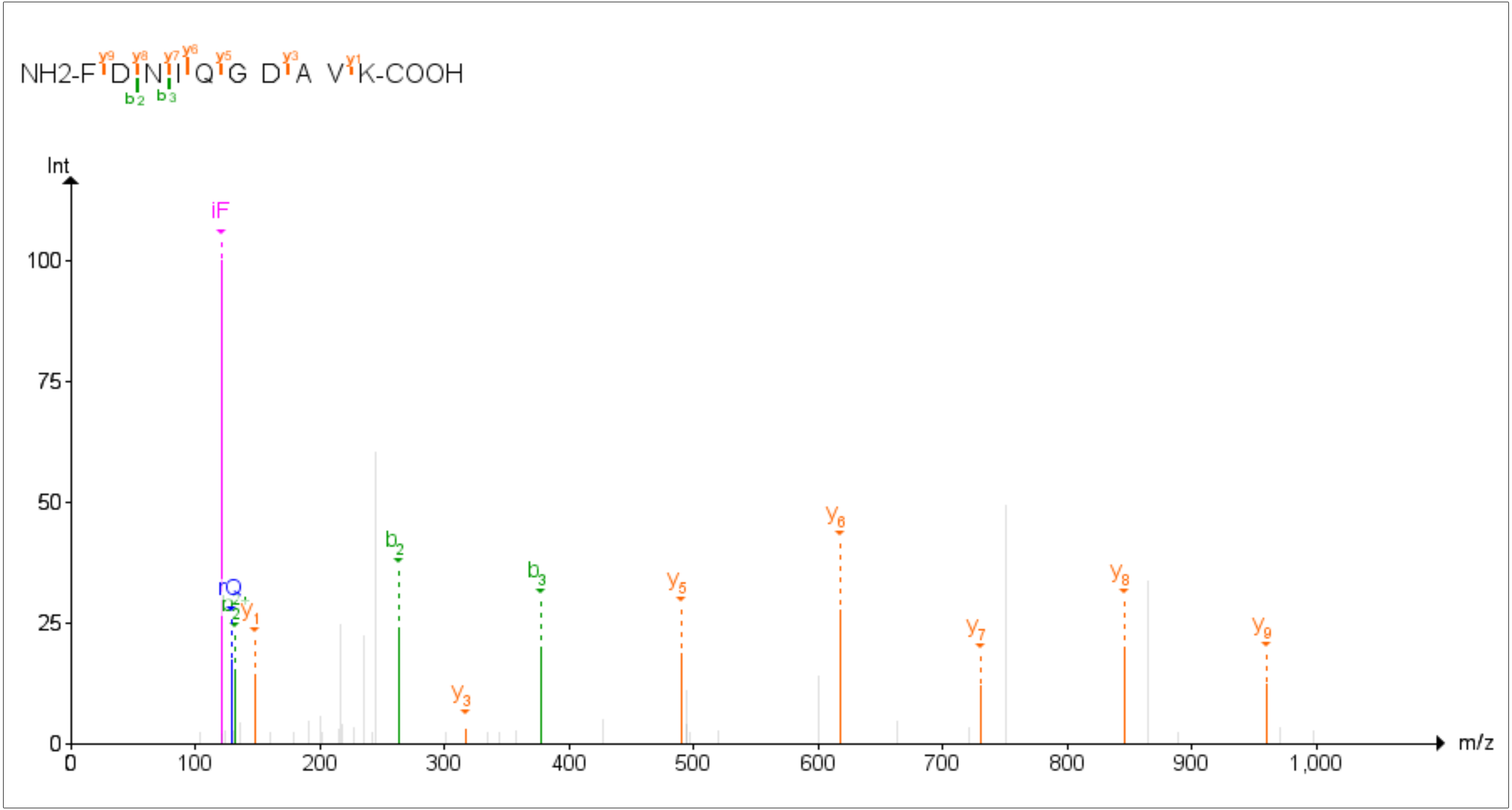

NH2-F D N I Q G D A V K-COOH
y9 y8 y7 y6 y5 y3 y1
b2 b3
Int
iF
rQ
y1
b2
y3
y5
y6
y7
y8
y9
0
25
50
75
100
0
100
200
300
400
500
600
700
800
900
1,000
m/z

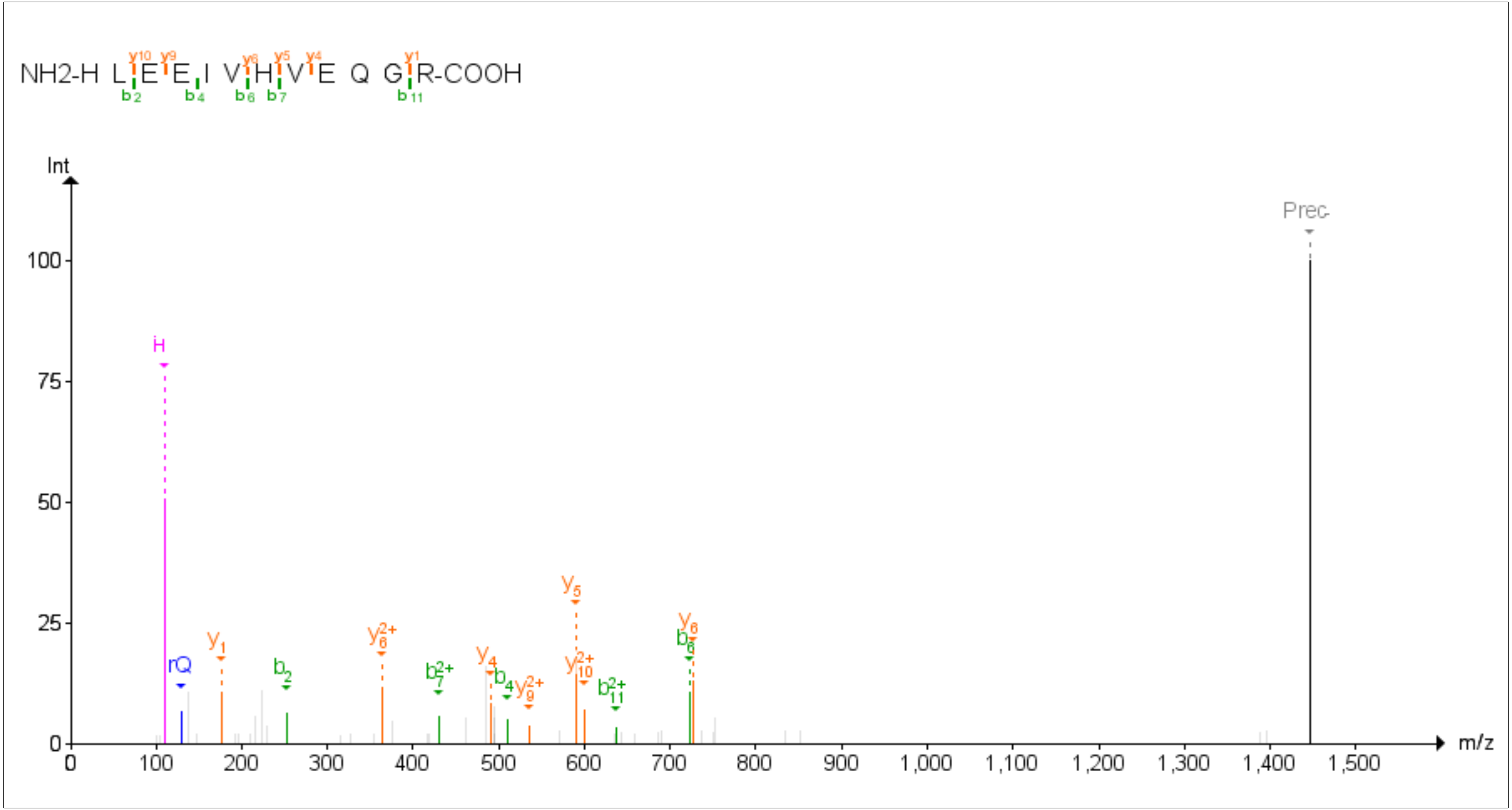

NH2-H L E E I V H V E Q G R-COOH
y10 y9 y6 y5 y4 y1
b2 b4 b6 b7 b11
Int
m/z
Prec.
H
rQ
y1
b2
y6 2+
b7 2+
y4
b4
y9 2+
y5
y10 2+
b11 2+
y6
b6
0
25
50
75
100
0
100
200
300
400
500
600
700
800
900
1,000
1,100
1,200
1,300
1,400
1,500

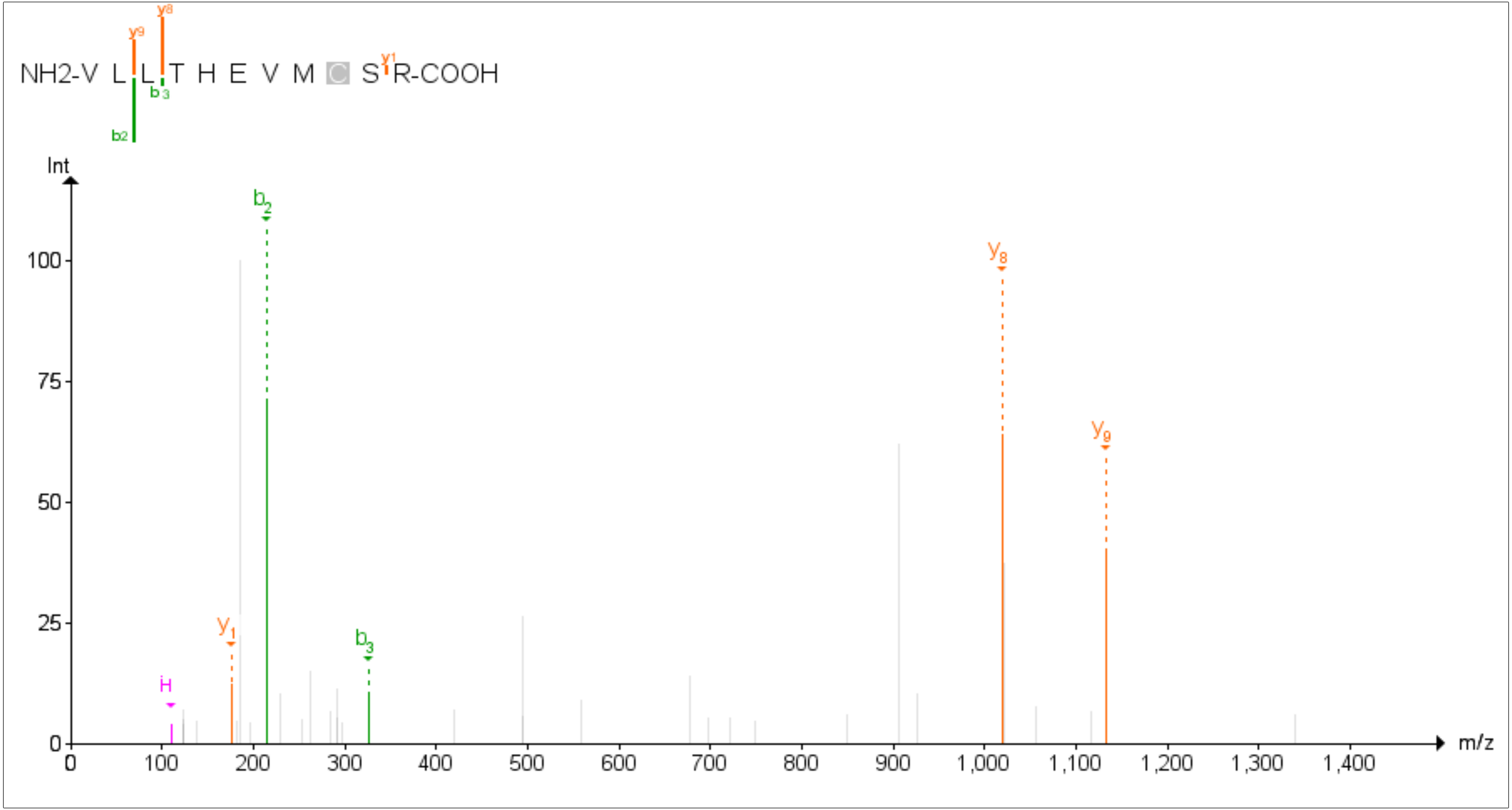

NH2-V L L T H E V M C S R-COOH
y9
y8
y1
b2
b3
Int
100
75
50
25
0
b2
y8
y9
y1
b3
H
0
100
200
300
400
500
600
700
800
900
1,000
1,100
1,200
1,300
1,400
m/z

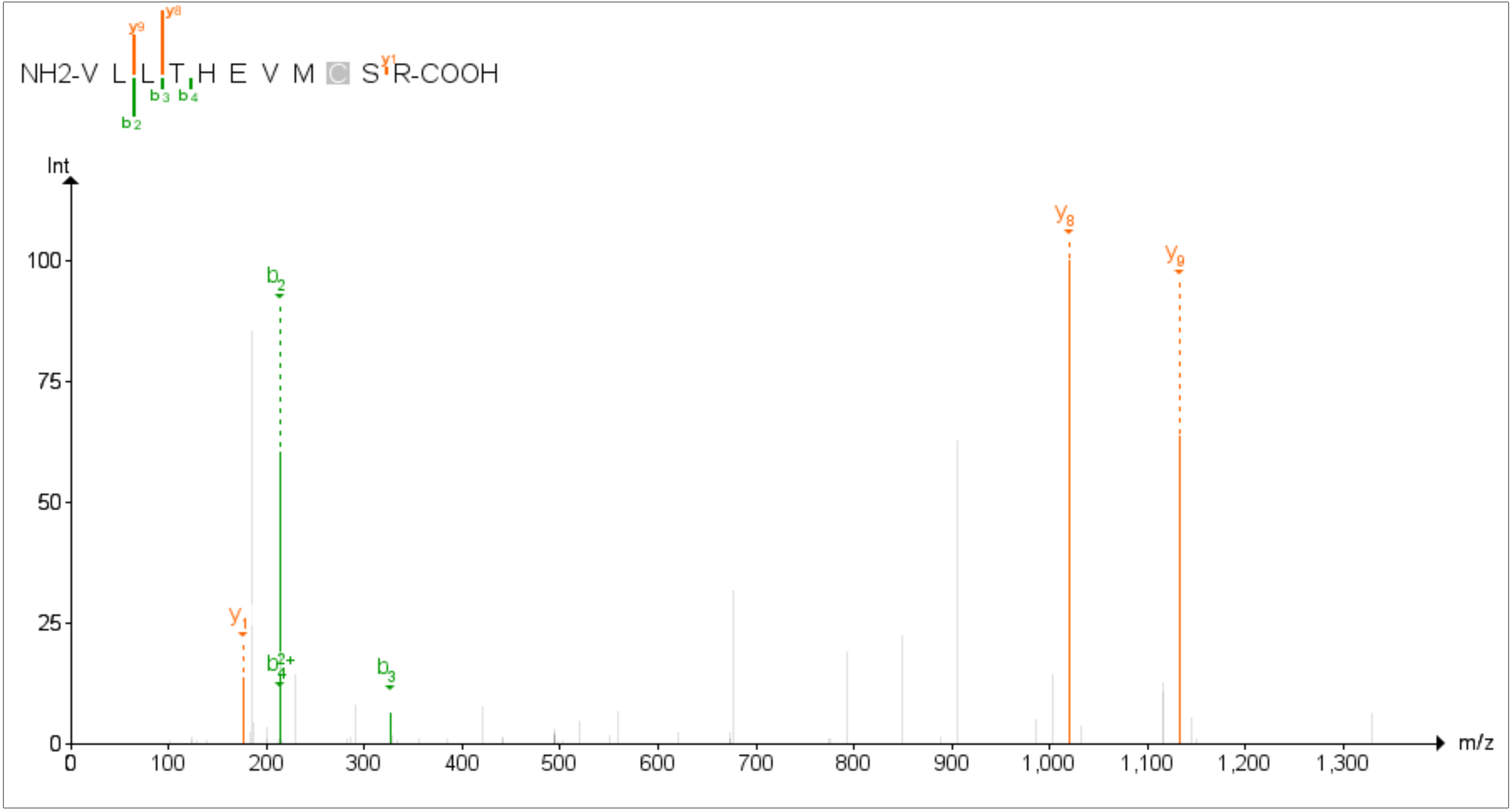

NH2-V L L T H E V M C S R-COOH
y9
y8
y1
b2
b3
b4
Int
100
75
50
25
0
y8
y9
b2
y1
b4 2+
b3
0
100
200
300
400
500
600
700
800
900
1,000
1,100
1,200
1,300
m/z

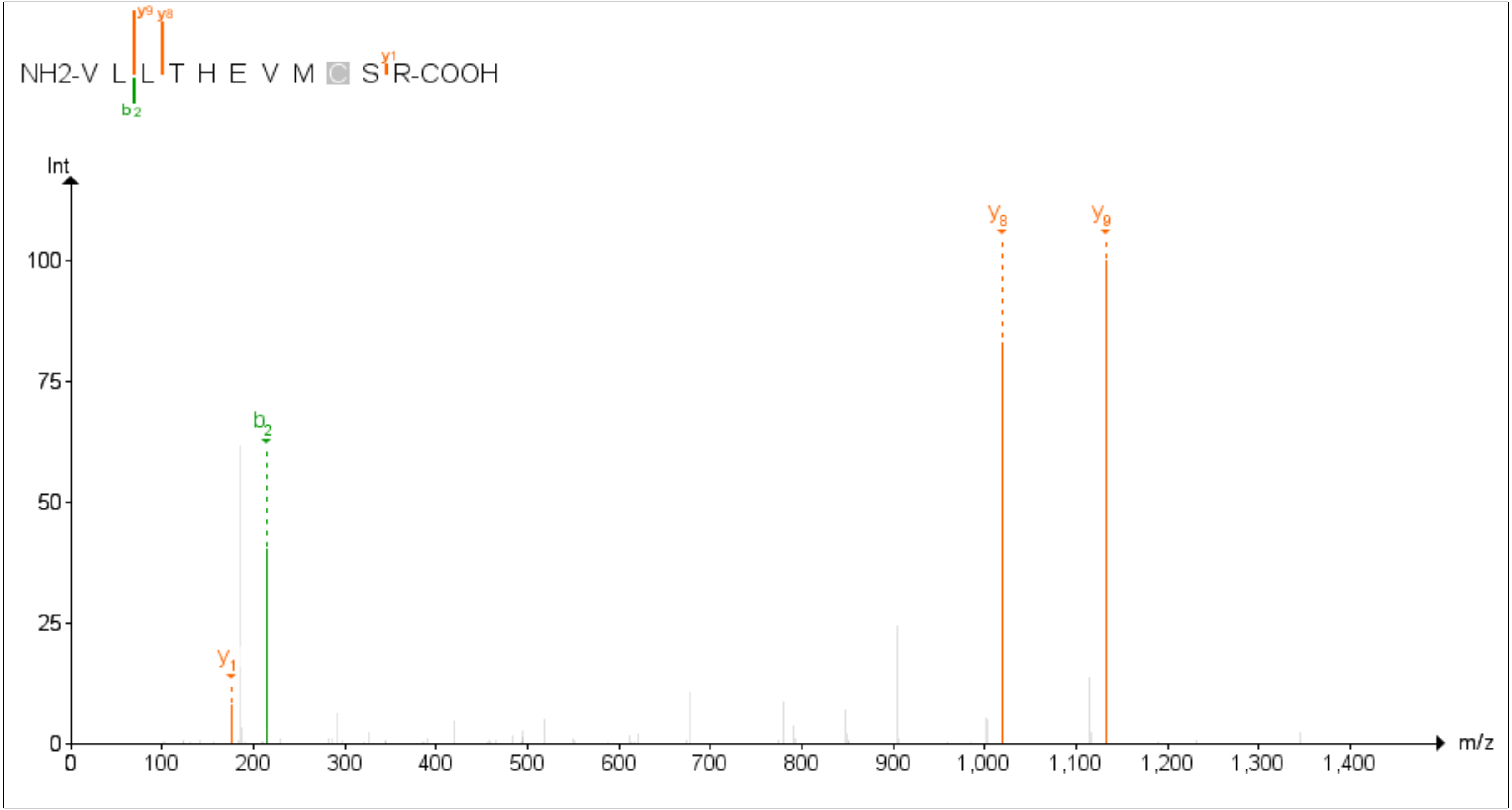

NH2-V L L T H E V M C S R-COOH
y9 y8 y1 b2
Int
100
75
50
25
0
y8
y9
b2
y1
0 100 200 300 400 500 600 700 800 900 1,000 1,100 1,200 1,300 1,400
m/z

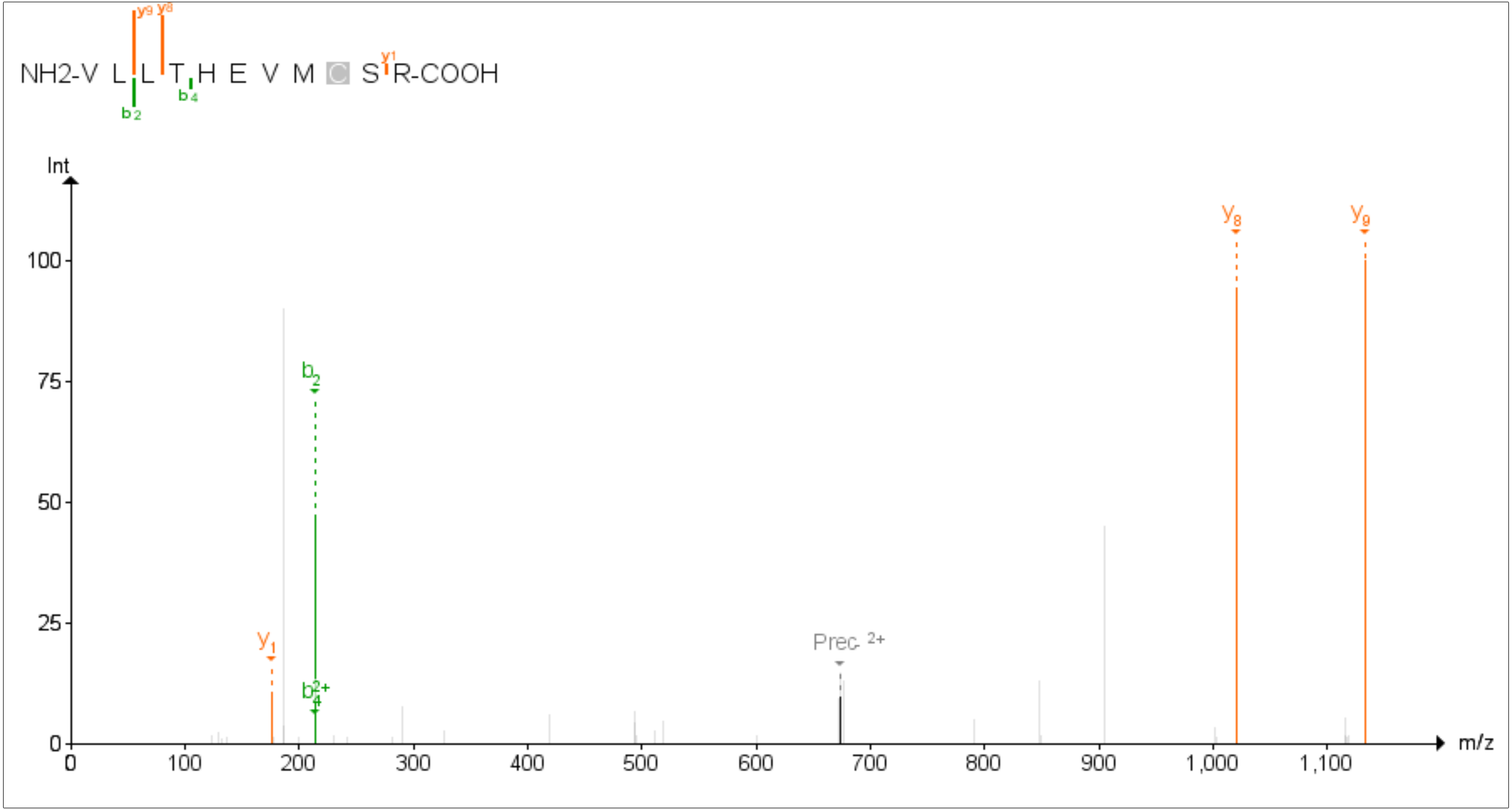

NH2-V L L T H E V M C S R-COOH
y9 y8
y1
b2
b4
Int
m/z
y8
y9
b2
y1
b4 2+
Prec. 2+
0
25
50
75
100
0
100
200
300
400
500
600
700
800
900
1,000
1,100

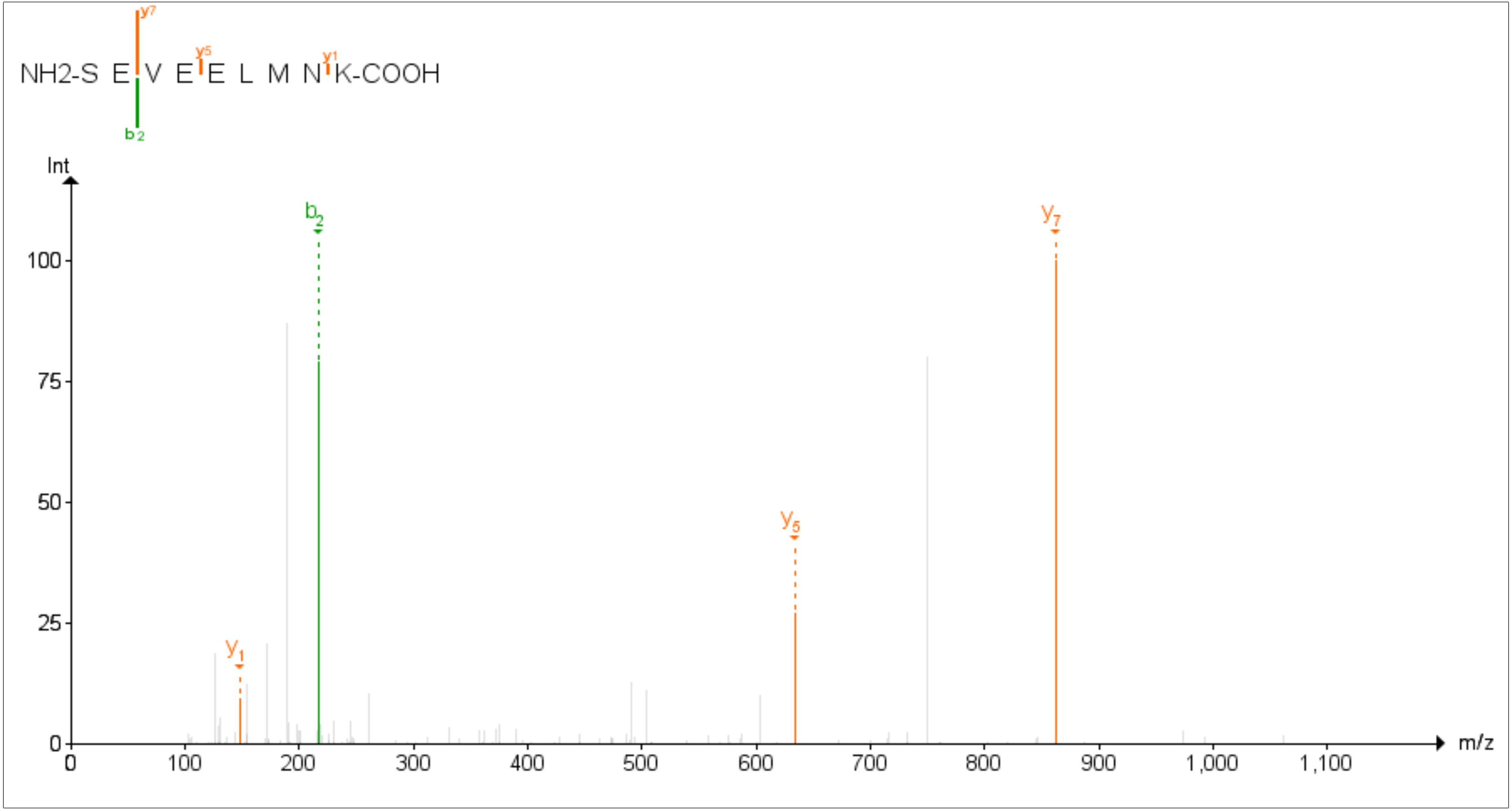

NH2-S E V E E L M N K-COOH
y7
y5
y1
b2
Int
m/z
0
25
50
75
100
0
100
200
300
400
500
600
700
800
900
1,000
1,100
b_2
y_7
y_5
y_1

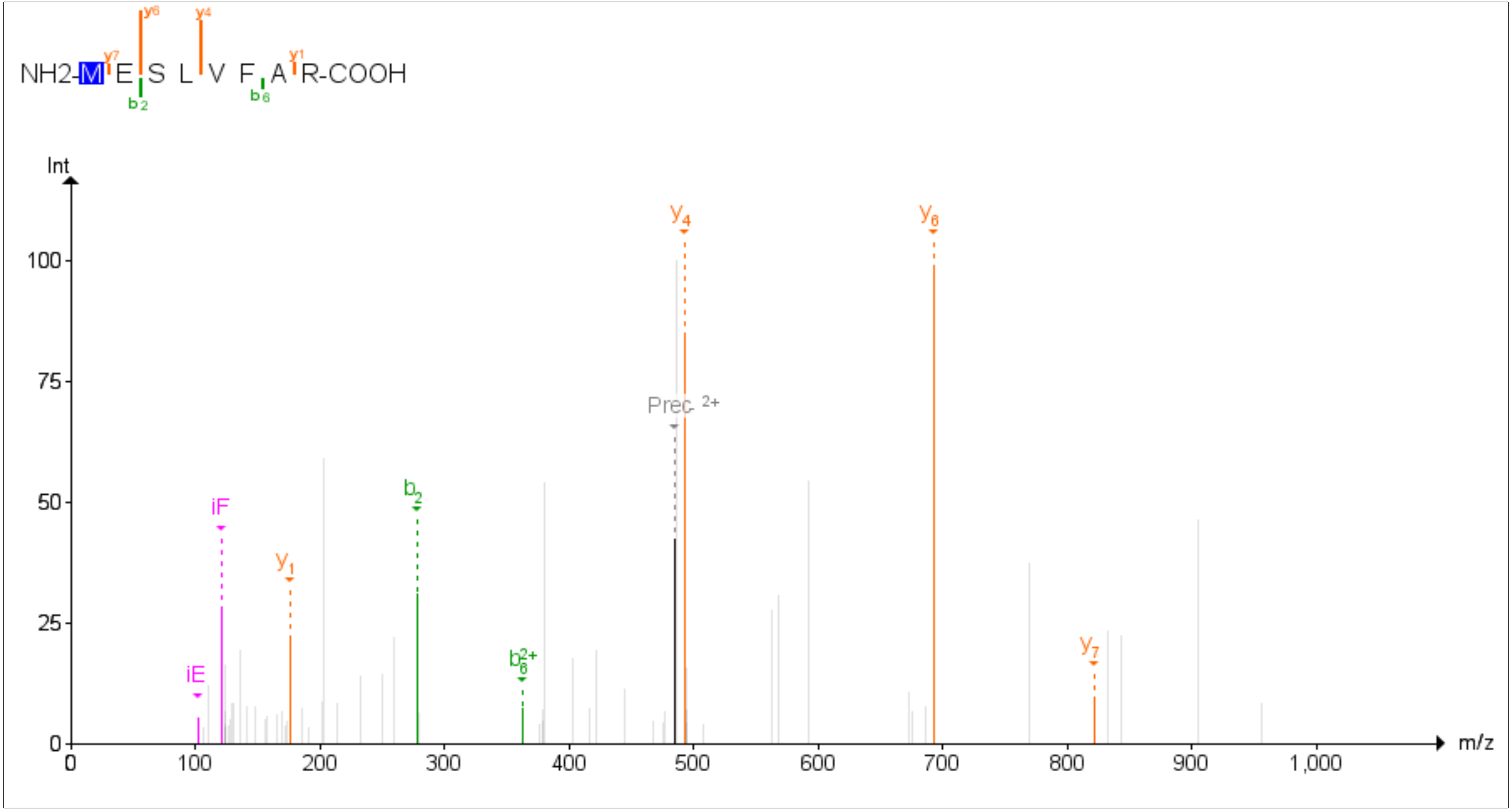

NH2-M E S L V F A R-COOH
y7
y6
y4
y1
b2
b6
Int
m/z
y4
y6
Prec. 2+
iF
b2
y1
iE
b6 2+
y7

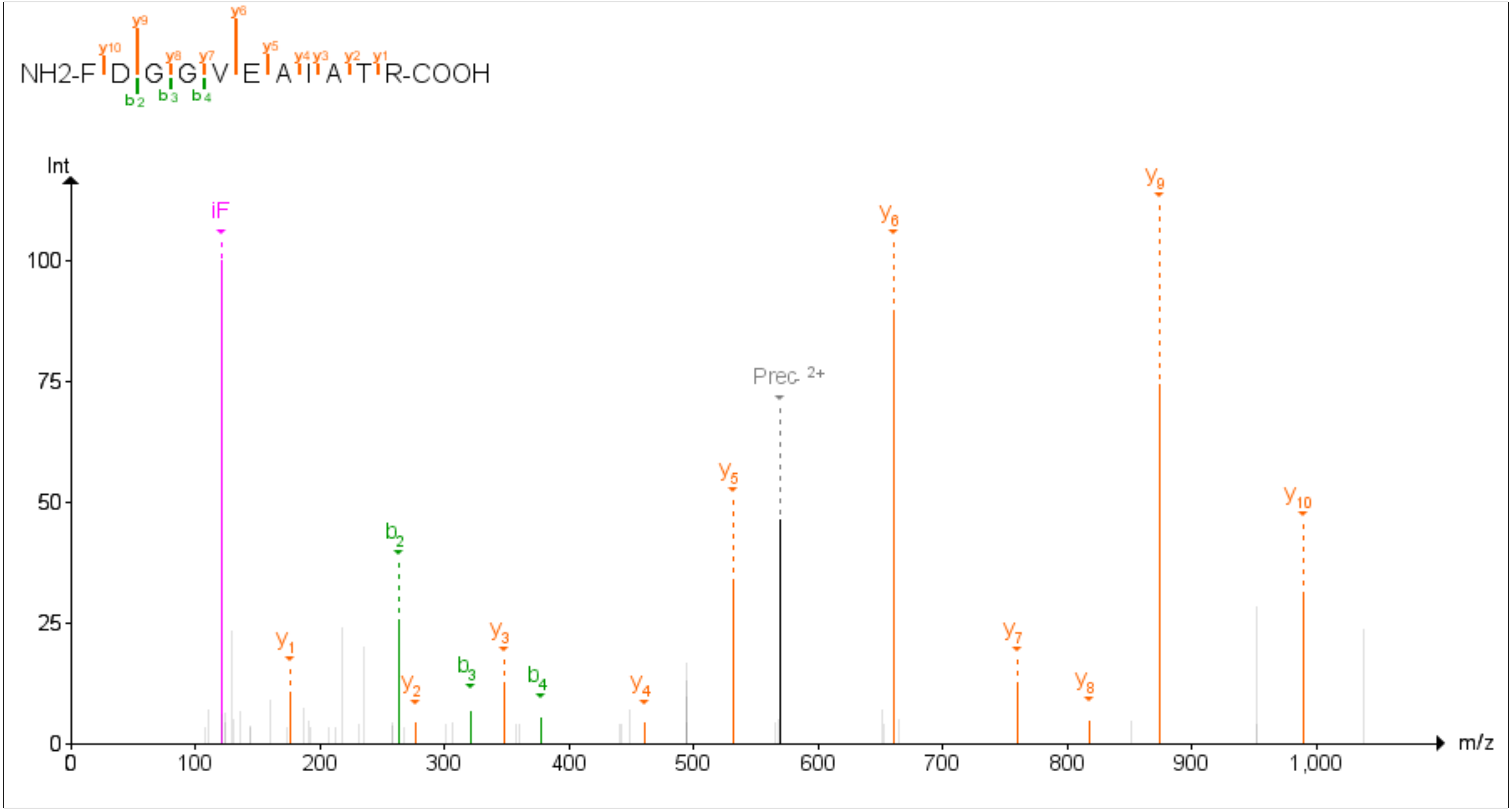

NH2-F D G G V E A I A T R-COOH
y10 y9 y8 y7 y6 y5 y4 y3 y2 y1
b2 b3 b4
Int
iF
Prec 2+
y1 y2 y3 y4 y5 y6 y7 y8 y9 y10
b2 b3 b4
0 25 50 75 100
0 100 200 300 400 500 600 700 800 900 1,000
m/z

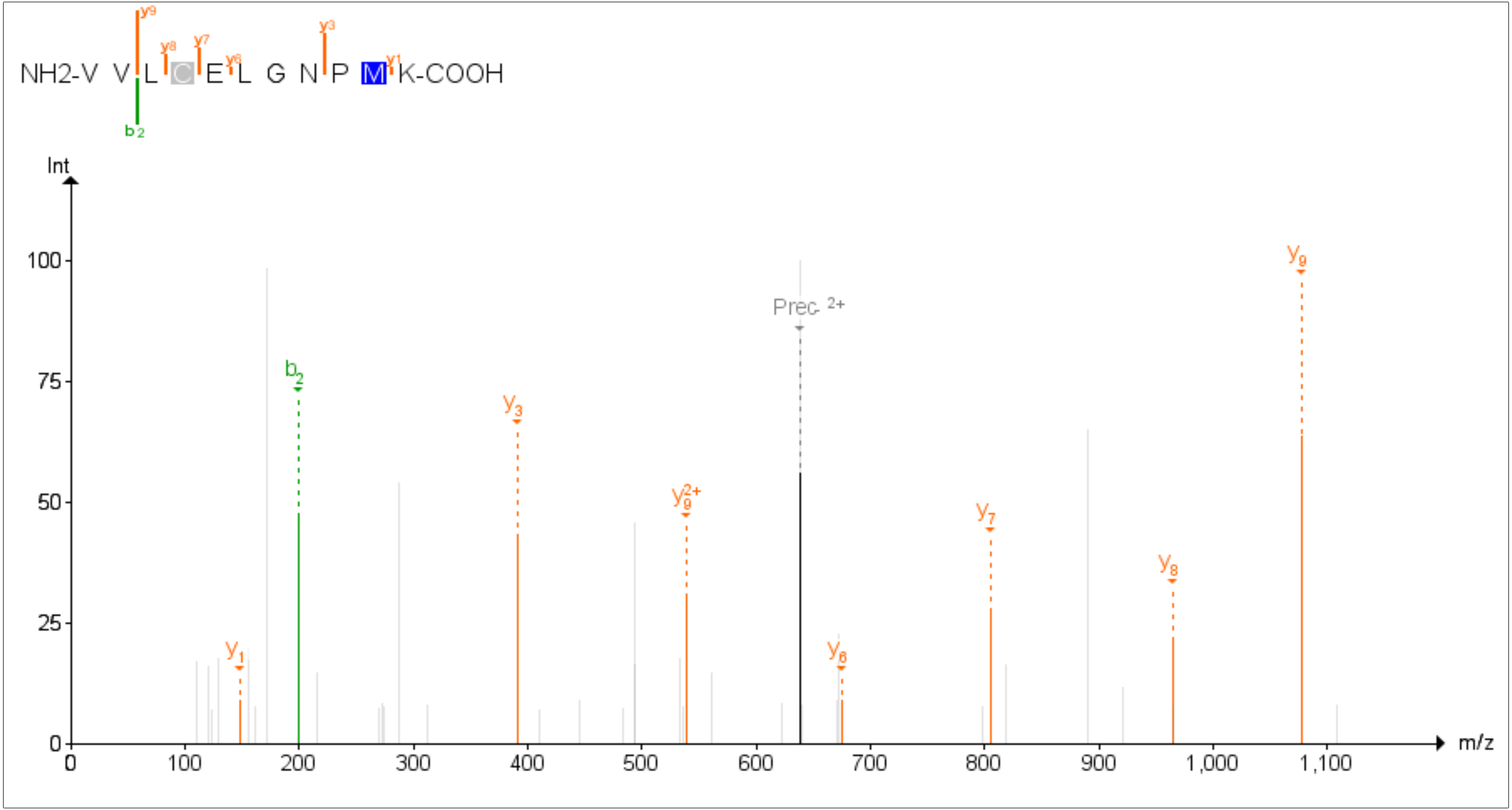
NH2-V V L C E L G N P M K-COOH
y9
y8
y7
y6
y3
y1
b 2
Int
m/z
Prec 2+
b_2
y_1
y_3
y_9^{2+}
y_6
y_7
y_8
y_9
0
25
50
75
100
0
100
200
300
400
500
600
700
800
900
1,000
1,100

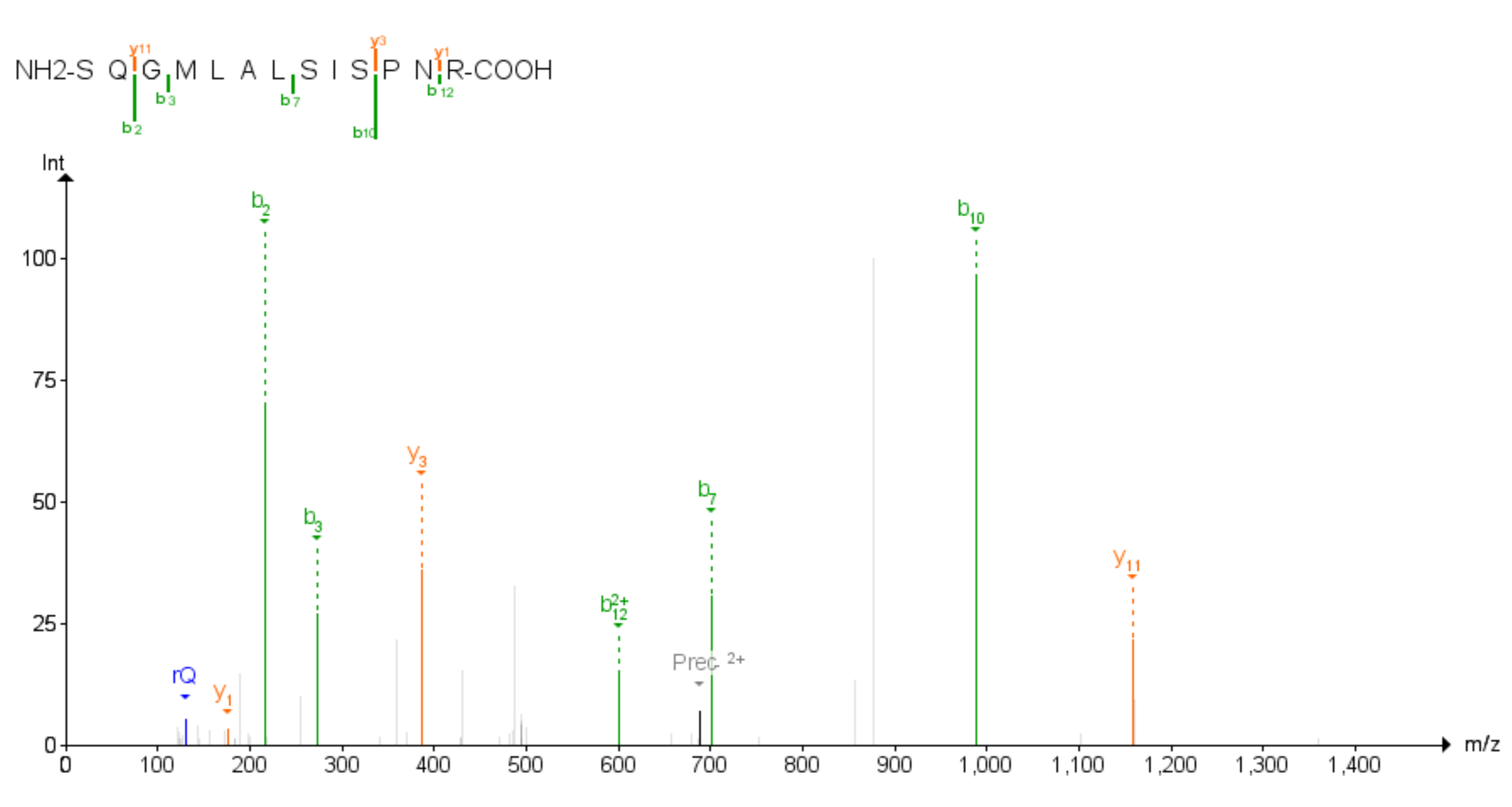
NH2-S Q G M L A L S I S P N R-COOH
y11
y3
y1
b 2
b 3
b 7
b10
b 12
Int
100
75
50
25
0
b2
b3
y3
b7
b10
y11
b2+ 12
Prec 2+
rQ
y1
0
100
200
300
400
500
600
700
800
900
1,000
1,100
1,200
1,300
1,400
m/z

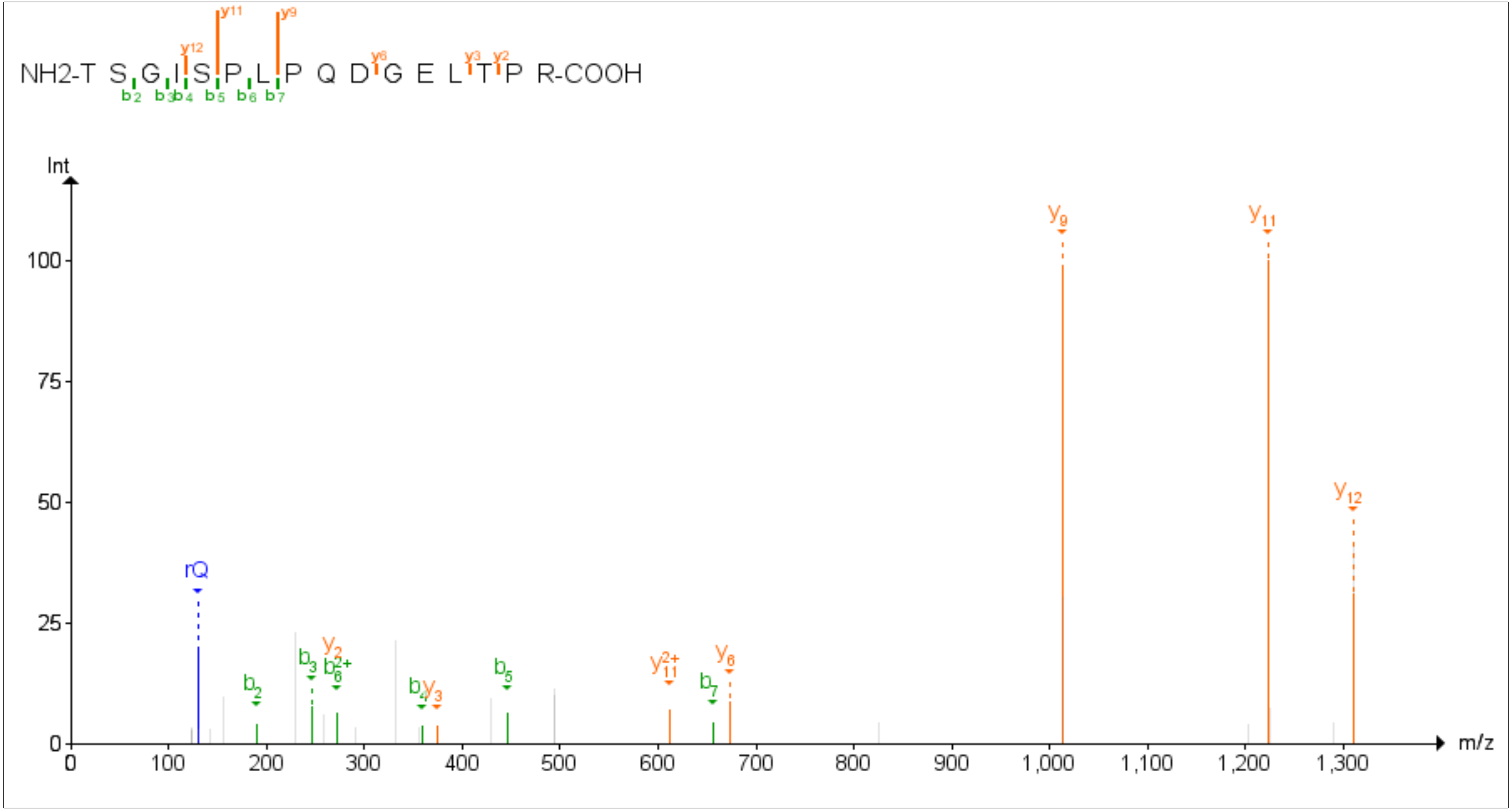

NH2-T S G I S P L P Q D G E L T P R-COOH
y11
y9
y12
y6
y3
y2
b2
b3
b4
b5
b6
b7
Int
100
75
50
25
0
m/z
0
100
200
300
400
500
600
700
800
900
1,000
1,100
1,200
1,300
rQ

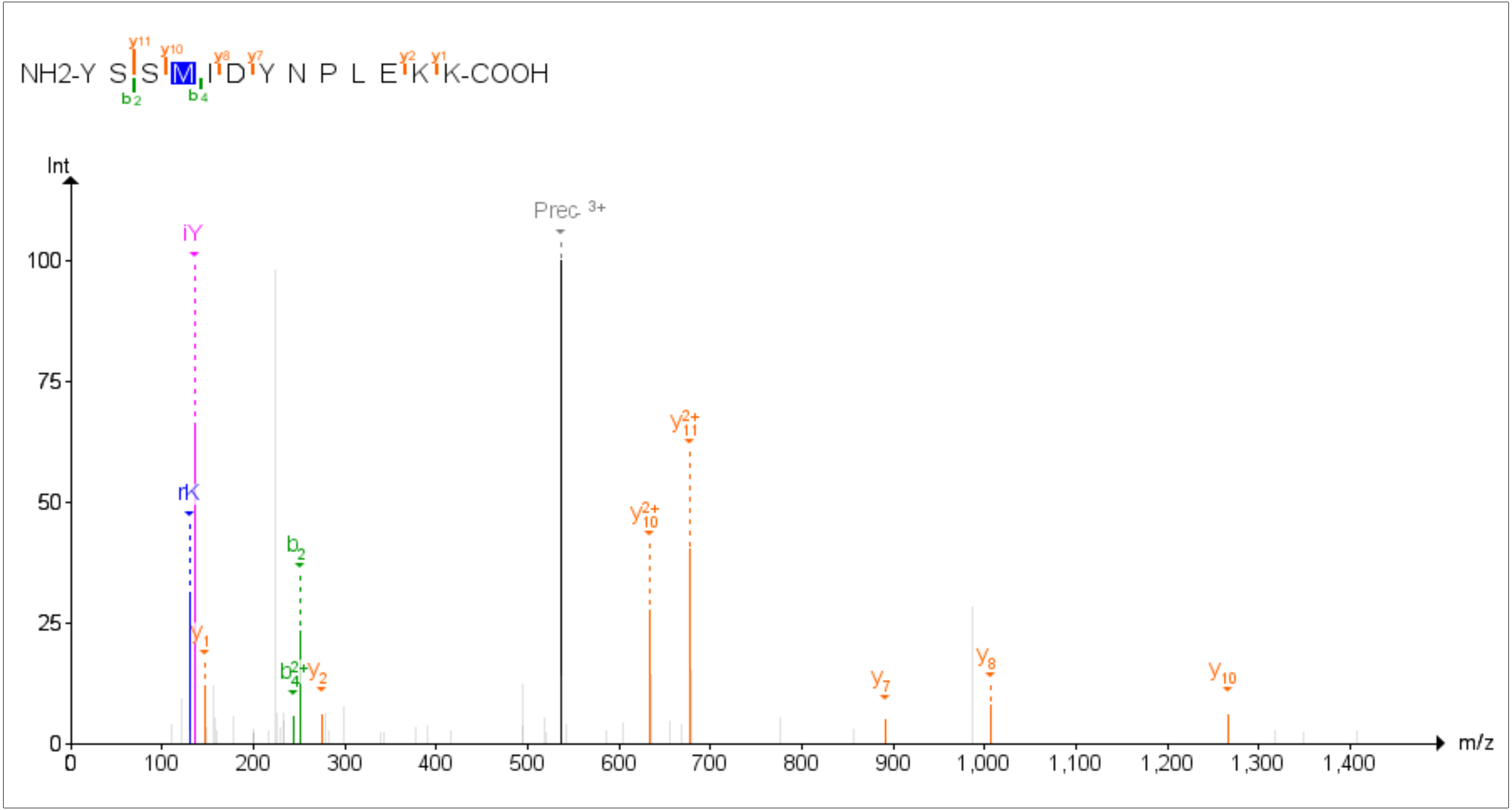

NH2-Y S S M I D Y N P L E K K-COOH
y11
y10
y8
y7
y2
y1
b2
b4
Int
m/z
Prec 3+
iY
rK
b2
y1
b4 2+
y2
y10 2+
y11 2+
y7
y8
y10
0
25
50
75
100
0
100
200
300
400
500
600
700
800
900
1,000
1,100
1,200
1,300
1,400

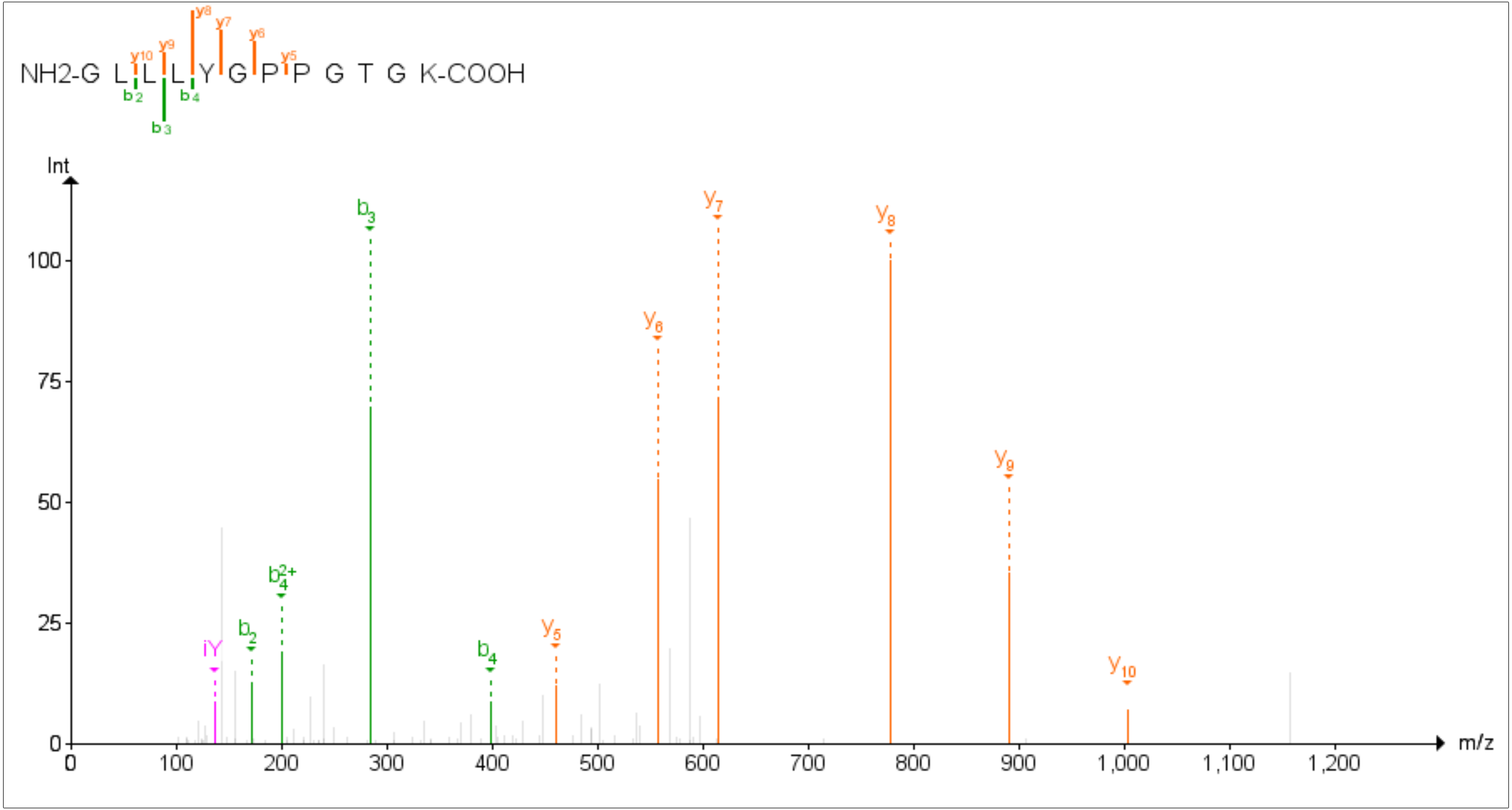

NH2-G L L L Y G P P G T G K-COOH
y10 y9 y8 y7 y6 y5
b2 b3 b4
Int
100
75
50
25
0
b3
y7
y8
y6
y9
b4 2+
b2
iY
b4
y5
y10
0 100 200 300 400 500 600 700 800 900 1,000 1,100 1,200
m/z

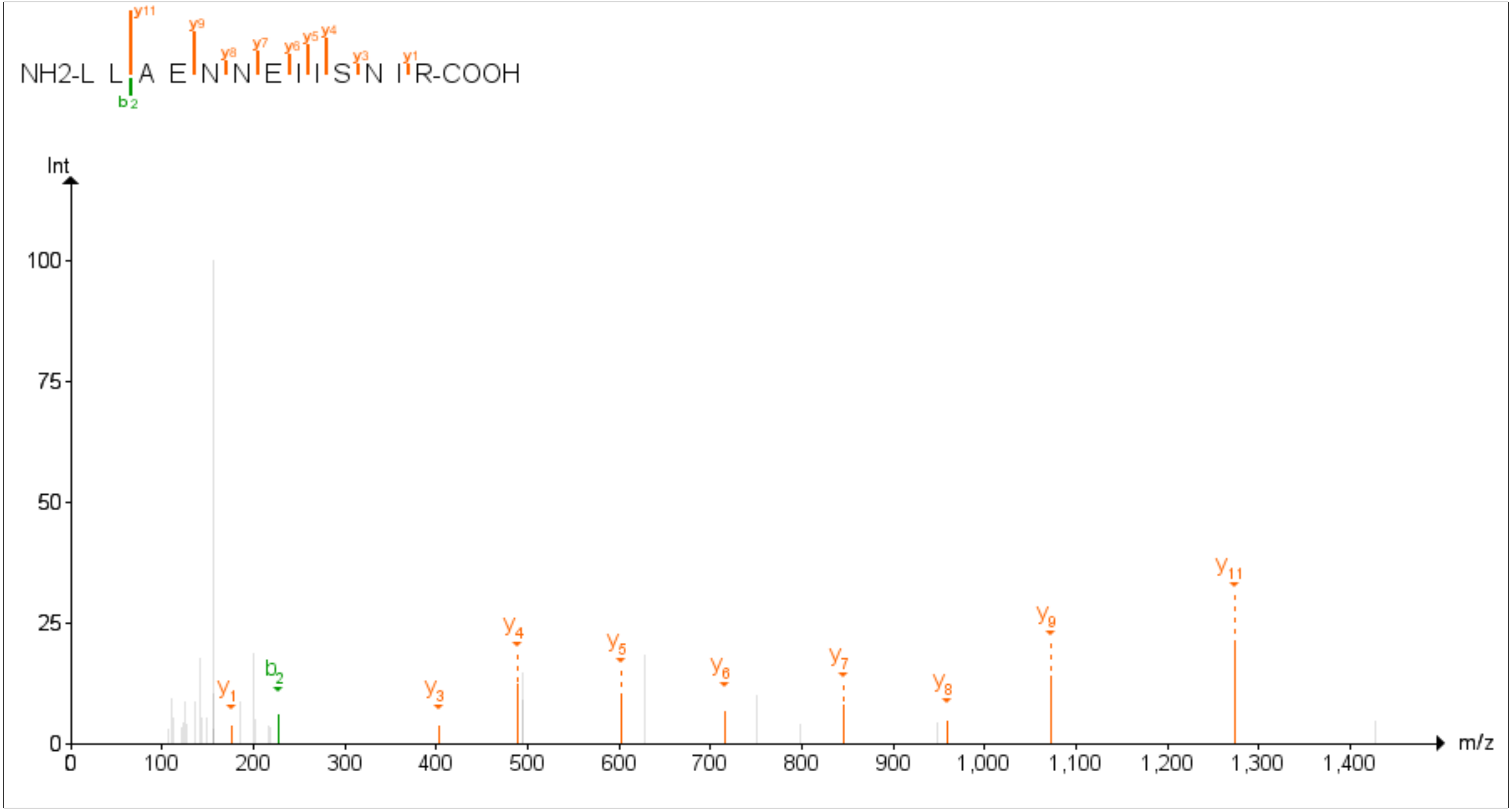

NH2-L L A E N N E I I S N I R-COOH
y11
y9
y8
y7
y6
y5
y4
y3
y1
b2
Int
100
75
50
25
0
m/z
0
100
200
300
400
500
600
700
800
900
1,000
1,100
1,200
1,300
1,400

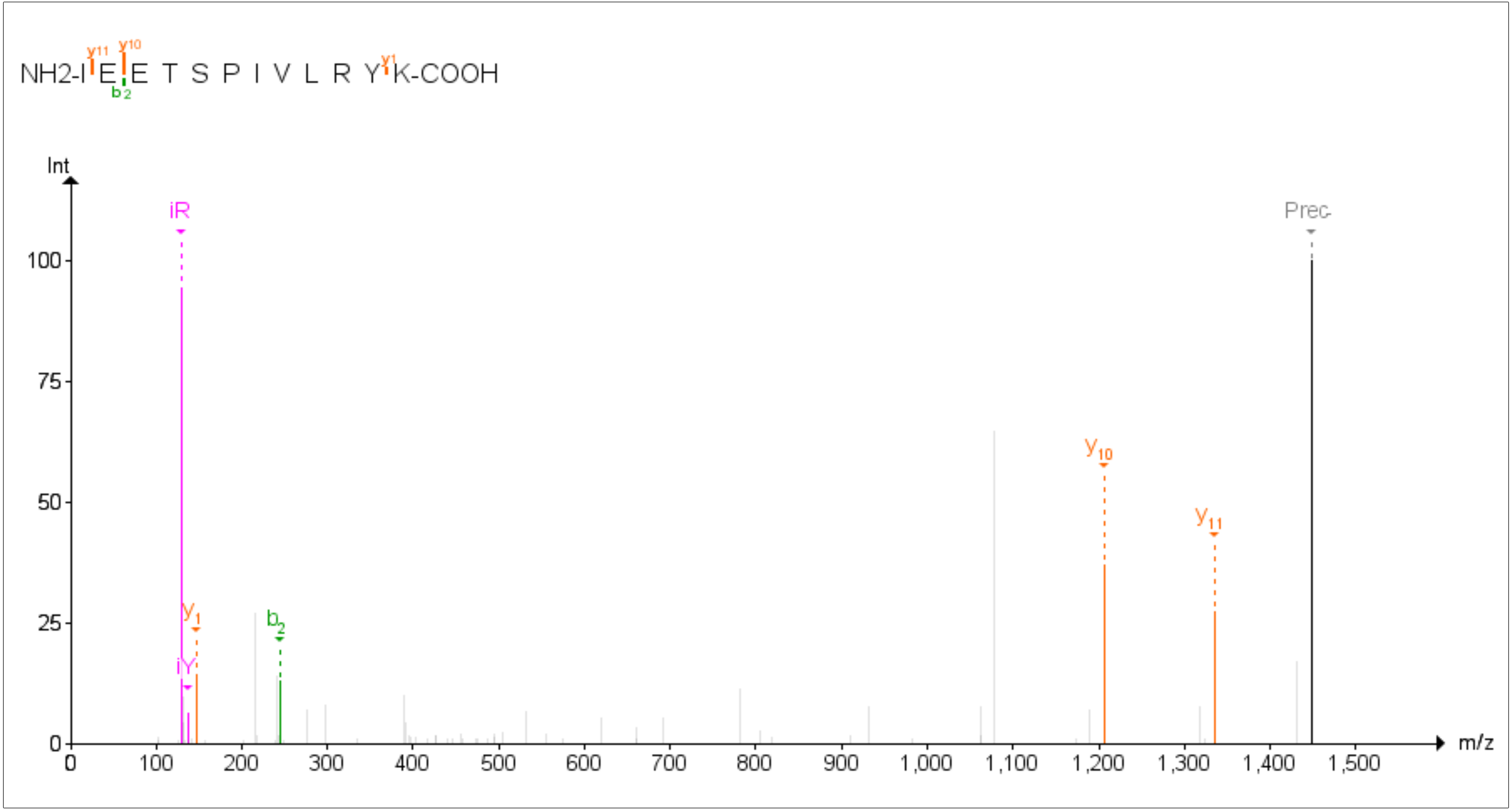

NH2-I E E T S P I V L R Y K-COOH
y11
y10
y1
b2
Int
iR
Prec
y10
y11
y1
b2
iY
100
75
50
25
0
0
100
200
300
400
500
600
700
800
900
1,000
1,100
1,200
1,300
1,400
1,500
m/z

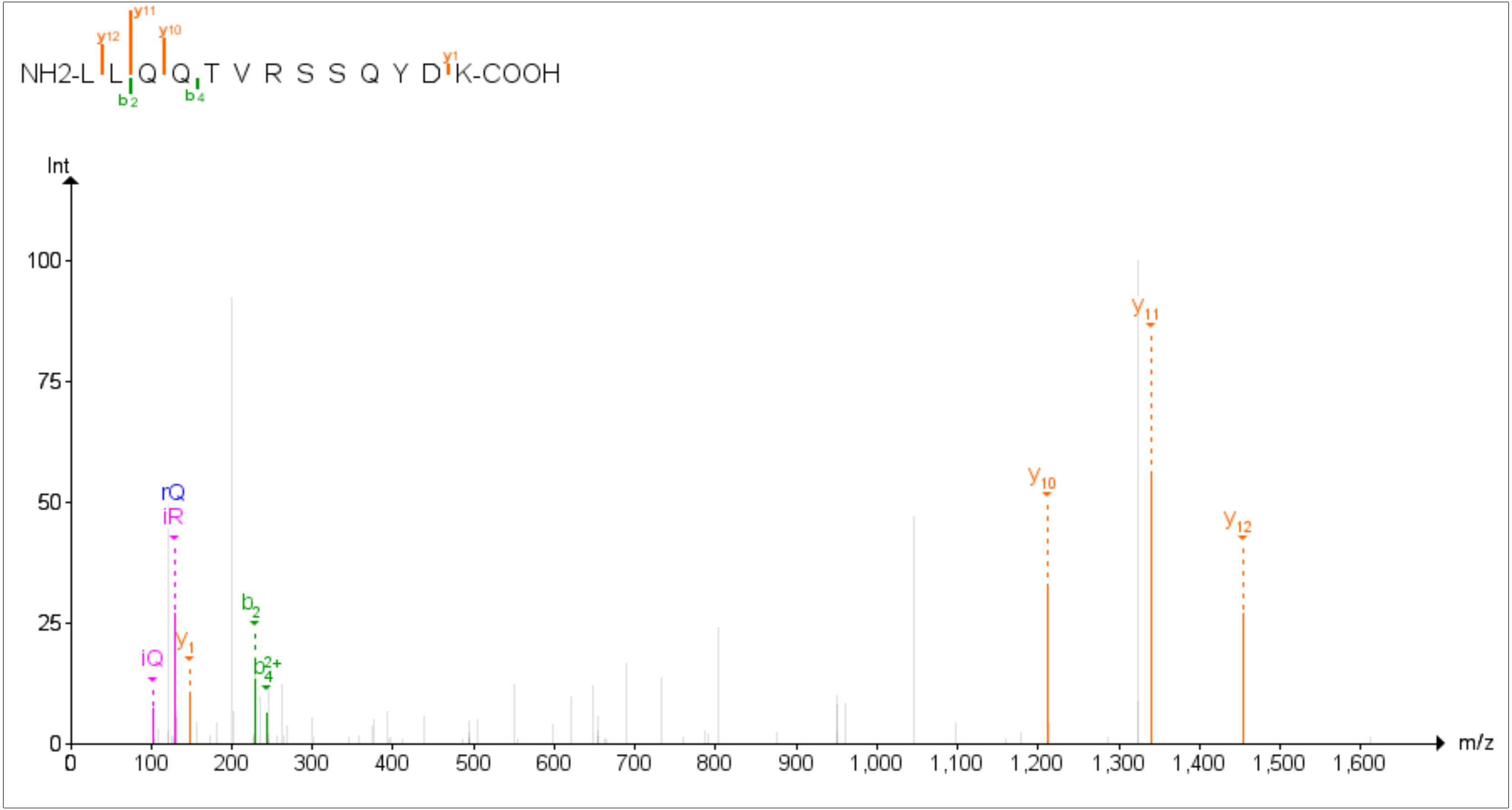

NH2-L L Q Q T V R S S Q Y D K-COOH
y12
y11
y10
y1
b2
b4
Int
m/z
rQ
iR
iQ
y1
b2
b4 2+
y10
y11
y12

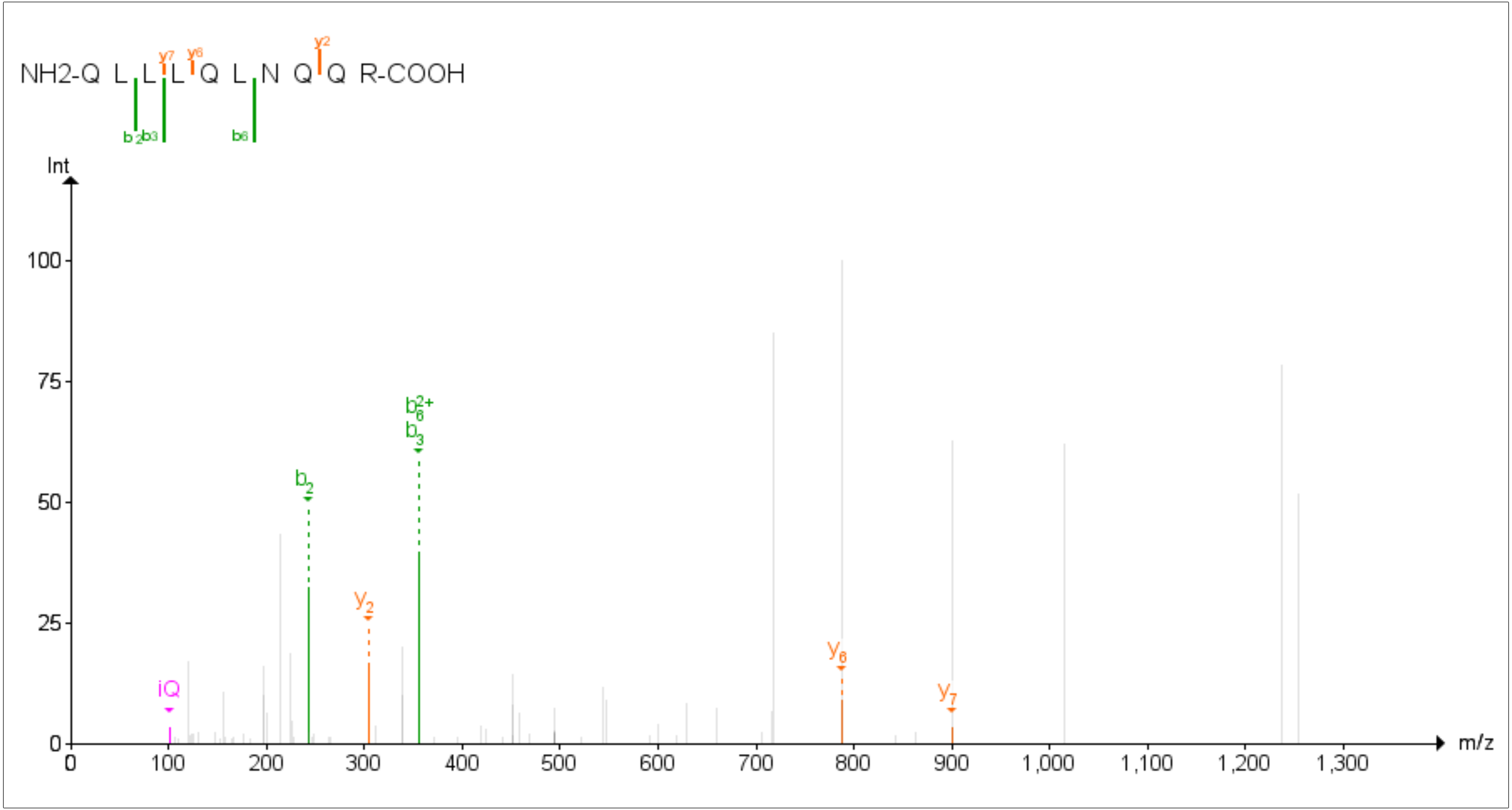
NH2-Q L L L Q L N Q Q R-COOH
y7
y6
y2
b2
b3
b6
Int
100
75
50
25
0
b6 2+
b3
b2
y2
iQ
y6
y7
0
100
200
300
400
500
600
700
800
900
1,000
1,100
1,200
1,300
m/z

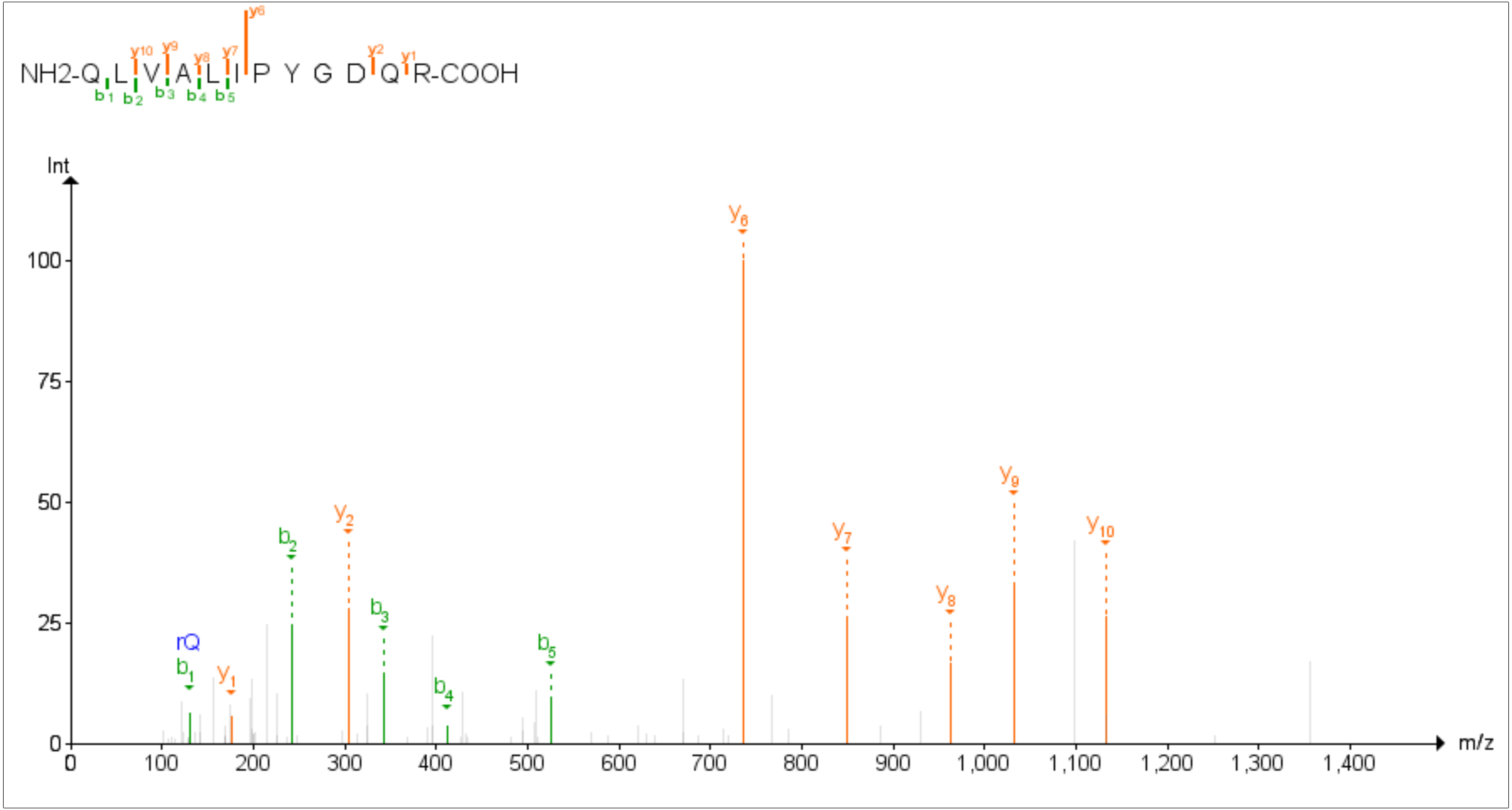

NH2-Q L V A L I P Y G D Q R-COOH
y10 y9 y8 y7 y6 y2 y1
b1 b2 b3 b4 b5
Int
100
75
50
25
0
y6
y9
y10
y7
y8
y2
b2
b3
b5
b4
rQ
b1
y1
0 100 200 300 400 500 600 700 800 900 1,000 1,100 1,200 1,300 1,400
m/z

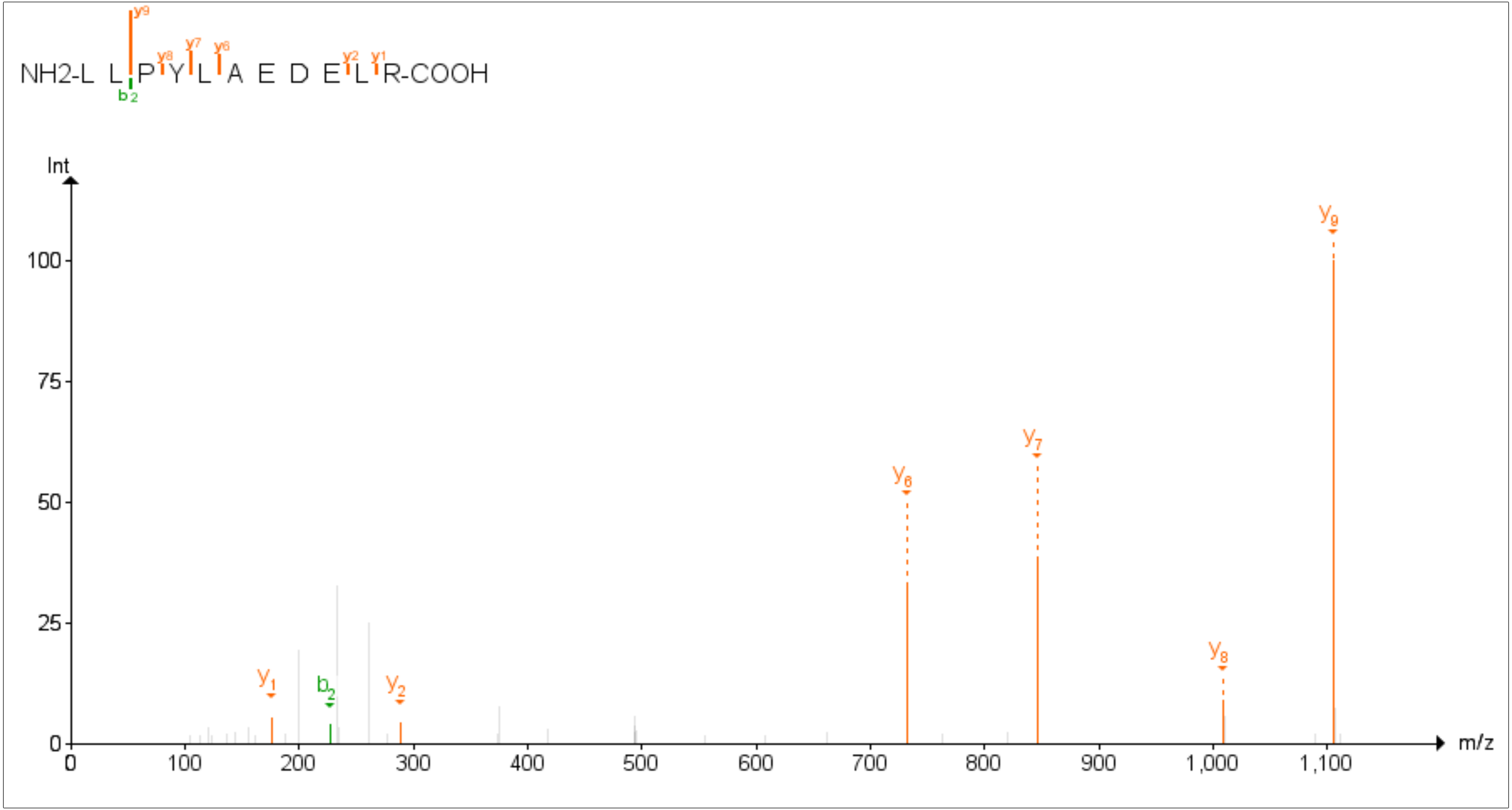
NH2-L L P Y L A E D E L R-COOH
y9
y8
y7
y6
y2
y1
b2
Int
100
75
50
25
0
y9
y7
y6
y8
y1
b2
y2
0
100
200
300
400
500
600
700
800
900
1,000
1,100
m/z

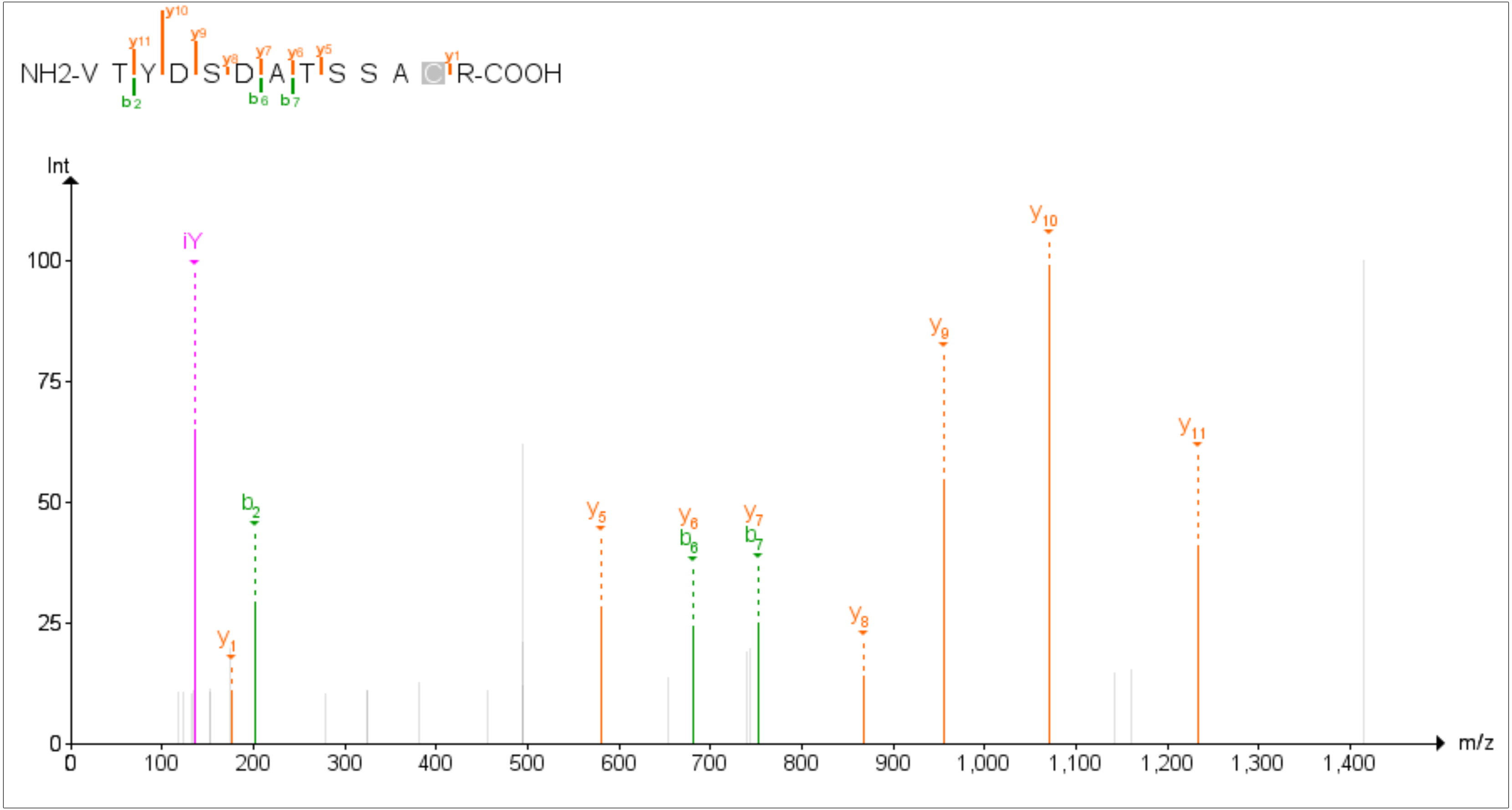

NH2-V T Y D S D A T S S A C R-COOH
y10
y11
y9
y8
y7
y6
y5
y1
b2
b6
b7
Int
iY
y10
y9
y11
b2
y5
y6
b6
y7
b7
y8
y1
100
75
50
25
0
0
100
200
300
400
500
600
700
800
900
1,000
1,100
1,200
1,300
1,400
m/z

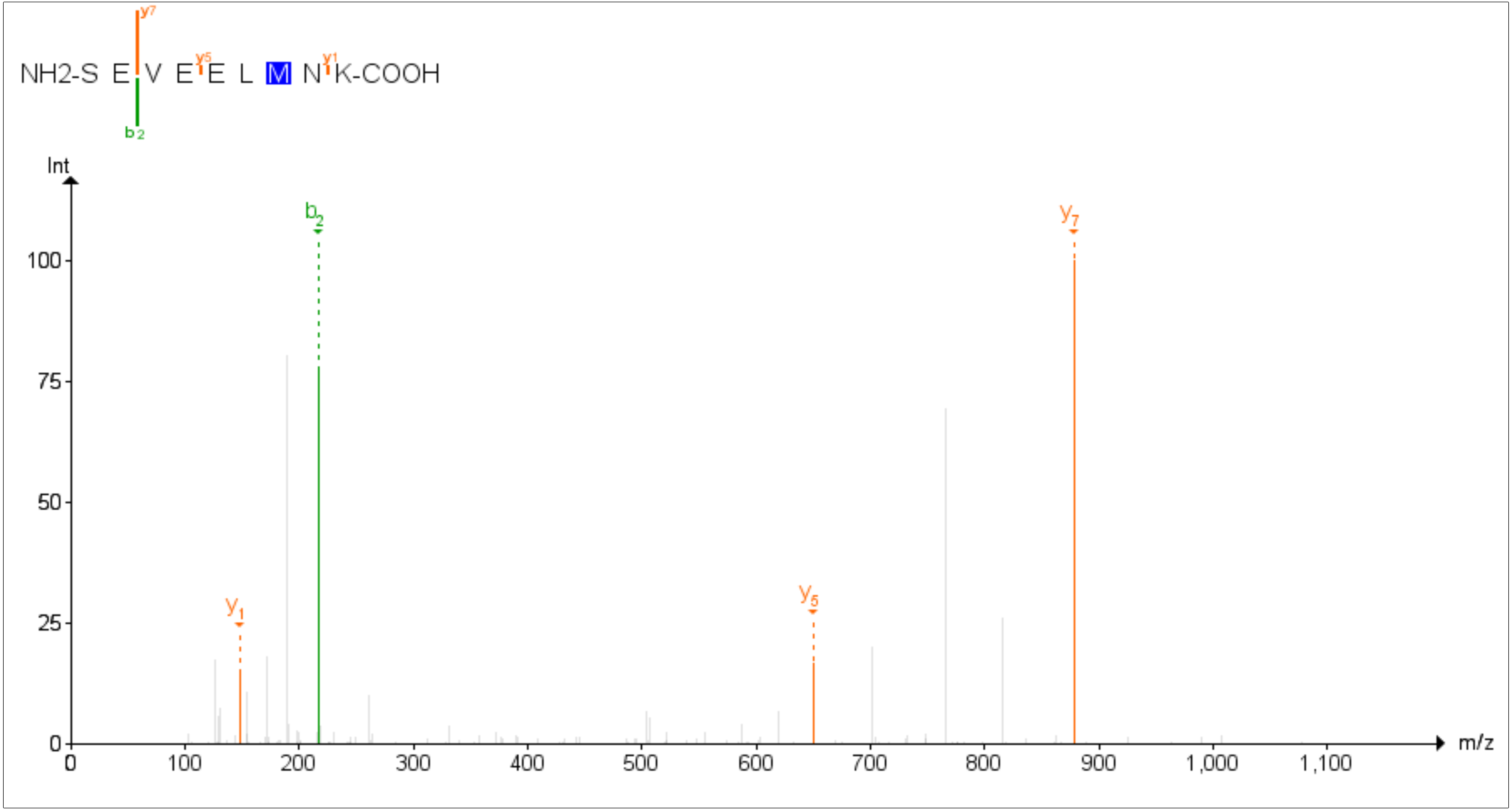

NH2-S E V E E L M N K-COOH
y7
y5
y1
b2
Int
100
75
50
25
0
b2
y7
y1
y5
m/z
0
100
200
300
400
500
600
700
800
900
1,000
1,100

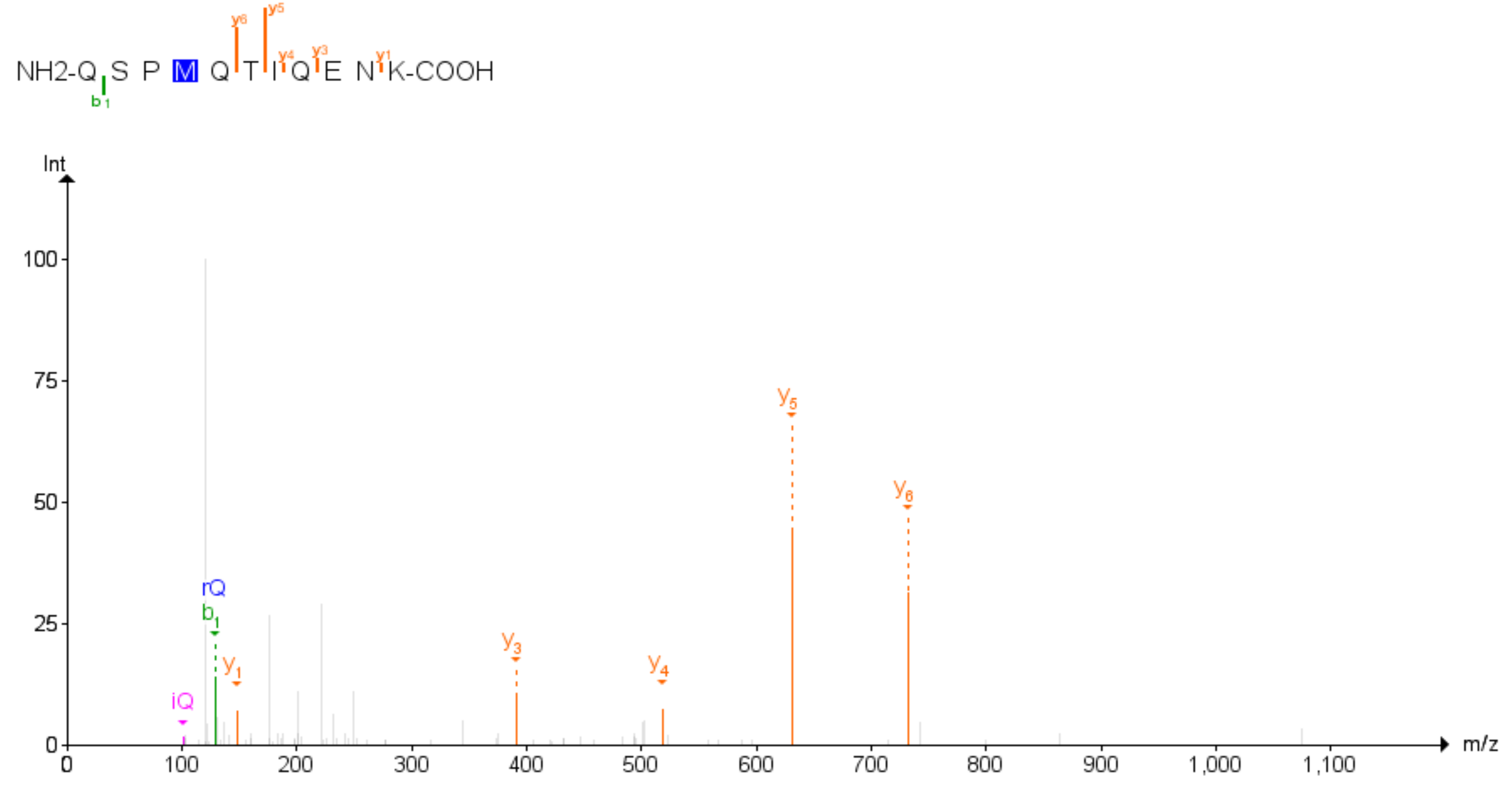

NH2-Q S P M Q T I Q E N K-COOH
b1
y6
y5
y4
y3
y1
Int
100
75
50
25
0
rQ
b1
iQ
y1
y3
y4
y5
y6
0
100
200
300
400
500
600
700
800
900
1,000
1,100
m/z

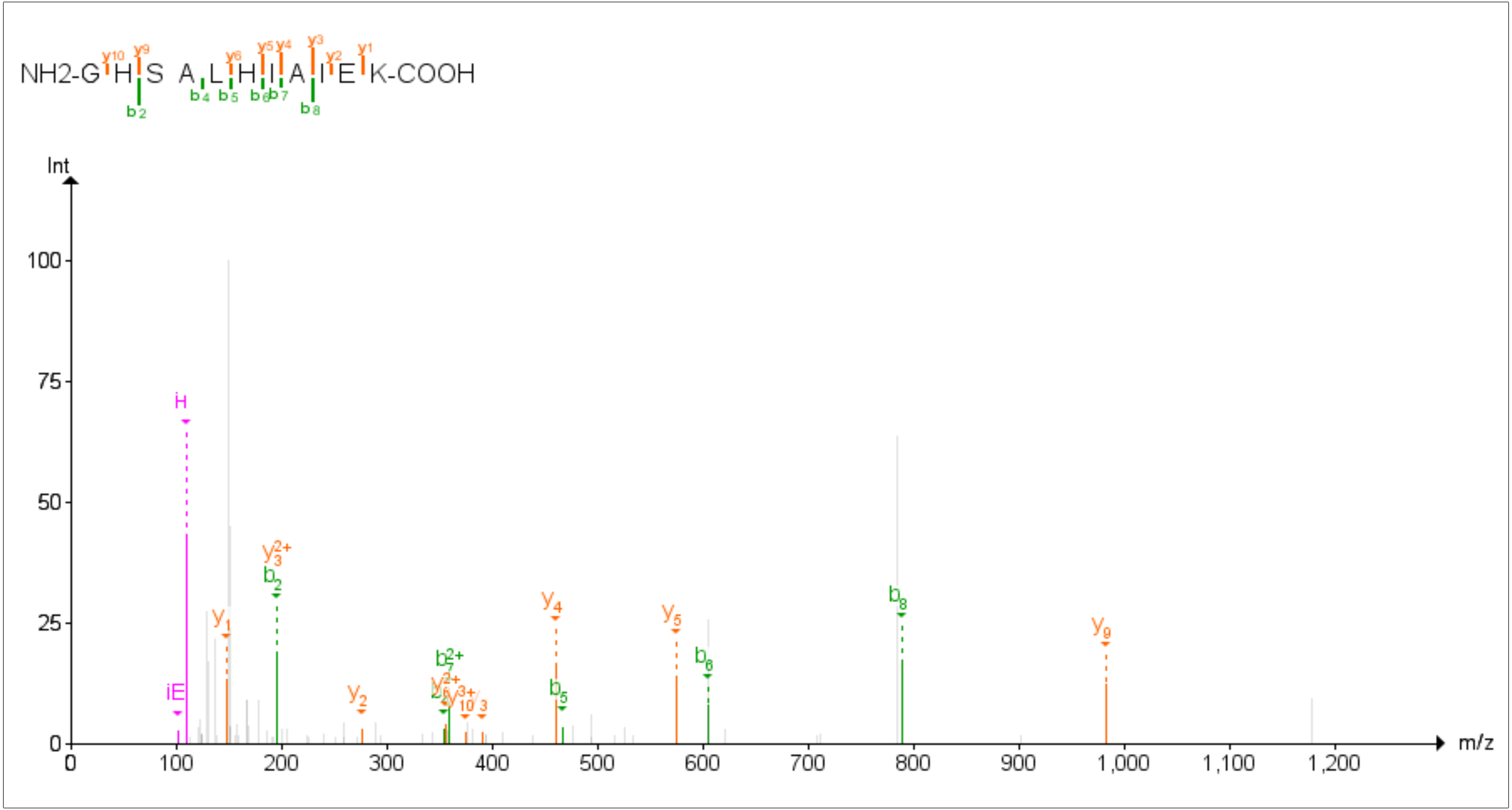

NH2-G H S A L H I A I E K-COOH
y10 y9 y6 y5 y4 y3 y2 y1
b2 b4 b5 b6 b7 b8
Int
100
75
50
25
0
iH
iE
y1
y_3^{2+}
b2
y2
b_7^{2+}
y4
b5
y5
b6
b8
y9
m/z
0
100
200
300
400
500
600
700
800
900
1,000
1,100
1,200

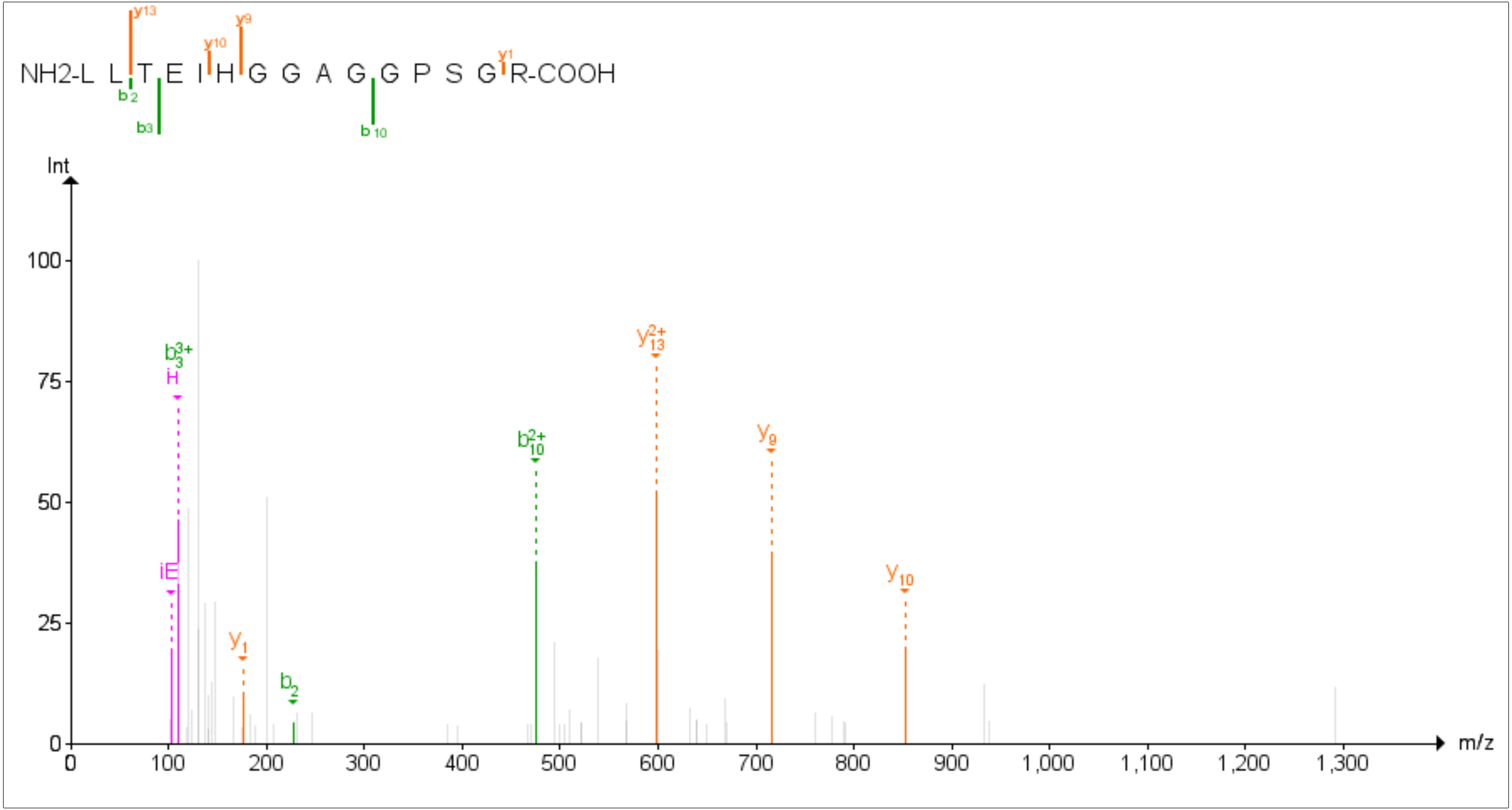

NH2-L L T E I H G G A G G P S G R-COOH
y13
y10
y9
y1
b2
b3
b10
Int
m/z
b_3^{3+}
iH
iE
y_1
b_2
b_{10}^{2+}
y_{13}^{2+}
y_9
y_{10}

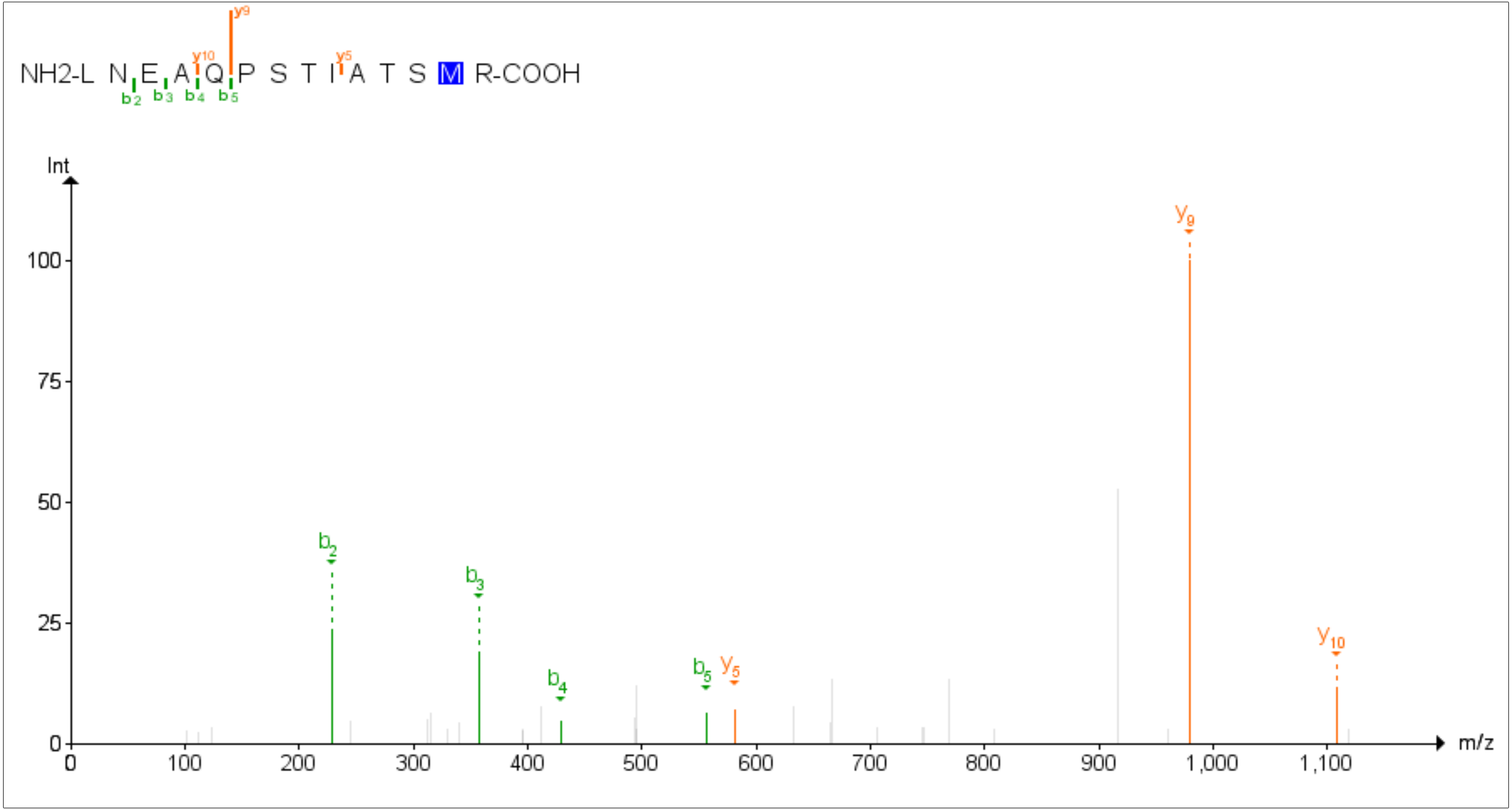

NH2-L N E A Q P S T I A T S M R-COOH
y9
y10
y5
b2
b3
b4
b5
Int
100
75
50
25
0
0
100
200
300
400
500
600
700
800
900
1,000
1,100
m/z
y9
b2
b3
b4
b5
y5
y10

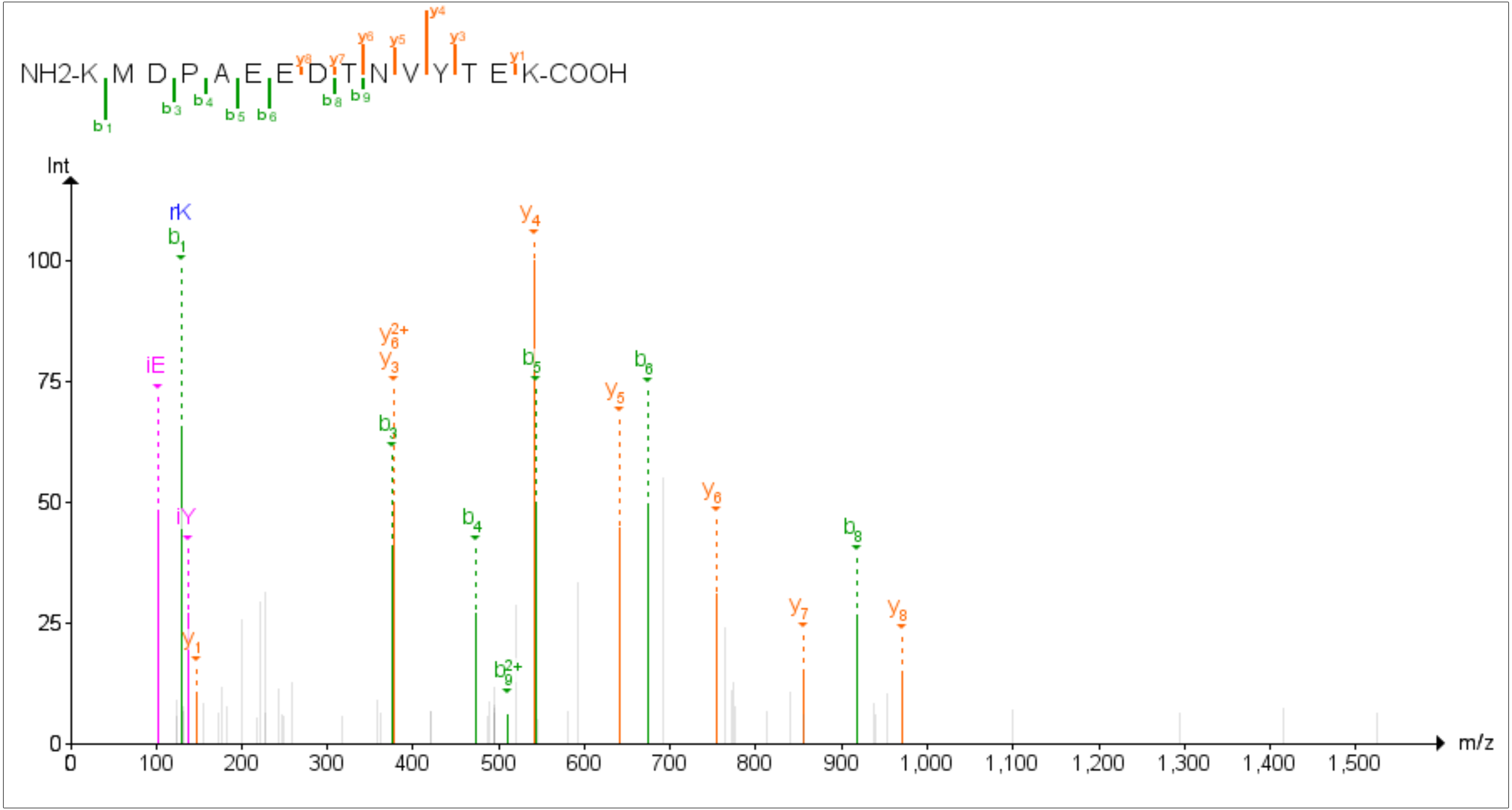

NH2-K M D P A E E D T N V Y T E K-COOH
y8 y7 y6 y5 y4 y3 y1
b1 b3 b4 b5 b6 b8 b9
Int
rK
b1
iE
iY
y1
y6 2+
y3
b3
b4
b9 2+
y4
b5
y5
b6
y6
y7
b8
y8
100
75
50
25
0
0 100 200 300 400 500 600 700 800 900 1,000 1,100 1,200 1,300 1,400 1,500
m/z

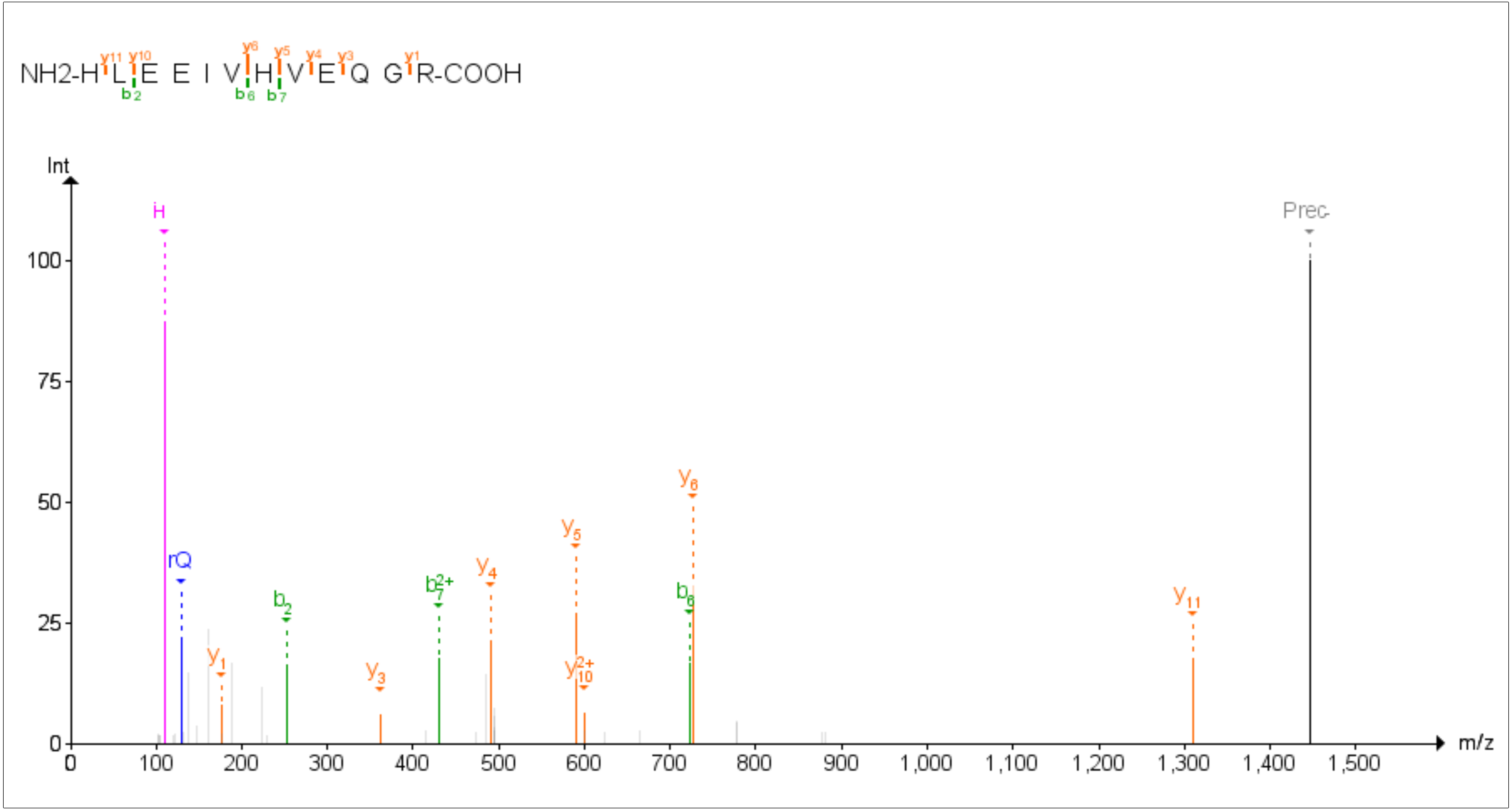
NH2-H L E E I V H V E Q G R-COOH
y11 y10 y6 y5 y4 y3 y1
b2 b6 b7
Int
H
rQ
y1
b2
y3
b7 2+
y4
y5
y10 2+
y6
b6
y11
Prec
100
75
50
25
0
0 100 200 300 400 500 600 700 800 900 1,000 1,100 1,200 1,300 1,400 1,500
m/z

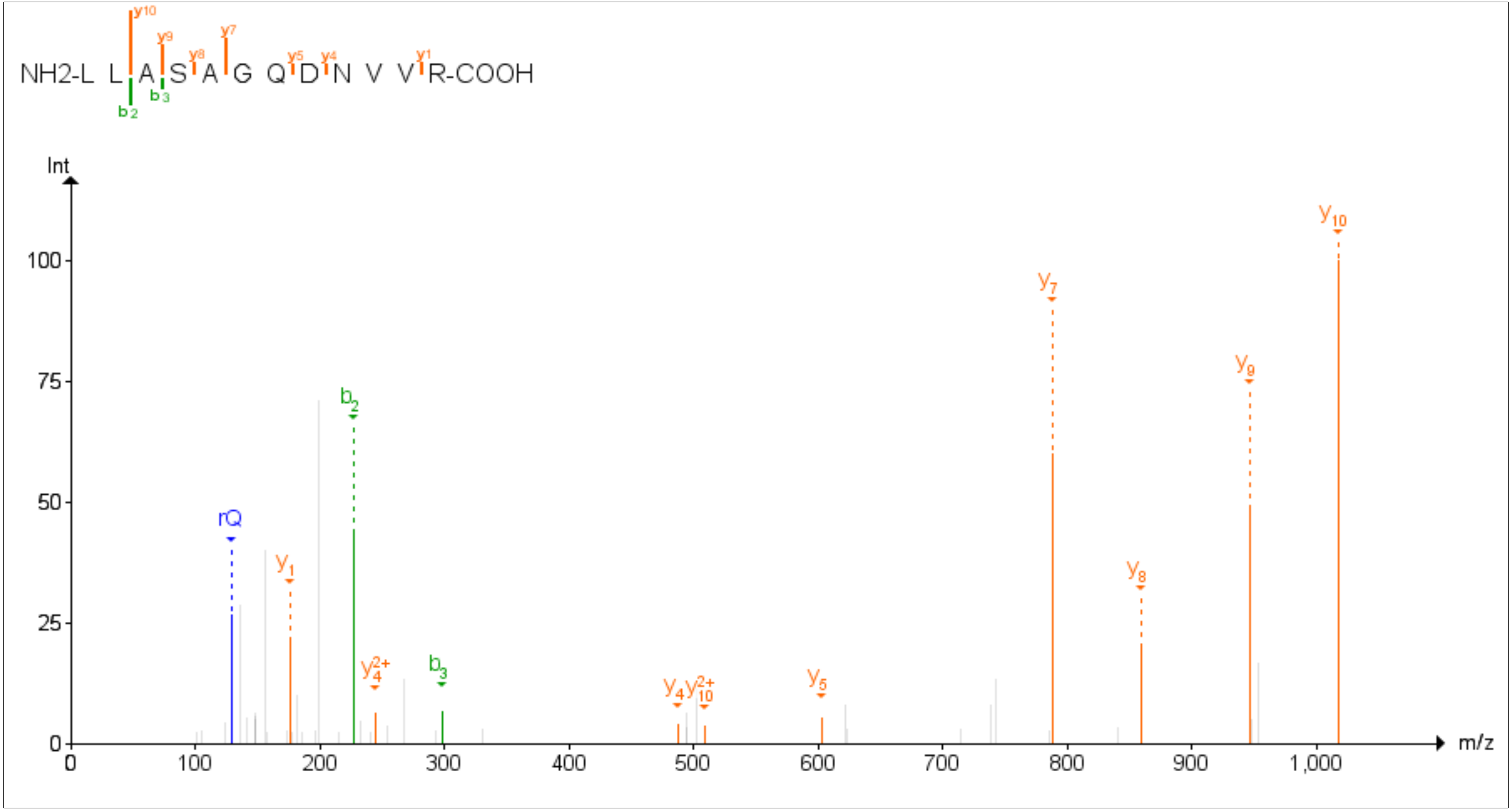

NH2-L L A S A G Q D N V V R-COOH
y10
y9
y8
y7
y5
y4
y1
b2
b3
Int
m/z
0
25
50
75
100
0
100
200
300
400
500
600
700
800
900
1,000
rQ
y1
b2
y_4^{2+}
b3
y4
y_{10}^{2+}
y5
y7
y8
y9
y10

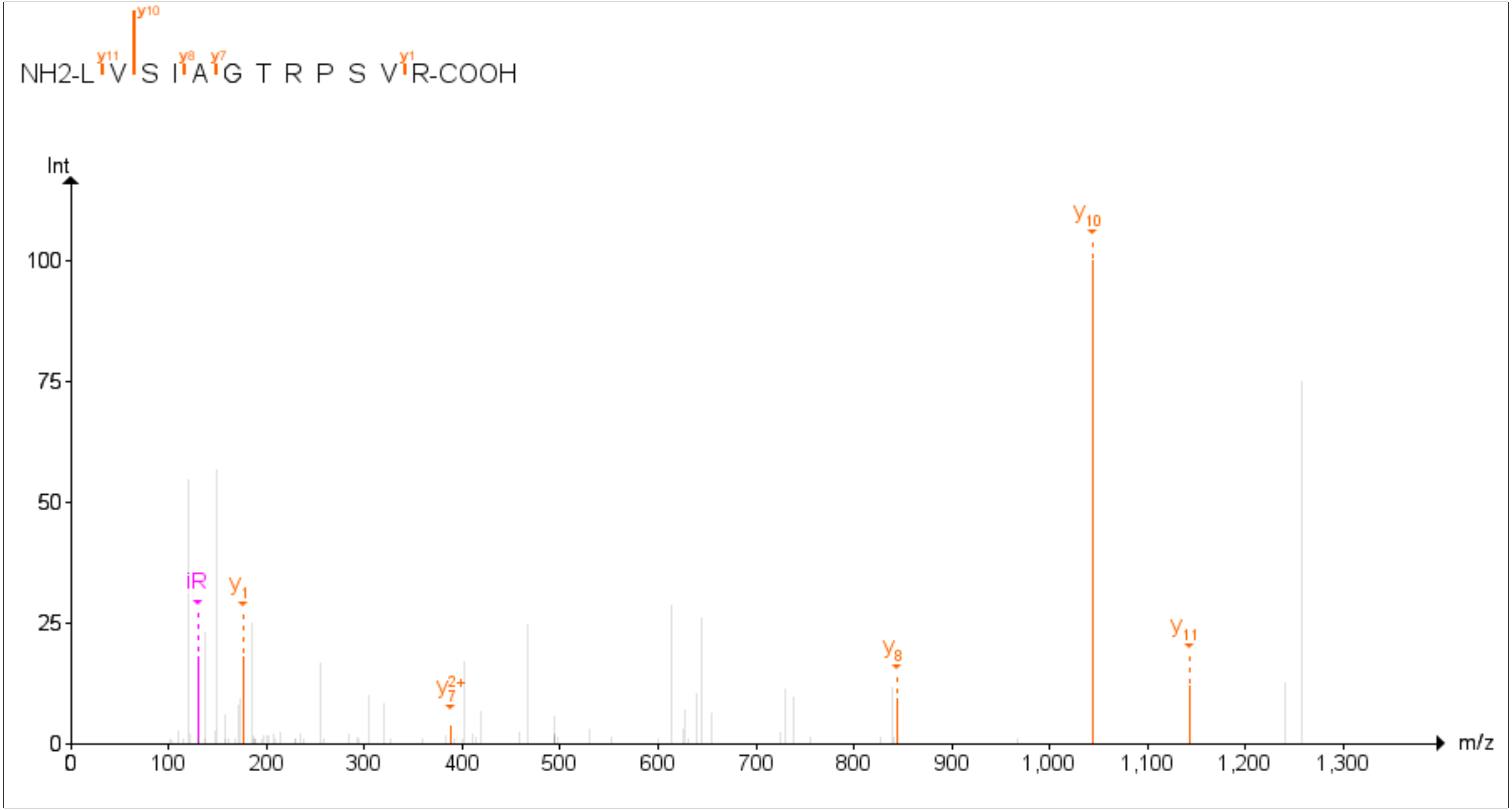

NH2-L V S I A G T R P S V R-COOH
y11 y10 y8 y7 y1
Int
100
75
50
25
0
iR
y1
y7 2+
y8
y10
y11
0 100 200 300 400 500 600 700 800 900 1,000 1,100 1,200 1,300
m/z

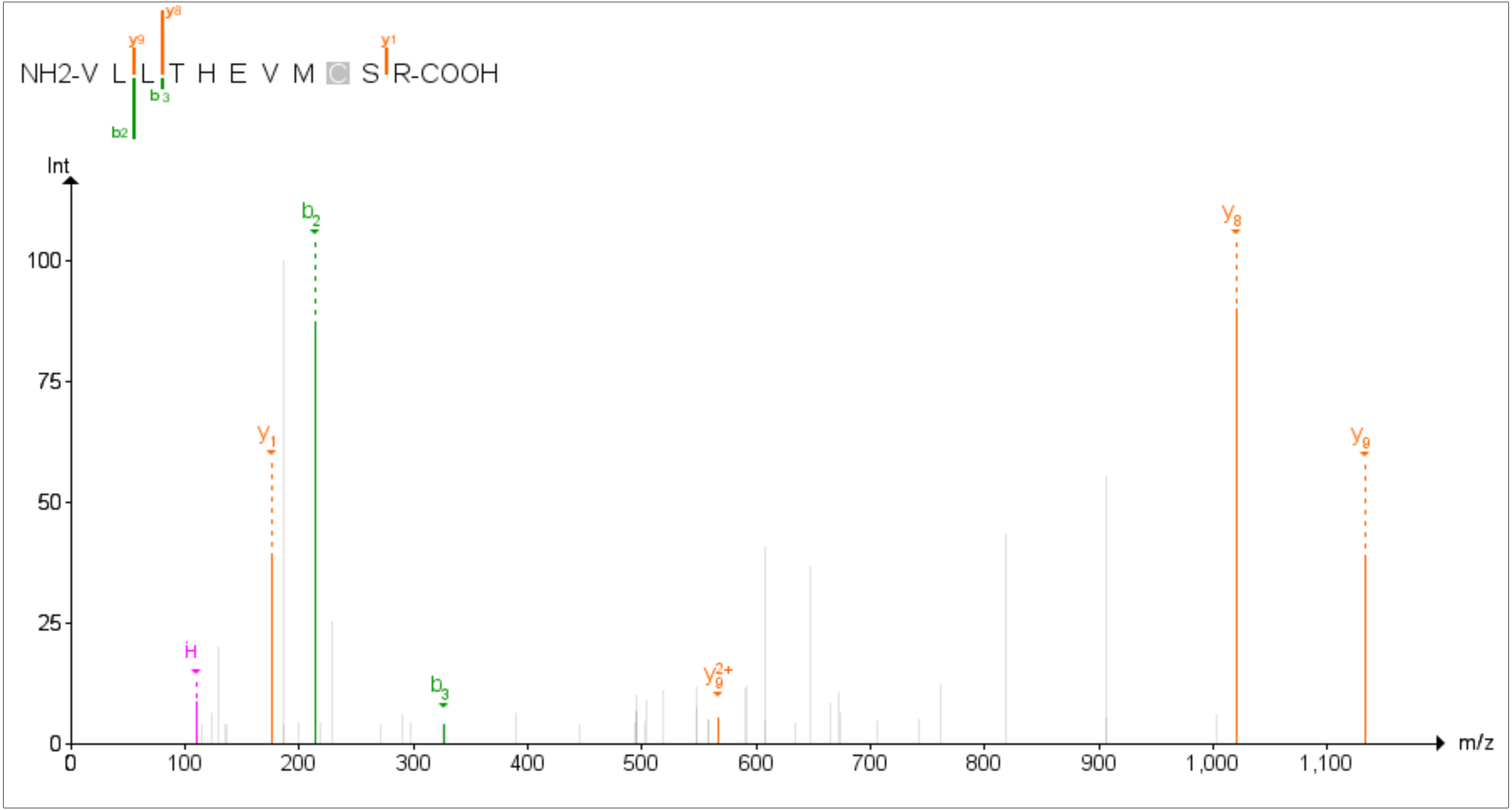

NH2-V L L T H E V M C S R-COOH
y9
y8
y1
b2
b3
Int
100
75
50
25
0
b2
y8
y1
y9
H
b3
y9 2+
0
100
200
300
400
500
600
700
800
900
1,000
1,100
m/z

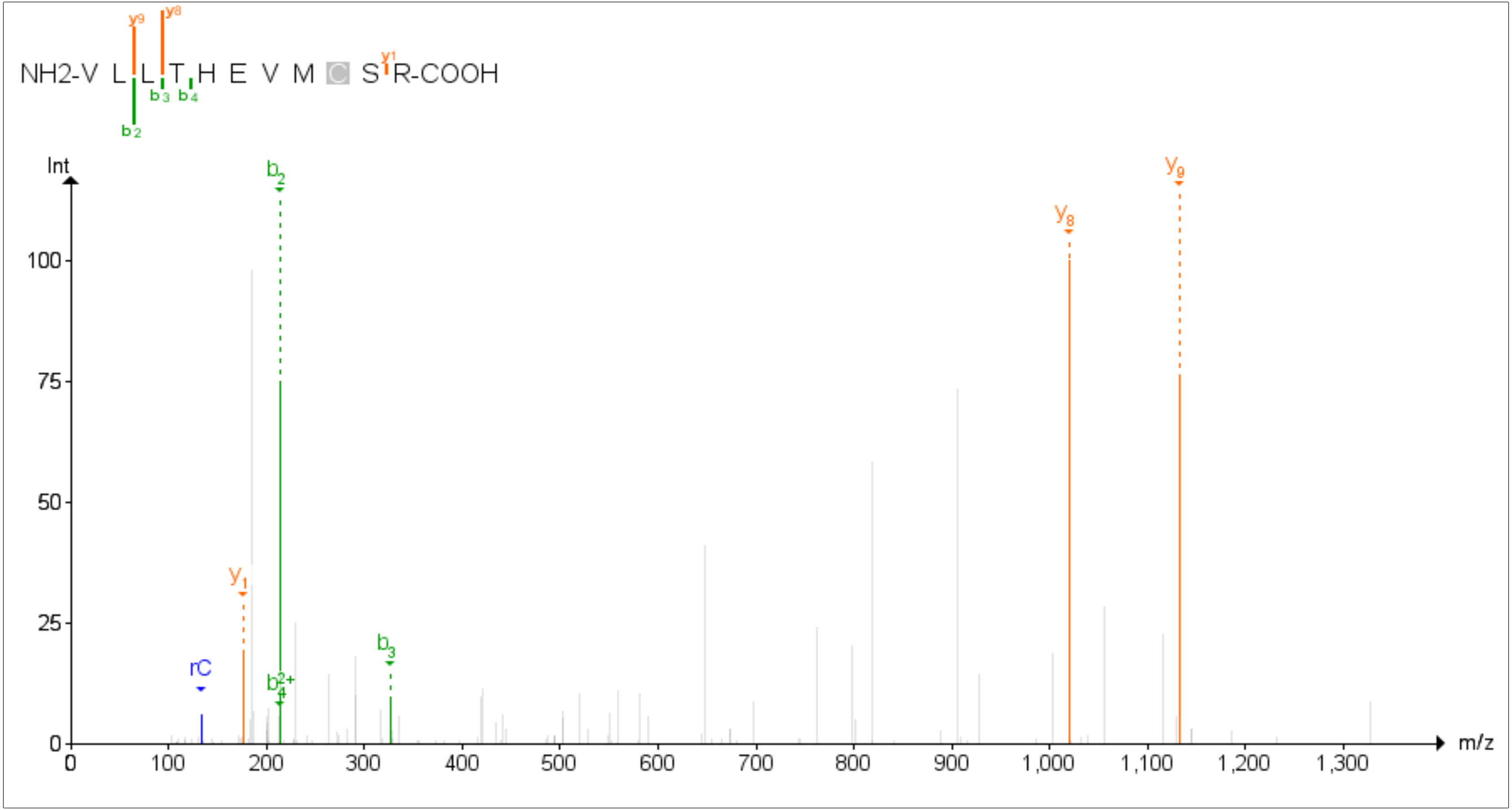

NH2-V L L T H E V M C S R-COOH
y9
y8
y1
b2
b3
b4
Int
m/z
100
75
50
25
0
100
200
300
400
500
600
700
800
900
1,000
1,100
1,200
1,300
rC
y1
b2
b3
b4 2+
y8
y9

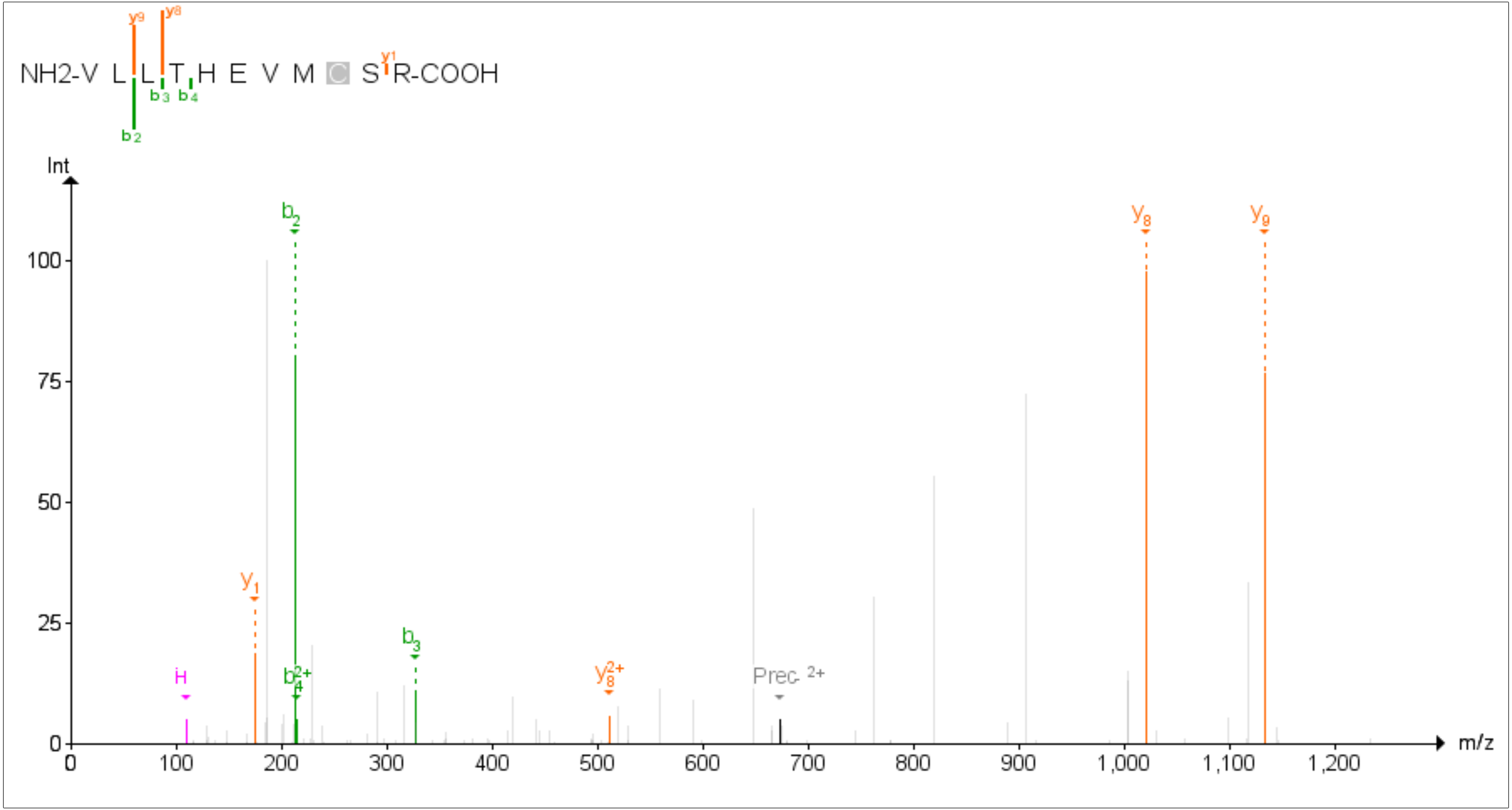

NH2-V L L T H E V M C S R-COOH
y9
y8
y1
b2
b3
b4
Int
100
75
50
25
0
b2
y8
y9
y1
H
b4 2+
b3
y8 2+
Prec 2+
0
100
200
300
400
500
600
700
800
900
1,000
1,100
1,200
m/z

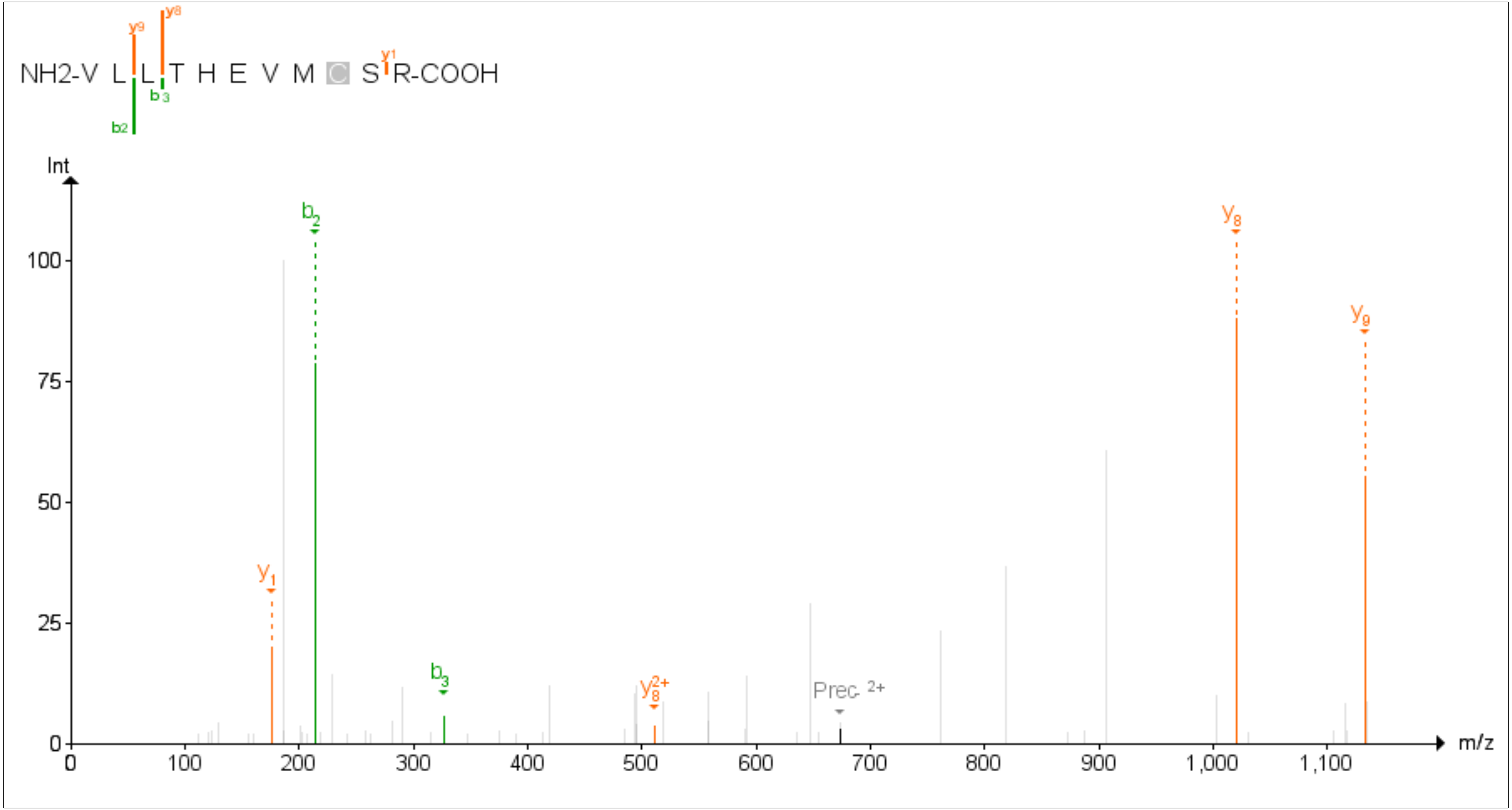
NH2-V L L T H E V M C S R-COOH
y9
y8
y1
b2
b3
Int
100
75
50
25
0
b2
y8
y9
y1
b3
y8 2+
Prec. 2+
0
100
200
300
400
500
600
700
800
900
1,000
1,100
m/z

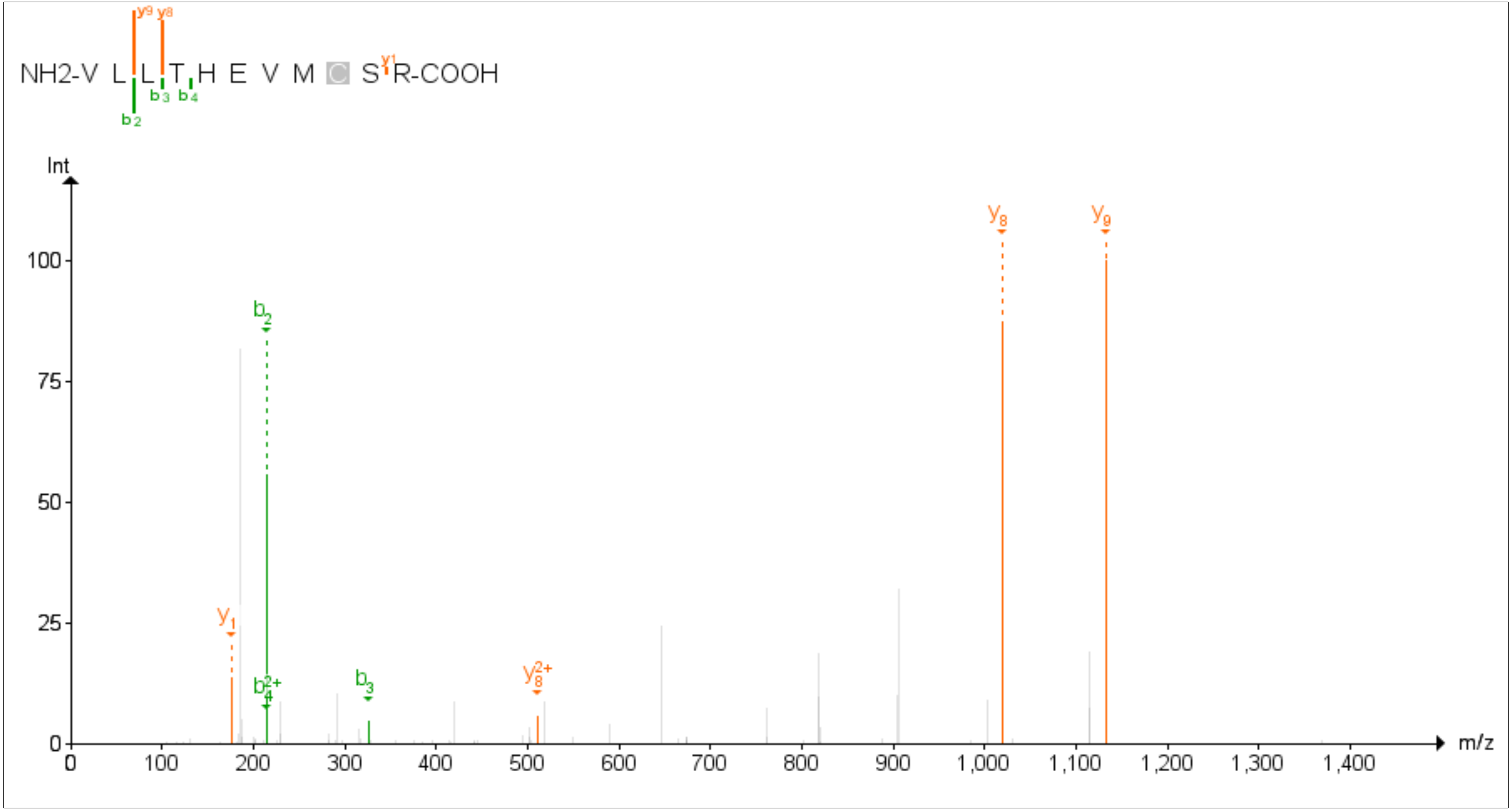

NH2-V L L T H E V M C S R-COOH
y9 y8
b2 b3 b4
y1
Int
100
75
50
25
0
y8
y9
b2
y1
b4 2+
b3
y8 2+
0 100 200 300 400 500 600 700 800 900 1,000 1,100 1,200 1,300 1,400
m/z

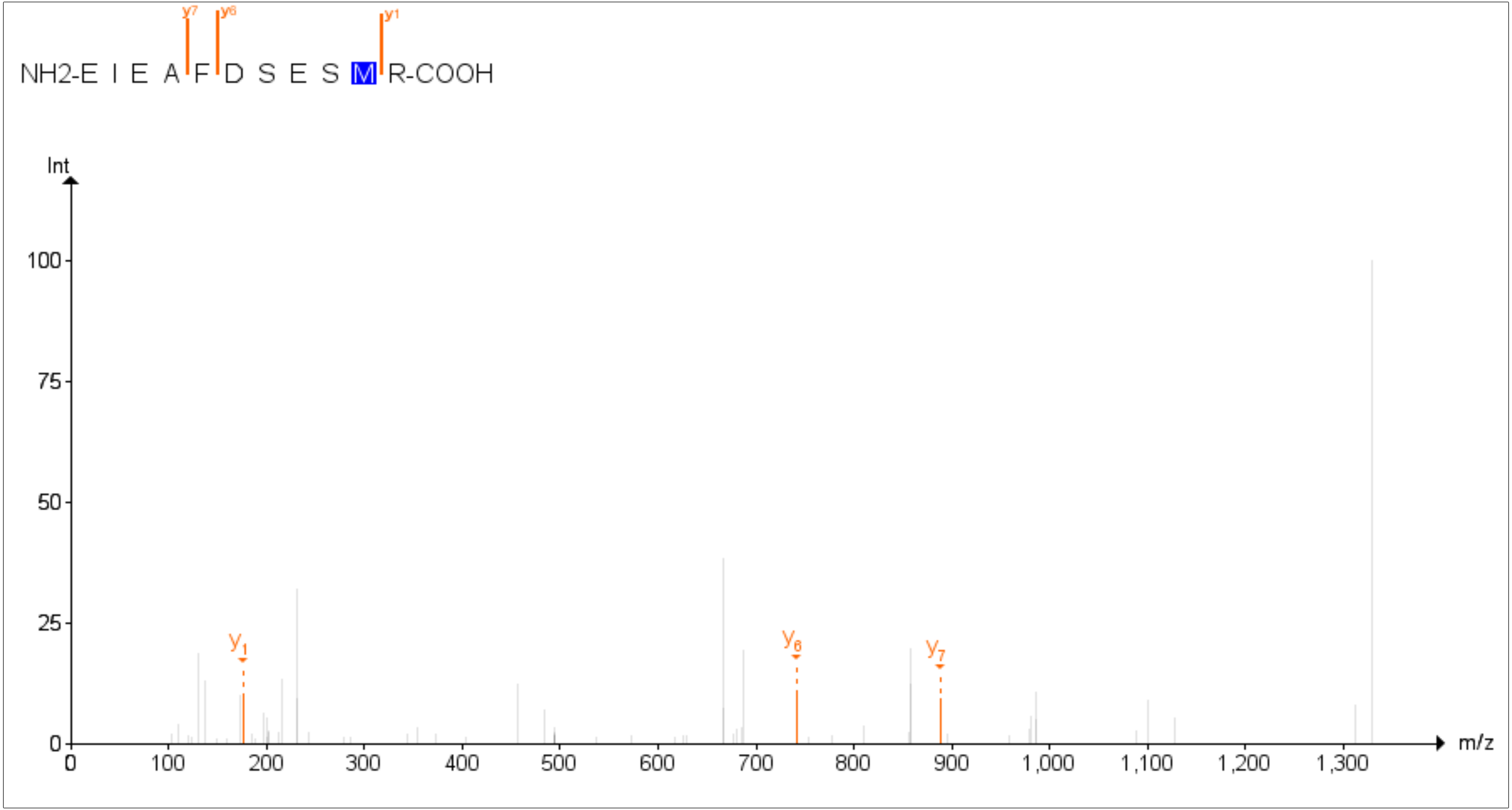
NH2-E I E A F D S E S M R-COOH
y7
y6
y1
Int
100
75
50
25
0
y1
y6
y7
0
100
200
300
400
500
600
700
800
900
1,000
1,100
1,200
1,300
m/z

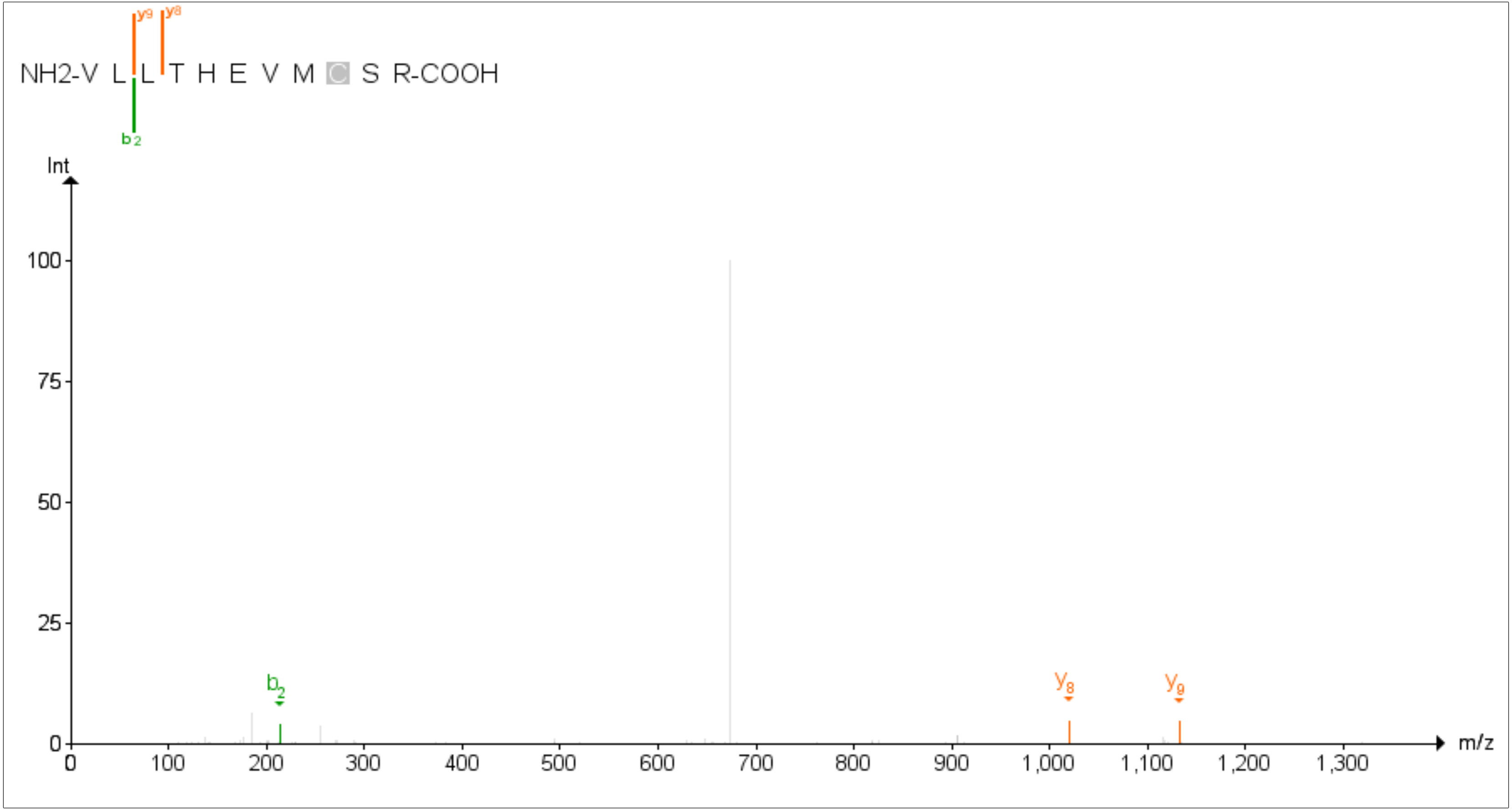

y9
y8
NH2-V L L T H E V M C S R-COOH
b2
Int
100
75
50
25
0
b2
y8
y9
0
100
200
300
400
500
600
700
800
900
1,000
1,100
1,200
1,300
m/z

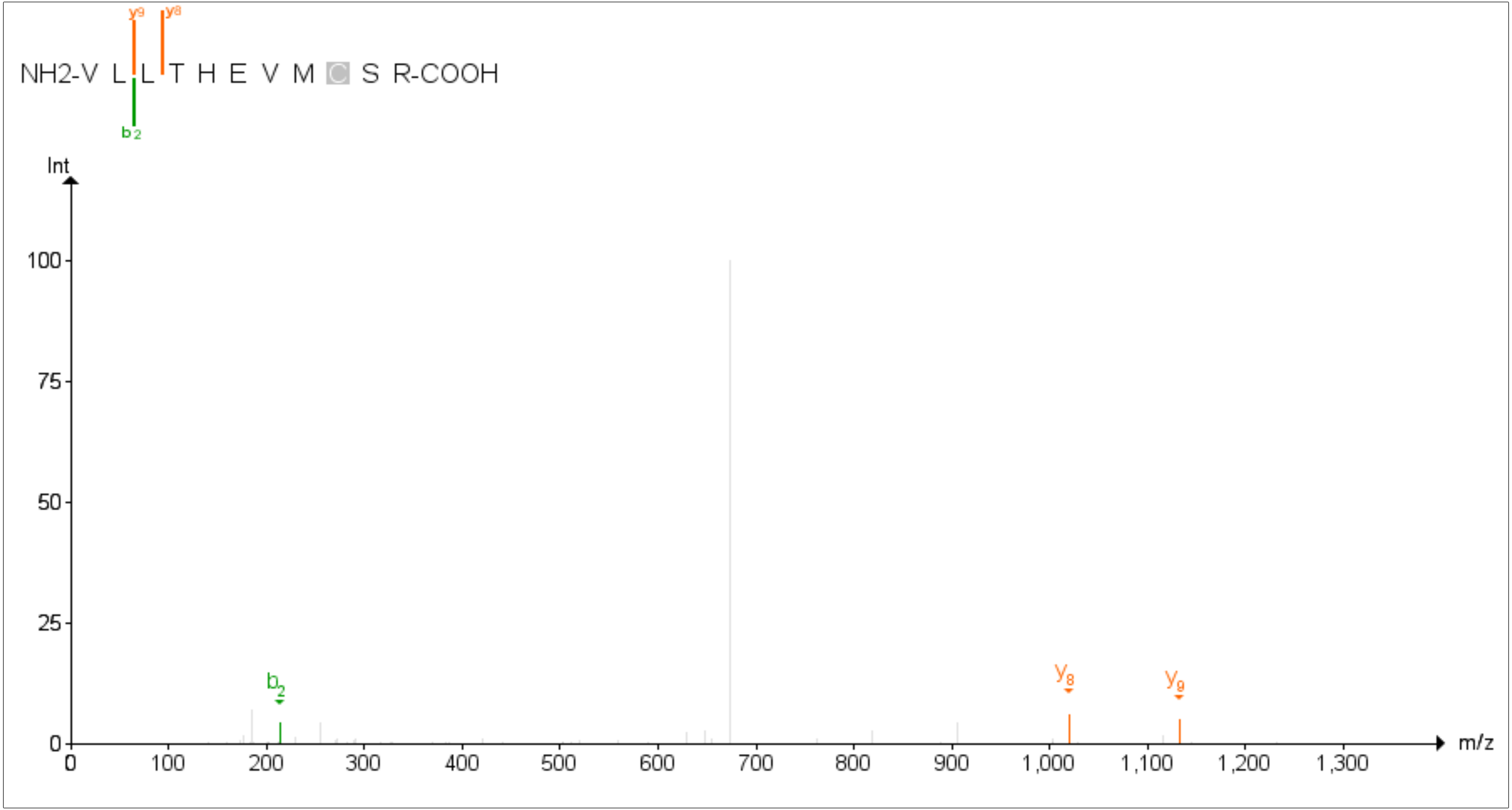
NH2-V L L T H E V M C S R-COOH
y9
y8
b2
Int
100
75
50
25
0
b2
y8
y9
0
100
200
300
400
500
600
700
800
900
1,000
1,100
1,200
1,300
m/z

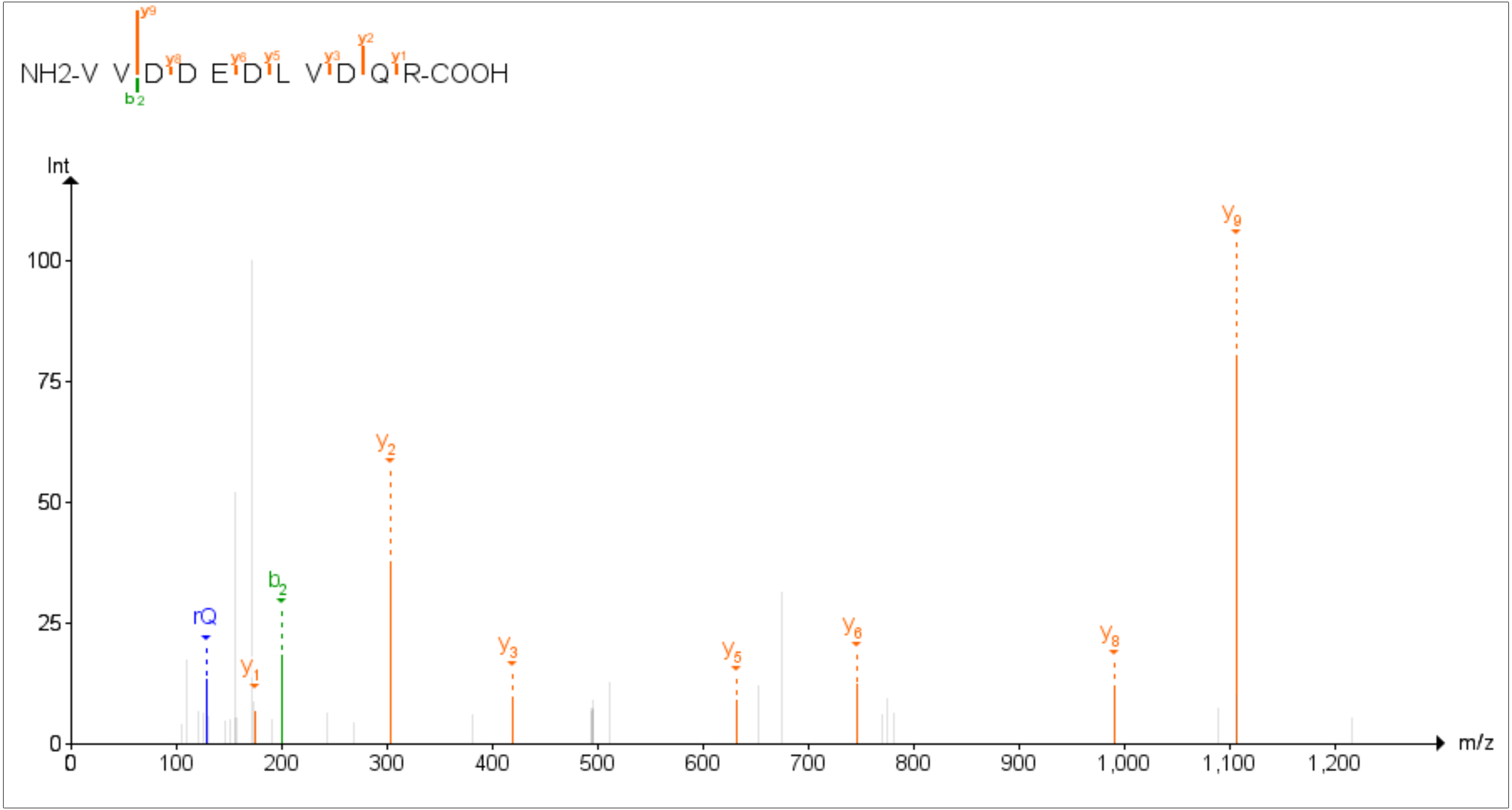
NH2-V V D D E D L V D Q R-COOH
y9 y8 y6 y5 y3 y2 y1
b2
Int
100
75
50
25
0
rQ
y1
b2
y2
y3
y5
y6
y8
y9
0 100 200 300 400 500 600 700 800 900 1,000 1,100 1,200
m/z

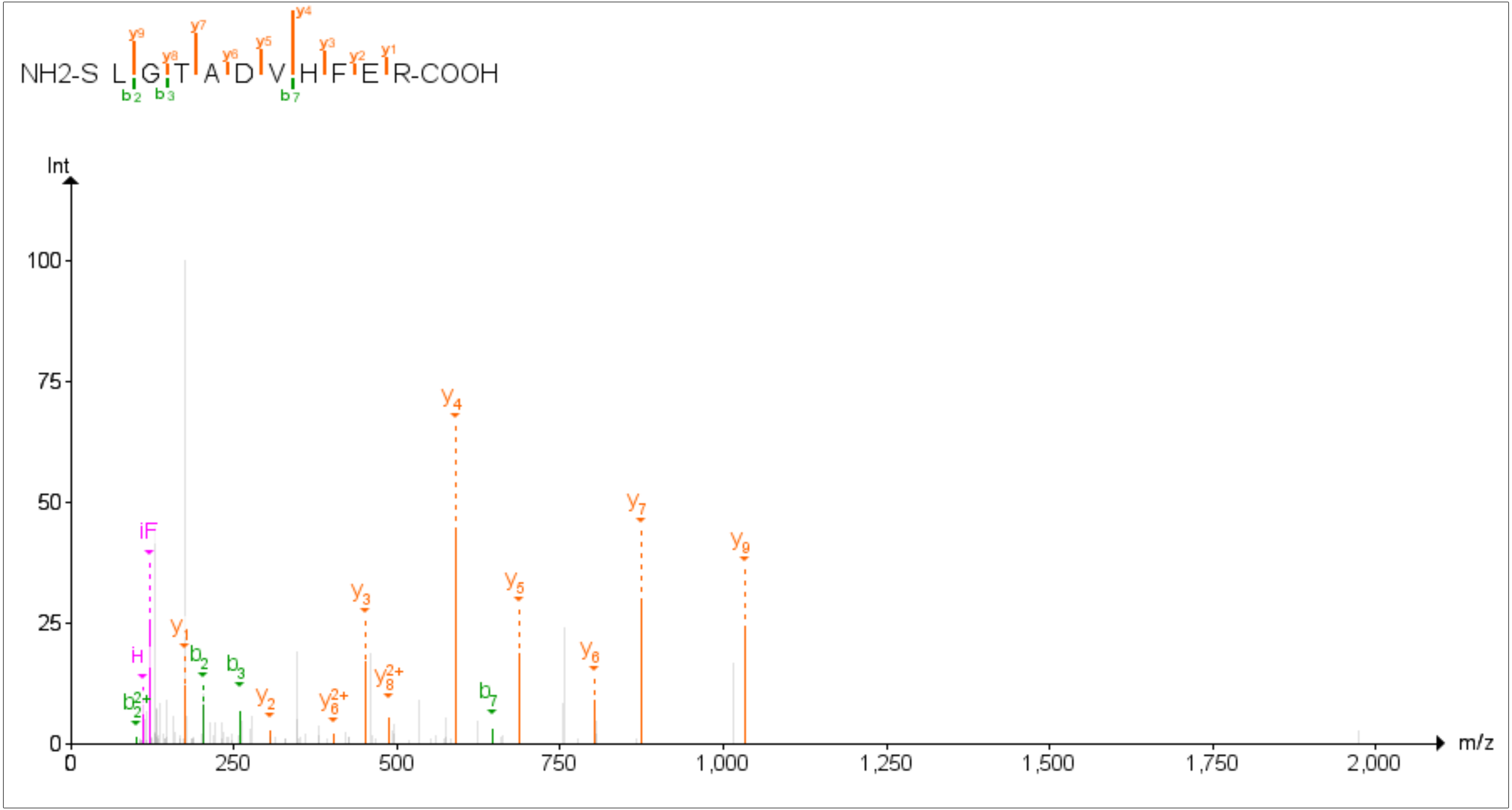

NH2-S L G T A D V H F E R-COOH
y9 y8 y7 y6 y5 y4 y3 y2 y1
b2 b3 b7
Int
100
75
50
25
0
0
250
500
750
1,000
1,250
1,500
1,750
2,000
m/z
iF
iH
y1
b2
b3
y2
y3
y4
y5
y6
y7
y9
b7

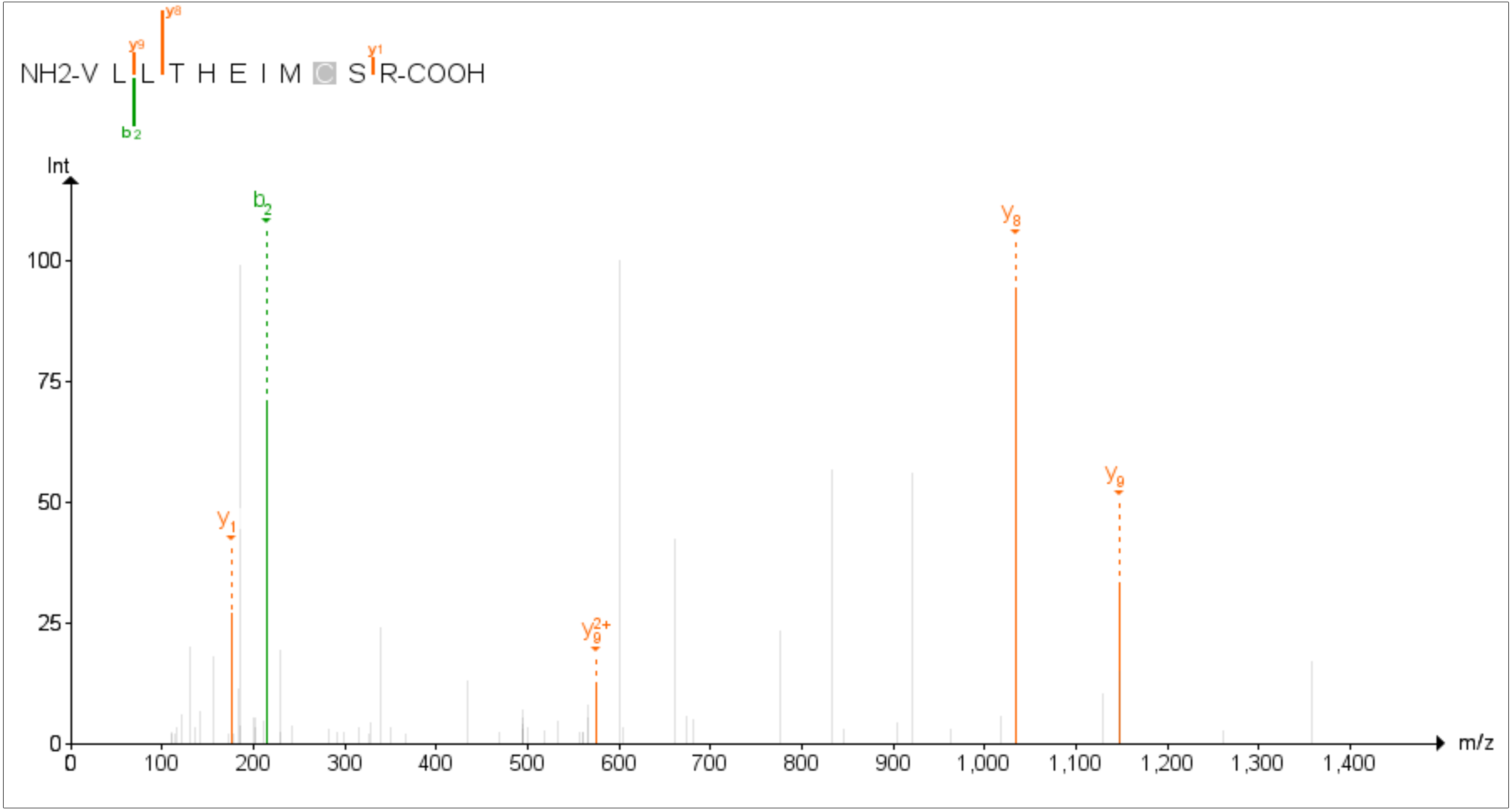

NH2-V L L T H E I M C S R-COOH
y9
y8
y1
b2
Int
100
75
50
25
0
0
100
200
300
400
500
600
700
800
900
1,000
1,100
1,200
1,300
1,400
m/z
b_2
y_1
y_9^{2+}
y_8
y_9

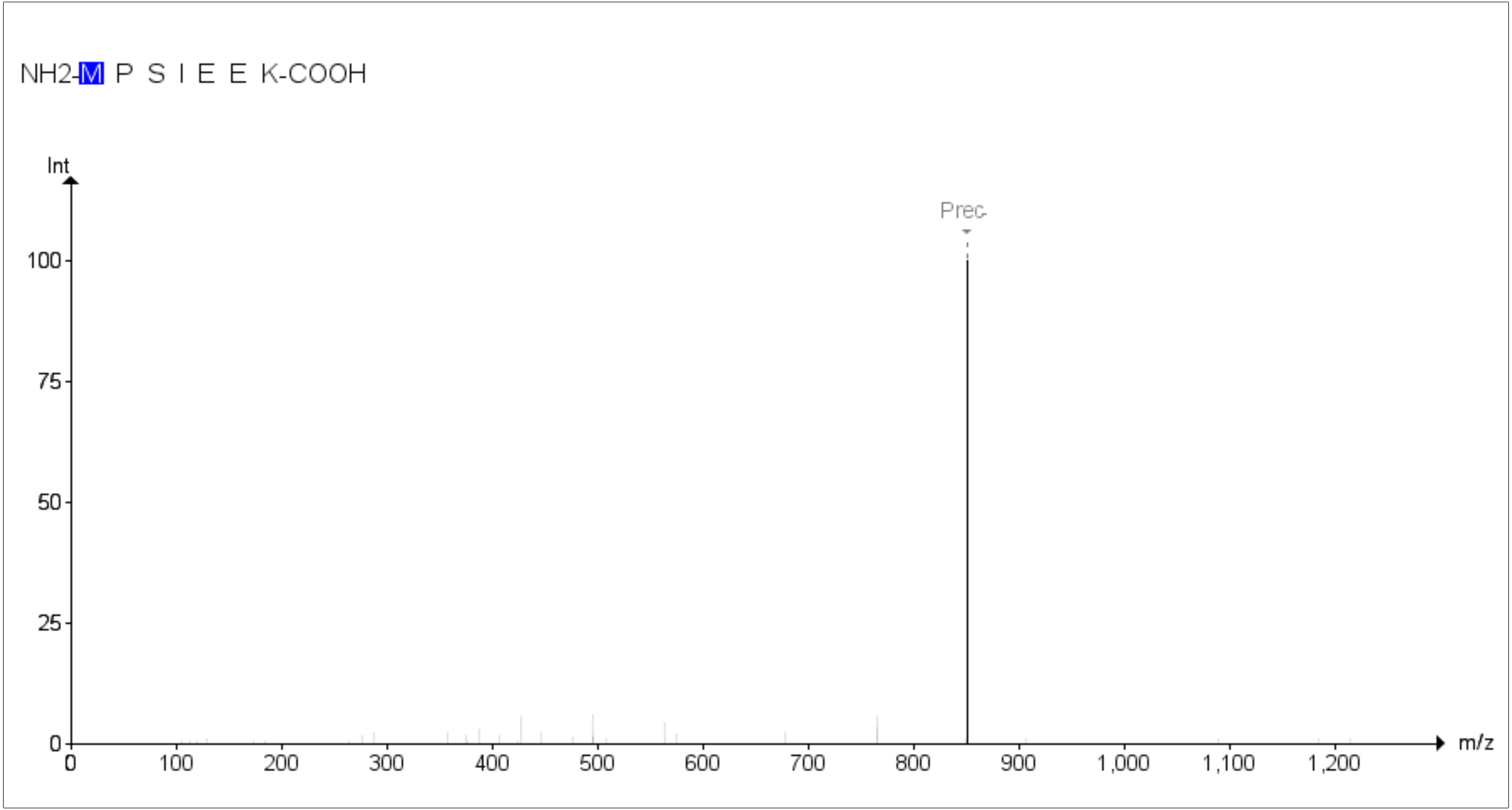
NH2-M P S I E E K-COOH
Int
100
75
50
25
0
Prec
m/z
0
100
200
300
400
500
600
700
800
900
1,000
1,100
1,200

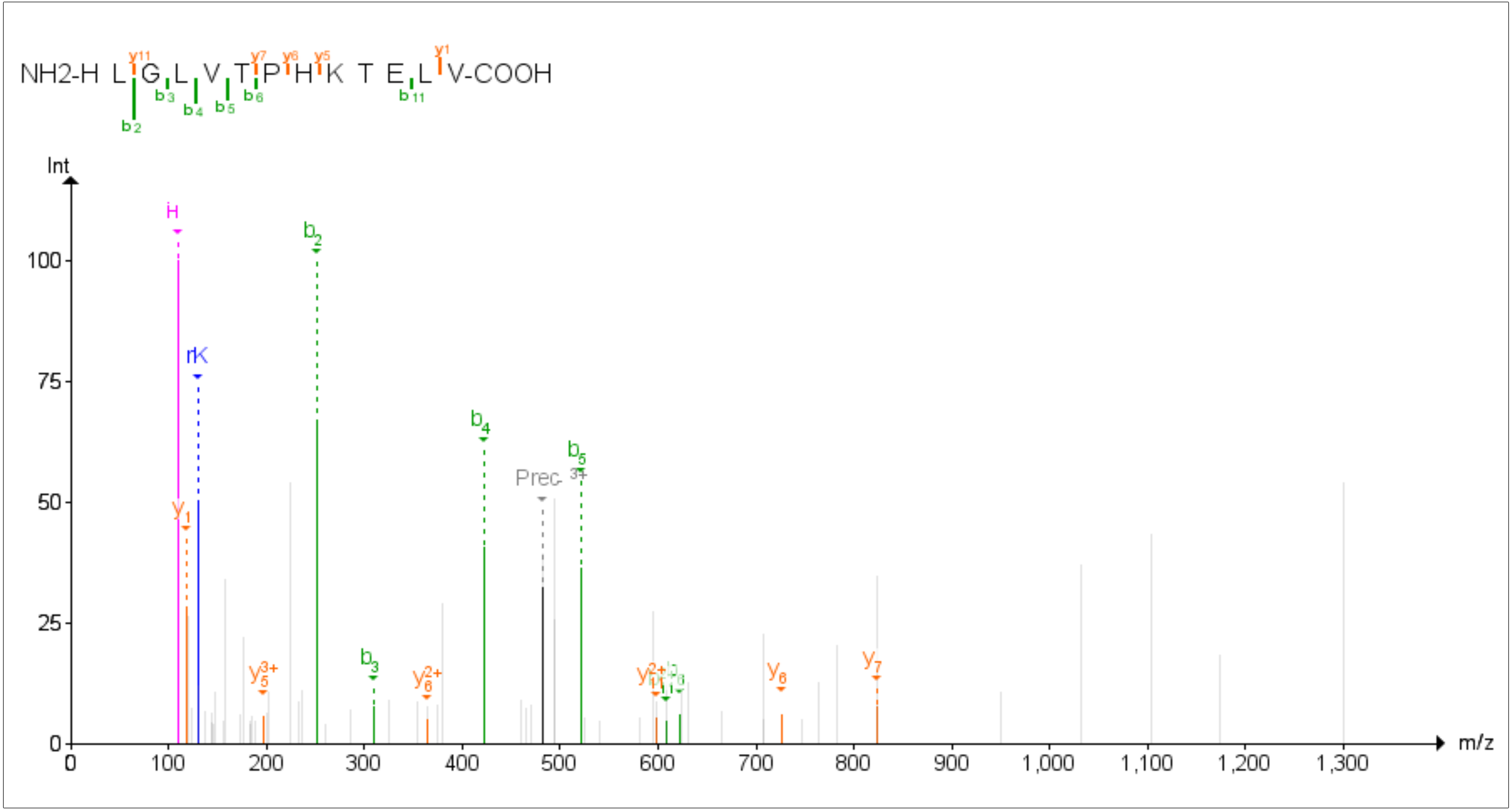

NH2-H L G L V T P H K T E L V-COOH
y11 y7 y6 y5 y1
b2 b3 b4 b5 b6 b11
Int
iH
b2
rK
b4
b5
Prec 3+
y1
y5 3+
b3
y6 2+
y11 2+
b11
b6
y6
y7
0
25
50
75
100
0
100
200
300
400
500
600
700
800
900
1,000
1,100
1,200
1,300
m/z

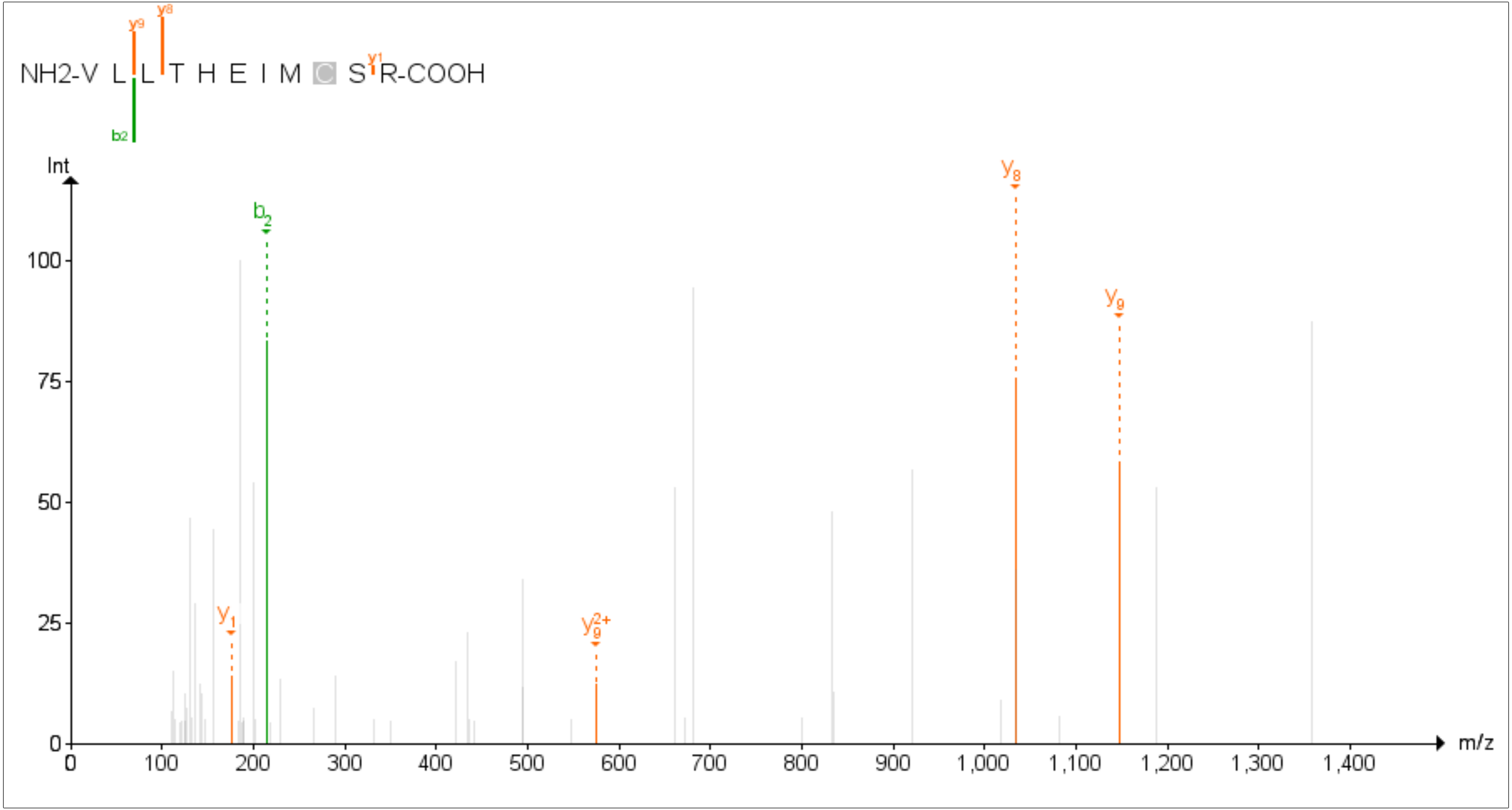

NH2-V L L T H E I M C S R-COOH
y9
y8
y1
b2
Int
m/z
100
75
50
25
0
0
100
200
300
400
500
600
700
800
900
1,000
1,100
1,200
1,300
1,400
b2
y8
y9
y1
y9 2+

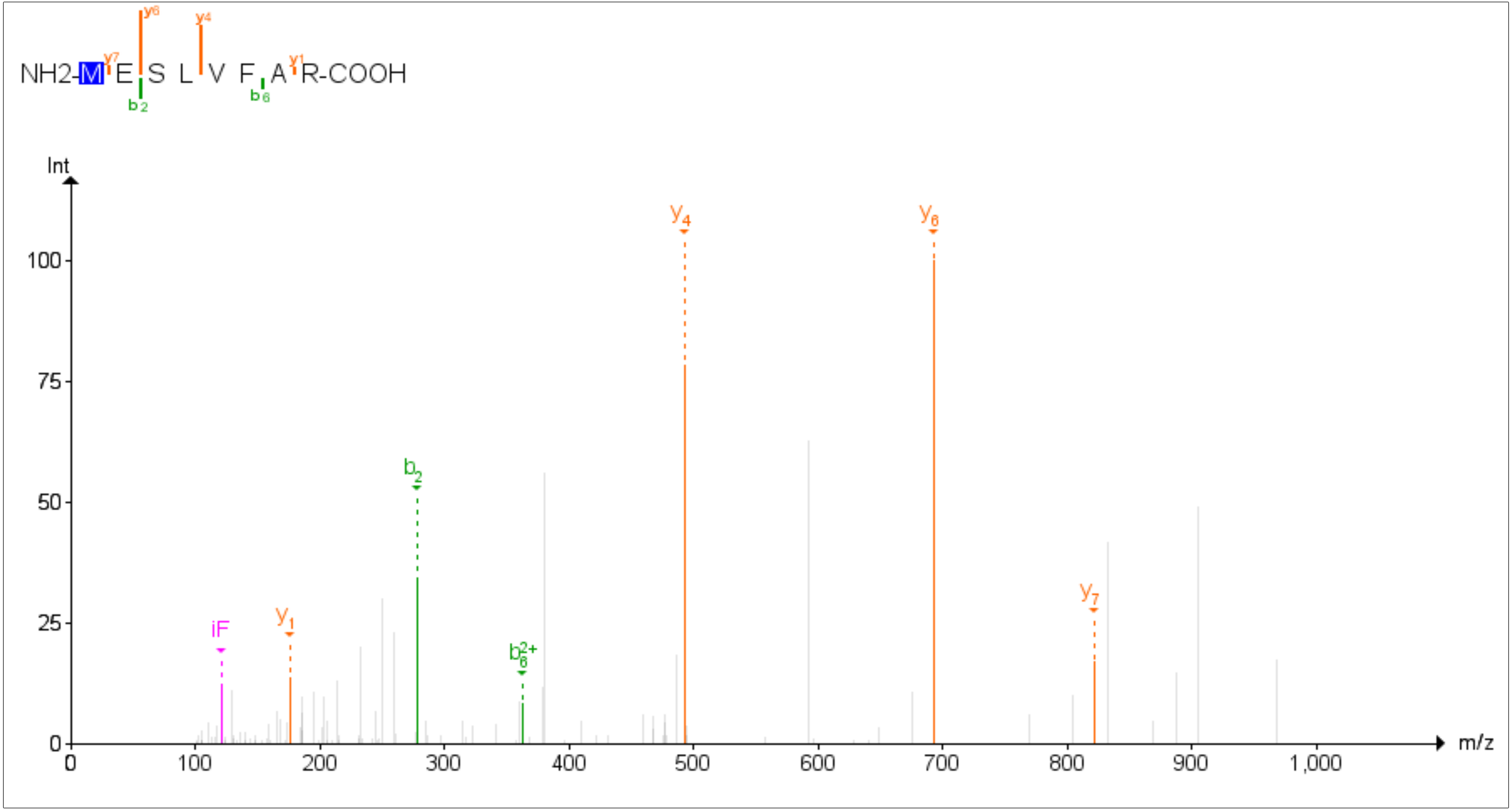

NH2-M E S L V F A R-COOH
y7
y6
y4
y1
b2
b6
Int
100
75
50
25
0
y4
y6
b2
iF
y1
b6 2+
y7
0
100
200
300
400
500
600
700
800
900
1,000
m/z

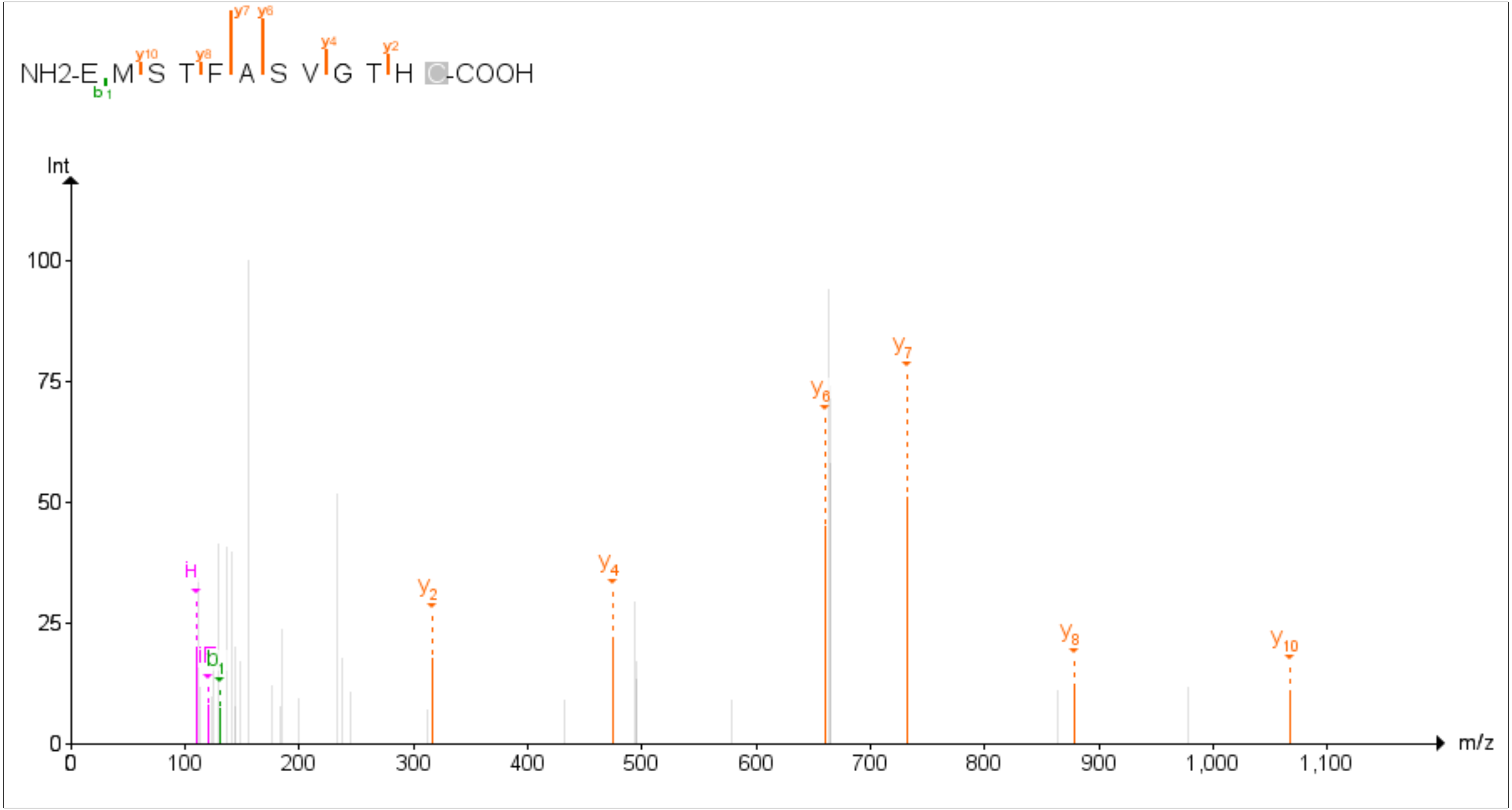

NH2-E M S T F A S V G T H C-COOH
b1
y10
y8
y7
y6
y4
y2
Int
m/z
100
75
50
25
0
0
100
200
300
400
500
600
700
800
900
1,000
1,100
H
b1
y2
y4
y6
y7
y8
y10

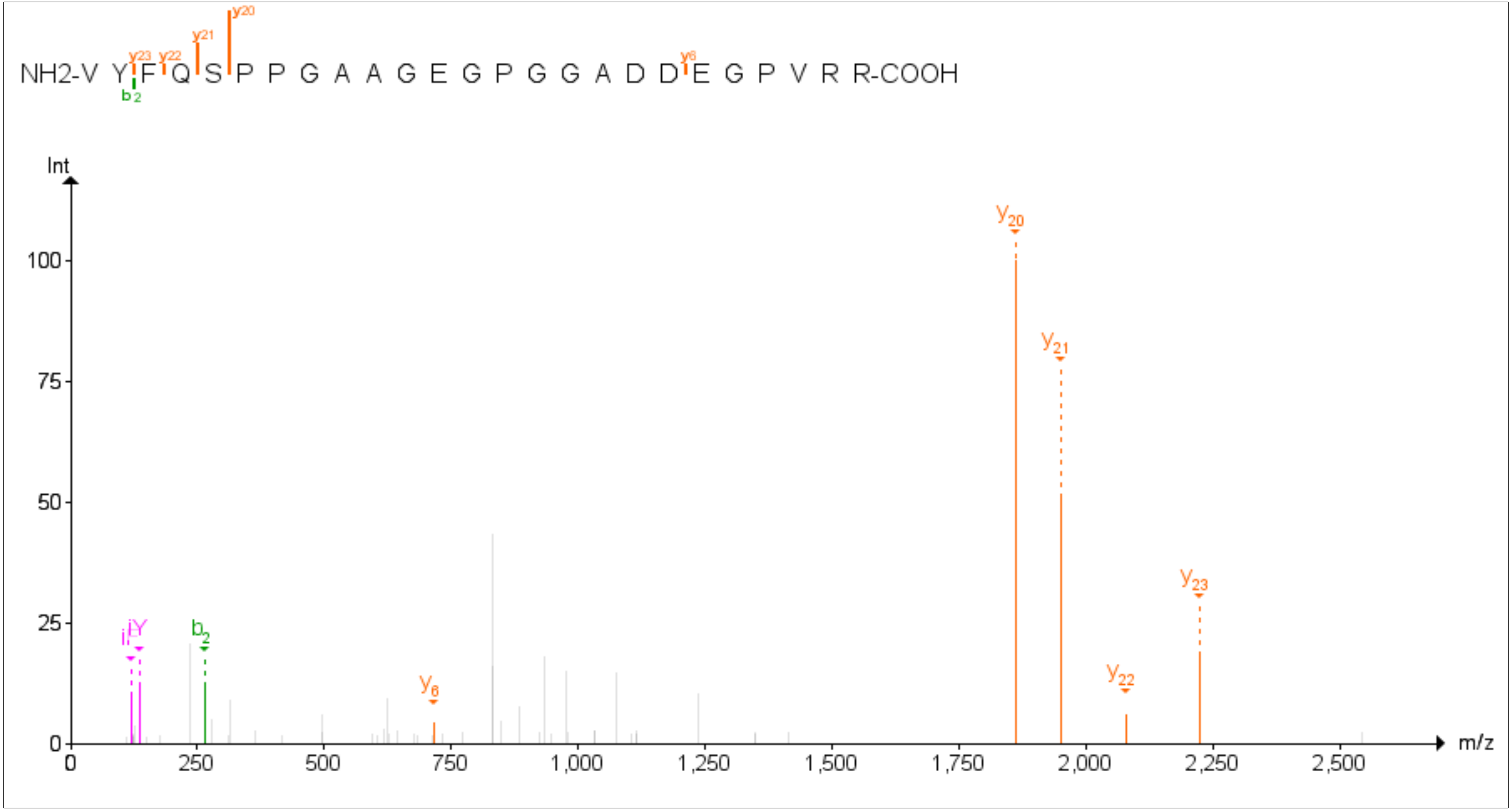
NH2-V Y F Q S P P G A A G E G P G G A D D E G P V R R-COOH
y20
y21
y23
y22
y6
b2
Int
100
75
50
25
0
iY
b2
y6
y20
y21
y22
y23
0
250
500
750
1,000
1,250
1,500
1,750
2,000
2,250
2,500
m/z

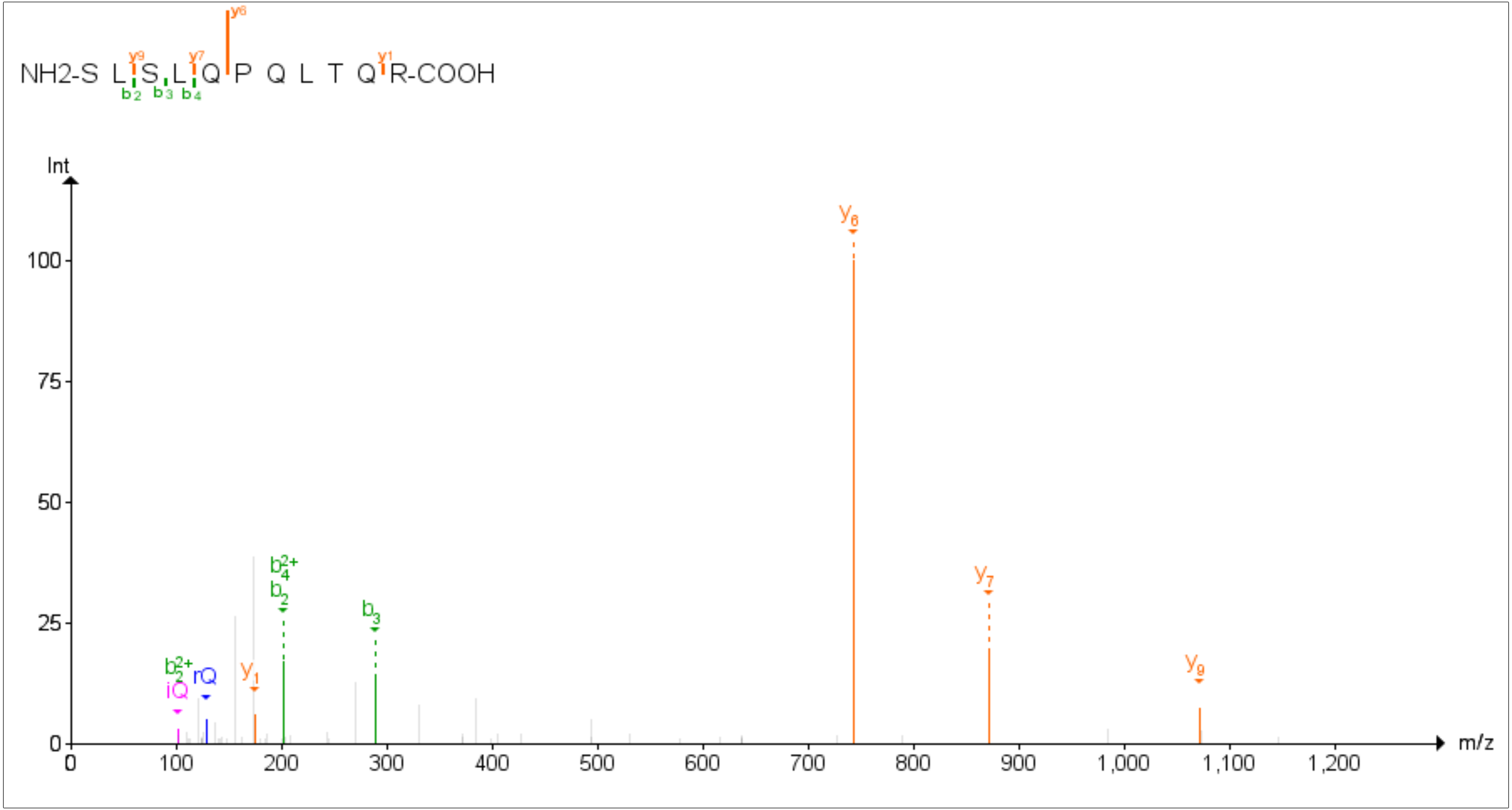

NH2-S L S L Q P Q L T Q R-COOH
y9 y7 y6 y1
b2 b3 b4
Int
100
75
50
25
0
y6
y7
y9
b4 2+
b2
b3
b2 2+
iQ
rQ
y1
0 100 200 300 400 500 600 700 800 900 1,000 1,100 1,200
m/z

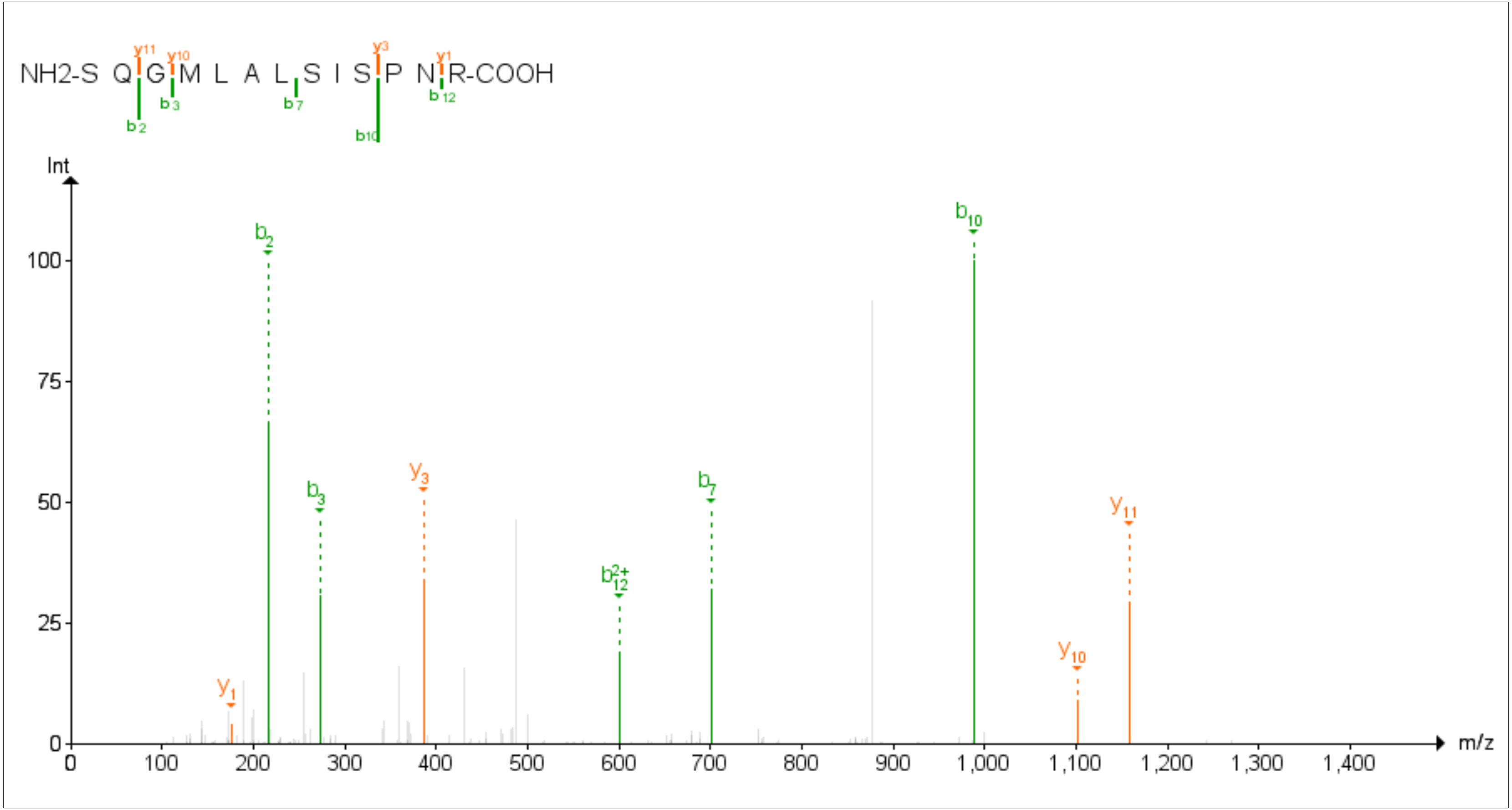

NH2-S Q G M L A L S I S P N R-COOH
y11
y10
y3
y1
b2
b3
b7
b10
b12
Int
m/z
b2
b10
b3
y3
b7
b12 2+
y11
y10
y1
0
25
50
75
100
0
100
200
300
400
500
600
700
800
900
1,000
1,100
1,200
1,300
1,400

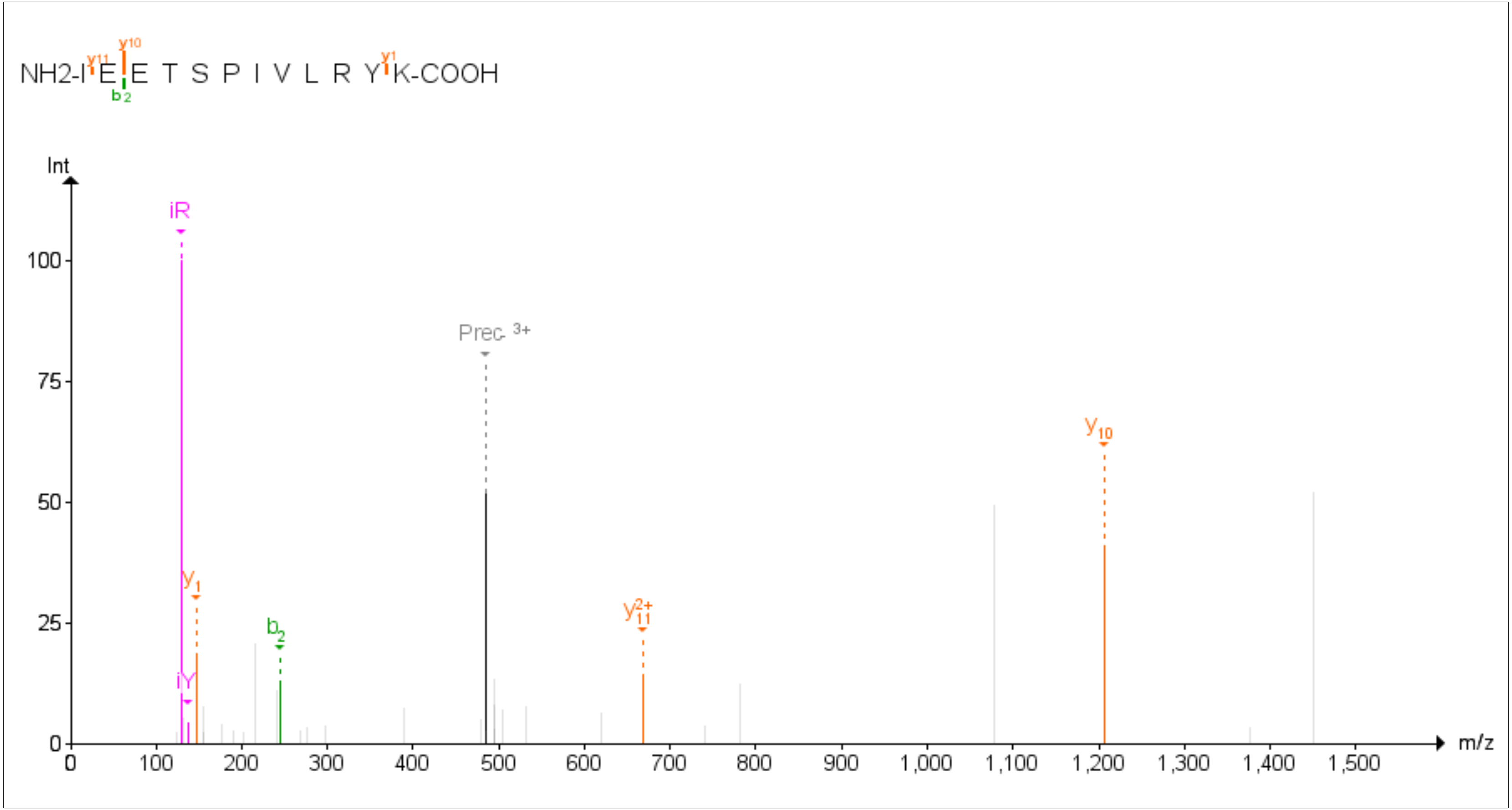
NH2-I E E T S P I V L R Y K-COOH
y11
y10
b2
y1
Int
iR
Prec 3+
y10
y1
b2
iY
y11 2+
m/z
0
25
50
75
100
0
100
200
300
400
500
600
700
800
900
1,000
1,100
1,200
1,300
1,400
1,500

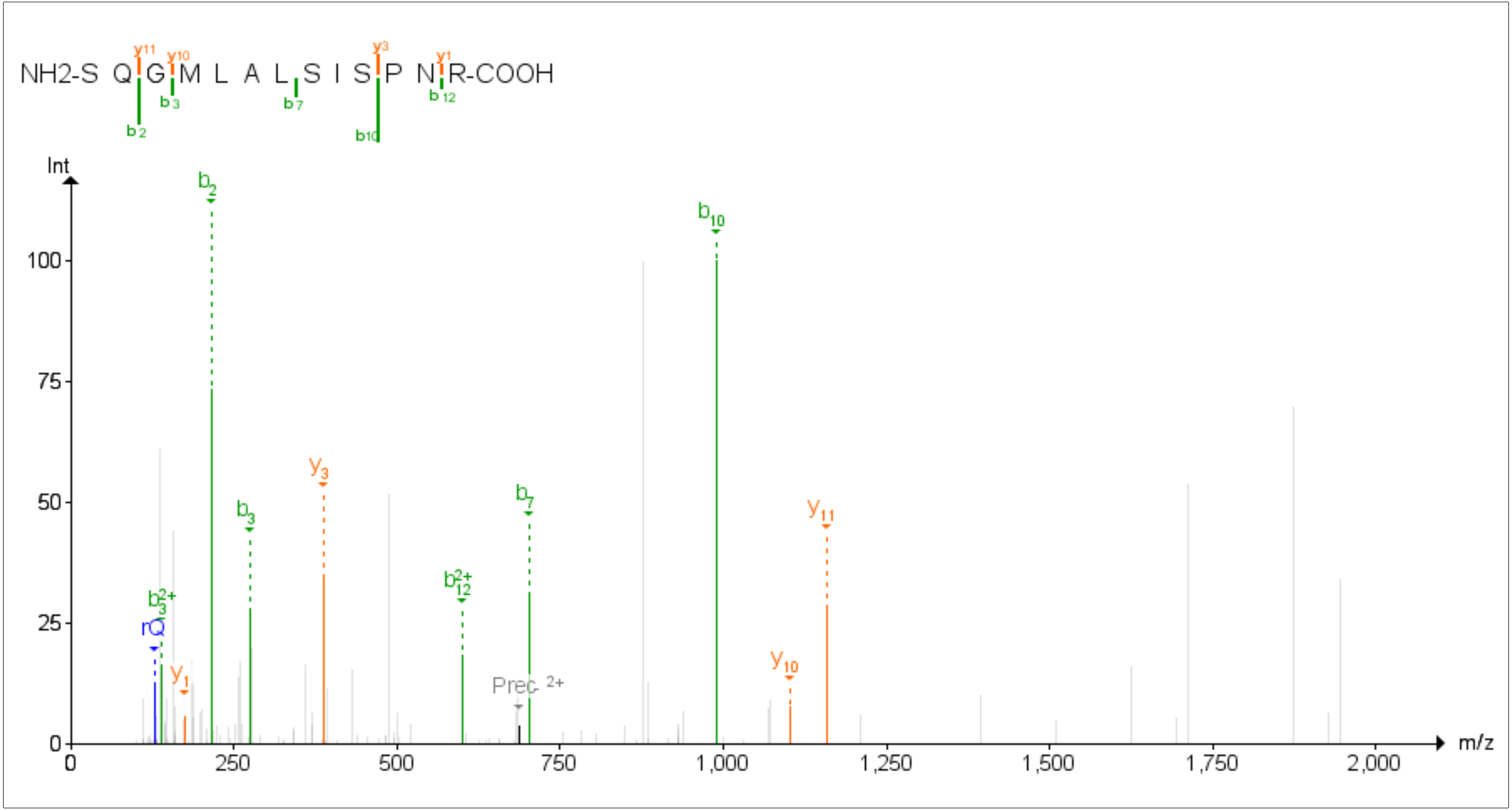

NH2-S Q G M L A L S I S P N R-COOH
y11 y10 y3 y1
b2 b3 b7 b10 b12
Int
m/z
b2
b10
y3
b3
b7
y11
b12 2+
b3 2+
rQ
y1
Prec. 2+
y10
0
25
50
75
100
0
250
500
750
1,000
1,250
1,500
1,750
2,000

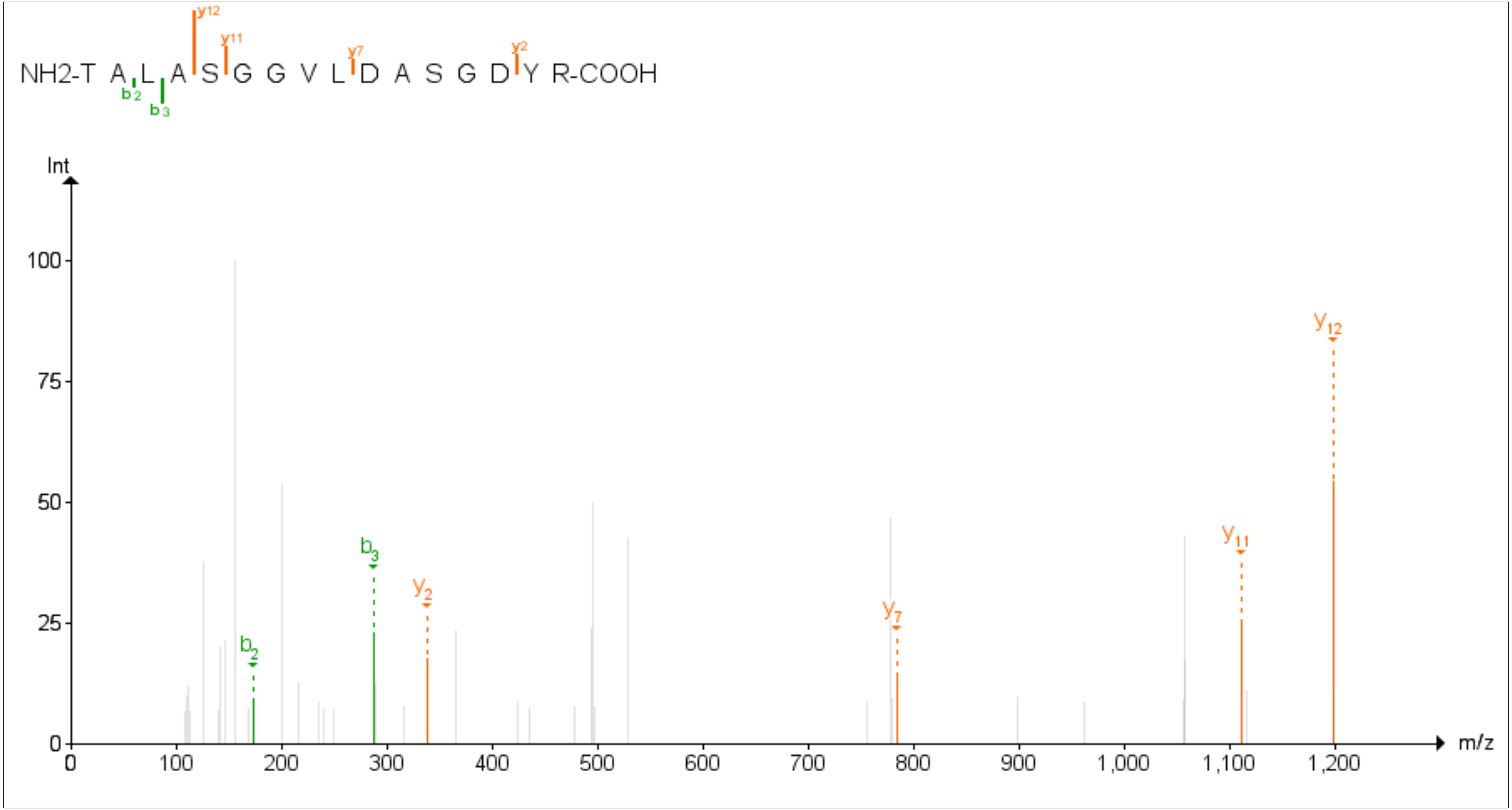

NH2-T A L A S G G V L D A S G D Y R-COOH
y12
y11
y7
y2
b2
b3
Int
100
75
50
25
0
m/z
0
100
200
300
400
500
600
700
800
900
1,000
1,100
1,200
b2
b3
y2
y7
y11
y12

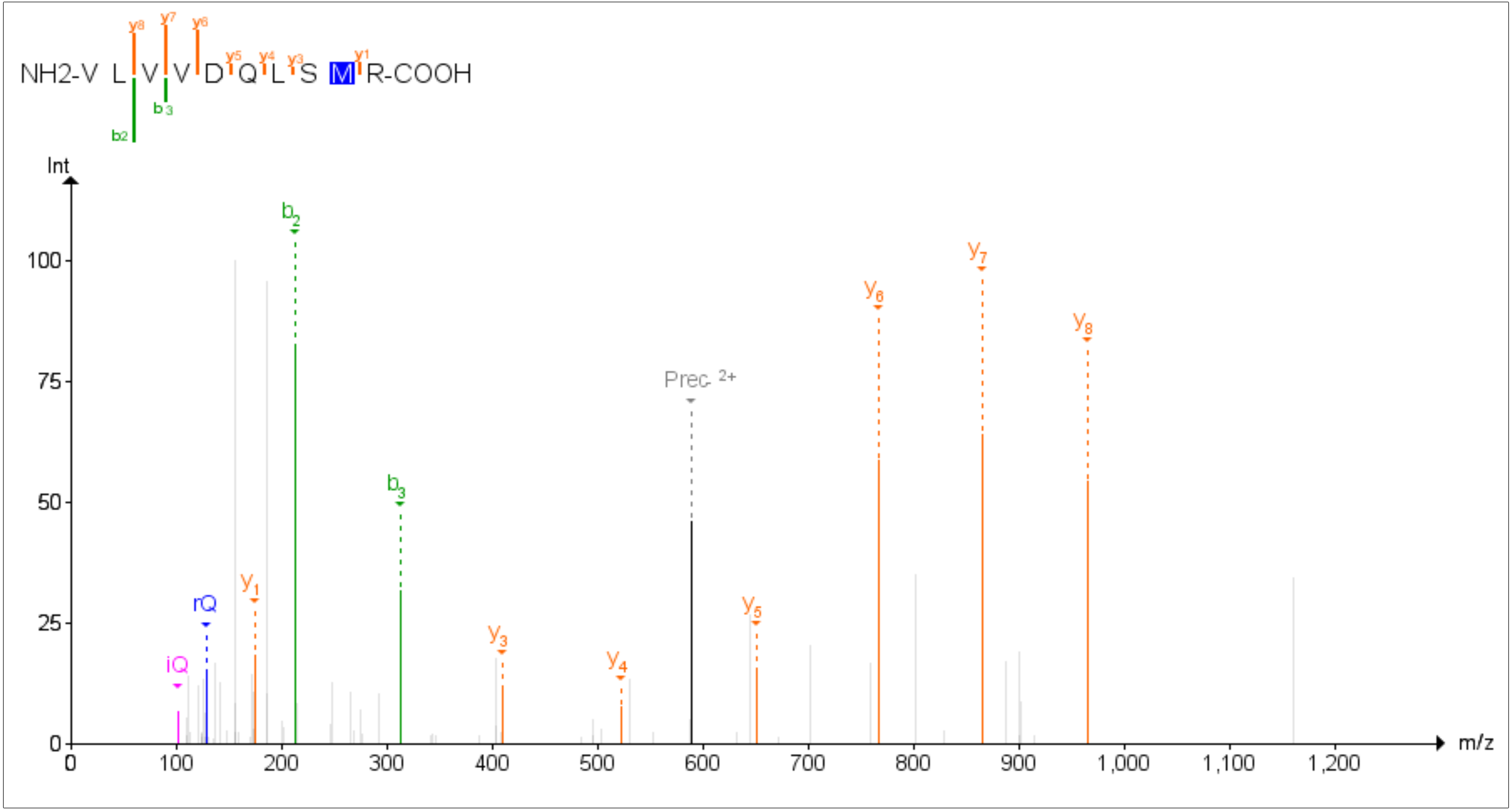
NH2-V L V V D Q L S M R-COOH
y8 y7 y6 y5 y4 y3 y1
b2 b3
Int
100
75
50
25
0
b2
b3
y1
rQ
iQ
y3
y4
Prec 2+
y5
y6
y7
y8
0 100 200 300 400 500 600 700 800 900 1,000 1,100 1,200
m/z

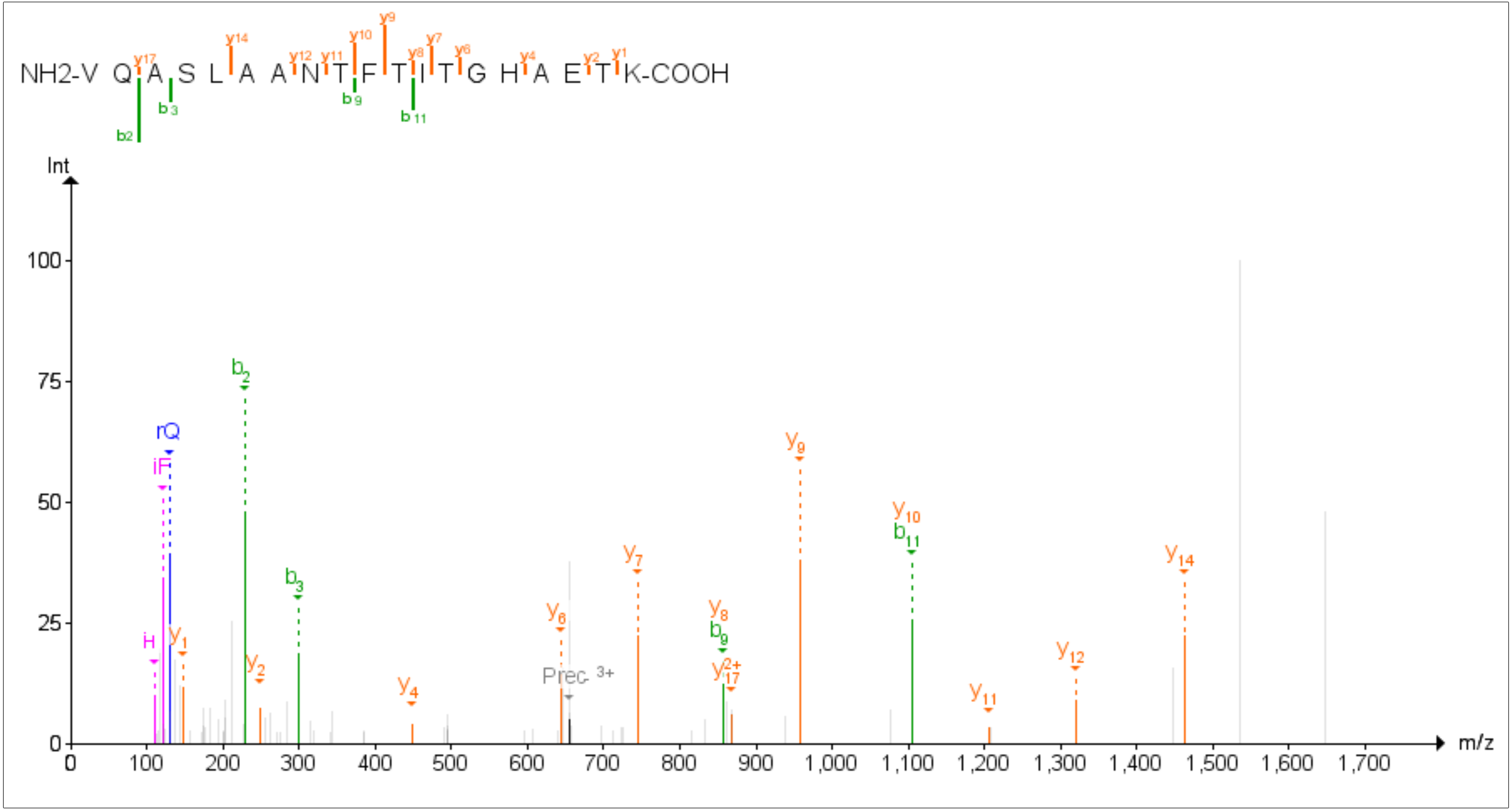

NH2-V Q A S L A A N T F T I T G H A E T K-COOH
Int
m/z
rQ
iF
H
b2
b3
b9
b11
y1
y2
y4
y6
y7
y8
y9
y10
y11
y12
y14
y17 2+
Prec 3+

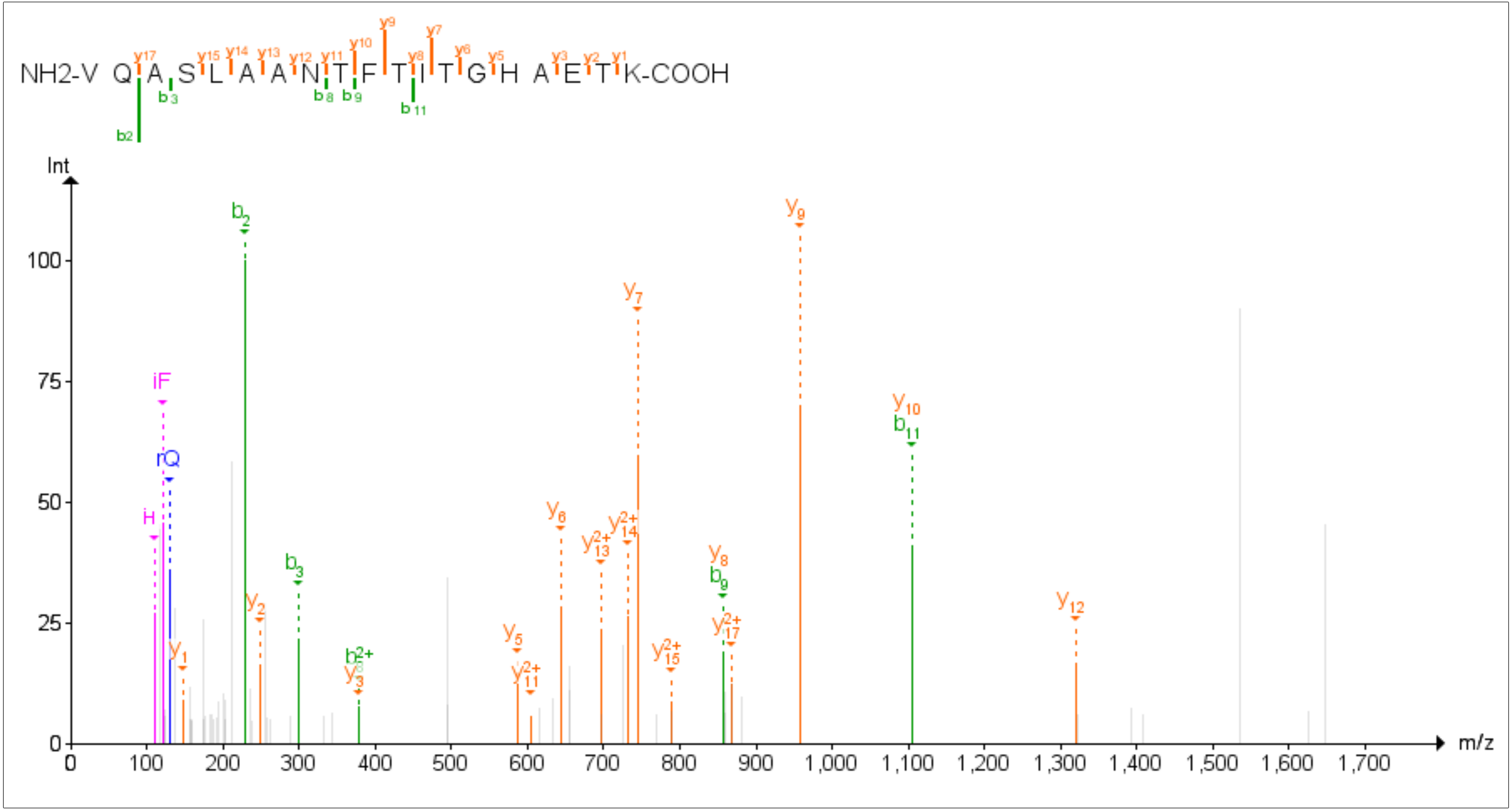

NH2-V Q A S L A A N T F T I T G H A E T K-COOH
y17 y15 y14 y13 y12 y11 y10 y9 y8 y7 y6 y5 y3 y2 y1
b2 b3 b8 b9 b11
Int
m/z
b2
y9
y7
iF
rQ
iH
y10
b11
y6
y13 2+
y14 2+
b3
y8
b9
y17 2+
y2
y1
y12
y5
y11 2+
y15 2+
b6 2+
y3

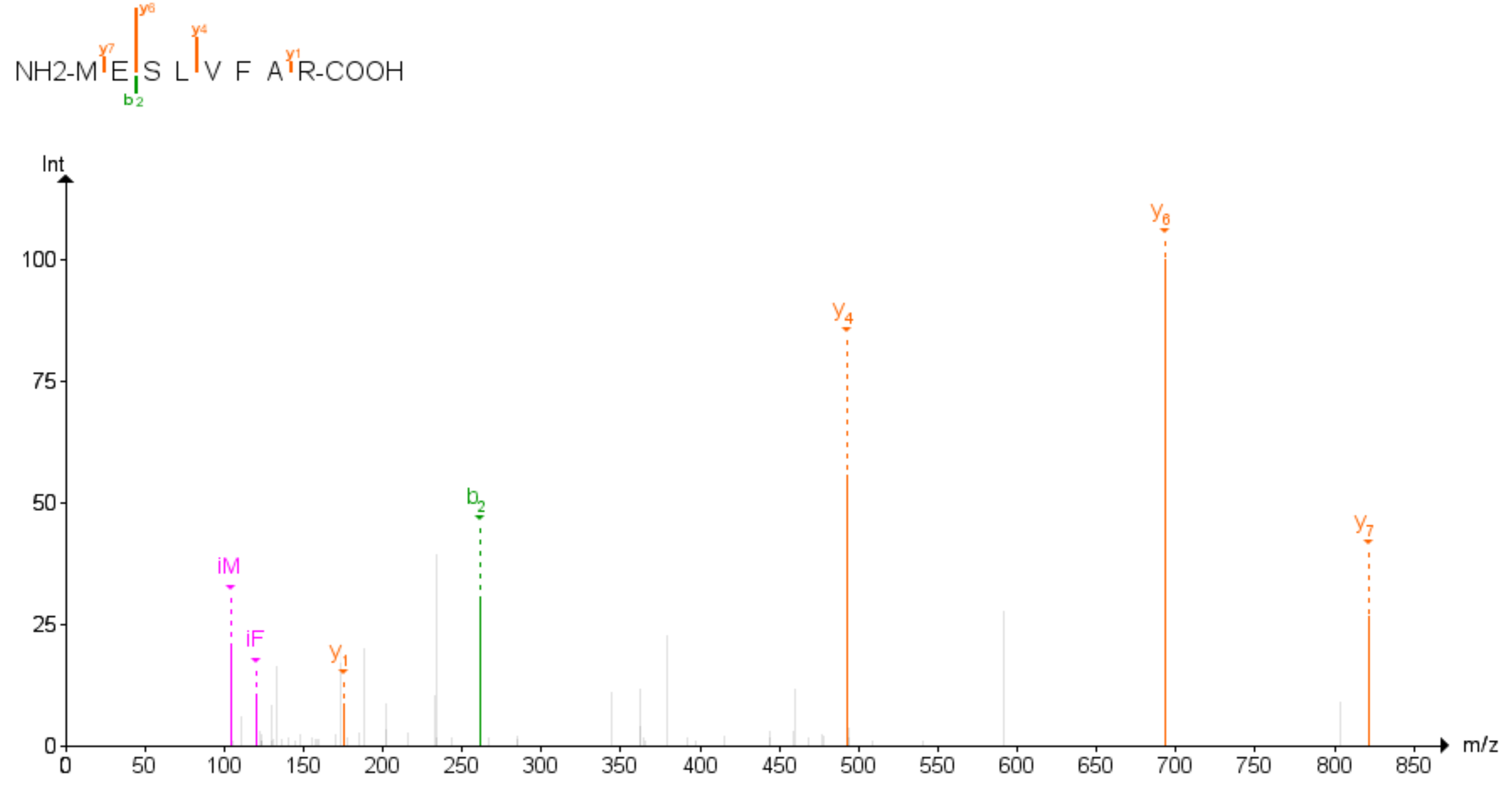

NH2-M E S L V F A R-COOH
y7
y6
y4
y1
b2
Int
m/z
100
75
50
25
0
0
50
100
150
200
250
300
350
400
450
500
550
600
650
700
750
800
850
iM
iF
y1
b2
y4
y6
y7

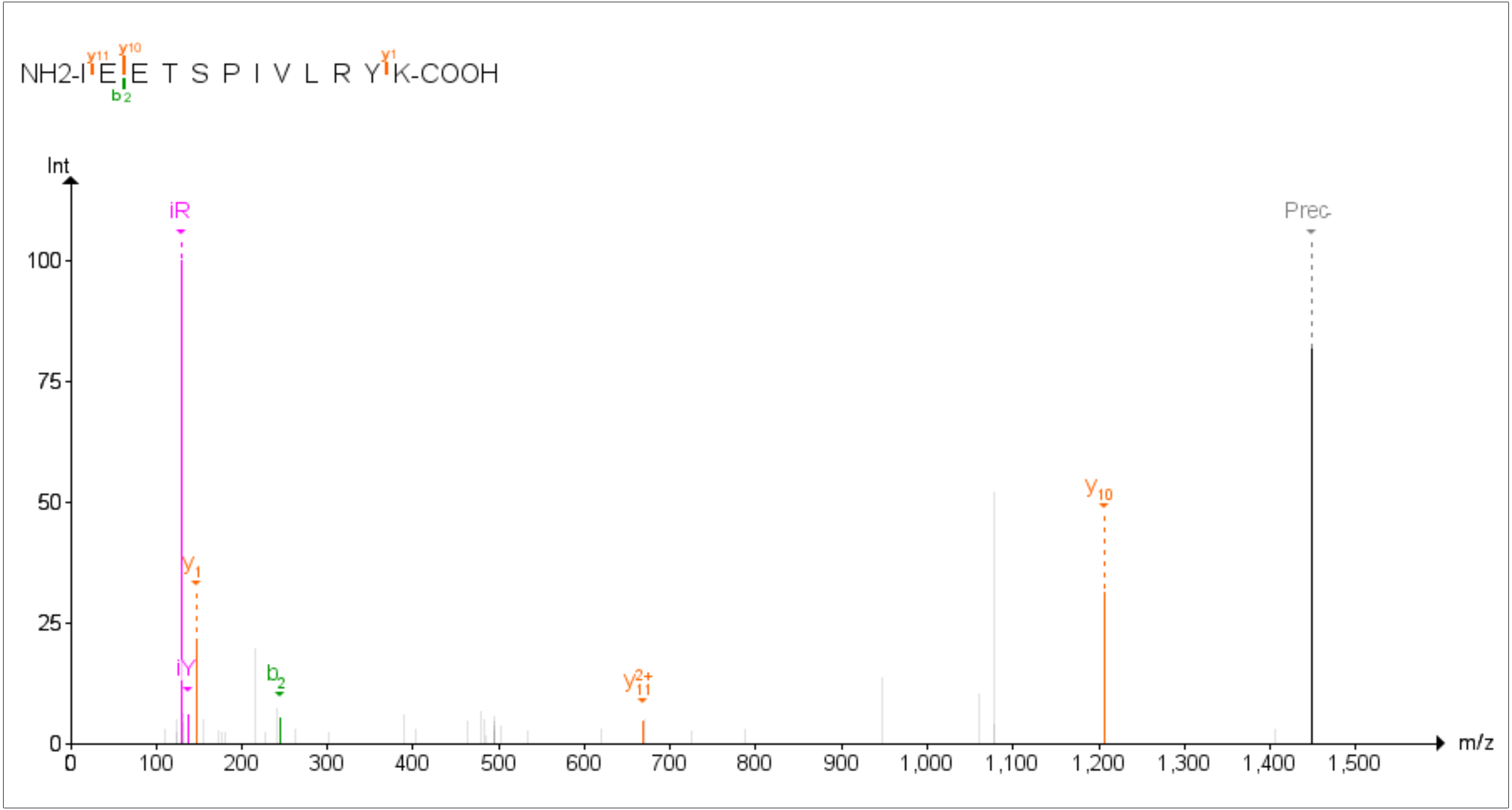

NH2-I E E T S P I V L R Y K-COOH
y11
y10
b2
y1
Int
iR
Prec
100
75
50
25
0
y1
iY
b2
y_{11}^{2+}
y_{10}
0
100
200
300
400
500
600
700
800
900
1,000
1,100
1,200
1,300
1,400
1,500
m/z

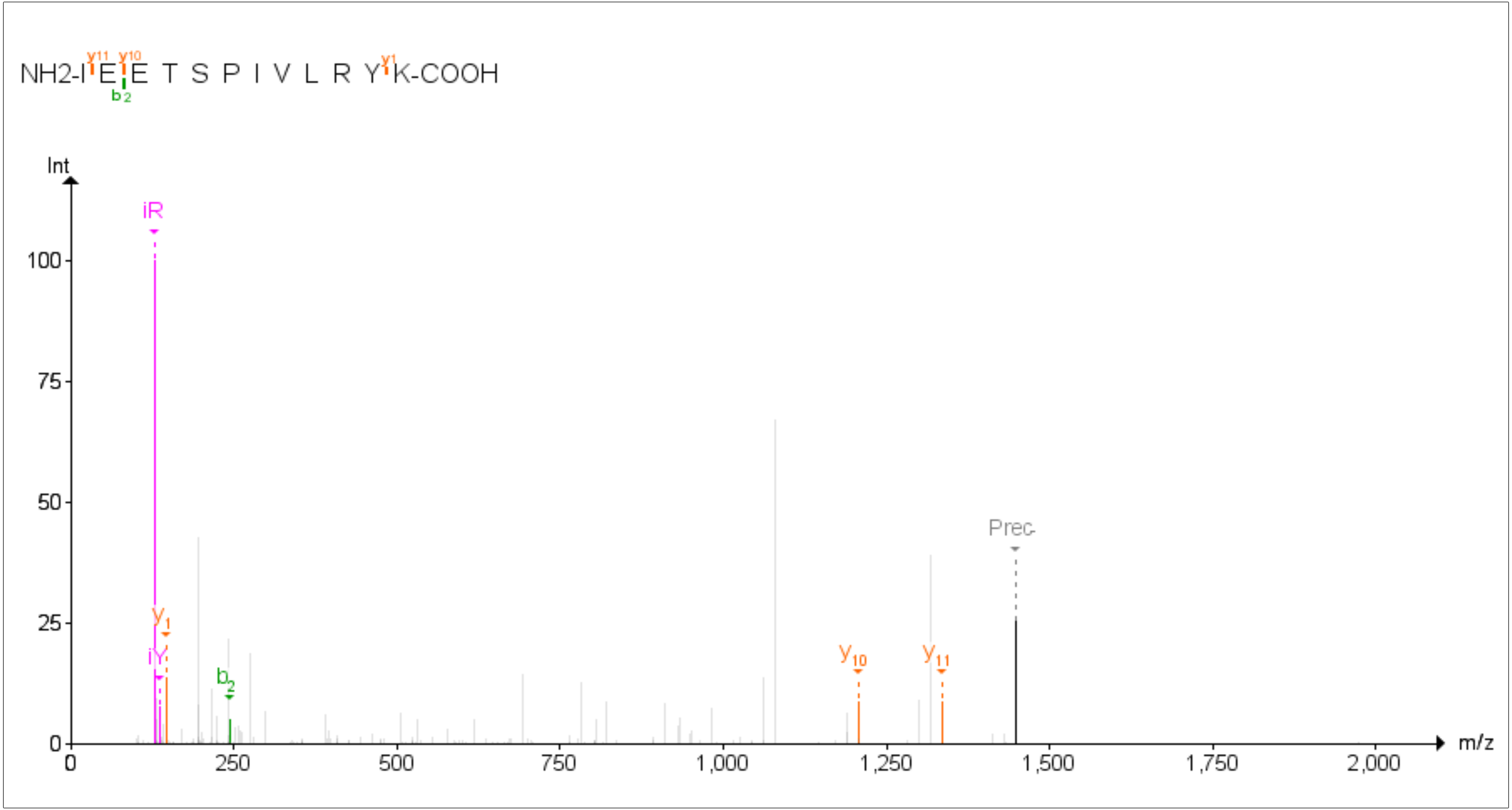
NH2-I E E T S P I V L R Y K-COOH
y11
y10
b2
y1
Int
iR
y1
iY
b2
y10
y11
Prec
m/z
0
25
50
75
100
0
250
500
750
1,000
1,250
1,500
1,750
2,000

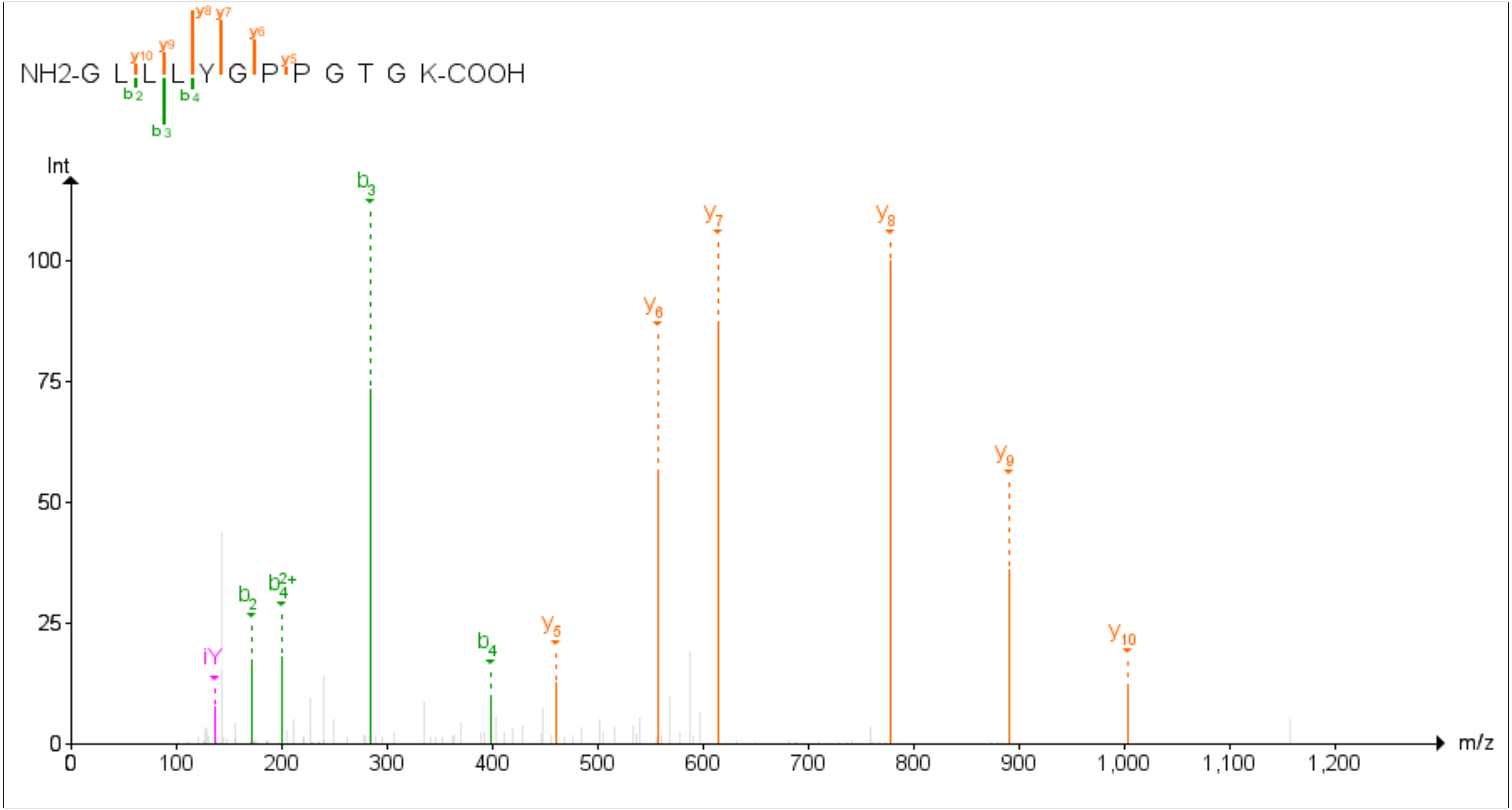

NH2-G L L L Y G P P G T G K-COOH
y10 y9 y8 y7 y6 y5
b2 b3 b4
Int
100
75
50
25
0
b3
y7
y8
y6
y9
b2
b4 2+
iY
b4
y5
y10
0 100 200 300 400 500 600 700 800 900 1,000 1,100 1,200
m/z

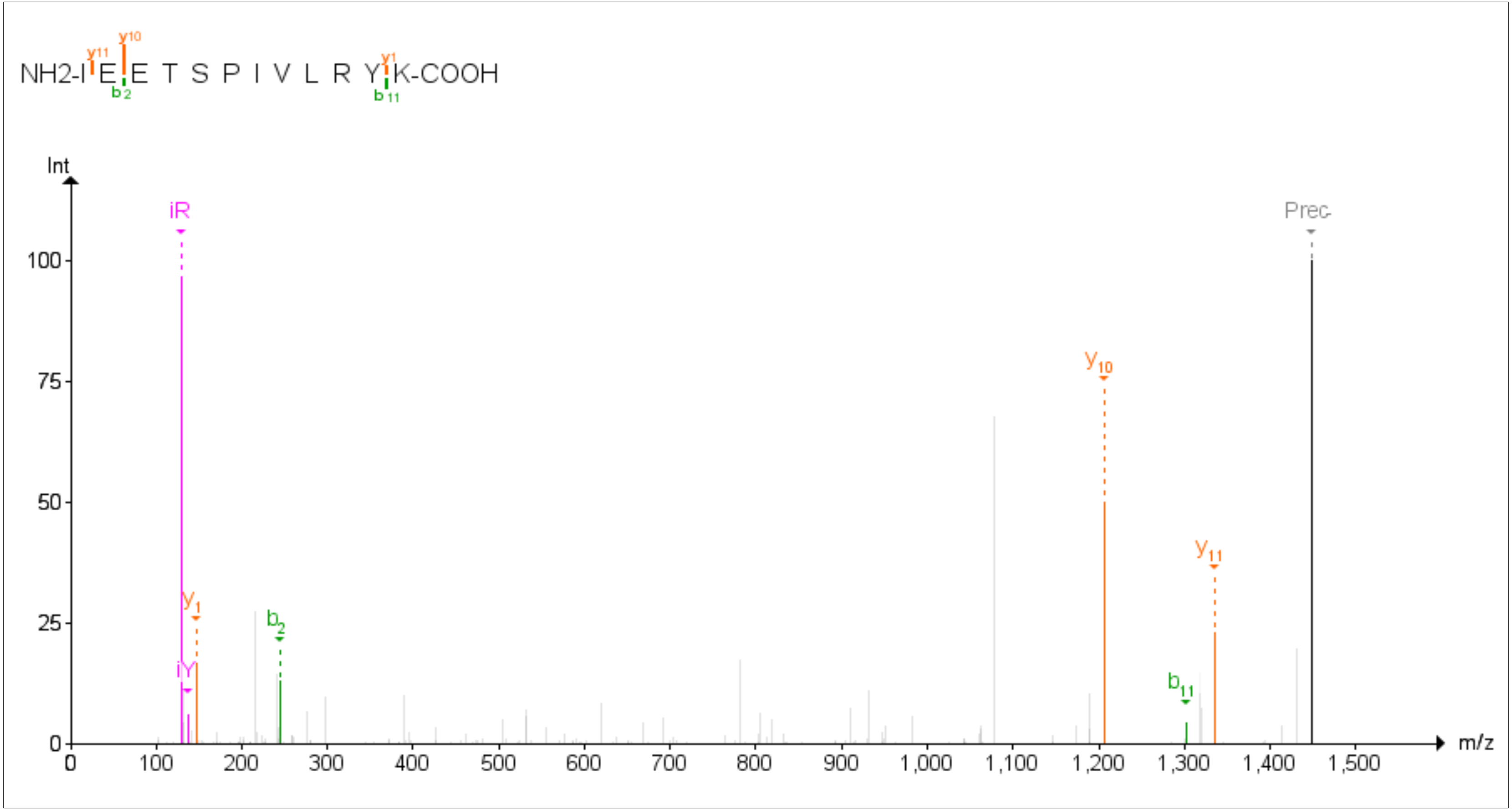

NH2-I E E T S P I V L R Y K-COOH
y11
y10
b2
y1
b11
Int
iR
Prec-
y10
y11
y1
iY
b2
b11
100
75
50
25
0
0
100
200
300
400
500
600
700
800
900
1,000
1,100
1,200
1,300
1,400
1,500
m/z

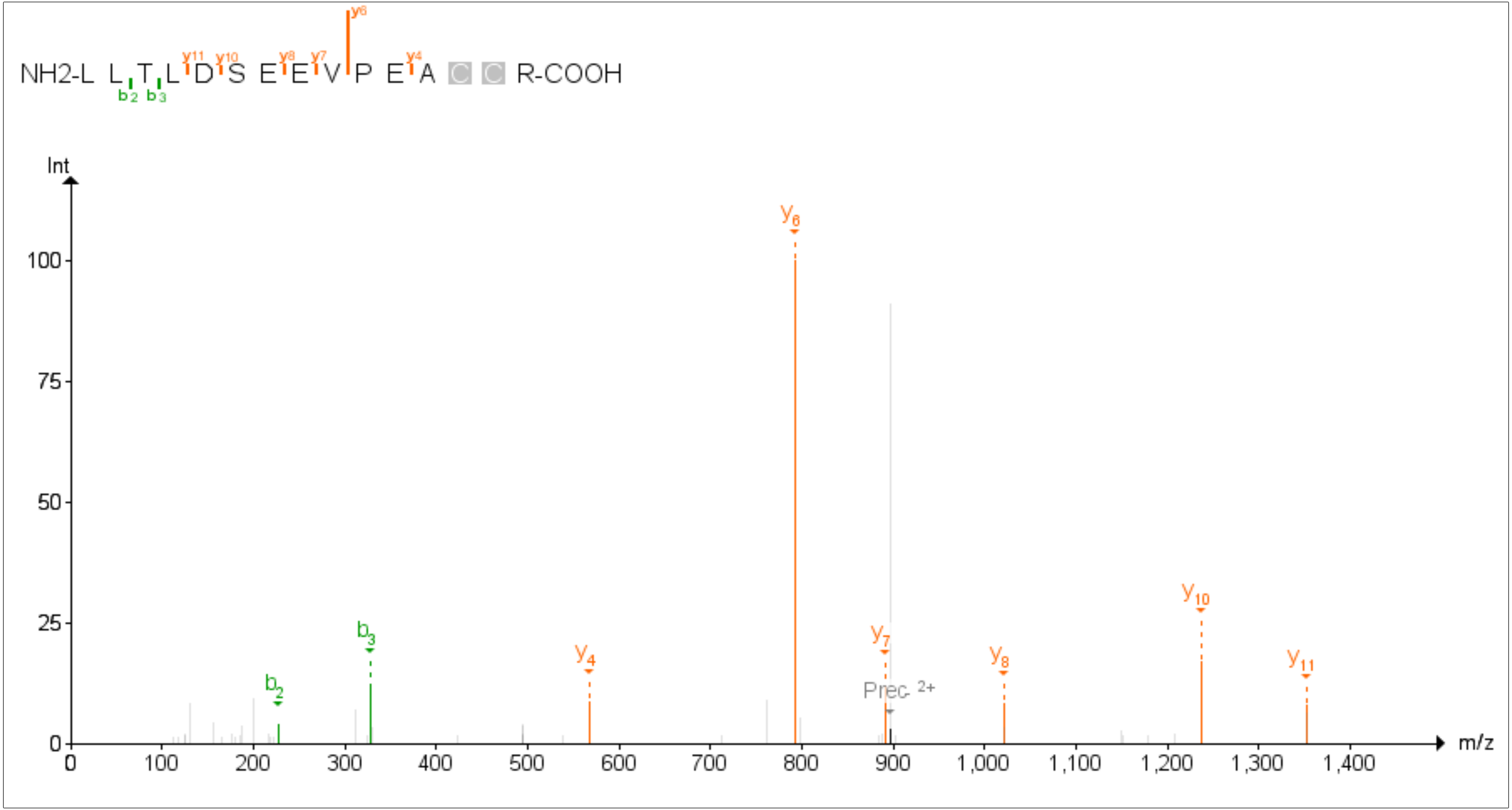

NH2-L L T L D S E E V P E A C C R-COOH
b2 b3 y11 y10 y8 y7 y6 y4
Int
100
75
50
25
0
b2
b3
y4
y6
y7
Prec 2+
y8
y10
y11
0 100 200 300 400 500 600 700 800 900 1,000 1,100 1,200 1,300 1,400
m/z

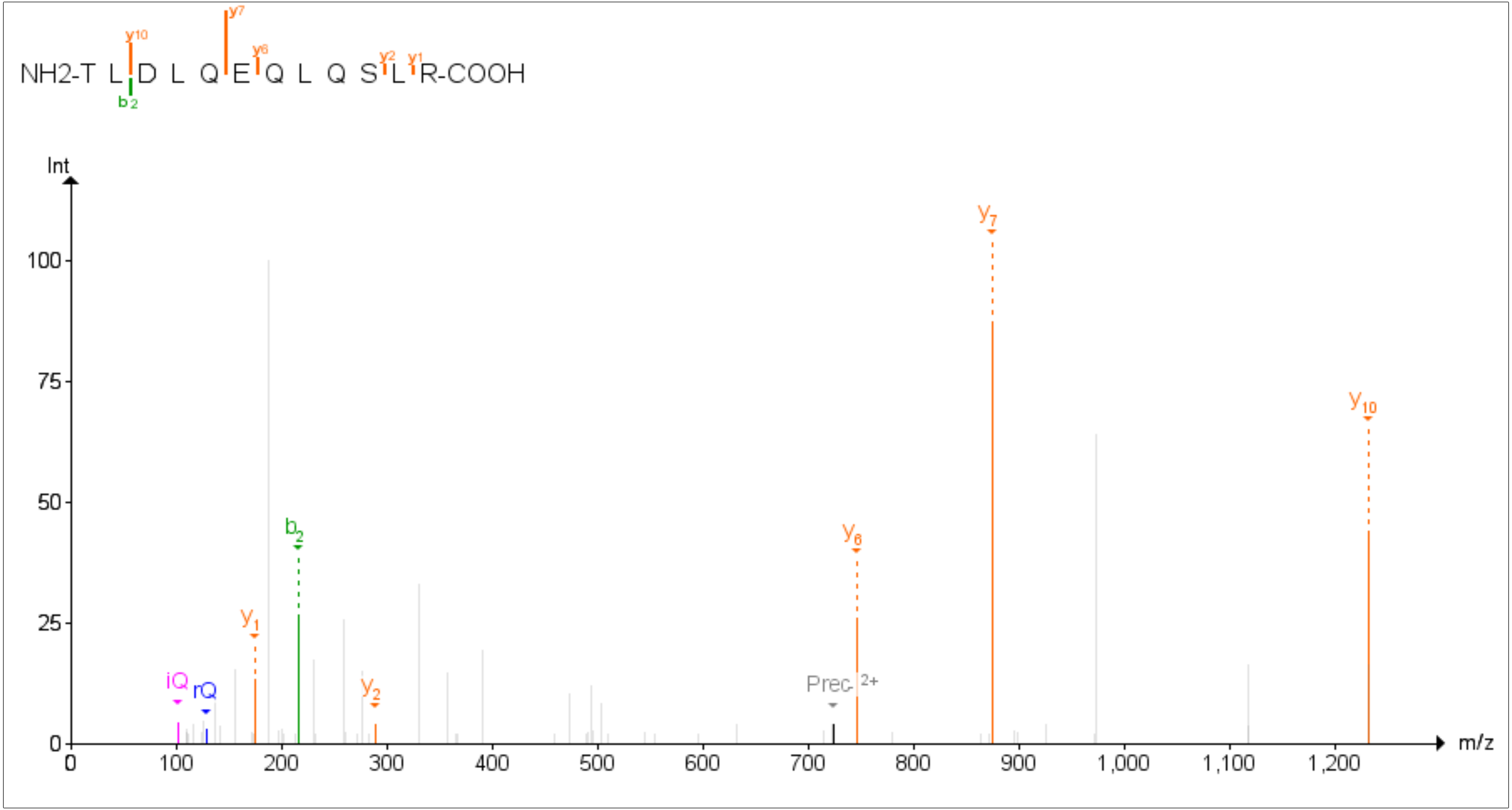

NH2-T L D L Q E Q L Q S L R-COOH
y10
y7
y6
y2
y1
b2
Int
100
75
50
25
0
y7
y10
b2
y6
y1
iQ
rQ
y2
Prec 2+
0
100
200
300
400
500
600
700
800
900
1,000
1,100
1,200
m/z

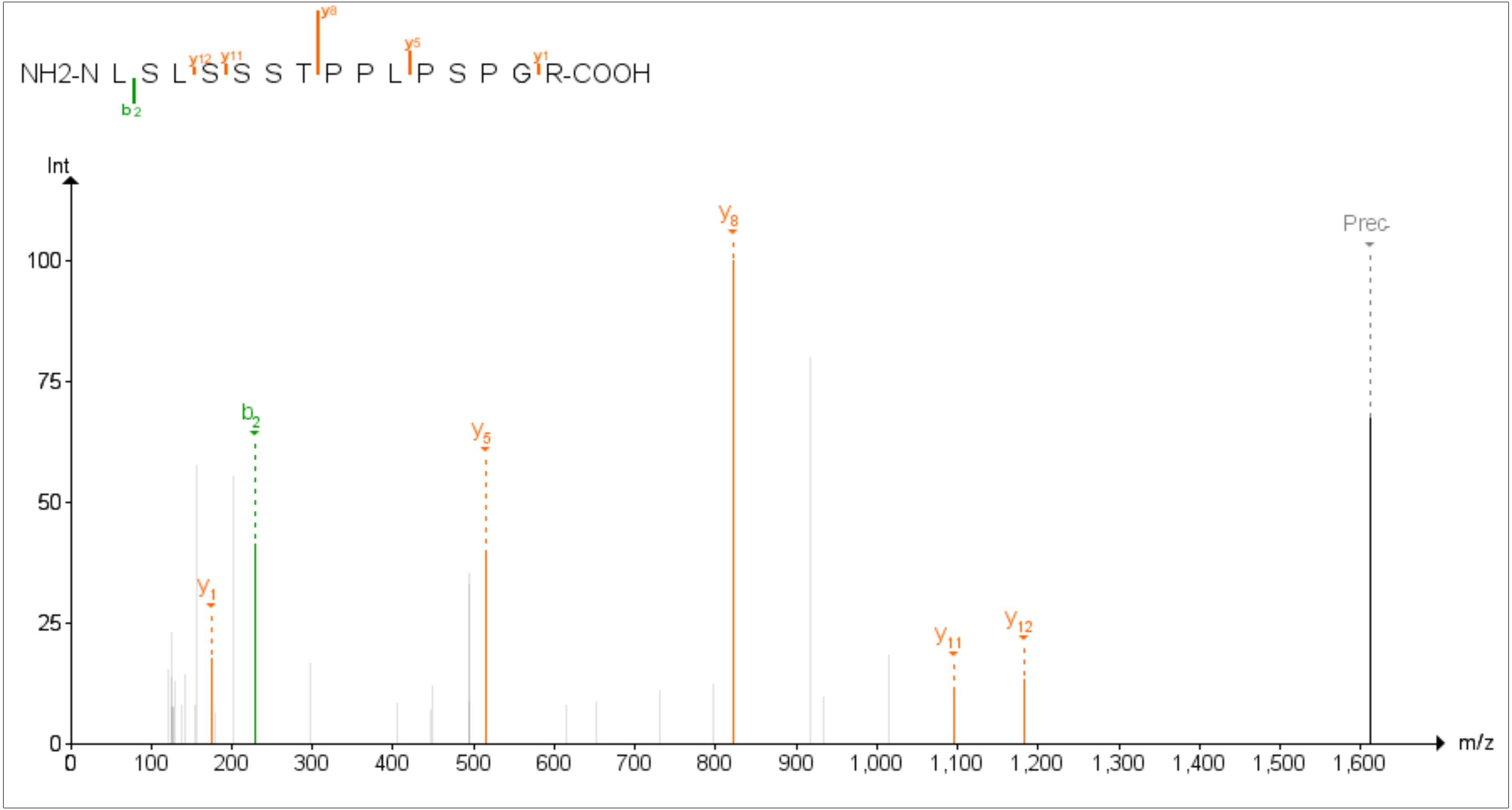

NH2-N L S L S S S T P P L P S P G R-COOH
b2
y12
y11
y8
y5
y1
Int
100
75
50
25
0
m/z
0
100
200
300
400
500
600
700
800
900
1,000
1,100
1,200
1,300
1,400
1,500
1,600
Prec

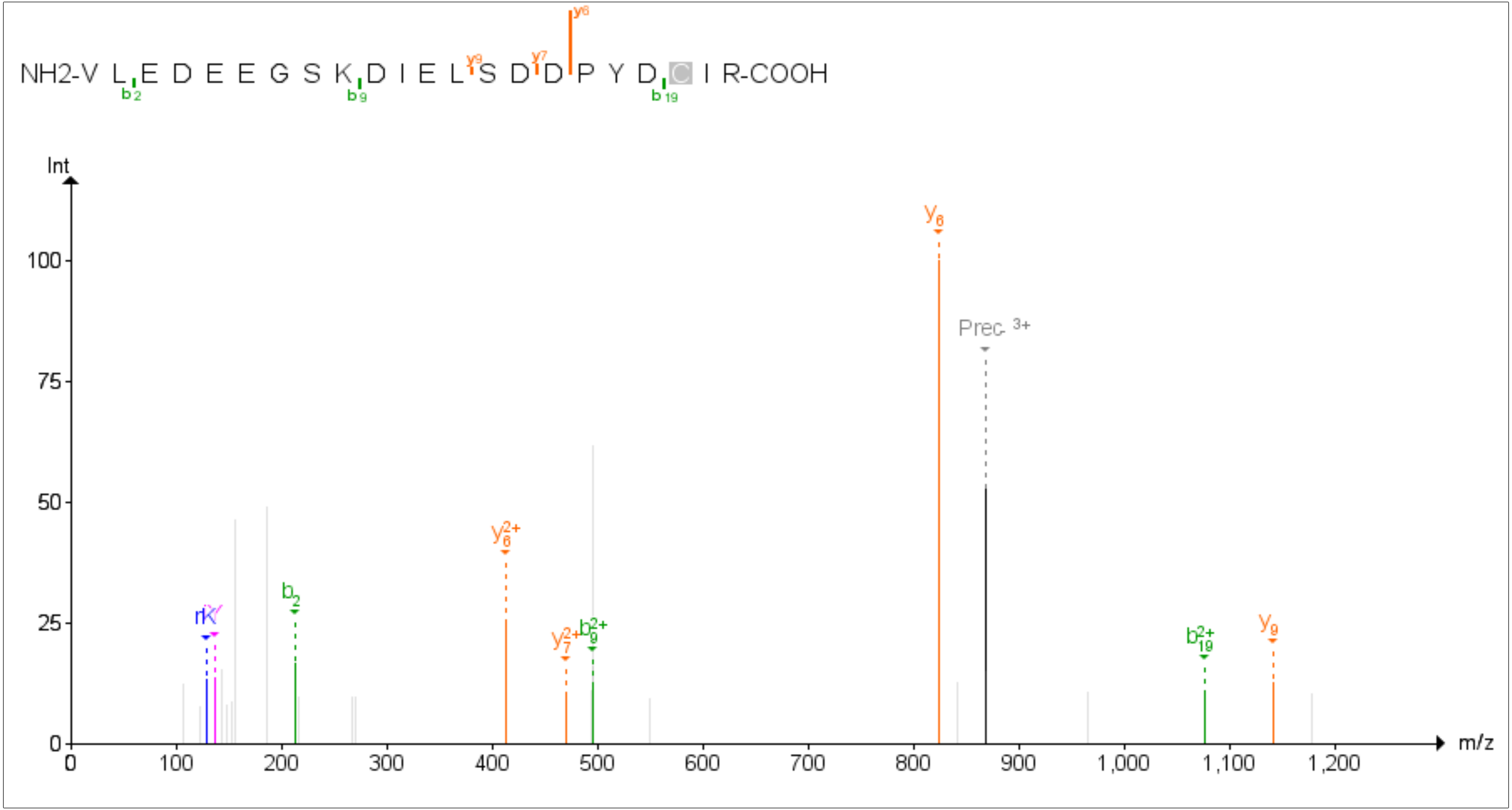

NH2-V L E D E E G S K D I E L S D D P Y D C I R-COOH
b2
b9
b19
y9
y7
y6
Int
100
75
50
25
0
m/z
0
100
200
300
400
500
600
700
800
900
1,000
1,100
1,200
y6
Prec 3+
y6 2+
b2
y7 2+
b9 2+
b19 2+
y9

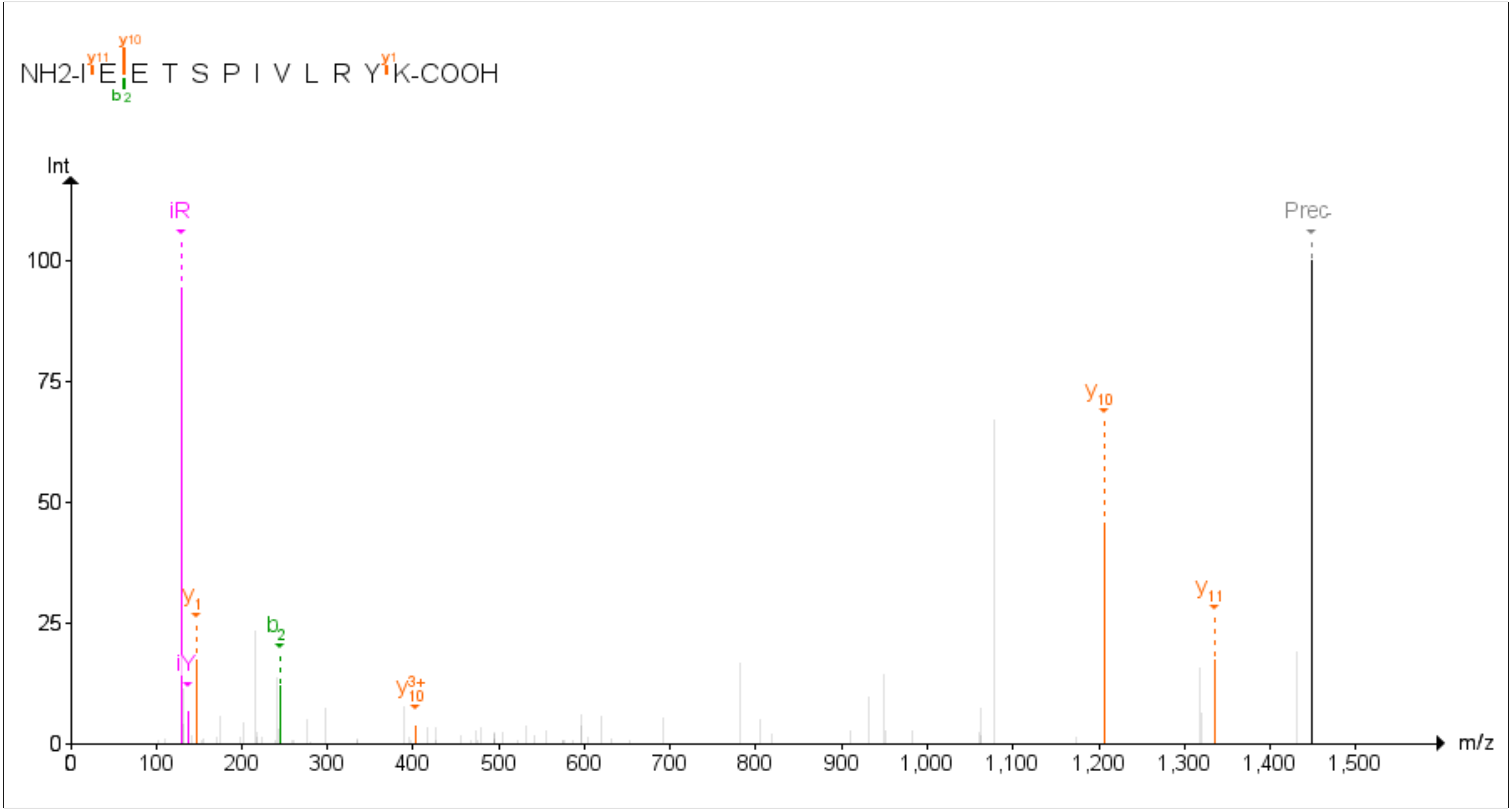

NH2-I E E T S P I V L R Y K-COOH
y11
y10
b2
y1
Int
iR
Prec
y10
y11
y1
iY
b2
y10 3+
100
75
50
25
0
0
100
200
300
400
500
600
700
800
900
1,000
1,100
1,200
1,300
1,400
1,500
m/z

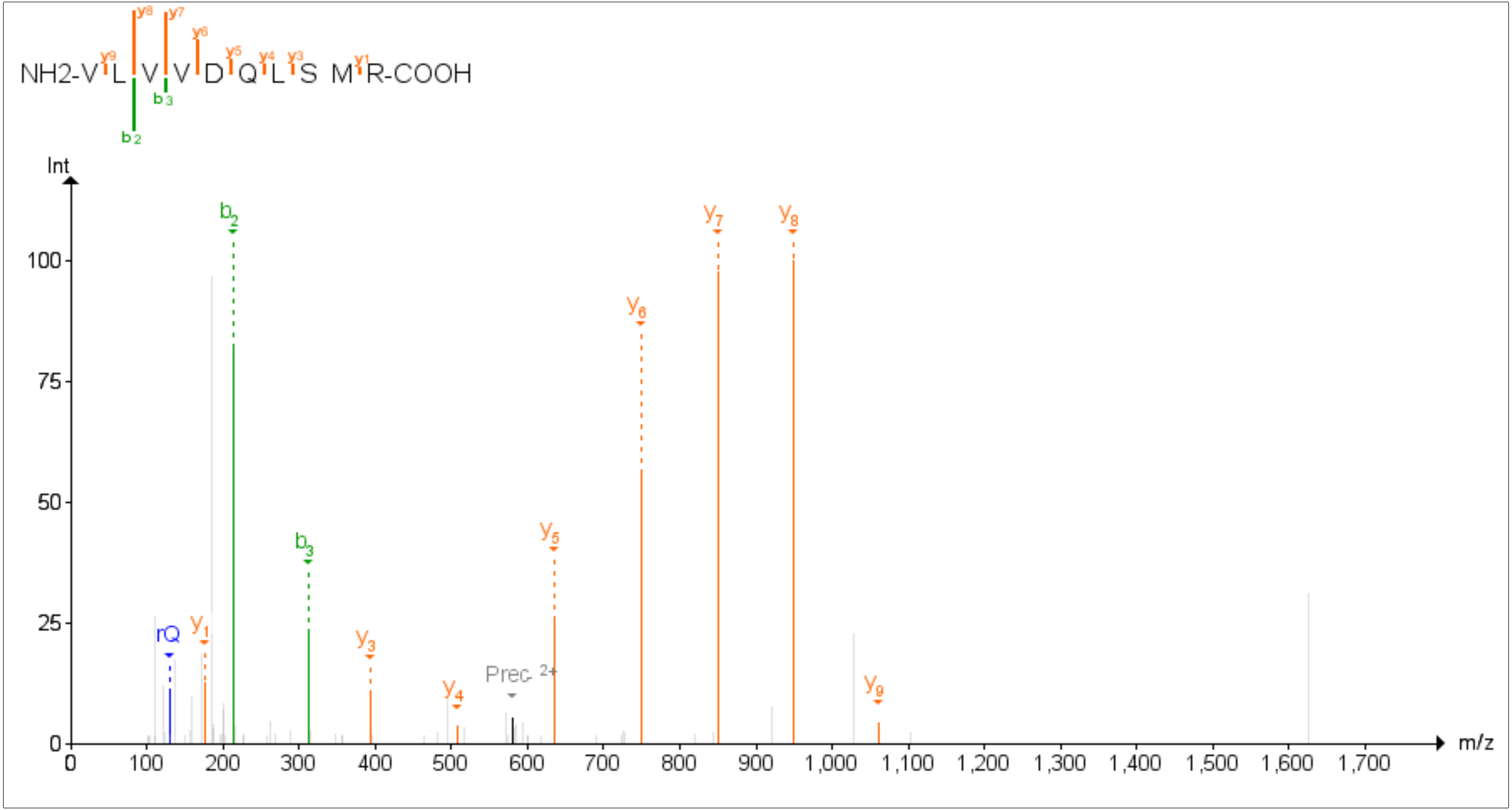

NH2-V L V V D Q L S M R-COOH
y9 y8 y7 y6 y5 y4 y3 y1
b2 b3
Int
100
75
50
25
0
b2
y7
y8
y6
y5
b3
rQ
y1
y3
y4
Prec 2+
y9
0 100 200 300 400 500 600 700 800 900 1,000 1,100 1,200 1,300 1,400 1,500 1,600 1,700
m/z

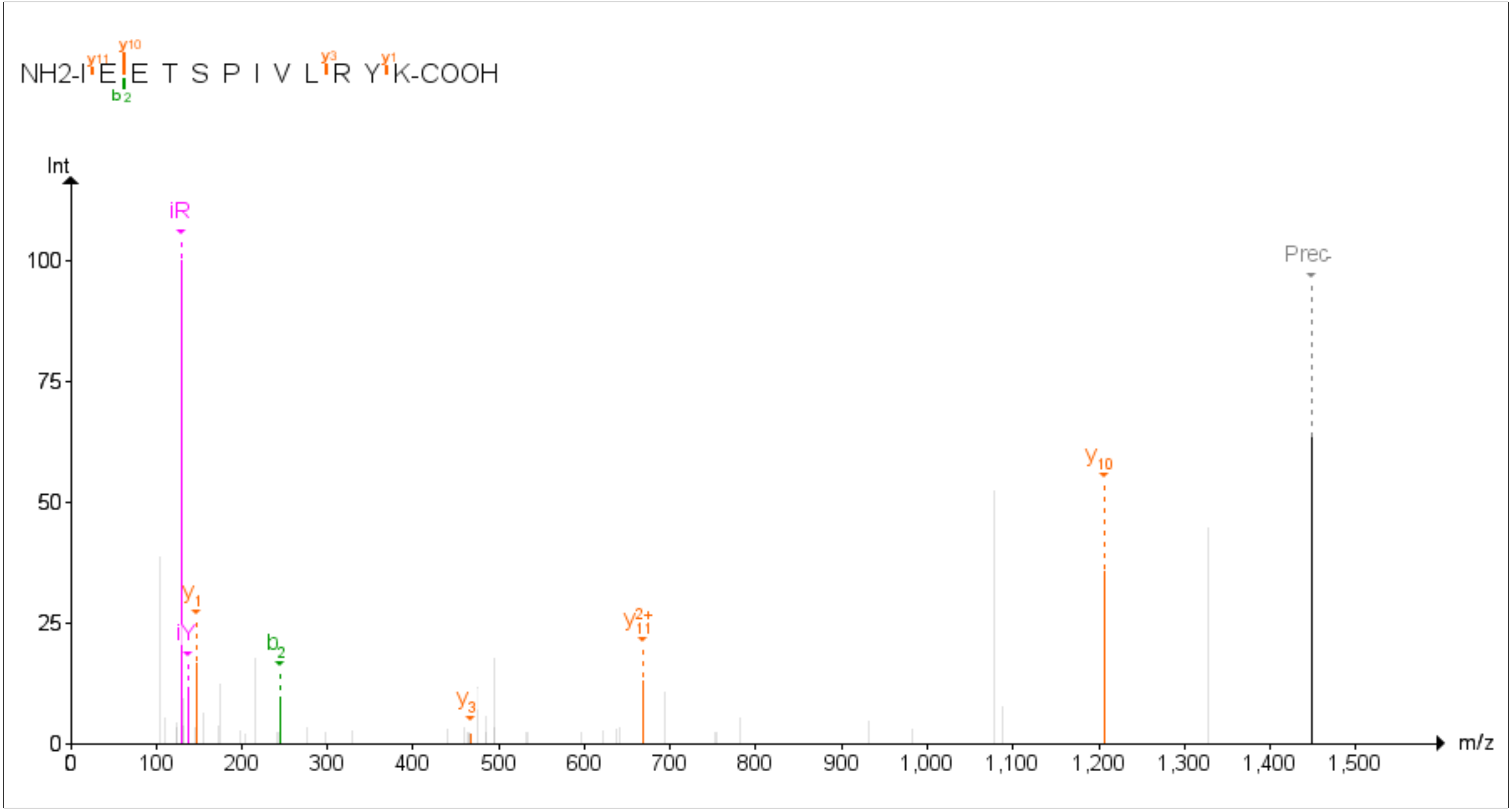

NH2-I E E T S P I V L R Y K-COOH
y11
y10
b2
y3
y1
Int
iR
Prec
y10
y1
iY
b2
y3
y11 2+
100
75
50
25
0
0
100
200
300
400
500
600
700
800
900
1,000
1,100
1,200
1,300
1,400
1,500
m/z

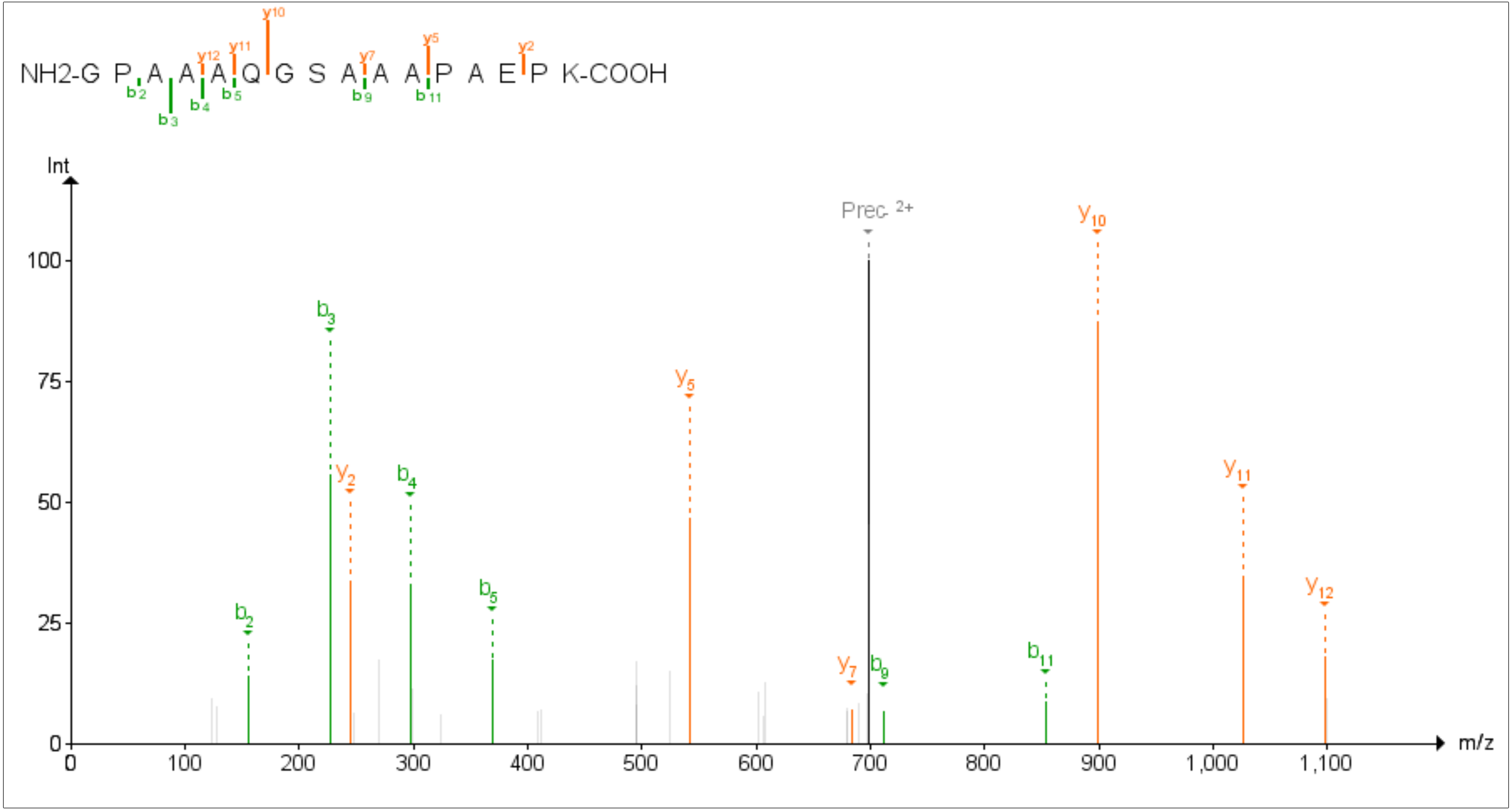

NH2-G P A A A Q G S A A A P A E P K-COOH
y10
y12
y11
y7
y5
y2
b2
b3
b4
b5
b9
b11
Int
Prec. 2+
100
75
50
25
0
m/z
0
100
200
300
400
500
600
700
800
900
1,000
1,100

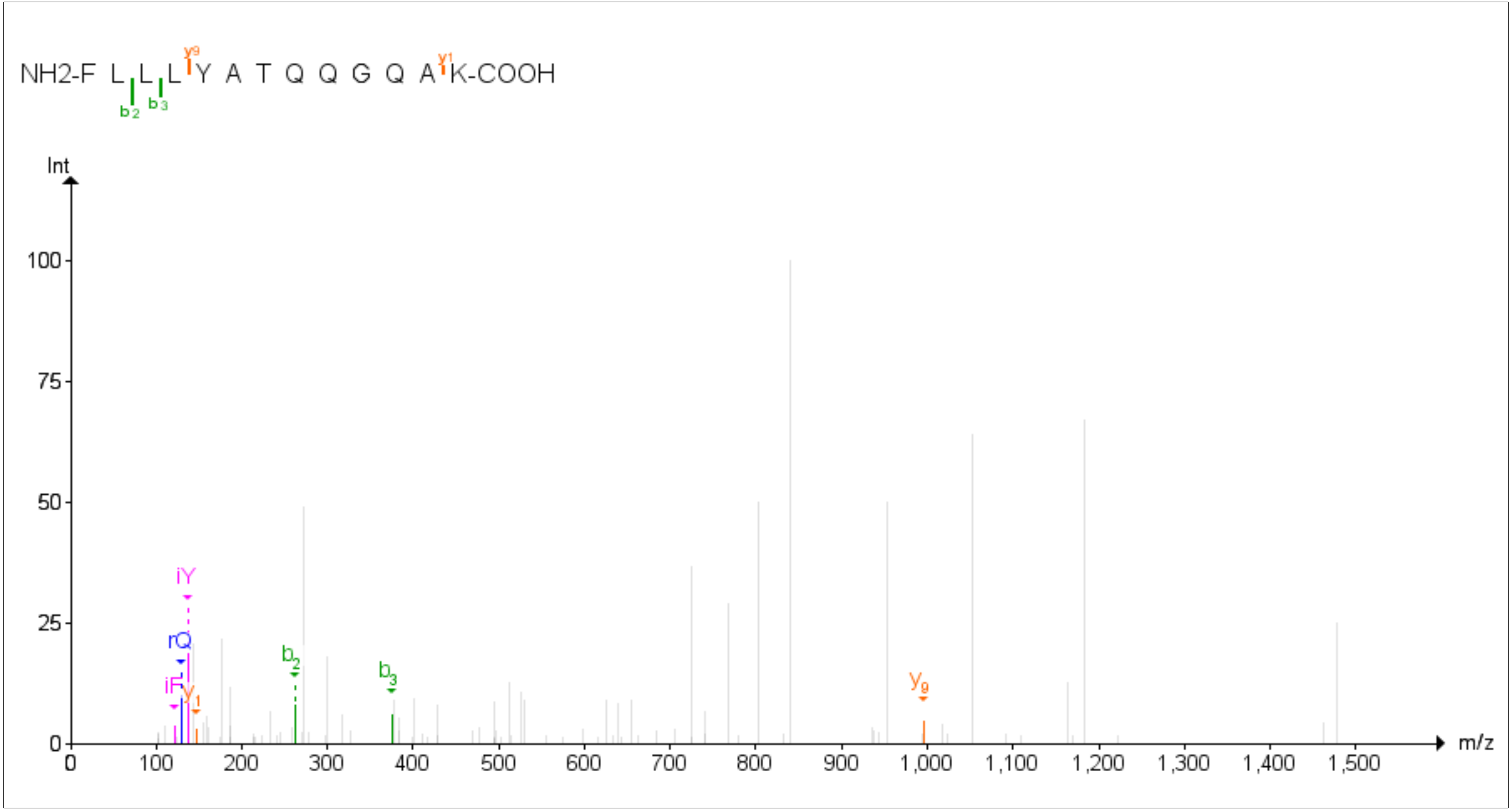

NH2-F L L L Y A T Q Q G Q A K-COOH
y9
y1
b2
b3
Int
100
75
50
25
0
iY
rQ
iF
y1
b2
b3
y9
0
100
200
300
400
500
600
700
800
900
1,000
1,100
1,200
1,300
1,400
1,500
m/z

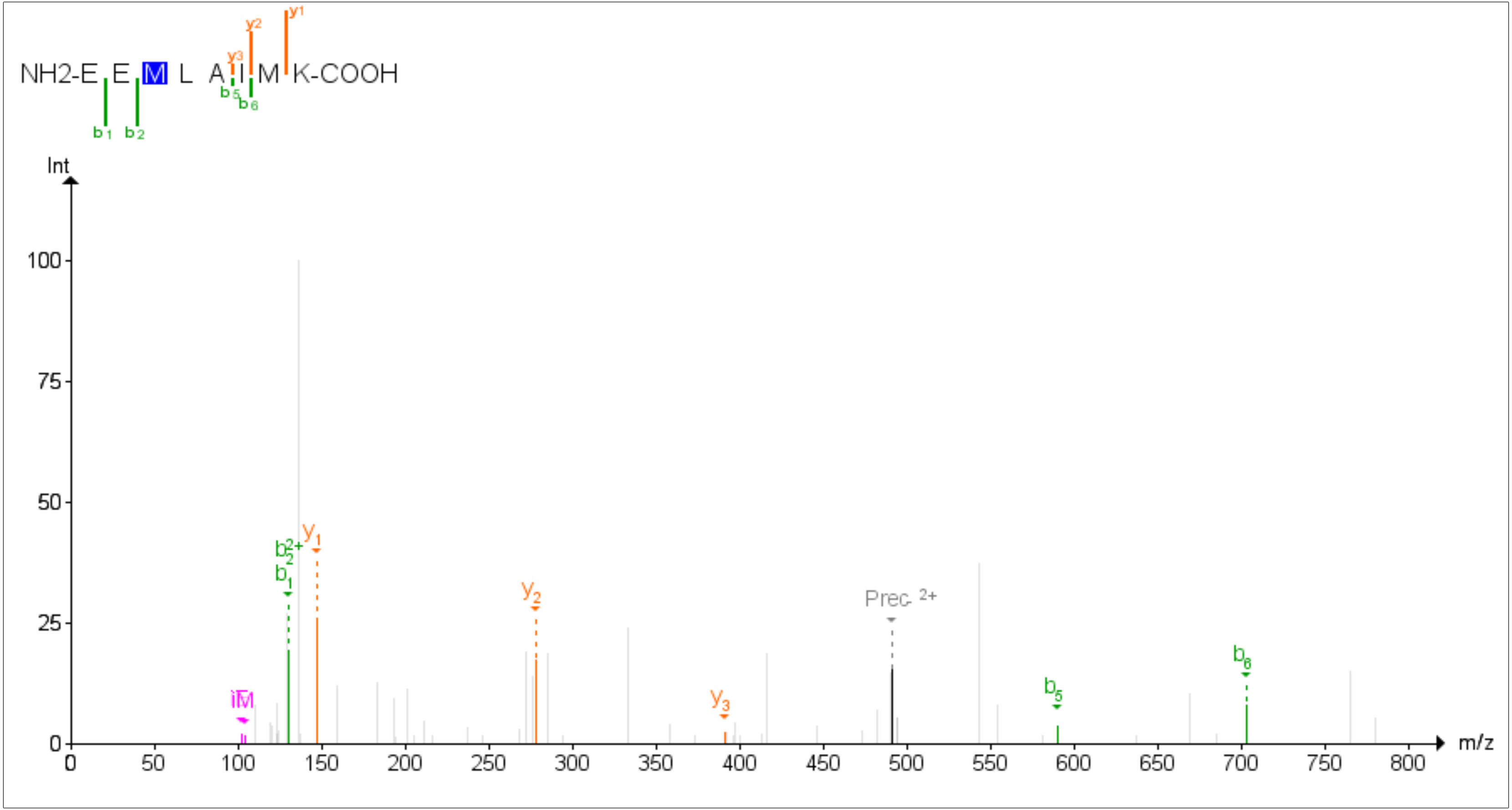

NH2-E E M L A I M K-COOH
y1
y2
y3
b1
b2
b5
b6
Int
100
75
50
25
0
m/z
0
50
100
150
200
250
300
350
400
450
500
550
600
650
700
750
800
iM
b1
b2 2+
y1
y2
y3
Prec 2+
b5
b6

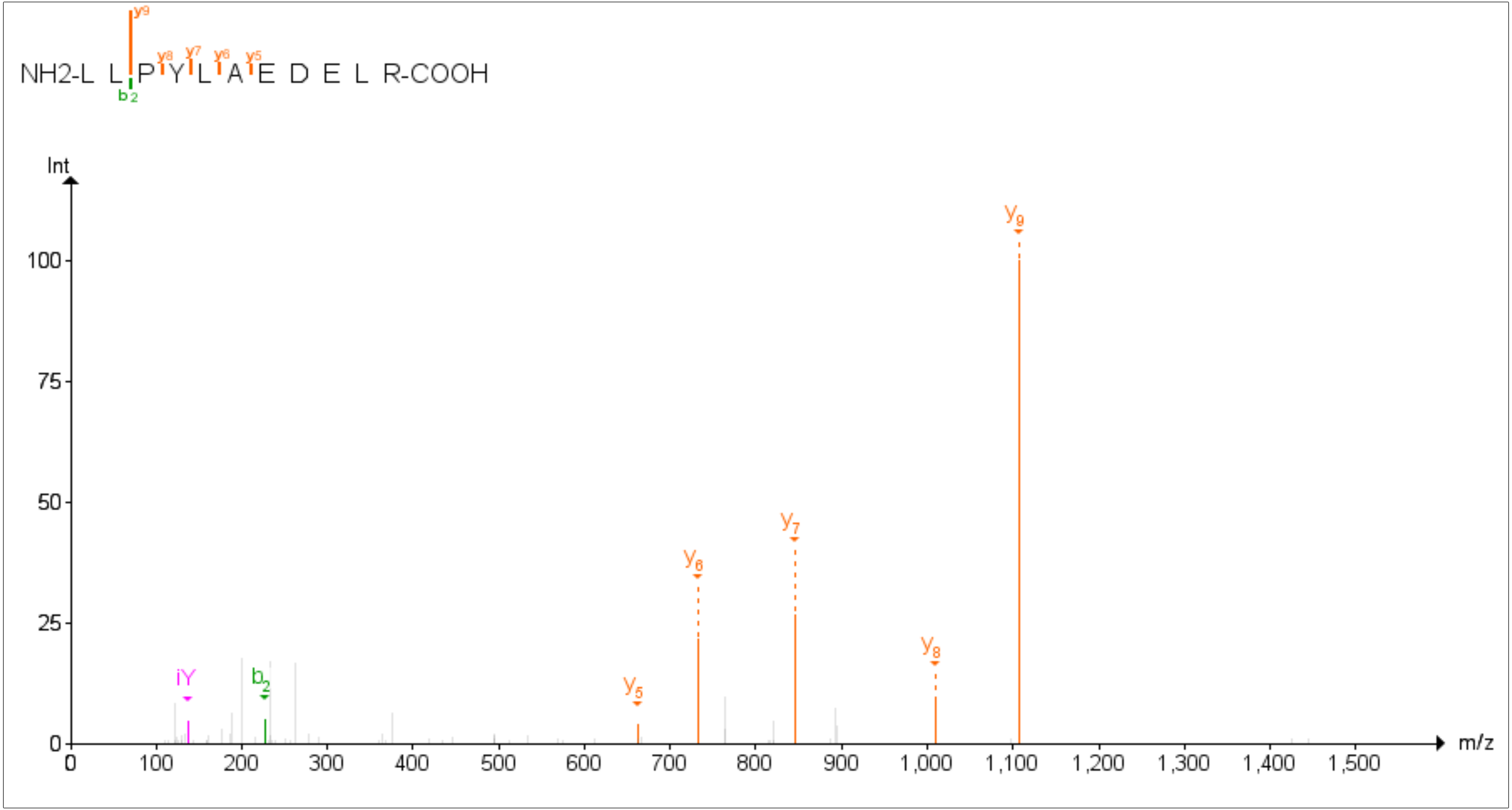

NH2-L L P Y L A E D E L R-COOH
y9
y8
y7
y6
y5
b2
Int
100
75
50
25
0
iY
b2
y5
y6
y7
y8
y9
0
100
200
300
400
500
600
700
800
900
1,000
1,100
1,200
1,300
1,400
1,500
m/z

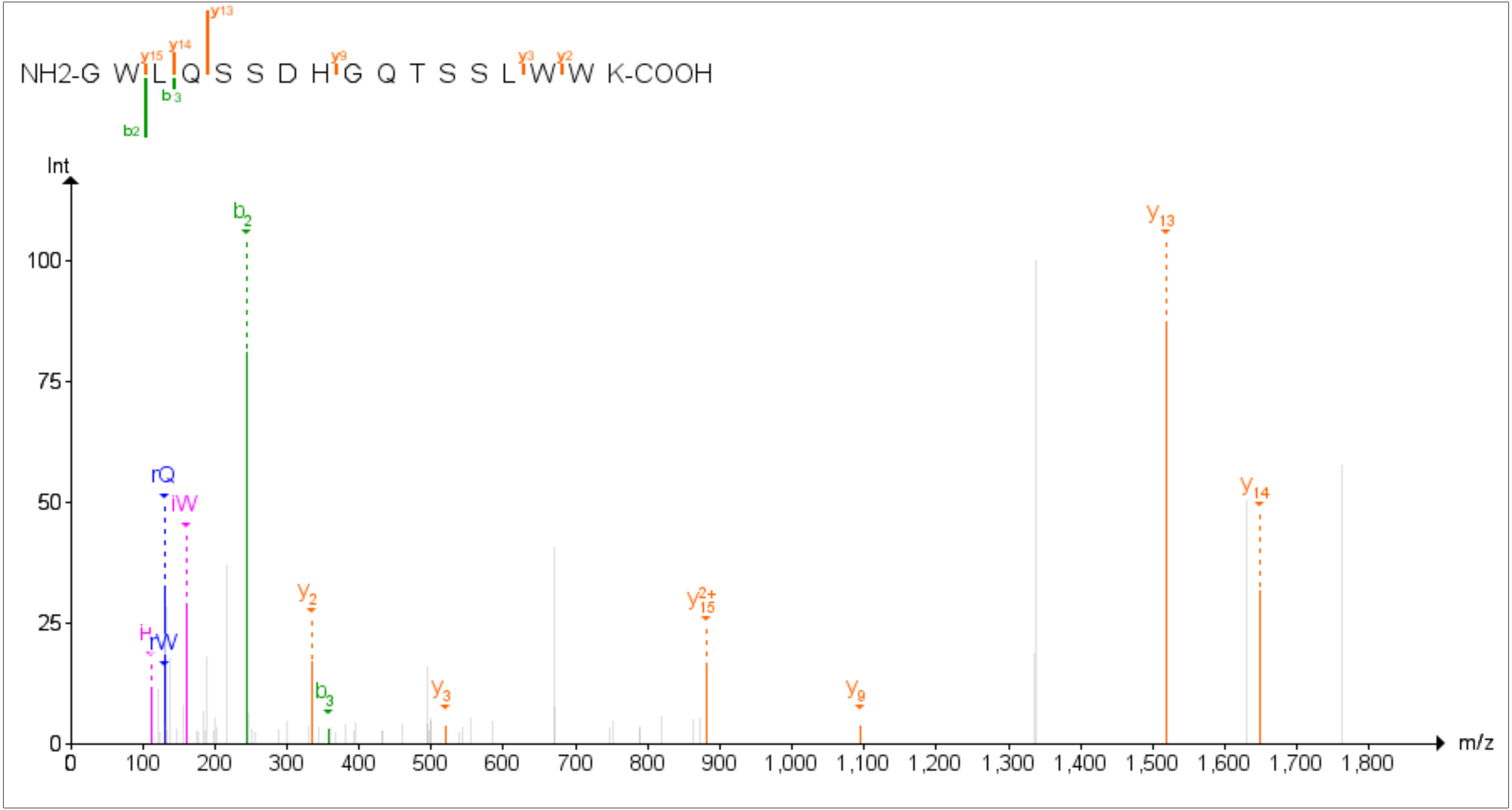

NH2-G W L Q S S D H G Q T S S L W W K-COOH
y13
y14
y15
y9
y3
y2
b3
b2
Int
m/z
rQ
iW
iH
rW
b2
y13
y14
y2
y15 2+
b3
y3
y9
0
25
50
75
100
0
100
200
300
400
500
600
700
800
900
1,000
1,100
1,200
1,300
1,400
1,500
1,600
1,700
1,800

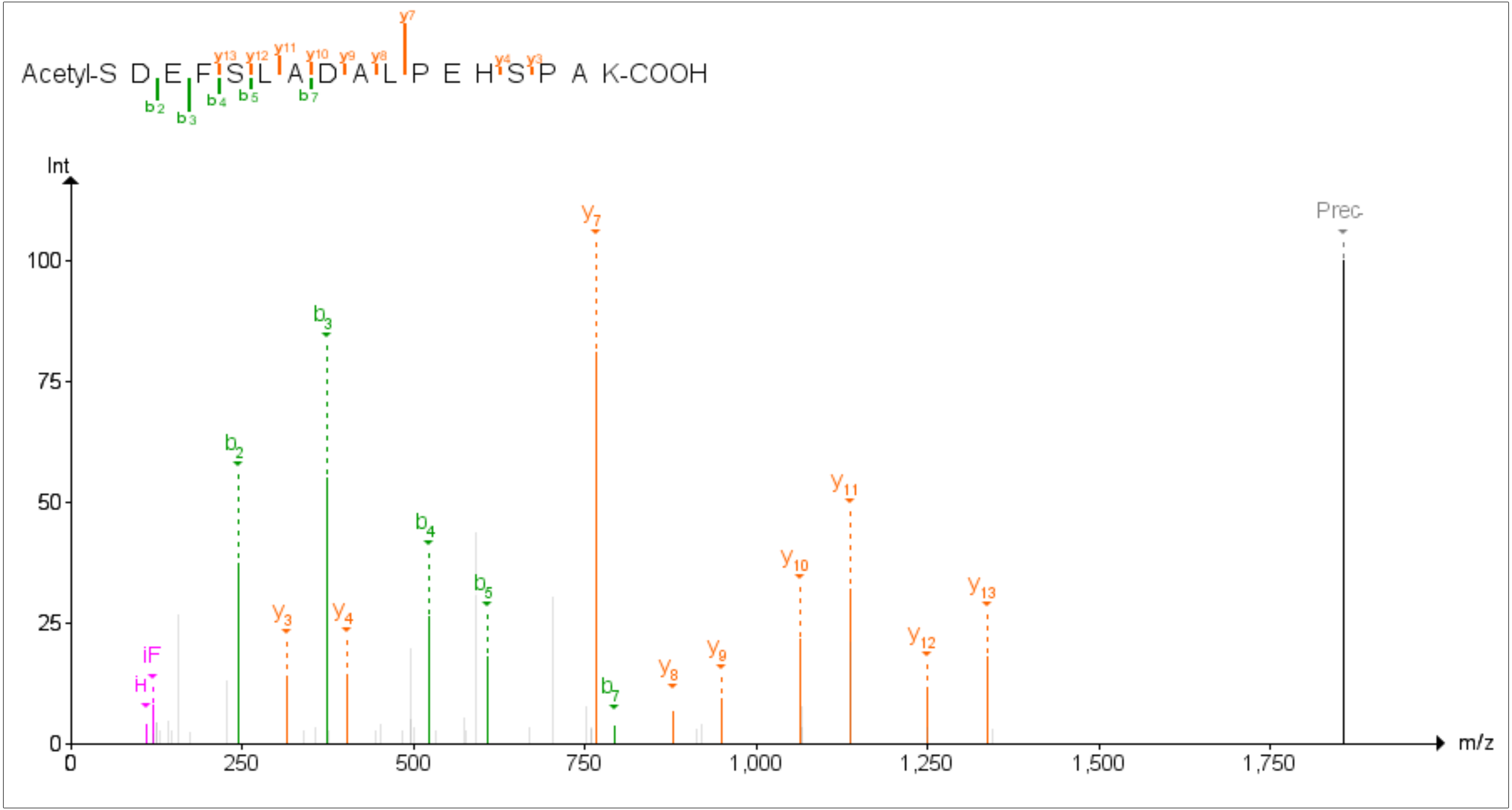

Acetyl-S D E F S L A D A L P E H S P A K-COOH
b2 b3 b4 b5 b7
y13 y12 y11 y10 y9 y8 y7 y4 y3
Int
m/z
Prec
0
25
50
75
100
250
500
750
1,000
1,250
1,500
1,750
iF
iH

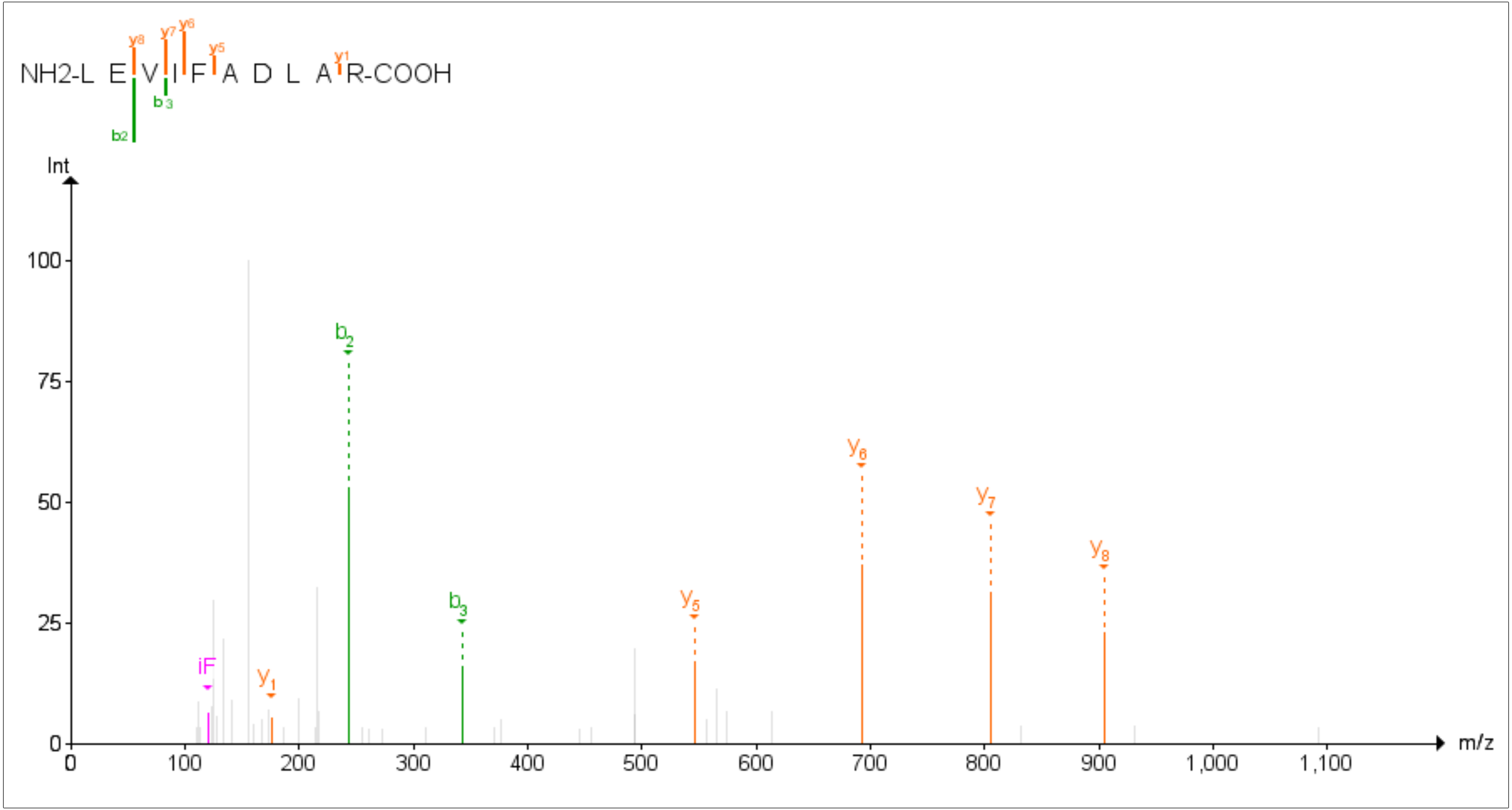

NH2-L E V I F A D L A R-COOH
y8
y7
y6
y5
y1
b2
b3
Int
100
75
50
25
0
b2
b3
y6
y7
y8
y5
iF
y1
m/z
0
100
200
300
400
500
600
700
800
900
1,000
1,100

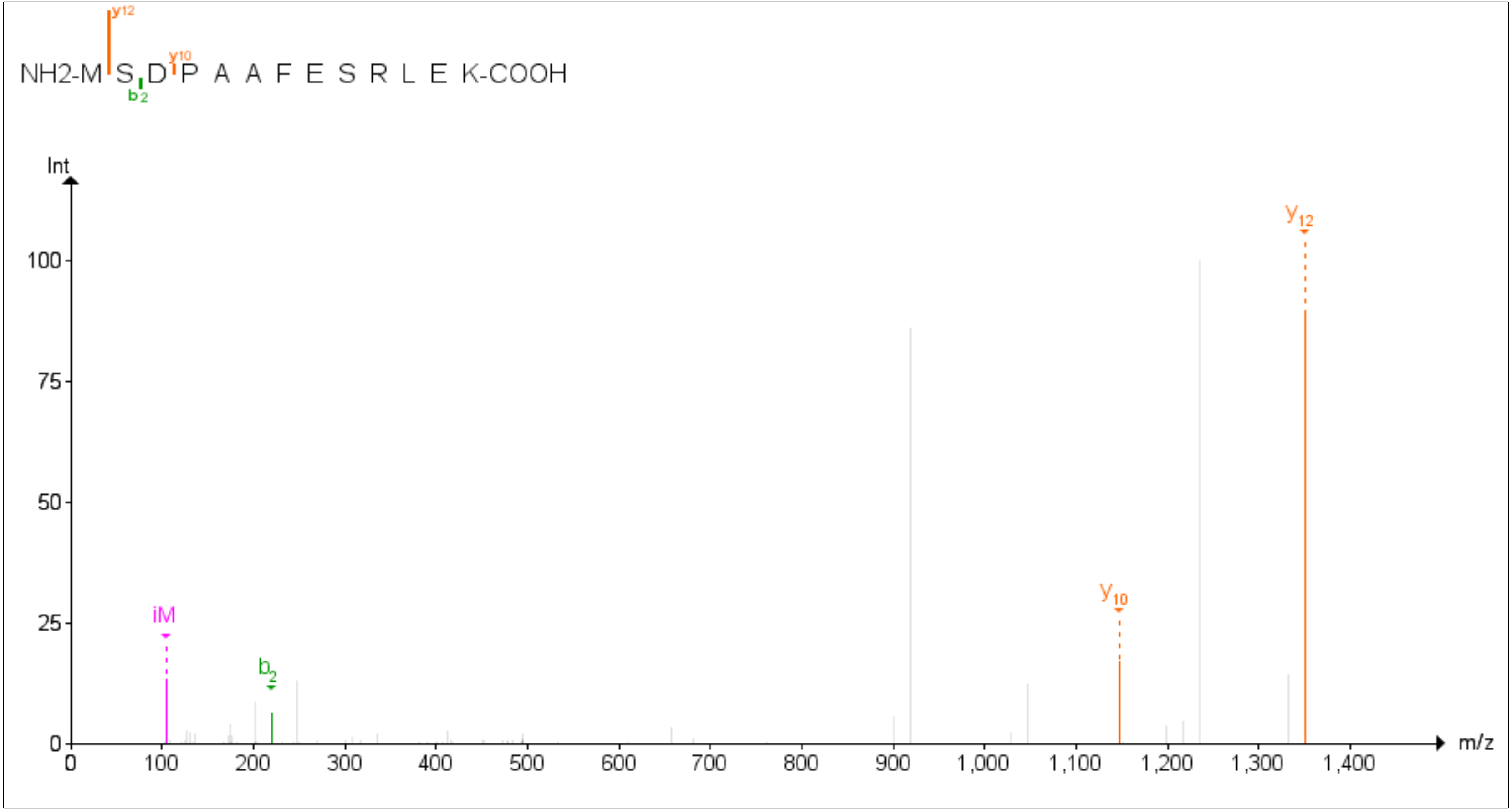
NH2-M S D P A A F E S R L E K-COOH
y12
y10
b2
Int
100
75
50
25
0
iM
b2
y10
y12
0
100
200
300
400
500
600
700
800
900
1,000
1,100
1,200
1,300
1,400
m/z

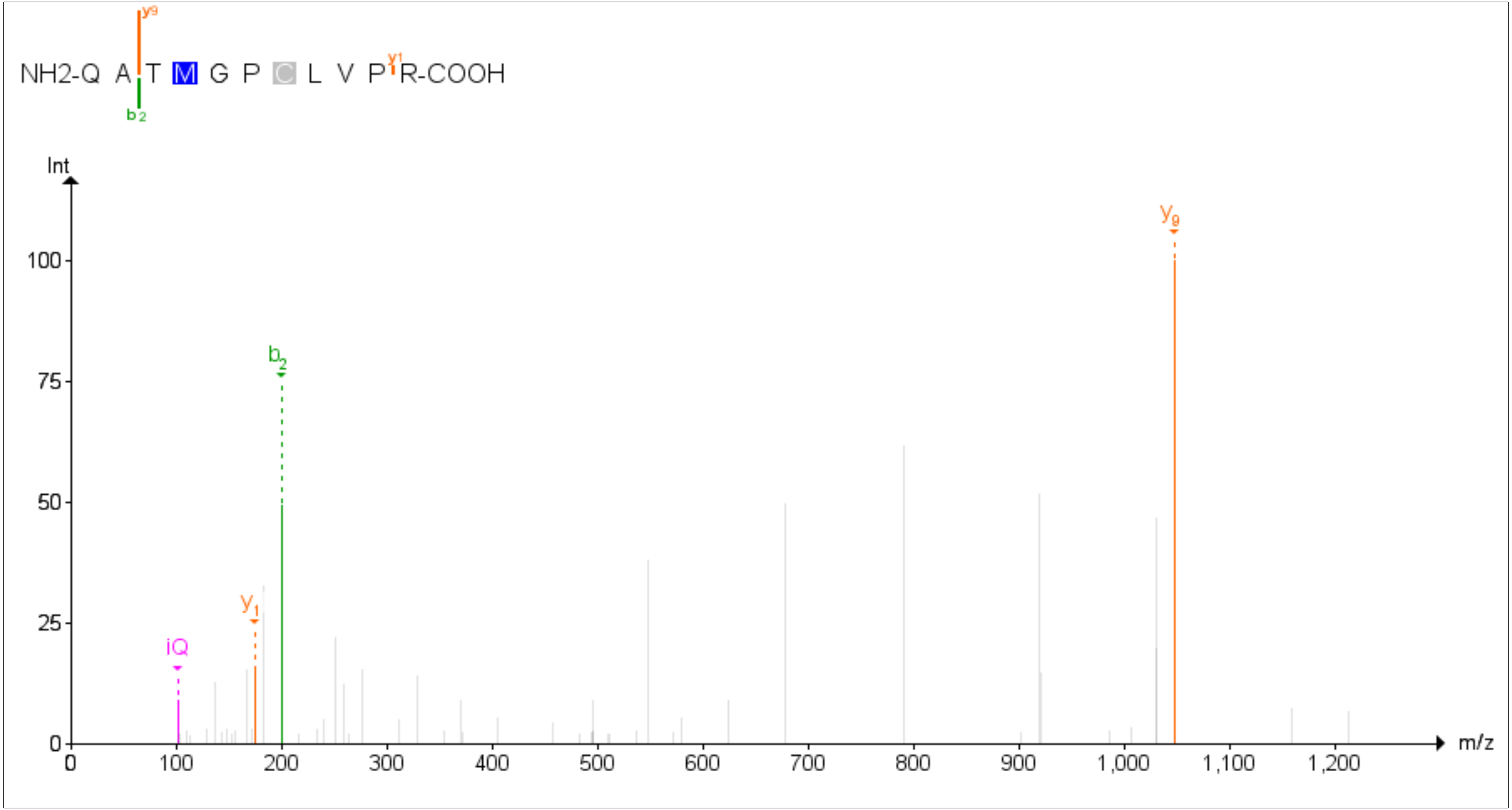

NH2-Q A T M G P C L V P R-COOH
y9
b2
y1
Int
100
75
50
25
0
m/z
0
100
200
300
400
500
600
700
800
900
1,000
1,100
1,200
iQ

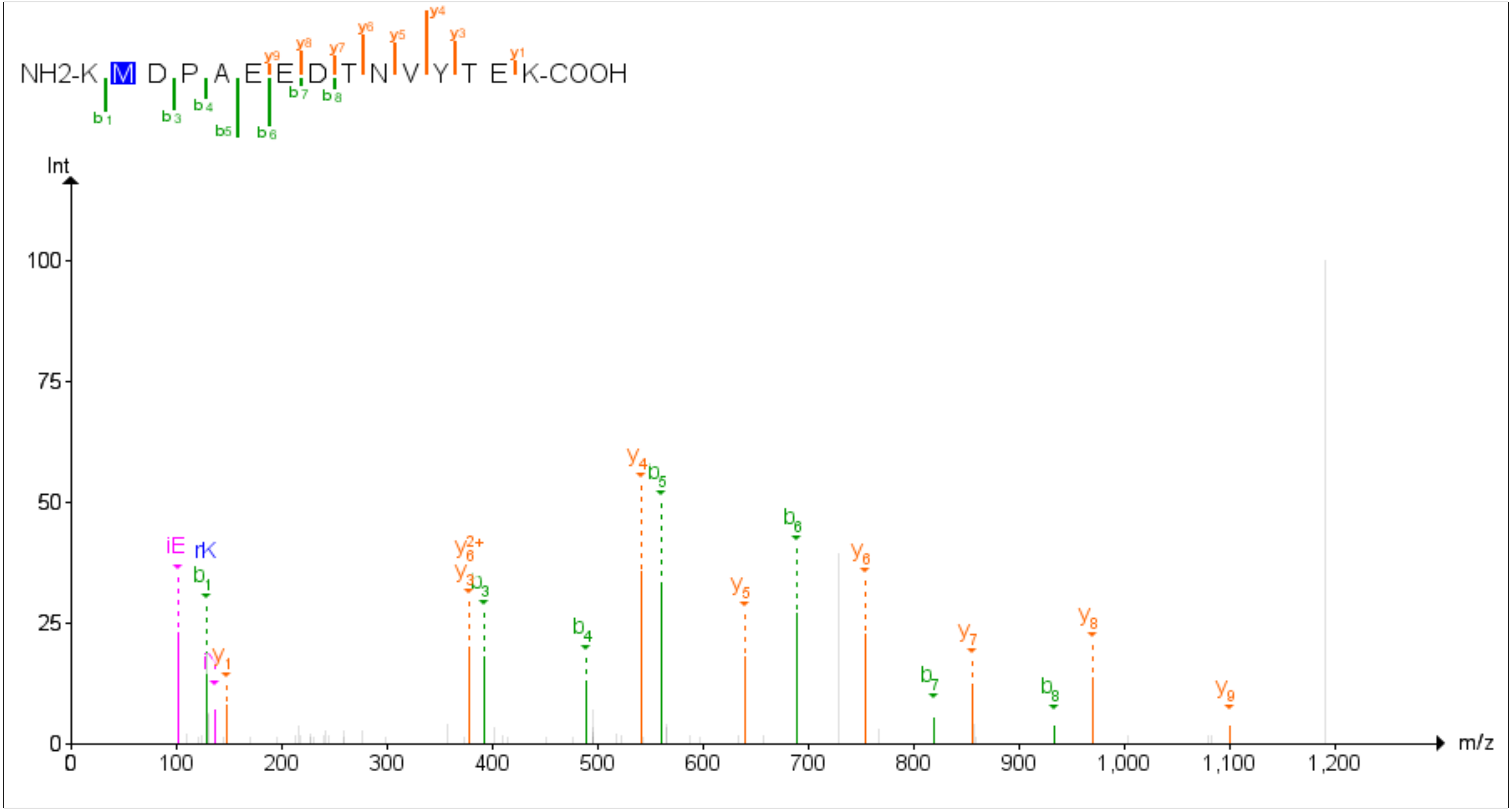

NH2-K M D P A E E D T N V Y T E K-COOH
b1 b3 b4 b5 b6 b7 b8
y9 y8 y7 y6 y5 y4 y3 y1
Int
100
75
50
25
0
iE
rK
b1
y1
y6 2+
y3
b3
b4
y4
b5
b6
y5
y6
b7
y7
b8
y8
y9
0 100 200 300 400 500 600 700 800 900 1,000 1,100 1,200
m/z

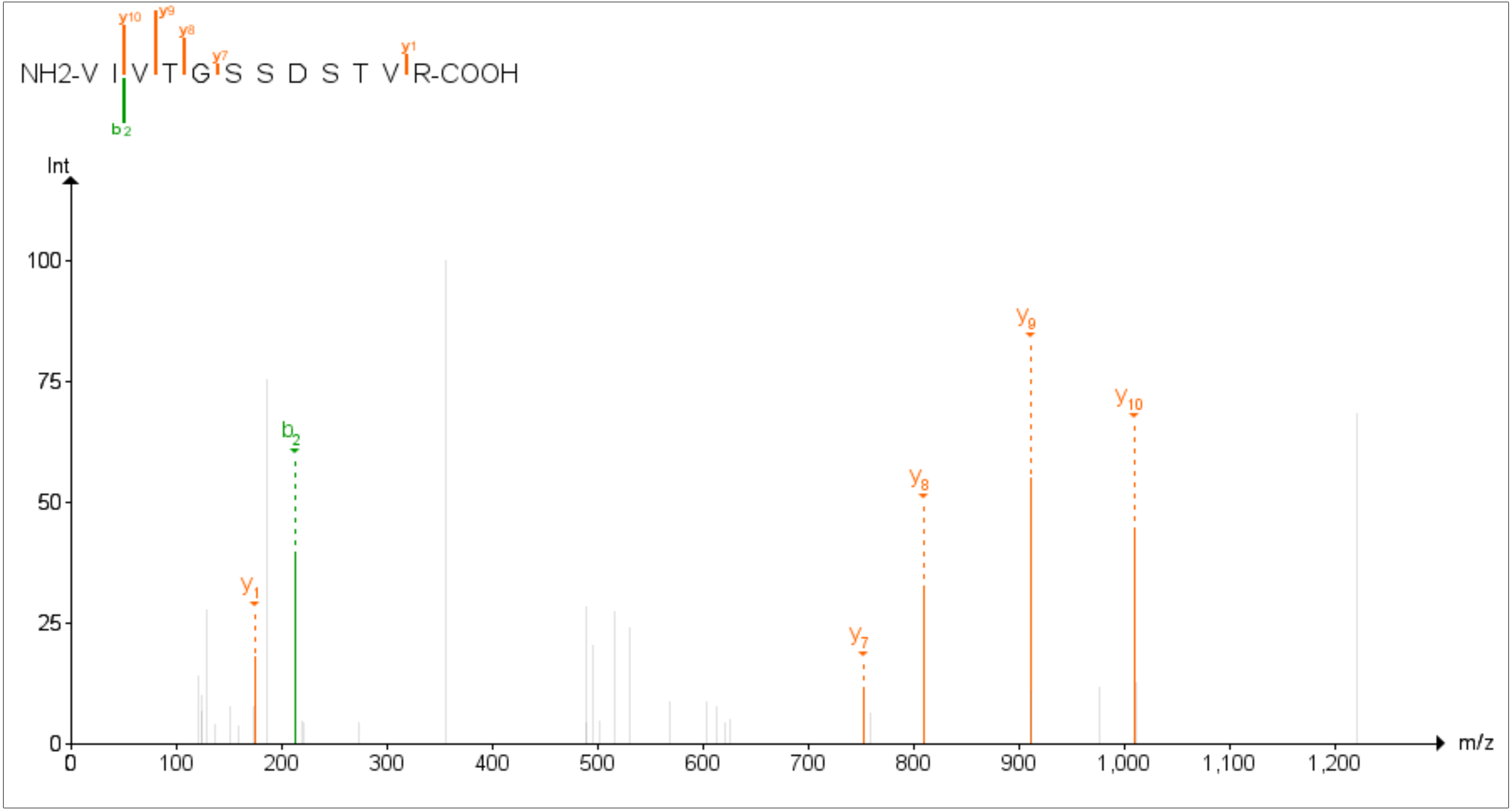

NH2-V I V T G S S D S T V R-COOH
y10
y9
y8
y7
y1
b2
Int
100
75
50
25
0
y9
y10
b2
y8
y1
y7
0
100
200
300
400
500
600
700
800
900
1,000
1,100
1,200
m/z

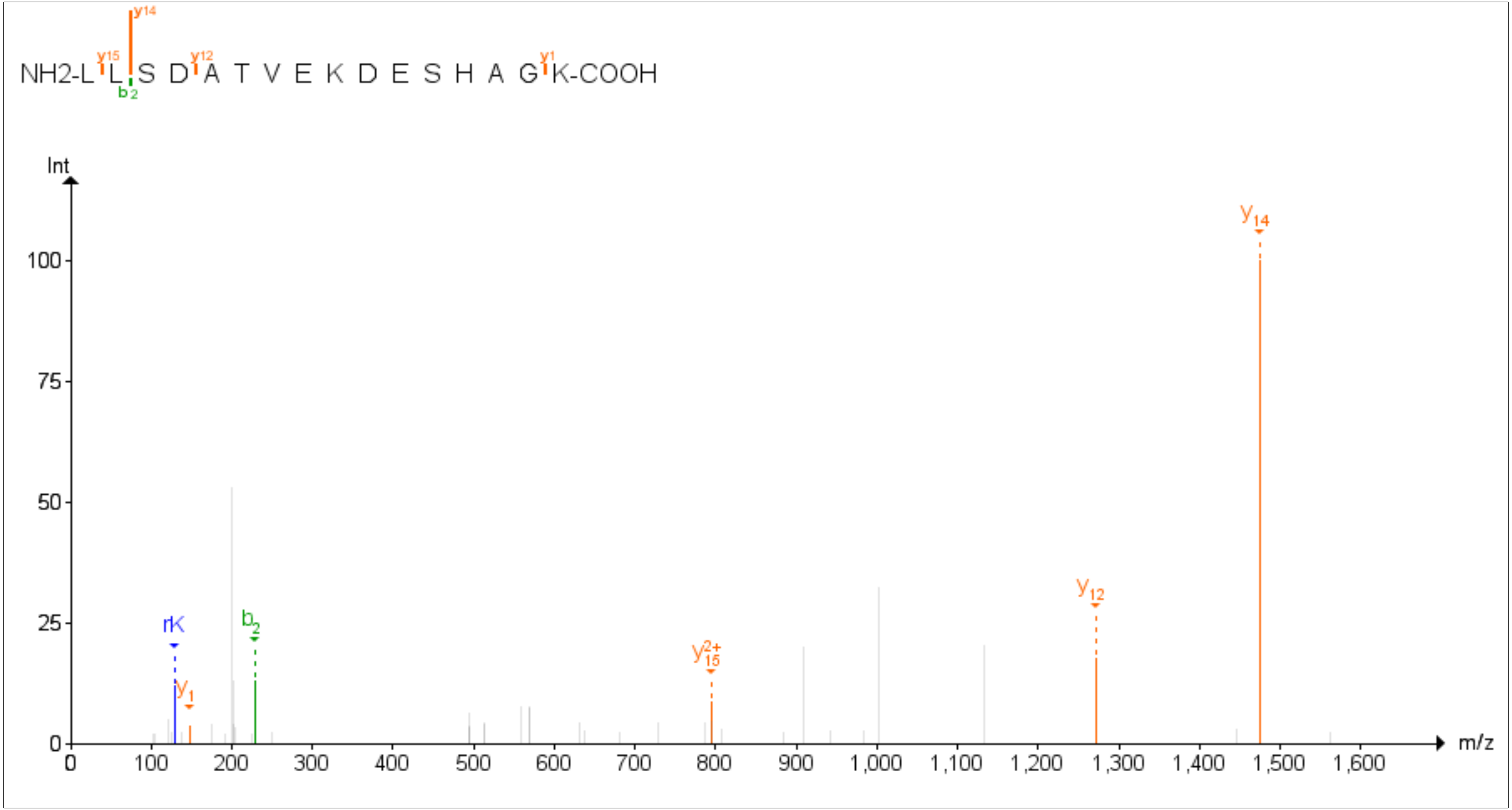

NH2-L L S D A T V E K D E S H A G K-COOH
y15
y14
y12
y1
b2
Int
100
75
50
25
0
rK
y1
b2
y15 2+
y12
y14
0
100
200
300
400
500
600
700
800
900
1,000
1,100
1,200
1,300
1,400
1,500
1,600
m/z

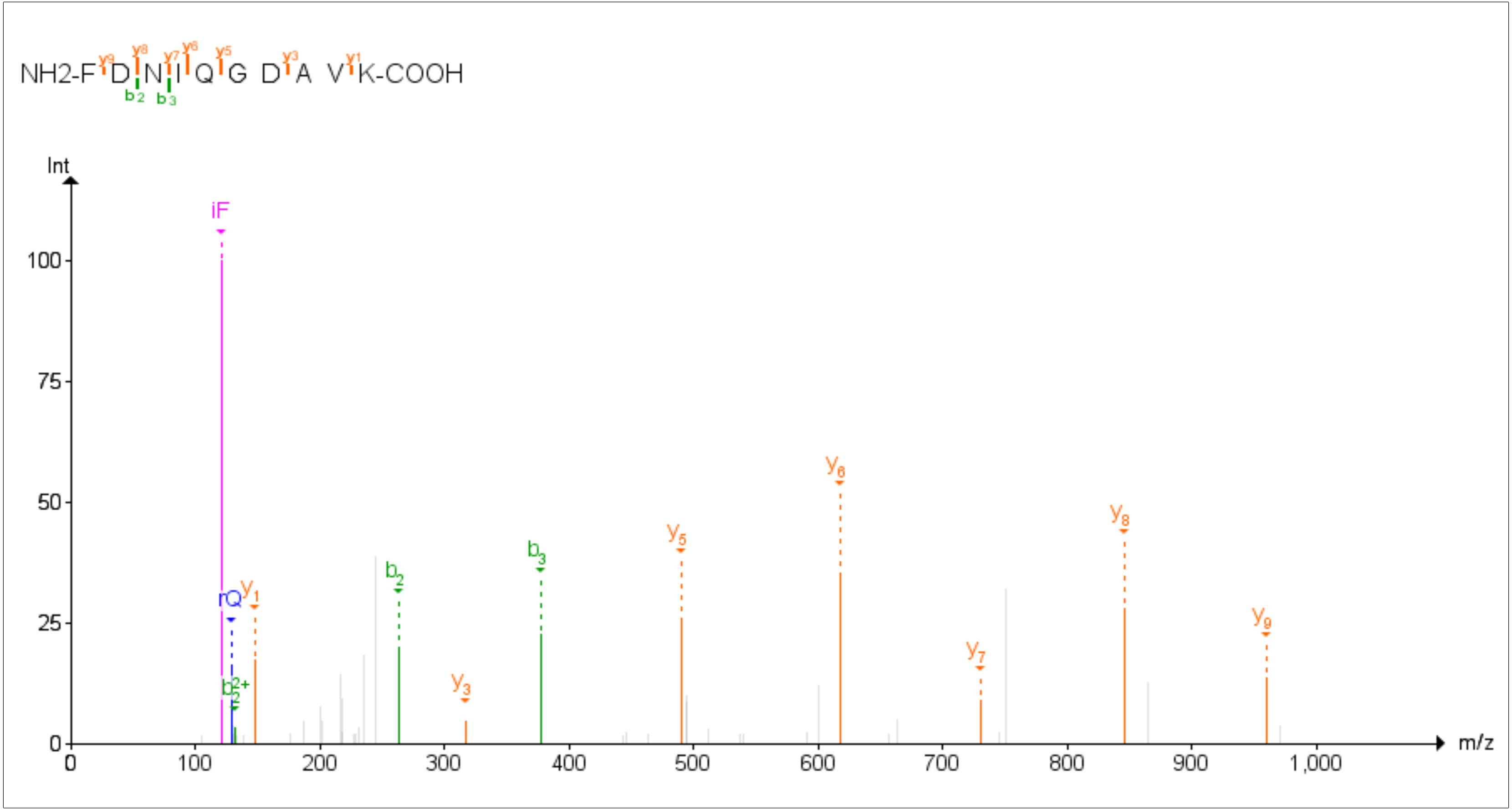

NH2-F D N I Q G D A V K-COOH
y9 y8 y7 y6 y5 y3 y1
b2 b3
Int
m/z
iF
rQ
y1
b2
b3
y3
y5
y6
y7
y8
y9
0
25
50
75
100
0
100
200
300
400
500
600
700
800
900
1,000

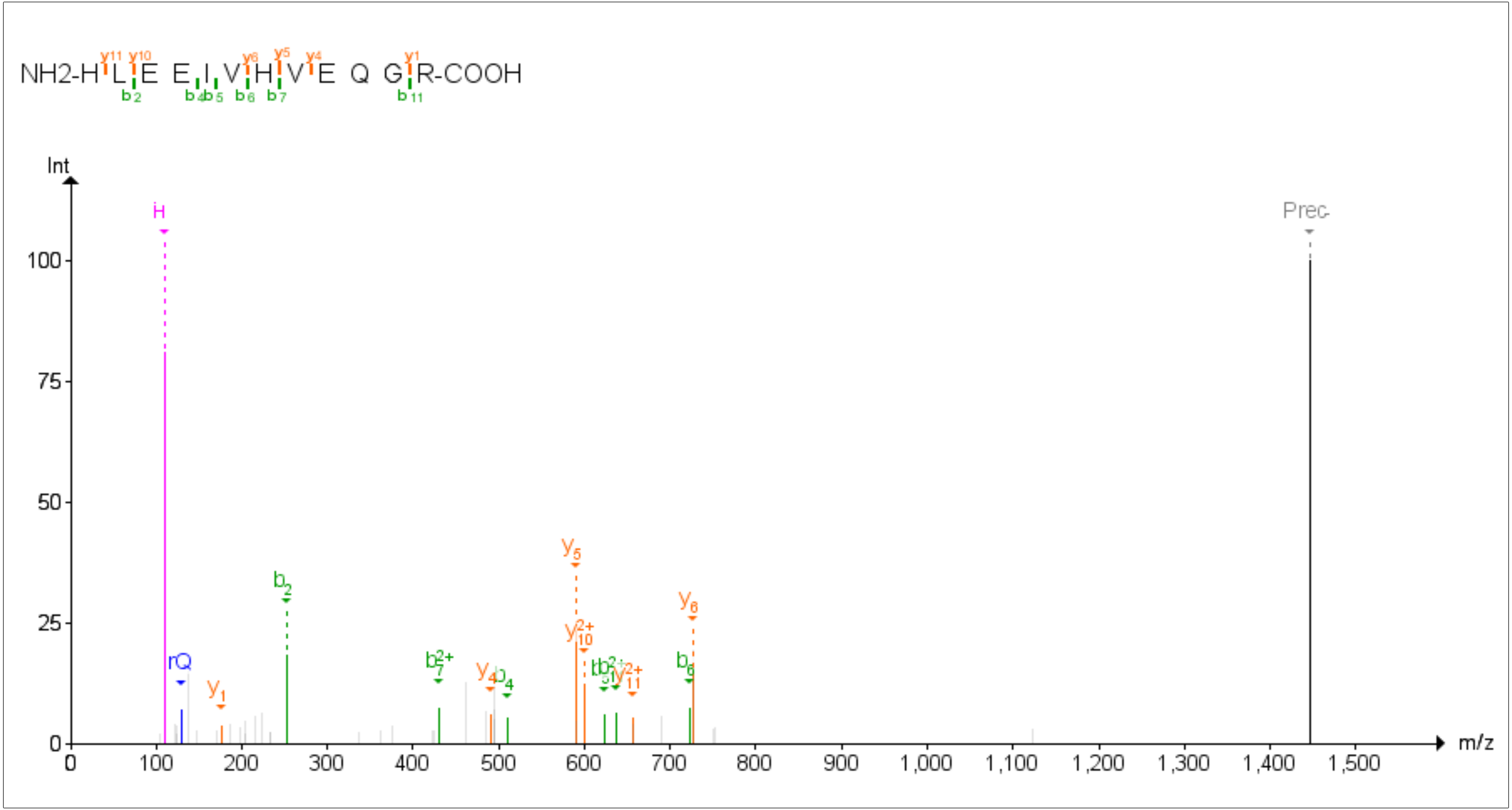
NH2-H L E E I V H V E Q G R-COOH
Int
Prec
m/z
0
25
50
75
100
0
100
200
300
400
500
600
700
800
900
1,000
1,100
1,200
1,300
1,400
1,500

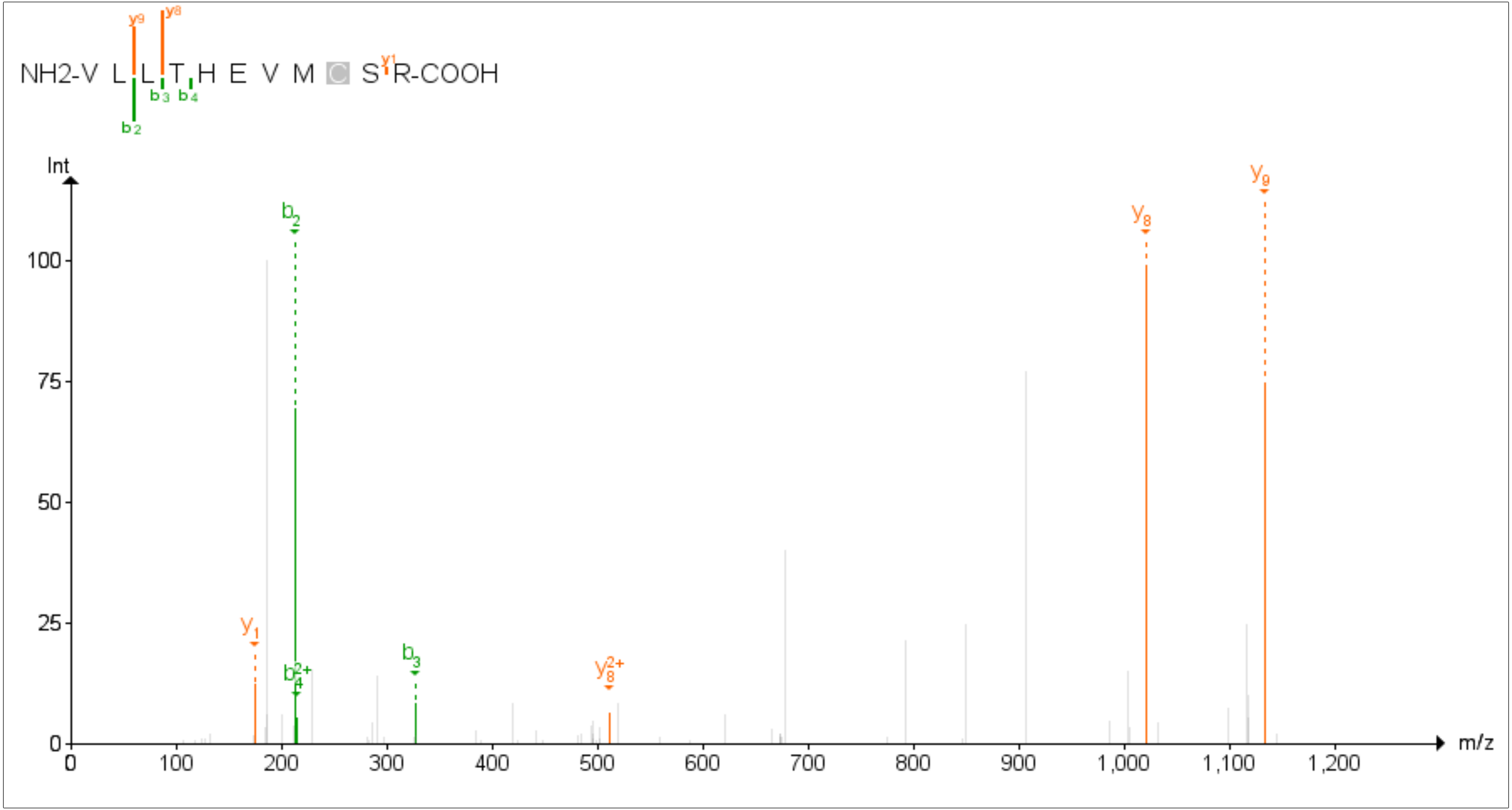

NH2-V L L T H E V M C S R-COOH
y9
y8
y1
b2
b3
b4
Int
m/z
0
25
50
75
100
100
200
300
400
500
600
700
800
900
1,000
1,100
1,200

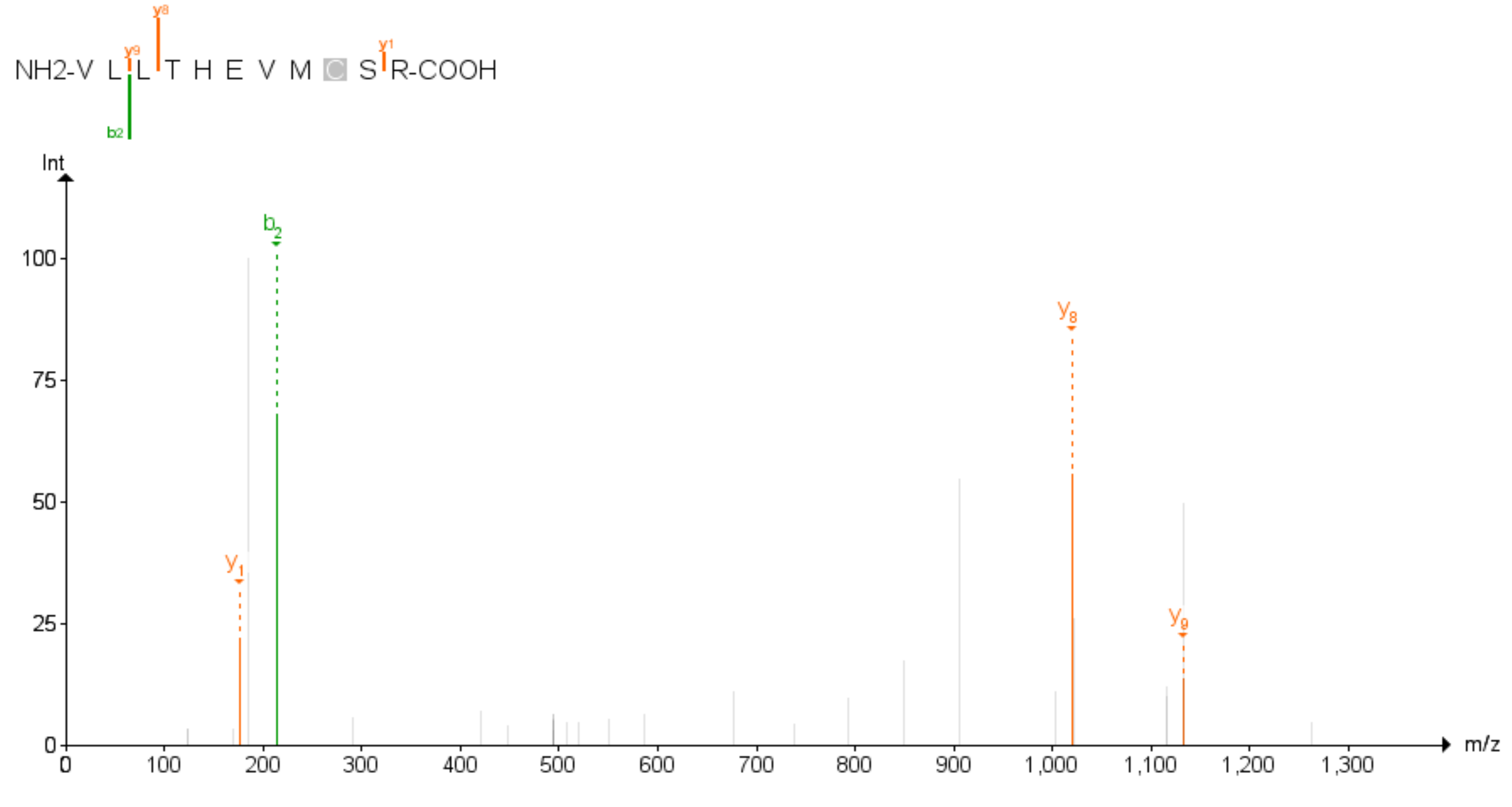

NH2-V L L T H E V M C S R-COOH
y8
y9
y1
b2
Int
100
75
50
25
0
b2
y1
y8
y9
0
100
200
300
400
500
600
700
800
900
1,000
1,100
1,200
1,300
m/z

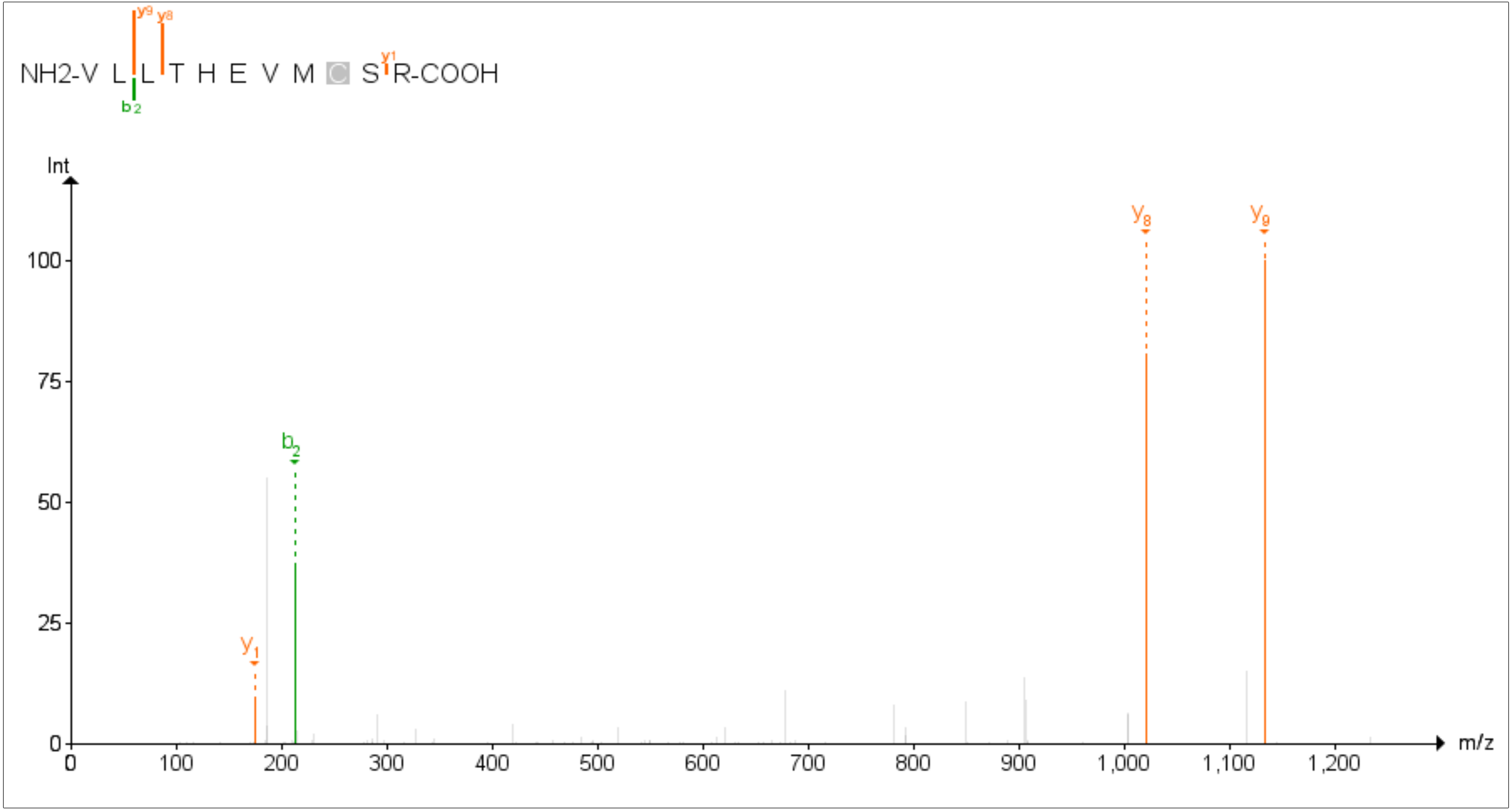

NH2-V L L T H E V M C S R-COOH
y9
y8
y1
b2
Int
100
75
50
25
0
m/z
0
100
200
300
400
500
600
700
800
900
1,000
1,100
1,200
y8
y9
b2
y1

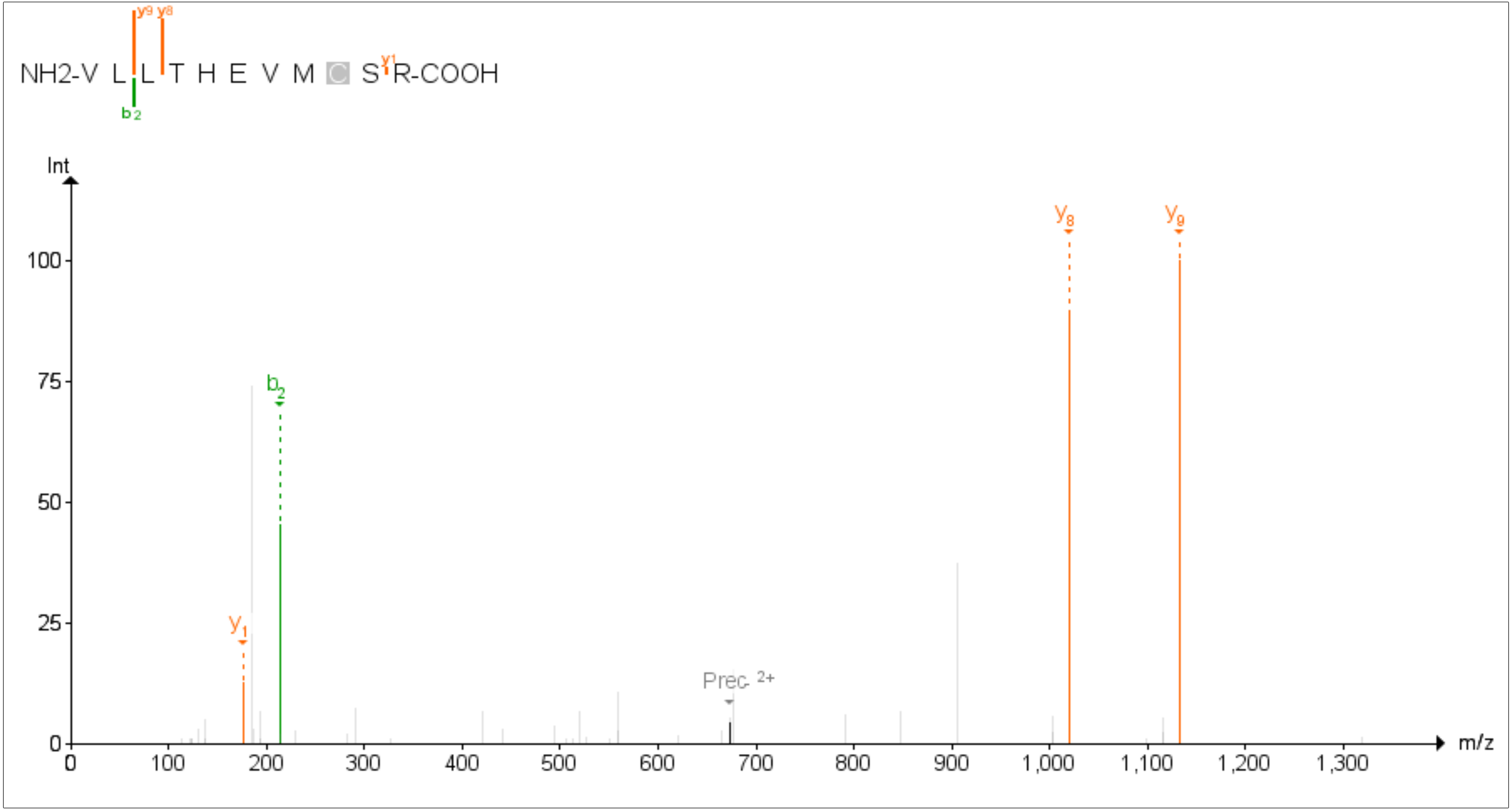

NH2-V L L T H E V M C S R-COOH
y9 y8
y1
b2
Int
100
75
50
25
0
y8
y9
b2
y1
Prec 2+
0
100
200
300
400
500
600
700
800
900
1,000
1,100
1,200
1,300
m/z

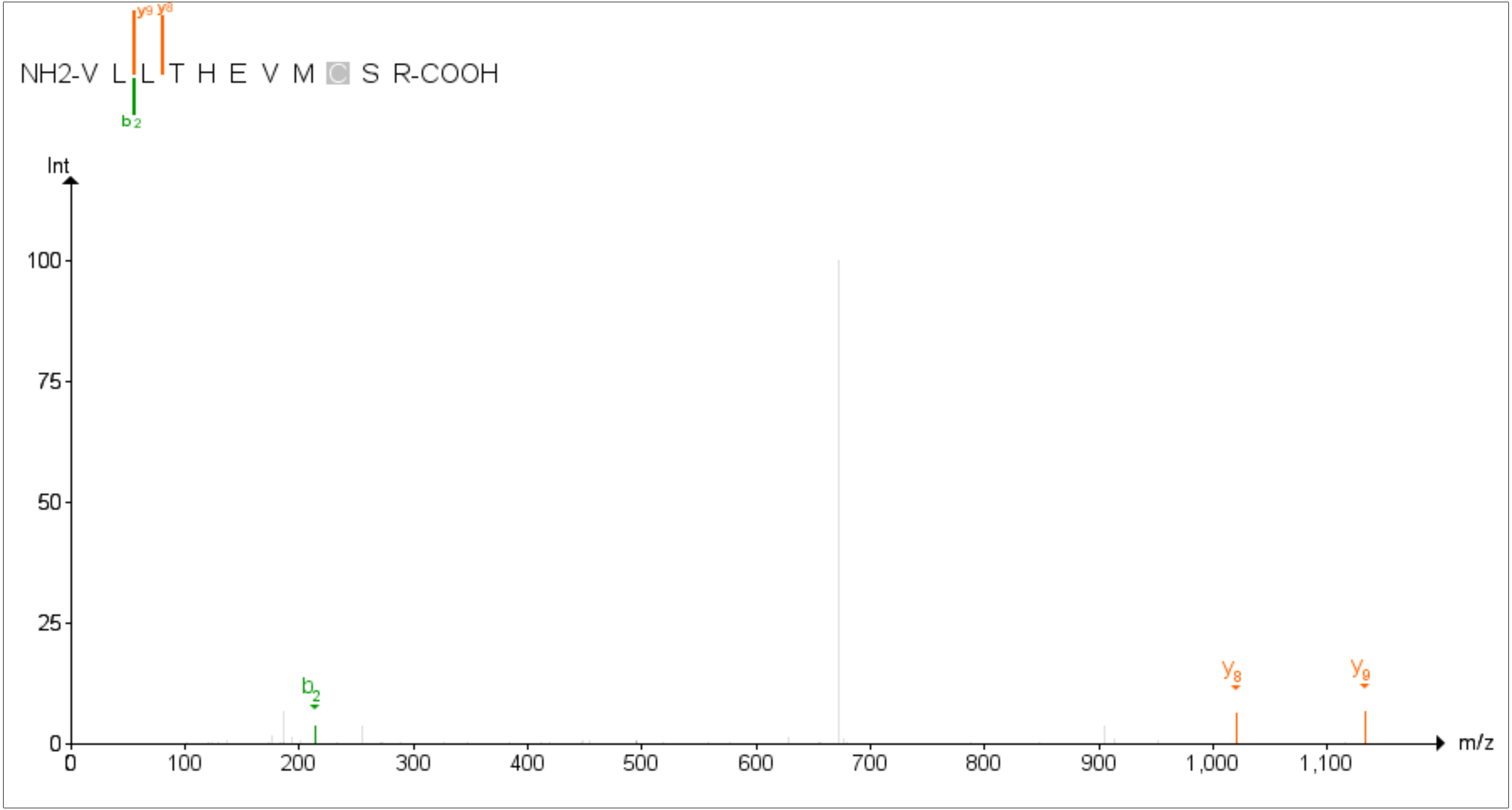

y9 y8
NH2-V L L T H E V M C S R-COOH
b2
Int
100
75
50
25
0
b2
y8
y9
0
100
200
300
400
500
600
700
800
900
1,000
1,100
m/z

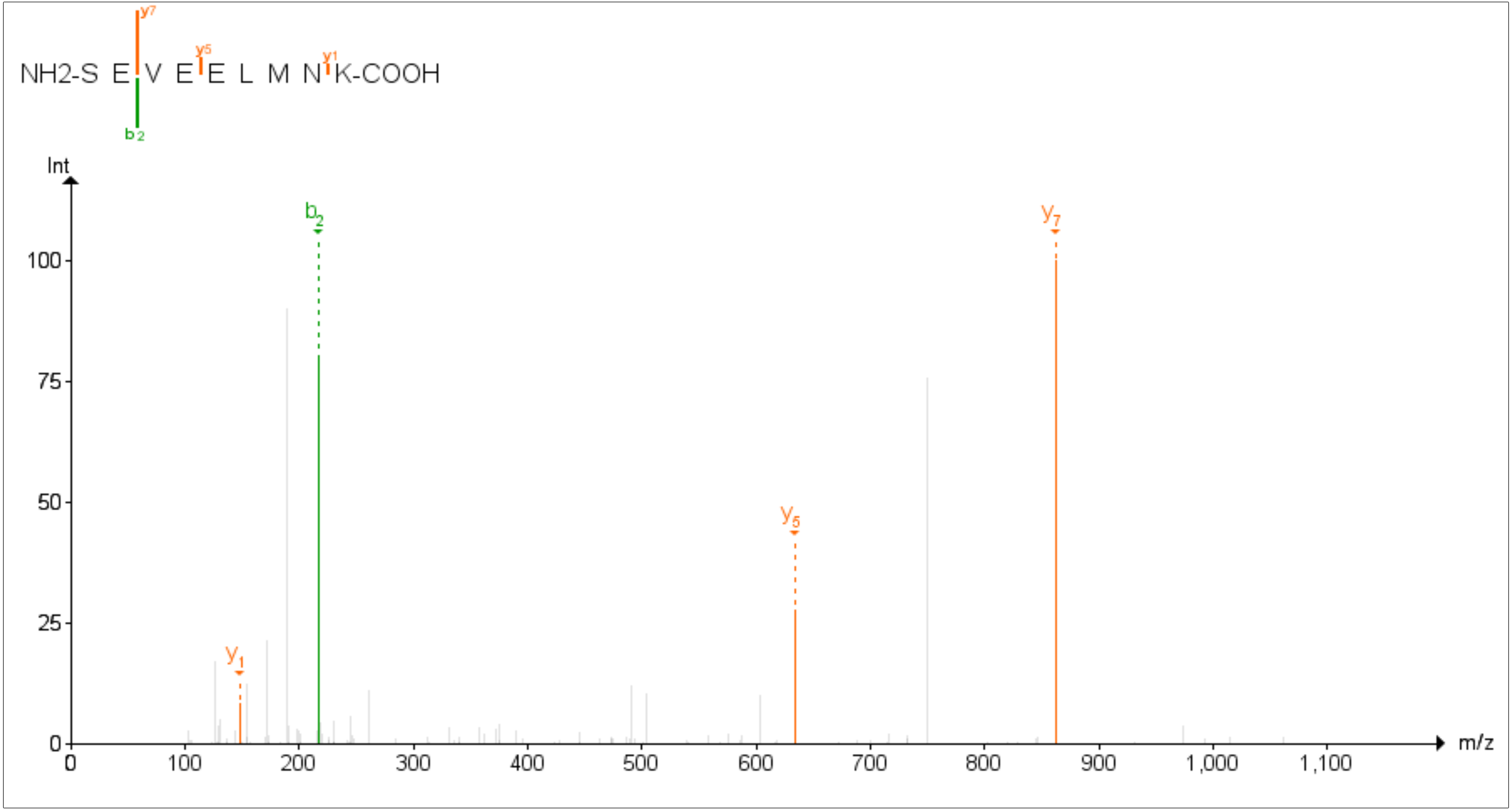

NH2-S E V E E L M N K-COOH
y7
y5
y1
b2
Int
100
75
50
25
0
m/z
0
100
200
300
400
500
600
700
800
900
1,000
1,100

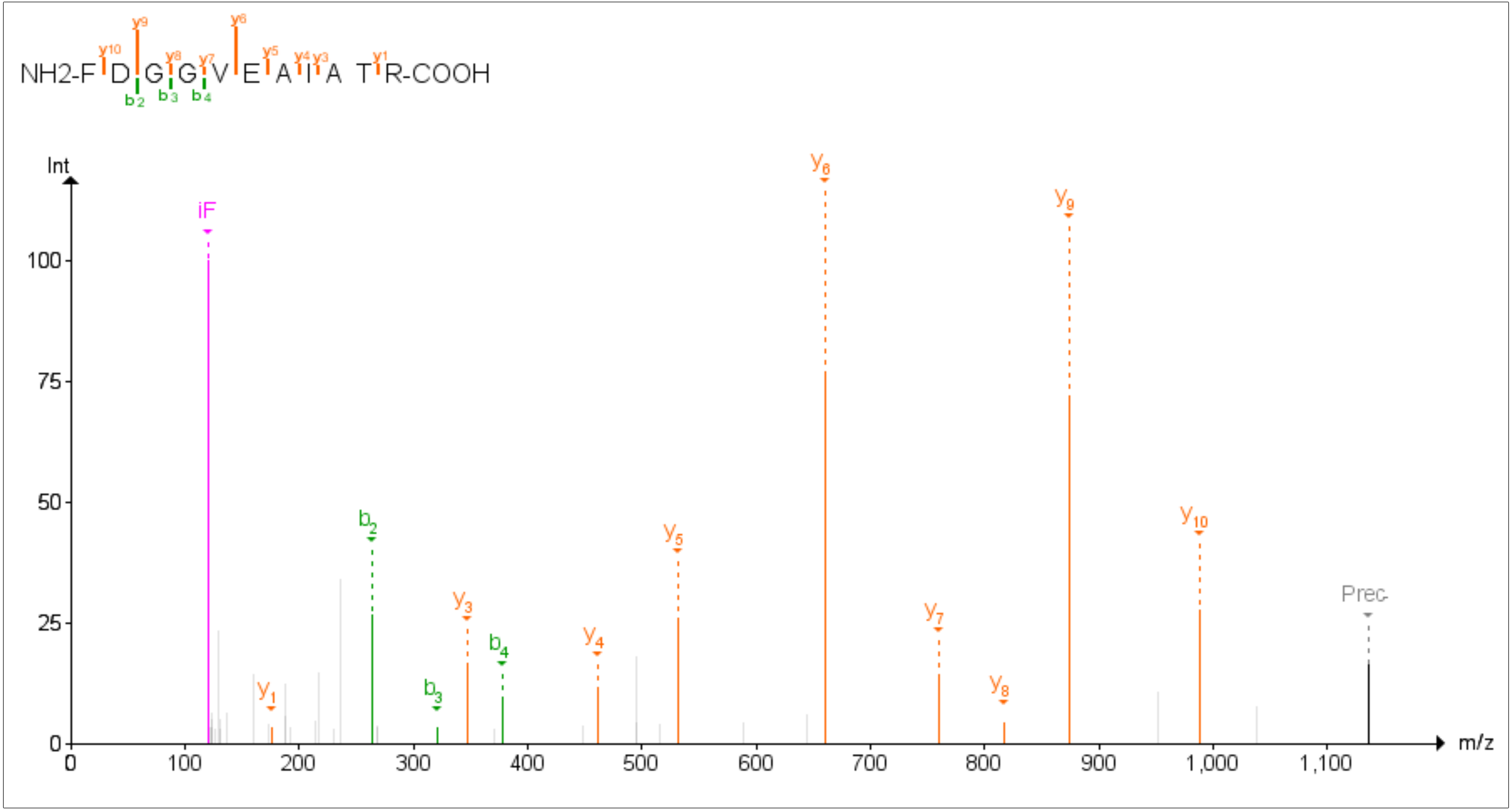

NH2-F D G G V E A I A T R-COOH
y10 y9 y8 y7 y6 y5 y4 y3 y1
b2 b3 b4
Int
iF
y6
y9
b2
y5
y10
y3
b4
y4
y7
y1
b3
y8
Prec
0
25
50
75
100
0
100
200
300
400
500
600
700
800
900
1,000
1,100
m/z

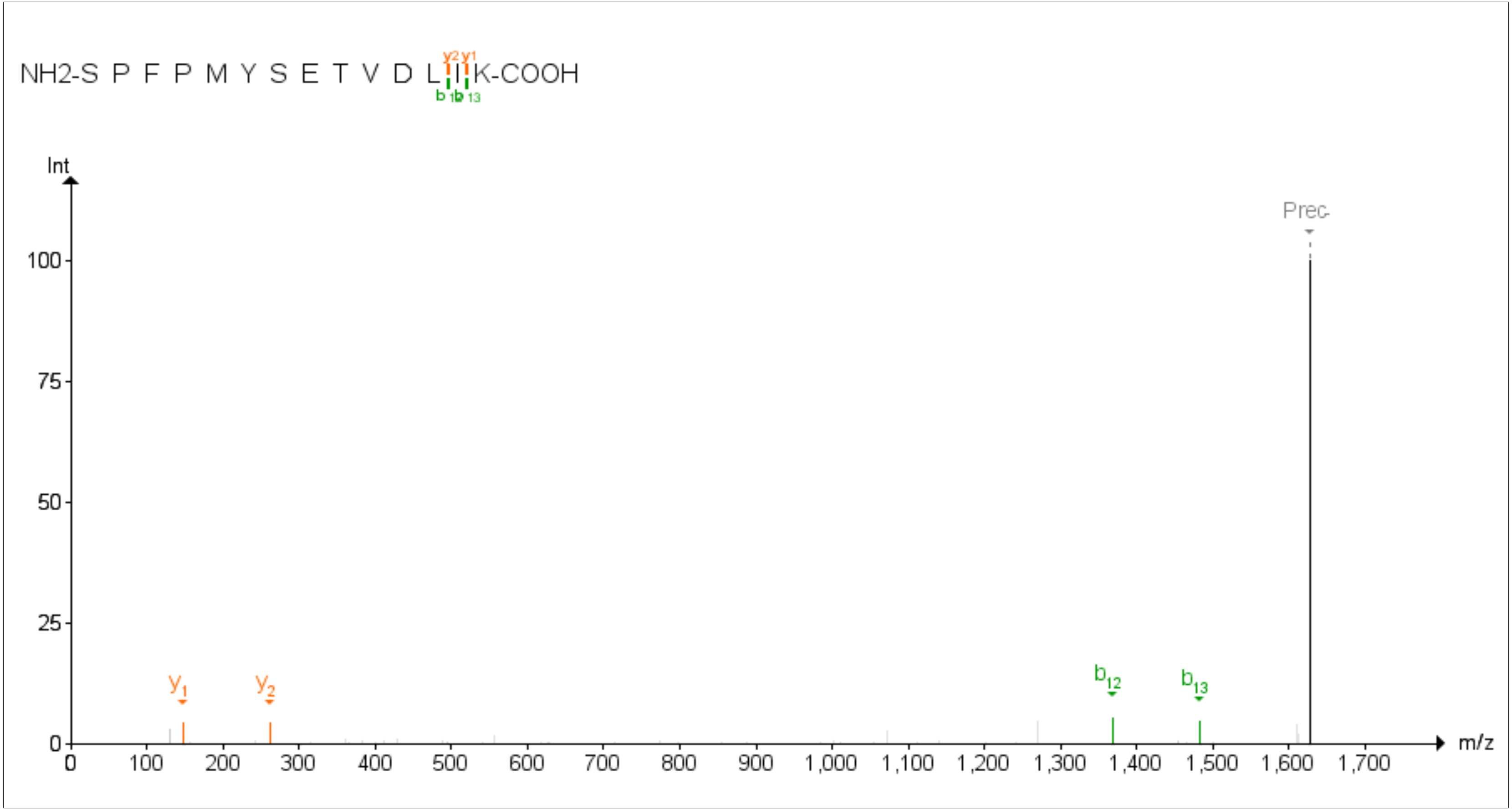
NH2-S P F P M Y S E T V D L I I K-COOH
y2 y1
b 12 b 13
Int
100
75
50
25
0
Prec
y1
y2
b12
b13
0 100 200 300 400 500 600 700 800 900 1,000 1,100 1,200 1,300 1,400 1,500 1,600 1,700
m/z

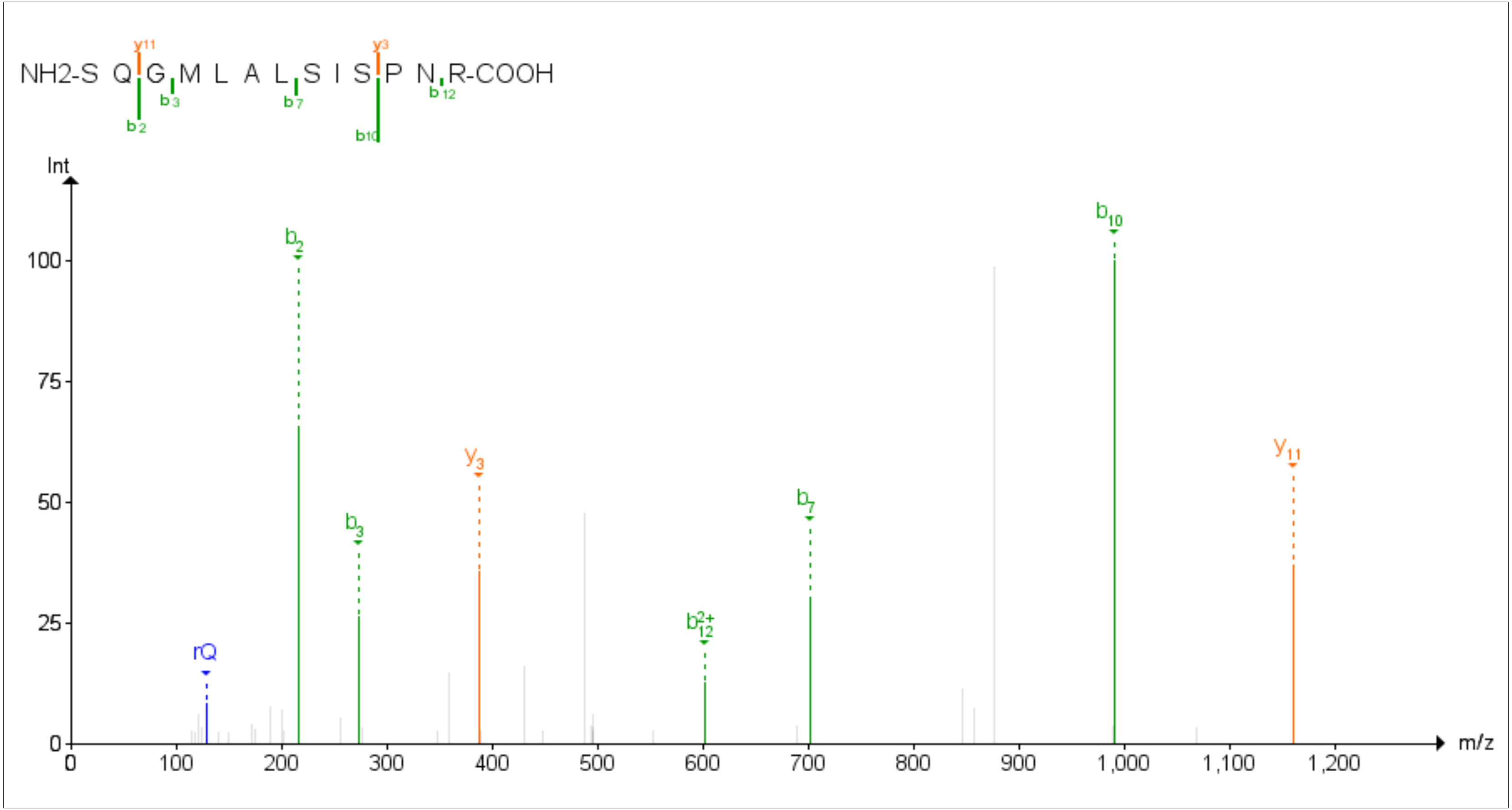

NH2-S Q G M L A L S I S P N R-COOH
y11
y3
b2
b3
b7
b10
b12
Int
100
75
50
25
0
rQ
b2
b3
y3
b12 2+
b7
b10
y11
0
100
200
300
400
500
600
700
800
900
1,000
1,100
1,200
m/z

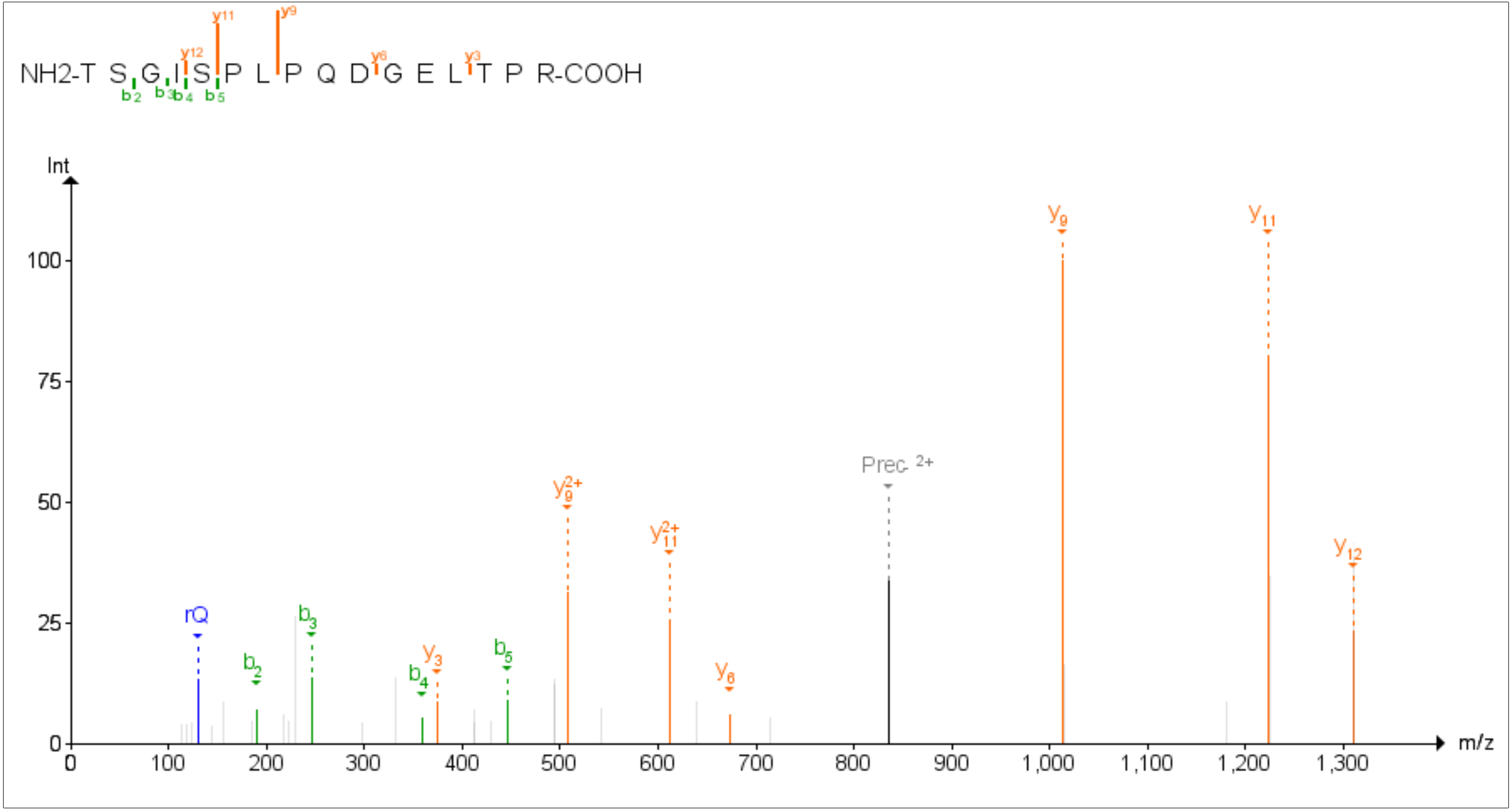
NH2-T S G I S P L P Q D G E L T P R-COOH
b2 b3 b4 b5
y12 y11 y9 y6 y3
Int
m/z
rQ
b2
b3
b4
b5
y3
y6
y9 2+
y11 2+
Prec 2+
y9
y11
y12
0 25 50 75 100
0 100 200 300 400 500 600 700 800 900 1,000 1,100 1,200 1,300

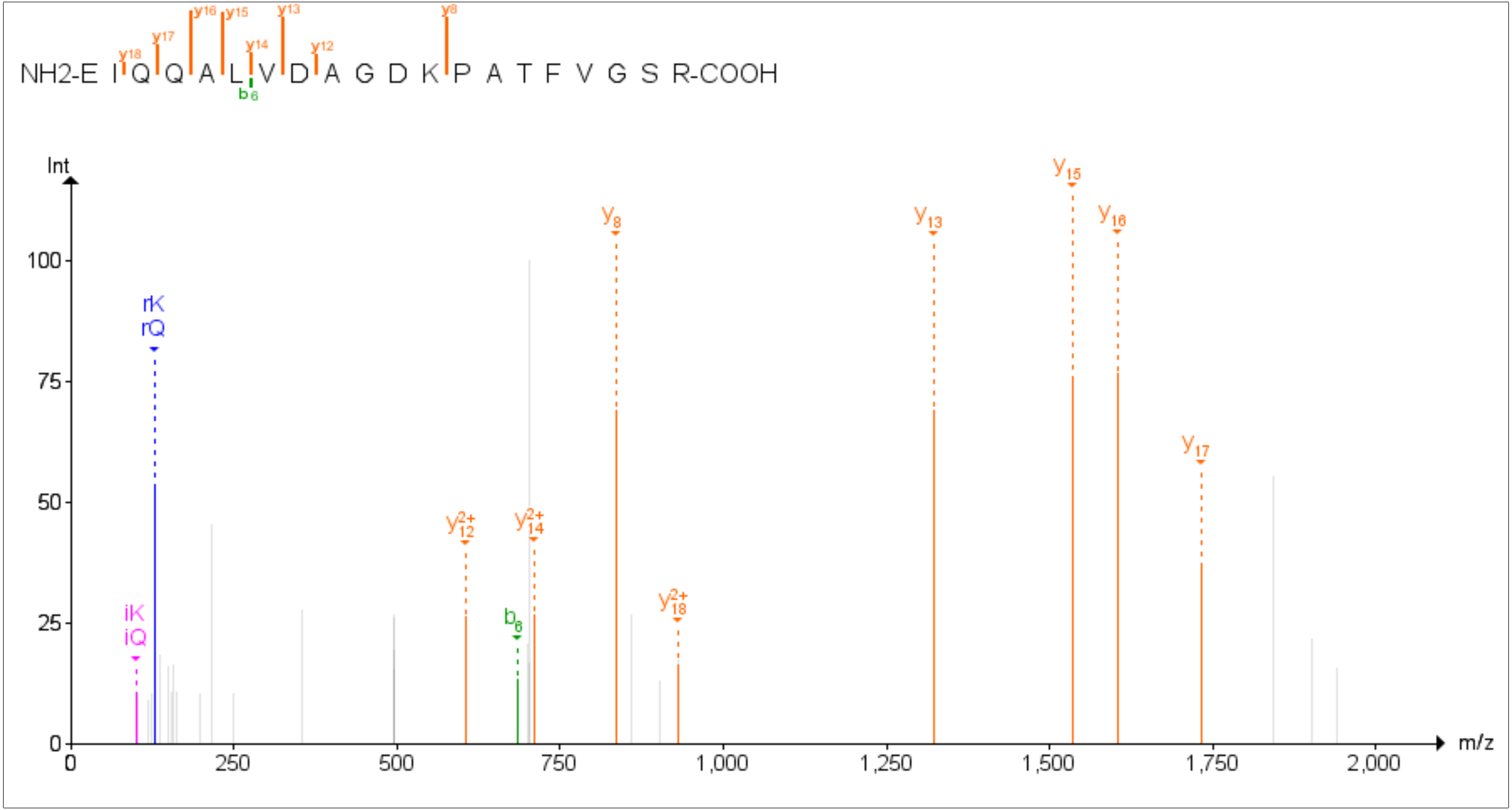

NH2-E I Q Q A L V D A G D K P A T F V G S R-COOH
y18
y17
y16
y15
y14
y13
y12
y8
b6
Int
100
75
50
25
0
rK
rQ
iK
iQ
y_{12}^{2+}
y_{14}^{2+}
b_6
y_8
y_{18}^{2+}
y_{13}
y_{15}
y_{16}
y_{17}
0
250
500
750
1,000
1,250
1,500
1,750
2,000
m/z

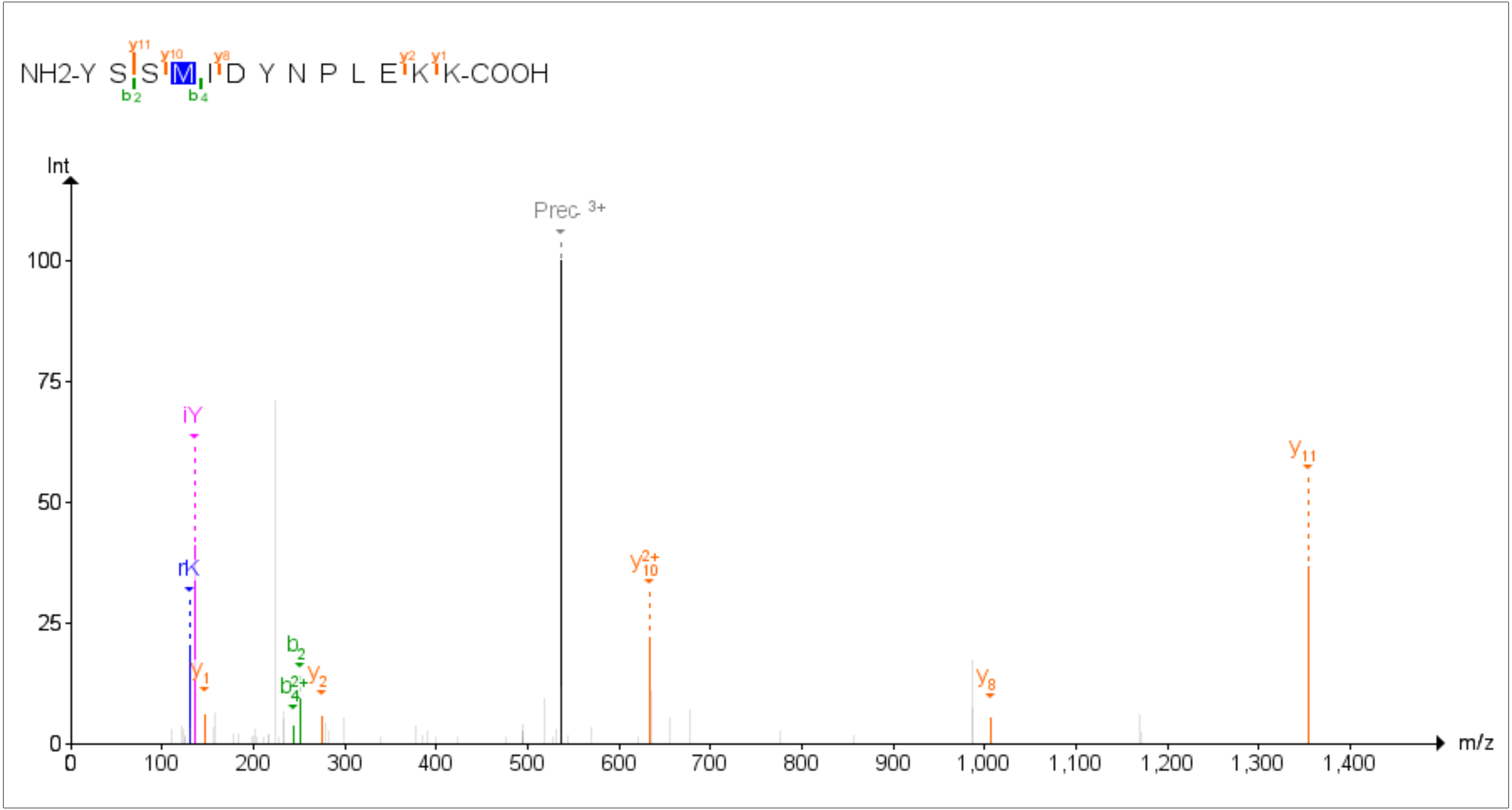

NH2-Y S S M I D Y N P L E K K-COOH
y11
y10
y8
y2
y1
b2
b4
Int
Prec 3+
iY
rK
y1
b2
b4 2+
y2
y10 2+
y8
y11
100
75
50
25
0
0
100
200
300
400
500
600
700
800
900
1,000
1,100
1,200
1,300
1,400
m/z

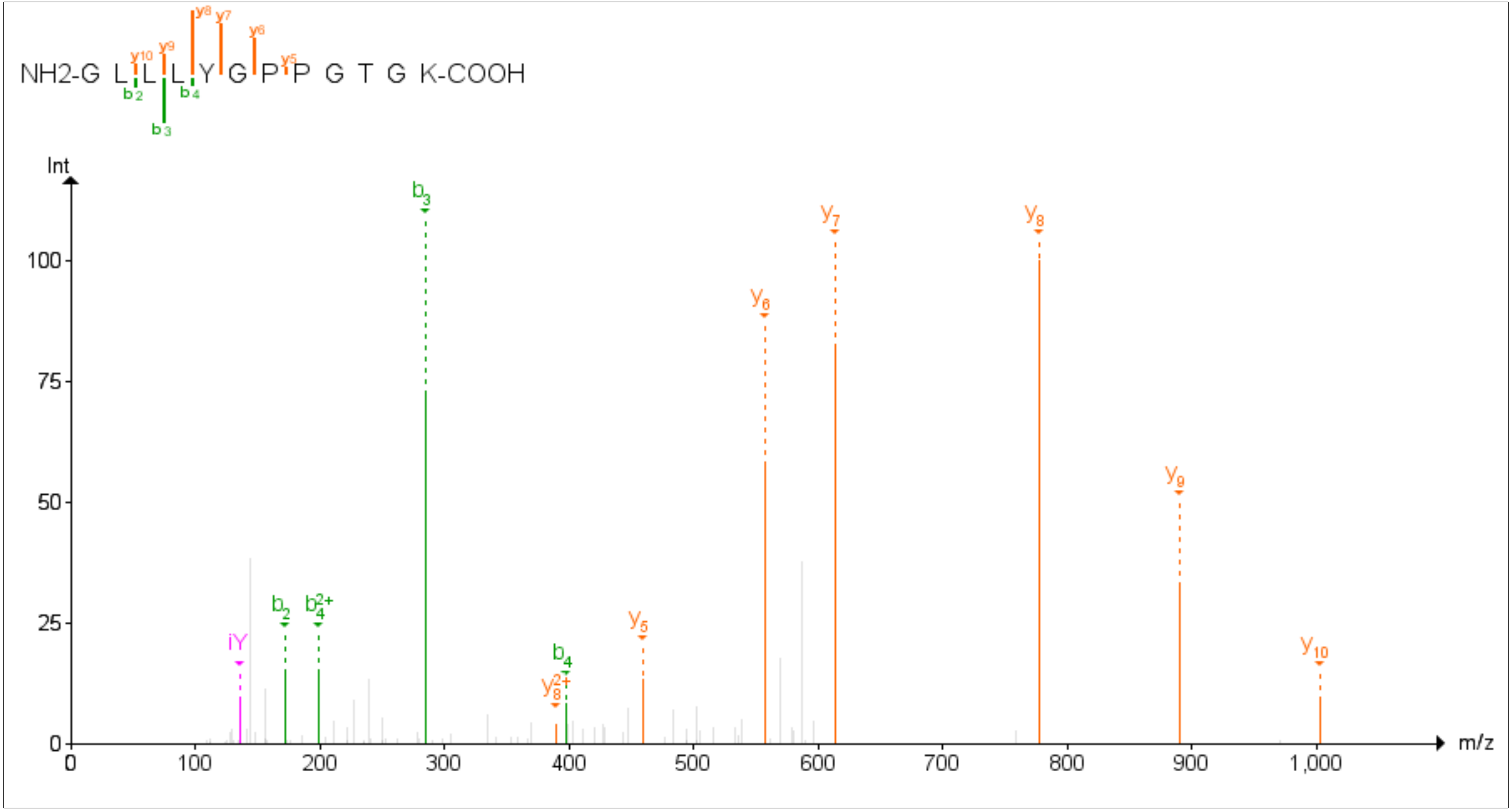
NH2-G L L L Y G P P G T G K-COOH
y10 y9 y8 y7 y6 y5
b2 b3 b4
Int
100
75
50
25
0
m/z
0 100 200 300 400 500 600 700 800 900 1,000
iY
b2
b4 2+
b3
y8 2+
b4
y5
y6
y7
y8
y9
y10

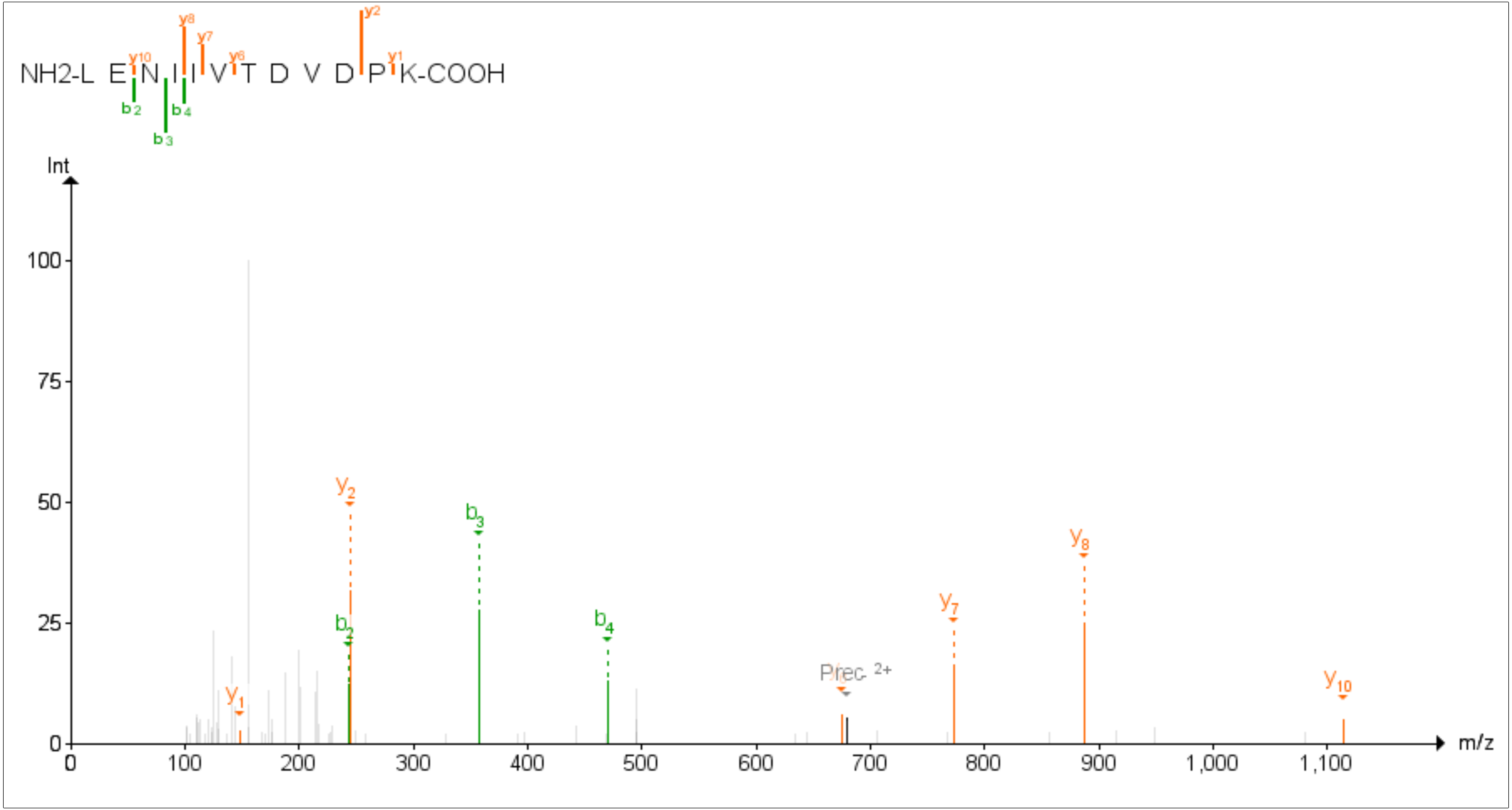

NH2-L E N I I V T D V D P K-COOH
y10
y8
y7
y6
y2
y1
b2
b3
b4
Int
100
75
50
25
0
y_2
b_3
y_8
y_7
b_2
b_4
y_1
Prec- 2+
y_{10}
0
100
200
300
400
500
600
700
800
900
1,000
1,100
m/z

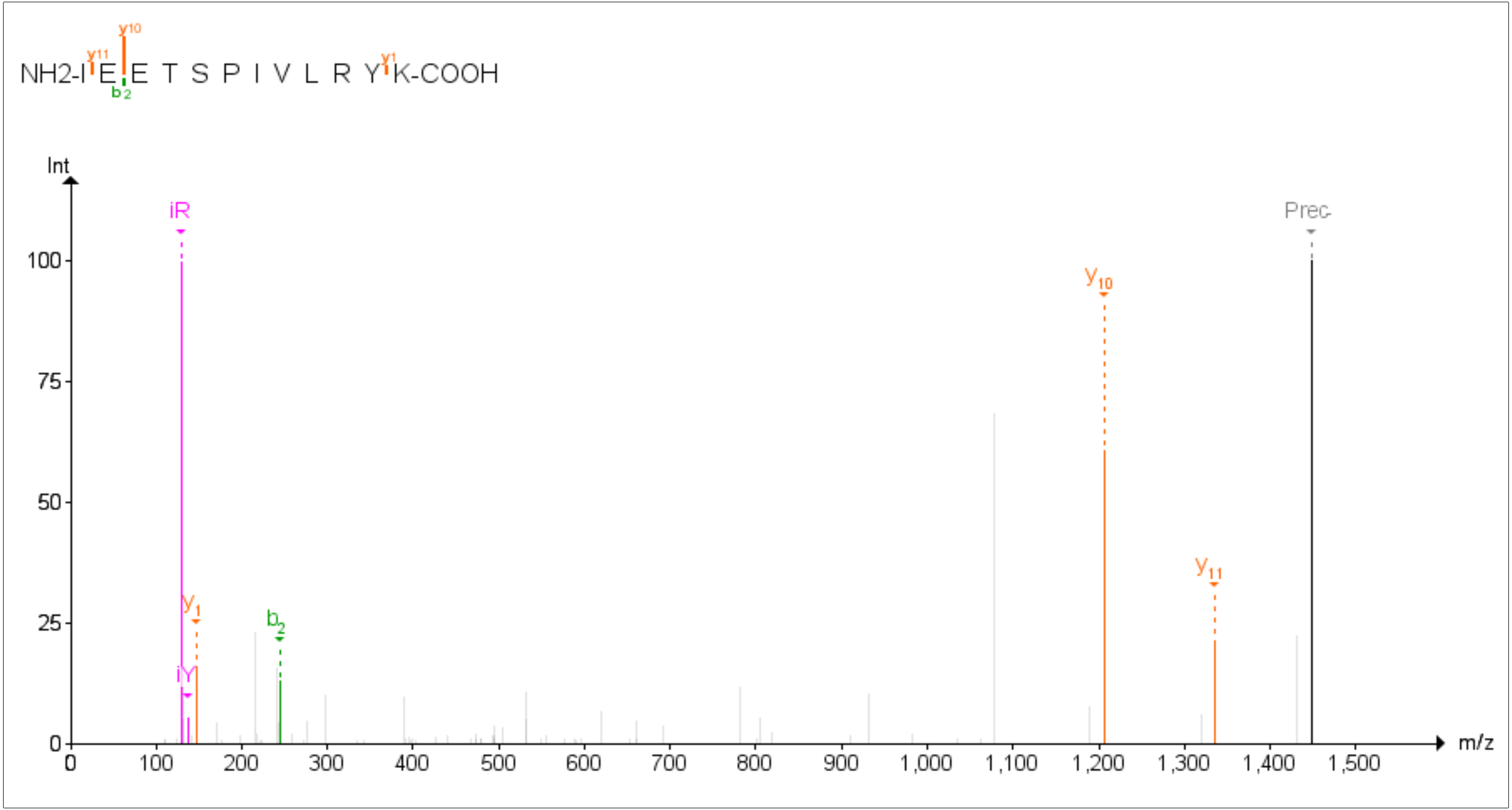

NH2-I E E T S P I V L R Y K-COOH
y11
y10
b2
y1
Int
iR
Prec
y10
y11
y1
b2
iY
100
75
50
25
0
0
100
200
300
400
500
600
700
800
900
1,000
1,100
1,200
1,300
1,400
1,500
m/z

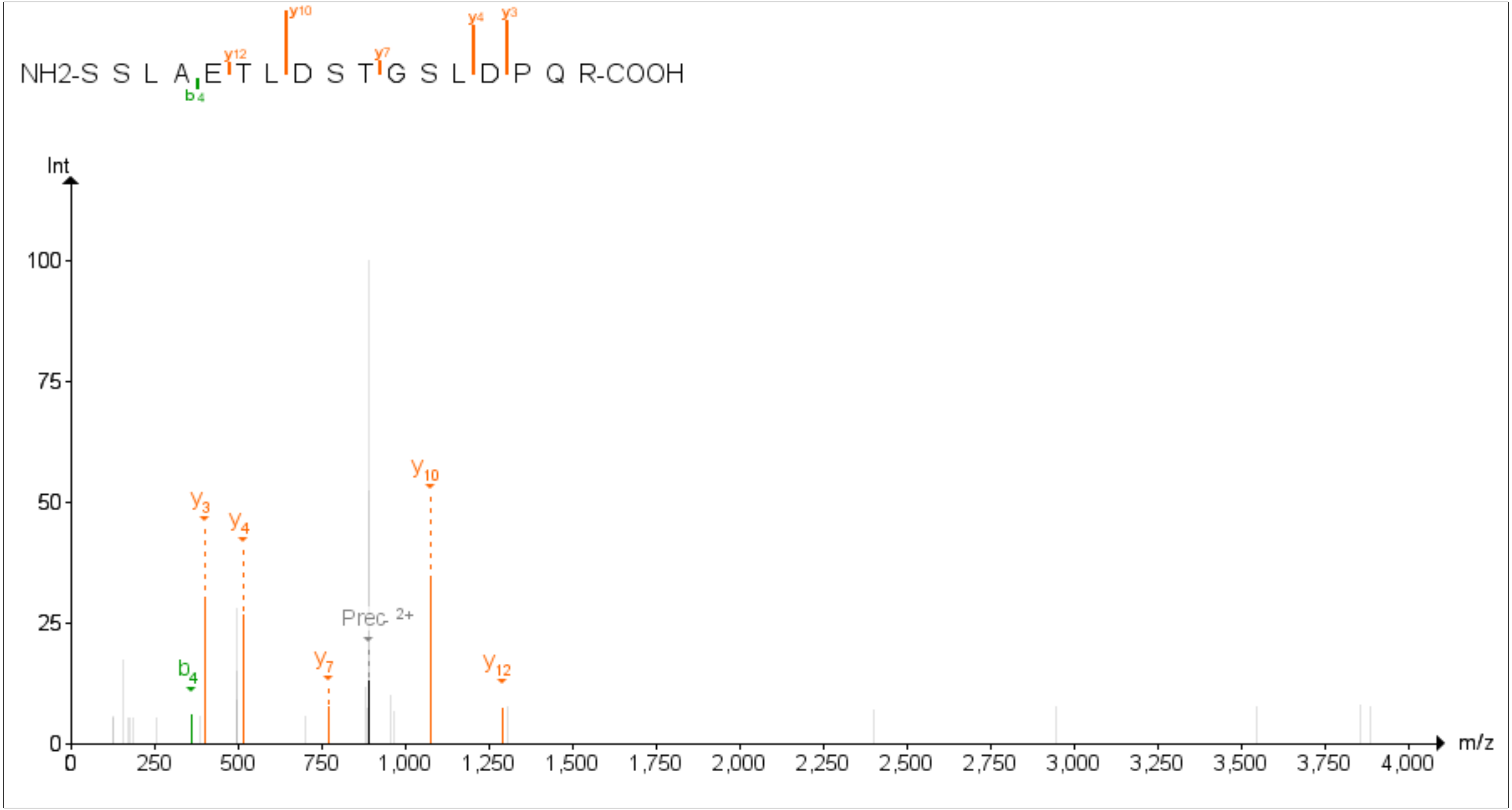

NH2-S S L A E T L D S T G S L D P Q R-COOH
y10
y4
y3
y12
y7
b4
Int
100
75
50
25
0
y3
y4
y10
Prec 2+
b4
y7
y12
0
250
500
750
1,000
1,250
1,500
1,750
2,000
2,250
2,500
2,750
3,000
3,250
3,500
3,750
4,000
m/z

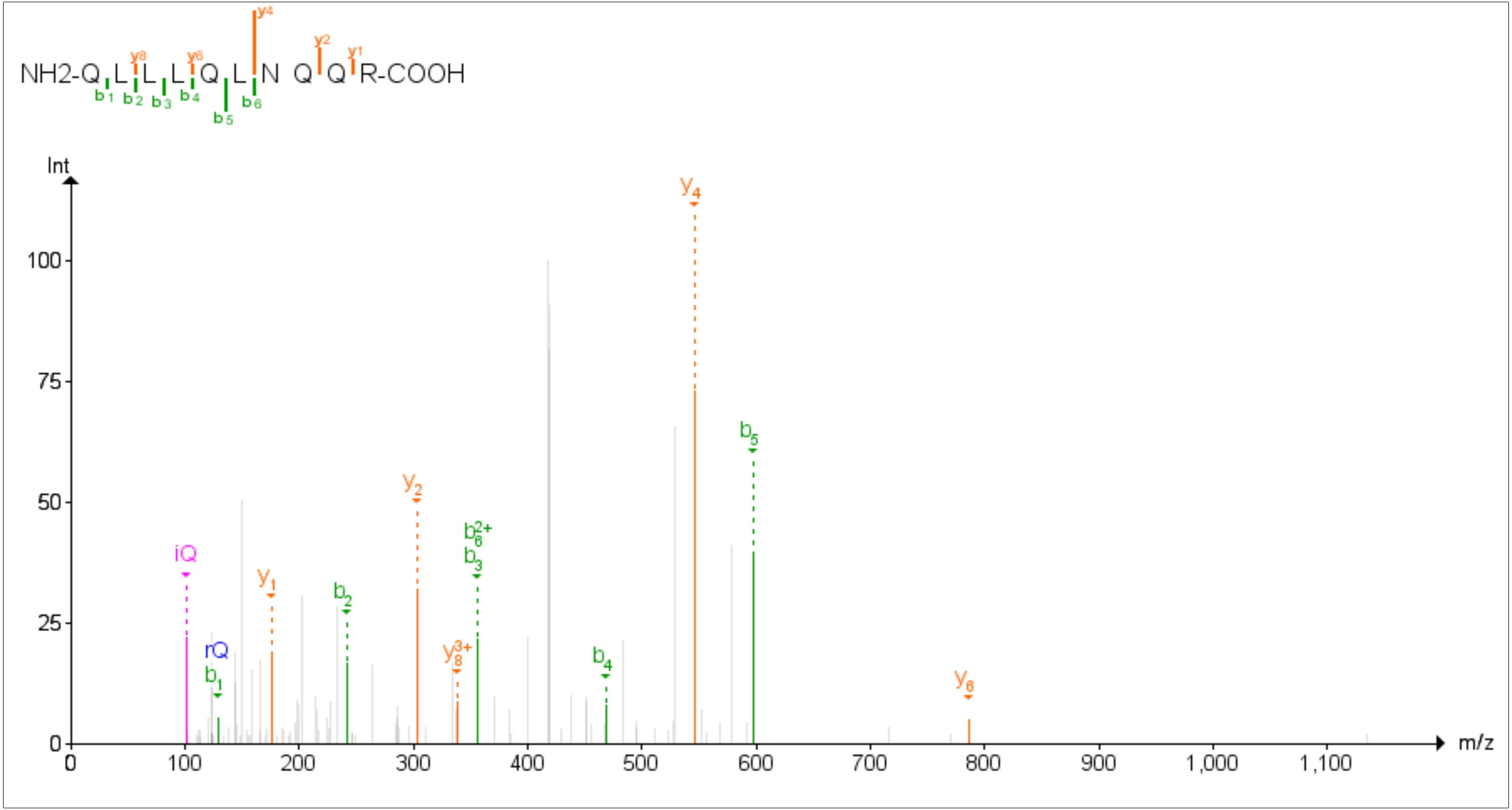
NH2-Q L L L Q L N Q Q R-COOH
b1 b2 b3 b4 b5 b6
y8 y6 y4 y2 y1
Int
m/z
iQ
rQ
b1
y1
b2
y2
y8 3+
b6 2+
b3
b4
y4
b5
y6

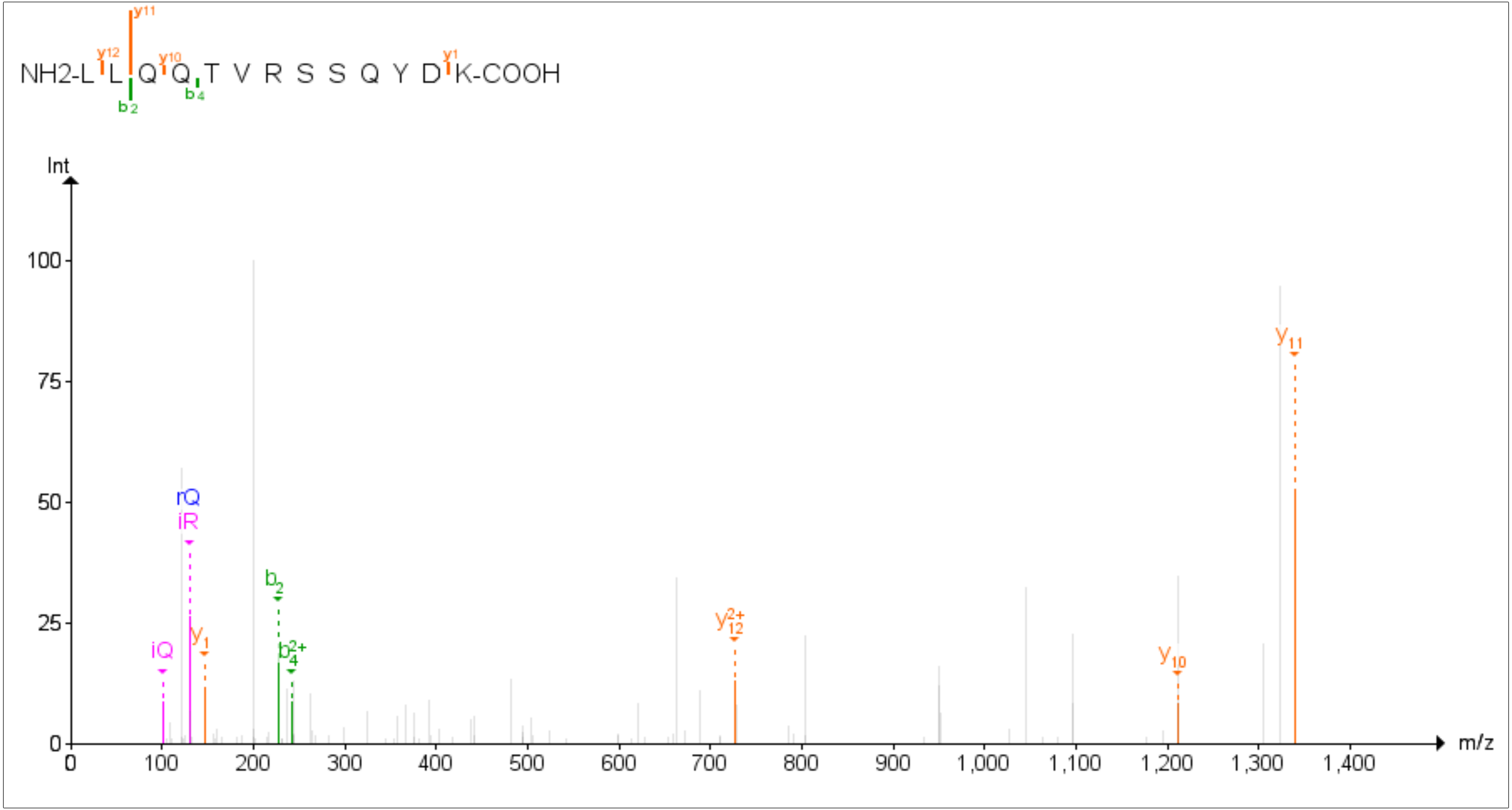

NH2-L L Q Q T V R S S Q Y D K-COOH
y12 y11 y10 y1
b2 b4
Int
m/z
rQ
iR
iQ
y1
b2
b4 2+
y12 2+
y10
y11

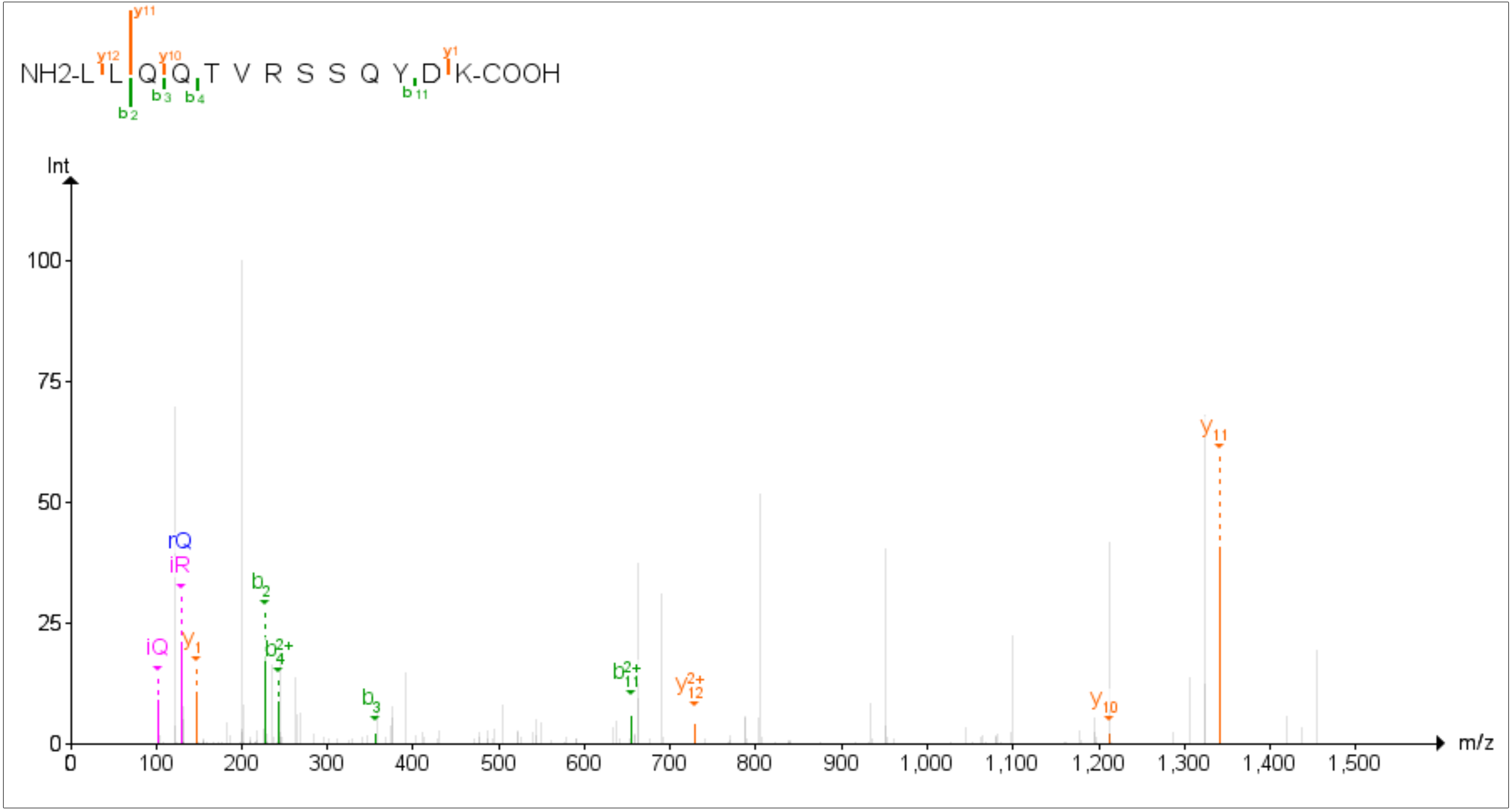

NH2-L L Q Q T V R S S Q Y D K-COOH
y12
y11
y10
y1
b2
b3
b4
b11
Int
m/z
rQ
iR
iQ
y1
b2
b4 2+
b3
b11 2+
y12 2+
y10
y11

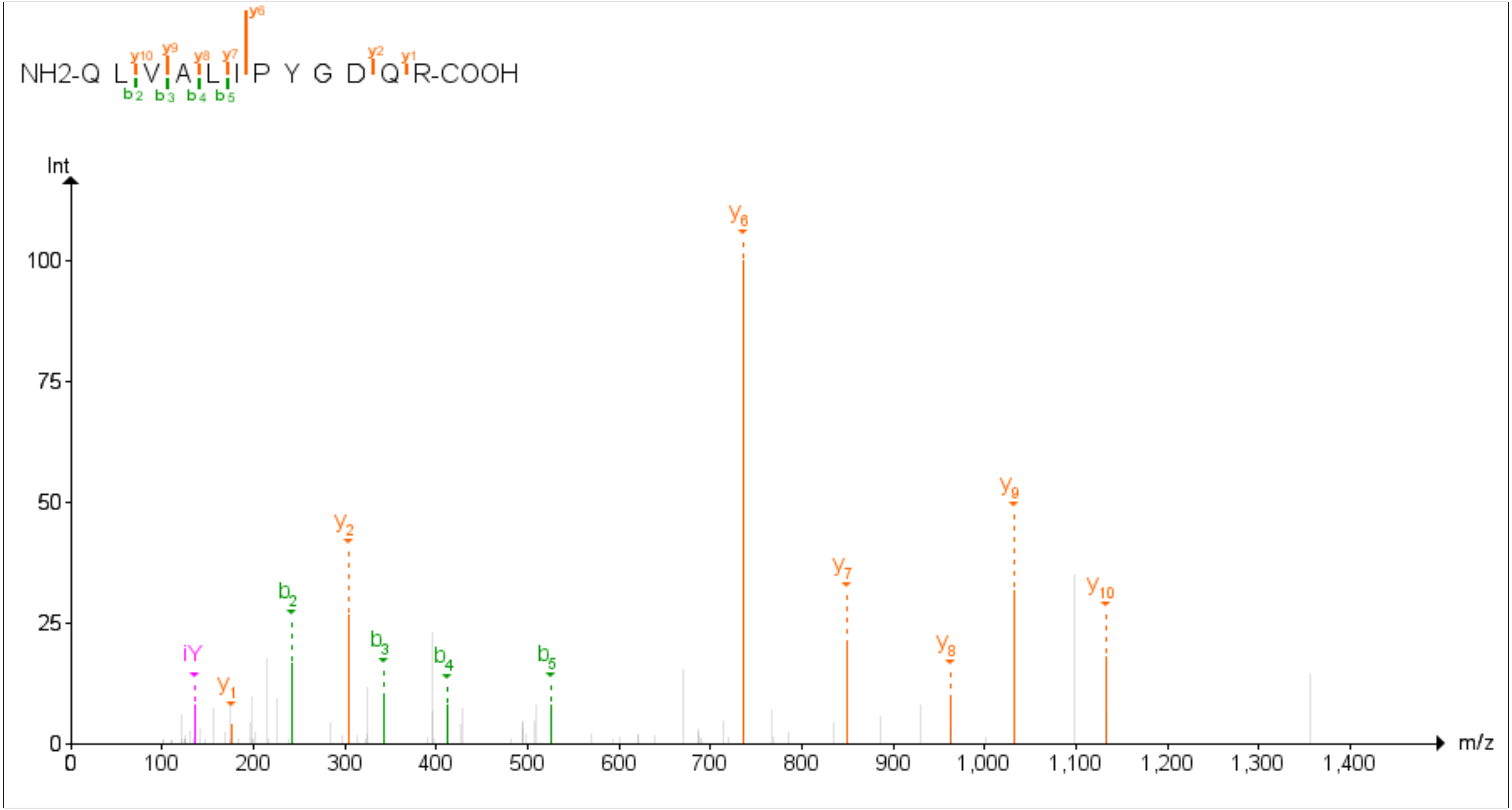

NH2-Q L I V A L I P Y G D Q R-COOH
y10 y9 y8 y7 y6 y2 y1
b2 b3 b4 b5
Int
100
75
50
25
0
iY
y1
b2
y2
b3
b4
b5
y6
y7
y8
y9
y10
0 100 200 300 400 500 600 700 800 900 1,000 1,100 1,200 1,300 1,400
m/z

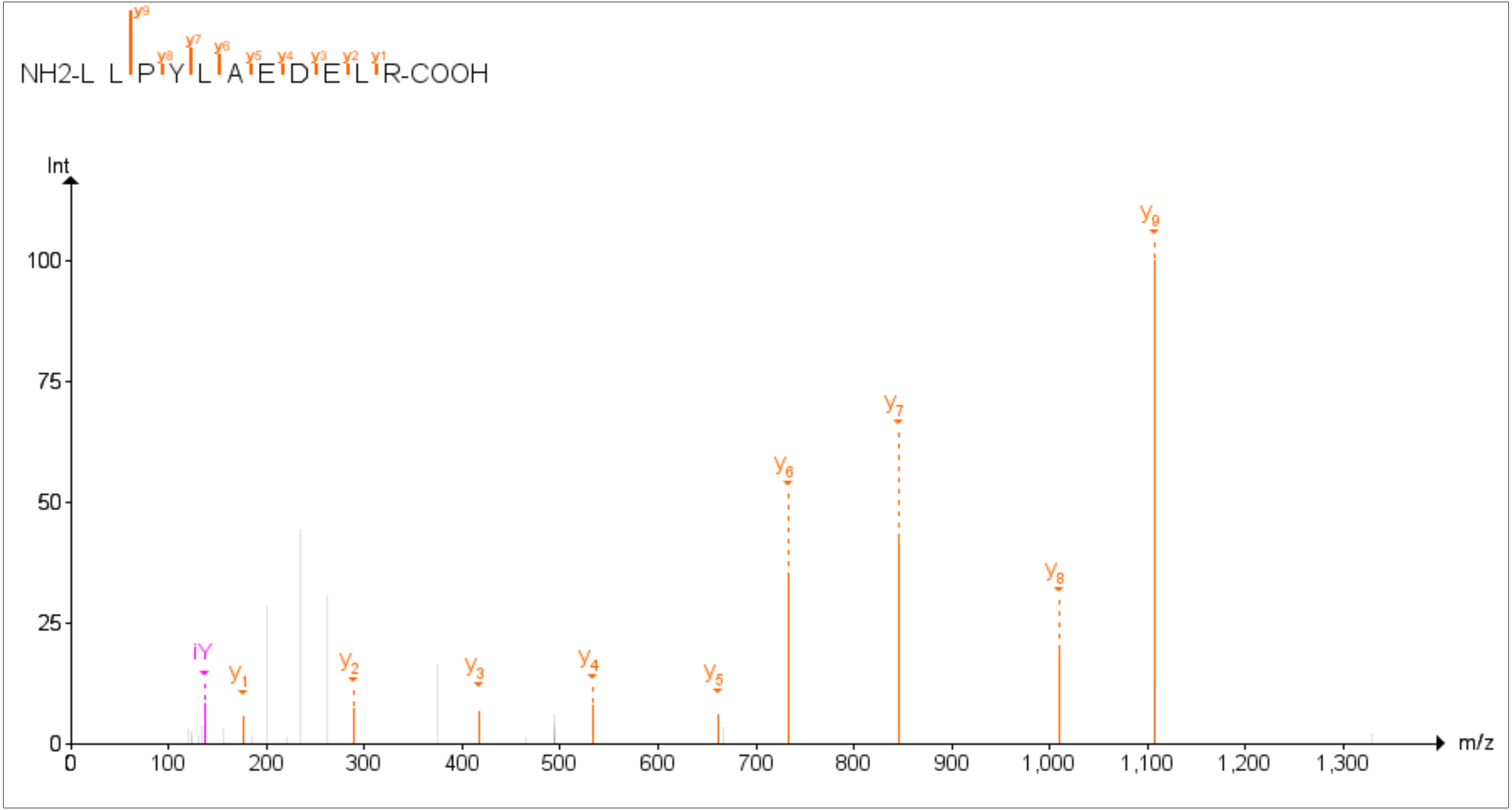

NH2-L L P Y L A E D E L R-COOH
y9 y8 y7 y6 y5 y4 y3 y2 y1
Int
100
75
50
25
0
iY
y1 y2 y3 y4 y5 y6 y7 y8 y9
0 100 200 300 400 500 600 700 800 900 1,000 1,100 1,200 1,300
m/z

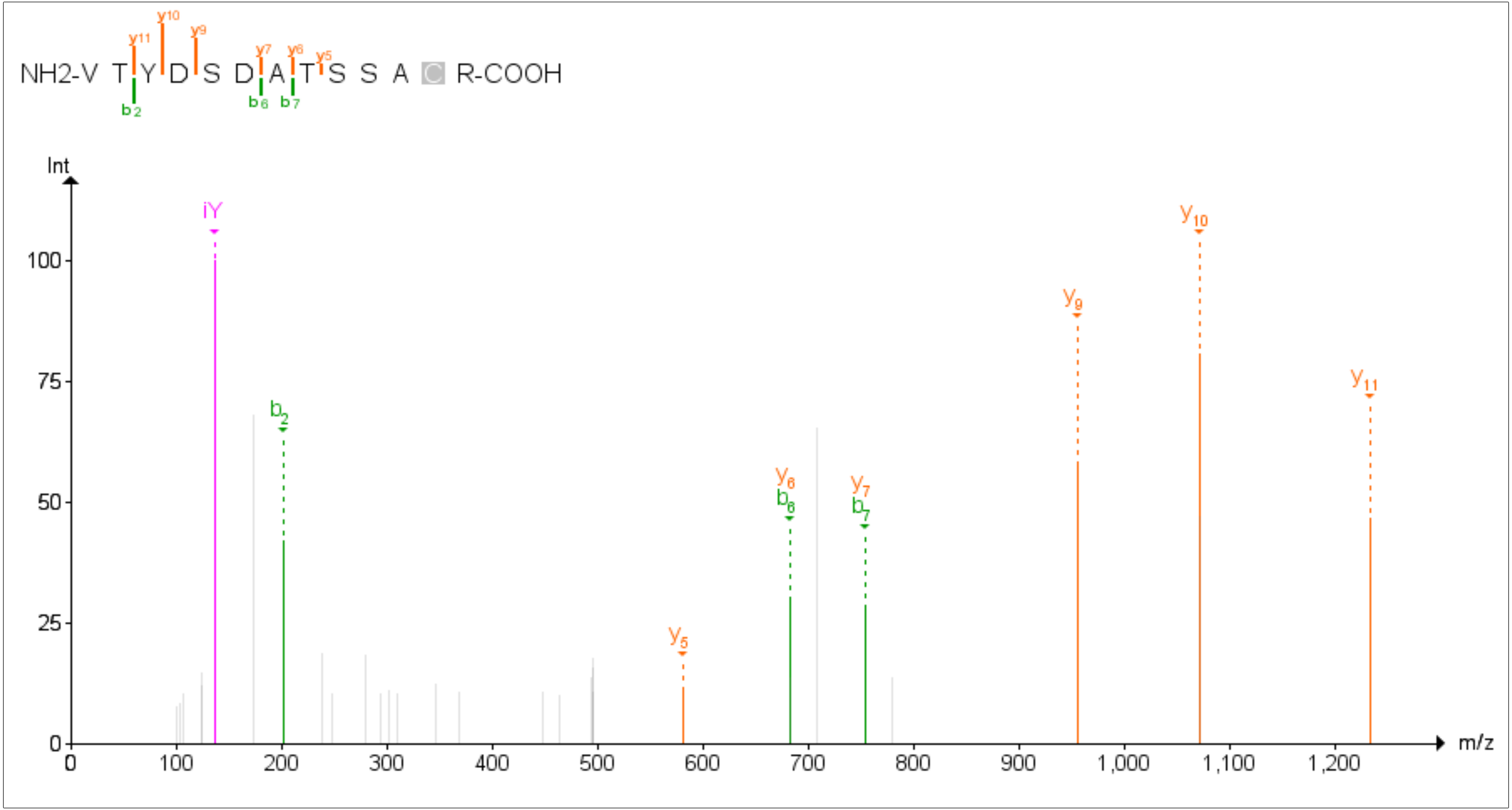

NH2-V T Y D S D A T S S A C R-COOH
y11 y10 y9 y7 y6 y5
b2 b6 b7
Int
iY
b2
y5
y6
b6
y7
b7
y9
y10
y11
0
25
50
75
100
0 100 200 300 400 500 600 700 800 900 1,000 1,100 1,200
m/z

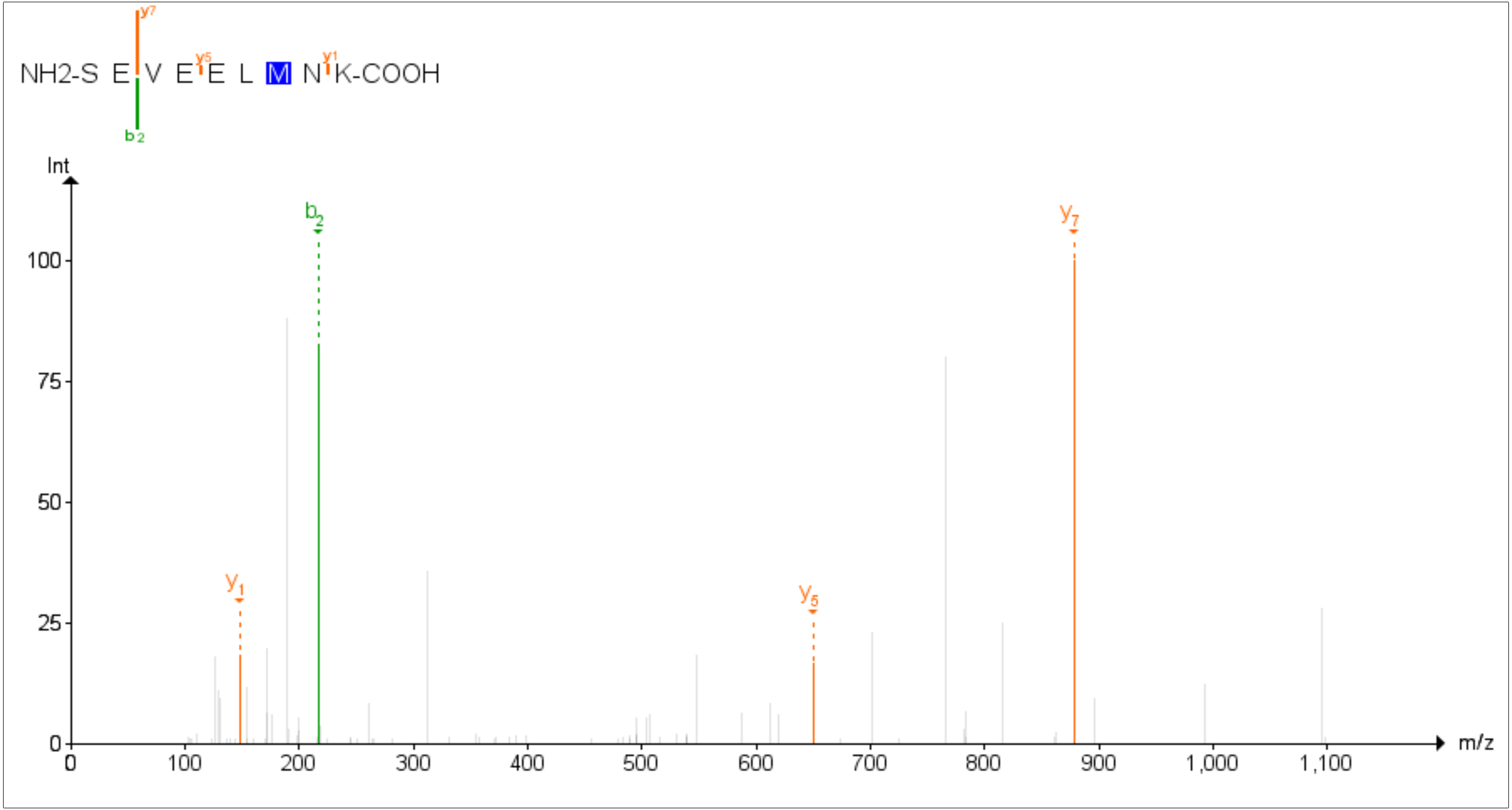

NH2-S E V E E L M N K-COOH
y7
y5
y1
b2
Int
100
75
50
25
0
b2
y7
y1
y5
0
100
200
300
400
500
600
700
800
900
1,000
1,100
m/z

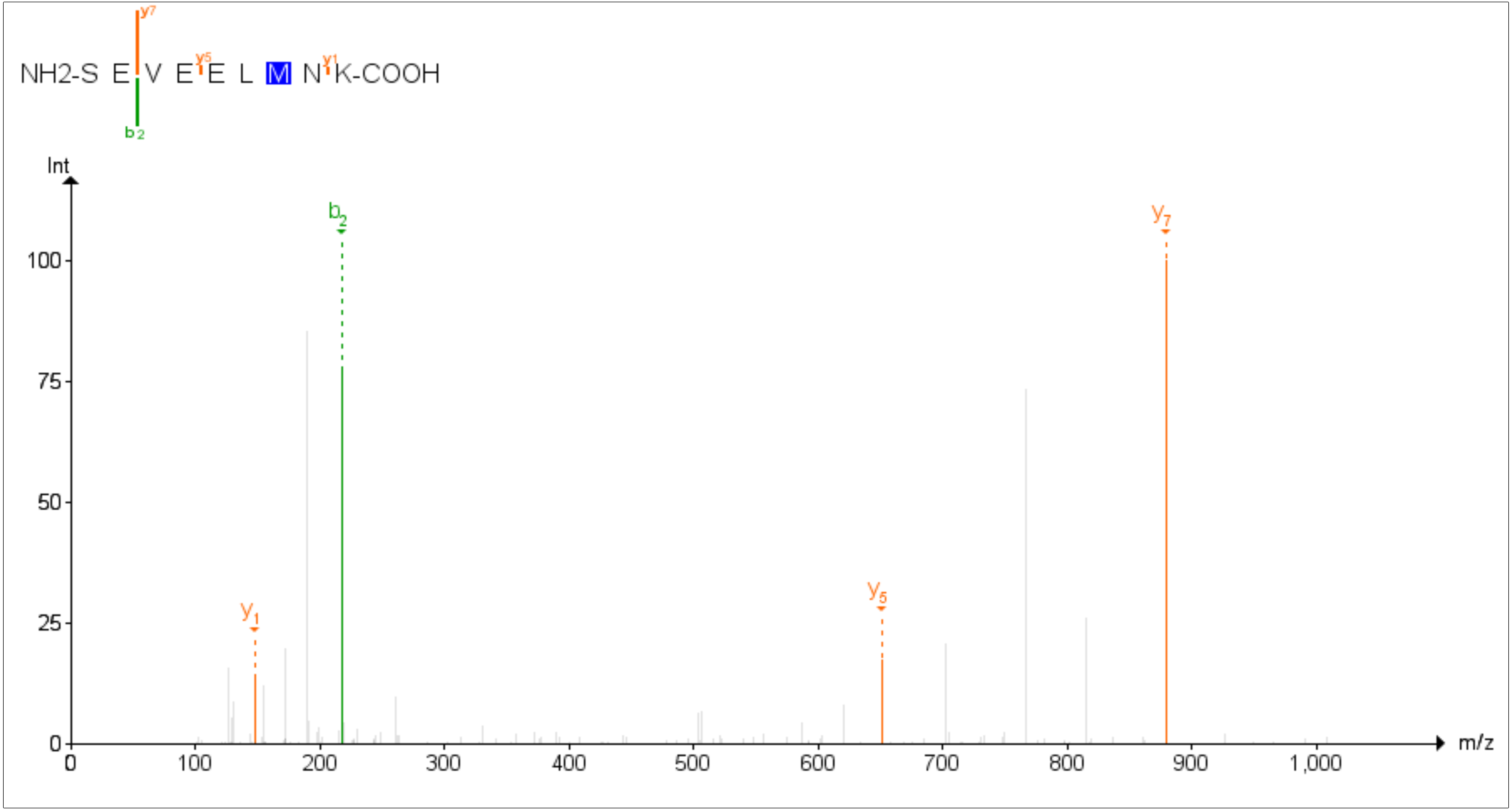

NH2-S E V E E L M N K-COOH
y7
y5
y1
b 2
Int
100
75
50
25
0
b2
y7
y5
y1
0
100
200
300
400
500
600
700
800
900
1,000
m/z

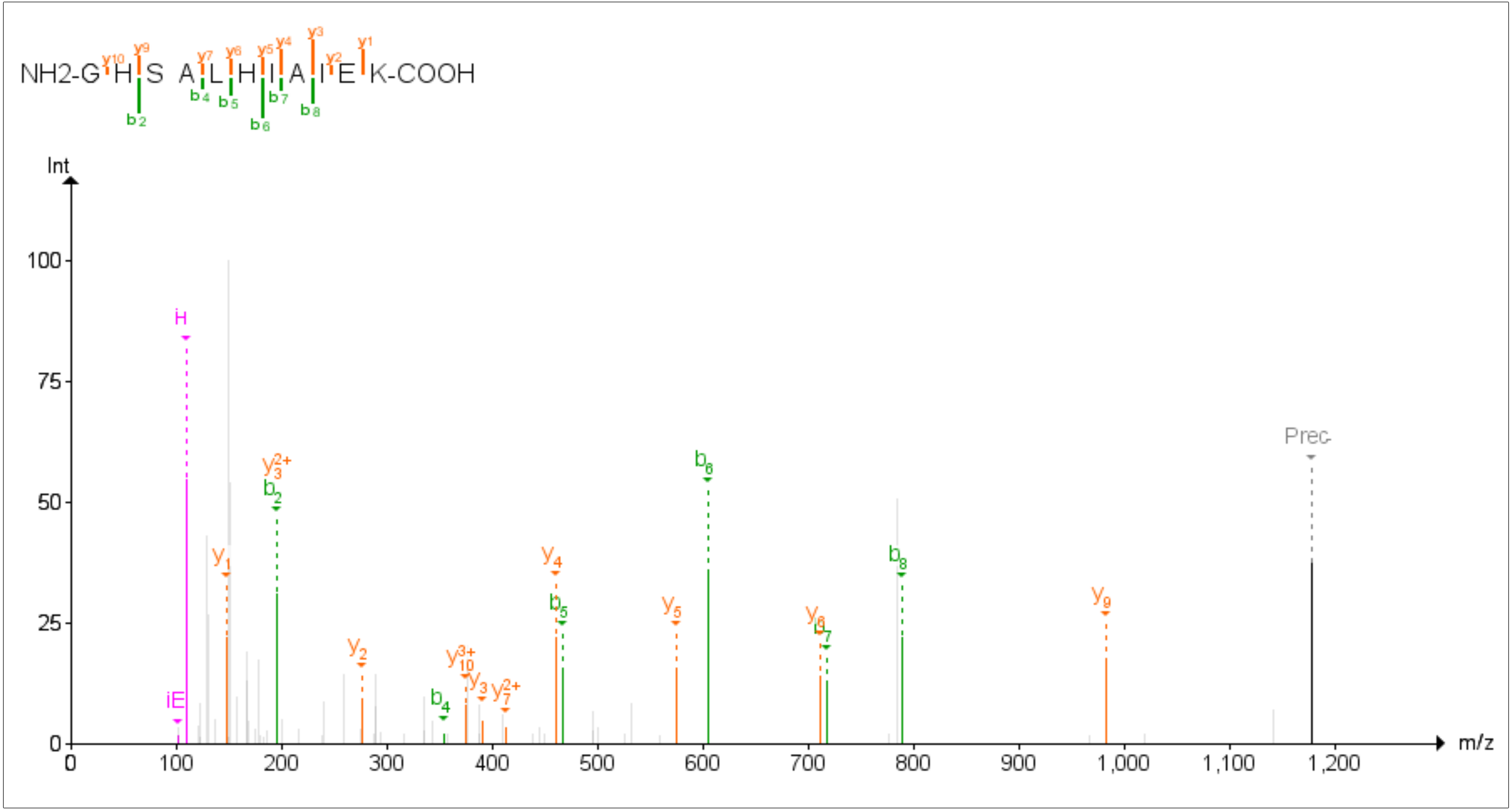

NH2-G H S A L H I A I E K-COOH
y10 y9 y7 y6 y5 y4 y3 y2 y1
b2 b4 b5 b6 b7 b8
Int
100
75
50
25
0
m/z
0
100
200
300
400
500
600
700
800
900
1,000
1,100
1,200
iH
iE
y1
y3 2+
b2
y2
b4
y10 3+
y3
y7 2+
y4
b5
y5
b6
y6
b7
b8
y9
Prec

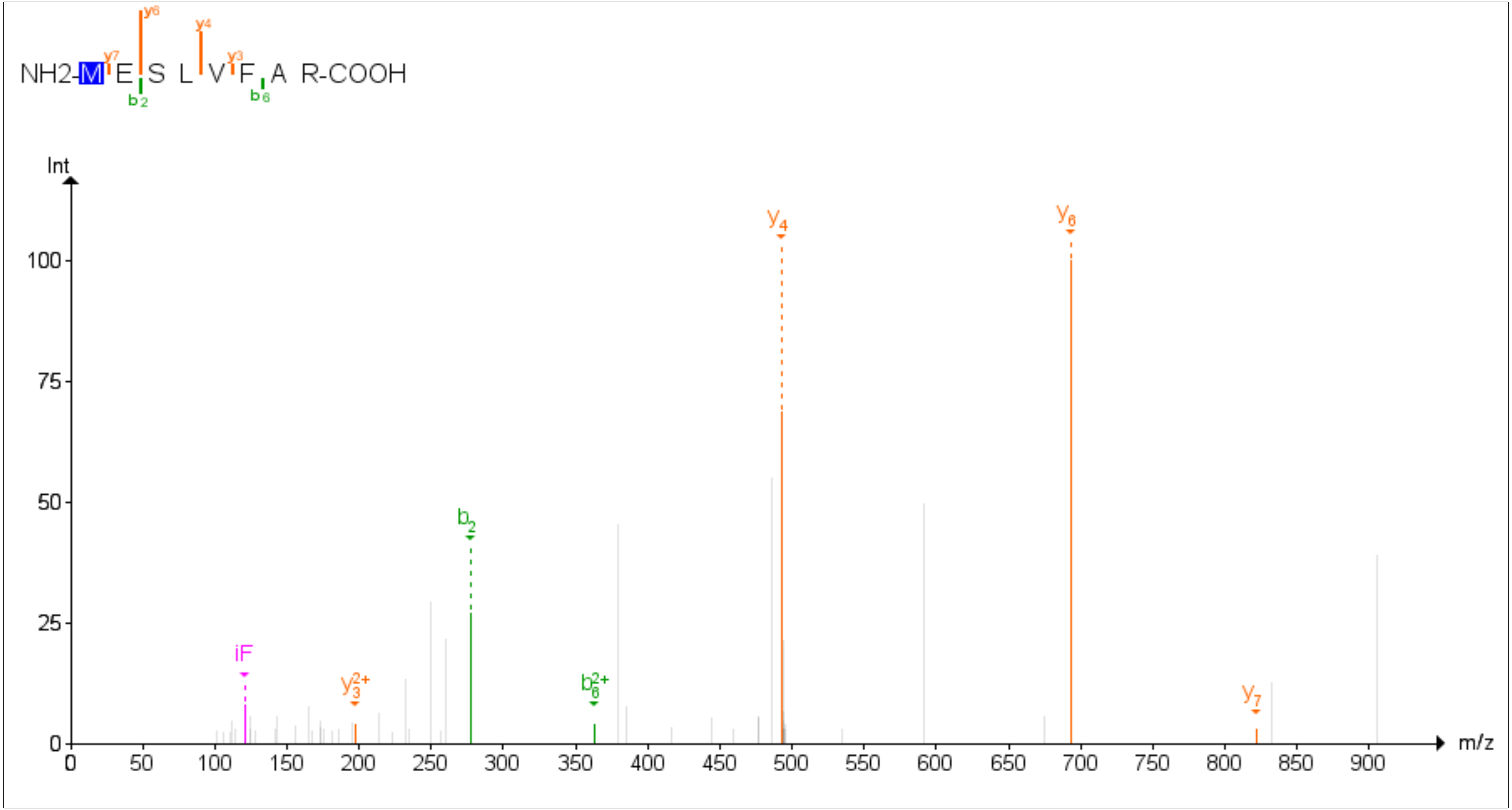

NH2-M E S L V F A R-COOH
y7
y6
y4
y3
b2
b6
Int
100
75
50
25
0
y4
y6
b2
iF
y_3^{2+}
b_6^{2+}
y7
0
50
100
150
200
250
300
350
400
450
500
550
600
650
700
750
800
850
900
m/z

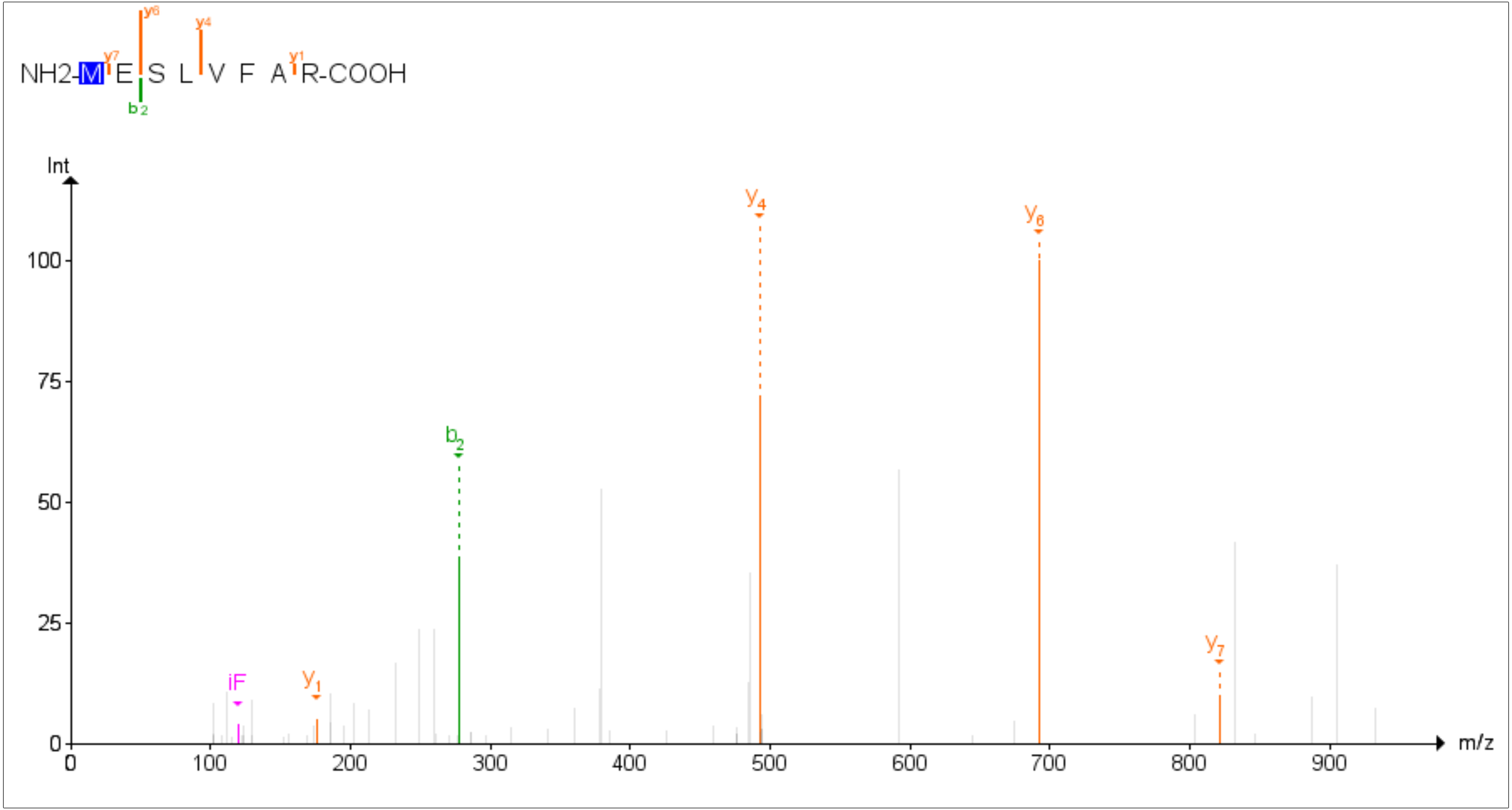

NH2-M E S L V F A R-COOH
y6
y4
y7
y1
b 2
Int
100
75
50
25
0
y4
y6
b2
y7
iF
y1
0
100
200
300
400
500
600
700
800
900
m/z

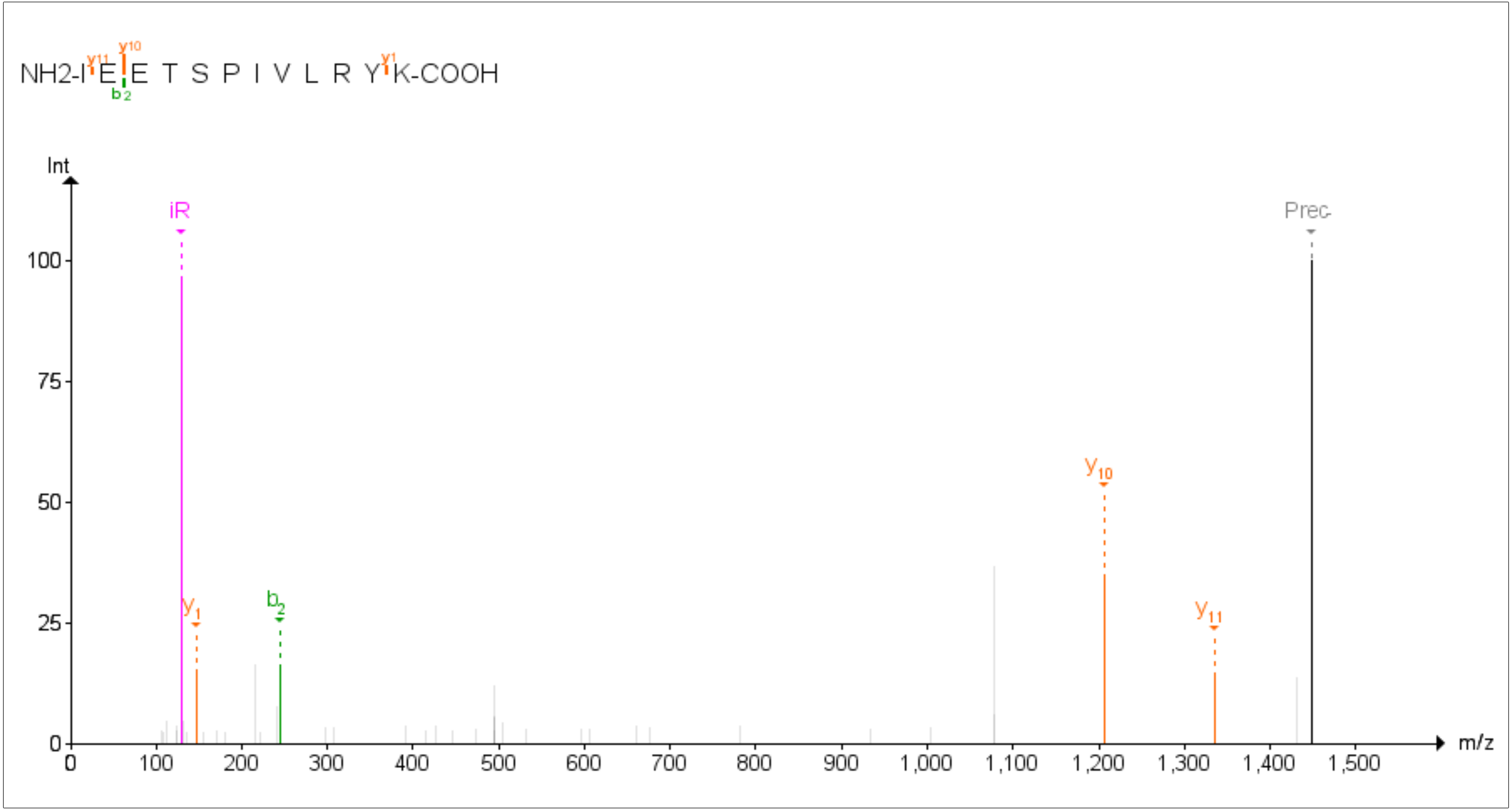
NH2-I E E T S P I V L R Y K-COOH
y11
y10
b2
y1
Int
iR
Prec
y10
y1
b2
y11
100
75
50
25
0
m/z
0
100
200
300
400
500
600
700
800
900
1,000
1,100
1,200
1,300
1,400
1,500

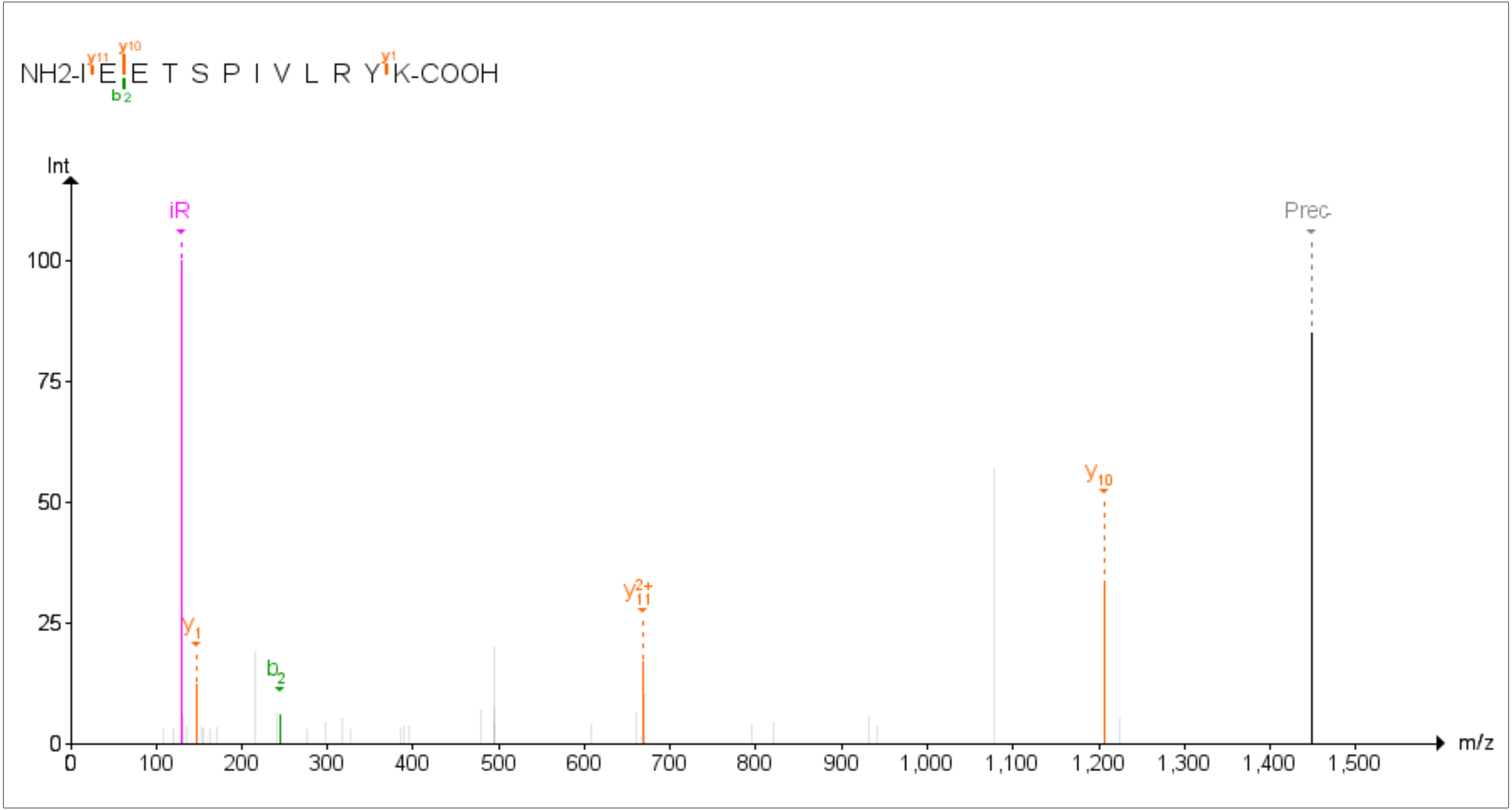

NH2-I E E T S P I V L R Y K-COOH
y11
y10
b2
y1
Int
iR
Prec
y1
b2
y11 2+
y10
100
75
50
25
0
0
100
200
300
400
500
600
700
800
900
1,000
1,100
1,200
1,300
1,400
1,500
m/z

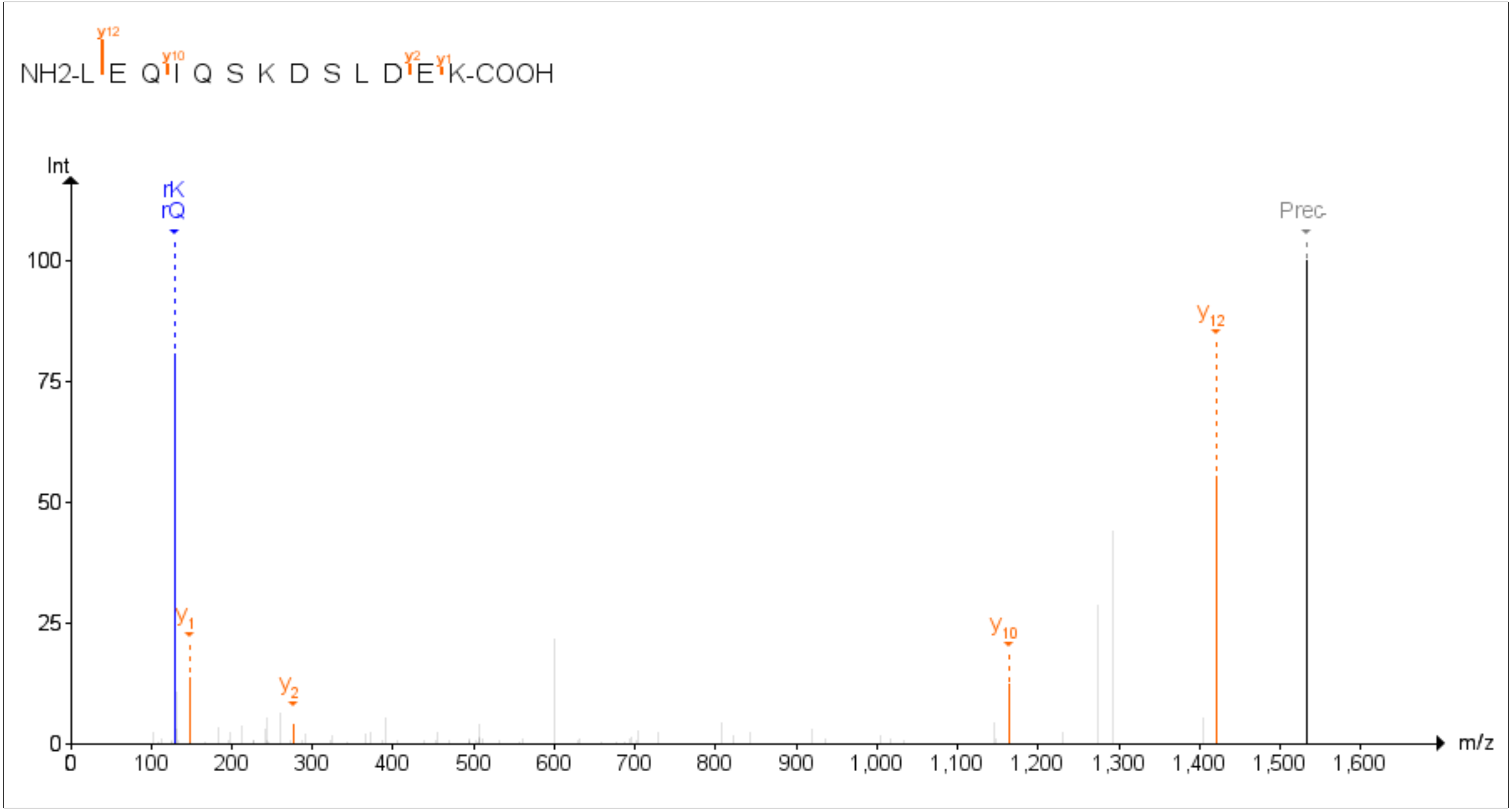

NH2-L E Q I Q S K D S L D E K-COOH
y12
y10
y2
y1
Int
rK
rQ
Prec
y12
y1
y2
y10
100
75
50
25
0
0
100
200
300
400
500
600
700
800
900
1,000
1,100
1,200
1,300
1,400
1,500
1,600
m/z

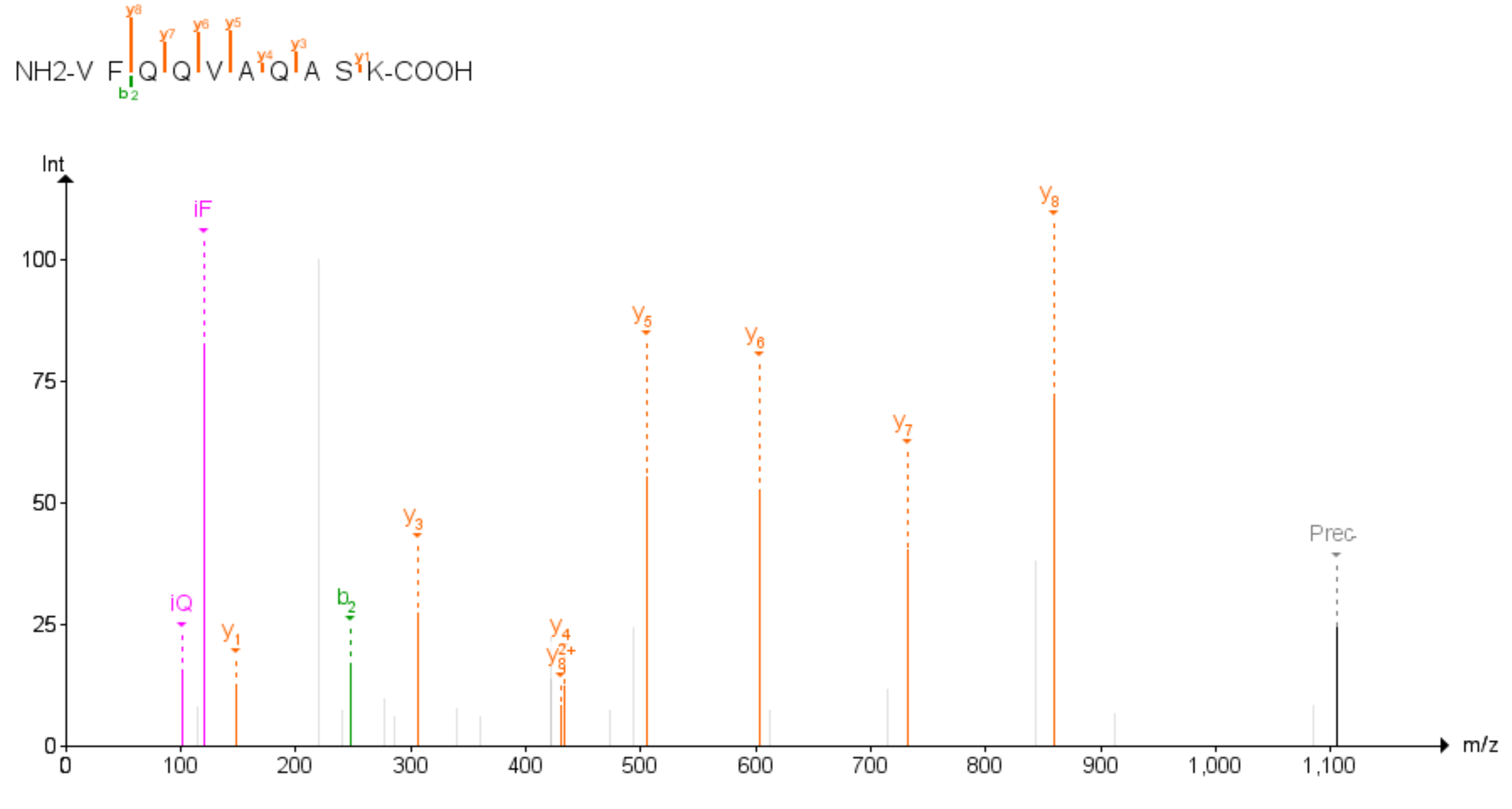

NH2-V F Q Q V A Q A S K-COOH
y8
y7
y6
y5
y4
y3
y1
b2
Int
100
75
50
25
0
iF
iQ
y1
b2
y3
y4
y8 2+
y5
y6
y7
y8
Prec
0
100
200
300
400
500
600
700
800
900
1,000
1,100
m/z

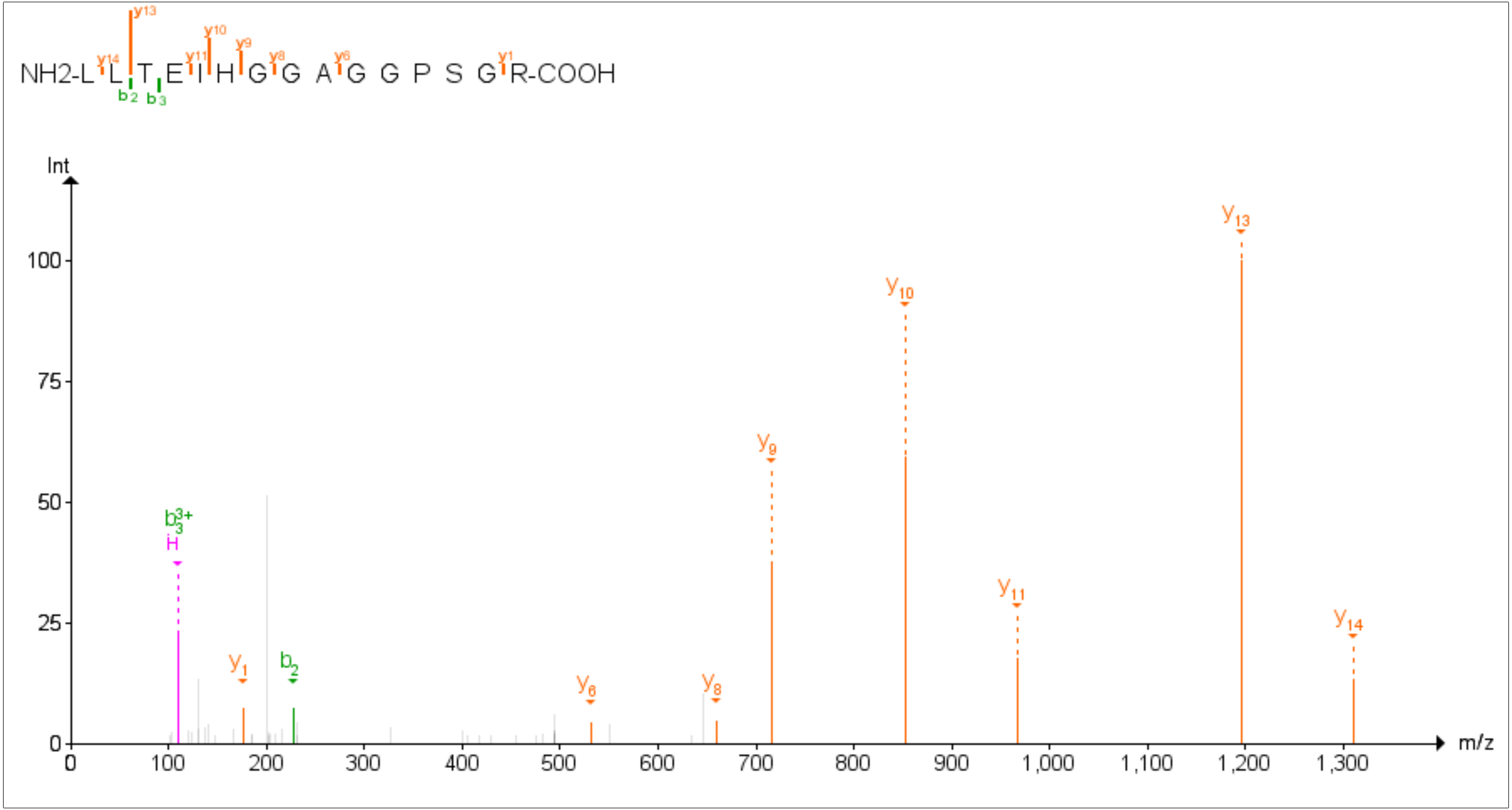

NH2-L L T E I H G G A G G P S G R-COOH
y14
y13
y11
y10
y9
y8
y6
y1
b2
b3
Int
m/z
100
75
50
25
0
0
100
200
300
400
500
600
700
800
900
1,000
1,100
1,200
1,300
b3 3+
H
y1
b2
y6
y8
y9
y10
y11
y13
y14

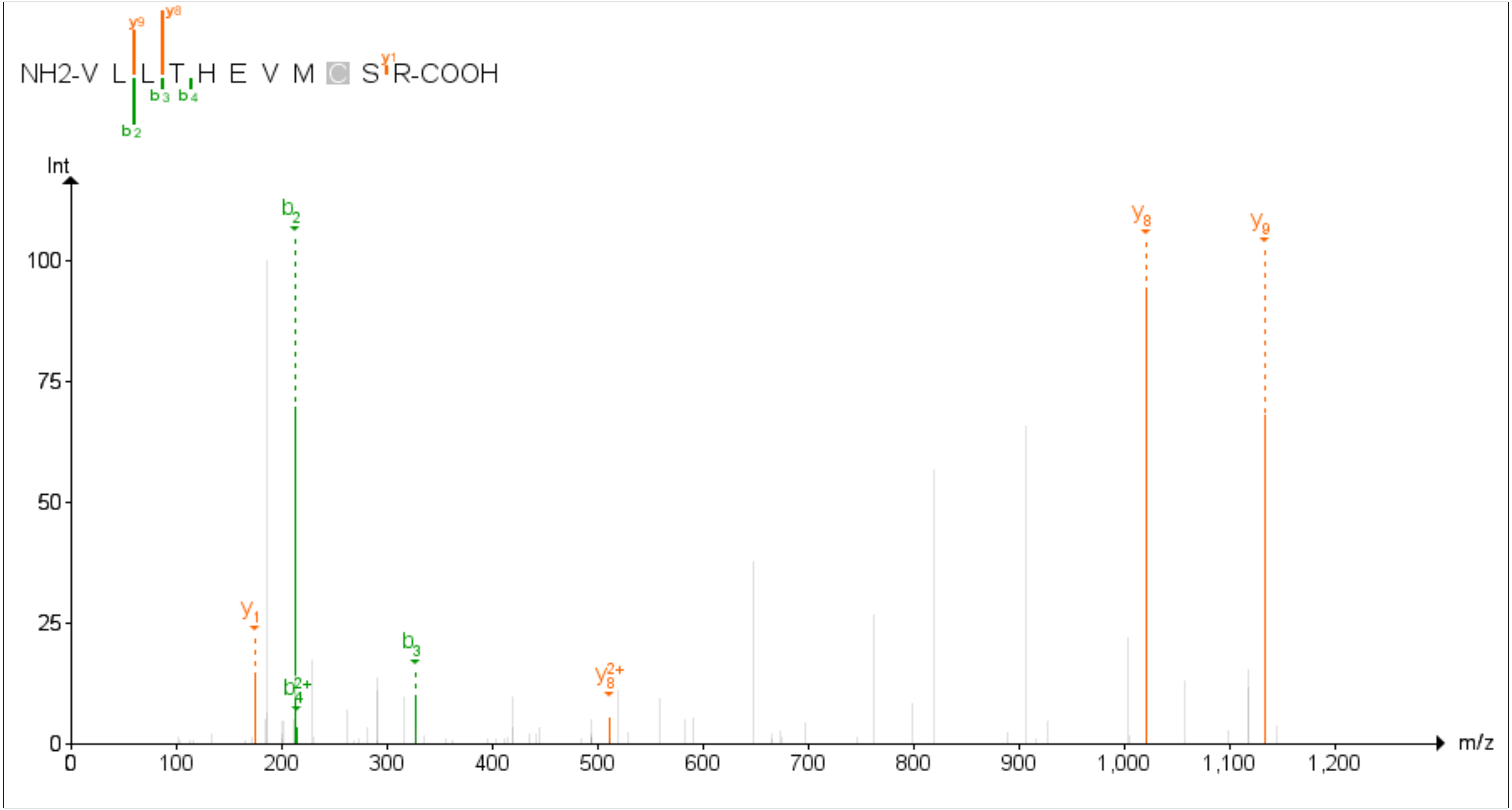
NH2-V L L T H E V M C S R-COOH
y9 y8 y1
b2 b3 b4
Int
100
75
50
25
0
b2
y8
y9
y1
b3
b_4^{2+}
y_8^{2+}
0 100 200 300 400 500 600 700 800 900 1,000 1,100 1,200
m/z

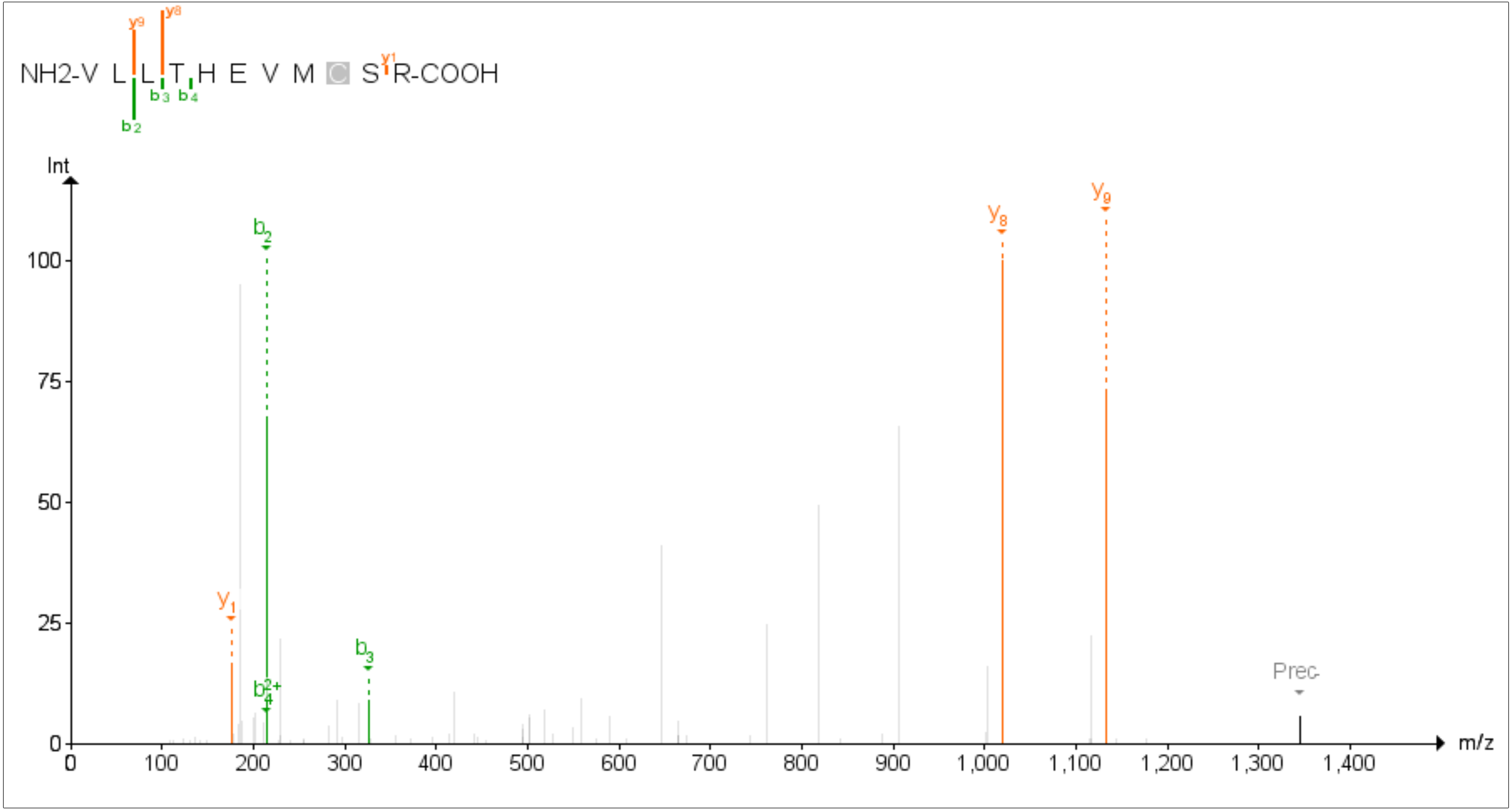
NH2-V L L T H E V M C S R-COOH
y9 y8 y1
b2 b3 b4
Int
100
75
50
25
0
b2
y8
y9
y1
b3
b4 2+
Prec
0 100 200 300 400 500 600 700 800 900 1,000 1,100 1,200 1,300 1,400
m/z

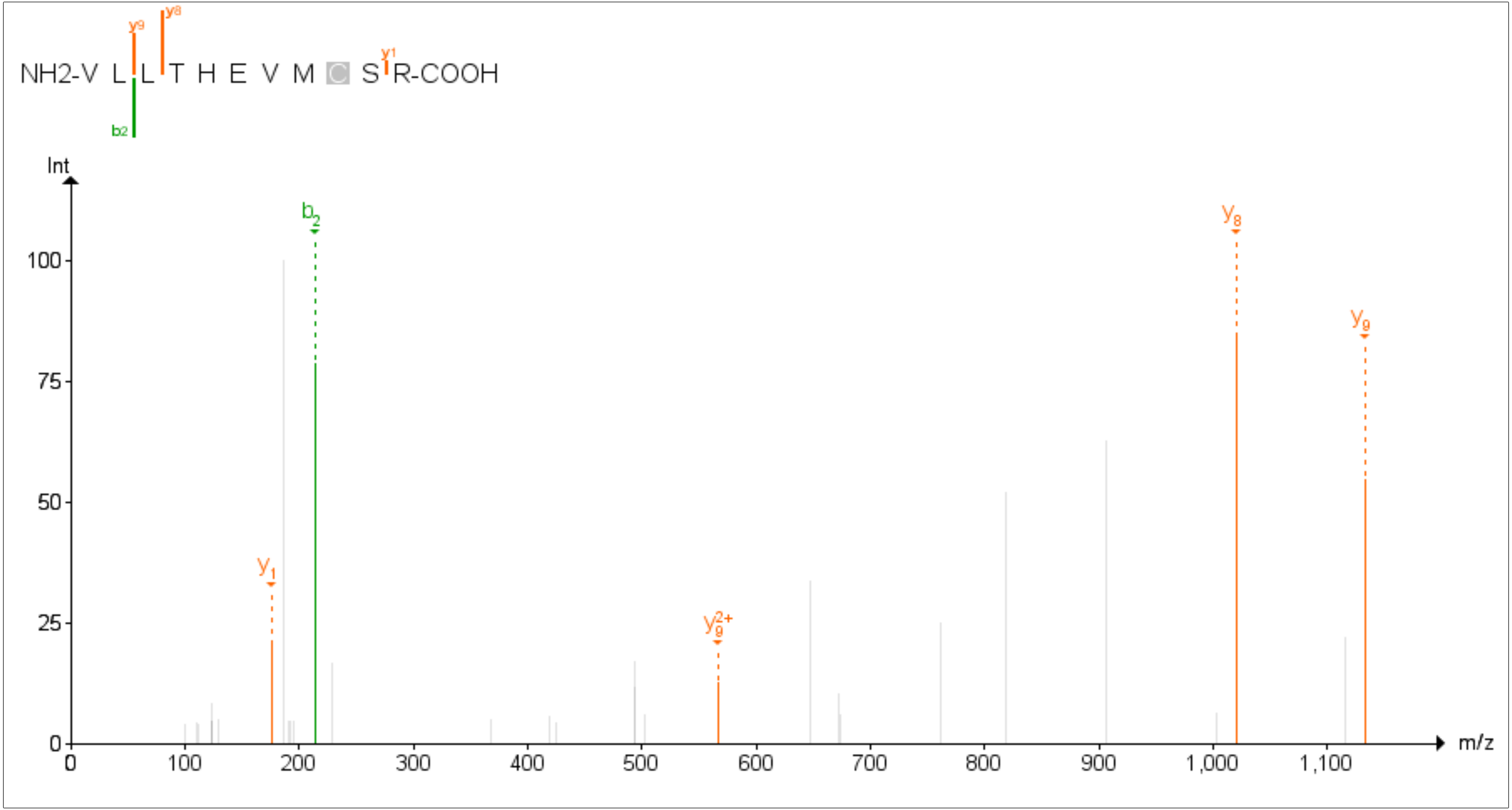

NH2-V L L T H E V M C S R-COOH
y9
y8
y1
b2
Int
100
75
50
25
0
b2
y8
y9
y1
y9 2+
0
100
200
300
400
500
600
700
800
900
1,000
1,100
m/z

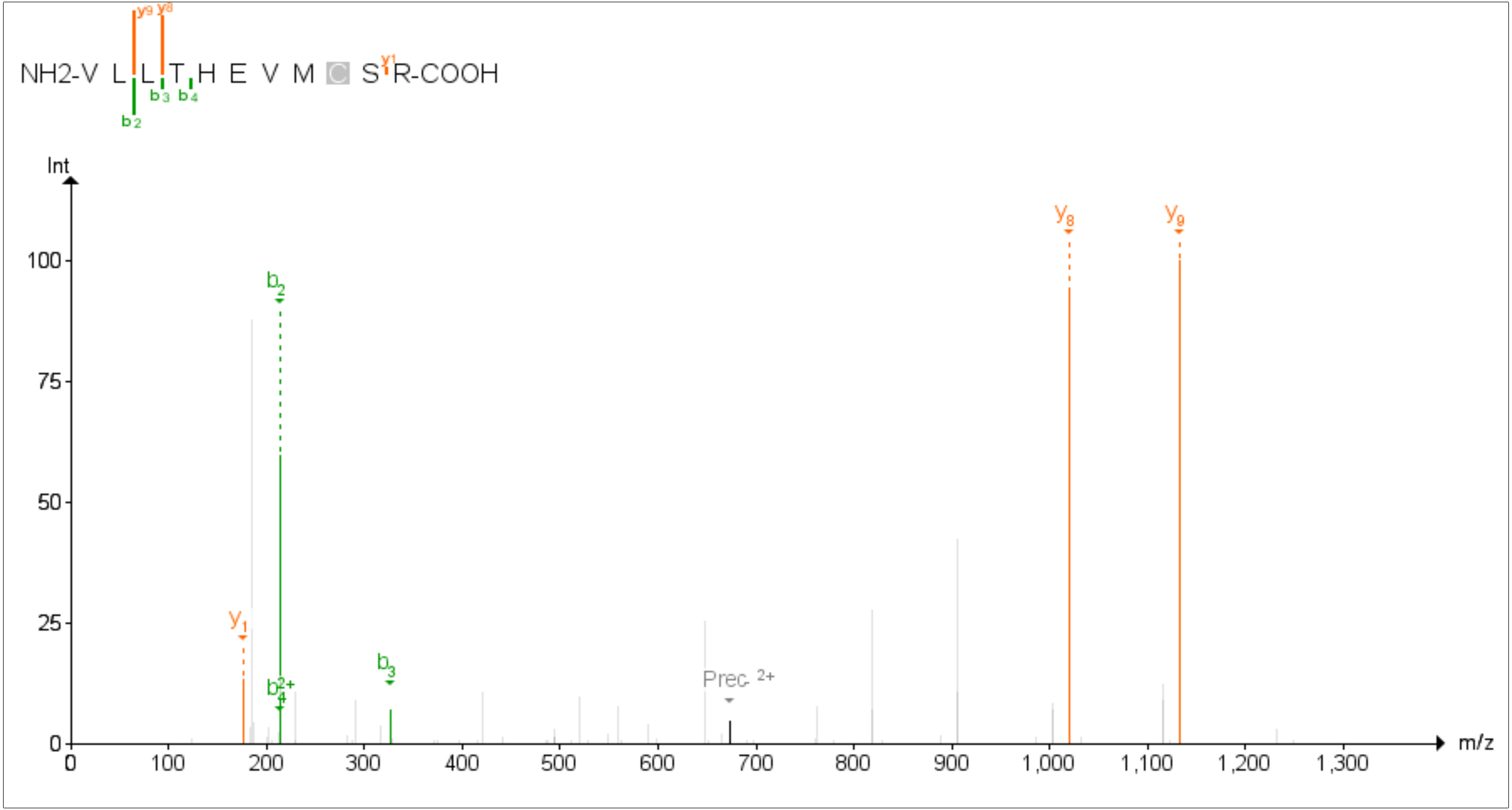

NH2-V L L T H E V M C S R-COOH
y9 y8
b 2
b 3
b 4
y1
Int
100
75
50
25
0
y8
y9
b2
y1
b4 2+
b3
Prec. 2+
0
100
200
300
400
500
600
700
800
900
1,000
1,100
1,200
1,300
m/z

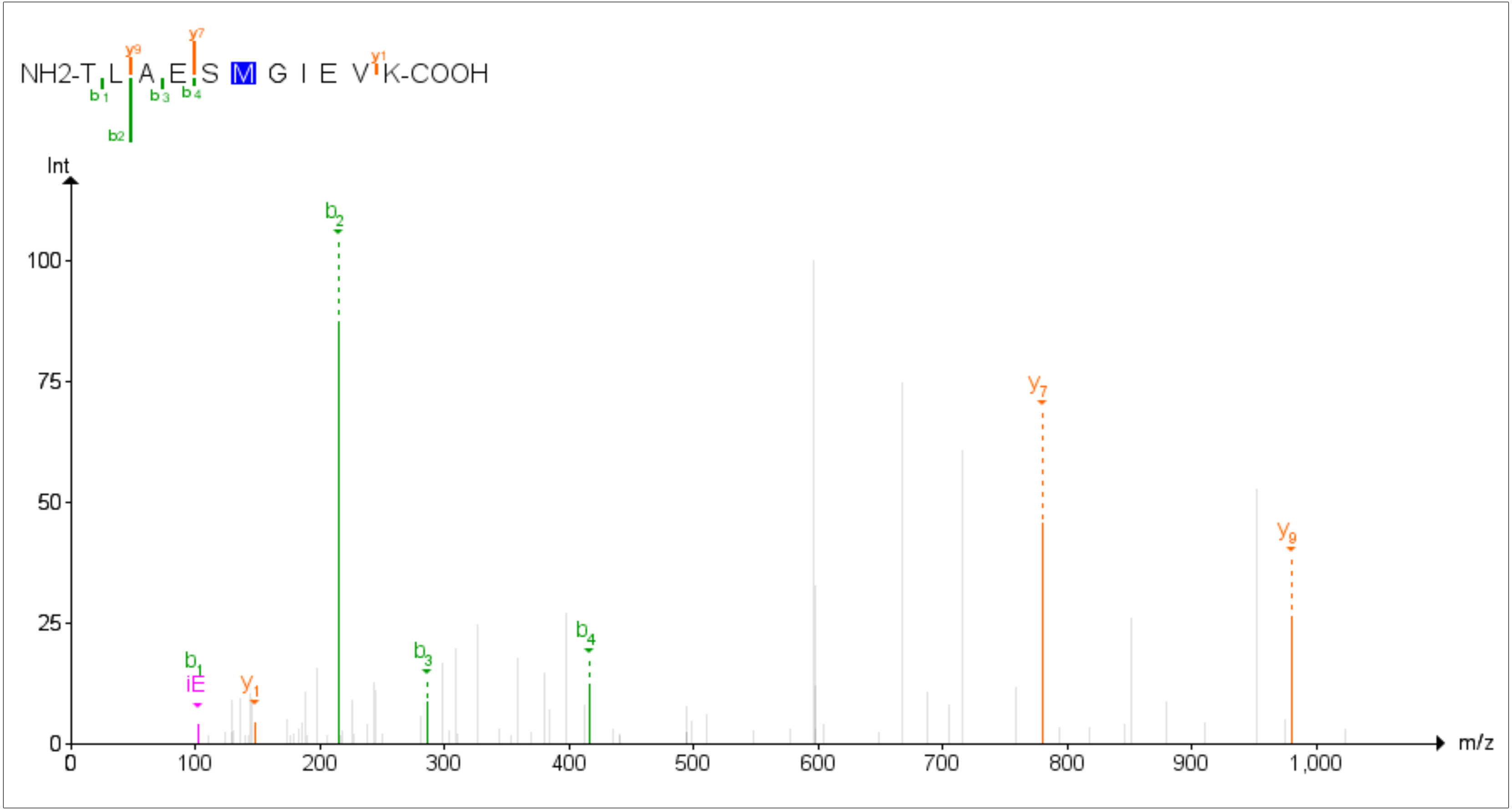

NH2-T L A E S M G I E V K-COOH
b1
b2
b3
b4
y9
y7
y1
Int
m/z
100
75
50
25
0
0
100
200
300
400
500
600
700
800
900
1,000
b2
b1
iE
y1
b3
b4
y7
y9

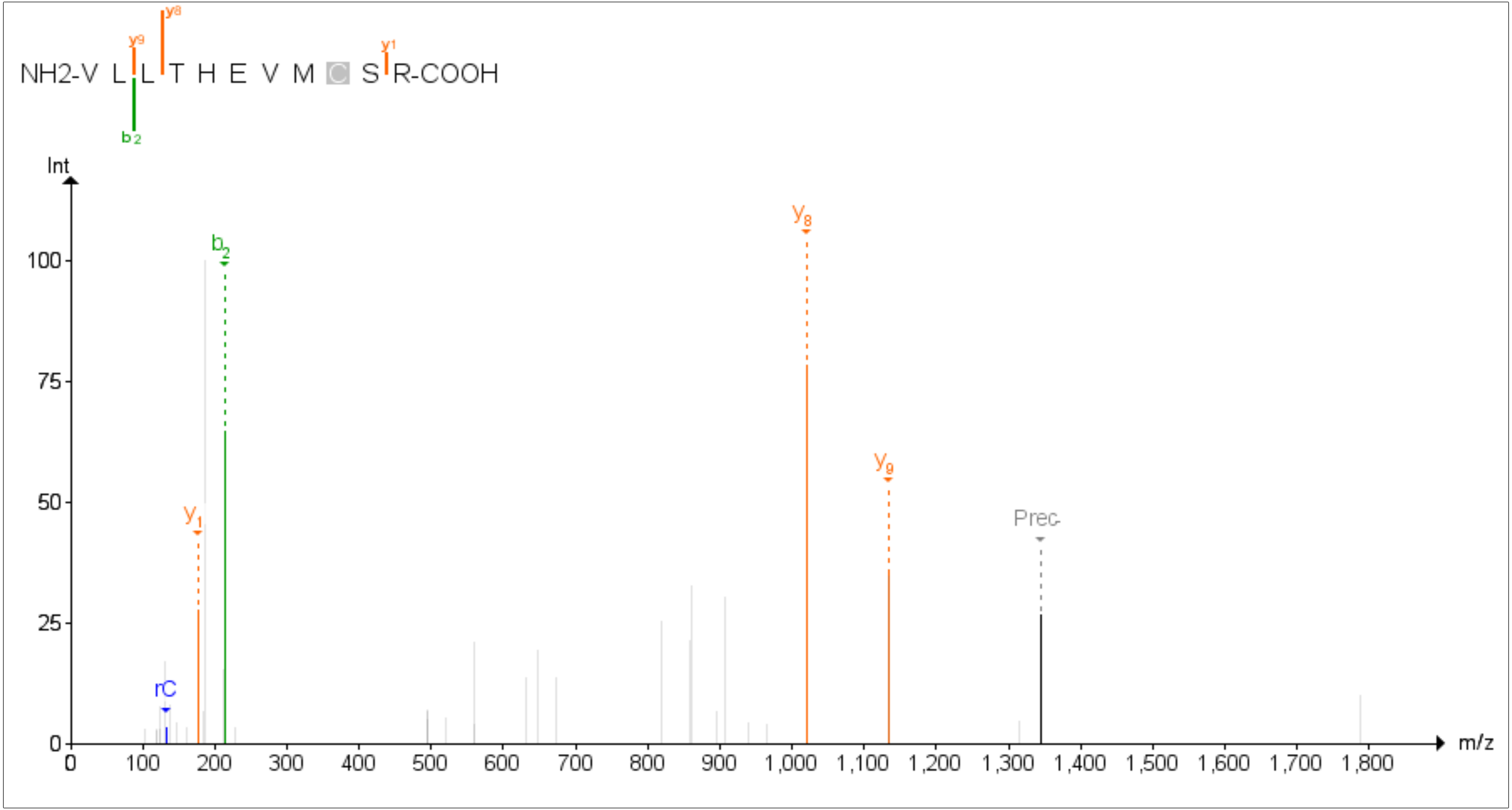

NH2-V L L T H E V M C S R-COOH
y9
y8
y1
b2
Int
100
75
50
25
0
b2
y8
y9
y1
Prec
rC
0 100 200 300 400 500 600 700 800 900 1,000 1,100 1,200 1,300 1,400 1,500 1,600 1,700 1,800
m/z

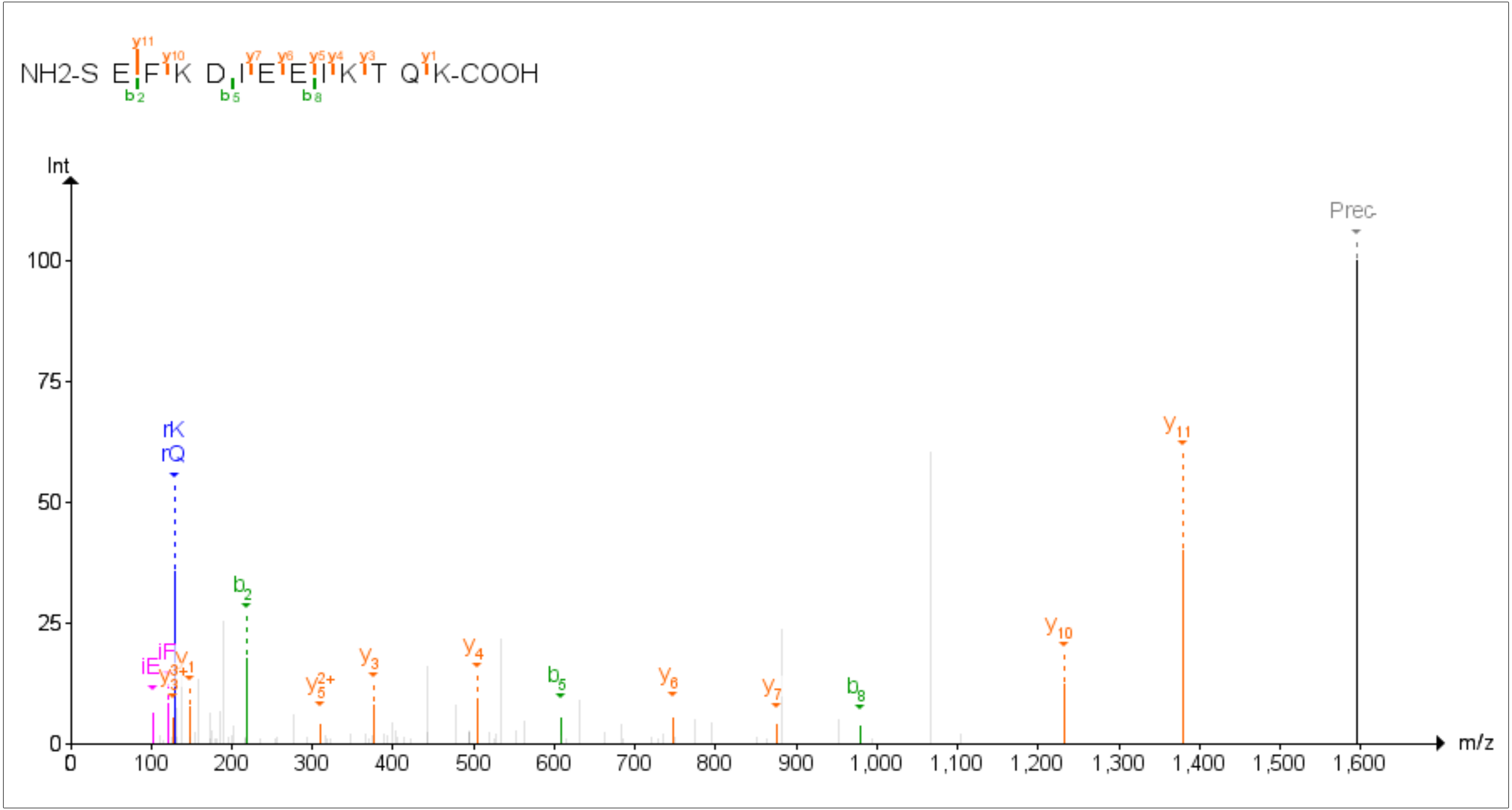

NH2-S E F K D I E E I K T Q K-COOH
y11 y10 y7 y6 y5 y4 y3 y1
b2 b5 b8
Int
m/z
Prec.
rK
rQ
b2
iE
iF
y1
y3
y3 2+
y5 2+
y3
y4
b5
y6
y7
b8
y10
y11

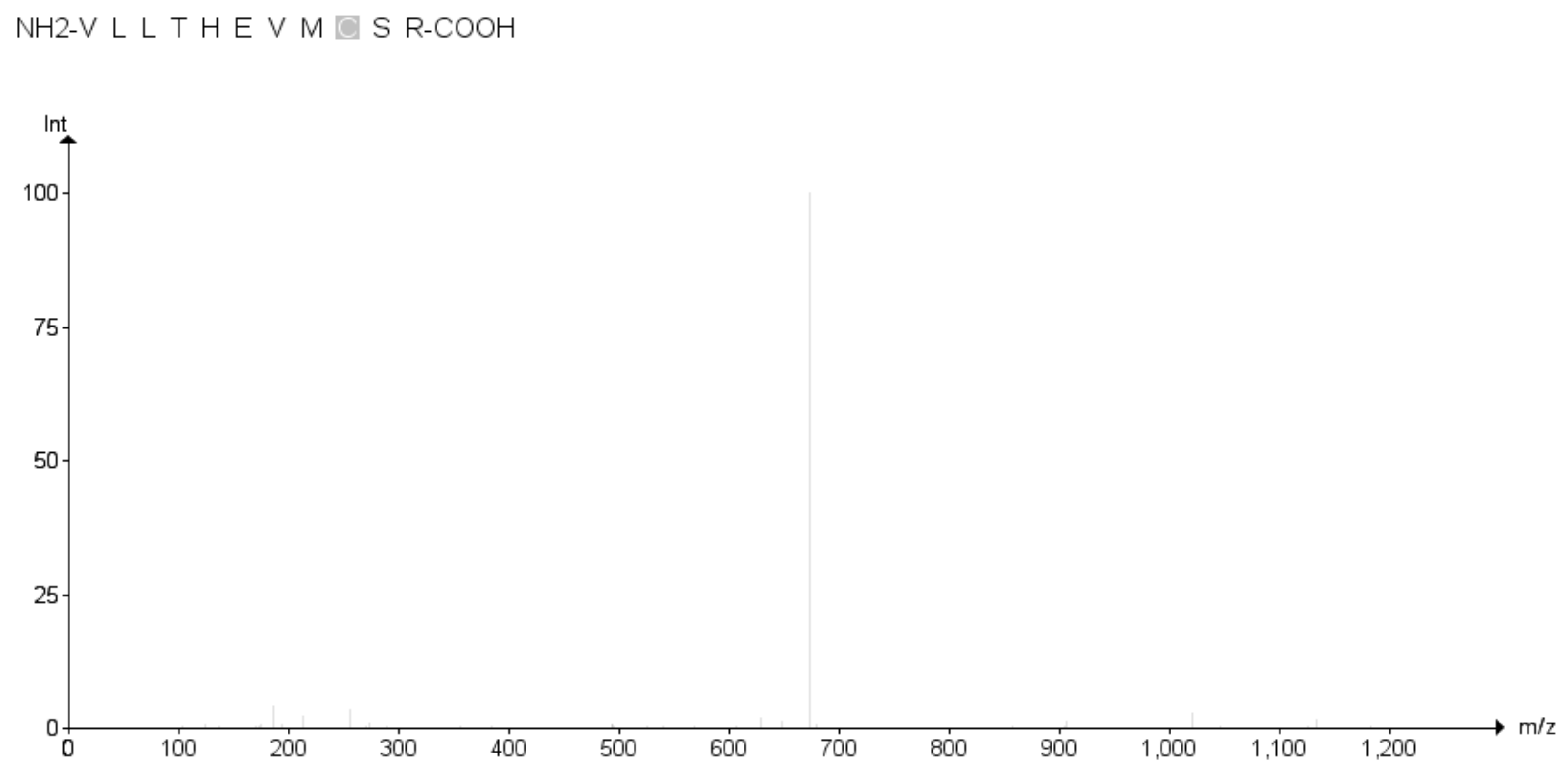
NH2-V L L T H E V M C S R-COOH
Int
100
75
50
25
0
0
100
200
300
400
500
600
700
800
900
1,000
1,100
1,200
m/z

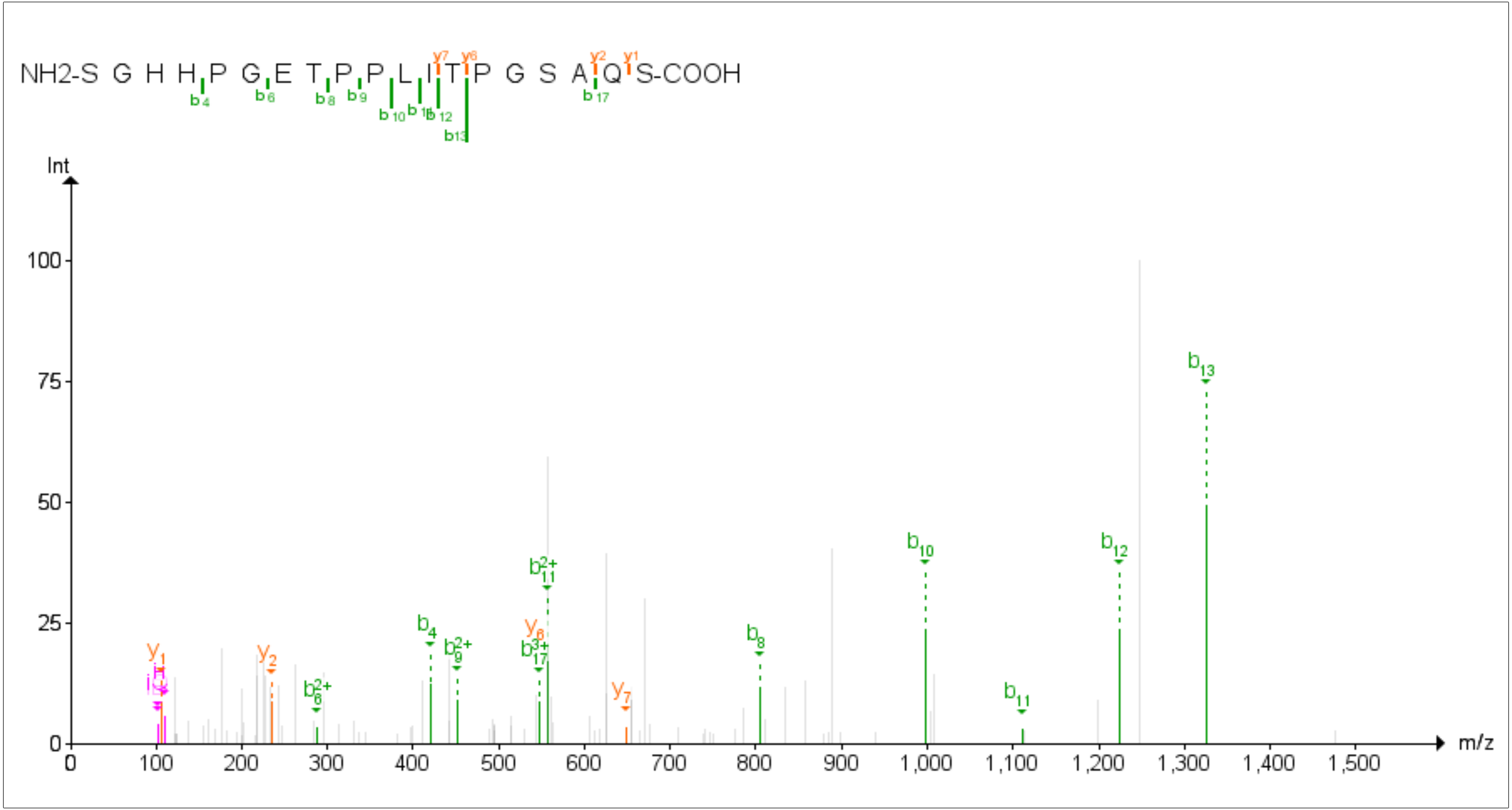

NH2-S G H H P G E T P P L I T P G S A Q S-COOH
Int
m/z
b13
b10
b12
b11
b8
b4
y1
y2
y7
y6

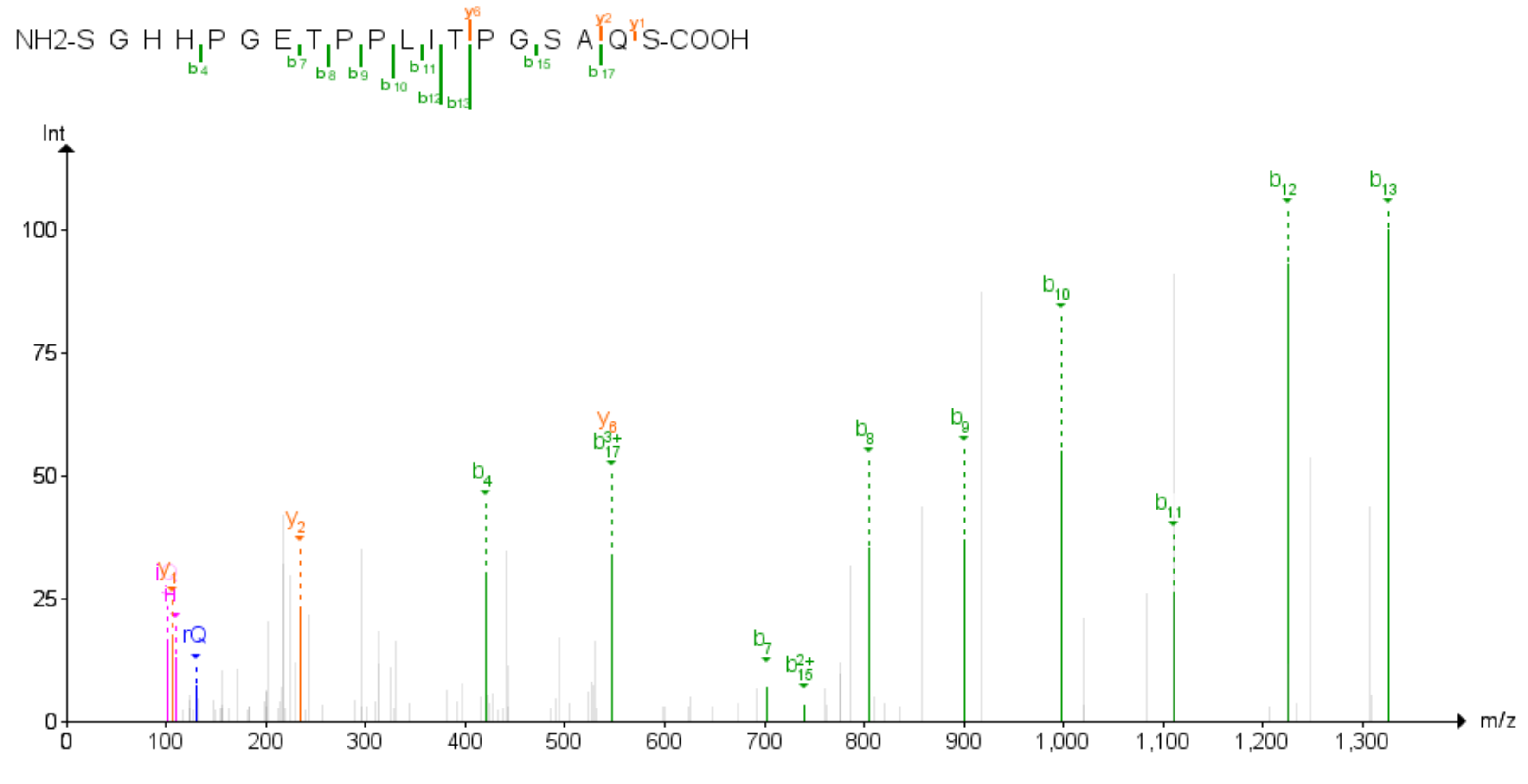
NH2-S G H H P G E T P P L I T P G S A Q S-COOH
y6 y2 y1
b4 b7 b8 b9 b10 b11 b12 b13 b15 b17
Int
m/z
0 25 50 75 100
0 100 200 300 400 500 600 700 800 900 1,000 1,100 1,200 1,300
y1
iH
rQ
y2
b4
y6
b17 3+
b7
b15 2+
b8
b9
b10
b11
b12
b13

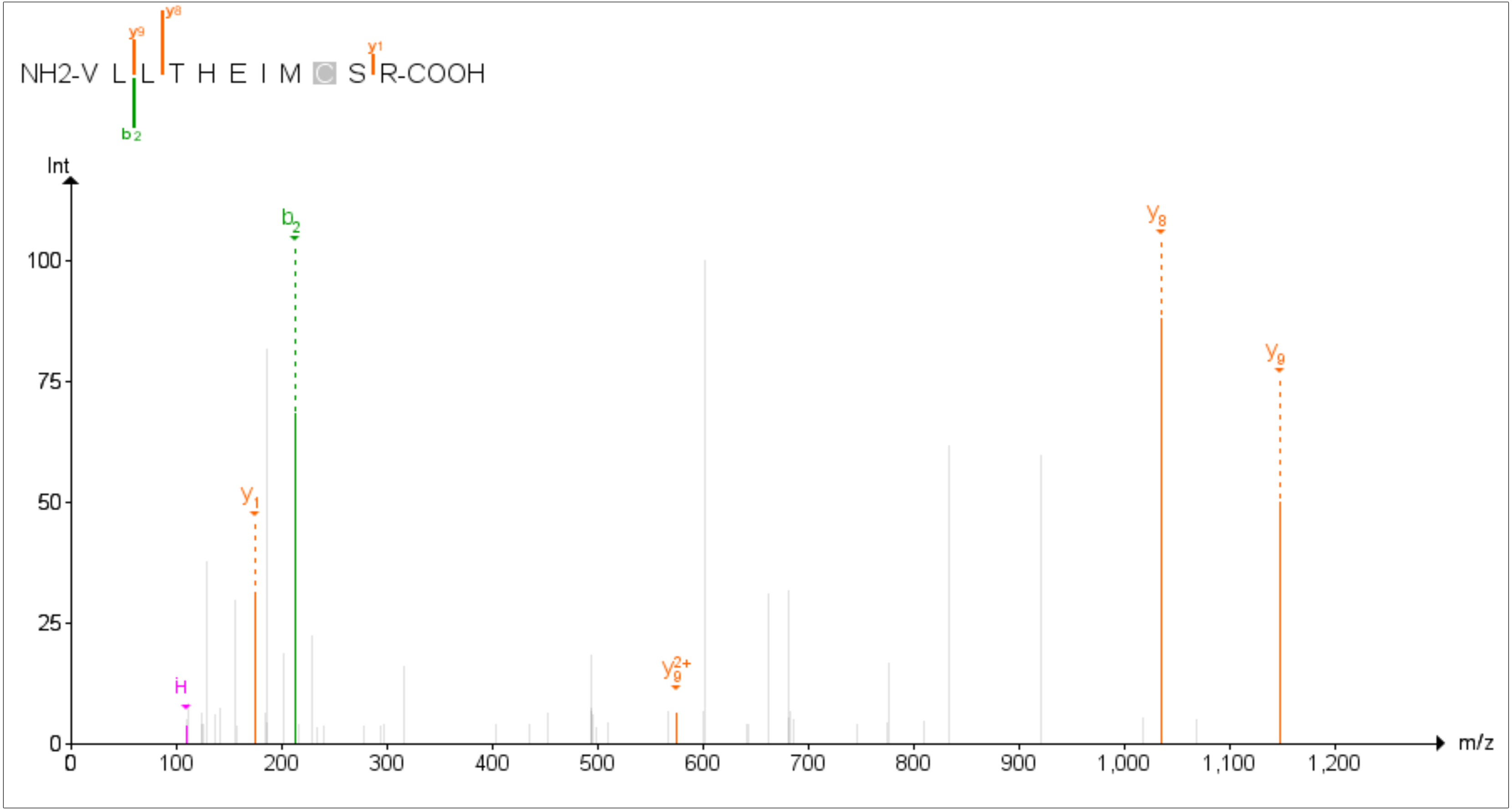

NH2-V L L T H E I M C S R-COOH
y9
y8
y1
b2
Int
100
75
50
25
0
b2
y8
y9
y1
H
y9 2+
0
100
200
300
400
500
600
700
800
900
1,000
1,100
1,200
m/z

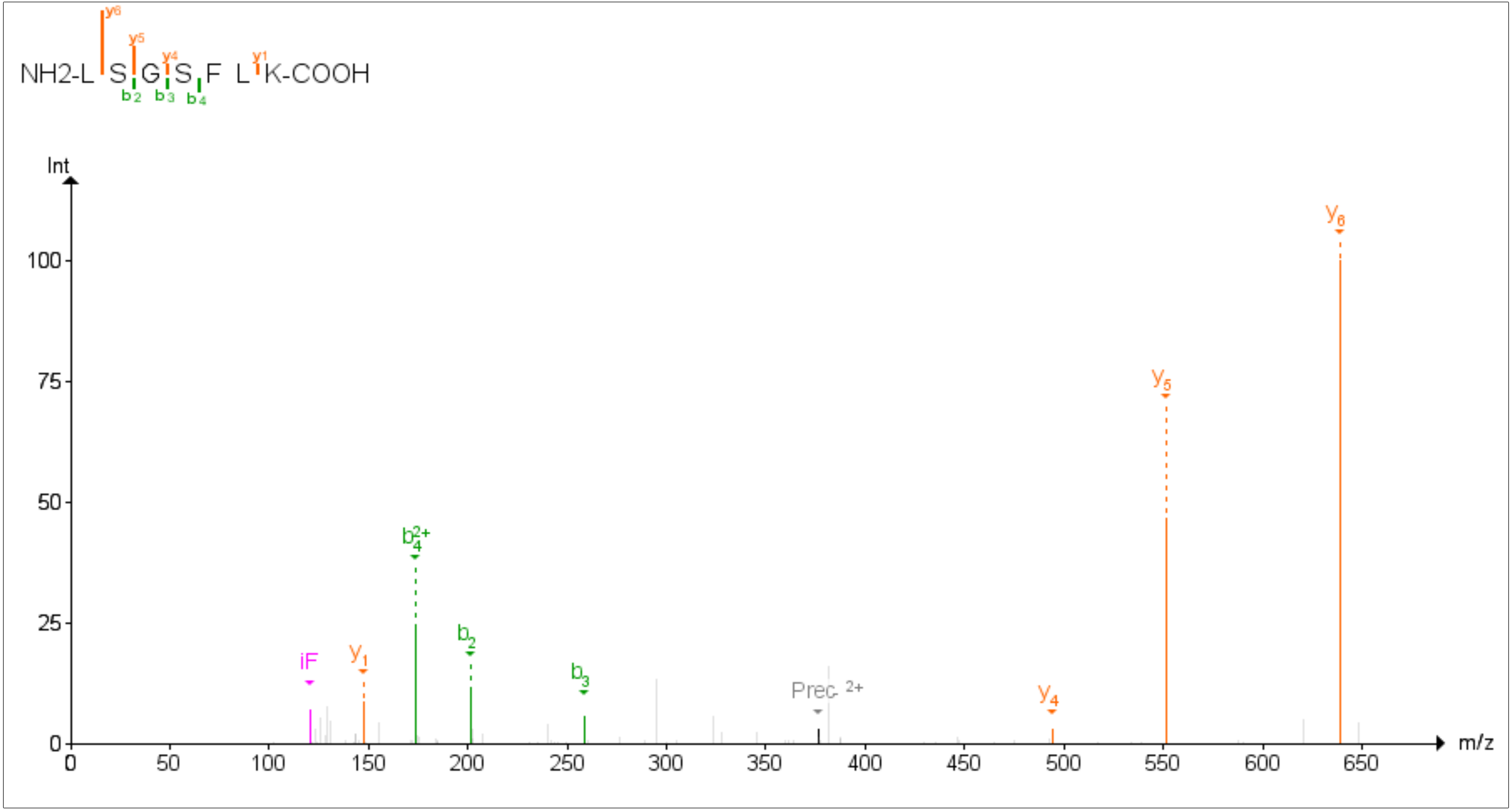

NH2-L S G S F L K-COOH
y6
y5
y4
y1
b2
b3
b4
Int
m/z
y6
y5
y4
y1
b$_4^{2+}$
b2
b3
iF
Prec. 2+
0
25
50
75
100
0
50
100
150
200
250
300
350
400
450
500
550
600
650

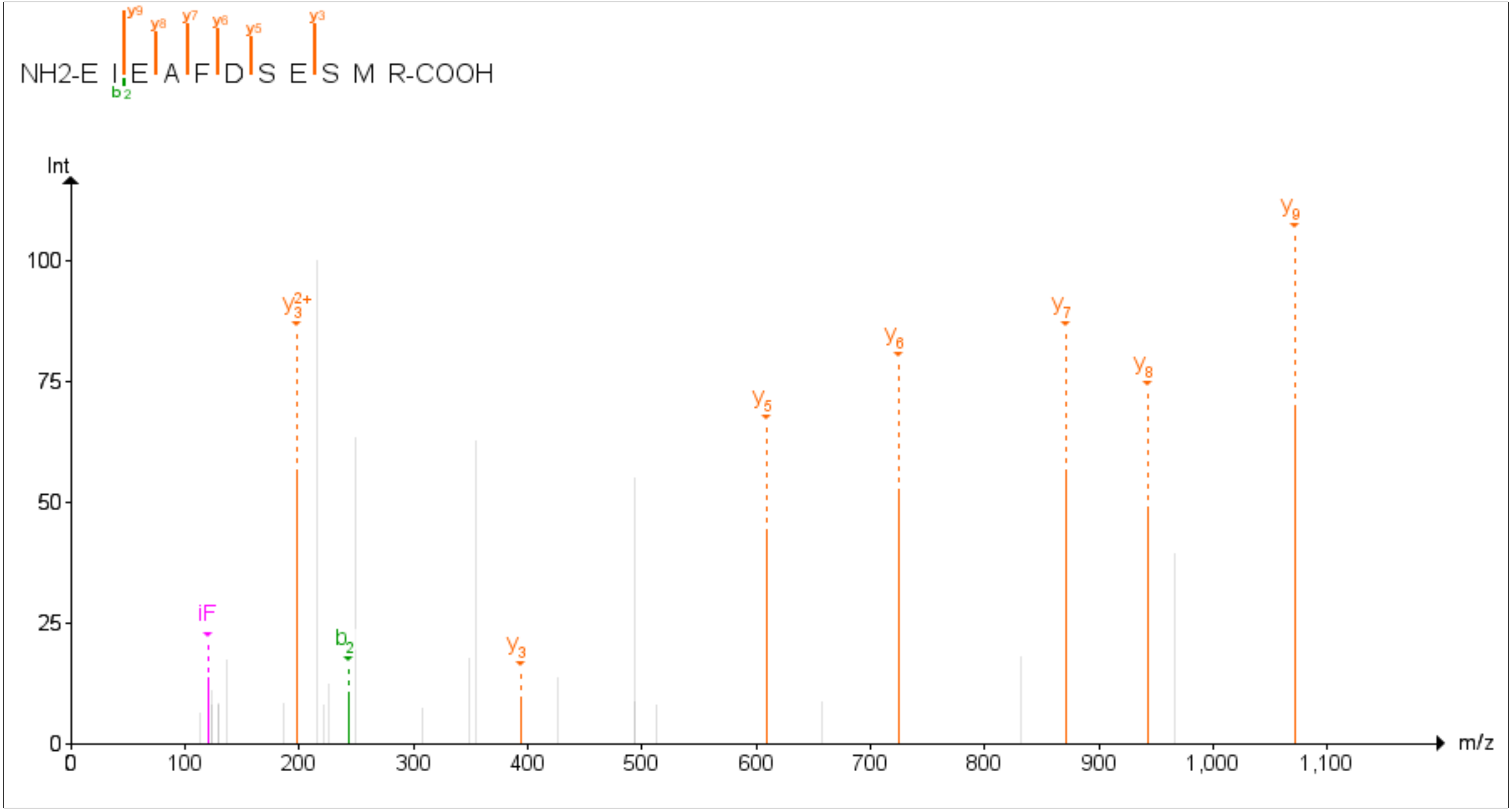

NH2-E I E A F D S E S M R-COOH
y9
y8
y7
y6
y5
y3
b2
Int
100
75
50
25
0
y3 2+
y9
y7
y6
y8
y5
iF
b2
y3
m/z
0
100
200
300
400
500
600
700
800
900
1,000
1,100

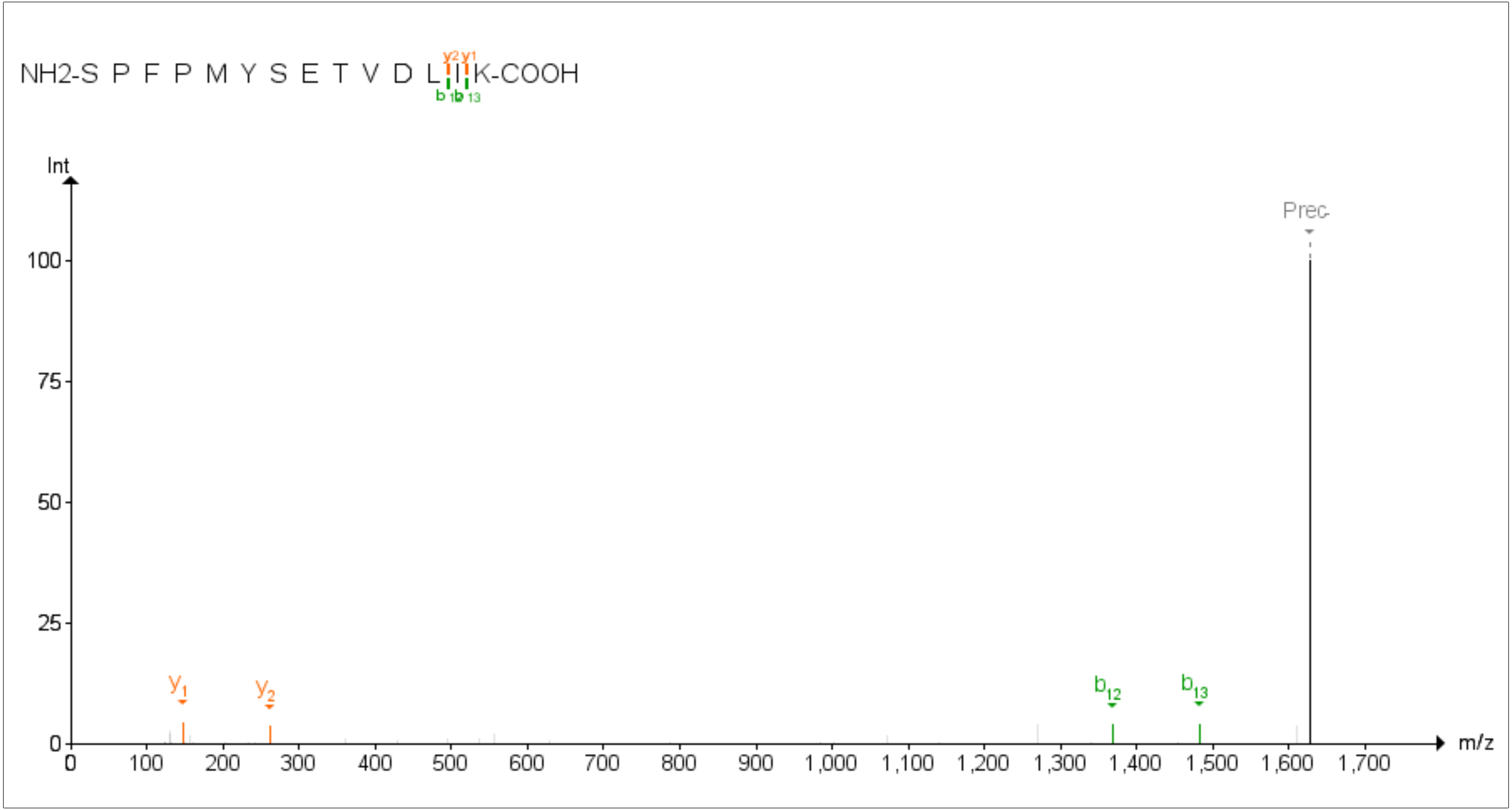

NH2-S P F P M Y S E T V D L I I K-COOH
y2 y1
b 12 b 13
Int
Prec
100
75
50
25
0
y1
y2
b12
b13
0 100 200 300 400 500 600 700 800 900 1,000 1,100 1,200 1,300 1,400 1,500 1,600 1,700
m/z

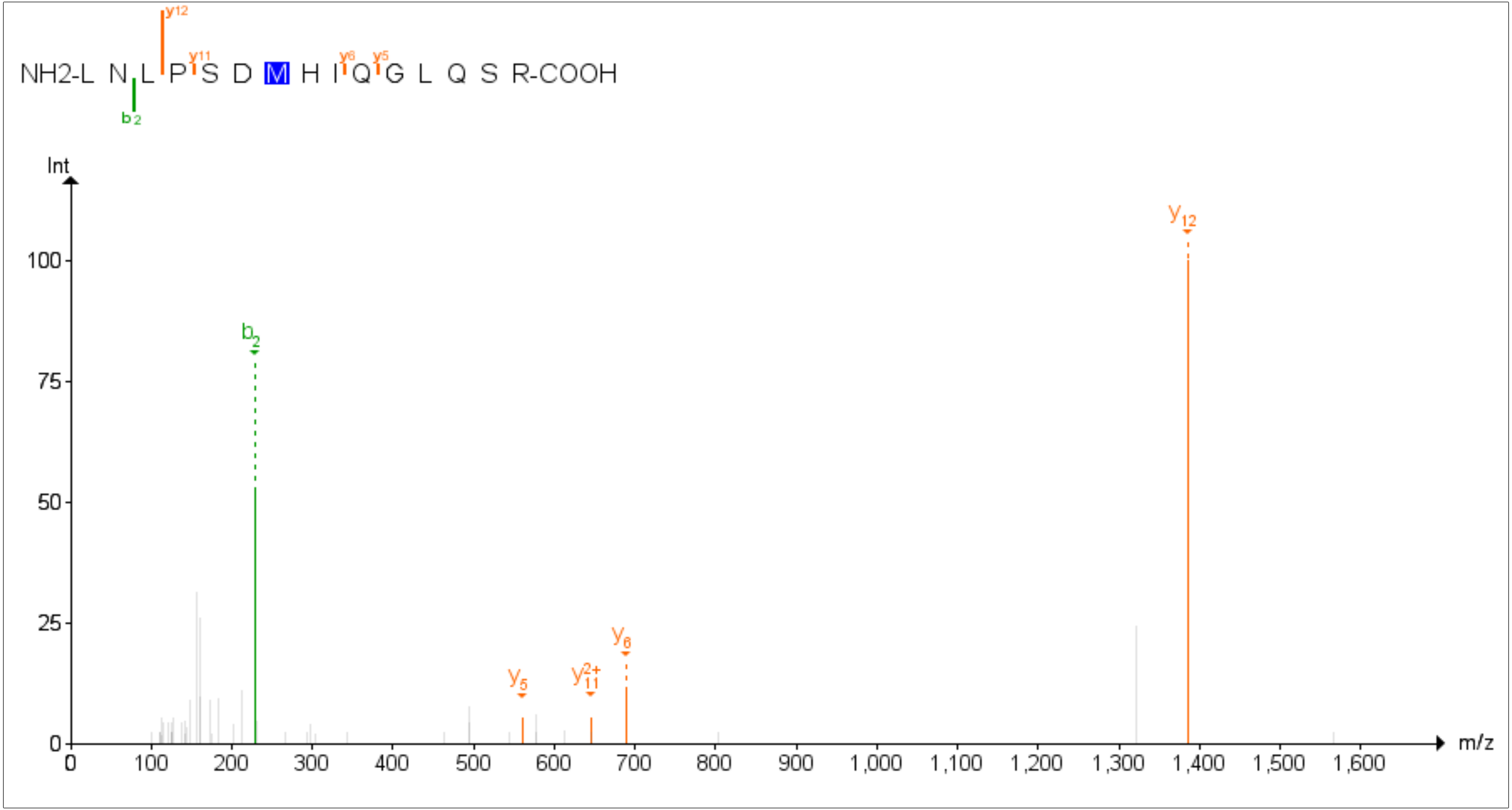
NH2-L N L P S D M H I Q G L Q S R-COOH
y12
y11
y6
y5
b2
Int
100
75
50
25
0
y12
b2
y5
y11 2+
y6
0
100
200
300
400
500
600
700
800
900
1,000
1,100
1,200
1,300
1,400
1,500
1,600
m/z

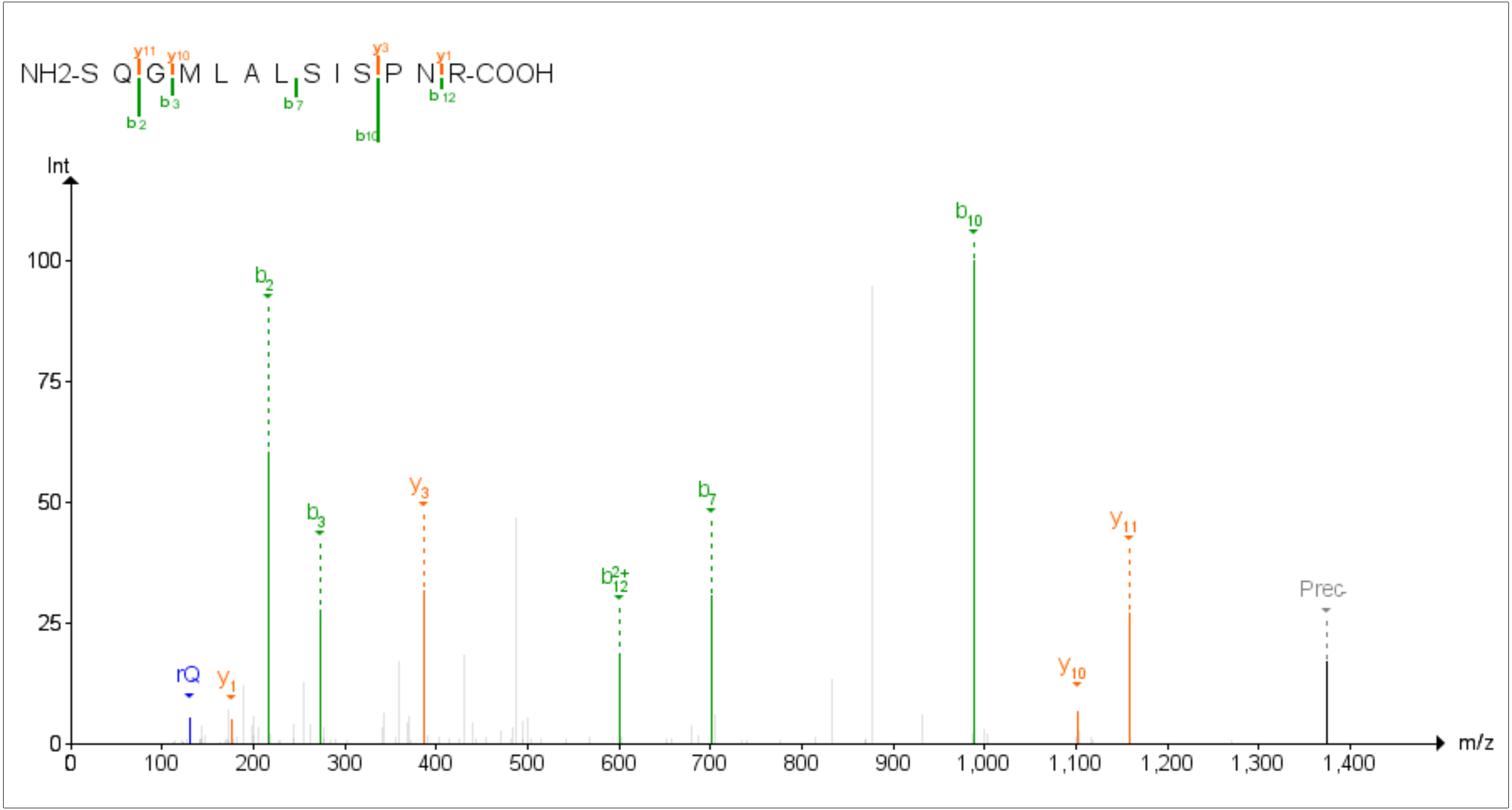
NH2-S Q G M L A L S I S P N R-COOH
y11
y10
y3
y1
b2
b3
b7
b10
b12
Int
100
75
50
25
0
b2
b3
y3
b7
b10
b12 2+
y11
y10
rQ
y1
Prec
0
100
200
300
400
500
600
700
800
900
1,000
1,100
1,200
1,300
1,400
m/z

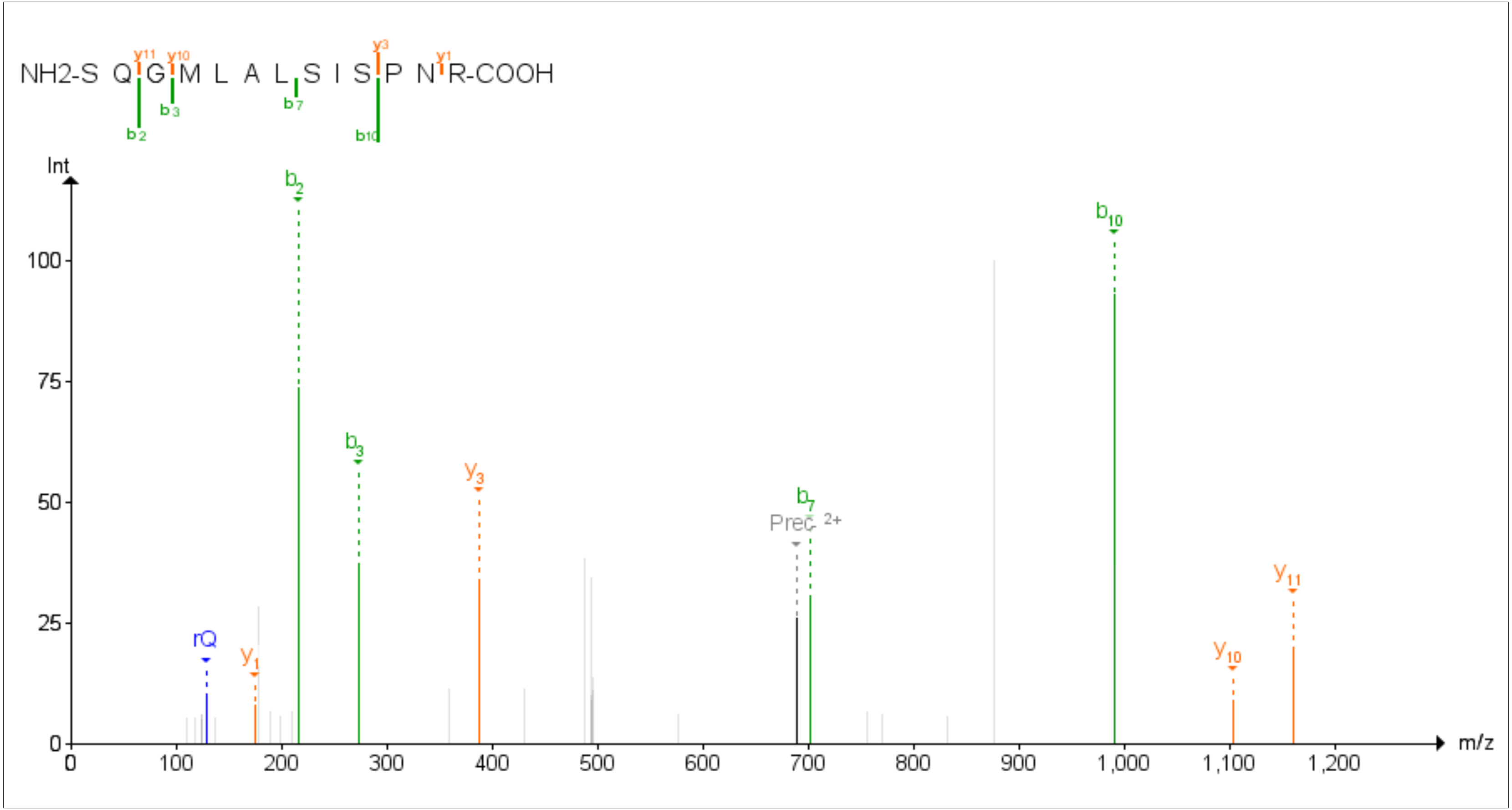

NH2-S Q G M L A L S I S P N R-COOH
y11 y10 y3 y1
b2 b3 b7 b10
Int
m/z
rQ
y1
b2
b3
y3
Prec 2+
b7
b10
y10
y11
0
25
50
75
100
0
100
200
300
400
500
600
700
800
900
1,000
1,100
1,200

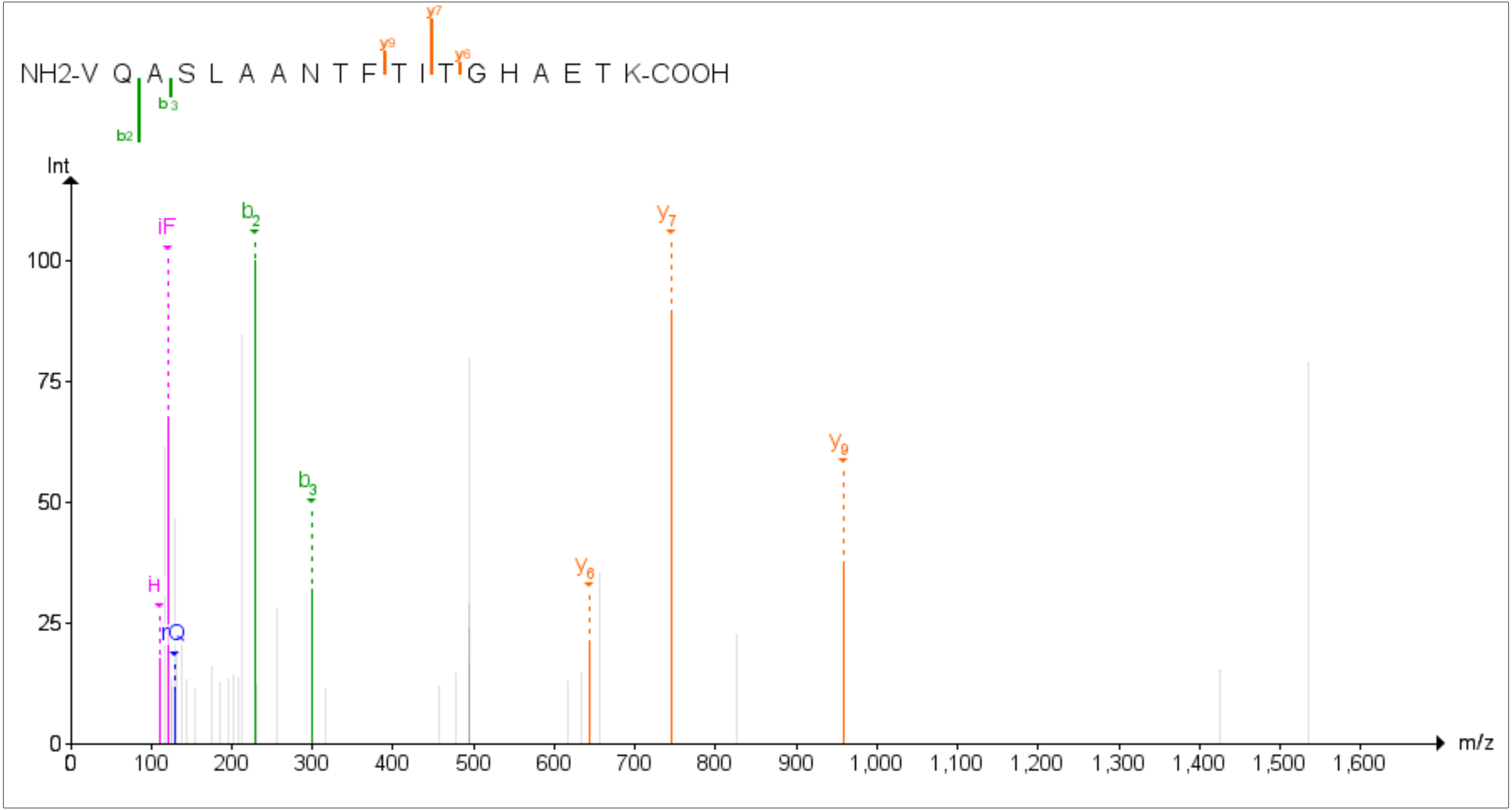

NH2-V Q A S L A A N T F T I T G H A E T K-COOH
y7
y9
y6
b2
b3
Int
iF
b2
y7
y9
b3
y6
iH
rQ
100
75
50
25
0
0
100
200
300
400
500
600
700
800
900
1,000
1,100
1,200
1,300
1,400
1,500
1,600
m/z

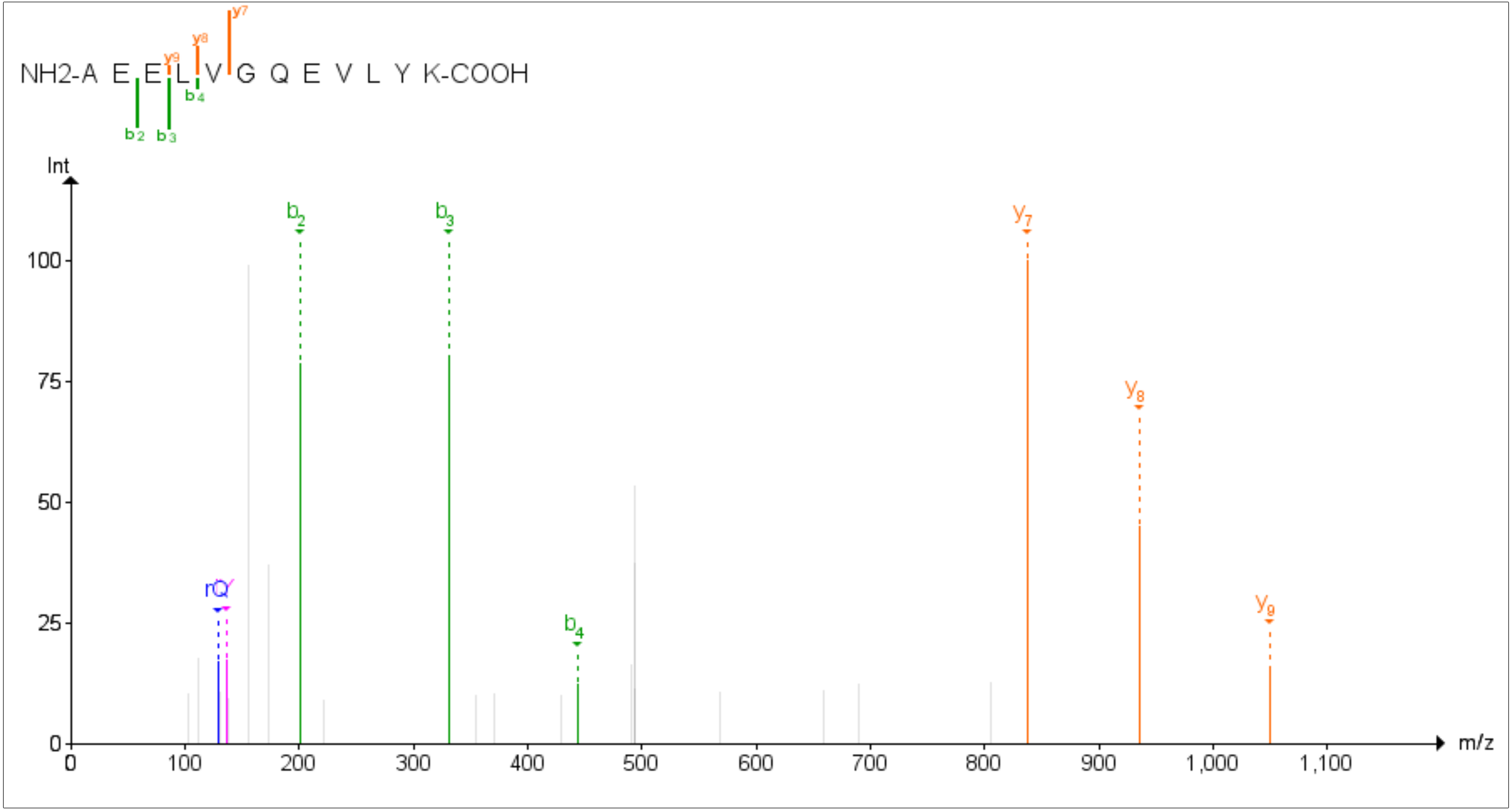
NH2-A E E L V G Q E V L Y K-COOH
y7
y8
y9
b2
b3
b4
Int
100
75
50
25
0
b2
b3
y7
y8
y9
b4
rQ
0
100
200
300
400
500
600
700
800
900
1,000
1,100
m/z

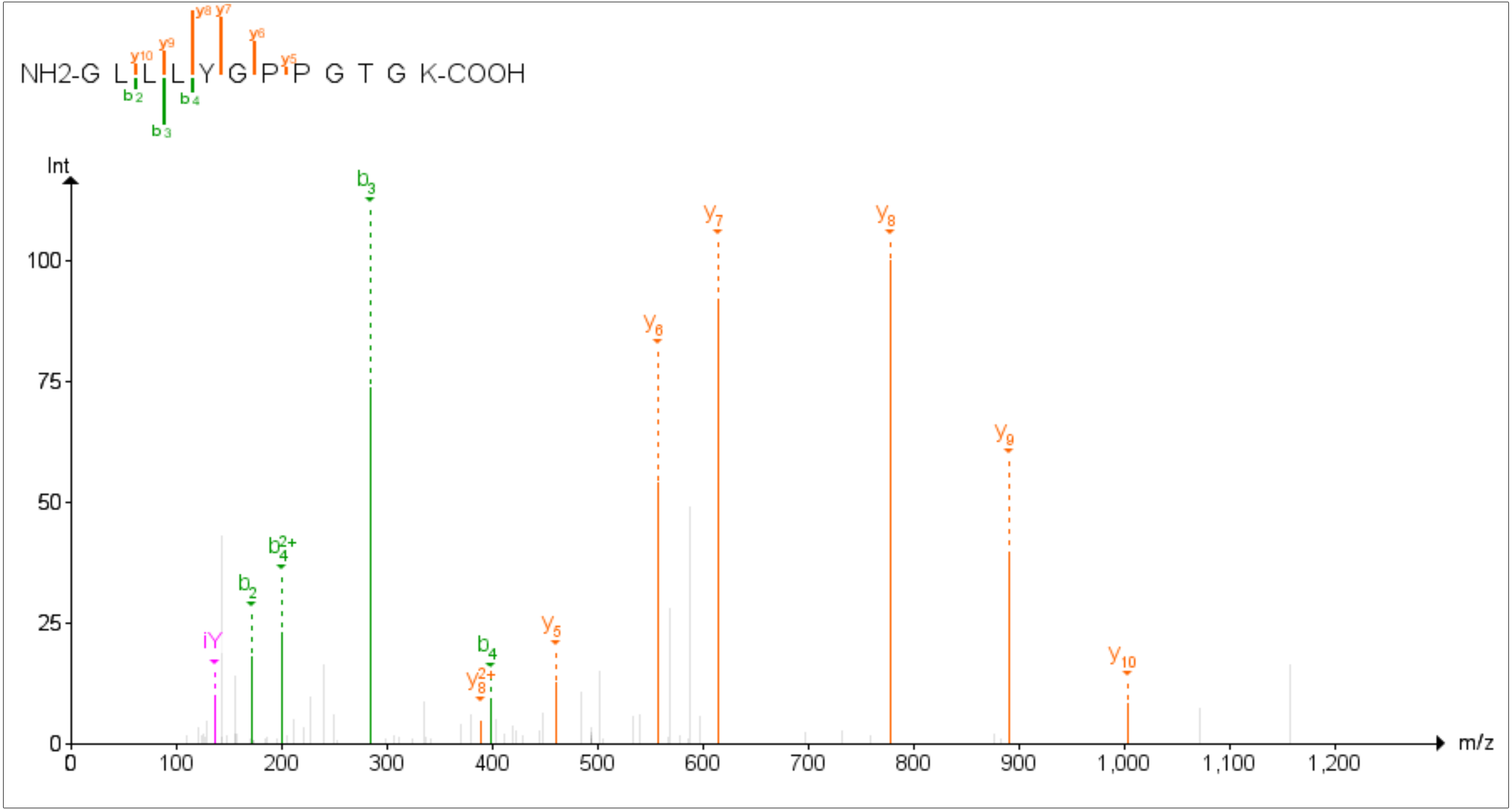

NH2-G L L L Y G P P G T G K-COOH
y10 y9 y8 y7 y6 y5
b2 b3 b4
Int
m/z
100
75
50
25
0
0 100 200 300 400 500 600 700 800 900 1,000 1,100 1,200
iY
b2
b4 2+
b3
y8 2+
b4
y5
y6
y7
y8
y9
y10

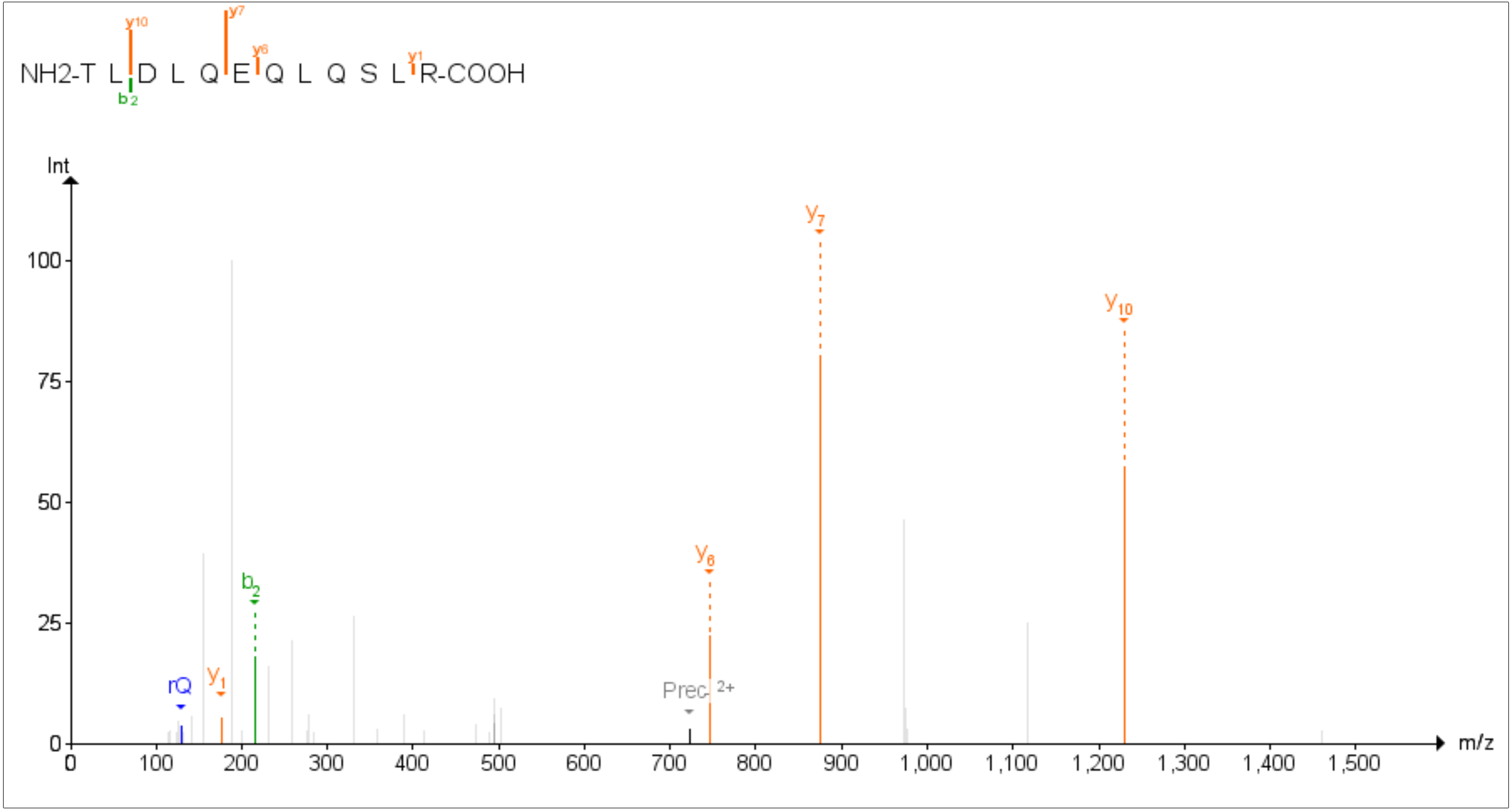

NH2-T L D L Q E Q L Q S L R-COOH
y10
y7
y6
y1
b2
Int
100
75
50
25
0
y7
y10
y6
b2
rQ
y1
Prec 2+
0
100
200
300
400
500
600
700
800
900
1,000
1,100
1,200
1,300
1,400
1,500
m/z

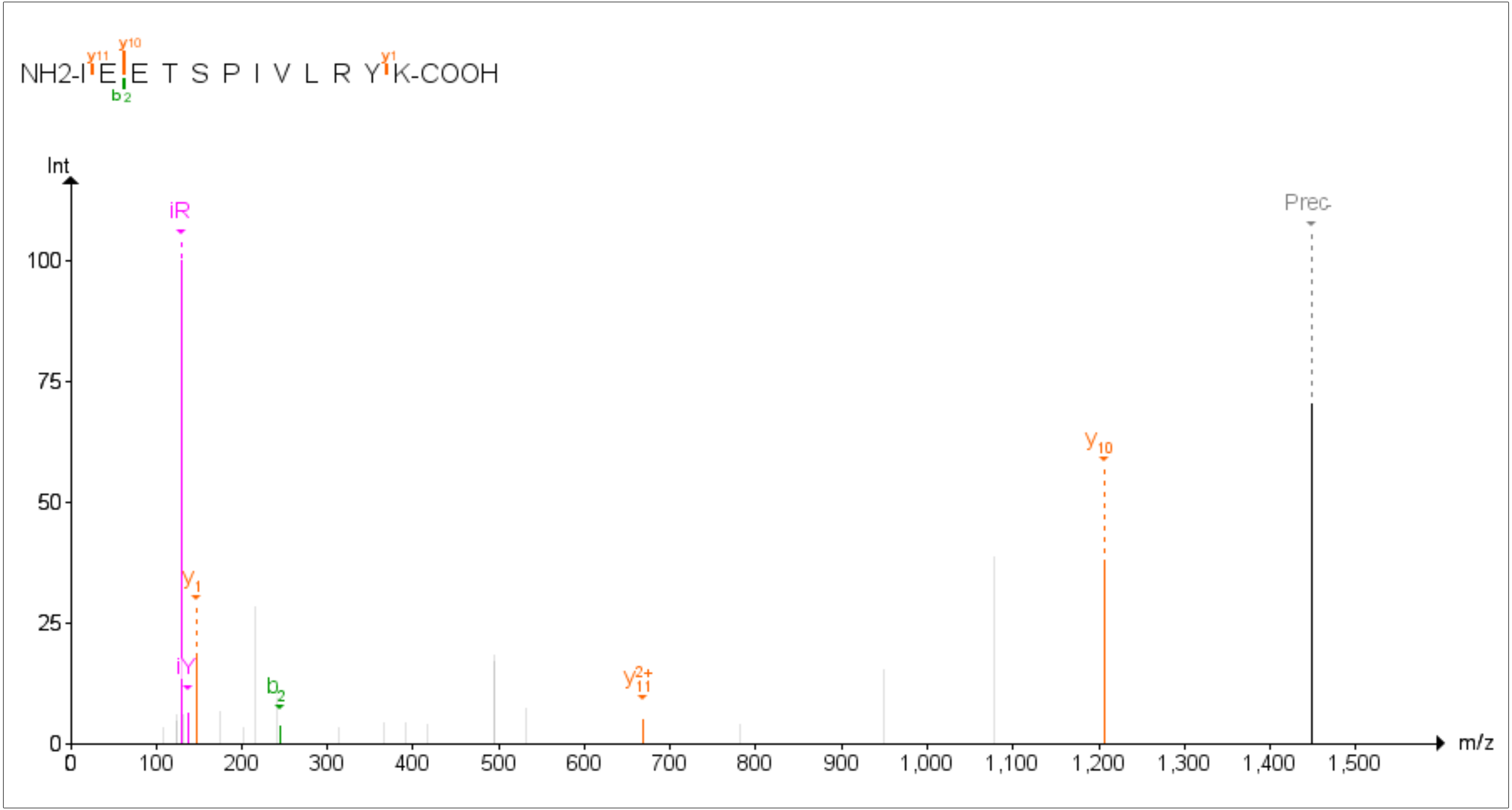

NH2-I E E T S P I V L R Y K-COOH
y11
y10
b2
y1
Int
iR
Prec-
100
75
50
25
0
y10
y1
iY
b2
y_{11}^{2+}
0
100
200
300
400
500
600
700
800
900
1,000
1,100
1,200
1,300
1,400
1,500
m/z

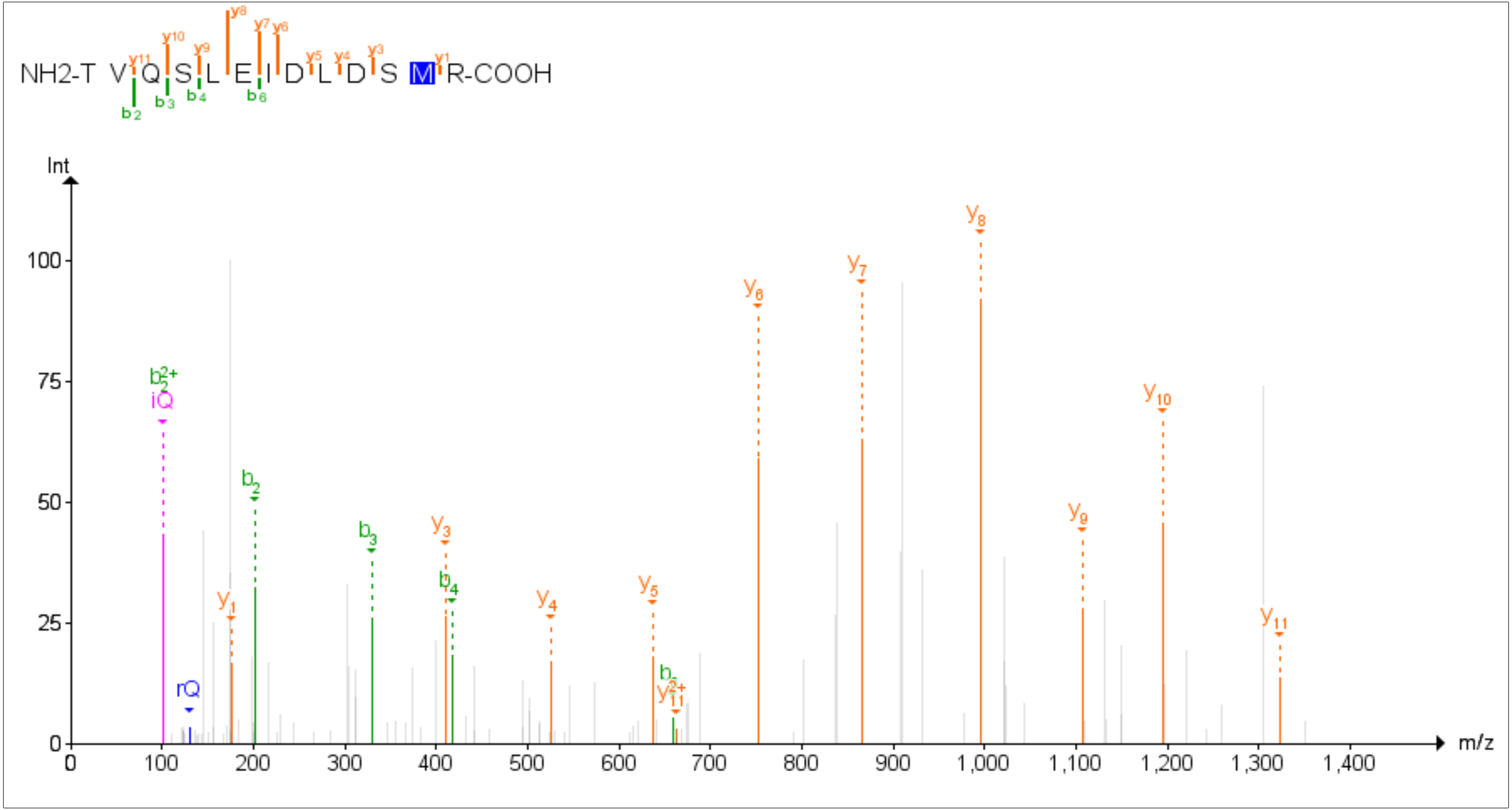

NH2-T V Q S L E I D L D S M R-COOH
y11 y10 y9 y8 y7 y6 y5 y4 y3 y1
b2 b3 b4 b6
Int
m/z
0 25 50 75 100
0 100 200 300 400 500 600 700 800 900 1,000 1,100 1,200 1,300 1,400
b2 2+
iQ
rQ
y1
b2
b3
y3
b4
y4
y5
b5
y11 2+
y6
y7
y8
y9
y10
y11

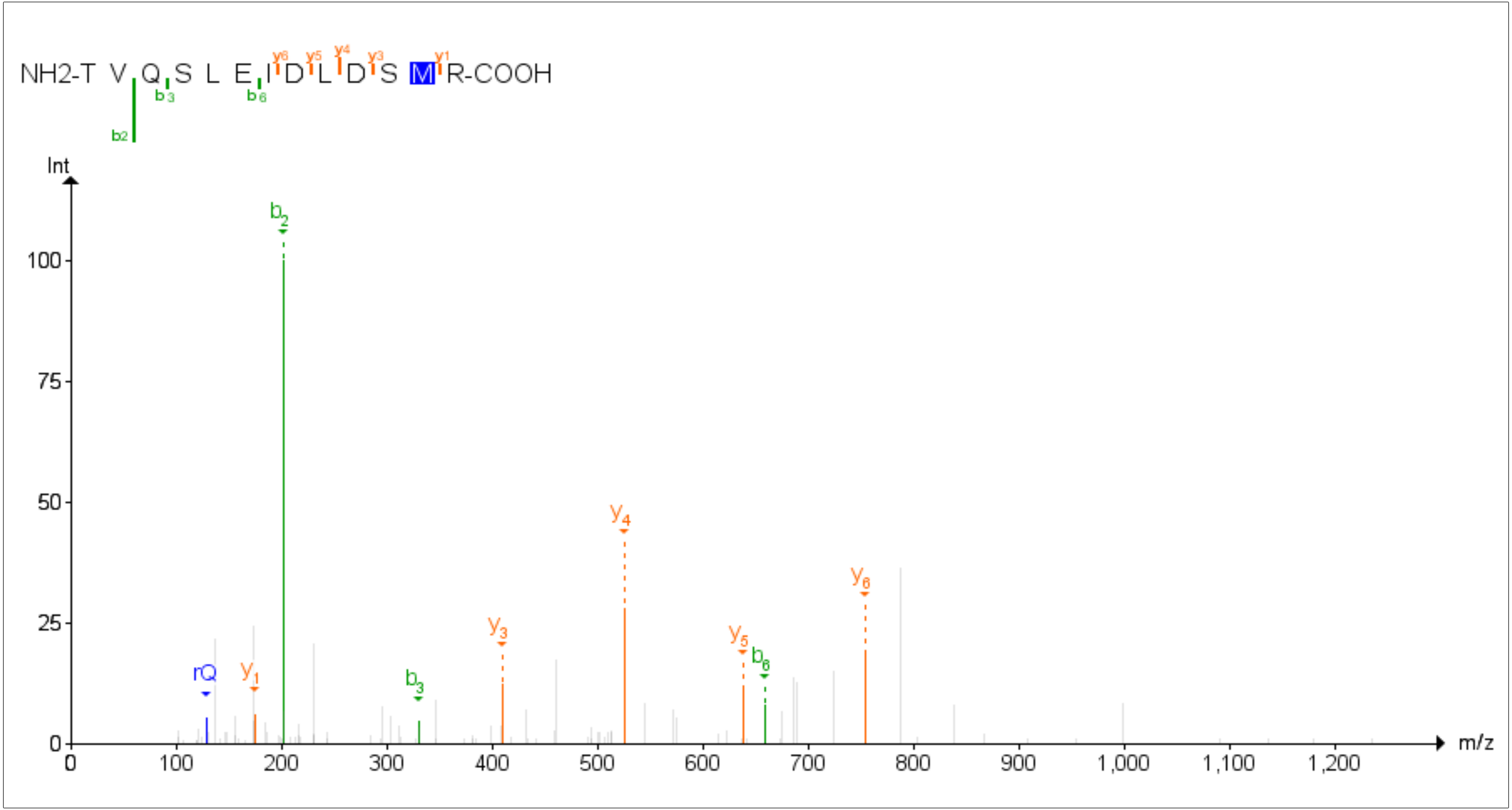

NH2-T V Q S L E I D L D S M R-COOH
b2
b3
b6
y6
y5
y4
y3
y1
Int
100
75
50
25
0
b2
y4
y6
y3
y5
b6
rQ
y1
b3
0
100
200
300
400
500
600
700
800
900
1,000
1,100
1,200
m/z

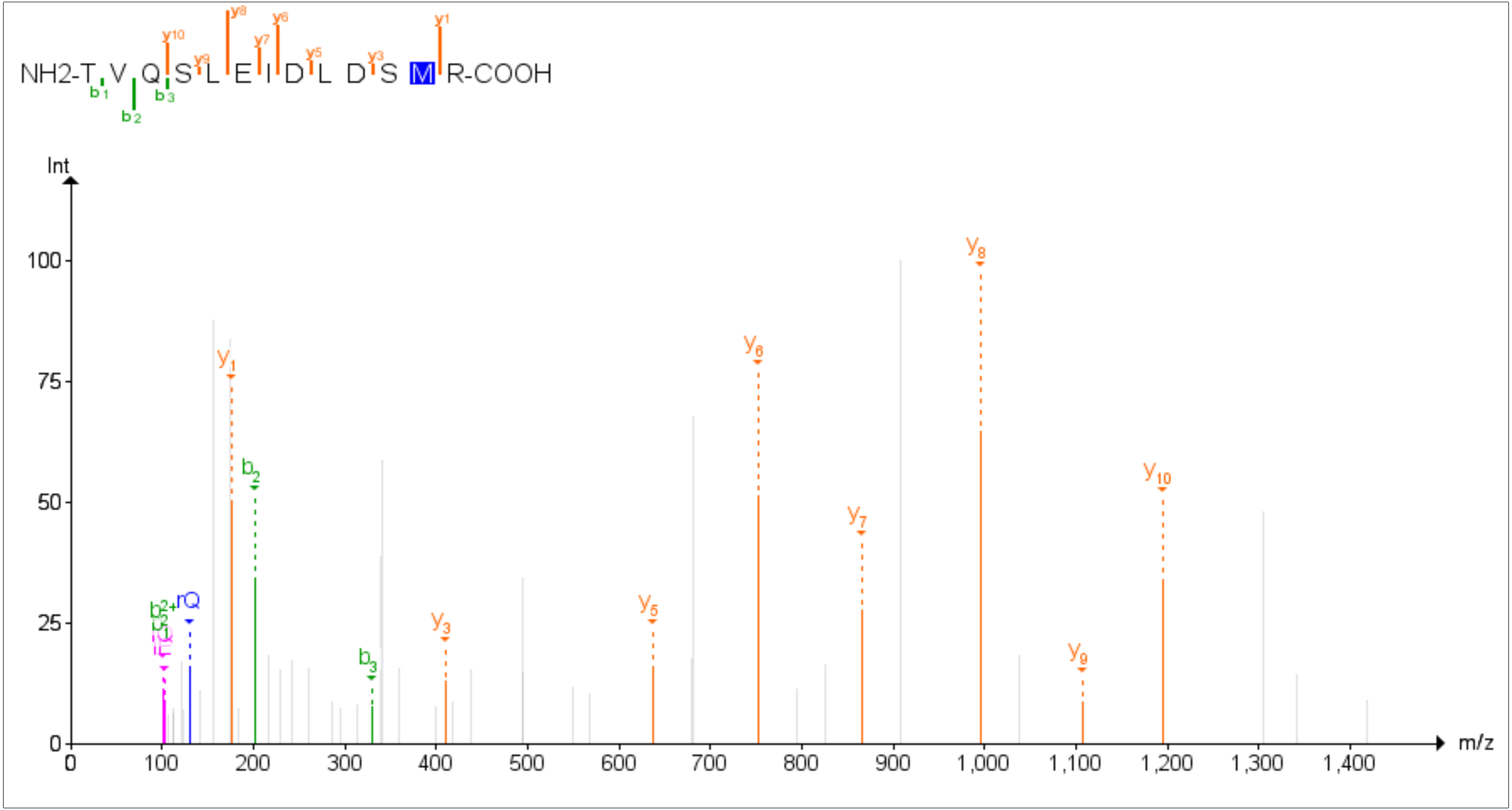

NH2-T V Q S L E I D L D S M R-COOH
b1
b2
b3
y10
y9
y8
y7
y6
y5
y3
y1
Int
m/z
0
25
50
75
100
0
100
200
300
400
500
600
700
800
900
1,000
1,100
1,200
1,300
1,400
b2
b3
rQ
y1
y3
y5
y6
y7
y8
y9
y10

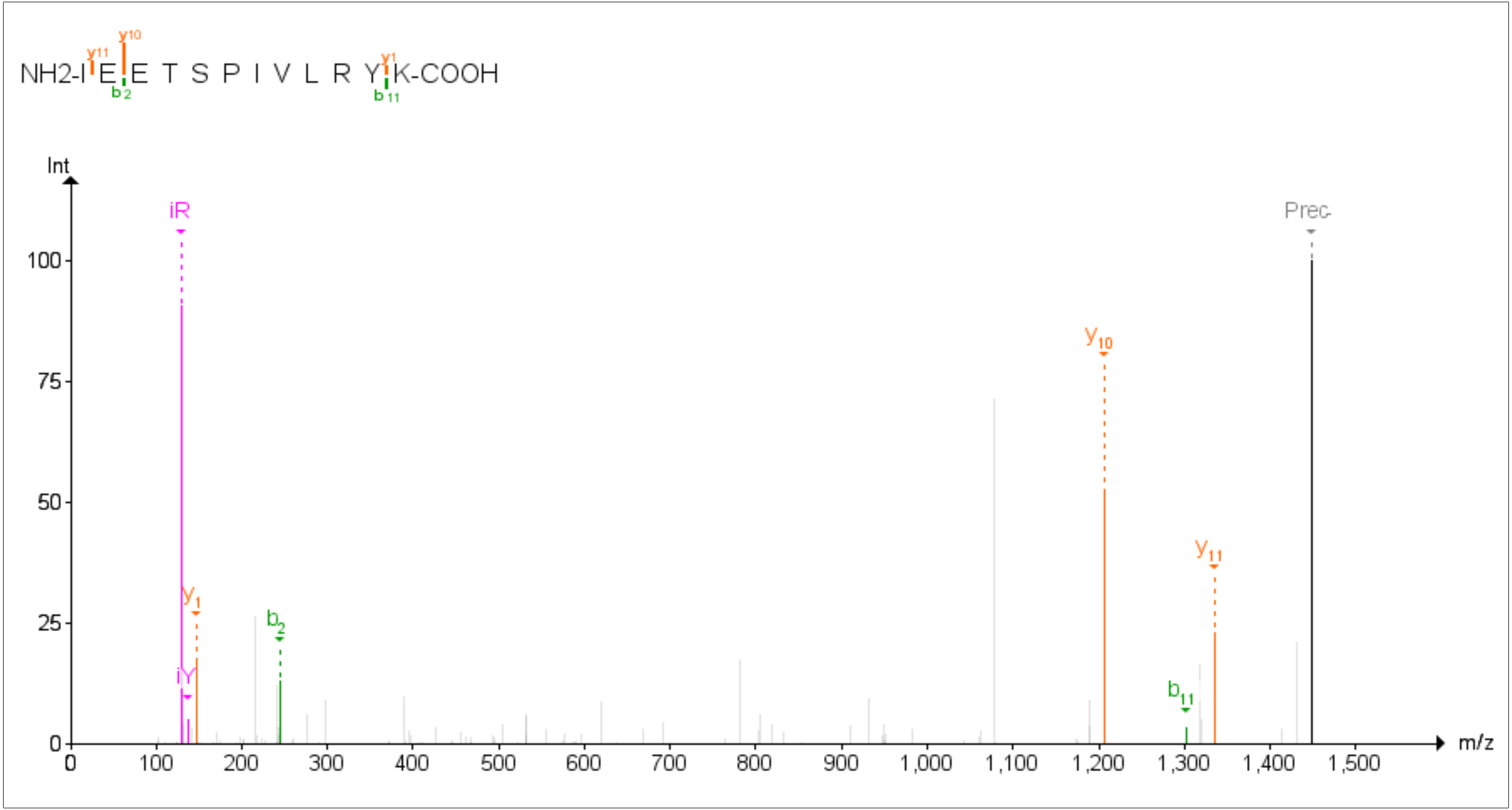

NH2-I E E T S P I V L R Y K-COOH
y11
y10
b2
y1
b11
Int
iR
Prec
y10
y11
y1
b2
iY
b11
0
25
50
75
100
0
100
200
300
400
500
600
700
800
900
1,000
1,100
1,200
1,300
1,400
1,500
m/z

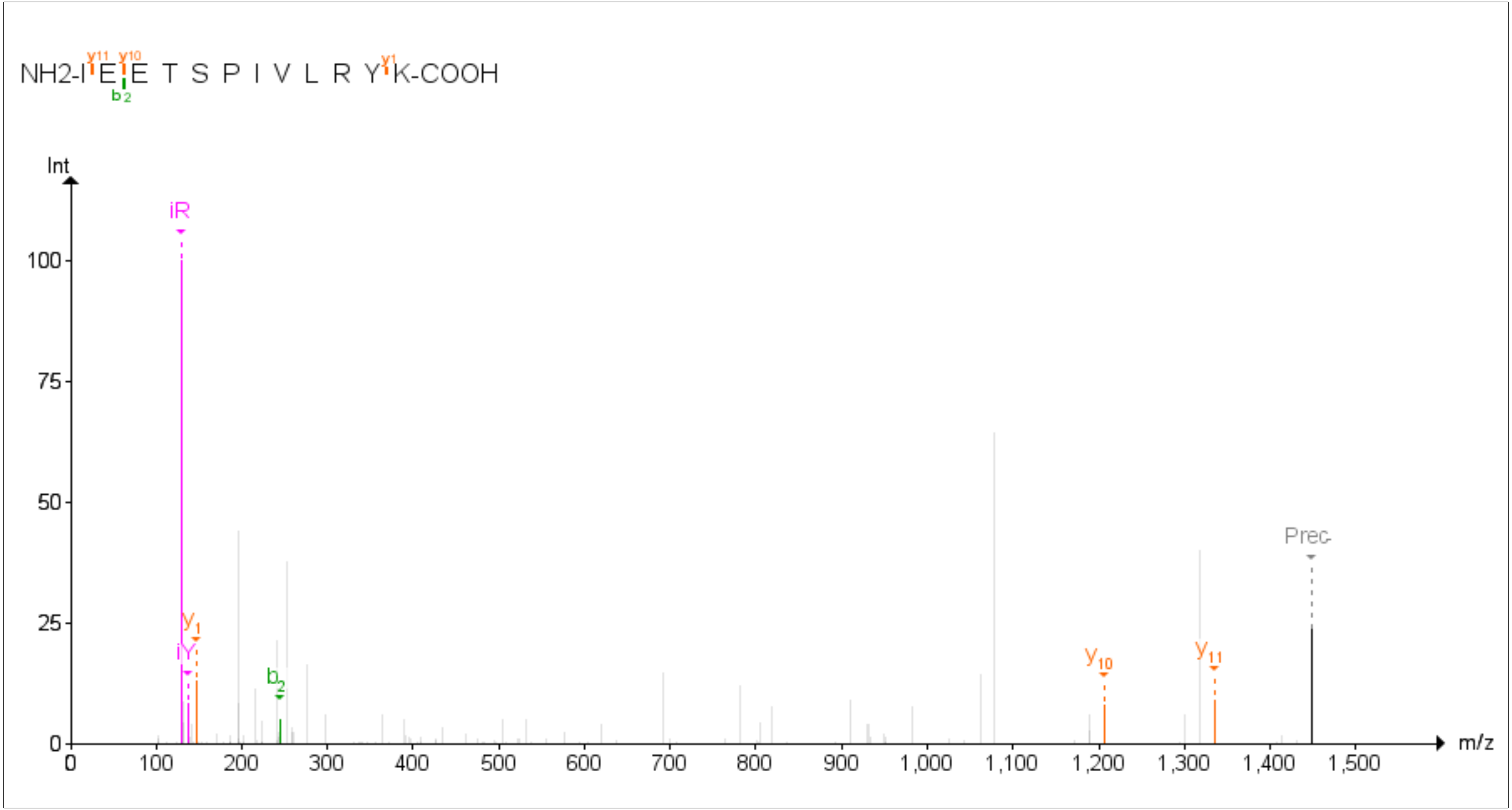

NH2-I E E T S P I V L R Y K-COOH
y11
y10
y1
b2
Int
iR
100
75
50
25
0
y1
iY
b2
y10
y11
Prec
0
100
200
300
400
500
600
700
800
900
1,000
1,100
1,200
1,300
1,400
1,500
m/z

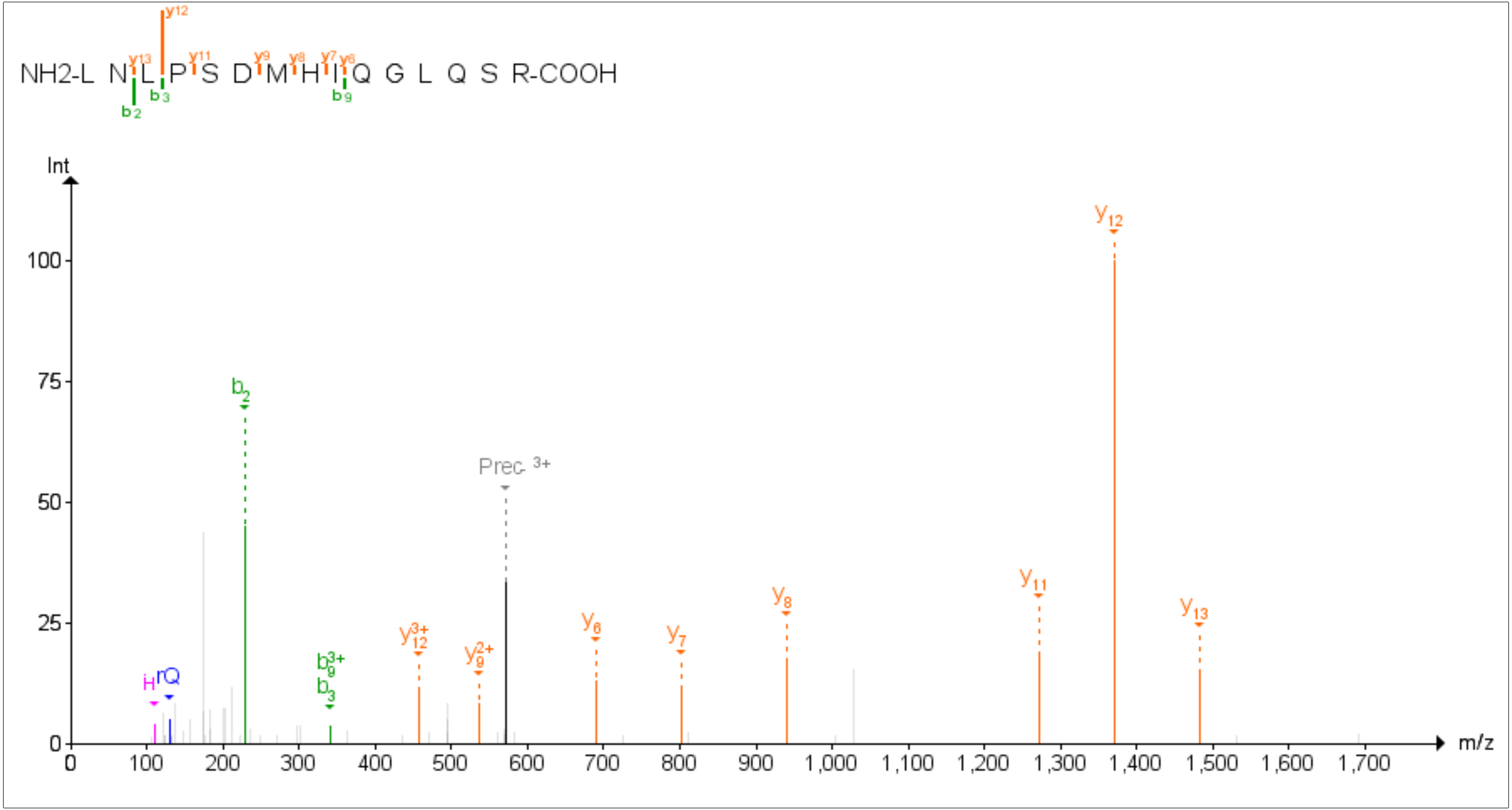
NH2-L N L P S D M H I Q G L Q S R-COOH
y12
y13
y11
y9
y8
y7
y6
b2
b3
b9
Int
m/z
Prec 3+
b2
y12
y11
y13
y8
y6
y7
y12 3+
y9 2+
b9 3+
b3
H
rQ
0
25
50
75
100
0
100
200
300
400
500
600
700
800
900
1,000
1,100
1,200
1,300
1,400
1,500
1,600
1,700

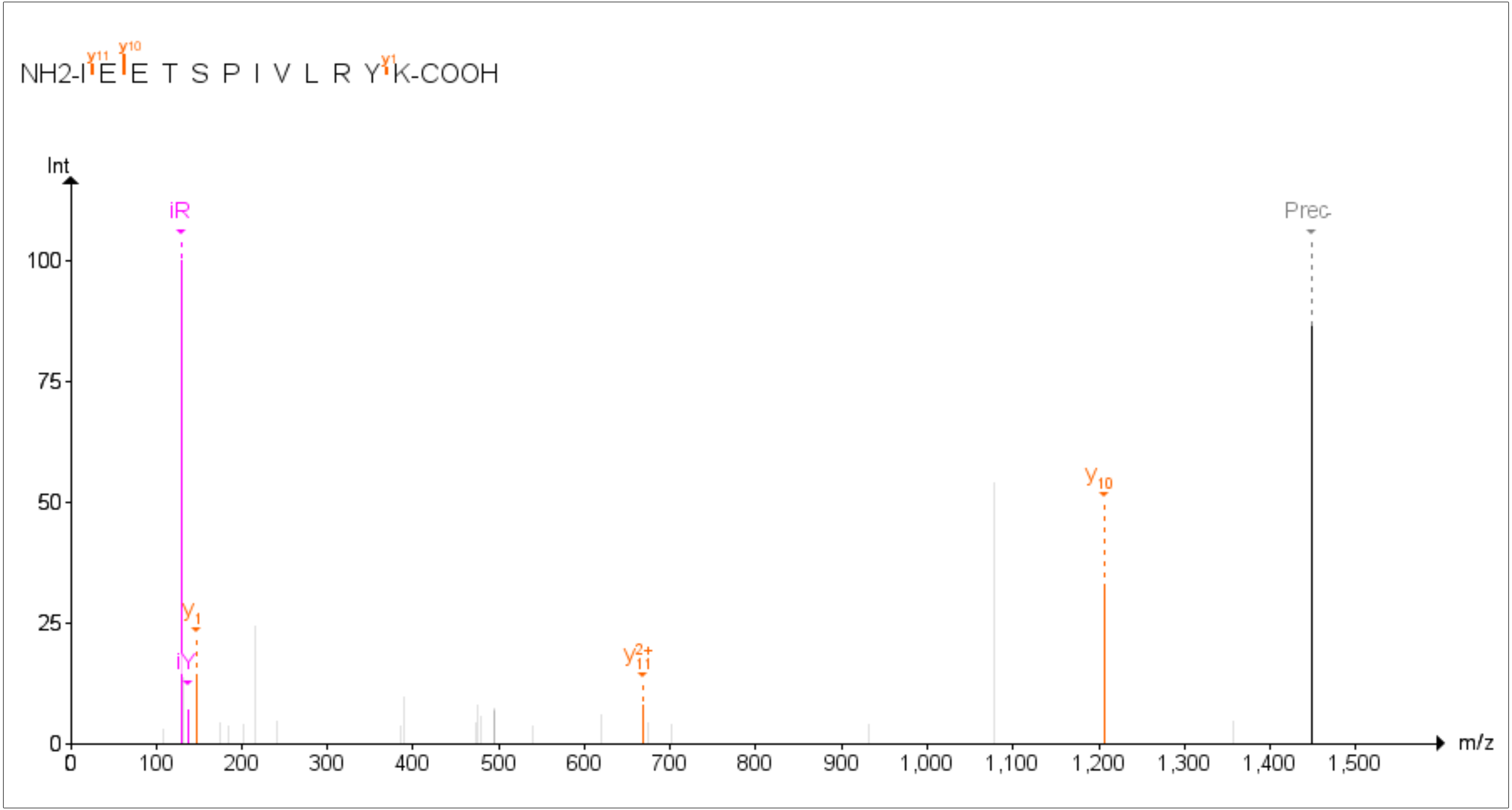

NH2-I E E T S P I V L R Y K-COOH
y11
y10
y1
Int
iR
Prec
100
75
50
25
0
y1
iY
y11 2+
y10
0
100
200
300
400
500
600
700
800
900
1,000
1,100
1,200
1,300
1,400
1,500
m/z

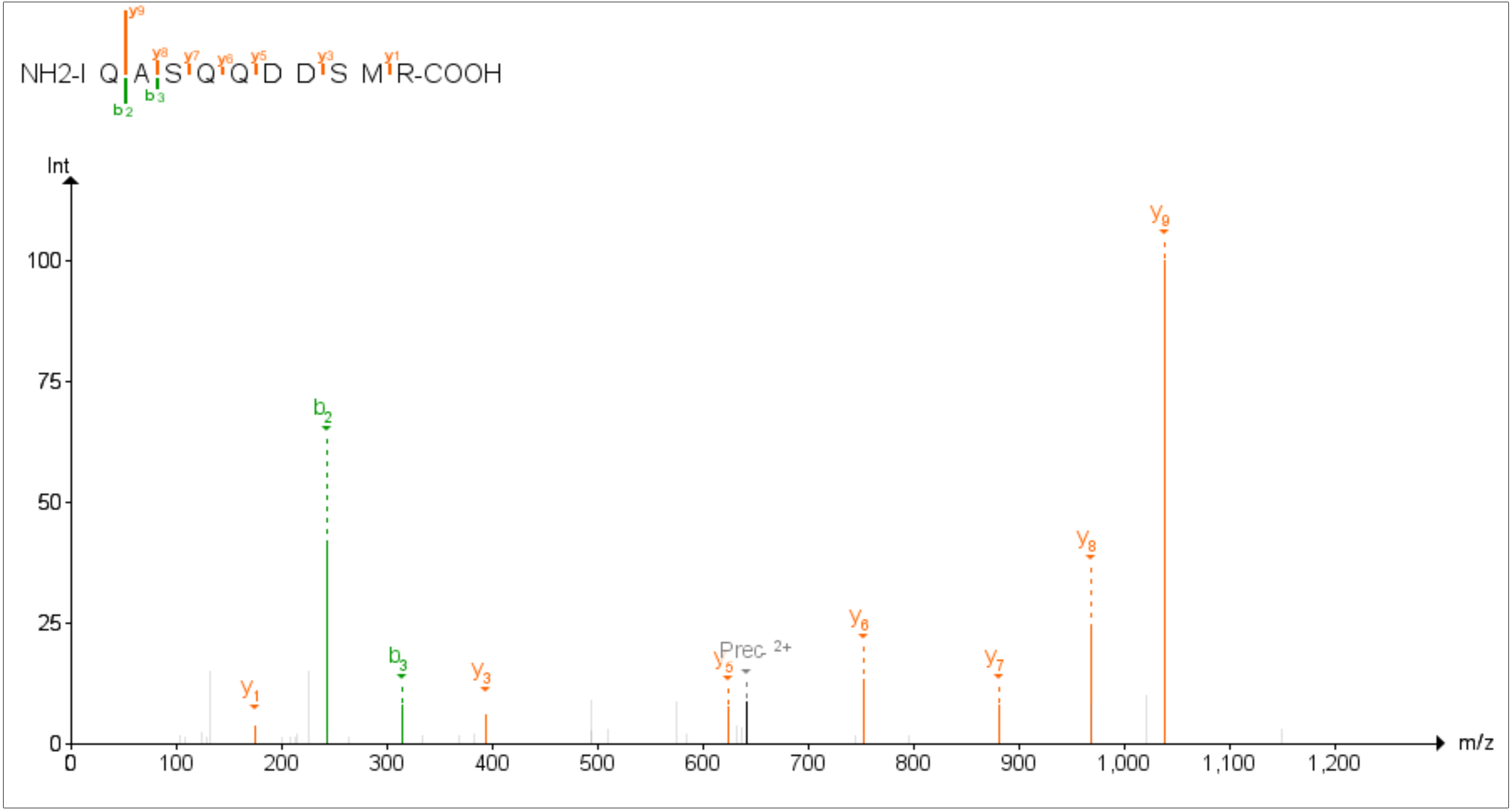

NH2-I Q A S Q Q D D S M R-COOH
y9
y8
y7
y6
y5
y3
y1
b2
b3
Int
100
75
50
25
0
m/z
0
100
200
300
400
500
600
700
800
900
1,000
1,100
1,200
y9
b2
y8
y6
Prec 2+
y5
y7
b3
y3
y1

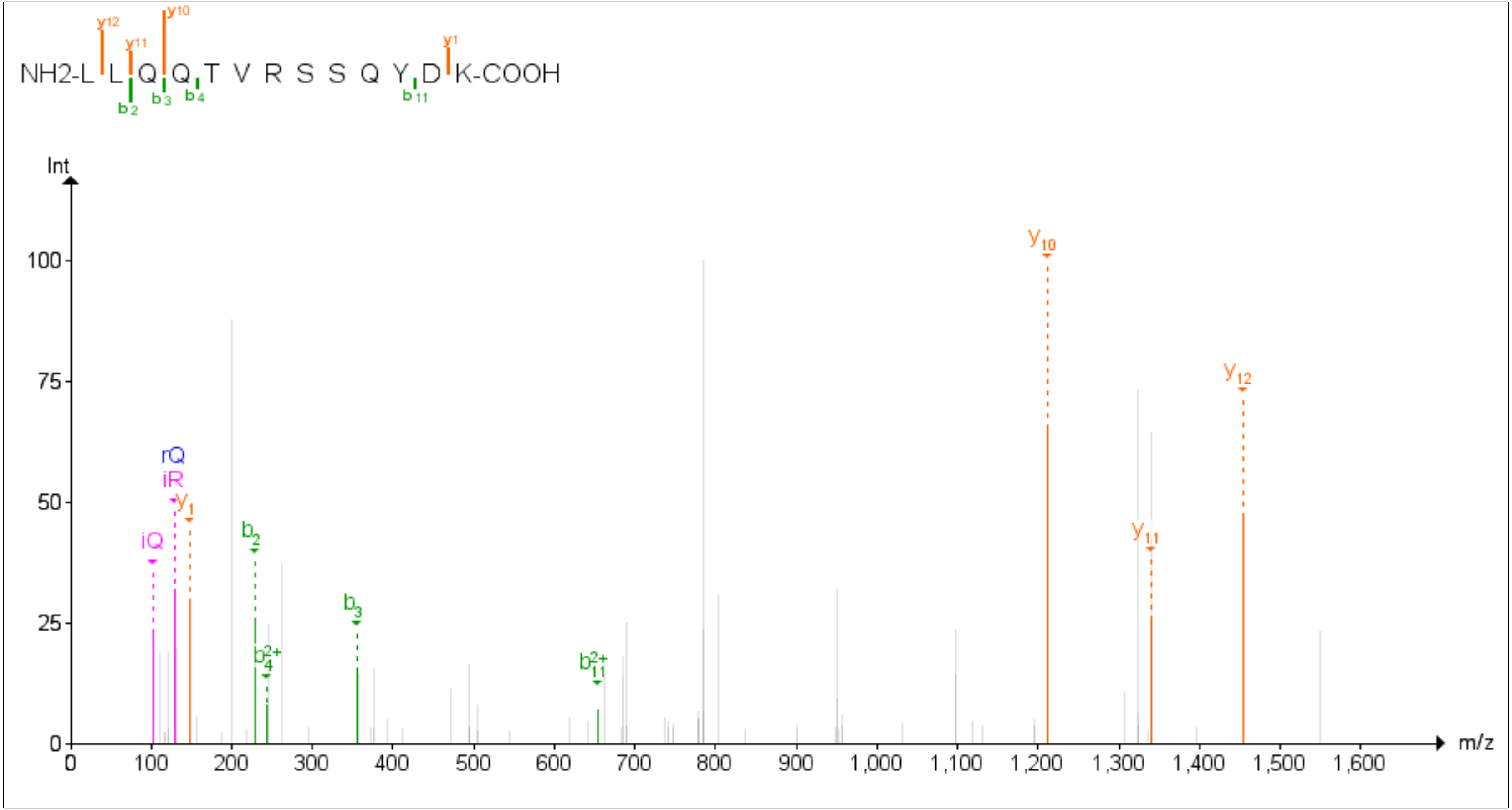
NH2-L L Q Q T V R S S Q Y D K-COOH
y12
y11
y10
y1
b2
b3
b4
b11
Int
100
75
50
25
0
rQ
iR
iQ
y1
b2
b2+4
b3
b2+11
y10
y12
y11
m/z
0
100
200
300
400
500
600
700
800
900
1,000
1,100
1,200
1,300
1,400
1,500
1,600

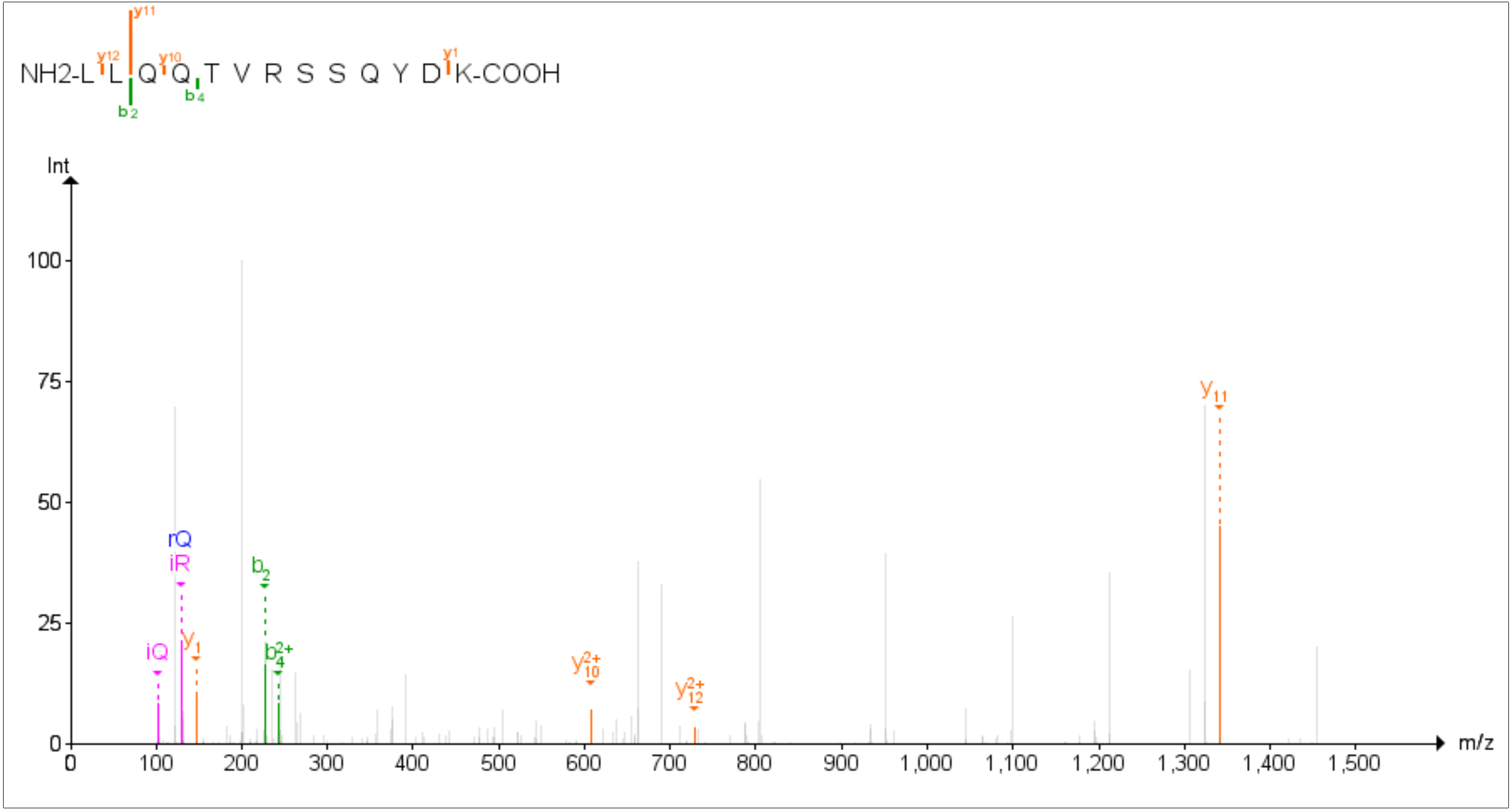

NH2-L L Q Q T V R S S Q Y D K-COOH
y11
y12
y10
y1
b2
b4
Int
100
75
50
25
0
rQ
iR
iQ
y1
b2
b4 2+
y10 2+
y12 2+
y11
0
100
200
300
400
500
600
700
800
900
1,000
1,100
1,200
1,300
1,400
1,500
m/z

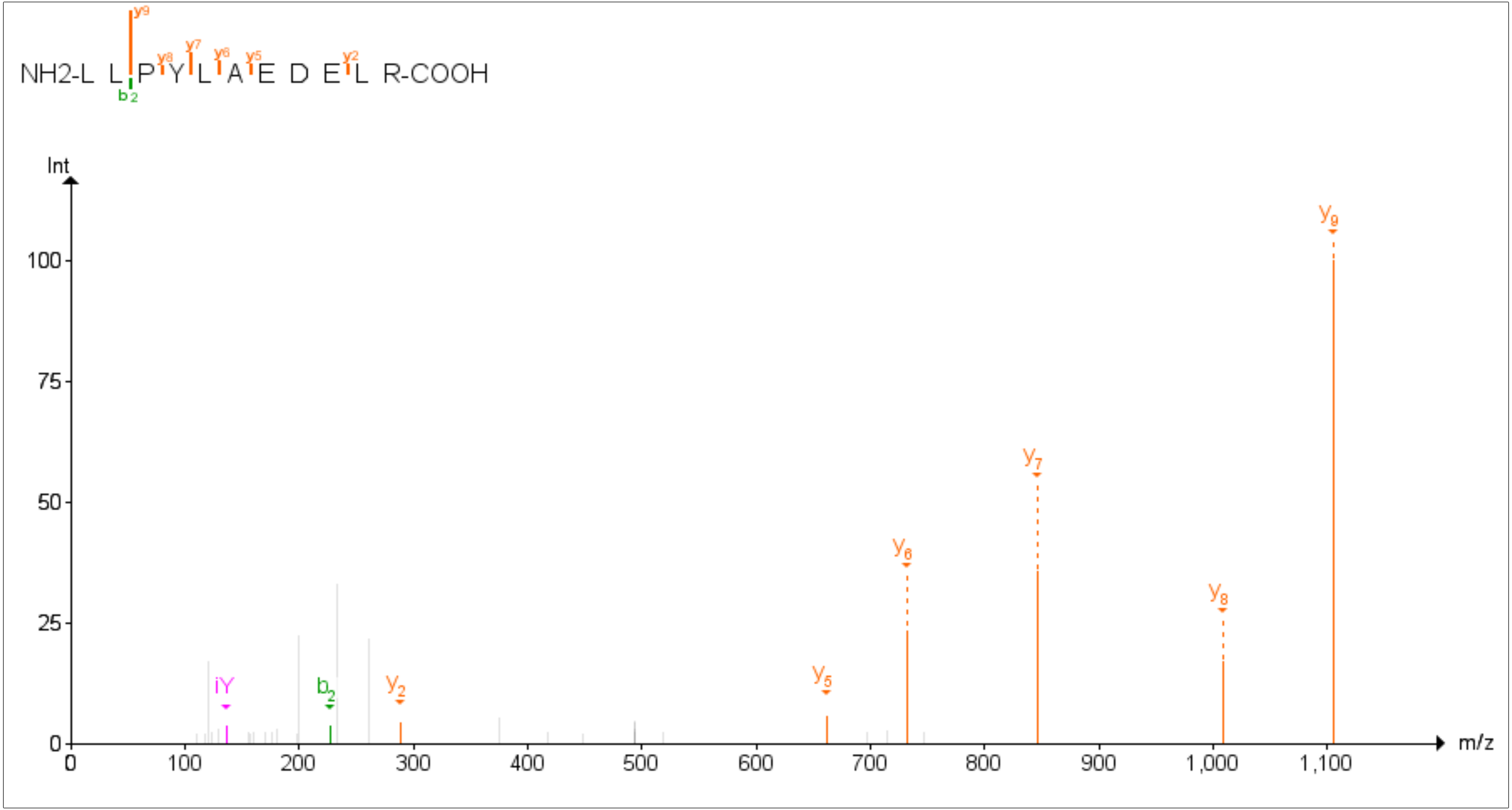

NH2-L L P Y L A E D E L R-COOH
b2
y9
y8
y7
y6
y5
y2
Int
100
75
50
25
0
iY
b2
y2
y5
y6
y7
y8
y9
0
100
200
300
400
500
600
700
800
900
1,000
1,100
m/z

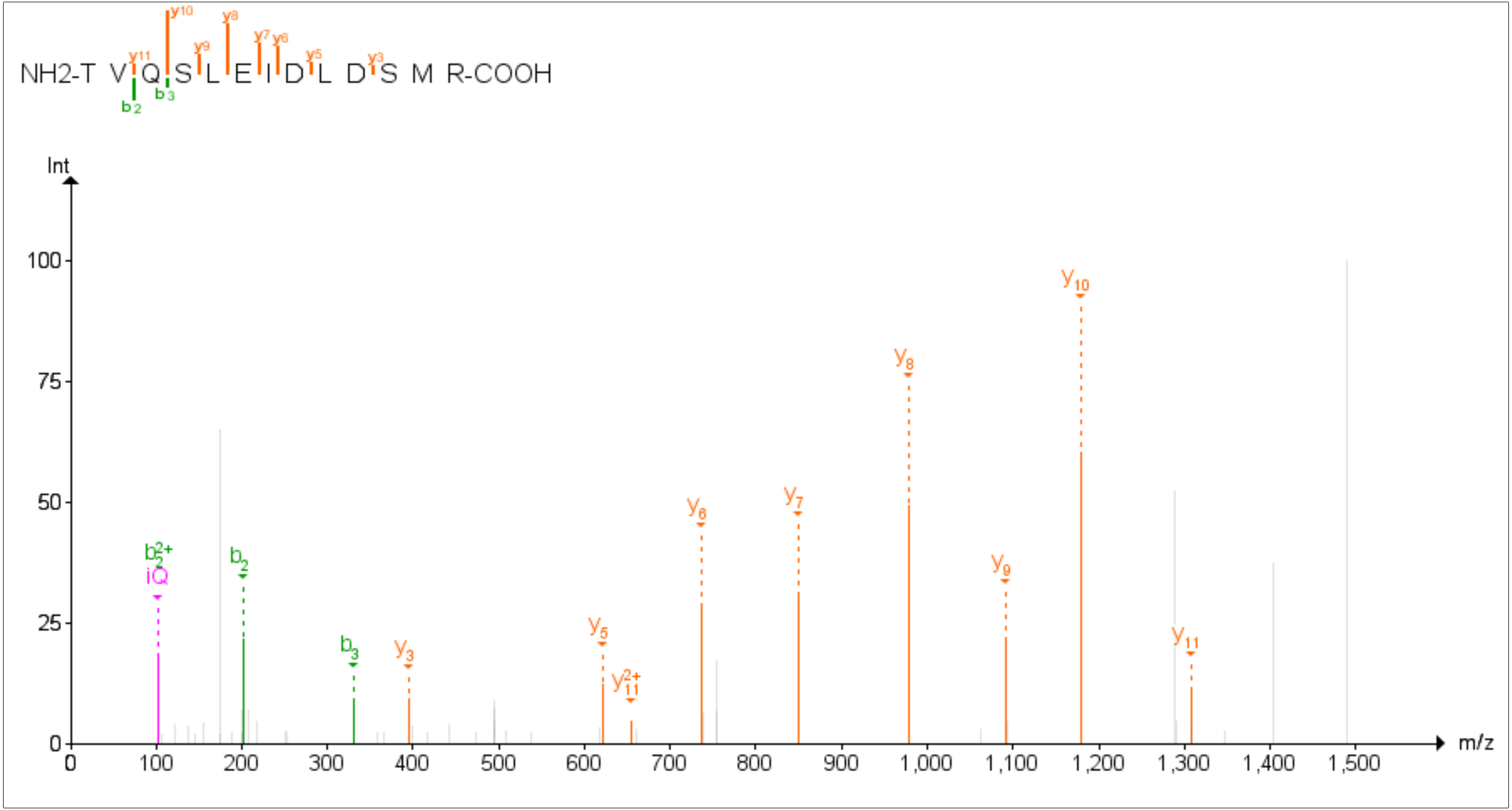

NH2-T V Q S L E I D L D S M R-COOH
y10
y8
y7
y6
y9
y11
y5
y3
b2
b3
Int
100
75
50
25
0
m/z
0
100
200
300
400
500
600
700
800
900
1,000
1,100
1,200
1,300
1,400
1,500
iQ
b2 2+
b2
b3
y3
y5
y11 2+
y6
y7
y8
y9
y10
y11

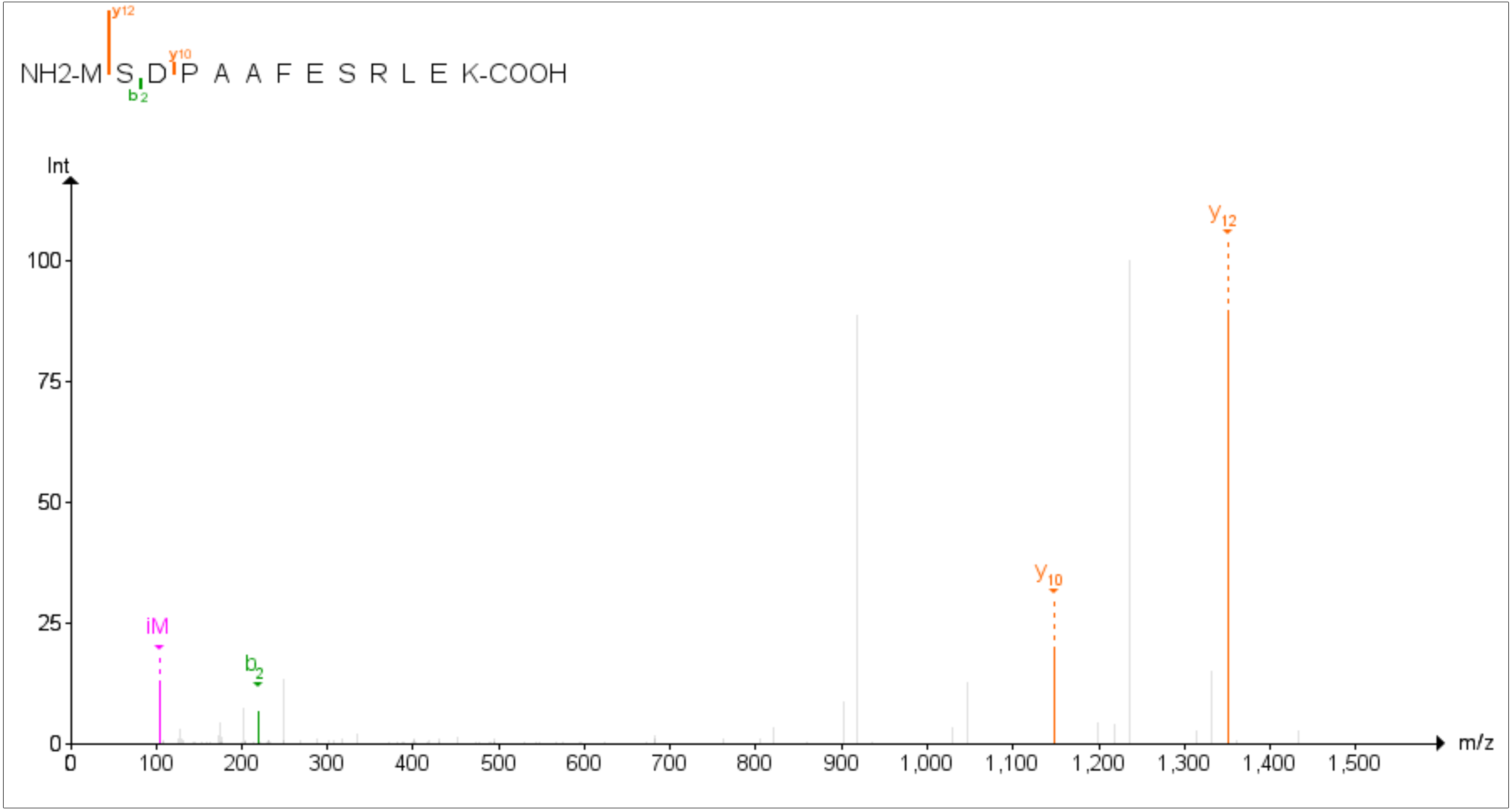

NH2-M S D P A A F E S R L E K-COOH
y12
y10
b2
Int
100
75
50
25
0
iM
b2
y10
y12
0
100
200
300
400
500
600
700
800
900
1,000
1,100
1,200
1,300
1,400
1,500
m/z

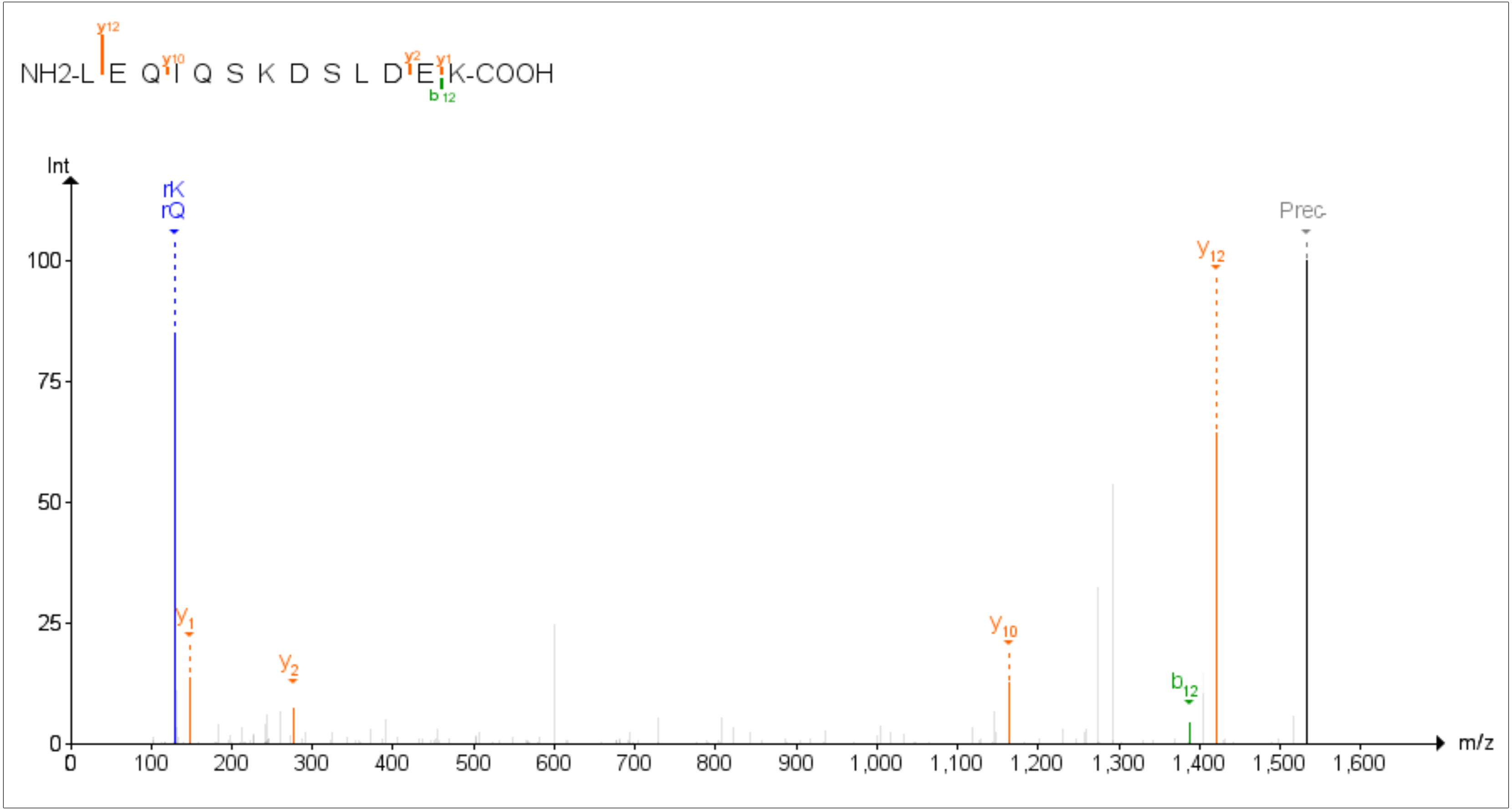

NH2-L E Q I Q S K D S L D E K-COOH
y12
y10
y2
y1
b12
Int
m/z
rK
rQ
Prec
y12
y1
y2
y10
b12
100
75
50
25
0
0
100
200
300
400
500
600
700
800
900
1,000
1,100
1,200
1,300
1,400
1,500
1,600

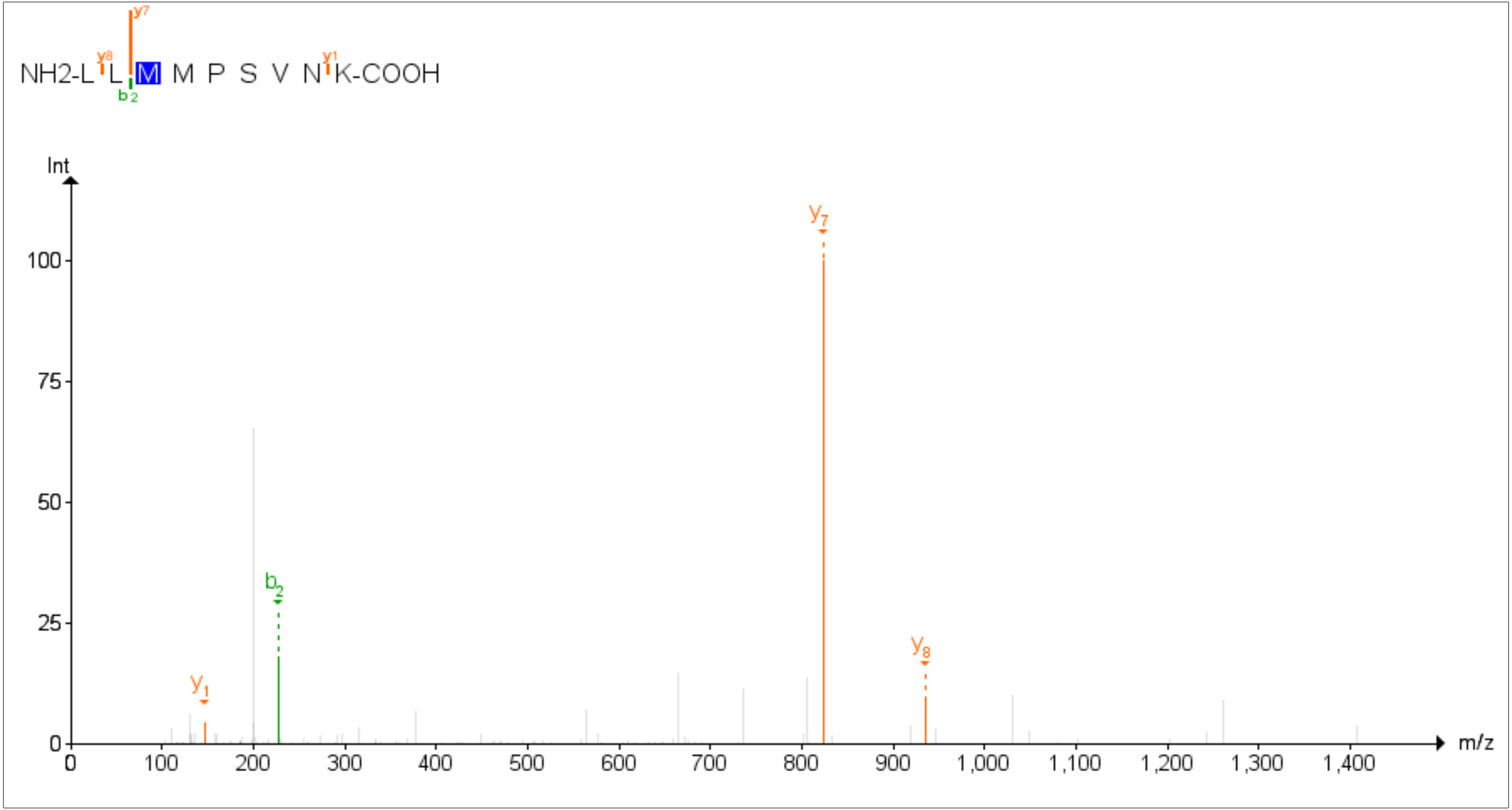
NH2-L L M M P S V N K-COOH
y7
y8
y1
b2
Int
100
75
50
25
0
y7
y8
y1
b2
0
100
200
300
400
500
600
700
800
900
1,000
1,100
1,200
1,300
1,400
m/z

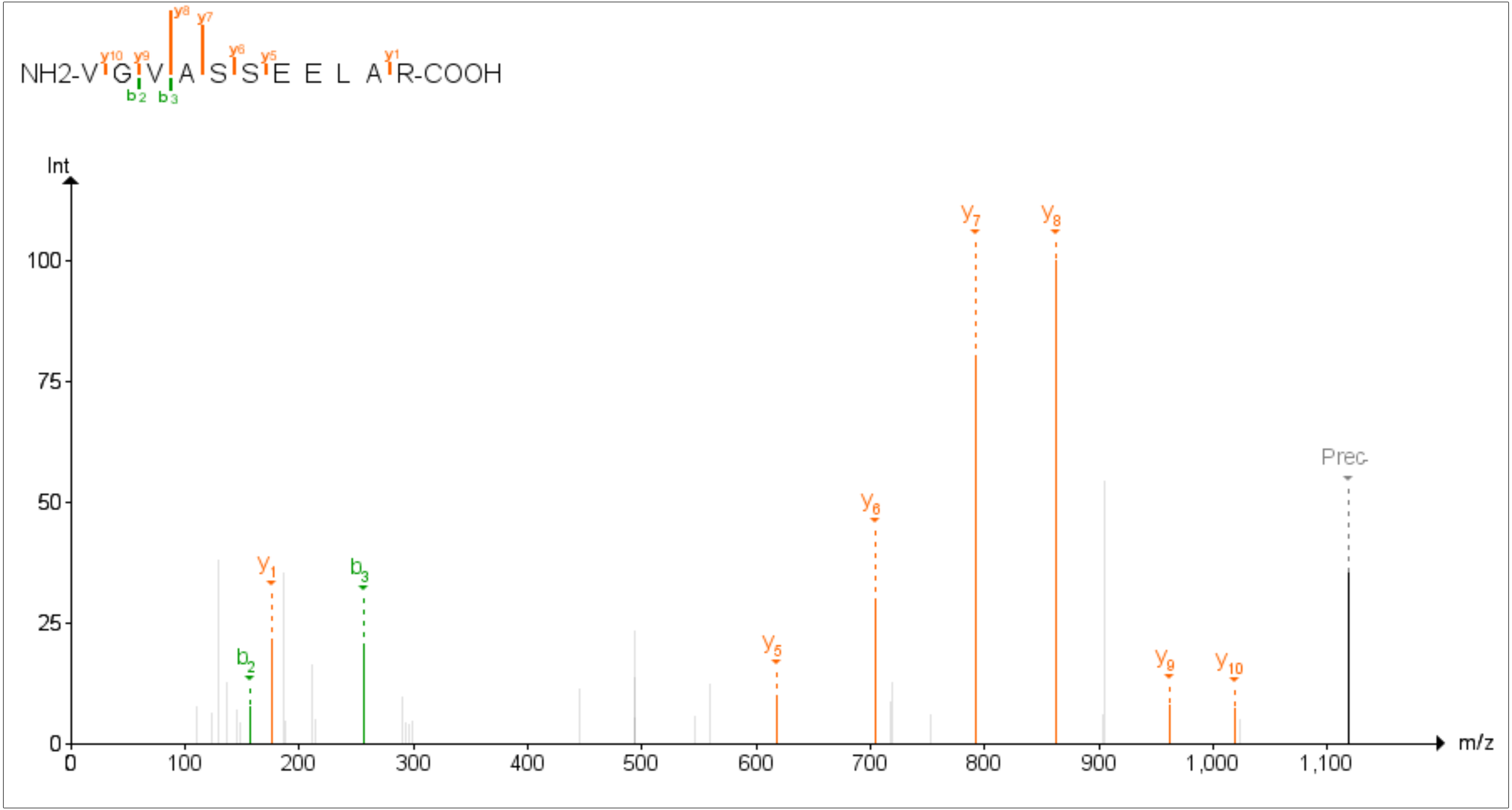
NH2-V G V A S S E E L A R-COOH
y10 y9 y8 y7 y6 y5 y1
b2 b3
Int
m/z
0
25
50
75
100
100
200
300
400
500
600
700
800
900
1,000
1,100
y1
b2
b3
y5
y6
y7
y8
y9
y10
Prec.

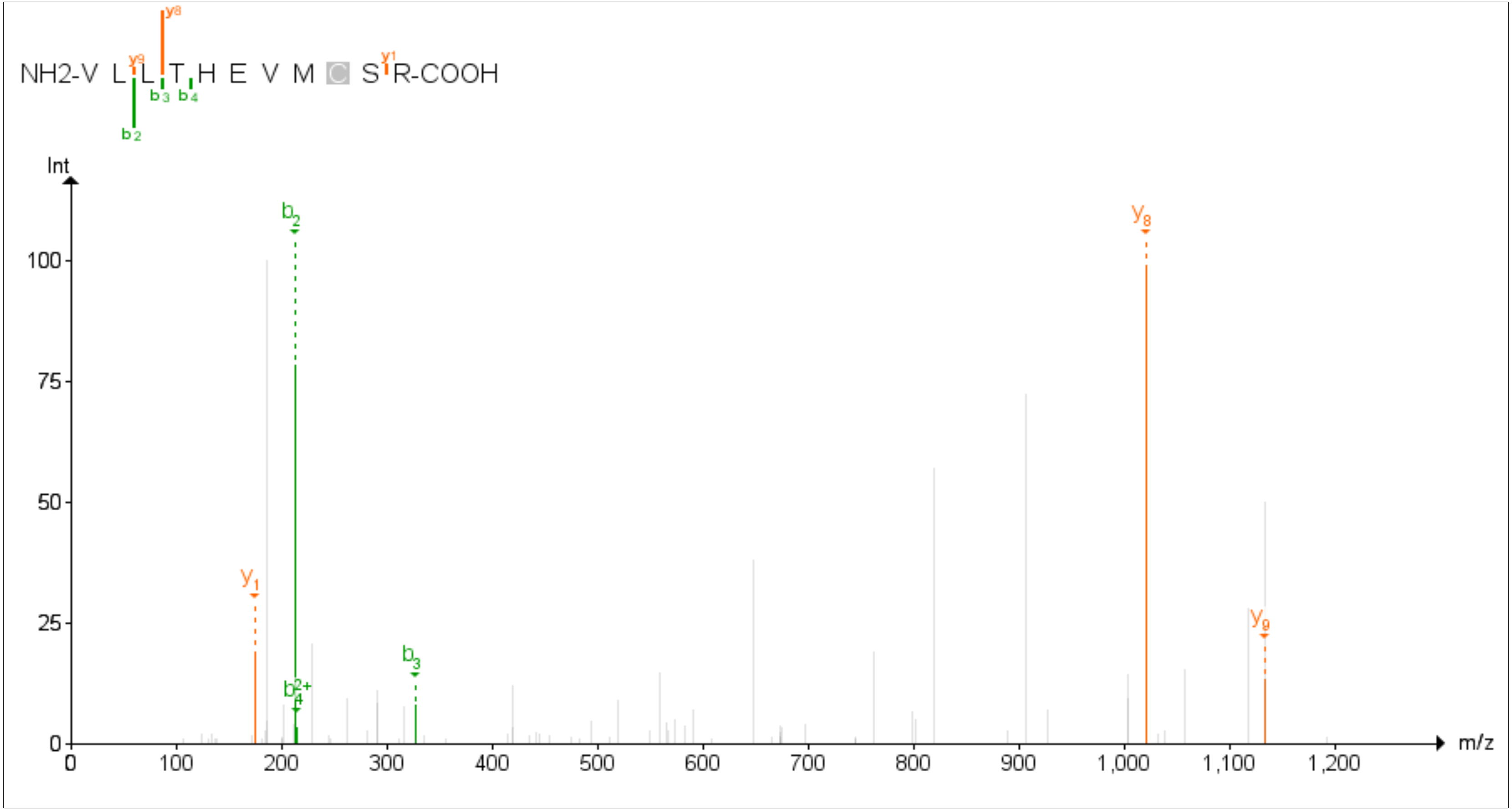

NH2-V L L T H E V M C S R-COOH
y9
y8
y1
b2
b3
b4
Int
b2
y8
y1
y9
b3
b4 2+
100
75
50
25
0
0
100
200
300
400
500
600
700
800
900
1,000
1,100
1,200
m/z

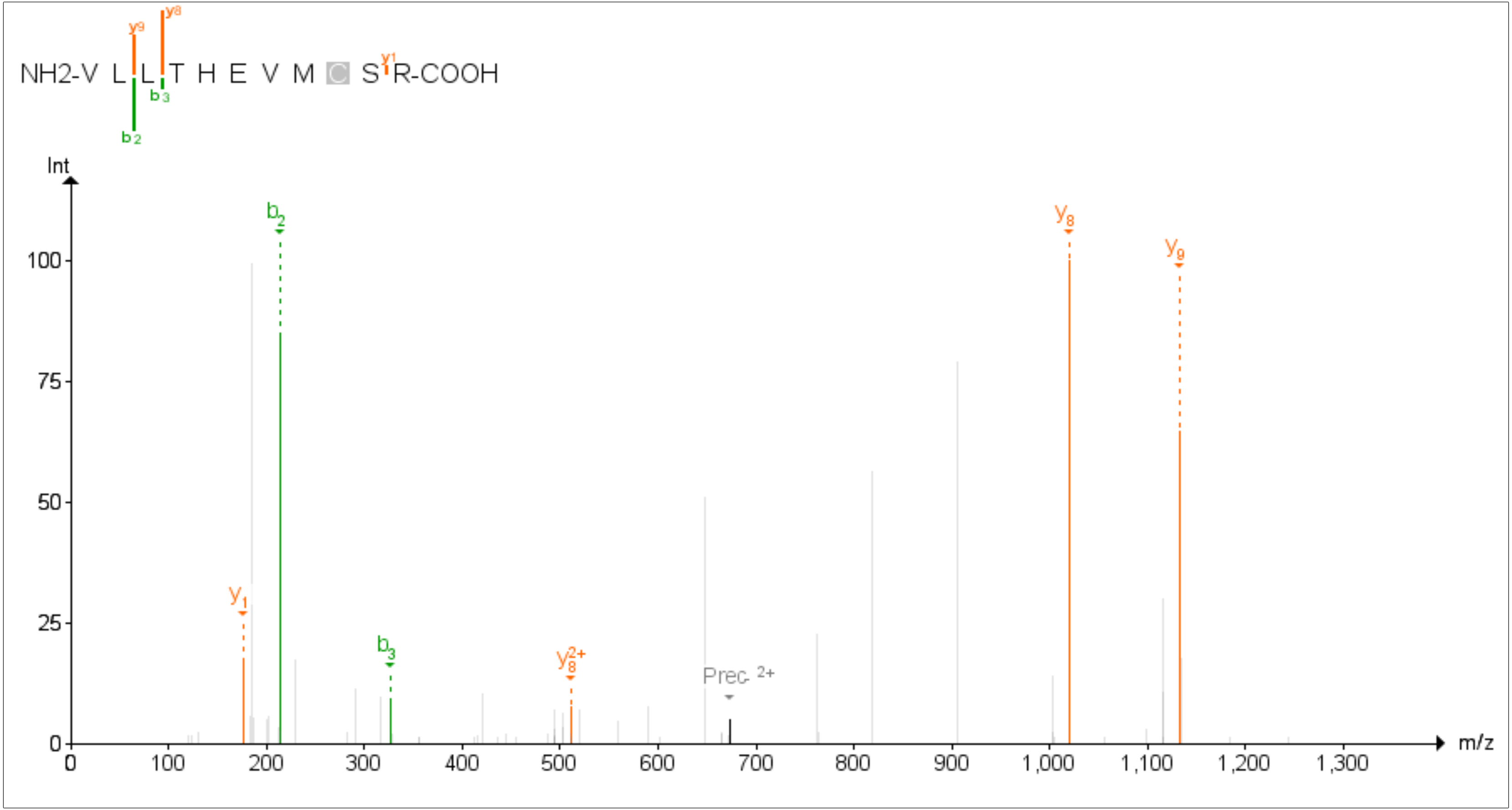

NH2-V L L T H E V M C S R-COOH
y9
y8
y1
b2
b3
Int
m/z
Prec 2+

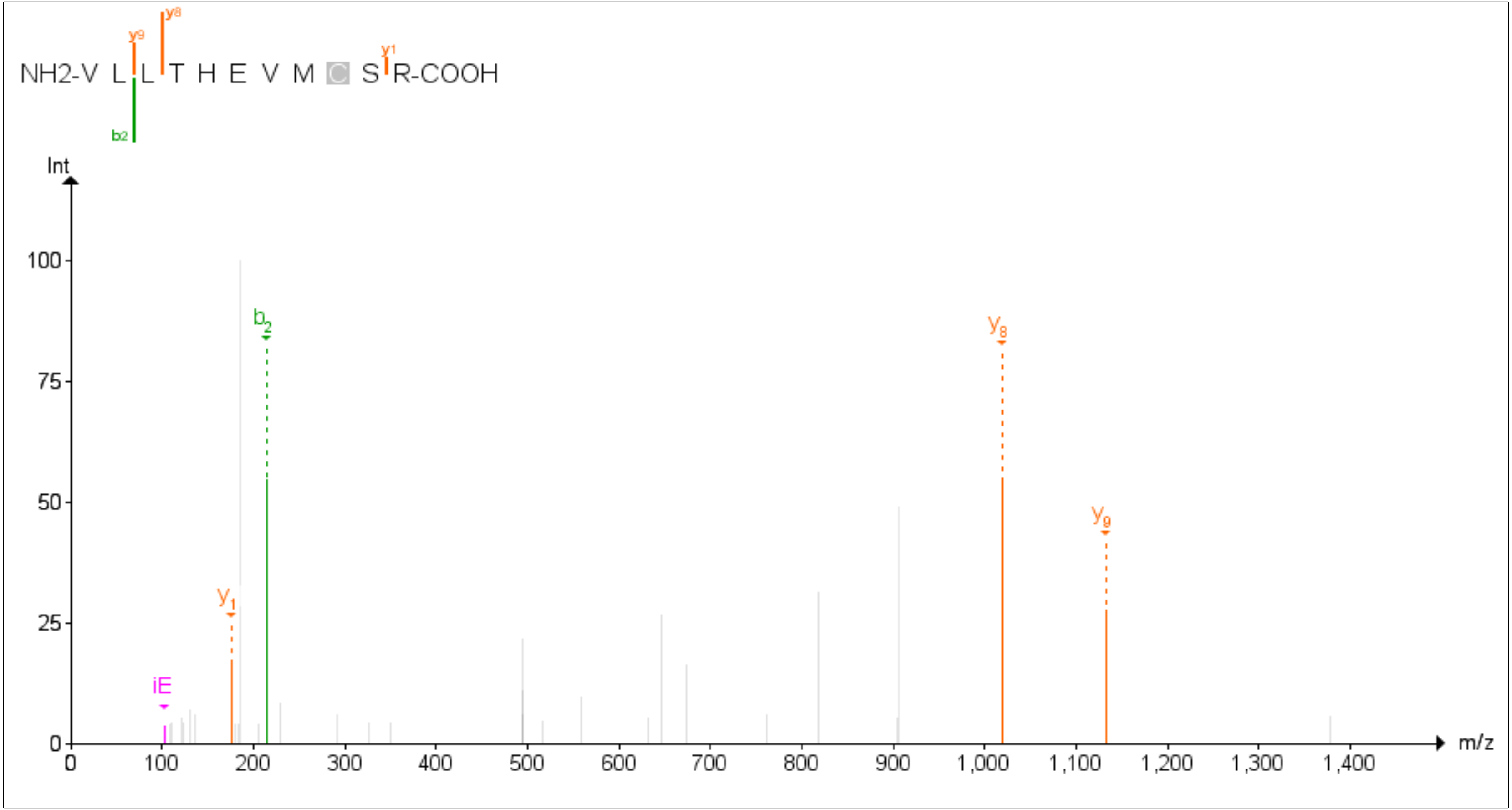

NH2-V L L T H E V M C S R-COOH
y9
y8
y1
b2
Int
100
75
50
25
0
b2
y8
y9
y1
iE
0
100
200
300
400
500
600
700
800
900
1,000
1,100
1,200
1,300
1,400
m/z

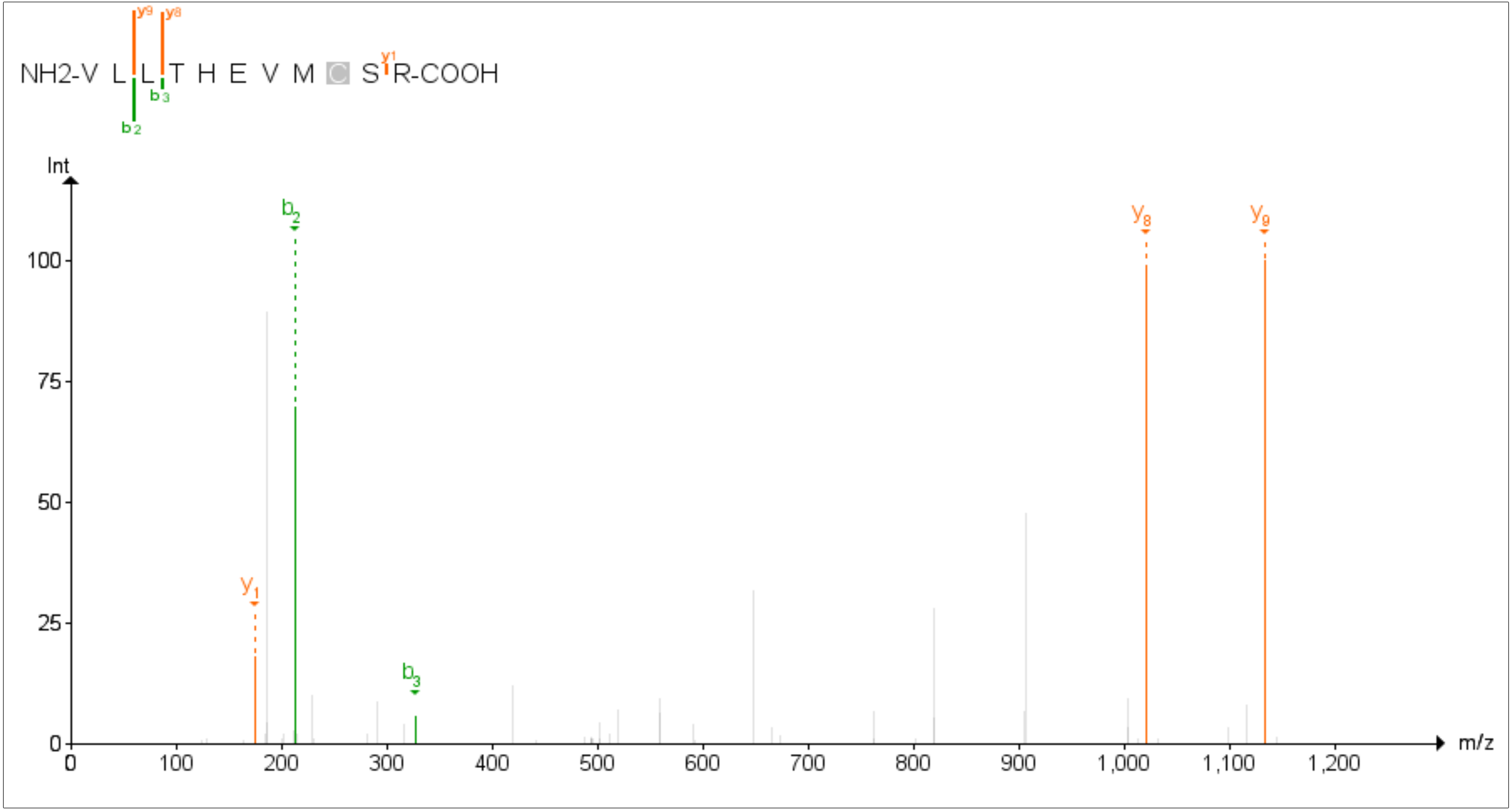

NH2-V L L T H E V M C S R-COOH
y9
y8
y1
b2
b3
Int
100
75
50
25
0
m/z
0
100
200
300
400
500
600
700
800
900
1,000
1,100
1,200

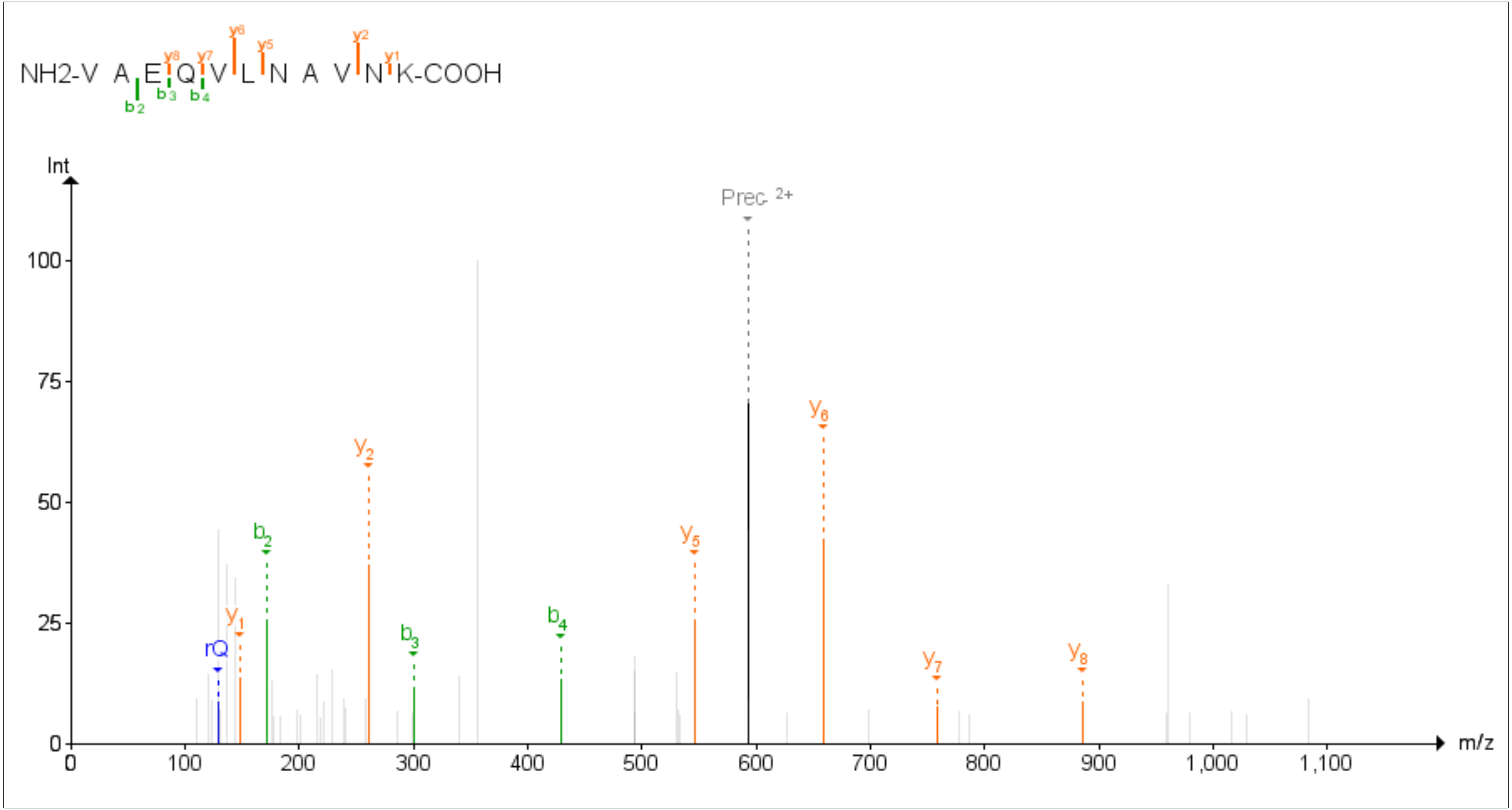

NH2-V A E Q V L N A V N K-COOH
y8 y7 y6 y5 y2 y1
b2 b3 b4
Int
m/z
Prec 2+
rQ
y1
b2
y2
b3
b4
y5
y6
y7
y8
0
25
50
75
100
0
100
200
300
400
500
600
700
800
900
1,000
1,100

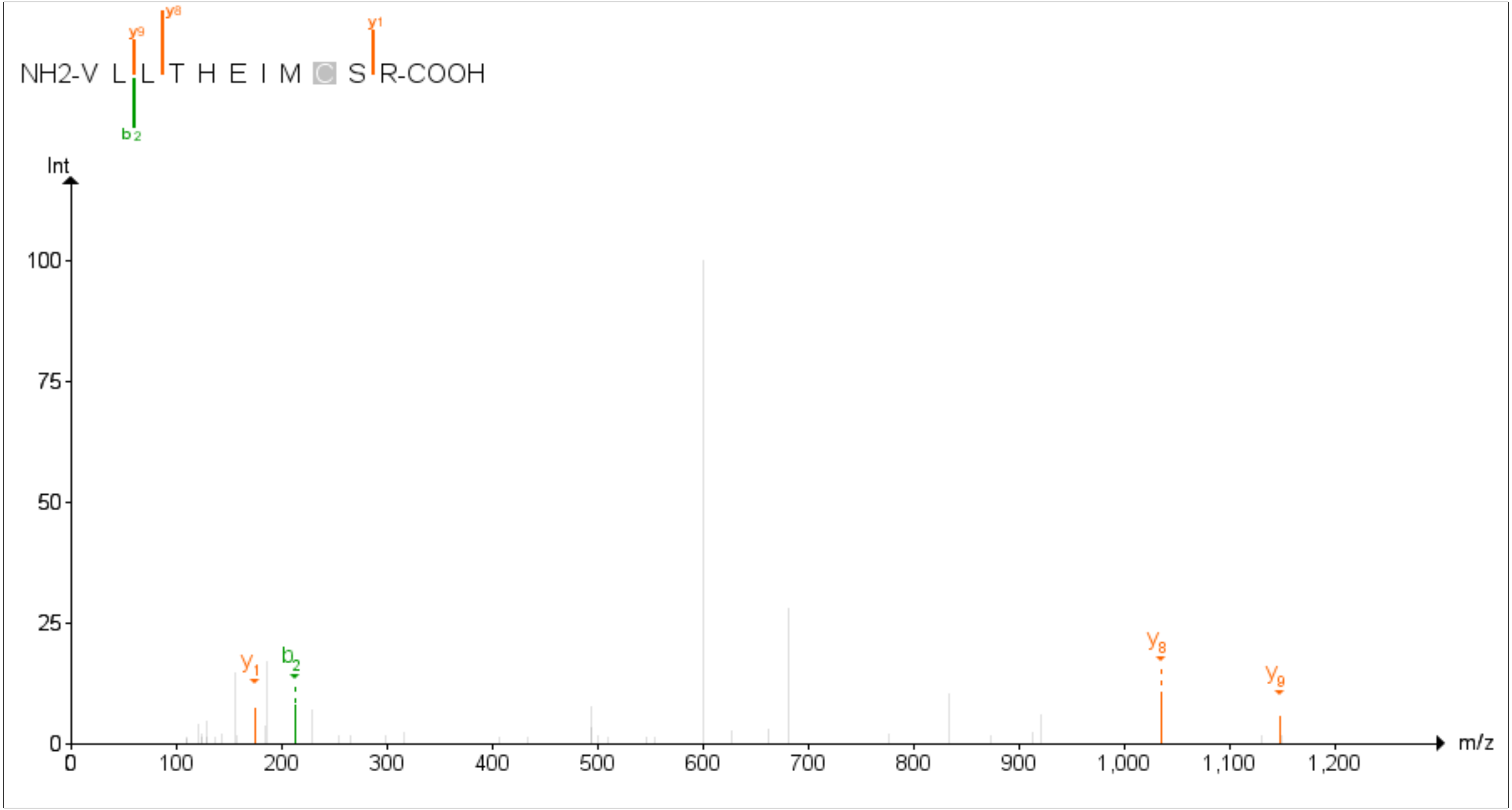

NH2-V L L T H E I M C S R-COOH
y9
y8
y1
b2
Int
100
75
50
25
0
m/z
0
100
200
300
400
500
600
700
800
900
1,000
1,100
1,200
y1
b2
y8
y9

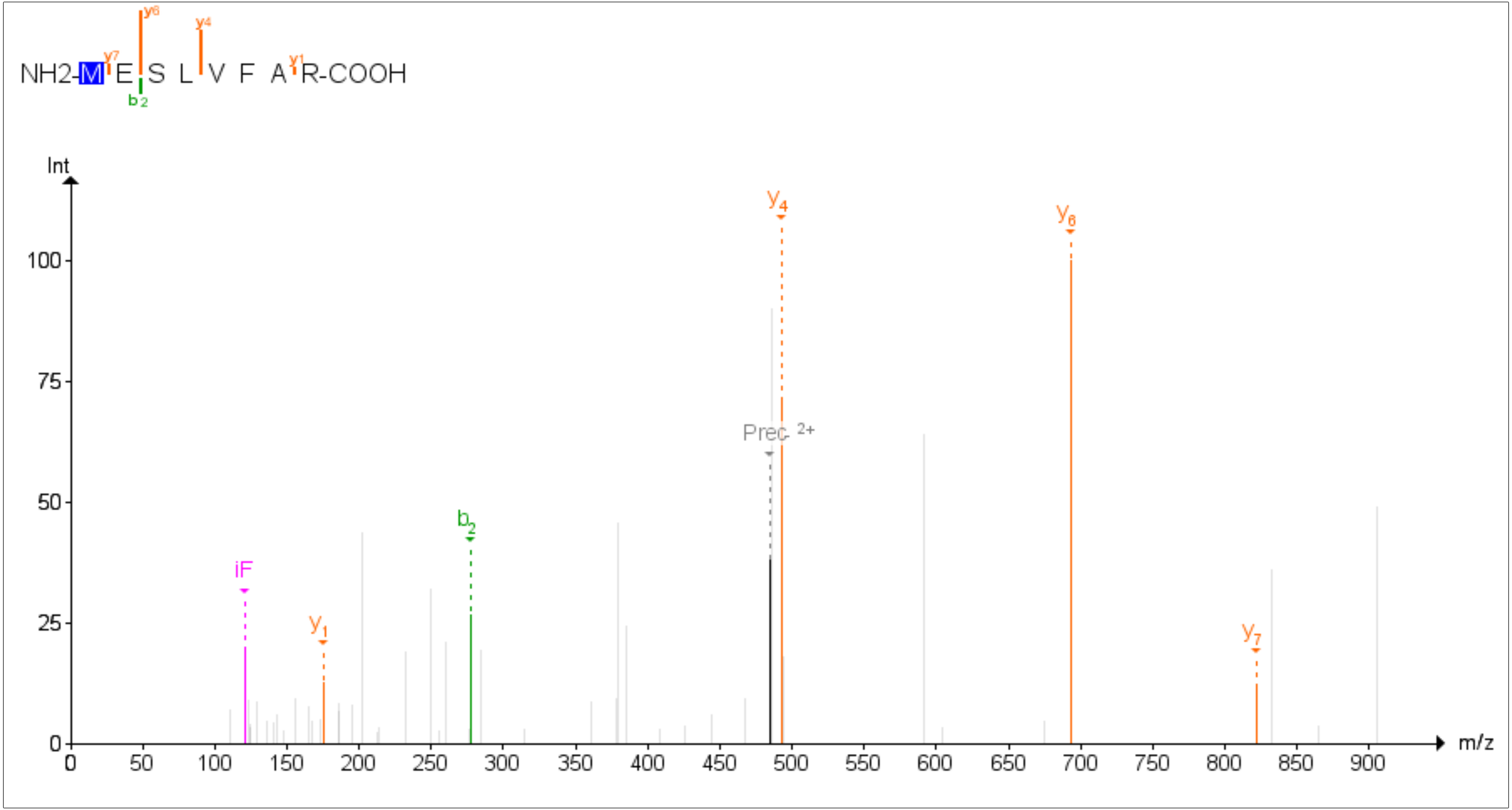

NH2-M E S L V F A R-COOH
y6
y4
y7
y1
b2
Int
m/z
iF
y1
b2
Prec 2+
y4
y6
y7
0
25
50
75
100
0 50 100 150 200 250 300 350 400 450 500 550 600 650 700 750 800 850 900

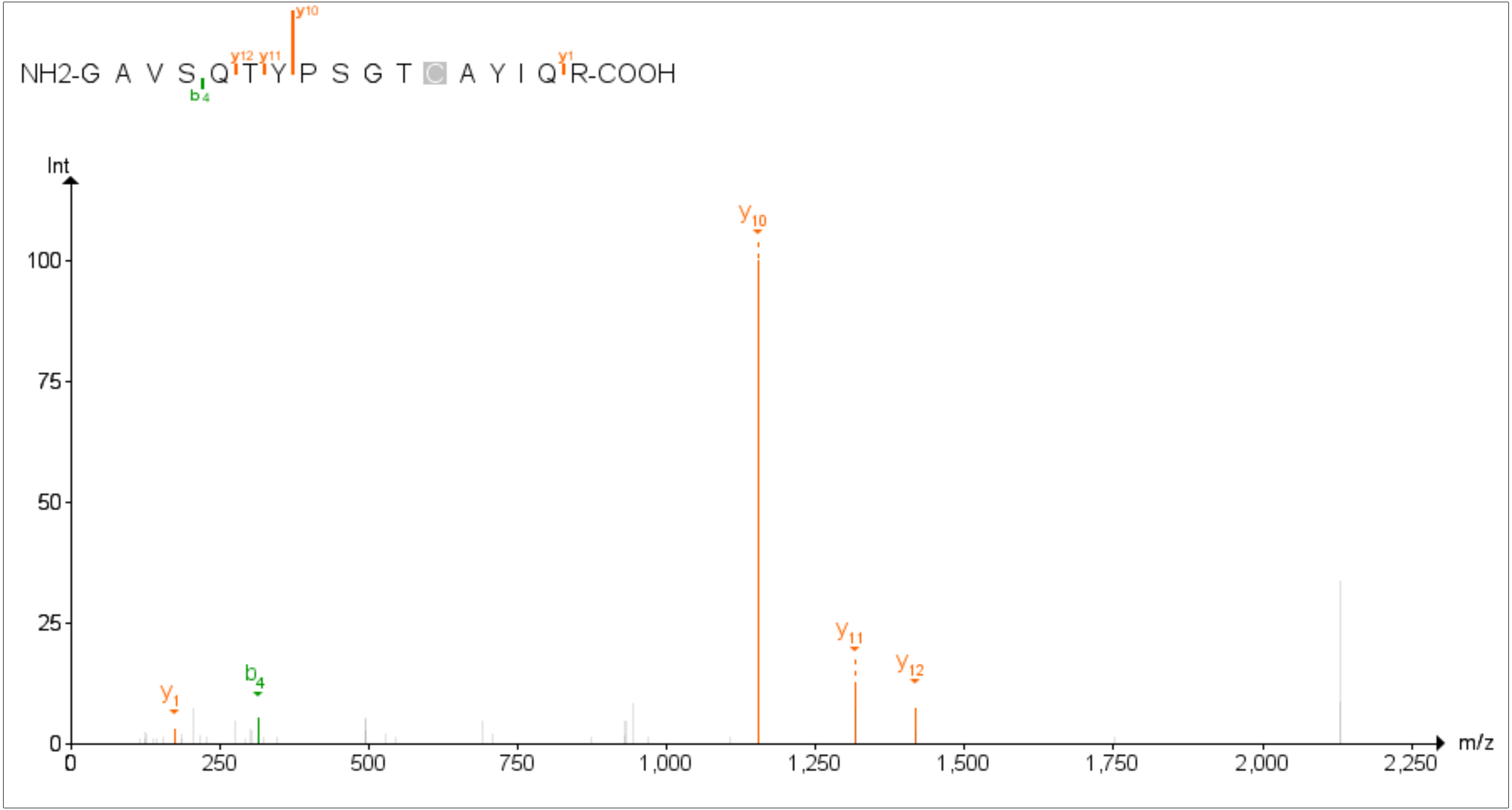

NH2-G A V S Q T Y P S G T C A Y I Q R-COOH
b4
y12
y11
y10
y1
Int
100
75
50
25
0
y10
y11
y12
y1
b4
0
250
500
750
1,000
1,250
1,500
1,750
2,000
2,250
m/z

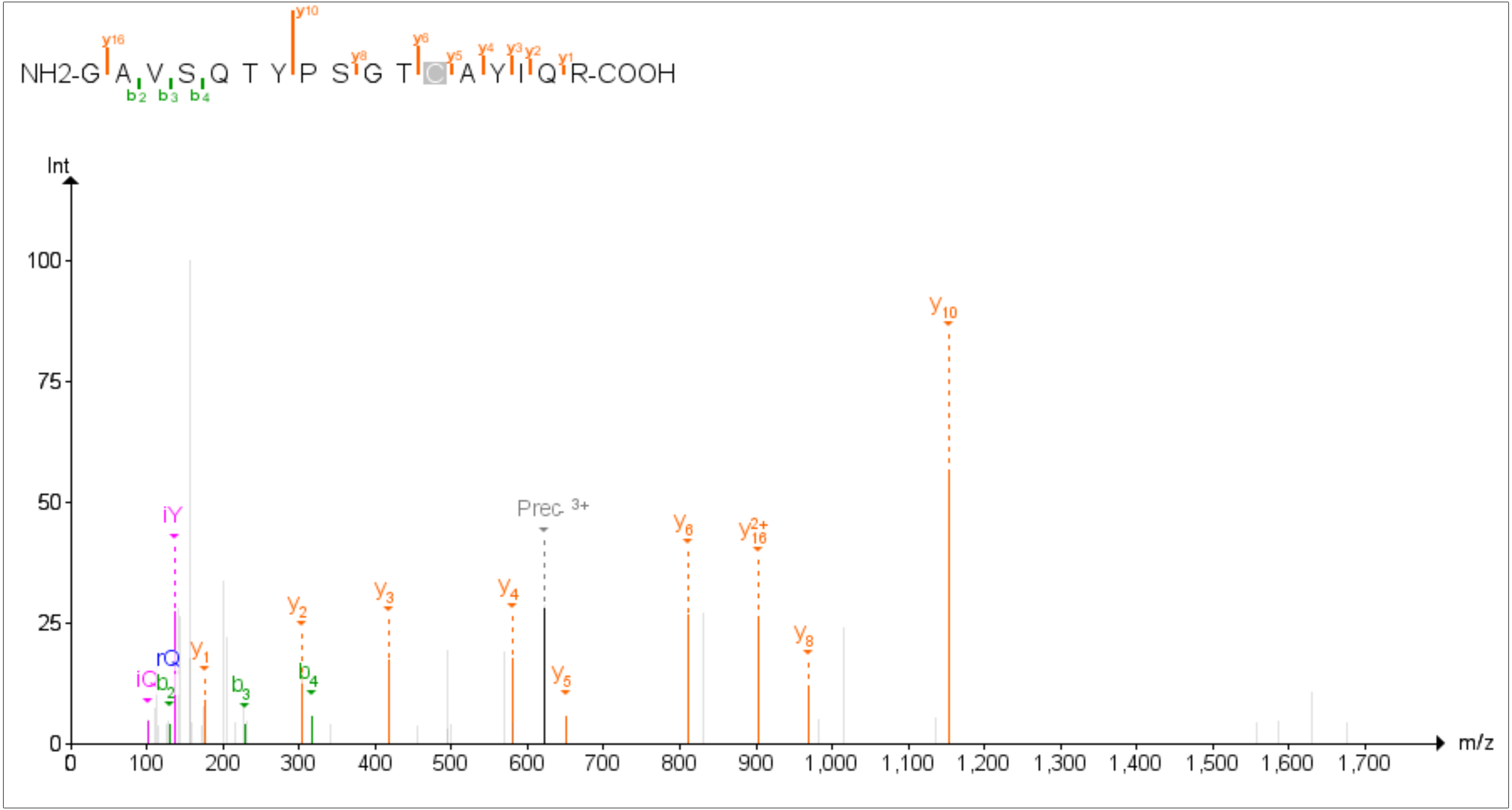

NH2-G A V S Q T Y P S G T C A Y I Q R-COOH
y16 y10 y8 y6 y5 y4 y3 y2 y1
b2 b3 b4
Int
m/z
Prec 3+
iY
rQ
iQ
y10
y6
y16 2+
y8
y5
y4
y3
y2
y1
b2
b3
b4

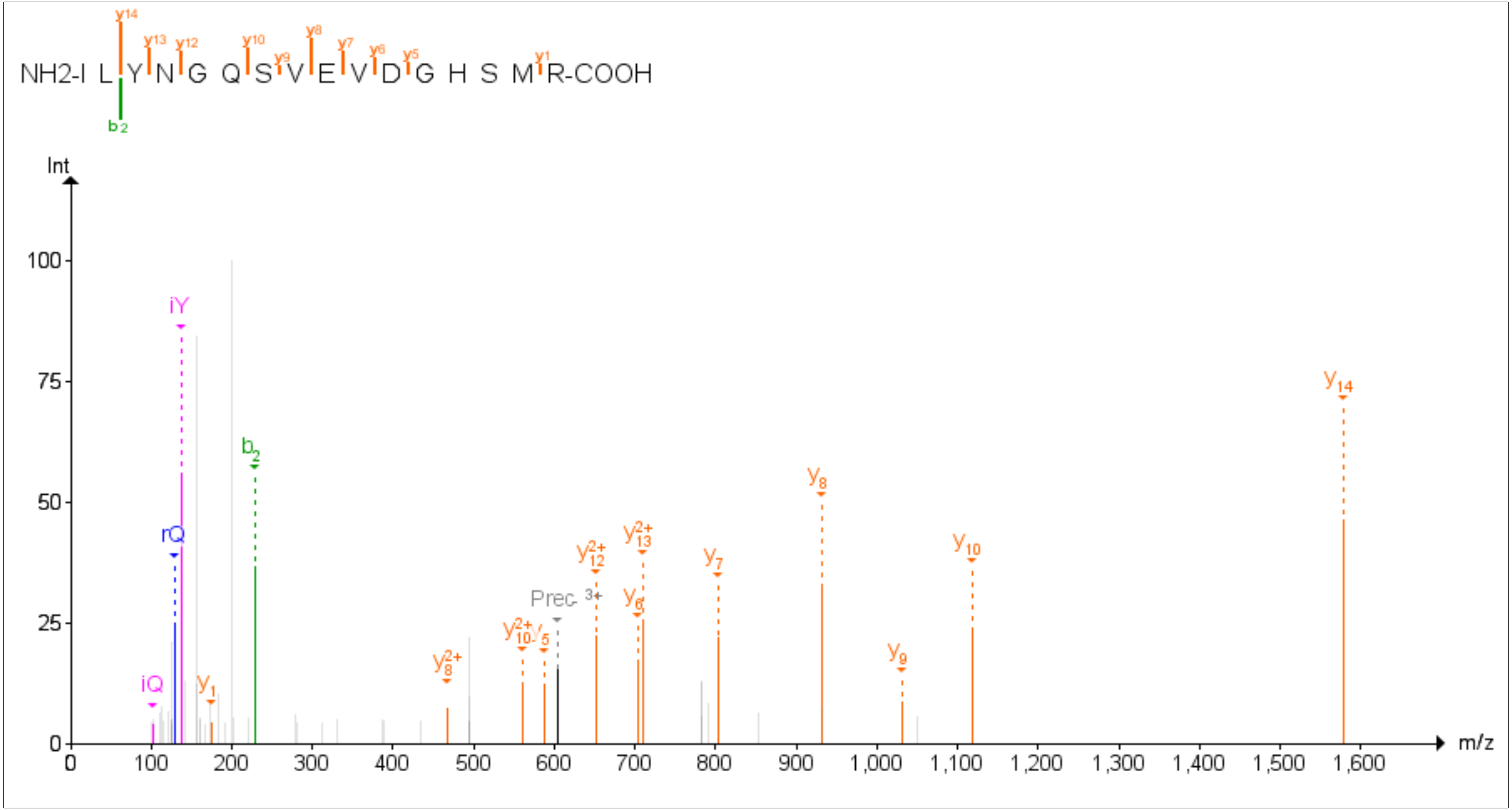

NH2-I L Y N G Q S V E V D G H S M R-COOH
y14 y13 y12 y10 y9 y8 y7 y6 y5 y1
b2
Int
100
75
50
25
0
iY
b2
rQ
iQ
y1
y14
y8
y2+ 12
y2+ 13
y7
y10
Prec 3+
y6
y2+ 10
y5
y2+ 8
y9
0 100 200 300 400 500 600 700 800 900 1,000 1,100 1,200 1,300 1,400 1,500 1,600
m/z

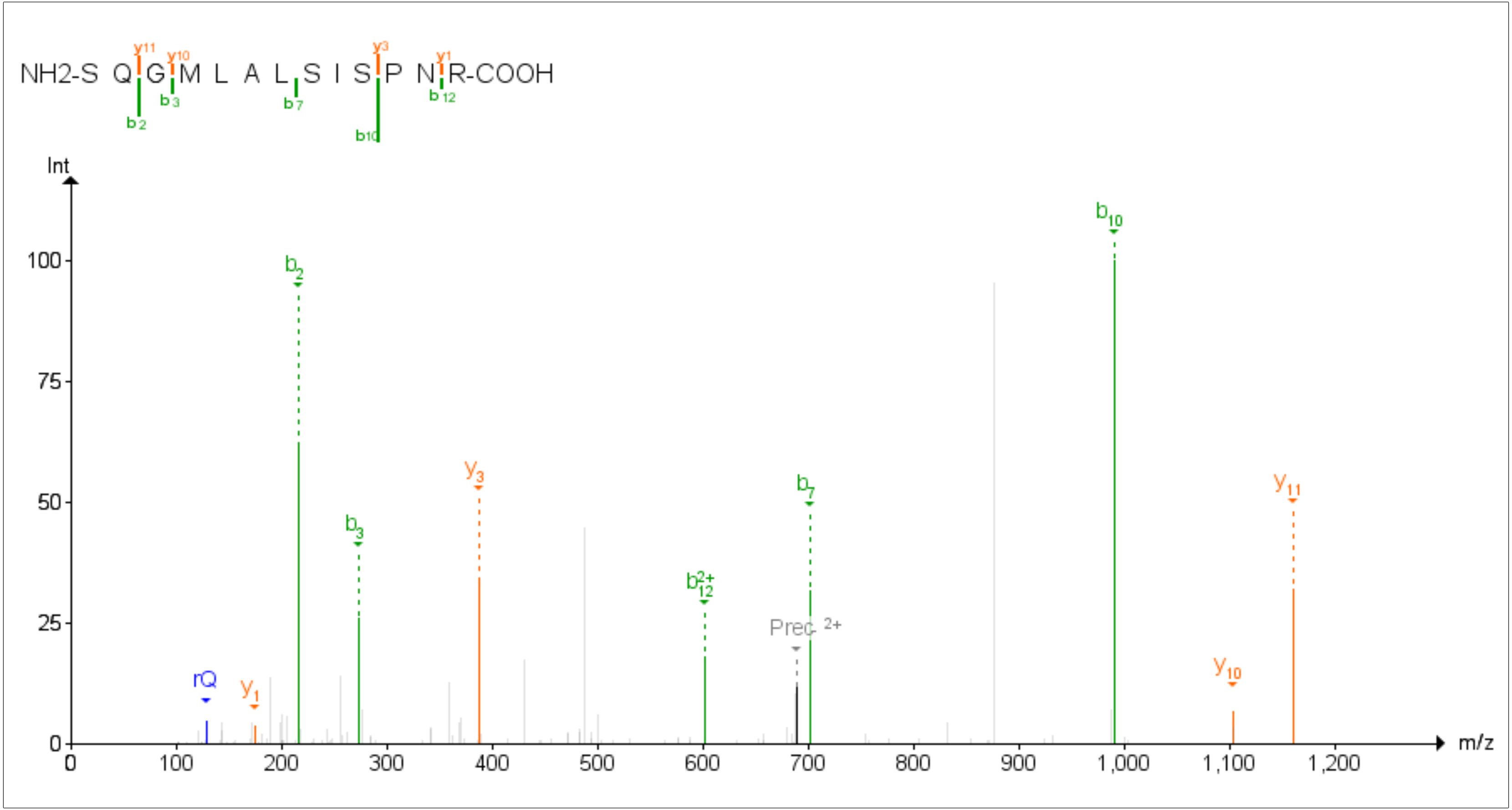

NH2-S Q G M L A L S I S P N R-COOH
y11
y10
y3
y1
b2
b3
b7
b10
b12
Int
100
75
50
25
0
b2
b3
y3
b7
b10
y11
y10
y1
rQ
Prec 2+
m/z
0
100
200
300
400
500
600
700
800
900
1,000
1,100
1,200

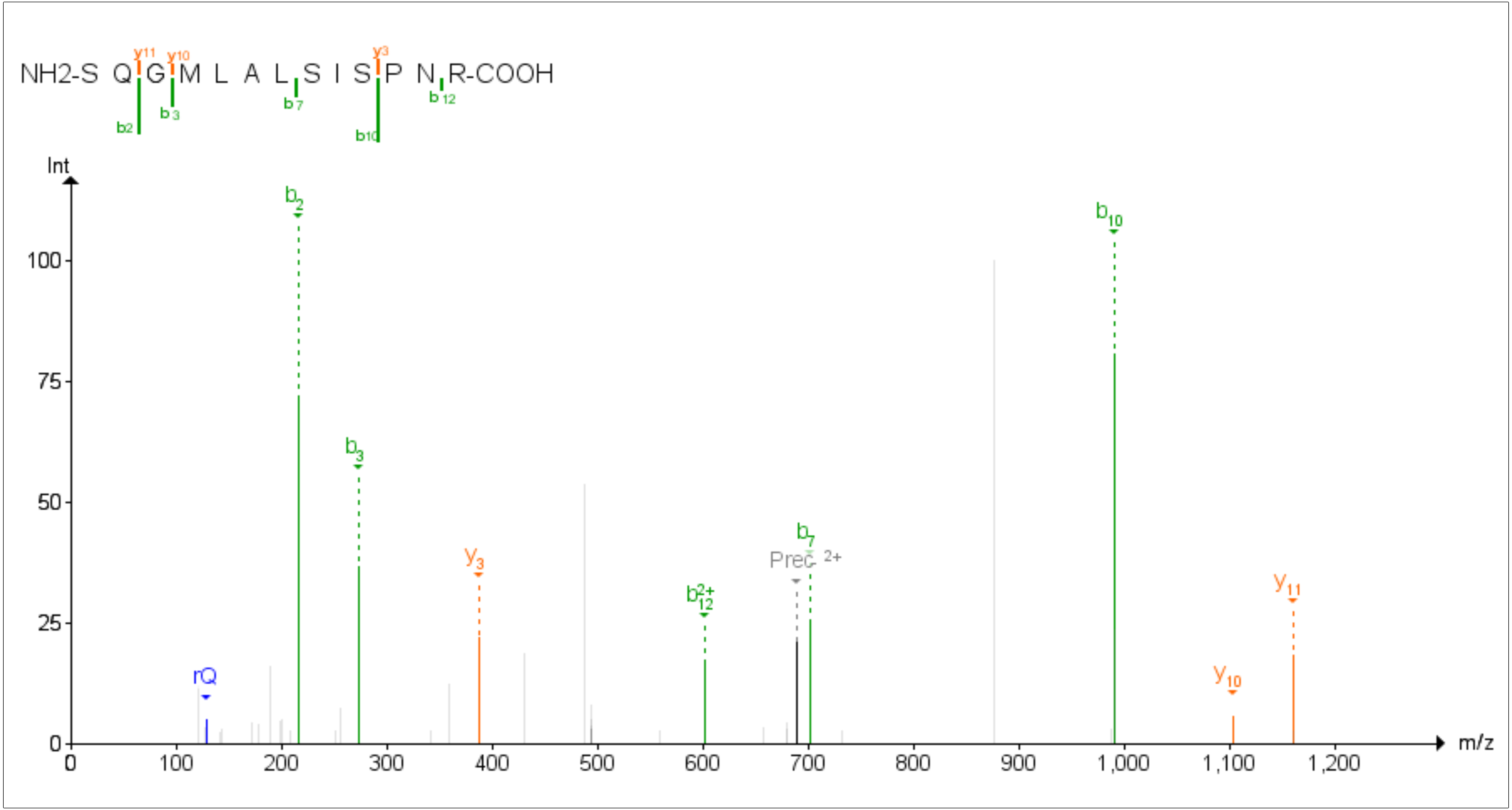

NH2-S Q G M L A L S I S P N R-COOH
y11
y10
y3
b2
b3
b7
b10
b12
Int
m/z
b2
b10
b3
y3
b12 2+
Prec 2+
b7
y11
y10
rQ
0
25
50
75
100
0
100
200
300
400
500
600
700
800
900
1,000
1,100
1,200

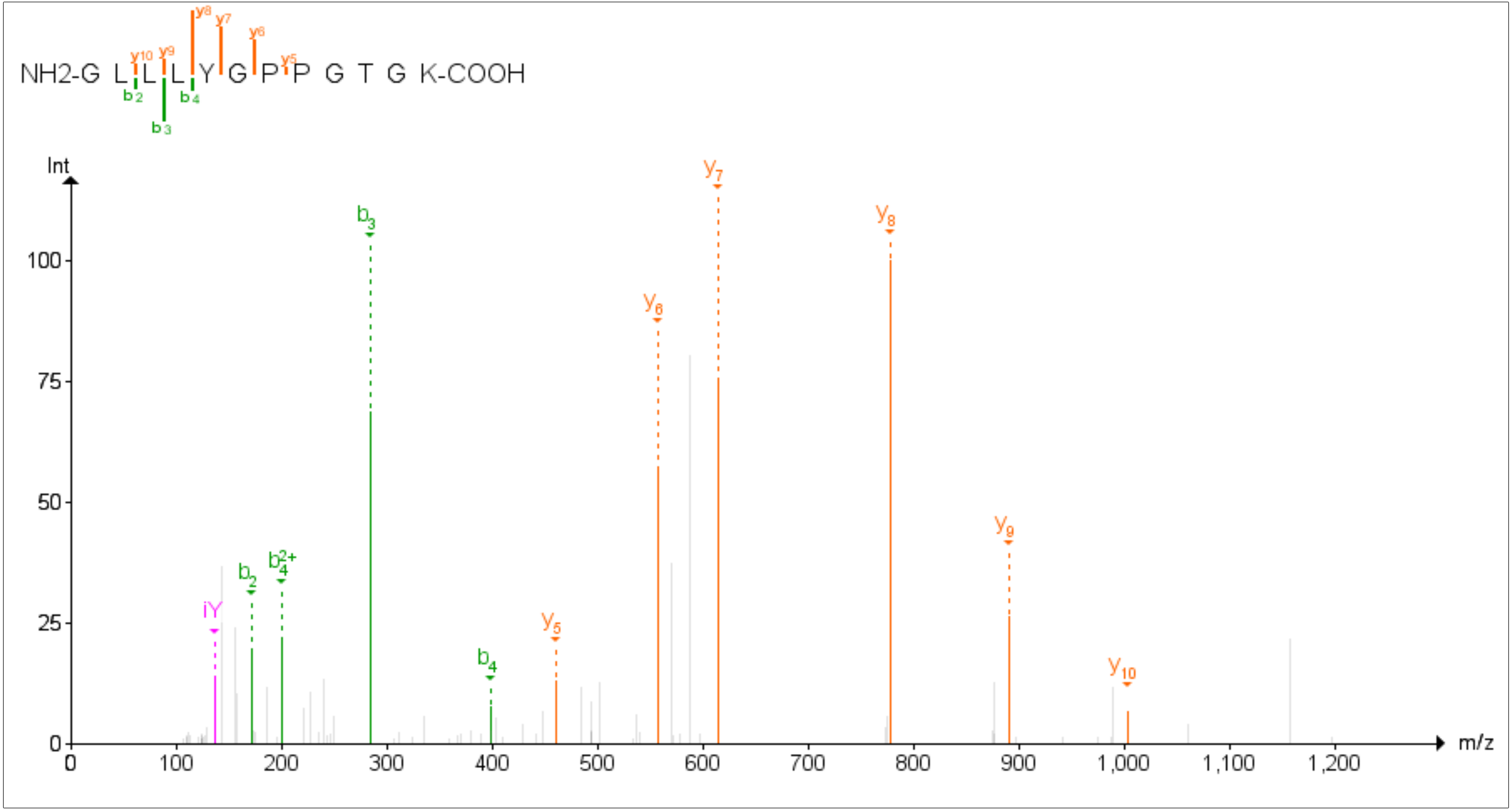

NH2-G L L L Y G P P G T G K-COOH
y10 y9 y8 y7 y6 y5
b2 b3 b4
Int
100
75
50
25
0
b3
y7
y8
y6
y9
y10
y5
b4
b2
b4 2+
iY
0
100
200
300
400
500
600
700
800
900
1,000
1,100
1,200
m/z

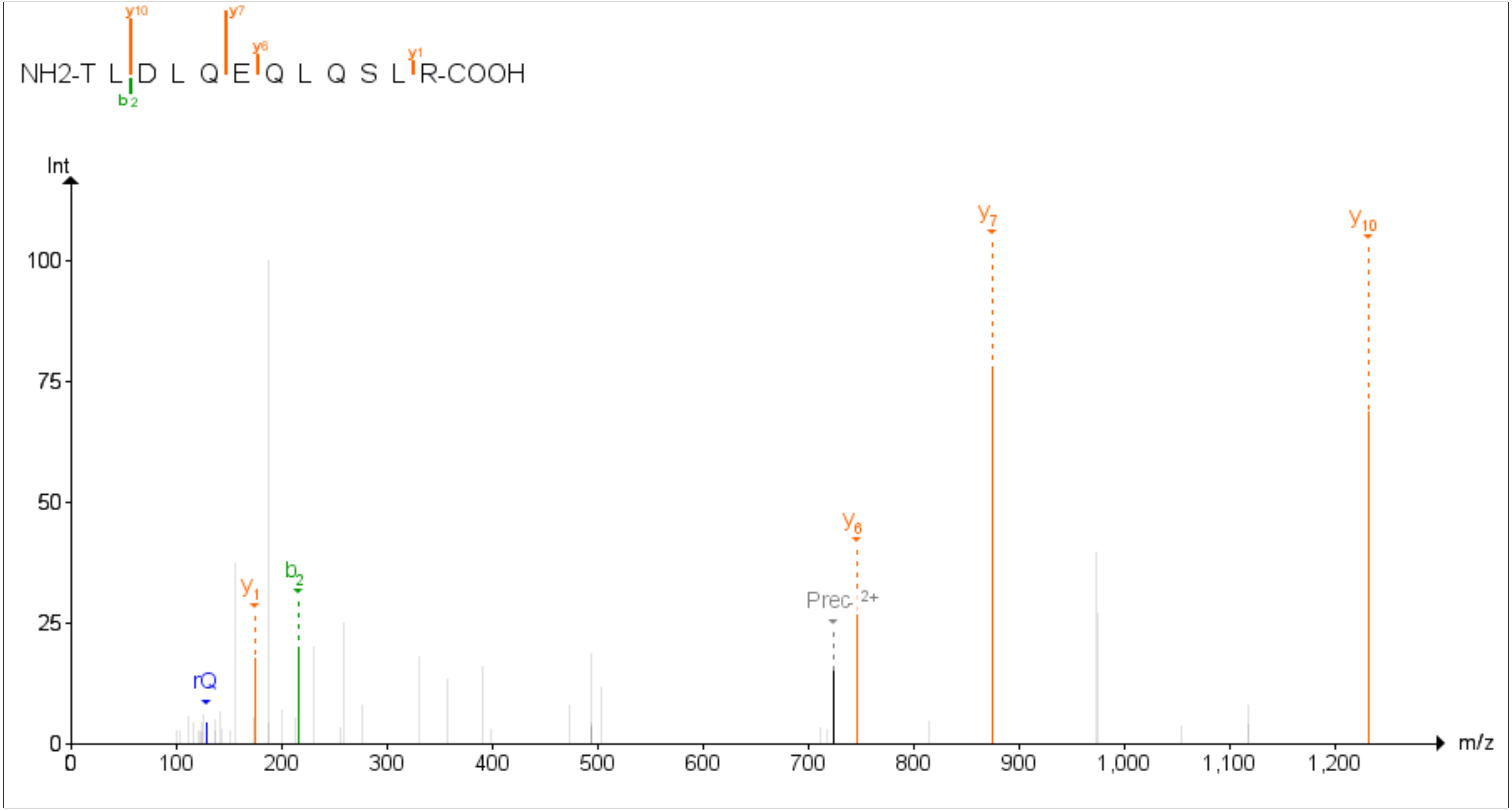

y10
y7
y6
y1
NH2-T L D L Q E Q L Q S L R-COOH
b2
Int
100
75
50
25
0
y7
y10
y6
Prec 2+
y1
b2
rQ
0
100
200
300
400
500
600
700
800
900
1,000
1,100
1,200
m/z

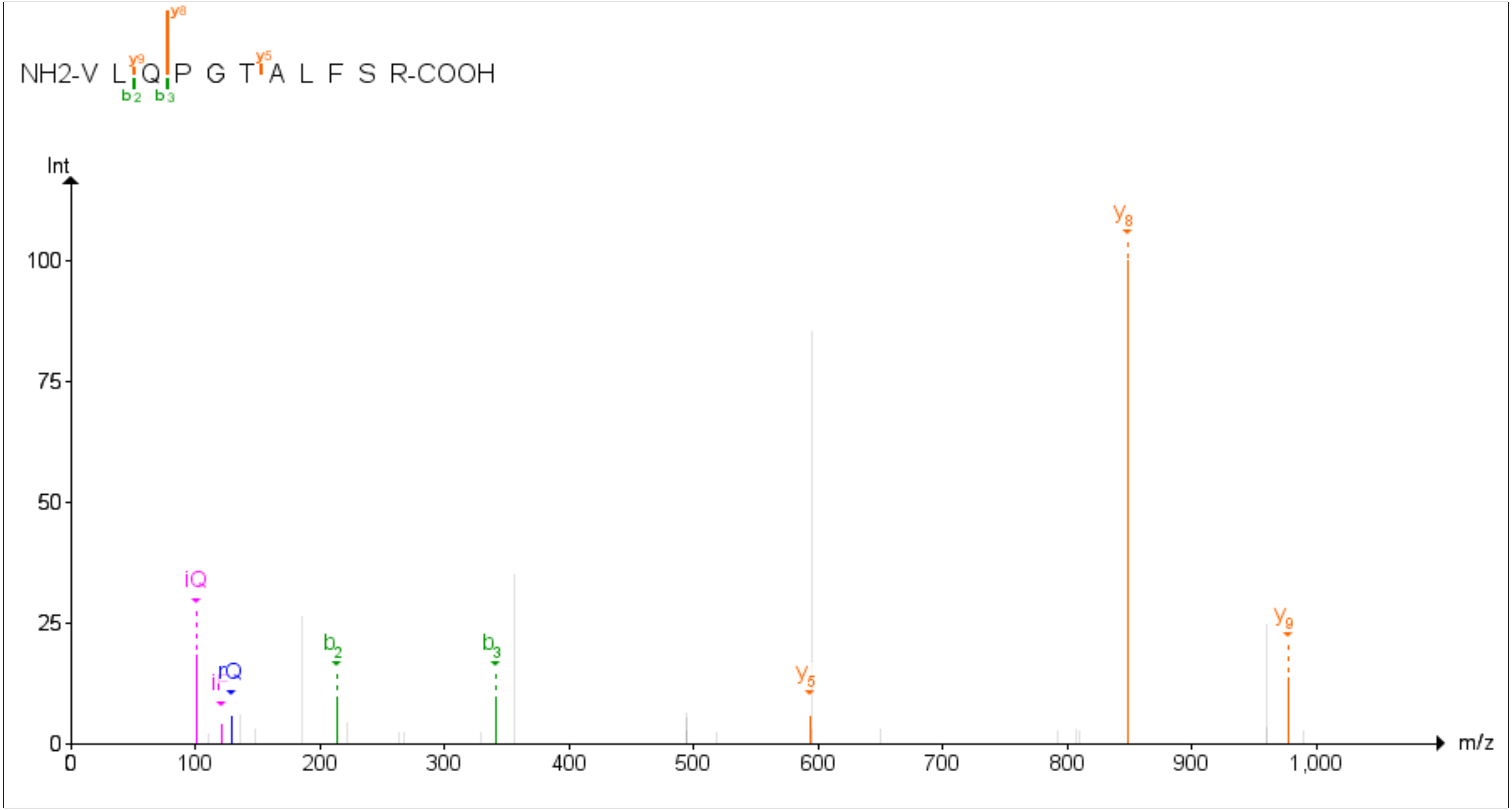

NH2-V L Q P G T A L F S R-COOH
y8
y9
y5
b2
b3
Int
100
75
50
25
0
iQ
iF
rQ
b2
b3
y5
y8
y9
0
100
200
300
400
500
600
700
800
900
1,000
m/z

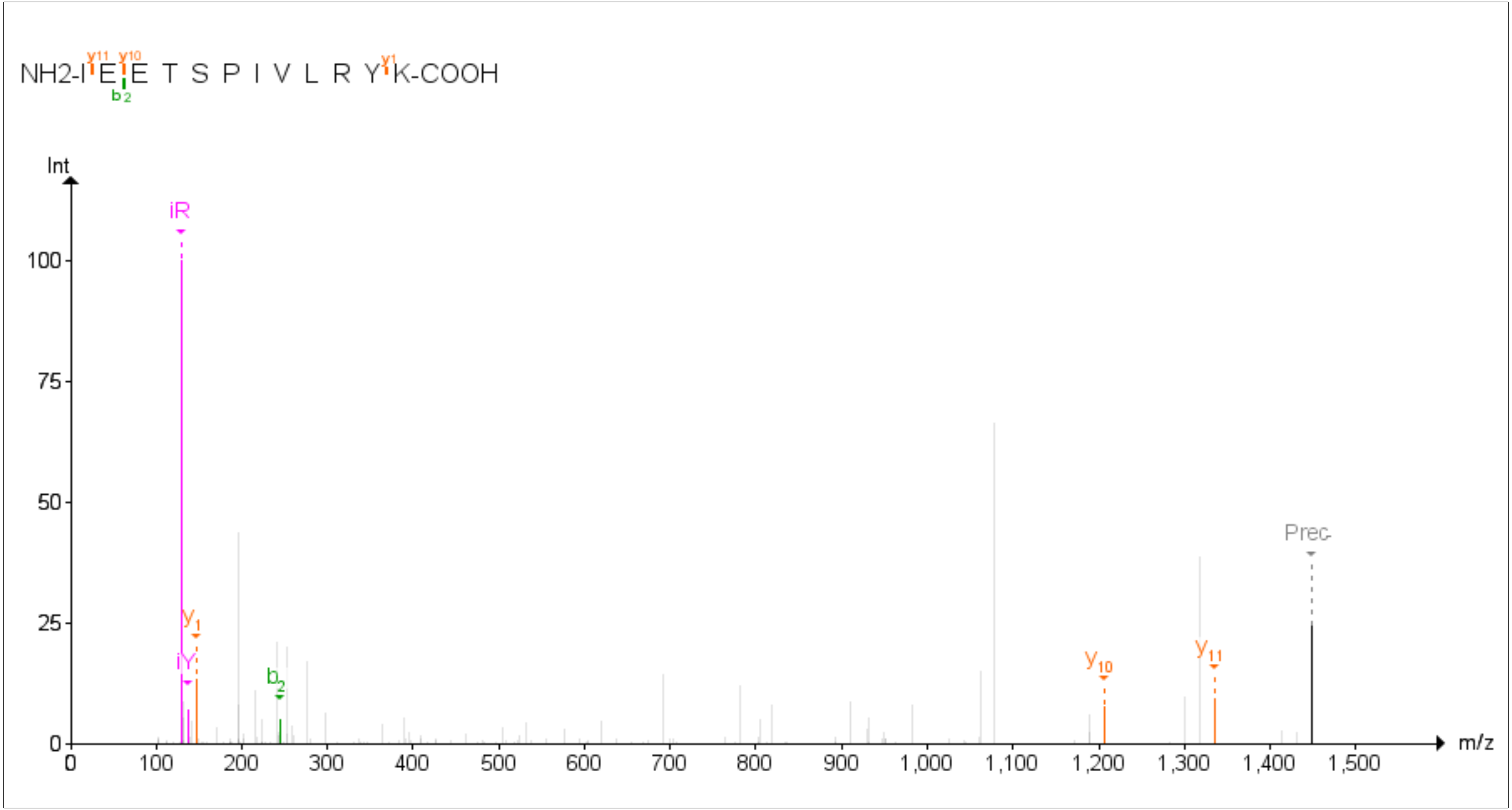

NH2-I E E T S P I V L R Y K-COOH
y11 y10 y1
b2
Int
iR
y1
iY
b2
y10
y11
Prec
0
25
50
75
100
0
100
200
300
400
500
600
700
800
900
1,000
1,100
1,200
1,300
1,400
1,500
m/z

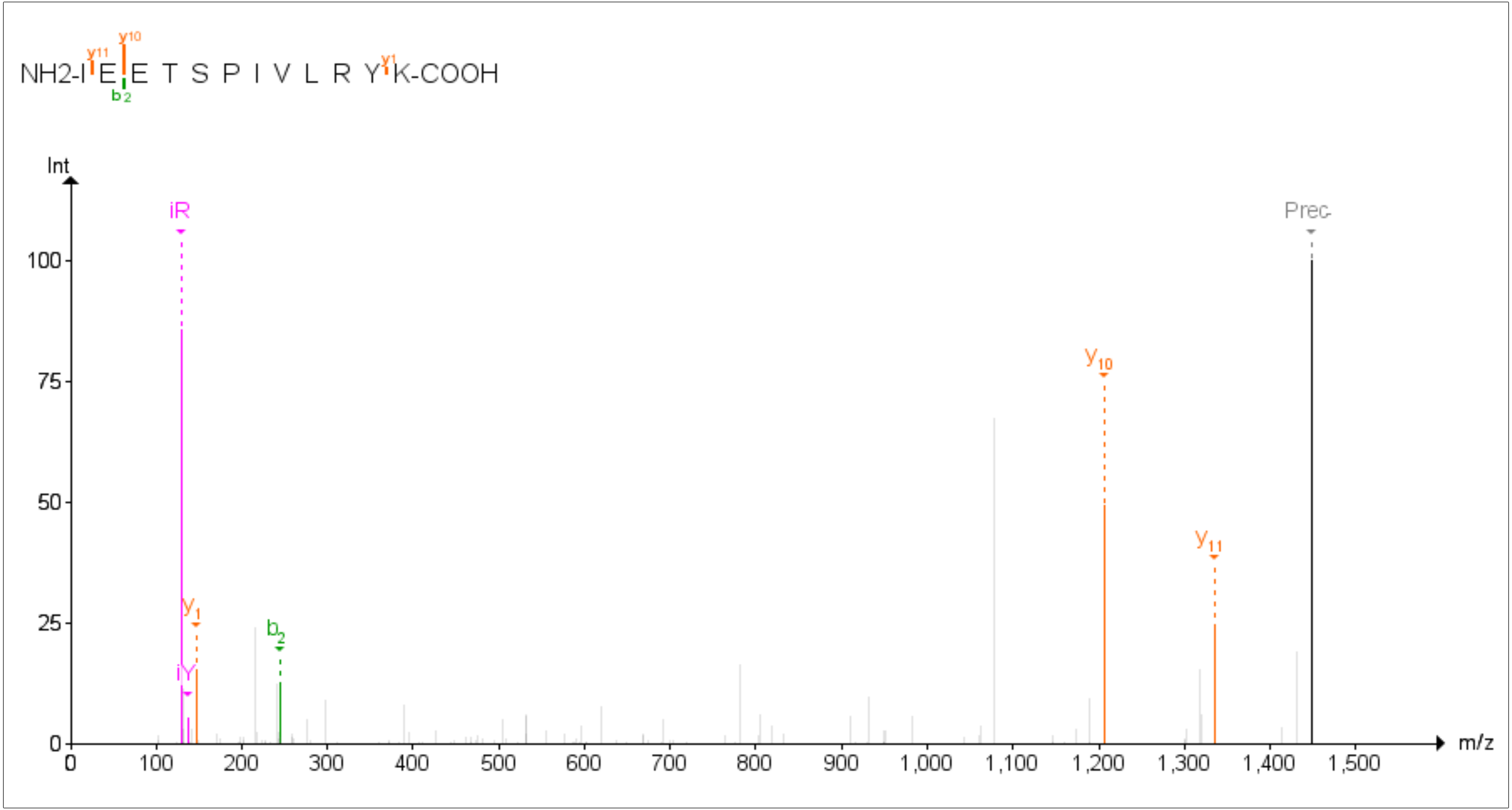

NH2-I E E T S P I V L R Y K-COOH
y11
y10
y1
b2
Int
iR
Prec.
y10
y11
y1
iY
b2
100
75
50
25
0
0
100
200
300
400
500
600
700
800
900
1,000
1,100
1,200
1,300
1,400
1,500
m/z

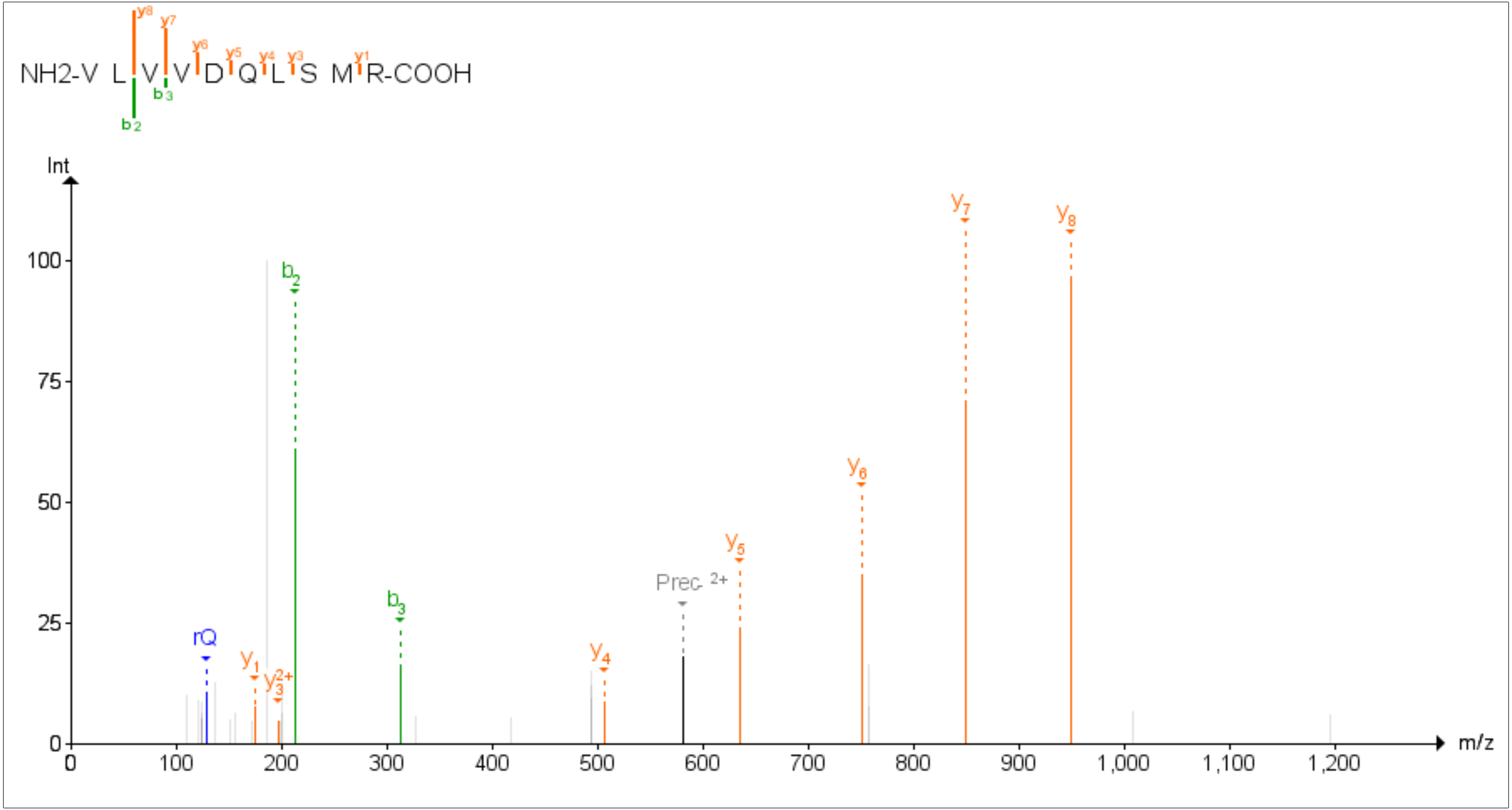

NH2-V L V V D Q L S M R-COOH
y8
y7
y6
y5
y4
y3
y1
b2
b3
Int
100
75
50
25
0
b2
y7
y8
y6
y5
Prec 2+
b3
rQ
y1
y3 2+
y4
0
100
200
300
400
500
600
700
800
900
1,000
1,100
1,200
m/z

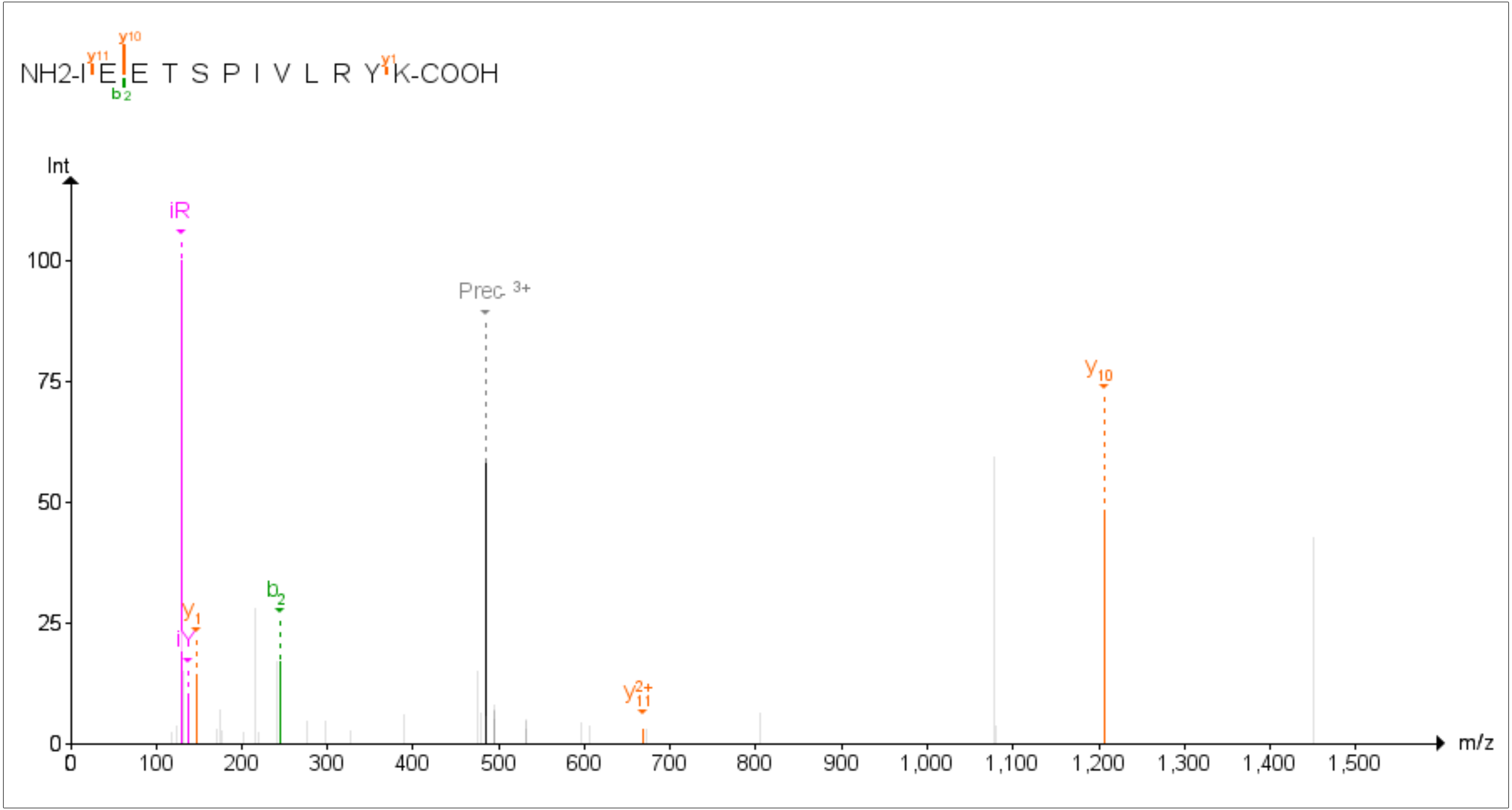
NH2-I E E T S P I V L R Y K-COOH
y11
y10
b2
y1
Int
iR
100
75
50
25
0
Prec 3+
y10
b2
y1
iY
y11 2+
0
100
200
300
400
500
600
700
800
900
1,000
1,100
1,200
1,300
1,400
1,500
m/z

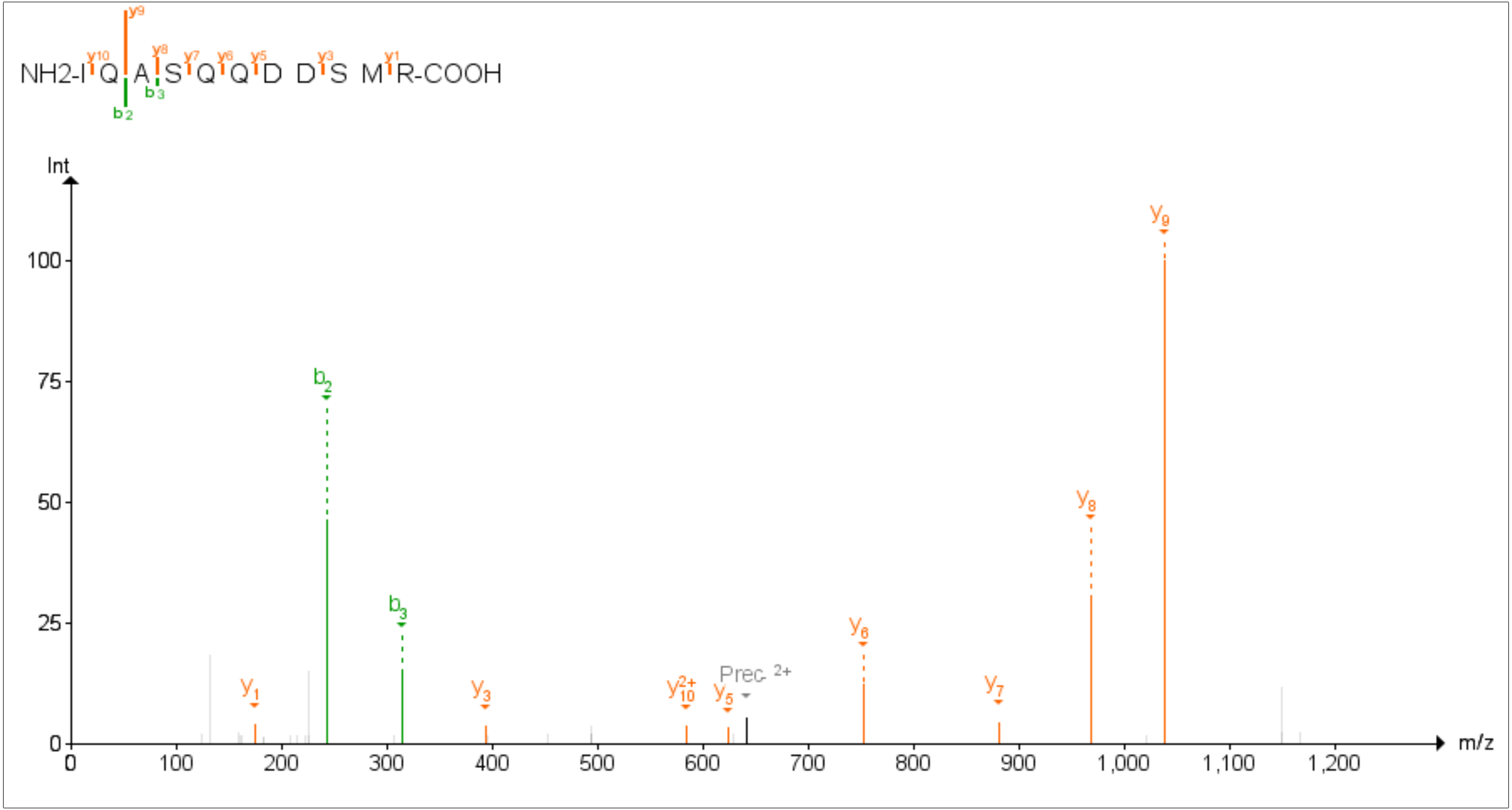

NH2-I Q A S Q Q D D S M R-COOH
y9
y10
y8
y7
y6
y5
y3
y1
b2
b3
Int
100
75
50
25
0
b2
b3
y1
y3
y10 2+
y5
Prec. 2+
y6
y7
y8
y9
m/z
0
100
200
300
400
500
600
700
800
900
1,000
1,100
1,200

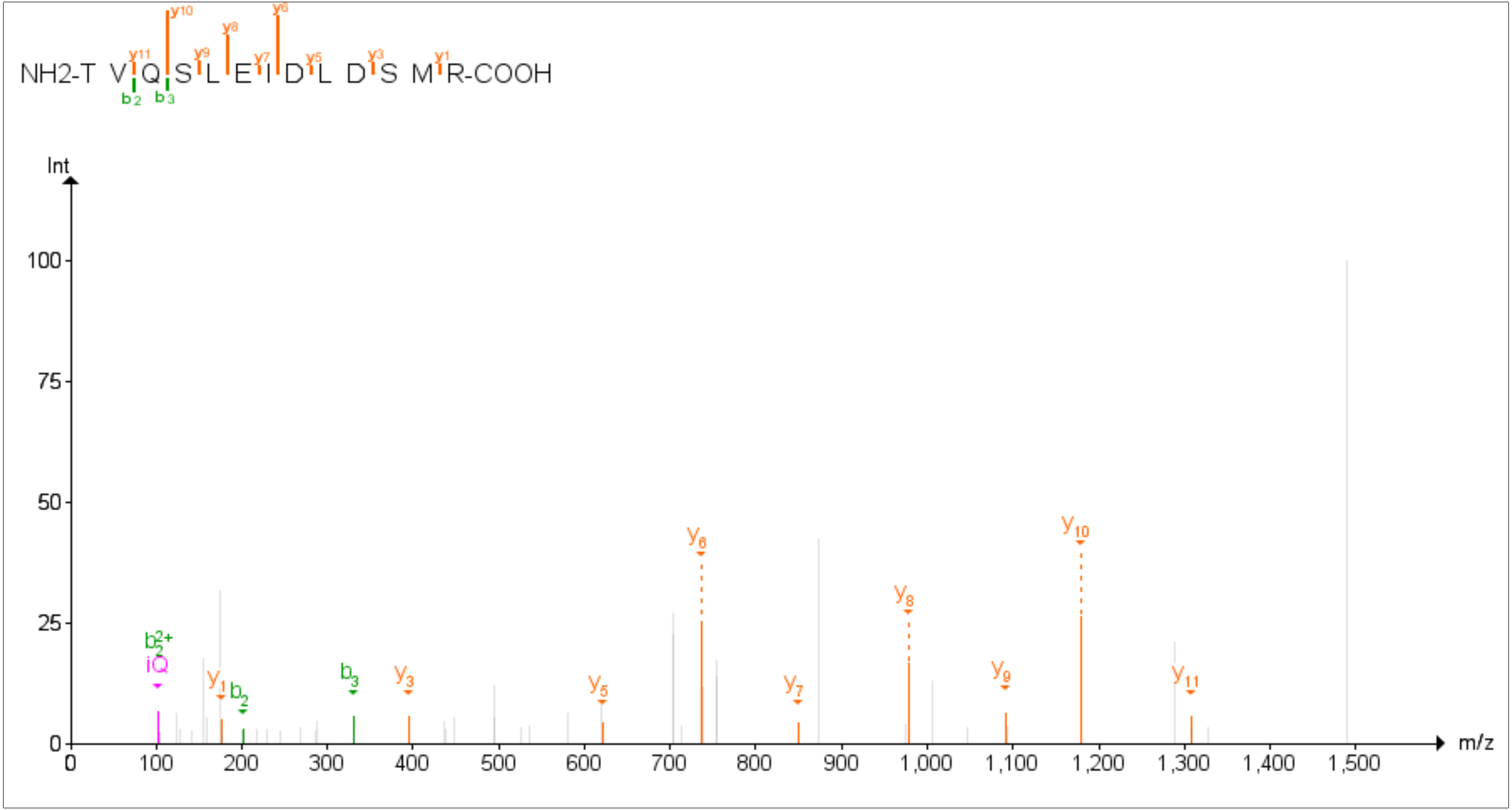

NH2-T V Q S L E I D L D S M R-COOH
y11 y10 y9 y8 y7 y6 y5 y3 y1
b2 b3
Int
100
75
50
25
0
m/z
0 100 200 300 400 500 600 700 800 900 1,000 1,100 1,200 1,300 1,400 1,500
b_2^{2+}
iQ
y_1 b_2 b_3 y_3 y_5 y_6 y_7 y_8 y_9 y_{10} y_{11}

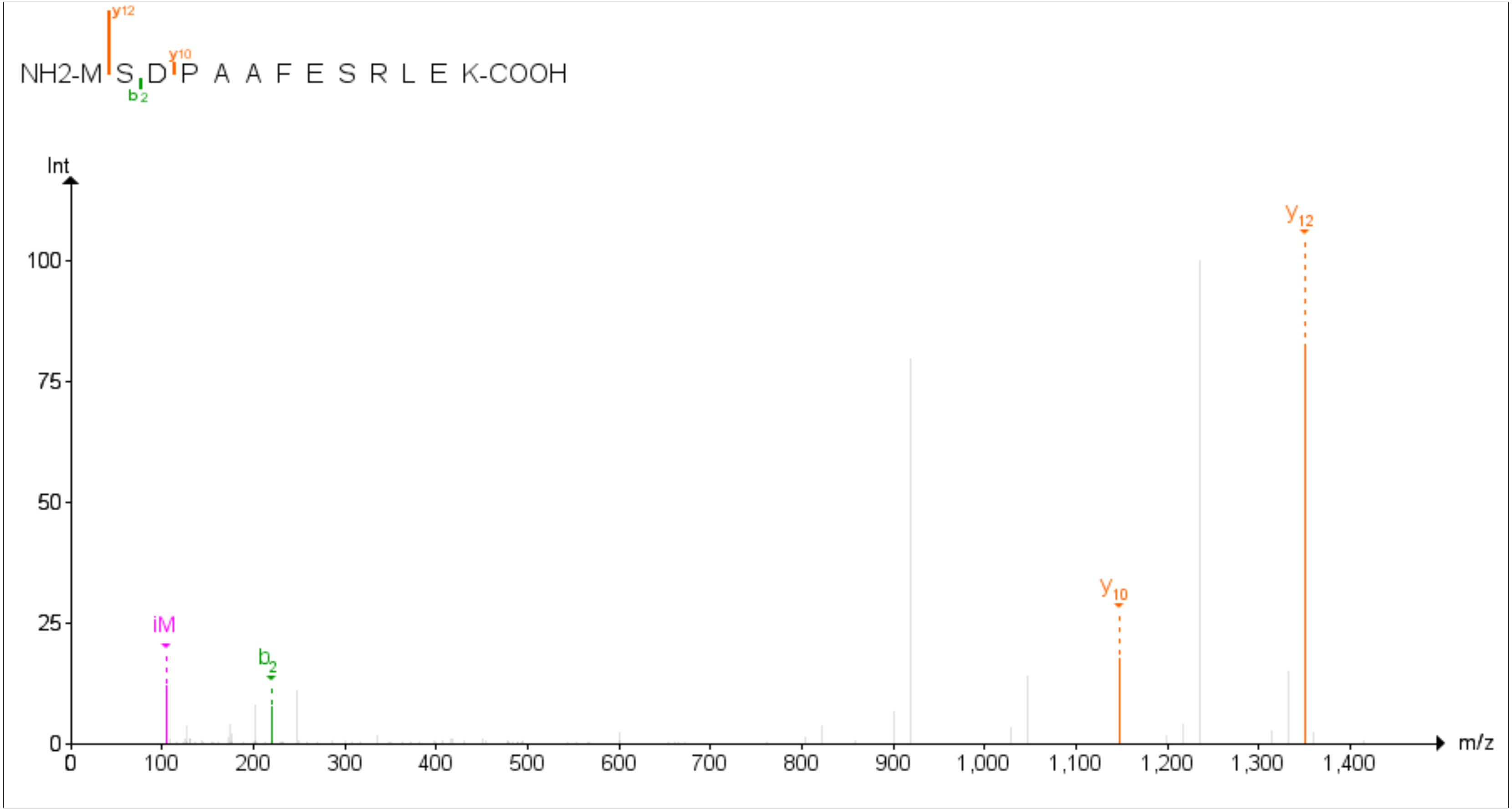

NH2-M S D P A A F E S R L E K-COOH
y12
y10
b2
Int
100
75
50
25
0
iM
b2
y10
y12
m/z
0
100
200
300
400
500
600
700
800
900
1,000
1,100
1,200
1,300
1,400

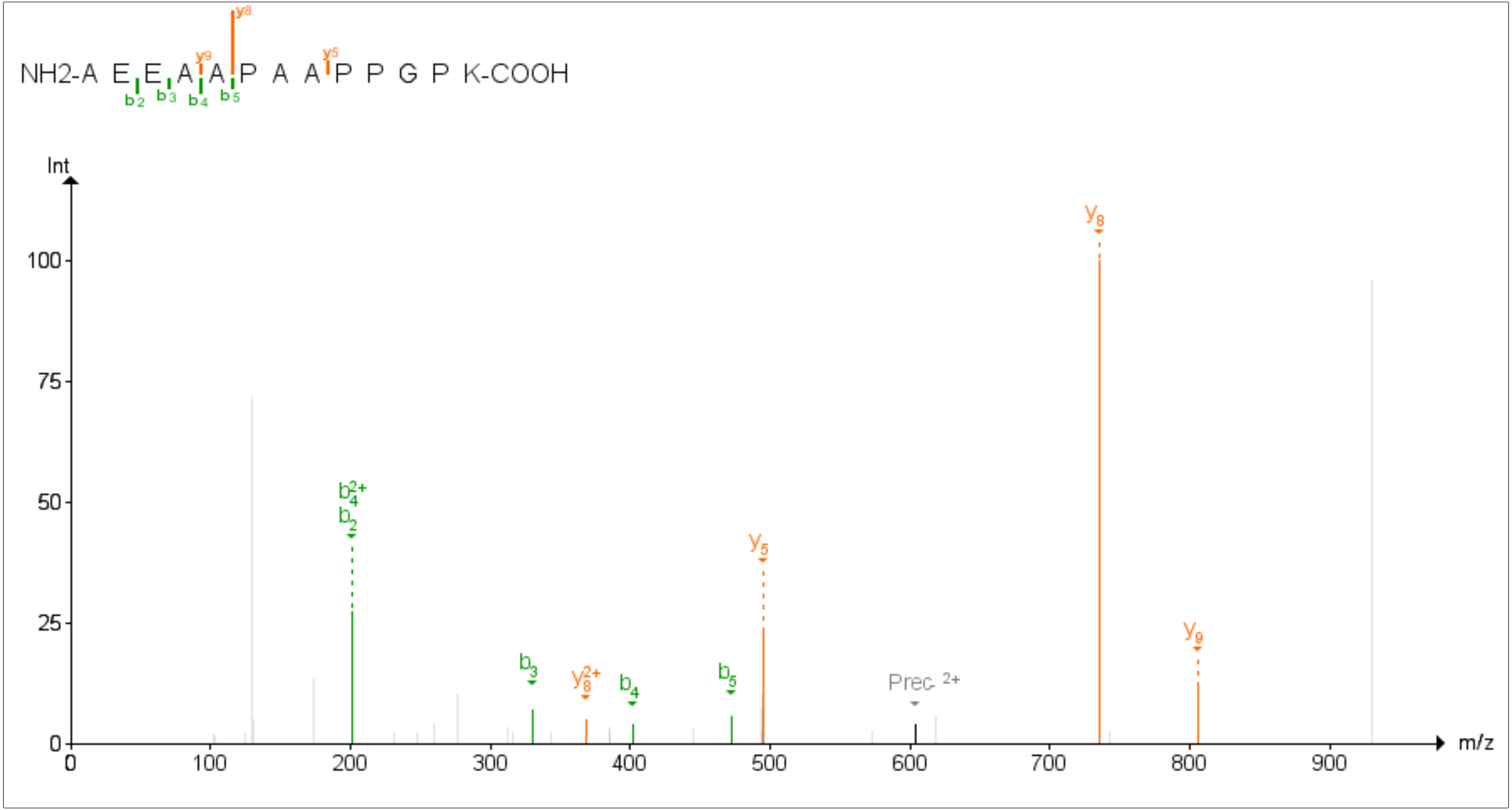
NH2-A E E A A P A A P P G P K-COOH
y8
y9
y5
b2
b3
b4
b5
Int
100
75
50
25
0
m/z
0
100
200
300
400
500
600
700
800
900
b_4^{2+}
b_2
b_3
y_8^{2+}
b_4
b_5
y_5
Prec. 2+
y_8
y_9

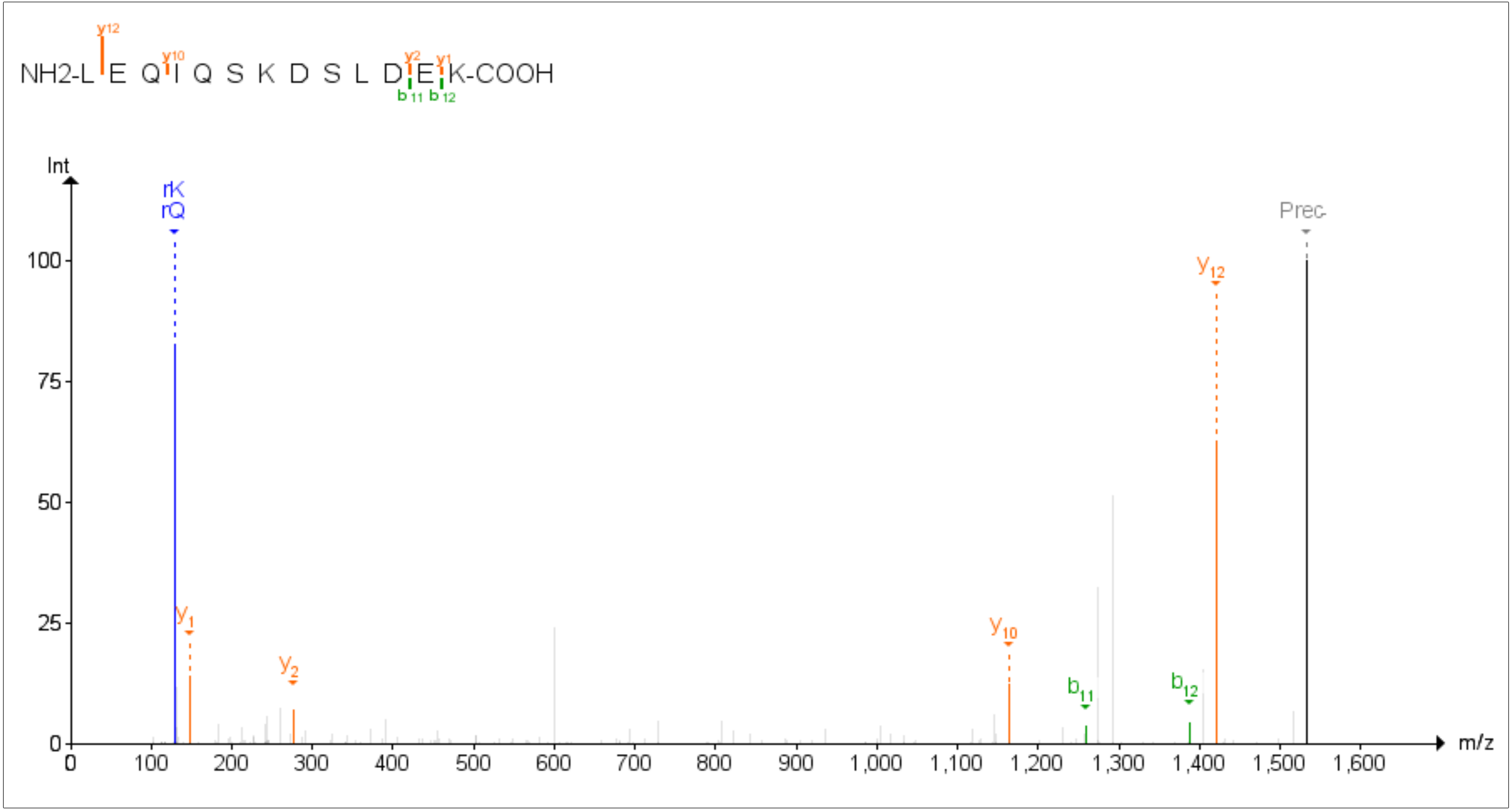

NH2-L E Q I Q S K D S L D E K-COOH
y12
y10
y2
y1
b 11
b 12
Int
rK
rQ
Prec
y12
y1
y2
y10
b11
b12
100
75
50
25
0
0
100
200
300
400
500
600
700
800
900
1,000
1,100
1,200
1,300
1,400
1,500
1,600
m/z

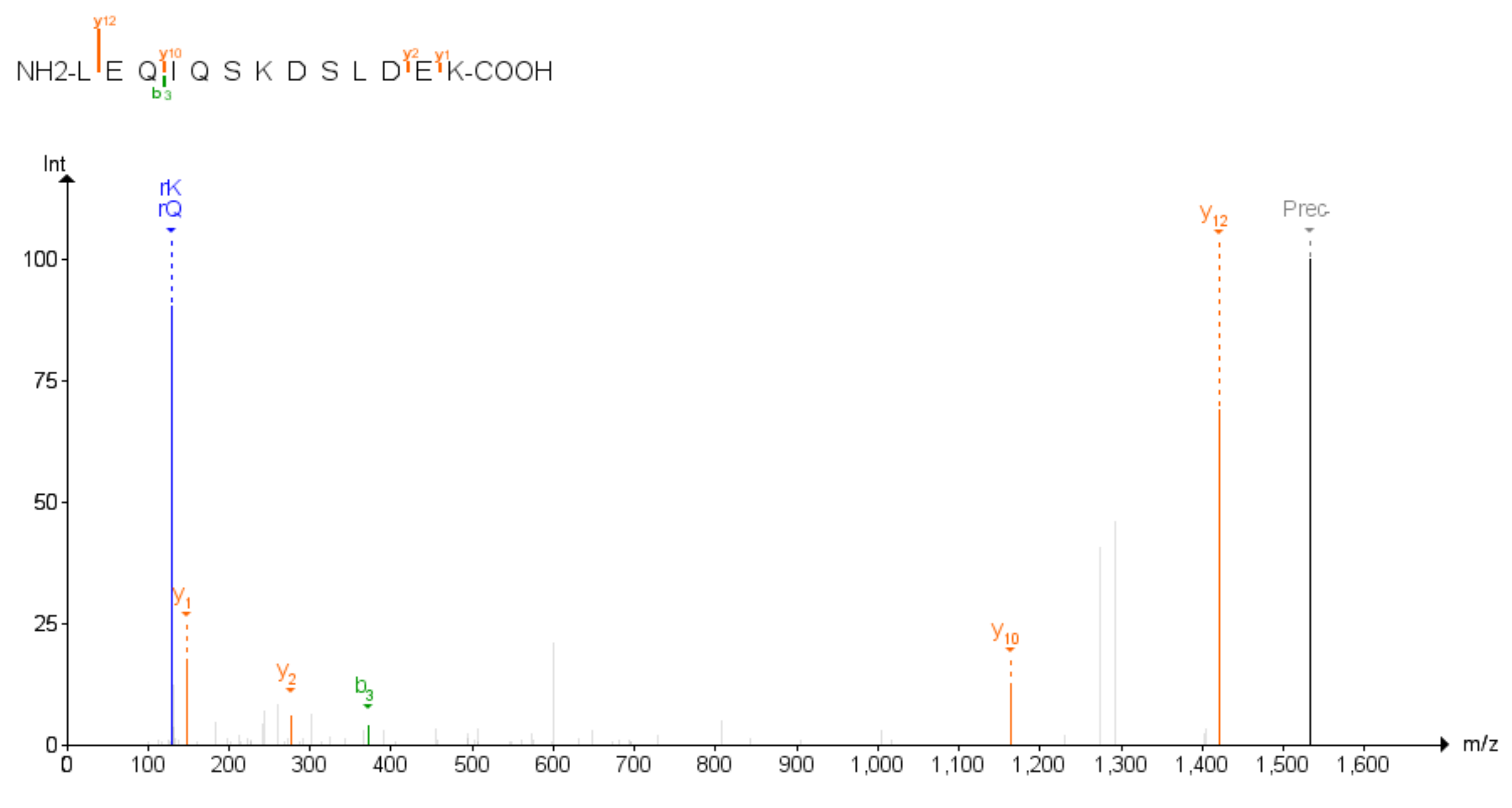

NH2-L E Q I Q S K D S L D E K-COOH
y12
y10
y2
y1
b3
Int
rK
rQ
Prec
100
75
50
25
0
y1
y2
b3
y10
y12
0
100
200
300
400
500
600
700
800
900
1,000
1,100
1,200
1,300
1,400
1,500
1,600
m/z

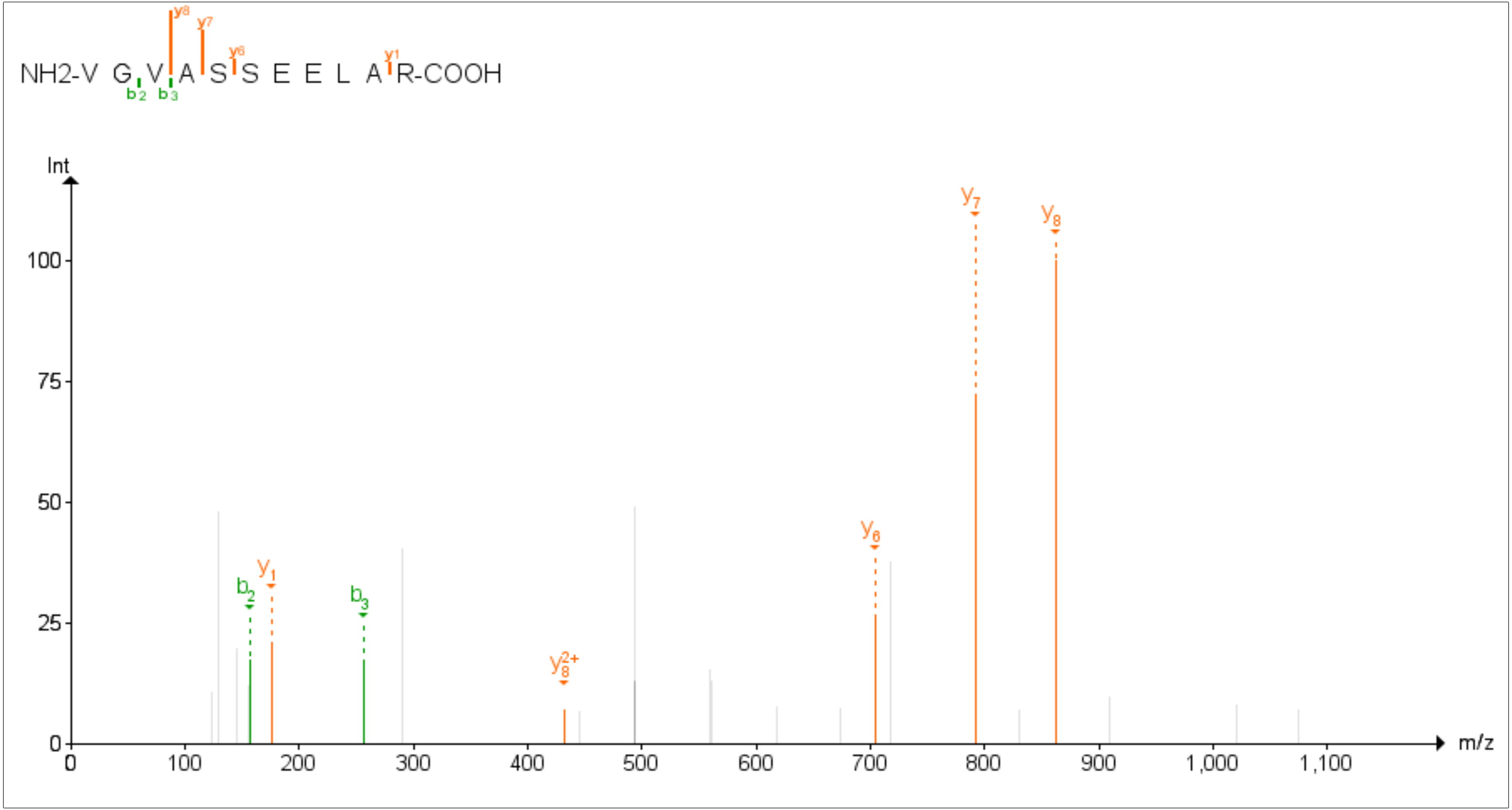

NH2-V G V A S S E E L A R-COOH
y8
y7
y6
y1
b2
b3
Int
100
75
50
25
0
y7
y8
y6
y1
b2
b3
y8 2+
0
100
200
300
400
500
600
700
800
900
1,000
1,100
m/z

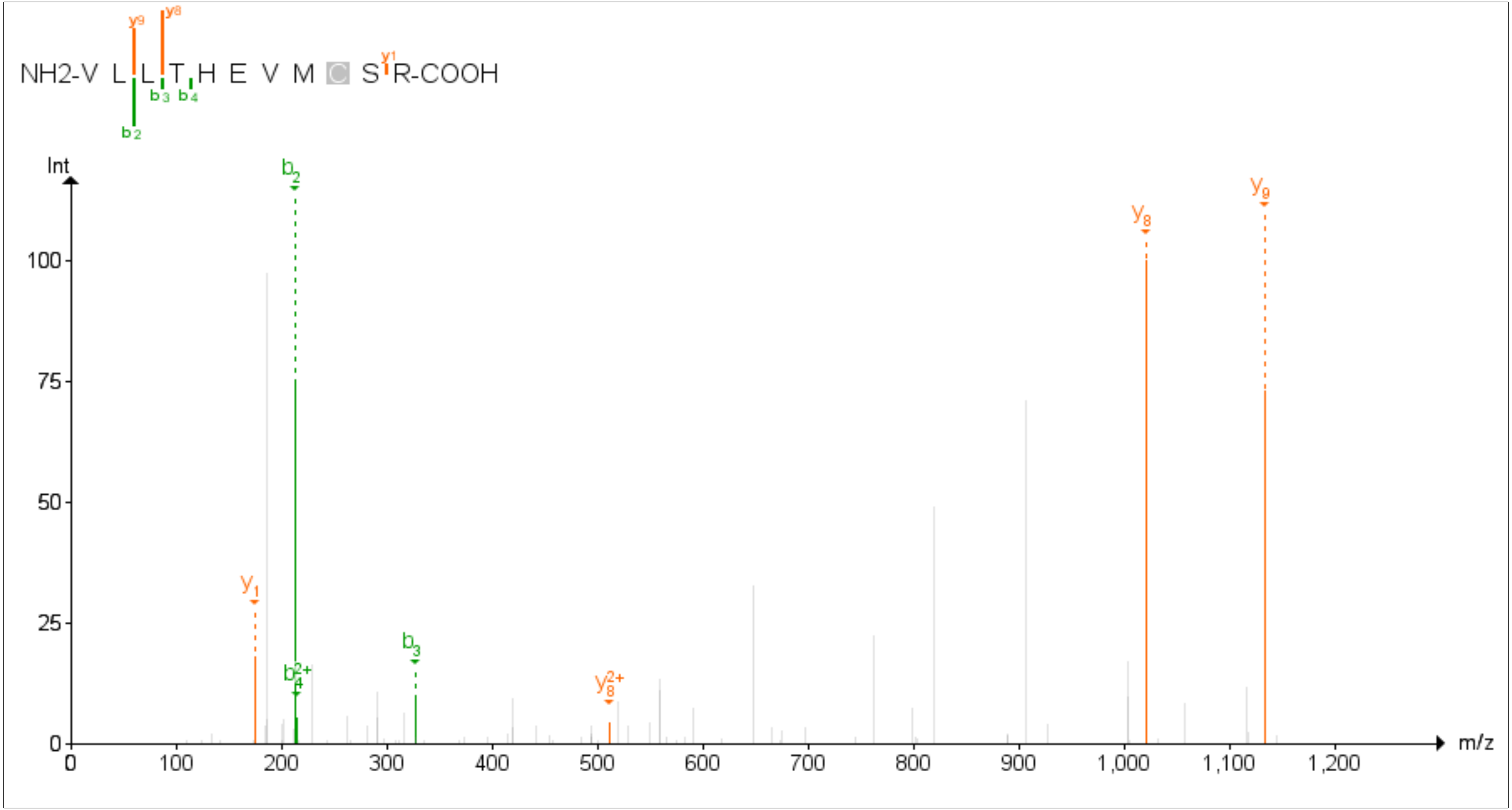

NH2-V L L T H E V M C S R-COOH
y9
y8
y1
b2
b3
b4
Int
100
75
50
25
0
y1
b2
b4 2+
b3
y8 2+
y8
y9
0
100
200
300
400
500
600
700
800
900
1,000
1,100
1,200
m/z

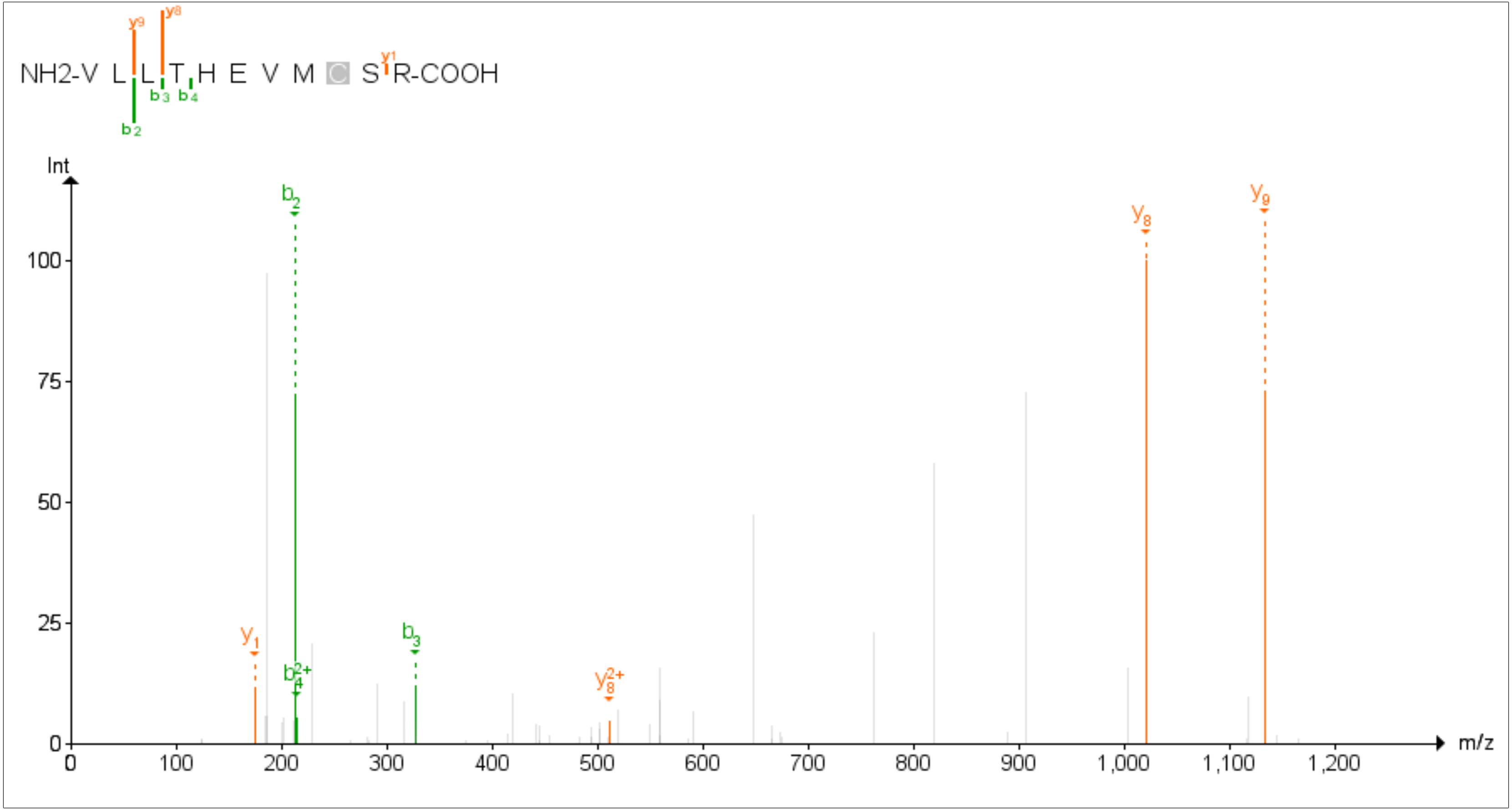

NH2-V L L T H E V M C S R-COOH
y9
y8
y1
b2
b3
b4
Int
100
75
50
25
0
b2
y8
y9
y1
b3
b4 2+
y8 2+
0
100
200
300
400
500
600
700
800
900
1,000
1,100
1,200
m/z

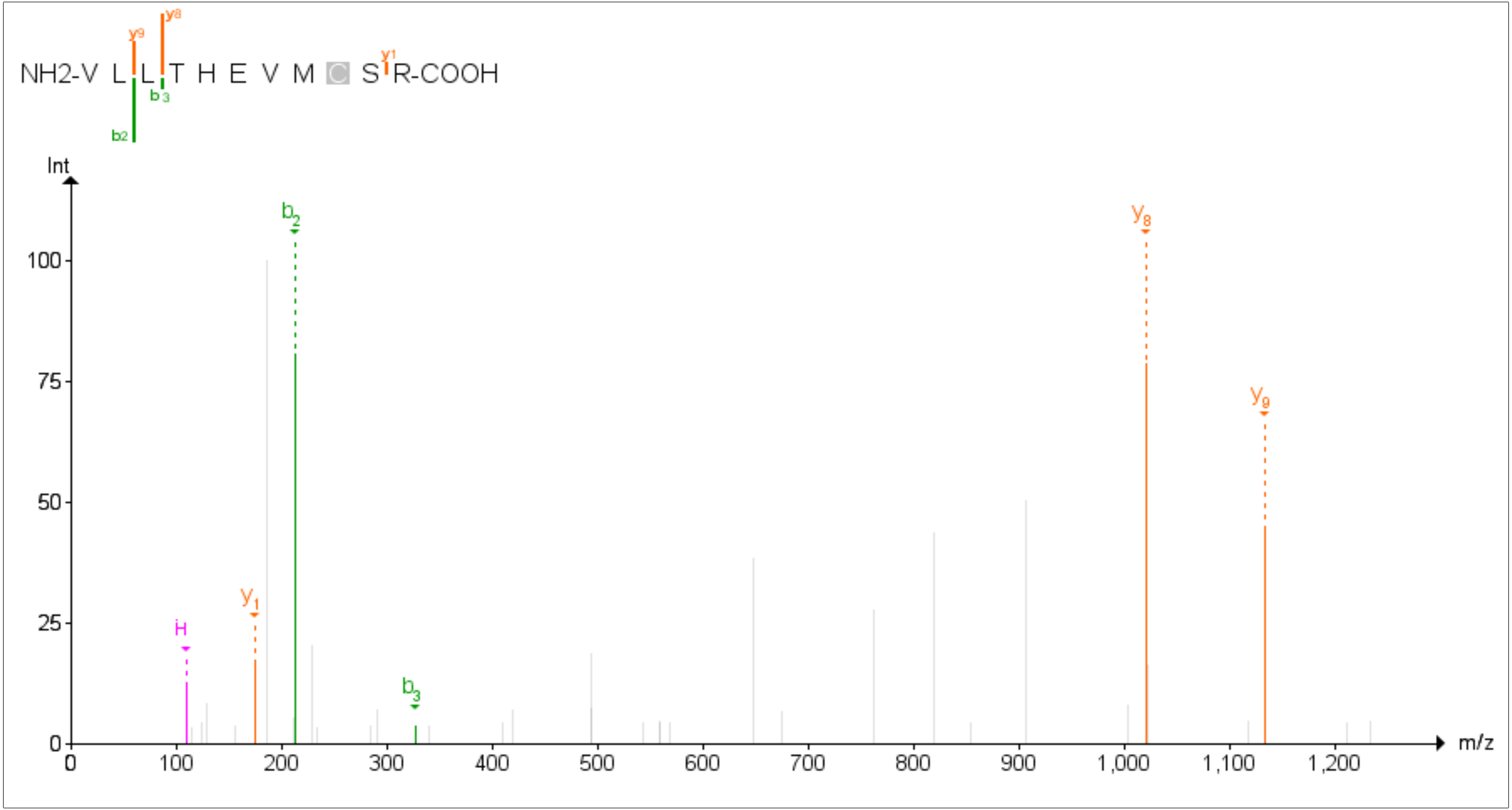

NH2-V L L T H E V M C S R-COOH
y9
y8
y1
b2
b3
Int
100
75
50
25
0
b2
y8
y9
y1
H
b3
0
100
200
300
400
500
600
700
800
900
1,000
1,100
1,200
m/z

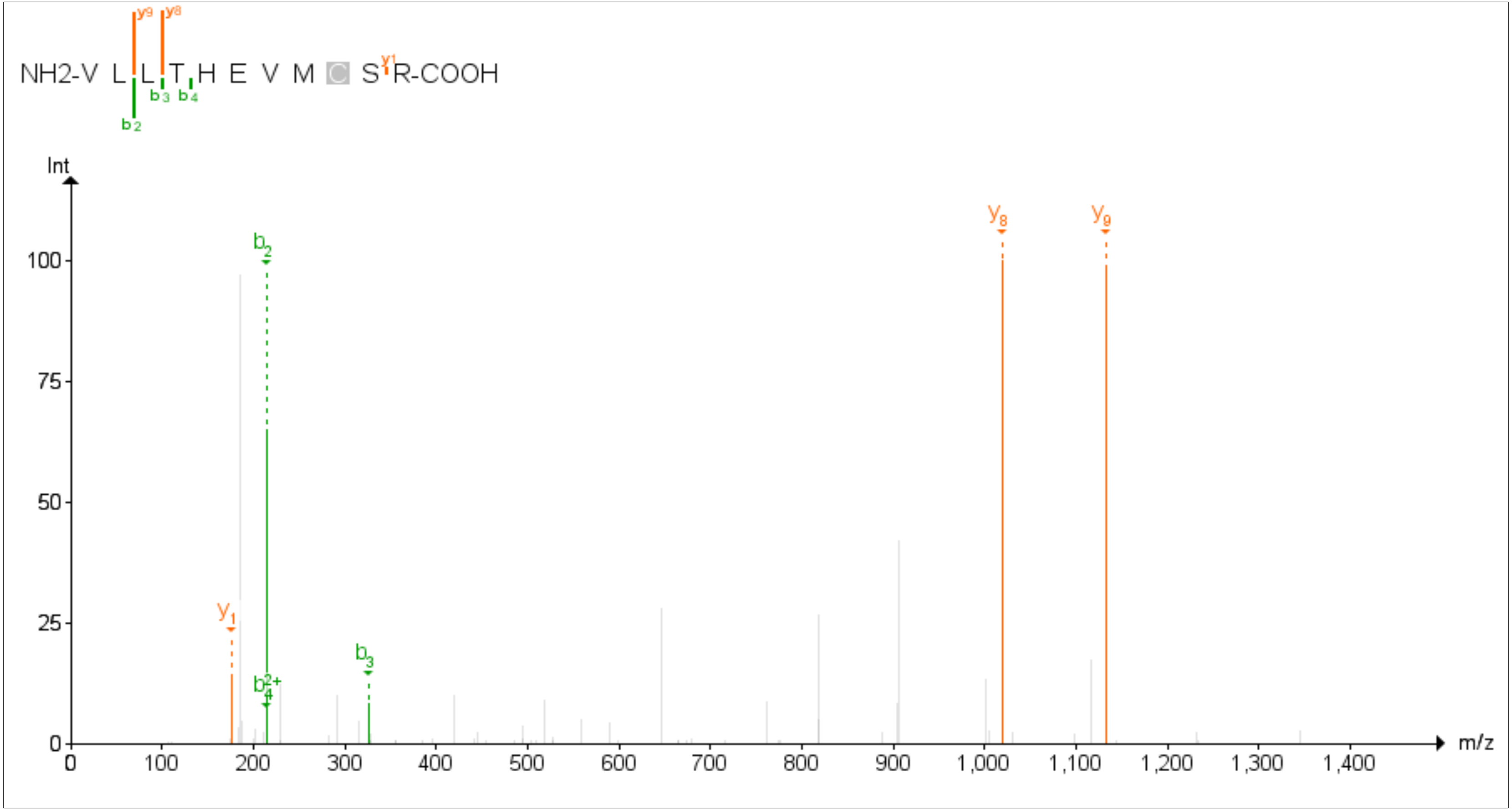

NH2-V L L T H E V M C S R-COOH
y9 y8 y1
b2 b3 b4
Int
100
75
50
25
0
b2
y8
y9
y1
b3
b4 2+
0 100 200 300 400 500 600 700 800 900 1,000 1,100 1,200 1,300 1,400
m/z

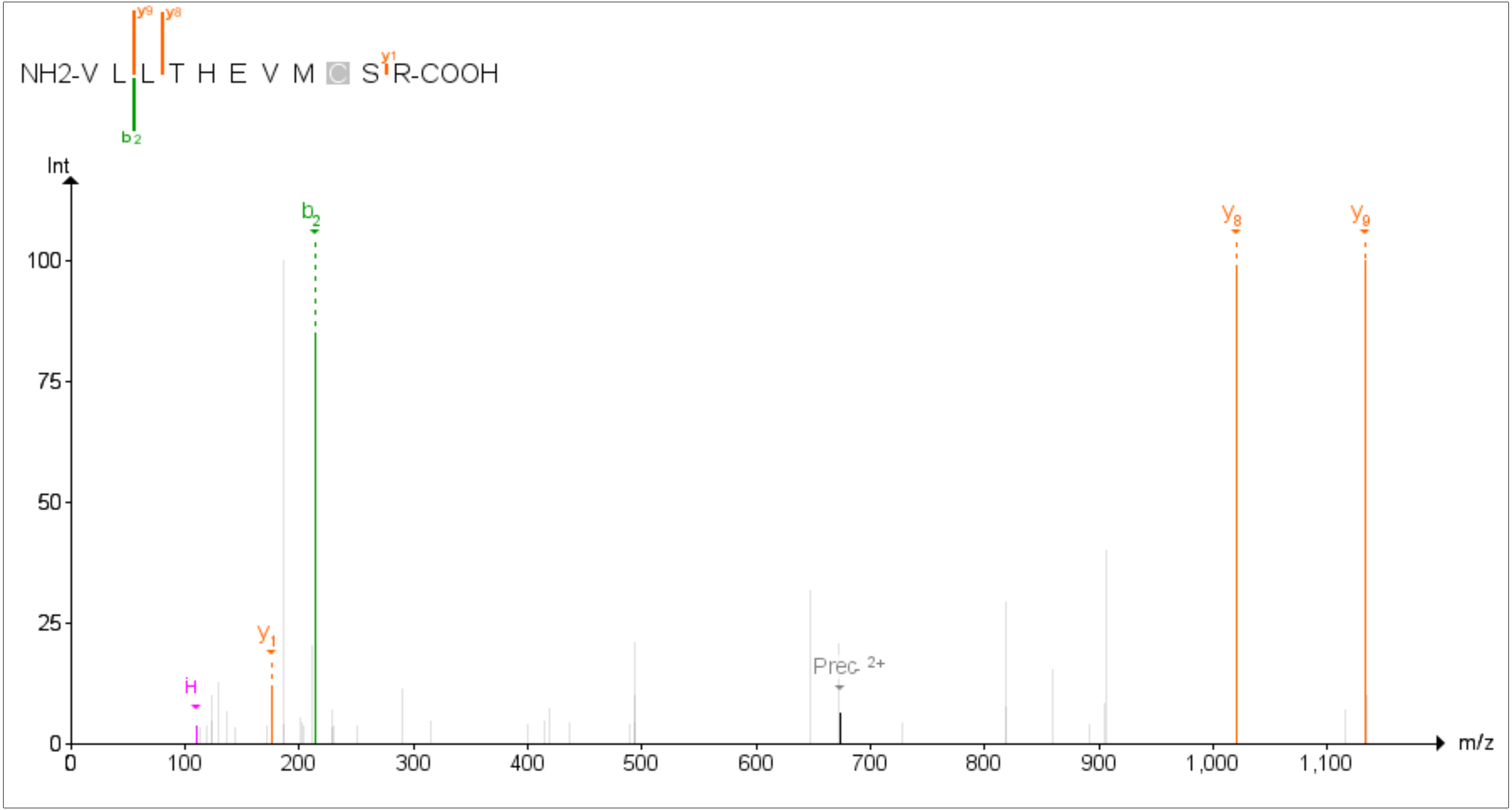

NH2-V L L T H E V M C S R-COOH
y9
y8
y1
b2
Int
100
75
50
25
0
b2
y8
y9
y1
H
Prec 2+
0
100
200
300
400
500
600
700
800
900
1,000
1,100
m/z

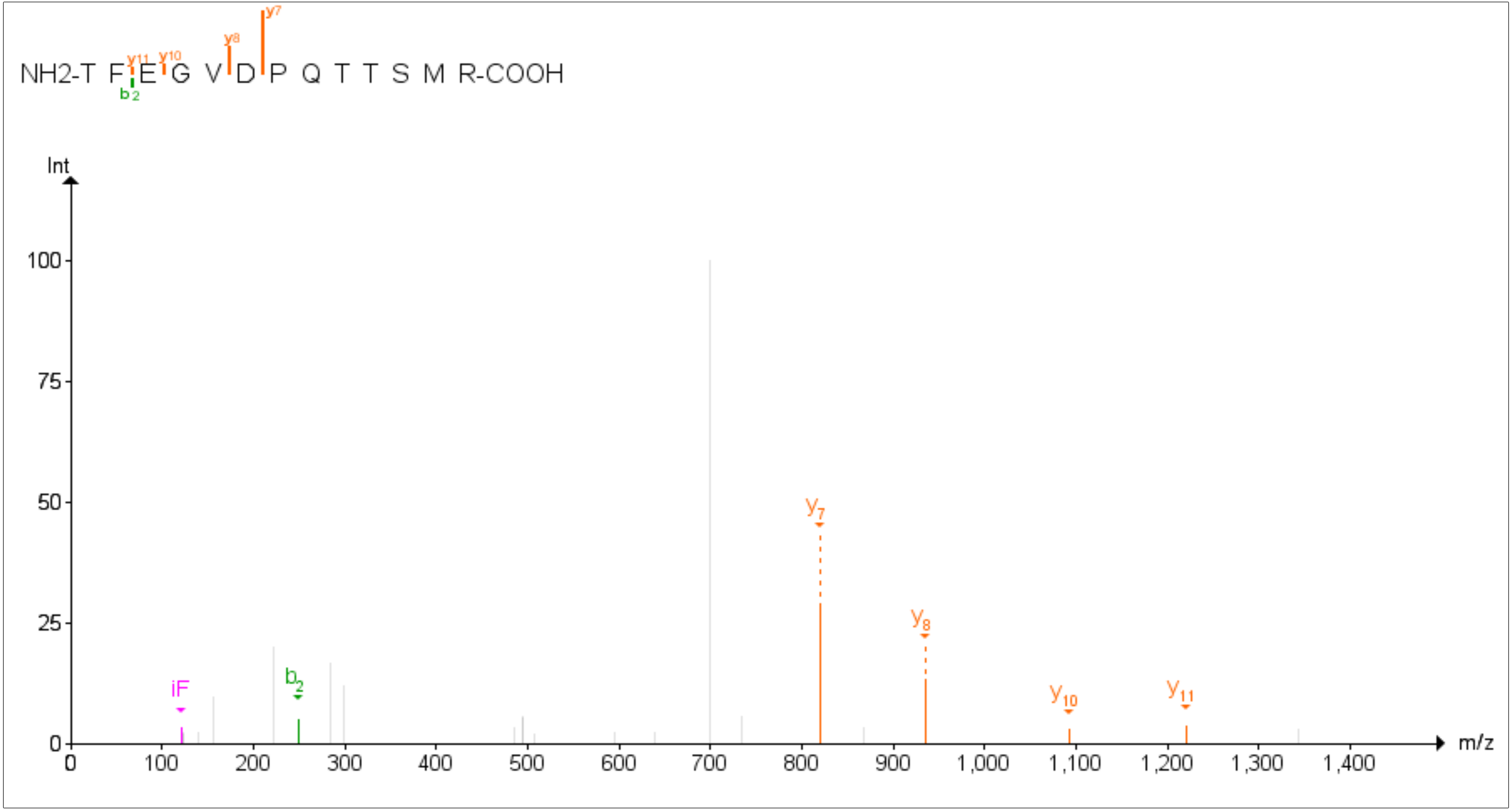

NH2-T F E G V D P Q T T S M R-COOH
y7
y8
y11
y10
b2
Int
100
75
50
25
0
y7
y8
y10
y11
iF
b2
0
100
200
300
400
500
600
700
800
900
1,000
1,100
1,200
1,300
1,400
m/z

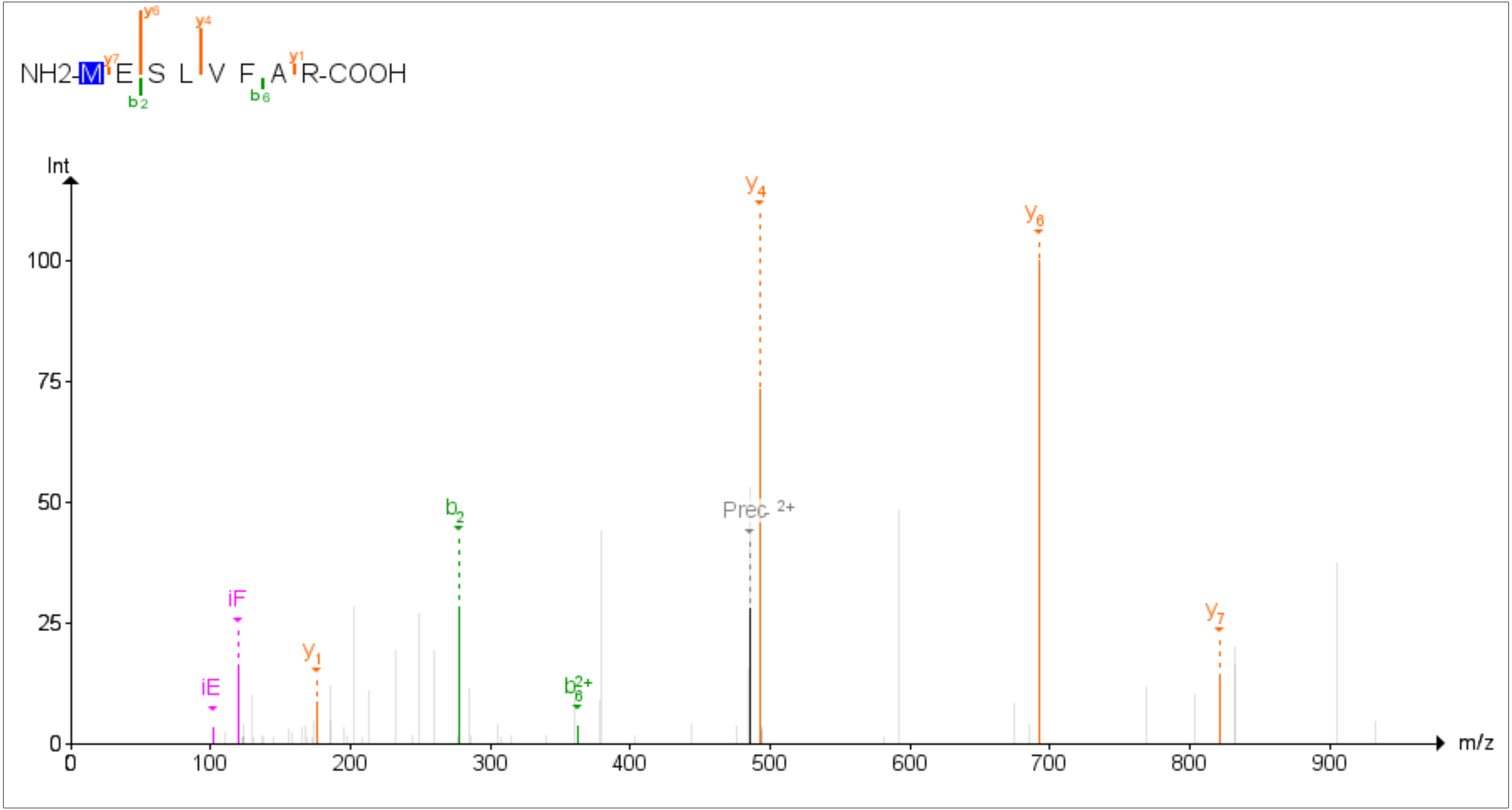

NH2-M E S L V F A R-COOH
y6
y7
y4
y1
b2
b6
Int
m/z
y4
y6
y7
y1
b2
b6 2+
Prec. 2+
iF
iE

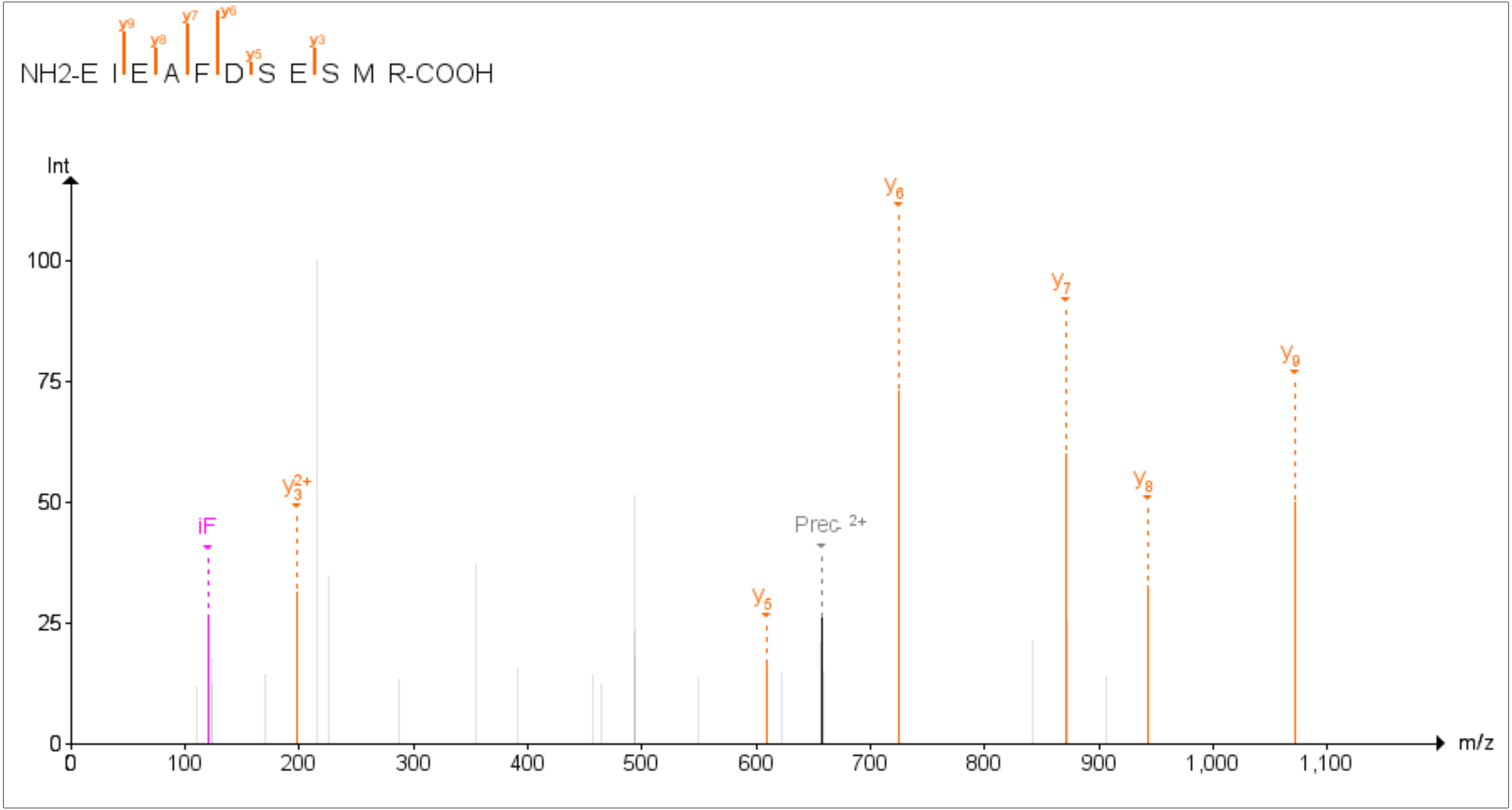

NH2-E I E A F D S E S M R-COOH
y9
y8
y7
y6
y5
y3
Int
100
75
50
25
0
iF
y_3^{2+}
y_5
Prec. 2+
y_6
y_7
y_8
y_9
0
100
200
300
400
500
600
700
800
900
1,000
1,100
m/z

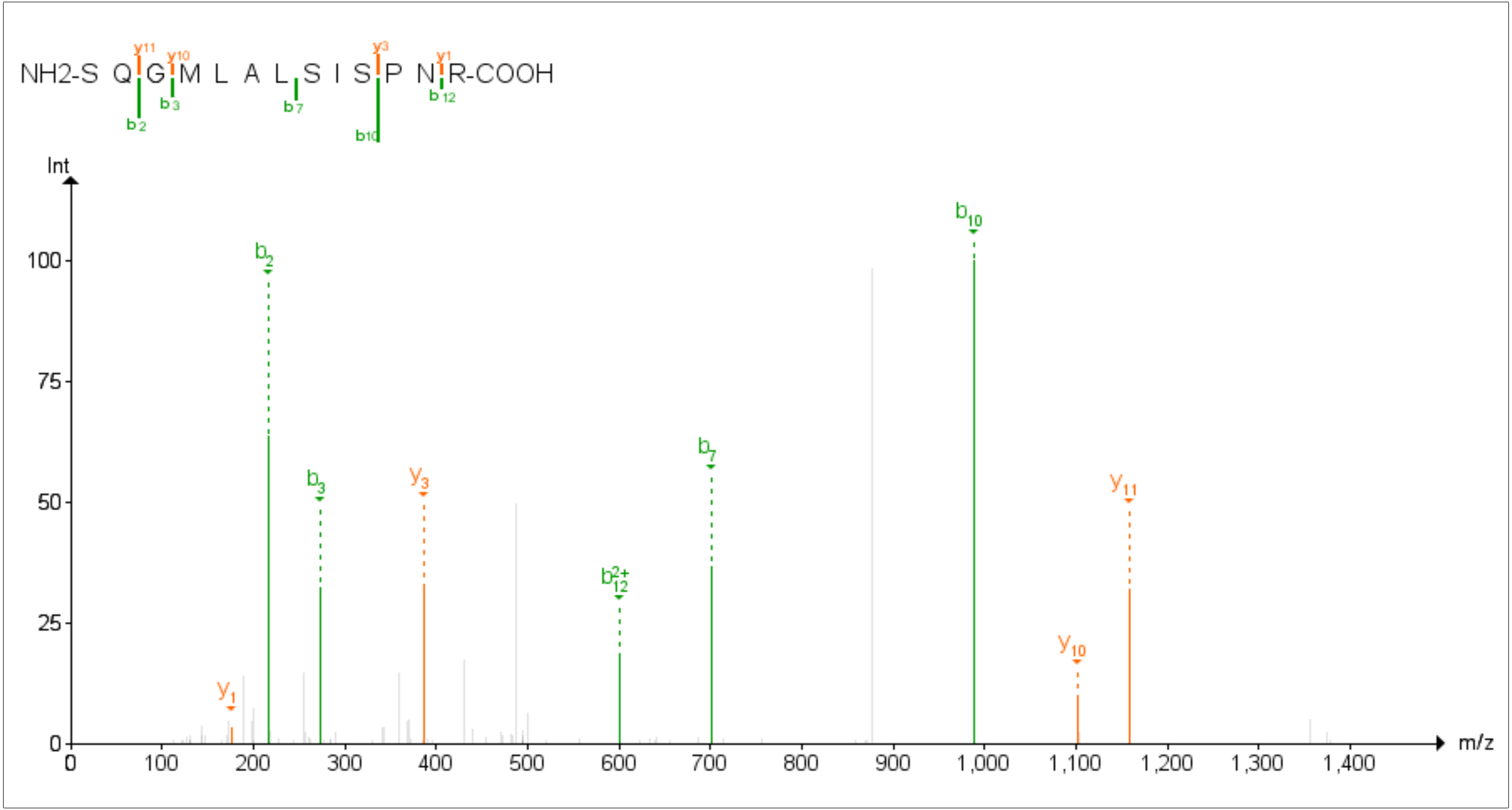

NH2-S Q G M L A L S I S P N R-COOH
y11
y10
y3
y1
b2
b3
b7
b10
b12
Int
100
75
50
25
0
m/z
0
100
200
300
400
500
600
700
800
900
1,000
1,100
1,200
1,300
1,400
b2
b3
y3
y1
b12 2+
b7
b10
y10
y11

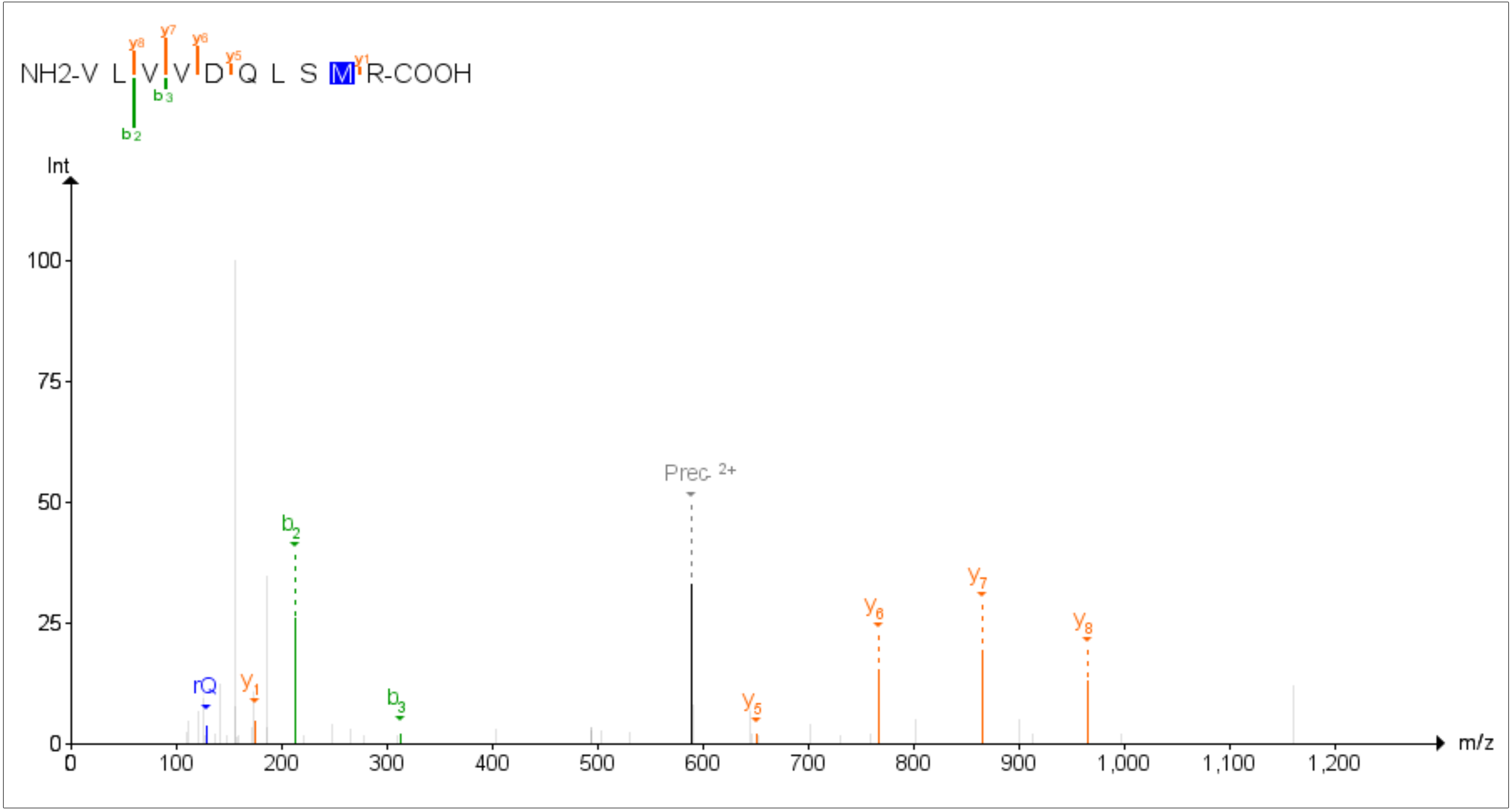

NH2-V L V V D Q L S M R-COOH
y8
y7
y6
y5
y1
b2
b3
Int
100
75
50
25
0
Prec 2+
b2
b3
rQ
y1
y5
y6
y7
y8
0
100
200
300
400
500
600
700
800
900
1,000
1,100
1,200
m/z

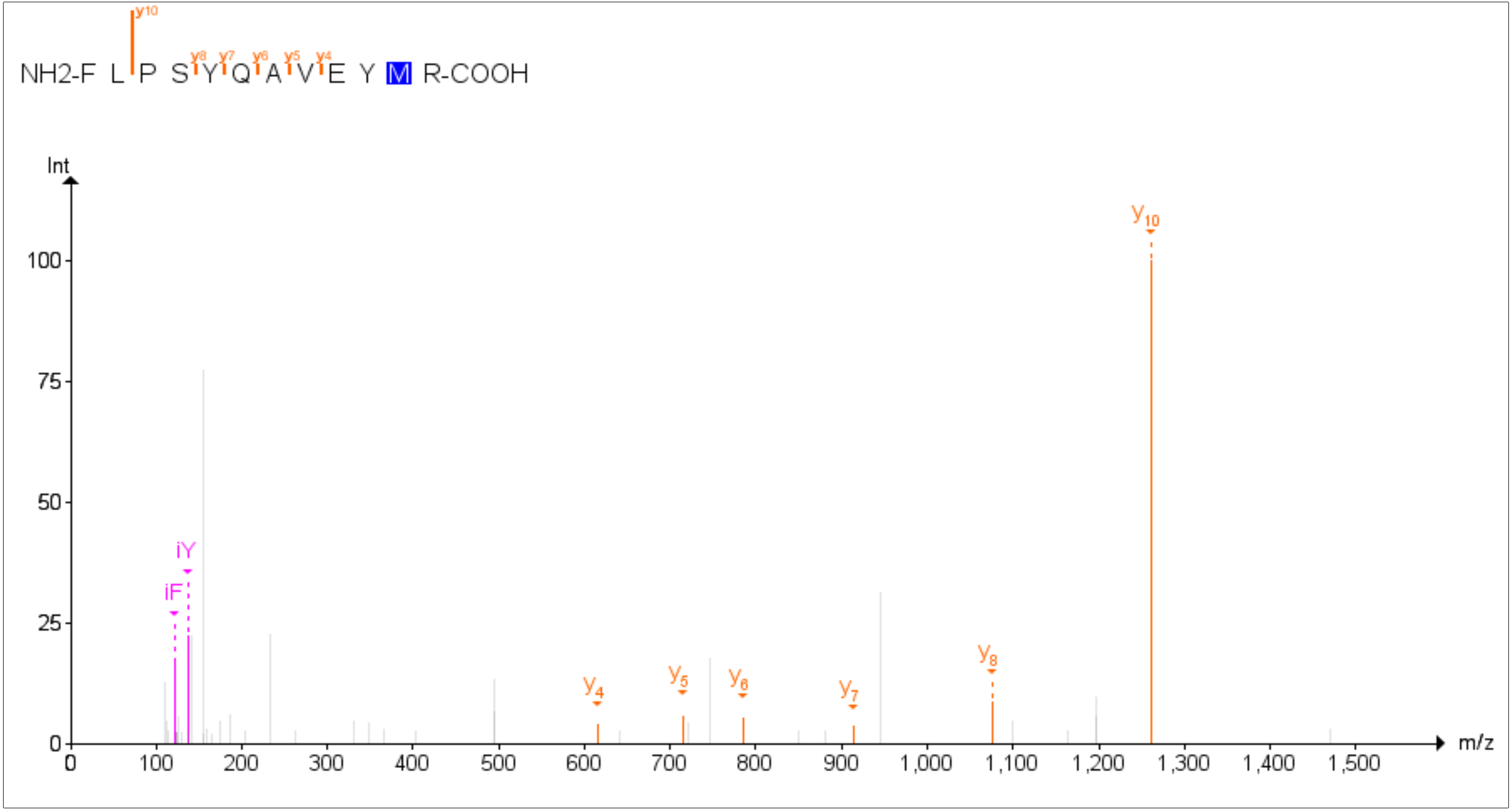

NH2-F L P S Y Q A V E Y M R-COOH
y10
y8
y7
y6
y5
y4
Int
m/z
100
75
50
25
0
0
100
200
300
400
500
600
700
800
900
1,000
1,100
1,200
1,300
1,400
1,500
y10
iY
iF
y4
y5
y6
y7
y8

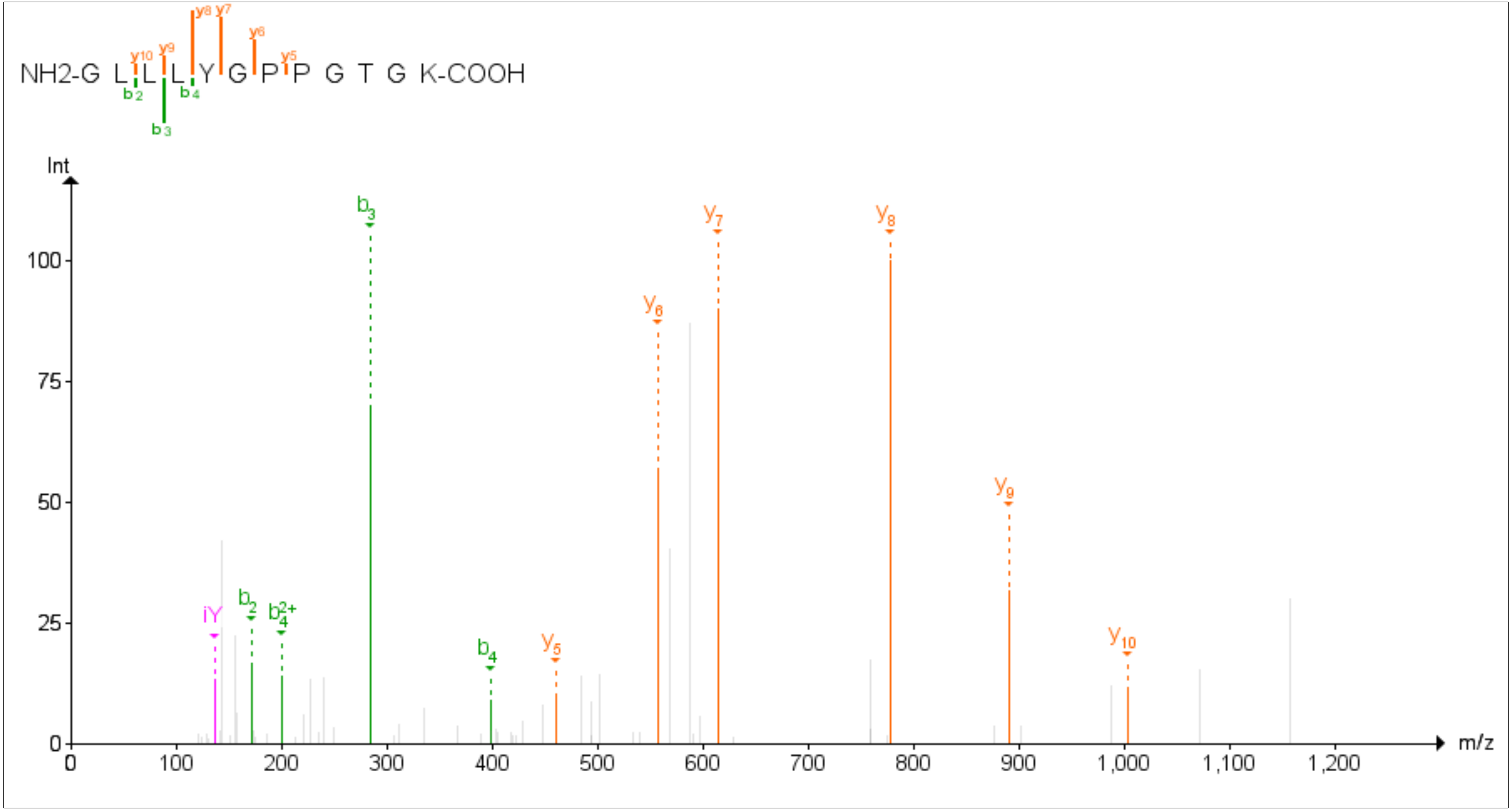

NH2-G L L L Y G P P G T G K-COOH
y10 y9 y8 y7 y6 y5
b2 b3 b4
Int
m/z
iY
b2
b4 2+
b3
b4
y5
y6
y7
y8
y9
y10
0
25
50
75
100
0 100 200 300 400 500 600 700 800 900 1,000 1,100 1,200

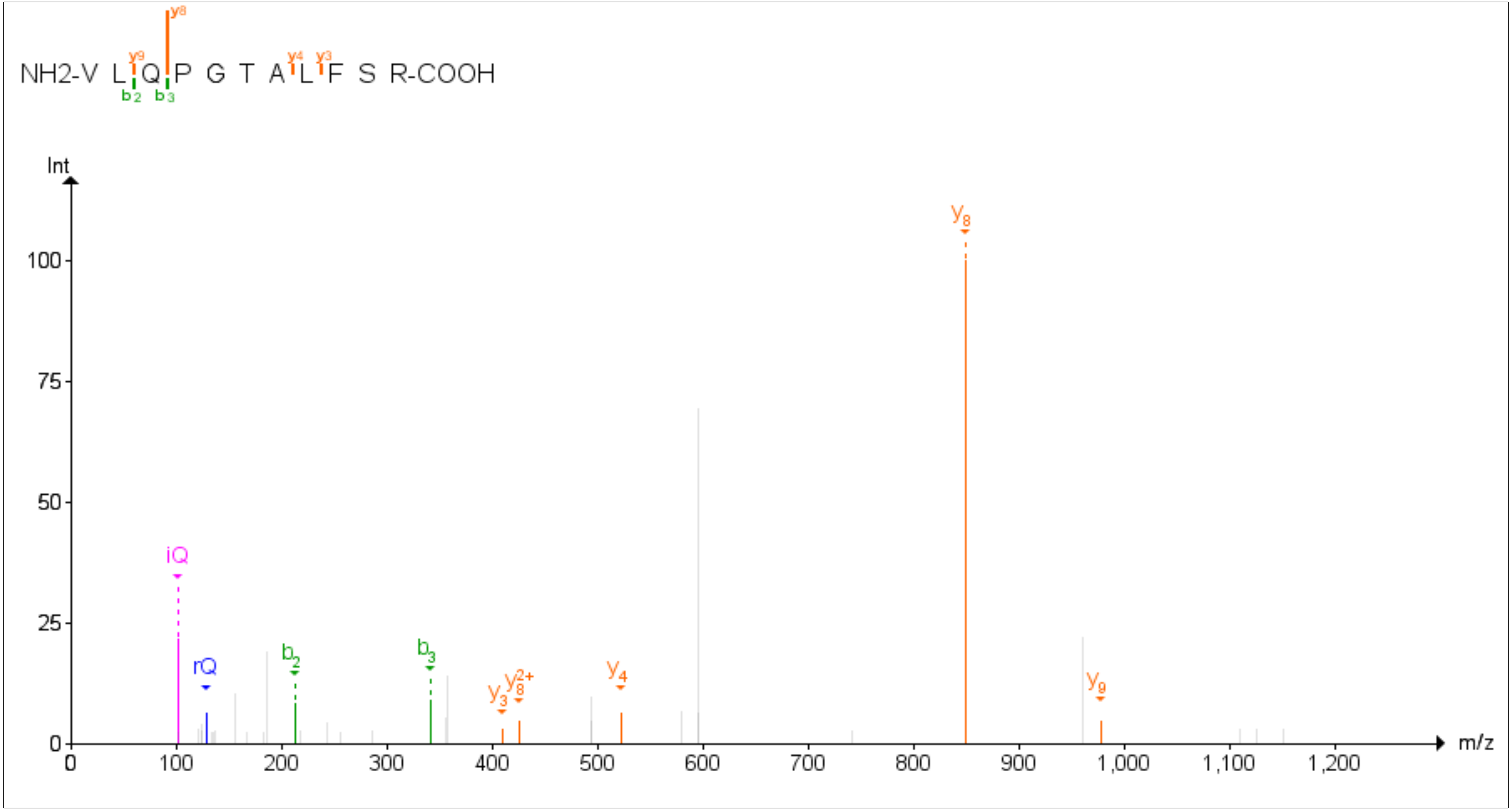

NH2-V L Q P G T A L F S R-COOH
y9
y8
y4
y3
b2
b3
Int
100
75
50
25
0
y8
iQ
rQ
b2
b3
y3
y8 2+
y4
y9
0
100
200
300
400
500
600
700
800
900
1,000
1,100
1,200
m/z

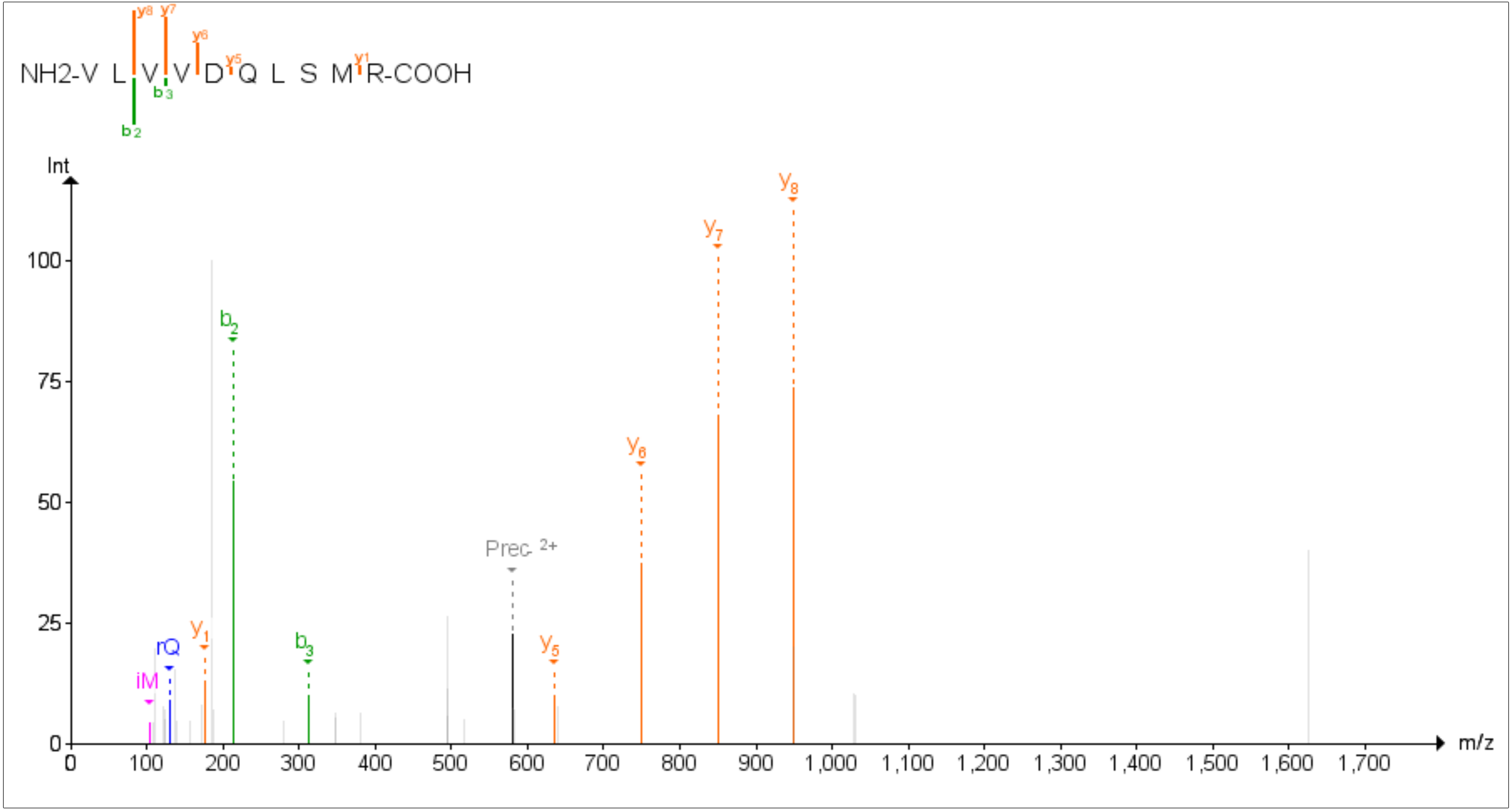

NH2-V L V V D Q L S M R-COOH
y8
y7
y6
y5
y1
b2
b3
Int
100
75
50
25
0
b2
y8
y7
y6
Prec 2+
rQ
y1
iM
b3
y5
0
100
200
300
400
500
600
700
800
900
1,000
1,100
1,200
1,300
1,400
1,500
1,600
1,700
m/z

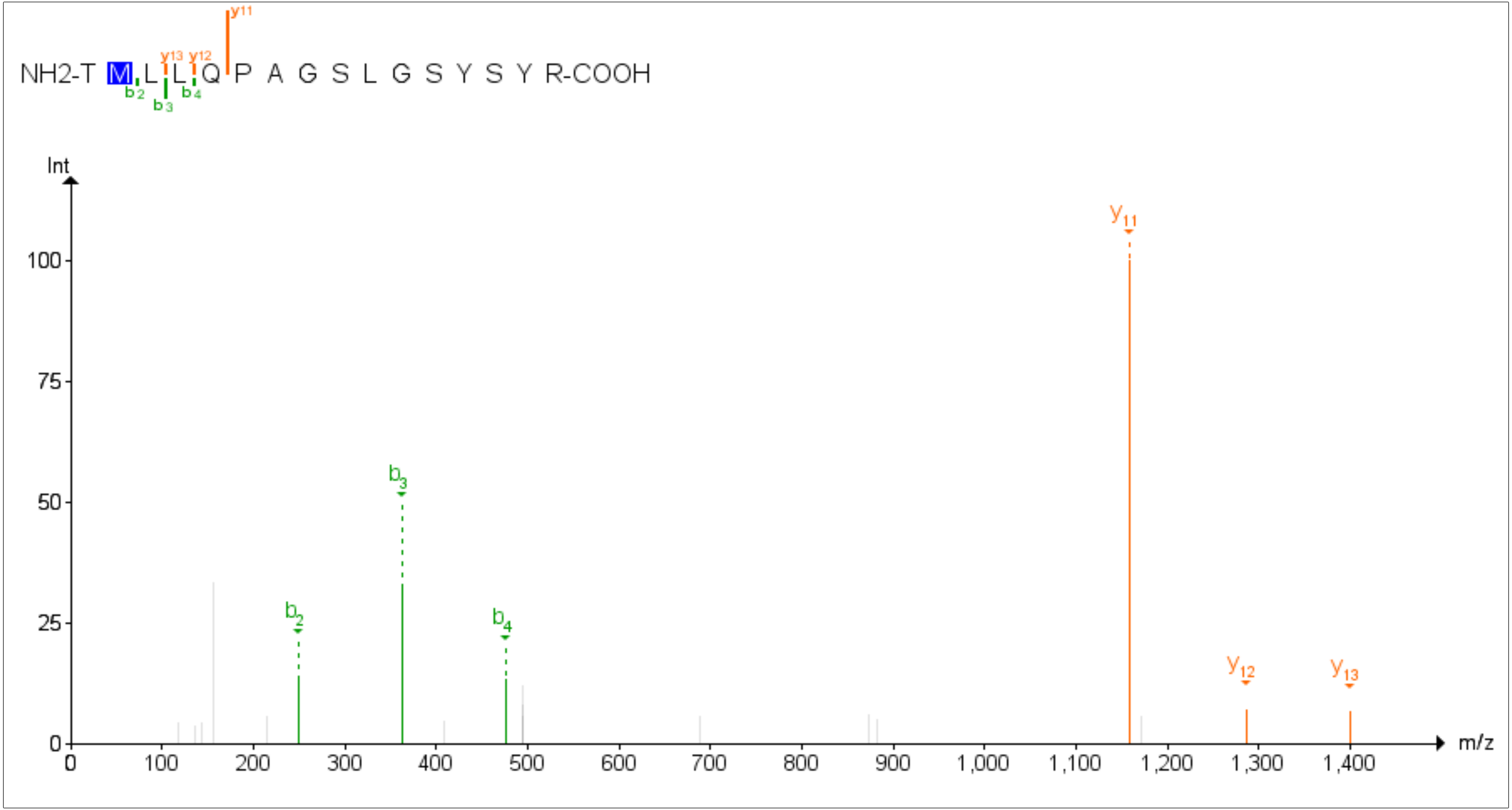

NH2-T M L L Q P A G S L G S Y S Y R-COOH
y11
y13
y12
b2
b3
b4
Int
100
75
50
25
0
y11
b3
b2
b4
y12
y13
m/z
0
100
200
300
400
500
600
700
800
900
1,000
1,100
1,200
1,300
1,400

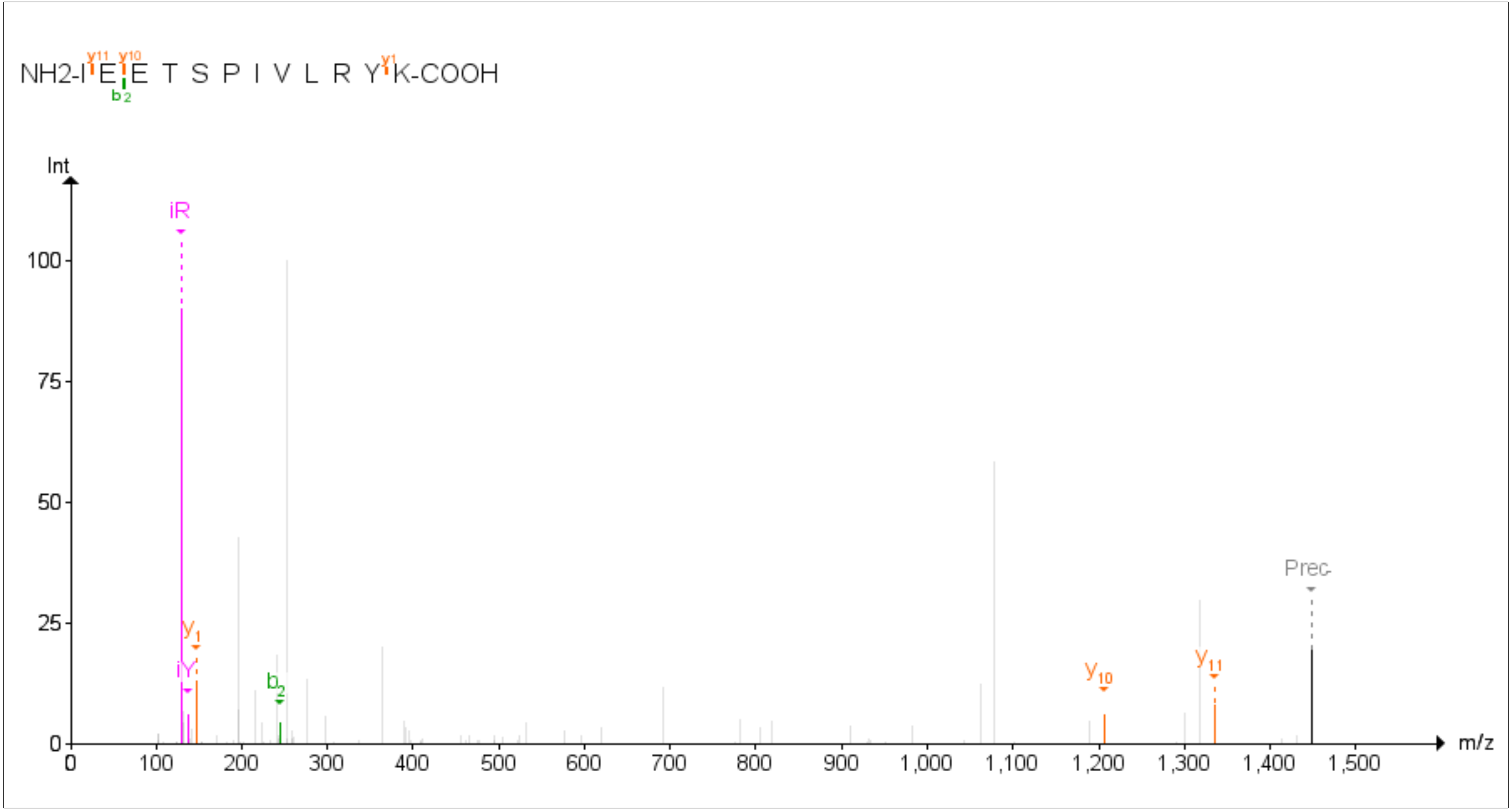

NH2-I E E T S P I V L R Y K-COOH
y11
y10
y1
b2
Int
iR
iY
y1
b2
y10
y11
Prec
100
75
50
25
0
0
100
200
300
400
500
600
700
800
900
1,000
1,100
1,200
1,300
1,400
1,500
m/z

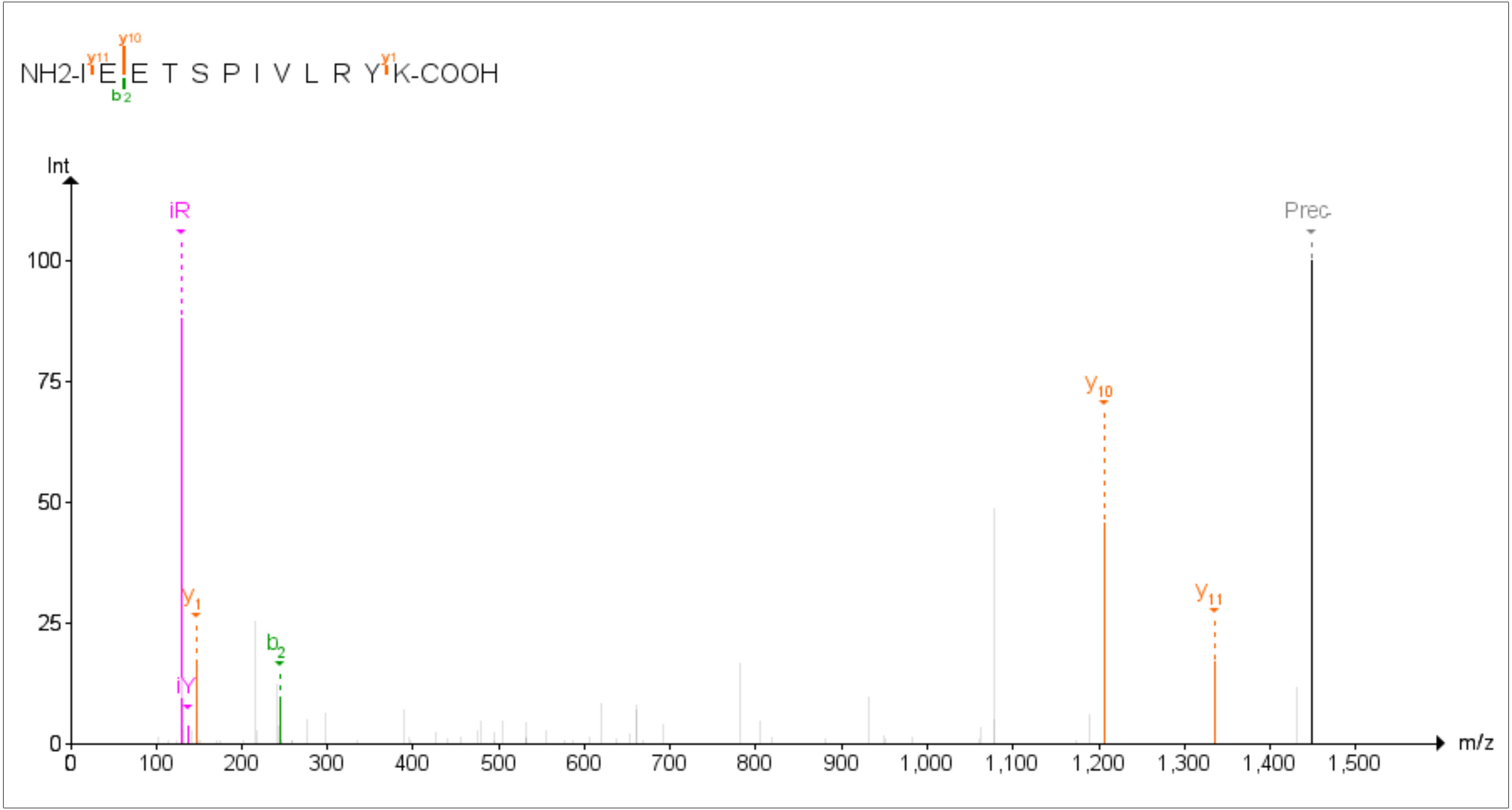

NH2-I E E T S P I V L R Y K-COOH
y11
y10
b2
y1
Int
100
75
50
25
0
iR
iY
y1
b2
y10
y11
Prec
0
100
200
300
400
500
600
700
800
900
1,000
1,100
1,200
1,300
1,400
1,500
m/z

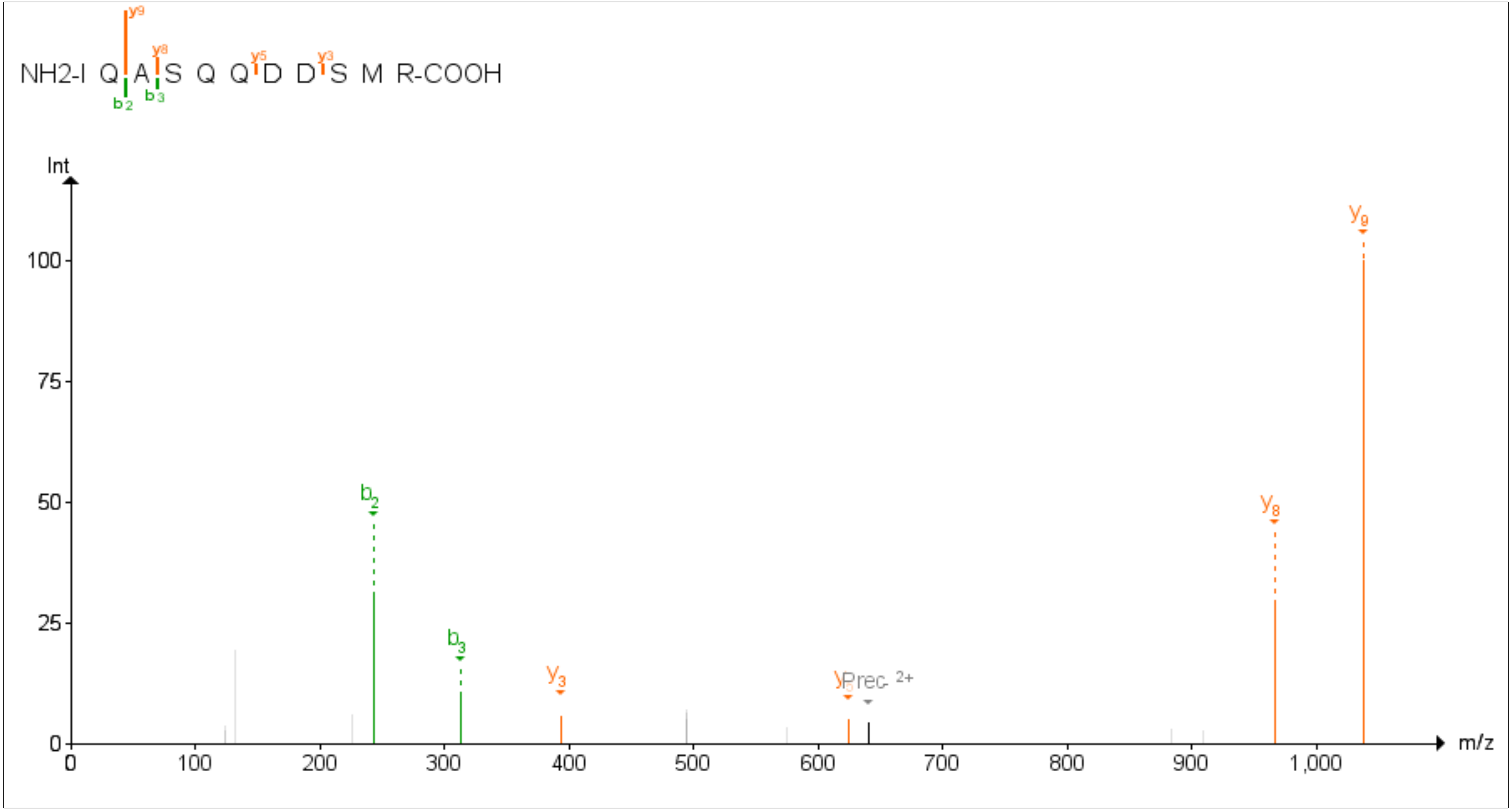

NH2-I Q A S Q Q D D S M R-COOH
y9
y8
y5
y3
b2
b3
Int
100
75
50
25
0
m/z
0
100
200
300
400
500
600
700
800
900
1,000
y9
y8
b2
b3
y3
Prec. 2+

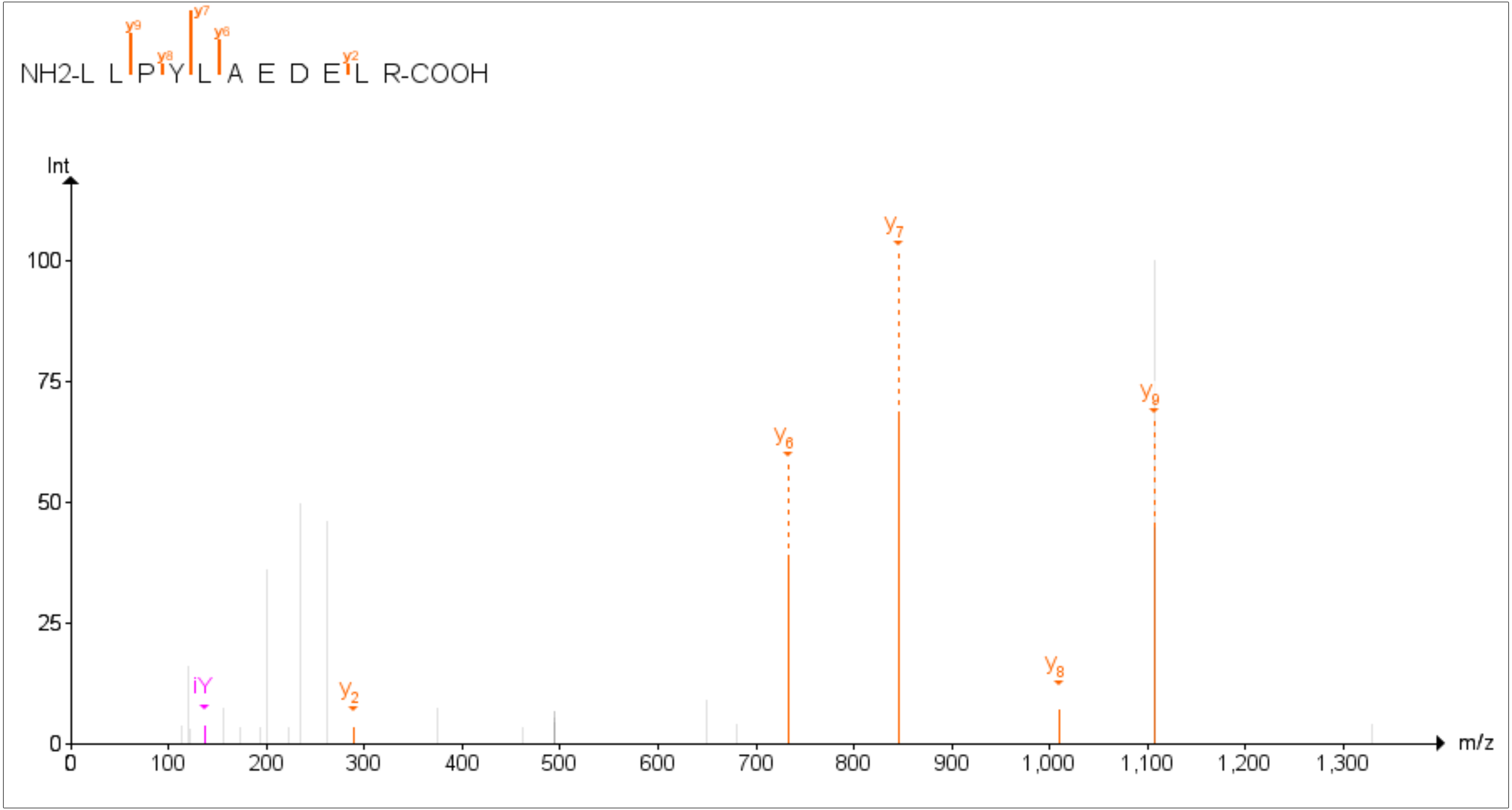
NH2-L L P Y L A E D E L R-COOH
y9 y8 y7 y6 y2
Int
100
75
50
25
0
y7
y6
y9
y8
iY
y2
0 100 200 300 400 500 600 700 800 900 1,000 1,100 1,200 1,300
m/z

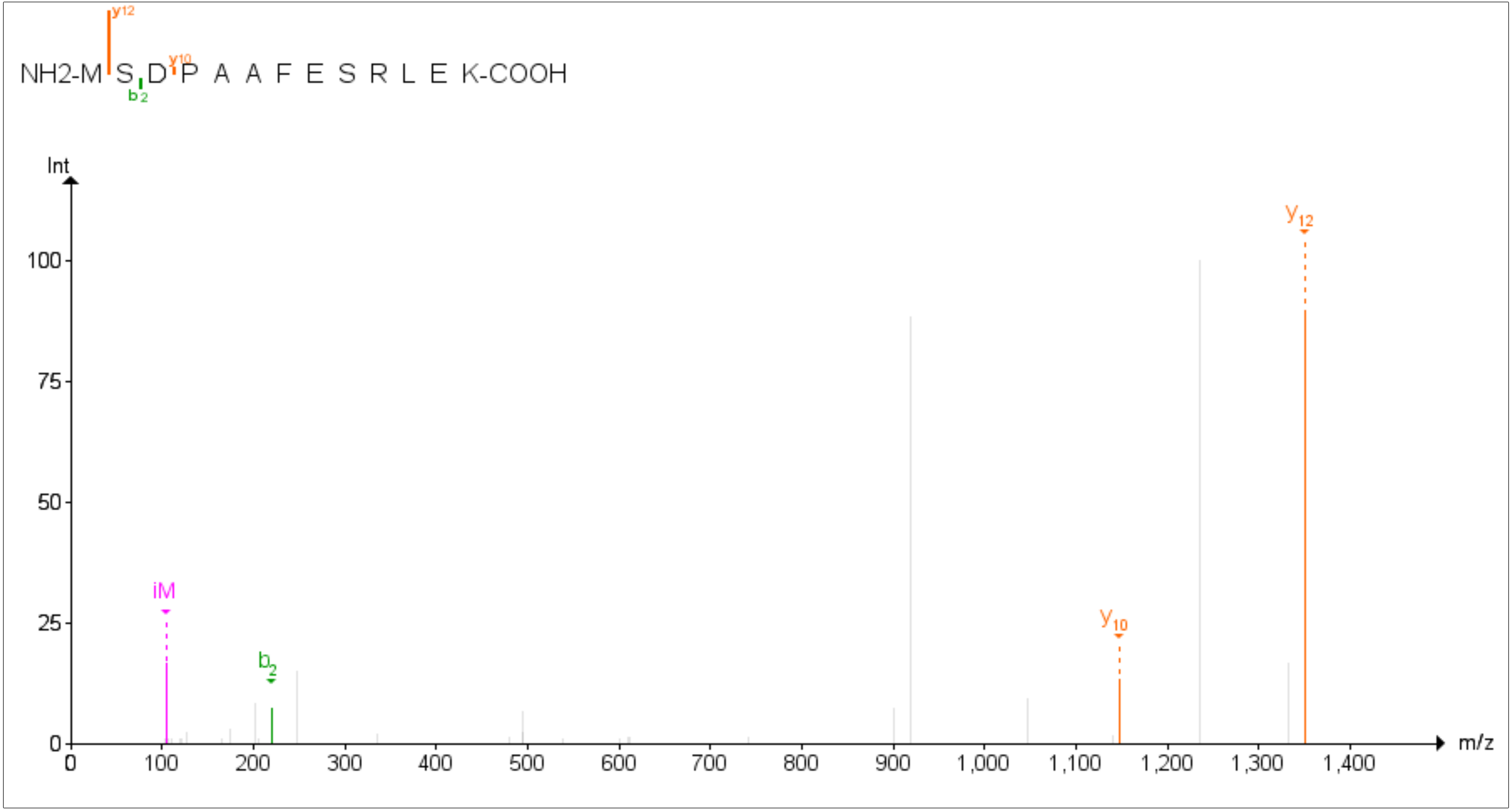

y12
y10
NH2-M S D P A A F E S R L E K-COOH
b2
Int
100
75
50
25
0
iM
b2
y10
y12
0
100
200
300
400
500
600
700
800
900
1,000
1,100
1,200
1,300
1,400
m/z

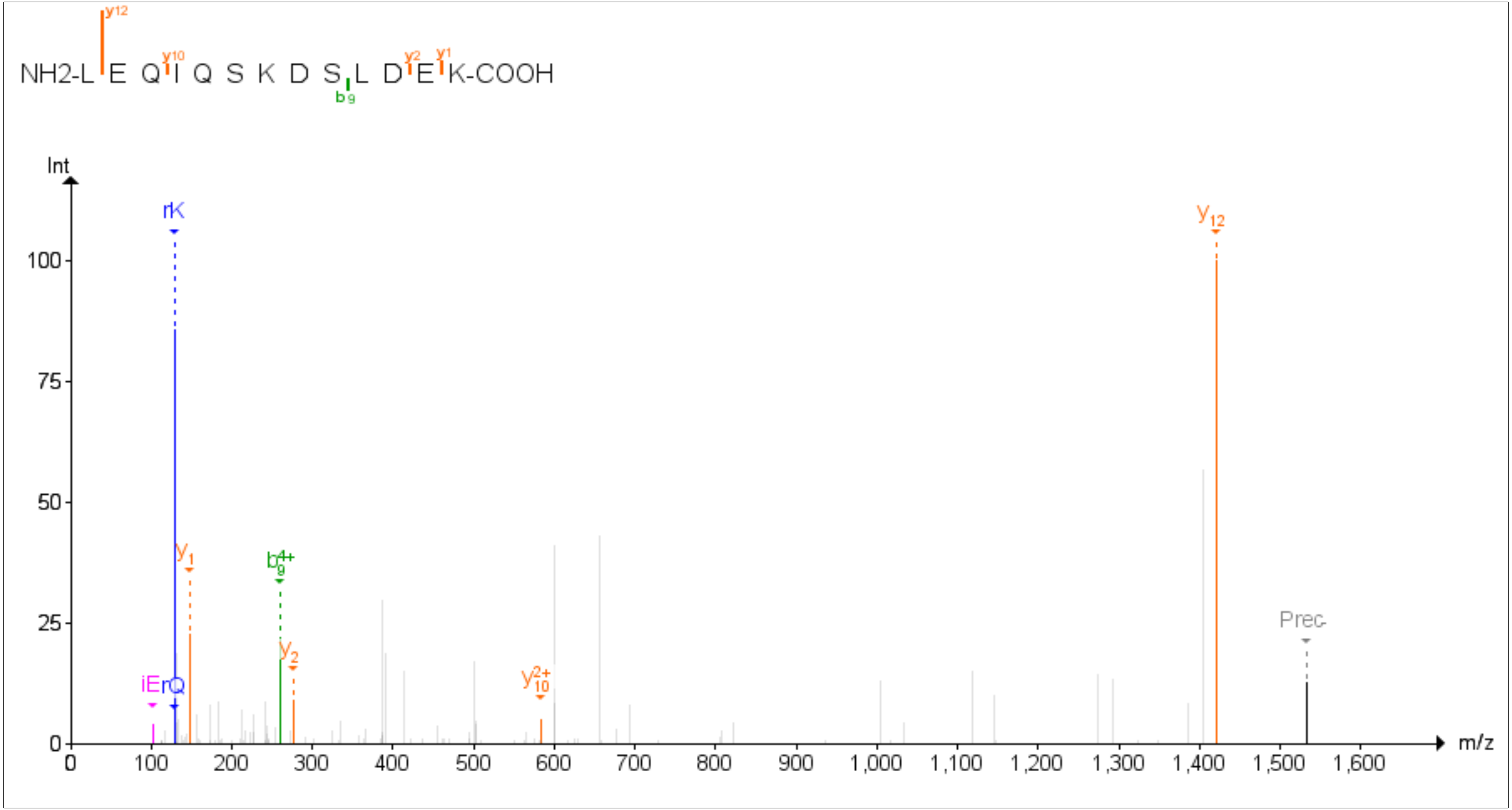

NH2-L E Q I Q S K D S L D E K-COOH
y12
y10
y2
y1
b9
Int
100
75
50
25
0
rK
y12
y1
b9 4+
y2
iE
rQ
y10 2+
Prec
0
100
200
300
400
500
600
700
800
900
1,000
1,100
1,200
1,300
1,400
1,500
1,600
m/z

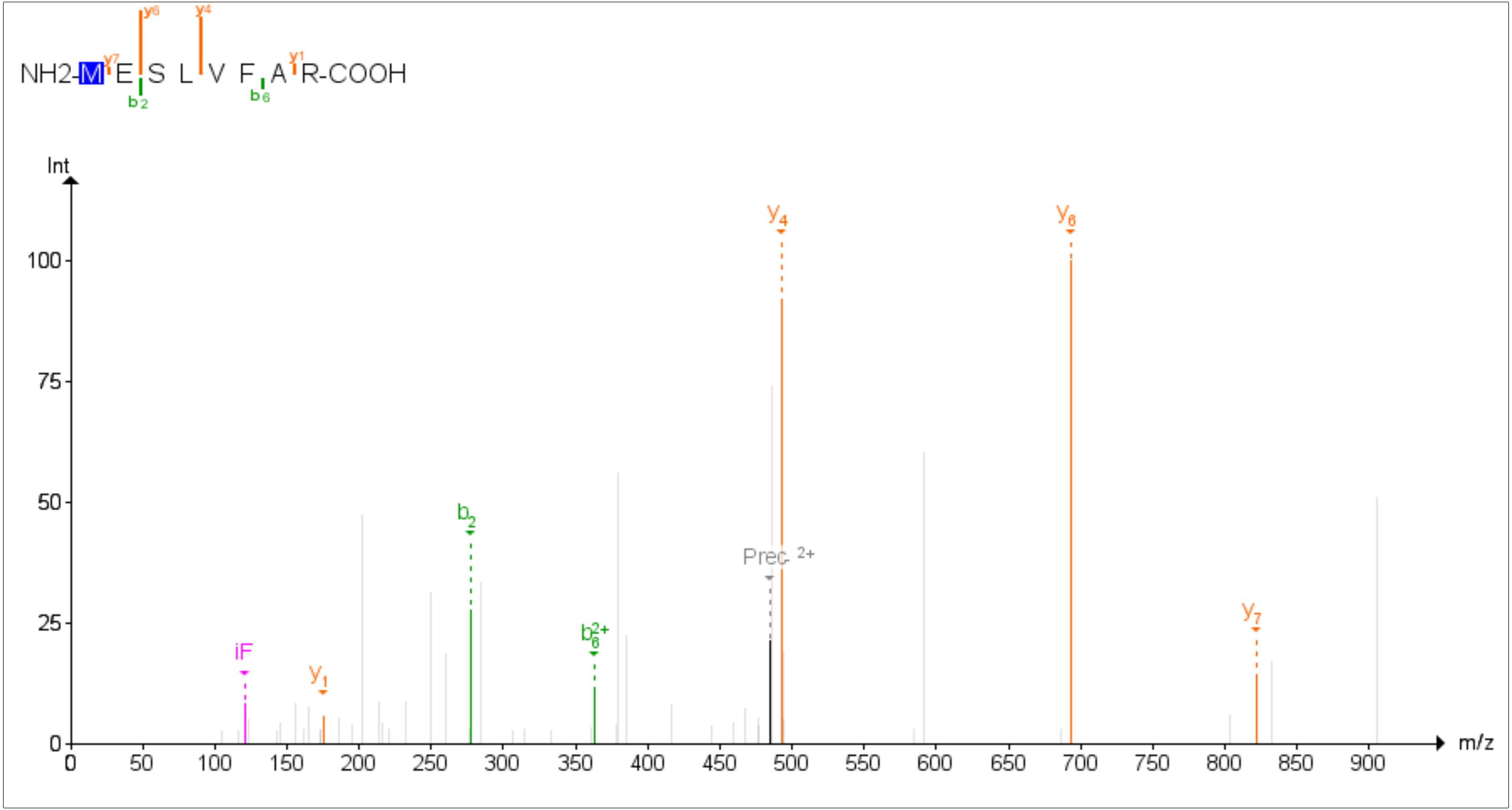

NH2-M E S L V F A R-COOH
y7
y6
y4
y1
b2
b6
Int
100
75
50
25
0
y4
y6
b2
Prec 2+
y7
iF
y1
b6 2+
0
50
100
150
200
250
300
350
400
450
500
550
600
650
700
750
800
850
900
m/z

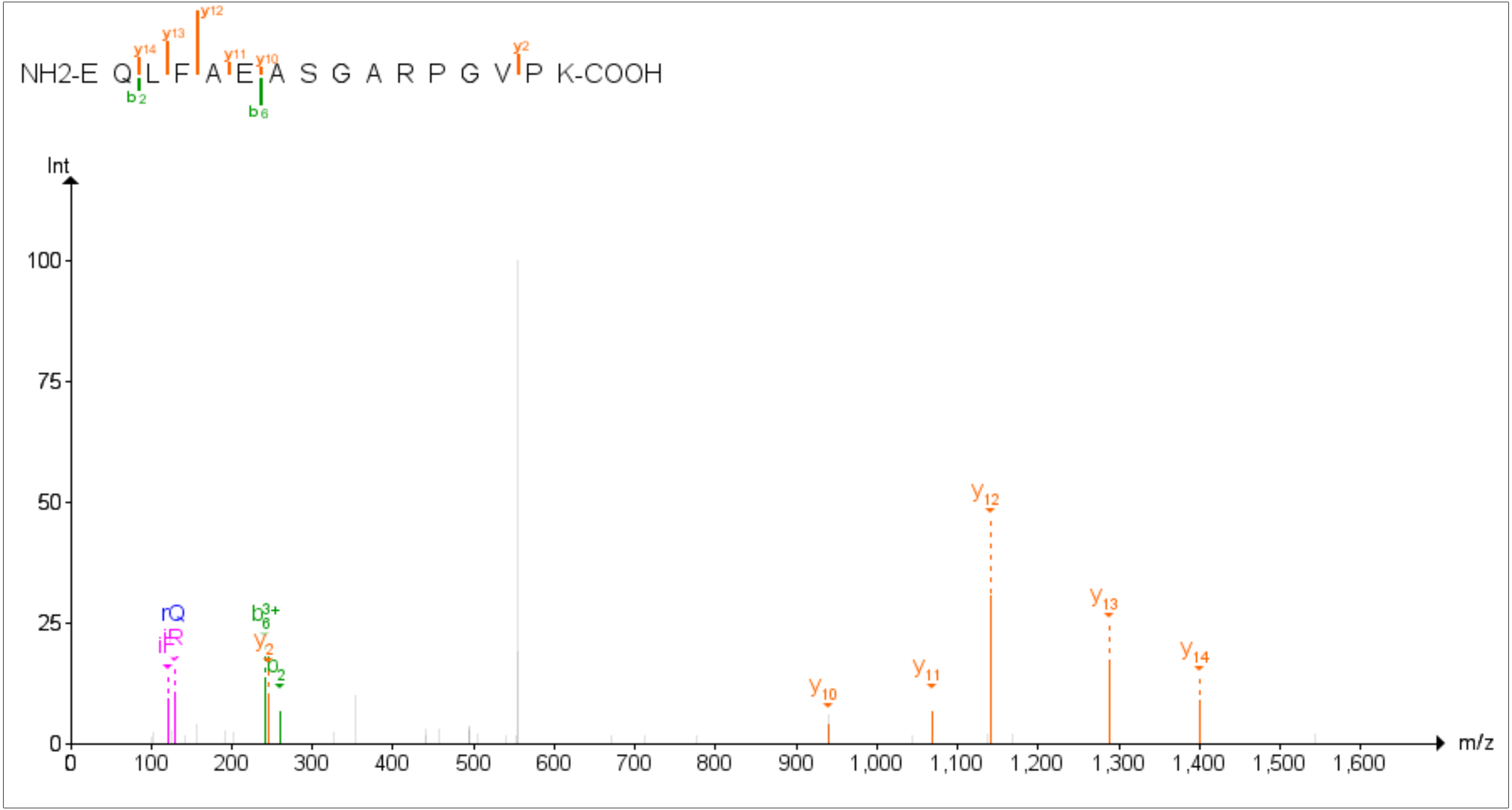

NH2-E Q L F A E A S G A R P G V P K-COOH
y14
y13
y12
y11
y10
y2
b2
b6
Int
100
75
50
25
0
rQ
iR
iF
b_6^{3+}
y_2
b_2
y_{10}
y_{11}
y_{12}
y_{13}
y_{14}
0
100
200
300
400
500
600
700
800
900
1,000
1,100
1,200
1,300
1,400
1,500
1,600
m/z

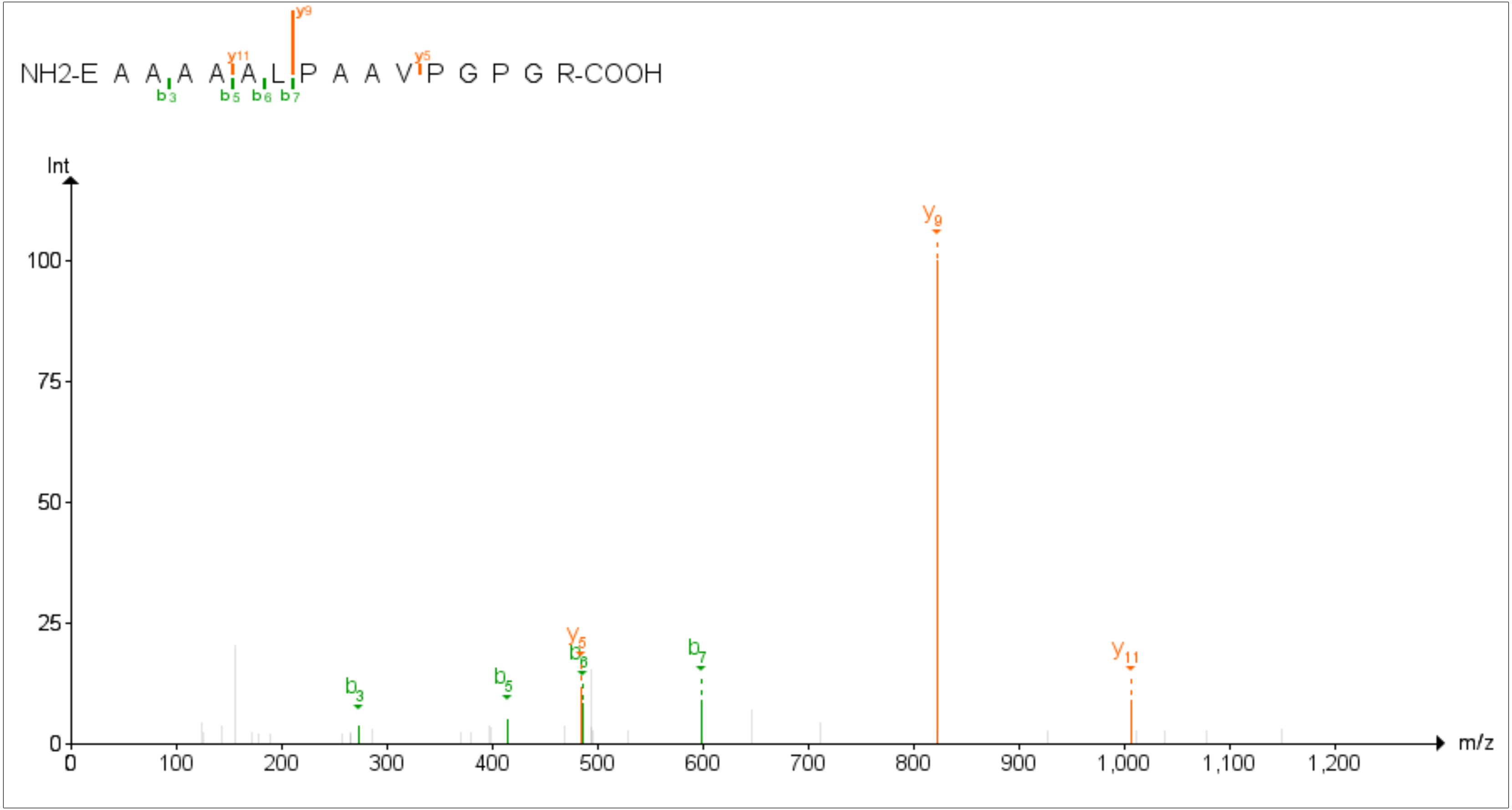

NH2-E A A A A A L P A A V P G P G R-COOH
y9
y11
y5
b3
b5
b6
b7
Int
100
75
50
25
0
m/z
0
100
200
300
400
500
600
700
800
900
1,000
1,100
1,200

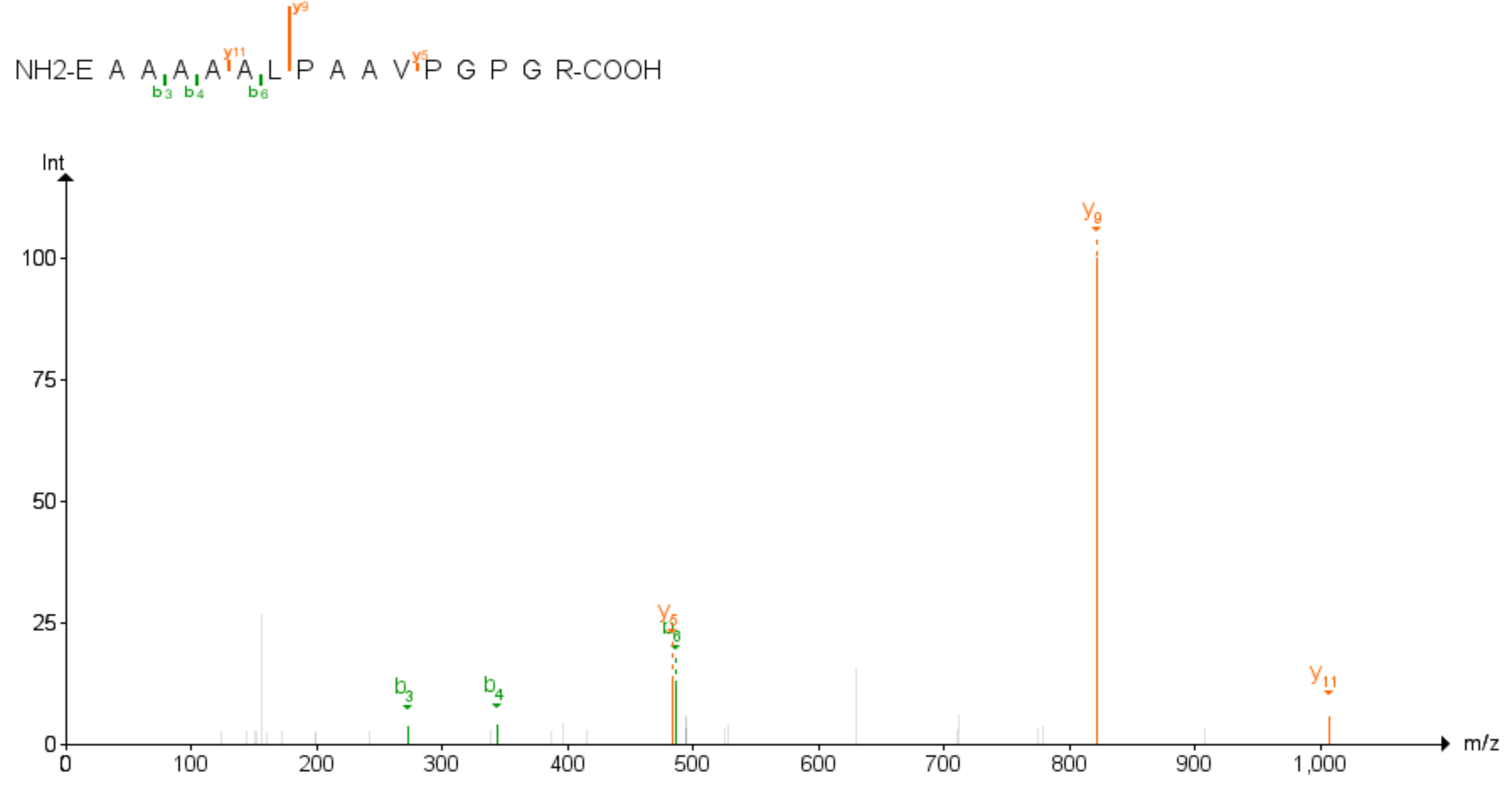
NH2-E A A A A A L P A A V P G P G R-COOH
y9
y11
y5
b3
b4
b6
Int
100
75
50
25
0
m/z
0
100
200
300
400
500
600
700
800
900
1,000
y9
y5
b6
b3
b4
y11

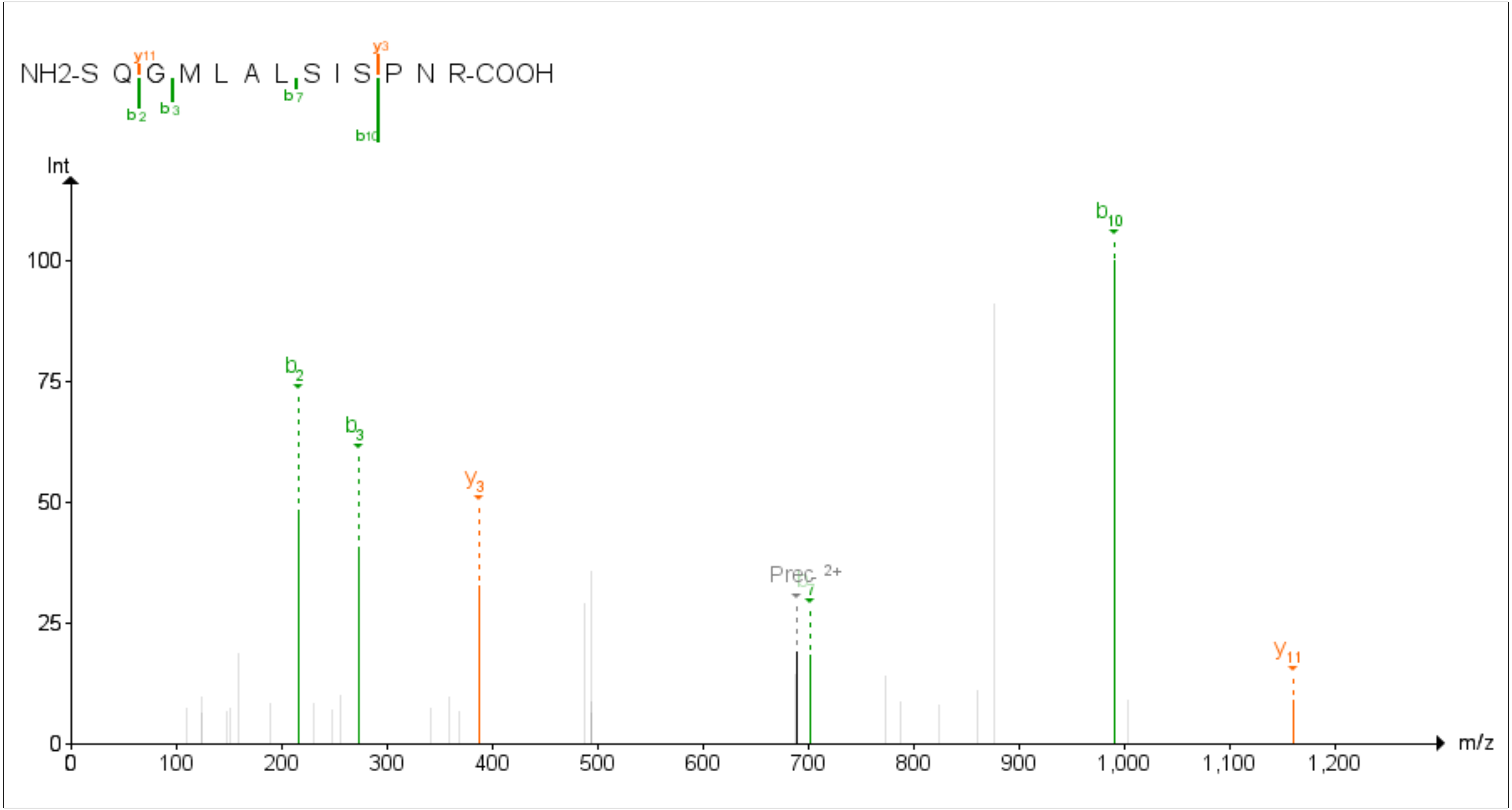

NH2-S Q G M L A L S I S P N R-COOH
y11
y3
b2
b3
b7
b10
Int
100
75
50
25
0
b10
b2
b3
y3
Prec 2+
b7
y11
0
100
200
300
400
500
600
700
800
900
1,000
1,100
1,200
m/z

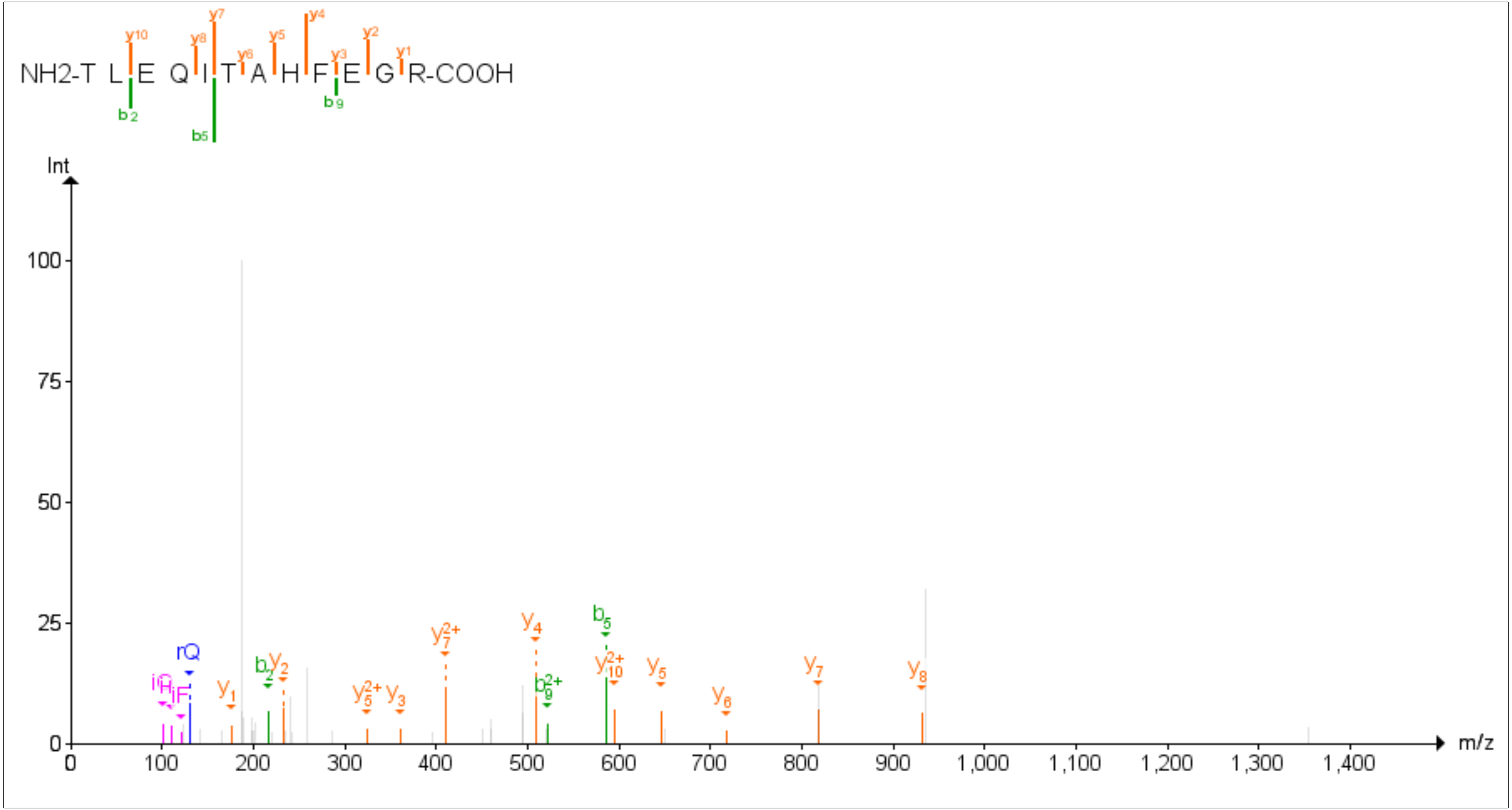

NH2-T L E Q I T A H F E G R-COOH
Int
m/z

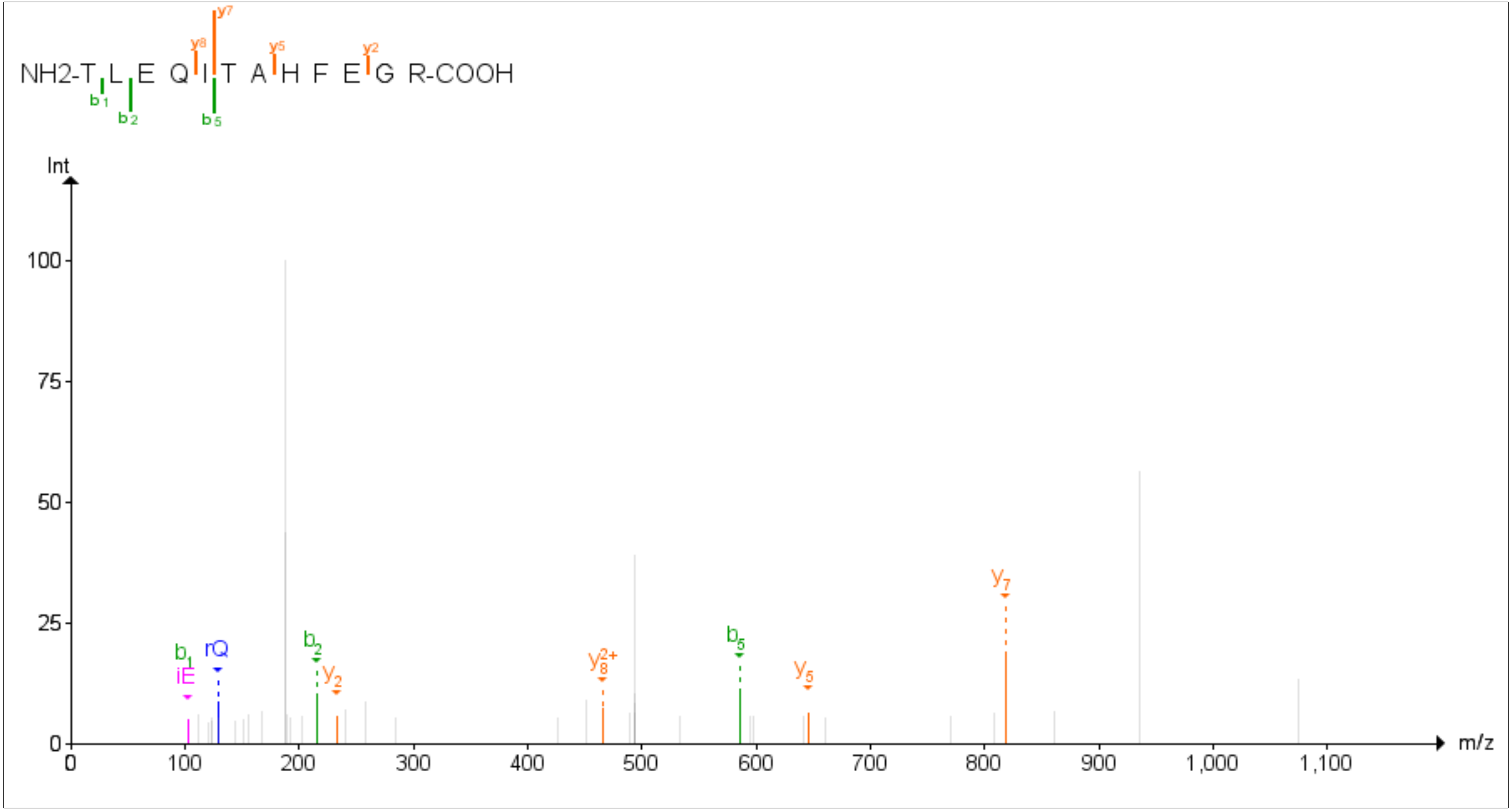

NH2-T L E Q I T A H F E G R-COOH
y7
y8
y5
y2
b1
b2
b5
Int
100
75
50
25
0
b1
iE
rQ
b2
y2
y8 2+
b5
y5
y7
0
100
200
300
400
500
600
700
800
900
1,000
1,100
m/z

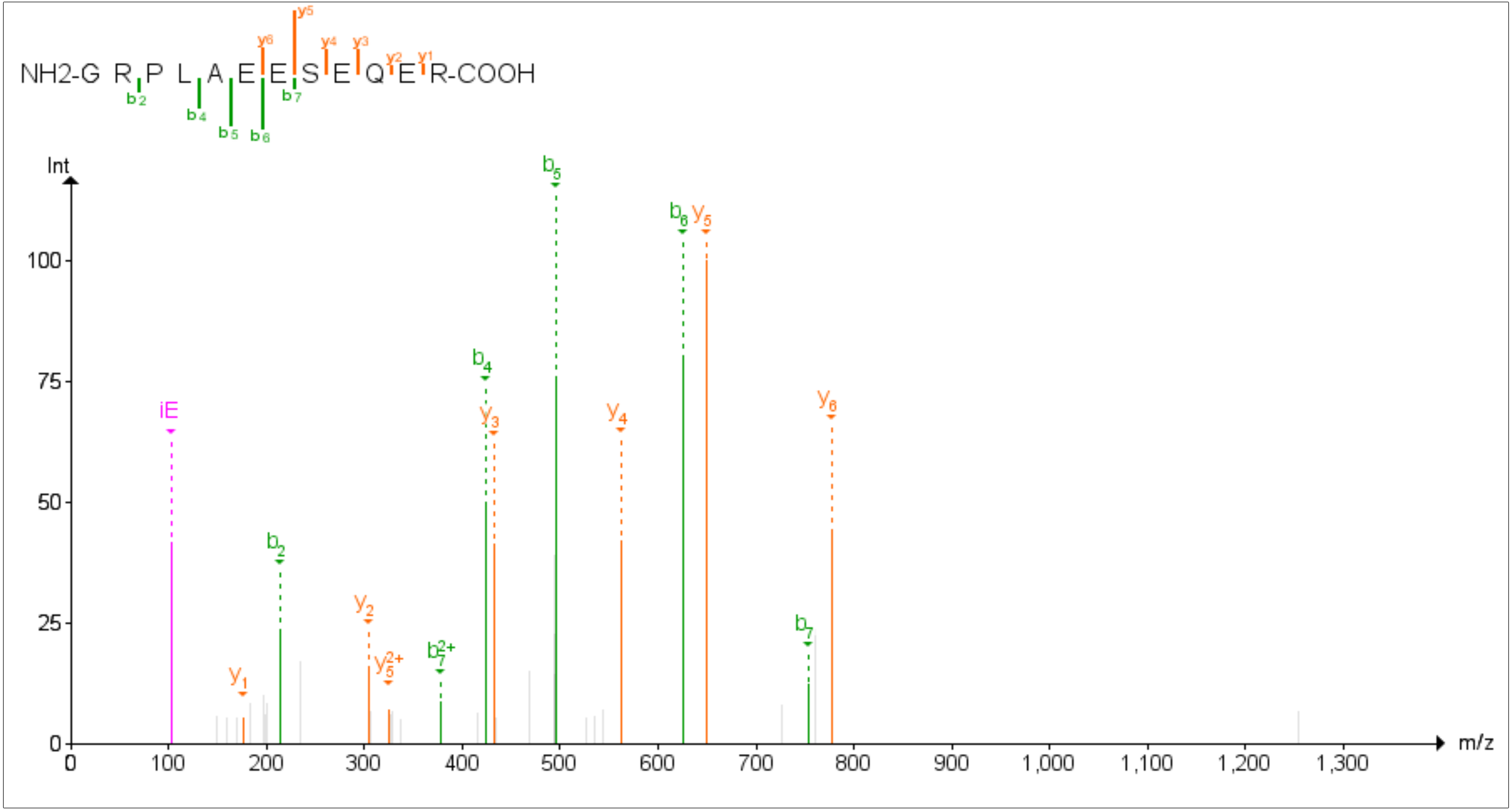

NH2-G R P L A E E S E Q E R-COOH
b2
b4
b5
b6
b7
y6
y5
y4
y3
y2
y1
Int
100
75
50
25
0
m/z
0
100
200
300
400
500
600
700
800
900
1,000
1,100
1,200
1,300
iE
y1
b2
y2
y5 2+
b7 2+
b4
y3
b5
y4
b6
y5
b7
y6

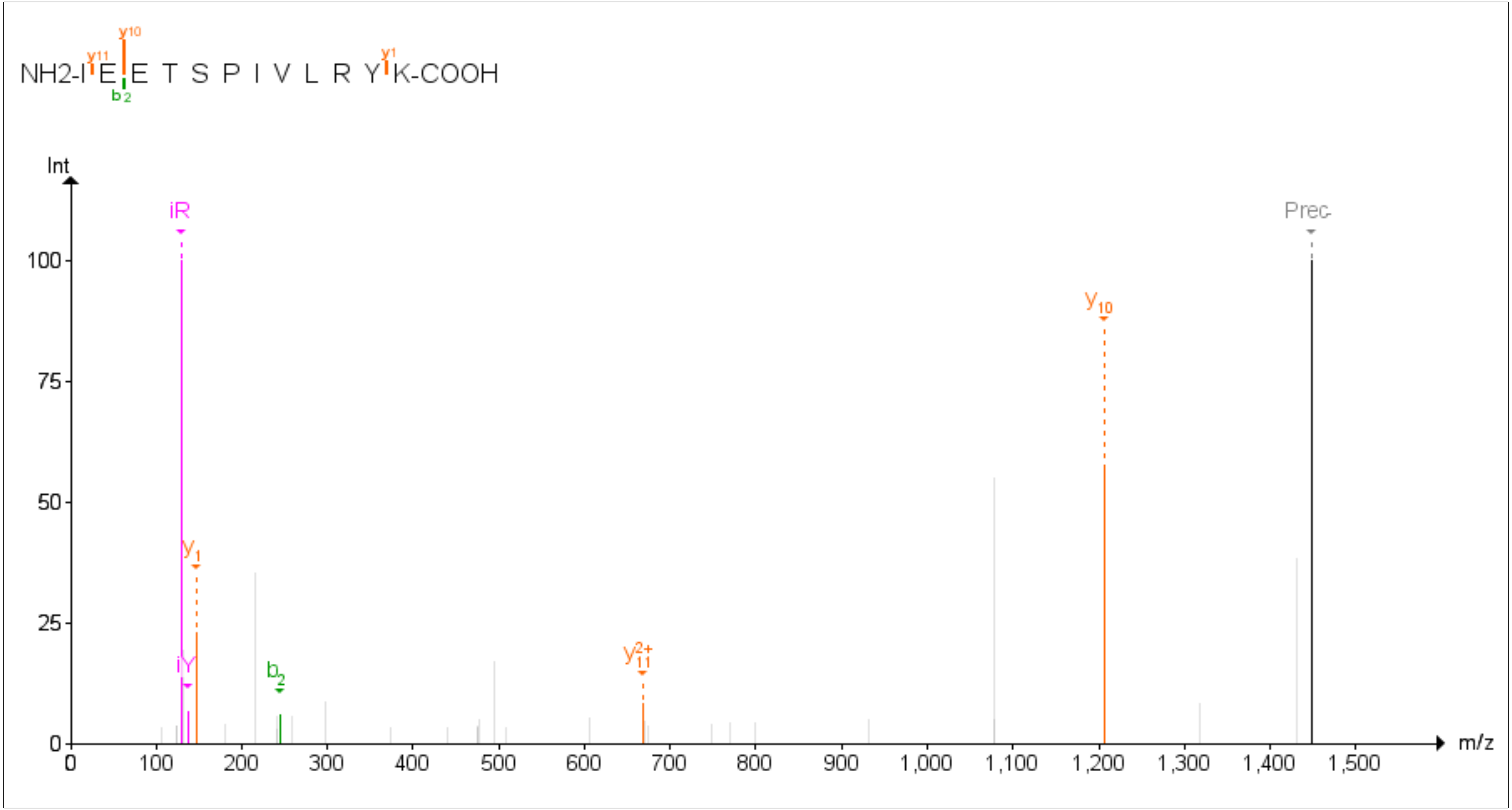
NH2-I E E T S P I V L R Y K-COOH
y11
y10
b2
y1
Int
m/z
iR
iY
y1
b2
y_{11}^{2+}
y10
Prec.
0
25
50
75
100
0
100
200
300
400
500
600
700
800
900
1,000
1,100
1,200
1,300
1,400
1,500

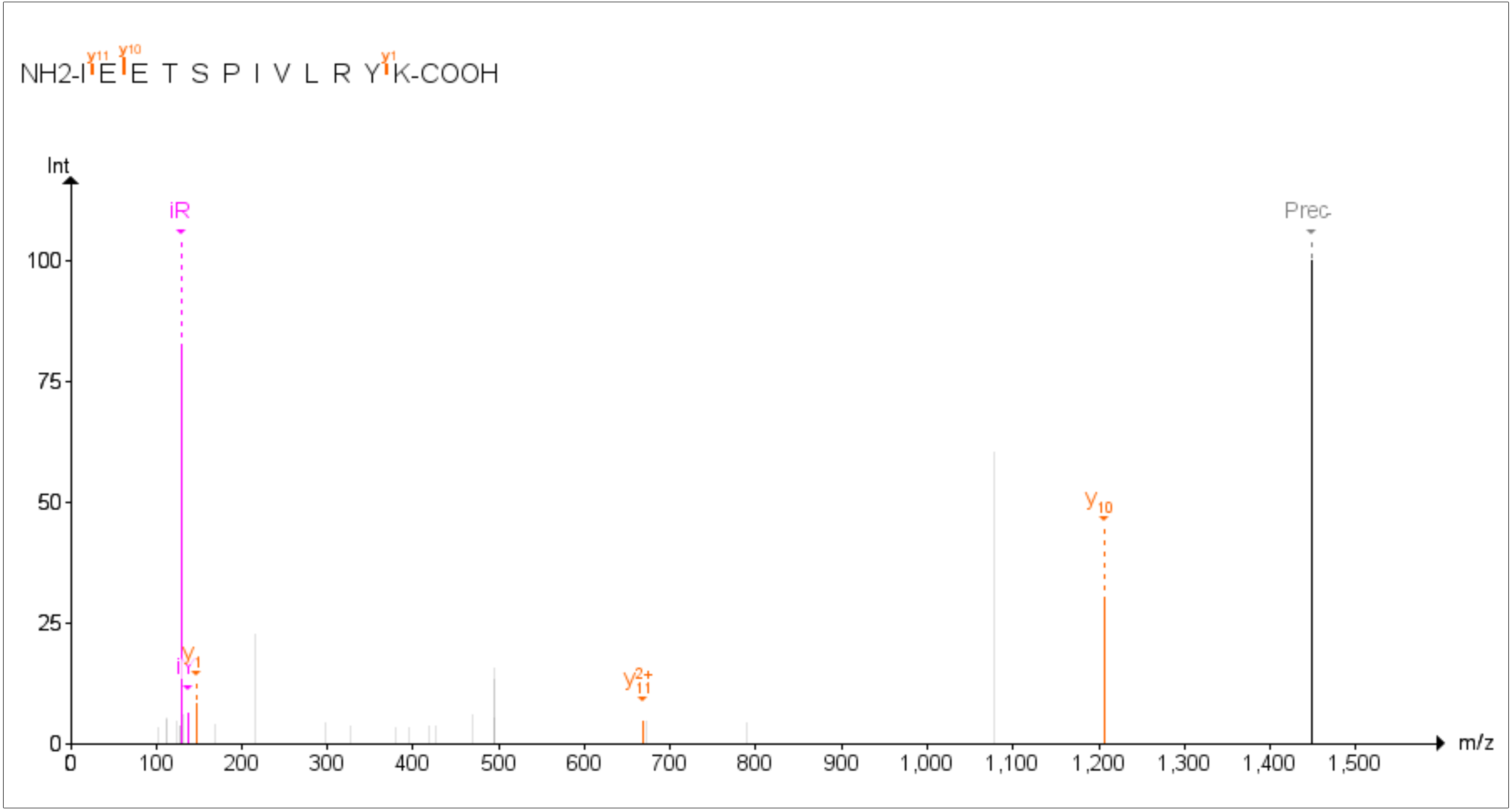

NH2-I E E T S P I V L R Y K-COOH
y11
y10
y1
Int
iR
Prec
y10
y2+ 11
y1
iY
100
75
50
25
0
0
100
200
300
400
500
600
700
800
900
1,000
1,100
1,200
1,300
1,400
1,500
m/z

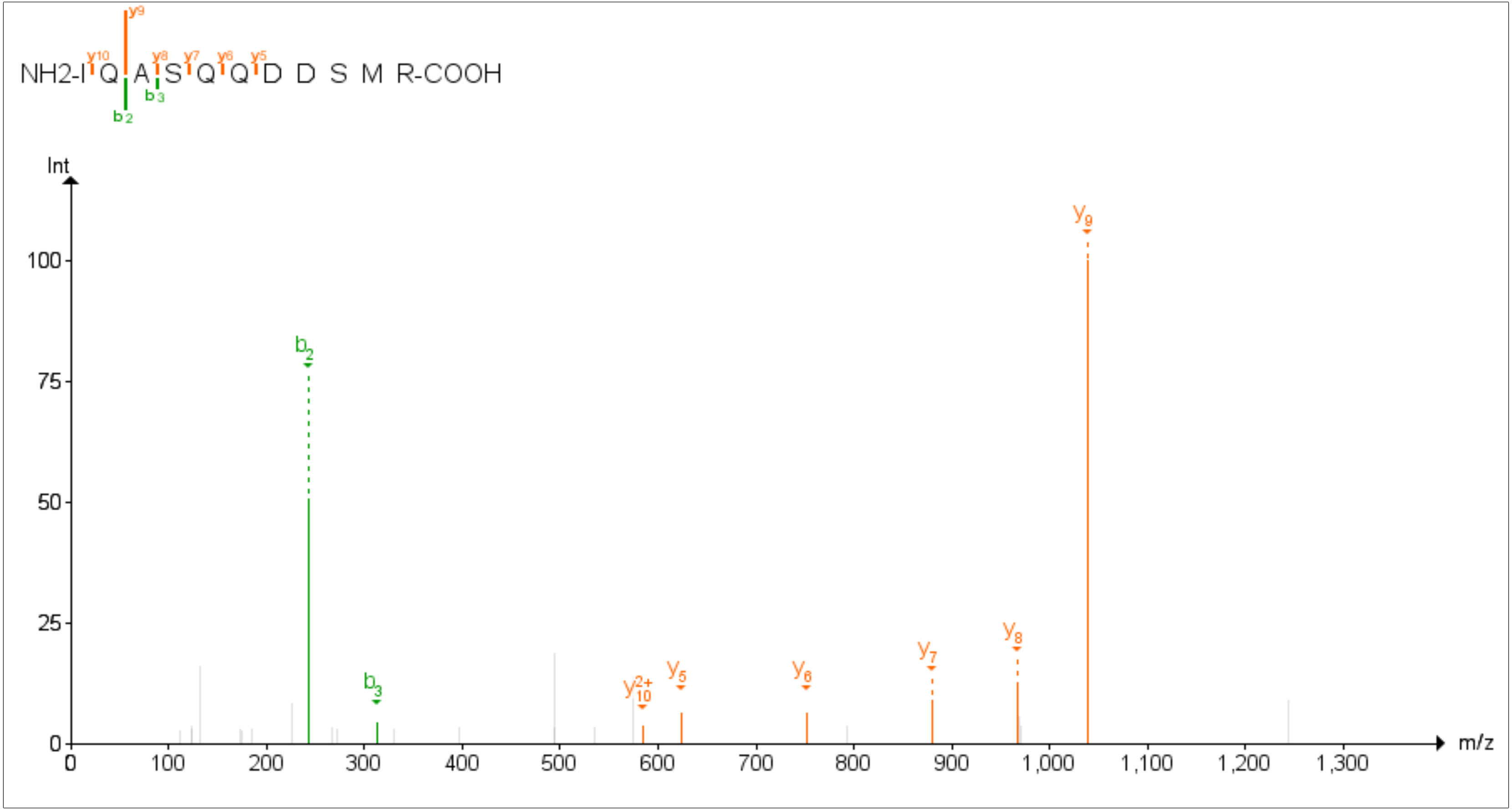

NH2-I Q A S Q Q D D S M R-COOH
y9
y10
y8
y7
y6
y5
b2
b3
Int
100
75
50
25
0
y9
b2
b3
y10 2+
y5
y6
y7
y8
0
100
200
300
400
500
600
700
800
900
1,000
1,100
1,200
1,300
m/z

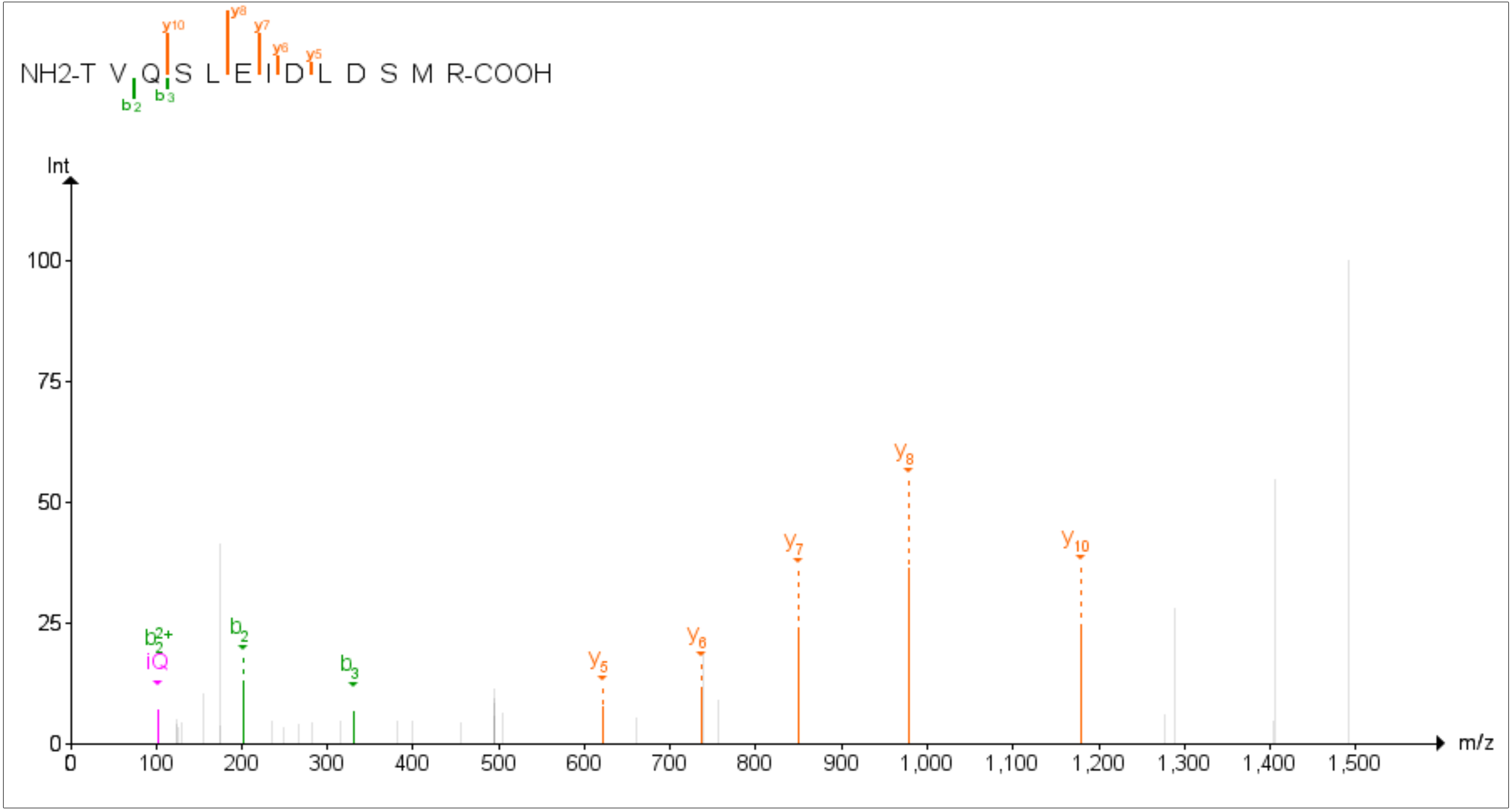

NH2-T V Q S L E I D L D S M R-COOH
y10
y8
y7
y6
y5
b2
b3
Int
m/z
100
75
50
25
0
0
100
200
300
400
500
600
700
800
900
1,000
1,100
1,200
1,300
1,400
1,500
b2 2+
iQ
b2
b3
y5
y6
y7
y8
y10

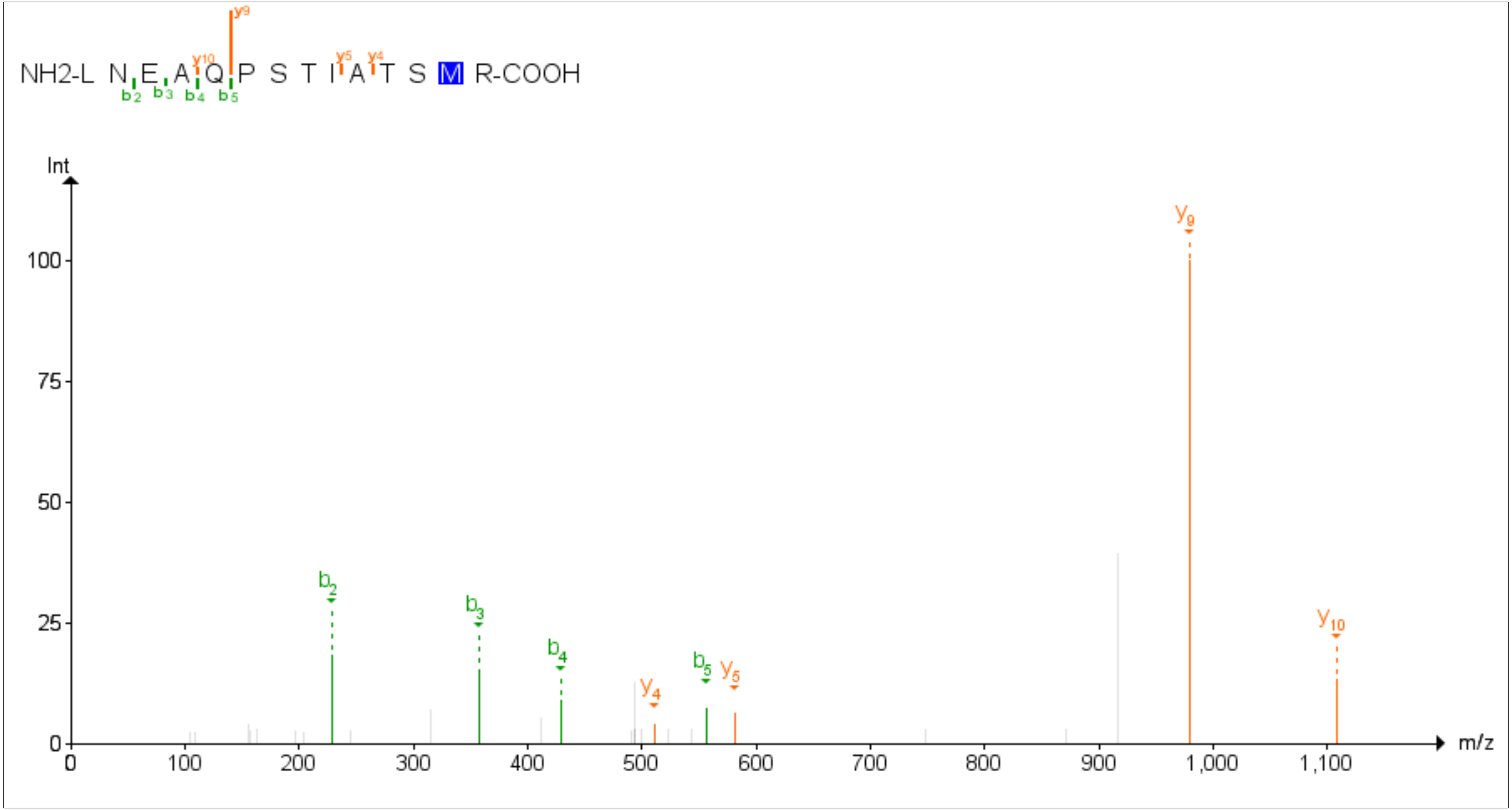

NH2-L N E A Q P S T I A T S M R-COOH
b2 b3 b4 b5
y10 y9 y5 y4
Int
100
75
50
25
0
b2
b3
b4
y4
b5
y5
y9
y10
0 100 200 300 400 500 600 700 800 900 1,000 1,100
m/z

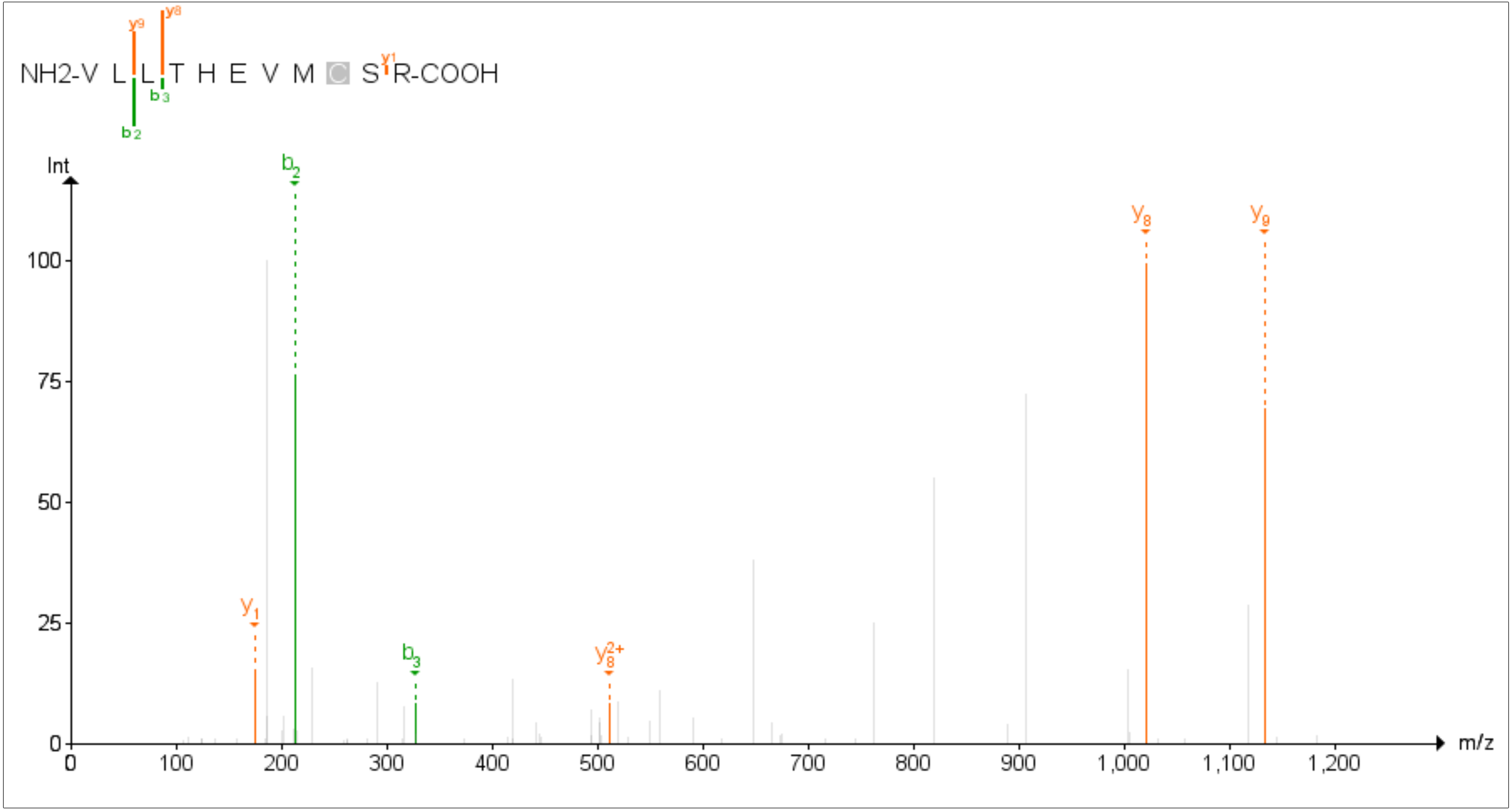

NH2-V L L T H E V M C S R-COOH
y9
y8
y1
b2
b3
Int
m/z
y1
b2
b3
y8 2+
y8
y9
0
25
50
75
100
0
100
200
300
400
500
600
700
800
900
1,000
1,100
1,200

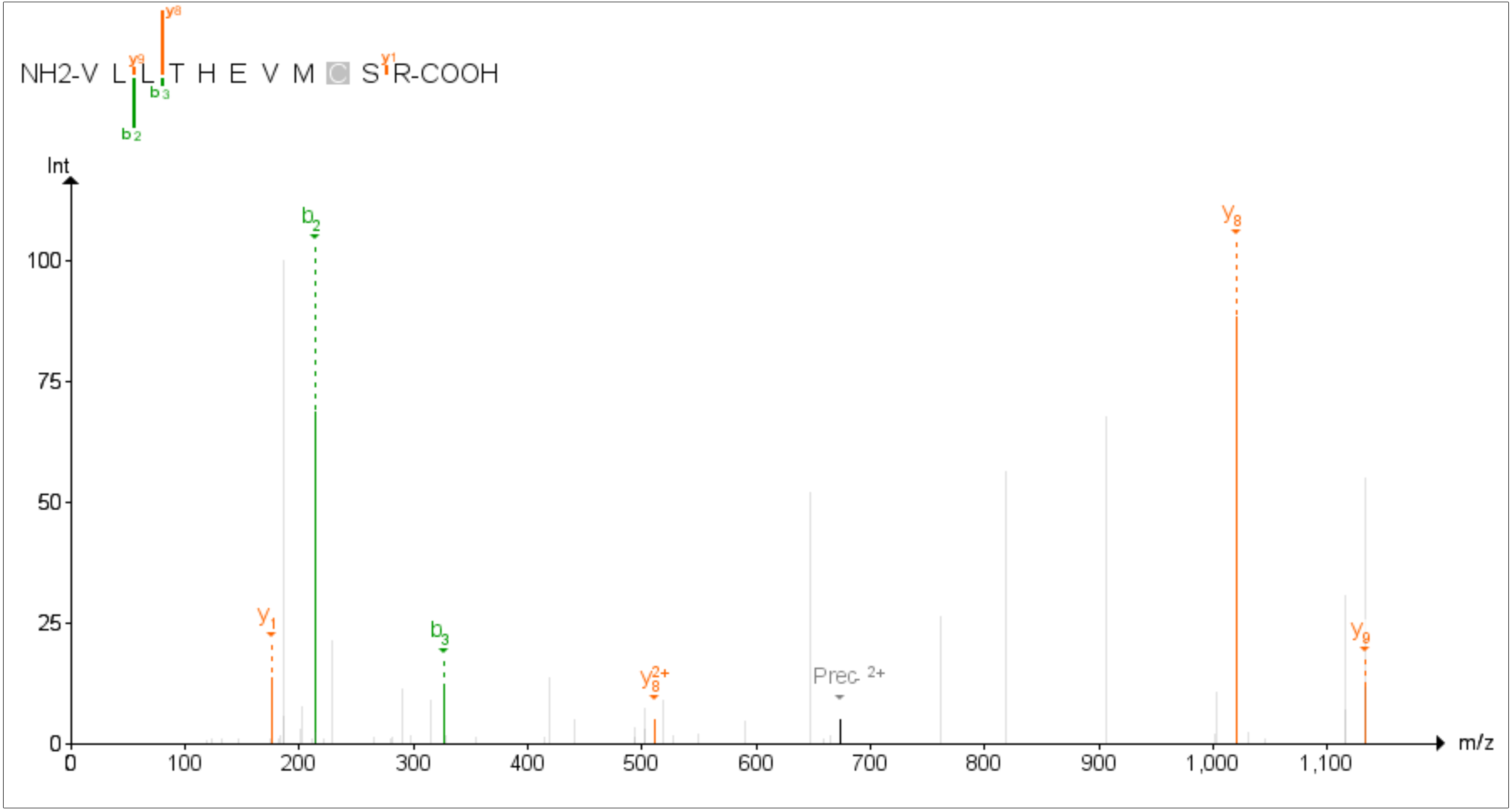

NH2-V L L T H E V M C S R-COOH
y8
y9
y1
b3
b2
Int
100
75
50
25
0
b2
y8
y1
b3
y8 2+
Prec. 2+
y9
m/z
0
100
200
300
400
500
600
700
800
900
1,000
1,100

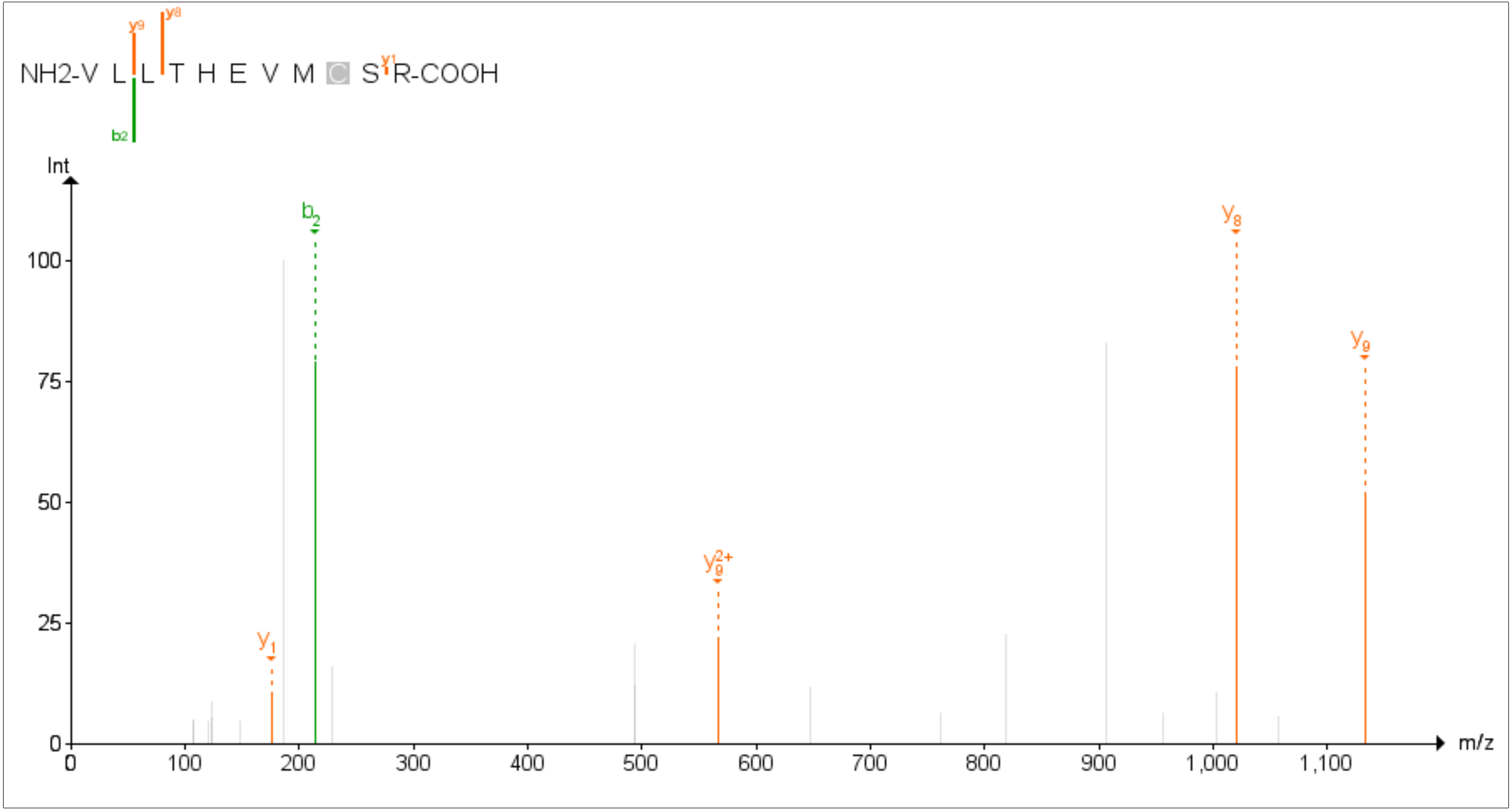
NH2-V L L T H E V M C S R-COOH
y9
y8
y1
b2
Int
100
75
50
25
0
b2
y8
y9
y1
y9 2+
0
100
200
300
400
500
600
700
800
900
1,000
1,100
m/z

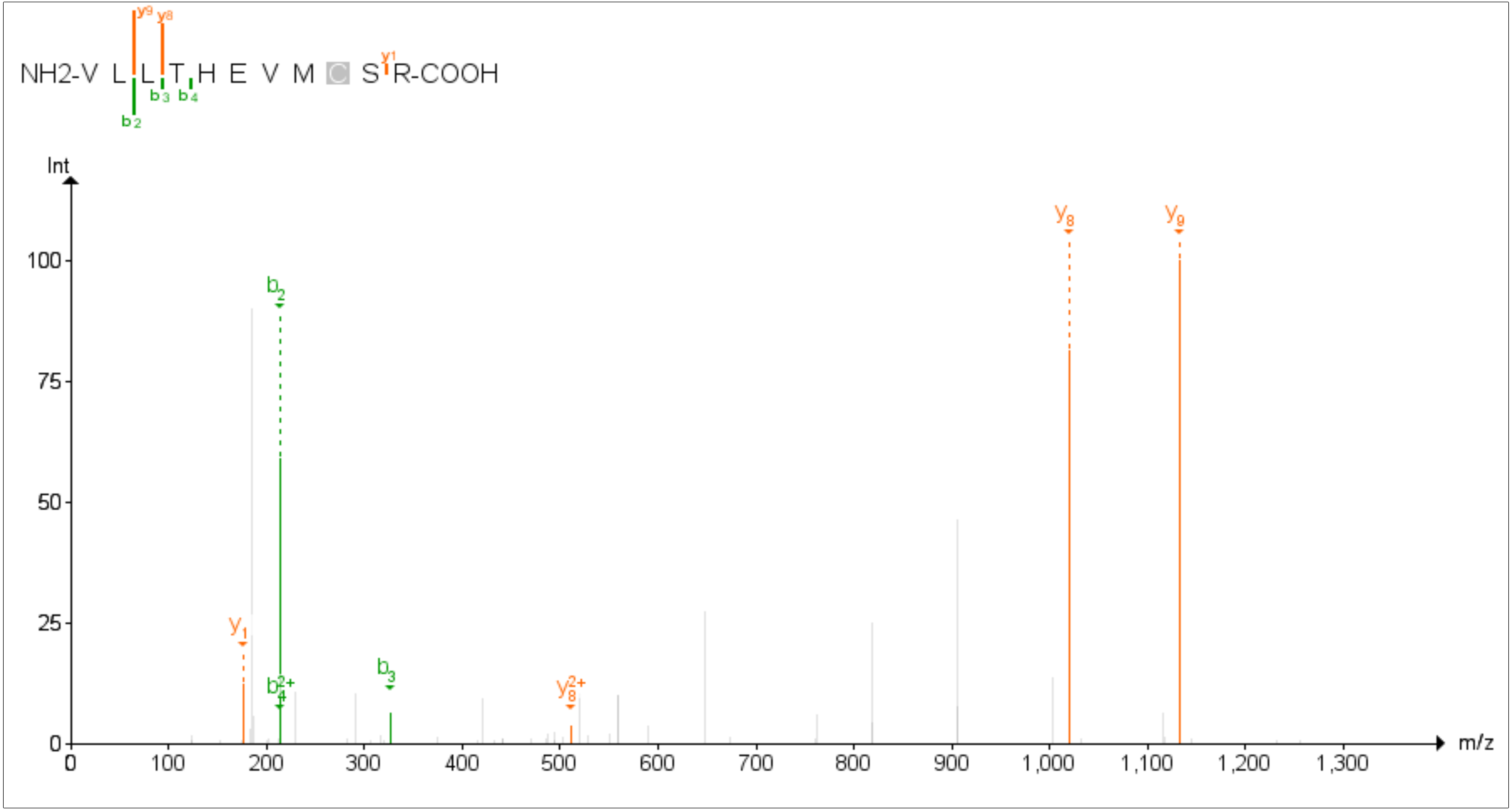

NH2-V L L T H E V M C S R-COOH
y9 y8 y1
b2 b3 b4
Int
100
75
50
25
0
y8
y9
b2
y1
b4 2+
b3
y8 2+
0 100 200 300 400 500 600 700 800 900 1,000 1,100 1,200 1,300
m/z

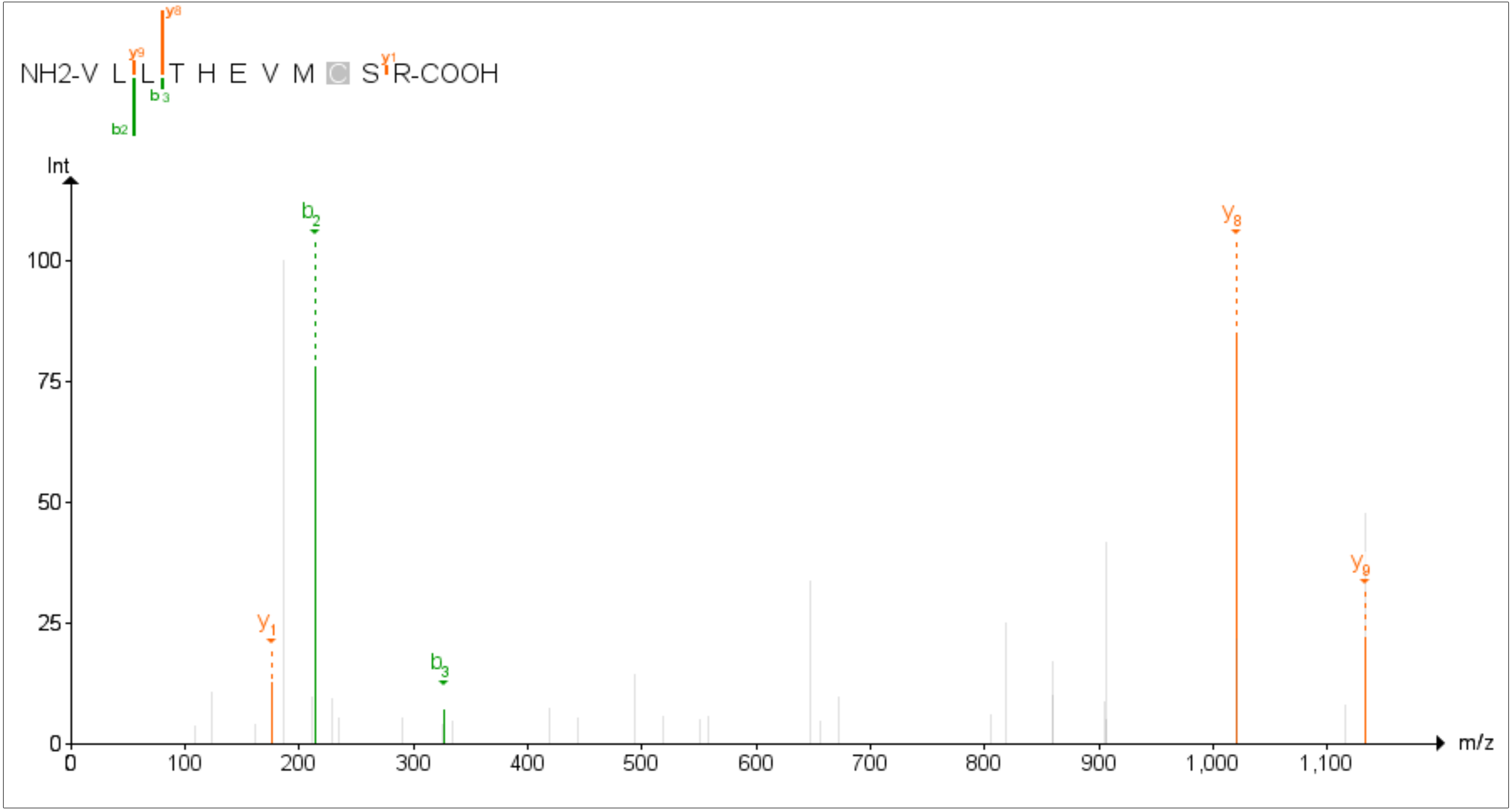

NH2-V L L T H E V M C S R-COOH
y8
y9
y1
b2
b3
Int
100
75
50
25
0
b2
y8
y9
y1
b3
0
100
200
300
400
500
600
700
800
900
1,000
1,100
m/z

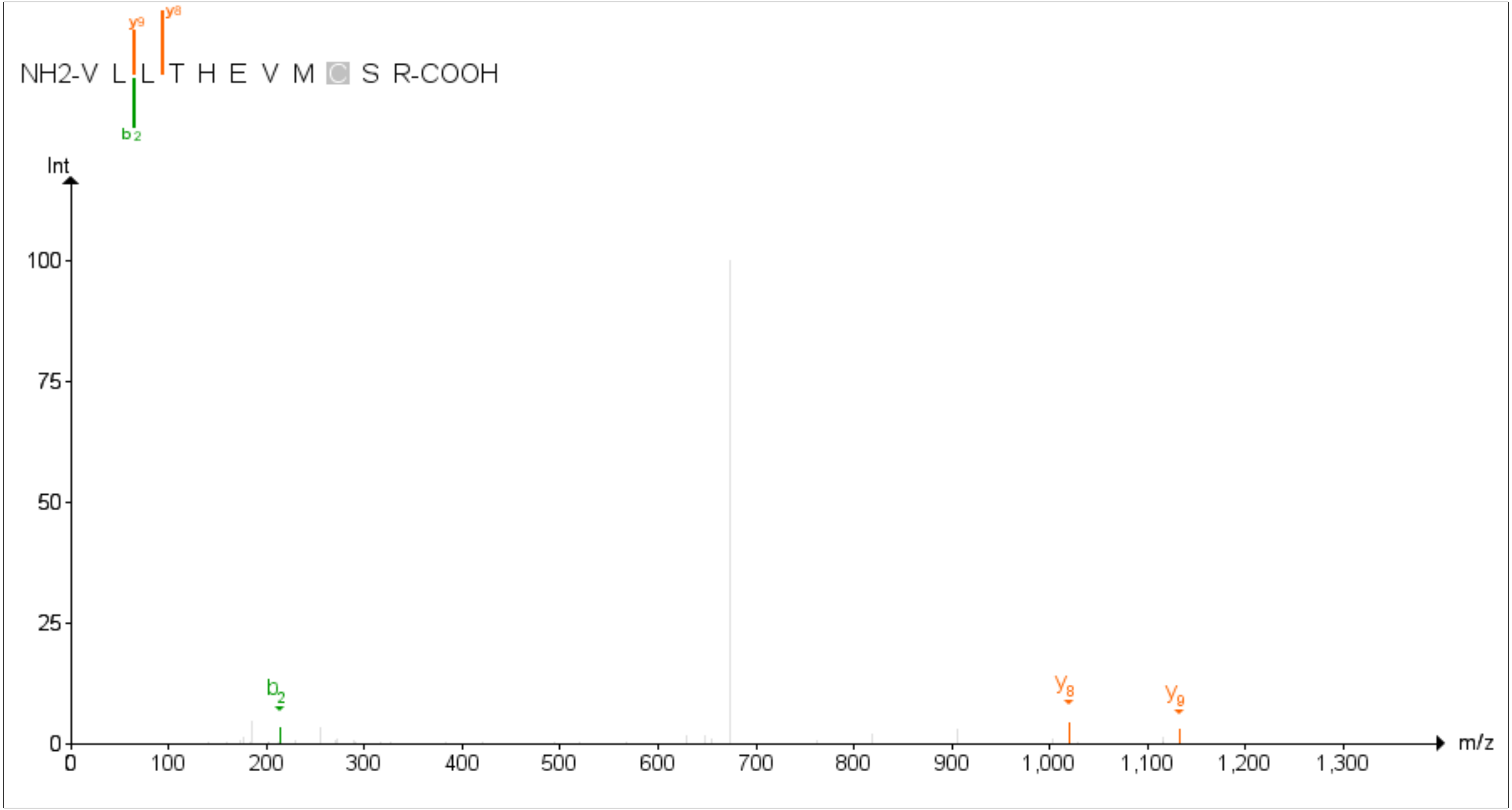
NH2-V L L T H E V M C S R-COOH
y9
y8
b2
Int
100
75
50
25
0
b2
y8
y9
0
100
200
300
400
500
600
700
800
900
1,000
1,100
1,200
1,300
m/z

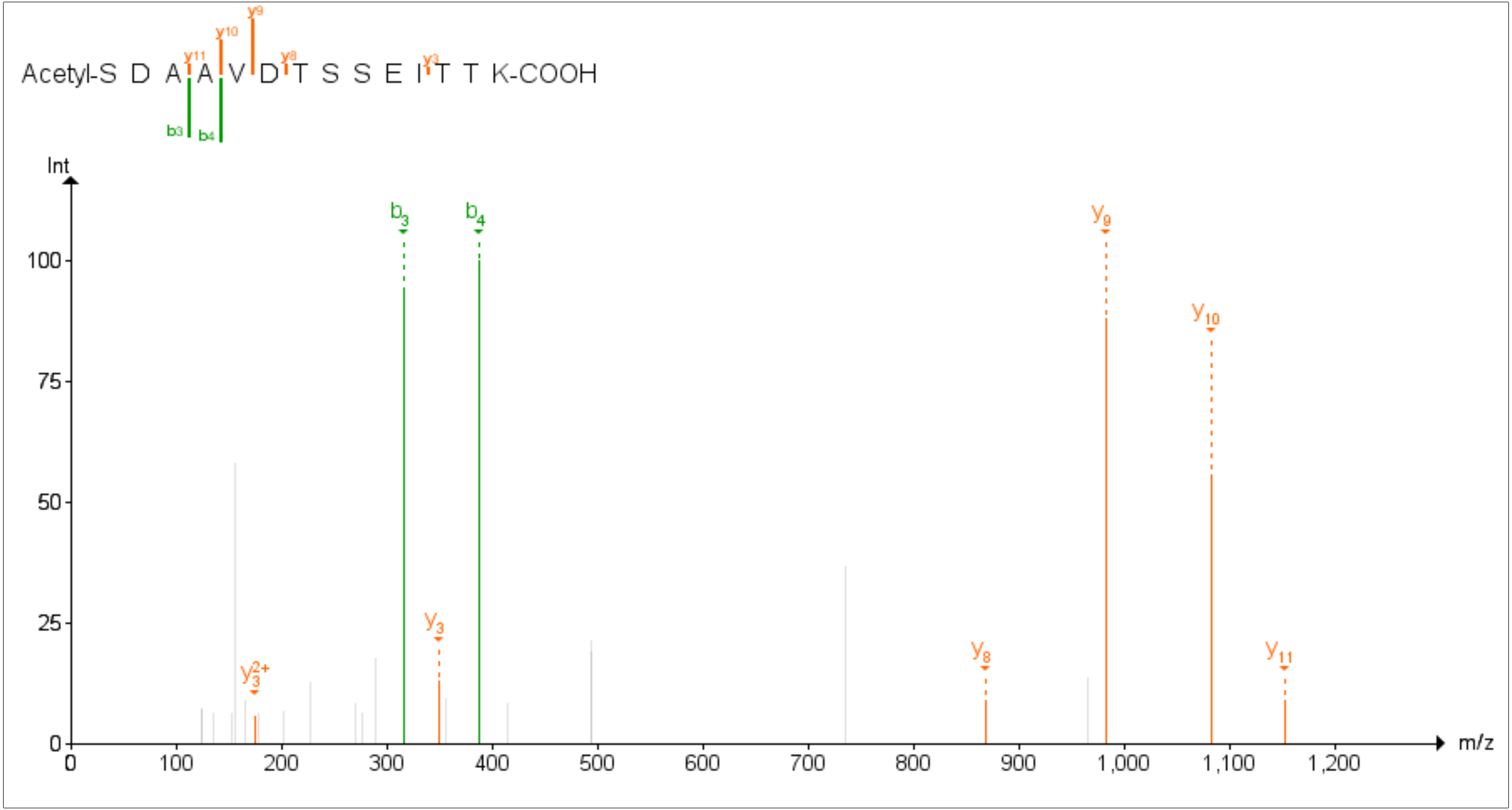

Acetyl-S D A A V D T S S E I T T K-COOH
y11 y10 y9 y8 y3
b3 b4
Int
100
75
50
25
0
b3 b4 y9 y10 y3 y8 y11
y3 2+
0 100 200 300 400 500 600 700 800 900 1,000 1,100 1,200
m/z

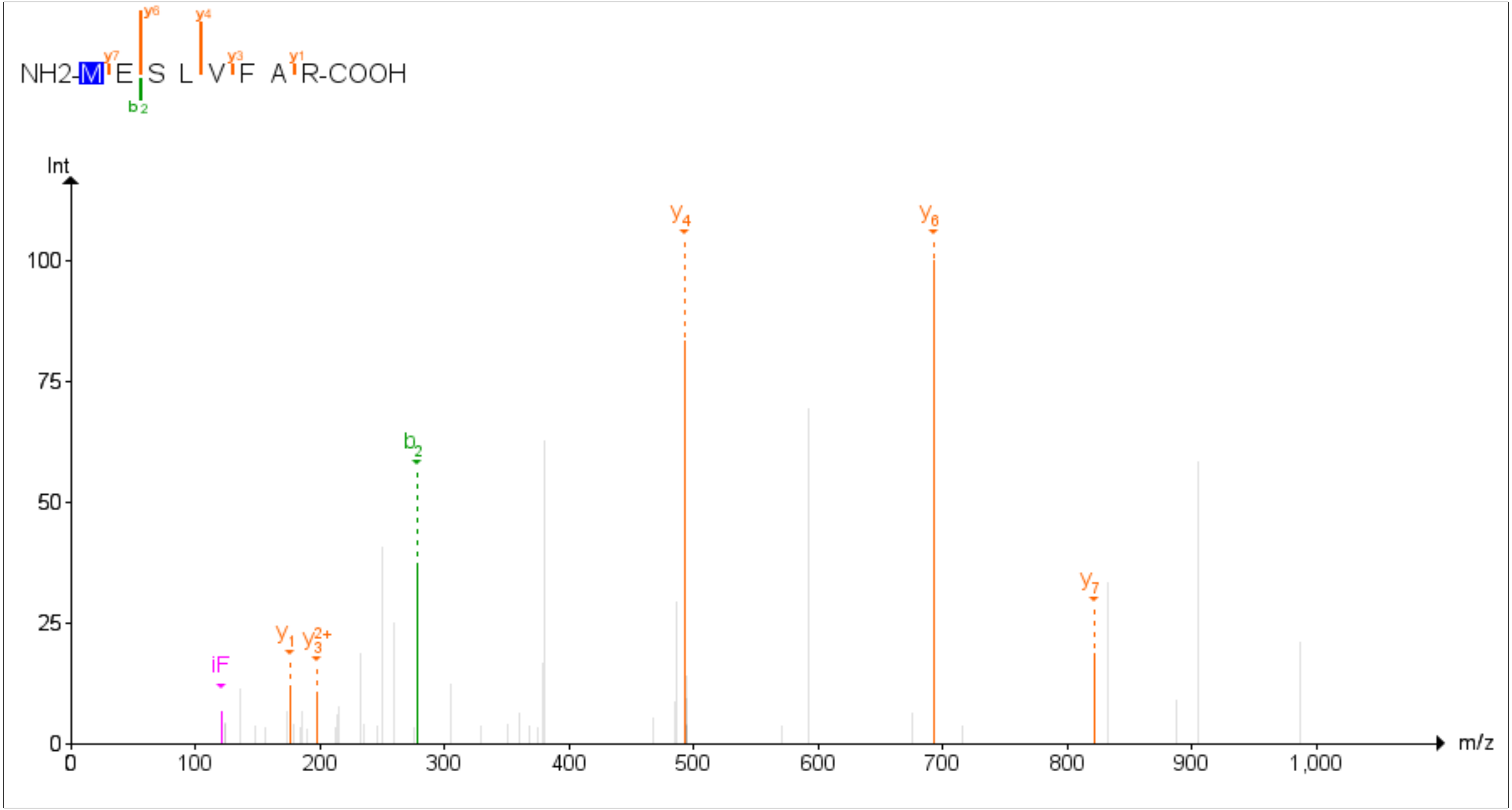

NH2-M E S L V F A R-COOH
y6
y4
y7
y3
y1
b2
Int
100
75
50
25
0
y4
y6
b2
y7
iF
y1
y3 2+
0
100
200
300
400
500
600
700
800
900
1,000
m/z

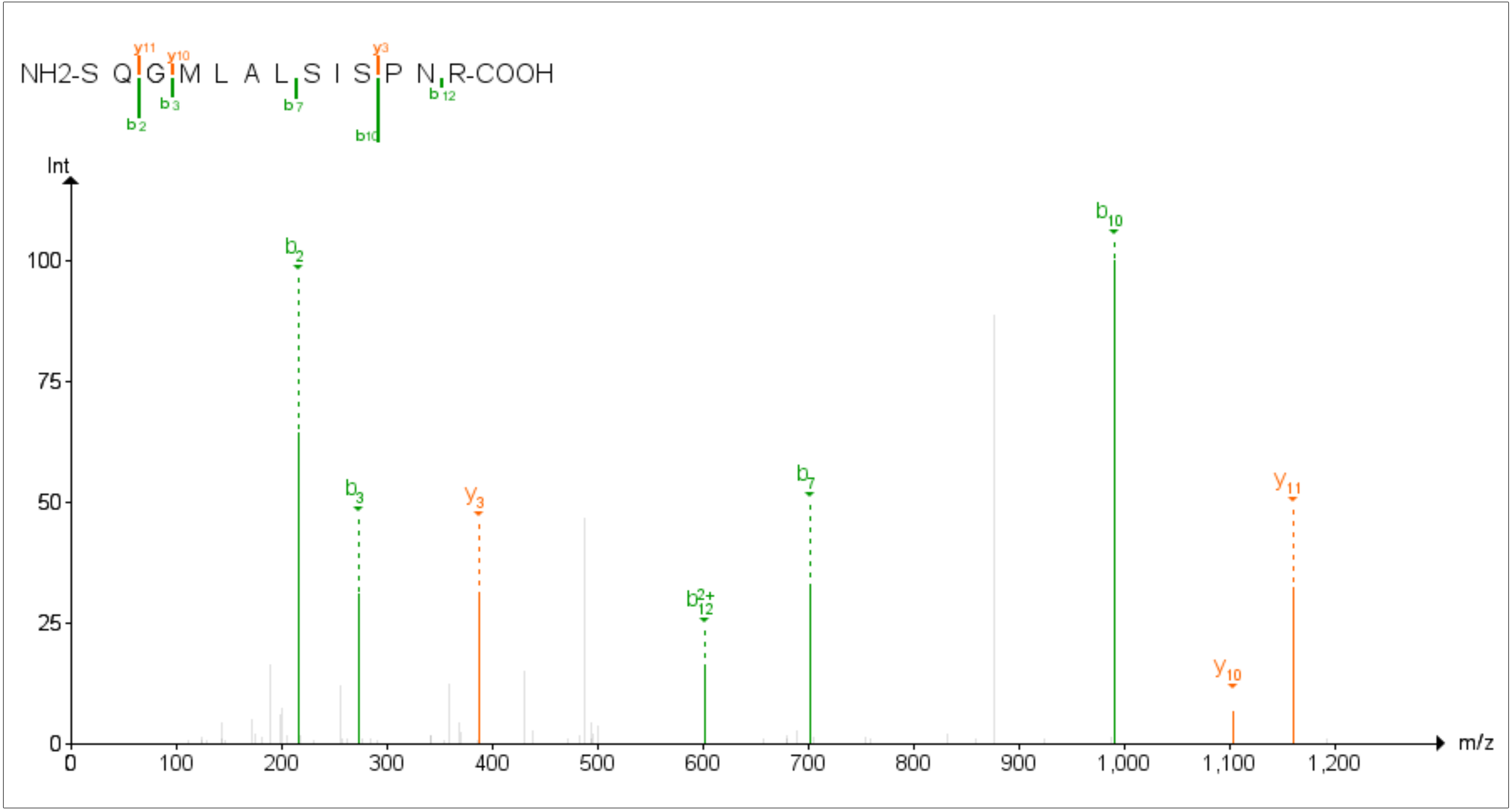
NH2-S Q G M L A L S I S P N R-COOH
y11 y10 y3
b2 b3 b7 b10 b12
Int
m/z
b2
b3
y3
b_{12}^{2+}
b7
b10
y10
y11
0
25
50
75
100
0
100
200
300
400
500
600
700
800
900
1,000
1,100
1,200

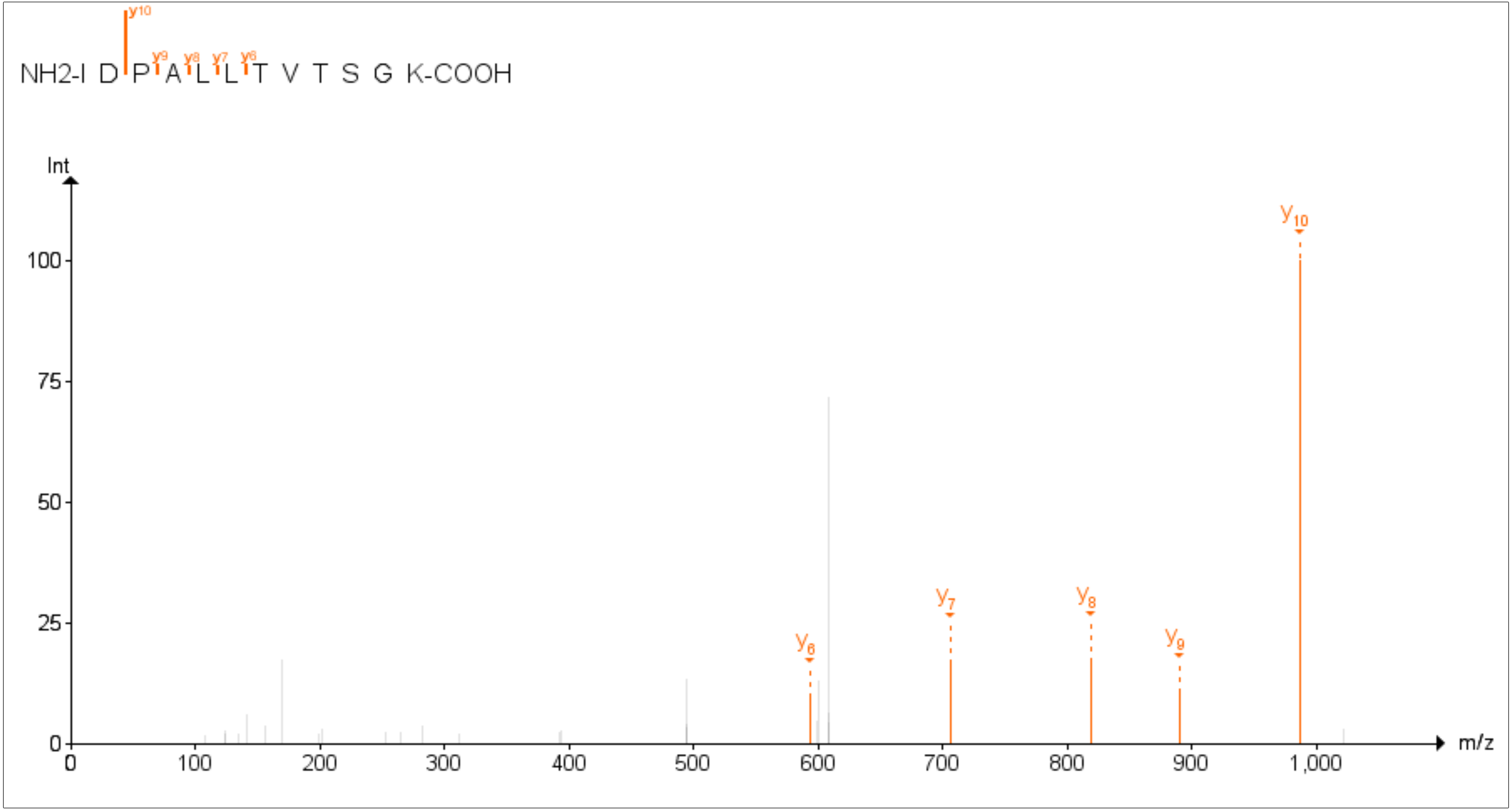

NH2-I D P A L L T V T S G K-COOH
y10
y9
y8
y7
y6
Int
m/z
0
25
50
75
100
0
100
200
300
400
500
600
700
800
900
1,000

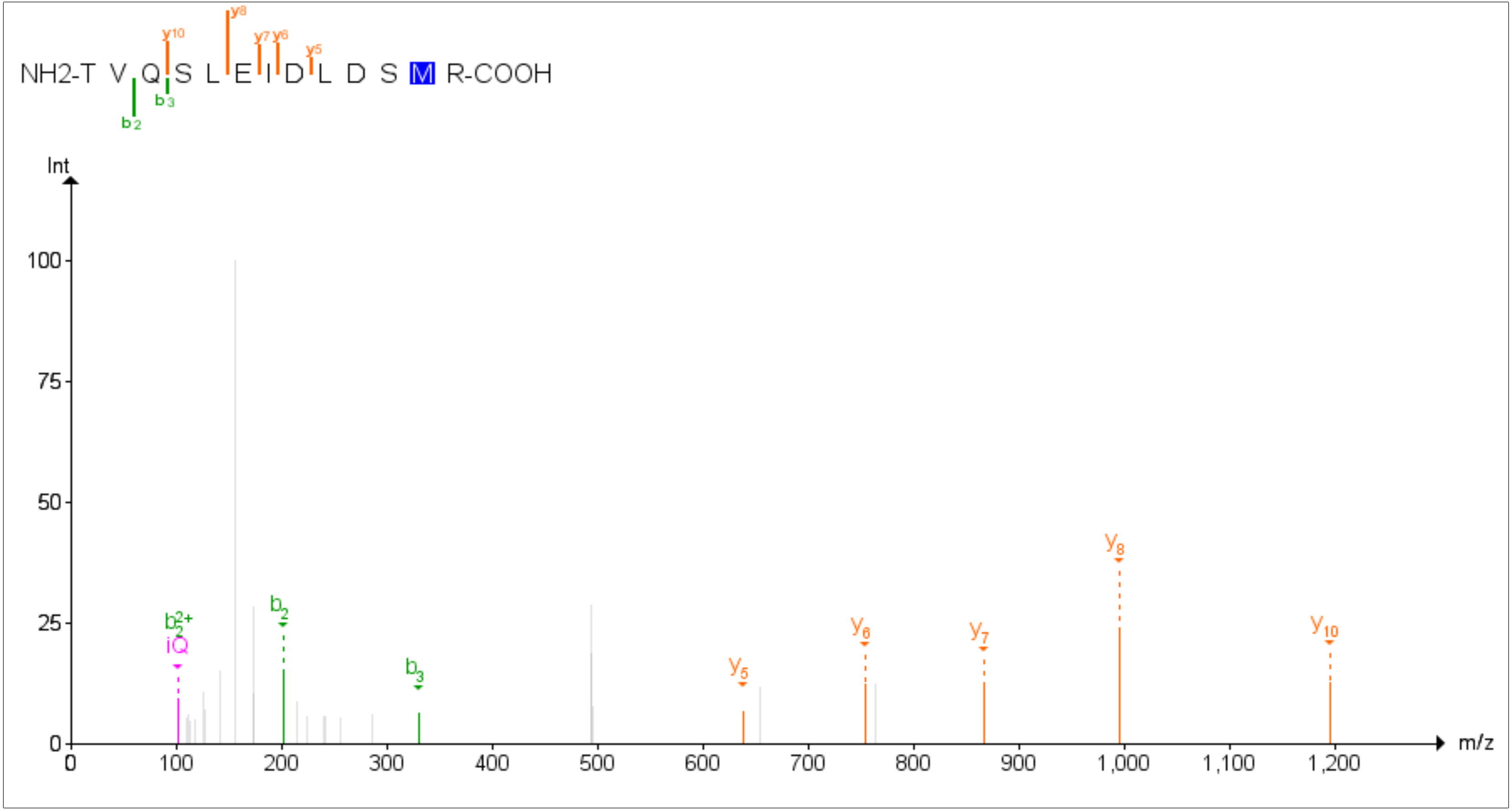

NH2-T V Q S L E I D L D S M R-COOH
y8
y10
y7 y6
y5
b2
b3
Int
100
75
50
25
0
b2 2+
iQ
b2
b3
y5
y6
y7
y8
y10
0
100
200
300
400
500
600
700
800
900
1,000
1,100
1,200
m/z

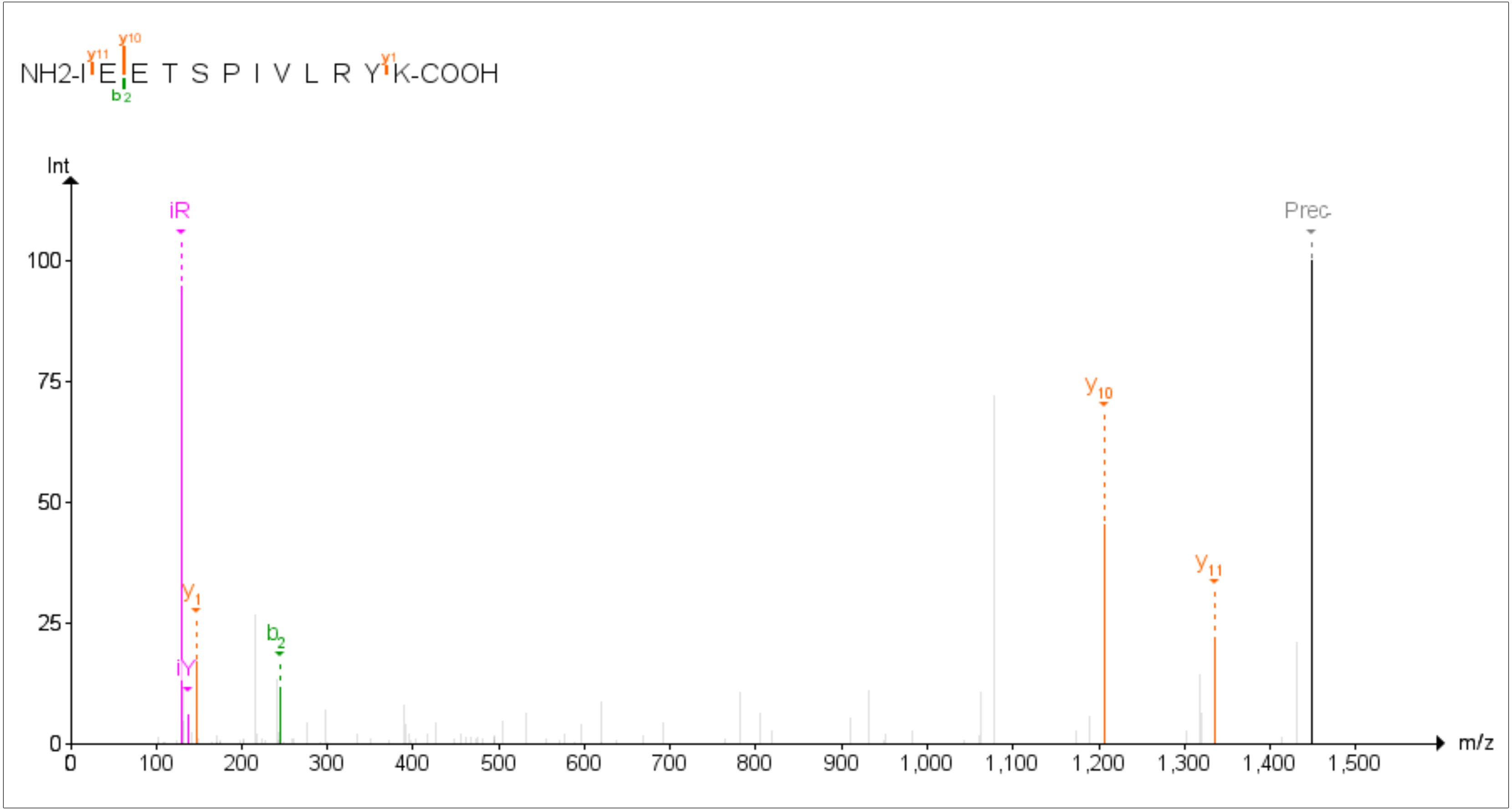

NH2-I E E T S P I V L R Y K-COOH
y11
y10
b2
y1
Int
iR
Prec
y10
y11
y1
iY
b2
100
75
50
25
0
0
100
200
300
400
500
600
700
800
900
1,000
1,100
1,200
1,300
1,400
1,500
m/z

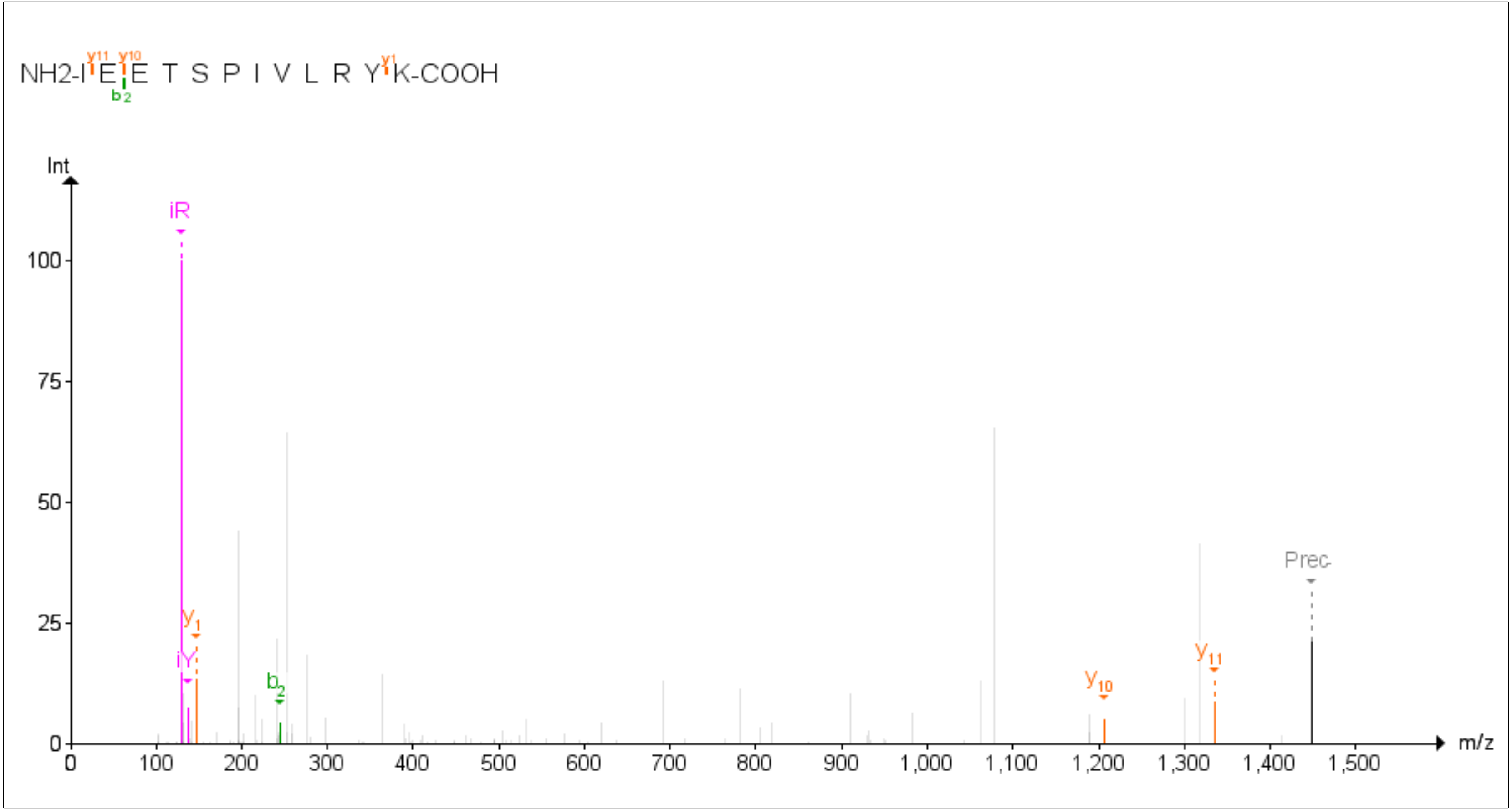

NH2-I E E T S P I V L R Y K-COOH
y11
y10
y1
b2
Int
iR
y1
iY
b2
y10
y11
Prec
100
75
50
25
0
0
100
200
300
400
500
600
700
800
900
1,000
1,100
1,200
1,300
1,400
1,500
m/z

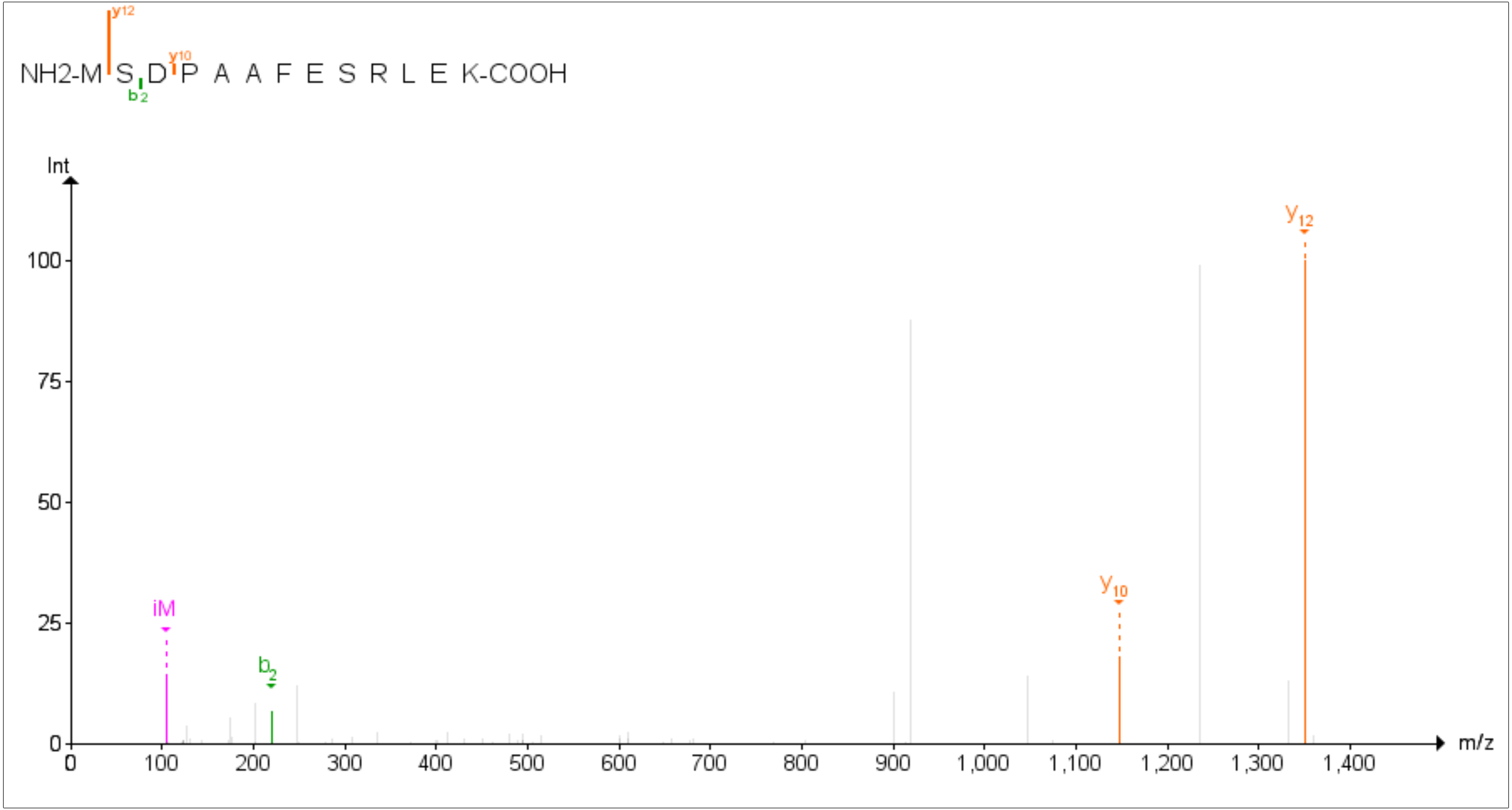

NH2-M S D P A A F E S R L E K-COOH
y12
y10
b2
Int
100
75
50
25
0
iM
b2
y10
y12
0
100
200
300
400
500
600
700
800
900
1,000
1,100
1,200
1,300
1,400
m/z

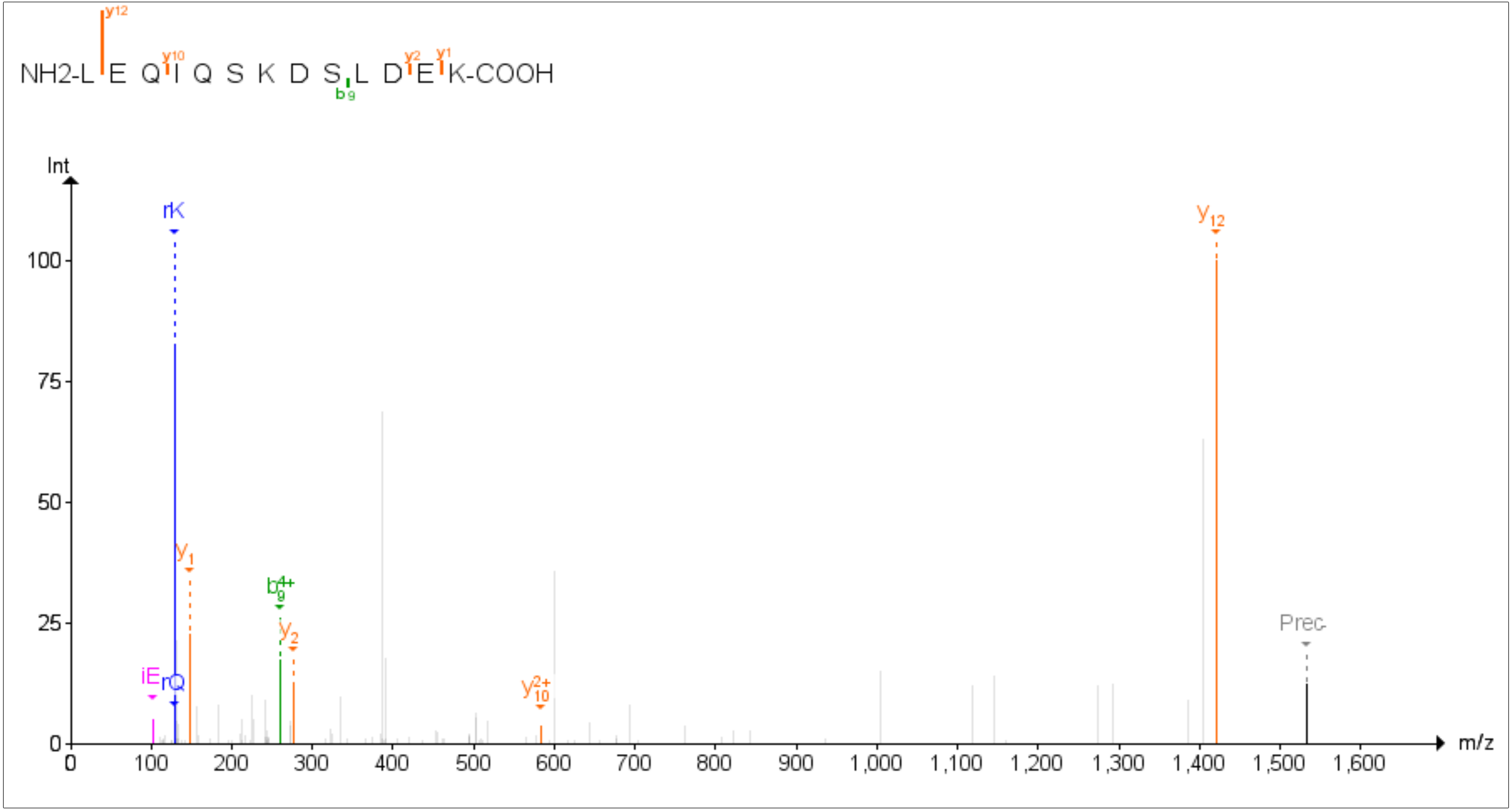

NH2-L E Q I Q S K D S L D E K-COOH
y12
y10
y2
y1
b9
Int
100
75
50
25
0
rK
y12
y1
b9 4+
y2
iE
rQ
y10 2+
Prec
0
100
200
300
400
500
600
700
800
900
1,000
1,100
1,200
1,300
1,400
1,500
1,600
m/z

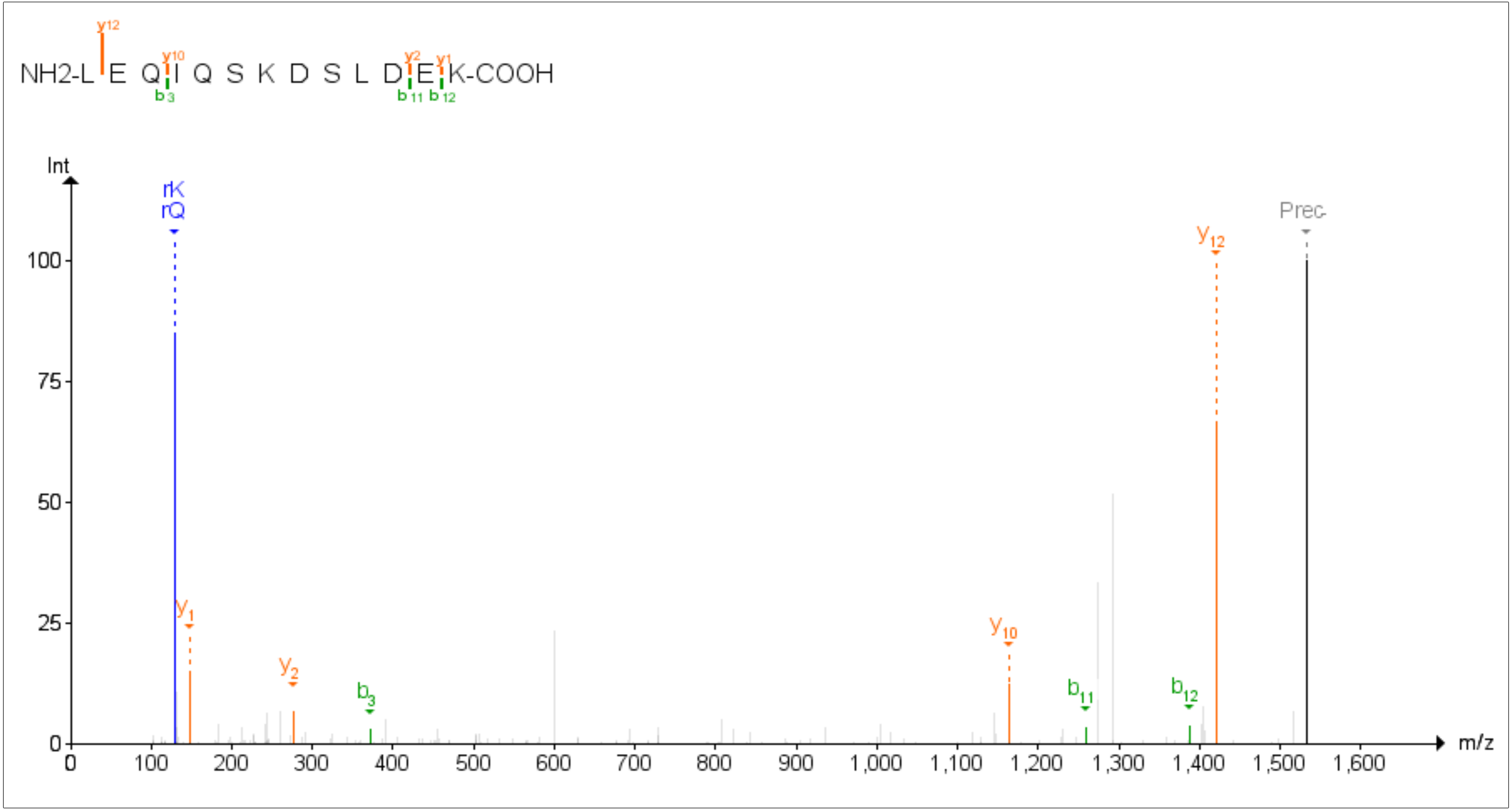

NH2-L E Q I Q S K D S L D E K-COOH
y12
y10
y2
y1
b3
b11
b12
Int
rK
rQ
Prec.
y12
y1
y2
b3
y10
b11
b12
100
75
50
25
0
0
100
200
300
400
500
600
700
800
900
1,000
1,100
1,200
1,300
1,400
1,500
1,600
m/z

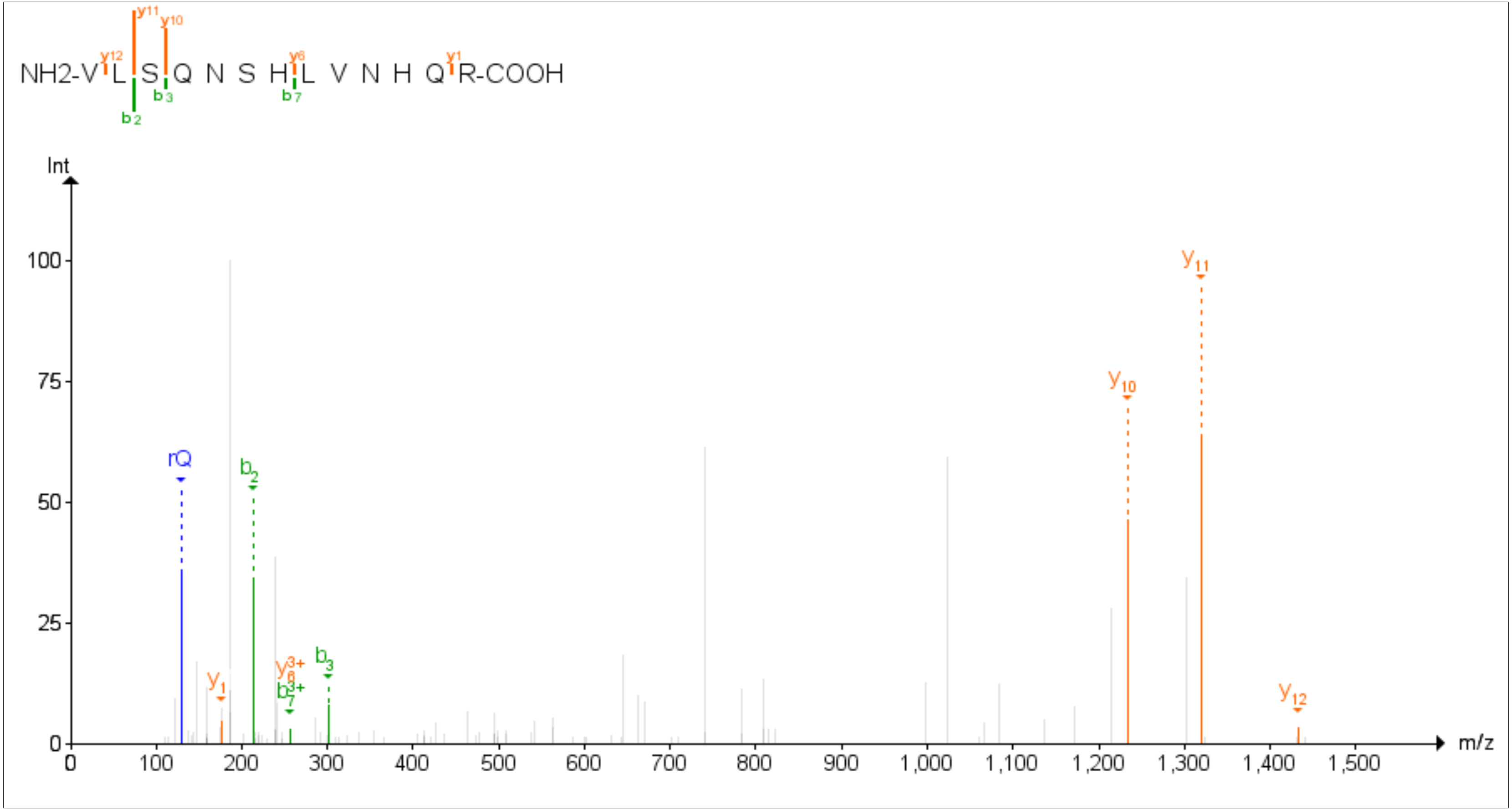

NH2-V L S Q N S H L V N H Q R-COOH
y12
y11
y10
y6
y1
b2
b3
b7
Int
100
75
50
25
0
rQ
b2
y1
y6 3+
b7 3+
b3
y10
y11
y12
0
100
200
300
400
500
600
700
800
900
1,000
1,100
1,200
1,300
1,400
1,500
m/z

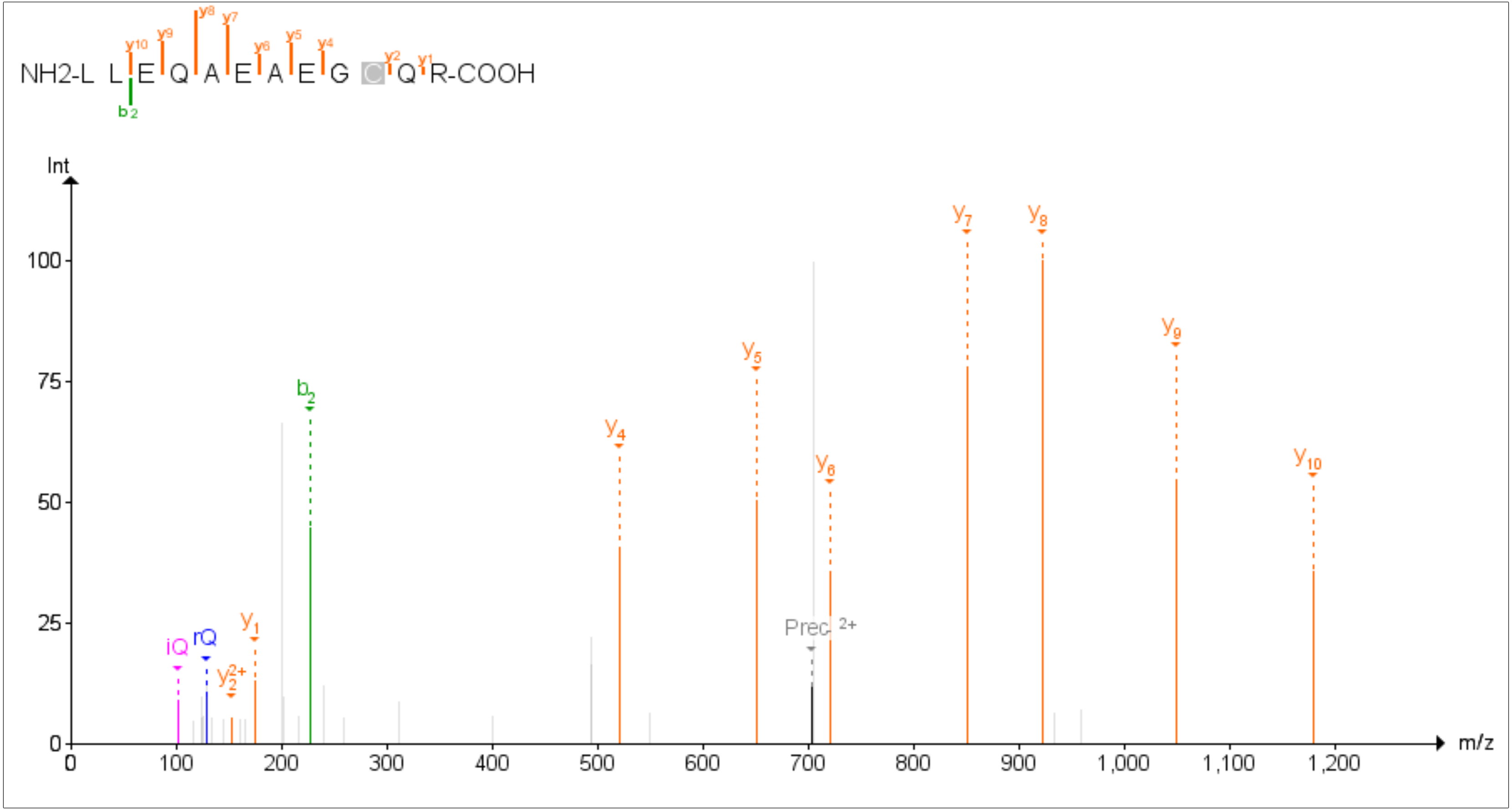
NH2-L L E Q A E A E G C Q R-COOH
y10 y9 y8 y7 y6 y5 y4 y2 y1
b2
Int
100
75
50
25
0
0 100 200 300 400 500 600 700 800 900 1,000 1,100 1,200
m/z
iQ
rQ
y1
y2 2+
b2
y4
y5
y6
Prec 2+
y7
y8
y9
y10

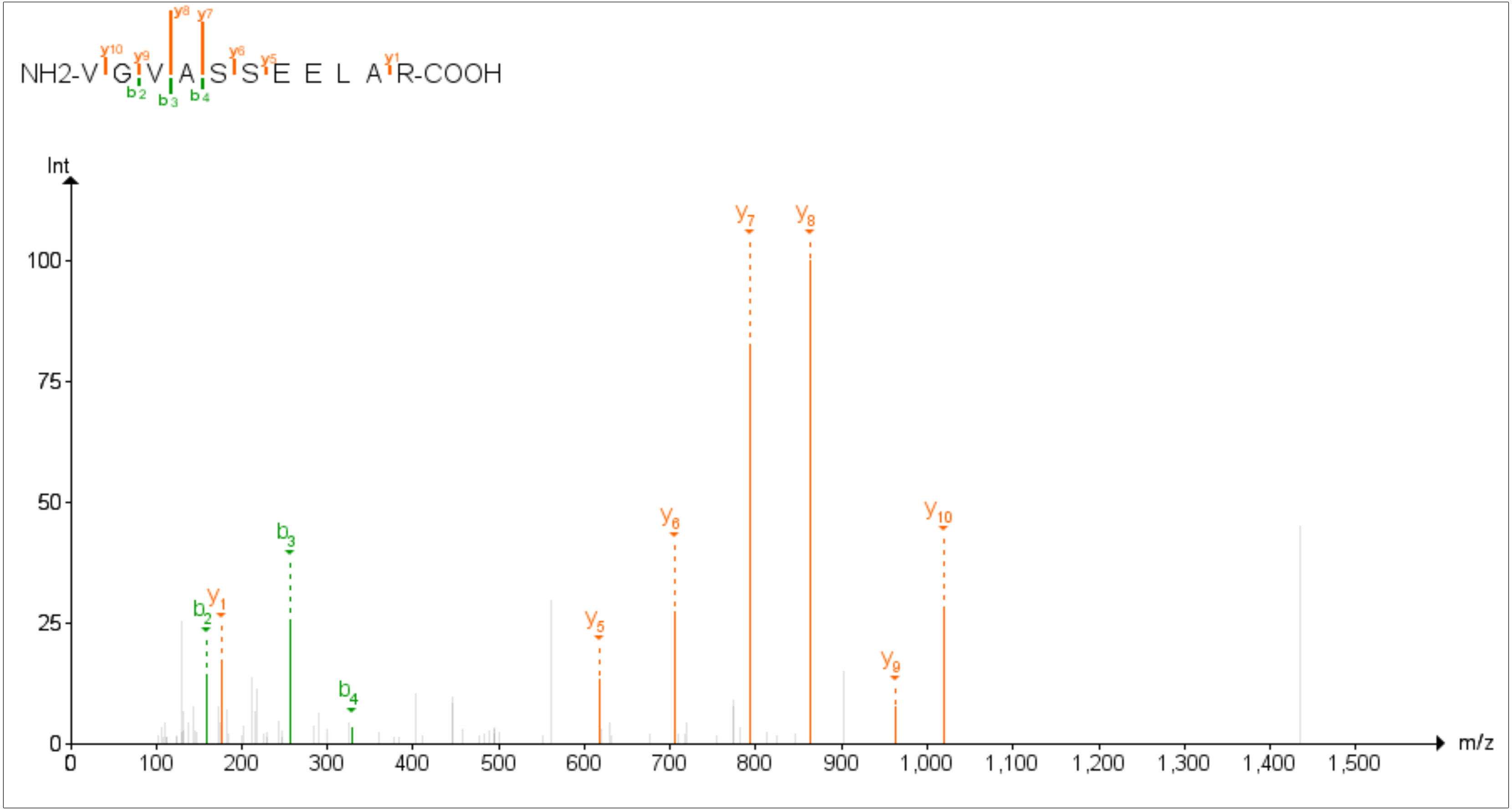

NH2-V G V A S S E E L A R-COOH
y10 y9 y8 y7 y6 y5 y1
b2 b3 b4
Int
100
75
50
25
0
y7 y8 y6 y10 b3 y1 b2 y5 y9 b4
0 100 200 300 400 500 600 700 800 900 1,000 1,100 1,200 1,300 1,400 1,500
m/z

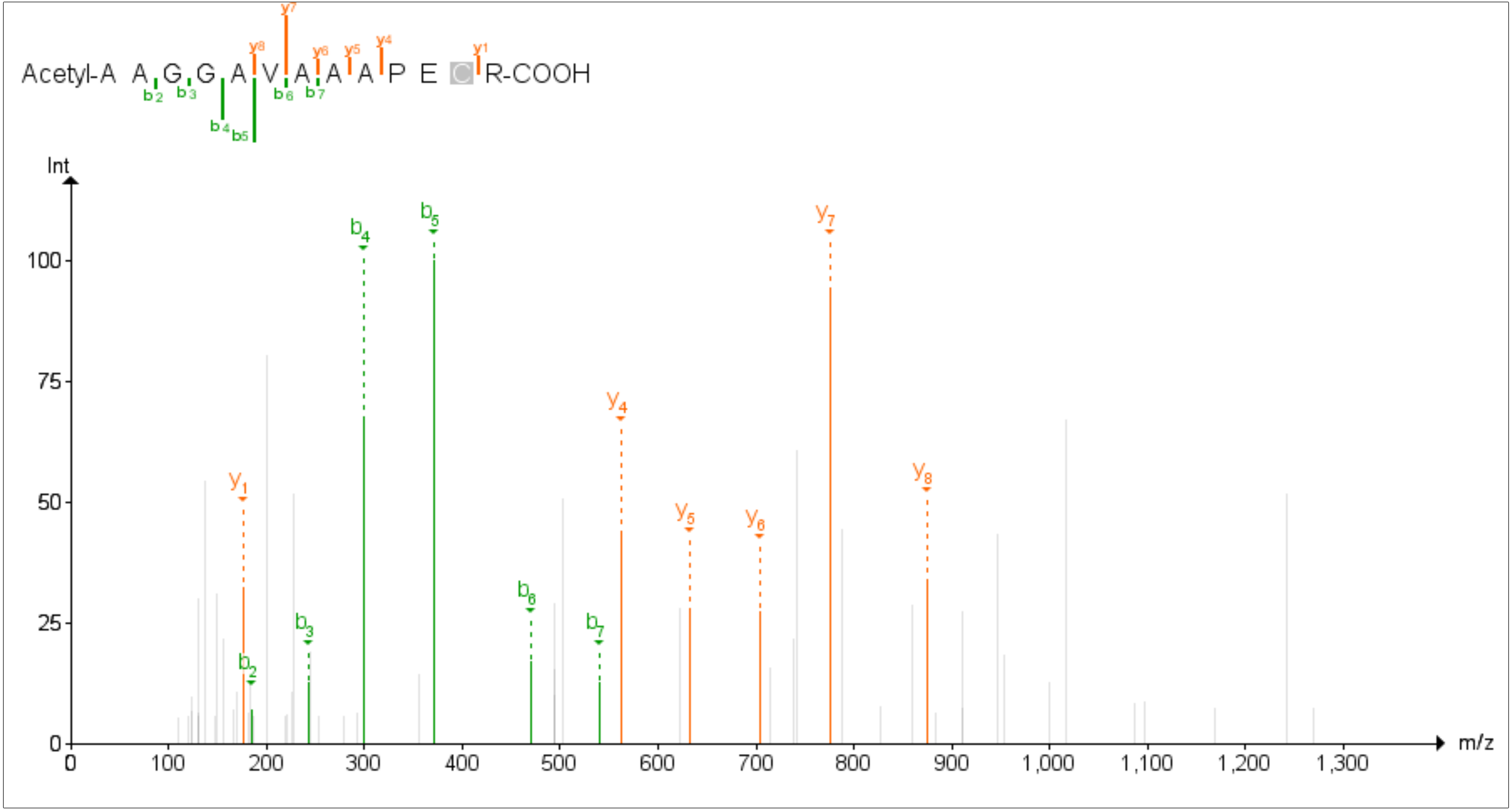

Acetyl-A A G G A V A A A A P E C R-COOH
b2 b3 b4 b5 b6 b7
y8 y7 y6 y5 y4 y1
Int
m/z
0 25 50 75 100
0 100 200 300 400 500 600 700 800 900 1,000 1,100 1,200 1,300

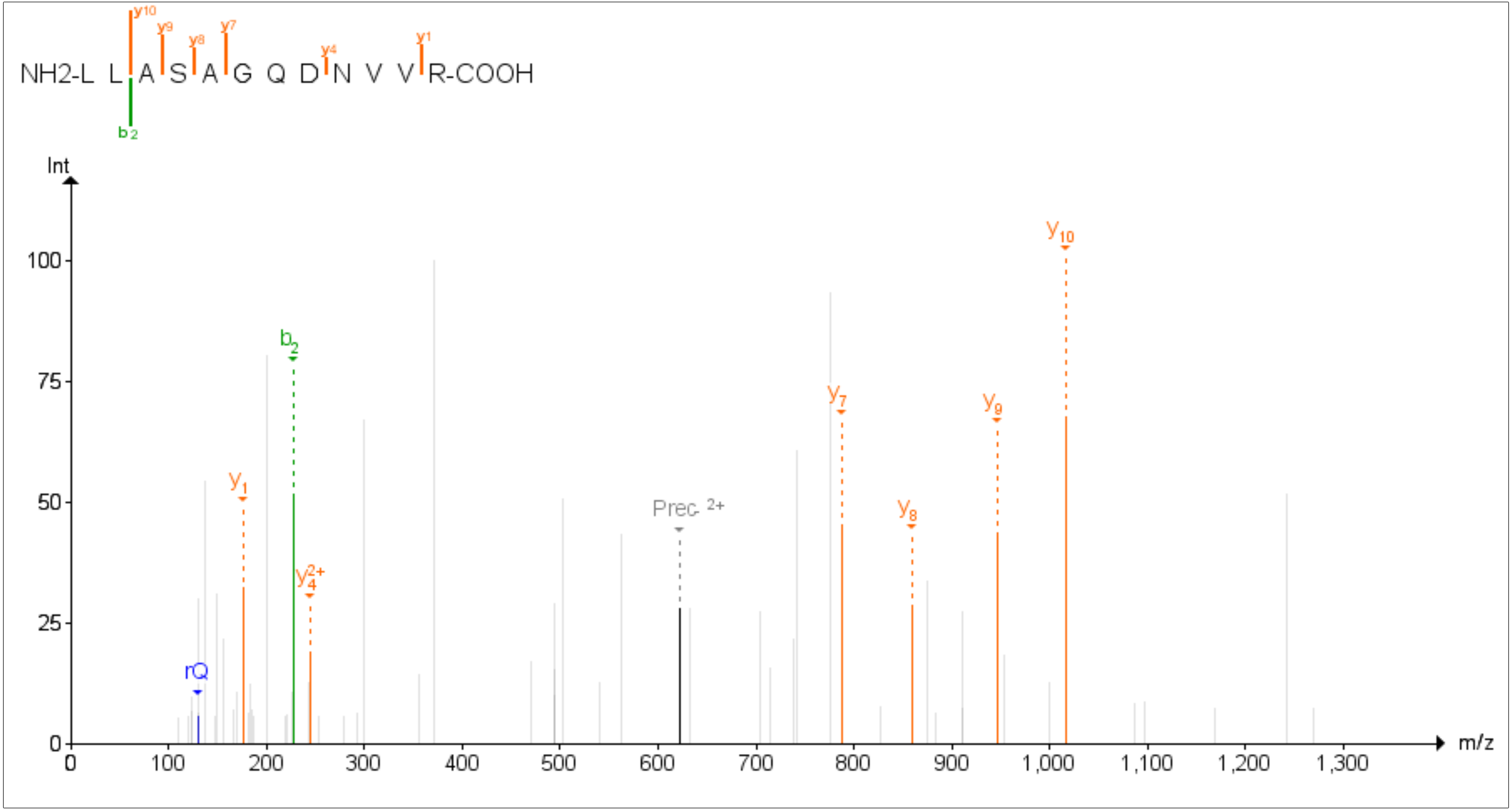

NH2-L L A S A G Q D N V V R-COOH
y10
y9
y8
y7
y4
y1
b2
Int
100
75
50
25
0
m/z
0
100
200
300
400
500
600
700
800
900
1,000
1,100
1,200
1,300
rQ
y1
b2
y4 2+
Prec 2+
y7
y8
y9
y10

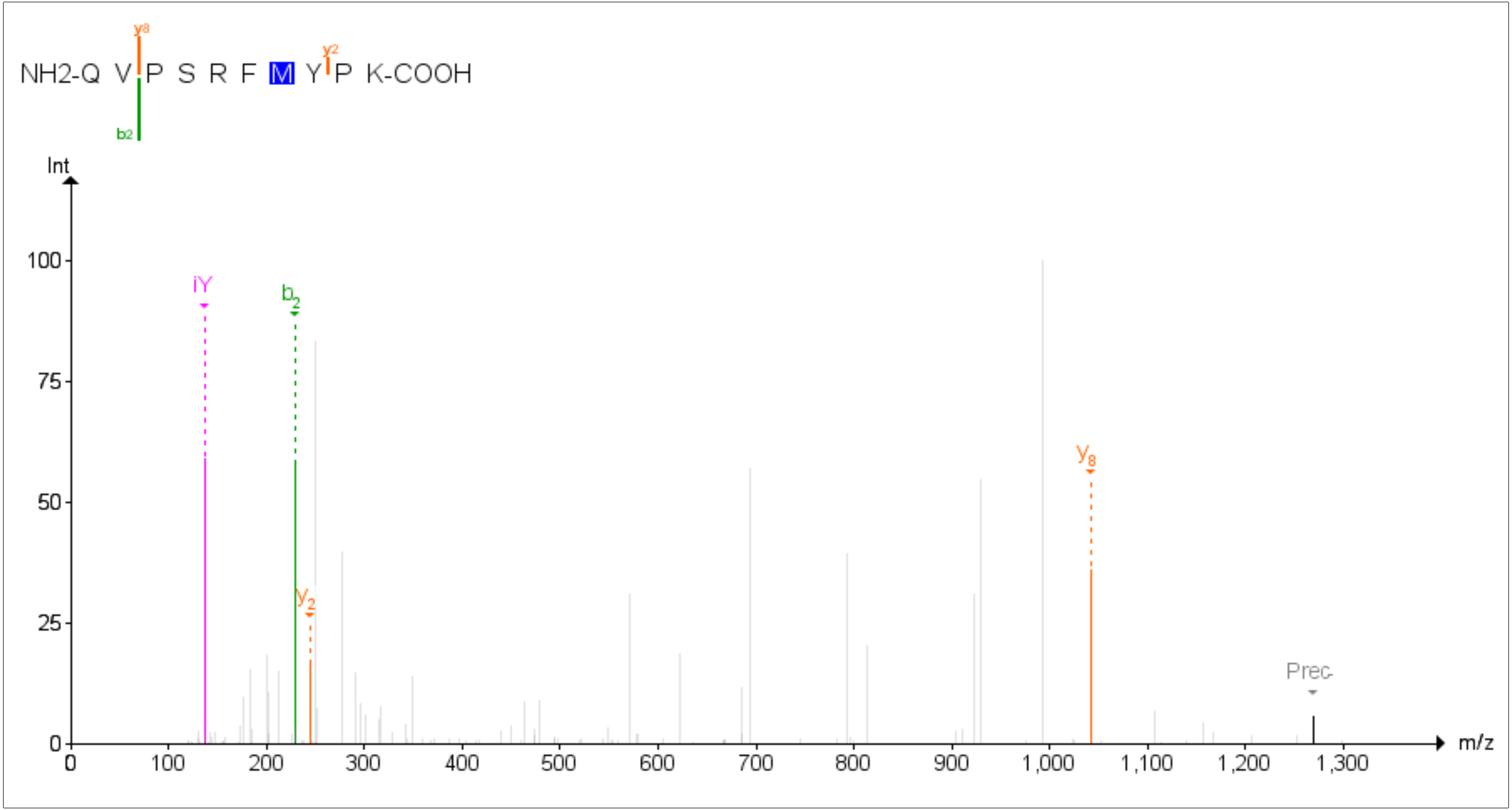

NH2-Q V P S R F M Y P K-COOH
y8
y2
b2
Int
100
75
50
25
0
iY
b2
y2
y8
Prec.
0
100
200
300
400
500
600
700
800
900
1,000
1,100
1,200
1,300
m/z

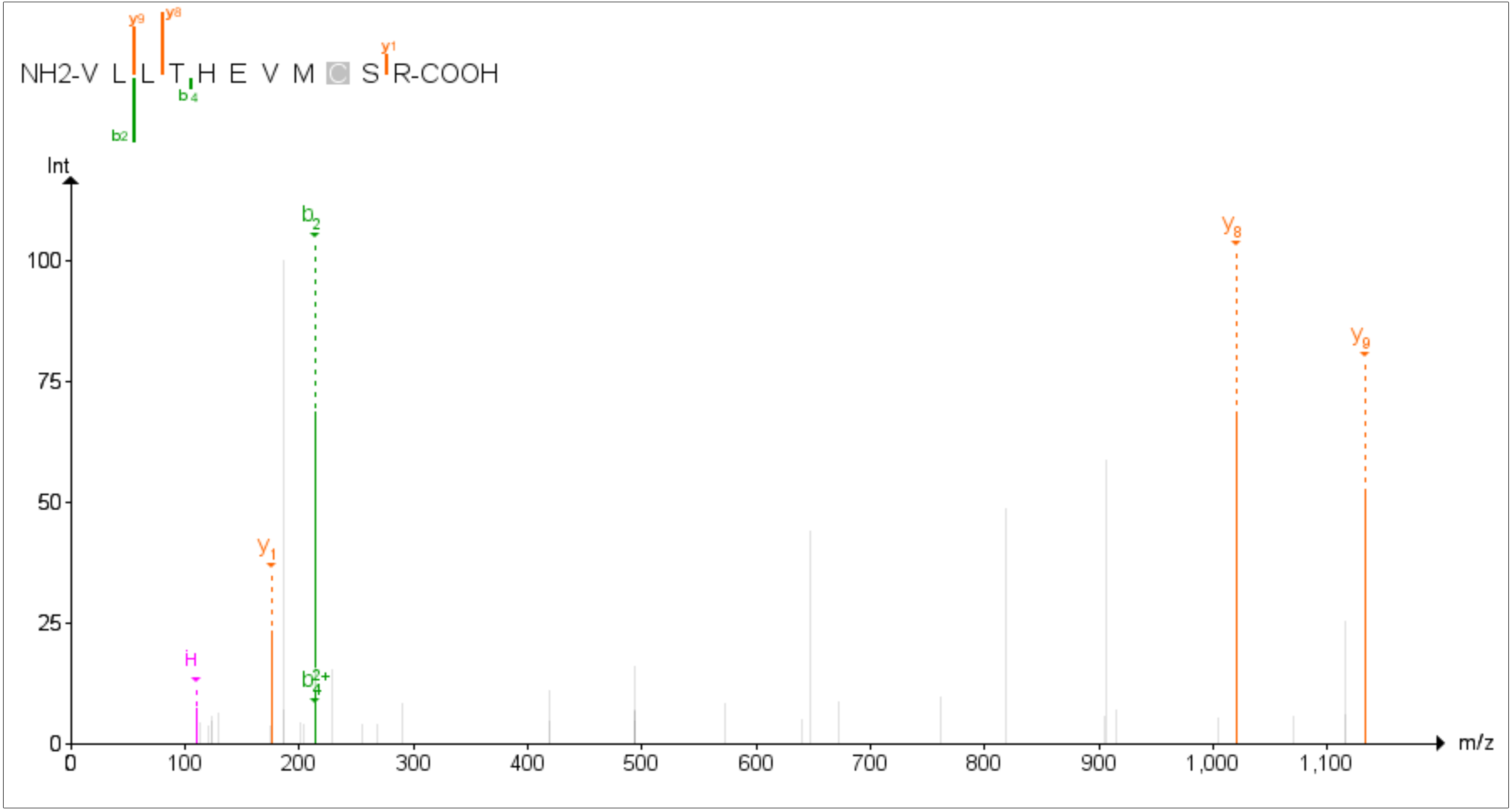
NH2-V L L T H E V M C S R-COOH
y9
y8
y1
b2
b4
Int
m/z
b2
y8
y9
y1
H
b4 2+
0
25
50
75
100
0
100
200
300
400
500
600
700
800
900
1,000
1,100

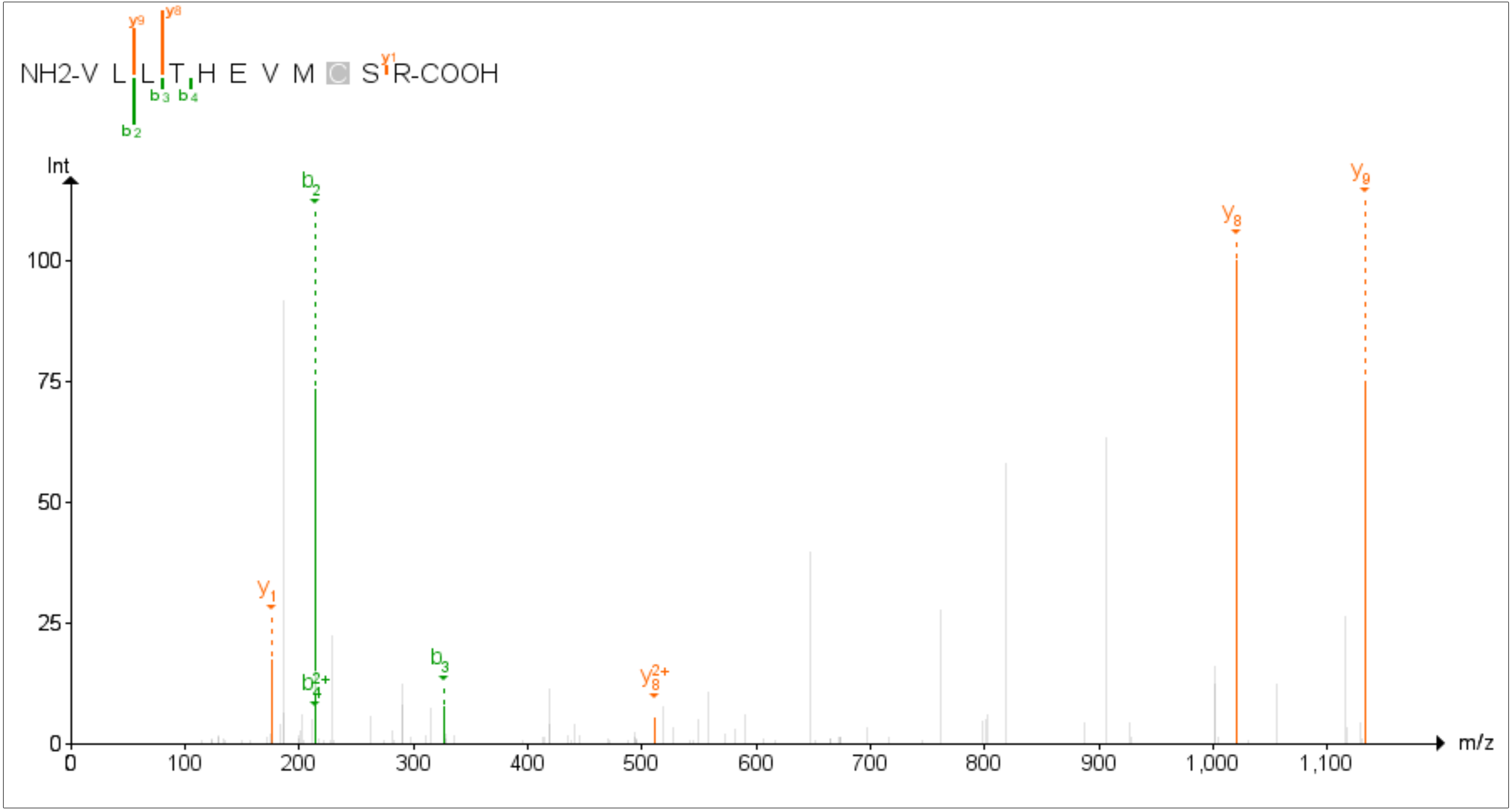

NH2-V L L T H E V M C S R-COOH
y9
y8
y1
b2
b3
b4
Int
100
75
50
25
0
m/z
0
100
200
300
400
500
600
700
800
900
1,000
1,100
b_2
y_8
y_9
y_1
b_4^{2+}
b_3
y_8^{2+}

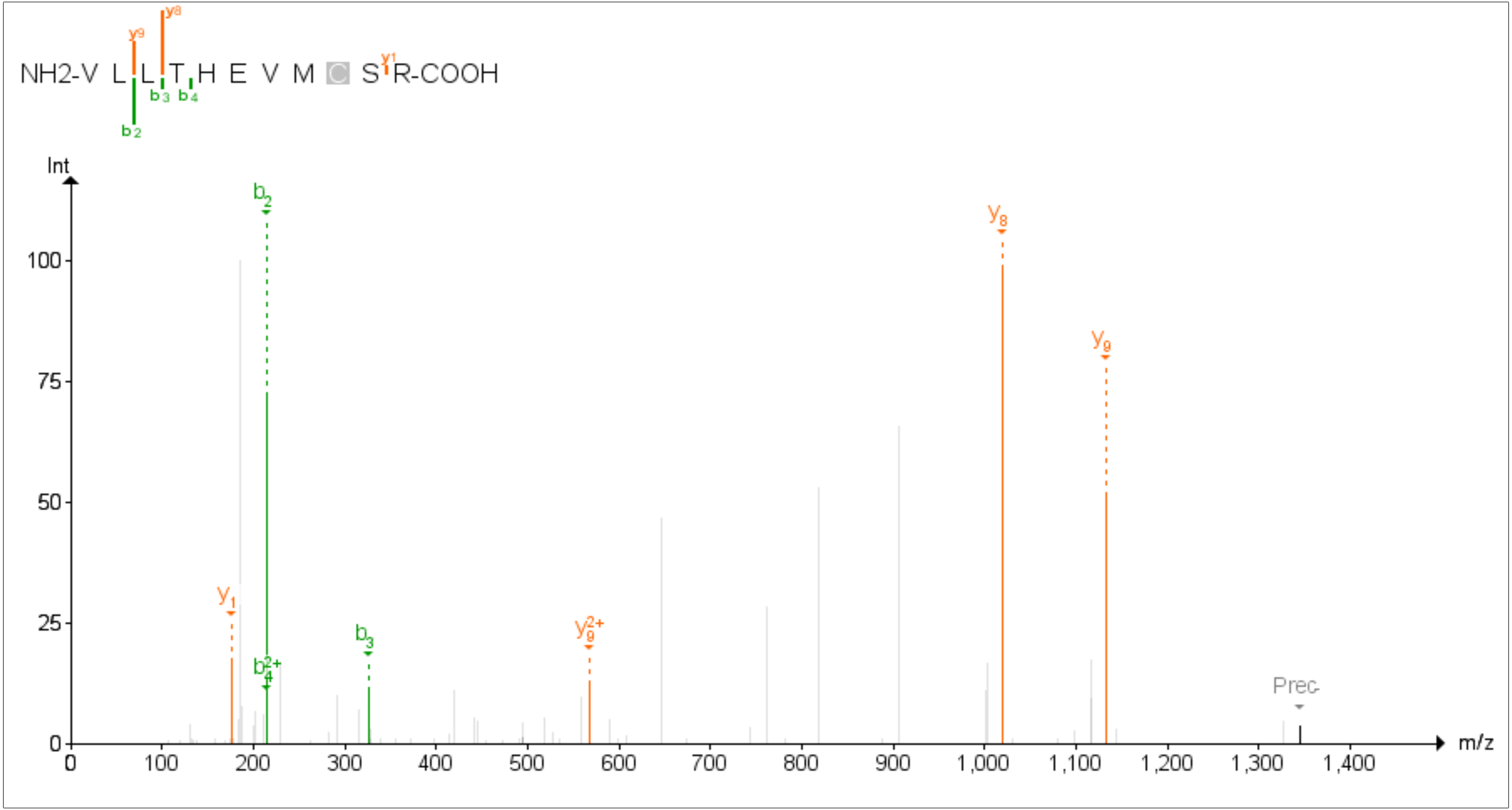

NH2-V L L T H E V M C S R-COOH
y9
y8
y1
b2
b3
b4
Int
100
75
50
25
0
b2
y8
y9
y1
b4 2+
b3
y9 2+
Prec.
m/z
0
100
200
300
400
500
600
700
800
900
1,000
1,100
1,200
1,300
1,400

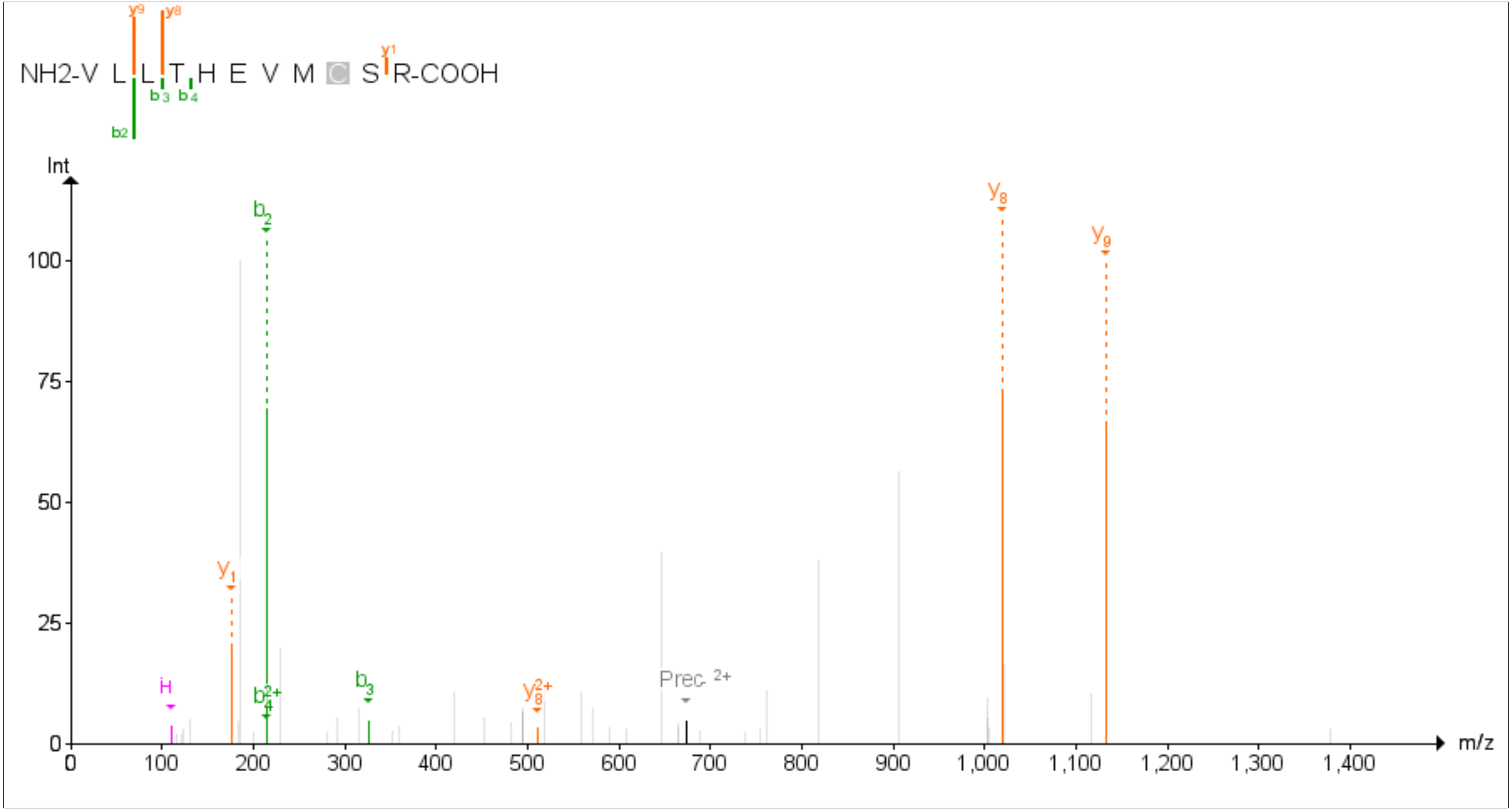

NH2-V L L T H E V M C S R-COOH
y9
y8
y1
b2
b3
b4
Int
m/z
b2
y8
y9
y1
H
b4 2+
b3
y8 2+
Prec. 2+
0
25
50
75
100
0
100
200
300
400
500
600
700
800
900
1,000
1,100
1,200
1,300
1,400

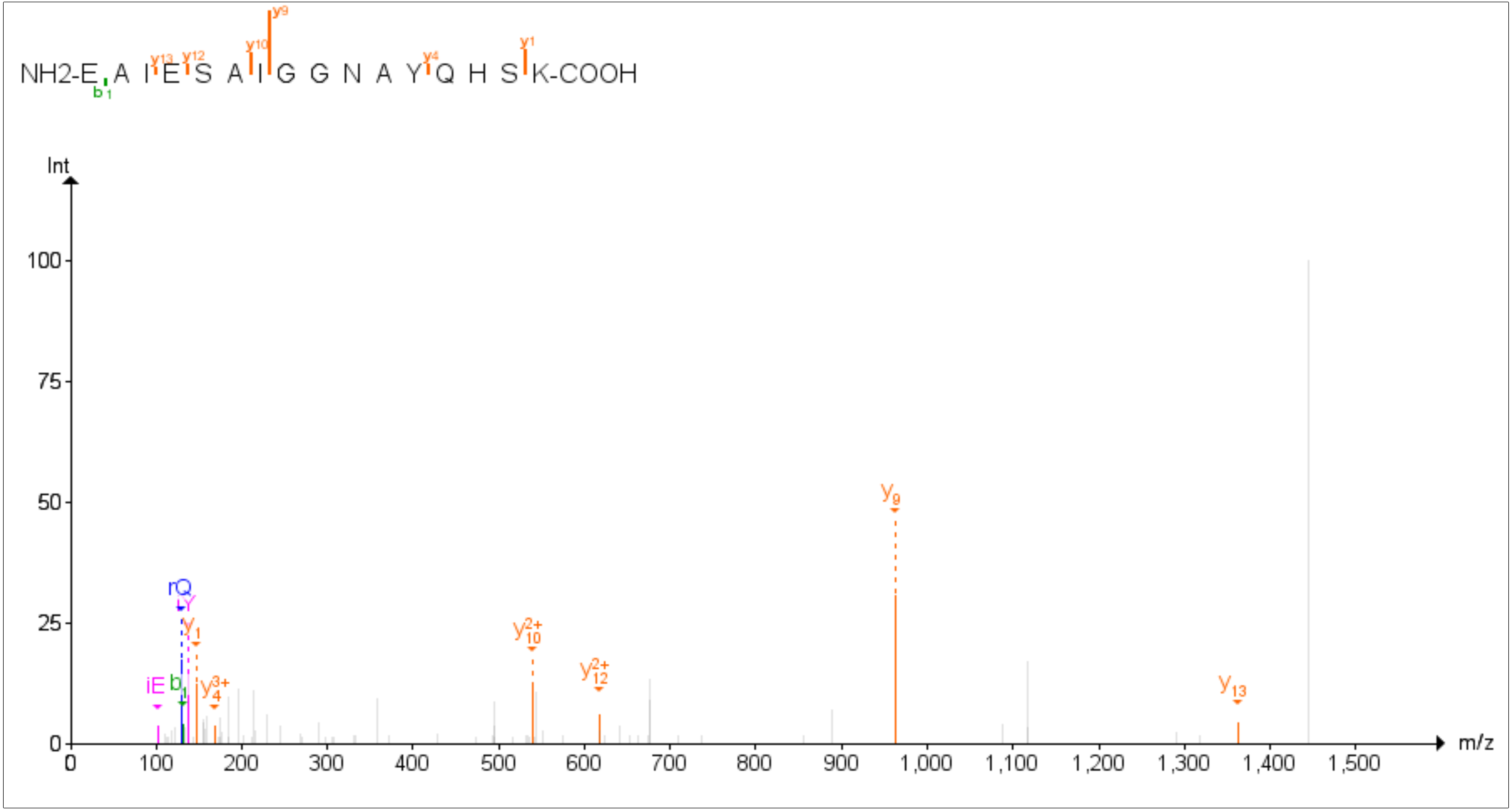
NH2-E A I E S A I G G N A Y Q H S K-COOH
b1
y13 y12 y10 y9 y4 y1
Int
100
75
50
25
0
m/z
0 100 200 300 400 500 600 700 800 900 1,000 1,100 1,200 1,300 1,400 1,500
rQ
iY
y1
iE
b1
y4 3+
y10 2+
y12 2+
y9
y13

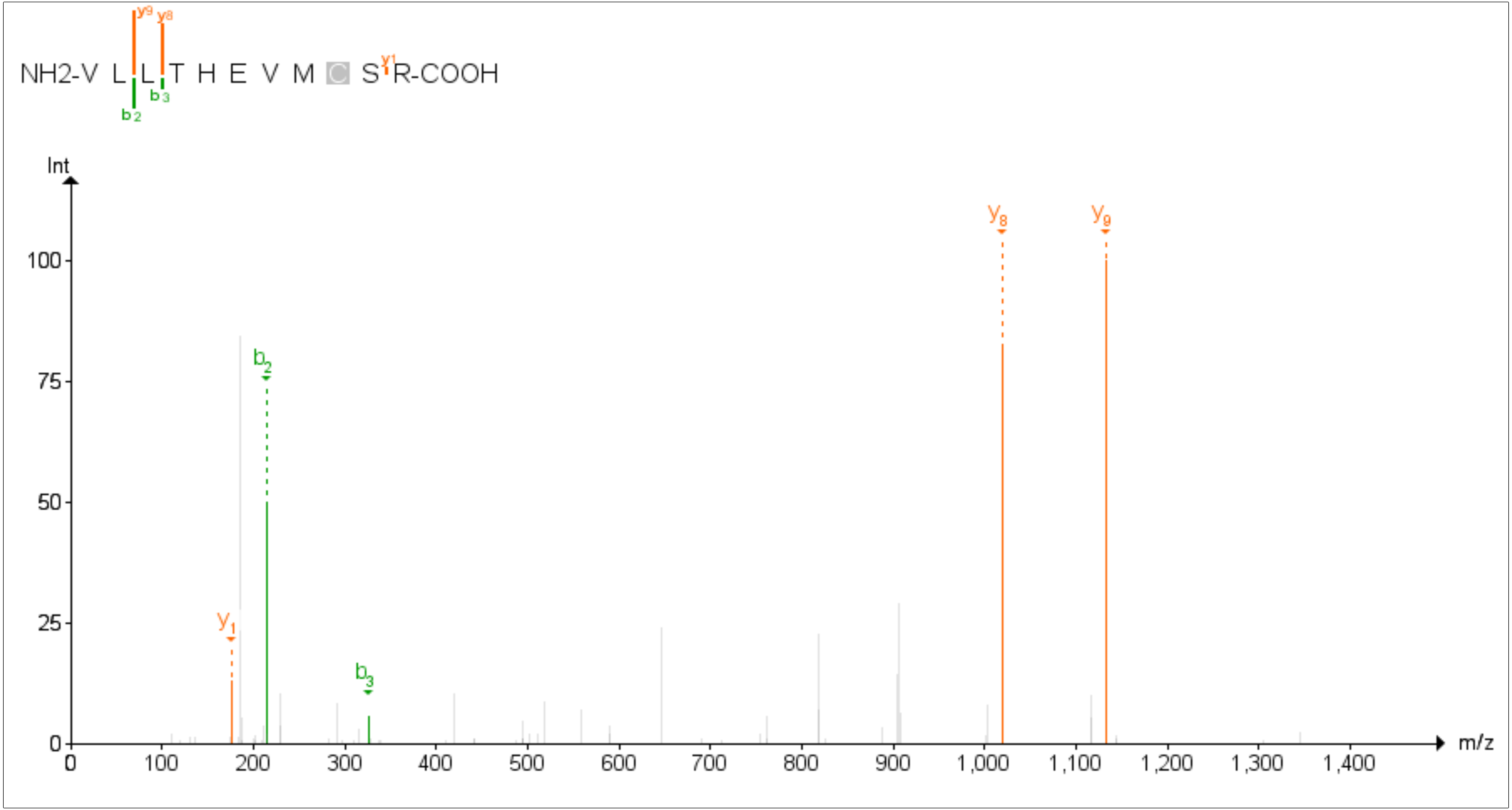
NH2-V L L T H E V M C S R-COOH
y9
y8
y1
b2
b3
Int
100
75
50
25
0
y8
y9
b2
y1
b3
0
100
200
300
400
500
600
700
800
900
1,000
1,100
1,200
1,300
1,400
m/z

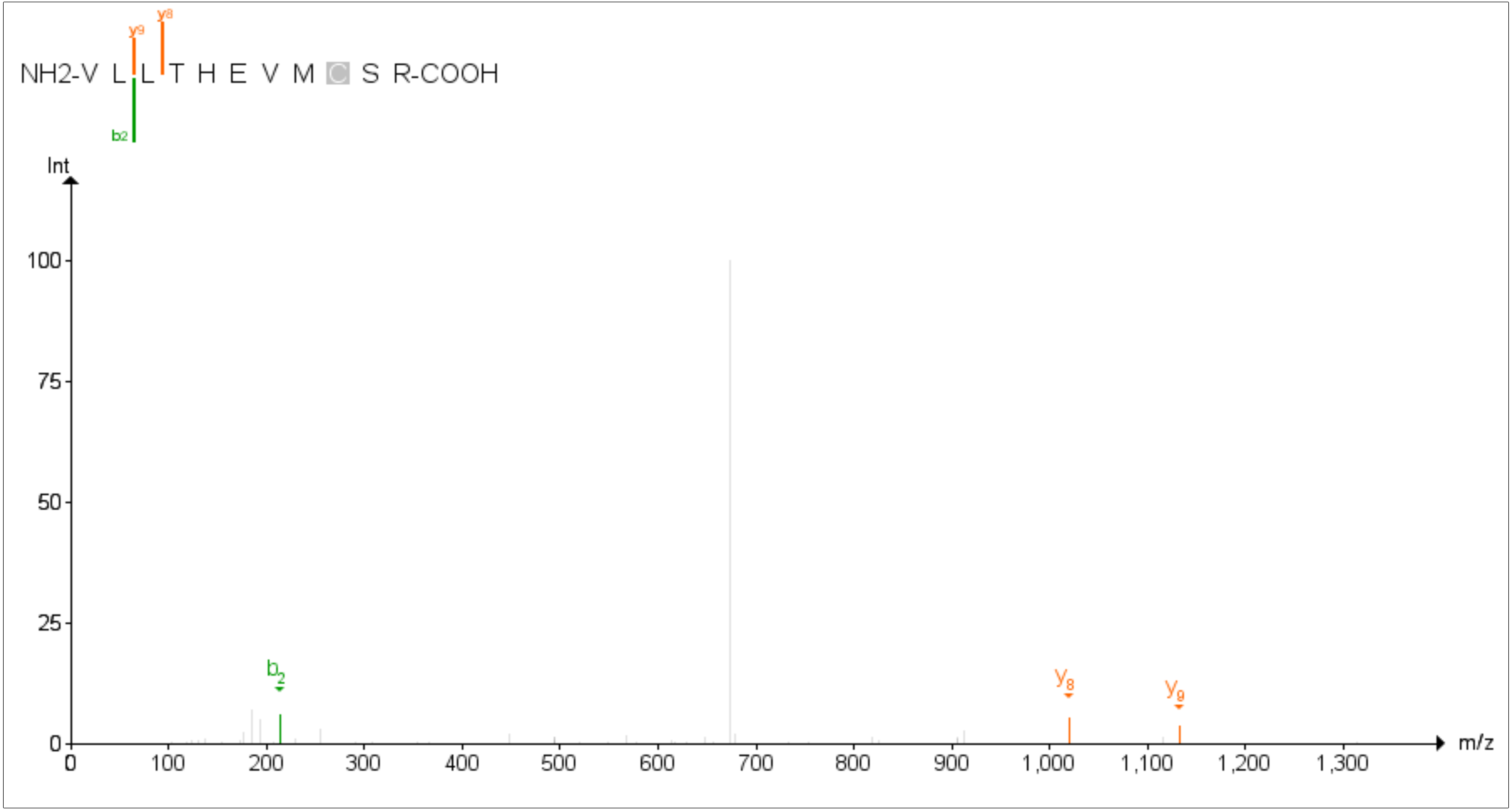

NH2-V L L T H E V M C S R-COOH
y9
y8
b2
Int
100
75
50
25
0
b2
y8
y9
0
100
200
300
400
500
600
700
800
900
1,000
1,100
1,200
1,300
m/z

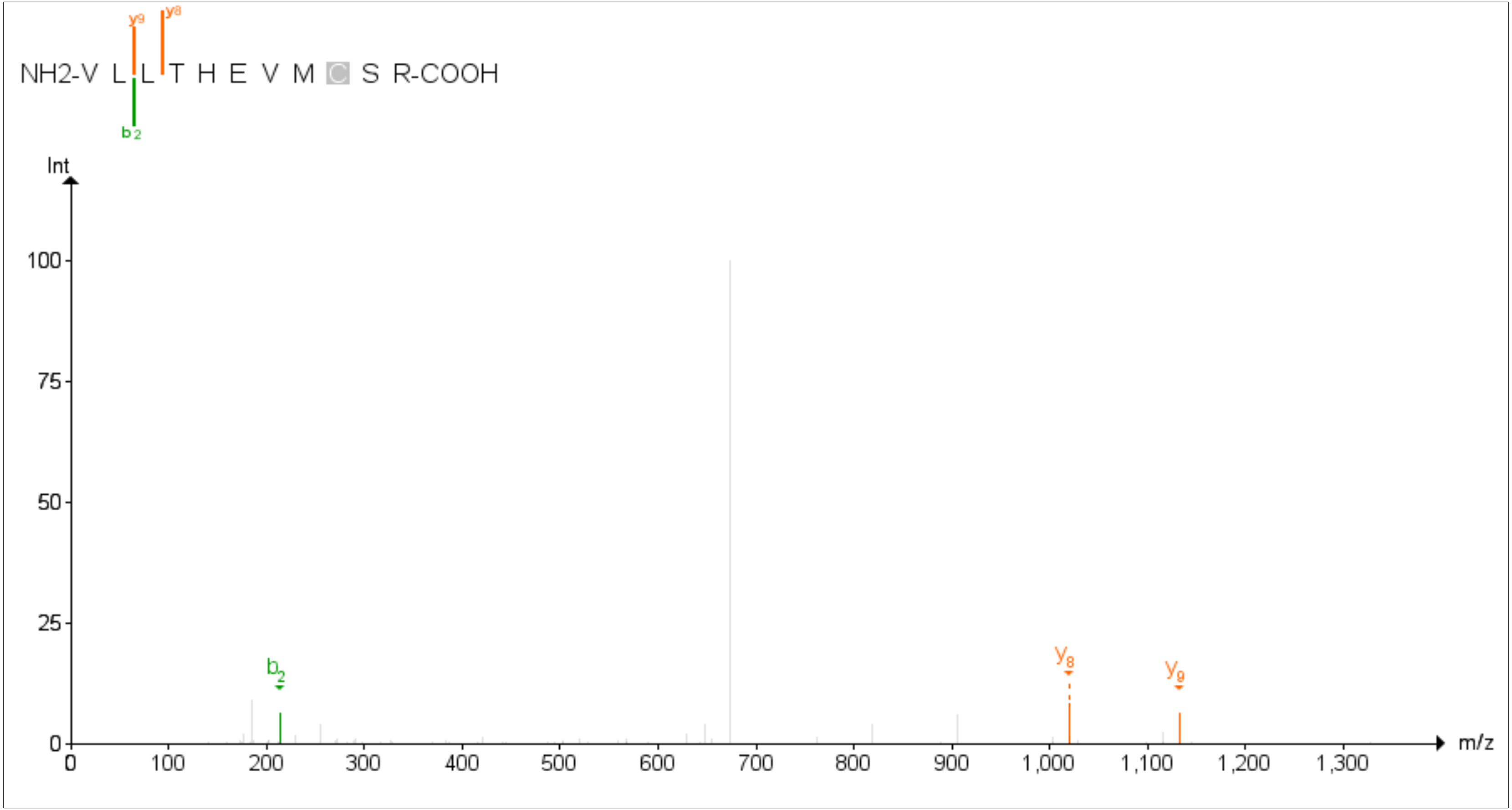

NH2-V L L T H E V M C S R-COOH
y9
y8
b2
Int
100
75
50
25
0
b2
y8
y9
0
100
200
300
400
500
600
700
800
900
1,000
1,100
1,200
1,300
m/z

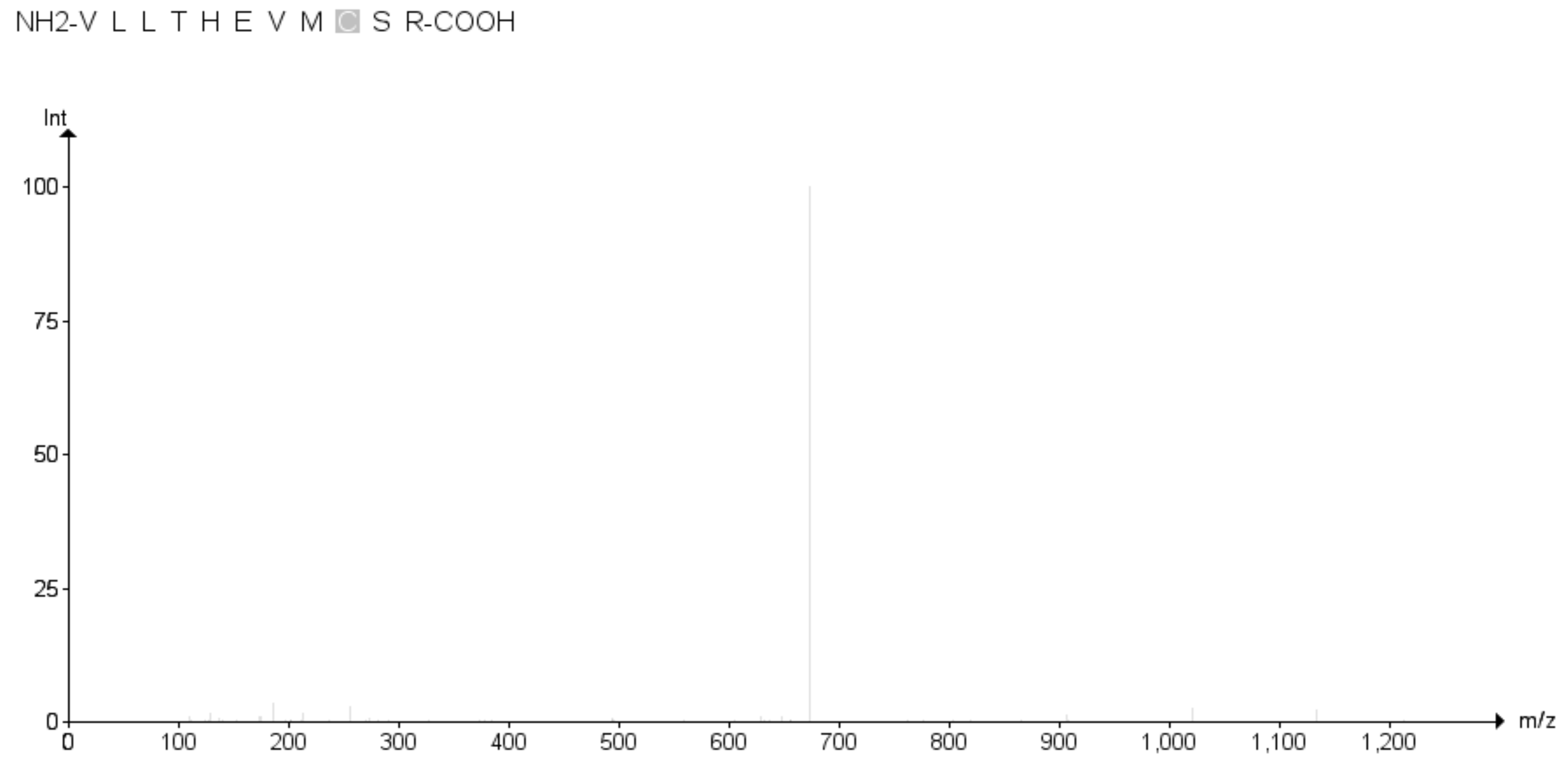

NH2-V L L T H E V M C S R-COOH
Int
100
75
50
25
0
m/z
0
100
200
300
400
500
600
700
800
900
1,000
1,100
1,200

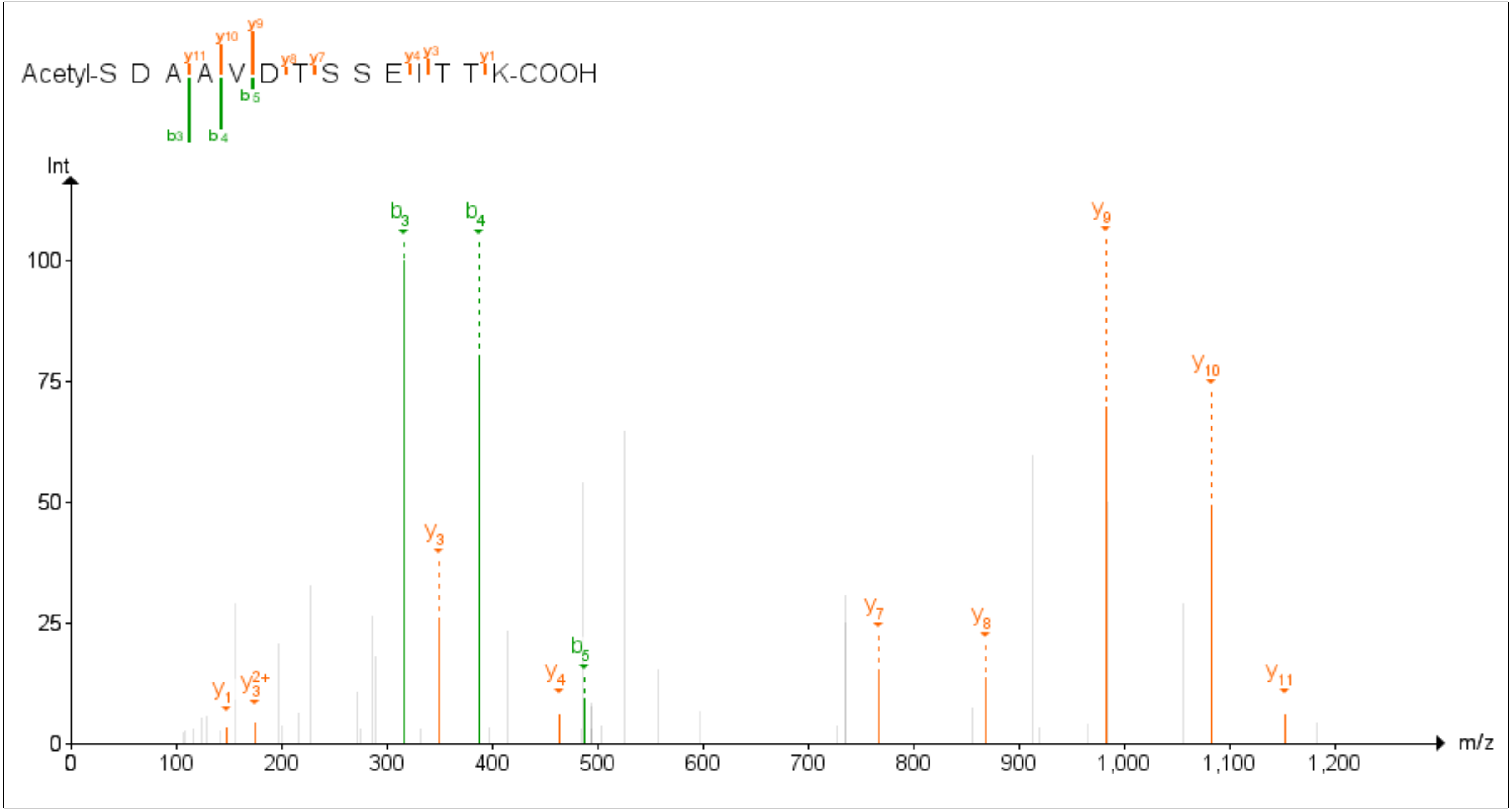

Acetyl-S D A A V D T S S E I T T K-COOH
y11
y10
y9
y8
y7
y4
y3
y1
b3
b4
b5
Int
100
75
50
25
0
m/z
0
100
200
300
400
500
600
700
800
900
1,000
1,100
1,200
b3
b4
y9
y10
y3
y7
y8
b5
y4
y1
y3 2+
y11

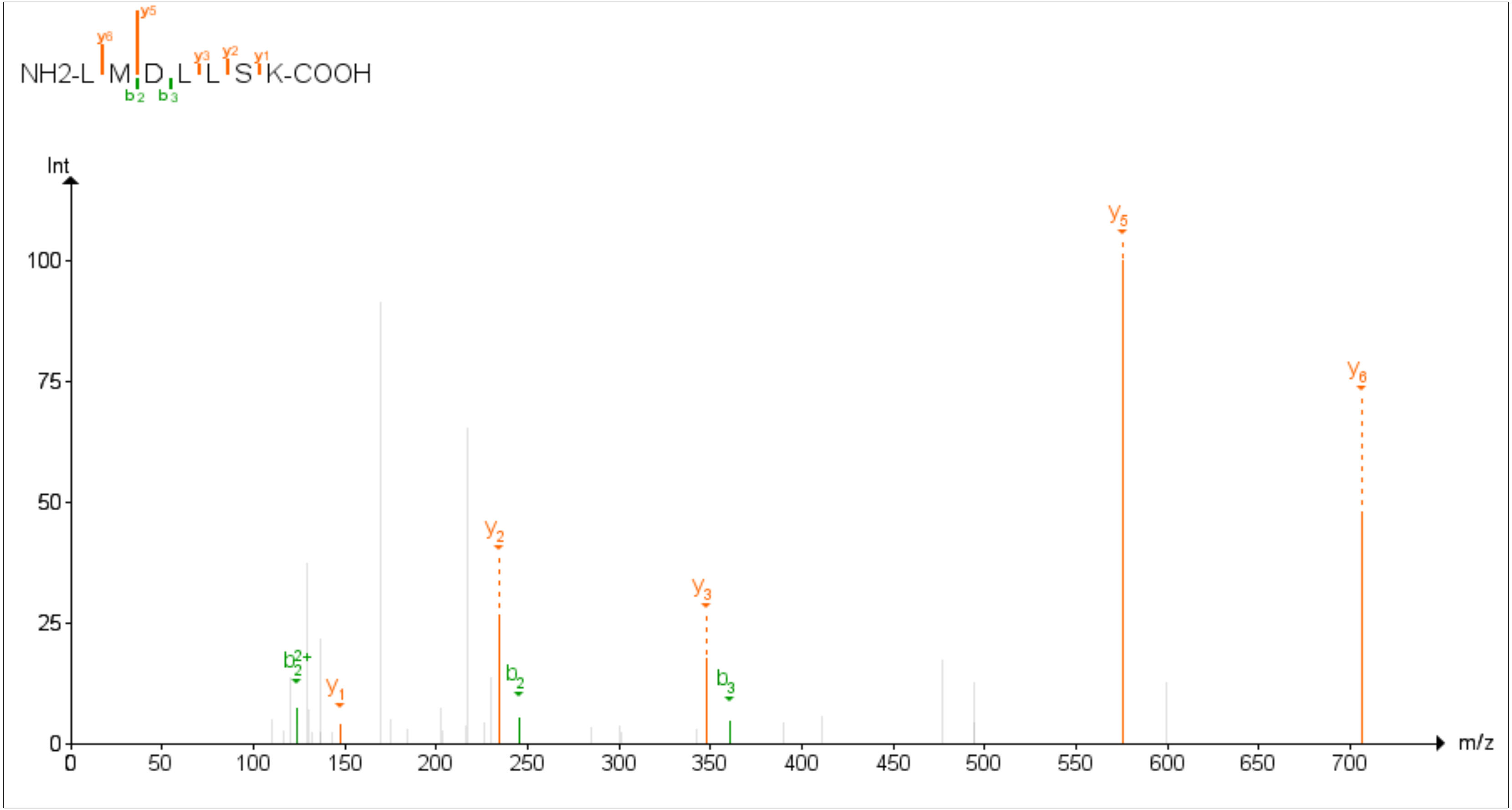

NH2-L M D L L S K-COOH
y6
y5
y3
y2
y1
b2
b3
Int
100
75
50
25
0
y5
y6
y2
y3
b2 2+
y1
b2
b3
0
50
100
150
200
250
300
350
400
450
500
550
600
650
700
m/z

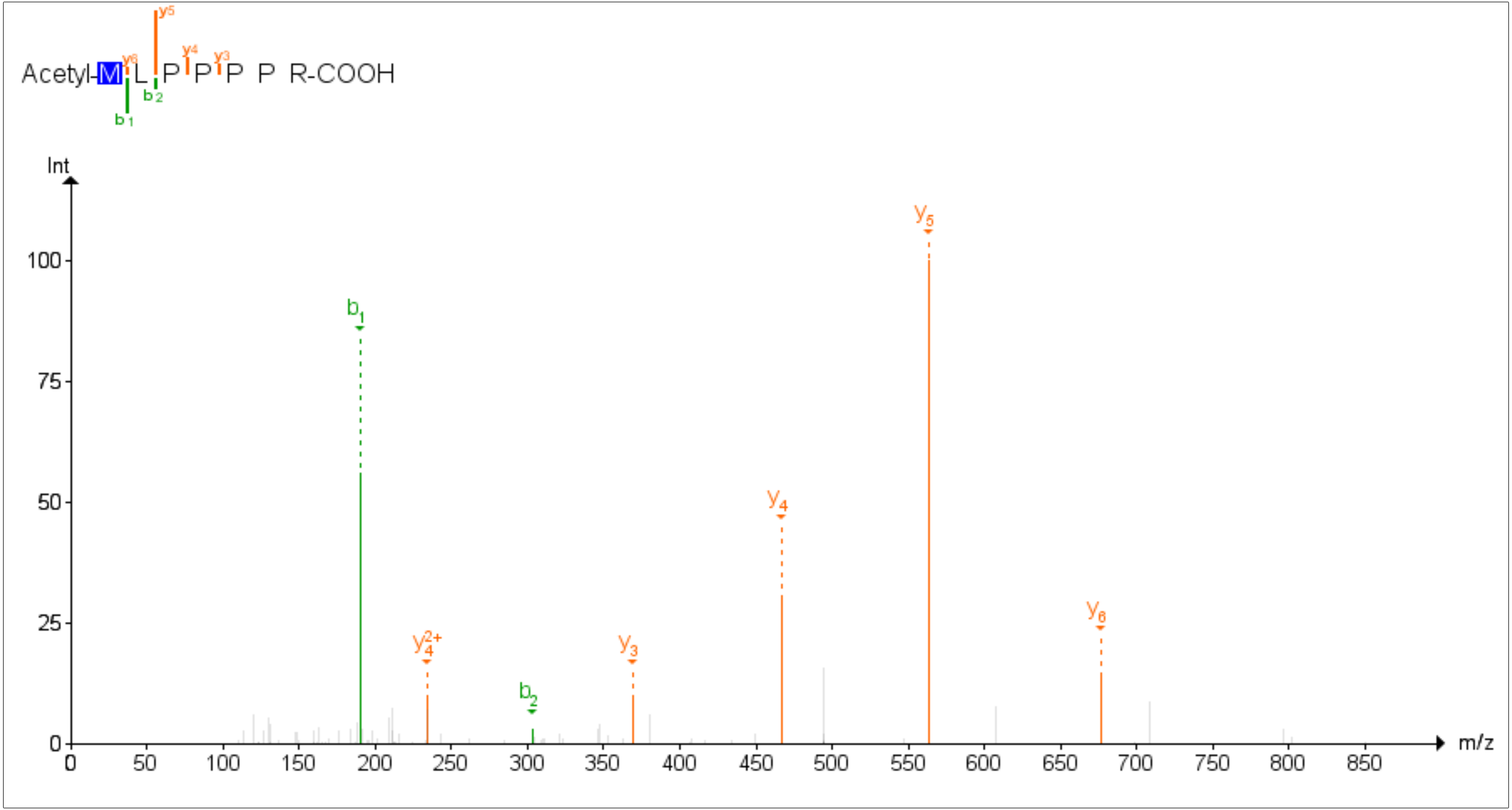

Acetyl-M L P P P P R-COOH
y5
y6
y4
y3
b 1
b 2
Int
100
75
50
25
0
b1
y4 2+
b2
y3
y4
y5
y6
0
50
100
150
200
250
300
350
400
450
500
550
600
650
700
750
800
850
m/z

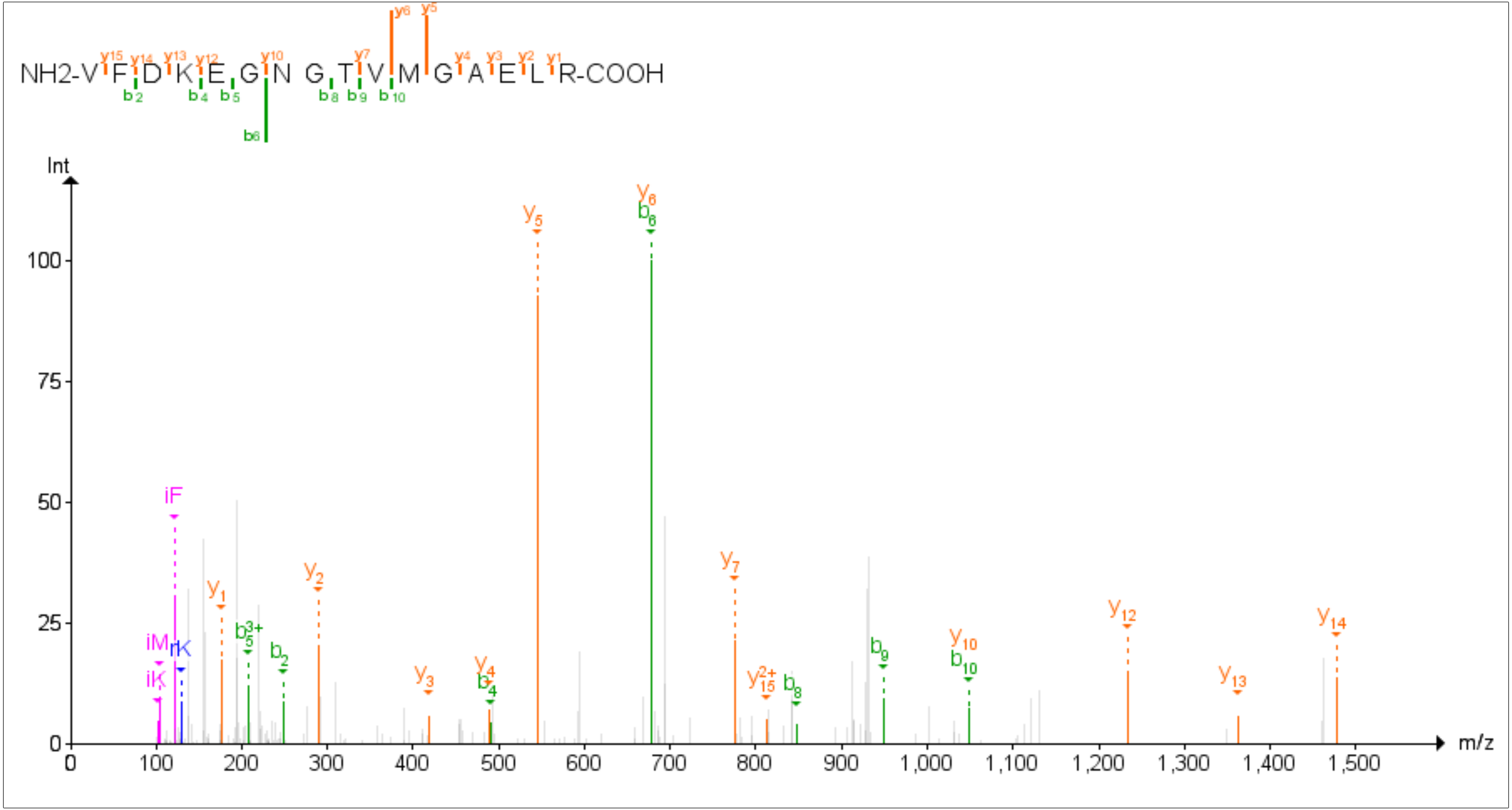

NH2-V F D K E G N G T V M G A E L R-COOH
y15 y14 y13 y12 y10 y7 y6 y5 y4 y3 y2 y1
b2 b4 b5 b6 b8 b9 b10
Int
0
25
50
75
100
m/z
0 100 200 300 400 500 600 700 800 900 1,000 1,100 1,200 1,300 1,400 1,500
iF
iM
iK
rK
y1
y2
y3
y4
y5
y6
y7
y10
y12
y13
y14
y15 2+
b5 3+
b2
b4
b6
b8
b9
b10

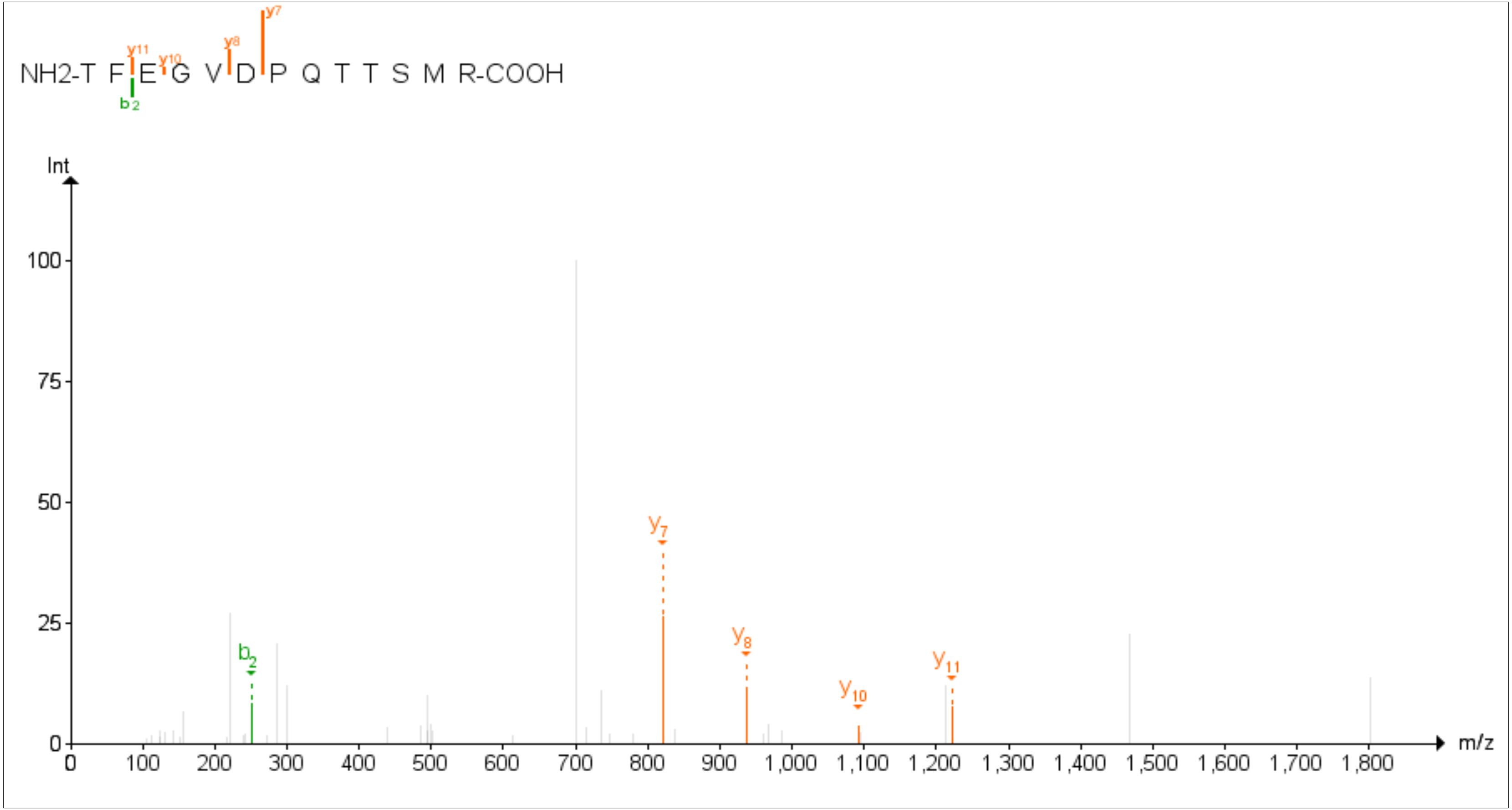

NH2-T F E G V D P Q T T S M R-COOH
y11
y10
y8
y7
b2
Int
100
75
50
25
0
m/z
0
100
200
300
400
500
600
700
800
900
1,000
1,100
1,200
1,300
1,400
1,500
1,600
1,700
1,800
y7
y8
y10
y11
b2

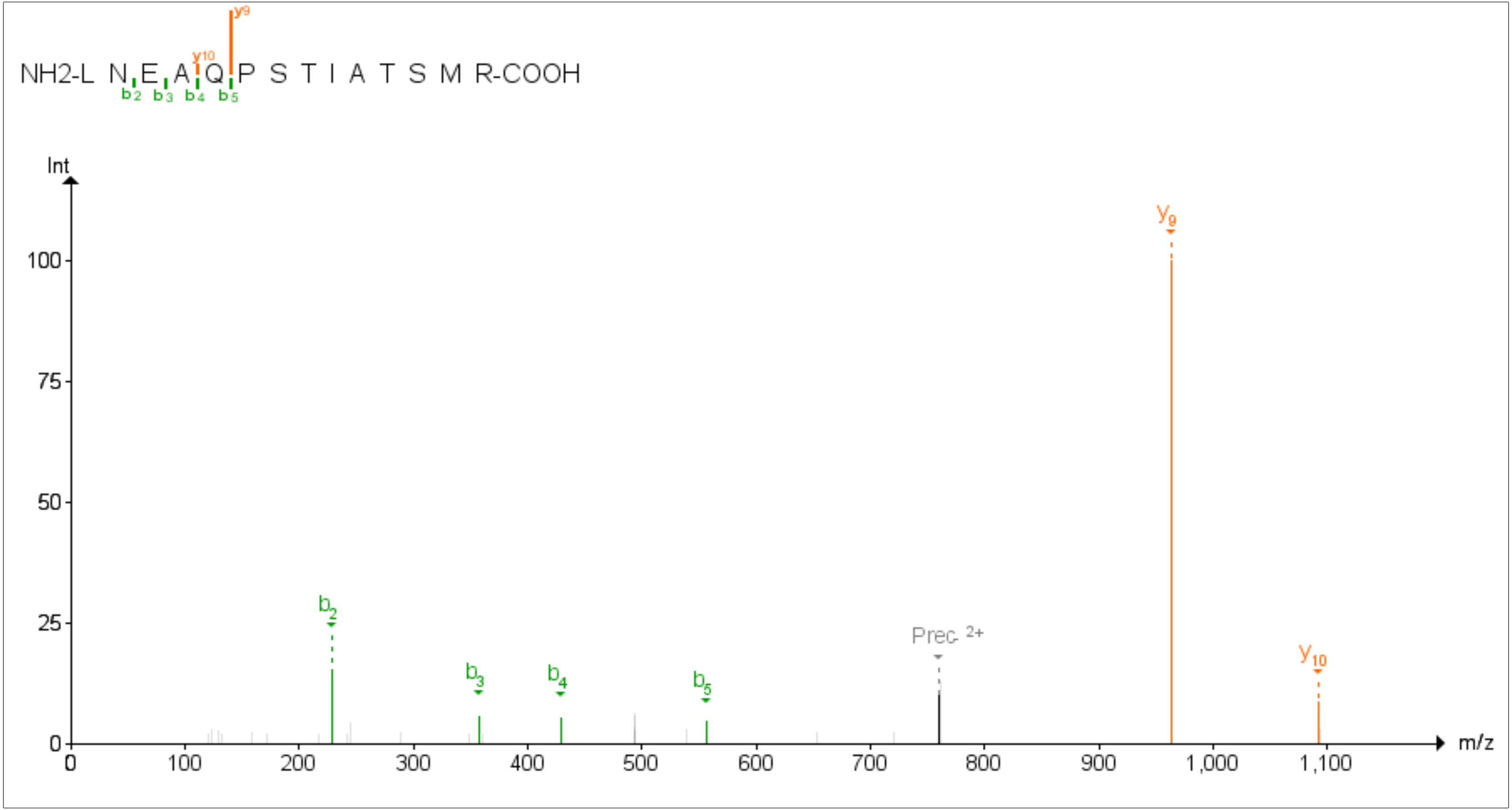

NH2-L N E A Q P S T I A T S M R-COOH
y9
y10
b2
b3
b4
b5
Int
100
75
50
25
0
y9
b2
b3
b4
b5
Prec 2+
y10
0
100
200
300
400
500
600
700
800
900
1,000
1,100
m/z

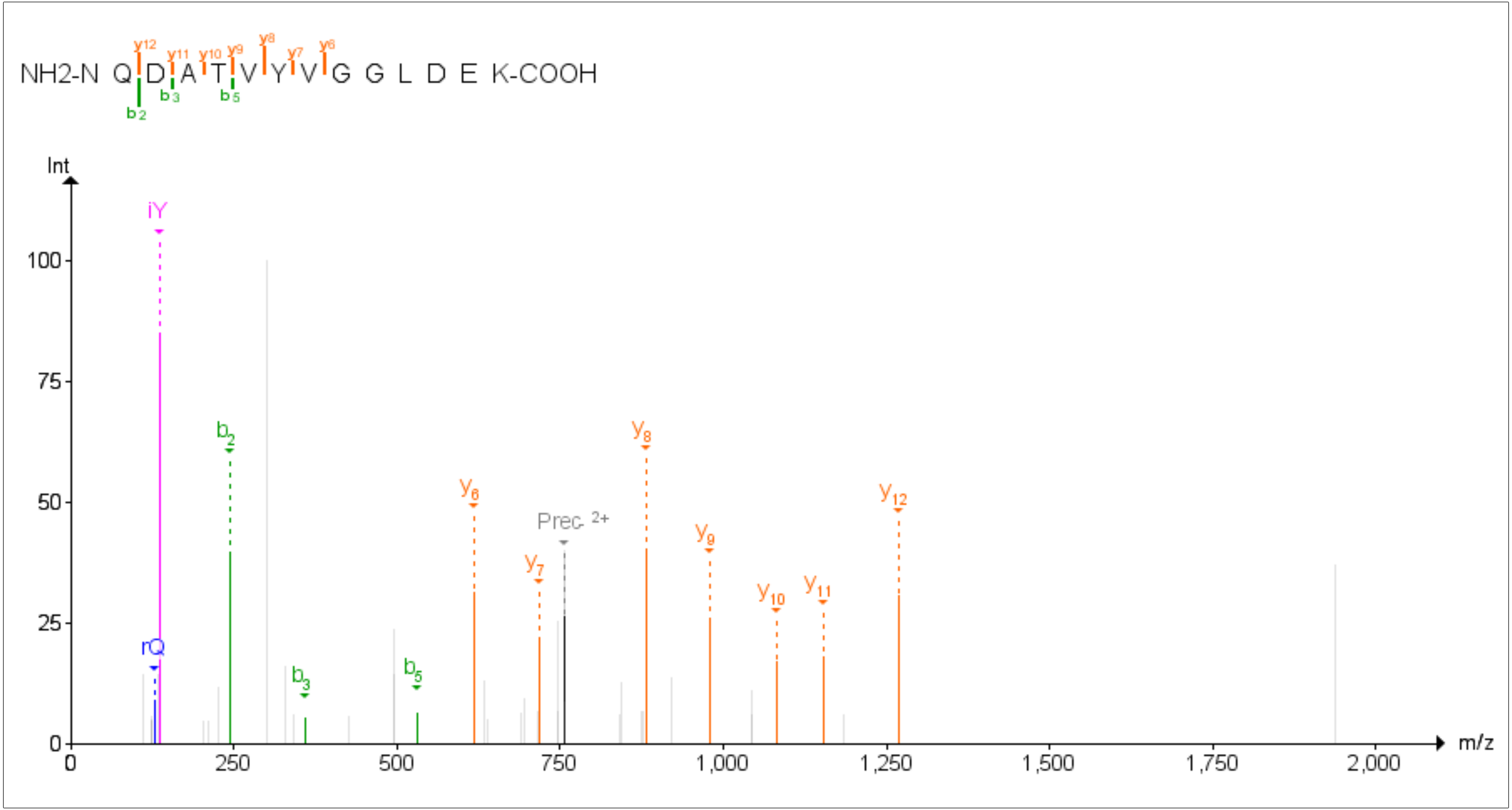
NH2-N Q D A T V Y V G G L D E K-COOH
y12 y11 y10 y9 y8 y7 y6
b2 b3 b5
Int
iY
rQ
b2
b3
b5
y6
y7
Prec 2+
y8
y9
y10
y11
y12
100
75
50
25
0
250
500
750
1,000
1,250
1,500
1,750
2,000
m/z

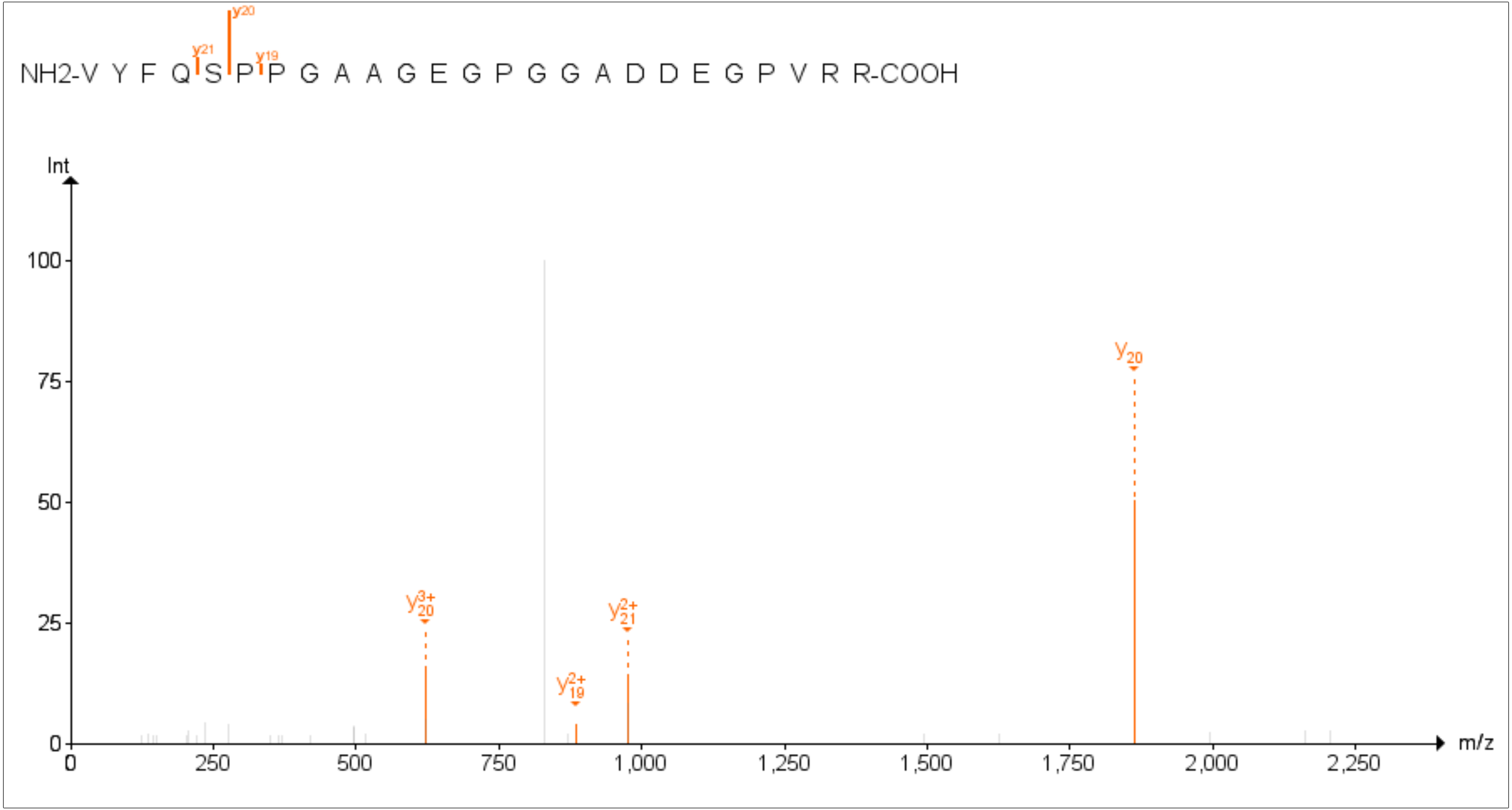

NH2-V Y F Q S P P G A A G E G P G G A D D E G P V R R-COOH
y20
y21
y19
Int
100
75
50
25
0
m/z
0
250
500
750
1,000
1,250
1,500
1,750
2,000
2,250
y20 3+
y19 2+
y21 2+
y20

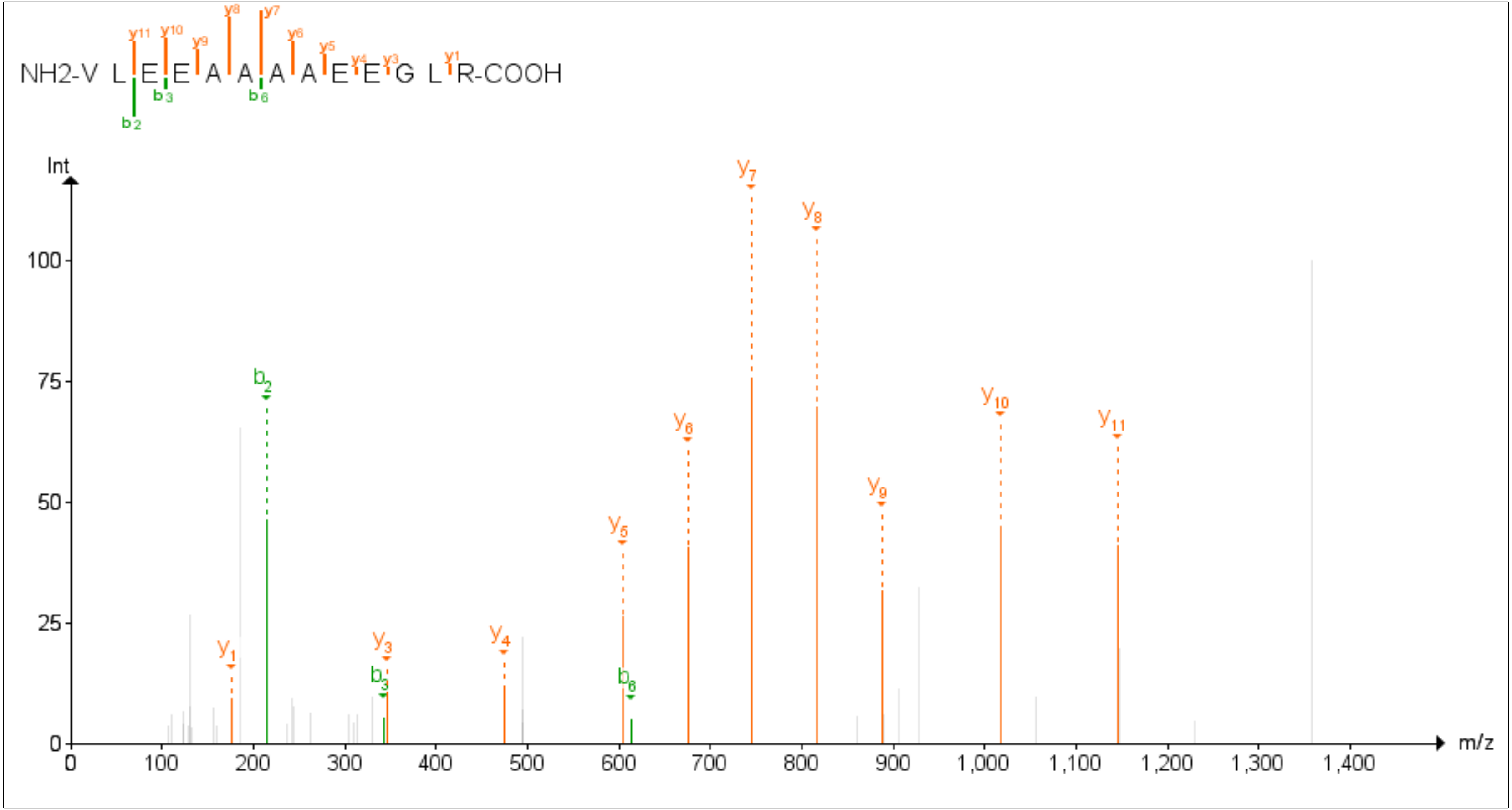

NH2-V L E E A A A A E E G L R-COOH
y11 y10 y9 y8 y7 y6 y5 y4 y3 y1
b2 b3 b6
Int
100
75
50
25
0
m/z
0 100 200 300 400 500 600 700 800 900 1,000 1,100 1,200 1,300 1,400

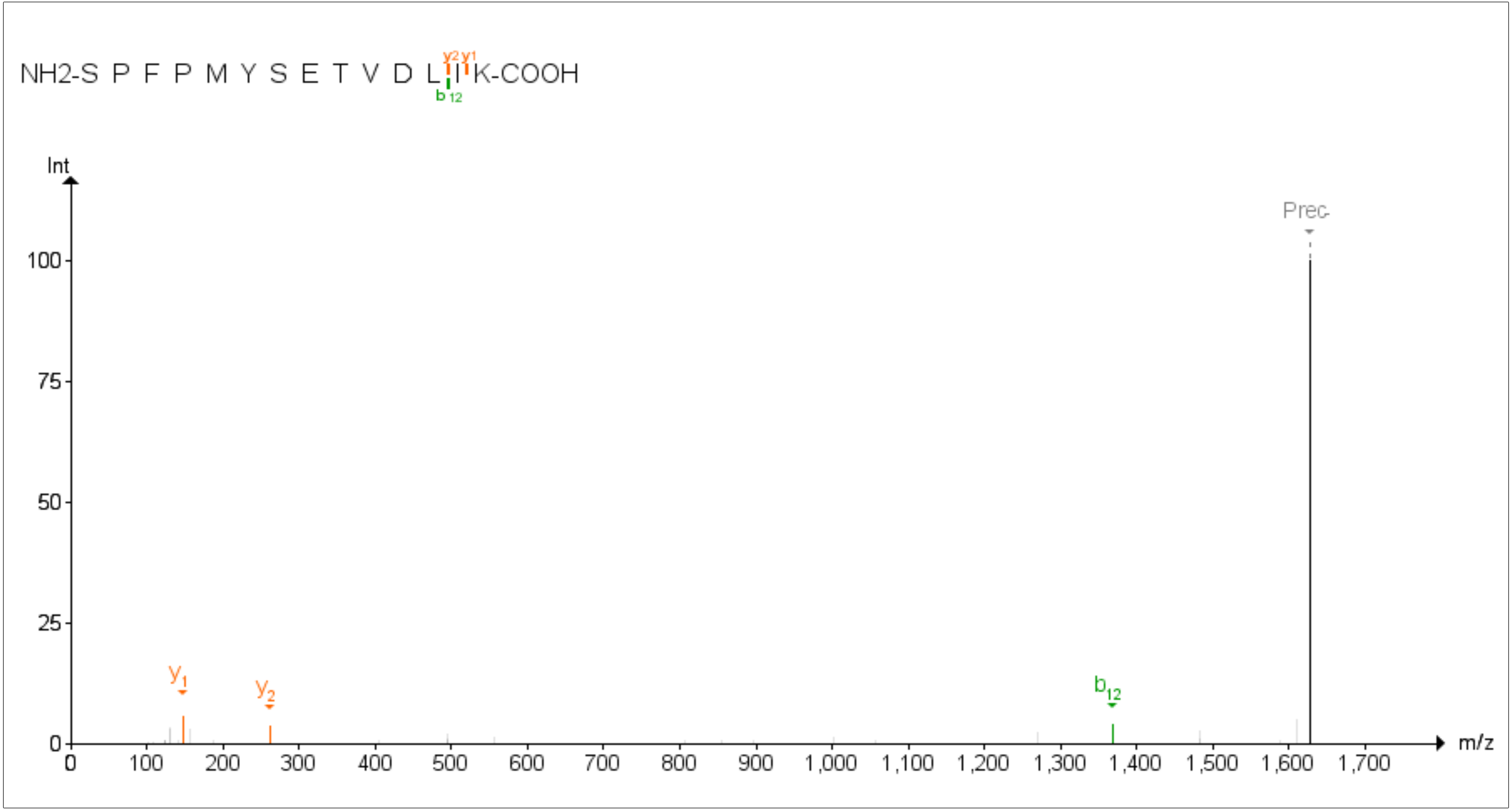

NH2-S P F P M Y S E T V D L I K-COOH
y2 y1
b12
Int
Prec
100
75
50
25
0
y1
y2
b12
0 100 200 300 400 500 600 700 800 900 1,000 1,100 1,200 1,300 1,400 1,500 1,600 1,700
m/z

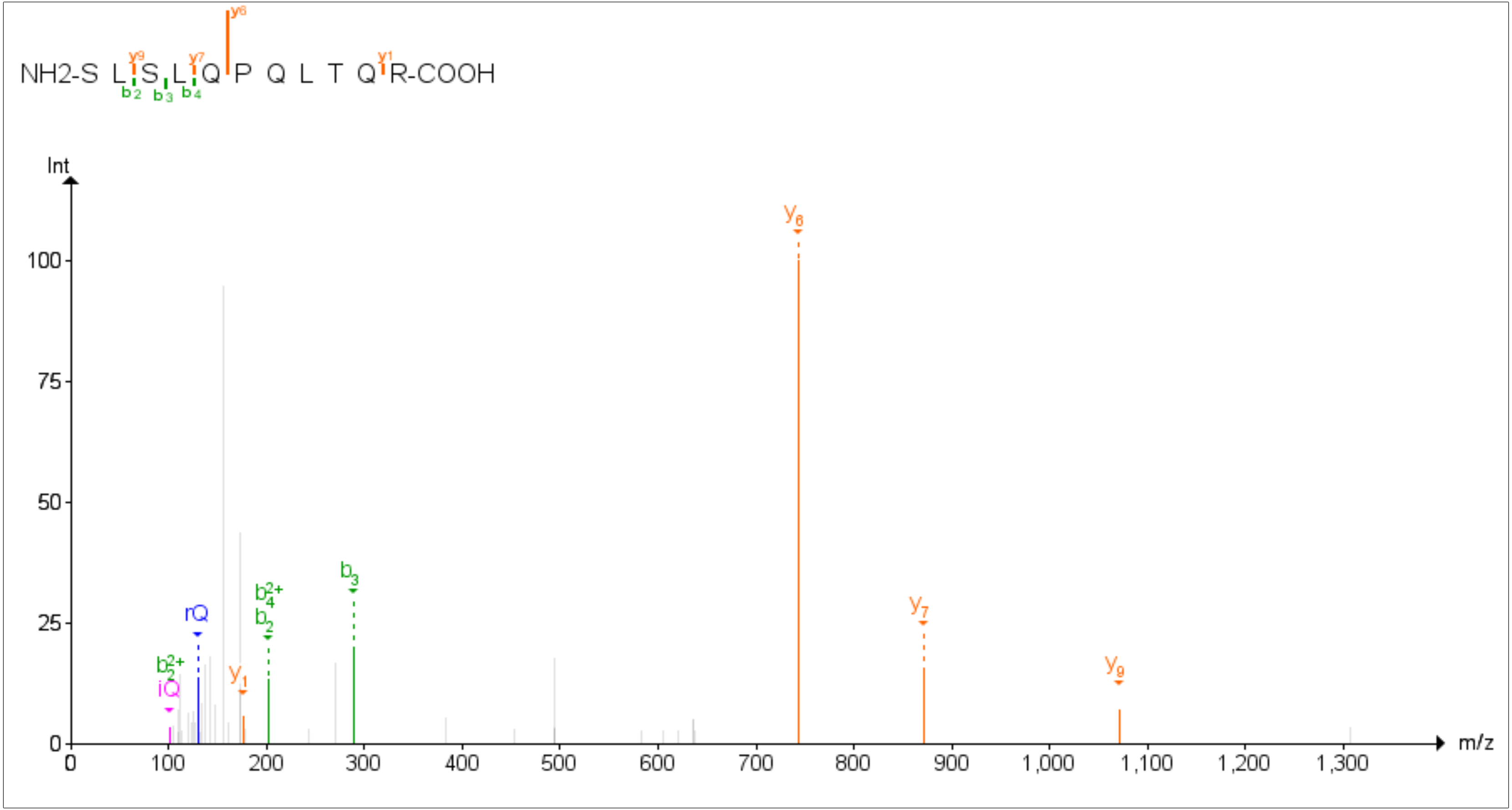

NH2-S L S L Q P Q L T Q R-COOH
y9
y7
y6
y1
b2
b3
b4
Int
100
75
50
25
0
y6
y7
y9
b3
b2
rQ
y1
iQ
0
100
200
300
400
500
600
700
800
900
1,000
1,100
1,200
1,300
m/z

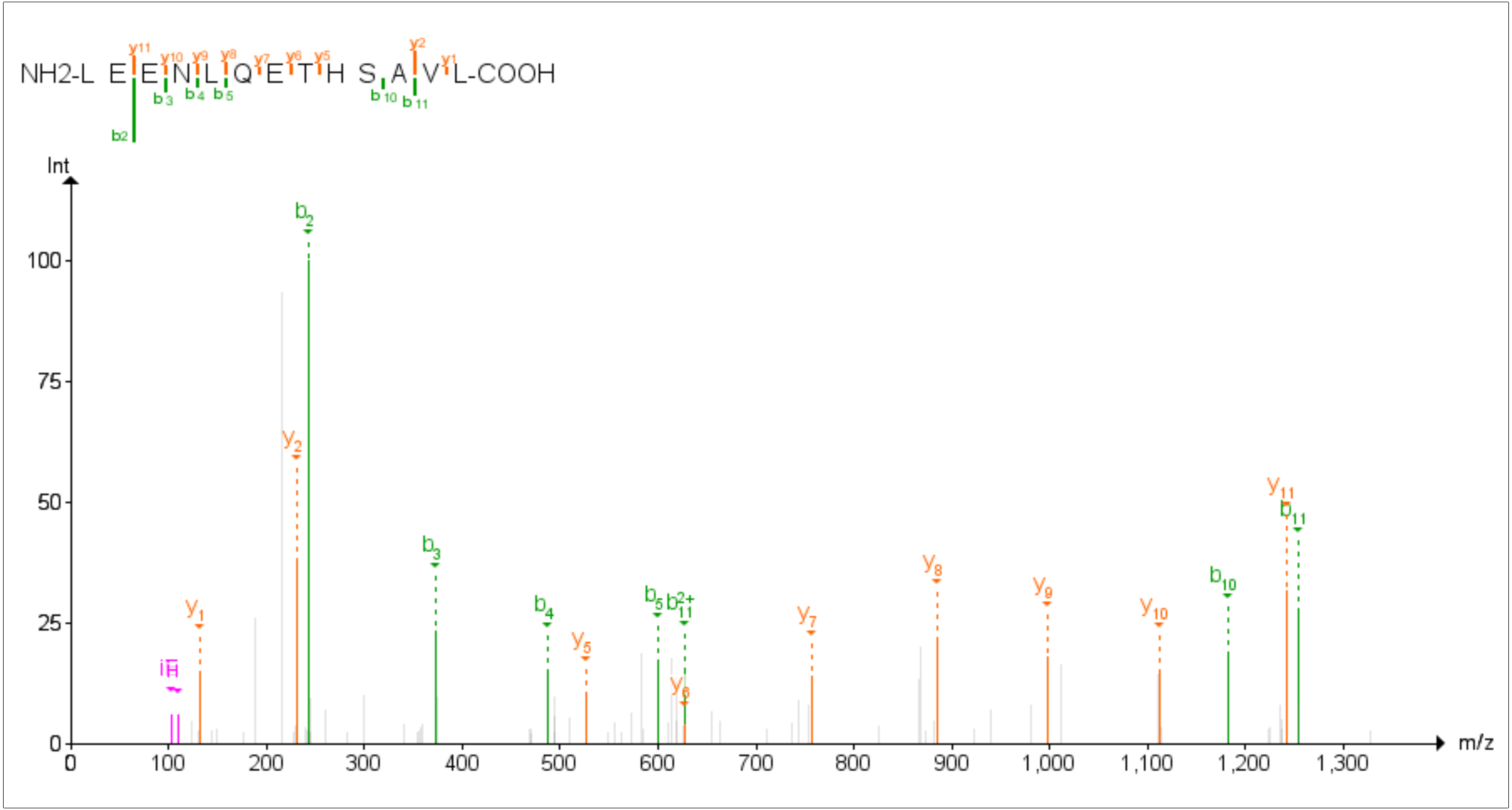
NH2-L E E N L Q E T H S A V L-COOH
y11 y10 y9 y8 y7 y6 y5 y2 y1
b2 b3 b4 b5 b10 b11
Int
100
75
50
25
0
b2
y2
b3
y1
iH
b4
y5
b5
b2+ 11
y6
y7
y8
y9
y10
b10
y11
b11
0 100 200 300 400 500 600 700 800 900 1,000 1,100 1,200 1,300
m/z

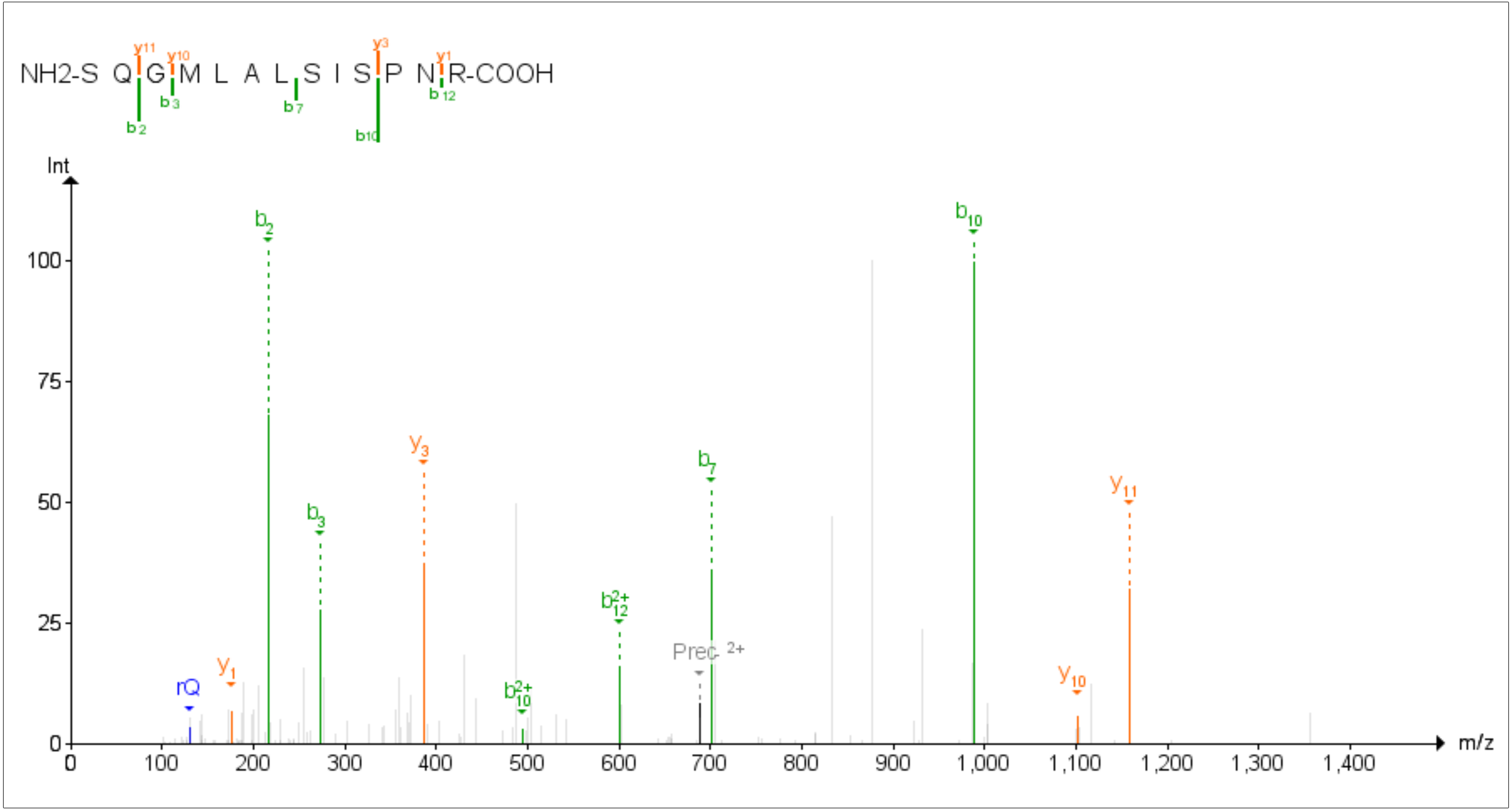
NH2-S Q G M L A L S I S P N R-COOH
y11
y10
y3
y1
b 2
b 3
b 7
b10
b 12
Int
100
75
50
25
0
b2
b3
y3
b7
b10
y11
y10
y1
rQ
b^{2+}_{10}
b^{2+}_{12}
Prec 2+
0
100
200
300
400
500
600
700
800
900
1,000
1,100
1,200
1,300
1,400
m/z

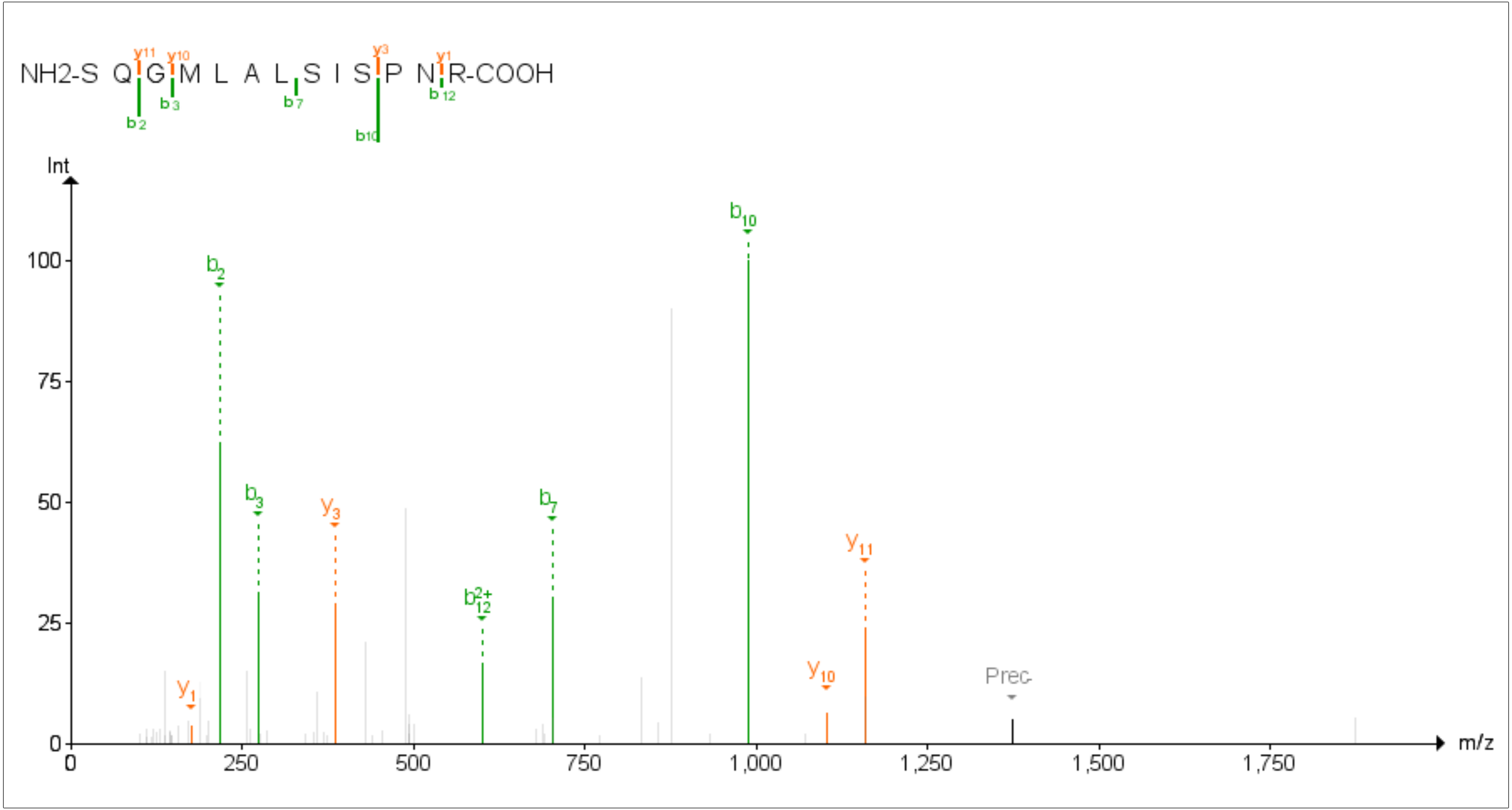
NH2-S Q G M L A L S I S P N R-COOH
y11
y10
y3
y1
b2
b3
b7
b10
b12
Int
m/z
b2
b3
y3
b7
b10
y11
y10
y1
Prec
100
75
50
25
0
250
500
750
1,000
1,250
1,500
1,750

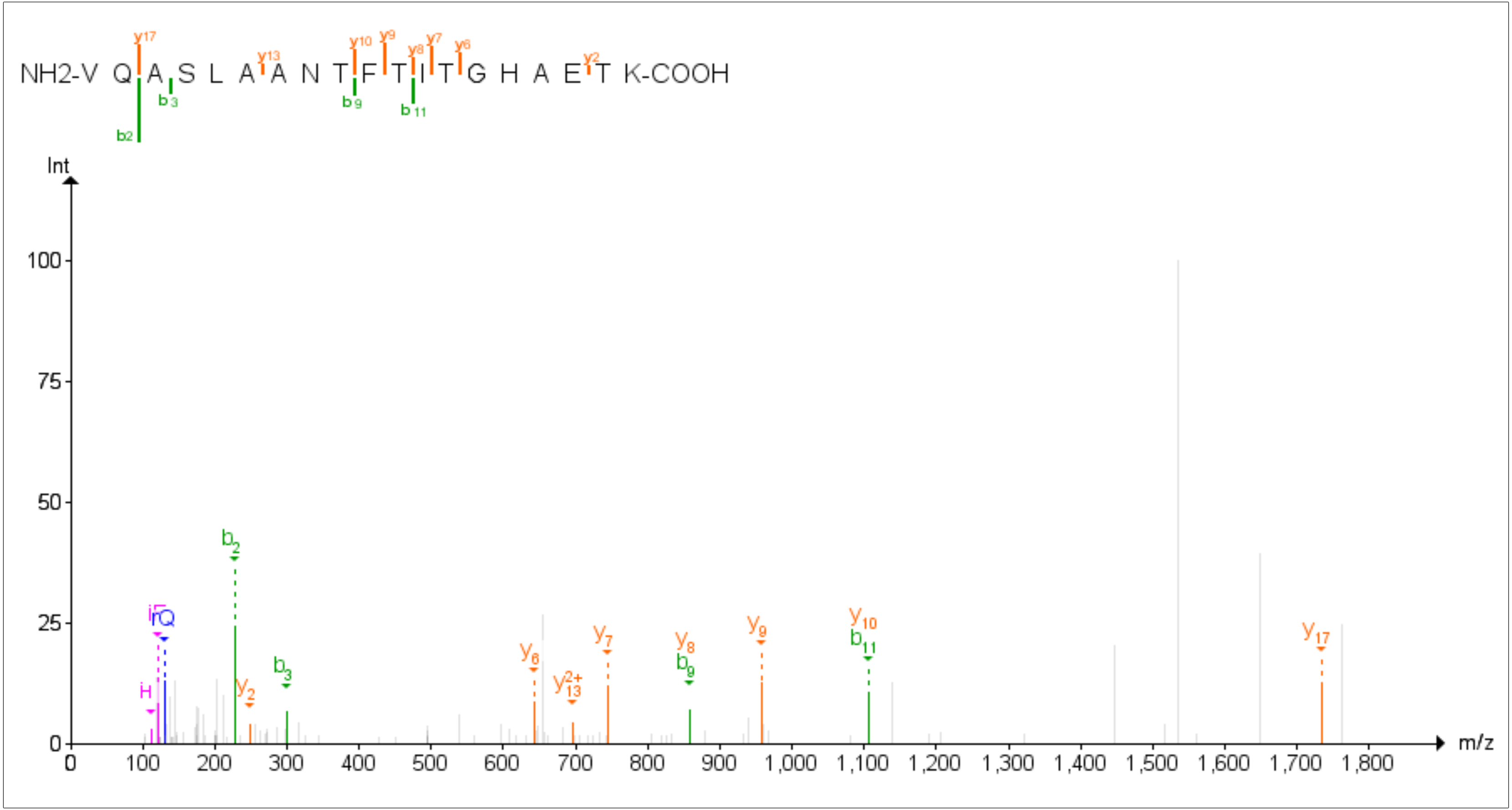

NH2-V Q A S L A A N T F T I T G H A E T K-COOH
y17 y13 y10 y9 y8 y7 y6 y2
b2 b3 b9 b11
Int
100
75
50
25
0
m/z
0 100 200 300 400 500 600 700 800 900 1,000 1,100 1,200 1,300 1,400 1,500 1,600 1,700 1,800
iH
iF
rQ
b2
y2
b3
y6
y13 2+
y7
y8
b9
y9
y10
b11
y17

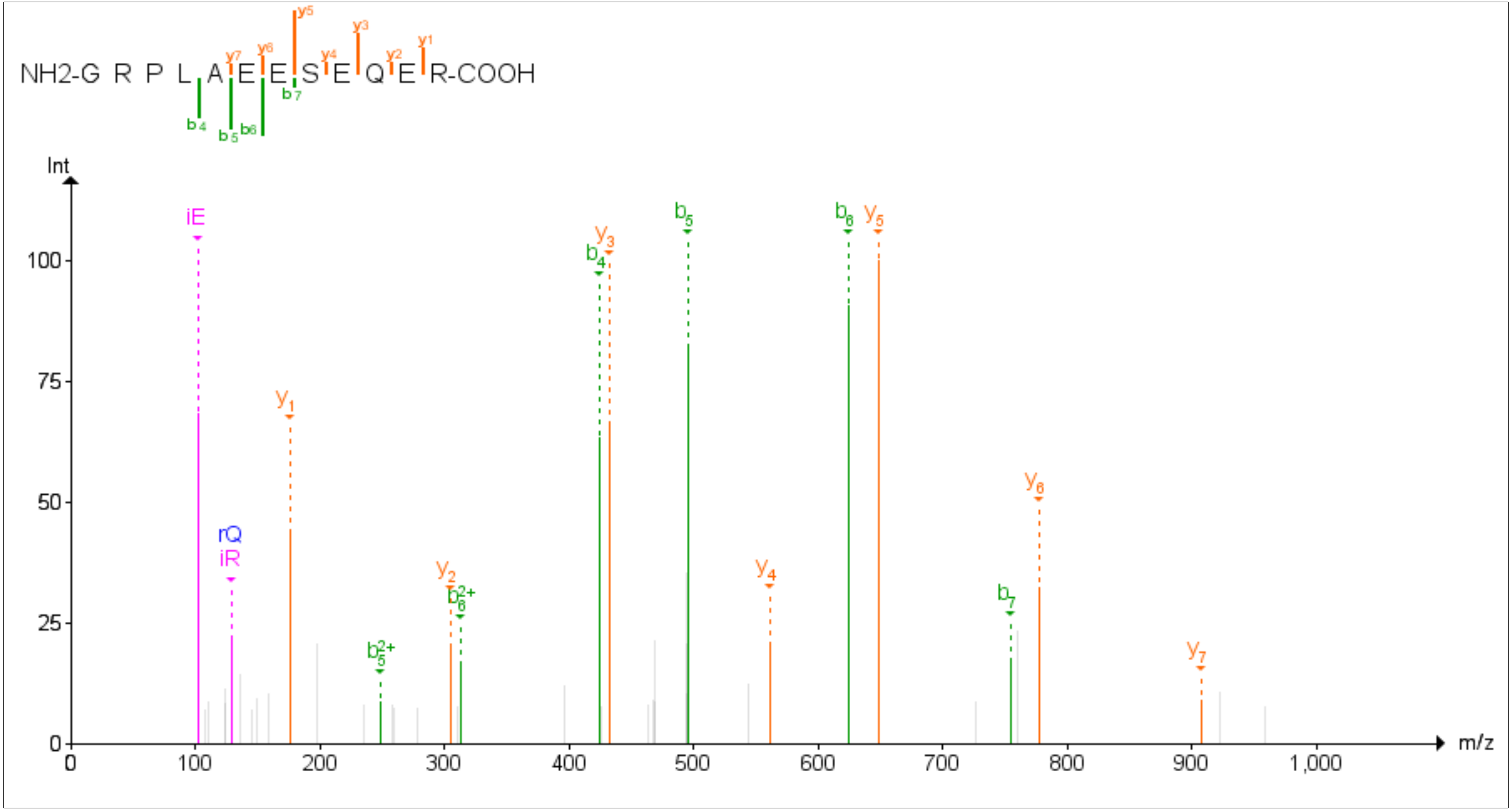

NH2-G R P L A E E S E Q E R-COOH
y5
y3
y7
y6
y4
y2
y1
b4
b5
b6
b7
Int
iE
rQ
iR
y1
b5 2+
y2
b6 2+
b4
y3
b5
y4
b6
y5
b7
y6
y7
0
25
50
75
100
0
100
200
300
400
500
600
700
800
900
1,000
m/z

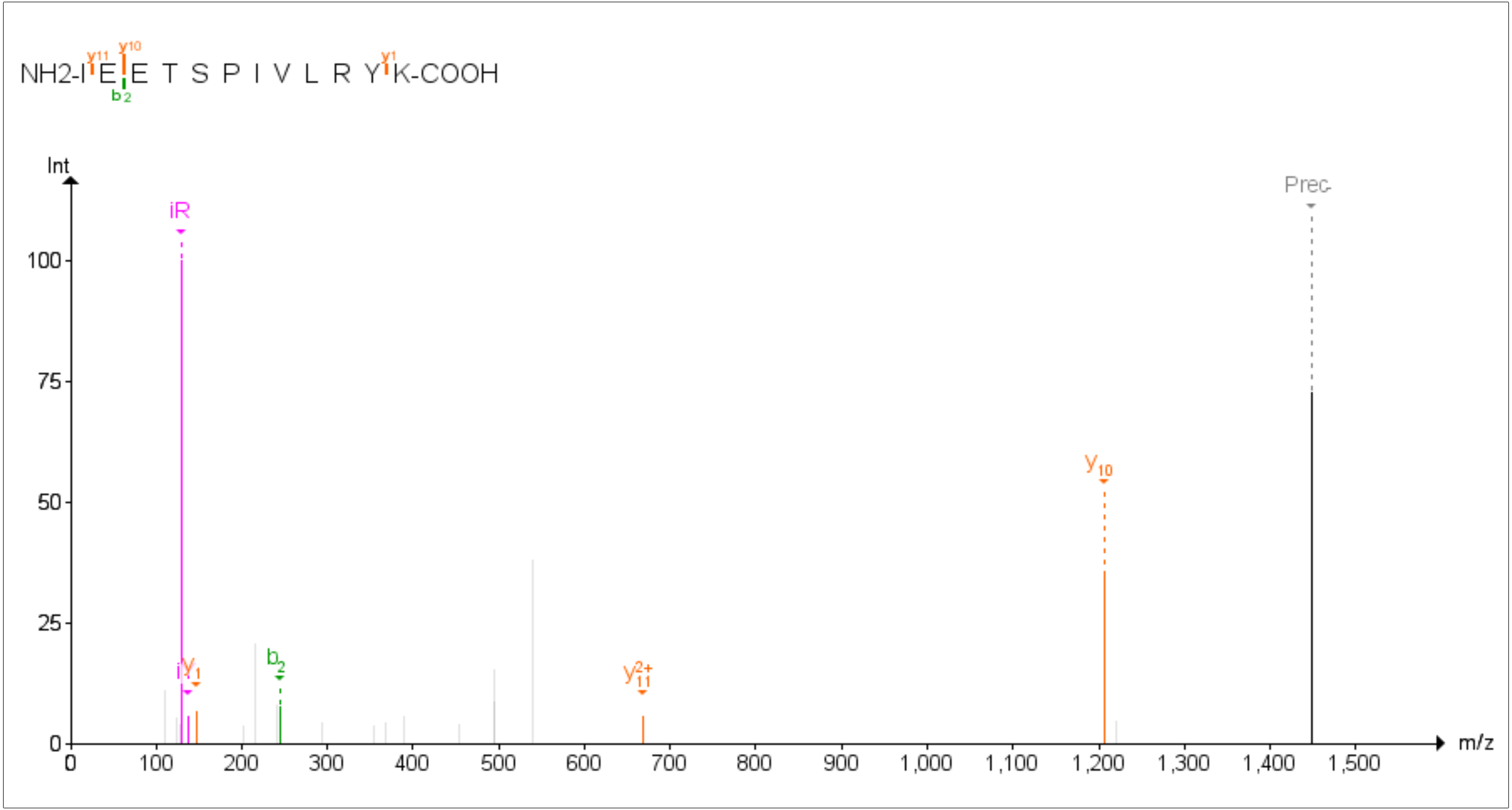
NH2-I E E T S P I V L R Y K-COOH
y11
y10
y1
b2
Int
iR
Prec
y10
y2+11
b2
y1
0
25
50
75
100
0
100
200
300
400
500
600
700
800
900
1,000
1,100
1,200
1,300
1,400
1,500
m/z

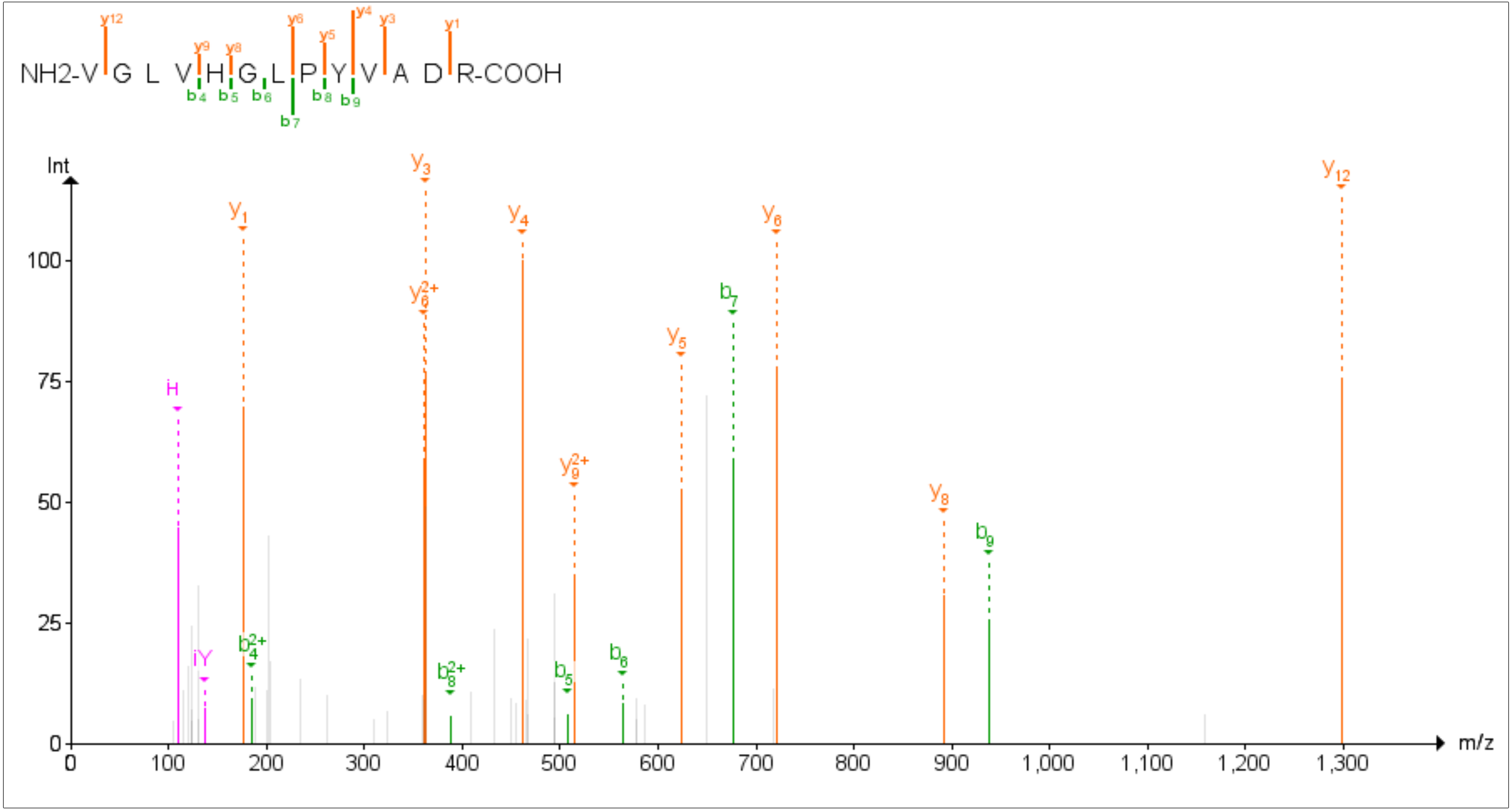

NH2-V G L V H G L P Y V A D R-COOH
y12 y9 y8 y6 y5 y4 y3 y1
b4 b5 b6 b7 b8 b9
Int
m/z
iH
iY
y1
y3
y4
y6
y12
y5
b7
y8
b9
0
25
50
75
100
0
100
200
300
400
500
600
700
800
900
1,000
1,100
1,200
1,300

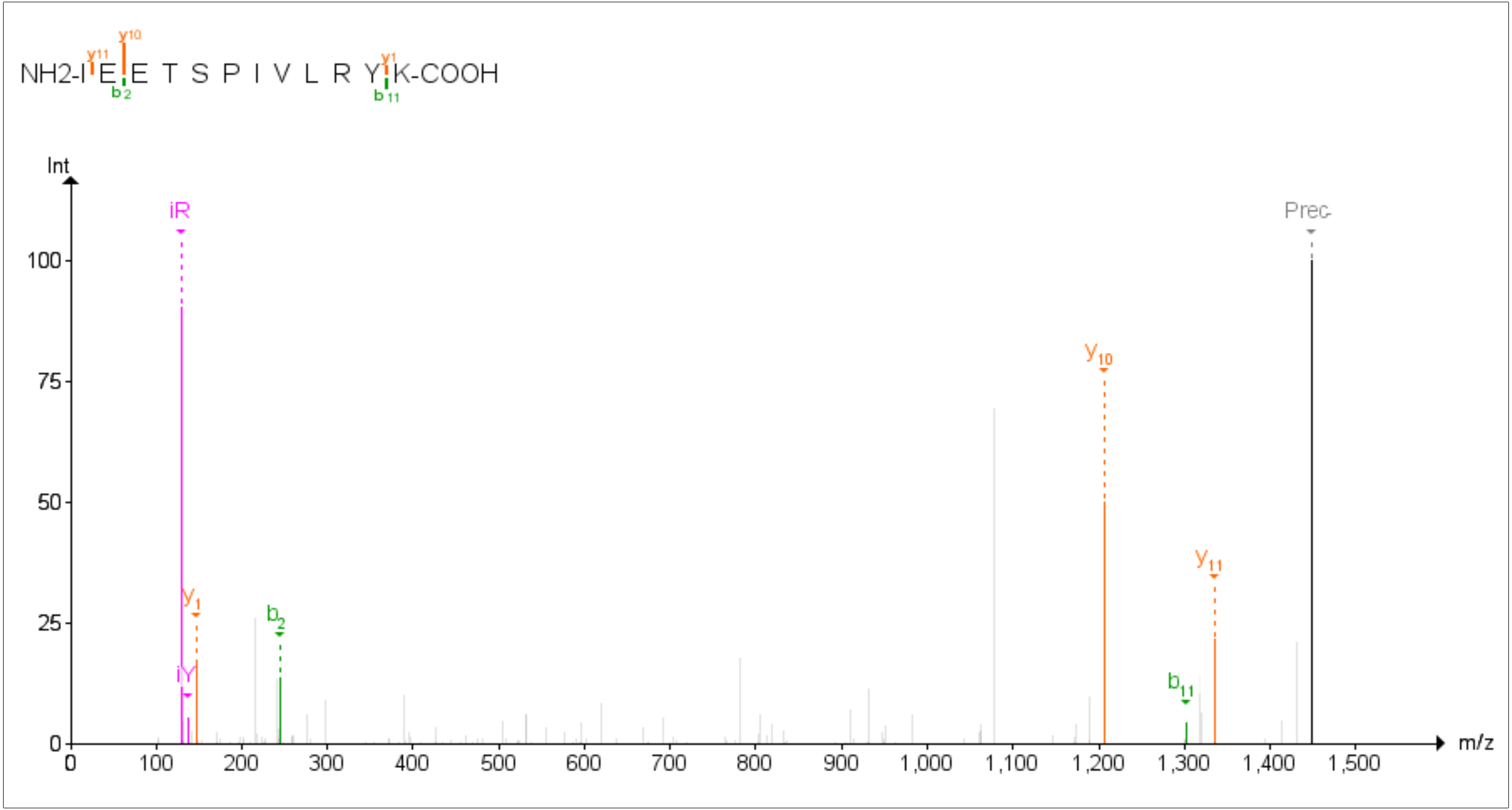

NH2-I E E T S P I V L R Y K-COOH
y11
y10
b2
y1
b11
Int
iR
Prec
y10
y11
y1
b2
iY
b11
100
75
50
25
0
0
100
200
300
400
500
600
700
800
900
1,000
1,100
1,200
1,300
1,400
1,500
m/z

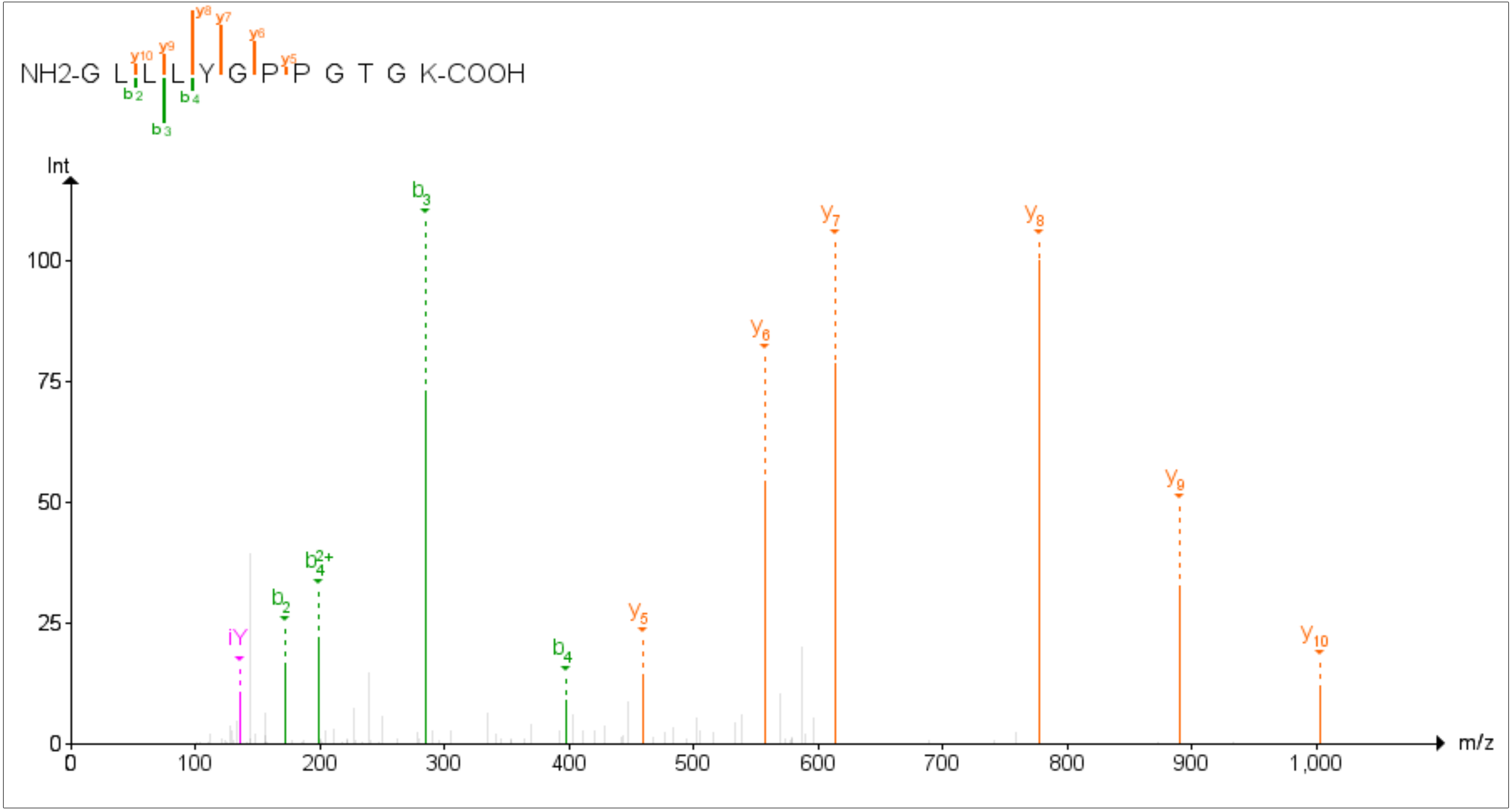

NH2-G L L L Y G P P G T G K-COOH
y10
y9
y8
y7
y6
y5
b2
b3
b4
Int
m/z
b3
y7
y8
y6
y9
b4 2+
b2
iY
b4
y5
y10
0
25
50
75
100
0
100
200
300
400
500
600
700
800
900
1,000

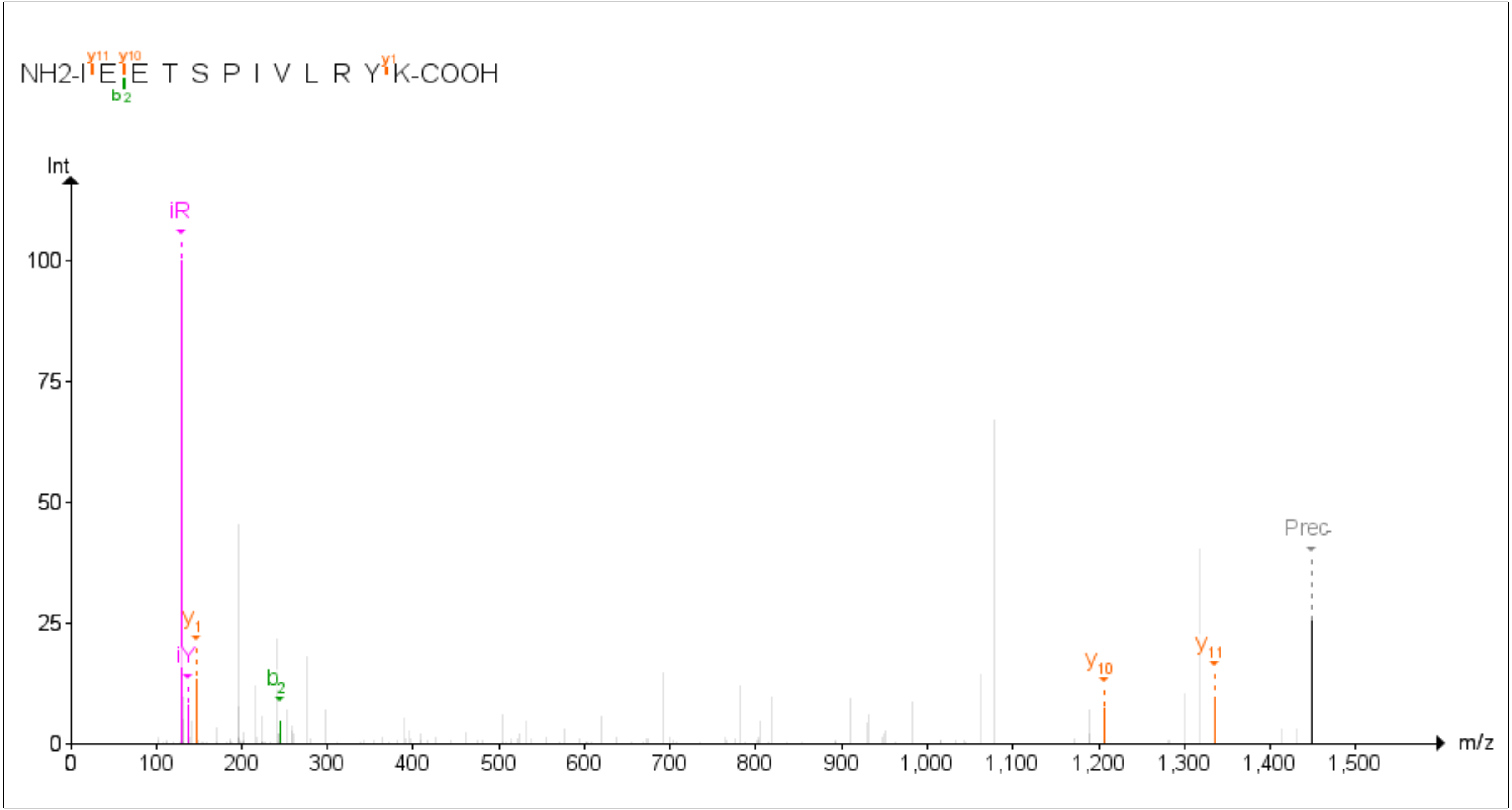
NH2-I E E T S P I V L R Y K-COOH
y11
y10
y1
b2
Int
iR
100
75
50
25
0
y1
iY
b2
y10
y11
Prec-
0
100
200
300
400
500
600
700
800
900
1,000
1,100
1,200
1,300
1,400
1,500
m/z

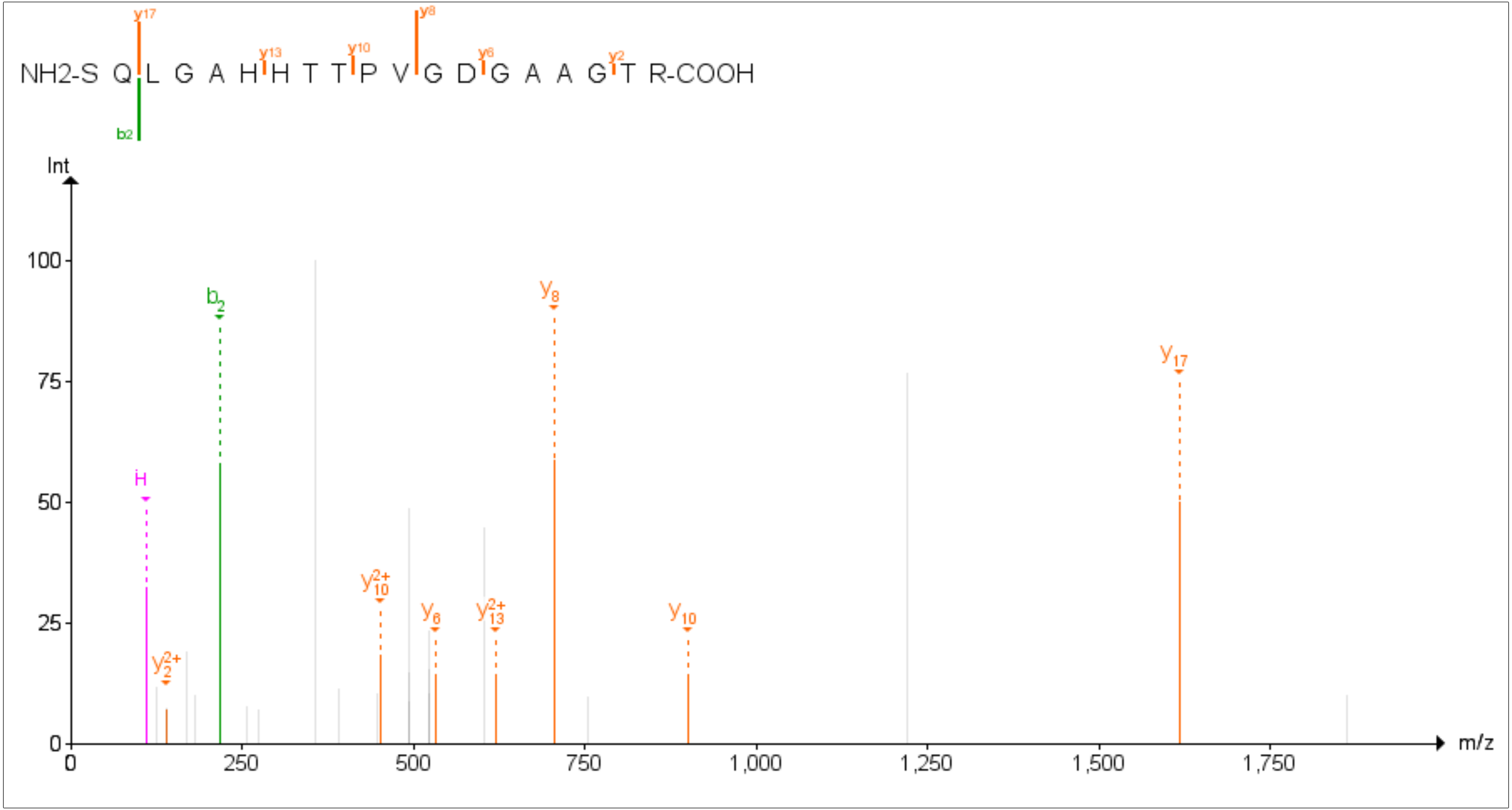

NH2-S Q L G A H H T T P V G D G A A G T R-COOH
y17
y13
y10
y8
y6
y2
b2
Int
100
75
50
25
0
Ḣ
y_2^{2+}
b_2
y_{10}^{2+}
y_6
y_{13}^{2+}
y_8
y_{10}
y_{17}
0
250
500
750
1,000
1,250
1,500
1,750
m/z

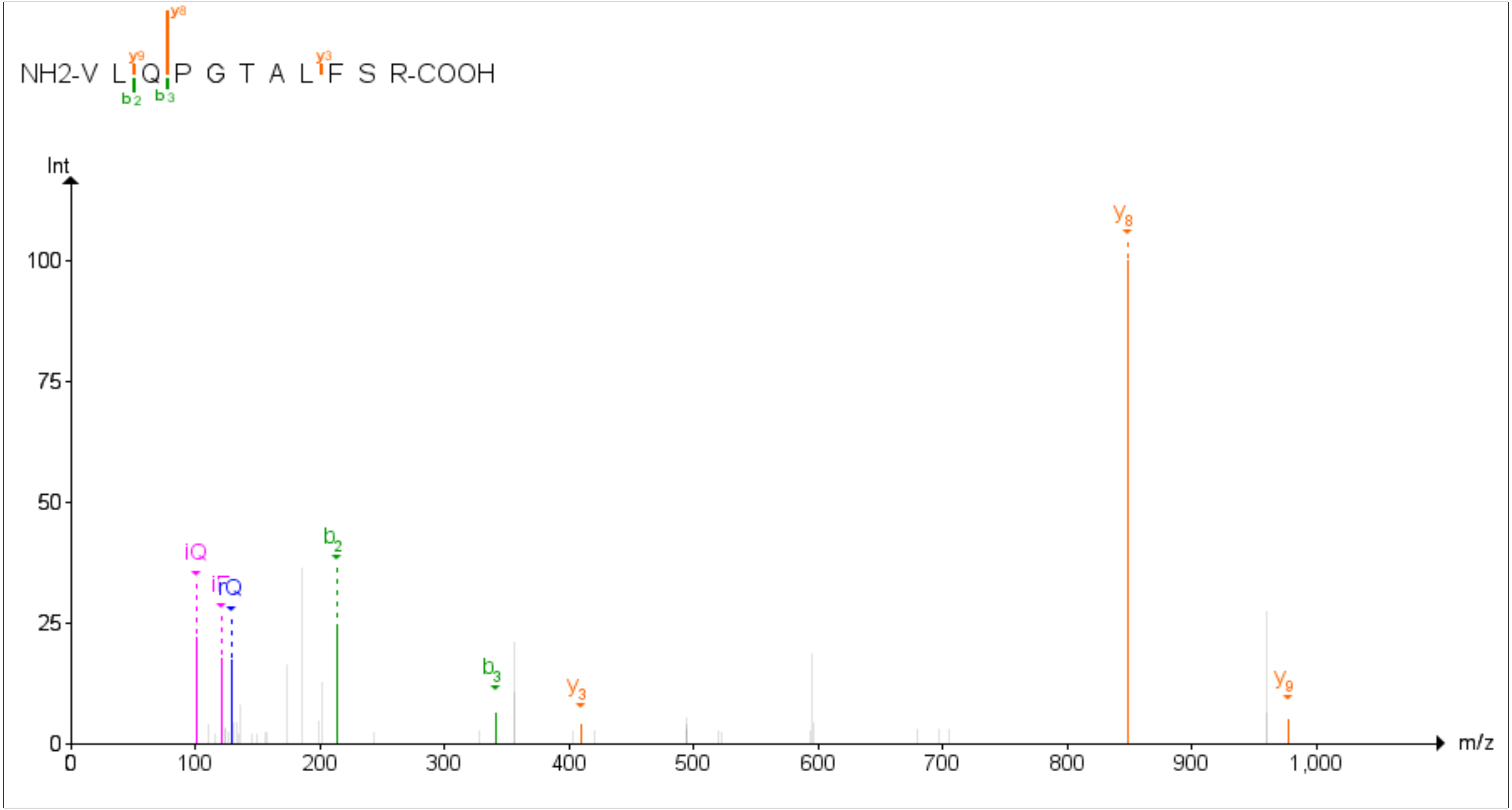

NH2-V L Q P G T A L F S R-COOH
y8
y9
y3
b2
b3
Int
100
75
50
25
0
iQ
iF
rQ
b2
b3
y3
y8
y9
0
100
200
300
400
500
600
700
800
900
1,000
m/z

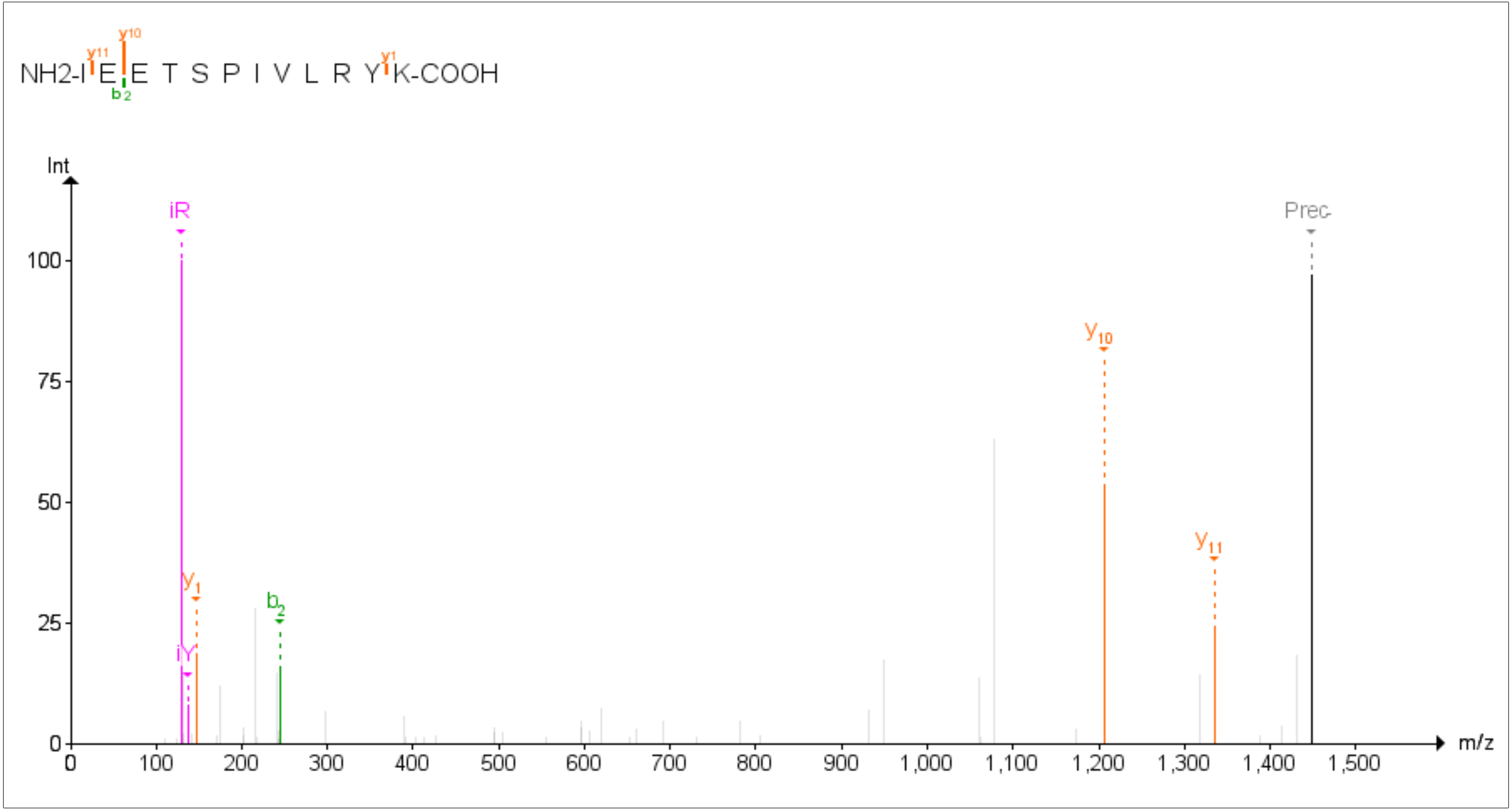

NH2-I E E T S P I V L R Y K-COOH
y11
y10
y1
b2
Int
iR
Prec
y10
y11
y1
b2
iY
100
75
50
25
0
0
100
200
300
400
500
600
700
800
900
1,000
1,100
1,200
1,300
1,400
1,500
m/z

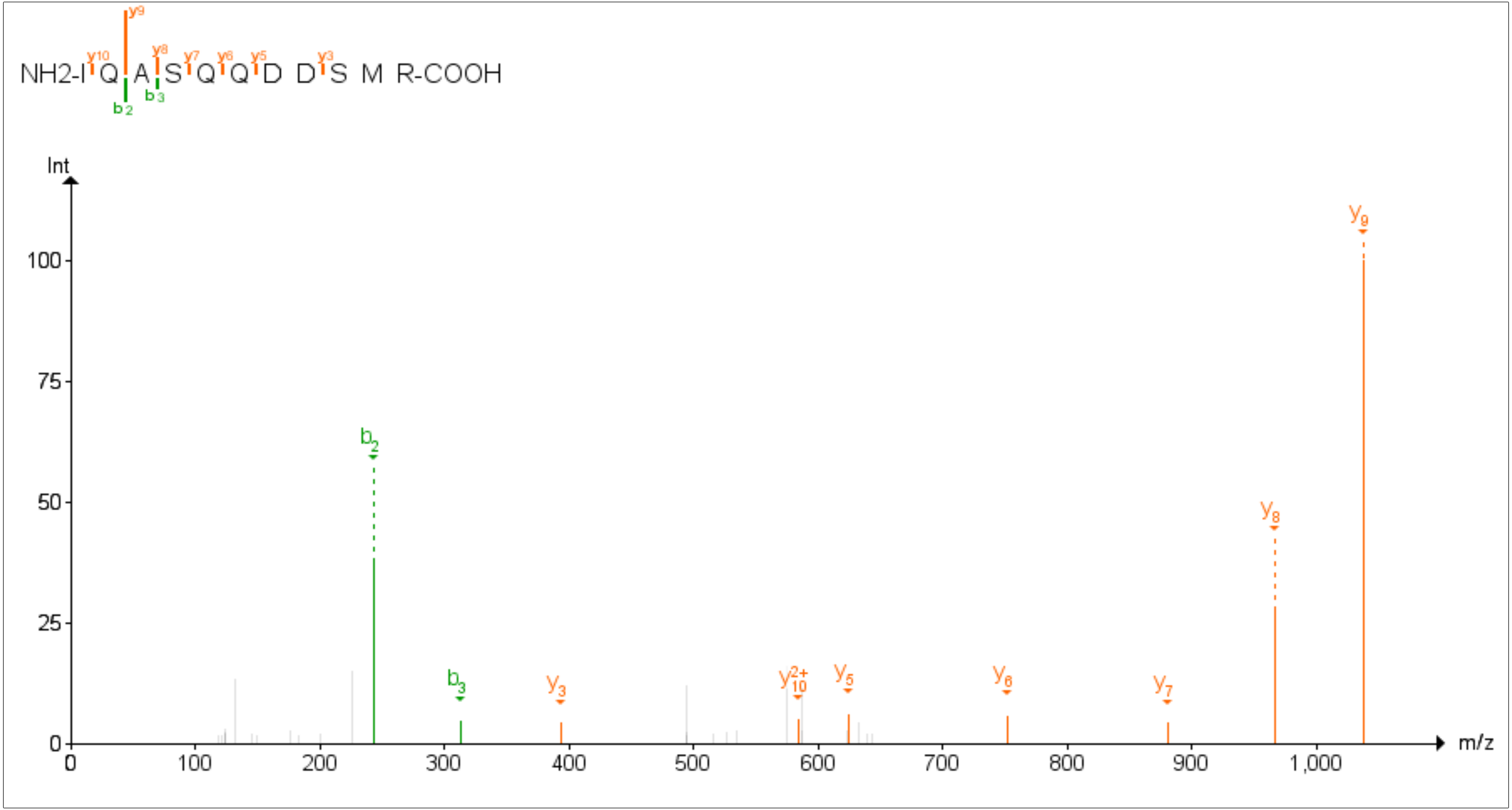

NH2-I Q A S Q Q D D S M R-COOH
y10 y9 y8 y7 y6 y5 y3
b2 b3
Int
100
75
50
25
0
m/z
0
100
200
300
400
500
600
700
800
900
1,000
y9
b2
y8
b3
y3
y10 2+
y5
y6
y7

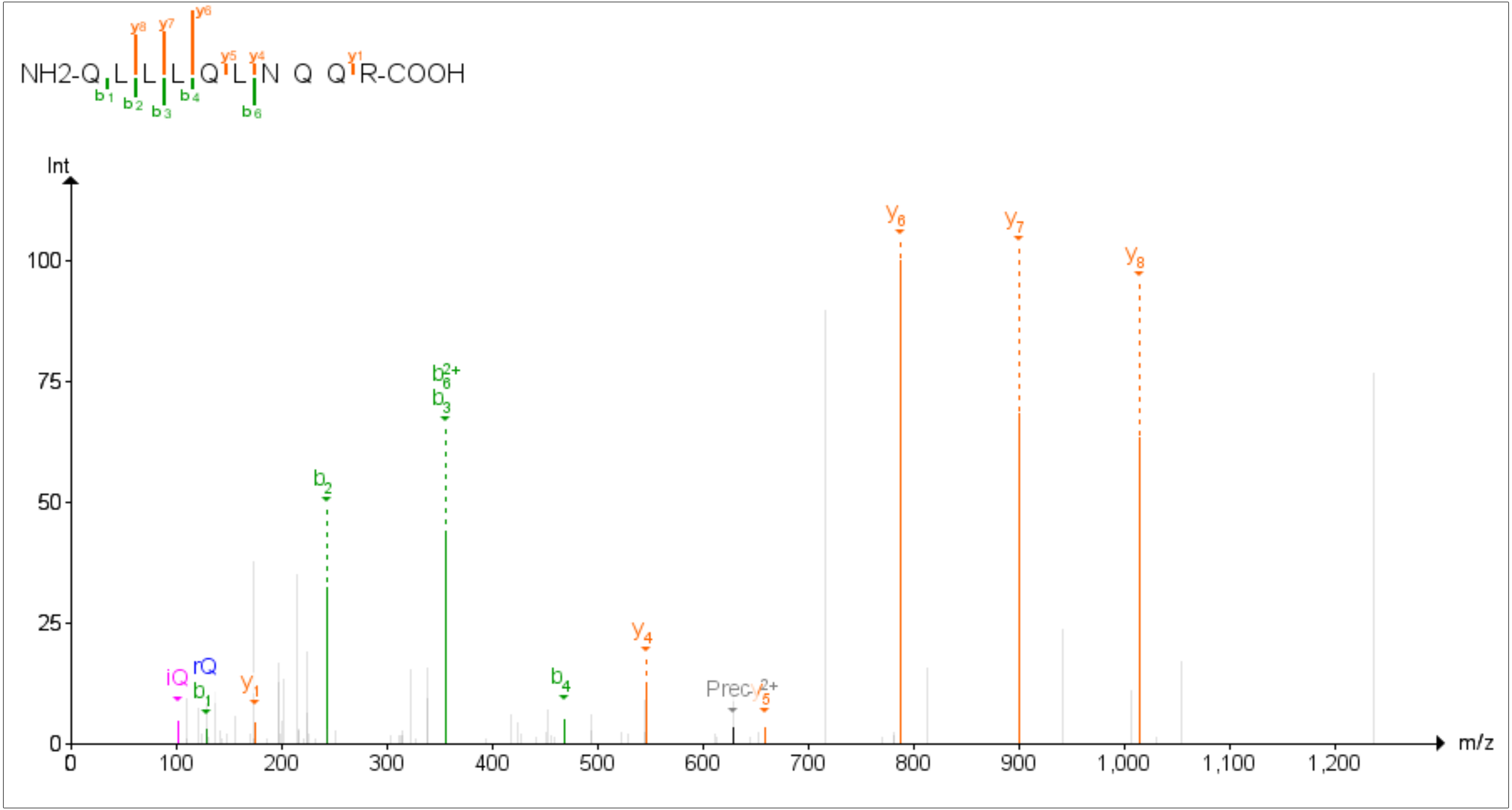

NH2-Q L L L L Q L N Q Q R-COOH
y8 y7 y6 y5 y4 y1
b1 b2 b3 b4 b6
Int
100
75
50
25
0
y6
y7
y8
b6 2+
b3
b2
y4
b4
iQ
rQ
b1
y1
Prec 2+
y5
m/z
0
100
200
300
400
500
600
700
800
900
1,000
1,100
1,200

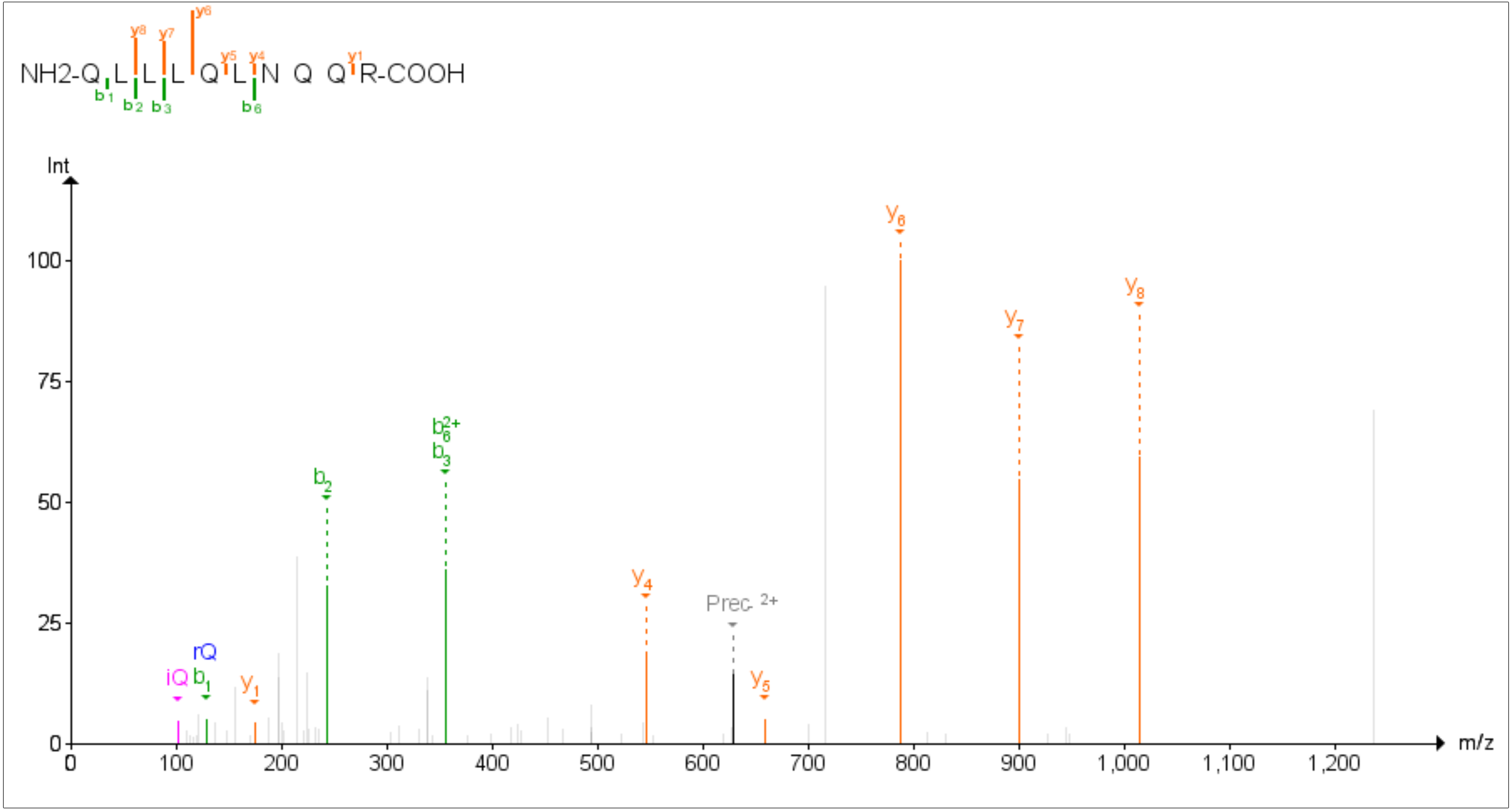

NH2-Q L L L Q L N Q Q R-COOH
y8 y7 y6 y5 y4 y1
b1 b2 b3 b6
Int
100
75
50
25
0
y6
y8
y7
b6 2+
b3
b2
y4
Prec 2+
rQ
iQ b1
y1
y5
0 100 200 300 400 500 600 700 800 900 1,000 1,100 1,200
m/z

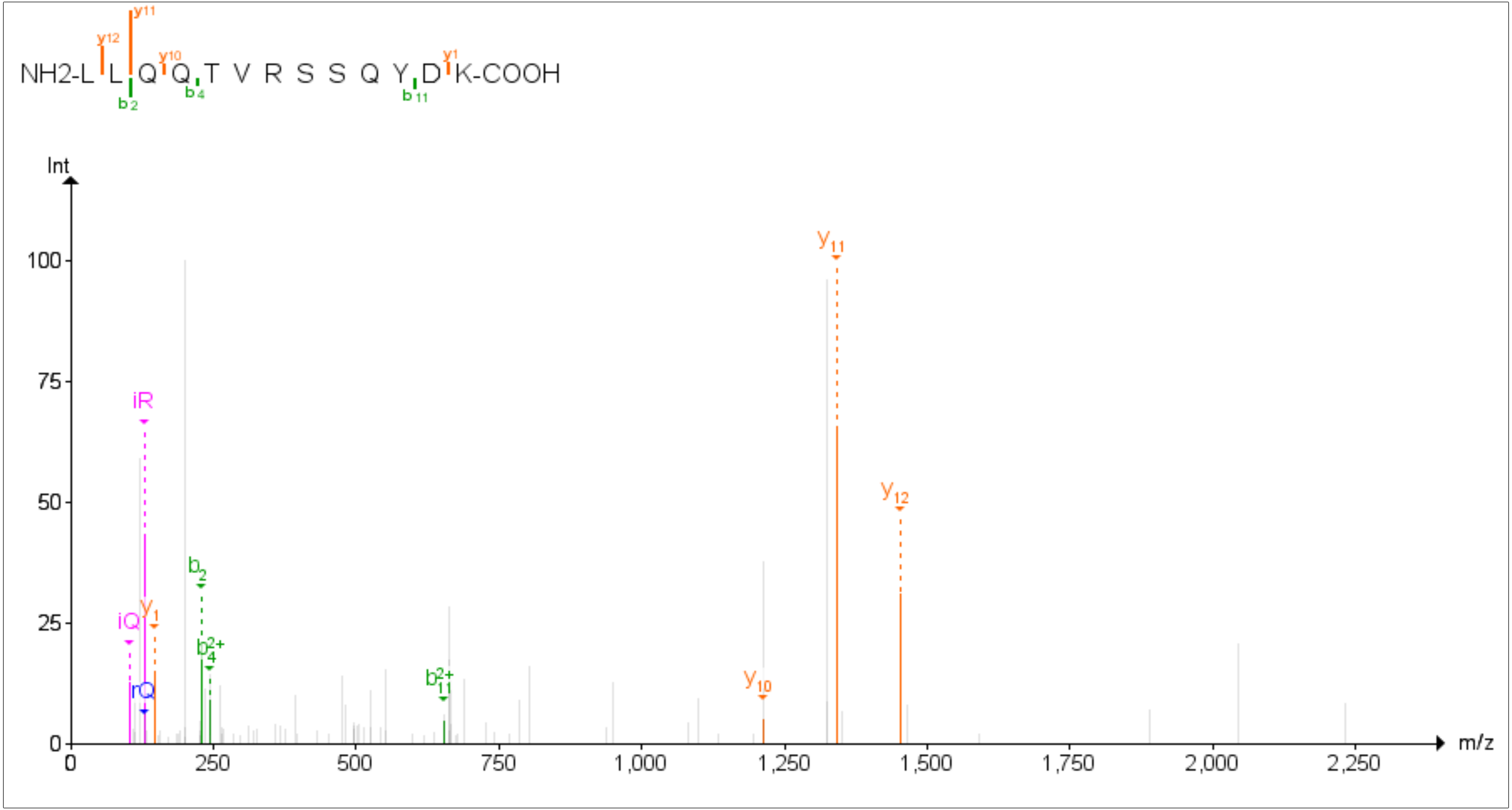
NH2-L L Q Q T V R S S Q Y D K-COOH
y12
y11
y10
y1
b 2
b 4
b 11
Int
100
75
50
25
0
iR
iQ
y1
rQ
b2
b4 2+
b11 2+
y10
y11
y12
0
250
500
750
1,000
1,250
1,500
1,750
2,000
2,250
m/z

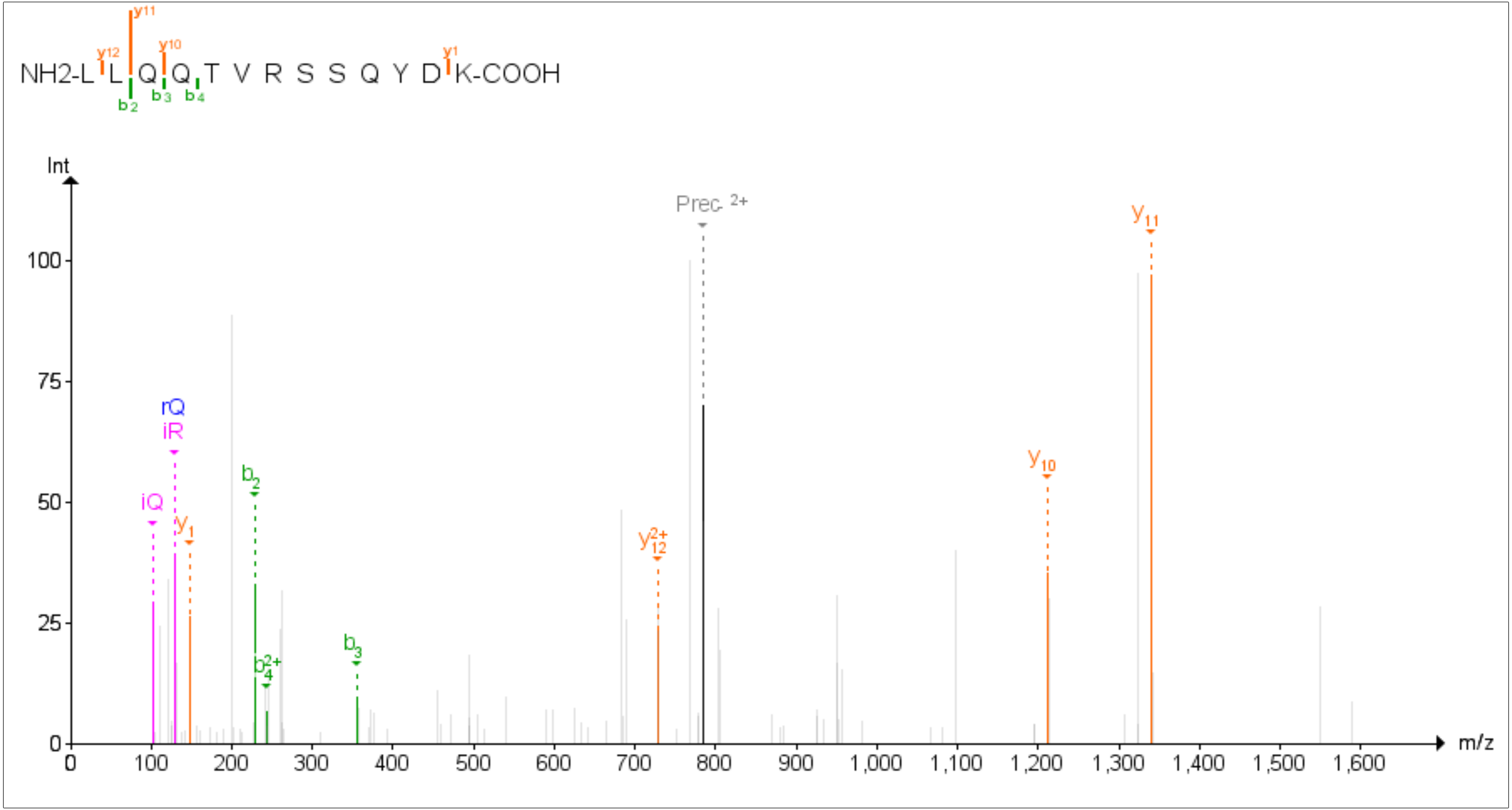

NH2-L L Q Q T V R S S Q Y D K-COOH
y12
y11
y10
y1
b2
b3
b4
Int
Prec 2+
y11
y10
rQ
iR
iQ
y1
b2
y12 2+
b4 2+
b3
100
75
50
25
0
0
100
200
300
400
500
600
700
800
900
1,000
1,100
1,200
1,300
1,400
1,500
1,600
m/z

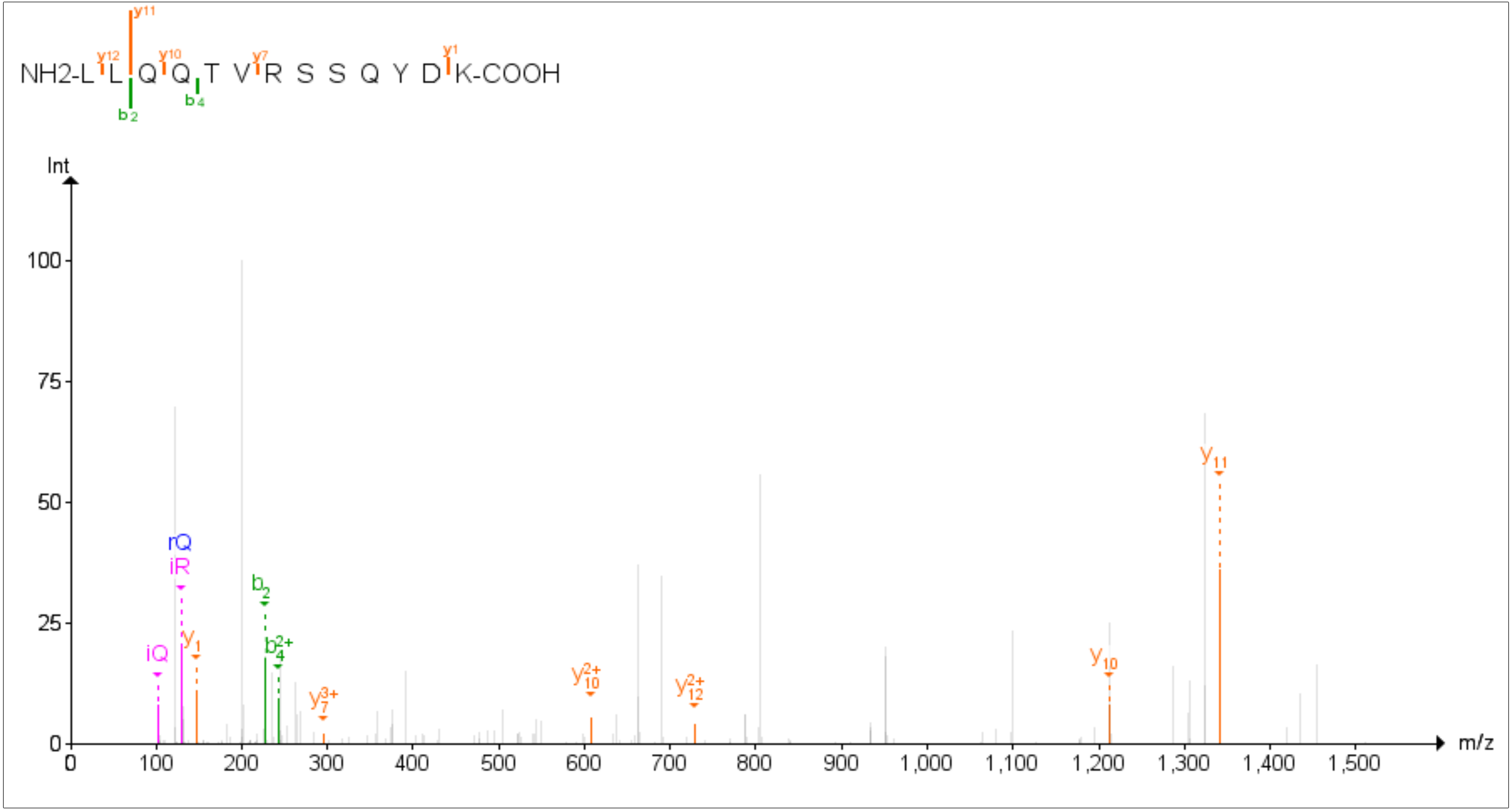

NH2-L L Q Q T V R S S Q Y D K-COOH
y11
y12
y10
y7
y1
b2
b4
Int
100
75
50
25
0
rQ
iR
iQ
y1
b2
b4 2+
y7 3+
y10 2+
y12 2+
y10
y11
0
100
200
300
400
500
600
700
800
900
1,000
1,100
1,200
1,300
1,400
1,500
m/z

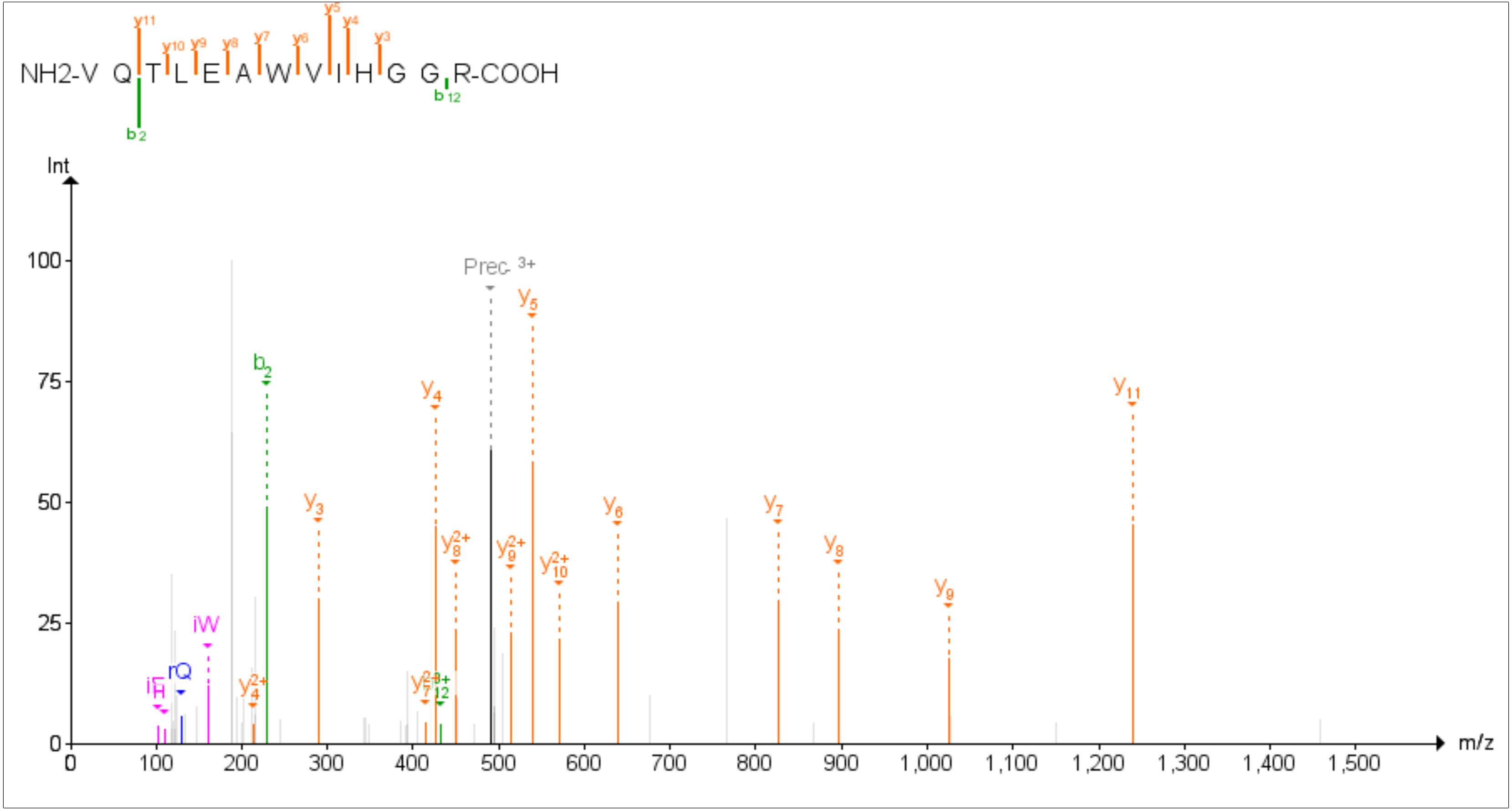

NH2-V Q T L E A W V I H G G R-COOH
y11 y10 y9 y8 y7 y6 y5 y4 y3
b 2
b 12
Int
m/z
Prec 3+

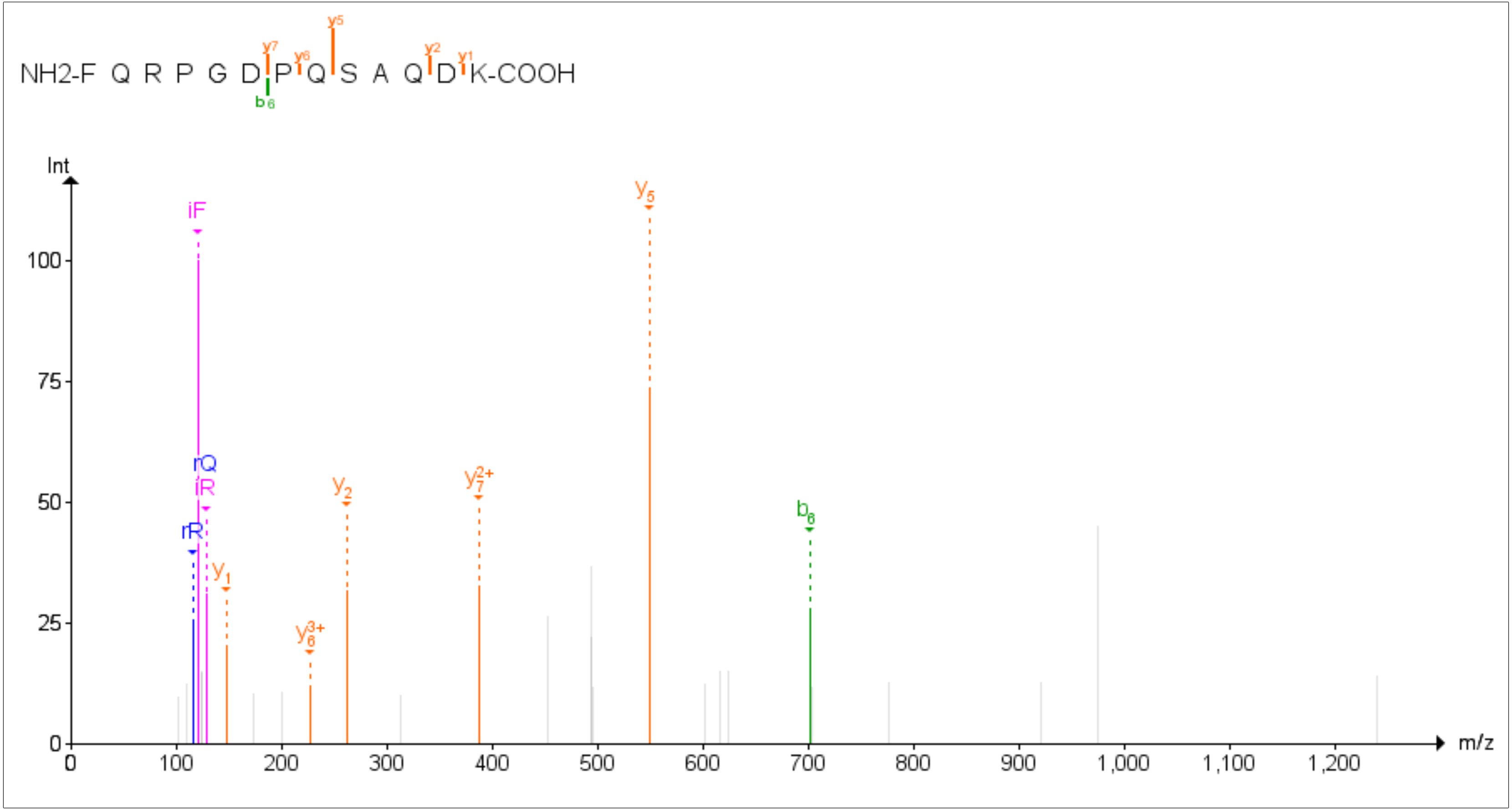
NH2-F Q R P G D P Q S A Q D K-COOH
y7 y6 y5 y2 y1
b6
Int
100
75
50
25
0
iF
rQ
iR
rR
y1
y2
y6 3+
y7 2+
y5
b6
0 100 200 300 400 500 600 700 800 900 1,000 1,100 1,200
m/z

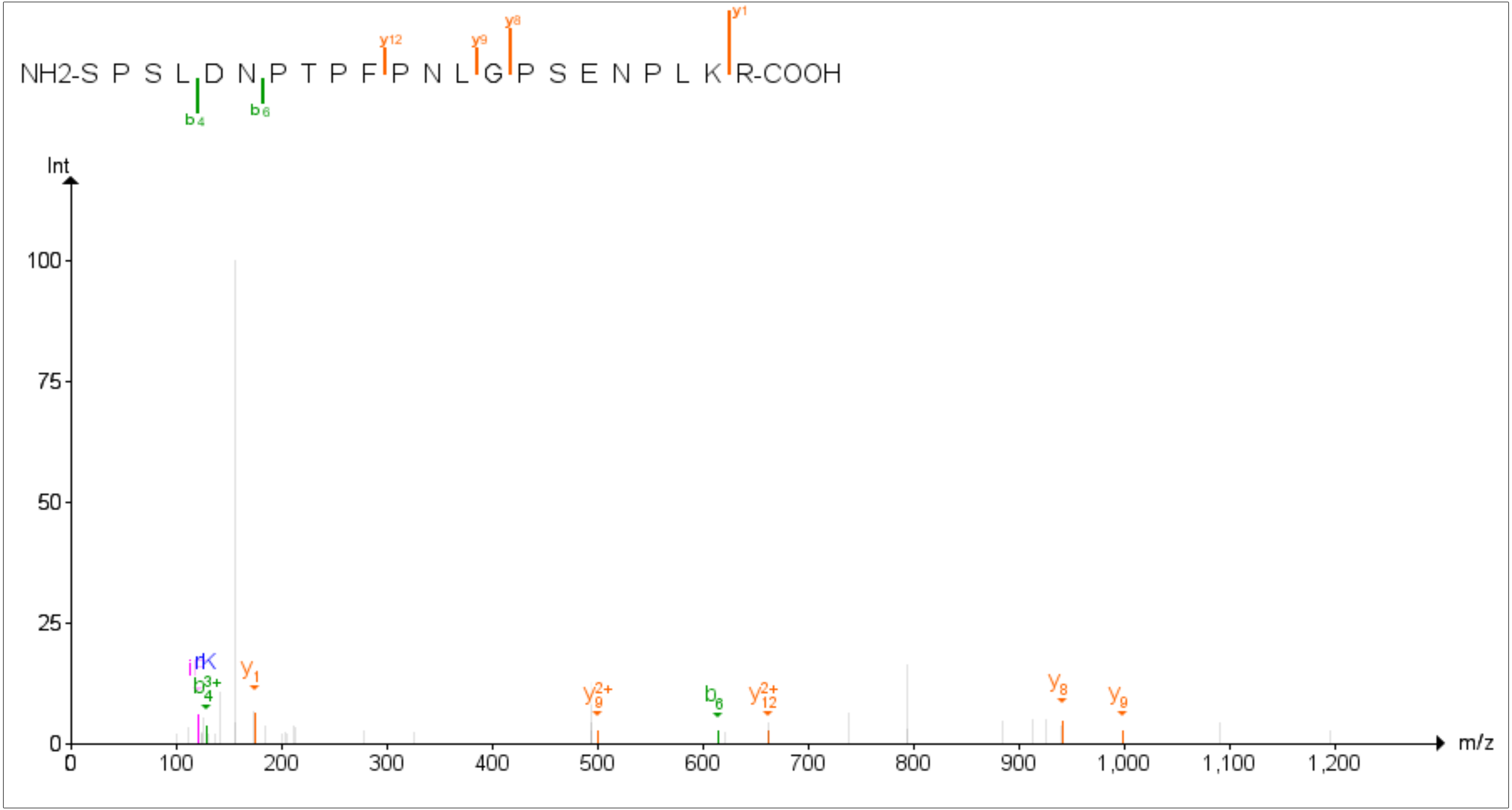
NH2-S P S L D N P T P F P N L G P S E N P L K R-COOH
b4
b6
y12
y9
y8
y1
Int
100
75
50
25
0
m/z
0
100
200
300
400
500
600
700
800
900
1,000
1,100
1,200
rK
b4 3+
y1
y9 2+
b6
y12 2+
y8
y9

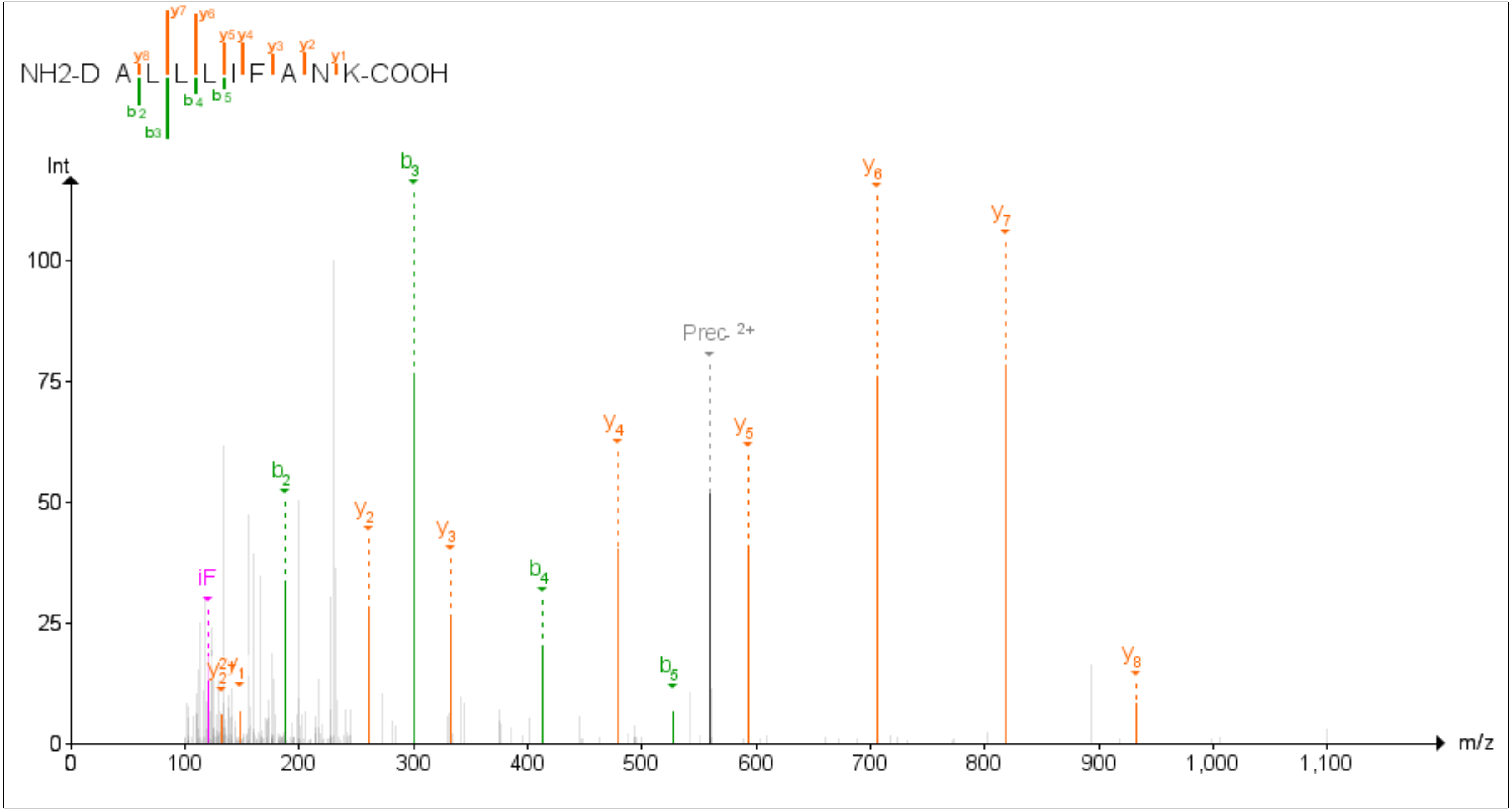

NH2-D A L L L I F A N K-COOH
y8 y7 y6 y5 y4 y3 y2 y1
b2 b3 b4 b5
Int
m/z
Prec 2+
iF

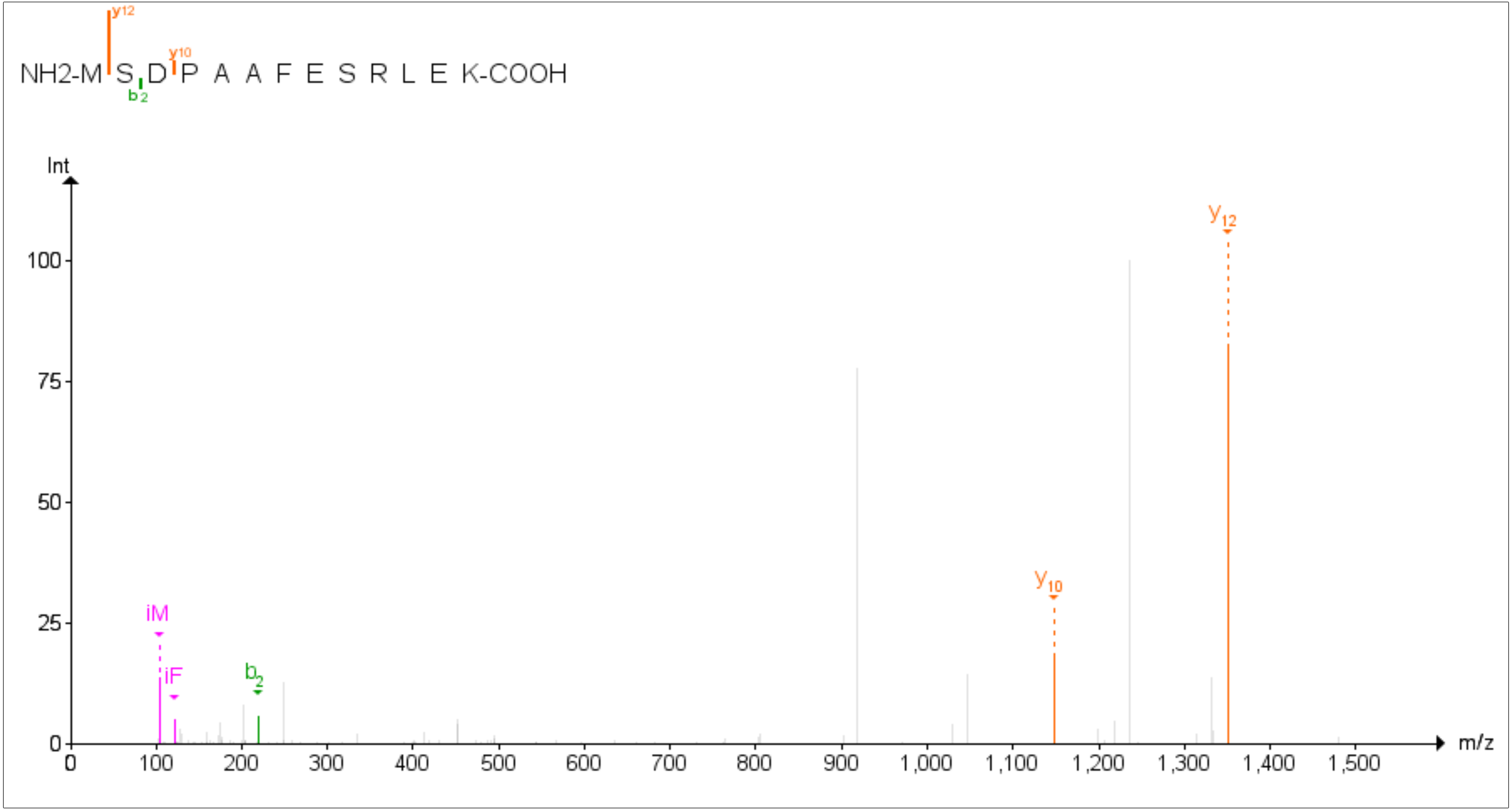

NH2-M S D P A A F E S R L E K-COOH
y12
y10
b2
Int
100
75
50
25
0
iM
iF
b2
y10
y12
0
100
200
300
400
500
600
700
800
900
1,000
1,100
1,200
1,300
1,400
1,500
m/z

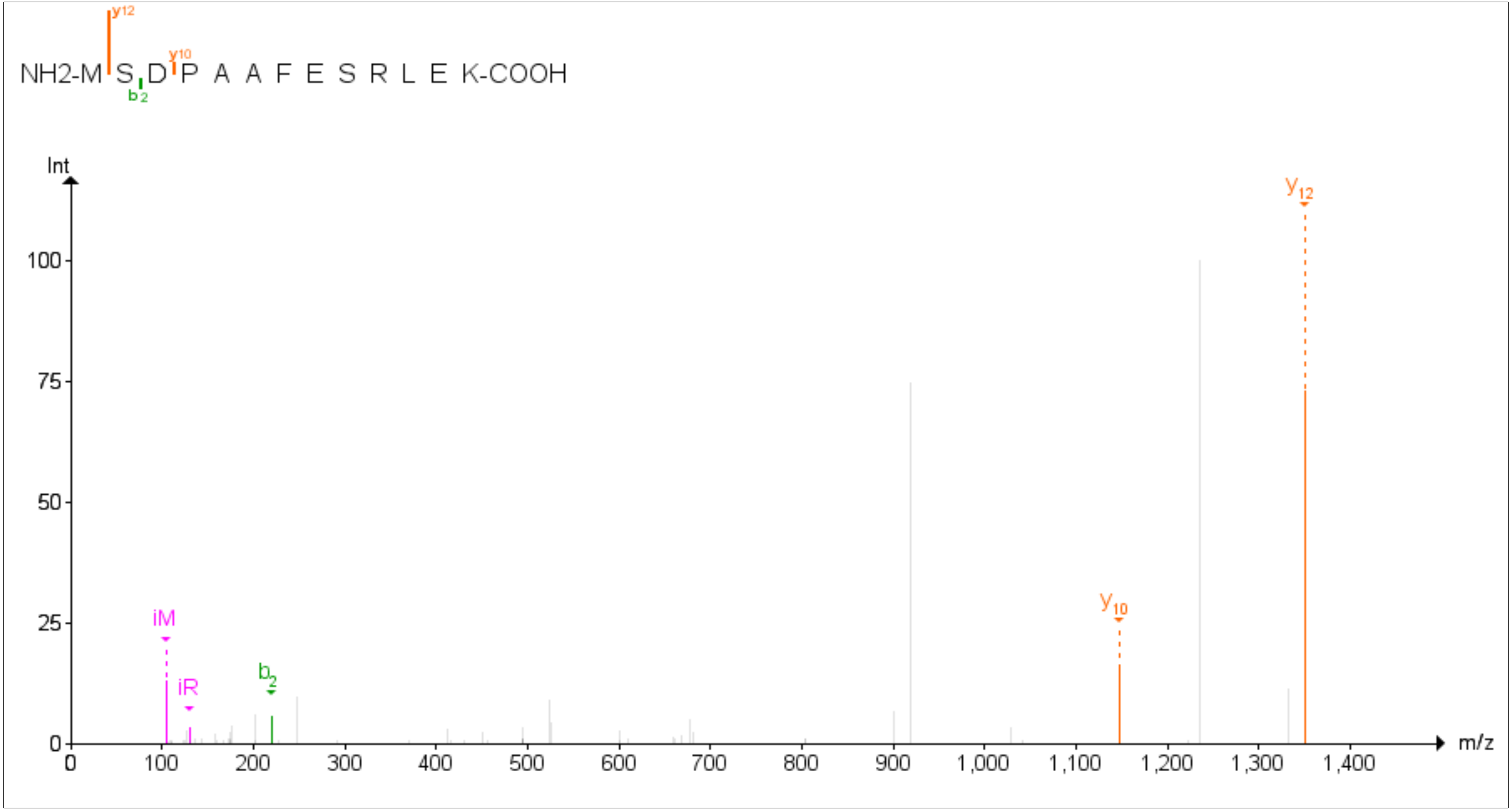

NH2-M S D P A A F E S R L E K-COOH
y12
y10
b2
Int
100
75
50
25
0
m/z
0
100
200
300
400
500
600
700
800
900
1,000
1,100
1,200
1,300
1,400
iM
iR
b2
y10
y12

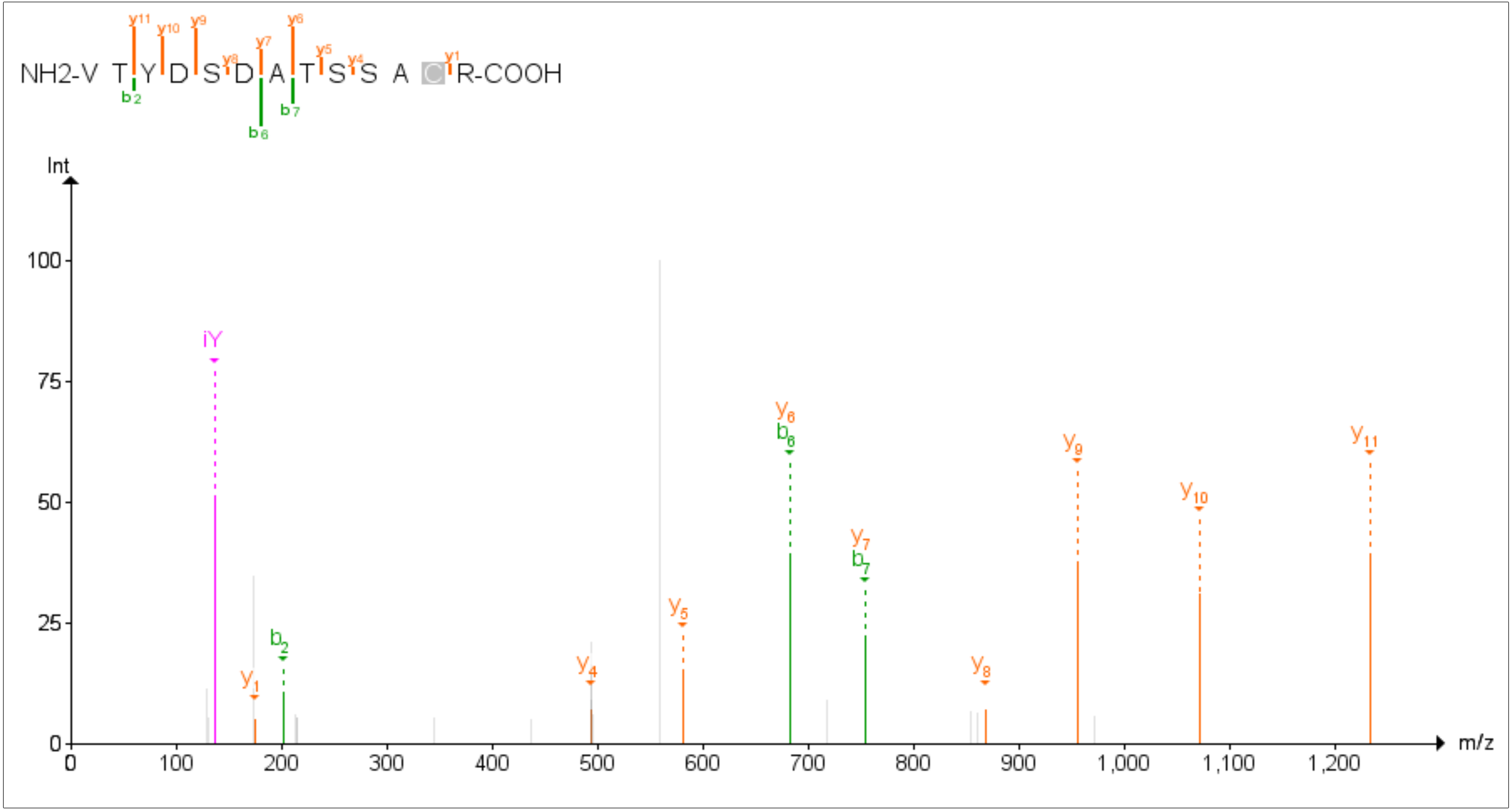

NH2-V T Y D S D A T S S A C R-COOH
y11 y10 y9 y8 y7 y6 y5 y4 y1
b2 b6 b7
Int
100
75
50
25
0
iY
y1
b2
y4
y5
y6
b6
y7
b7
y8
y9
y10
y11
0 100 200 300 400 500 600 700 800 900 1,000 1,100 1,200
m/z

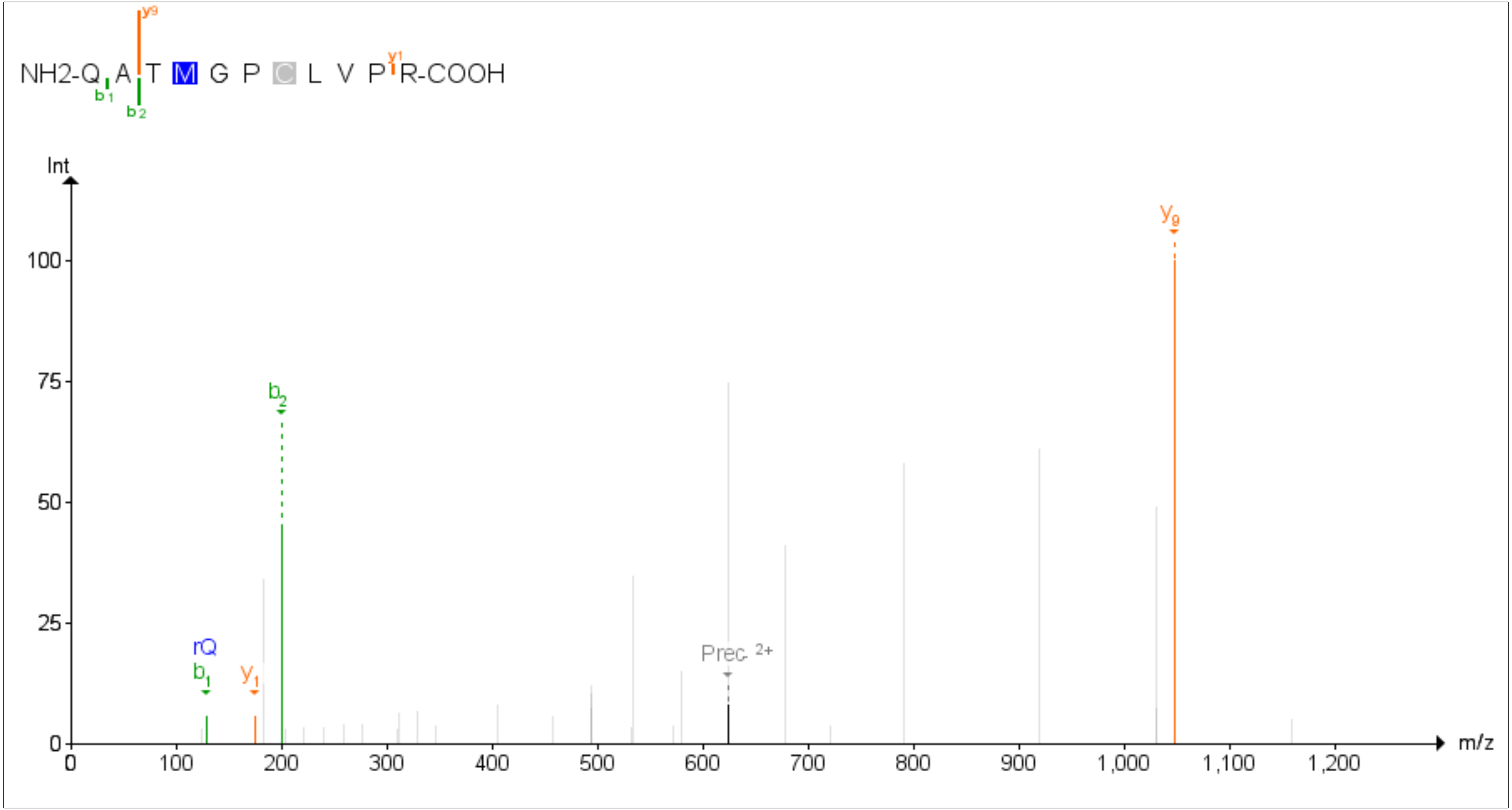

NH2-Q A T M G P C L V P R-COOH
y9
b1
b2
y1
Int
100
75
50
25
0
rQ
b1
y1
b2
Prec 2+
y9
0
100
200
300
400
500
600
700
800
900
1,000
1,100
1,200
m/z

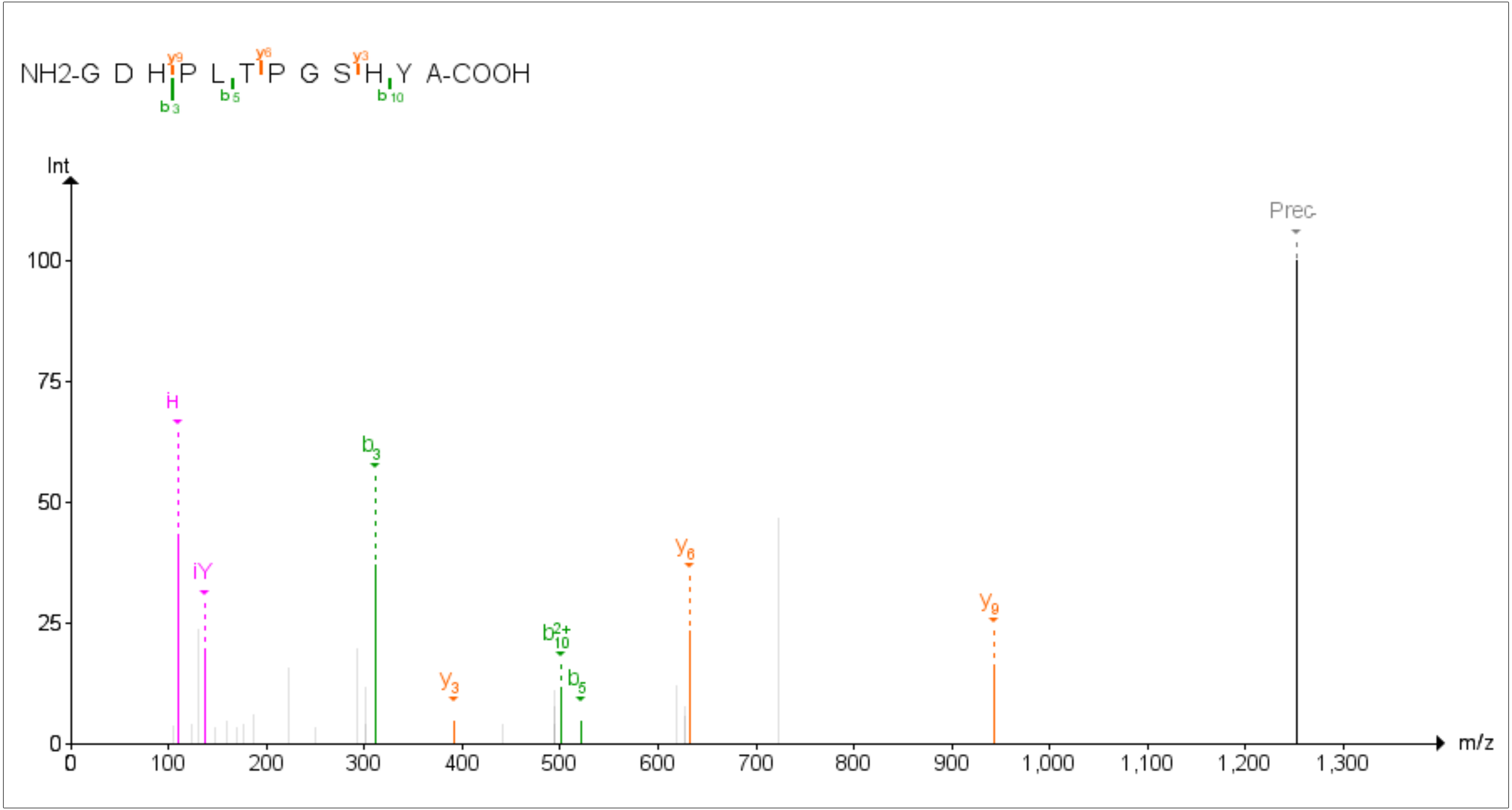

NH2-G D H P L T P G S H Y A-COOH
y9
y6
y3
b3
b5
b10
Int
100
75
50
25
0
Prec.
iH
iY
b3
y3
b10 2+
b5
y6
y9
0
100
200
300
400
500
600
700
800
900
1,000
1,100
1,200
1,300
m/z

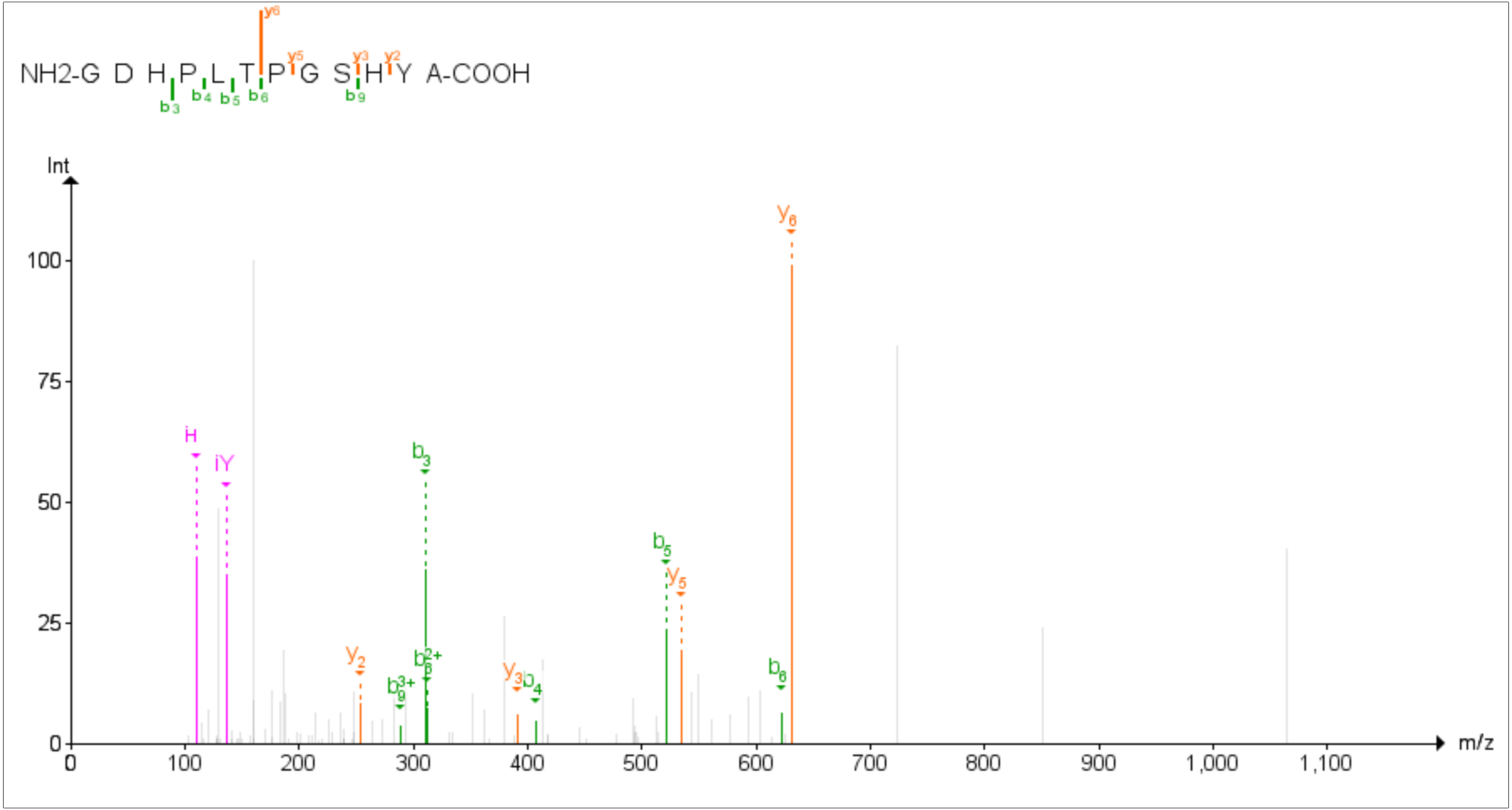

NH2-G D H P L T P G S H Y A-COOH
b3 b4 b5 b6 b9
y6 y5 y3 y2
Int
m/z
iH
iY
b3
b5
y5
y6
y2
b9 3+
b6 2+
y3
b4
b6
0
25
50
75
100
0
100
200
300
400
500
600
700
800
900
1,000
1,100

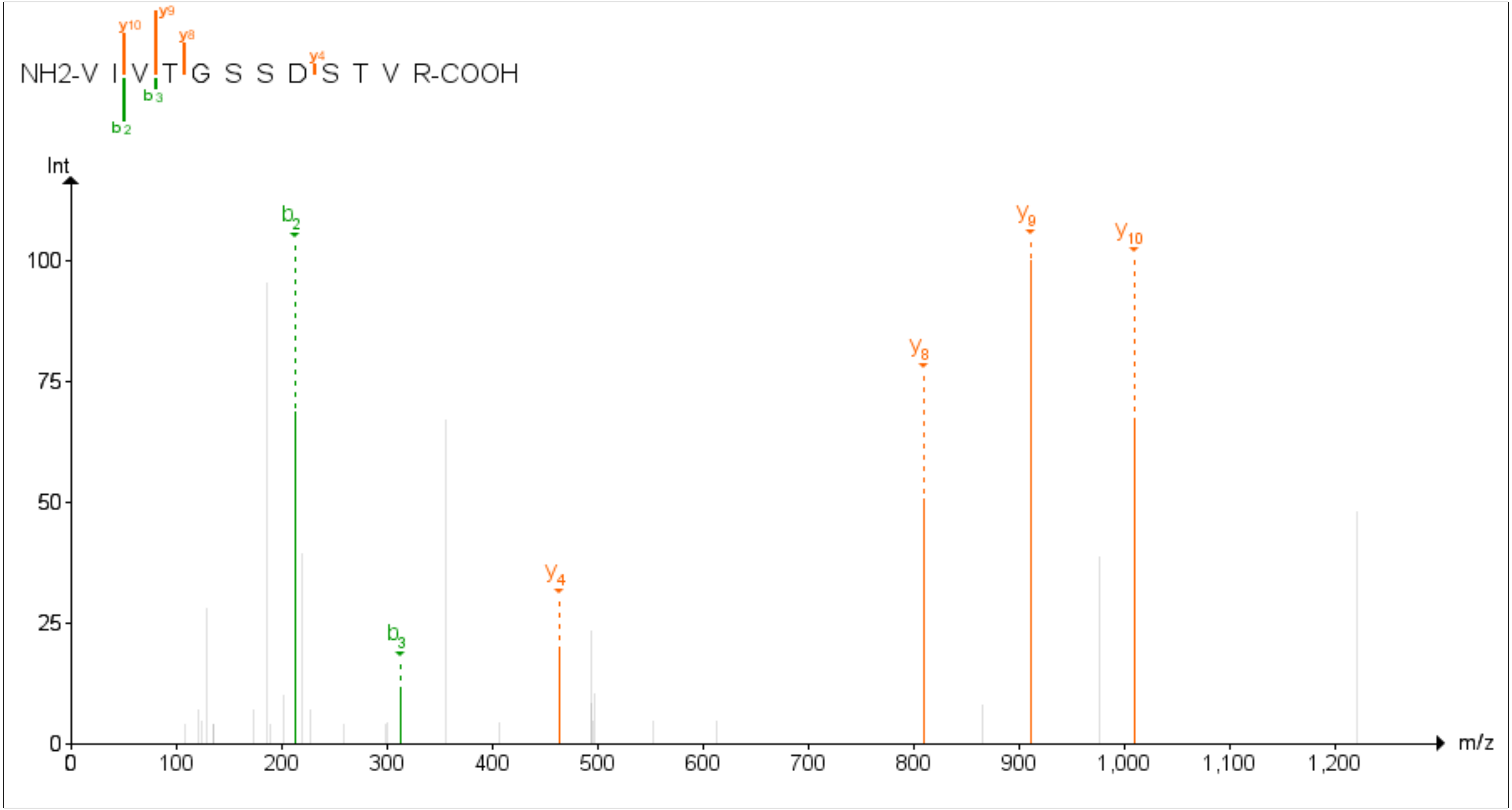

NH2-V I V T G S S D S T V R-COOH
y10
y9
y8
y4
b2
b3
Int
0
25
50
75
100
m/z
0
100
200
300
400
500
600
700
800
900
1,000
1,100
1,200

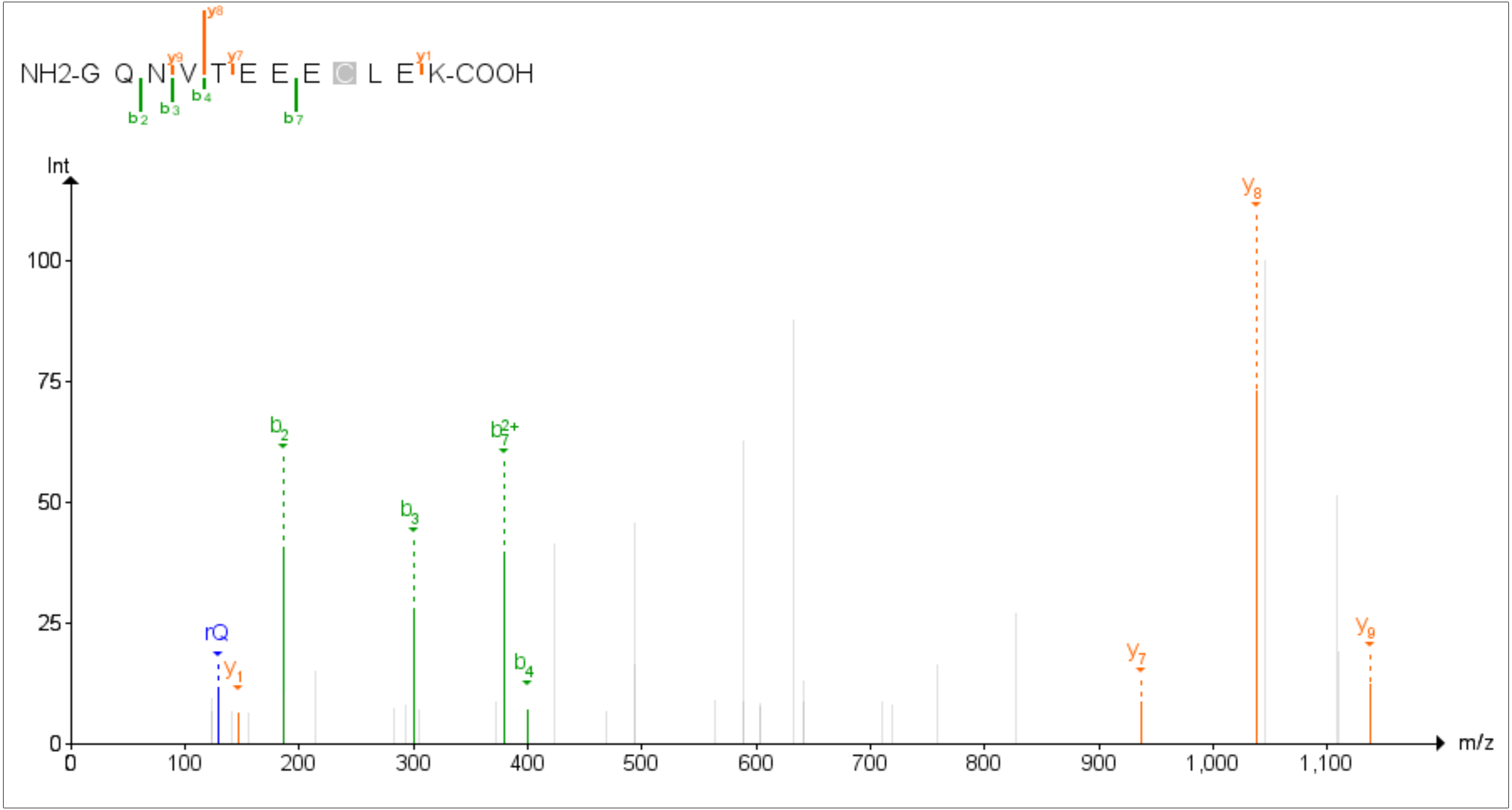
NH2-G Q N V T E E E C L E K-COOH
y9
y8
y7
y1
b2
b3
b4
b7
Int
100
75
50
25
0
rQ
y1
b2
b3
b7 2+
b4
y7
y8
y9
0
100
200
300
400
500
600
700
800
900
1,000
1,100
m/z

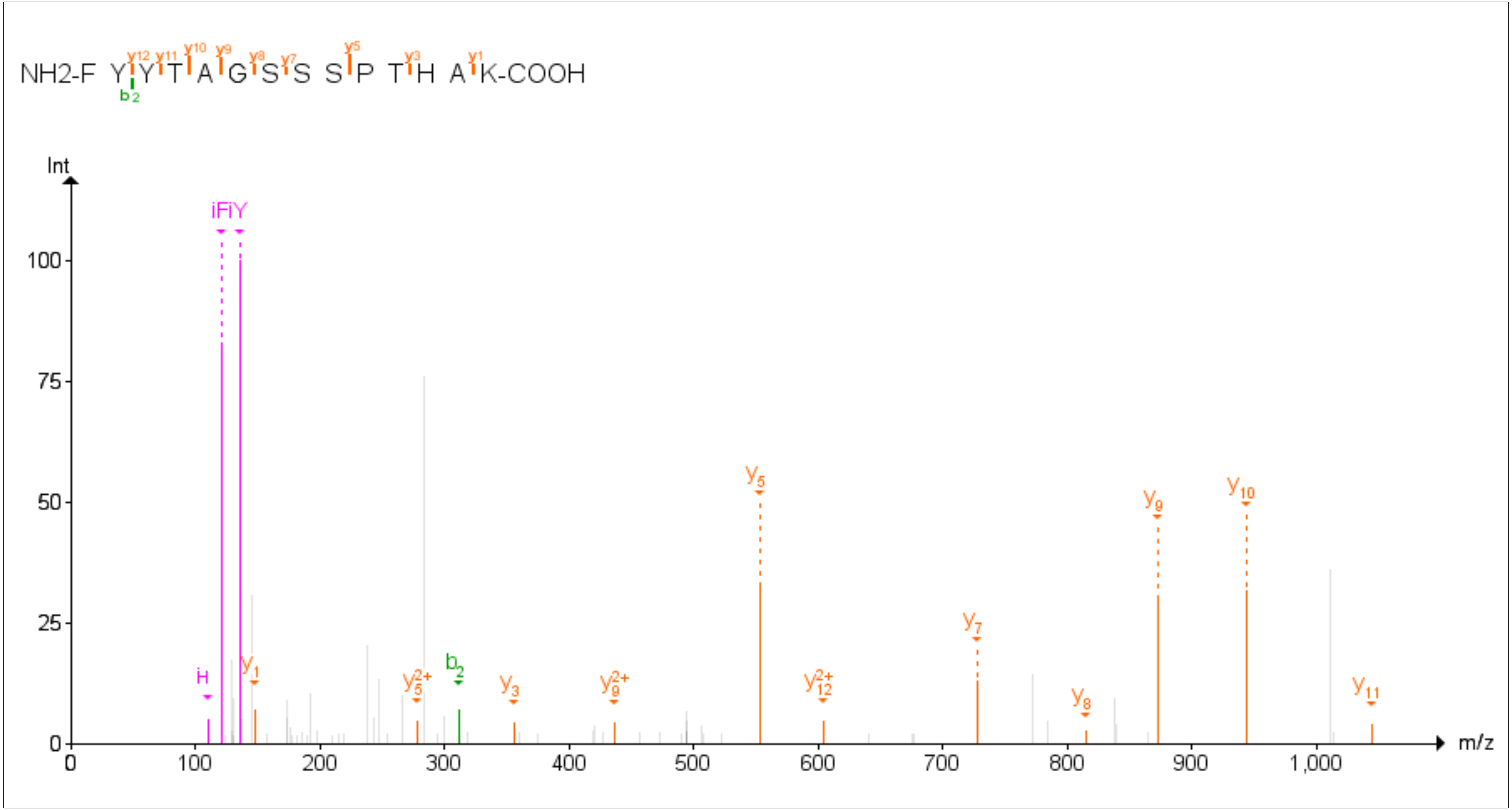

NH2-F Y Y T A G S S S P T H A K-COOH
y12 y11 y10 y9 y8 y7 y5 y3 y1
b2
Int
m/z
iFiY
H
y1
y5 2+
b2
y3
y9 2+
y5
y12 2+
y7
y8
y9
y10
y11
0
25
50
75
100
0
100
200
300
400
500
600
700
800
900
1,000

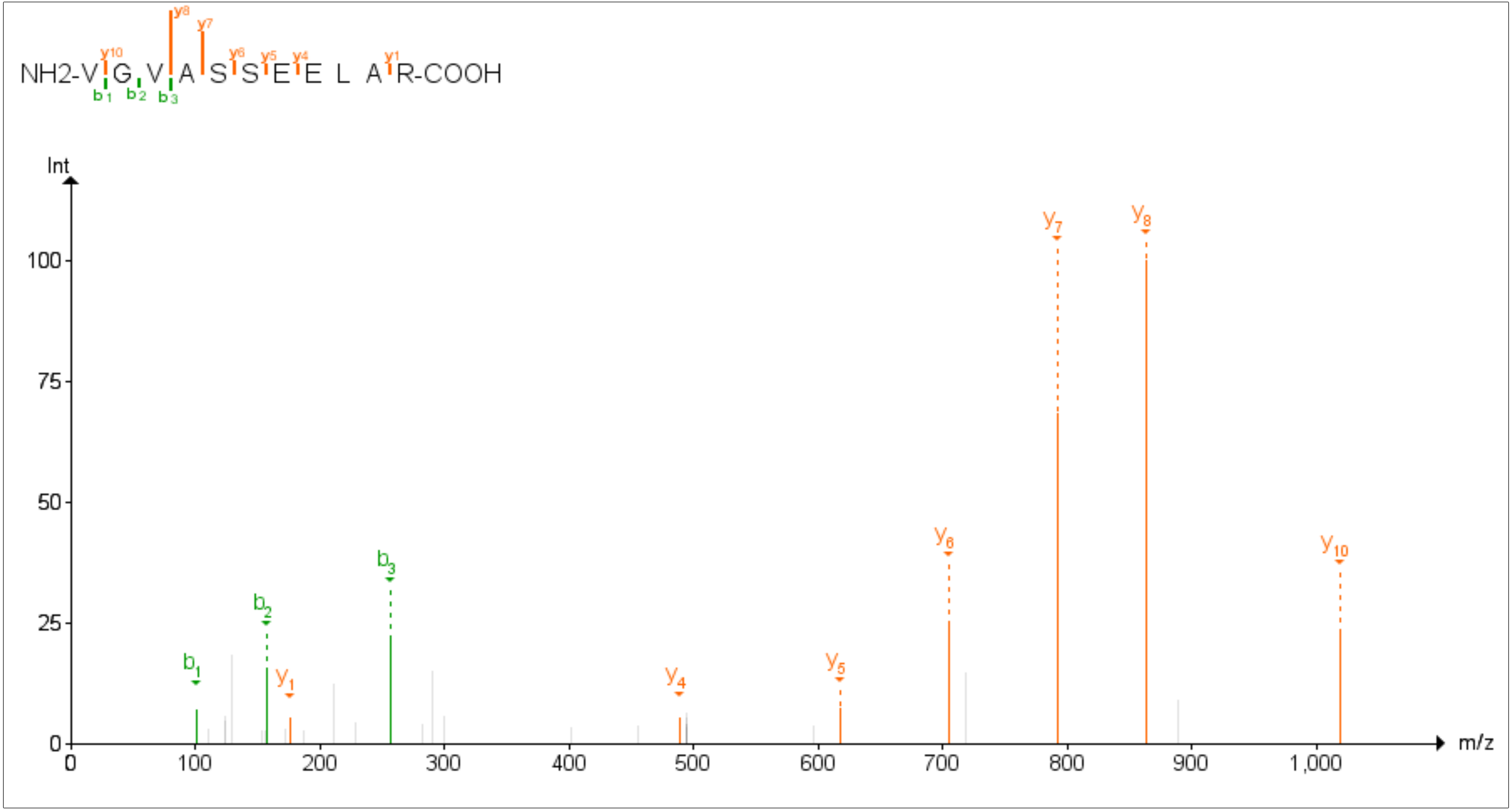
NH2-V G V A S S E E L A R-COOH
y10 y8 y7 y6 y5 y4 y1
b1 b2 b3
Int
100
75
50
25
0
m/z
0 100 200 300 400 500 600 700 800 900 1,000
b1 b2 b3 y1 y4 y5 y6 y7 y8 y10

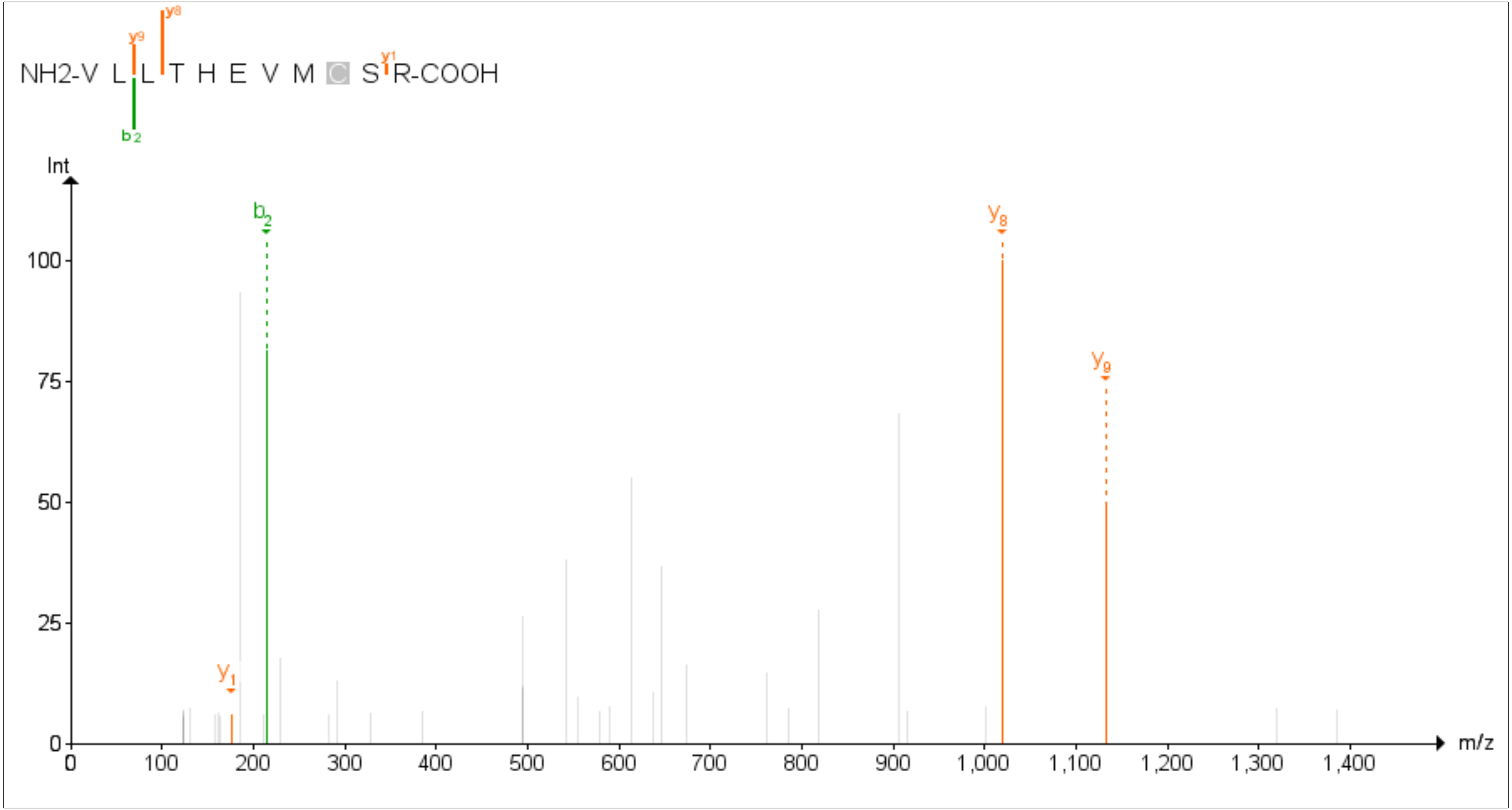
NH2-V L L T H E V M C S R-COOH
y9
y8
y1
b 2
Int
b2
y8
y9
y1
100
75
50
25
0
0
100
200
300
400
500
600
700
800
900
1,000
1,100
1,200
1,300
1,400
m/z

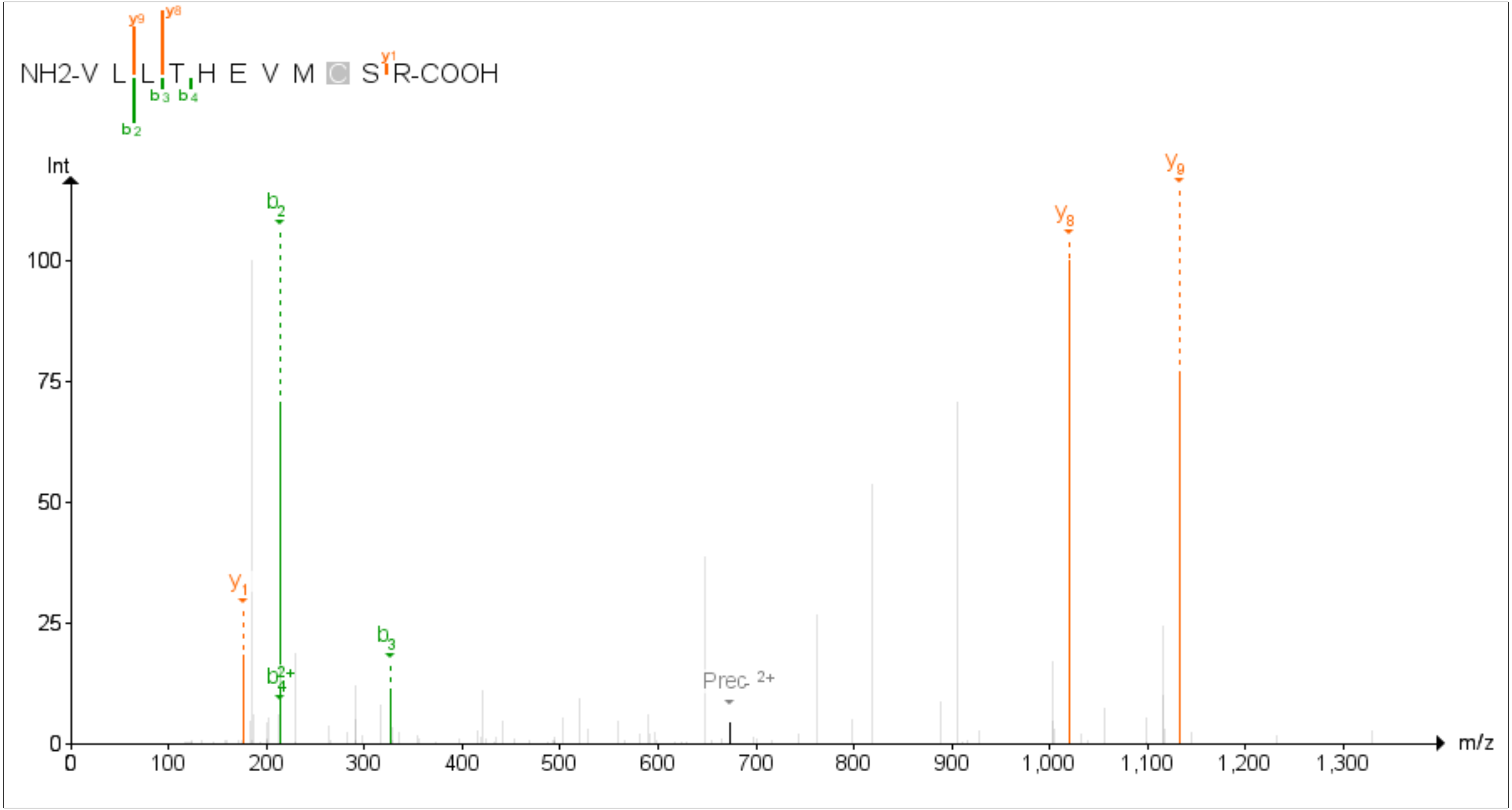

NH2-V L L T H E V M C S R-COOH
y9
y8
y1
b2
b3
b4
Int
m/z
b2
y8
y9
y1
b3
b4 2+
Prec 2+
0
25
50
75
100
0
100
200
300
400
500
600
700
800
900
1,000
1,100
1,200
1,300

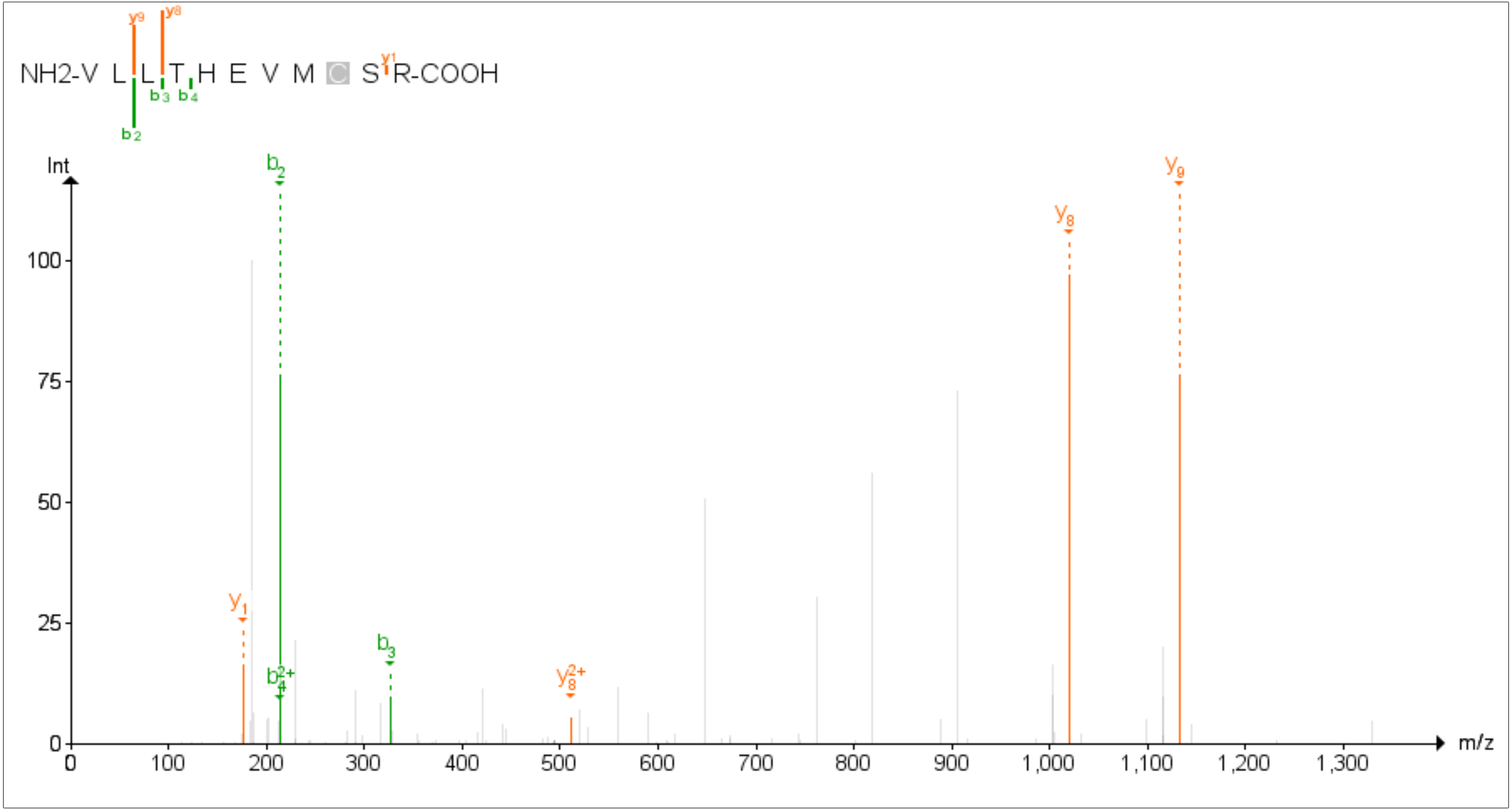

NH2-V L L T H E V M C S R-COOH
y9
y8
y1
b2
b3
b4
Int
100
75
50
25
0
b2
y9
y8
y1
b4 2+
b3
y8 2+
0
100
200
300
400
500
600
700
800
900
1,000
1,100
1,200
1,300
m/z

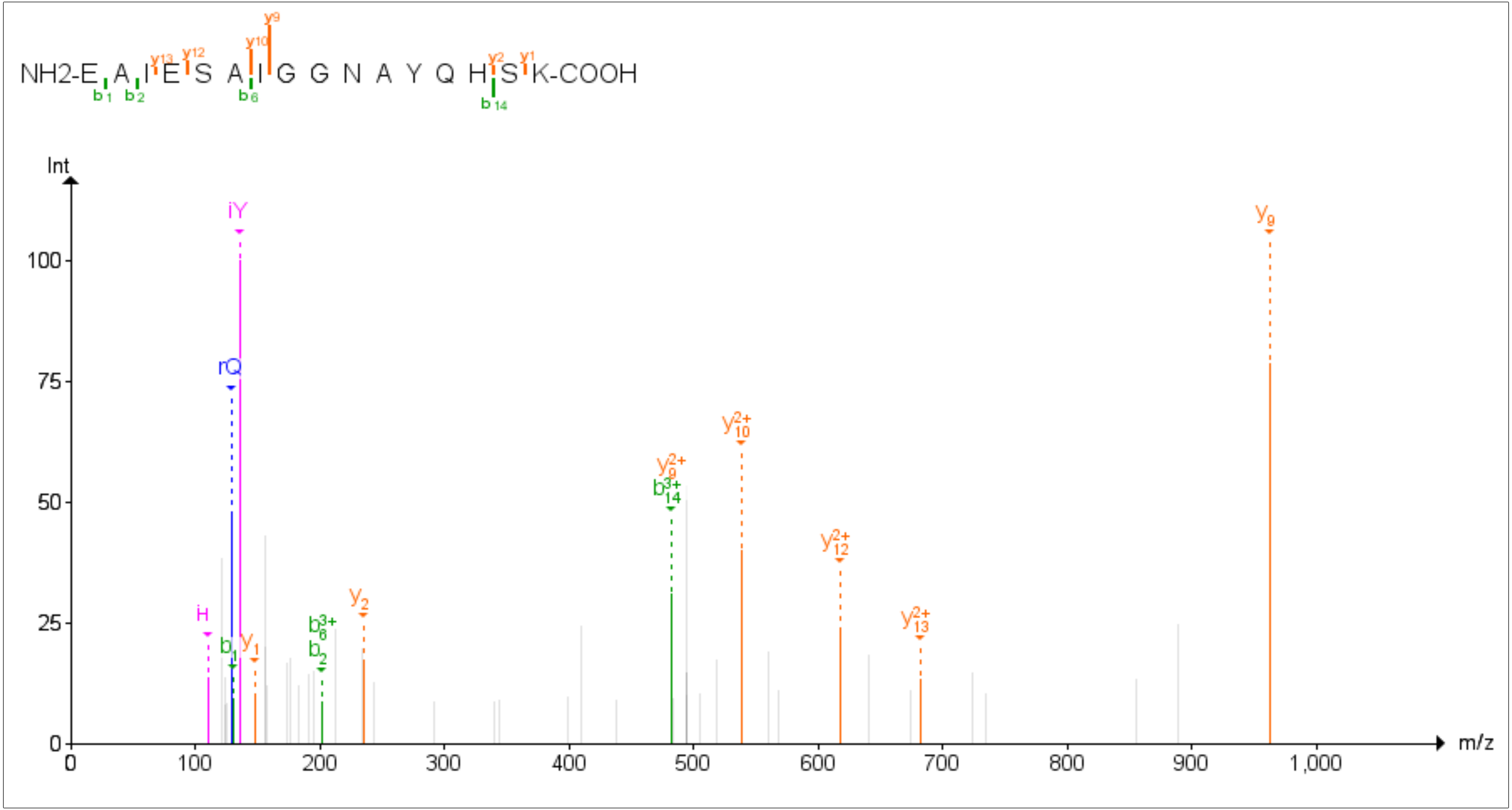
NH2-E A I E S A I G G N A Y Q H S K-COOH
b1 b2 b6 b14
y13 y12 y10 y9 y2 y1
Int
100
75
50
25
0
iY
rQ
iH
b1
y1
b6 3+
b2
y2
y9 2+
b14 3+
y10 2+
y12 2+
y13 2+
y9
0 100 200 300 400 500 600 700 800 900 1,000
m/z

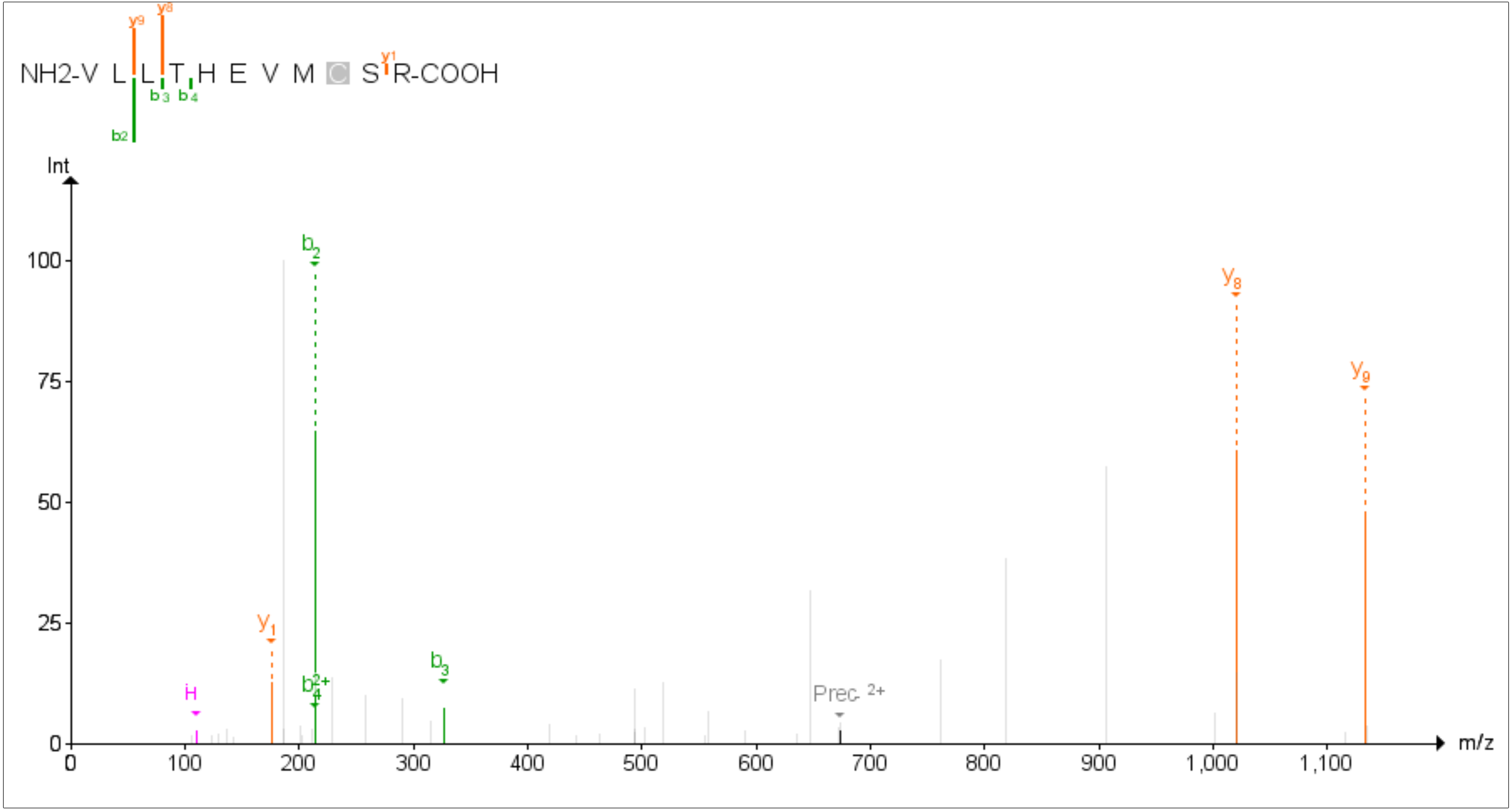

NH2-V L L T H E V M C S R-COOH
y9
y8
y1
b2
b3
b4
Int
100
75
50
25
0
b2
y8
y9
y1
H
b4 2+
b3
Prec- 2+
0
100
200
300
400
500
600
700
800
900
1,000
1,100
m/z

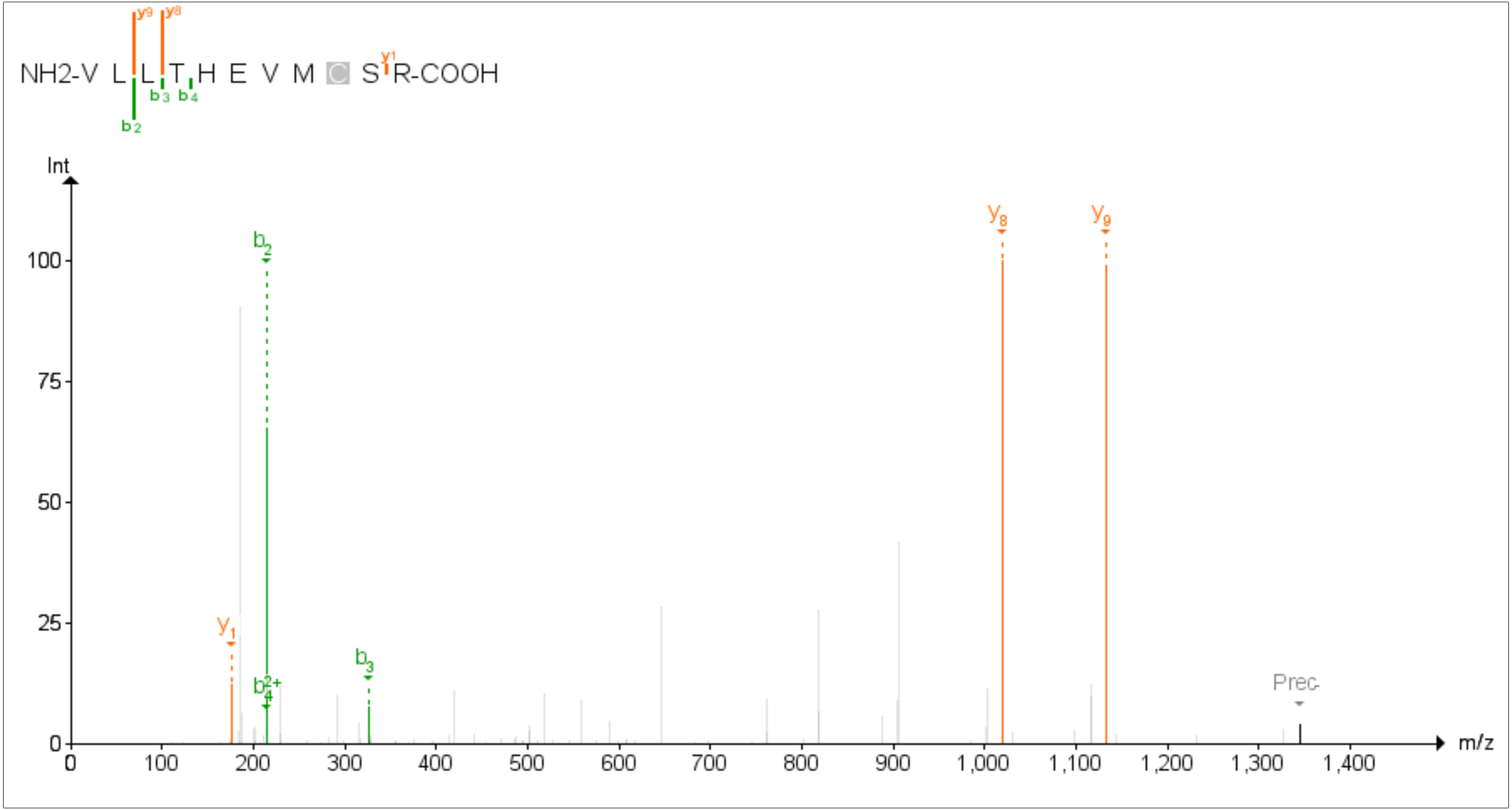
NH2-V L L T H E V M C S R-COOH
y9
y8
y1
b2
b3
b4
Int
100
75
50
25
0
b2
y8
y9
y1
b3
b_4^{2+}
Prec
0
100
200
300
400
500
600
700
800
900
1,000
1,100
1,200
1,300
1,400
m/z

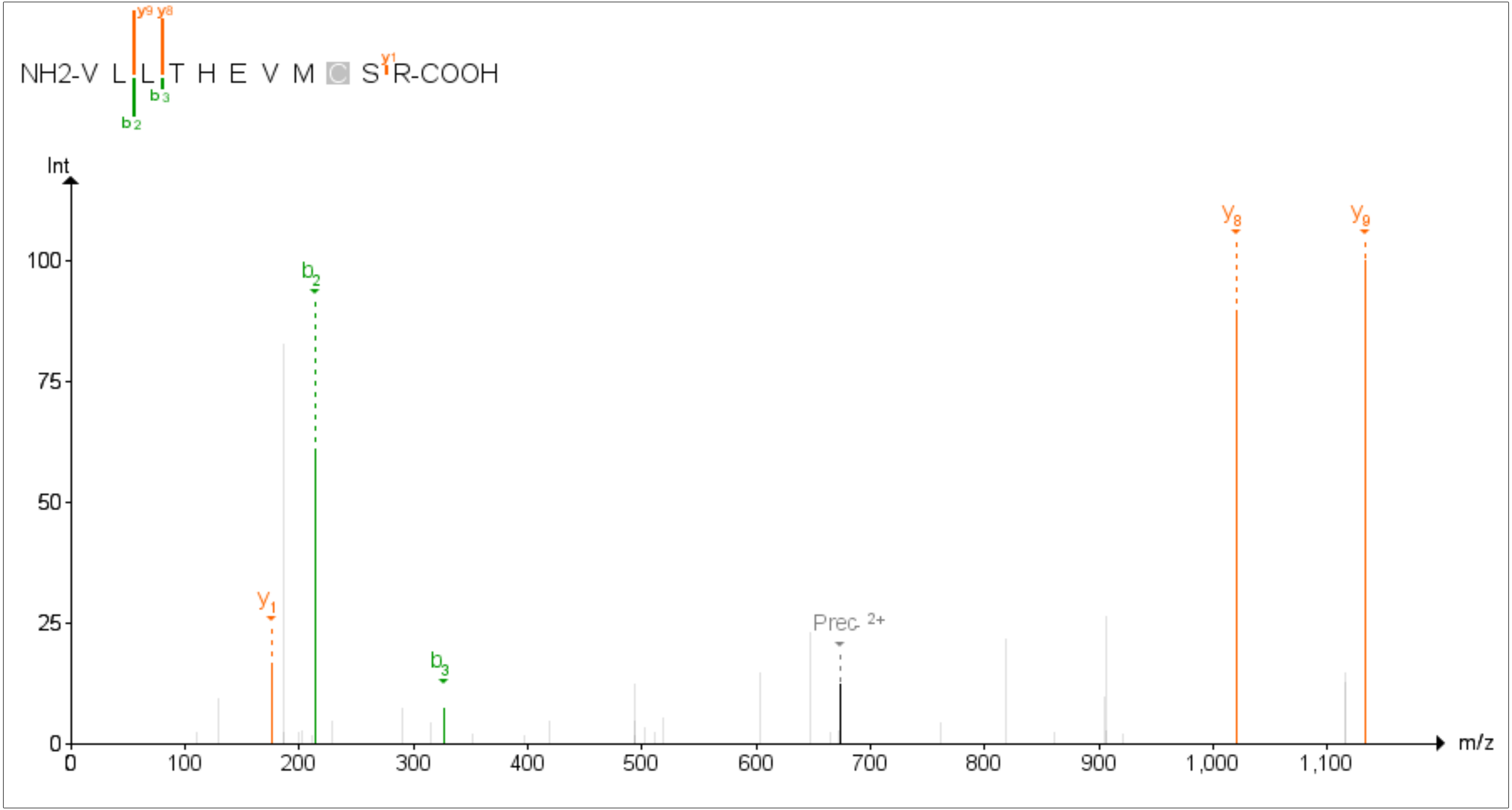

NH2-V L L T H E V M C S R-COOH
y9 y8 y1
b2 b3
Int
100
75
50
25
0
b2
y8
y9
y1
b3
Prec. 2+
m/z
0
100
200
300
400
500
600
700
800
900
1,000
1,100

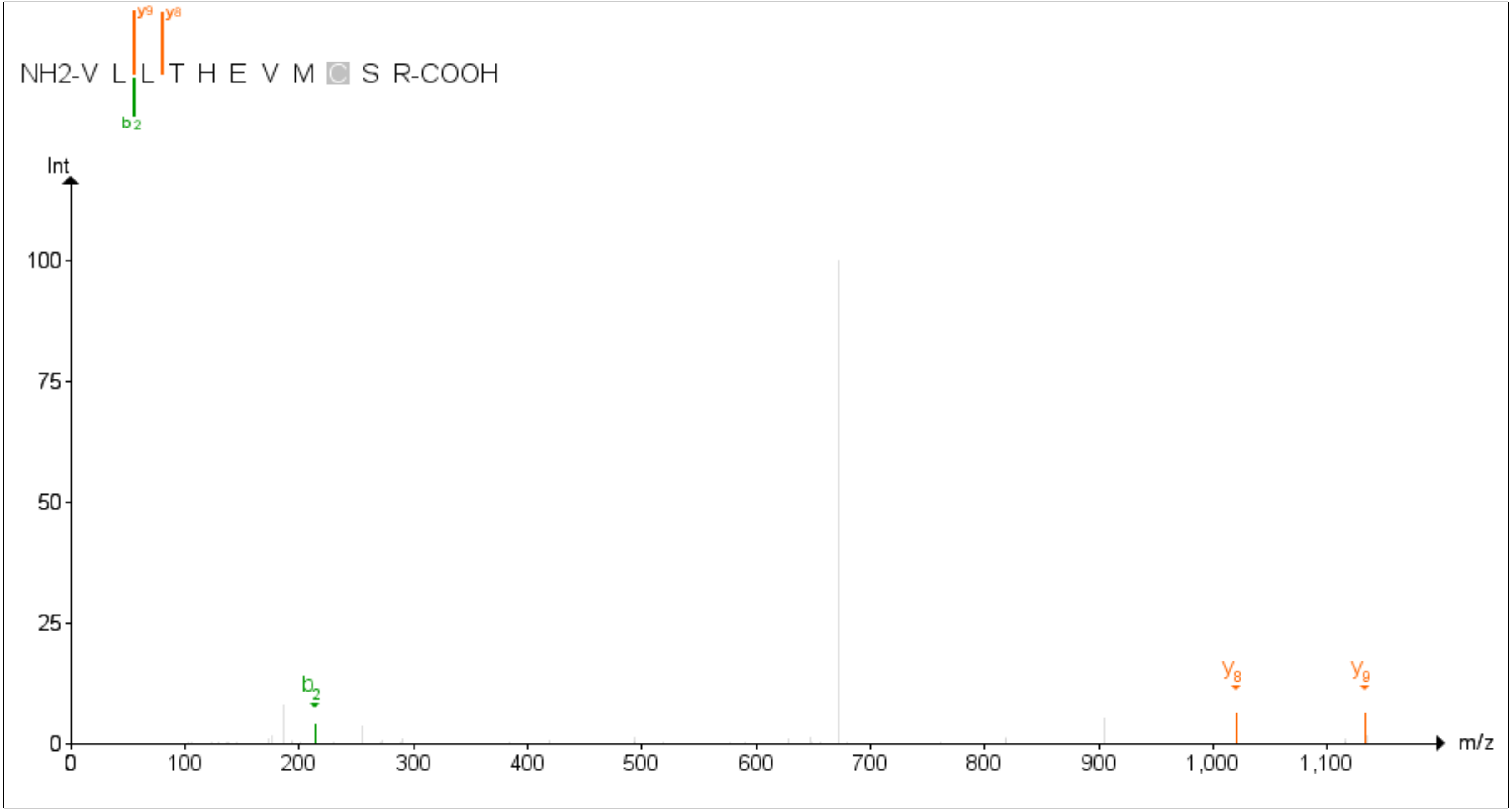
y9
y8
NH2-V L L T H E V M C S R-COOH
b2
Int
100
75
50
25
0
b2
y8
y9
0
100
200
300
400
500
600
700
800
900
1,000
1,100
m/z

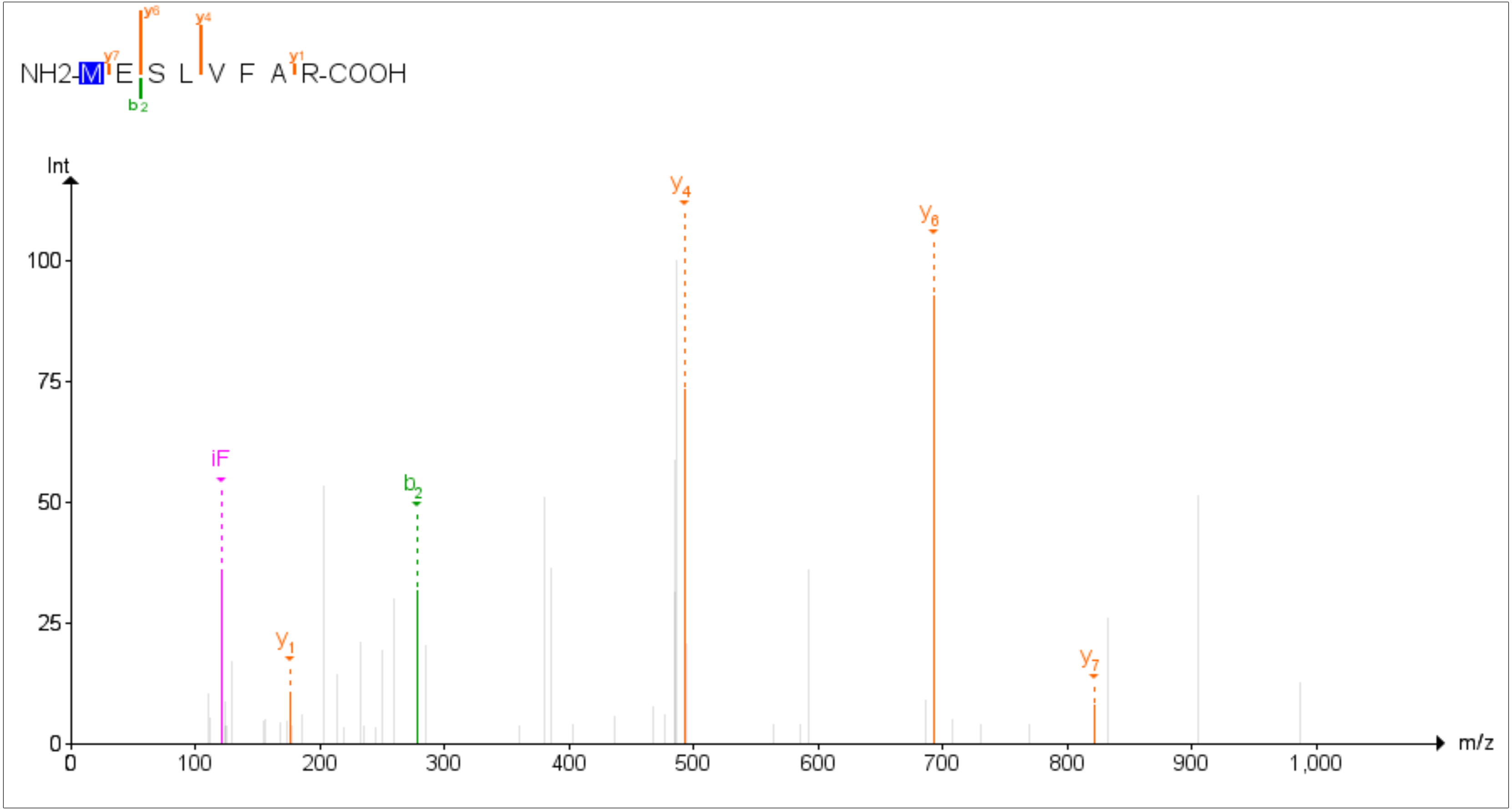

NH2-M E S L V F A R-COOH
y7
y6
y4
y1
b2
Int
m/z
iF
y1
b2
y4
y6
y7
0
25
50
75
100
0
100
200
300
400
500
600
700
800
900
1,000

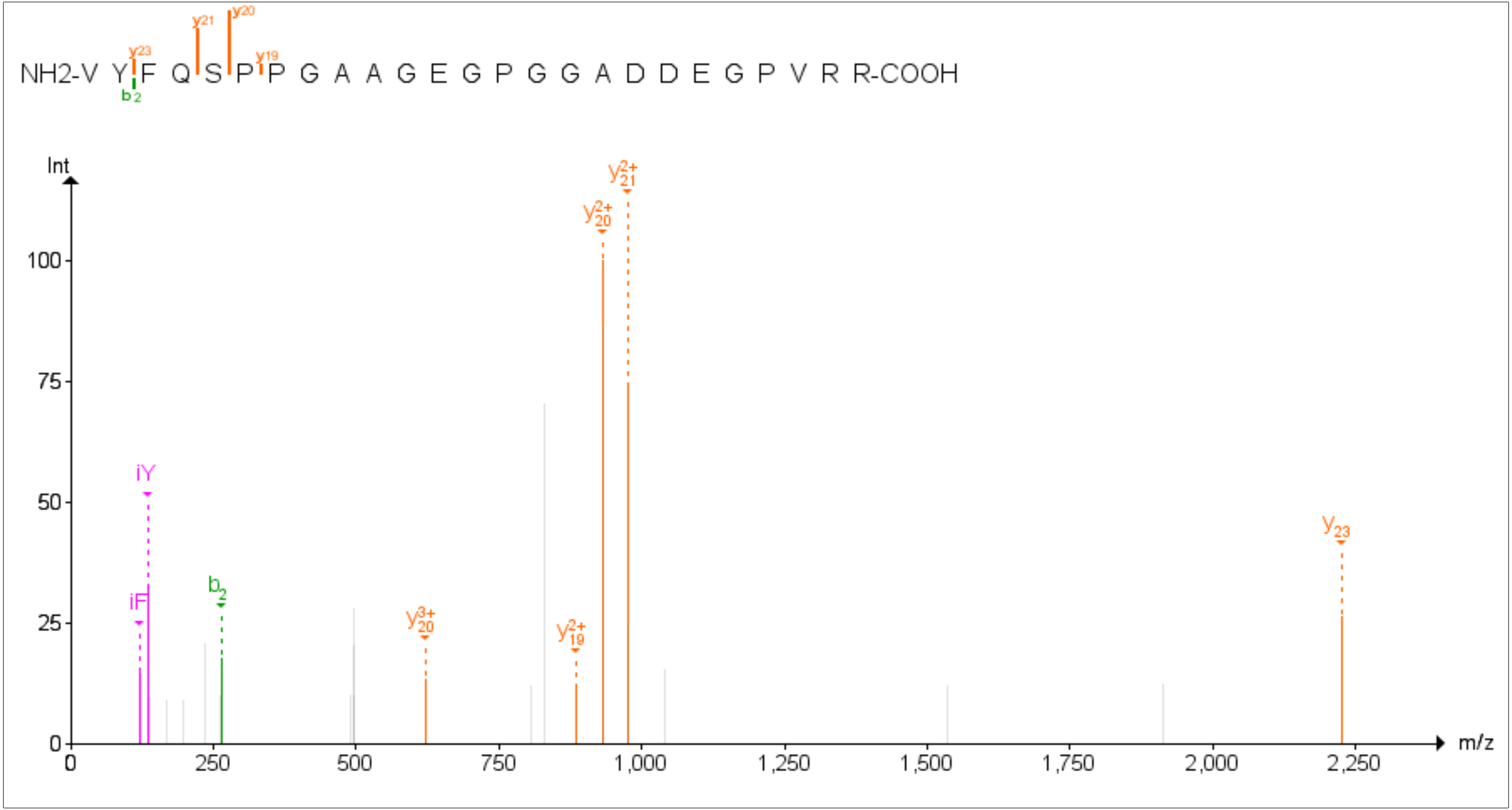

NH2-V Y F Q S P P G A A G E G P G G A D D E G P V R R-COOH
y23
y21
y20
y19
b2
Int
100
75
50
25
0
iY
iF
b2
y20 3+
y19 2+
y20 2+
y21 2+
y23
0
250
500
750
1,000
1,250
1,500
1,750
2,000
2,250
m/z

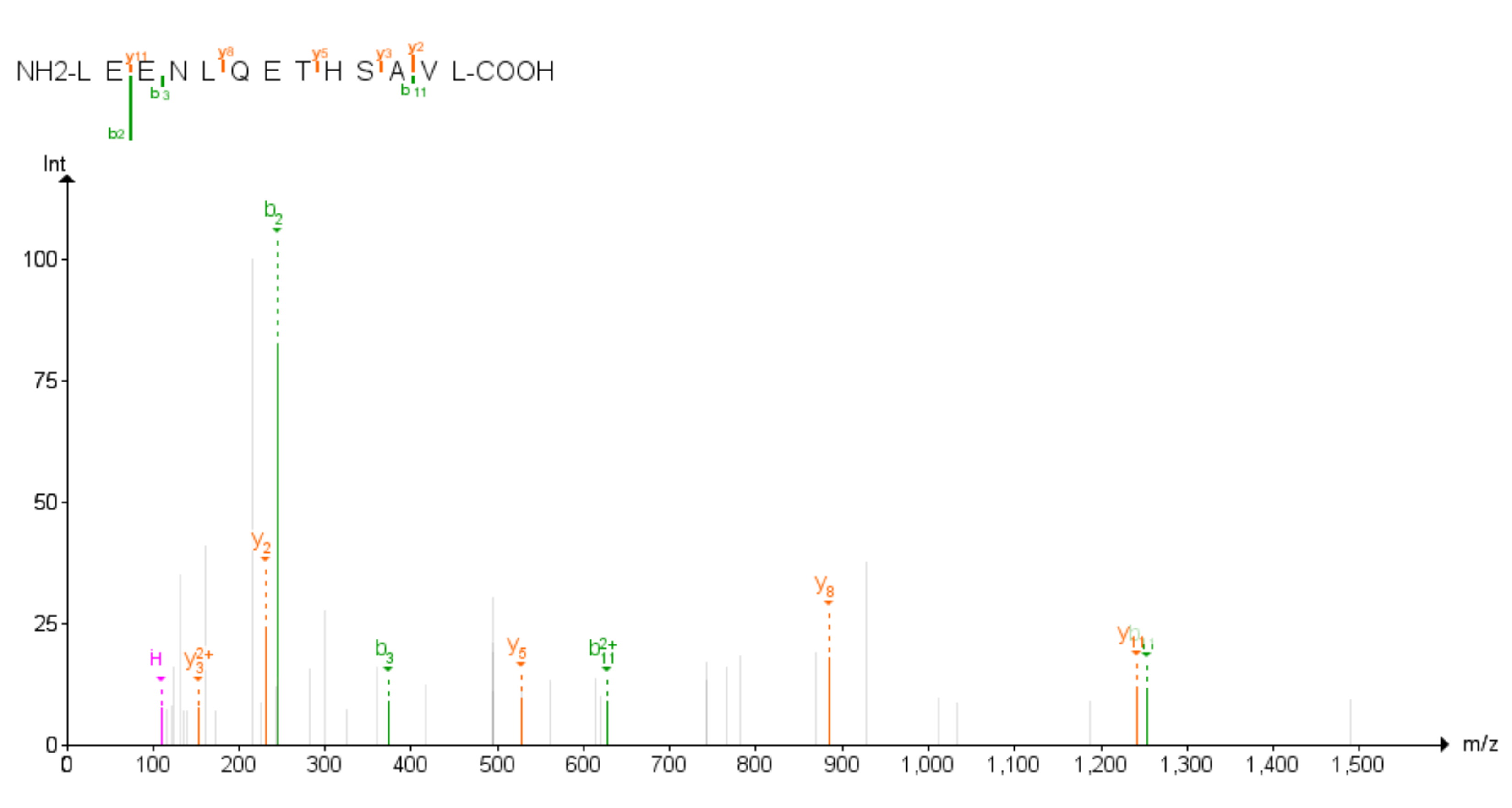

NH2-L E E N L Q E T H S A V L-COOH
y11 y8 y5 y3 y2
b2 b3 b11
Int
100
75
50
25
0
b2
y2
y8
H
y3 2+
b3
y5
b11 2+
y11
b11
0
100
200
300
400
500
600
700
800
900
1,000
1,100
1,200
1,300
1,400
1,500
m/z

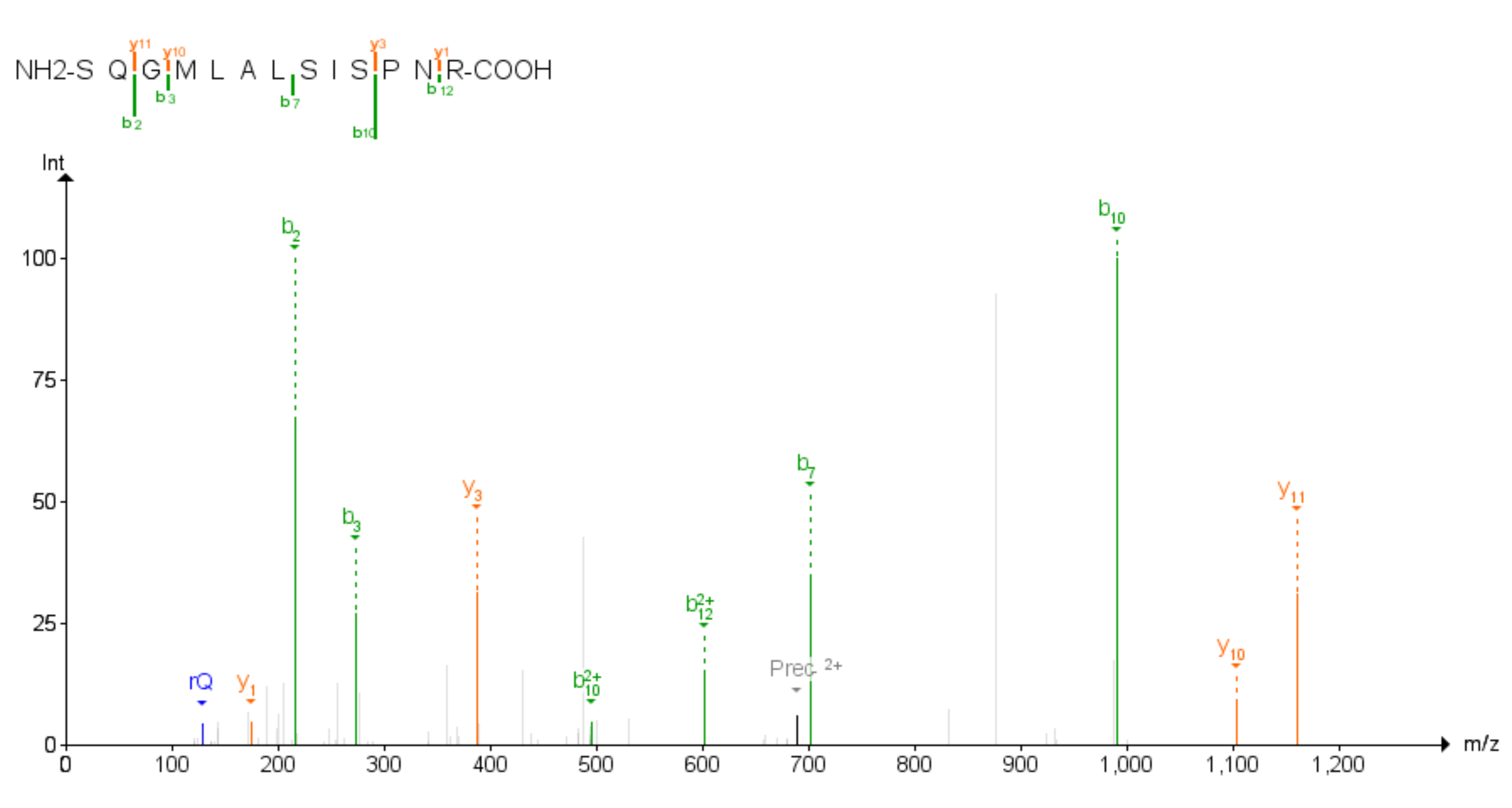
NH2-S Q G M L A L S I S P N R-COOH
y11
y10
y3
y1
b2
b3
b7
b10
b12
Int
100
75
50
25
0
m/z
0
100
200
300
400
500
600
700
800
900
1,000
1,100
1,200
rQ
y1
b2
b3
y3
b2+ 10
b2+ 12
Prec 2+
b7
b10
y10
y11

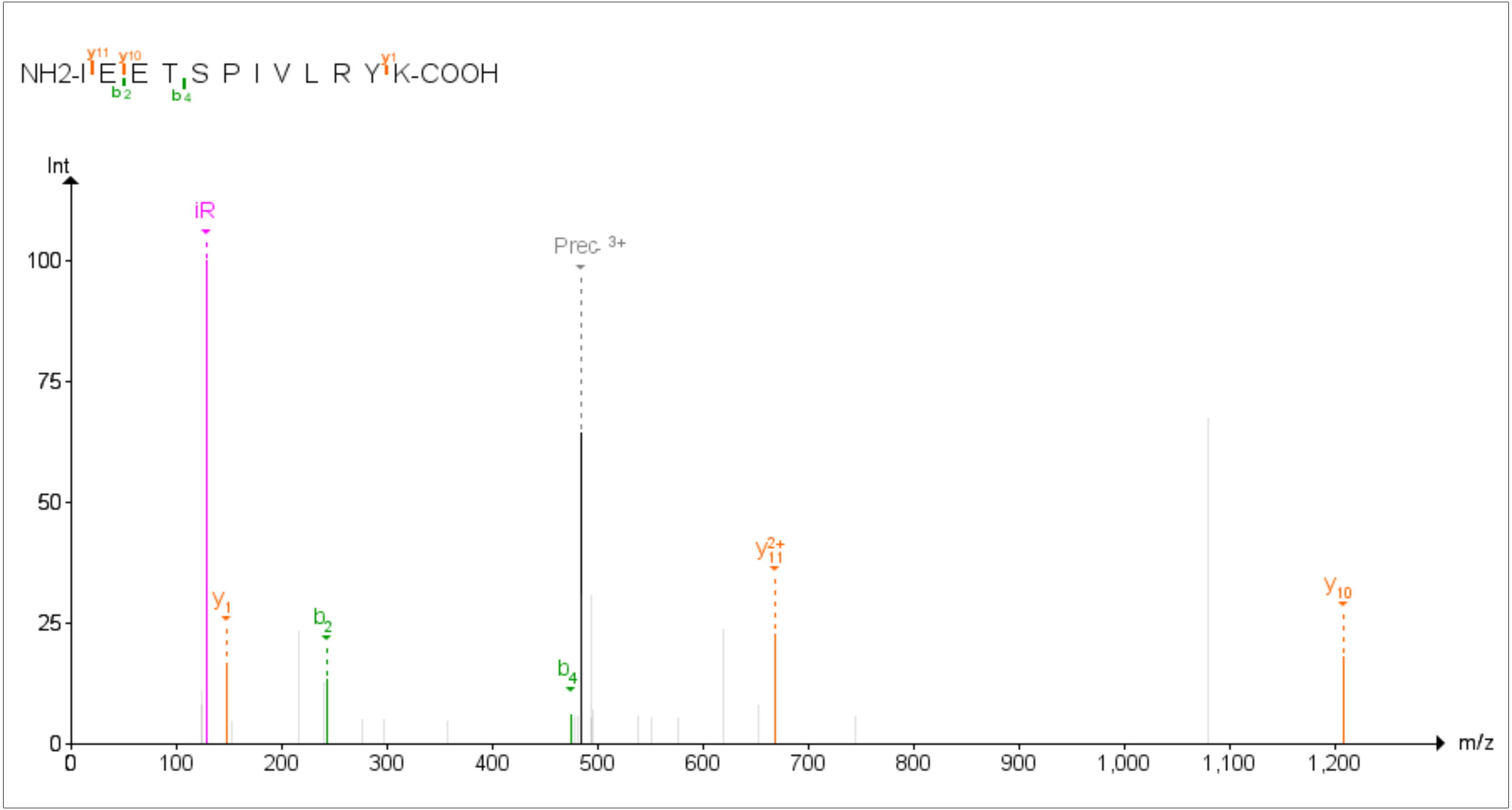

NH2-I E E T S P I V L R Y K-COOH
y11
y10
y1
b2
b4
Int
iR
Prec 3+
y1
b2
b4
y11 2+
y10
100
75
50
25
0
0
100
200
300
400
500
600
700
800
900
1,000
1,100
1,200
m/z

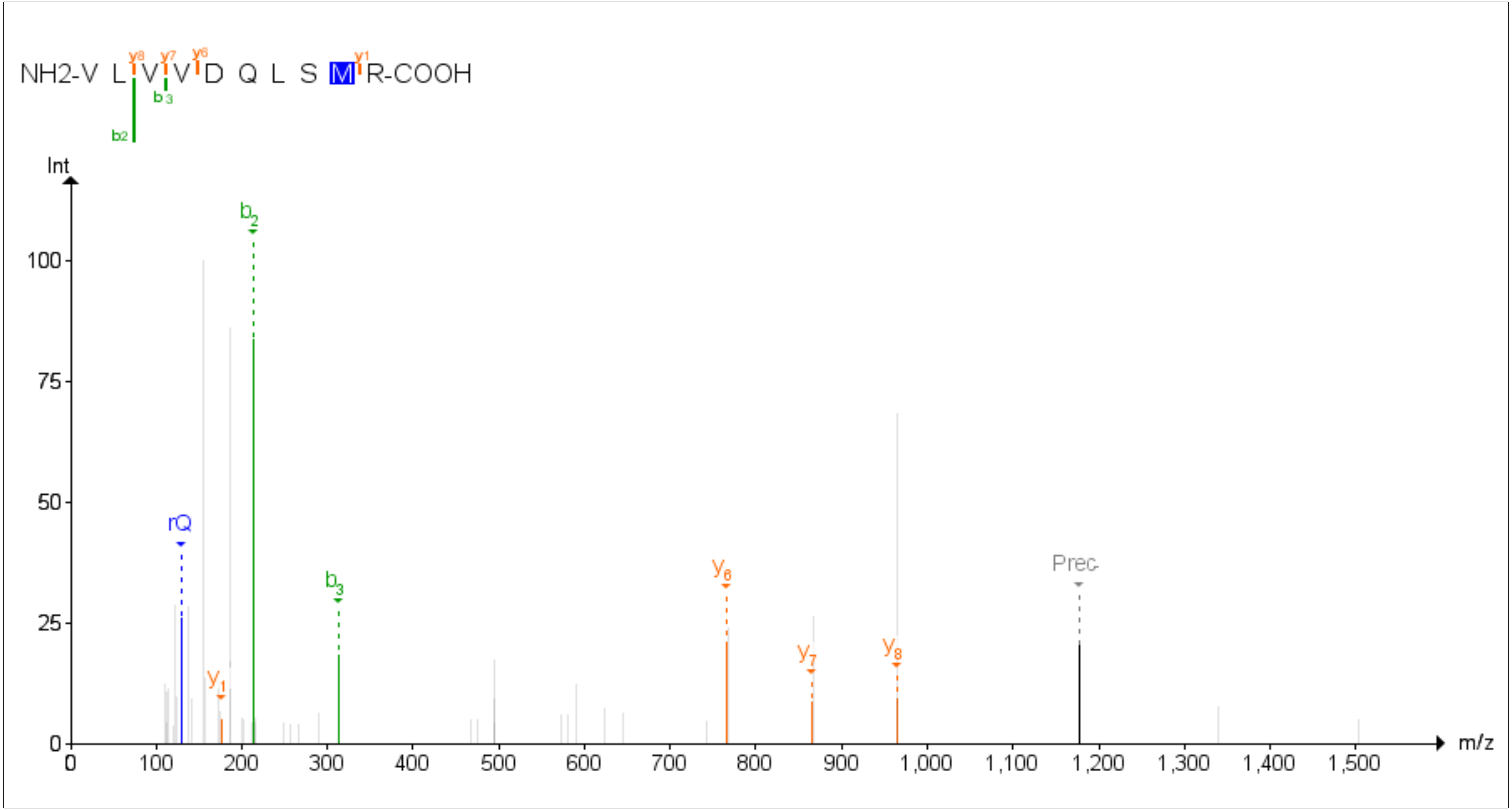

NH2-V L V V D Q L S M R-COOH
y8
y7
y6
y1
b2
b3
Int
100
75
50
25
0
rQ
b2
b3
y1
y6
y7
y8
Prec
0
100
200
300
400
500
600
700
800
900
1,000
1,100
1,200
1,300
1,400
1,500
m/z

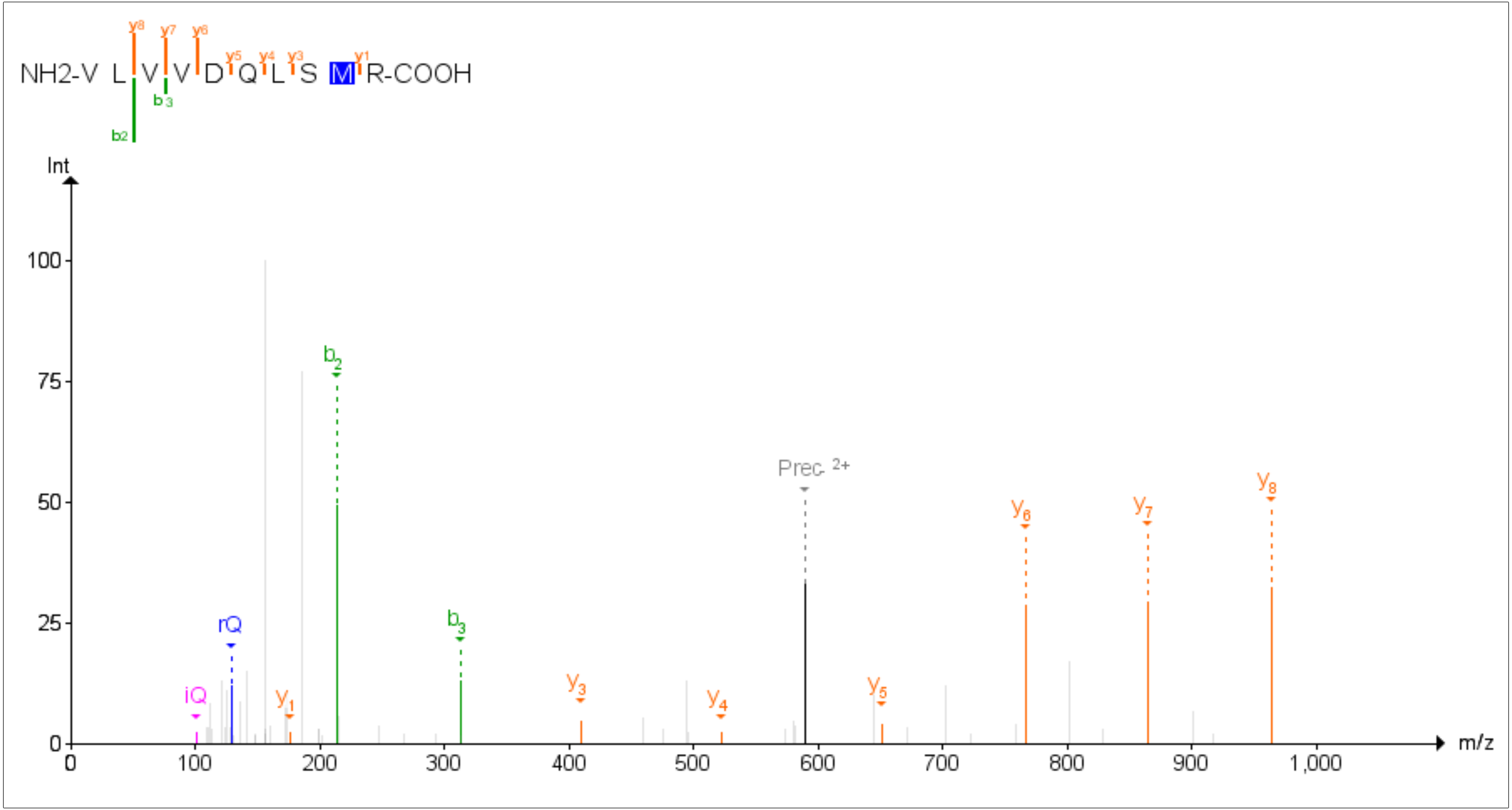
NH2-V L V V D Q L S M R-COOH
y8 y7 y6 y5 y4 y3 y1
b2 b3
Int
100
75
50
25
0
0 100 200 300 400 500 600 700 800 900 1,000
m/z
iQ
rQ
y1
b2
b3
y3
y4
Prec 2+
y5
y6
y7
y8

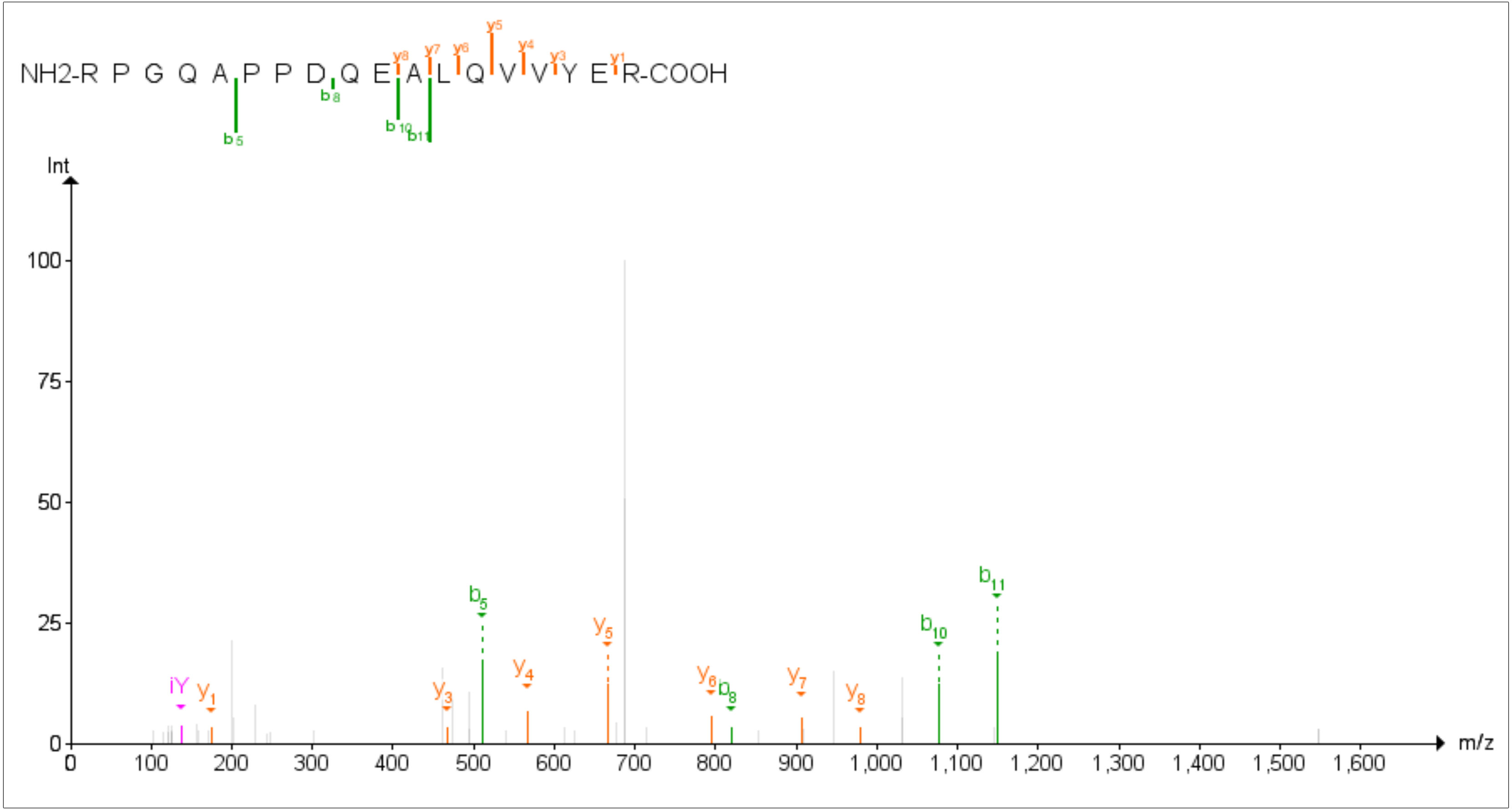
NH2-R P G Q A P P D Q E A L Q V V Y E R-COOH
y8 y7 y6 y5 y4 y3 y1
b5 b8 b10 b11
Int
100
75
50
25
0
iY
y1 y3 b5 y4 y5 y6 b8 y7 y8 b10 b11
0 100 200 300 400 500 600 700 800 900 1,000 1,100 1,200 1,300 1,400 1,500 1,600
m/z

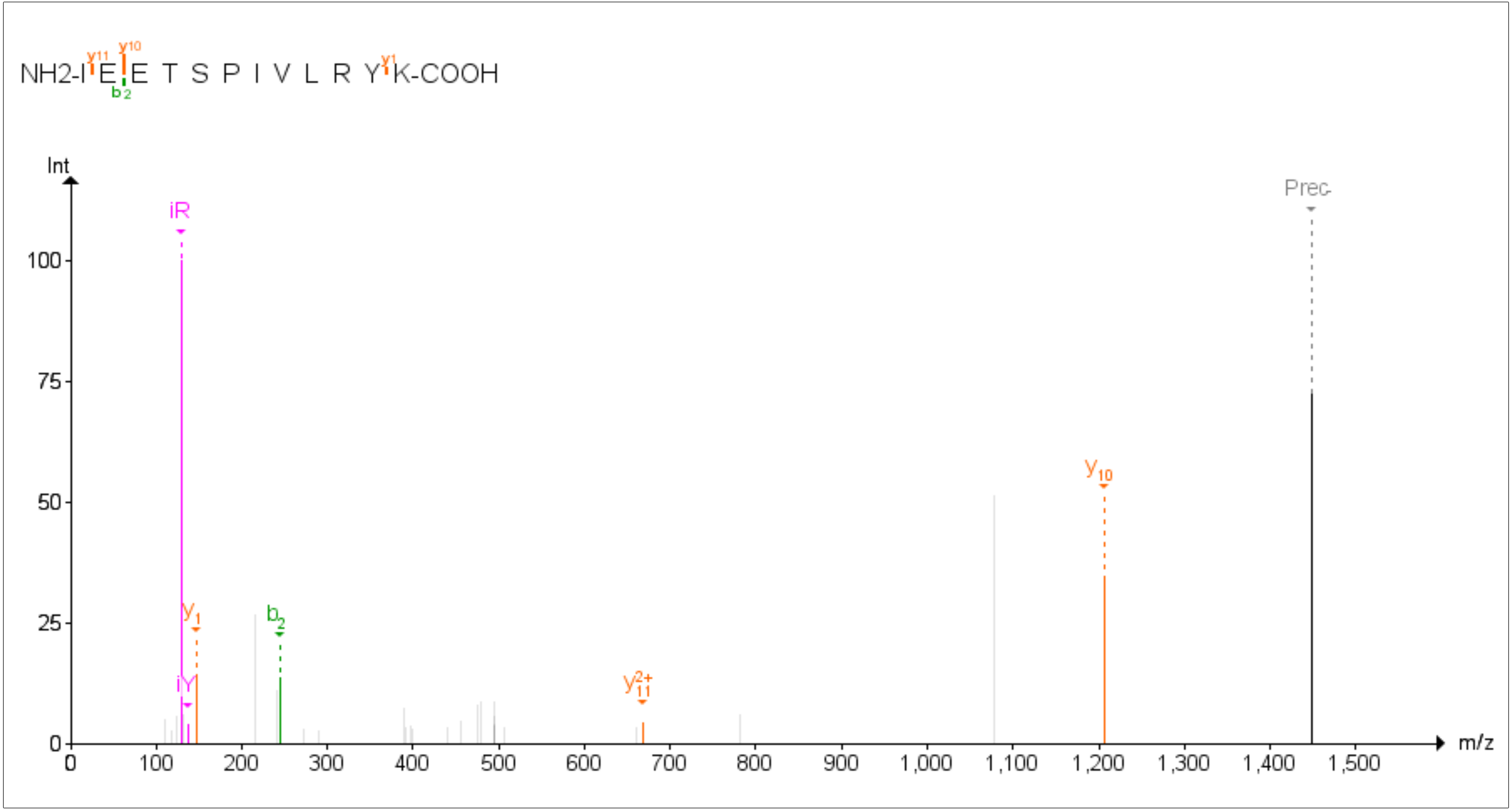

NH2-I E E T S P I V L R Y K-COOH
y11
y10
y1
b2
Int
m/z
iR
iY
y1
b2
y11 2+
y10
Prec
0
25
50
75
100
0
100
200
300
400
500
600
700
800
900
1,000
1,100
1,200
1,300
1,400
1,500

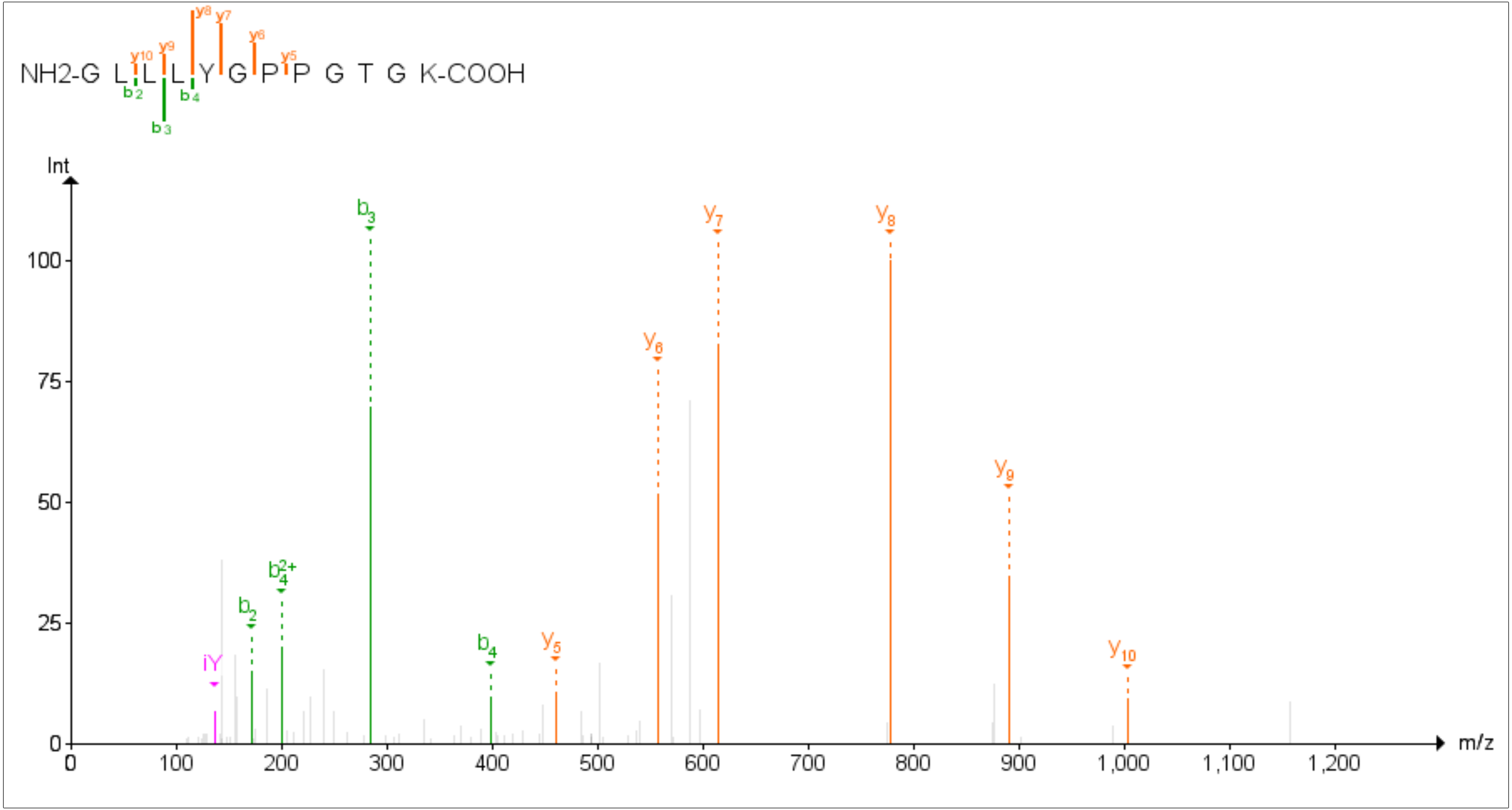

NH2-G L L L Y G P P G T G K-COOH
y10 y9 y8 y7 y6 y5
b2 b3 b4
Int
0
25
50
75
100
m/z
0
100
200
300
400
500
600
700
800
900
1,000
1,100
1,200
iY
b_2
b_4^{2+}
b_3
b_4
y_5
y_6
y_7
y_8
y_9
y_{10}

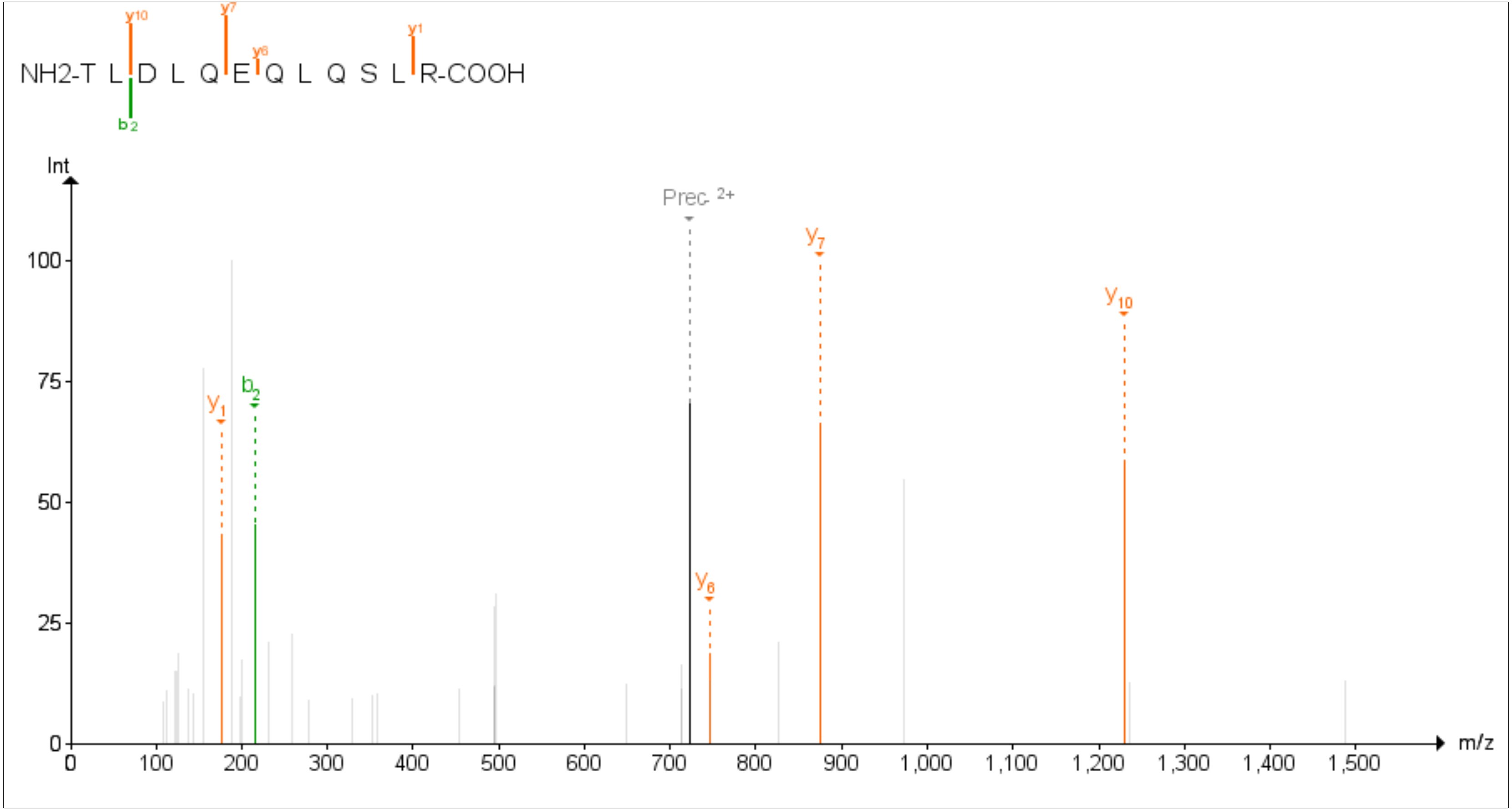

y10
y7
y6
y1
NH2-T L D L Q E Q L Q S L R-COOH
b2
Int
Prec. 2+
y7
y10
y1
b2
y6
100
75
50
25
0
0
100
200
300
400
500
600
700
800
900
1,000
1,100
1,200
1,300
1,400
1,500
m/z

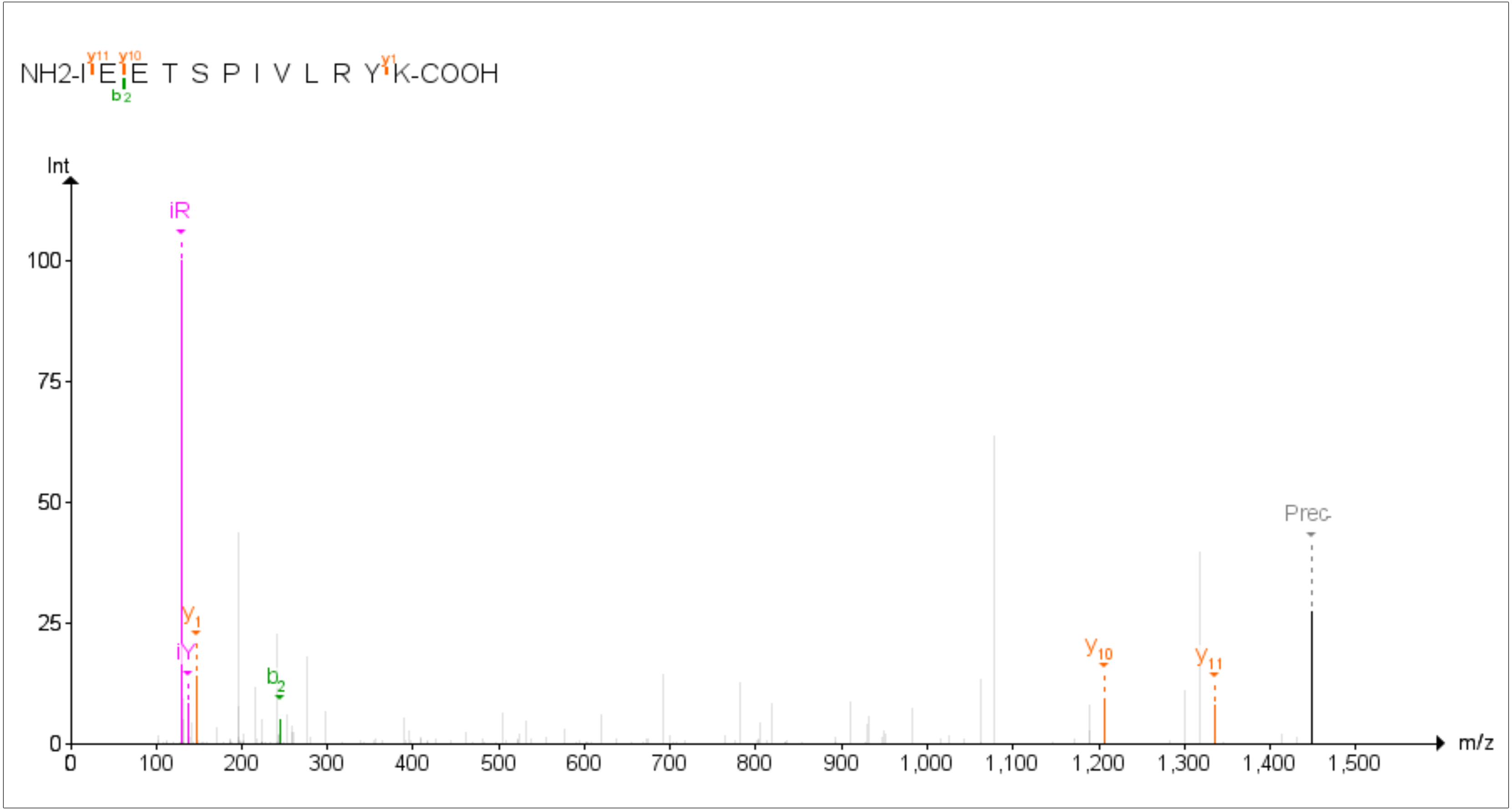

NH2-I E E T S P I V L R Y K-COOH
y11
y10
y1
b2
Int
iR
y1
iY
b2
y10
y11
Prec
100
75
50
25
0
0
100
200
300
400
500
600
700
800
900
1,000
1,100
1,200
1,300
1,400
1,500
m/z

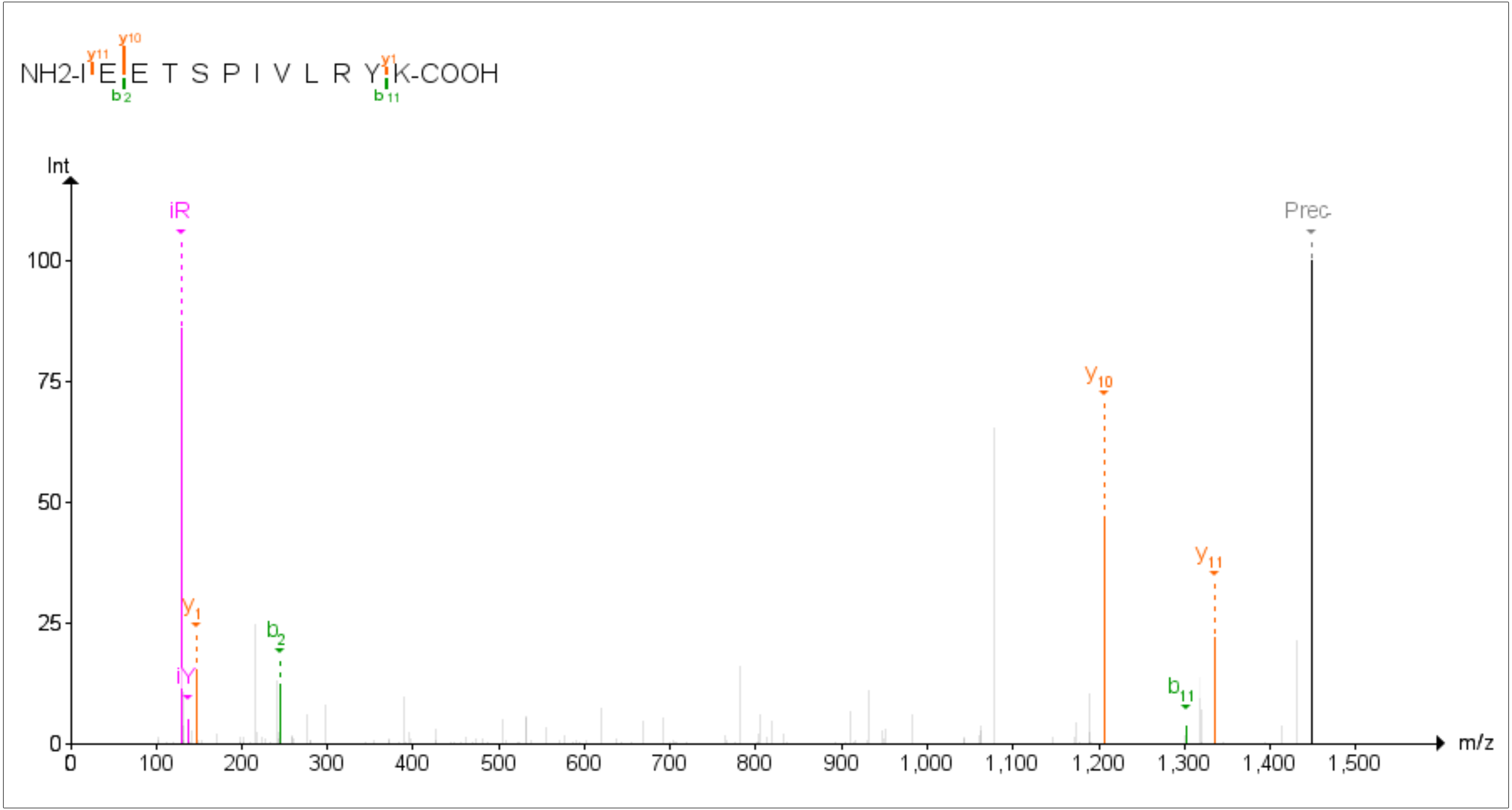

NH2-I E E T S P I V L R Y K-COOH
y11
y10
y1
b2
b11
Int
iR
Prec
y10
y11
y1
iY
b2
b11
100
75
50
25
0
0
100
200
300
400
500
600
700
800
900
1,000
1,100
1,200
1,300
1,400
1,500
m/z

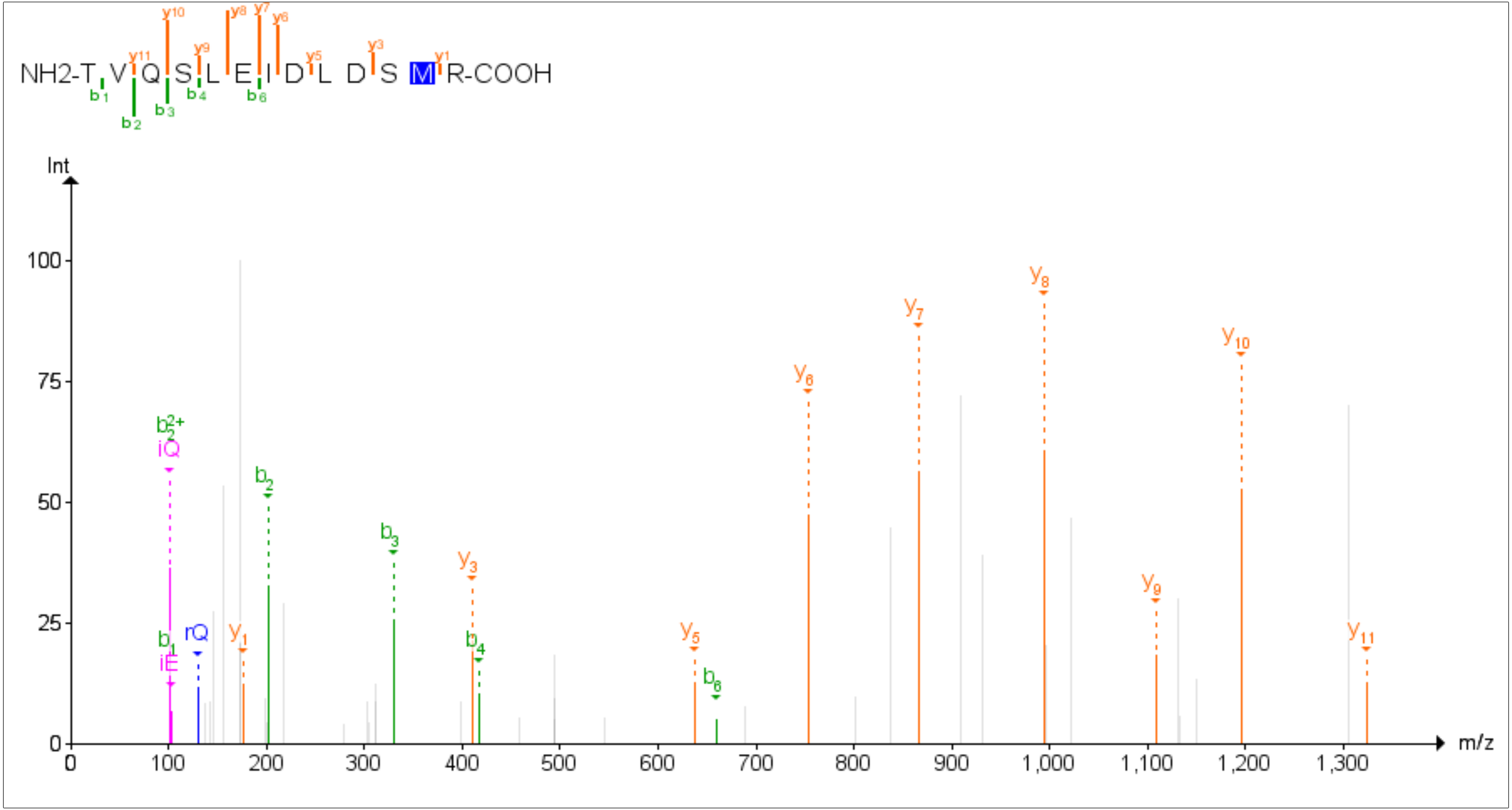

NH2-T V Q S L E I D L D S M R-COOH
y10 y8 y7 y6 y11 y9 y5 y3 y1
b1 b2 b3 b4 b6
Int
m/z
100
75
50
25
0
0 100 200 300 400 500 600 700 800 900 1,000 1,100 1,200 1,300
b2 2+
iQ
b2
b3
y3
b1
iE
rQ
y1
b4
y5
b6
y6
y7
y8
y10
y9
y11

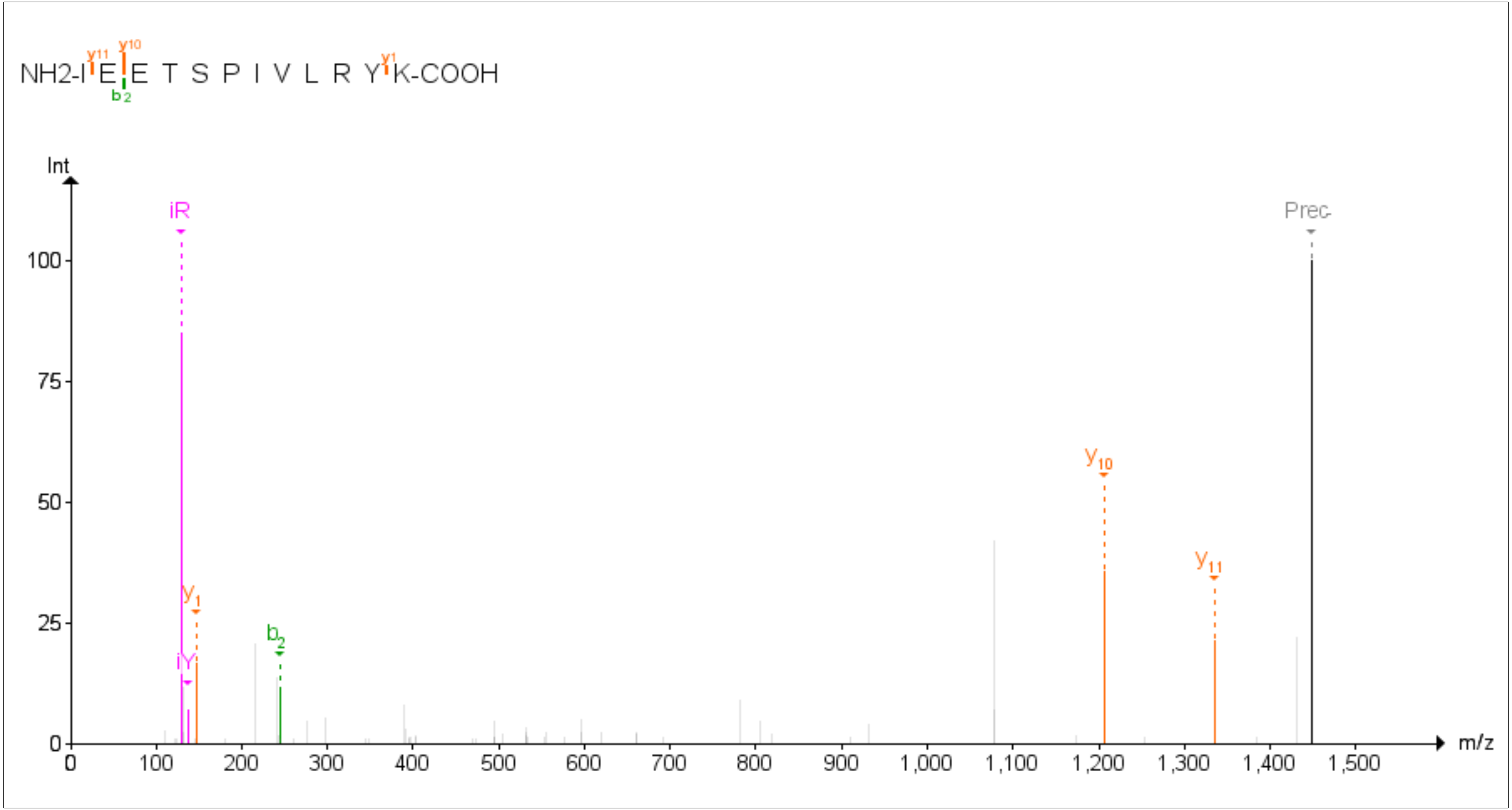

NH2-I E E T S P I V L R Y K-COOH
y11
y10
b2
y1
Int
m/z
iR
Prec
y10
y11
y1
iY
b2
0
25
50
75
100
0
100
200
300
400
500
600
700
800
900
1,000
1,100
1,200
1,300
1,400
1,500

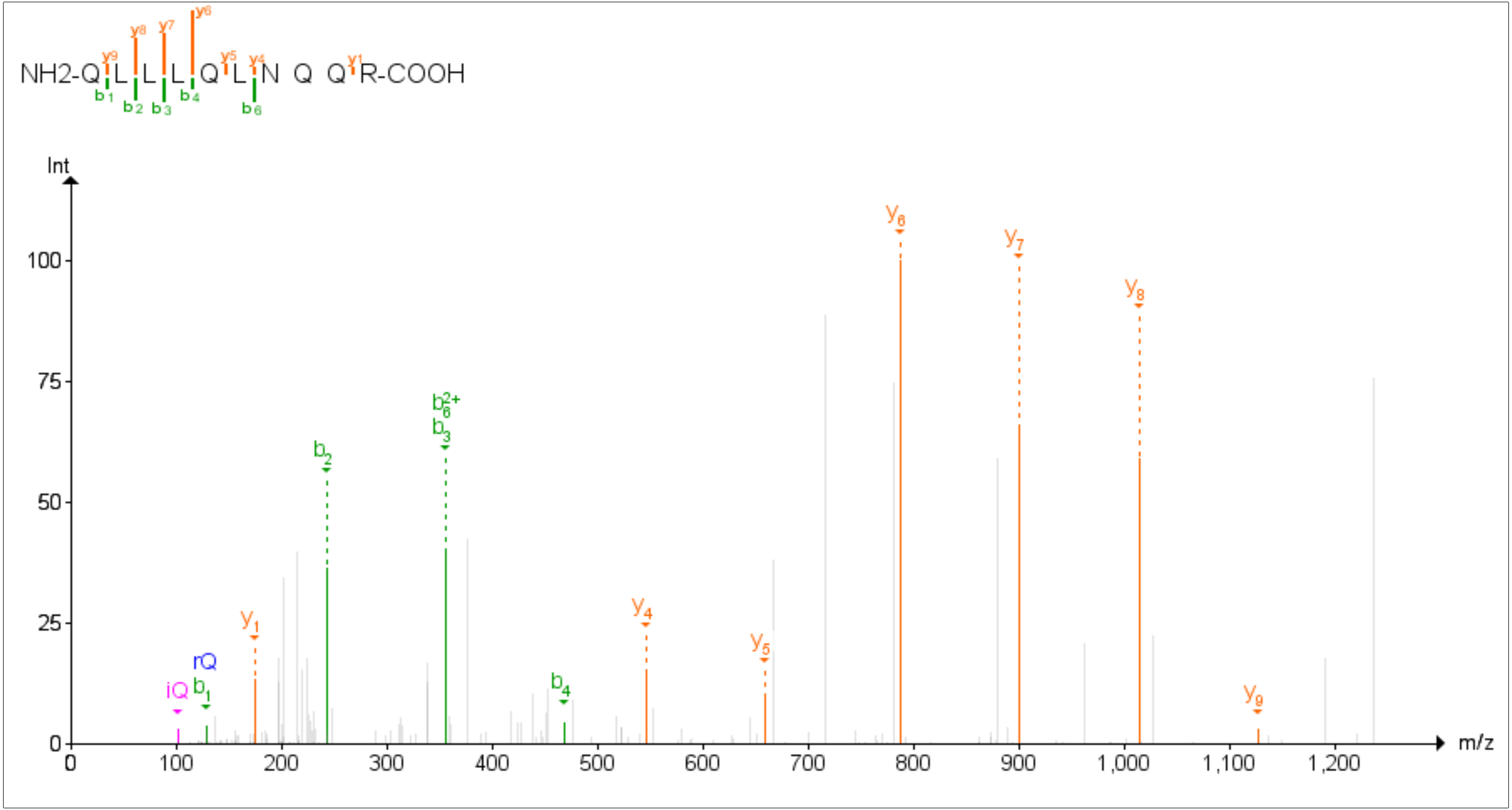

NH2-Q L L L Q L N Q Q R-COOH
y9 y8 y7 y6 y5 y4 y1
b1 b2 b3 b4 b6
Int
100
75
50
25
0
y6
y7
y8
b6 2+
b3
b2
y1
y4
y5
y9
rQ
iQ
b1
b4
0
100
200
300
400
500
600
700
800
900
1,000
1,100
1,200
m/z

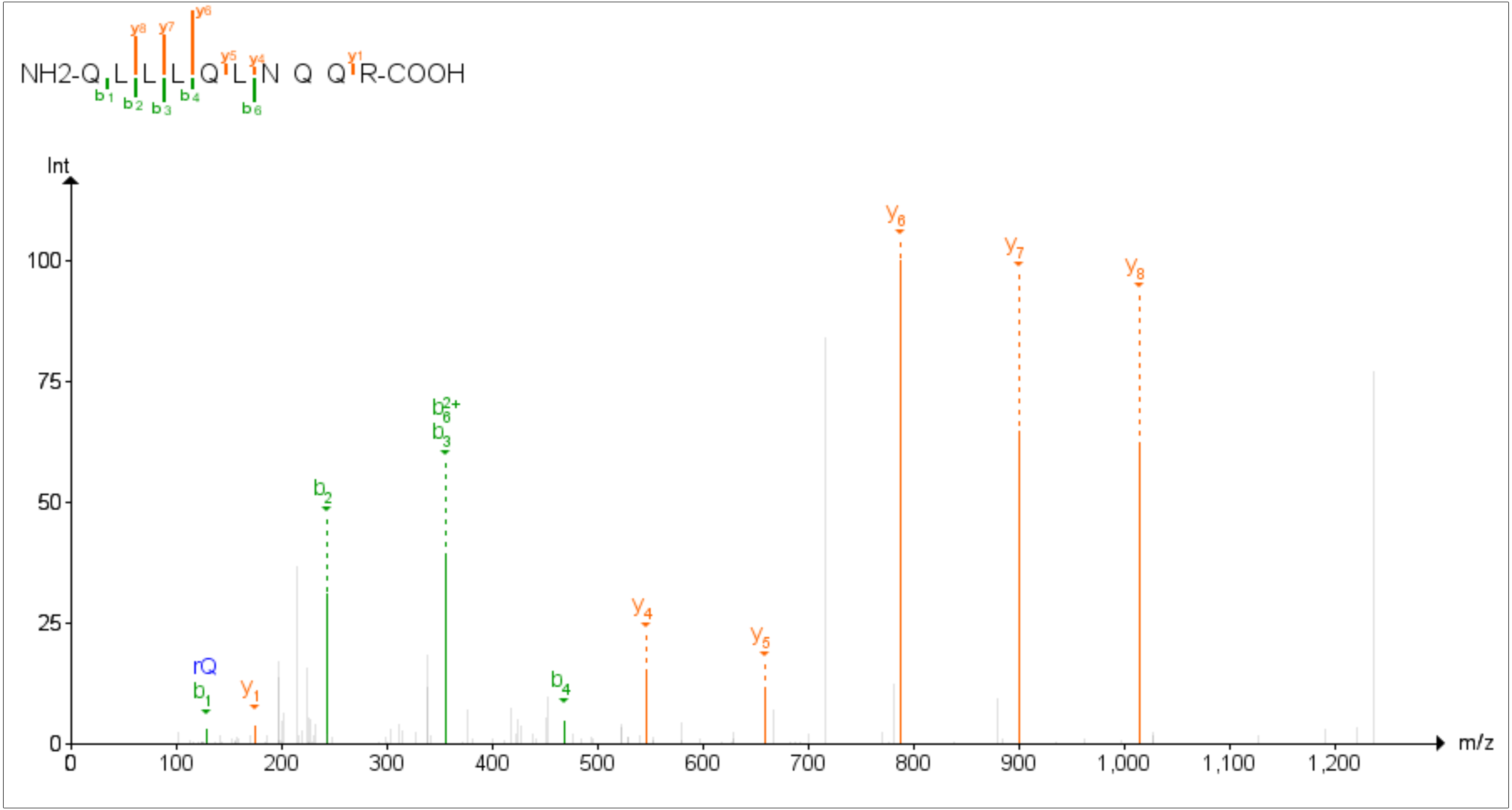

NH2-Q L L L Q L N Q Q R-COOH
y8 y7 y6 y5 y4 y1
b1 b2 b3 b4 b6
Int
100
75
50
25
0
m/z
0 100 200 300 400 500 600 700 800 900 1,000 1,100 1,200
rQ
b1
y1
b2
b6 2+
b3
b4
y4
y5
y6
y7
y8

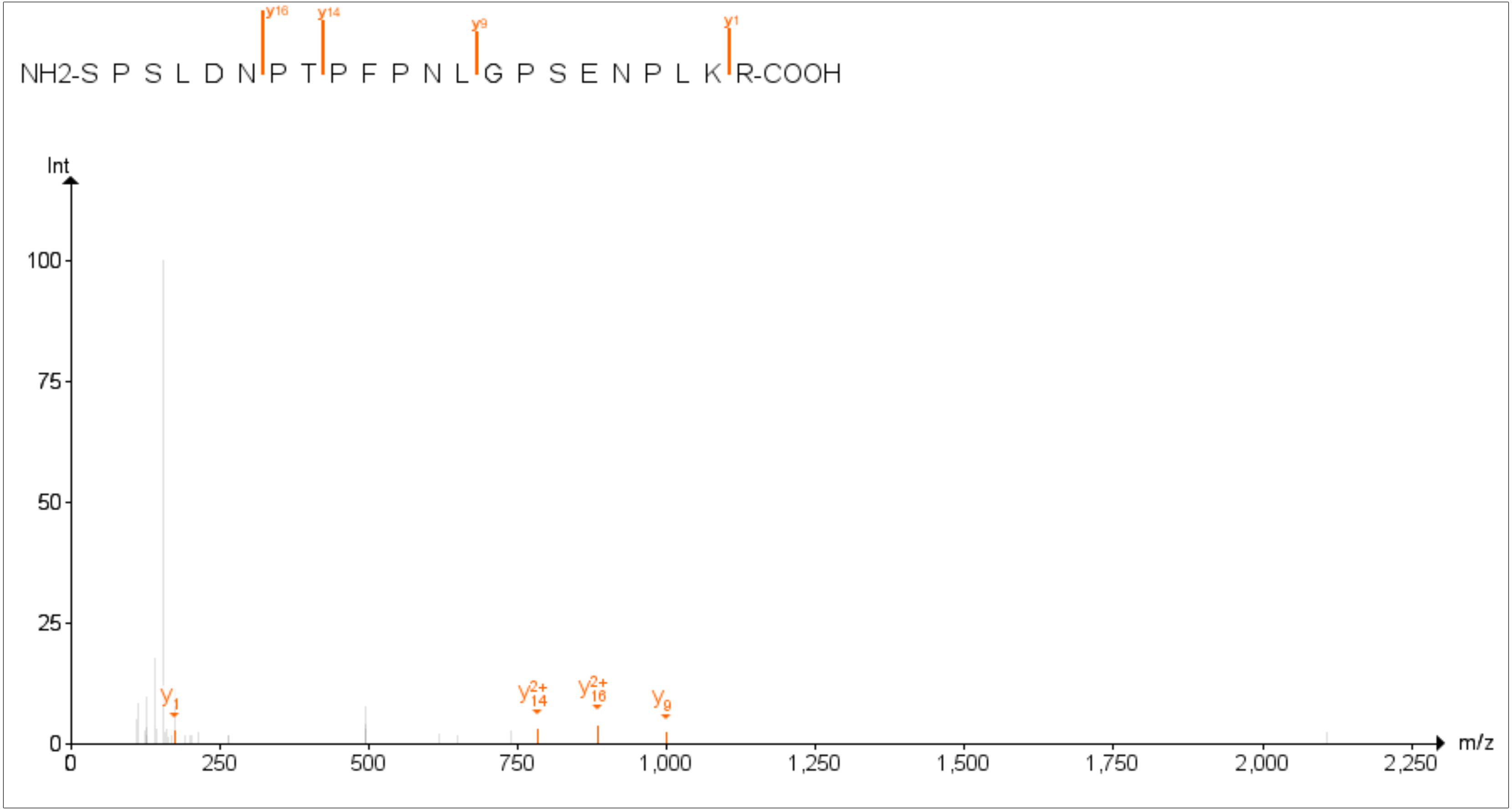

NH2-S P S L D N P T P F P N L G P S E N P L K R-COOH
y16
y14
y9
y1
Int
100
75
50
25
0
y1
y14 2+
y16 2+
y9
0
250
500
750
1,000
1,250
1,500
1,750
2,000
2,250
m/z

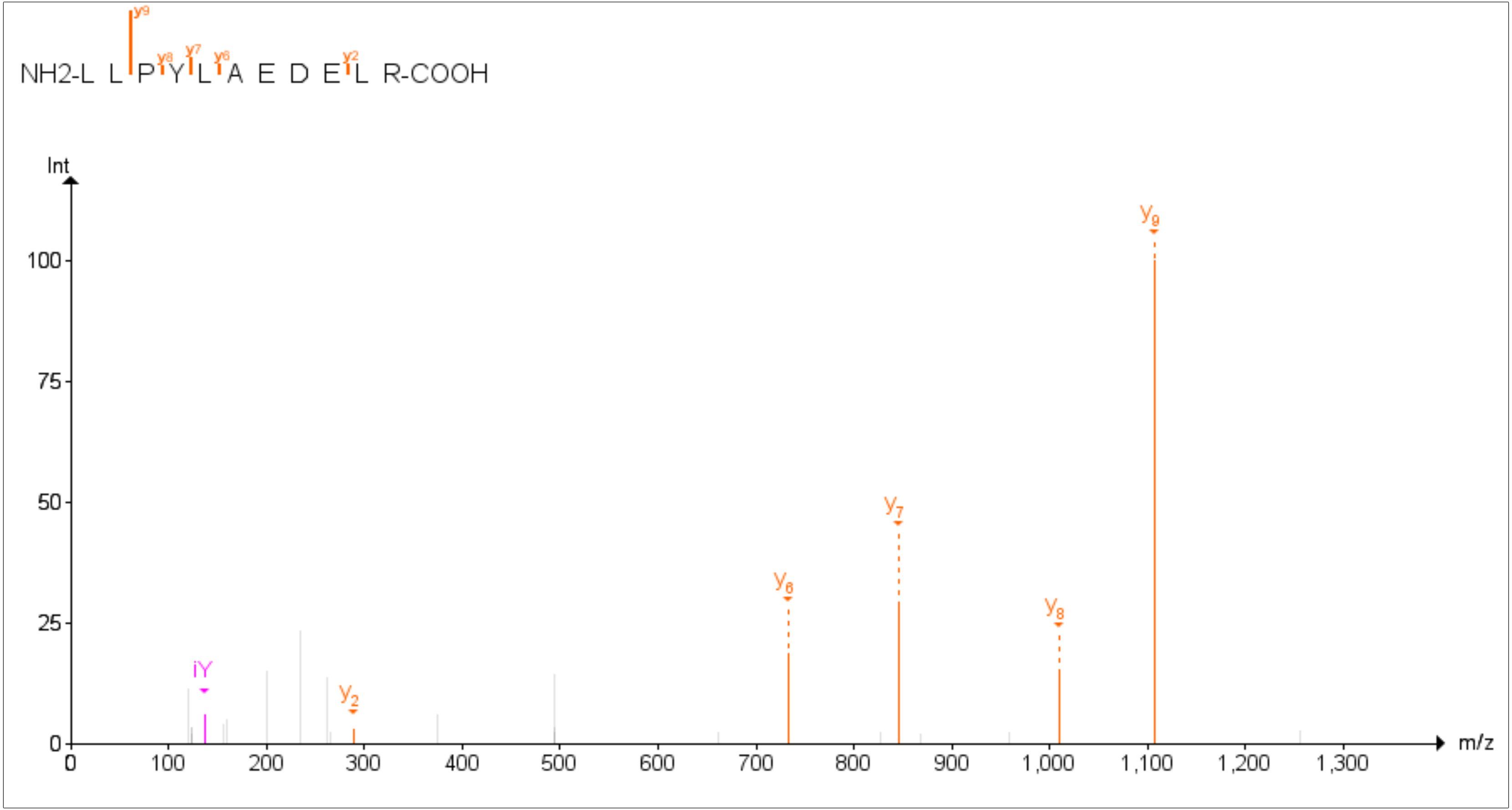

NH2-L L P Y L A E D E L R-COOH
y9
y8
y7
y6
y2
Int
100
75
50
25
0
iY
y2
y6
y7
y8
y9
0
100
200
300
400
500
600
700
800
900
1,000
1,100
1,200
1,300
m/z

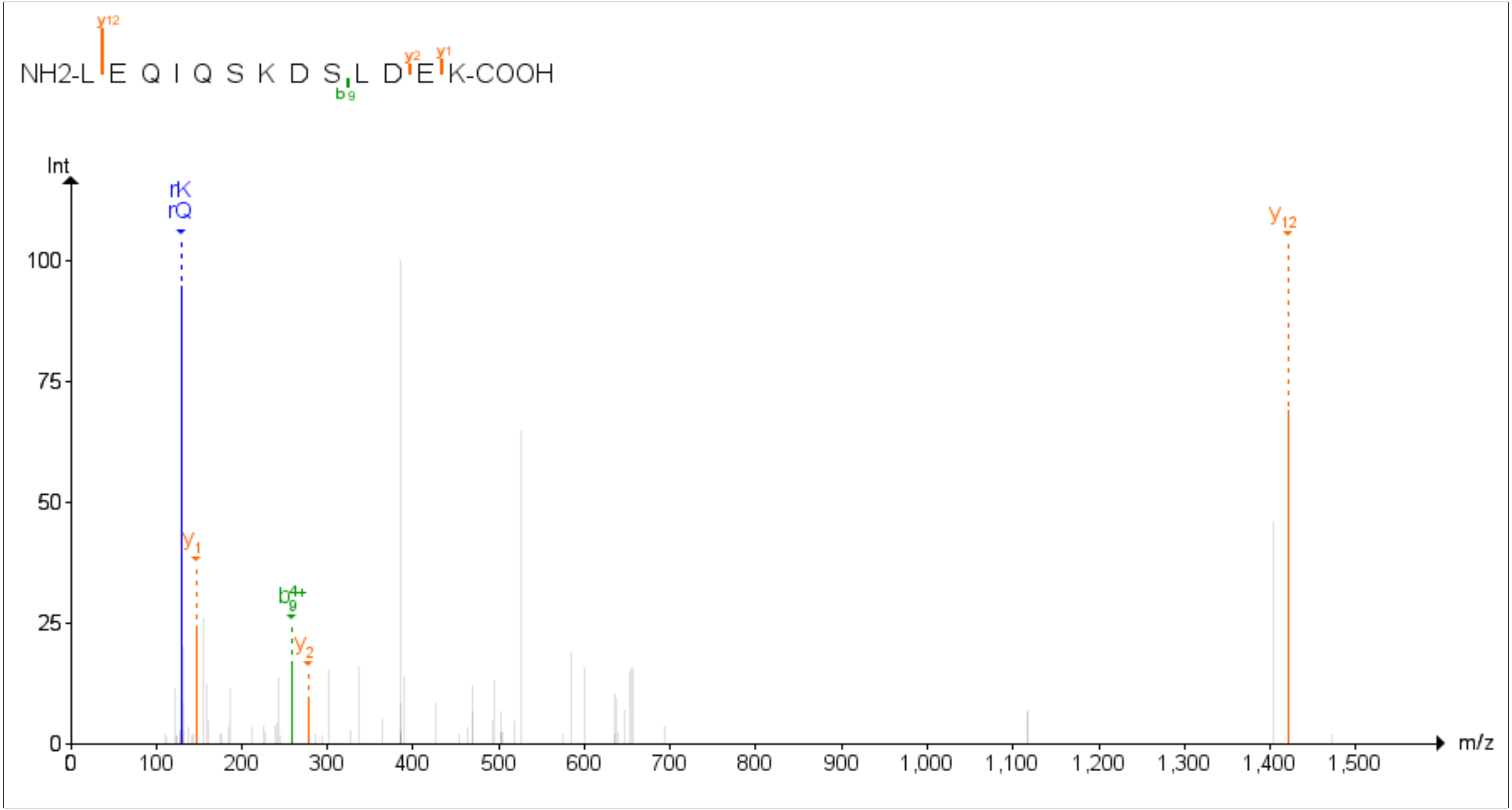

NH2-L E Q I Q S K D S L D E K-COOH
y12
y2
y1
b9
Int
rK
rQ
y12
y1
b9++
y2
100
75
50
25
0
0
100
200
300
400
500
600
700
800
900
1,000
1,100
1,200
1,300
1,400
1,500
m/z

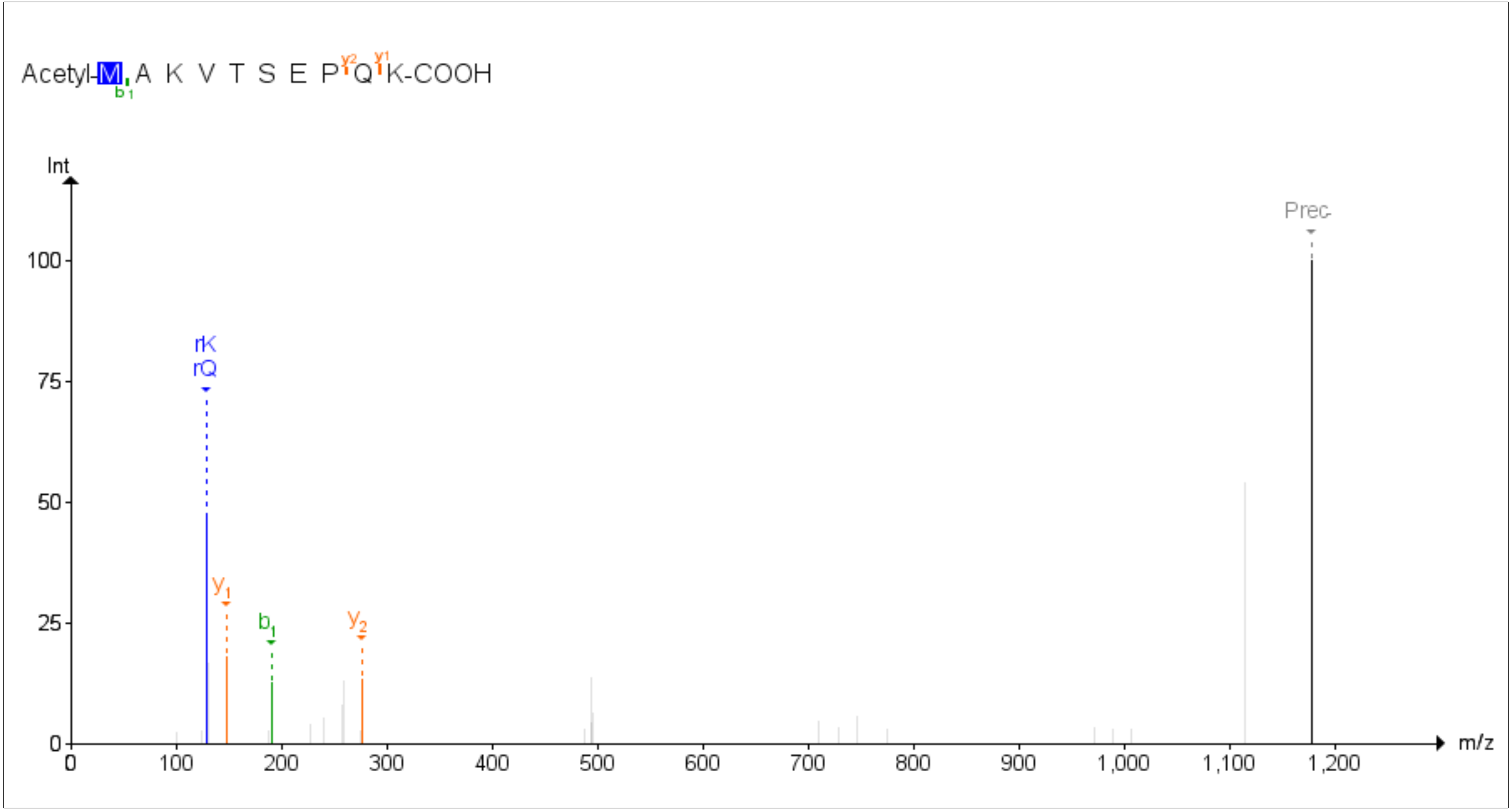

Acetyl-M A K V T S E P Q K-COOH
b1
y2
y1
Int
100
75
50
25
0
rK
rQ
y1
b1
y2
Prec
0
100
200
300
400
500
600
700
800
900
1,000
1,100
1,200
m/z

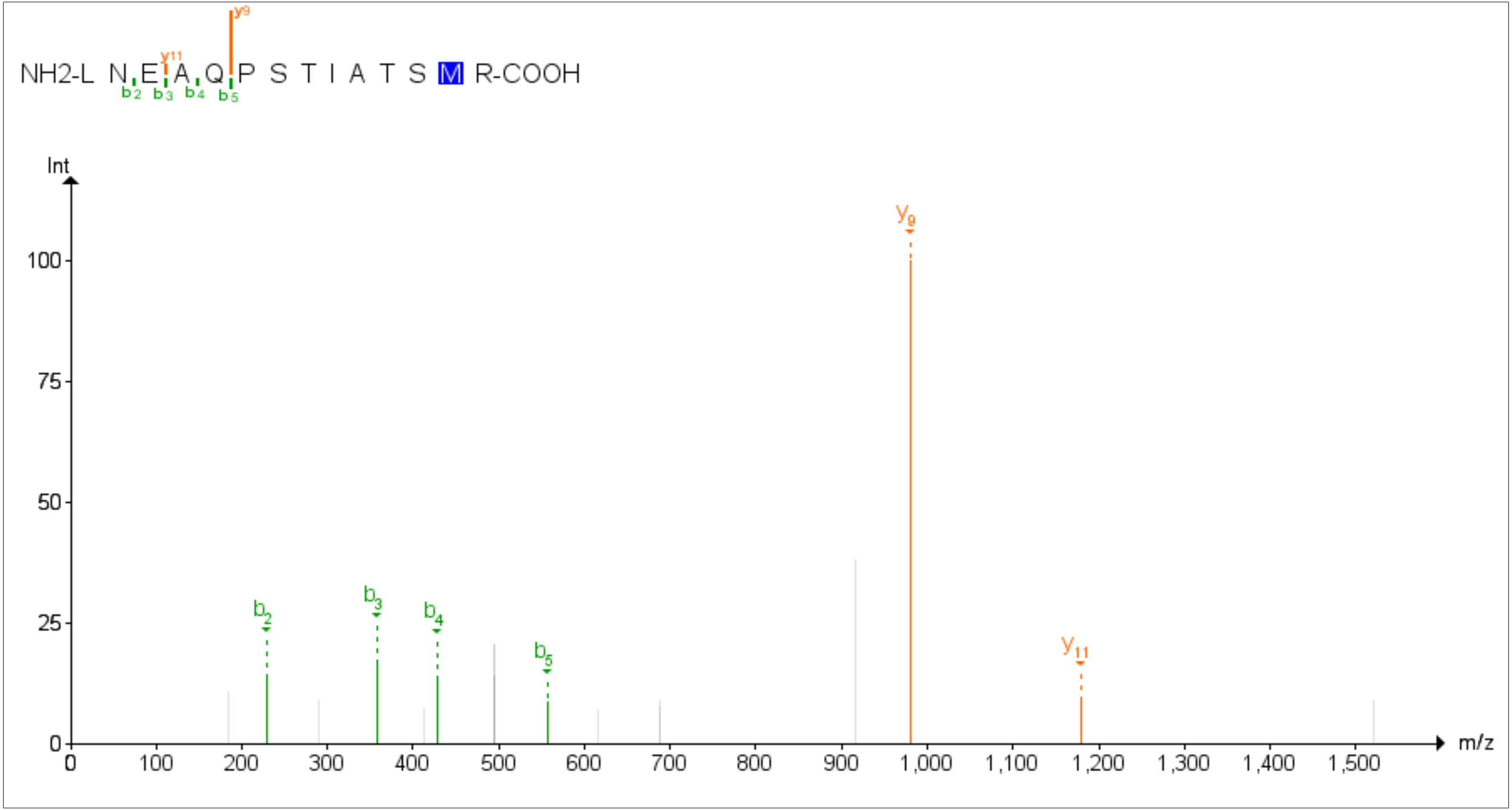

NH2-L N E A Q P S T I A T S M R-COOH
y9
y11
b2
b3
b4
b5
Int
100
75
50
25
0
m/z
0
100
200
300
400
500
600
700
800
900
1,000
1,100
1,200
1,300
1,400
1,500

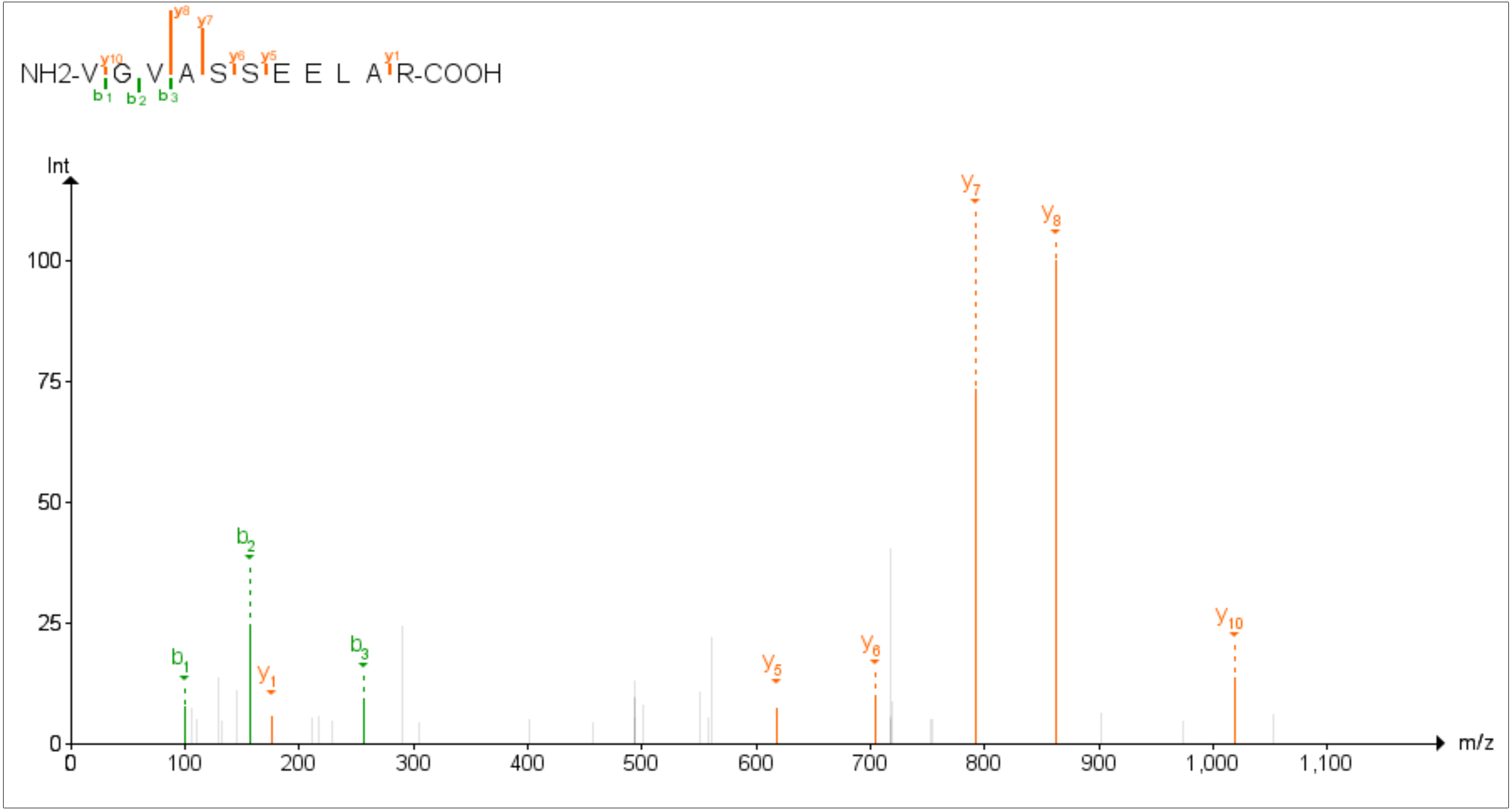

NH2-V G V A S S E E L A R-COOH
y10 y8 y7 y6 y5 y1
b1 b2 b3
Int
100
75
50
25
0
0 100 200 300 400 500 600 700 800 900 1,000 1,100
m/z
b1 b2 b3 y1 y5 y6 y7 y8 y10

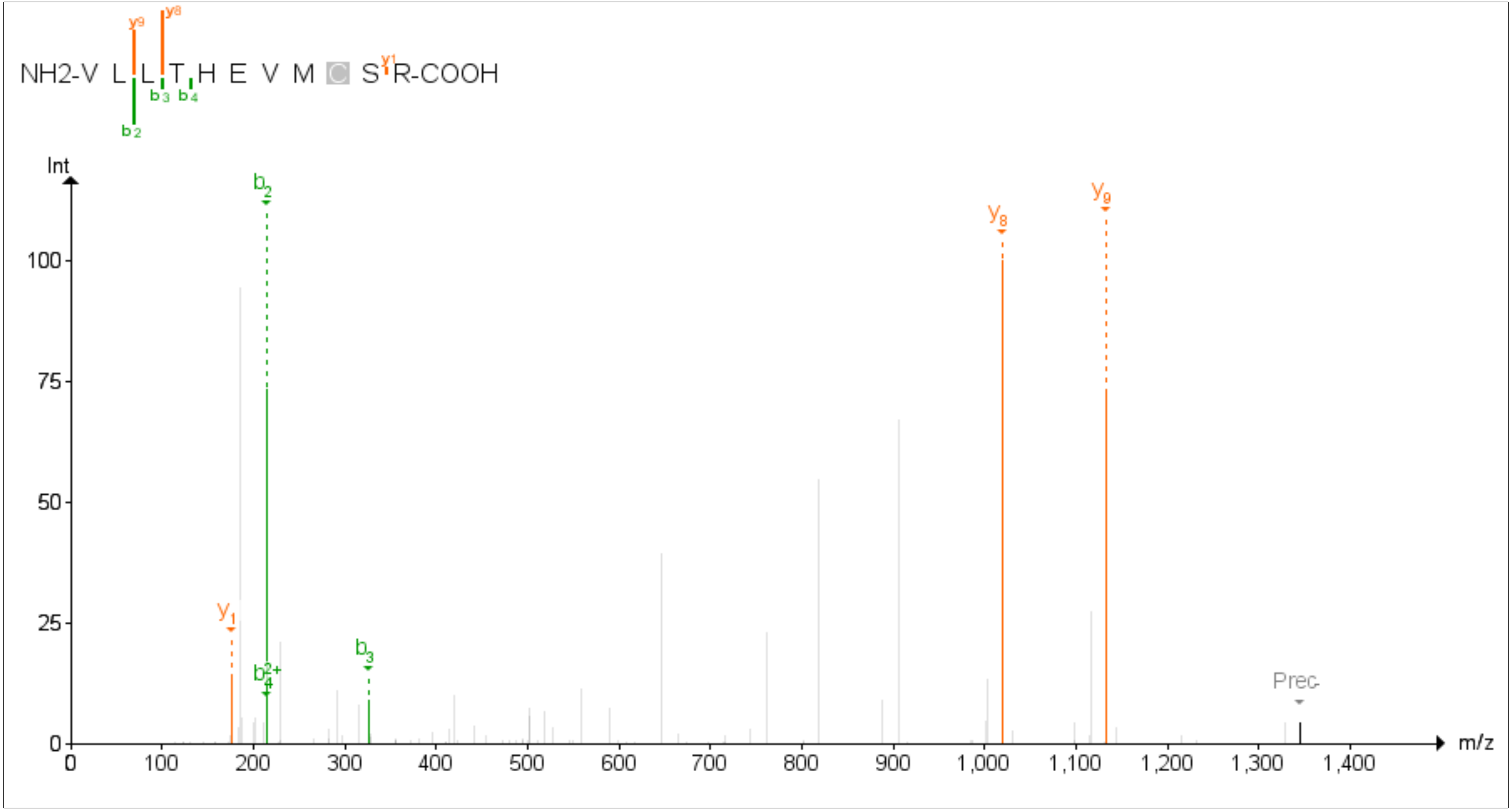

NH2-V L L T H E V M C S R-COOH
y9
y8
y1
b2
b3
b4
Int
100
75
50
25
0
b2
y8
y9
y1
b4 2+
b3
Prec.
0
100
200
300
400
500
600
700
800
900
1,000
1,100
1,200
1,300
1,400
m/z

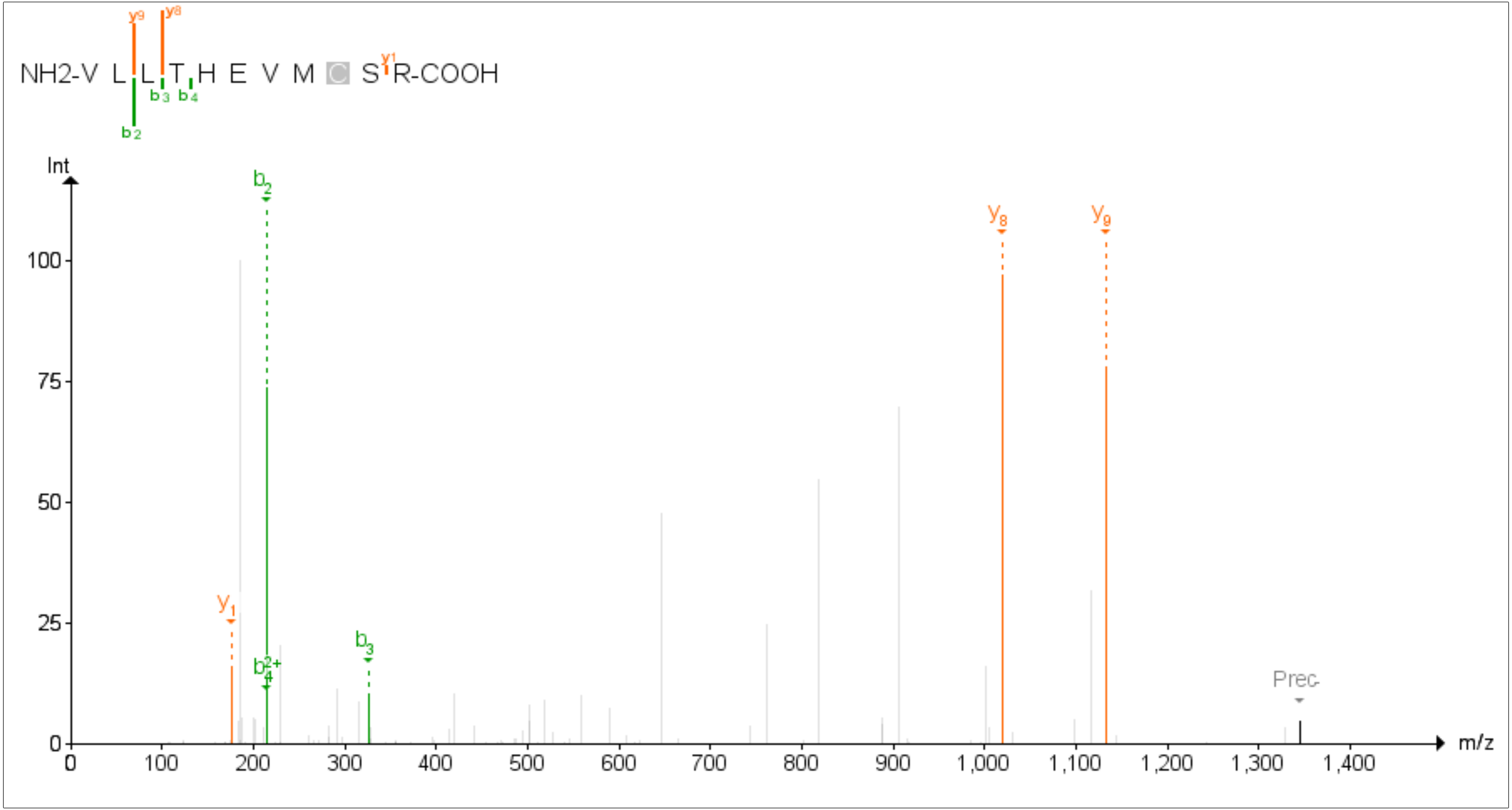

NH2-V L L T H E V M C S R-COOH
y9
y8
y1
b2
b3
b4
Int
100
75
50
25
0
b2
y8
y9
y1
b4 2+
b3
Prec.
m/z
0
100
200
300
400
500
600
700
800
900
1,000
1,100
1,200
1,300
1,400

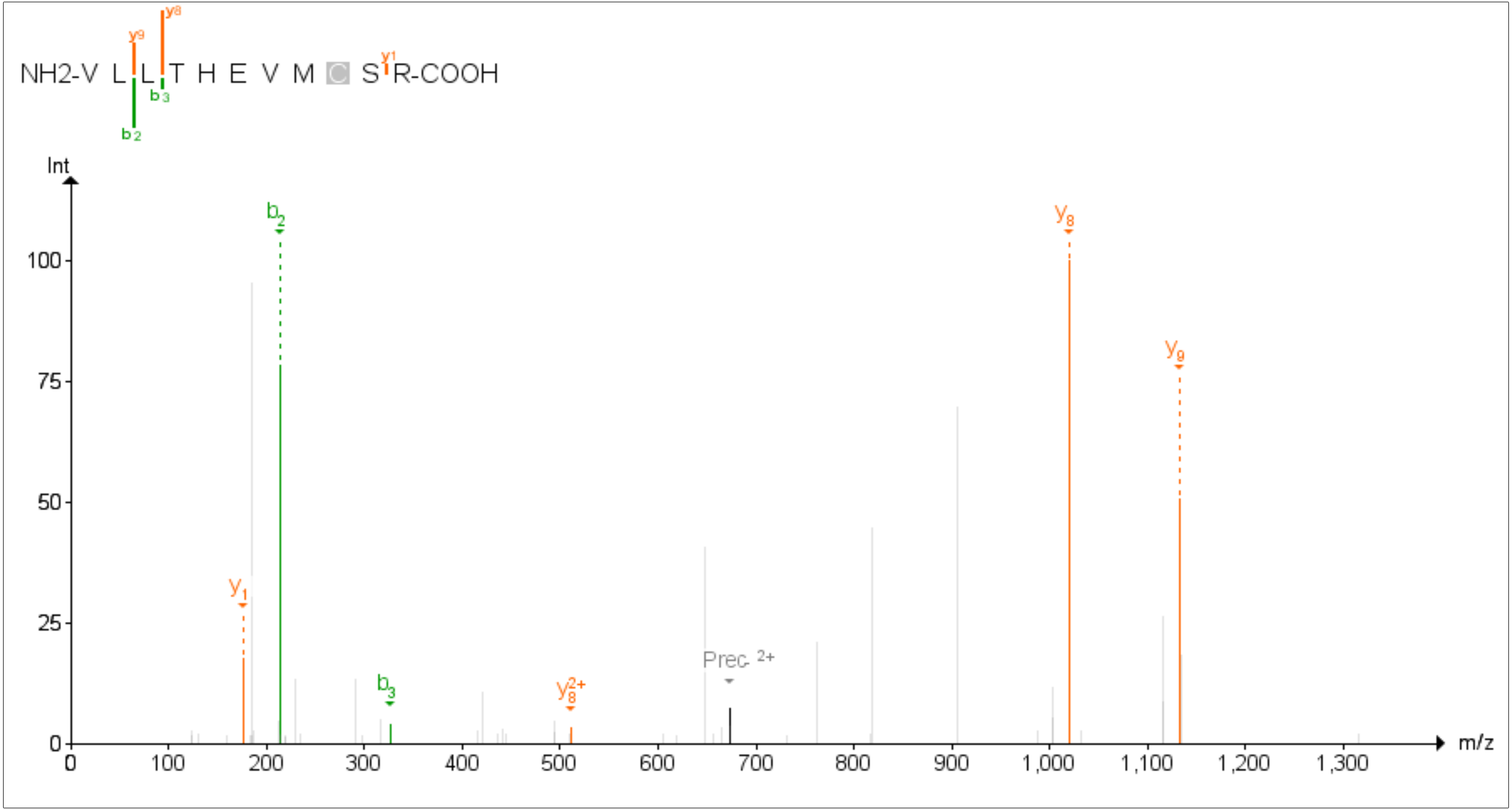

NH2-V L L T H E V M C S R-COOH
y9
y8
y1
b2
b3
Int
m/z
Prec. 2+
0
25
50
75
100
0
100
200
300
400
500
600
700
800
900
1,000
1,100
1,200
1,300

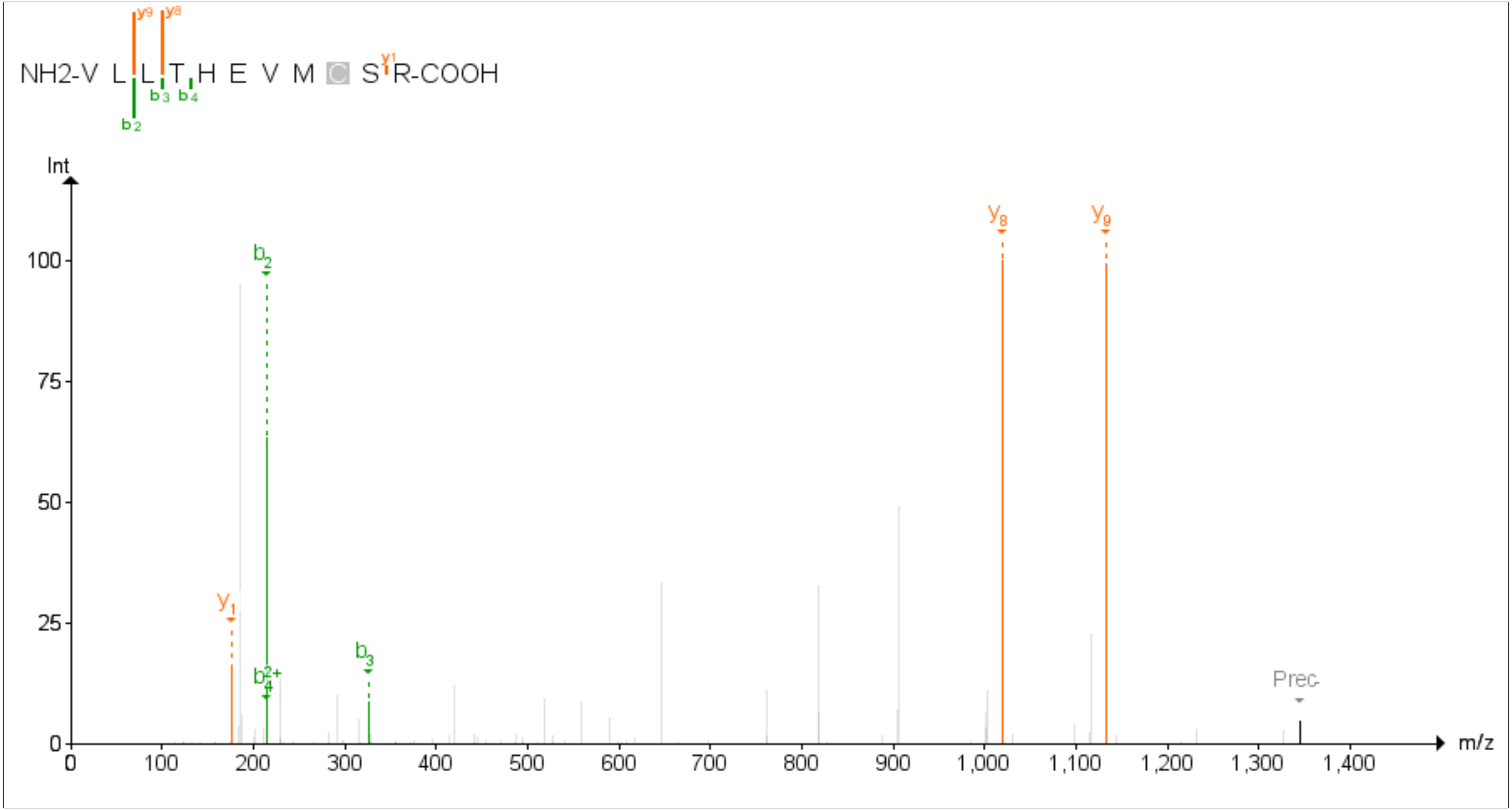

NH2-V L L T H E V M C S R-COOH
y9
y8
y1
b2
b3
b4
Int
100
75
50
25
0
y8
y9
b2
y1
b4 2+
b3
Prec
0
100
200
300
400
500
600
700
800
900
1,000
1,100
1,200
1,300
1,400
m/z

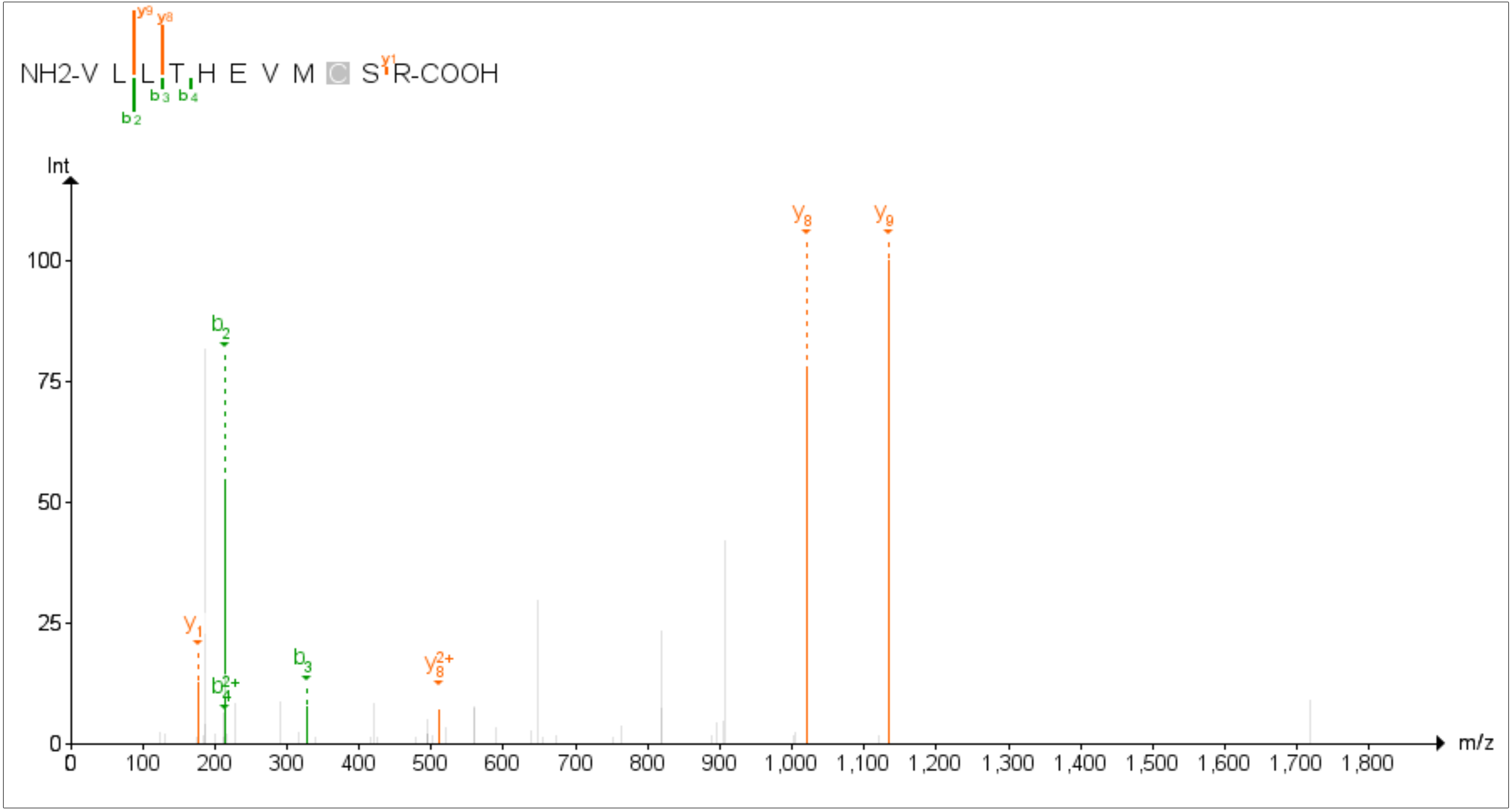
NH2-V L L T H E V M C S R-COOH
y9
y8
b2
b3
b4
y1
Int
100
75
50
25
0
m/z
0 100 200 300 400 500 600 700 800 900 1,000 1,100 1,200 1,300 1,400 1,500 1,600 1,700 1,800
y8
y9
b2
y1
b4 2+
b3
y8 2+

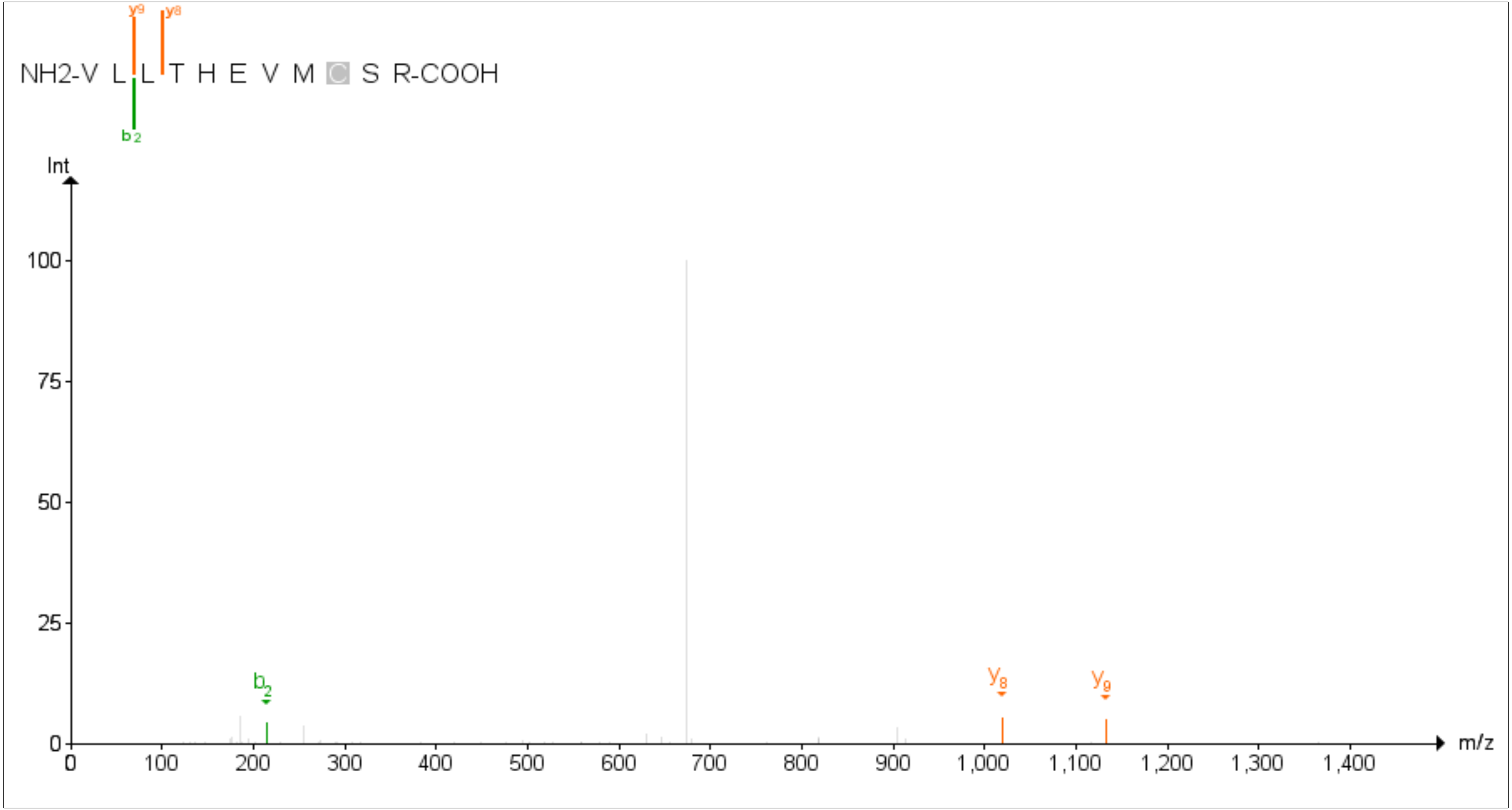

NH2-V L L T H E V M C S R-COOH
y9
y8
b2
Int
100
75
50
25
0
b2
y8
y9
0
100
200
300
400
500
600
700
800
900
1,000
1,100
1,200
1,300
1,400
m/z

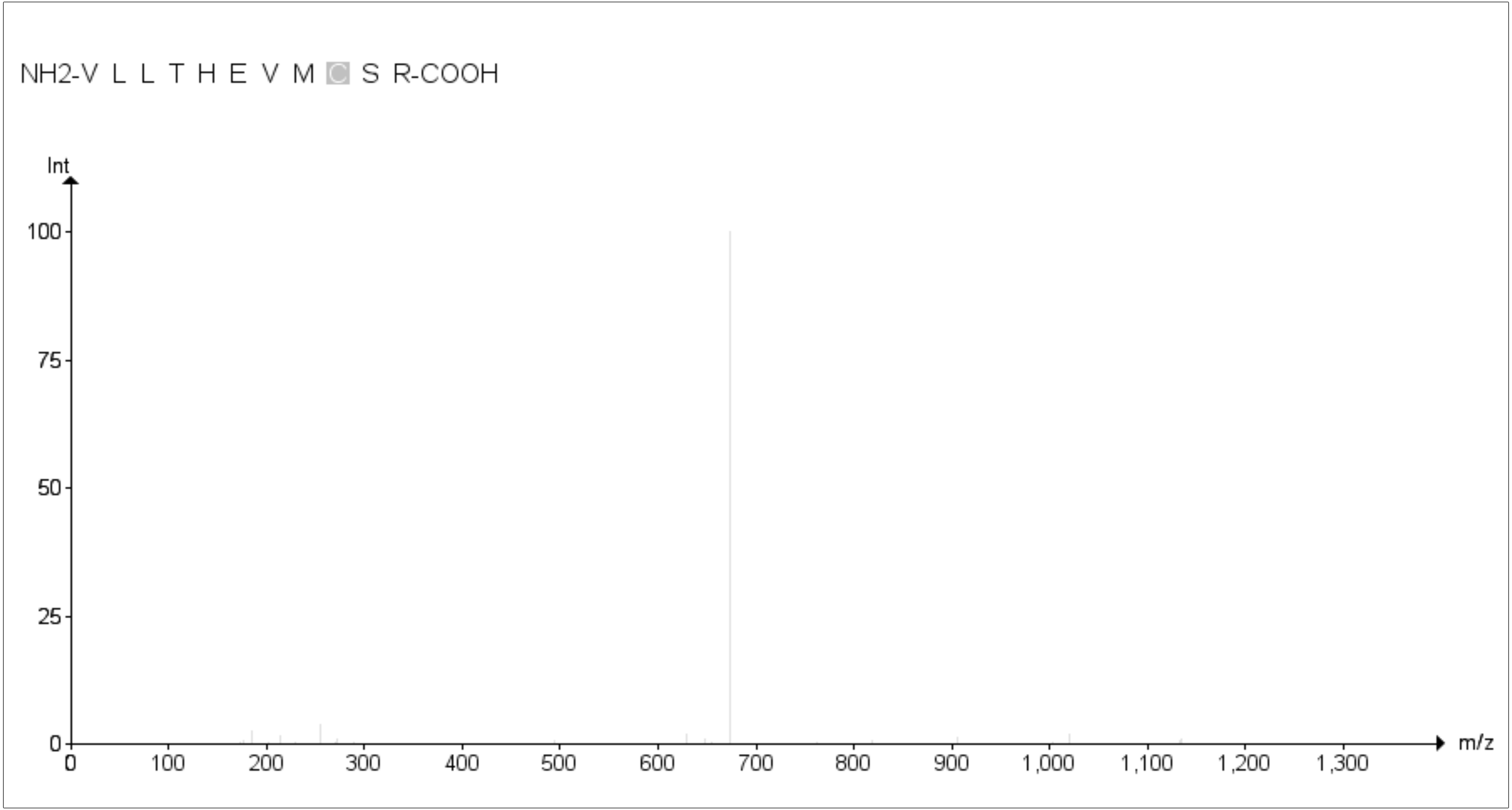
NH2-V L L T H E V M C S R-COOH
Int
100
75
50
25
0
0
100
200
300
400
500
600
700
800
900
1,000
1,100
1,200
1,300
m/z

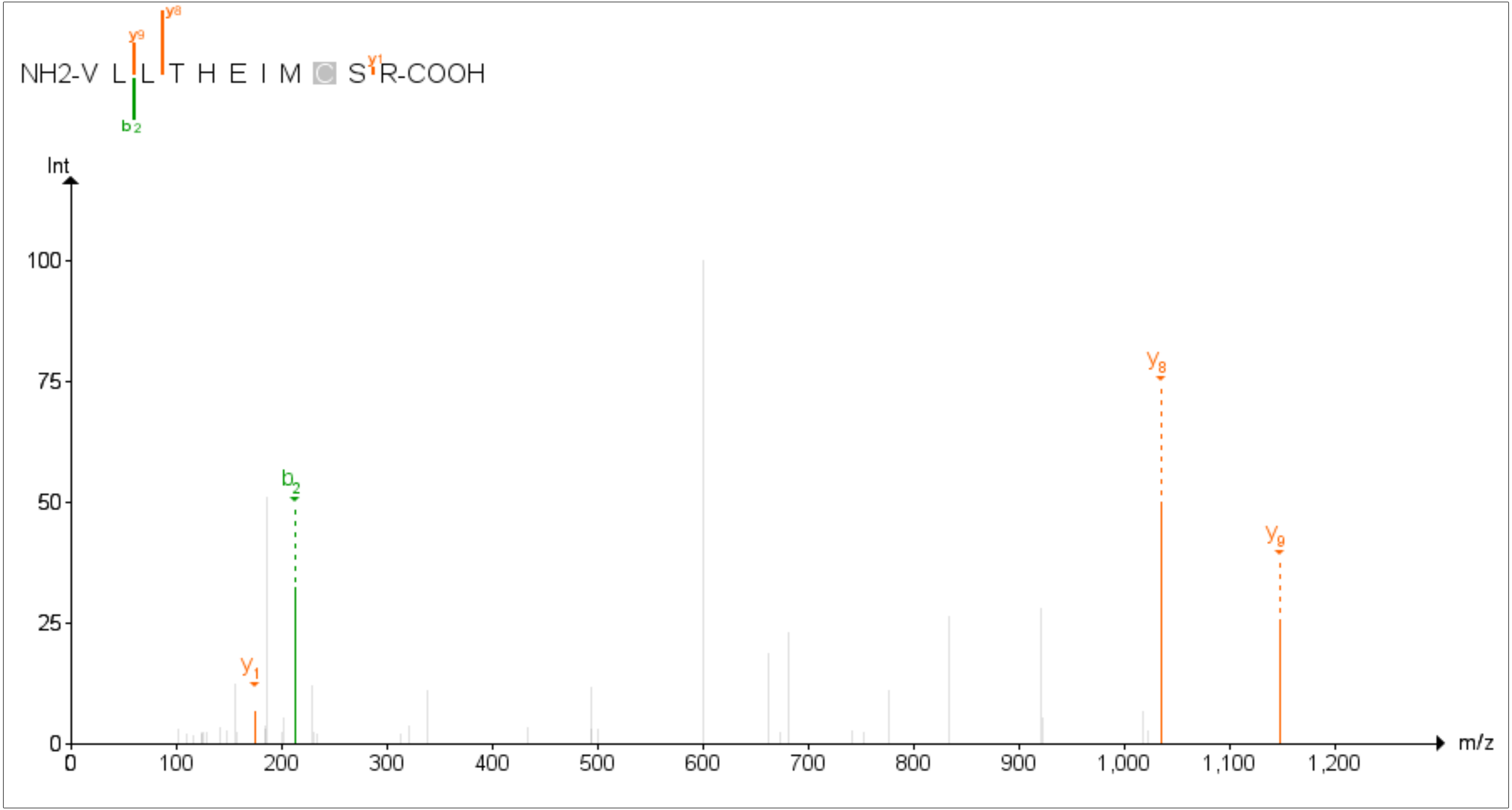

NH2-V L L T H E I M C S R-COOH
y9
y8
y1
b2
Int
100
75
50
25
0
m/z
0
100
200
300
400
500
600
700
800
900
1,000
1,100
1,200
y1
b2
y8
y9

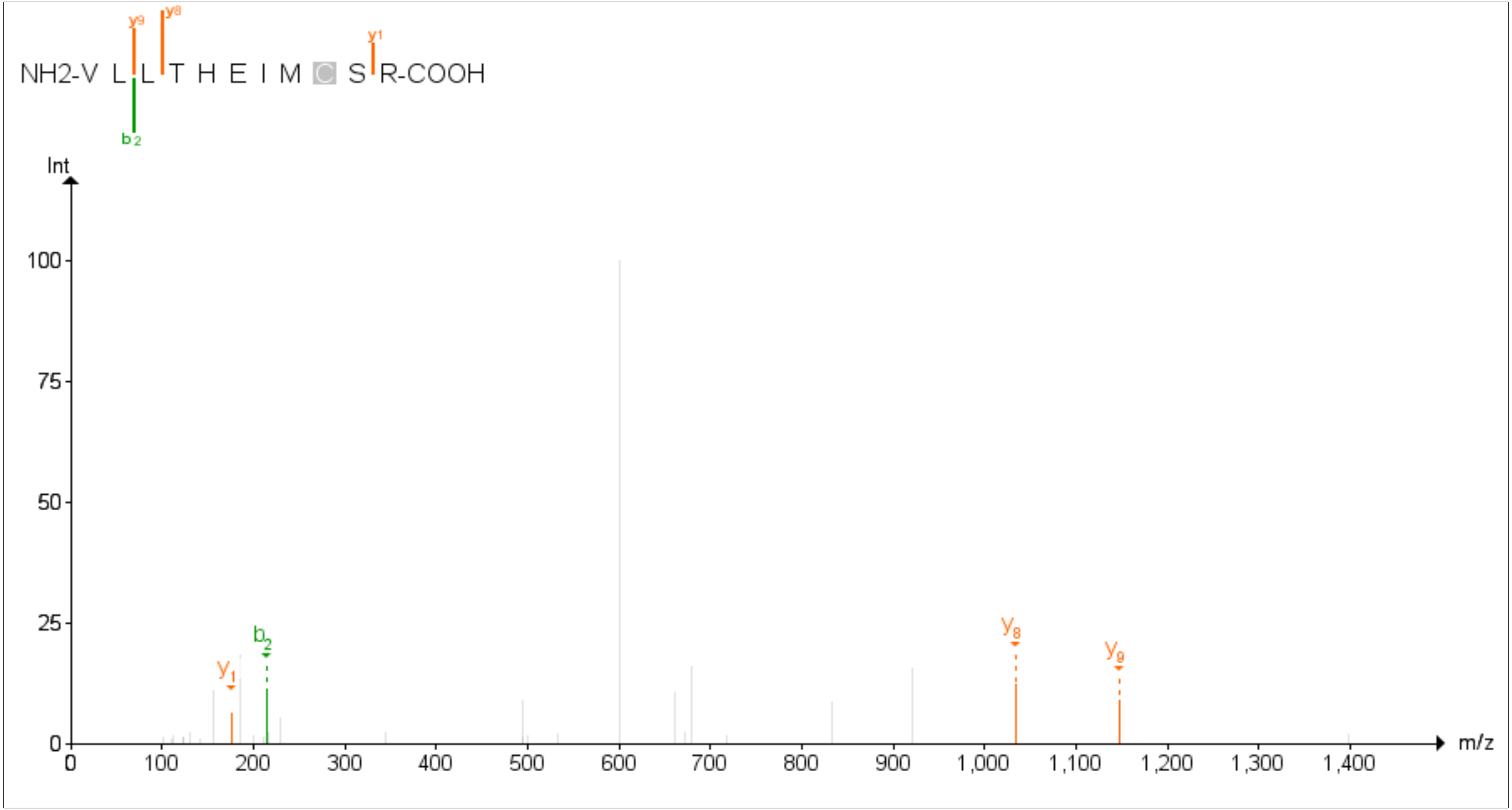

NH2-V L L T H E I M C S R-COOH
y9
y8
y1
b2
Int
100
75
50
25
0
m/z
0
100
200
300
400
500
600
700
800
900
1,000
1,100
1,200
1,300
1,400
y1
b2
y8
y9

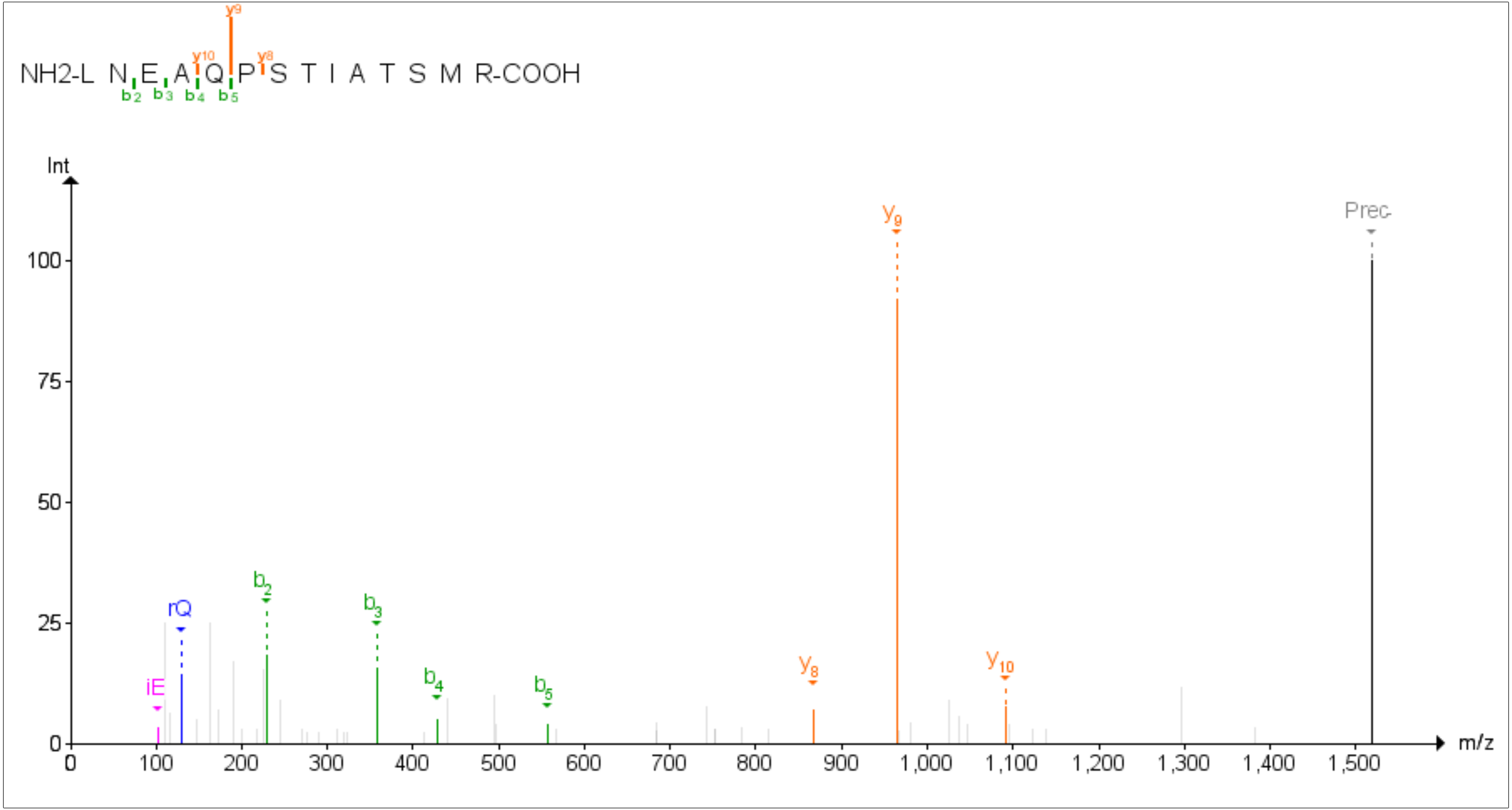

NH2-L N E A Q P S T I A T S M R-COOH
y9
y10
y8
b2 b3 b4 b5
Int
m/z
Prec
rQ
iE
0
25
50
75
100
0
100
200
300
400
500
600
700
800
900
1,000
1,100
1,200
1,300
1,400
1,500

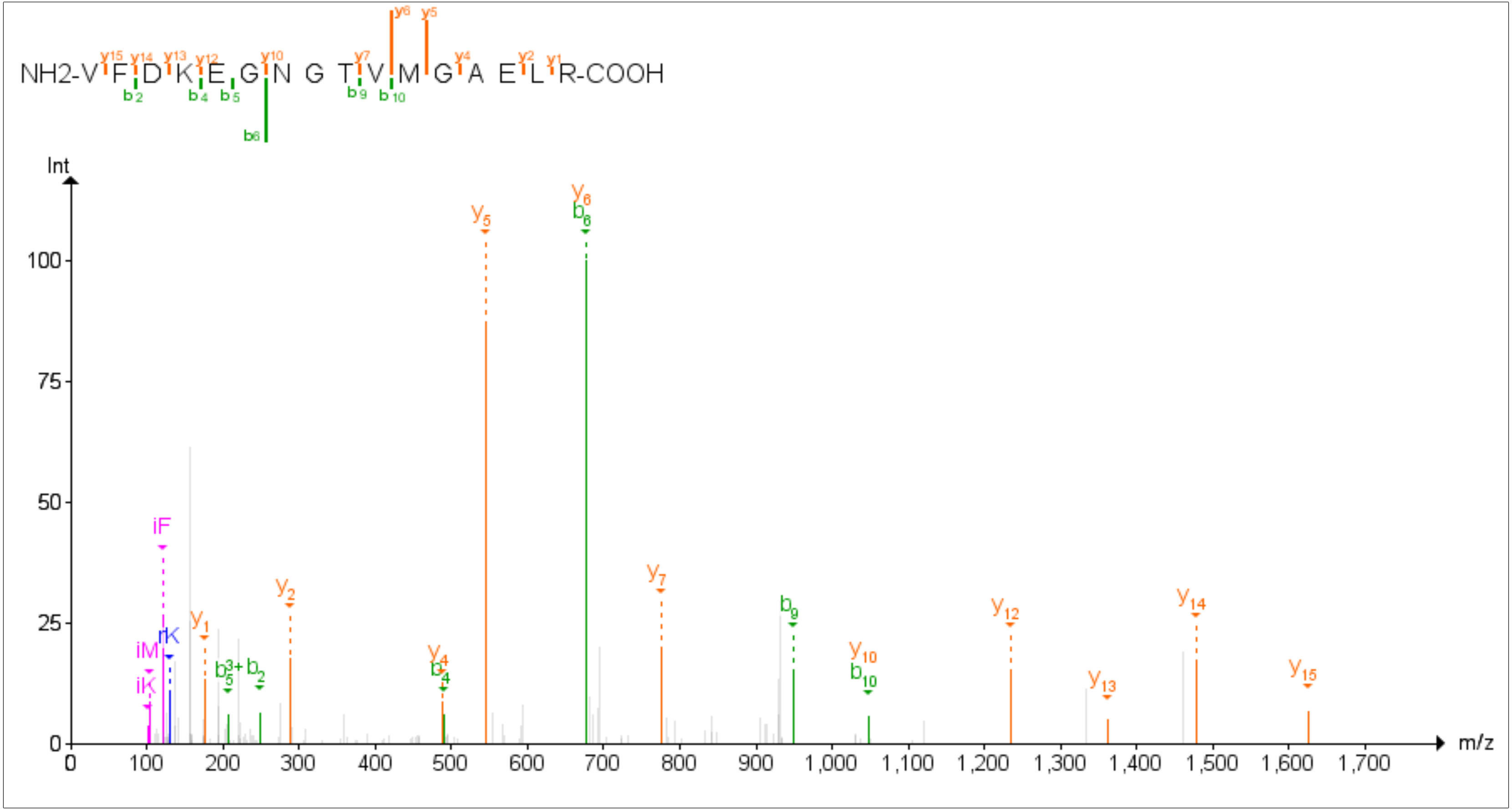
NH2-V F D K E G N G T V M G A E L R-COOH
y15 y14 y13 y12 y10 y7 y6 y5 y4 y2 y1
b2 b4 b5 b6 b9 b10
Int
m/z
iF
iM
rK
iK
y1
y2
b5 3+
b2
y4
b4
y5
y6
b6
y7
b9
y10
b10
y12
y13
y14
y15

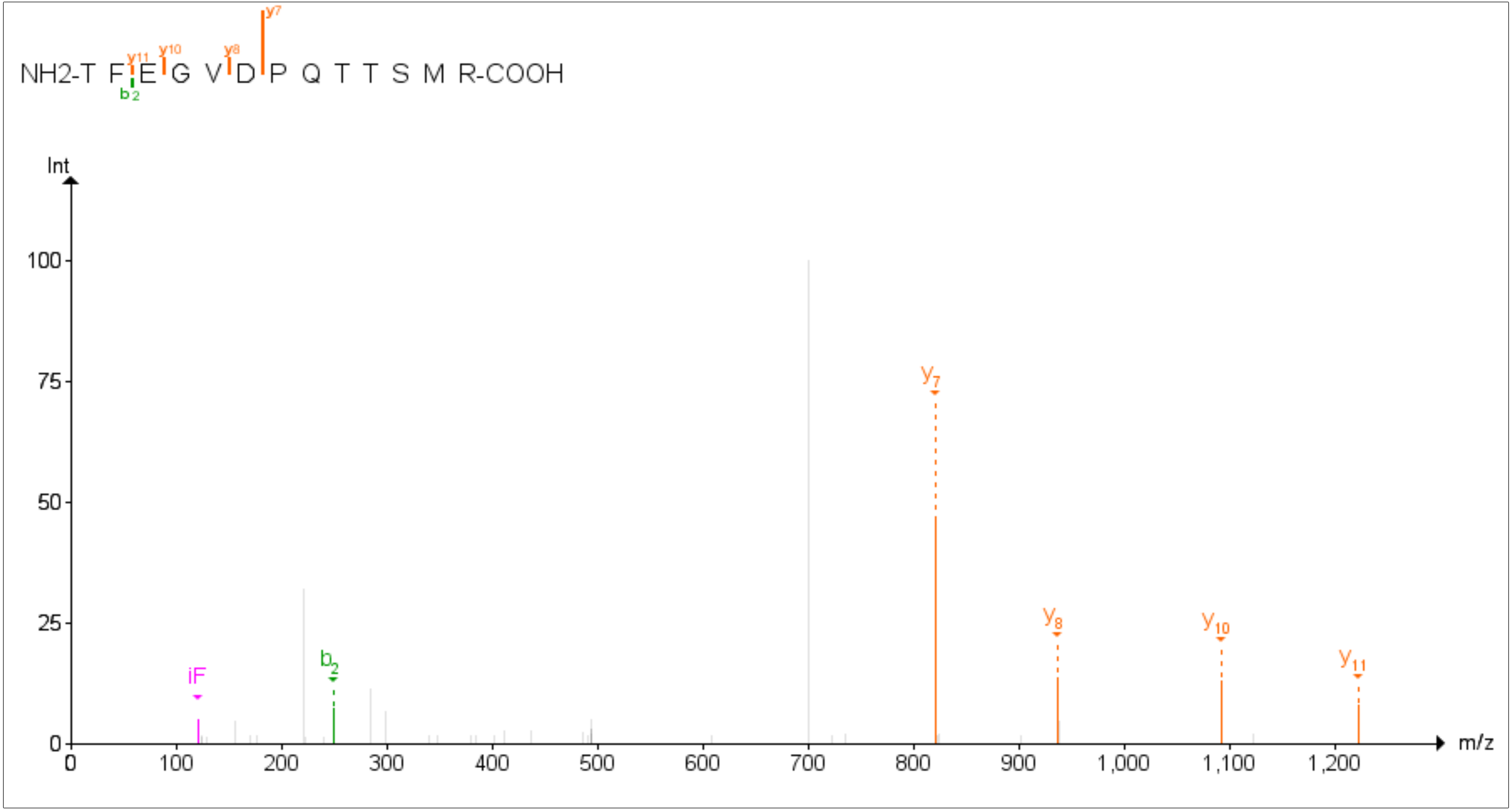

NH2-T F E G V D P Q T T S M R-COOH
y7
y11
y10
y8
b2
Int
100
75
50
25
0
iF
b2
y7
y8
y10
y11
0
100
200
300
400
500
600
700
800
900
1,000
1,100
1,200
m/z

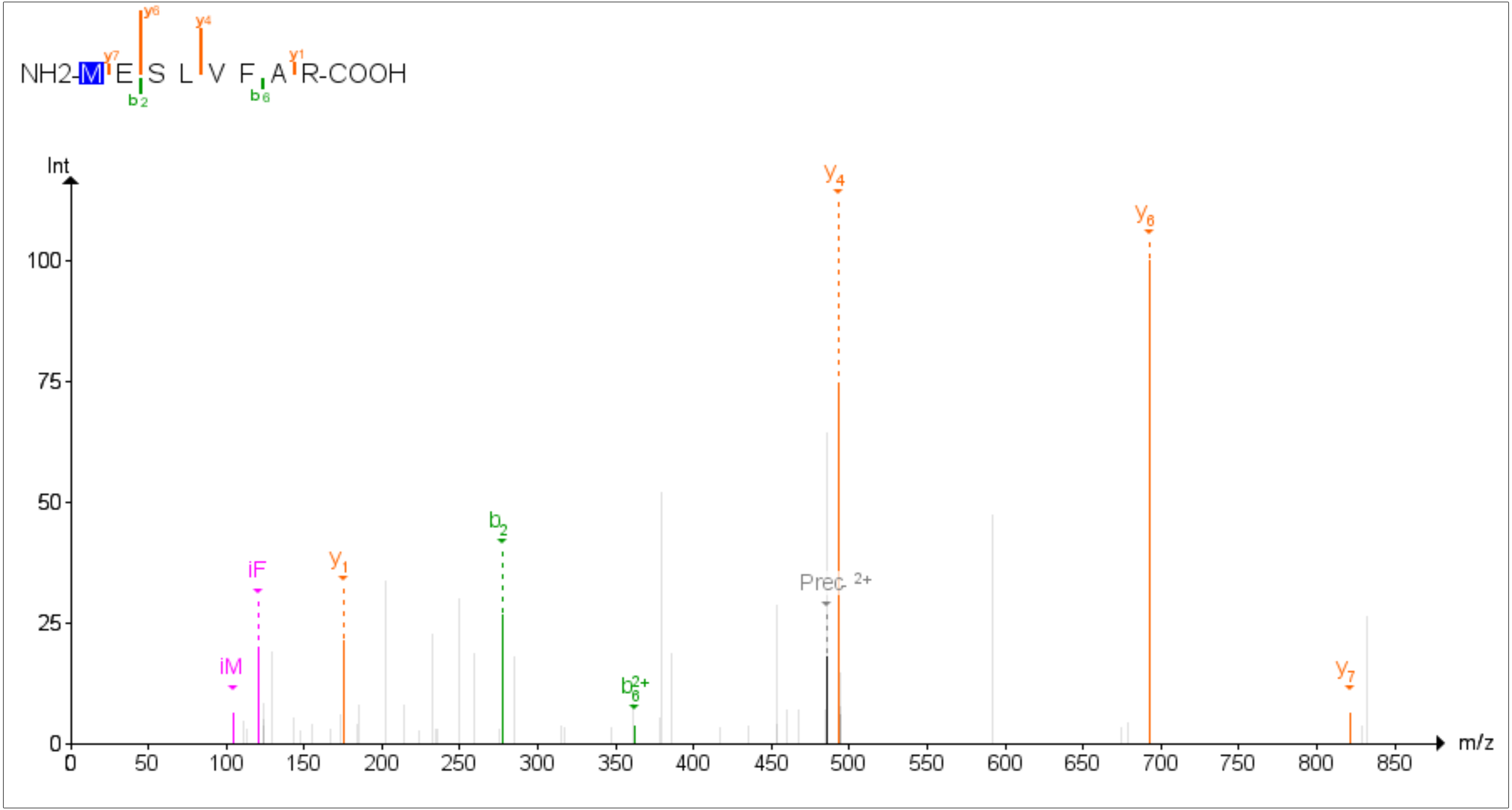

NH2-M E S L V F A R-COOH
y6
y4
y7
y1
b2
b6
Int
m/z
y4
y6
b2
iF
y1
Prec. 2+
iM
b6 2+
y7
0
25
50
75
100
0
50
100
150
200
250
300
350
400
450
500
550
600
650
700
750
800
850

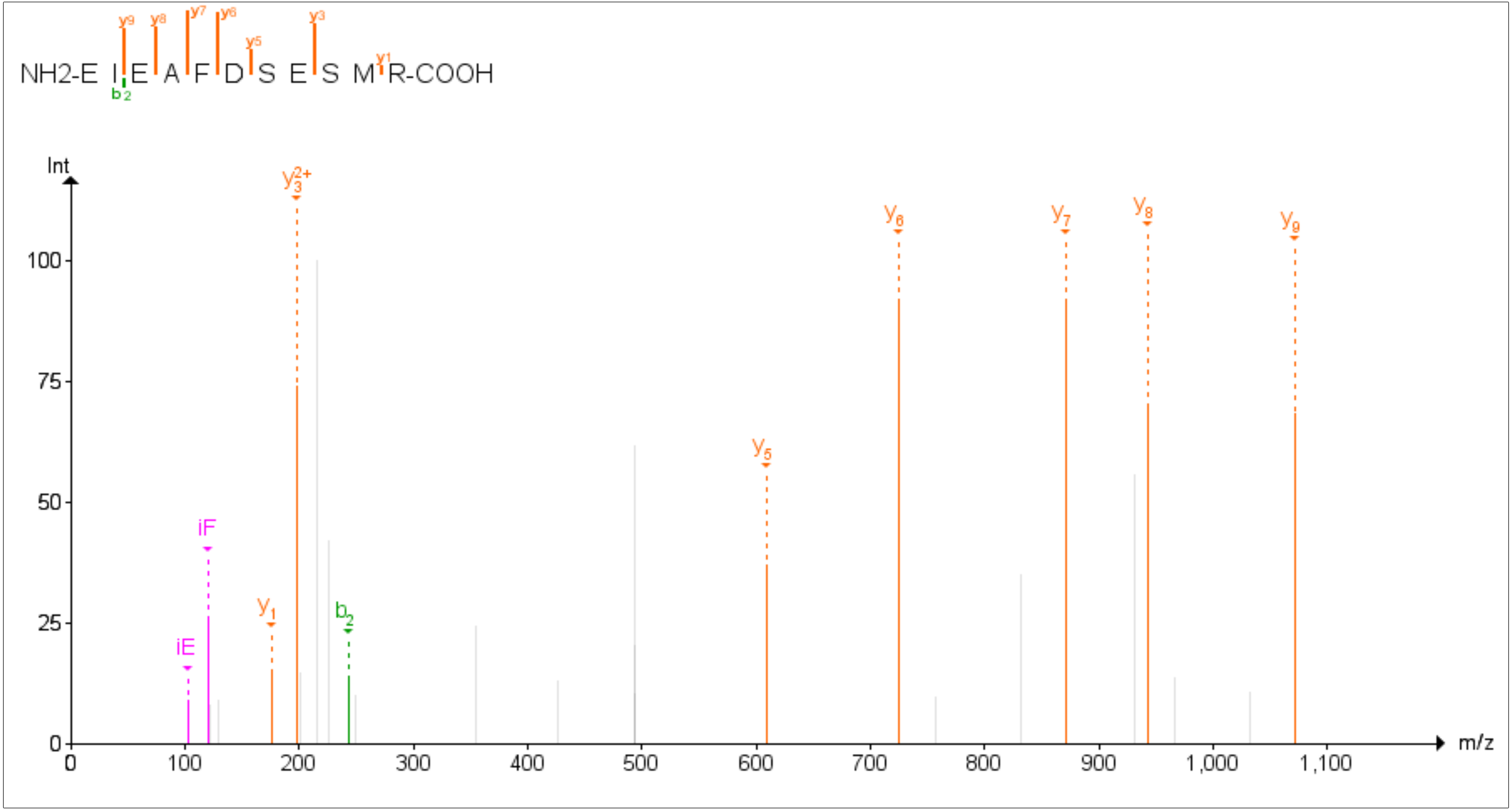

NH2-E I E A F D S E S M R-COOH
y9 y8 y7 y6 y5 y3 y1
b2
Int
100
75
50
25
0
y_3^{2+}
y_6
y_7
y_8
y_9
y_5
iF
iE
y_1
b_2
0 100 200 300 400 500 600 700 800 900 1,000 1,100
m/z

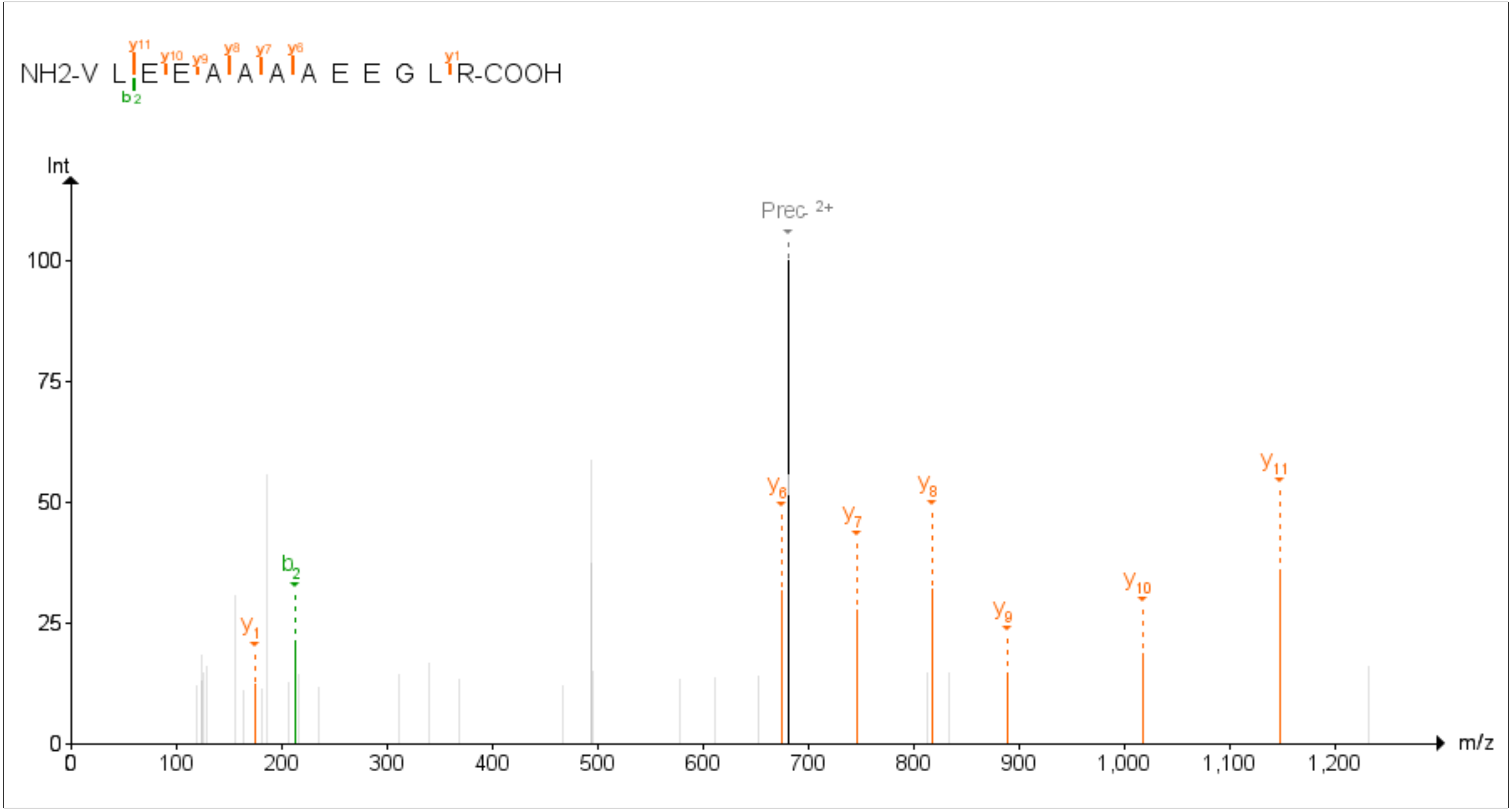

NH2-V L E E A A A A E E G L R-COOH
y11 y10 y9 y8 y7 y6 y1
b2
Int
Prec 2+
100
75
50
25
0
y1
b2
y6
y7
y8
y9
y10
y11
0
100
200
300
400
500
600
700
800
900
1,000
1,100
1,200
m/z

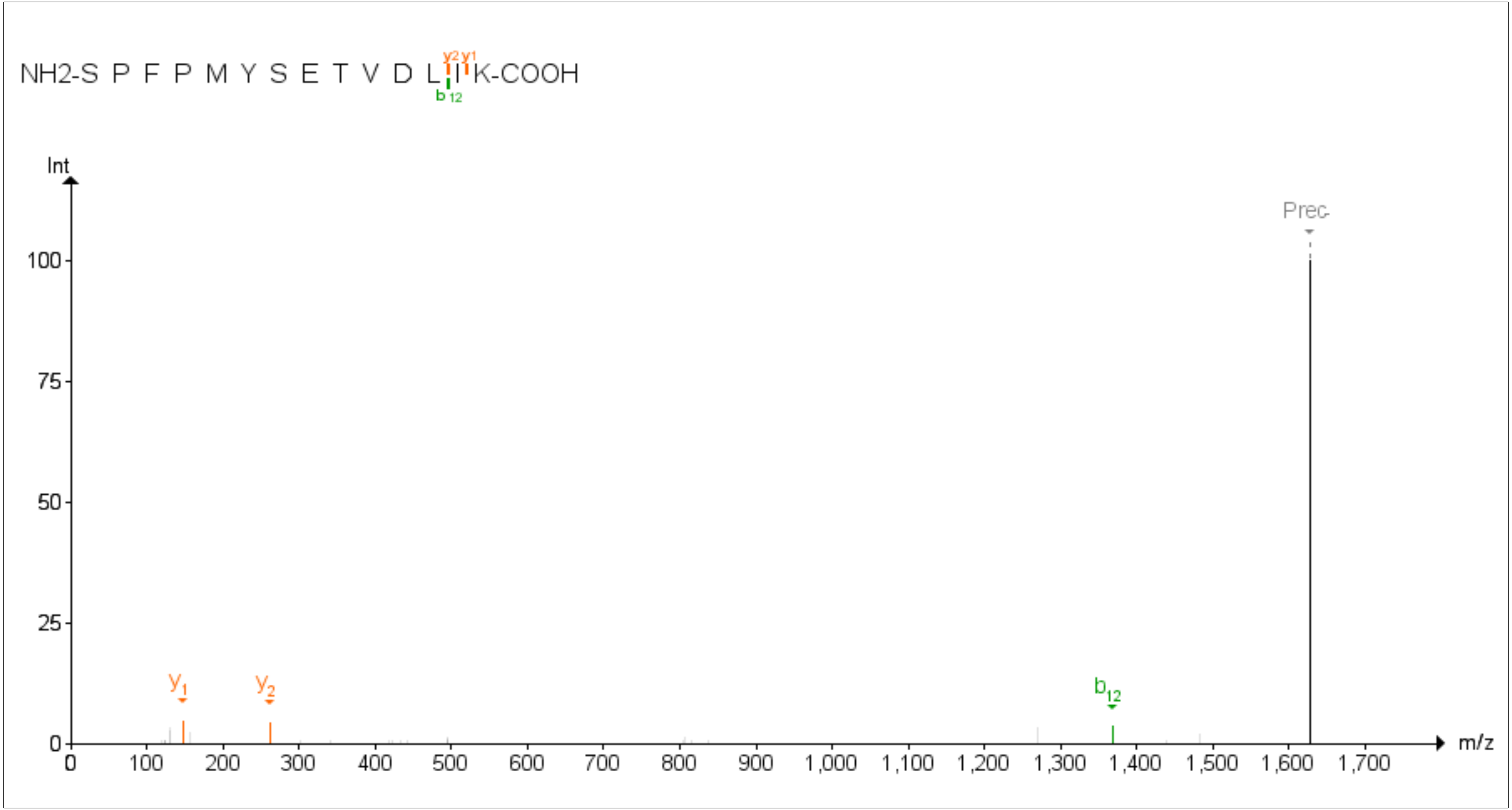

NH2-S P F P M Y S E T V D L I K-COOH
y2 y1
b 12
Int
Prec-
100
75
50
25
0
y1
y2
b12
0 100 200 300 400 500 600 700 800 900 1,000 1,100 1,200 1,300 1,400 1,500 1,600 1,700
m/z

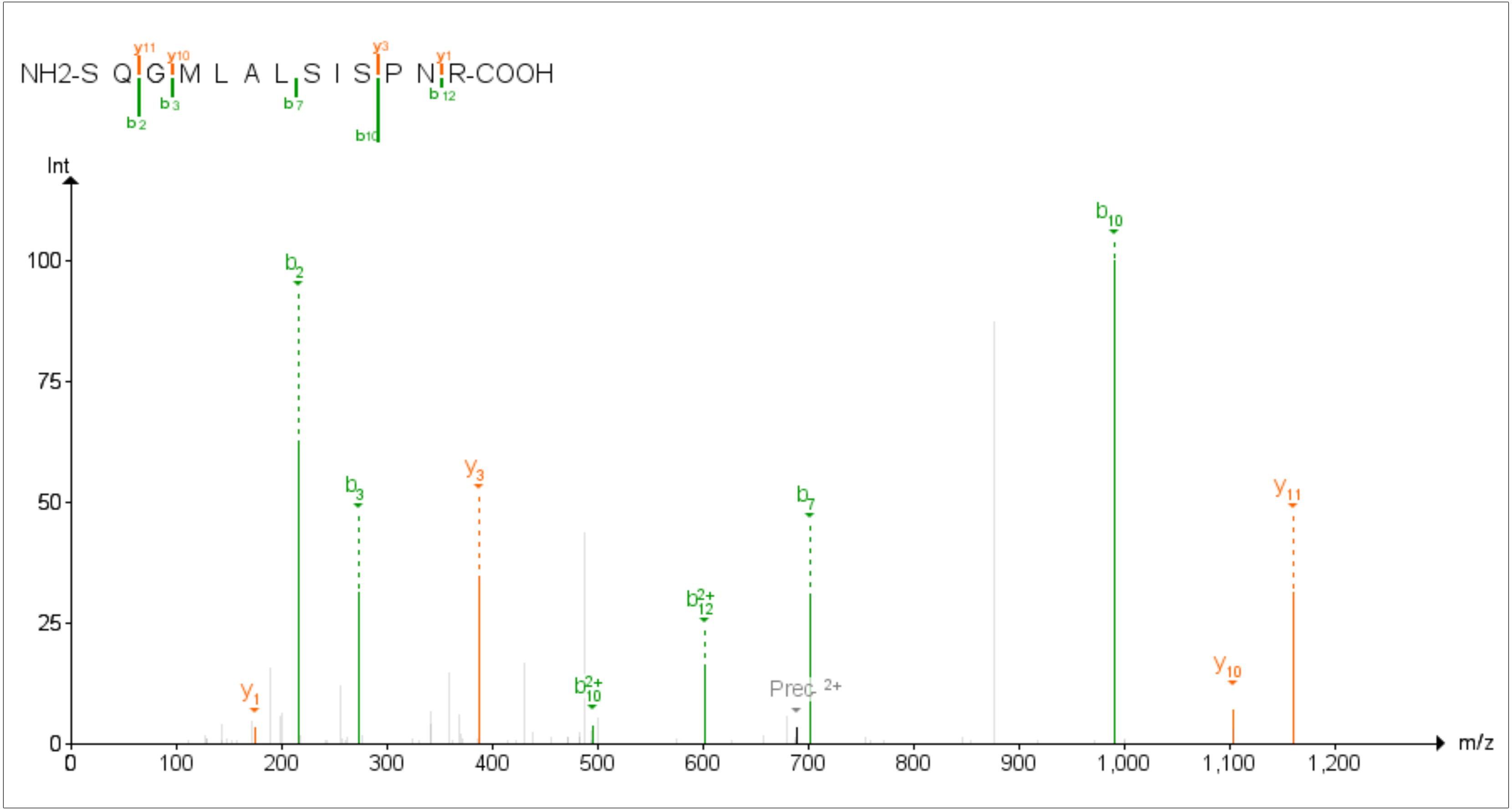

NH2-S Q G M L A L S I S P N R-COOH
y11 y10 y3 y1
b2 b3 b7 b10 b12
Int
100
75
50
25
0
b2
b3
y3
y1
b10 2+
b12 2+
Prec 2+
b7
b10
y10
y11
0 100 200 300 400 500 600 700 800 900 1,000 1,100 1,200
m/z

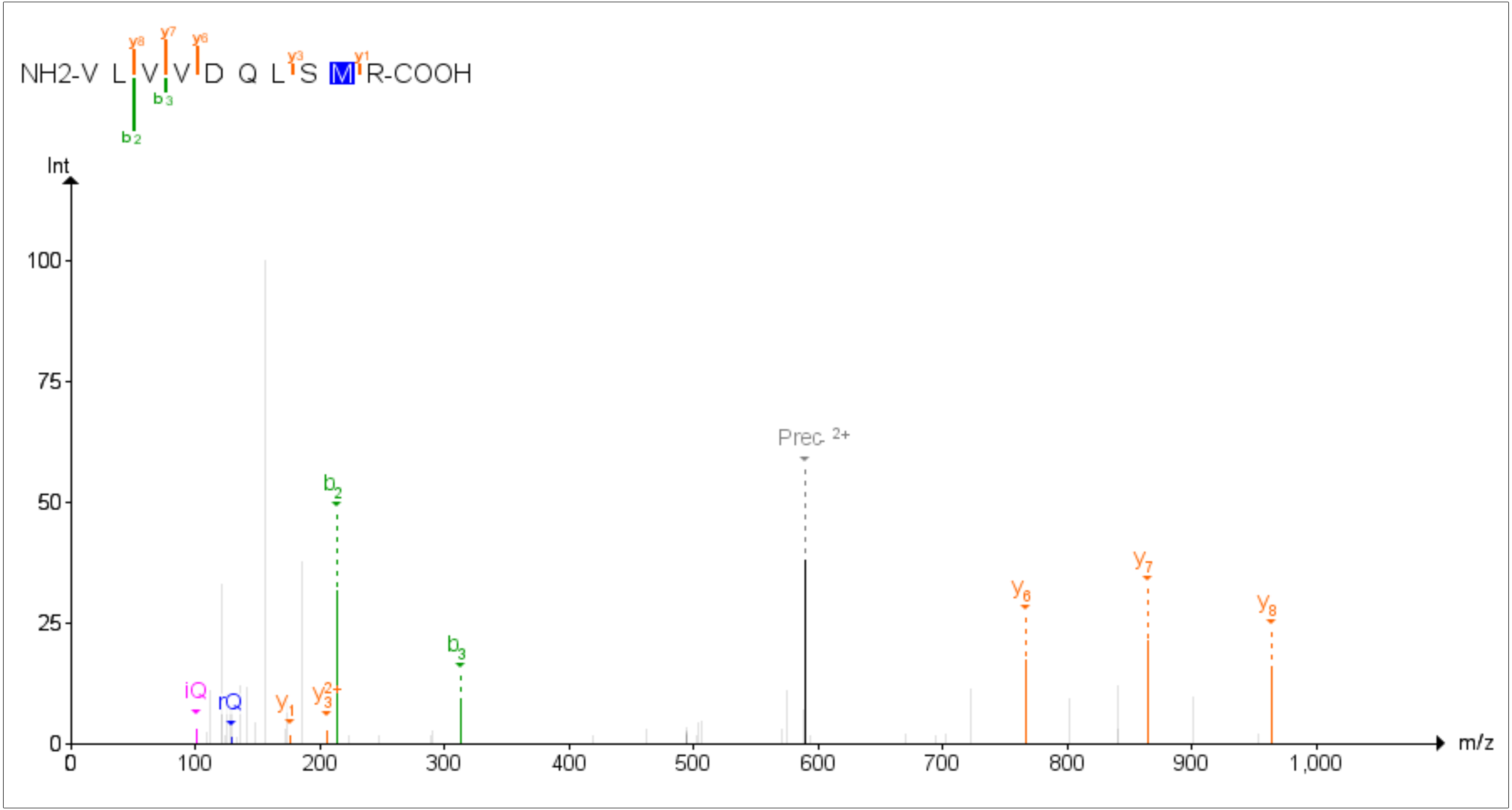

NH2-V L V V D Q L S M R-COOH
y8
y7
y6
y3
y1
b2
b3
Int
100
75
50
25
0
Prec 2+
b2
b3
y6
y7
y8
iQ
rQ
y1
y3 2+
0
100
200
300
400
500
600
700
800
900
1,000
m/z

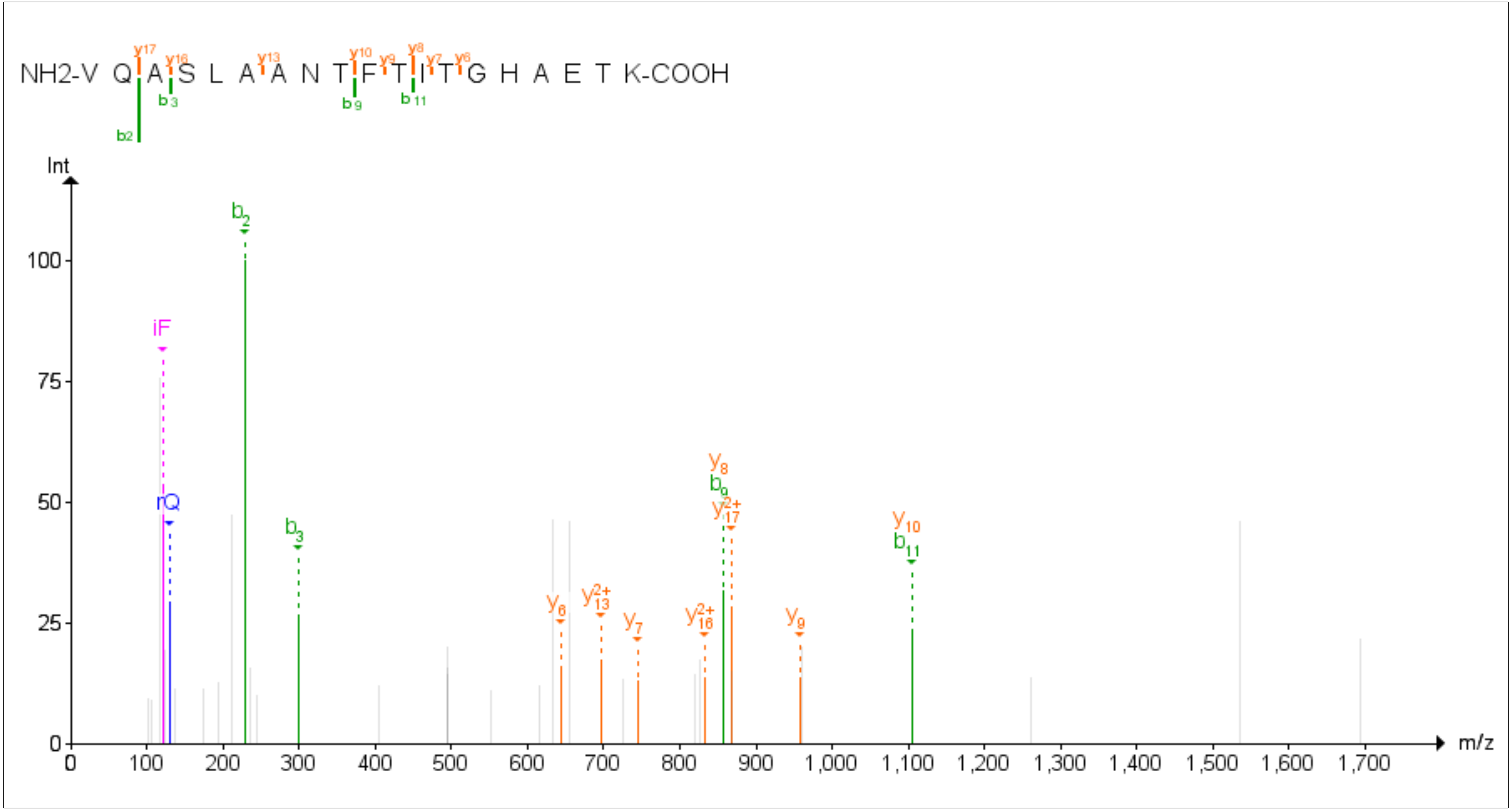

NH2-V Q A S L A A N T F T I T G H A E T K-COOH
y17
y16
y13
y10
y9
y8
y7
y6
b2
b3
b9
b11
Int
iF
rQ
100
75
50
25
0
m/z
0
100
200
300
400
500
600
700
800
900
1,000
1,100
1,200
1,300
1,400
1,500
1,600
1,700

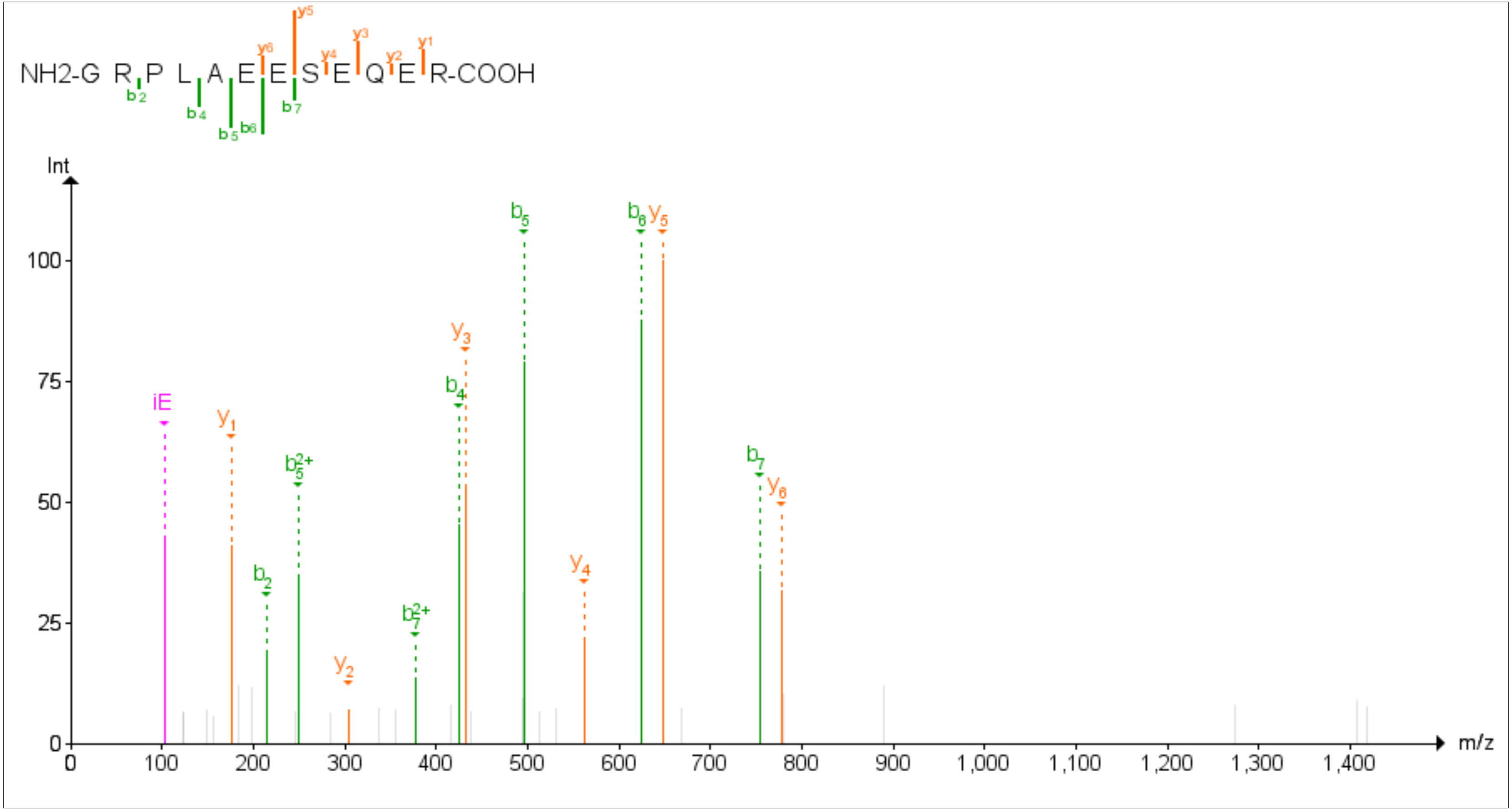

NH2-G R P L A E E S E Q E R-COOH
y5 y6 y4 y3 y2 y1
b2 b4 b5 b6 b7
Int
100
75
50
25
0
iE
y1
b2
b5 2+
y2
b7 2+
b4
y3
b5
y4
b6
y5
b7
y6
0
100
200
300
400
500
600
700
800
900
1,000
1,100
1,200
1,300
1,400
m/z

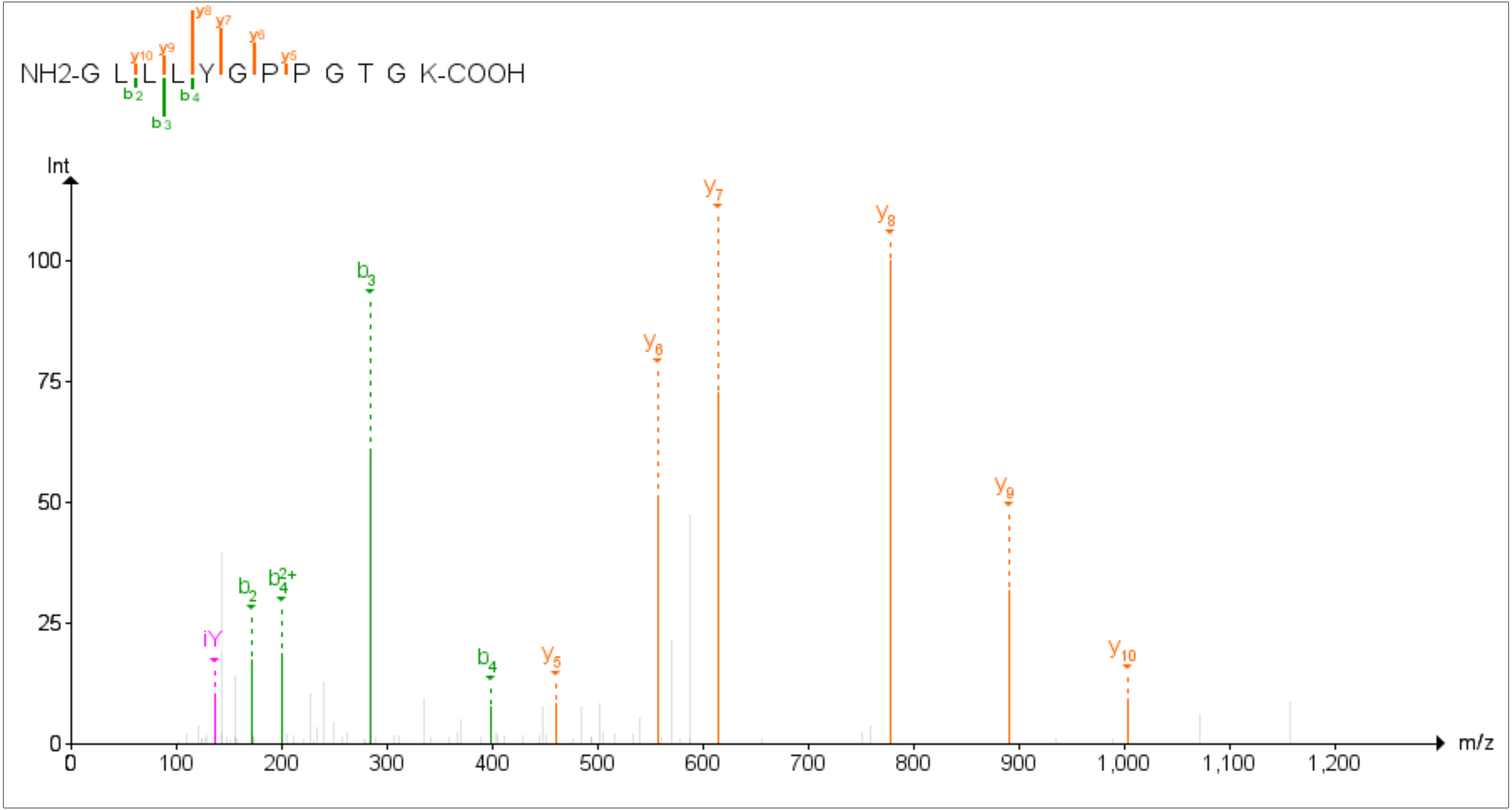

NH2-G L L L Y G P P G T G K-COOH
y10 y9 y8 y7 y6 y5
b2 b3 b4
Int
100
75
50
25
0
iY
b2
b4 2+
b3
b4
y5
y6
y7
y8
y9
y10
0 100 200 300 400 500 600 700 800 900 1,000 1,100 1,200
m/z

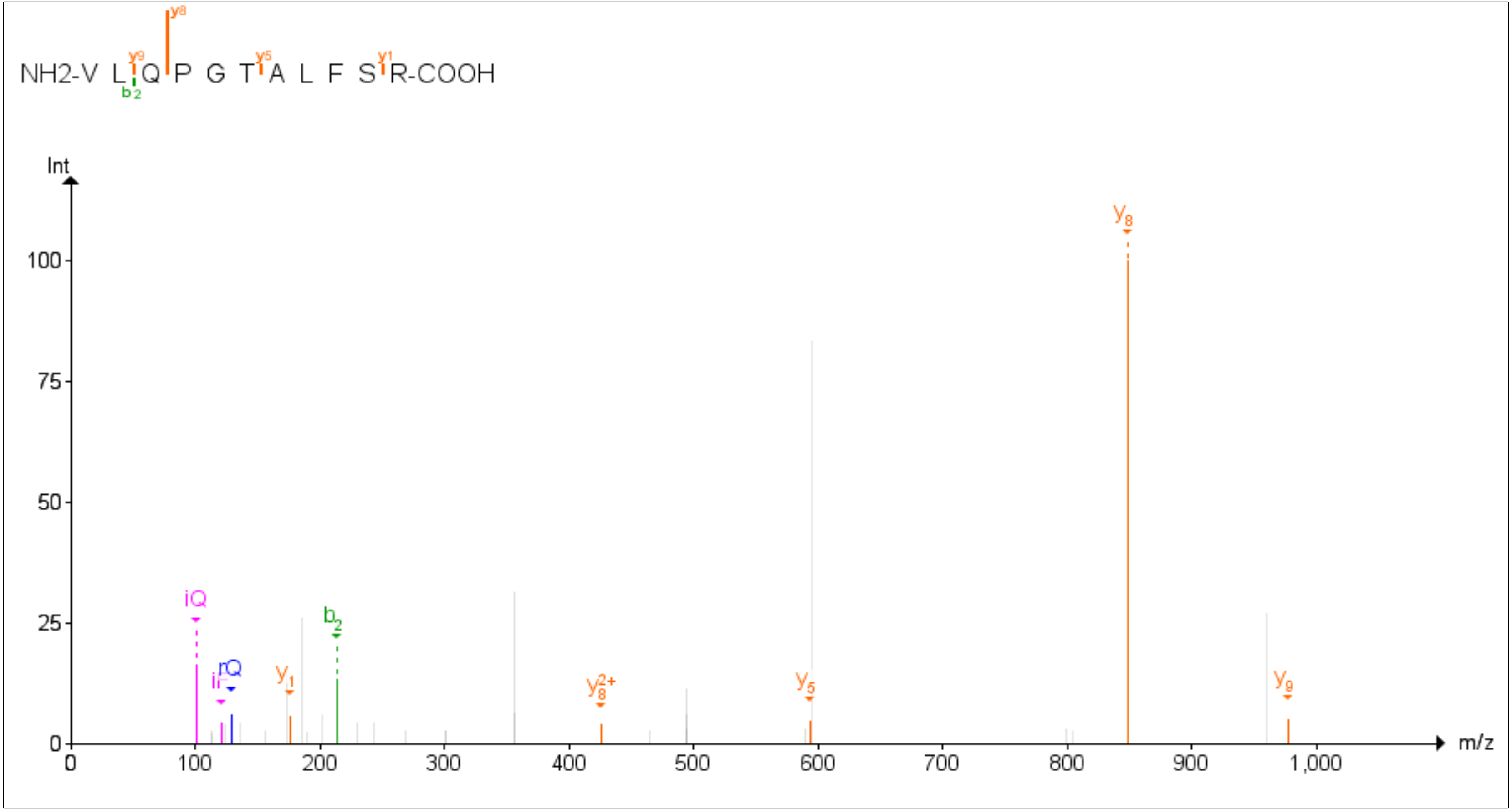

NH2-V L Q P G T A L F S R-COOH
y8
y9
y5
y1
b2
Int
100
75
50
25
0
iQ
iF
rQ
y1
b2
y8 2+
y5
y8
y9
0
100
200
300
400
500
600
700
800
900
1,000
m/z

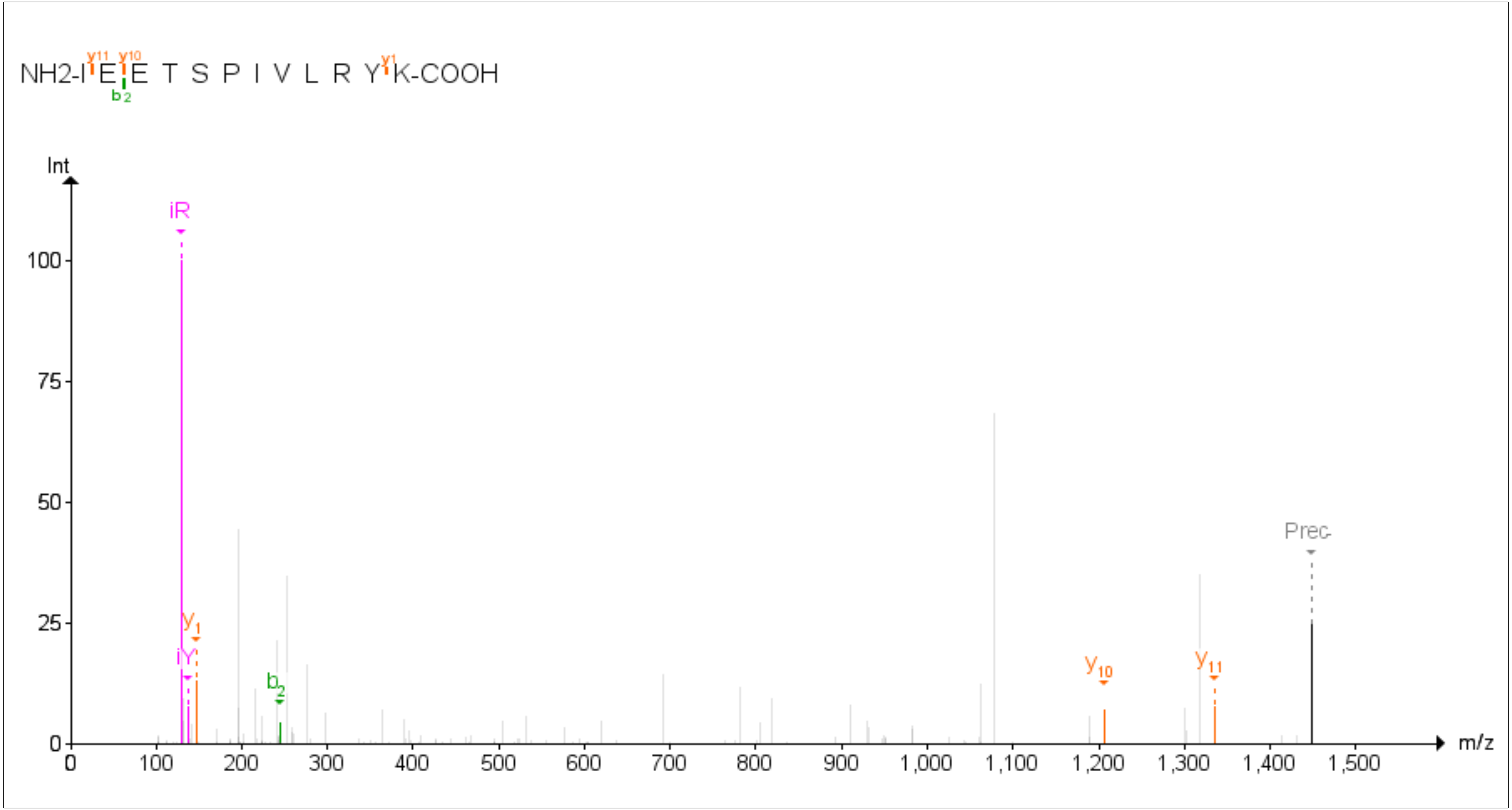

NH2-I E E T S P I V L R Y K-COOH
y11
y10
y1
b2
Int
iR
y1
iY
b2
y10
y11
Prec
m/z
0
25
50
75
100
0
100
200
300
400
500
600
700
800
900
1,000
1,100
1,200
1,300
1,400
1,500

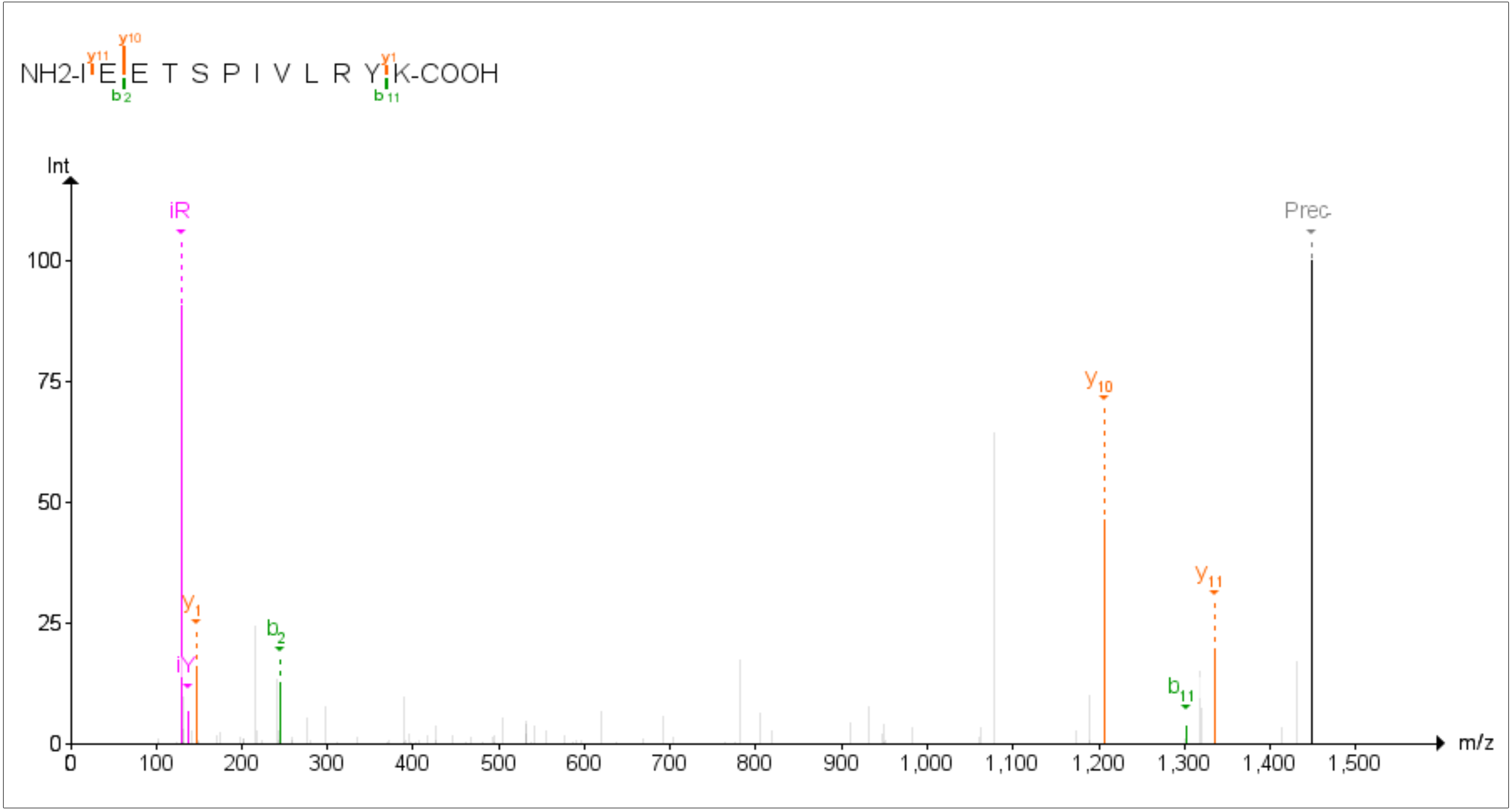

NH2-I E E T S P I V L R Y K-COOH
y11
y10
y1
b2
b11
Int
iR
Prec
y10
y11
y1
iY
b2
b11
100
75
50
25
0
0
100
200
300
400
500
600
700
800
900
1,000
1,100
1,200
1,300
1,400
1,500
m/z

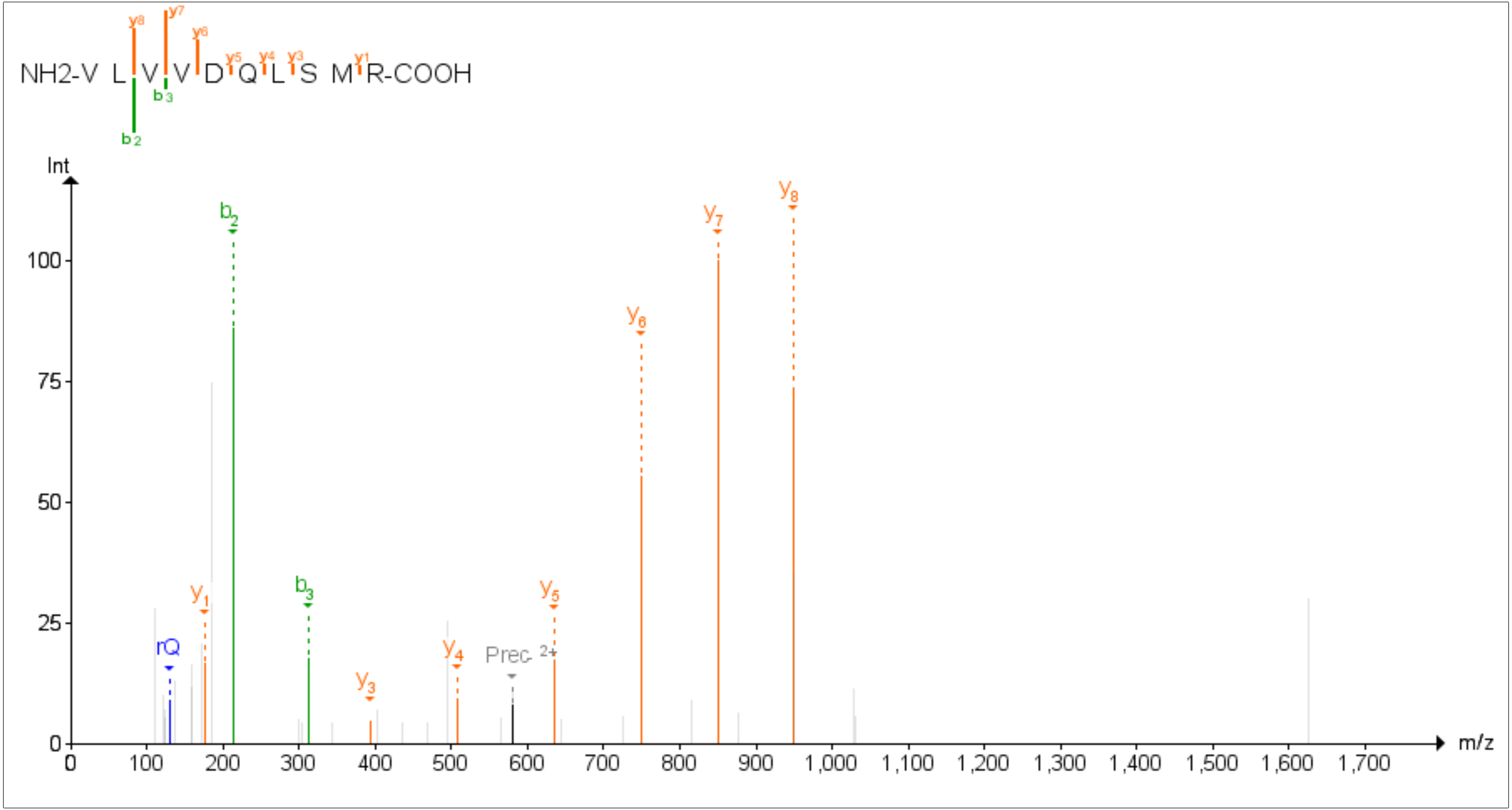

NH2-V L V V D Q L S M R-COOH
y8 y7 y6 y5 y4 y3 y1
b2 b3
Int
m/z
b2
y8
y7
y6
b3
y1
y5
rQ
y4
Prec- 2+
y3
0
25
50
75
100
0
100
200
300
400
500
600
700
800
900
1,000
1,100
1,200
1,300
1,400
1,500
1,600
1,700

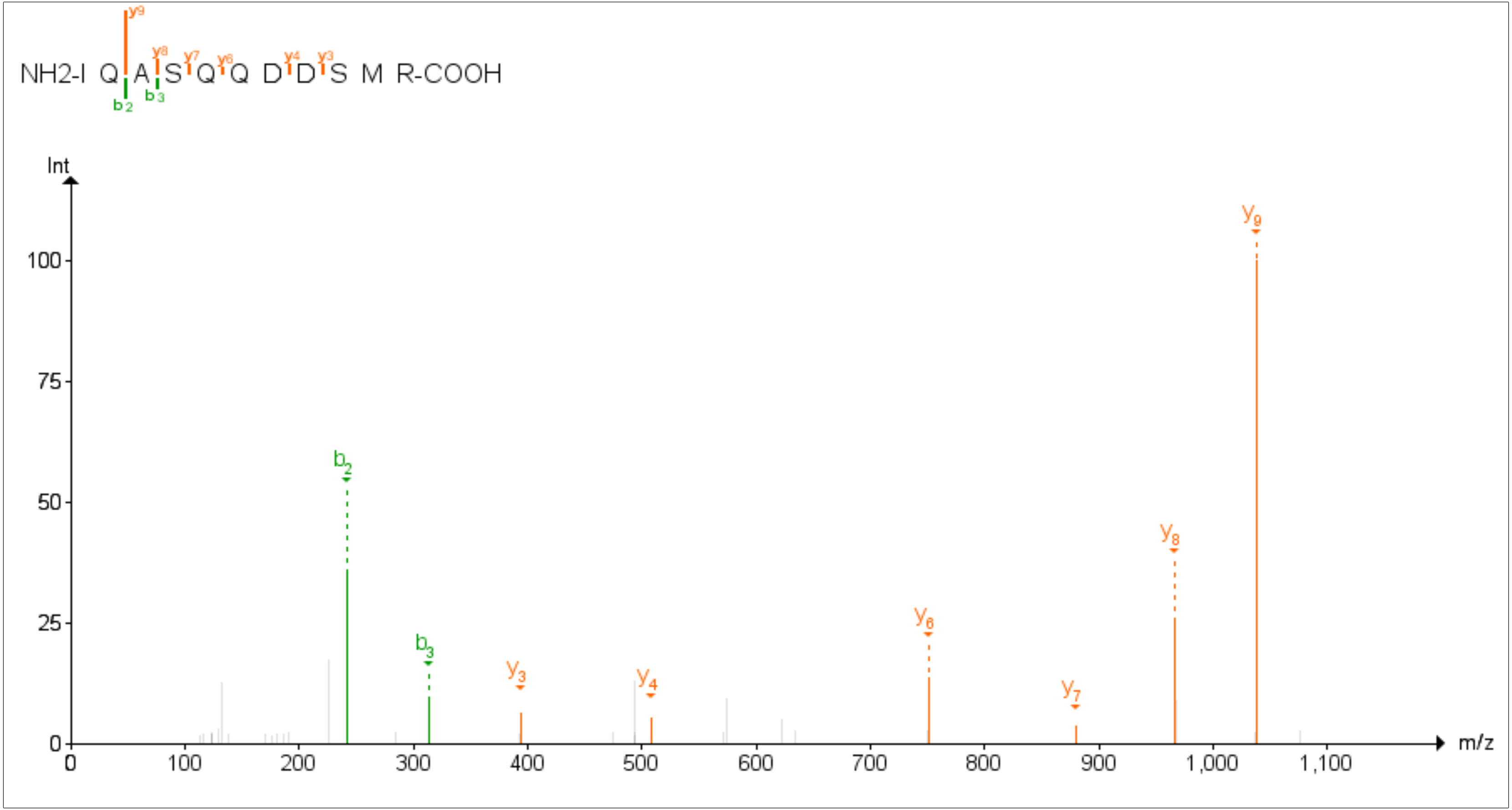

NH2-I Q A S Q Q D D S M R-COOH
y9
y8
y7
y6
y4
y3
b2
b3
Int
100
75
50
25
0
b2
b3
y3
y4
y6
y7
y8
y9
0
100
200
300
400
500
600
700
800
900
1,000
1,100
m/z

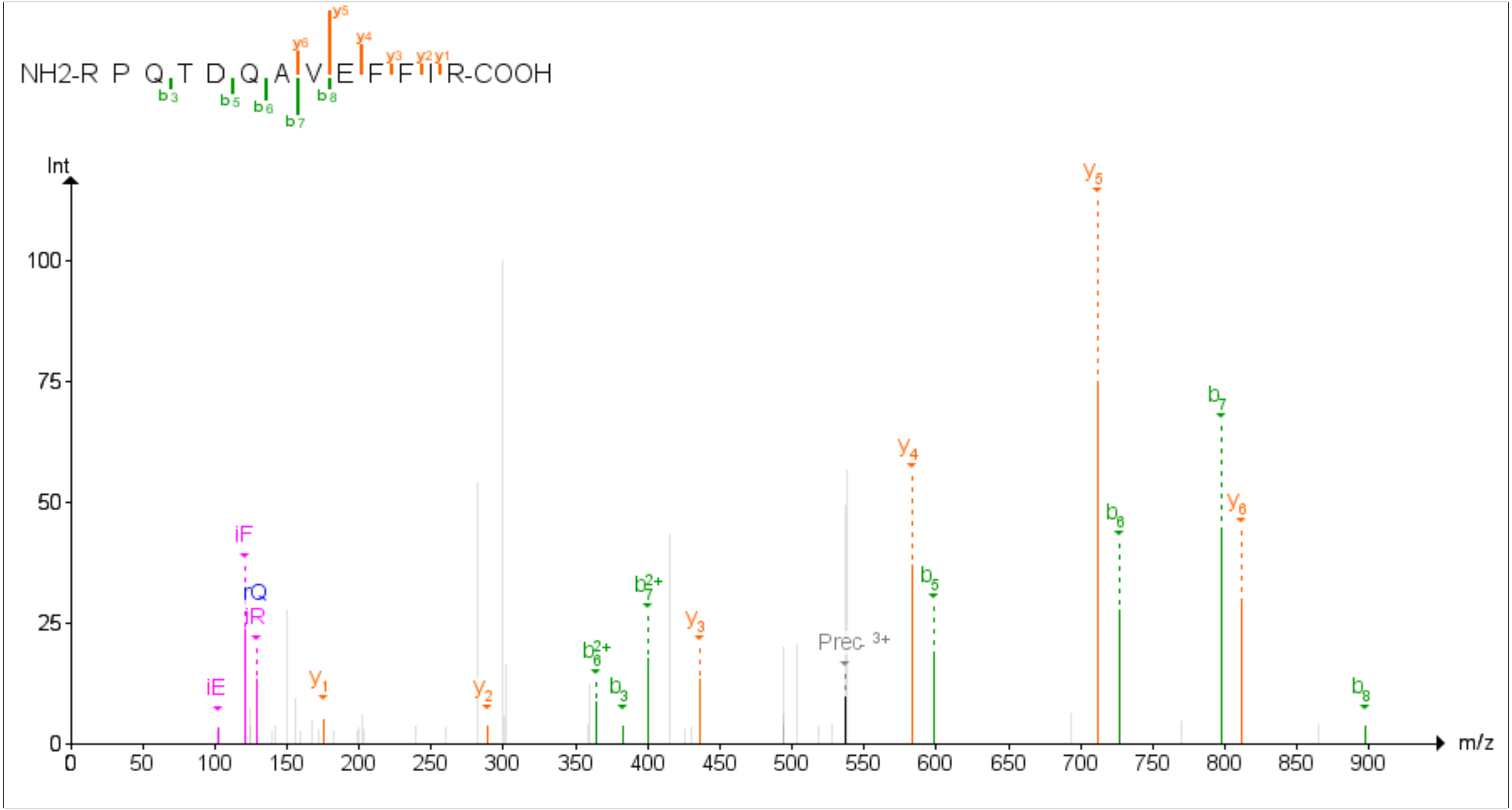

NH2-R P Q T D Q A V E F F I R-COOH
y5
y6
y4
y3
y2
y1
b3
b5
b6
b7
b8
Int
100
75
50
25
0
m/z
0
50
100
150
200
250
300
350
400
450
500
550
600
650
700
750
800
850
900
y5
b7
y4
b6
y6
b5
iF
rQ
iR
b7 2+
y3
b6 2+
Prec 3+
iE
y1
y2
b3
b8

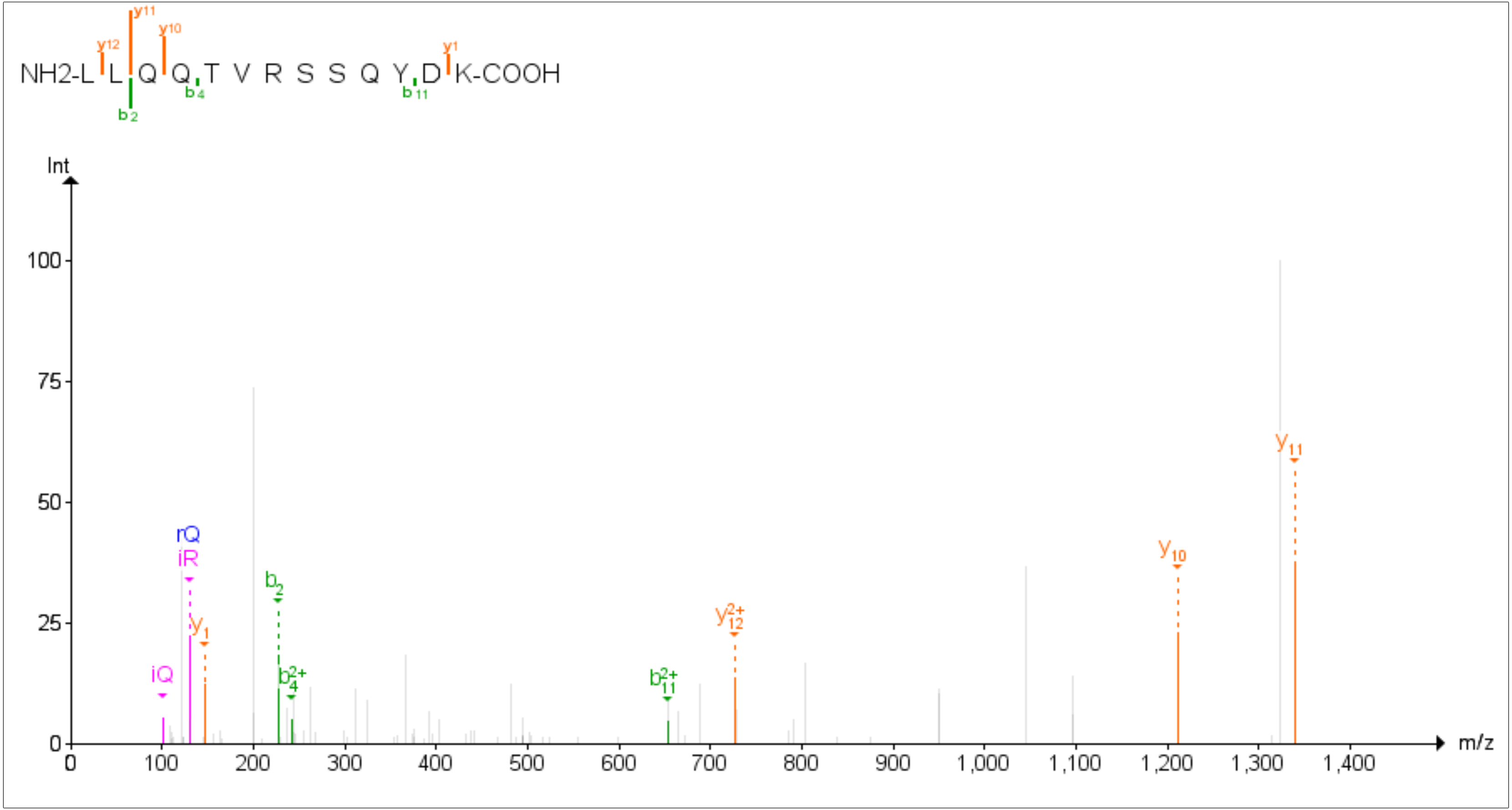

NH2-L L Q Q T V R S S Q Y D K-COOH
y12
y11
y10
y1
b2
b4
b11
Int
100
75
50
25
0
rQ
iR
iQ
y1
b2
b4 2+
b11 2+
y12 2+
y10
y11
0
100
200
300
400
500
600
700
800
900
1,000
1,100
1,200
1,300
1,400
m/z

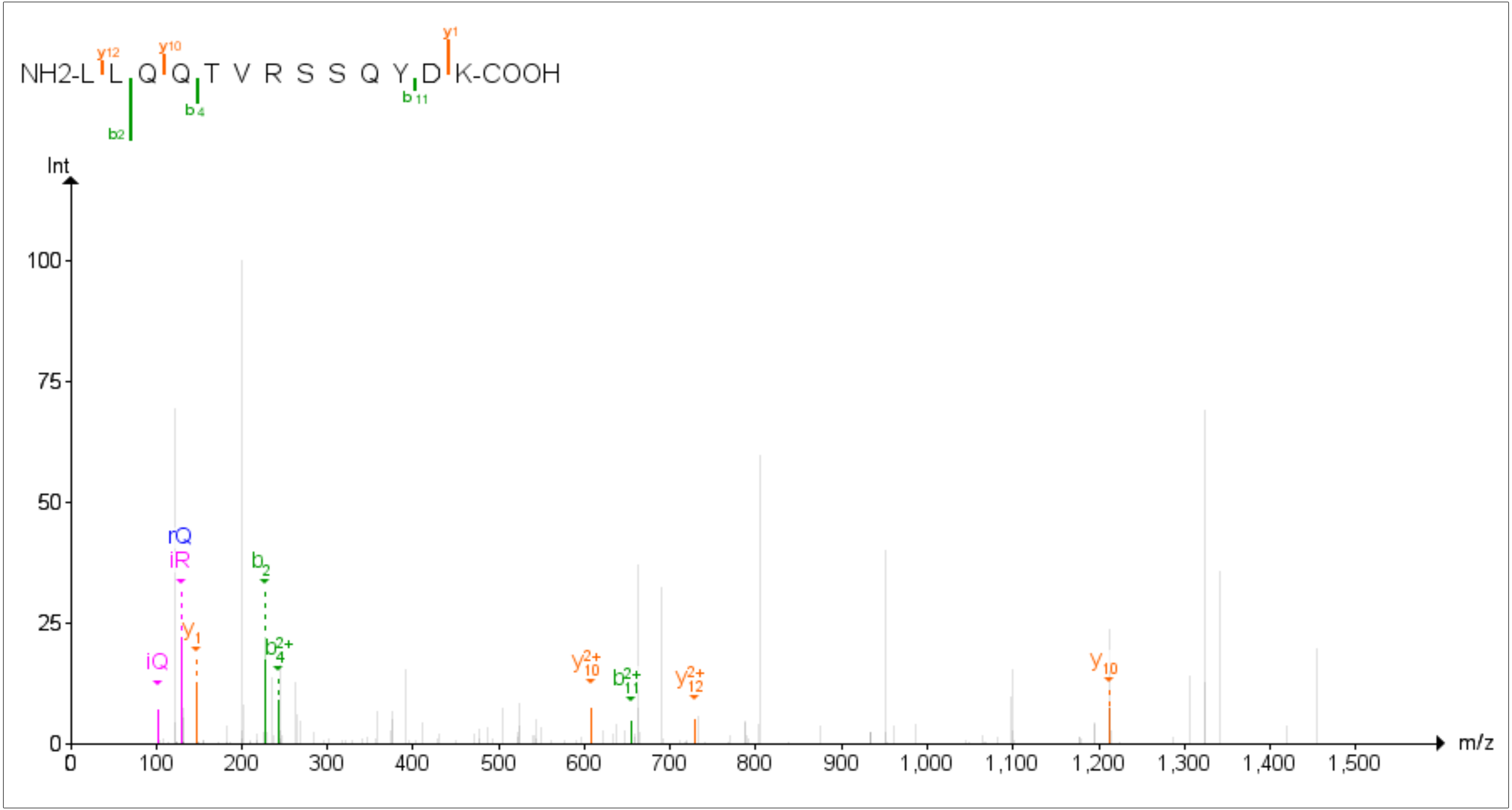

NH2-L L Q Q T V R S S Q Y D K-COOH
y12
y10
y1
b2
b4
b11
Int
100
75
50
25
0
rQ
iR
iQ
y1
b2
b4 2+
y10 2+
b11 2+
y12 2+
y10
0
100
200
300
400
500
600
700
800
900
1,000
1,100
1,200
1,300
1,400
1,500
m/z

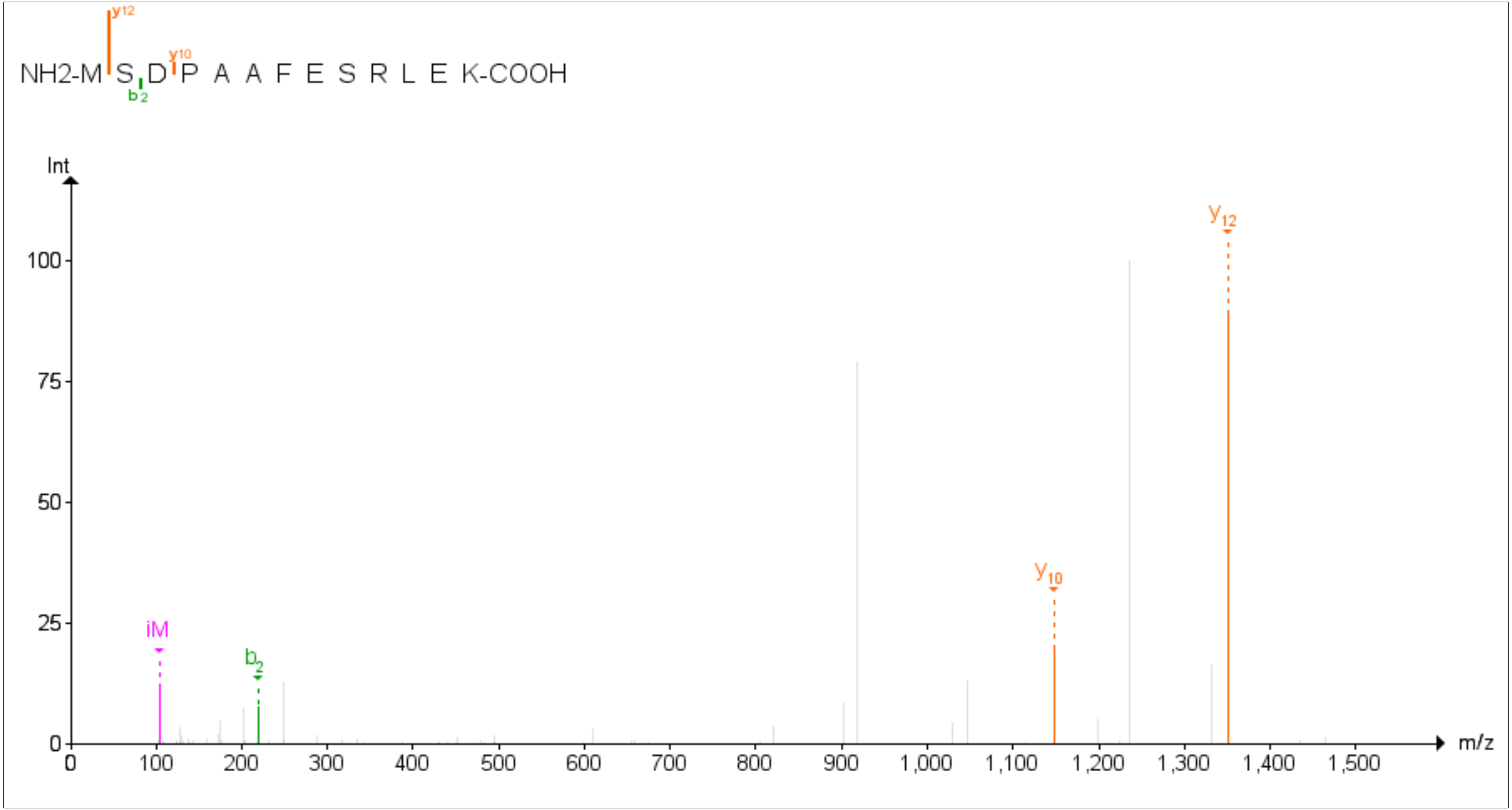

y12
y10
NH2-M S D P A A F E S R L E K-COOH
b2
Int
100
75
50
25
0
iM
b2
y10
y12
0
100
200
300
400
500
600
700
800
900
1,000
1,100
1,200
1,300
1,400
1,500
m/z

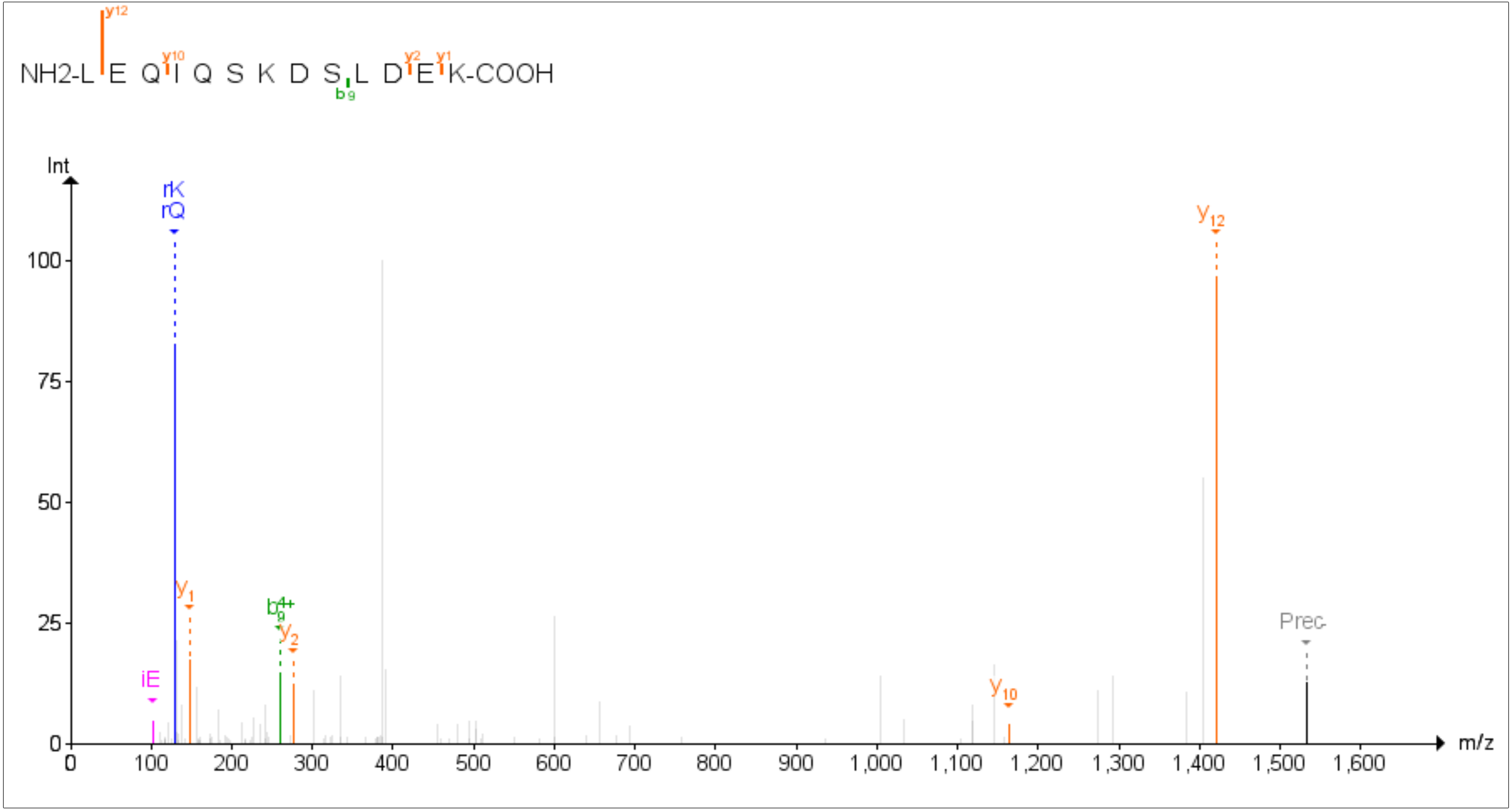

NH2-L E Q I Q S K D S L D E K-COOH
y12
y10
y2
y1
b9
Int
rK
rQ
y12
100
75
50
25
0
y1
b9 4+
y2
iE
y10
Prec
0
100
200
300
400
500
600
700
800
900
1,000
1,100
1,200
1,300
1,400
1,500
1,600
m/z

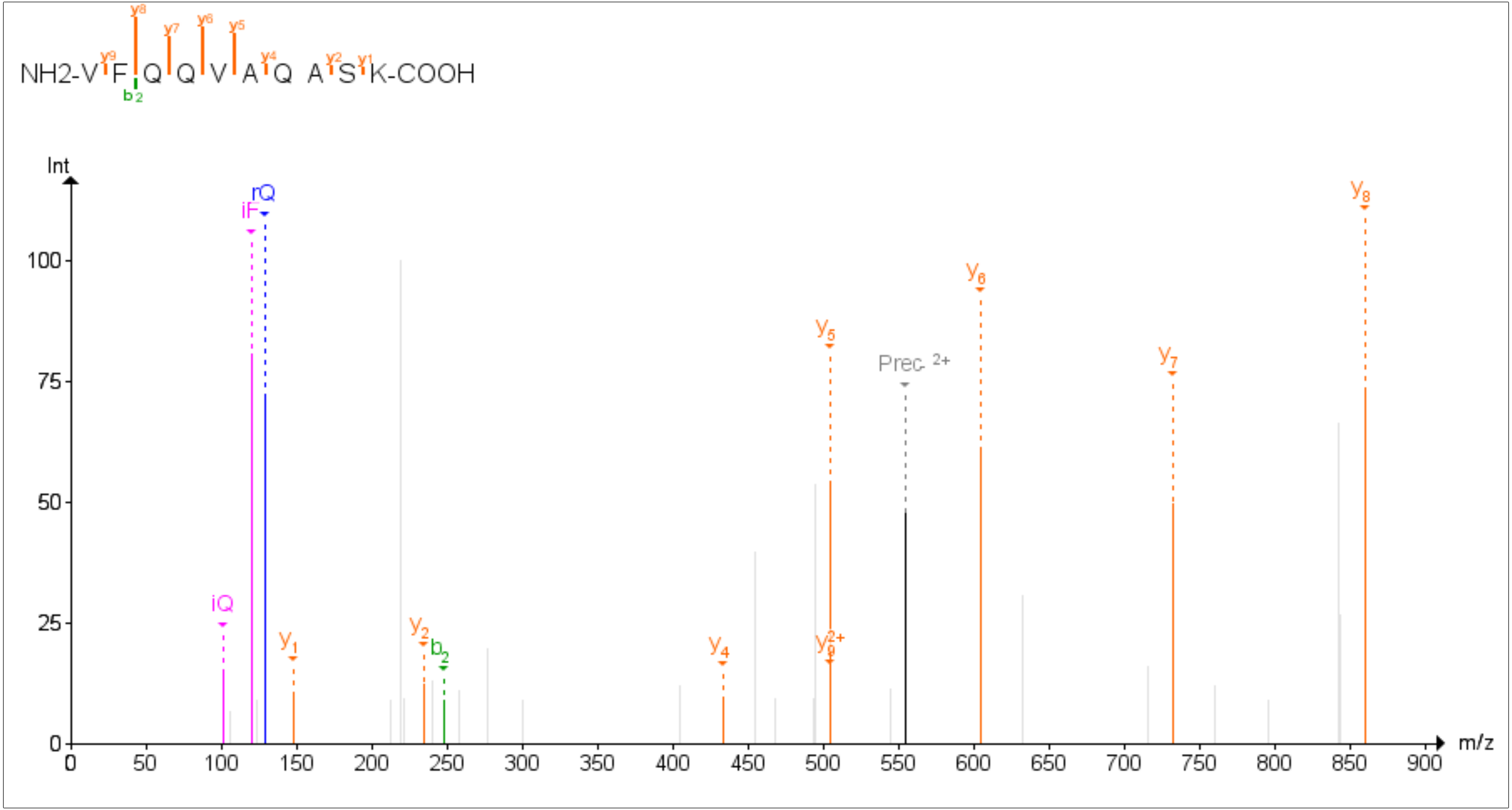

NH2-V F Q Q V A Q A S K-COOH
y9 y8 y7 y6 y5 y4 y2 y1
b2
Int
100
75
50
25
0
iQ
iF
nQ
y1
y2
b2
y4
y5
y9 2+
Prec 2+
y6
y7
y8
0 50 100 150 200 250 300 350 400 450 500 550 600 650 700 750 800 850 900
m/z

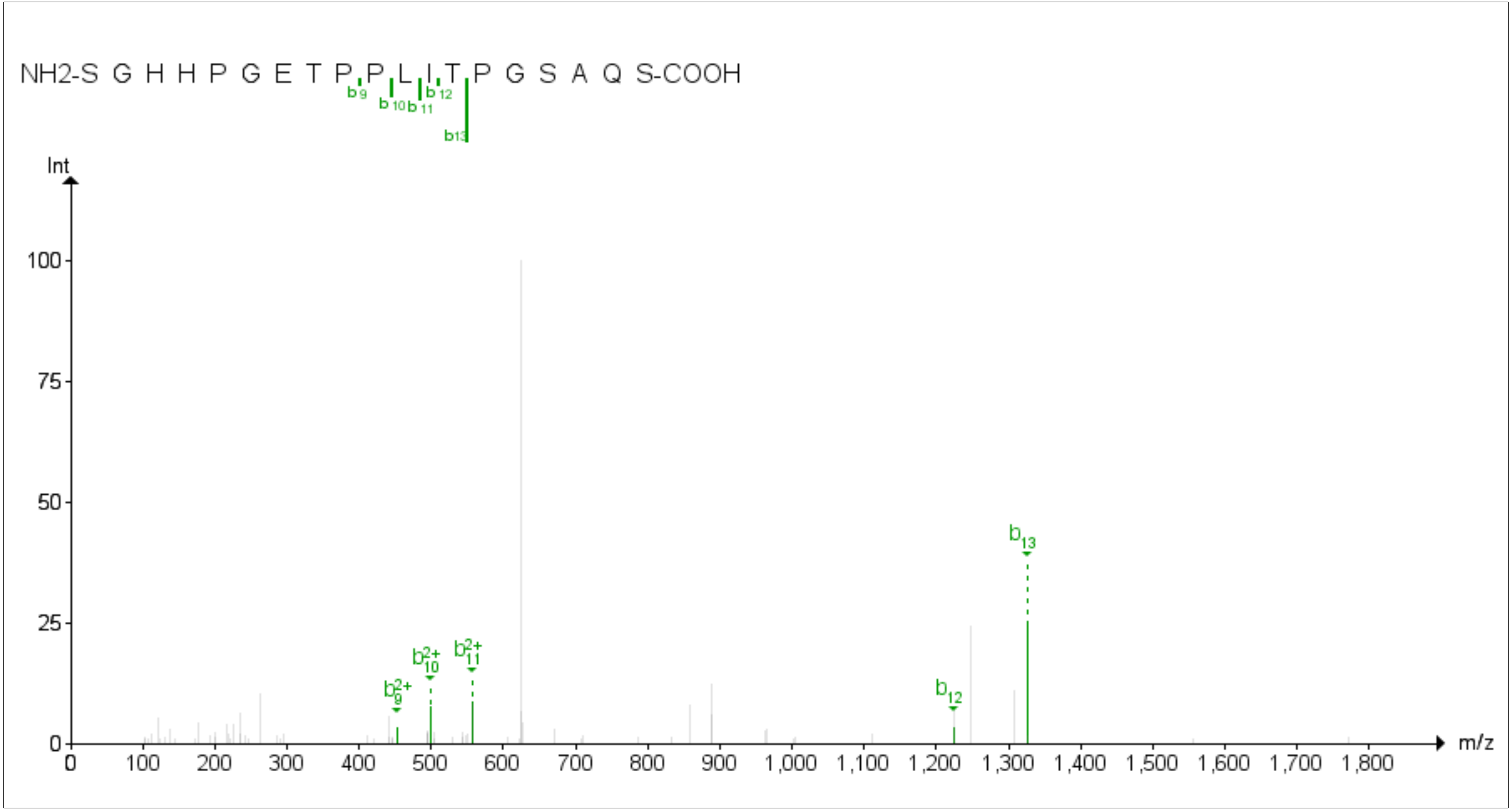

NH2-S G H H P G E T P P L I T P G S A Q S-COOH
b9
b10
b11
b12
b13
Int
100
75
50
25
0
b_{13}
b_{9}^{2+}
b_{10}^{2+}
b_{11}^{2+}
b_{12}
0
100
200
300
400
500
600
700
800
900
1,000
1,100
1,200
1,300
1,400
1,500
1,600
1,700
1,800
m/z

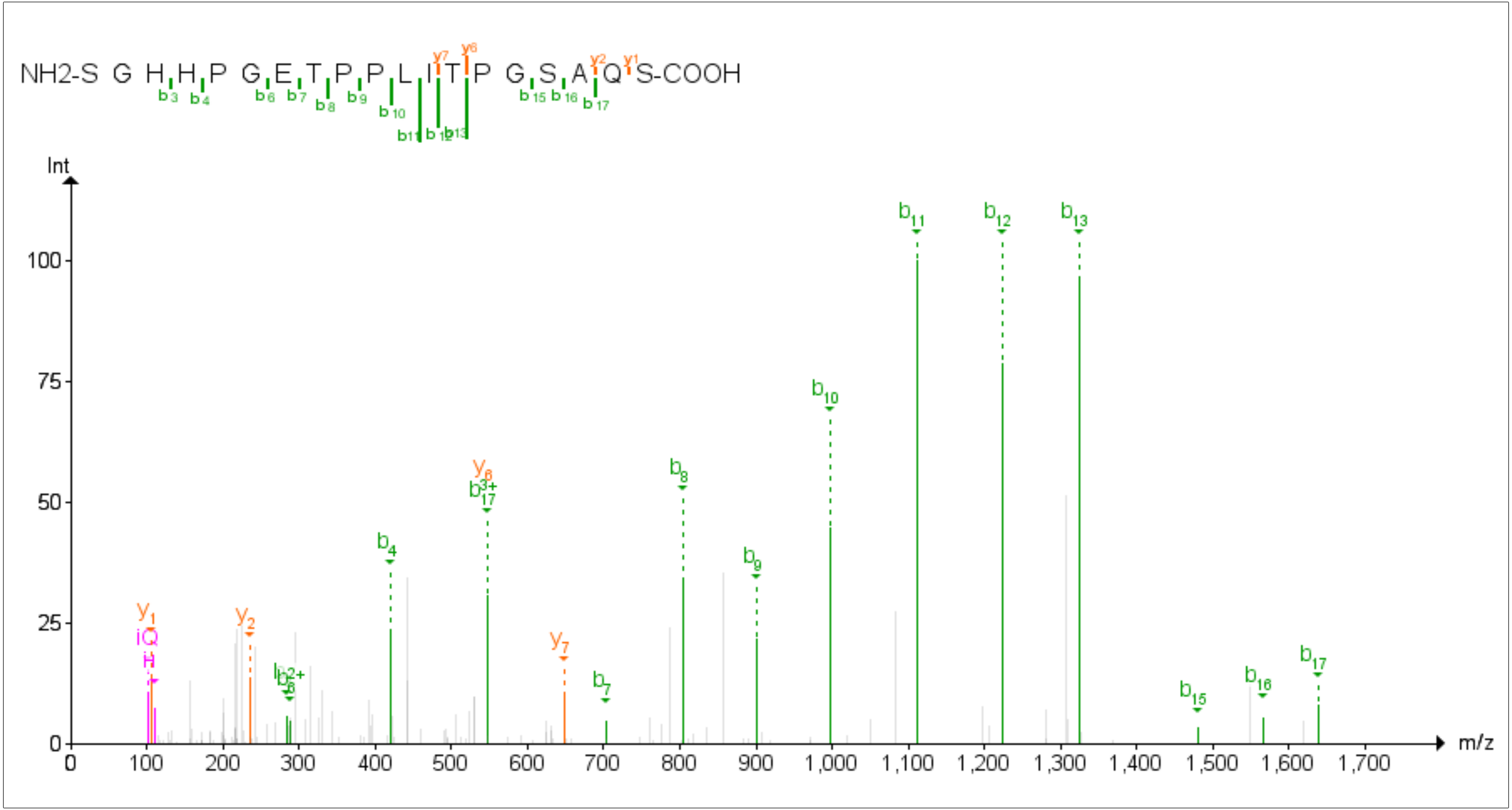

NH2-S G H H P G E T P P L I T P G S A Q S-COOH
Int
m/z
0
25
50
75
100
200
300
400
500
600
700
800
900
1,000
1,100
1,200
1,300
1,400
1,500
1,600
1,700

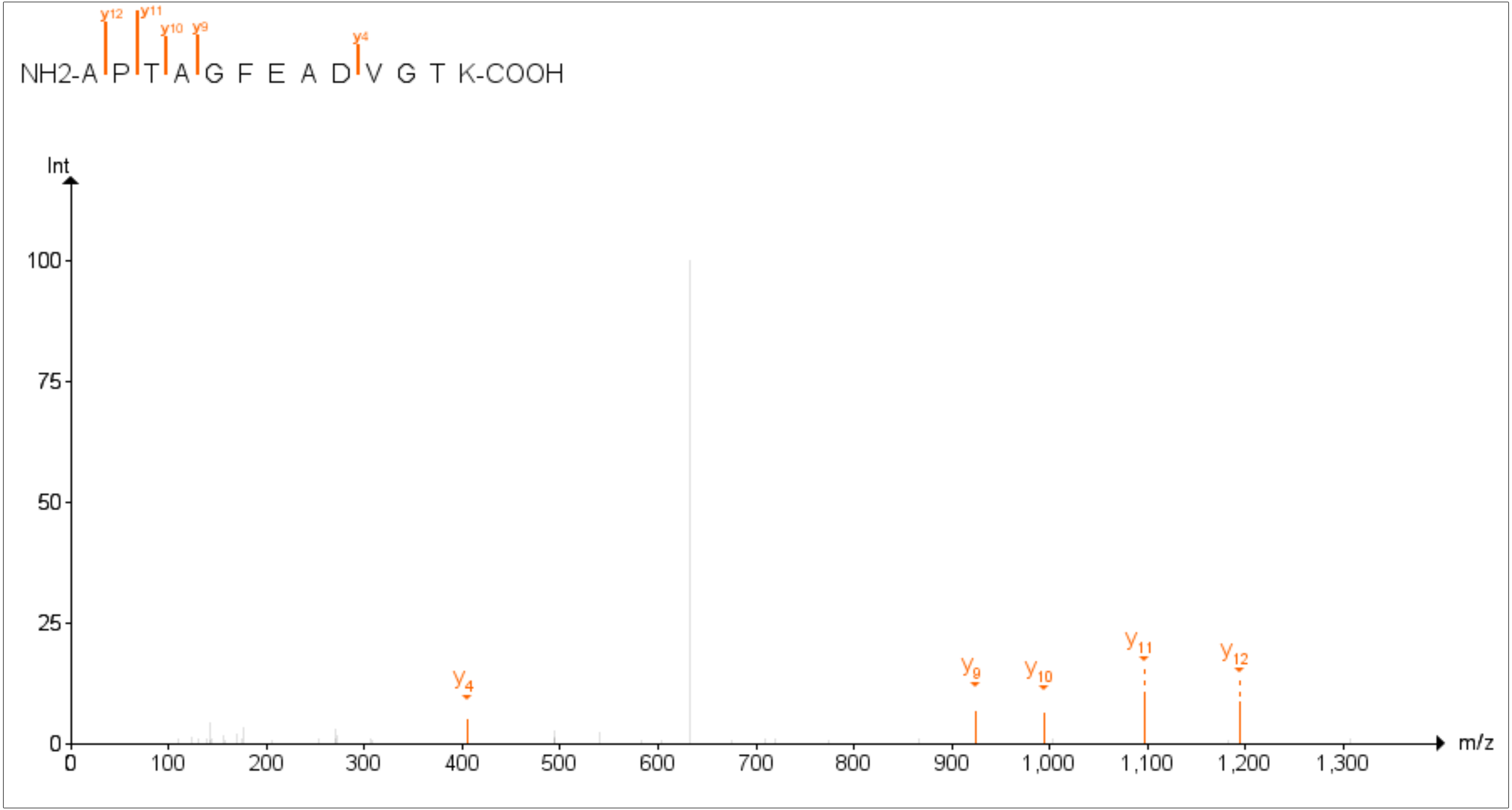

NH2-A P T A G F E A D V G T K-COOH
y12
y11
y10
y9
y4
Int
100
75
50
25
0
m/z
0
100
200
300
400
500
600
700
800
900
1,000
1,100
1,200
1,300

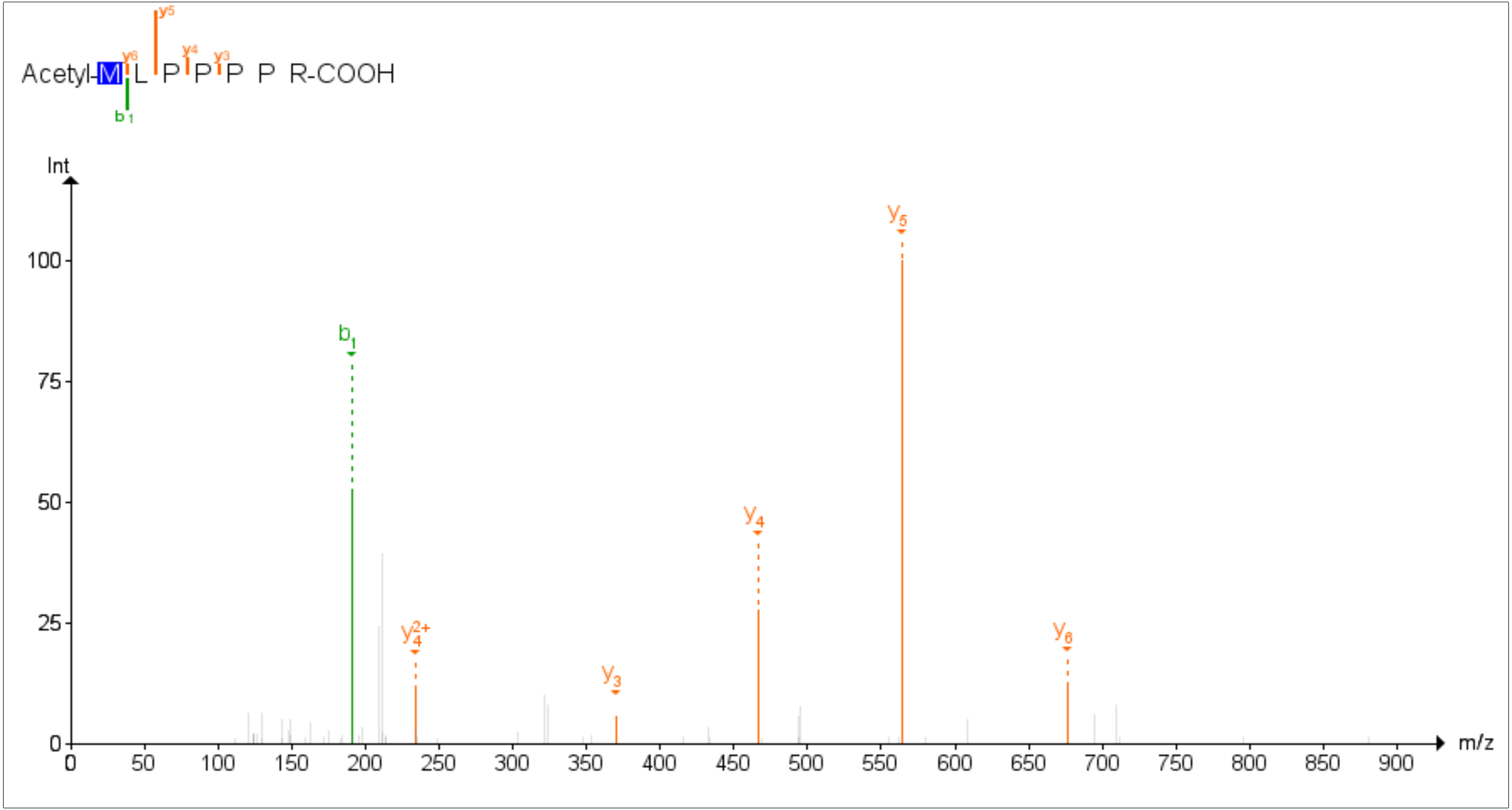

Acetyl-M L P P P P R-COOH
y5
y6
y4
y3
b 1
Int
100
75
50
25
0
b1
y4 2+
y3
y4
y5
y6
0
50
100
150
200
250
300
350
400
450
500
550
600
650
700
750
800
850
900
m/z

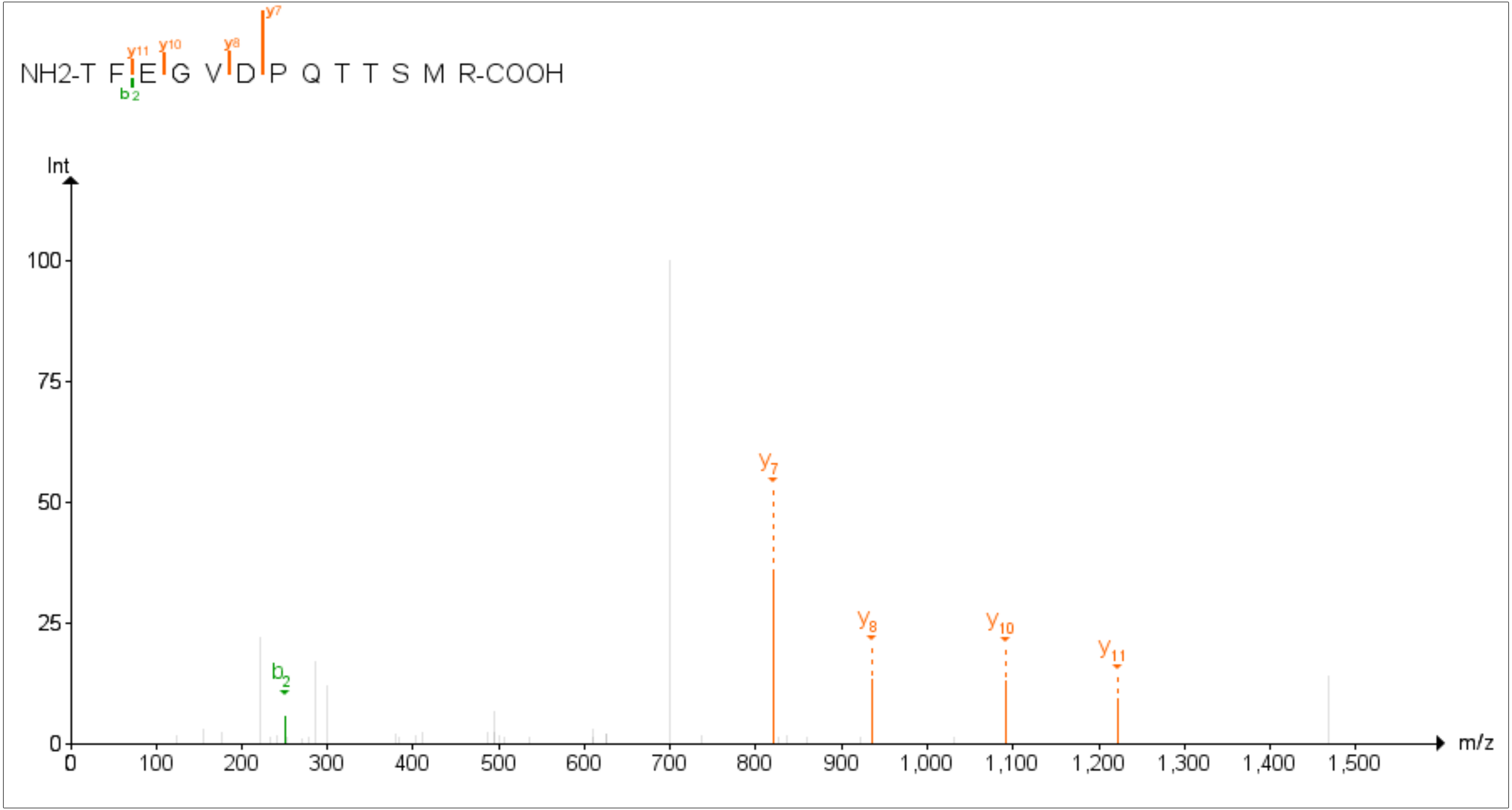
NH2-T F E G V D P Q T T S M R-COOH
y11
y10
y8
y7
b2
Int
100
75
50
25
0
y7
y8
y10
y11
b2
0
100
200
300
400
500
600
700
800
900
1,000
1,100
1,200
1,300
1,400
1,500
m/z

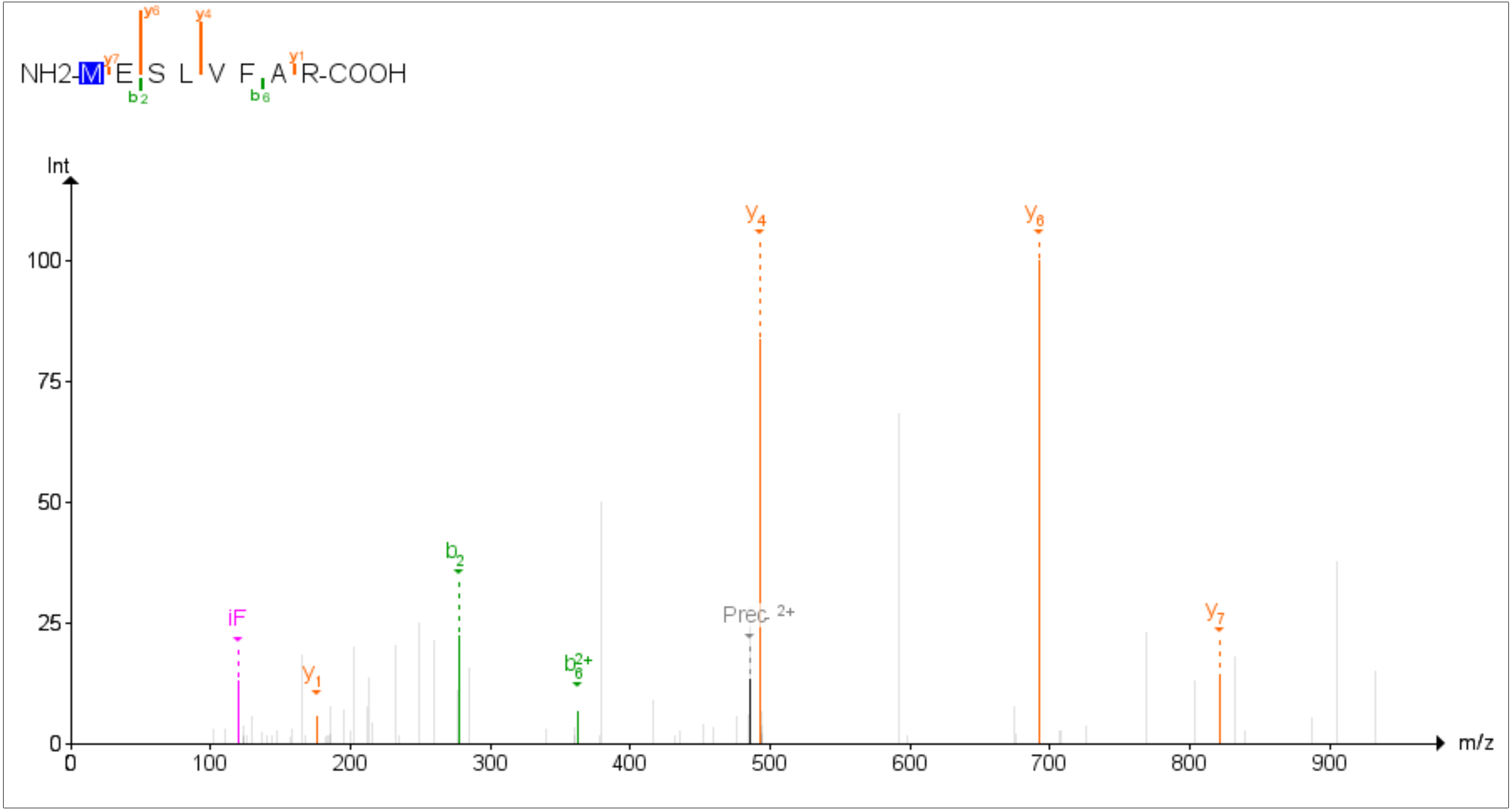

NH2-M E S L V F A R-COOH
y7
y6
y4
y1
b2
b6
Int
100
75
50
25
0
y4
y6
b2
iF
y1
b6 2+
Prec. 2+
y7
0
100
200
300
400
500
600
700
800
900
m/z

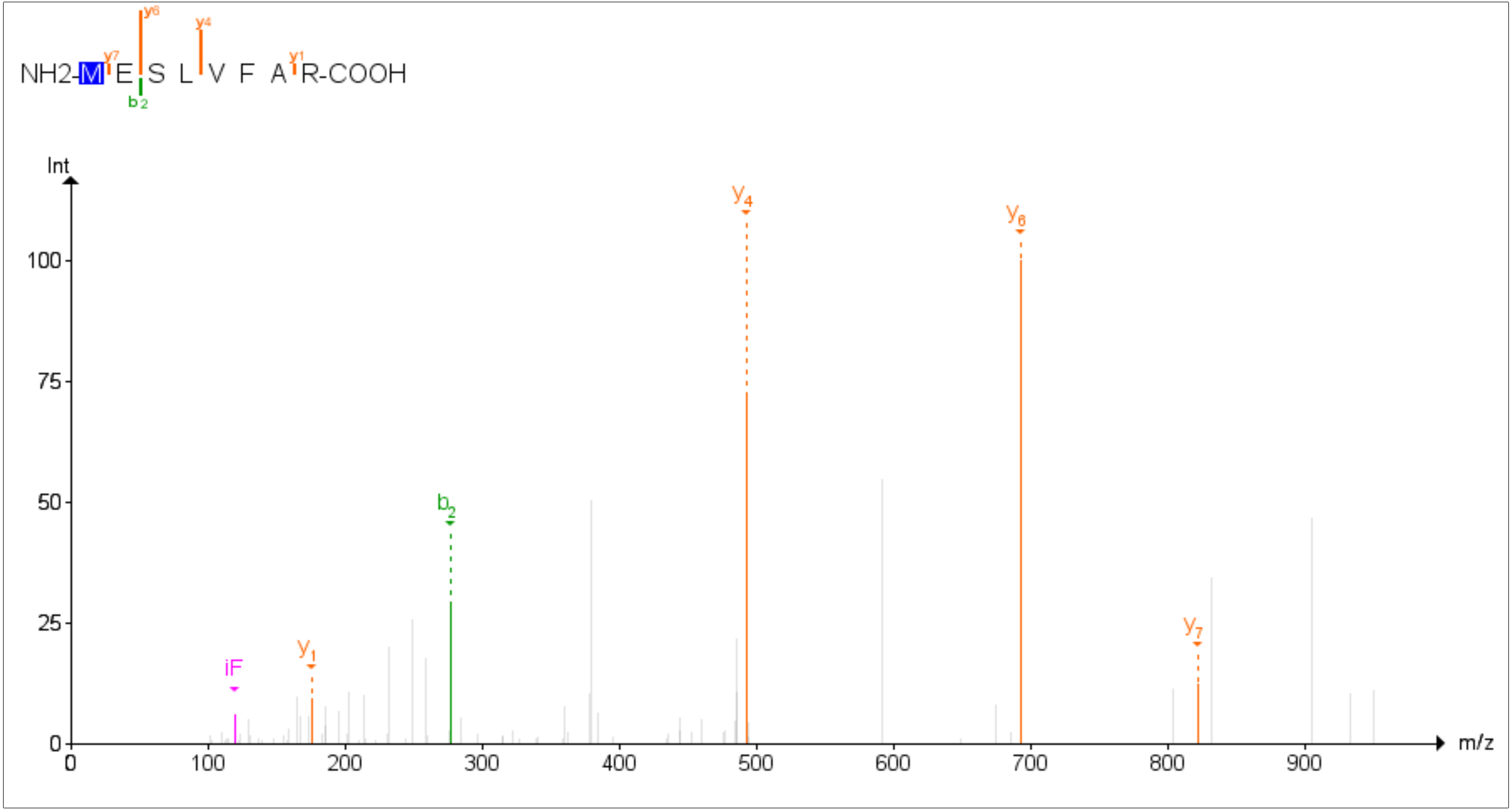

NH2-M E S L V F A R-COOH
y7
y6
y4
y1
b2
Int
100
75
50
25
0
y4
y6
b2
y7
y1
iF
0
100
200
300
400
500
600
700
800
900
m/z

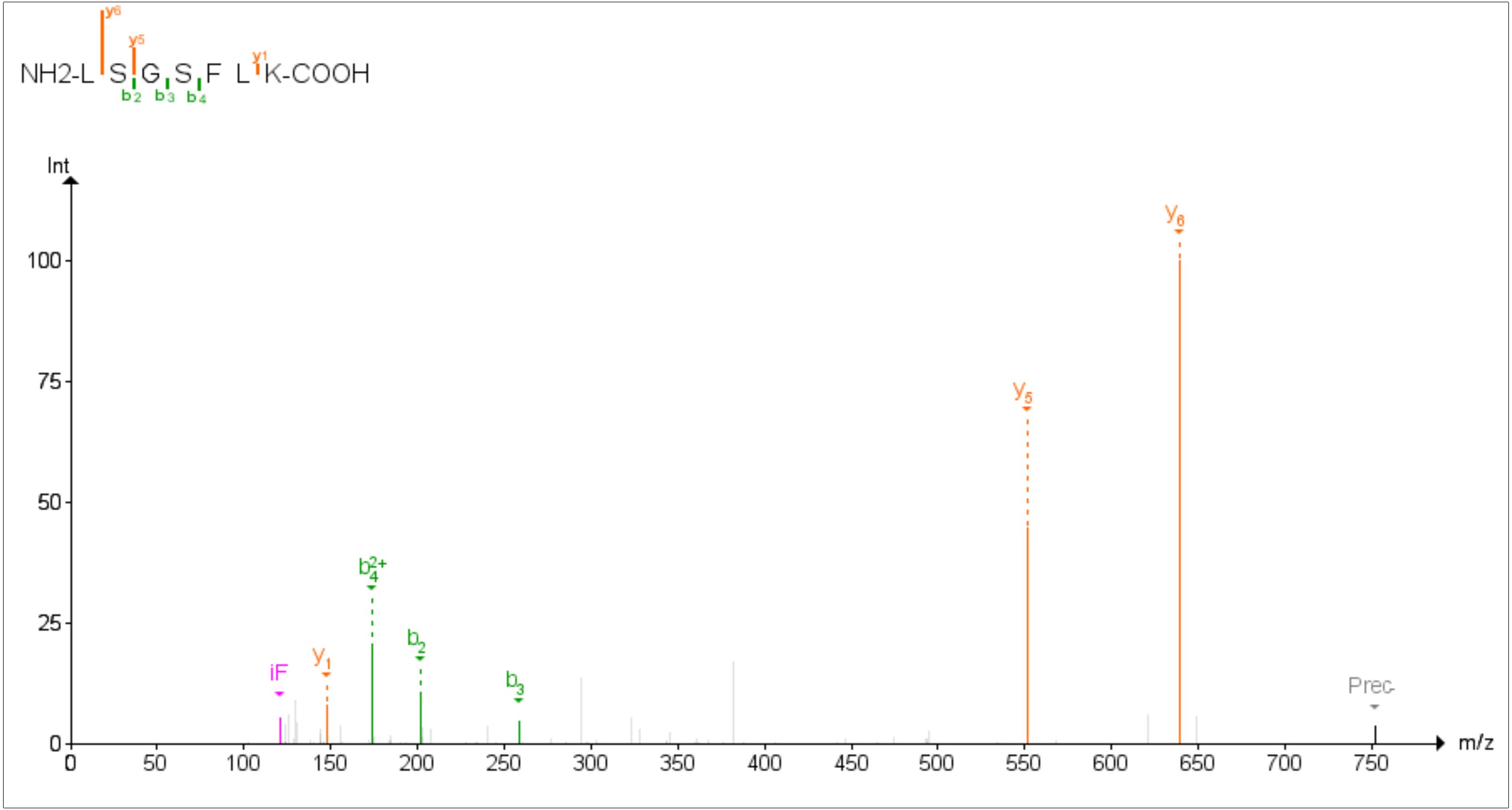
NH2-L S G S F L K-COOH
y6
y5
y1
b2
b3
b4
Int
100
75
50
25
0
y6
y5
b4 2+
b2
y1
iF
b3
Prec
0
50
100
150
200
250
300
350
400
450
500
550
600
650
700
750
m/z

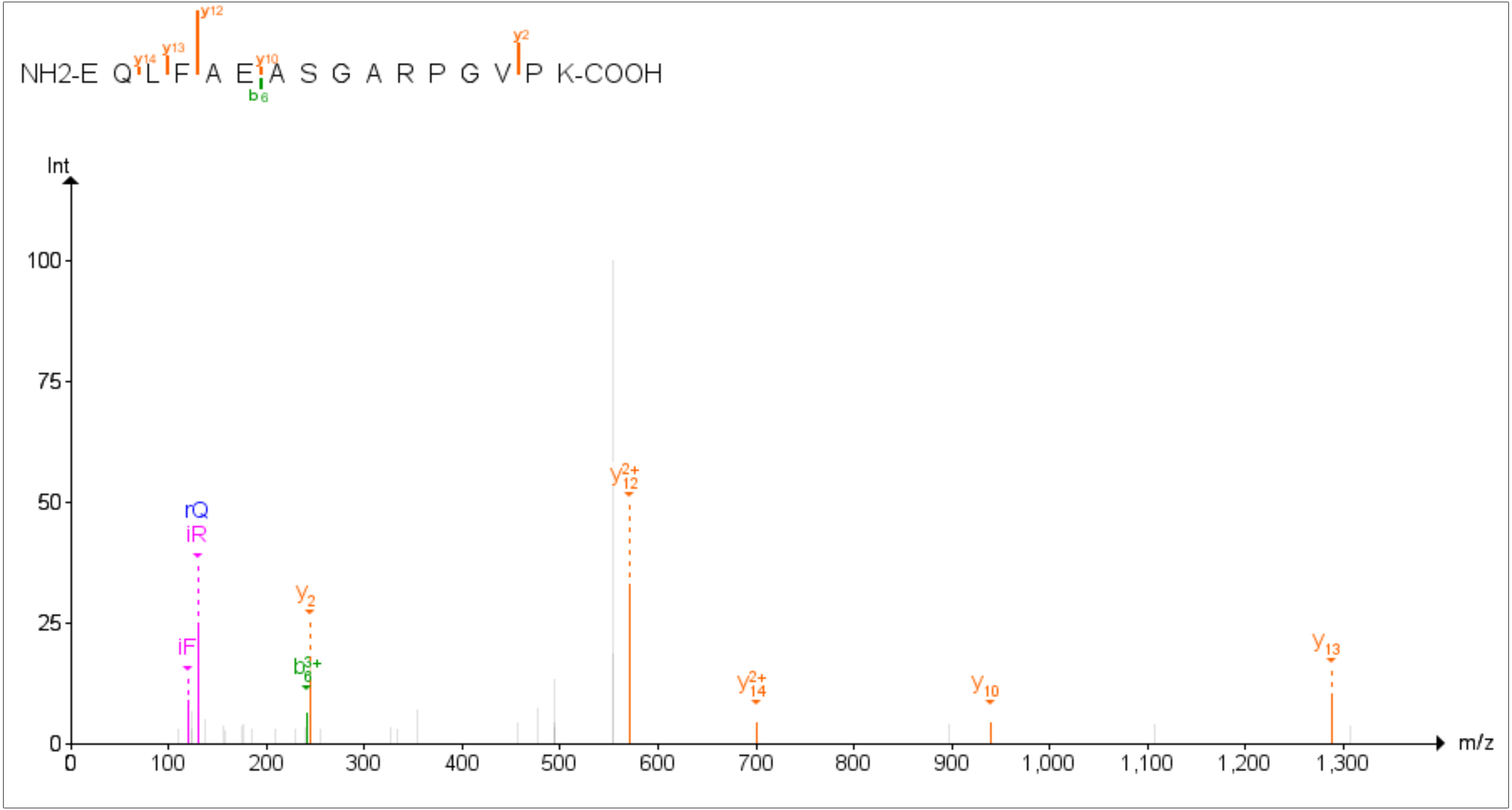

NH2-E Q L F A E A S G A R P G V P K-COOH
y14
y13
y12
y10
y2
b6
Int
100
75
50
25
0
rQ
iR
iF
y2
b6 3+
y12 2+
y14 2+
y10
y13
0
100
200
300
400
500
600
700
800
900
1,000
1,100
1,200
1,300
m/z

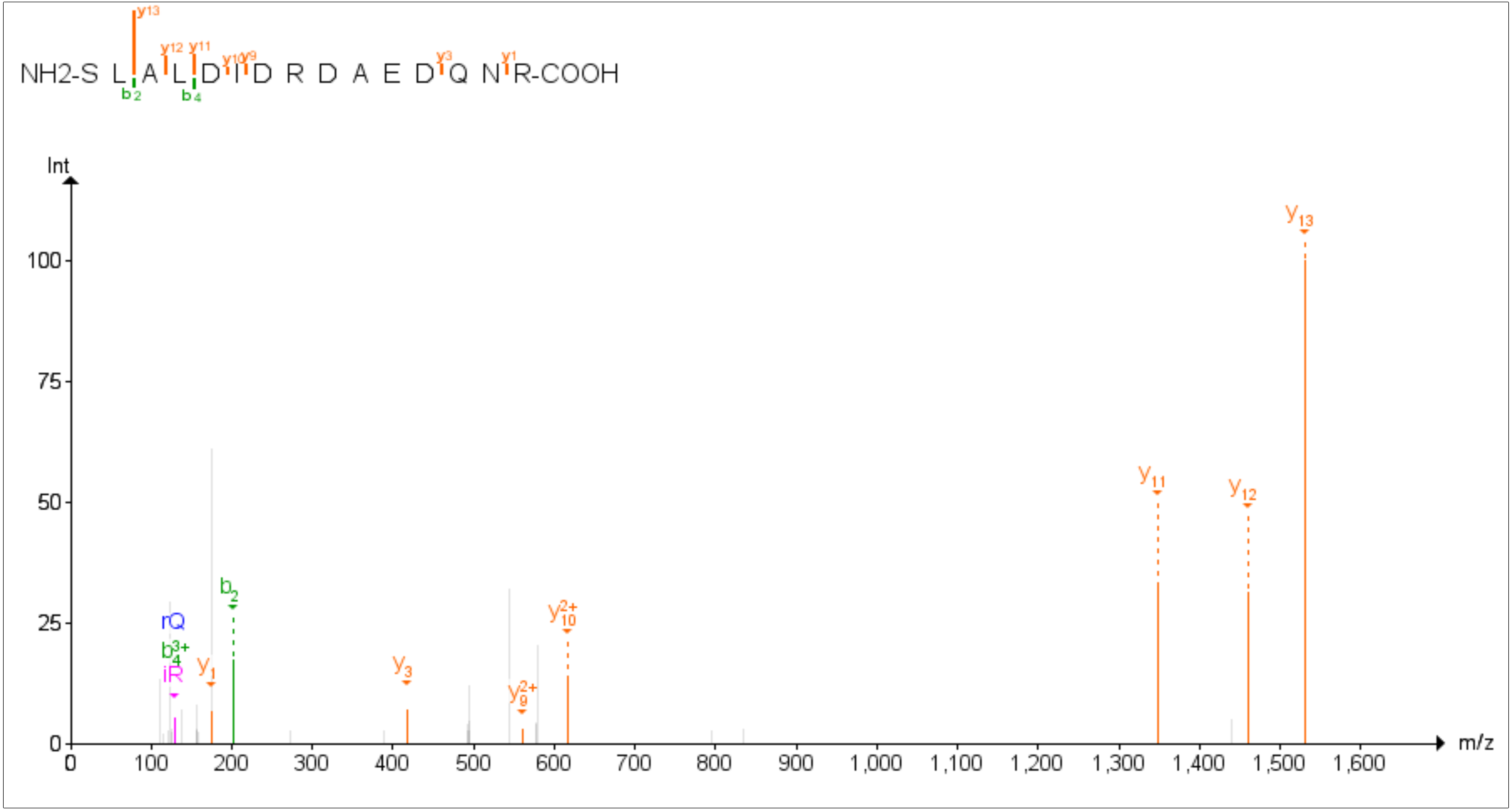
NH2-S L A L D I D R D A E D Q N R-COOH
y13
y12
y11
y10
y9
y3
y1
b2
b4
Int
100
75
50
25
0
m/z
0
100
200
300
400
500
600
700
800
900
1,000
1,100
1,200
1,300
1,400
1,500
1,600
y13
y11
y12
b2
rQ
b4 3+
iR
y1
y3
y9 2+
y10 2+

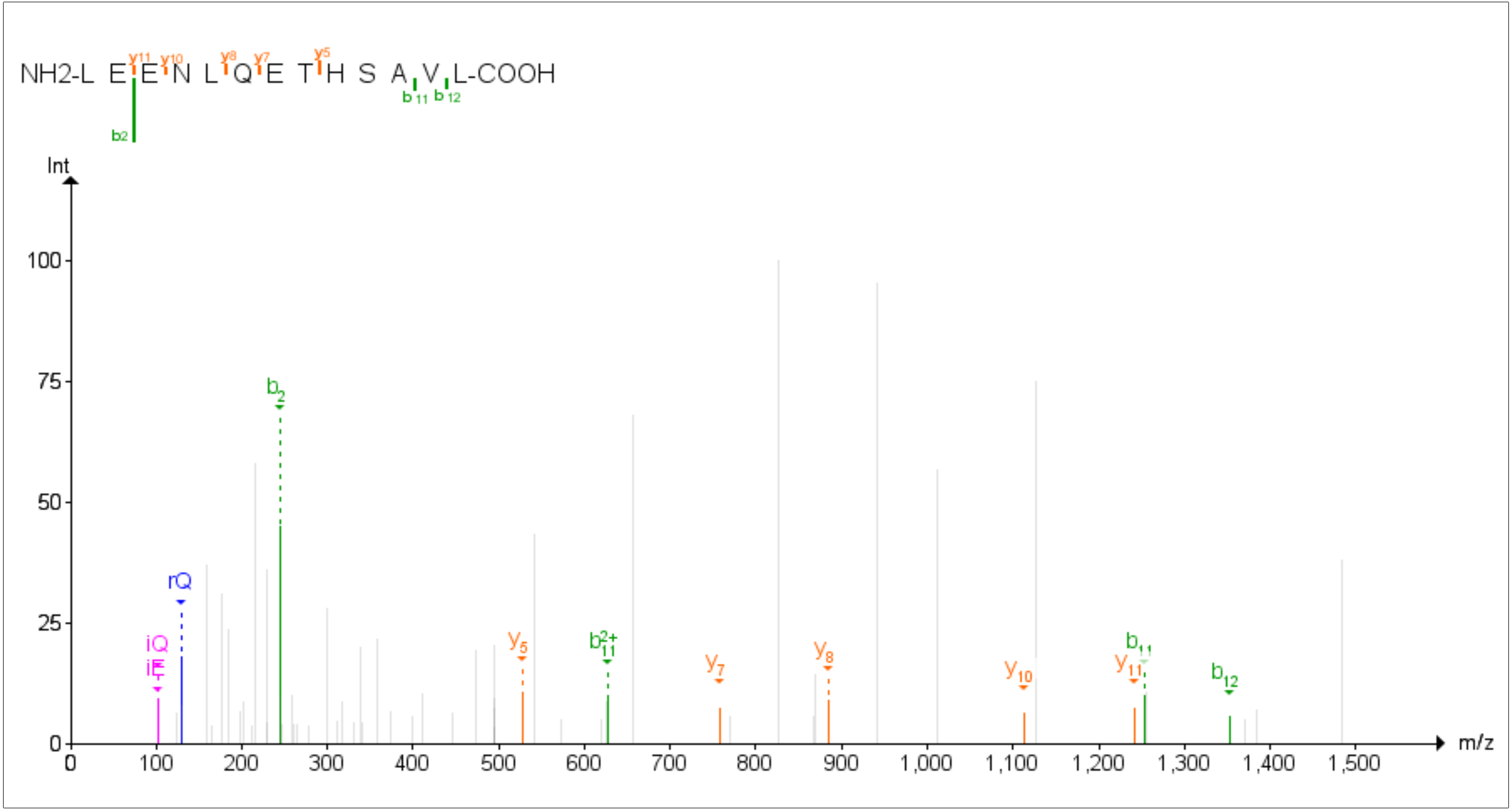

NH2-L E E N L Q E T H S A V L-COOH
y11 y10 y8 y7 y5
b2 b 11 b 12
Int
m/z
0
25
50
75
100
0 100 200 300 400 500 600 700 800 900 1,000 1,100 1,200 1,300 1,400 1,500
iQ
iE
rQ
b2
y5
b2+ 11
y7
y8
y10
y11
b11
b12

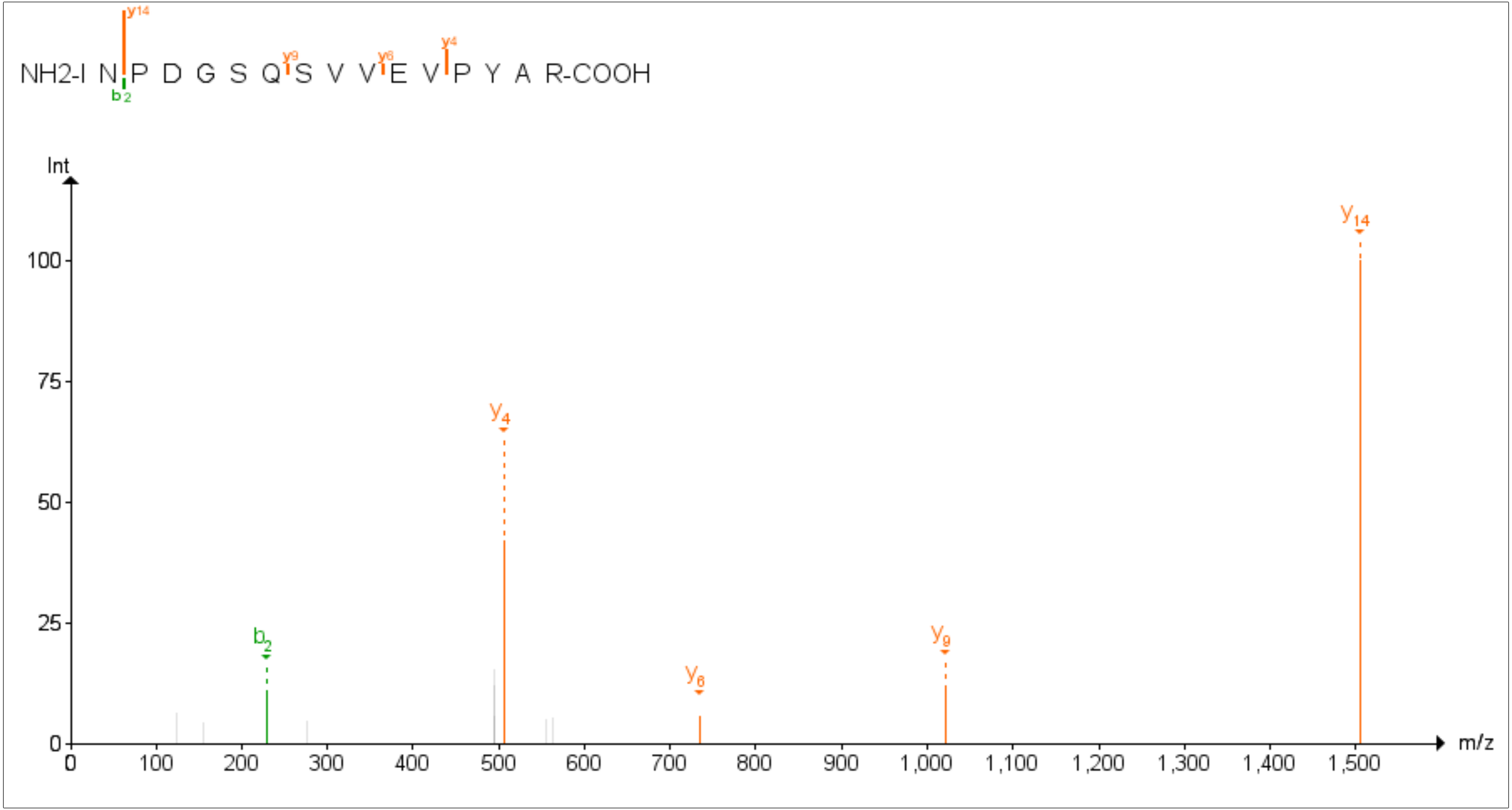

NH2-I N P D G S Q S V V E V P Y A R-COOH
y14
b2
y9
y6
y4
Int
100
75
50
25
0
m/z
0
100
200
300
400
500
600
700
800
900
1,000
1,100
1,200
1,300
1,400
1,500
y14
y4
b2
y6
y9

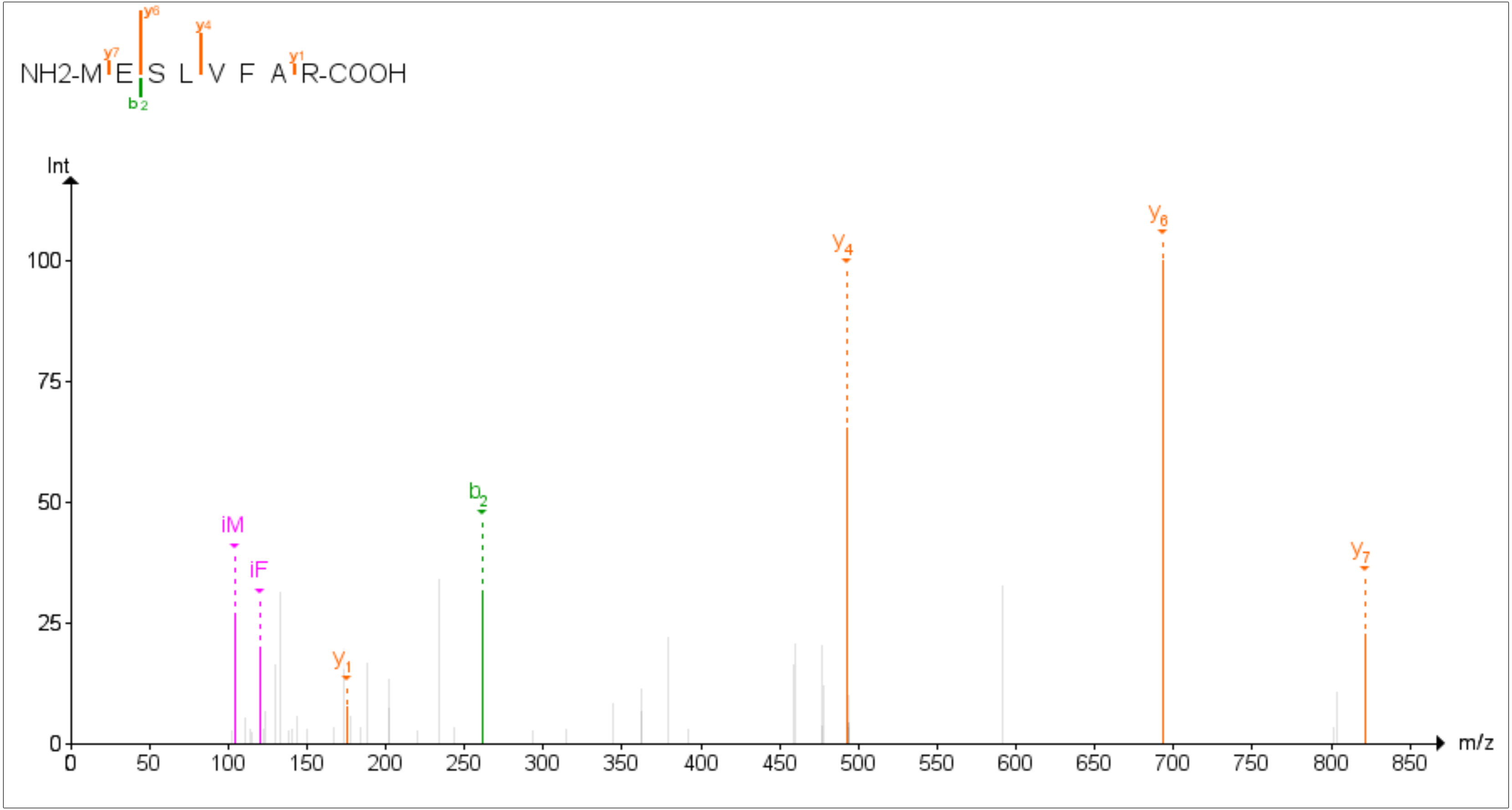

NH2-M E S L V F A R-COOH
y7
y6
y4
y1
b2
Int
100
75
50
25
0
iM
iF
y1
b2
y4
y6
y7
0
50
100
150
200
250
300
350
400
450
500
550
600
650
700
750
800
850
m/z

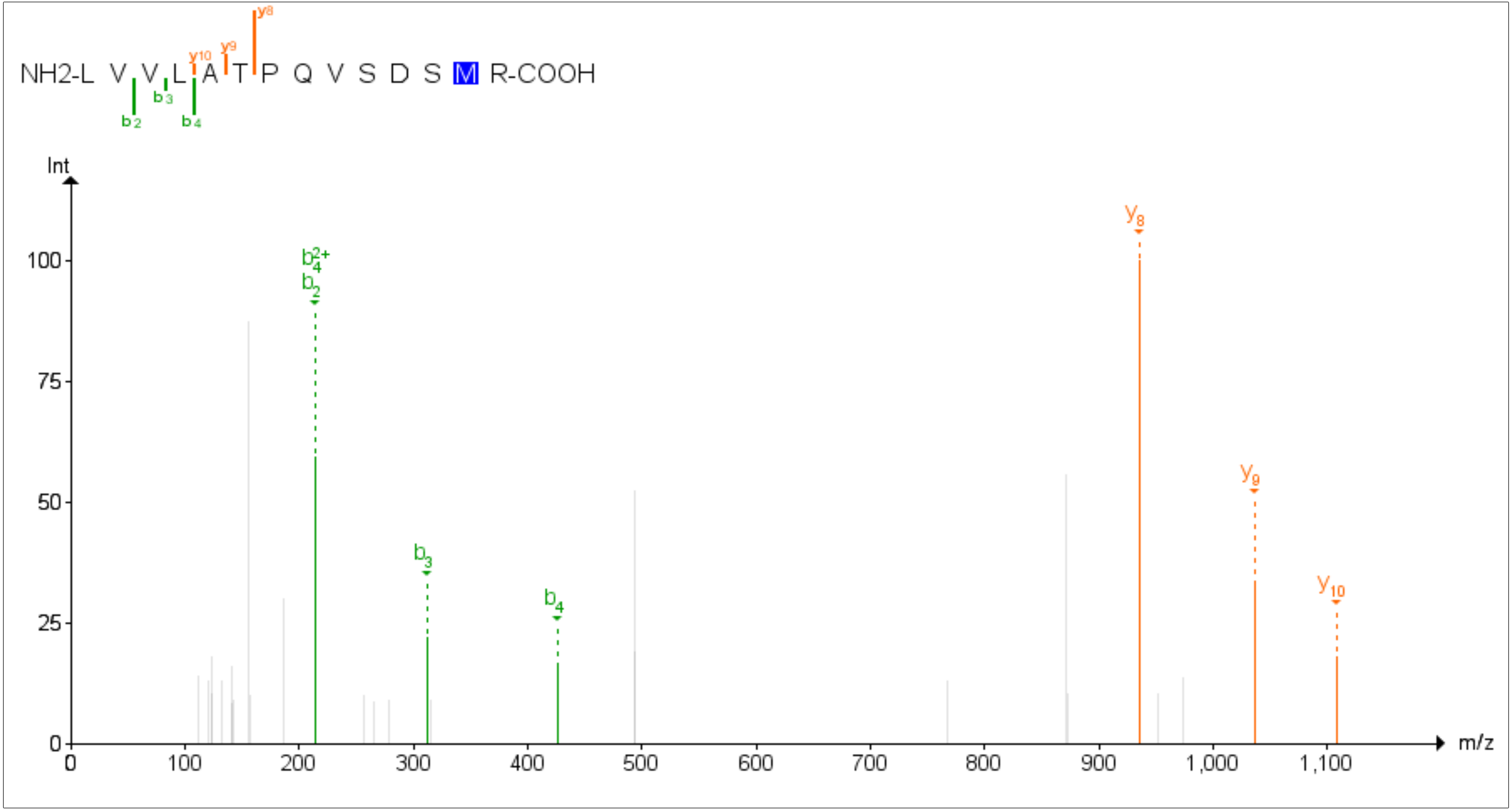

NH2-L V V L A T P Q V S D S M R-COOH
y8
y9
y10
b2
b3
b4
Int
100
75
50
25
0
b4 2+
b2
b3
b4
y8
y9
y10
0
100
200
300
400
500
600
700
800
900
1,000
1,100
m/z

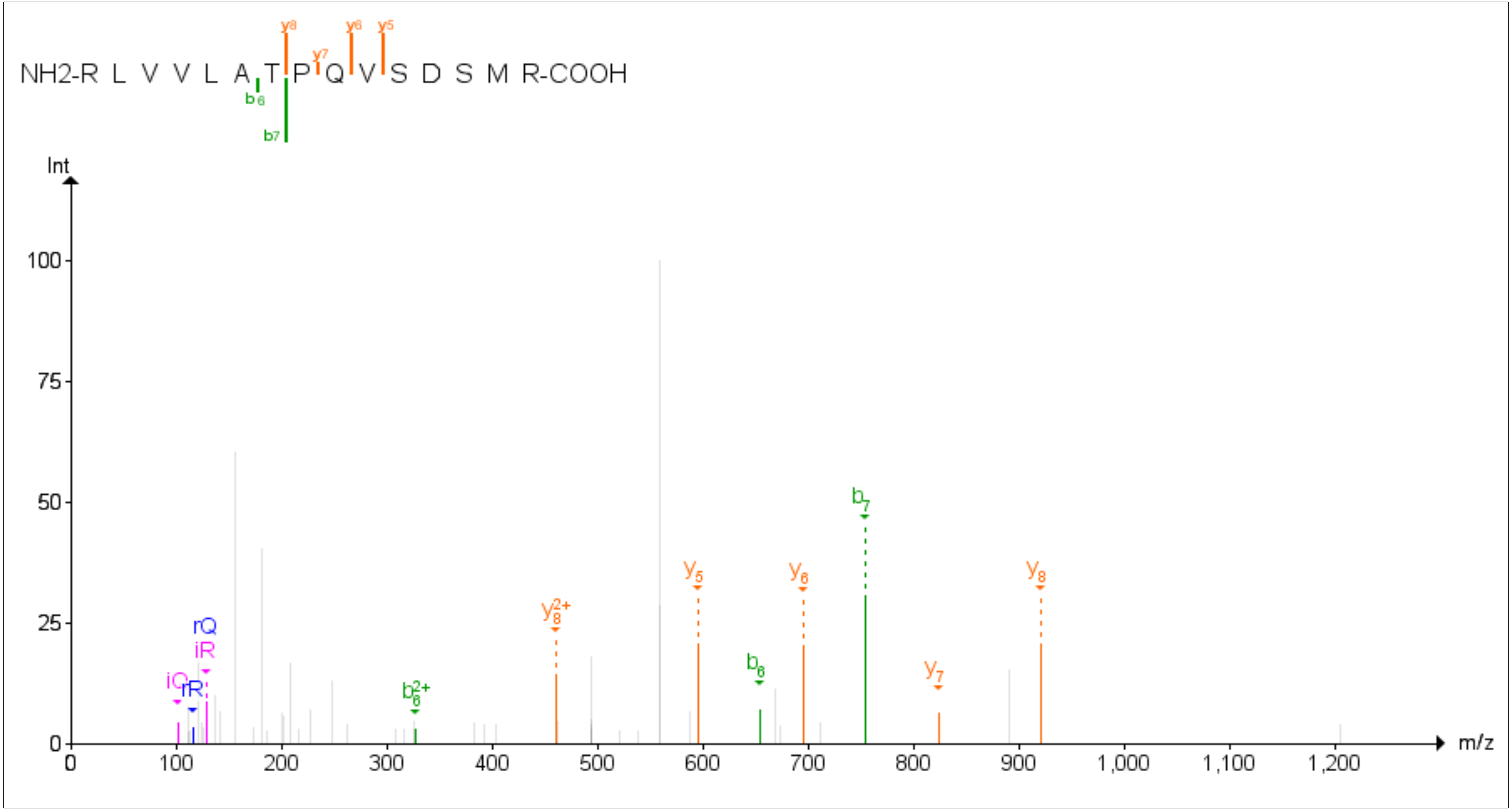

NH2-R L V V L A T P Q V S D S M R-COOH
y8
y7
y6
y5
b6
b7
Int
100
75
50
25
0
m/z
0
100
200
300
400
500
600
700
800
900
1,000
1,100
1,200
rQ
iR
iQ
rR
b6 2+
y8 2+
y5
b6
y6
b7
y7
y8

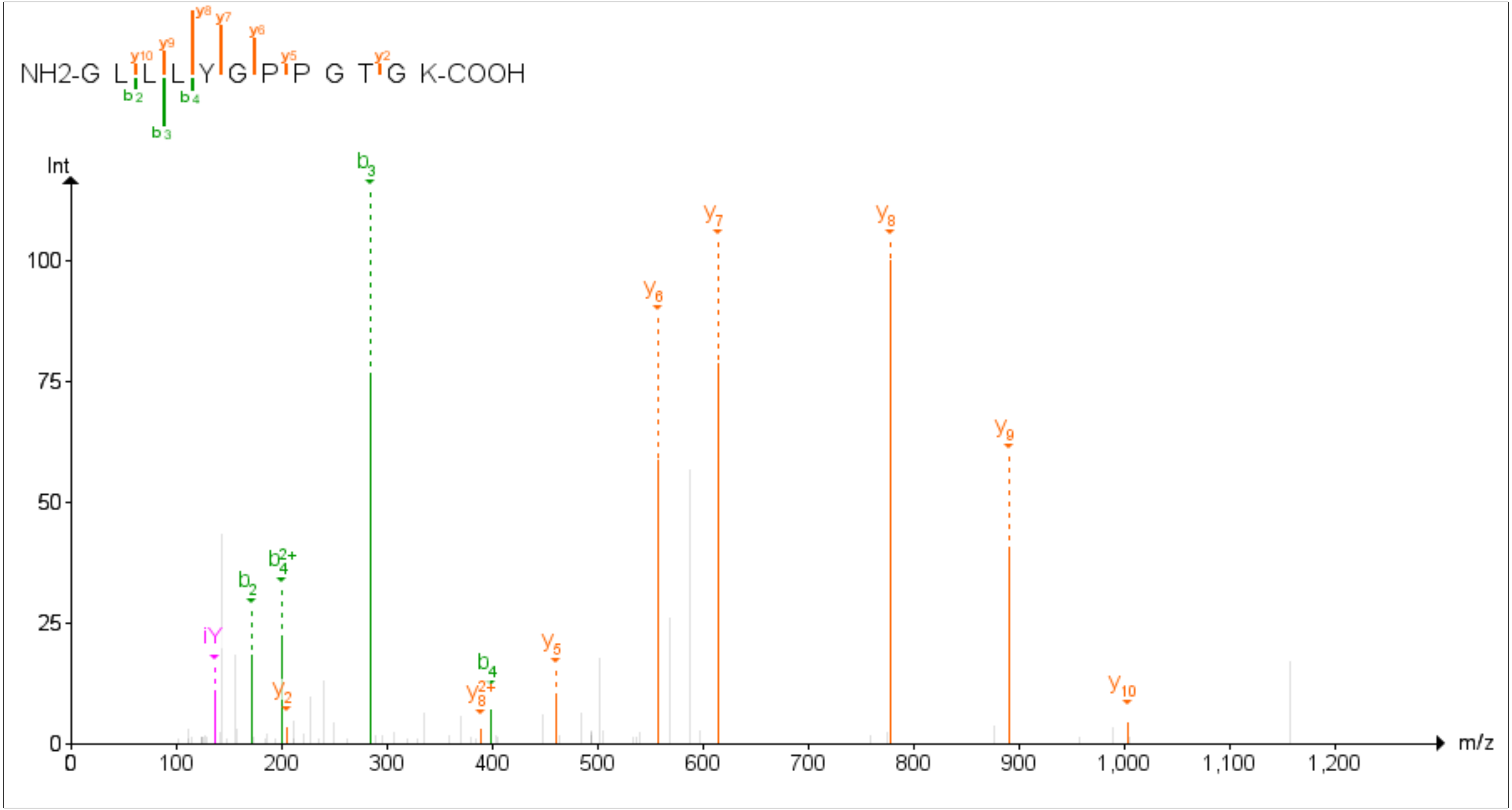
NH2-G L L L Y G P P G T G K-COOH
y10 y9 y8 y7 y6 y5 y2
b2 b3 b4
Int
m/z
b3
y7
y8
y6
y9
b2
b_4^{2+}
iY
y2
y_8^{2+}
b4
y5
y10
0
25
50
75
100
0
100
200
300
400
500
600
700
800
900
1,000
1,100
1,200

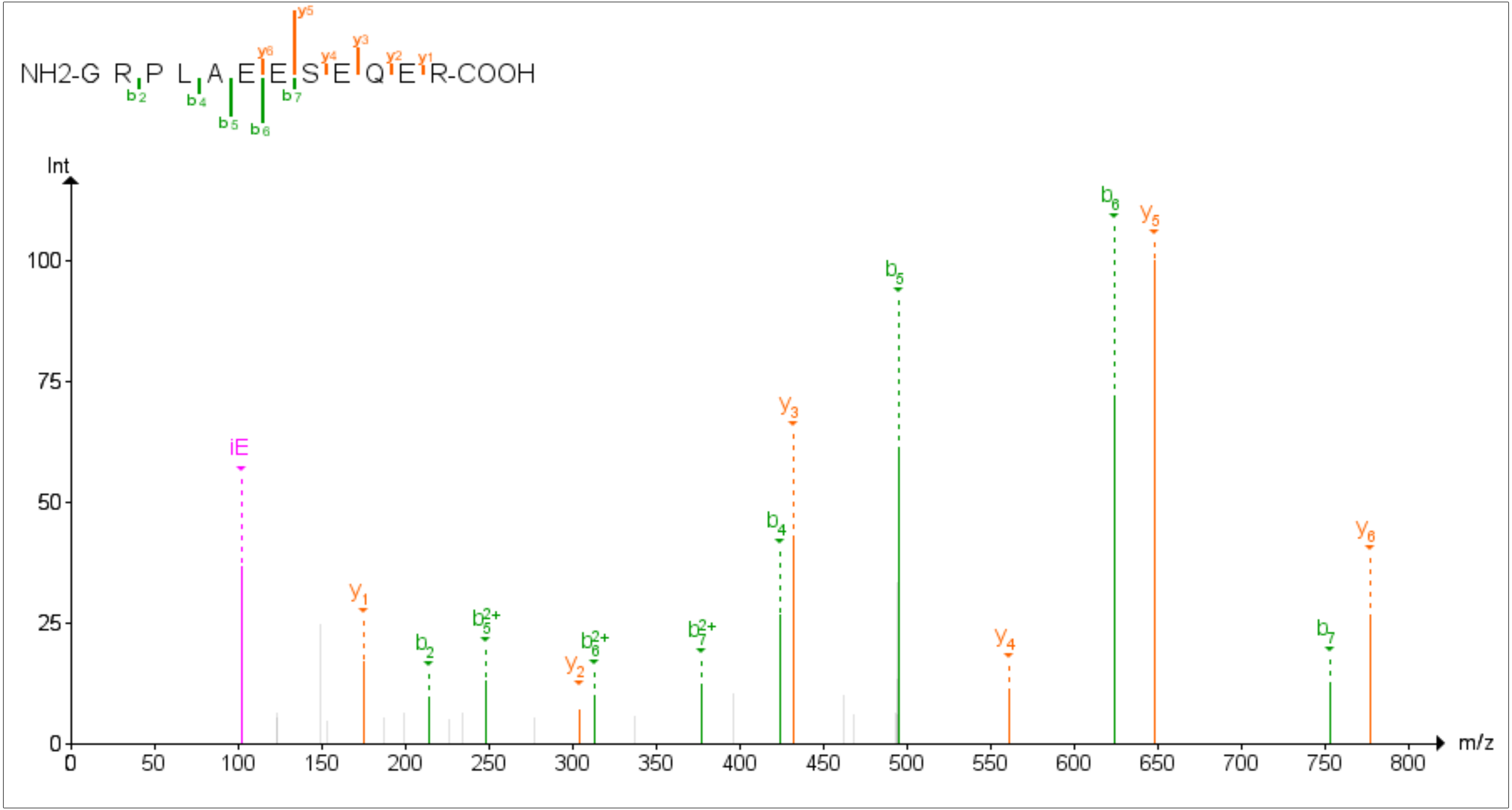

NH2-G R P L A E E S E Q E R-COOH
Int
m/z

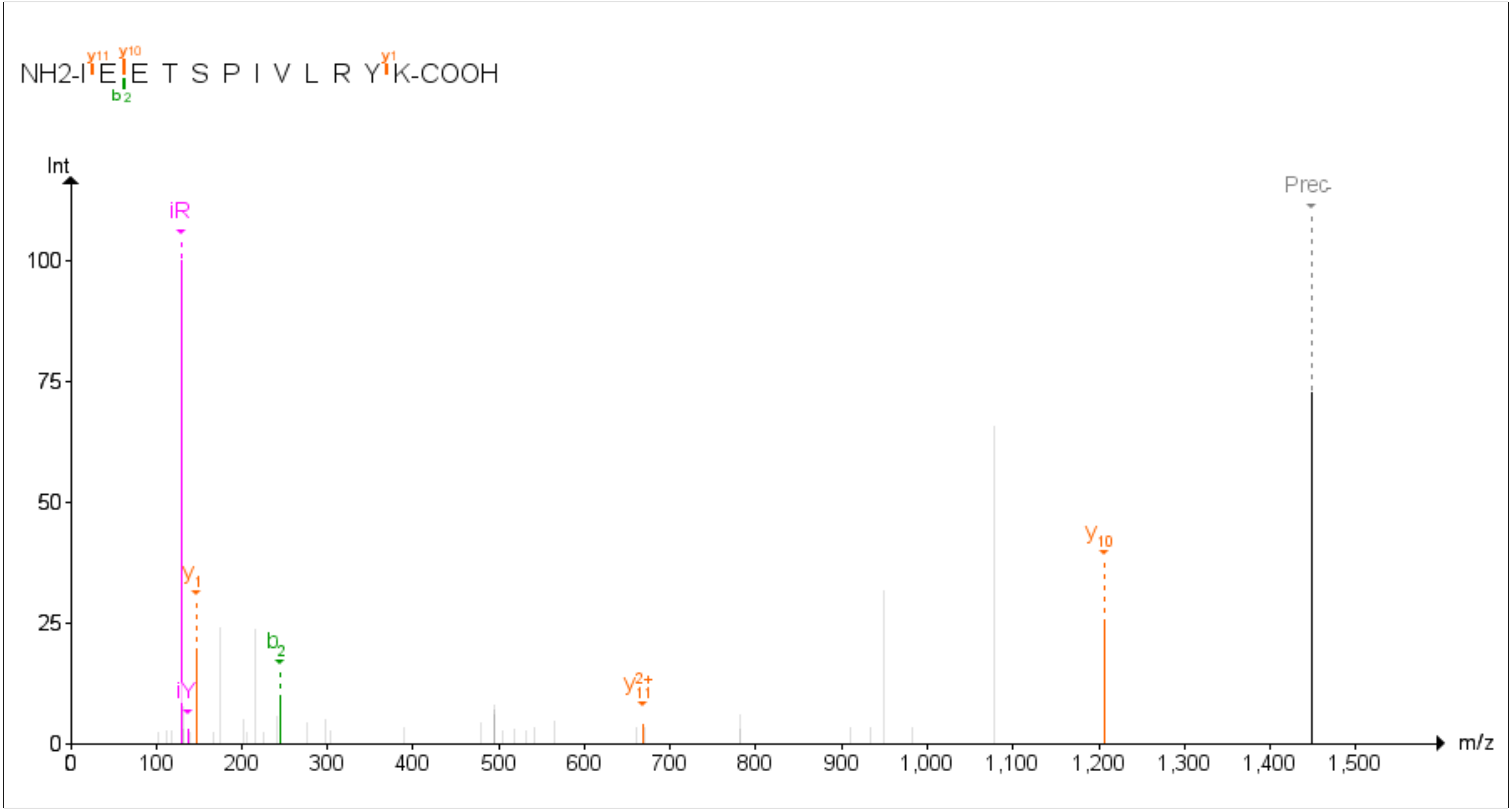

NH2-I E E T S P I V L R Y K-COOH
y11
y10
b2
y1
Int
m/z
iR
iY
y1
b2
y11 2+
y10
Prec-
0
25
50
75
100
0
100
200
300
400
500
600
700
800
900
1,000
1,100
1,200
1,300
1,400
1,500

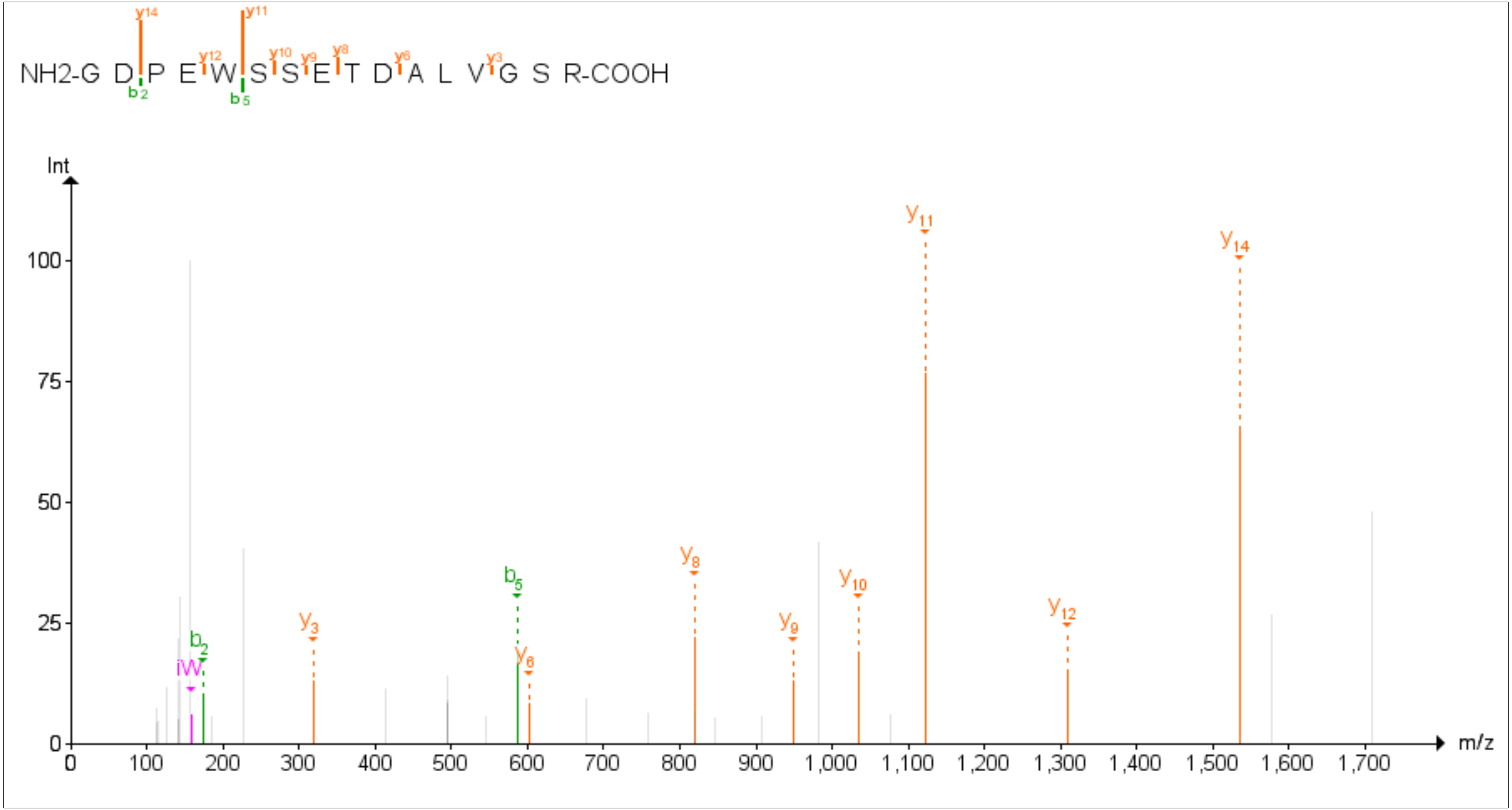

NH2-G D P E W S S E T D A L V G S R-COOH
y14
y11
y12
y10
y9
y8
y6
y3
b2
b5
Int
100
75
50
25
0
y11
y14
y8
b5
y10
y9
y12
y3
b2
iW
y6
m/z
0
100
200
300
400
500
600
700
800
900
1,000
1,100
1,200
1,300
1,400
1,500
1,600
1,700

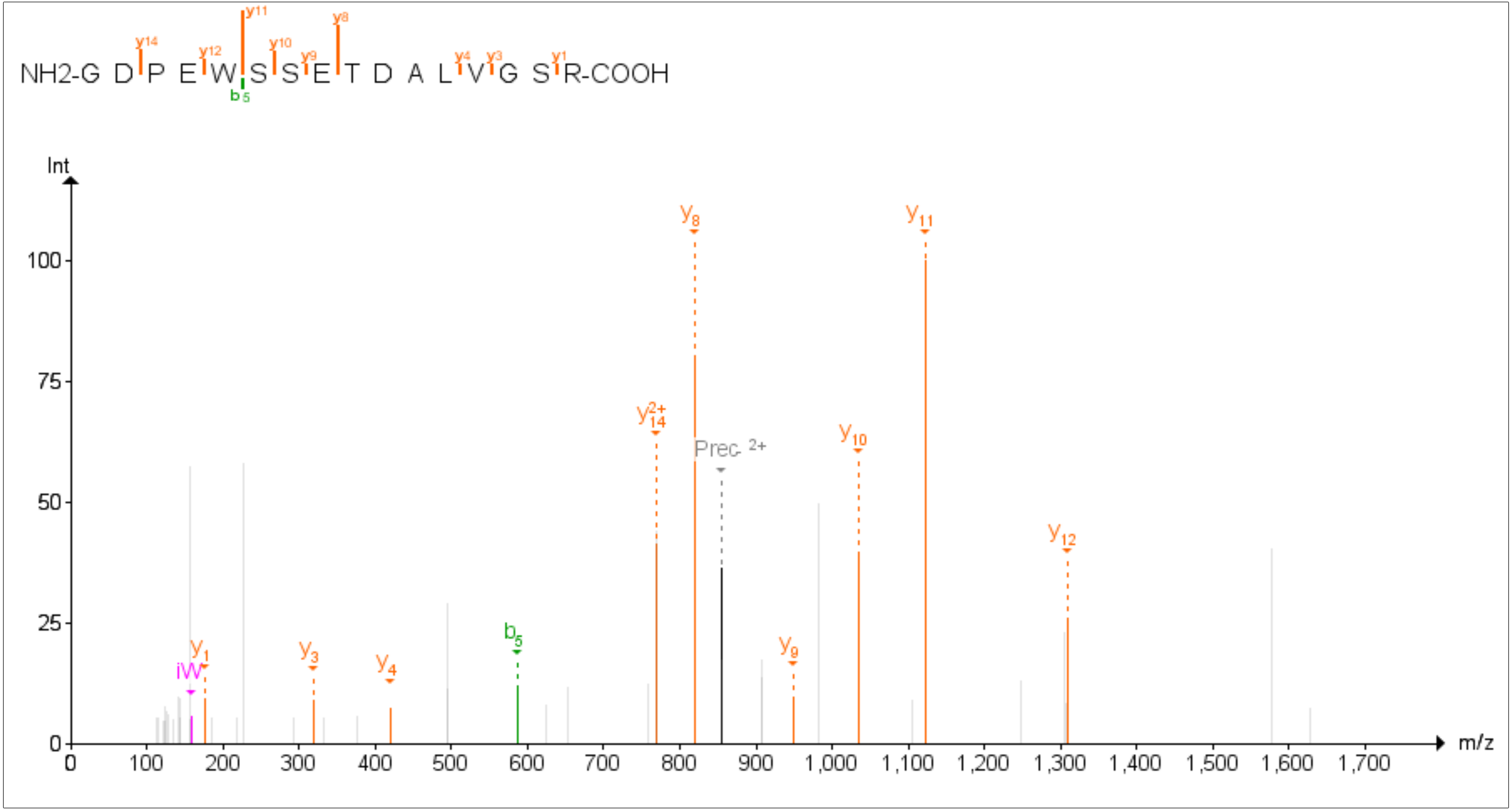

NH2-G D P E W S S E T D A L V G S R-COOH
y14 y12 y11 y10 y9 y8 y4 y3 y1
b5
Int
m/z
y8
y11
y14 2+
Prec 2+
y10
y12
y1
iW
y3
y4
b5
y9
0
25
50
75
100
0
100
200
300
400
500
600
700
800
900
1,000
1,100
1,200
1,300
1,400
1,500
1,600
1,700

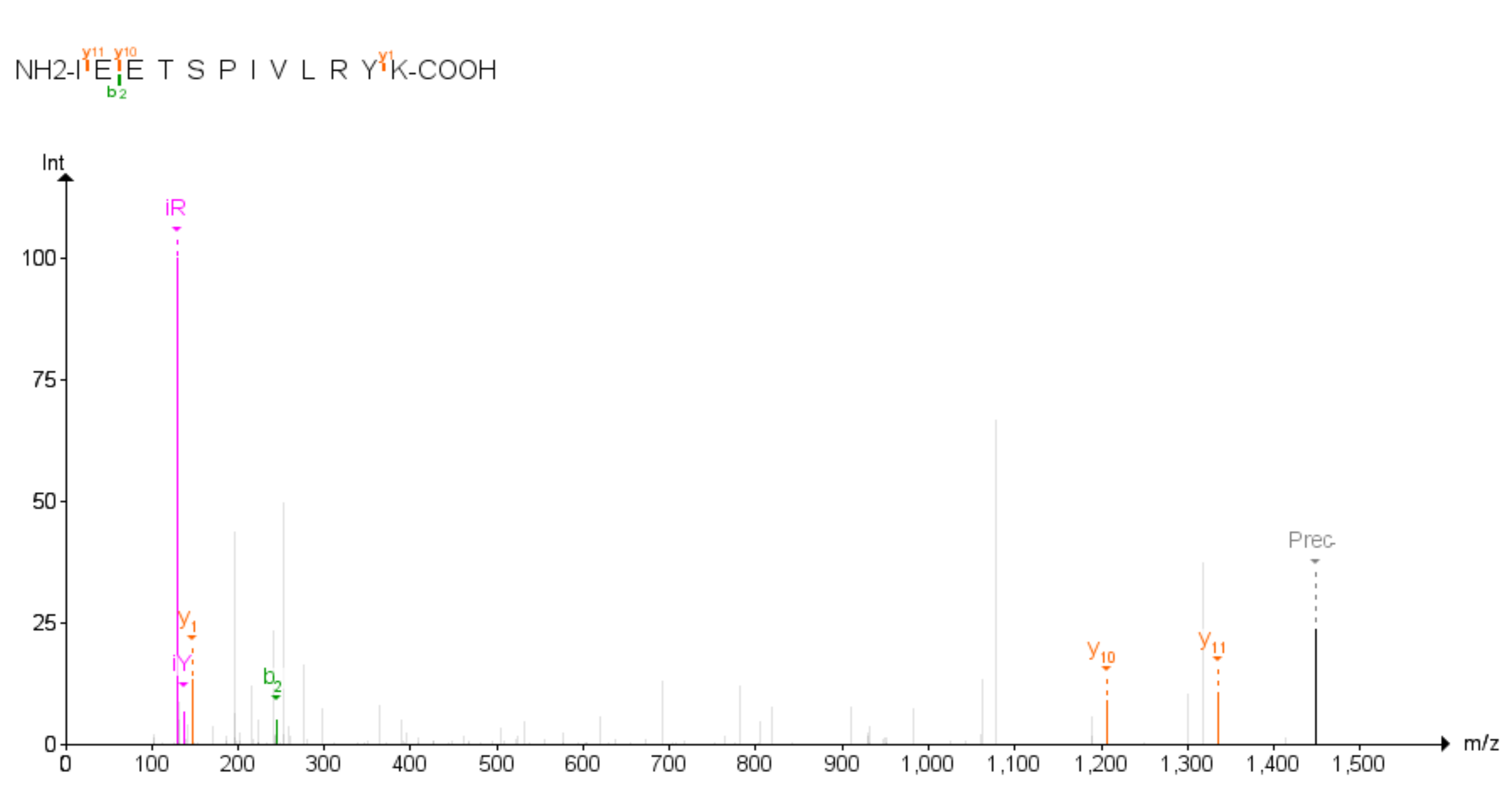

NH2-I E E T S P I V L R Y K-COOH
y11
y10
y1
b2
Int
iR
iY
y1
b2
y10
y11
Prec
m/z
0
25
50
75
100
0
100
200
300
400
500
600
700
800
900
1,000
1,100
1,200
1,300
1,400
1,500

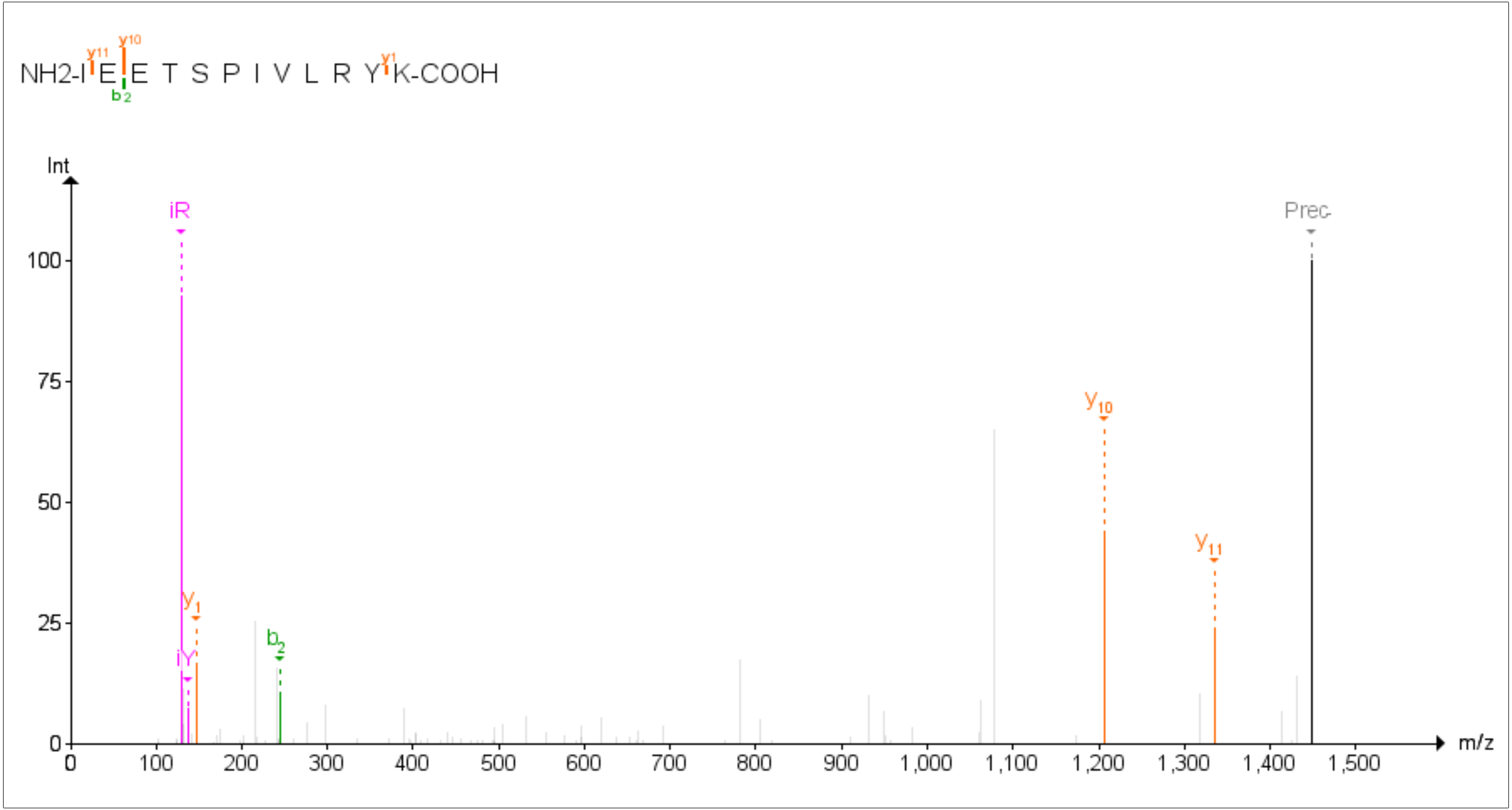

NH2-I E E T S P I V L R Y K-COOH
y11
y10
y1
b2
Int
iR
Prec
y10
y11
y1
iY
b2
100
75
50
25
0
0
100
200
300
400
500
600
700
800
900
1,000
1,100
1,200
1,300
1,400
1,500
m/z

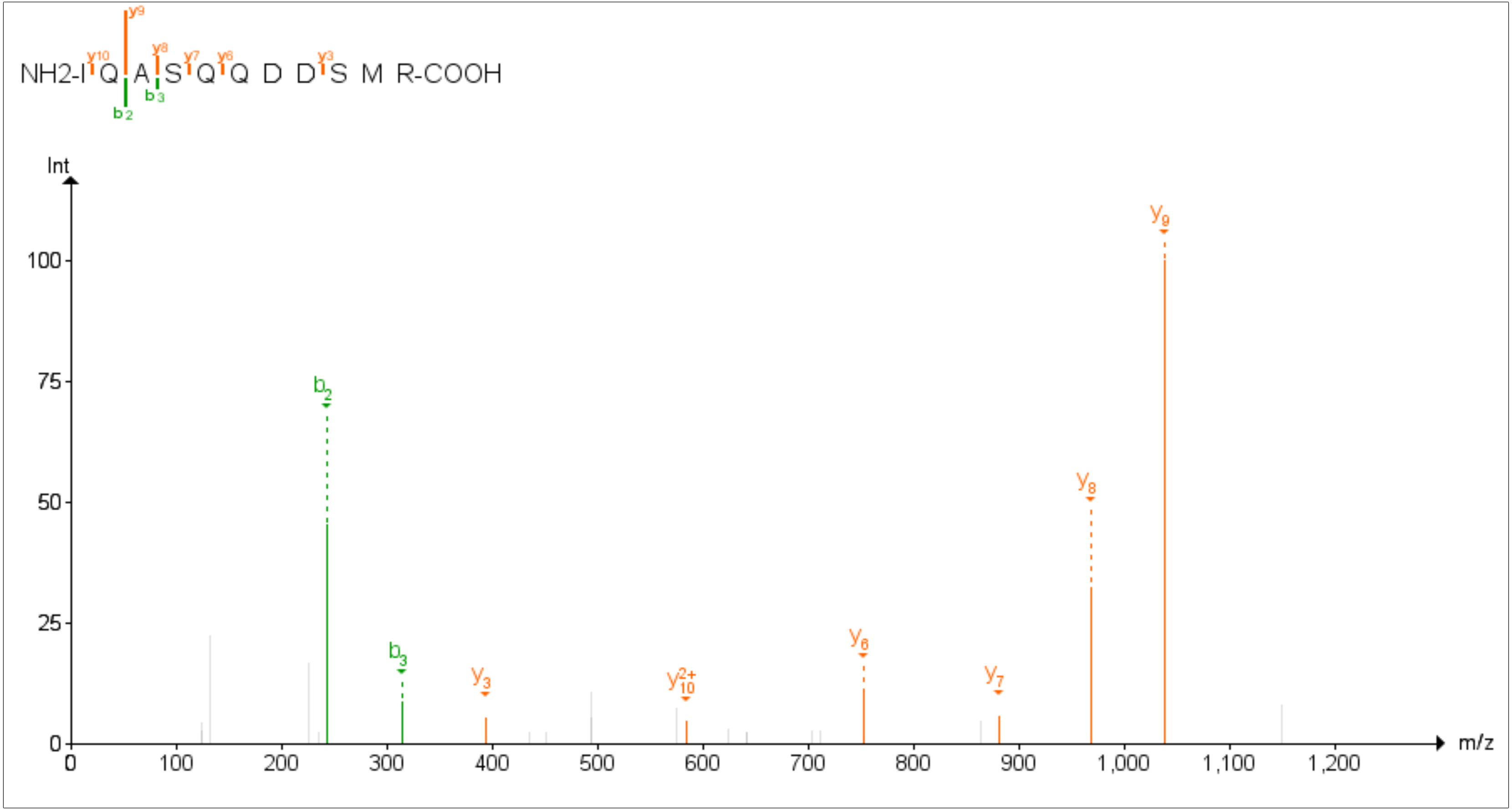

NH2-I Q A S Q Q D D S M R-COOH
y10 y9 y8 y7 y6 y3
b2 b3
Int
100
75
50
25
0
m/z
0 100 200 300 400 500 600 700 800 900 1,000 1,100 1,200
b2
b3
y3
y10 2+
y6
y7
y8
y9

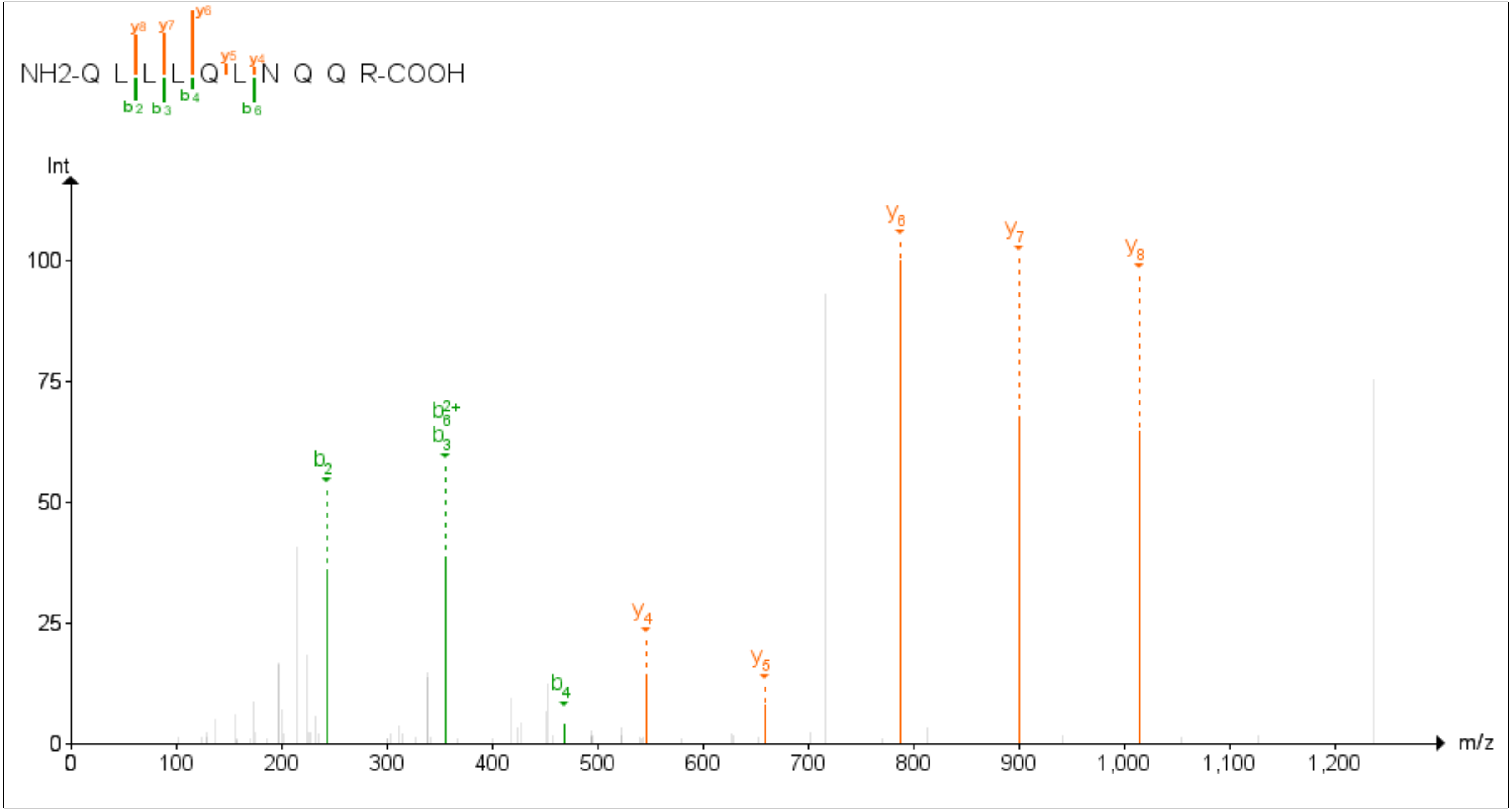

NH2-Q L L L Q L N Q Q R-COOH
y8 y7 y6 y5 y4
b2 b3 b4 b6
Int
100
75
50
25
0
b2
b6 2+
b3
b4
y4
y5
y6
y7
y8
0 100 200 300 400 500 600 700 800 900 1,000 1,100 1,200
m/z

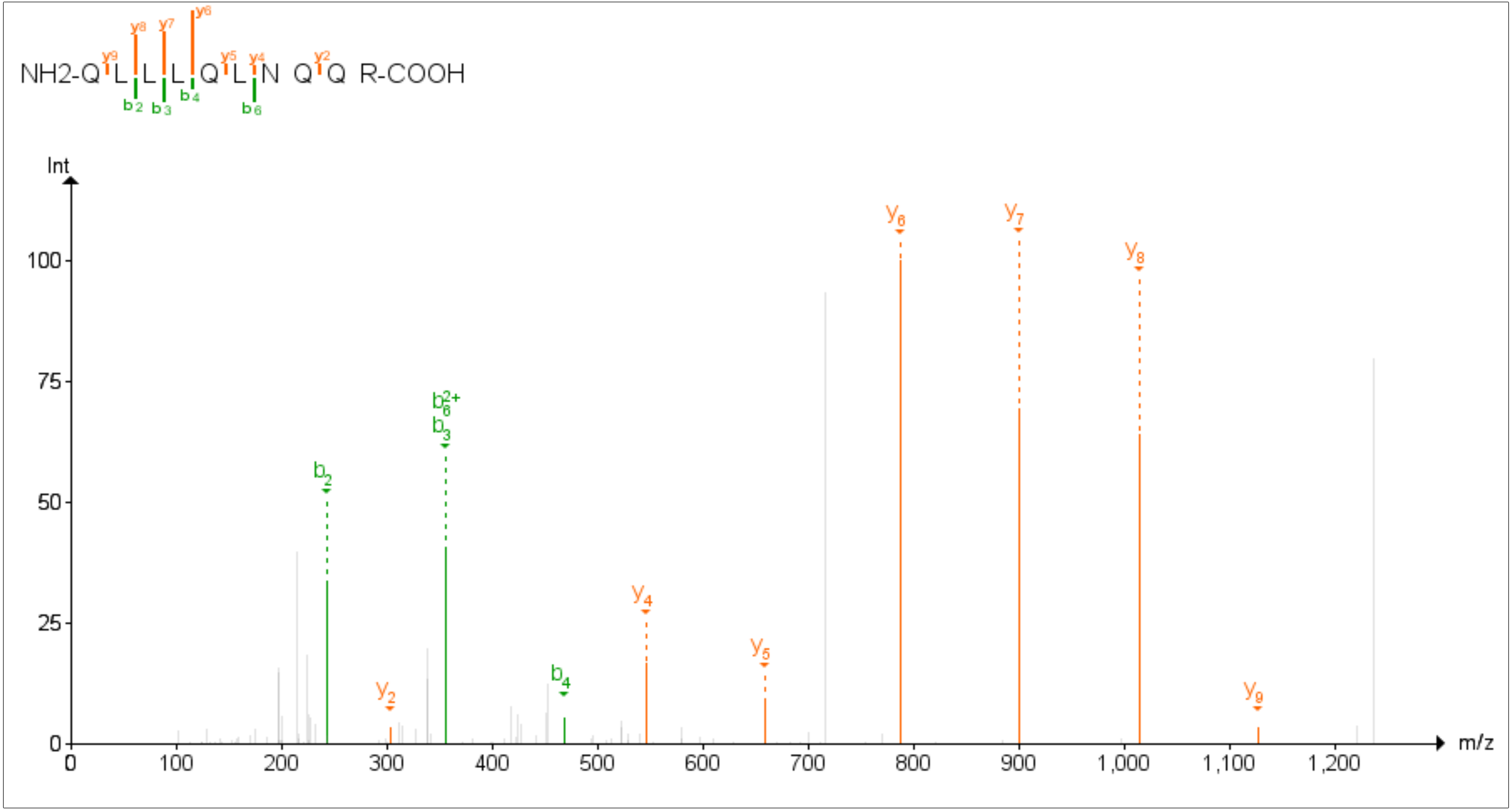
NH2-Q L L L Q L N Q Q R-COOH
y9 y8 y7 y6 y5 y4 y2
b2 b3 b4 b6
Int
m/z
0
25
50
75
100
200
300
400
500
600
700
800
900
1,000
1,100
1,200
b2
b3
b6 2+
b4
y2
y4
y5
y6
y7
y8
y9

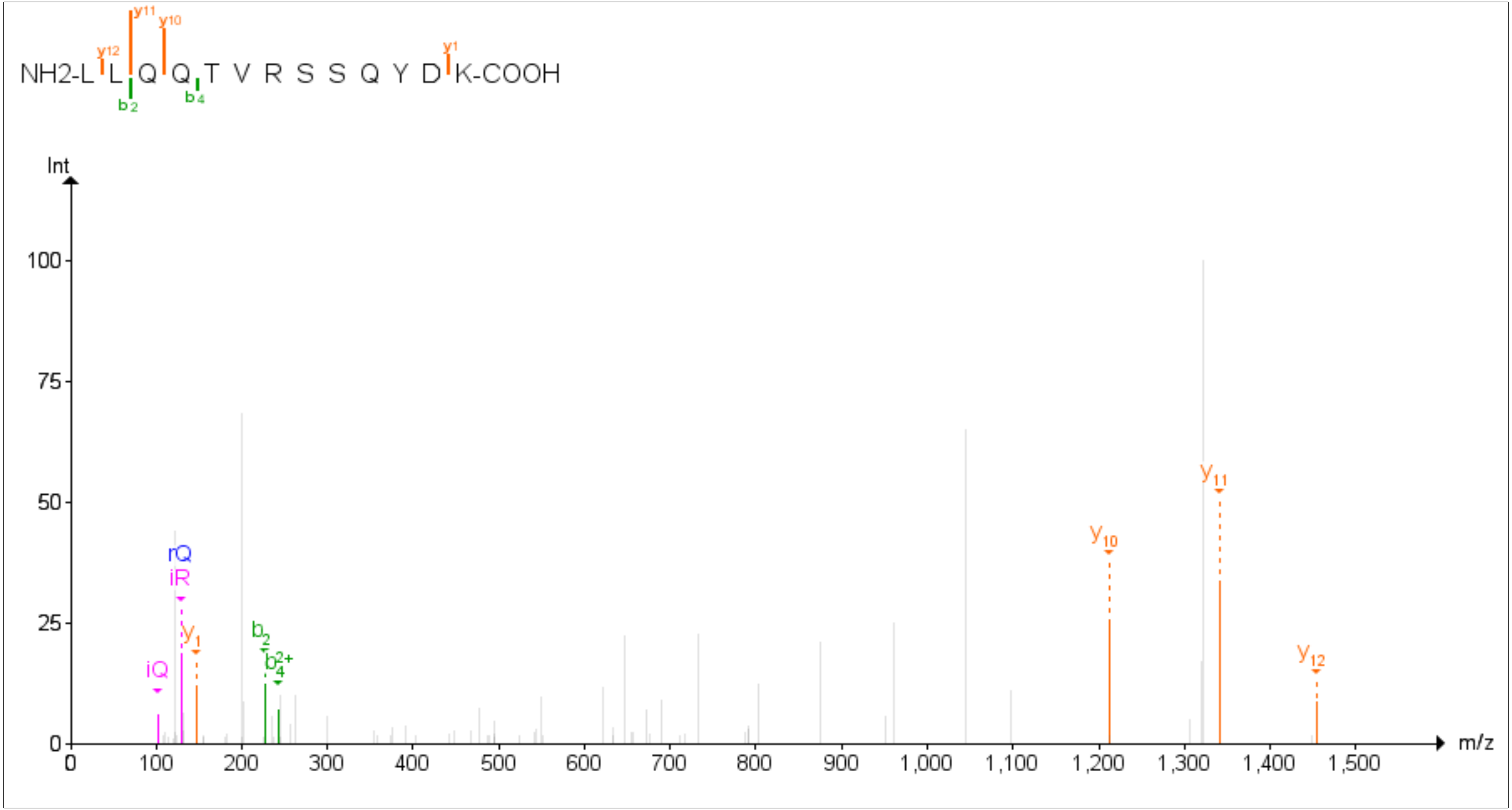

NH2-L L Q Q T V R S S Q Y D K-COOH
y12
y11
y10
y1
b2
b4
Int
100
75
50
25
0
m/z
0
100
200
300
400
500
600
700
800
900
1,000
1,100
1,200
1,300
1,400
1,500
rQ
iR
iQ
y1
b2
b4 2+
y10
y11
y12

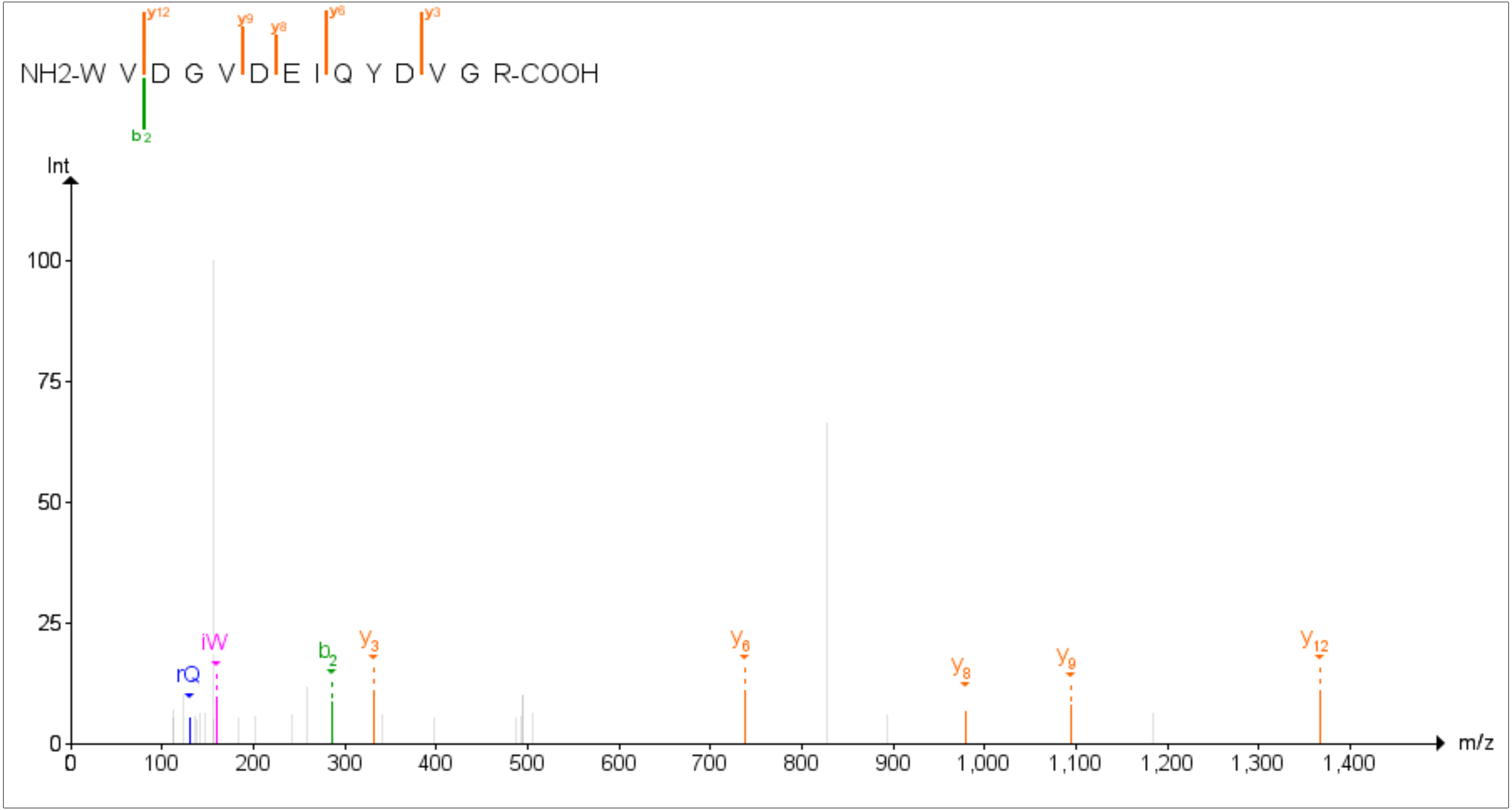

NH2-W V D G V D E I Q Y D V G R-COOH
y12
y9
y8
y6
y3
b2
Int
100
75
50
25
0
rQ
iW
b2
y3
y6
y8
y9
y12
0
100
200
300
400
500
600
700
800
900
1,000
1,100
1,200
1,300
1,400
m/z

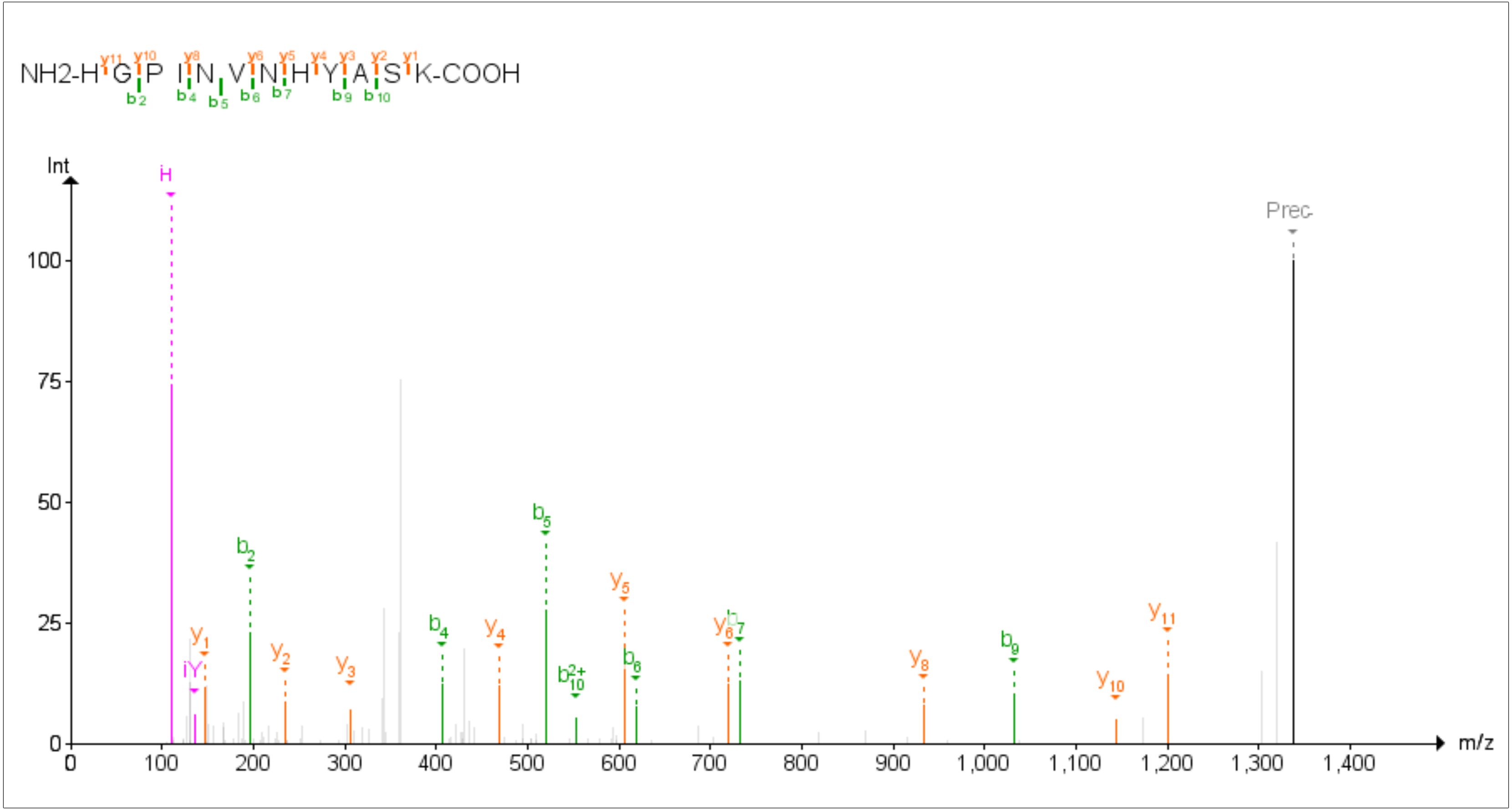

NH2-H G P I N V N H Y A S K-COOH
y11 y10 y8 y6 y5 y4 y3 y2 y1
b2 b4 b5 b6 b7 b9 b10
Int
iH
Prec
iY
y1
b2
y2
y3
b4
y4
b5
b10 2+
y5
b6
y6
b7
y8
b9
y10
y11
100
75
50
25
0
0
100
200
300
400
500
600
700
800
900
1,000
1,100
1,200
1,300
1,400
m/z

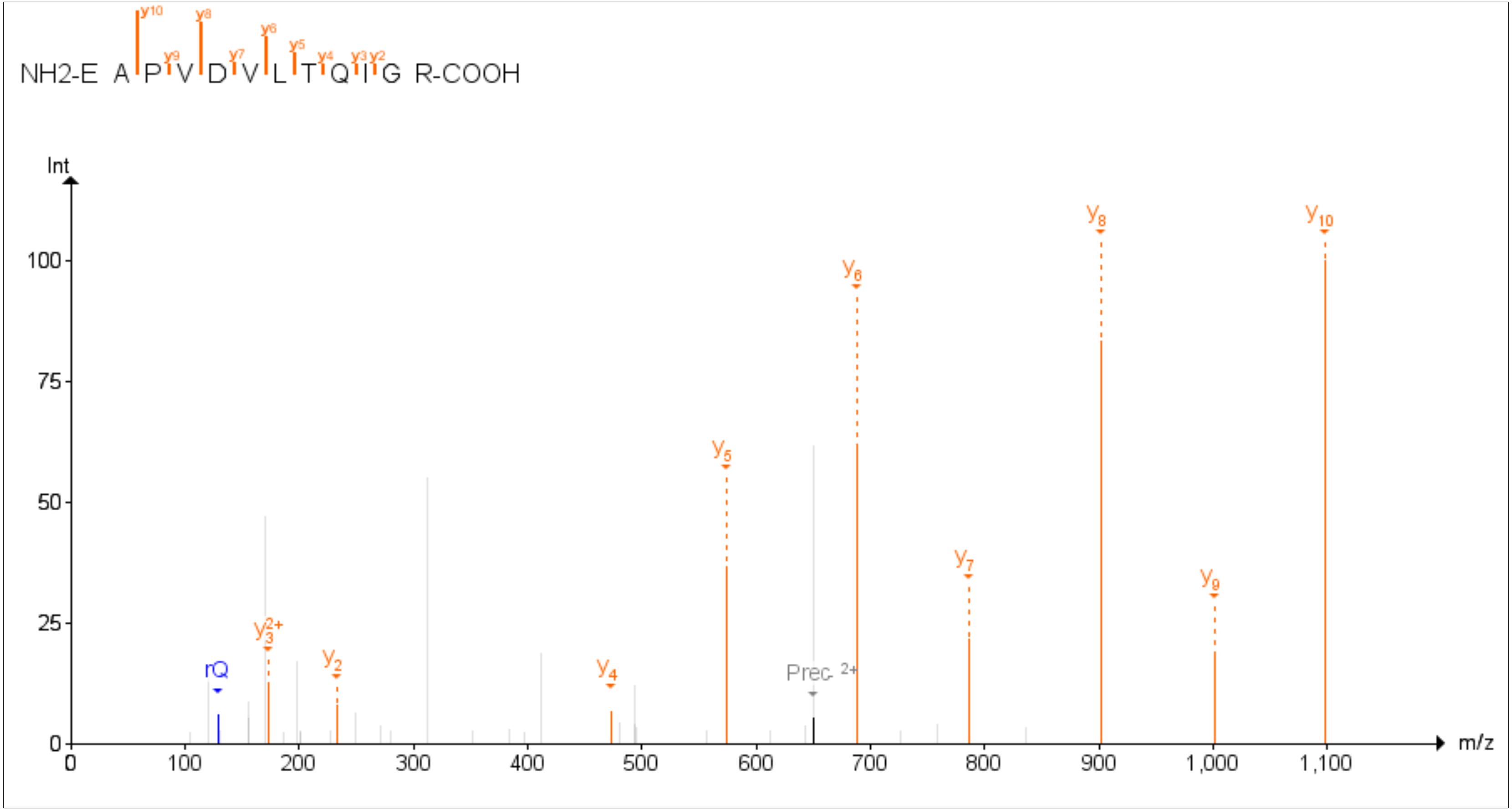

NH2-E A P V D V L T Q I G R-COOH
y10 y9 y8 y7 y6 y5 y4 y3 y2
Int
100
75
50
25
0
m/z
0 100 200 300 400 500 600 700 800 900 1,000 1,100
rQ
y_3^{2+}
y_2
y_4
y_5
y_6
Prec- 2+
y_7
y_8
y_9
y_{10}

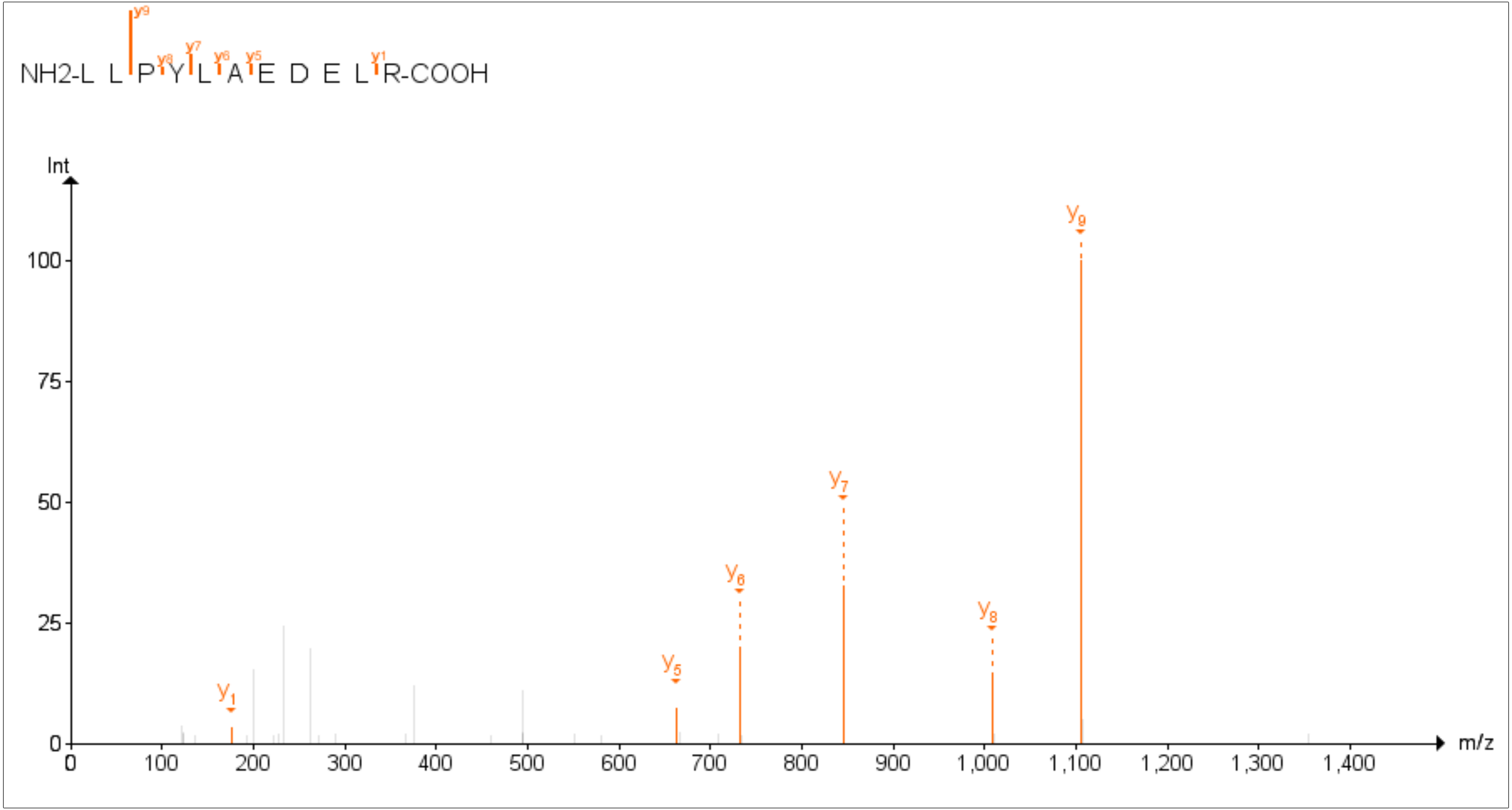

NH2-L L P Y L A E D E L R-COOH
y9
y8
y7
y6
y5
y1
Int
100
75
50
25
0
m/z
0
100
200
300
400
500
600
700
800
900
1,000
1,100
1,200
1,300
1,400
y1
y5
y6
y7
y8
y9

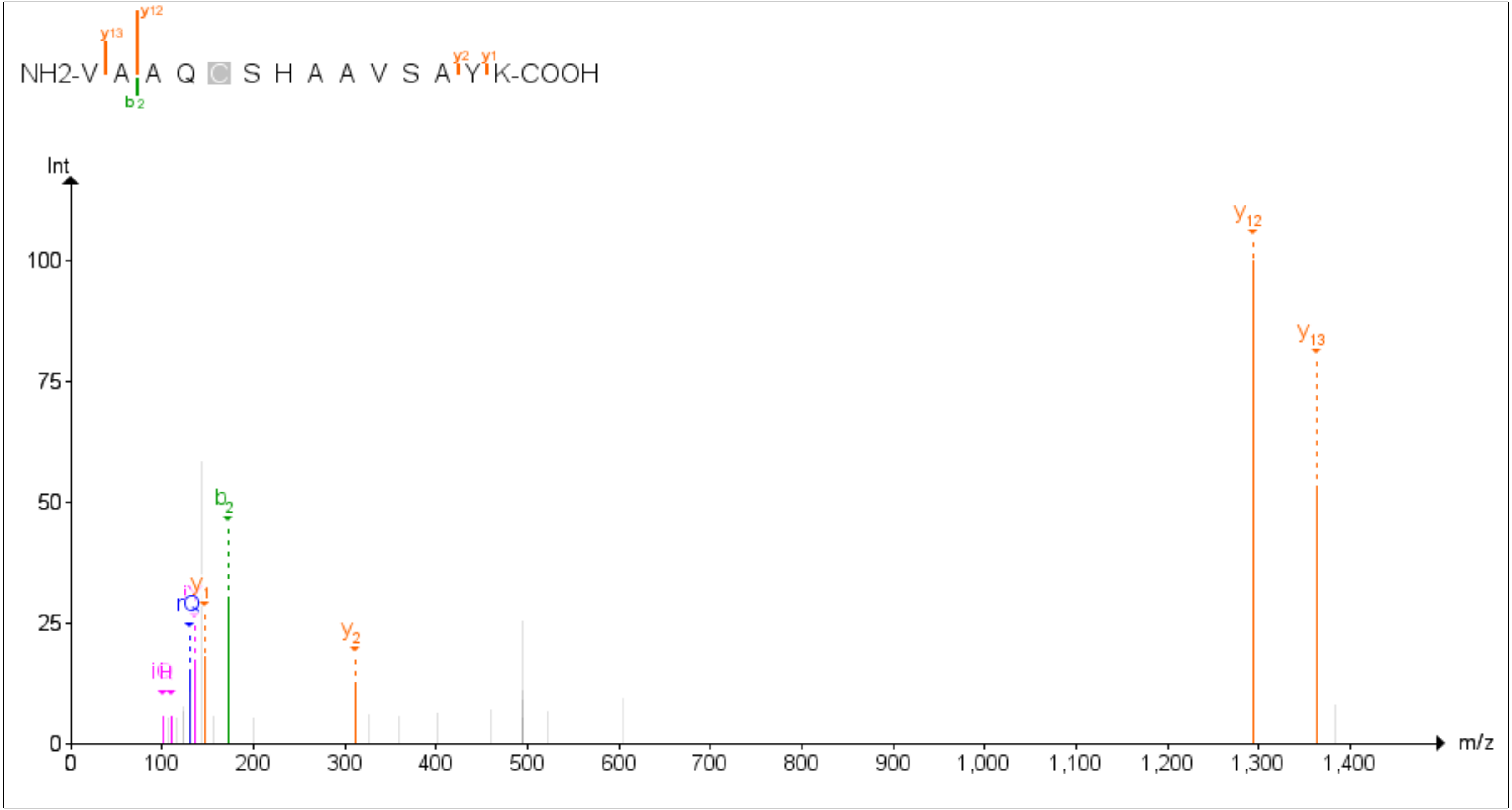

NH2-V A A Q C S H A A V S A Y K-COOH
y13
y12
b2
y2
y1
Int
100
75
50
25
0
y12
y13
b2
y1
rQ
iH
y2
0
100
200
300
400
500
600
700
800
900
1,000
1,100
1,200
1,300
1,400
m/z

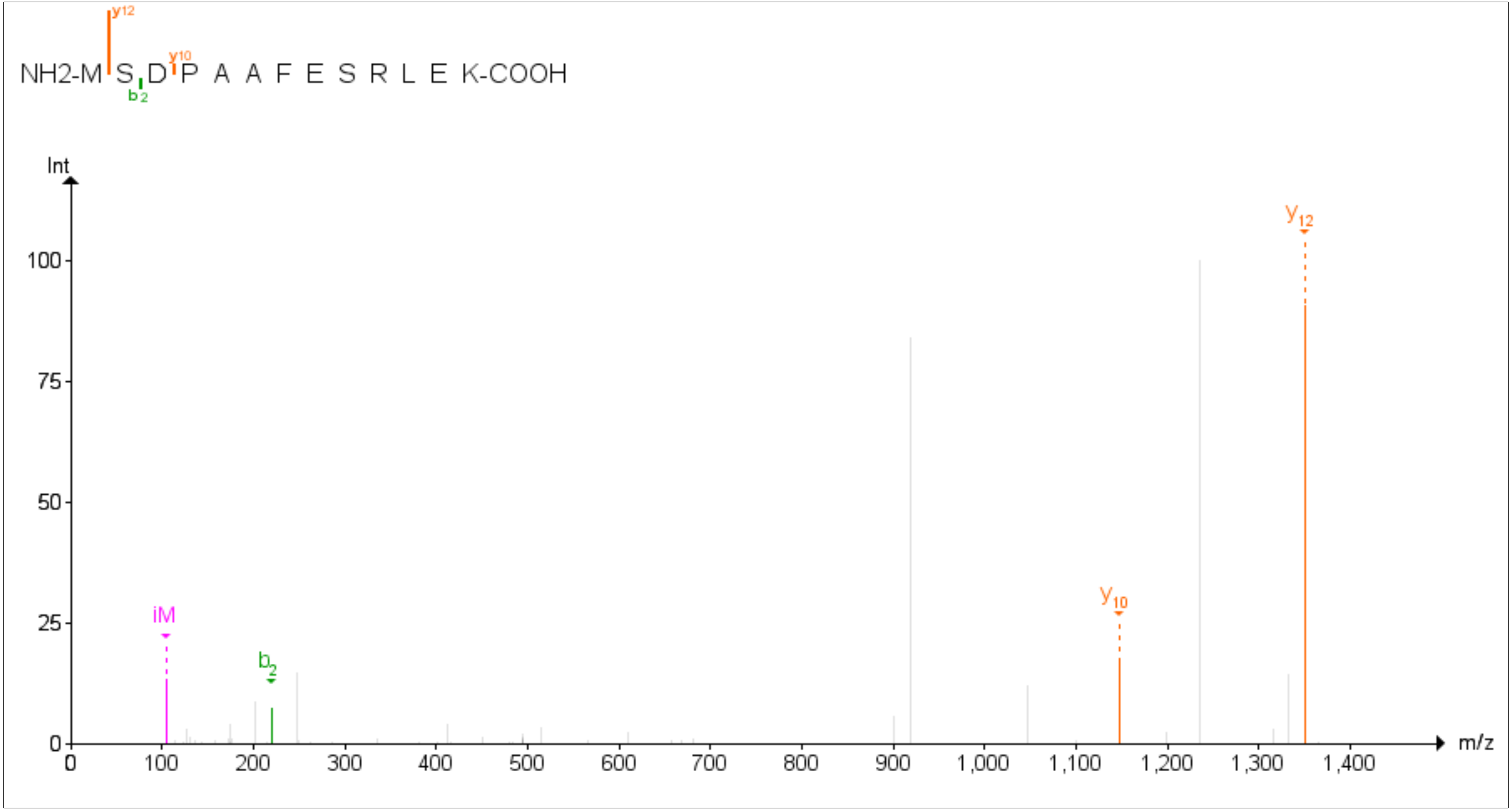

NH2-M S D P A A F E S R L E K-COOH
y12
y10
b2
Int
100
75
50
25
0
iM
y12
y10
b2
0
100
200
300
400
500
600
700
800
900
1,000
1,100
1,200
1,300
1,400
m/z

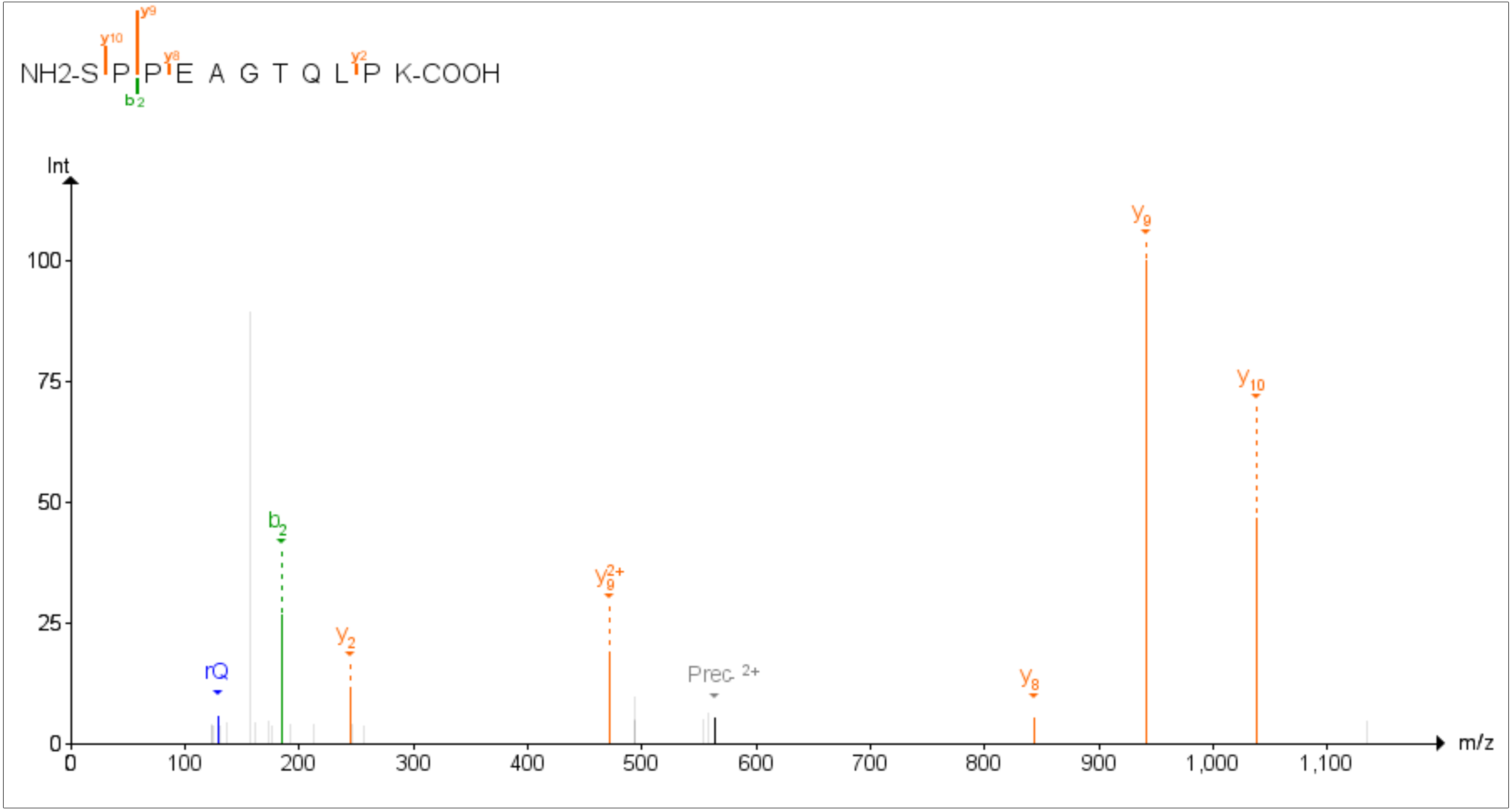

NH2-S P P E A G T Q L P K-COOH
y10
y9
y8
y2
b2
Int
100
75
50
25
0
b2
rQ
y2
y9 2+
Prec 2+
y8
y9
y10
0
100
200
300
400
500
600
700
800
900
1,000
1,100
m/z

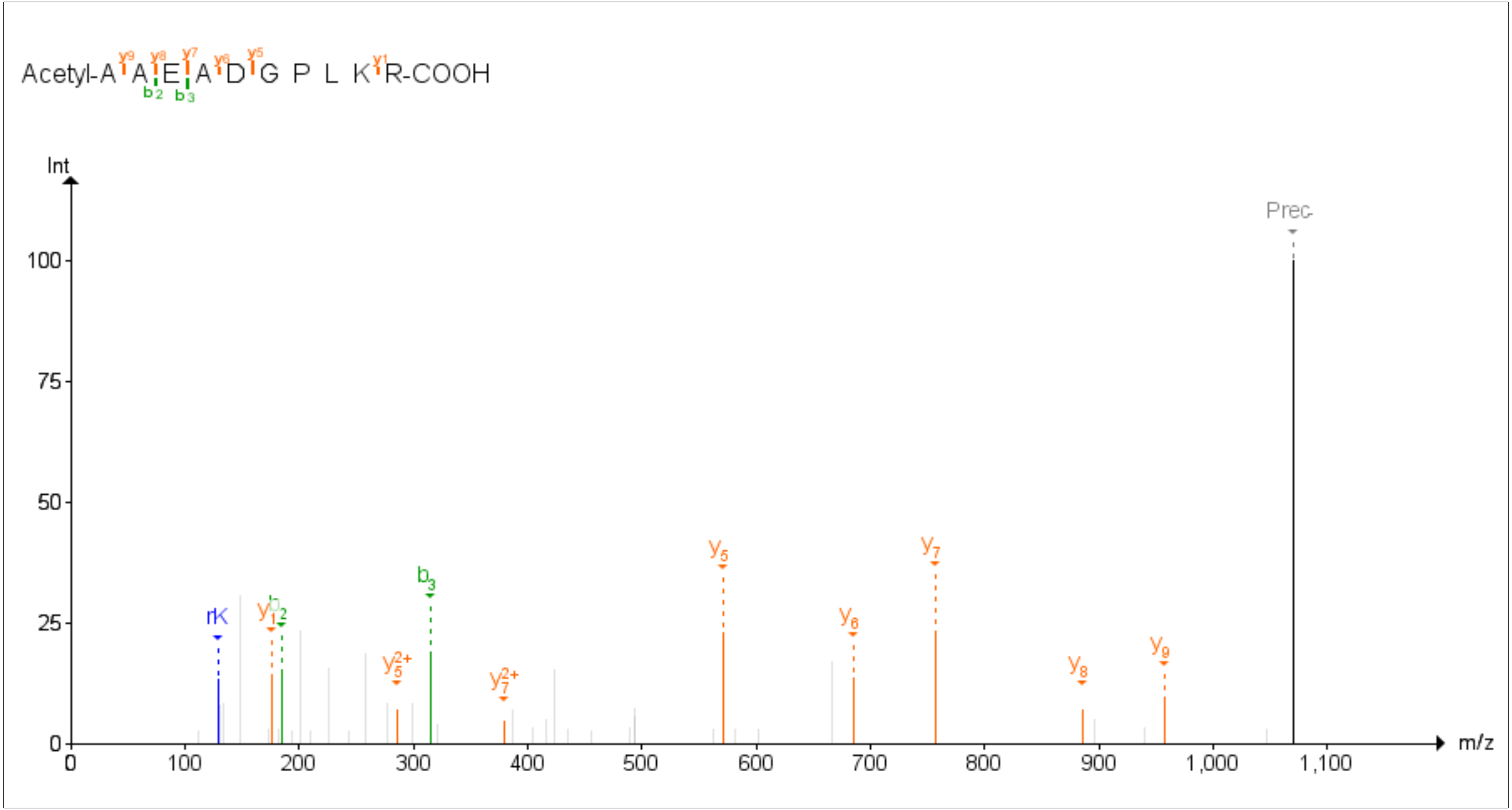

Acetyl-A A E A D G P L K R-COOH
Int
Prec
m/z

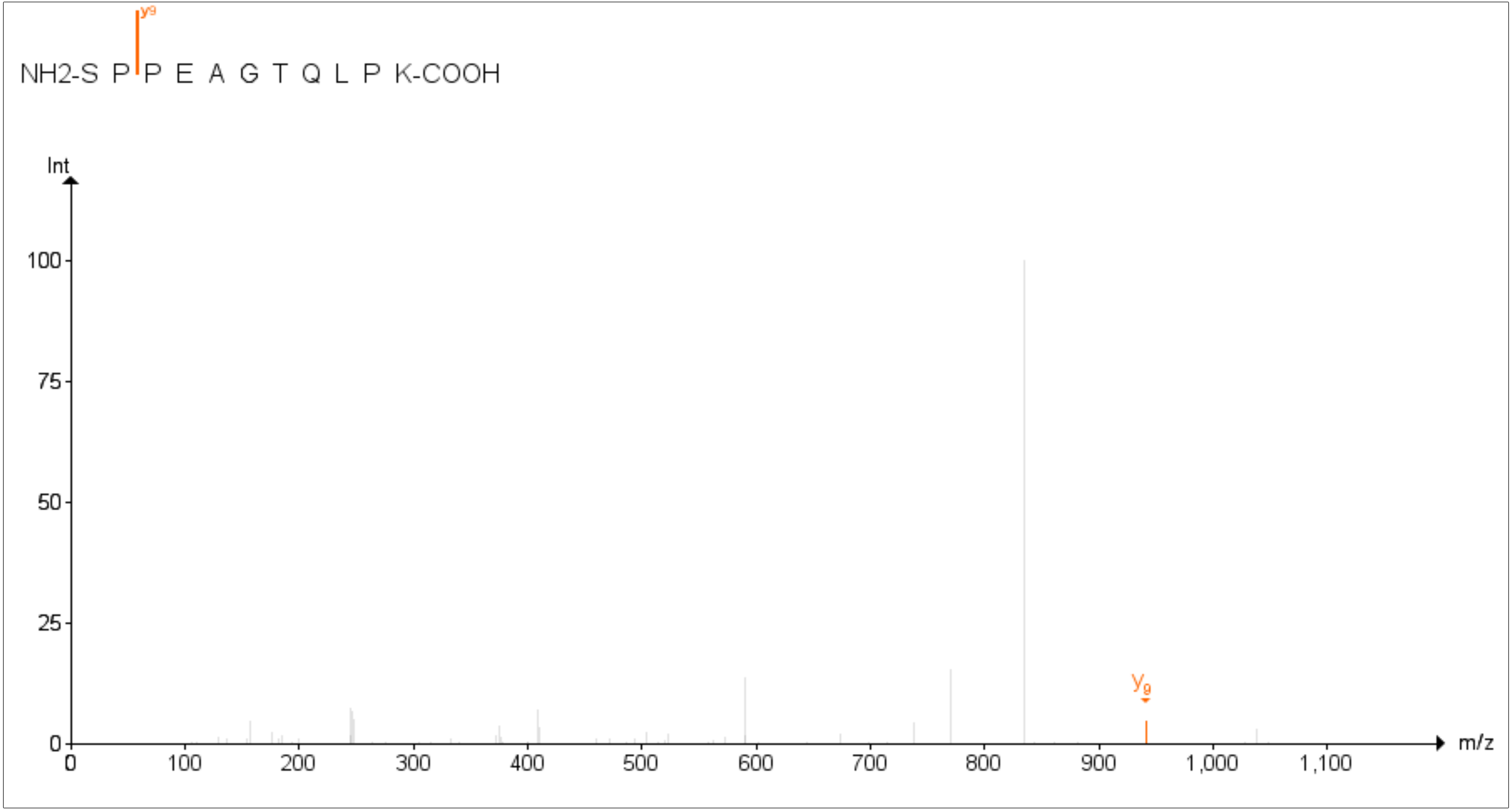

y9
NH2-S P P E A G T Q L P K-COOH
Int
100
75
50
25
0
y9
0
100
200
300
400
500
600
700
800
900
1,000
1,100
m/z

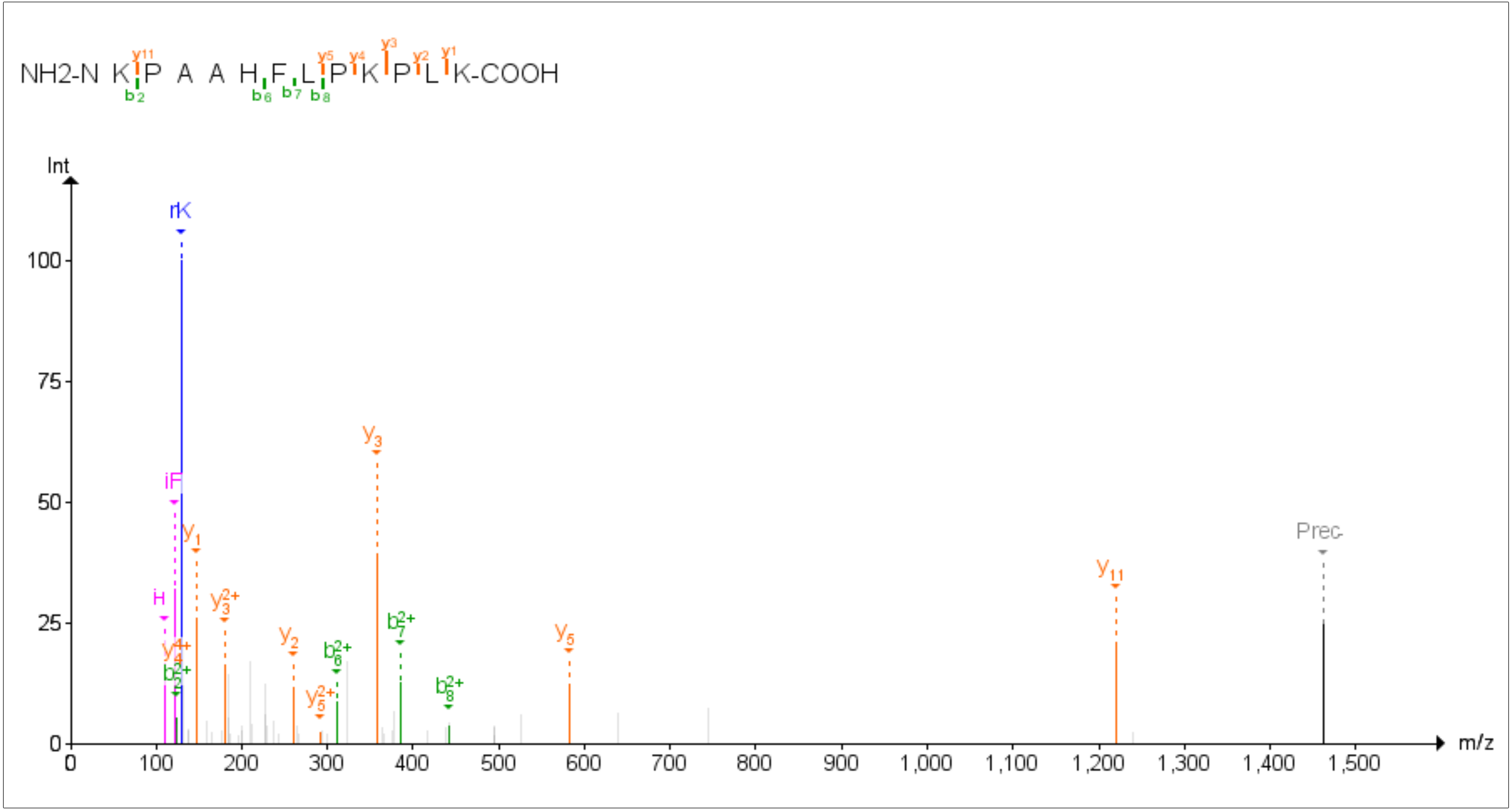

NH2-N K P A A H F L P K P L K-COOH
y11 y5 y4 y3 y2 y1
b2 b6 b7 b8
Int
rK
iF
iH
y1
y3
y11
Prec
y2
y5
m/z
0
25
50
75
100
0
100
200
300
400
500
600
700
800
900
1,000
1,100
1,200
1,300
1,400
1,500

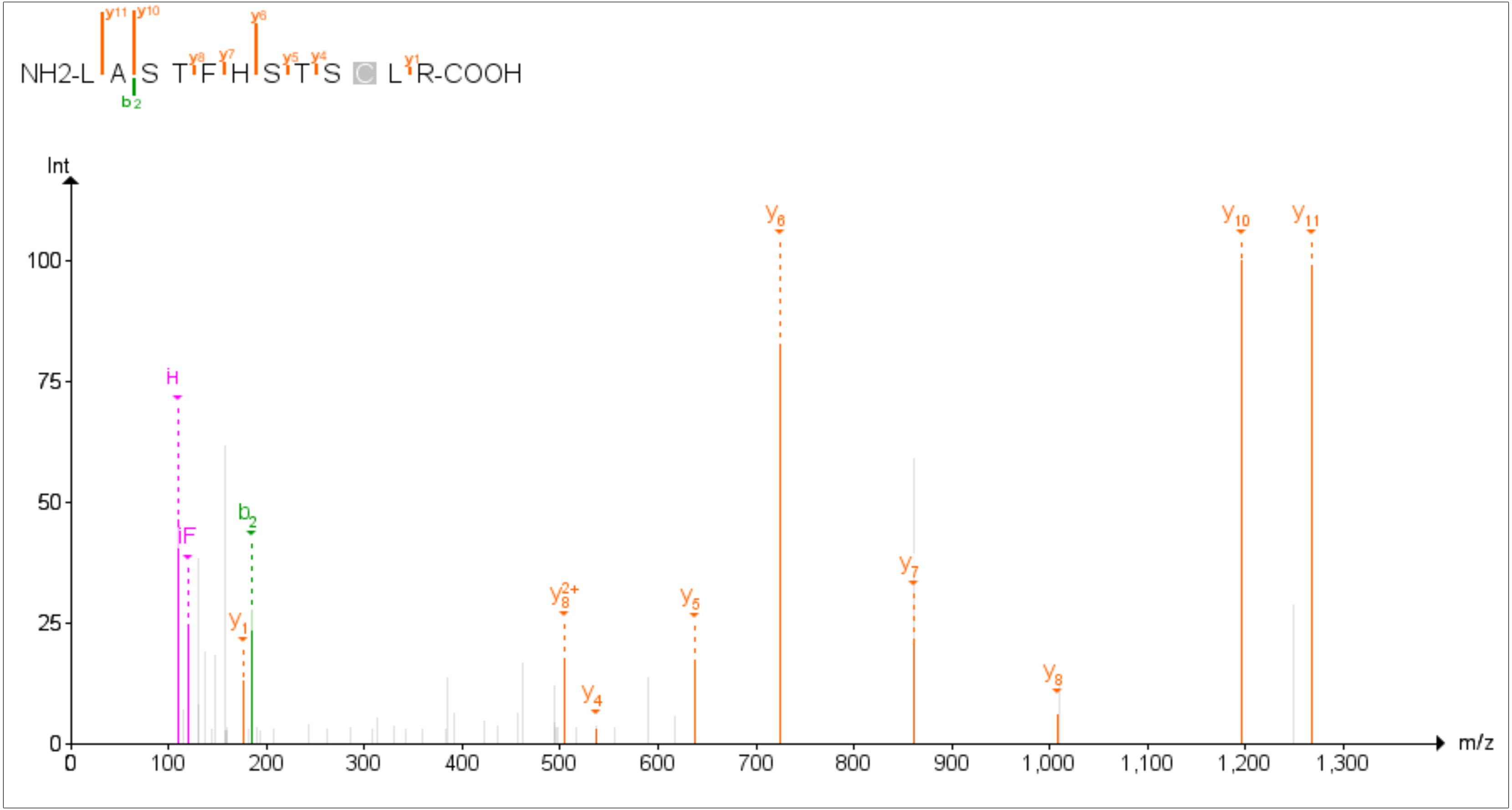
NH2-L A S T F H S T S C L R-COOH
y11 y10 y8 y7 y6 y5 y4 y1
b2
Int
100
75
50
25
0
iH
iF
b2
y1
y8 2+
y4
y5
y6
y7
y8
y10
y11
0 100 200 300 400 500 600 700 800 900 1,000 1,100 1,200 1,300
m/z

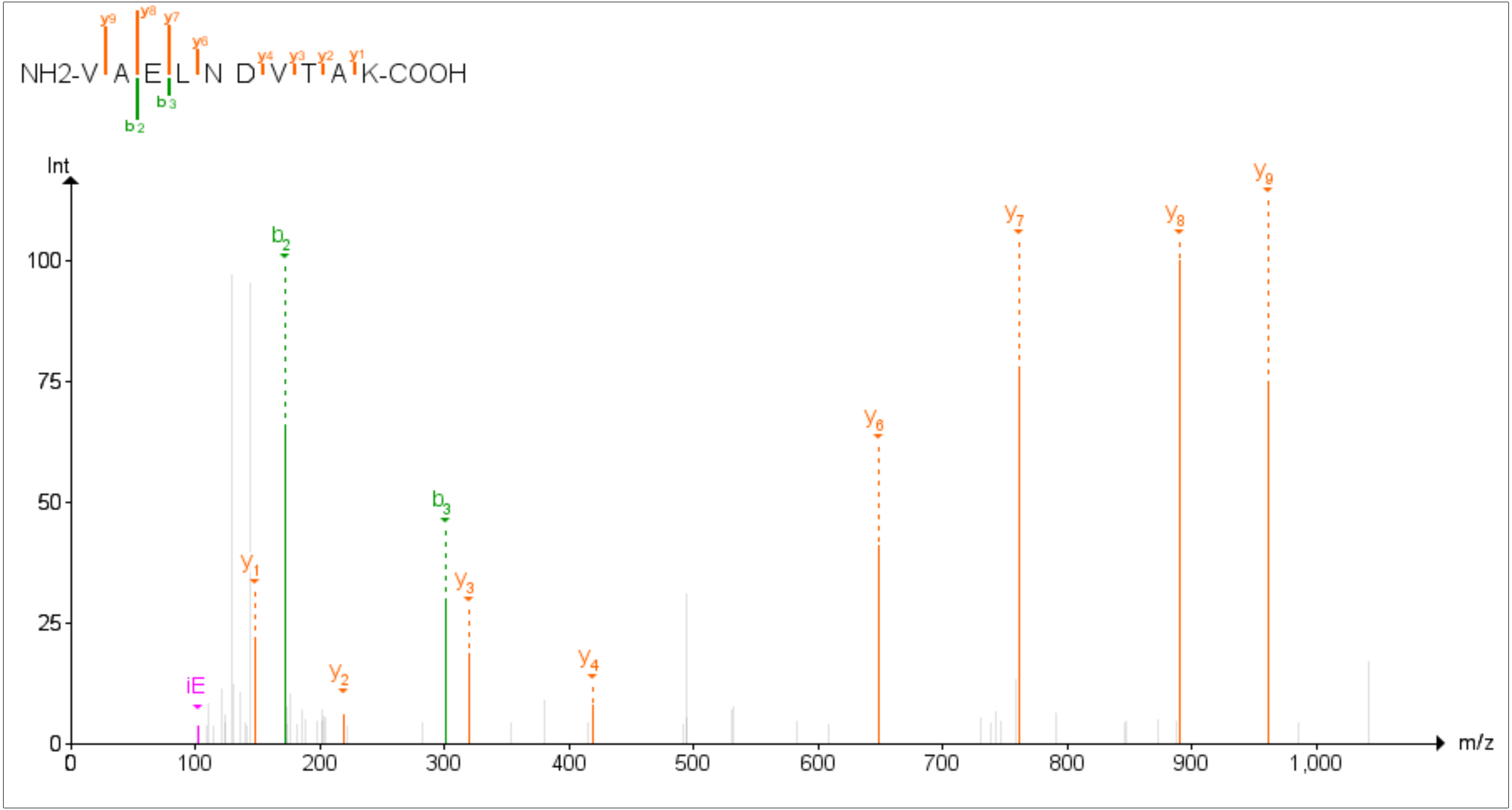

NH2-V A E L N D V T A K-COOH
y9 y8 y7 y6 y4 y3 y2 y1
b2 b3
Int
100
75
50
25
0
m/z
0
100
200
300
400
500
600
700
800
900
1,000
iE
y1
b2
y2
b3
y3
y4
y6
y7
y8
y9

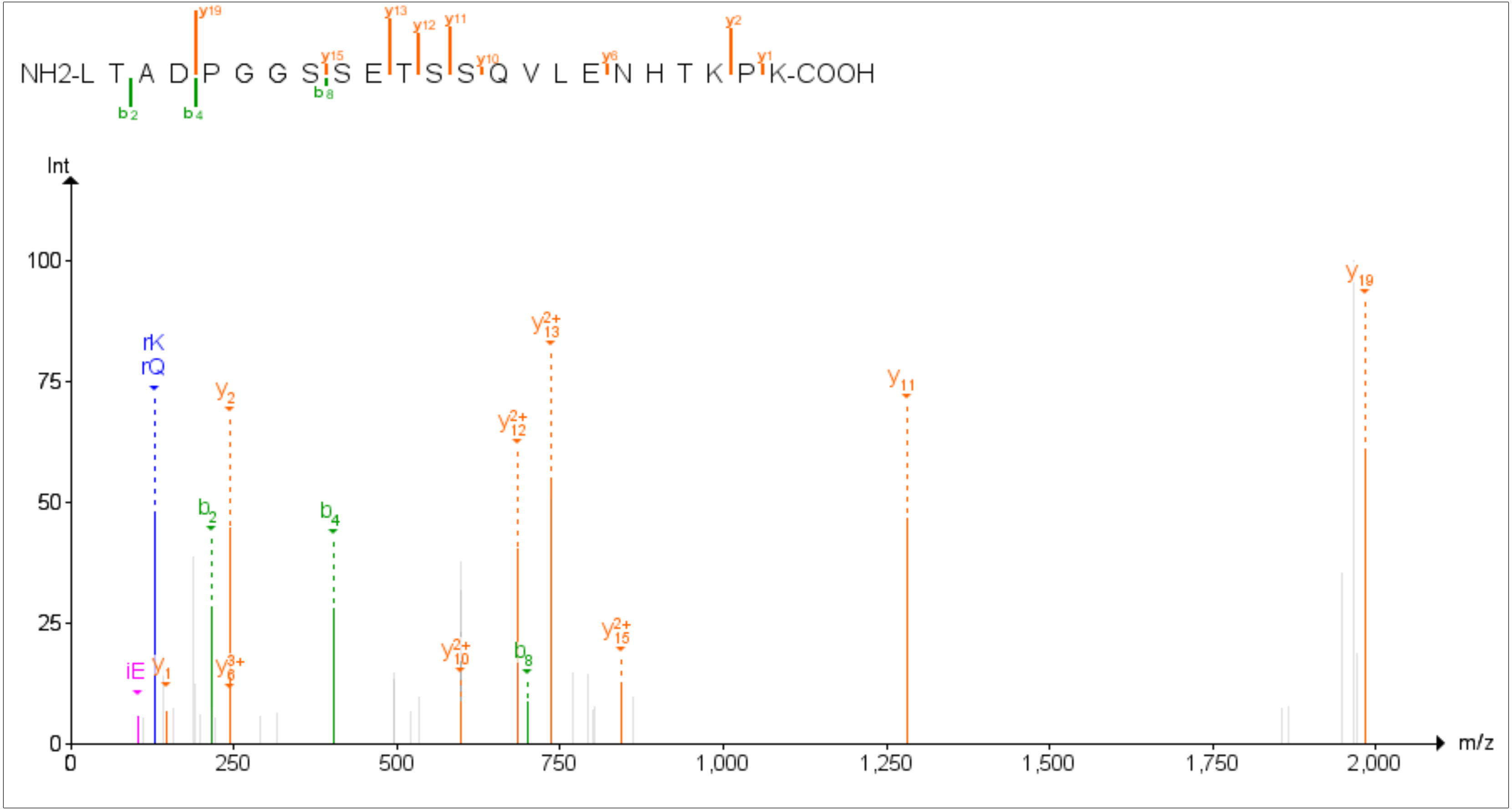

NH2-L T A D P G G S S E T S S Q V L E N H T K P K-COOH
b2 b4 b8
y19 y15 y13 y12 y11 y10 y6 y2 y1
Int
100
75
50
25
0
0 250 500 750 1,000 1,250 1,500 1,750 2,000
m/z
rK
rQ
iE
y1
b2
y2
y6 3+
b4
y10 2+
y12 2+
b8
y13 2+
y15 2+
y11
y19

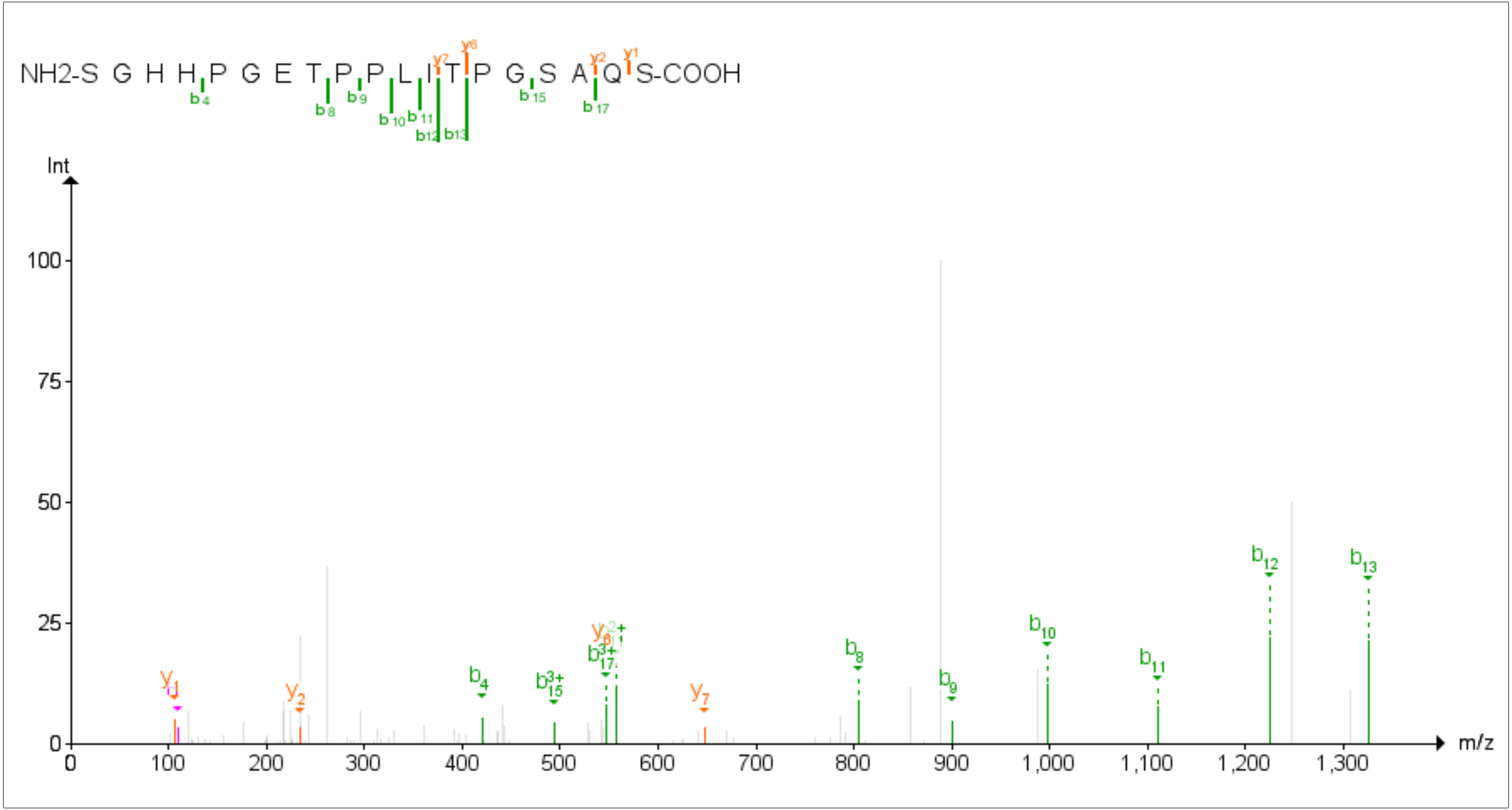
NH2-S G H H P G E T P P L I T P G S A Q S-COOH
b4
b8
b9
b10
b11
b12
b13
b15
b17
y7
y6
y2
y1
Int
100
75
50
25
0
m/z
0
100
200
300
400
500
600
700
800
900
1,000
1,100
1,200
1,300
y1
y2
b4
b$^{3+}_{15}$
b$^{3+}_{17}$
y6
b$^{2+}_{11}$
y7
b8
b9
b10
b11
b12
b13

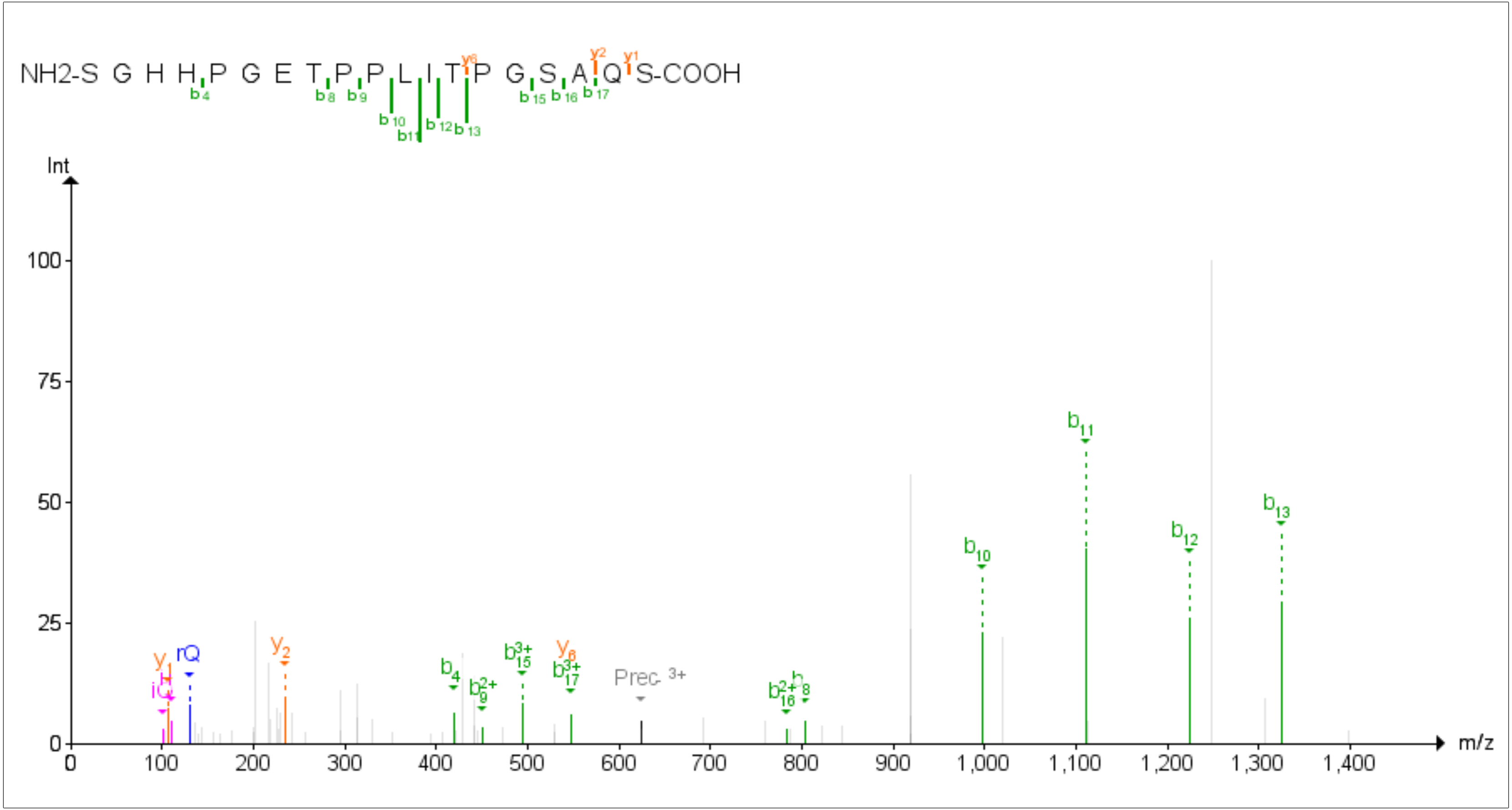

NH2-S G H H P G E T P P L I T P G S A Q S-COOH
b4 b8 b9 b10 b11 b12 b13 b15 b16 b17
y6 y2 y1
Int
100
75
50
25
0
m/z
0 100 200 300 400 500 600 700 800 900 1,000 1,100 1,200 1,300 1,400
y1
iQ
rQ
y2
b4
b9 2+
b15 3+
y6
b17 3+
Prec 3+
b16 2+
b8
b10
b11
b12
b13

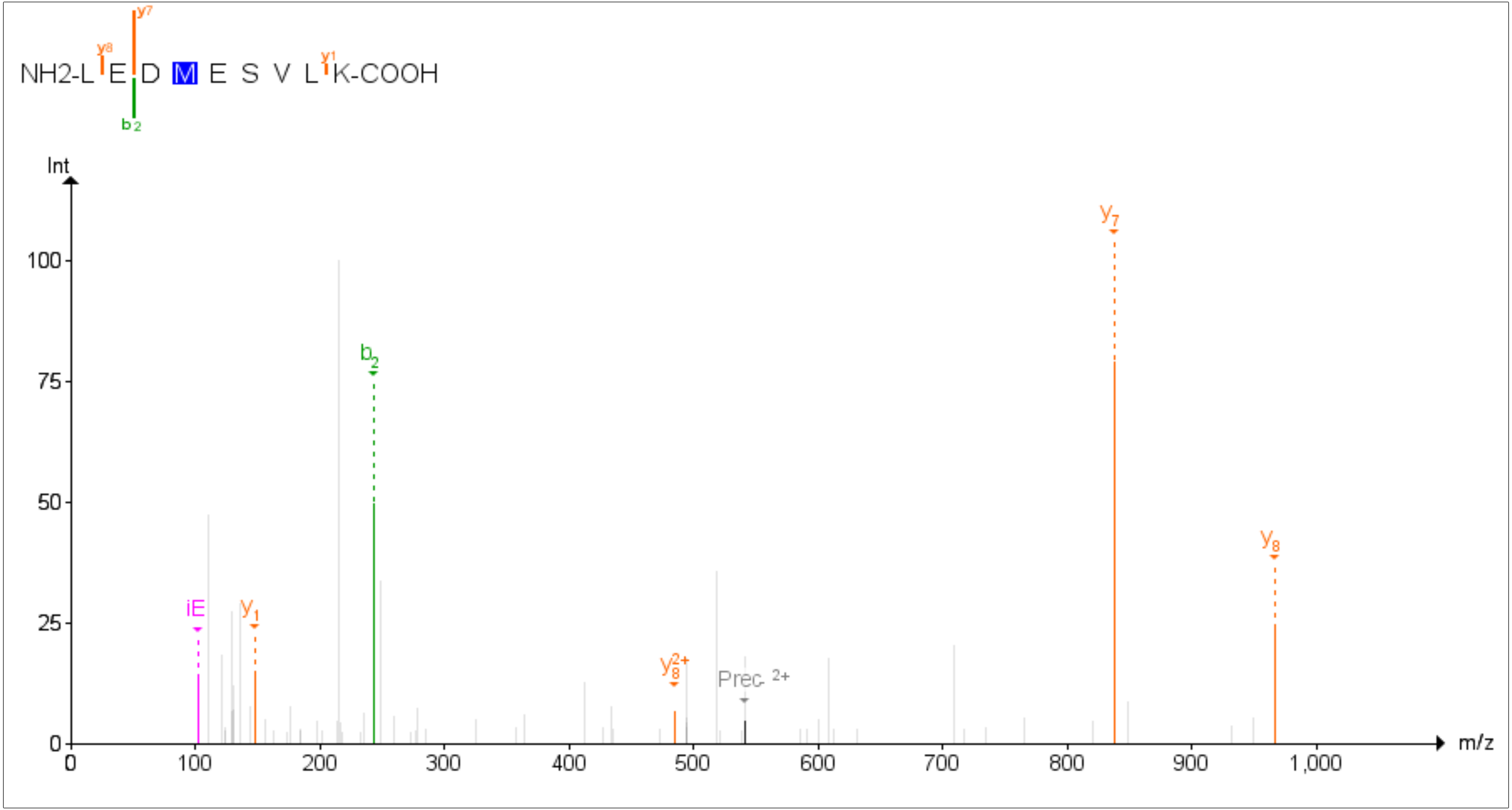

NH2-L E D M E S V L K-COOH
y8
y7
y1
b2
Int
100
75
50
25
0
iE
y1
b2
y8 2+
Prec 2+
y7
y8
0
100
200
300
400
500
600
700
800
900
1,000
m/z

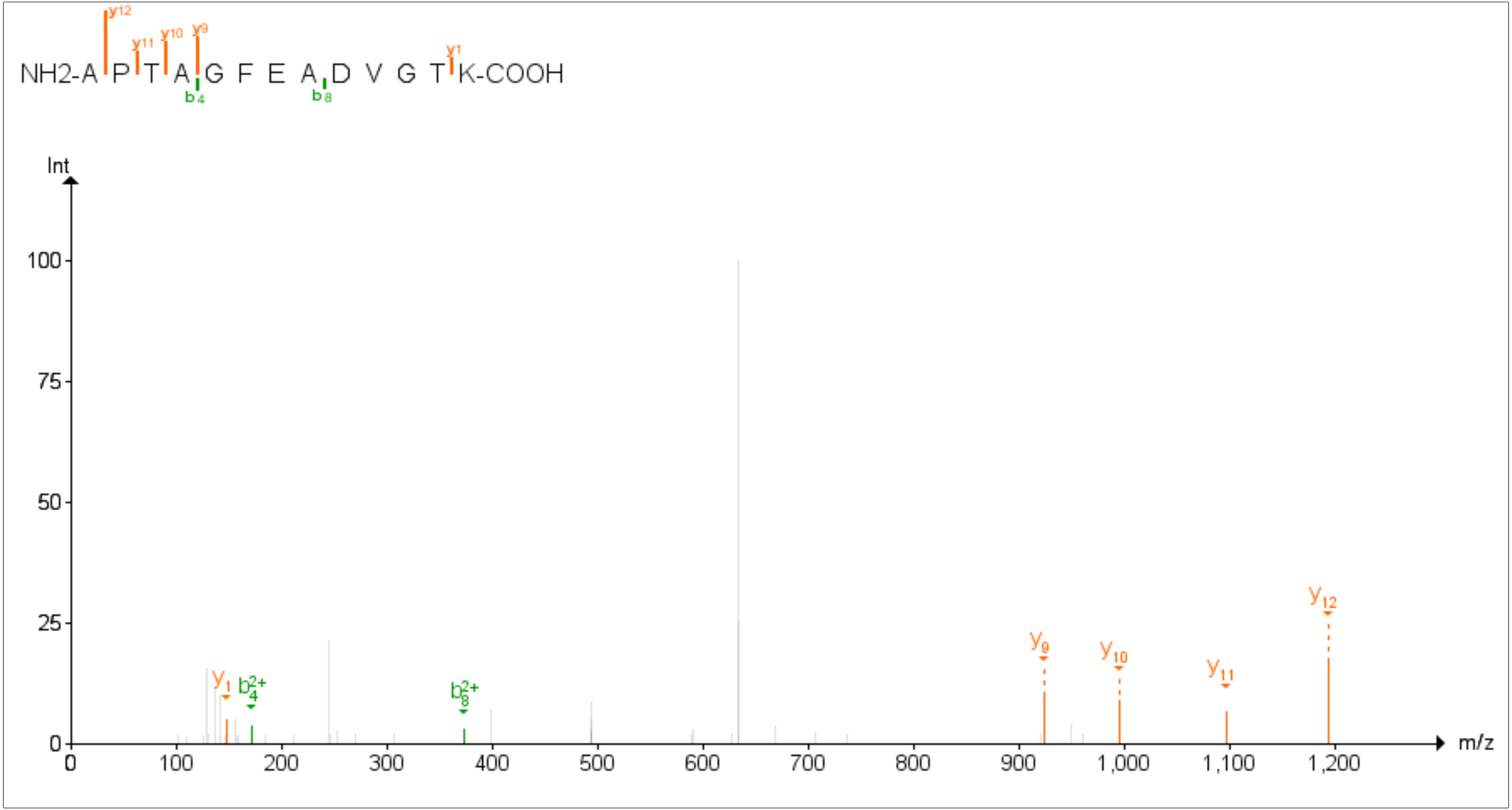

NH2-A P T A G F E A D V G T K-COOH
y12
y11
y10
y9
y1
b4
b8
Int
100
75
50
25
0
y1
b_4^{2+}
b_8^{2+}
y9
y10
y11
y12
0
100
200
300
400
500
600
700
800
900
1,000
1,100
1,200
m/z

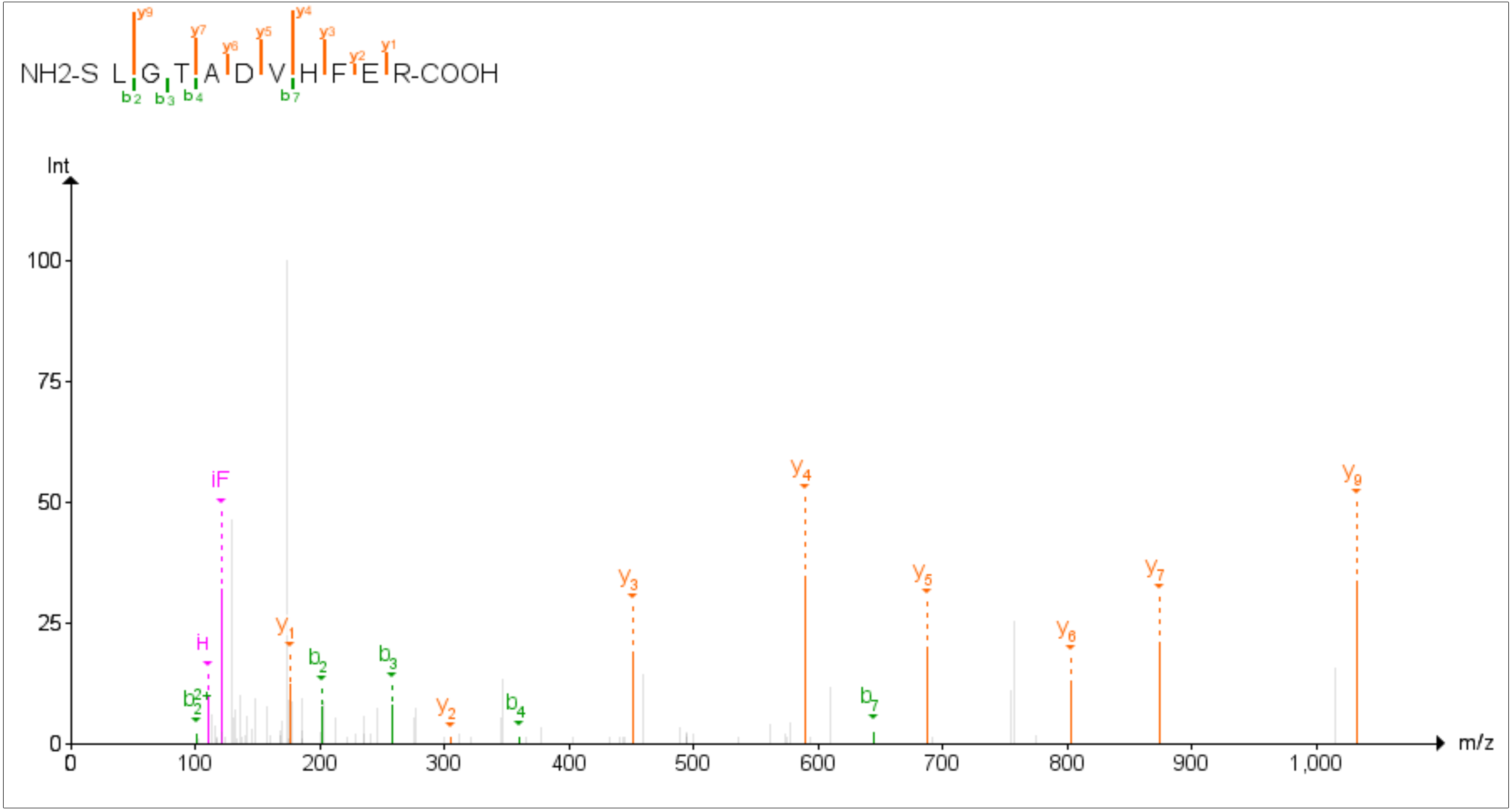

NH2-S L G T A D V H F E R-COOH
y9 y7 y6 y5 y4 y3 y2 y1
b2 b3 b4 b7
Int
100
75
50
25
0
iF
iH
b2 2+
y1
b2
b3
y2
b4
y3
y4
b7
y5
y6
y7
y9
0 100 200 300 400 500 600 700 800 900 1,000
m/z

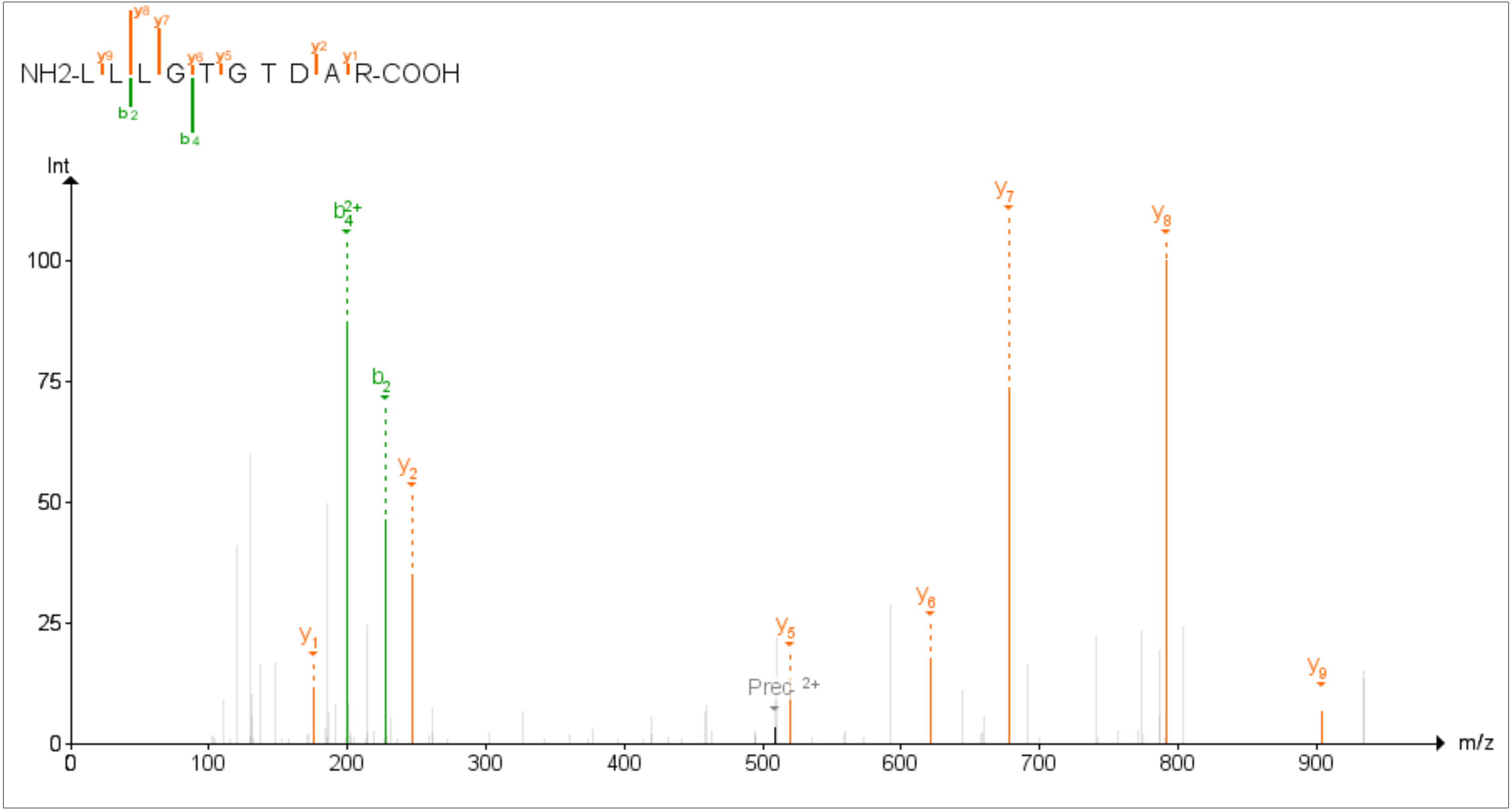

NH2-L L L G T G T D A R-COOH
y9 y8 y7 y6 y5 y2 y1
b2 b4
Int
100
75
50
25
0
b_4^{2+}
b_2
y_1
y_2
y_5
y_6
y_7
y_8
y_9
Prec. 2+
0 100 200 300 400 500 600 700 800 900
m/z

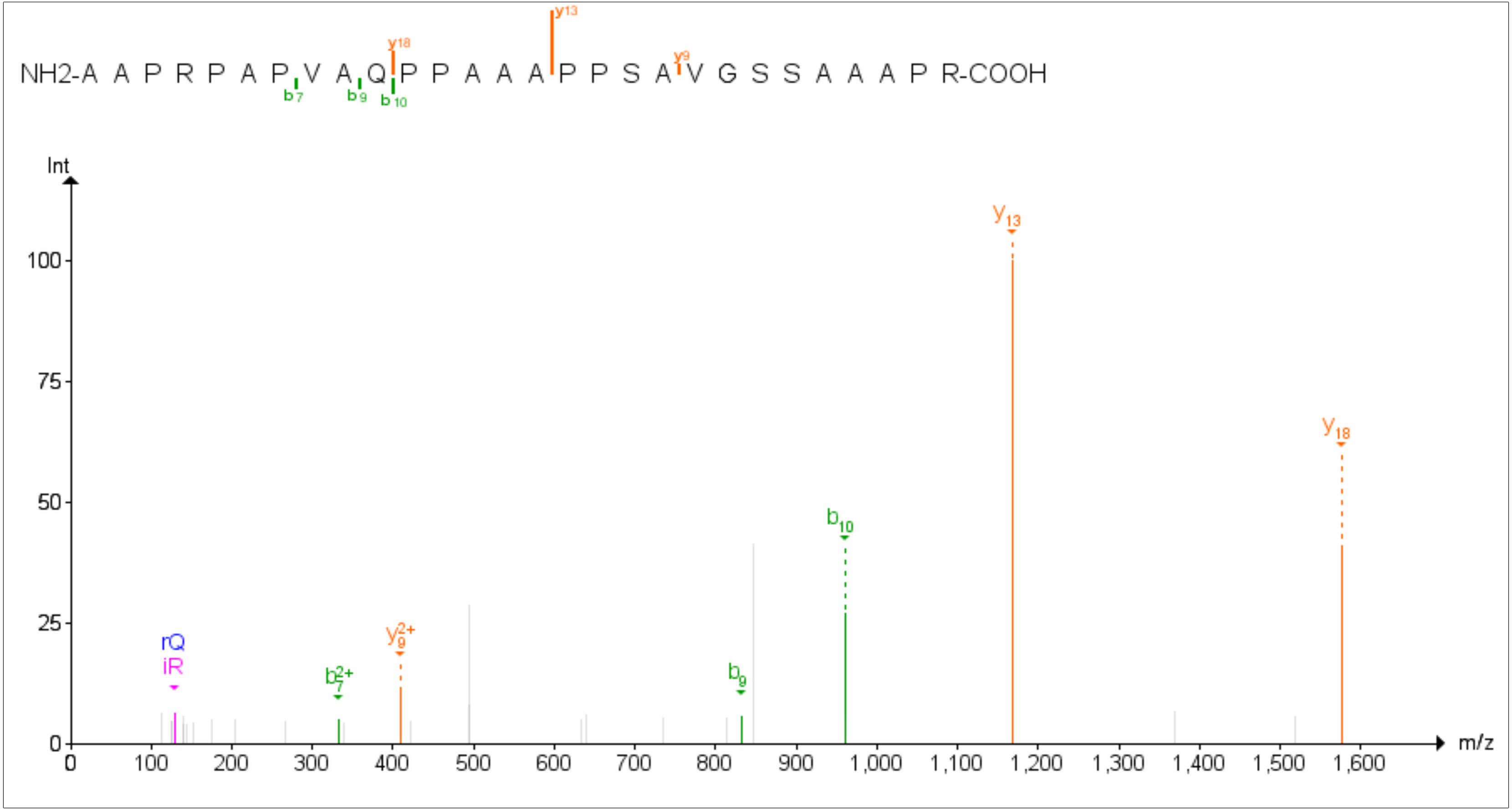

NH2-A A P R P A P V A Q P P A A A P P S A V G S S A A A P R-COOH
y13
y18
y9
b7
b9
b10
Int
100
75
50
25
0
y13
y18
b10
b9
rQ
iR
b7 2+
y9 2+
0
100
200
300
400
500
600
700
800
900
1,000
1,100
1,200
1,300
1,400
1,500
1,600
m/z

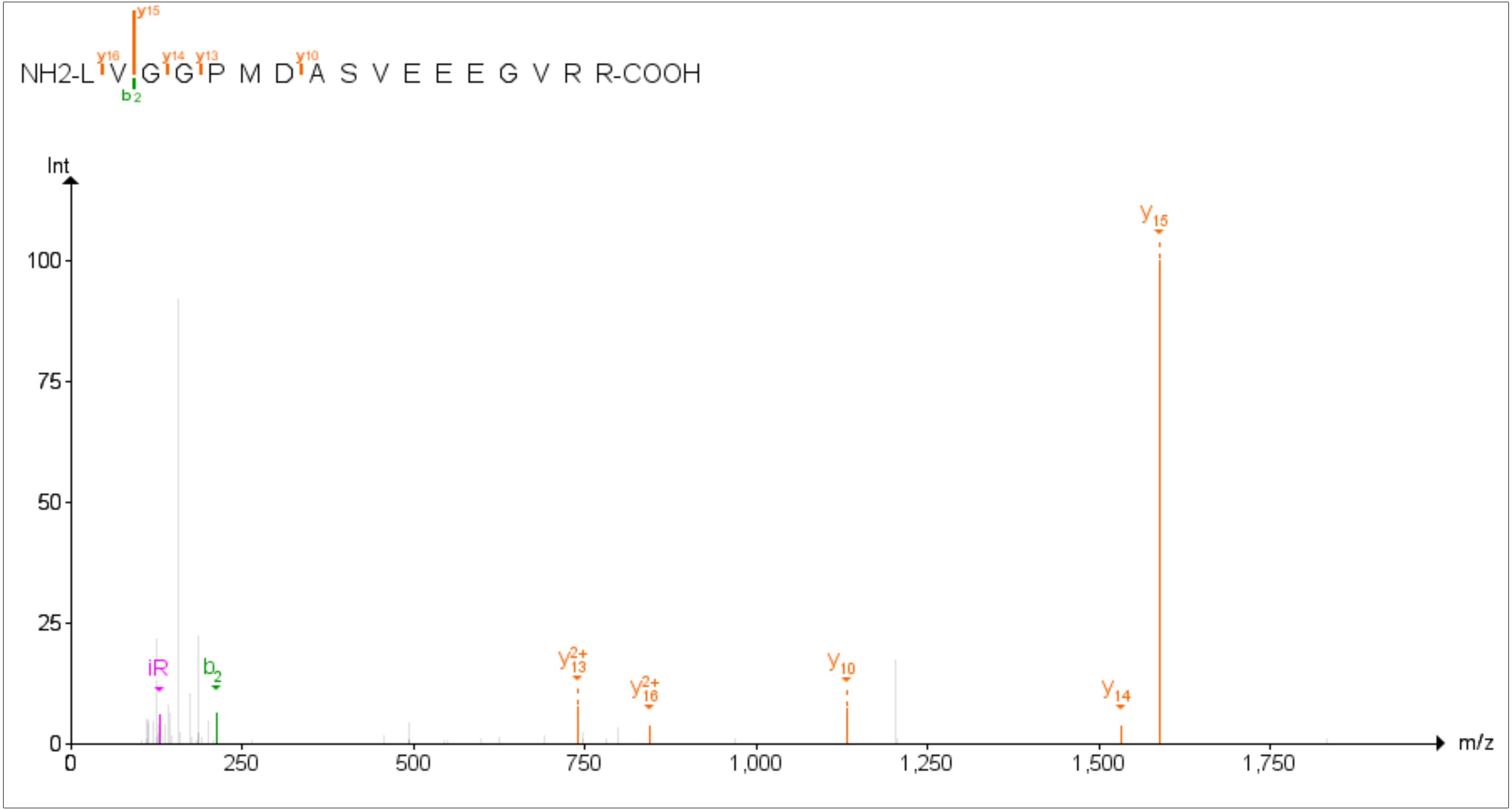

NH2-L V G G P M D A S V E E E G V R R-COOH
y15
y16
y14
y13
y10
b2
Int
100
75
50
25
0
iR
b2
y13 2+
y16 2+
y10
y14
y15
0
250
500
750
1,000
1,250
1,500
1,750
m/z

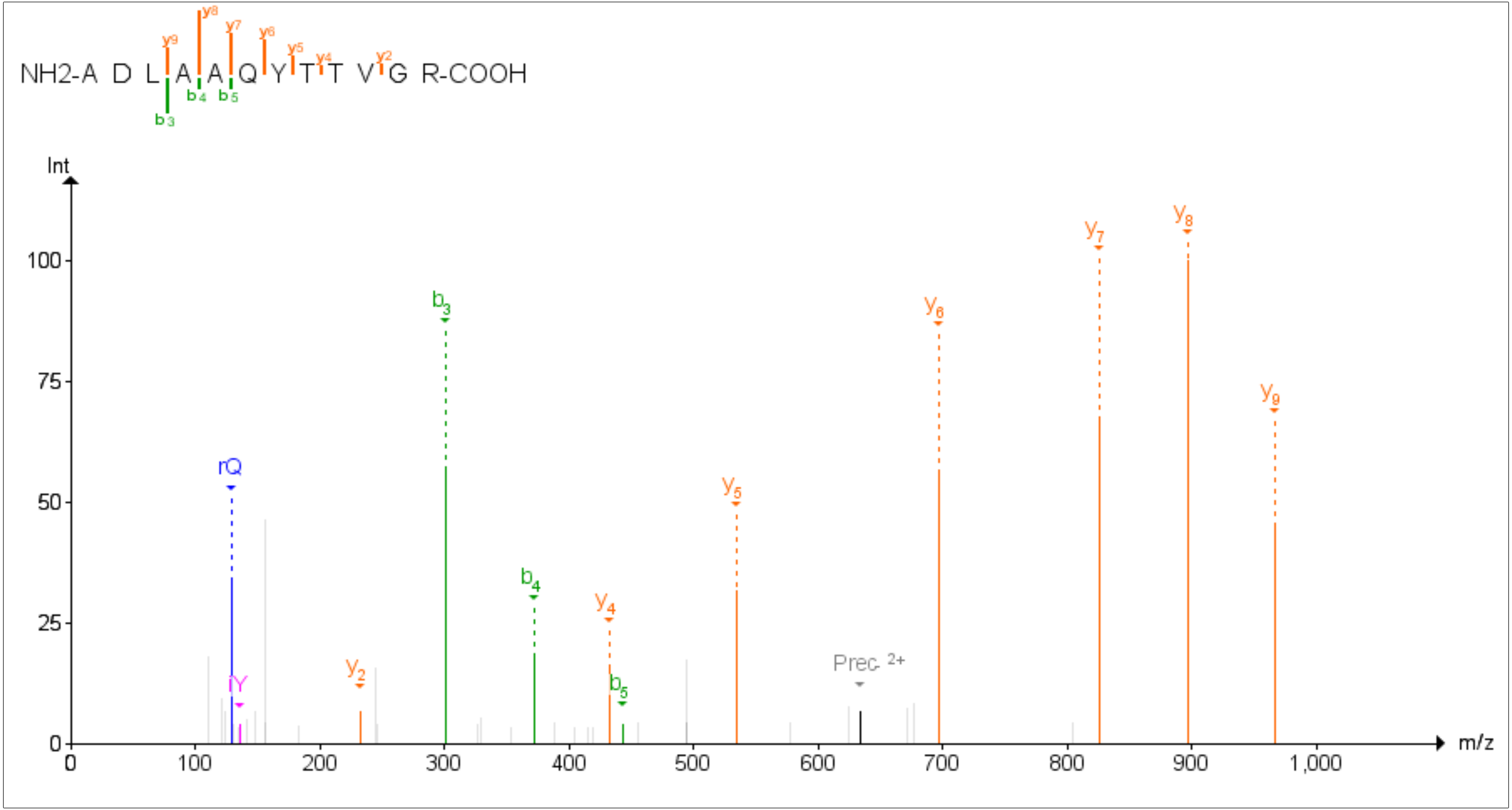

NH2-A D L A A Q Y T T V G R-COOH
y9 y8 y7 y6 y5 y4 y2
b3 b4 b5
Int
100
75
50
25
0
rQ
iY
y2
b3
b4
y4
b5
y5
Prec 2+
y6
y7
y8
y9
0
100
200
300
400
500
600
700
800
900
1,000
m/z

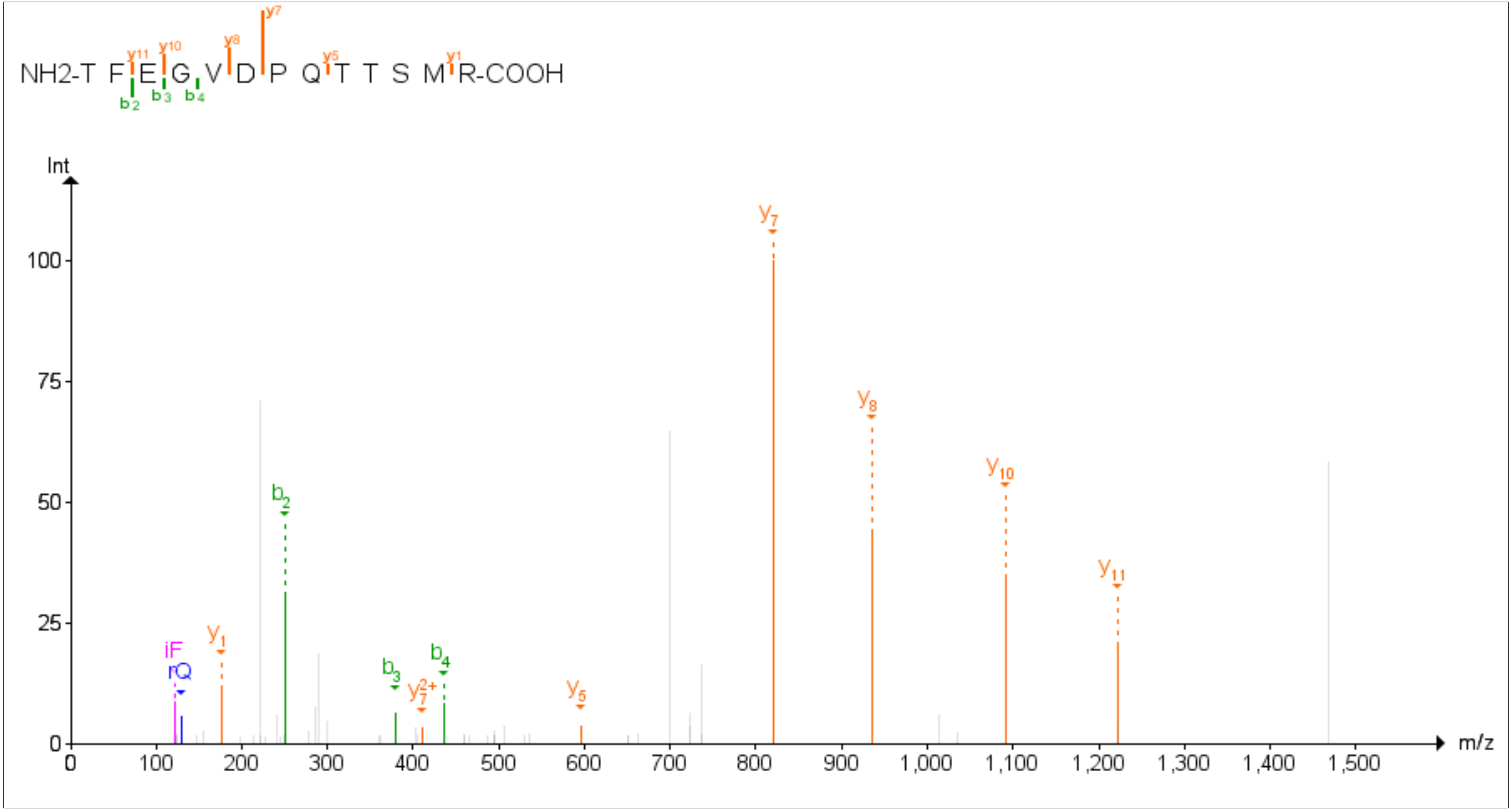

NH2-T F E G V D P Q T T S M R-COOH
y11 y10 y8 y7 y5 y1
b2 b3 b4
Int
0
25
50
75
100
m/z
0 100 200 300 400 500 600 700 800 900 1,000 1,100 1,200 1,300 1,400 1,500
iF
rQ
y1
b2
b3
y7 2+
b4
y5
y7
y8
y10
y11

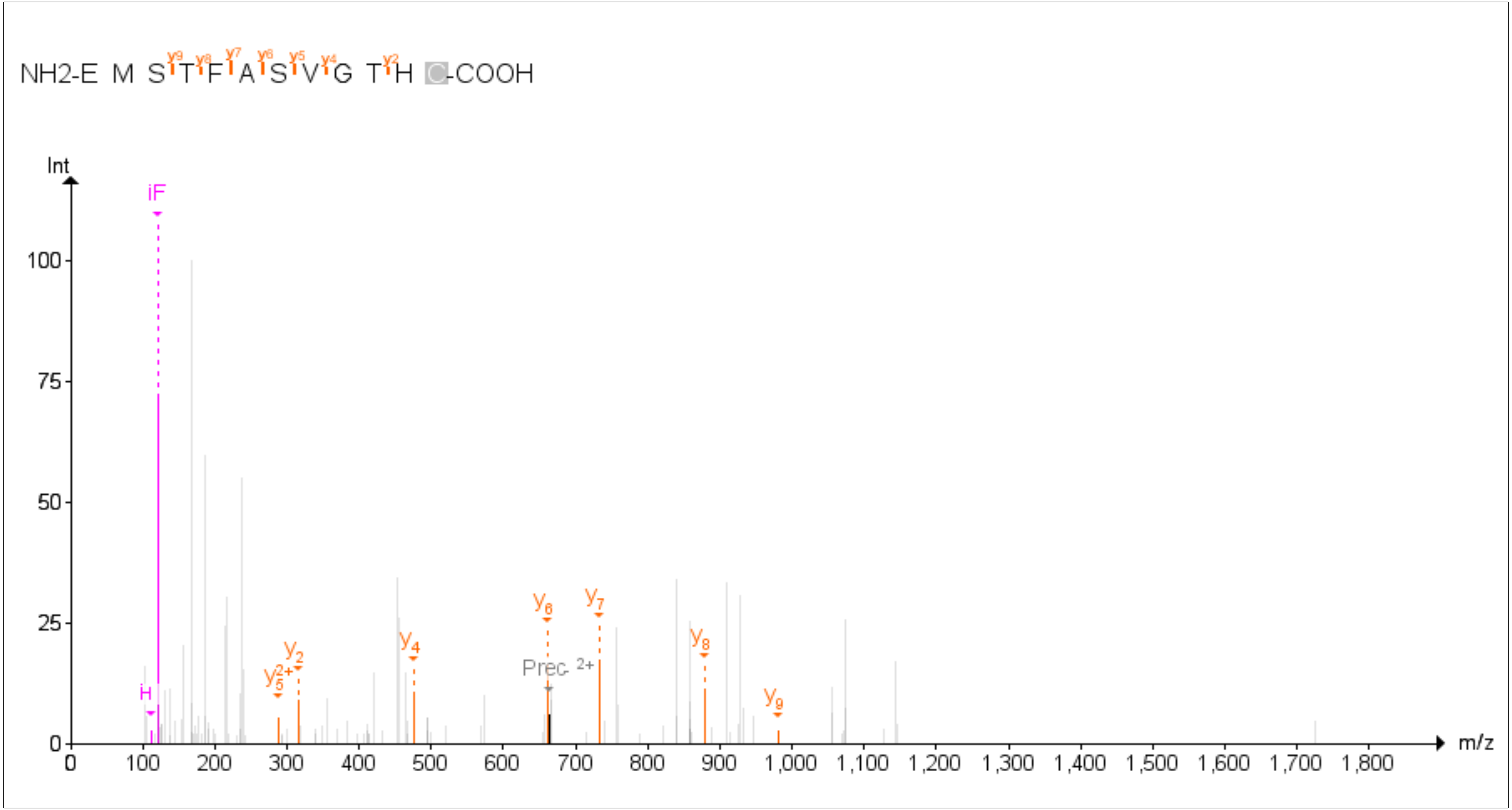

NH2-E M S T F A S V G T H C-COOH
y9 y8 y7 y6 y5 y4 y2
Int
iF
H
y2
y5 2+
y4
y6
y7
Prec 2+
y8
y9
m/z
0
25
50
75
100
0 100 200 300 400 500 600 700 800 900 1,000 1,100 1,200 1,300 1,400 1,500 1,600 1,700 1,800

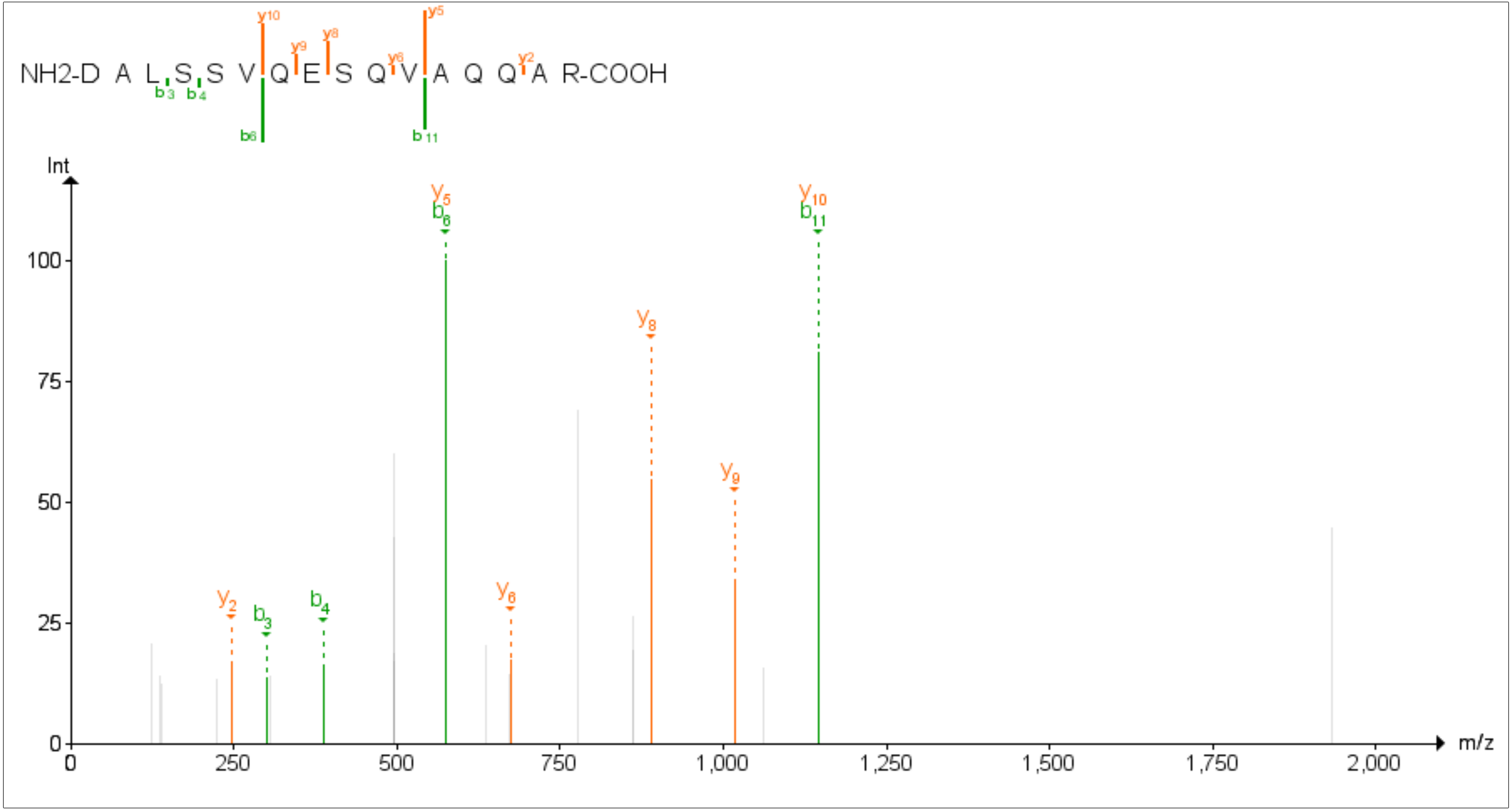

NH2-D A L S S V Q E S Q V A Q Q A R-COOH
y10
y9
y8
y6
y5
y2
b3
b4
b6
b11
Int
100
75
50
25
0
y5
b6
y10
b11
y8
y9
y2
b3
b4
y6
0
250
500
750
1,000
1,250
1,500
1,750
2,000
m/z

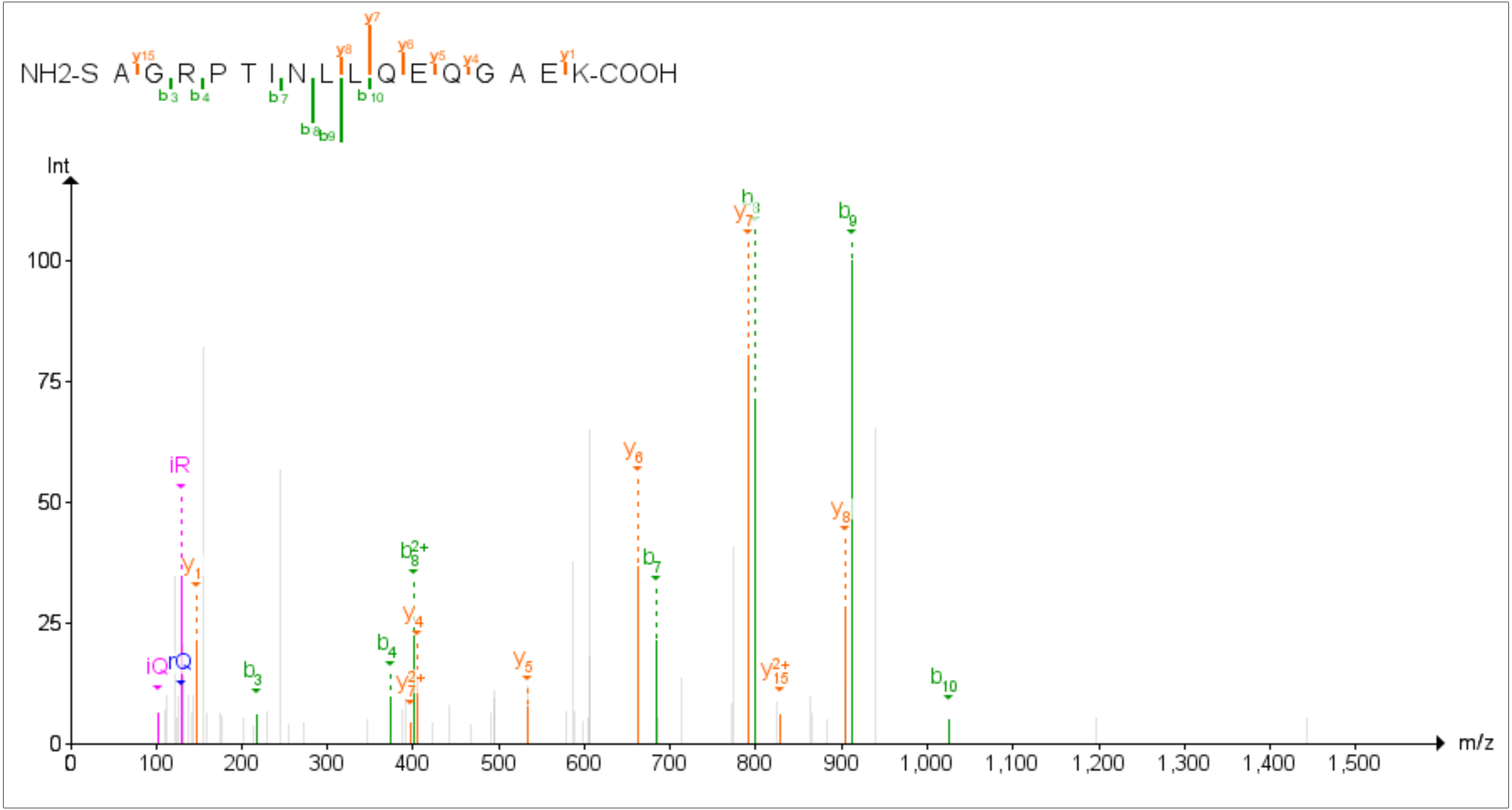

NH2-S A G R P T I N L L Q E Q G A E K-COOH
y15
y8
y7
y6
y5
y4
y1
b3
b4
b7
b8
b9
b10
Int
100
75
50
25
0
iR
iQ
rQ
y1
b3
b4
b8 2+
y4
y7 2+
y5
y6
b7
y7
b8
y15 2+
y8
b9
b10
m/z
0
100
200
300
400
500
600
700
800
900
1,000
1,100
1,200
1,300
1,400
1,500

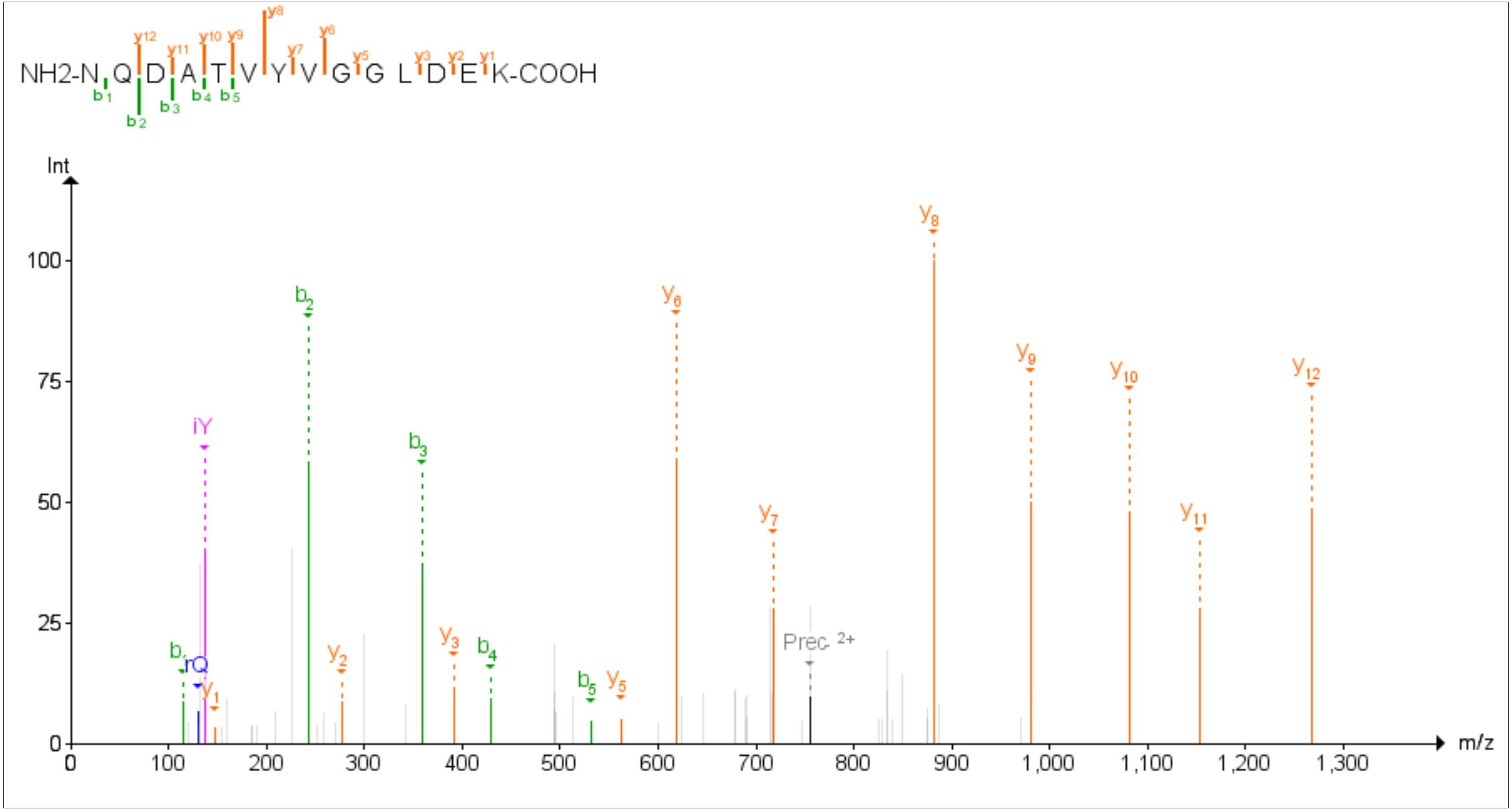

NH2-N Q D A T V Y V G G L D E K-COOH
b1
b2
b3
b4
b5
y12
y11
y10
y9
y8
y7
y6
y5
y3
y2
y1
Int
m/z
0
25
50
75
100
0
100
200
300
400
500
600
700
800
900
1,000
1,100
1,200
1,300
iY
rQ
Prec 2+

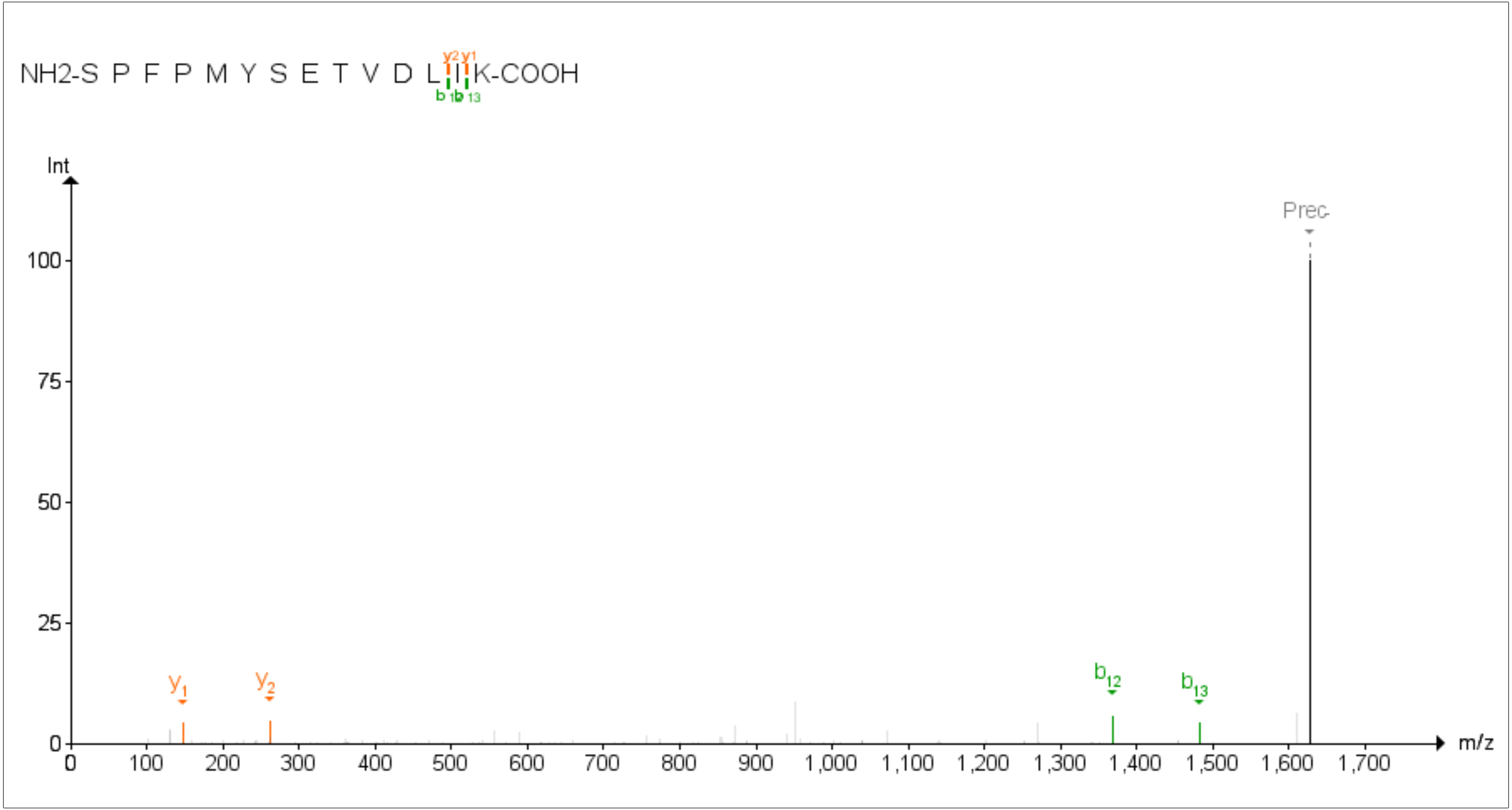
NH2-S P F P M Y S E T V D L I I K-COOH
y2 y1
b 12 b 13
Int
Prec
100
75
50
25
0
y1
y2
b12
b13
0 100 200 300 400 500 600 700 800 900 1,000 1,100 1,200 1,300 1,400 1,500 1,600 1,700
m/z

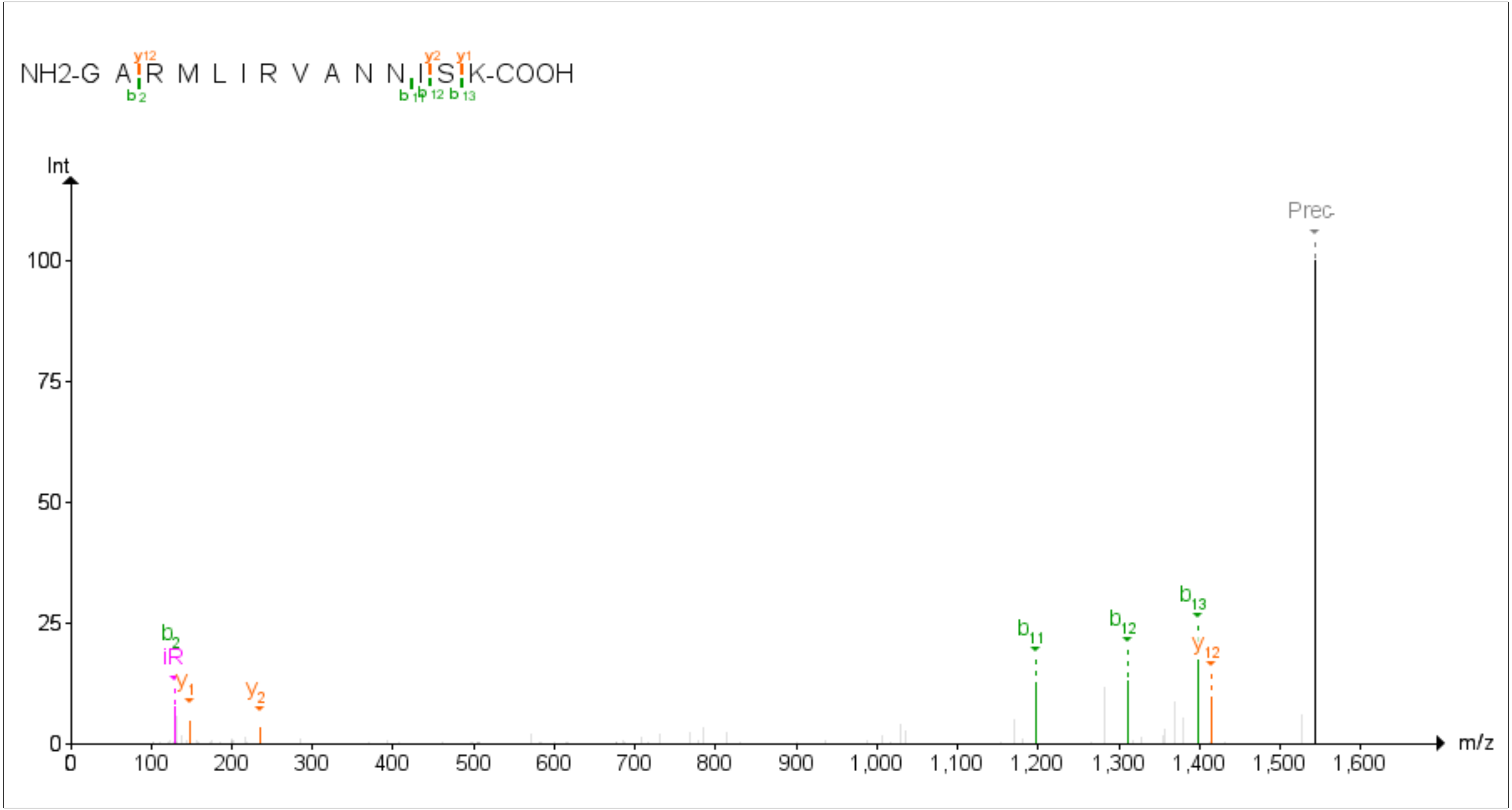

NH2-G A R M L I R V A N N I I S K-COOH
y12
y2
y1
b 2
b 11
b 12
b 13
Int
Prec
100
75
50
25
0
b2
iR
y1
y2
b11
b12
b13
y12
0
100
200
300
400
500
600
700
800
900
1,000
1,100
1,200
1,300
1,400
1,500
1,600
m/z

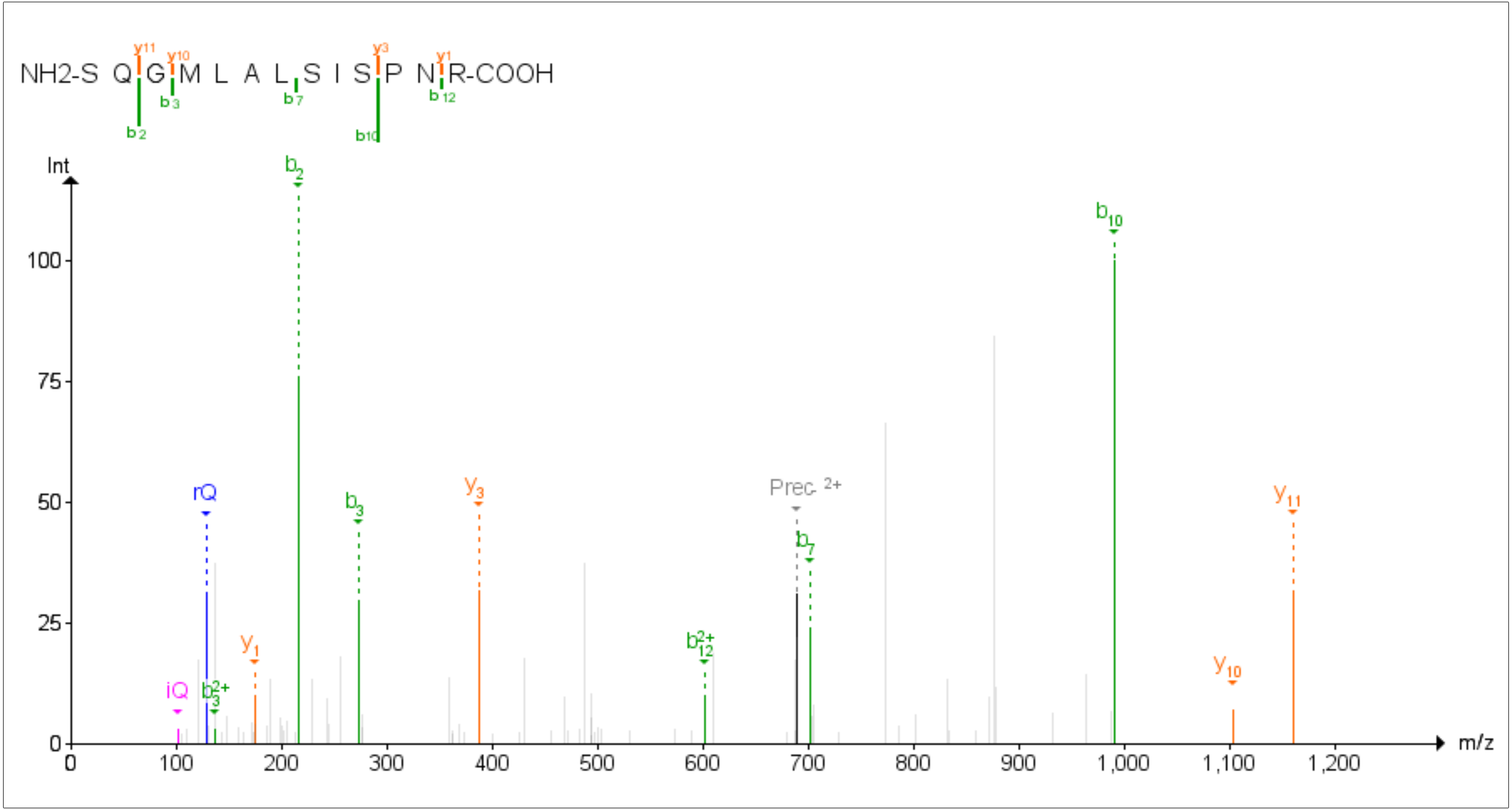

NH2-S Q G M L A L S I S P N R-COOH
y11
y10
y3
y1
b2
b3
b7
b10
b12
Int
m/z
b2
b10
rQ
b3
y3
Prec 2+
b7
y11
b12 2+
y10
y1
iQ
b3 2+

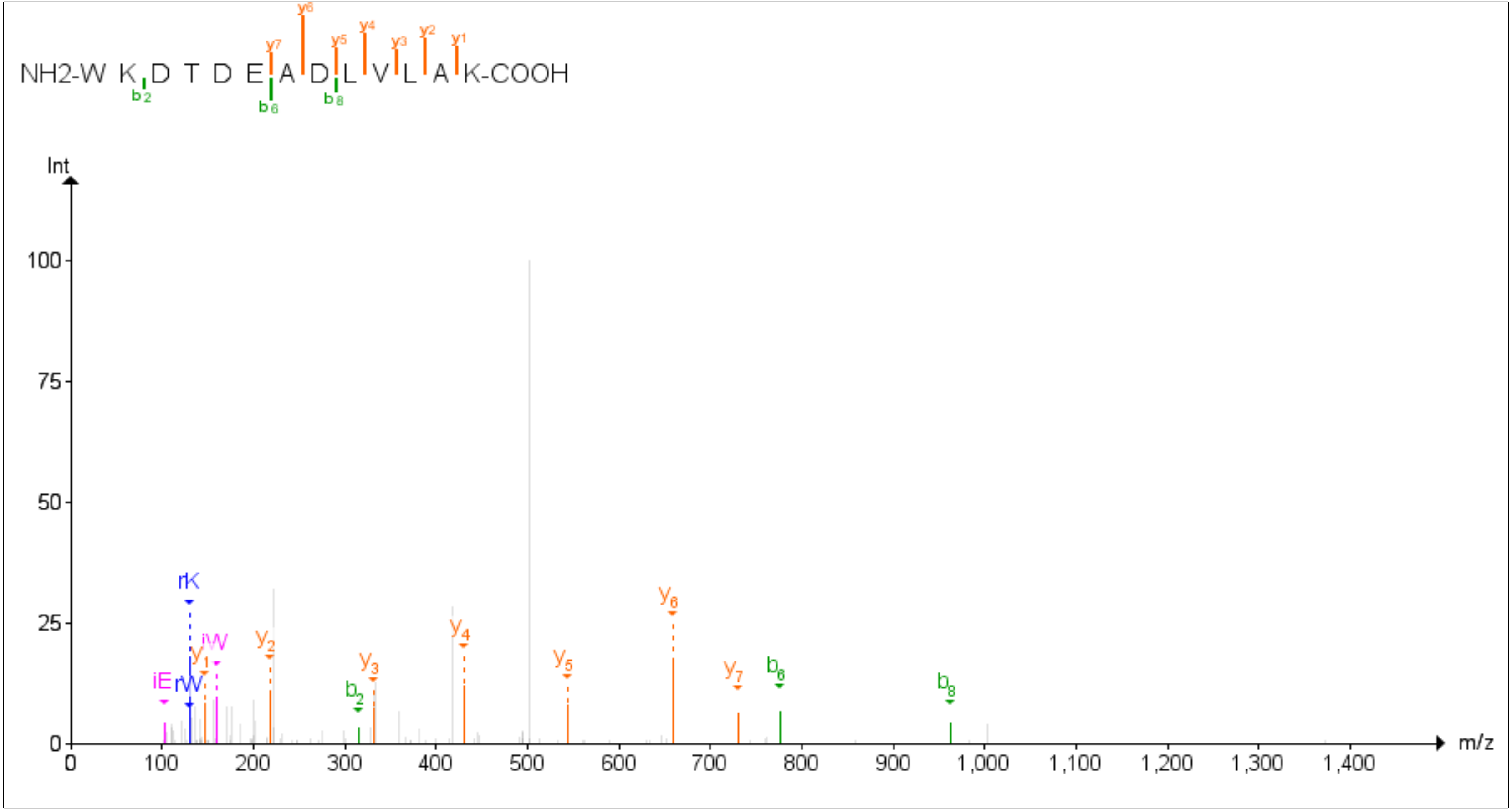

NH2-W K D T D E A D L V L A K-COOH
y7 y6 y5 y4 y3 y2 y1
b2 b6 b8
Int
100
75
50
25
0
rK
iW
y1
iE
rW
y2
b2
y3
y4
y5
y6
y7
b6
b8
0 100 200 300 400 500 600 700 800 900 1,000 1,100 1,200 1,300 1,400
m/z

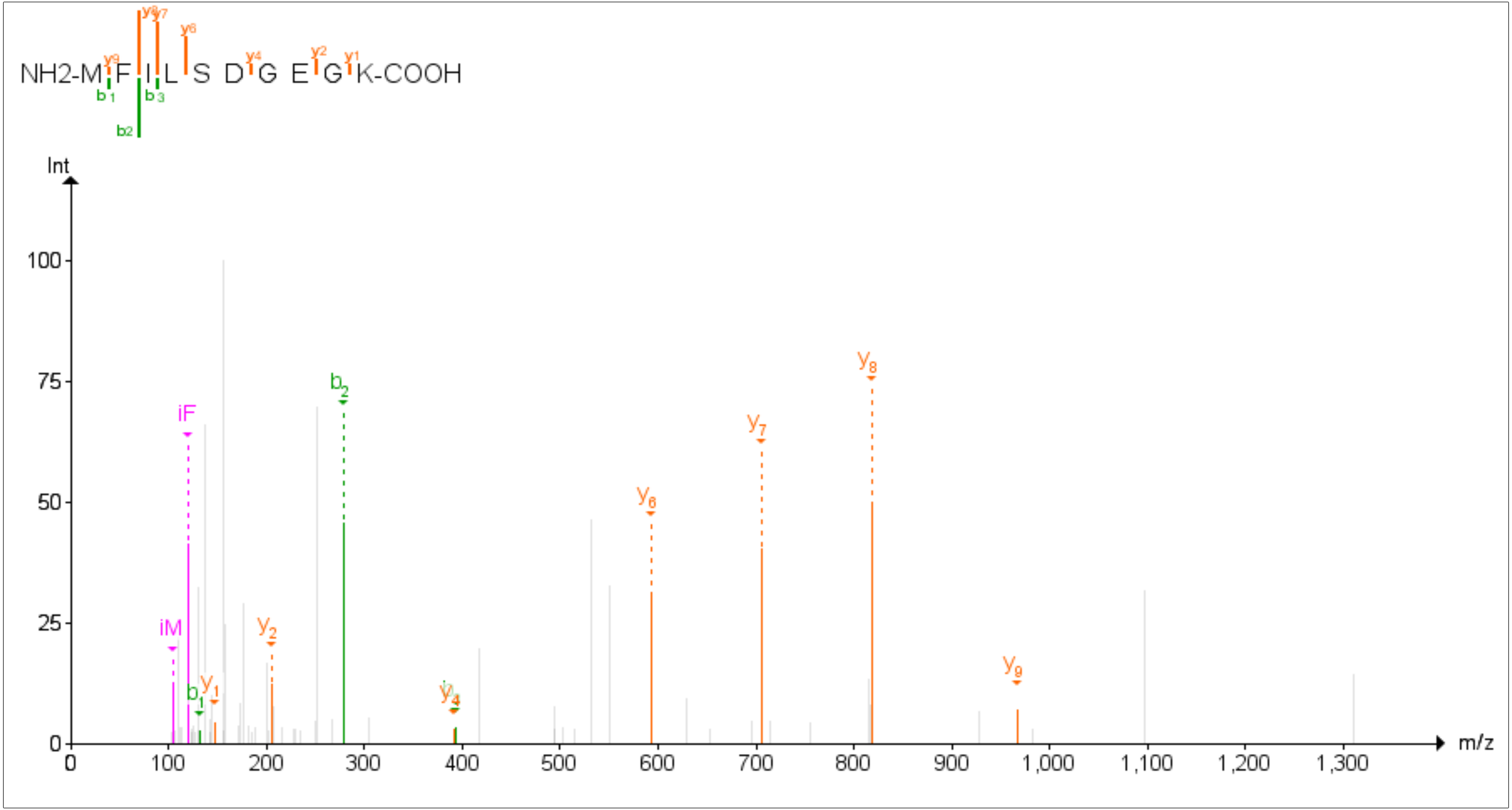

NH2-M F I L S D G E G K-COOH
Int
m/z
iF
iM
b2
b1
y1
y2
y4
y6
y7
y8
y9

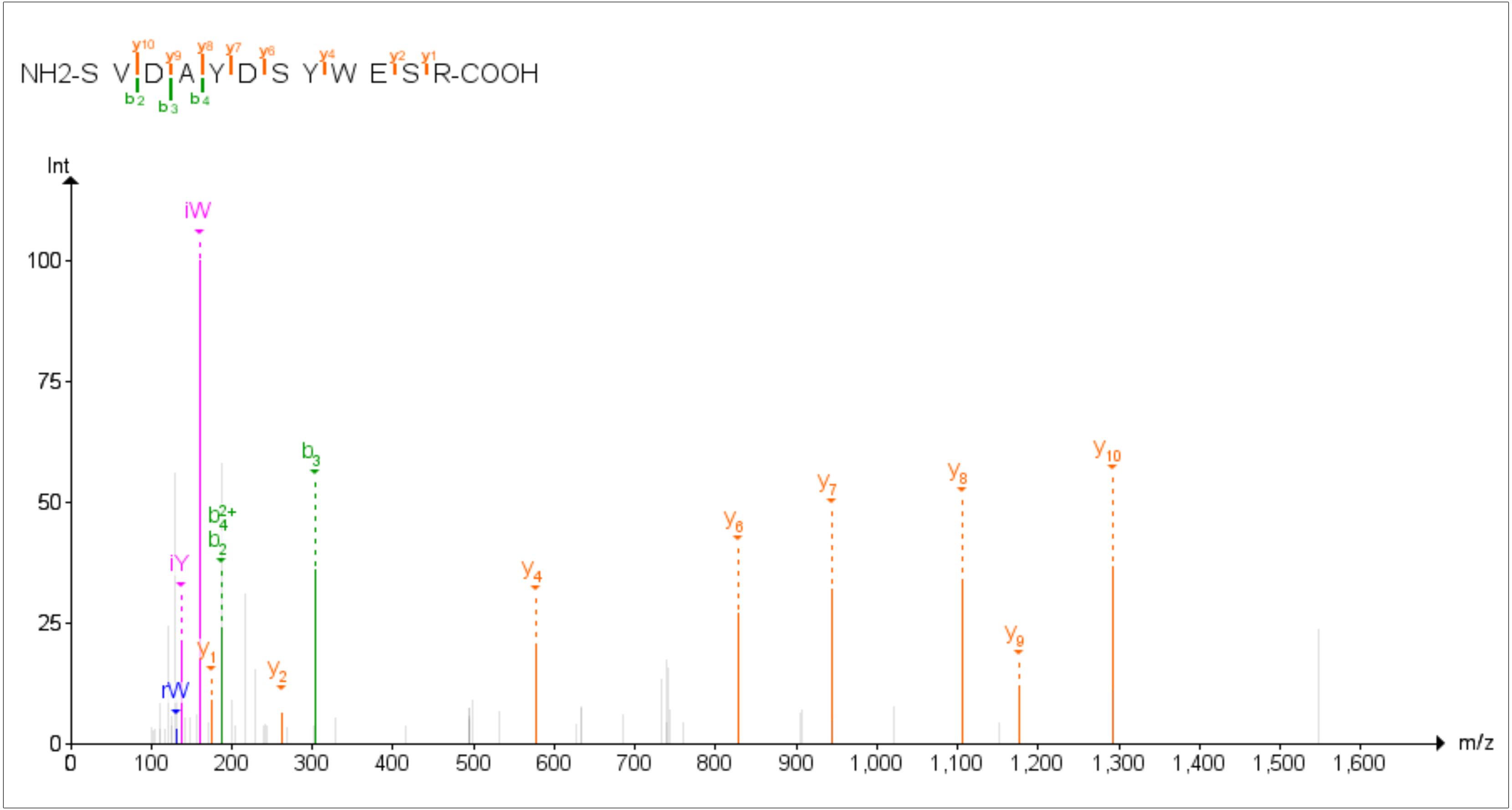

NH2-S V D A Y D S Y W E S R-COOH
y10 y9 y8 y7 y6 y4 y2 y1
b2 b3 b4
Int
iW
iY
rW
y1
y2
b3
b4 2+
b2
y4
y6
y7
y8
y9
y10
0
25
50
75
100
0
100
200
300
400
500
600
700
800
900
1,000
1,100
1,200
1,300
1,400
1,500
1,600
m/z

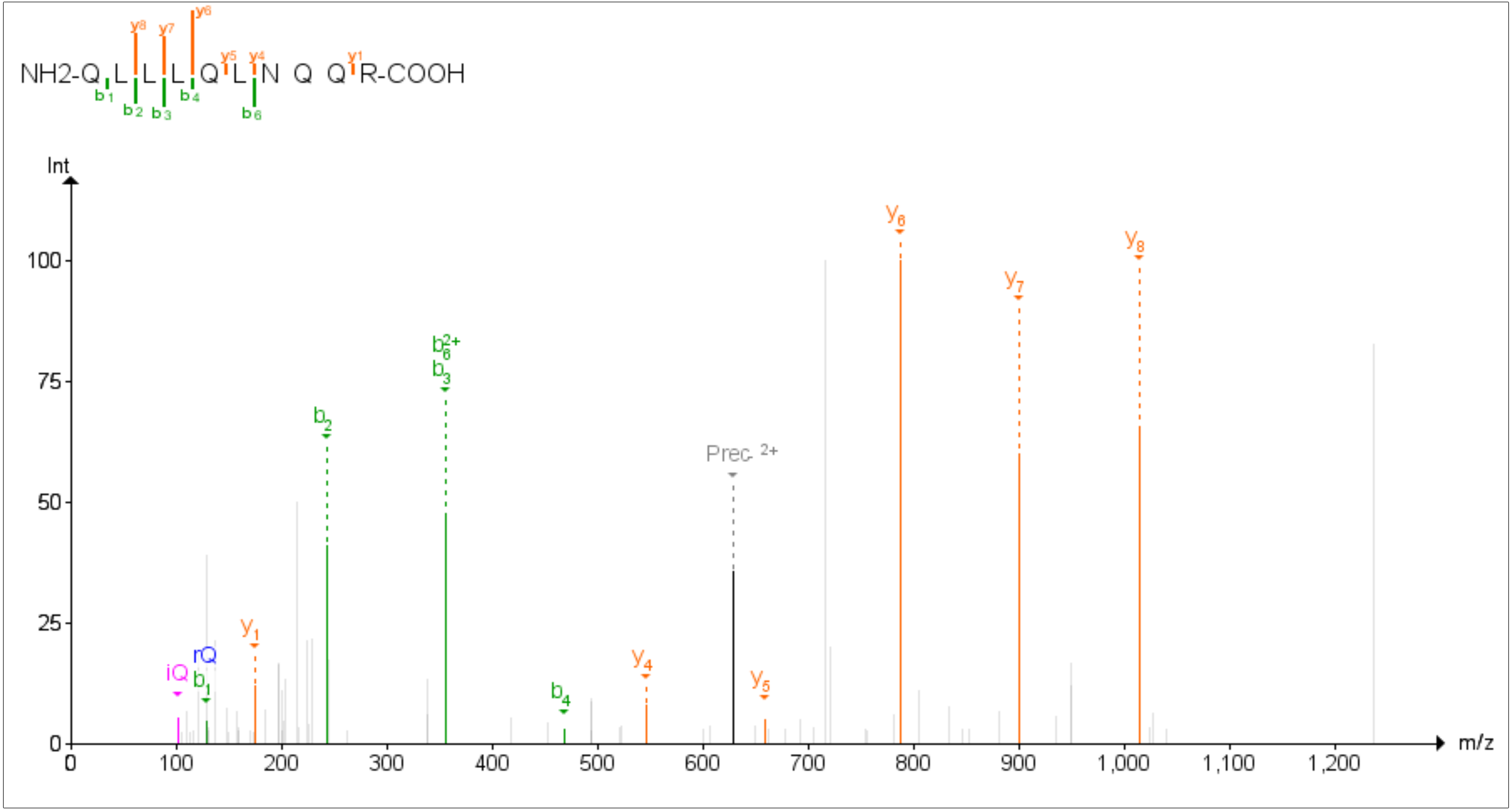

NH2-Q L L L Q L N Q Q R-COOH
Int
m/z
Prec 2+
iQ
rQ

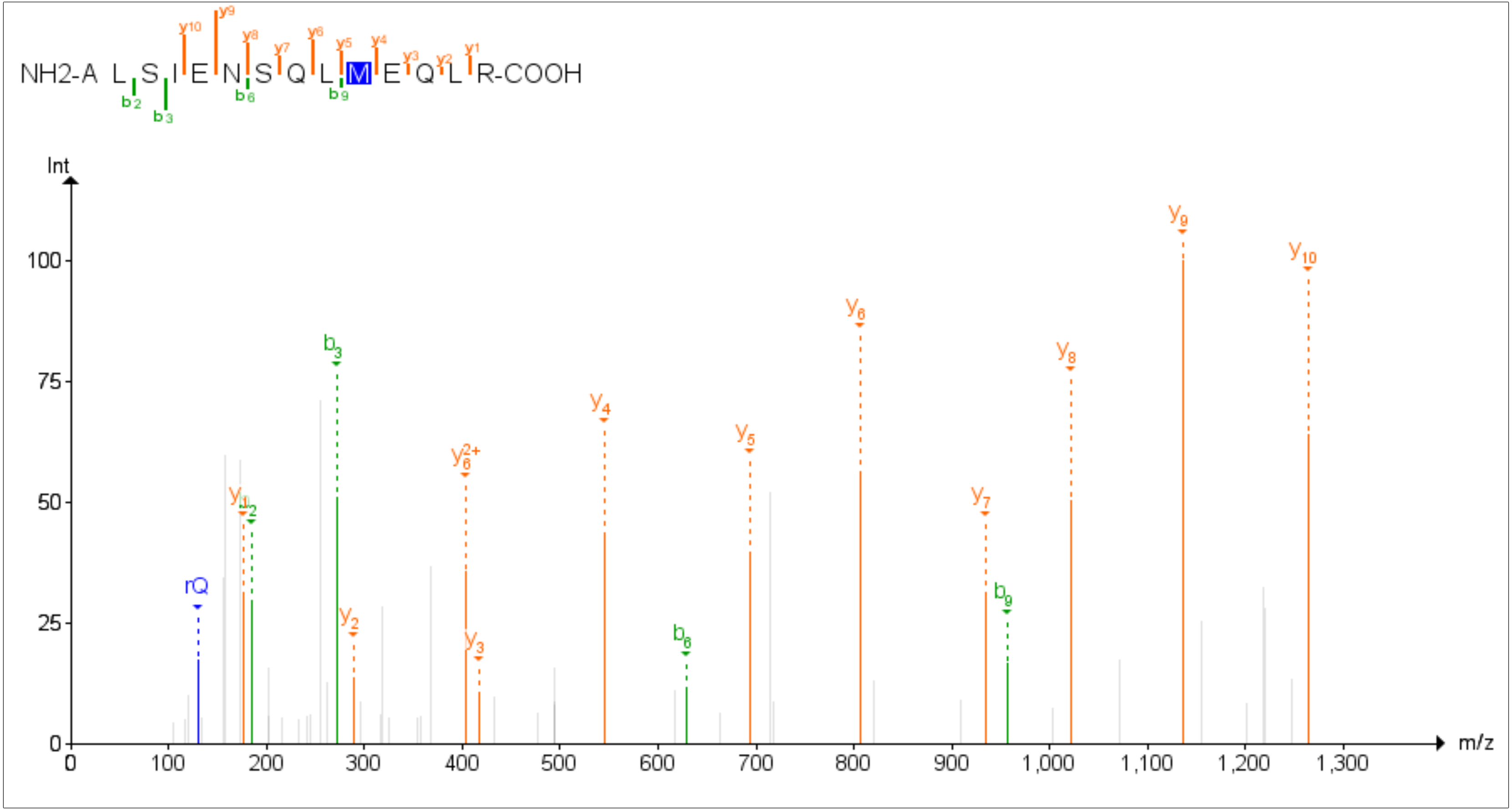

NH2-A L S I E N S Q L M E Q L R-COOH
y10 y9 y8 y7 y6 y5 y4 y3 y2 y1
b2 b3 b6 b9
Int
m/z
0
25
50
75
100
0
100
200
300
400
500
600
700
800
900
1,000
1,100
1,200
1,300
rQ
y1
b2
b3
y2
y3
y4
y5
y6
y7
y8
y9
y10
b6
b9

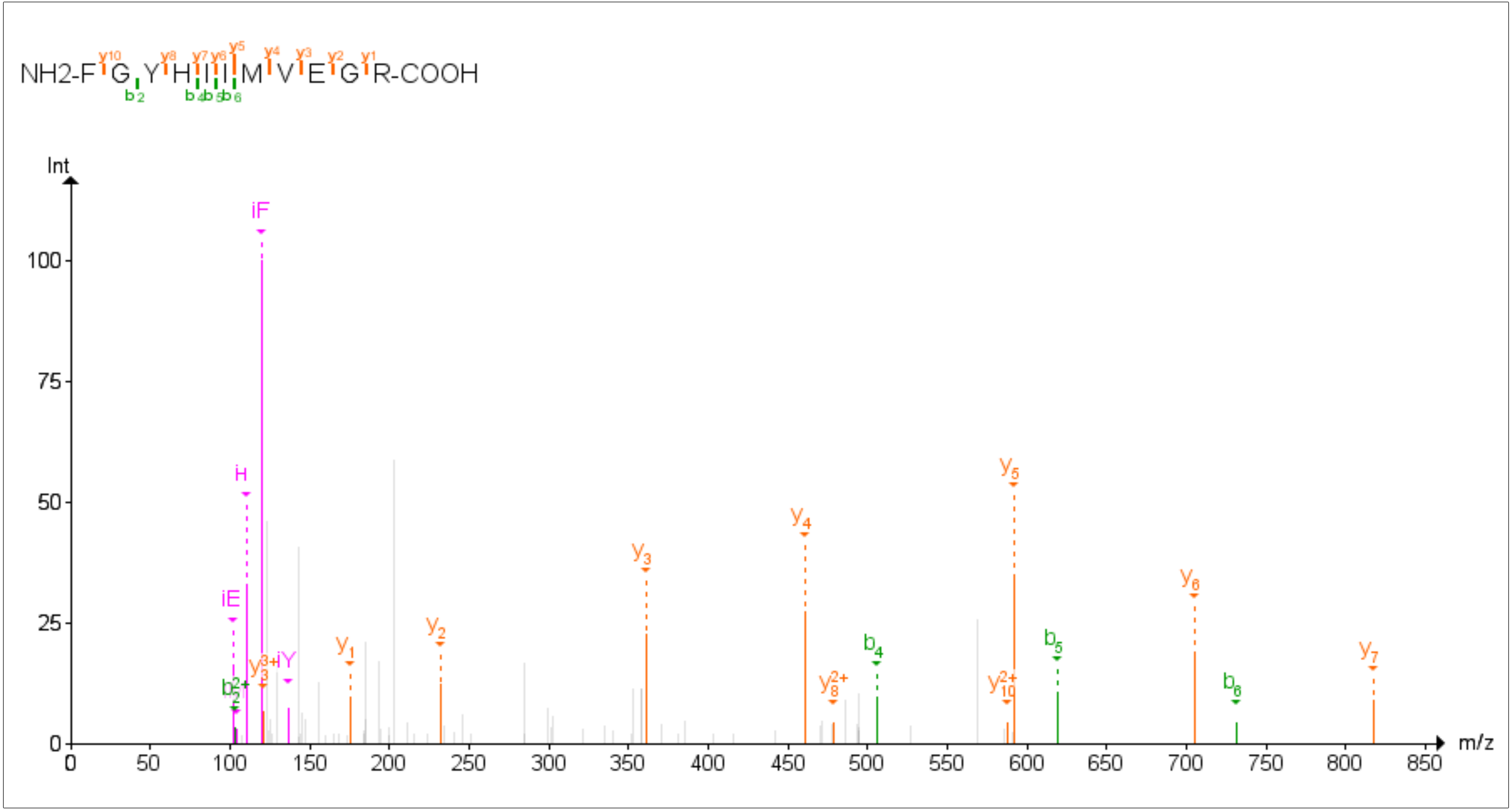

NH2-FGYHIIIMVEGR-COOH
y10 y8 y7 y6 y5 y4 y3 y2 y1
b2 b4 b5 b6
Int
m/z
iF
iH
iE
iY
y1
y2
y3
y4
y5
y6
y7
b4
b5
b6

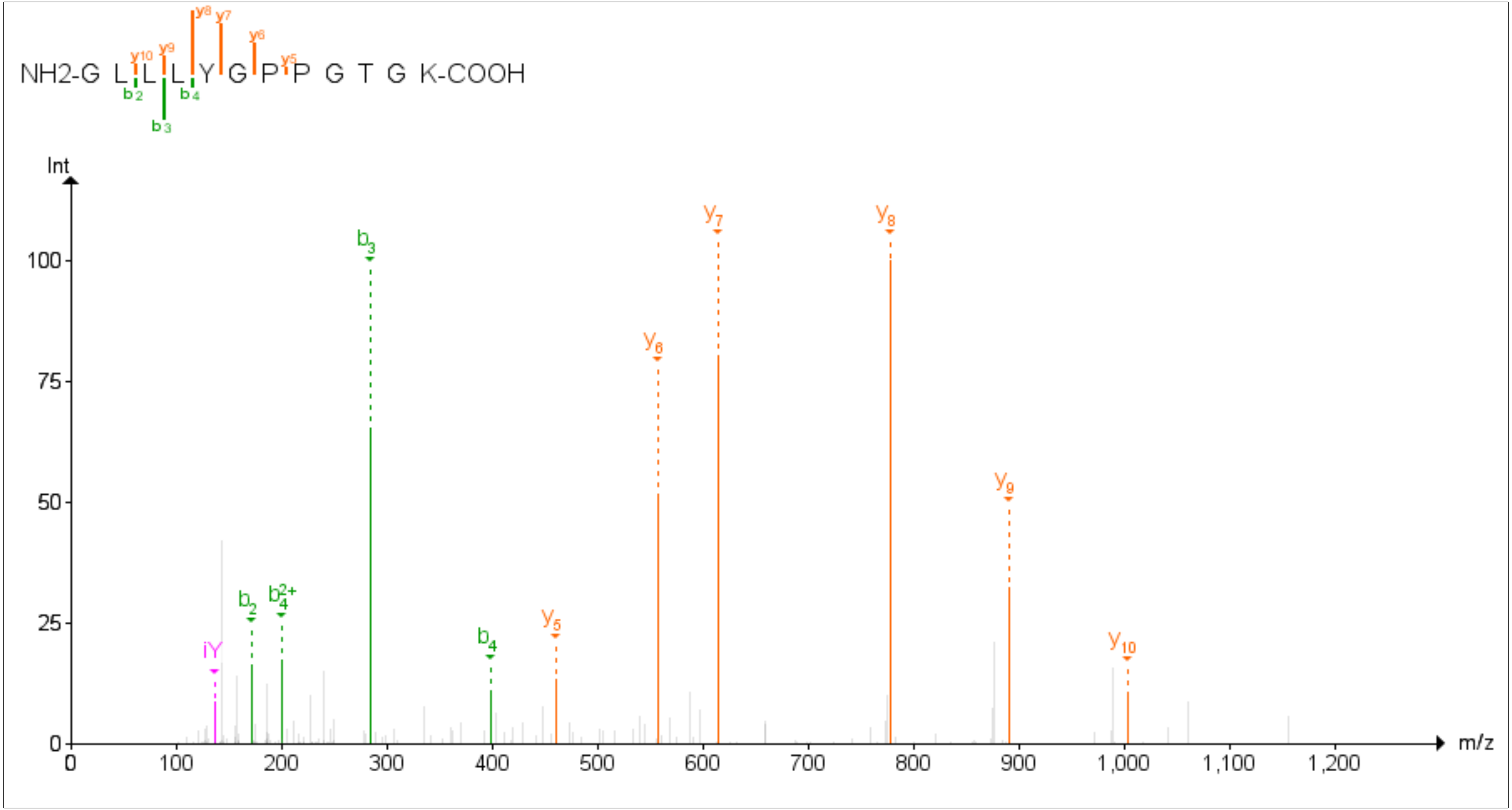

NH2-G L L L Y G P P G T G K-COOH
y10 y9 y8 y7 y6 y5
b2 b3 b4
Int
100
75
50
25
0
b3
y7
y8
y6
y9
y10
y5
b4
b2
b_4^{2+}
iY
0 100 200 300 400 500 600 700 800 900 1,000 1,100 1,200
m/z

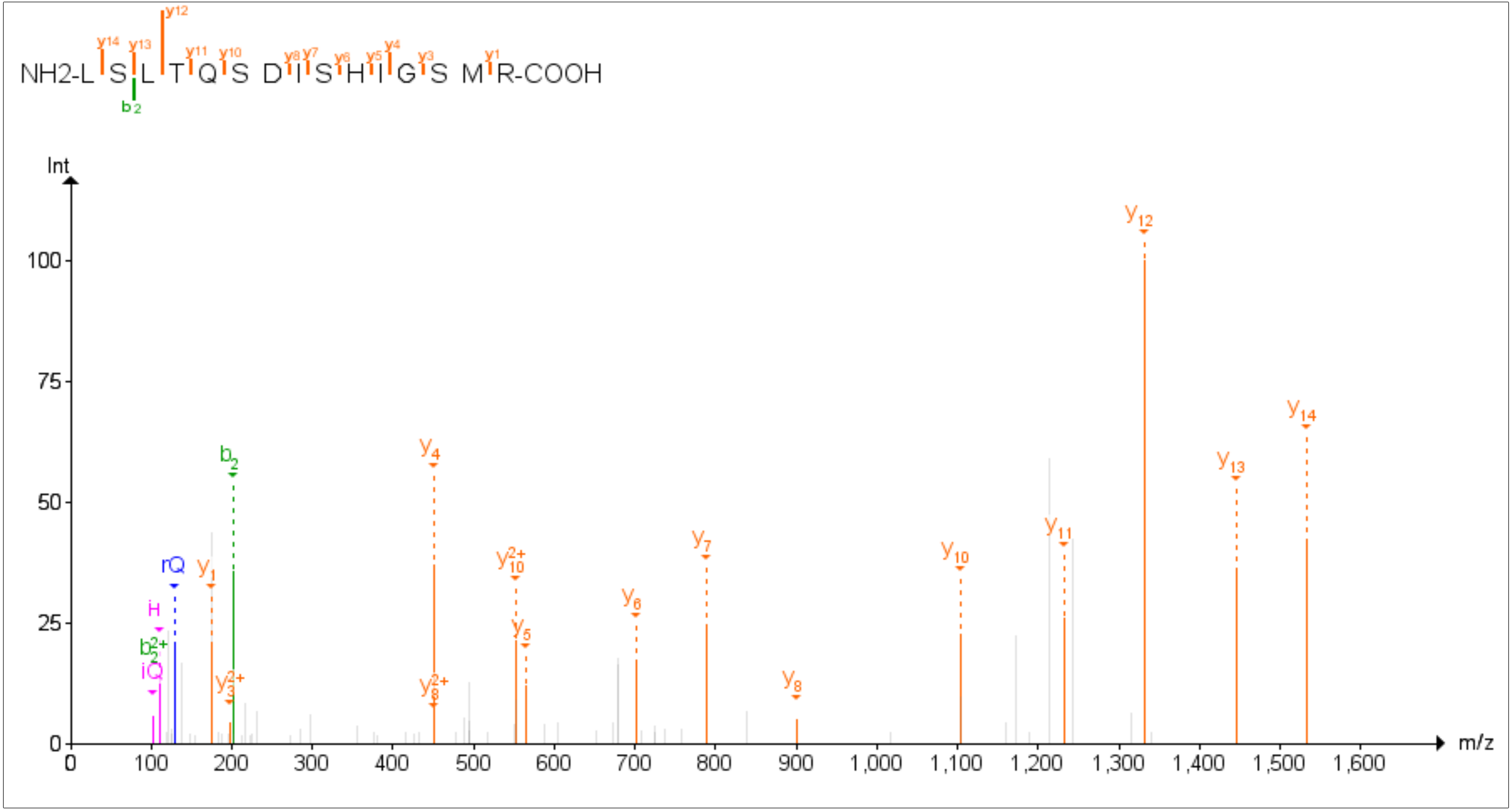

NH2-L S L T Q S D I S H I G S M R-COOH
y14 y13 y12 y11 y10 y8 y7 y6 y5 y4 y3 y1
b2
Int
m/z
0
25
50
75
100
0
100
200
300
400
500
600
700
800
900
1,000
1,100
1,200
1,300
1,400
1,500
1,600
y12
y14
y13
y11
y10
y7
y6
y5
y8
y4
b2
y10 2+
y8 2+
y3 2+
y1
rQ
H
b2 2+
iQ

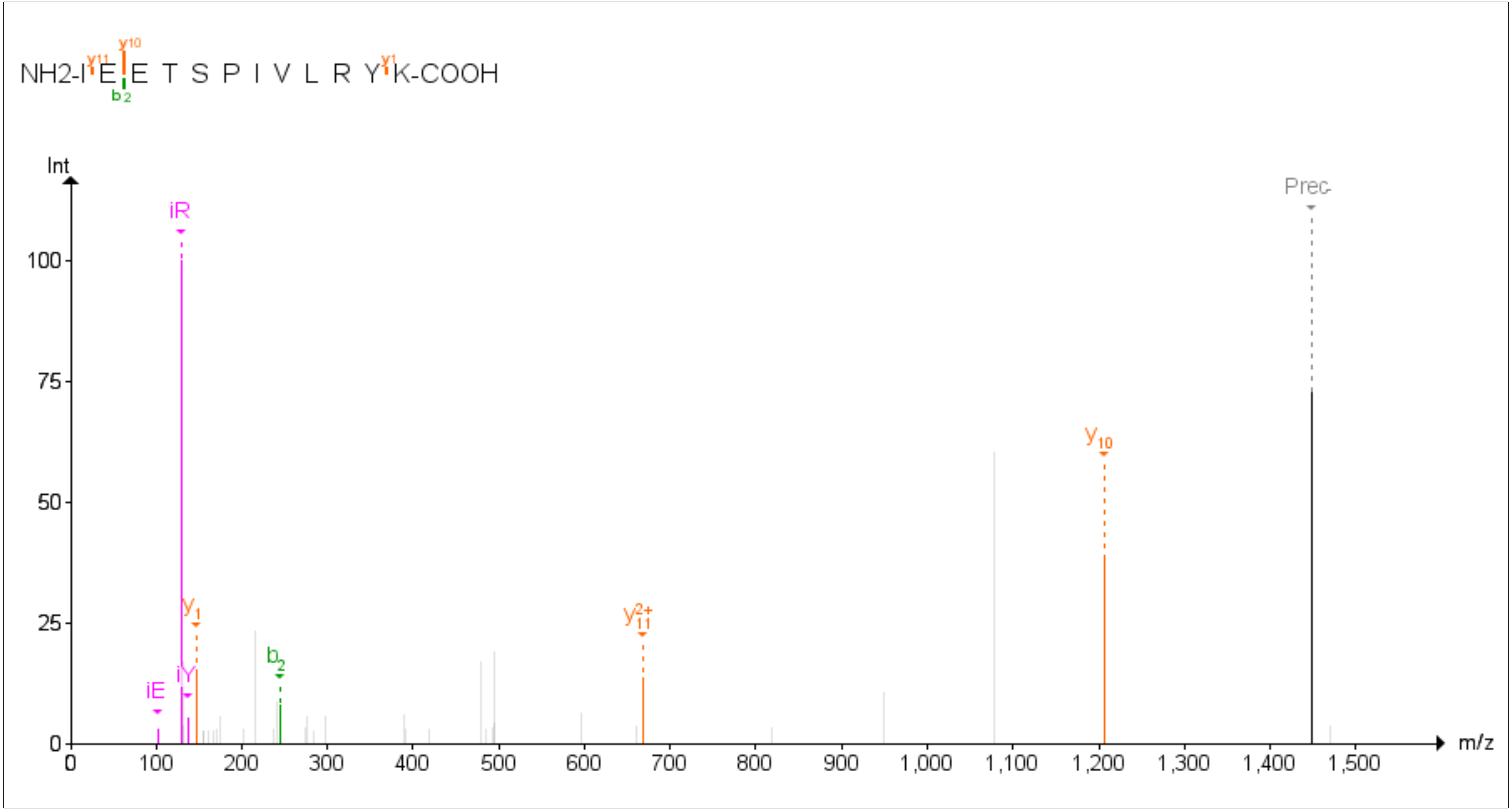

NH2-I E E T S P I V L R Y K-COOH
y11
y10
y1
b2
Int
100
75
50
25
0
iR
y1
iY
iE
b2
y11 2+
y10
Prec
0
100
200
300
400
500
600
700
800
900
1,000
1,100
1,200
1,300
1,400
1,500
m/z

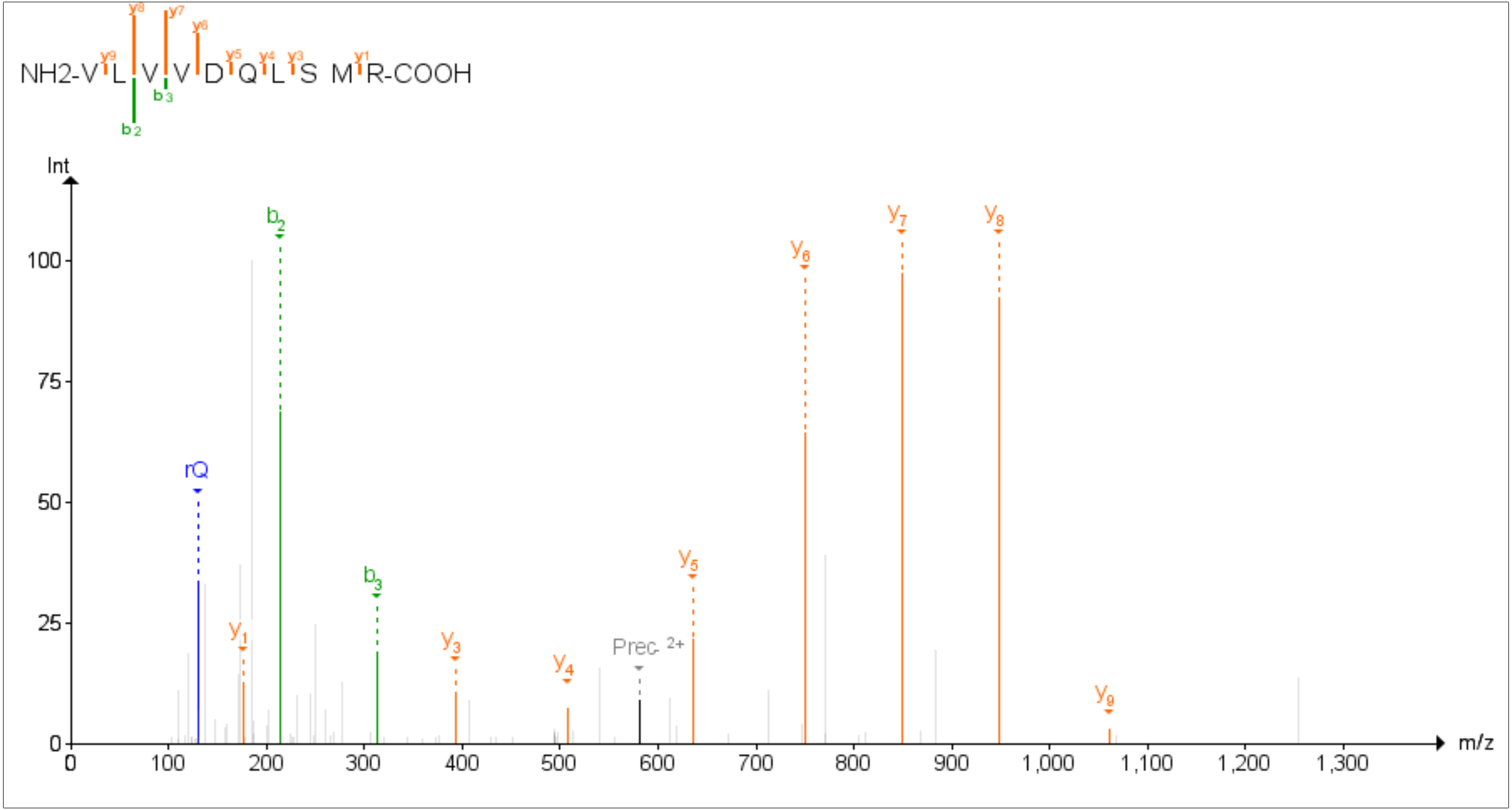

NH2-V L V V D Q L S M R-COOH
y9 y8 y7 y6 y5 y4 y3 y1
b2 b3
Int
100
75
50
25
0
rQ
b2
b3
y1
y3
y4
Prec 2+
y5
y6
y7
y8
y9
0 100 200 300 400 500 600 700 800 900 1,000 1,100 1,200 1,300
m/z

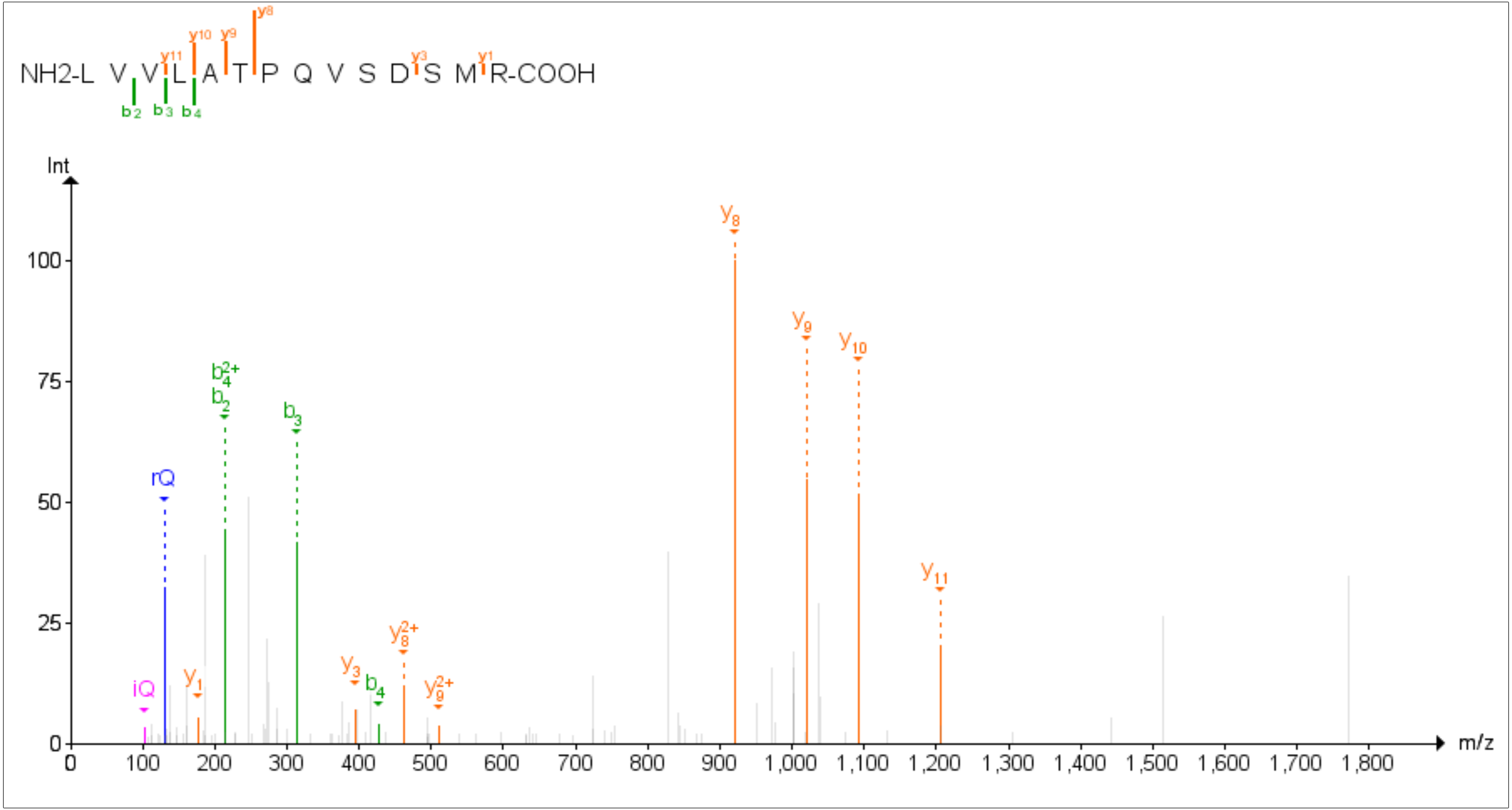
NH2-L V V L A T P Q V S D S M R-COOH
y11 y10 y9 y8 y3 y1
b2 b3 b4
Int
m/z
0
25
50
75
100
0 100 200 300 400 500 600 700 800 900 1,000 1,100 1,200 1,300 1,400 1,500 1,600 1,700 1,800
y8
y9
y10
y11
b4 2+
b2
b3
rQ
iQ
y1
y3
b4
y8 2+
y9 2+

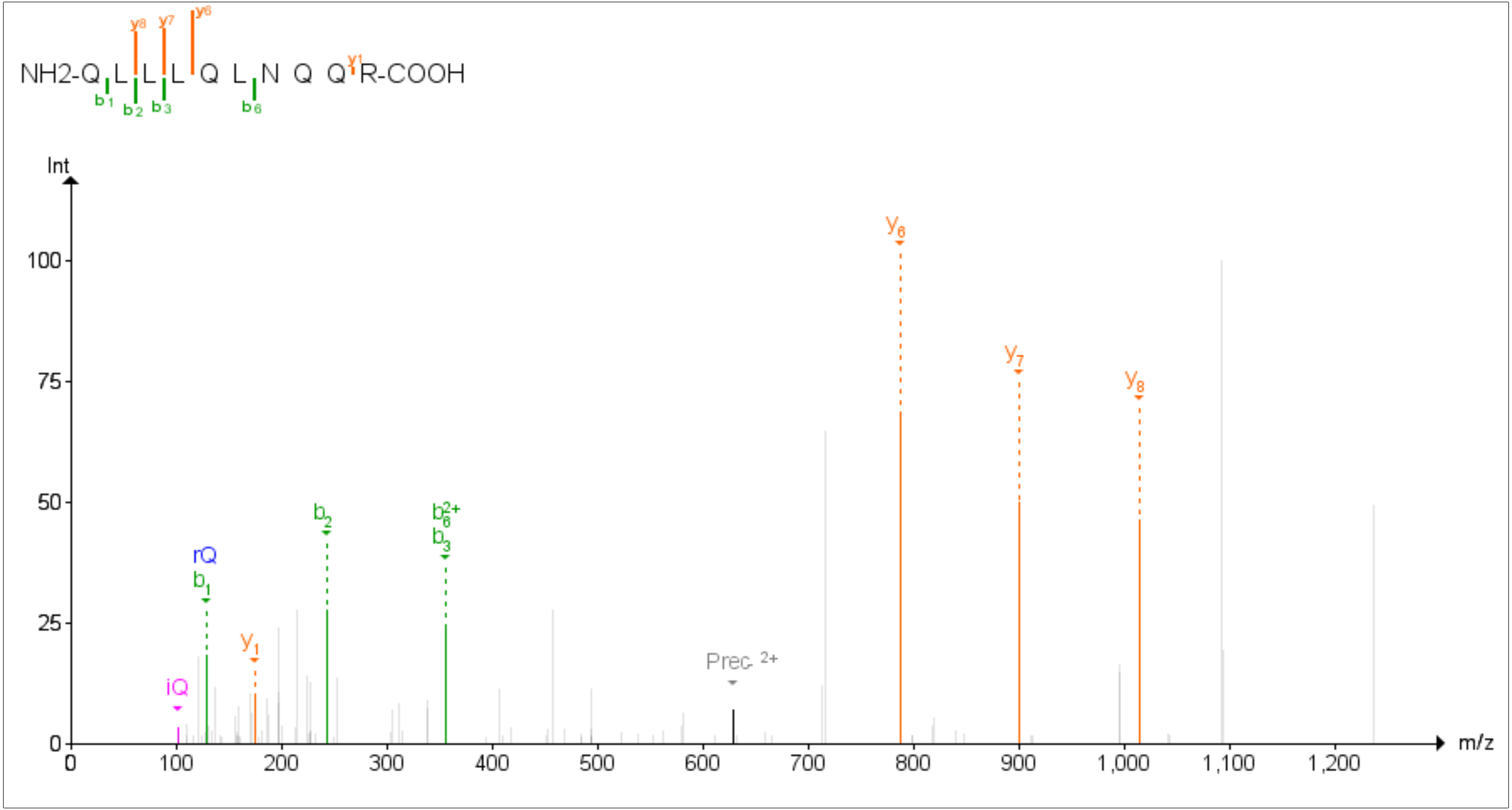

NH2-Q L L L Q L N Q Q R-COOH
y8 y7 y6 y1
b1 b2 b3 b6
Int
m/z
y6
y7
y8
b2
b6 2+
b3
rQ
b1
y1
iQ
Prec. 2+
0
25
50
75
100
0
100
200
300
400
500
600
700
800
900
1,000
1,100
1,200

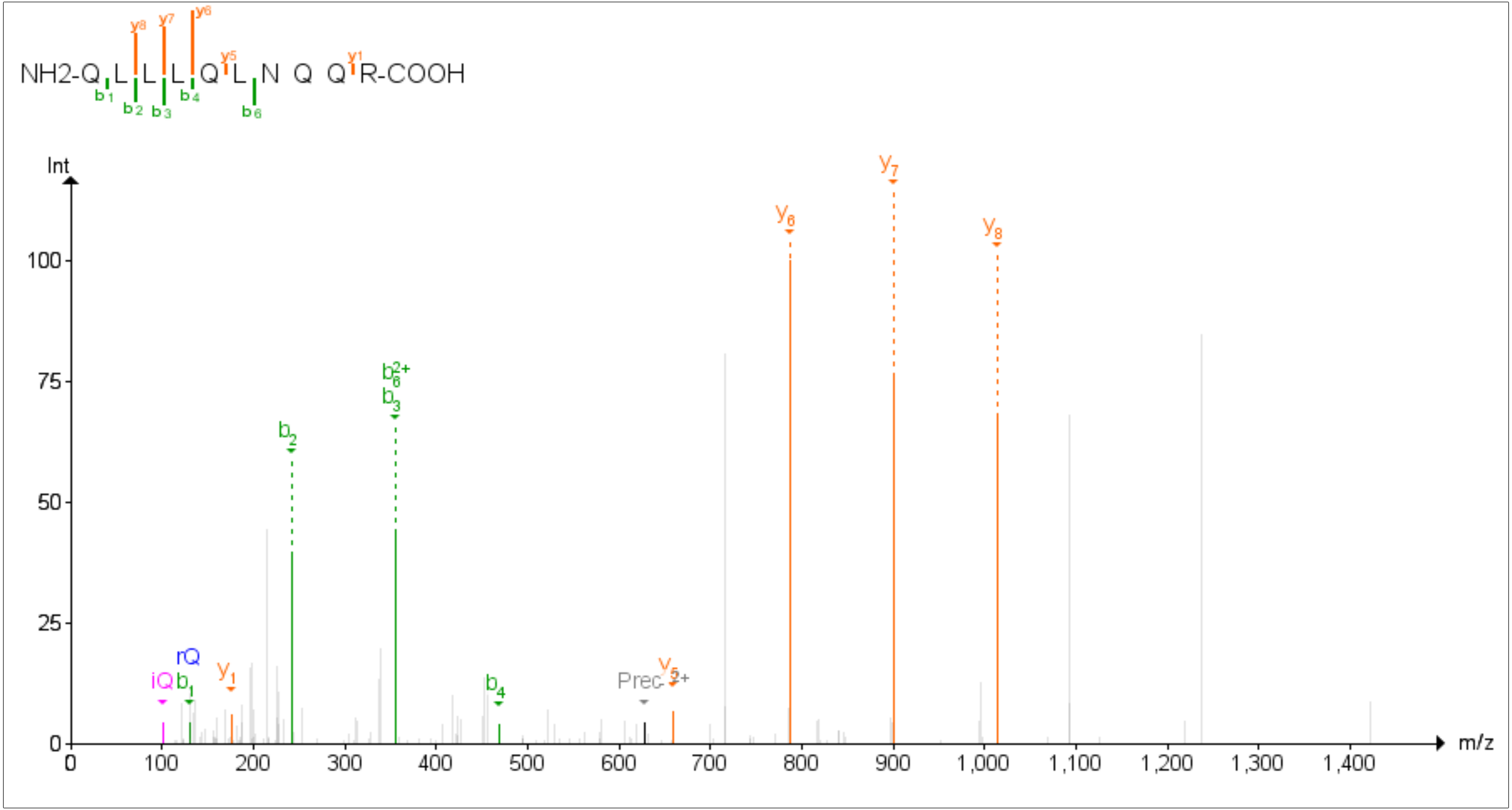
NH2-Q L L L Q L N Q Q R-COOH
y8 y7 y6 y5 y1
b1 b2 b3 b4 b6
Int
m/z
y7
y6
y8
b6 2+
b3
b2
rQ
iQ b1
y1
b4
Prec 2+
y5
0
25
50
75
100
0 100 200 300 400 500 600 700 800 900 1,000 1,100 1,200 1,300 1,400

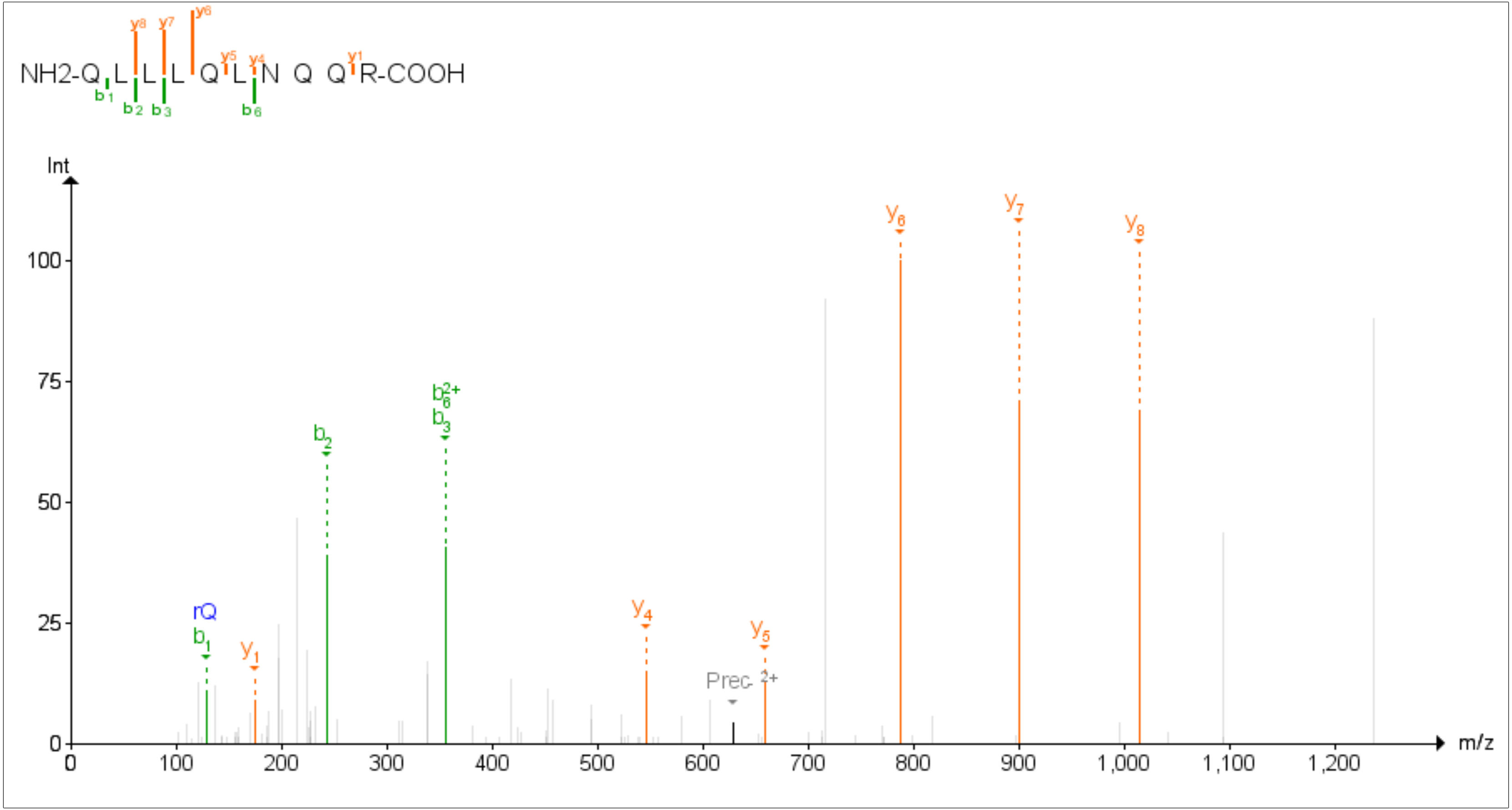

NH2-Q L L L Q L N Q Q R-COOH
y8 y7 y6 y5 y4 y1
b1 b2 b3 b6
Int
m/z
rQ
b1
y1
b2
b6 2+
b3
y4
Prec. 2+
y5
y6
y7
y8

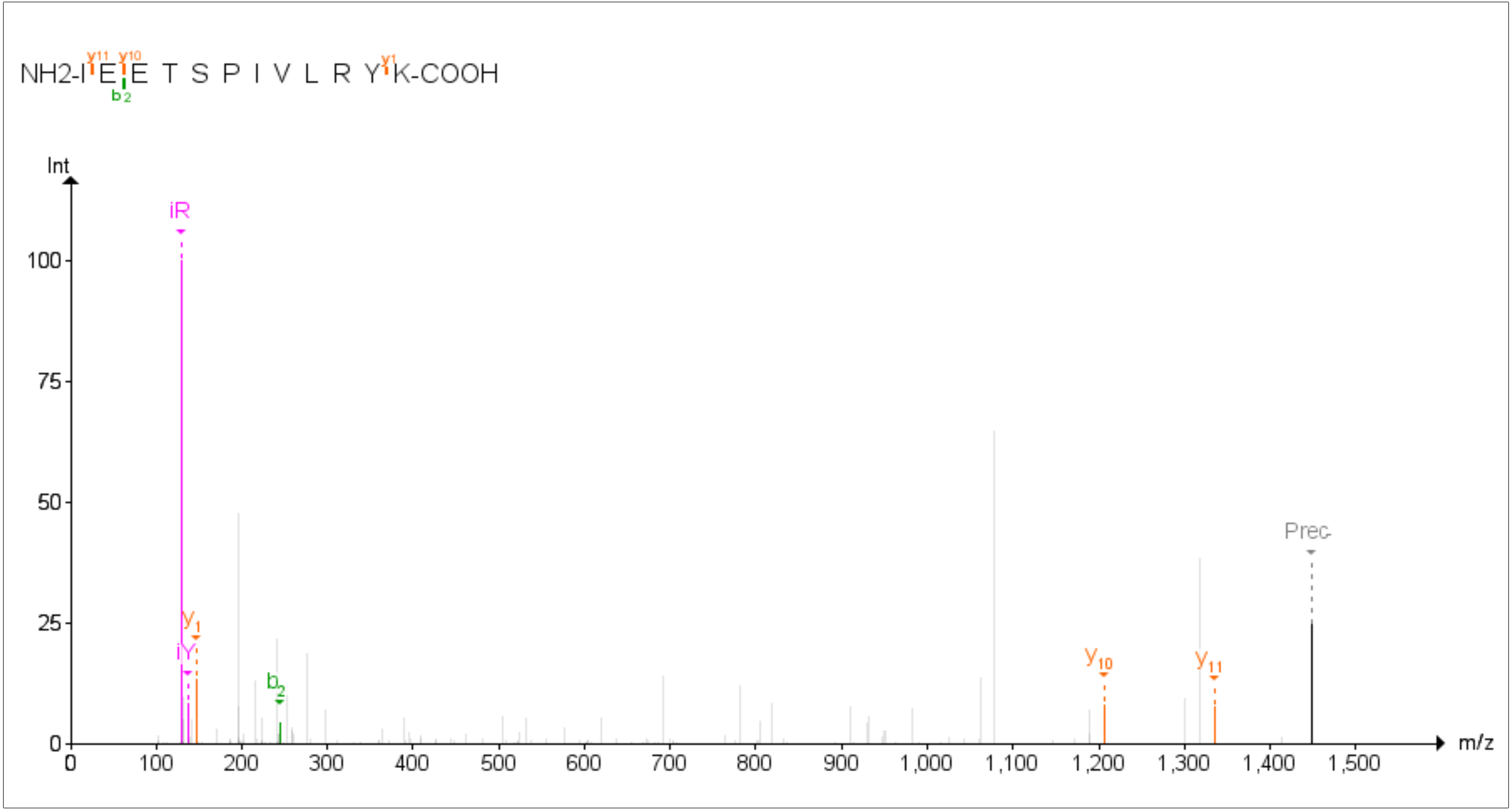

NH2-I E E T S P I V L R Y K-COOH
y11
y10
y1
b2
Int
iR
y1
iY
b2
y10
y11
Prec
100
75
50
25
0
0
100
200
300
400
500
600
700
800
900
1,000
1,100
1,200
1,300
1,400
1,500
m/z

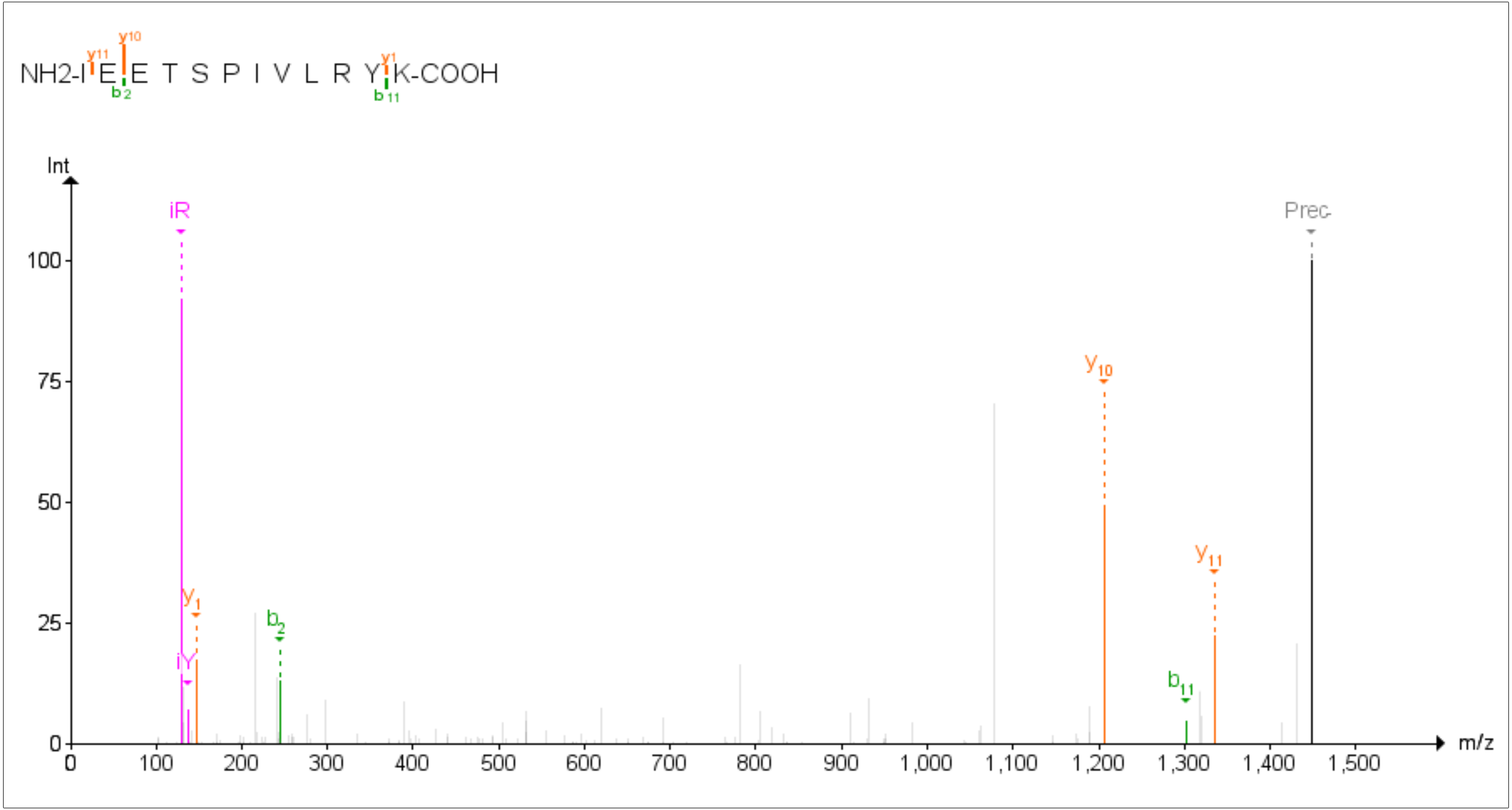
NH2-I E E T S P I V L R Y K-COOH
y11
y10
y1
b2
b11
Int
iR
Prec
y10
y11
y1
iY
b2
b11
100
75
50
25
0
0
100
200
300
400
500
600
700
800
900
1,000
1,100
1,200
1,300
1,400
1,500
m/z

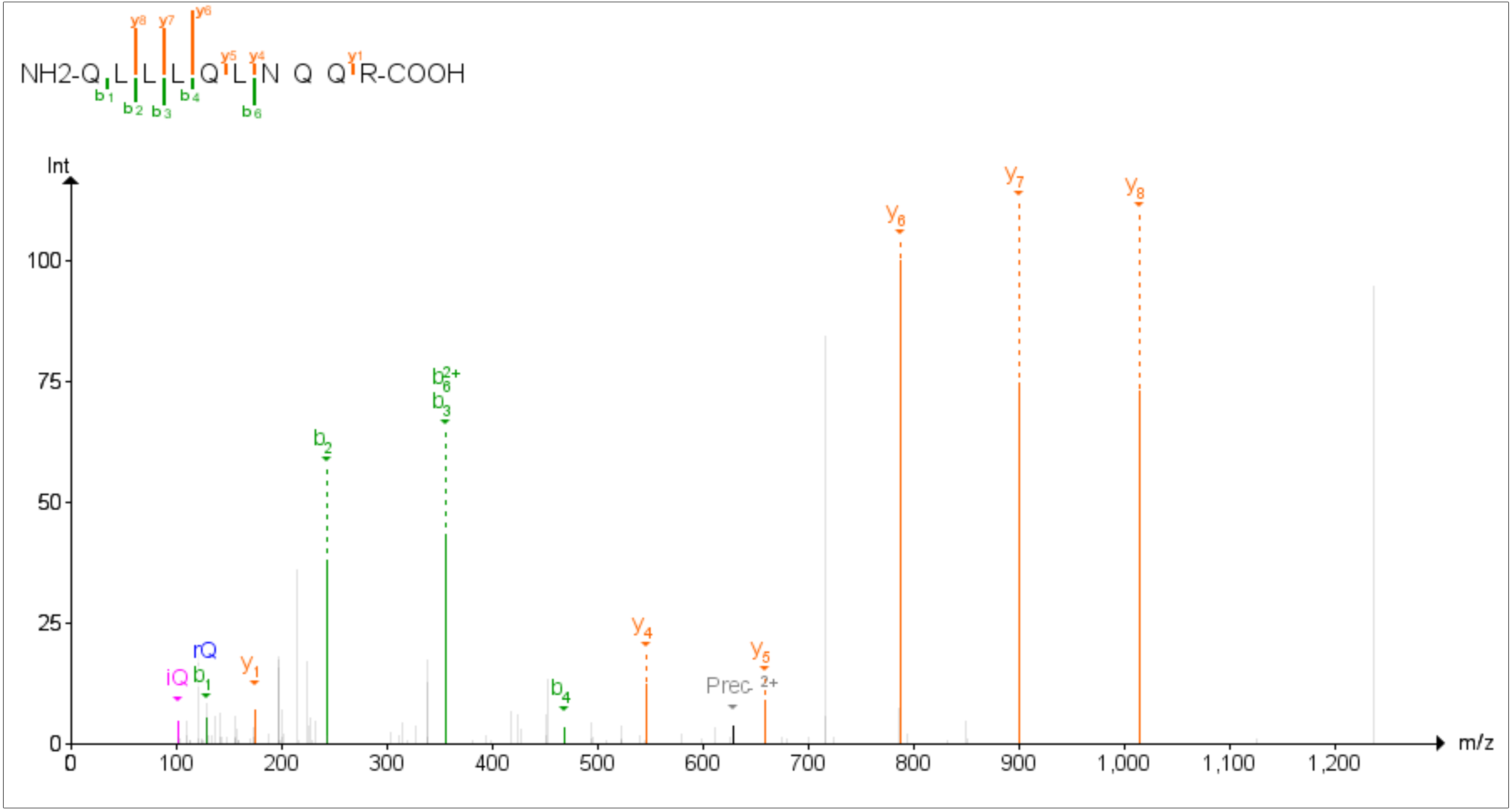

NH2-Q L L L Q L N Q Q R-COOH
y8 y7 y6 y5 y4 y1
b1 b2 b3 b4 b6
Int
m/z
y6
y7
y8
b6 2+
b3
b2
y4
y5
Prec 2+
b4
y1
rQ
iQ
b1
0
25
50
75
100
0
100
200
300
400
500
600
700
800
900
1,000
1,100
1,200

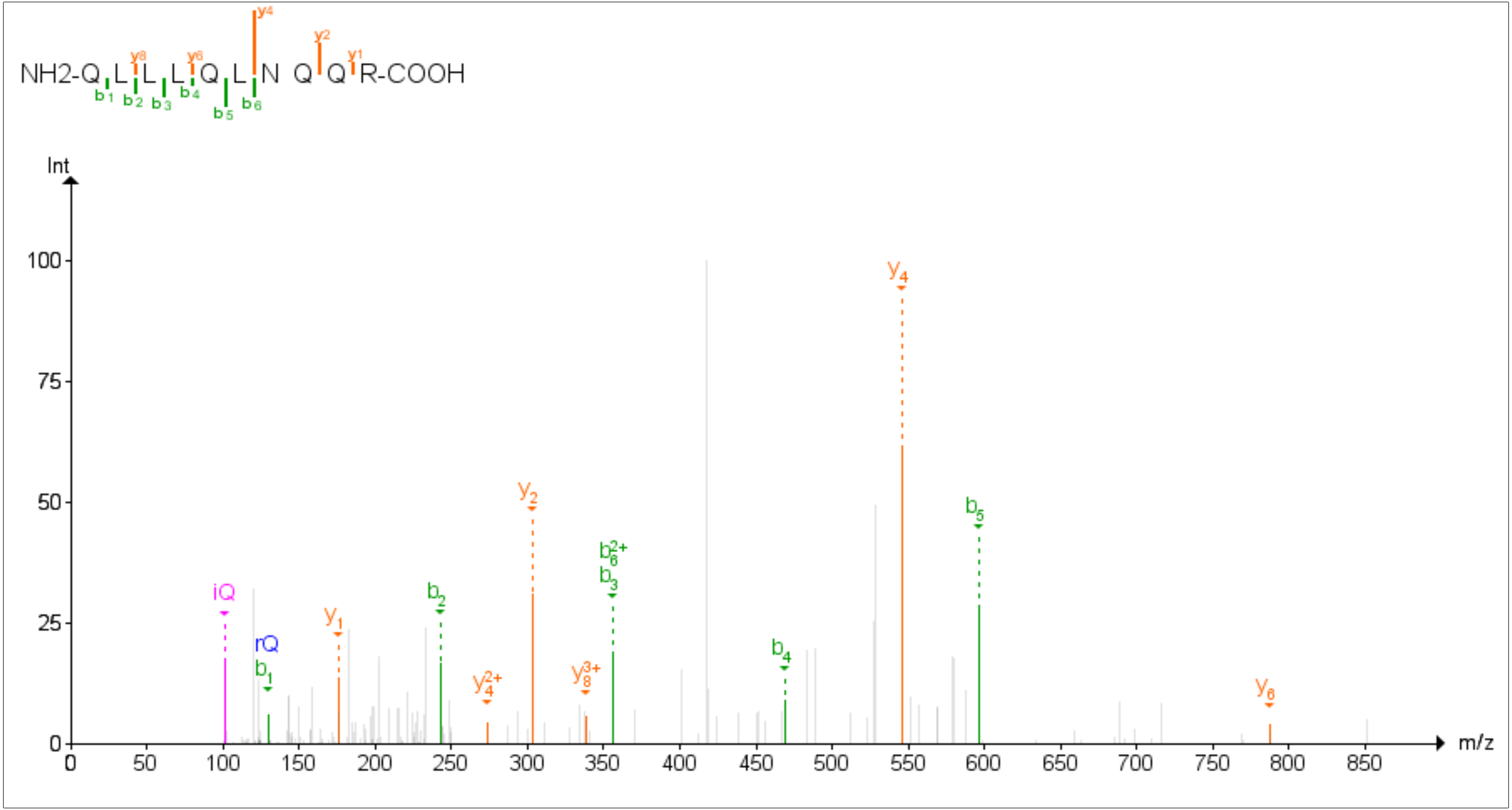

NH2-Q L L L Q L N Q Q R-COOH
y8 y6 y4 y2 y1
b1 b2 b3 b4 b5 b6
Int
100
75
50
25
0
y4
y2
b5
b6 2+
b3
iQ
rQ
b1
y1
b2
y4 2+
y8 3+
b4
y6
m/z
0 50 100 150 200 250 300 350 400 450 500 550 600 650 700 750 800 850

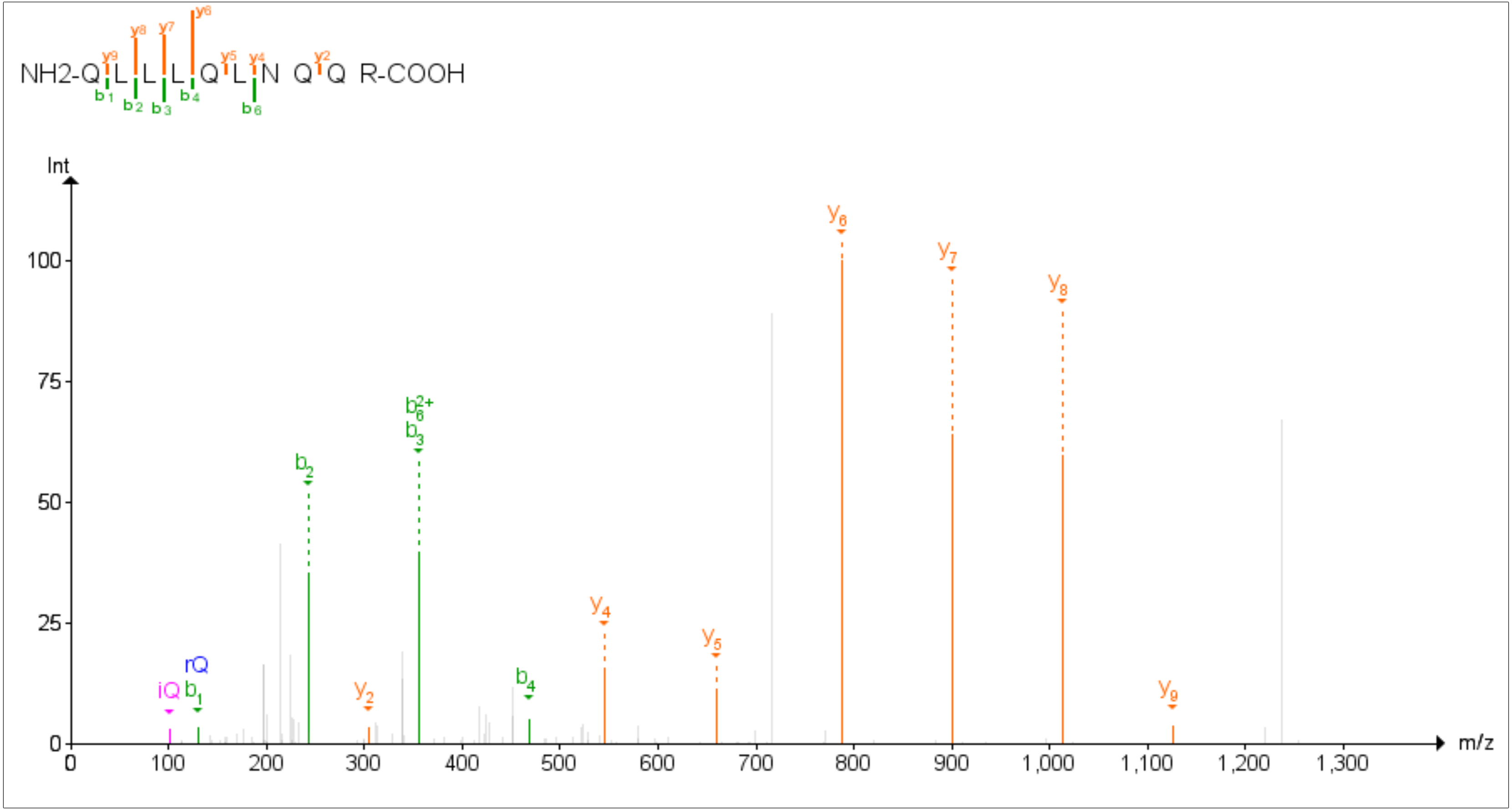
NH2-Q L L L Q L N Q Q R-COOH
y9 y8 y7 y6 y5 y4 y2
b1 b2 b3 b4 b6
Int
100
75
50
25
0
m/z
0 100 200 300 400 500 600 700 800 900 1,000 1,100 1,200 1,300
iQ
rQ
b1
b2
b3
b6 2+
b4
y2
y4
y5
y6
y7
y8
y9

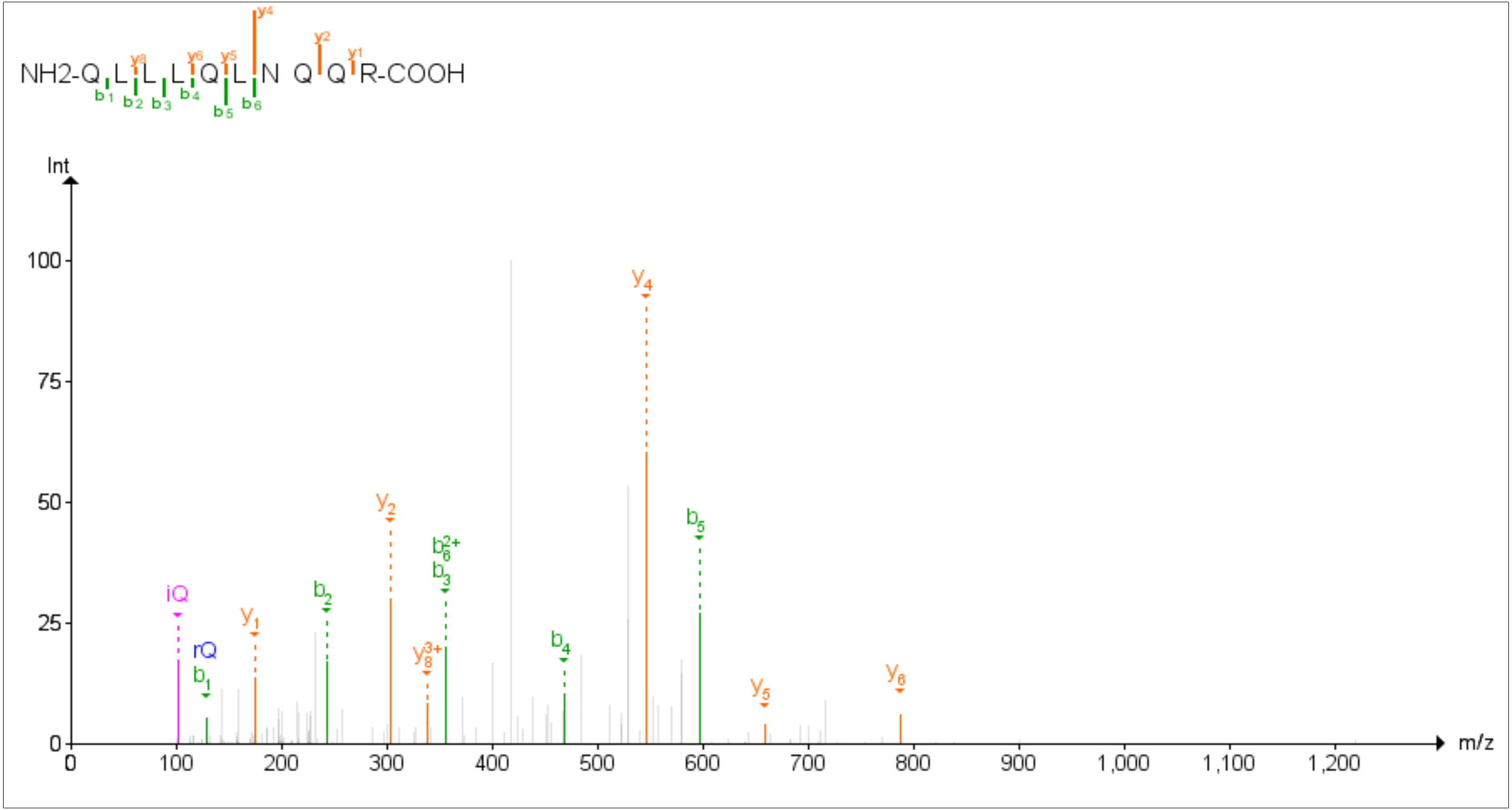

NH2-Q L L L Q L N Q Q R-COOH
b1 b2 b3 b4 b5 b6
y8 y6 y5 y4 y2 y1
Int
100
75
50
25
0
iQ
rQ
b1
y1
b2
y2
y8 3+
b6 2+
b3
b4
y4
b5
y5
y6
0 100 200 300 400 500 600 700 800 900 1,000 1,100 1,200
m/z

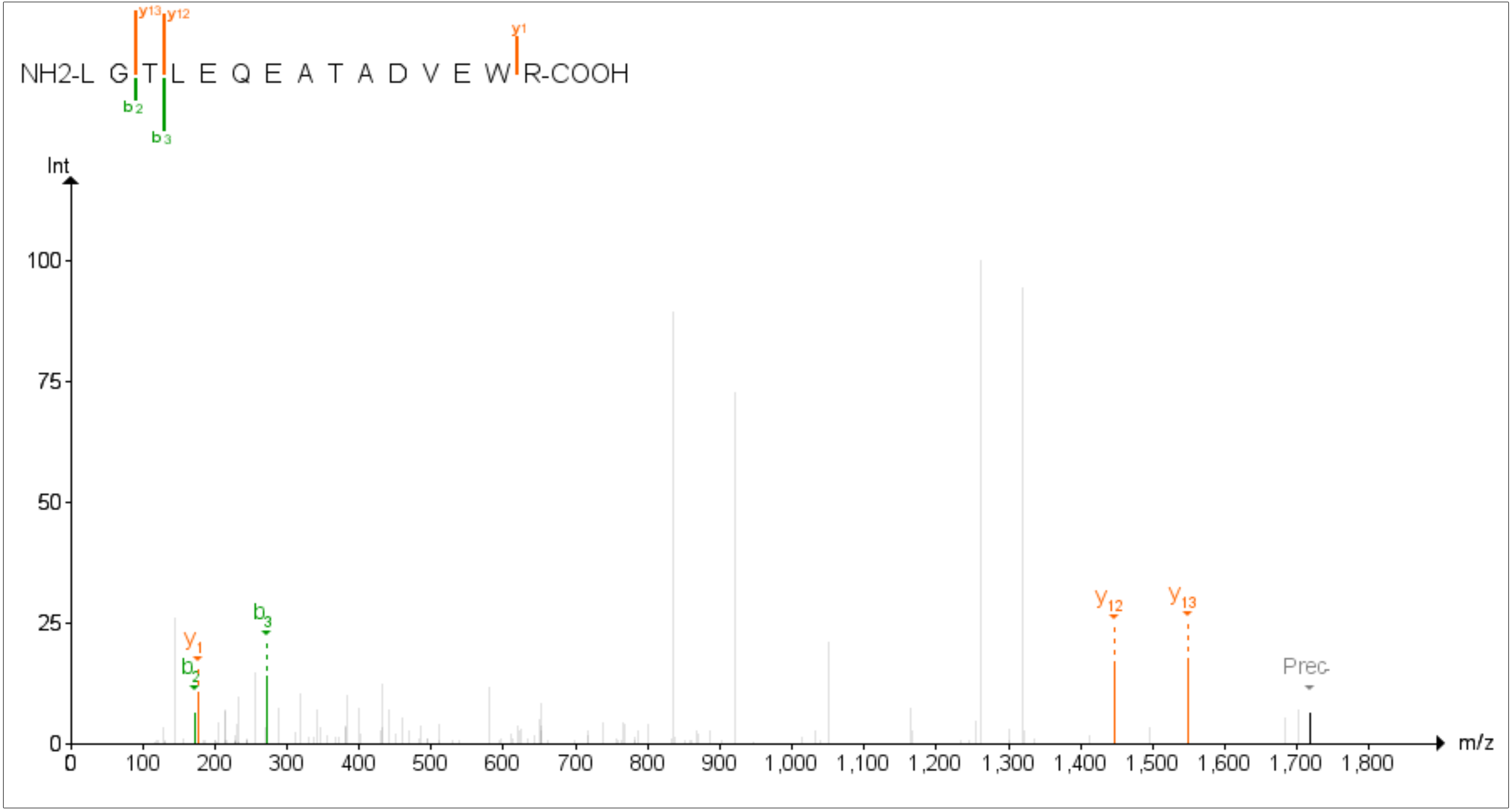

NH2-L G T L E Q E A T A D V E W R-COOH
y13
y12
y1
b2
b3
Int
100
75
50
25
0
m/z
0
100
200
300
400
500
600
700
800
900
1,000
1,100
1,200
1,300
1,400
1,500
1,600
1,700
1,800
y1
b2
b3
y12
y13
Prec

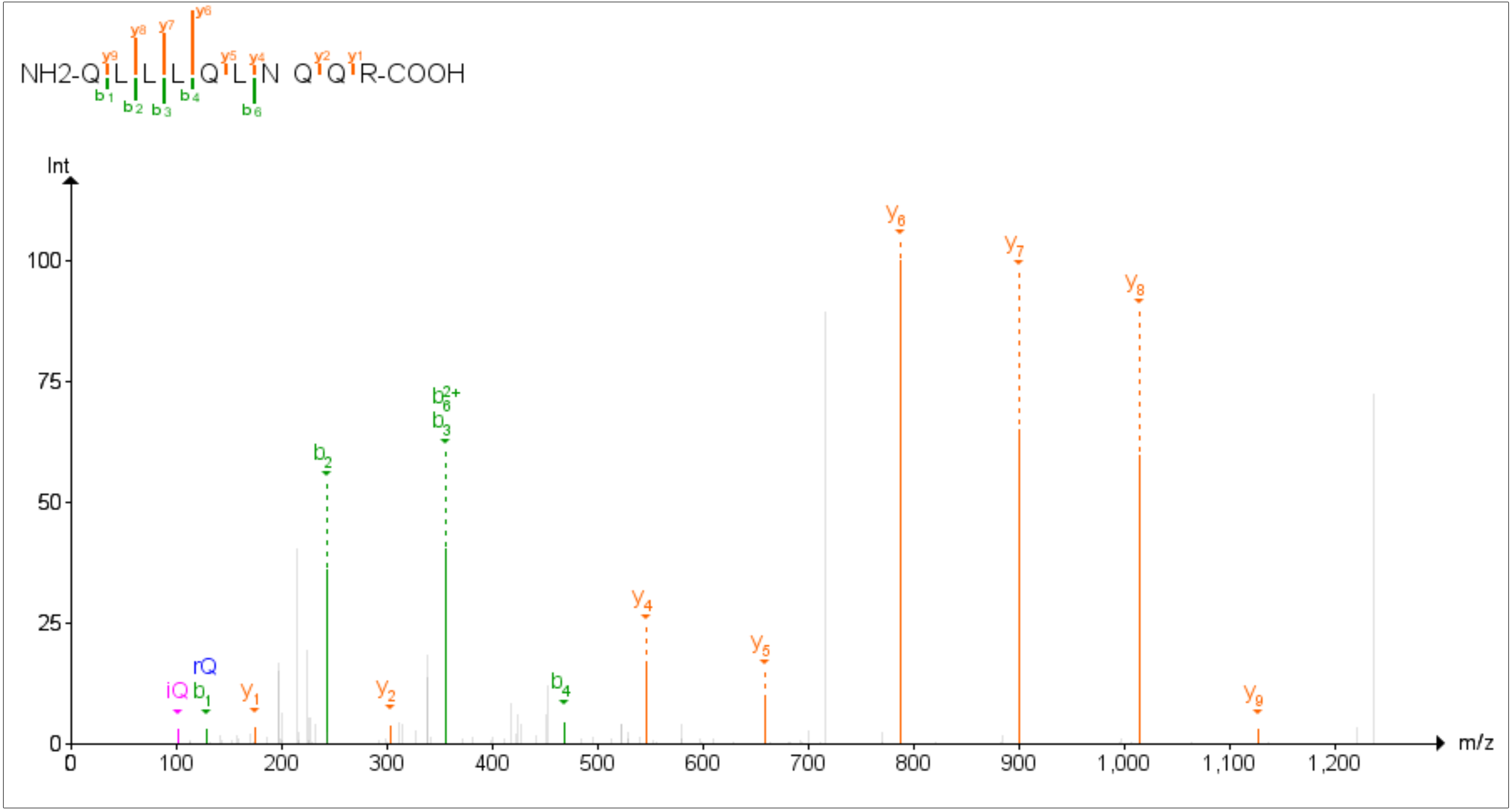

NH2-Q L L L Q L N Q Q R-COOH
y9 y8 y7 y6 y5 y4 y2 y1
b1 b2 b3 b4 b6
Int
100
75
50
25
0
y6
y7
y8
b6 2+
b3
b2
y4
y5
rQ
iQ
b1
y1
y2
b4
y9
0 100 200 300 400 500 600 700 800 900 1,000 1,100 1,200
m/z

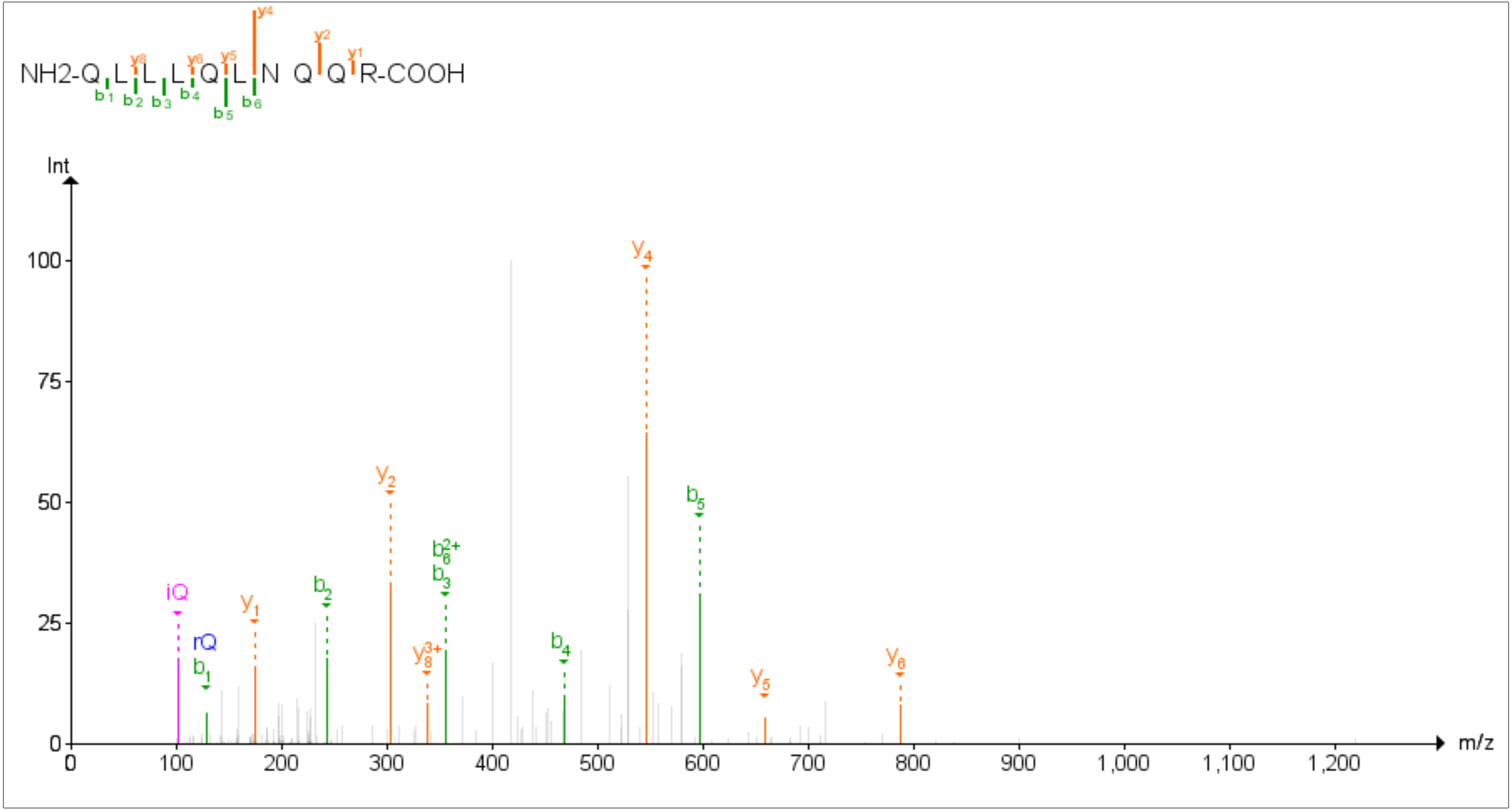

NH2-Q L L L Q L N Q Q R-COOH
y4
y2
y1
y8
y6
y5
b1 b2 b3 b4 b5 b6
Int
100
75
50
25
0
y4
y2
b5
b6 2+
b3
b2
iQ
y1
rQ
b1
y8 3+
b4
y5
y6
0 100 200 300 400 500 600 700 800 900 1,000 1,100 1,200
m/z

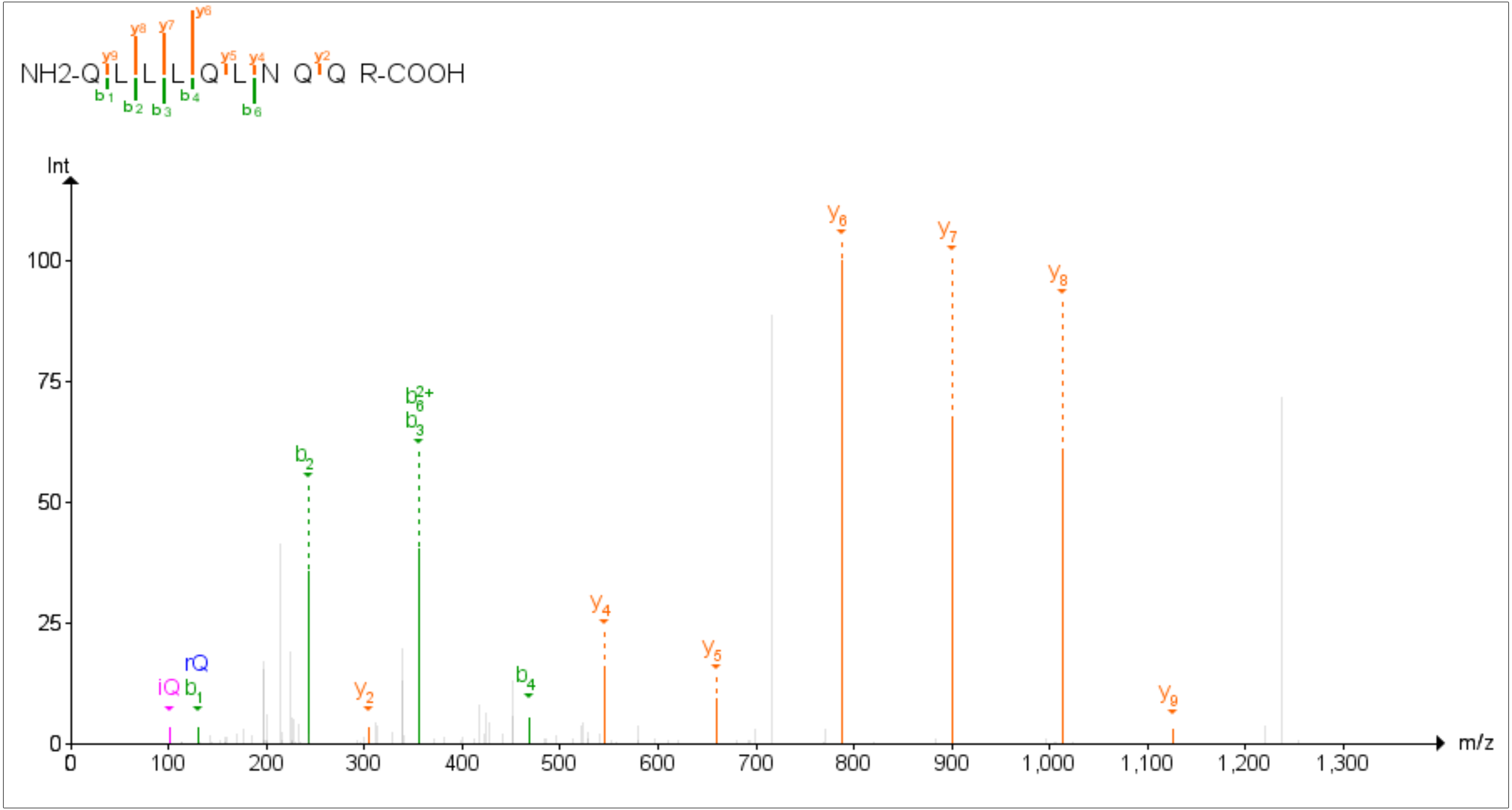

NH2-Q L L L Q L N Q Q R-COOH
y9 y8 y7 y6 y5 y4 y2
b1 b2 b3 b4 b6
Int
100
75
50
25
0
y6
y7
y8
b6 2+
b3
b2
y4
y5
b4
rQ
iQ
b1
y2
y9
0
100
200
300
400
500
600
700
800
900
1,000
1,100
1,200
1,300
m/z

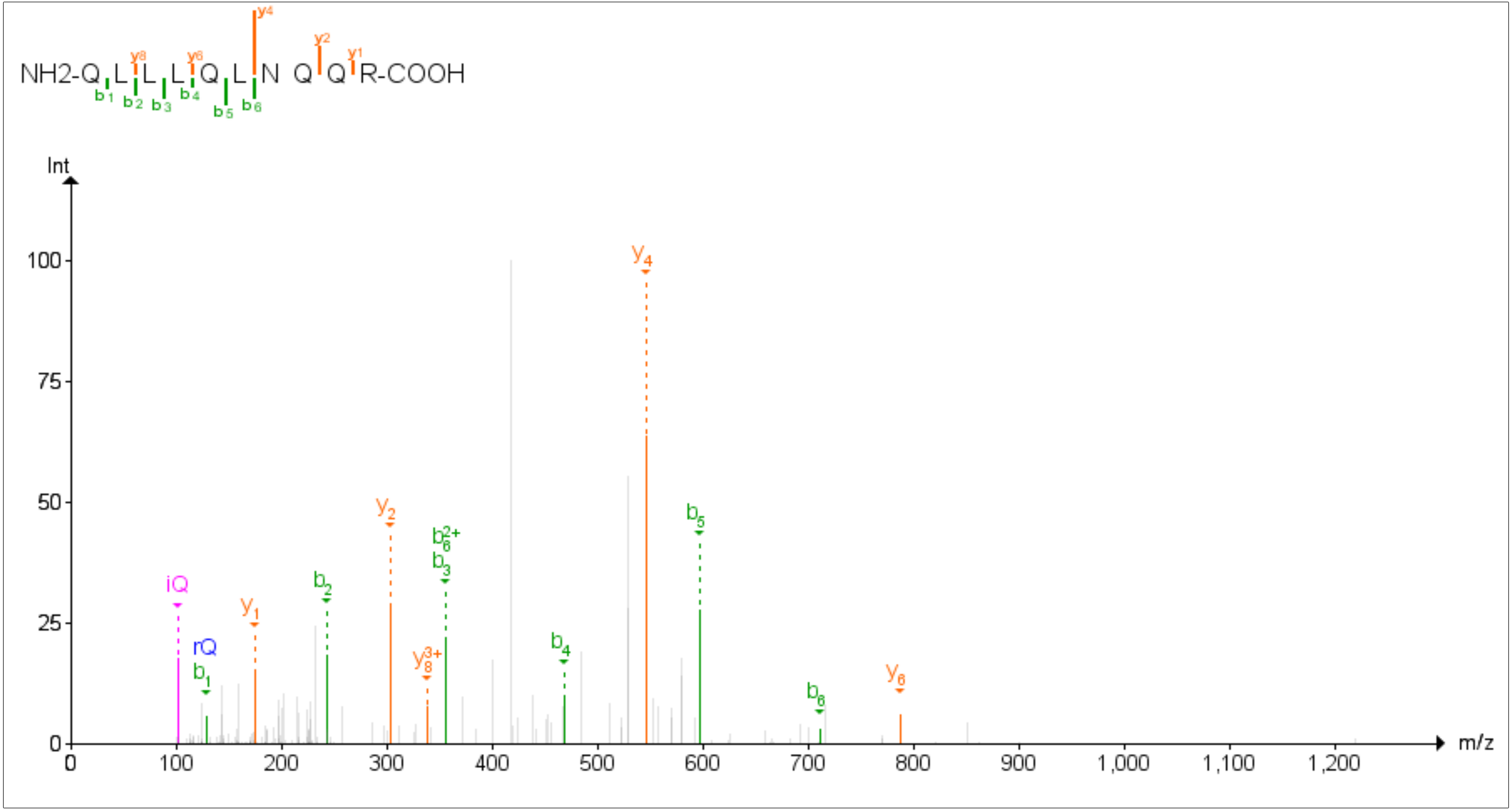
NH2-Q L L L Q L N Q Q R-COOH
b1 b2 b3 b4 b5 b6
y8 y6 y4 y2 y1
Int
m/z
iQ
rQ
b1
y1
b2
y2
b6 2+
b3
y8 3+
b4
y4
b5
b6
y6
0
25
50
75
100
0
100
200
300
400
500
600
700
800
900
1,000
1,100
1,200

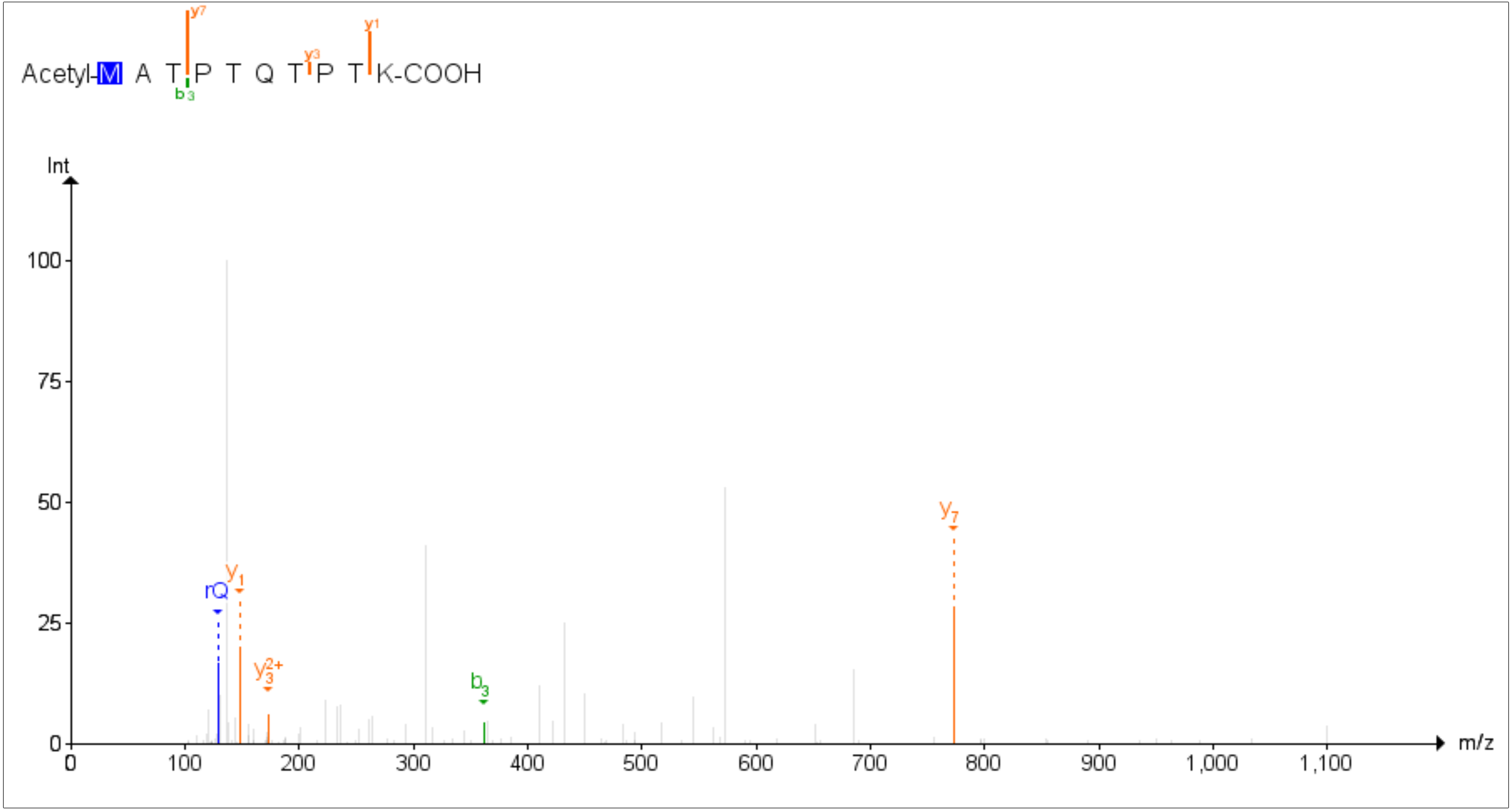
Acetyl-M A T P T Q T P T K-COOH
y7
y3
y1
b3
Int
100
75
50
25
0
rQ
y1
y2+3
b3
y7
0
100
200
300
400
500
600
700
800
900
1,000
1,100
m/z

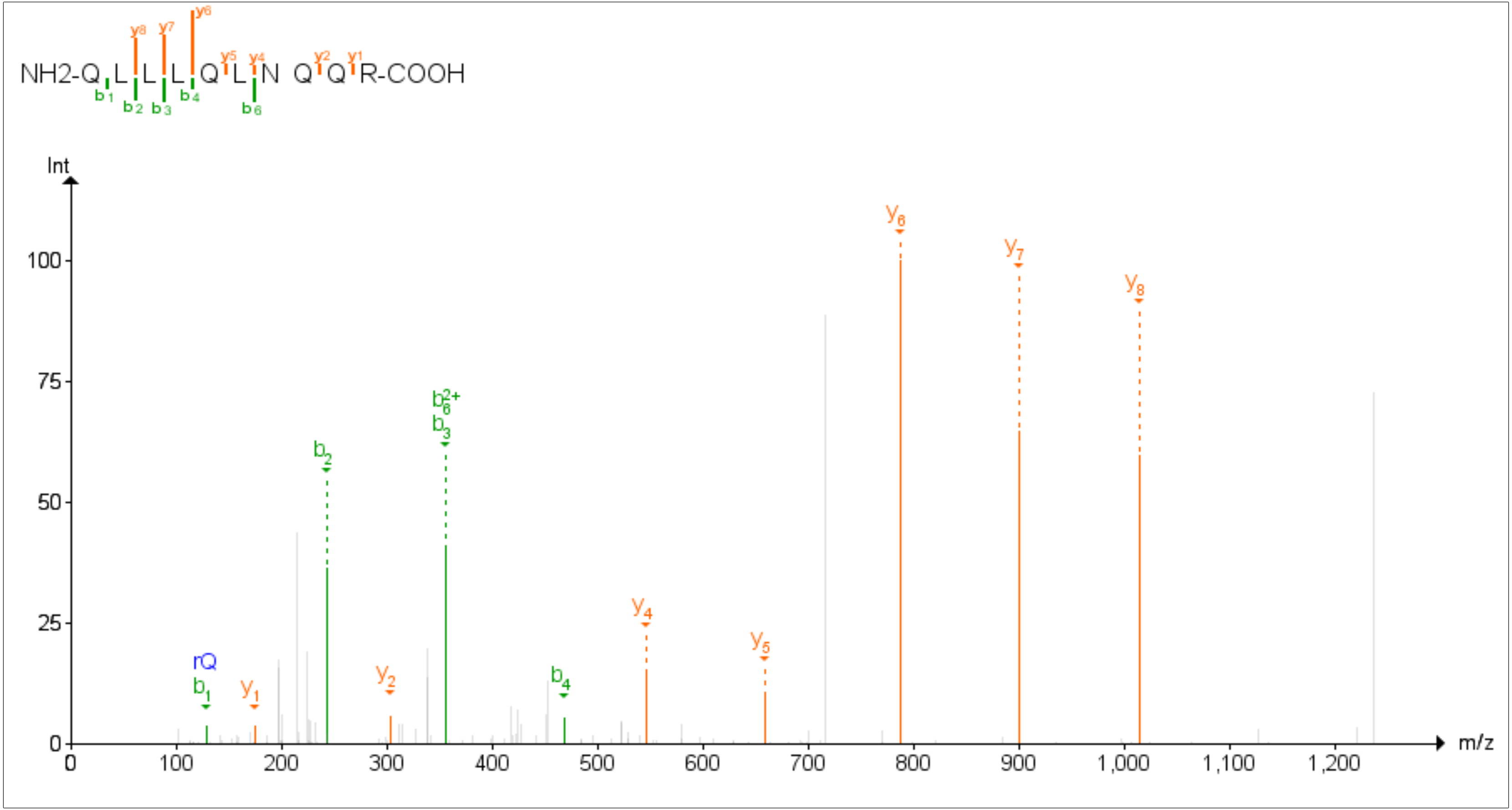

NH2-Q L L L Q L N Q Q R-COOH
b1 b2 b3 b4 b6
y8 y7 y6 y5 y4 y2 y1
Int
m/z
rQ
b1
y1
b2
b6 2+
b3
y2
b4
y4
y5
y6
y7
y8
0
25
50
75
100
0
100
200
300
400
500
600
700
800
900
1,000
1,100
1,200

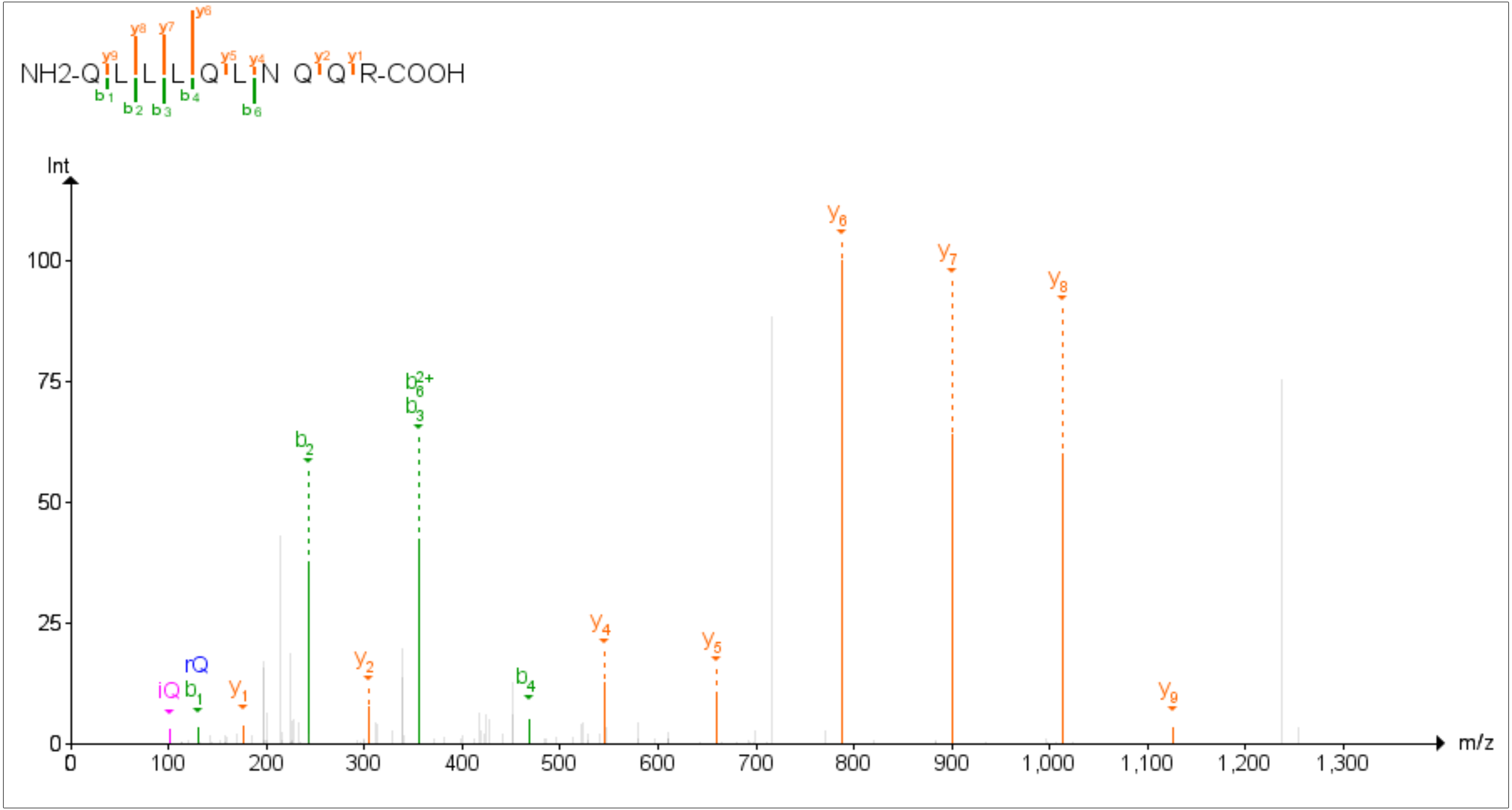
NH2-Q L L L Q L N Q Q R-COOH
y9 y8 y7 y6 y5 y4 y2 y1
b1 b2 b3 b4 b6
Int
m/z
y6
y7
y8
b6 2+
b3
b2
rQ
iQ
b1
y1
y2
b4
y4
y5
y9
0
25
50
75
100
0
100
200
300
400
500
600
700
800
900
1,000
1,100
1,200
1,300

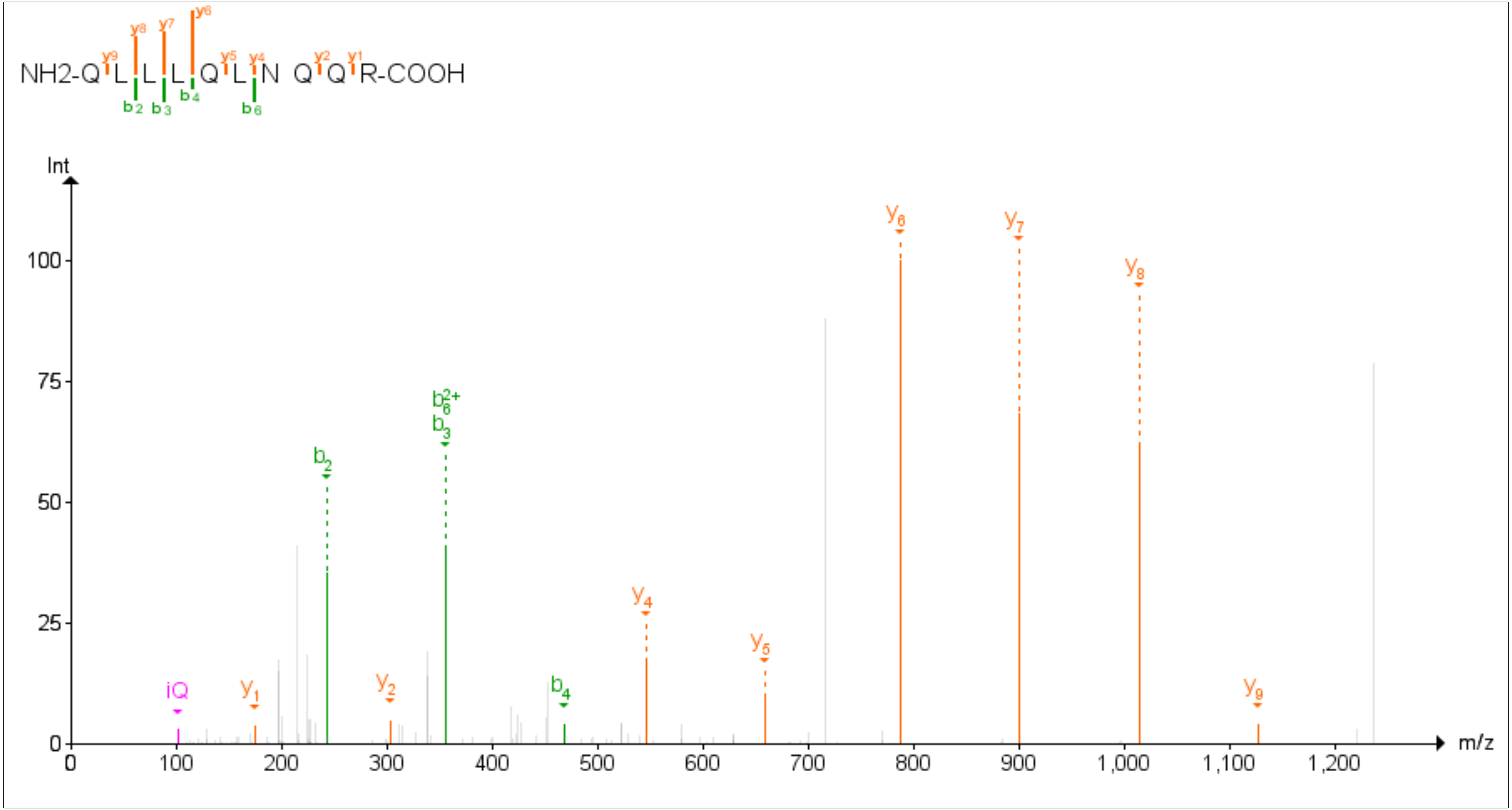

NH2-Q L L L Q L N Q Q R-COOH
y9 y8 y7 y6 y5 y4 y2 y1
b2 b3 b4 b6
Int
100
75
50
25
0
y6
y7
y8
b6 2+
b3
b2
y4
y5
iQ
y1
y2
b4
y9
0
100
200
300
400
500
600
700
800
900
1,000
1,100
1,200
m/z

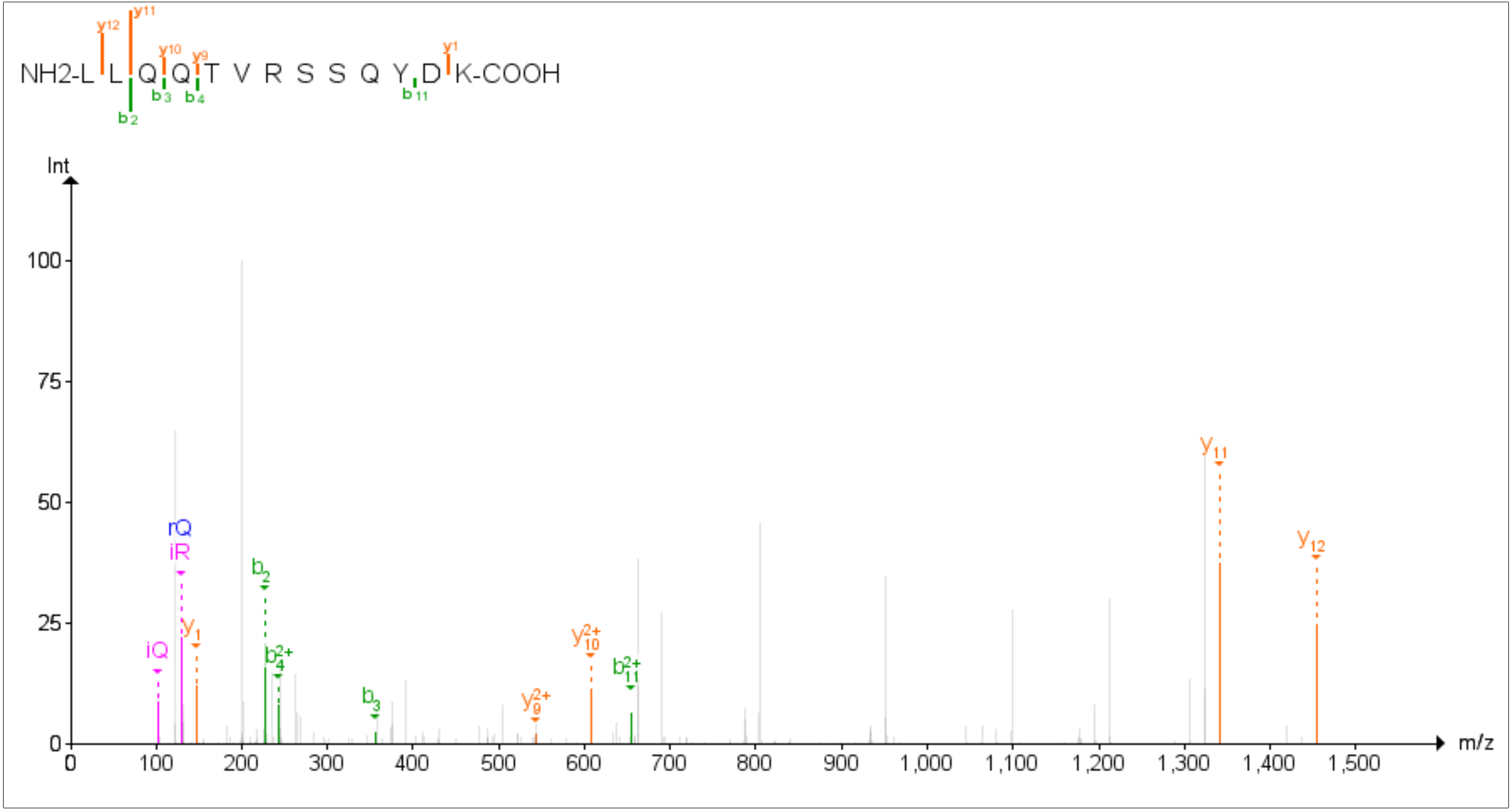

NH2-L L Q Q T V R S S Q Y D K-COOH
y12
y11
y10
y9
y1
b2
b3
b4
b11
Int
100
75
50
25
0
rQ
iR
iQ
y1
b2
b4 2+
b3
y9 2+
y10 2+
b11 2+
y11
y12
m/z
0
100
200
300
400
500
600
700
800
900
1,000
1,100
1,200
1,300
1,400
1,500

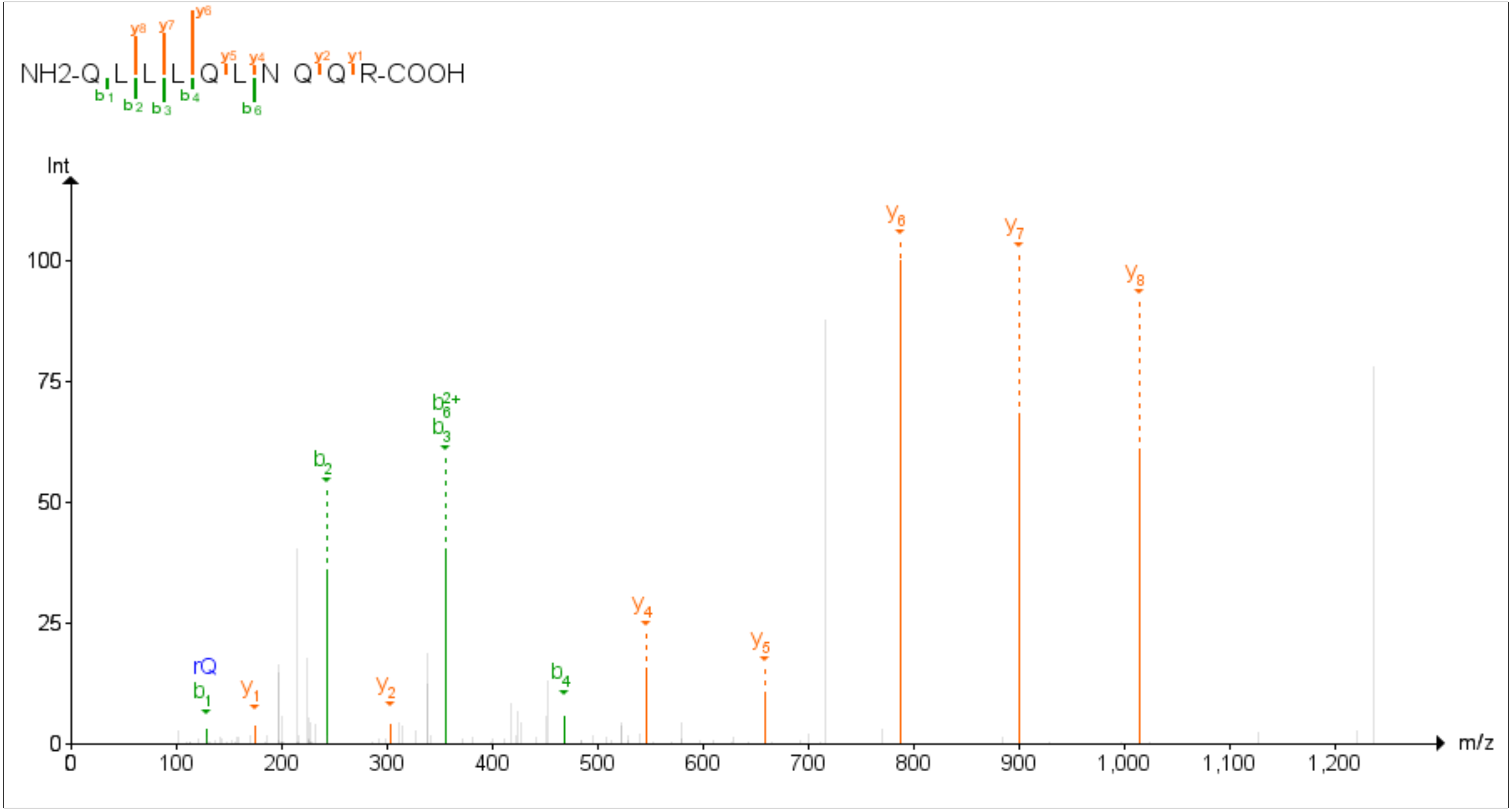

NH2-Q L L L L Q L N Q Q R-COOH
y8 y7 y6 y5 y4 y2 y1
b1 b2 b3 b4 b6
Int
100
75
50
25
0
y6
y7
y8
b6 2+
b3
b2
y4
y5
b4
rQ
b1
y1
y2
0 100 200 300 400 500 600 700 800 900 1,000 1,100 1,200
m/z

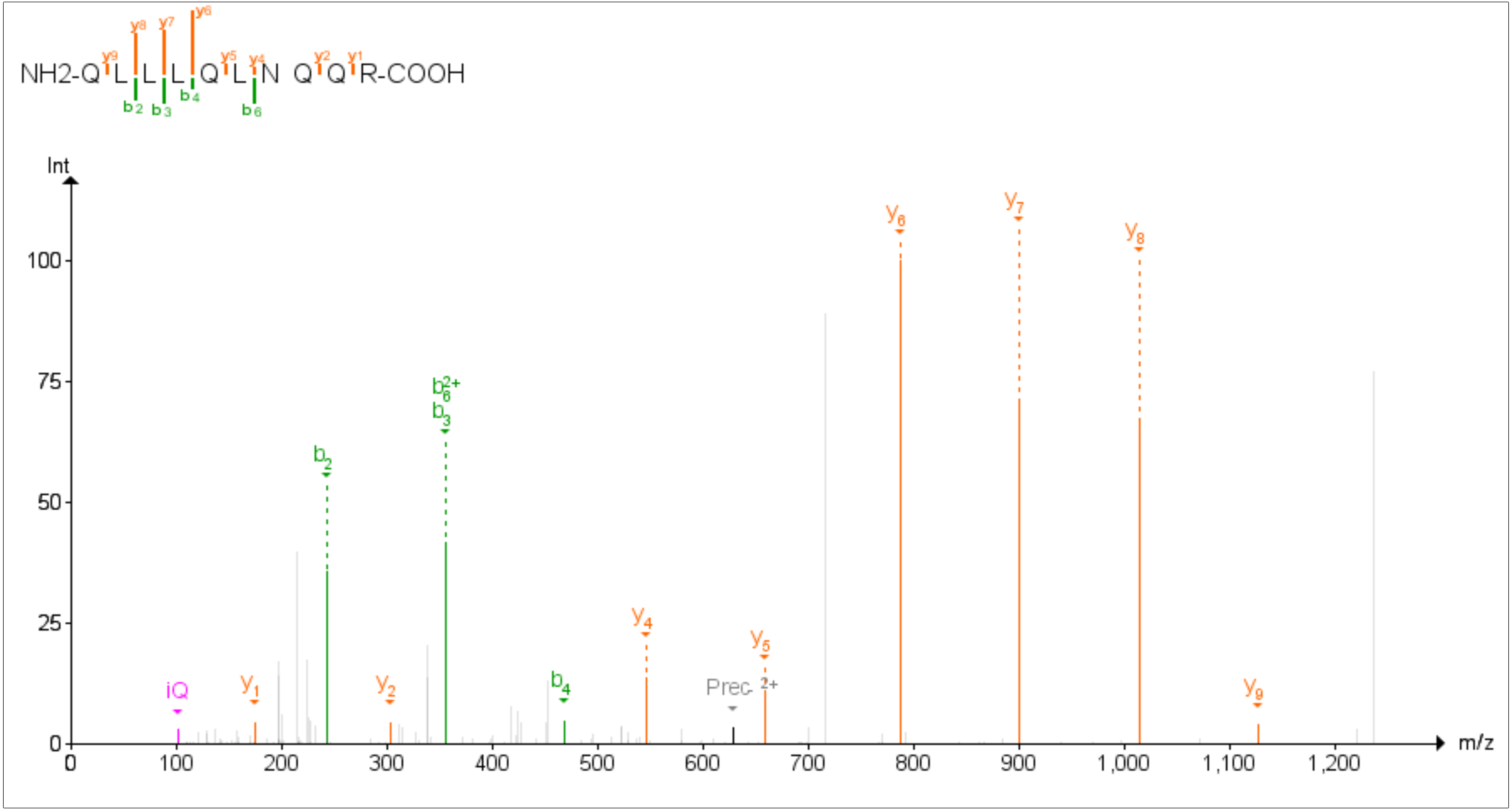

NH2-Q L L L Q L N Q Q R-COOH
y9 y8 y7 y6 y5 y4 y2 y1
b2 b3 b4 b6
Int
m/z
iQ
y1
y2
b2
b6 2+
b3
b4
y4
Prec. 2+
y5
y6
y7
y8
y9
0
25
50
75
100
0
100
200
300
400
500
600
700
800
900
1,000
1,100
1,200

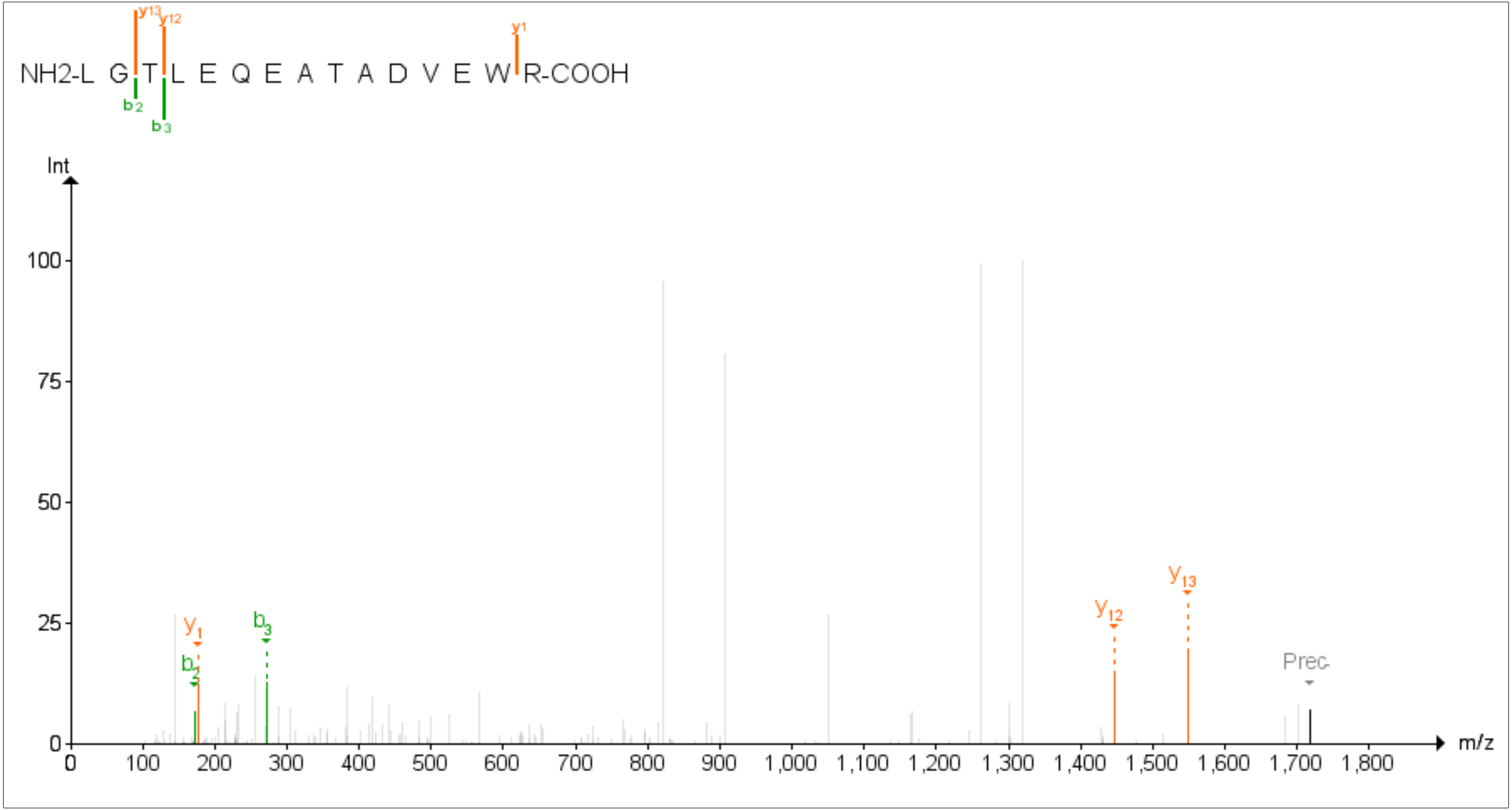

NH2-L G T L E Q E A T A D V E W R-COOH
y13
y12
y1
b2
b3
Int
100
75
50
25
0
y1
b2
b3
y12
y13
Prec
m/z
0
100
200
300
400
500
600
700
800
900
1,000
1,100
1,200
1,300
1,400
1,500
1,600
1,700
1,800

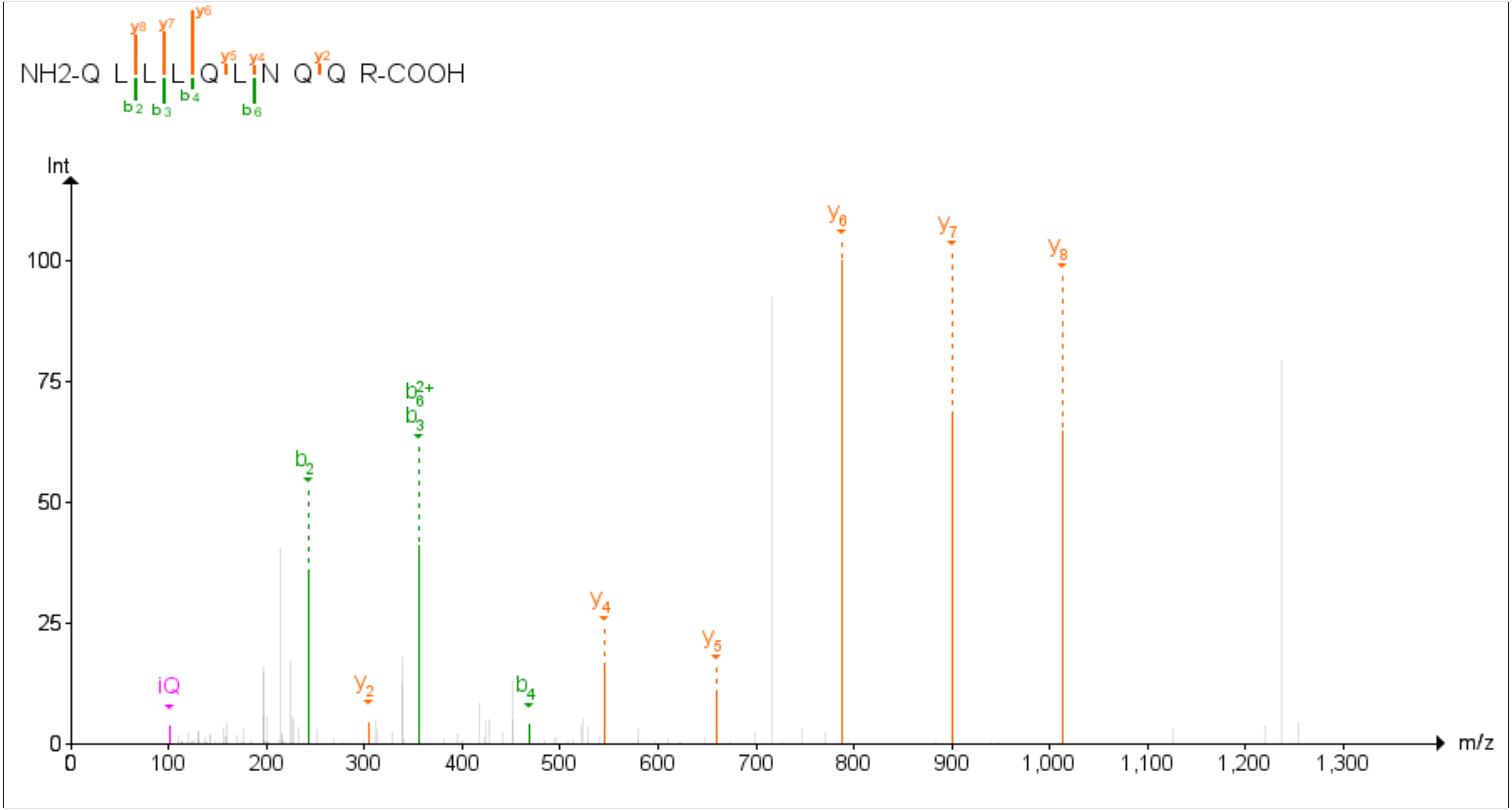

NH2-Q L L L Q L N Q Q R-COOH
y8 y7 y6 y5 y4 y2
b2 b3 b4 b6
Int
100
75
50
25
0
b2
b6 2+
b3
b4
iQ
y2
y4
y5
y6
y7
y8
0 100 200 300 400 500 600 700 800 900 1,000 1,100 1,200 1,300
m/z

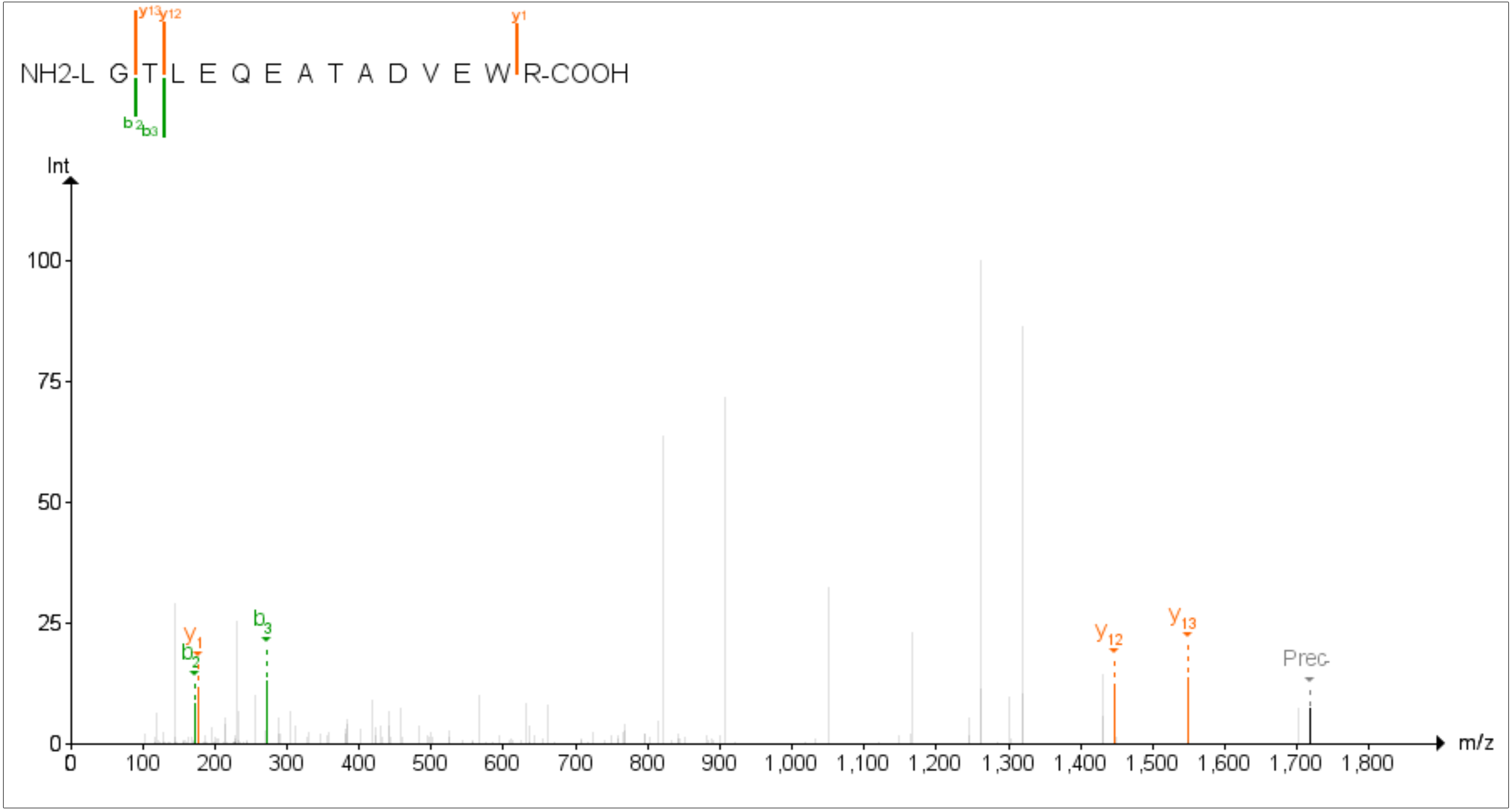

NH2-L G T L E Q E A T A D V E W R-COOH
y13
y12
y1
b2
b3
Int
m/z
100
75
50
25
0
0
100
200
300
400
500
600
700
800
900
1,000
1,100
1,200
1,300
1,400
1,500
1,600
1,700
1,800
y1
b2
b3
y12
y13
Prec.

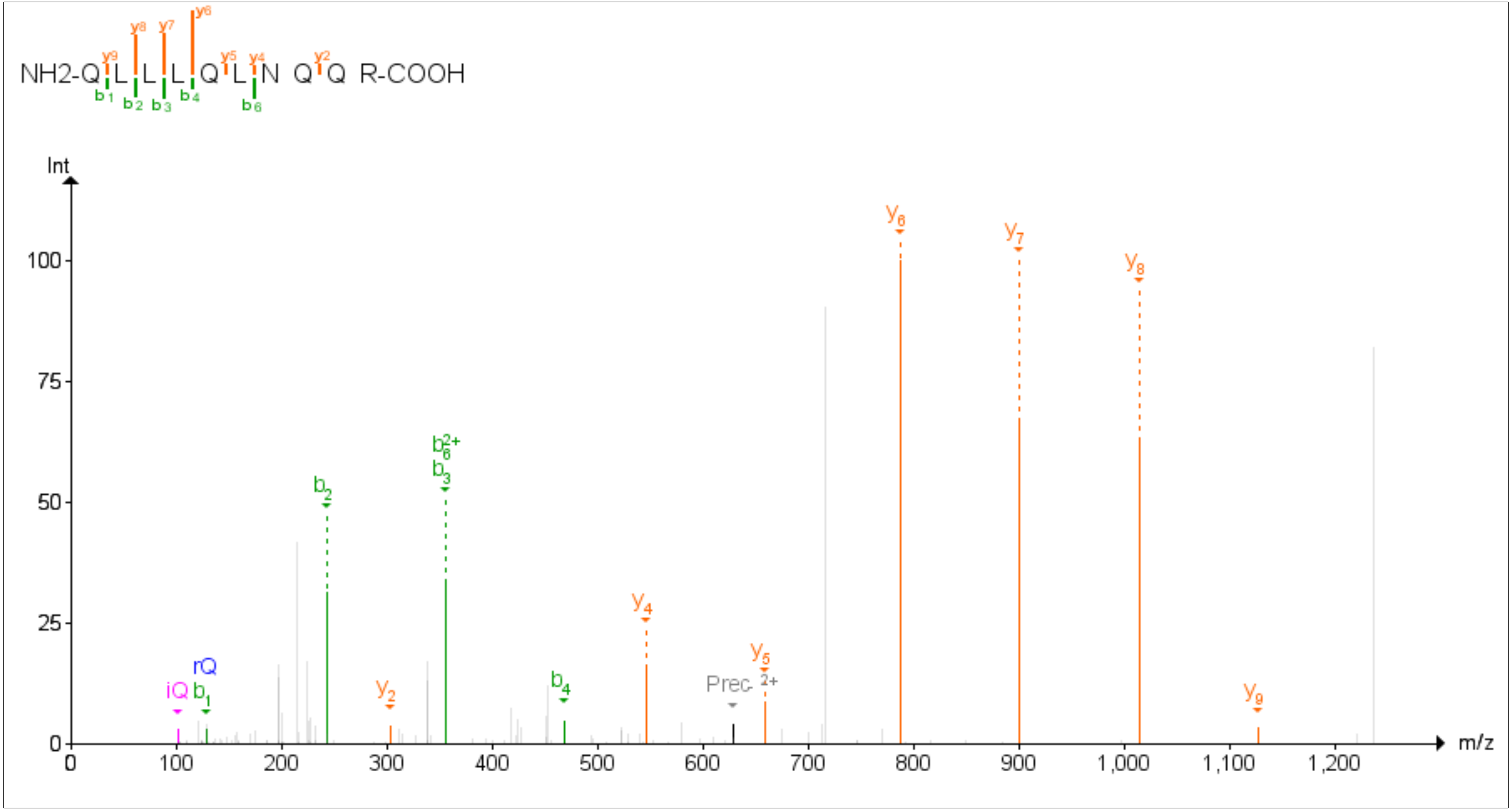

NH2-Q L L L Q L N Q Q R-COOH
y9 y8 y7 y6 y5 y4 y2
b1 b2 b3 b4 b6
Int
m/z
0
25
50
75
100
0
100
200
300
400
500
600
700
800
900
1,000
1,100
1,200
y6
y7
y8
y9
y4
y5
y2
b2
b6 2+
b3
b4
b1
rQ
iQ
Prec 2+

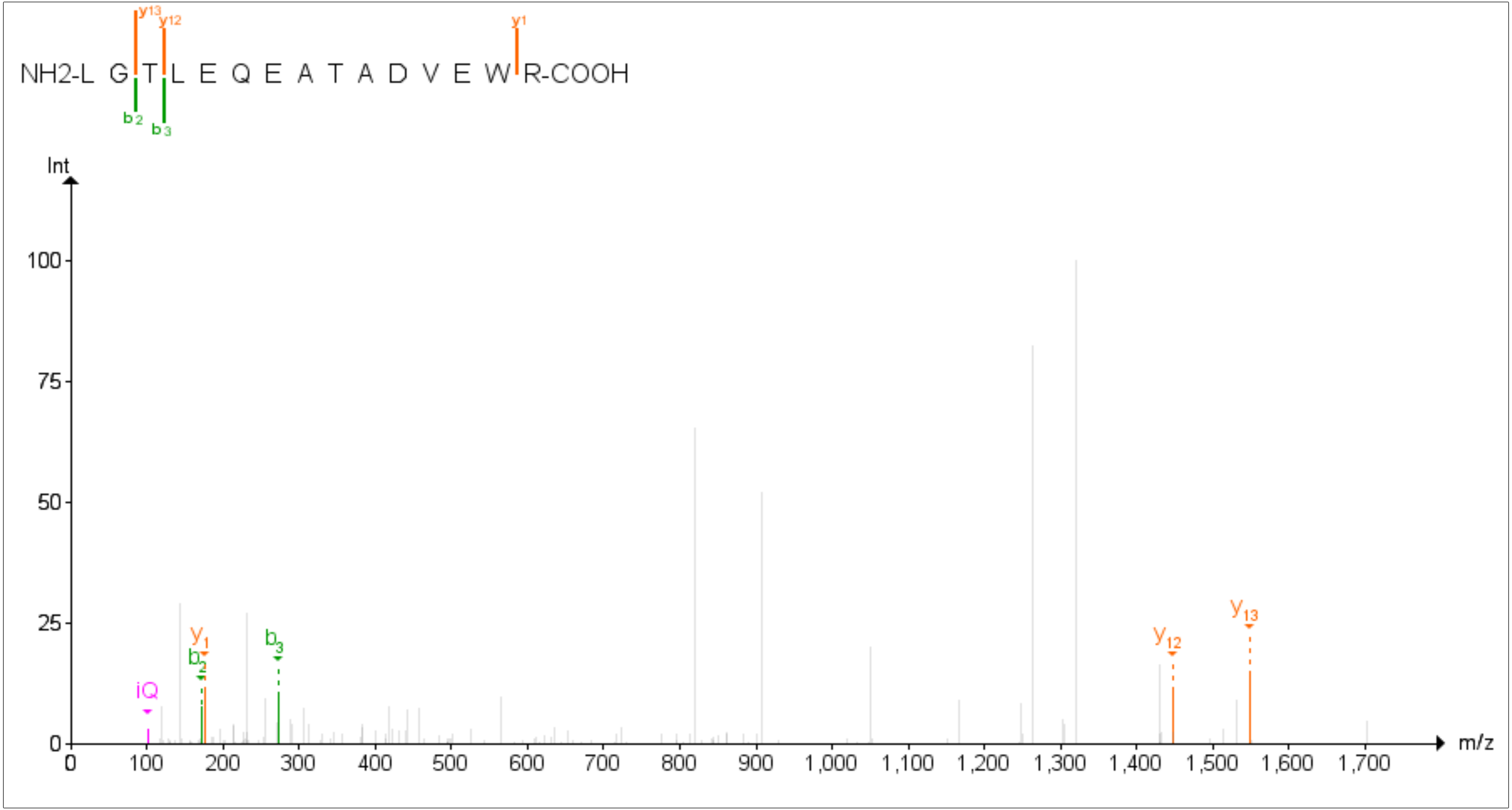

NH2-L G T L E Q E A T A D V E W R-COOH
y13
y12
y1
b2
b3
Int
100
75
50
25
0
0
100
200
300
400
500
600
700
800
900
1,000
1,100
1,200
1,300
1,400
1,500
1,600
1,700
m/z
iQ
y1
b2
b3
y12
y13

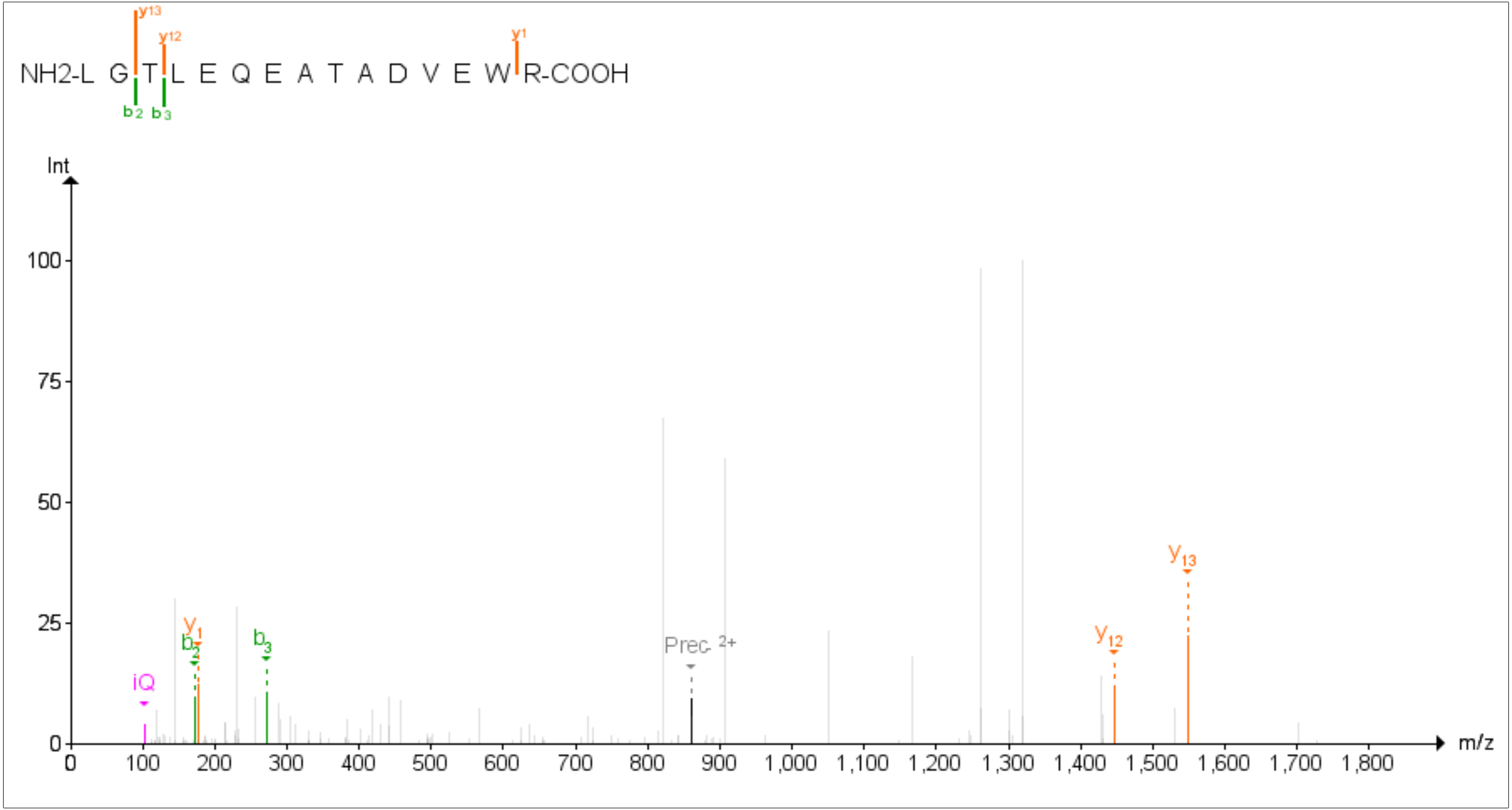

y13
y12
y1
NH2-L G T L E Q E A T A D V E W R-COOH
b2
b3
Int
100
75
50
25
0
y13
y12
y1
b2
b3
iQ
Prec 2+
0
100
200
300
400
500
600
700
800
900
1,000
1,100
1,200
1,300
1,400
1,500
1,600
1,700
1,800
m/z

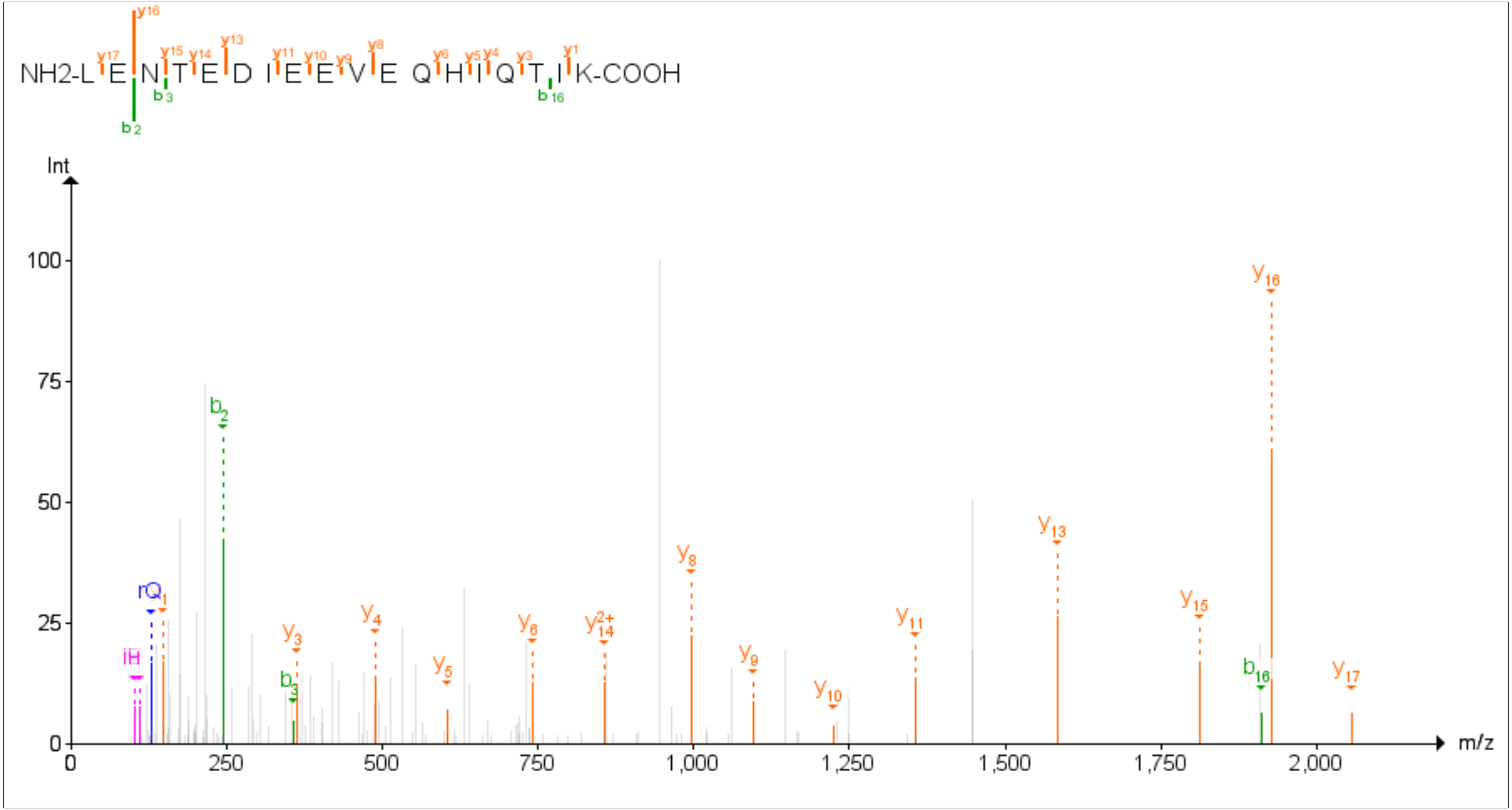
NH2-L E N T E D I E E V E Q H I Q T I K-COOH
Int
m/z
0
25
50
75
100
250
500
750
1,000
1,250
1,500
1,750
2,000

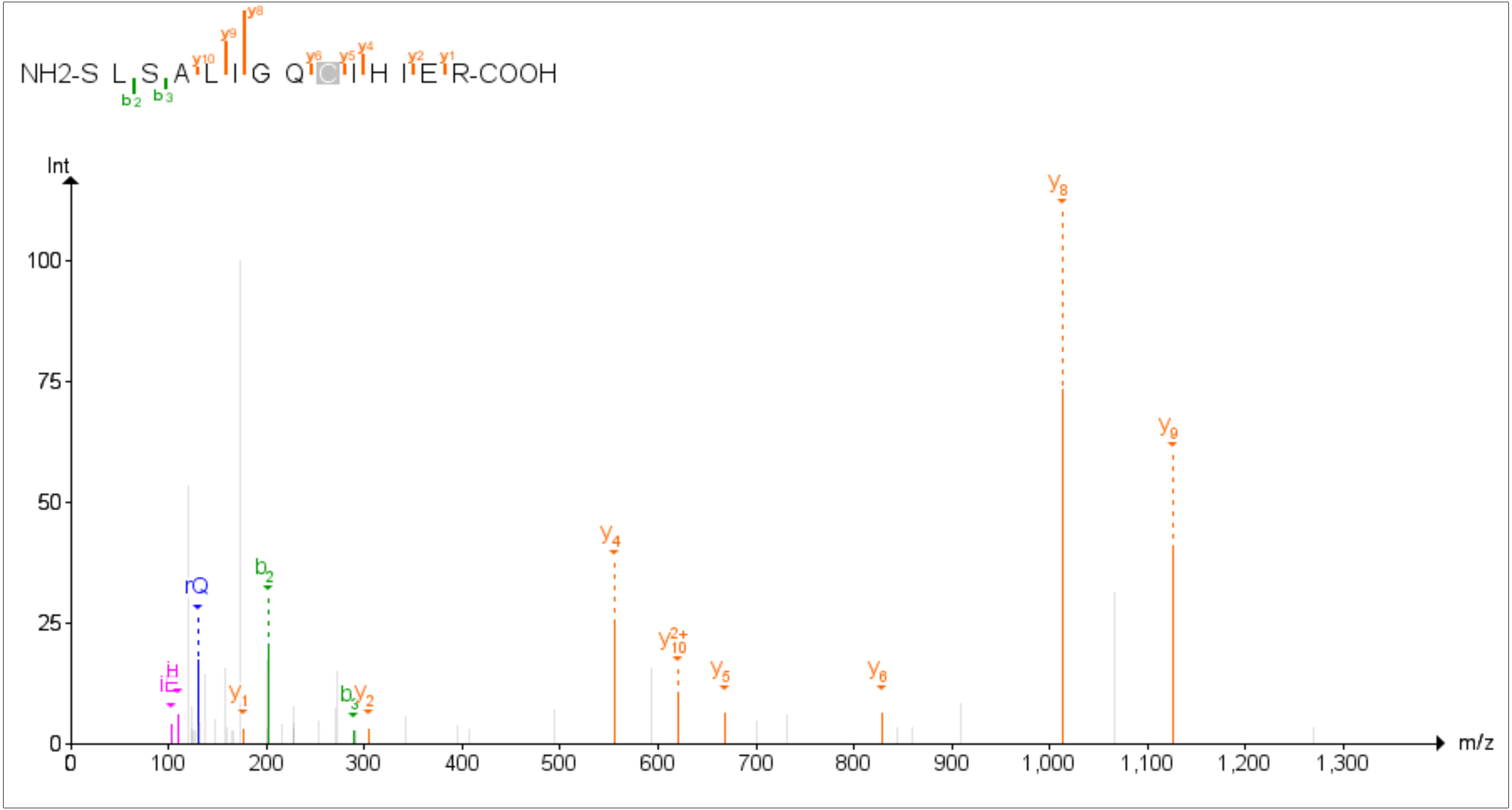

NH2-S L S A L I G Q C I H I E R-COOH
b2
b3
y10
y9
y8
y6
y5
y4
y2
y1
Int
100
75
50
25
0
rQ
b2
iL
iH
y1
b3
y2
y4
y10 2+
y5
y6
y8
y9
0
100
200
300
400
500
600
700
800
900
1,000
1,100
1,200
1,300
m/z

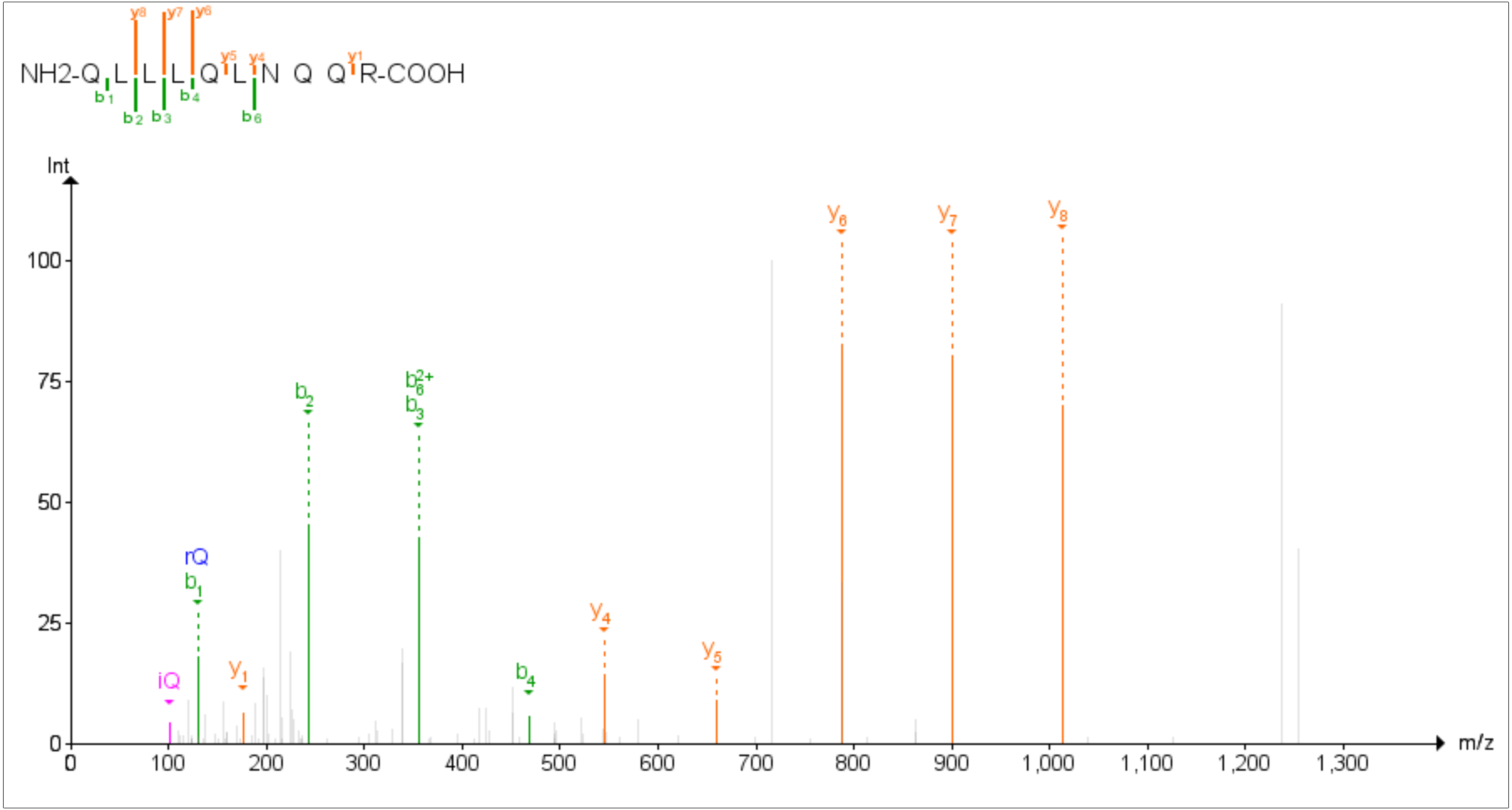

NH2-Q L L L Q L N Q Q R-COOH
y8 y7 y6 y5 y4 y1
b1 b2 b3 b4 b6
Int
100
75
50
25
0
y6 y7 y8
b2
b6 2+
b3
rQ
b1
iQ
y1
b4
y4
y5
0 100 200 300 400 500 600 700 800 900 1,000 1,100 1,200 1,300
m/z

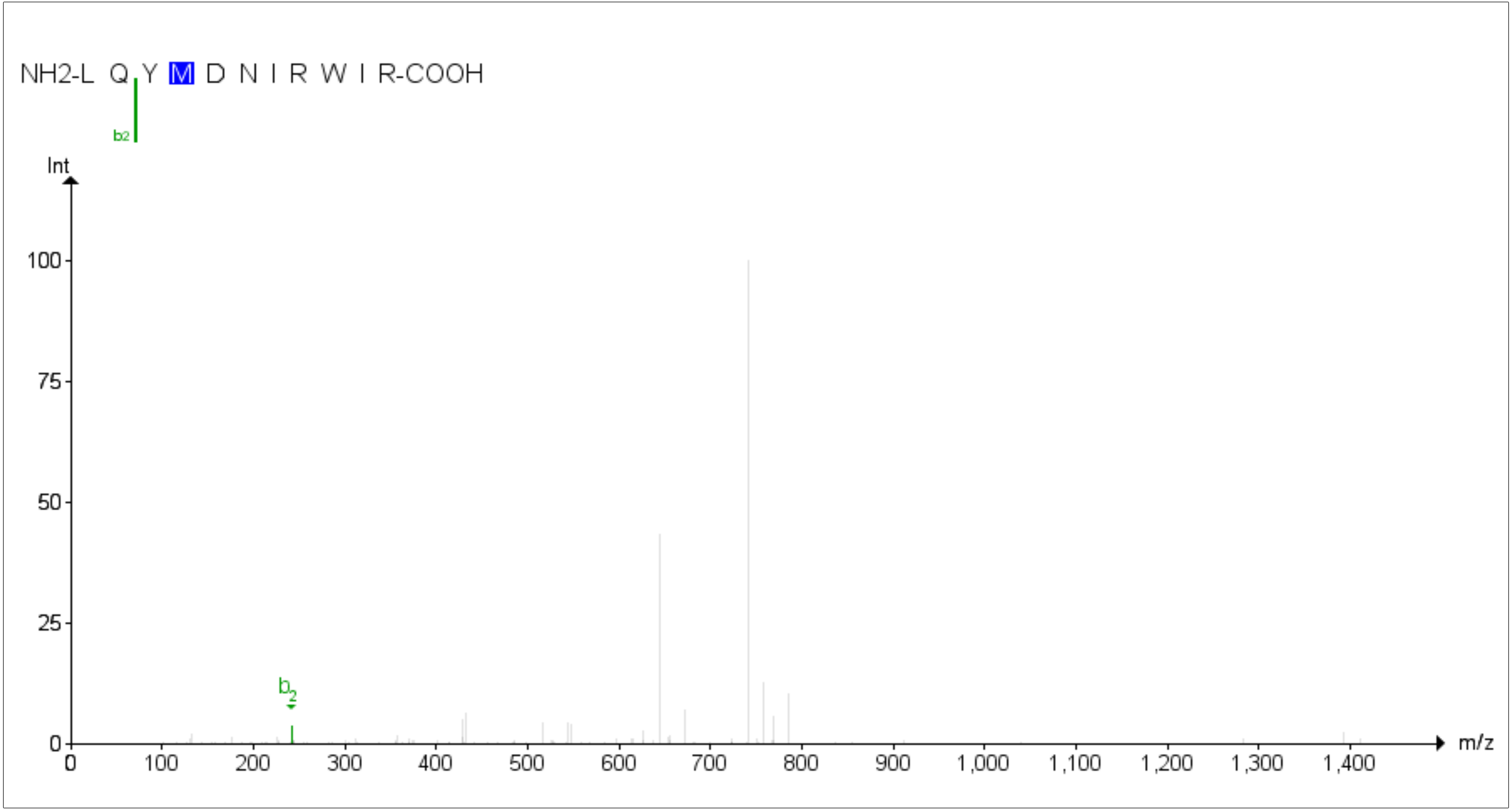

NH2-L Q Y M D N I R W I R-COOH
b2
Int
100
75
50
25
0
b2
0
100
200
300
400
500
600
700
800
900
1,000
1,100
1,200
1,300
1,400
m/z

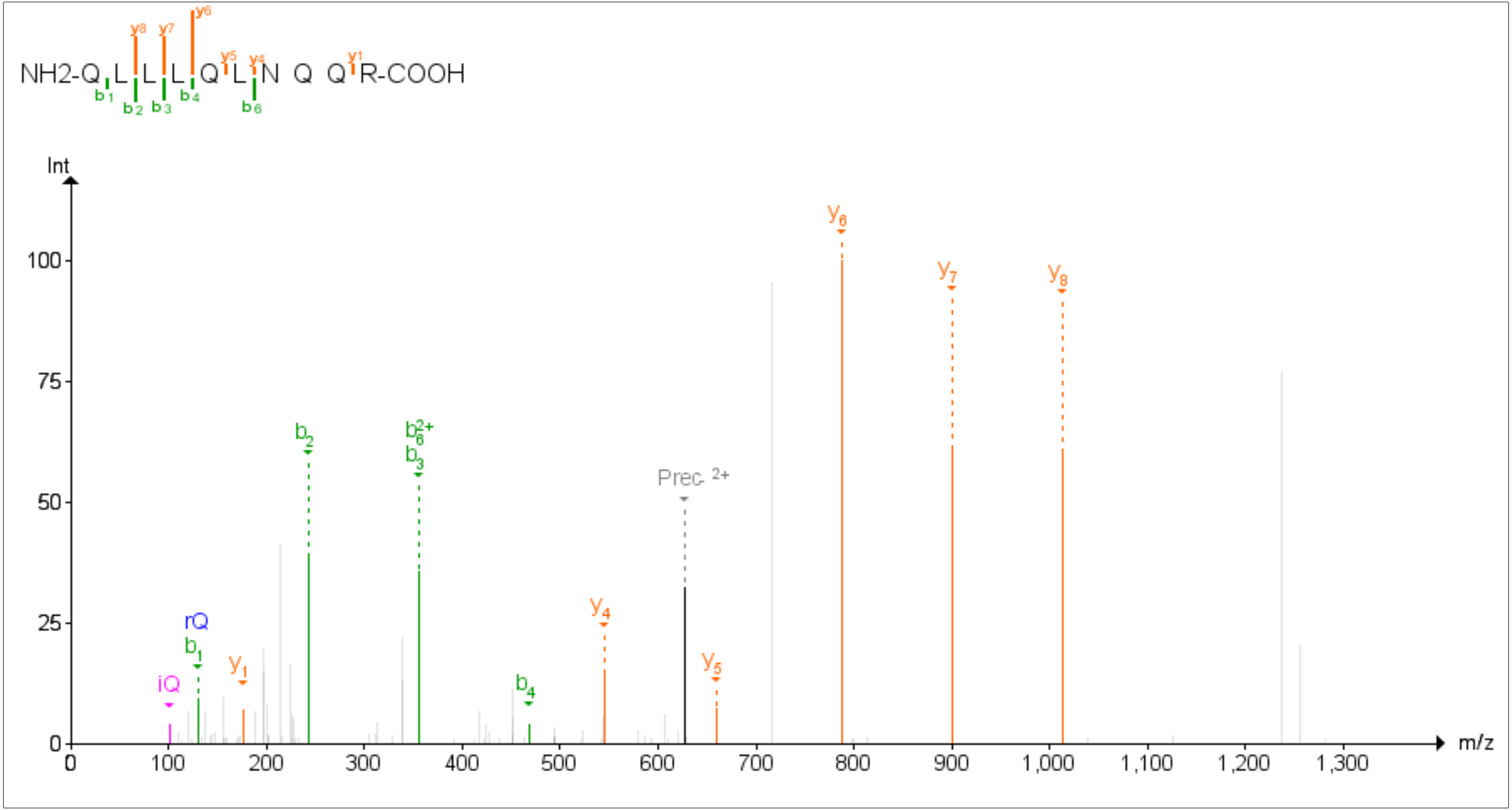

NH2-Q L L L Q L N Q Q R-COOH
y8 y7 y6 y5 y4 y1
b1 b2 b3 b4 b6
Int
100
75
50
25
0
b2
b6 2+
b3
Prec 2+
y6
y7
y8
y4
y5
b4
rQ
b1
y1
iQ
0
100
200
300
400
500
600
700
800
900
1,000
1,100
1,200
1,300
m/z

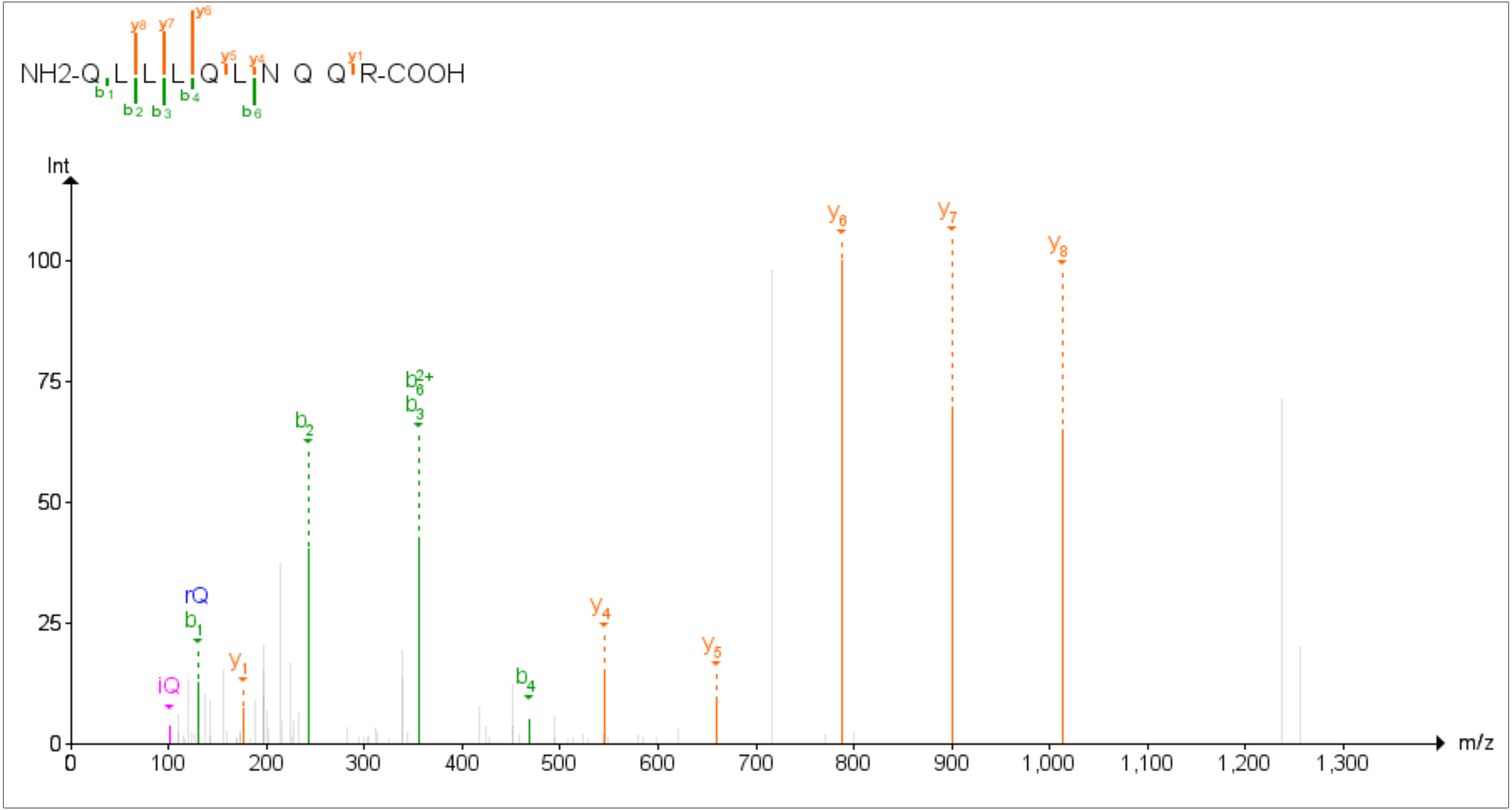

NH2-Q L L L Q L N Q Q R-COOH
y8 y7 y6 y5 y4 y1
b1 b2 b3 b4 b6
Int
m/z
y6
y7
y8
b6 2+
b3
b2
rQ
b1
iQ
y1
b4
y4
y5

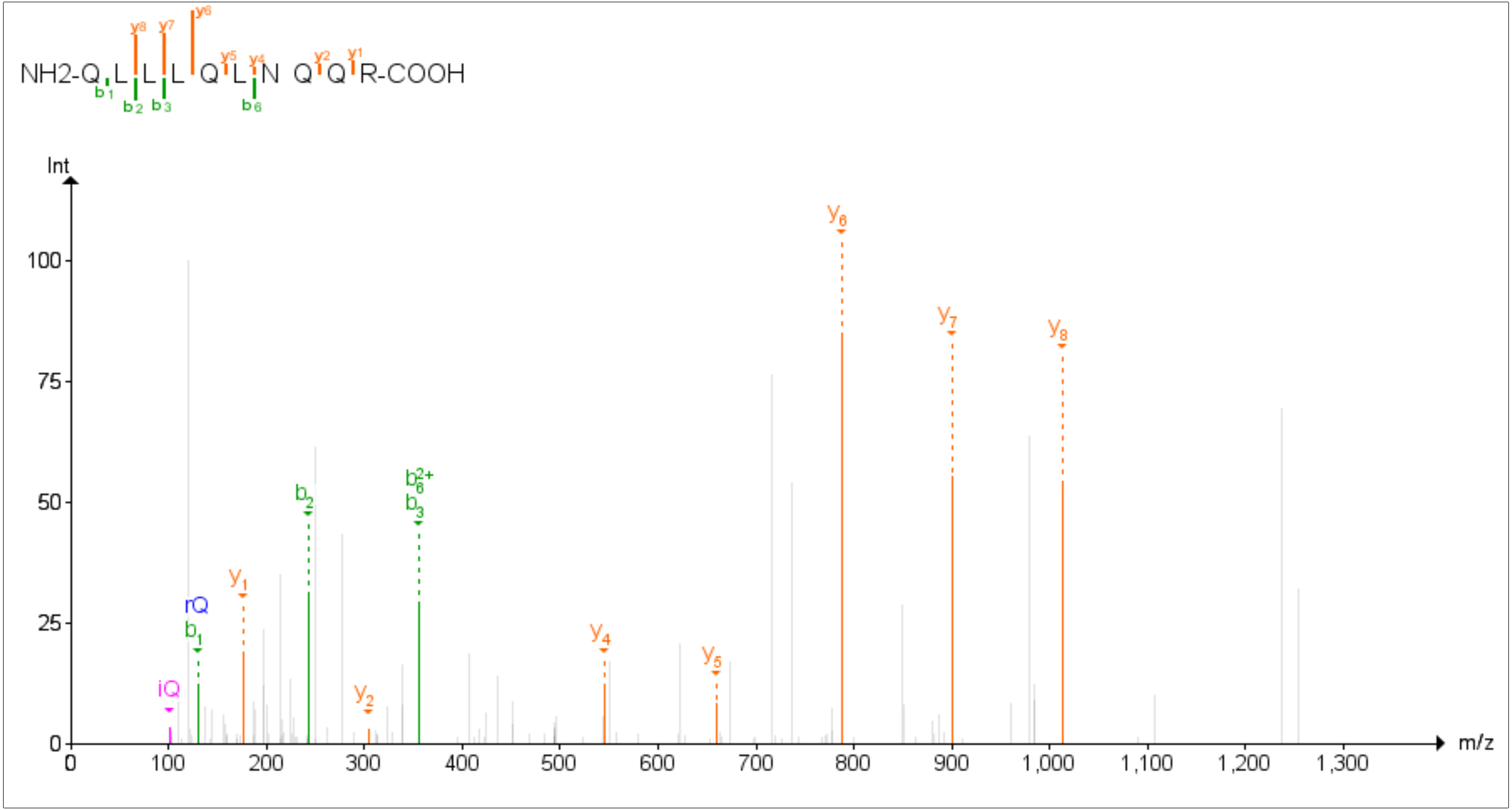

NH2-Q L L L Q L N Q Q R-COOH
y8 y7 y6 y5 y4 y2 y1
b1 b2 b3 b6
Int
100
75
50
25
0
y6
y7
y8
b2
b6 2+
b3
y1
rQ
b1
iQ
y2
y4
y5
0 100 200 300 400 500 600 700 800 900 1,000 1,100 1,200 1,300
m/z

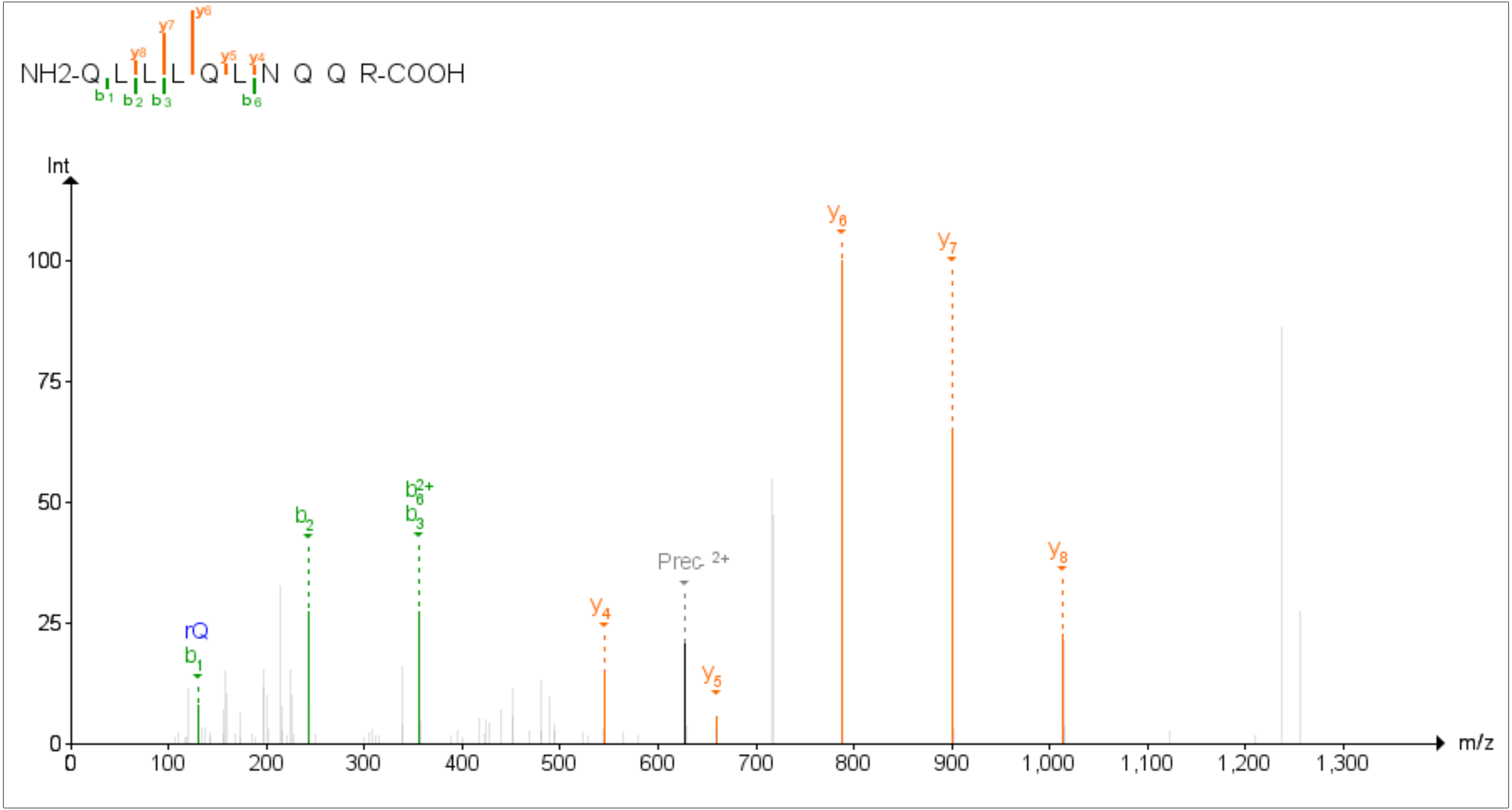

NH2-Q L L L Q L N Q Q R-COOH
y8 y7 y6 y5 y4
b1 b2 b3 b6
Int
100
75
50
25
0
rQ
b1
b2
b6 2+
b3
y4
Prec. 2+
y5
y6
y7
y8
0 100 200 300 400 500 600 700 800 900 1,000 1,100 1,200 1,300
m/z

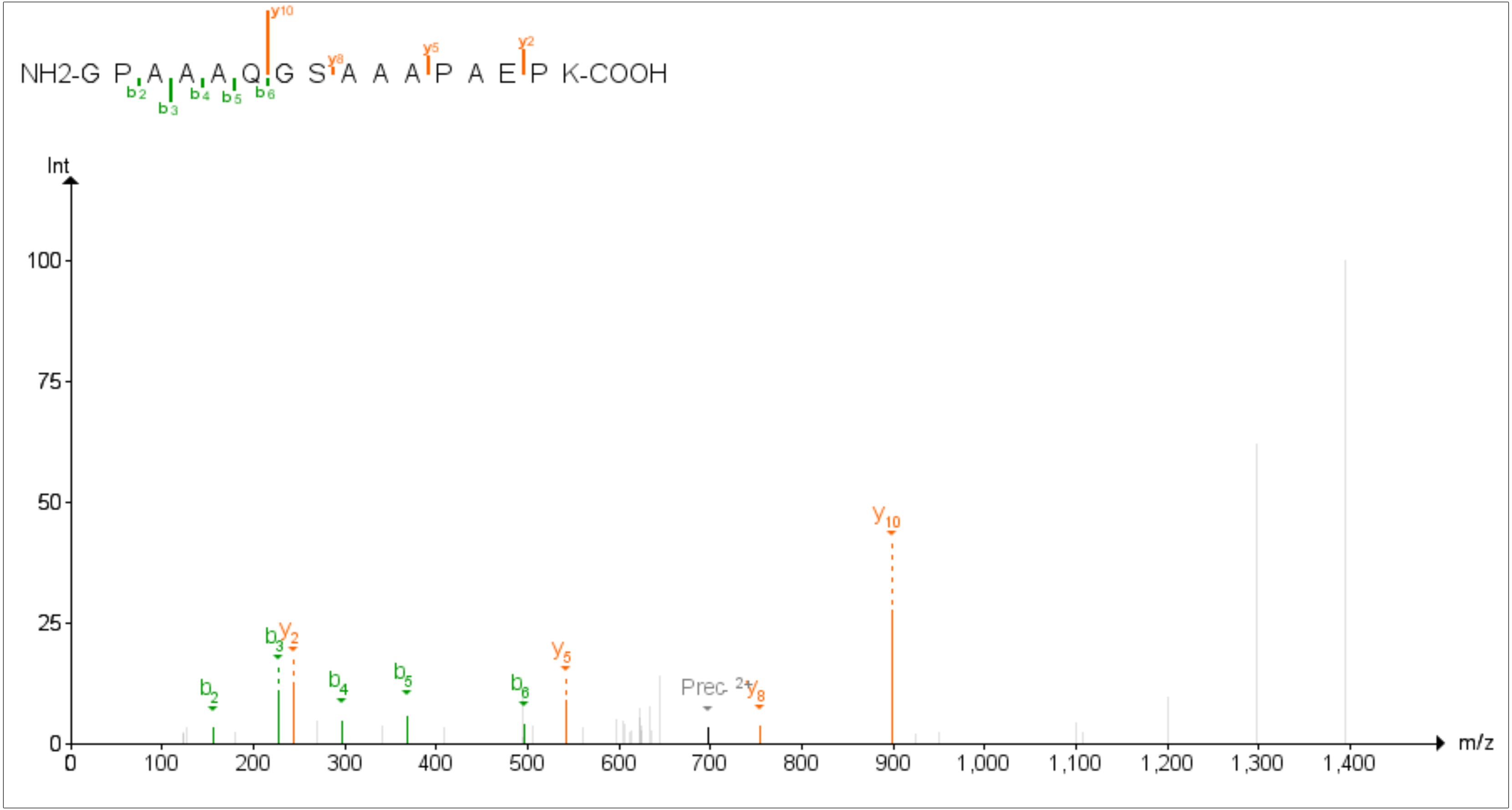

NH2-G P A A A Q G S A A A P A E P K-COOH
y10
y8
y5
y2
b2
b3
b4
b5
b6
Int
100
75
50
25
0
b2
b3
y2
b4
b5
b6
y5
Prec 2+
y8
y10
0
100
200
300
400
500
600
700
800
900
1,000
1,100
1,200
1,300
1,400
m/z

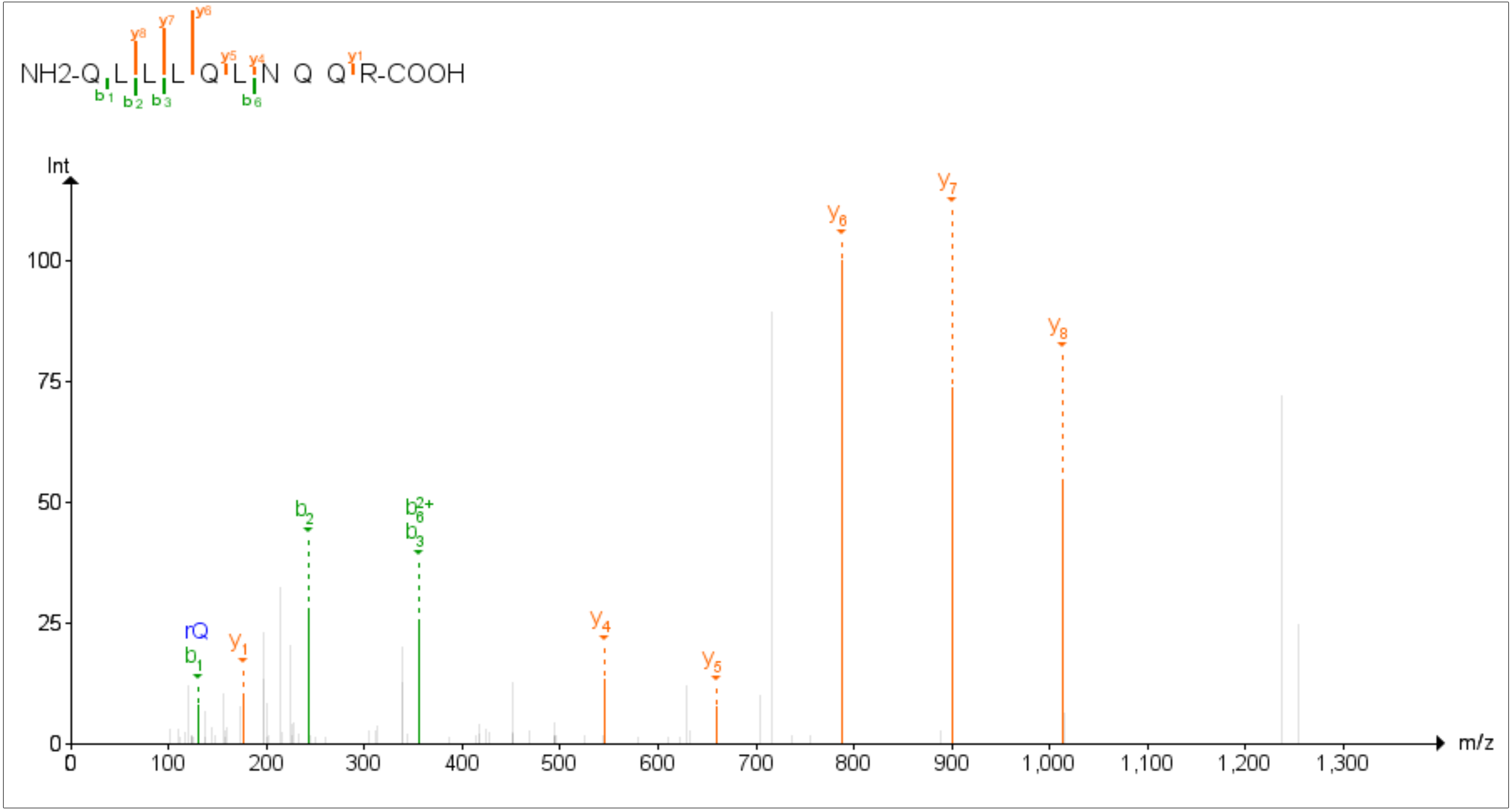

NH2-Q L L L Q L N Q Q R-COOH
y8 y7 y6 y5 y4 y1
b1 b2 b3 b6
Int
100
75
50
25
0
rQ
b1
y1
b2
b6 2+
b3
y4
y5
y6
y7
y8
0 100 200 300 400 500 600 700 800 900 1,000 1,100 1,200 1,300
m/z

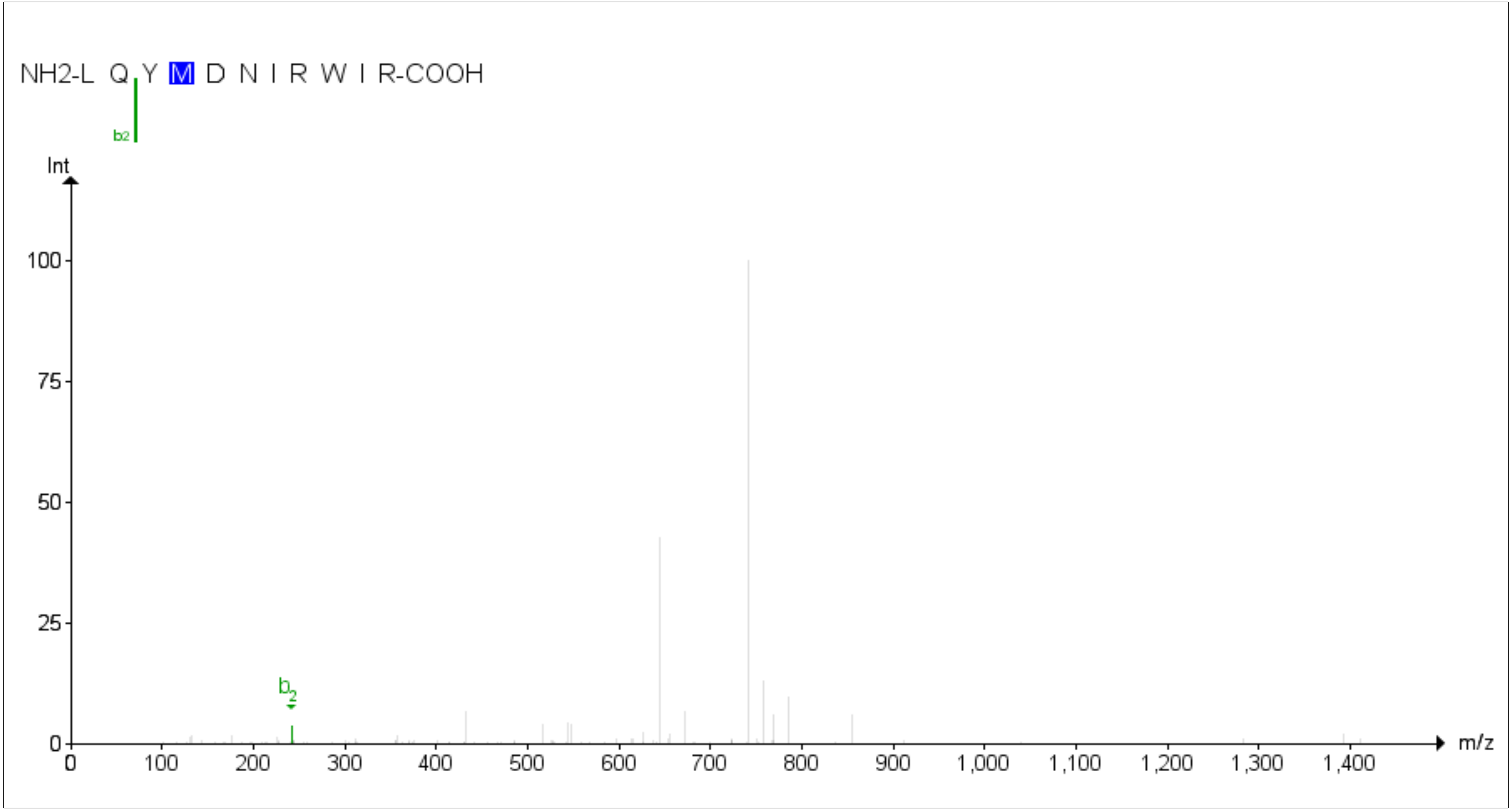
NH2-L Q Y M D N I R W I R-COOH
b2
Int
100
75
50
25
0
b2
0
100
200
300
400
500
600
700
800
900
1,000
1,100
1,200
1,300
1,400
m/z

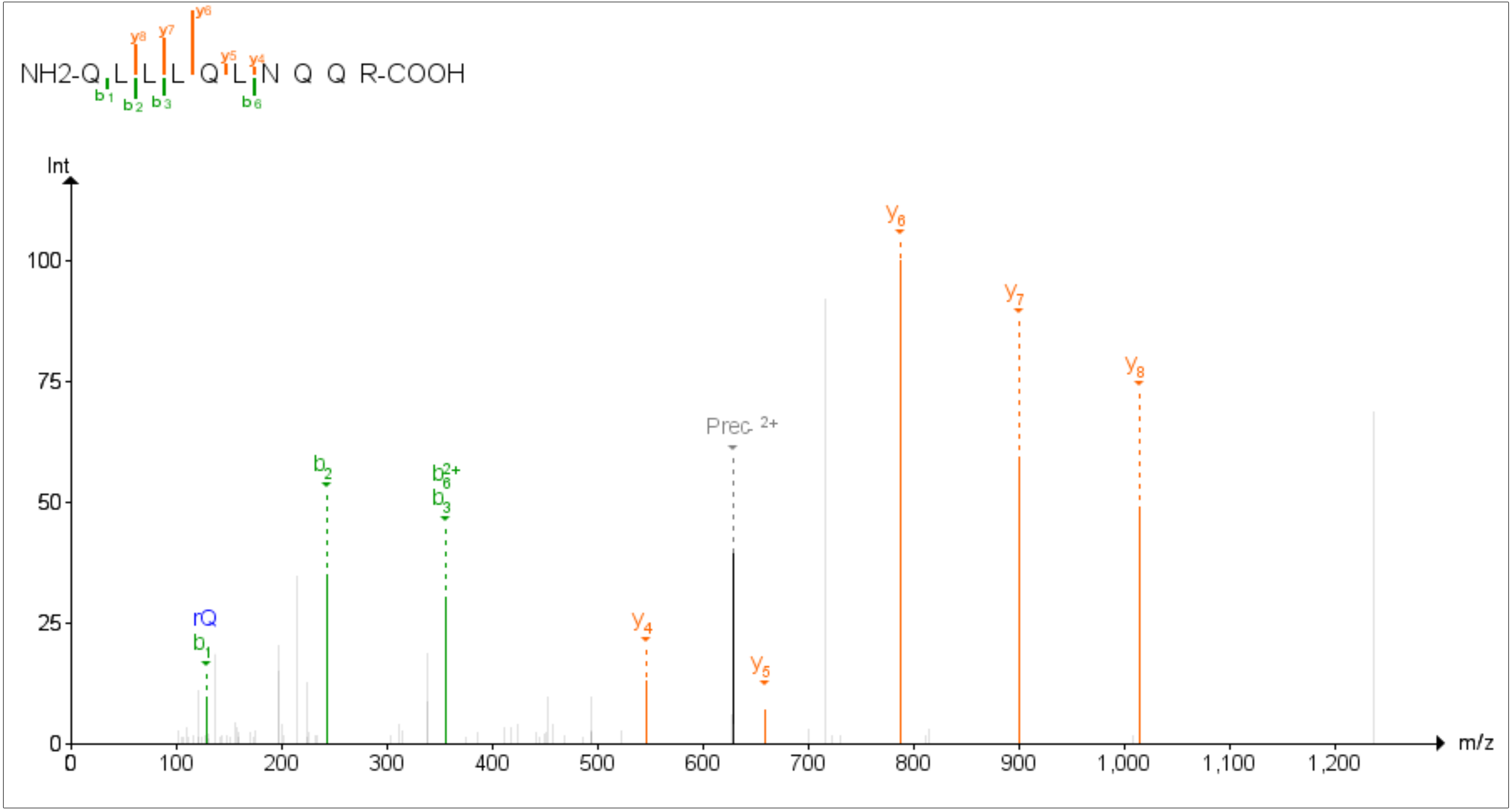

NH2-Q L L L Q L N Q Q R-COOH
y8 y7 y6 y5 y4
b1 b2 b3 b6
Int
m/z
y6
y7
y8
Prec 2+
b2
b6 2+
b3
rQ
b1
y4
y5
0 25 50 75 100
0 100 200 300 400 500 600 700 800 900 1,000 1,100 1,200

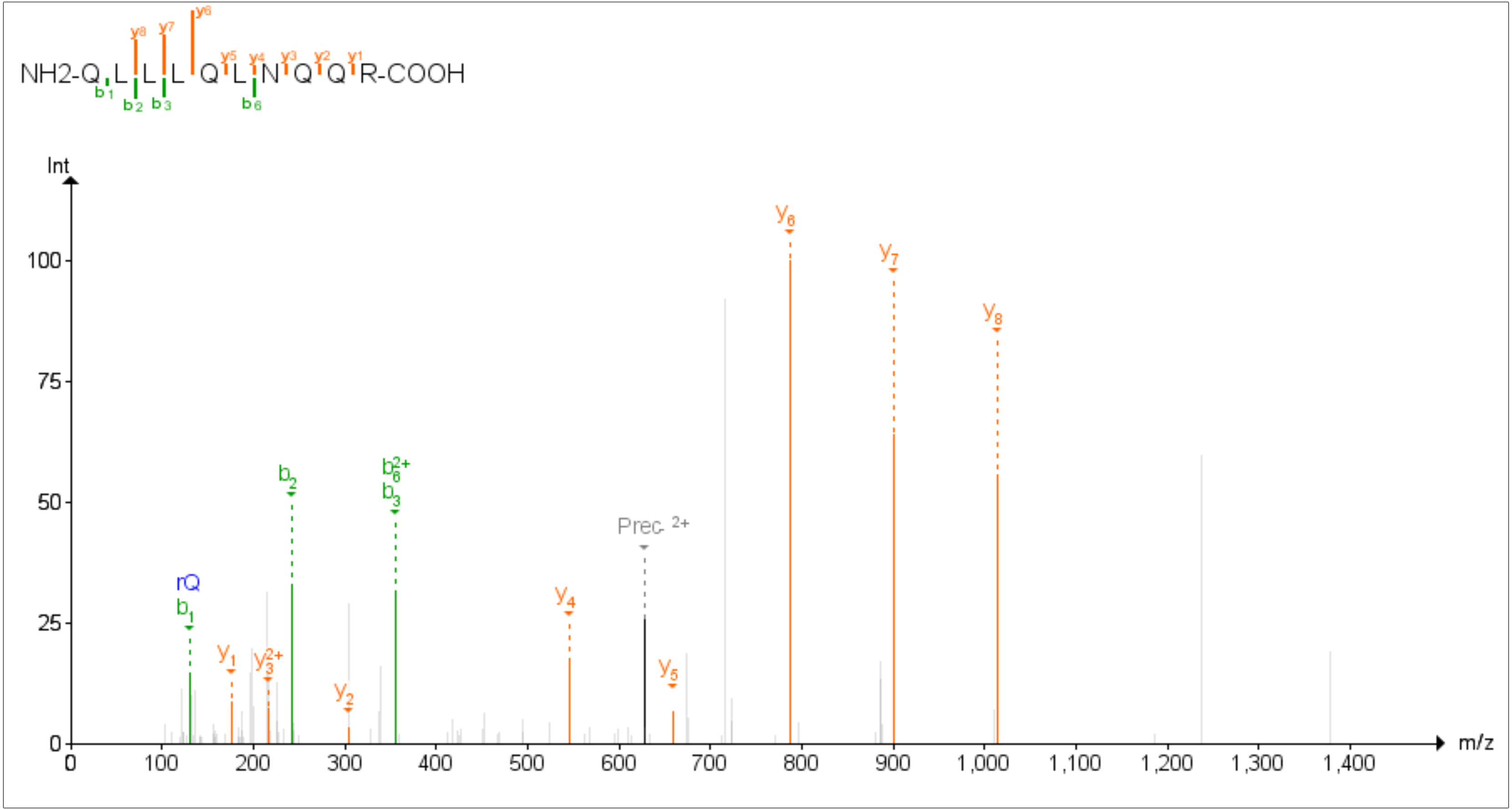
NH2-Q L L L Q L N Q Q R-COOH
y8 y7 y6 y5 y4 y3 y2 y1
b1 b2 b3 b6
Int
m/z
100
75
50
25
0
0 100 200 300 400 500 600 700 800 900 1,000 1,100 1,200 1,300 1,400
rQ
b1
y1
y3 2+
b2
y2
b6 2+
b3
y4
Prec. 2+
y5
y6
y7
y8

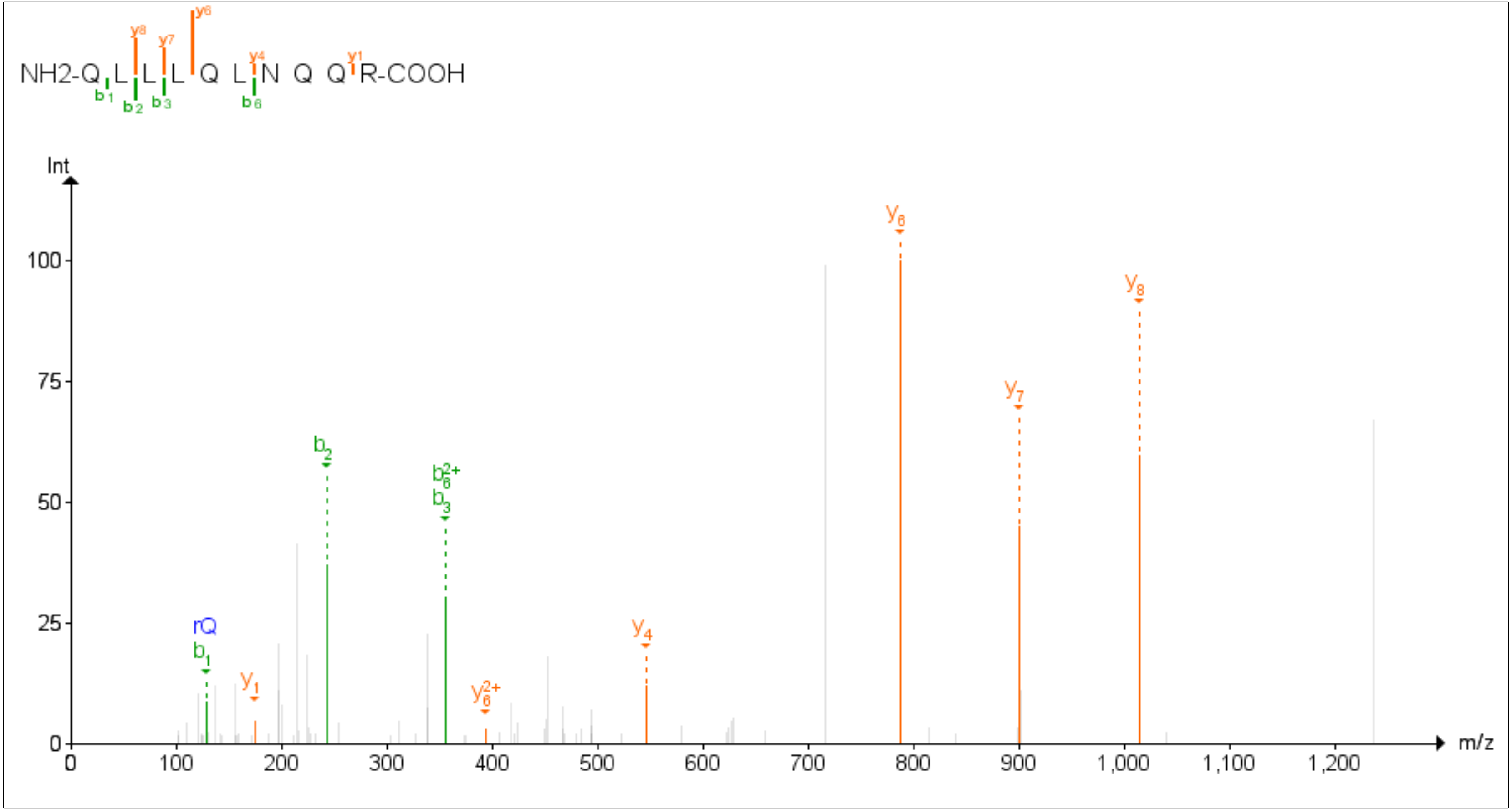

NH2-Q L L L Q L N Q Q R-COOH
y8 y7 y6 y4 y1
b1 b2 b3 b6
Int
m/z
y6
y8
y7
b2
b6 2+
b3
rQ
b1
y1
y6 2+
y4
0
25
50
75
100
0
100
200
300
400
500
600
700
800
900
1,000
1,100
1,200

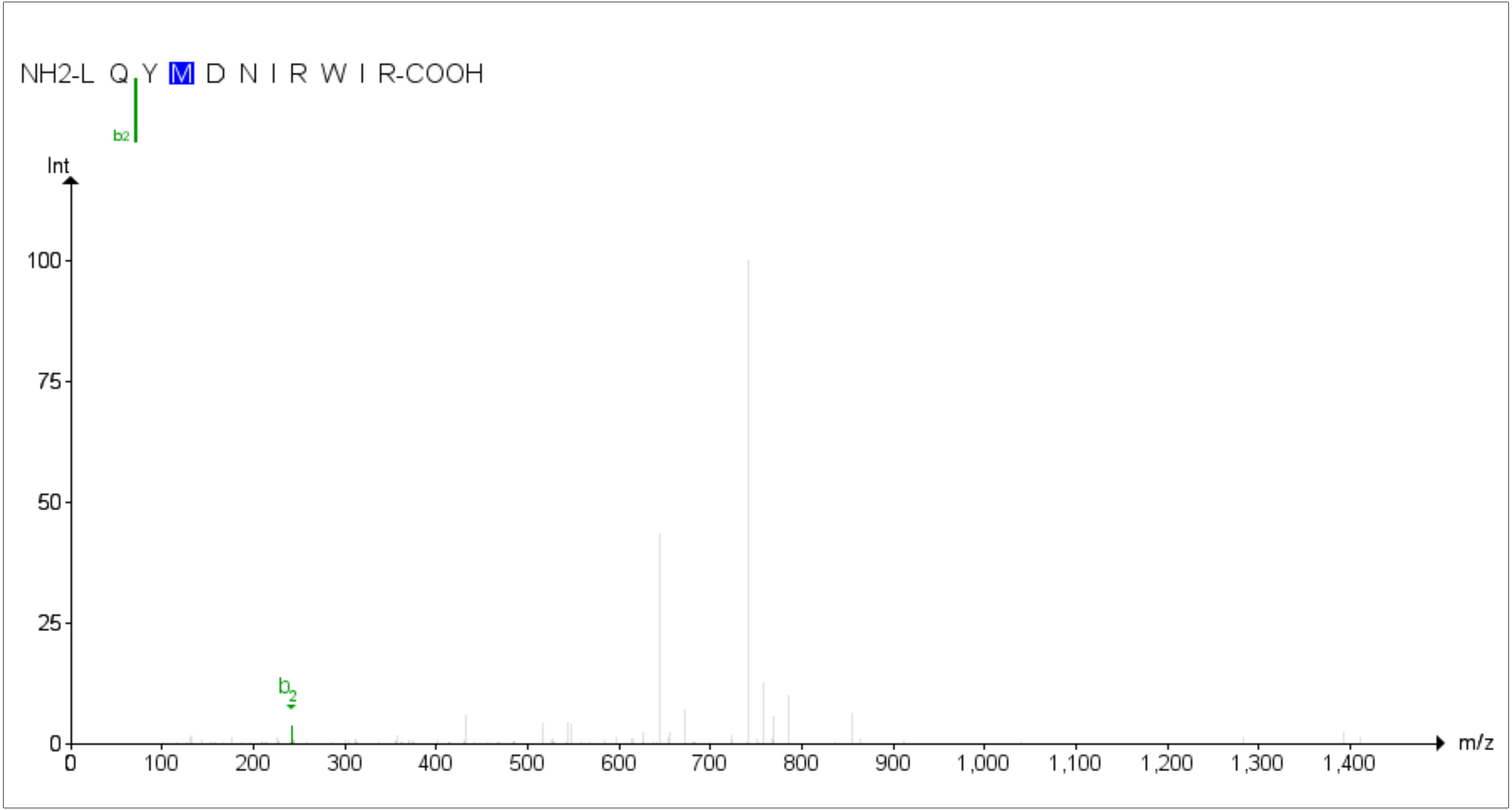

NH2-L Q Y M D N I R W I R-COOH
b2
Int
100
75
50
25
0
b2
0
100
200
300
400
500
600
700
800
900
1,000
1,100
1,200
1,300
1,400
m/z

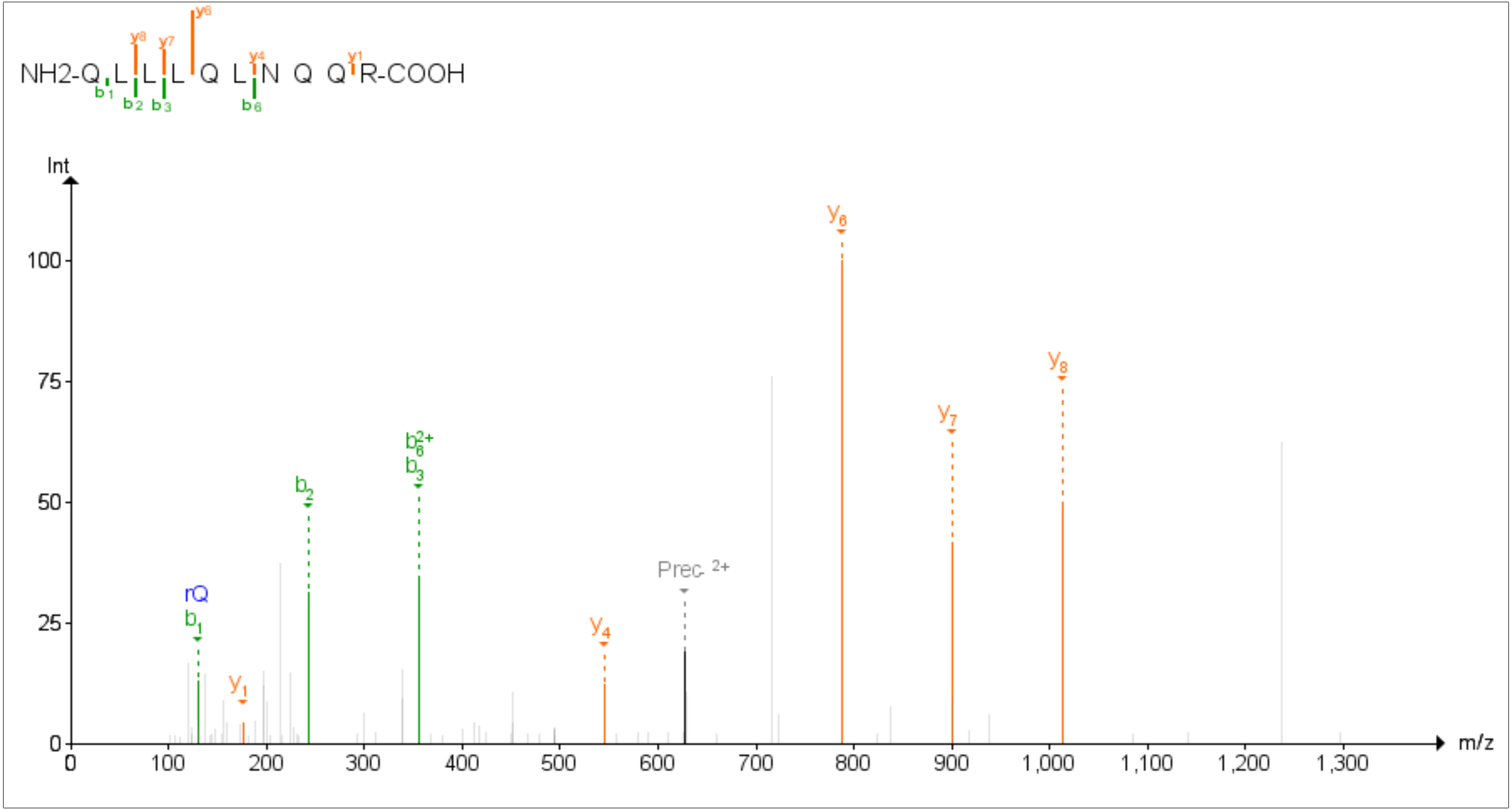

NH2-Q L L L Q L N Q Q R-COOH
b1 b2 b3 b6
y8 y7 y6 y4 y1
Int
100
75
50
25
0
rQ
b1
y1
b2
b6 2+
b3
y4
Prec 2+
y6
y7
y8
0 100 200 300 400 500 600 700 800 900 1,000 1,100 1,200 1,300
m/z

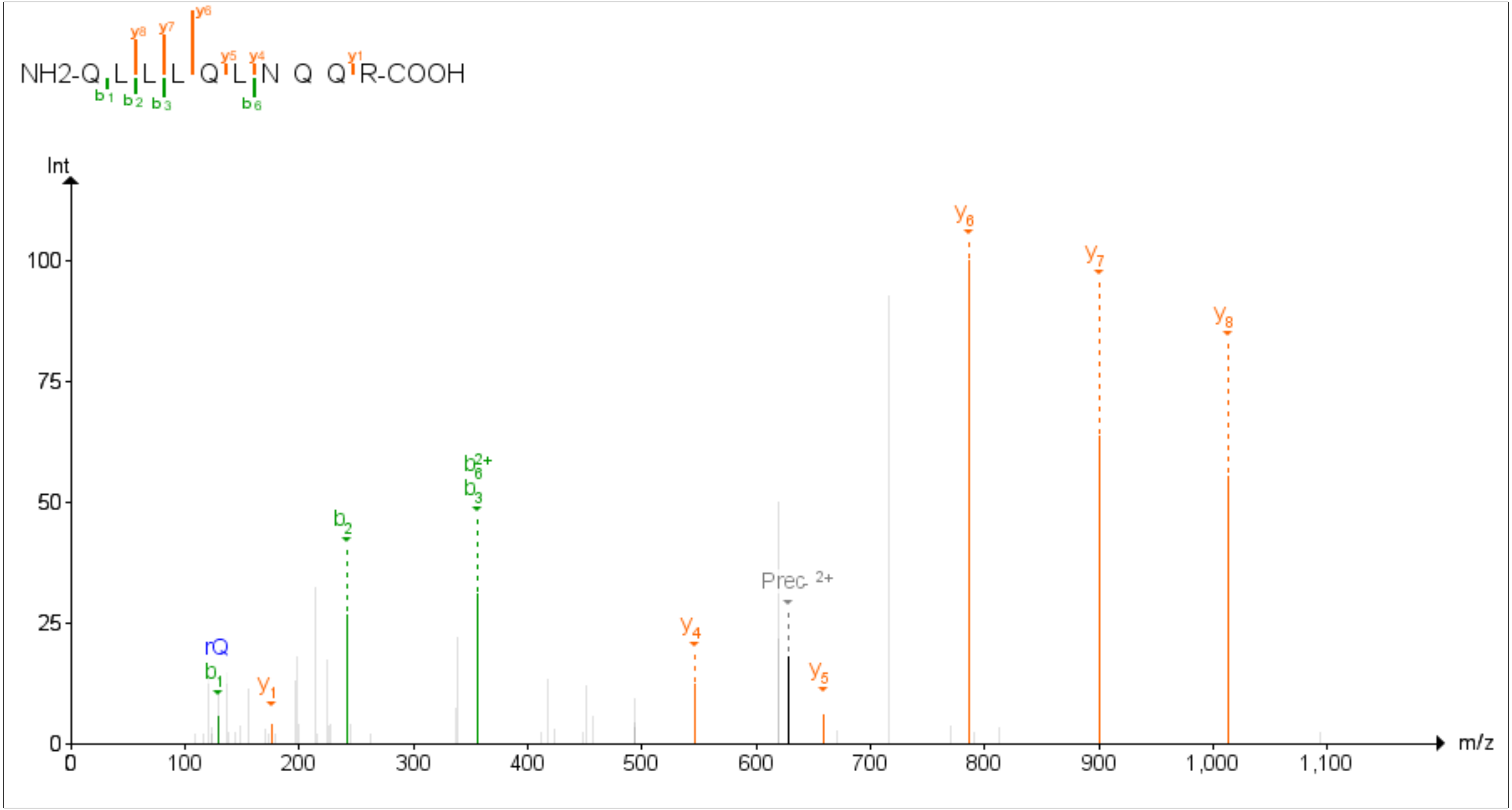

NH2-Q L L L Q L N Q Q R-COOH
b1 b2 b3 b6
y8 y7 y6 y5 y4 y1
Int
100
75
50
25
0
rQ
b1
y1
b2
b6 2+
b3
Prec 2+
y4
y5
y6
y7
y8
0 100 200 300 400 500 600 700 800 900 1,000 1,100
m/z

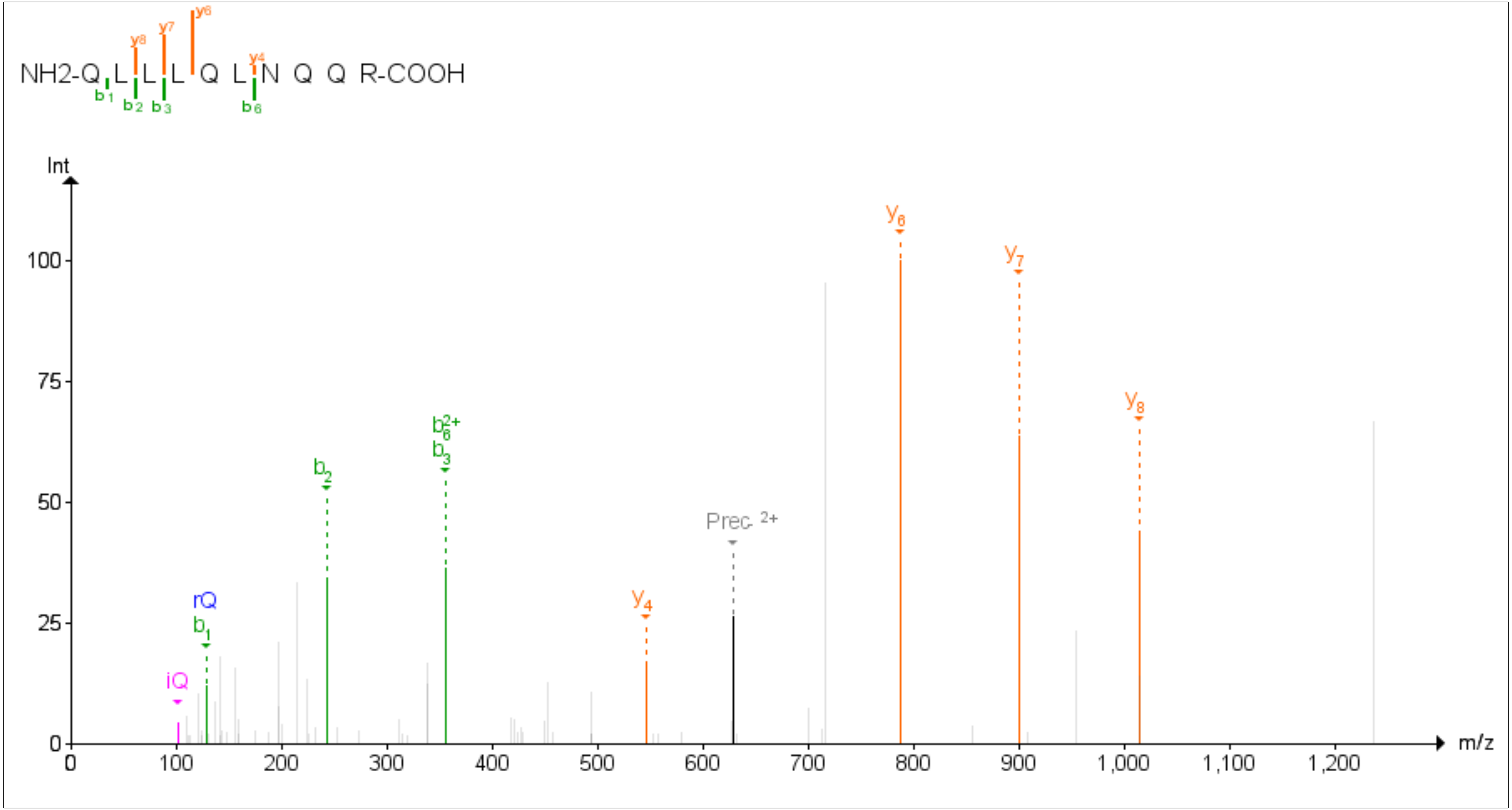

NH2-Q L L L Q L N Q Q R-COOH
Int
m/z
y6
y7
y8
y4
b1
b2
b3
b6
b6 2+
b3
b2
b1
rQ
iQ
y4
Prec 2+

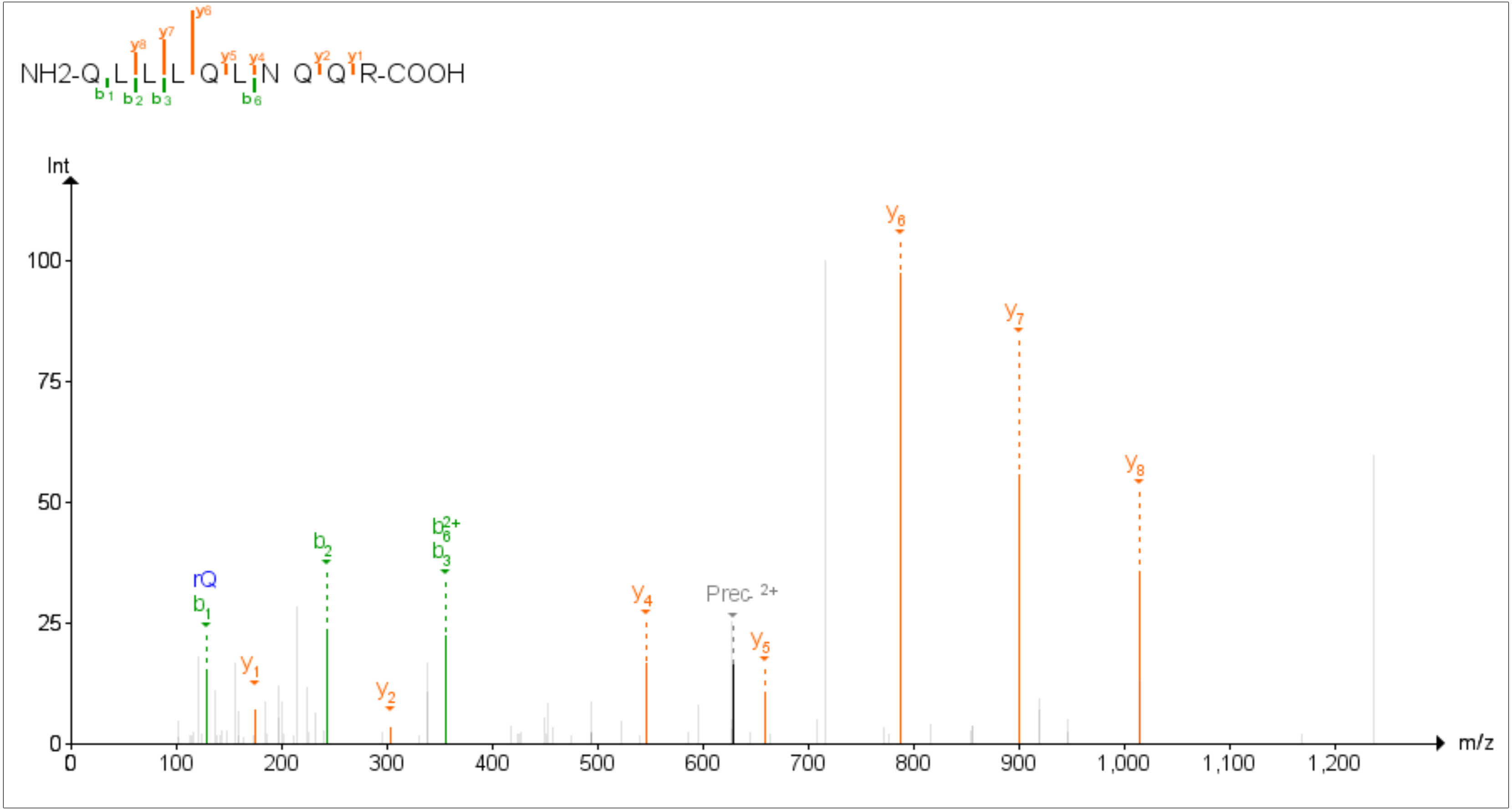

NH2-Q L L L Q L N Q Q R-COOH
y8 y7 y6 y5 y4 y2 y1
b1 b2 b3 b6
Int
100
75
50
25
0
rQ
b1
y1
y2
b2
b6 2+
b3
y4
Prec. 2+
y5
y6
y7
y8
0 100 200 300 400 500 600 700 800 900 1,000 1,100 1,200
m/z

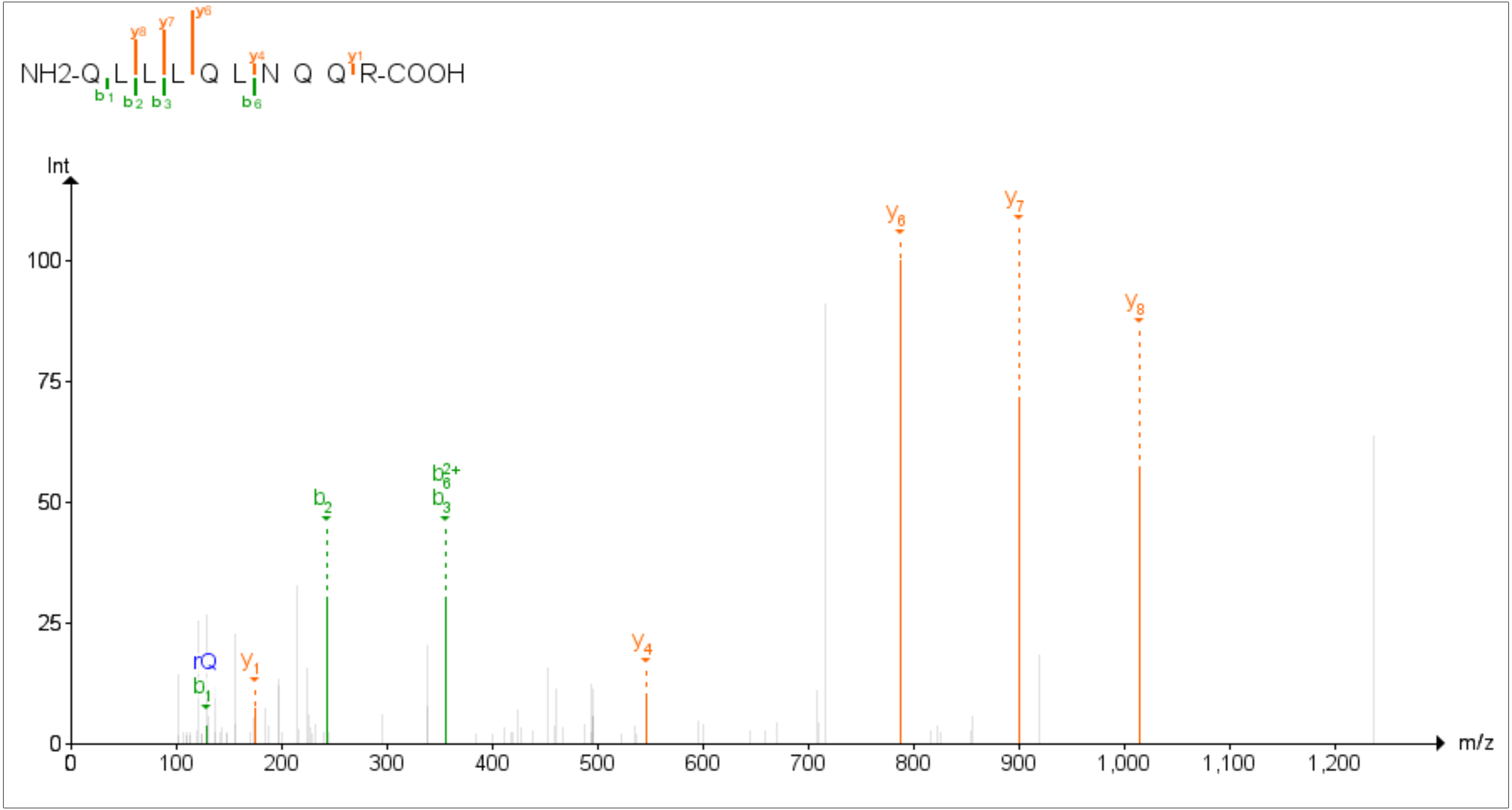

NH2-Q L L L Q L N Q Q R-COOH
y8 y7 y6 y4 y1
b1 b2 b3 b6
Int
100
75
50
25
0
rQ
b1
y1
b2
b6 2+
b3
y4
y6
y7
y8
0 100 200 300 400 500 600 700 800 900 1,000 1,100 1,200
m/z

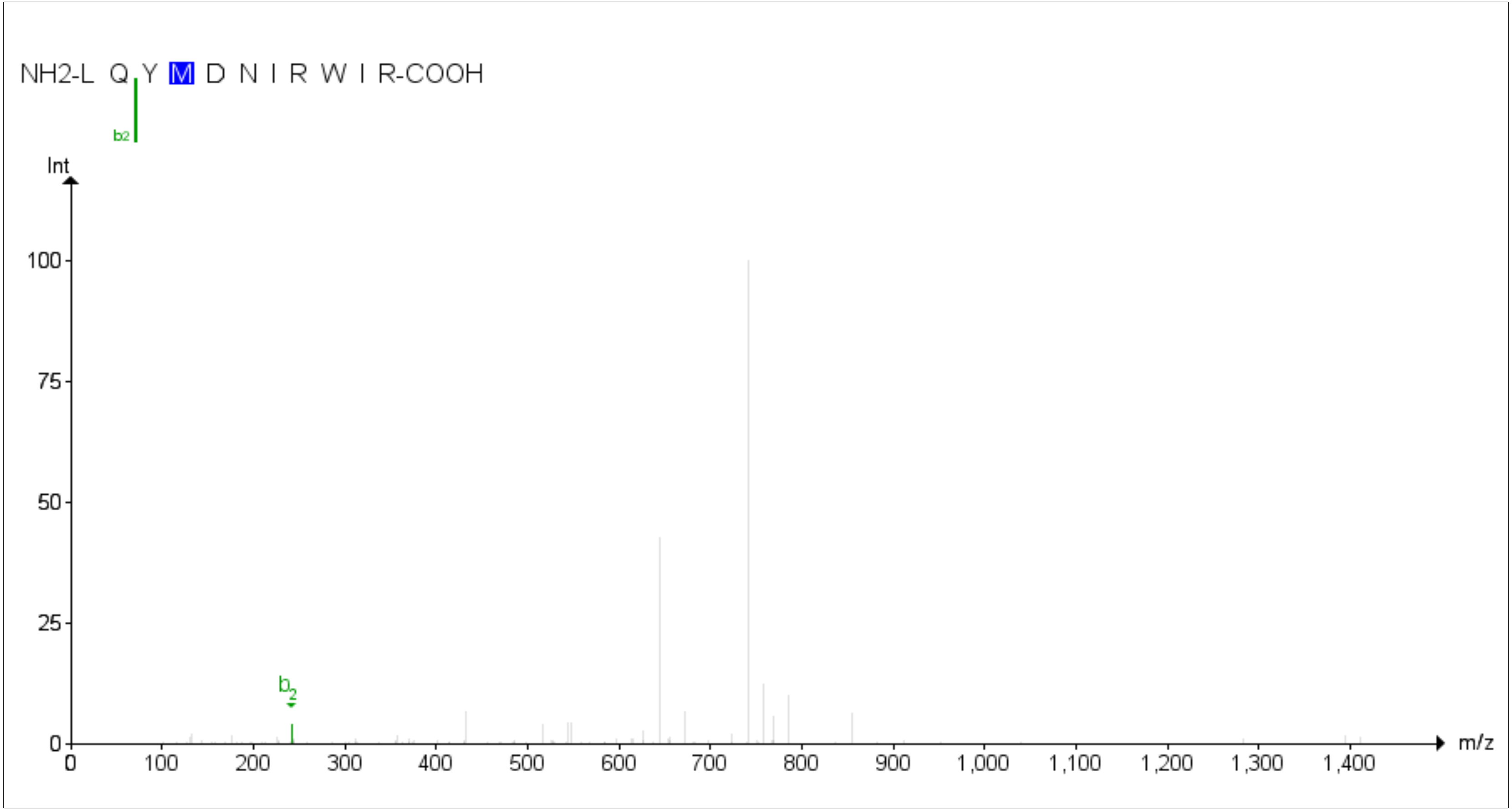
NH2-L Q Y M D N I R W I R-COOH
b2
Int
100
75
50
25
0
b2
0
100
200
300
400
500
600
700
800
900
1,000
1,100
1,200
1,300
1,400
m/z

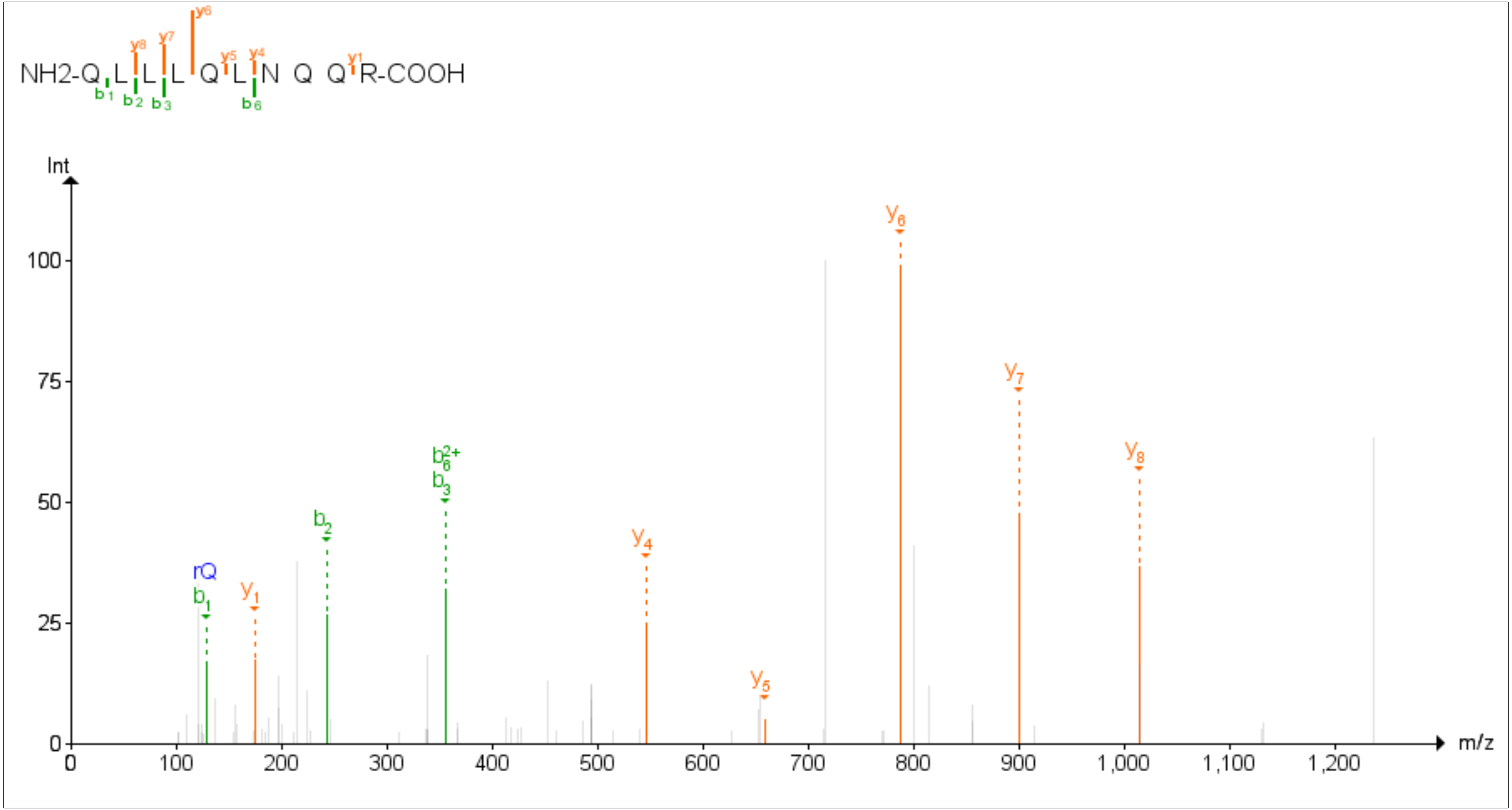
NH2-Q L L L Q L N Q Q R-COOH
y8 y7 y6 y5 y4 y1
b1 b2 b3 b6
Int
100
75
50
25
0
y6
y7
y8
b6 2+
b3
b2
y4
rQ
b1
y1
y5
0 100 200 300 400 500 600 700 800 900 1,000 1,100 1,200
m/z

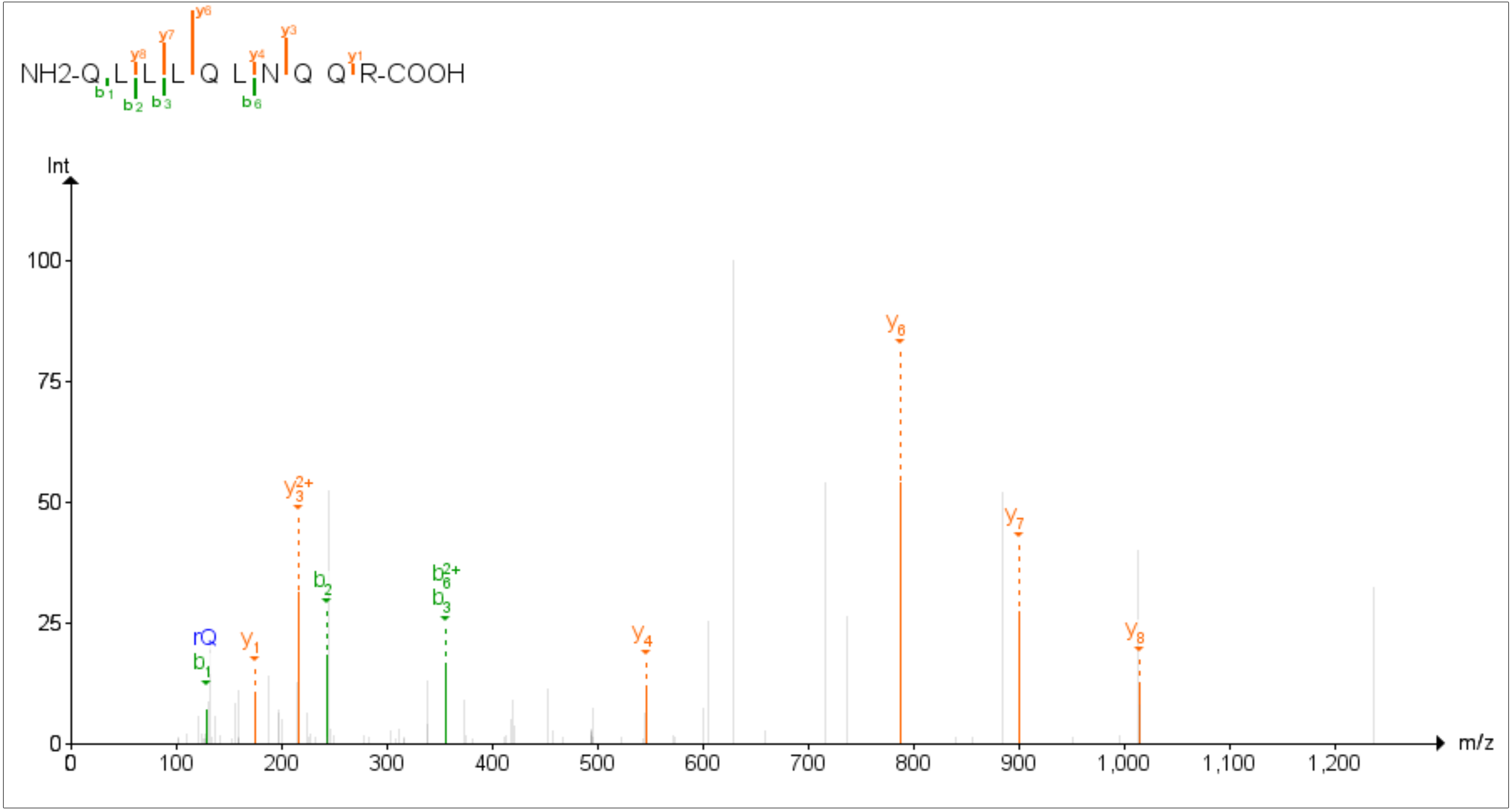

NH2-Q L L L Q L N Q Q R-COOH
y8 y7 y6 y4 y3 y1
b1 b2 b3 b6
Int
100
75
50
25
0
m/z
0
100
200
300
400
500
600
700
800
900
1,000
1,100
1,200
rQ
b1
y1
y3 2+
b2
b6 2+
b3
y4
y6
y7
y8

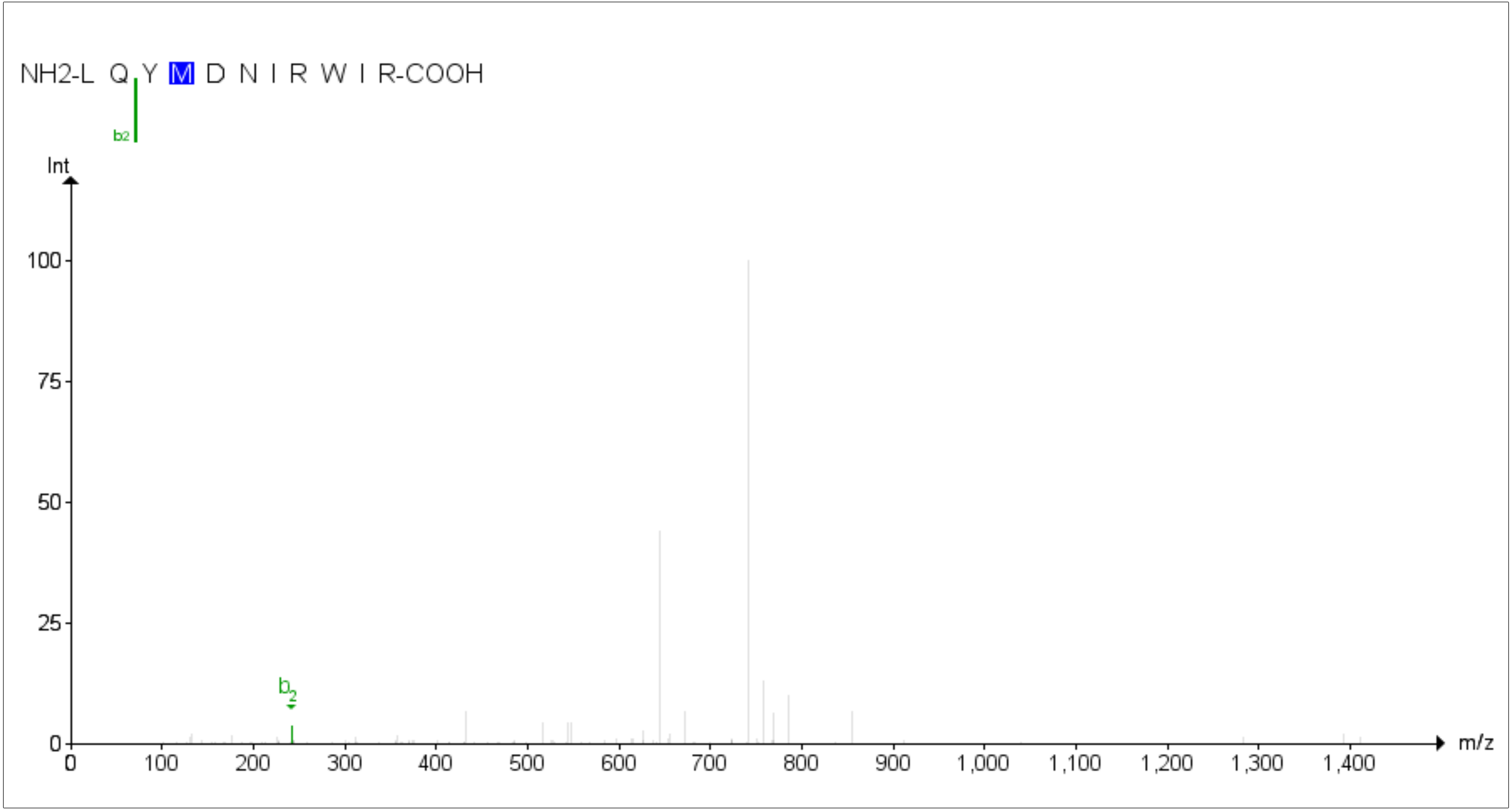
NH2-L Q Y M D N I R W I R-COOH
b2
Int
100
75
50
25
0
b2
0
100
200
300
400
500
600
700
800
900
1,000
1,100
1,200
1,300
1,400
m/z

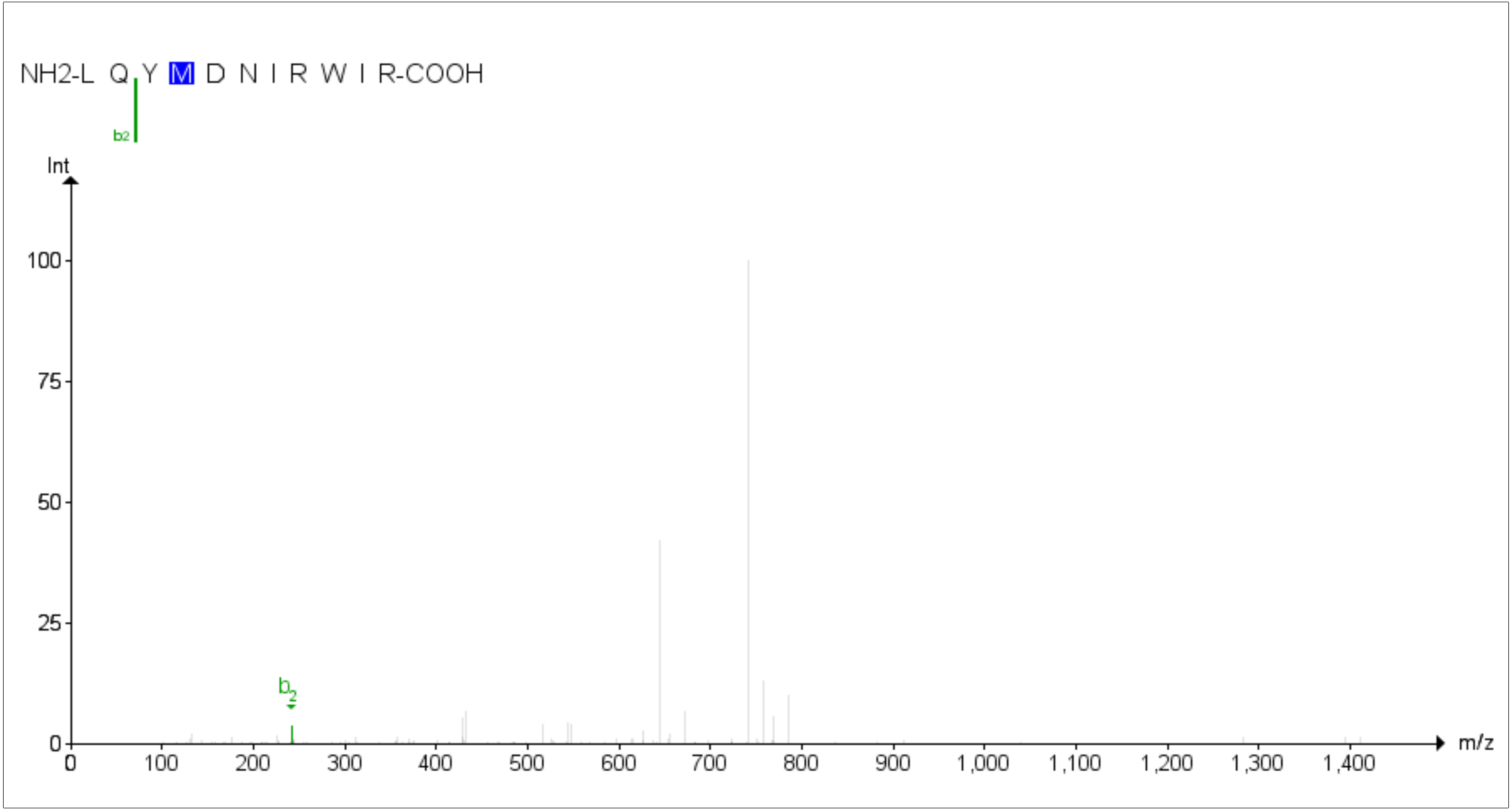

NH2-L Q Y M D N I R W I R-COOH
b2
Int
100
75
50
25
0
b2
m/z
0
100
200
300
400
500
600
700
800
900
1,000
1,100
1,200
1,300
1,400

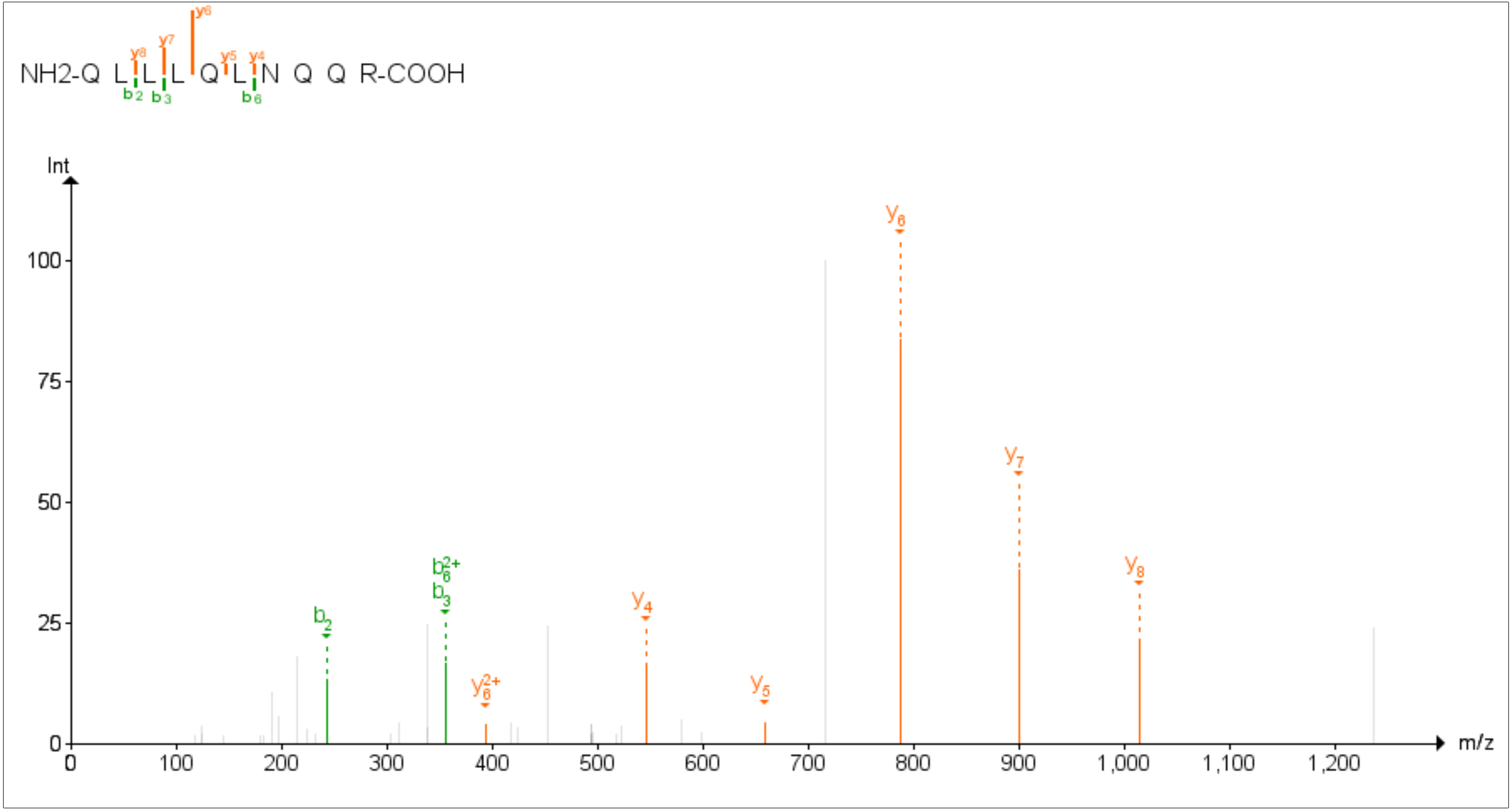

NH2-Q L L L Q L N Q Q R-COOH
Int
m/z
y6
y7
y8
y4
y5
b2
b3
b6

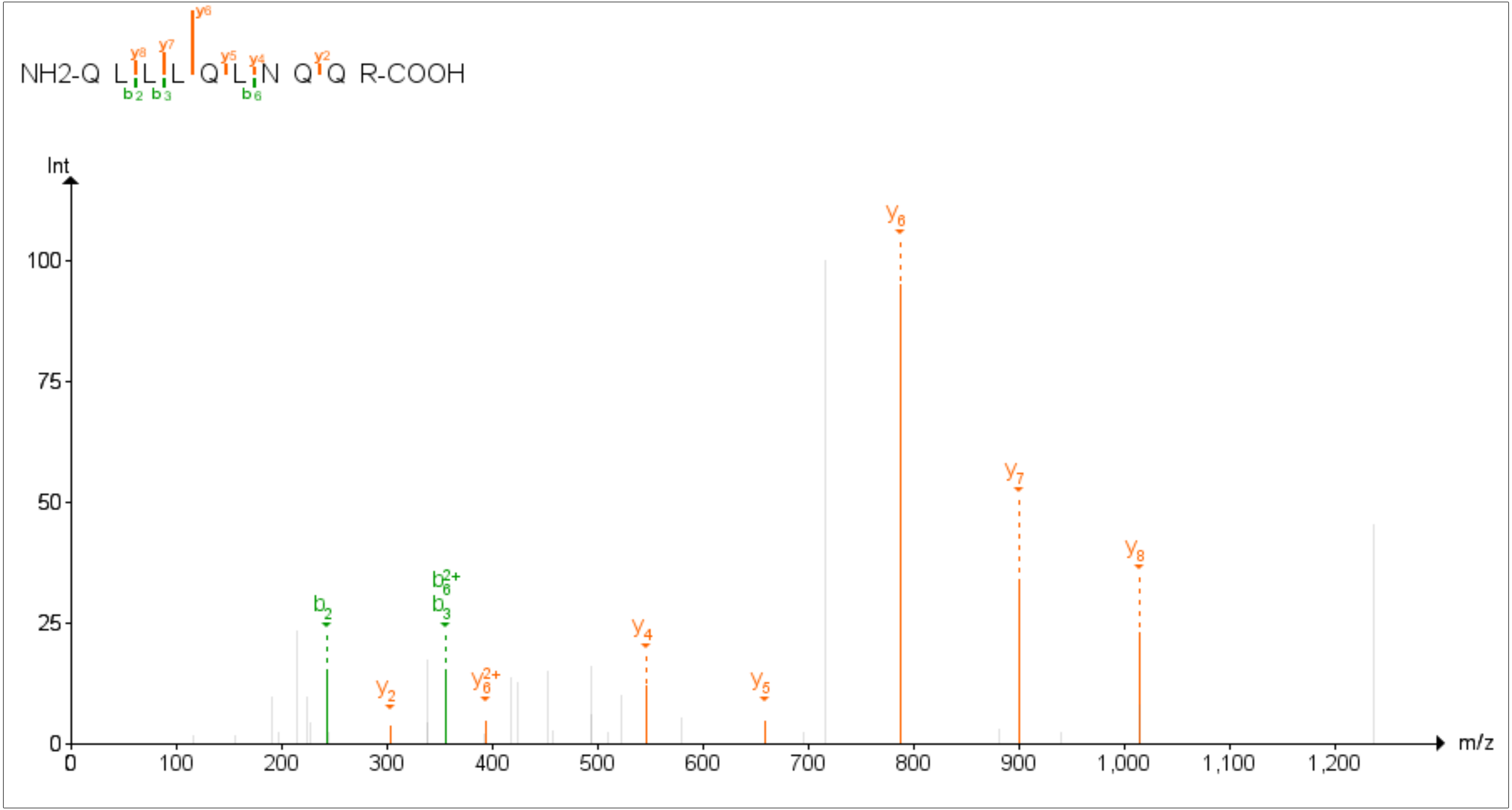

NH2-Q L L L Q L N Q Q R-COOH
y8 y7 y6 y5 y4 y2
b2 b3 b6
Int
m/z
100
75
50
25
0
0 100 200 300 400 500 600 700 800 900 1,000 1,100 1,200
y6
y7
y8
b2
b6 2+
b3
y2
y6 2+
y4
y5

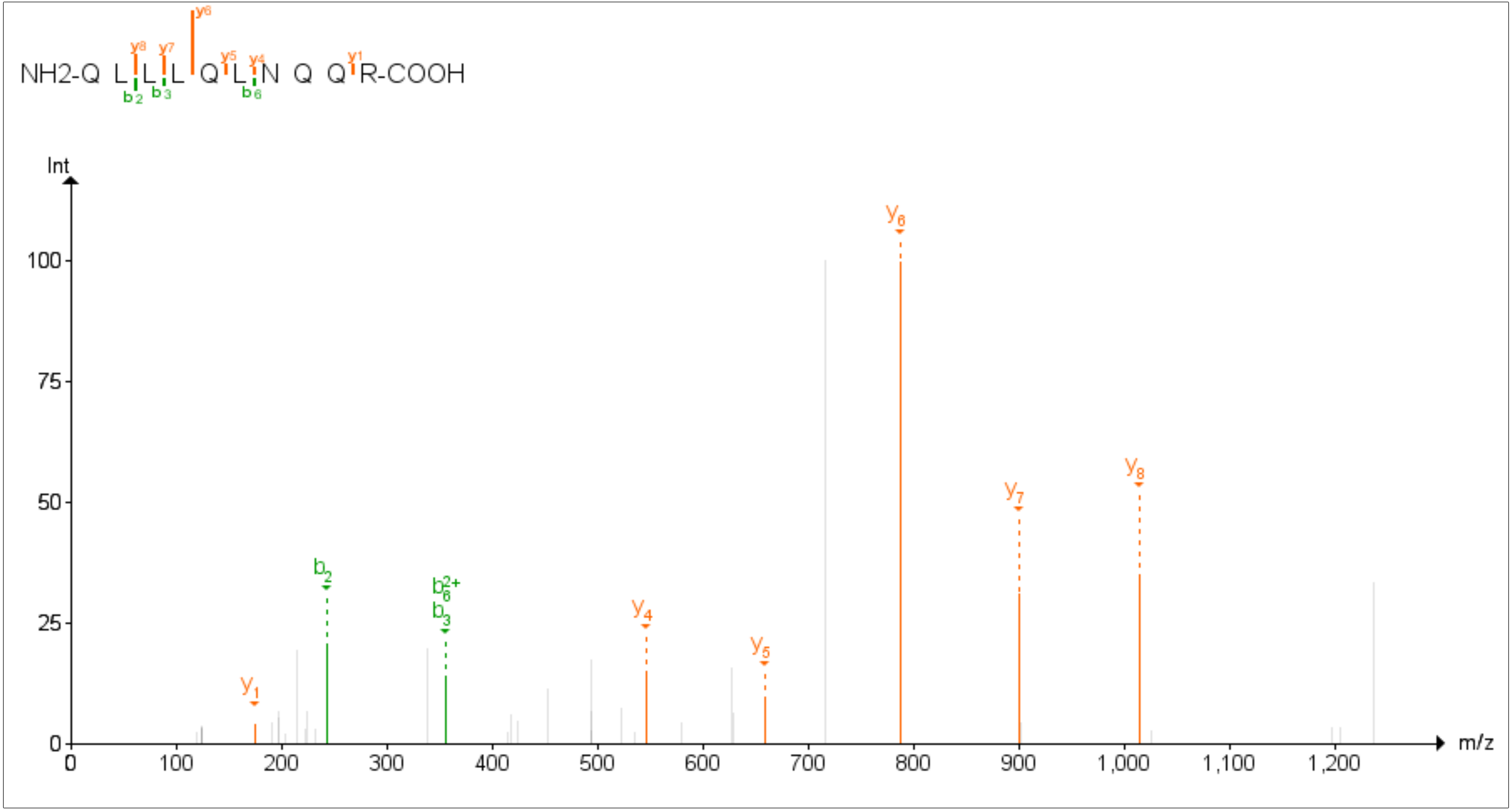
NH2-Q L L L Q L N Q Q R-COOH
y8 y7 y6 y5 y4 y1
b2 b3 b6
Int
100
75
50
25
0
m/z
0
100
200
300
400
500
600
700
800
900
1,000
1,100
1,200
y6
y8
y7
b2
b6 2+
b3
y4
y5
y1

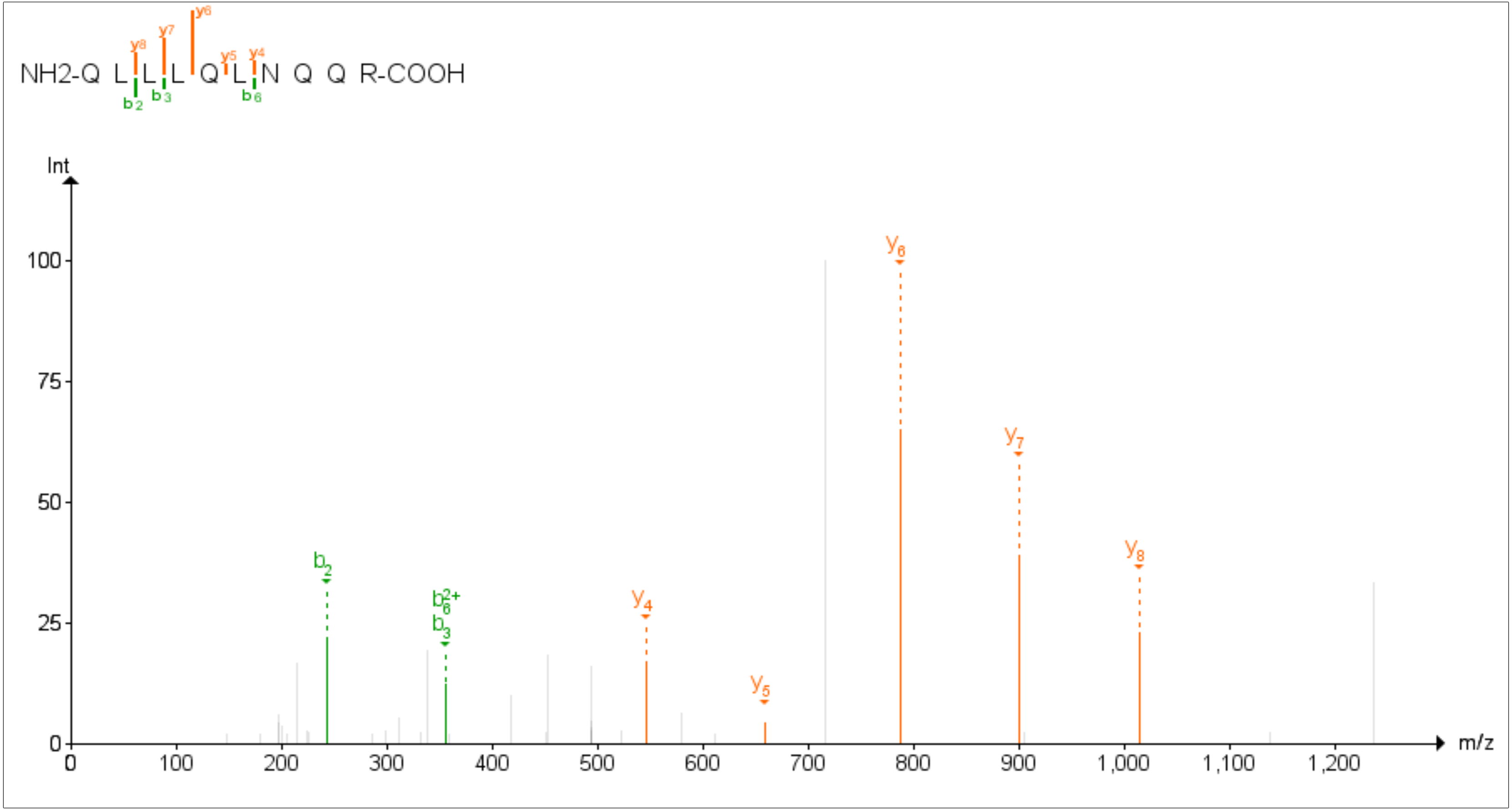
NH2-Q L L L Q L N Q Q R-COOH
y8 y7 y6 y5 y4
b2 b3 b6
Int
100
75
50
25
0
b2
b6 2+
b3
y4
y5
y6
y7
y8
m/z
0 100 200 300 400 500 600 700 800 900 1,000 1,100 1,200

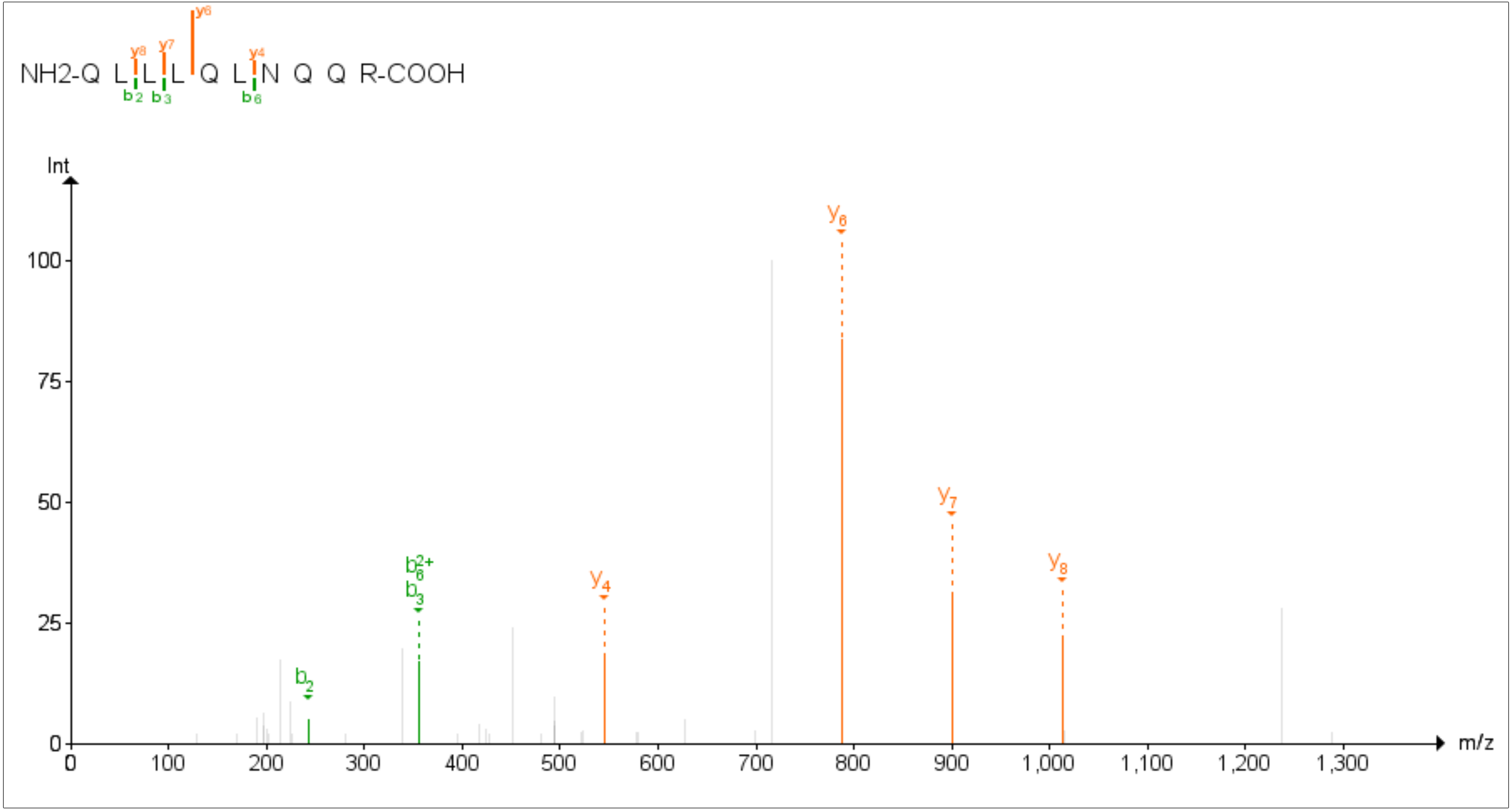

NH2-Q L L L Q L N Q Q R-COOH
y8 y7 y6 y4
b2 b3 b6
Int
100
75
50
25
0
y6
y7
y8
y4
b6 2+
b3
b2
0 100 200 300 400 500 600 700 800 900 1,000 1,100 1,200 1,300
m/z

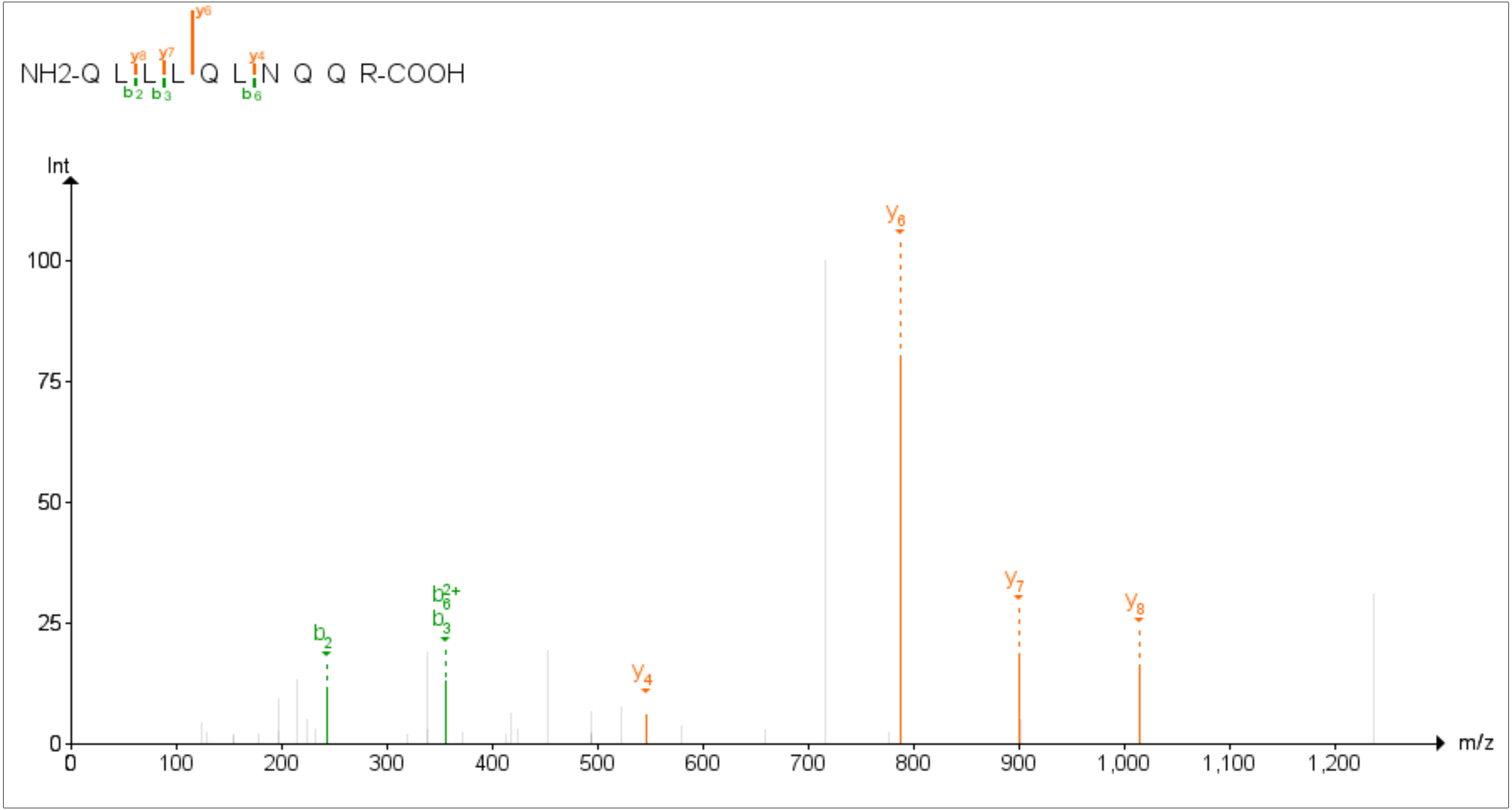
NH2-Q L L L Q L N Q Q R-COOH
y8 y7 y6 y4
b2 b3 b6
Int
100
75
50
25
0
y6
y7
y8
y4
b2
b6 2+
b3
0 100 200 300 400 500 600 700 800 900 1,000 1,100 1,200
m/z

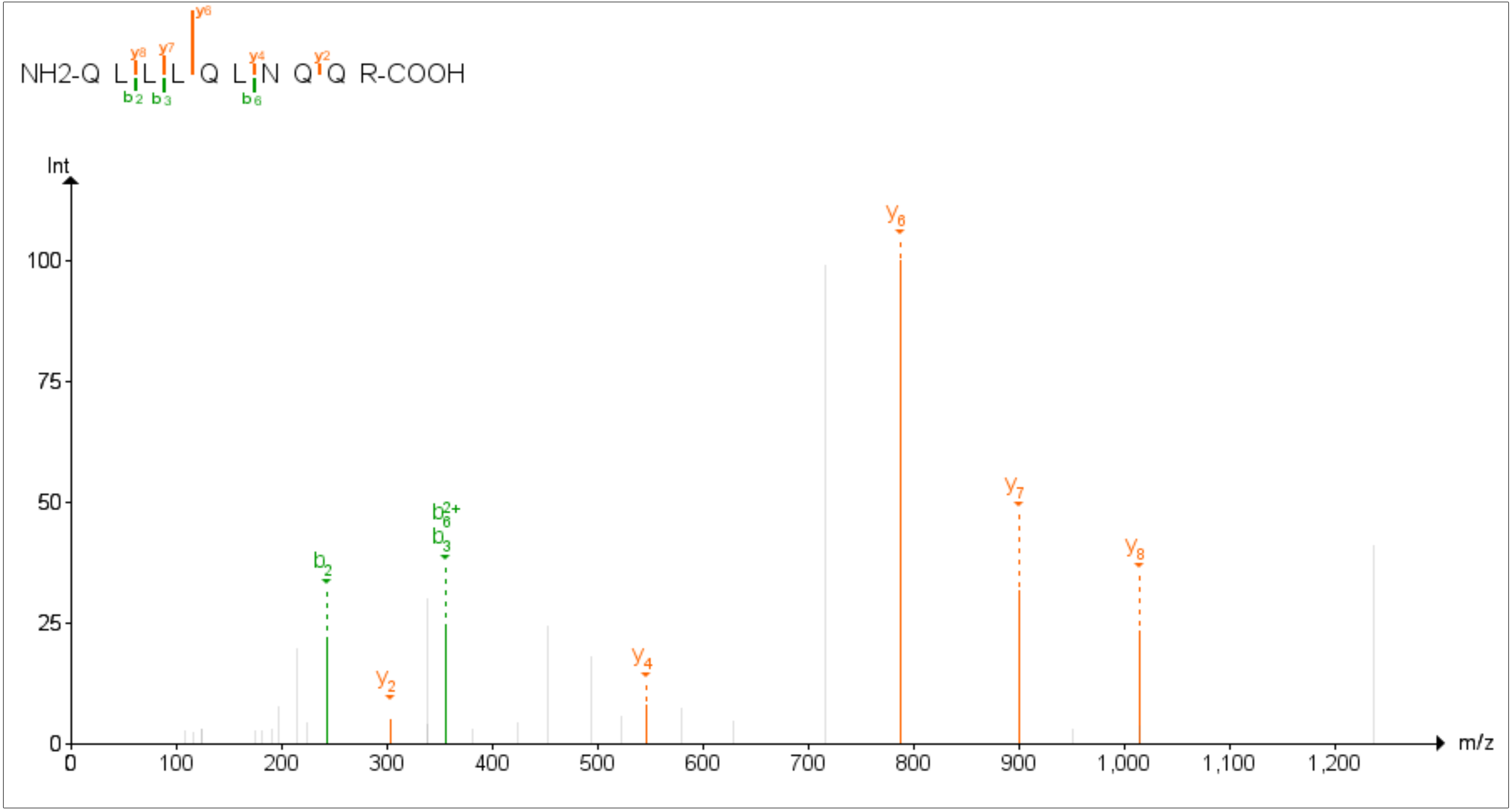

NH2-Q L L L Q L N Q Q R-COOH
y8 y7 y6 y4 y2
b2 b3 b6
Int
100
75
50
25
0
b2
y2
b6 2+
b3
y4
y6
y7
y8
0 100 200 300 400 500 600 700 800 900 1,000 1,100 1,200
m/z

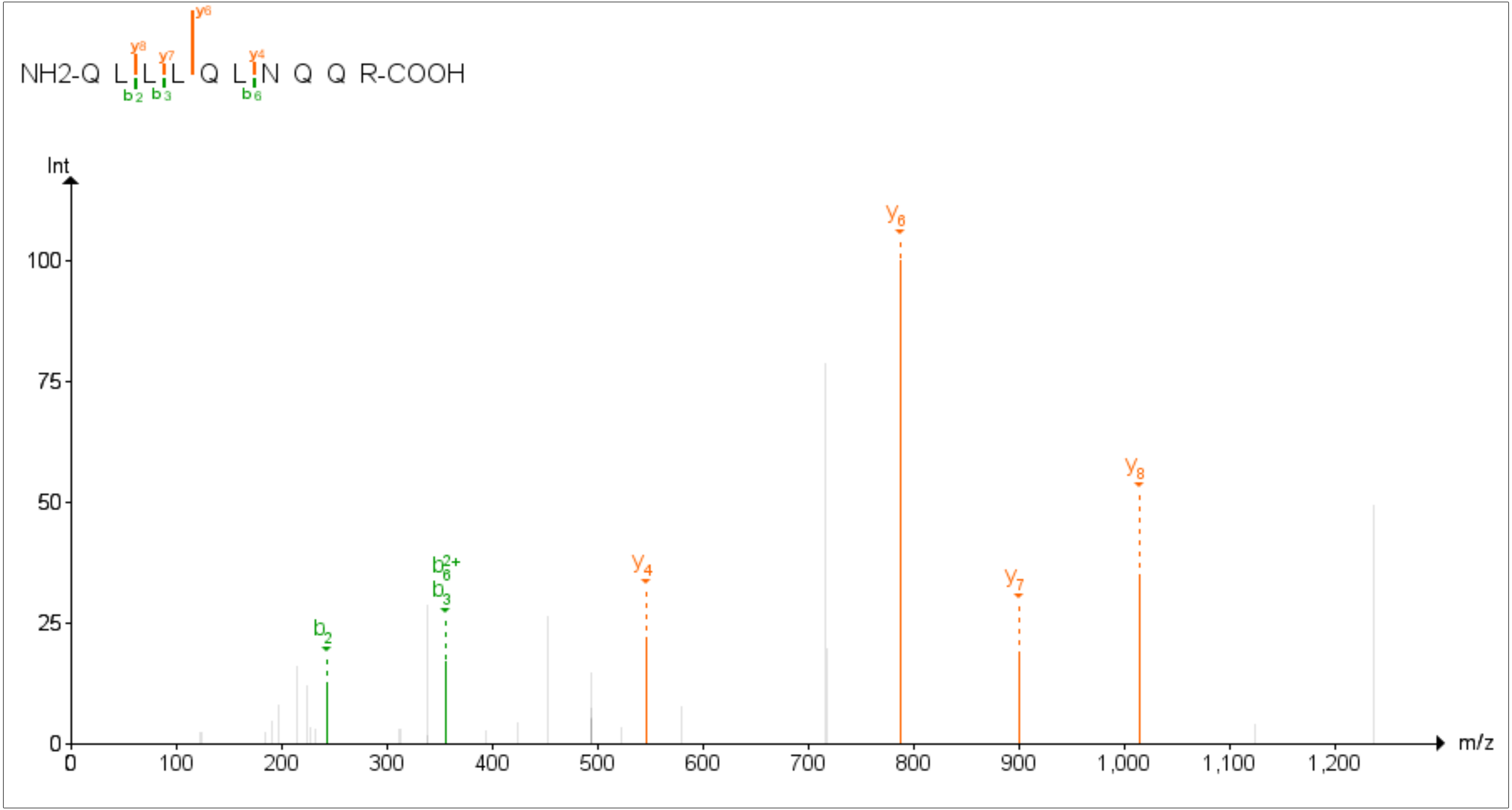

NH2-Q L L L Q L N Q Q R-COOH
y6
y8
y7
y4
b2
b3
b6
Int
100
75
50
25
0
m/z
0
100
200
300
400
500
600
700
800
900
1,000
1,100
1,200
b2
b6 2+
b3
y4
y6
y7
y8

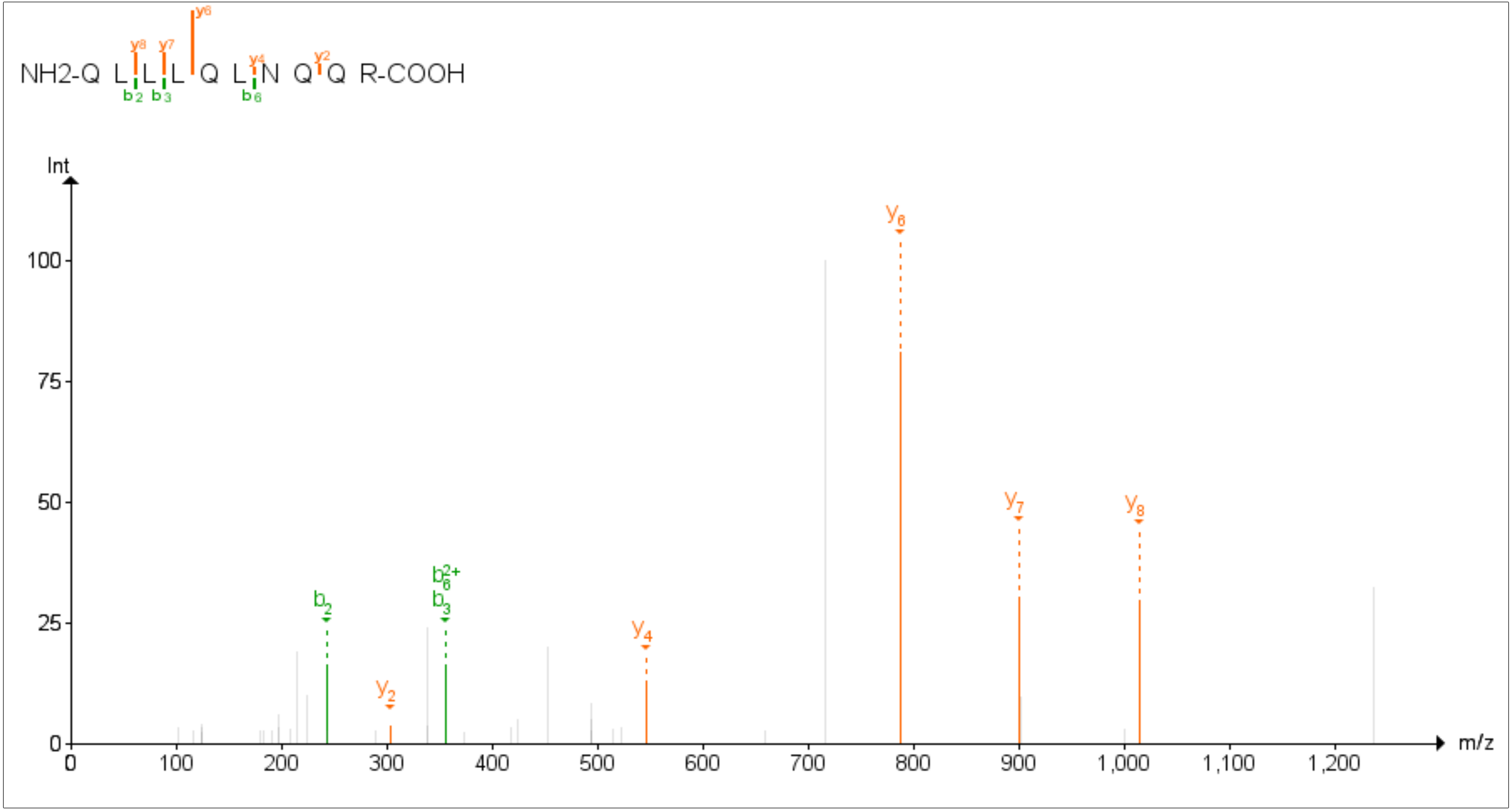

NH2-Q L L L Q L N Q Q R-COOH
y8 y7 y6 y4 y2
b2 b3 b6
Int
100
75
50
25
0
y6
y7
y8
b2
b6 2+
b3
y2
y4
0 100 200 300 400 500 600 700 800 900 1,000 1,100 1,200
m/z

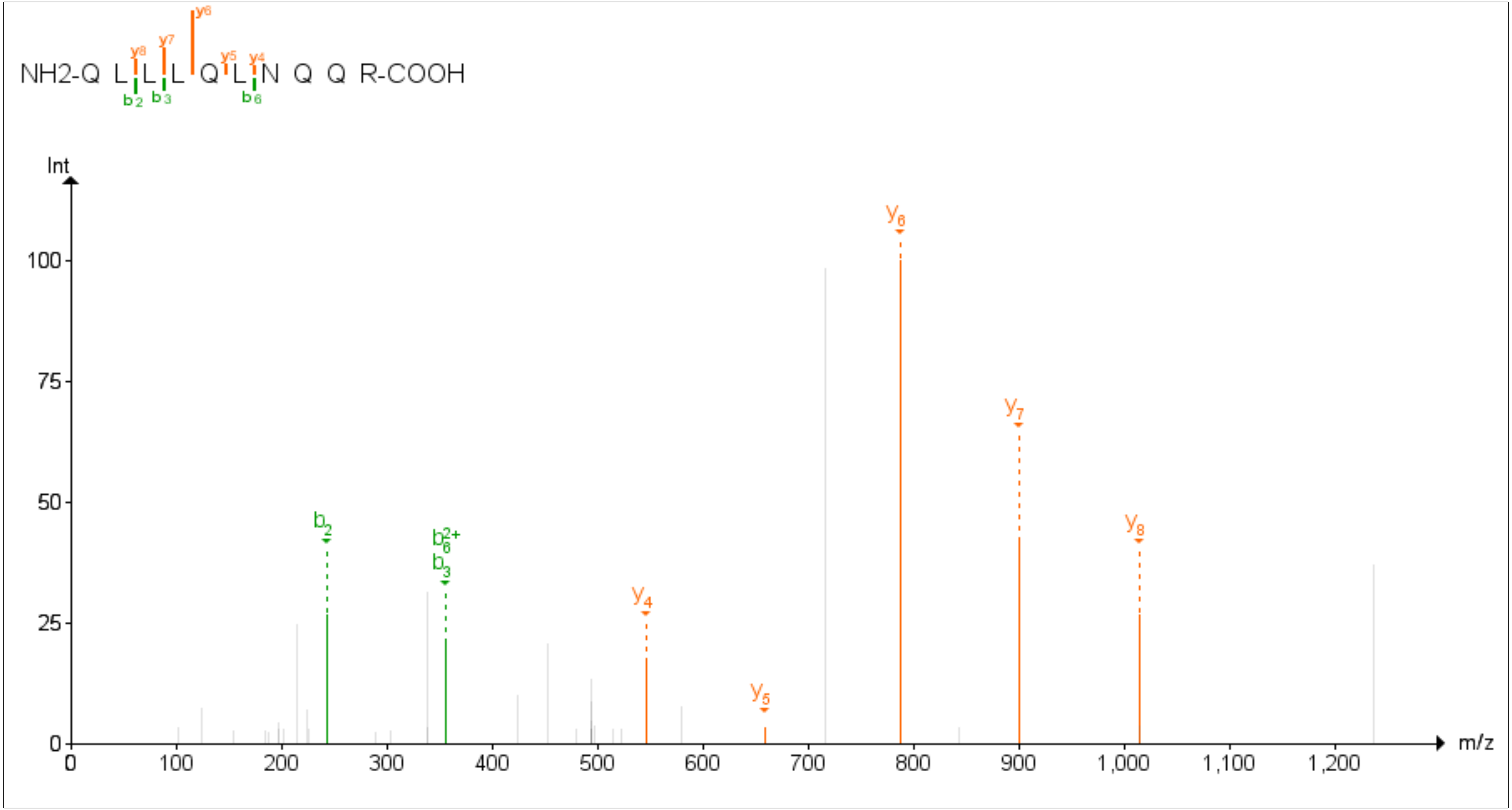
NH2-Q L L L Q L N Q Q R-COOH
y8 y7 y6 y5 y4
b2 b3 b6
Int
100
75
50
25
0
b2
b6 2+
b3
y4
y5
y6
y7
y8
0 100 200 300 400 500 600 700 800 900 1,000 1,100 1,200
m/z

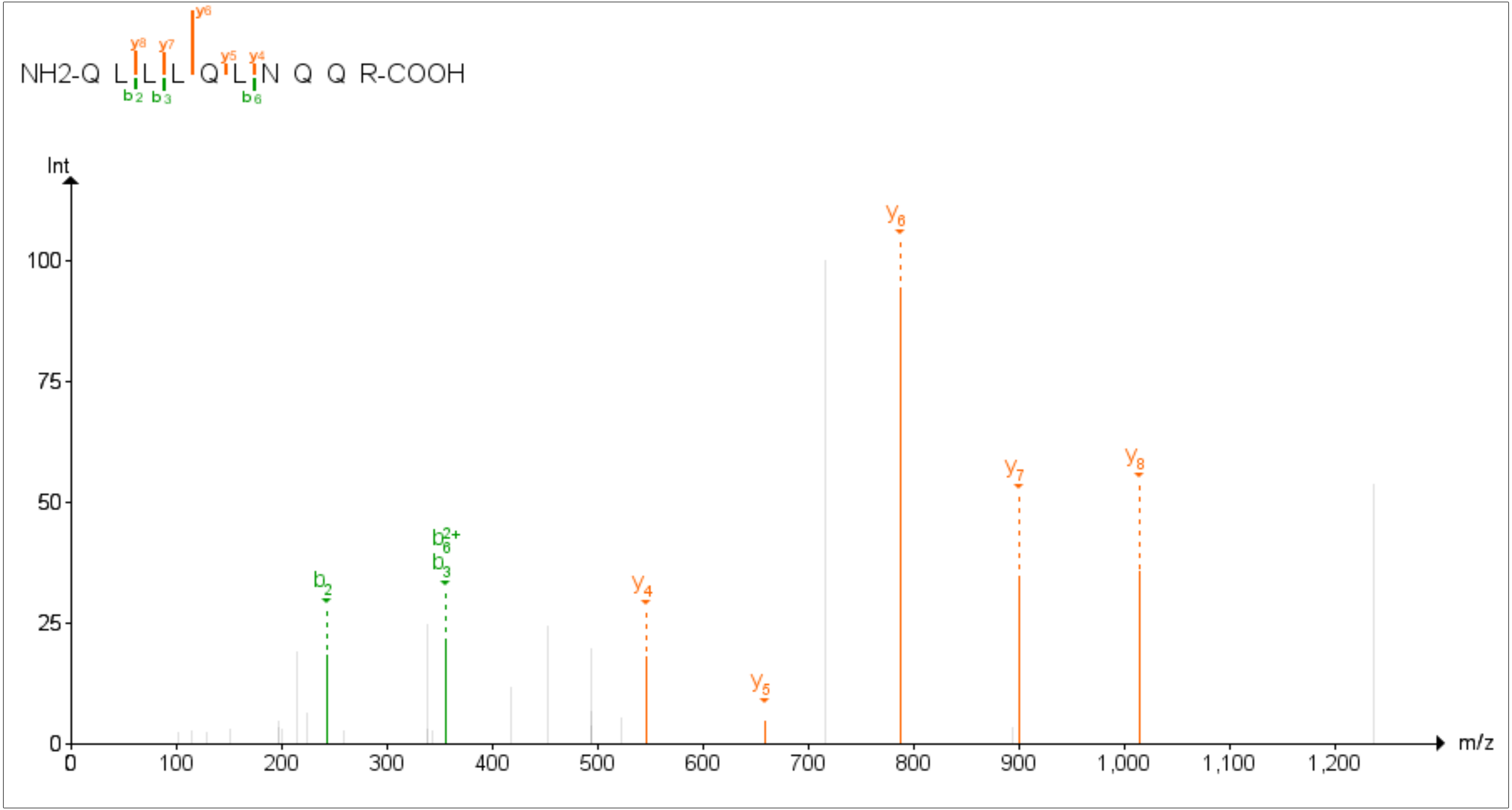

NH2-Q L L L Q L N Q Q R-COOH
y8 y7 y6 y5 y4
b2 b3 b6
Int
m/z
0
25
50
75
100
100
200
300
400
500
600
700
800
900
1,000
1,100
1,200
b2
b6 2+
b3
y4
y5
y6
y7
y8

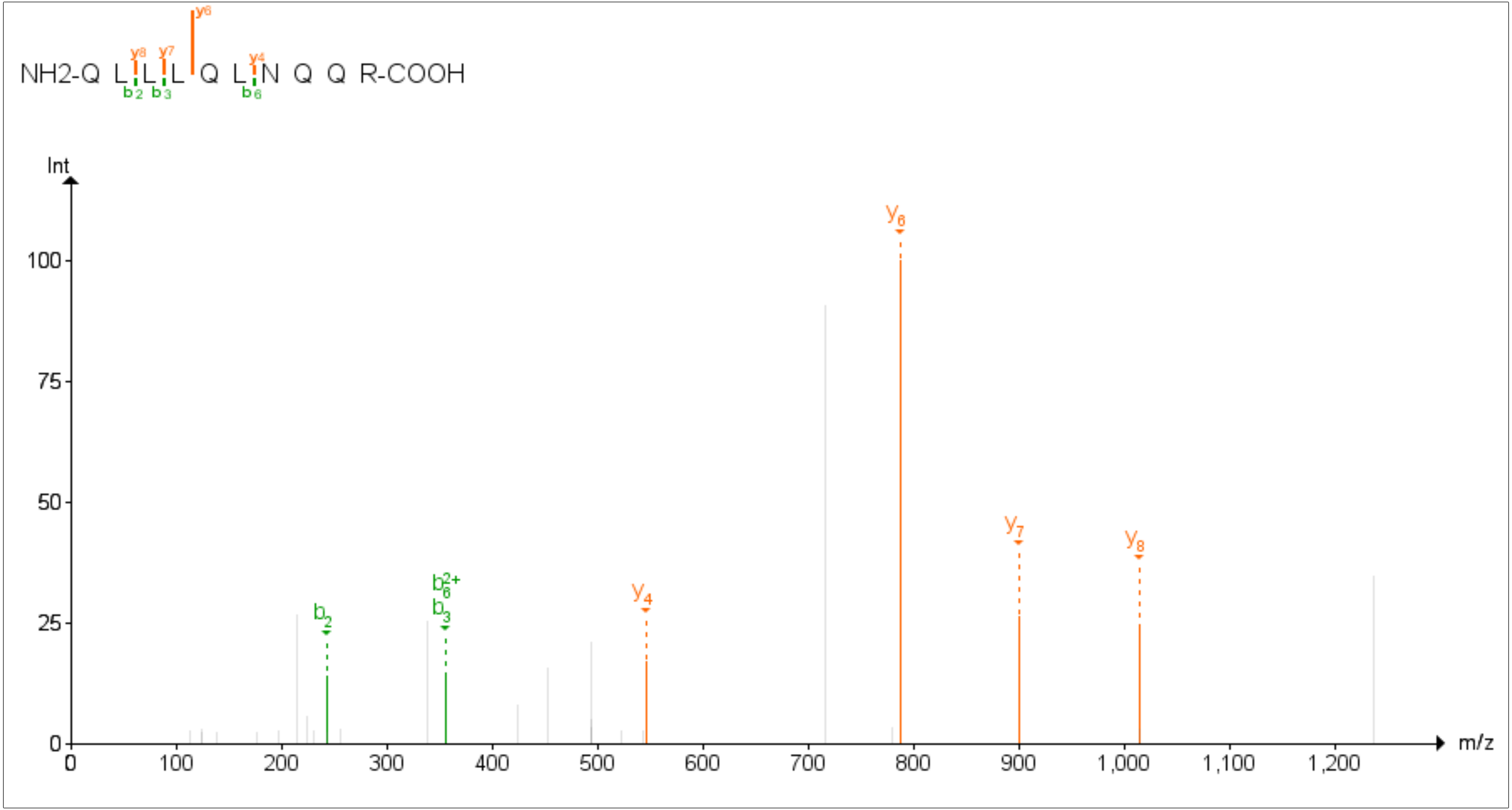

NH2-Q L L L Q L N Q Q R-COOH
y8 y7 y6 y4
b2 b3 b6
Int
100
75
50
25
0
y6
y7
y8
y4
b2
b6 2+
b3
0
100
200
300
400
500
600
700
800
900
1,000
1,100
1,200
m/z

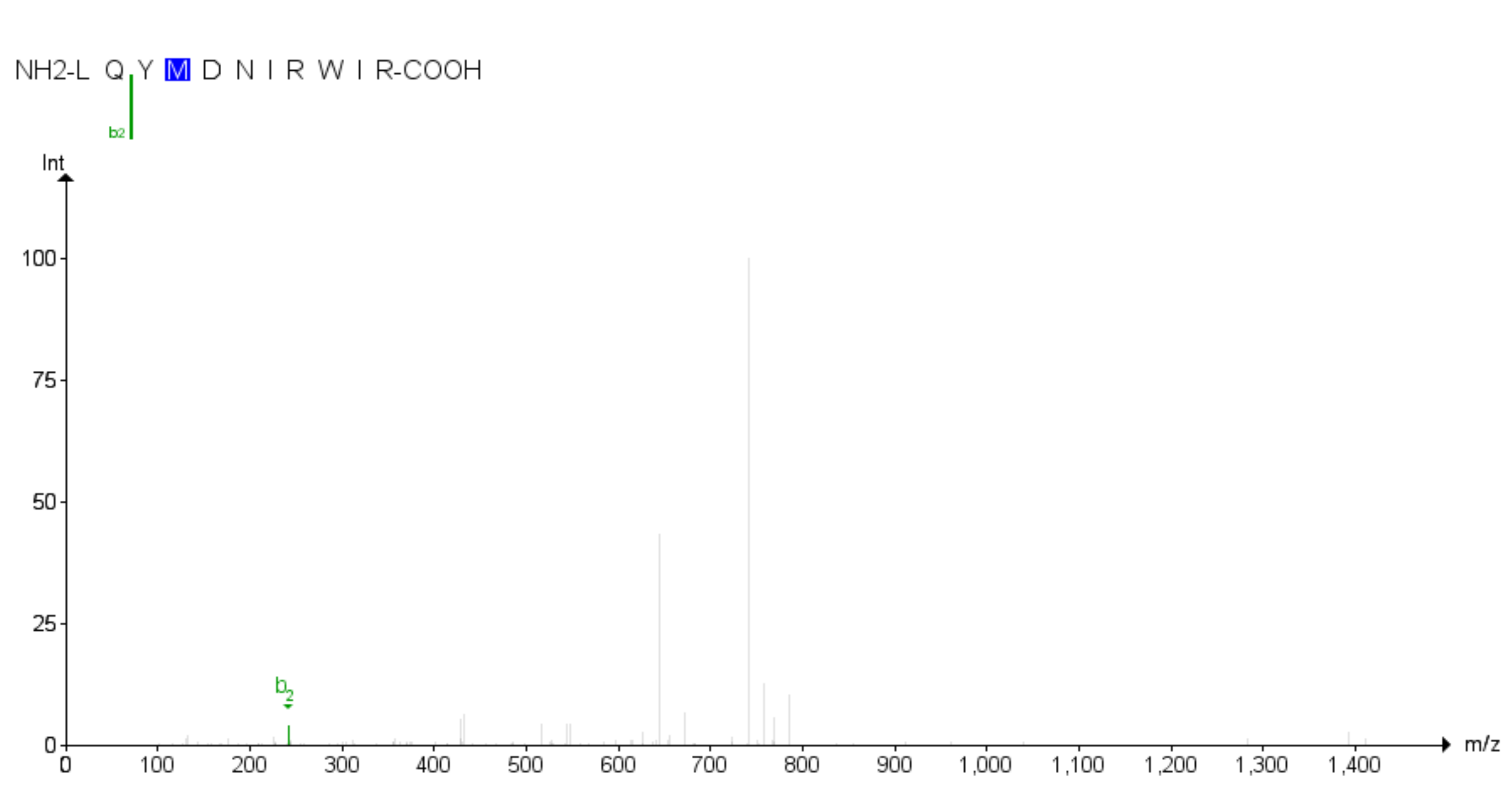

NH2-L Q Y M D N I R W I R-COOH
b2
Int
100
75
50
25
0
b2
0
100
200
300
400
500
600
700
800
900
1,000
1,100
1,200
1,300
1,400
m/z

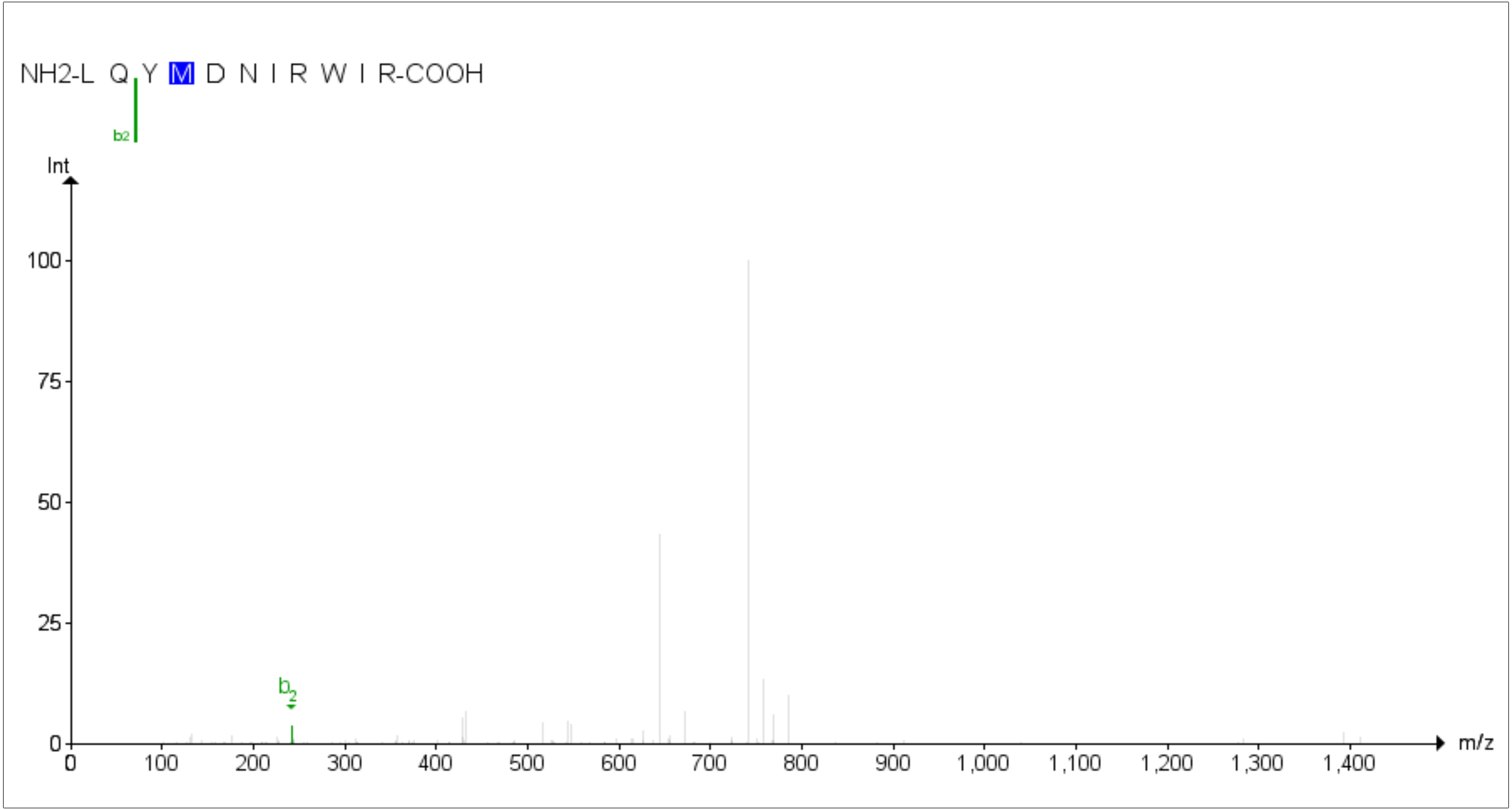

NH2-L Q Y M D N I R W I R-COOH
b2
Int
100
75
50
25
0
b_2
0
100
200
300
400
500
600
700
800
900
1,000
1,100
1,200
1,300
1,400
m/z

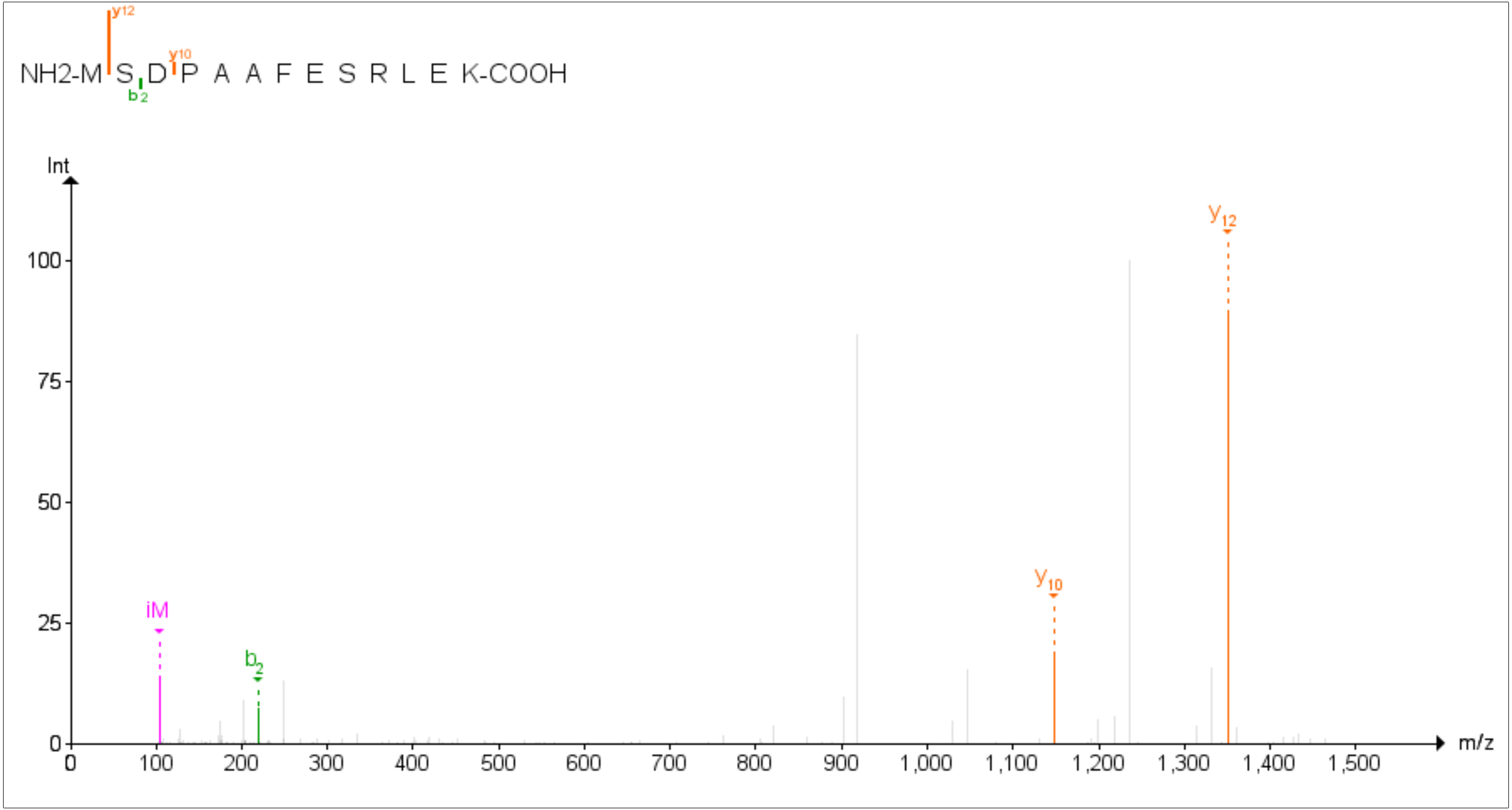

NH2-M S D P A A F E S R L E K-COOH
y12
y10
b2
Int
100
75
50
25
0
y12
y10
iM
b2
0
100
200
300
400
500
600
700
800
900
1,000
1,100
1,200
1,300
1,400
1,500
m/z

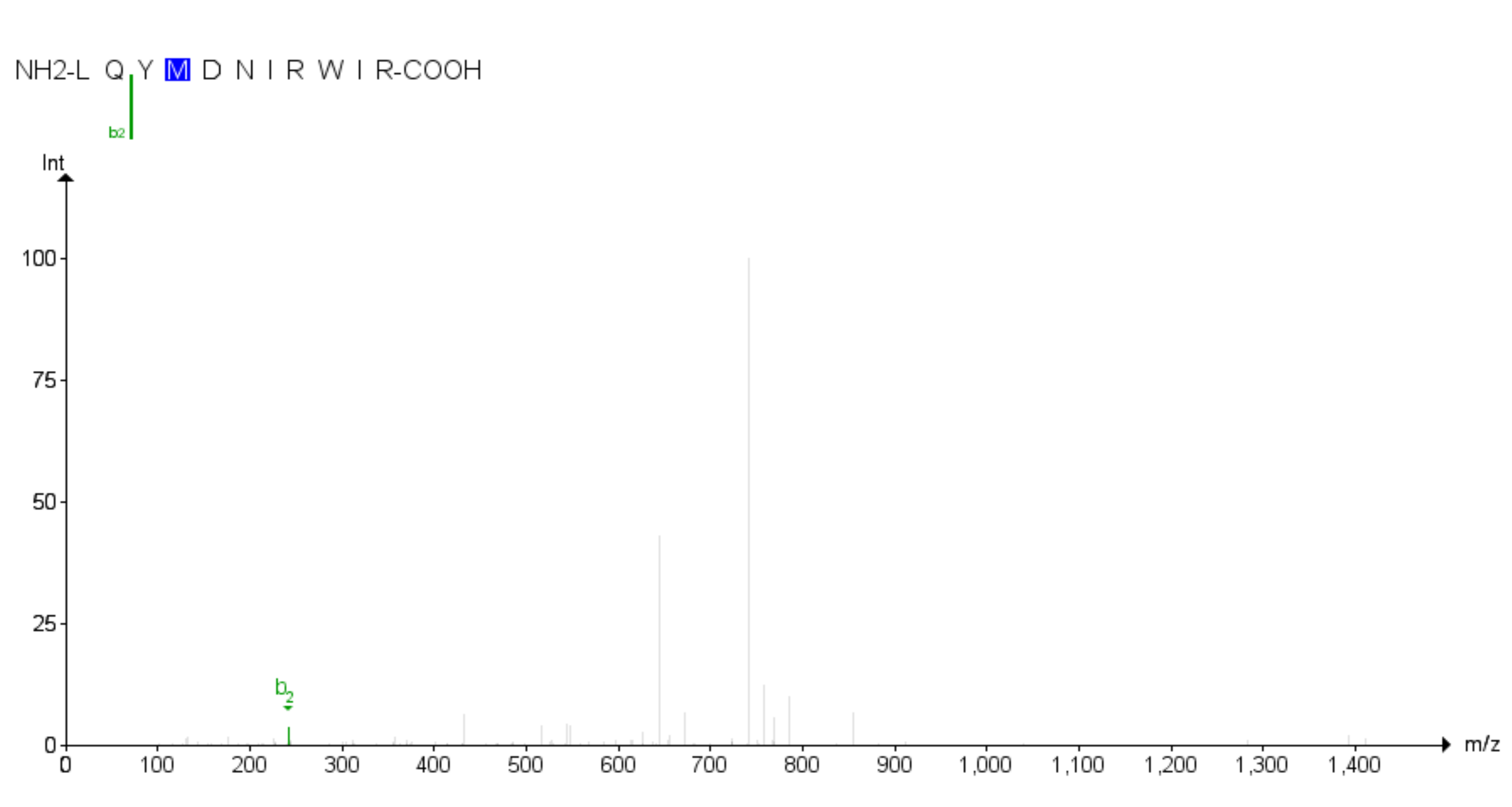

NH2-L Q Y M D N I R W I R-COOH
b2
Int
100
75
50
25
0
b2
m/z
0
100
200
300
400
500
600
700
800
900
1,000
1,100
1,200
1,300
1,400

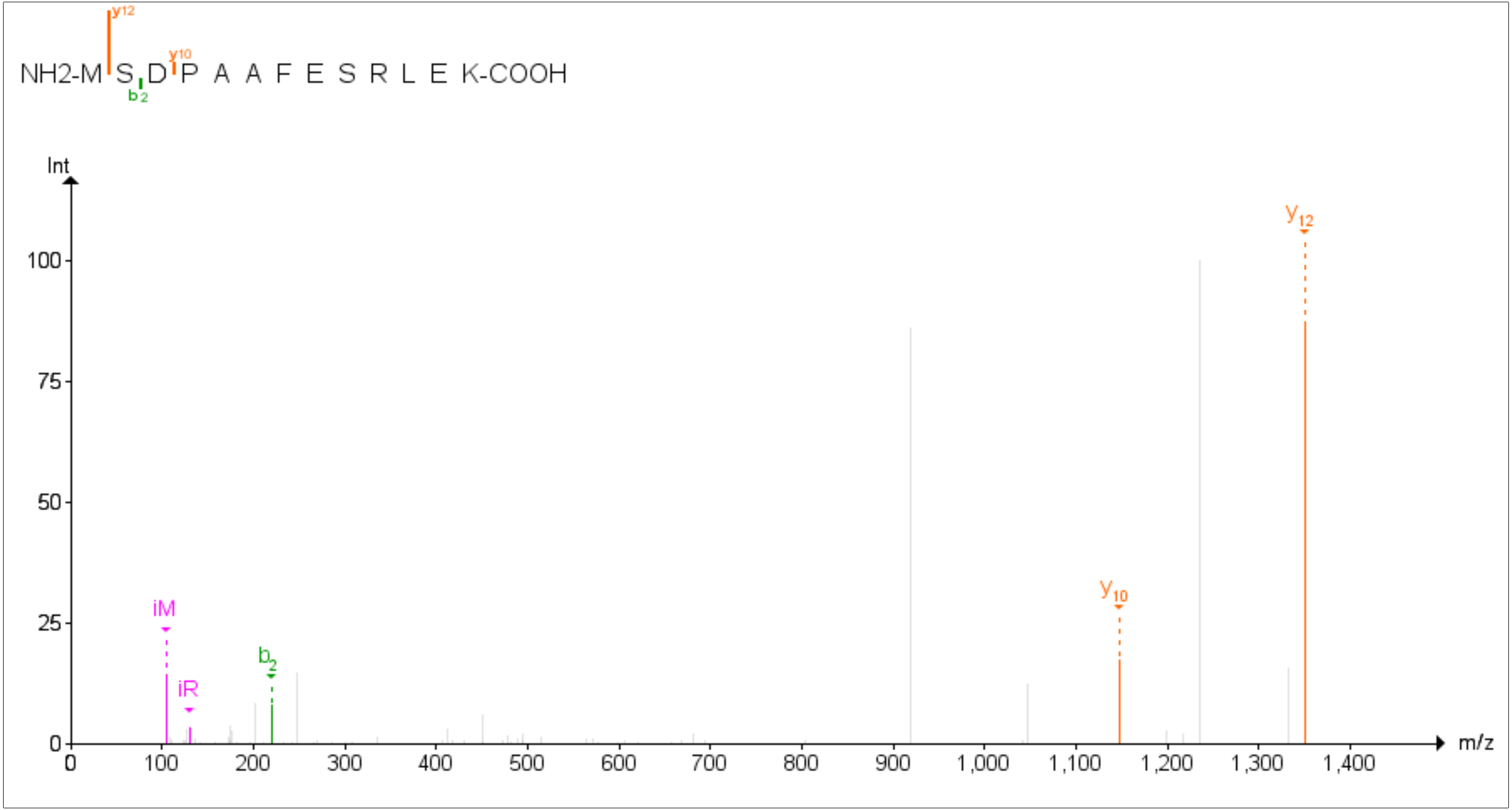
NH2-M S D P A A F E S R L E K-COOH
y12
y10
b2
Int
100
75
50
25
0
iM
iR
b2
y10
y12
m/z
0
100
200
300
400
500
600
700
800
900
1,000
1,100
1,200
1,300
1,400

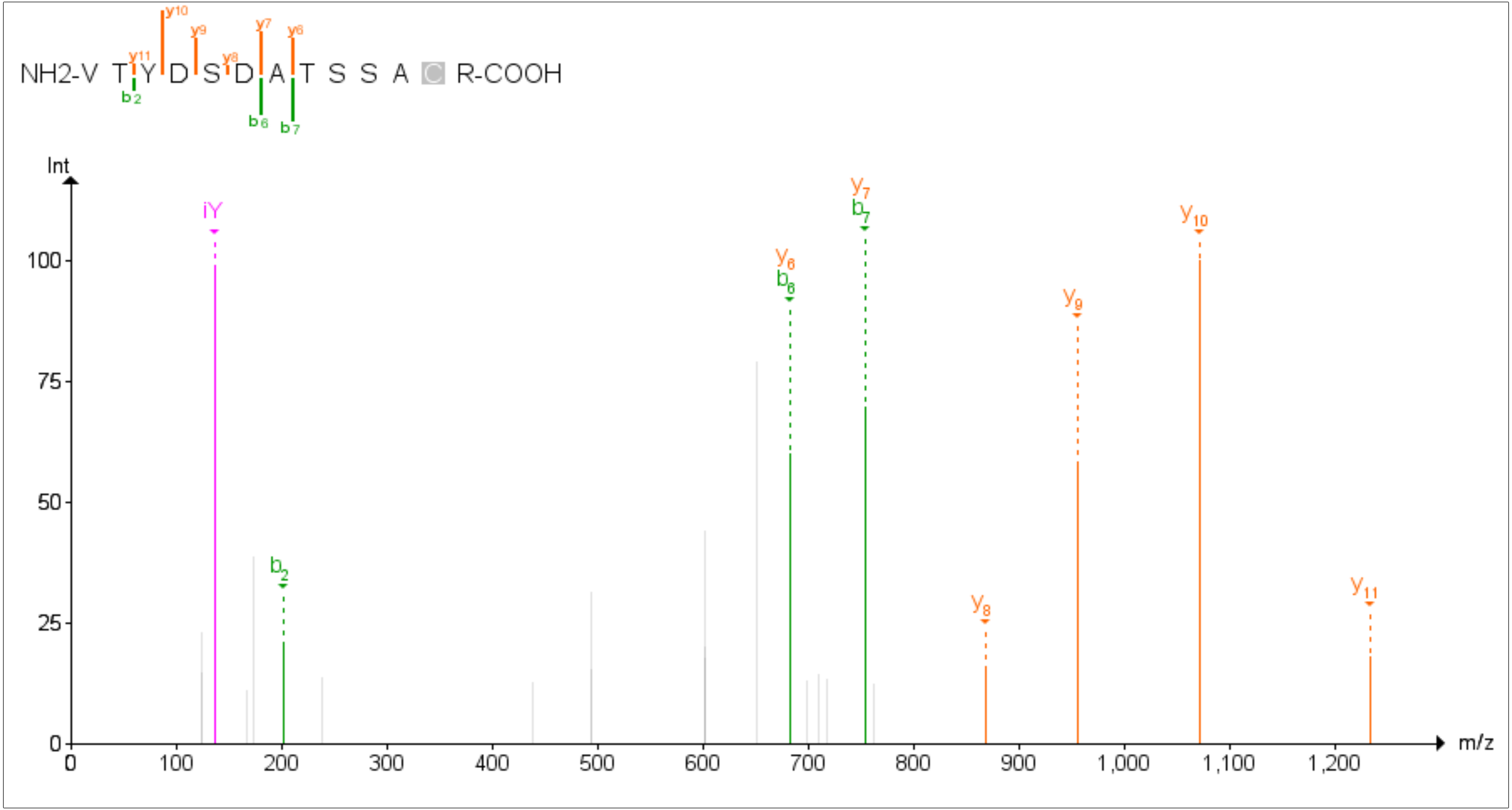

NH2-V T Y D S D A T S S A C R-COOH
y10
y9
y11
y8
y7
y6
b2
b6
b7
Int
iY
y7
b7
y10
y6
b6
y9
b2
y8
y11
100
75
50
25
0
0
100
200
300
400
500
600
700
800
900
1,000
1,100
1,200
m/z

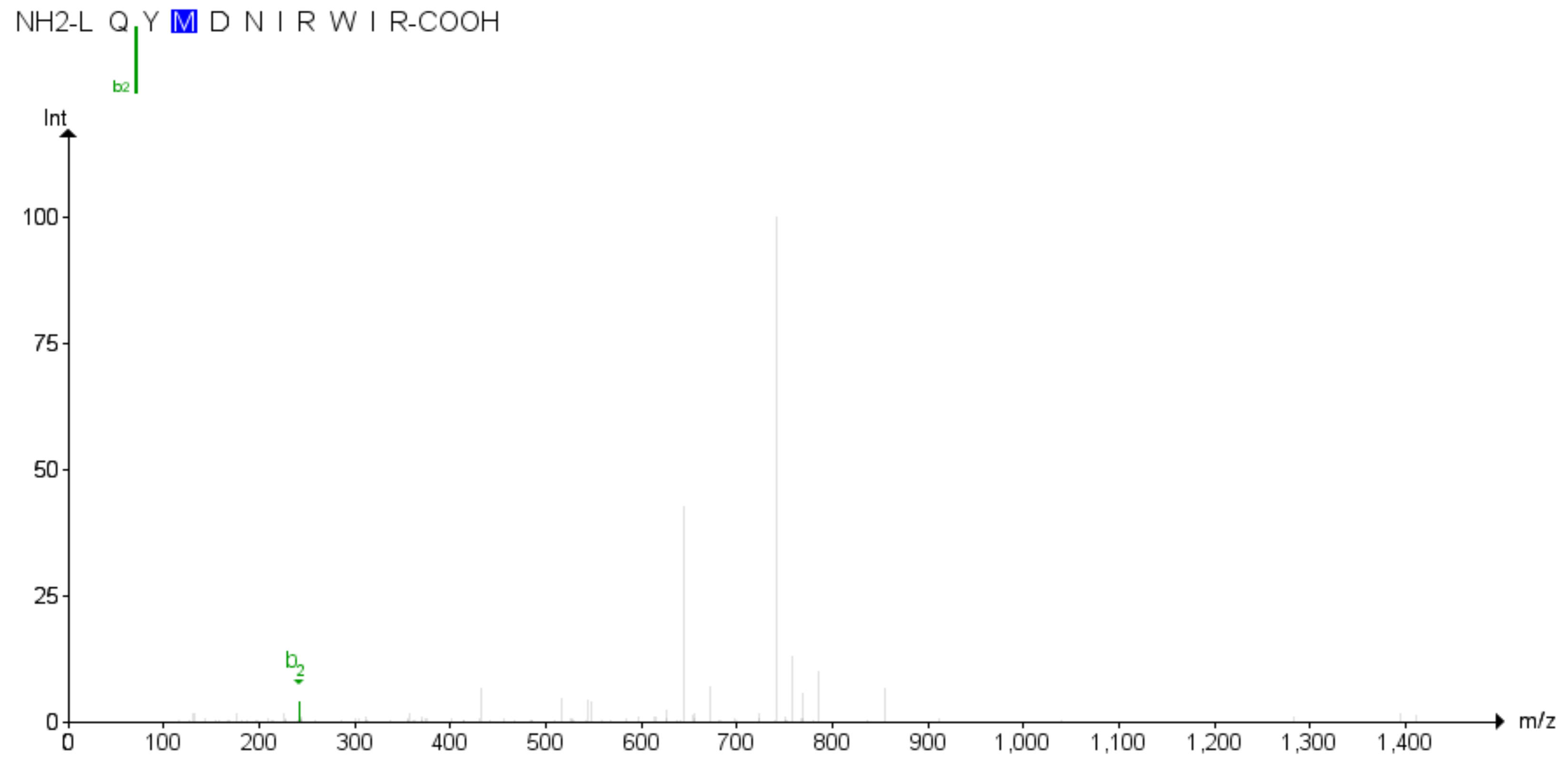

NH2-L Q Y M D N I R W I R-COOH
b2
Int
100
75
50
25
0
b2
0
100
200
300
400
500
600
700
800
900
1,000
1,100
1,200
1,300
1,400
m/z

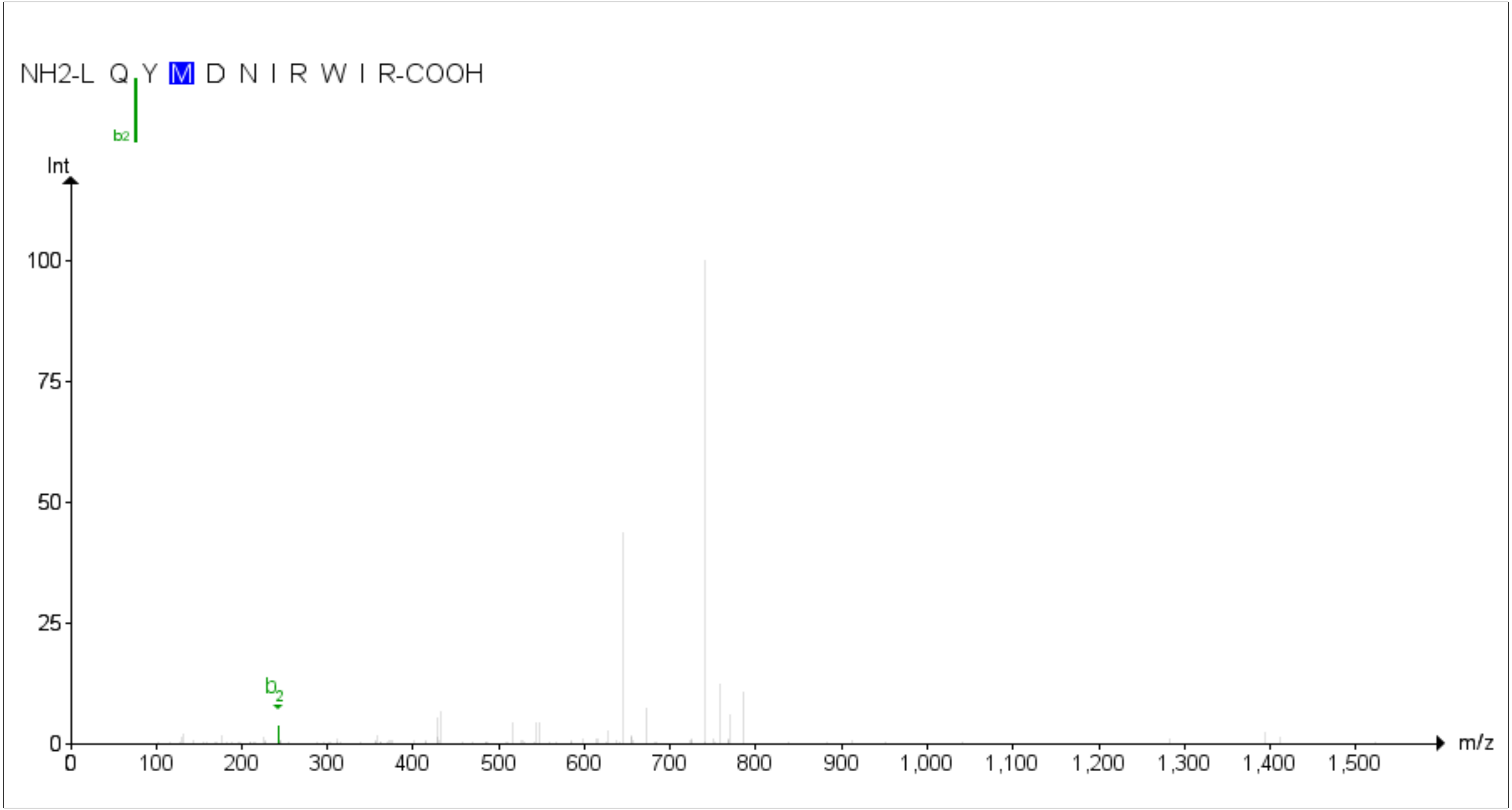
NH2-L Q Y M D N I R W I R-COOH
b2
Int
100
75
50
25
0
b2
0
100
200
300
400
500
600
700
800
900
1,000
1,100
1,200
1,300
1,400
1,500
m/z

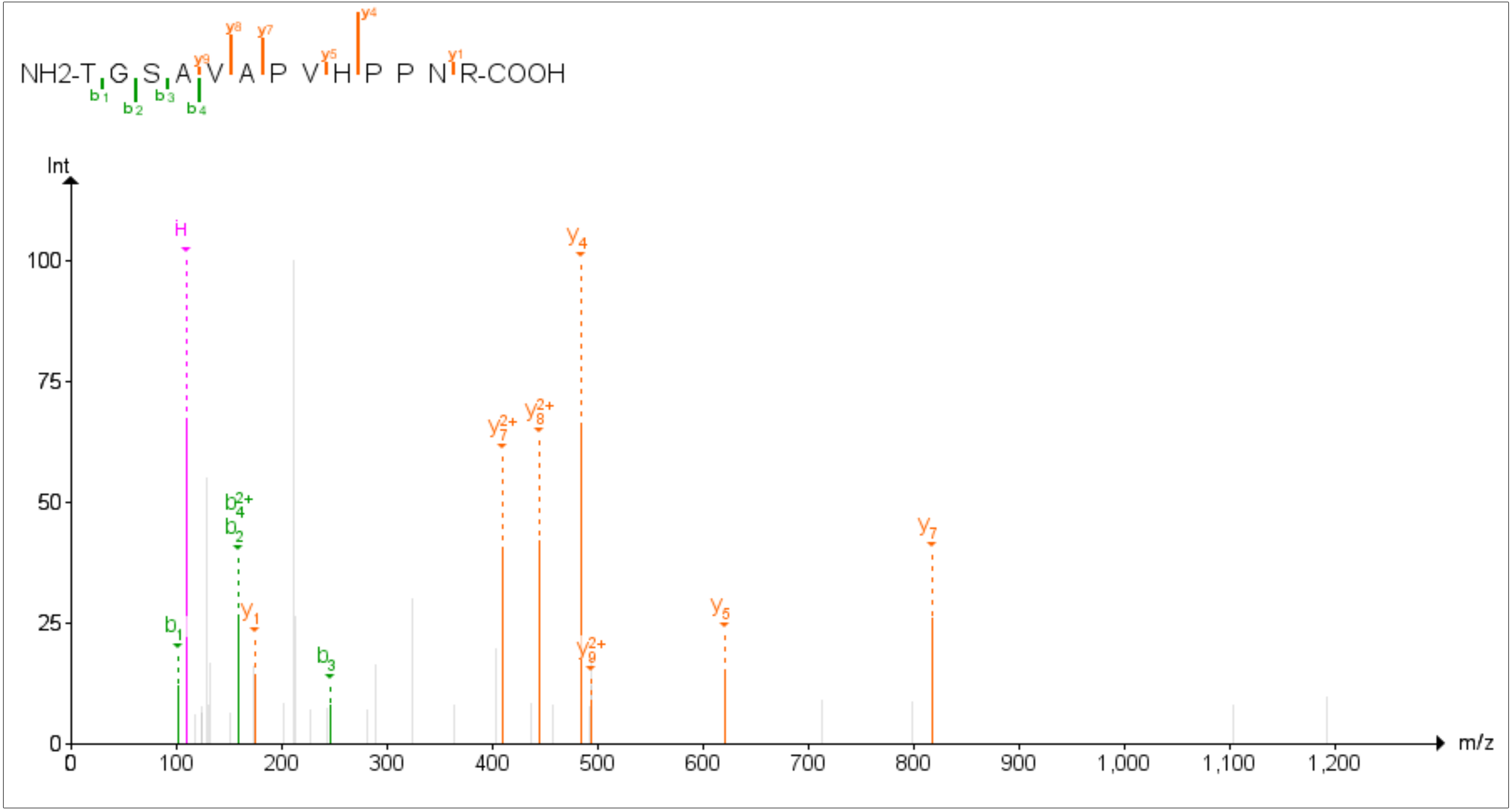

NH2-T G S A V A P V H P P N R-COOH
b1
b2
b3
b4
y9
y8
y7
y5
y4
y1
Int
100
75
50
25
0
H
b1
b2
b4 2+
y1
b3
y7 2+
y8 2+
y4
y9 2+
y5
y7
m/z
0
100
200
300
400
500
600
700
800
900
1,000
1,100
1,200

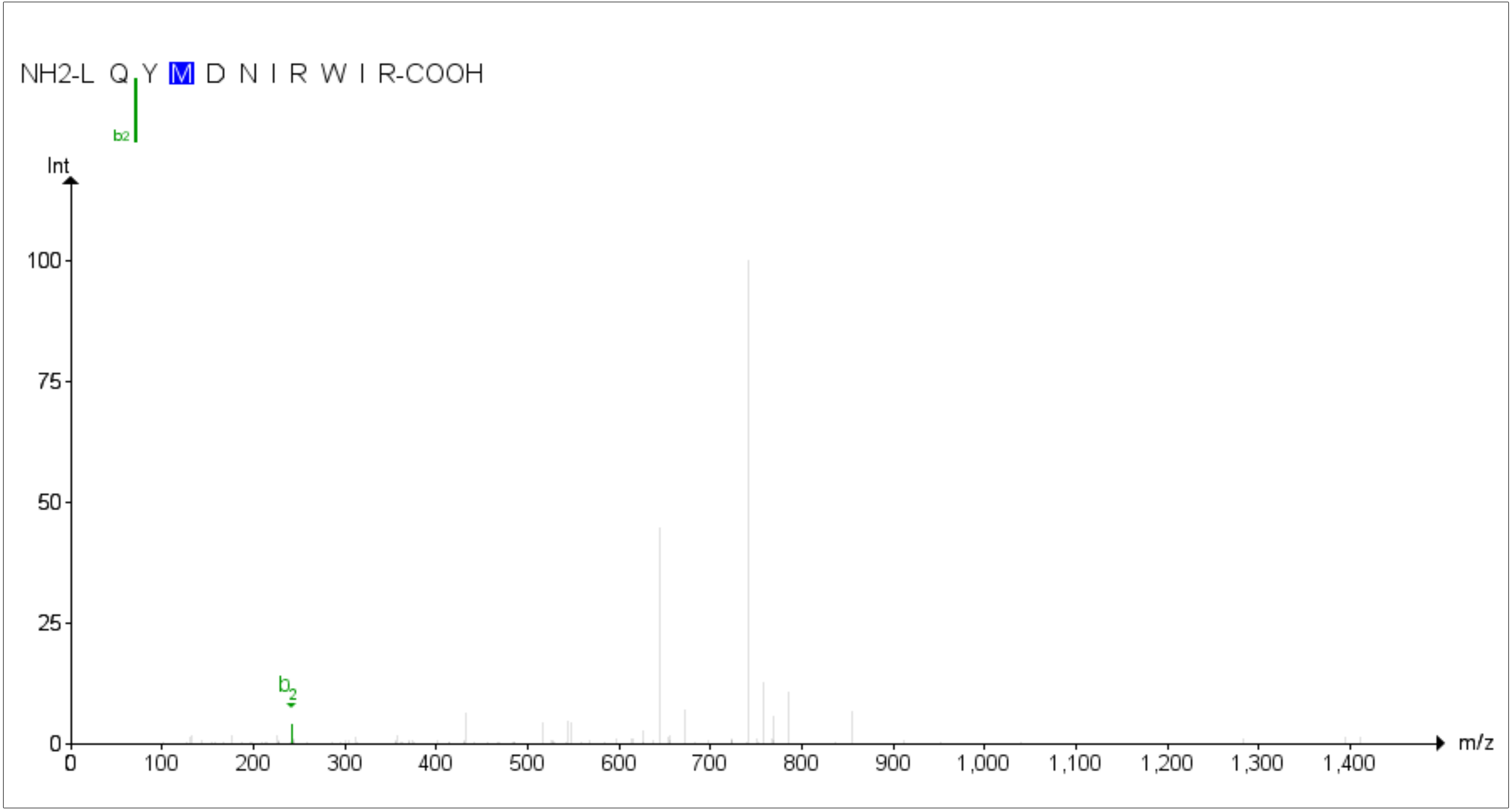
NH2-L Q Y M D N I R W I R-COOH
b2
Int
100
75
50
25
0
b2
0
100
200
300
400
500
600
700
800
900
1,000
1,100
1,200
1,300
1,400
m/z

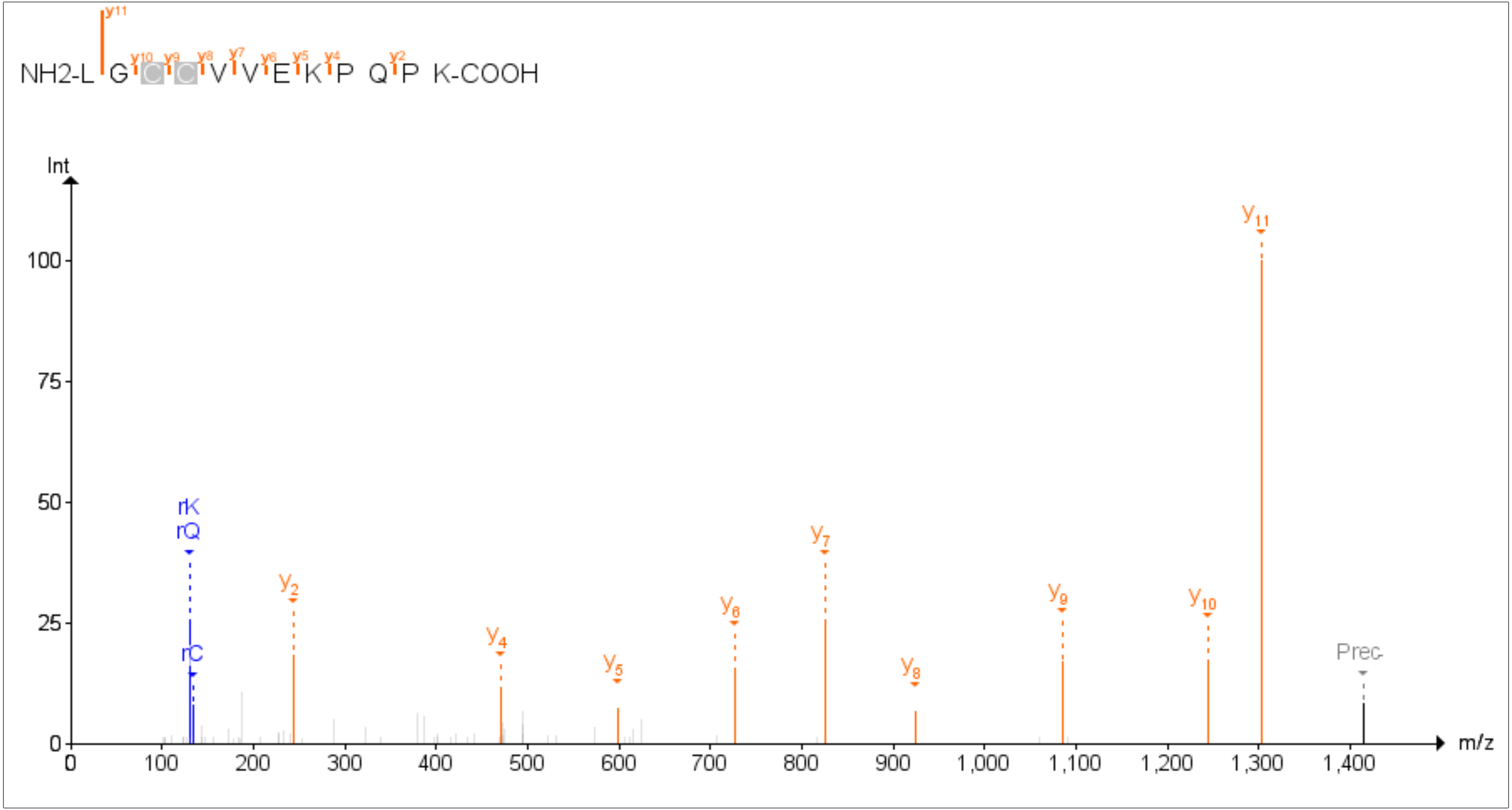
NH2-L G C C V V E K P Q P K-COOH
y11
y10
y9
y8
y7
y6
y5
y4
y2
Int
100
75
50
25
0
rK
rQ
rC
y_2
y_4
y_5
y_6
y_7
y_8
y_9
y_{10}
y_{11}
Prec
0
100
200
300
400
500
600
700
800
900
1,000
1,100
1,200
1,300
1,400
m/z

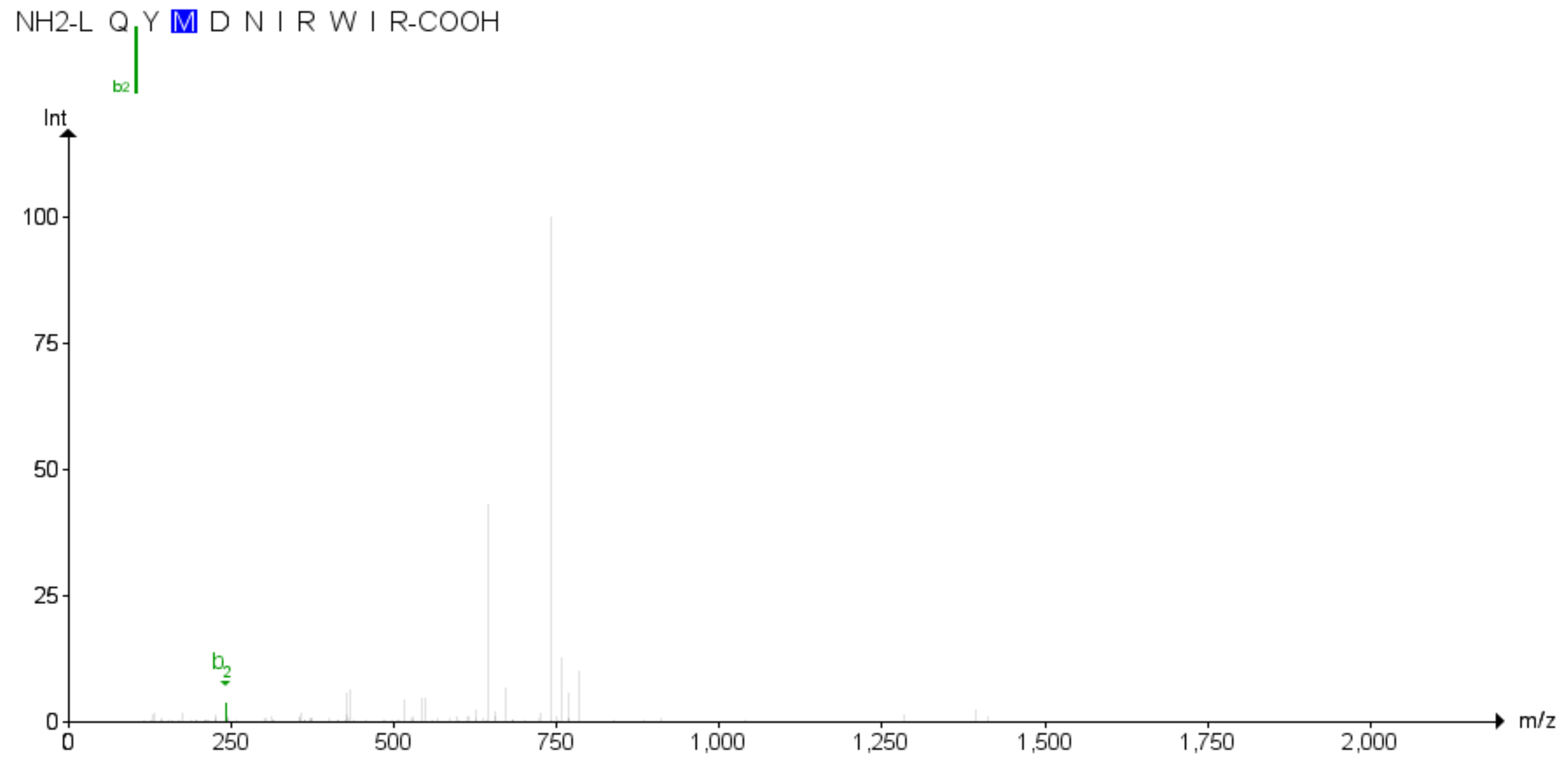
NH2-L Q Y M D N I R W I R-COOH
b2
Int
100
75
50
25
0
b2
0
250
500
750
1,000
1,250
1,500
1,750
2,000
m/z

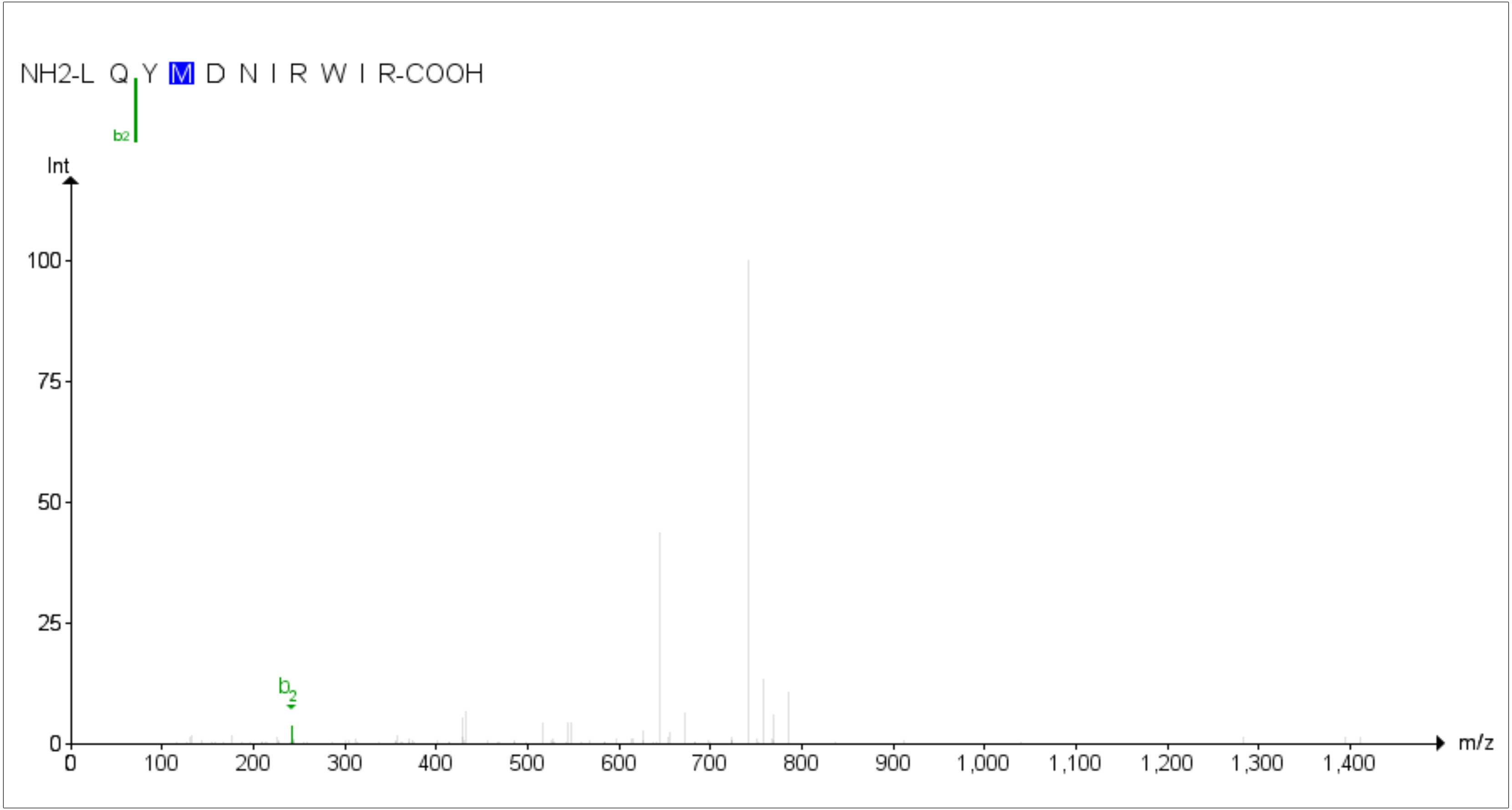

NH2-L Q Y M D N I R W I R-COOH
b2
Int
100
75
50
25
0
b2
0
100
200
300
400
500
600
700
800
900
1,000
1,100
1,200
1,300
1,400
m/z

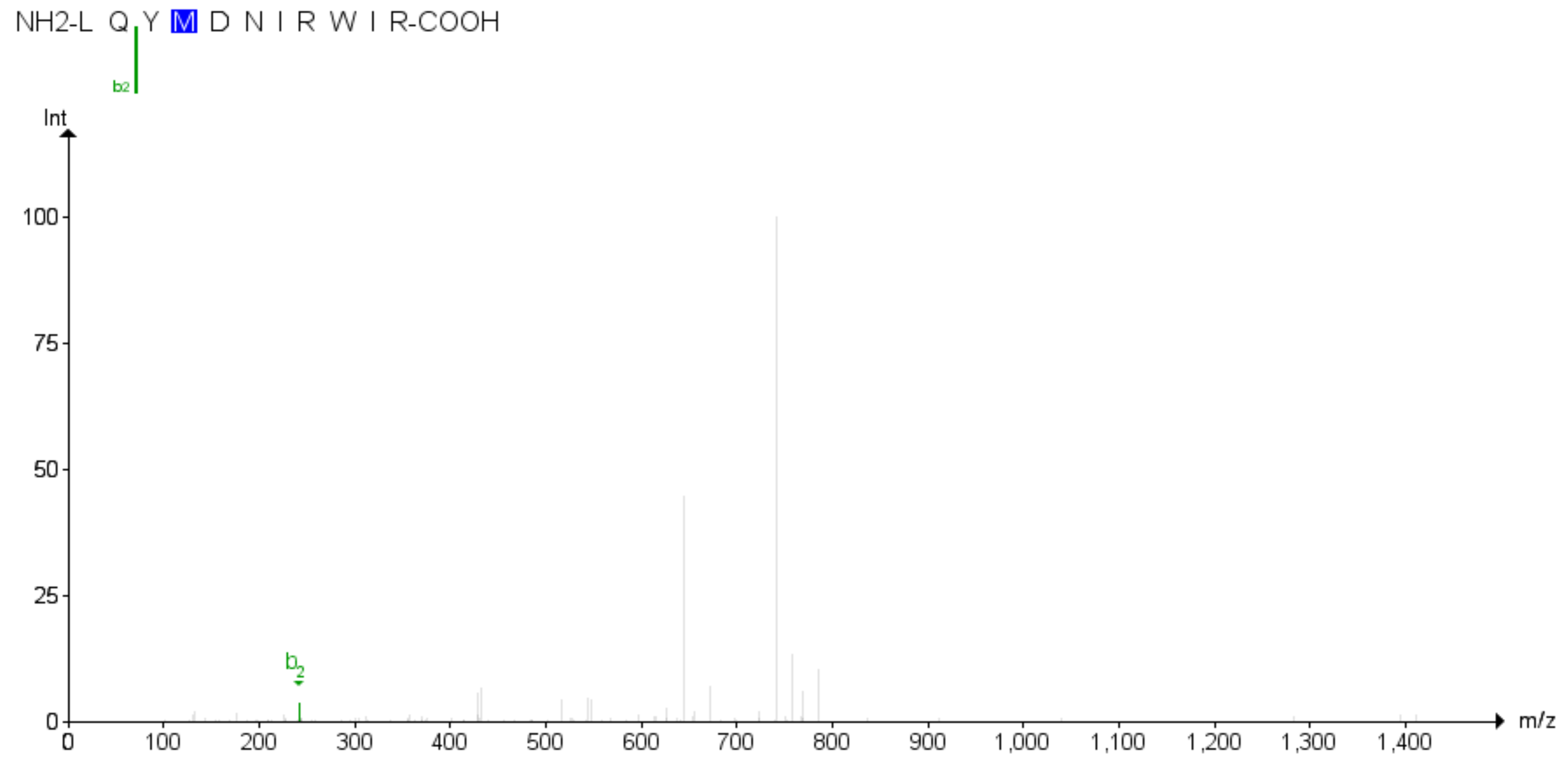

NH2-L Q Y M D N I R W I R-COOH
b2
Int
100
75
50
25
0
b2
0
100
200
300
400
500
600
700
800
900
1,000
1,100
1,200
1,300
1,400
m/z

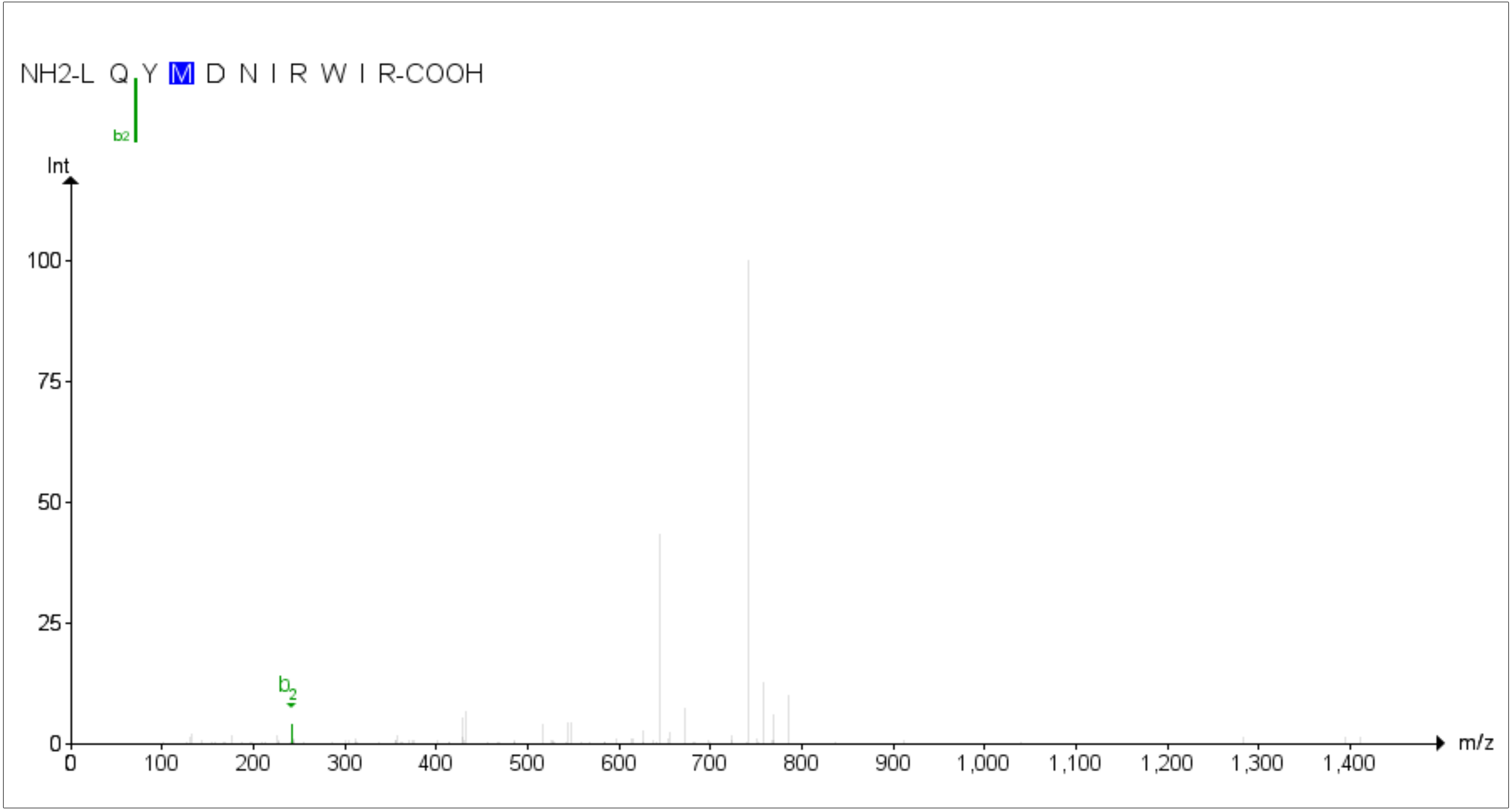
NH2-L Q Y M D N I R W I R-COOH
b2
Int
100
75
50
25
0
b2
0
100
200
300
400
500
600
700
800
900
1,000
1,100
1,200
1,300
1,400
m/z

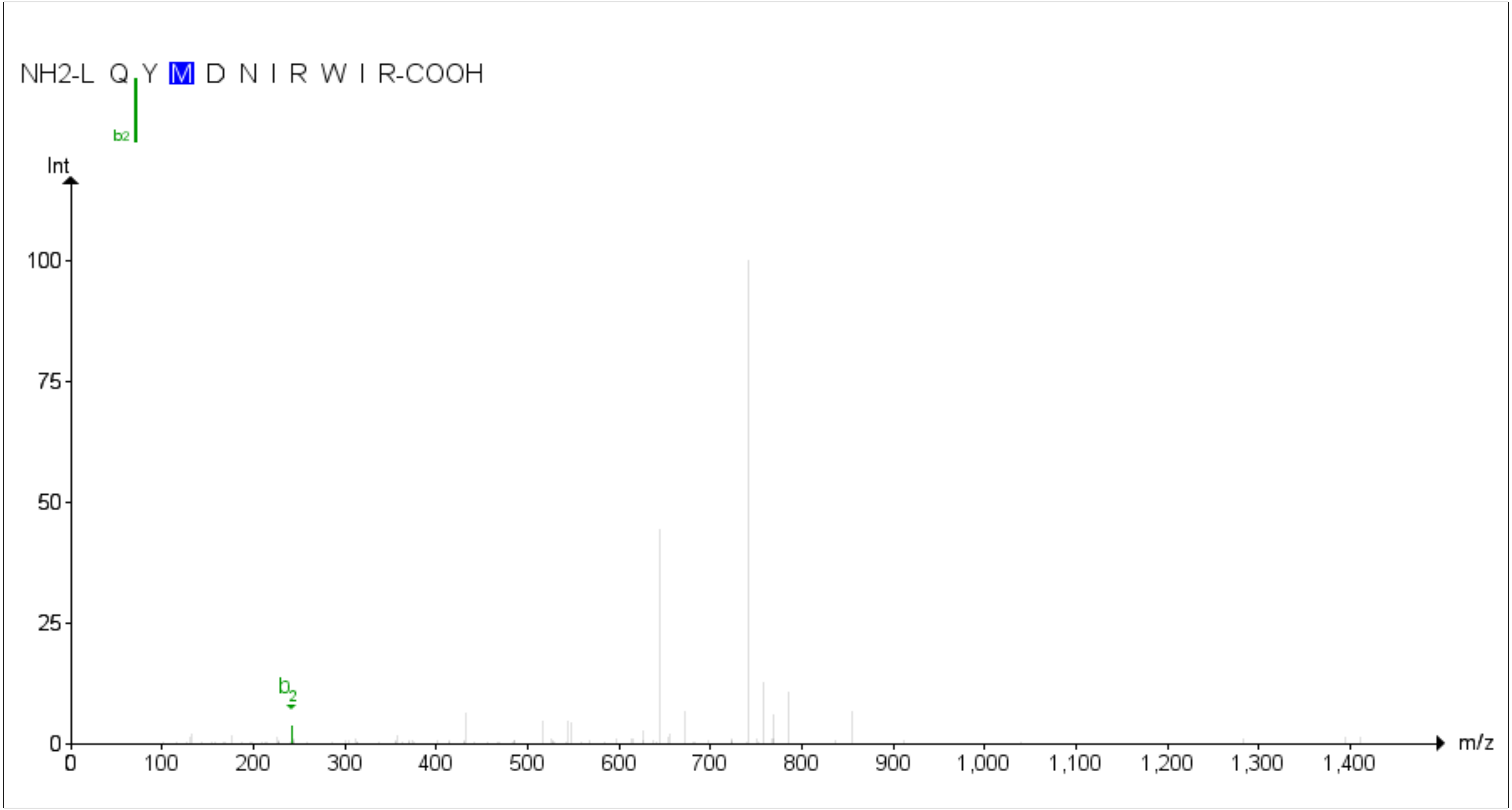

NH2-L Q Y M D N I R W I R-COOH
b2
Int
100
75
50
25
0
b2
m/z
0
100
200
300
400
500
600
700
800
900
1,000
1,100
1,200
1,300
1,400

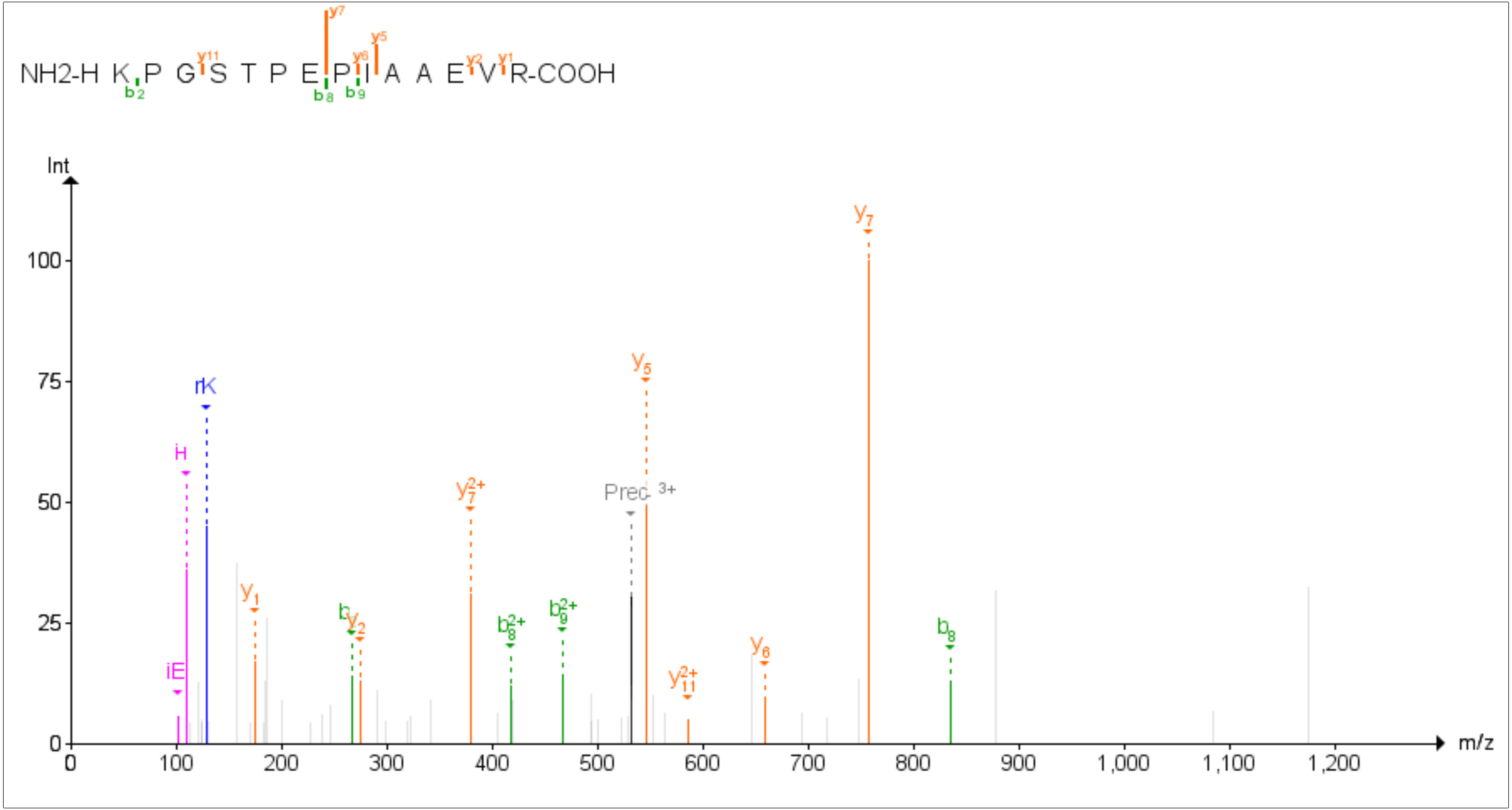

NH2-H K P G S T P E P I A A E V R-COOH
Int
m/z
0
25
50
75
100
100
200
300
400
500
600
700
800
900
1,000
1,100
1,200
y7
y5
y6
y11
y2
y1
b2
b8
b9
rK
iH
iE
Prec 3+

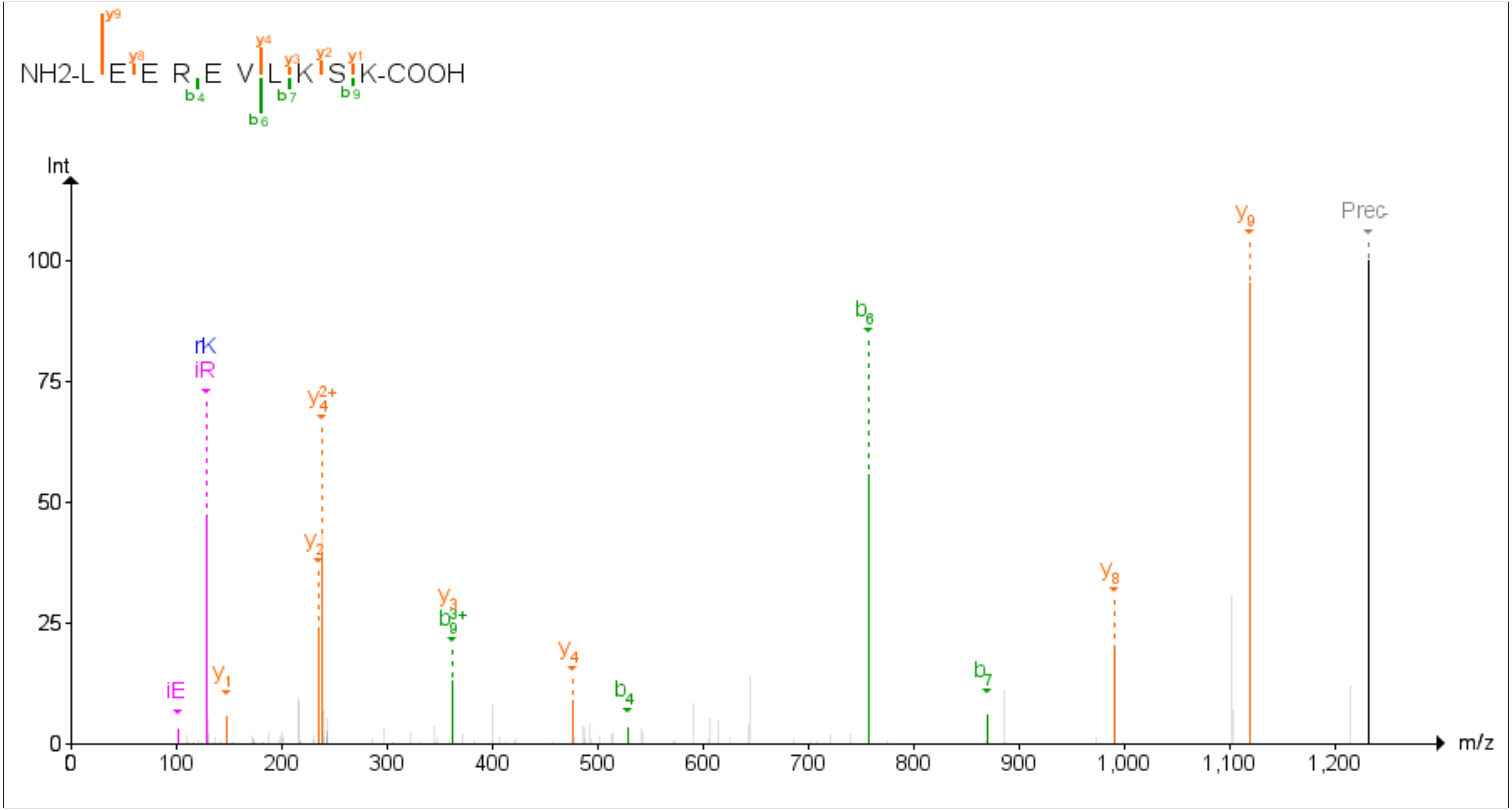

NH2-L E E R E V L K S K-COOH
y9 y8 y4 y3 y2 y1
b4 b6 b7 b9
Int
0
25
50
75
100
m/z
0 100 200 300 400 500 600 700 800 900 1,000 1,100 1,200
Prec
y9
b6
rK
iR
y4 2+
y2
y3
b9 3+
y4
b4
b7
y8
iE
y1

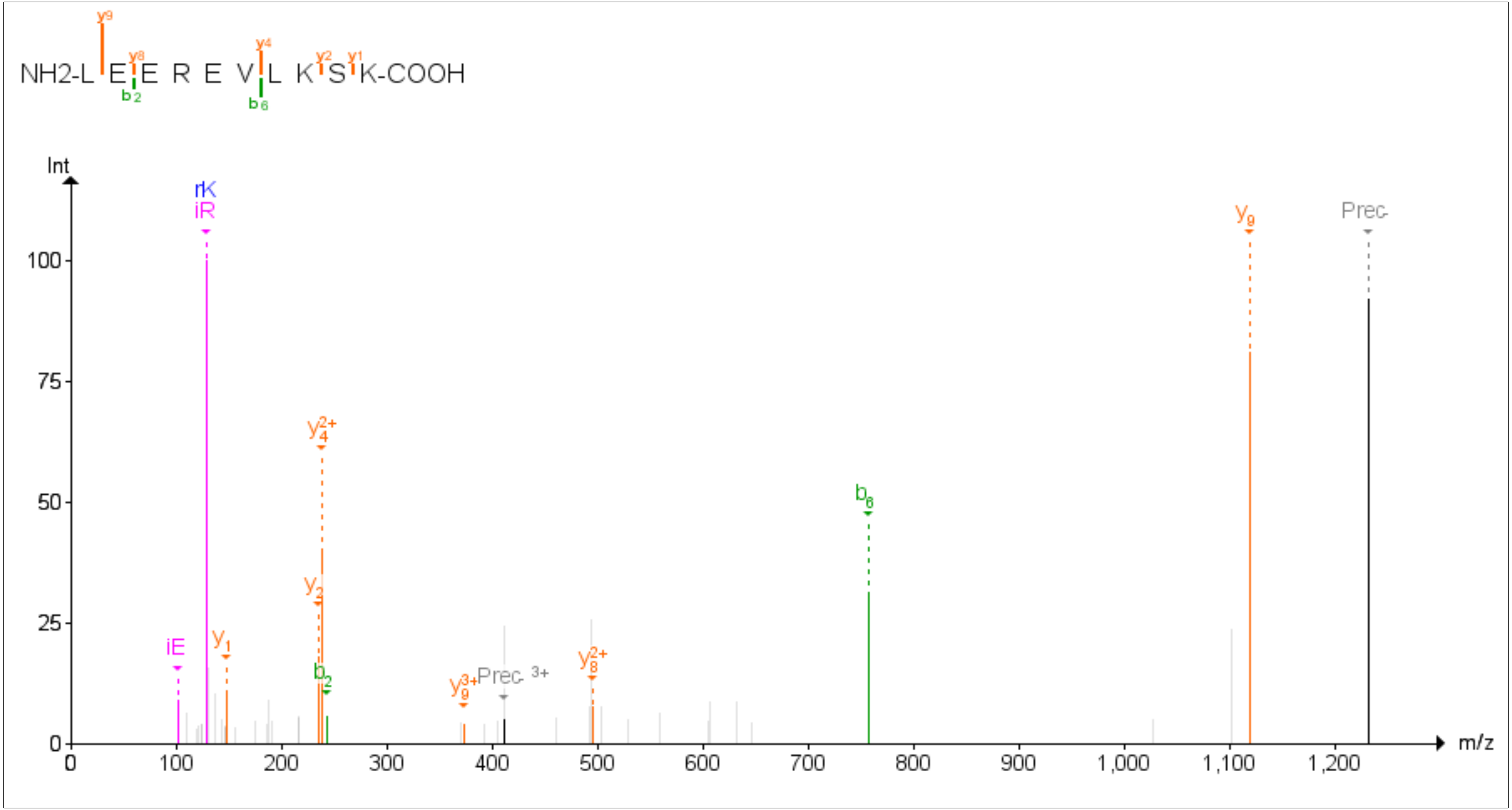

NH2-L E E R E V L K S K-COOH
y9
y8
y4
y2
y1
b2
b6
Int
rK
iR
y9
Prec
100
75
50
25
0
y4 2+
b6
y2
iE
y1
b2
y9 3+
Prec 3+
y8 2+
0
100
200
300
400
500
600
700
800
900
1,000
1,100
1,200
m/z

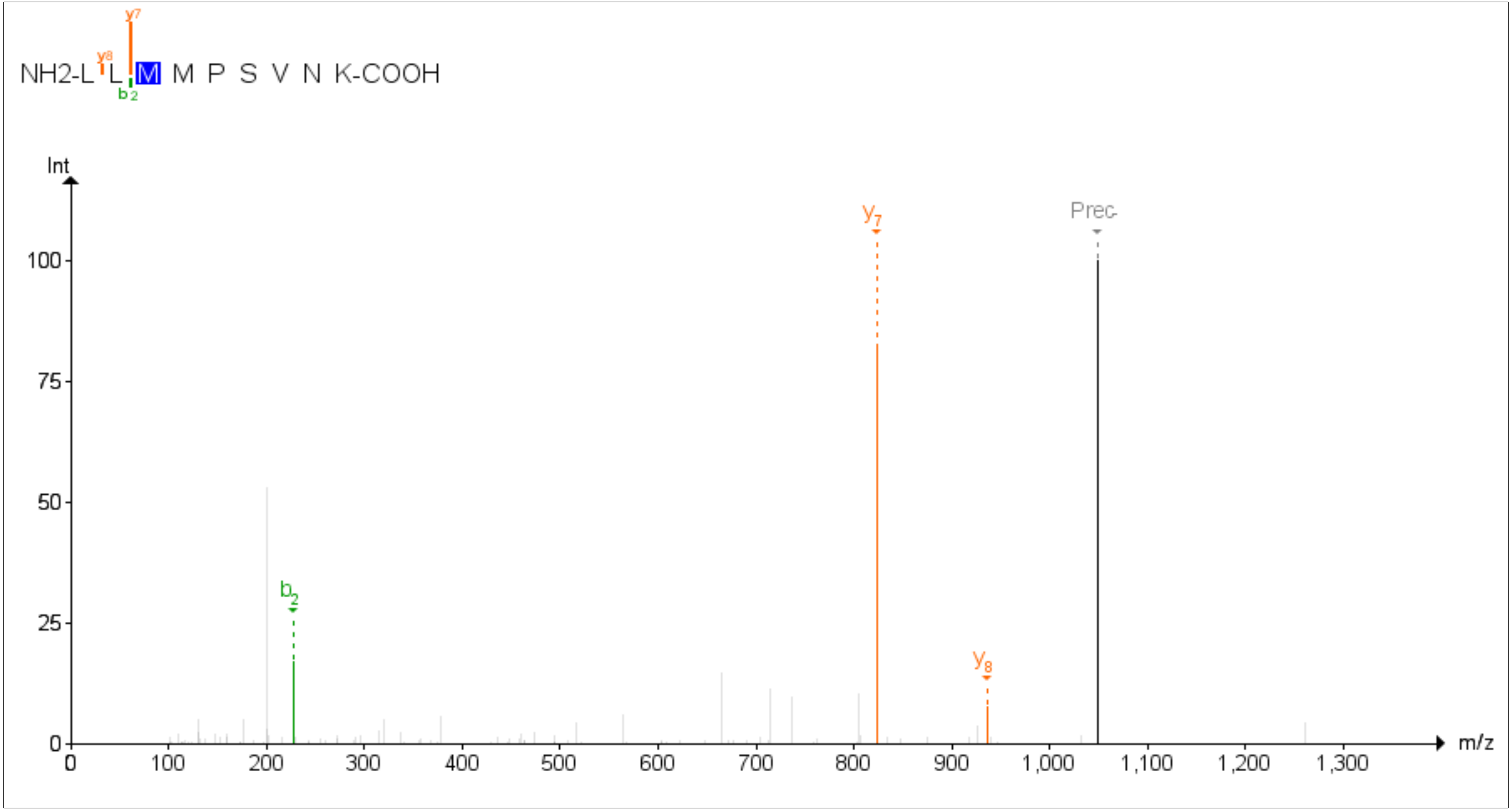

NH2-L L M M P S V N K-COOH
y7
y8
b2
Int
100
75
50
25
0
y7
Prec
b2
y8
0
100
200
300
400
500
600
700
800
900
1,000
1,100
1,200
1,300
m/z

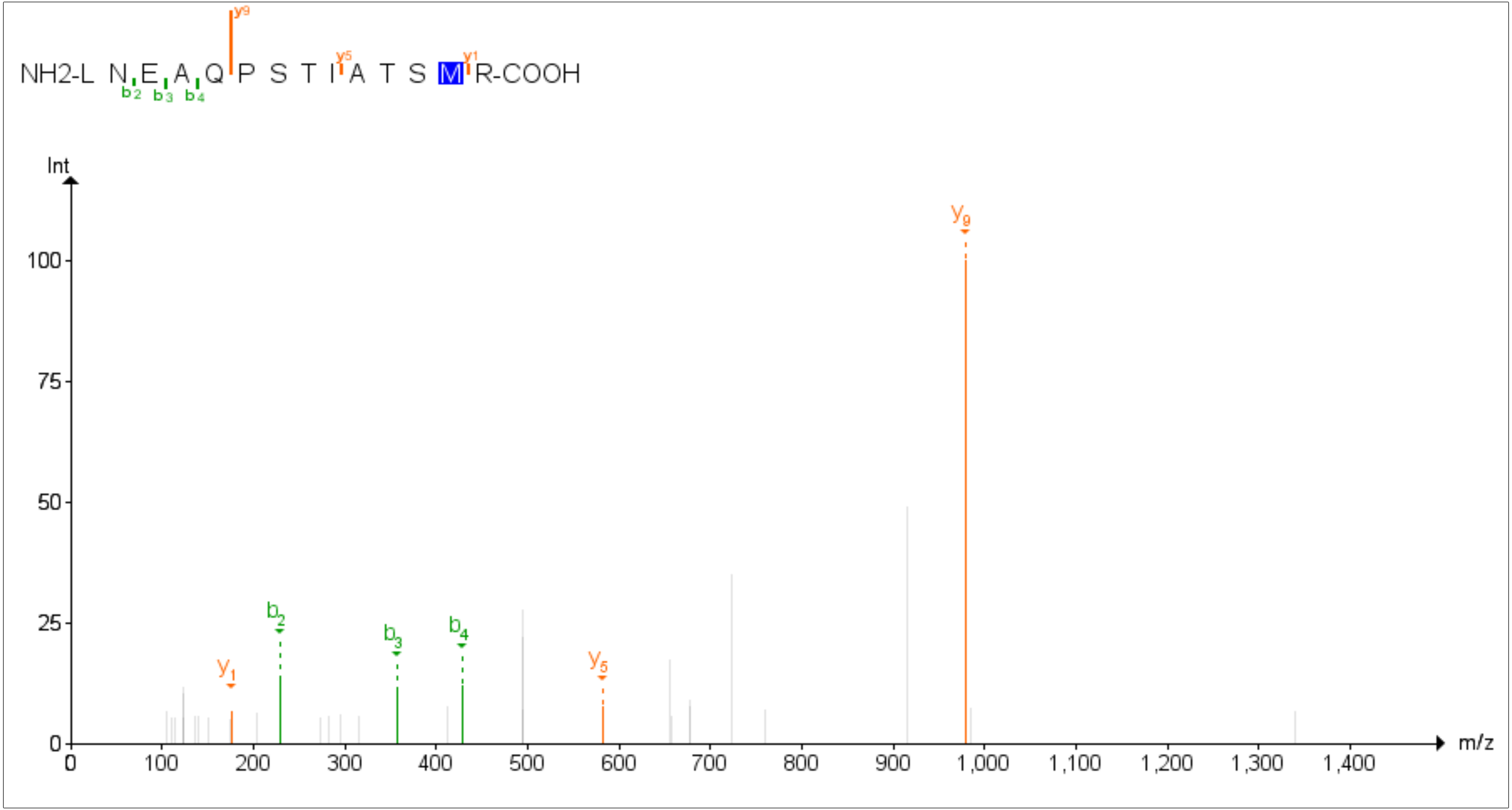

NH2-L N E A Q P S T I A T S M R-COOH
y9
y5
y1
b2
b3
b4
Int
100
75
50
25
0
m/z
0
100
200
300
400
500
600
700
800
900
1,000
1,100
1,200
1,300
1,400

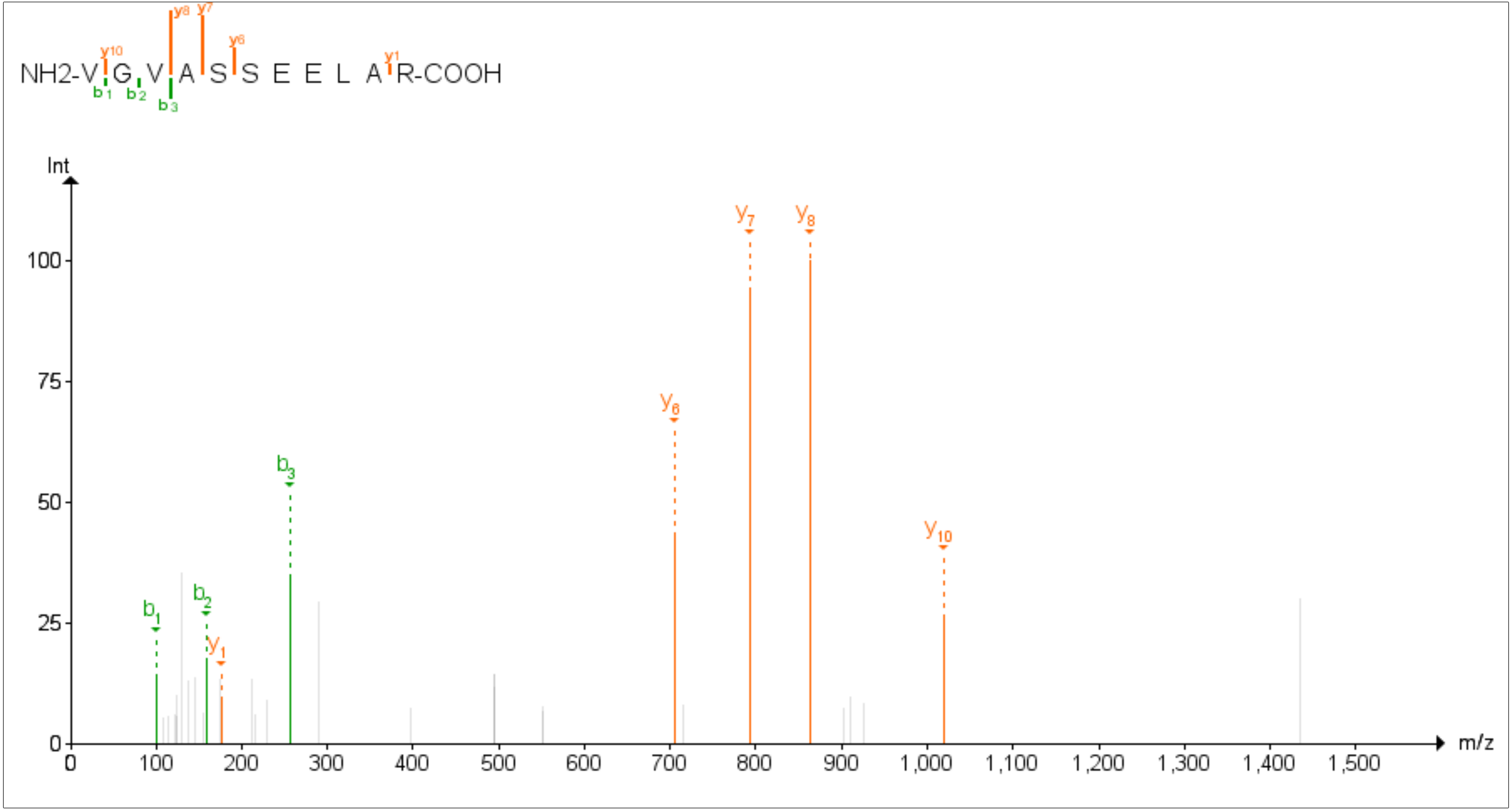

NH2-V G V A S S E E L A R-COOH
y10
y8
y7
y6
y1
b1
b2
b3
Int
100
75
50
25
0
m/z
0
100
200
300
400
500
600
700
800
900
1,000
1,100
1,200
1,300
1,400
1,500

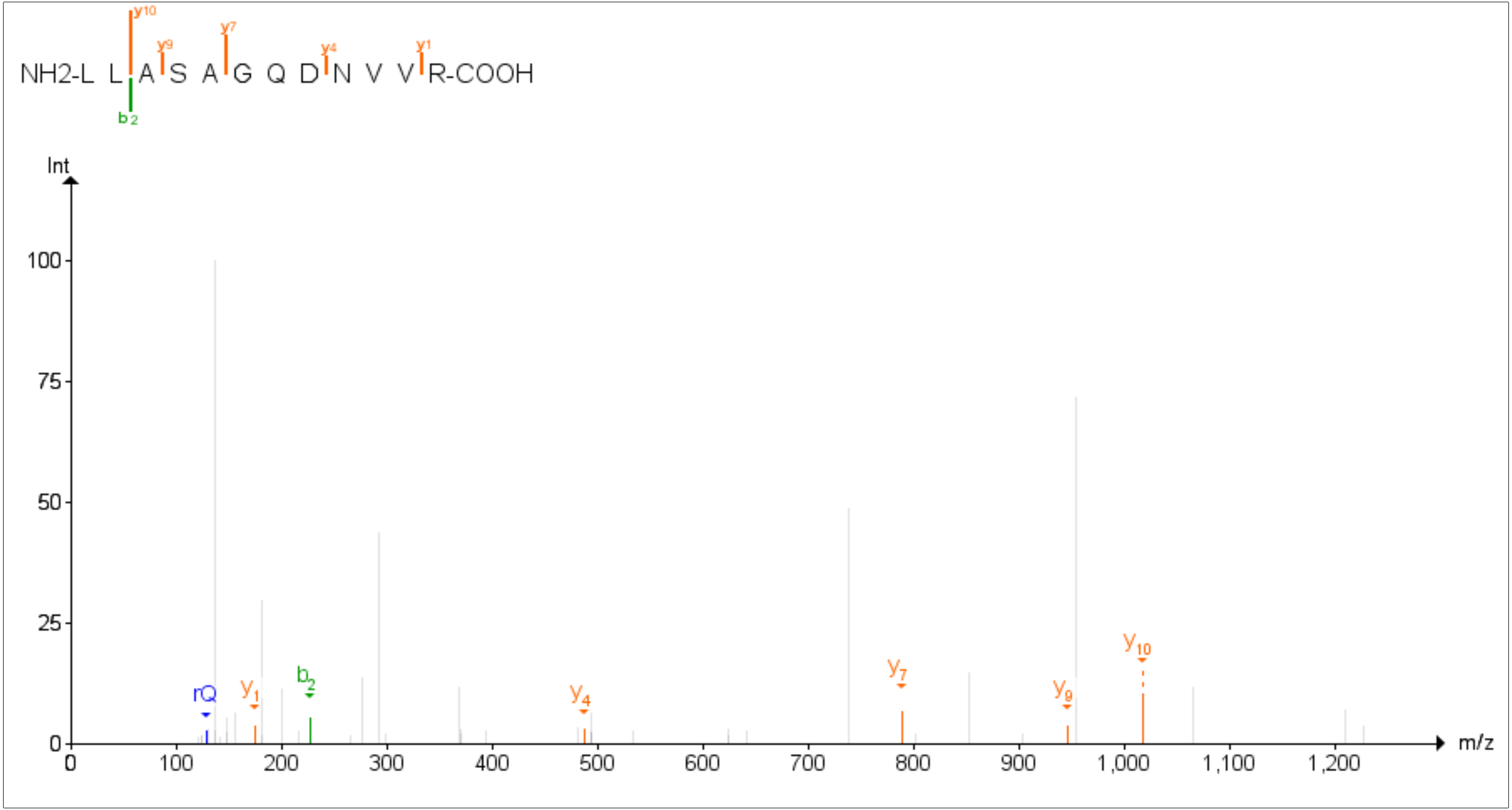

NH2-L L A S A G Q D N V V R-COOH
y10
y9
y7
y4
y1
b2
Int
100
75
50
25
0
rQ
y1
b2
y4
y7
y9
y10
0
100
200
300
400
500
600
700
800
900
1,000
1,100
1,200
m/z

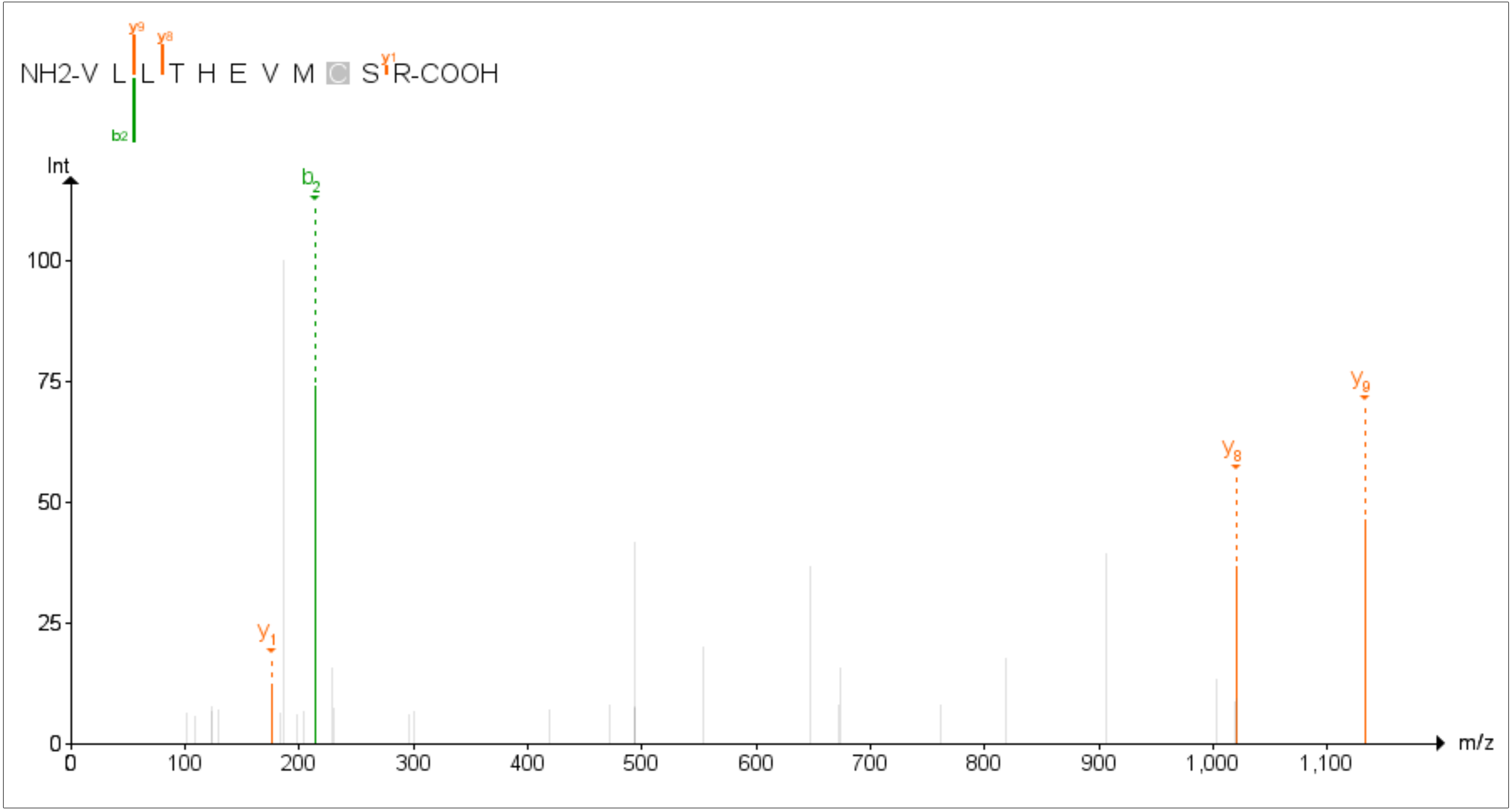

NH2-V L L T H E V M C S R-COOH
y9
y8
y1
b2
Int
m/z
100
75
50
25
0
0
100
200
300
400
500
600
700
800
900
1,000
1,100

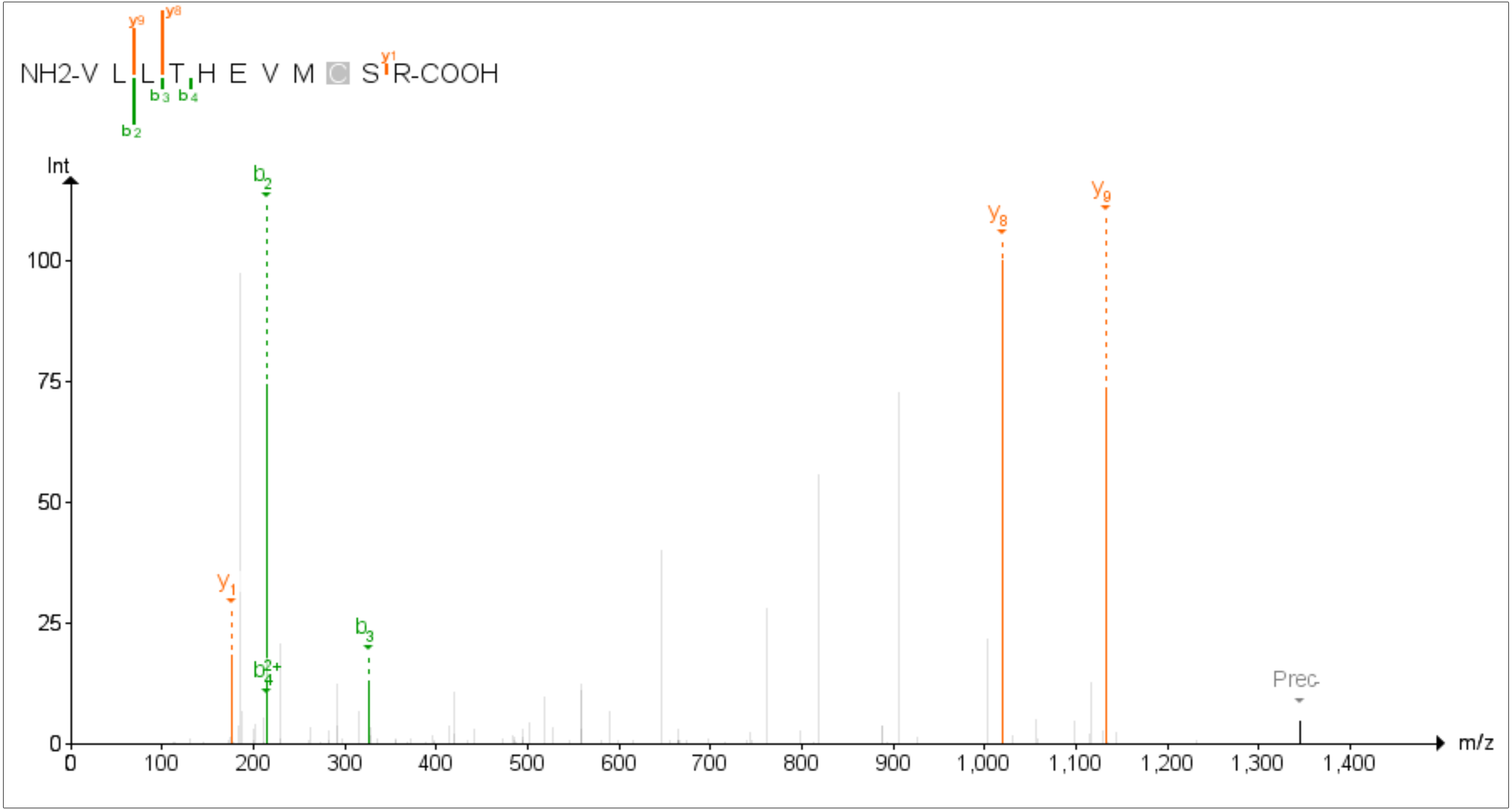

NH2-V L L T H E V M C S R-COOH
y9
y8
y1
b2
b3
b4
Int
100
75
50
25
0
y1
b2
b3
b4 2+
y8
y9
Prec.
0
100
200
300
400
500
600
700
800
900
1,000
1,100
1,200
1,300
1,400
m/z

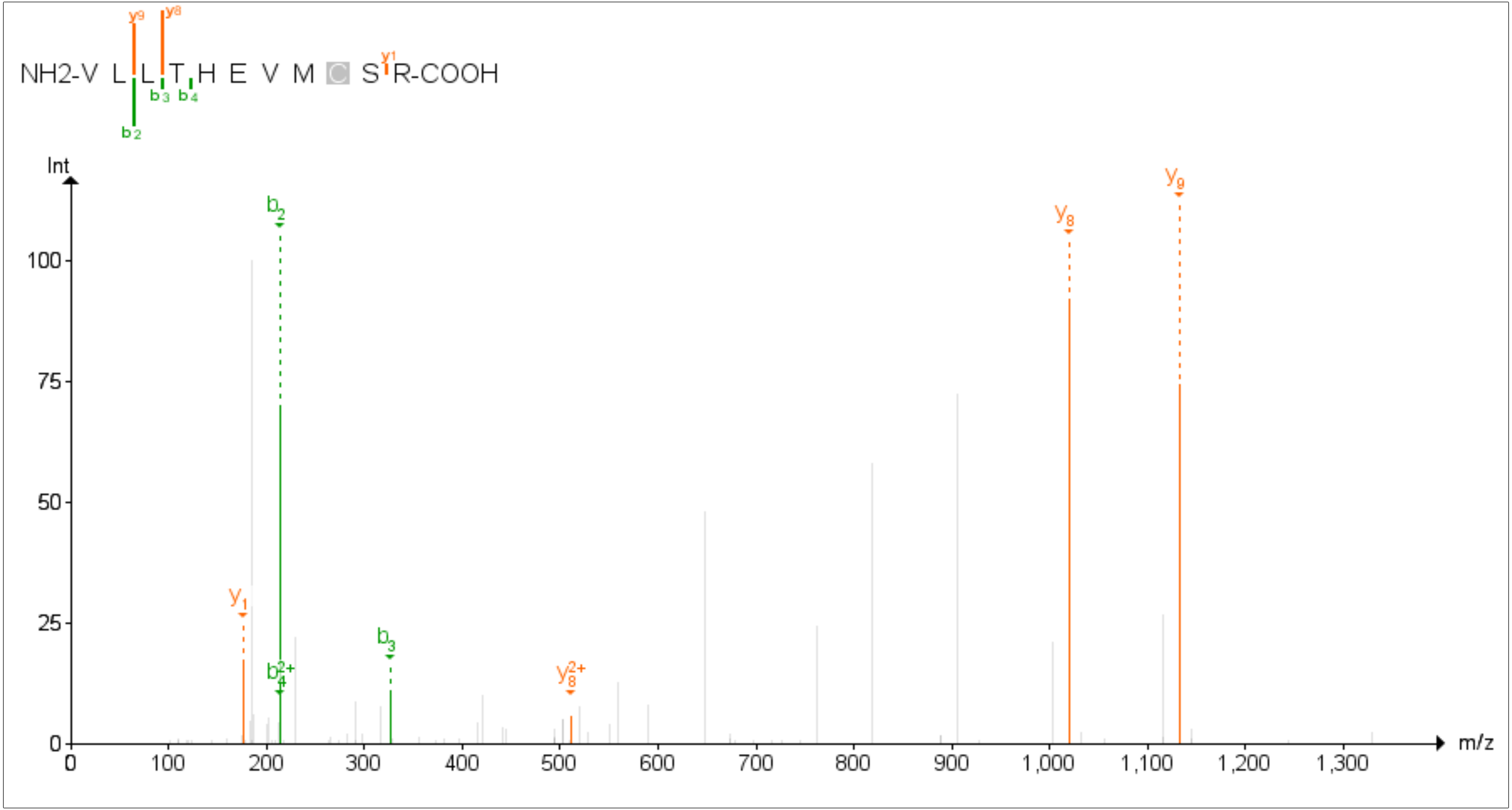

NH2-V L L T H E V M C S R-COOH
y9 y8 y1
b2 b3 b4
Int
100
75
50
25
0
b2
y8
y9
y1
b4 2+
b3
y8 2+
0 100 200 300 400 500 600 700 800 900 1,000 1,100 1,200 1,300
m/z

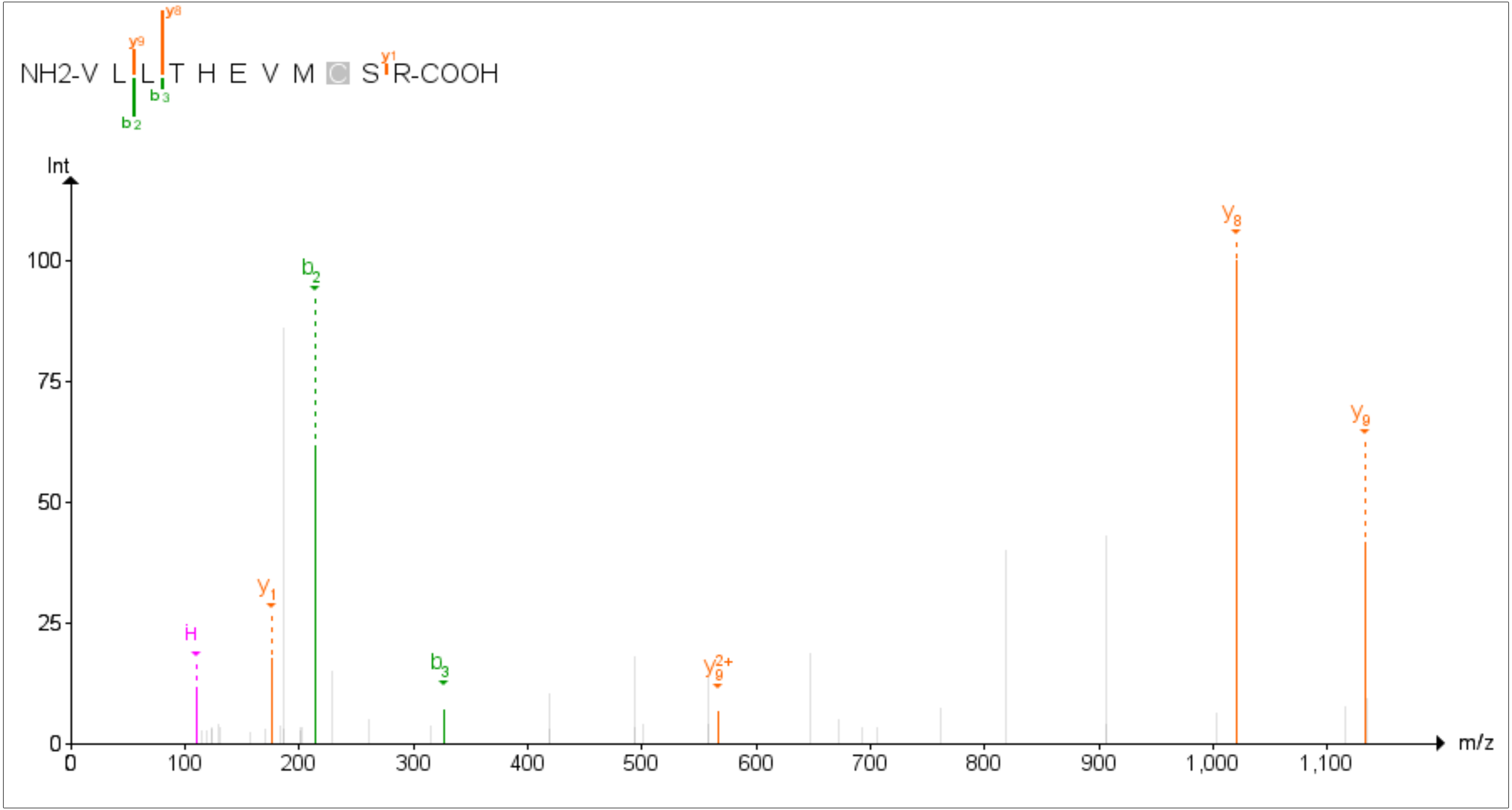

NH2-V L L T H E V M C S R-COOH
y9
y8
y1
b2
b3
Int
m/z
0
25
50
75
100
0
100
200
300
400
500
600
700
800
900
1,000
1,100
H
y1
b2
b3
y9 2+
y8
y9

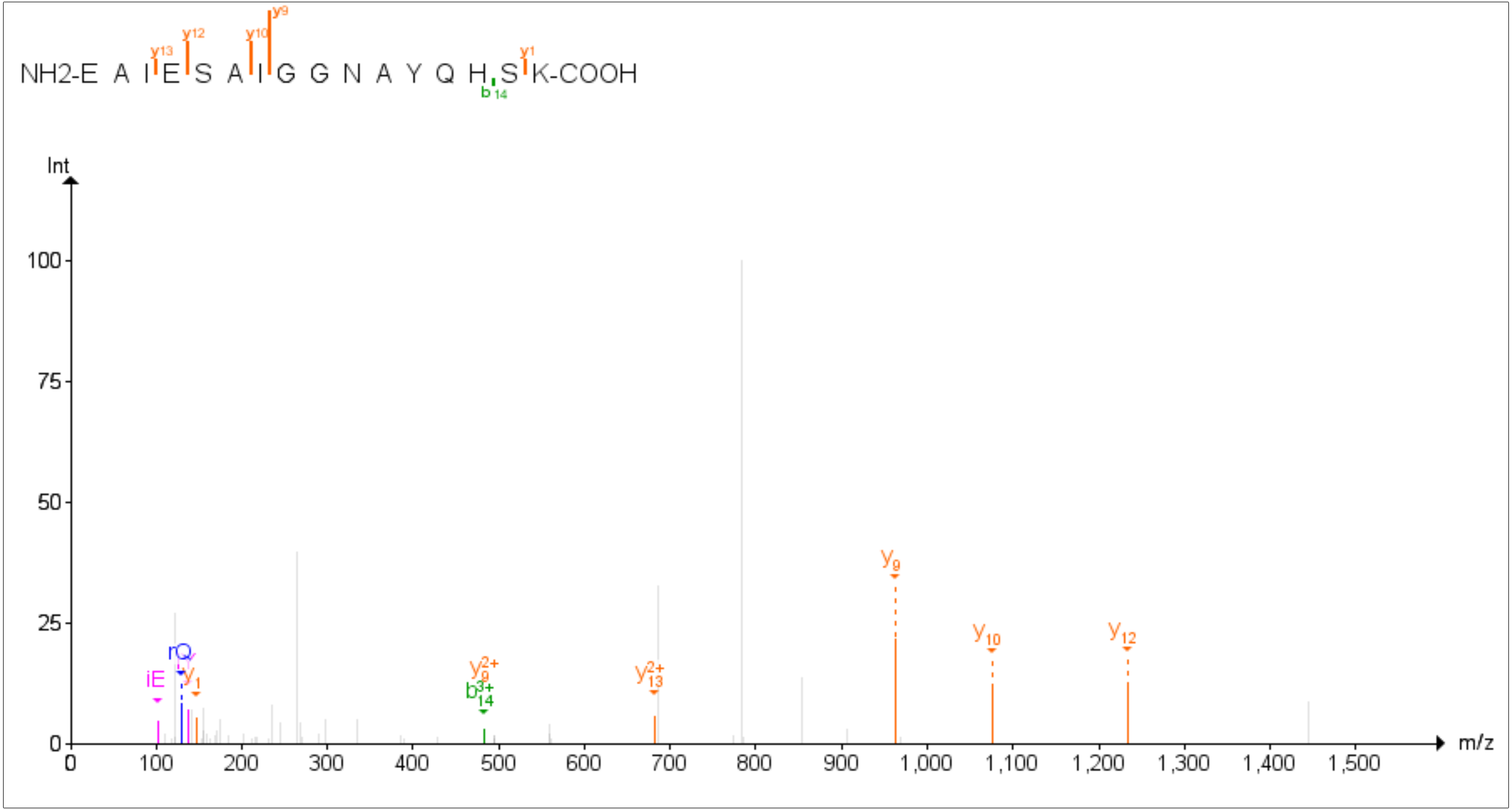
NH2-E A I E S A I G G N A Y Q H S K-COOH
y13
y12
y10
y9
y1
b14
Int
100
75
50
25
0
iE
rQ
y1
y9 2+
b14 3+
y13 2+
y9
y10
y12
0
100
200
300
400
500
600
700
800
900
1,000
1,100
1,200
1,300
1,400
1,500
m/z

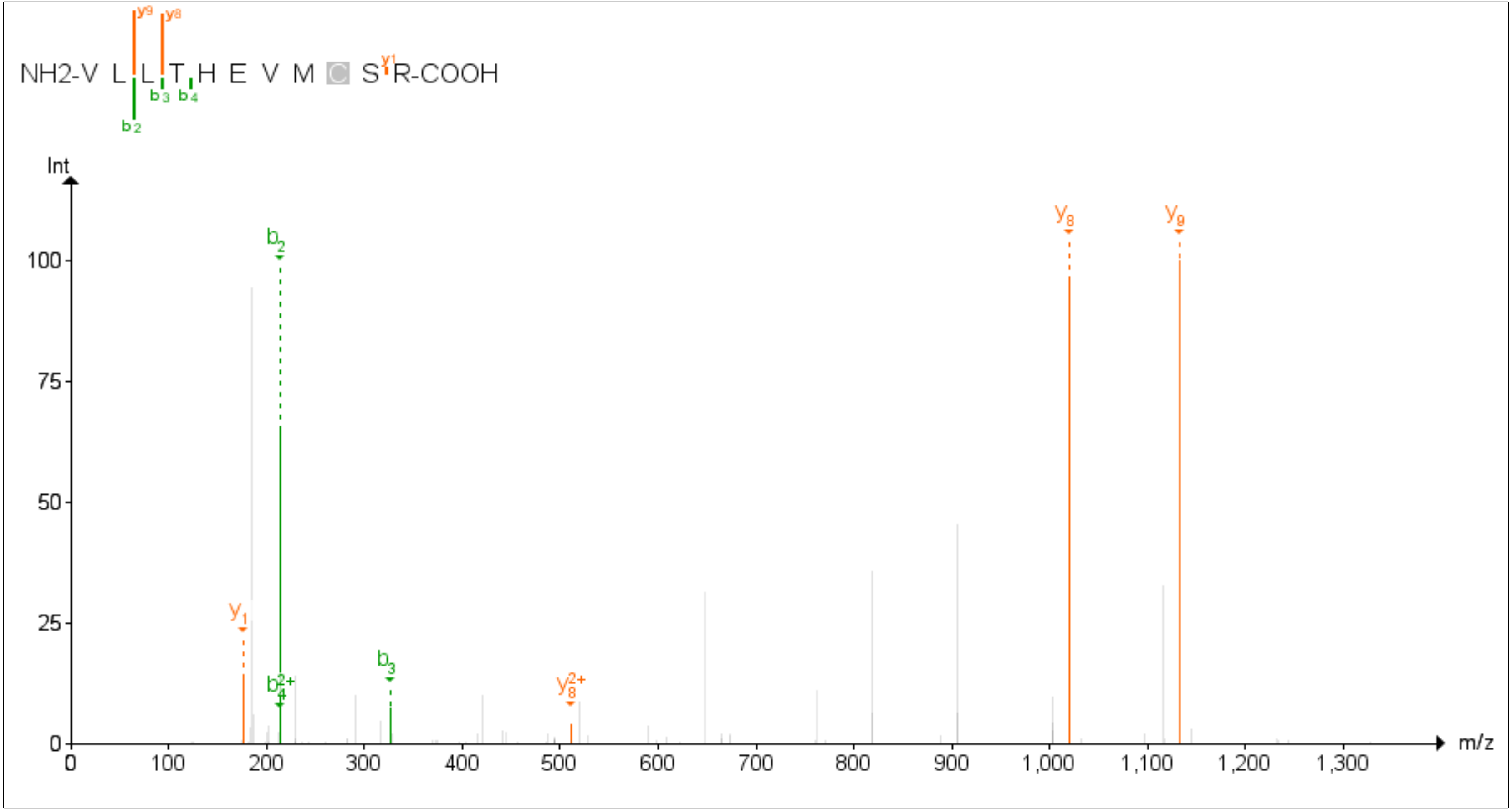
NH2-V L L T H E V M C S R-COOH
y9
y8
y1
b2
b3
b4
Int
100
75
50
25
0
y8
y9
b2
y1
b4 2+
b3
y8 2+
0
100
200
300
400
500
600
700
800
900
1,000
1,100
1,200
1,300
m/z

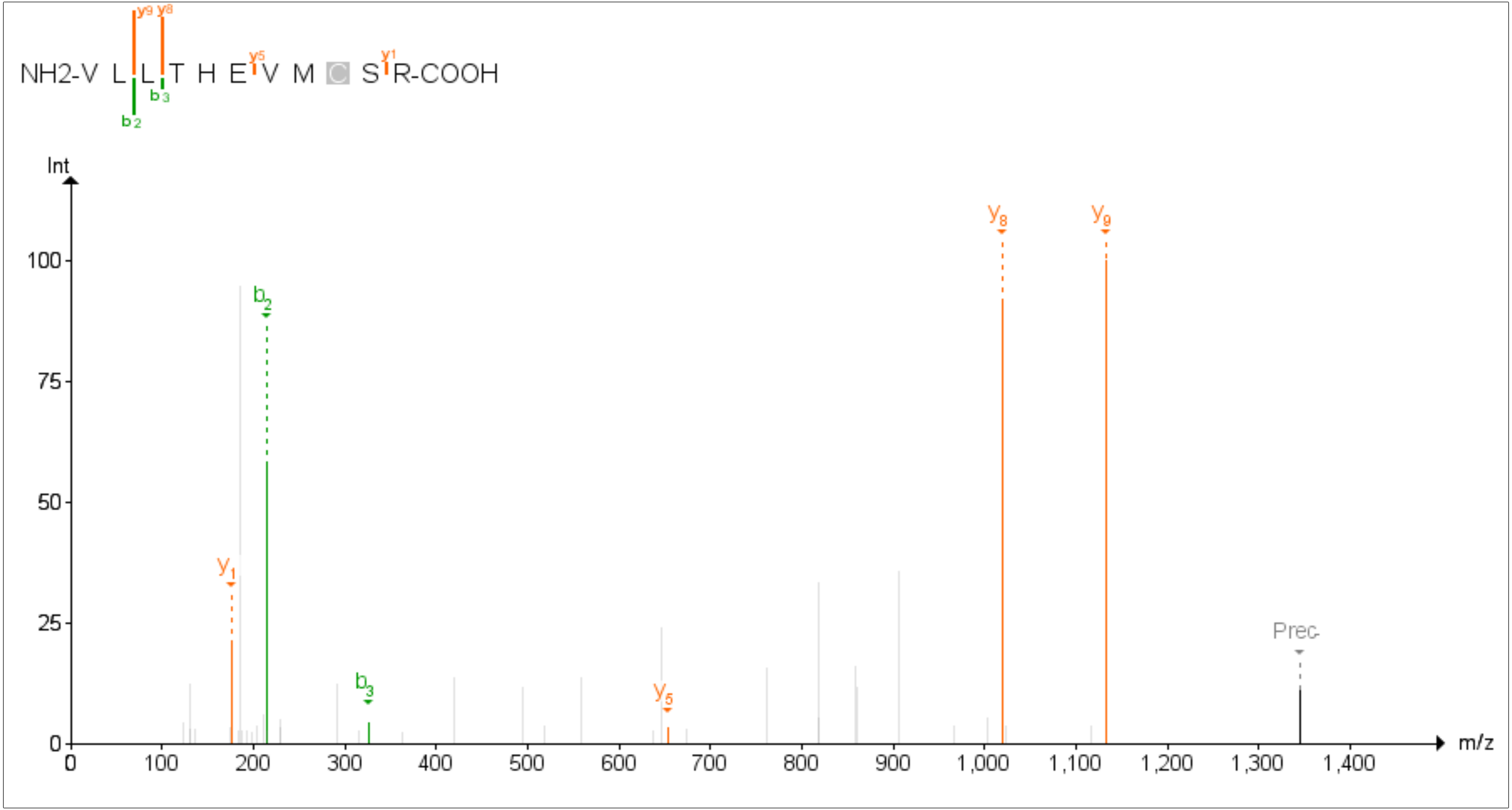

NH2-V L L T H E V M C S R-COOH
y9 y8 y5 y1
b2 b3
Int
100
75
50
25
0
y1
b2
b3
y5
y8
y9
Prec.
0 100 200 300 400 500 600 700 800 900 1,000 1,100 1,200 1,300 1,400
m/z

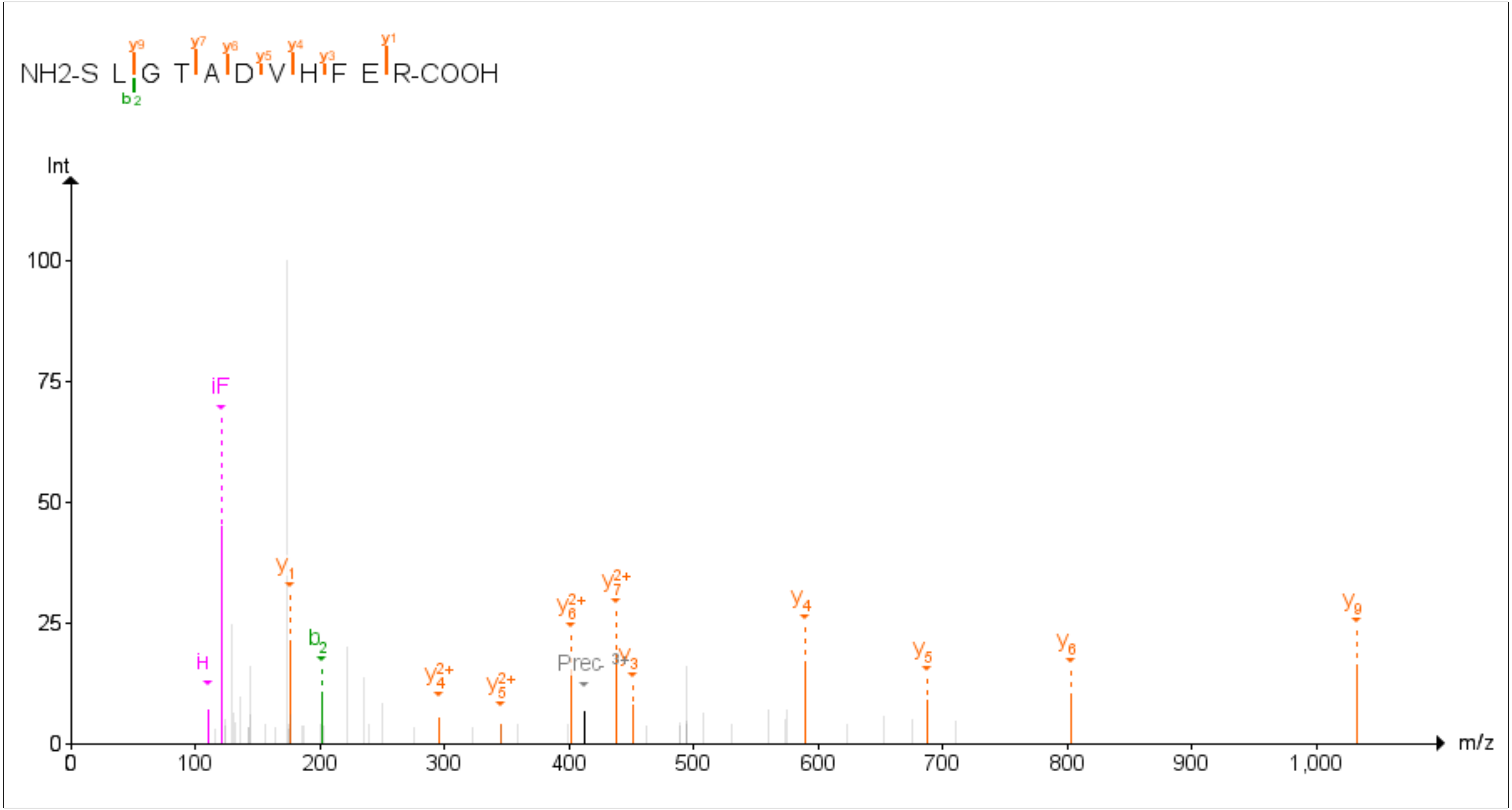

NH2-S L G T A D V H F E R-COOH
y9
y7
y6
y5
y4
y3
y1
b2
Int
100
75
50
25
0
iF
iH
y1
b2
y4 2+
y5 2+
y6 2+
y7 2+
Prec- 3+
y3
y4
y5
y6
y9
0
100
200
300
400
500
600
700
800
900
1,000
m/z

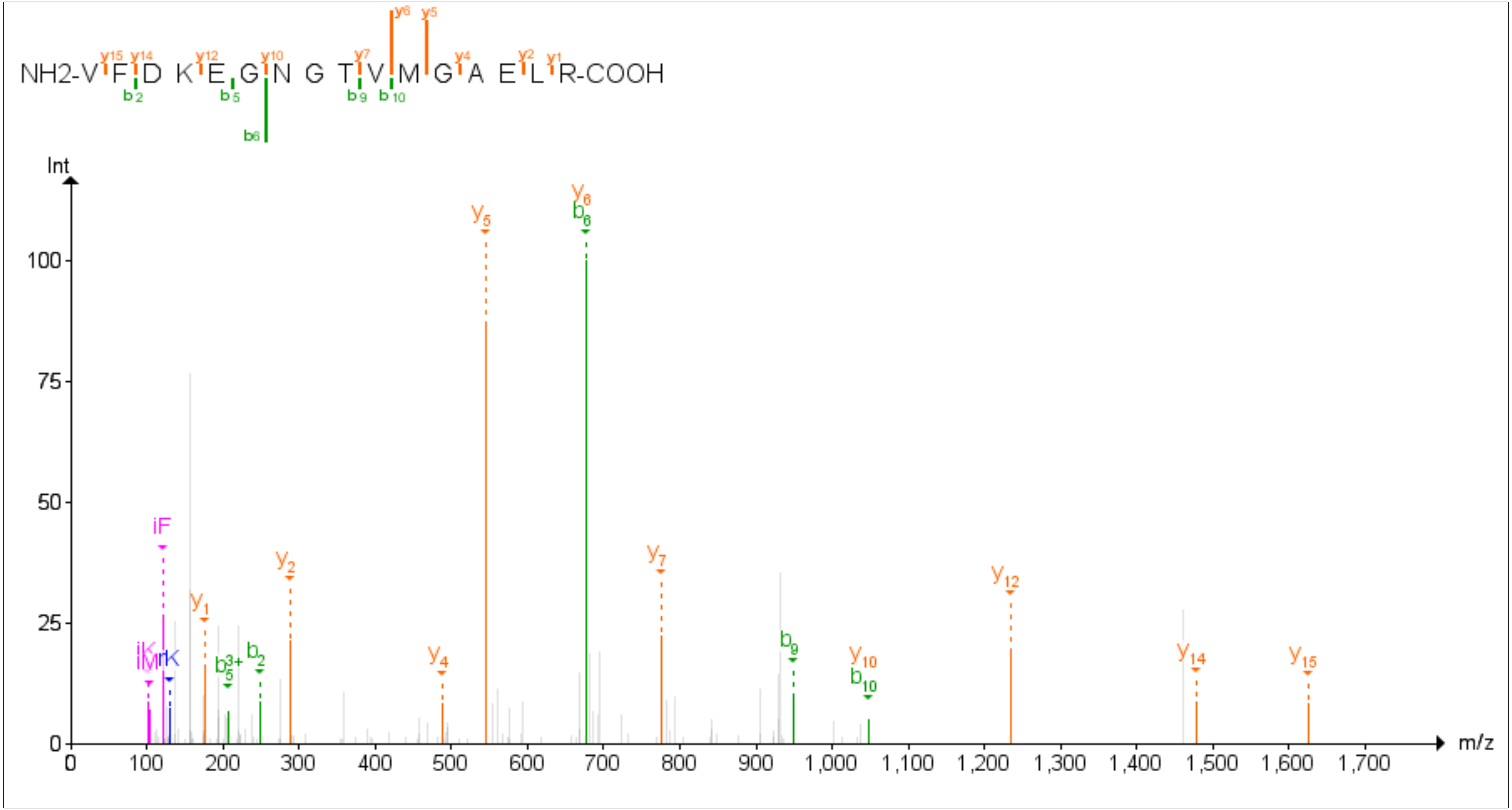

NH2-V F D K E G N G T V M G A E L R-COOH
Int
m/z

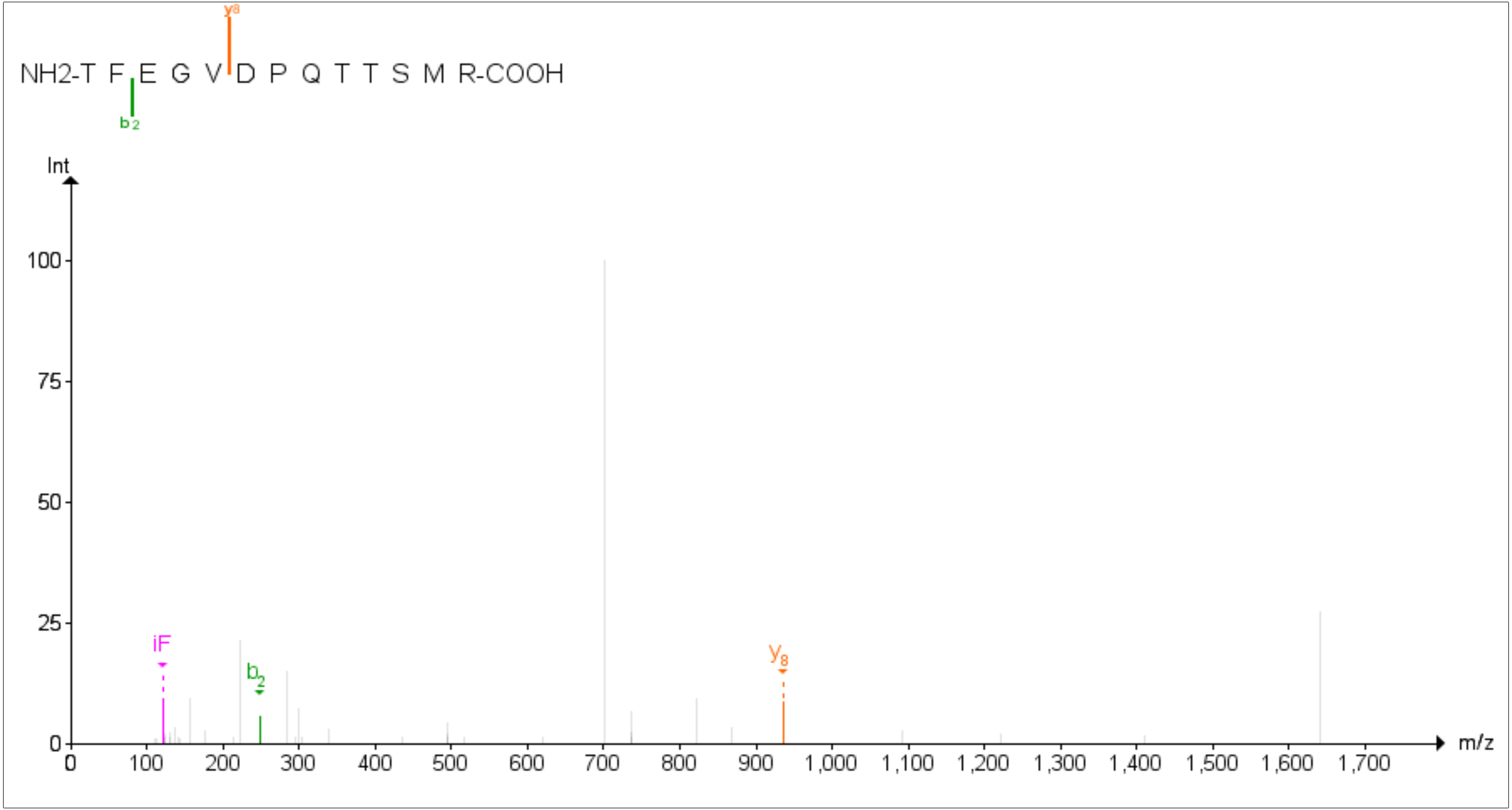

NH2-T F E G V D P Q T T S M R-COOH
y8
b2
Int
100
75
50
25
0
iF
b2
y8
0
100
200
300
400
500
600
700
800
900
1,000
1,100
1,200
1,300
1,400
1,500
1,600
1,700
m/z

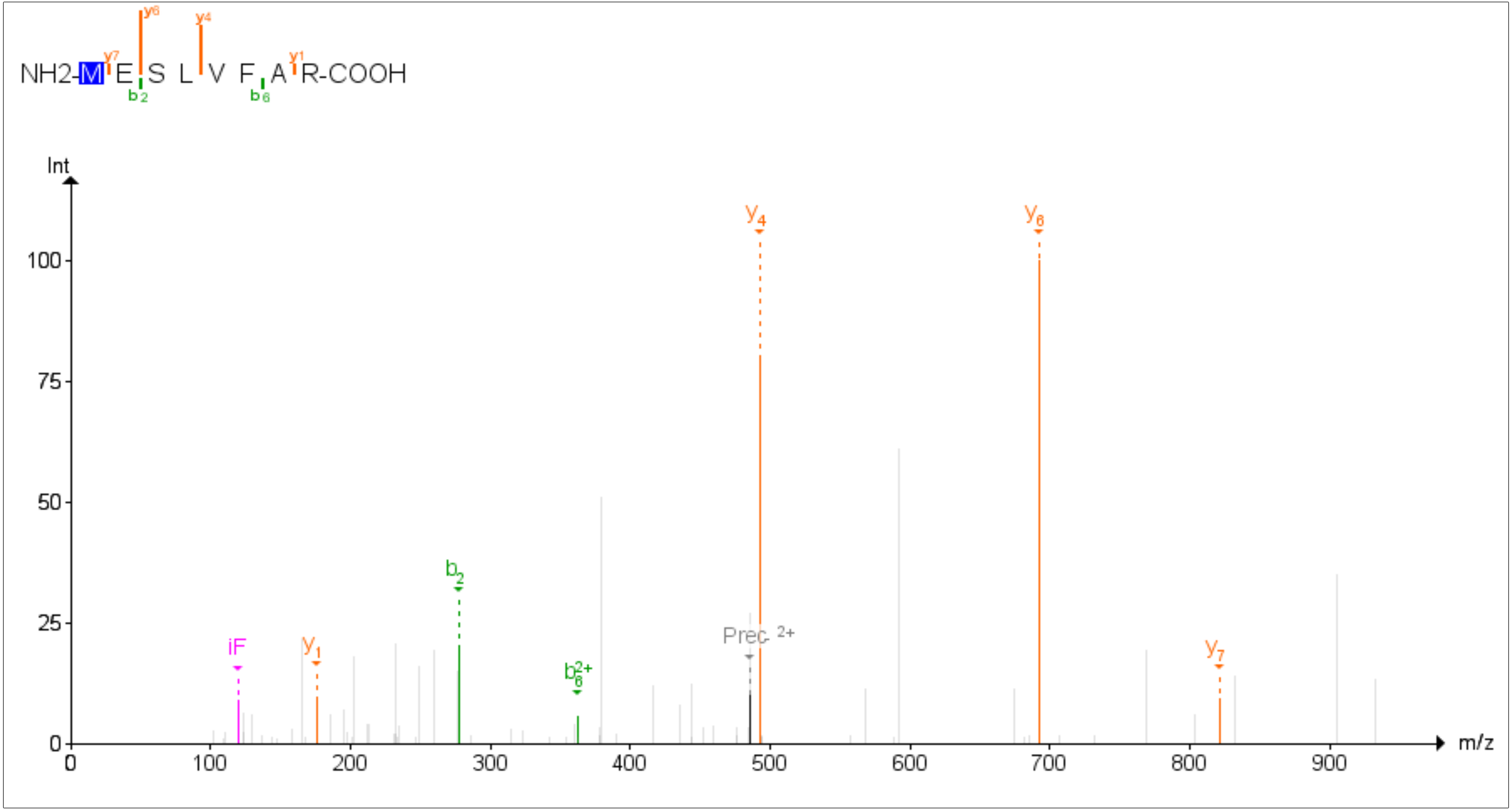

NH2-M E S L V F A R-COOH
y7
y6
y4
y1
b2
b6
Int
100
75
50
25
0
y4
y6
b2
iF
y1
b6 2+
Prec 2+
y7
0
100
200
300
400
500
600
700
800
900
m/z

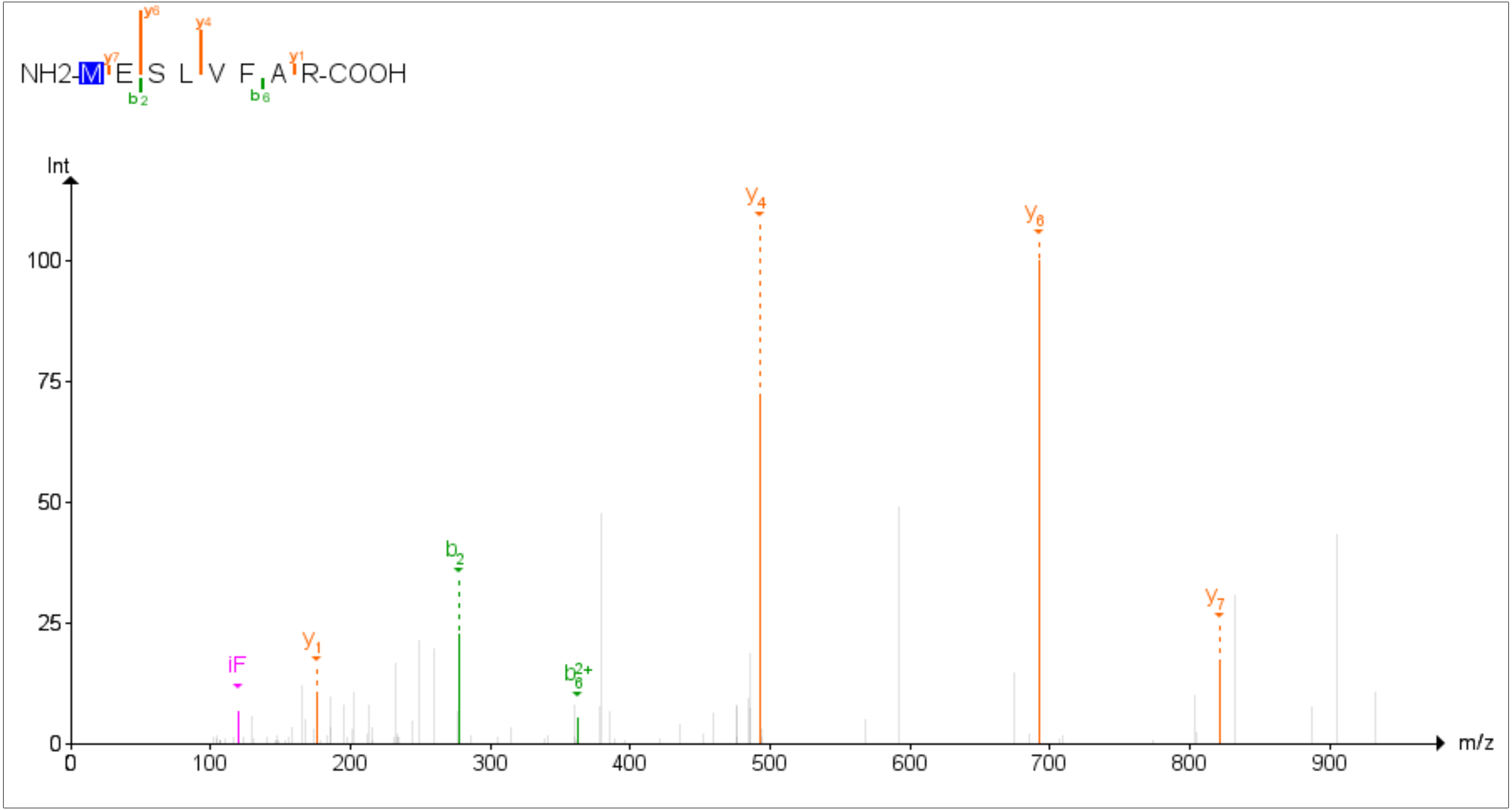
NH2-M E S L V F A R-COOH
y7
y6
y4
y1
b2
b6
Int
100
75
50
25
0
y4
y6
y7
b2
y1
iF
b6 2+
0
100
200
300
400
500
600
700
800
900
m/z

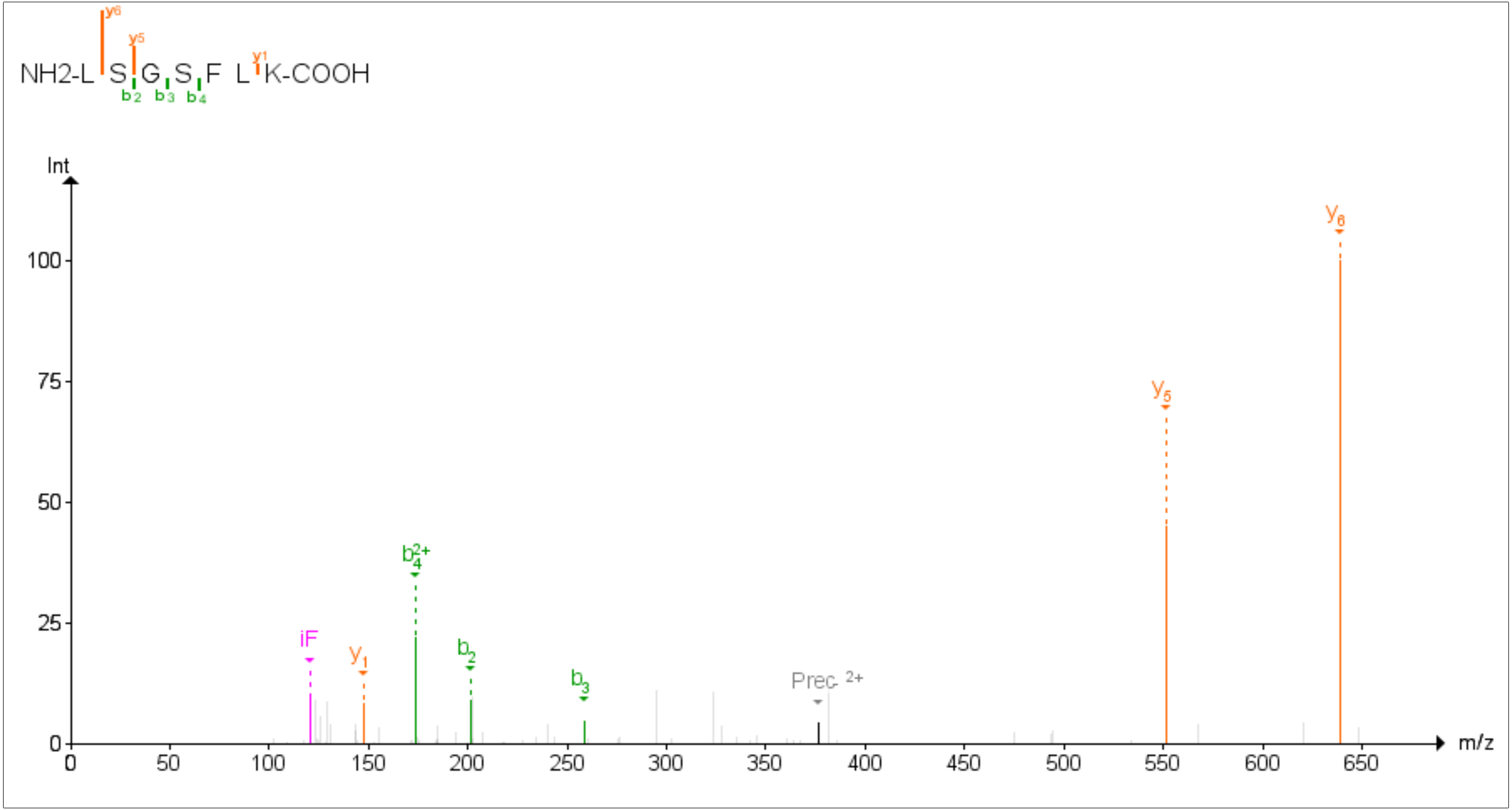

NH2-L S G S F L K-COOH
y6
y5
y1
b2
b3
b4
Int
100
75
50
25
0
y6
y5
b4 2+
iF
y1
b2
b3
Prec. 2+
0
50
100
150
200
250
300
350
400
450
500
550
600
650
m/z

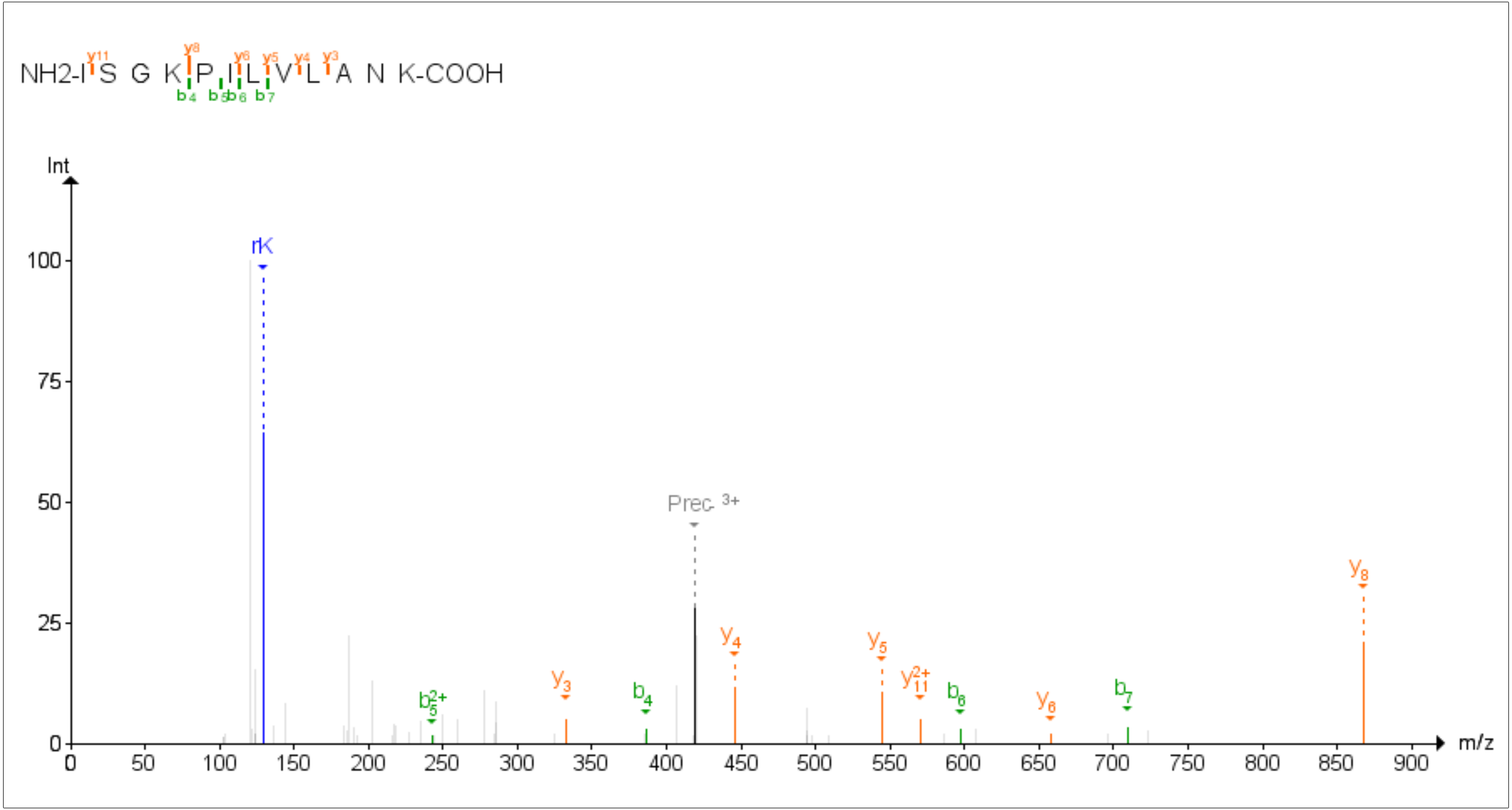
NH2-I S G K P I L V L A N K-COOH
y11 y8 y6 y5 y4 y3
b4 b5 b6 b7
Int
100
75
50
25
0
rK
Prec 3+
y3
b4
y4
y5
y11 2+
b6
y6
b7
y8
b5 2+
m/z
0 50 100 150 200 250 300 350 400 450 500 550 600 650 700 750 800 850 900

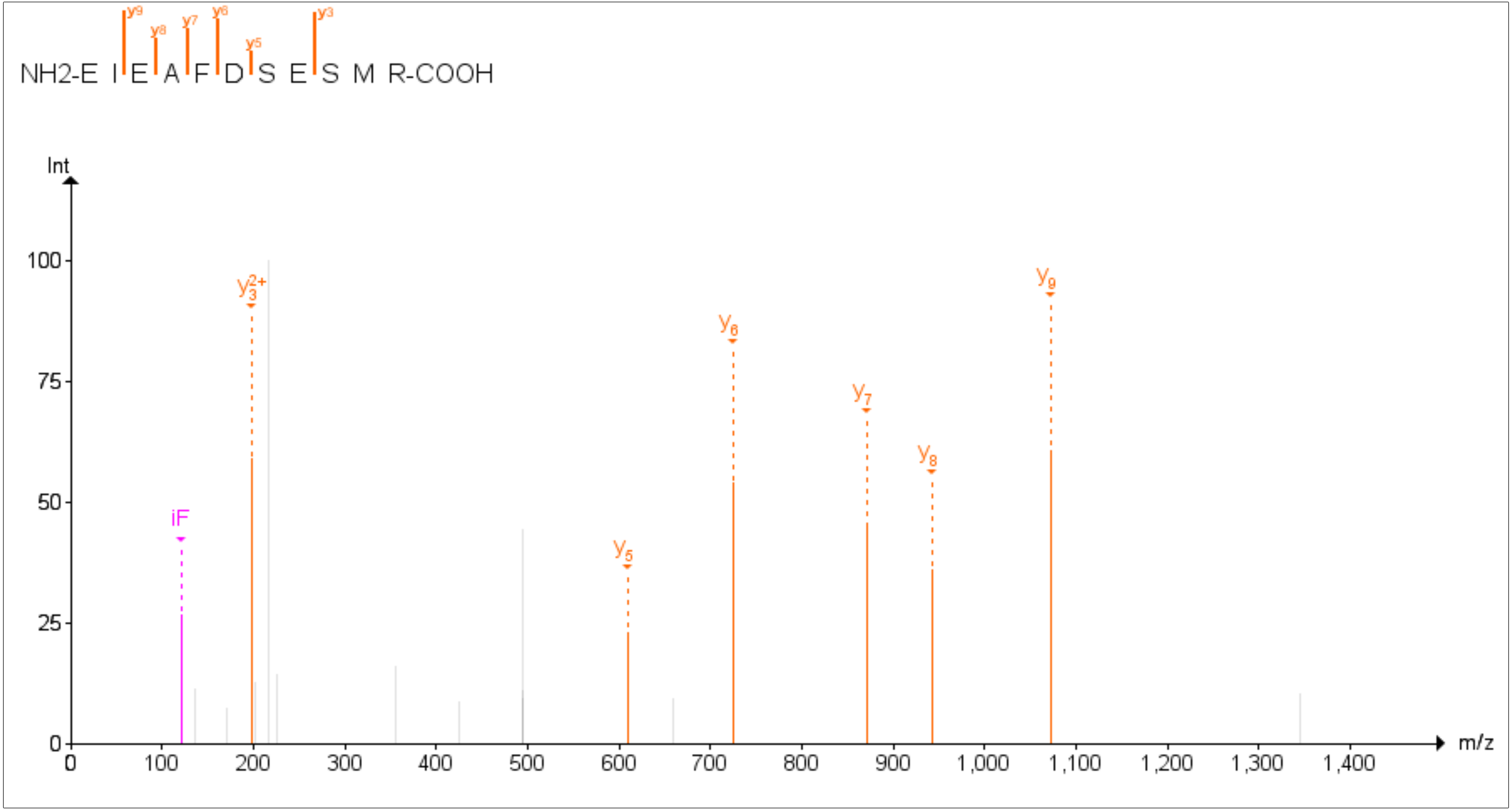
NH2-E I E A F D S E S M R-COOH
y9
y8
y7
y6
y5
y3
Int
100
75
50
25
0
y_3^{2+}
iF
y_5
y_6
y_7
y_8
y_9
0
100
200
300
400
500
600
700
800
900
1,000
1,100
1,200
1,300
1,400
m/z

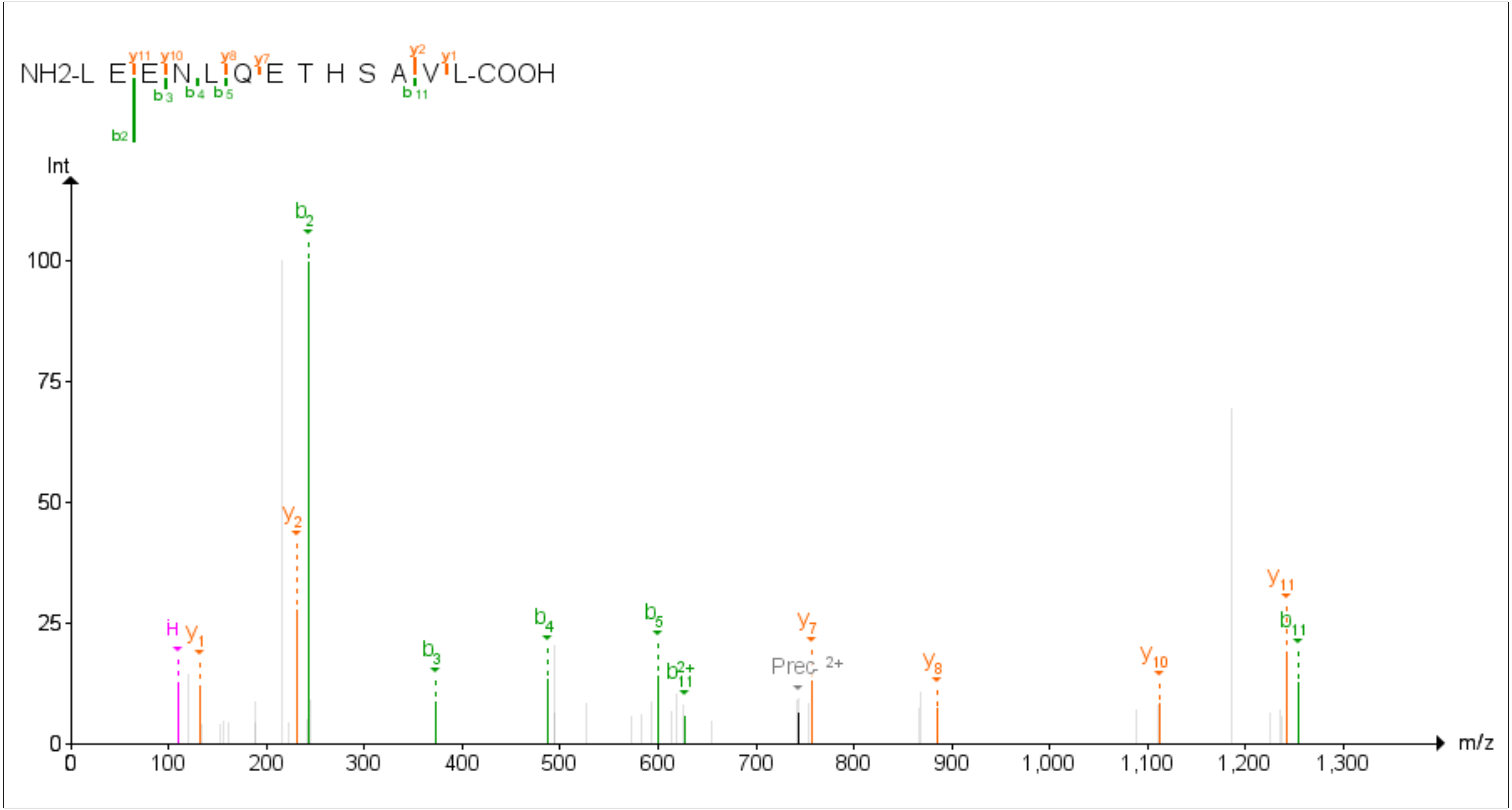

NH2-L E E N L Q E T H S A V L-COOH
y11 y10 y8 y7 y2 y1
b2 b3 b4 b5 b11
Int
100
75
50
25
0
b2
y2
H
y1
b3
b4
b5
b11 2+
Prec 2+
y7
y8
y10
y11
b11
0
100
200
300
400
500
600
700
800
900
1,000
1,100
1,200
1,300
m/z

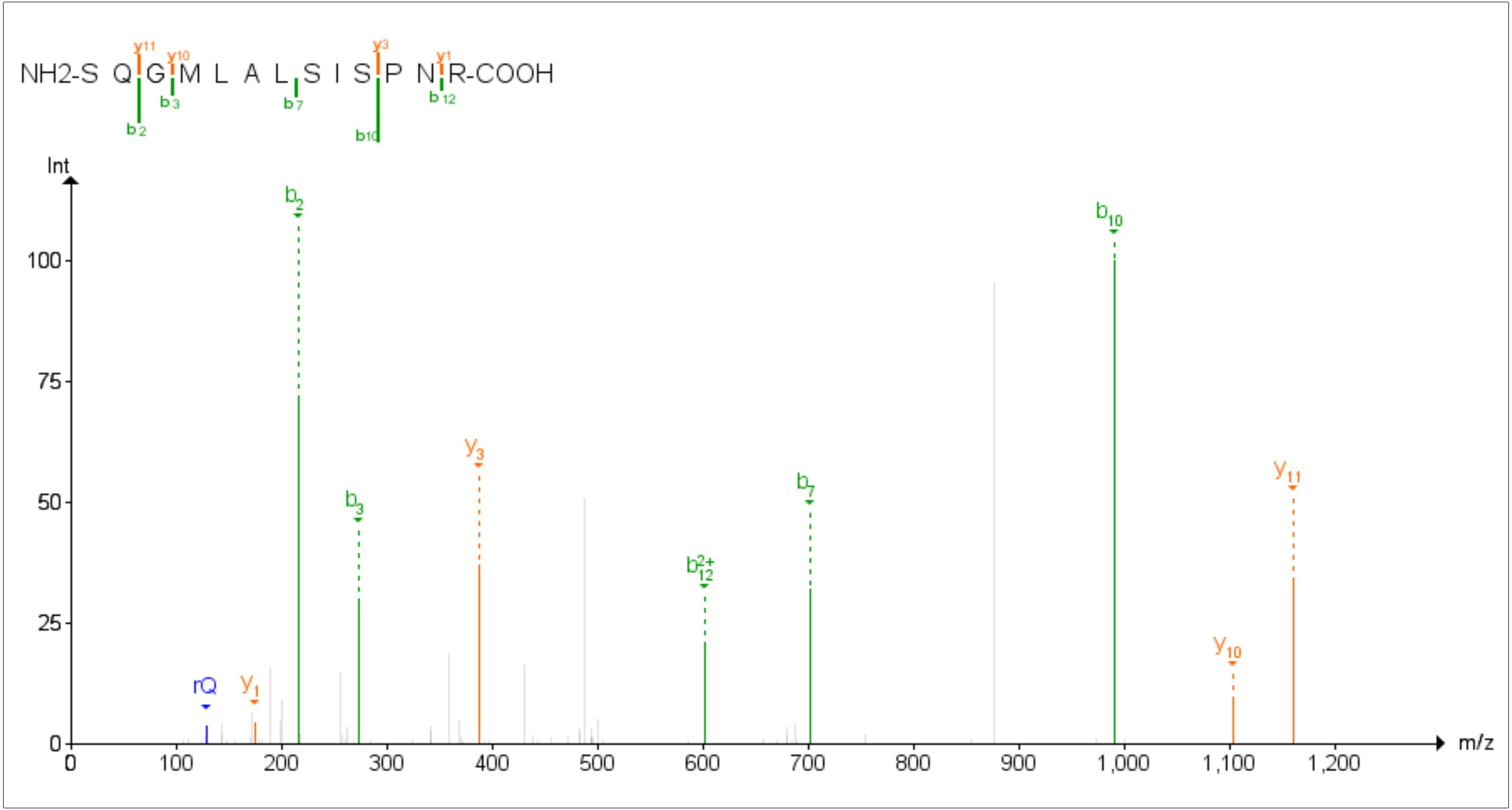

NH2-S Q G M L A L S I S P N R-COOH
y11
y10
y3
y1
b 2
b 3
b 7
b10
b 12
Int
100
75
50
25
0
b2
b10
y3
y11
b3
b7
b12 2+
y10
rQ
y1
0
100
200
300
400
500
600
700
800
900
1,000
1,100
1,200
m/z

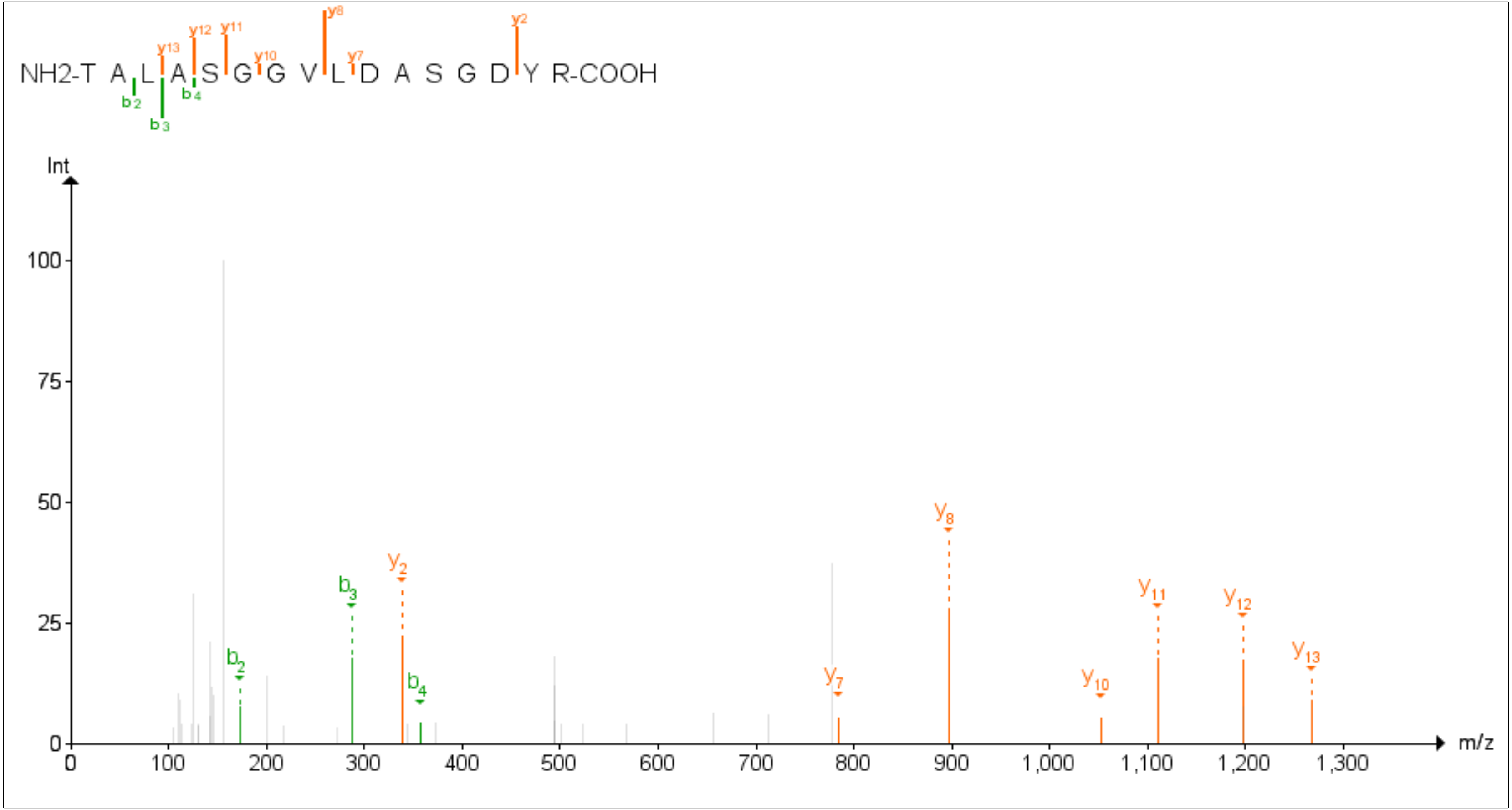

NH2-T A L A S G G V L D A S G D Y R-COOH
y13
y12
y11
y10
y8
y7
y2
b2
b3
b4
Int
100
75
50
25
0
m/z
0
100
200
300
400
500
600
700
800
900
1,000
1,100
1,200
1,300
b2
b3
y2
b4
y7
y8
y10
y11
y12
y13

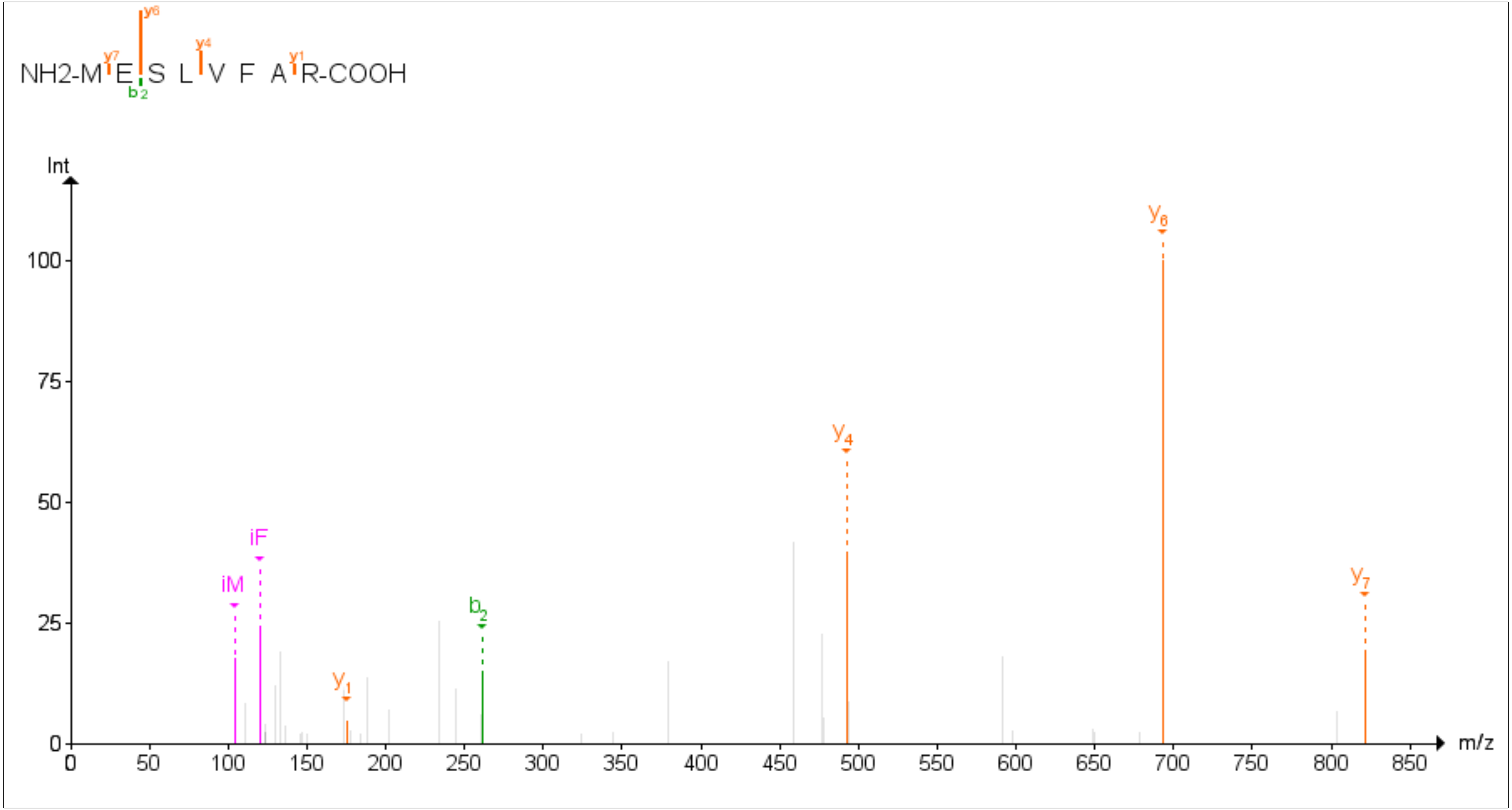

NH2-M E S L V F A R-COOH
y7
y6
y4
y1
b2
Int
m/z
0
25
50
75
100
0
50
100
150
200
250
300
350
400
450
500
550
600
650
700
750
800
850
iM
iF
y1
b2
y4
y6
y7

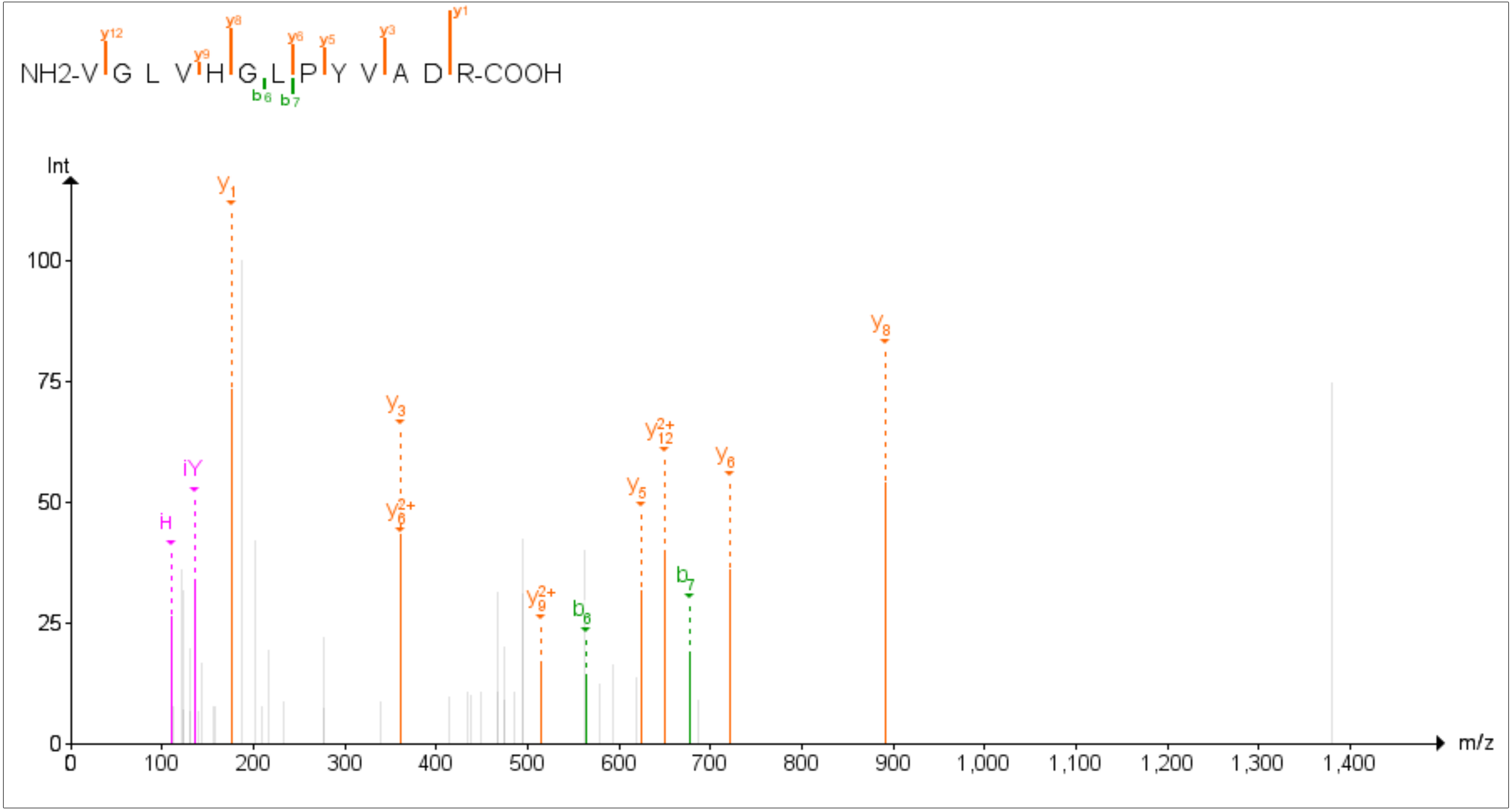
NH2-V G L V H G L P Y V A D R-COOH
y12 y9 y8 y6 y5 y3 y1
b6 b7
Int
m/z
y1
y3
y6 2+
iY
iH
y9 2+
b6
y5
y12 2+
b7
y6
y8
0
25
50
75
100
0 100 200 300 400 500 600 700 800 900 1,000 1,100 1,200 1,300 1,400

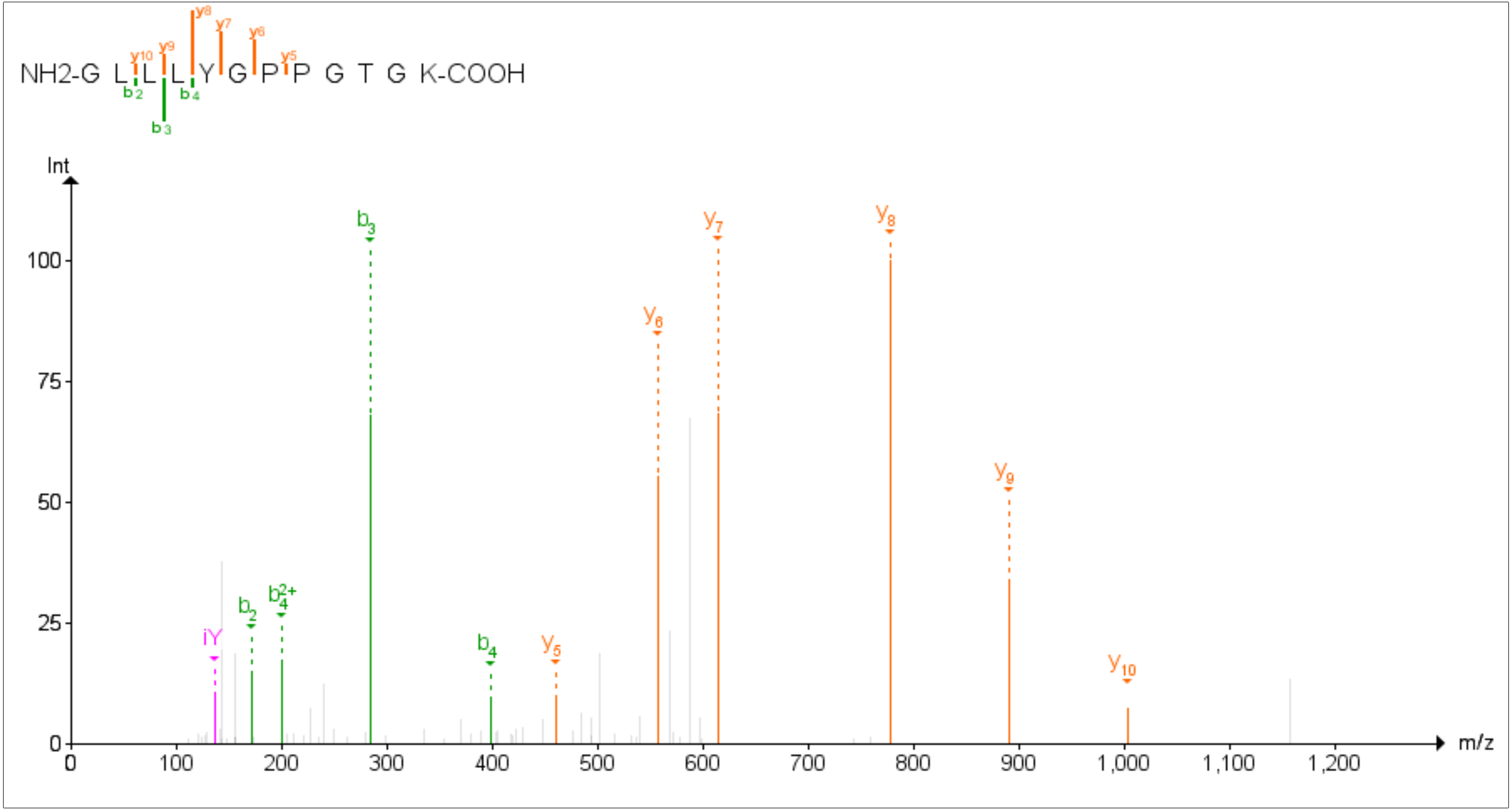

NH2-G L L L Y G P P G T G K-COOH
y10 y9 y8 y7 y6 y5
b2 b3 b4
Int
m/z
b3
y7
y8
y6
y9
b4
y5
y10
iY
b2
b4 2+
0
25
50
75
100
0
100
200
300
400
500
600
700
800
900
1,000
1,100
1,200

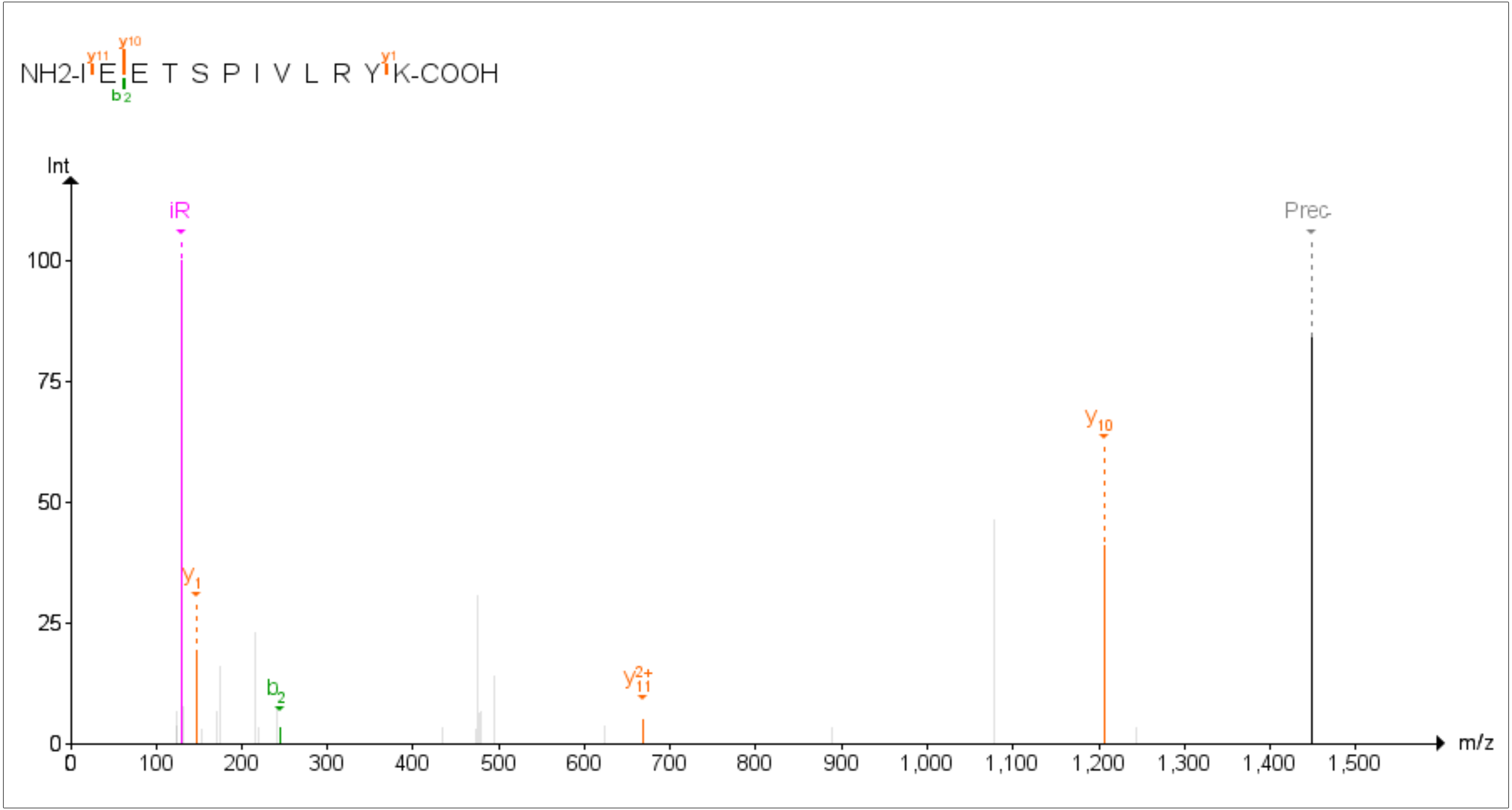

NH2-I E E T S P I V L R Y K-COOH
y11
y10
b2
y1
Int
iR
Prec.
y1
b2
y11 2+
y10
100
75
50
25
0
m/z
0
100
200
300
400
500
600
700
800
900
1,000
1,100
1,200
1,300
1,400
1,500

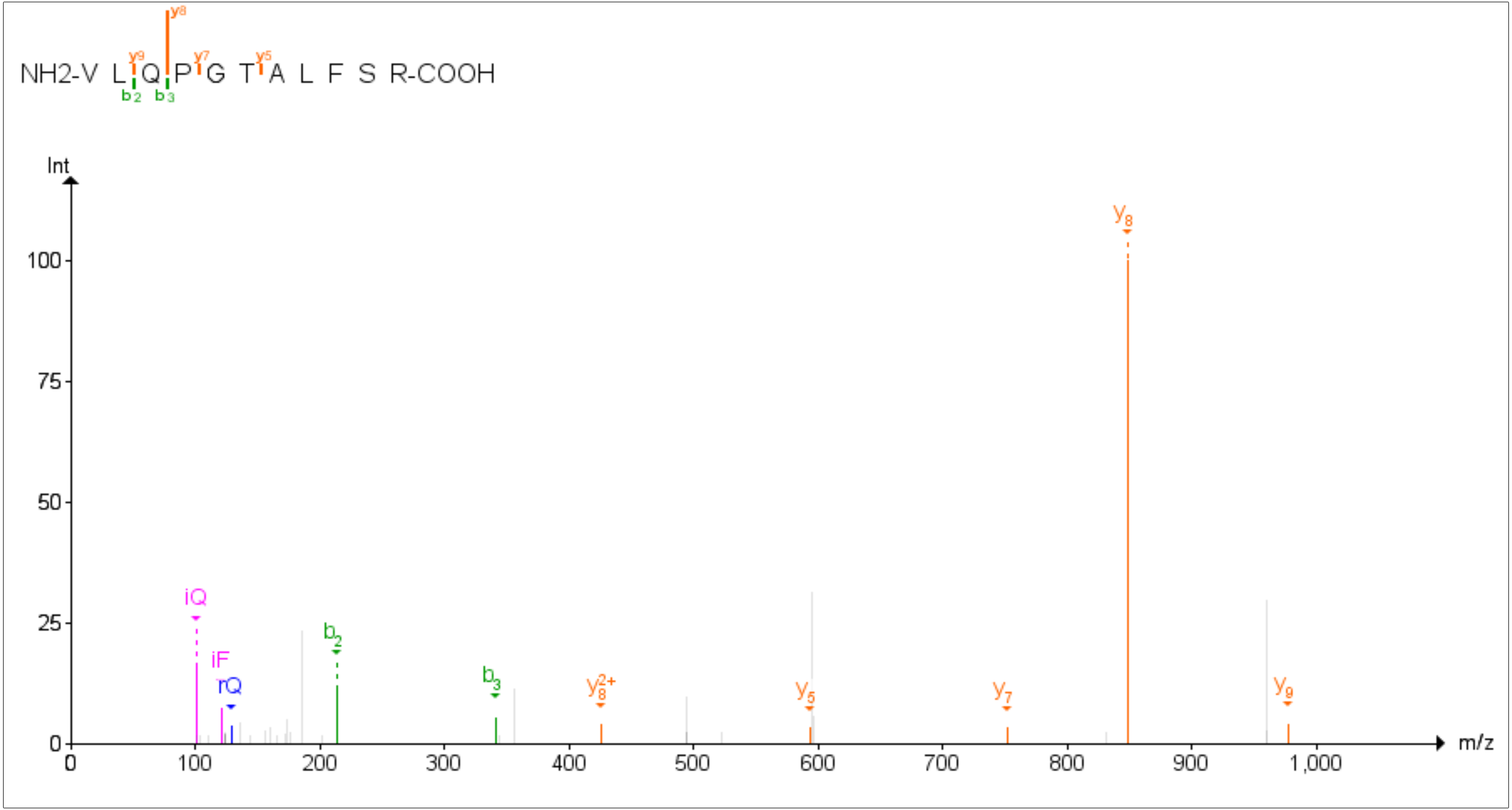

NH2-V L Q P G T A L F S R-COOH
y8
y9
y7
y5
b2
b3
Int
100
75
50
25
0
m/z
0
100
200
300
400
500
600
700
800
900
1,000
iQ
iF
rQ
b2
b3
y8 2+
y5
y7
y8
y9

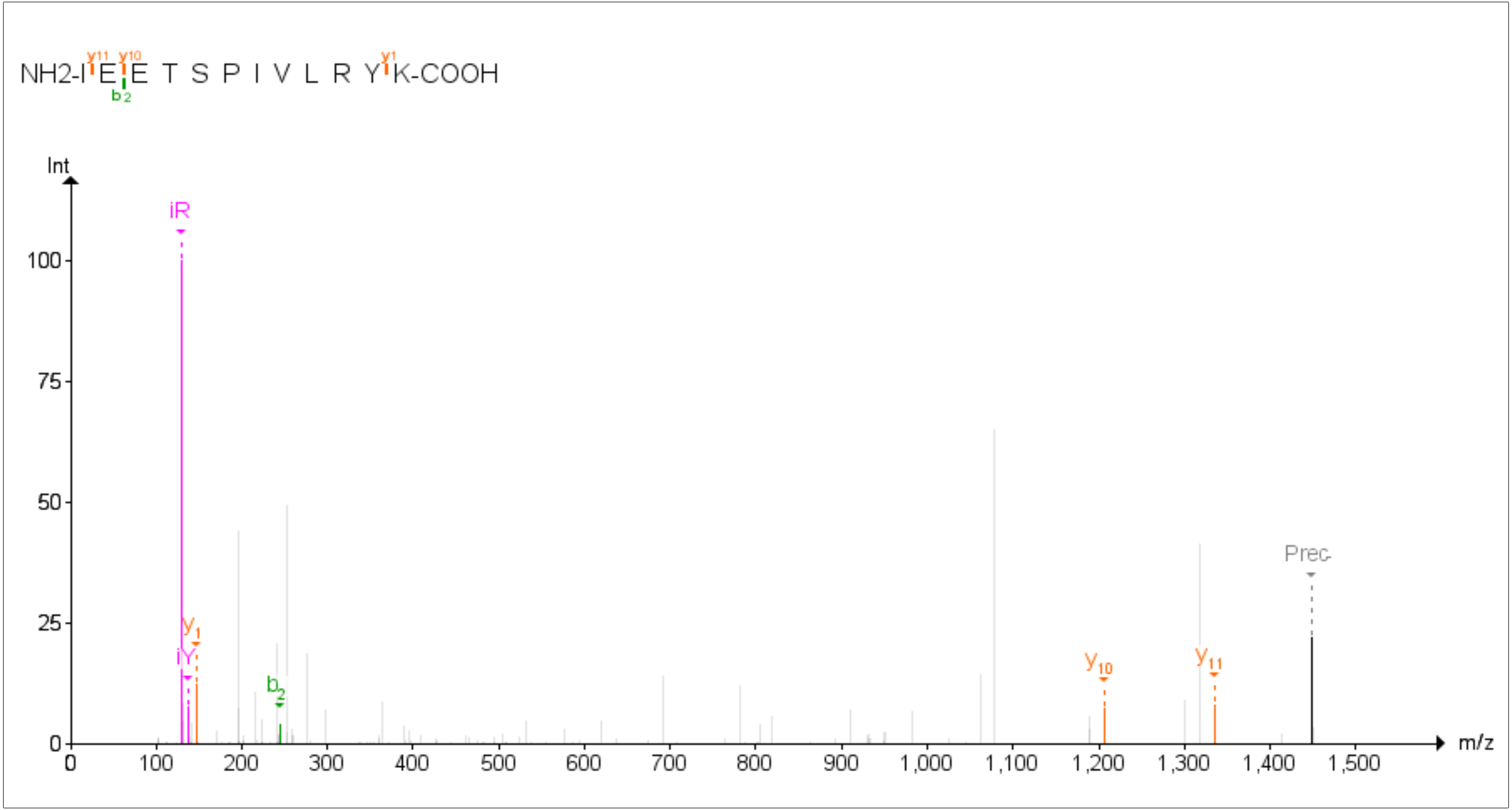

NH2-I E E T S P I V L R Y K-COOH
y11
y10
y1
b2
Int
iR
iY
y1
b2
y10
y11
Prec
0
25
50
75
100
0
100
200
300
400
500
600
700
800
900
1,000
1,100
1,200
1,300
1,400
1,500
m/z

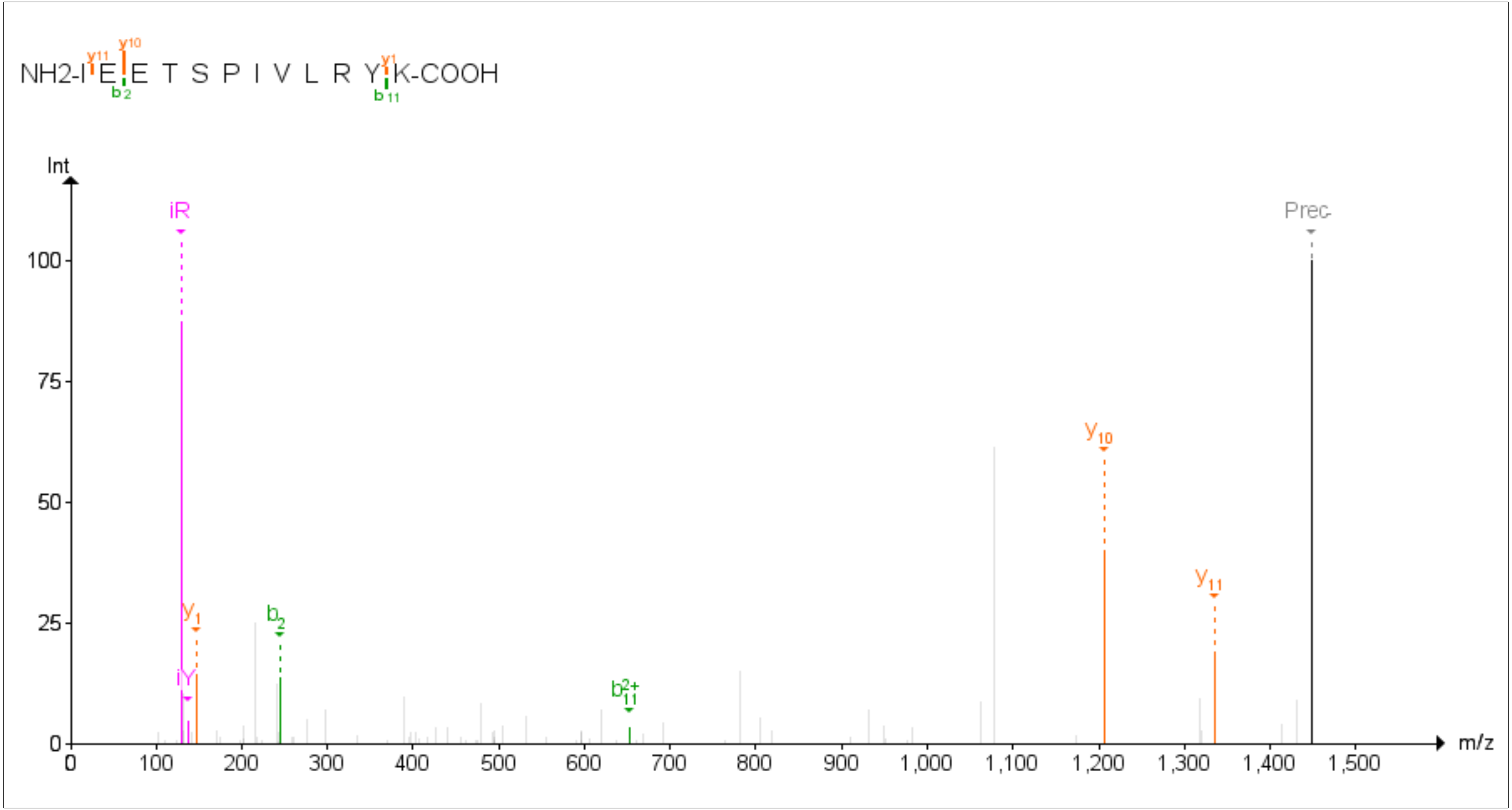

NH2-I E E T S P I V L R Y K-COOH
y11
y10
y1
b2
b11
Int
m/z
iR
iY
y1
b2
b11 2+
y10
y11
Prec
0
25
50
75
100
0
100
200
300
400
500
600
700
800
900
1,000
1,100
1,200
1,300
1,400
1,500

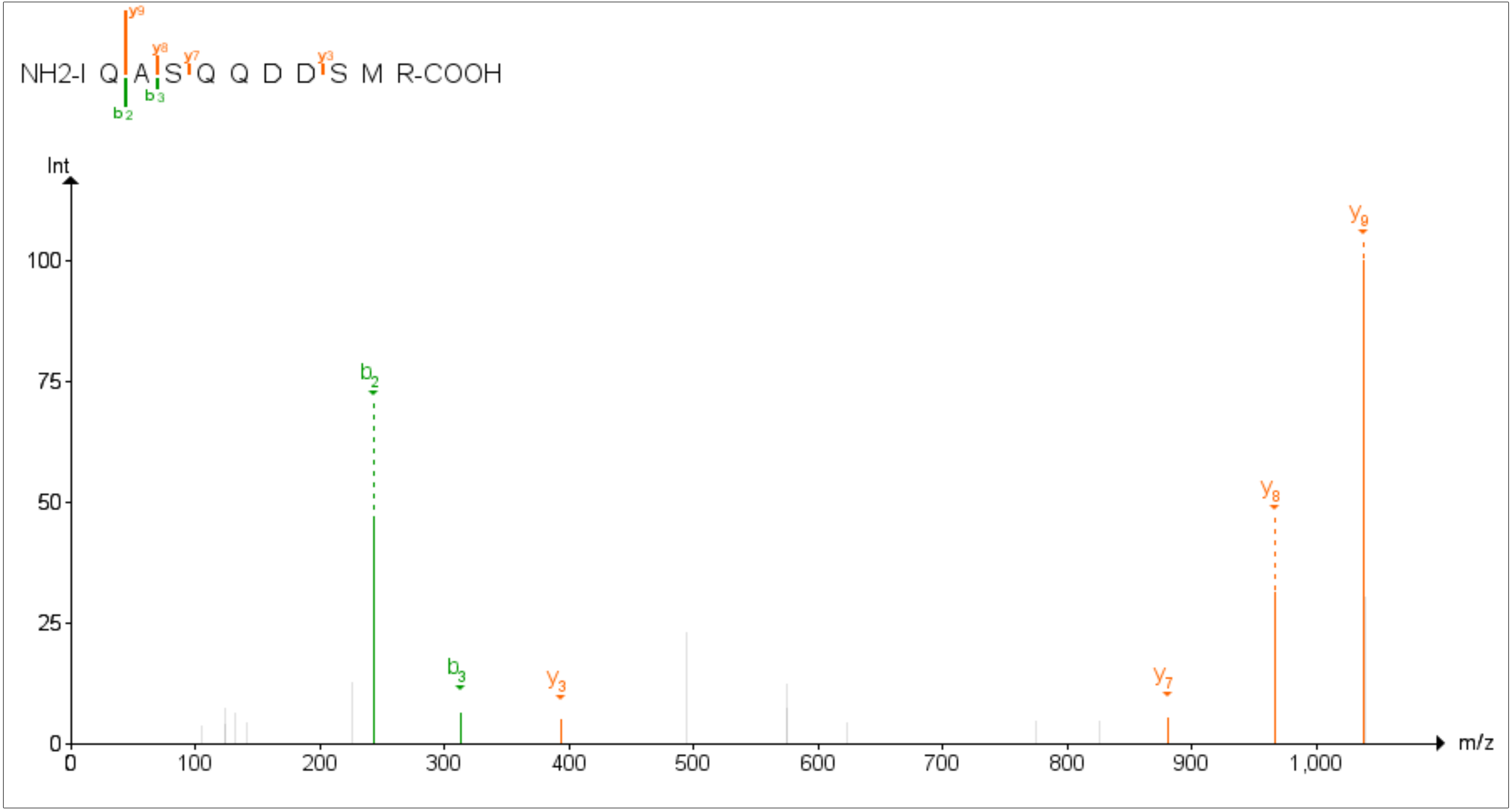

NH2-I Q A S Q Q D D S M R-COOH
y9
y8
y7
y3
b2
b3
Int
100
75
50
25
0
m/z
0
100
200
300
400
500
600
700
800
900
1,000

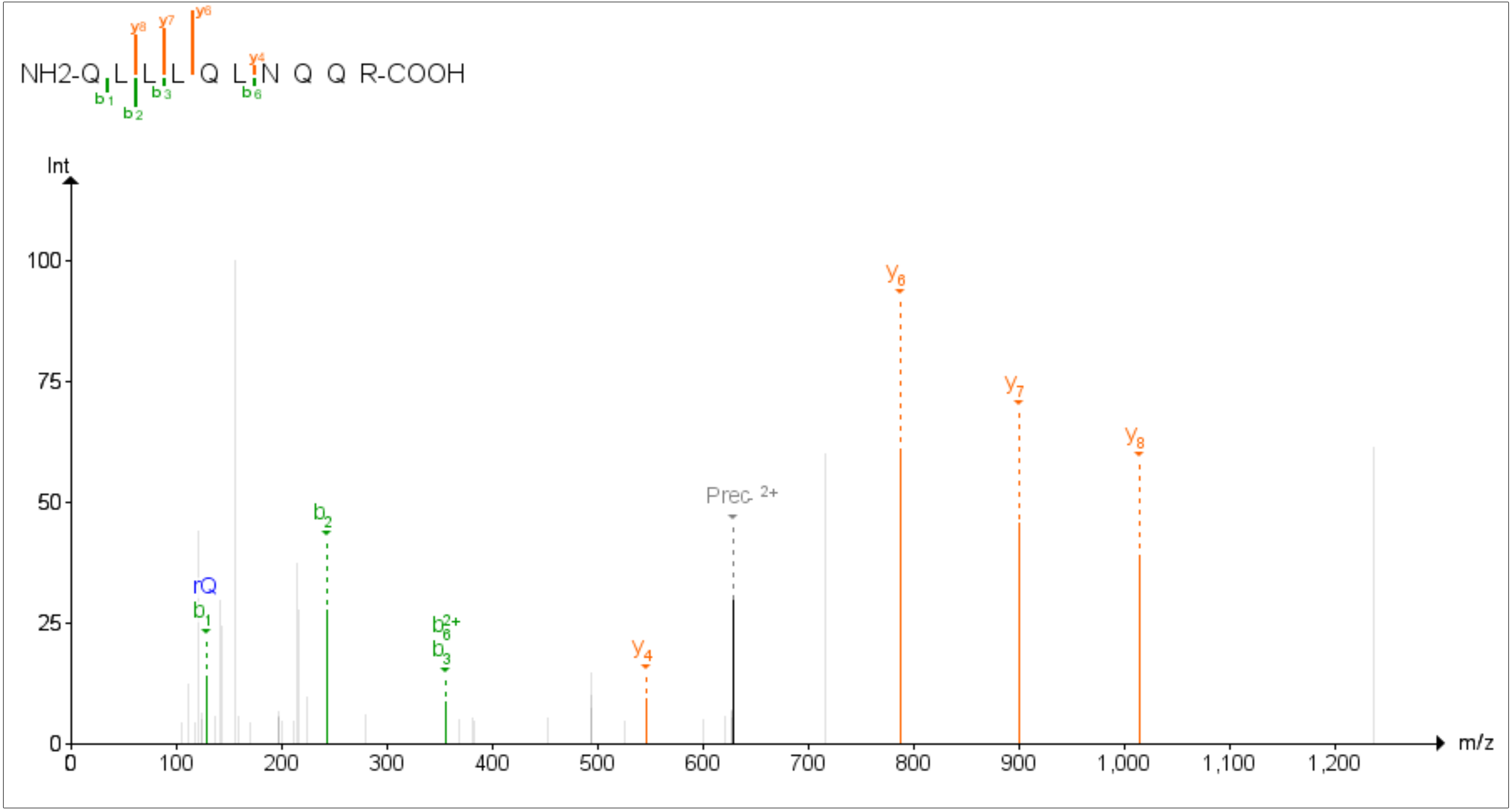

NH2-Q L L L Q L N Q Q R-COOH
y8
y7
y6
y4
b1
b2
b3
b6
Int
100
75
50
25
0
rQ
b1
b2
b6 2+
b3
Prec 2+
y4
y6
y7
y8
0
100
200
300
400
500
600
700
800
900
1,000
1,100
1,200
m/z

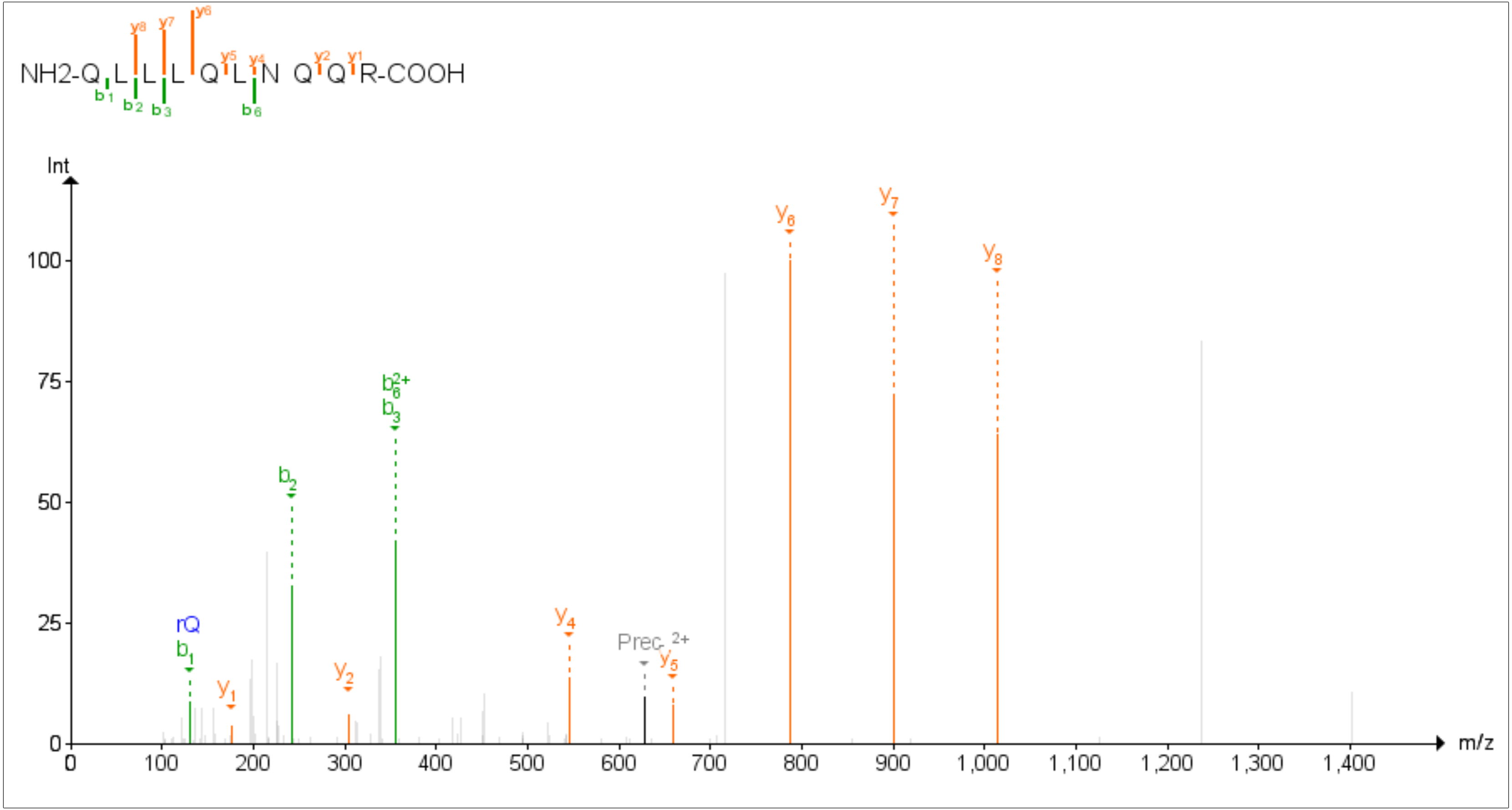

NH2-Q L L L Q L N Q Q R-COOH
y8
y7
y6
y5
y4
y2
y1
b1
b2
b3
b6
Int
m/z
rQ
Prec 2+

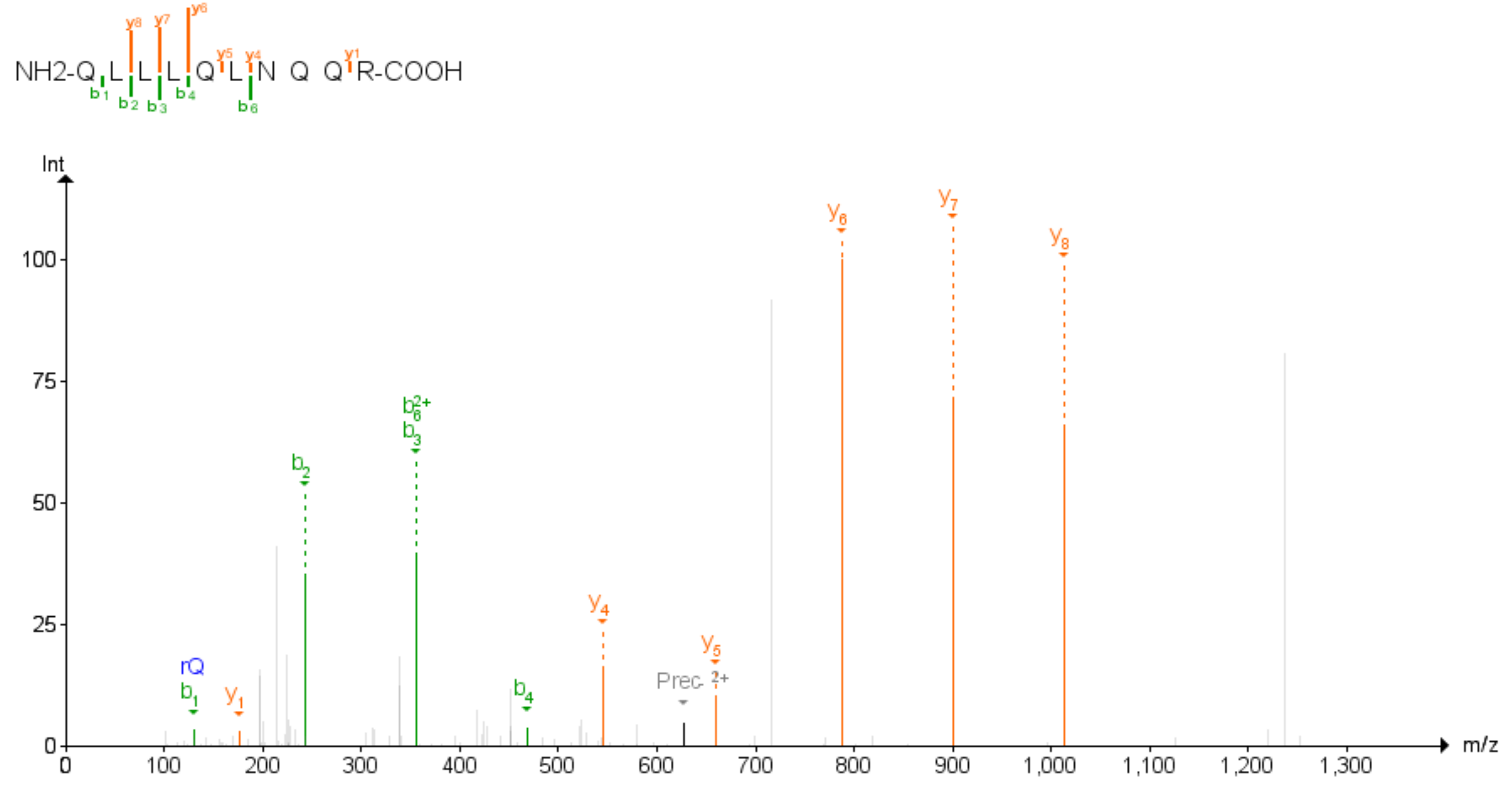

NH2-Q L L L Q L N Q Q R-COOH
y8 y7 y6 y5 y4 y1
b1 b2 b3 b4 b6
Int
100
75
50
25
0
rQ
b1
y1
b2
b6 2+
b3
b4
y4
Prec 2+
y5
y6
y7
y8
0 100 200 300 400 500 600 700 800 900 1,000 1,100 1,200 1,300
m/z

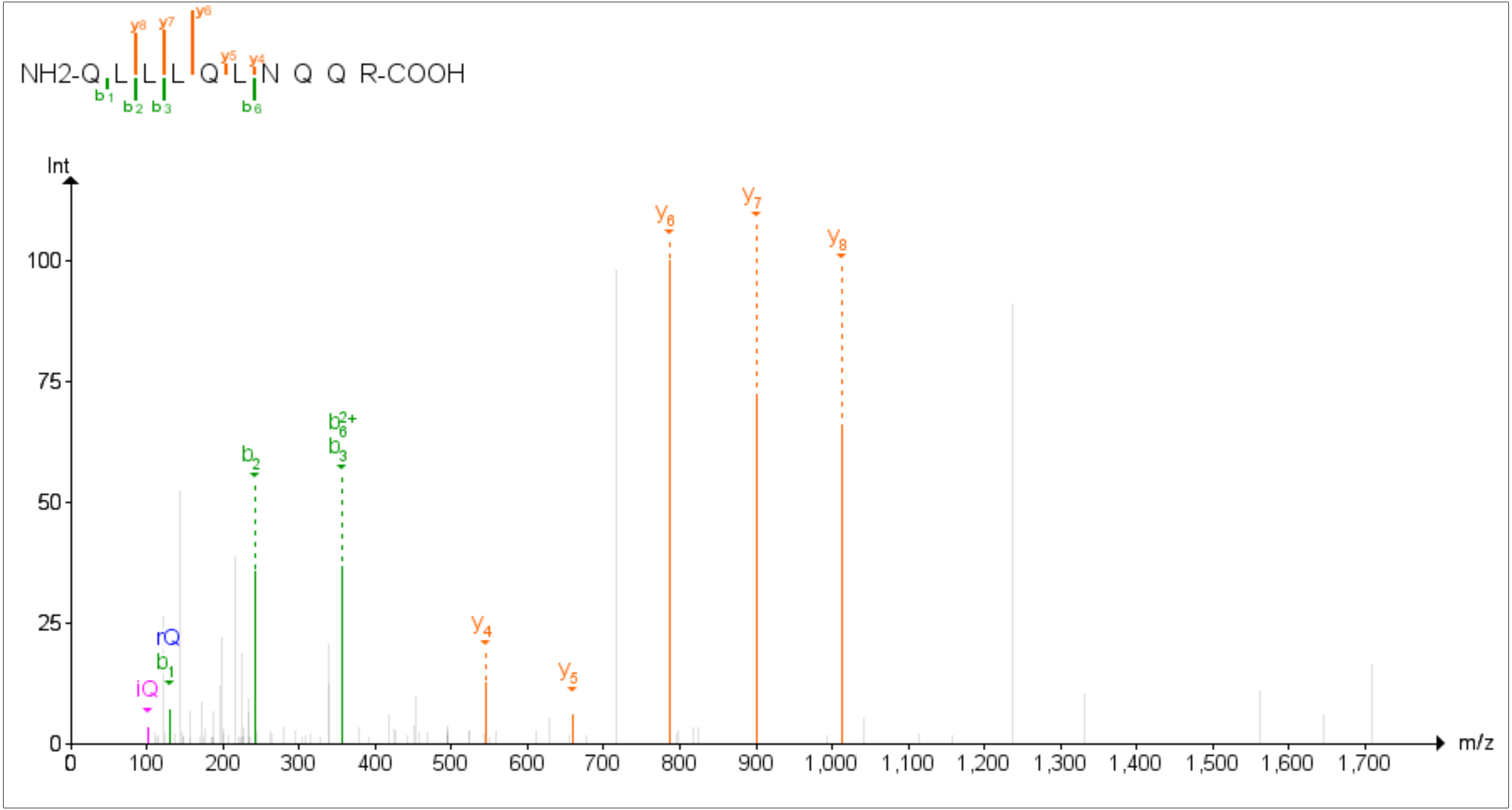

NH2-Q L L L Q L N Q Q R-COOH
y8 y7 y6 y5 y4
b1 b2 b3 b6
Int
100
75
50
25
0
y6
y7
y8
b$_6^{2+}$
b3
b2
y4
y5
rQ
b1
iQ
0 100 200 300 400 500 600 700 800 900 1,000 1,100 1,200 1,300 1,400 1,500 1,600 1,700
m/z

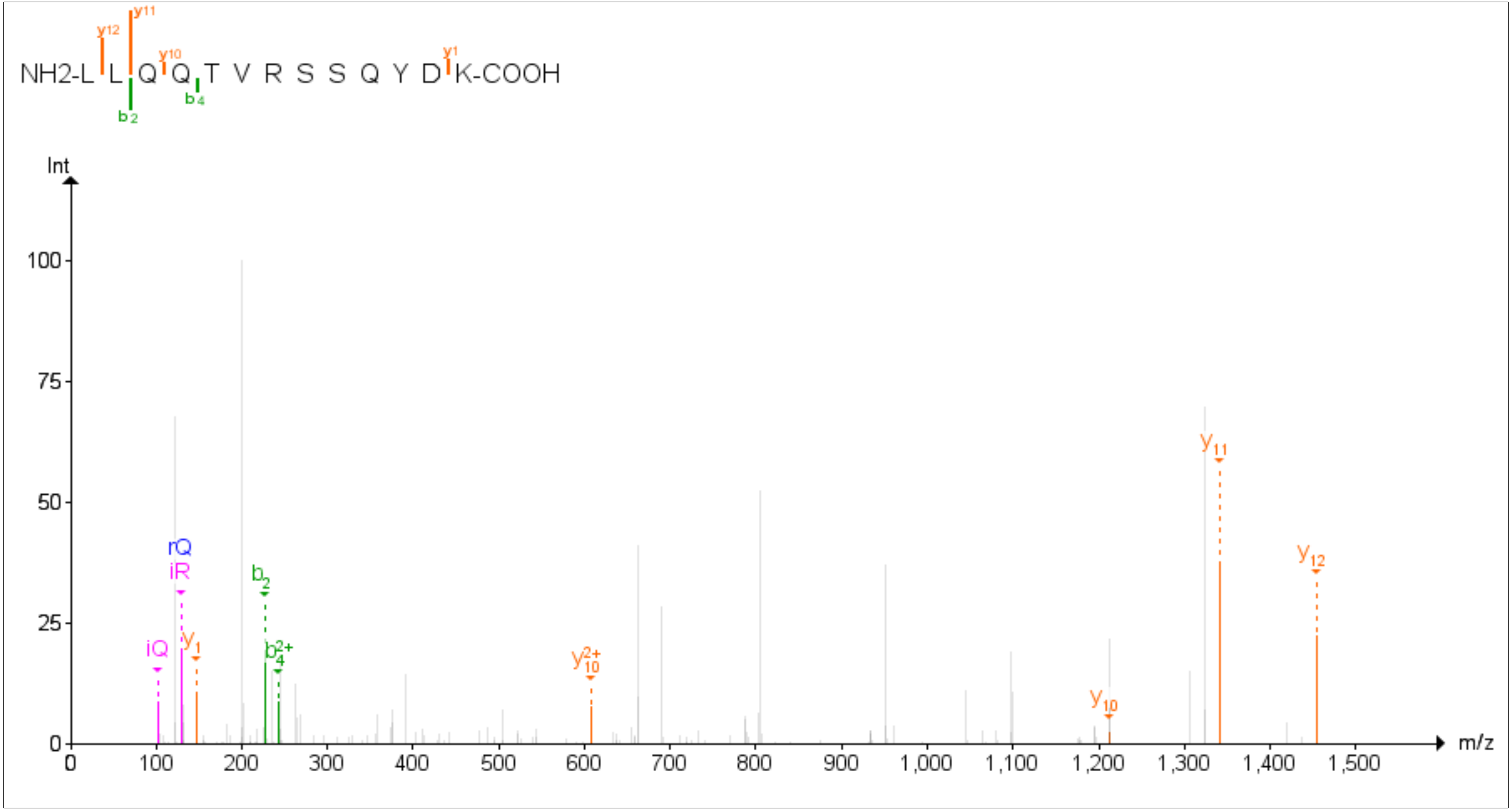

NH2-L L Q Q T V R S S Q Y D K-COOH
y12
y11
y10
y1
b2
b4
Int
100
75
50
25
0
rQ
iR
iQ
y1
b2
b4 2+
y10 2+
y10
y11
y12
0
100
200
300
400
500
600
700
800
900
1,000
1,100
1,200
1,300
1,400
1,500
m/z

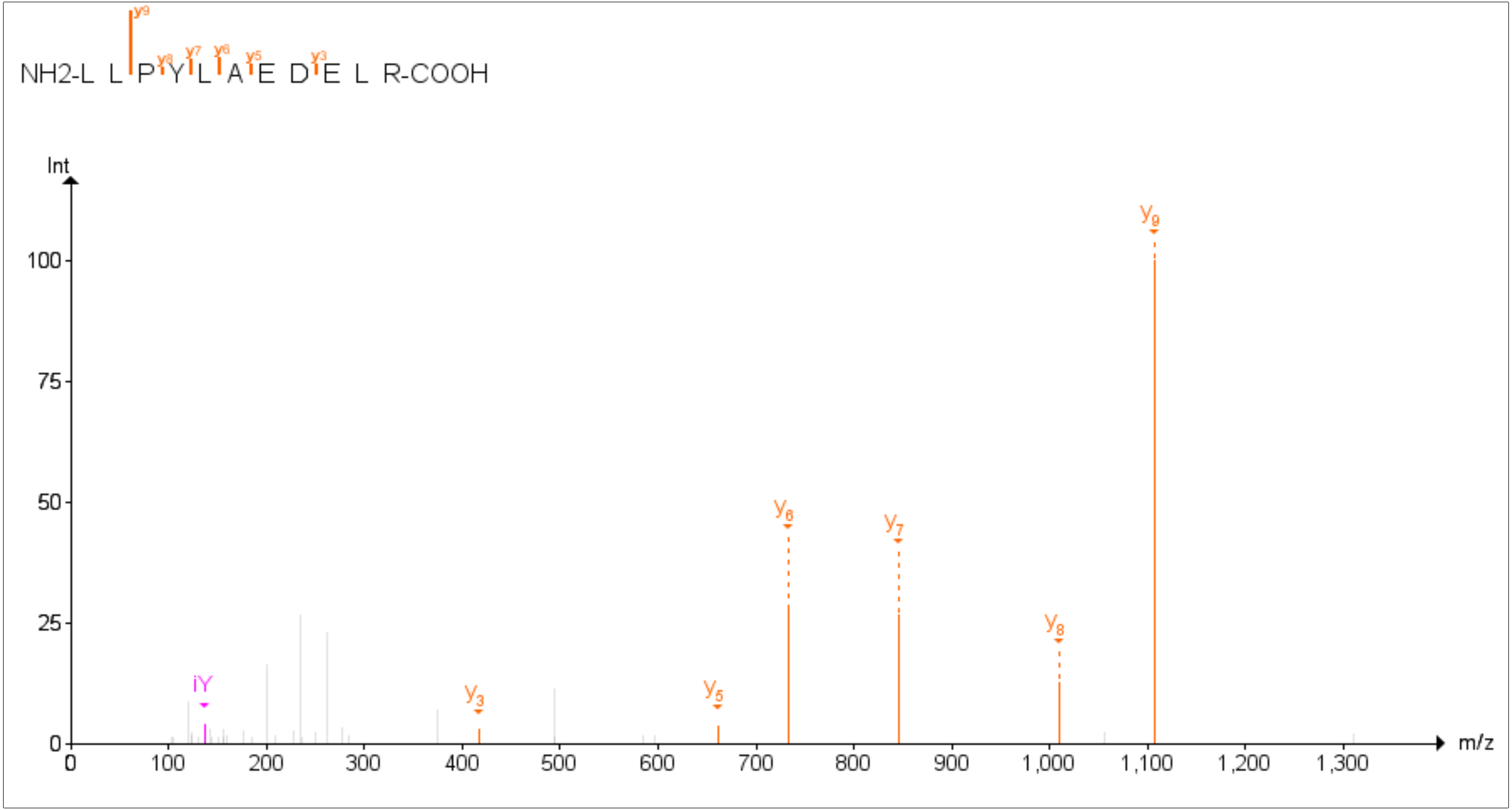

NH2-L L P Y L A E D E L R-COOH
y9
y8
y7
y6
y5
y3
Int
100
75
50
25
0
iY
y3
y5
y6
y7
y8
y9
0
100
200
300
400
500
600
700
800
900
1,000
1,100
1,200
1,300
m/z

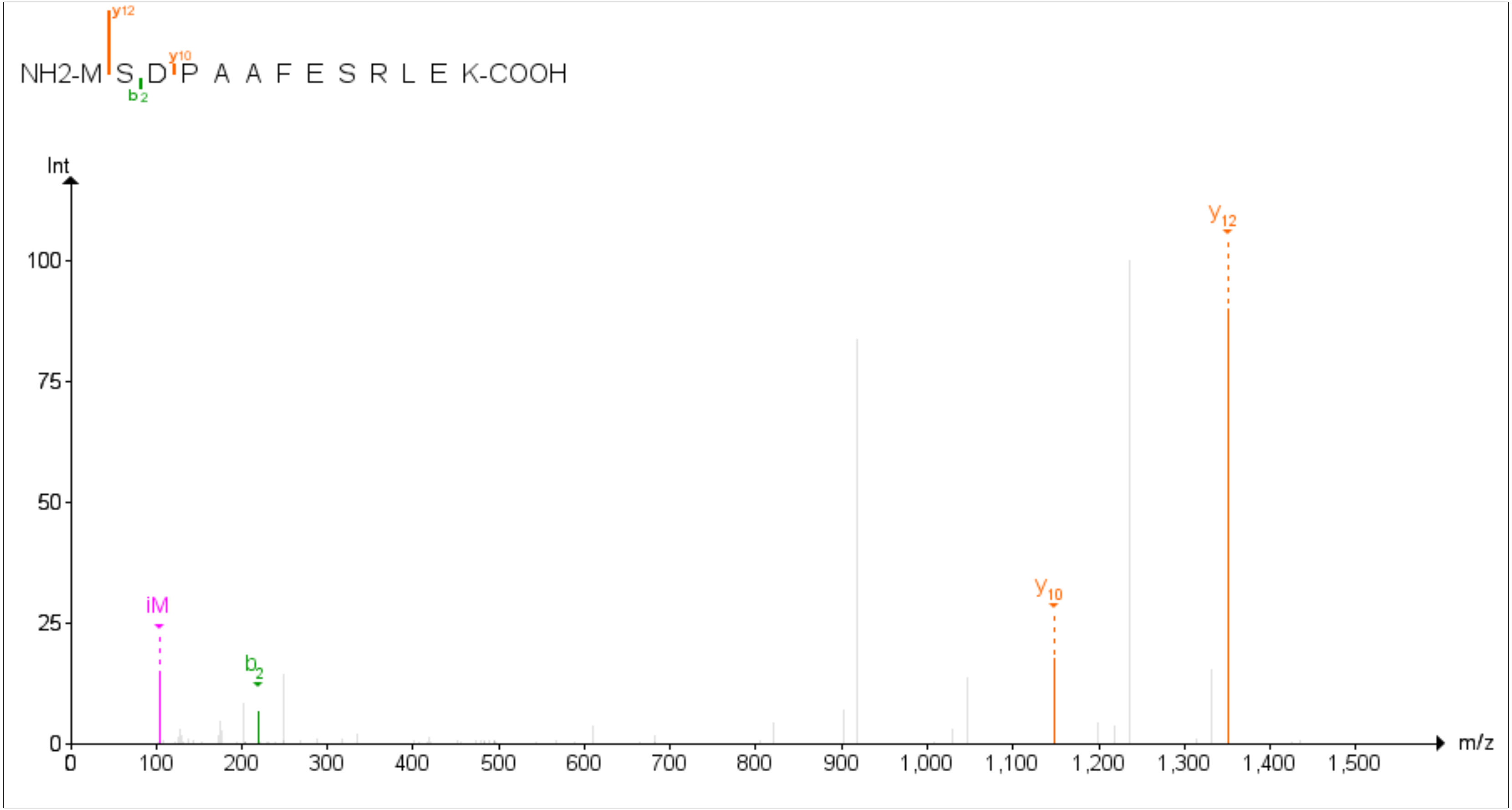

NH2-M S D P A A F E S R L E K-COOH
y12
y10
b2
Int
100
75
50
25
0
iM
b2
y10
y12
0
100
200
300
400
500
600
700
800
900
1,000
1,100
1,200
1,300
1,400
1,500
m/z

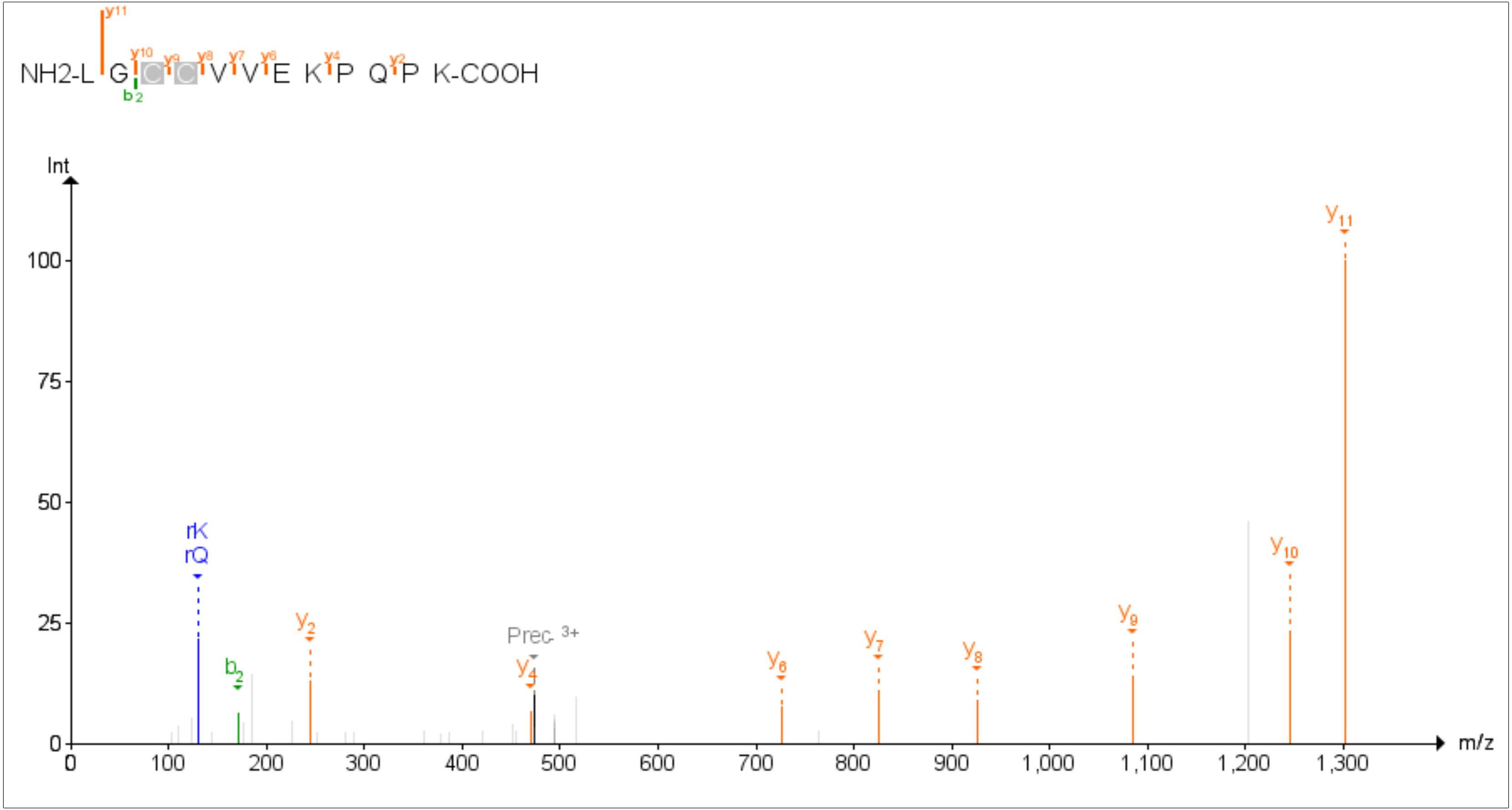

NH2-L G C C V V E K P Q P K-COOH
y11
y10
y9
y8
y7
y6
y4
y2
b2
Int
100
75
50
25
0
rK
rQ
b2
y2
Prec 3+
y4
y6
y7
y8
y9
y10
y11
0
100
200
300
400
500
600
700
800
900
1,000
1,100
1,200
1,300
m/z

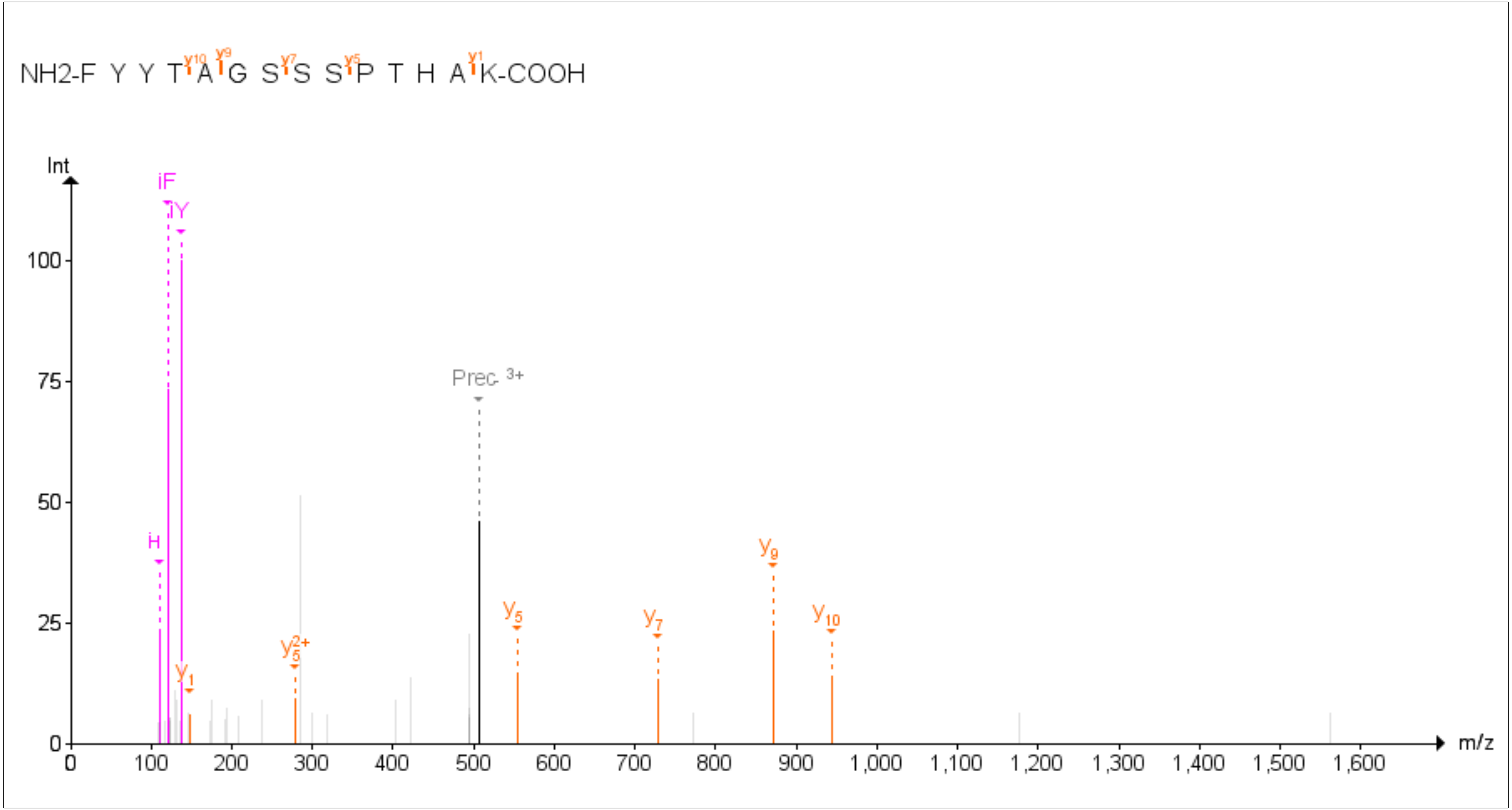

NH2-F Y Y T A G S S S P T H A K-COOH
y10
y9
y7
y5
y1
Int
iF
iY
iH
Prec 3+
y1
y5 2+
y5
y7
y9
y10
0
25
50
75
100
0
100
200
300
400
500
600
700
800
900
1,000
1,100
1,200
1,300
1,400
1,500
1,600
m/z

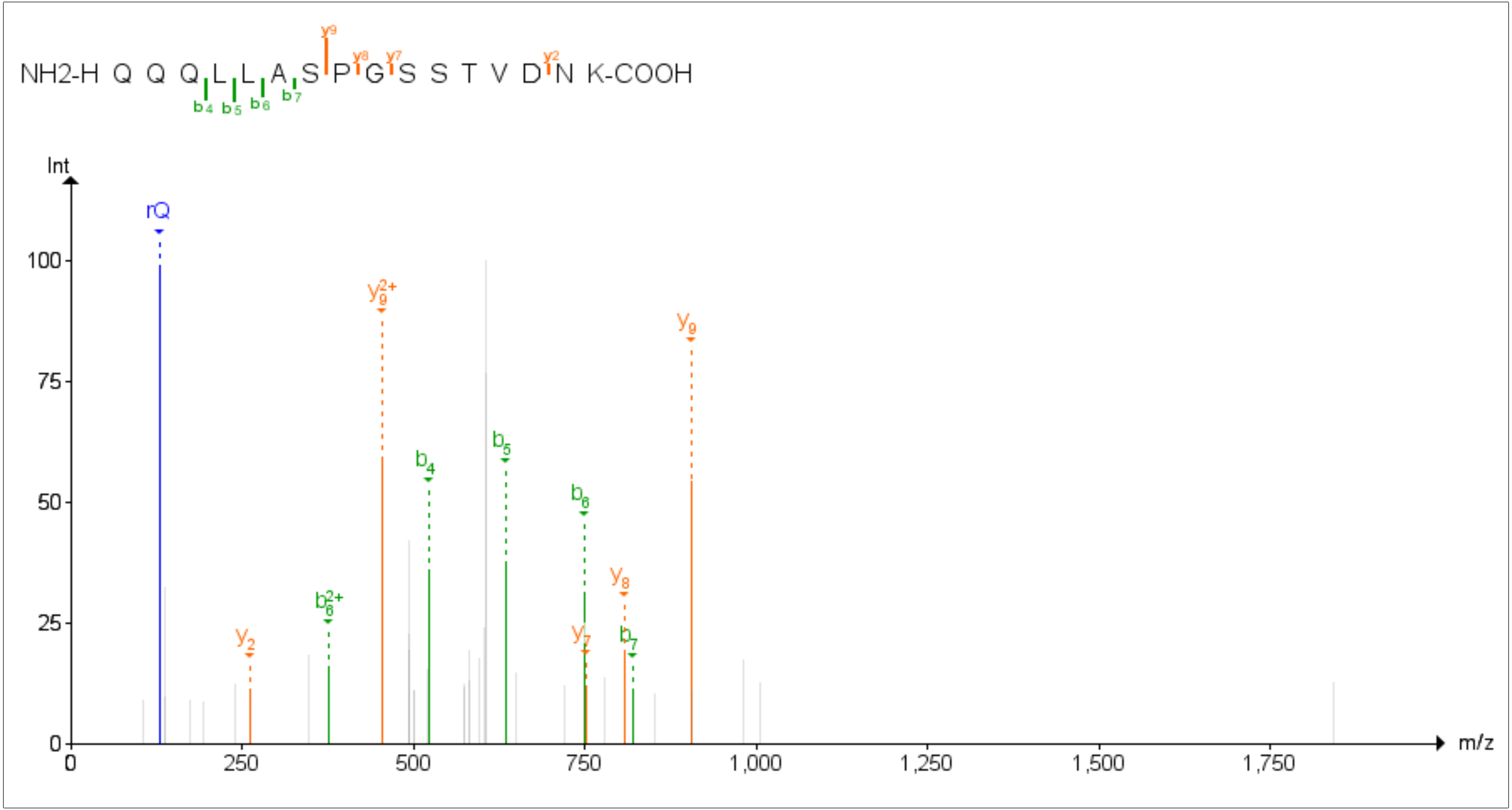

NH2-H Q Q Q L L A S P G S S T V D N K-COOH
b4 b5 b6 b7
y9 y8 y7 y2
Int
rQ
y9 2+
y9
b4
b5
b6
y8
y7
b7
b6 2+
y2
100
75
50
25
0
0
250
500
750
1,000
1,250
1,500
1,750
m/z

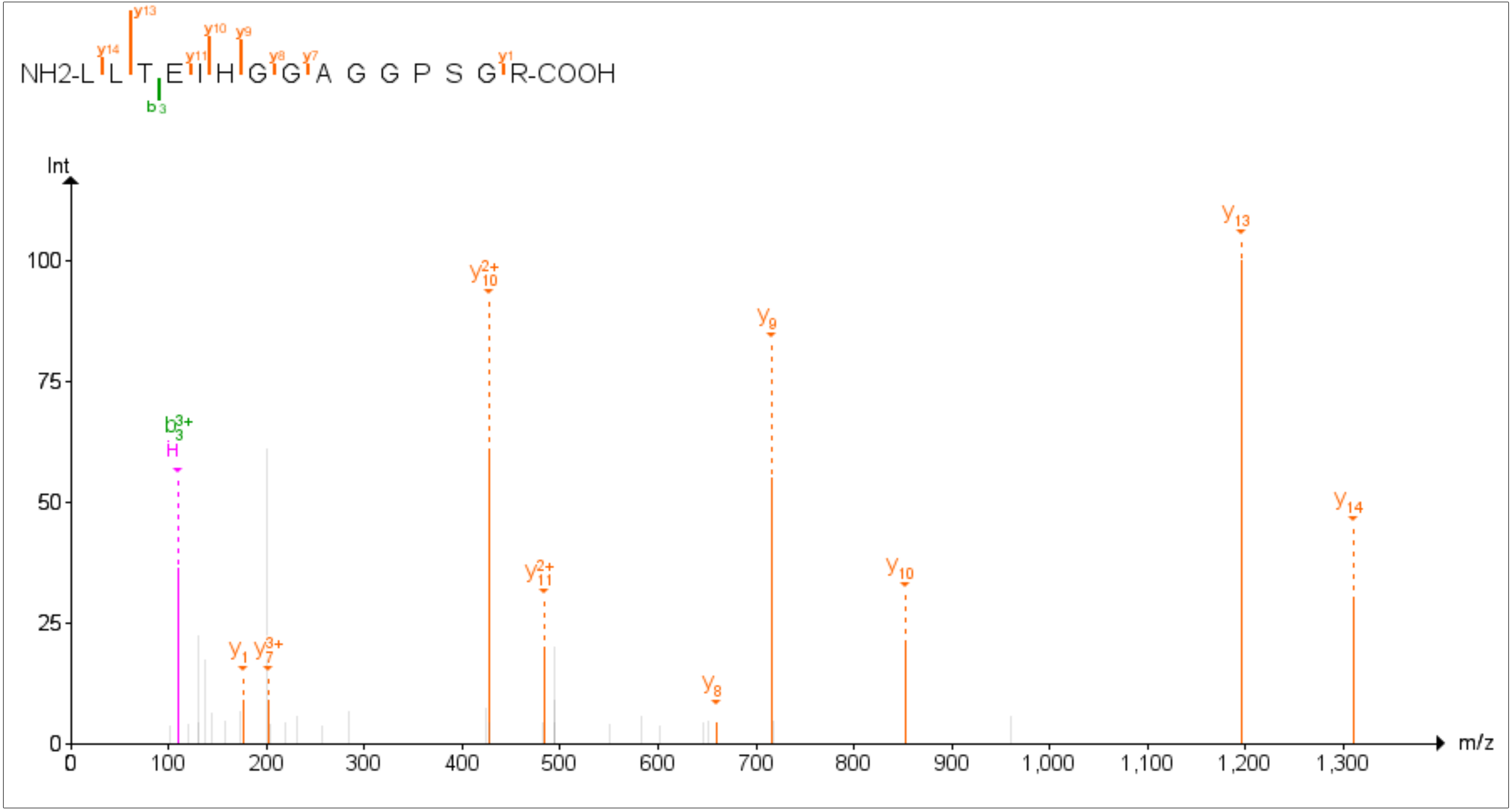

NH2-L L T E I H G G A G G P S G R-COOH
y14 y13 y11 y10 y9 y8 y7 y1
b3
Int
m/z
y13
y10 2+
y9
b3 3+
H
y14
y11 2+
y10
y1
y7 3+
y8
0
25
50
75
100
0
100
200
300
400
500
600
700
800
900
1,000
1,100
1,200
1,300

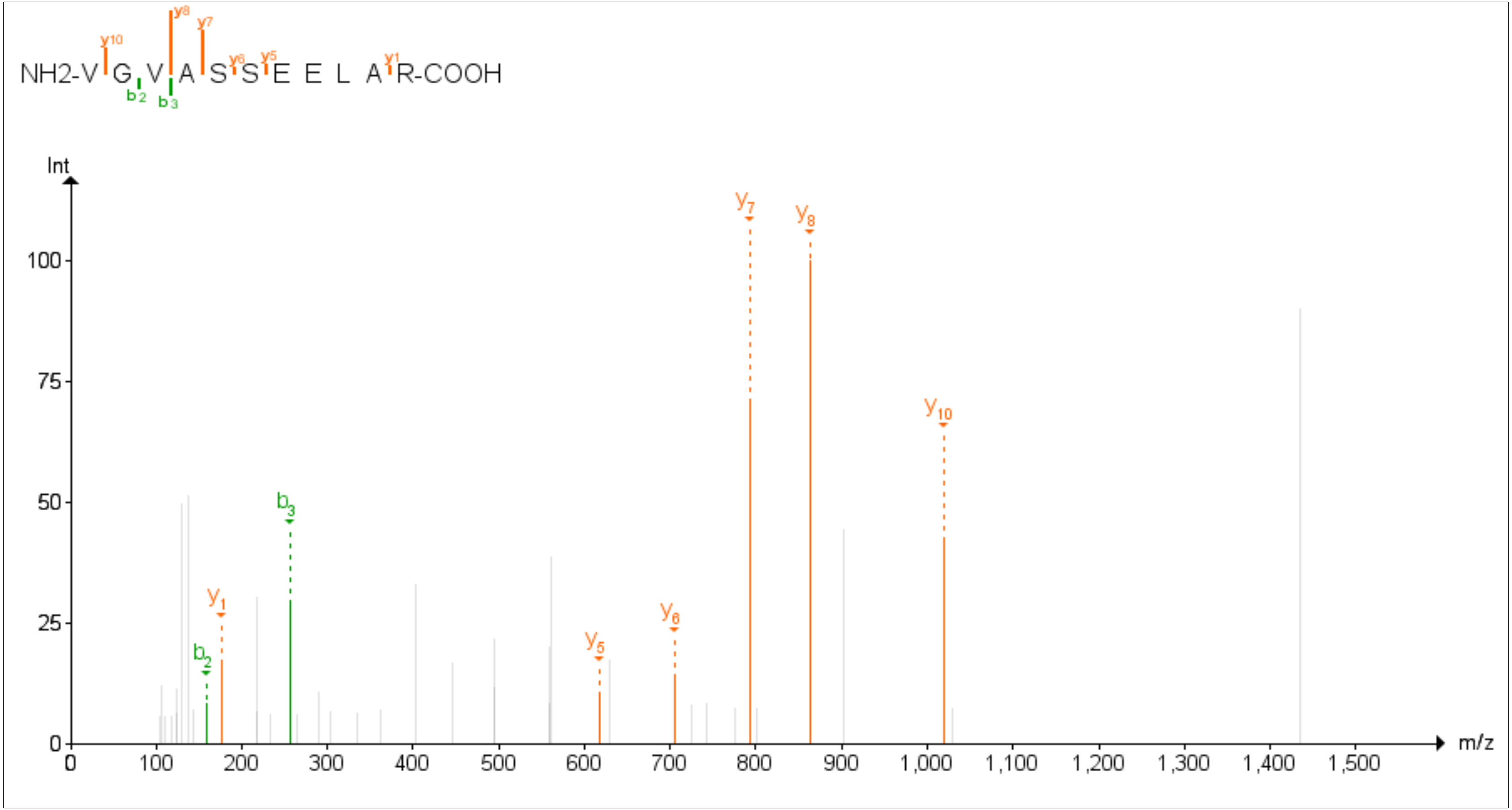

NH2-V G V A S S E E L A R-COOH
y10
y8
y7
y6
y5
y1
b2
b3
Int
100
75
50
25
0
m/z
0
100
200
300
400
500
600
700
800
900
1,000
1,100
1,200
1,300
1,400
1,500
y7
y8
y10
b3
y1
b2
y5
y6

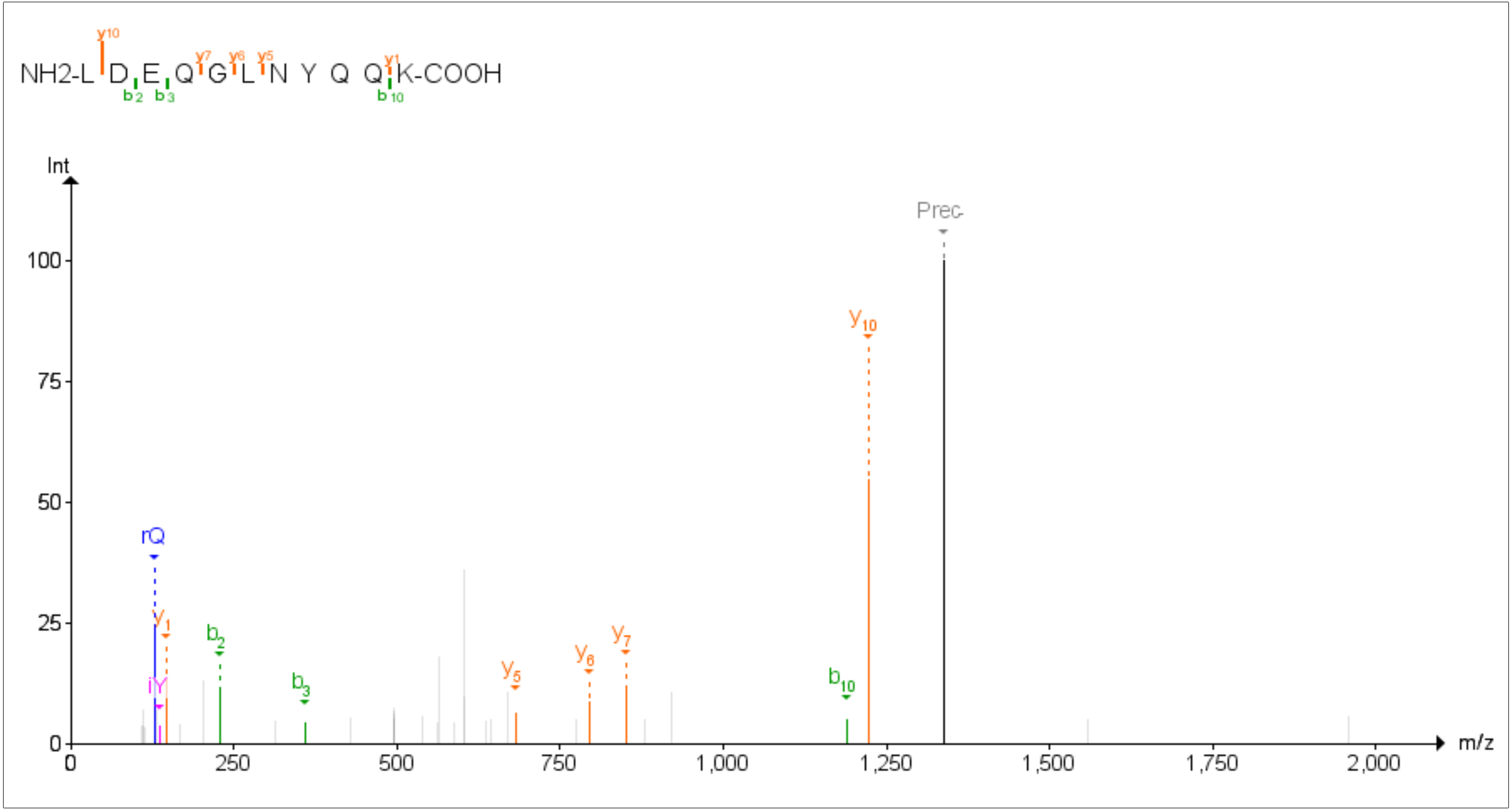

NH2-L D E Q G L N Y Q Q K-COOH
y10
y7
y6
y5
y1
b2
b3
b10
Int
100
75
50
25
0
Prec
y10
rQ
y1
iY
b2
b3
y5
y6
y7
b10
0
250
500
750
1,000
1,250
1,500
1,750
2,000
m/z

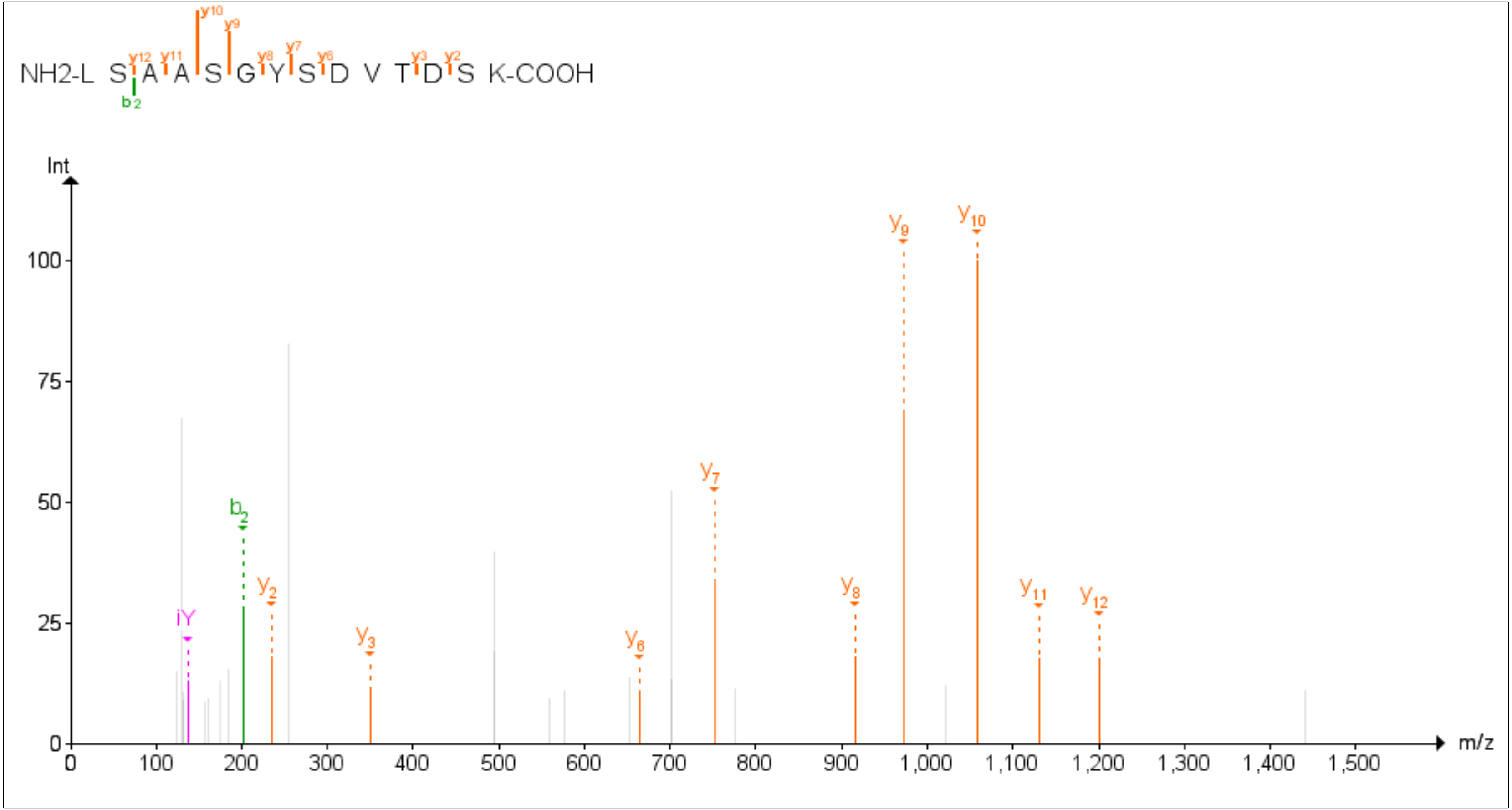

NH2-L S A A S G Y S D V T D S K-COOH
y12 y11 y10 y9 y8 y7 y6 y3 y2
b2
Int
100
75
50
25
0
m/z
0 100 200 300 400 500 600 700 800 900 1,000 1,100 1,200 1,300 1,400 1,500
iY
b2
y2
y3
y6
y7
y8
y9
y10
y11
y12

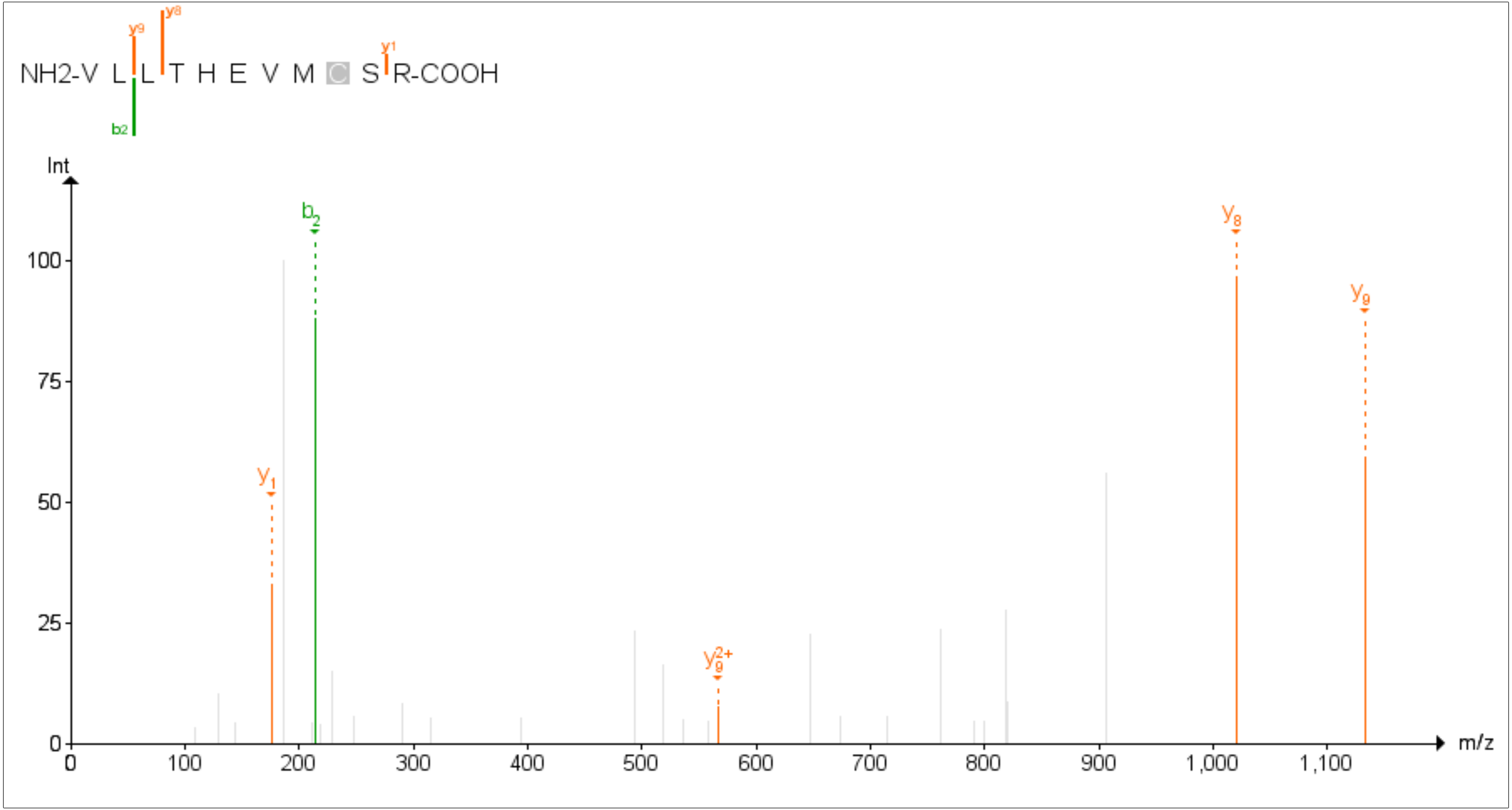
NH2-V L L T H E V M C S R-COOH
y9
y8
y1
b2
Int
100
75
50
25
0
b2
y8
y9
y1
y9 2+
0
100
200
300
400
500
600
700
800
900
1,000
1,100
m/z

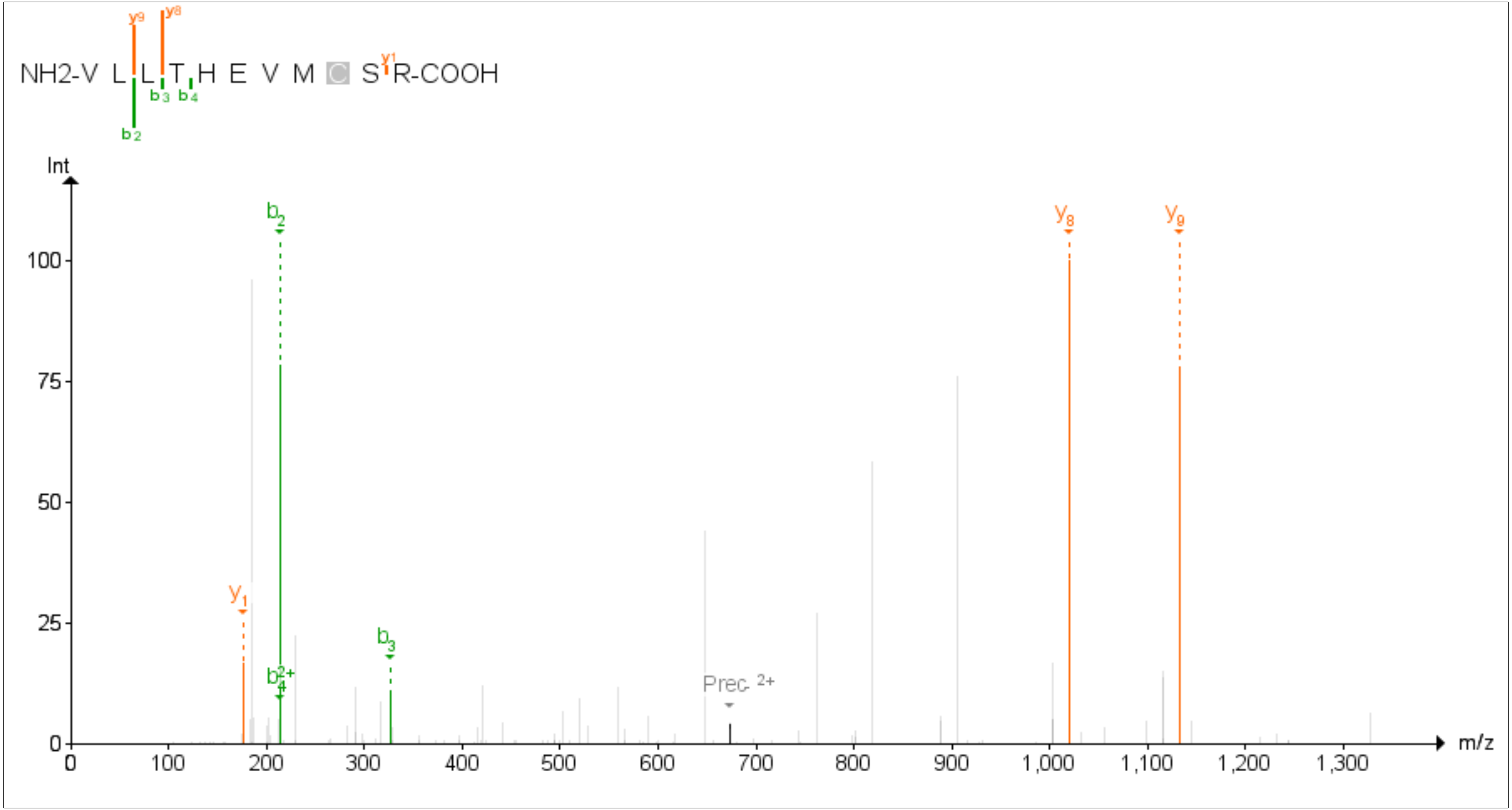

NH2-V L L T H E V M C S R-COOH
y9
y8
y1
b2
b3
b4
Int
100
75
50
25
0
b2
y8
y9
y1
b4 2+
b3
Prec 2+
m/z
0
100
200
300
400
500
600
700
800
900
1,000
1,100
1,200
1,300

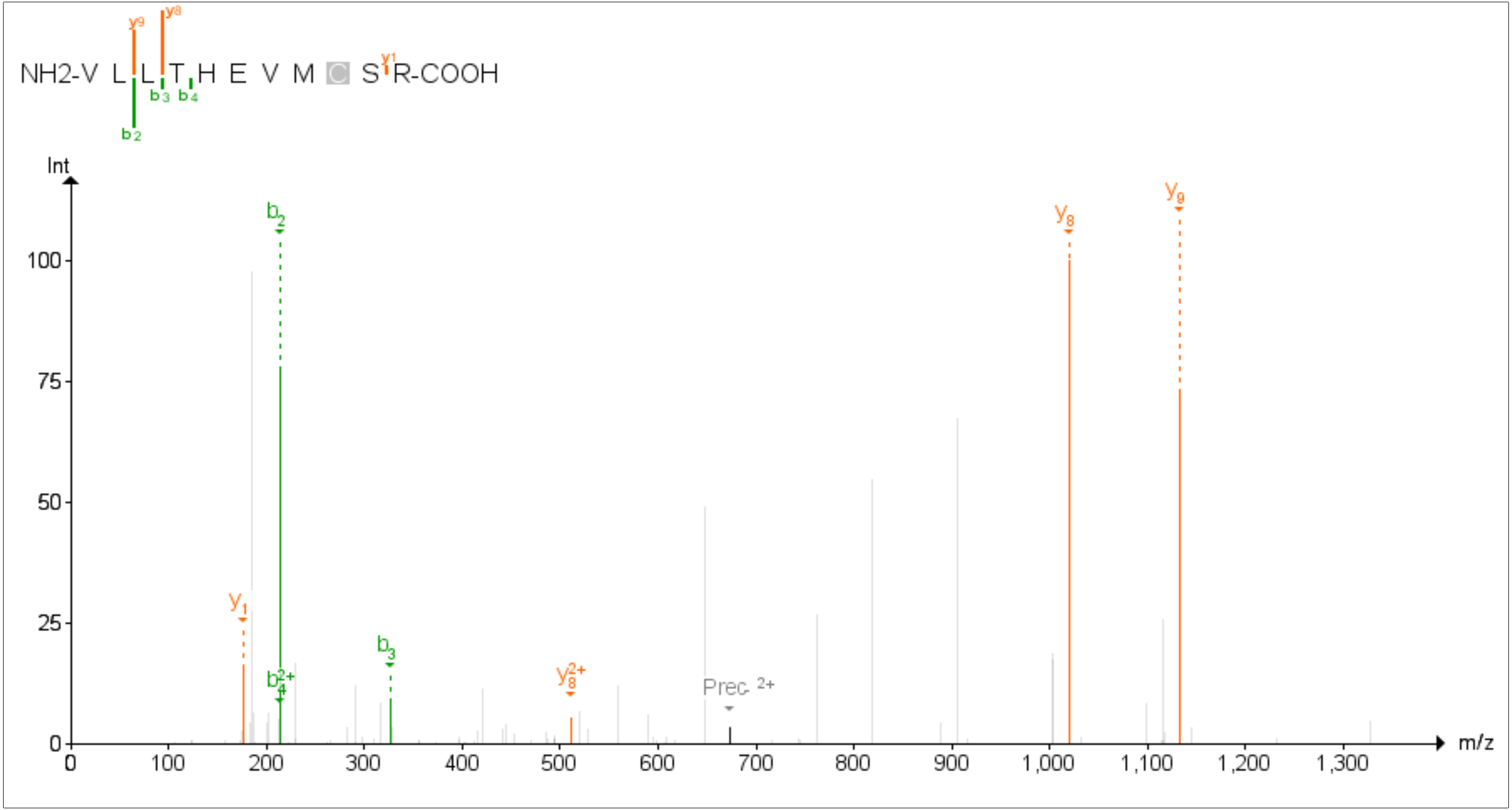

NH2-V L L T H E V M C S R-COOH
y9
y8
y1
b2
b3
b4
Int
100
75
50
25
0
b2
y8
y9
y1
b3
b_4^{2+}
y_8^{2+}
Prec. 2+
0
100
200
300
400
500
600
700
800
900
1,000
1,100
1,200
1,300
m/z

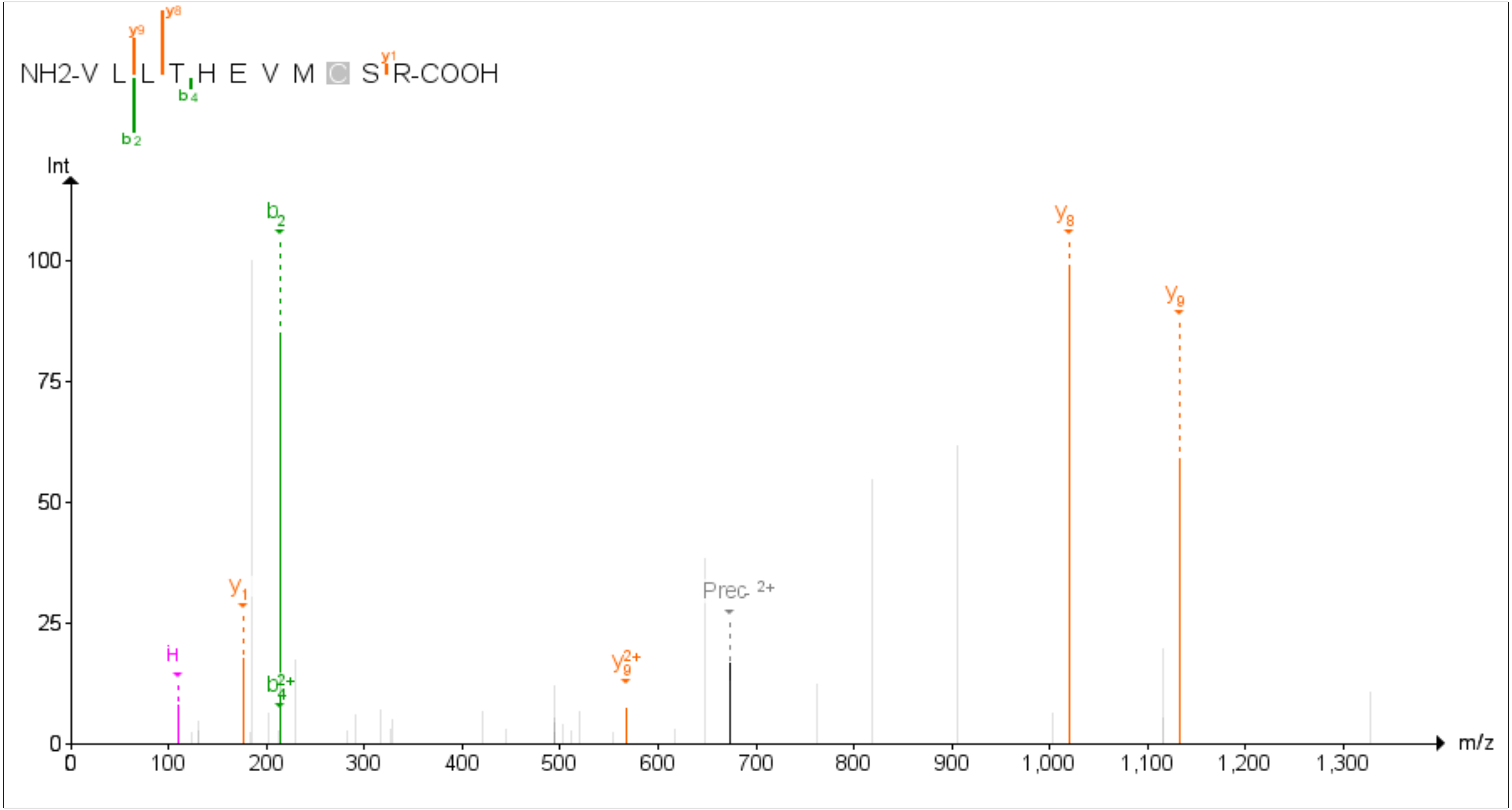

NH2-V L L T H E V M C S R-COOH
y9
y8
y1
b2
b4
Int
m/z
b2
y8
y9
y1
H
b4 2+
y9 2+
Prec 2+
100
75
50
25
0
0
100
200
300
400
500
600
700
800
900
1,000
1,100
1,200
1,300

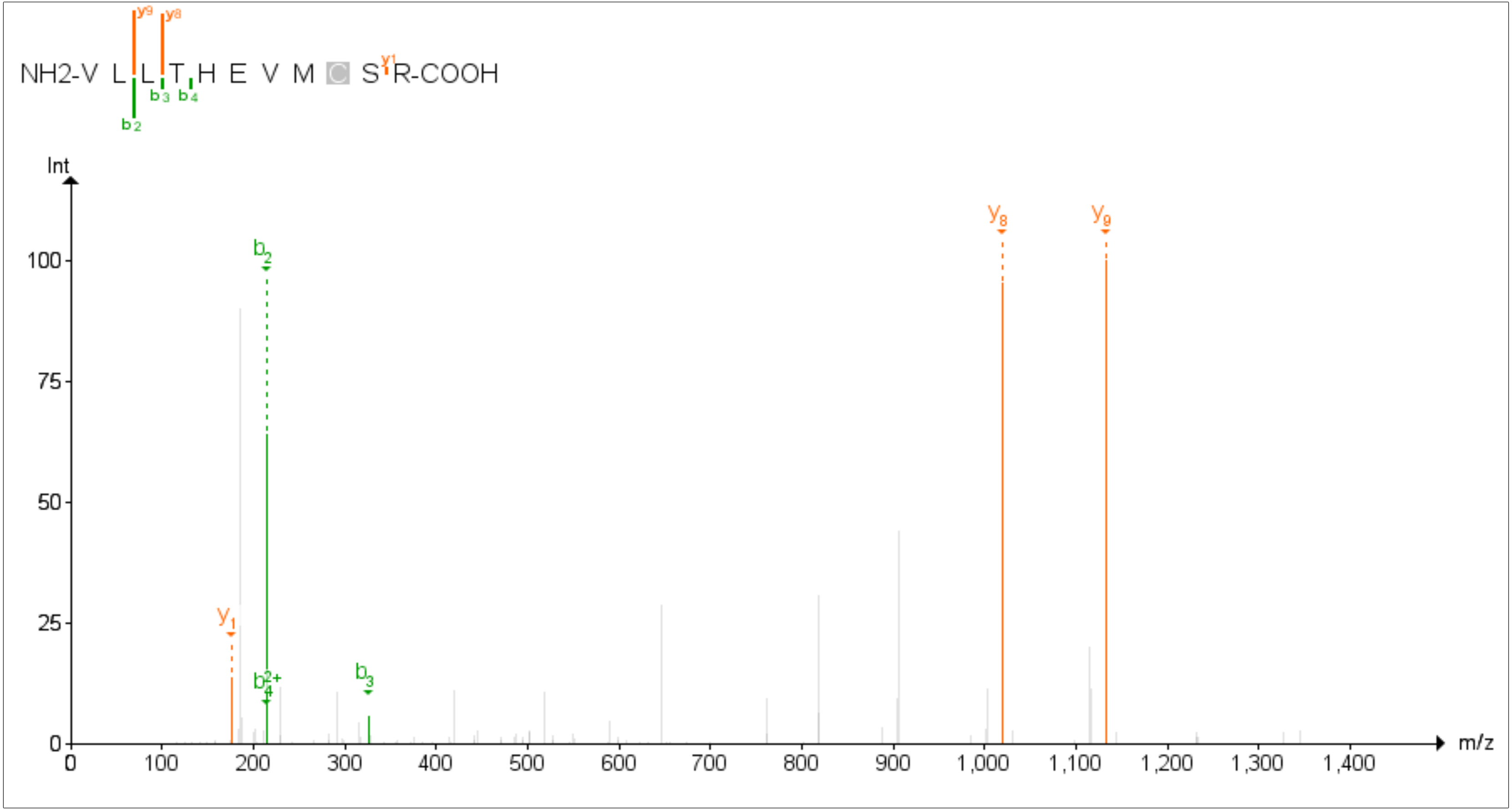

NH2-V L L T H E V M C S R-COOH
y9
y8
y1
b2
b3
b4
Int
100
75
50
25
0
y8
y9
b2
y1
b4 2+
b3
0
100
200
300
400
500
600
700
800
900
1,000
1,100
1,200
1,300
1,400
m/z

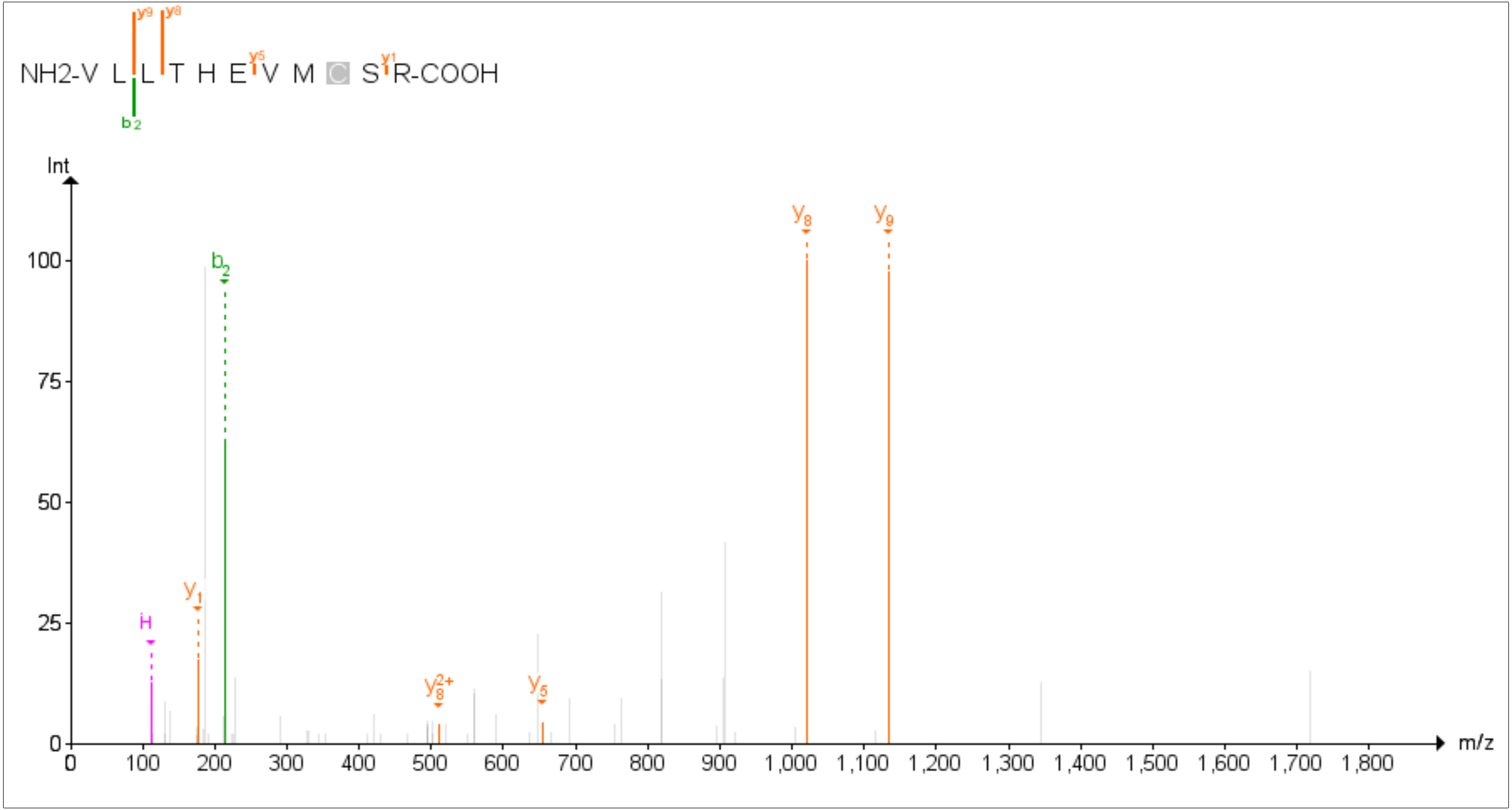

NH2-V L L T H E V M C S R-COOH
y9
y8
y5
y1
b2
Int
100
75
50
25
0
m/z
0
100
200
300
400
500
600
700
800
900
1,000
1,100
1,200
1,300
1,400
1,500
1,600
1,700
1,800
y8
y9
b2
y1
H
y8 2+
y5

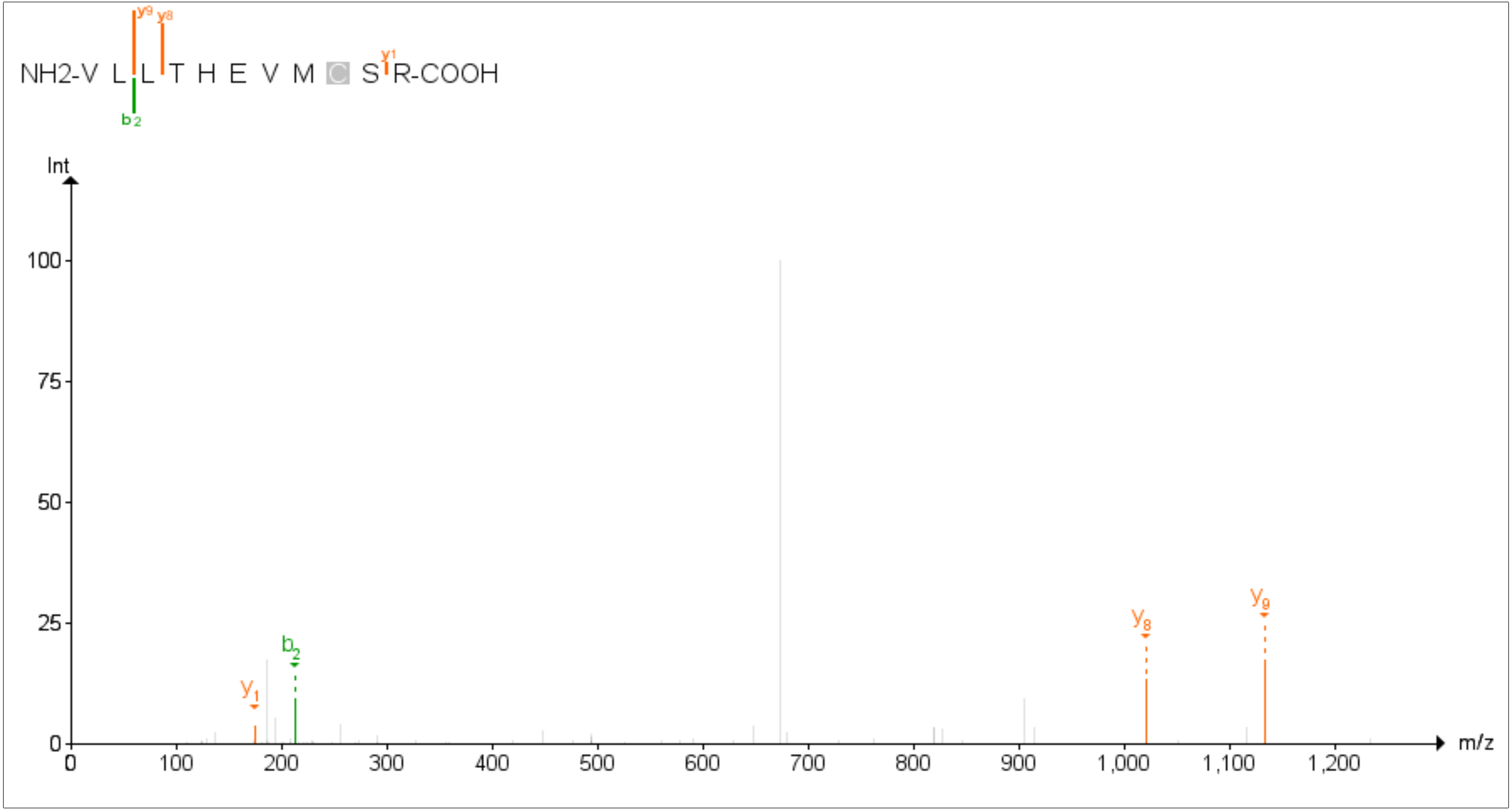
NH2-V L L T H E V M C S R-COOH
y9
y8
y1
b2
Int
100
75
50
25
0
0
100
200
300
400
500
600
700
800
900
1,000
1,100
1,200
m/z
y1
b2
y8
y9

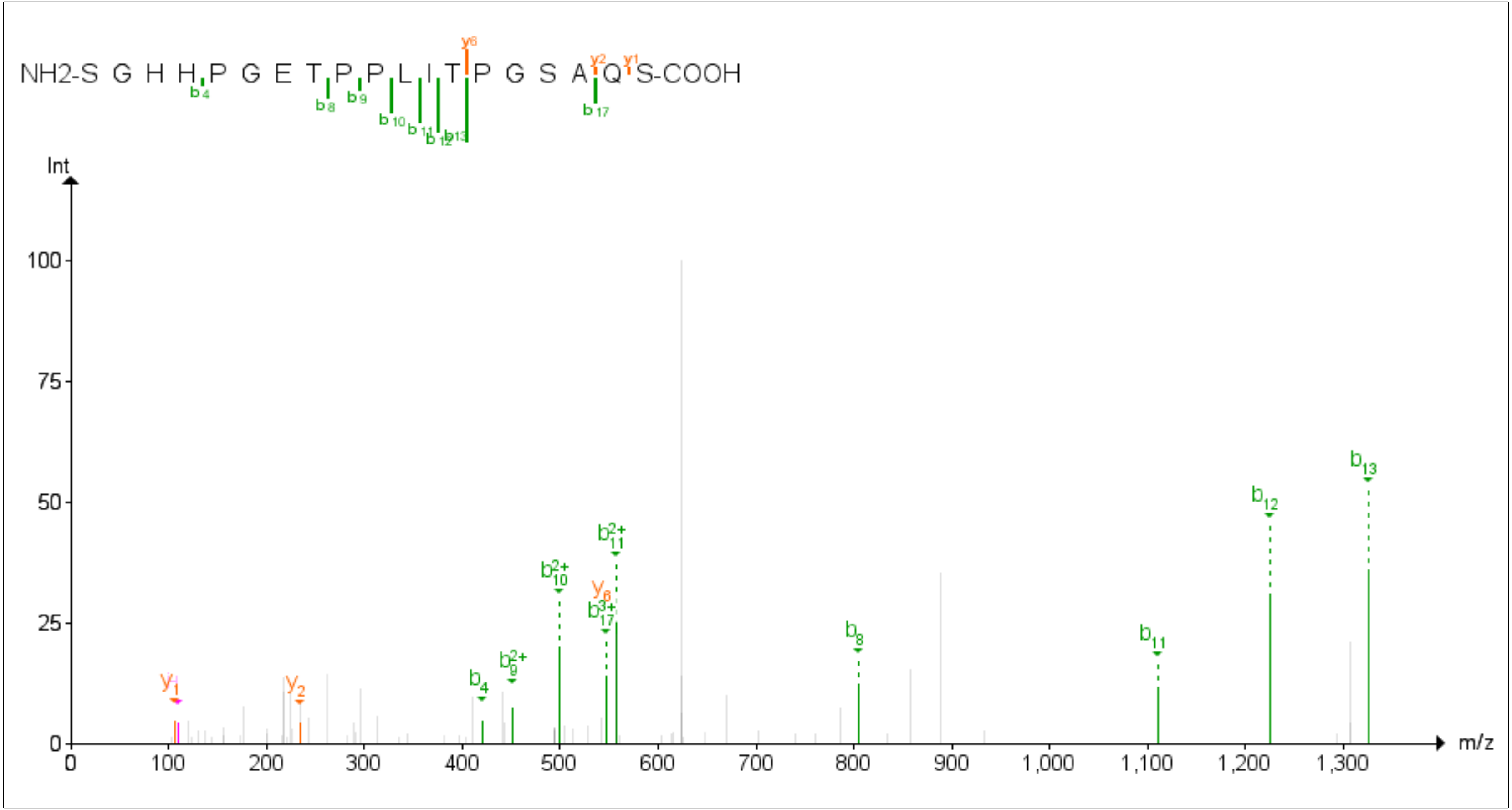

NH2-S G H H P G E T P P L I T P G S A Q S-COOH
Int
m/z
b4
b8
b9
b10
b11
b12
b13
b17
y6
y2
y1

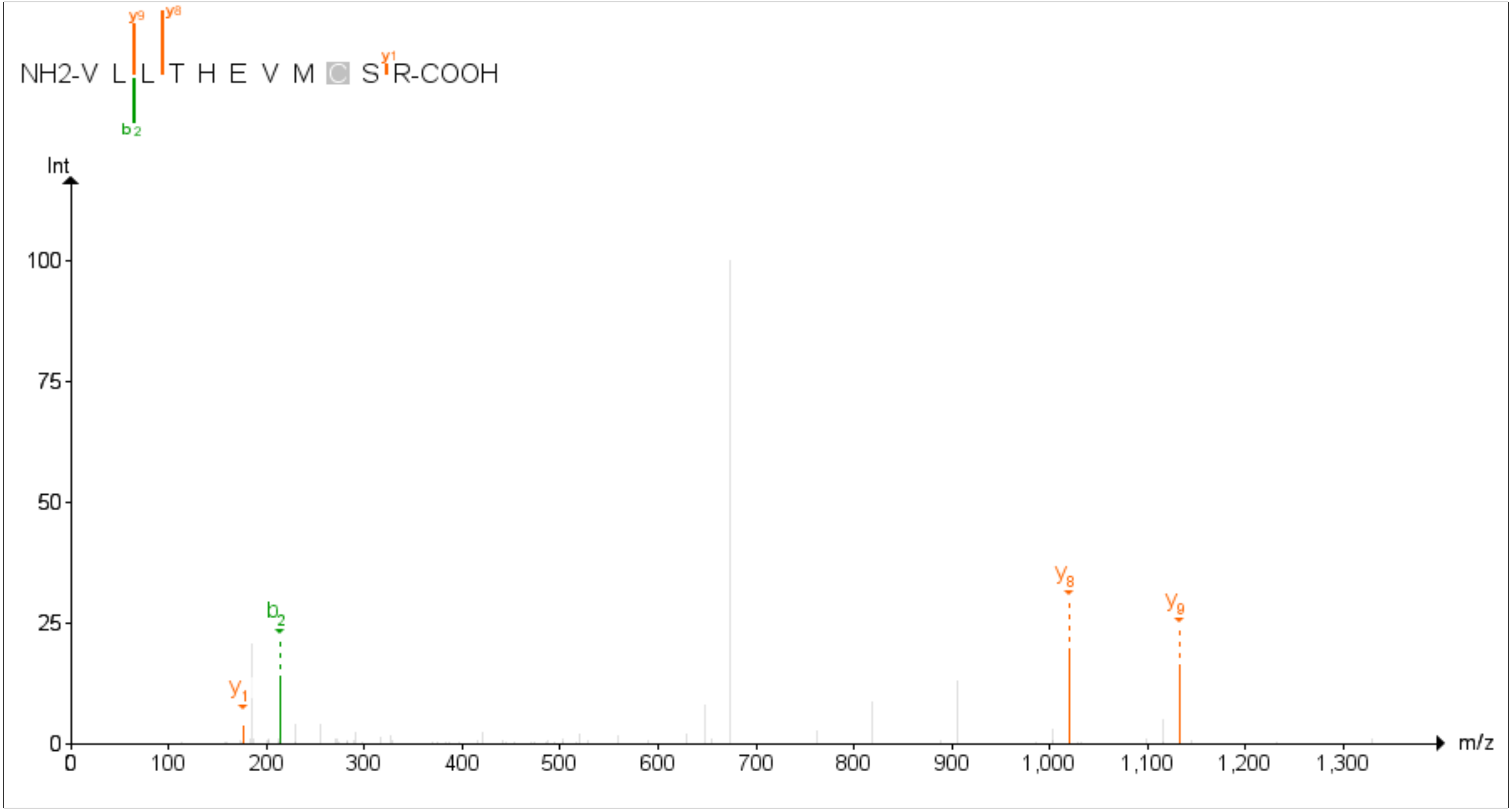

NH2-V L L T H E V M C S R-COOH
y9
y8
y1
b2
Int
100
75
50
25
0
m/z
0
100
200
300
400
500
600
700
800
900
1,000
1,100
1,200
1,300
y1
b2
y8
y9

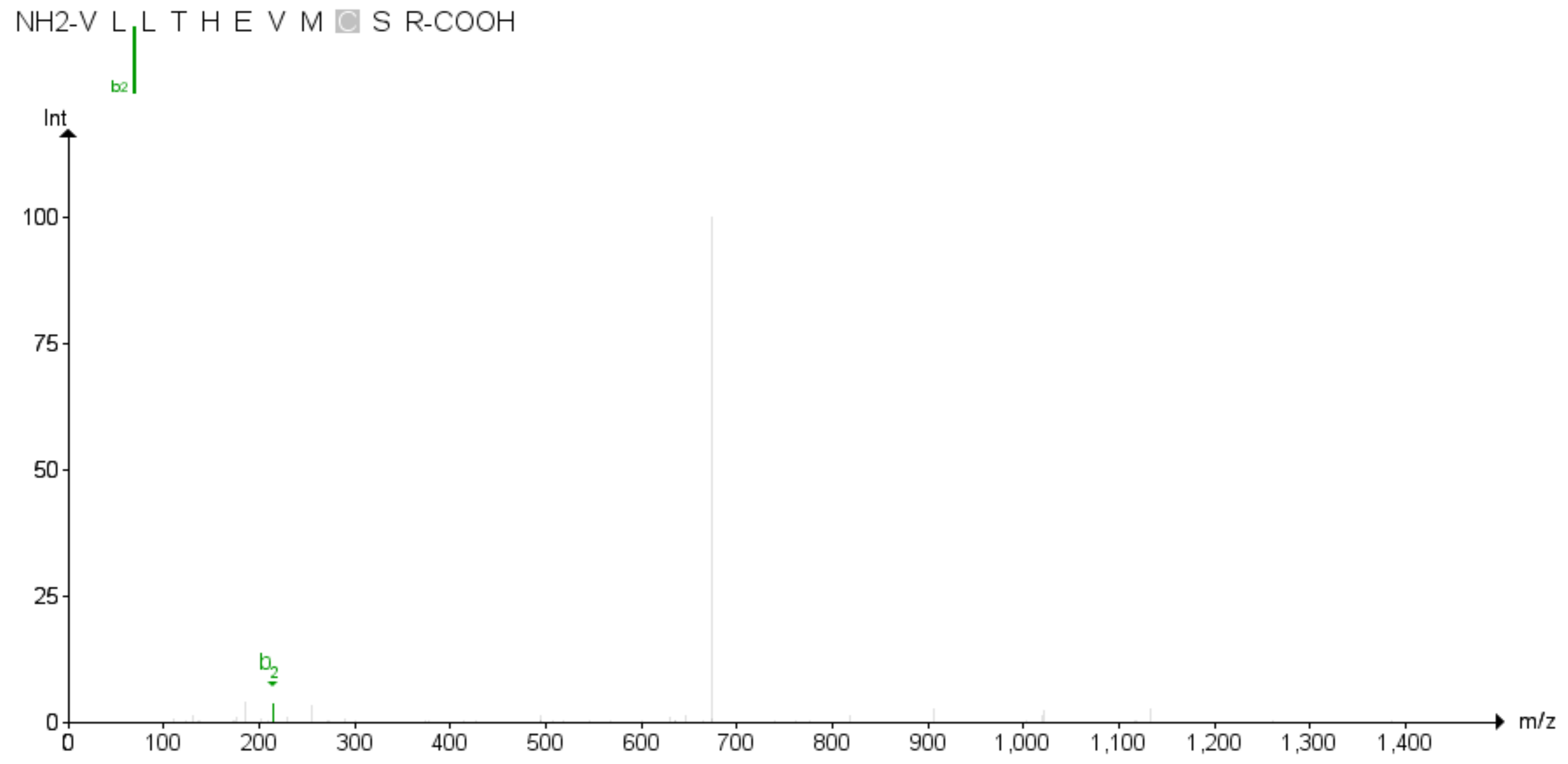
NH2-V L L T H E V M C S R-COOH
b2
Int
100
75
50
25
0
b2
0
100
200
300
400
500
600
700
800
900
1,000
1,100
1,200
1,300
1,400
m/z

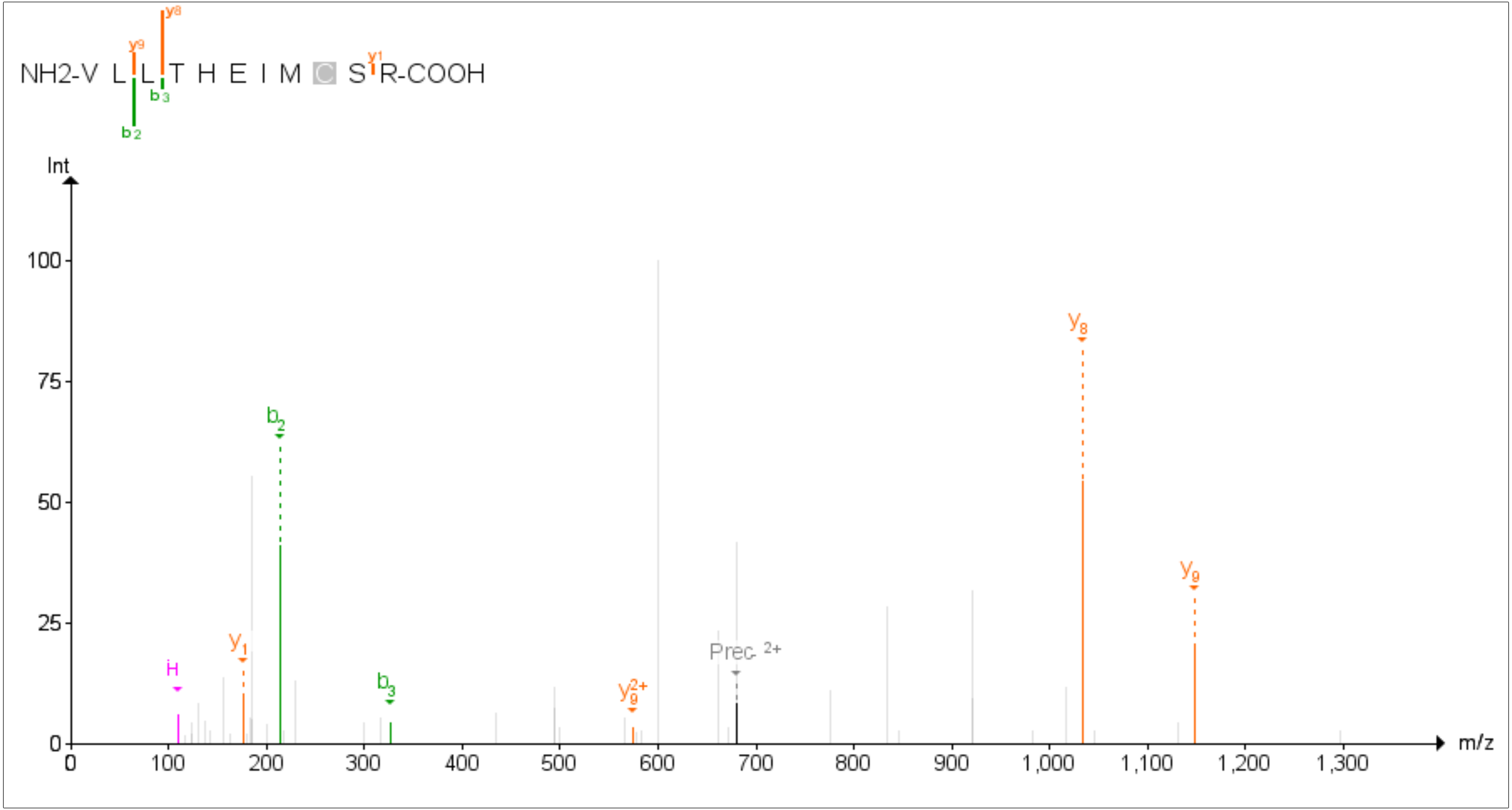

NH2-V L L T H E I M C S R-COOH
y9
y8
y1
b2
b3
Int
100
75
50
25
0
y8
b2
y9
y1
H
b3
y9 2+
Prec 2+
0
100
200
300
400
500
600
700
800
900
1,000
1,100
1,200
1,300
m/z

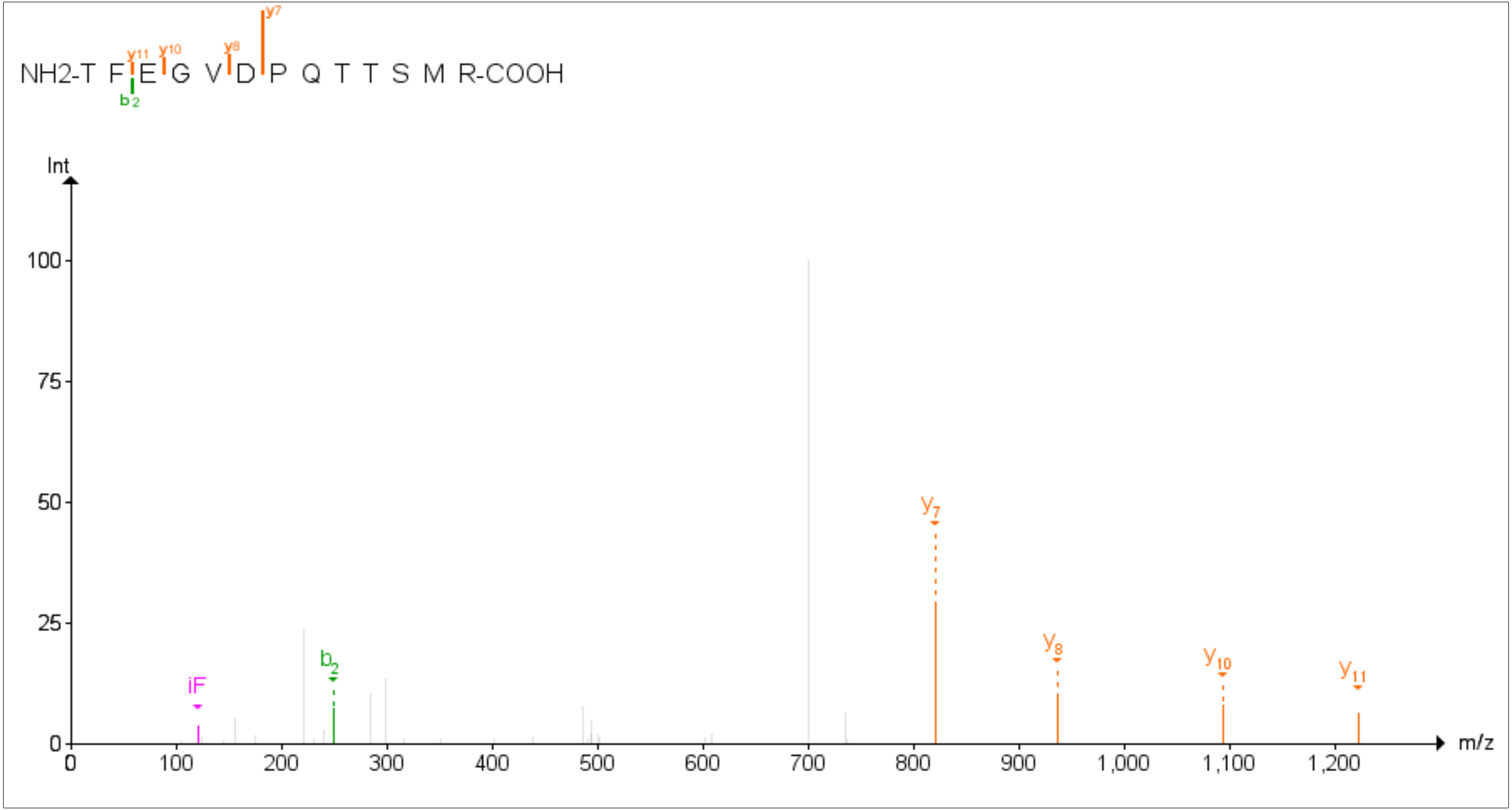

NH2-T F E G V D P Q T T S M R-COOH
y11
y10
y8
y7
b2
Int
100
75
50
25
0
iF
b2
y7
y8
y10
y11
0
100
200
300
400
500
600
700
800
900
1,000
1,100
1,200
m/z

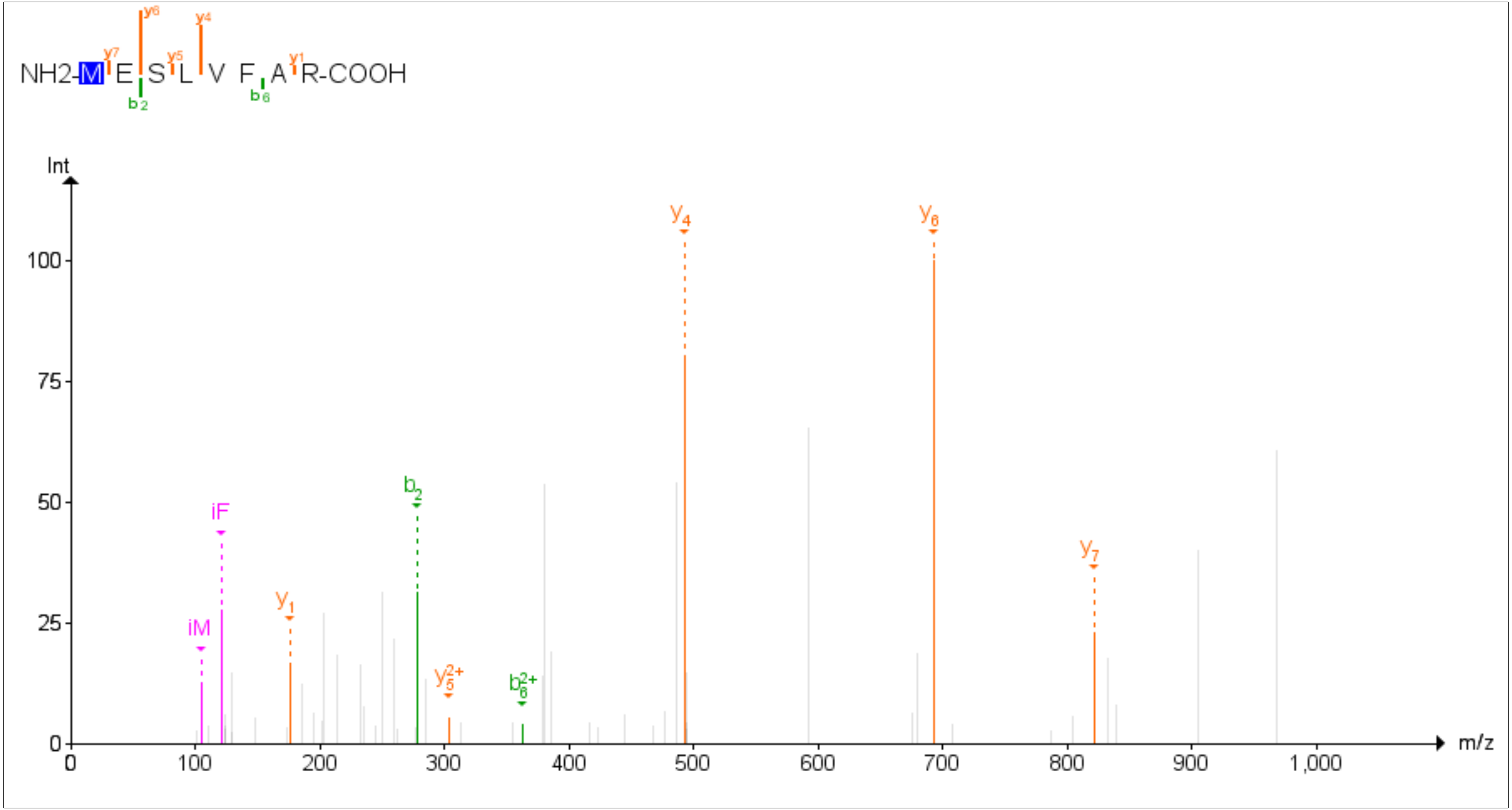
NH2-M E S L V F A R-COOH
y7
y6
y5
y4
y1
b2
b6
Int
100
75
50
25
0
y4
y6
y7
iF
iM
y1
b2
y5 2+
b6 2+
m/z
0
100
200
300
400
500
600
700
800
900
1,000

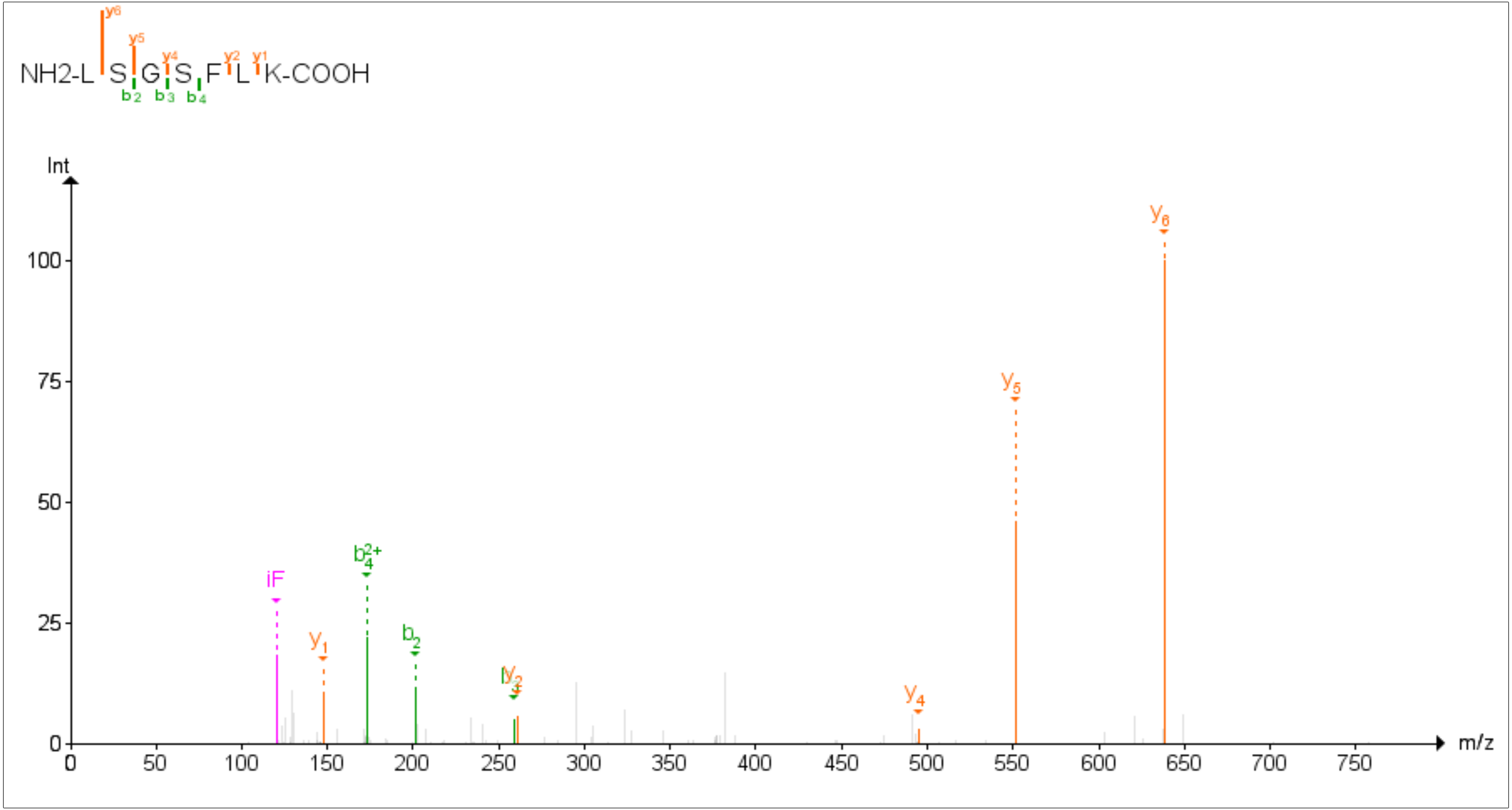

NH2-L S G S F L K-COOH
y6
y5
y4
y2
y1
b2
b3
b4
Int
m/z
iF
y1
b4 2+
b2
y2
y4
y5
y6
0
25
50
75
100
0
50
100
150
200
250
300
350
400
450
500
550
600
650
700
750

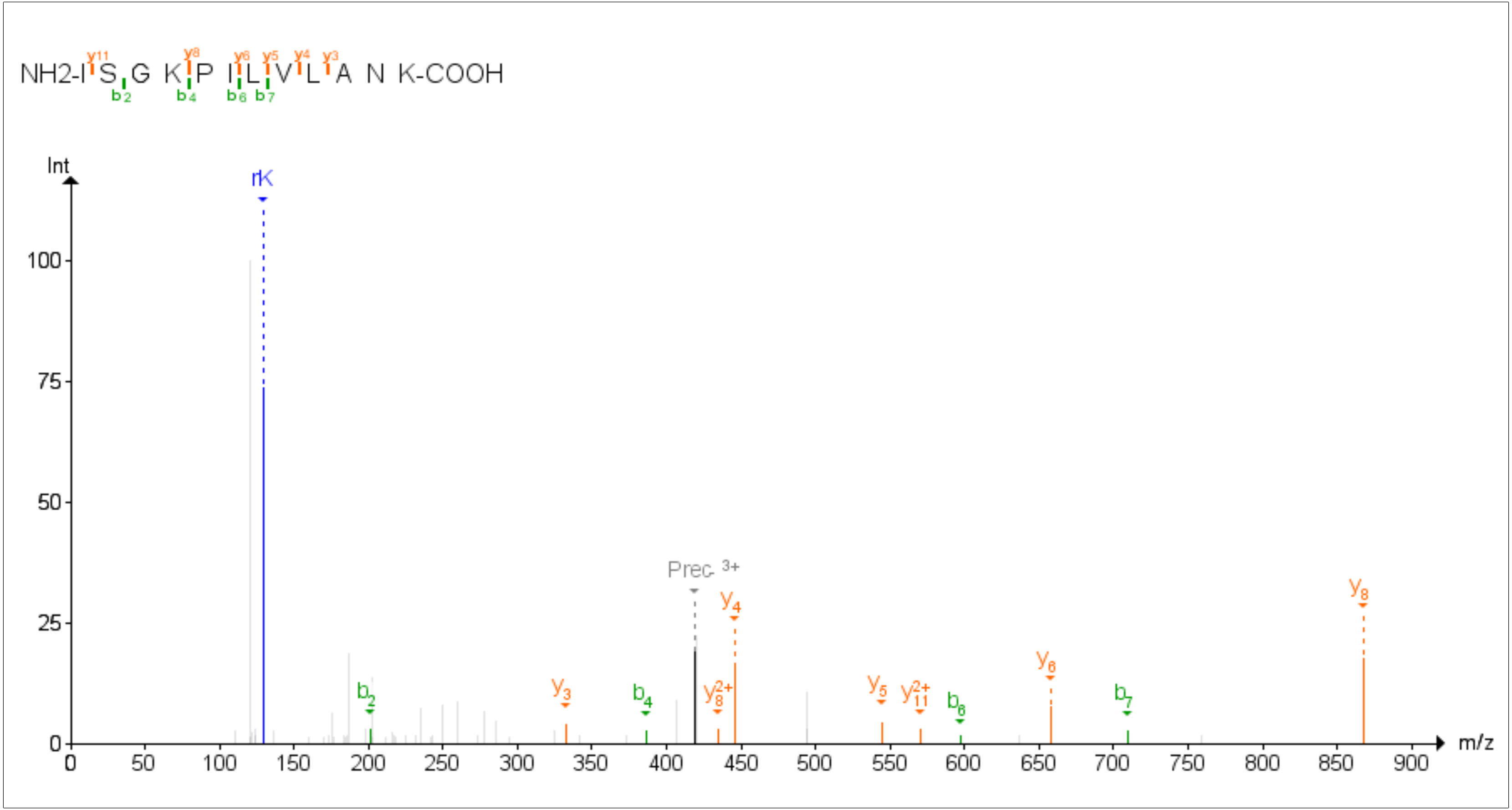

NH2-I S G K P I L V L A N K-COOH
y11 y8 y6 y5 y4 y3
b2 b4 b6 b7
Int
m/z
rK
Prec 3+
b2
y3
b4
y8 2+
y4
y5
y11 2+
b6
y6
b7
y8
0
25
50
75
100
0 50 100 150 200 250 300 350 400 450 500 550 600 650 700 750 800 850 900

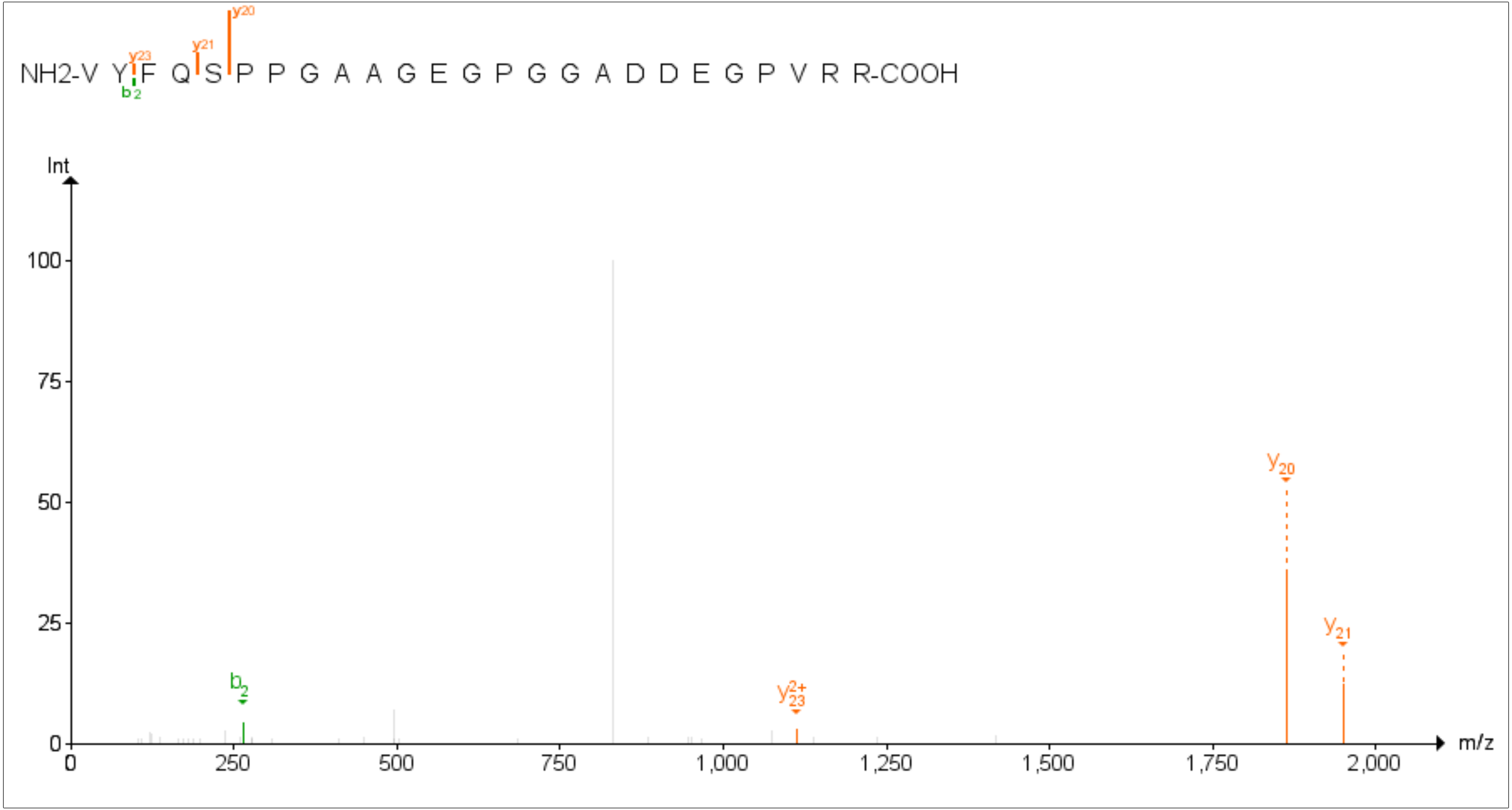
NH2-V Y F Q S P P G A A G E G P G G A D D E G P V R R-COOH
y20
y21
y23
b2
Int
100
75
50
25
0
0
250
500
750
1,000
1,250
1,500
1,750
2,000
m/z
y20
y21
y23 2+
b2

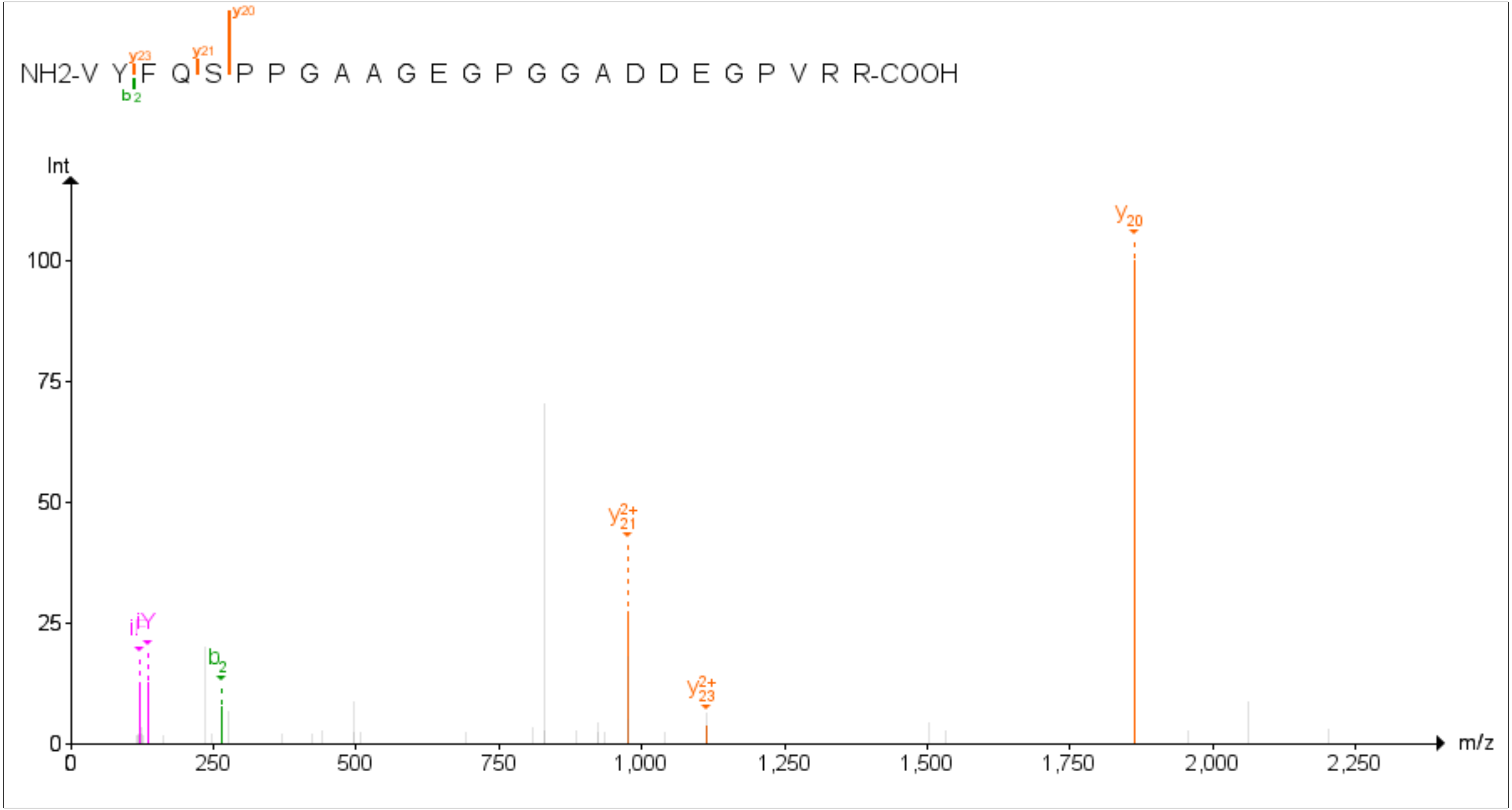

NH2-V Y F Q S P P G A A G E G P G G A D D E G P V R R-COOH
y20
y21
y23
b2
Int
100
75
50
25
0
y20
y21 2+
y23 2+
b2
iY
0
250
500
750
1,000
1,250
1,500
1,750
2,000
2,250
m/z

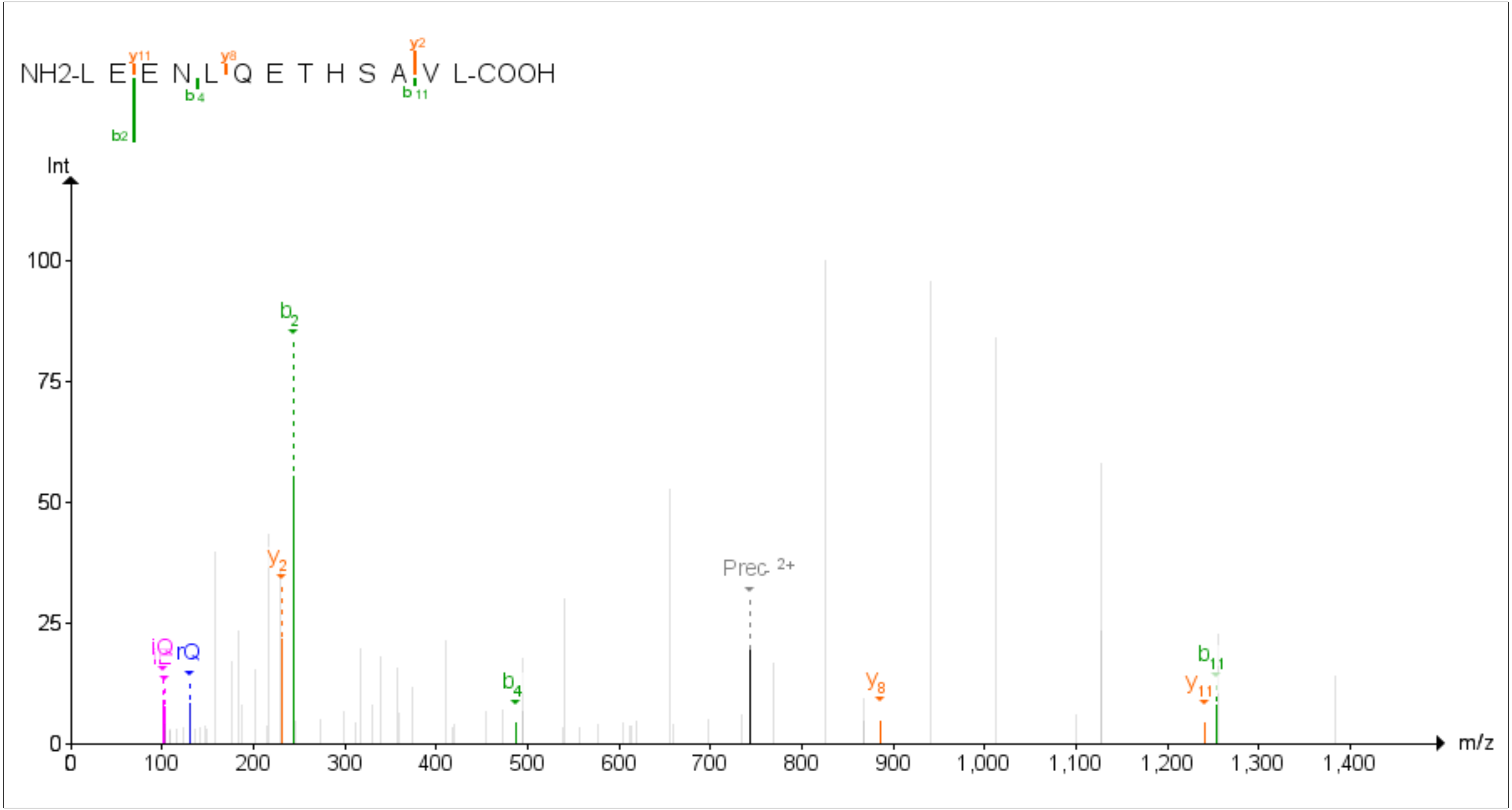
NH2-L E E N L Q E T H S A V L-COOH
y11 y8 y2
b2 b4 b11
Int
100
75
50
25
0
m/z
0 100 200 300 400 500 600 700 800 900 1,000 1,100 1,200 1,300 1,400
b2
y2
iQ
rQ
b4
Prec 2+
y8
y11
b11

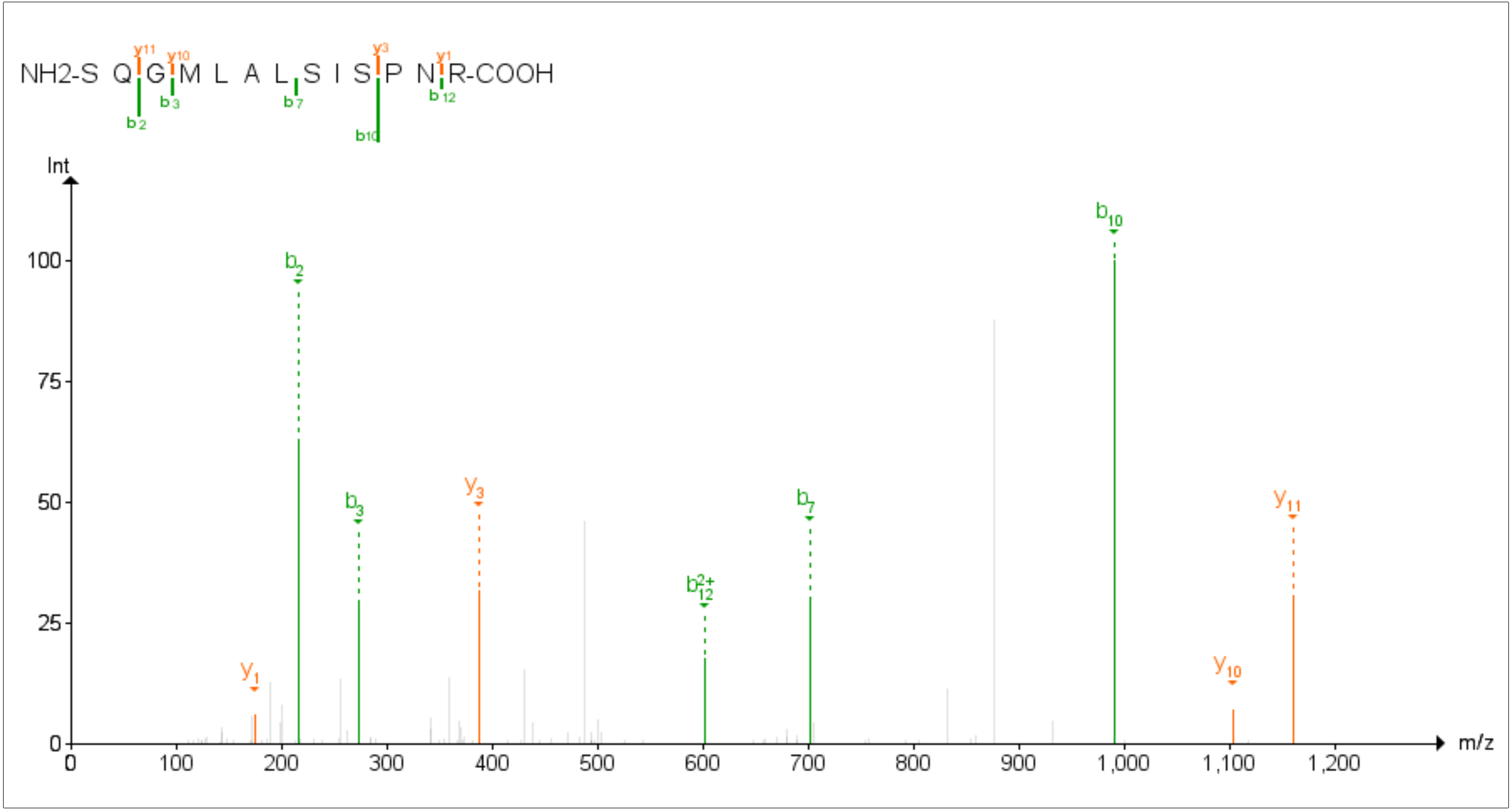

NH2-S Q G M L A L S I S P N R-COOH
y11
y10
y3
y1
b2
b3
b7
b10
b12
Int
100
75
50
25
0
b2
b3
y3
b7
b10
y1
y10
y11
b12 2+
0
100
200
300
400
500
600
700
800
900
1,000
1,100
1,200
m/z

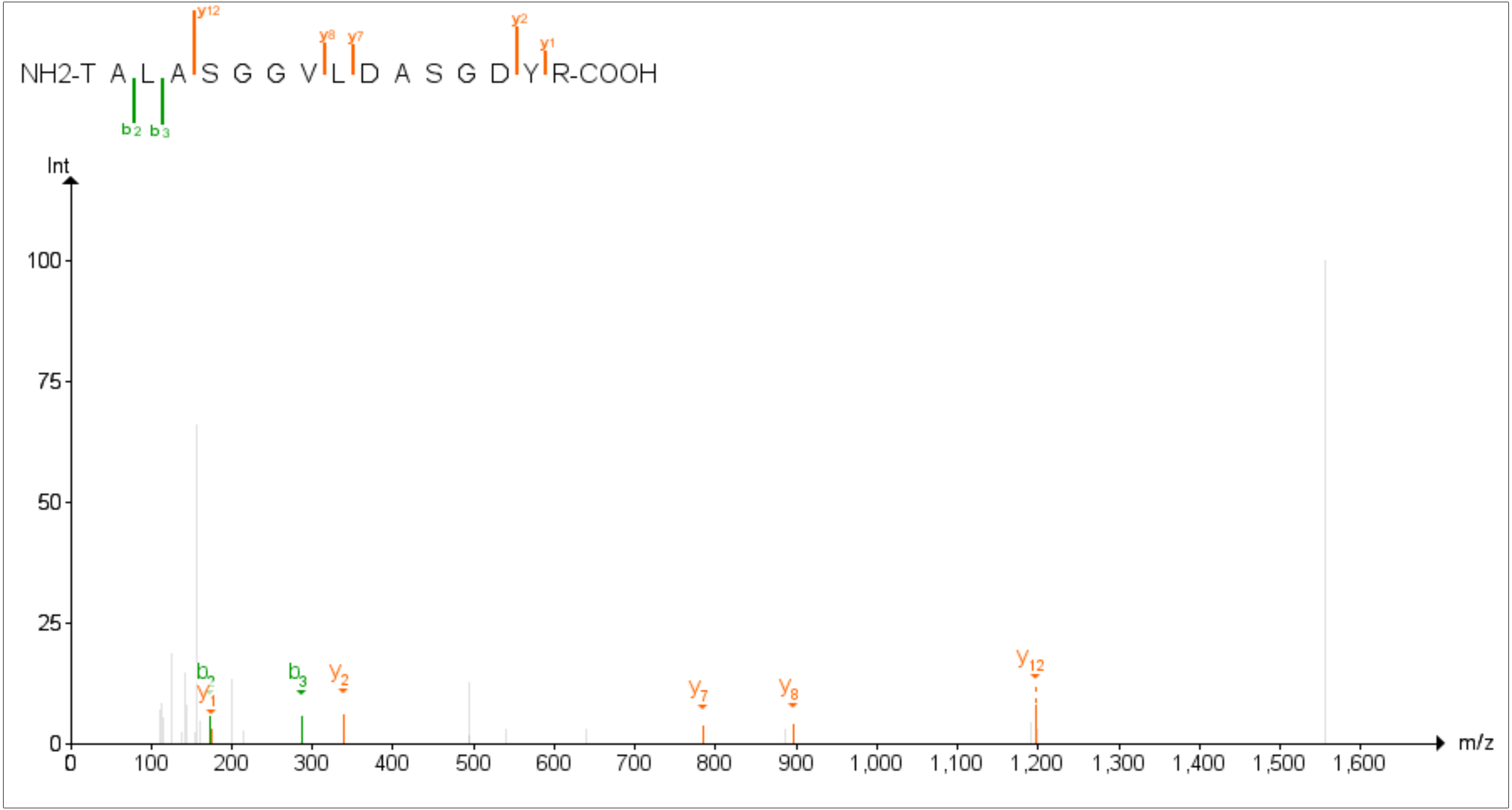

NH2-T A L A S G G V L D A S G D Y R-COOH
y12
y8
y7
y2
y1
b2
b3
Int
100
75
50
25
0
b2
y1
b3
y2
y7
y8
y12
0
100
200
300
400
500
600
700
800
900
1,000
1,100
1,200
1,300
1,400
1,500
1,600
m/z

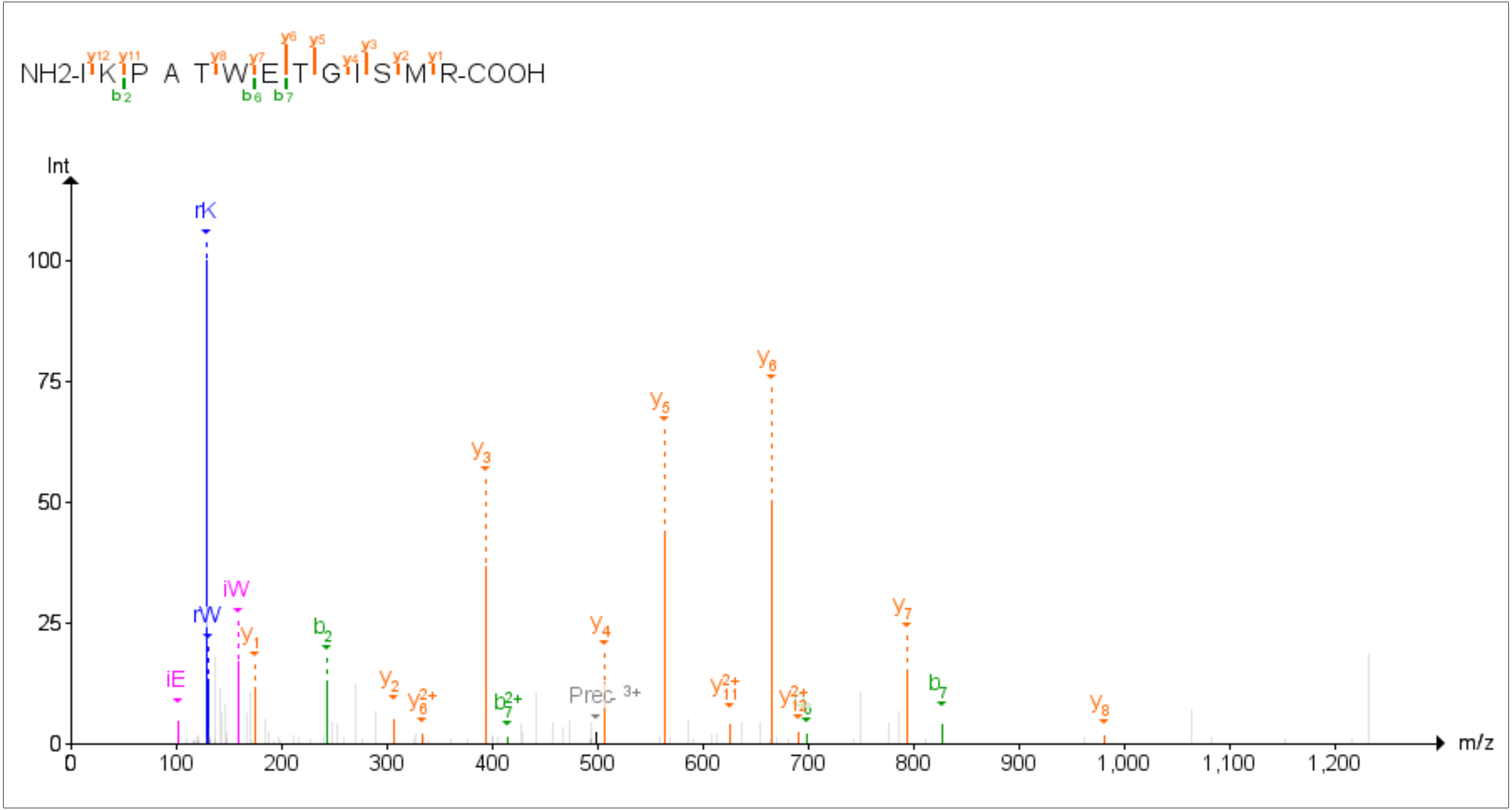

NH2-IKPATWETGISMR-COOH
Int
m/z
rK
rW
iW
iE
Prec 3+

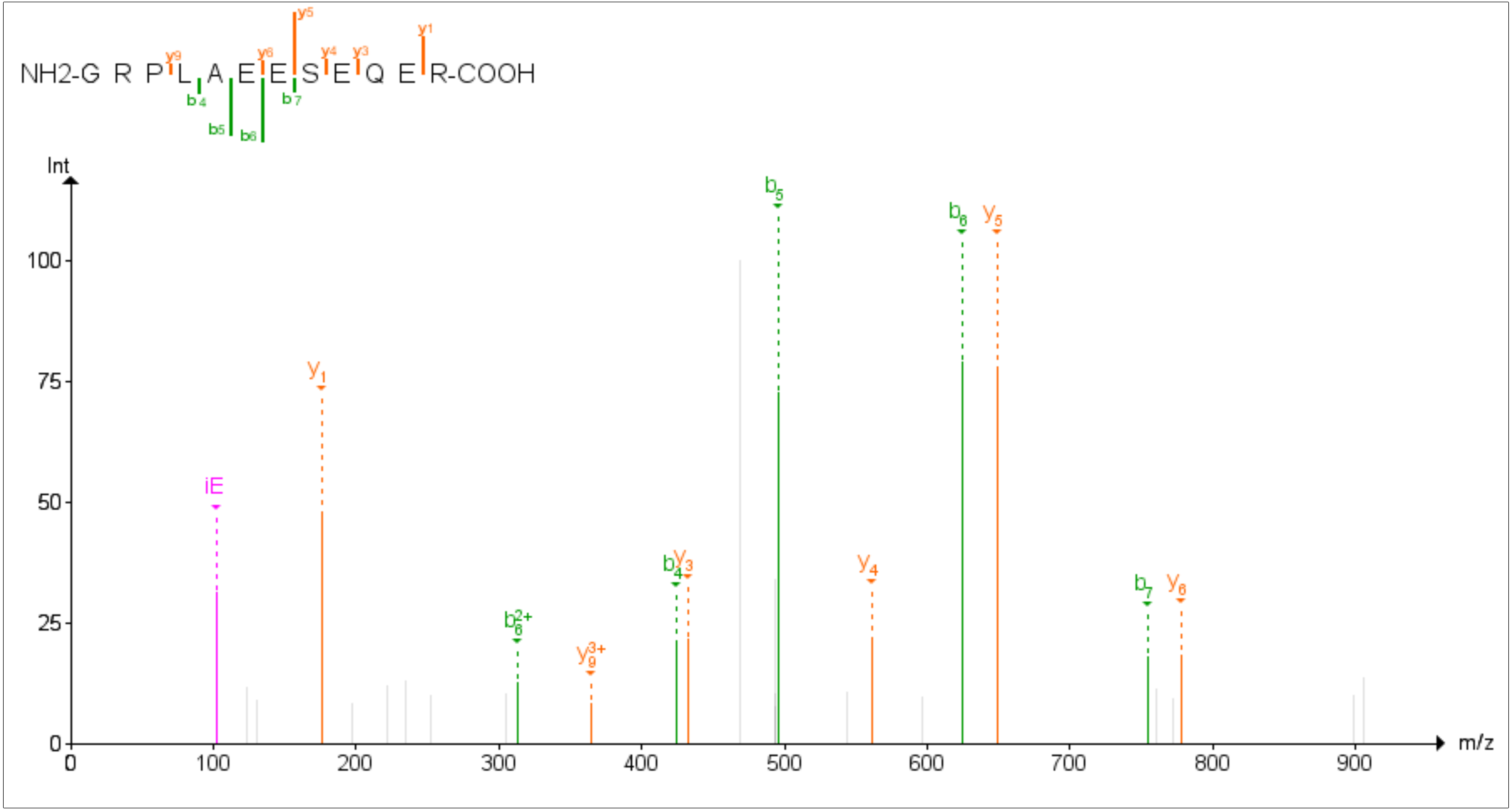

NH2-G R P L A E E S E Q E R-COOH
y9 y6 y5 y4 y3 y1
b4 b5 b6 b7
Int
100
75
50
25
0
0 100 200 300 400 500 600 700 800 900
m/z
iE
y1
b6 2+
y9 3+
b4 y3
b5
y4
b6 y5
b7 y6

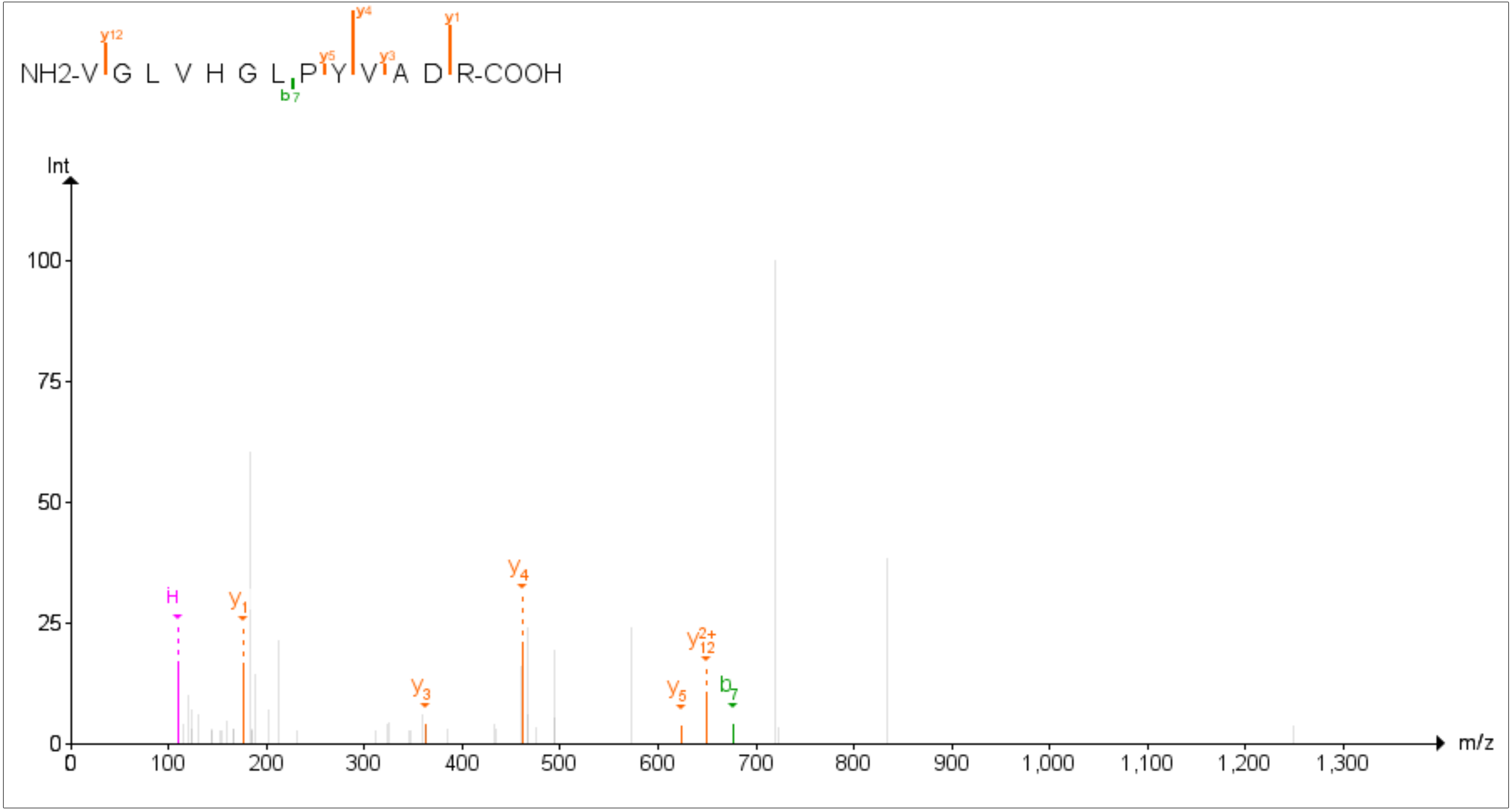

NH2-V G L V H G L P Y V A D R-COOH
y12
y5
y4
y3
y1
b7
Int
100
75
50
25
0
H
y1
y3
y4
y5
y12 2+
b7
0
100
200
300
400
500
600
700
800
900
1,000
1,100
1,200
1,300
m/z

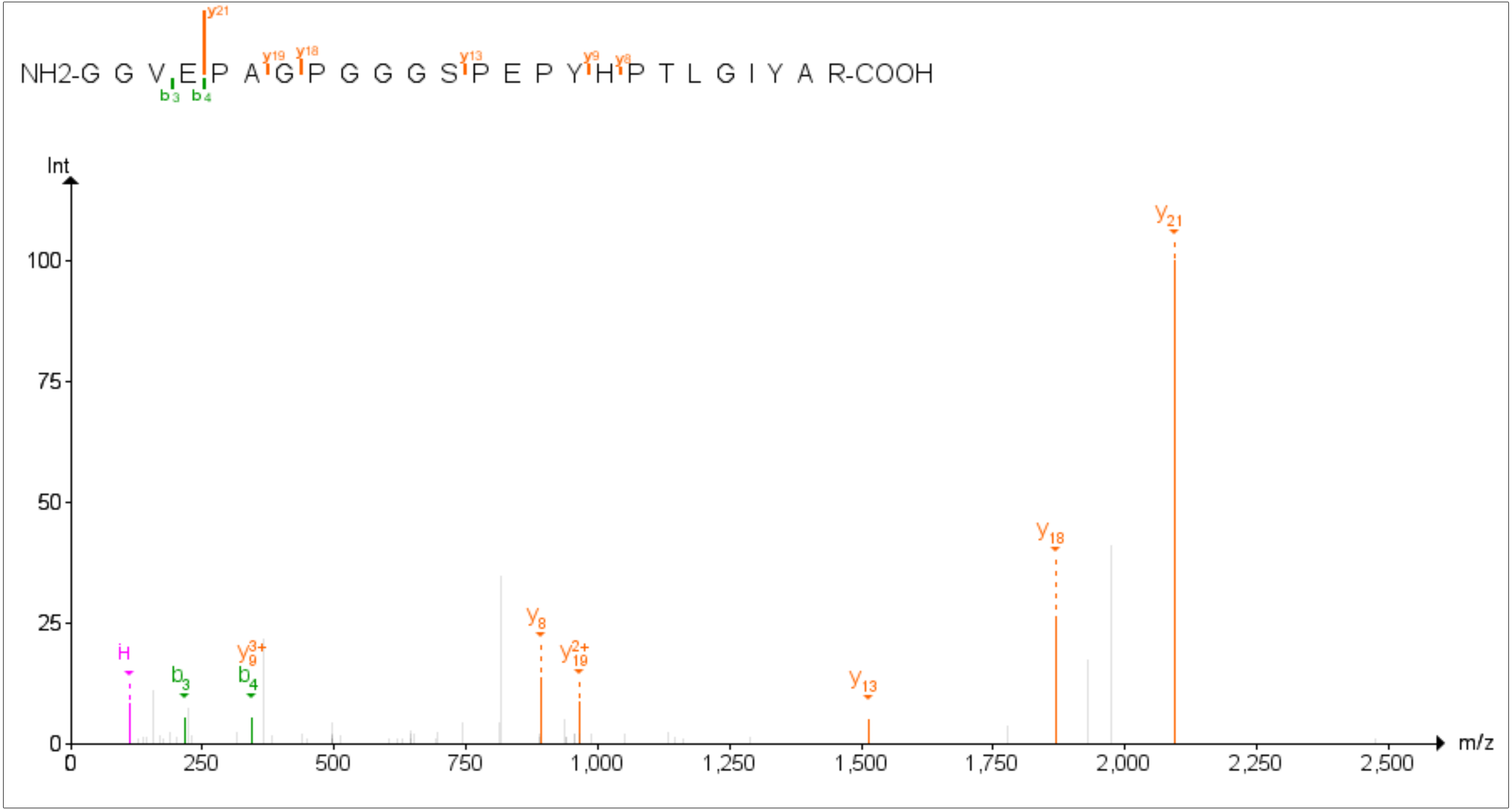
NH2-G G V E P A G P G G G S P E P Y H P T L G I Y A R-COOH
y21
y19
y18
y13
y9
y8
b3
b4
Int
100
75
50
25
0
y21
y18
y8
y19 2+
y13
H
b3
y9 3+
b4
0
250
500
750
1,000
1,250
1,500
1,750
2,000
2,250
2,500
m/z

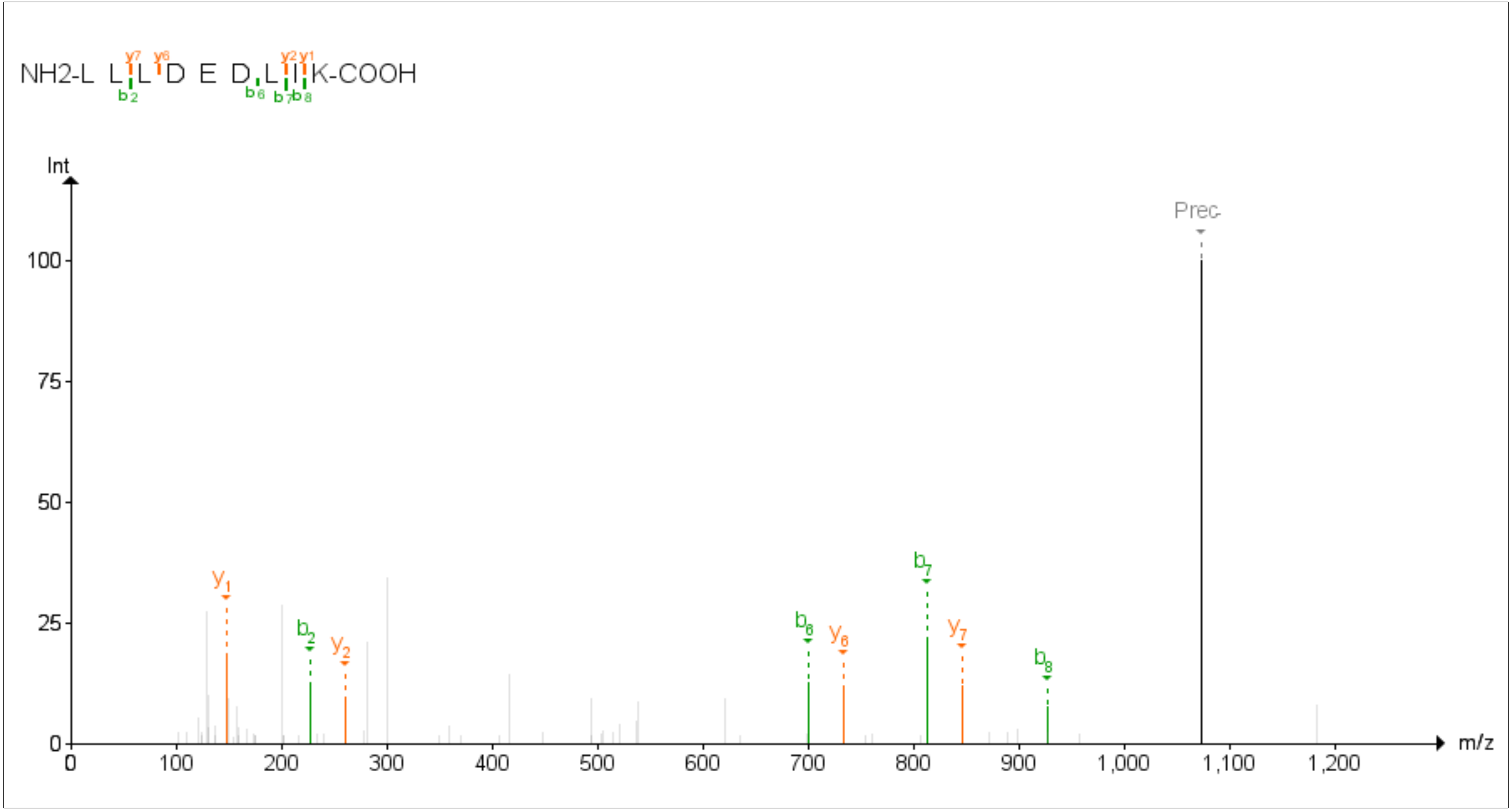
NH2-L L L D E D L I I K-COOH
y7 y6 y2 y1
b2 b6 b7 b8
Int
100
75
50
25
0
Prec
y1
b2
y2
b6
y6
b7
y7
b8
0 100 200 300 400 500 600 700 800 900 1,000 1,100 1,200
m/z

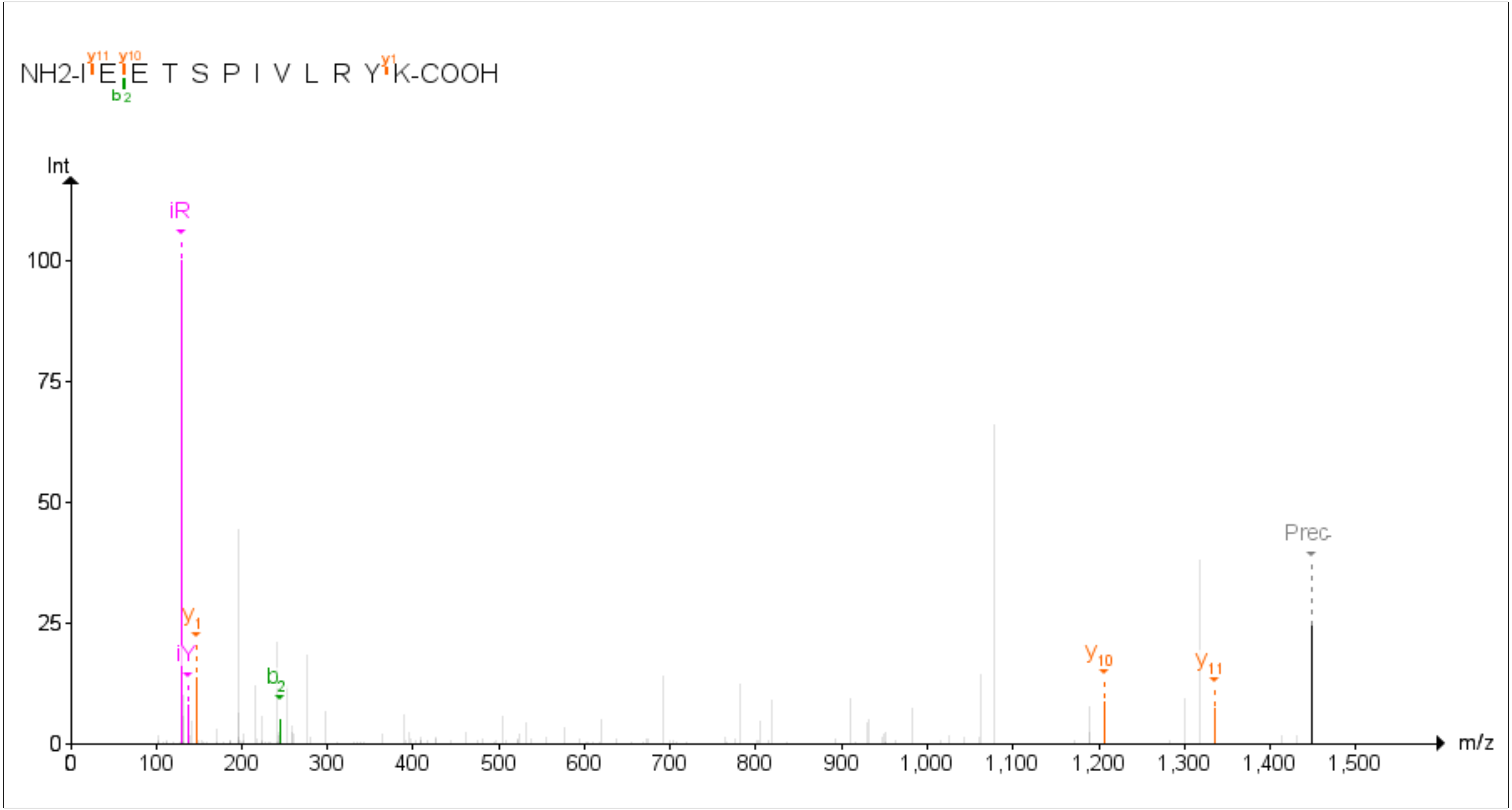

NH2-I E E T S P I V L R Y K-COOH
y11
y10
y1
b2
Int
iR
y1
iY
b2
y10
y11
Prec
100
75
50
25
0
0
100
200
300
400
500
600
700
800
900
1,000
1,100
1,200
1,300
1,400
1,500
m/z

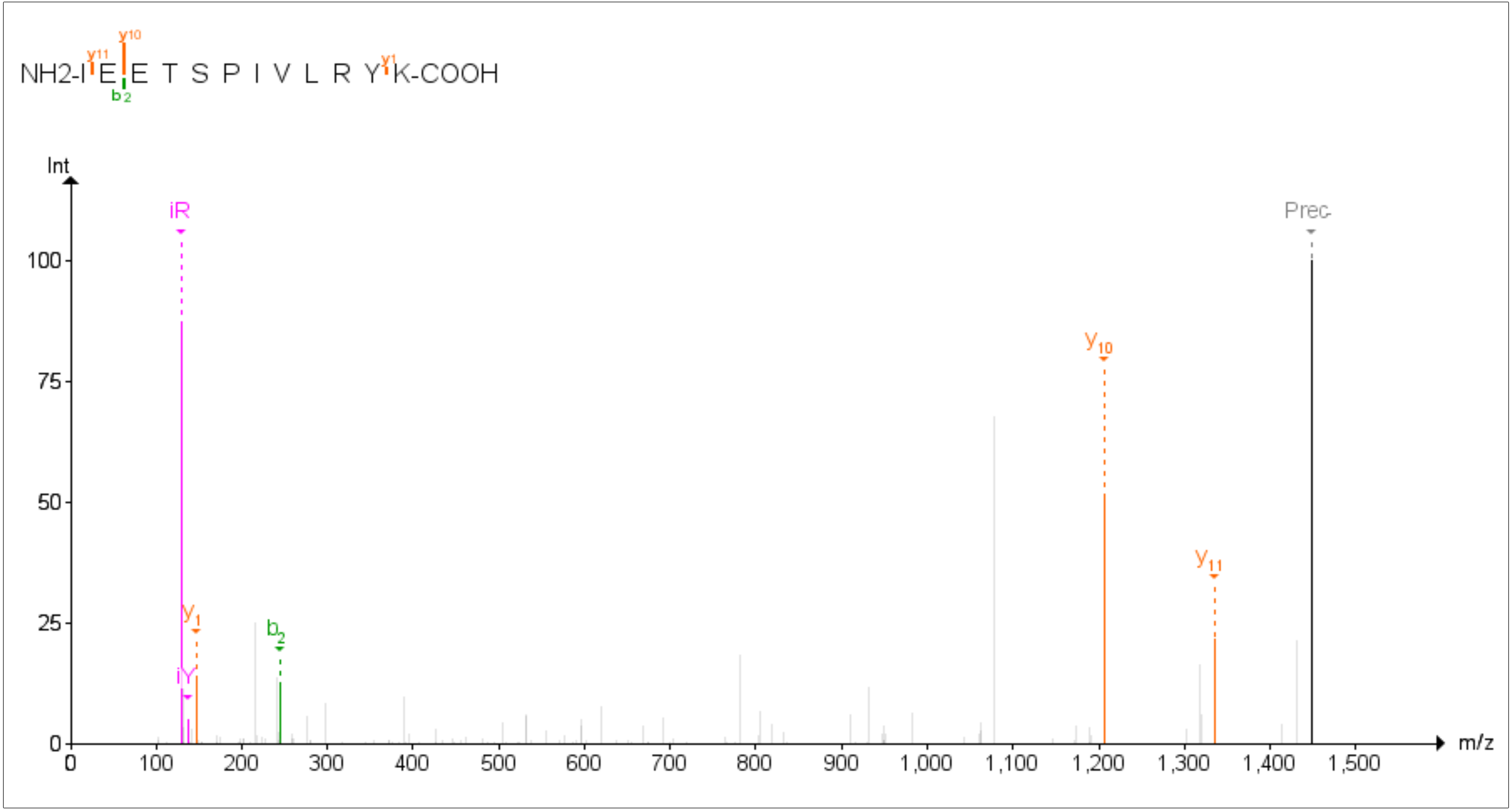

NH2-I E E T S P I V L R Y K-COOH
y11
y10
b2
y1
Int
iR
Prec
y10
y11
y1
iY
b2
100
75
50
25
0
0
100
200
300
400
500
600
700
800
900
1,000
1,100
1,200
1,300
1,400
1,500
m/z

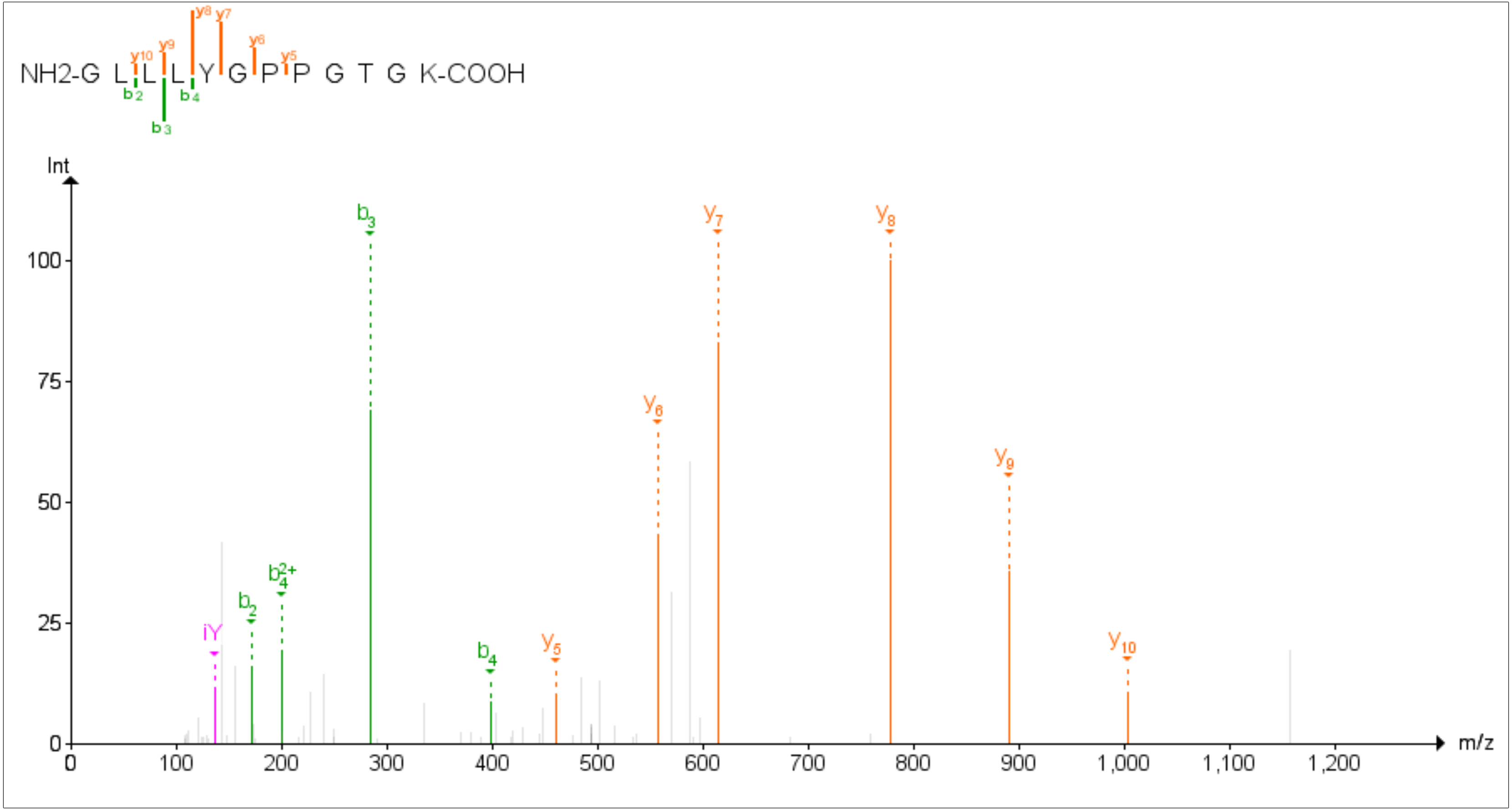

NH2-G L L L Y G P P G T G K-COOH
y10 y9 y8 y7 y6 y5
b2 b3 b4
Int
100
75
50
25
0
b3
y7
y8
y6
y9
b4 2+
b2
iY
b4
y5
y10
0 100 200 300 400 500 600 700 800 900 1,000 1,100 1,200
m/z

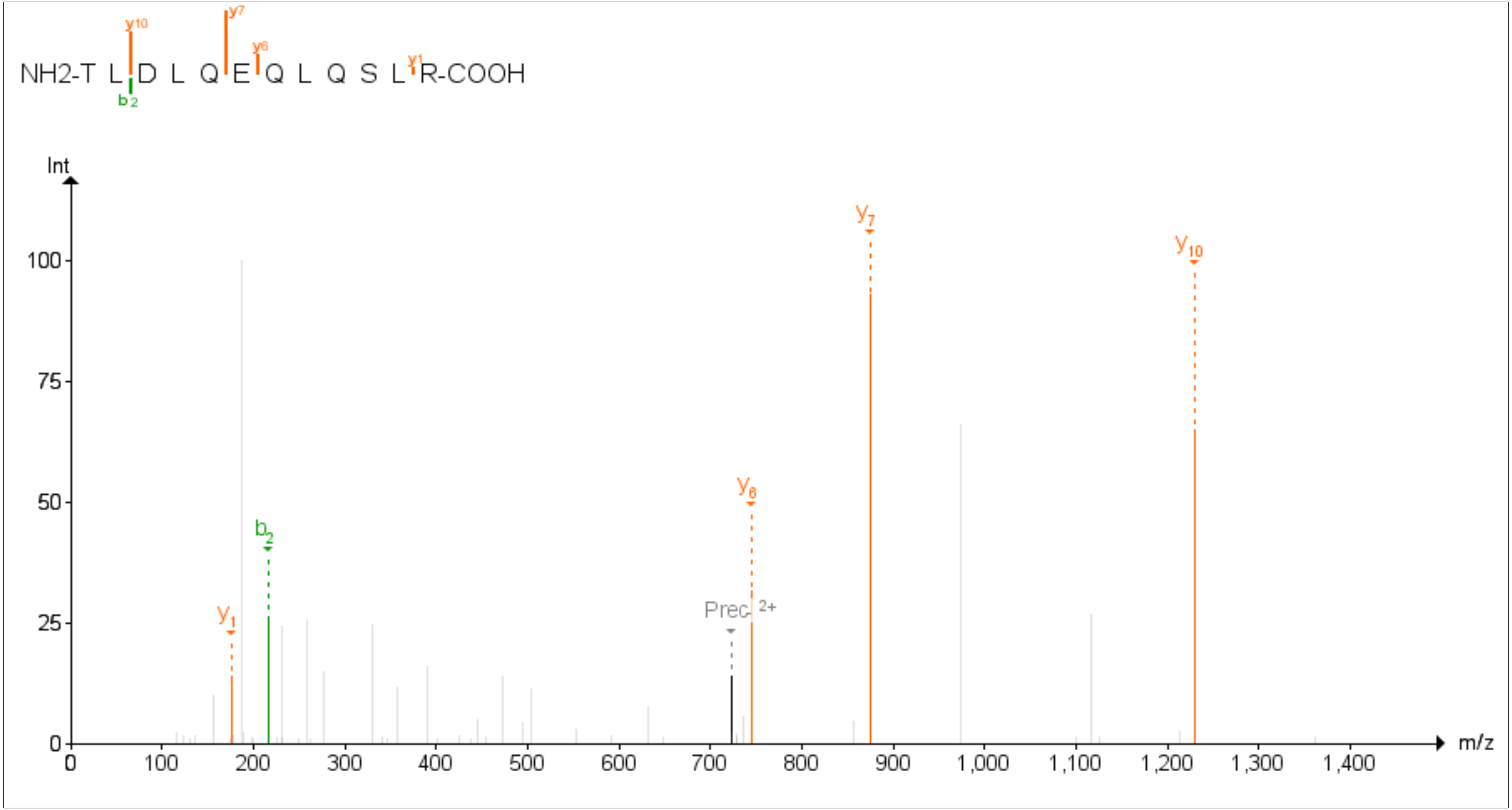
NH2-T L D L Q E Q L Q S L R-COOH
y10
y7
y6
y1
b2
Int
100
75
50
25
0
m/z
0
100
200
300
400
500
600
700
800
900
1,000
1,100
1,200
1,300
1,400
y7
y10
y6
b2
y1
Prec 2+

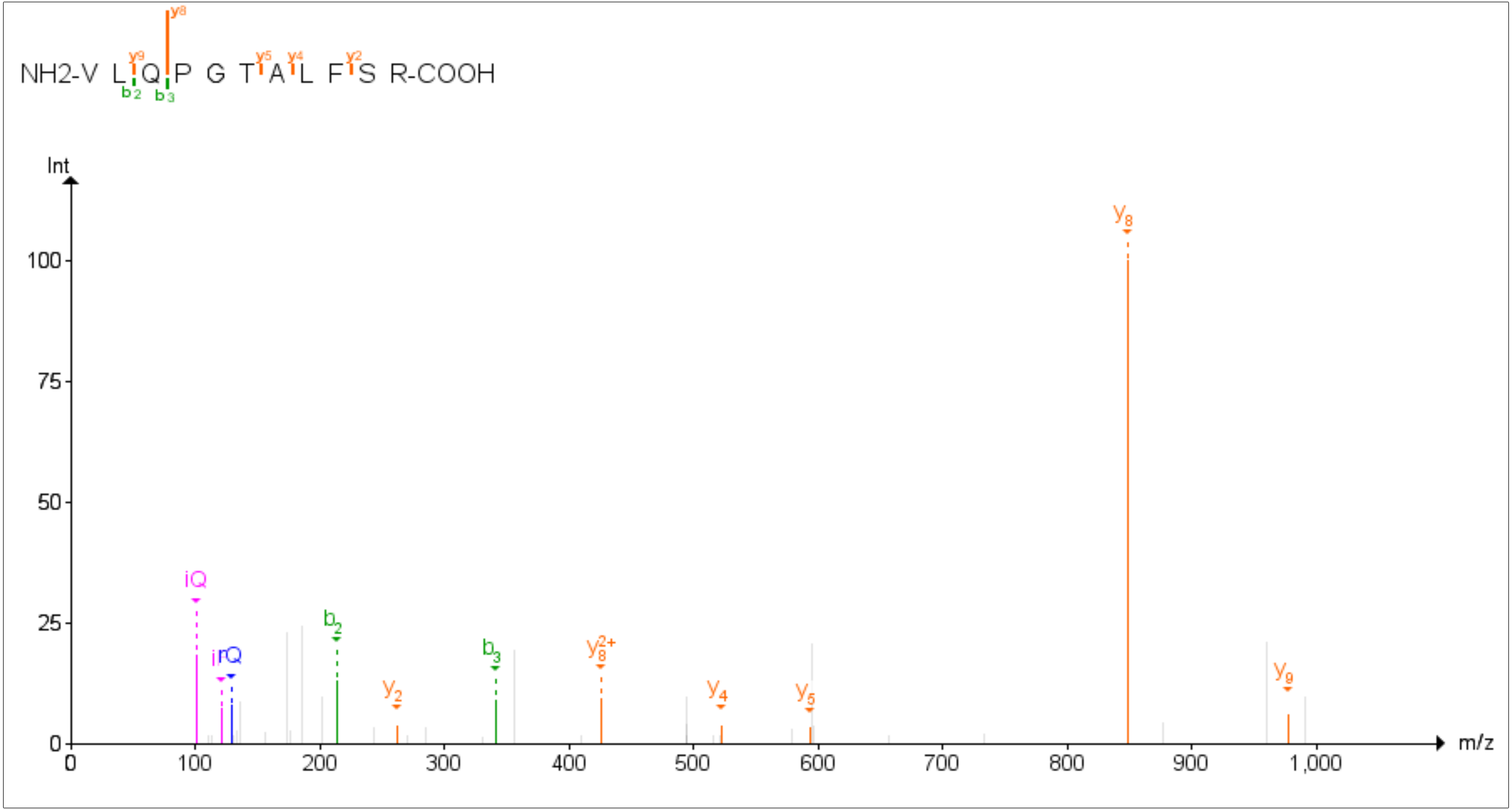

NH2-V L Q P G T A L F S R-COOH
y8
y9
y5
y4
y2
b2
b3
Int
100
75
50
25
0
y8
iQ
irQ
b2
b3
y2
y8 2+
y4
y5
y9
0
100
200
300
400
500
600
700
800
900
1,000
m/z

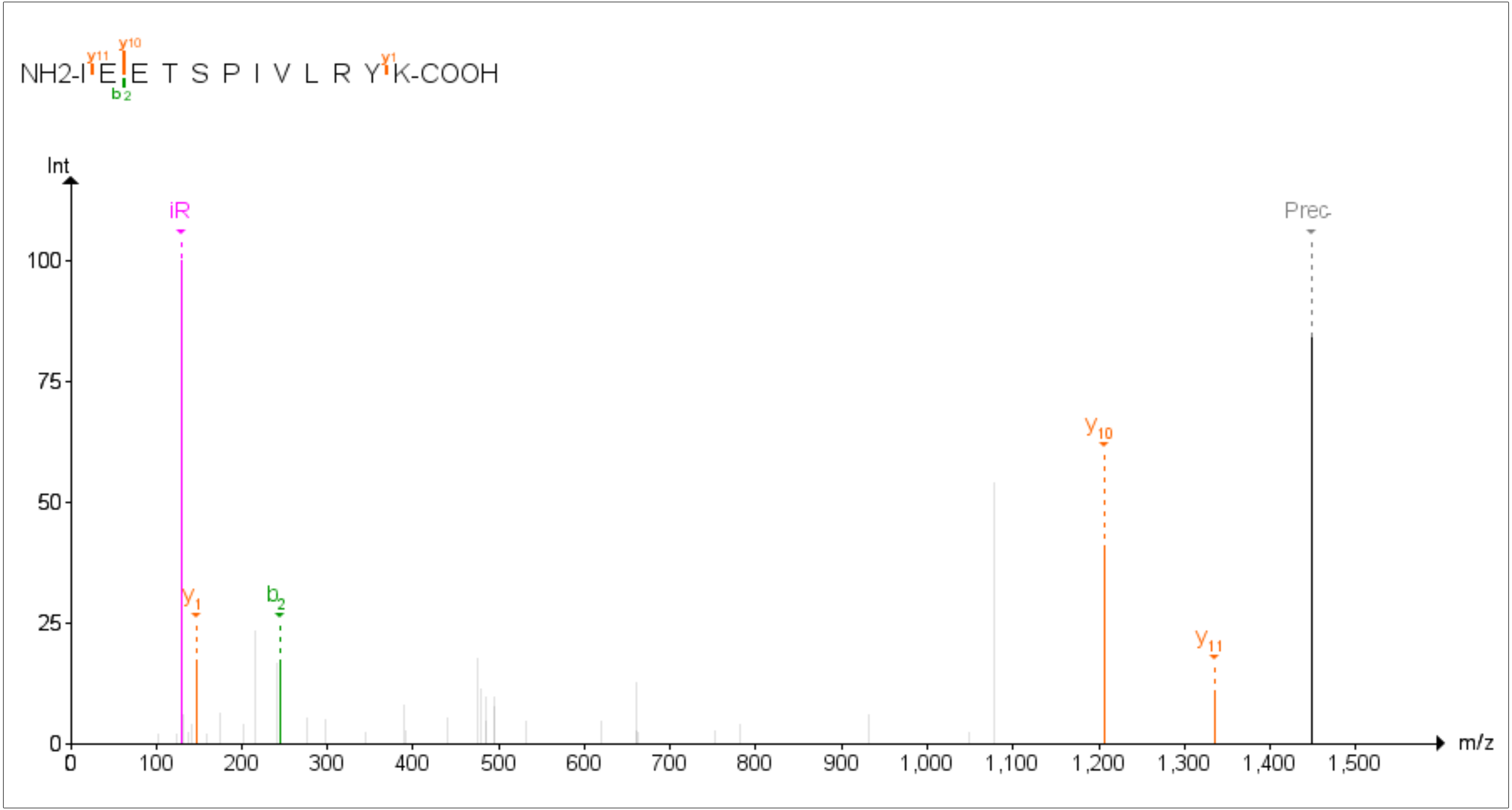

NH2-I E E T S P I V L R Y K-COOH
y11
y10
y1
b2
Int
m/z
iR
Prec
y1
b2
y10
y11
0
25
50
75
100
0
100
200
300
400
500
600
700
800
900
1,000
1,100
1,200
1,300
1,400
1,500

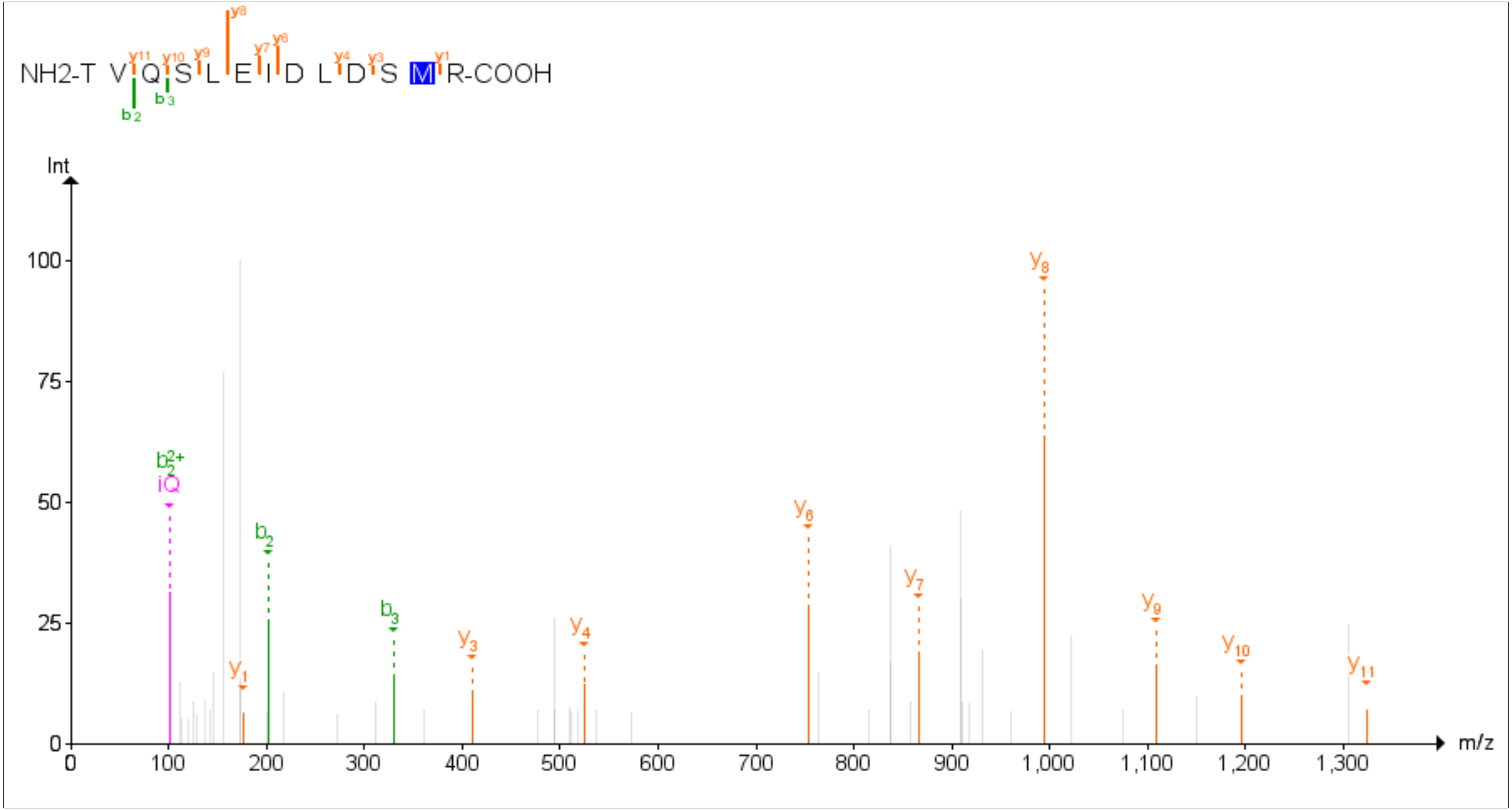

NH2-T V Q S L E I D L D S M R-COOH
y11 y10 y9 y8 y7 y6 y4 y3 y1
b2 b3
Int
m/z
iQ
b2 2+
b2
b3
y1
y3
y4
y6
y7
y8
y9
y10
y11

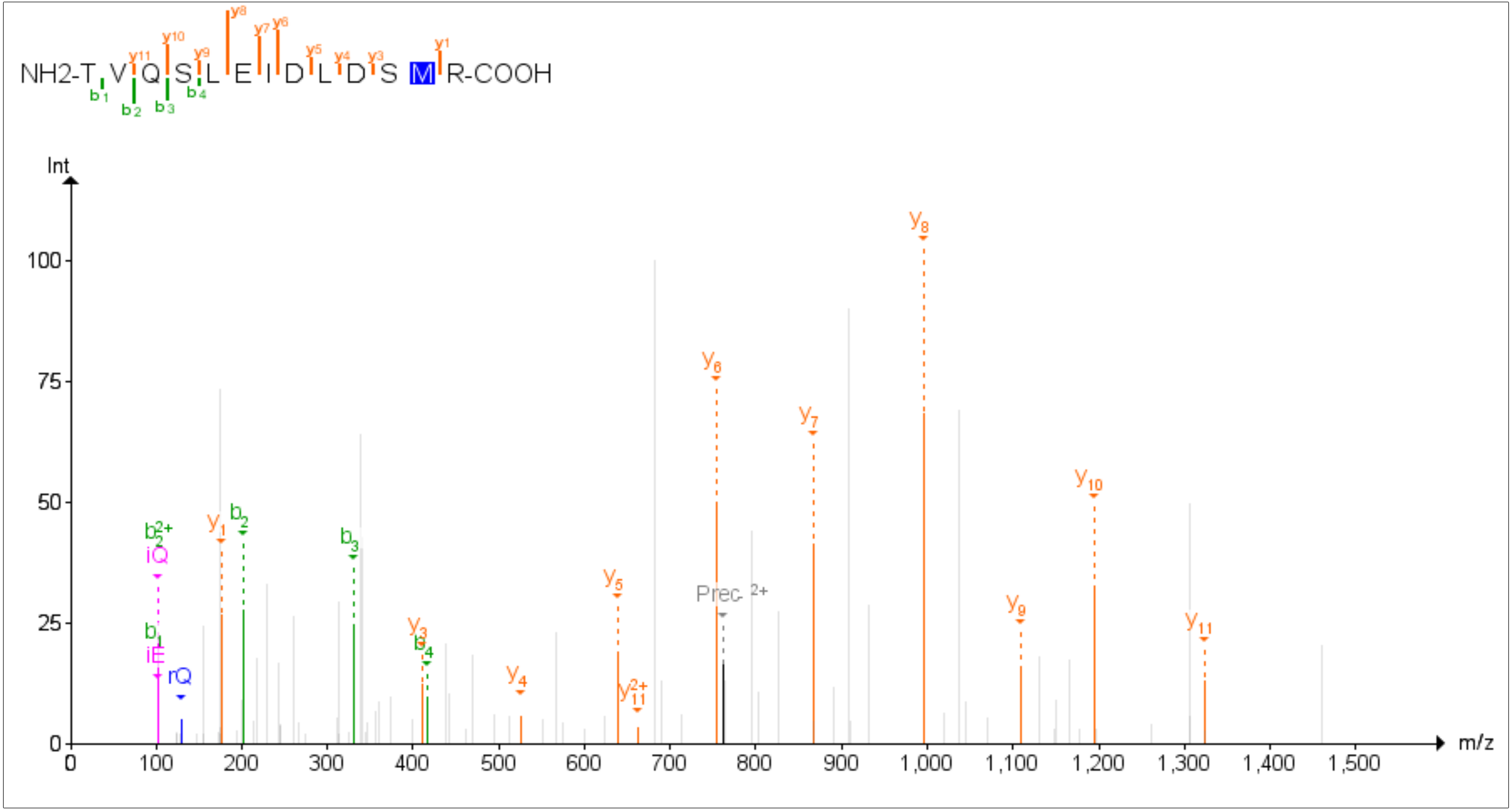

NH2-T V Q S L E I D L D S M R-COOH
b1 b2 b3 b4
y11 y10 y9 y8 y7 y6 y5 y4 y3 y1
Int
100
75
50
25
0
m/z
0 100 200 300 400 500 600 700 800 900 1,000 1,100 1,200 1,300 1,400 1,500
b2 2+
iQ
b1
iE
rQ
y1
b2
b3
y3
b4
y4
y5
y11 2+
Prec 2+
y6
y7
y8
y9
y10
y11

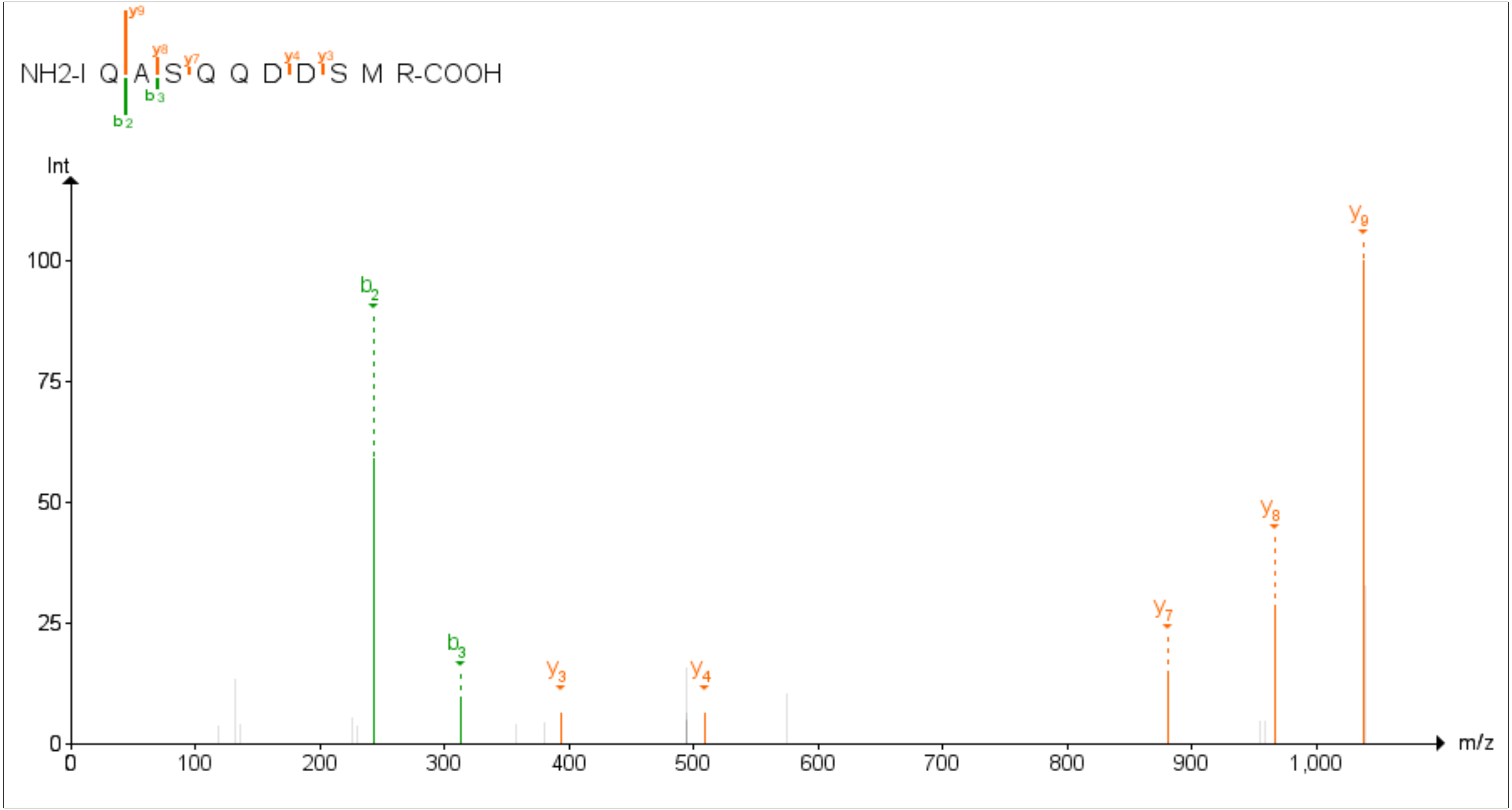

NH2-I Q A S Q Q D D S M R-COOH
y9
y8
y7
y4
y3
b2
b3
Int
100
75
50
25
0
m/z
0
100
200
300
400
500
600
700
800
900
1,000
b_2
b_3
y_3
y_4
y_7
y_8
y_9

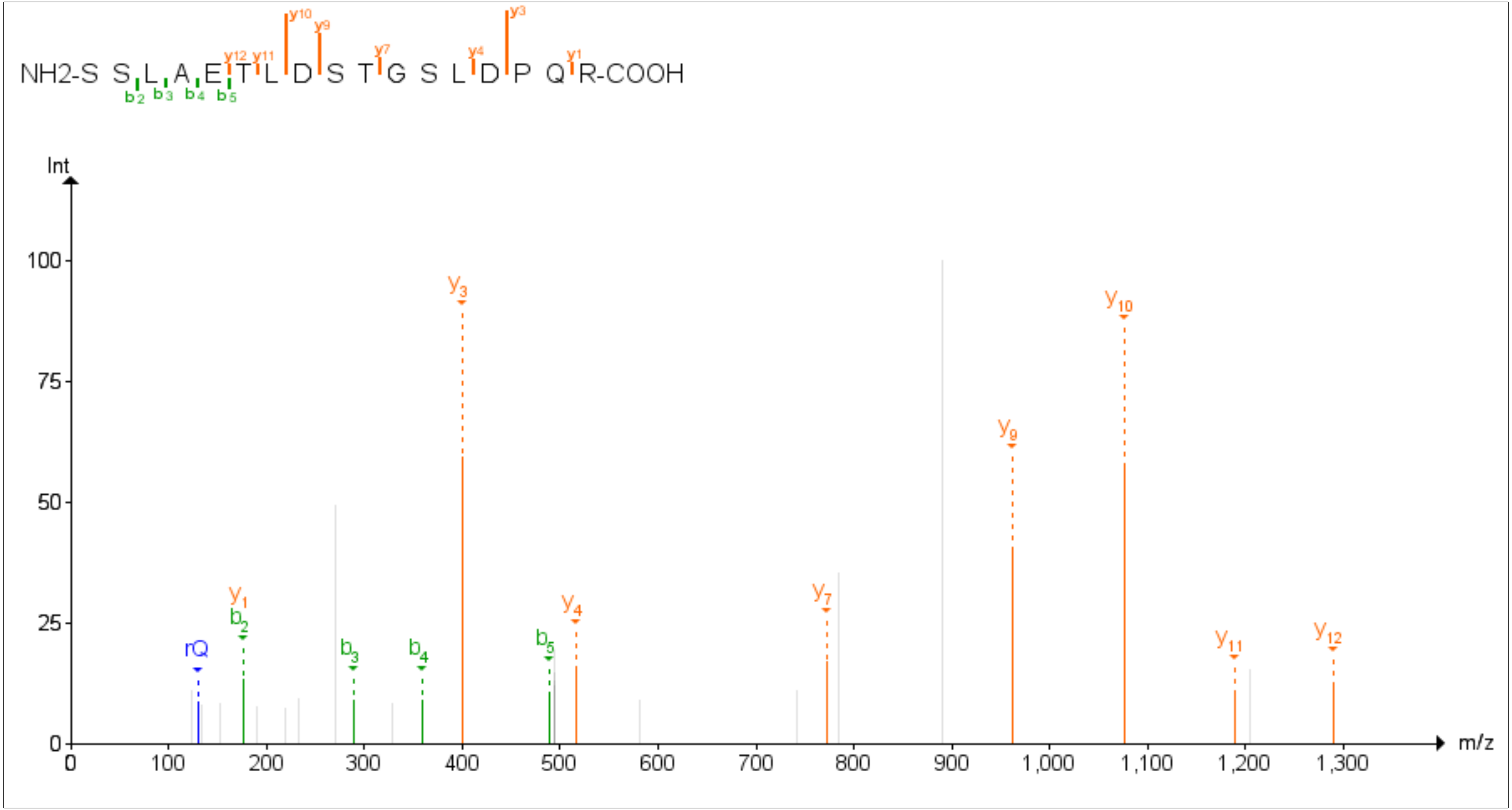

NH2-S S L A E T L D S T G S L D P Q R-COOH
b2 b3 b4 b5
y12 y11 y10 y9 y7 y4 y3 y1
Int
100
75
50
25
0
rQ
y1
b2
b3
b4
y3
b5
y4
y7
y9
y10
y11
y12
0 100 200 300 400 500 600 700 800 900 1,000 1,100 1,200 1,300
m/z

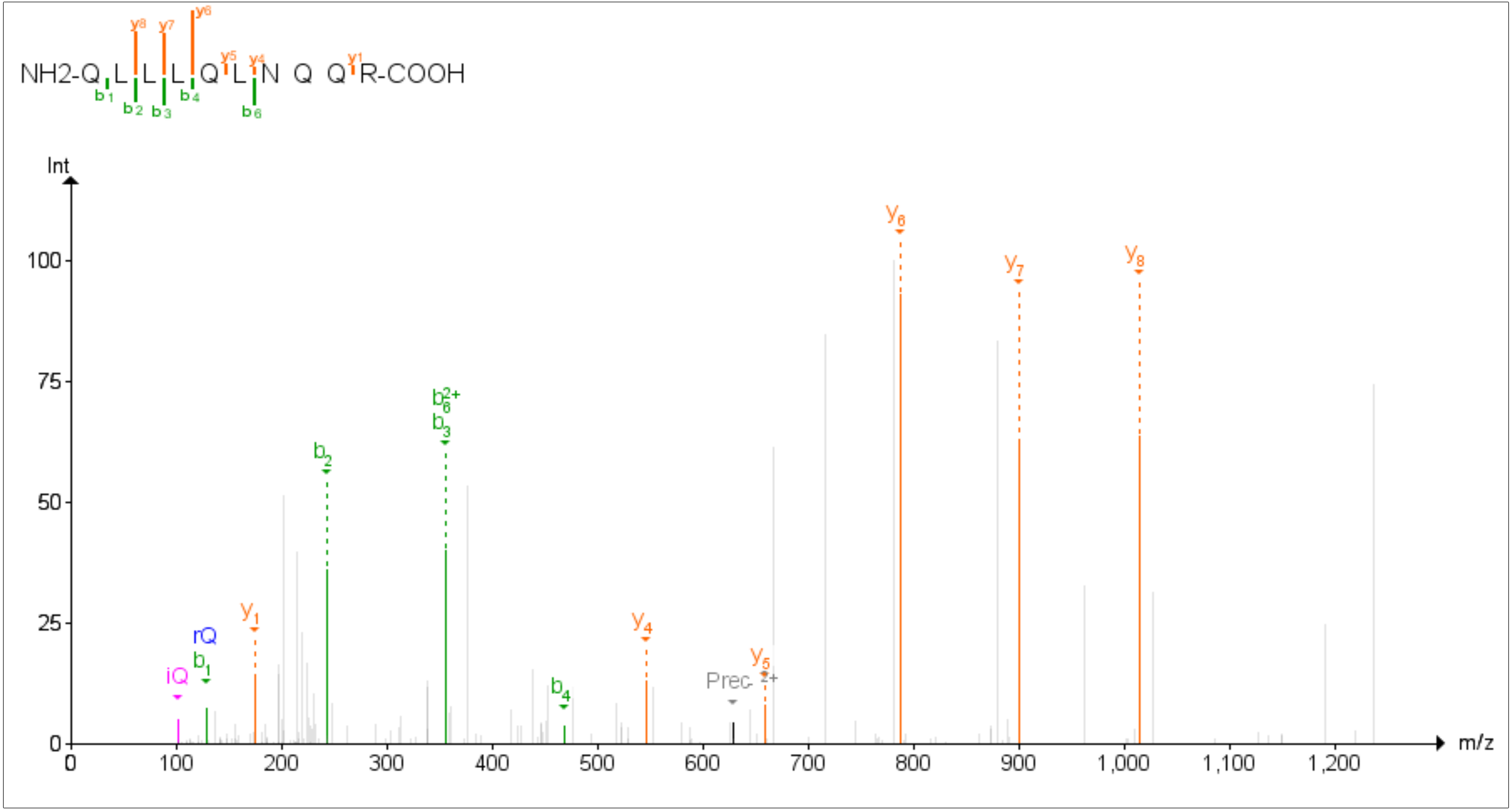

NH2-Q L L L Q L N Q Q R-COOH
y8 y7 y6 y5 y4 y1
b1 b2 b3 b4 b6
Int
100
75
50
25
0
m/z
0 100 200 300 400 500 600 700 800 900 1,000 1,100 1,200
iQ
rQ
b1
y1
b2
b6 2+
b3
b4
y4
Prec 2+
y5
y6
y7
y8

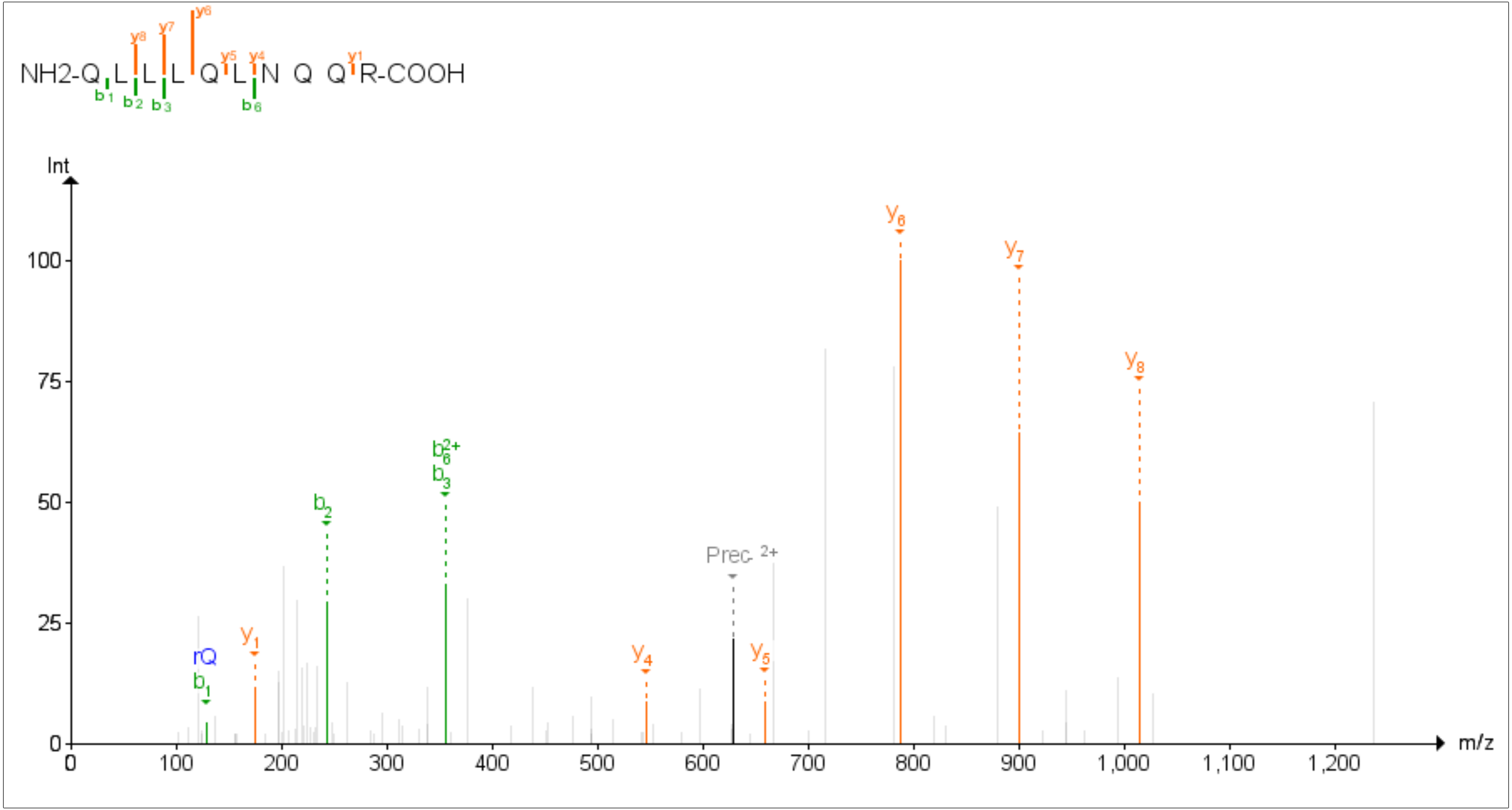

NH2-Q L L L Q L N Q Q R-COOH
y8 y7 y6 y5 y4 y1
b1 b2 b3 b6
Int
m/z
y6
y7
y8
b6 2+
b3
b2
Prec 2+
rQ
b1
y1
y4
y5

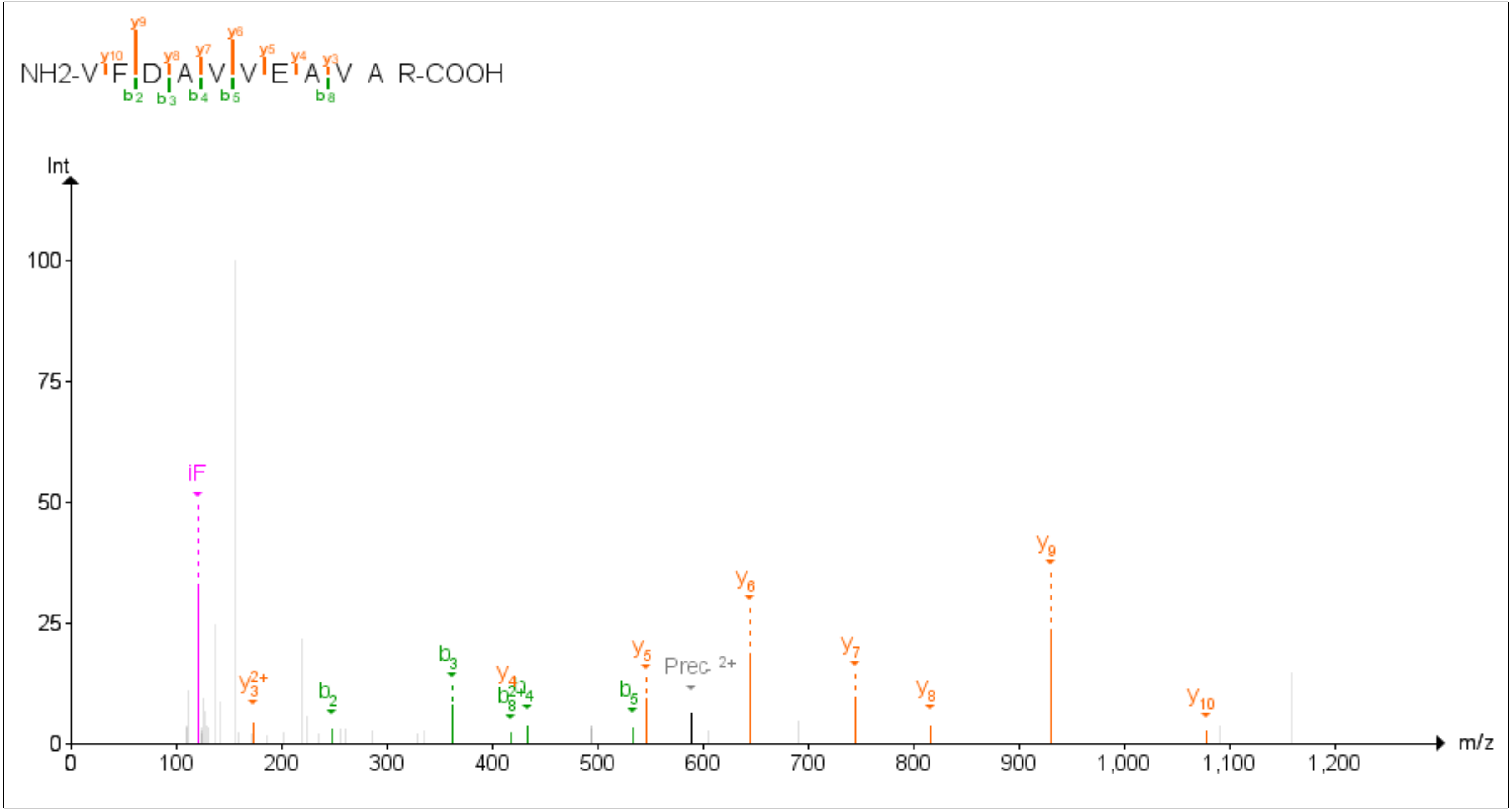
NH2-V F D A V V E A V A R-COOH
y10 y9 y8 y7 y6 y5 y4 y3
b2 b3 b4 b5 b8
Int
100
75
50
25
0
iF
y3 2+
b2
b3
y4
b8 2+
b5
y5
Prec. 2+
y6
y7
y8
y9
y10
0 100 200 300 400 500 600 700 800 900 1,000 1,100 1,200
m/z

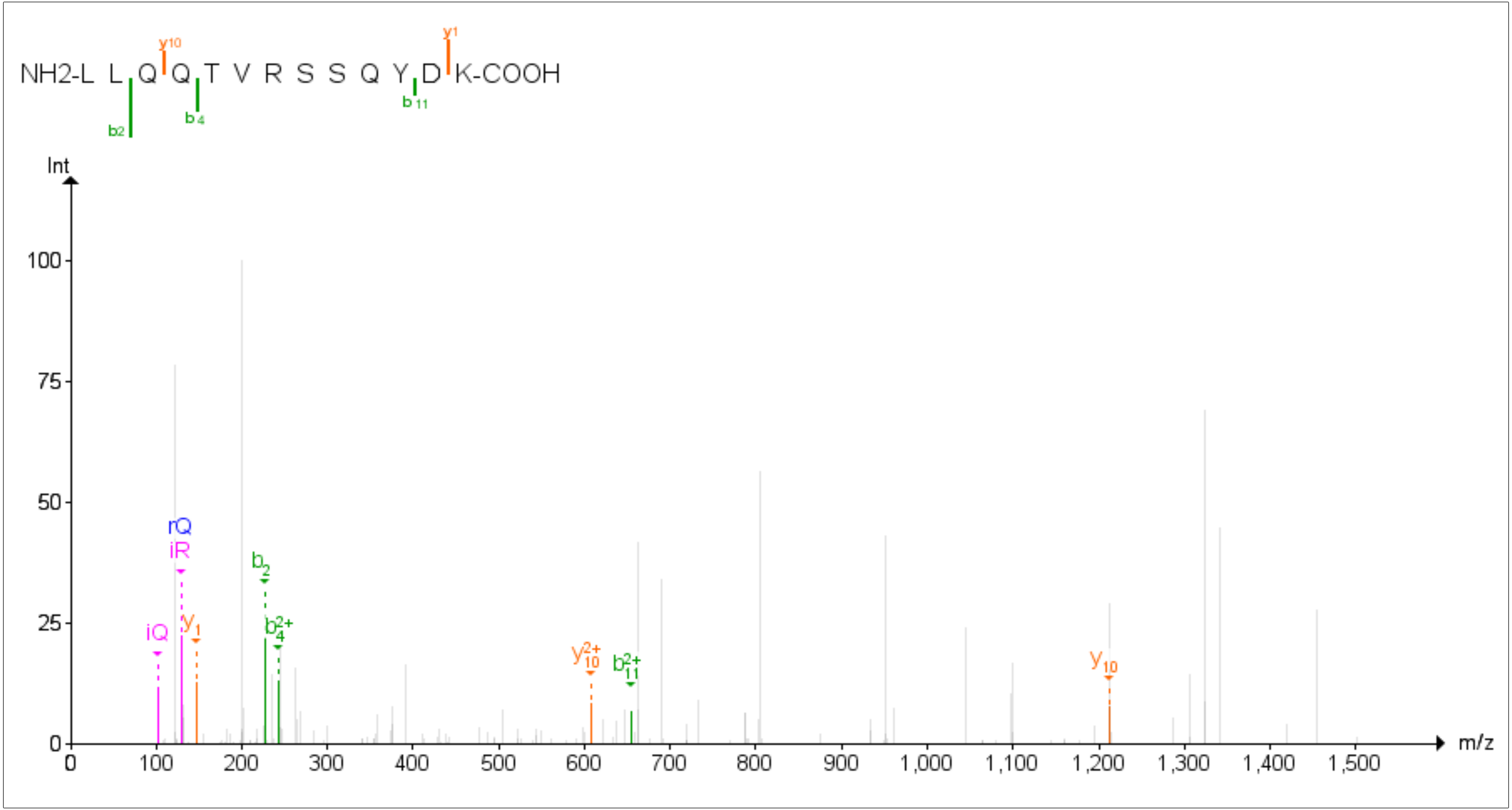
NH2-L L Q Q T V R S S Q Y D K-COOH
y10
y1
b2
b4
b11
Int
100
75
50
25
0
rQ
iR
iQ
y1
b2
b4 2+
y10 2+
b11 2+
y10
0
100
200
300
400
500
600
700
800
900
1,000
1,100
1,200
1,300
1,400
1,500
m/z

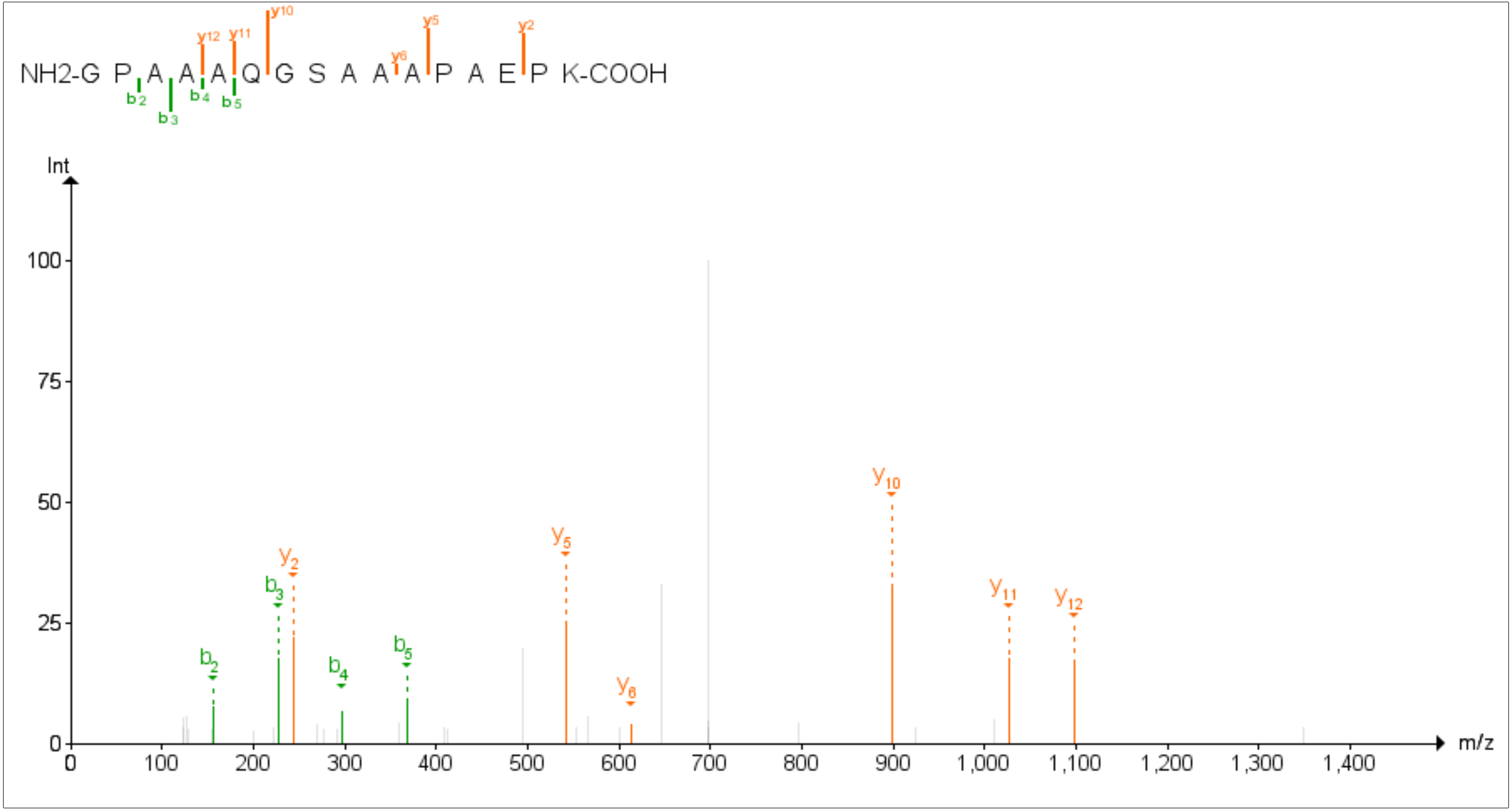

NH2-G P A A A Q G S A A A A P A E P K-COOH
b2
b3
b4
b5
y12
y11
y10
y6
y5
y2
Int
100
75
50
25
0
m/z
0
100
200
300
400
500
600
700
800
900
1,000
1,100
1,200
1,300
1,400

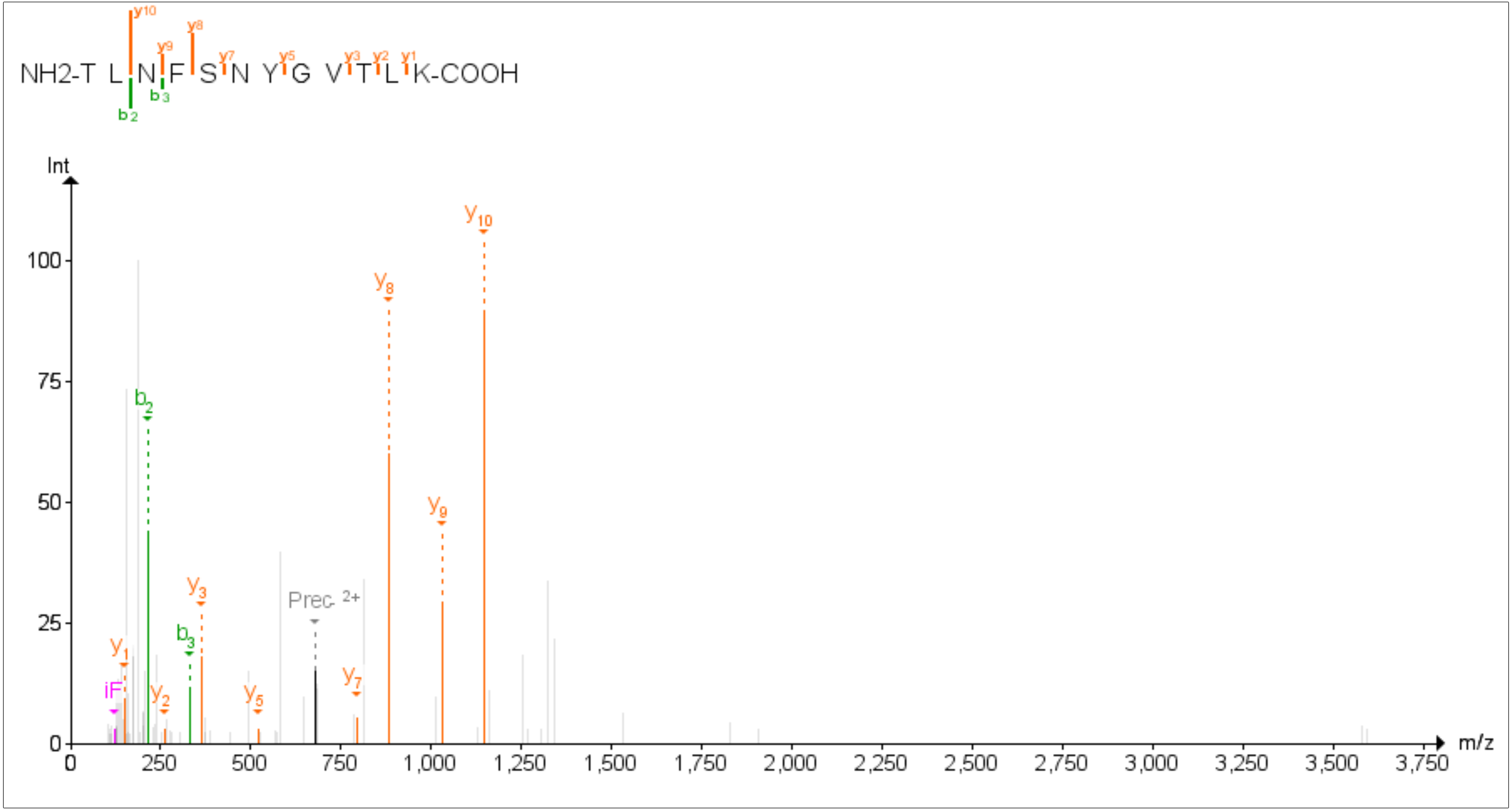

NH2-T L N F S N Y G V T L K-COOH
y10
y9
y8
y7
y5
y3
y2
y1
b2
b3
Int
m/z
Prec 2+
iF
0
25
50
75
100
0
250
500
750
1,000
1,250
1,500
1,750
2,000
2,250
2,500
2,750
3,000
3,250
3,500
3,750

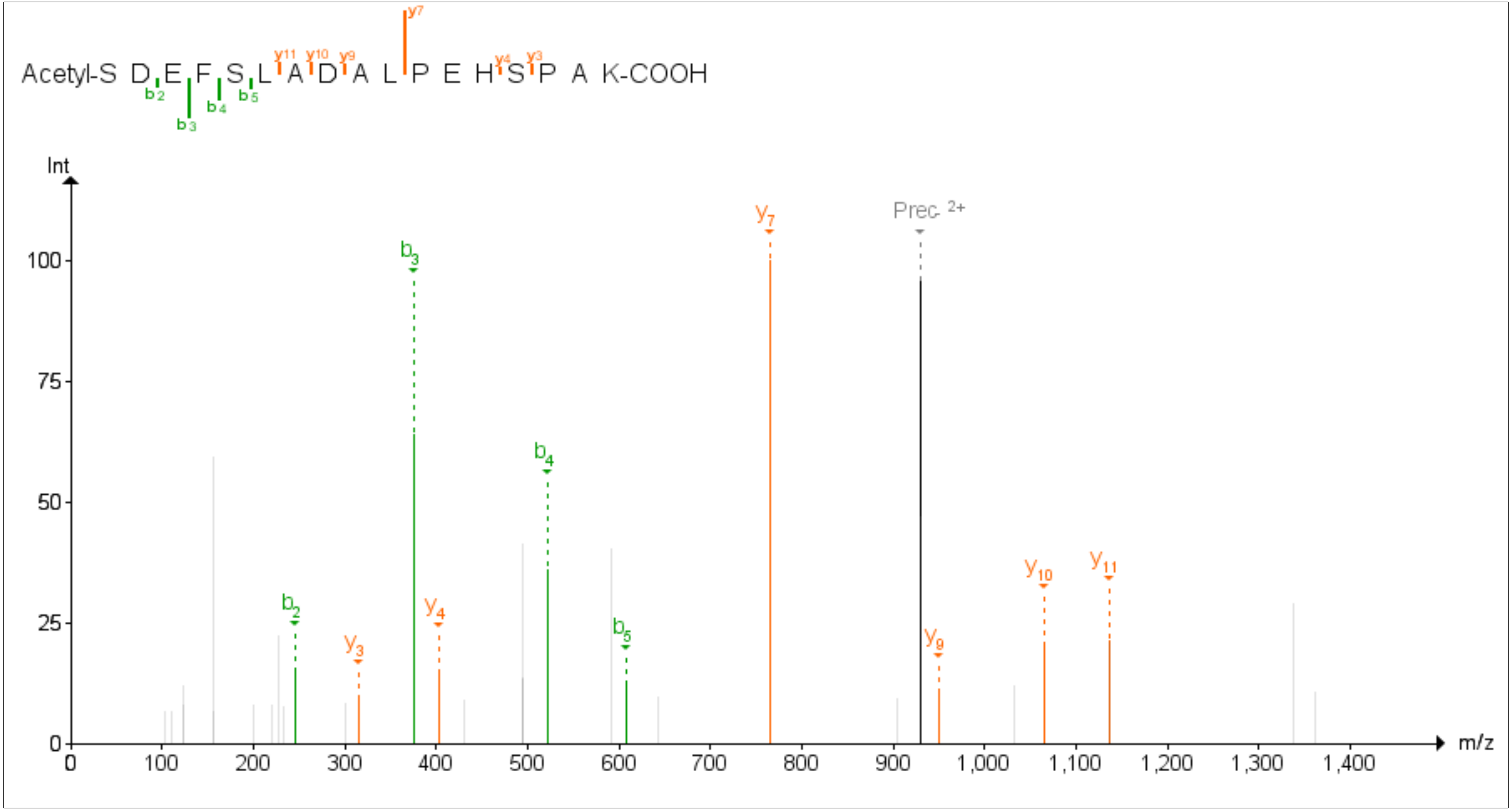

Acetyl-S D E F S L A D A L P E H S P A K-COOH
b2 b3 b4 b5
y11 y10 y9 y7 y4 y3
Int
m/z
Prec. 2+

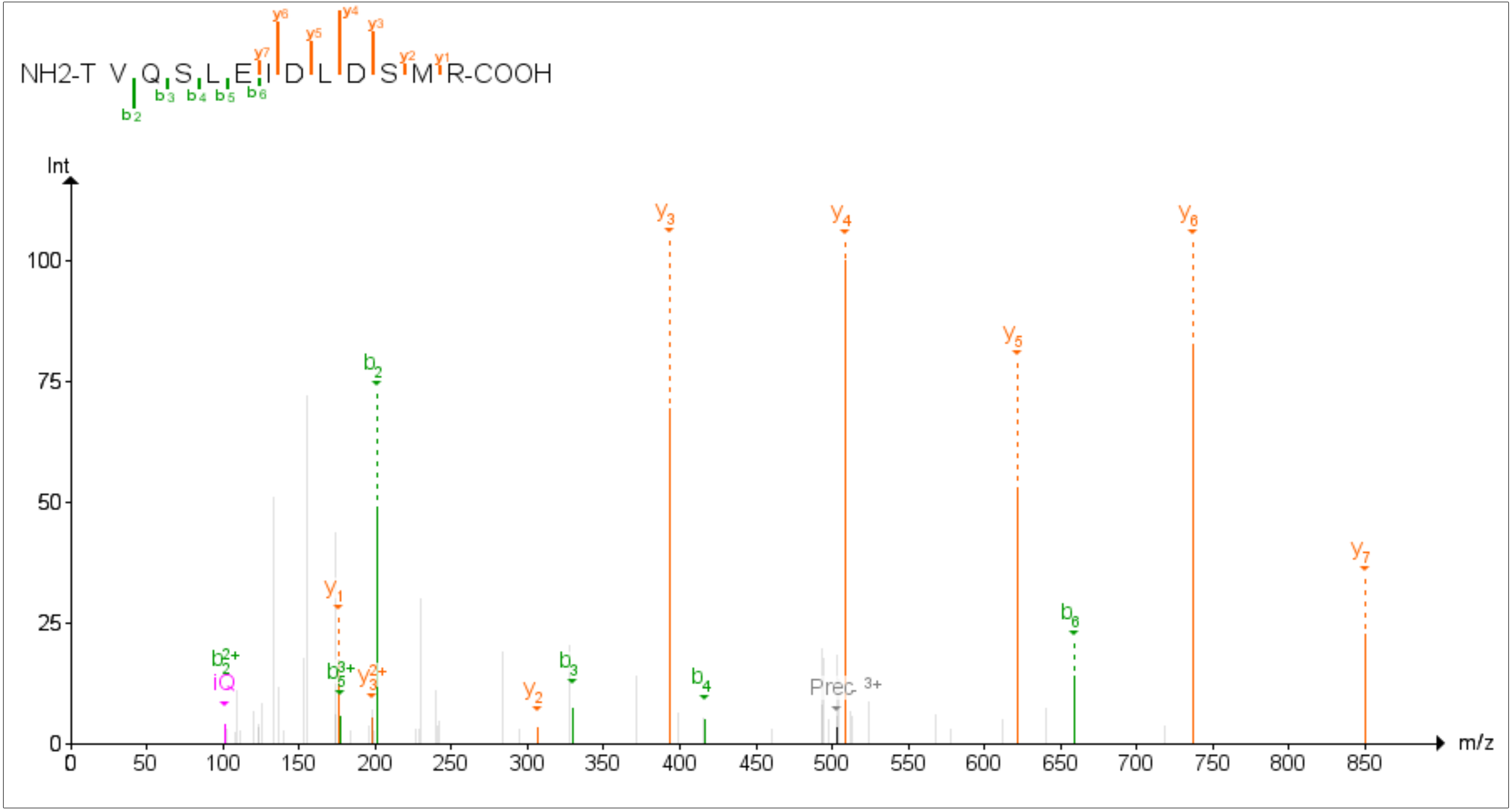
NH2-T V Q S L E I D L D S M R-COOH
y7 y6 y5 y4 y3 y2 y1
b2 b3 b4 b5 b6
Int
m/z
y3
y4
y6
y5
b2
y7
b6
y1
b2 2+
iQ
b5 3+
y3 2+
y2
b3
b4
Prec. 3+
0 25 50 75 100
0 50 100 150 200 250 300 350 400 450 500 550 600 650 700 750 800 850

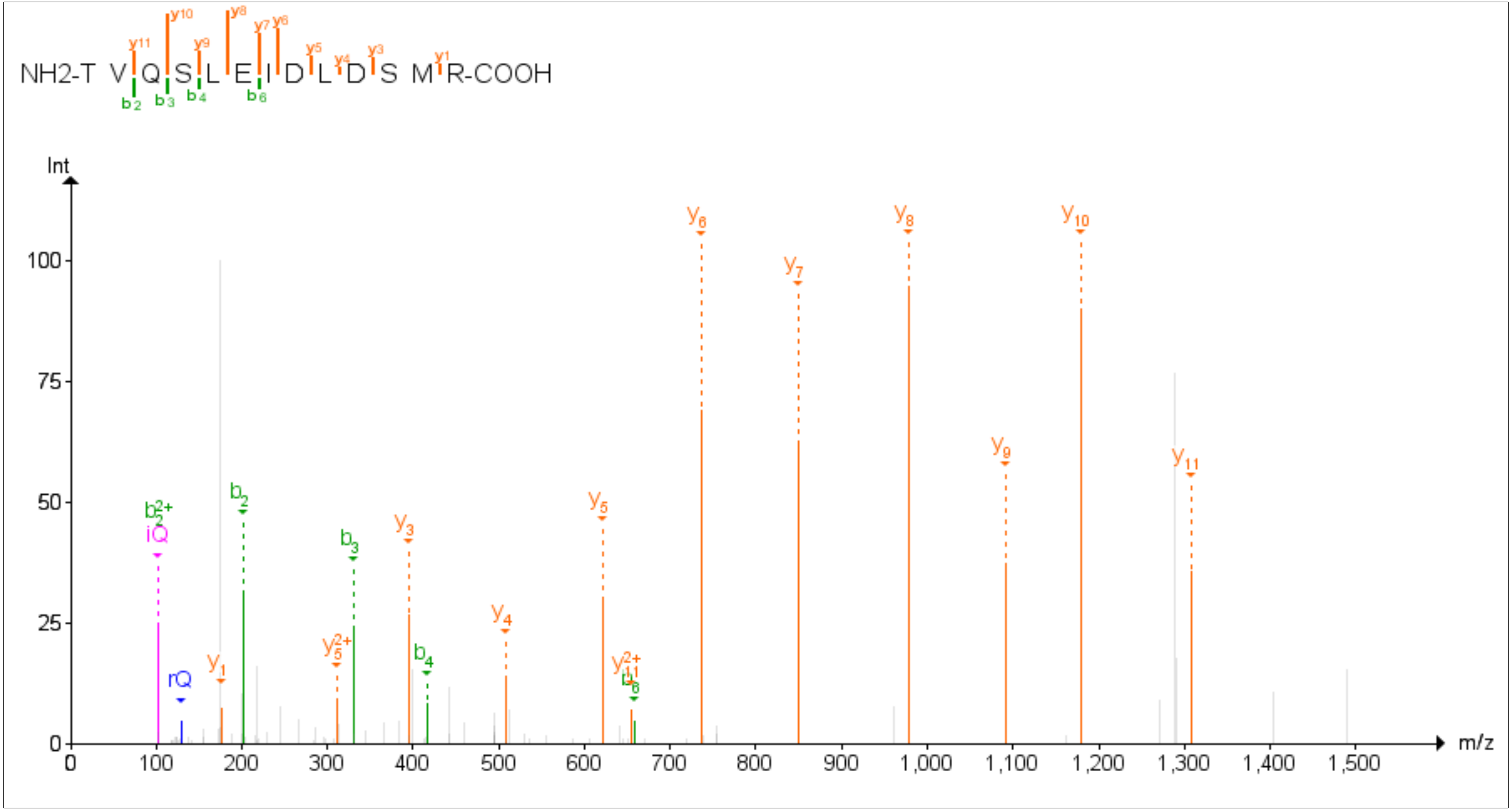

NH2-T V Q S L E I D L D S M R-COOH
y11 y10 y9 y8 y7 y6 y5 y4 y3 y1
b2 b3 b4 b6
Int
100
75
50
25
0
m/z
0 100 200 300 400 500 600 700 800 900 1,000 1,100 1,200 1,300 1,400 1,500
y6 y8 y10 y7 y9 y11 y5 y3 y4 y1 y5 2+ y11 2+ b2 b3 b4 b6 b2 2+ iQ rQ

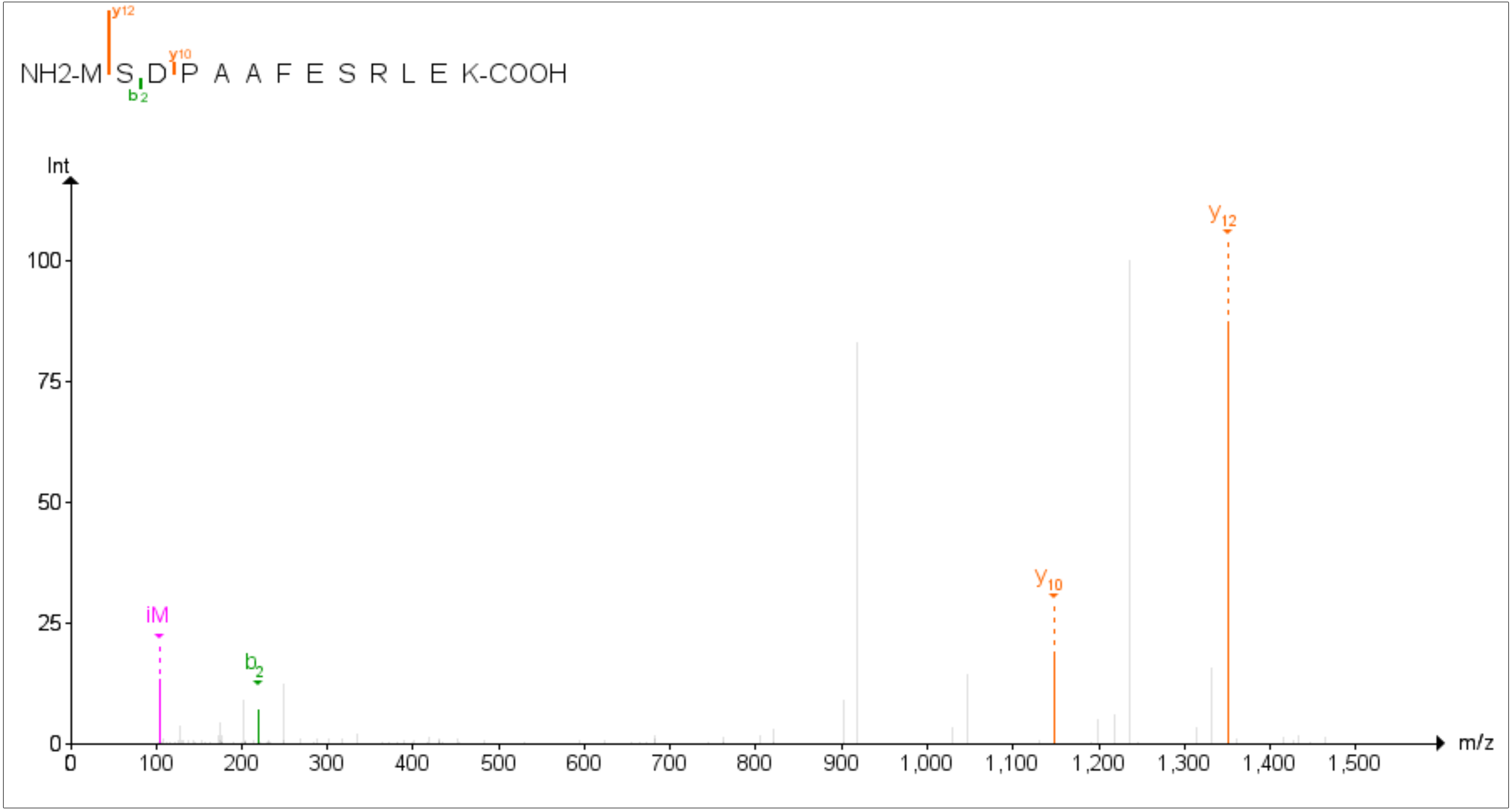

y12
y10
NH2-M S D P A A F E S R L E K-COOH
b2
Int
100
75
50
25
0
y12
y10
iM
b2
0 100 200 300 400 500 600 700 800 900 1,000 1,100 1,200 1,300 1,400 1,500
m/z

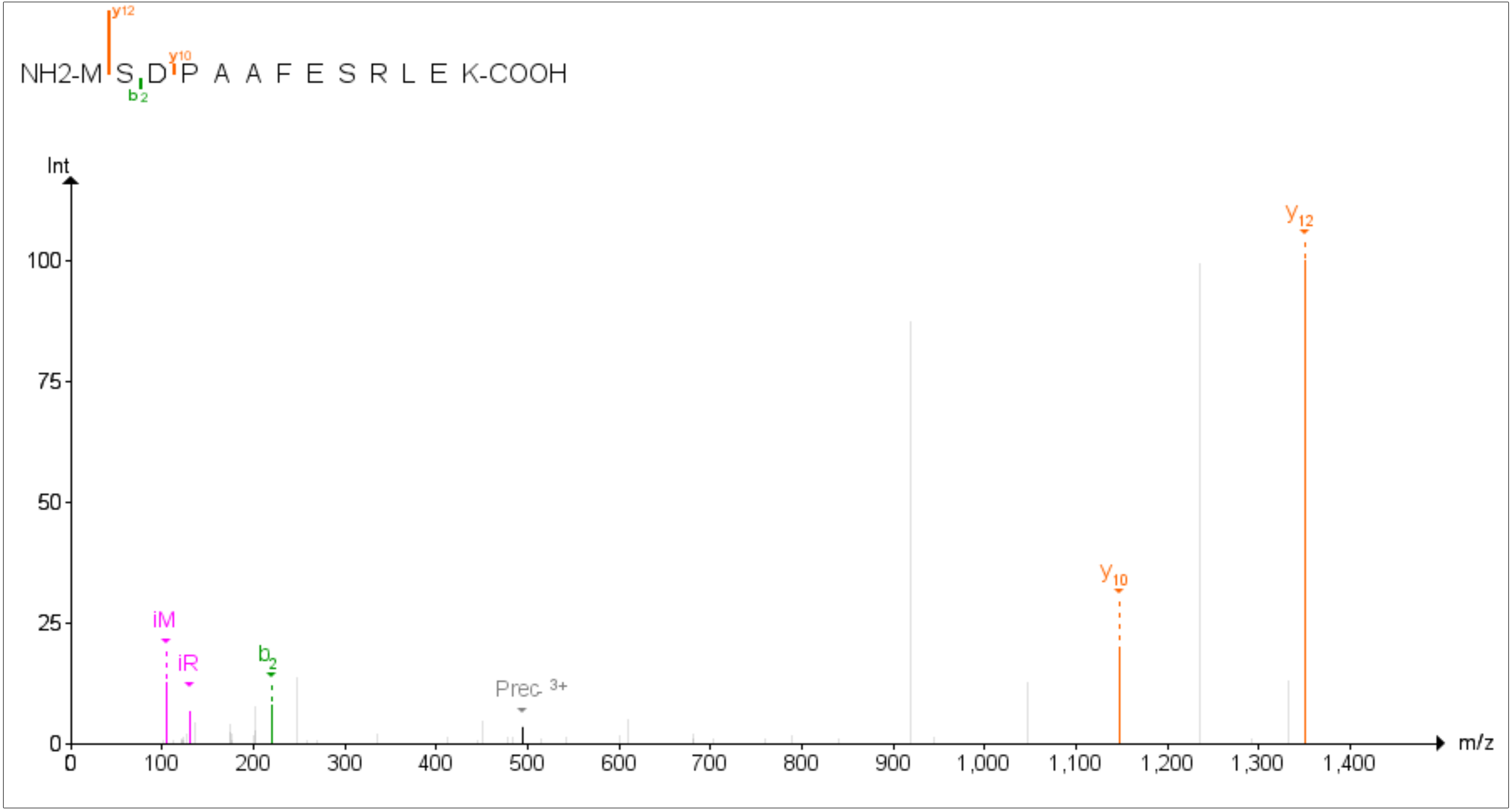

NH2-M S D P A A F E S R L E K-COOH
y12
y10
b2
Int
100
75
50
25
0
iM
iR
b2
Prec 3+
y10
y12
0
100
200
300
400
500
600
700
800
900
1,000
1,100
1,200
1,300
1,400
m/z

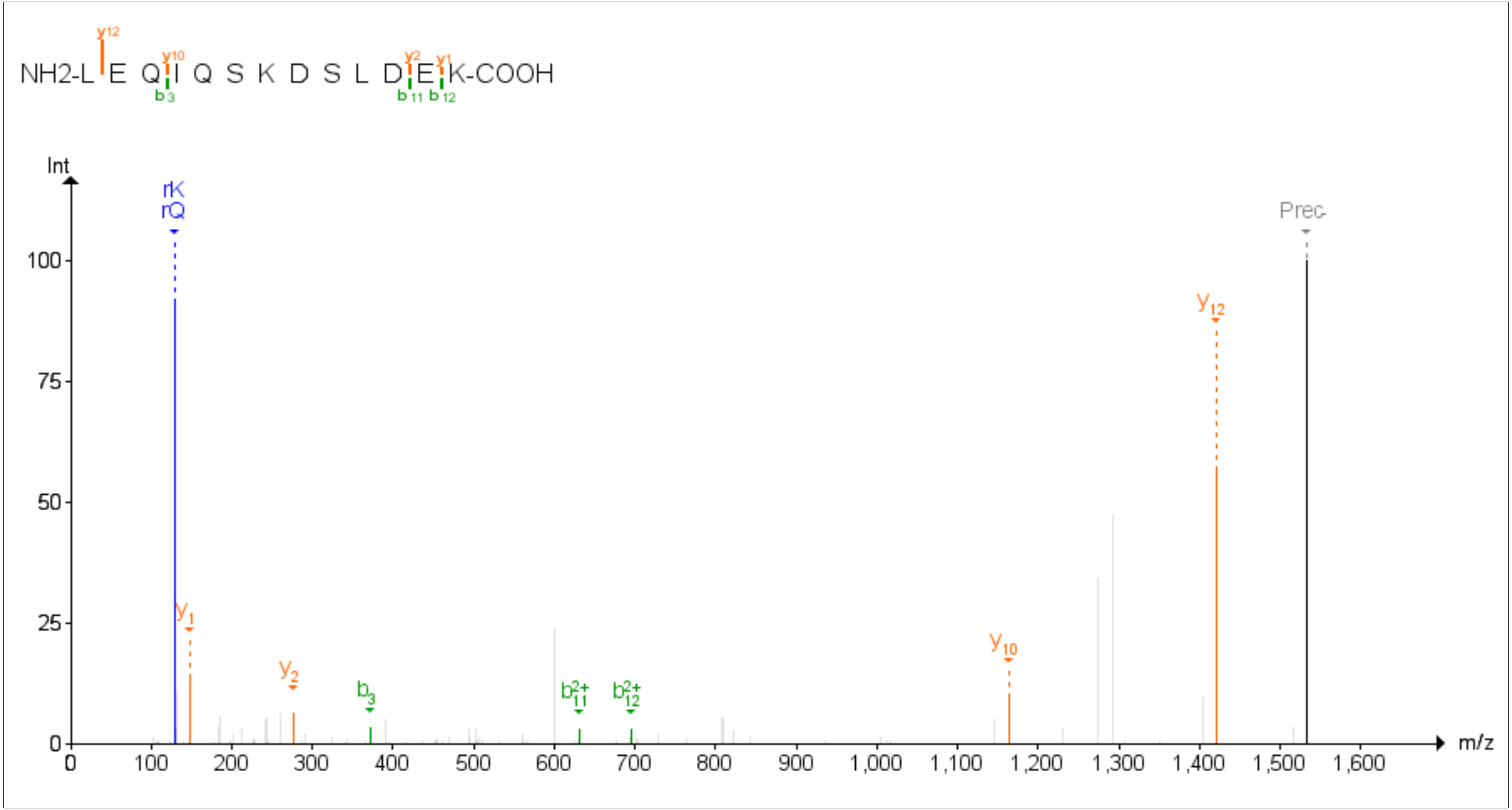

NH2-L E Q I Q S K D S L D E K-COOH
y12
y10
y2
y1
b3
b11
b12
Int
rK
rQ
Prec
y12
y1
y2
b3
b2+ 11
b2+ 12
y10
100
75
50
25
0
m/z
0
100
200
300
400
500
600
700
800
900
1,000
1,100
1,200
1,300
1,400
1,500
1,600

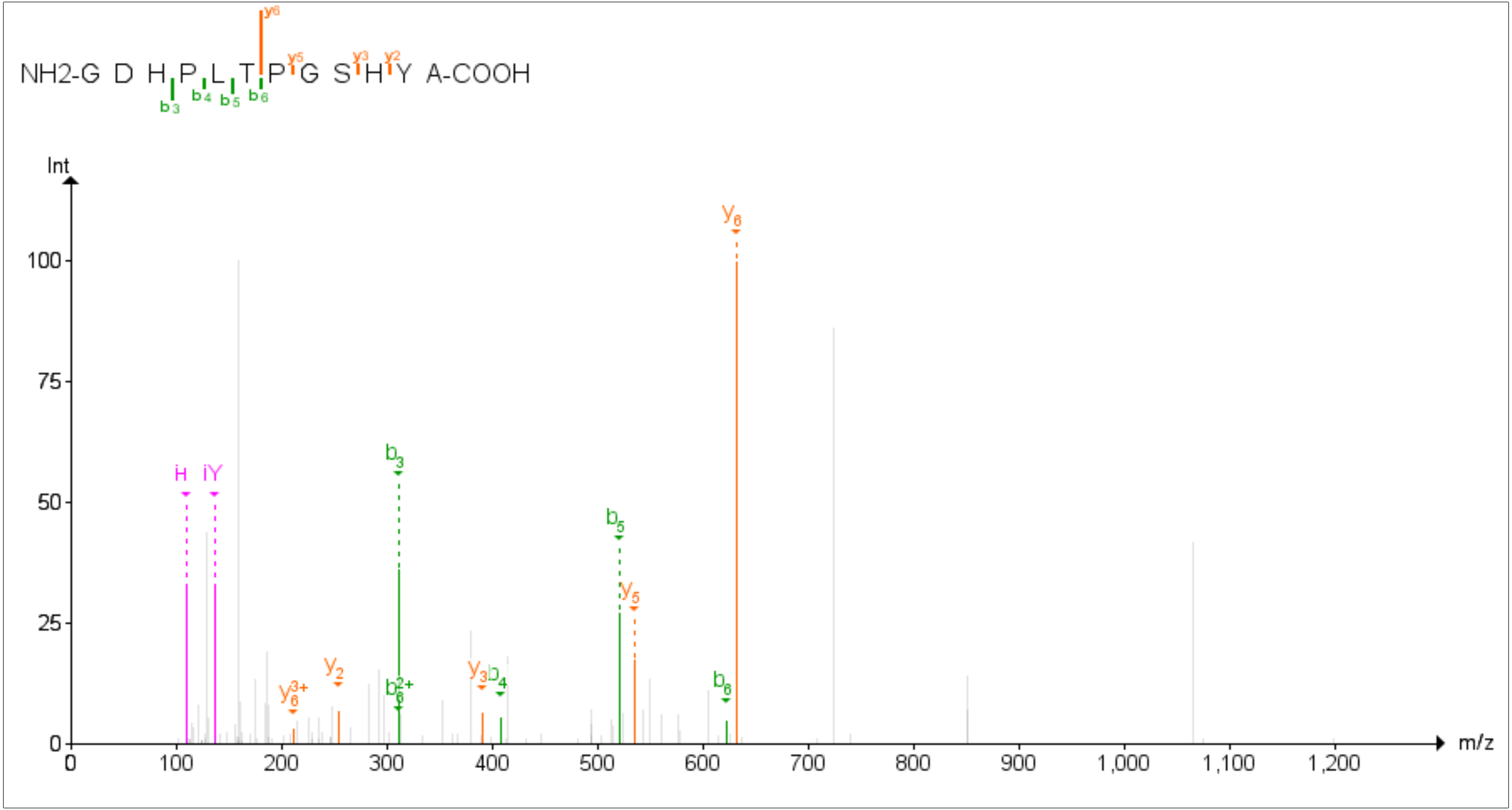

NH2-G D H P L T P G S H Y A-COOH
b3 b4 b5 b6
y6 y5 y3 y2
Int
m/z
0
25
50
75
100
0
100
200
300
400
500
600
700
800
900
1,000
1,100
1,200
iH
iY
b3
b5
y5
y6
y2
y3
b4
b6

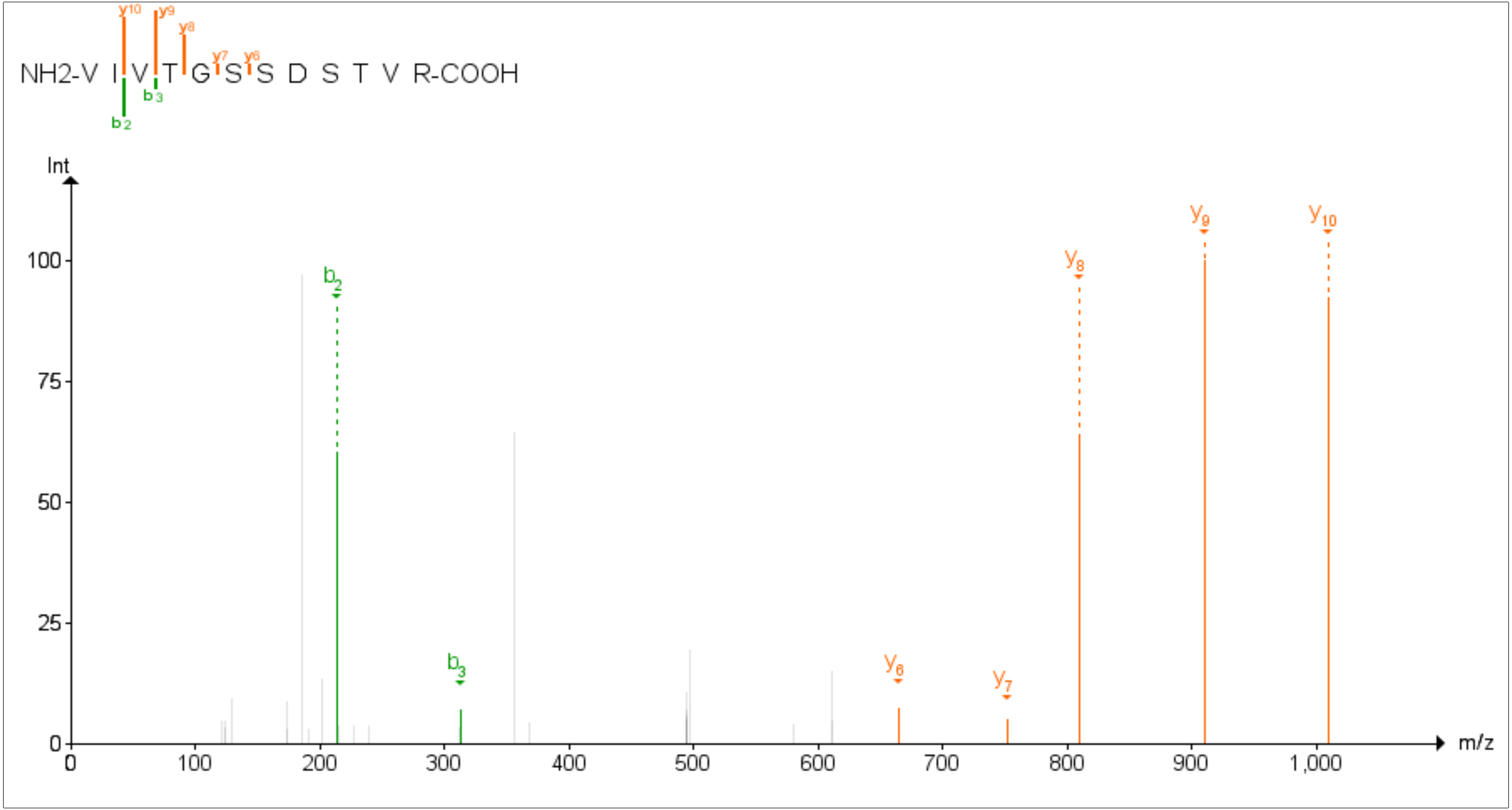

NH2-V I V T G S S D S T V R-COOH
y10 y9 y8 y7 y6
b2 b3
Int
100
75
50
25
0
0 100 200 300 400 500 600 700 800 900 1,000
m/z
b2
b3
y6
y7
y8
y9
y10

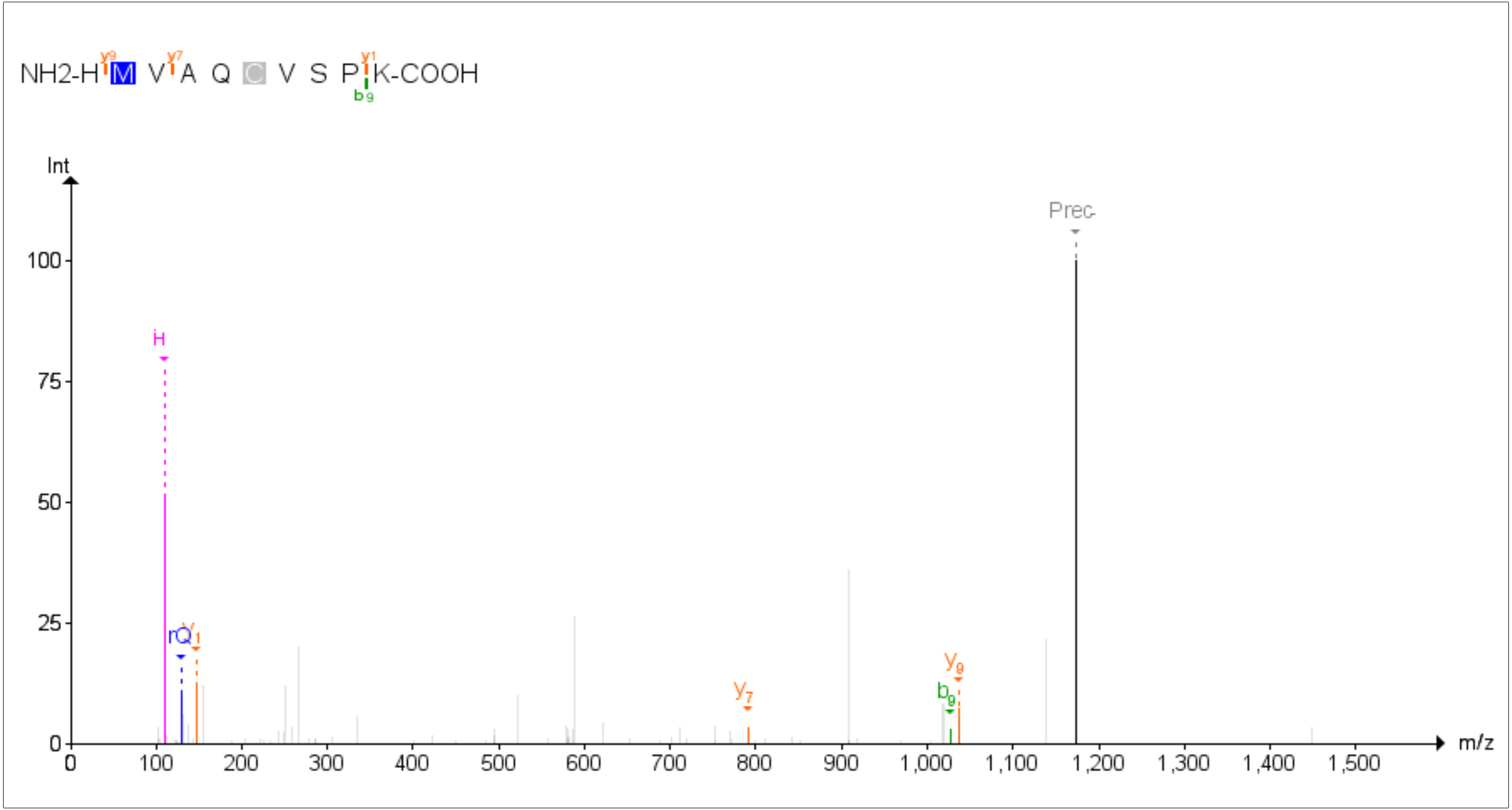

NH2-H M V A Q C V S P K-COOH
y9
y7
y1
b9
Int
Prec
H
rQ
y1
y7
b9
y9
100
75
50
25
0
0
100
200
300
400
500
600
700
800
900
1,000
1,100
1,200
1,300
1,400
1,500
m/z

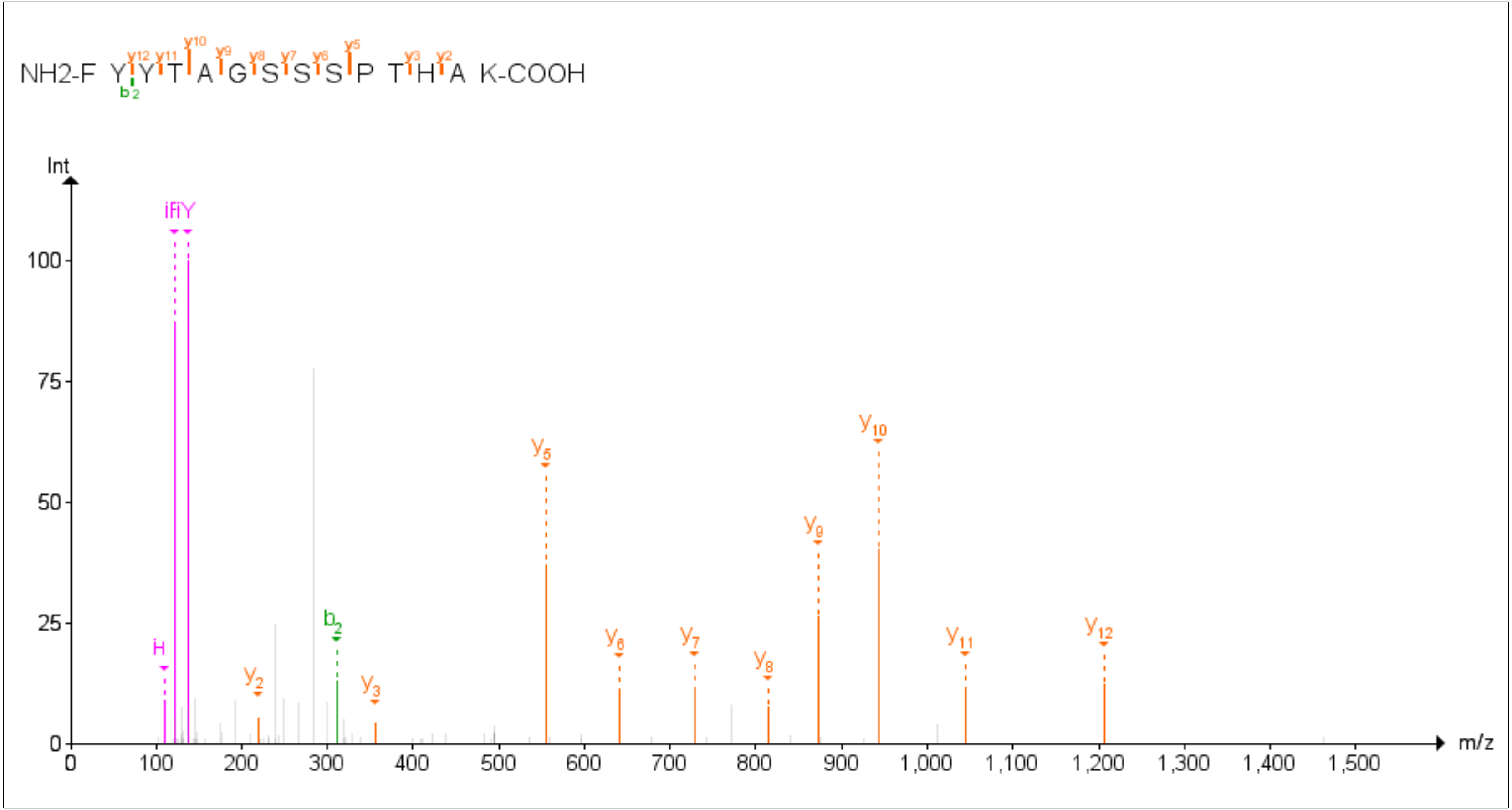
NH2-F Y Y T A G S S S P T H A K-COOH
b2
y12 y11 y10 y9 y8 y7 y6 y5 y3 y2
Int
m/z
iF
iY
iH
y2
b2
y3
y5
y6
y7
y8
y9
y10
y11
y12
0
25
50
75
100
0
100
200
300
400
500
600
700
800
900
1,000
1,100
1,200
1,300
1,400
1,500

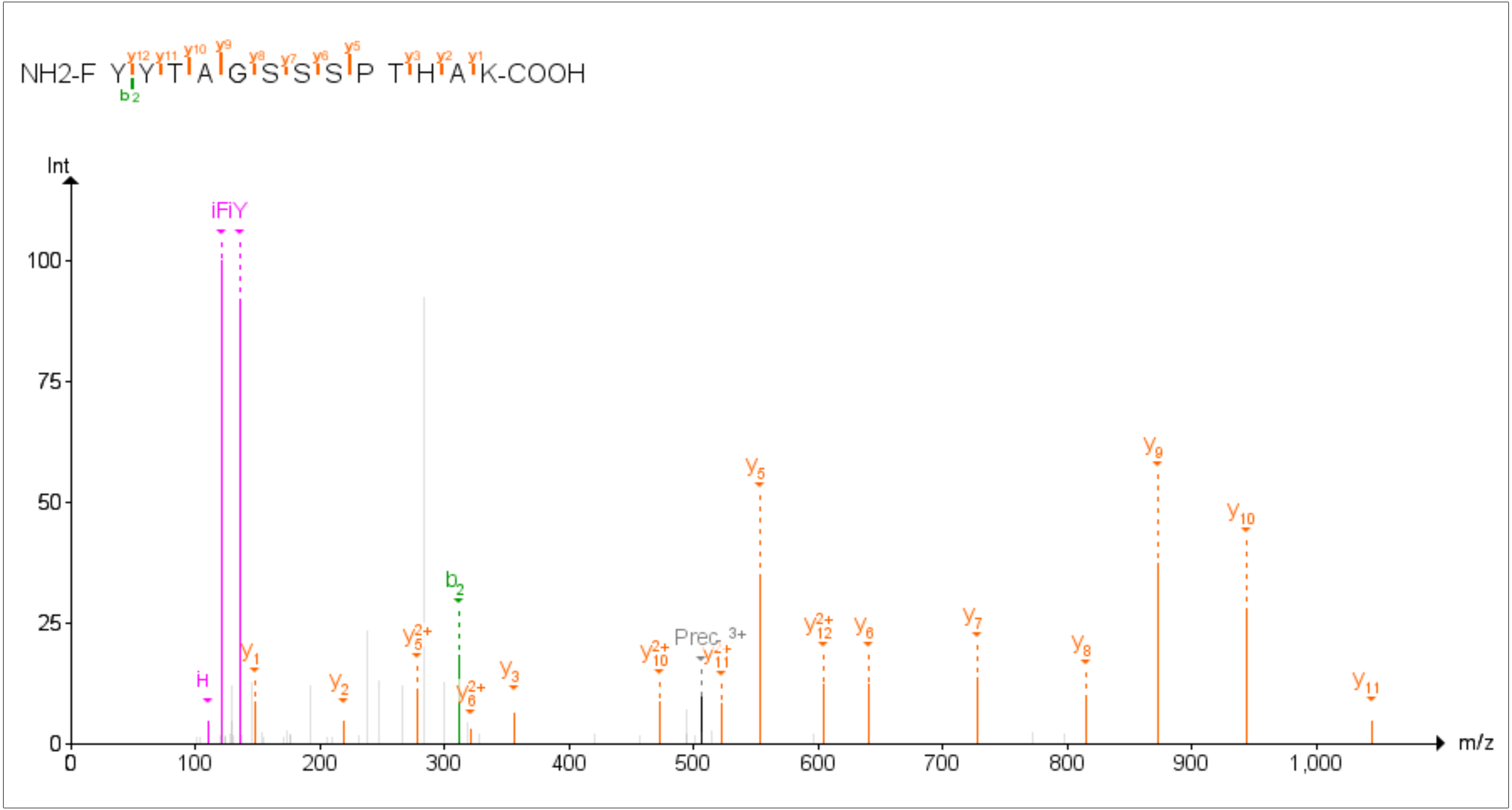
NH2-F Y Y T A G S S S P T H A K-COOH
y12 y11 y10 y9 y8 y7 y6 y5 y3 y2 y1
b2
Int
m/z
iFiY
iH
y1
y2
y5 2+
b2
y6 2+
y3
y10 2+
Prec. 3+
y11 2+
y5
y12 2+
y6
y7
y8
y9
y10
y11
0
25
50
75
100
0
100
200
300
400
500
600
700
800
900
1,000

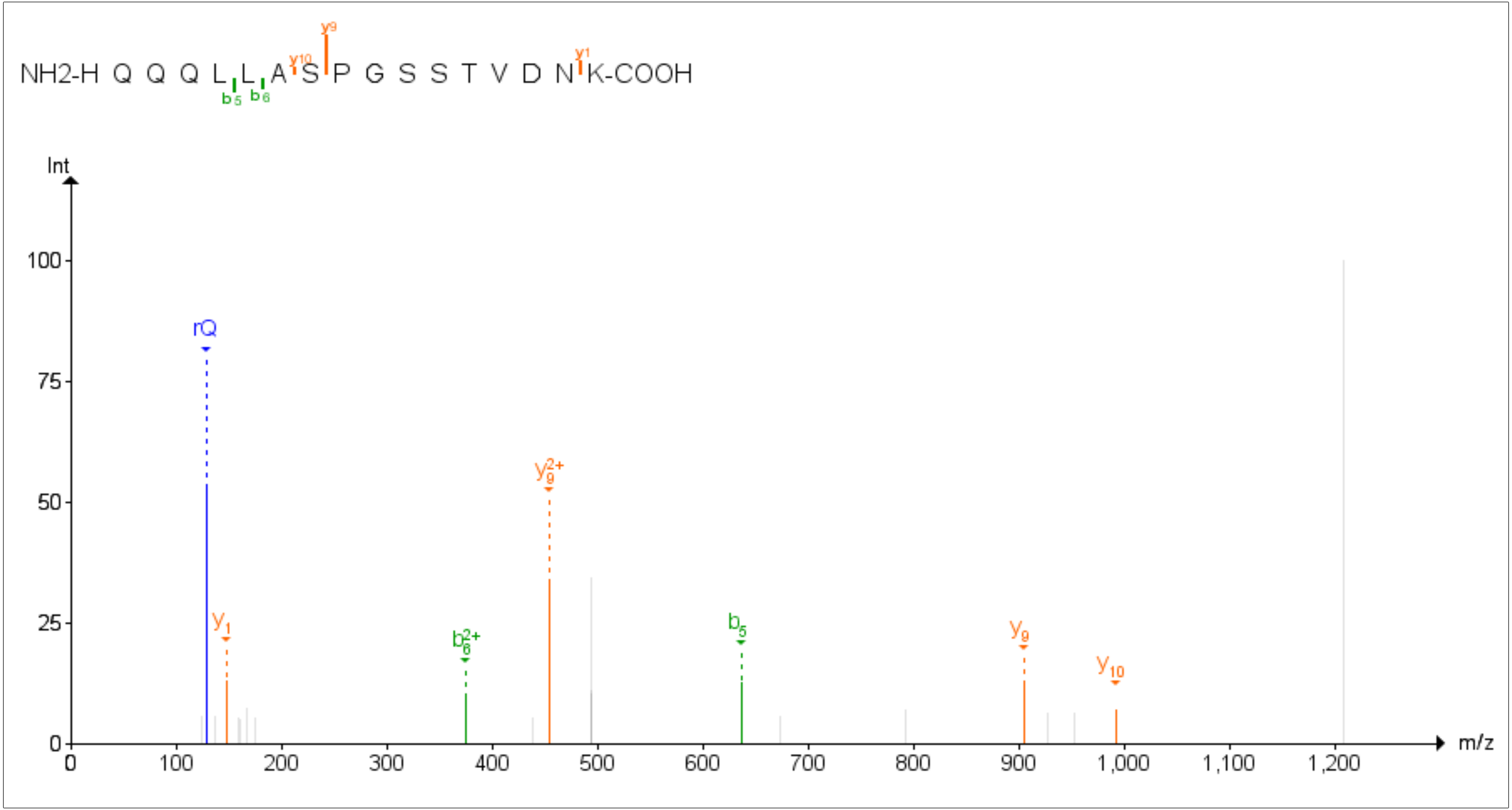

NH2-H Q Q Q L L A S P G S S T V D N K-COOH
y9
y10
y1
b5
b6
Int
100
75
50
25
0
rQ
y1
b6 2+
y9 2+
b5
y9
y10
0
100
200
300
400
500
600
700
800
900
1,000
1,100
1,200
m/z

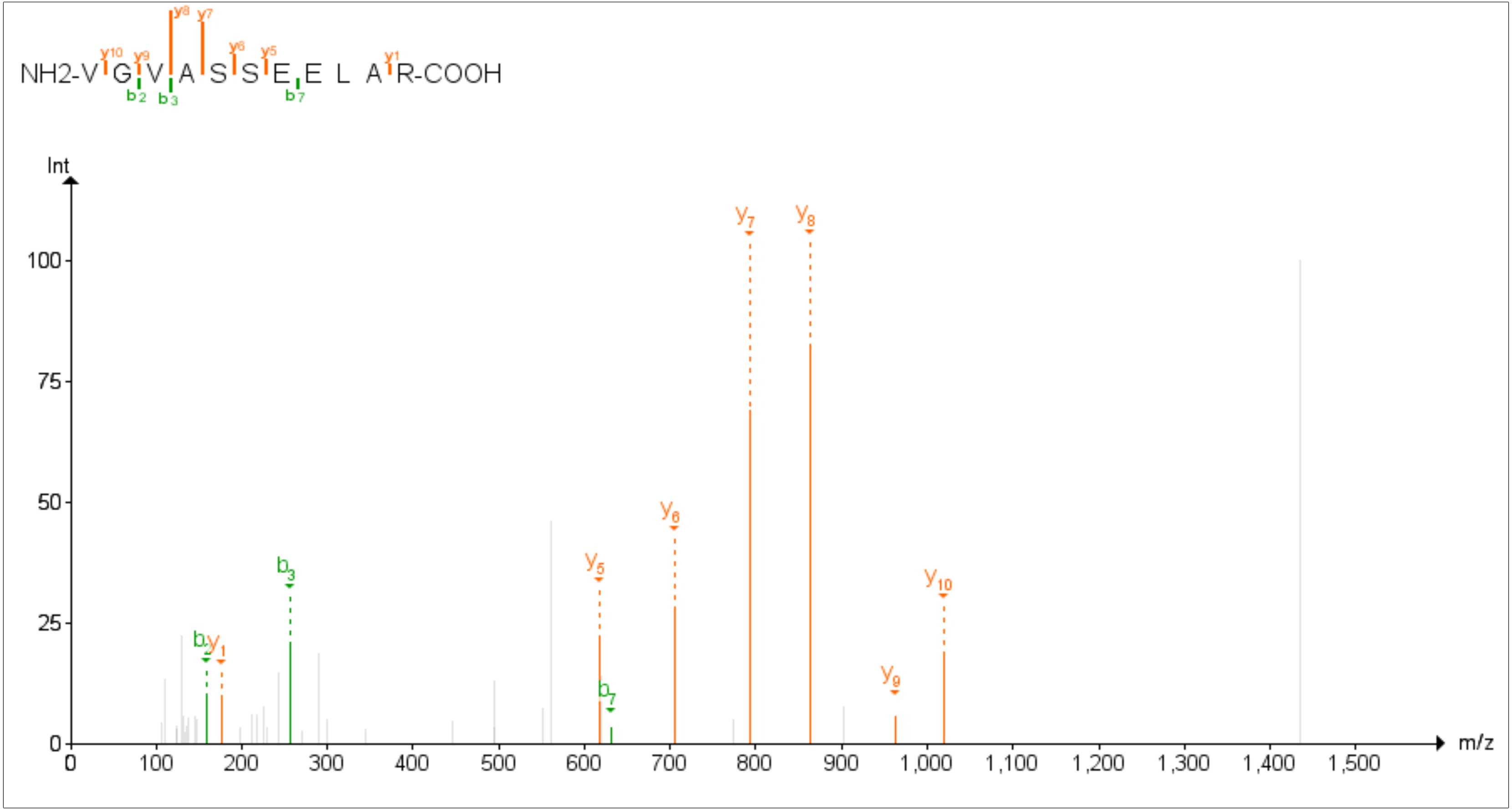

NH2-V G V A S S E E L A R-COOH
y10 y9 y8 y7 y6 y5 y1
b2 b3 b7
Int
100
75
50
25
0
m/z
0 100 200 300 400 500 600 700 800 900 1,000 1,100 1,200 1,300 1,400 1,500
y7 y8 y6 y5 y10 y9 b3 b2 y1 b7

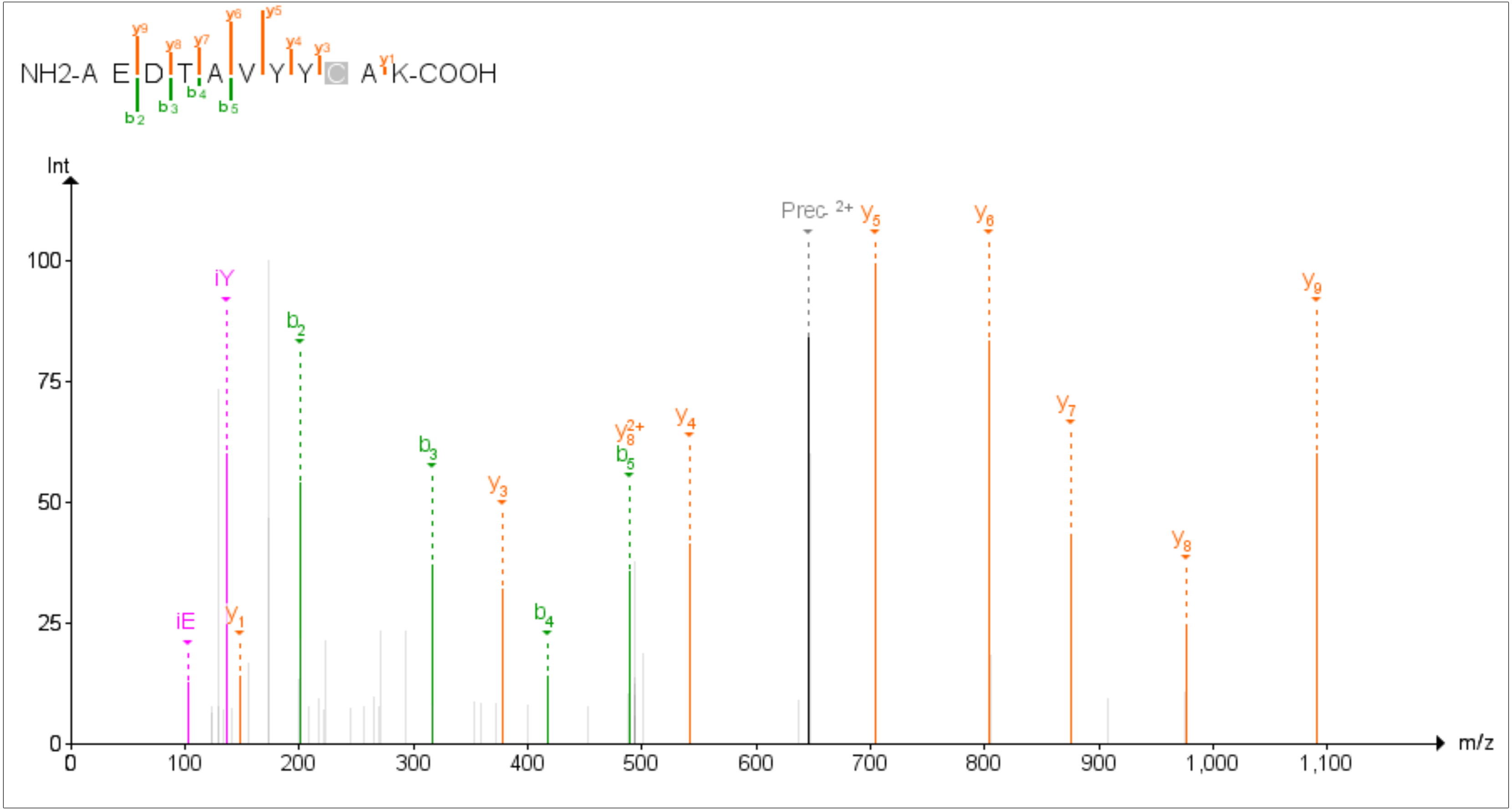

NH2-A E D T A V Y Y C A K-COOH
y9 y8 y7 y6 y5 y4 y3 y1
b2 b3 b4 b5
Int
m/z
Prec. 2+
iY
iE
y1
b2
b3
y3
b4
y8 2+
b5
y4
y5
y6
y7
y8
y9
0
25
50
75
100
0
100
200
300
400
500
600
700
800
900
1,000
1,100

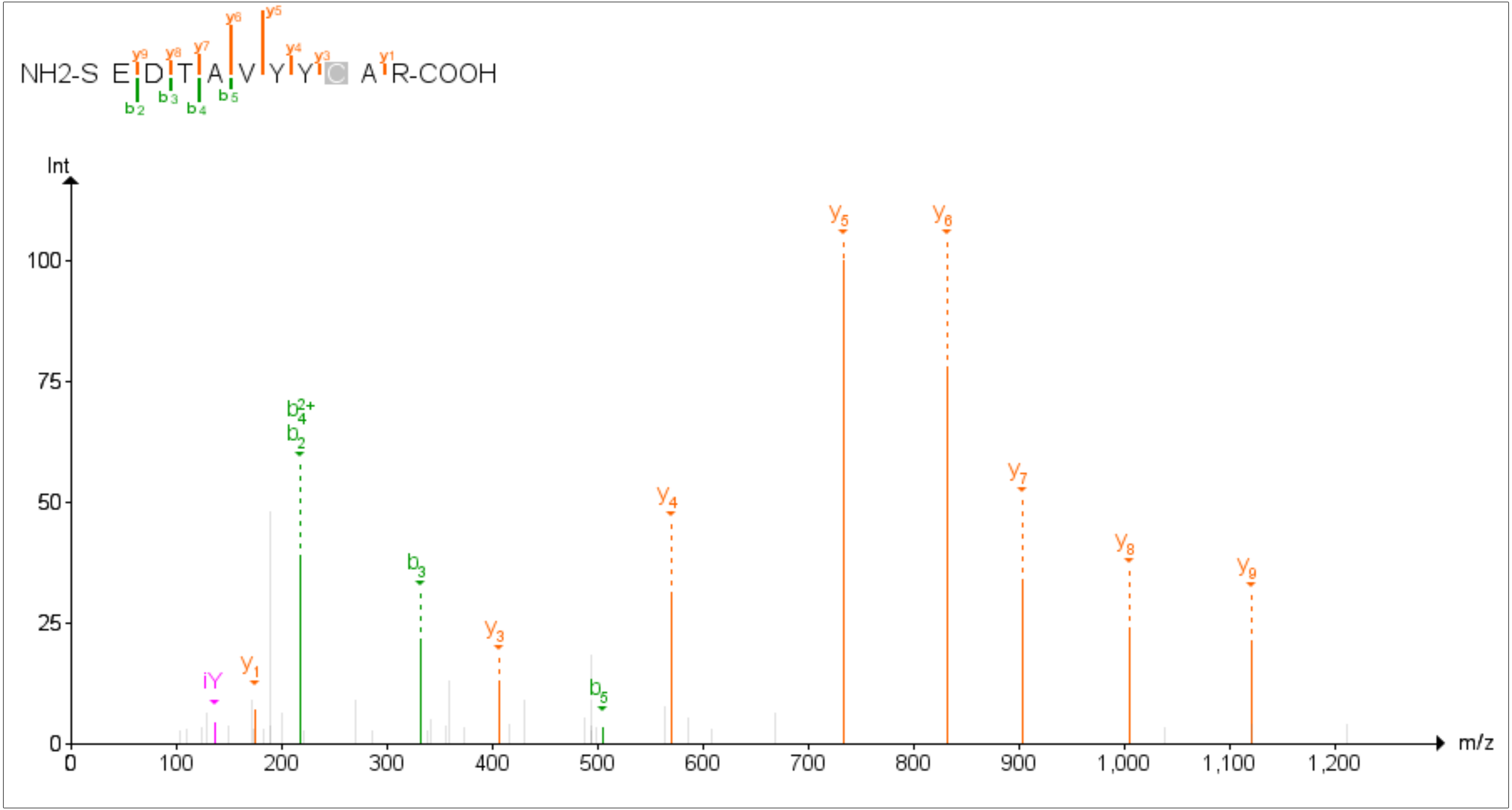

NH2-S E D T A V Y Y C A R-COOH
y9 y8 y7 y6 y5 y4 y3 y1
b2 b3 b4 b5
Int
100
75
50
25
0
b_4^{2+}
b_2
b_3
b_5
iY
y_1
y_3
y_4
y_5
y_6
y_7
y_8
y_9
0 100 200 300 400 500 600 700 800 900 1,000 1,100 1,200
m/z

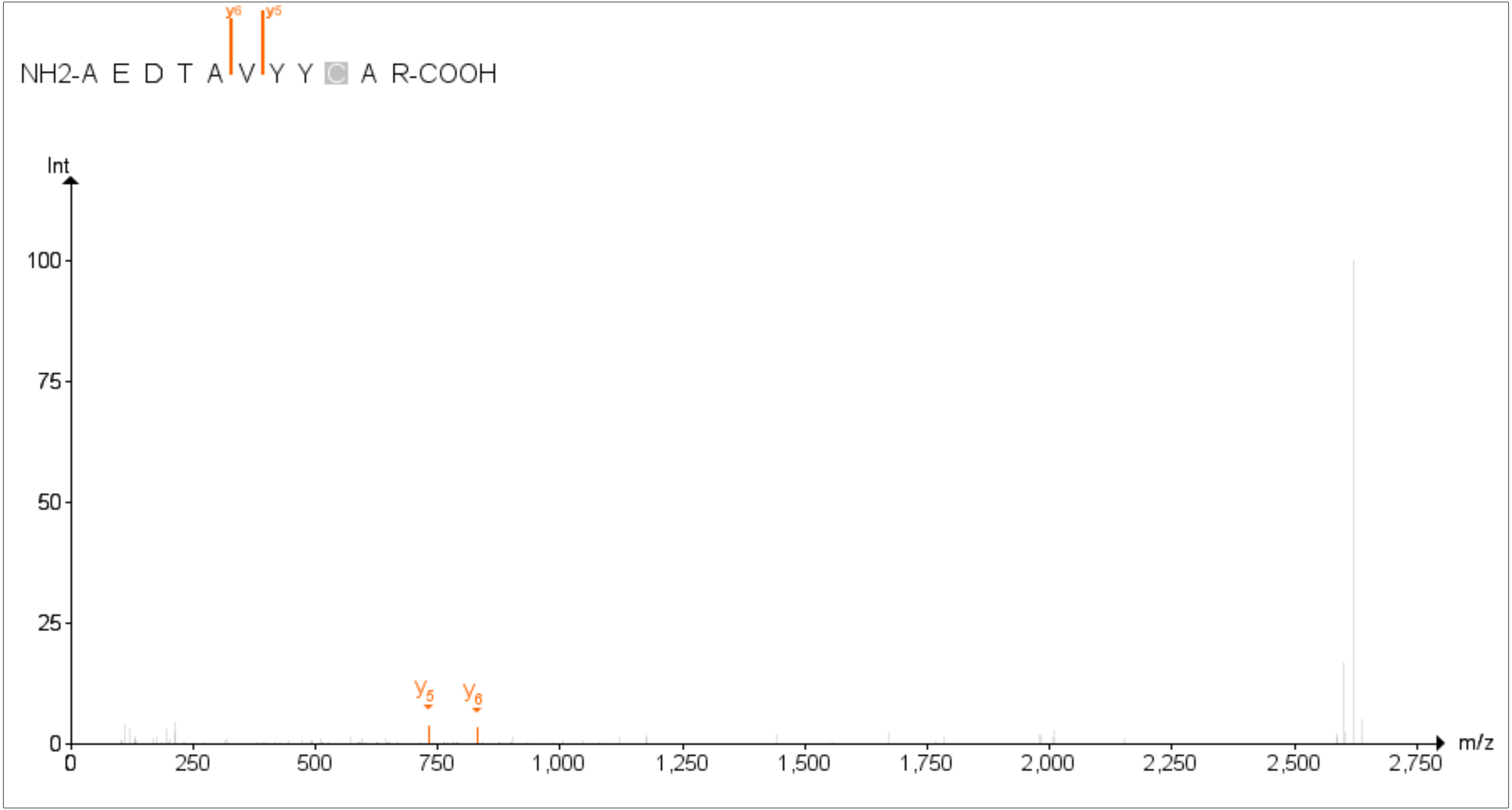

y6
y5
NH2-A E D T A V Y Y C A R-COOH
Int
100
75
50
25
0
y5
y6
0
250
500
750
1,000
1,250
1,500
1,750
2,000
2,250
2,500
2,750
m/z

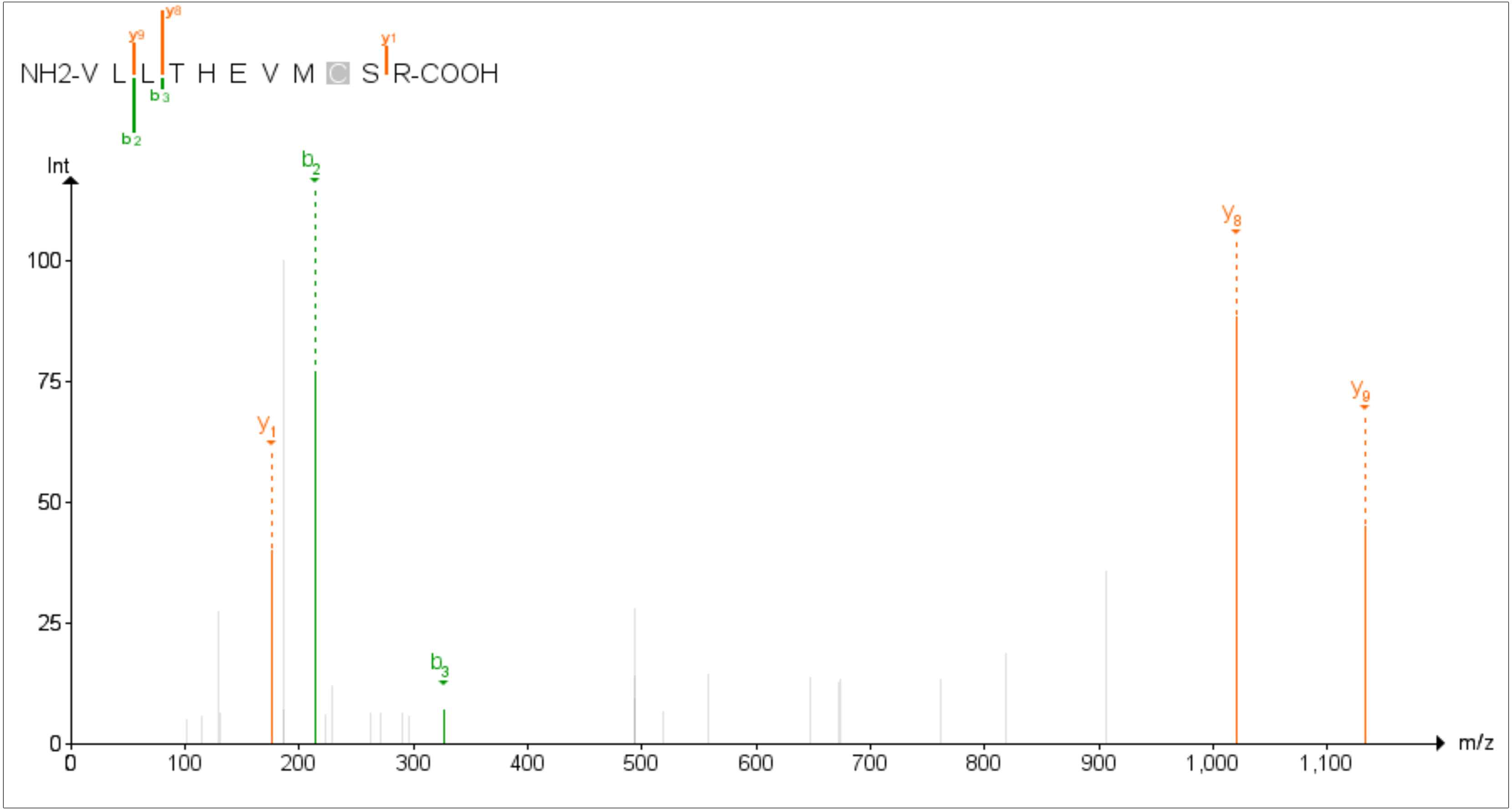

NH2-V L L T H E V M C S R-COOH
y9
y8
y1
b2
b3
Int
100
75
50
25
0
b2
y8
y9
y1
b3
0
100
200
300
400
500
600
700
800
900
1,000
1,100
m/z

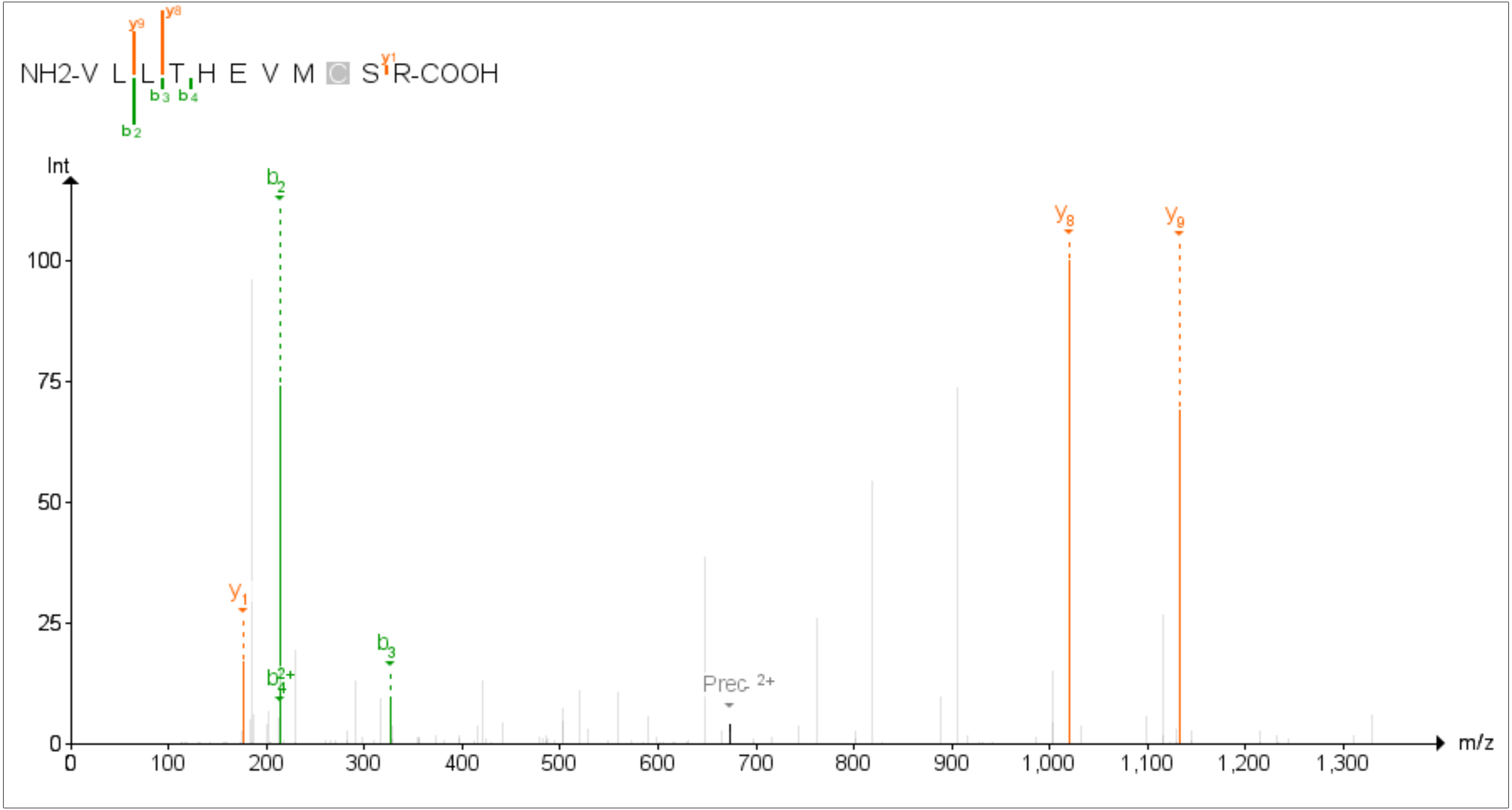

NH2-V L L T H E V M C S R-COOH
y9
y8
y1
b2
b3
b4
Int
100
75
50
25
0
b2
y8
y9
y1
b3
b4 2+
Prec 2+
0
100
200
300
400
500
600
700
800
900
1,000
1,100
1,200
1,300
m/z

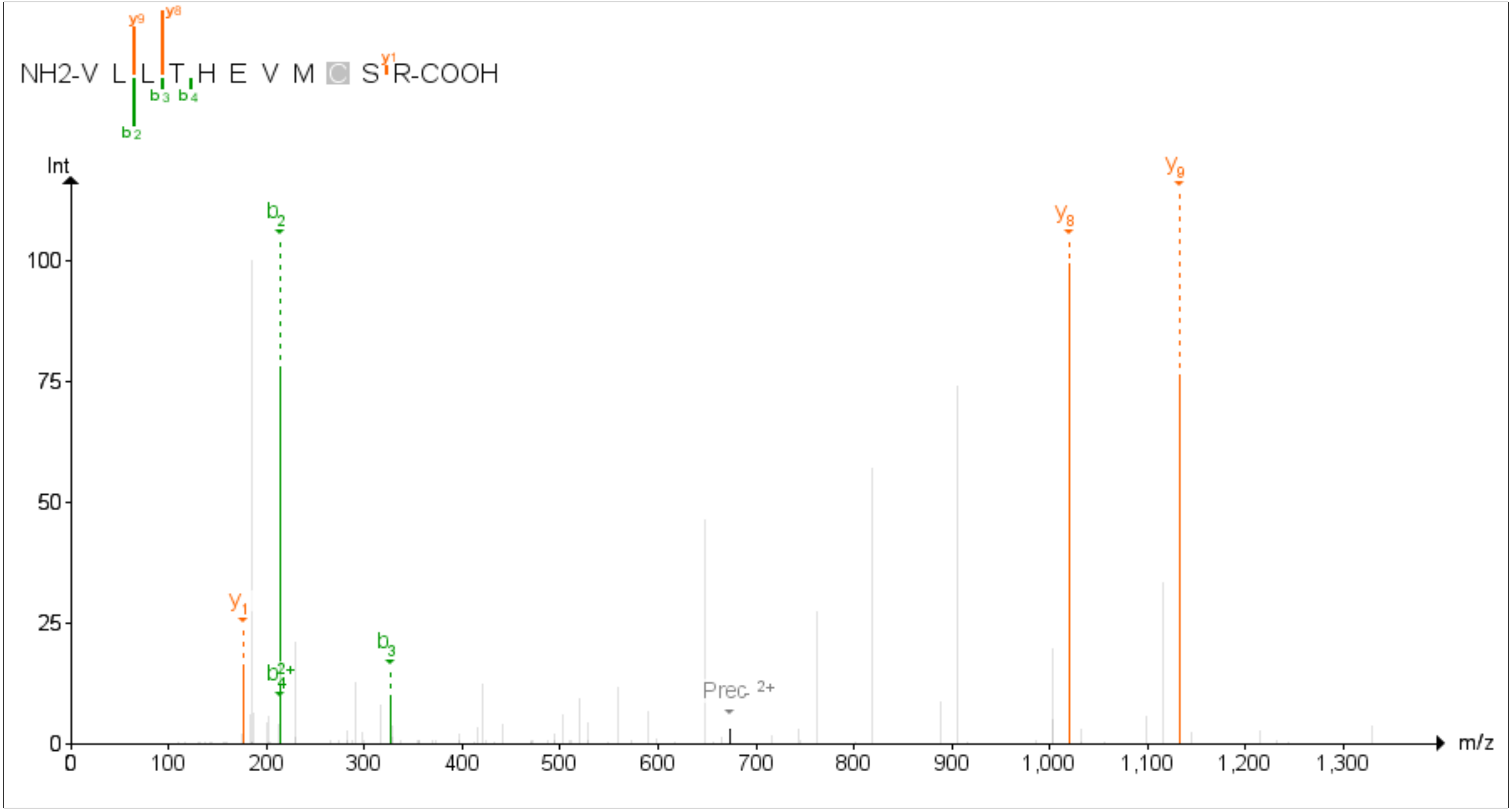

NH2-V L L T H E V M C S R-COOH
y9 y8 y1
b2 b3 b4
Int
100
75
50
25
0
b2
y8
y9
y1
b4 2+
b3
Prec 2+
0 100 200 300 400 500 600 700 800 900 1,000 1,100 1,200 1,300
m/z

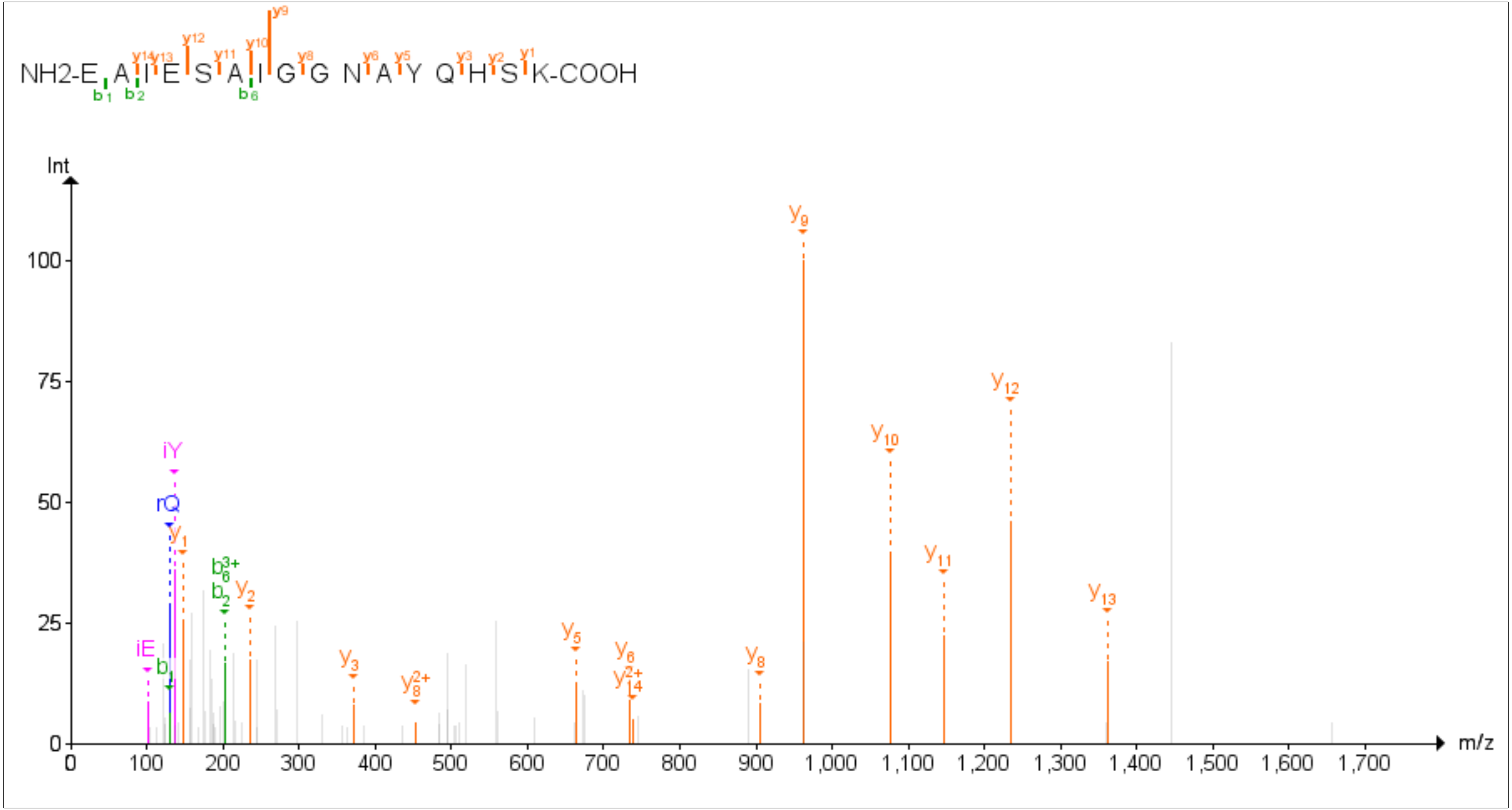
NH2-E A I I E S A I G G N A Y Q H S K-COOH
y14 y13 y12 y11 y10 y9 y8 y6 y5 y3 y2 y1
b1 b2 b6
Int
m/z
0
25
50
75
100
0 100 200 300 400 500 600 700 800 900 1,000 1,100 1,200 1,300 1,400 1,500 1,600 1,700
iY
rQ
y1
b6 3+
b2
y2
iE
b1
y3
y8 2+
y5
y6
y14 2+
y8
y9
y10
y11
y12
y13

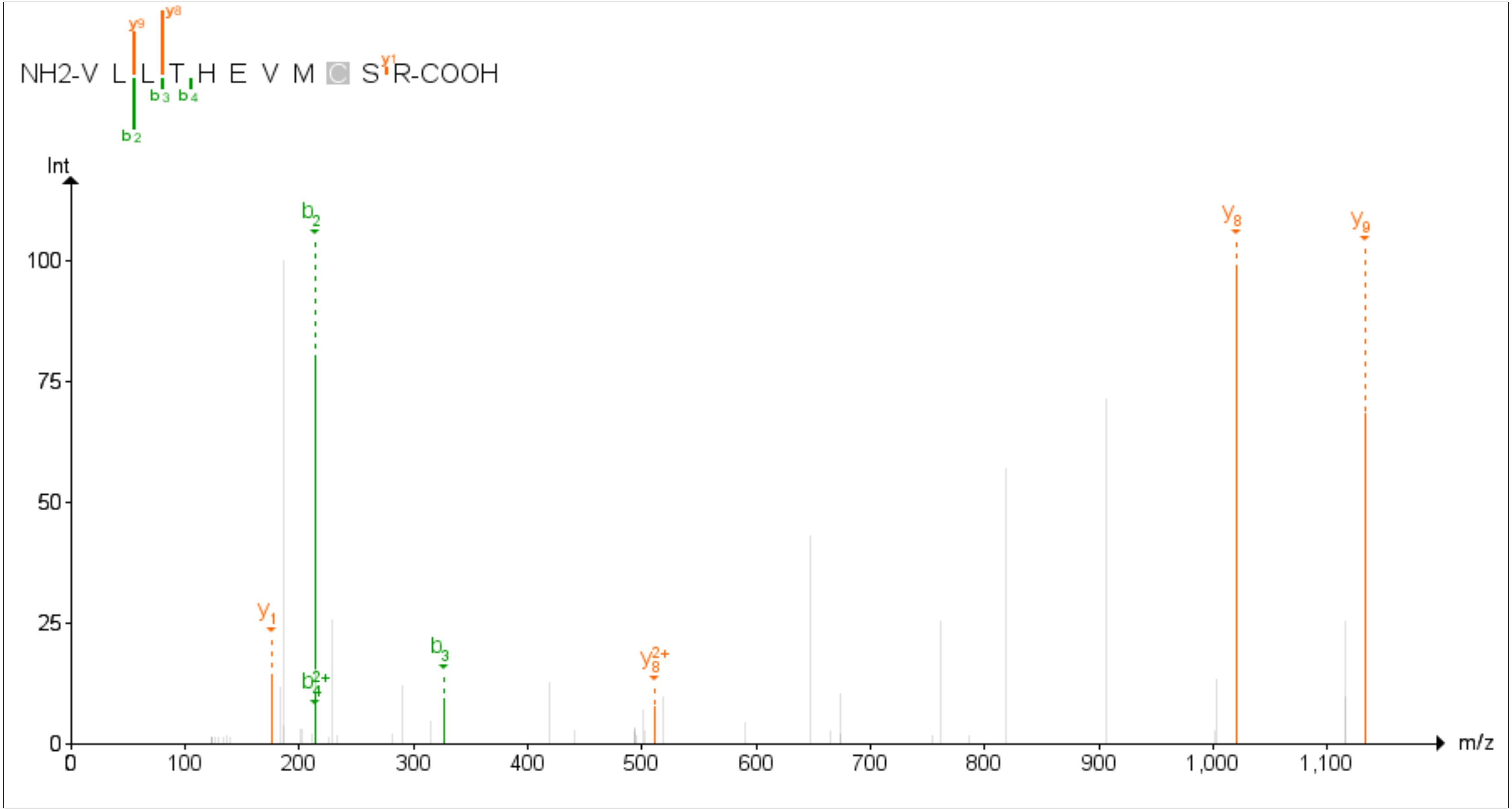
NH2-V L L T H E V M C S R-COOH
y9
y8
y1
b2
b3
b4
Int
m/z
0
25
50
75
100
0
100
200
300
400
500
600
700
800
900
1,000
1,100
b2
y1
b4 2+
b3
y8 2+
y8
y9

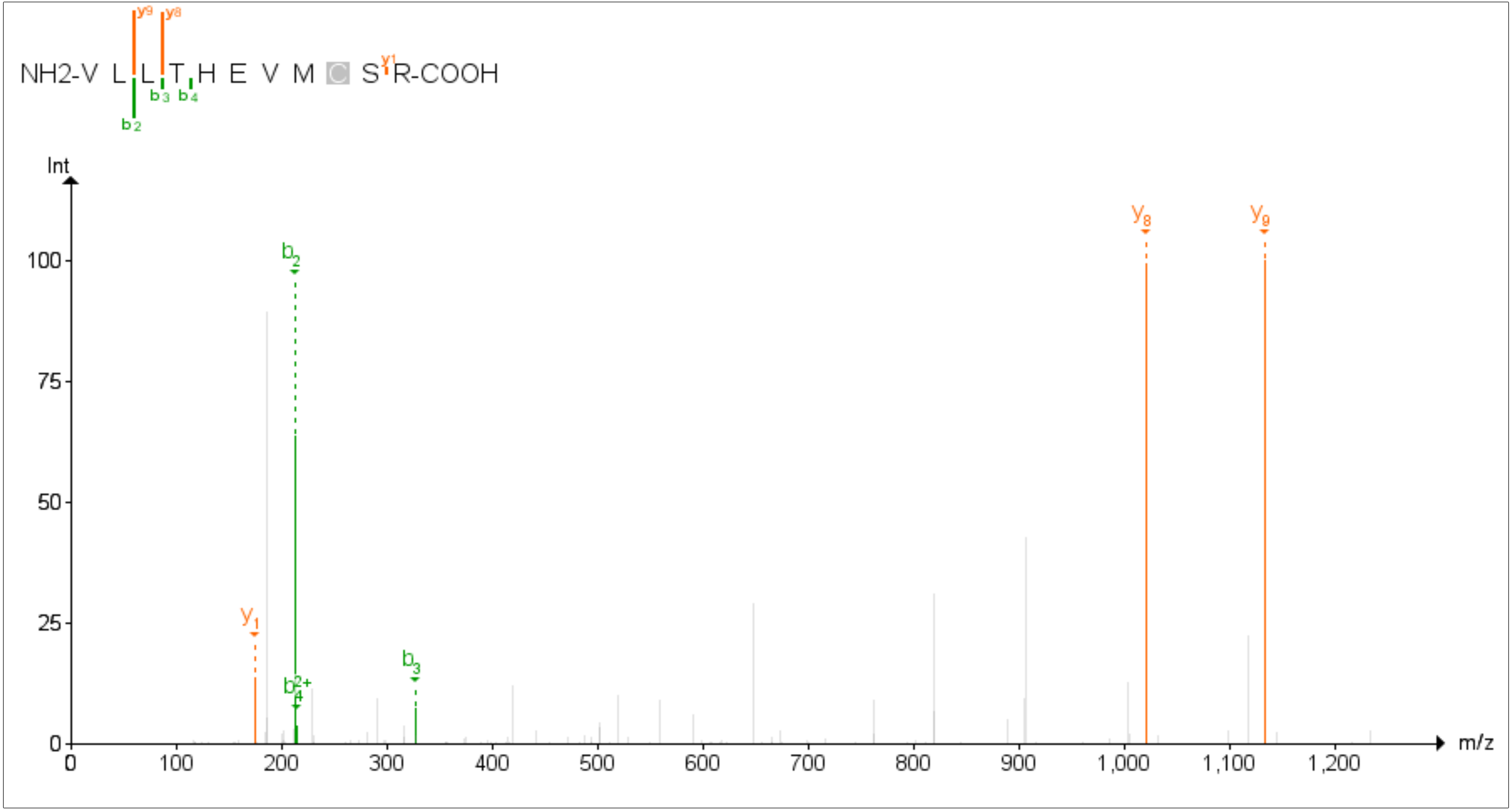

NH2-V L L T H E V M C S R-COOH
y9
y8
y1
b2
b3
b4
Int
100
75
50
25
0
m/z
0
100
200
300
400
500
600
700
800
900
1,000
1,100
1,200
y1
b2
b4 2+
b3
y8
y9

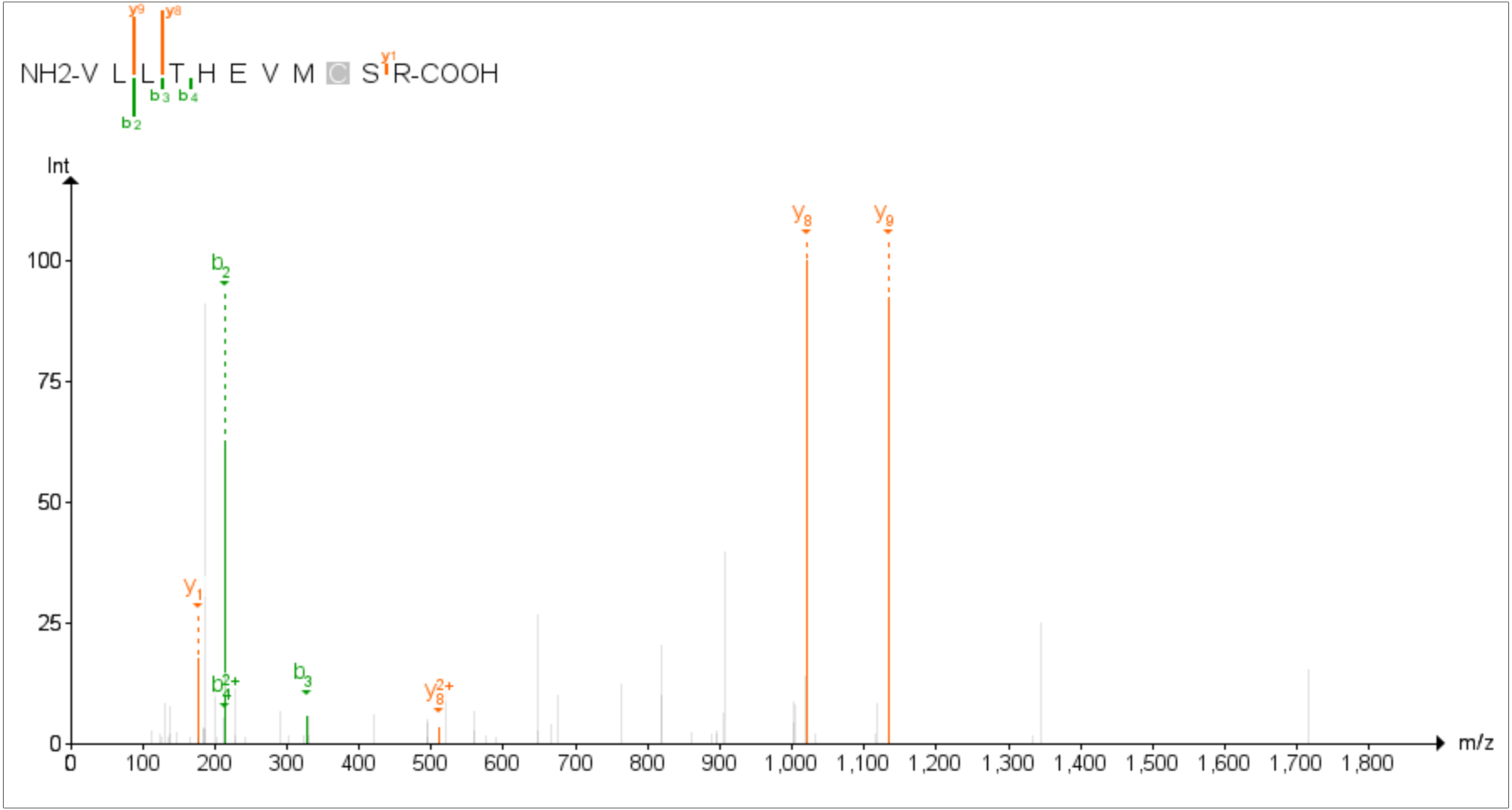

NH2-V L L T H E V M C S R-COOH
y9
y8
y1
b2
b3
b4
Int
100
75
50
25
0
y8
y9
b2
y1
b4 2+
b3
y8 2+
m/z
0
100
200
300
400
500
600
700
800
900
1,000
1,100
1,200
1,300
1,400
1,500
1,600
1,700
1,800

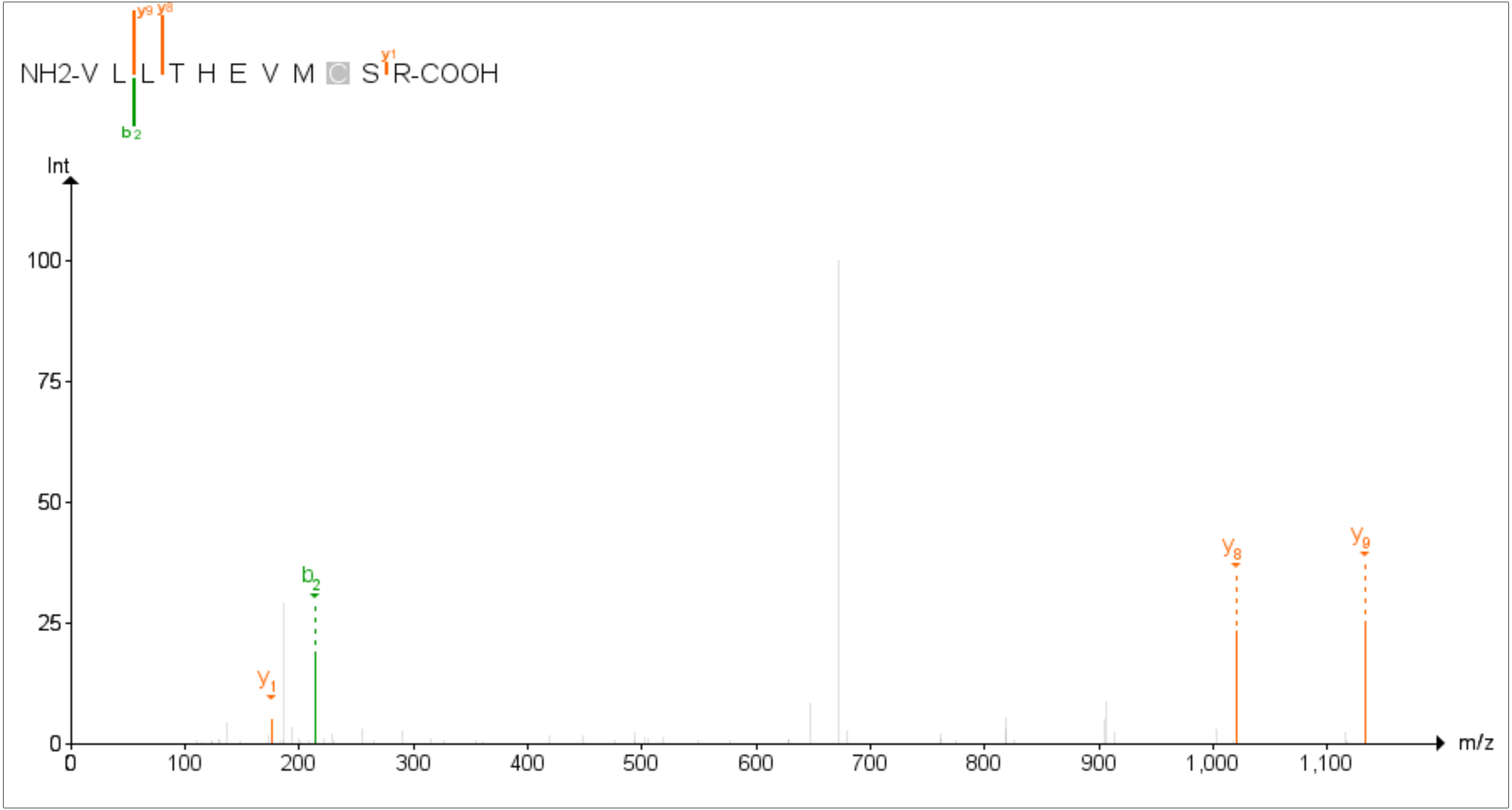

NH2-V L L T H E V M C S R-COOH
y9 y8
y1
b2
Int
100
75
50
25
0
m/z
0
100
200
300
400
500
600
700
800
900
1,000
1,100
y1
b2
y8
y9

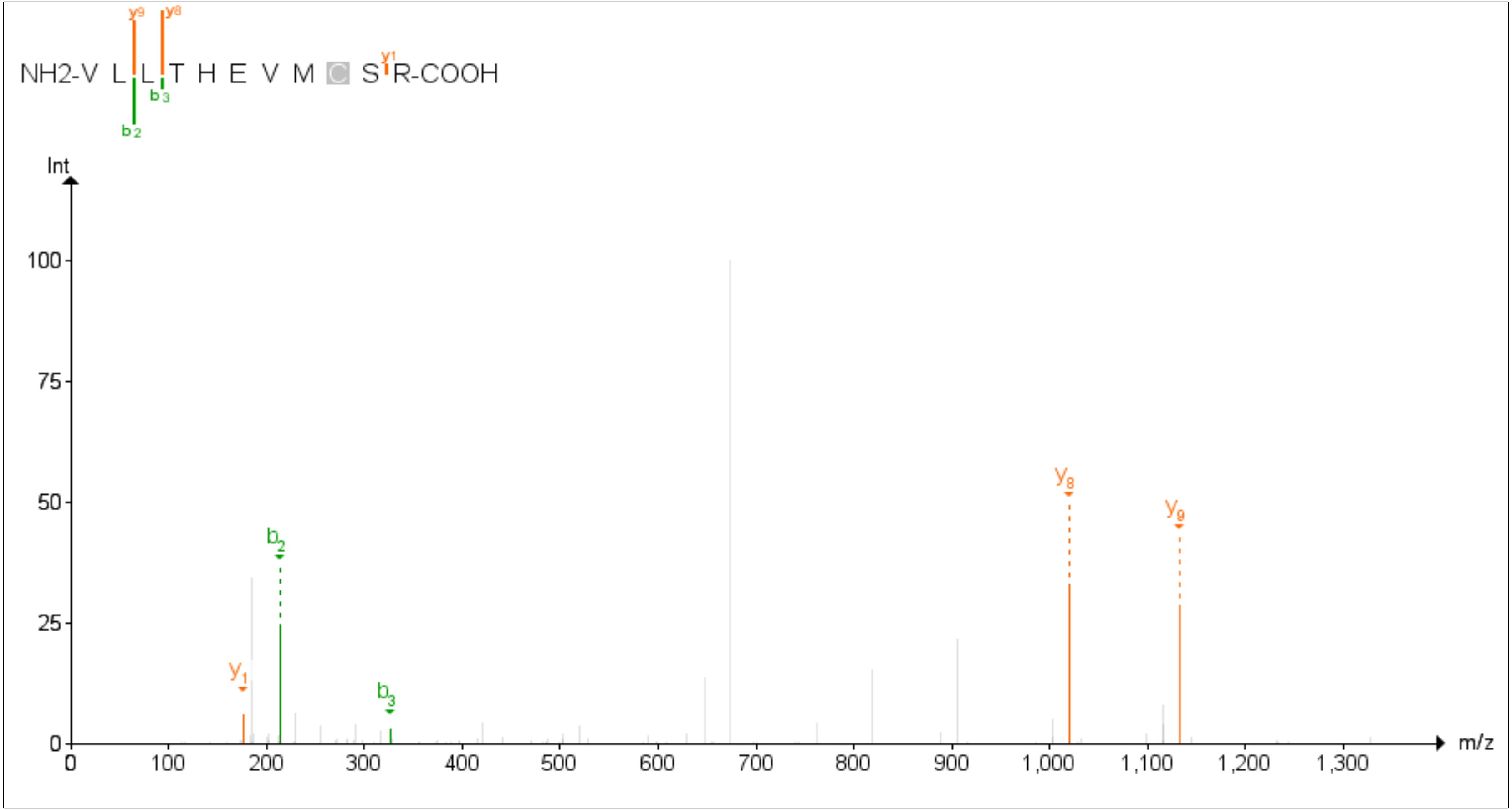

NH2-V L L T H E V M C S R-COOH
y9
y8
y1
b2
b3
Int
100
75
50
25
0
y1
b2
b3
y8
y9
0
100
200
300
400
500
600
700
800
900
1,000
1,100
1,200
1,300
m/z

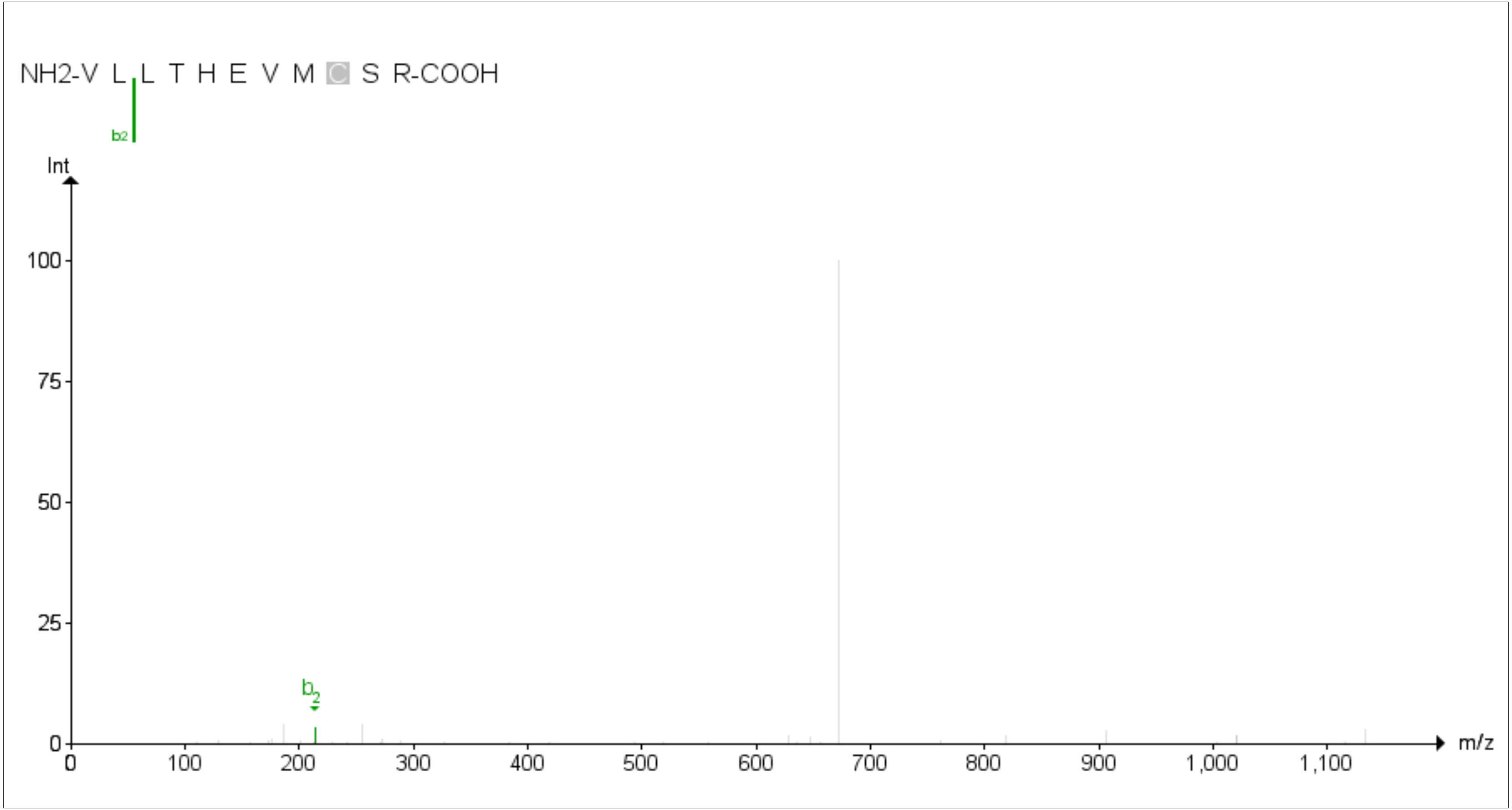

NH2-V L L T H E V M C S R-COOH
b2
Int
100
75
50
25
0
b2
0
100
200
300
400
500
600
700
800
900
1,000
1,100
m/z

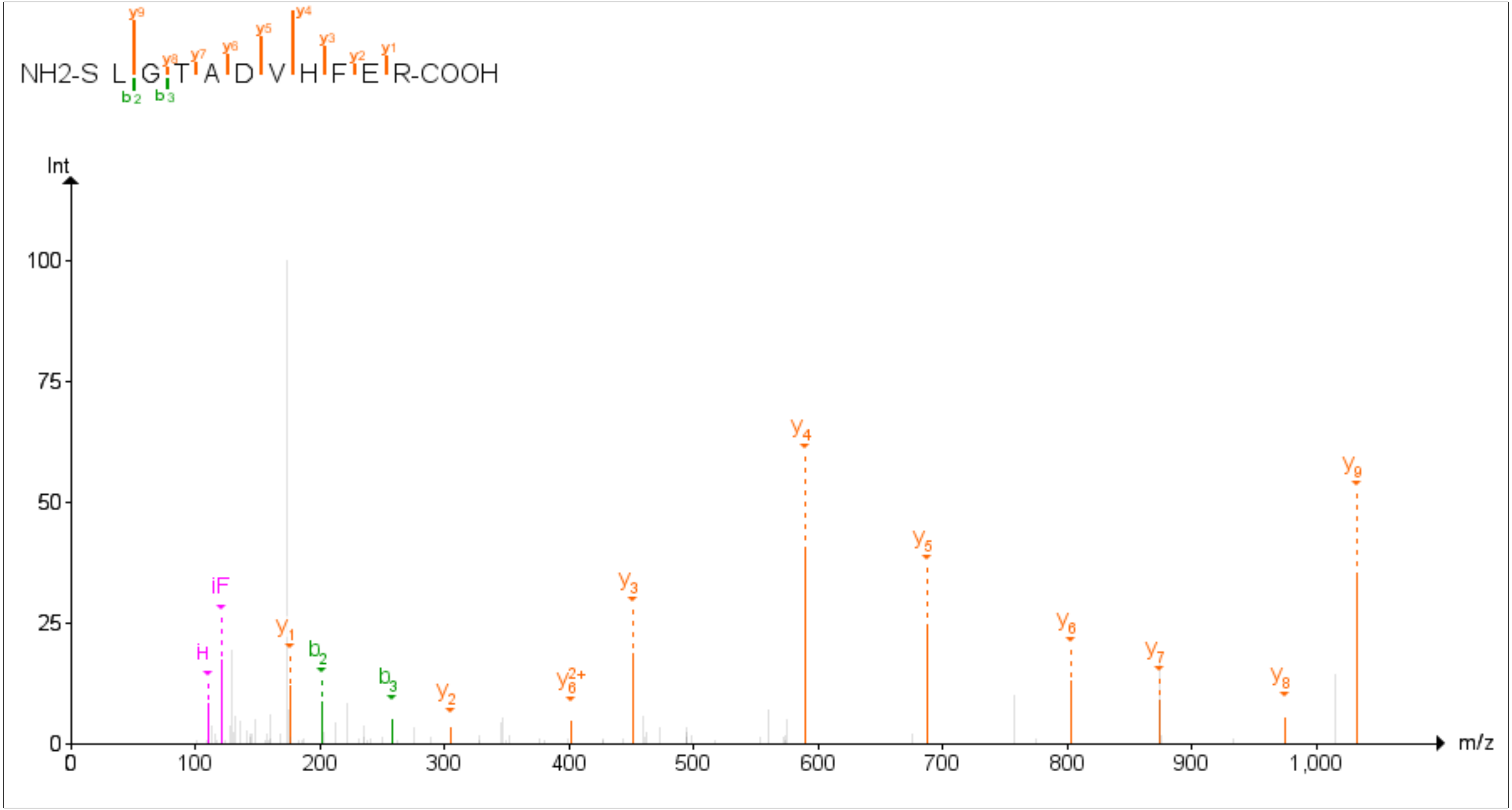

NH2-S L G T A D V H F E R-COOH
y9 y8 y7 y6 y5 y4 y3 y2 y1
b2 b3
Int
100
75
50
25
0
m/z
0 100 200 300 400 500 600 700 800 900 1,000
iH
iF
y1
b2
b3
y2
y6 2+
y3
y4
y5
y6
y7
y8
y9

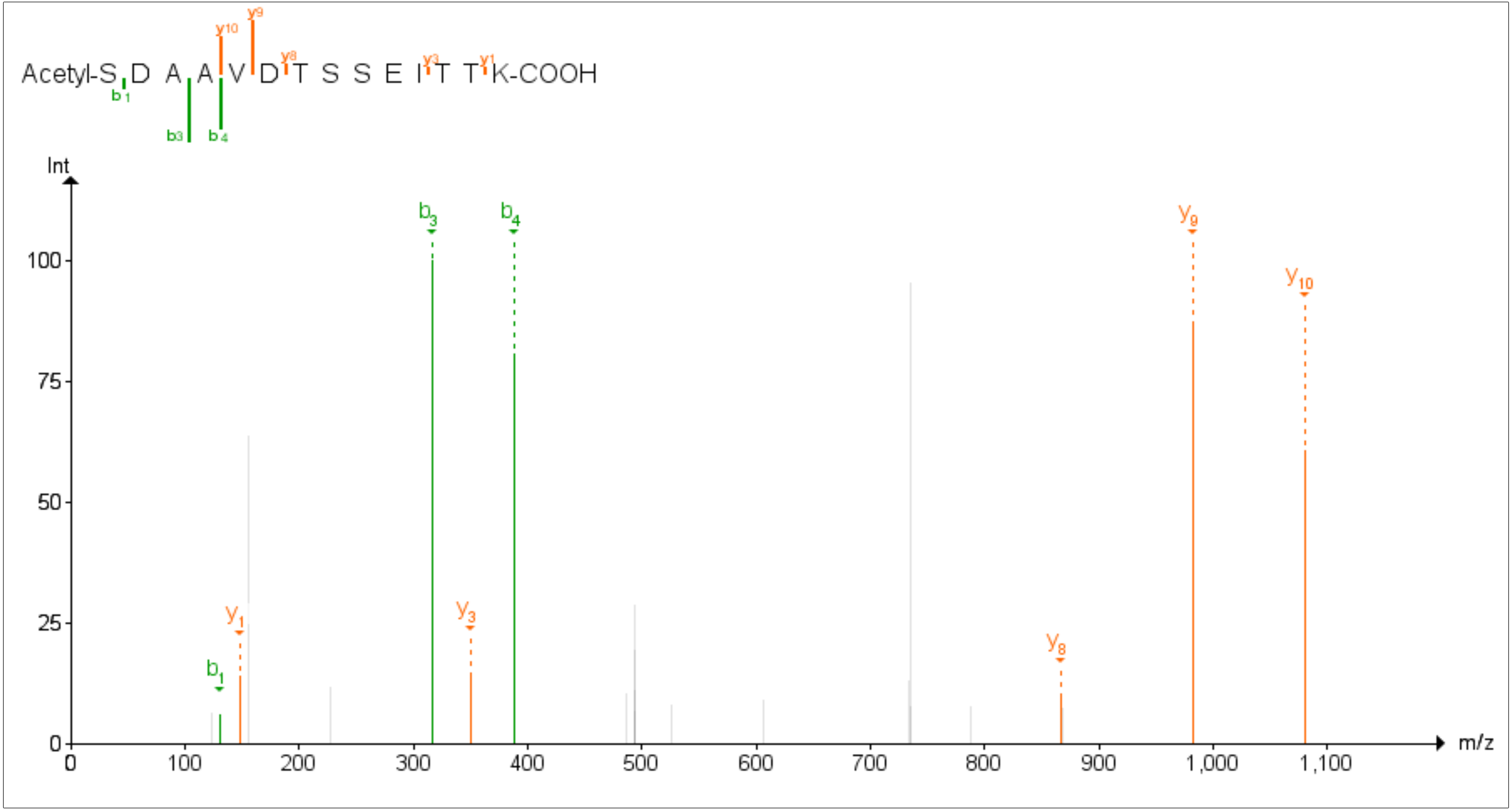

Acetyl-S D A A V D T S S E I T T K-COOH
b1
b3
b4
y10
y9
y8
y3
y1
Int
100
75
50
25
0
m/z
0
100
200
300
400
500
600
700
800
900
1,000
1,100

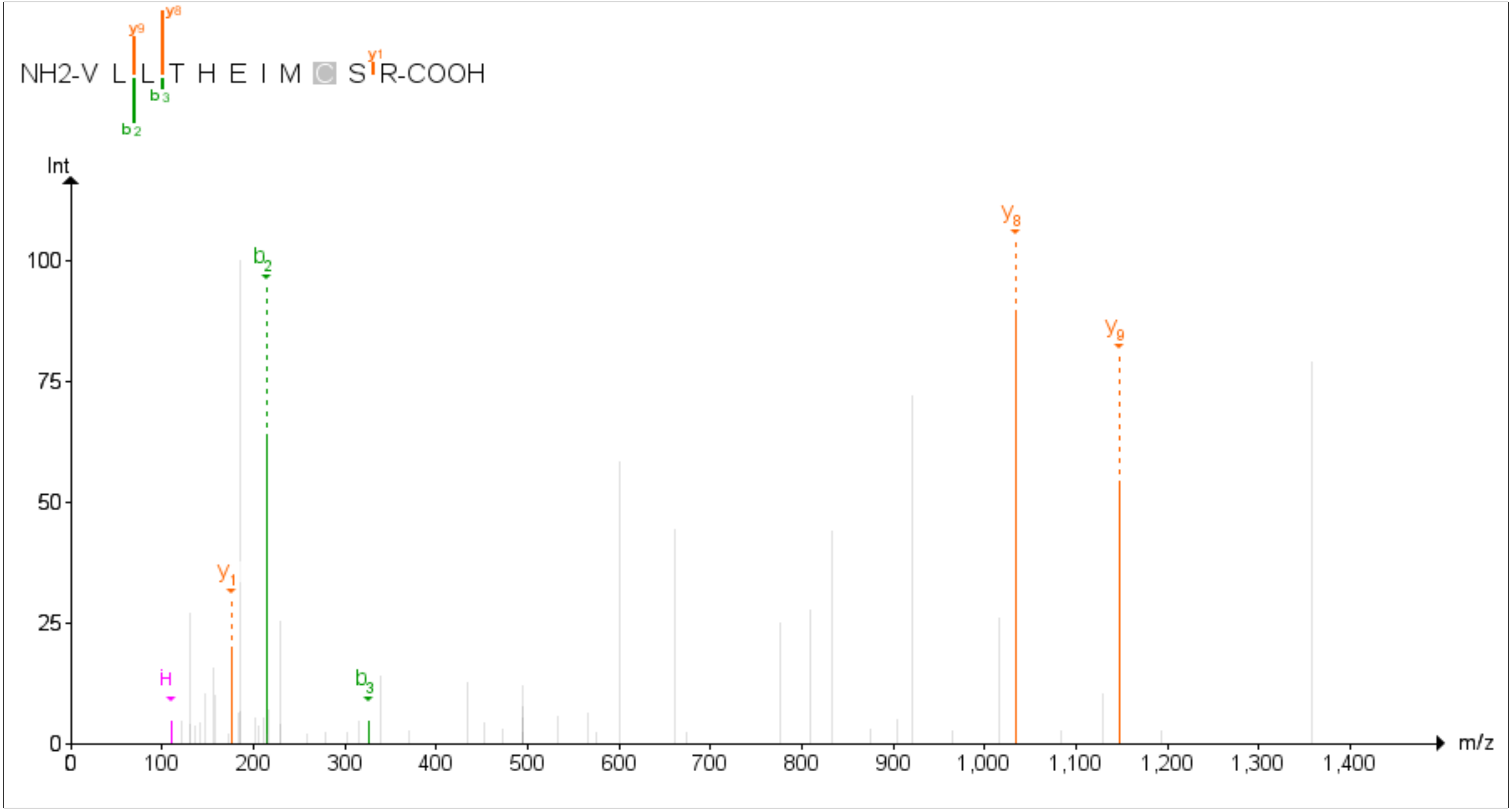

NH2-V L L T H E I M C S R-COOH
y9
y8
y1
b2
b3
Int
100
75
50
25
0
b2
y1
H
b3
y8
y9
0
100
200
300
400
500
600
700
800
900
1,000
1,100
1,200
1,300
1,400
m/z

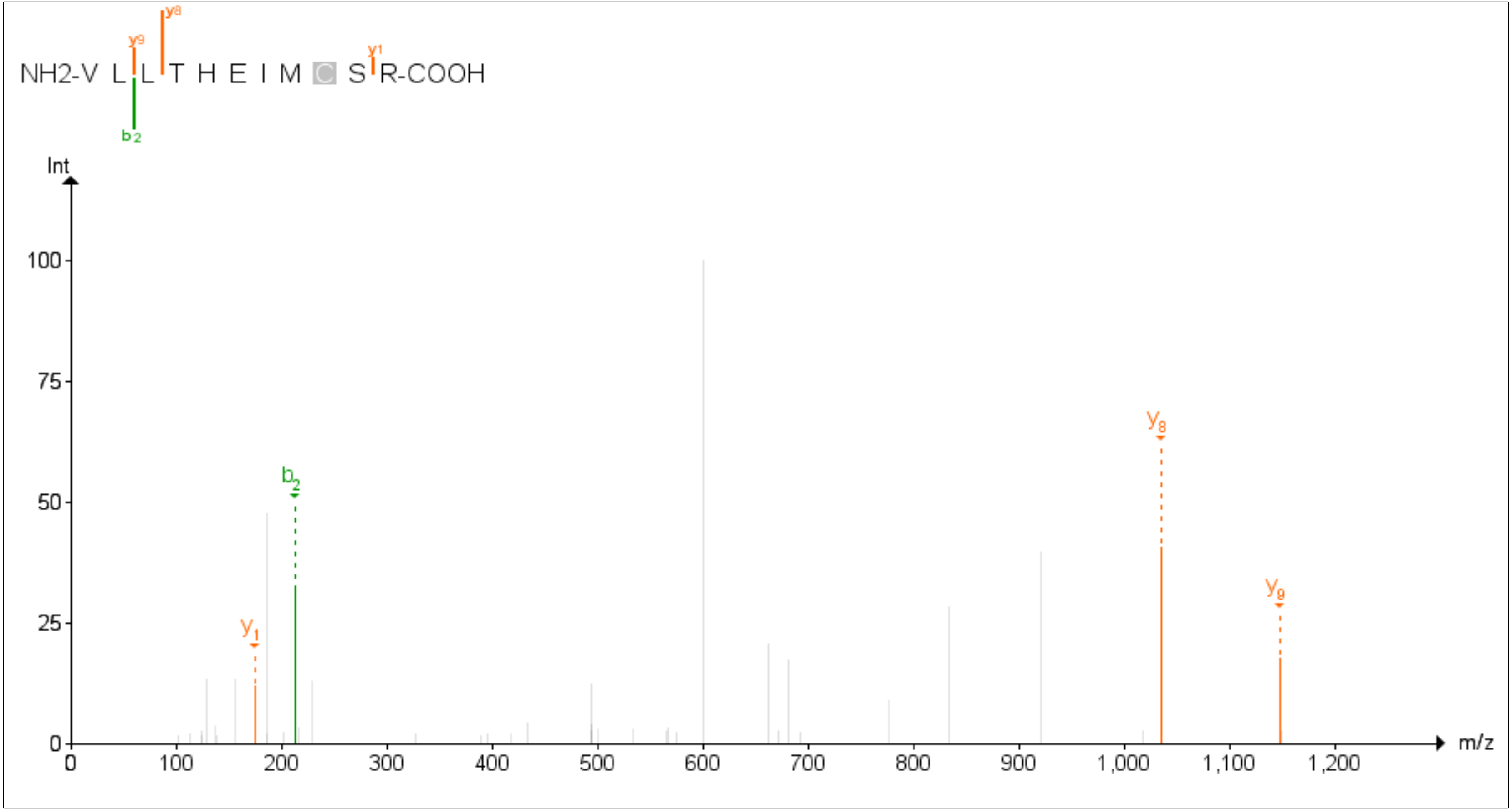

NH2-V L L T H E I M C S R-COOH
y9
y8
y1
b2
Int
100
75
50
25
0
y1
b2
y8
y9
0
100
200
300
400
500
600
700
800
900
1,000
1,100
1,200
m/z

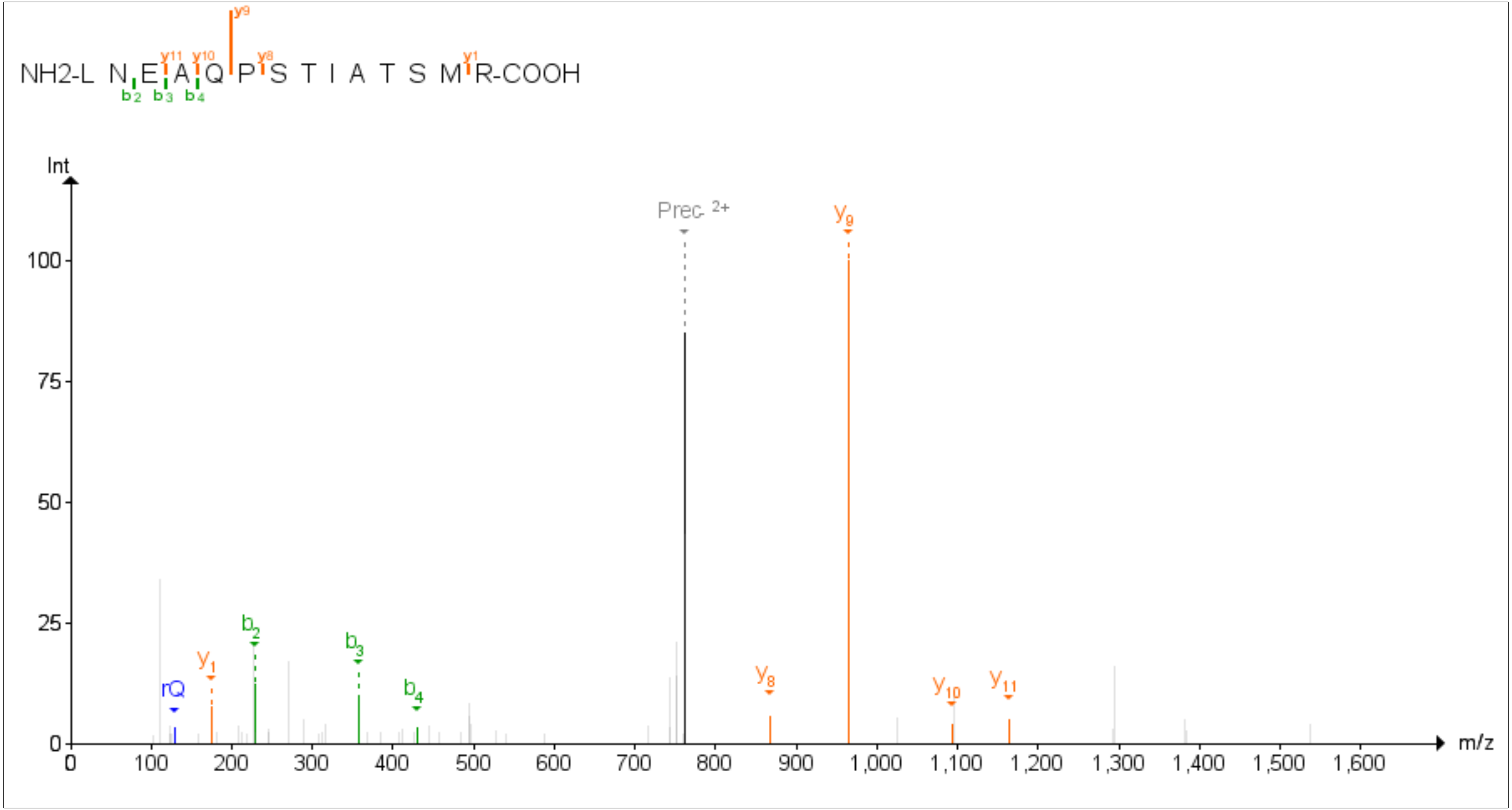

NH2-L N E A Q P S T I A T S M R-COOH
b2 b3 b4
y11 y10 y9 y8 y1
Int
100
75
50
25
0
Prec 2+
y9
y8
y10
y11
y1
b2
b3
b4
rQ
0 100 200 300 400 500 600 700 800 900 1,000 1,100 1,200 1,300 1,400 1,500 1,600
m/z

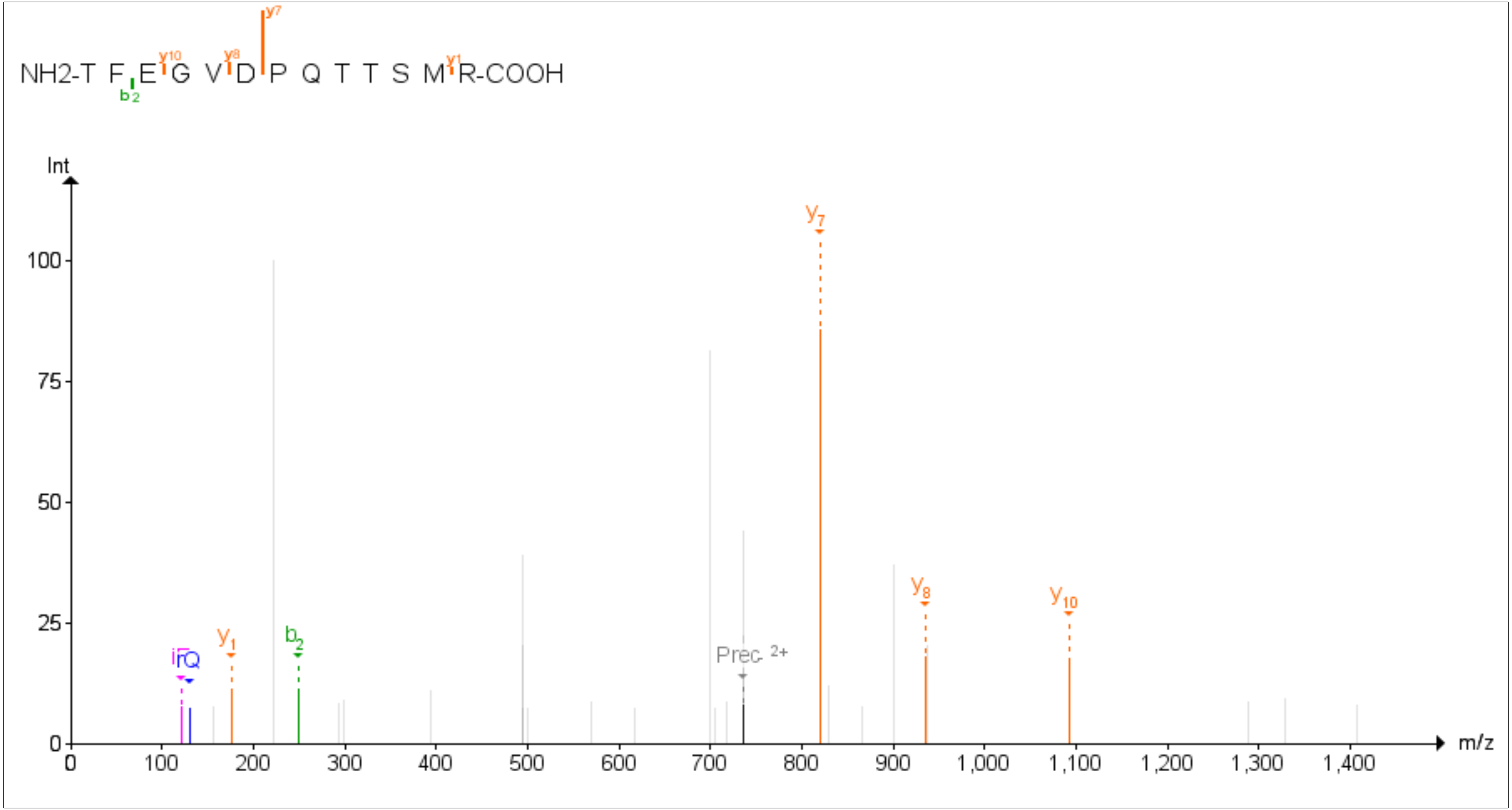

NH2-T F E G V D P Q T T S M R-COOH
b2
y10
y8
y7
y1
Int
100
75
50
25
0
m/z
0
100
200
300
400
500
600
700
800
900
1,000
1,100
1,200
1,300
1,400
y7
y8
y10
y1
b2
rQ
Prec 2+

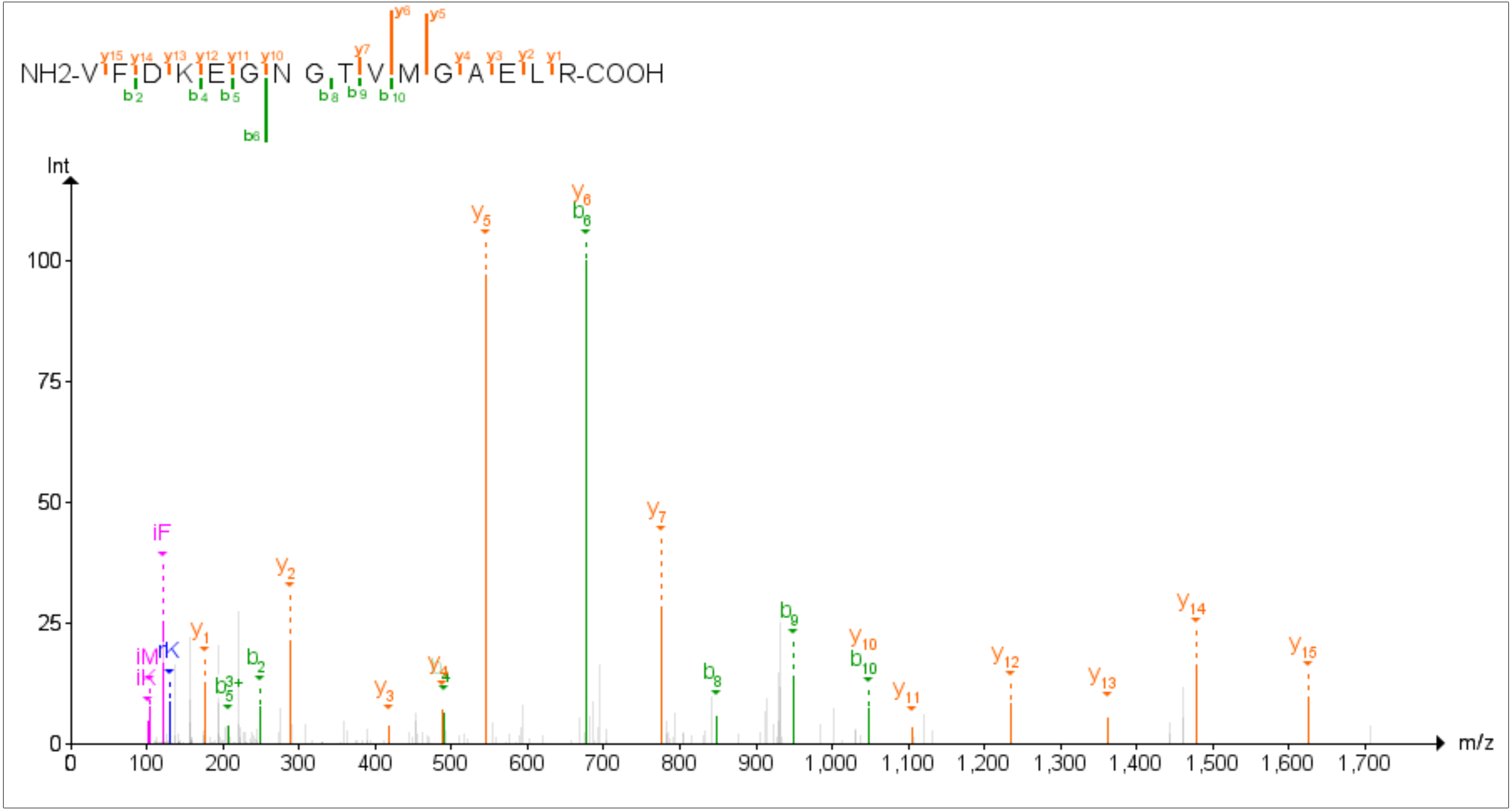
NH2-VFDKEGIN GTVMGAELR-COOH
y15 y14 y13 y12 y11 y10 y7 y6 y5 y4 y3 y2 y1
b2 b4 b5 b6 b8 b9 b10
Int
m/z
0
25
50
75
100
0
100
200
300
400
500
600
700
800
900
1,000
1,100
1,200
1,300
1,400
1,500
1,600
1,700
iF
iM
iK
rK
y1
b2
y2
y3
y4
y5
y6
b6
y7
b8
b9
y10
b10
y11
y12
y13
y14
y15

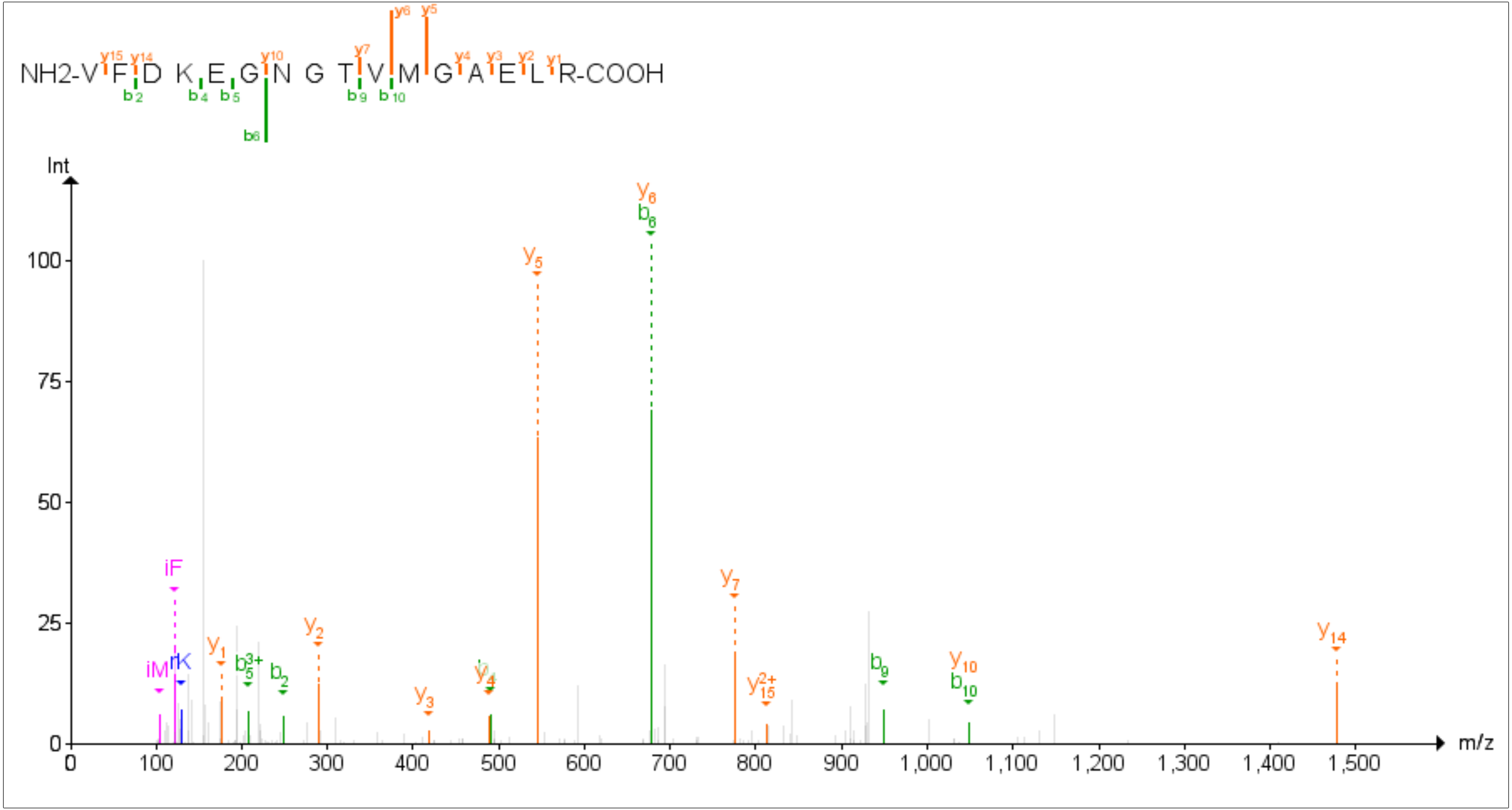

NH2-V F D K E G N G T V M G A E L R-COOH
y15 y14 y10 y7 y6 y5 y4 y3 y2 y1
b2 b4 b5 b6 b9 b10
Int
100
75
50
25
0
m/z
0 100 200 300 400 500 600 700 800 900 1,000 1,100 1,200 1,300 1,400 1,500
iF
iM
rK
y1
y2
y3
y4
y5
y6
b6
y7
y14
y10
b10
b9
b2

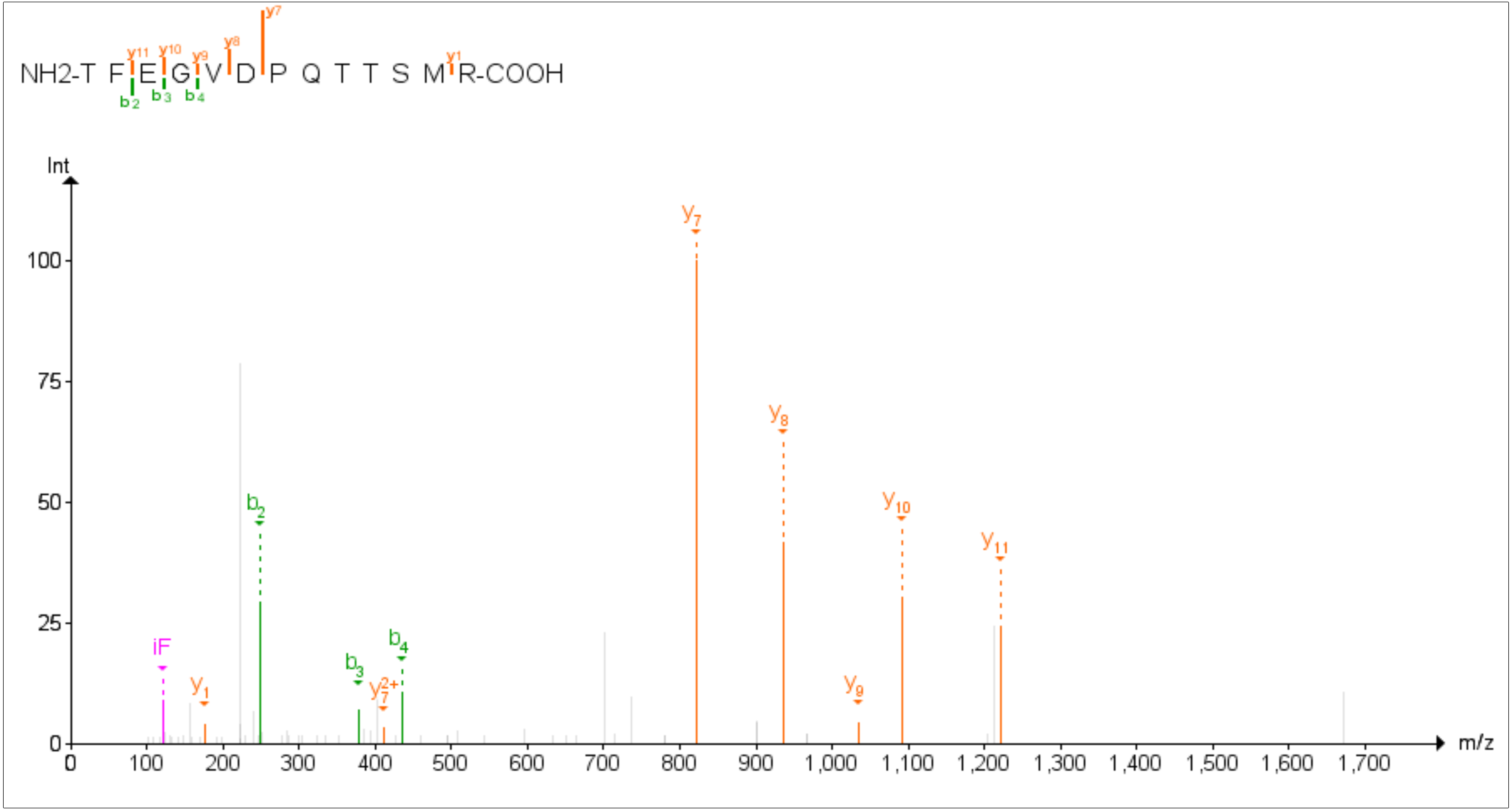
NH2-T F E G V D P Q T T S M R-COOH
y11 y10 y9 y8 y7 y1
b2 b3 b4
Int
m/z
y7
y8
y10
y11
b2
iF
y1
b3
b4
y9
0
25
50
75
100
0 100 200 300 400 500 600 700 800 900 1,000 1,100 1,200 1,300 1,400 1,500 1,600 1,700

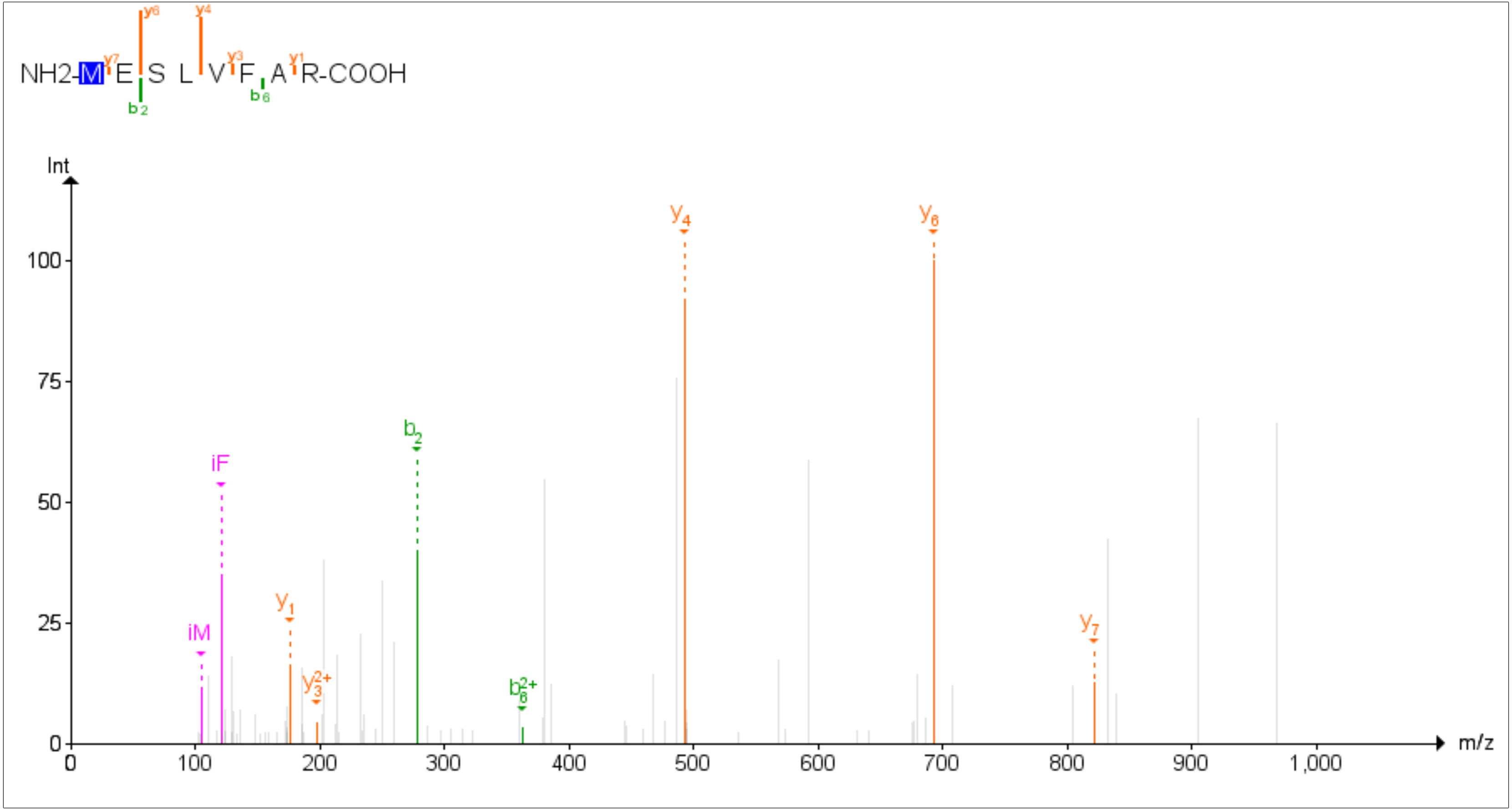

NH2-M E S L V F A R-COOH
y7 y6 y4 y3 y1
b2 b6
Int
m/z
y4
y6
b2
iF
iM
y1
y_3^{2+}
b_6^{2+}
y7
0
25
50
75
100
0
100
200
300
400
500
600
700
800
900
1,000

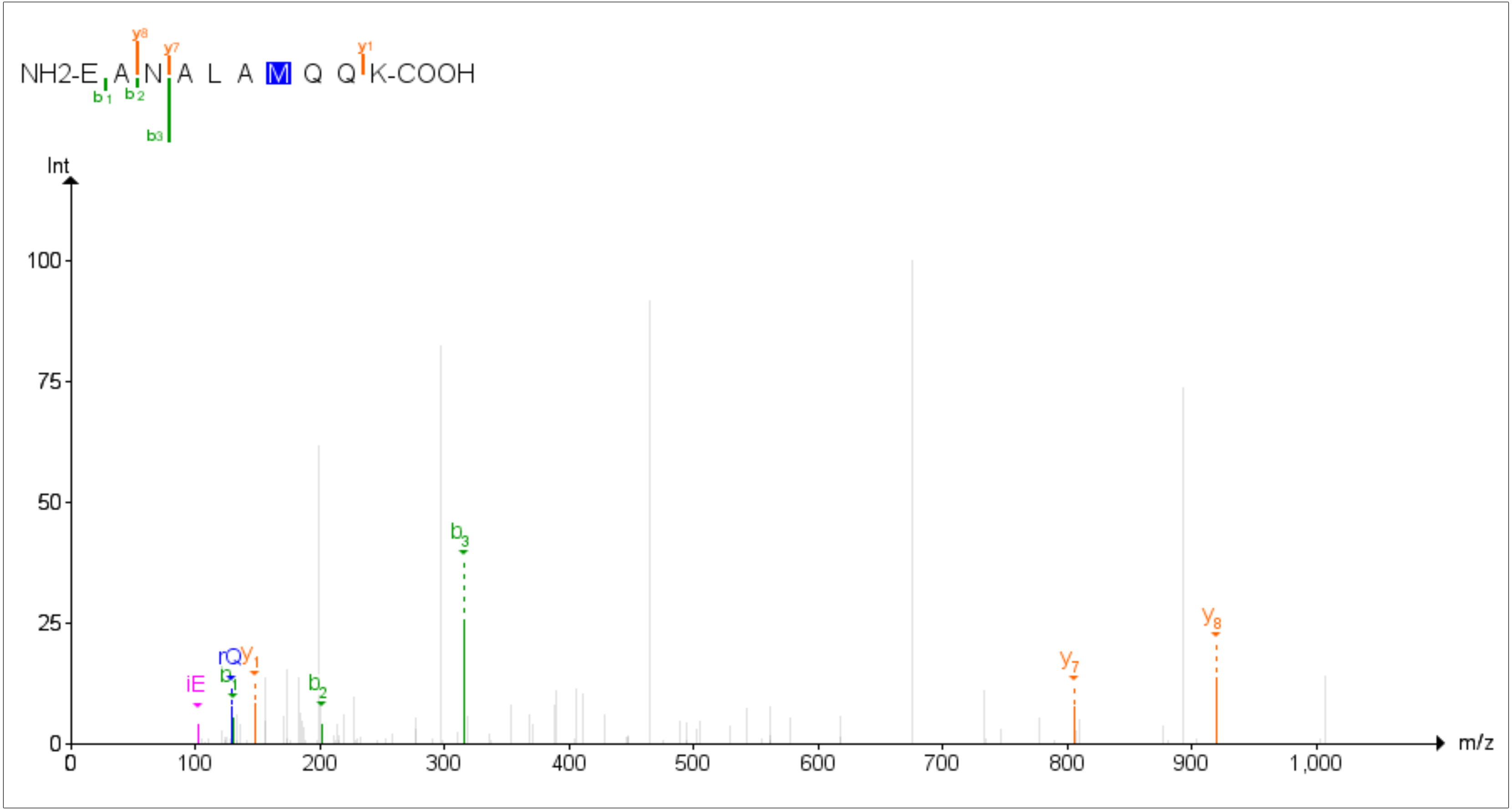

NH2-E A N A L A M Q Q K-COOH
y8 y7 y1
b1 b2 b3
Int
100
75
50
25
0
b3
y8
y7
rQ y1
iE
b1
b2
0 100 200 300 400 500 600 700 800 900 1,000
m/z

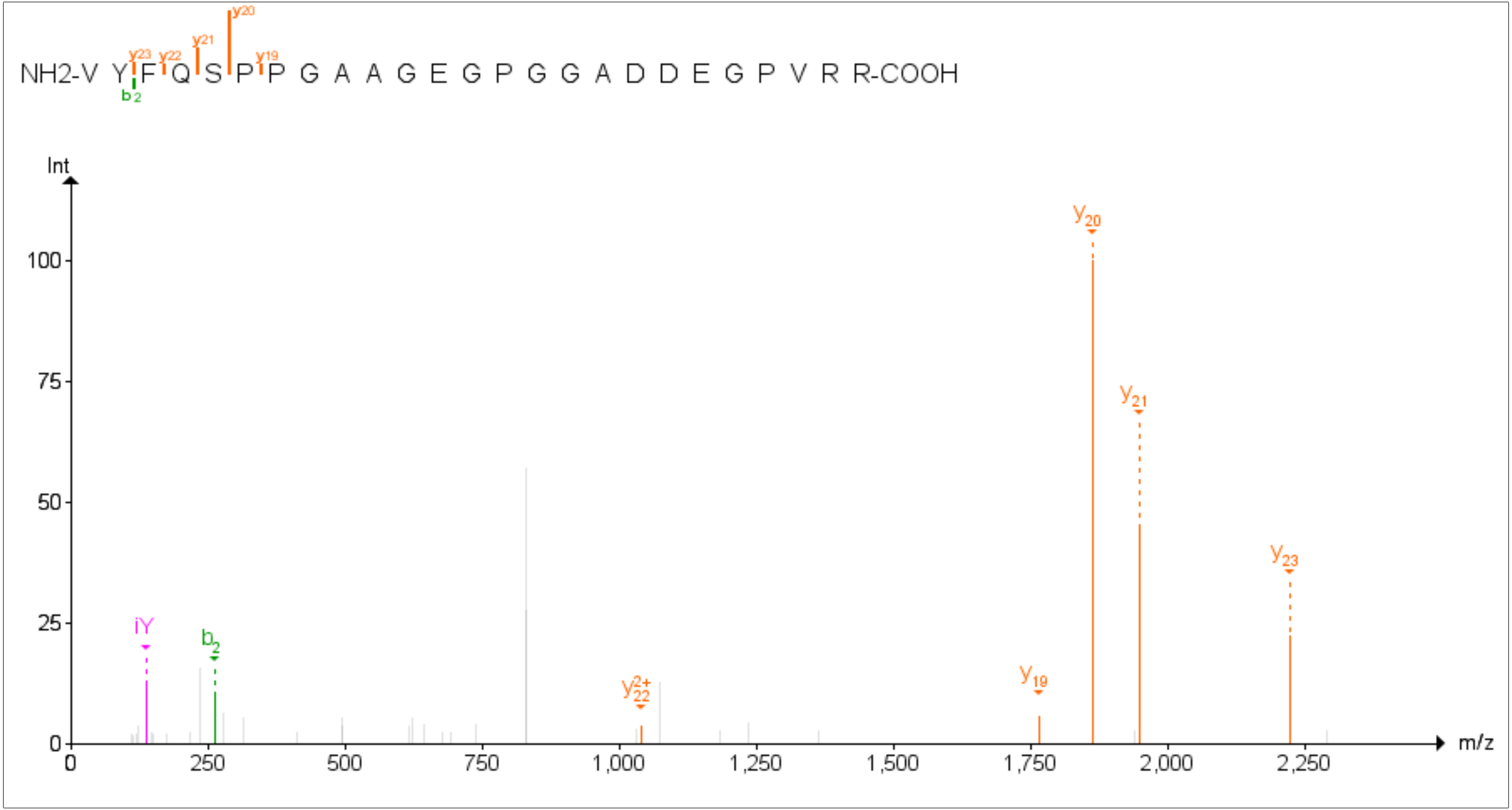

NH2-V Y F Q S P P G A A G E G P G G A D D E G P V R R-COOH
y23 y22 y21 y20 y19
b2
Int
100
75
50
25
0
iY
b2
y_{22}^{2+}
y_{19}
y_{20}
y_{21}
y_{23}
0
250
500
750
1,000
1,250
1,500
1,750
2,000
2,250
m/z

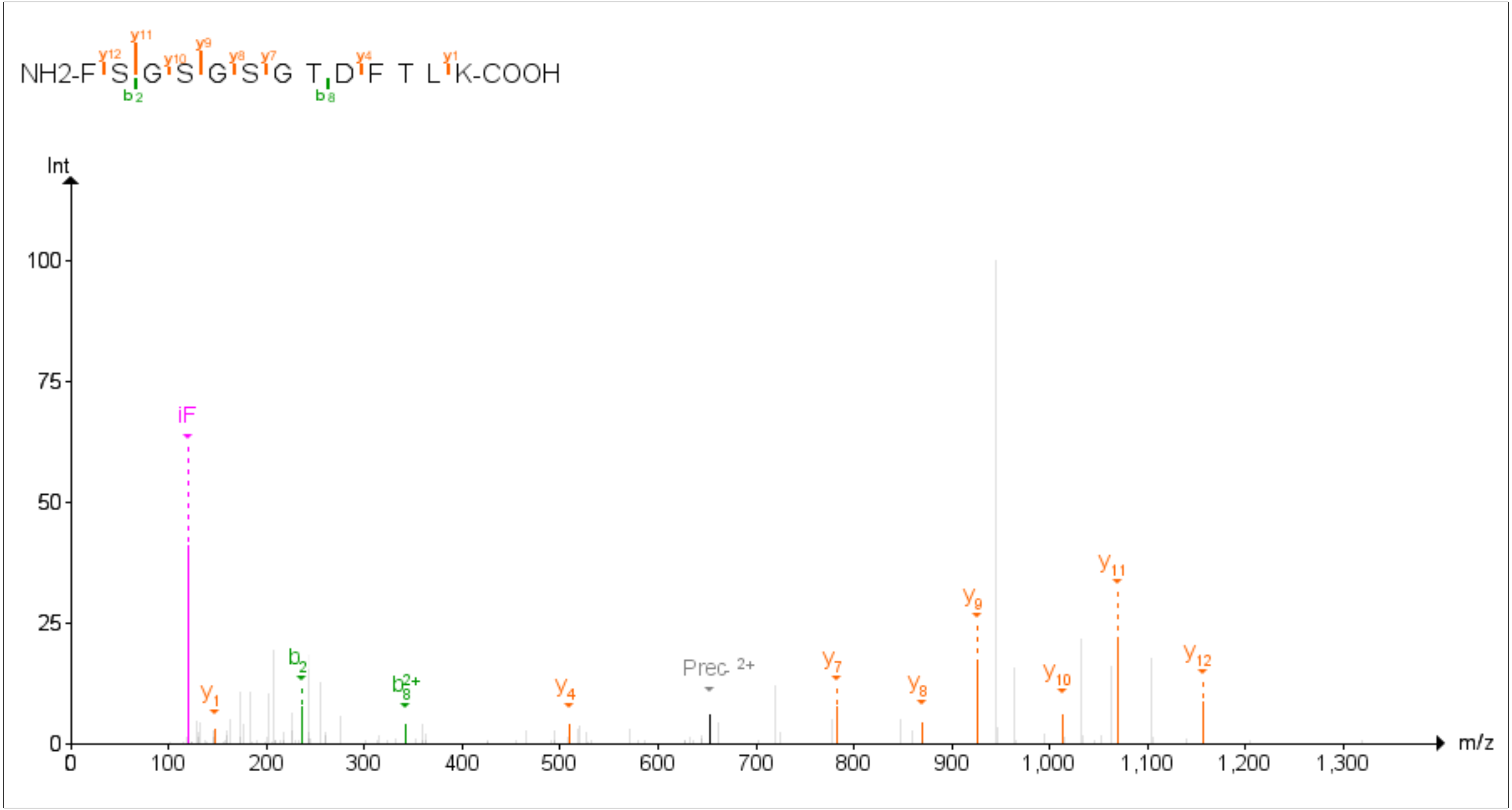

NH2-F S G S G S G T D F T L K-COOH
y12 y11 y10 y9 y8 y7 y4 y1
b2 b8
Int
100
75
50
25
0
iF
y1
b2
b_8^{2+}
y4
Prec 2+
y7
y8
y9
y10
y11
y12
m/z
0
100
200
300
400
500
600
700
800
900
1,000
1,100
1,200
1,300

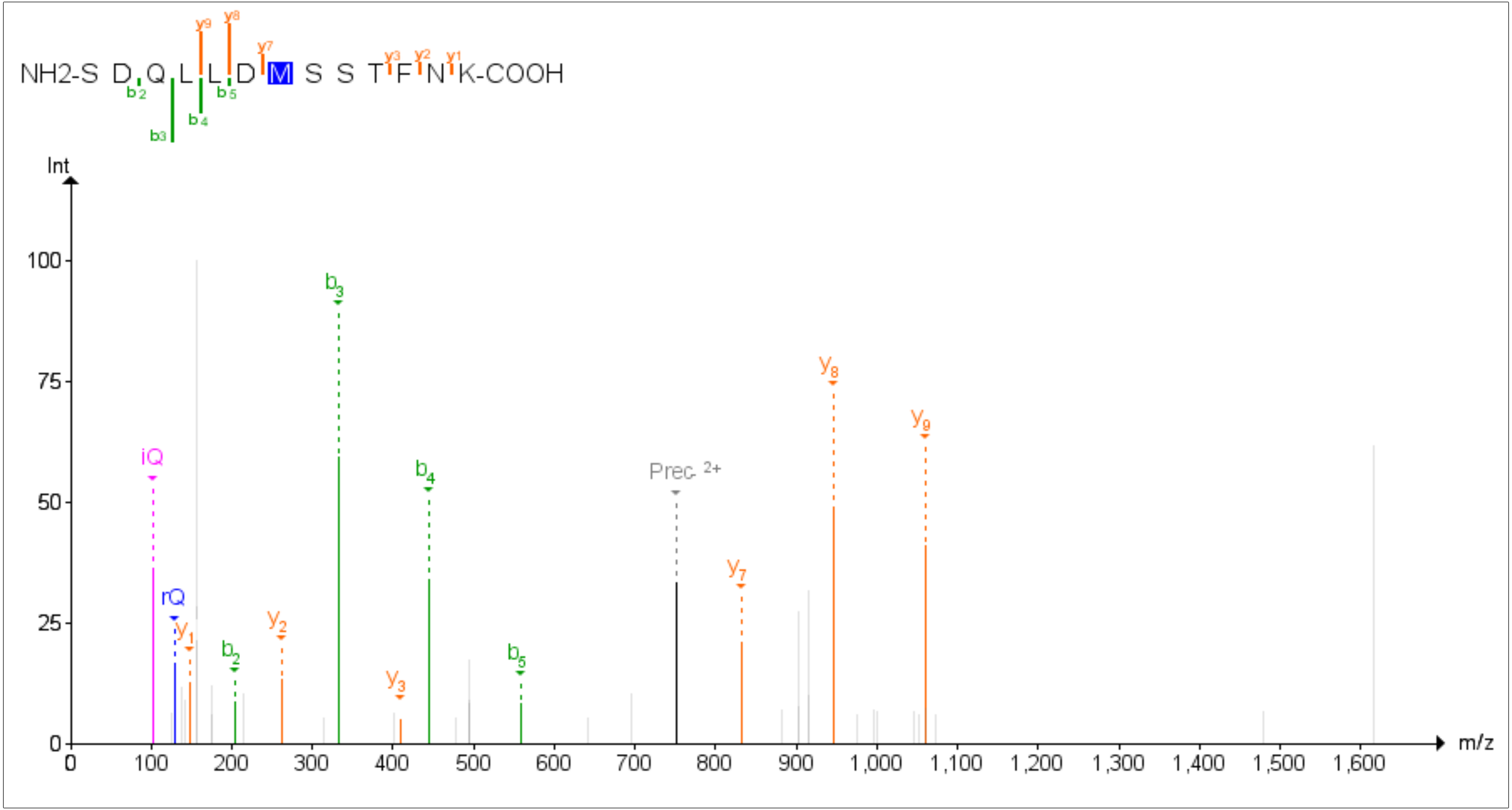

NH2-S D Q L L D M S S T F N K-COOH
y9 y8 y7 y3 y2 y1
b2 b3 b4 b5
Int
m/z
0 25 50 75 100
0 100 200 300 400 500 600 700 800 900 1,000 1,100 1,200 1,300 1,400 1,500 1,600
iQ
rQ
y1
b2
y2
b3
y3
b4
b5
Prec 2+
y7
y8
y9

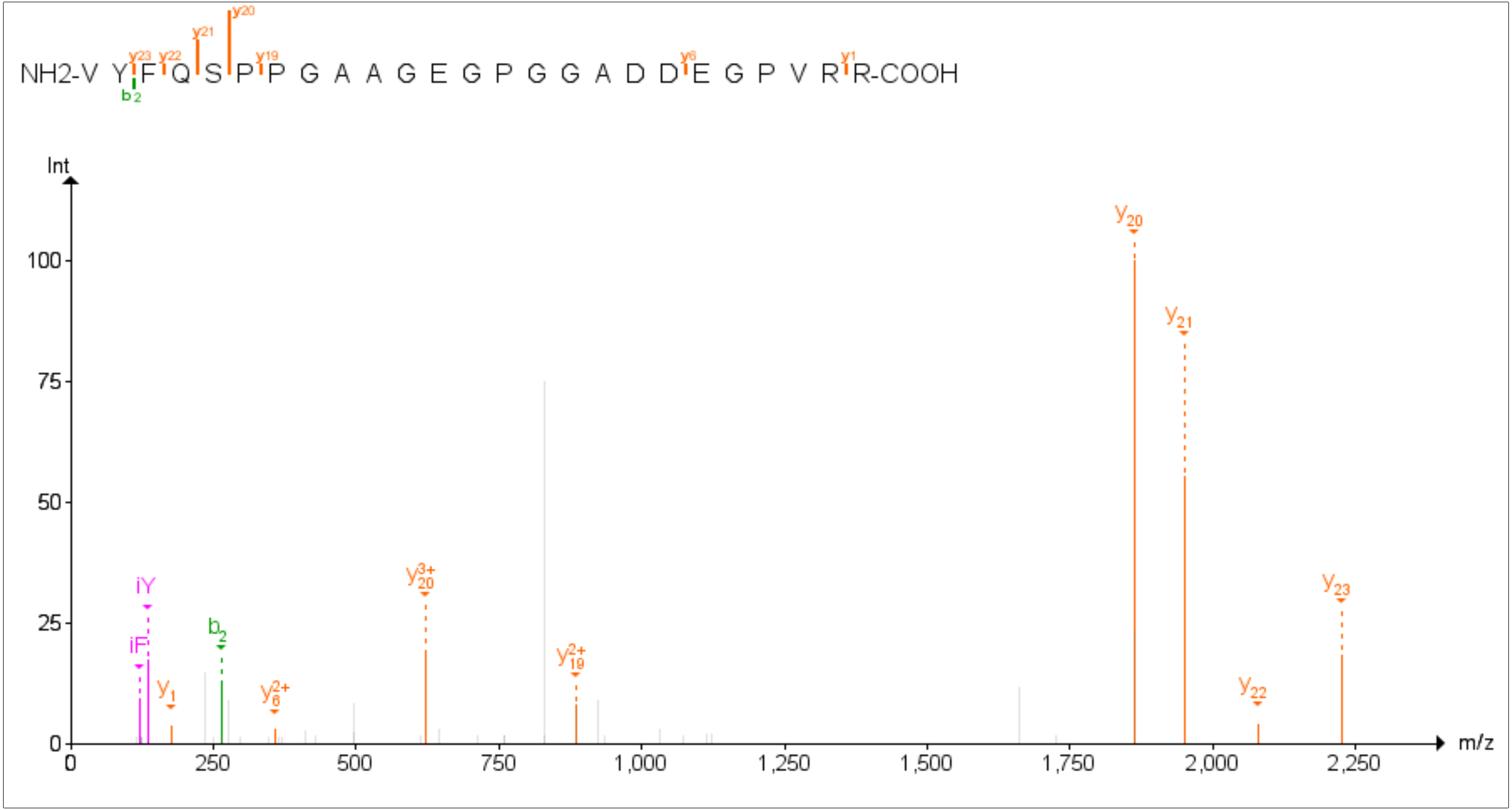

NH2-V Y F Q S P P G A A G E G P G G A D D E G P V R R-COOH
y23 y22 y21 y20 y19 y6 y1 b2
Int
100
75
50
25
0
iY
iF
b2
y1
y6 2+
y20 3+
y19 2+
y20
y21
y22
y23
0 250 500 750 1,000 1,250 1,500 1,750 2,000 2,250
m/z

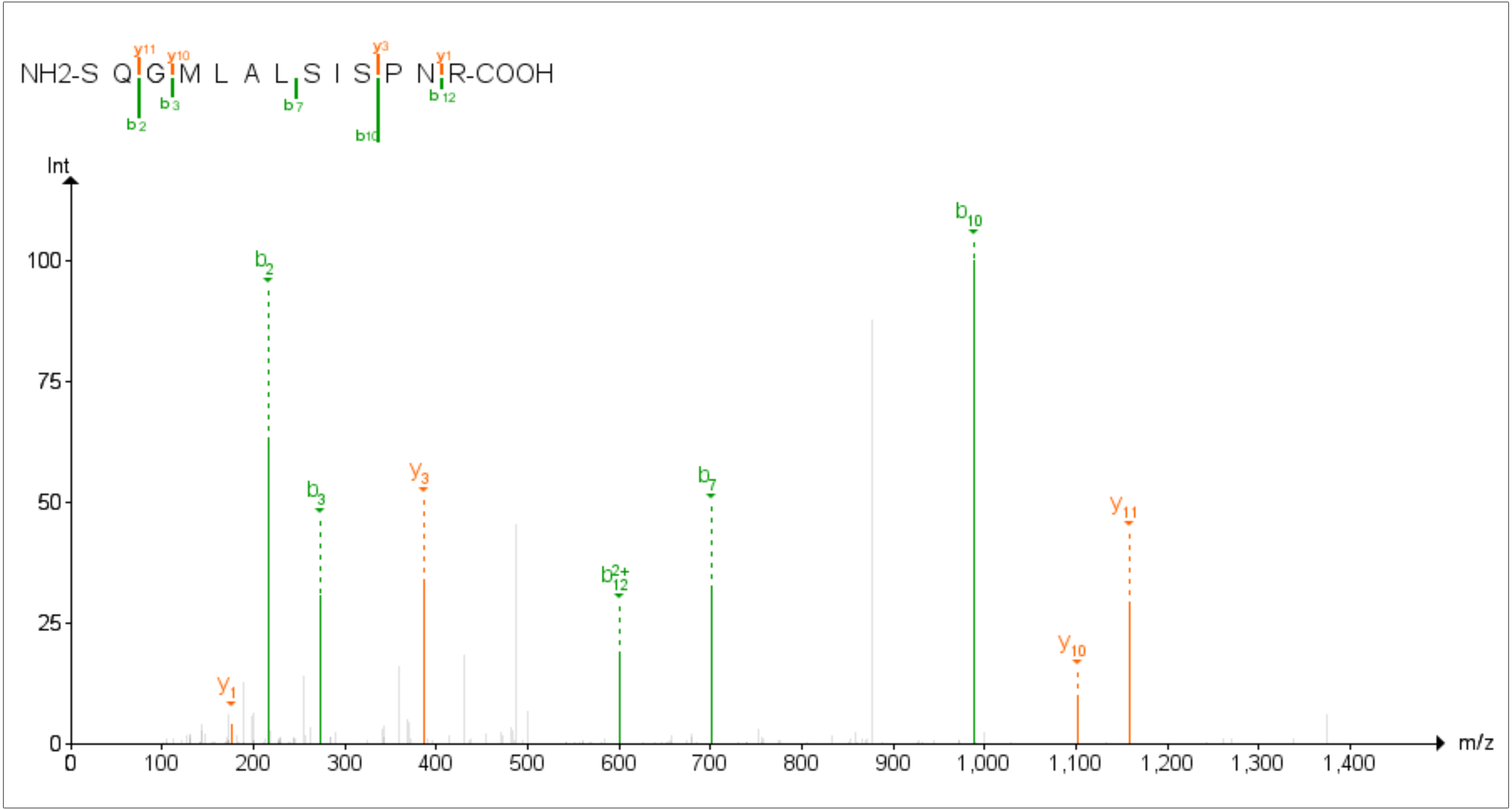

NH2-S Q G M L A L S I S P N R-COOH
y11
y10
y3
y1
b2
b3
b7
b10
b12
Int
100
75
50
25
0
b2
b3
y3
b7
b12 2+
b10
y1
y10
y11
0
100
200
300
400
500
600
700
800
900
1,000
1,100
1,200
1,300
1,400
m/z

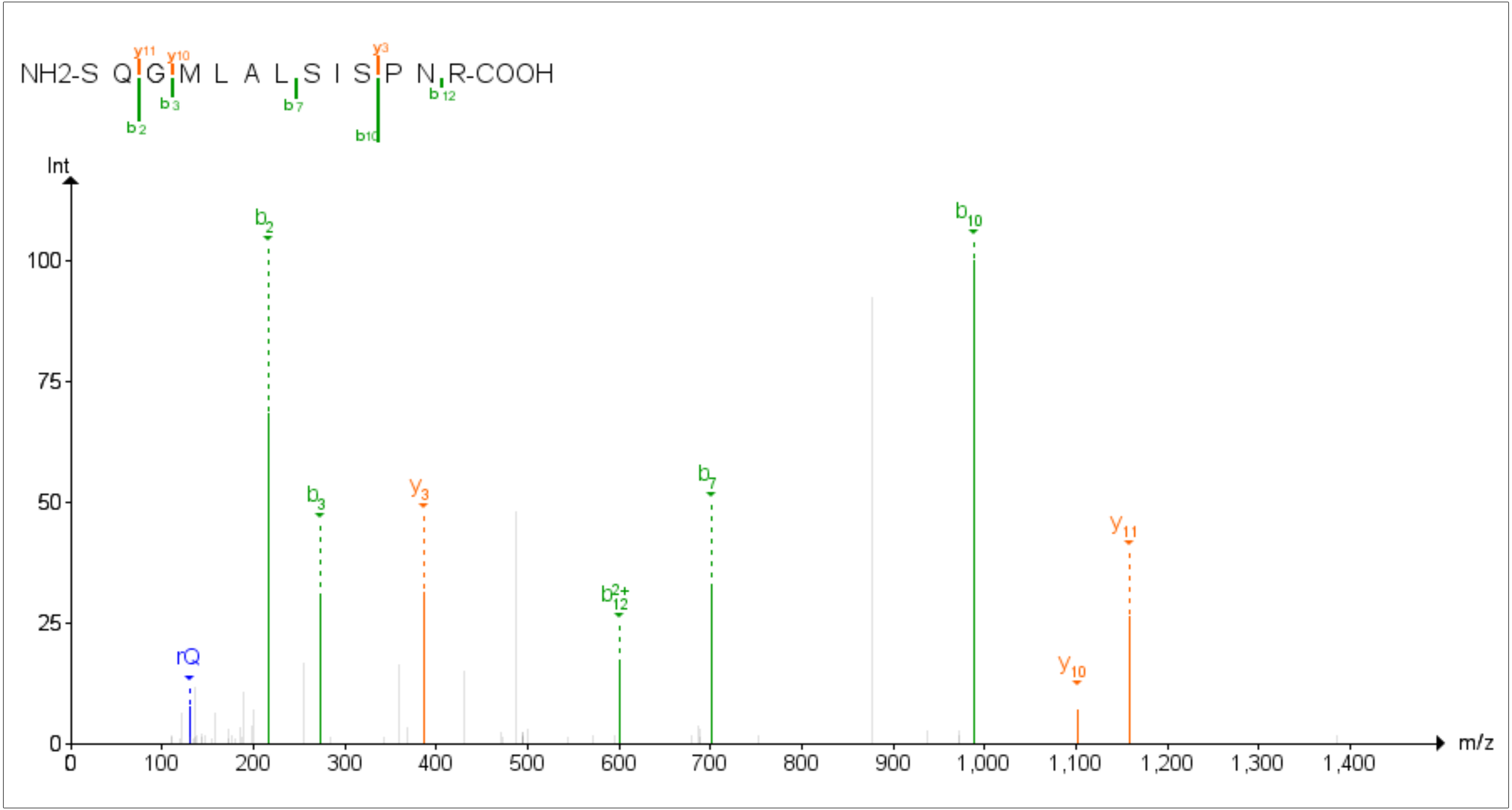

NH2-S Q G M L A L S I S P N R-COOH
y11
y10
y3
b2
b3
b7
b10
b12
Int
100
75
50
25
0
rQ
b2
b3
y3
b12 2+
b7
b10
y10
y11
m/z
0
100
200
300
400
500
600
700
800
900
1,000
1,100
1,200
1,300
1,400

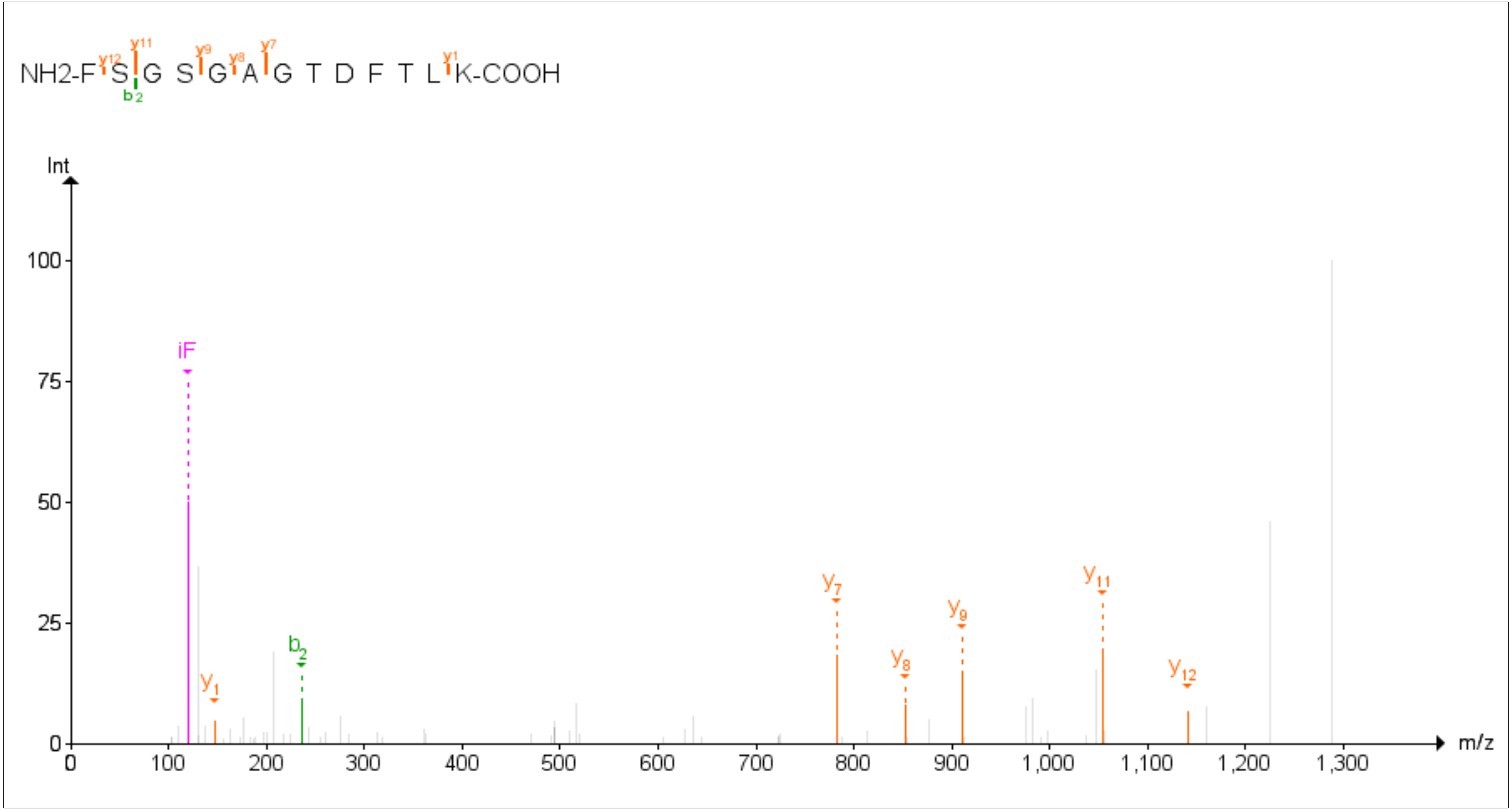

NH2-F S G S G A G T D F T L K-COOH
y12 y11 y9 y8 y7 y1
b2
Int
100
75
50
25
0
iF
y1
b2
y7
y8
y9
y11
y12
0 100 200 300 400 500 600 700 800 900 1,000 1,100 1,200 1,300
m/z

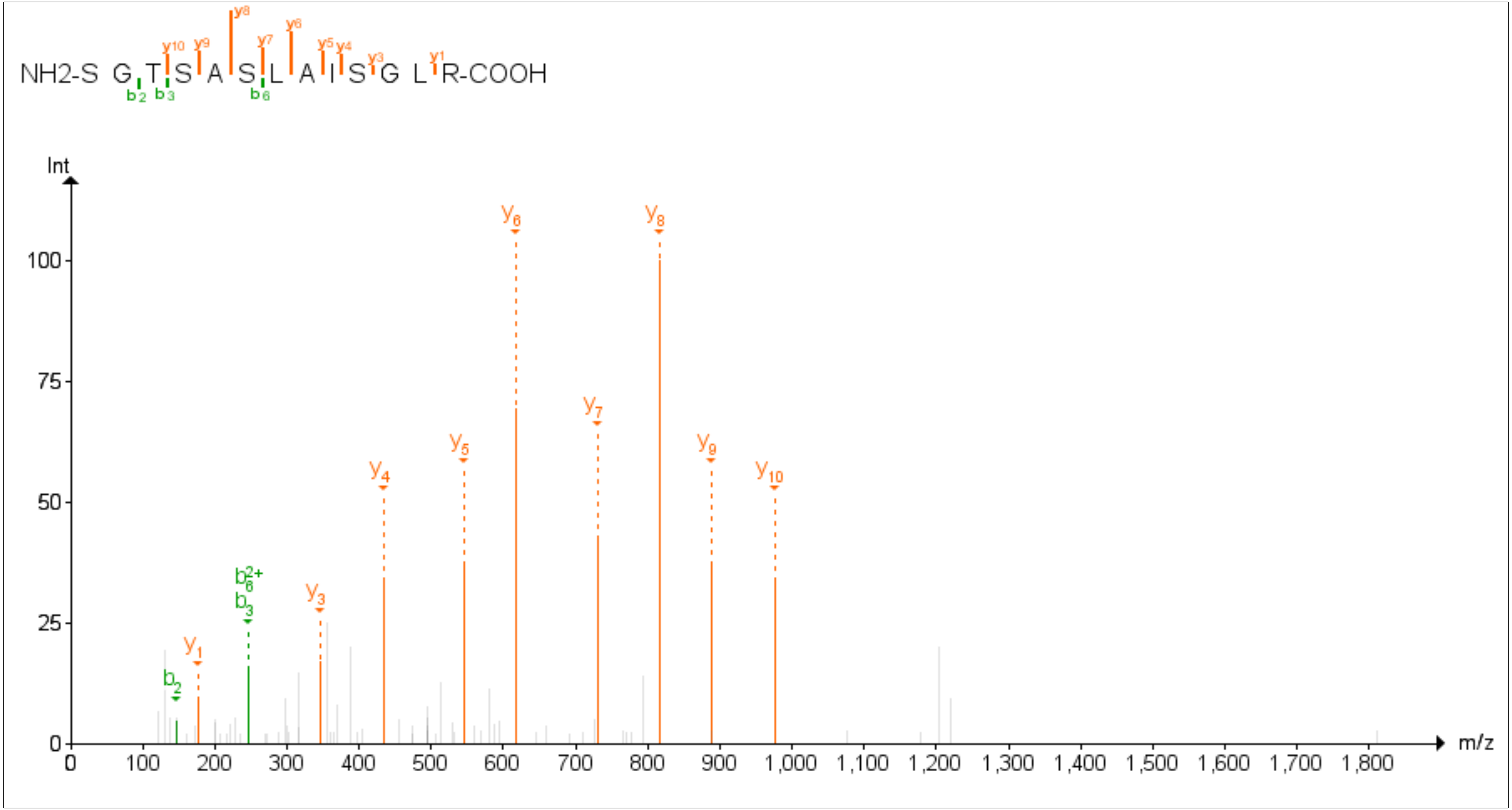
NH2-S G T S A S L A I S G L R-COOH
y10 y9 y8 y7 y6 y5 y4 y3 y1
b2 b3 b6
Int
100
75
50
25
0
y6
y8
y7
y5
y9
y4
y10
b6 2+
b3
y3
y1
b2
0 100 200 300 400 500 600 700 800 900 1,000 1,100 1,200 1,300 1,400 1,500 1,600 1,700 1,800
m/z

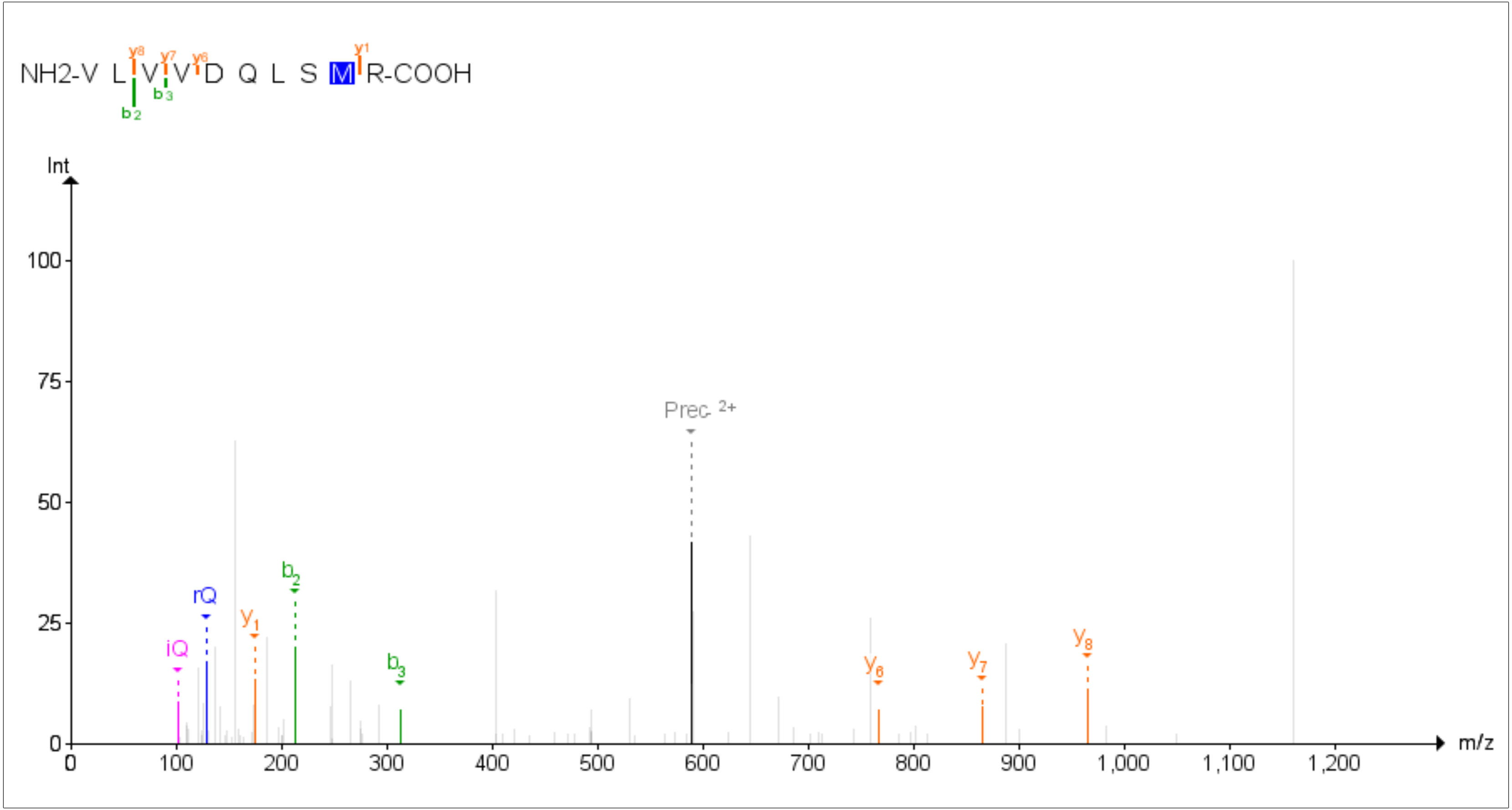
NH2-V L V V D Q L S M R-COOH
y8
y7
y6
y1
b2
b3
Int
100
75
50
25
0
Prec 2+
iQ
rQ
y1
b2
b3
y6
y7
y8
0
100
200
300
400
500
600
700
800
900
1,000
1,100
1,200
m/z

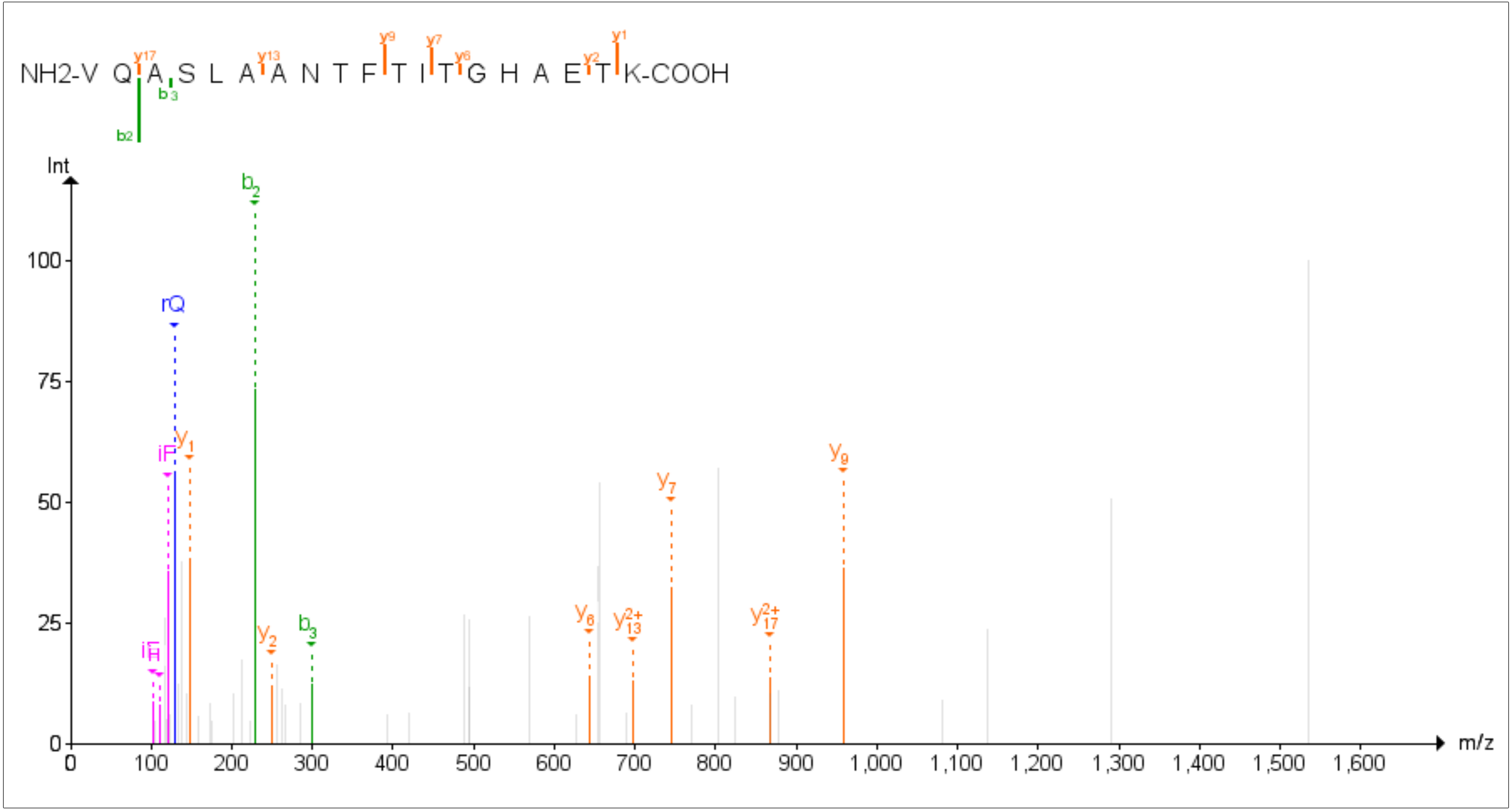

NH2-V Q A S L A A N T F T I T G H A E T K-COOH
y17
y13
y9
y7
y6
y2
y1
b2
b3
Int
m/z
rQ
iF
iH
y1
y2
b2
b3
y6
y13 2+
y7
y17 2+
y9
0
25
50
75
100
0
100
200
300
400
500
600
700
800
900
1,000
1,100
1,200
1,300
1,400
1,500
1,600

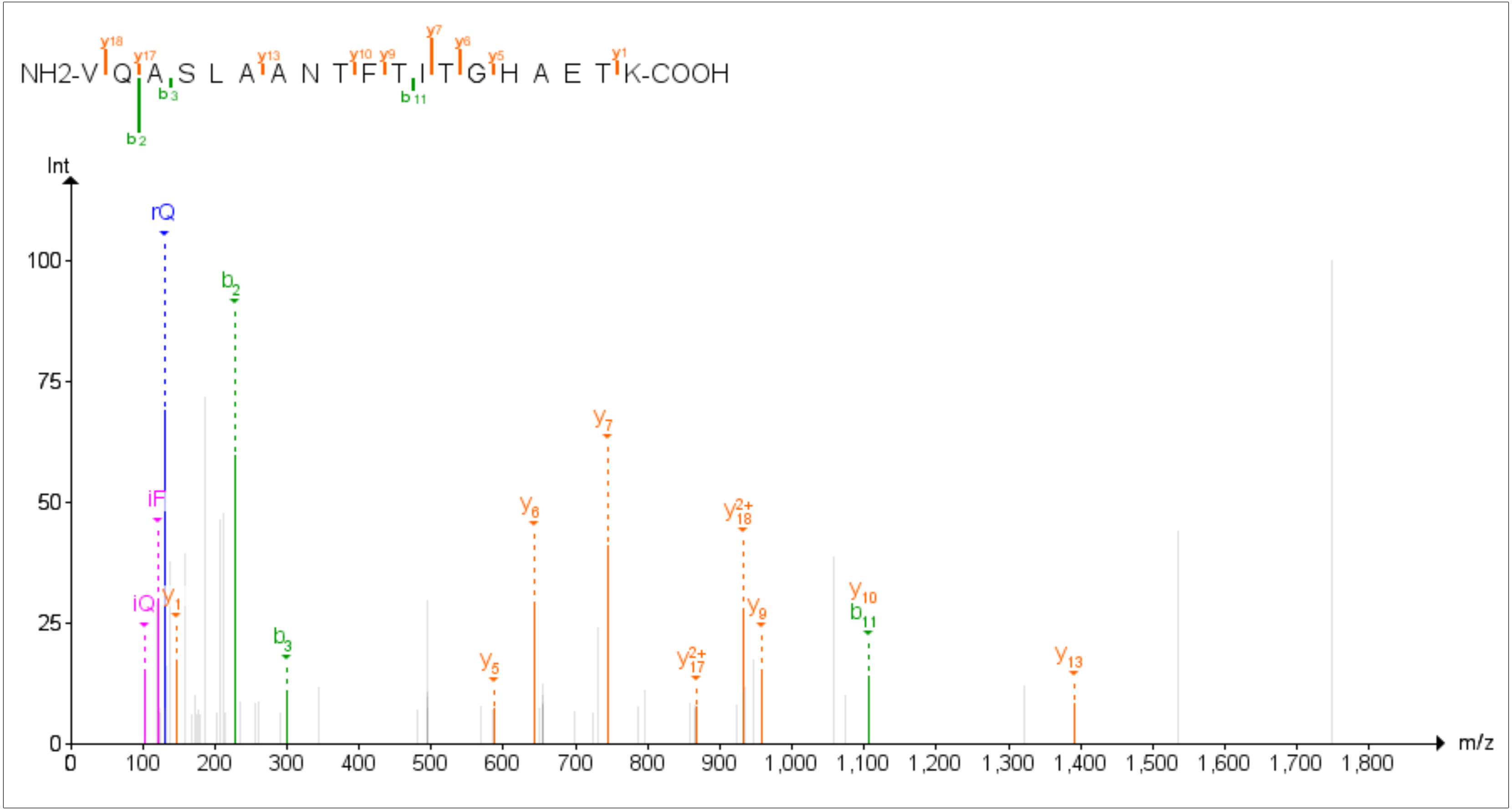

NH2-V Q A S L A A N T F T I T G H A E T K-COOH
y18 y17 y13 y10 y9 y7 y6 y5 y1
b2 b3 b11
Int
m/z
rQ
b2
iF
iQ
y1
b3
y5
y6
y7
y17 2+
y18 2+
y9
y10
b11
y13
0
25
50
75
100
0 100 200 300 400 500 600 700 800 900 1,000 1,100 1,200 1,300 1,400 1,500 1,600 1,700 1,800

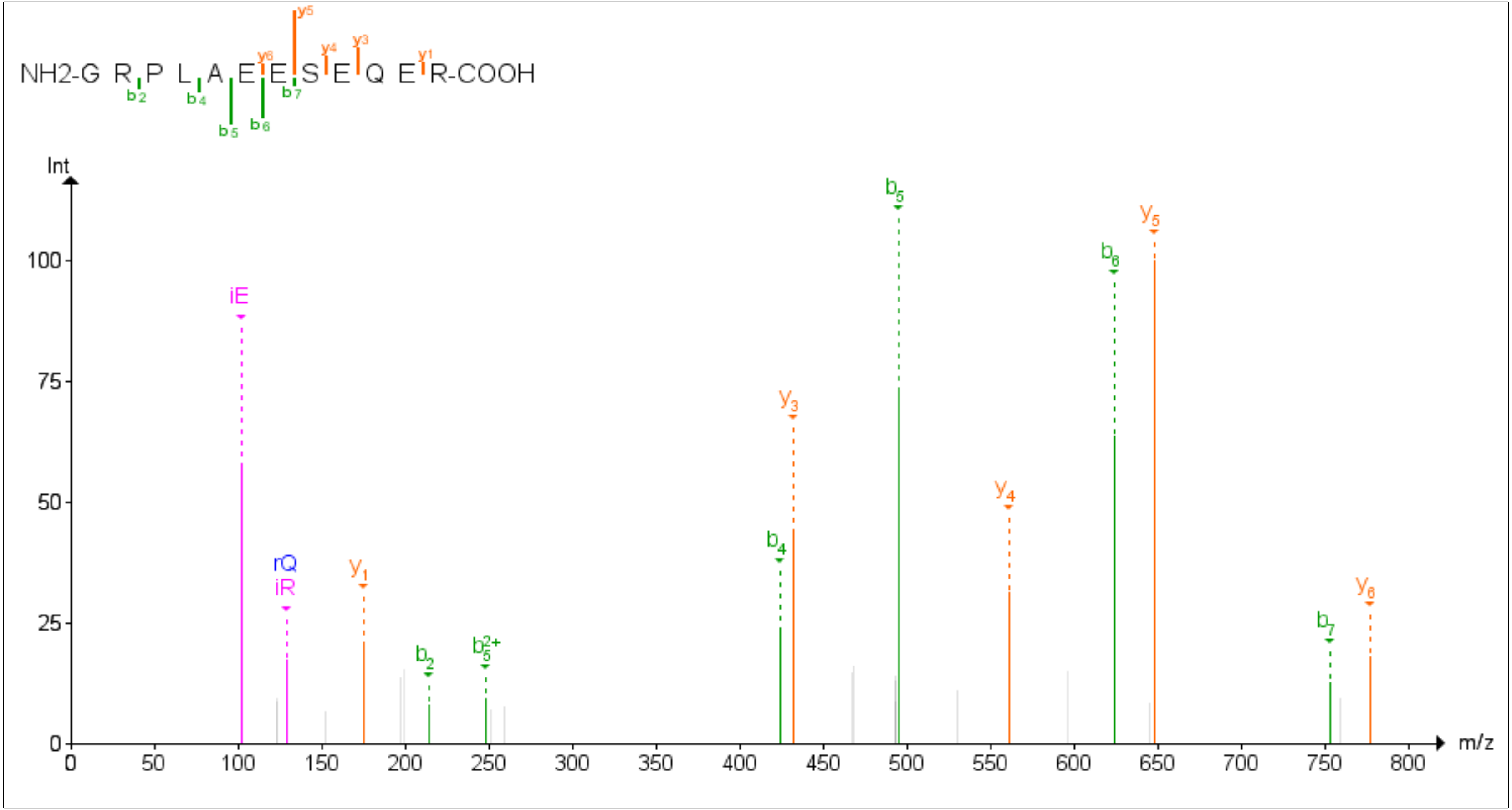

NH2-G R P L A E E S E Q E R-COOH
b2
b4
b5
b6
b7
y6
y5
y4
y3
y1
Int
100
75
50
25
0
iE
rQ
iR
y1
b2
b5 2+
b4
y3
b5
y4
b6
y5
b7
y6
0
50
100
150
200
250
300
350
400
450
500
550
600
650
700
750
800
m/z

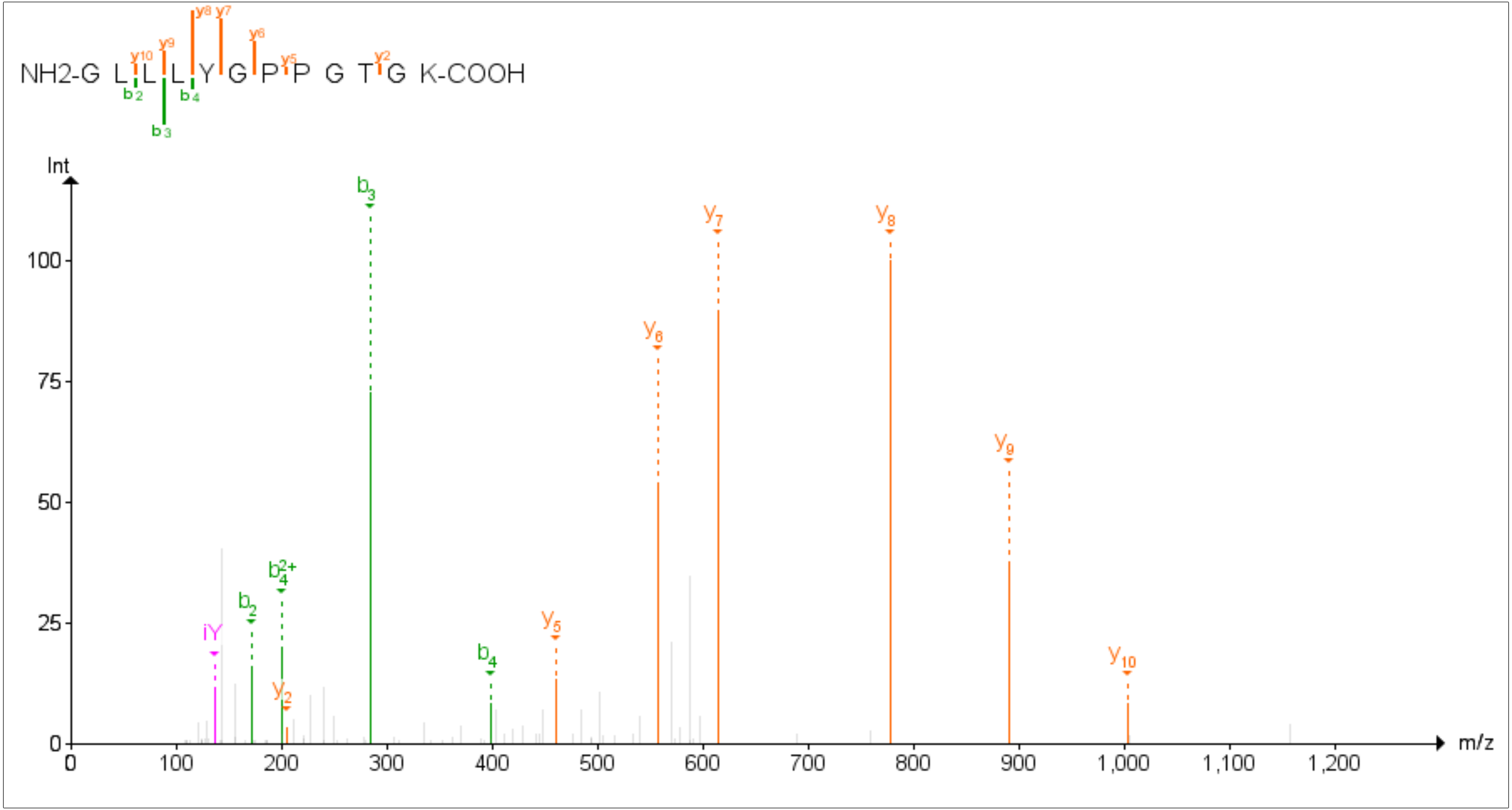

NH2-G L L L Y G P P G T G K-COOH
y10 y9 y8 y7 y6 y5 y2
b2 b3 b4
Int
100
75
50
25
0
b3
y7
y8
y6
y9
b4 2+
b2
iY
y5
b4
y2
y10
0 100 200 300 400 500 600 700 800 900 1,000 1,100 1,200
m/z

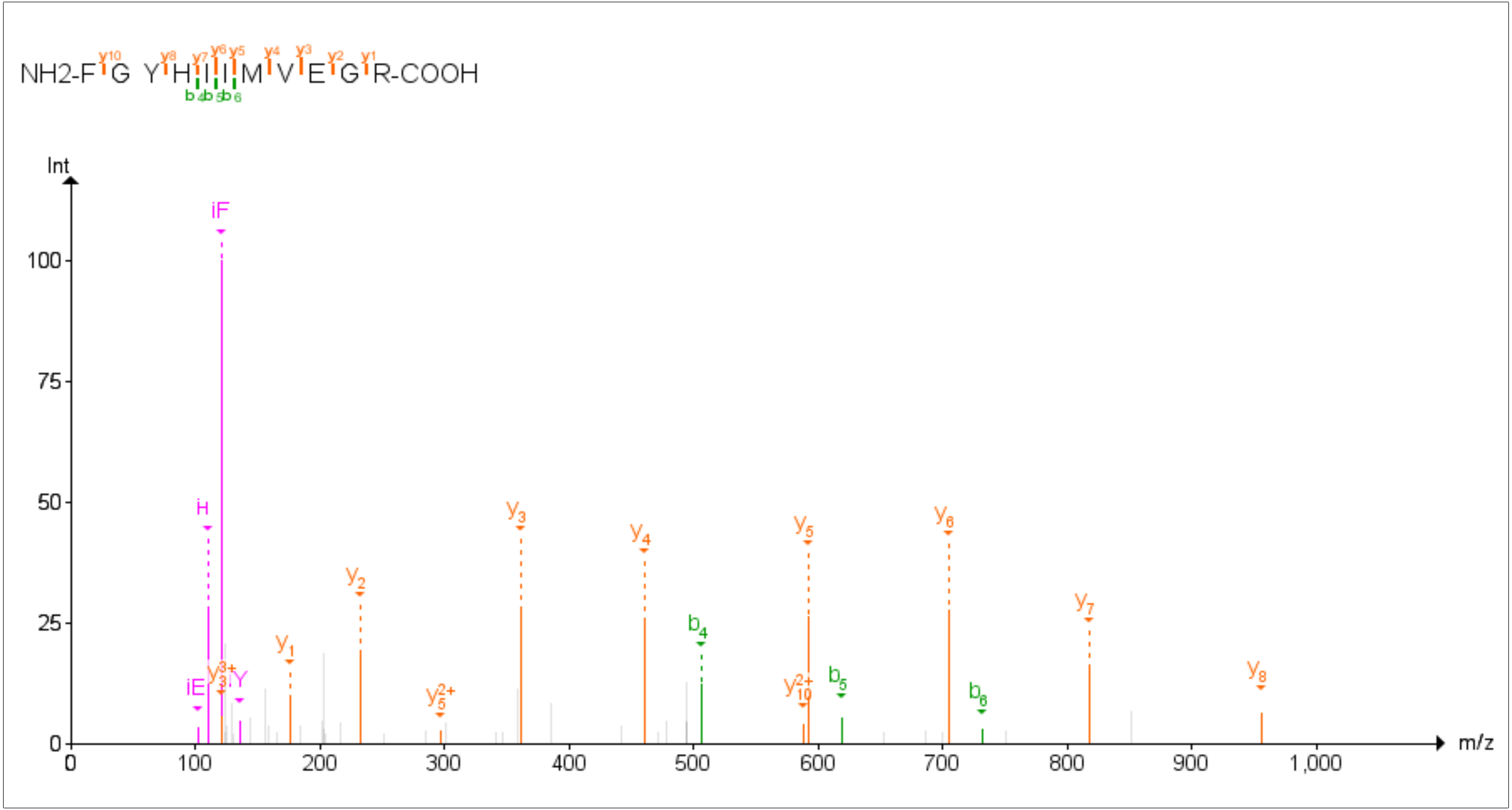

NH2-F G Y H I I M V E G R-COOH
y10
y8
y7
y6
y5
y4
y3
y2
y1
b4
b5
b6
Int
m/z
iF
iH
iE
iY
y1
y2
y3
y4
y5
y6
y7
y8
y3 3+
y5 2+
y10 2+
b4
b5
b6
0
25
50
75
100
0
100
200
300
400
500
600
700
800
900
1,000

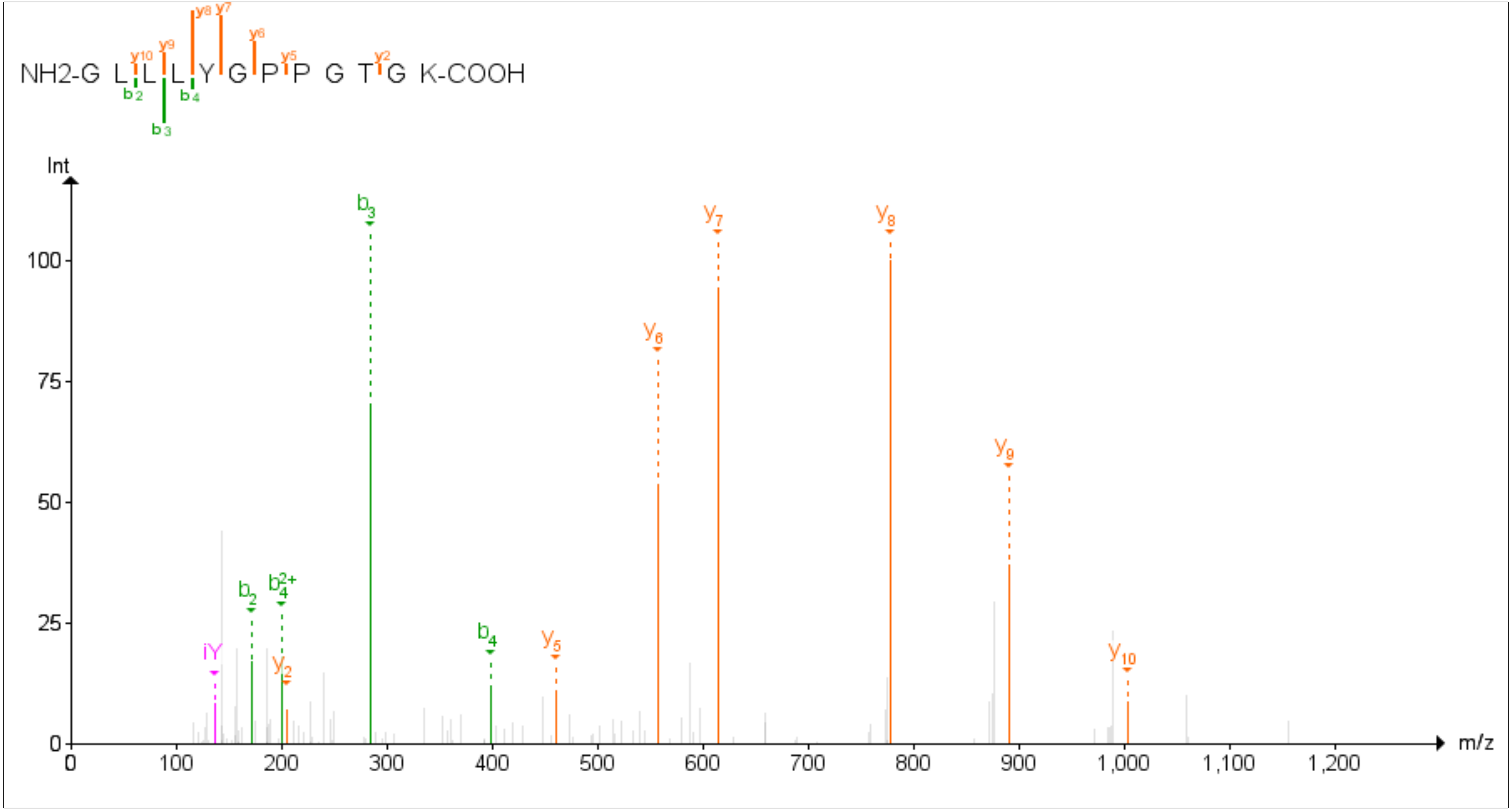

NH2-G L L L Y G P P G T G K-COOH
y10 y9 y8 y7 y6 y5 y2
b2 b3 b4
Int
100
75
50
25
0
b3
y7
y8
y6
y9
b2
b4 2+
iY
y2
b4
y5
y10
m/z
0
100
200
300
400
500
600
700
800
900
1,000
1,100
1,200

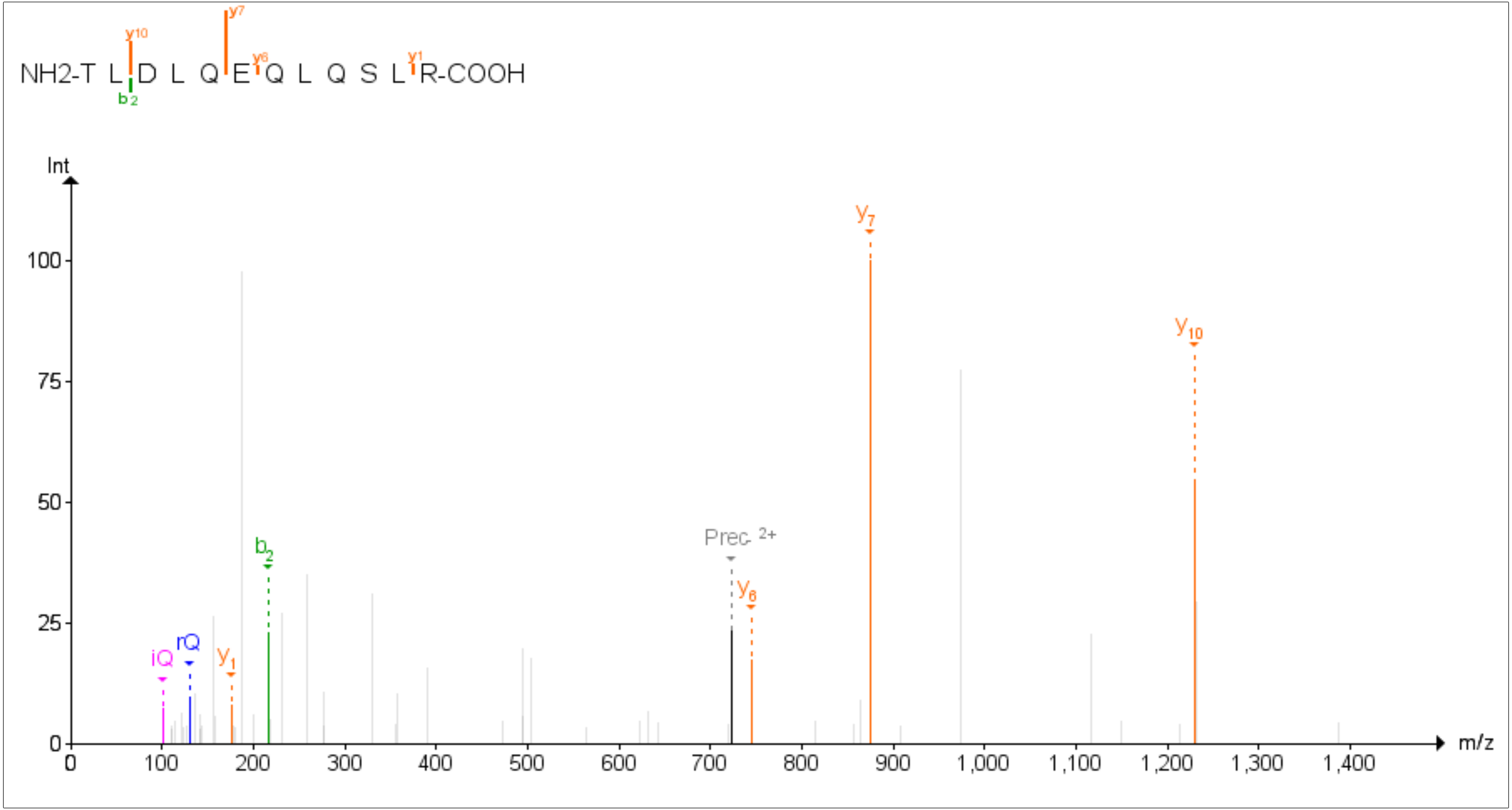

NH2-T L D L Q E Q L Q S L R-COOH
y10
y7
y6
y1
b2
Int
100
75
50
25
0
y7
y10
Prec 2+
y6
b2
iQ
rQ
y1
0
100
200
300
400
500
600
700
800
900
1,000
1,100
1,200
1,300
1,400
m/z

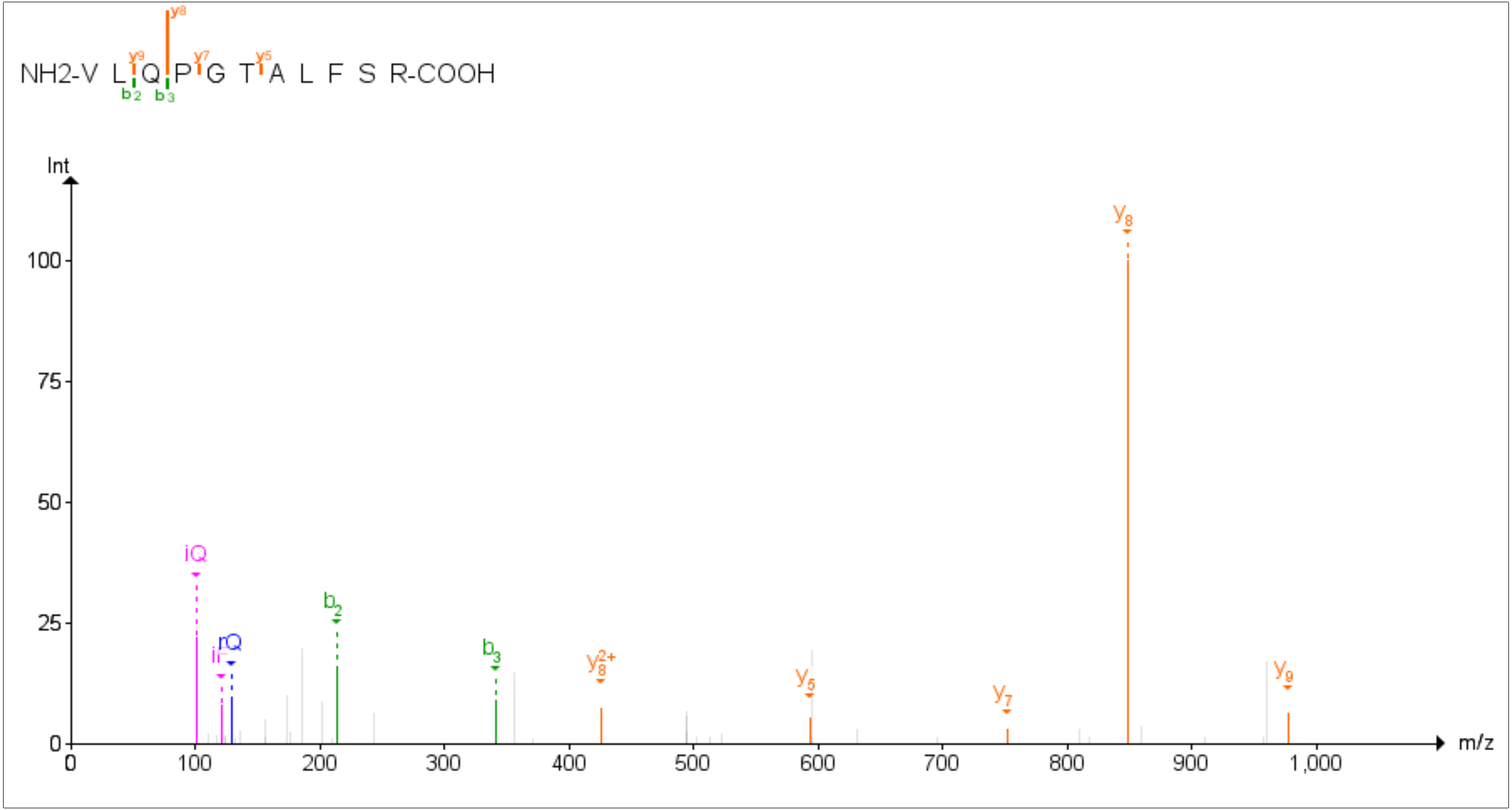

NH2-V L Q P G T A L F S R-COOH
y8
y9
y7
y5
b2
b3
Int
100
75
50
25
0
iQ
iF
rQ
b2
b3
y8 2+
y5
y7
y8
y9
m/z
0
100
200
300
400
500
600
700
800
900
1,000

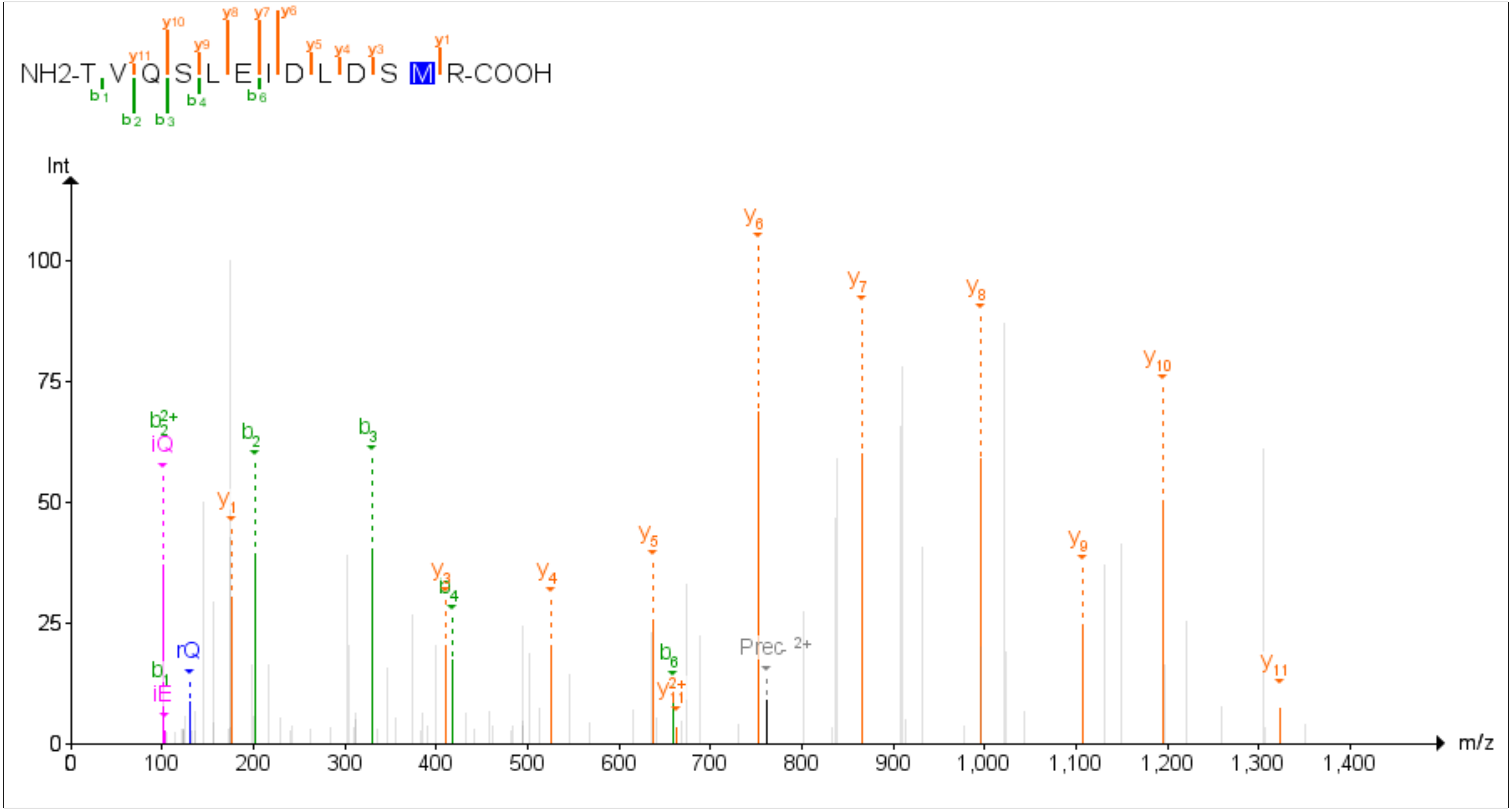

NH2-T V Q S L E I D L D S M R-COOH
Int
m/z
0
25
50
75
100
0
100
200
300
400
500
600
700
800
900
1,000
1,100
1,200
1,300
1,400
y6
y7
y8
y10
y9
y5
y4
y3
y1
y11
b2
b3
b4
b6
b1
iQ
iE
rQ
Prec. 2+

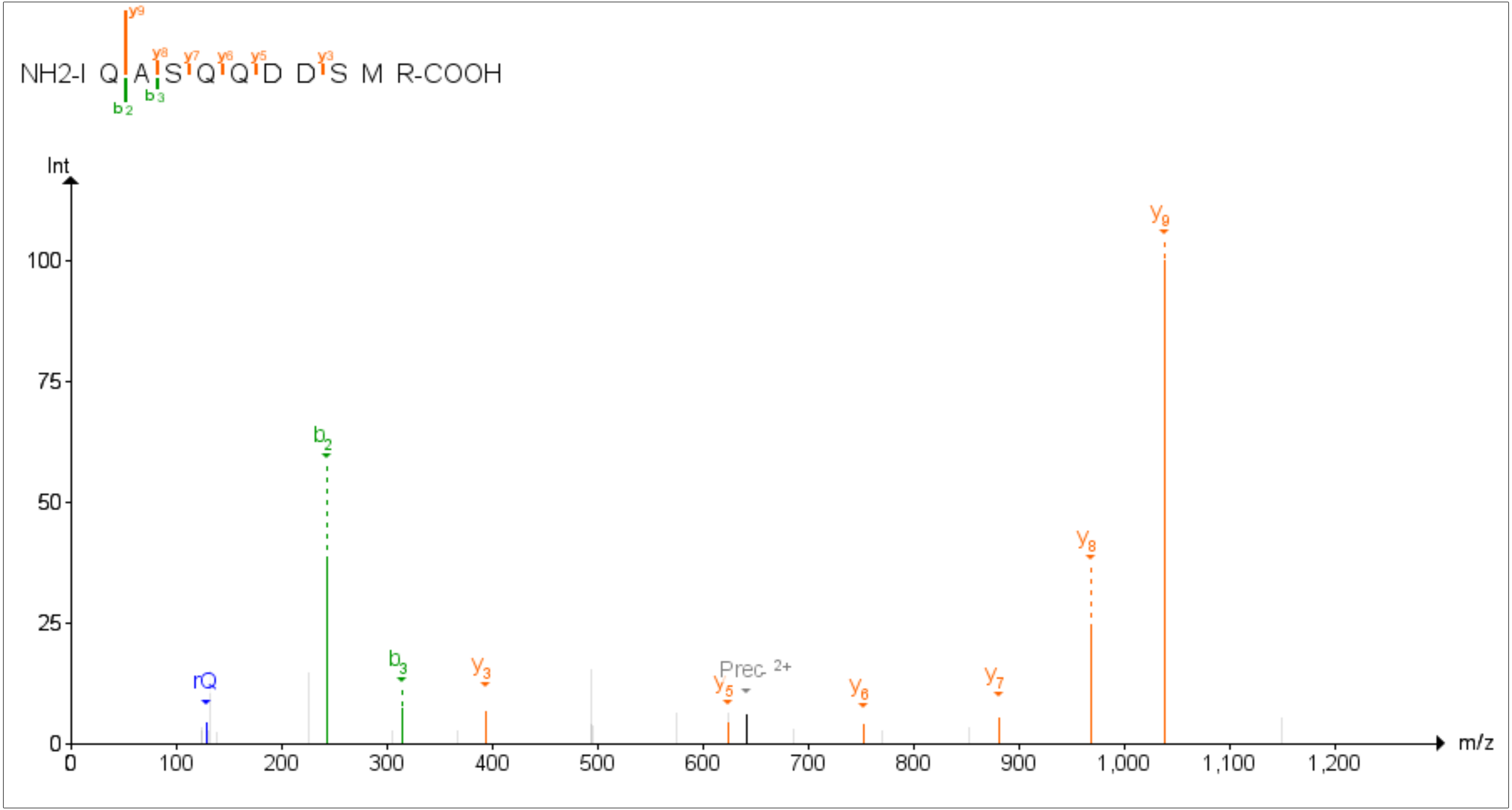

NH2-I Q A S Q Q D D S M R-COOH
y9
y8
y7
y6
y5
y3
b2
b3
Int
100
75
50
25
0
y9
b2
y8
rQ
b3
y3
Prec. 2+
y5
y6
y7
m/z
0
100
200
300
400
500
600
700
800
900
1,000
1,100
1,200

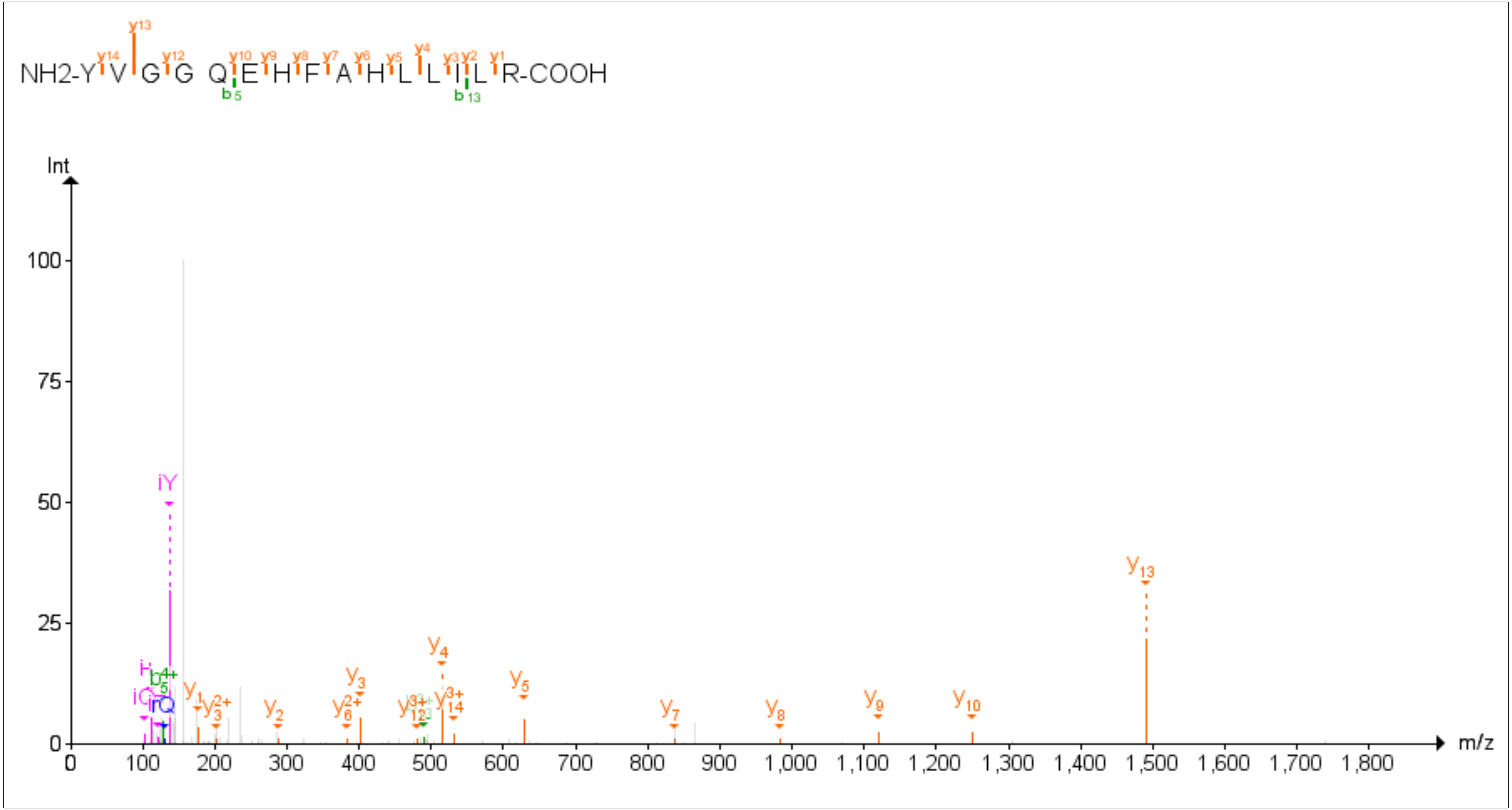

NH2-Y V G G Q E H F A H L L I L R-COOH
y14 y13 y12 y10 y9 y8 y7 y6 y5 y4 y3 y2 y1
b5 b13
Int
100
75
50
25
0
iY
y13
y4
y3
y5
y1
y2
y7
y8
y9
y10
m/z
0 100 200 300 400 500 600 700 800 900 1,000 1,100 1,200 1,300 1,400 1,500 1,600 1,700 1,800

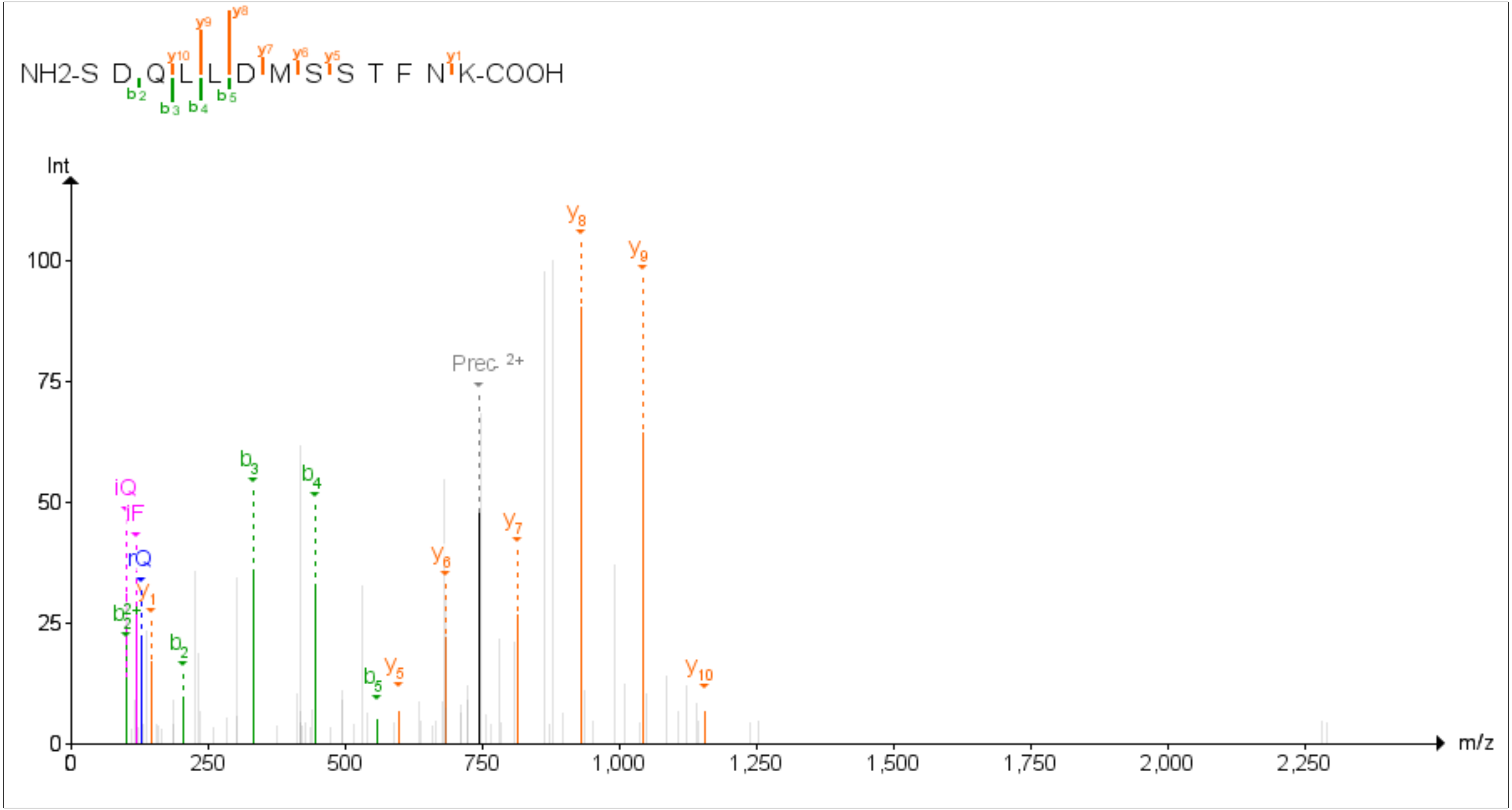

NH2-S D Q L L D M S S T F N K-COOH
b2 b3 b4 b5
y10 y9 y8 y7 y6 y5 y1
Int
100
75
50
25
0
Prec 2+
iQ
iF
rQ
b2 2+
y1
b2
b3
b4
b5
y5
y6
y7
y8
y9
y10
0 250 500 750 1,000 1,250 1,500 1,750 2,000 2,250
m/z

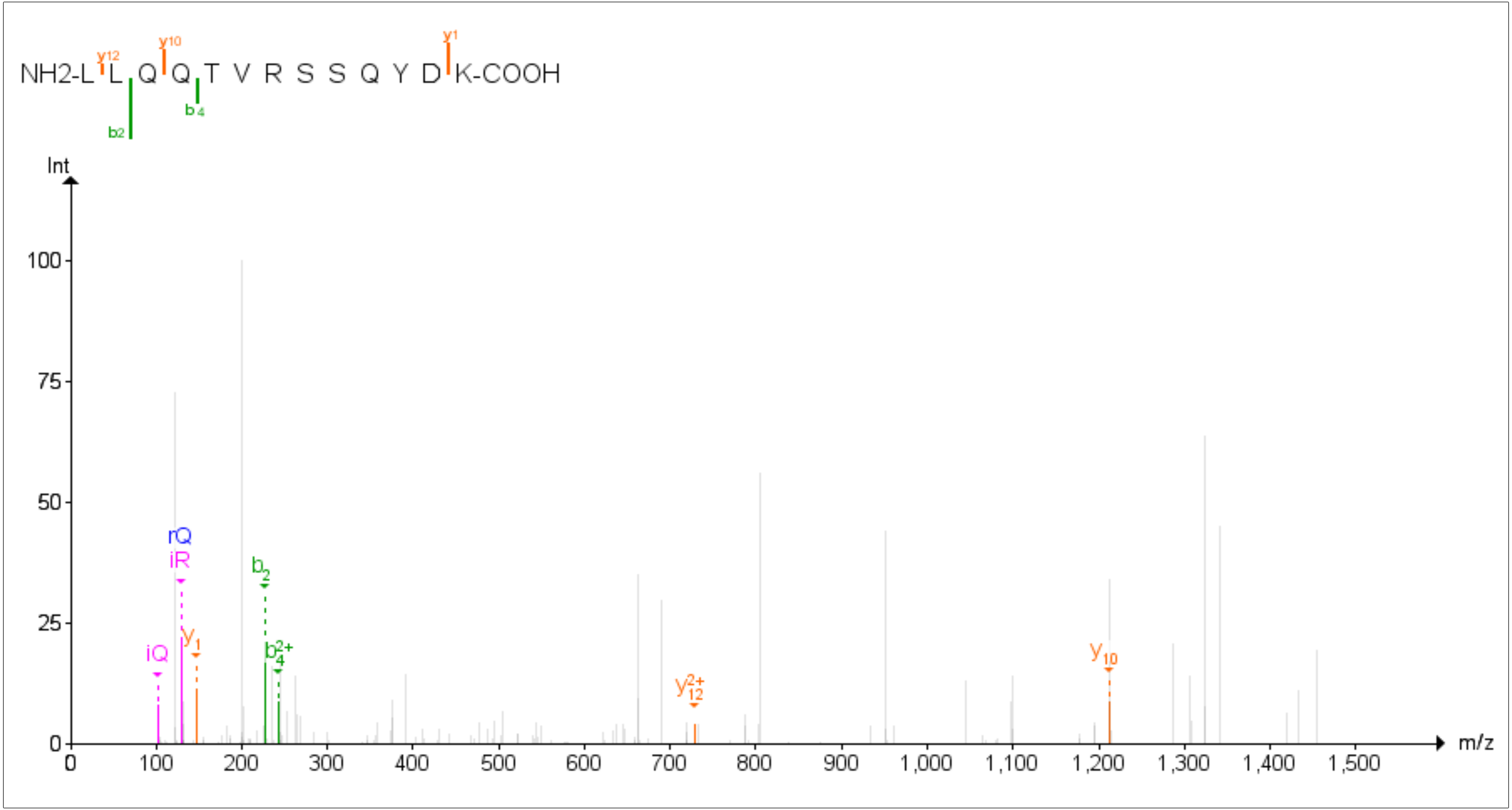

NH2-L L Q Q T V R S S Q Y D K-COOH
y12
y10
y1
b2
b4
Int
100
75
50
25
0
rQ
iR
iQ
y1
b2
b4 2+
y12 2+
y10
0
100
200
300
400
500
600
700
800
900
1,000
1,100
1,200
1,300
1,400
1,500
m/z

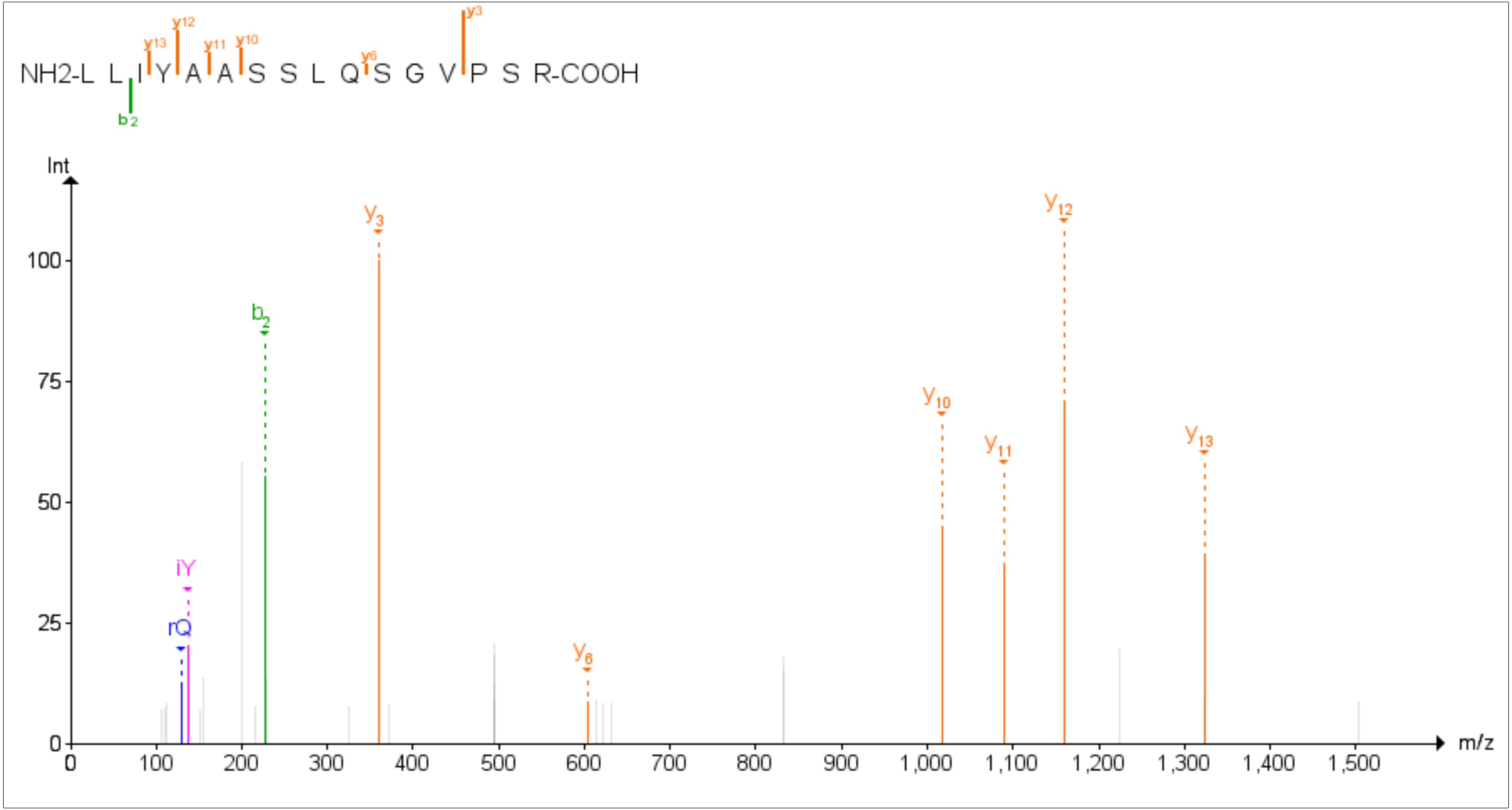
NH2-L L I Y A A S S L Q S G V P S R-COOH
b2
y13
y12
y11
y10
y6
y3
Int
m/z
100
75
50
25
0
0
100
200
300
400
500
600
700
800
900
1,000
1,100
1,200
1,300
1,400
1,500
iY
rQ
b2
y3
y6
y10
y11
y12
y13

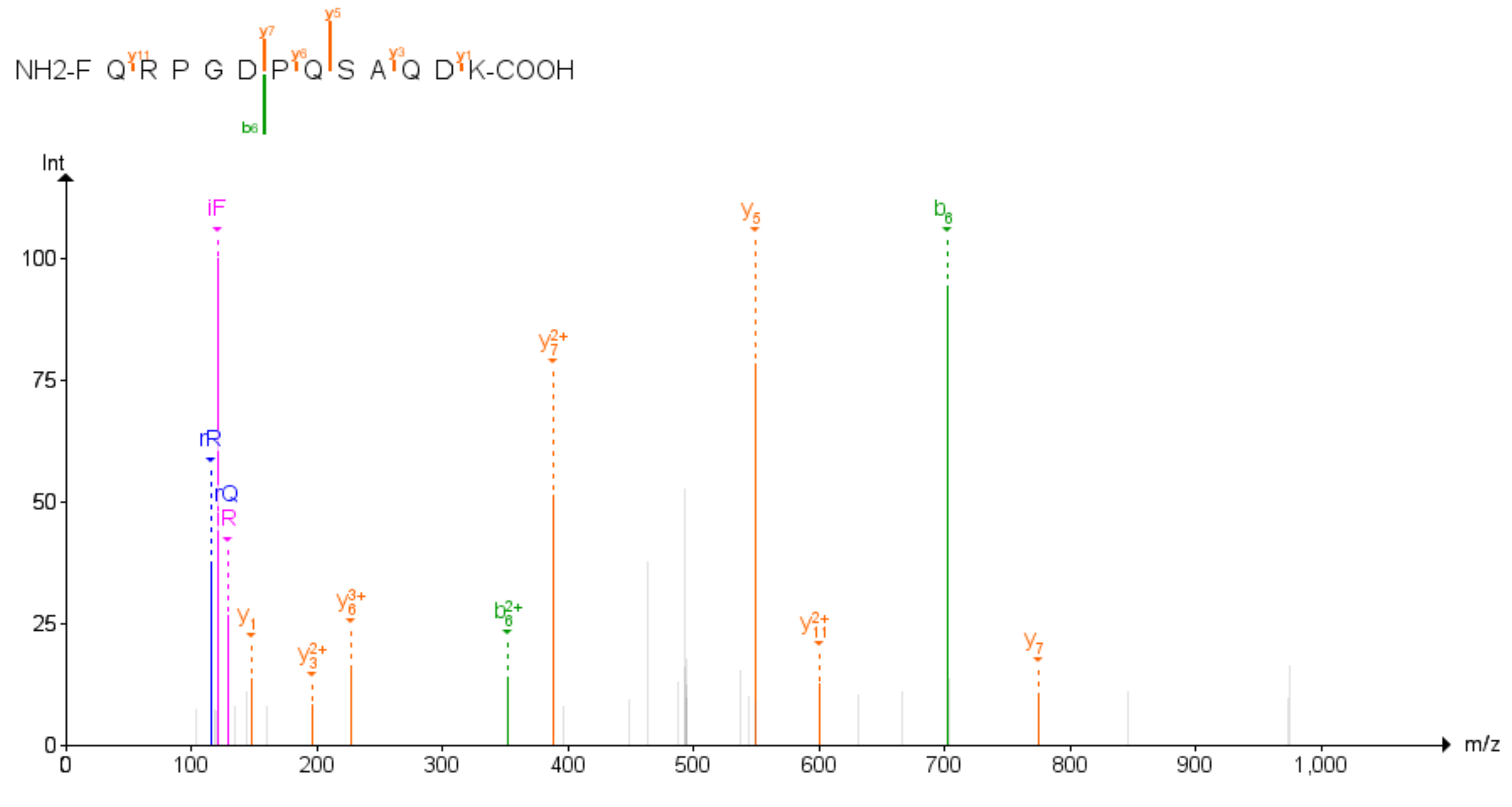

NH2-F Q R P G D P Q S A Q D K-COOH
y11
y7
y6
y5
y3
y1
b6
Int
iF
y5
b6
100
75
50
25
0
rR
rQ
iR
y1
y3 2+
y6 3+
b6 2+
y7 2+
y11 2+
y7
0
100
200
300
400
500
600
700
800
900
1,000
m/z

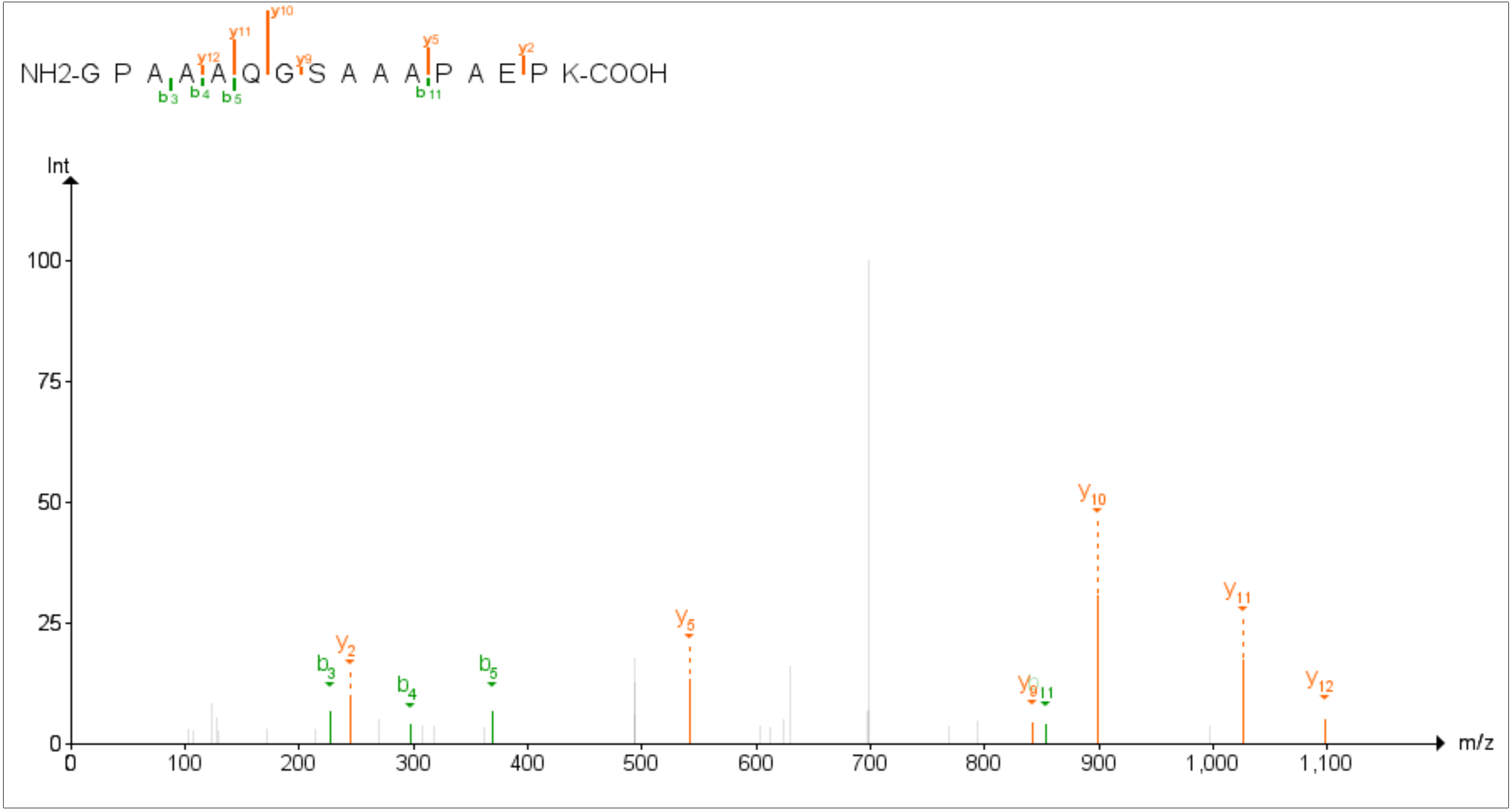

NH2-G P A A A Q G S A A A P A E P K-COOH
y10
y11
y12
y9
y5
y2
b3
b4
b5
b11
Int
100
75
50
25
0
m/z
0
100
200
300
400
500
600
700
800
900
1,000
1,100

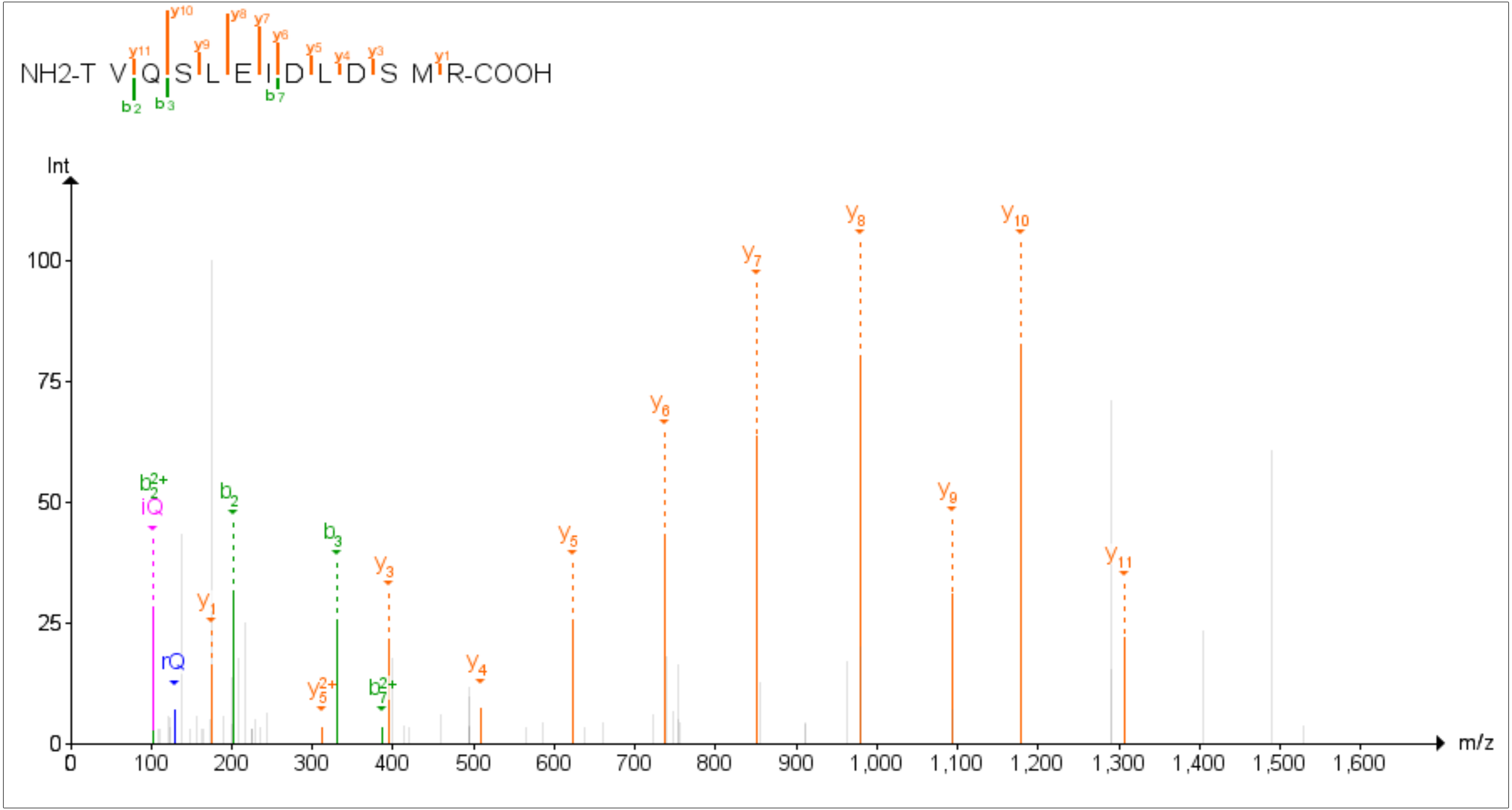

NH2-T V Q S L E I D L D S M R-COOH
y10 y8 y7 y6 y11 y9 y5 y4 y3 y1
b2 b3 b7
Int
100
75
50
25
0
b_2^{2+}
iQ
b_2
b_3
y_1
rQ
y_5^{2+}
b_7^{2+}
y_3
y_4
y_5
y_6
y_7
y_8
y_9
y_{10}
y_{11}
0 100 200 300 400 500 600 700 800 900 1,000 1,100 1,200 1,300 1,400 1,500 1,600
m/z

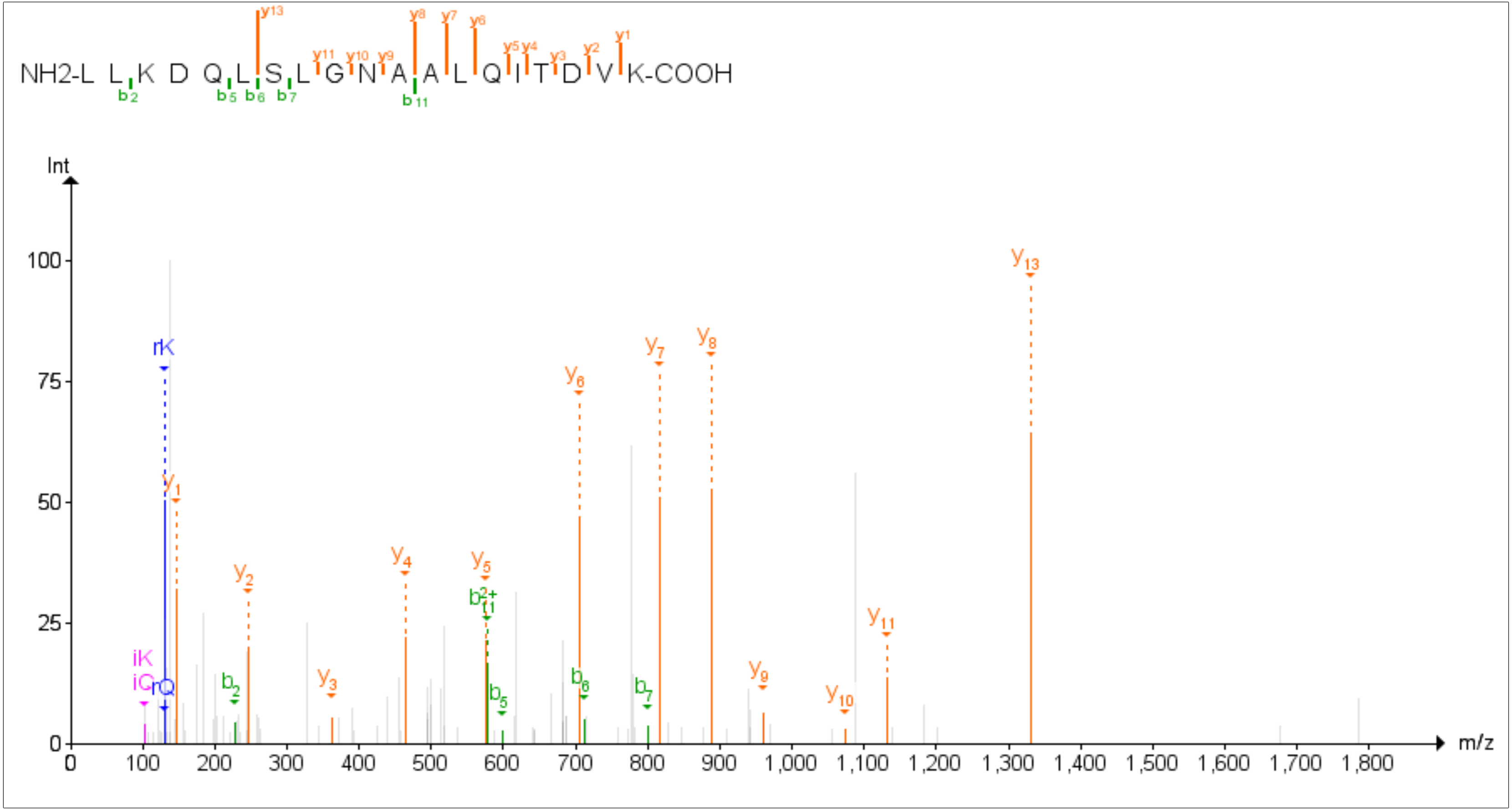
NH2-L L K D Q L S L G N A A L Q I T D V K-COOH
y13 y11 y10 y9 y8 y7 y6 y5 y4 y3 y2 y1
b2 b5 b6 b7 b11
Int
m/z
rK
y1
y2
y4
y5
y6
y7
y8
y13
y11
y9
y10
y3
b2
b5
b6
b7
iK
iQ
rQ

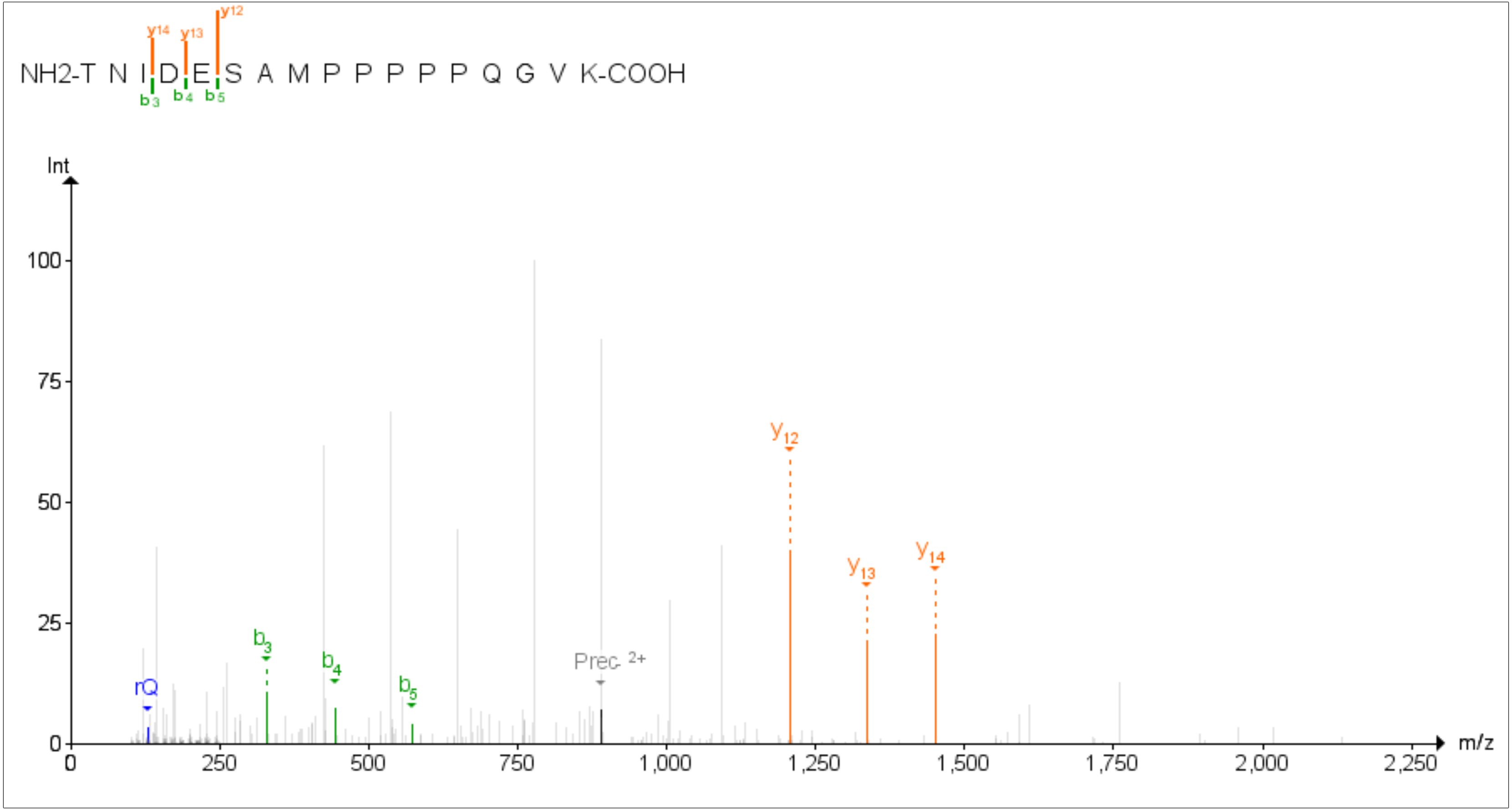

NH2-T N I D E S A M P P P P P Q G V K-COOH
y14 y13 y12
b3 b4 b5
Int
100
75
50
25
0
y12
y13
y14
b3
b4
b5
rQ
Prec- 2+
0 250 500 750 1,000 1,250 1,500 1,750 2,000 2,250
m/z

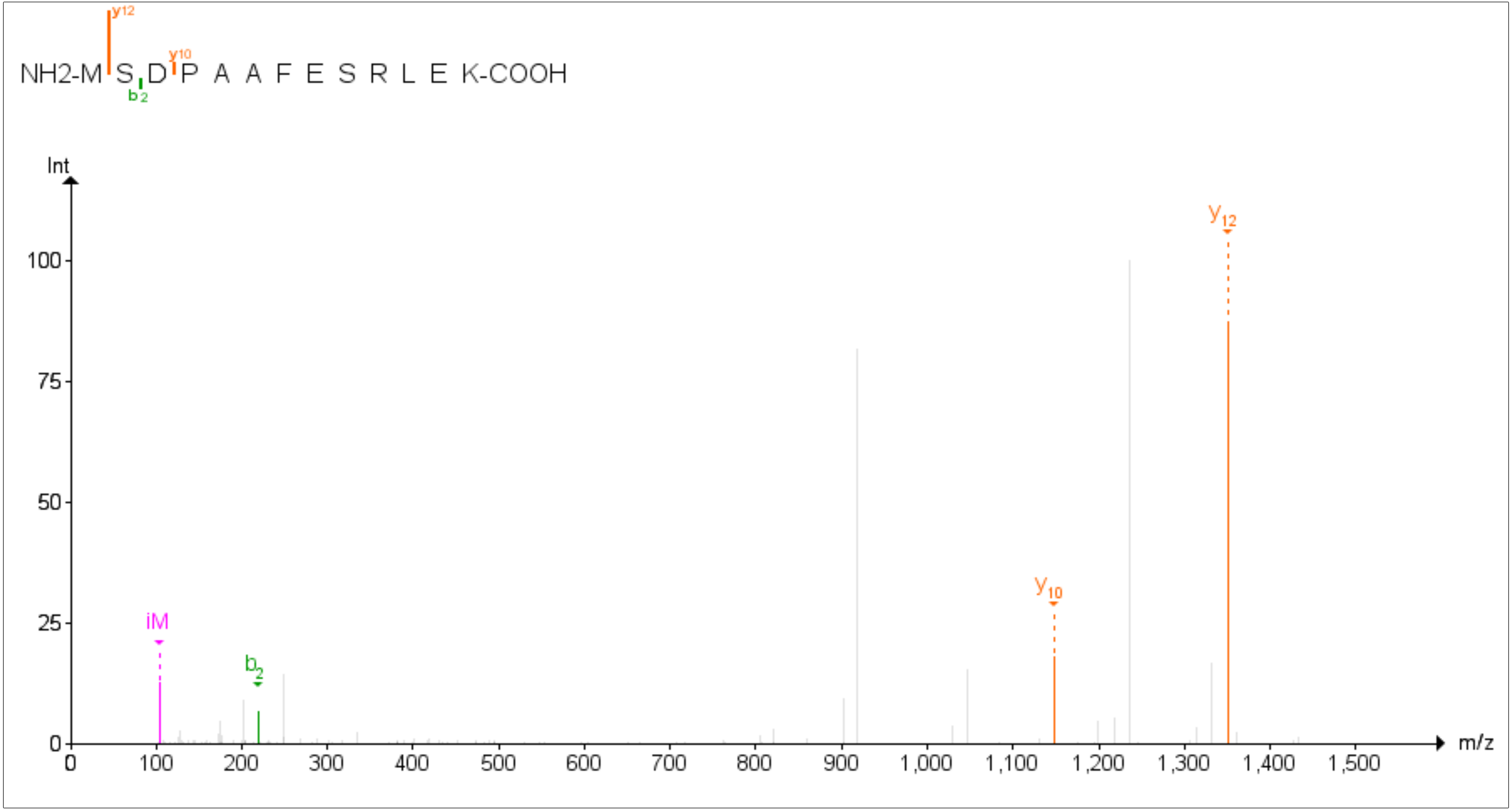
NH2-M S D P A A F E S R L E K-COOH
y12
y10
b2
Int
100
75
50
25
0
iM
b2
y10
y12
0
100
200
300
400
500
600
700
800
900
1,000
1,100
1,200
1,300
1,400
1,500
m/z

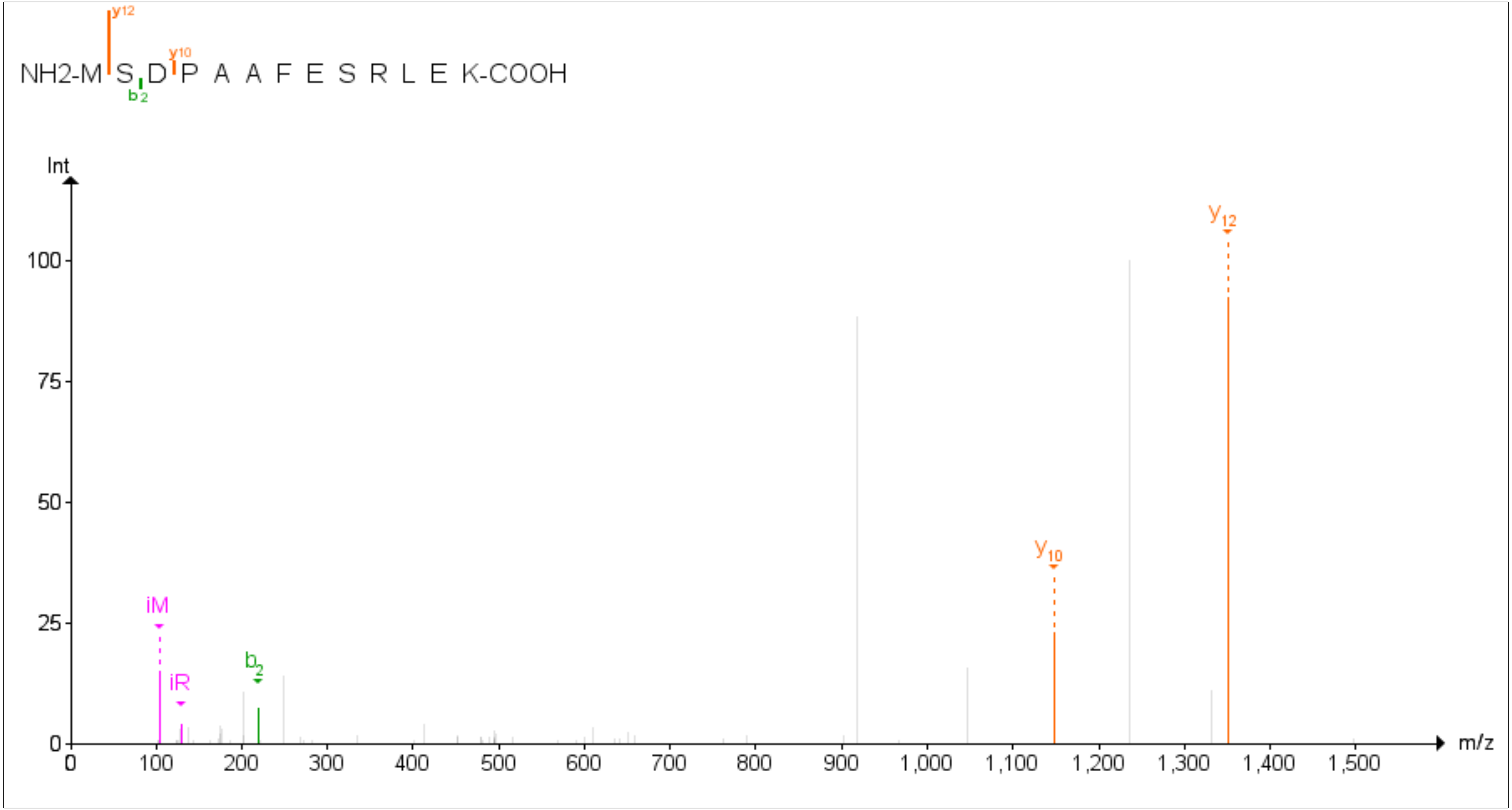

NH2-M S D P A A F E S R L E K-COOH
y12
y10
b2
Int
100
75
50
25
0
iM
iR
b2
y10
y12
0
100
200
300
400
500
600
700
800
900
1,000
1,100
1,200
1,300
1,400
1,500
m/z

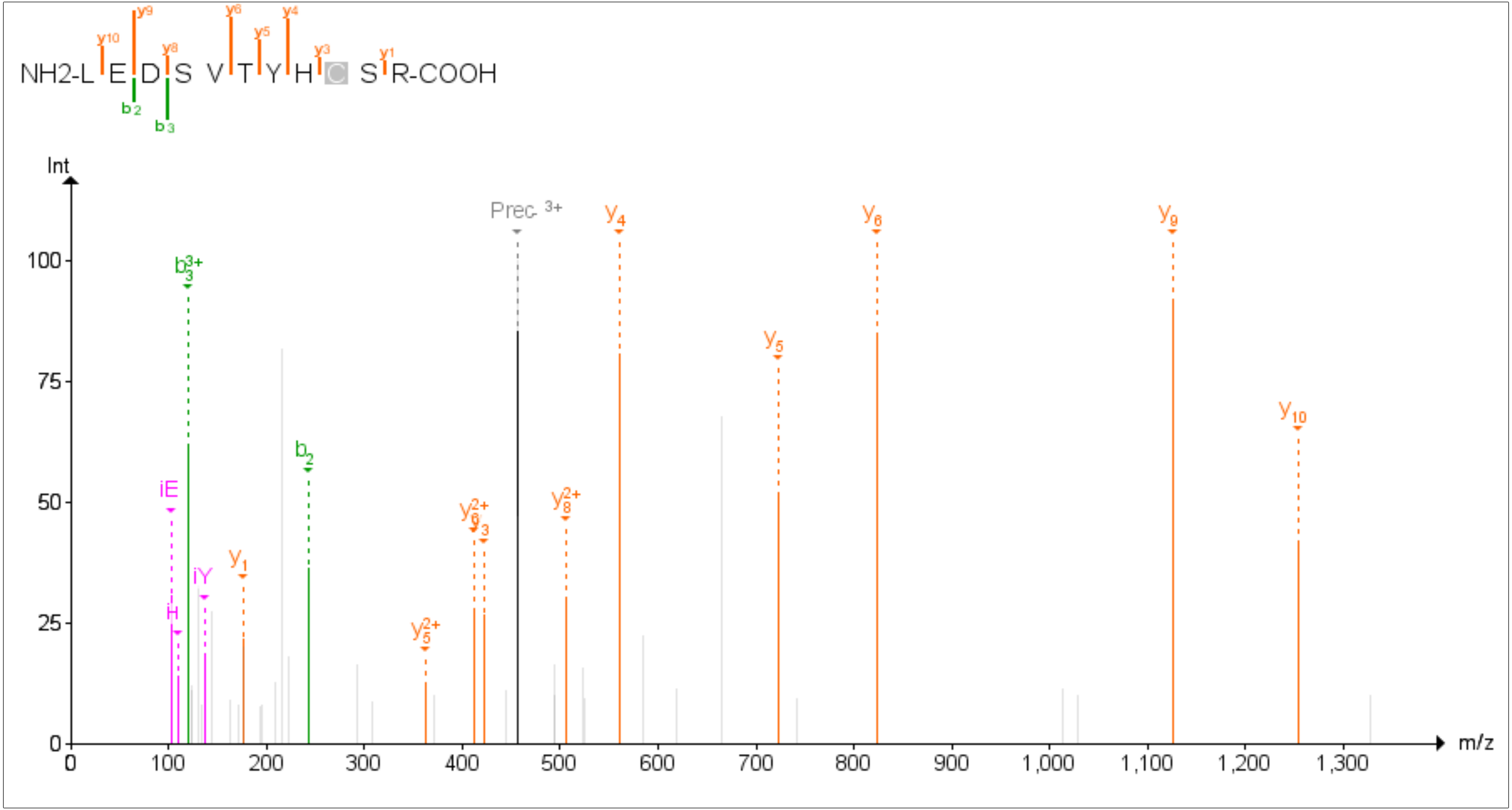

NH2-L E D S V T Y H C S R-COOH
y10 y9 y8 y6 y5 y4 y3 y1
b2 b3
Int
m/z
Prec. 3+
y4
y6
y9
b3 3+
y5
y10
b2
iE
y6 2+
y3
y8 2+
y1
iY
iH
y5 2+
0
25
50
75
100
0
100
200
300
400
500
600
700
800
900
1,000
1,100
1,200
1,300

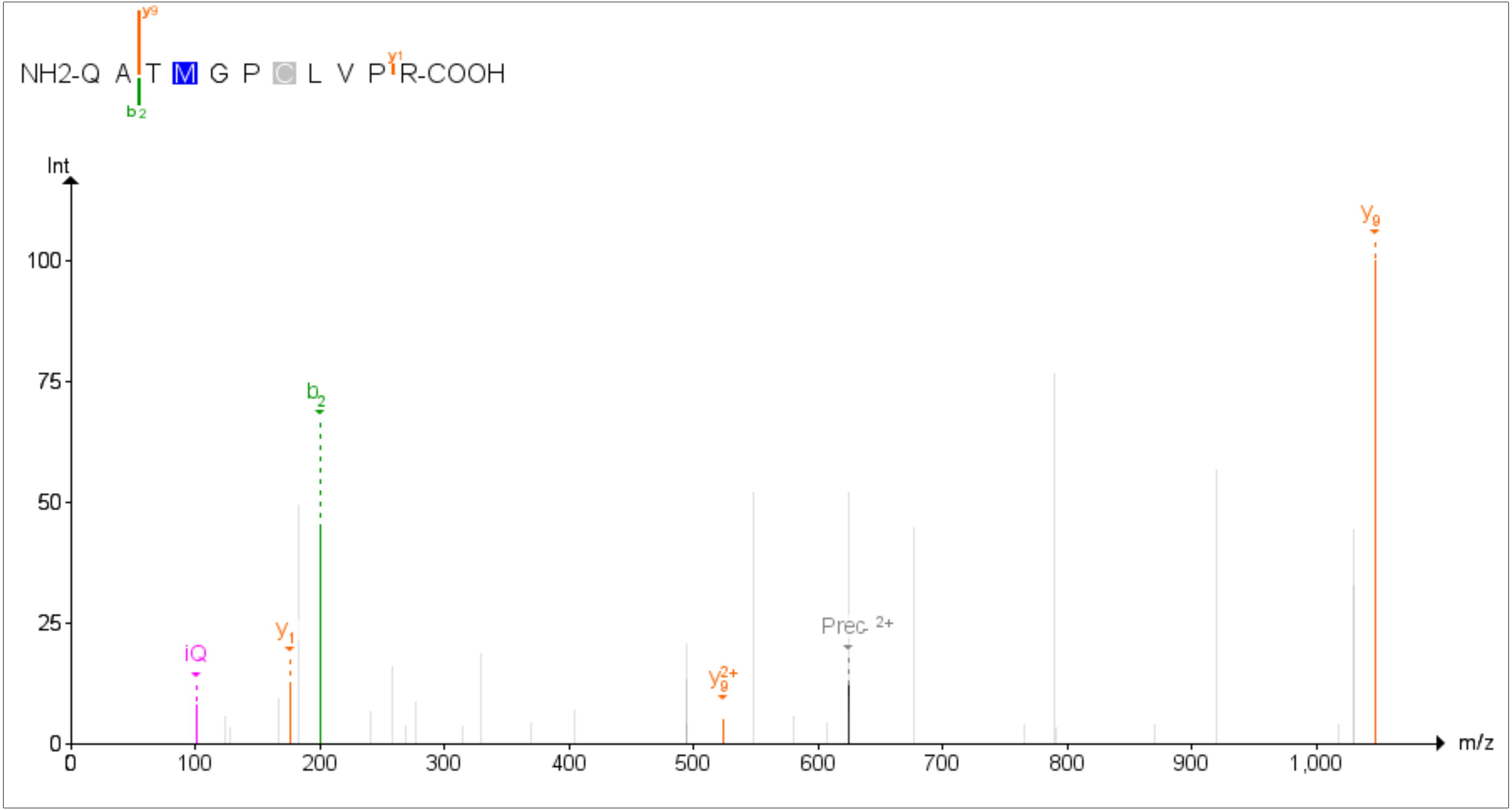

y9
NH2-Q A T M G P C L V P R-COOH
y1
b 2
Int
100
75
50
25
0
y9
b2
iQ
y1
y9 2+
Prec 2+
0
100
200
300
400
500
600
700
800
900
1,000
m/z

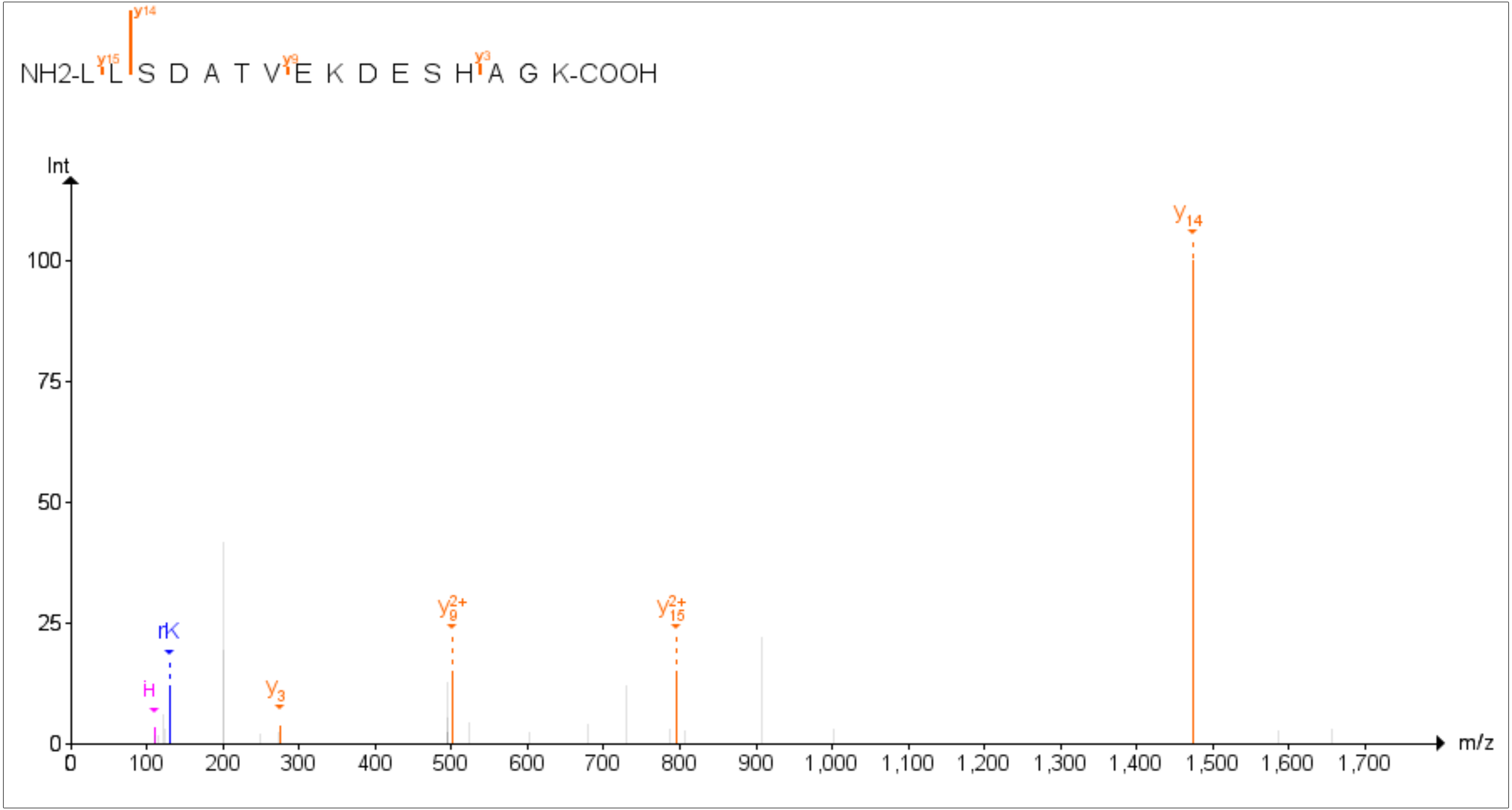

NH2-L L S D A T V E K D E S H A G K-COOH
y14
y15
y9
y3
Int
100
75
50
25
0
y14
y9 2+
y15 2+
rK
H
y3
0 100 200 300 400 500 600 700 800 900 1,000 1,100 1,200 1,300 1,400 1,500 1,600 1,700
m/z

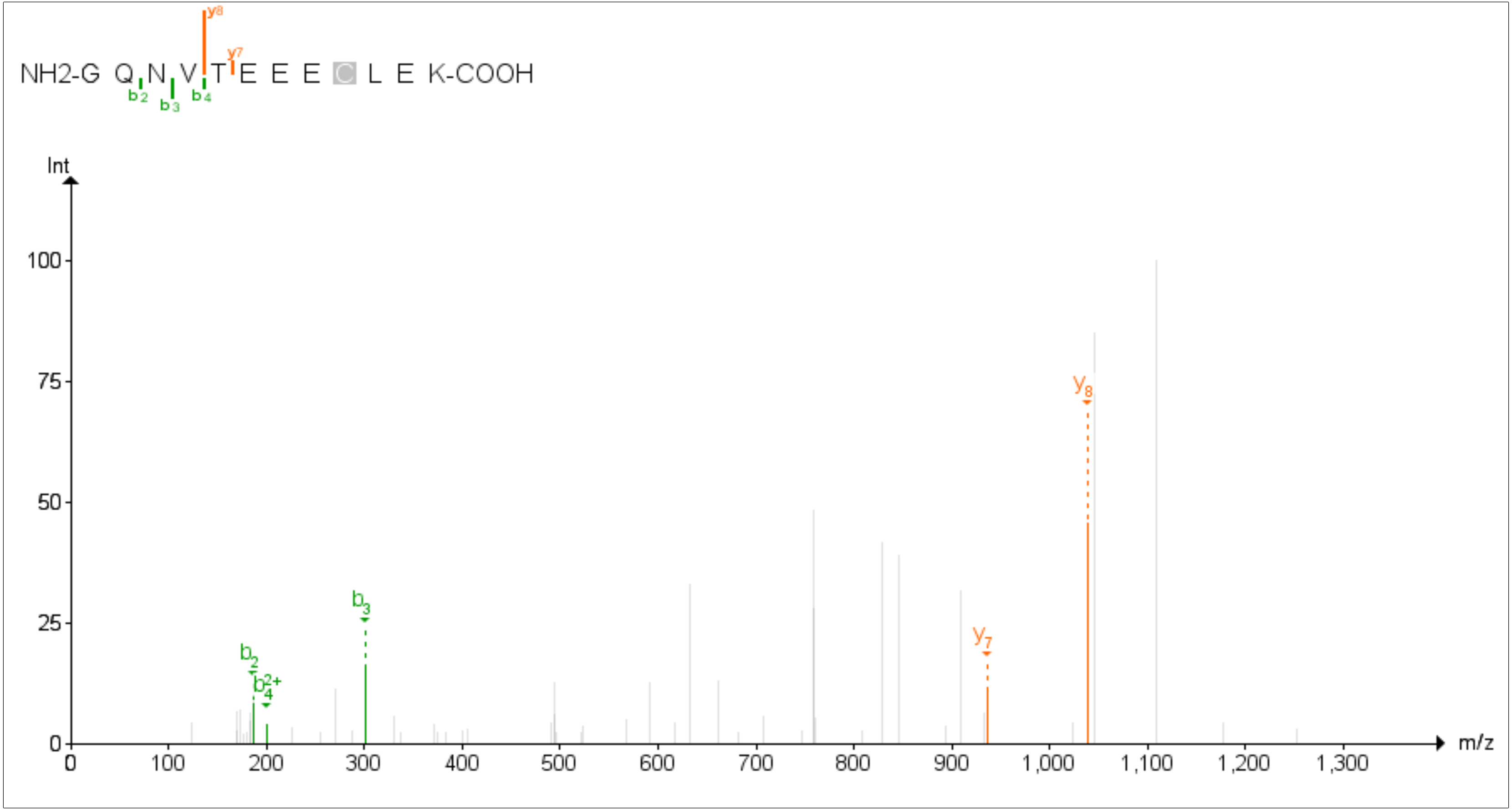

NH2-G Q N V T E E E C L E K-COOH
y8
y7
b2
b3
b4
Int
100
75
50
25
0
b3
b2
b_4^{2+}
y7
y8
0
100
200
300
400
500
600
700
800
900
1,000
1,100
1,200
1,300
m/z

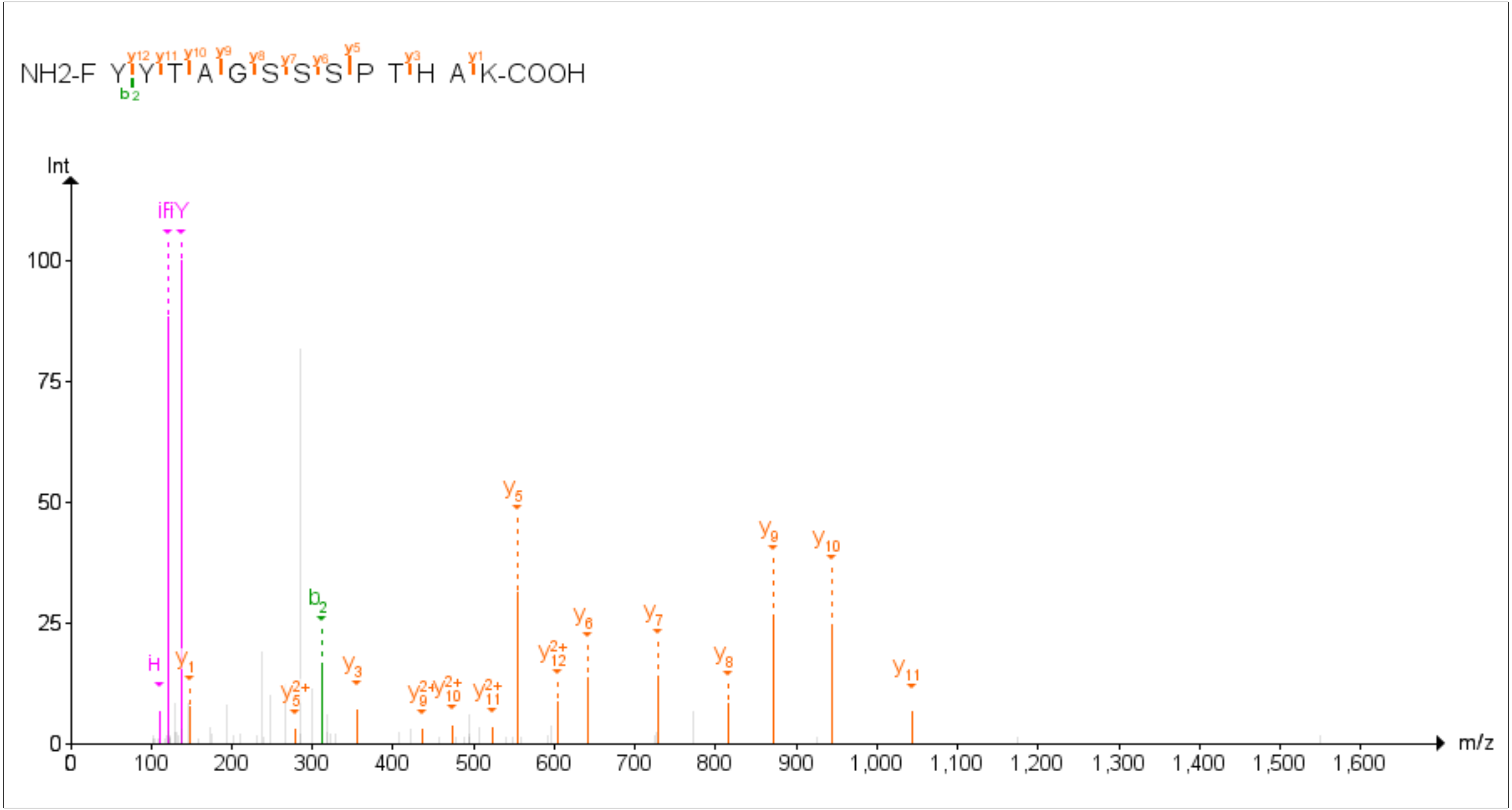

NH2-F Y Y T A G S S S P T H A K-COOH
y12 y11 y10 y9 y8 y7 y6 y5 y3 y1
b2
Int
m/z
0
25
50
75
100
0
100
200
300
400
500
600
700
800
900
1,000
1,100
1,200
1,300
1,400
1,500
1,600
iFiY
iH
y1
y5 2+
b2
y3
y9 2+
y10 2+
y11 2+
y5
y12 2+
y6
y7
y8
y9
y10
y11

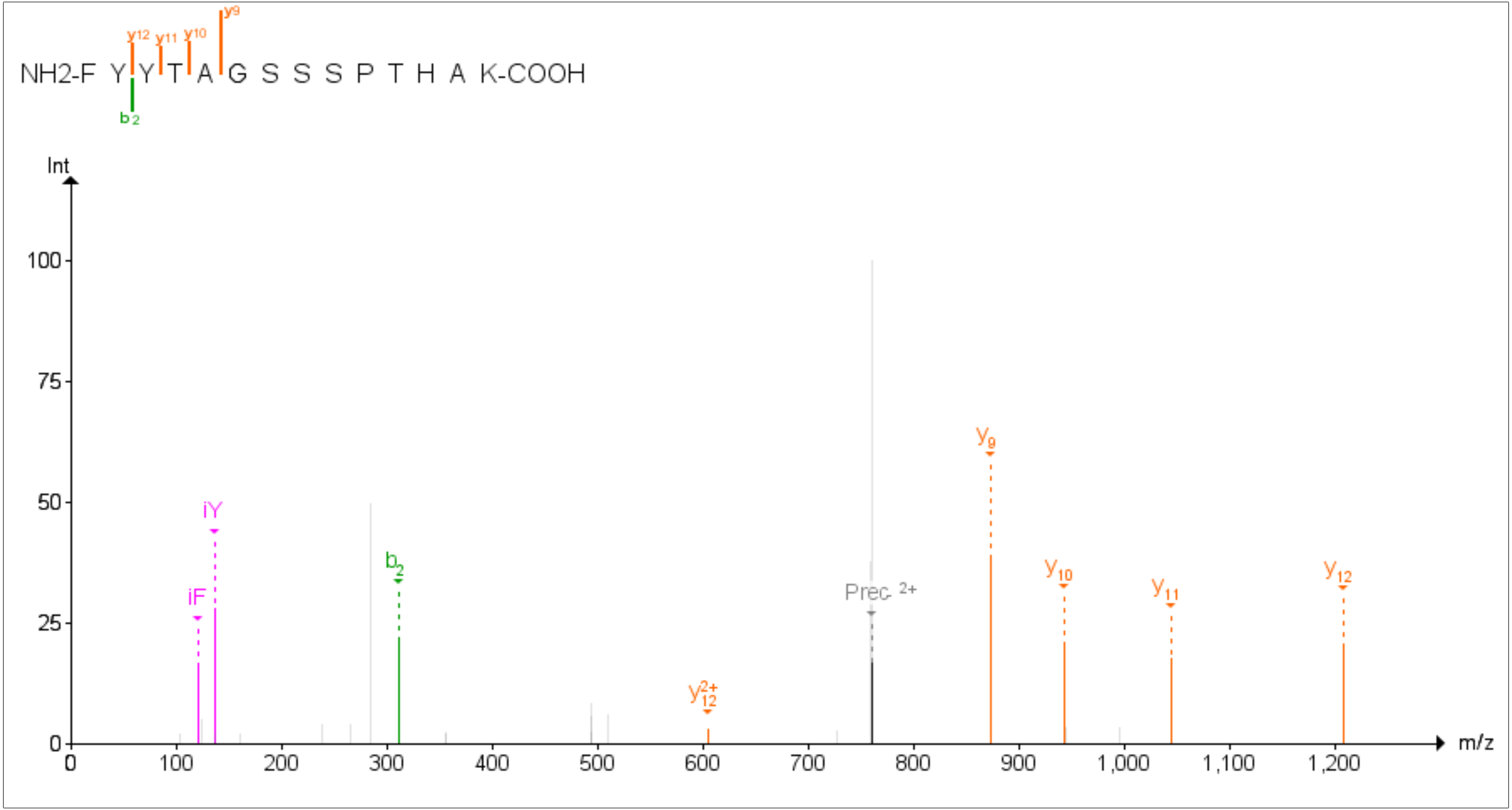

NH2-F Y Y T A G S S S P T H A K-COOH
y12 y11 y10 y9
b2
Int
100
75
50
25
0
iY
iF
b2
y12 2+
Prec 2+
y9
y10
y11
y12
0 100 200 300 400 500 600 700 800 900 1,000 1,100 1,200
m/z

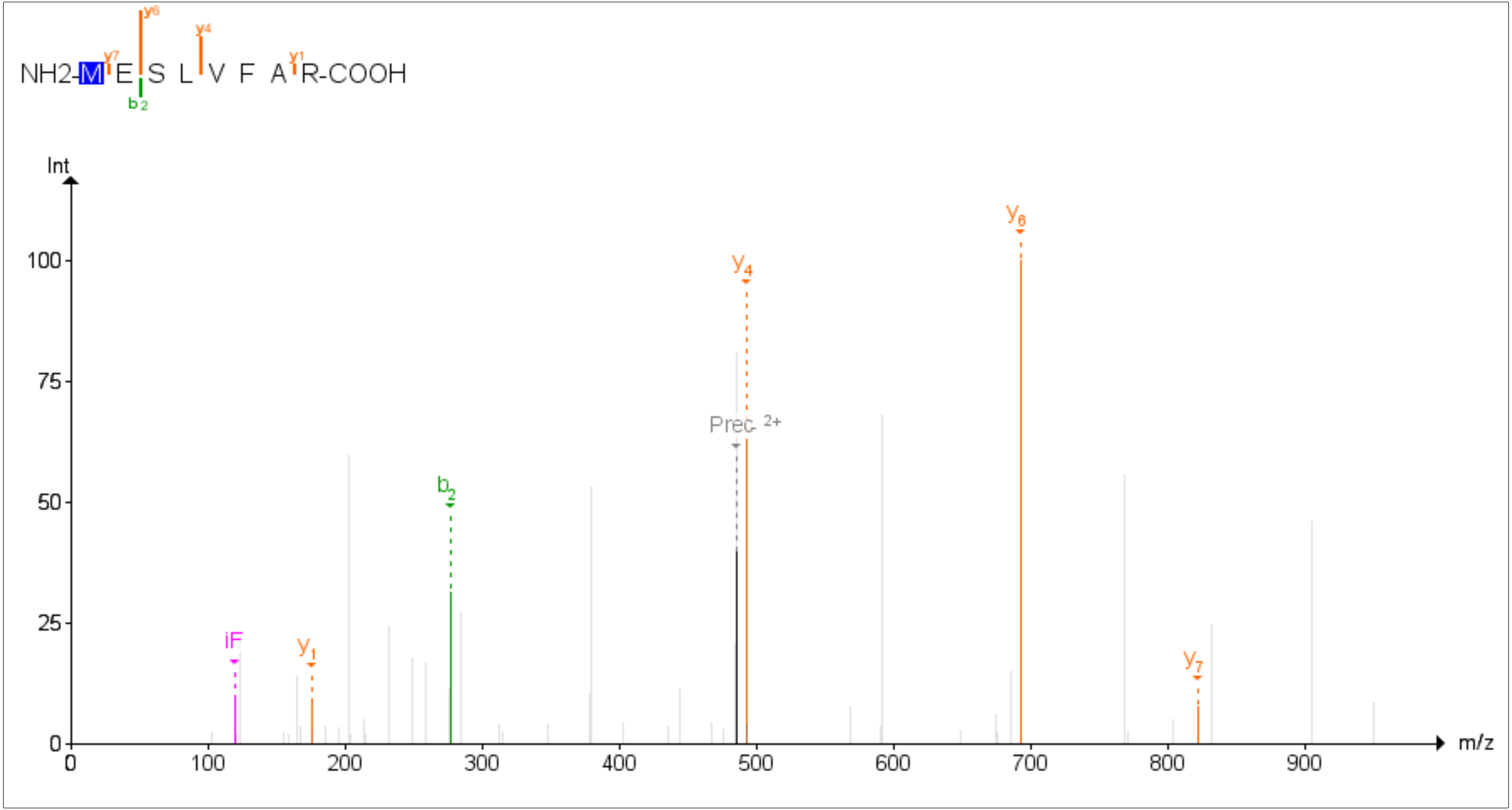

NH2-M E S L V F A R-COOH
y6
y4
y7
y1
b 2
Int
100
75
50
25
0
y6
y4
Prec 2+
b2
iF
y1
y7
0
100
200
300
400
500
600
700
800
900
m/z

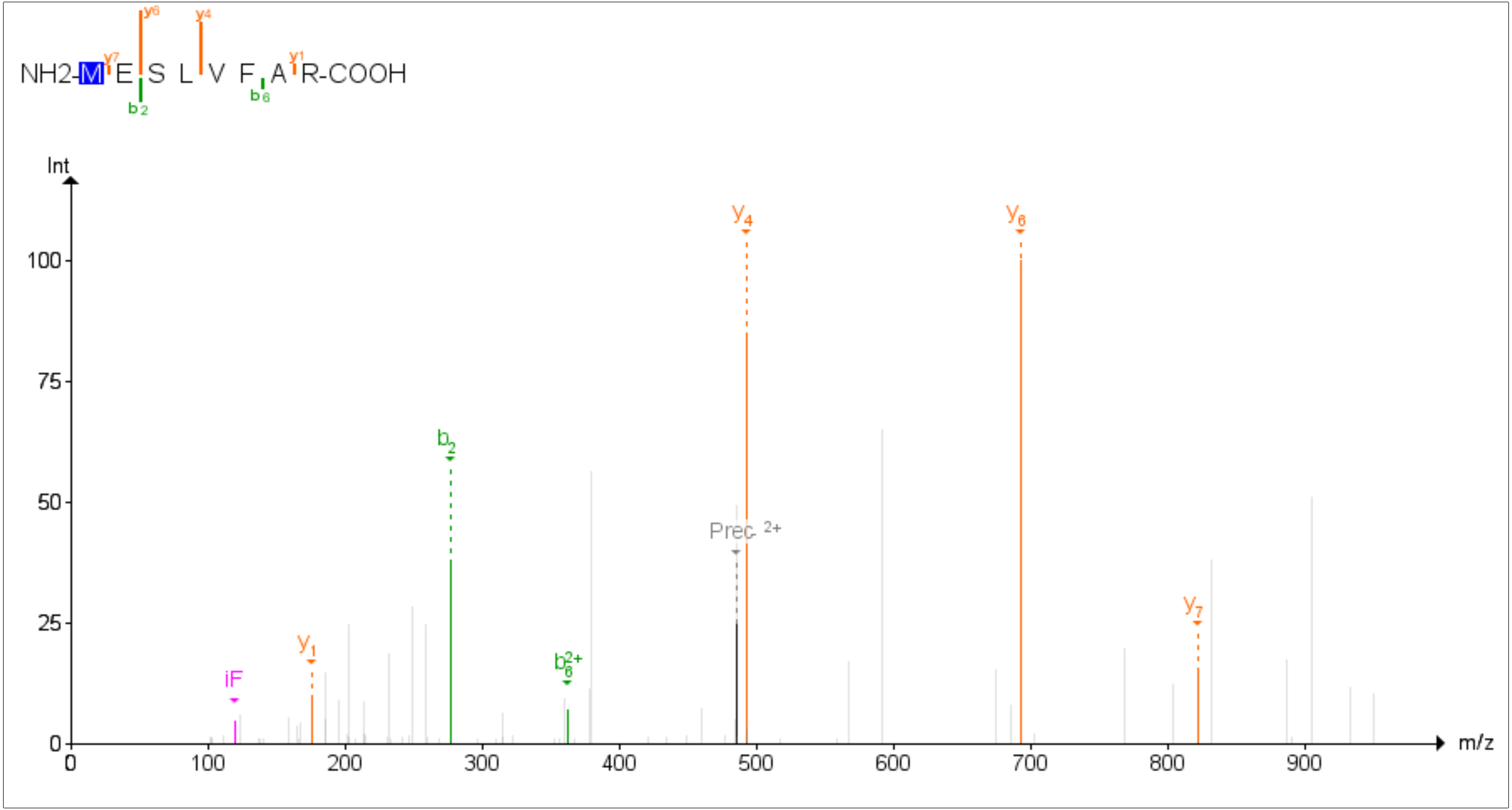

NH2-M E S L V F A R-COOH
y7
y6
y4
y1
b2
b6
Int
100
75
50
25
0
y4
y6
b2
Prec 2+
y7
y1
iF
b6 2+
0
100
200
300
400
500
600
700
800
900
m/z

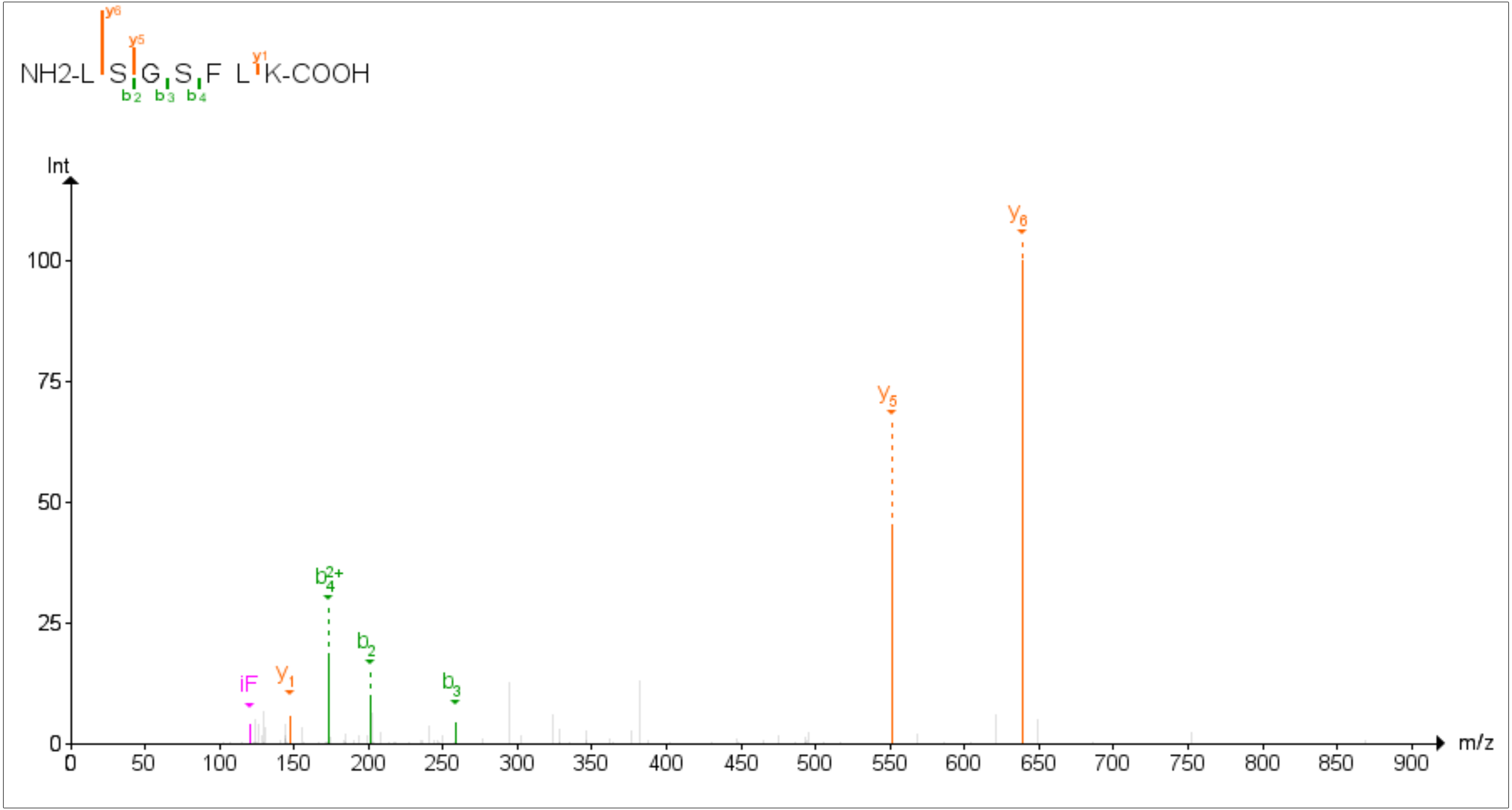
NH2-L S G S F L K-COOH
y6
y5
y1
b2
b3
b4
Int
100
75
50
25
0
m/z
0
50
100
150
200
250
300
350
400
450
500
550
600
650
700
750
800
850
900
y6
y5
b4 2+
b2
b3
y1
iF

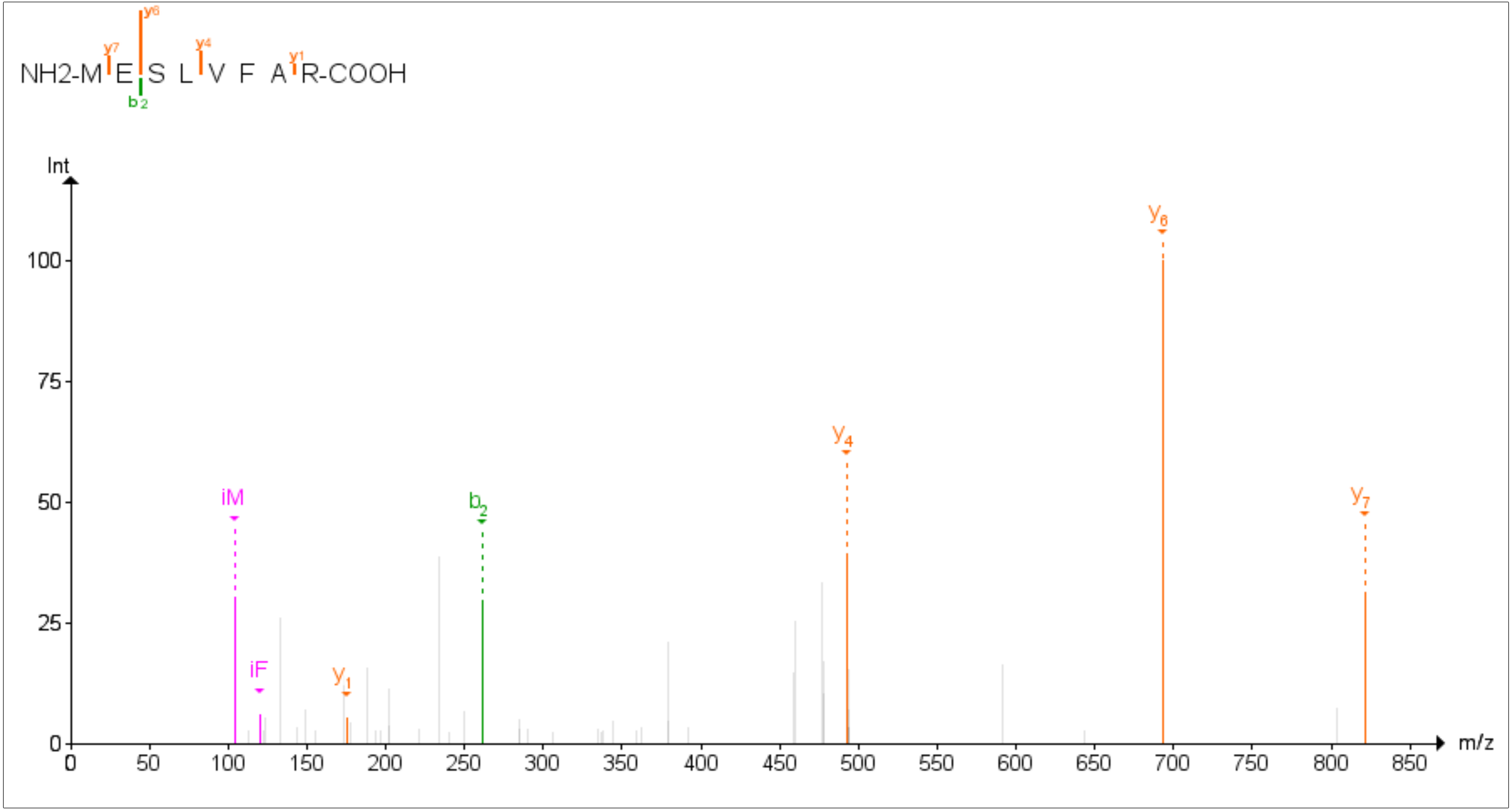

NH2-M E S L V F A R-COOH
y6
y7
y4
y1
b2
Int
100
75
50
25
0
iM
iF
y1
b2
y4
y6
y7
0
50
100
150
200
250
300
350
400
450
500
550
600
650
700
750
800
850
m/z

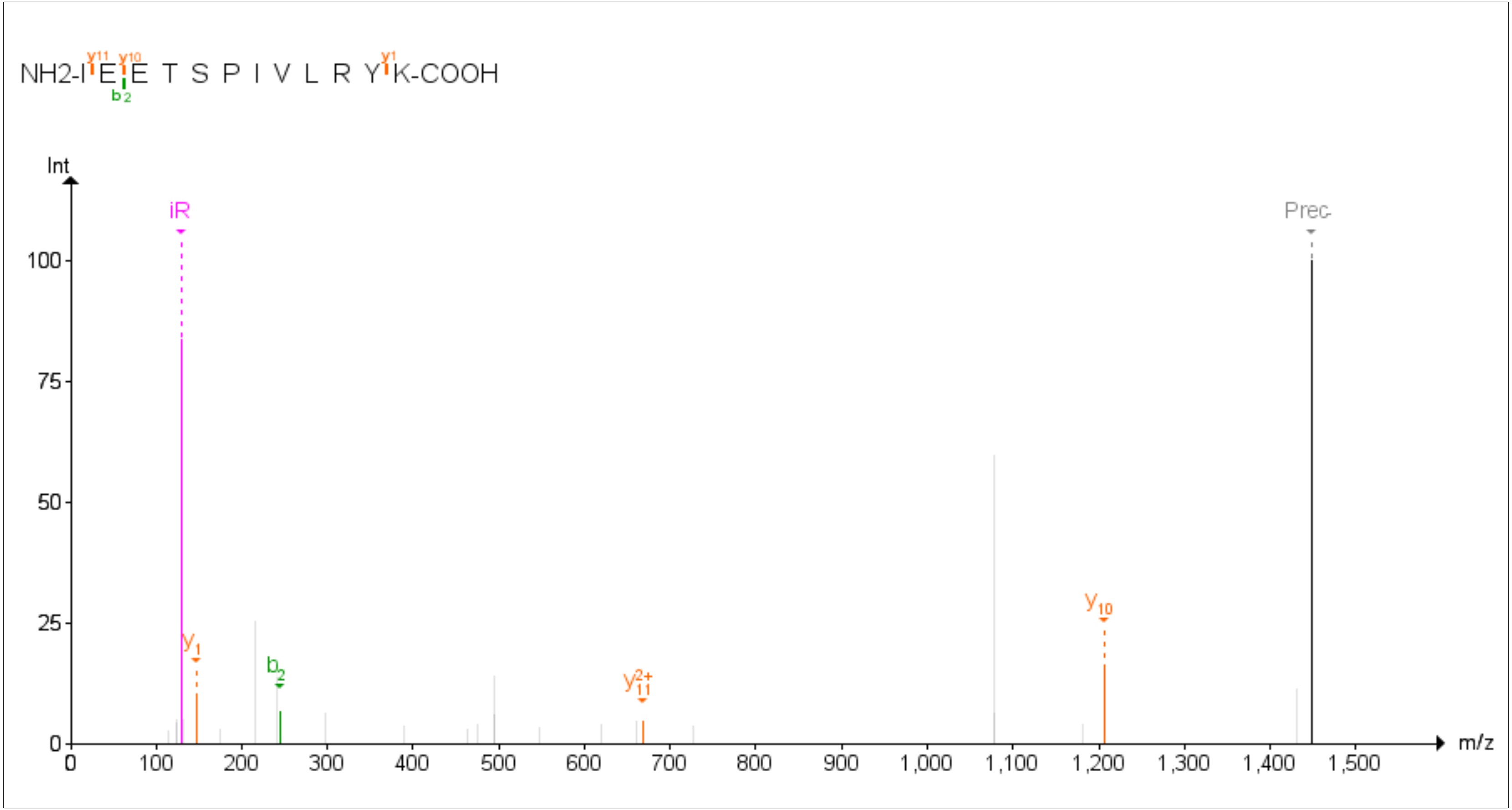

NH2-I E E T S P I V L R Y K-COOH
y11
y10
y1
b2
Int
iR
Prec
y1
b2
y11 2+
y10
100
75
50
25
0
0
100
200
300
400
500
600
700
800
900
1,000
1,100
1,200
1,300
1,400
1,500
m/z

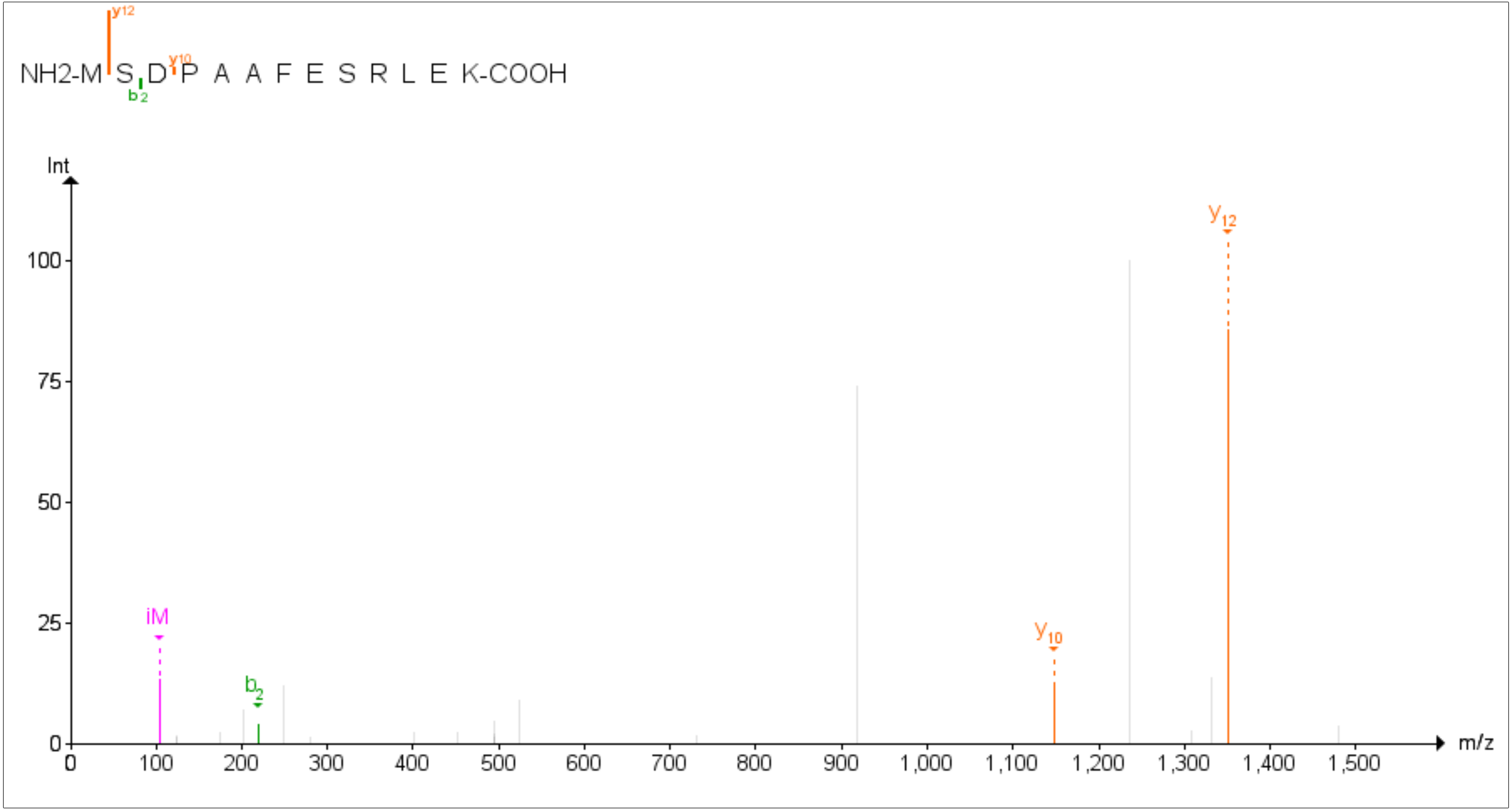

NH2-M S D P A A F E S R L E K-COOH
y12
y10
b2
Int
100
75
50
25
0
iM
b2
y10
y12
0
100
200
300
400
500
600
700
800
900
1,000
1,100
1,200
1,300
1,400
1,500
m/z

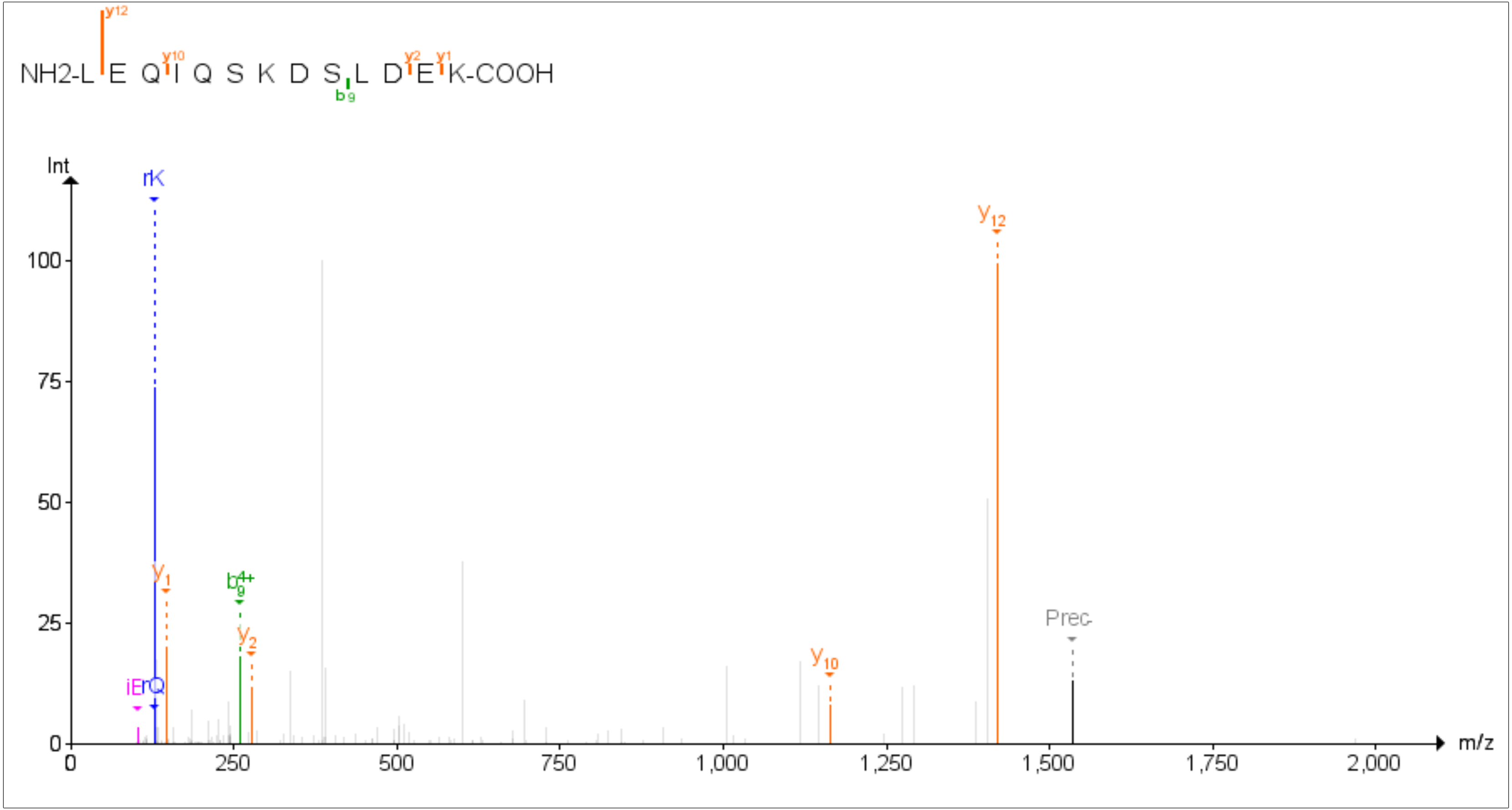

NH2-L E Q I Q S K D S L D E K-COOH
y12
y10
y2
y1
b9
Int
rK
y12
y1
b9 4+
y2
iE
rQ
y10
Prec
100
75
50
25
0
0
250
500
750
1,000
1,250
1,500
1,750
2,000
m/z

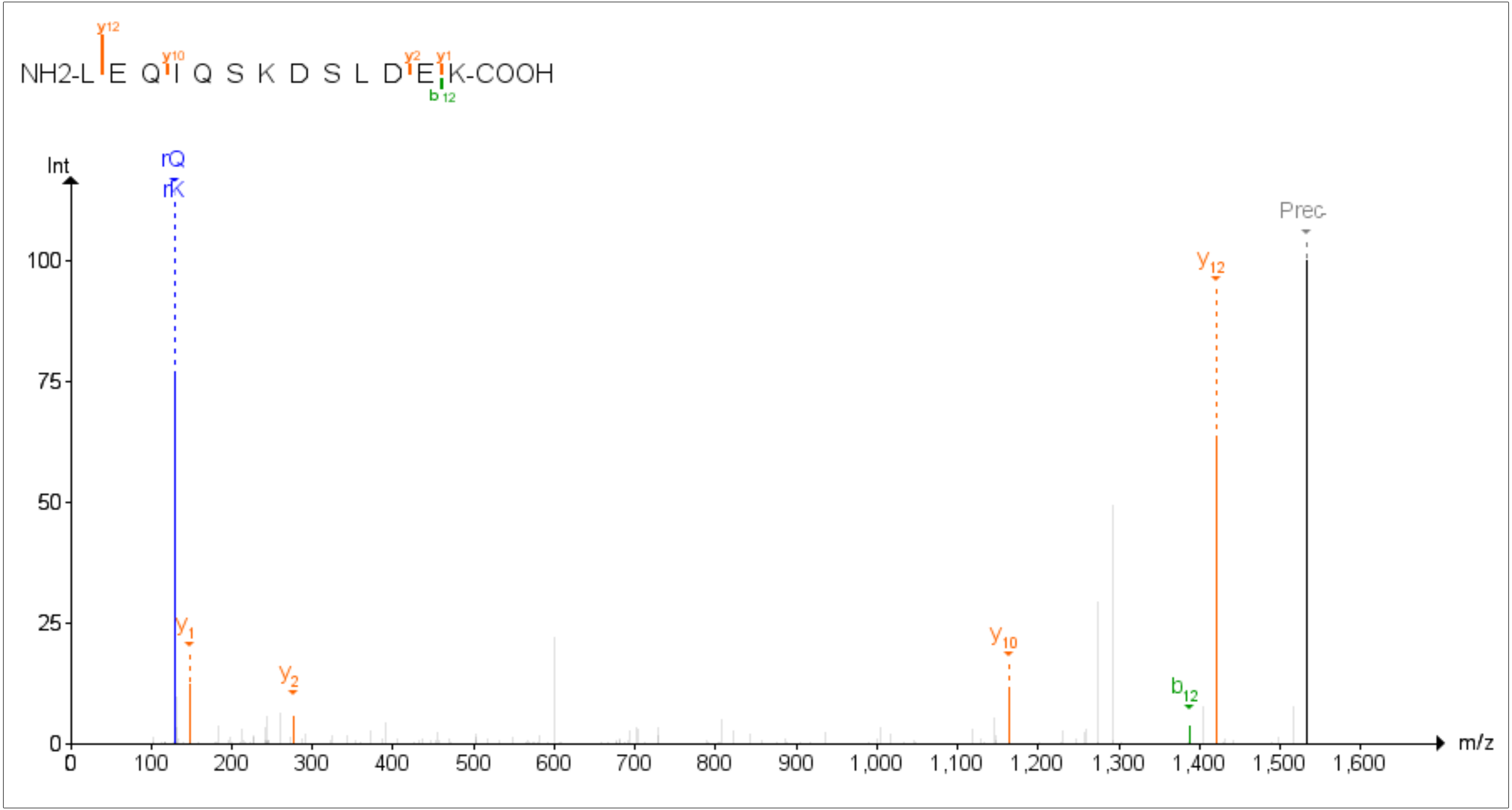

NH2-L E Q I Q S K D S L D E K-COOH
y12
y10
y2
y1
b12
Int
rQ
rK
Prec
y12
y1
y2
y10
b12
100
75
50
25
0
0
100
200
300
400
500
600
700
800
900
1,000
1,100
1,200
1,300
1,400
1,500
1,600
m/z

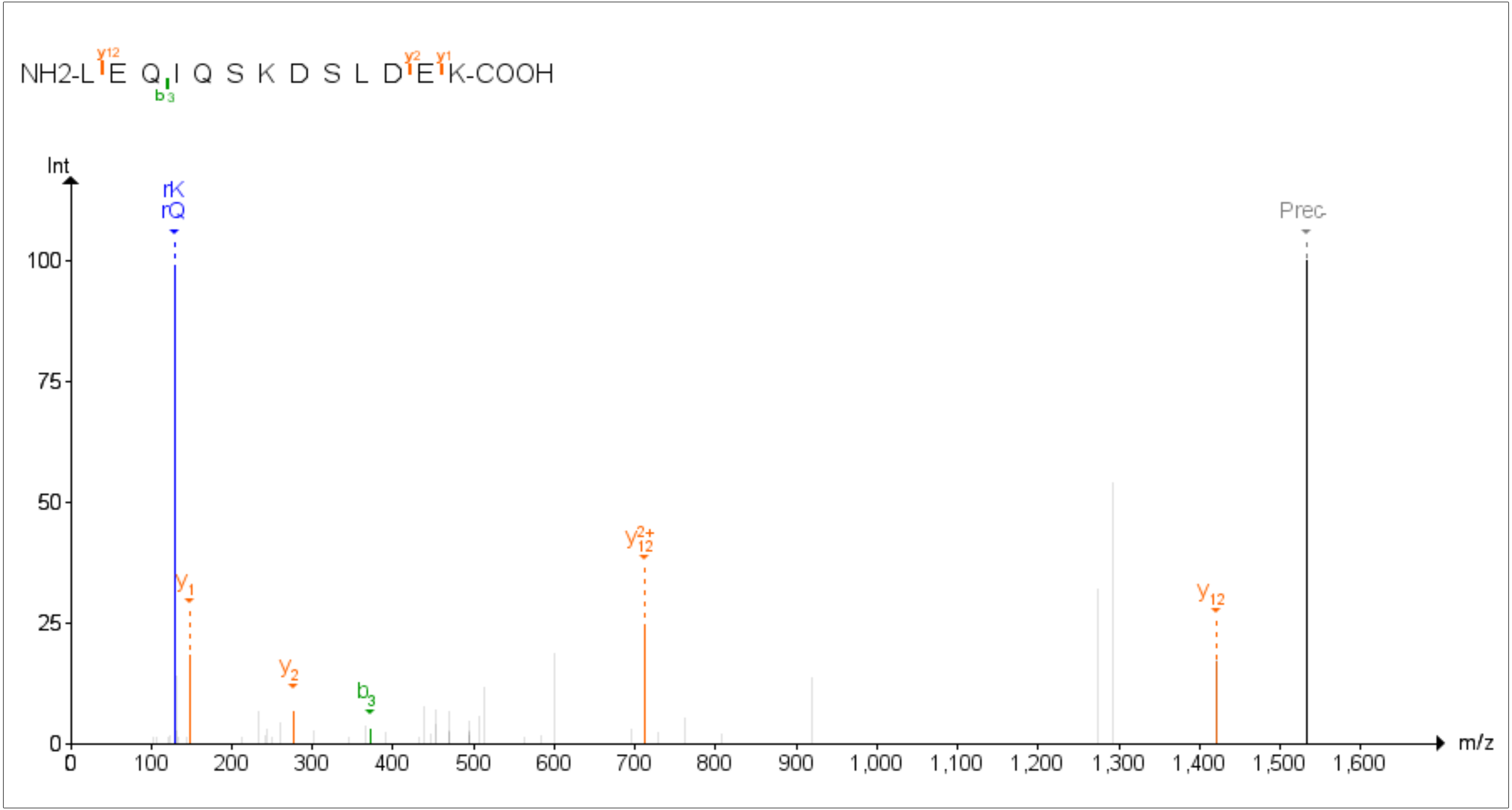

NH2-L E Q I Q S K D S L D E K-COOH
y12
b3
y2
y1
Int
rK
rQ
Prec
100
75
50
25
0
y_1
y_2
b_3
y_{12}^{2+}
y_{12}
0
100
200
300
400
500
600
700
800
900
1,000
1,100
1,200
1,300
1,400
1,500
1,600
m/z

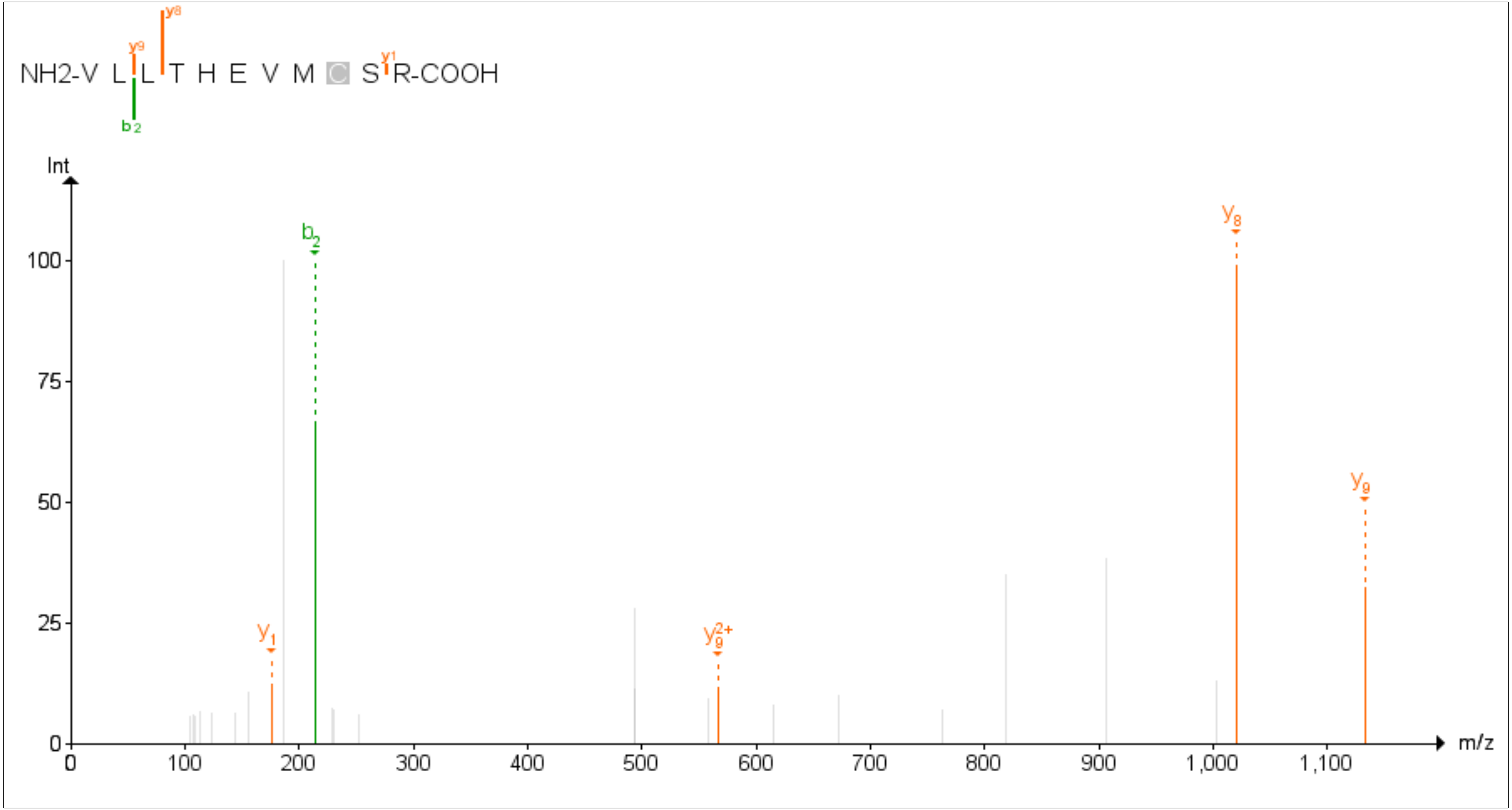

NH2-V L L T H E V M C S R-COOH
y8
y9
y1
b2
Int
100
75
50
25
0
m/z
0
100
200
300
400
500
600
700
800
900
1,000
1,100
y_8
b_2
y_9
y_1
y_9^{2+}

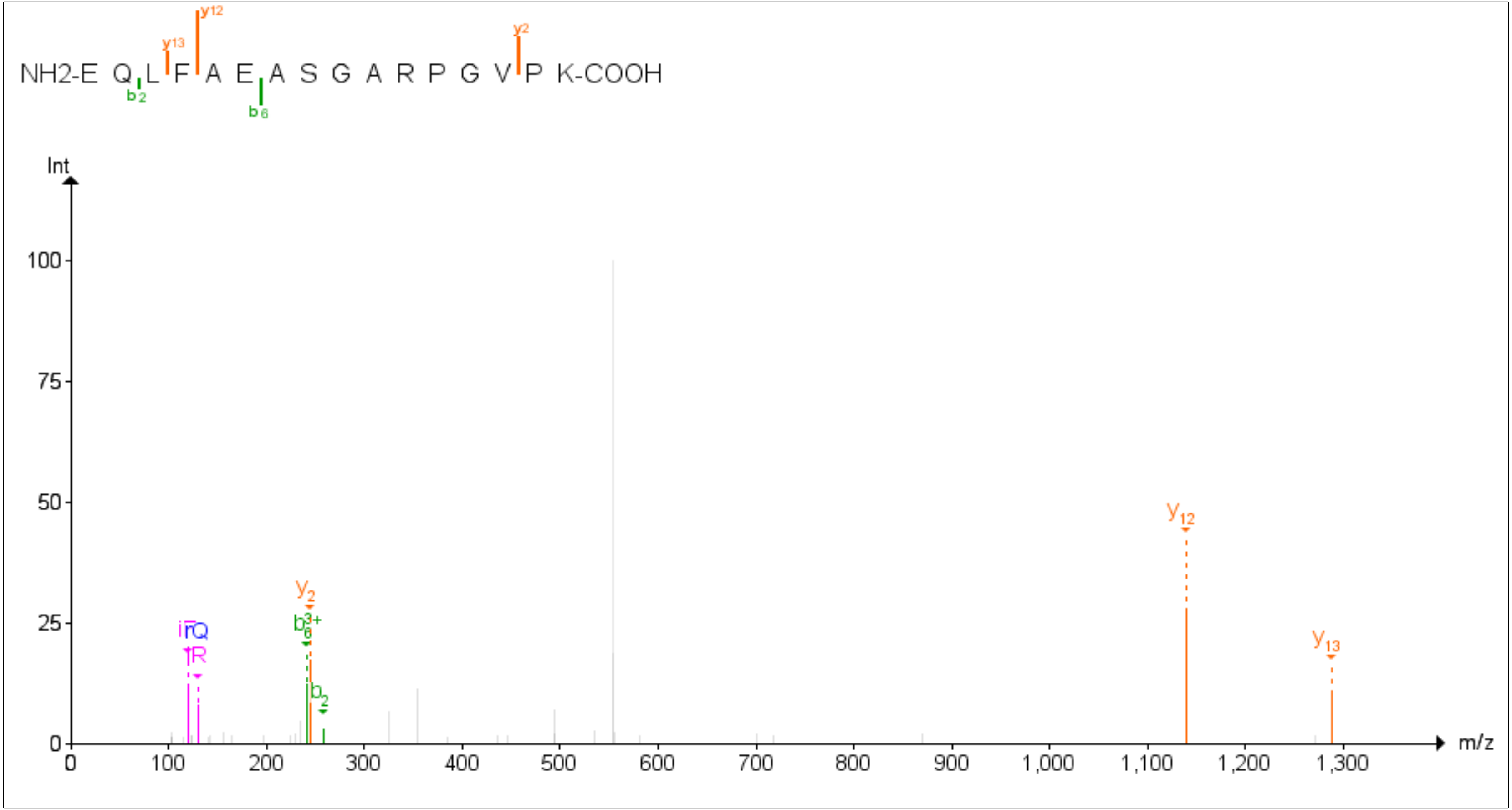
NH2-E Q L F A E A S G A R P G V P K-COOH
y12
y13
y2
b2
b6
Int
100
75
50
25
0
y12
y13
y2
b6 3+
b2
irQ
iR
0
100
200
300
400
500
600
700
800
900
1,000
1,100
1,200
1,300
m/z

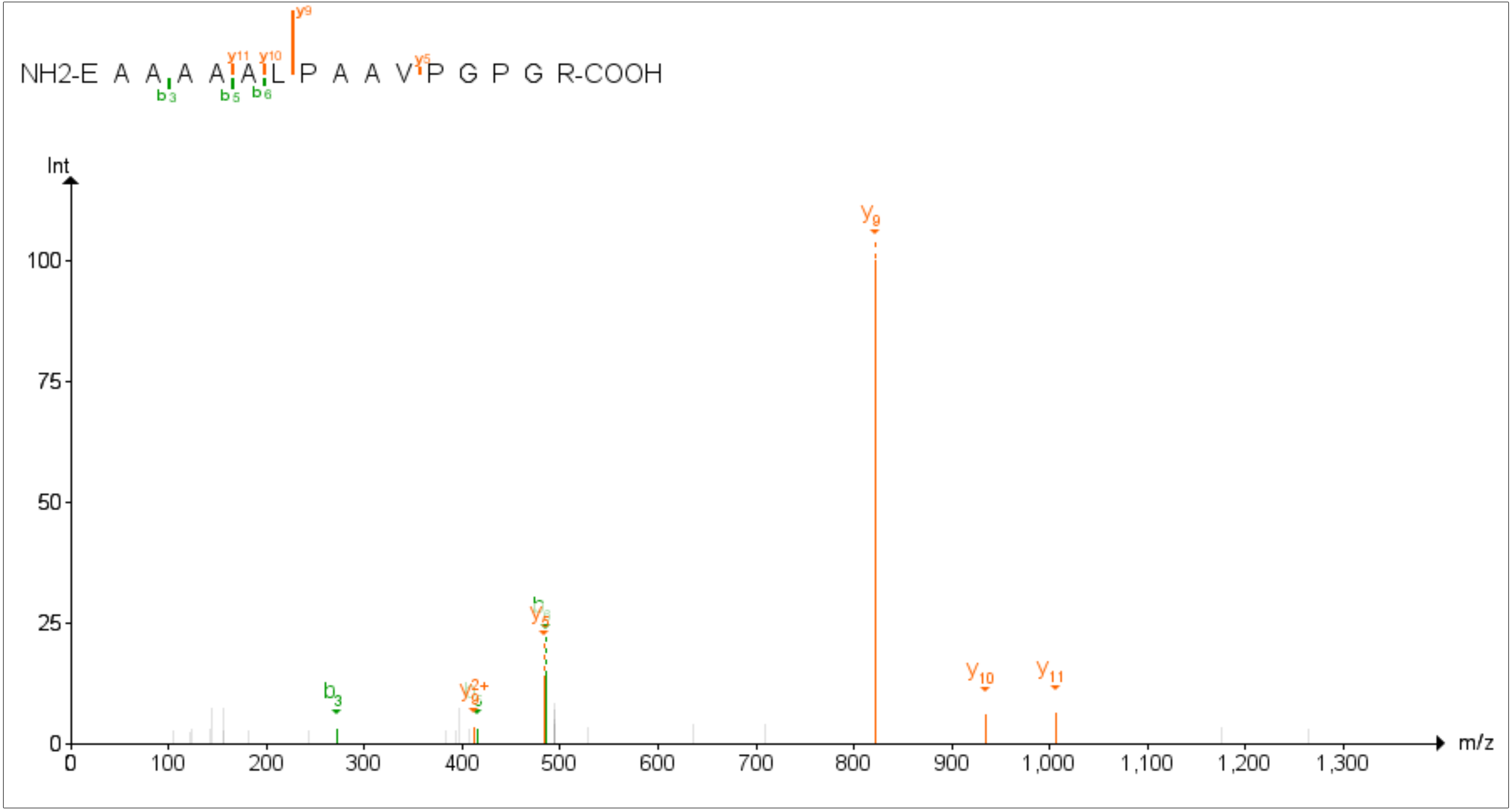

NH2-E A A A A A L P A A V P G P G R-COOH
Int
m/z
y9
y10
y11
y5
b3

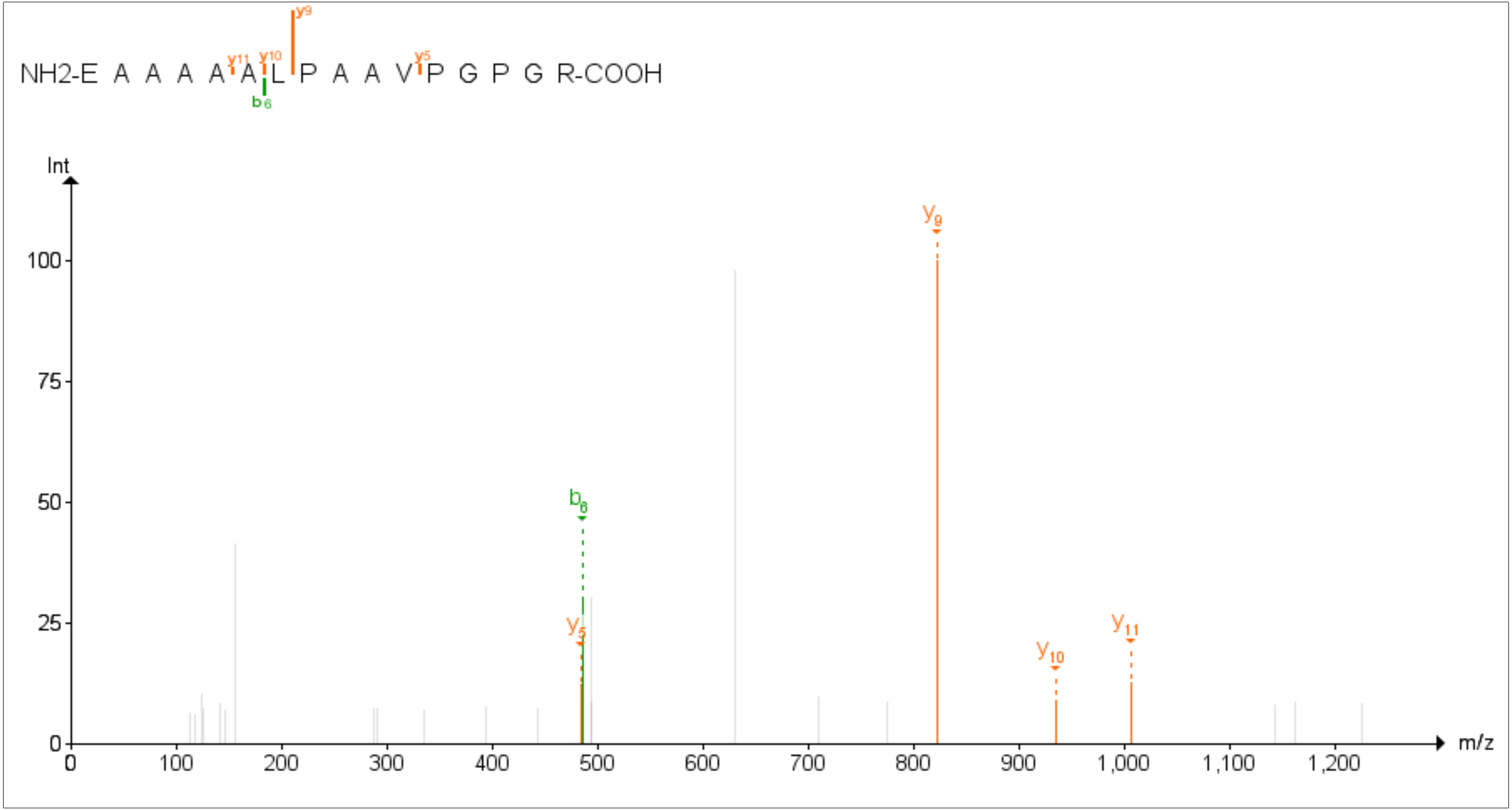

NH2-E A A A A A L P A A V P G P G R-COOH
y9
y11
y10
y5
b6
Int
100
75
50
25
0
m/z
0
100
200
300
400
500
600
700
800
900
1,000
1,100
1,200
y9
b6
y5
y10
y11

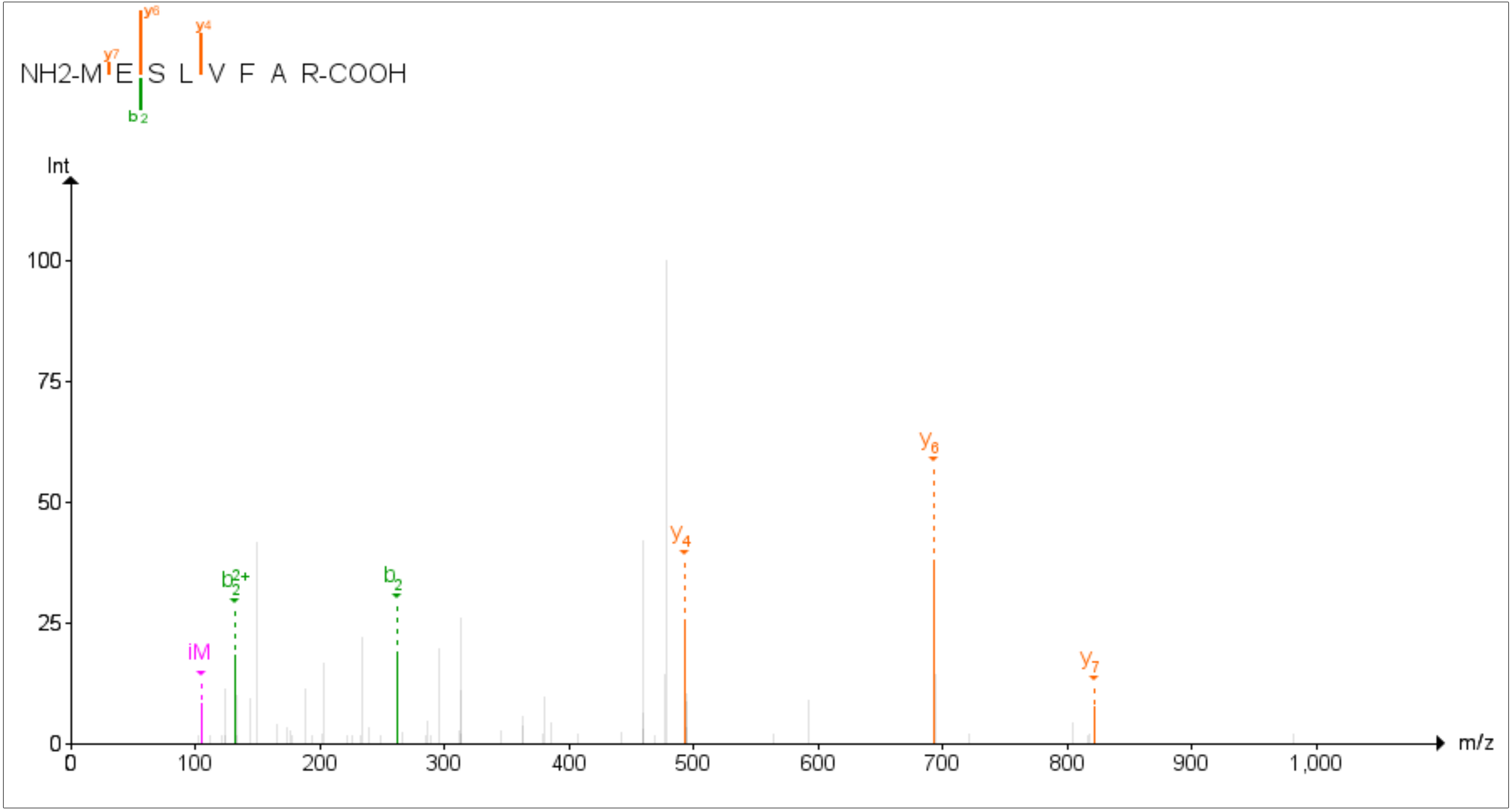

NH2-M E S L V F A R-COOH
y6
y4
y7
b2
Int
100
75
50
25
0
iM
b2 2+
b2
y4
y6
y7
0
100
200
300
400
500
600
700
800
900
1,000
m/z

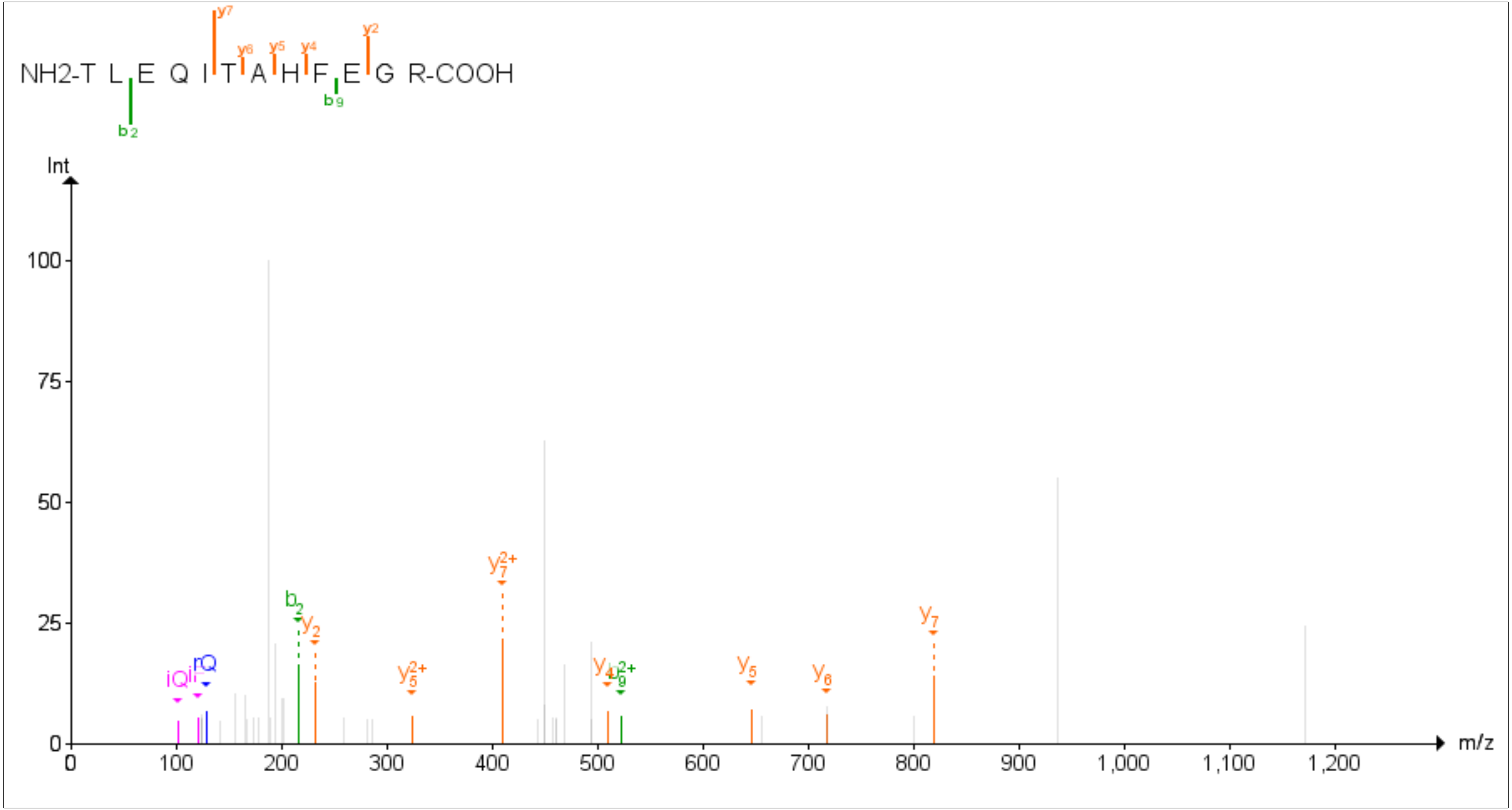

NH2-T L E Q I T A H F E G R-COOH
y7
y6
y5
y4
y2
b2
b9
Int
100
75
50
25
0
iQ
iF
rQ
b2
y2
y5 2+
y7 2+
y4
b9 2+
y5
y6
y7
0
100
200
300
400
500
600
700
800
900
1,000
1,100
1,200
m/z

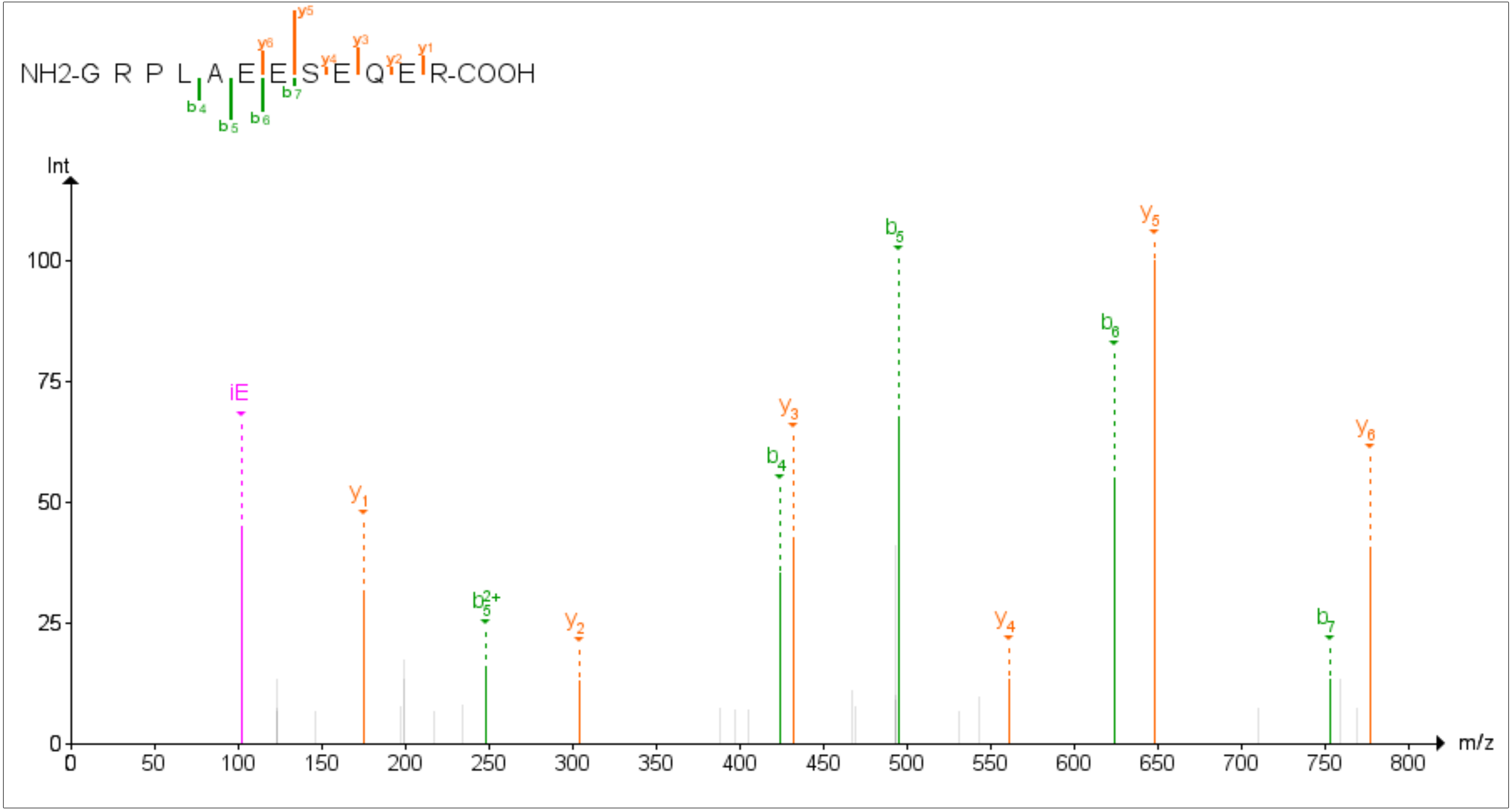

NH2-G R P L A E E S E Q E R-COOH
y5 y6 y3 y4 y2 y1
b4 b5 b6 b7
Int
100
75
50
25
0
iE
y1
b5 2+
y2
b4
y3
b5
y4
b6
y5
b7
y6
0 50 100 150 200 250 300 350 400 450 500 550 600 650 700 750 800
m/z

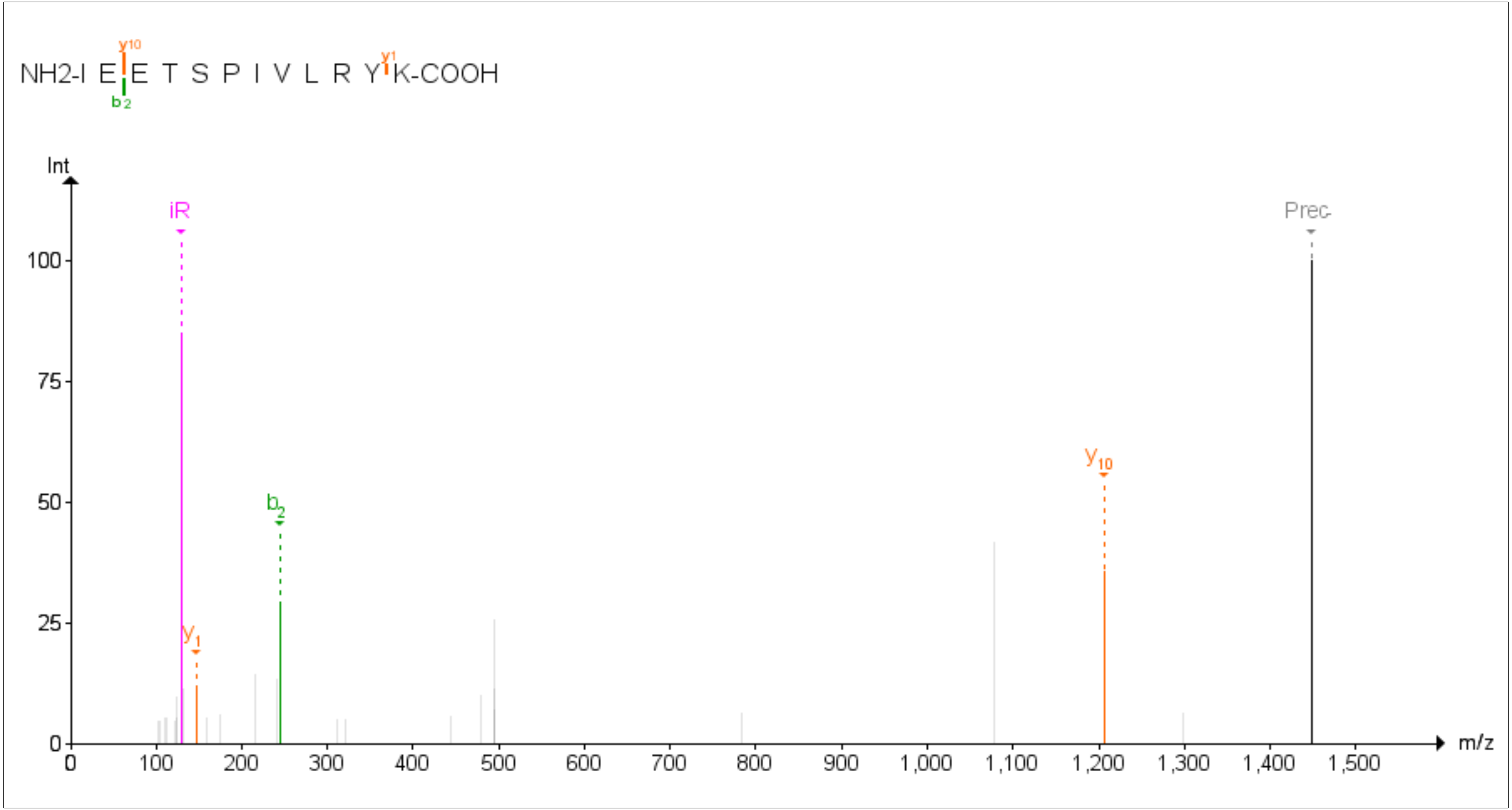

y10
y1
NH2-I E E T S P I V L R Y K-COOH
b2
Int
iR
Prec
100
75
50
25
0
y10
b2
y1
0
100
200
300
400
500
600
700
800
900
1,000
1,100
1,200
1,300
1,400
1,500
m/z

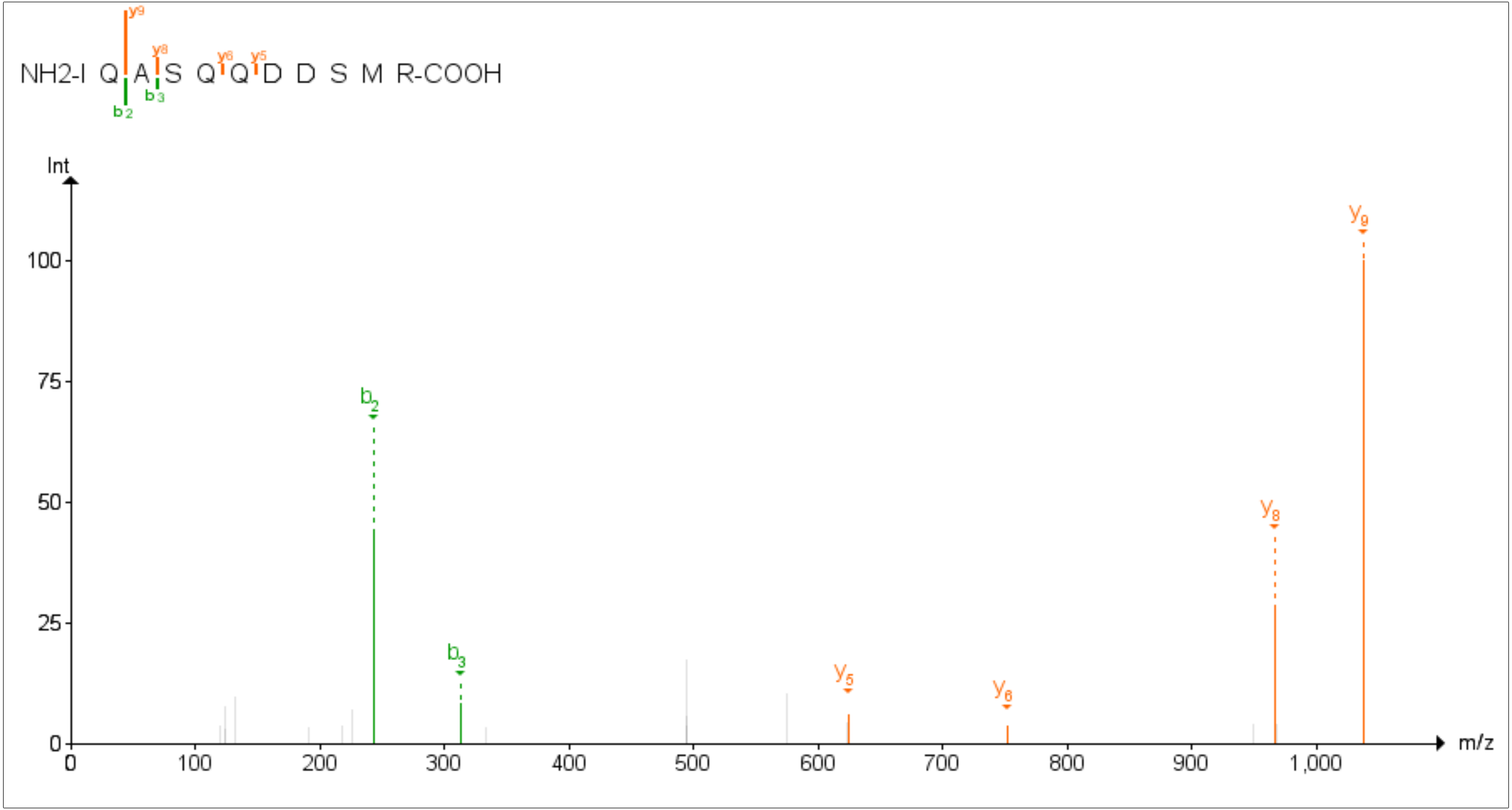

NH2-I Q A S Q Q D D S M R-COOH
y9
y8
y6
y5
b2
b3
Int
100
75
50
25
0
m/z
0
100
200
300
400
500
600
700
800
900
1,000

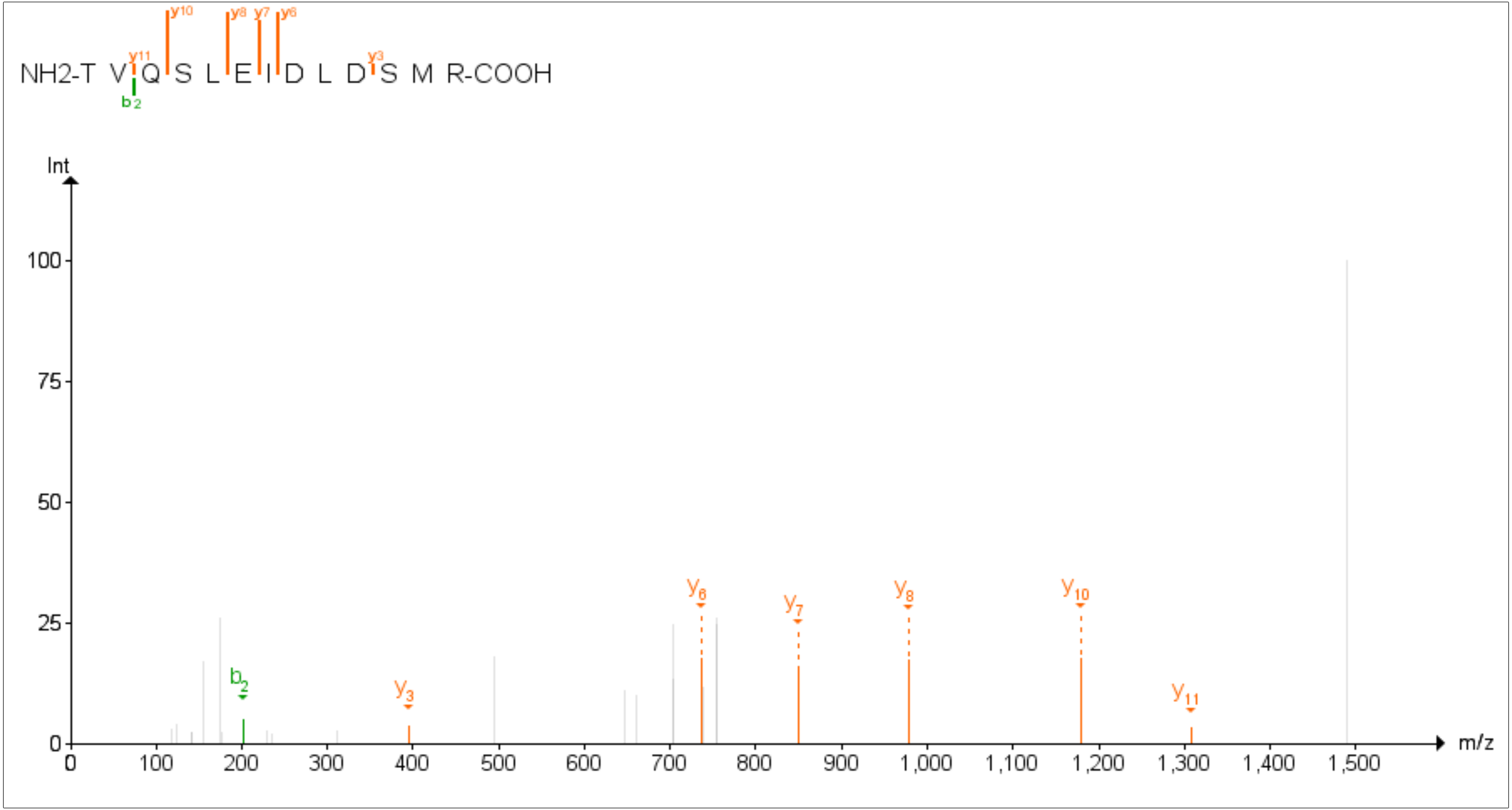
NH2-T V Q S L E I D L D S M R-COOH
y10 y8 y7 y6 y11 y3 b2
Int
m/z
y6 y7 y8 y10 y11 b2 y3
0 25 50 75 100
0 100 200 300 400 500 600 700 800 900 1,000 1,100 1,200 1,300 1,400 1,500

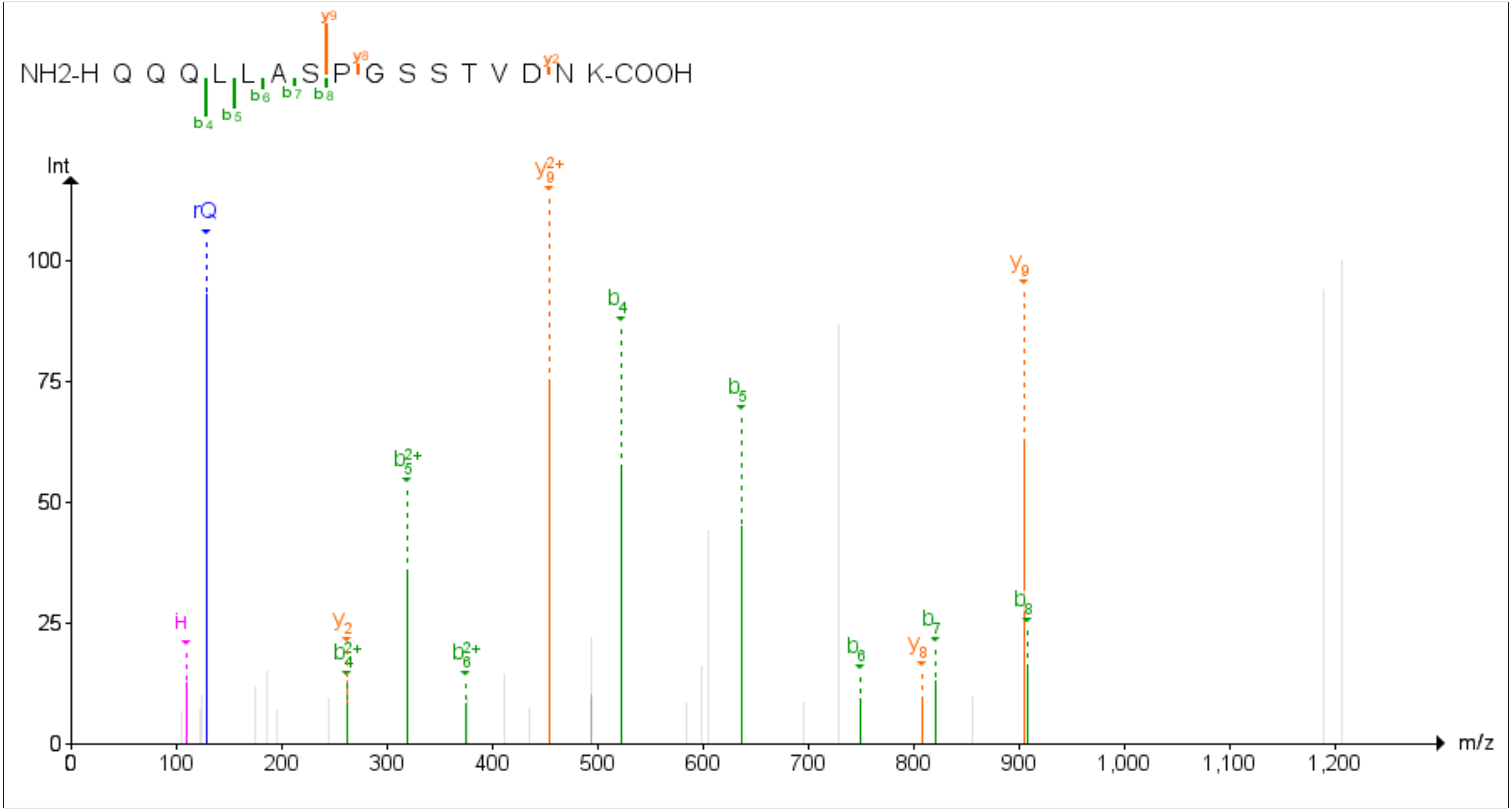

NH2-H Q Q Q L L A S P G S S T V D N K-COOH
y9
y8
y2
b4
b5
b6
b7
b8
Int
m/z
rQ
y9 2+
y9
b4
b5
b5 2+
b4 2+
b6 2+
y2
H
b6
y8
b7
b8
0
25
50
75
100
0
100
200
300
400
500
600
700
800
900
1,000
1,100
1,200

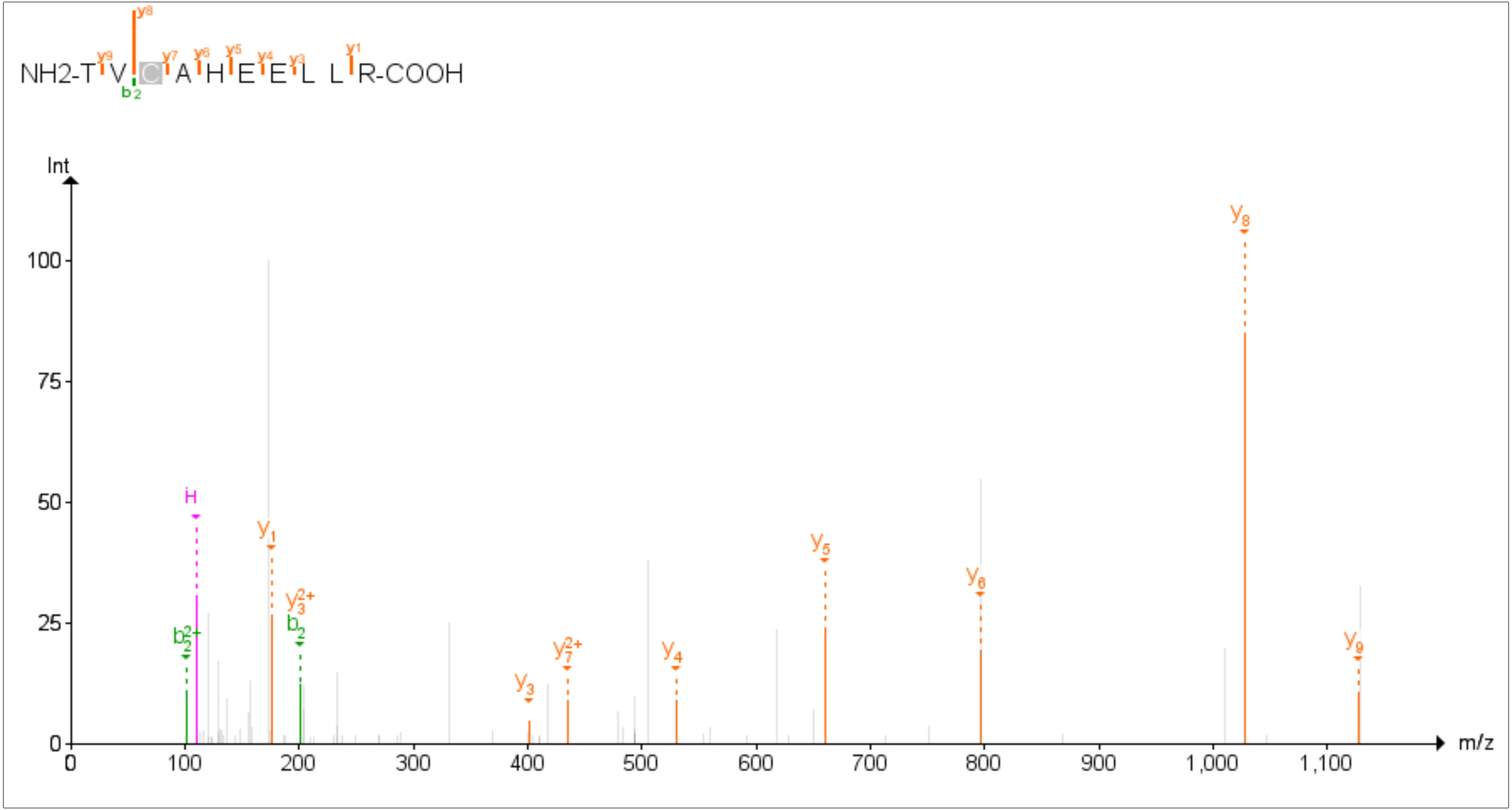

NH2-TVCAHEELLR-COOH
Int
m/z
y8
y9
y7
y6
y5
y4
y3
y1
b2
H
y1
b2 2+
y3 2+
b2
y3
y7 2+
y4
y5
y6
y8
y9

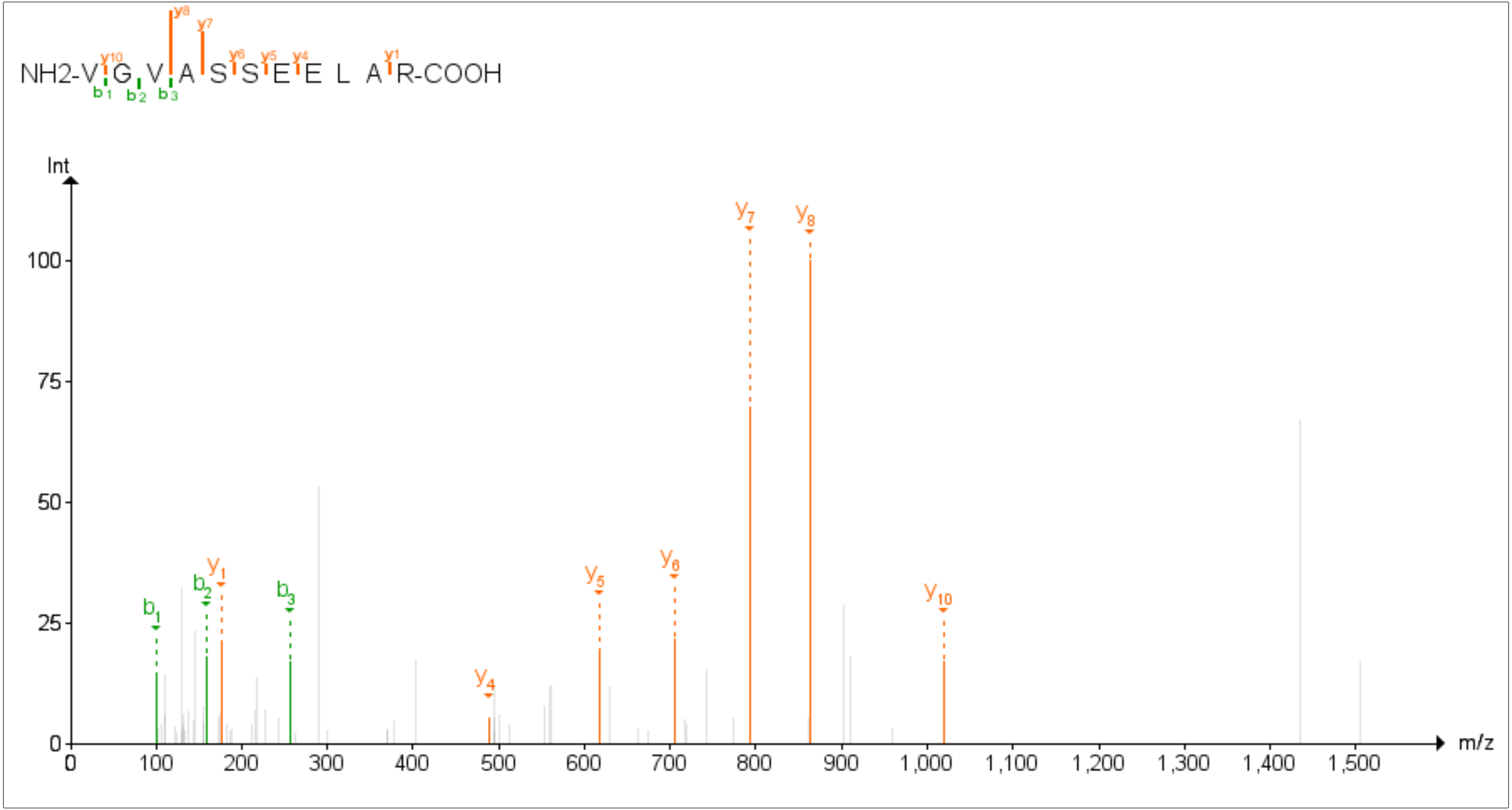

NH2-V G V A S S E E L A R-COOH
y10 y8 y7 y6 y5 y4 y1
b1 b2 b3
Int
m/z
0
25
50
75
100
0
100
200
300
400
500
600
700
800
900
1,000
1,100
1,200
1,300
1,400
1,500
b1
b2
y1
b3
y4
y5
y6
y7
y8
y10

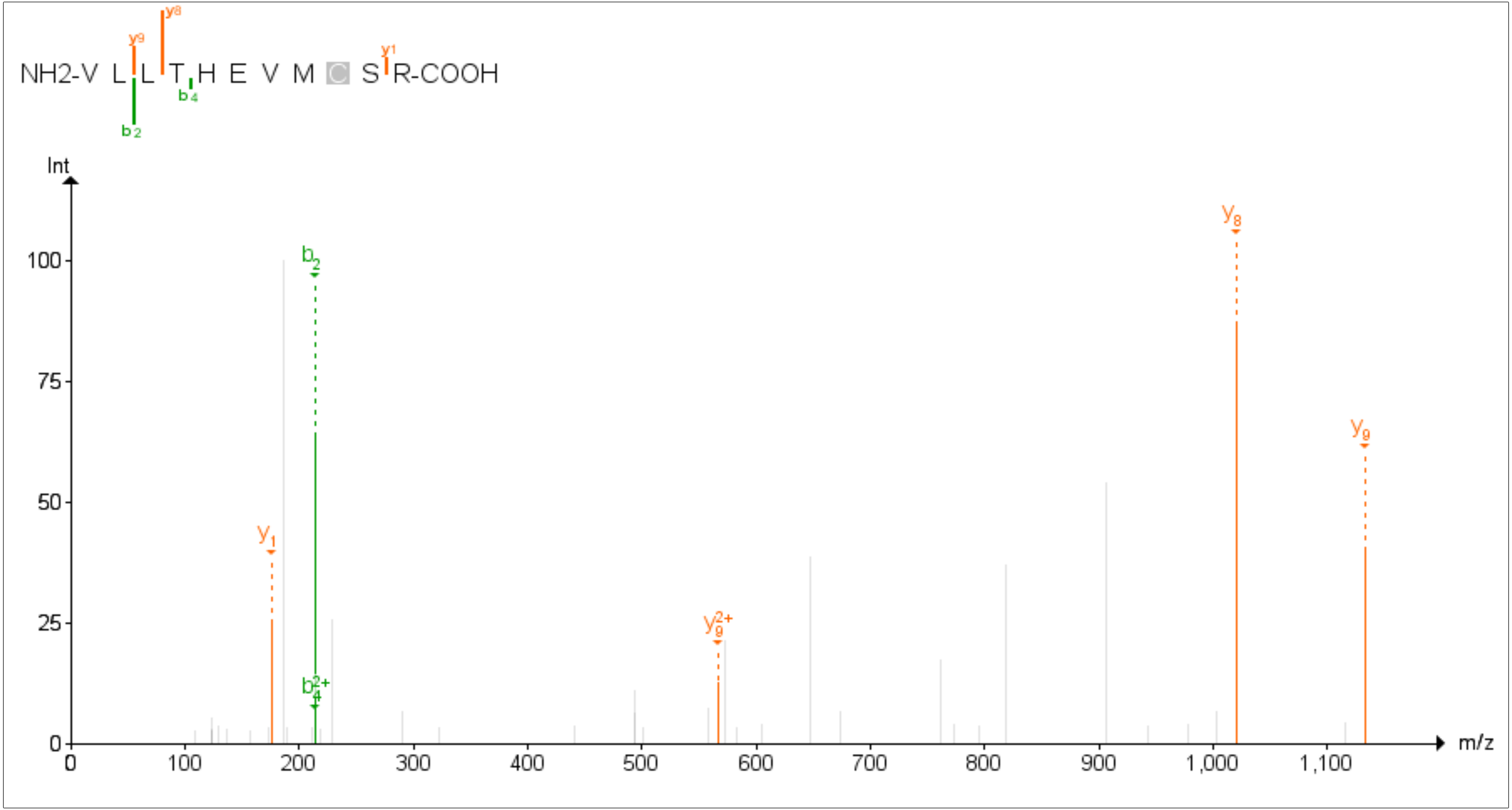

NH2-V L L T H E V M C S R-COOH
y8
y9
y1
b2
b4
Int
m/z
b2
y8
y9
y1
y9 2+
b4 2+
0
25
50
75
100
0
100
200
300
400
500
600
700
800
900
1,000
1,100

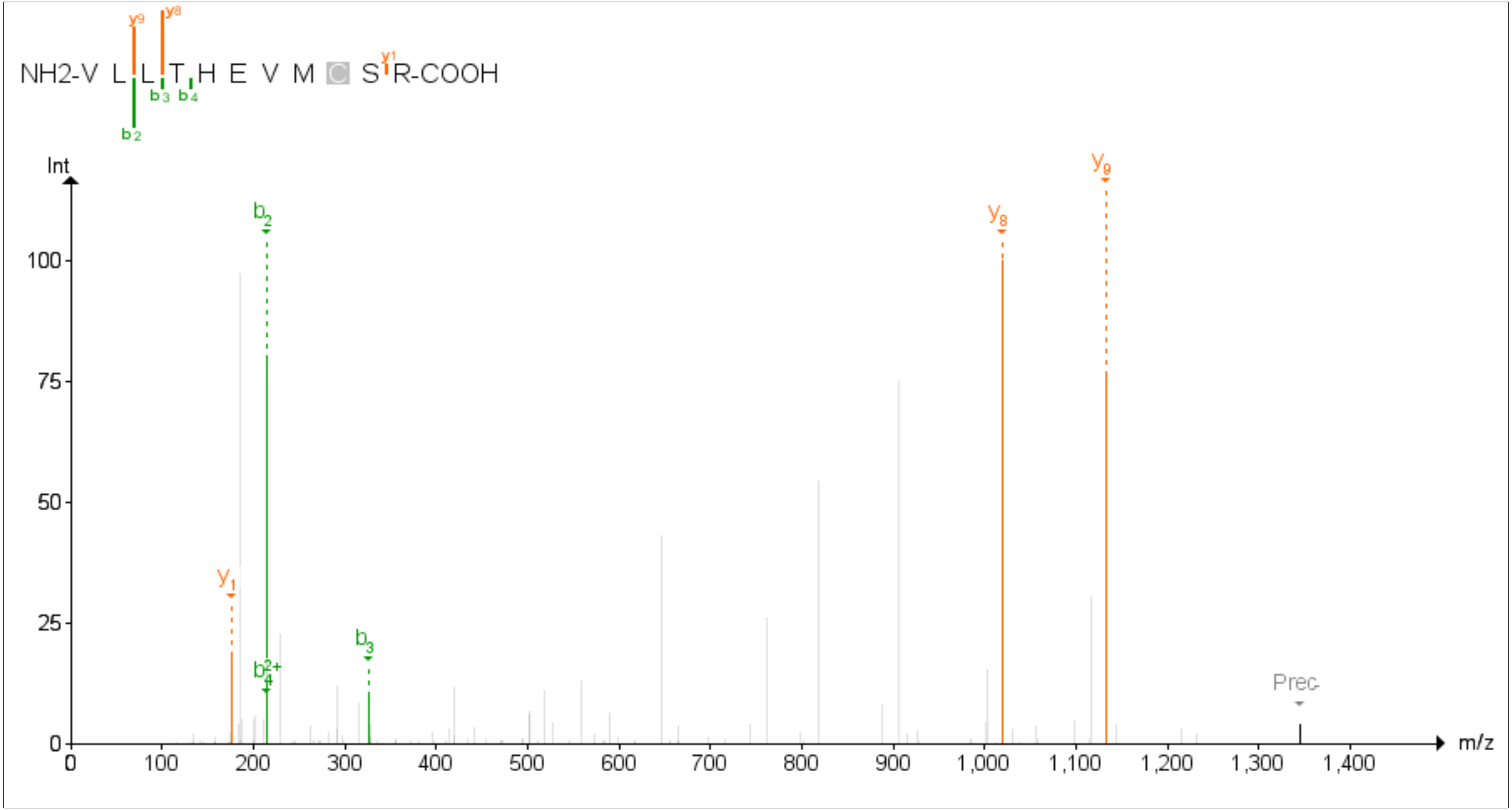

NH2-V L L T H E V M C S R-COOH
y9
y8
y1
b2
b3
b4
Int
100
75
50
25
0
b2
y8
y9
y1
b3
b4 2+
Prec.
0
100
200
300
400
500
600
700
800
900
1,000
1,100
1,200
1,300
1,400
m/z

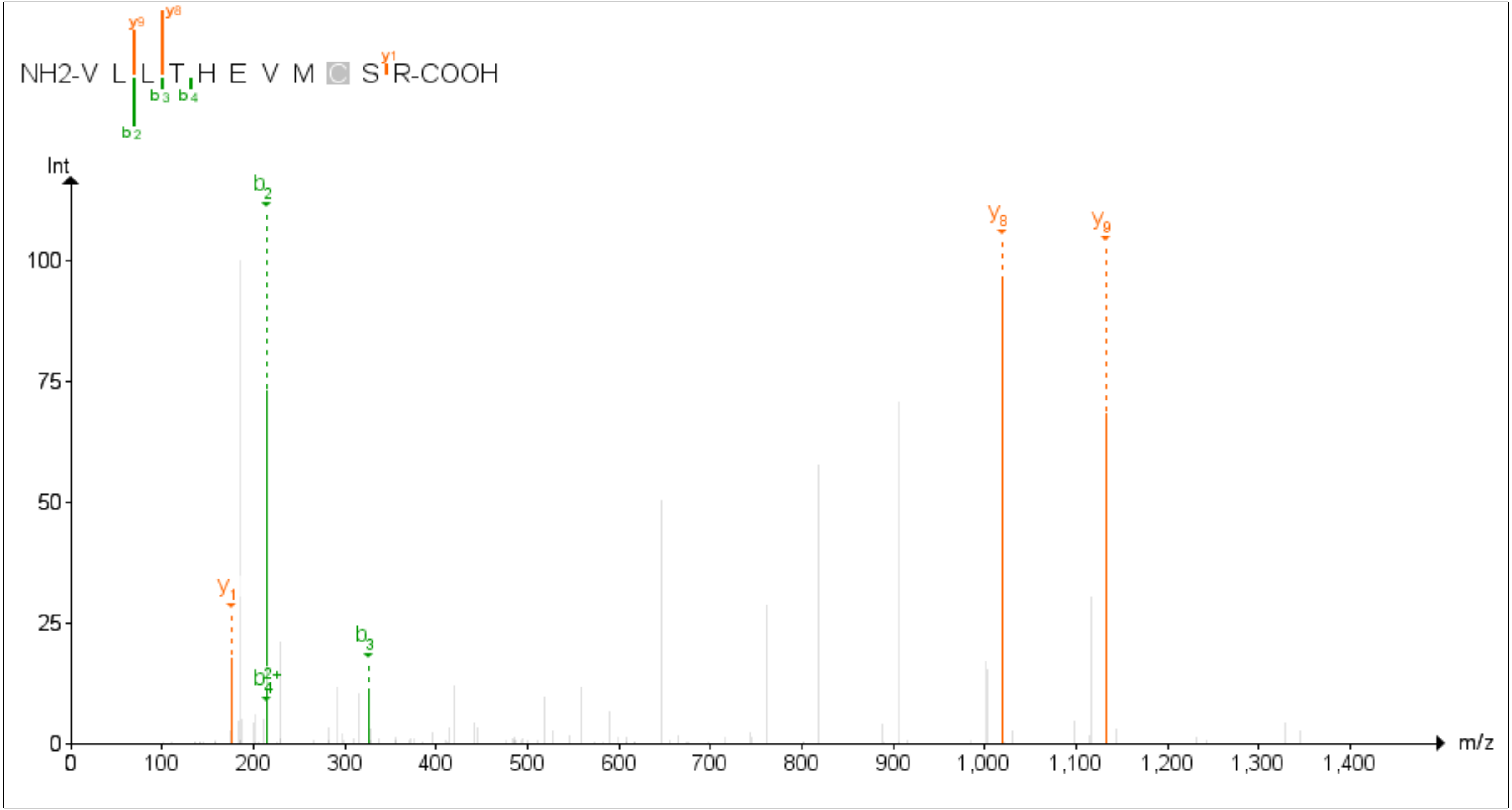

NH2-V L L T H E V M C S R-COOH
y9
y8
y1
b2
b3
b4
Int
100
75
50
25
0
b2
y8
y9
y1
b3
b4 2+
m/z
0
100
200
300
400
500
600
700
800
900
1,000
1,100
1,200
1,300
1,400

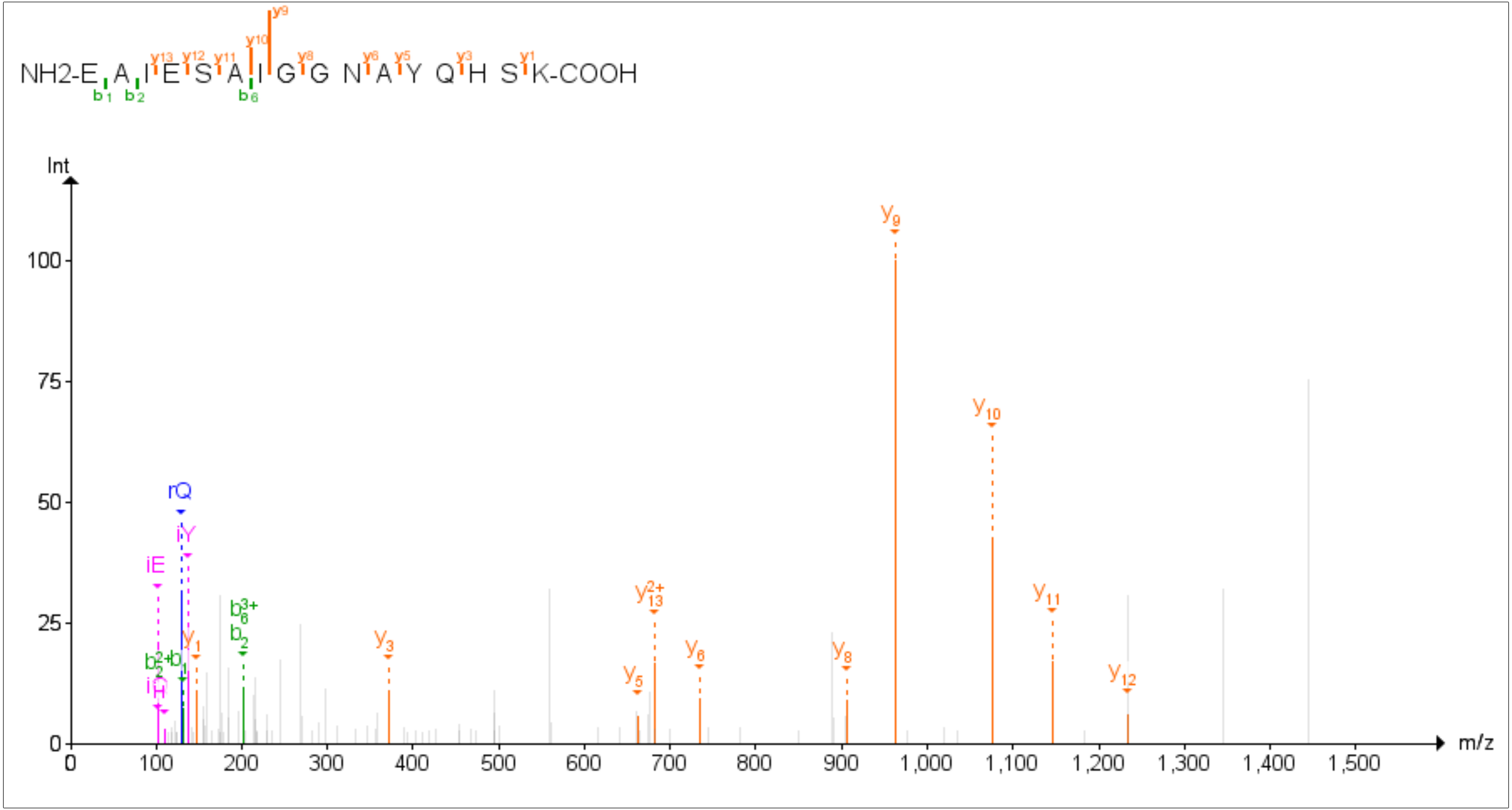

NH2-E A I E S A I G G N A Y Q H S K-COOH
b1 b2 b6
y13 y12 y11 y10 y9 y8 y6 y5 y3 y1
Int
m/z
0
25
50
75
100
0
100
200
300
400
500
600
700
800
900
1,000
1,100
1,200
1,300
1,400
1,500
rQ
iY
iE
iQ
iH
b2 2+ b1
b6 3+
b2
y1
y3
y5
y13 2+
y6
y8
y9
y10
y11
y12

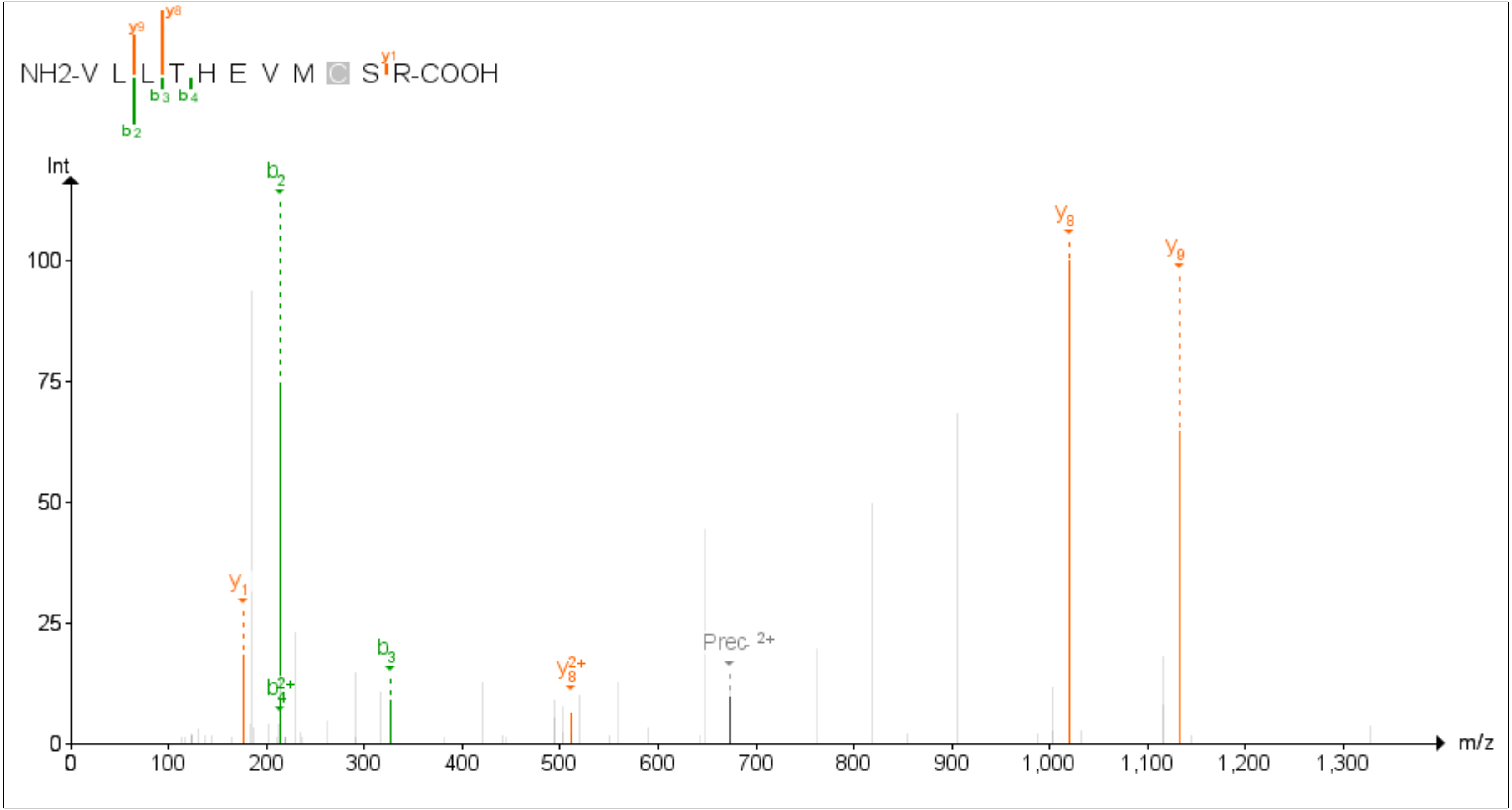

NH2-V L L T H E V M C S R-COOH
y9
y8
y1
b2
b3
b4
Int
100
75
50
25
0
b2
y8
y9
y1
b3
b4 2+
y8 2+
Prec. 2+
0
100
200
300
400
500
600
700
800
900
1,000
1,100
1,200
1,300
m/z

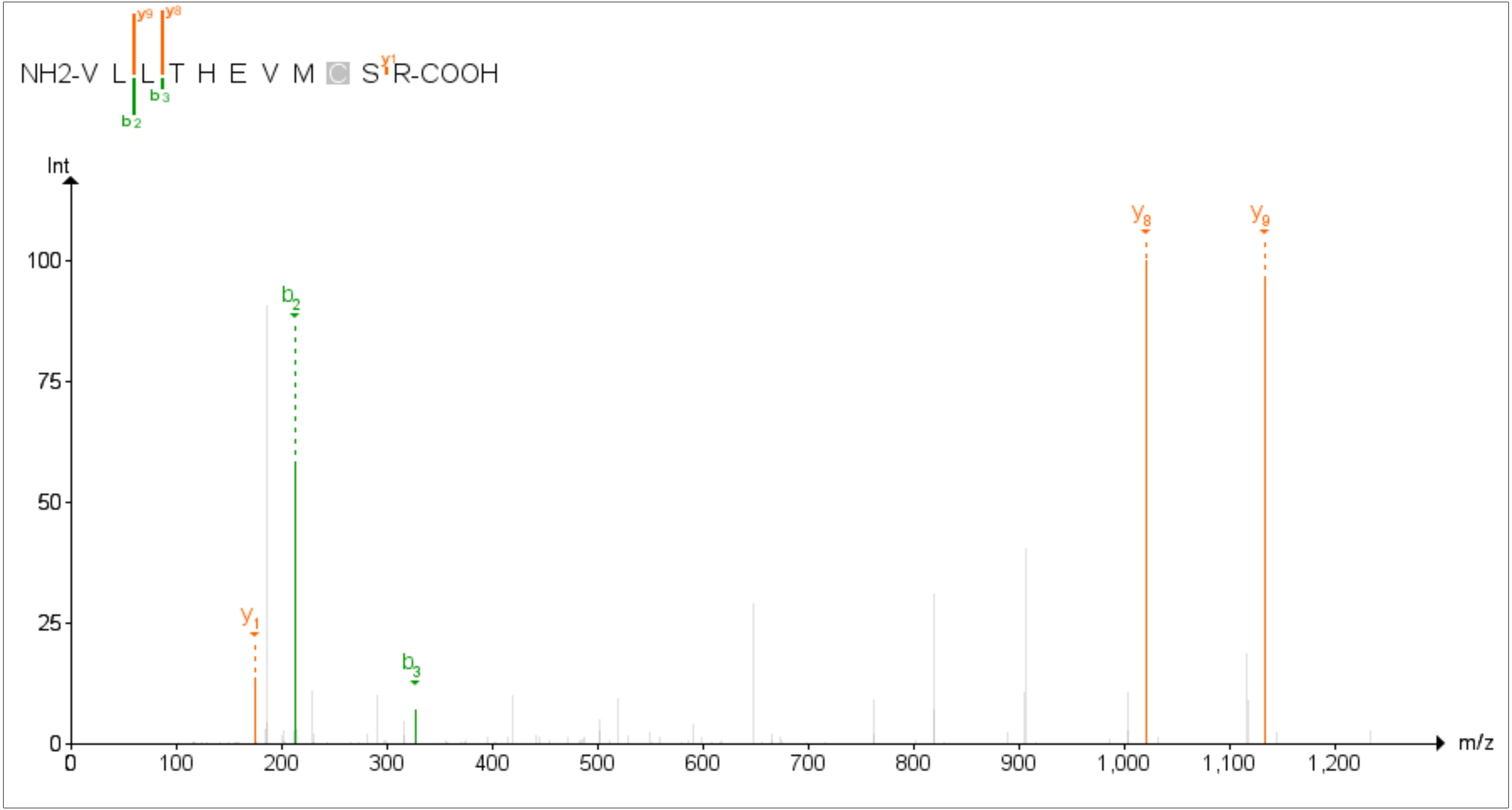

NH2-V L L T H E V M C S R-COOH
y9
y8
y1
b2
b3
Int
100
75
50
25
0
y8
y9
b2
y1
b3
0
100
200
300
400
500
600
700
800
900
1,000
1,100
1,200
m/z

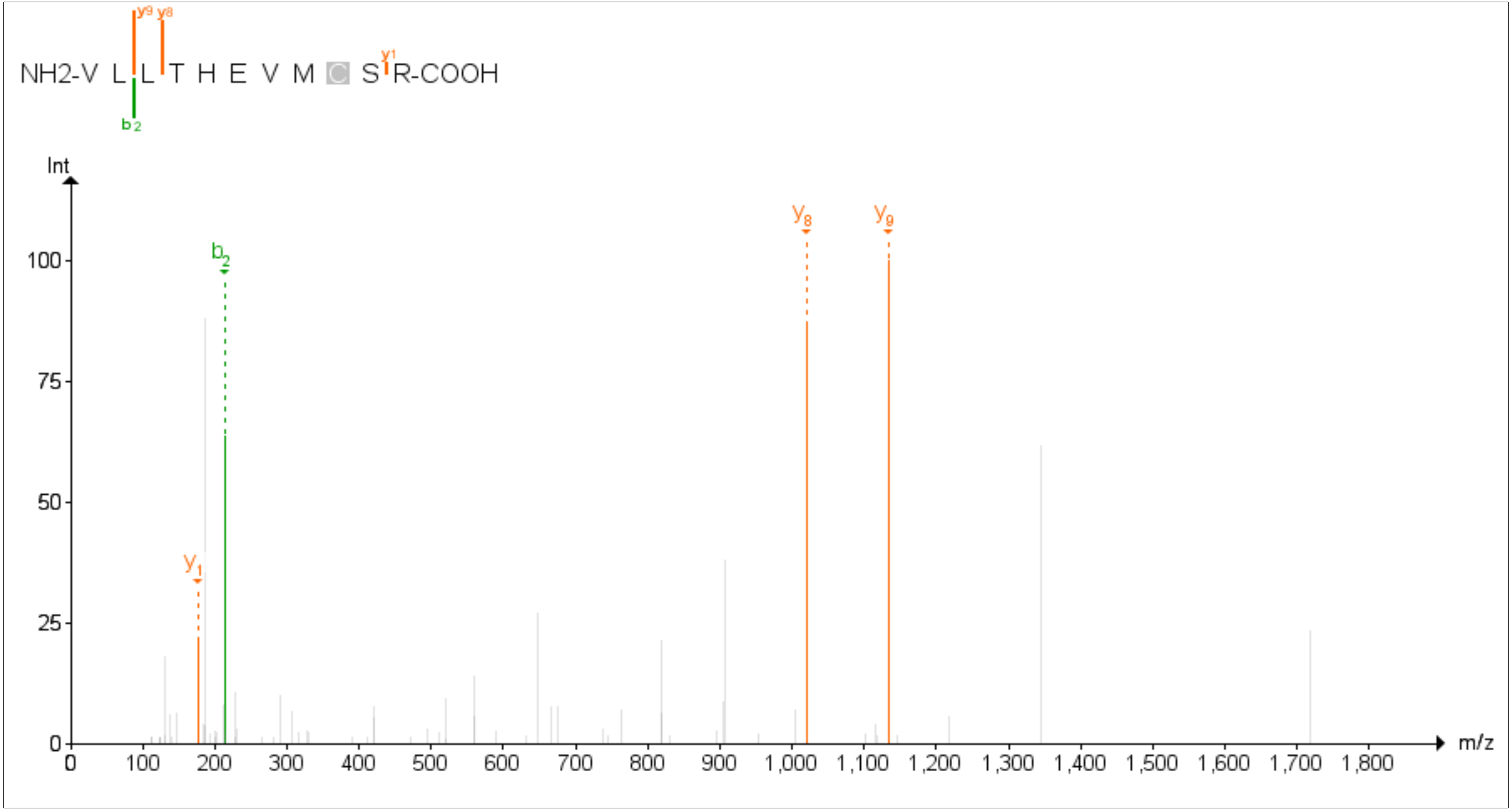

NH2-V L L T H E V M C S R-COOH
y9 y8
b2
y1
Int
100
75
50
25
0
y8
y9
b2
y1
0 100 200 300 400 500 600 700 800 900 1,000 1,100 1,200 1,300 1,400 1,500 1,600 1,700 1,800
m/z

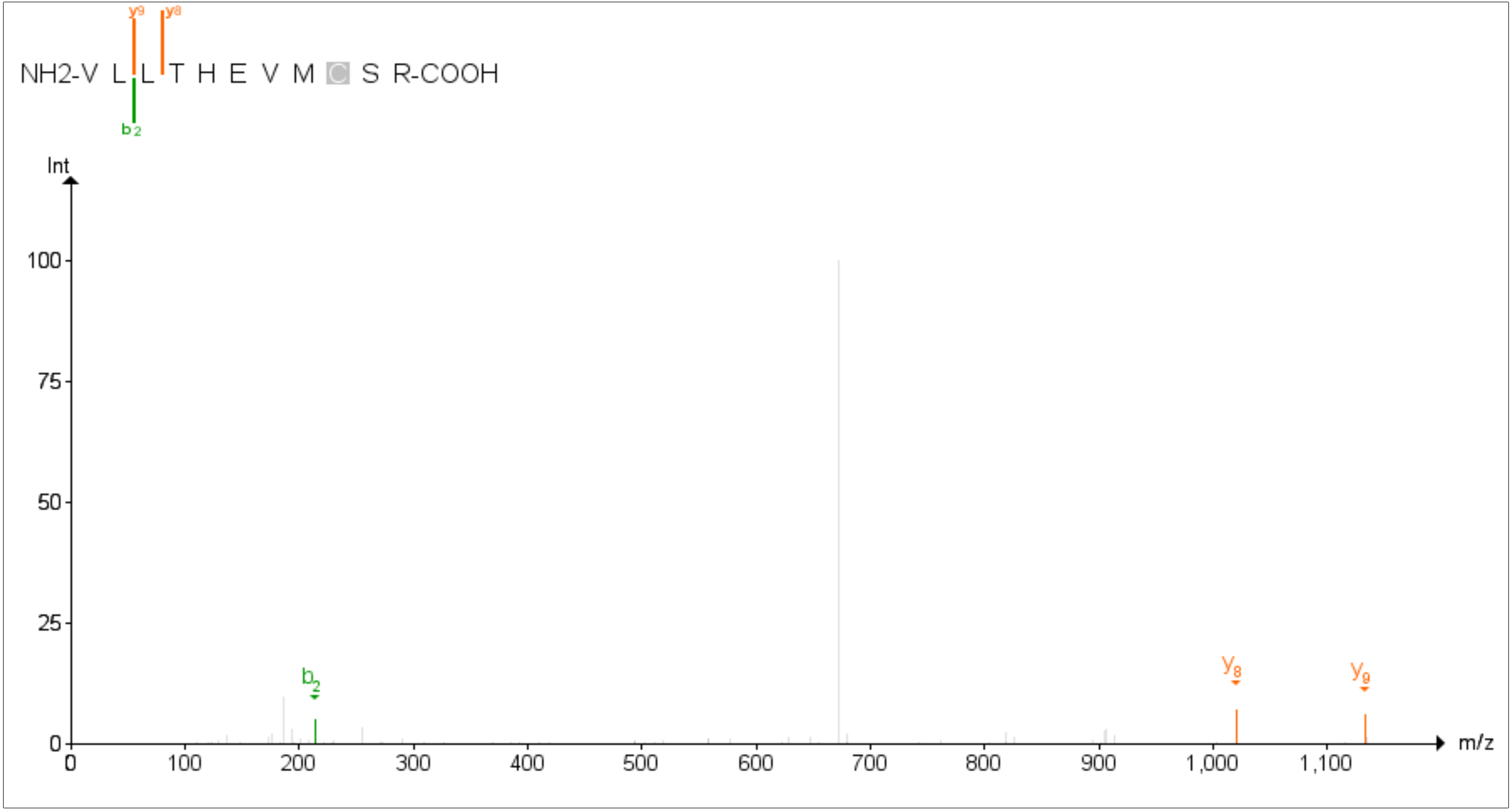
y9
y8
NH2-V L L T H E V M C S R-COOH
b2
Int
100
75
50
25
0
b2
y8
y9
0
100
200
300
400
500
600
700
800
900
1,000
1,100
m/z

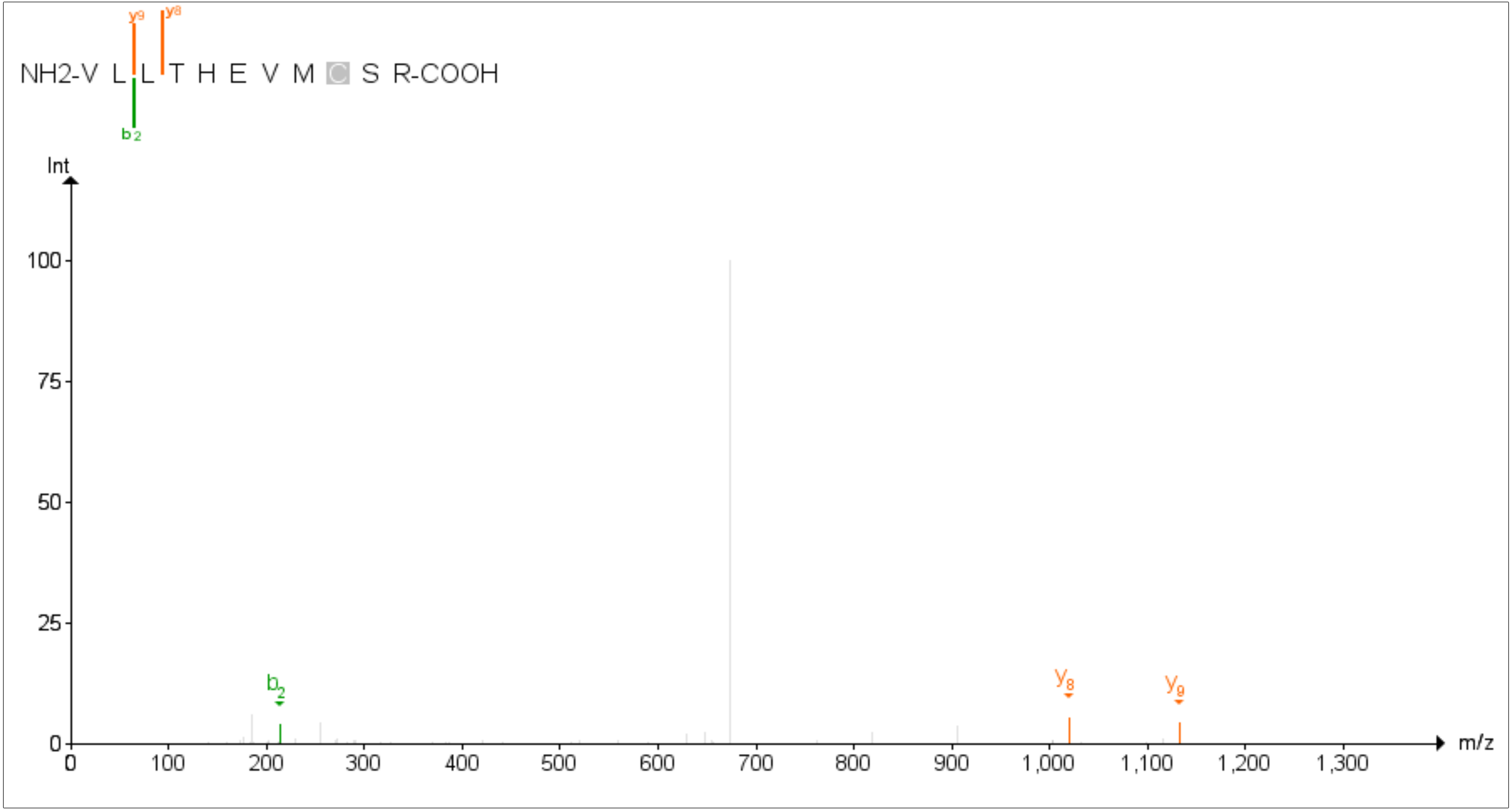

NH2-V L L T H E V M C S R-COOH
y9
y8
b2
Int
100
75
50
25
0
b2
y8
y9
0
100
200
300
400
500
600
700
800
900
1,000
1,100
1,200
1,300
m/z

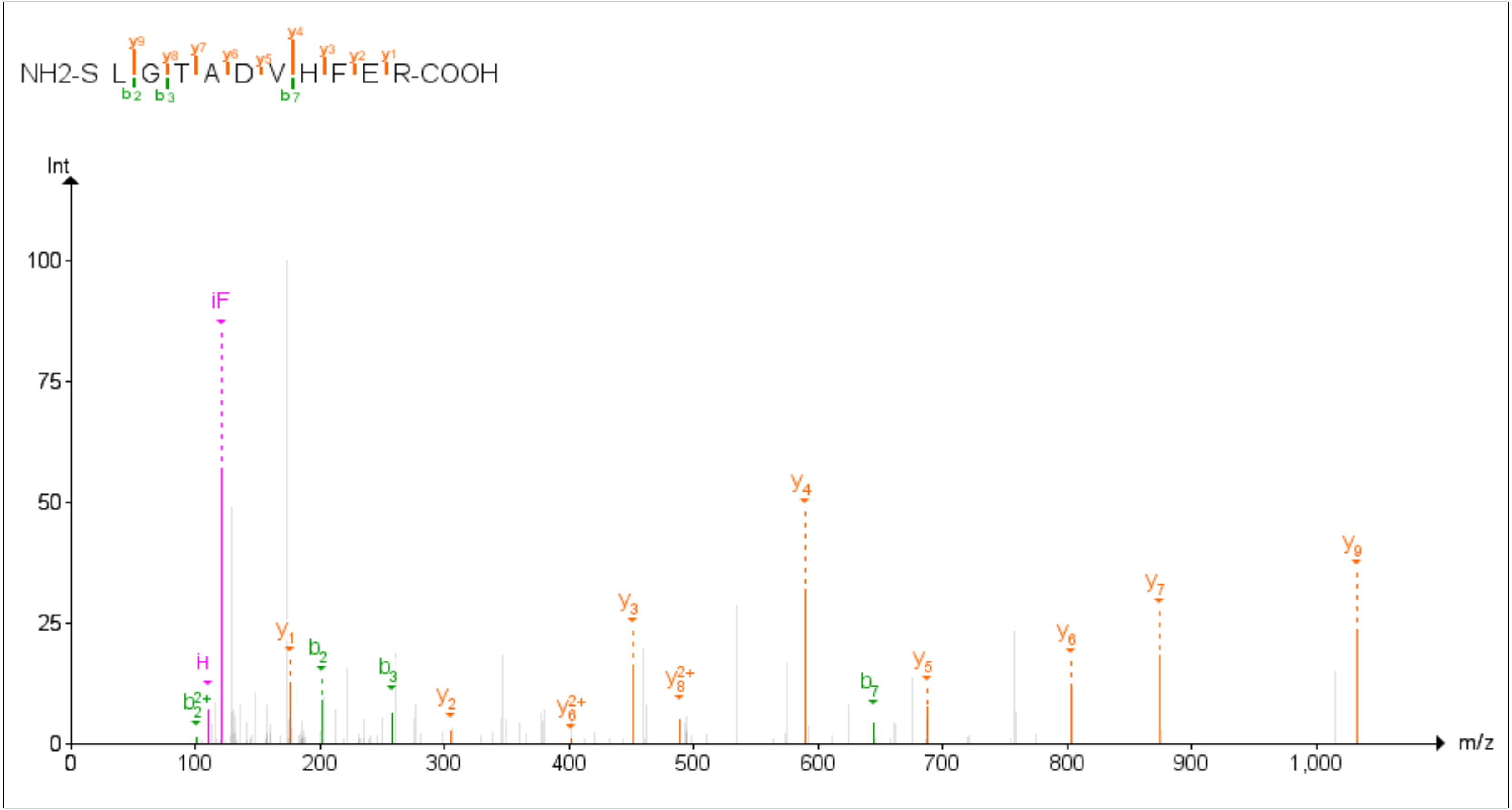

NH2-S L G T A D V H F E R-COOH
y9 y8 y7 y6 y5 y4 y3 y2 y1
b2 b3 b7
Int
100
75
50
25
0
iF
iH
b_2^{2+}
y_1
b_2
b_3
y_2
y_6^{2+}
y_3
y_8^{2+}
y_4
b_7
y_5
y_6
y_7
y_9
0 100 200 300 400 500 600 700 800 900 1,000
m/z

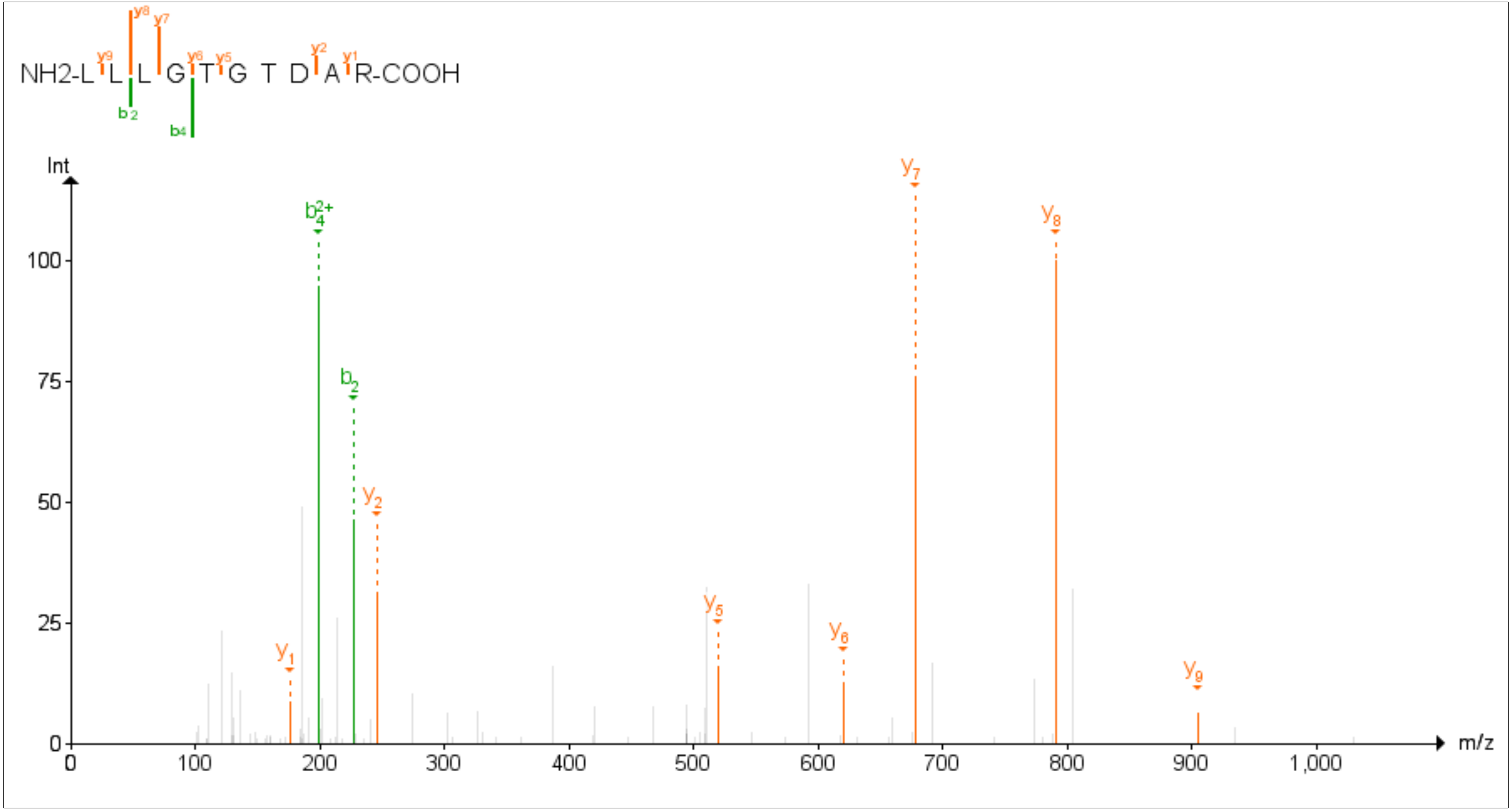

NH2-L L L G T G T D A R-COOH
y9
y8
y7
y6
y5
y2
y1
b2
b4
Int
100
75
50
25
0
b4 2+
b2
y2
y1
y5
y6
y7
y8
y9
0
100
200
300
400
500
600
700
800
900
1,000
m/z

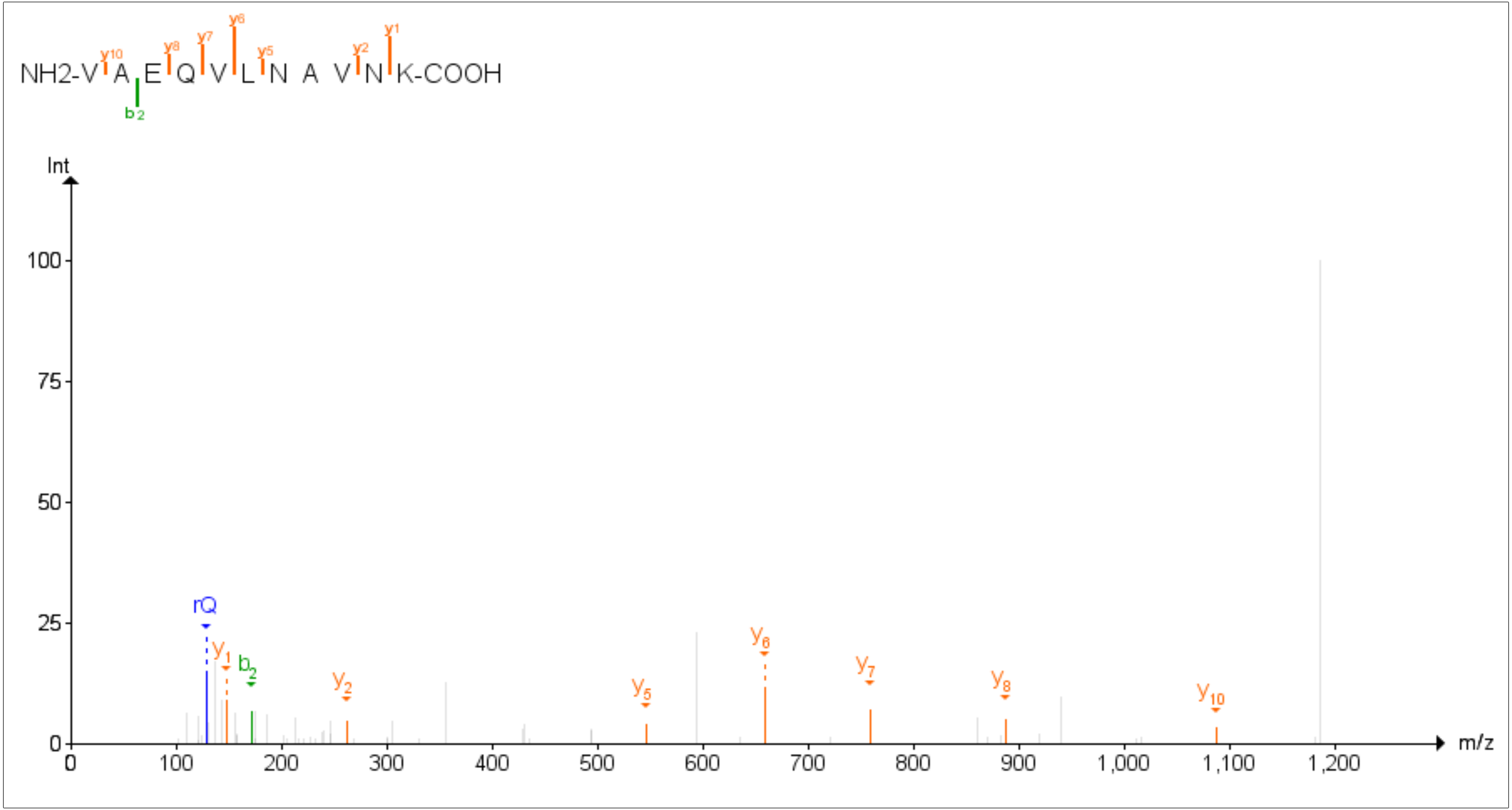

NH2-V A E Q V L N A V N K-COOH
y10 y8 y7 y6 y5 y2 y1
b2
Int
100
75
50
25
0
rQ
y1
b2
y2
y5
y6
y7
y8
y10
0 100 200 300 400 500 600 700 800 900 1,000 1,100 1,200
m/z

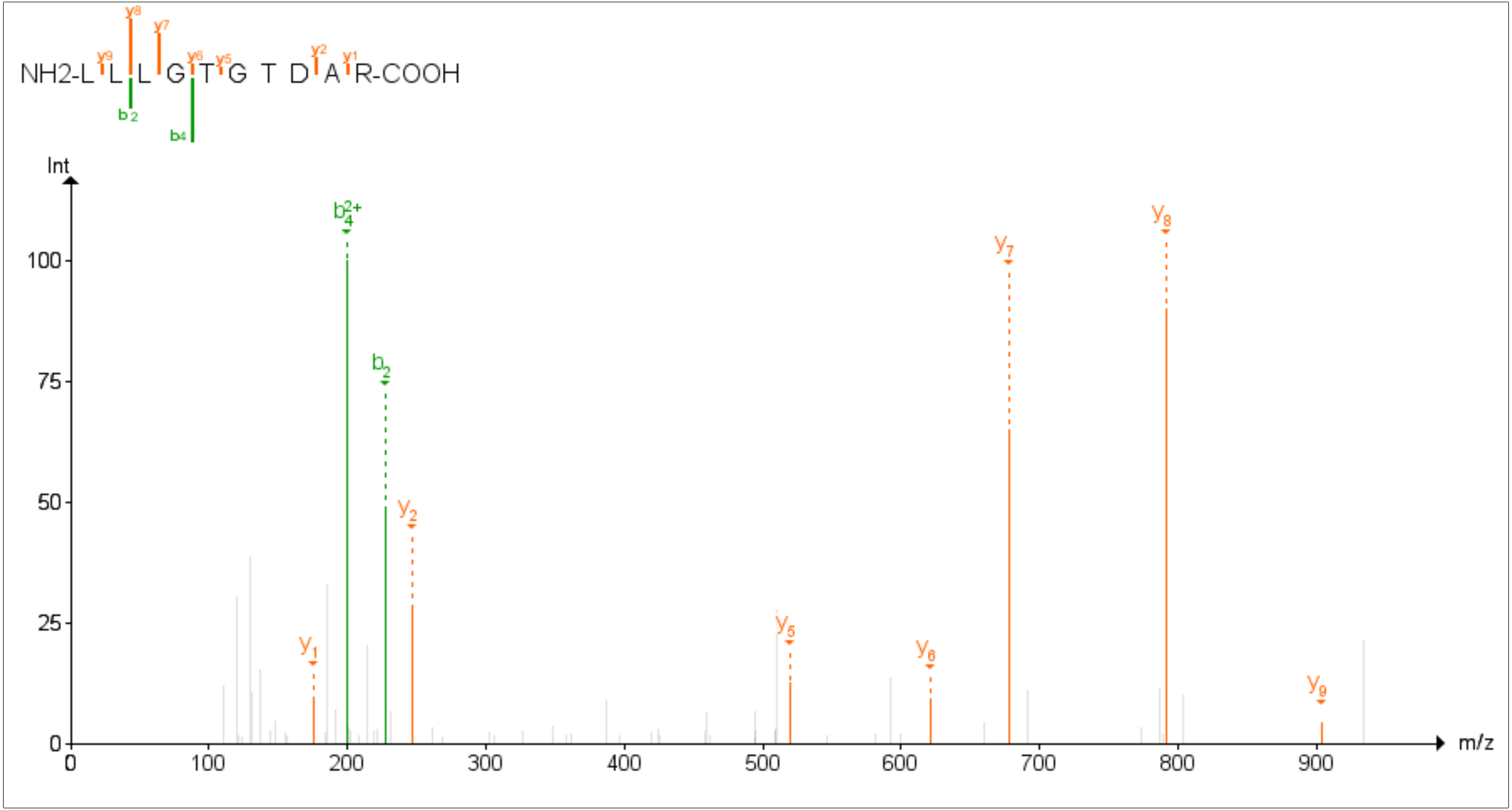

NH2-L L L G T G T D A R-COOH
y9 y8 y7 y6 y5 y2 y1
b2 b4
Int
m/z
0
25
50
75
100
0
100
200
300
400
500
600
700
800
900
b4 2+
b2
y1
y2
y5
y6
y7
y8
y9

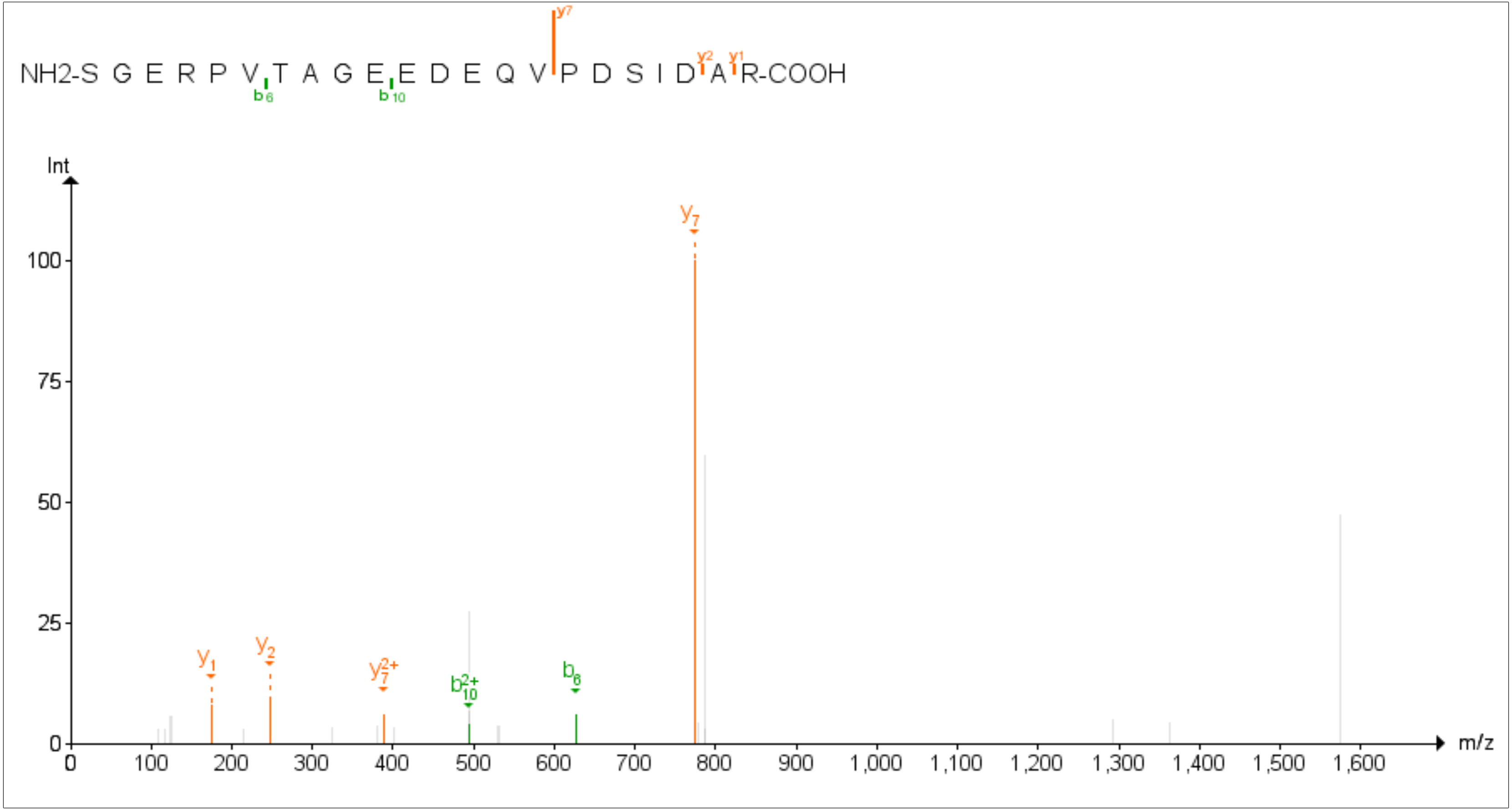

NH2-S G E R P V T A G E E D E Q V P D S I D A R-COOH
y7
y2
y1
b6
b10
Int
100
75
50
25
0
y7
y1
y2
y_7^{2+}
b_{10}^{2+}
b6
m/z
0
100
200
300
400
500
600
700
800
900
1,000
1,100
1,200
1,300
1,400
1,500
1,600

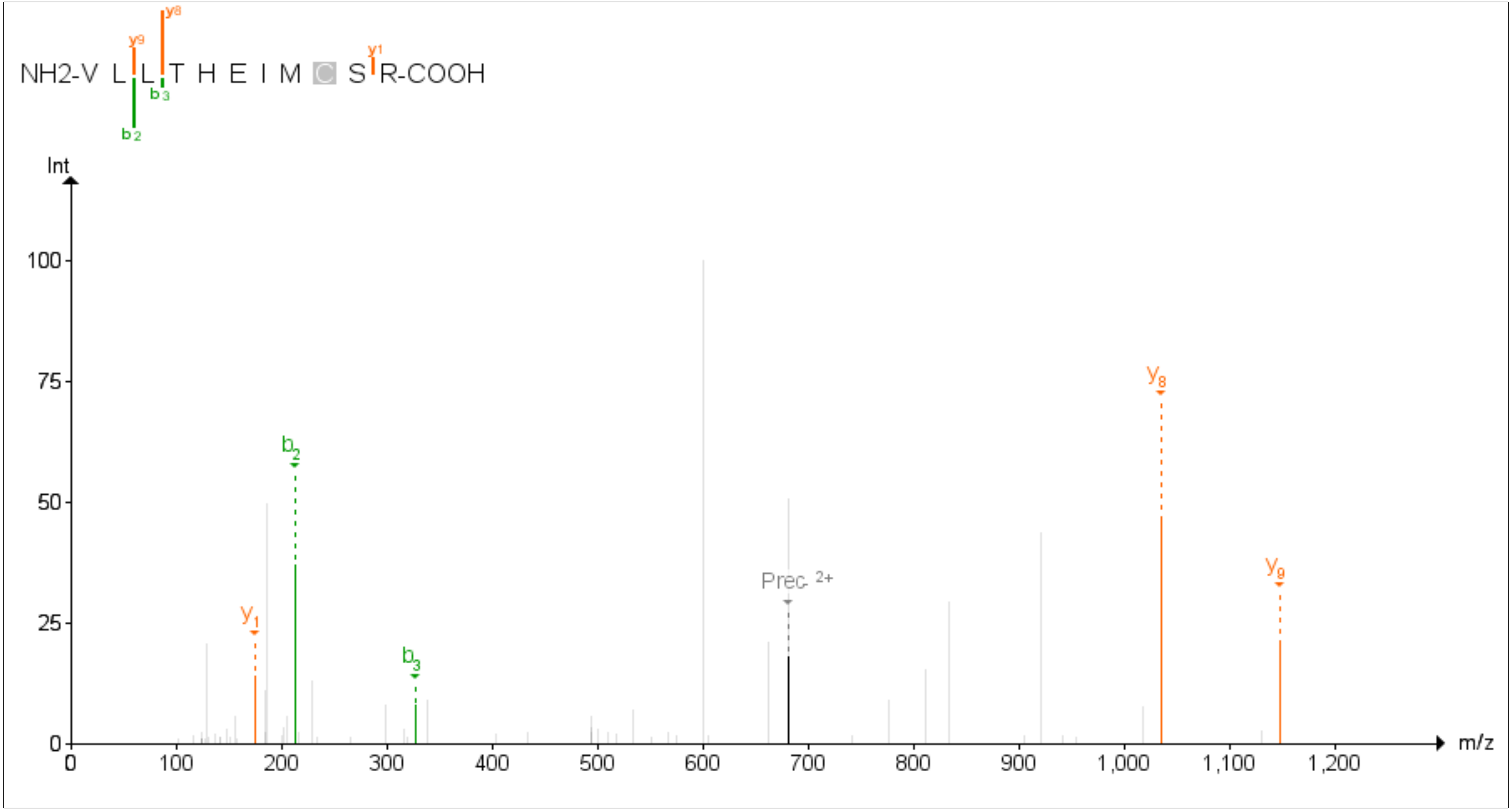
NH2-V L L T H E I M C S R-COOH
y9
y8
y1
b2
b3
Int
100
75
50
25
0
m/z
0
100
200
300
400
500
600
700
800
900
1,000
1,100
1,200
y1
b2
b3
Prec. 2+
y8
y9

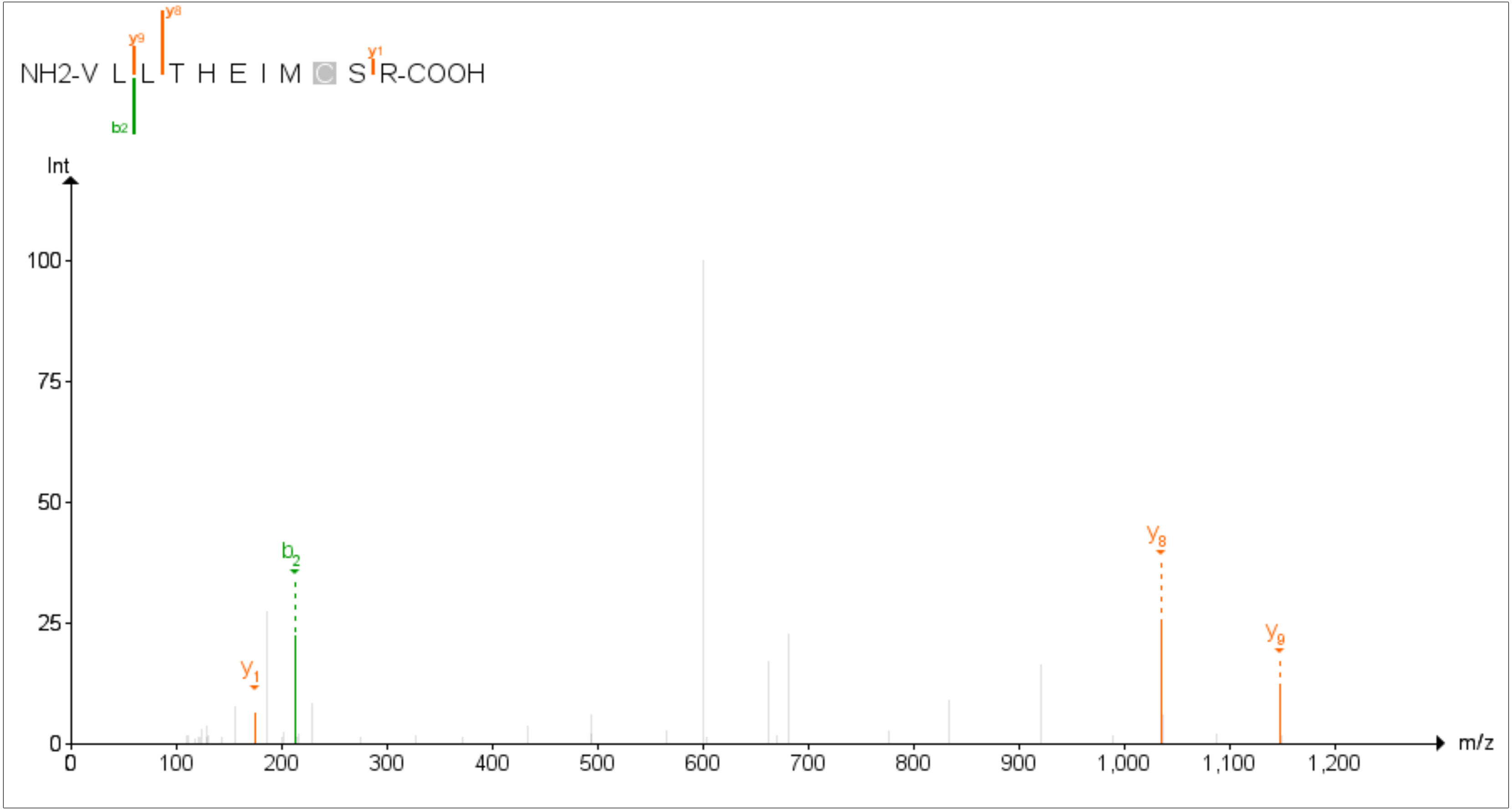

NH2-V L L T H E I M C S R-COOH
y9
y8
y1
b2
Int
100
75
50
25
0
y1
b2
y8
y9
0
100
200
300
400
500
600
700
800
900
1,000
1,100
1,200
m/z

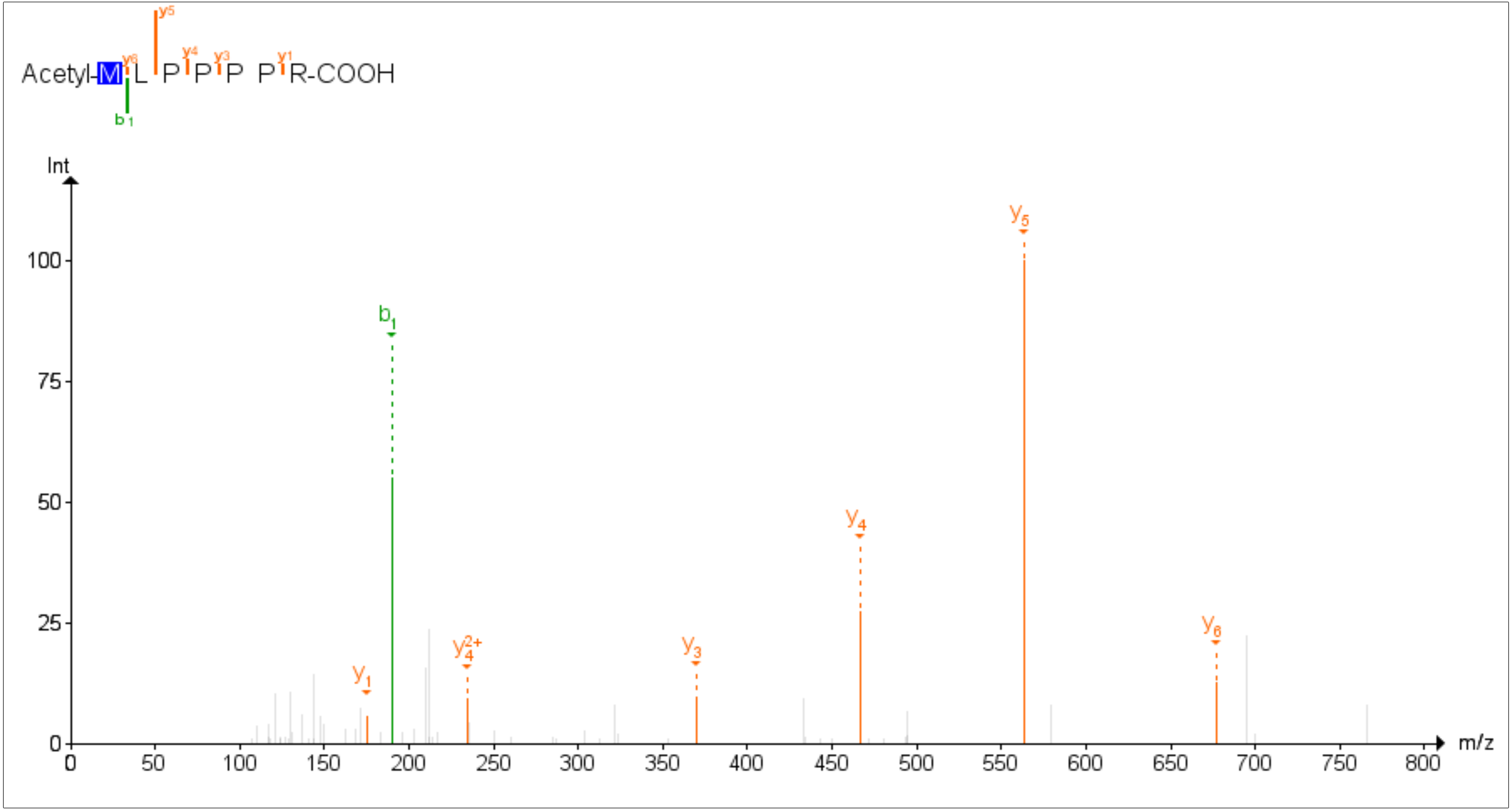

Acetyl-M L P P P P R-COOH
y6
y5
y4
y3
y1
b1
Int
100
75
50
25
0
b1
y5
y4
y6
y1
y4 2+
y3
0 50 100 150 200 250 300 350 400 450 500 550 600 650 700 750 800
m/z

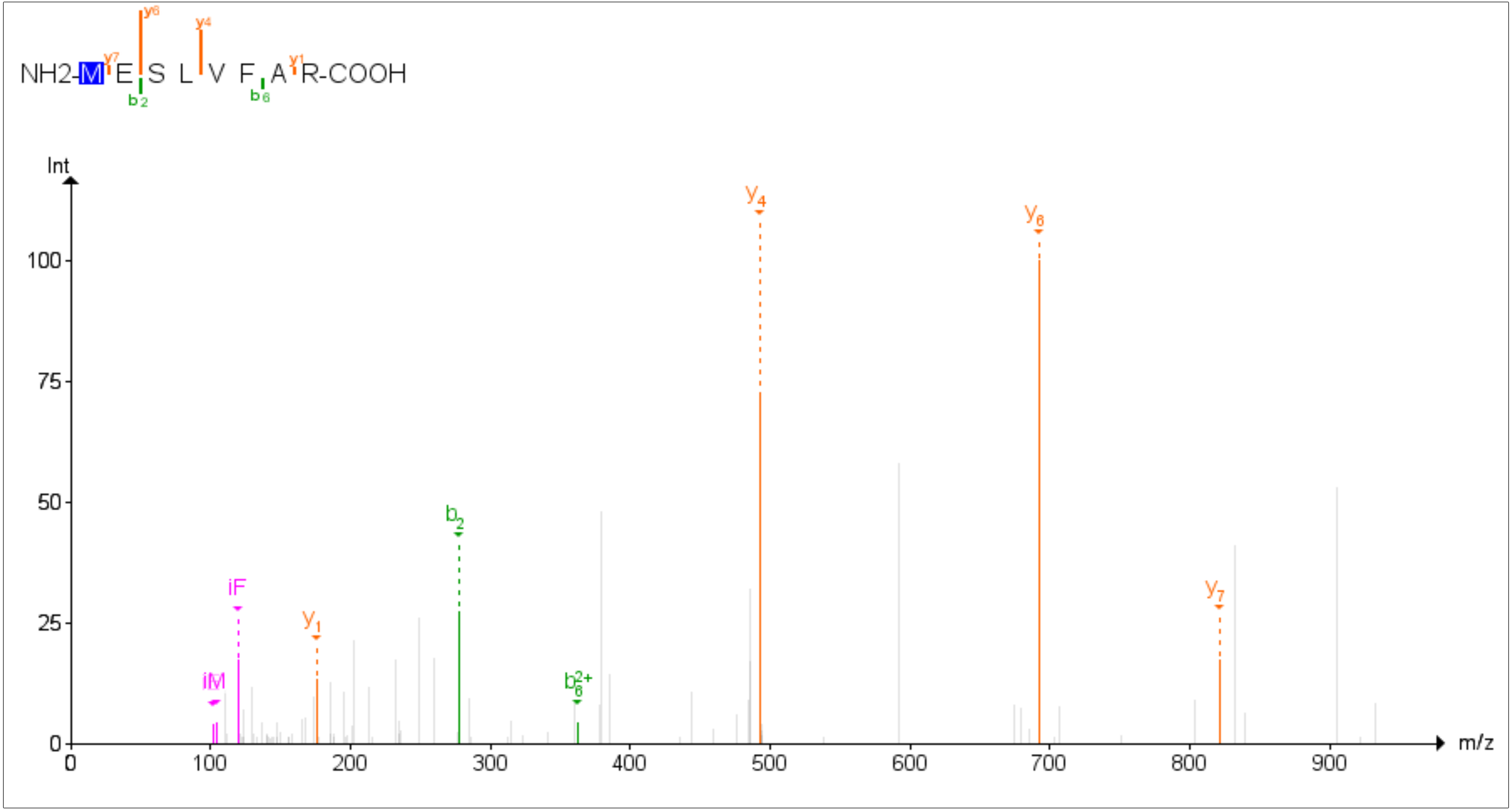

NH2-M E S L V F A R-COOH
y7
y6
y4
y1
b2
b6
Int
m/z
0
25
50
75
100
200
300
400
500
600
700
800
900
iM
iF
y1
b2
b6 2+
y4
y6
y7

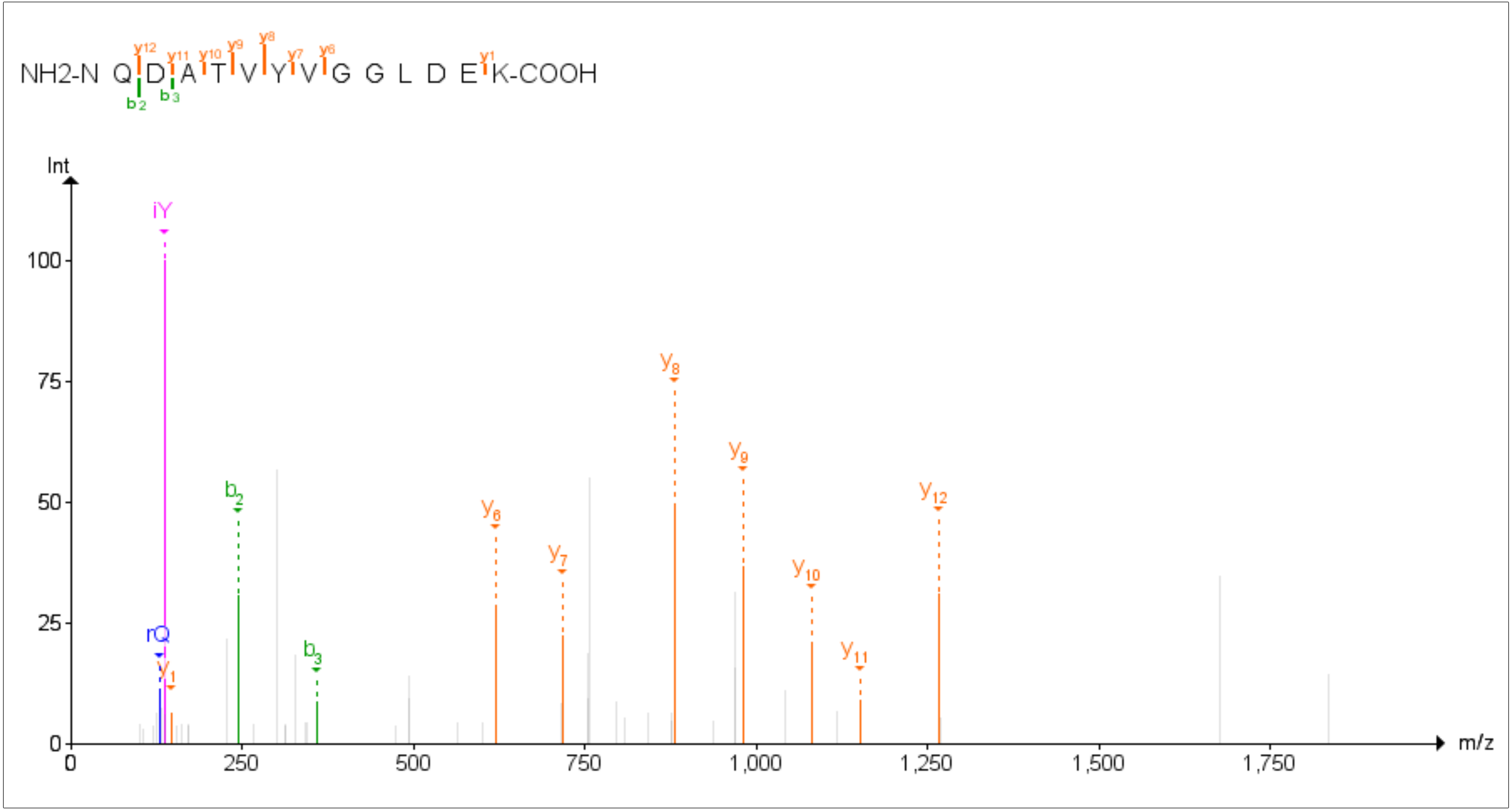

NH2-N Q D A T V Y V G G L D E K-COOH
y12 y11 y10 y9 y8 y7 y6 y1
b2 b3
Int
m/z
iY
rQ
y1
b2
b3
y6
y7
y8
y9
y10
y11
y12
0
25
50
75
100
0
250
500
750
1,000
1,250
1,500
1,750

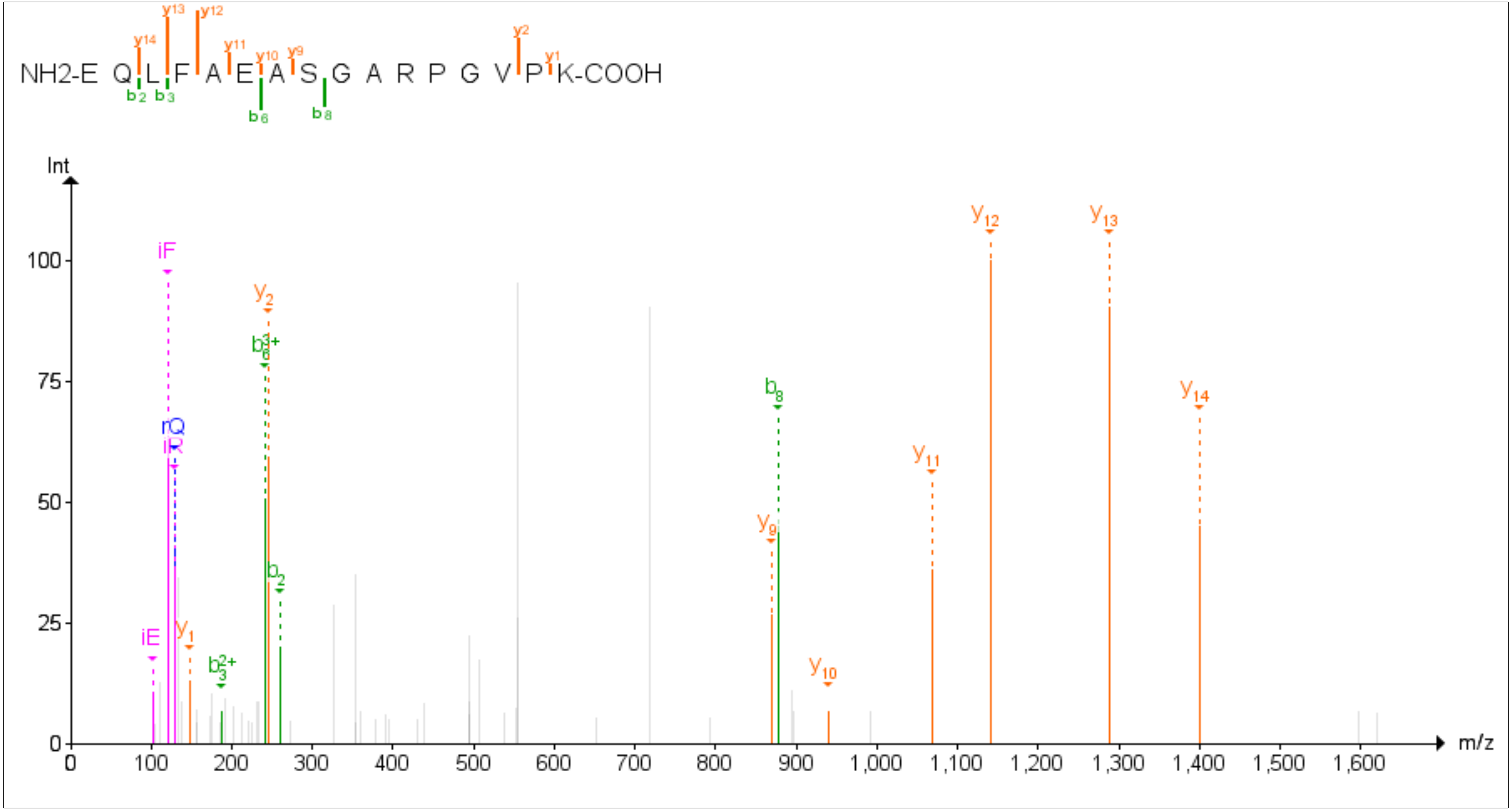

NH2-E Q L F A E A S G A R P G V P K-COOH
y14 y13 y12 y11 y10 y9 y2 y1
b2 b3 b6 b8
Int
m/z
iF
y2
b6 3+
b8
y12
y13
y14
y11
rQ
iR
y9
b2
iE
y1
b3 2+
y10

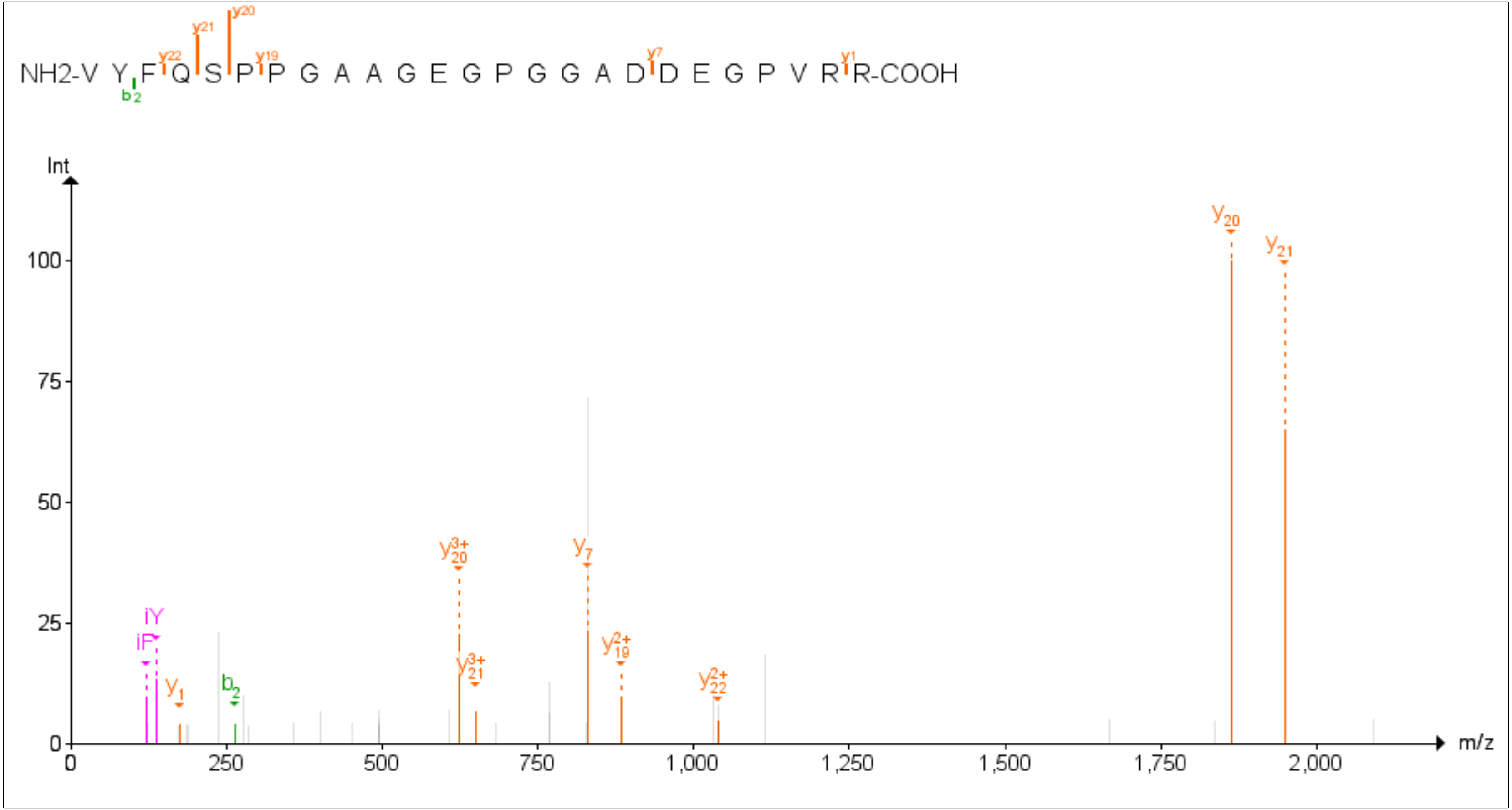

NH2-V Y F Q S P P G A A G E G P G G A D D E G P V R R-COOH
b2
y22
y21
y20
y19
y7
y1
Int
m/z
iY
iF
y1
b2
y20 3+
y21 3+
y7
y19 2+
y22 2+
y20
y21

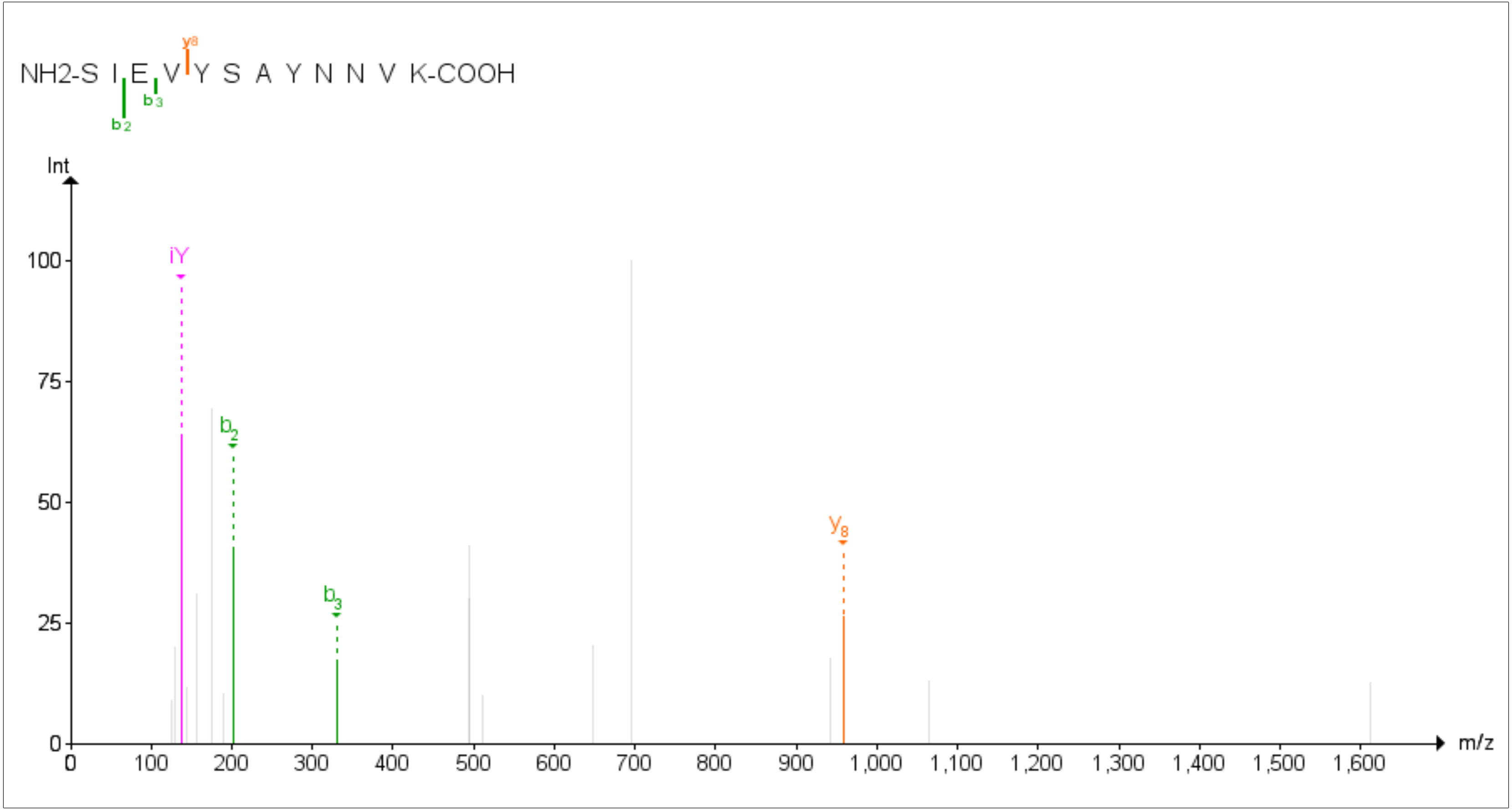

NH2-S I E V Y S A Y N N V K-COOH
y8
b2
b3
Int
100
75
50
25
0
iY
b2
b3
y8
0
100
200
300
400
500
600
700
800
900
1,000
1,100
1,200
1,300
1,400
1,500
1,600
m/z

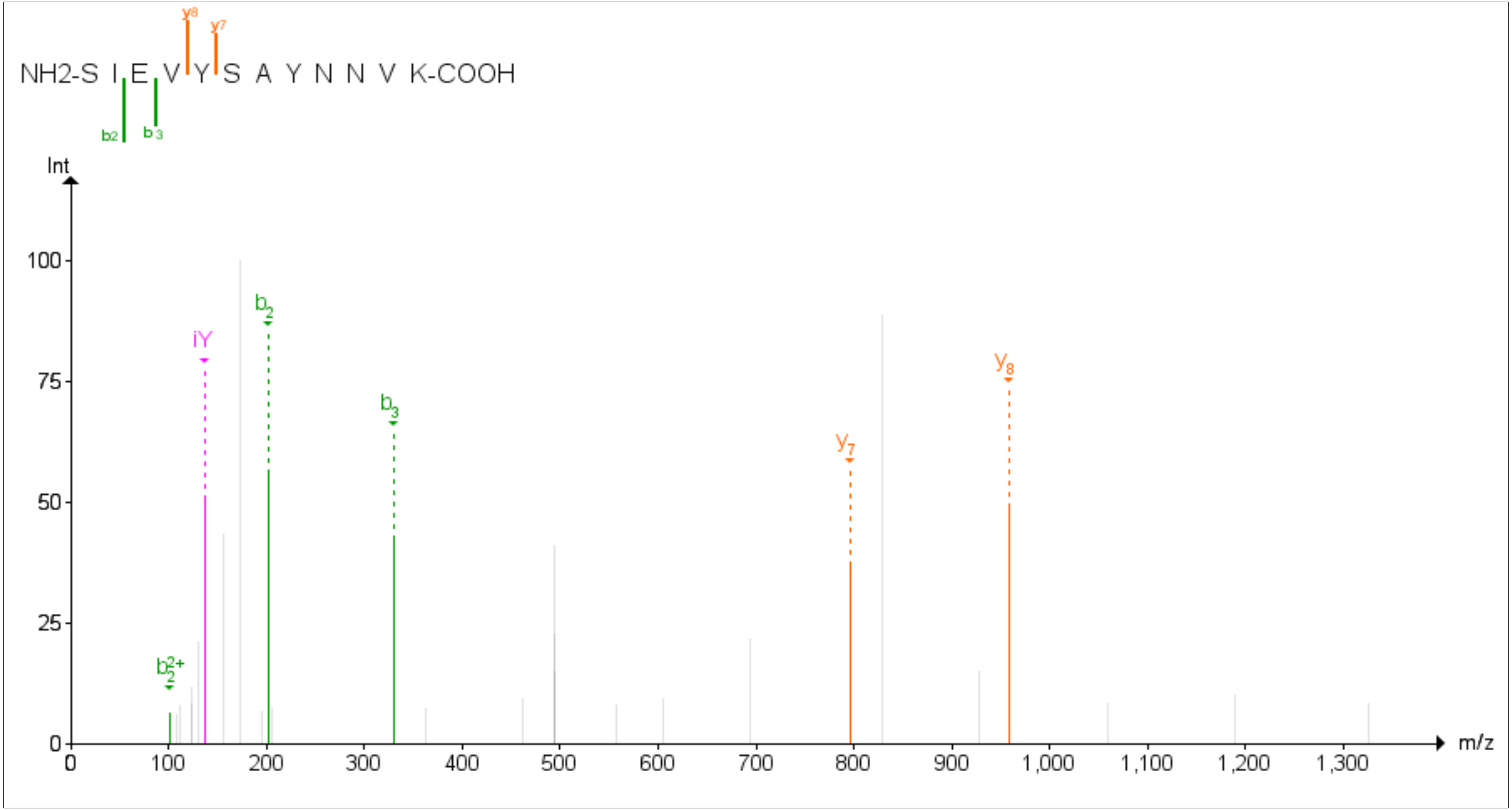

NH2-S I E V Y S A Y N N V K-COOH
y8
y7
b2
b3
Int
100
75
50
25
0
iY
b2
b3
b2 2+
y7
y8
0
100
200
300
400
500
600
700
800
900
1,000
1,100
1,200
1,300
m/z

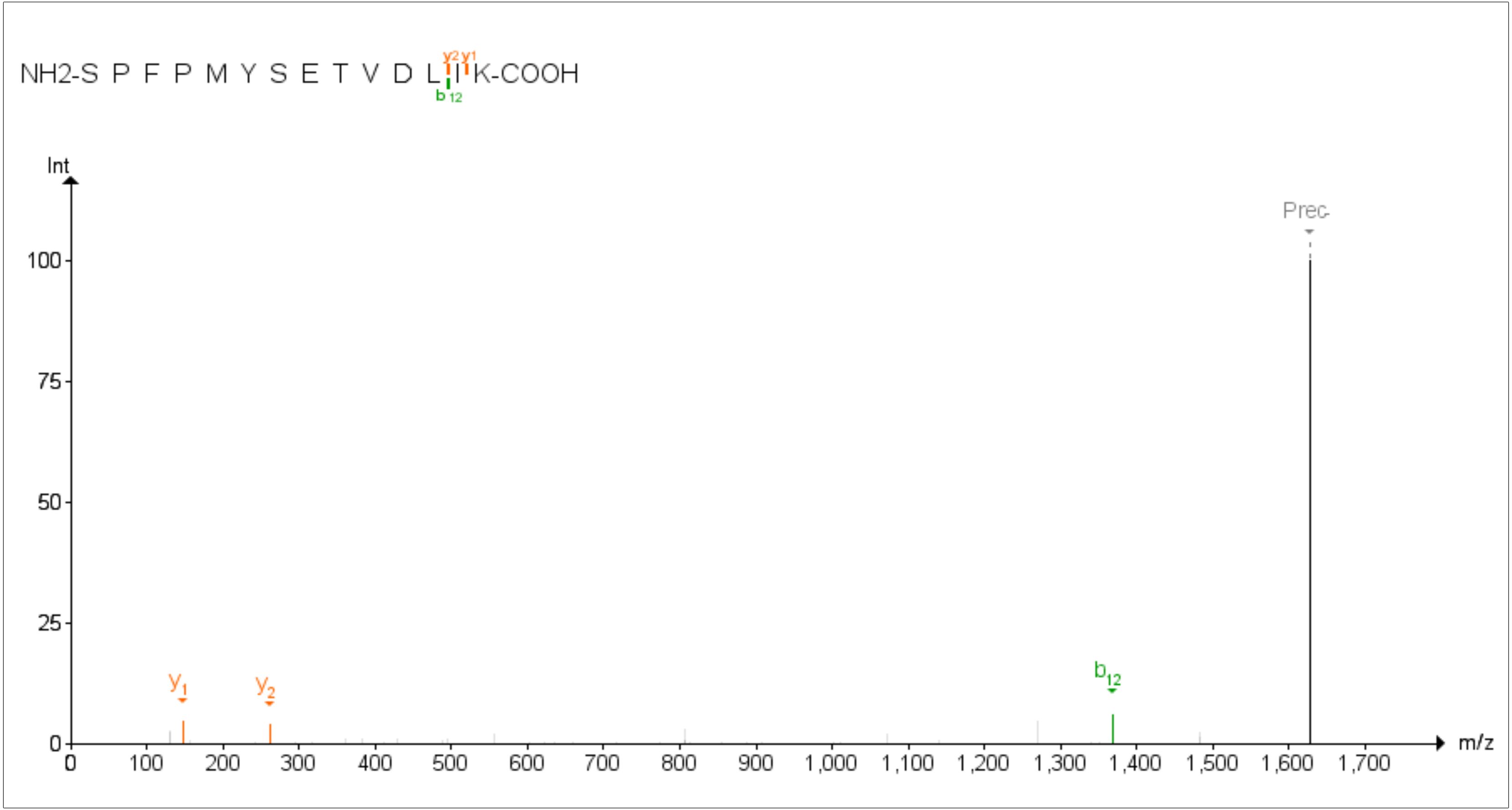

NH2-S P F P M Y S E T V D L I I K-COOH
y2 y1
b 12
Int
Prec
100
75
50
25
0
y1
y2
b12
0
100
200
300
400
500
600
700
800
900
1,000
1,100
1,200
1,300
1,400
1,500
1,600
1,700
m/z

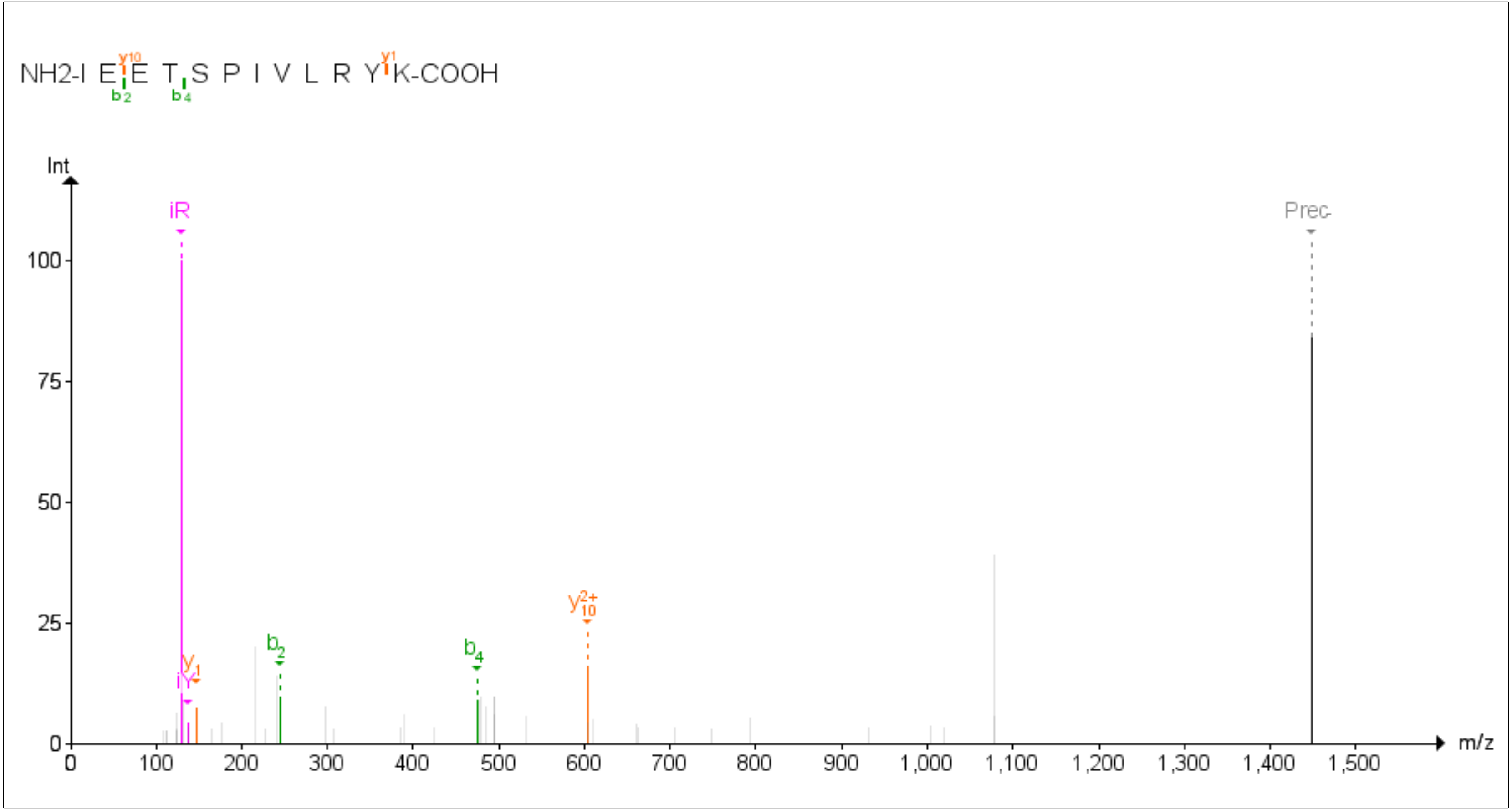

NH2-I E E T S P I V L R Y K-COOH
y10
y1
b2
b4
Int
iR
Prec.
y1
iY
b2
b4
y10 2+
m/z
0
25
50
75
100
0
100
200
300
400
500
600
700
800
900
1,000
1,100
1,200
1,300
1,400
1,500

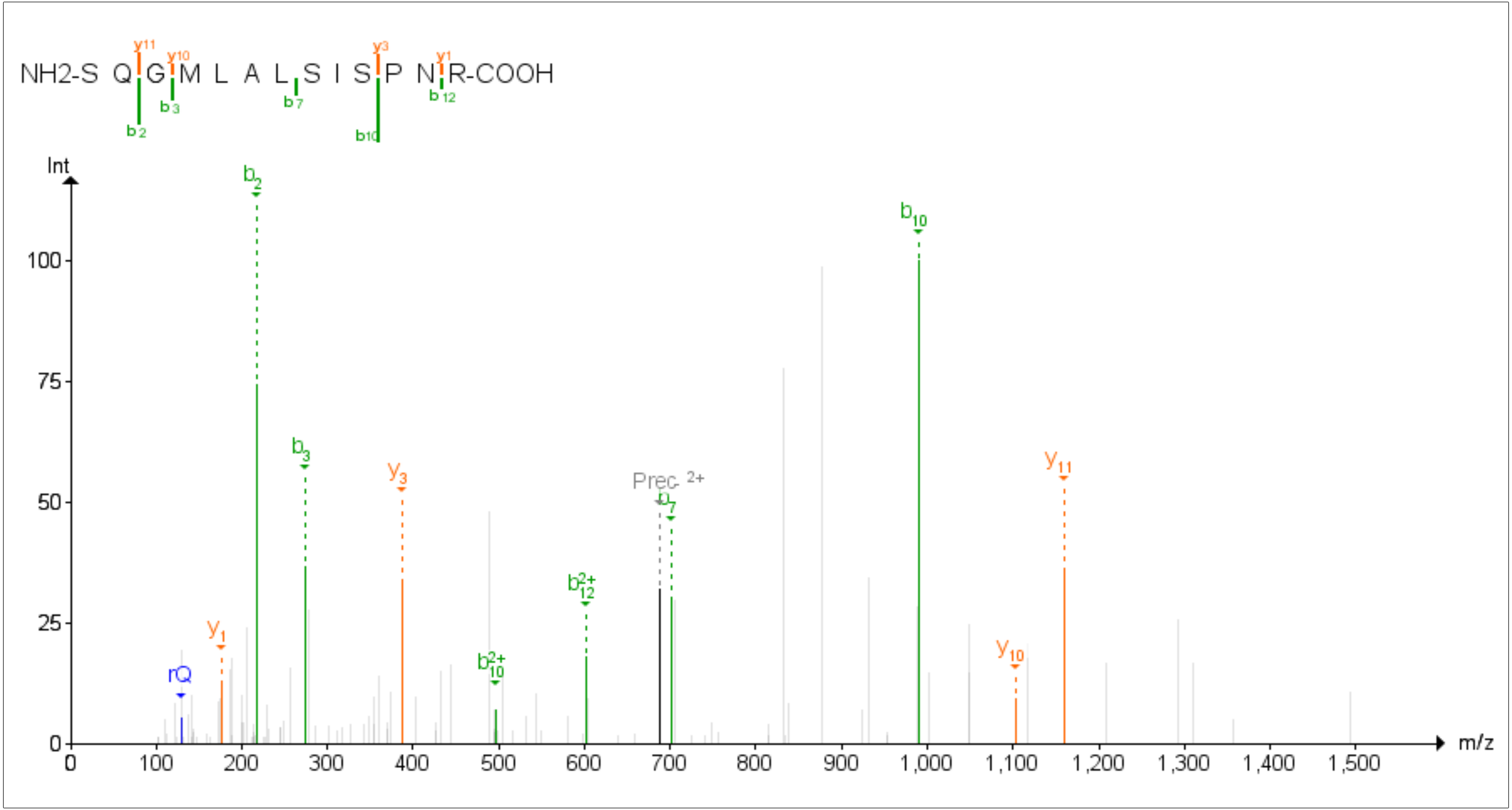

NH2-S Q G M L A L S I S P N R-COOH
y11
y10
y3
y1
b2
b3
b7
b10
b12
Int
100
75
50
25
0
b2
b10
b3
y3
y11
Prec 2+
b7
b2+ 12
b2+ 10
y10
y1
rQ
0
100
200
300
400
500
600
700
800
900
1,000
1,100
1,200
1,300
1,400
1,500
m/z

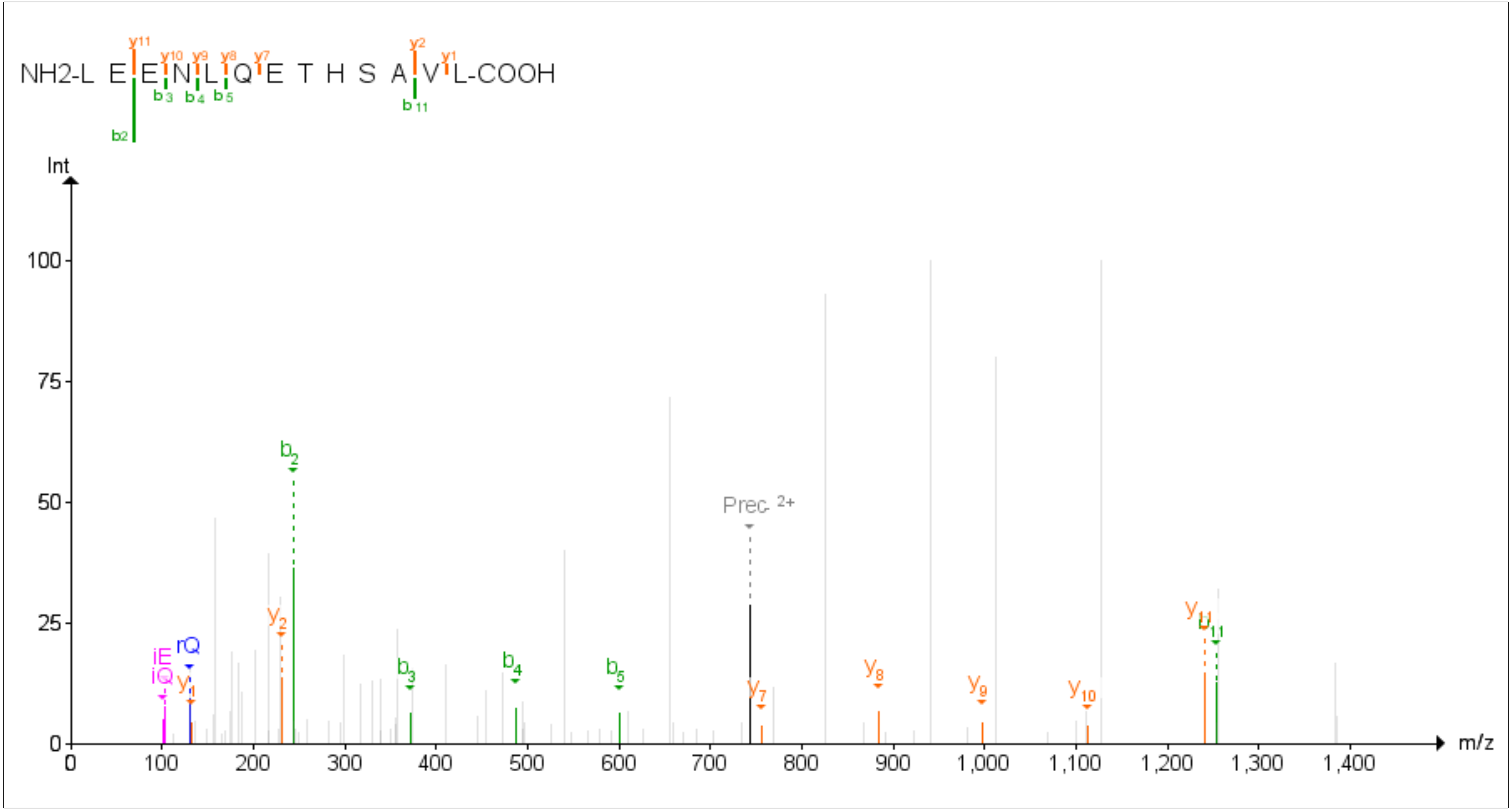

NH2-L E E N L Q E T H S A V L-COOH
y11 y10 y9 y8 y7 y2 y1
b2 b3 b4 b5 b11
Int
100
75
50
25
0
m/z
0
100
200
300
400
500
600
700
800
900
1,000
1,100
1,200
1,300
1,400
b2
Prec 2+
iE
iQ
rQ
y1
y2
b3
b4
b5
y7
y8
y9
y10
y11
b11

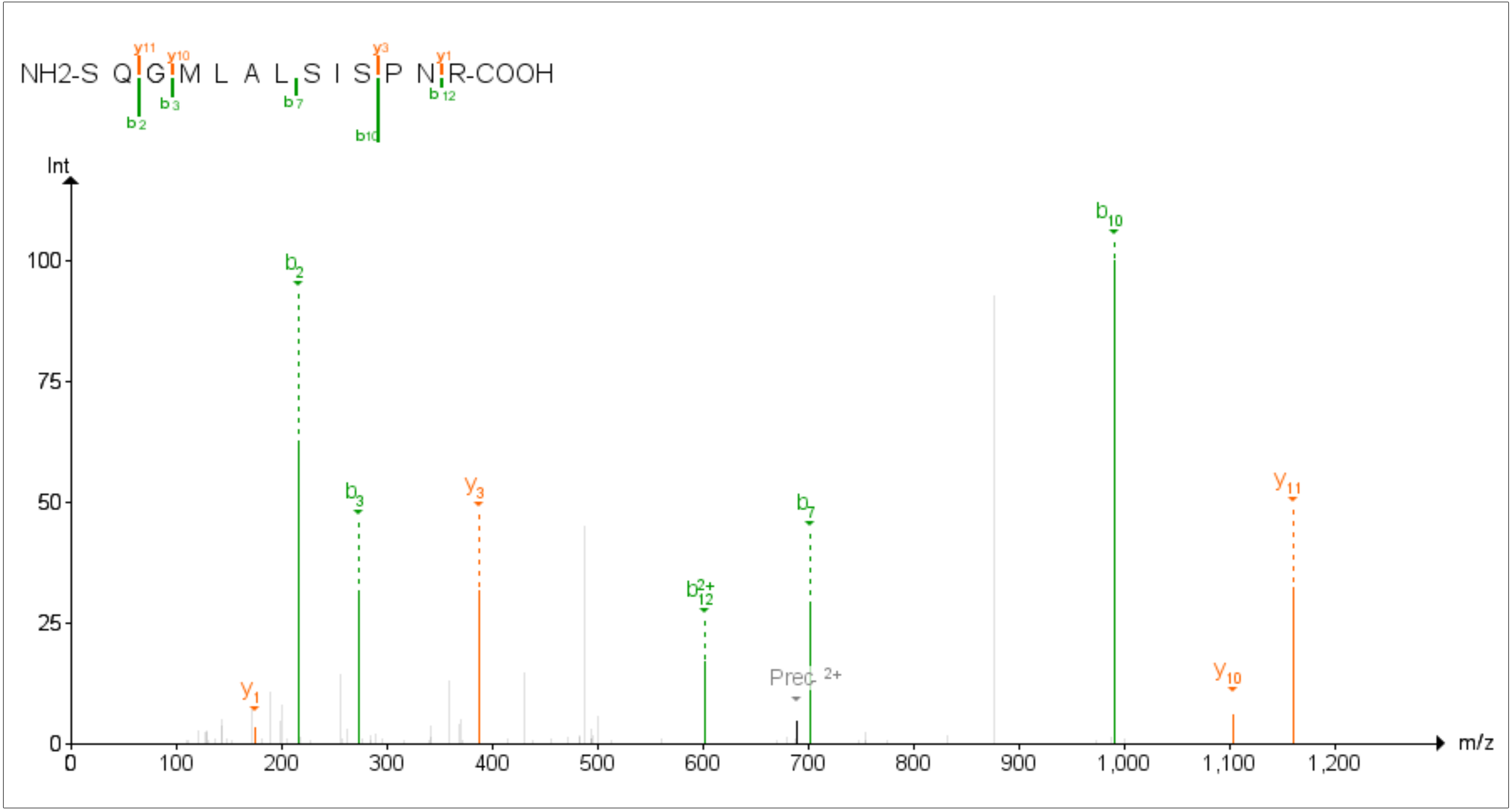

NH2-S Q G M L A L S I S P N R-COOH
y11 y10 y3 y1
b2 b3 b7 b10 b12
Int
m/z
b10
b2
b3
y3
b7
y11
b12 2+
Prec. 2+
y10
y1
0
25
50
75
100
0
100
200
300
400
500
600
700
800
900
1,000
1,100
1,200

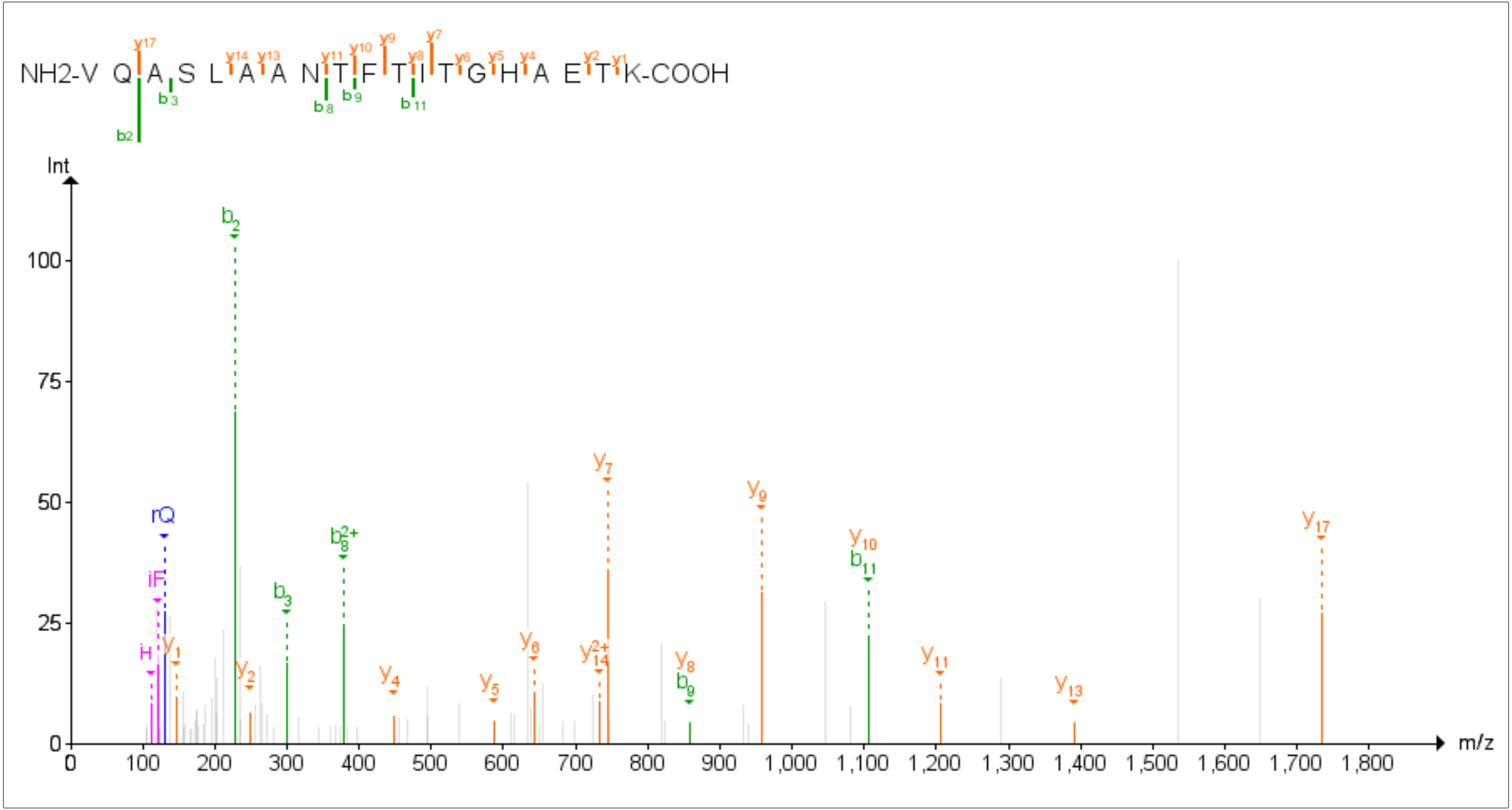
NH2-V Q A S L A A N T F T I T G H A E T K-COOH
Int
m/z
b2
b3
b8
b9
b11
y17
y14
y13
y11
y10
y9
y8
y7
y6
y5
y4
y2
y1
rQ
iF
H
0
25
50
75
100
0
100
200
300
400
500
600
700
800
900
1,000
1,100
1,200
1,300
1,400
1,500
1,600
1,700
1,800

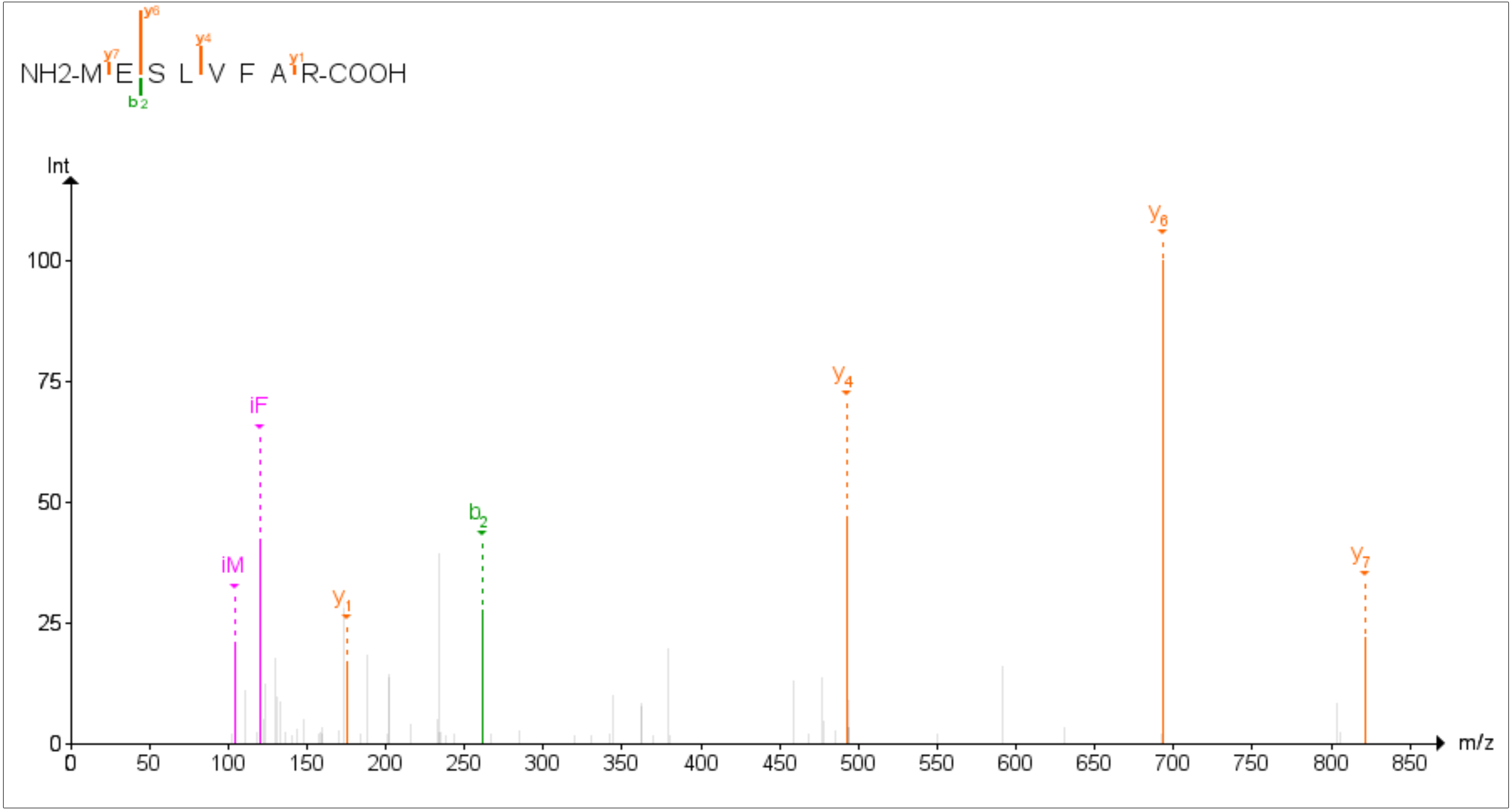

NH2-M E S L V F A R-COOH
y7
y6
y4
y1
b2
Int
100
75
50
25
0
iF
iM
y1
b2
y4
y6
y7
0
50
100
150
200
250
300
350
400
450
500
550
600
650
700
750
800
850
m/z

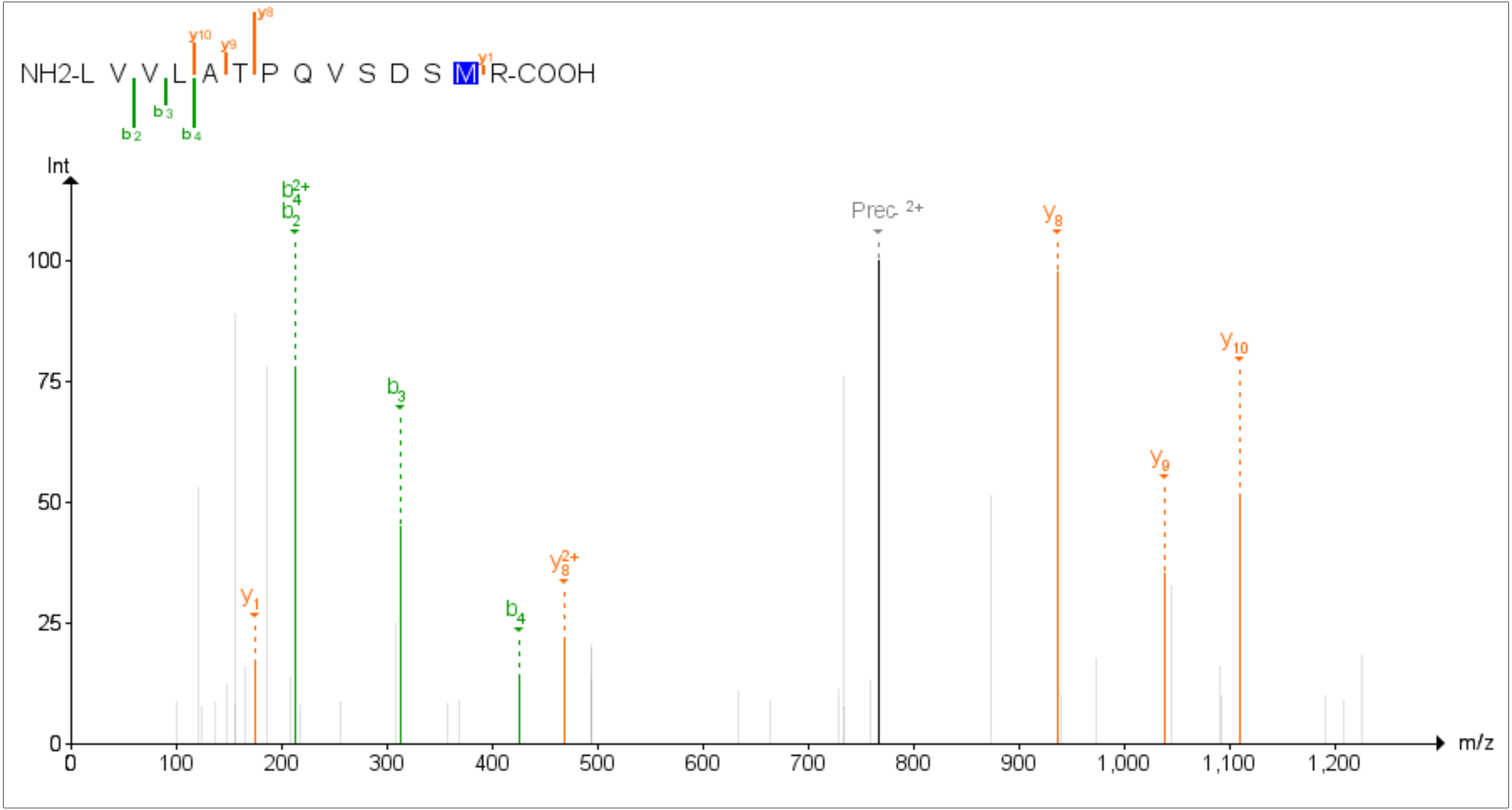

NH2-L V V L A T P Q V S D S M R-COOH
b2
b3
b4
y10
y9
y8
y1
Int
m/z
Prec 2+
b_4^{2+}
b_2
b_3
b_4
y_1
y_8^{2+}
y_8
y_9
y_{10}
0
25
50
75
100
0
100
200
300
400
500
600
700
800
900
1,000
1,100
1,200

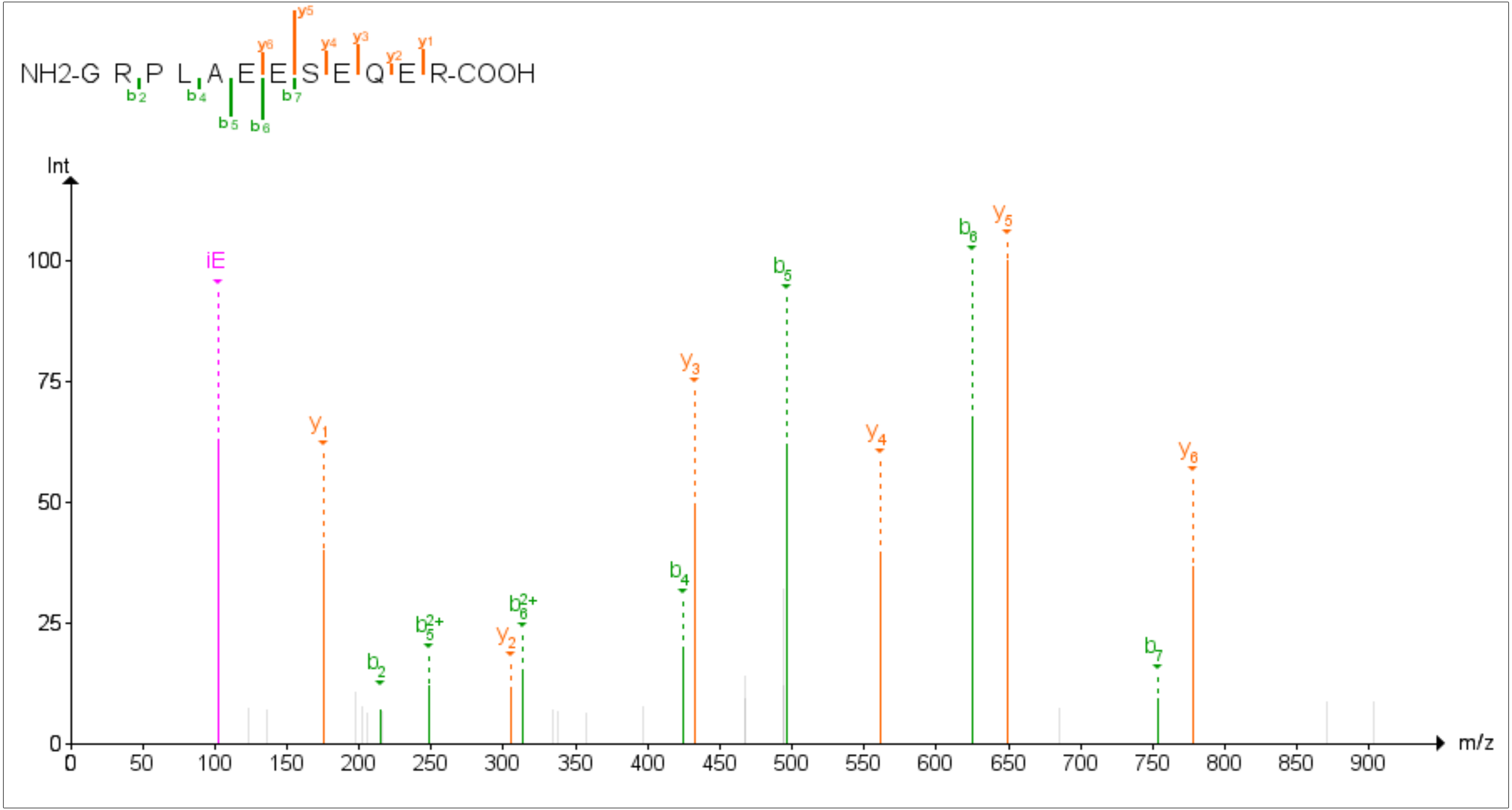

NH2-G R P L A E E S E Q E R-COOH
b2 b4 b5 b6 b7
y6 y5 y4 y3 y2 y1
Int
iE
y1
b2
b5 2+
y2
b6 2+
b4
y3
b5
y4
b6
y5
b7
y6
m/z
0 25 50 75 100
0 50 100 150 200 250 300 350 400 450 500 550 600 650 700 750 800 850 900

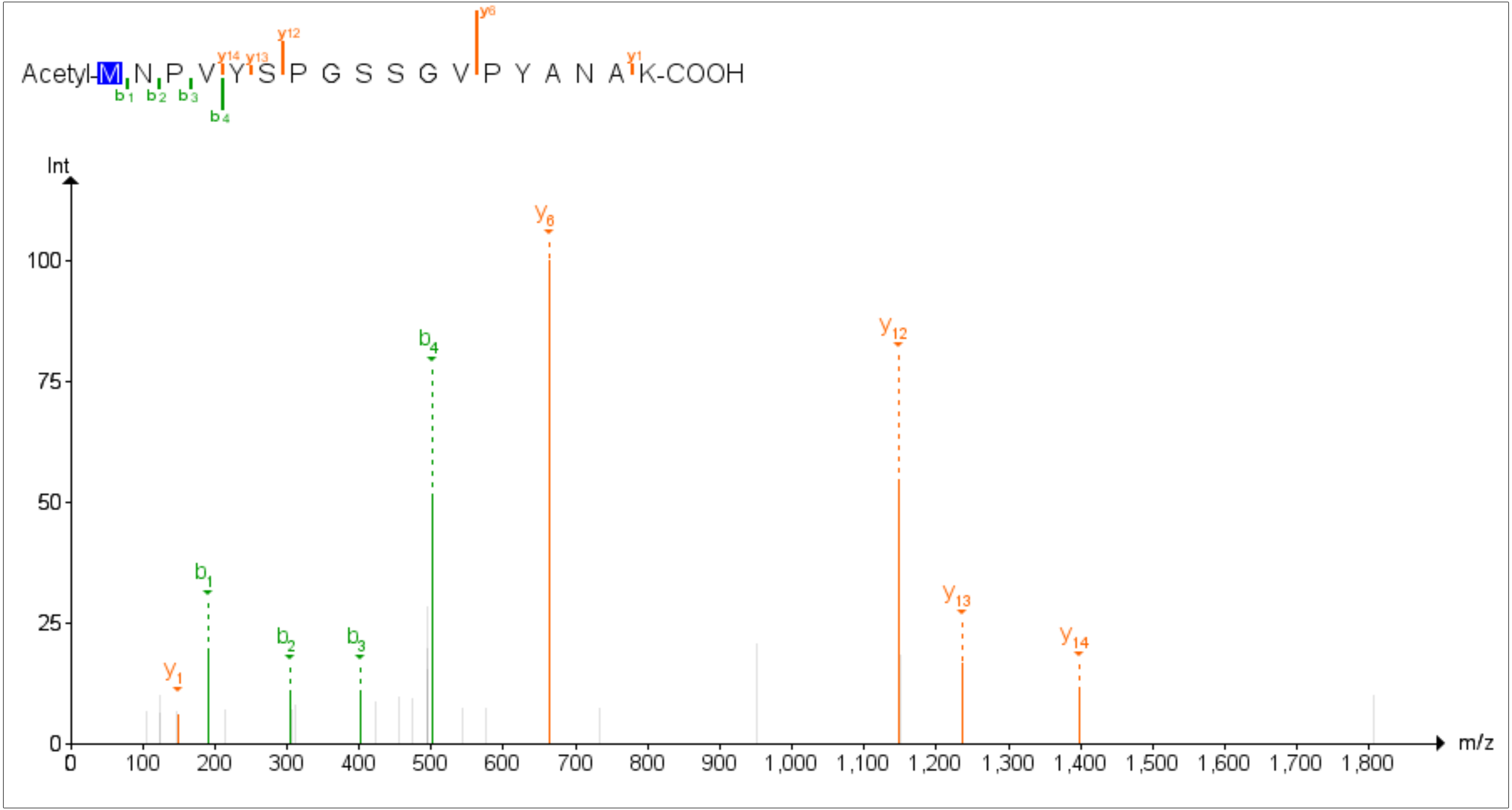

Acetyl-M N P V Y S P G S S G V P Y A N A K-COOH
b1 b2 b3 b4
y14 y13 y12 y6 y1
Int
100
75
50
25
0
y6
b4
y12
b1
y13
y1
b2
b3
y14
0 100 200 300 400 500 600 700 800 900 1,000 1,100 1,200 1,300 1,400 1,500 1,600 1,700 1,800
m/z

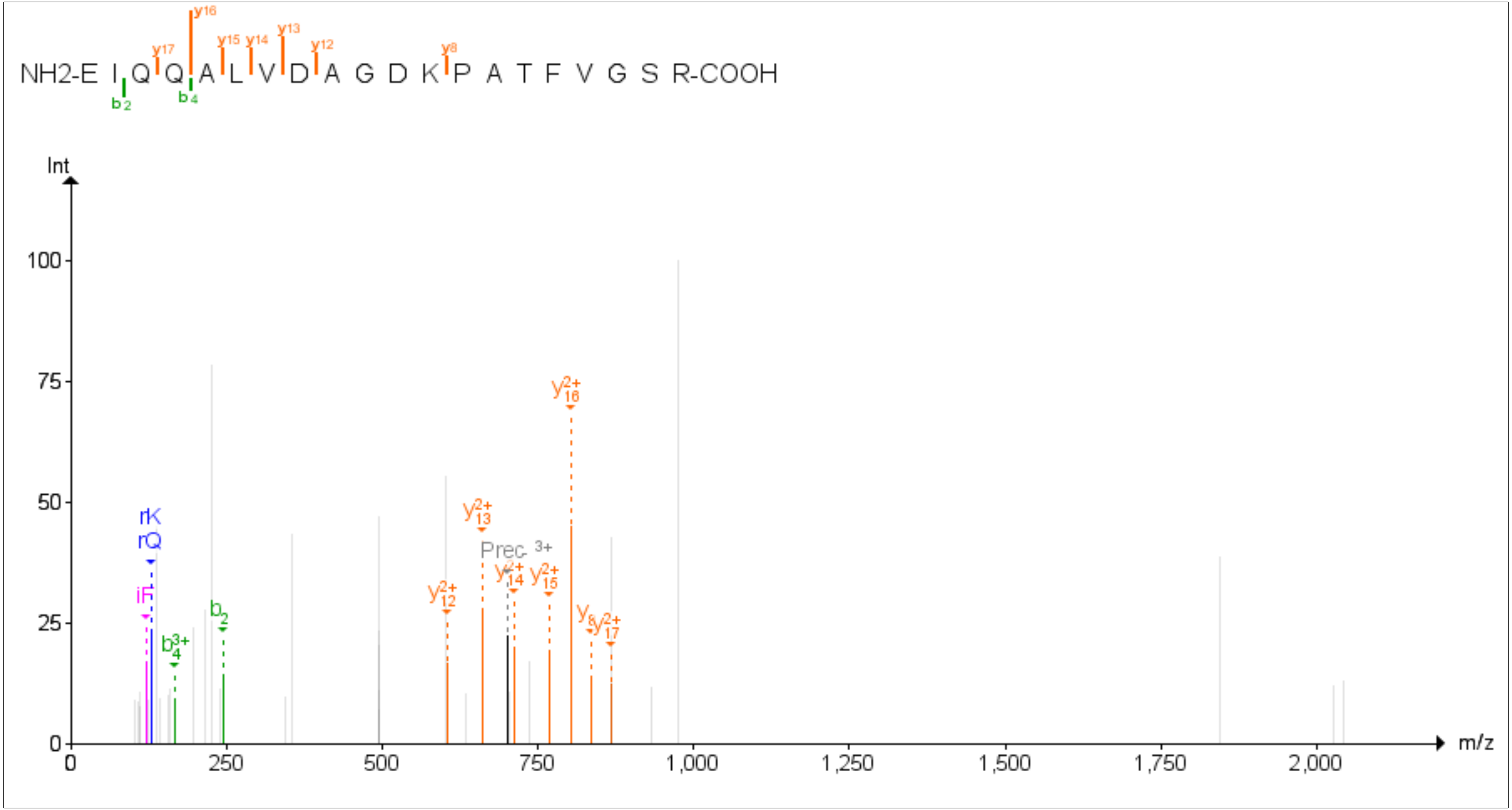

NH2-E I Q Q A L V D A G D K P A T F V G S R-COOH
y16
y17
y15
y14
y13
y12
y8
b2
b4
Int
100
75
50
25
0
rK
rQ
iF
b2
b4 3+
y12 2+
y13 2+
Prec 3+
y14 2+
y15 2+
y16 2+
y8
y17 2+
0
250
500
750
1,000
1,250
1,500
1,750
2,000
m/z

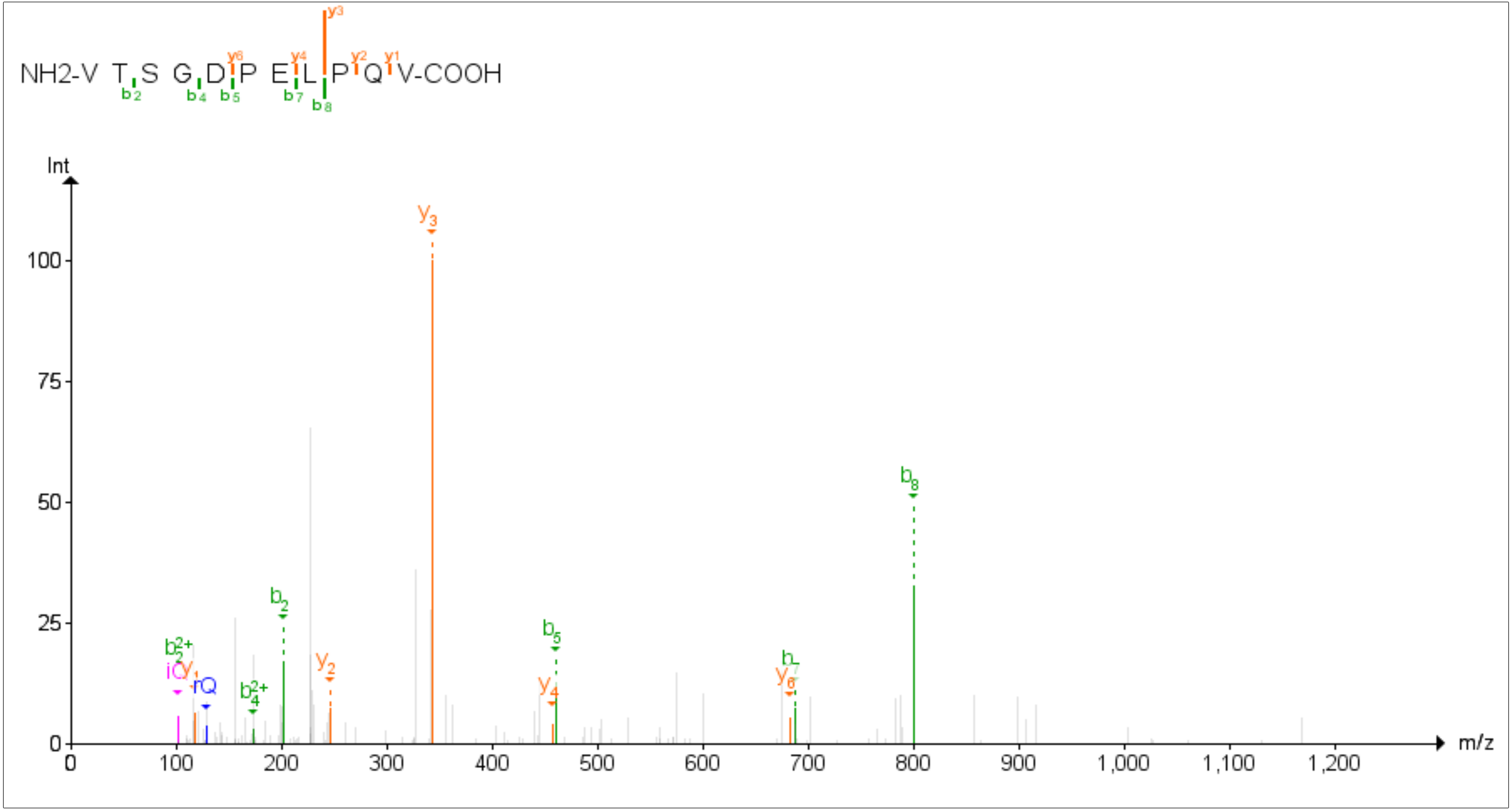

NH2-V T S G D P E L P Q V-COOH
b2
b4
b5
b7
b8
y6
y4
y3
y2
y1
Int
100
75
50
25
0
m/z
0
100
200
300
400
500
600
700
800
900
1,000
1,100
1,200
y3
b8
b2
b5
y2
y4
y6
b7
iQ
y1
rQ
b2 2+
b4 2+

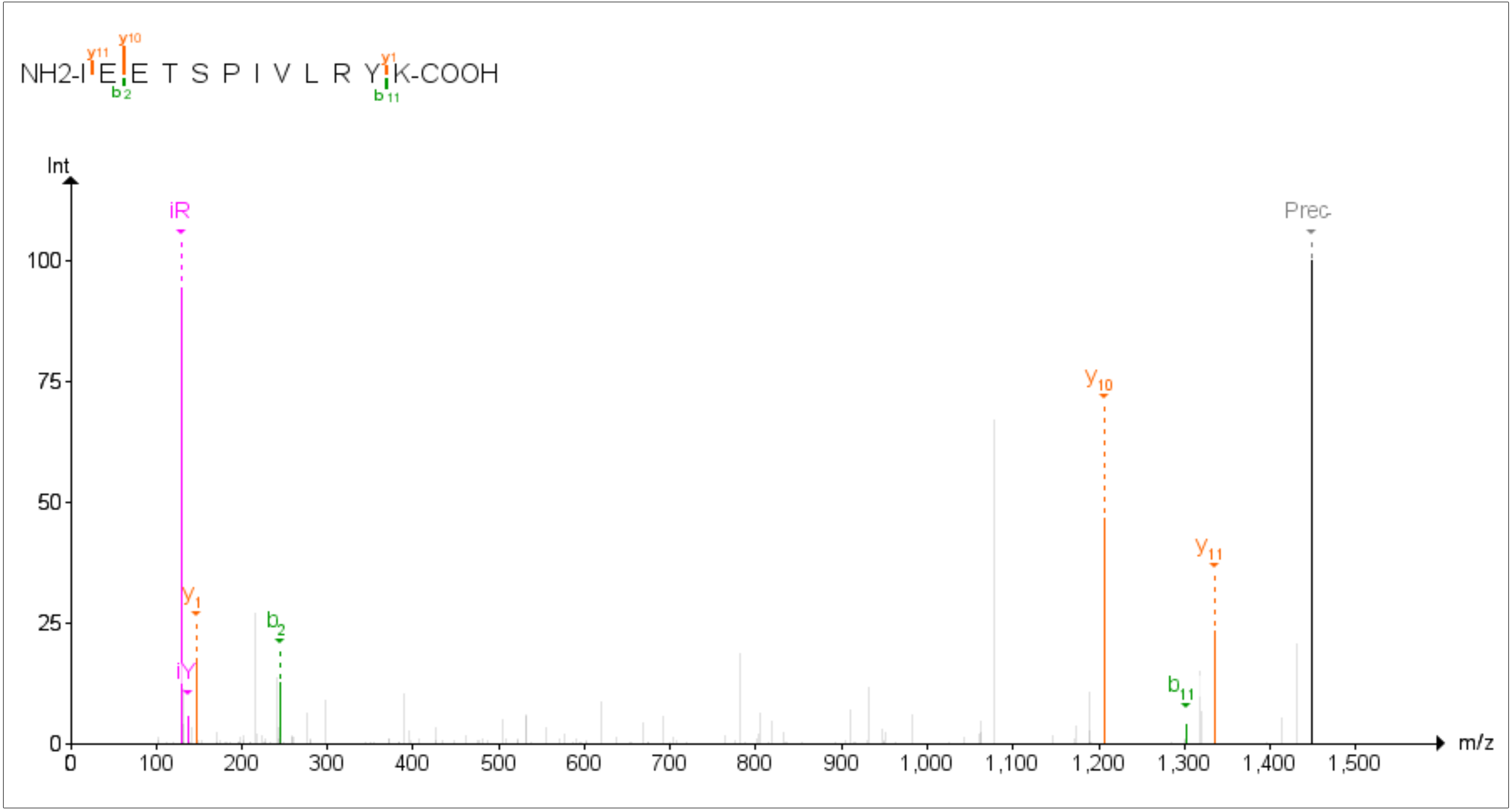

NH2-I E E T S P I V L R Y K-COOH
y11
y10
b2
y1
b11
Int
iR
Prec
100
75
50
25
0
y10
y11
y1
b2
iY
b11
0
100
200
300
400
500
600
700
800
900
1,000
1,100
1,200
1,300
1,400
1,500
m/z

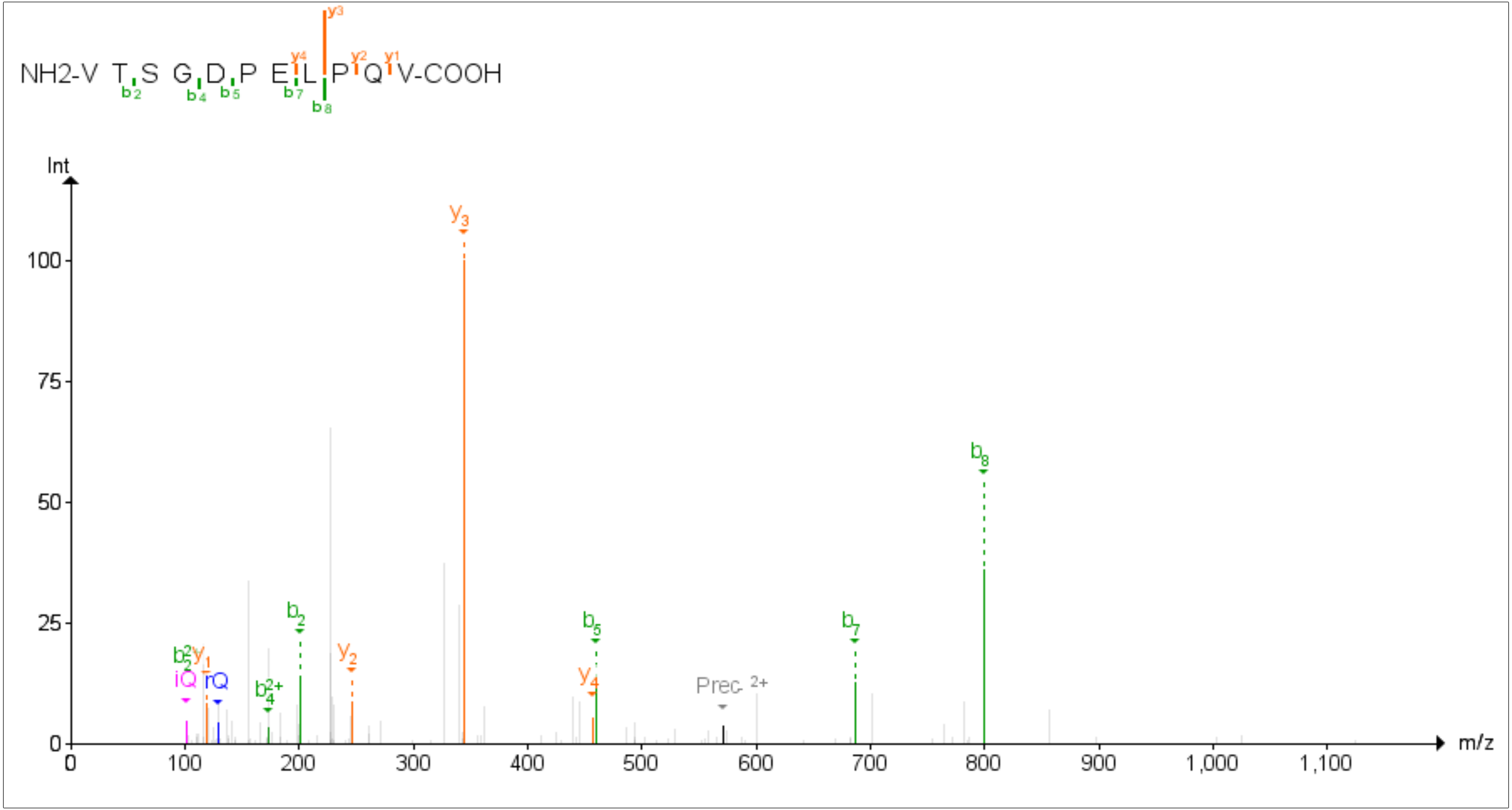

NH2-V T S G D P E I L P Q V-COOH
y3 y4 y2 y1
b2 b4 b5 b7 b8
Int
m/z
0
25
50
75
100
0
100
200
300
400
500
600
700
800
900
1,000
1,100
y3
b8
b2
b5
b7
y2
y4
y1
iQ
rQ
Prec 2+

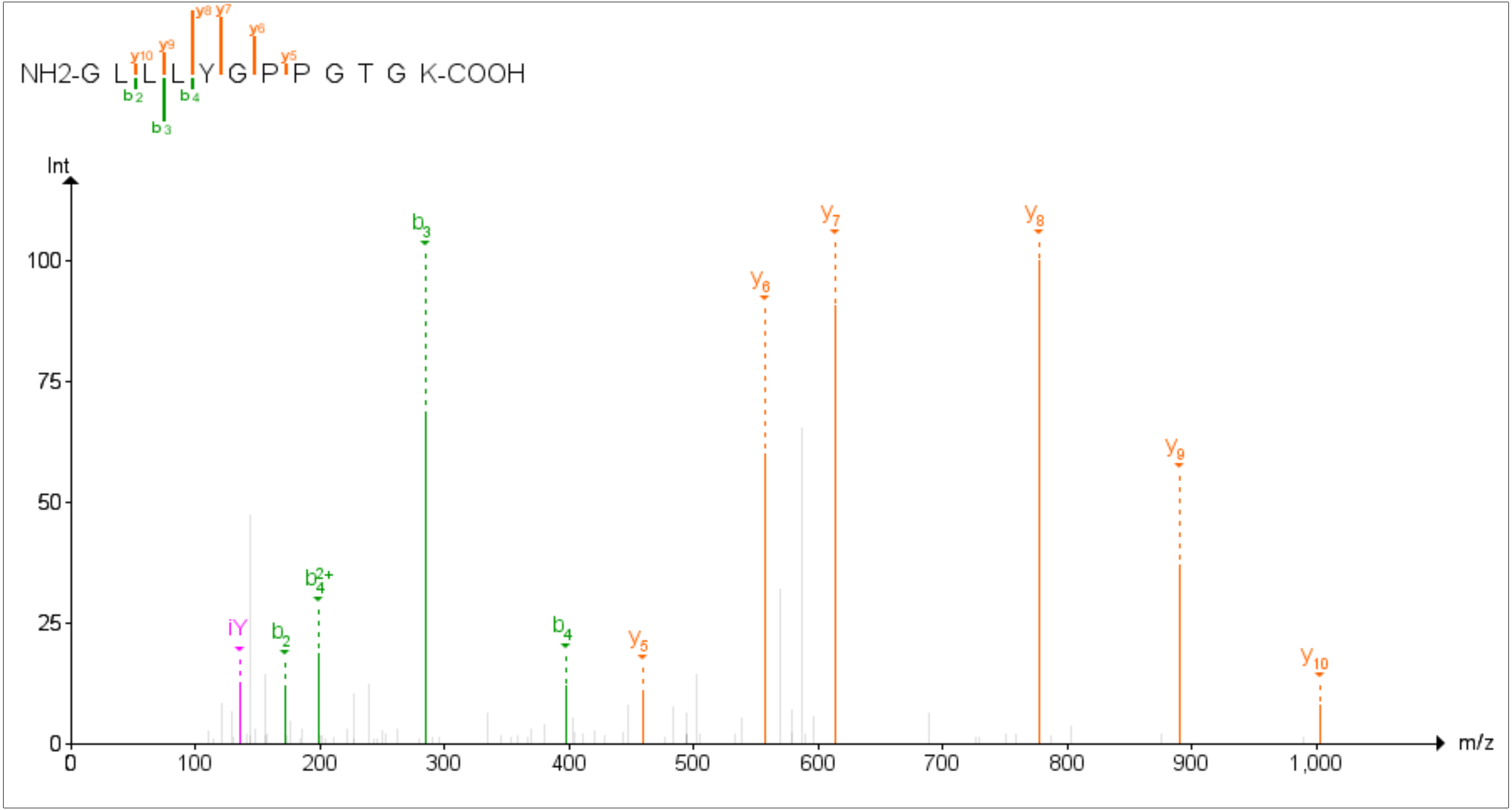

NH2-G L L L Y G P P G T G K-COOH
y10 y9 y8 y7 y6 y5
b2 b3 b4
Int
100
75
50
25
0
iY
b2
b4 2+
b3
b4
y5
y6
y7
y8
y9
y10
0 100 200 300 400 500 600 700 800 900 1,000
m/z

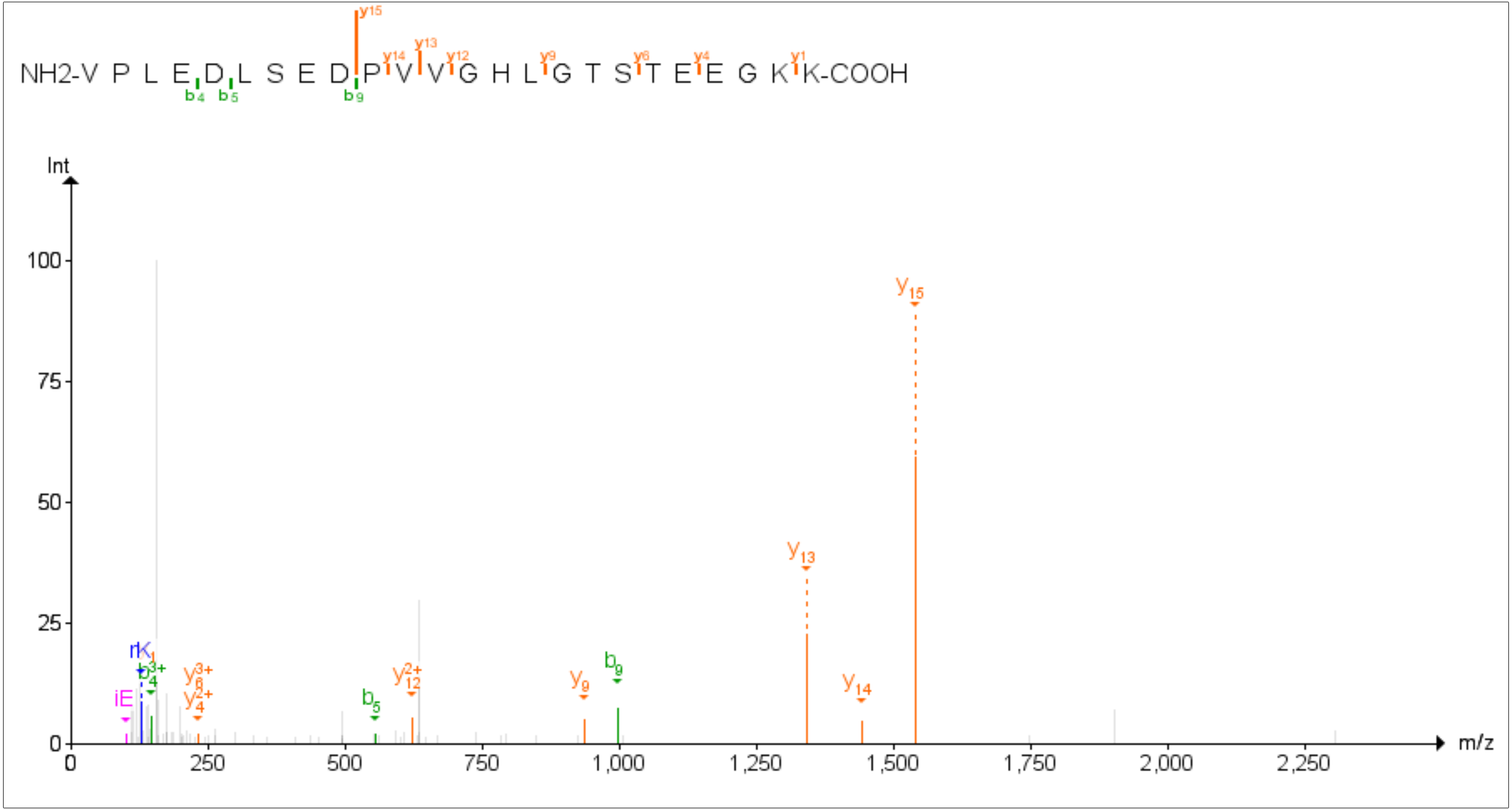

NH2-V P L E D L S E D P V V G H L G T S T E E G K K-COOH
b4 b5 b9
y15 y14 y13 y12 y9 y6 y4 y1
Int
100
75
50
25
0
m/z
0
250
500
750
1,000
1,250
1,500
1,750
2,000
2,250
y15
y13
y14
b9
y9
y12 2+
b5
y6 3+
y4 2+
b4 3+
rK1
iE

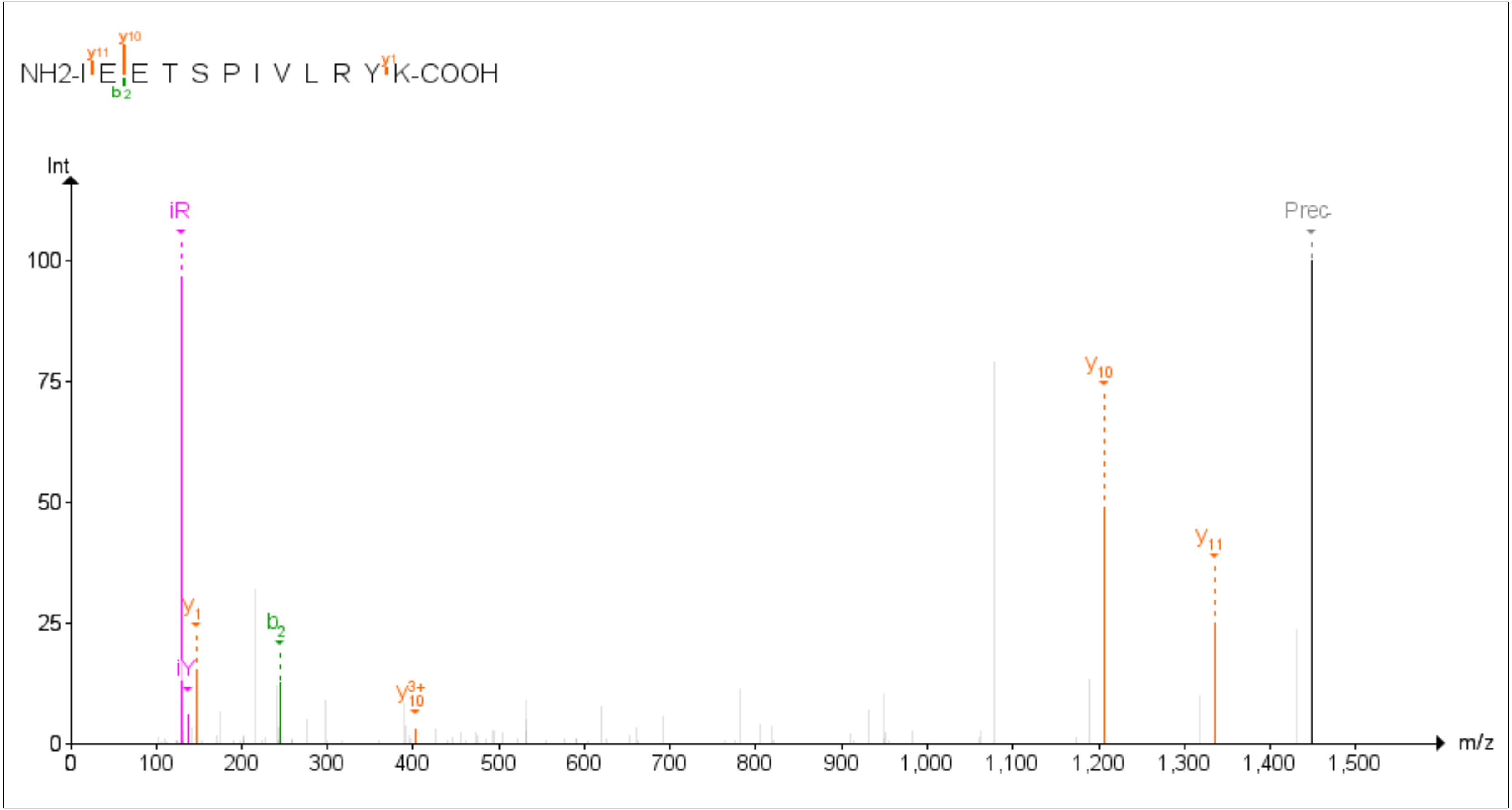

NH2-I E E T S P I V L R Y K-COOH
y11
y10
b2
y1
Int
iR
Prec
100
75
50
25
0
y10
y11
y1
iY
b2
y10 3+
0
100
200
300
400
500
600
700
800
900
1,000
1,100
1,200
1,300
1,400
1,500
m/z

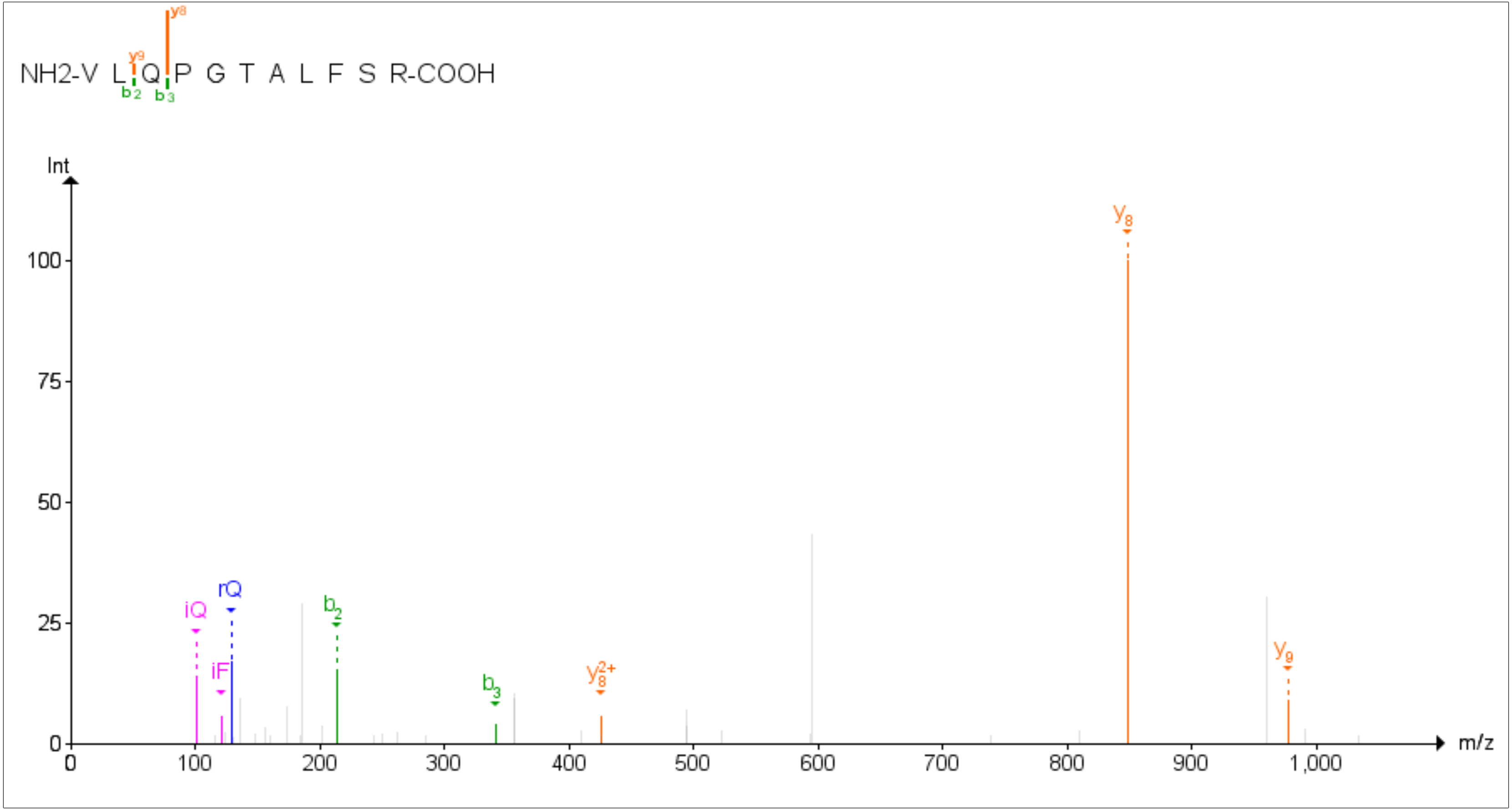

NH2-V L Q P G T A L F S R-COOH
y8
y9
b2
b3
Int
100
75
50
25
0
m/z
0
100
200
300
400
500
600
700
800
900
1,000
y8
y9
y8 2+
b2
b3
rQ
iQ
iF

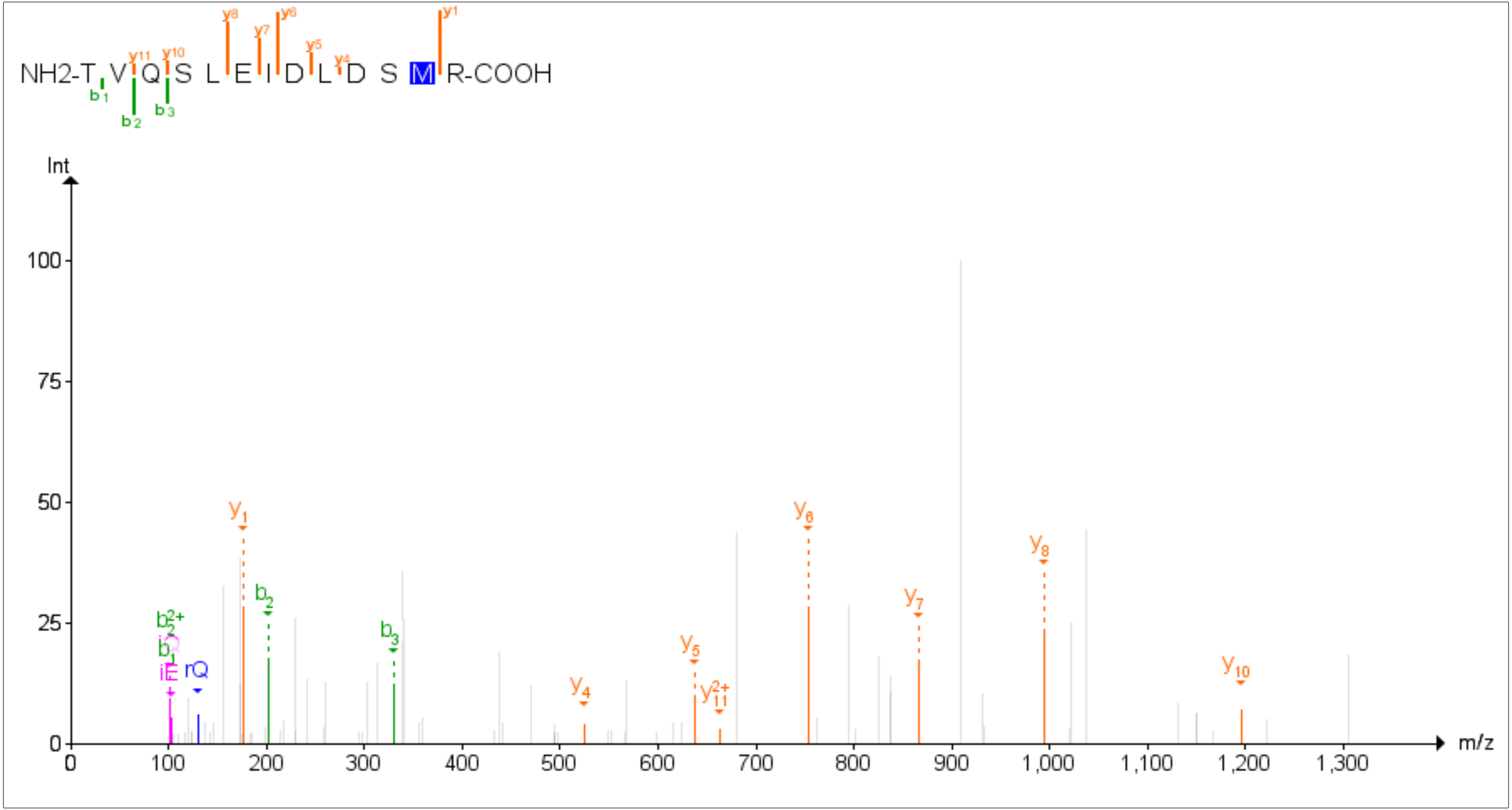

NH2-T V Q S L E I D L D S M R-COOH
y11 y10 y8 y7 y6 y5 y4 y1
b1 b2 b3
Int
100
75
50
25
0
y1
b2
b2 2+
iQ
b1
iE
rQ
b3
y4
y5
y11 2+
y6
y7
y8
y10
m/z
0 100 200 300 400 500 600 700 800 900 1,000 1,100 1,200 1,300

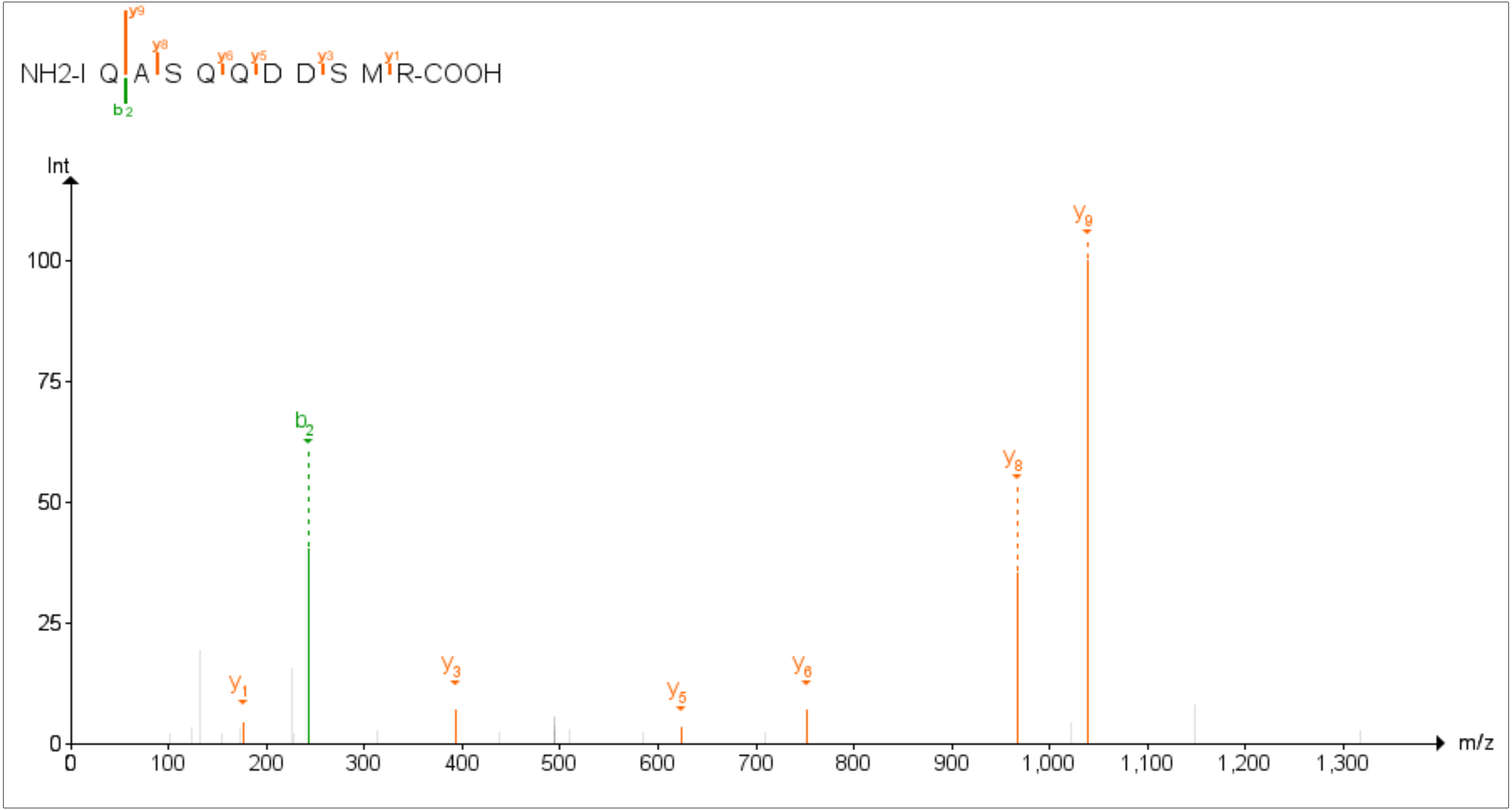
NH2-I Q A S Q Q D D S M R-COOH
y9
y8
y6
y5
y3
y1
b2
Int
100
75
50
25
0
y9
b2
y8
y1
y3
y5
y6
0
100
200
300
400
500
600
700
800
900
1,000
1,100
1,200
1,300
m/z

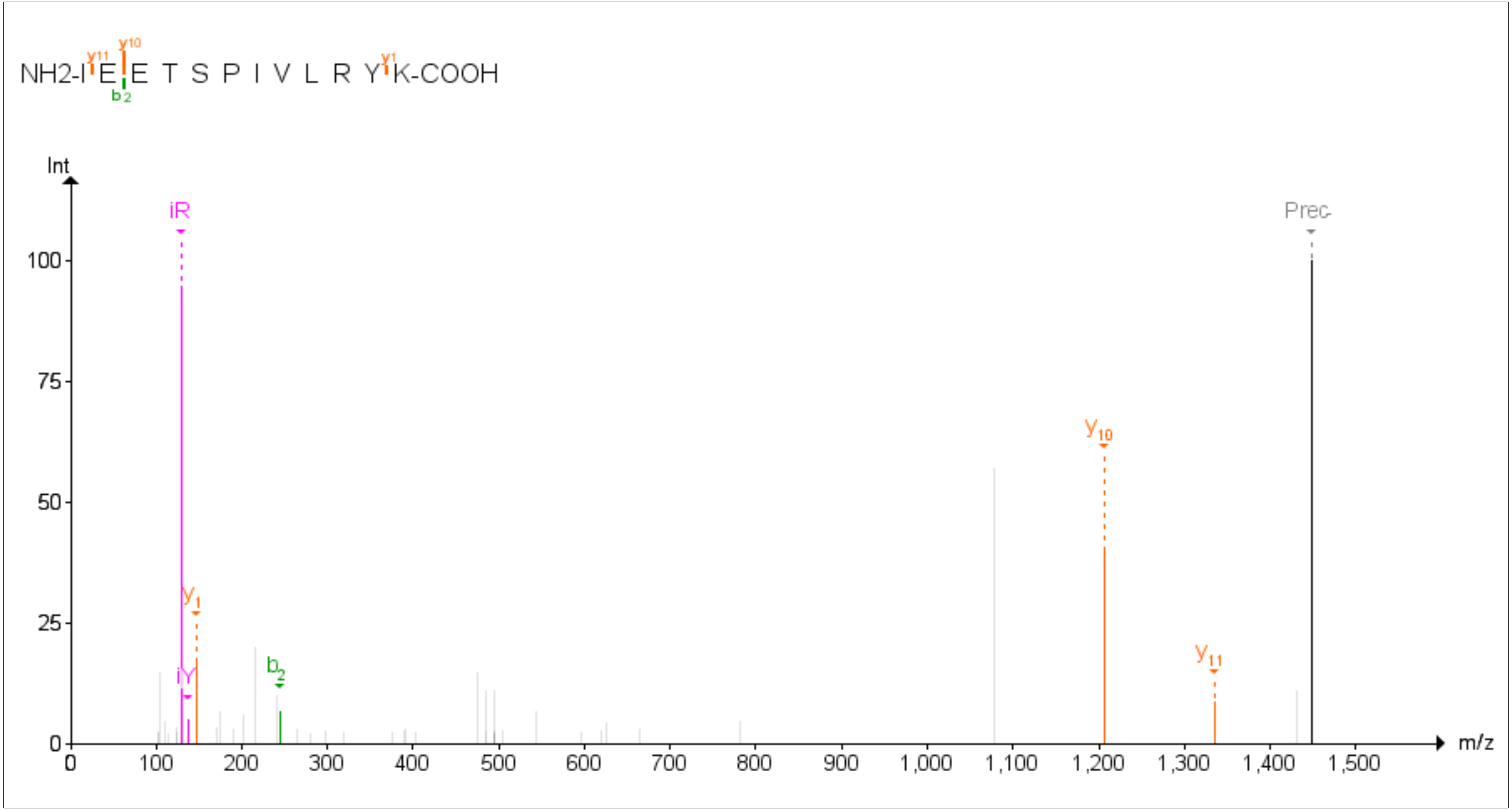

NH2-I E E T S P I V L R Y K-COOH
y11
y10
b2
y1
Int
m/z
iR
iY
y1
b2
y10
y11
Prec
0
25
50
75
100
0
100
200
300
400
500
600
700
800
900
1,000
1,100
1,200
1,300
1,400
1,500

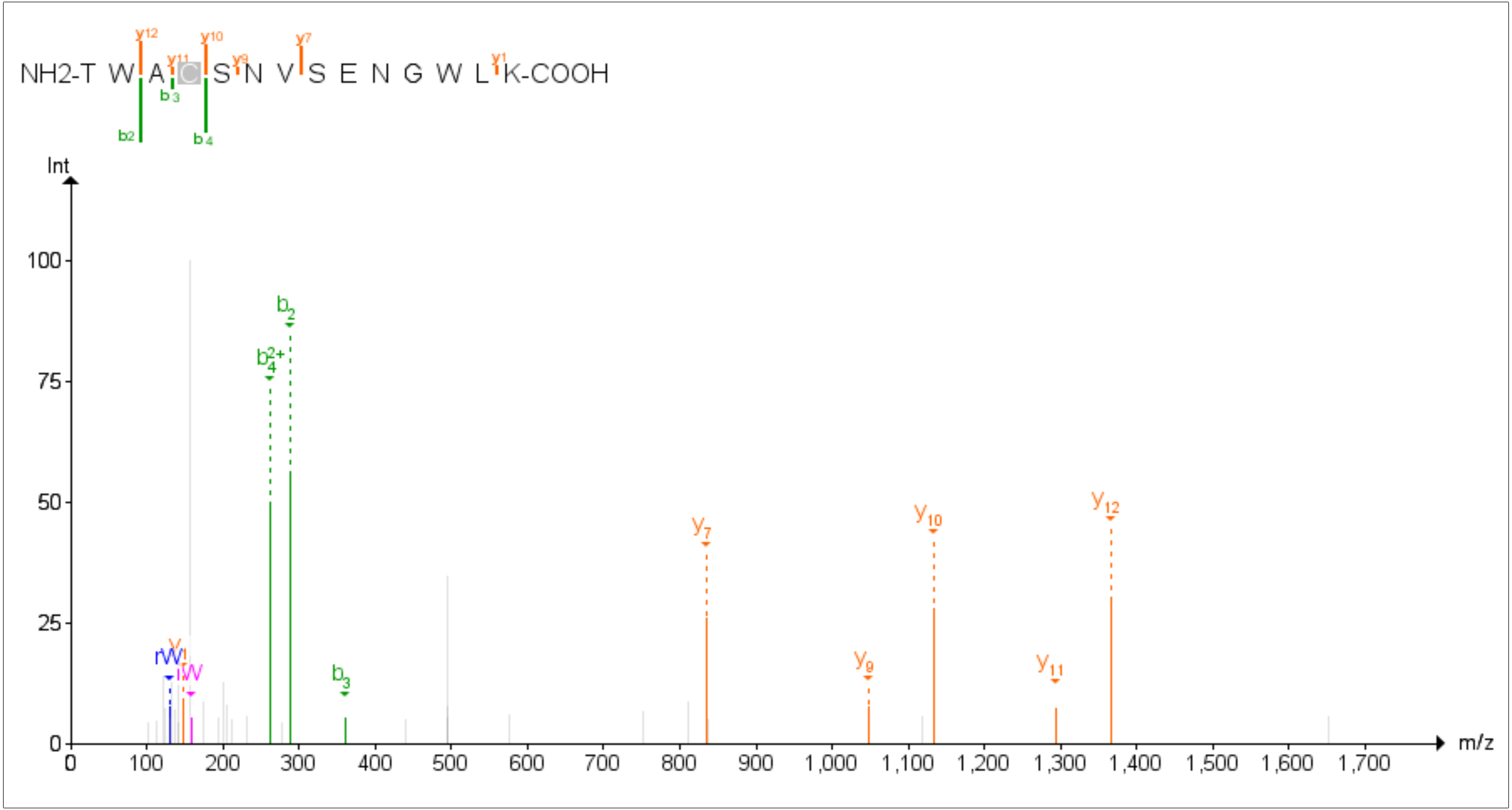
NH2-T W A C S N V S E N G W L K-COOH
y12
y11
y10
y9
y7
y1
b2
b3
b4
Int
100
75
50
25
0
b2
b4 2+
y7
y10
y12
y9
y11
b3
rW
y1
iW
m/z
0
100
200
300
400
500
600
700
800
900
1,000
1,100
1,200
1,300
1,400
1,500
1,600
1,700

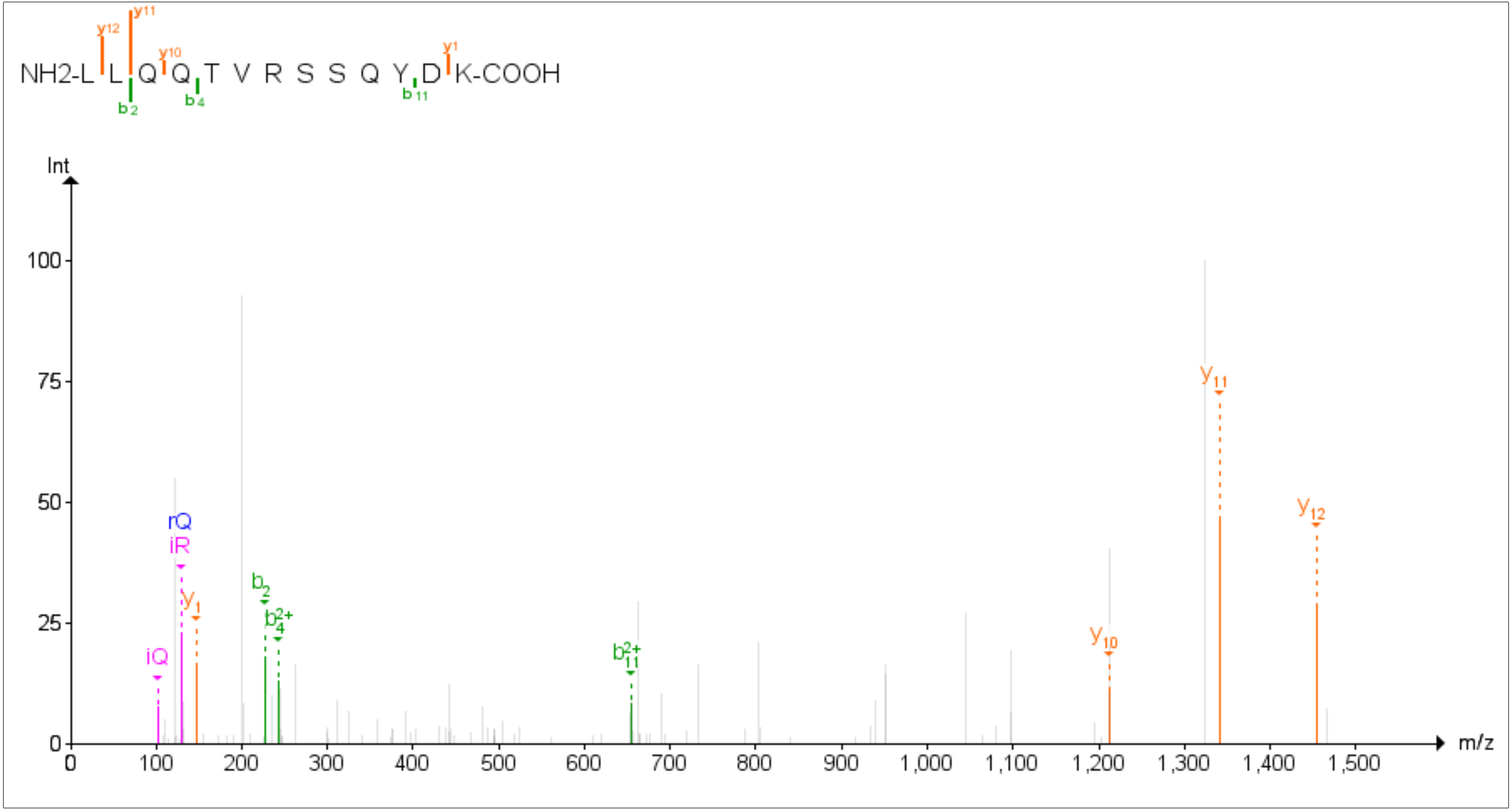

NH2-L L Q Q T V R S S Q Y D K-COOH
y12 y11 y10 y1
b2 b4 b11
Int
100
75
50
25
0
rQ
iR
iQ
y1
b2
b4 2+
b11 2+
y10
y11
y12
0 100 200 300 400 500 600 700 800 900 1,000 1,100 1,200 1,300 1,400 1,500
m/z

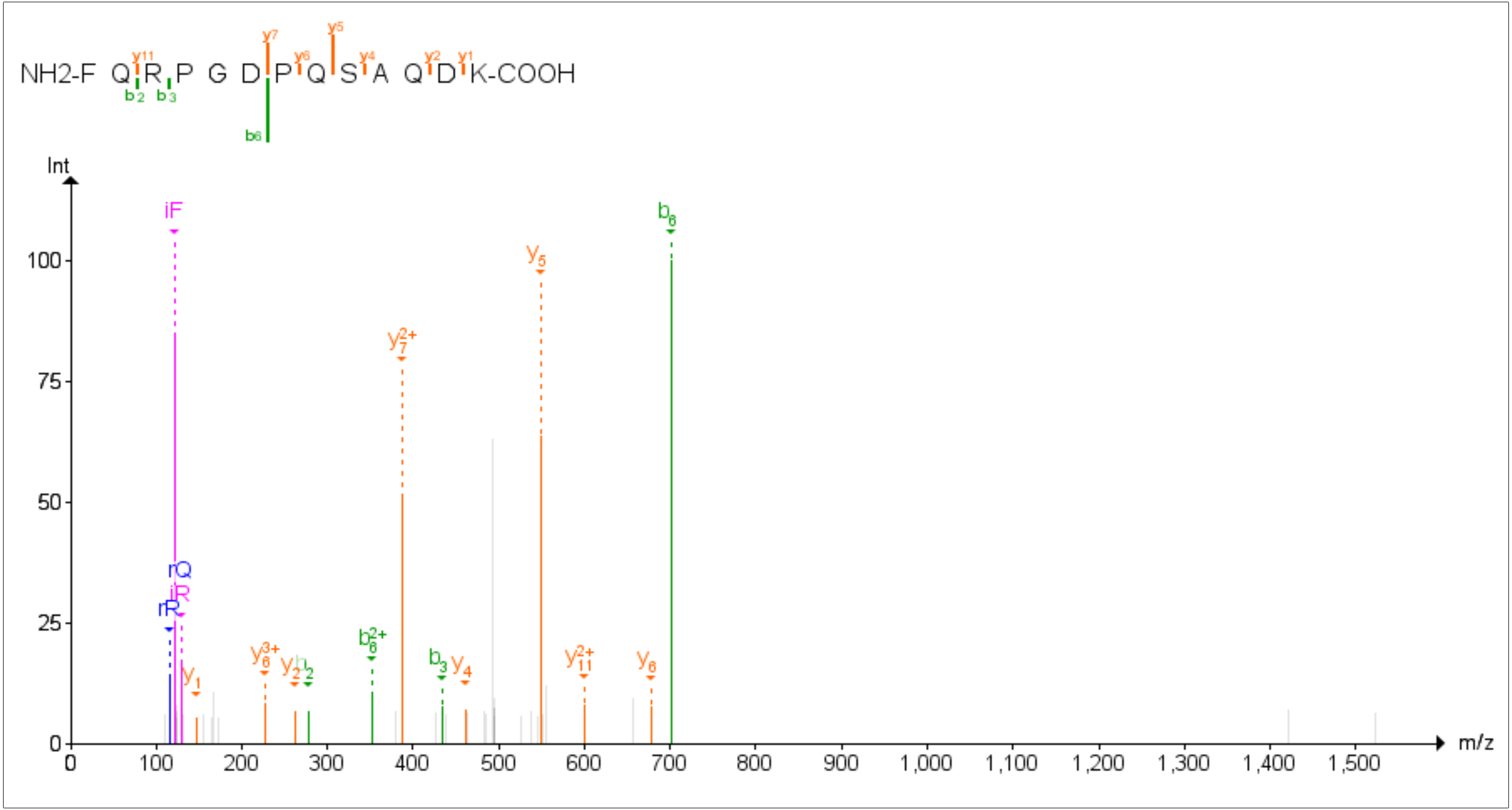

NH2-F Q R P G D P Q S A Q D K-COOH
Int
m/z
iF
rQ
iR
rR
b_6
y_5
y_7^{2+}
y_1
y_6^{3+}
y_2
b_2
b_6^{2+}
b_3
y_4
y_{11}^{2+}
y_6

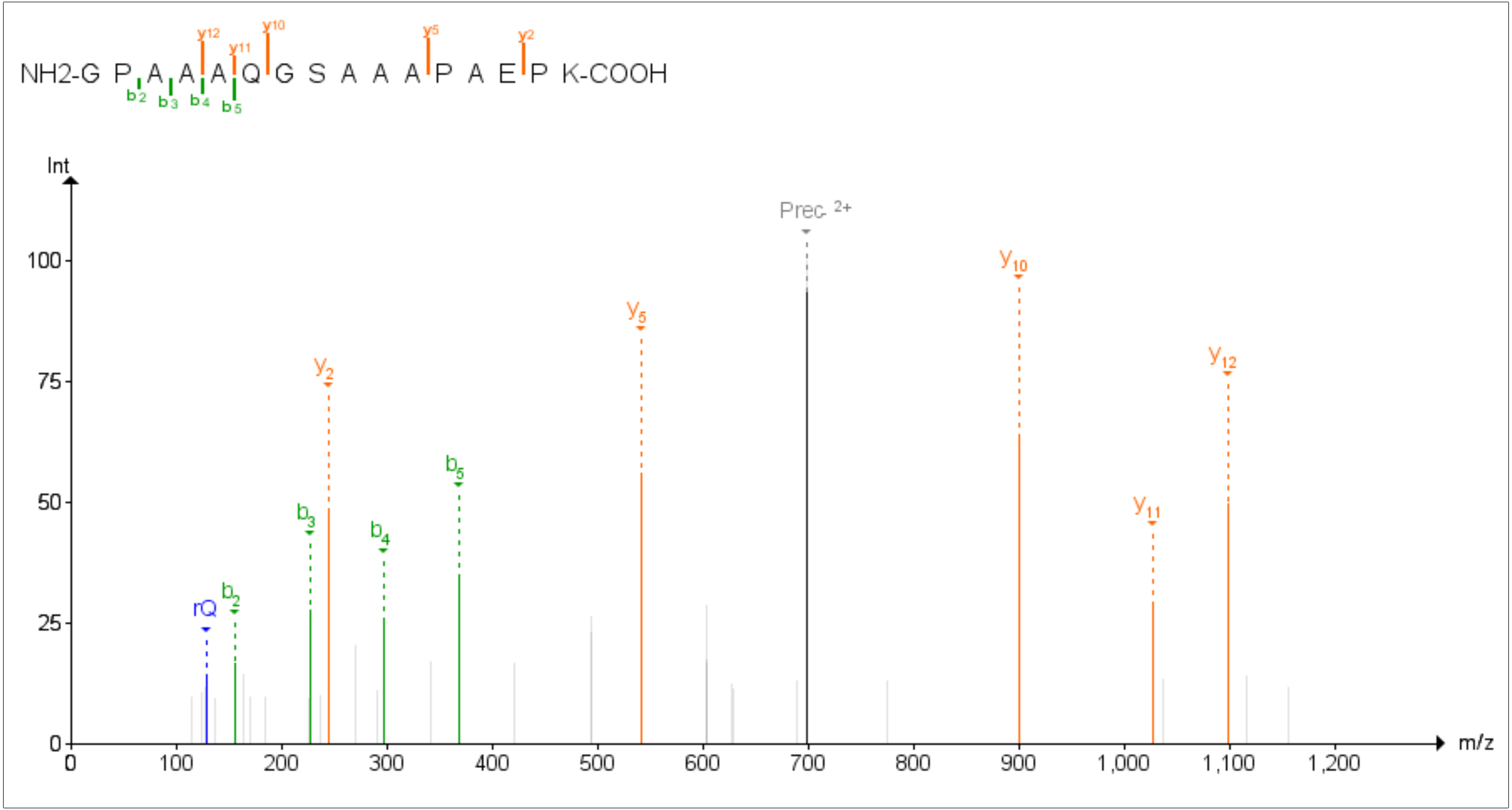

NH2-G P A A A Q G S A A A P A E P K-COOH
y12 y11 y10 y5 y2
b2 b3 b4 b5
Int
m/z
Prec. 2+
y10
y5
y2
y12
y11
b5
b3
b4
b2
rQ
0
25
50
75
100
0
100
200
300
400
500
600
700
800
900
1,000
1,100
1,200

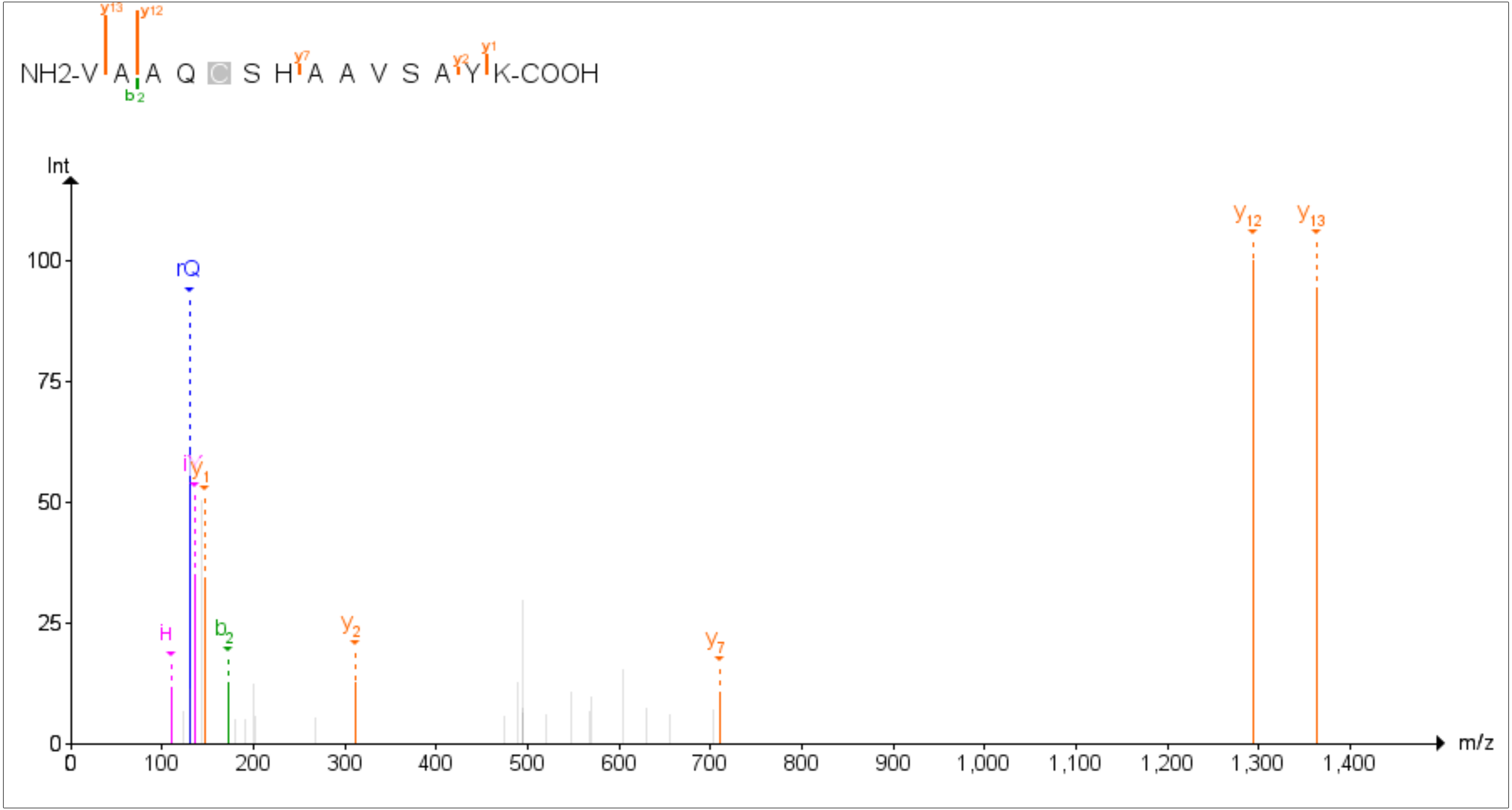

NH2-V A A Q C S H A A V S A Y K-COOH
y13
y12
y7
y2
y1
b2
Int
100
75
50
25
0
rQ
iY
y1
iH
b2
y2
y7
y12
y13
0
100
200
300
400
500
600
700
800
900
1,000
1,100
1,200
1,300
1,400
m/z

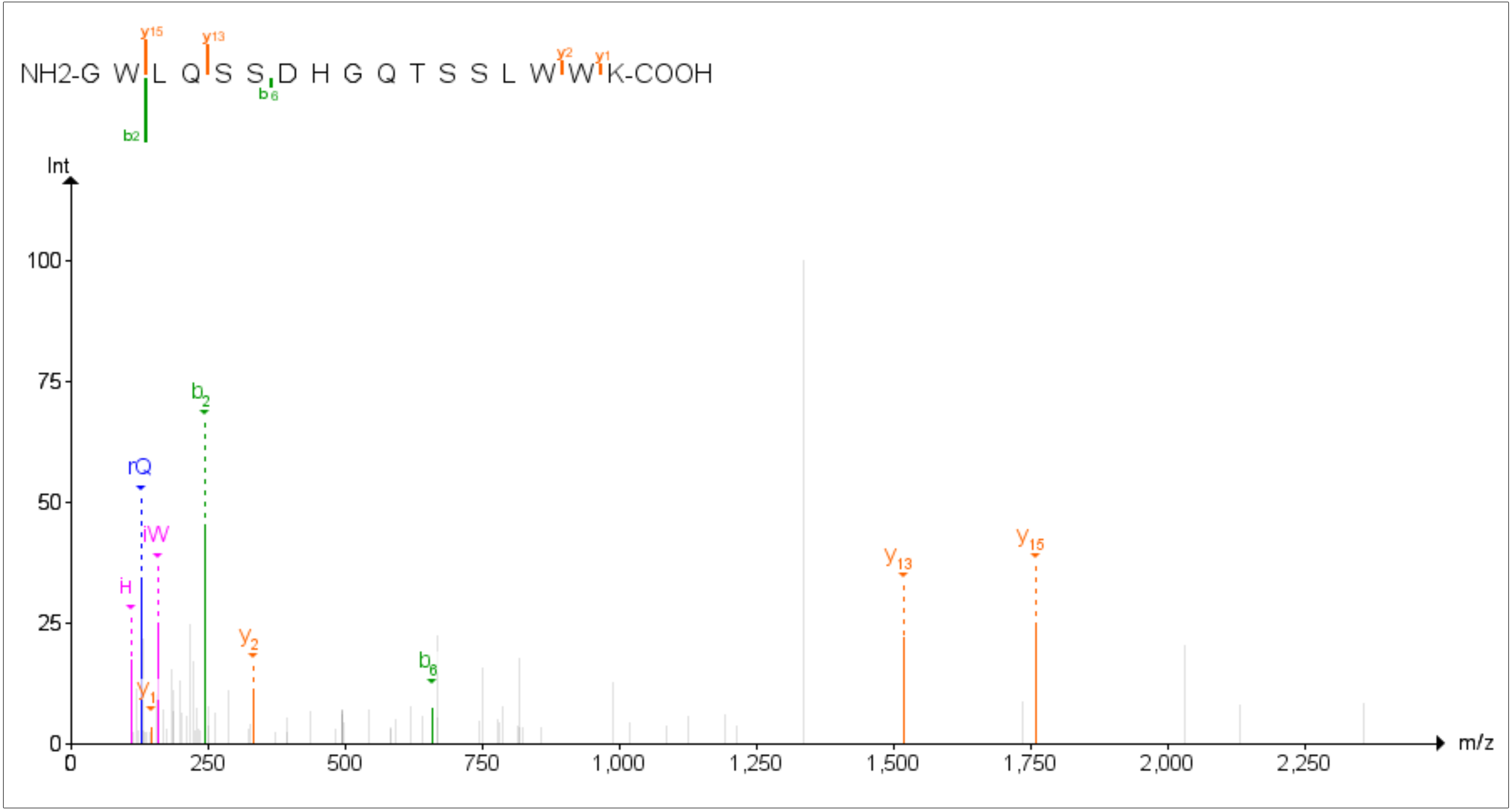

NH2-G W L Q S S D H G Q T S S L W W K-COOH
y15
y13
y2
y1
b2
b6
Int
100
75
50
25
0
rQ
iW
iH
b2
y2
y1
b6
y13
y15
0
250
500
750
1,000
1,250
1,500
1,750
2,000
2,250
m/z

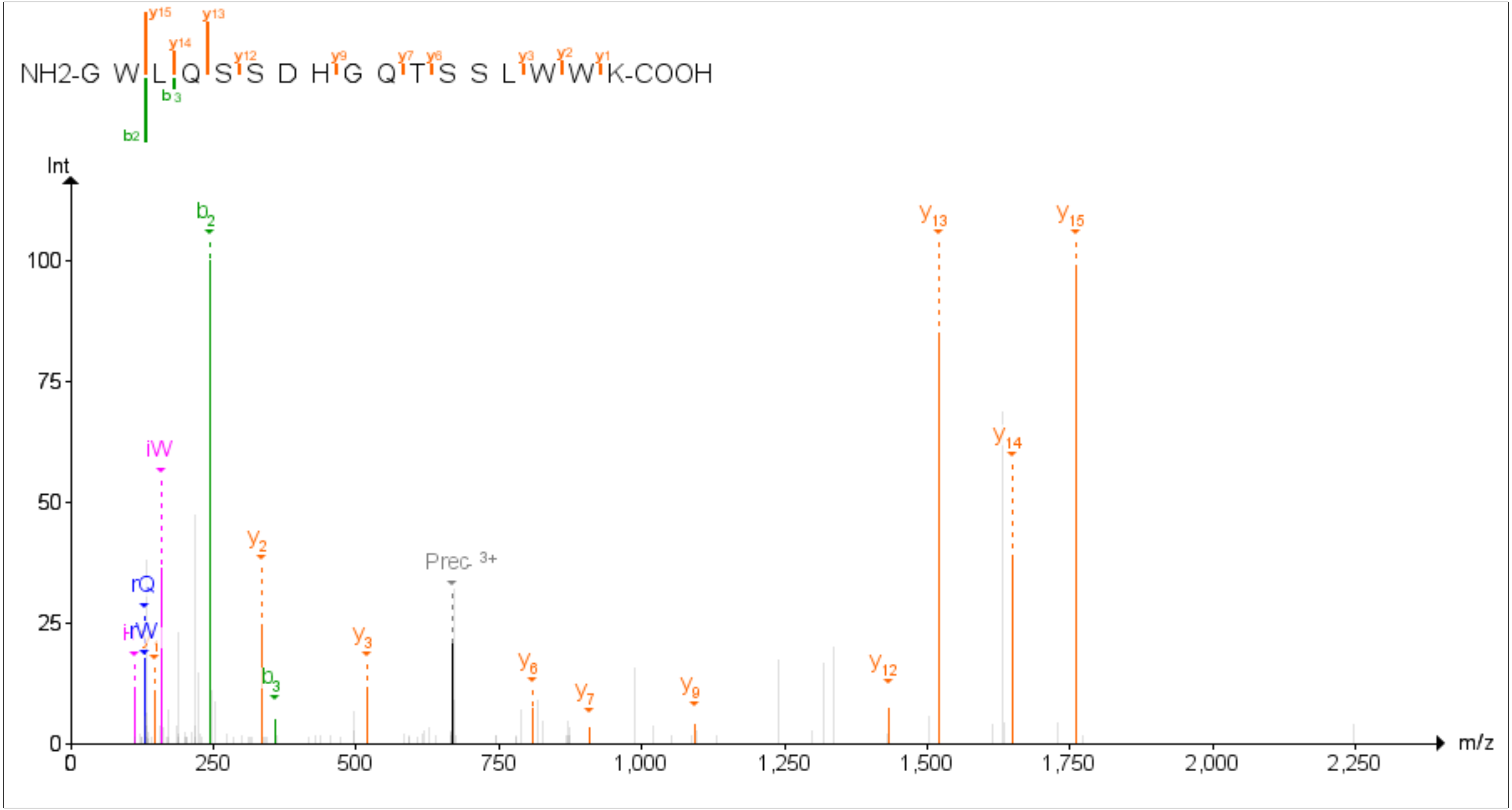

NH2-G W L Q S S D H G Q T S S L W W K-COOH
y15 y14 y13 y12 y9 y7 y6 y3 y2 y1
b2 b3
Int
100
75
50
25
0
b2
y13
y15
y14
iW
y2
Prec 3+
rQ
i-rW
y3
b3
y6
y7
y9
y12
0 250 500 750 1,000 1,250 1,500 1,750 2,000 2,250
m/z

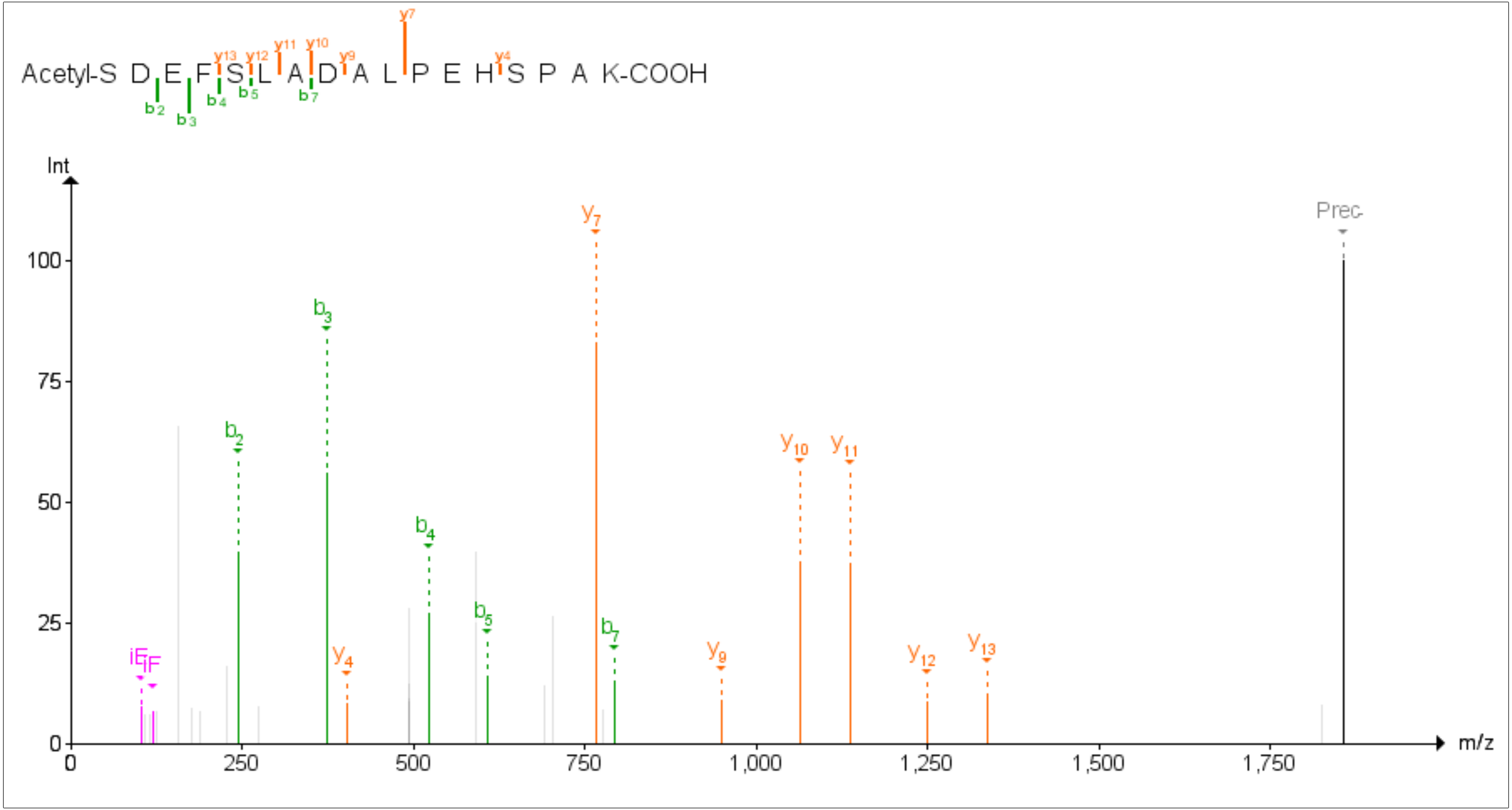

Acetyl-S D E F S L A D A L P E H S P A K-COOH
y7
y13 y12 y11 y10 y9 y4
b2 b3 b4 b5 b7
Int
100
75
50
25
0
m/z
0
250
500
750
1,000
1,250
1,500
1,750
Prec
y7
b3
b2
y10
y11
b4
b5
b7
iE
iF
y4
y9
y12
y13

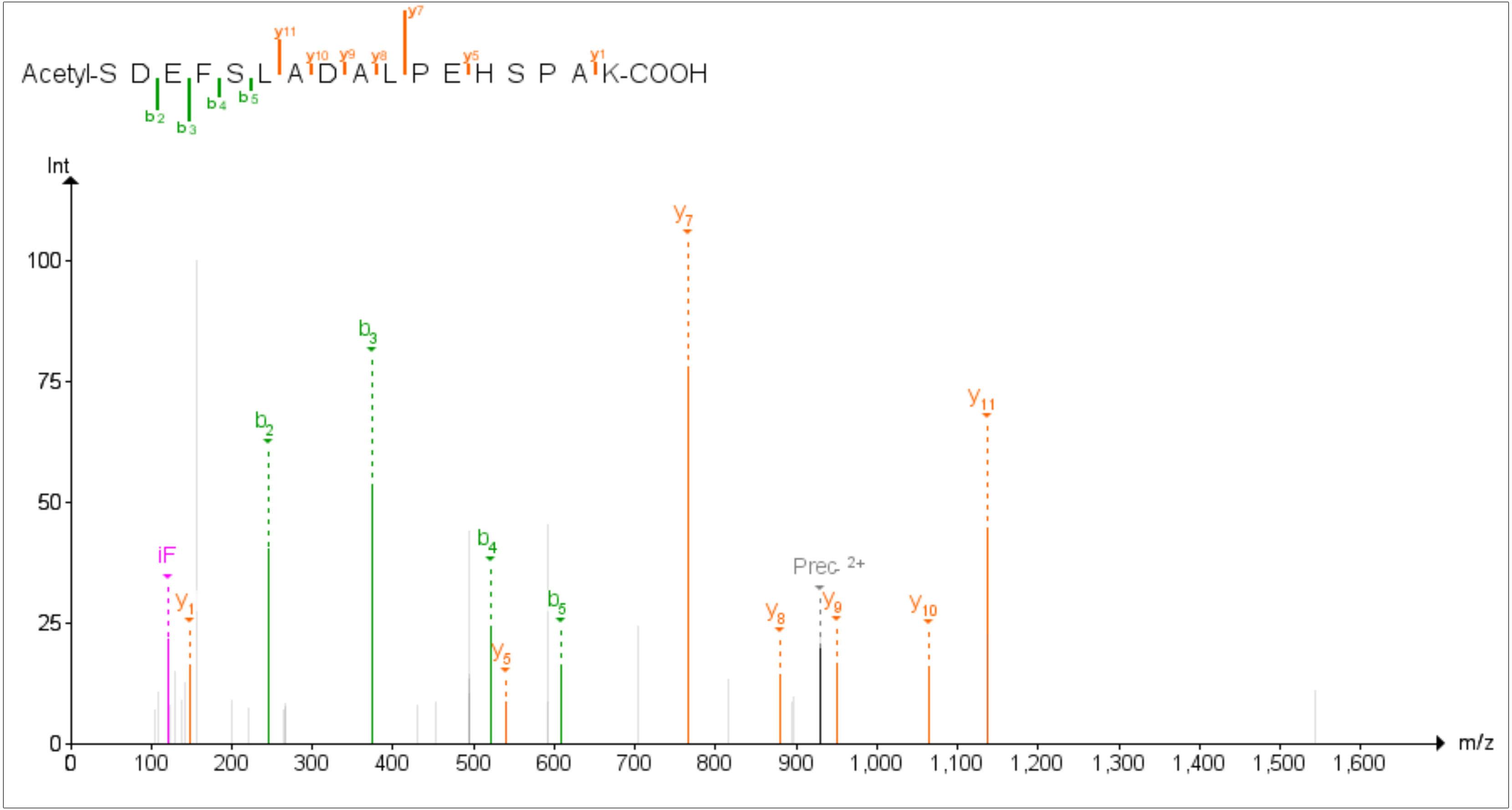

Acetyl-S D E F S L A D A L P E H S P A K-COOH
y11 y10 y9 y8 y7 y5 y1
b2 b3 b4 b5
Int
100
75
50
25
0
iF
y1
b2
b3
b4
b5
y5
y7
y8
Prec. 2+
y9
y10
y11
0 100 200 300 400 500 600 700 800 900 1,000 1,100 1,200 1,300 1,400 1,500 1,600
m/z

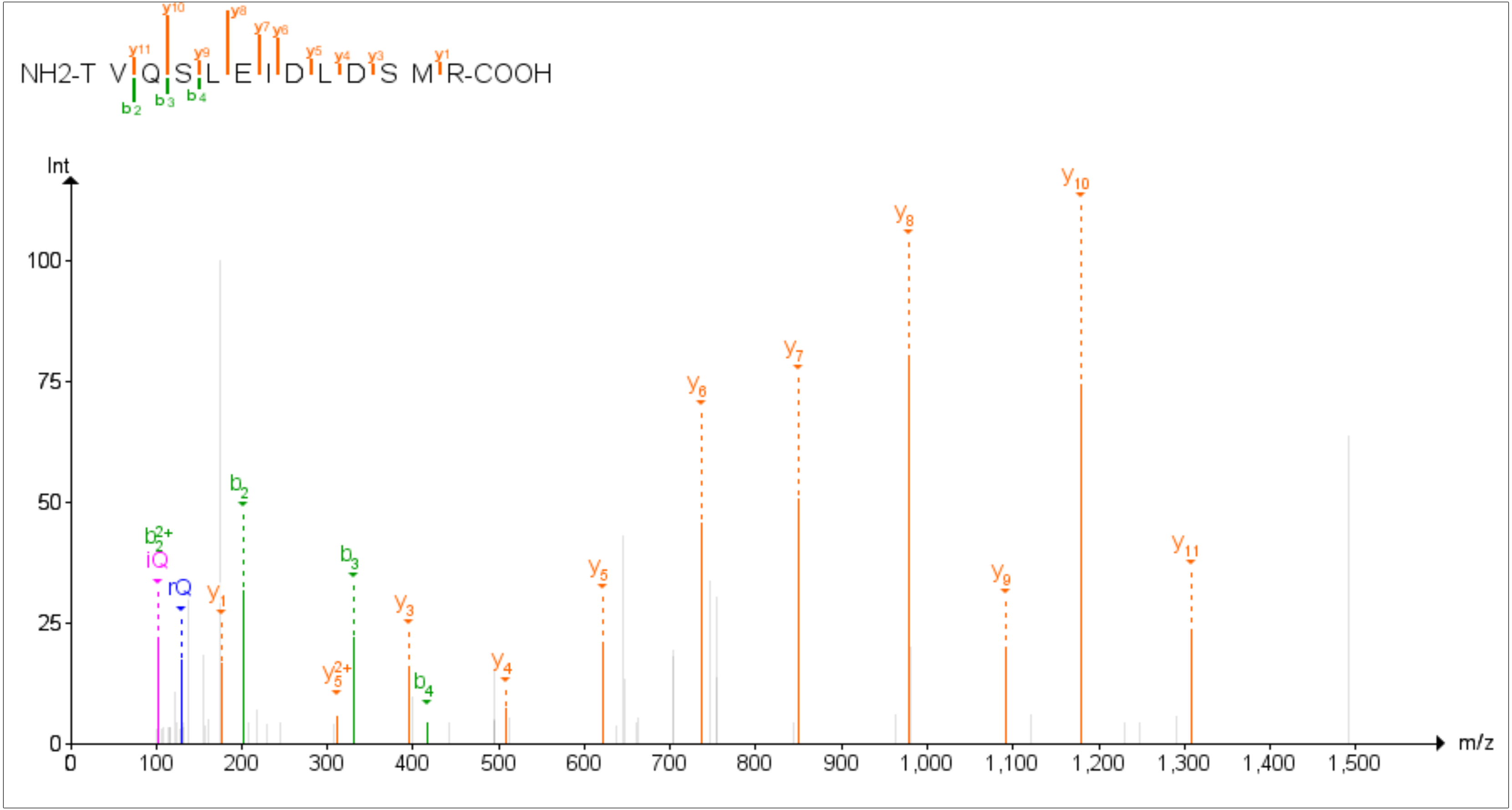

NH2-T V Q S L E I D L D S M R-COOH
y11 y10 y9 y8 y7 y6 y5 y4 y3 y1
b2 b3 b4
Int
100
75
50
25
0
m/z
0
100
200
300
400
500
600
700
800
900
1,000
1,100
1,200
1,300
1,400
1,500
b$_2^{2+}$
iQ
rQ
y$_1$
b$_2$
y$_5^{2+}$
b$_3$
y$_3$
b$_4$
y$_4$
y$_5$
y$_6$
y$_7$
y$_8$
y$_9$
y$_{10}$
y$_{11}$

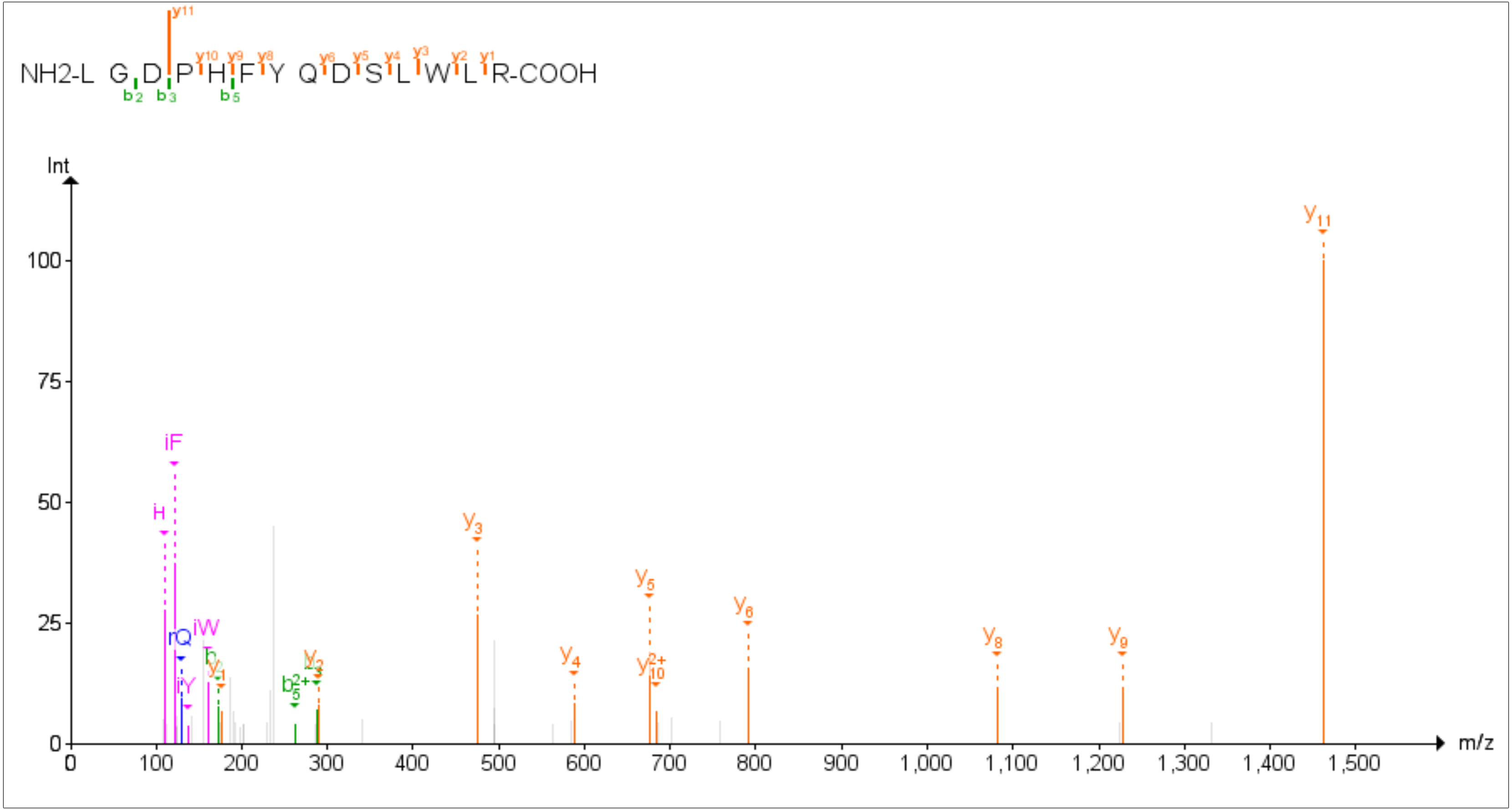

NH2-L G D P H F Y Q D S L W L R-COOH
Int
m/z
y11
y3
y5
y6
y4
y8
y9
iF
iH
iW
rQ
iY

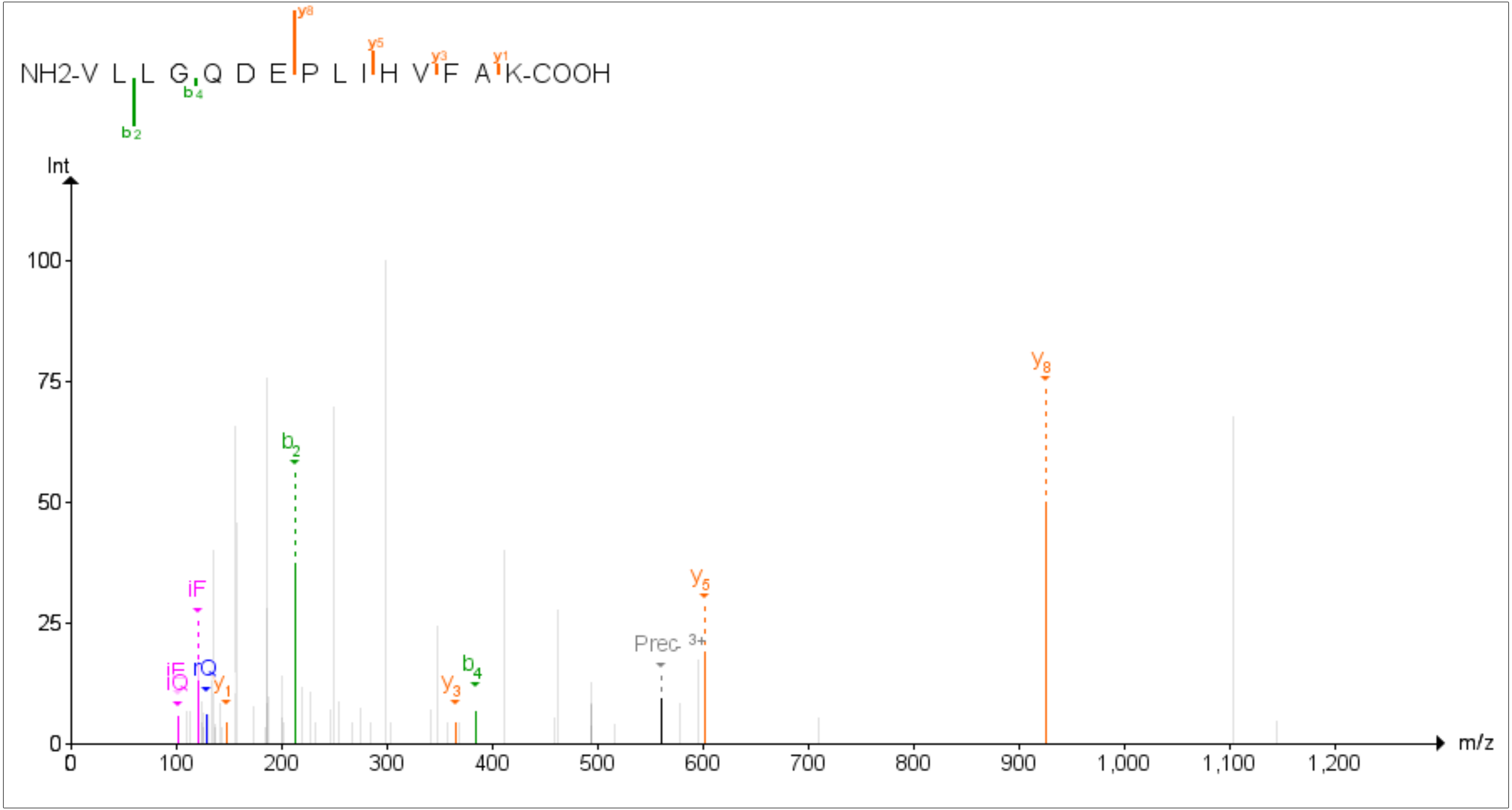

NH2-V L L G Q D E P L I H V F A K-COOH
b2
b4
y8
y5
y3
y1
Int
100
75
50
25
0
b2
y8
iF
iF
iQ
rQ
y1
y3
b4
Prec- 3+
y5
0
100
200
300
400
500
600
700
800
900
1,000
1,100
1,200
m/z

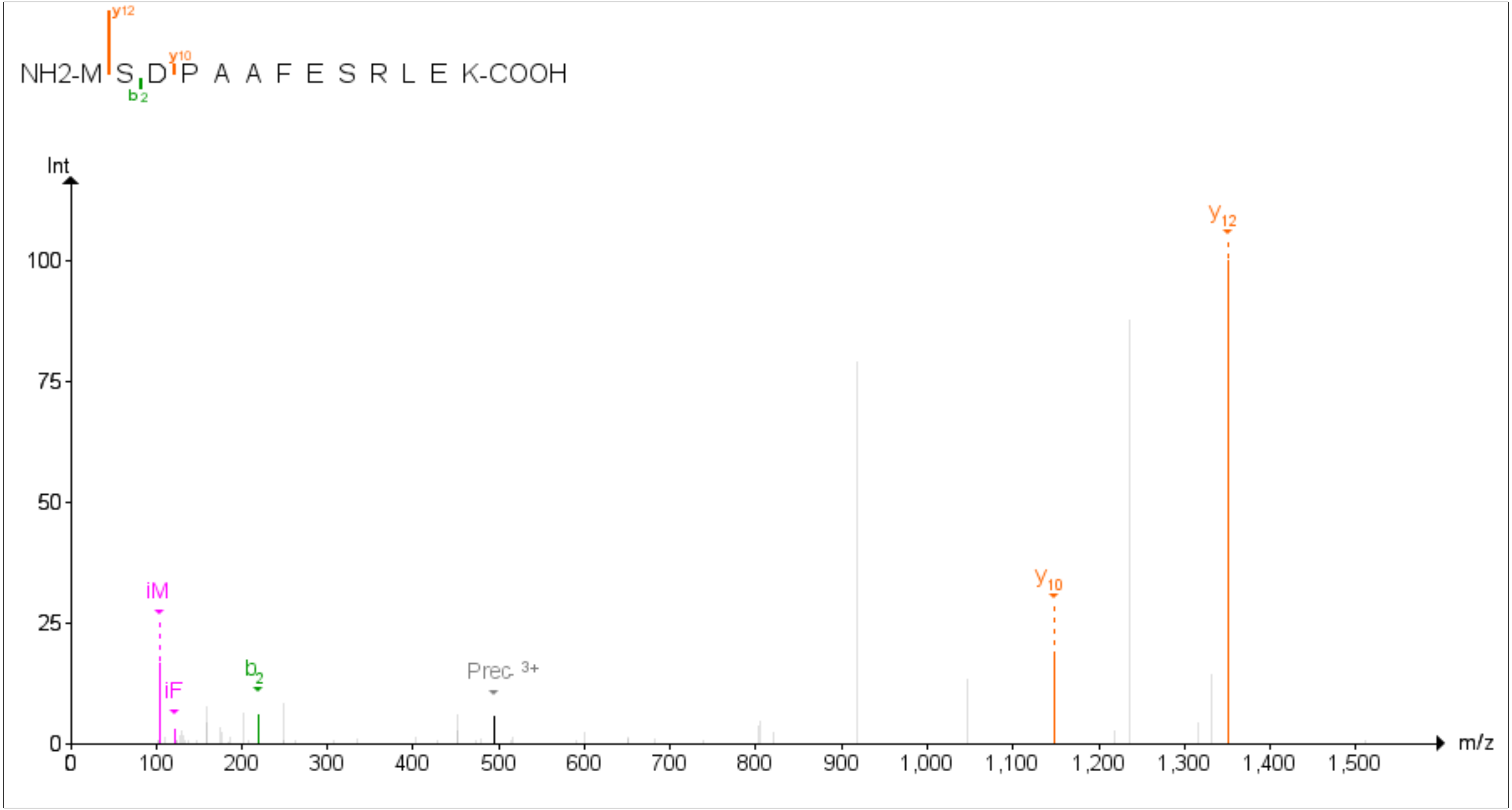

y12
y10
NH2-M S D P A A F E S R L E K-COOH
b2
Int
100
75
50
25
0
y12
y10
iM
iF
b2
Prec 3+
0
100
200
300
400
500
600
700
800
900
1,000
1,100
1,200
1,300
1,400
1,500
m/z

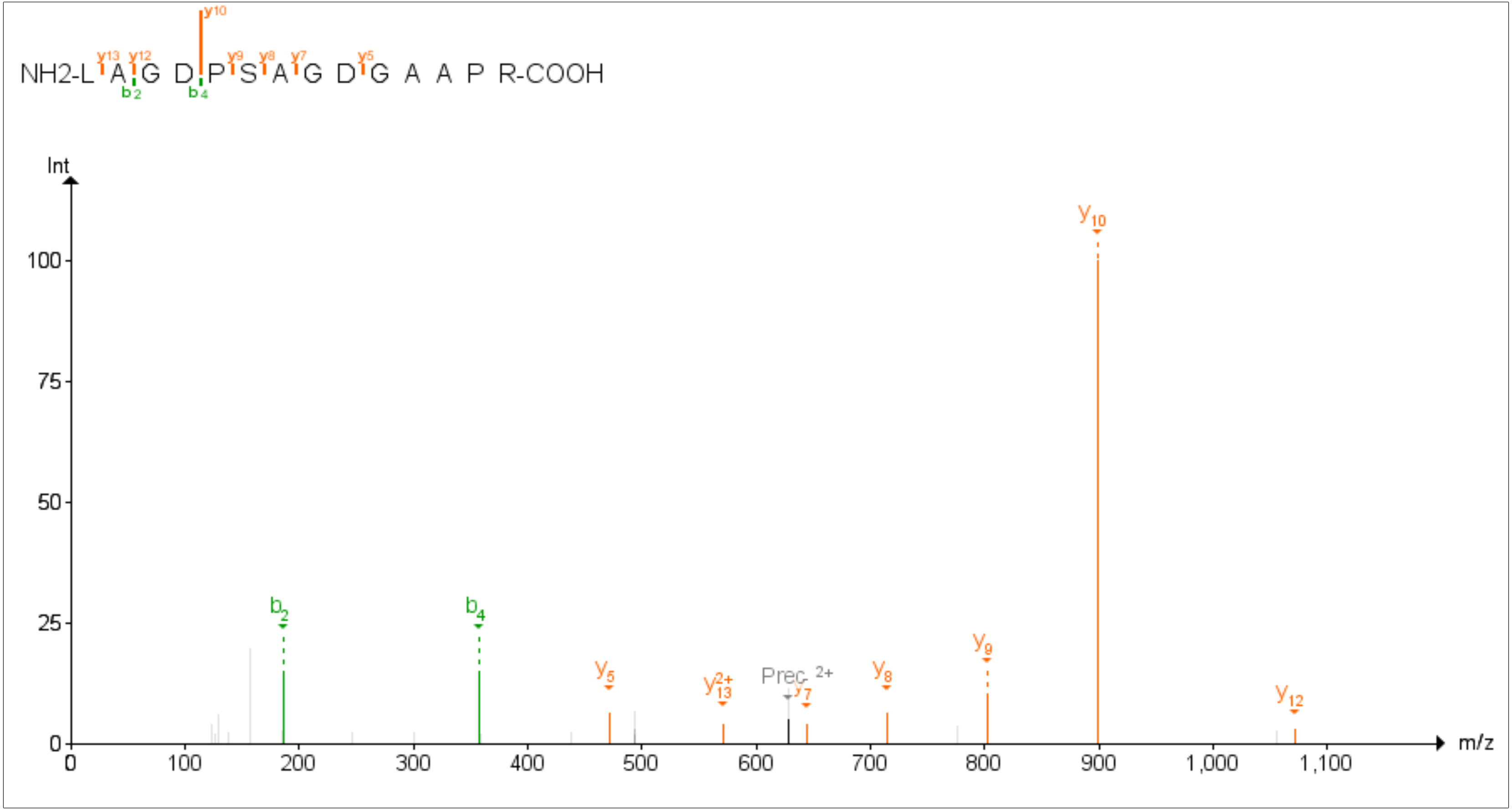

NH2-L A G D P S A G D G A A P R-COOH
y13 y12 y10 y9 y8 y7 y5
b2 b4
Int
m/z
0
25
50
75
100
0 100 200 300 400 500 600 700 800 900 1,000 1,100
b2
b4
y5
y2+13
Prec. 2+
y7
y8
y9
y10
y12

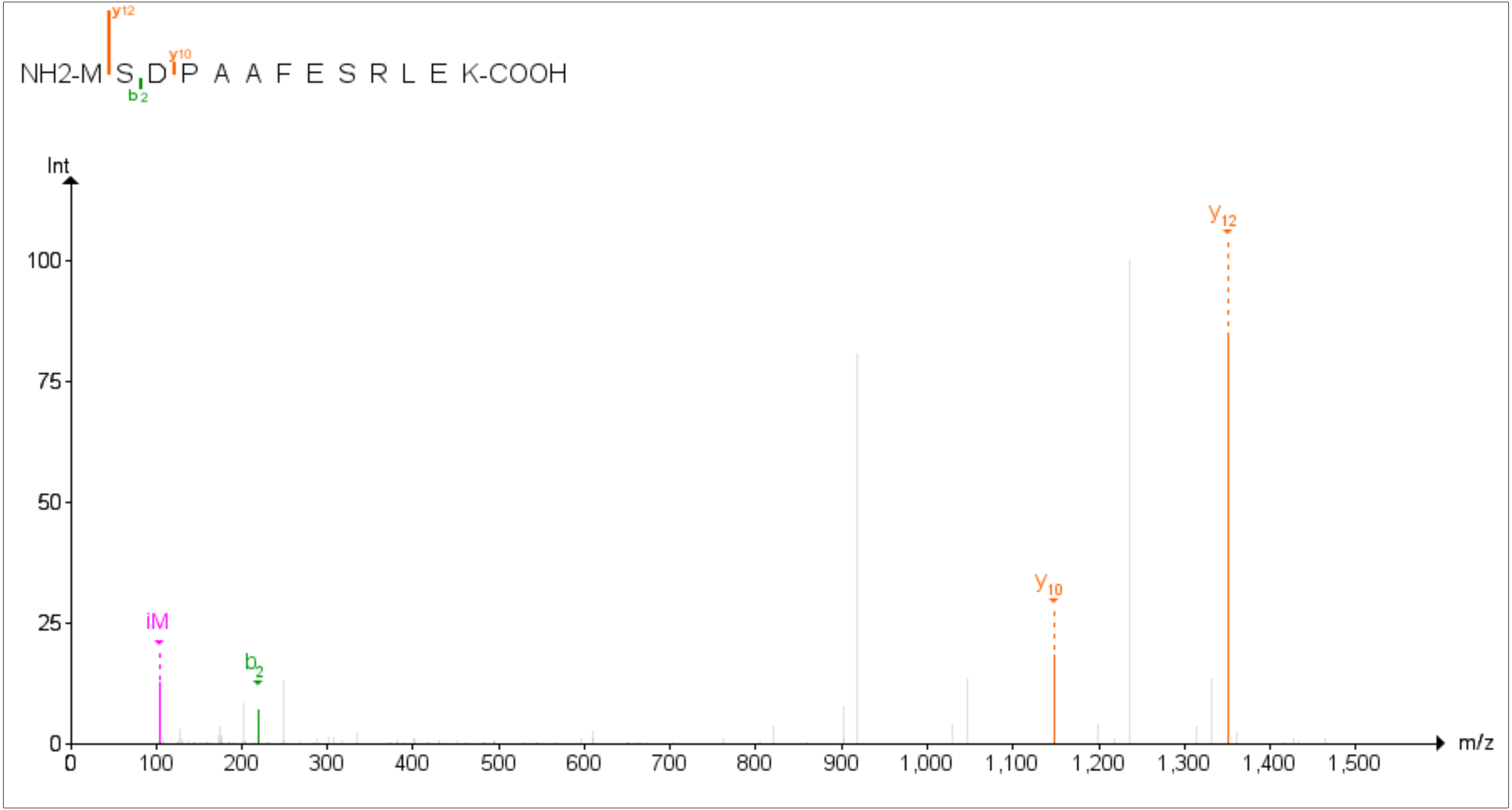

NH2-M S D P A A F E S R L E K-COOH
y12
y10
b2
Int
100
75
50
25
0
iM
b2
y10
y12
0
100
200
300
400
500
600
700
800
900
1,000
1,100
1,200
1,300
1,400
1,500
m/z

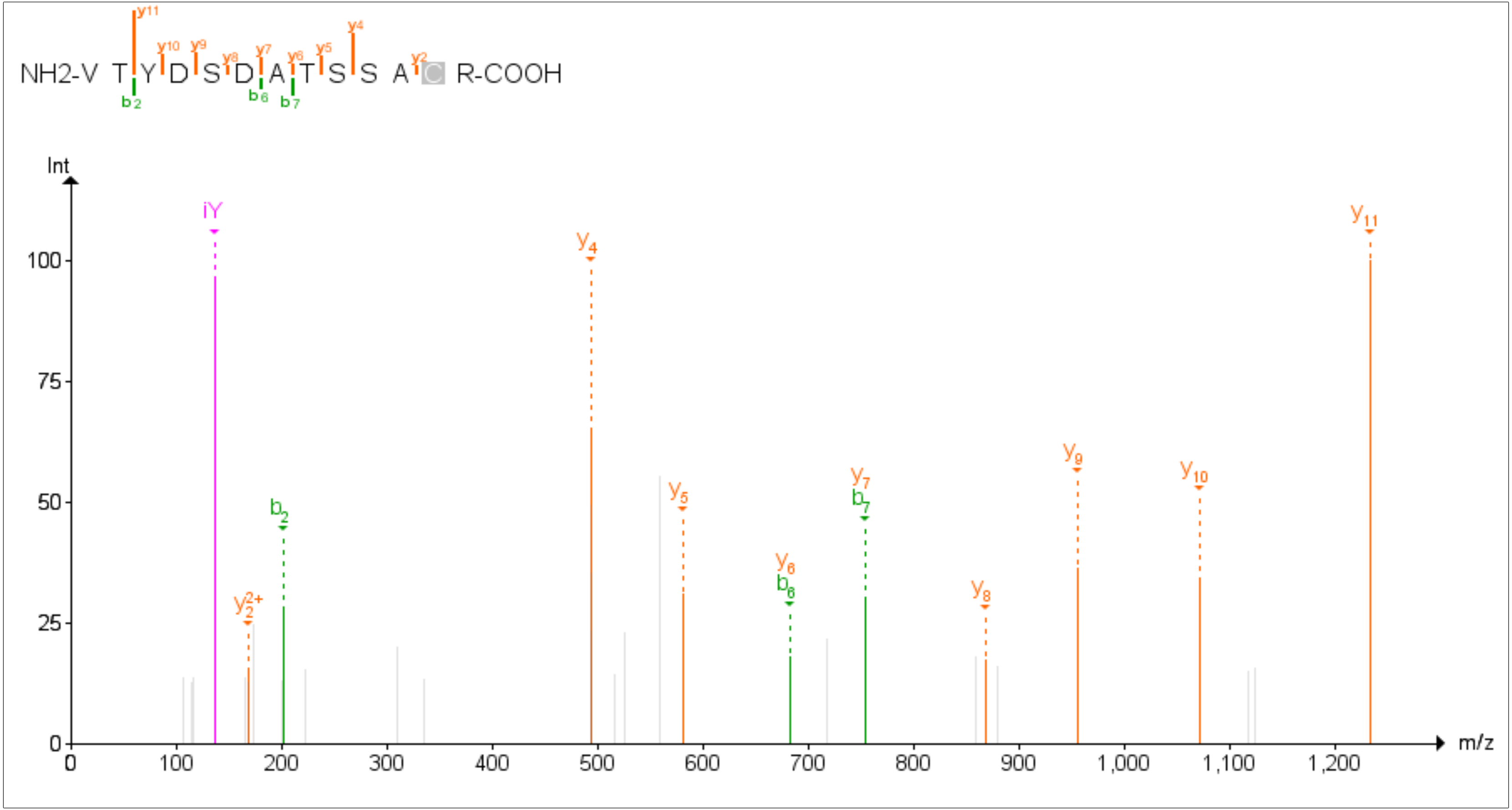

NH2-V T Y D S D A T S S A C R-COOH
y11 y10 y9 y8 y7 y6 y5 y4 y2
b2 b6 b7
Int
iY
y4
y11
b2
y5
y7
b7
y9
y10
y6
b6
y8
100
75
50
25
0
0 100 200 300 400 500 600 700 800 900 1,000 1,100 1,200
m/z

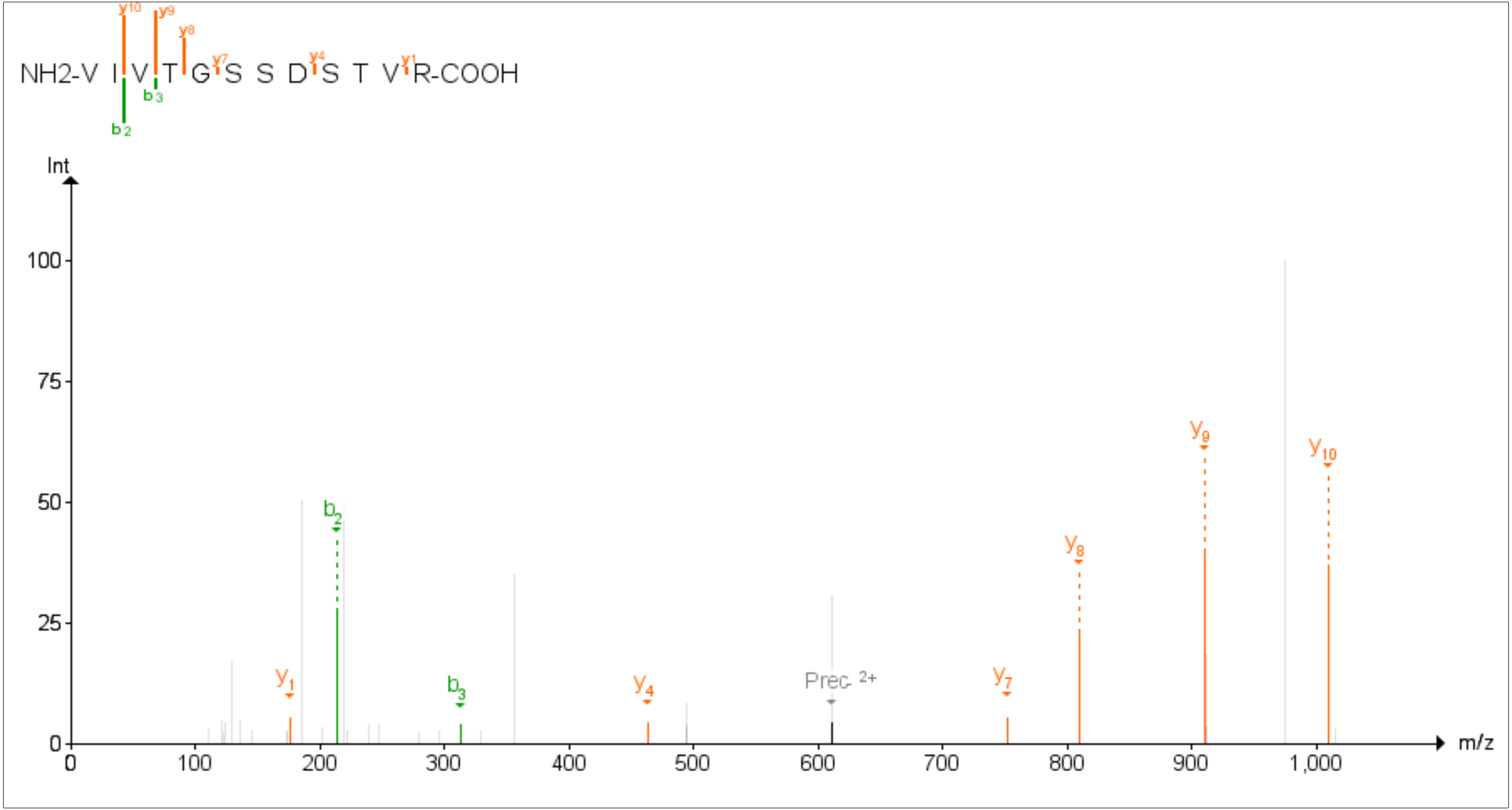

NH2-V I V T G S S D S T V R-COOH
y10
y9
y8
y7
y4
y1
b2
b3
Int
100
75
50
25
0
m/z
0
100
200
300
400
500
600
700
800
900
1,000
y1
b2
b3
y4
Prec- 2+
y7
y8
y9
y10

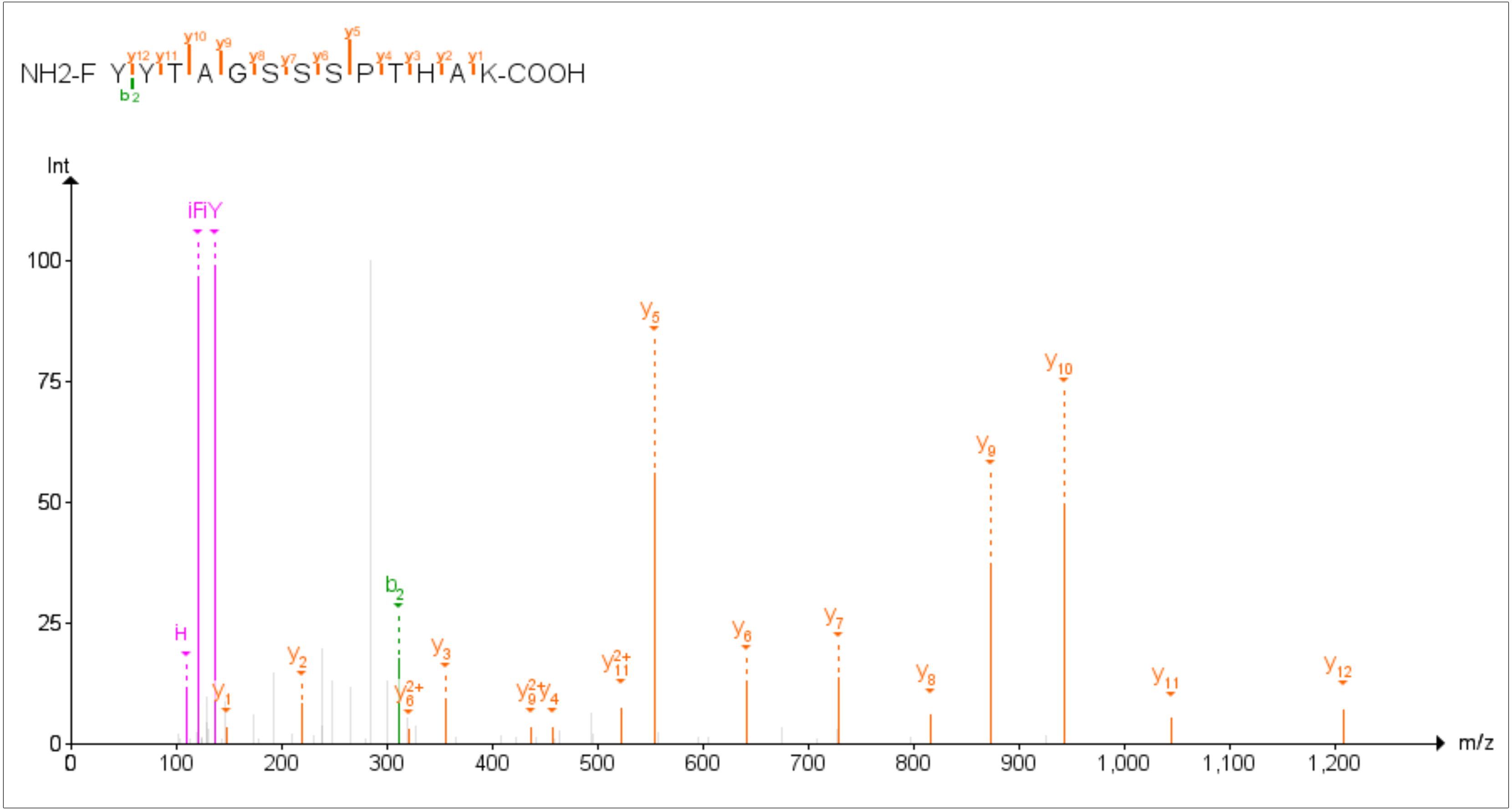

NH2-F Y Y T A G S S S P T H A K-COOH
y12 y11 y10 y9 y8 y7 y6 y5 y4 y3 y2 y1
b2
Int
m/z
iFiY
H
y1
y2
b2
y3
y6 2+
y9 2+
y4
y11 2+
y5
y6
y7
y8
y9
y10
y11
y12

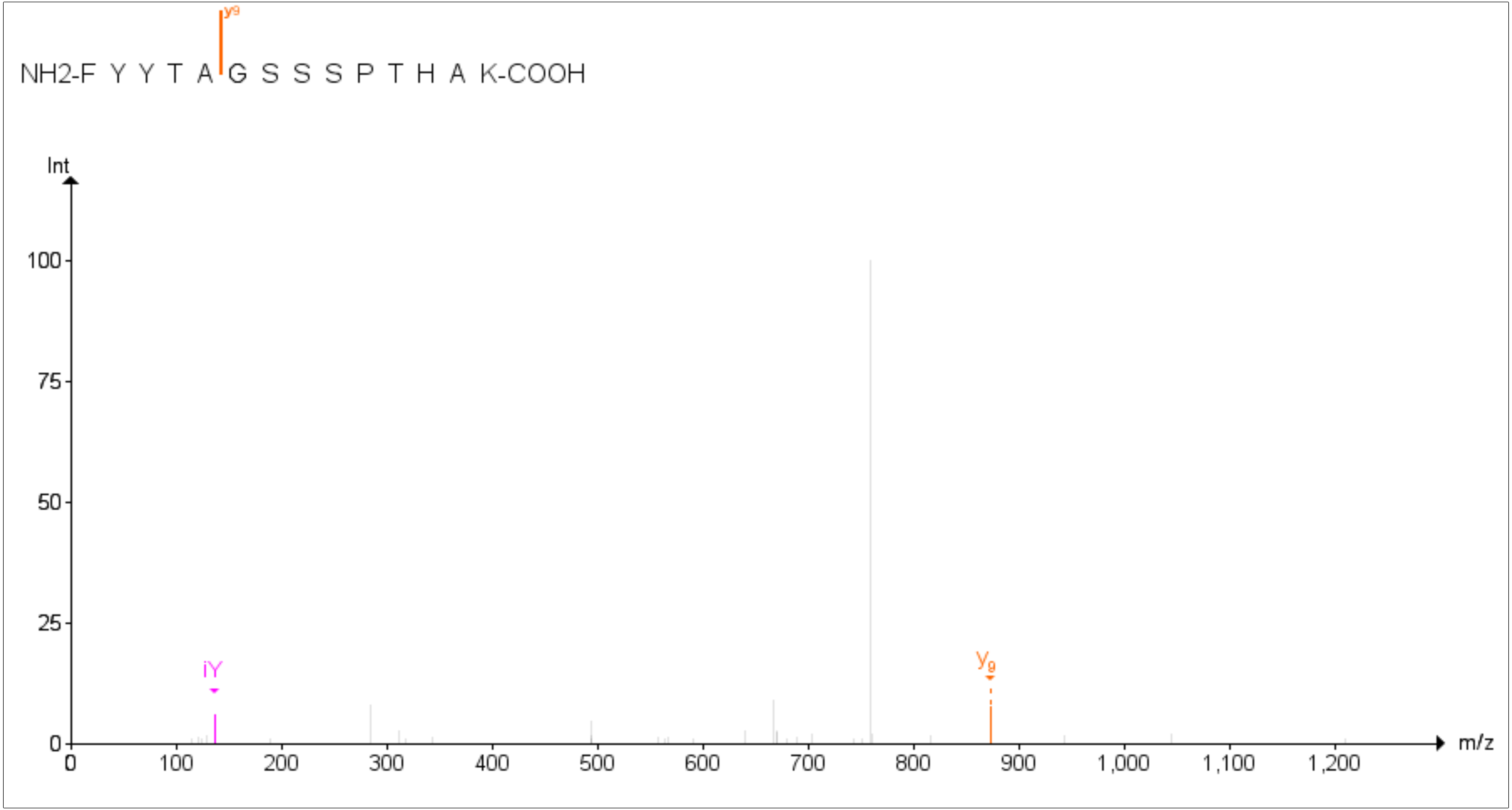

y9
NH2-F Y Y T A G S S S P T H A K-COOH
Int
100
75
50
25
0
iY
y9
0
100
200
300
400
500
600
700
800
900
1,000
1,100
1,200
m/z

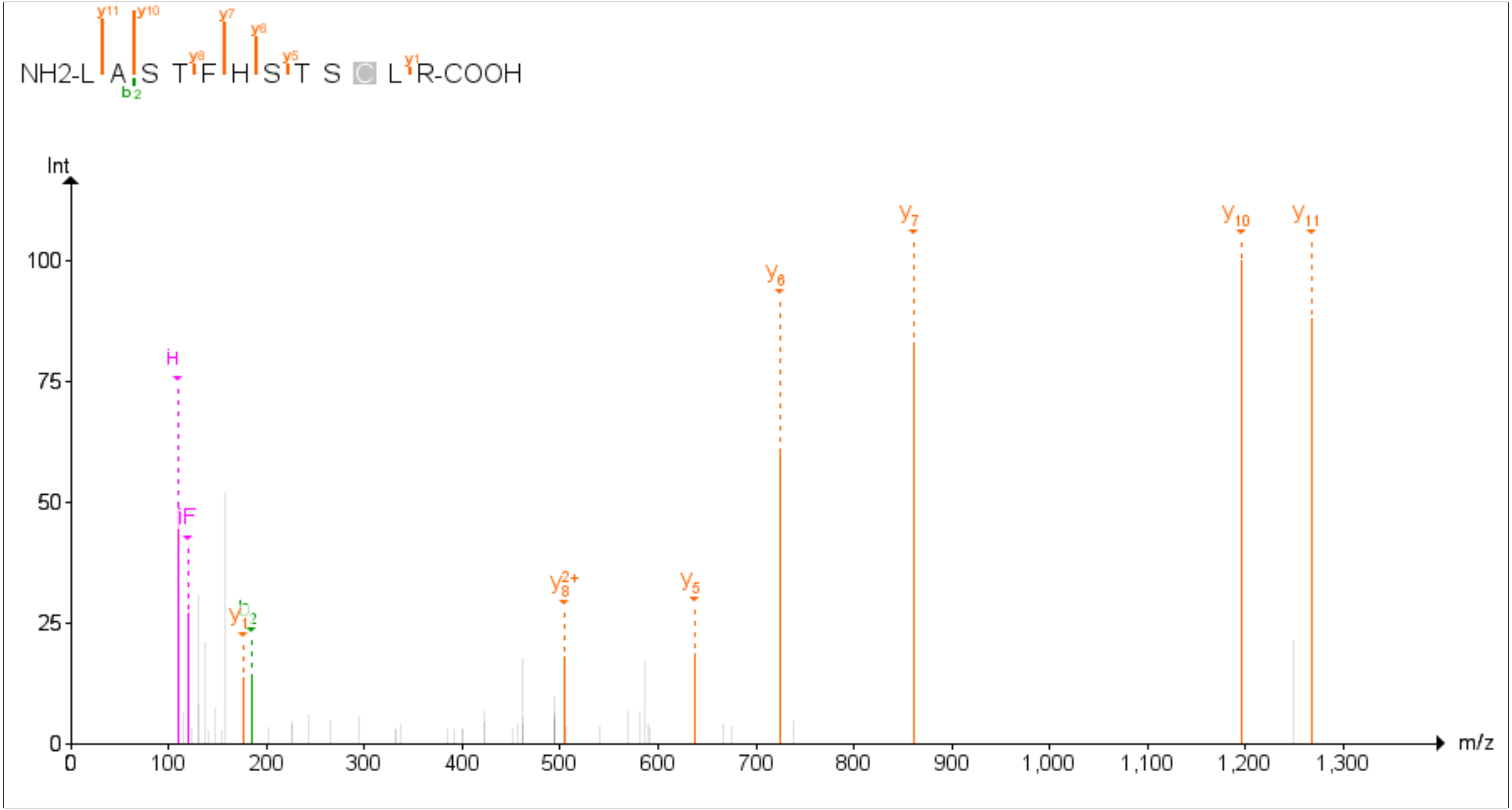

NH2-L A S T F H S T S C L R-COOH
y11
y10
y8
y7
y6
y5
y1
b2
Int
100
75
50
25
0
H
IF
y1
b2
y8 2+
y5
y6
y7
y10
y11
0
100
200
300
400
500
600
700
800
900
1,000
1,100
1,200
1,300
m/z

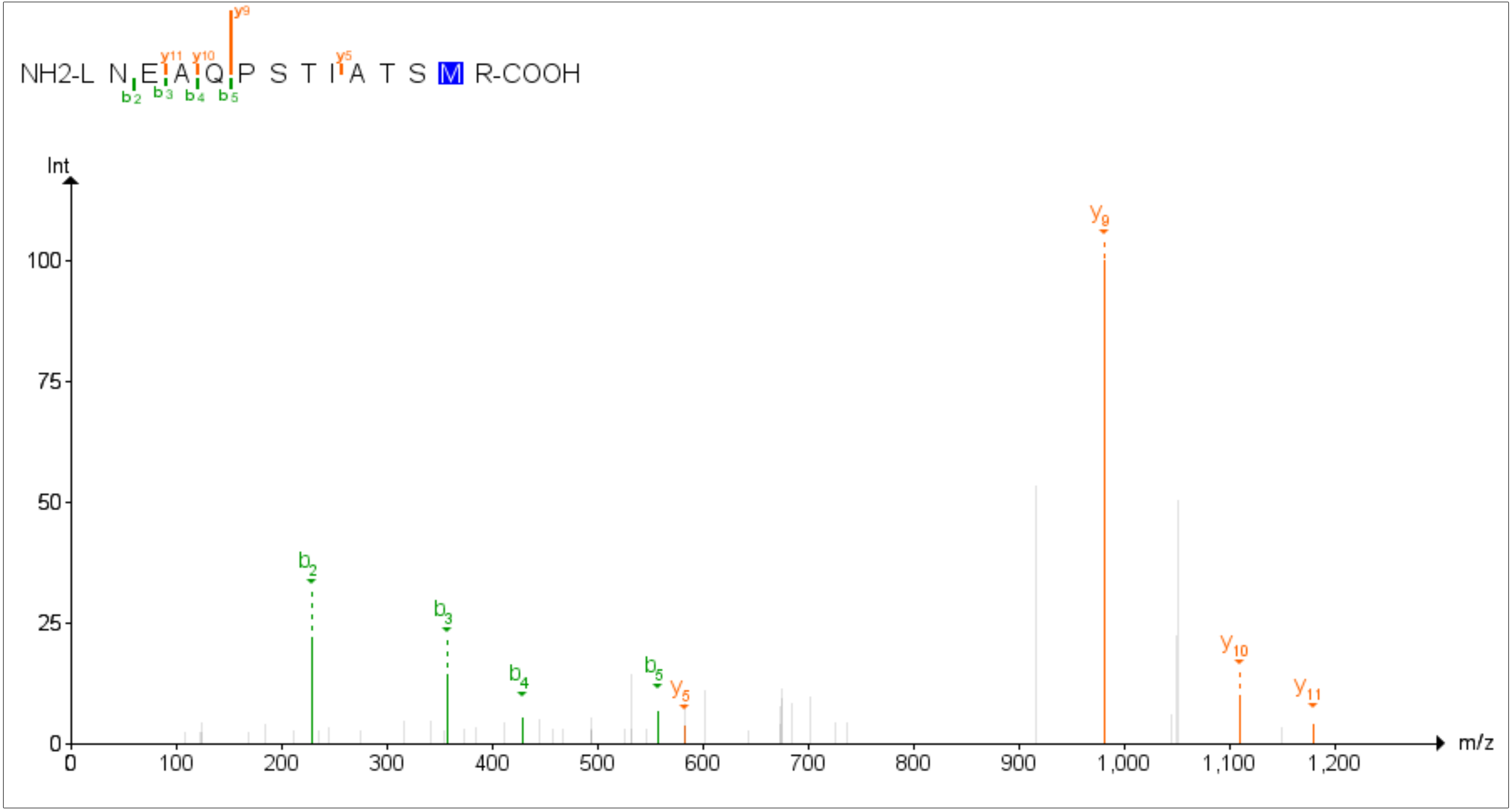

NH2-L N E A Q P S T I A T S M R-COOH
y9
y11
y10
y5
b2
b3
b4
b5
Int
100
75
50
25
0
y9
b2
b3
b4
b5
y5
y10
y11
m/z
0
100
200
300
400
500
600
700
800
900
1,000
1,100
1,200

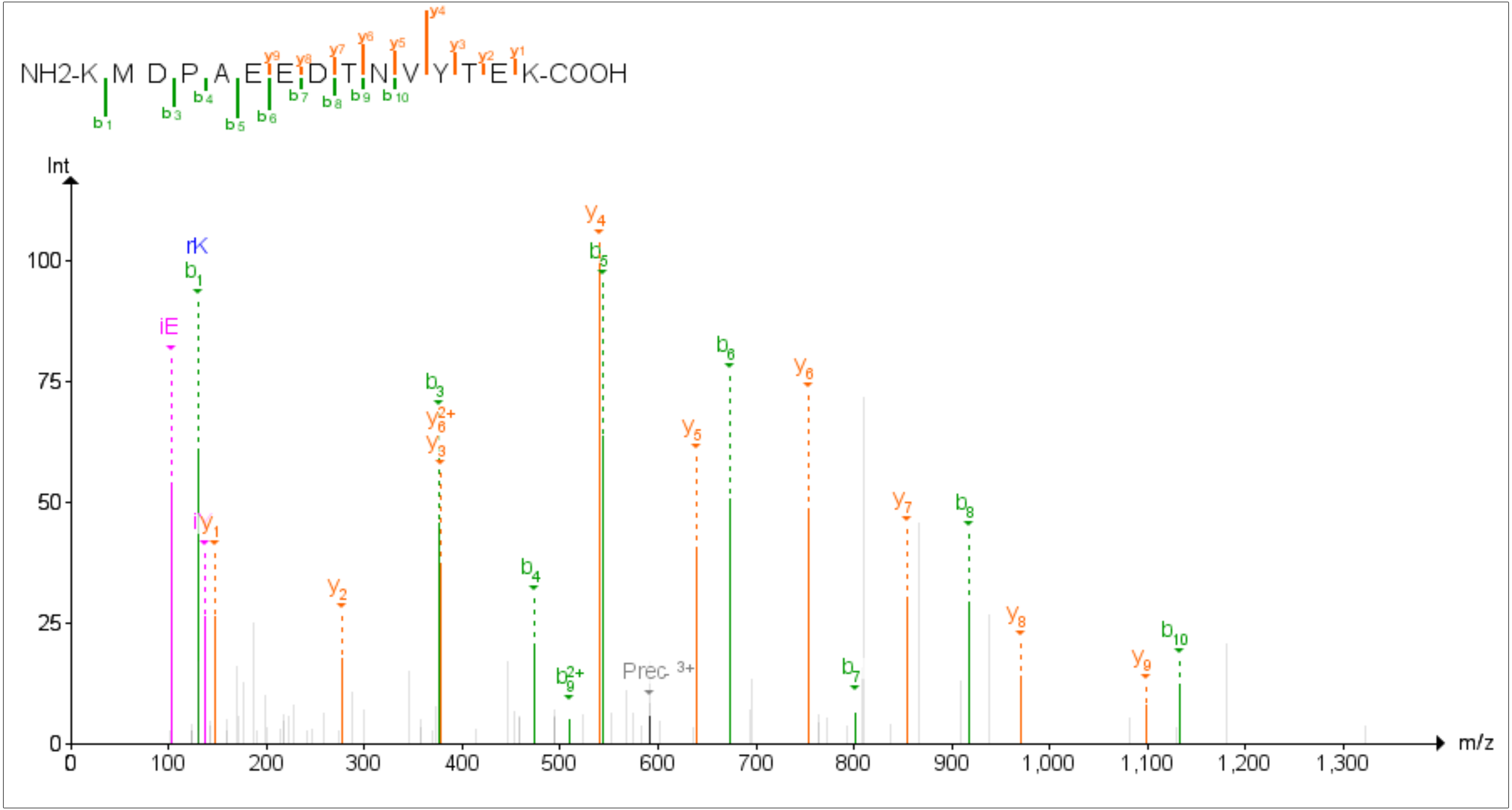

NH2-K M D P A E E D T N V Y T E K-COOH
y4 y9 y8 y7 y6 y5 y3 y2 y1
b1 b3 b4 b5 b6 b7 b8 b9 b10
Int
100
75
50
25
0
rK
b1
iE
iY
y1
y2
b3
y6 2+
y3
b4
b9 2+
y4
b5
Prec 3+
y5
b6
y6
b7
y7
b8
y8
y9
b10
0
100
200
300
400
500
600
700
800
900
1,000
1,100
1,200
1,300
m/z

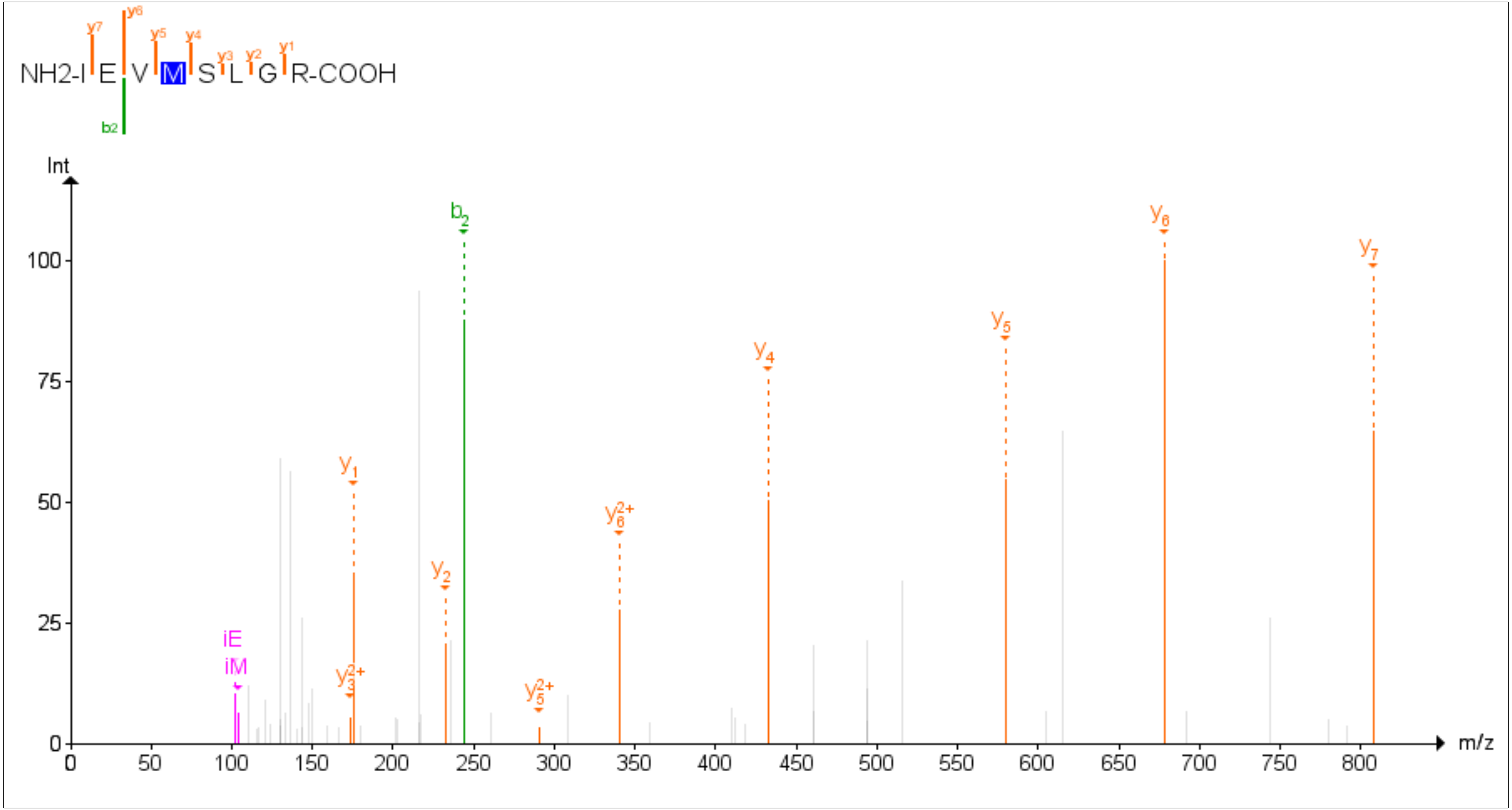

NH2-I E V M S L G R-COOH
y7
y6
y5
y4
y3
y2
y1
b2
Int
m/z
b2
y6
y7
y5
y4
y1
y6 2+
y2
y3 2+
y5 2+
iE
iM
0
25
50
75
100
0
50
100
150
200
250
300
350
400
450
500
550
600
650
700
750
800

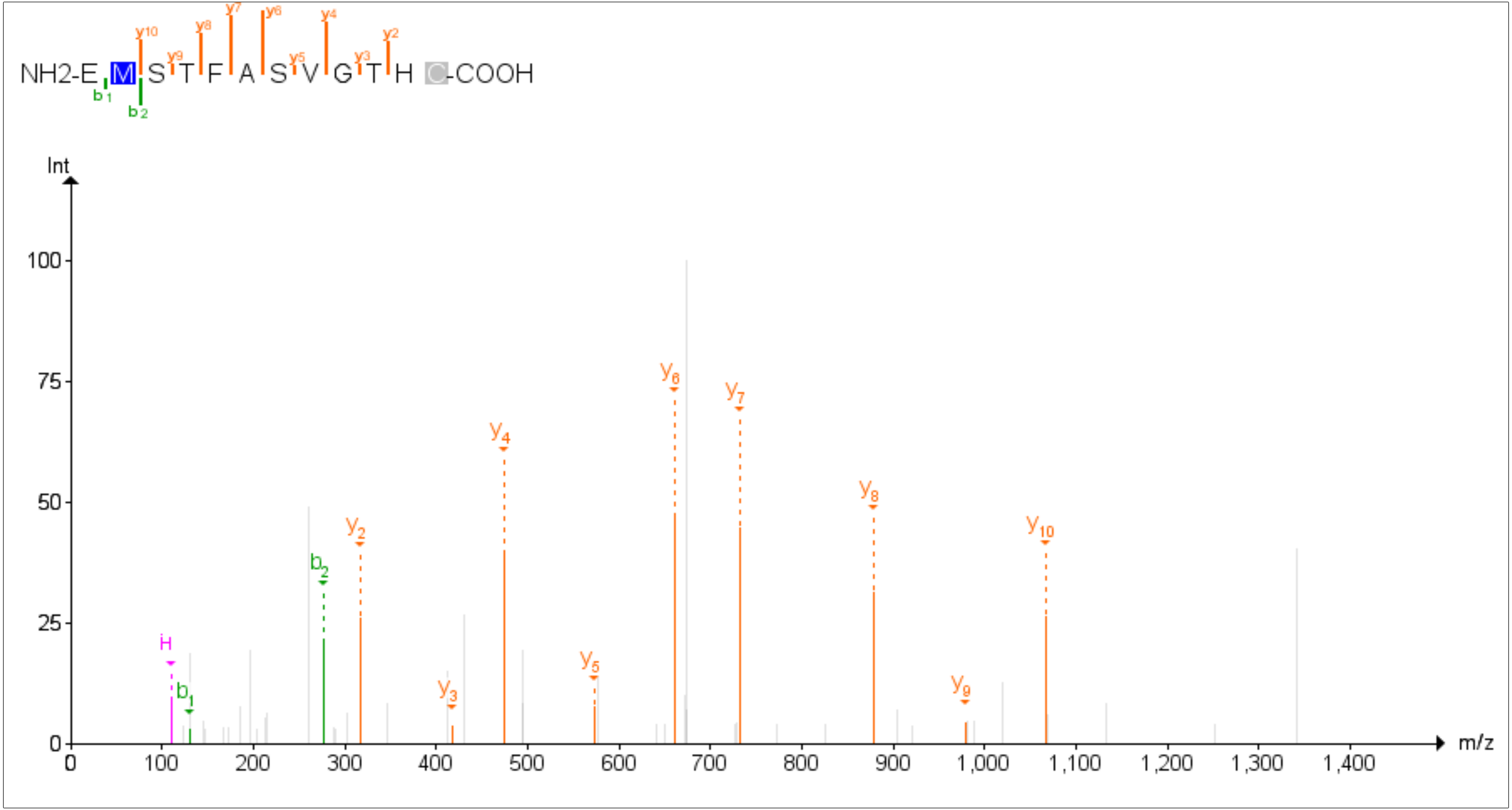

NH2-E M S T F A S V G T H C-COOH
y10 y9 y8 y7 y6 y5 y4 y3 y2
b1 b2
Int
100
75
50
25
0
y6
y7
y4
y8
y2
y10
b2
H
b1
y3
y5
y9
0 100 200 300 400 500 600 700 800 900 1,000 1,100 1,200 1,300 1,400
m/z

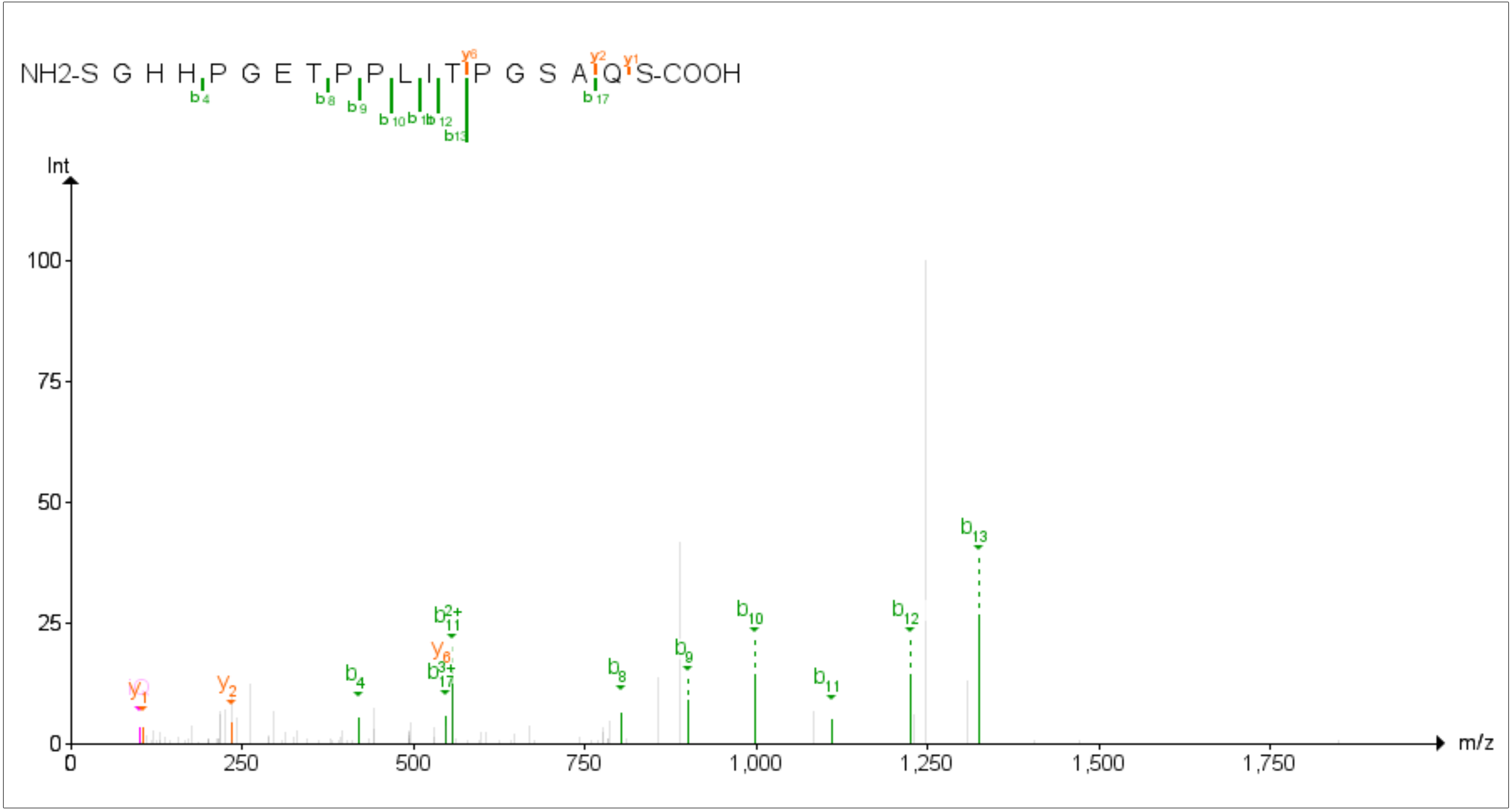
NH2-S G H H P G E T P P L I T P G S A Q S-COOH
y6 y2 y1
b4 b8 b9 b10 b11 b12 b13 b17
Int
100
75
50
25
0
0 250 500 750 1,000 1,250 1,500 1,750
m/z
y1 y2 b4 b3+17 y6 b2+11 b8 b9 b10 b11 b12 b13

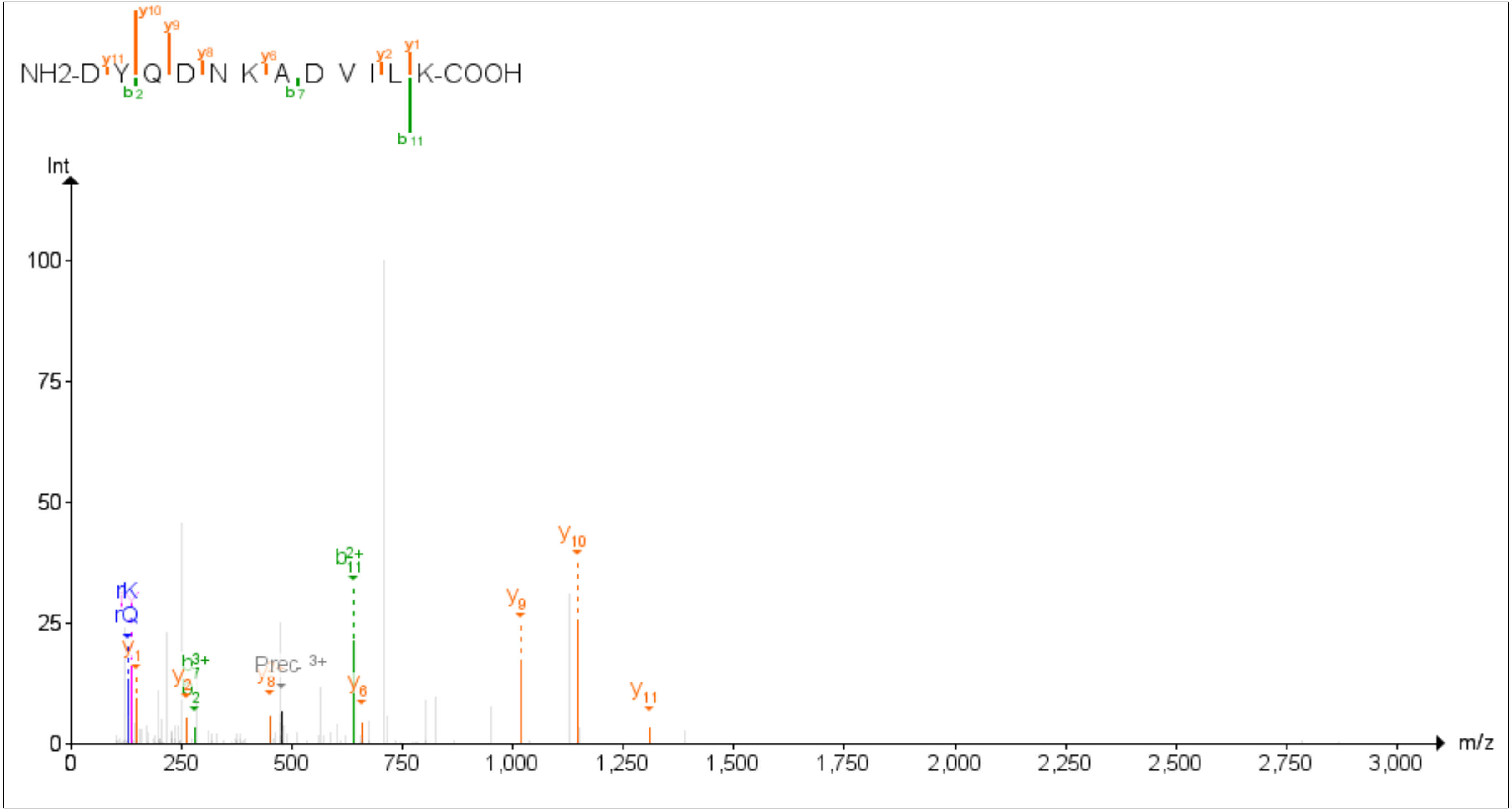

NH2-D Y Q D N K A D V I L K-COOH
y11
y10
y9
y8
y6
y2
y1
b 2
b 7
b 11
Int
100
75
50
25
0
m/z
0
250
500
750
1,000
1,250
1,500
1,750
2,000
2,250
2,500
2,750
3,000
rK
rQ
y1
y2
b2
Prec- 3+
y8
b11 2+
y6
y9
y10
y11

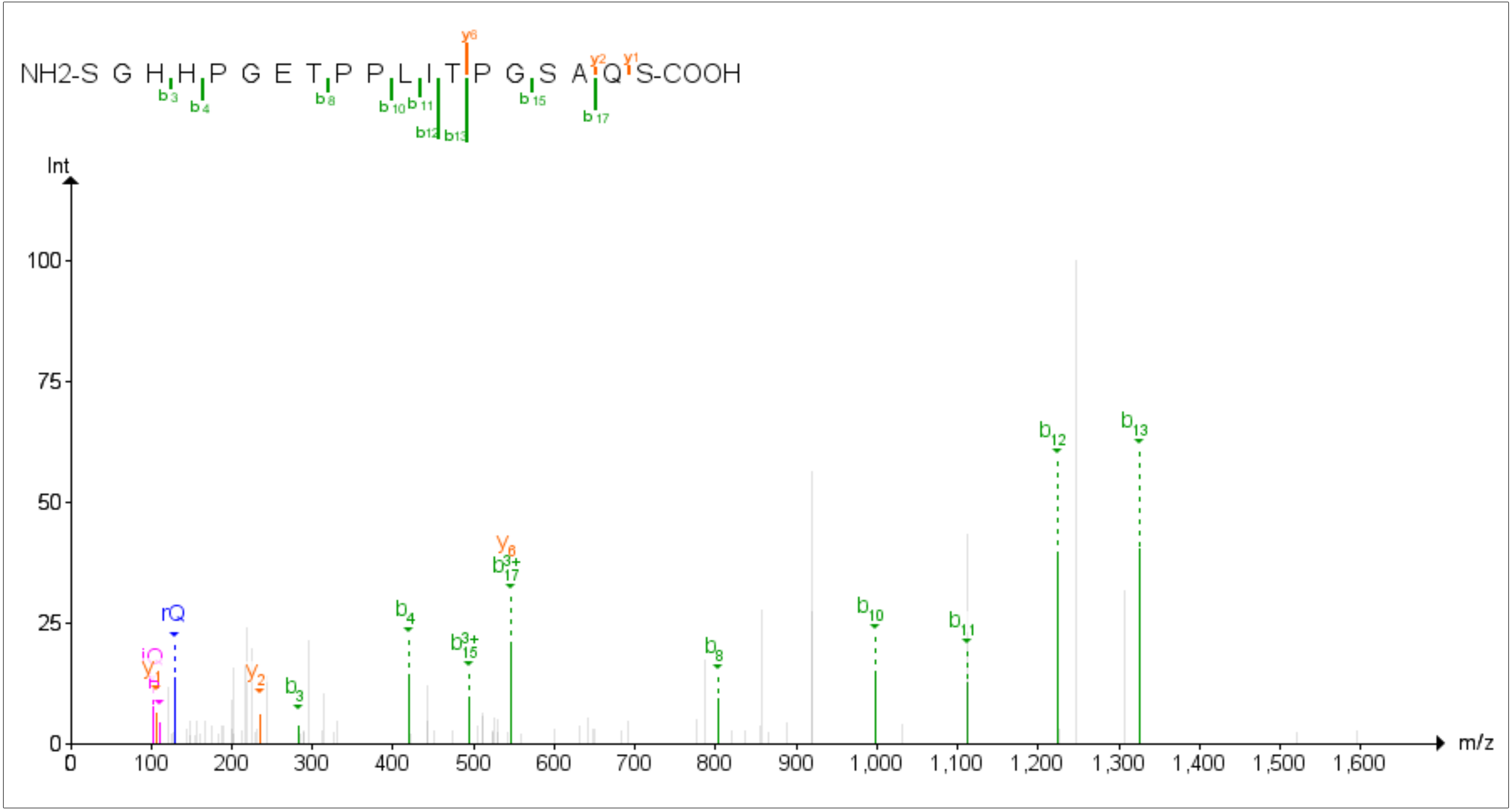
NH2-S G H H P G E T P P L I T P G S A Q S-COOH
b3 b4 b8 b10 b11 b12 b13 b15 b17
y6 y2 y1
Int
100
75
50
25
0
m/z
0 100 200 300 400 500 600 700 800 900 1,000 1,100 1,200 1,300 1,400 1,500 1,600
rQ
iQ
y1
y2
b3
b4
b3+ 15
y6
b3+ 17
b8
b10
b11
b12
b13

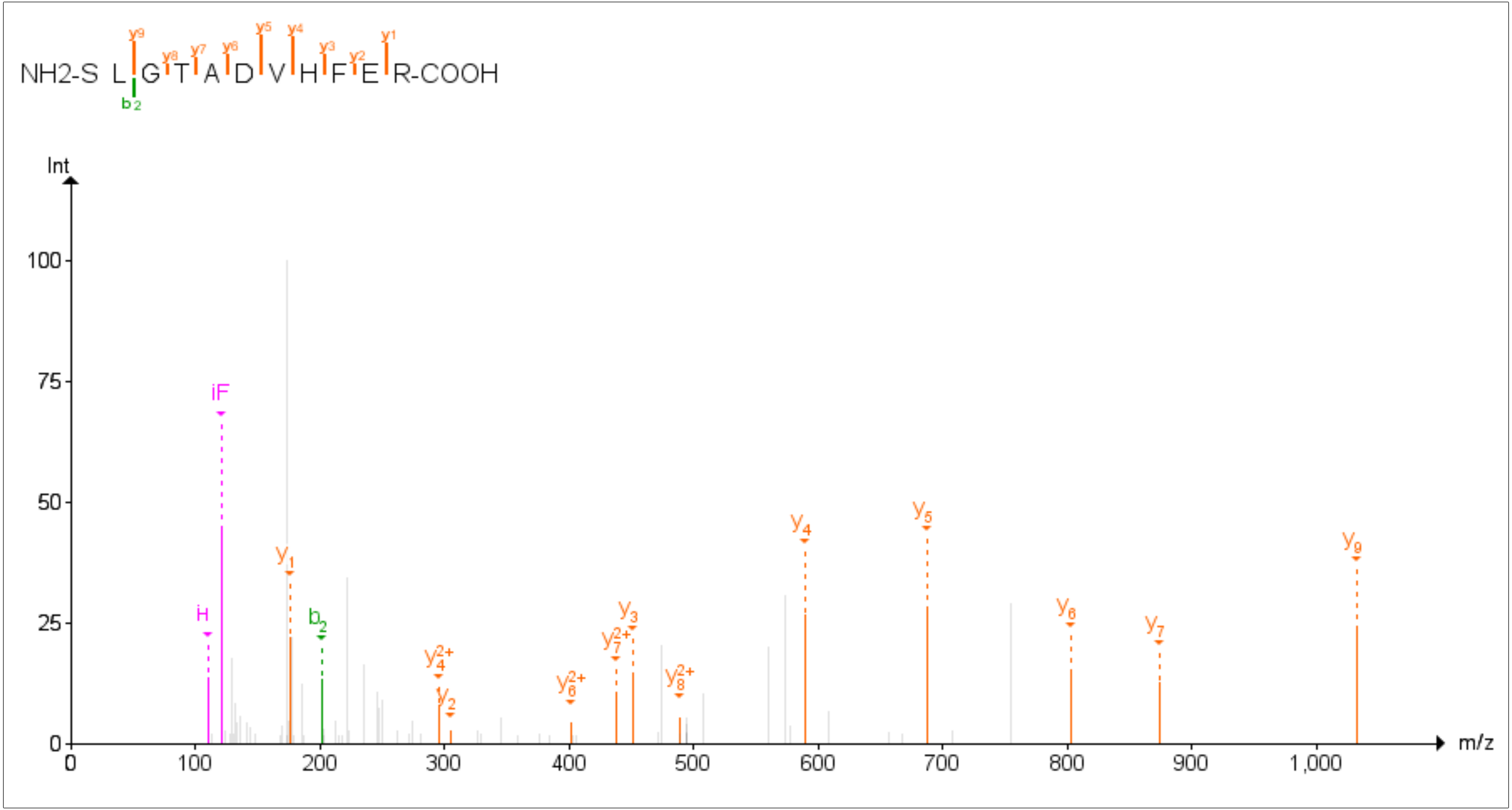

NH2-S L G T A D V H F E R-COOH
y9 y8 y7 y6 y5 y4 y3 y2 y1
b2
Int
100
75
50
25
0
iF
H
y1
b2
y4 2+
y2
y6 2+
y7 2+
y3
y8 2+
y4
y5
y6
y7
y9
0
100
200
300
400
500
600
700
800
900
1,000
m/z

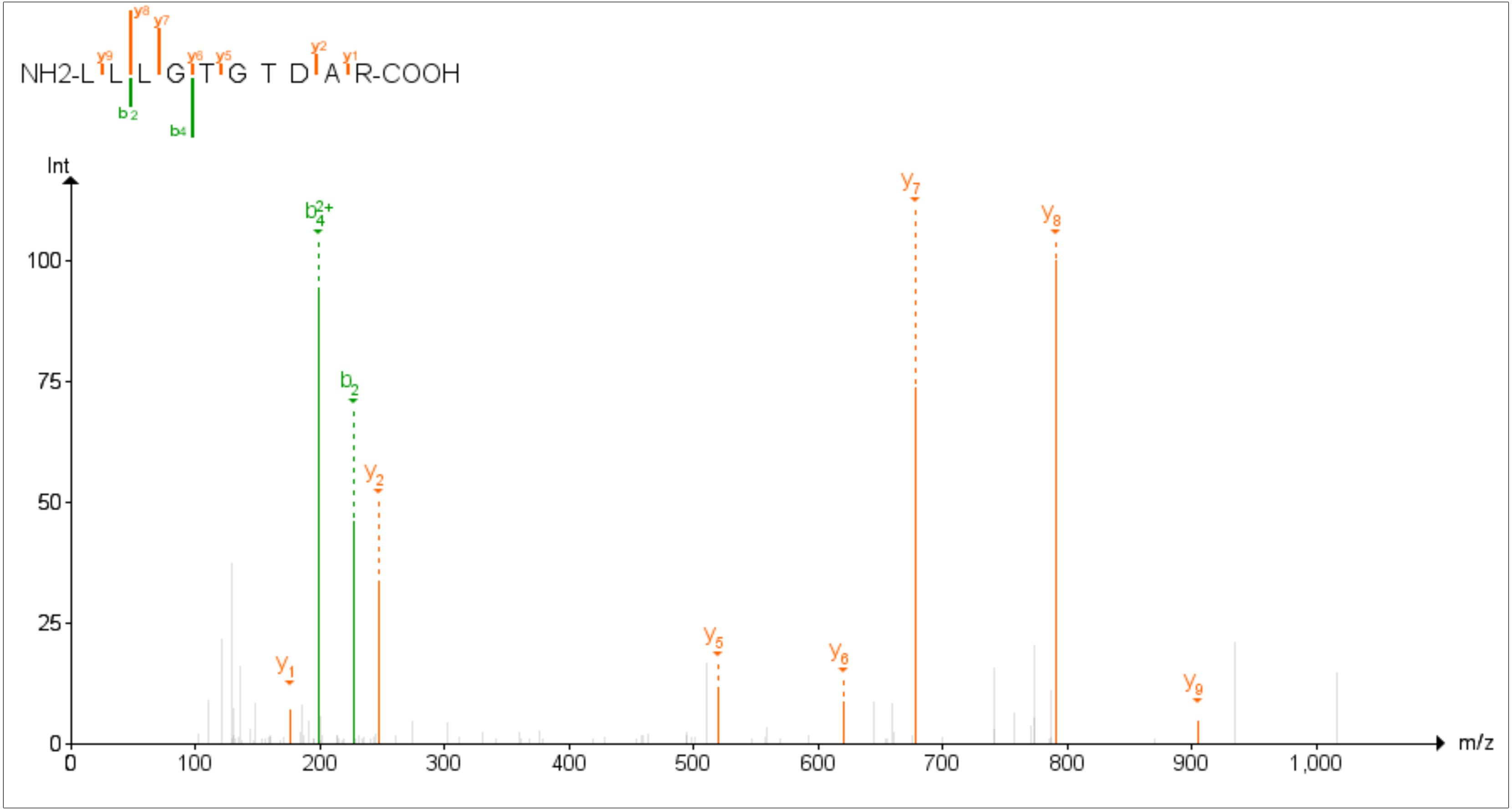

NH2-L L L G T G T D A R-COOH
y9 y8 y7 y6 y5 y2 y1
b2 b4
Int
100
75
50
25
0
m/z
0 100 200 300 400 500 600 700 800 900 1,000
b4 2+
b2
y2
y1
y5
y6
y7
y8
y9

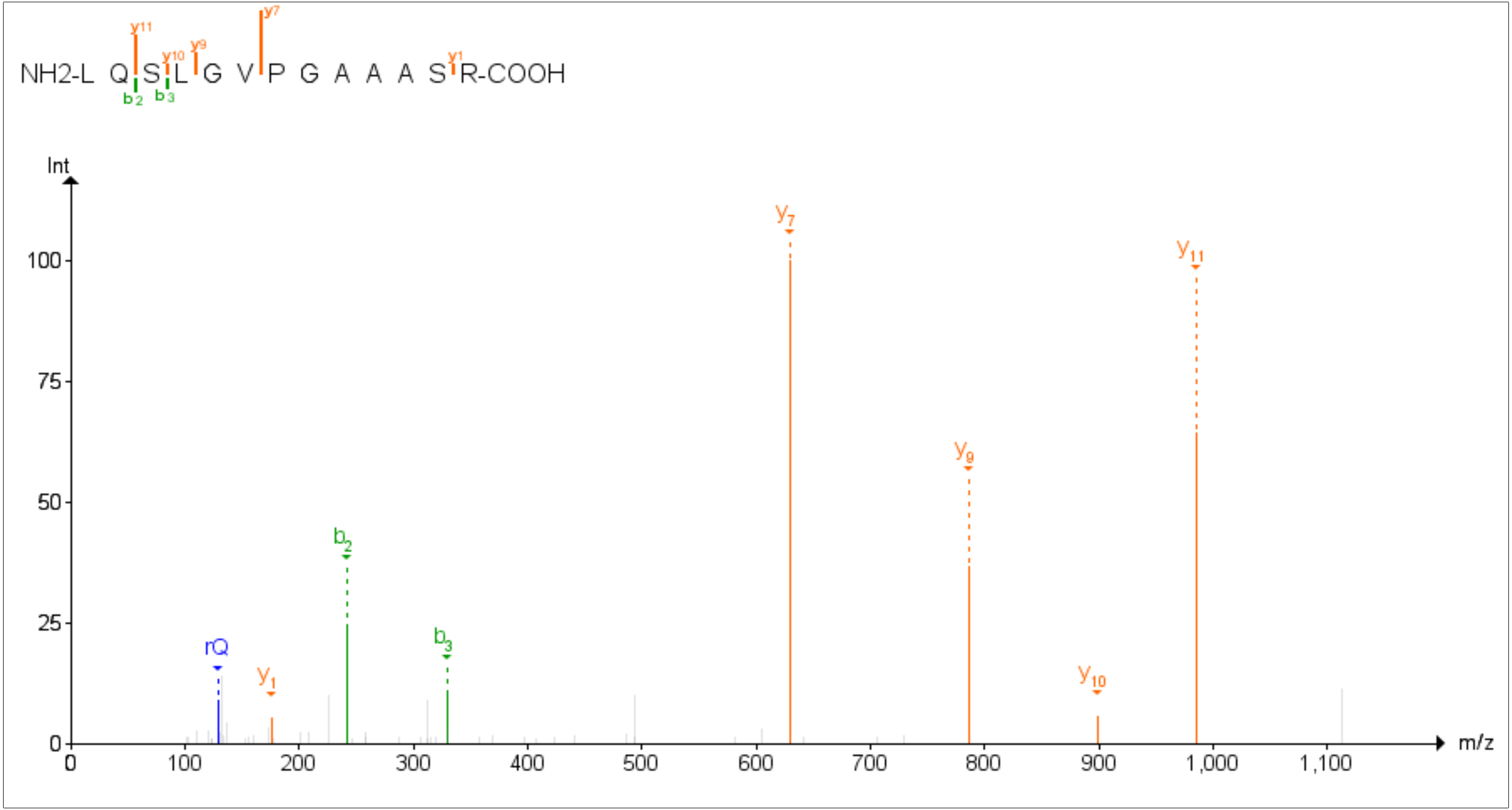
NH2-L Q S L G V P G A A A S R-COOH
y11
y10
y9
y7
y1
b2
b3
Int
100
75
50
25
0
y7
y11
y9
b2
b3
rQ
y1
y10
0
100
200
300
400
500
600
700
800
900
1,000
1,100
m/z

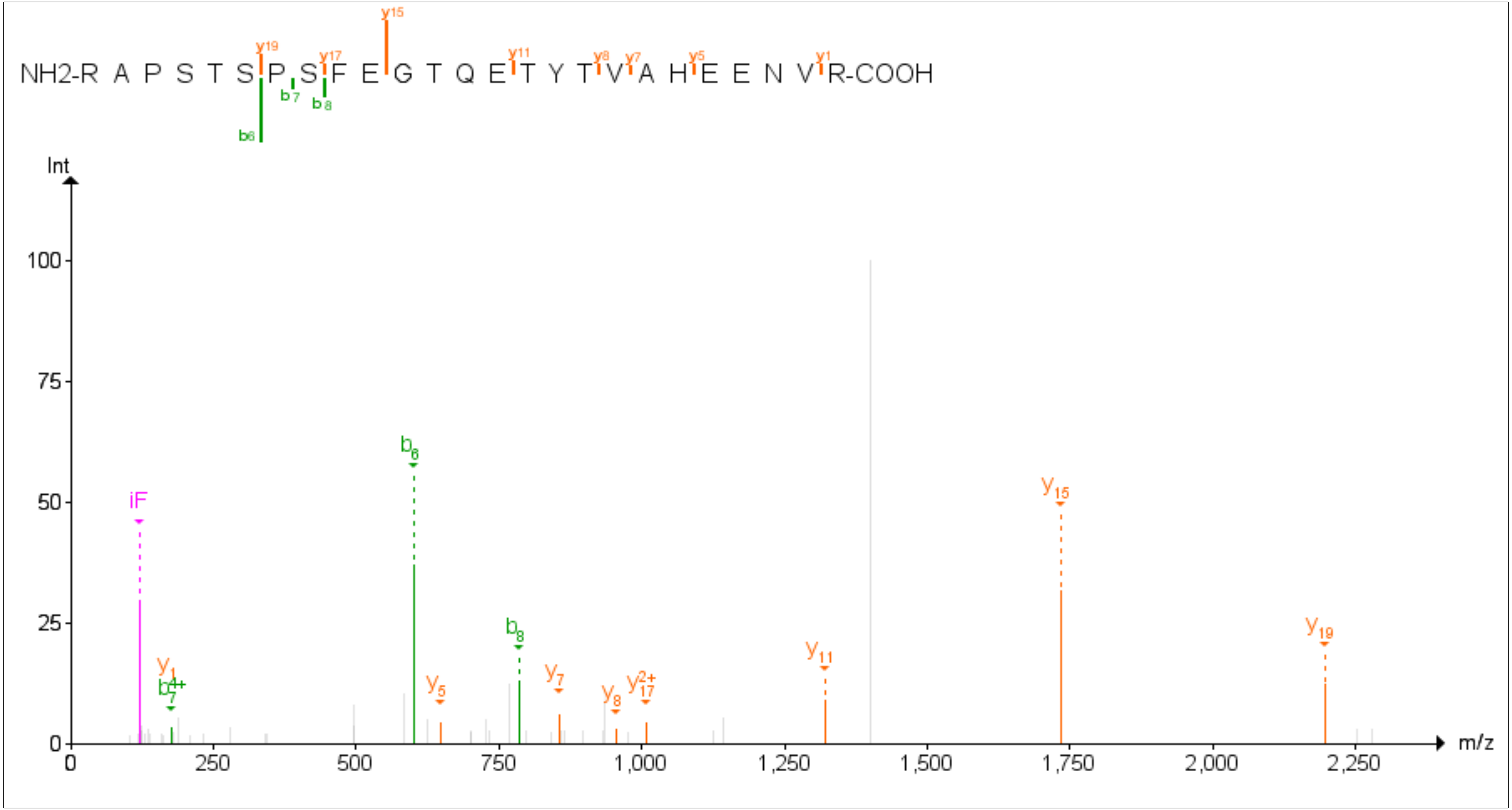
NH2-R A P S T S P S F E G T Q E T Y T V A H E E N V R-COOH
y19 y17 y15 y11 y8 y7 y5 y1
b6 b7 b8
Int
100
75
50
25
0
iF
b_6
b_8
y_1
b_7^{4+}
y_5
y_7
y_8
y_{17}^{2+}
y_{11}
y_{15}
y_{19}
0
250
500
750
1,000
1,250
1,500
1,750
2,000
2,250
m/z

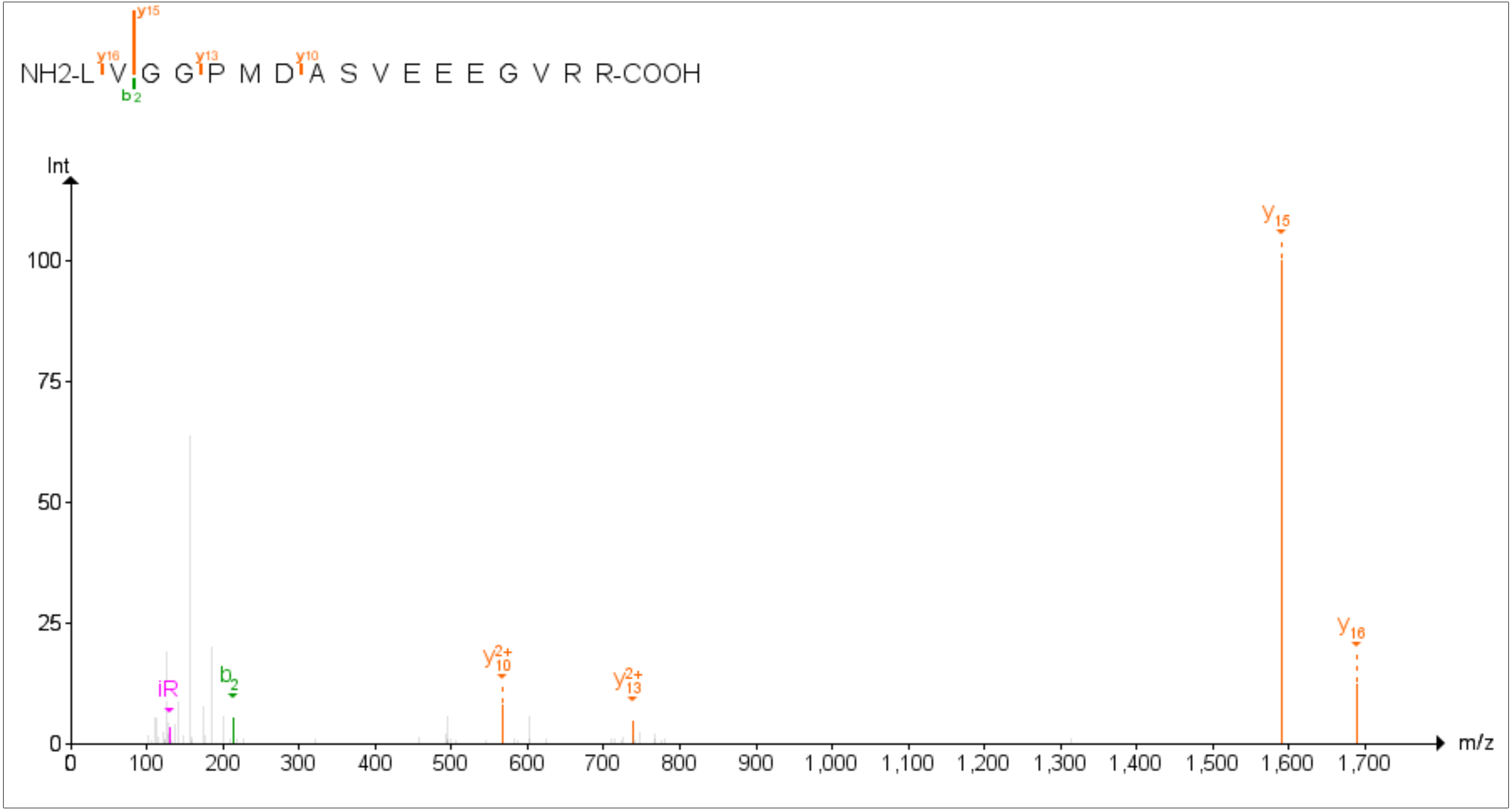

NH2-L V G G P M D A S V E E E G V R R-COOH
y15
y16
y13
y10
b2
Int
100
75
50
25
0
iR
b2
y10 2+
y13 2+
y15
y16
0
100
200
300
400
500
600
700
800
900
1,000
1,100
1,200
1,300
1,400
1,500
1,600
1,700
m/z

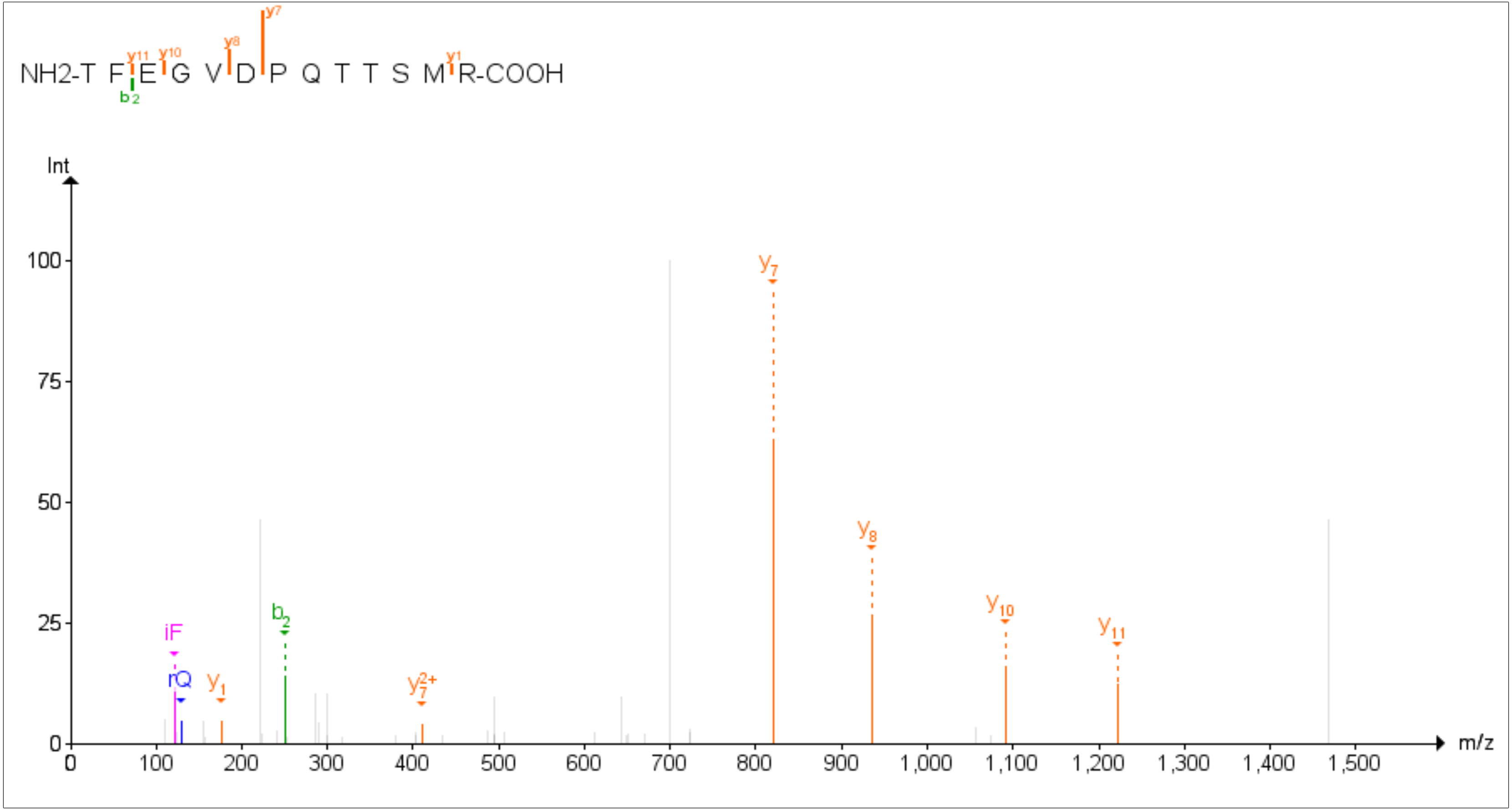

NH2-T F E G V D P Q T T S M R-COOH
y11 y10 y8 y7 y1 b2
Int
100
75
50
25
0
y7
y8
y10
y11
b2
iF
rQ
y1
y7 2+
0 100 200 300 400 500 600 700 800 900 1,000 1,100 1,200 1,300 1,400 1,500
m/z

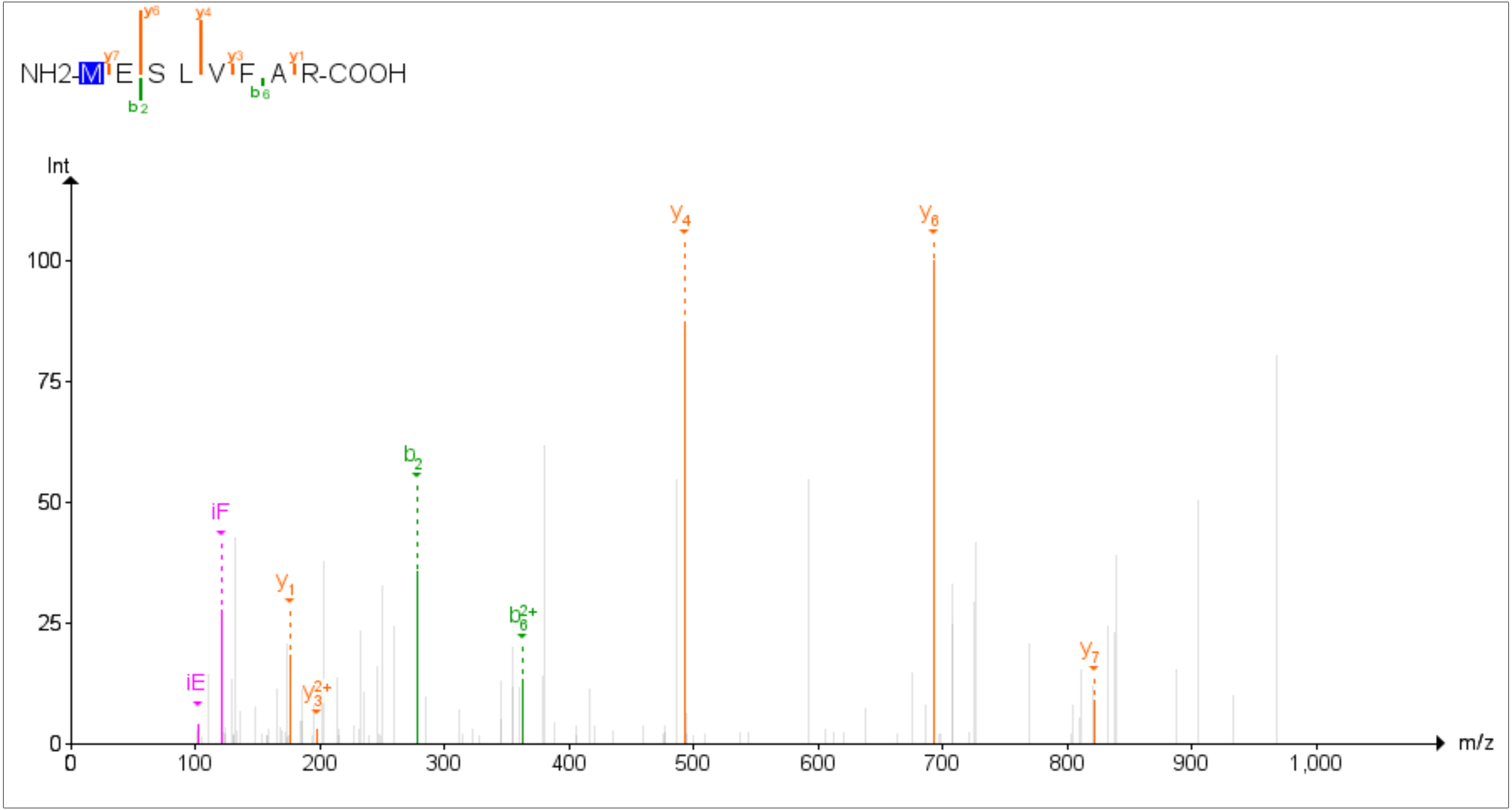

NH2-M E S L V F A R-COOH
y6
y4
y7
y3
y1
b2
b6
Int
100
75
50
25
0
y4
y6
b2
iF
y1
b6 2+
iE
y3 2+
y7
m/z
0
100
200
300
400
500
600
700
800
900
1,000

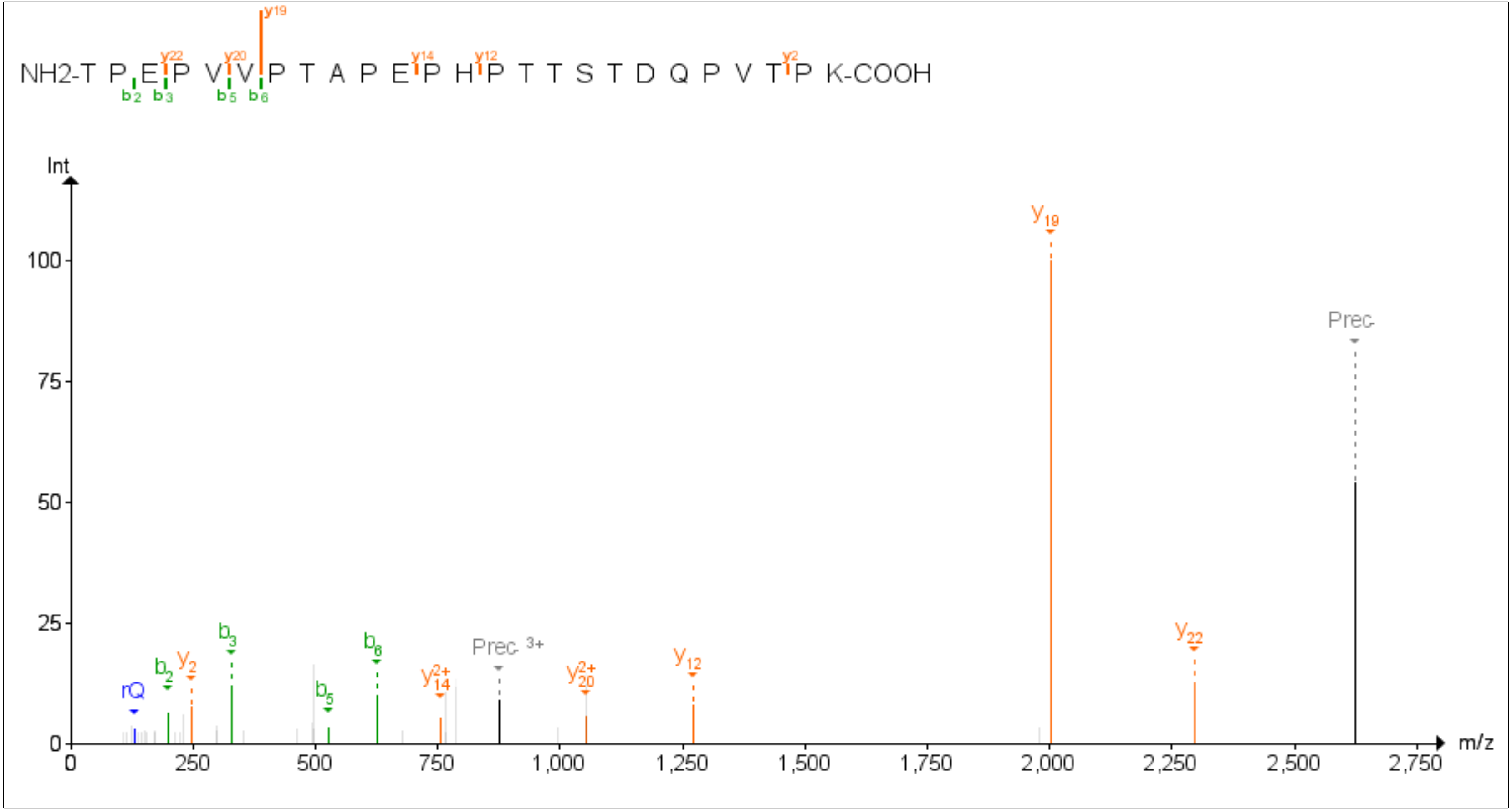

NH2-T P E P V V P T A P E P H P T T S T D Q P V T P K-COOH
y19
y22
y20
y14
y12
y2
b2
b3
b5
b6
Int
100
75
50
25
0
y19
Prec-
y22
y12
y20 2+
Prec- 3+
y14 2+
b6
b5
b3
y2
b2
rQ
0
250
500
750
1,000
1,250
1,500
1,750
2,000
2,250
2,500
2,750
m/z

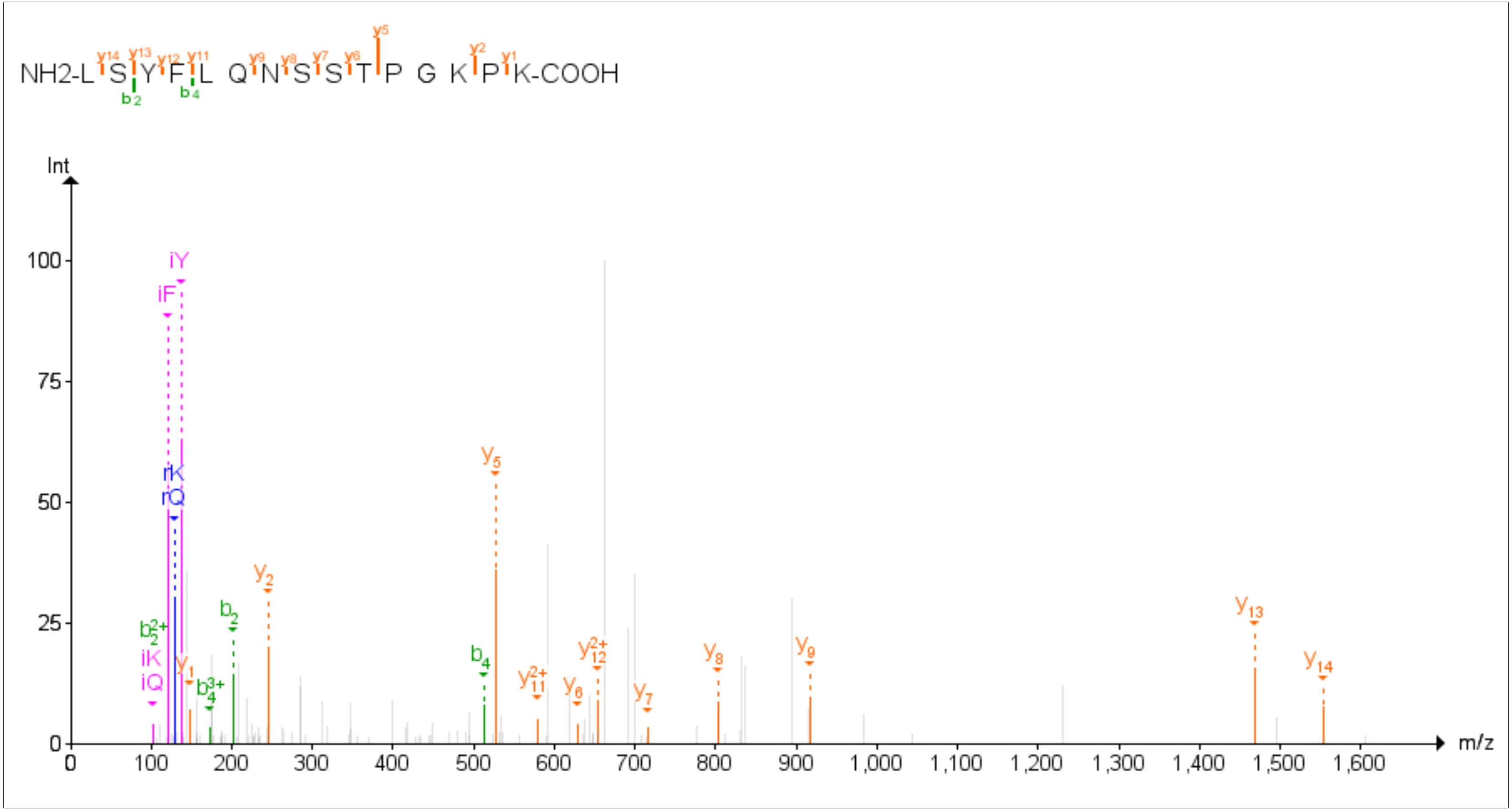

NH2-L S Y F L Q N S S T P G K P K-COOH
y14 y13 y12 y11 y9 y8 y7 y6 y5 y2 y1
b2 b4
Int
m/z
100
75
50
25
0
0 100 200 300 400 500 600 700 800 900 1,000 1,100 1,200 1,300 1,400 1,500 1,600
iY
iF
rK
rQ
b2 2+
iK
iQ
y1
b4 3+
b2
y2
y5
b4
y11 2+
y6
y12 2+
y7
y8
y9
y13
y14

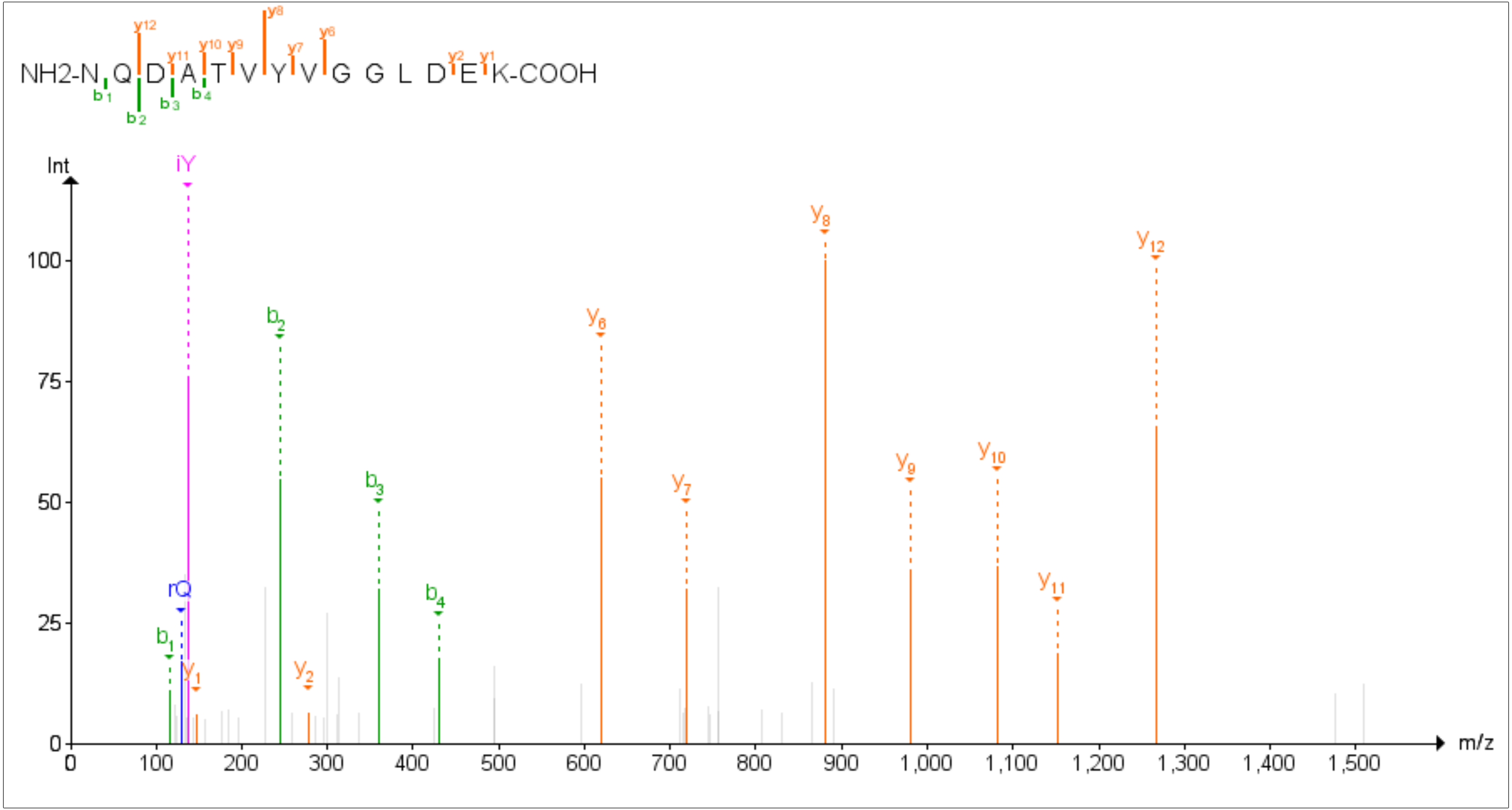
NH2-N Q D A T V Y V G G L D E K-COOH
y12 y11 y10 y9 y8 y7 y6 y2 y1
b1 b2 b3 b4
Int
iY
rQ
b1
y1
y2
b2
b3
b4
y6
y7
y8
y9
y10
y11
y12
0
25
50
75
100
0
100
200
300
400
500
600
700
800
900
1,000
1,100
1,200
1,300
1,400
1,500
m/z

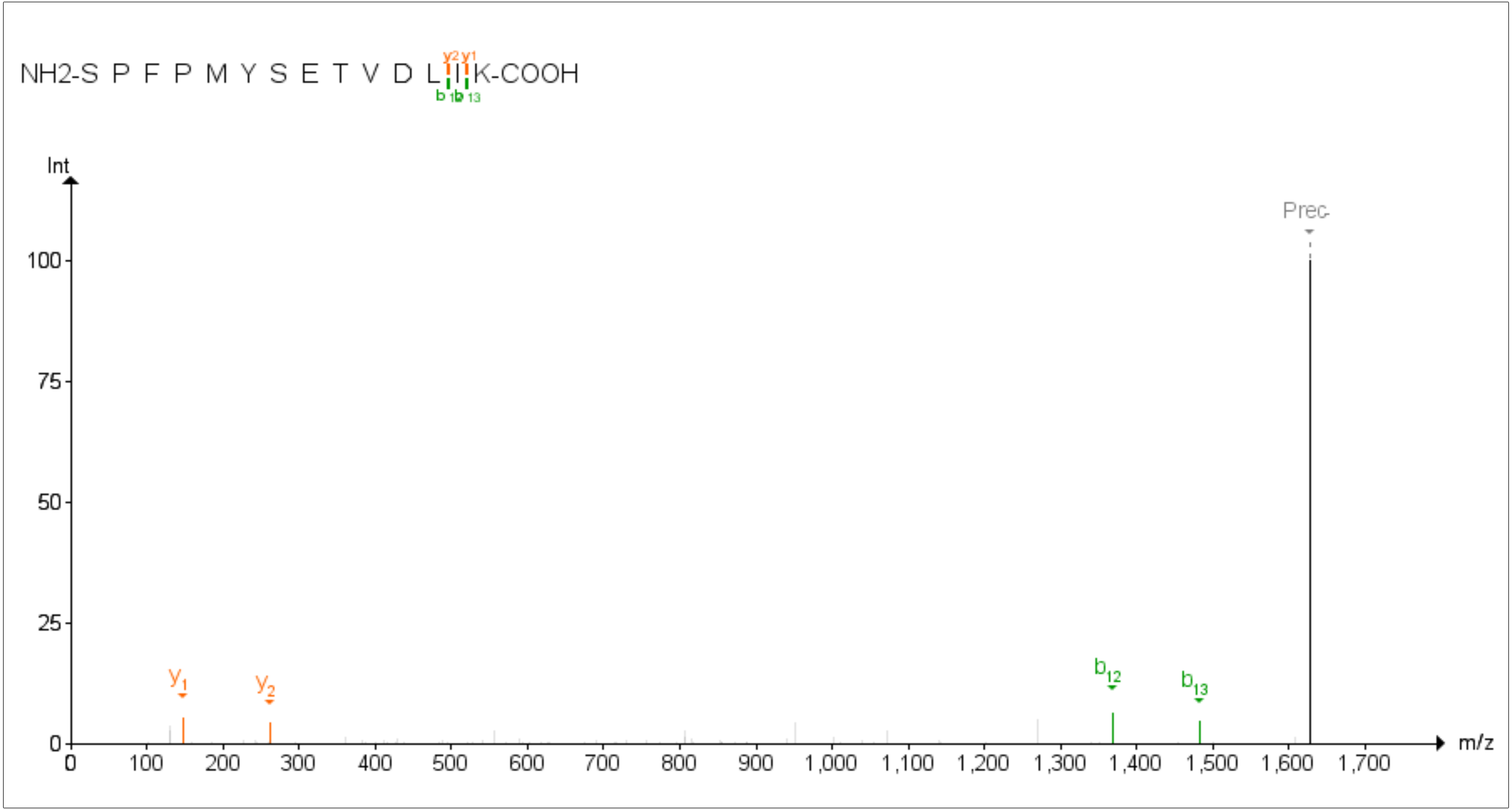
NH2-S P F P M Y S E T V D L I I K-COOH
y2 y1
b 12 b 13
Int
100
75
50
25
0
Prec
y1
y2
b12
b13
m/z
0 100 200 300 400 500 600 700 800 900 1,000 1,100 1,200 1,300 1,400 1,500 1,600 1,700

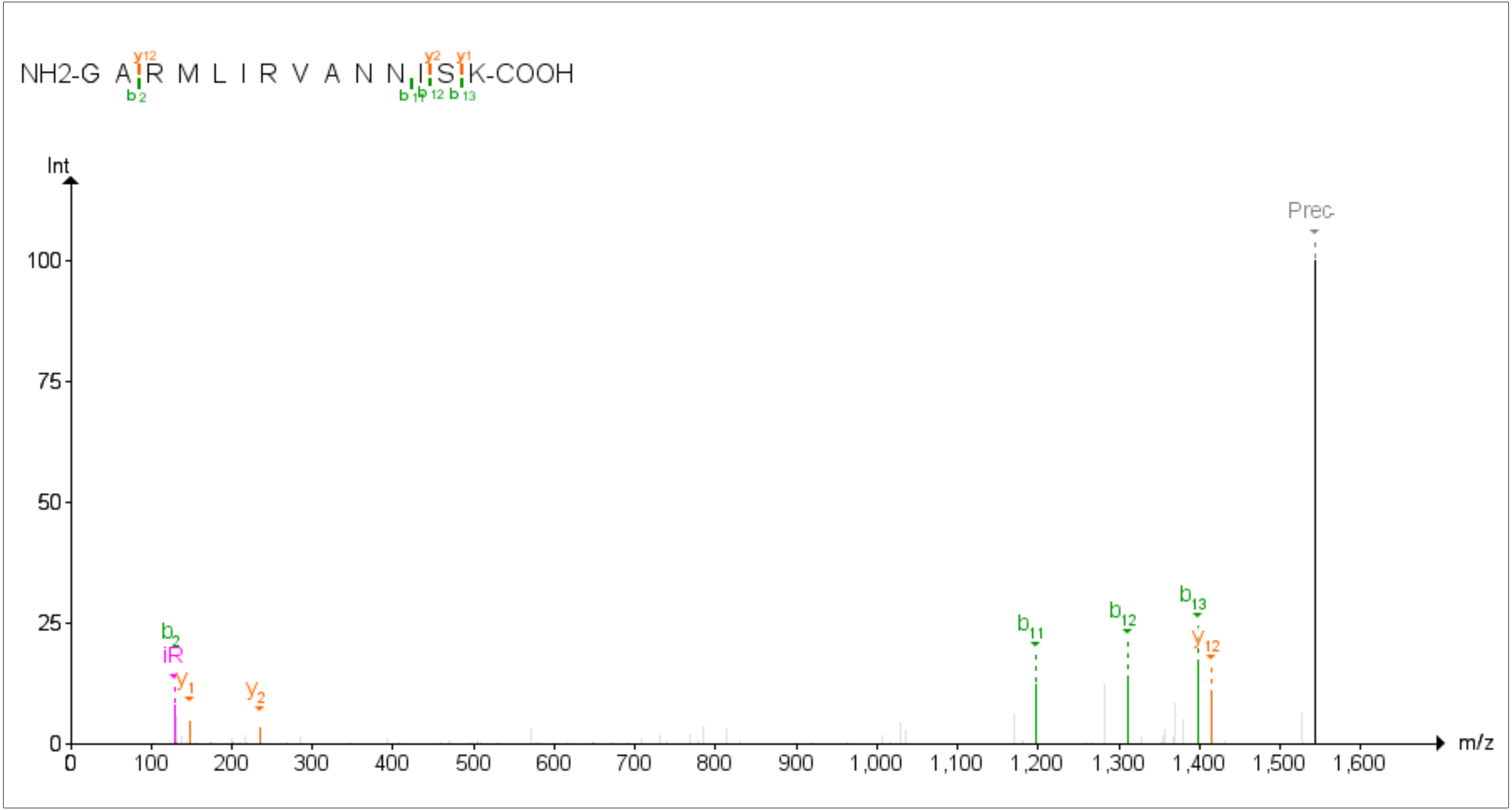

NH2-G A R M L I R V A N N I S K-COOH
y12 y2 y1
b2 b11 b12 b13
Int
m/z
Prec
b2
iR
y1
y2
b11
b12
b13
y12
0
25
50
75
100
0
100
200
300
400
500
600
700
800
900
1,000
1,100
1,200
1,300
1,400
1,500
1,600

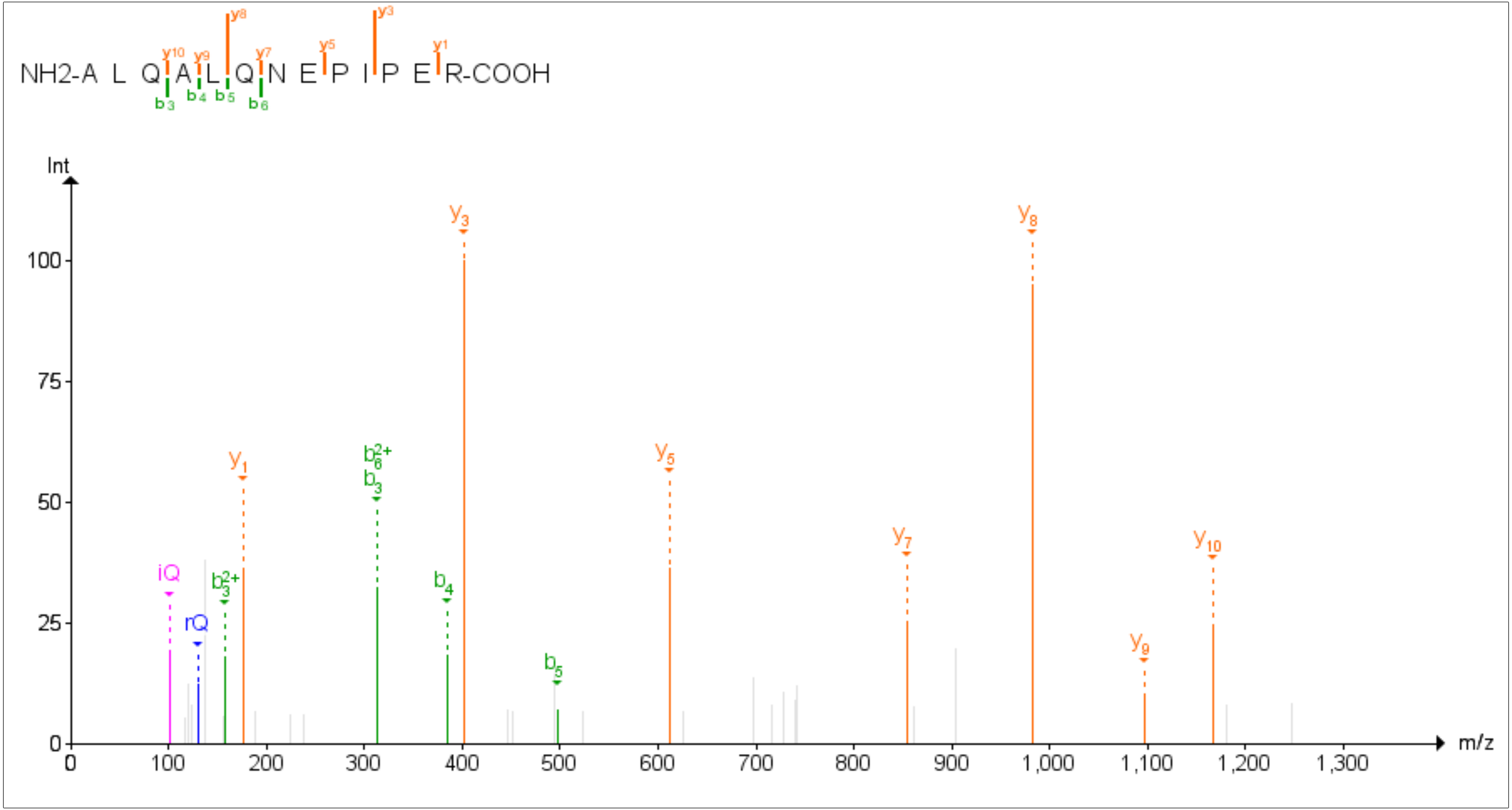

NH2-A L Q A L Q N E P I P E R-COOH
y10 y9 y8 y7 y5 y3 y1
b3 b4 b5 b6
Int
100
75
50
25
0
y3
y8
y1
b6 2+
b3
y5
y7
y10
iQ
b3 2+
b4
rQ
b5
y9
0 100 200 300 400 500 600 700 800 900 1,000 1,100 1,200 1,300
m/z

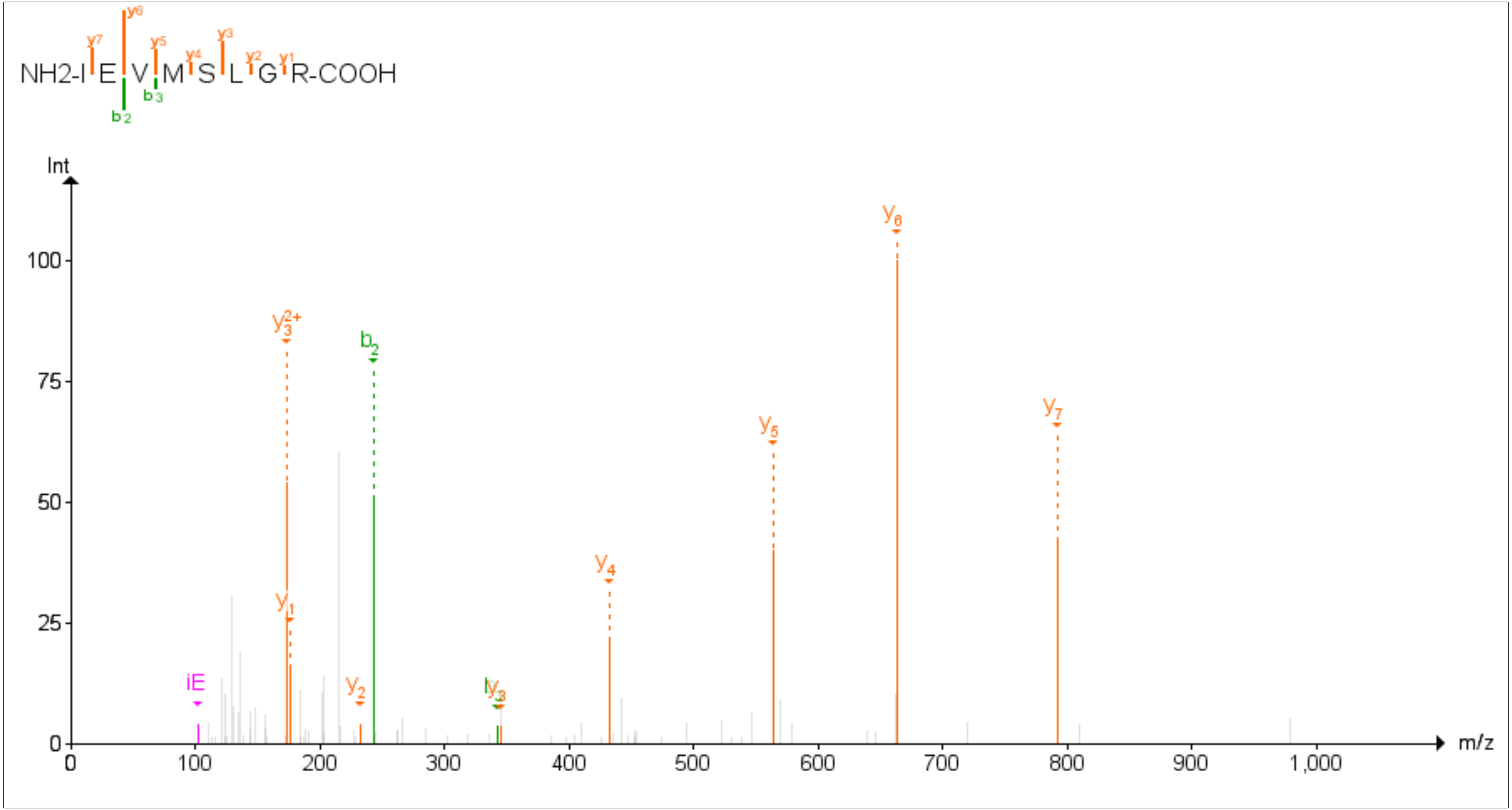

NH2-I E V M S L G R-COOH
y7 y6 y5 y4 y3 y2 y1
b2 b3
Int
m/z
0
25
50
75
100
0
100
200
300
400
500
600
700
800
900
1,000
iE
y1
y3 2+
y2
b2
b3
y3
y4
y5
y6
y7

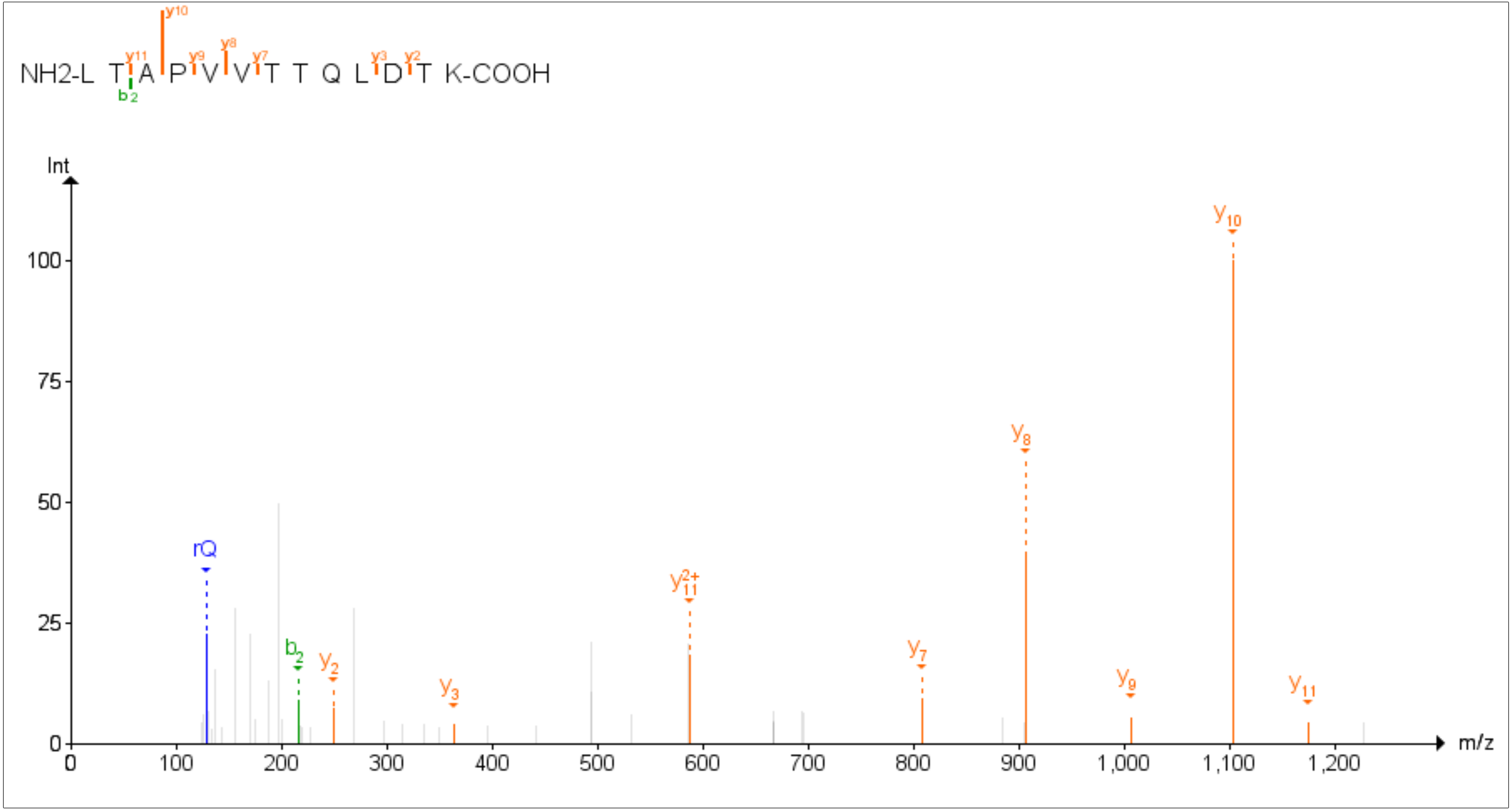

NH2-L T A P V V T T Q L D T K-COOH
y10
y11
y9
y8
y7
y3
y2
b2
Int
100
75
50
25
0
rQ
b2
y2
y3
y11 2+
y7
y8
y9
y10
y11
0
100
200
300
400
500
600
700
800
900
1,000
1,100
1,200
m/z

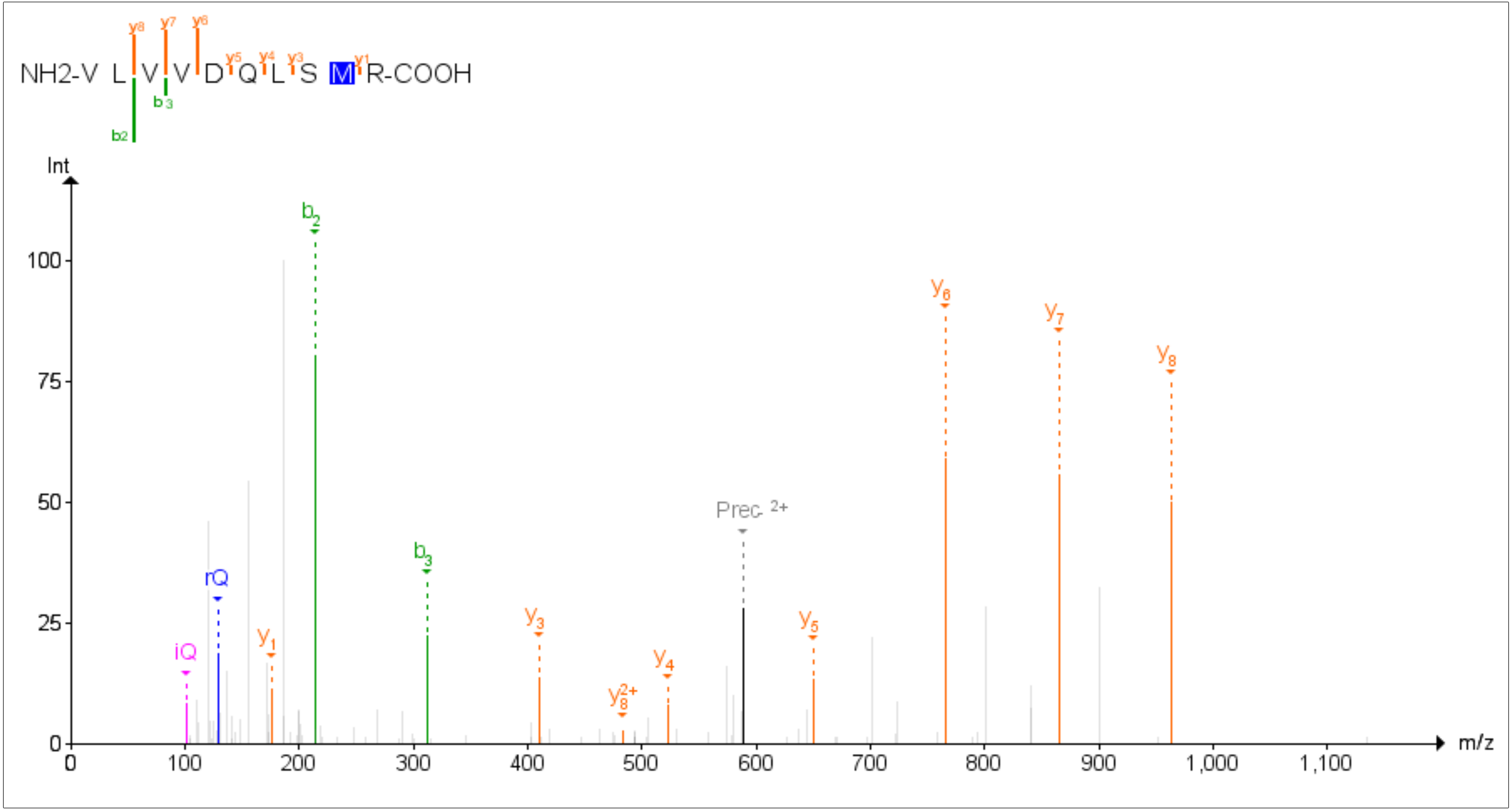

NH2-V L V V D Q L S M R-COOH
y8 y7 y6 y5 y4 y3 y1
b2 b3
Int
m/z
b2
b3
rQ
iQ
y1
y3
y8 2+
y4
Prec 2+
y5
y6
y7
y8
0
25
50
75
100
0
100
200
300
400
500
600
700
800
900
1,000
1,100

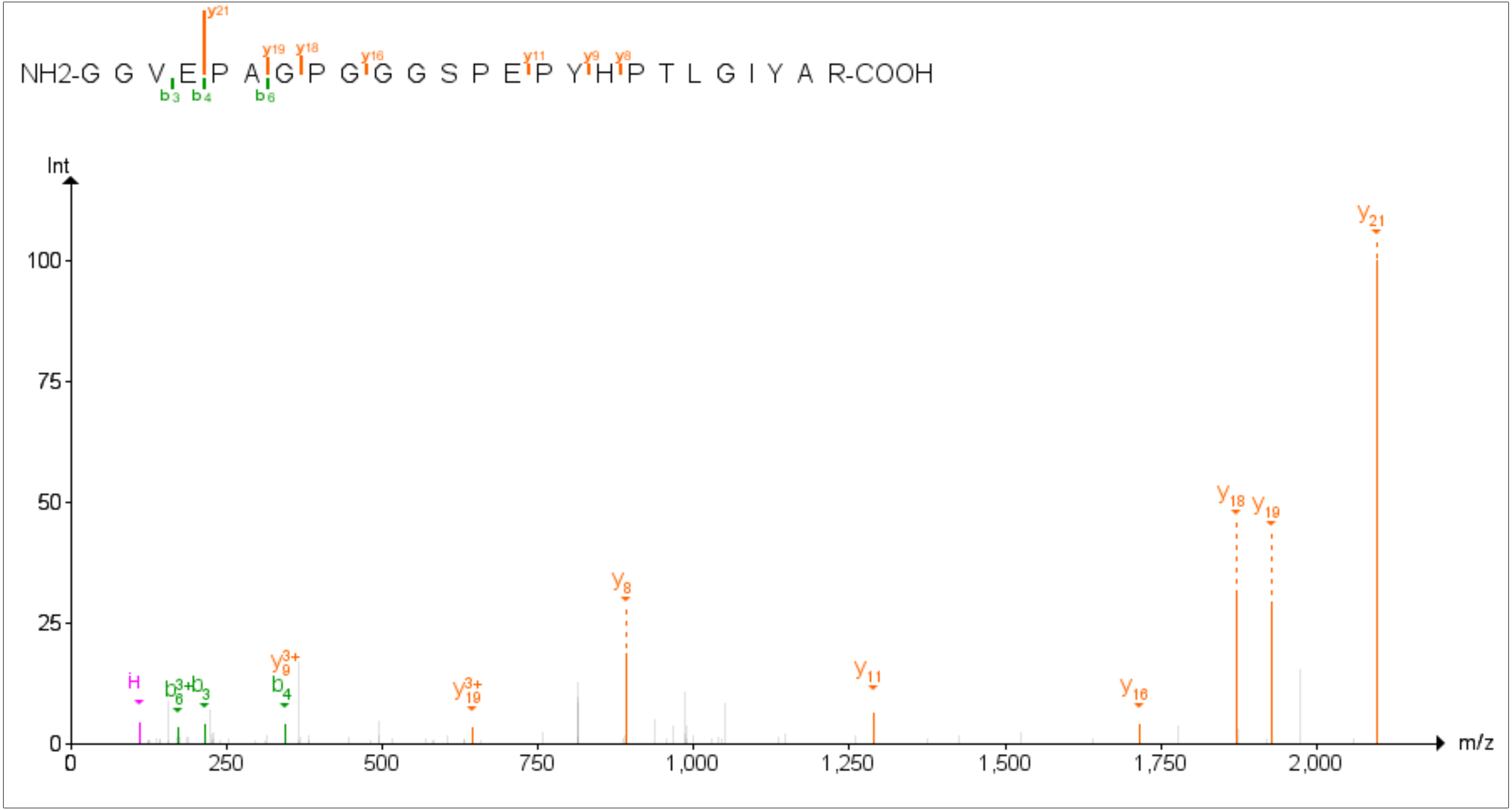

NH2-G G V E P A G P G G G S P E P Y H P T L G I Y A R-COOH
y21
y19
y18
y16
y11
y9
y8
b3
b4
b6
Int
100
75
50
25
0
y21
y18
y19
y8
H
b6 3+ +b3
y9 3+
b4
y19 3+
y11
y16
m/z
0
250
500
750
1,000
1,250
1,500
1,750
2,000

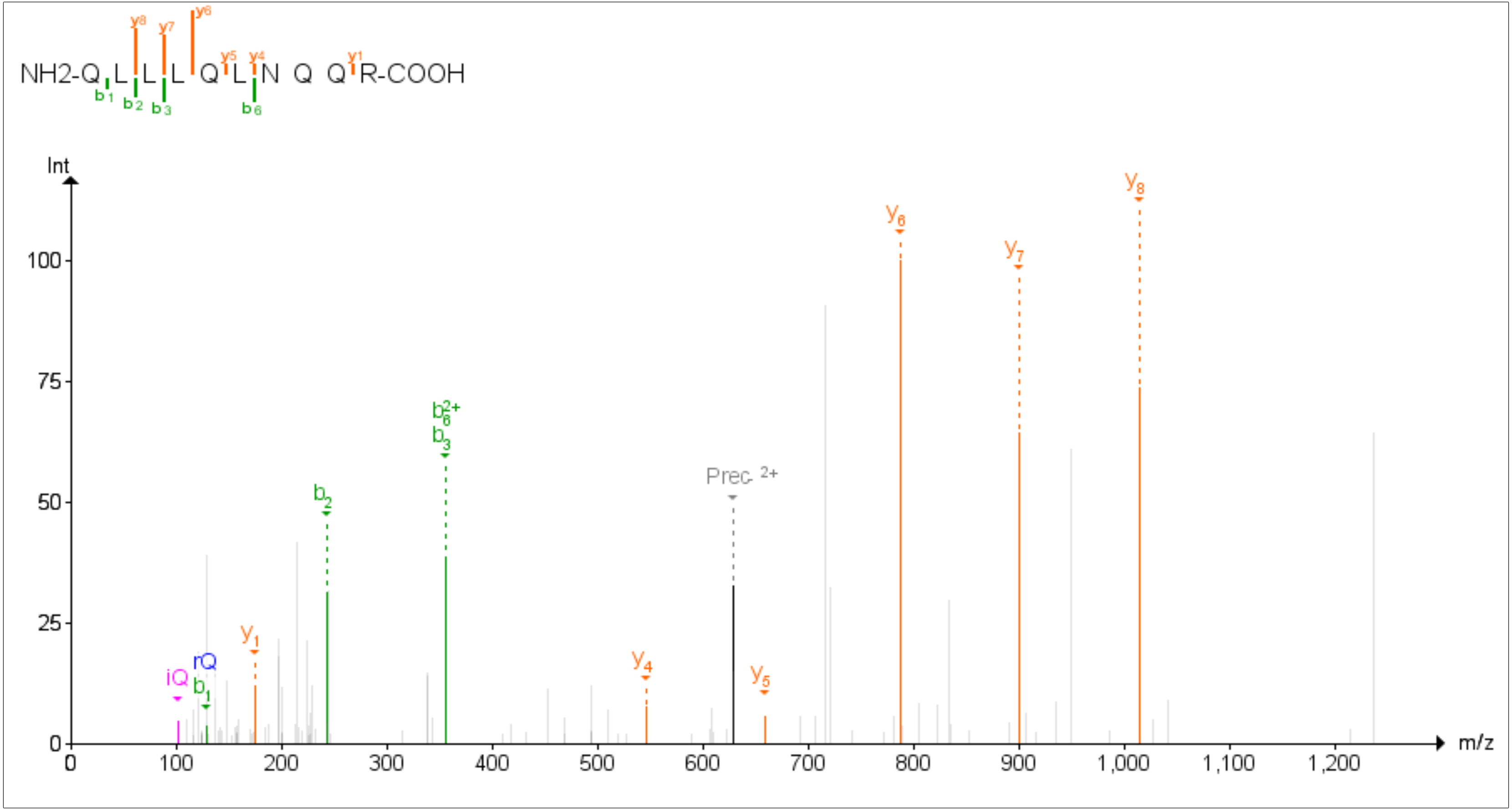

NH2-Q L L L Q L N Q Q R-COOH
y8 y7 y6 y5 y4 y1
b1 b2 b3 b6
Int
m/z
0
25
50
75
100
0
100
200
300
400
500
600
700
800
900
1,000
1,100
1,200
iQ
rQ
b1
y1
b2
b6 2+
b3
Prec 2+
y4
y5
y6
y7
y8

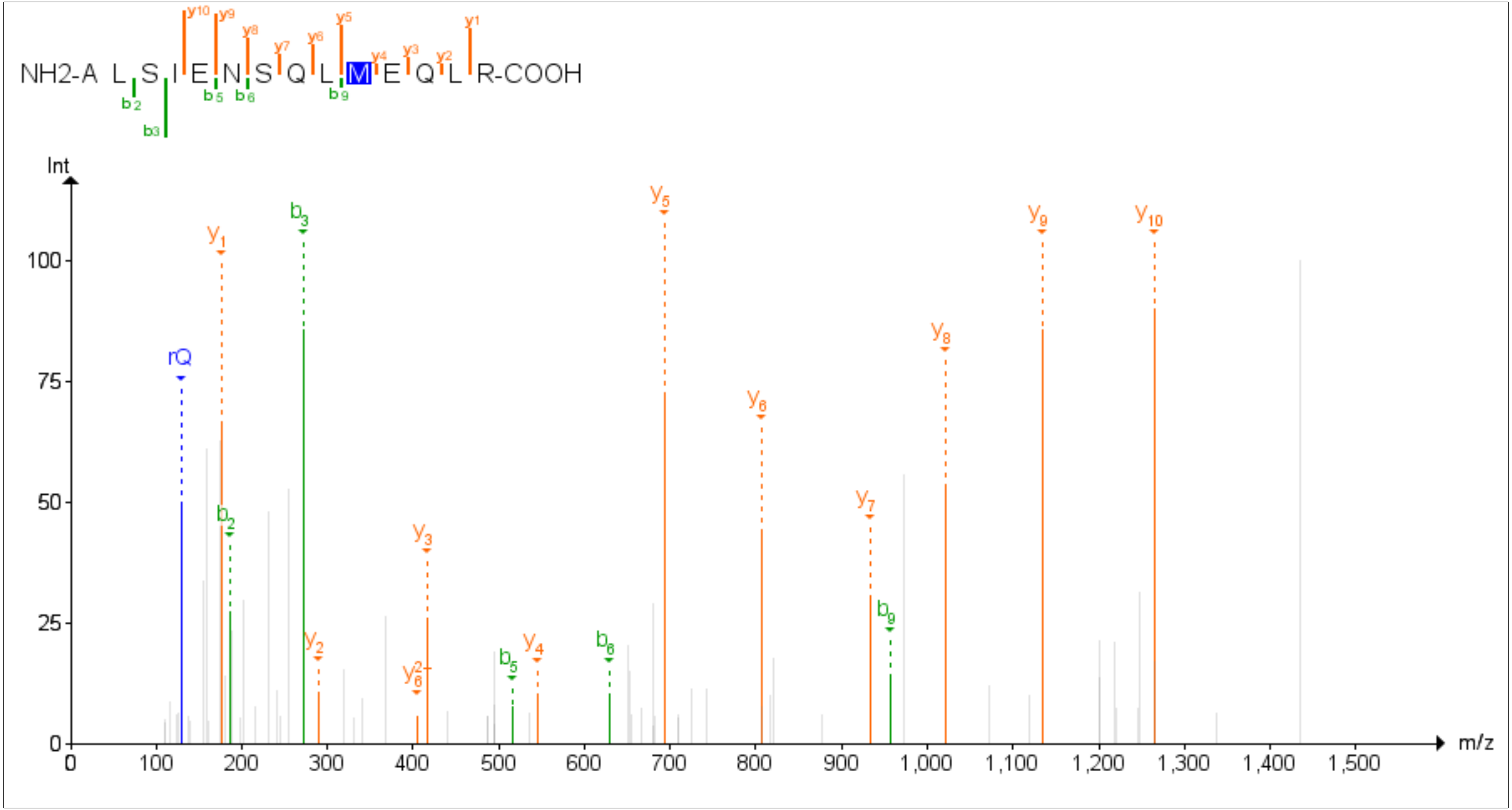

NH2-A L S I E N S Q L M E Q L R-COOH
y10 y9 y8 y7 y6 y5 y4 y3 y2 y1
b2 b3 b5 b6 b9
Int
rQ
y1
b2
b3
y2
y3
y6 2+
b5
y4
b6
y5
y6
y7
b9
y8
y9
y10
100
75
50
25
0
100
200
300
400
500
600
700
800
900
1,000
1,100
1,200
1,300
1,400
1,500
m/z

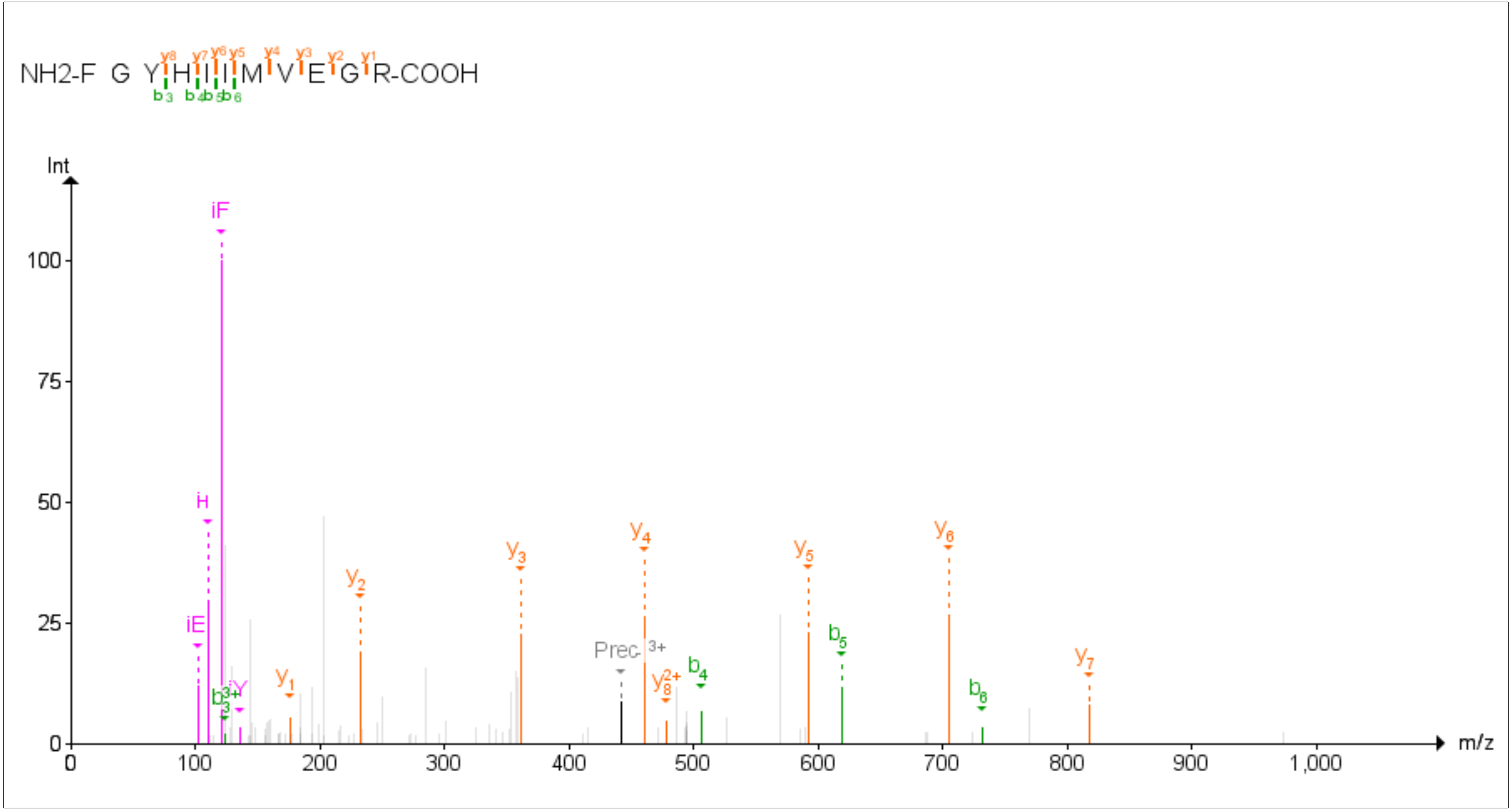
NH2-F G Y H I I M V E G R-COOH
y8 y7 y6 y5 y4 y3 y2 y1
b3 b4 b5 b6
Int
100
75
50
25
0
iF
H
iE
b3 3+
iY
y1
y2
y3
Prec 3+
y4
y8 2+
b4
y5
b5
y6
b6
y7
0
100
200
300
400
500
600
700
800
900
1,000
m/z

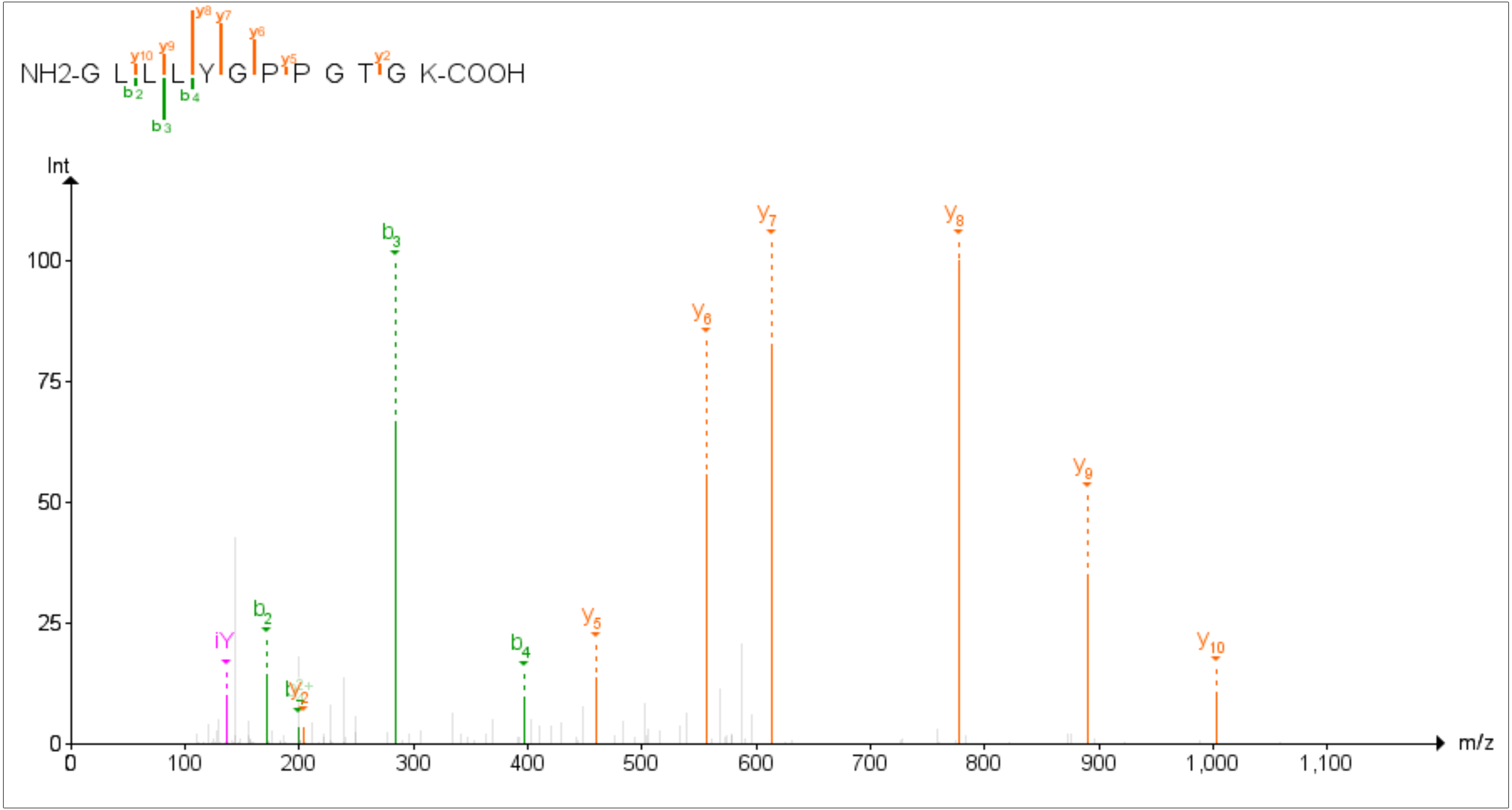

NH2-G L L L Y G P P G T G K-COOH
y10 y9 y8 y7 y6 y5 y2
b2 b3 b4
Int
0
25
50
75
100
m/z
0
100
200
300
400
500
600
700
800
900
1,000
1,100
iY
b2
b3
b4
y5
y6
y7
y8
y9
y10
y2

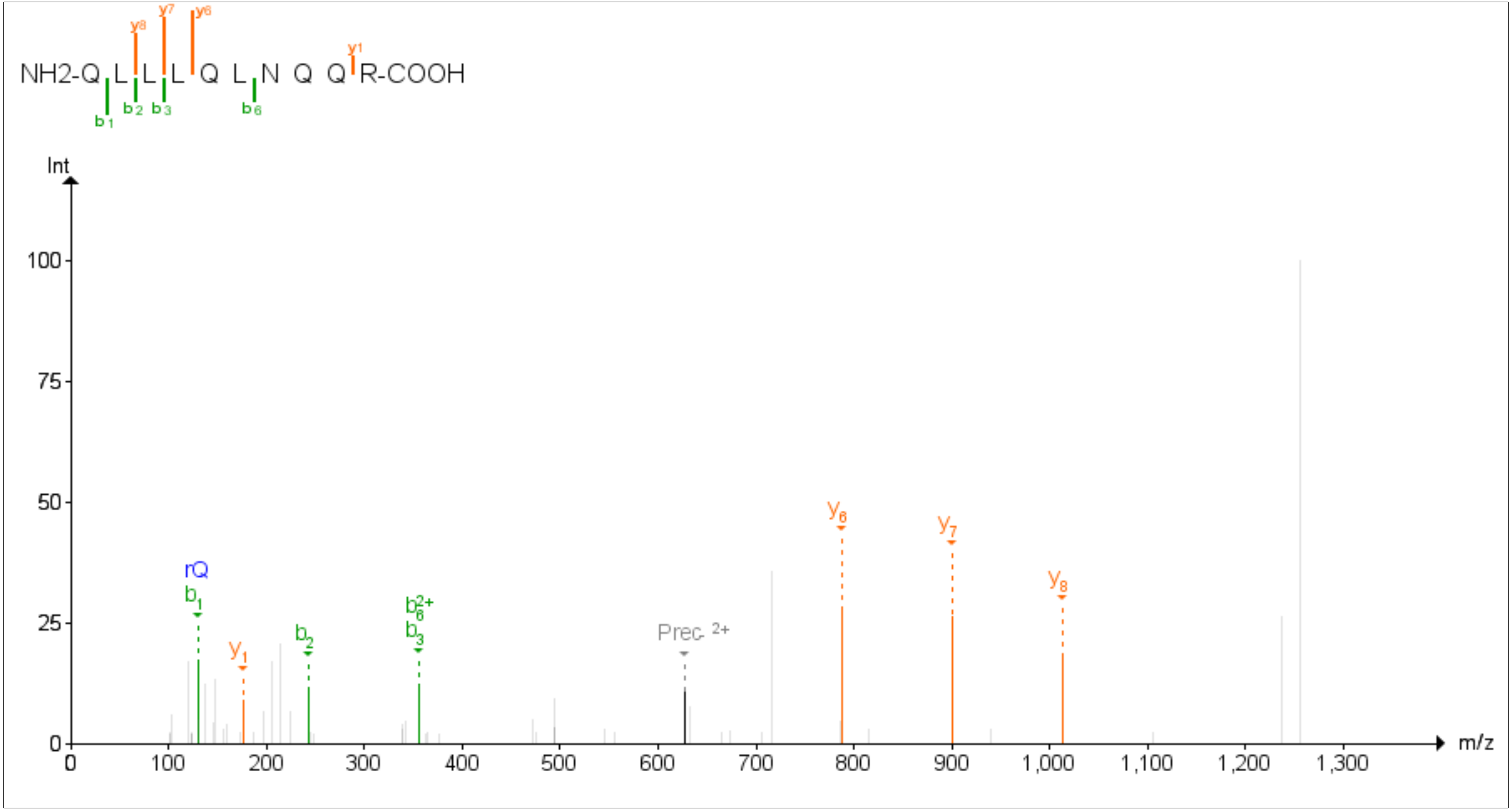

NH2-Q L L L Q L N Q Q R-COOH
y8 y7 y6 y1
b1 b2 b3 b6
Int
100
75
50
25
0
rQ
b1
y1
b2
b2+6
b3
Prec 2+
y6
y7
y8
0 100 200 300 400 500 600 700 800 900 1,000 1,100 1,200 1,300
m/z

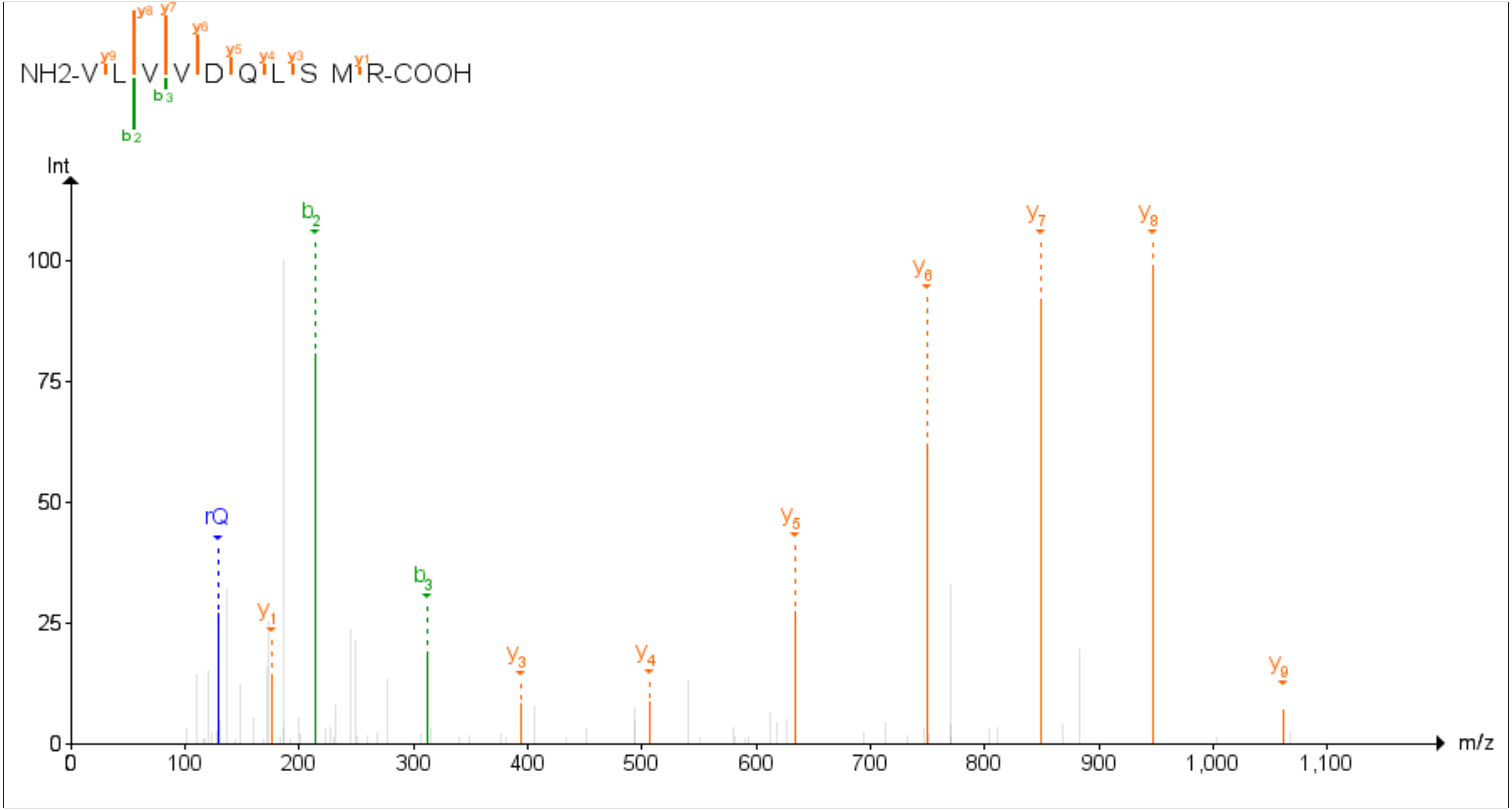

NH2-V L V V D Q L S M R-COOH
y9 y8 y7 y6 y5 y4 y3 y1
b2 b3
Int
100
75
50
25
0
rQ
b2
b3
y1
y3
y4
y5
y6
y7
y8
y9
0 100 200 300 400 500 600 700 800 900 1,000 1,100
m/z

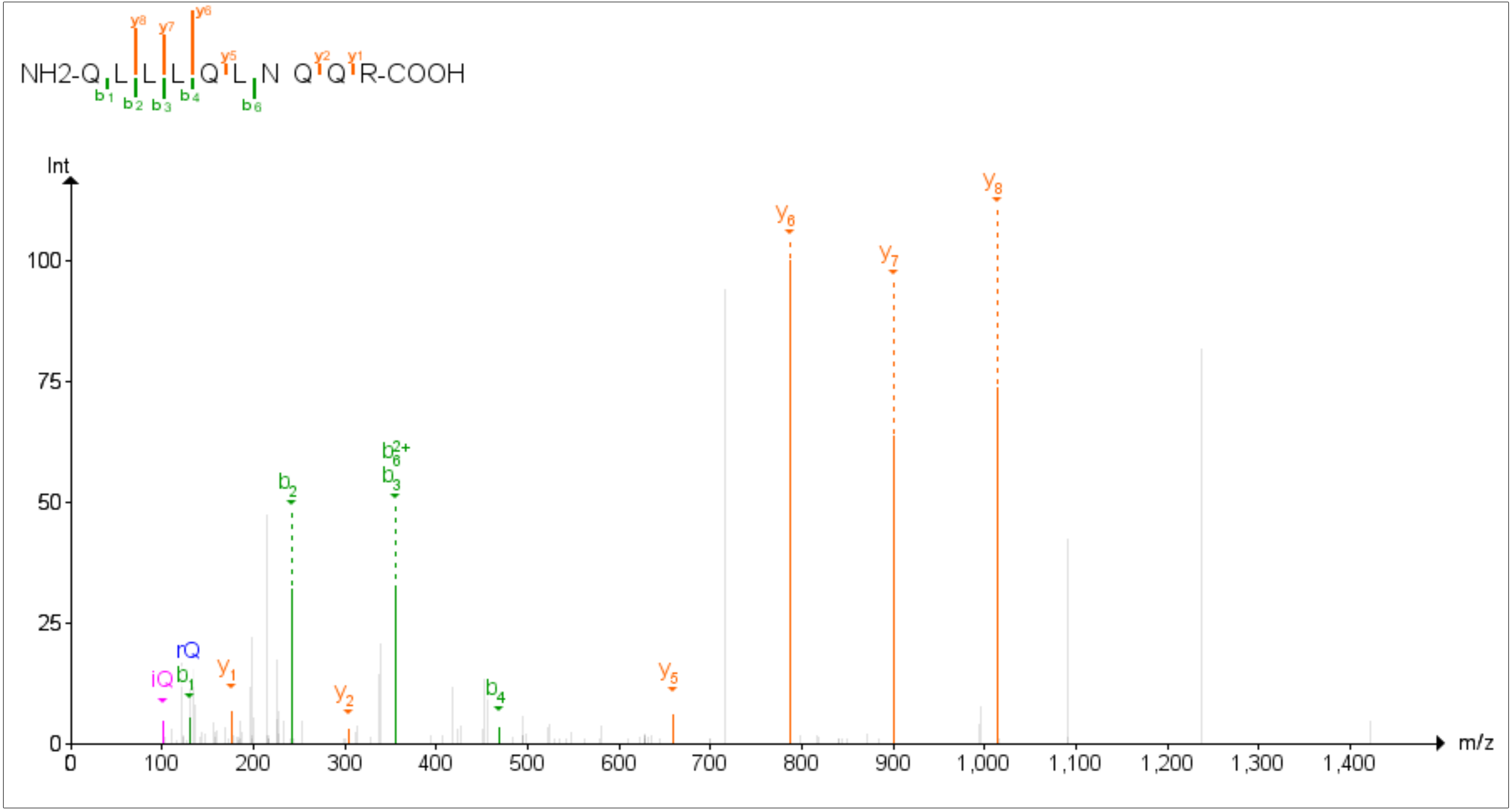
NH2-Q L L L Q L N Q Q R-COOH
y8 y7 y6 y5 y2 y1
b1 b2 b3 b4 b6
Int
100
75
50
25
0
m/z
0 100 200 300 400 500 600 700 800 900 1,000 1,100 1,200 1,300 1,400
iQ
rQ
b1
y1
b2
y2
b6 2+
b3
b4
y5
y6
y7
y8

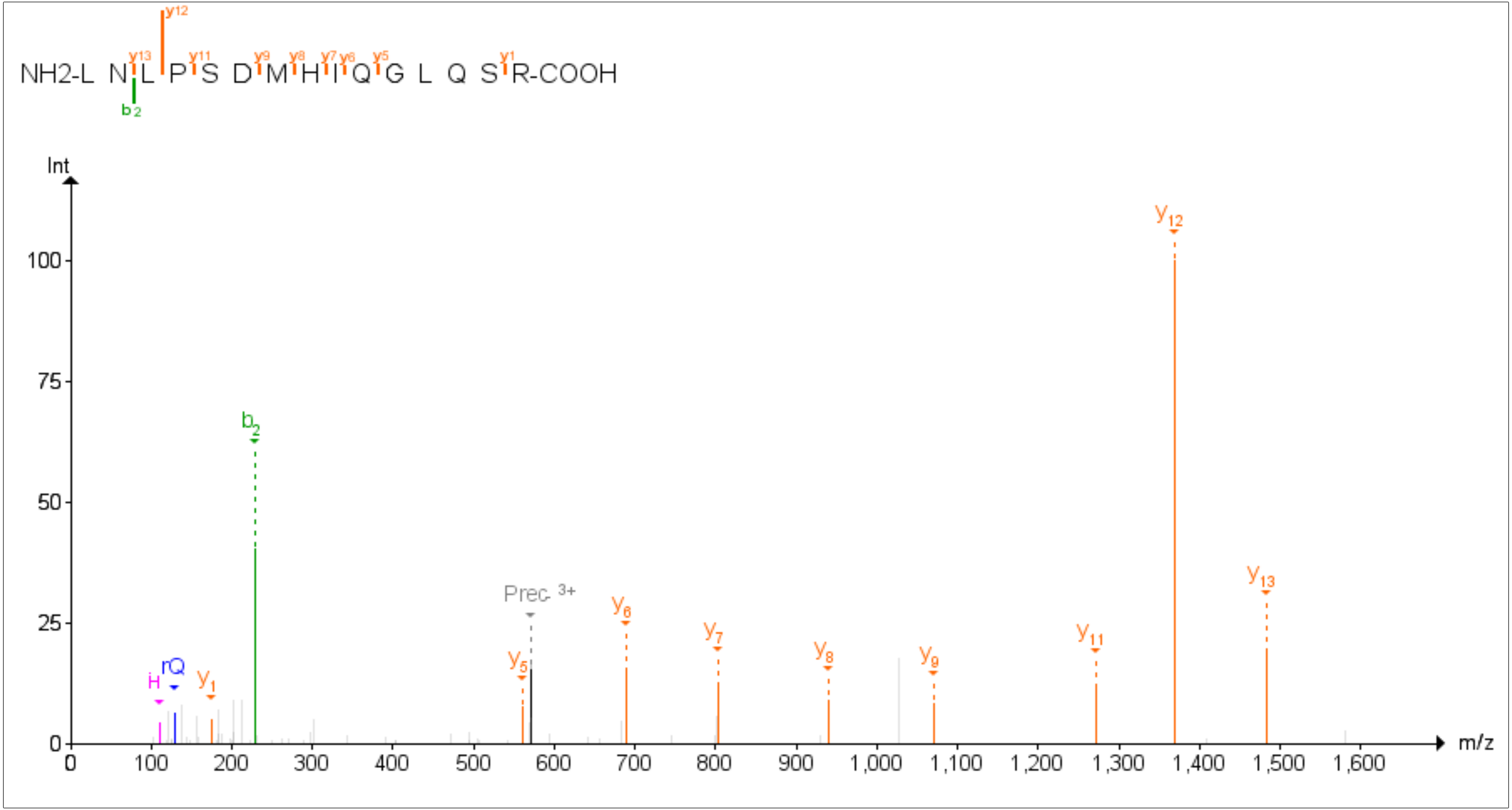

NH2-L N L P S D M H I Q G L Q S R-COOH
y12
y13
y11
y9
y8
y7
y6
y5
y1
b2
Int
100
75
50
25
0
m/z
0
100
200
300
400
500
600
700
800
900
1,000
1,100
1,200
1,300
1,400
1,500
1,600
Prec 3+

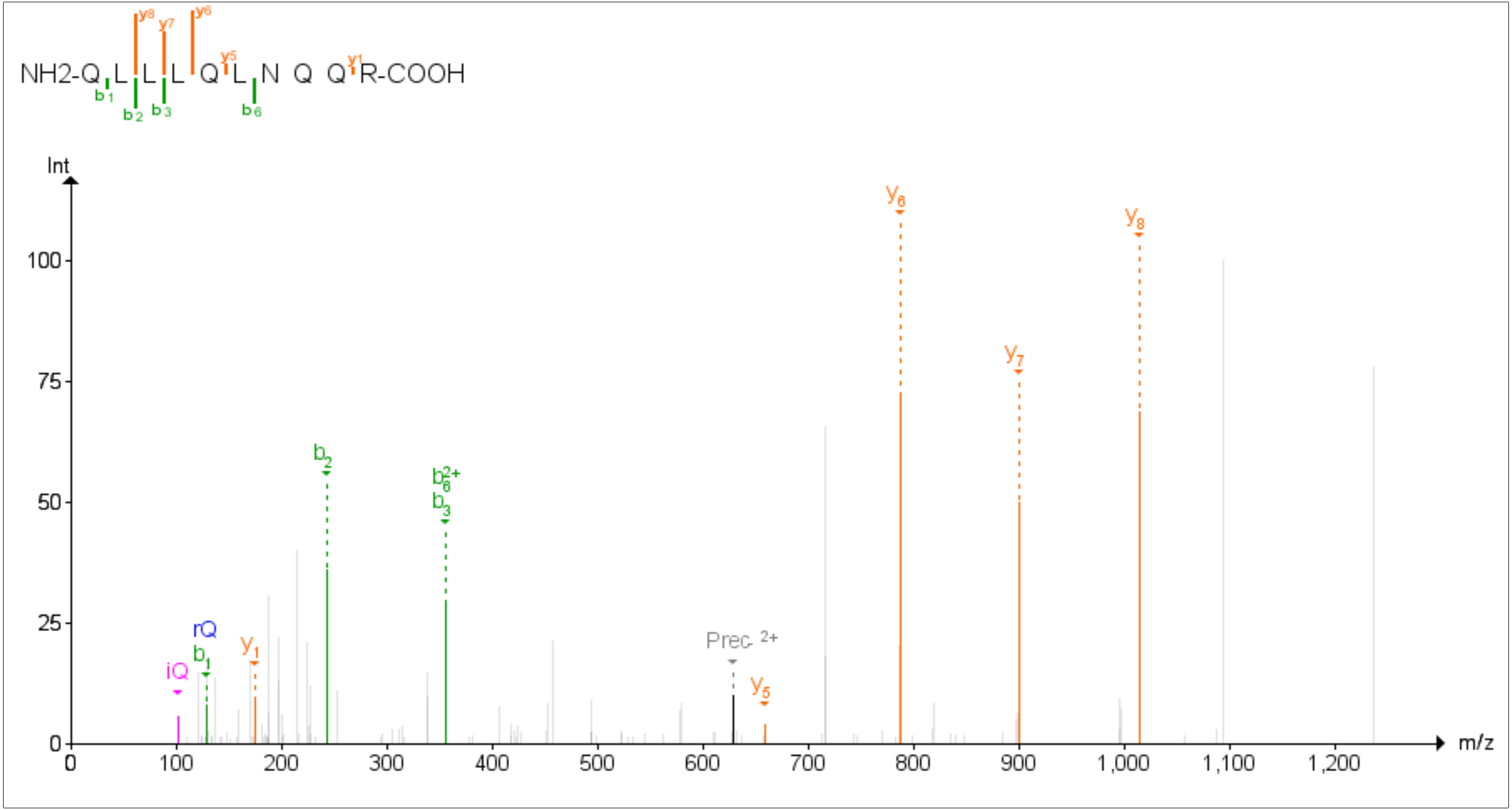

NH2-Q L L L Q L N Q Q R-COOH
y8 y7 y6 y5 y1
b1 b2 b3 b6
Int
100
75
50
25
0
y6
y8
y7
b2
b6 2+
b3
rQ
b1
iQ
y1
Prec 2+
y5
0 100 200 300 400 500 600 700 800 900 1,000 1,100 1,200
m/z

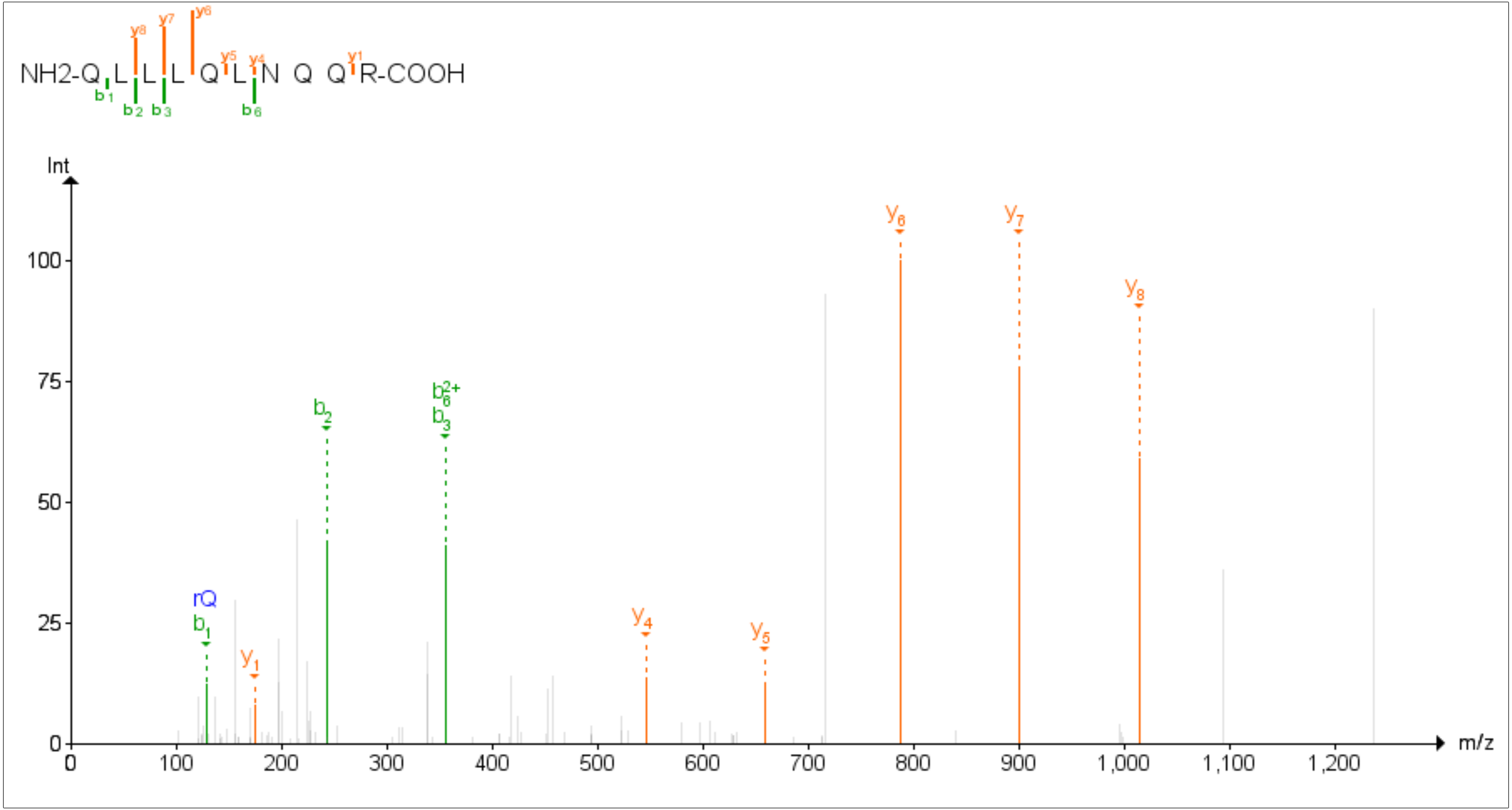

NH2-Q L L L Q L N Q Q R-COOH
b1
b2
b3
b6
y8
y7
y6
y5
y4
y1
Int
100
75
50
25
0
b2
b6 2+
b3
rQ
b1
y1
y4
y5
y6
y7
y8
0
100
200
300
400
500
600
700
800
900
1,000
1,100
1,200
m/z

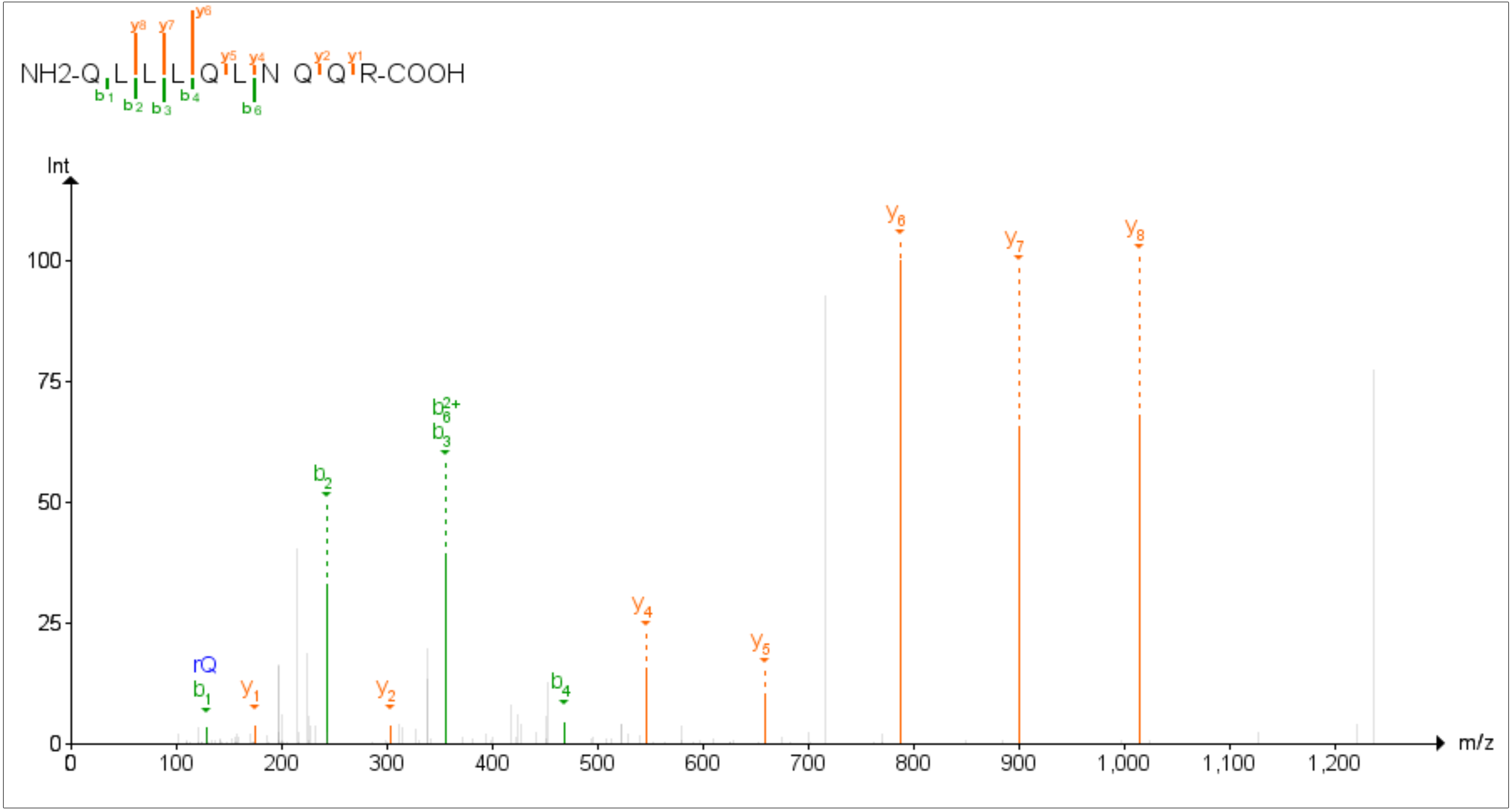
NH2-Q L L L Q L N Q Q R-COOH
y8 y7 y6 y5 y4 y2 y1
b1 b2 b3 b4 b6
Int
100
75
50
25
0
m/z
0 100 200 300 400 500 600 700 800 900 1,000 1,100 1,200
rQ
b1
y1
y2
b2
b6 2+
b3
b4
y4
y5
y6
y7
y8

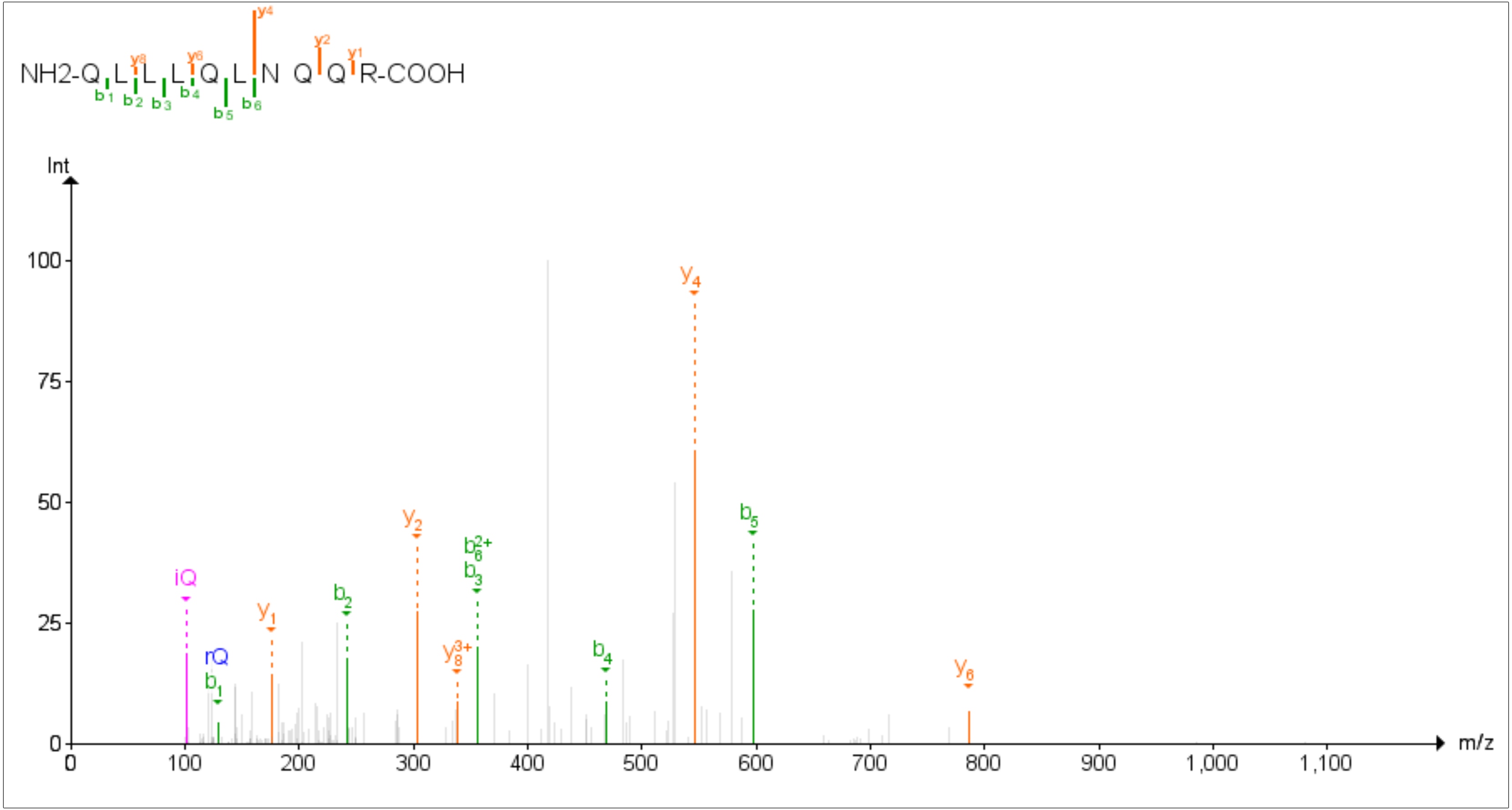

NH2-Q L L L Q L N Q Q R-COOH
b1 b2 b3 b4 b5 b6
y8 y6 y4 y2 y1
Int
m/z
iQ
rQ
b1
y1
b2
y2
b6 2+
b3
y8 3+
b4
y4
b5
y6

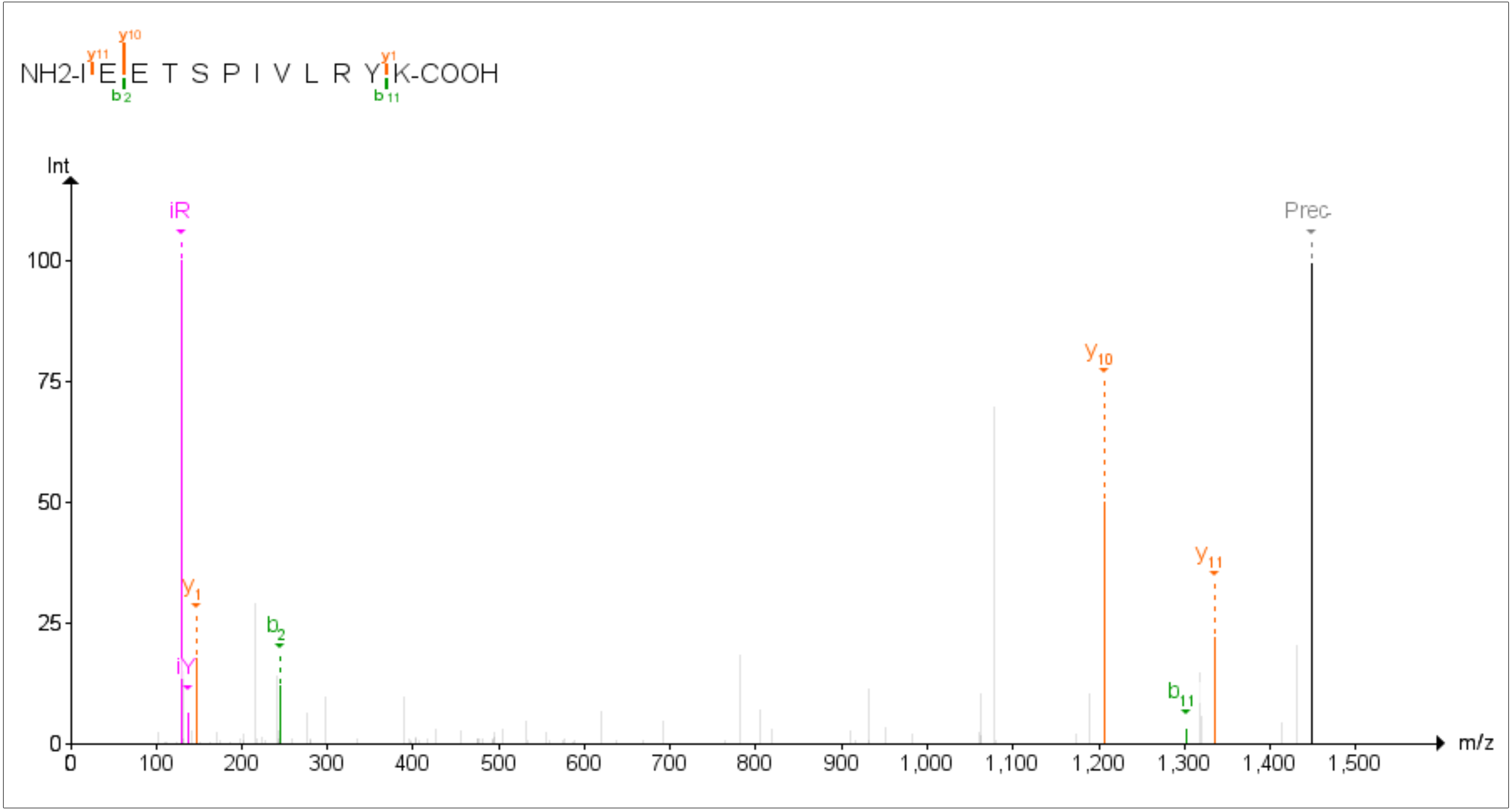

NH2-I E E T S P I V L R Y K-COOH
y11
y10
y1
b2
b11
Int
iR
Prec
100
75
50
25
0
y10
y11
y1
iY
b2
b11
0
100
200
300
400
500
600
700
800
900
1,000
1,100
1,200
1,300
1,400
1,500
m/z

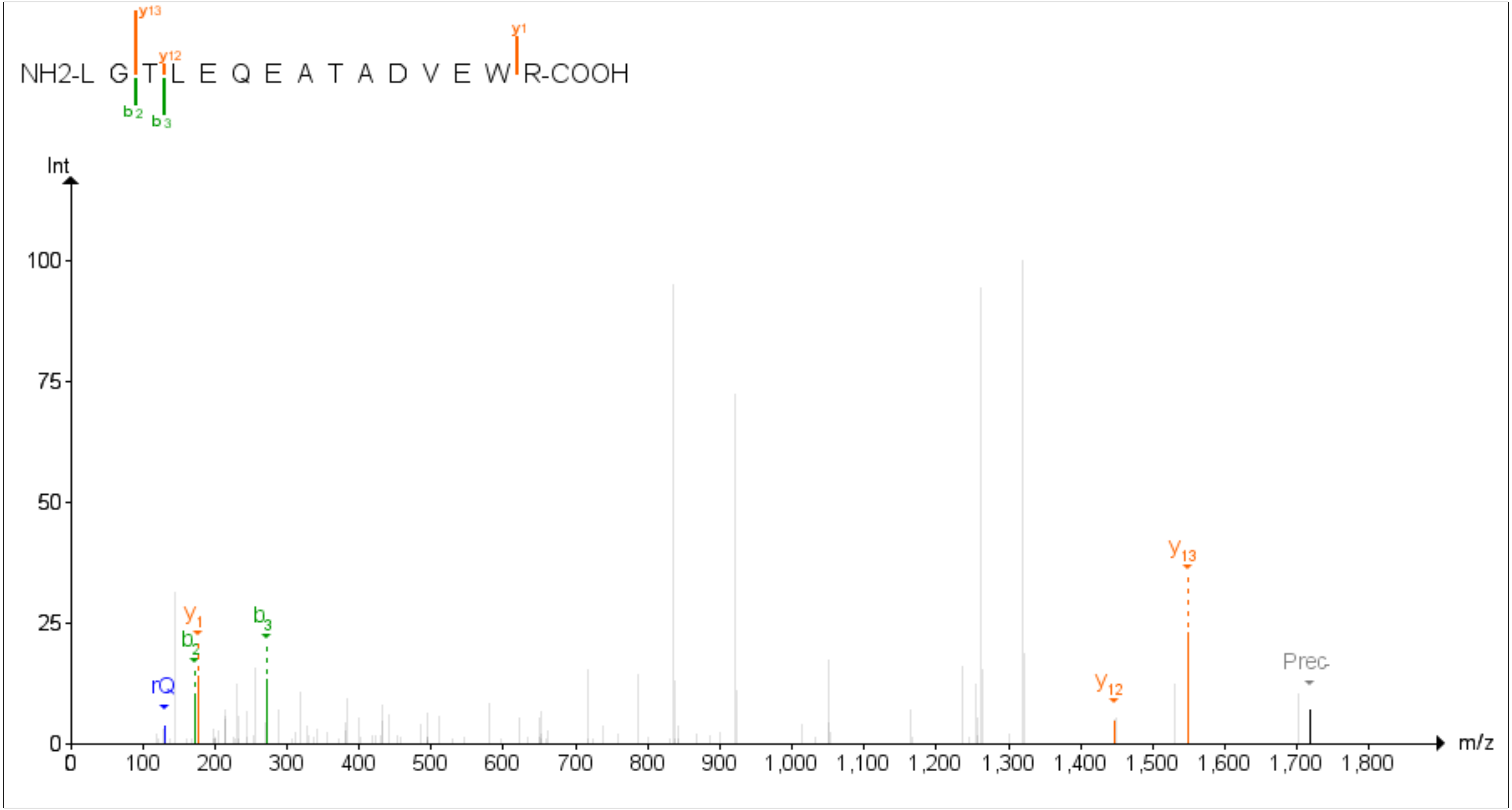

NH2-L G T L E Q E A T A D V E W R-COOH
y13
y12
y1
b2
b3
Int
100
75
50
25
0
rQ
y1
b2
b3
y13
y12
Prec
m/z
0
100
200
300
400
500
600
700
800
900
1,000
1,100
1,200
1,300
1,400
1,500
1,600
1,700
1,800

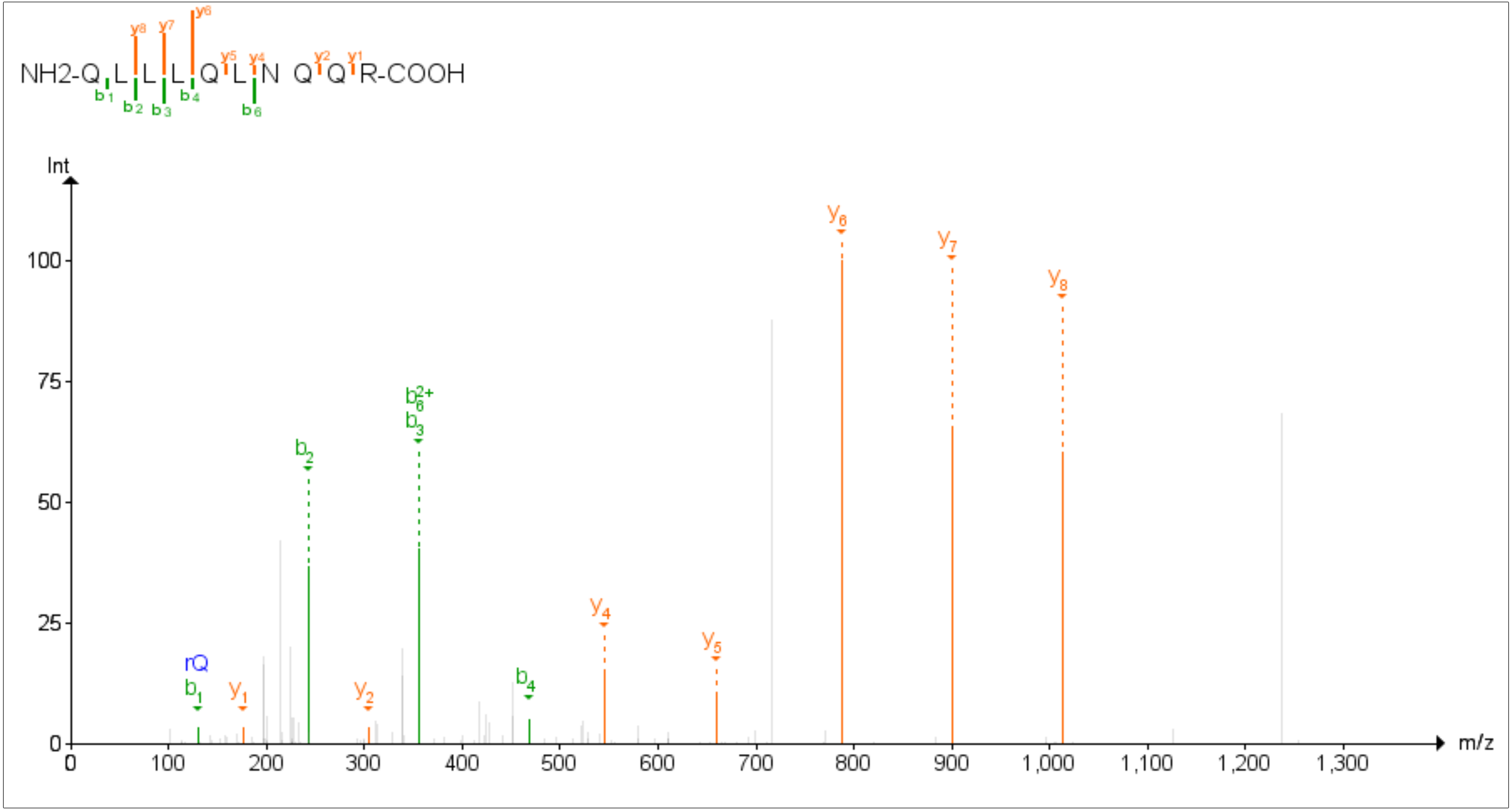

NH2-Q L L L Q L N Q Q R-COOH
y8 y7 y6 y5 y4 y2 y1
b1 b2 b3 b4 b6
Int
100
75
50
25
0
rQ
b1
y1
b2
y2
b6 2+
b3
b4
y4
y5
y6
y7
y8
0
100
200
300
400
500
600
700
800
900
1,000
1,100
1,200
1,300
m/z

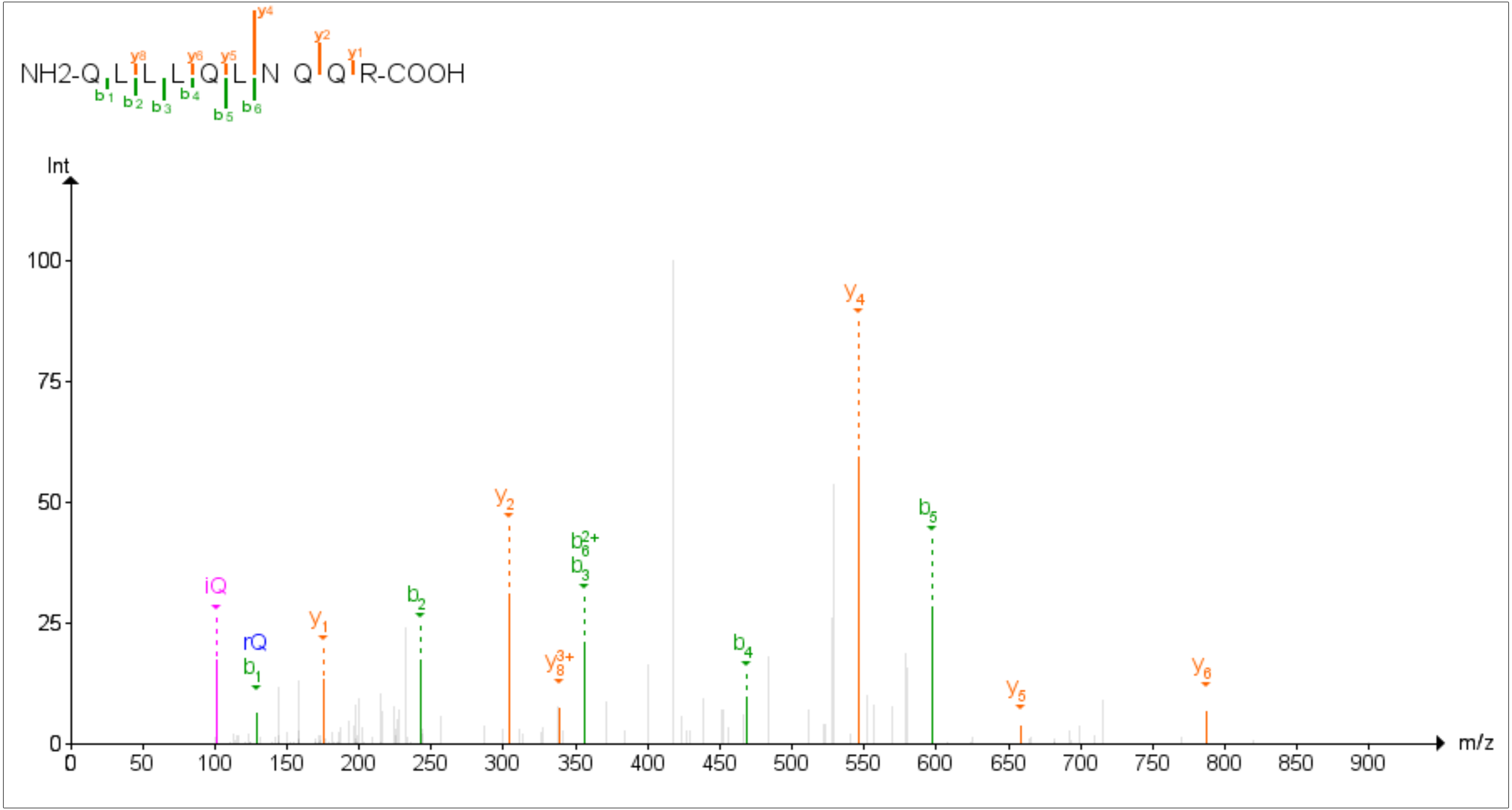

NH2-Q L L L L Q L N Q Q R-COOH
y8 y6 y5 y4 y2 y1
b1 b2 b3 b4 b5 b6
Int
100
75
50
25
0
iQ
rQ
b1
y1
b2
y2
y8 3+
b6 2+
b3
b4
y4
b5
y5
y6
0 50 100 150 200 250 300 350 400 450 500 550 600 650 700 750 800 850 900
m/z

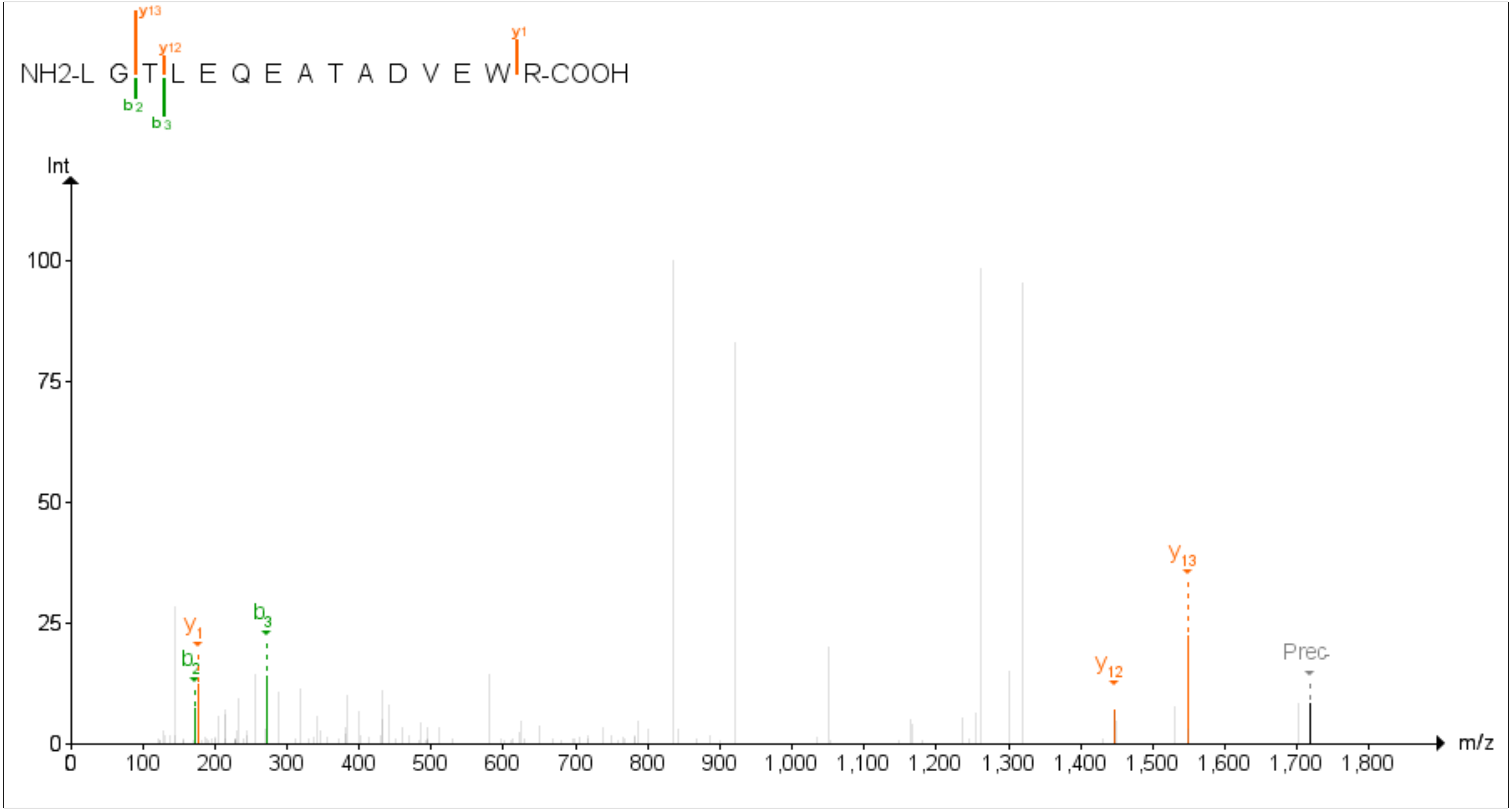

NH2-L G T L E Q E A T A D V E W R-COOH
y13
y12
y1
b2
b3
Int
100
75
50
25
0
y1
b2
b3
y12
y13
Prec
m/z
0
100
200
300
400
500
600
700
800
900
1,000
1,100
1,200
1,300
1,400
1,500
1,600
1,700
1,800

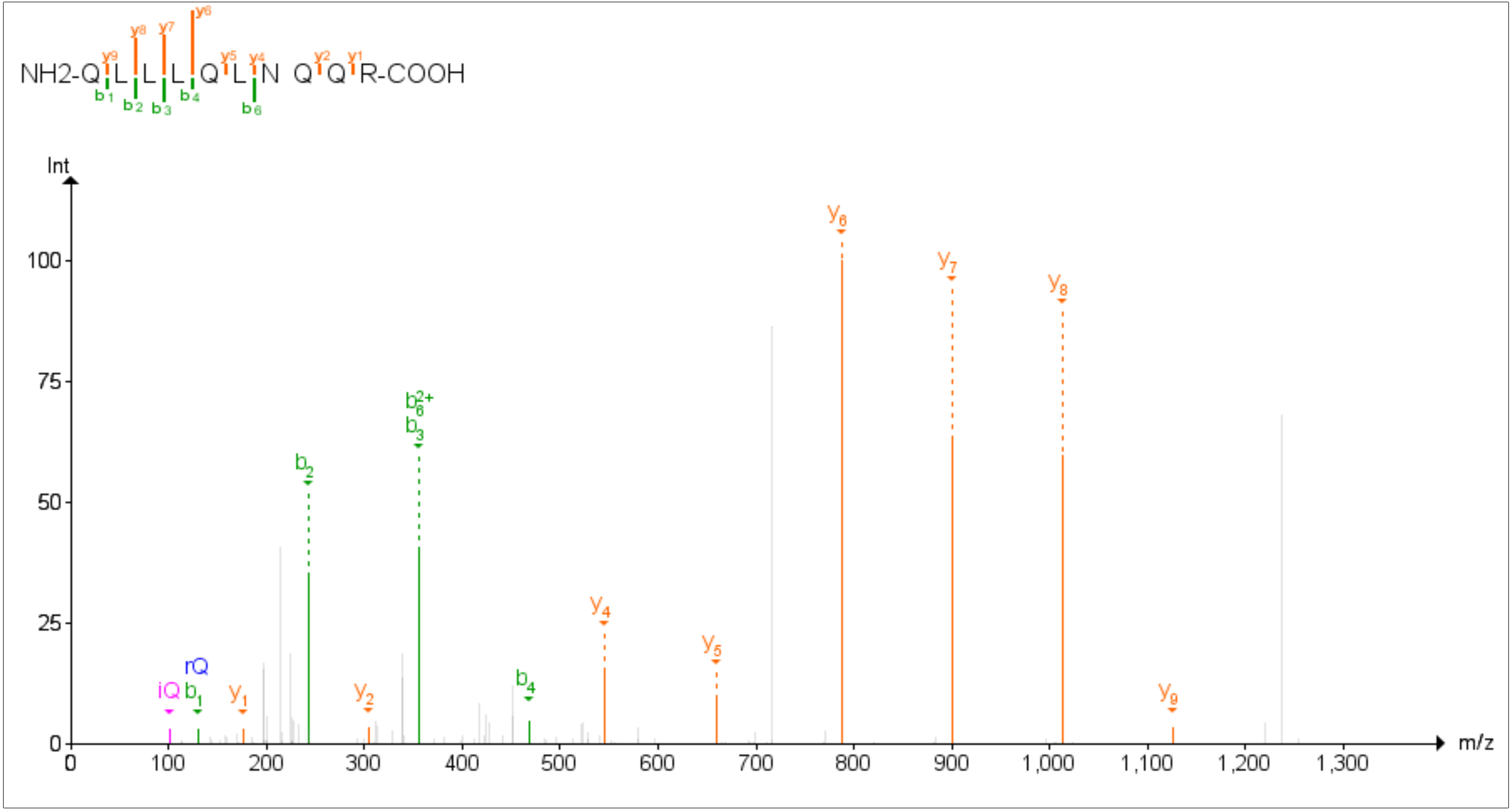

NH2-Q L L L Q L N Q Q R-COOH
y9 y8 y7 y6 y5 y4 y2 y1
b1 b2 b3 b4 b6
Int
100
75
50
25
0
m/z
0 100 200 300 400 500 600 700 800 900 1,000 1,100 1,200 1,300
y_6
y_7
y_8
b_6^{2+}
b_3
b_2
y_4
y_5
b_4
rQ
iQ
b_1
y_1
y_2
y_9

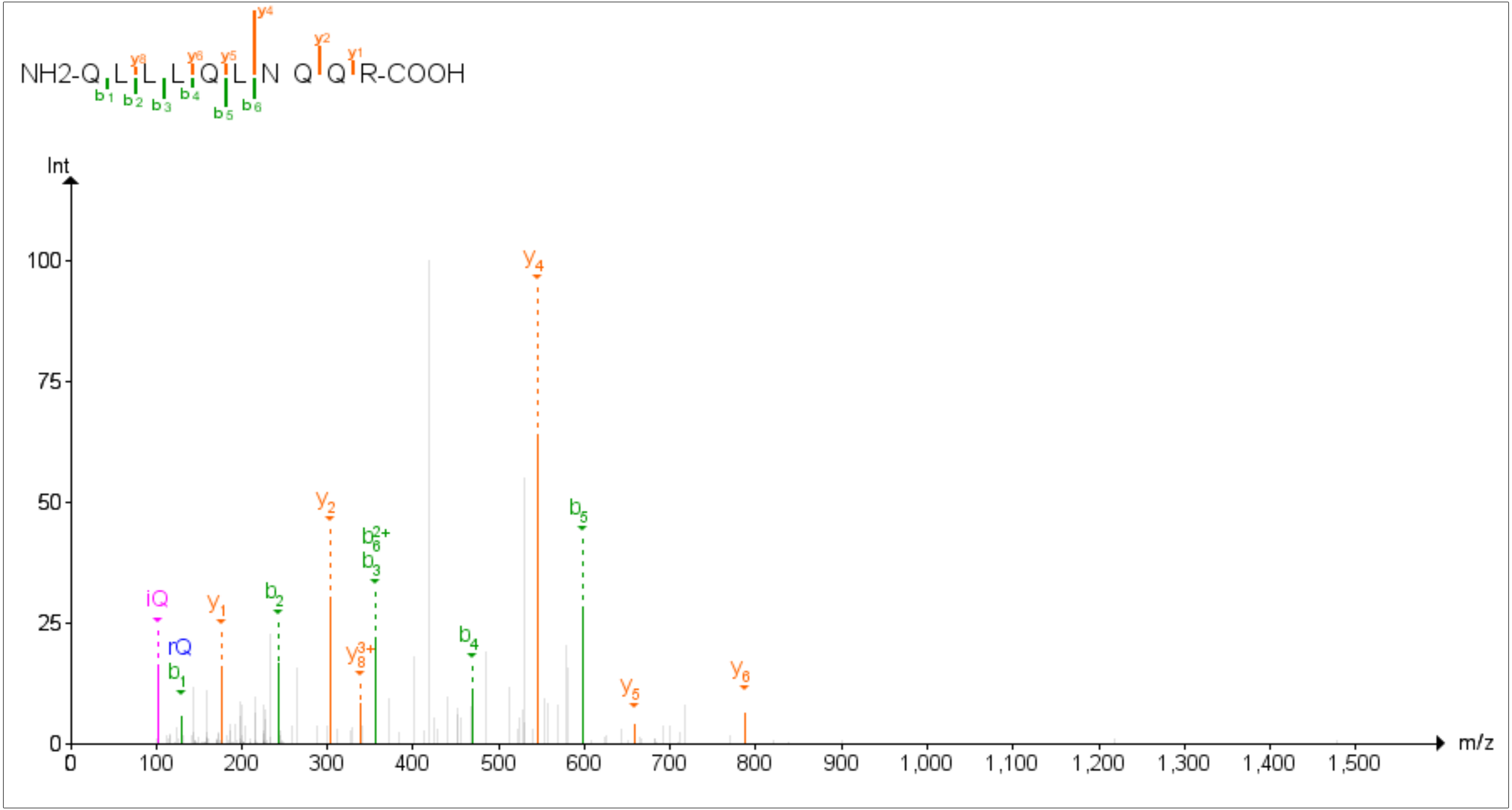

NH2-Q L L L Q L N Q Q R-COOH
b1 b2 b3 b4 b5 b6
y8 y6 y5 y4 y2 y1
Int
m/z
iQ
rQ
b1
y1
b2
y2
y8 3+
b6 2+
b3
b4
y4
b5
y5
y6

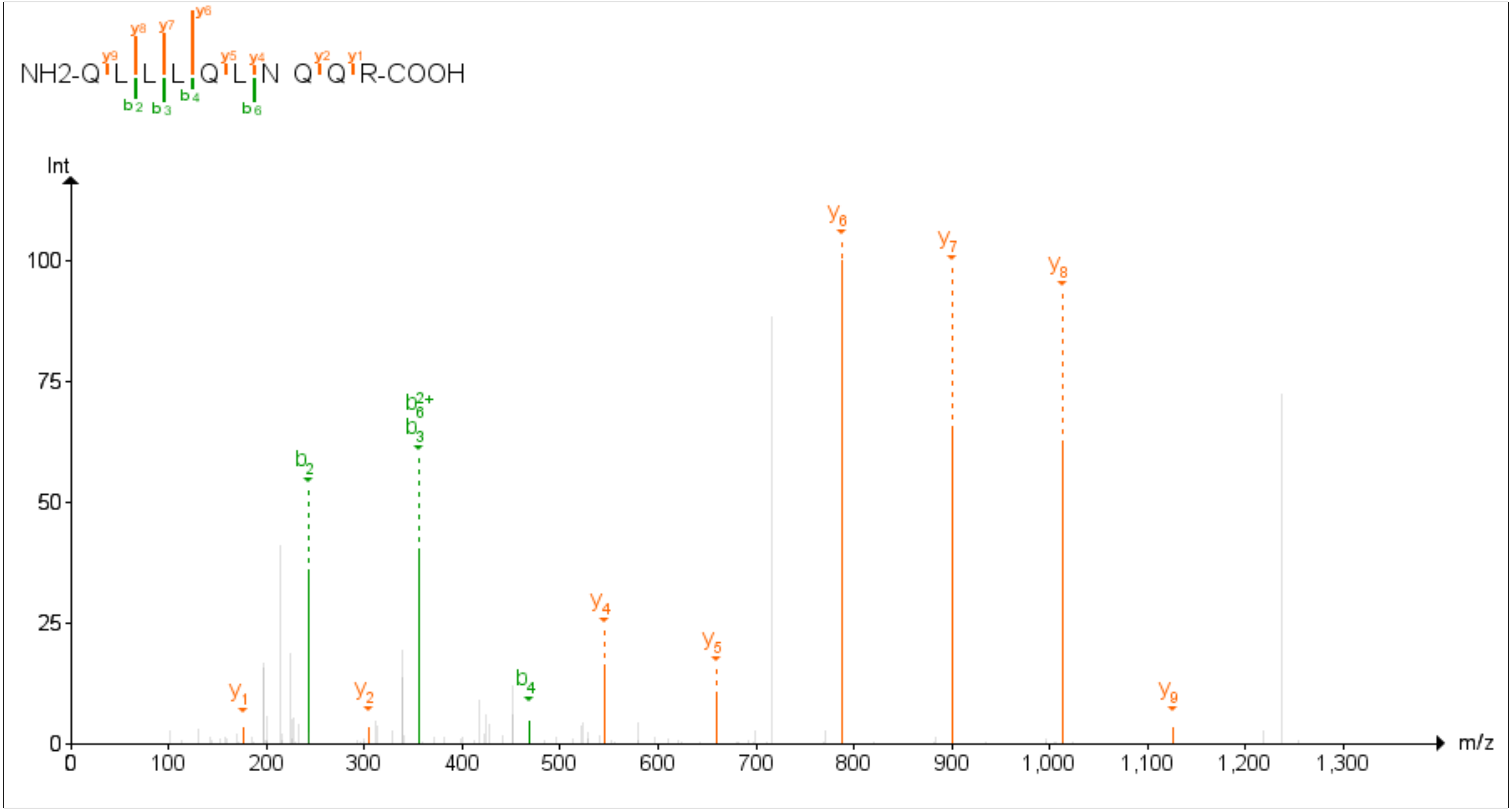

NH2-Q L L L Q L N Q Q R-COOH
y9 y8 y7 y6 y5 y4 y2 y1
b2 b3 b4 b6
Int
100
75
50
25
0
m/z
0
100
200
300
400
500
600
700
800
900
1,000
1,100
1,200
1,300
y6
y7
y8
b6 2+
b3
b2
y4
y5
b4
y1
y2
y9

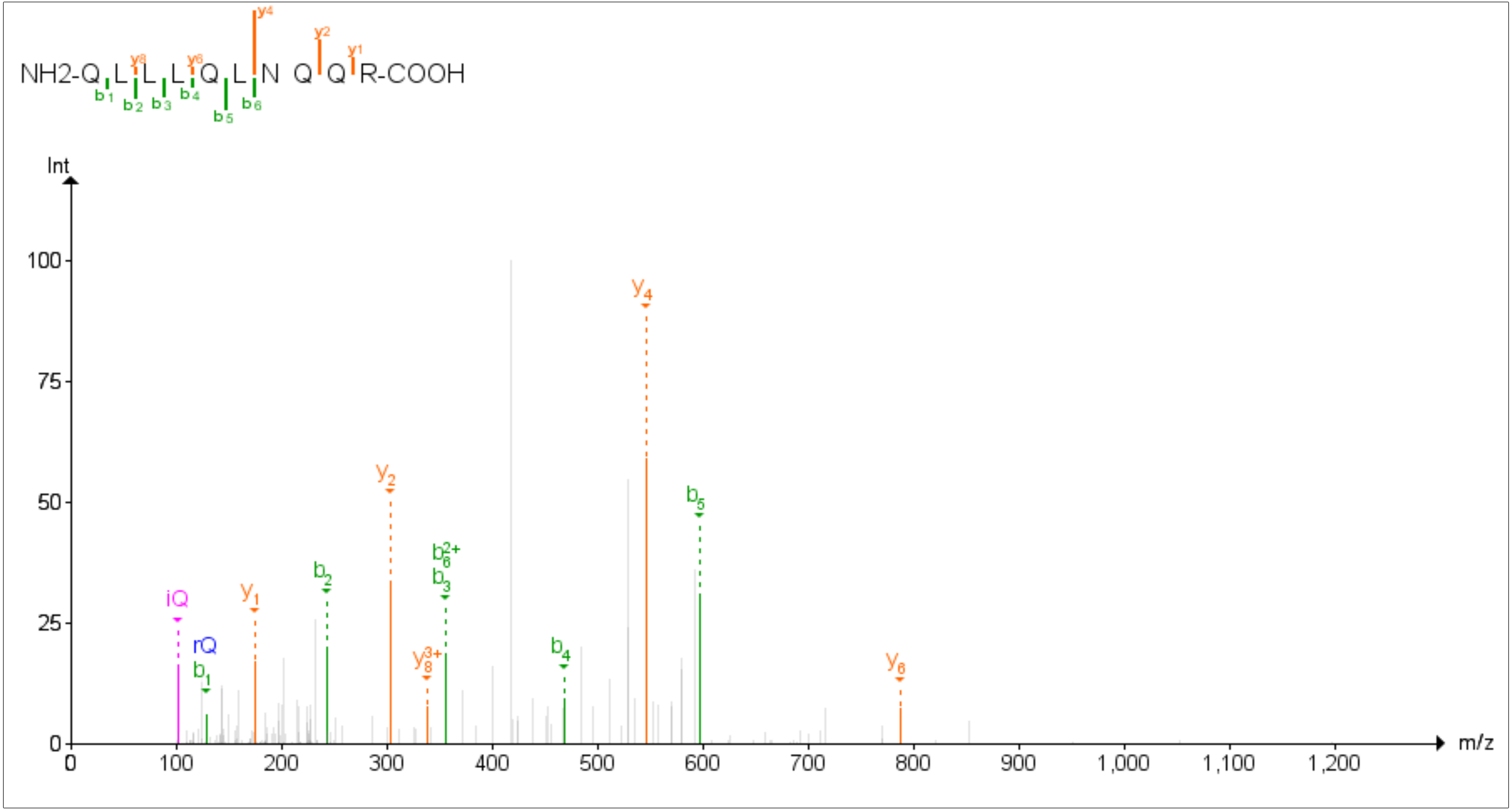

NH2-Q L L L Q L N Q Q R-COOH
y8 y6 y4 y2 y1
b1 b2 b3 b4 b5 b6
Int
100
75
50
25
0
iQ
rQ
b1
y1
b2
y2
y8 3+
b6 2+
b3
b4
y4
b5
y6
0 100 200 300 400 500 600 700 800 900 1,000 1,100 1,200
m/z

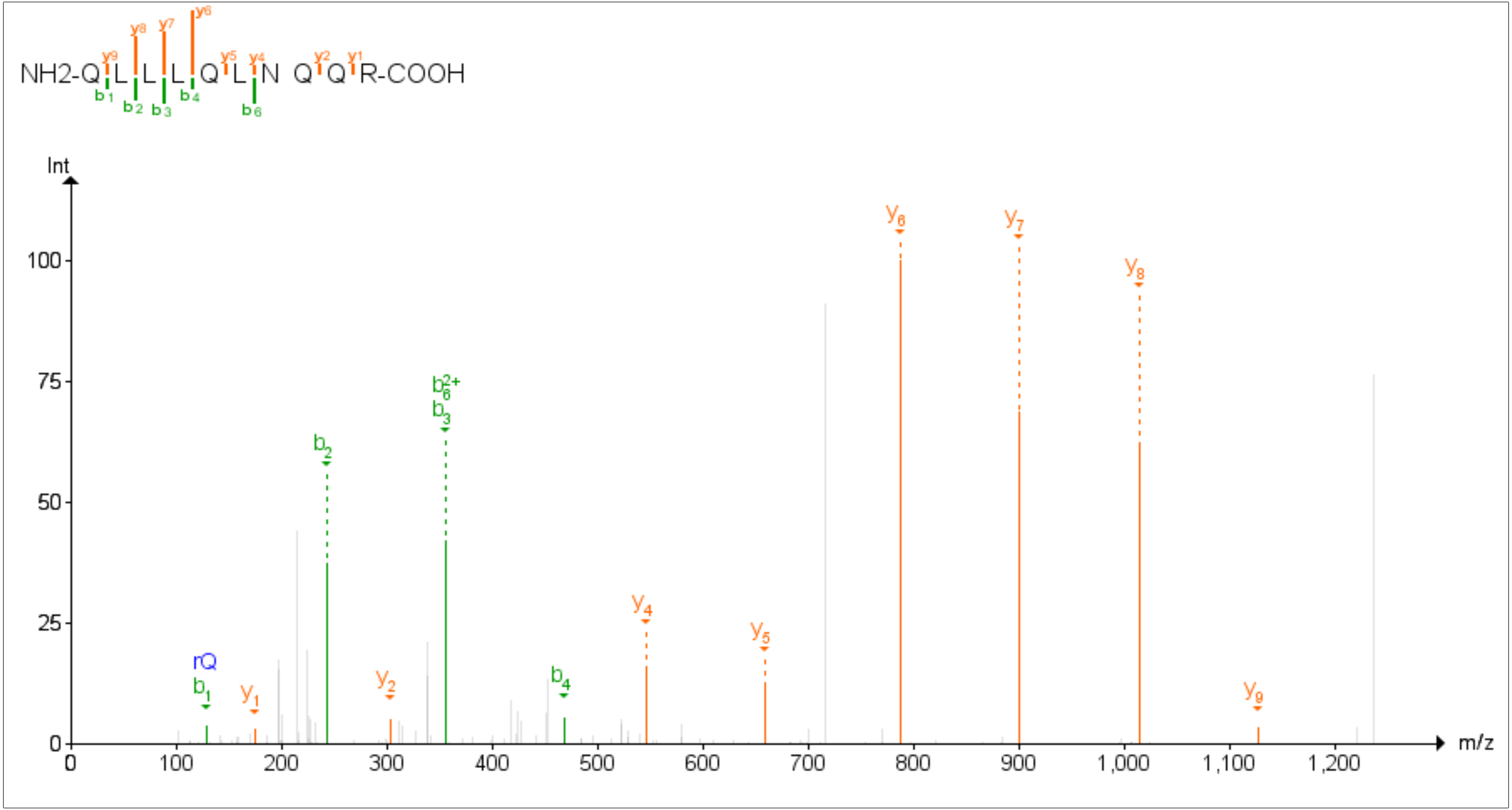

NH2-Q L L L Q L N Q Q R-COOH
y9 y8 y7 y6 y5 y4 y2 y1
b1 b2 b3 b4 b6
Int
0
25
50
75
100
m/z
0
100
200
300
400
500
600
700
800
900
1,000
1,100
1,200
rQ
b1
y1
b2
y2
b6 2+
b3
b4
y4
y5
y6
y7
y8
y9

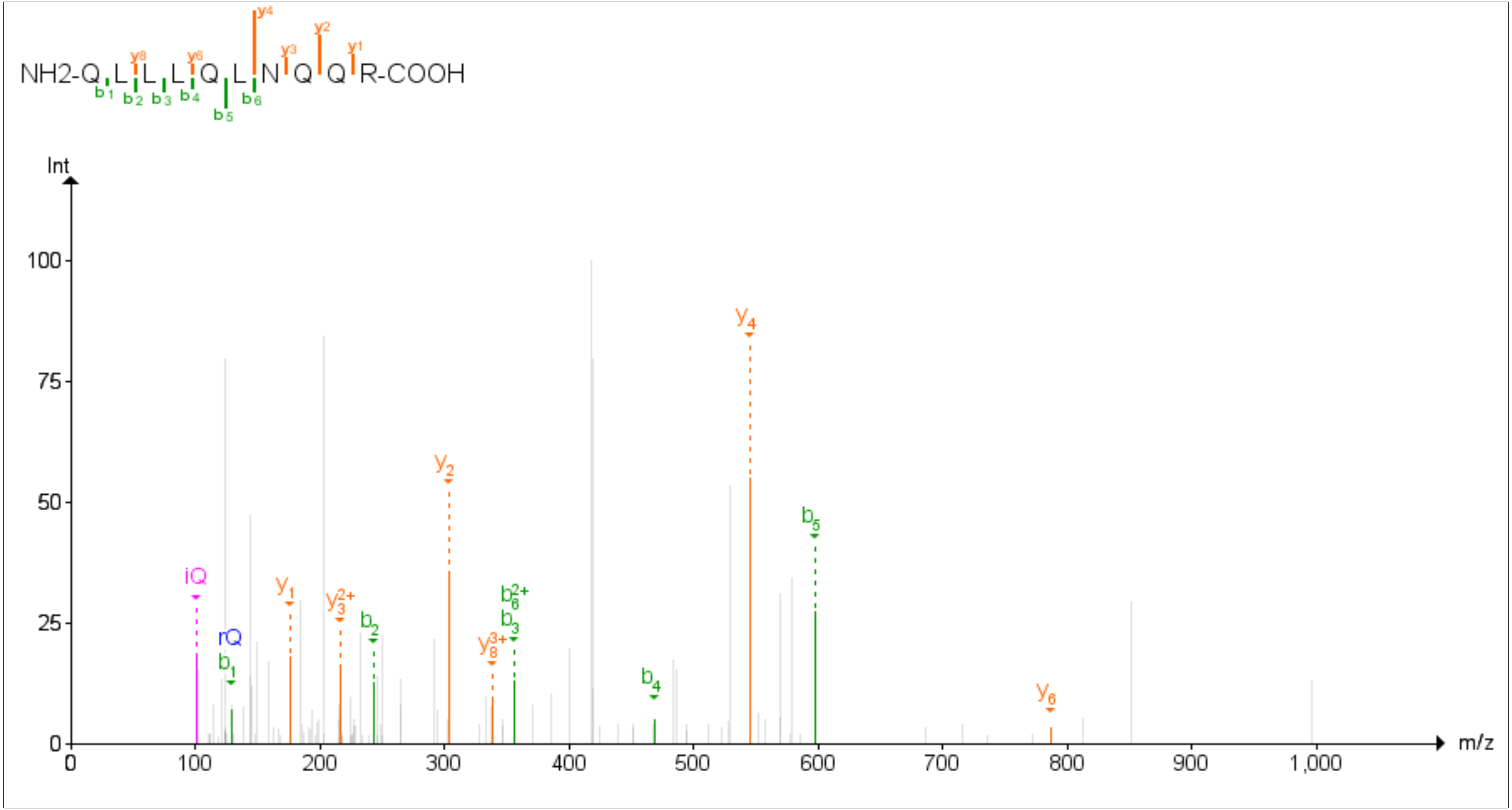
NH2-Q L L L L Q L N Q Q R-COOH
b1 b2 b3 b4 b5 b6
y8 y6 y4 y3 y2 y1
Int
100
75
50
25
0
iQ
rQ
b1
y1
y3 2+
b2
y2
y8 3+
b6 2+
b3
b4
y4
b5
y6
0 100 200 300 400 500 600 700 800 900 1,000
m/z

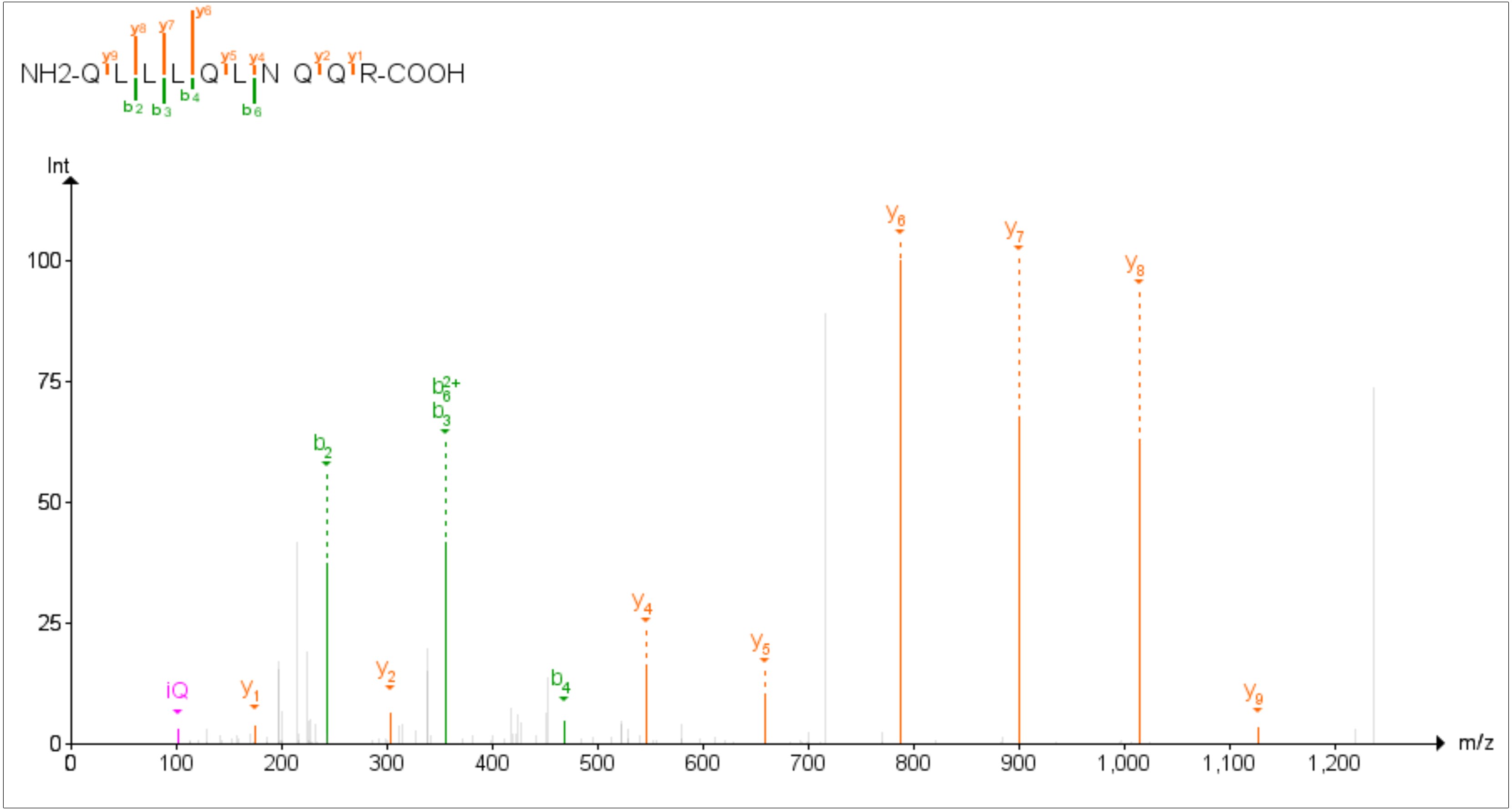

NH2-Q L L L Q L N Q Q R-COOH
y9 y8 y7 y6 y5 y4 y2 y1
b2 b3 b4 b6
Int
100
75
50
25
0
iQ
y1
b2
y2
b6 2+
b3
b4
y4
y5
y6
y7
y8
y9
0
100
200
300
400
500
600
700
800
900
1,000
1,100
1,200
m/z

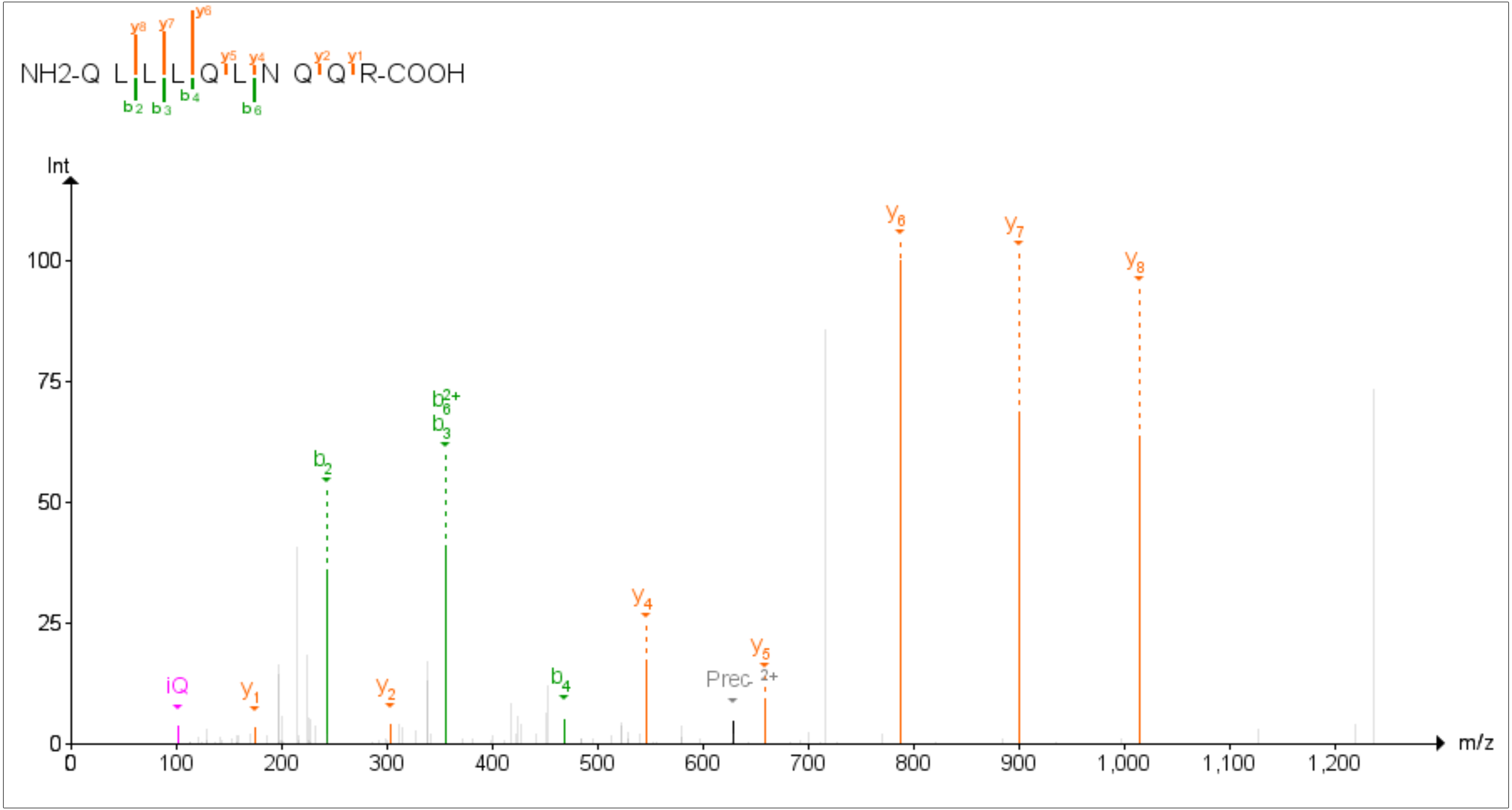

NH2-Q L L L Q L N Q Q R-COOH
y8 y7 y6 y5 y4 y2 y1
b2 b3 b4 b6
Int
m/z
iQ
y1
y2
b2
b3
b_6^{2+}
b4
y4
Prec 2+
y5
y6
y7
y8
0
25
50
75
100
0
100
200
300
400
500
600
700
800
900
1,000
1,100
1,200

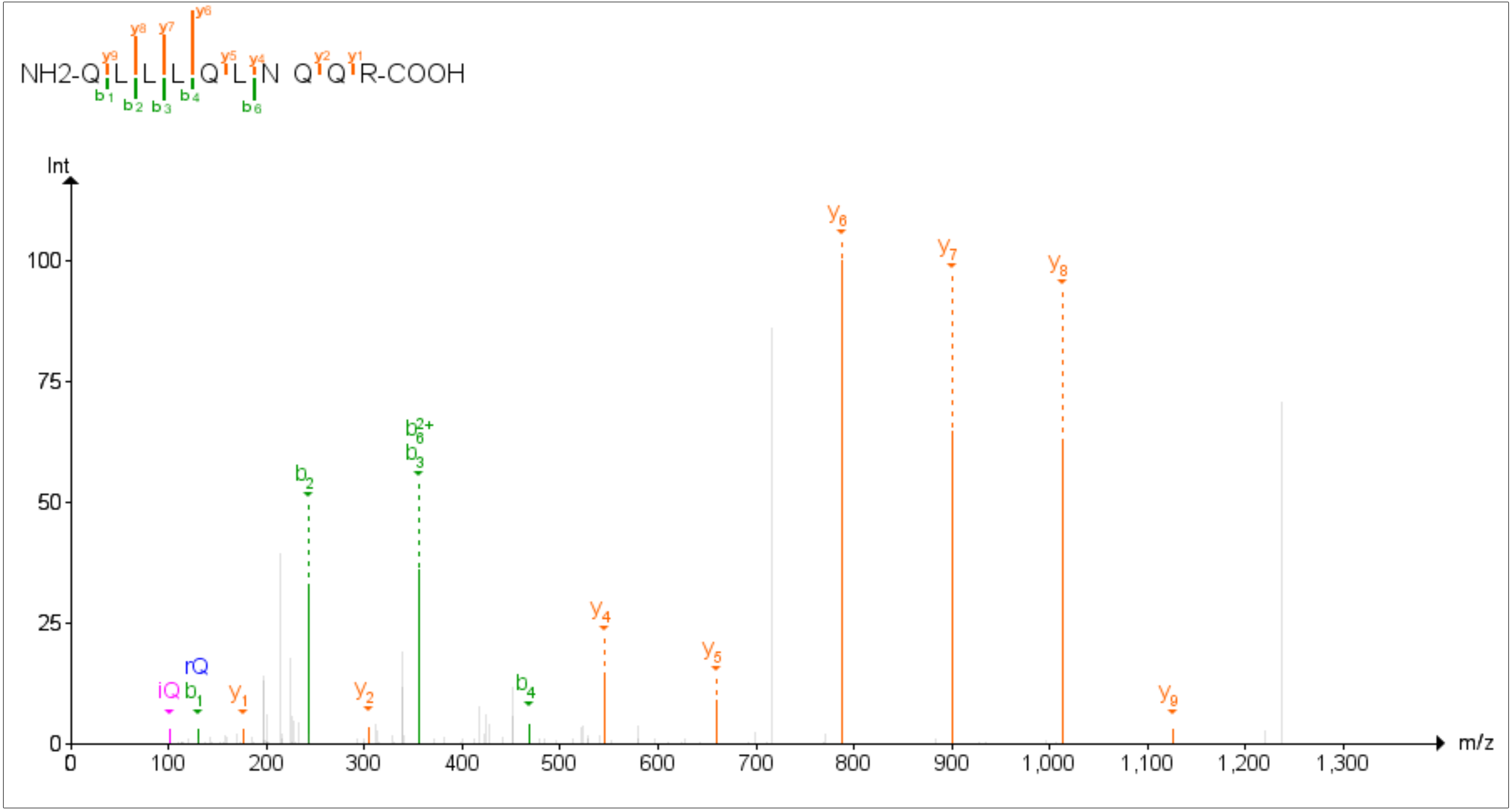

NH2-Q L L L Q L N Q Q R-COOH
y9 y8 y7 y6 y5 y4 y2 y1
b1 b2 b3 b4 b6
Int
m/z
100
75
50
25
0
0 100 200 300 400 500 600 700 800 900 1,000 1,100 1,200 1,300
y6
y7
y8
b2
b6 2+
b3
y4
y5
b4
y9
iQ
rQ
b1
y1
y2

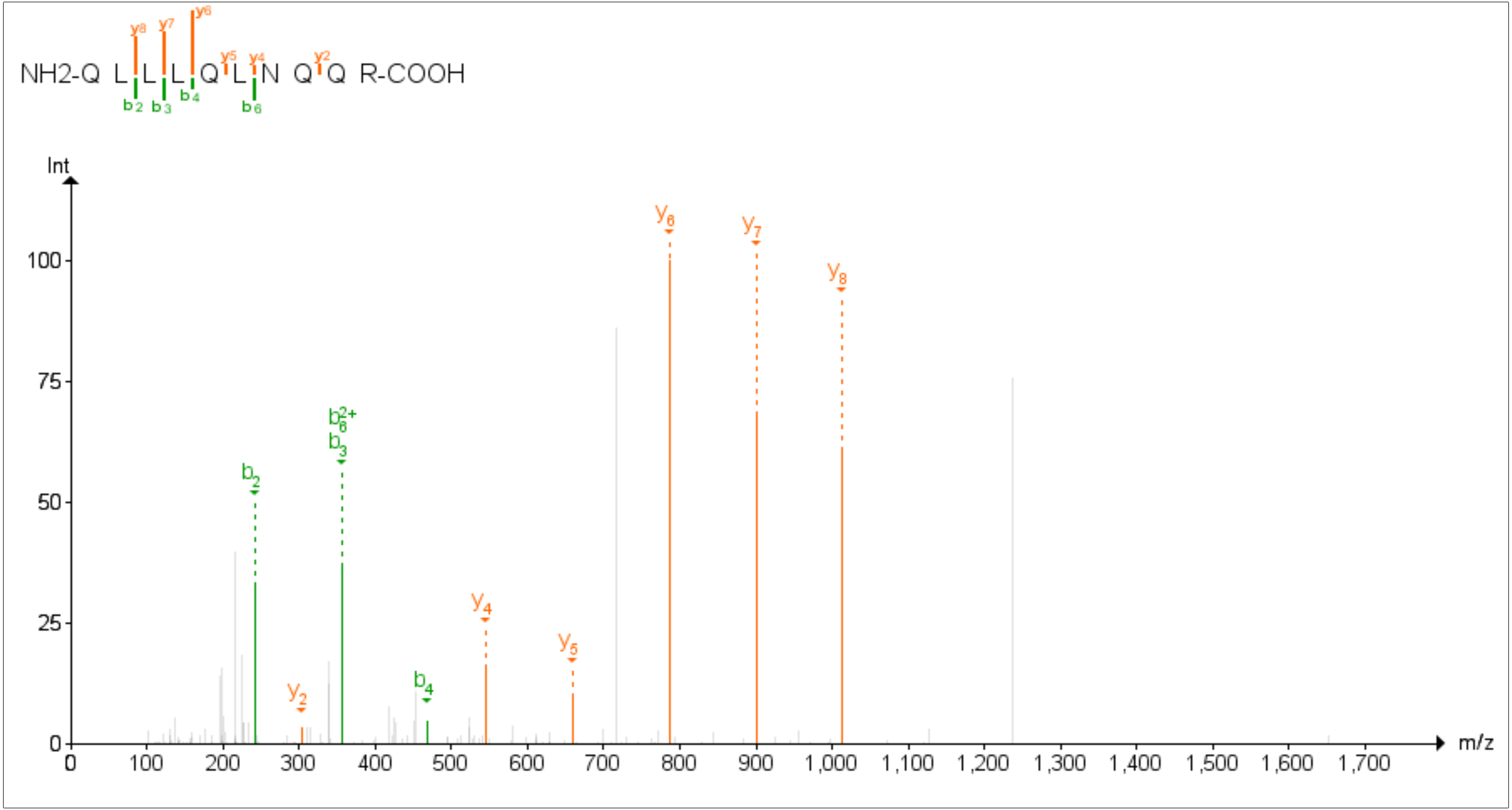

NH2-Q L L L Q L N Q Q R-COOH
y8 y7 y6 y5 y4 y2
b2 b3 b4 b6
Int
m/z
0
25
50
75
100
0
100
200
300
400
500
600
700
800
900
1,000
1,100
1,200
1,300
1,400
1,500
1,600
1,700
b2
b6 2+
b3
y2
b4
y4
y5
y6
y7
y8

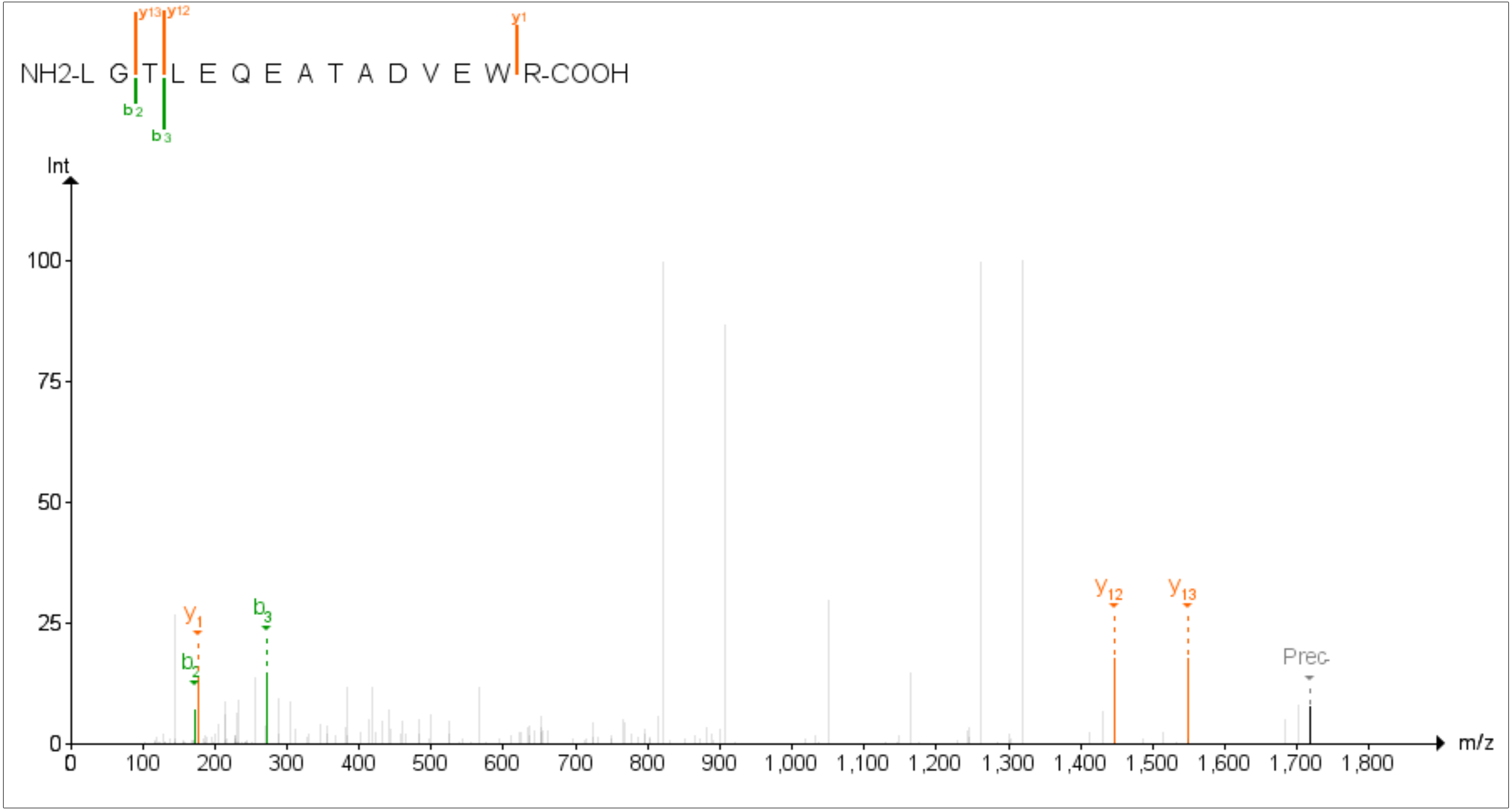

NH2-L G T L E Q E A T A D V E W R-COOH
y13
y12
y1
b2
b3
Int
100
75
50
25
0
y1
b2
b3
y12
y13
Prec.
0
100
200
300
400
500
600
700
800
900
1,000
1,100
1,200
1,300
1,400
1,500
1,600
1,700
1,800
m/z

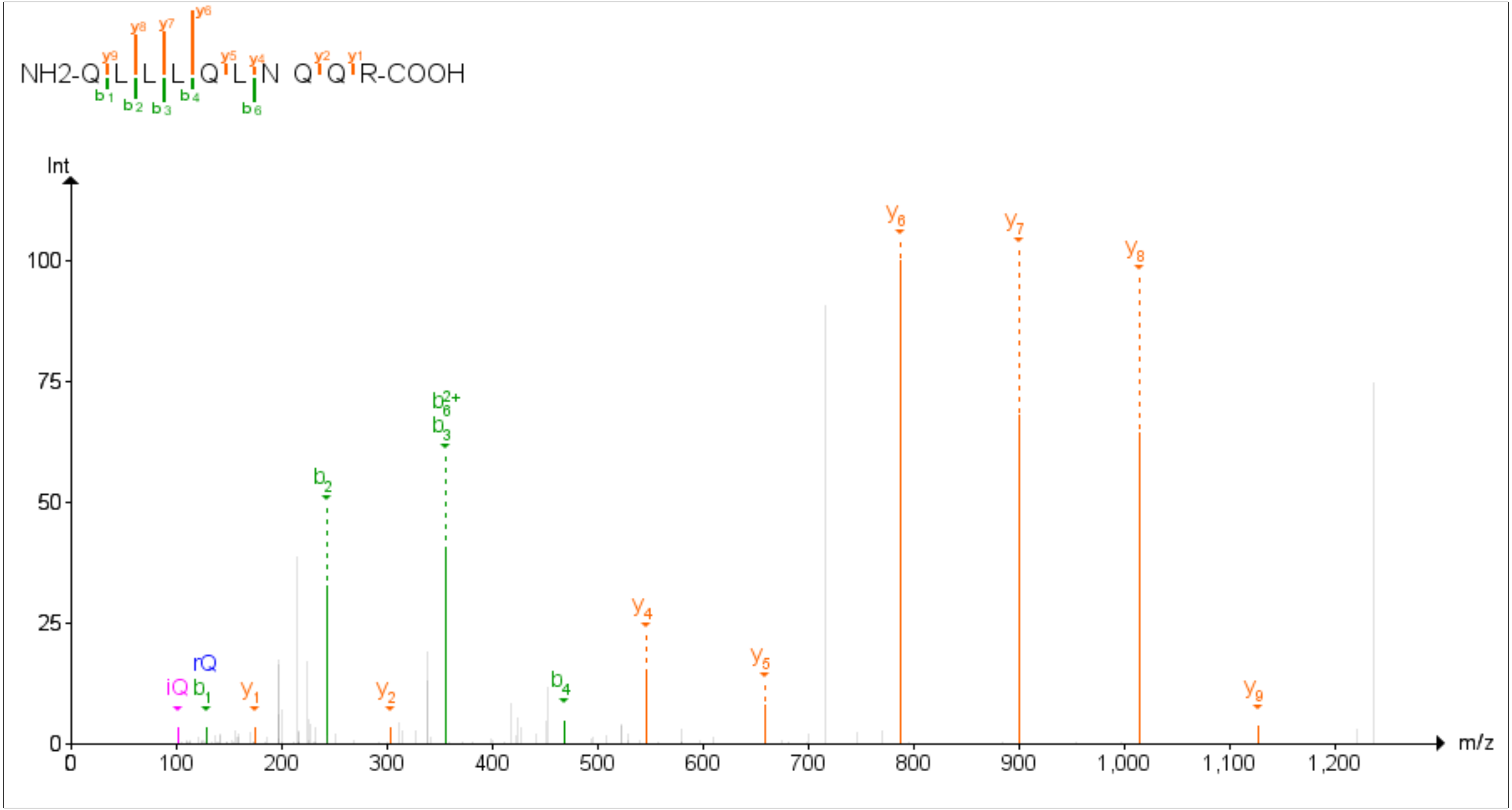

NH2-Q L L L Q L N Q Q R-COOH
y9 y8 y7 y6 y5 y4 y2 y1
b1 b2 b3 b4 b6
Int
100
75
50
25
0
m/z
0
100
200
300
400
500
600
700
800
900
1,000
1,100
1,200
y_6
y_7
y_8
b_6^{2+}
b_3
b_2
y_4
y_5
rQ
iQ
b_1
y_1
y_2
b_4
y_9

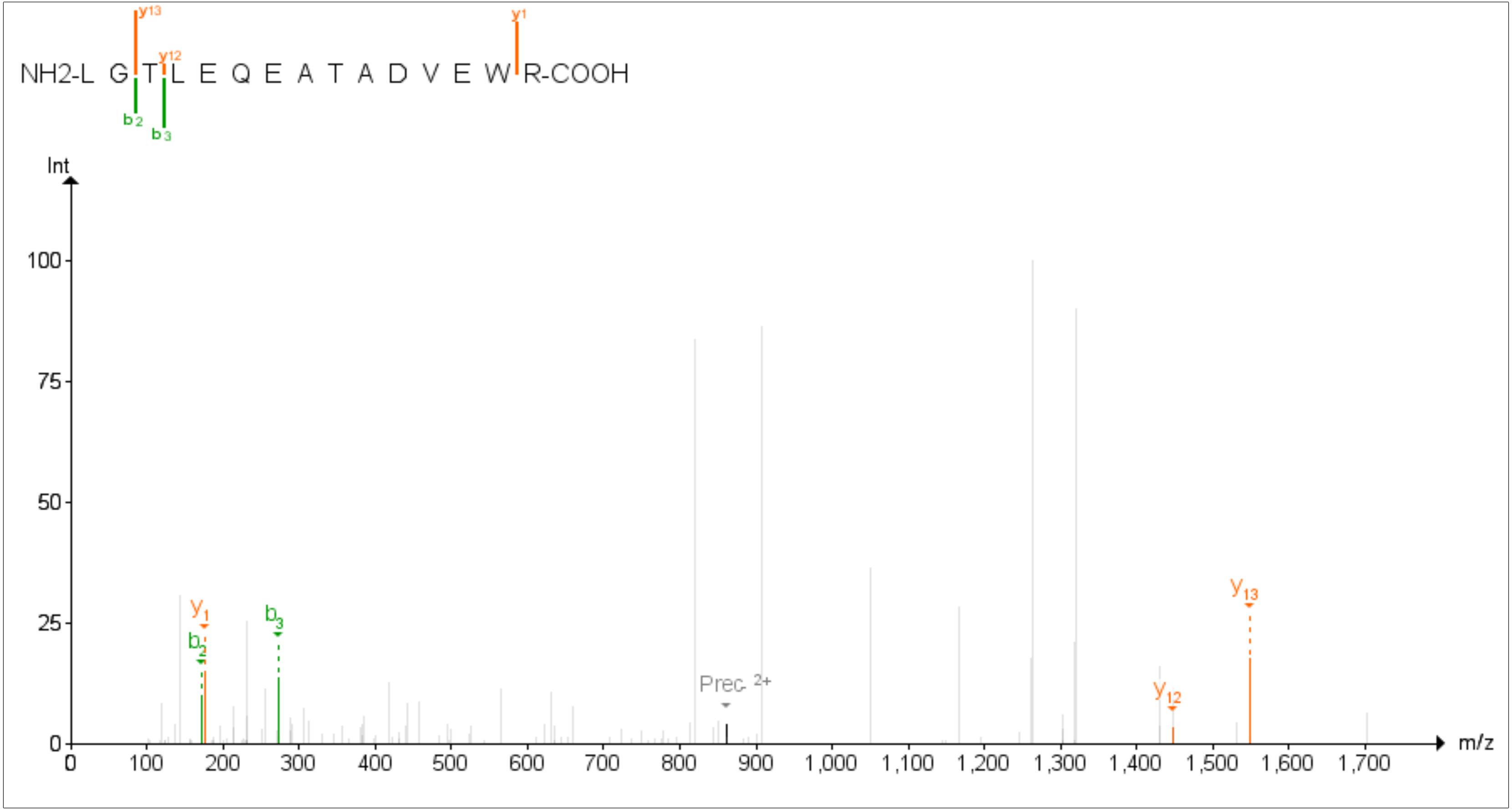

NH2-L G T L E Q E A T A D V E W R-COOH
y13
y12
y1
b2
b3
Int
100
75
50
25
0
y1
b2
b3
Prec 2+
y12
y13
0
100
200
300
400
500
600
700
800
900
1,000
1,100
1,200
1,300
1,400
1,500
1,600
1,700
m/z

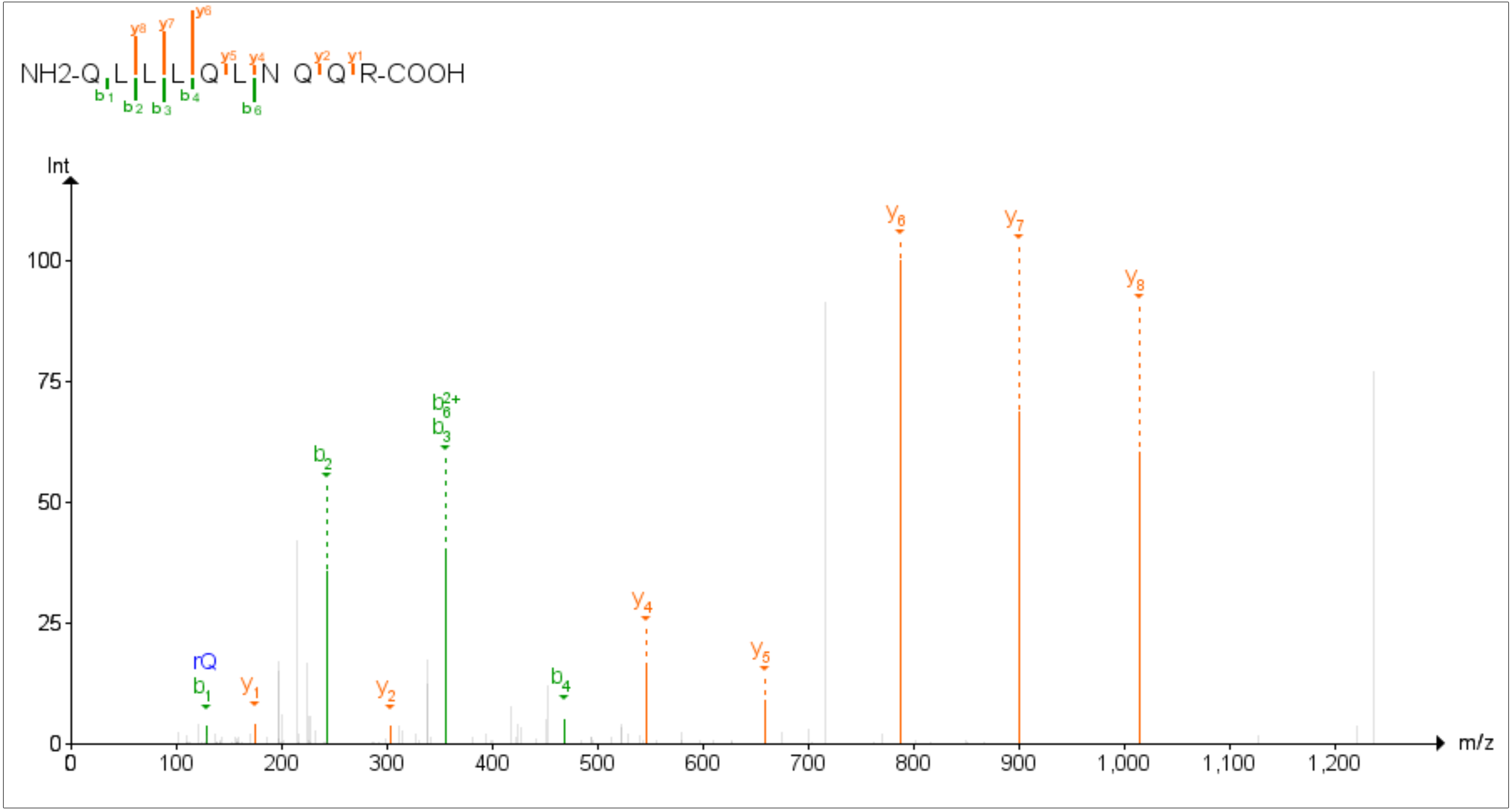

NH2-Q L L L Q L N Q Q R-COOH
y8 y7 y6 y5 y4 y2 y1
b1 b2 b3 b4 b6
Int
m/z
100
75
50
25
0
0 100 200 300 400 500 600 700 800 900 1,000 1,100 1,200
rQ
b1
y1
y2
b2
b6 2+
b3
b4
y4
y5
y6
y7
y8

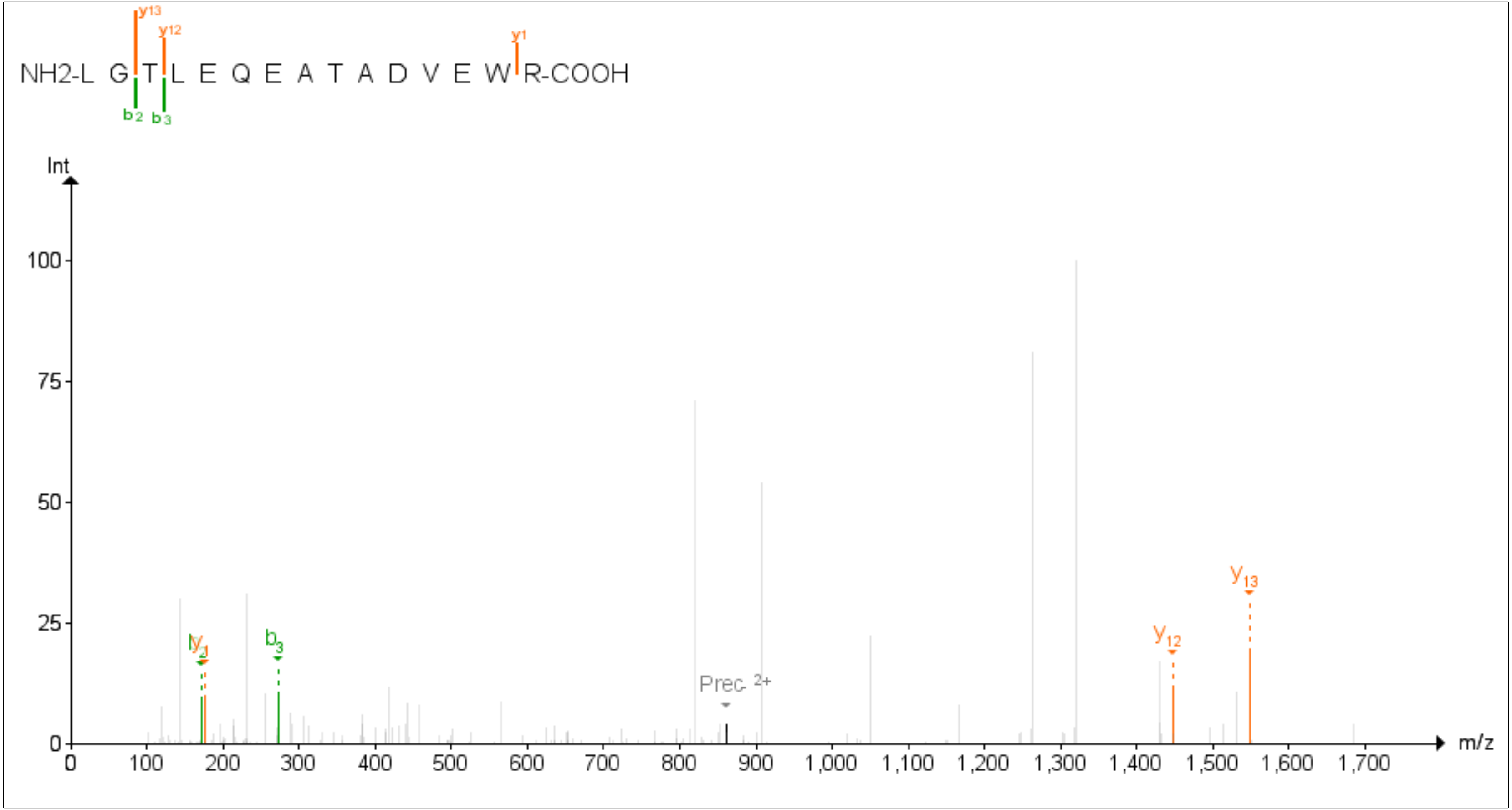

y13
y12
y1
NH2-L G T L E Q E A T A D V E W R-COOH
b2
b3
Int
100
75
50
25
0
y1
b3
Prec 2+
y12
y13
0
100
200
300
400
500
600
700
800
900
1,000
1,100
1,200
1,300
1,400
1,500
1,600
1,700
m/z

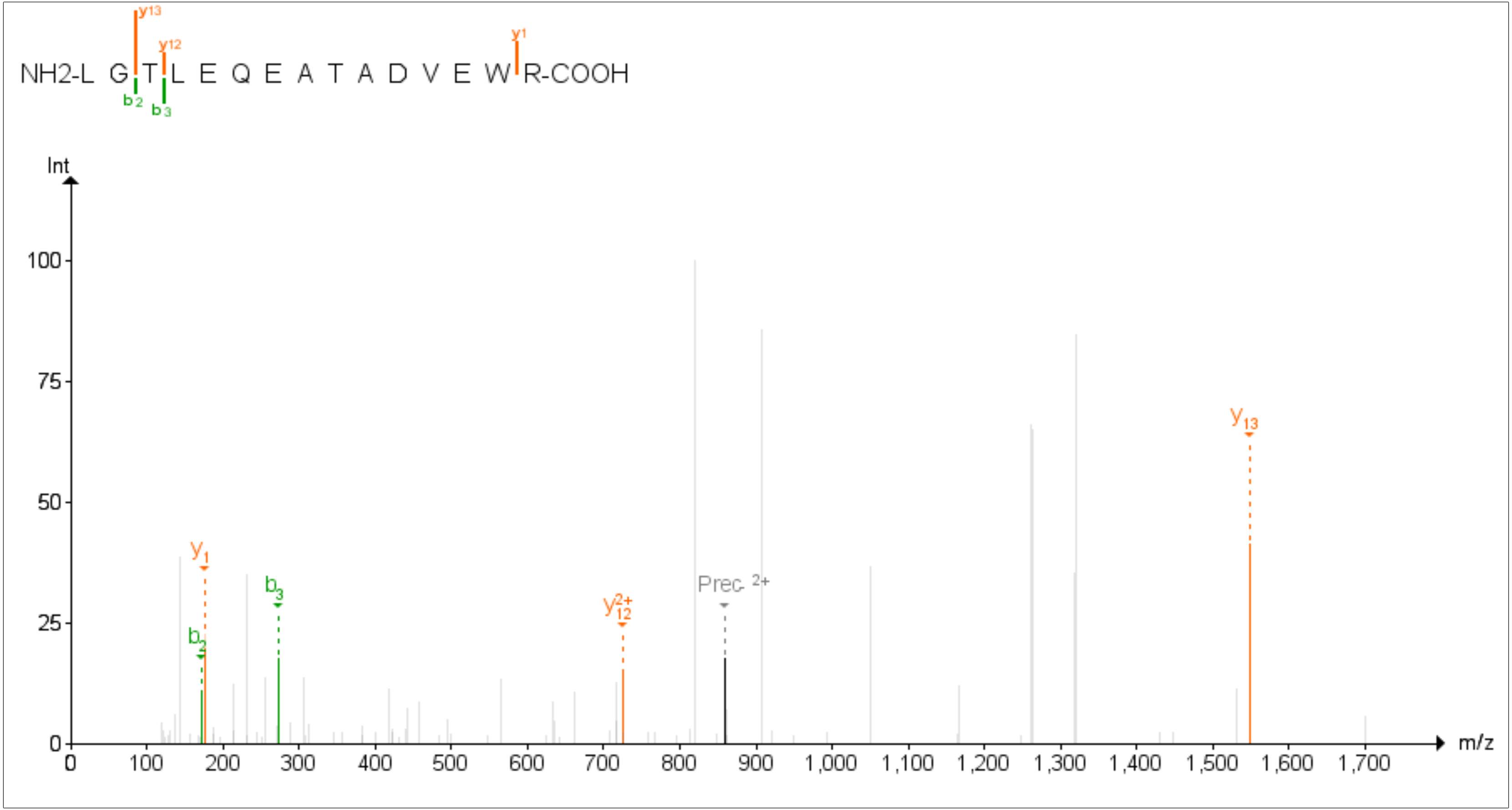

NH2-L G T L E Q E A T A D V E W R-COOH
y13
y12
y1
b 2
b 3
Int
100
75
50
25
0
y1
b3
b2
y12 2+
Prec 2+
y13
0
100
200
300
400
500
600
700
800
900
1,000
1,100
1,200
1,300
1,400
1,500
1,600
1,700
m/z

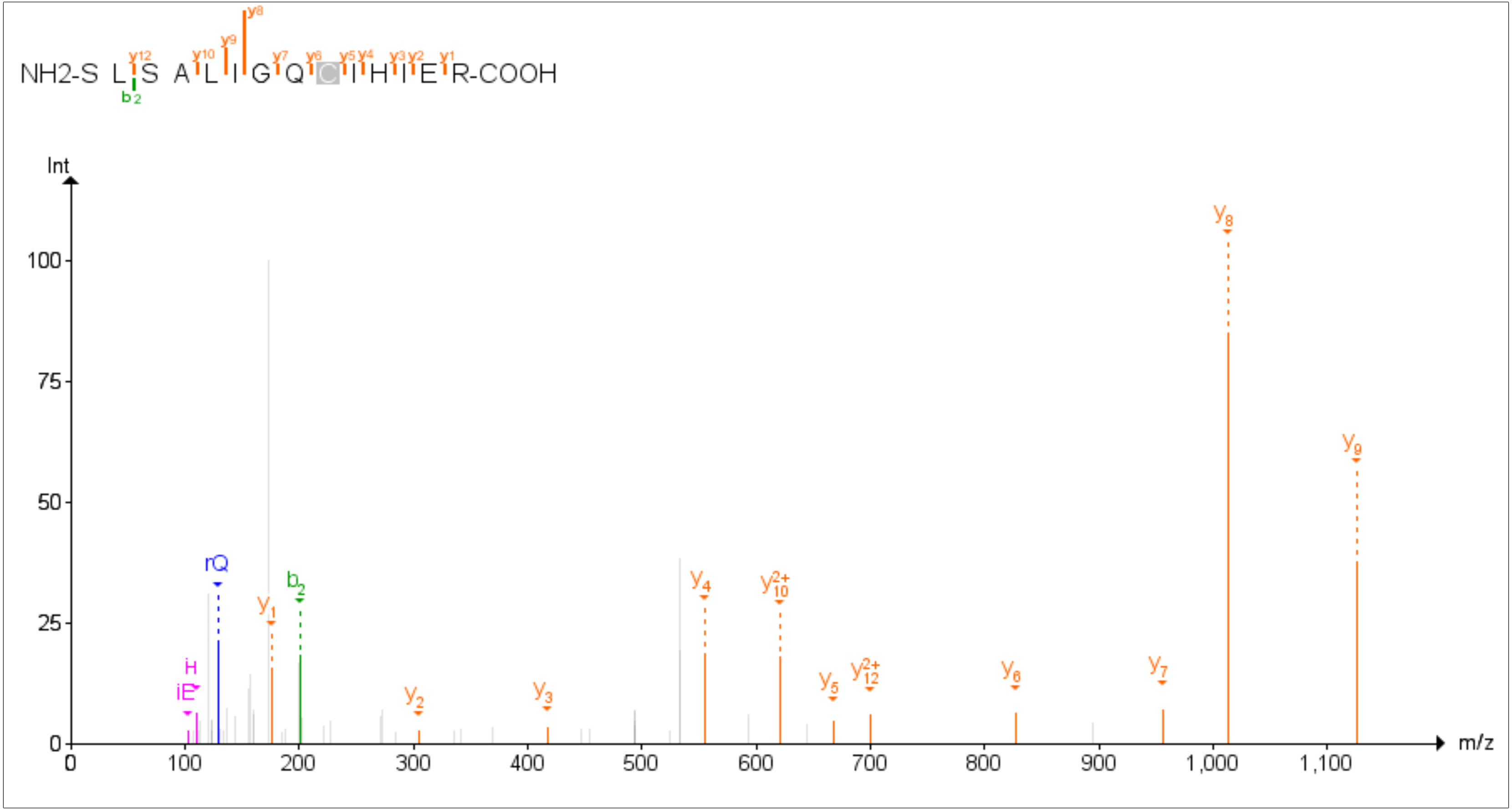

NH2-S L S A L I G Q C I H I E R-COOH
y12 y10 y9 y8 y7 y6 y5 y4 y3 y2 y1
b2
Int
100
75
50
25
0
y8
y9
rQ
b2
y1
iH
iE
y2
y3
y4
y10 2+
y5
y12 2+
y6
y7
0
100
200
300
400
500
600
700
800
900
1,000
1,100
m/z

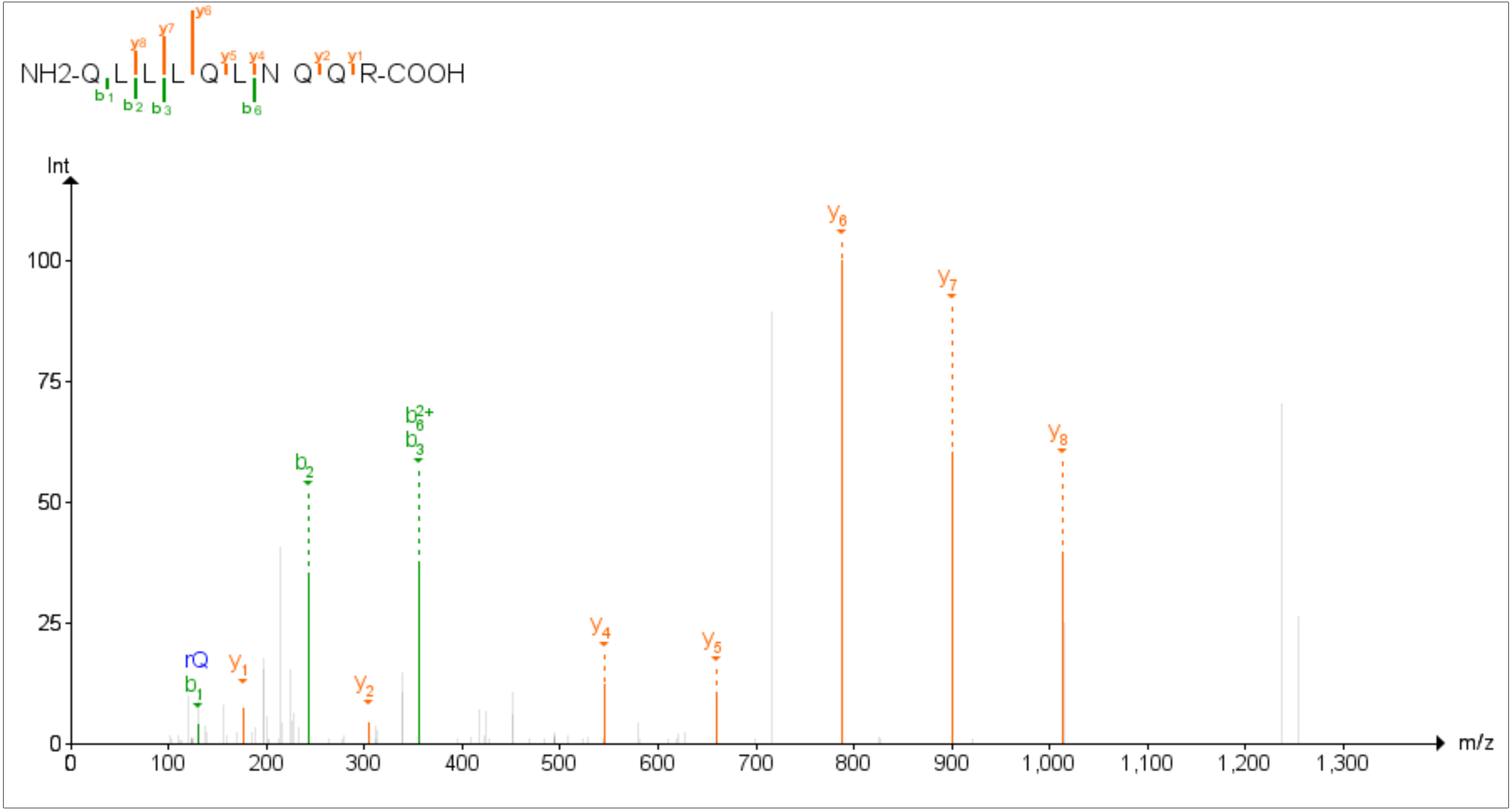

NH2-Q L L L Q L N Q Q R-COOH
y8 y7 y6 y5 y4 y2 y1
b1 b2 b3 b6
Int
100
75
50
25
0
rQ
b1
y1
b2
y2
b6 2+
b3
y4
y5
y6
y7
y8
0
100
200
300
400
500
600
700
800
900
1,000
1,100
1,200
1,300
m/z

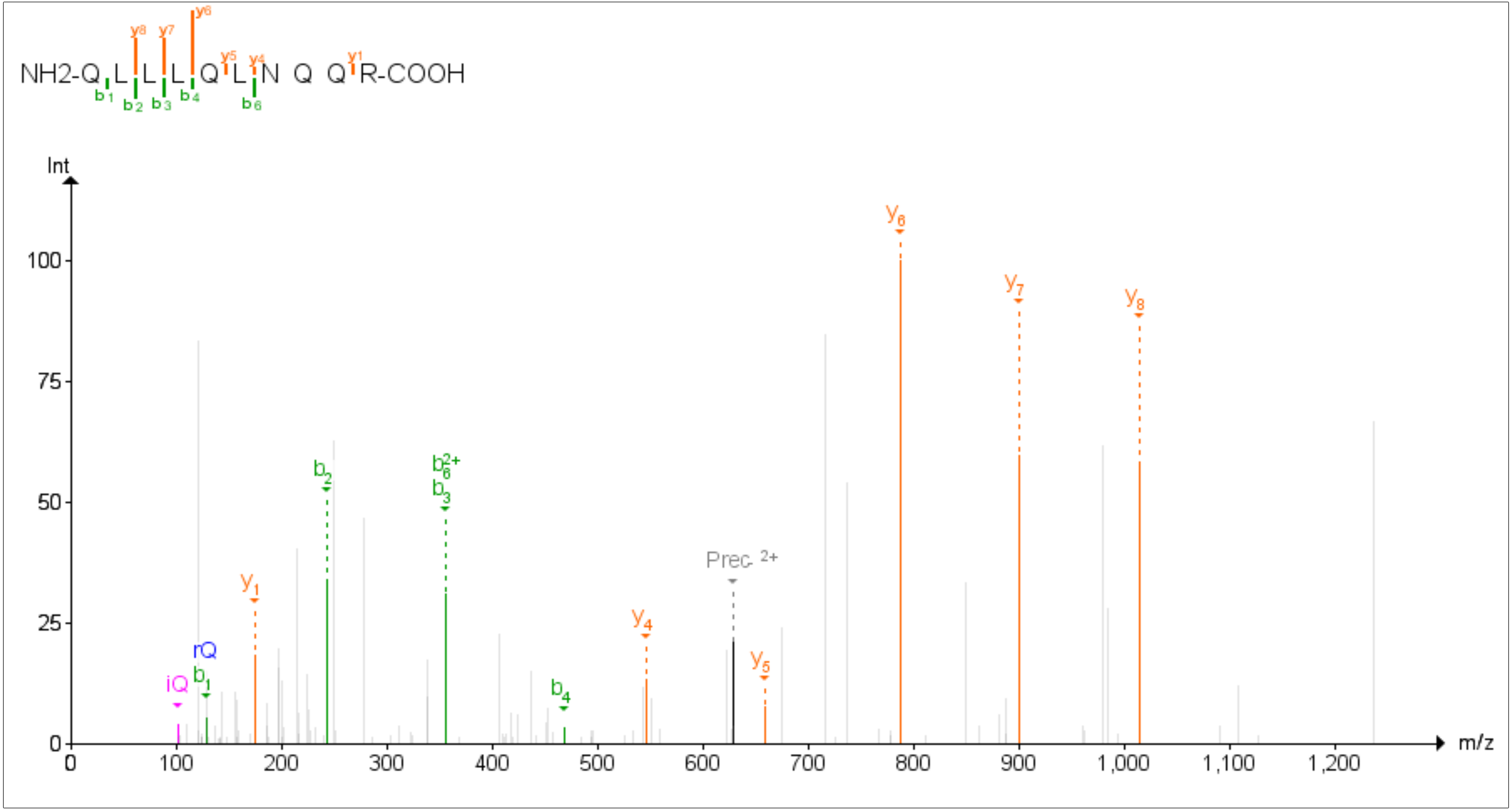

NH2-Q L L L L Q L N Q Q R-COOH
y8 y7 y6 y5 y4 y1
b1 b2 b3 b4 b6
Int
m/z
y6
y7
y8
b2
b6 2+
b3
y1
rQ
iQ
b1
b4
y4
Prec. 2+
y5
0
25
50
75
100
0
100
200
300
400
500
600
700
800
900
1,000
1,100
1,200

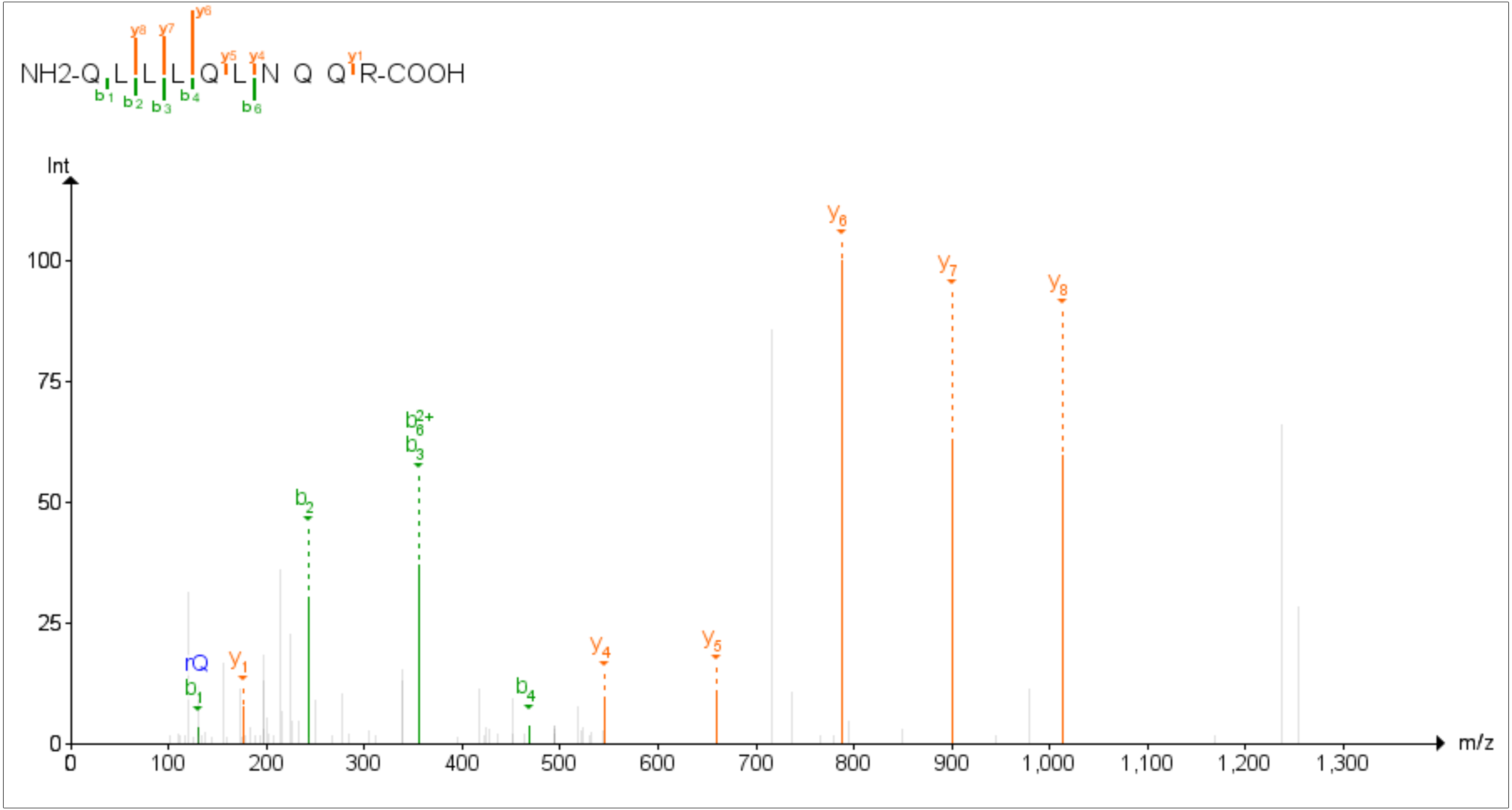

NH2-Q L L L Q L N Q Q R-COOH
y8 y7 y6 y5 y4 y1
b1 b2 b3 b4 b6
Int
m/z
y6
y7
y8
b6 2+
b3
b2
rQ
b1
y1
b4
y4
y5
0
25
50
75
100
0
100
200
300
400
500
600
700
800
900
1,000
1,100
1,200
1,300

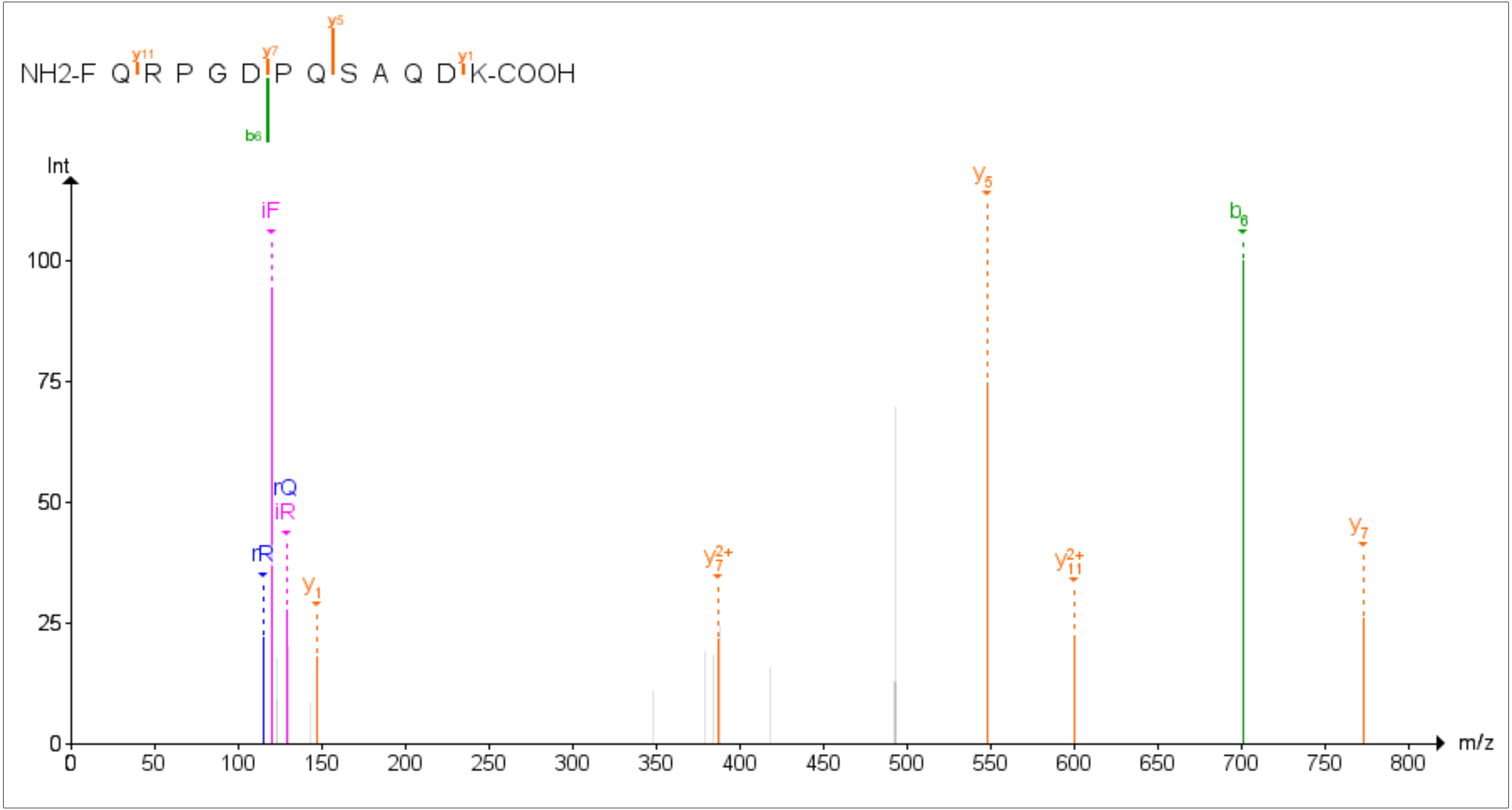

NH2-F Q R P G D P Q S A Q D K-COOH
y11
y7
y5
y1
b6
Int
iF
rQ
iR
rR
y1
y7 2+
y5
y11 2+
b6
y7
100
75
50
25
0
0 50 100 150 200 250 300 350 400 450 500 550 600 650 700 750 800
m/z

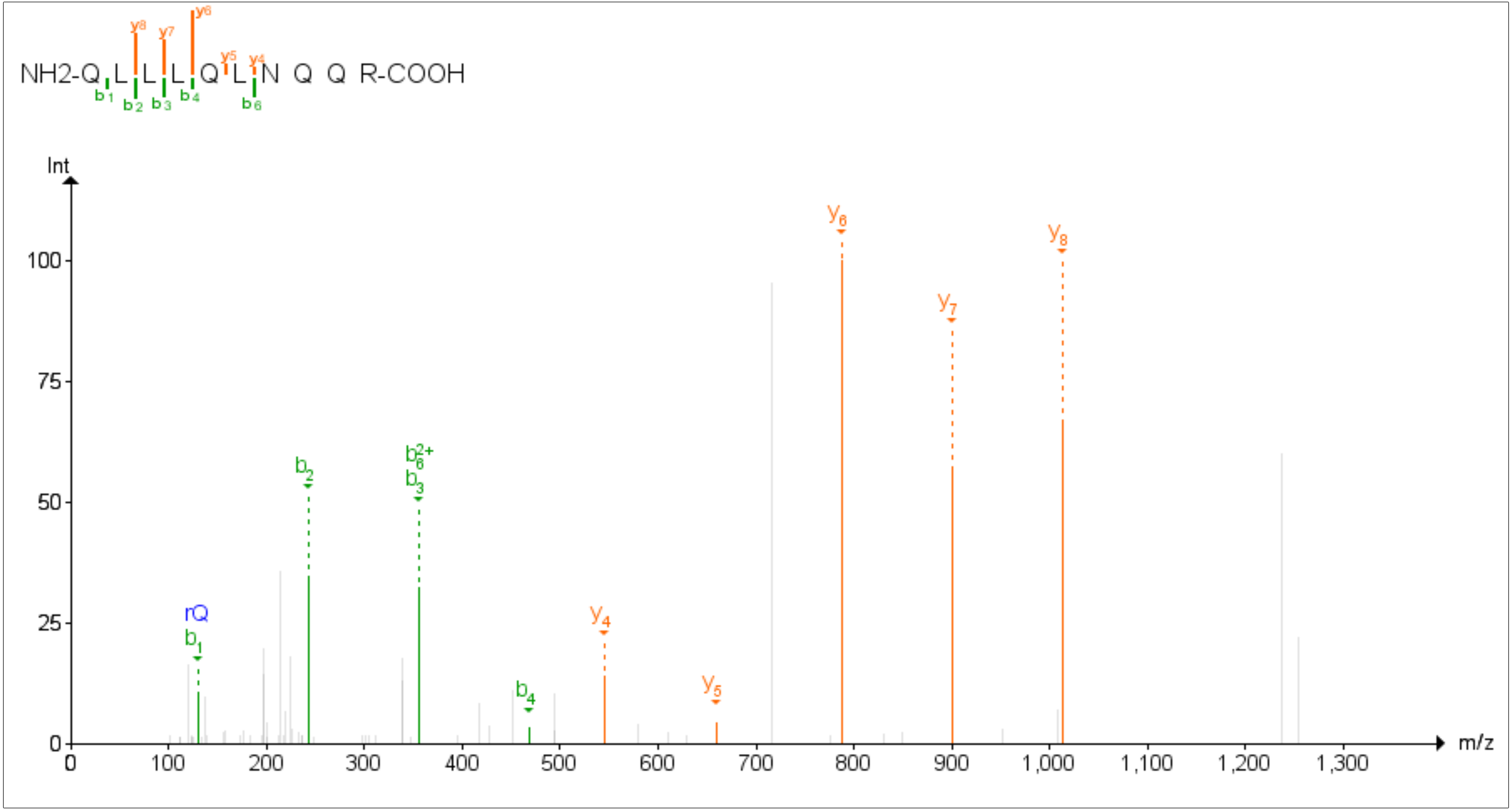

NH2-Q L L L Q L N Q Q R-COOH
Int
m/z
y6
y8
y7
b2
b6 2+
b3
rQ
b1
b4
y4
y5
0
25
50
75
100
0
100
200
300
400
500
600
700
800
900
1,000
1,100
1,200
1,300

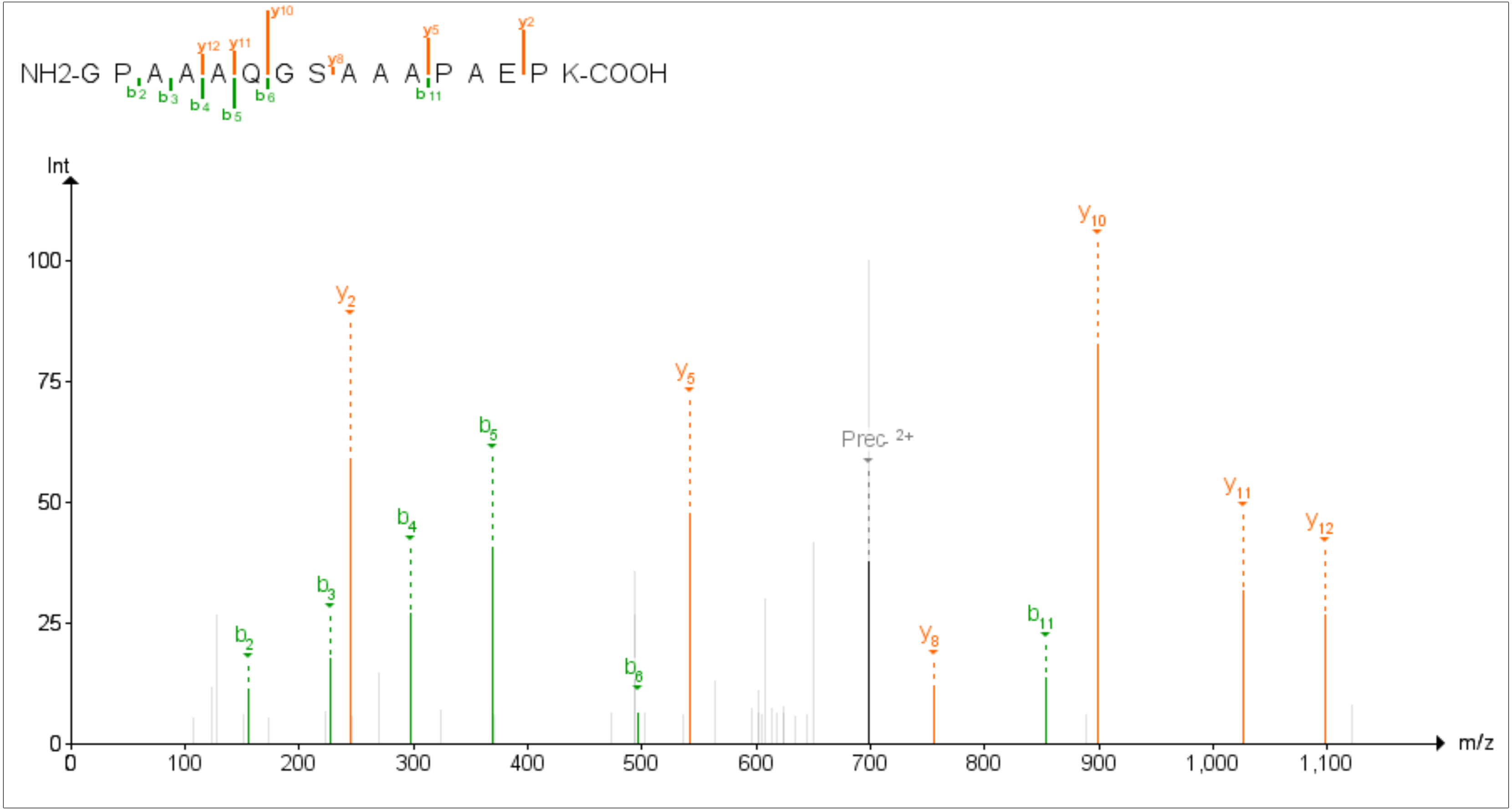

NH2-G P A A A A Q G S A A A P A E P K-COOH
y10
y12
y11
y5
y2
y8
b2
b3
b4
b5
b6
b11
Int
100
75
50
25
0
y2
y10
y5
b5
b4
b3
b2
b6
Prec. 2+
y8
b11
y11
y12
0
100
200
300
400
500
600
700
800
900
1,000
1,100
m/z

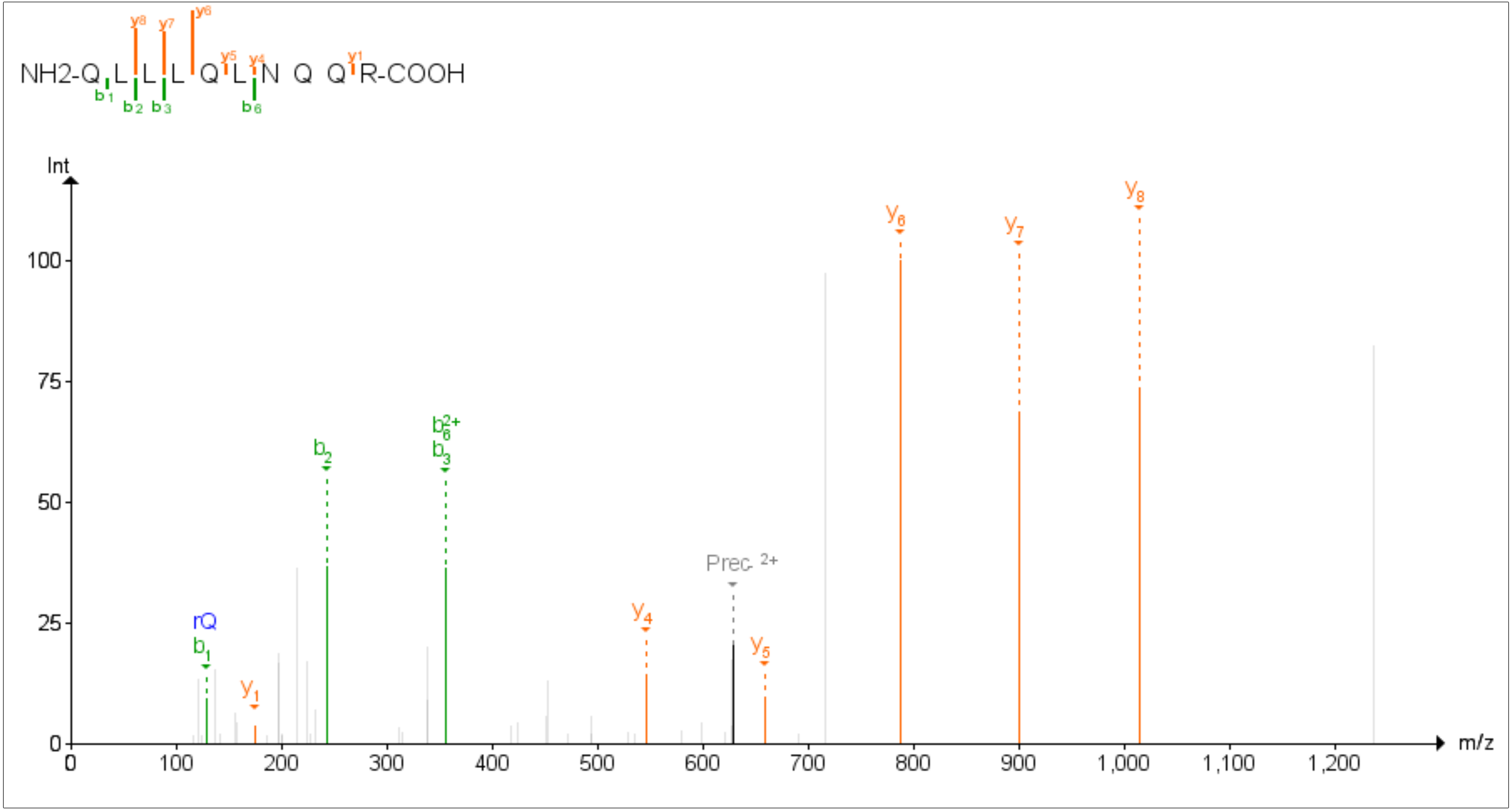

NH2-Q L L L Q L N Q Q R-COOH
y8 y7 y6 y5 y4 y1
b1 b2 b3 b6
Int
100
75
50
25
0
rQ
b1
y1
b2
b6 2+
b3
y4
Prec. 2+
y5
y6
y7
y8
0 100 200 300 400 500 600 700 800 900 1,000 1,100 1,200
m/z

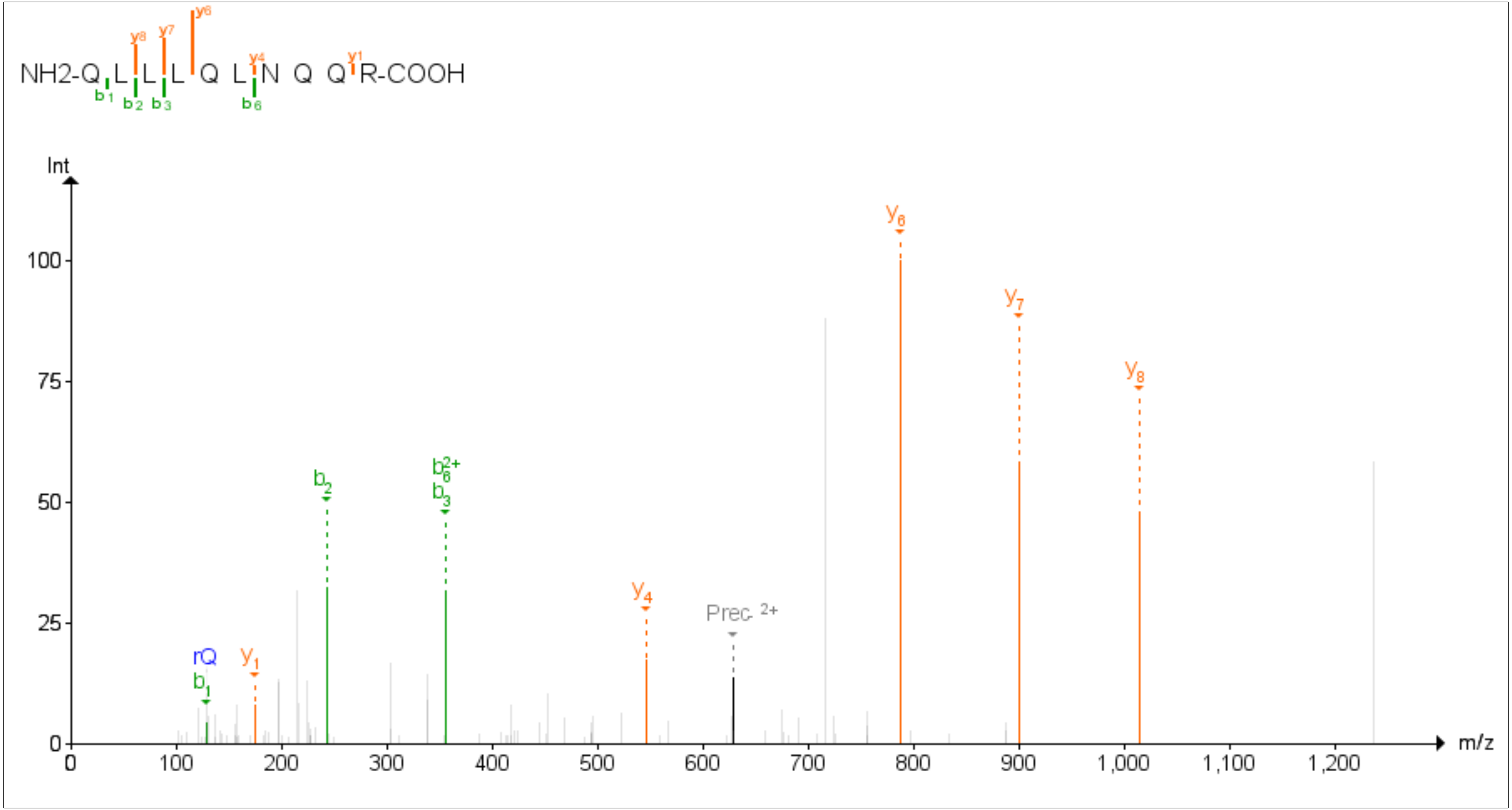

NH2-Q L L L Q L N Q Q R-COOH
b1 b2 b3 b6
y8 y7 y6 y4 y1
Int
m/z
rQ
b1
y1
b2
b6 2+
b3
y4
Prec 2+
y6
y7
y8

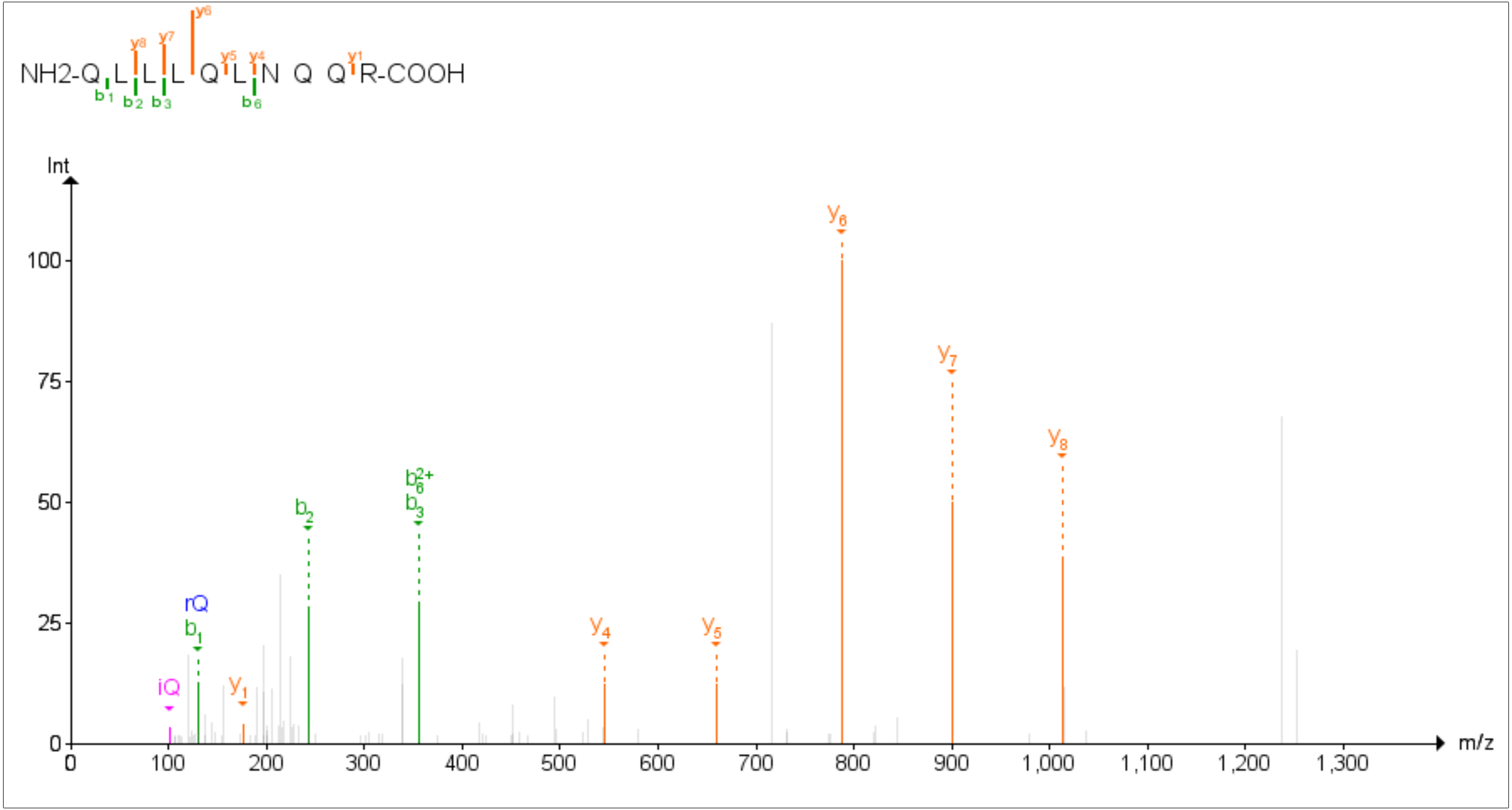

NH2-Q L L L Q L N Q Q R-COOH
y8 y7 y6 y5 y4 y1
b1 b2 b3 b6
Int
100
75
50
25
0
y6
y7
y8
b_6^{2+}
b_3
b_2
rQ
b_1
iQ
y_1
y_4
y_5
0 100 200 300 400 500 600 700 800 900 1,000 1,100 1,200 1,300
m/z

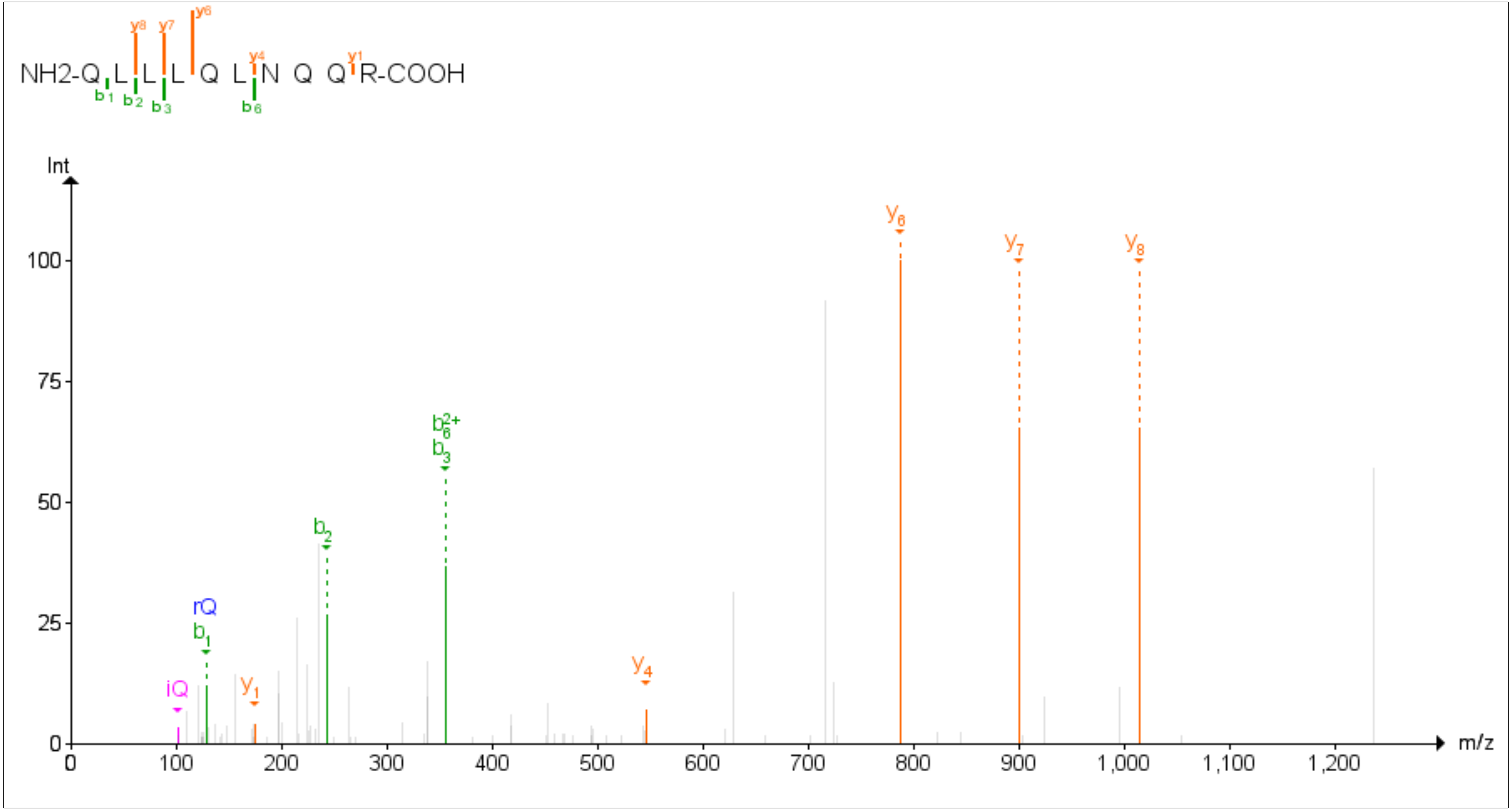

NH2-Q L L L Q L N Q Q R-COOH
y8 y7 y6 y4 y1
b1 b2 b3 b6
Int
100
75
50
25
0
m/z
0 100 200 300 400 500 600 700 800 900 1,000 1,100 1,200
y6
y7
y8
b6 2+
b3
b2
rQ
b1
iQ
y1
y4

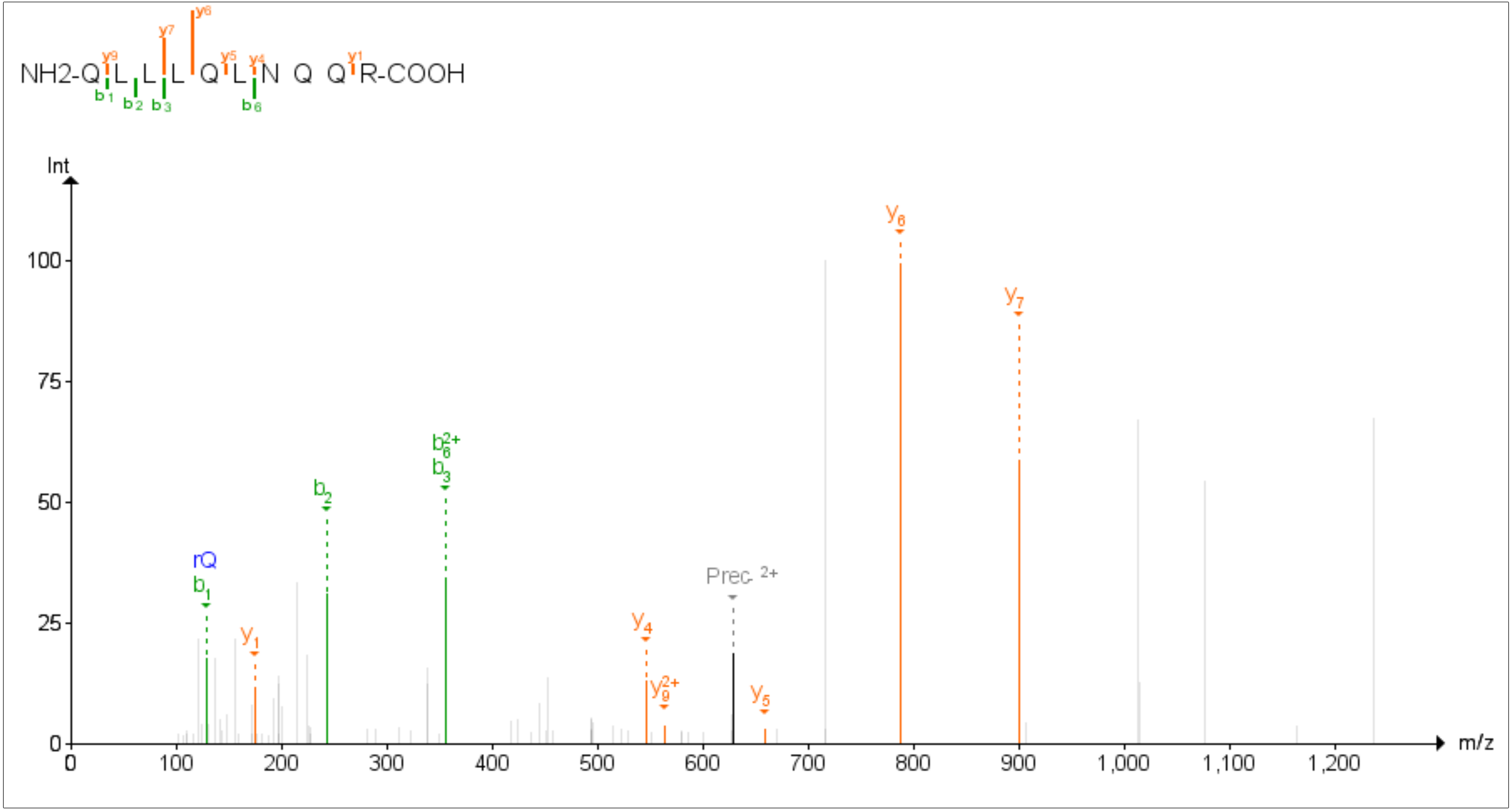

NH2-Q L L L Q L N Q Q R-COOH
y9 y7 y6 y5 y4 y1
b1 b2 b3 b6
Int
100
75
50
25
0
y6
y7
b6 2+
b3
b2
rQ
b1
y1
Prec. 2+
y4
y9 2+
y5
0
100
200
300
400
500
600
700
800
900
1,000
1,100
1,200
m/z

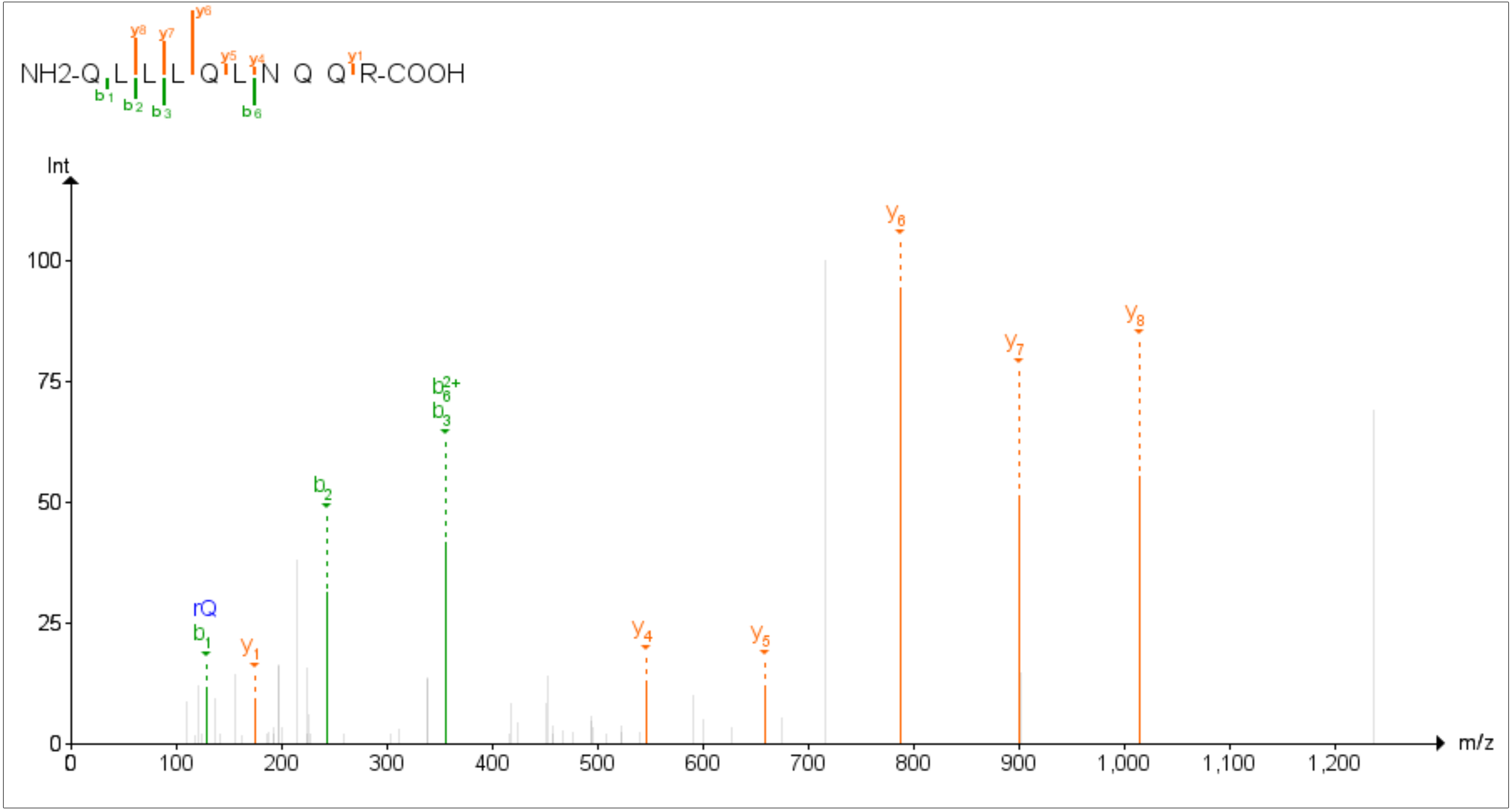

NH2-Q L L L Q L N Q Q R-COOH
y8 y7 y6 y5 y4 y1
b1 b2 b3 b6
Int
m/z
rQ
b1
y1
b2
b3
y4
y5
y6
y7
y8

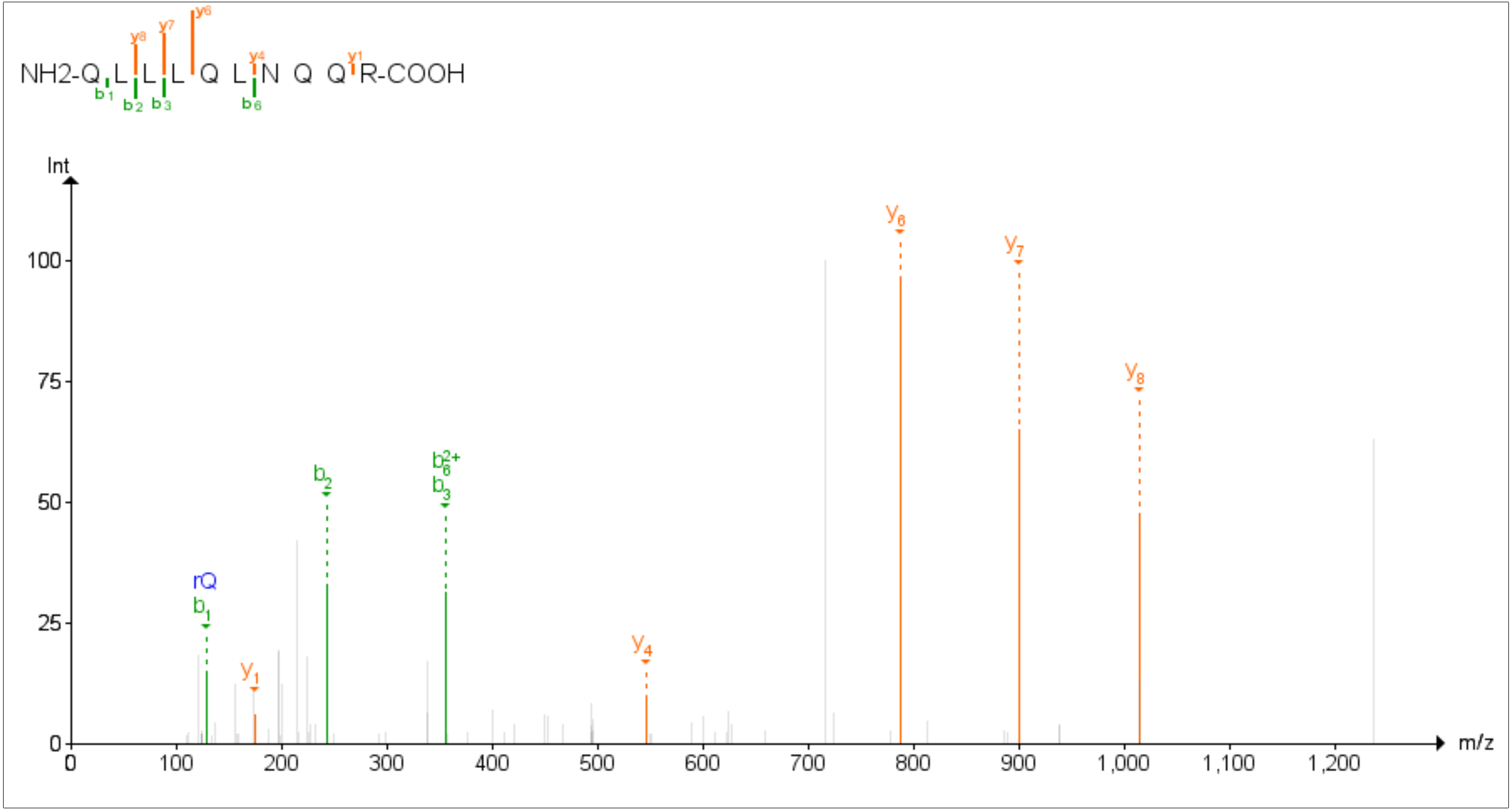

NH2-Q L L L Q L N Q Q R-COOH
y8 y7 y6 y4 y1
b1 b2 b3 b6
Int
100
75
50
25
0
y6
y7
y8
b6 2+
b3
b2
rQ
b1
y1
y4
0 100 200 300 400 500 600 700 800 900 1,000 1,100 1,200
m/z

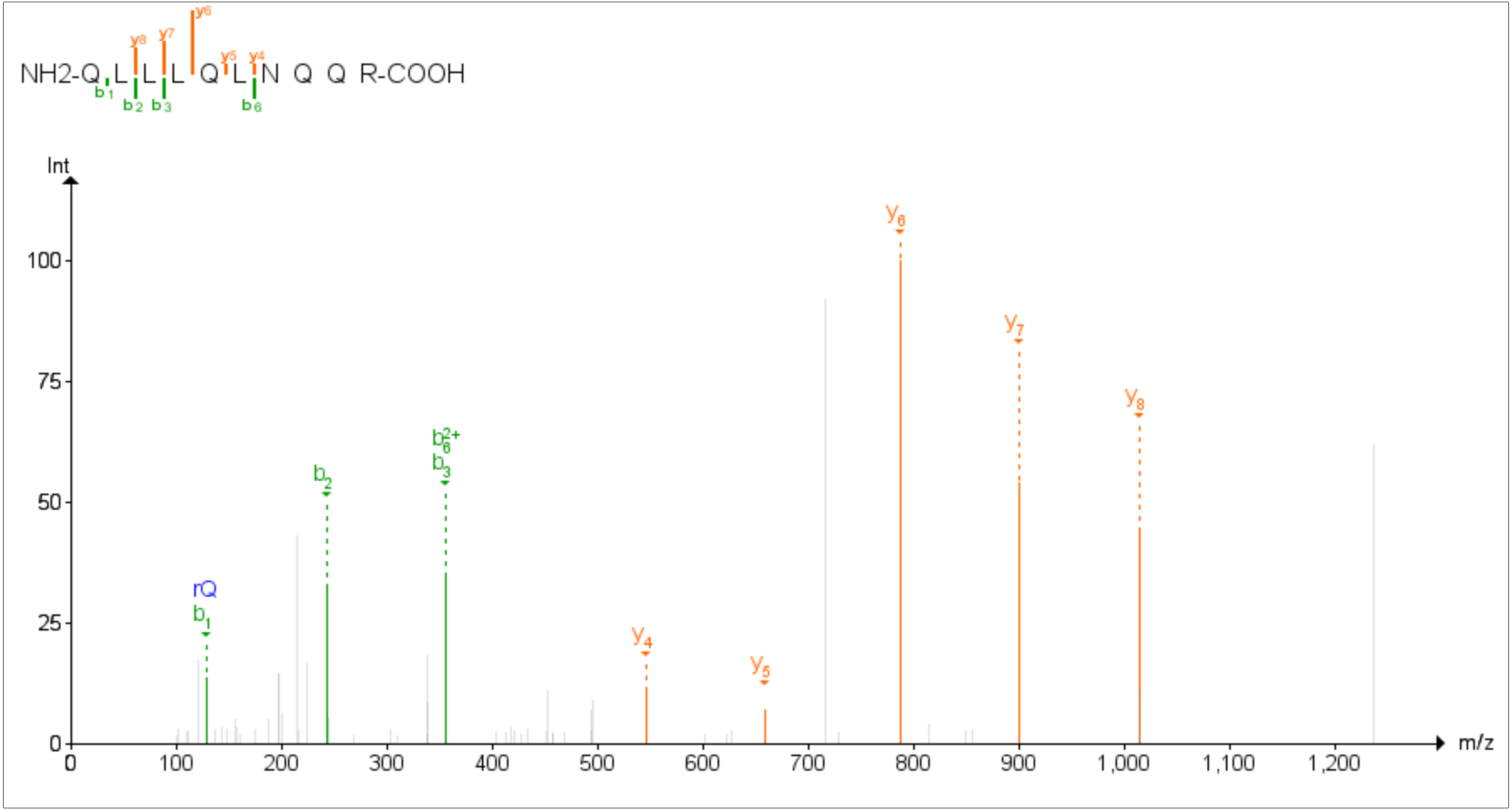

NH2-Q L L L Q L N Q Q R-COOH
y8
y7
y6
y5
y4
b1
b2
b3
b6
Int
m/z
rQ
b1
b2
b_6^{2+}
b3
y4
y5
y6
y7
y8
0
25
50
75
100
0
100
200
300
400
500
600
700
800
900
1,000
1,100
1,200

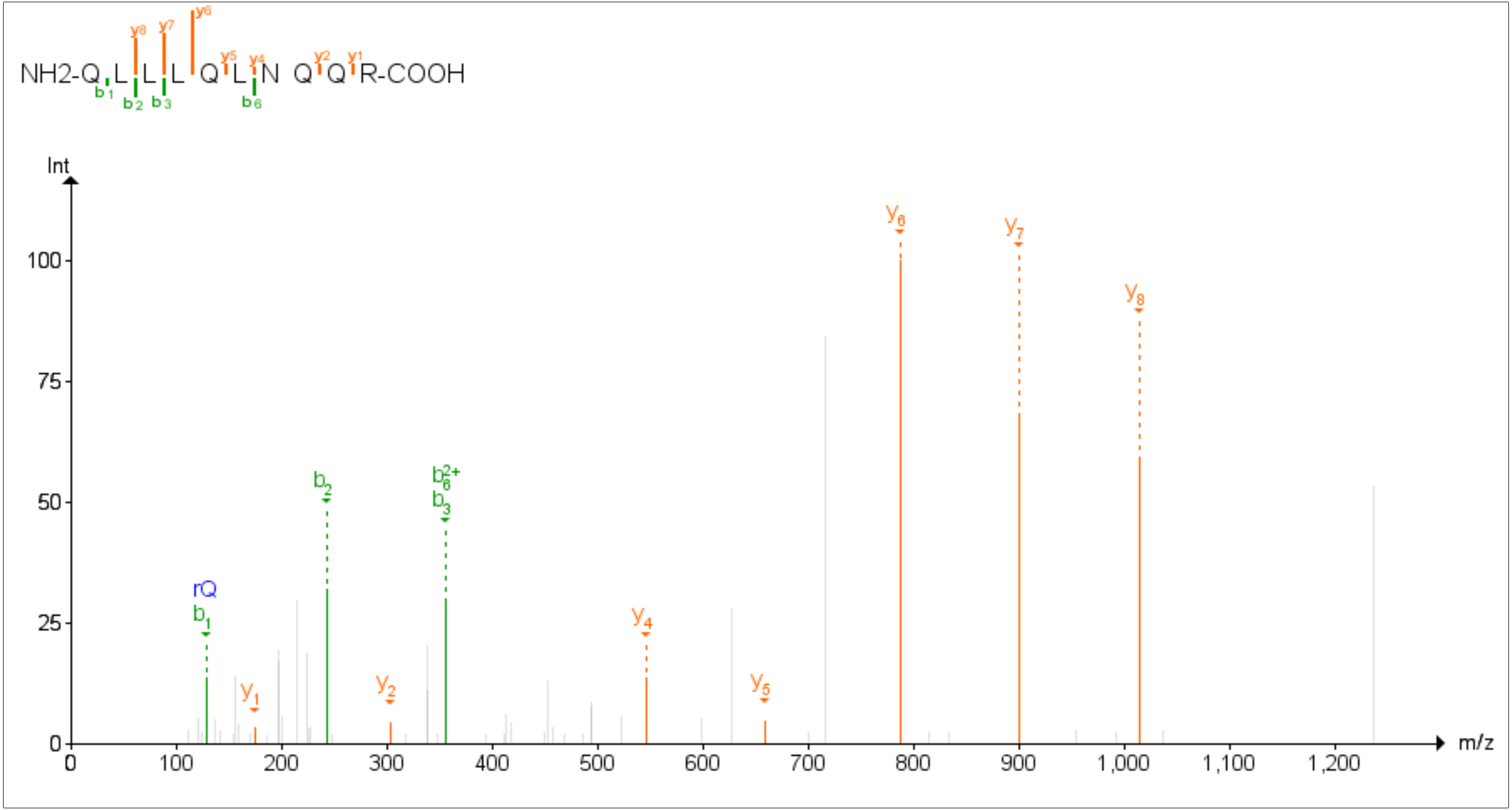

NH2-QLLLLQLNQQR-COOH
b1 b2 b3 b6
y8 y7 y6 y5 y4 y2 y1
Int
100
75
50
25
0
rQ
b1
y1
b2
y2
b6 2+
b3
y4
y5
y6
y7
y8
0 100 200 300 400 500 600 700 800 900 1,000 1,100 1,200
m/z

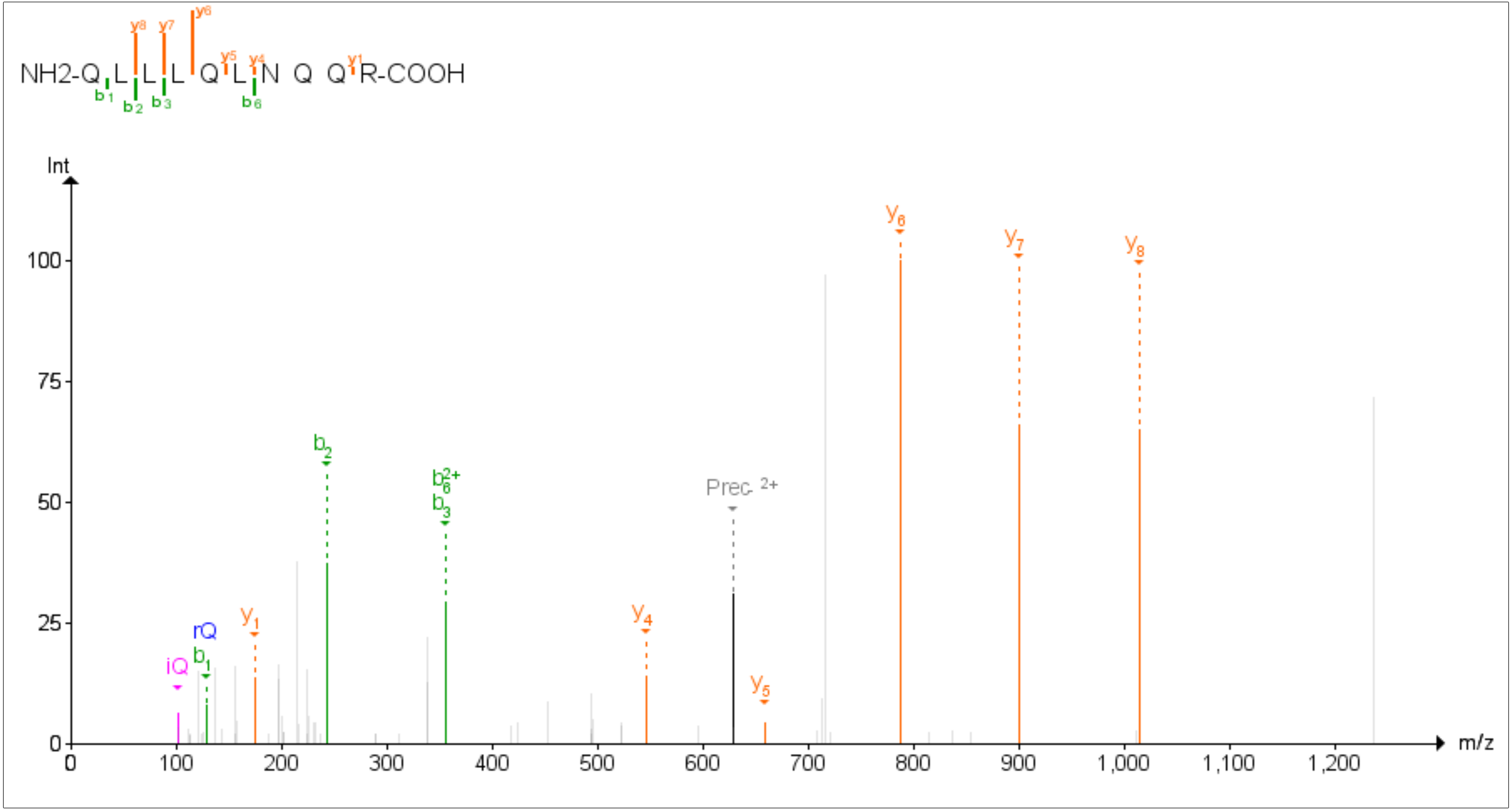

NH2-Q L L L Q L N Q Q R-COOH
y8 y7 y6 y5 y4 y1
b1 b2 b3 b6
Int
100
75
50
25
0
m/z
0 100 200 300 400 500 600 700 800 900 1,000 1,100 1,200
y6
y7
y8
b2
b6 2+
b3
Prec. 2+
y1
y4
y5
rQ
b1
iQ

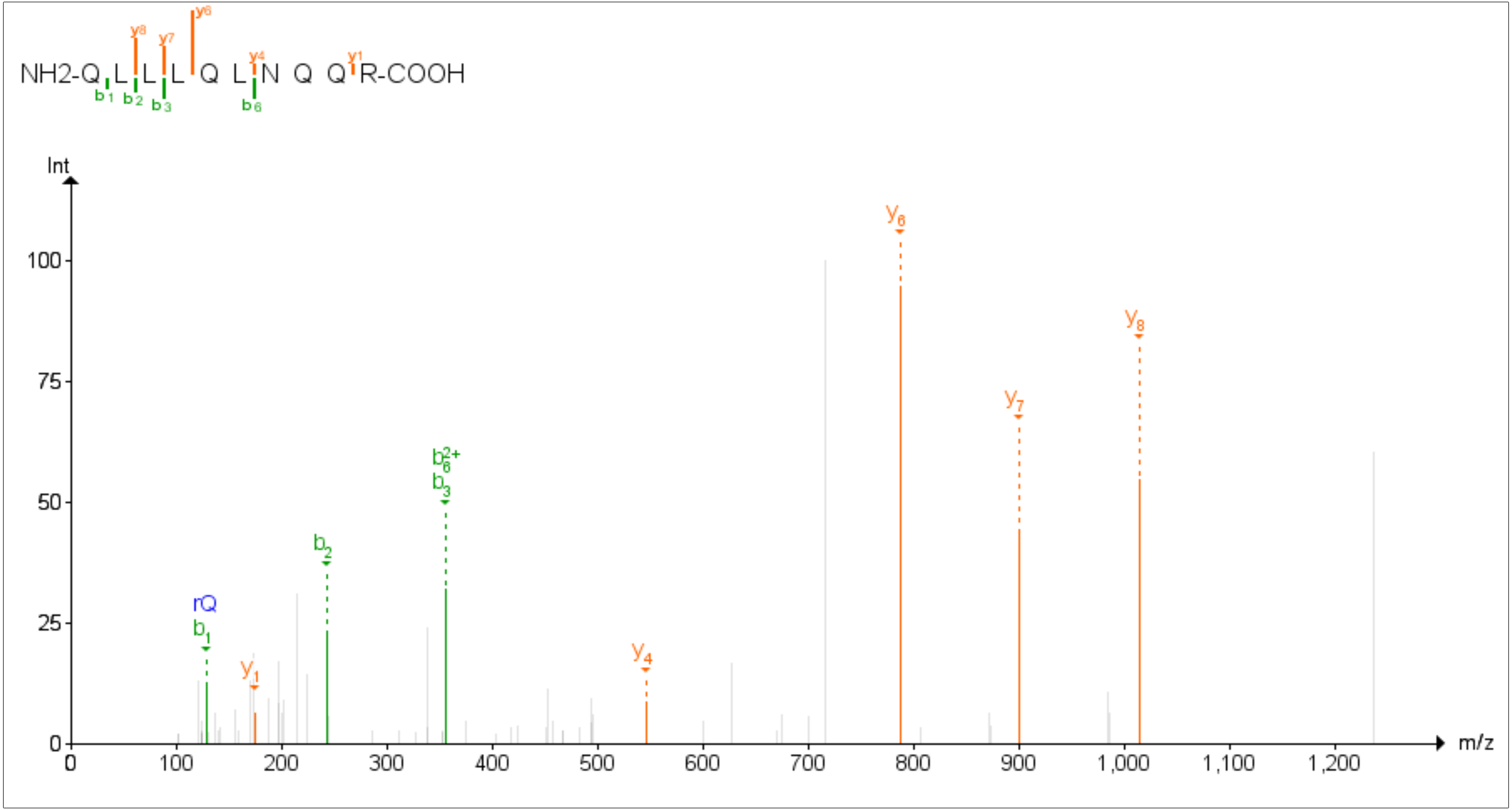

NH2-Q L L L Q L N Q Q R-COOH
y8
y7
y6
y4
y1
b1
b2
b3
b6
Int
100
75
50
25
0
y6
y8
y7
b6 2+
b3
b2
rQ
b1
y1
y4
m/z
0
100
200
300
400
500
600
700
800
900
1,000
1,100
1,200

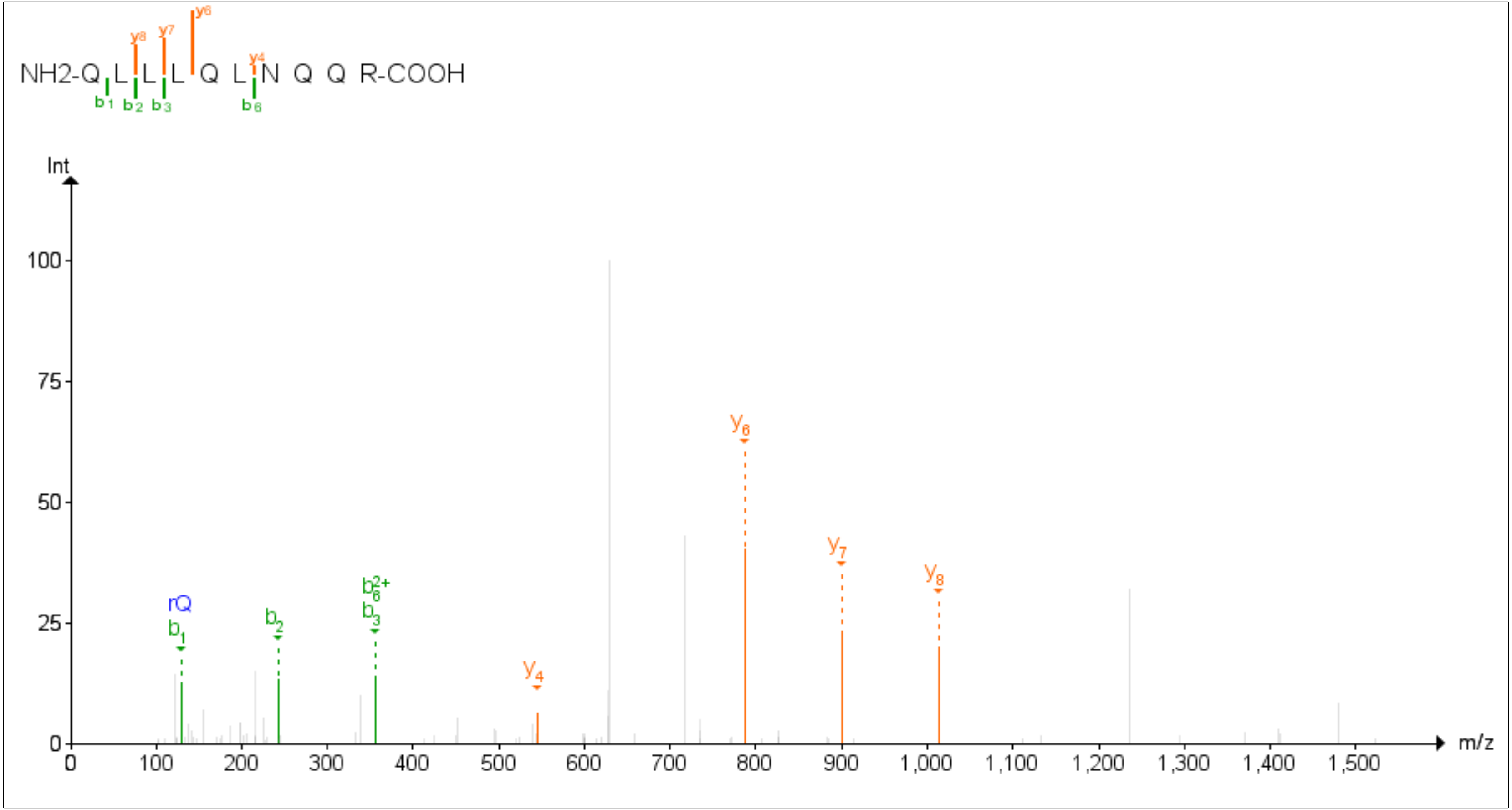

NH2-Q L L L Q L N Q Q R-COOH
y8 y7 y6 y4
b1 b2 b3 b6
Int
100
75
50
25
0
rQ
b1
b2
b6 2+
b3
y4
y6
y7
y8
0 100 200 300 400 500 600 700 800 900 1,000 1,100 1,200 1,300 1,400 1,500
m/z

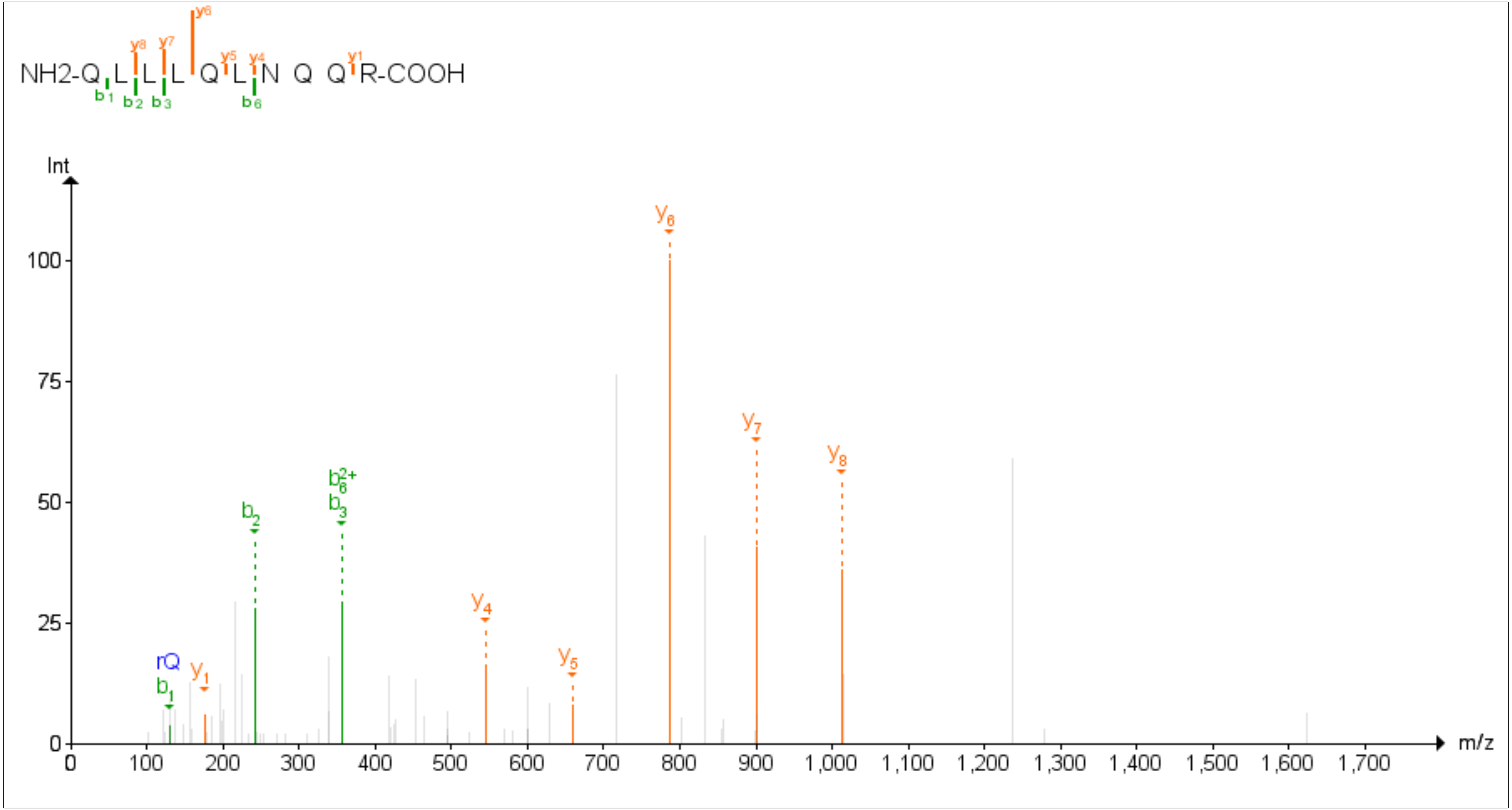

NH2-Q L L L Q L N Q Q R-COOH
y8 y7 y6 y5 y4 y1
b1 b2 b3 b6
Int
m/z
y6
y7
y8
y4
y5
y1
b2
b6 2+
b3
b1
rQ
0
25
50
75
100
0
100
200
300
400
500
600
700
800
900
1,000
1,100
1,200
1,300
1,400
1,500
1,600
1,700

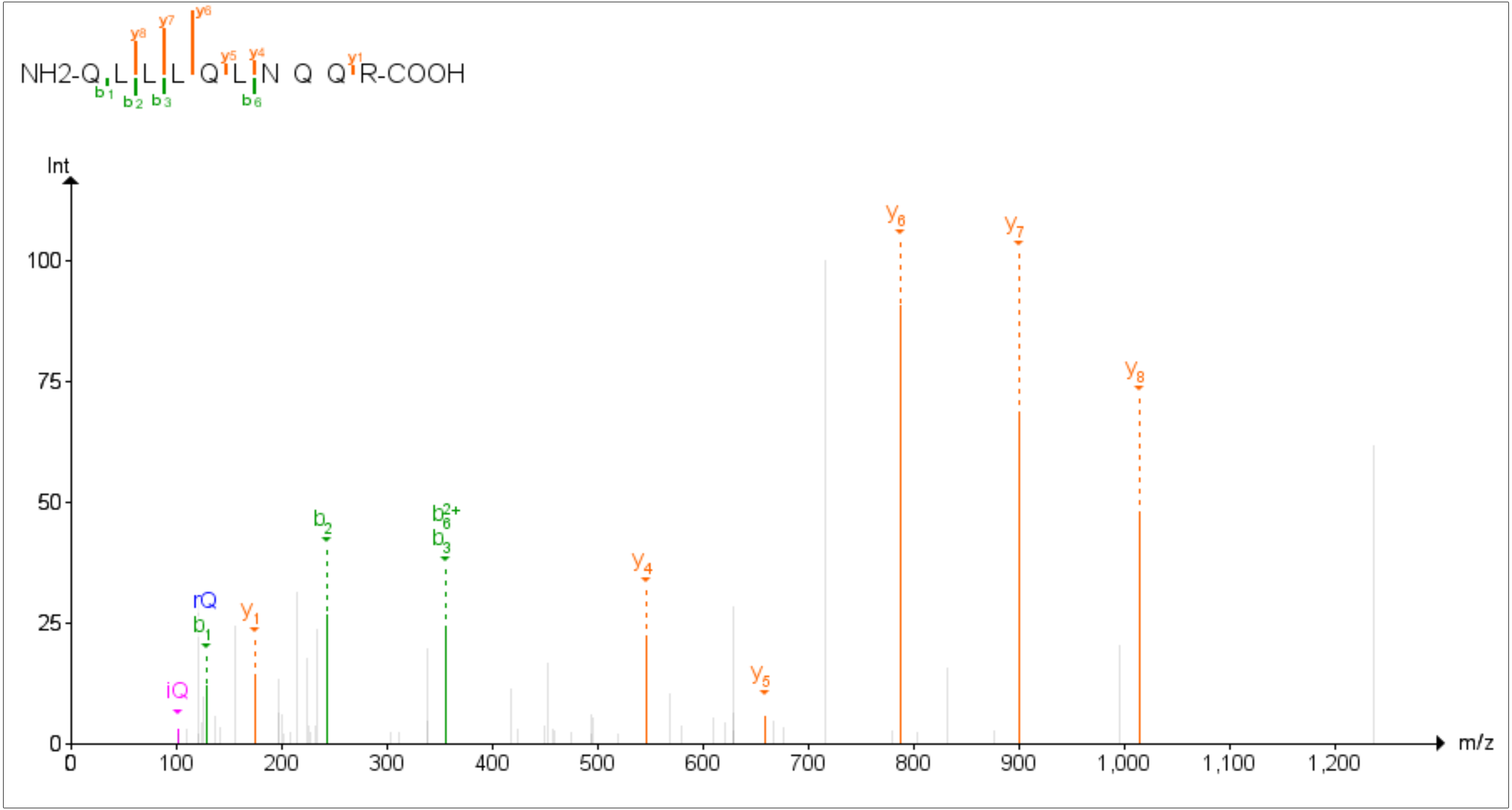

NH2-Q L L L Q L N Q Q R-COOH
y8 y7 y6 y5 y4 y1
b1 b2 b3 b6
Int
100
75
50
25
0
y6
y7
y8
b2
b6 2+
b3
y4
rQ
b1
y1
iQ
y5
m/z
0
100
200
300
400
500
600
700
800
900
1,000
1,100
1,200

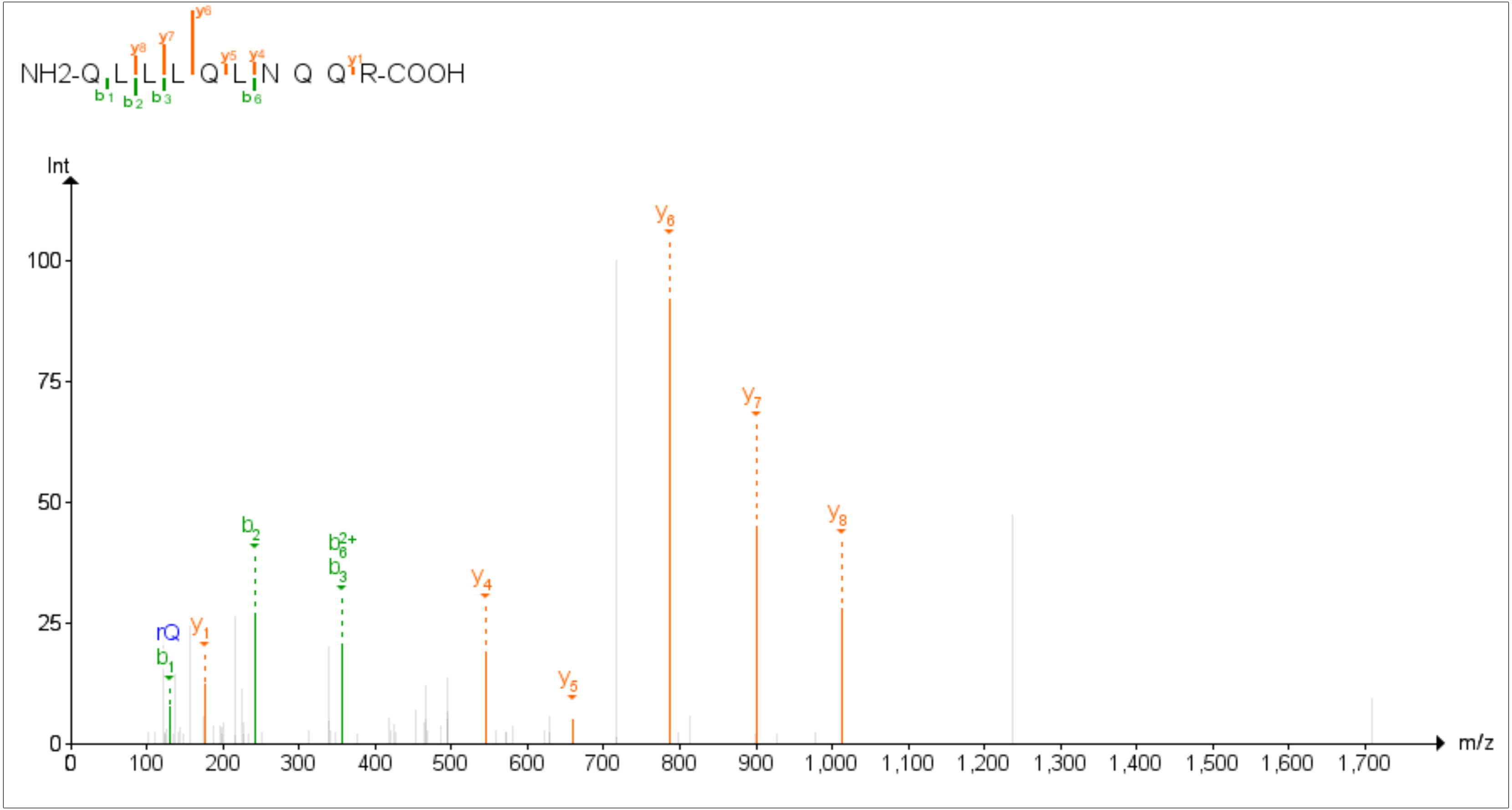

NH2-Q L L L Q L N Q Q R-COOH
y8 y7 y6 y5 y4 y1
b1 b2 b3 b6
Int
m/z
rQ
y1
b1
b2
b6 2+
b3
y4
y5
y6
y7
y8

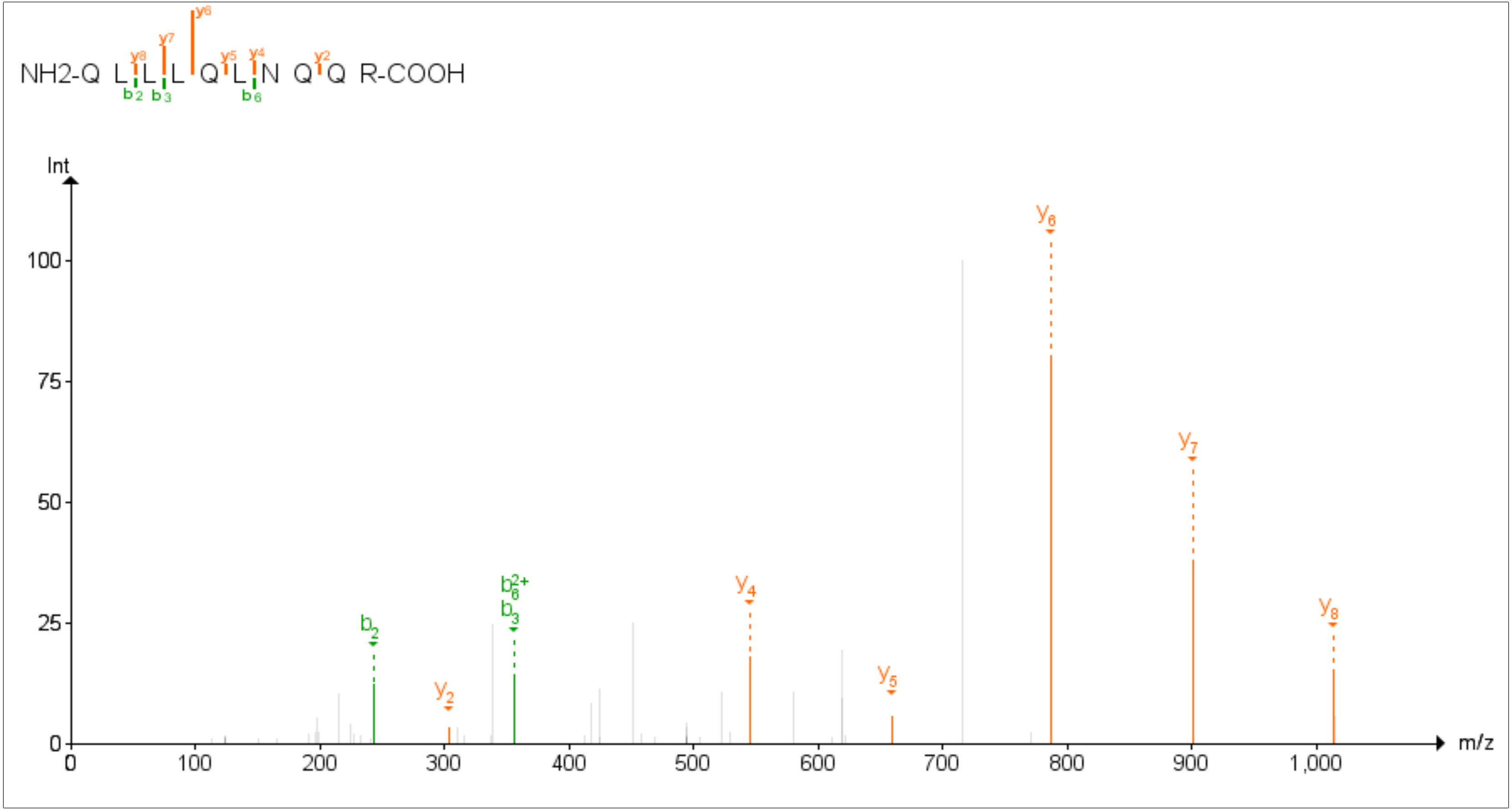

NH2-Q L L L Q L N Q Q R-COOH
y8 y7 y6 y5 y4 y2
b2 b3 b6
Int
100
75
50
25
0
m/z
0
100
200
300
400
500
600
700
800
900
1,000
y6
y7
y8
y4
y5
y2
b2
b6 2+
b3

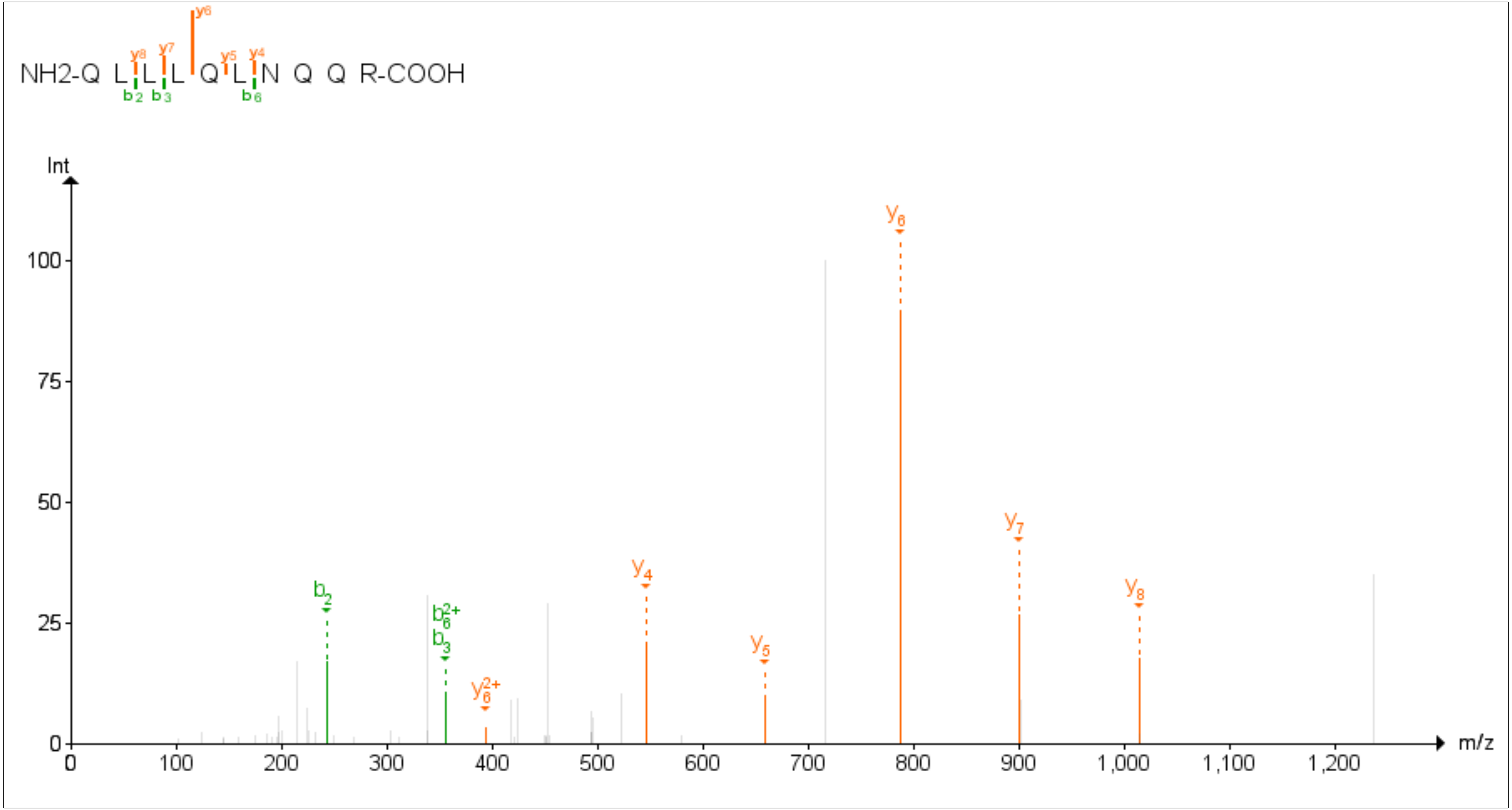

NH2-Q L L L Q L N Q Q R-COOH
y8 y7 y6 y5 y4
b2 b3 b6
Int
m/z
0
25
50
75
100
0
100
200
300
400
500
600
700
800
900
1,000
1,100
1,200
b2
b6 2+
b3
y6 2+
y4
y5
y6
y7
y8

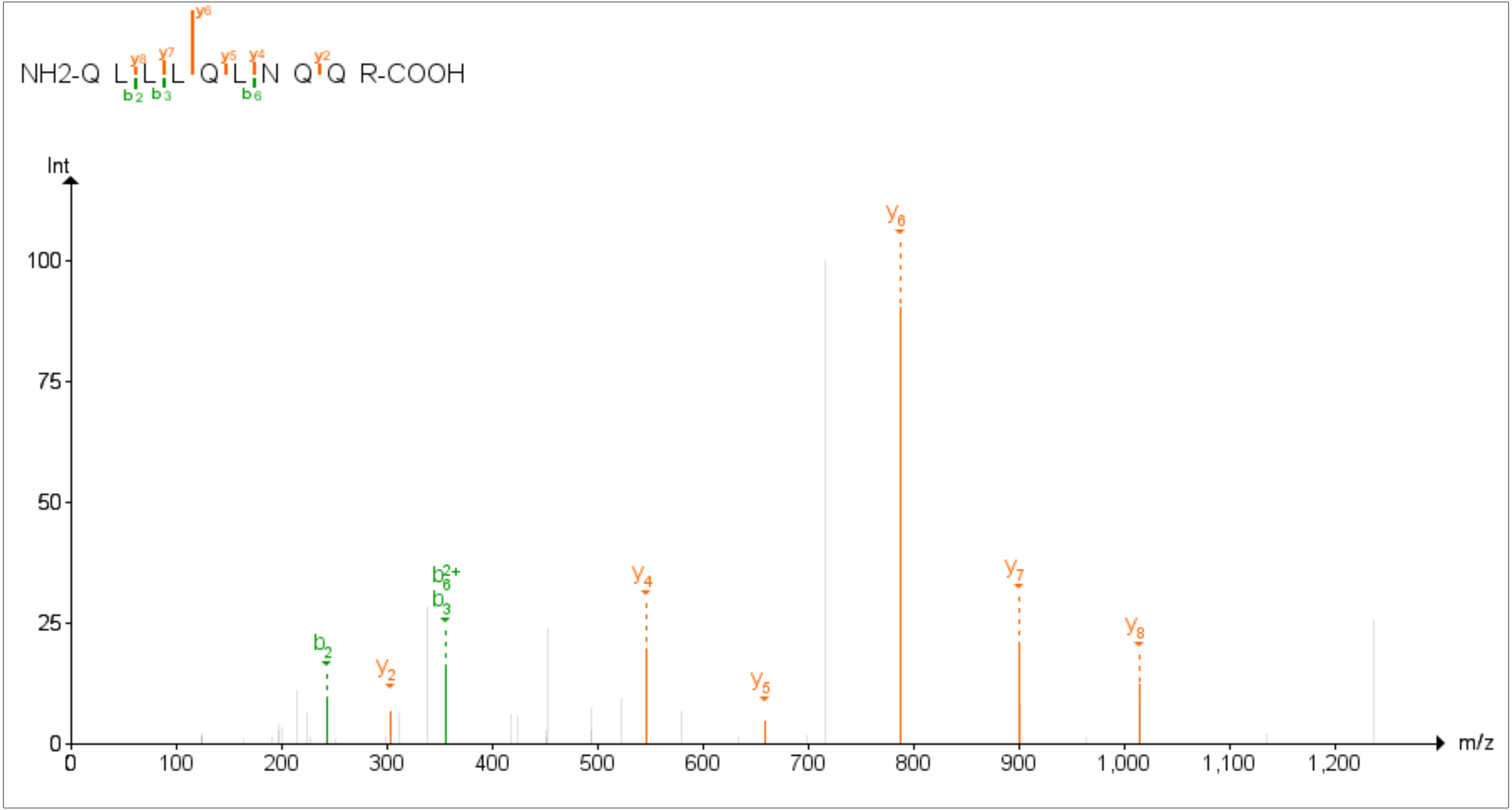

NH2-Q L L L Q L N Q Q R-COOH
y8 y7 y6 y5 y4 y2
b2 b3 b6
Int
m/z
y6
y4
y7
y8
y5
y2
b2
b3
b6 2+
0 25 50 75 100
0 100 200 300 400 500 600 700 800 900 1,000 1,100 1,200

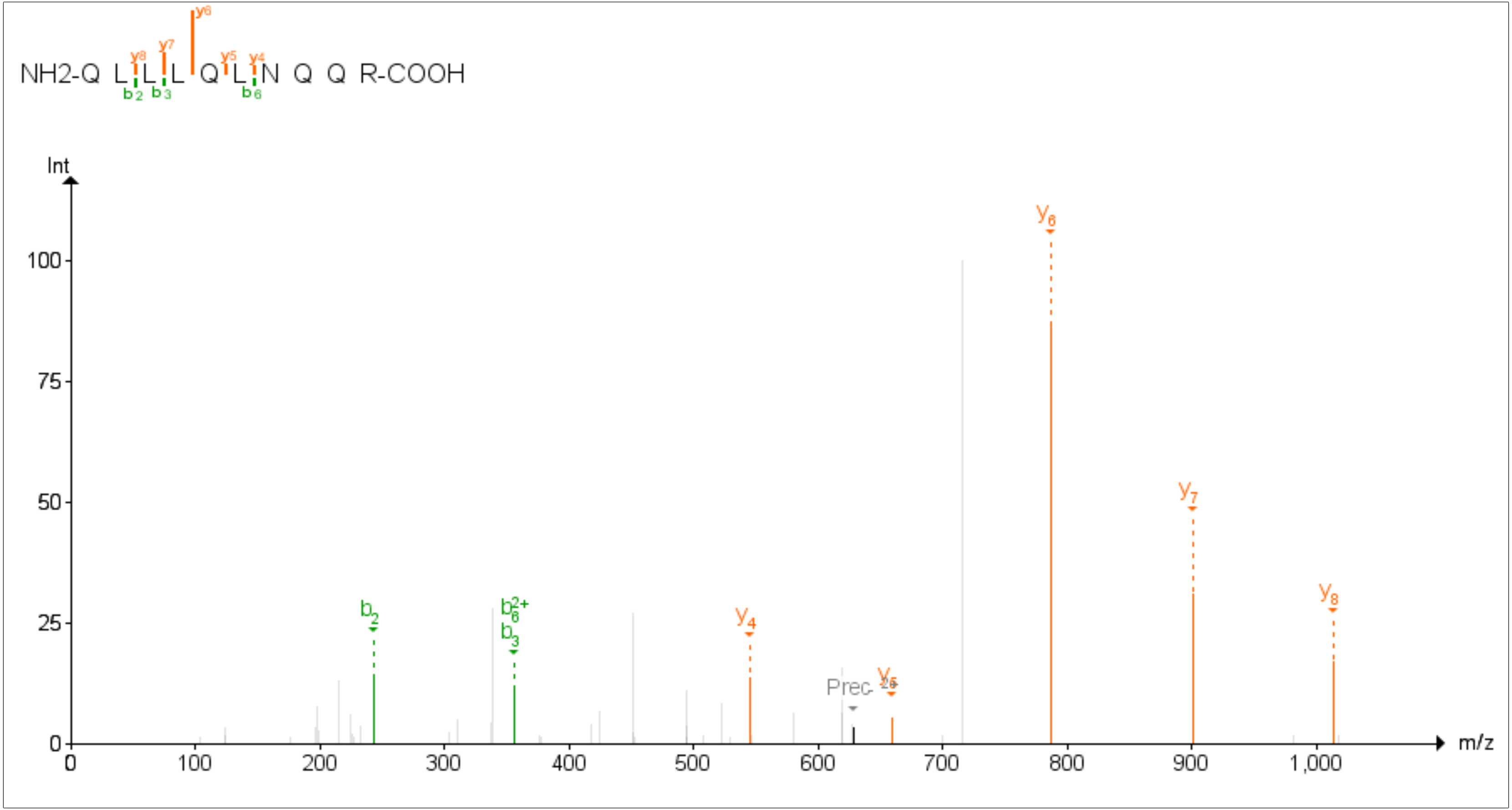

NH2-Q L L L Q L N Q Q R-COOH
y8 y7 y6 y5 y4
b2 b3 b6
Int
100
75
50
25
0
b_2
b_6^{2+}
b_3
y_4
Prec
y_5
y_6
y_7
y_8
0
100
200
300
400
500
600
700
800
900
1,000
m/z

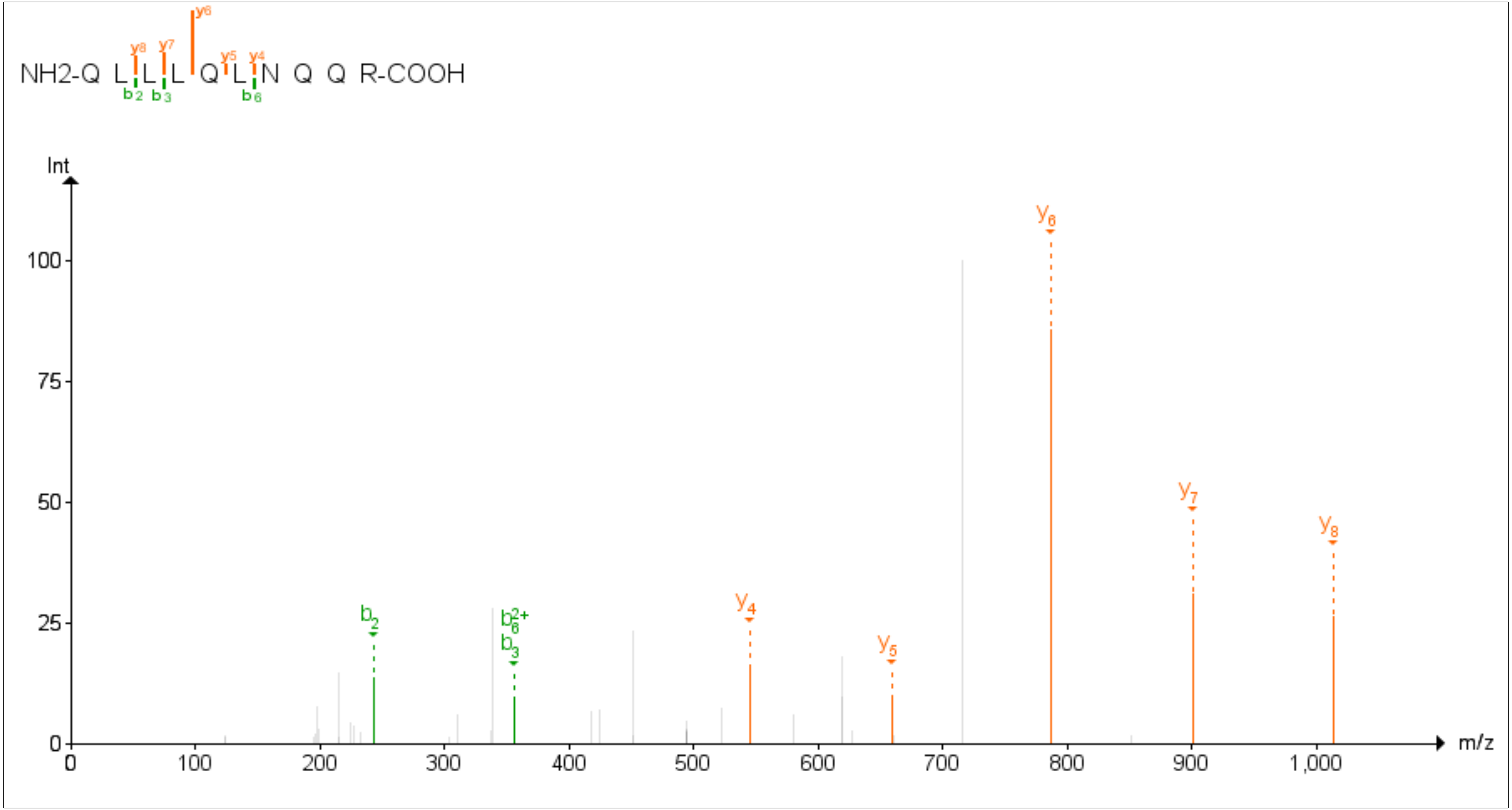

NH2-Q L L L Q L N Q Q R-COOH
y8
y7
y6
y5
y4
b2
b3
b6
Int
100
75
50
25
0
m/z
0
100
200
300
400
500
600
700
800
900
1,000
b2
b6 2+
b3
y4
y5
y6
y7
y8

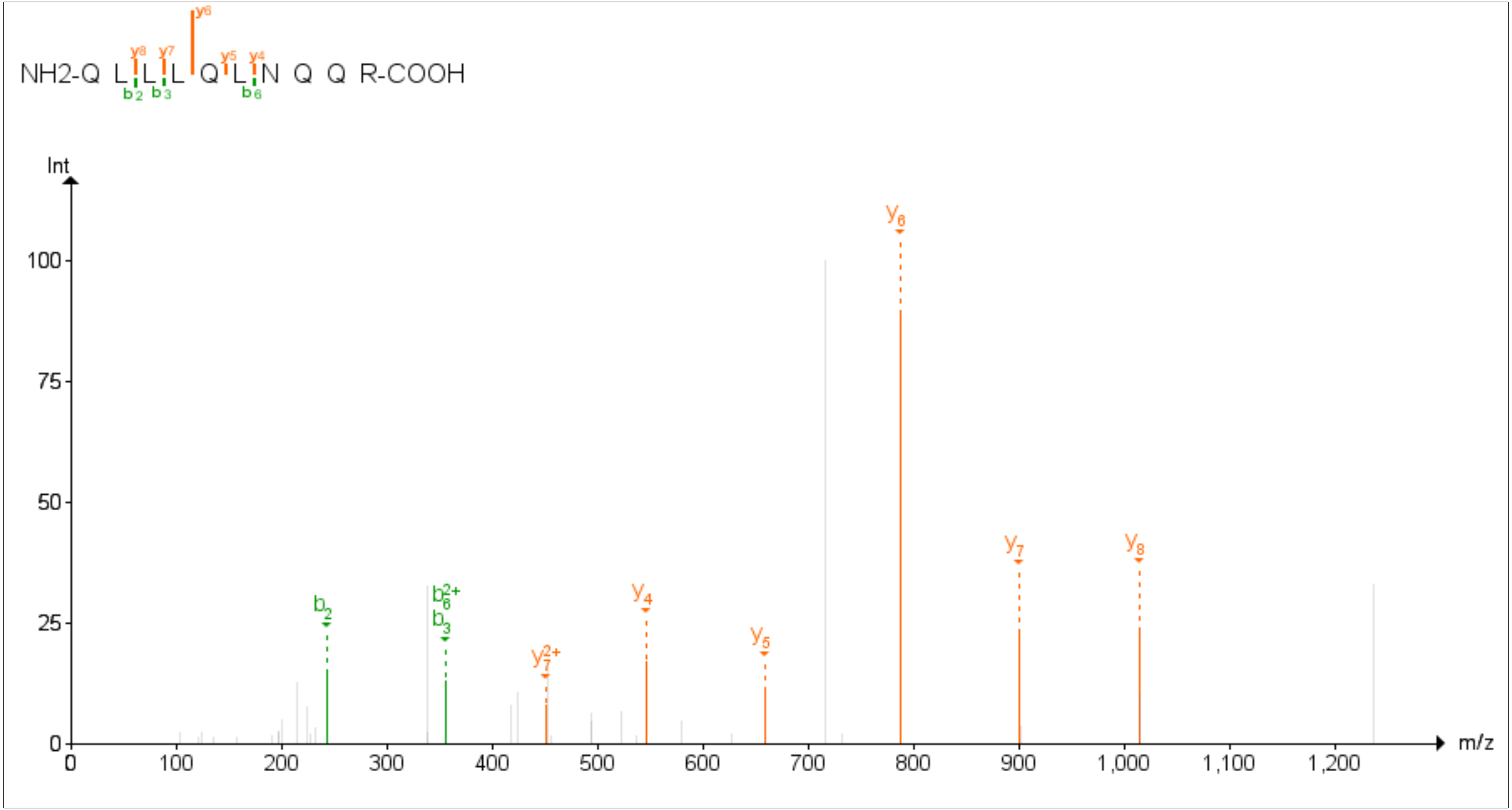

NH2-Q L L L Q L N Q Q R-COOH
y8 y7 y6 y5 y4
b2 b3 b6
Int
100
75
50
25
0
m/z
0 100 200 300 400 500 600 700 800 900 1,000 1,100 1,200
b_2
b_6^{2+}
b_3
y_7^{2+}
y_4
y_5
y_6
y_7
y_8

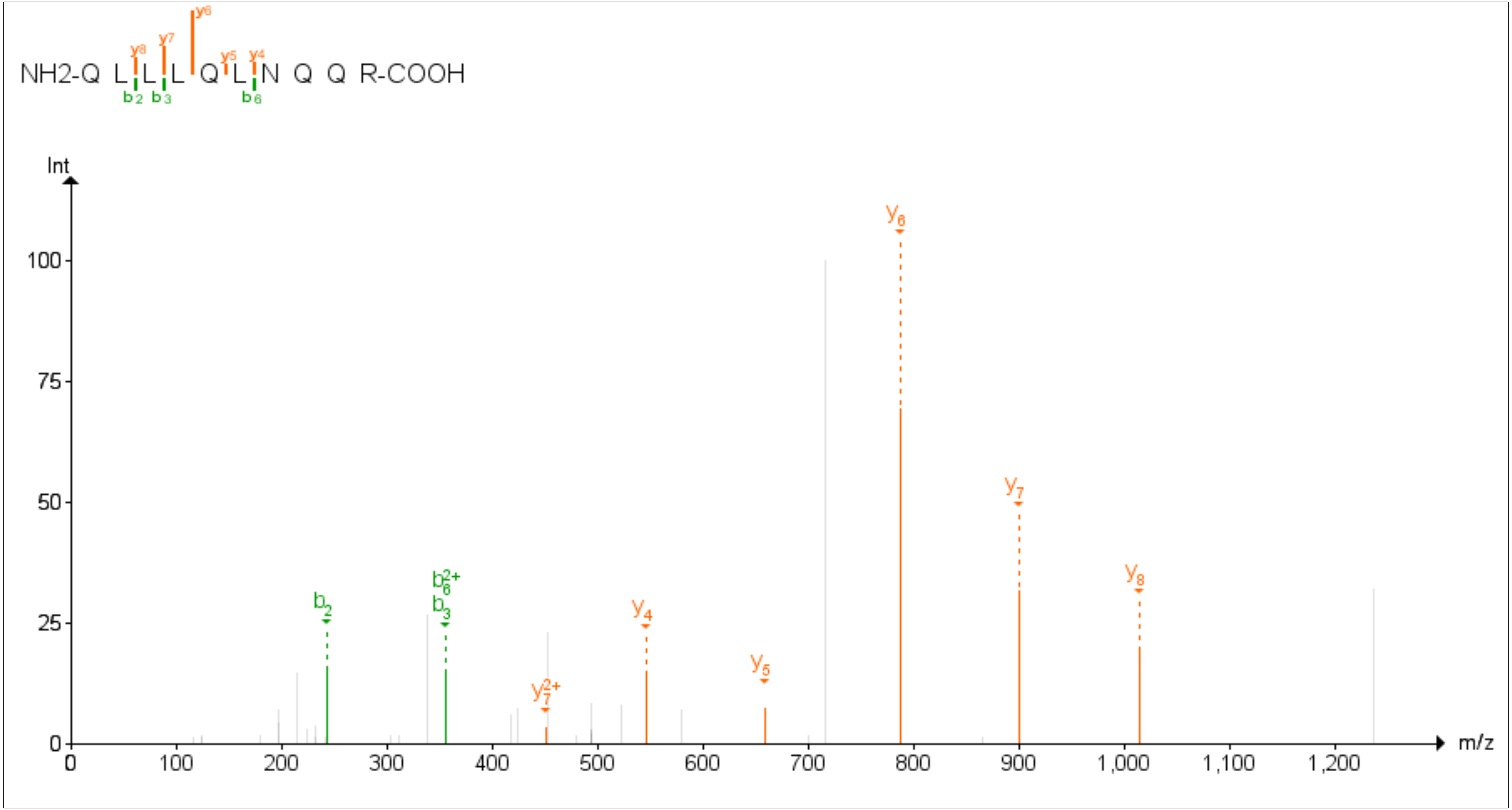

NH2-Q L L L Q L N Q Q R-COOH
y8 y7 y6 y5 y4
b2 b3 b6
Int
100
75
50
25
0
0 100 200 300 400 500 600 700 800 900 1,000 1,100 1,200
m/z
b2
b6 2+
b3
y7 2+
y4
y5
y6
y7
y8

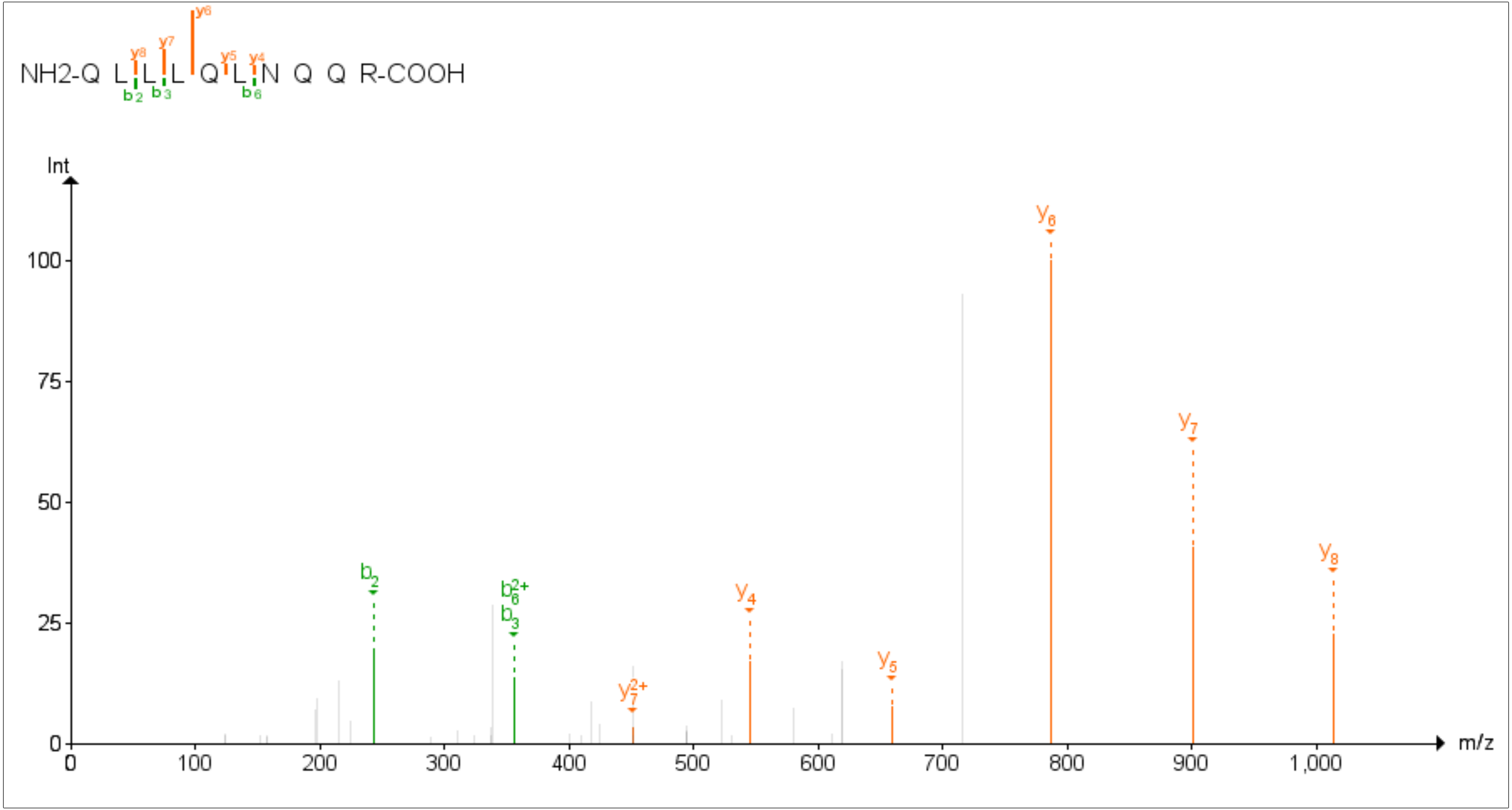

NH2-Q L L L Q L N Q Q R-COOH
y8 y7 y6 y5 y4
b2 b3 b6
Int
100
75
50
25
0
m/z
0
100
200
300
400
500
600
700
800
900
1,000
b2
b6 2+
b3
y7 2+
y4
y5
y6
y7
y8

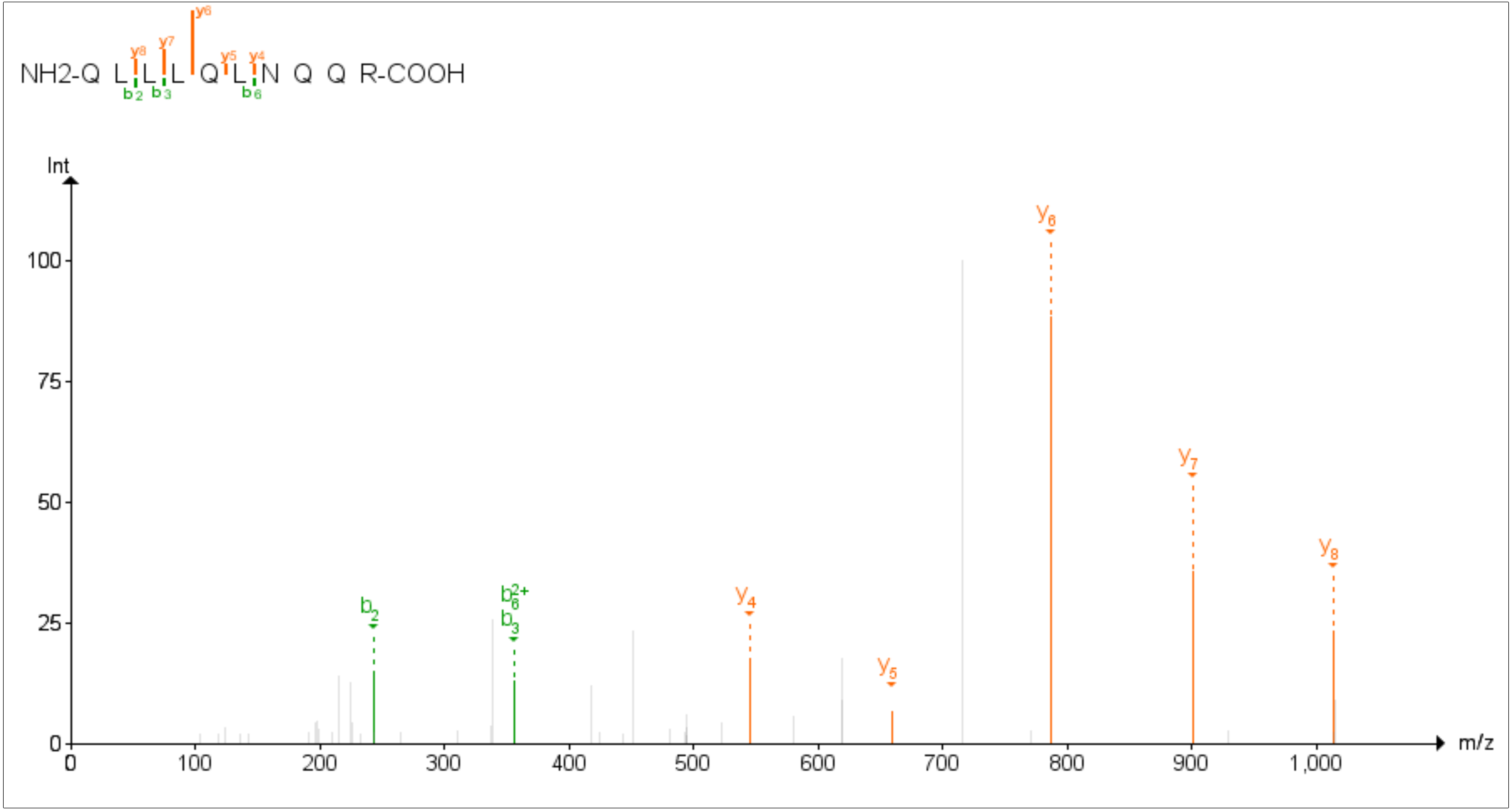

NH2-Q L L L Q L N Q Q R-COOH
y8 y7 y6 y5 y4
b2 b3 b6
Int
100
75
50
25
0
y6
y7
y8
y4
y5
b2
b6 2+
b3
0 100 200 300 400 500 600 700 800 900 1,000
m/z

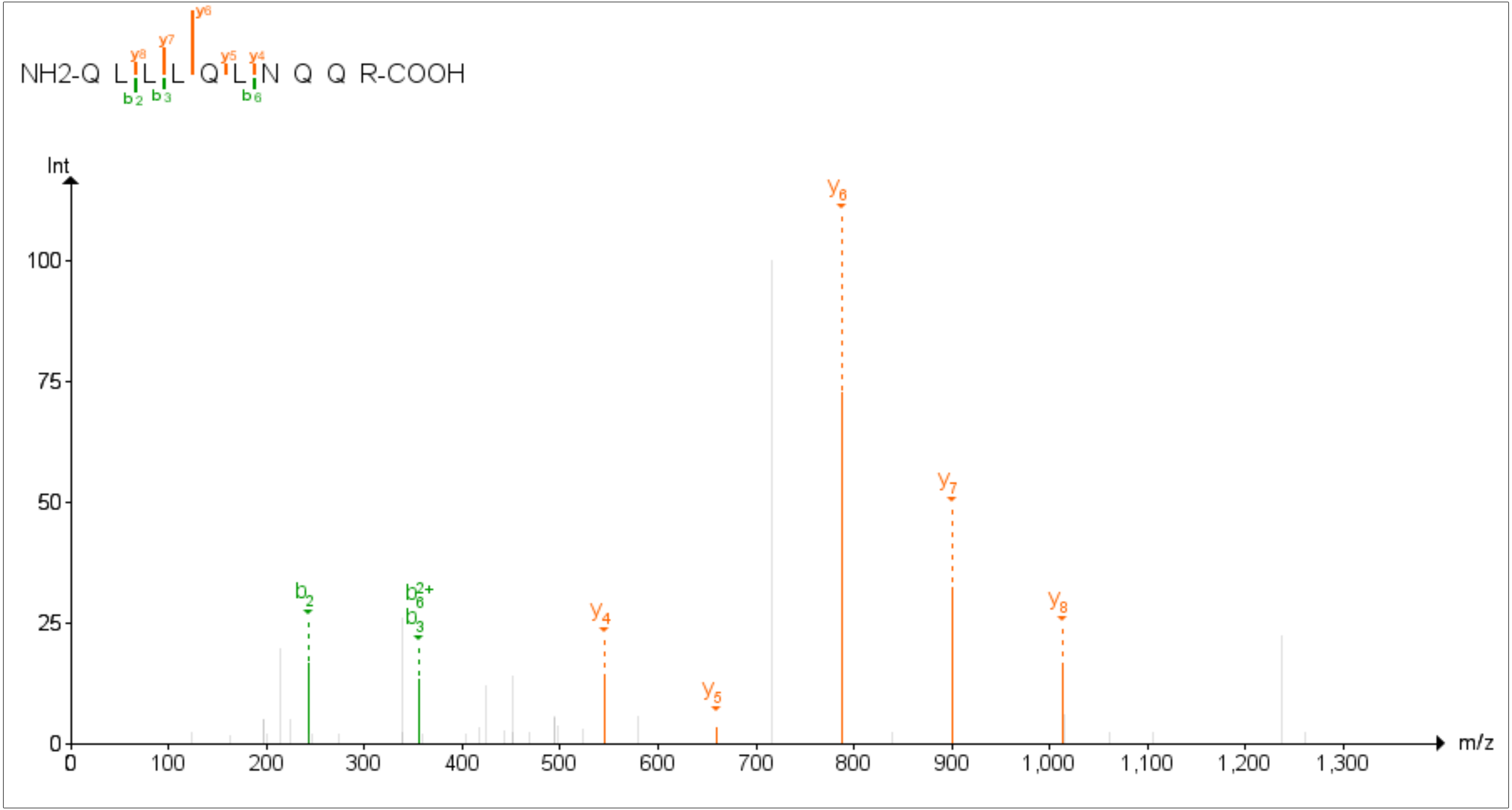

NH2-Q L L L Q L N Q Q R-COOH
y8 y7 y6 y5 y4
b2 b3 b6
Int
100
75
50
25
0
b2
b6 2+
b3
y4
y5
y6
y7
y8
0 100 200 300 400 500 600 700 800 900 1,000 1,100 1,200 1,300
m/z

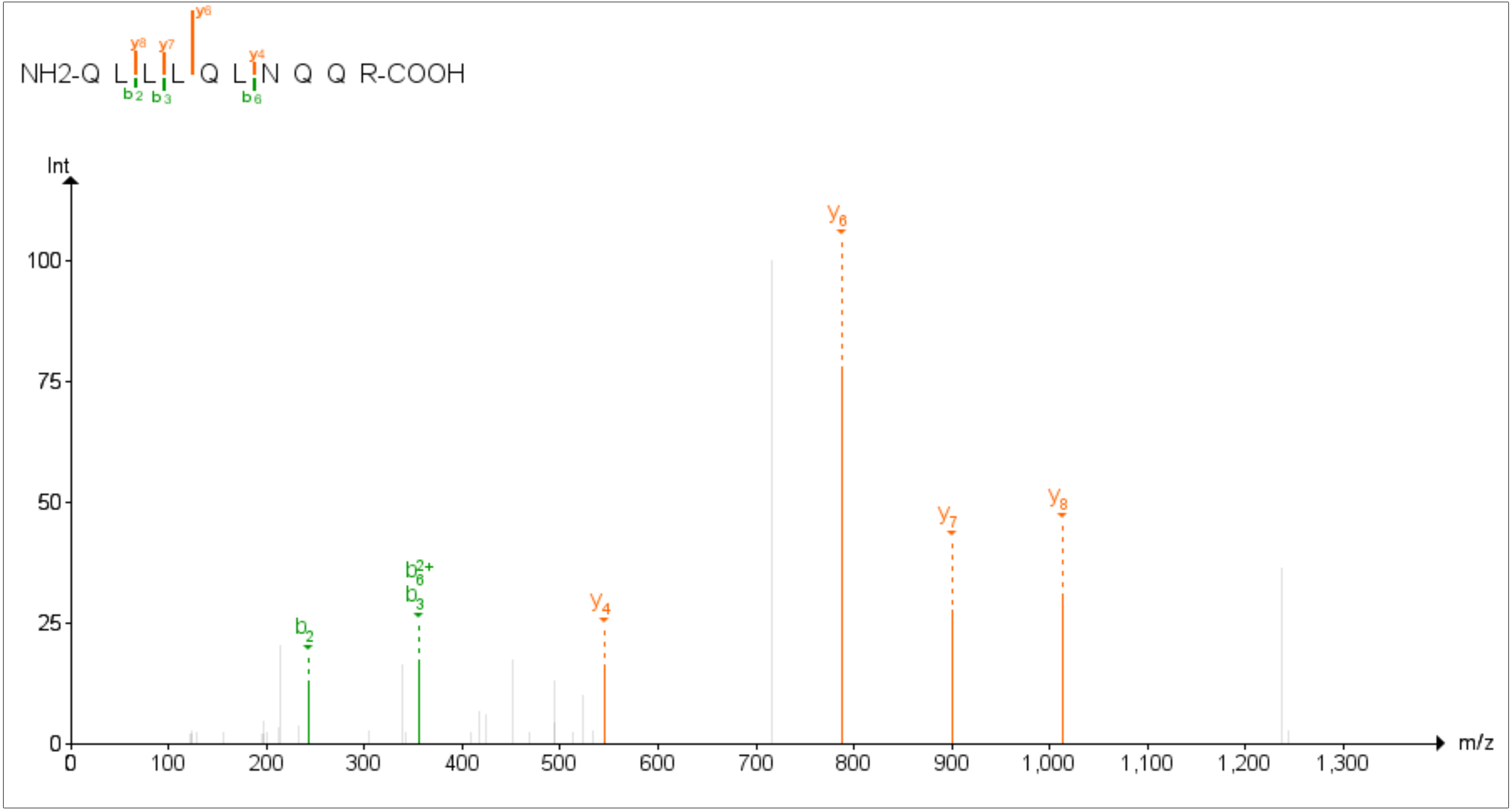

NH2-Q L L L Q L N Q Q R-COOH
y8 y7 y6 y4
b2 b3 b6
Int
100
75
50
25
0
y6
y7
y8
y4
b2
b6 2+
b3
m/z
0 100 200 300 400 500 600 700 800 900 1,000 1,100 1,200 1,300

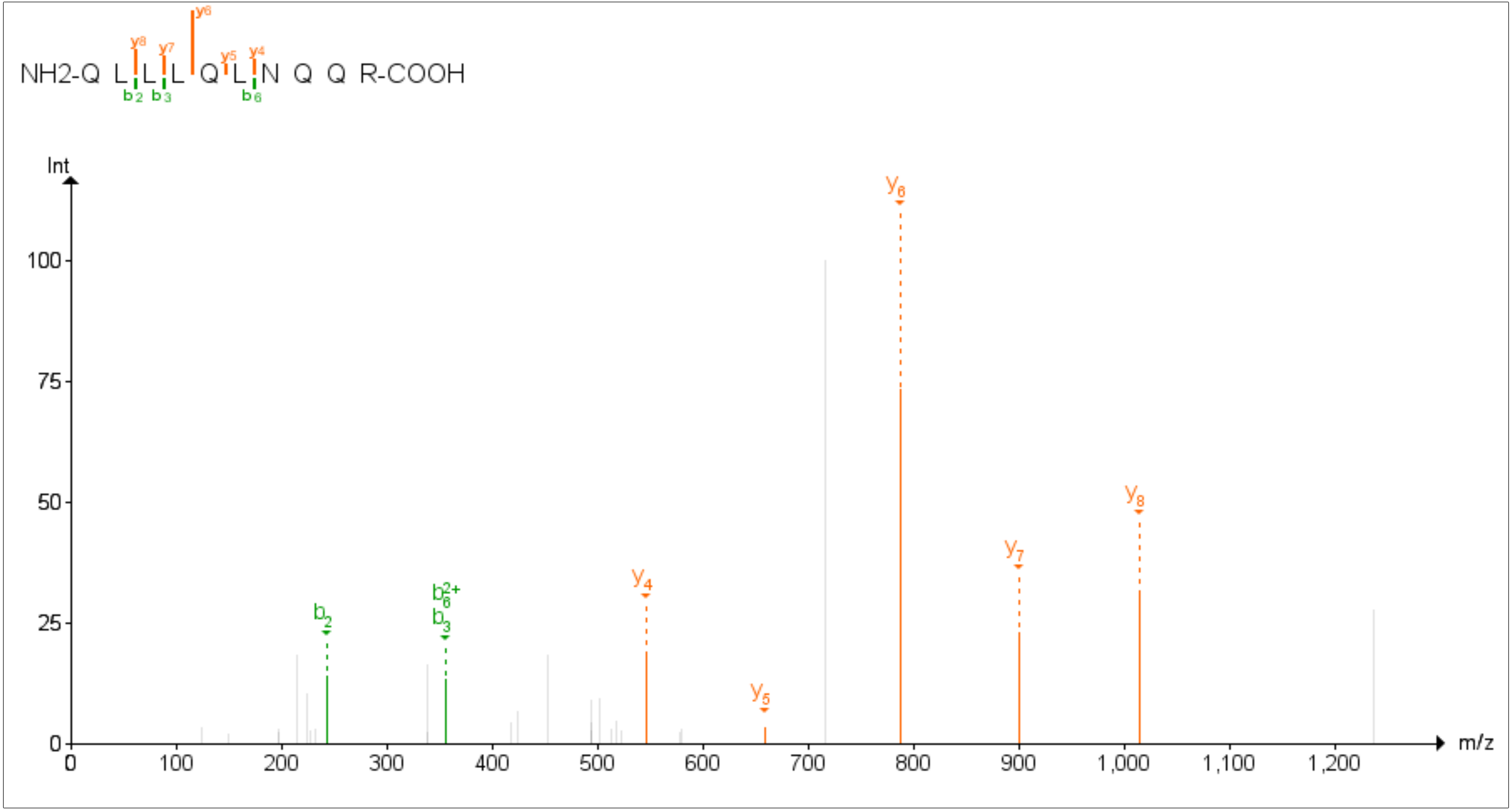
NH2-Q L L L Q L N Q Q R-COOH
y8 y7 y6 y5 y4
b2 b3 b6
Int
100
75
50
25
0
m/z
0 100 200 300 400 500 600 700 800 900 1,000 1,100 1,200
b2
b6 2+
b3
y4
y5
y6
y7
y8

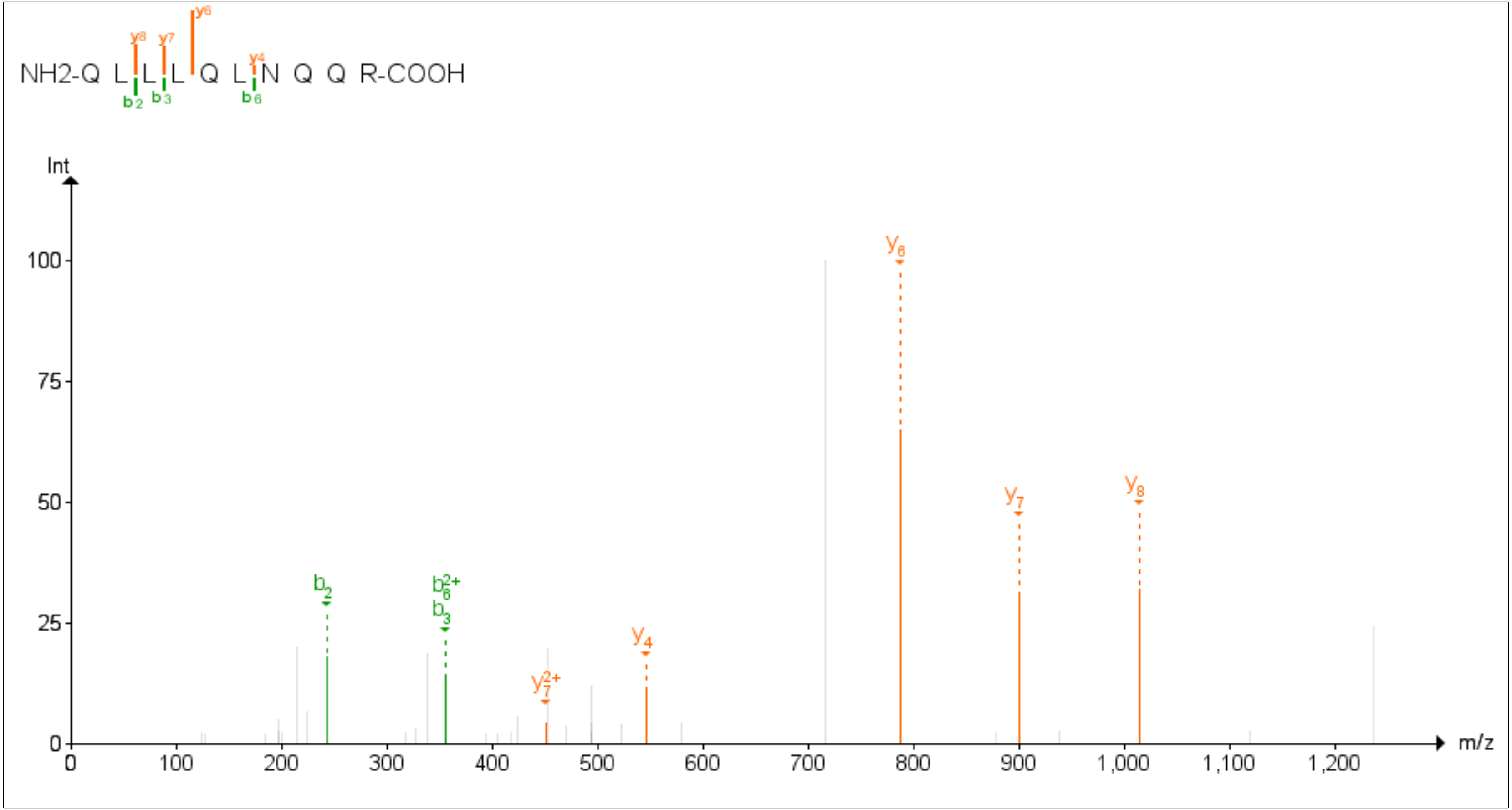

NH2-Q L L L Q L N Q Q R-COOH
y8
y7
y6
y4
b2
b3
b6
Int
100
75
50
25
0
y6
y7
y8
b2
b6 2+
b3
y7 2+
y4
0
100
200
300
400
500
600
700
800
900
1,000
1,100
1,200
m/z

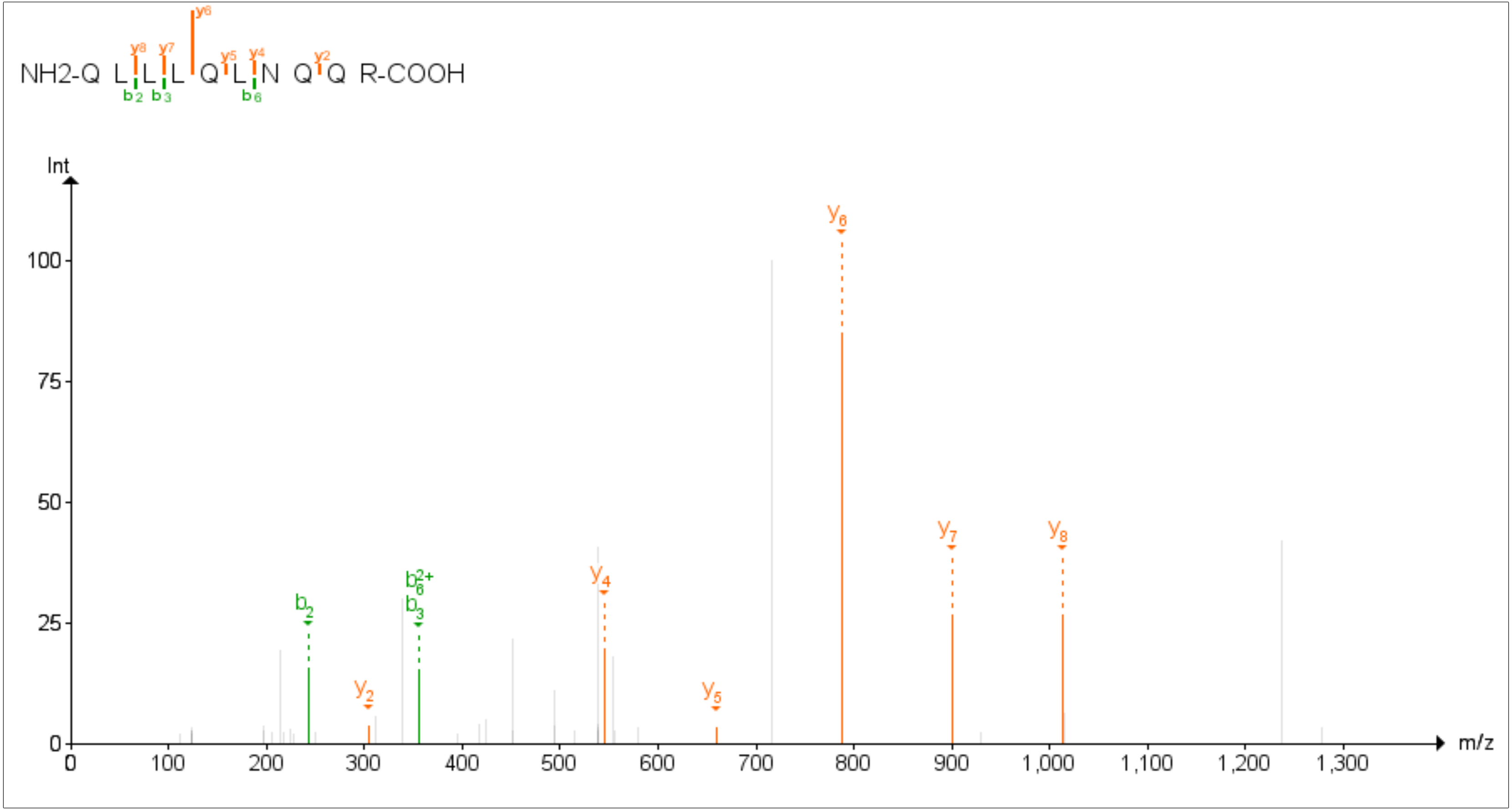

NH2-Q L L L Q L N Q Q R-COOH
y8 y7 y6 y5 y4 y2
b2 b3 b6
Int
100
75
50
25
0
y6
y7
y8
y4
b6 2+
b3
b2
y2
y5
0 100 200 300 400 500 600 700 800 900 1,000 1,100 1,200 1,300
m/z

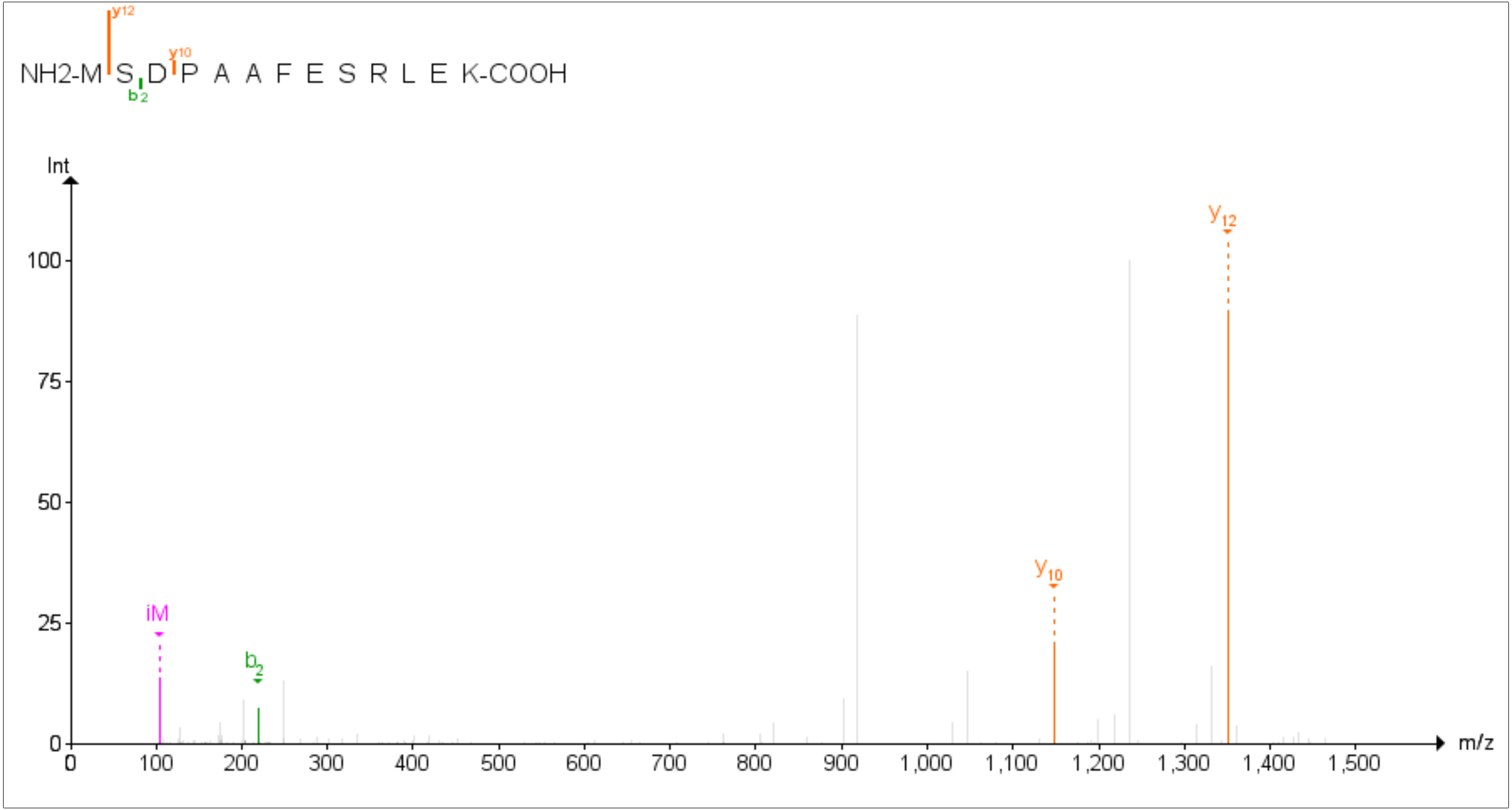

NH2-M S D P A A F E S R L E K-COOH
y12
y10
b2
Int
100
75
50
25
0
iM
b2
y10
y12
0
100
200
300
400
500
600
700
800
900
1,000
1,100
1,200
1,300
1,400
1,500
m/z

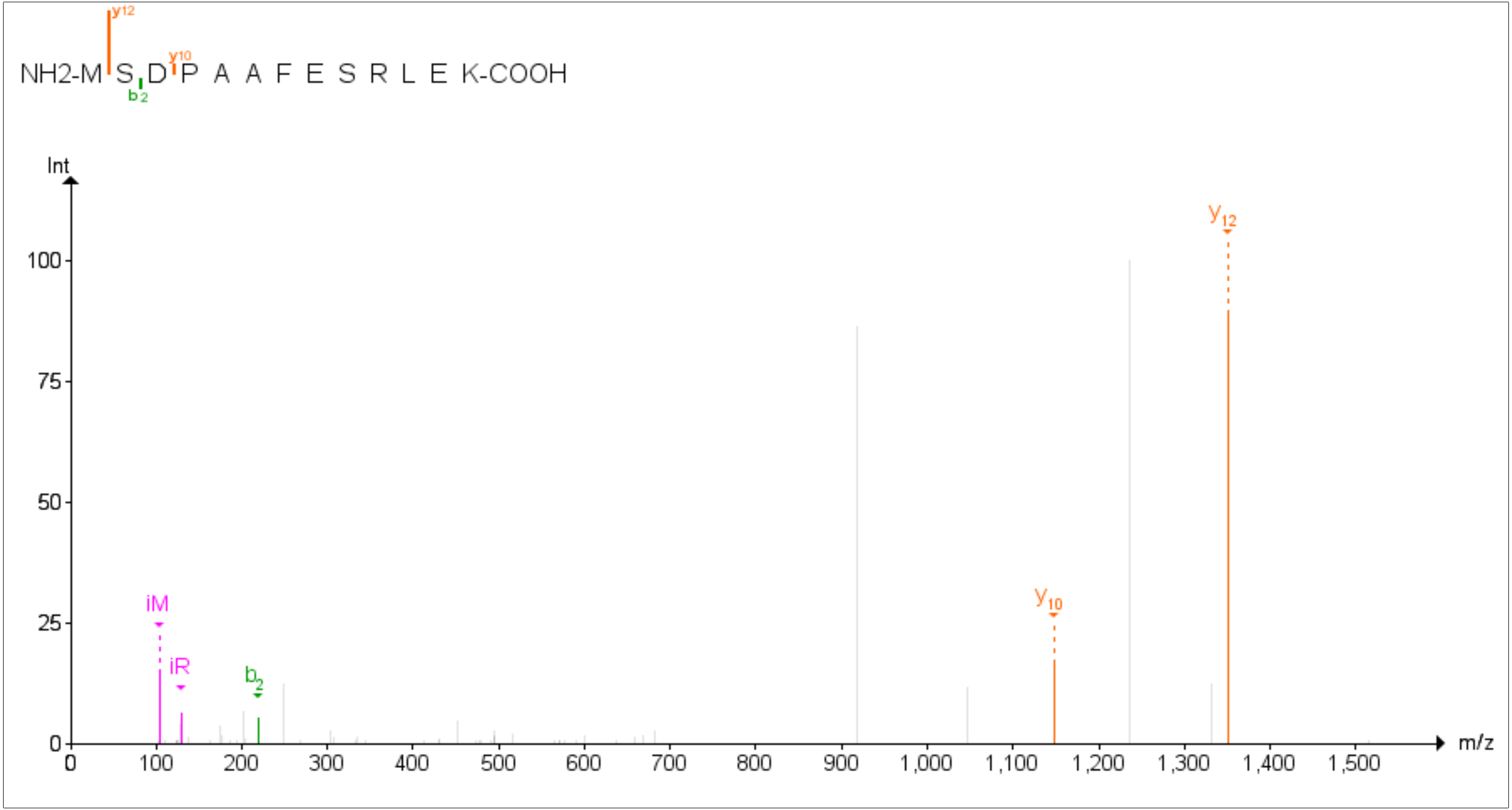

y12
y10
NH2-M S D P A A F E S R L E K-COOH
b2
Int
100
75
50
25
0
iM
iR
b2
y10
y12
0
100
200
300
400
500
600
700
800
900
1,000
1,100
1,200
1,300
1,400
1,500
m/z

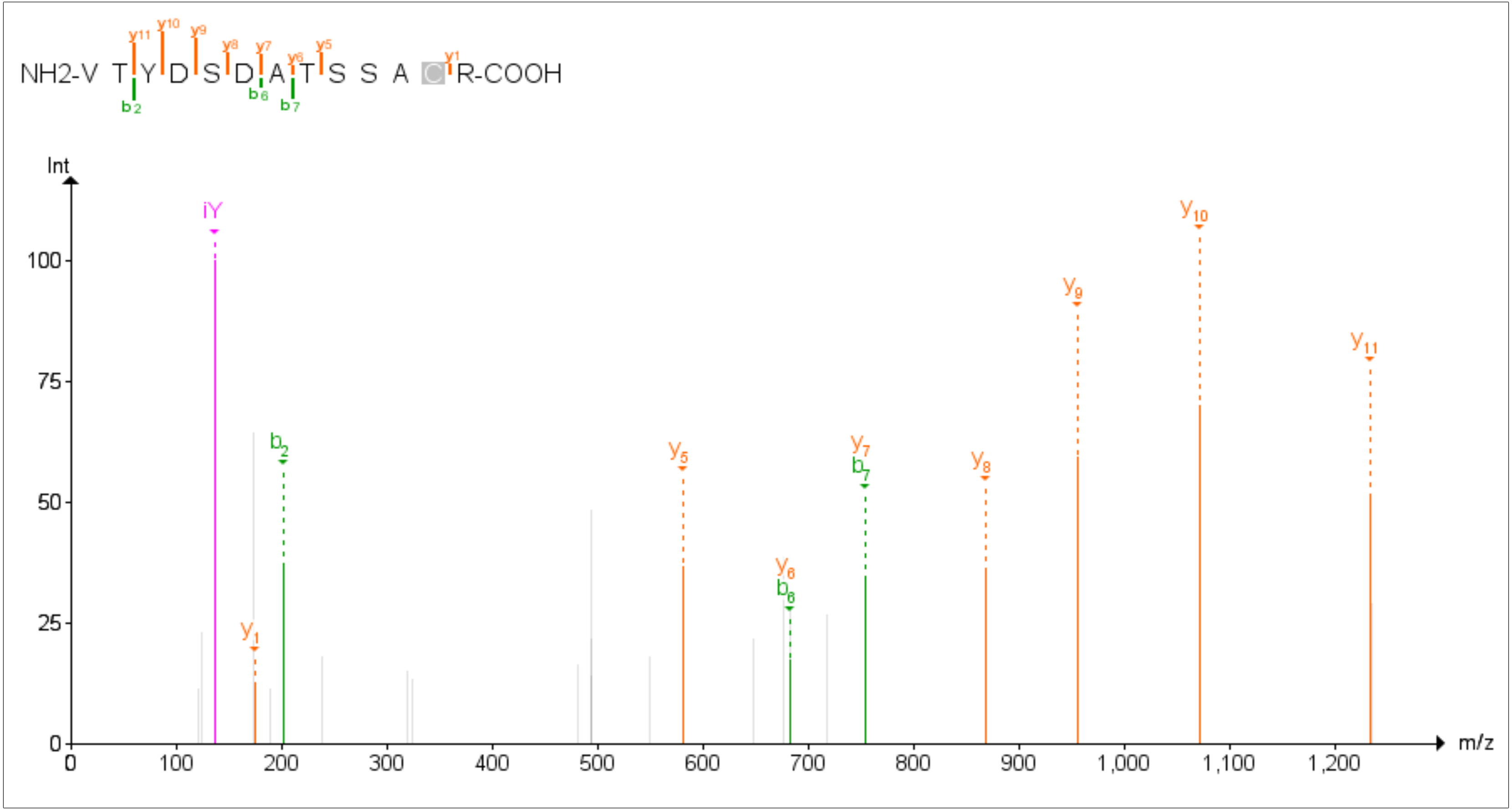
NH2-V T Y D S D A T S S A C R-COOH
y11 y10 y9 y8 y7 y6 y5 y1
b2 b6 b7
Int
iY
y10
y9
y11
b2
y5
y7
b7
y8
y6
b6
y1
100
75
50
25
0
0 100 200 300 400 500 600 700 800 900 1,000 1,100 1,200
m/z

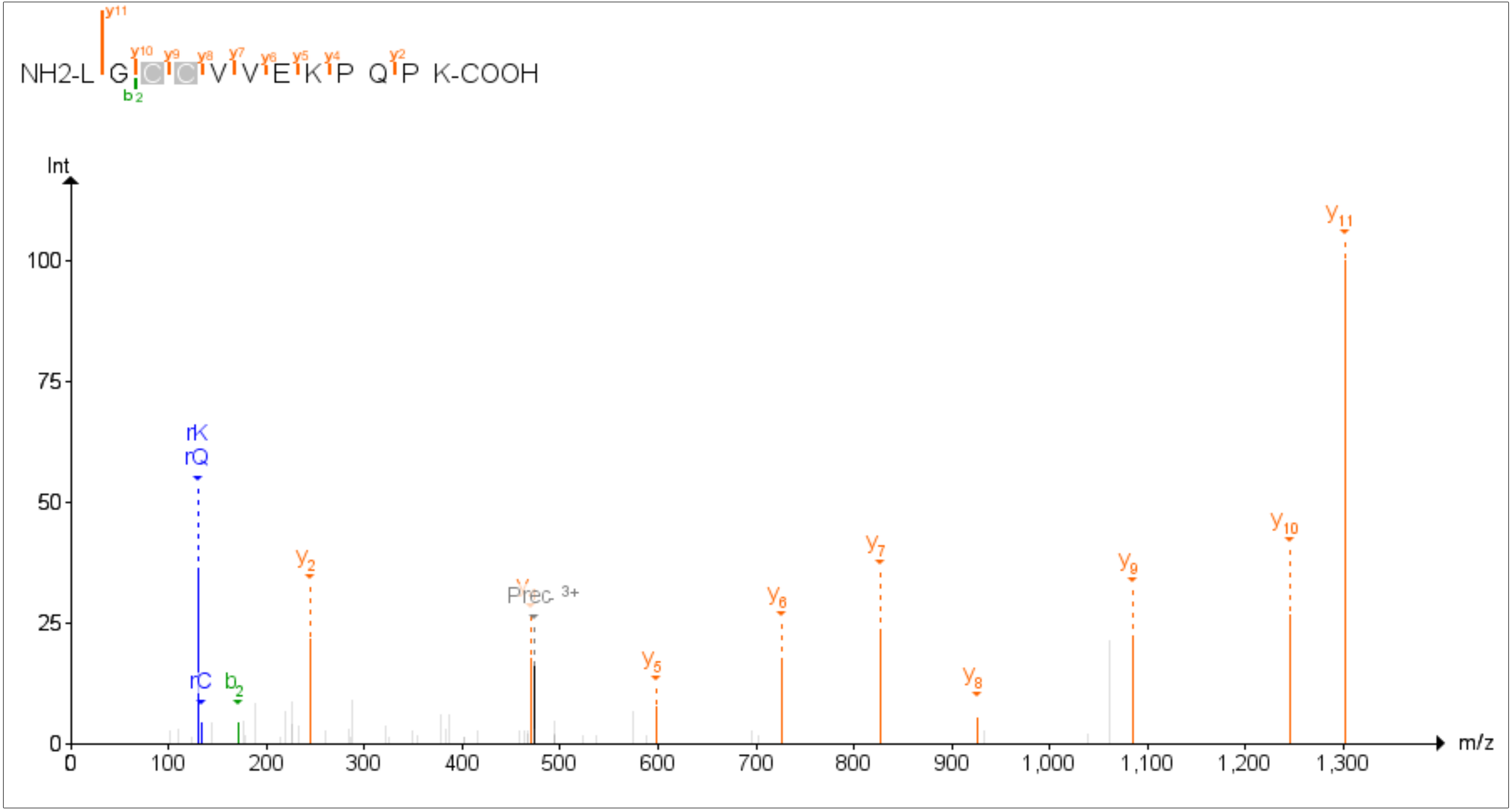

NH2-L G C C V V E K P Q P K-COOH
y11
y10
y9
y8
y7
y6
y5
y4
y2
b2
Int
100
75
50
25
0
rK
rQ
rC
b2
y2
Prec 3+
y5
y6
y7
y8
y9
y10
y11
m/z
0
100
200
300
400
500
600
700
800
900
1,000
1,100
1,200
1,300

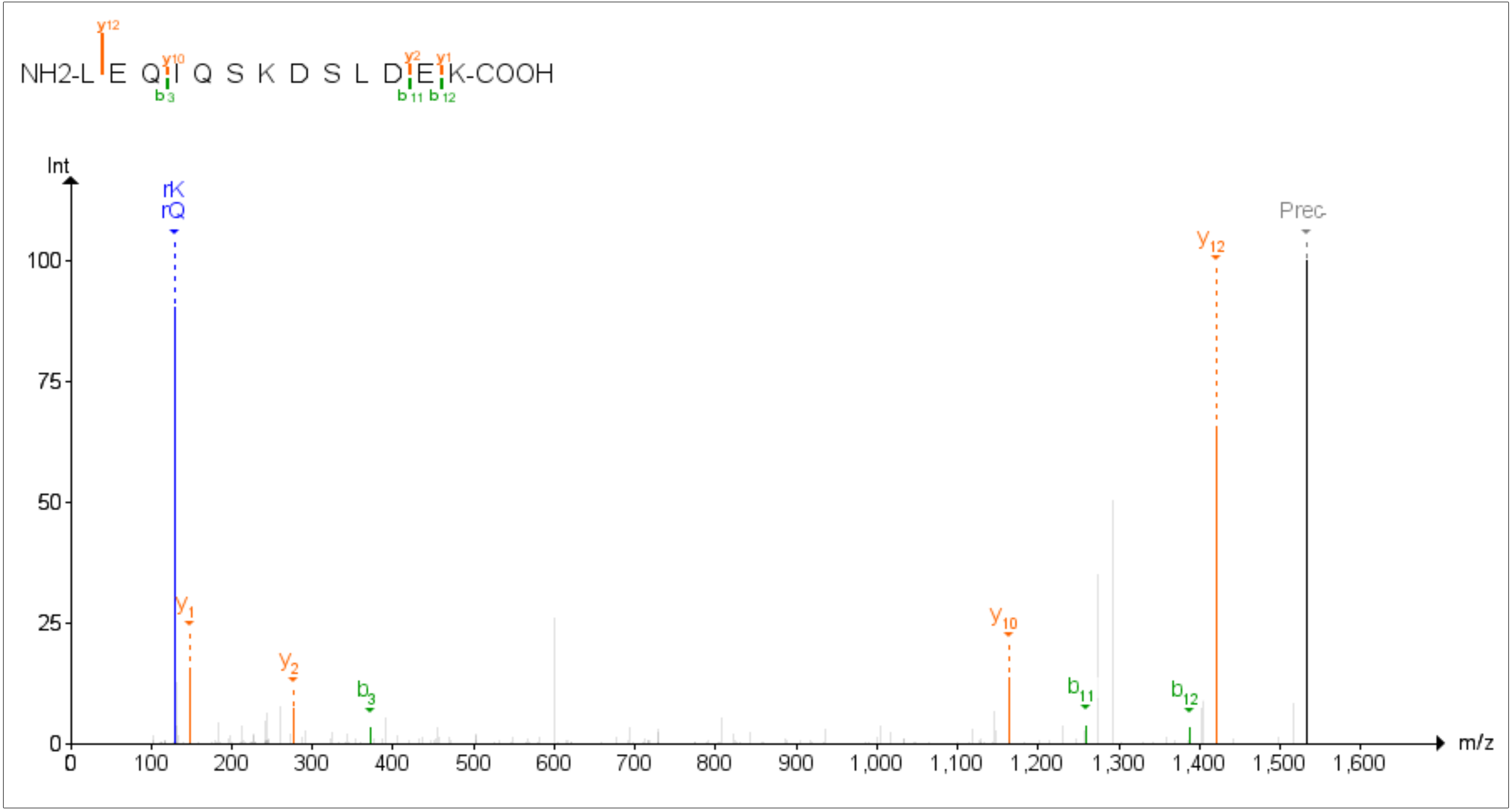

NH2-L E Q I Q S K D S L D E K-COOH
y12
y10
y2
y1
b3
b11
b12
Int
rK
rQ
Prec
y12
y1
y2
b3
y10
b11
b12
100
75
50
25
0
0
100
200
300
400
500
600
700
800
900
1,000
1,100
1,200
1,300
1,400
1,500
1,600
m/z

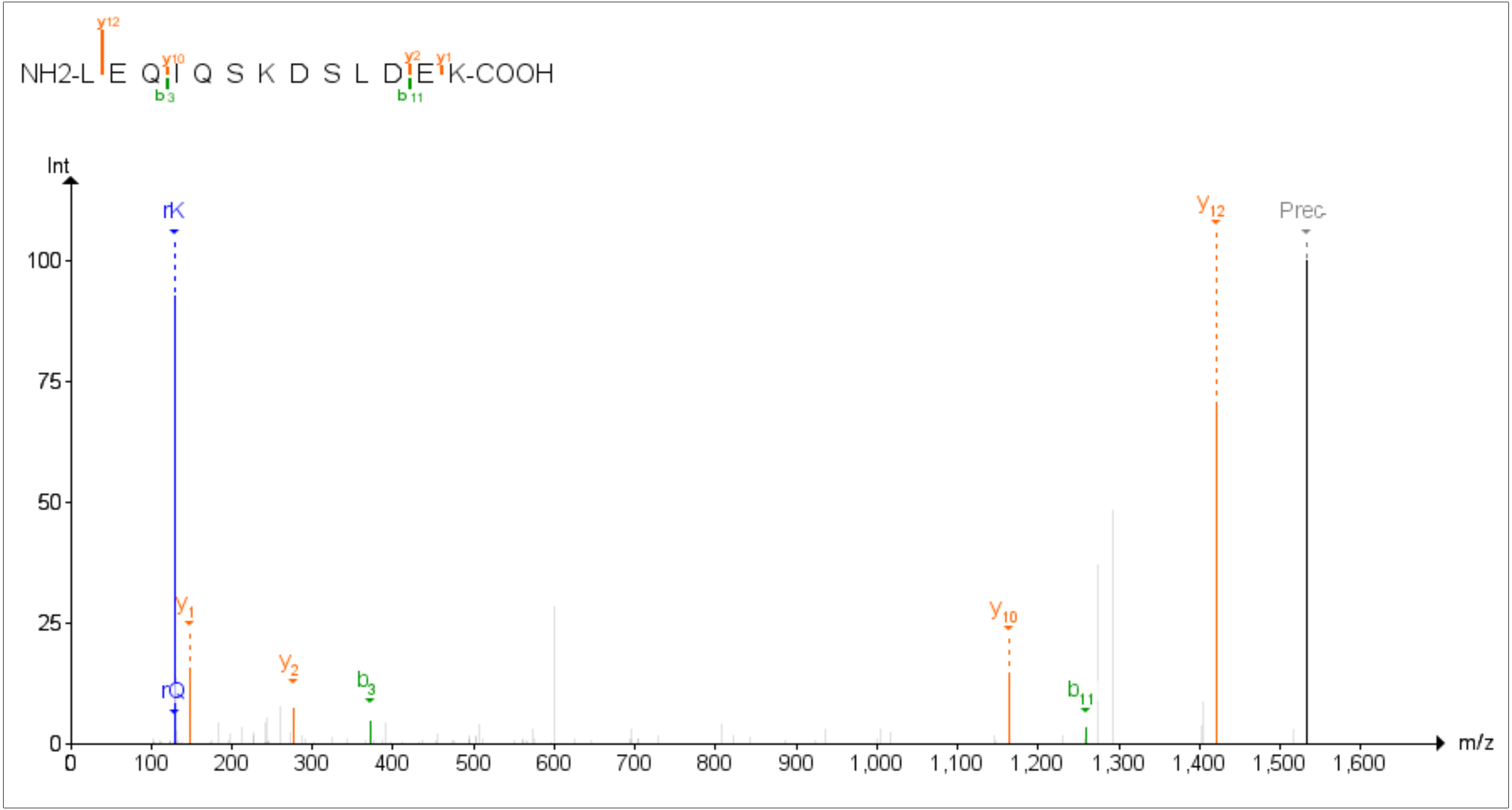
NH2-L E Q I Q S K D S L D E K-COOH
y12
y10
y2
y1
b3
b11
Int
100
75
50
25
0
rK
rQ
y1
y2
b3
y10
b11
y12
Prec
0
100
200
300
400
500
600
700
800
900
1,000
1,100
1,200
1,300
1,400
1,500
1,600
m/z

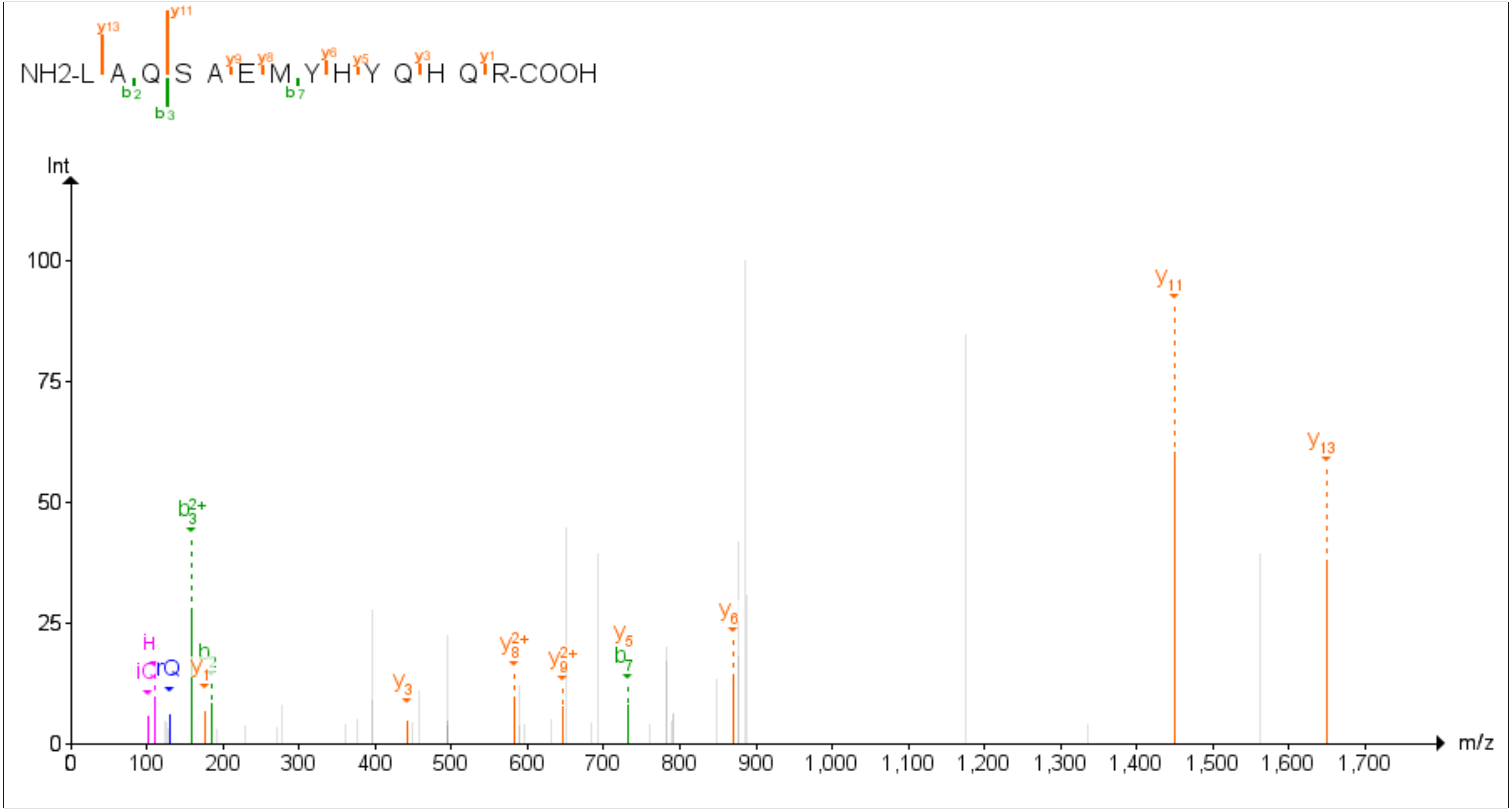

NH2-L A Q S A E M Y H Y Q H Q R-COOH
y13
y11
y9
y8
y6
y5
y3
y1
b2
b3
b7
Int
100
75
50
25
0
m/z
0
100
200
300
400
500
600
700
800
900
1,000
1,100
1,200
1,300
1,400
1,500
1,600
1,700
y11
y13
b3 2+
y6
y5
b7
y8 2+
y9 2+
y3
iH
iQ
rQ
y1

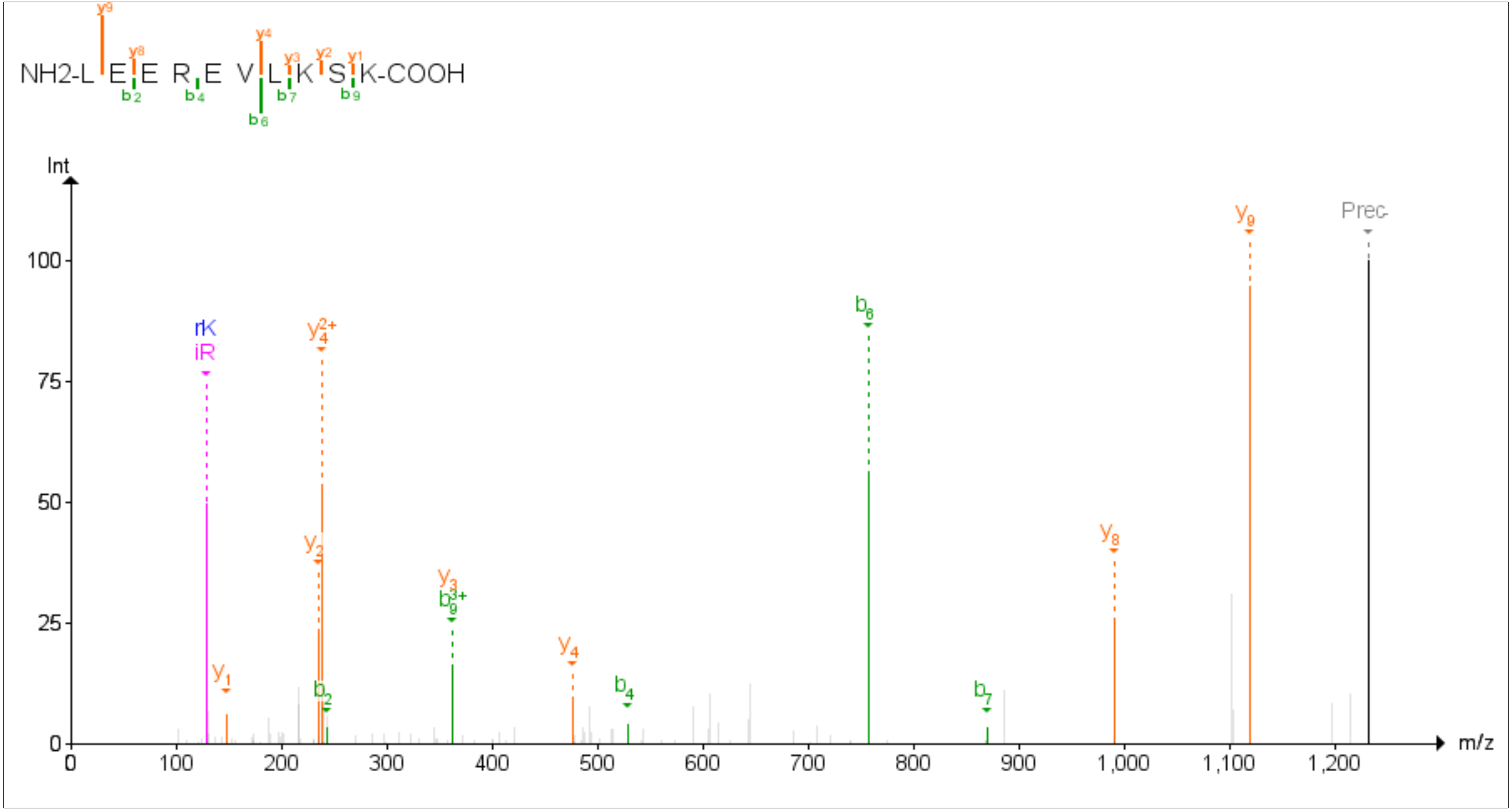
NH2-L E E R E V L K S K-COOH
y9 y8 y4 y3 y2 y1
b2 b4 b6 b7 b9
Int
m/z
Prec.
y9
b6
rK
iR
y_4^{2+}
y2
y3
b_9^{3+}
y1
b2
y4
b4
b7
y8

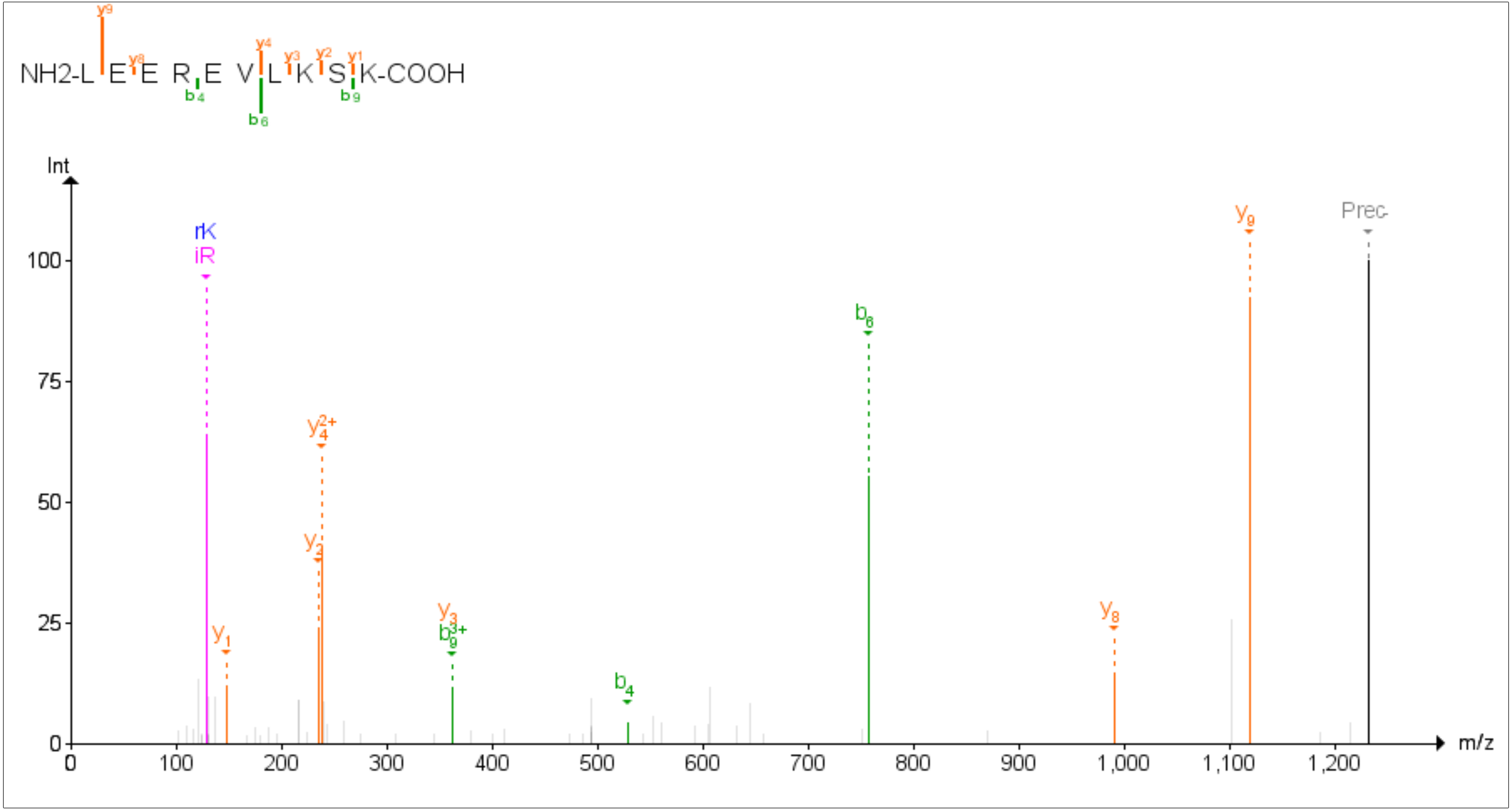
NH2-L E E R E V L K S K-COOH
y9
y8
y4
y3
y2
y1
b4
b6
b9
Int
m/z
rK
iR
Prec
0
25
50
75
100
200
300
400
500
600
700
800
900
1,000
1,100
1,200

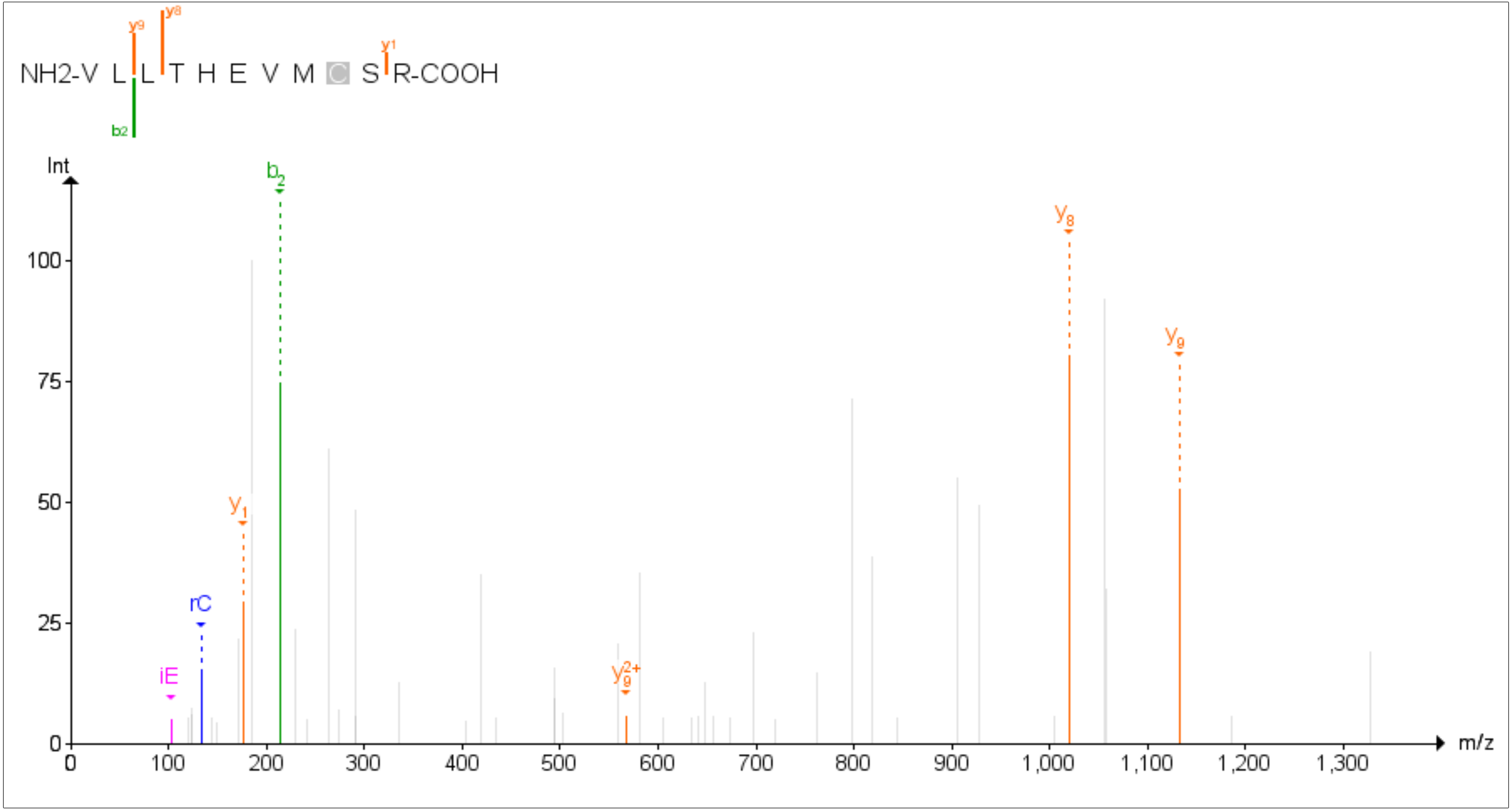
NH2-V L L T H E V M C S R-COOH
y9
y8
y1
b2
Int
100
75
50
25
0
b2
y8
y9
y1
rC
iE
y9 2+
0
100
200
300
400
500
600
700
800
900
1,000
1,100
1,200
1,300
m/z

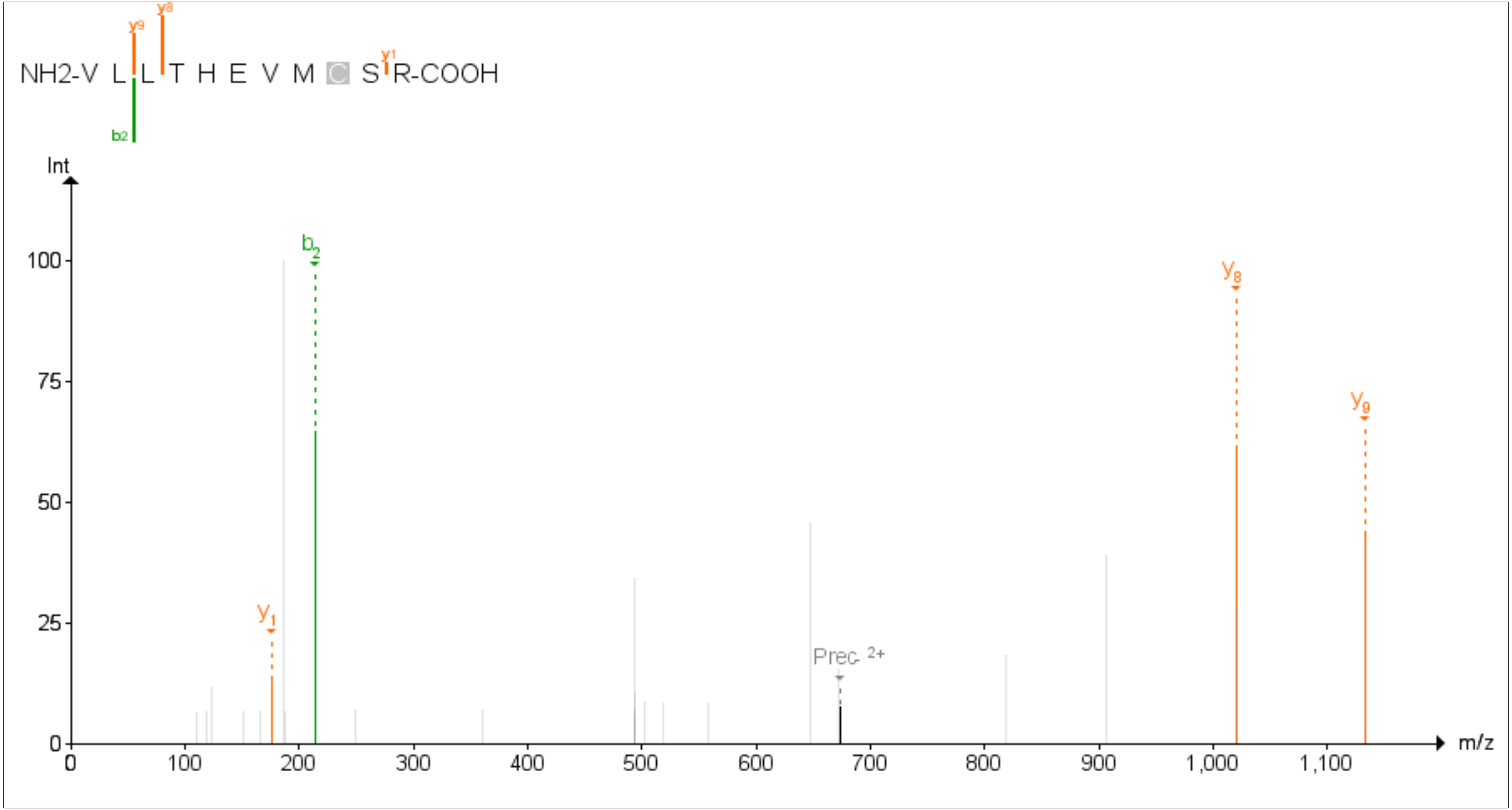

NH2-V L L T H E V M C S R-COOH
y9
y8
y1
b2
Int
100
75
50
25
0
b2
y8
y9
y1
Prec 2+
0
100
200
300
400
500
600
700
800
900
1,000
1,100
m/z

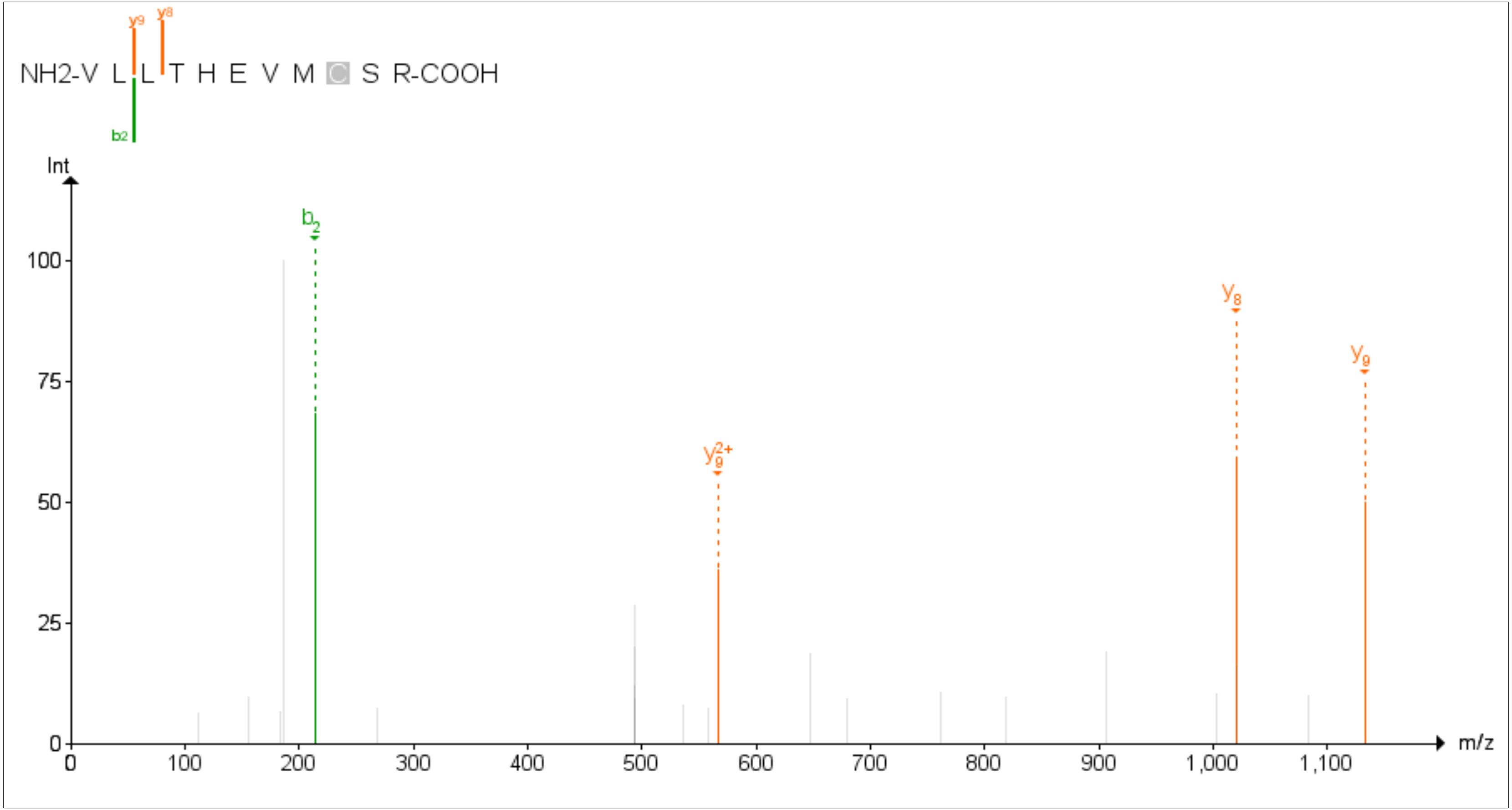

y9
y8
NH2-V L L T H E V M C S R-COOH
b2
Int
b2
y8
y9
y9 2+
100
75
50
25
0
0
100
200
300
400
500
600
700
800
900
1,000
1,100
m/z

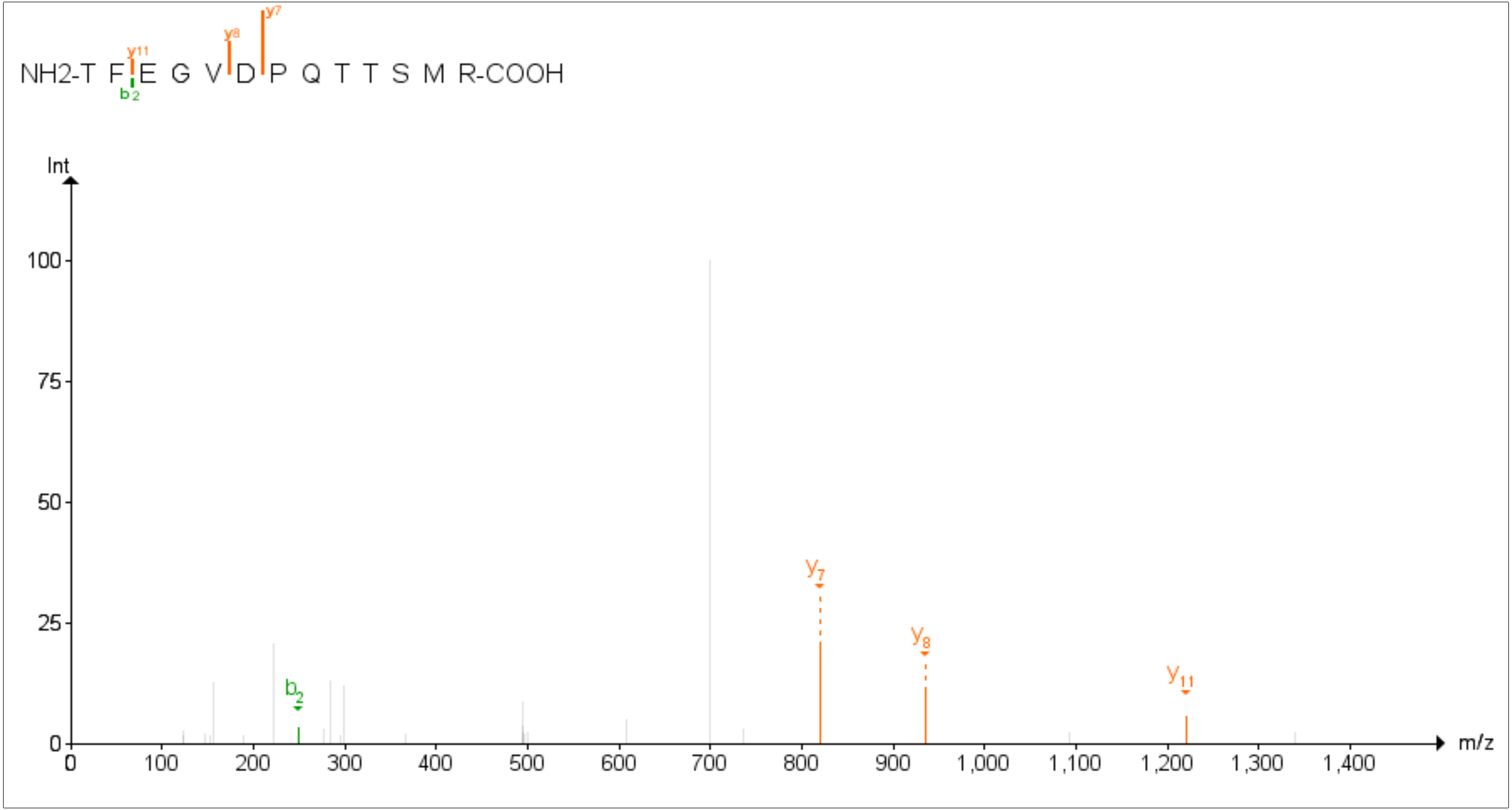

NH2-T F E G V D P Q T T S M R-COOH
y11
y8
y7
b2
Int
100
75
50
25
0
m/z
0
100
200
300
400
500
600
700
800
900
1,000
1,100
1,200
1,300
1,400
y7
y8
y11
b2

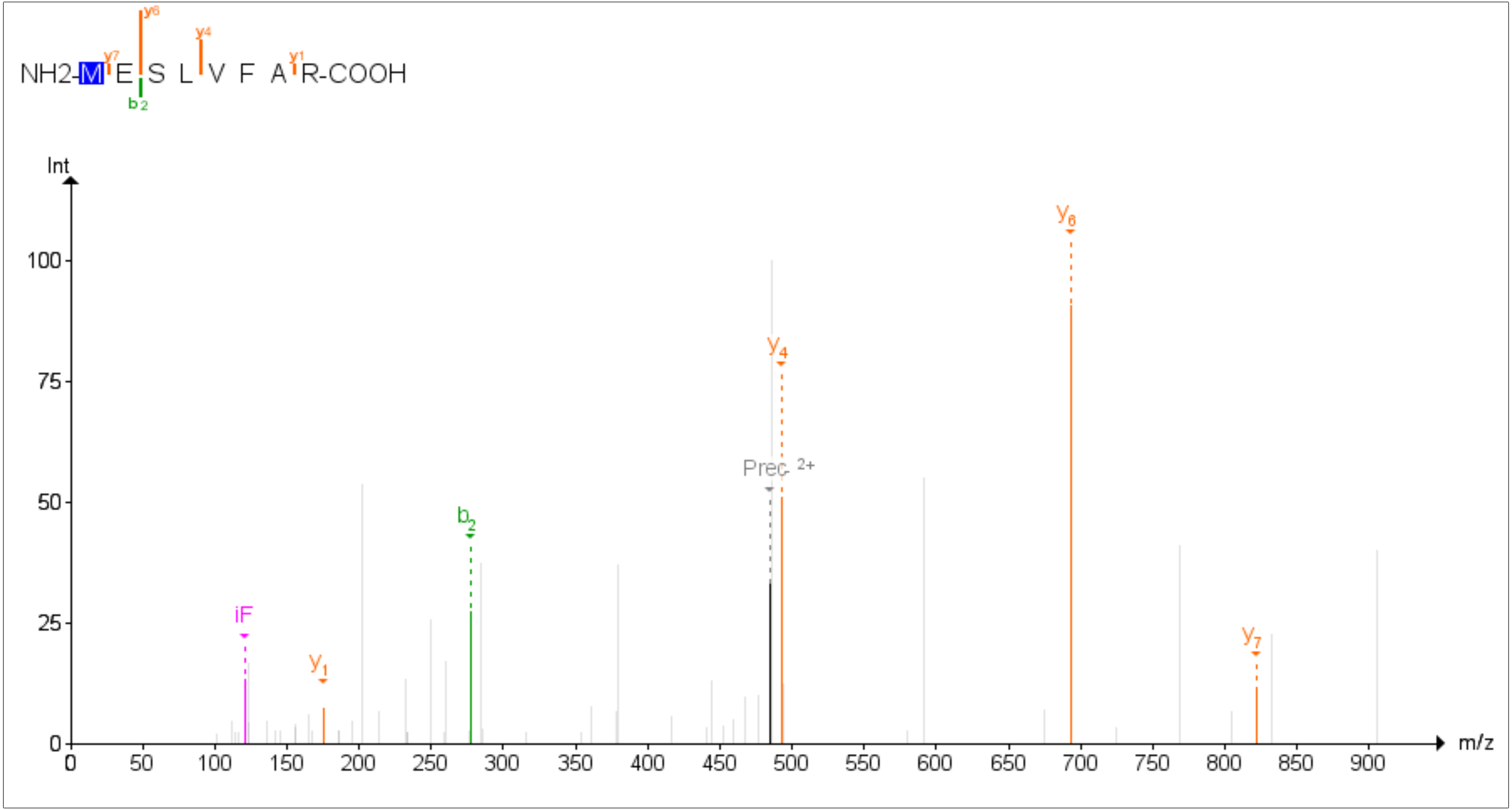

NH2-M E S L V F A R-COOH
y7
y6
y4
y1
b2
Int
m/z
100
75
50
25
0
y6
y4
Prec 2+
b2
iF
y1
y7
0
50
100
150
200
250
300
350
400
450
500
550
600
650
700
750
800
850
900

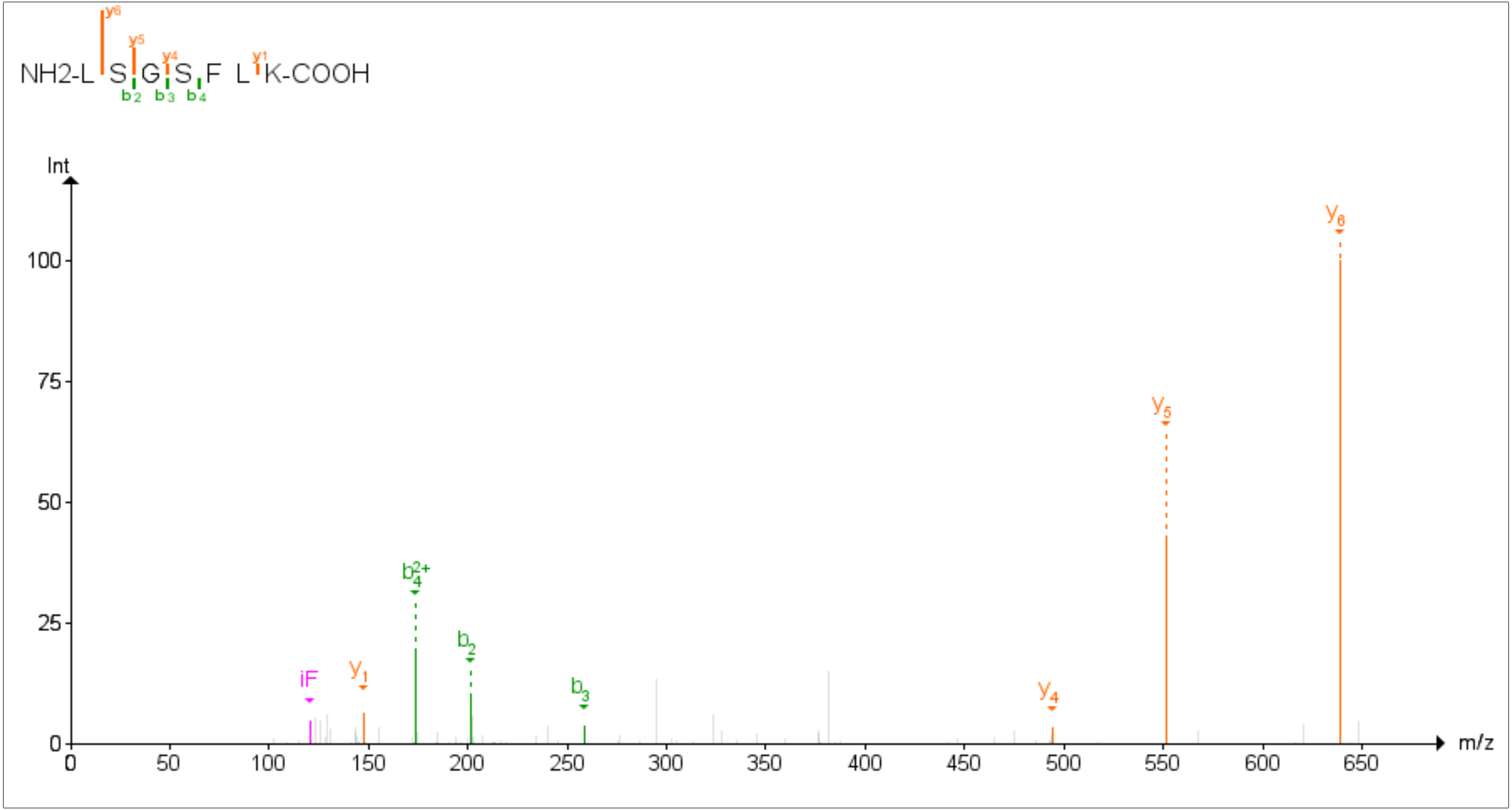

NH2-L S G S F L K-COOH
y6
y5
y4
y1
b2
b3
b4
Int
m/z
0
25
50
75
100
0
50
100
150
200
250
300
350
400
450
500
550
600
650
iF
y1
b4 2+
b2
b3
y4
y5
y6

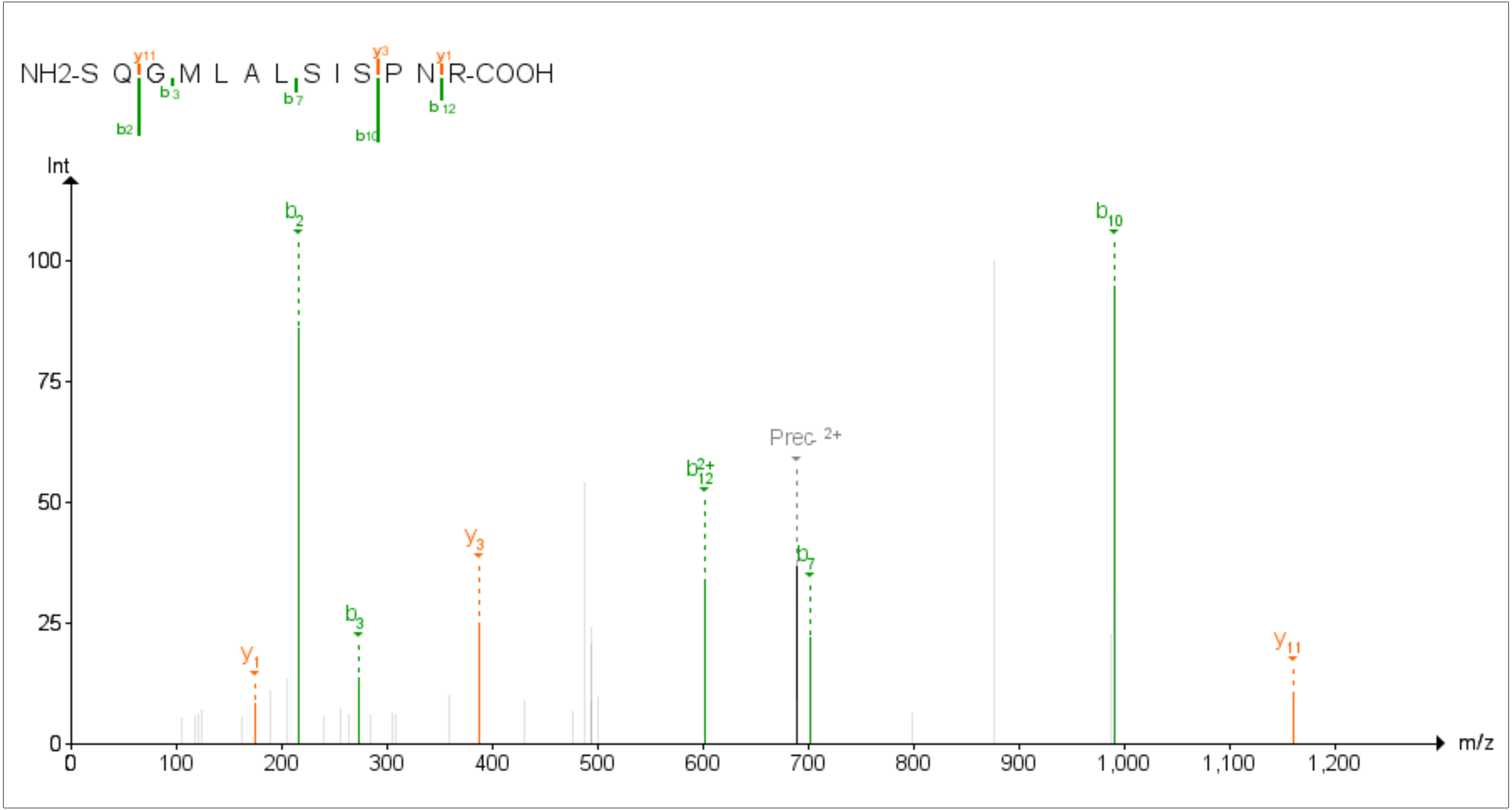

NH2-S Q G M L A L S I S P N R-COOH
y11
y3
y1
b2
b3
b7
b10
b12
Int
100
75
50
25
0
b2
b10
Prec 2+
b12 2+
y3
b7
b3
y1
y11
0
100
200
300
400
500
600
700
800
900
1,000
1,100
1,200
m/z

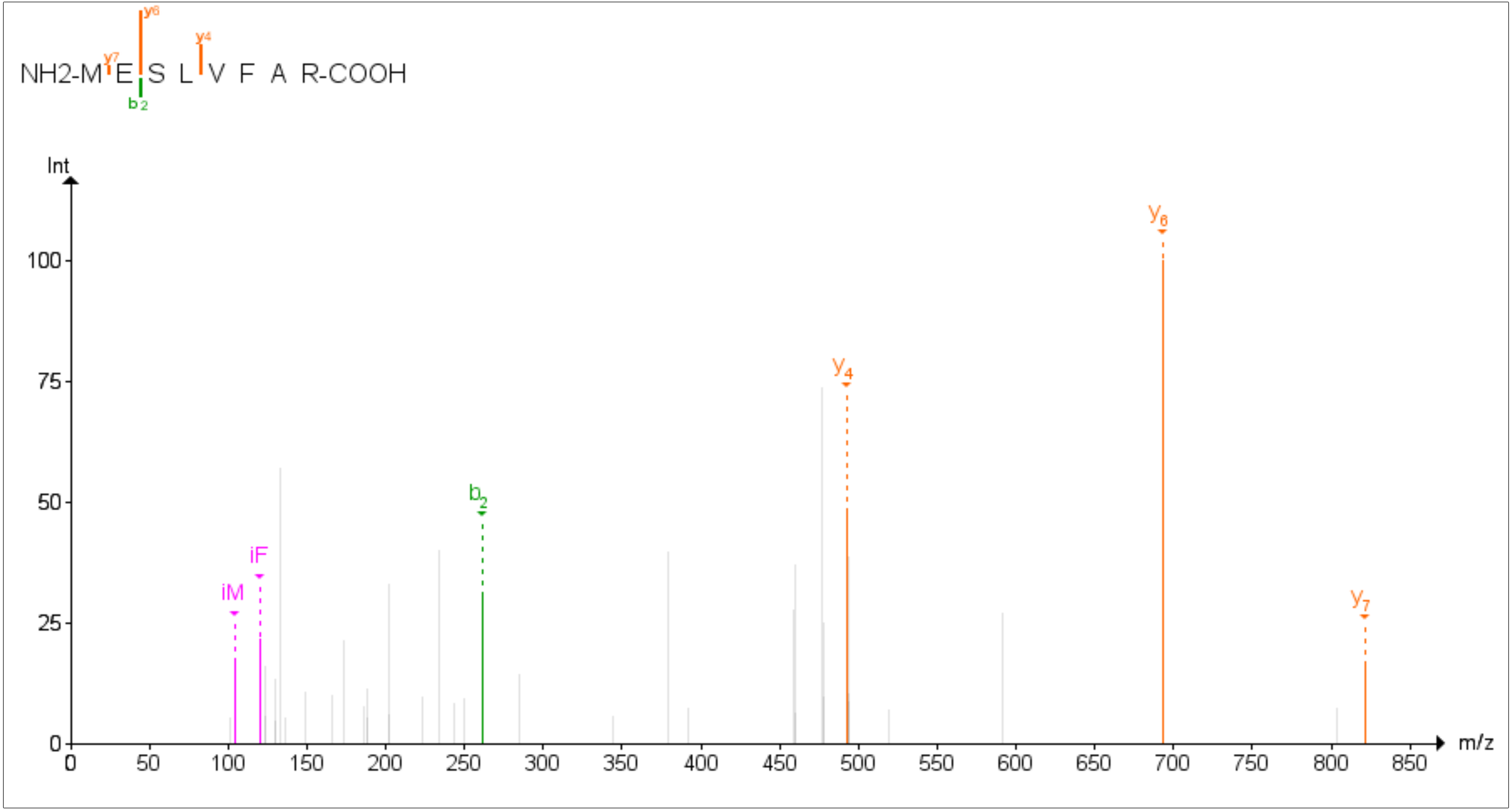

NH2-M E S L V F A R-COOH
y6
y7
y4
b2
Int
100
75
50
25
0
y6
y4
b2
iF
iM
y7
0 50 100 150 200 250 300 350 400 450 500 550 600 650 700 750 800 850
m/z

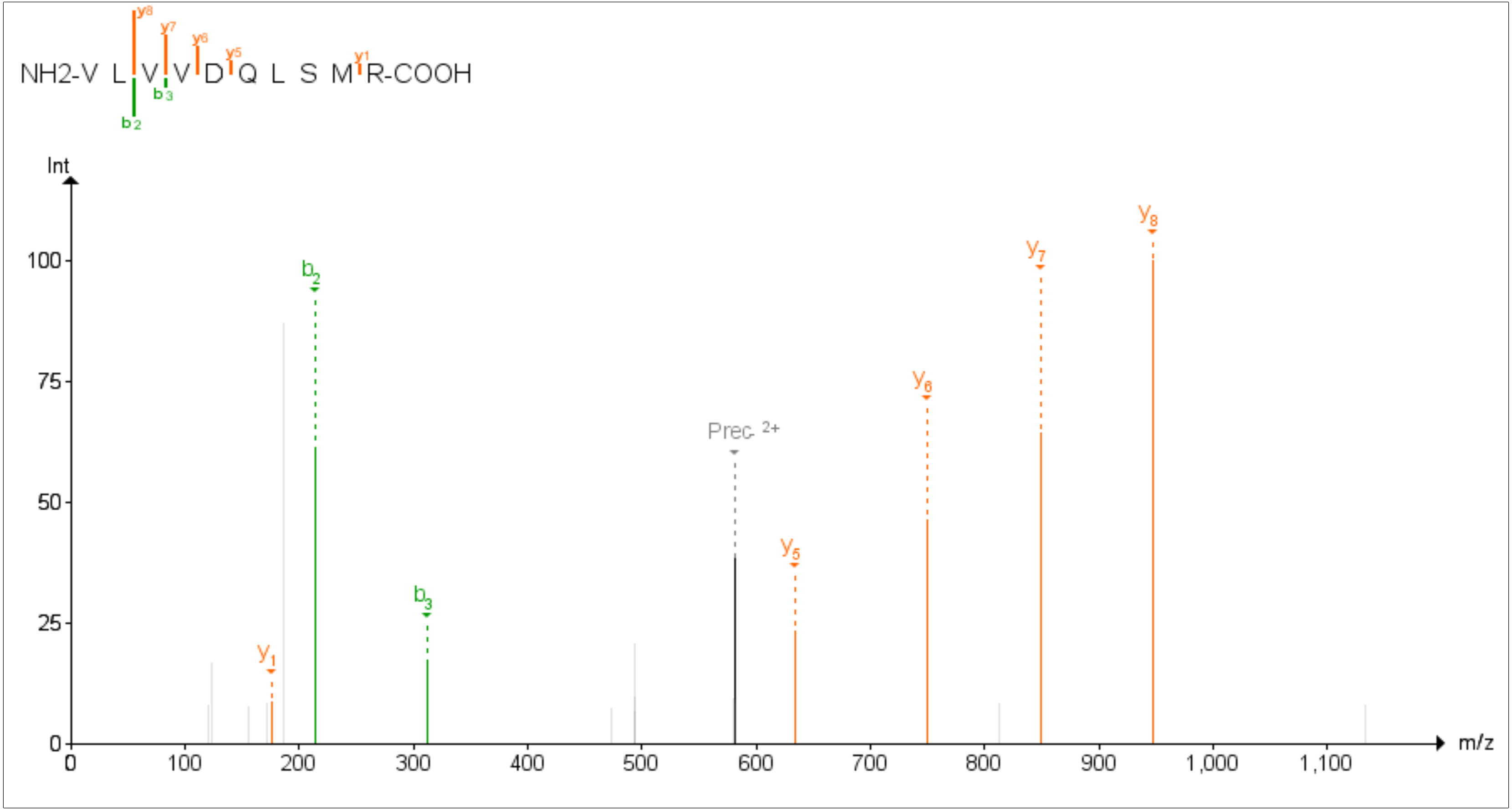

NH2-V L V V D Q L S M R-COOH
y8
y7
y6
y5
y1
b2
b3
Int
100
75
50
25
0
b2
b3
y1
Prec 2+
y5
y6
y7
y8
0
100
200
300
400
500
600
700
800
900
1,000
1,100
m/z

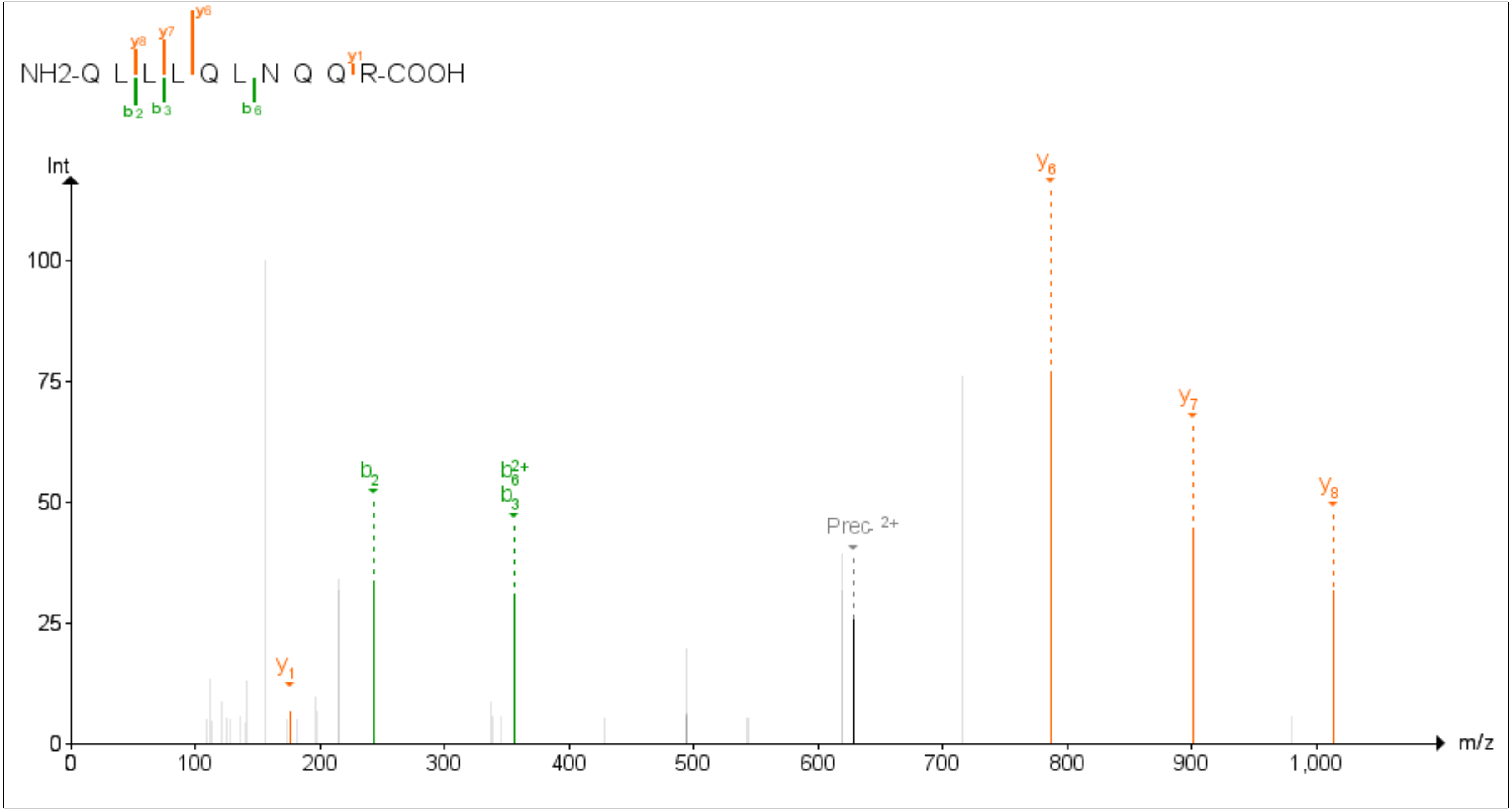

NH2-Q L L L Q L N Q Q R-COOH
y8
y7
y6
y1
b2
b3
b6
Int
y6
y7
y8
y1
b2
b6 2+
b3
Prec. 2+
m/z
0
25
50
75
100
0
100
200
300
400
500
600
700
800
900
1,000

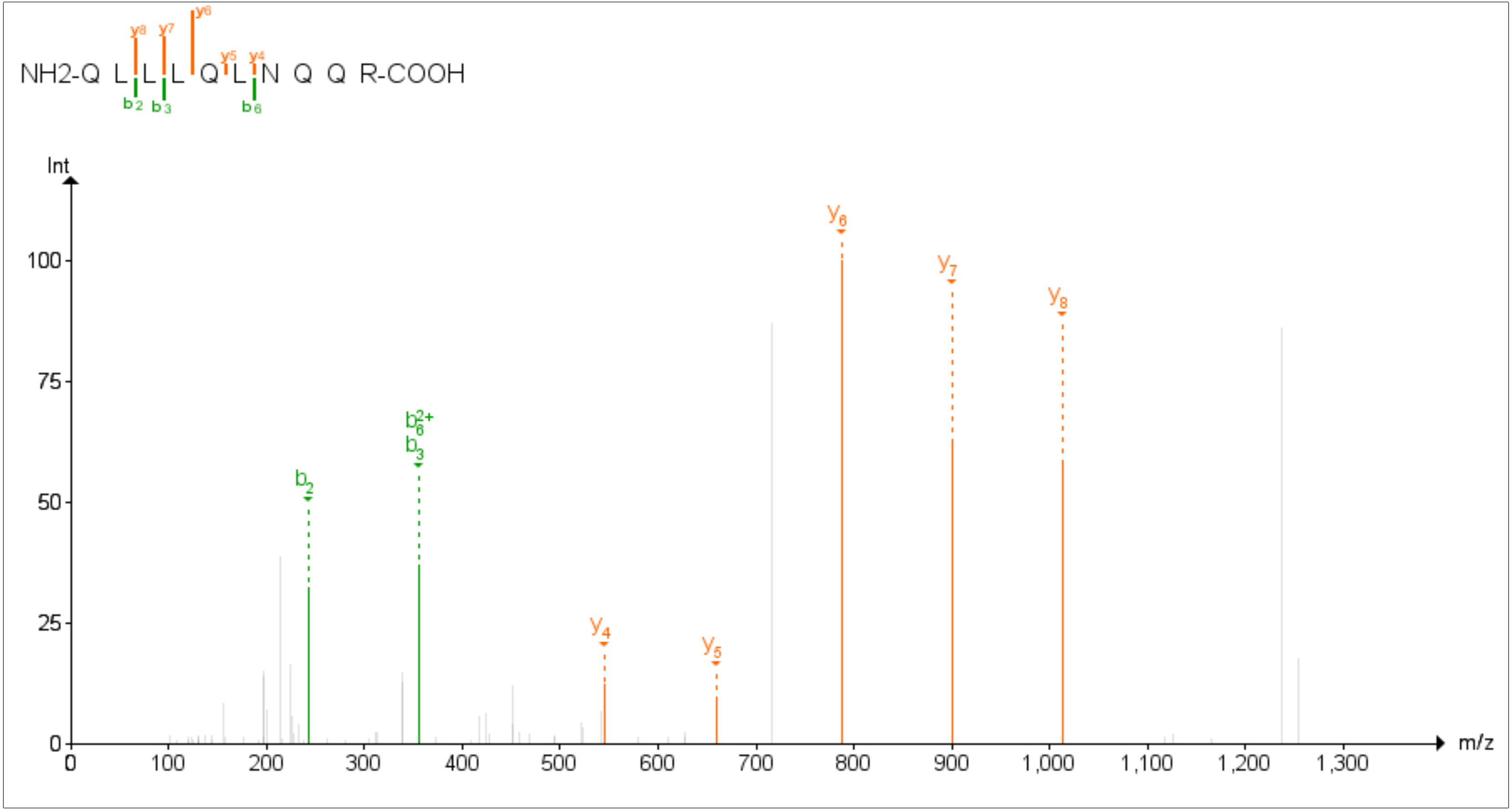

NH2-Q L L L Q L N Q Q R-COOH
y8 y7 y6 y5 y4
b2 b3 b6
Int
100
75
50
25
0
b2
b6 2+
b3
y4
y5
y6
y7
y8
0 100 200 300 400 500 600 700 800 900 1,000 1,100 1,200 1,300
m/z

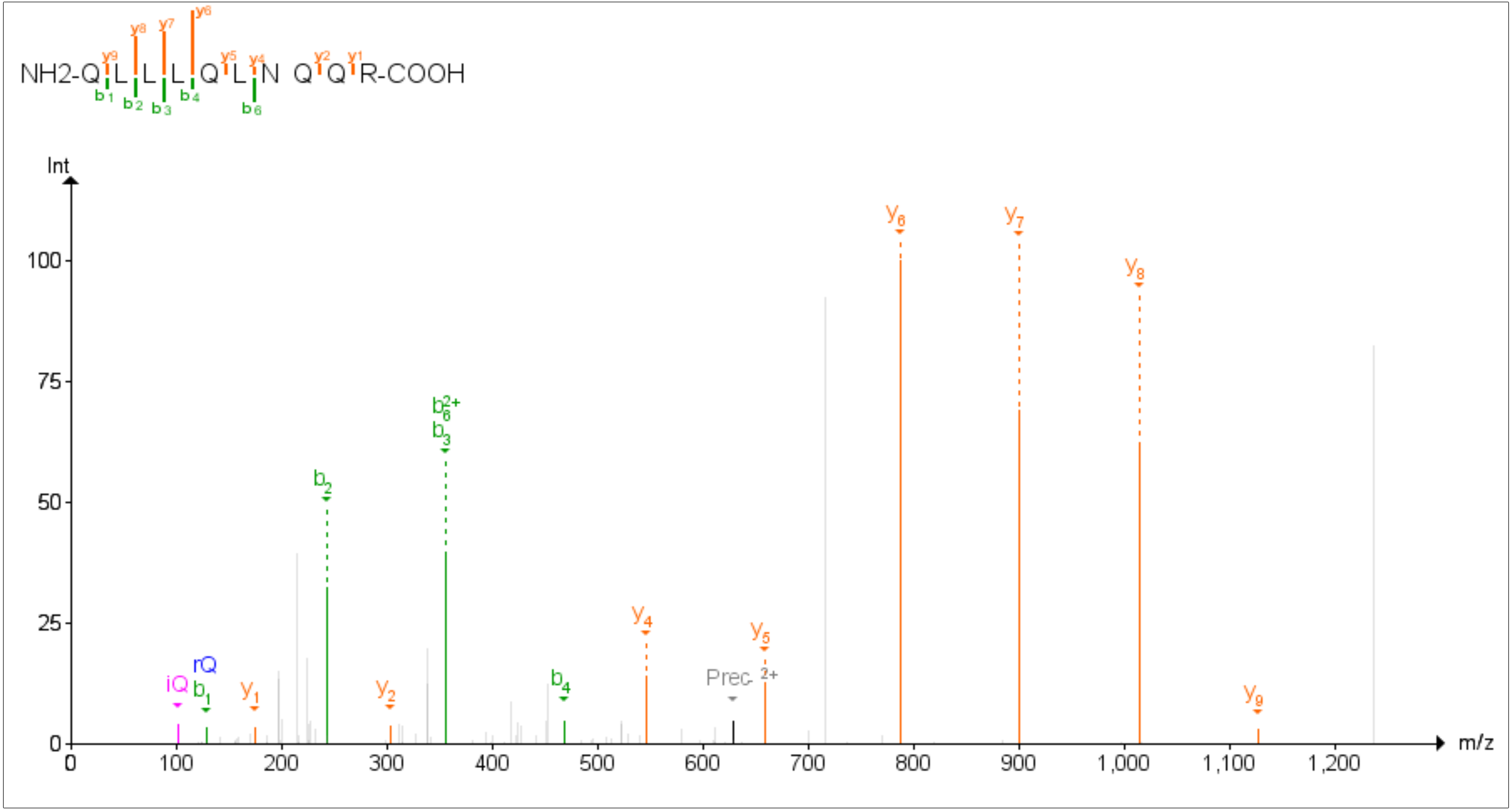

NH2-Q L L L Q L N Q Q R-COOH
Int
m/z
100
75
50
25
0
0
100
200
300
400
500
600
700
800
900
1,000
1,100
1,200
y6
y7
y8
b6 2+
b3
b2
y4
y5
Prec 2+
b4
rQ
iQ
b1
y1
y2
y9

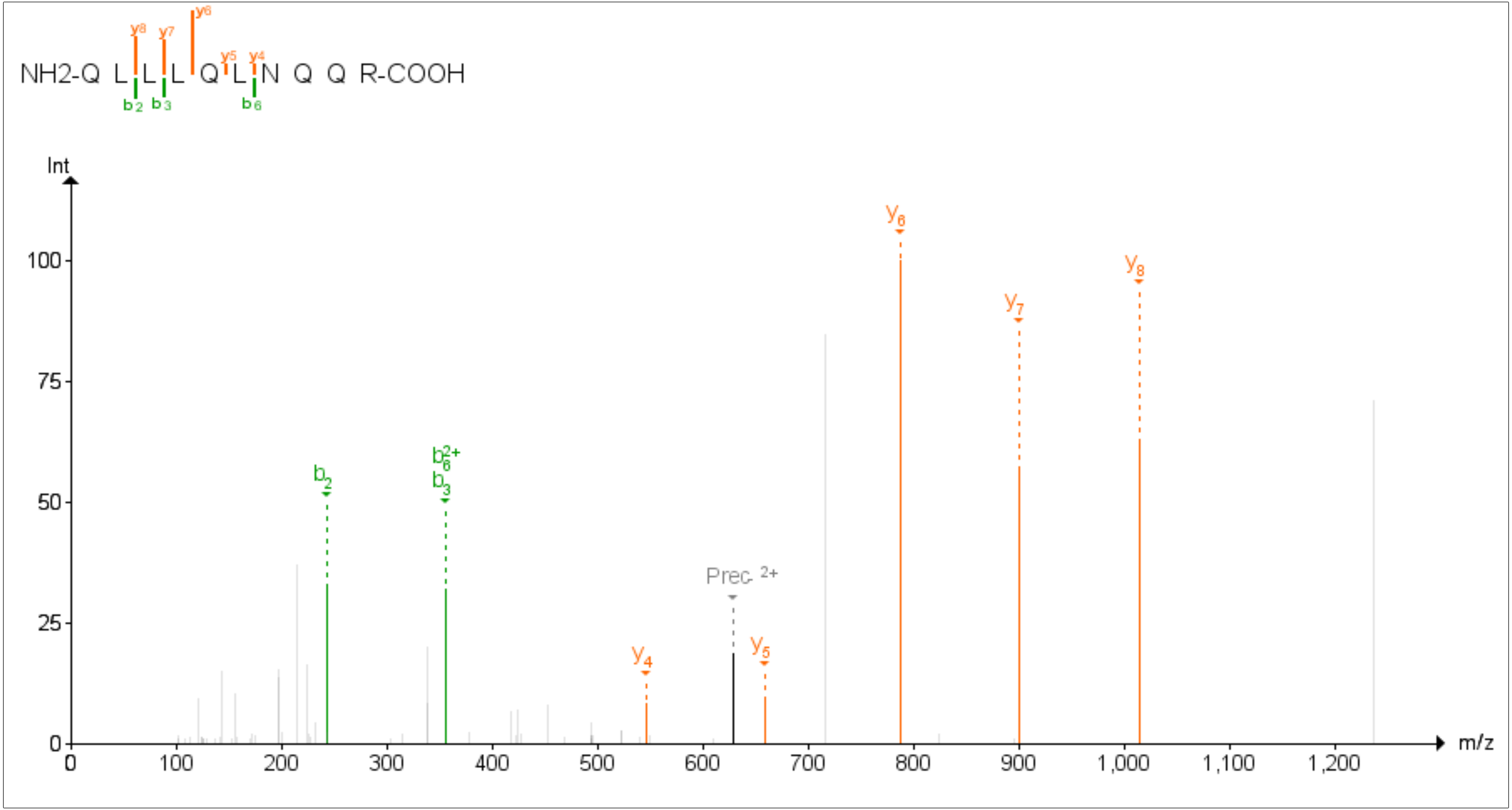
NH2-Q L L L Q L N Q Q R-COOH
y8 y7 y6 y5 y4
b2 b3 b6
Int
100
75
50
25
0
y_6
y_7
y_8
b_2
b_6^{2+}
b_3
Prec 2+
y_4
y_5
0 100 200 300 400 500 600 700 800 900 1,000 1,100 1,200
m/z

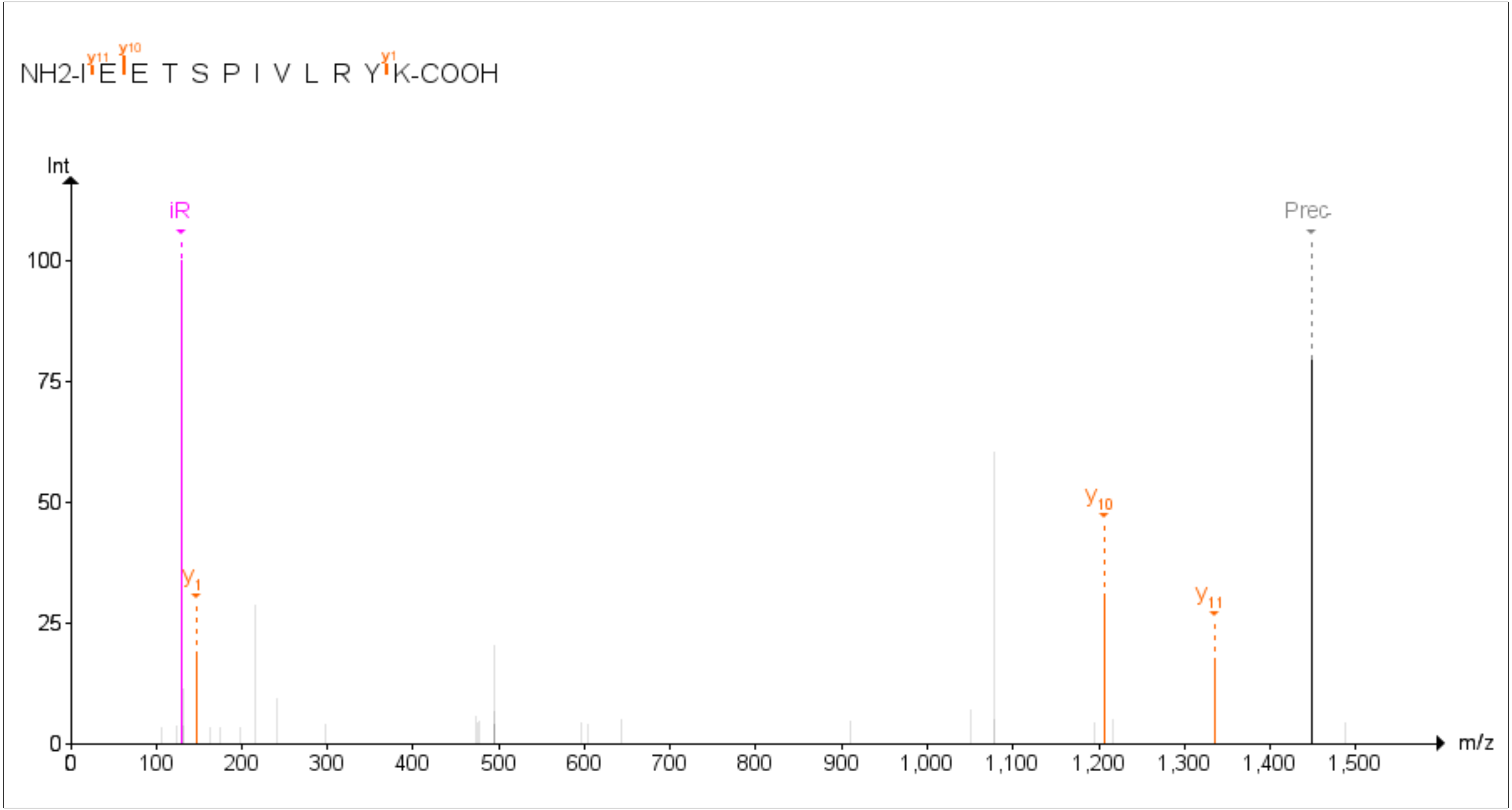
NH2-I E E T S P I V L R Y K-COOH
y11
y10
y1
Int
iR
Prec.
100
75
50
25
0
y1
y10
y11
0
100
200
300
400
500
600
700
800
900
1,000
1,100
1,200
1,300
1,400
1,500
m/z

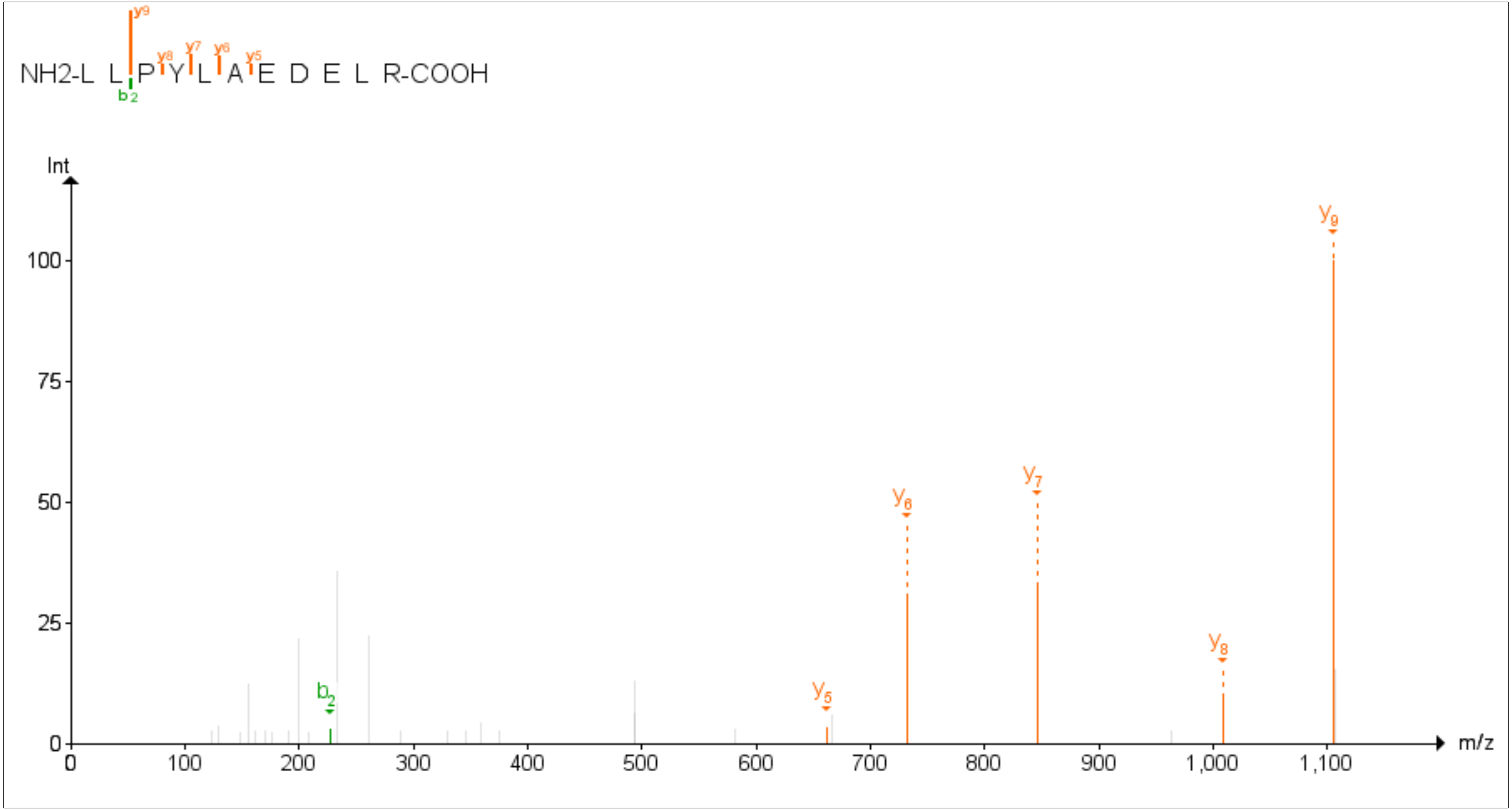

NH2-L L P Y L A E D E L R-COOH
y9
y8
y7
y6
y5
b2
Int
100
75
50
25
0
m/z
0
100
200
300
400
500
600
700
800
900
1,000
1,100
y9
y7
y6
y8
y5
b2

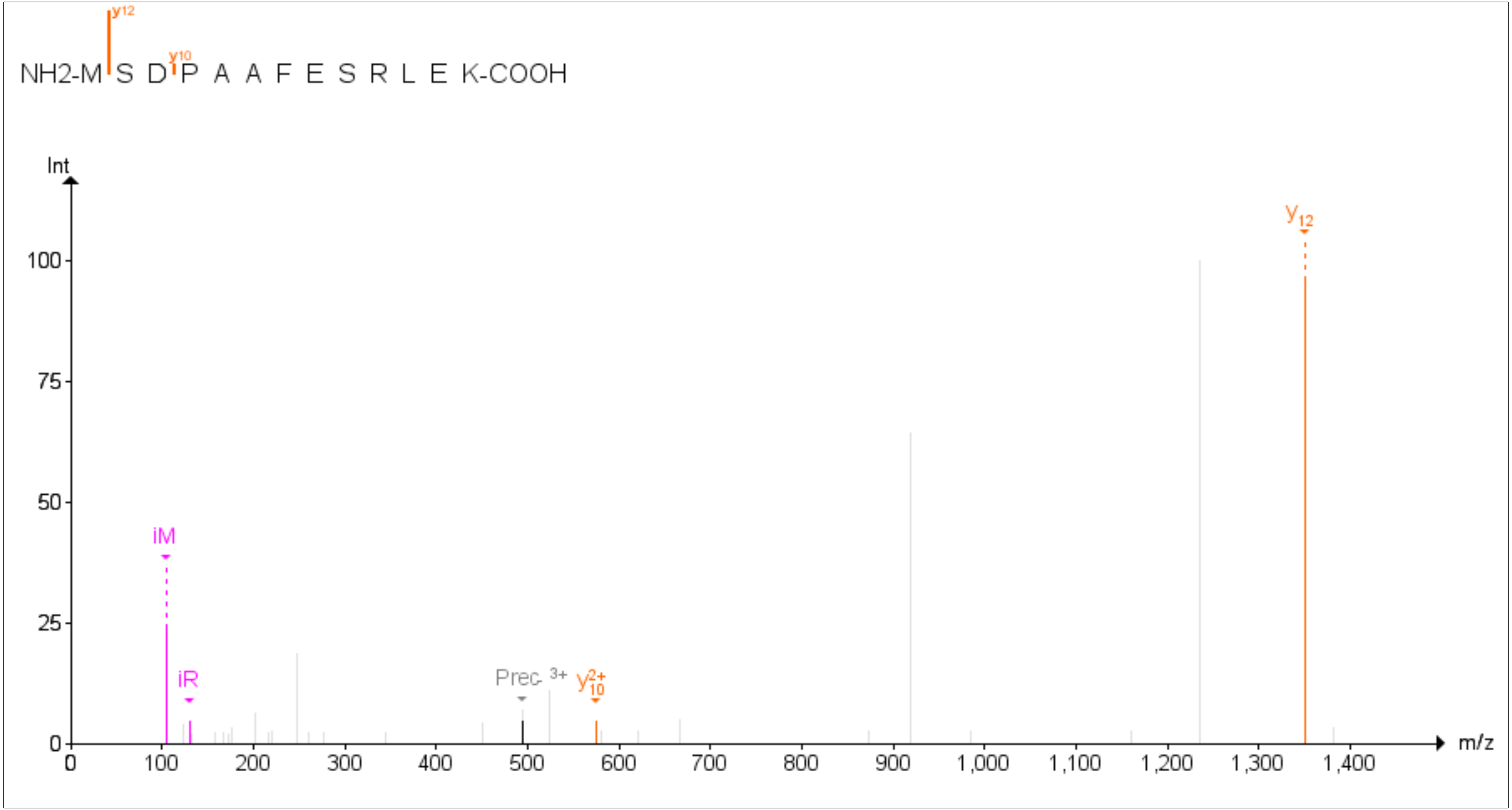

NH2-M S D P A A F E S R L E K-COOH
y12
y10
Int
100
75
50
25
0
iM
iR
Prec 3+
y10 2+
y12
0
100
200
300
400
500
600
700
800
900
1,000
1,100
1,200
1,300
1,400
m/z

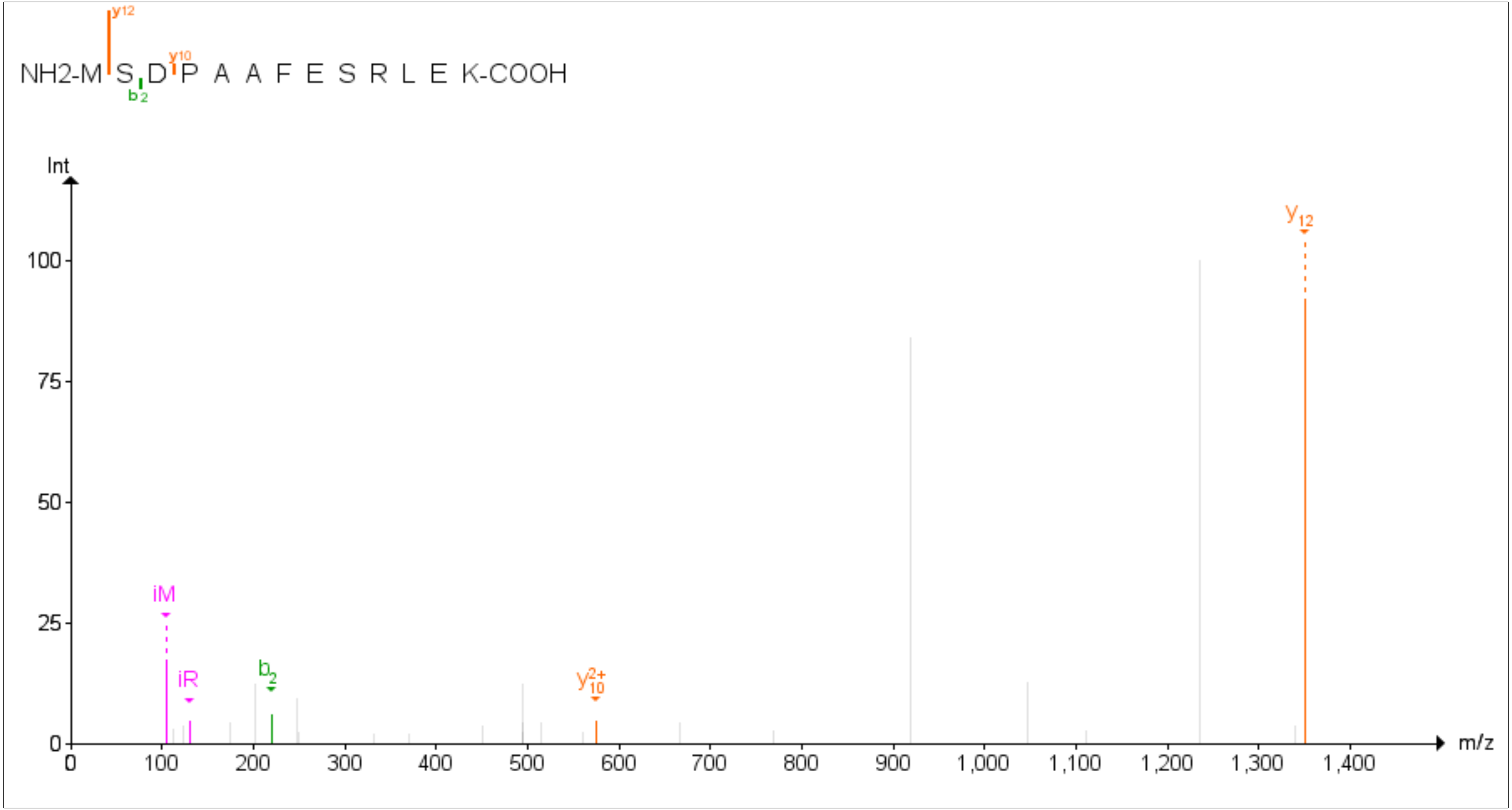

NH2-M S D P A A F E S R L E K-COOH
y12
y10
b2
Int
100
75
50
25
0
iM
iR
b_2
y_{10}^{2+}
y_{12}
0 100 200 300 400 500 600 700 800 900 1,000 1,100 1,200 1,300 1,400
m/z

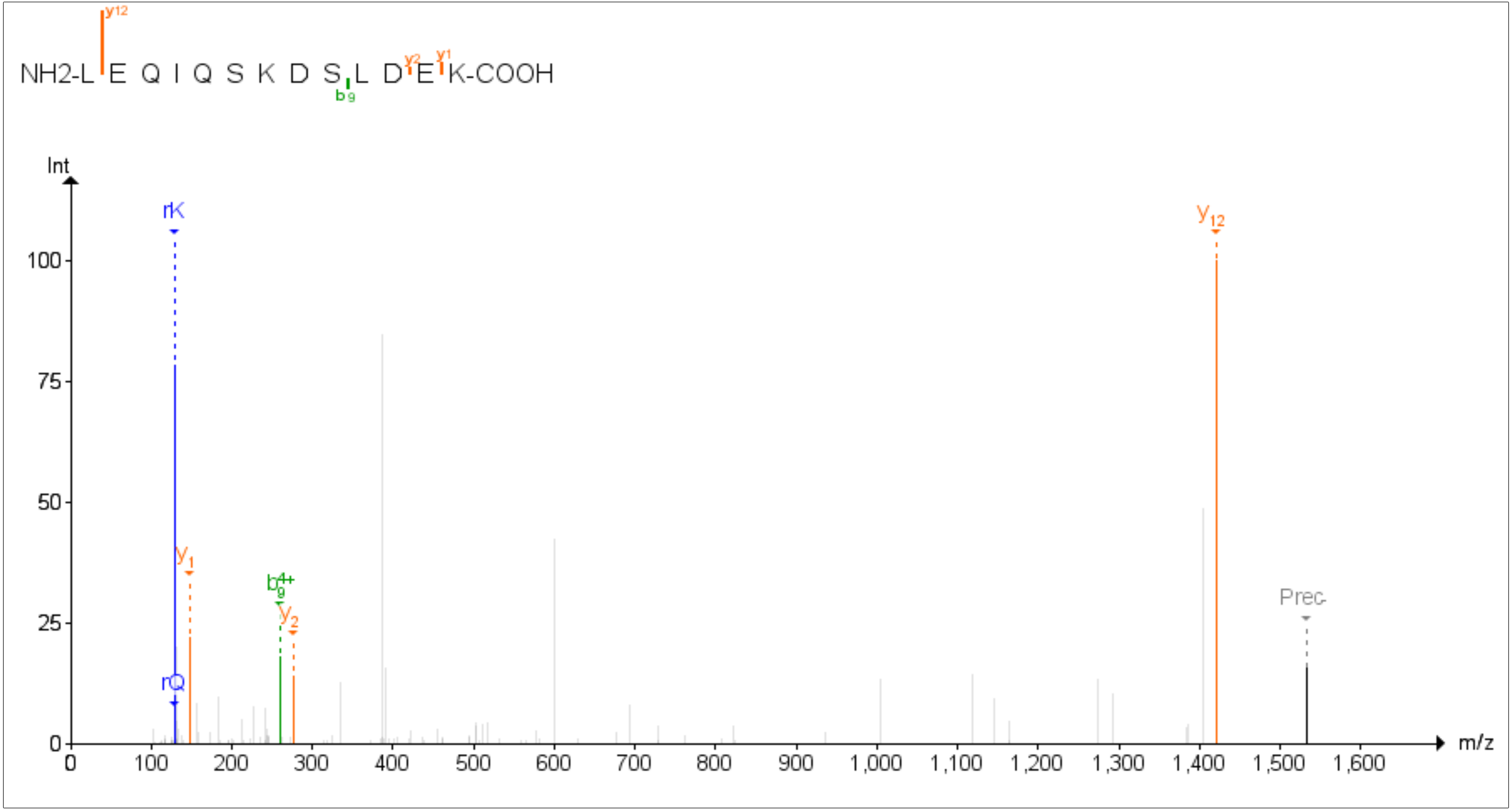

NH2-L E Q I Q S K D S L D E K-COOH
y12
y2
y1
b9
Int
rK
y12
100
75
50
y1
b9 4+
y2
Prec
25
rQ
0
0
100
200
300
400
500
600
700
800
900
1,000
1,100
1,200
1,300
1,400
1,500
1,600
m/z

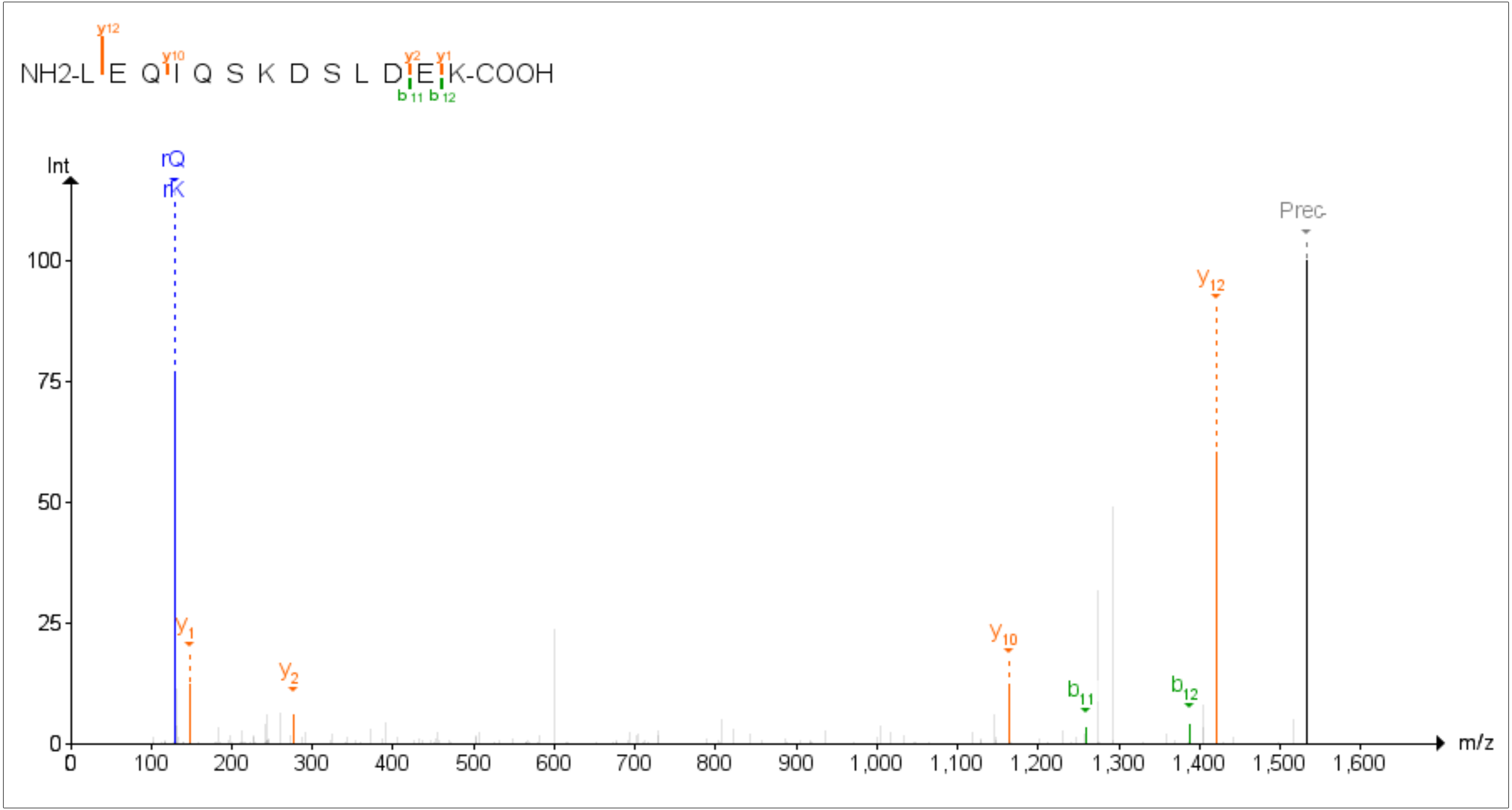

NH2-L E Q I Q S K D S L D E K-COOH
y12
y10
y2
y1
b 11
b 12
Int
m/z
rQ
rK
Prec
y1
y2
y10
b11
b12
y12
0
25
50
75
100
0
100
200
300
400
500
600
700
800
900
1,000
1,100
1,200
1,300
1,400
1,500
1,600

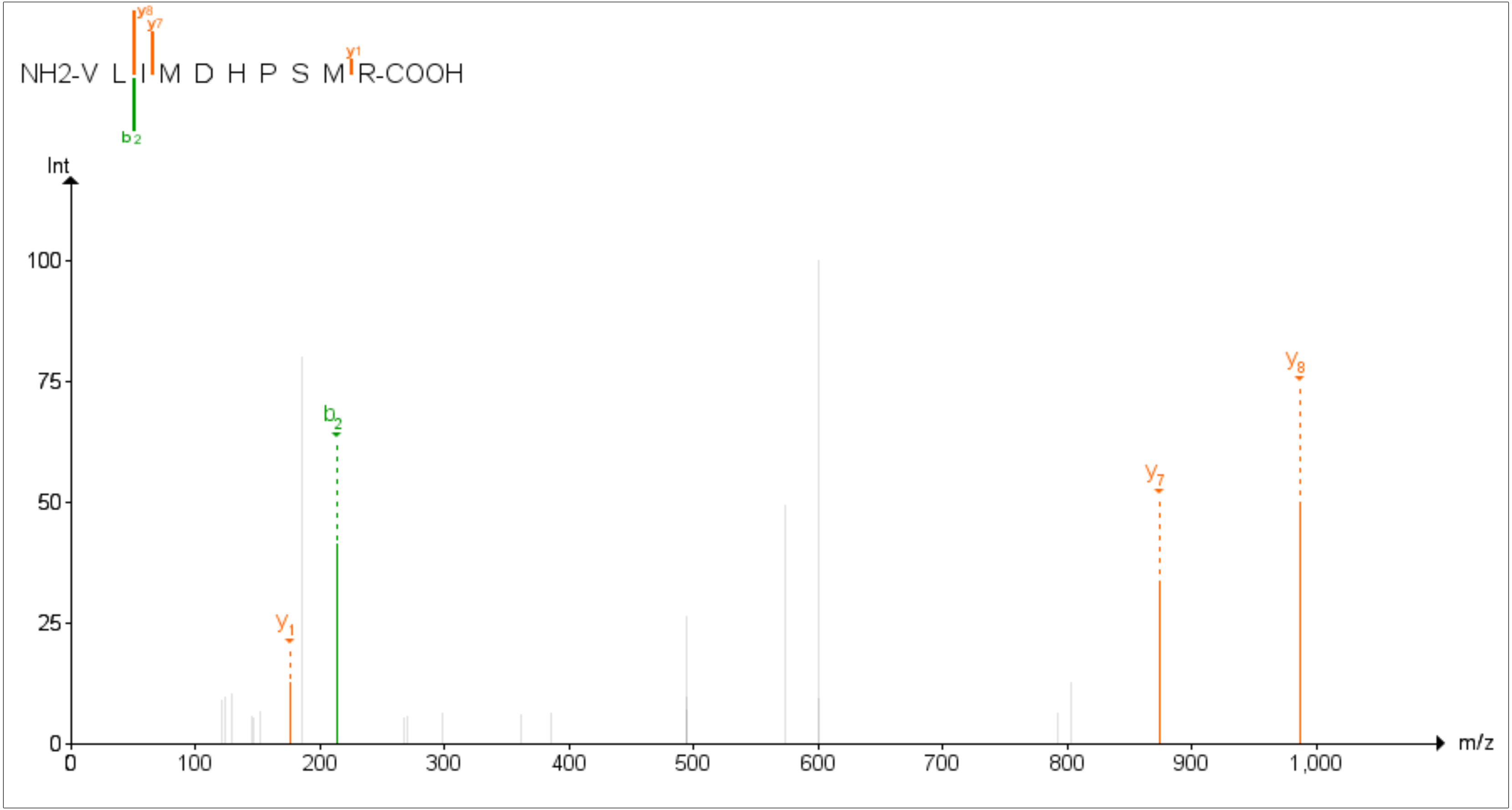

NH2-V L I M D H P S M R-COOH
y8
y7
y1
b2
Int
100
75
50
25
0
0
100
200
300
400
500
600
700
800
900
1,000
m/z
y1
b2
y7
y8

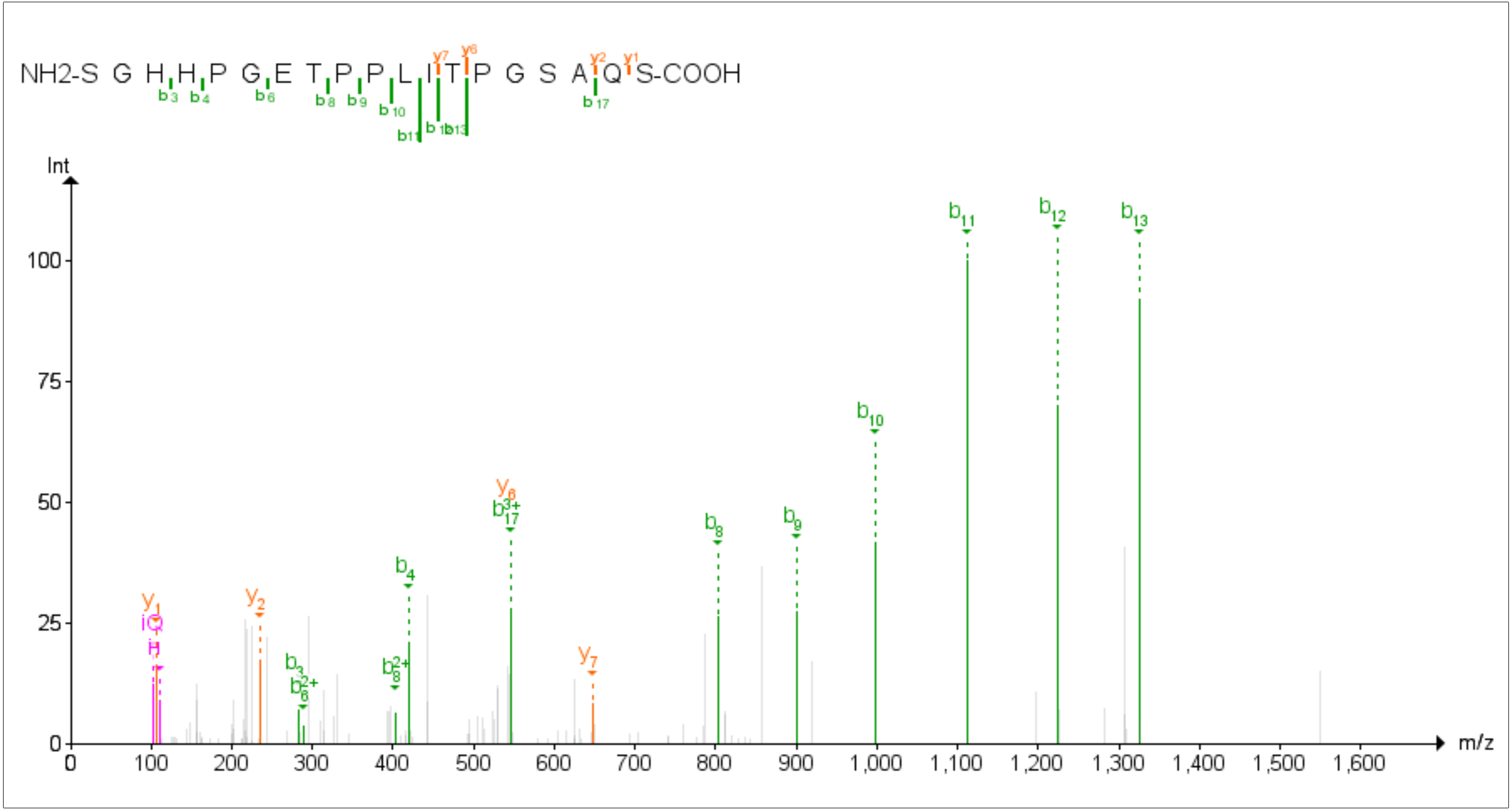

NH2-S G H H P G E T P P L I T P G S A Q S-COOH
y7 y6 y2 y1
b3 b4 b6 b8 b9 b10 b11 b12 b13 b17
Int
m/z

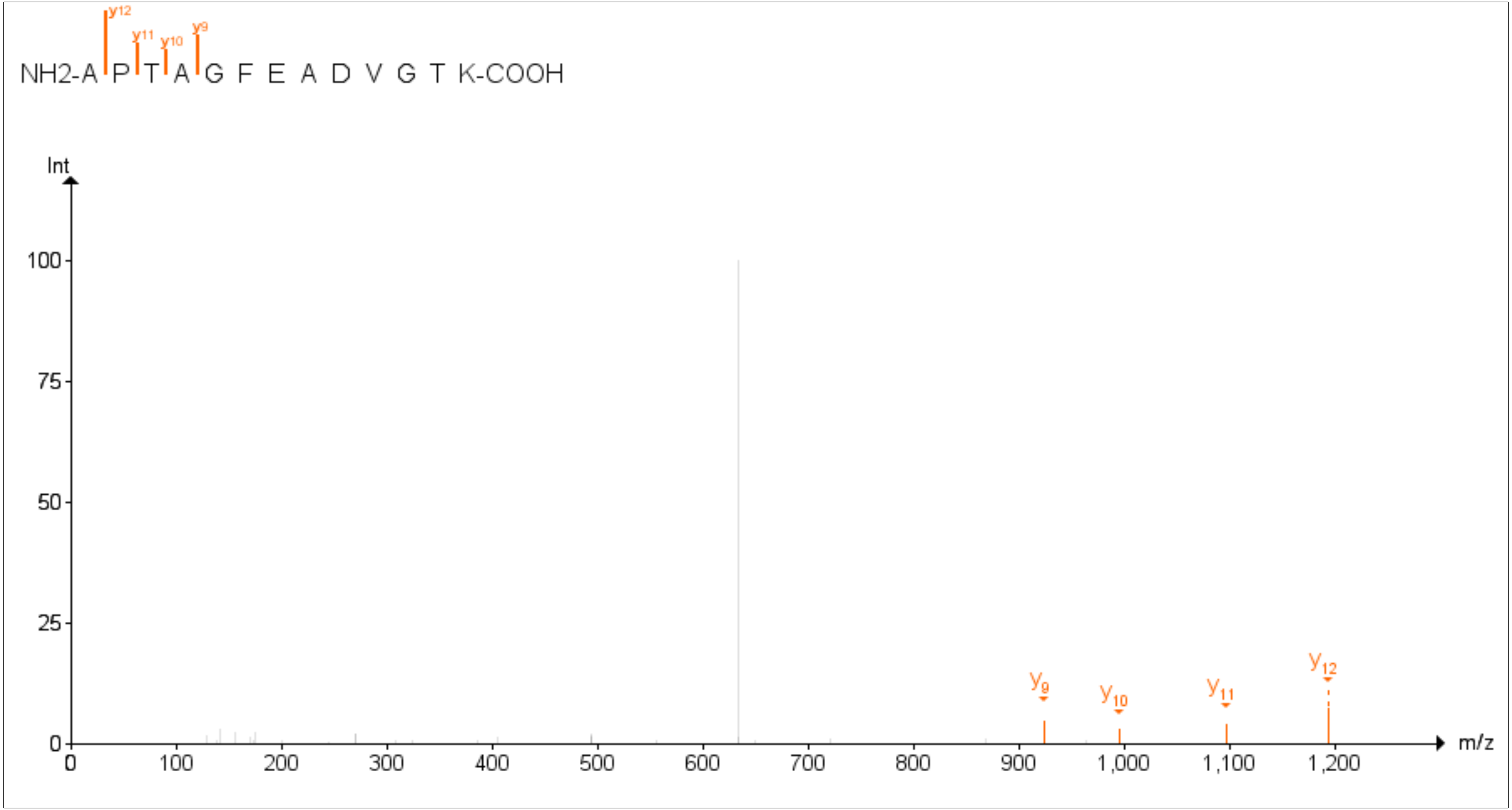

NH2-A P T A G F E A D V G T K-COOH
y12
y11
y10
y9
Int
100
75
50
25
0
m/z
0
100
200
300
400
500
600
700
800
900
1,000
1,100
1,200

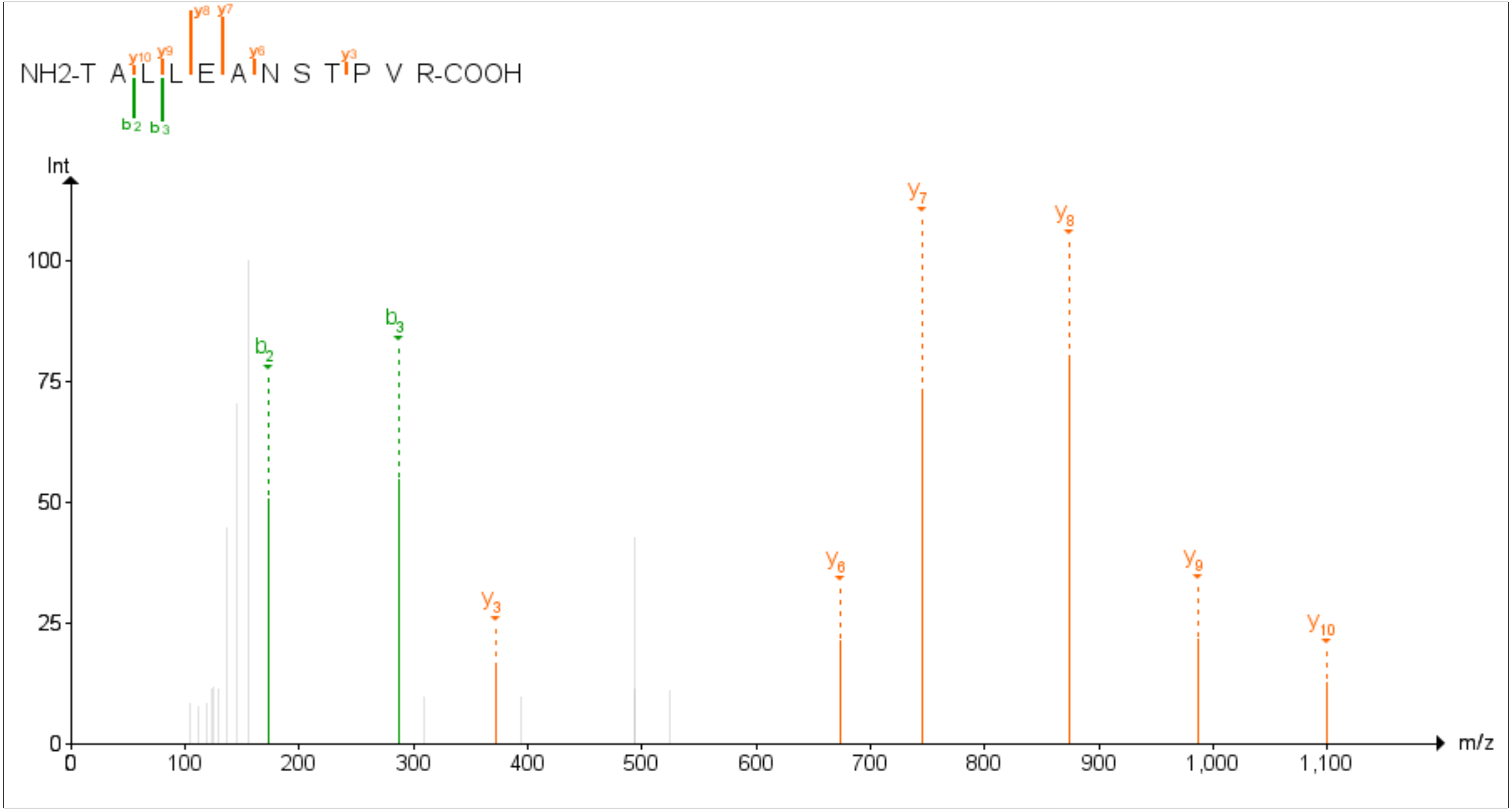

NH2-T A L L E A N S T P V R-COOH
y10 y9 y8 y7 y6 y3
b2 b3
Int
100
75
50
25
0
b2
b3
y3
y6
y7
y8
y9
y10
0 100 200 300 400 500 600 700 800 900 1,000 1,100
m/z

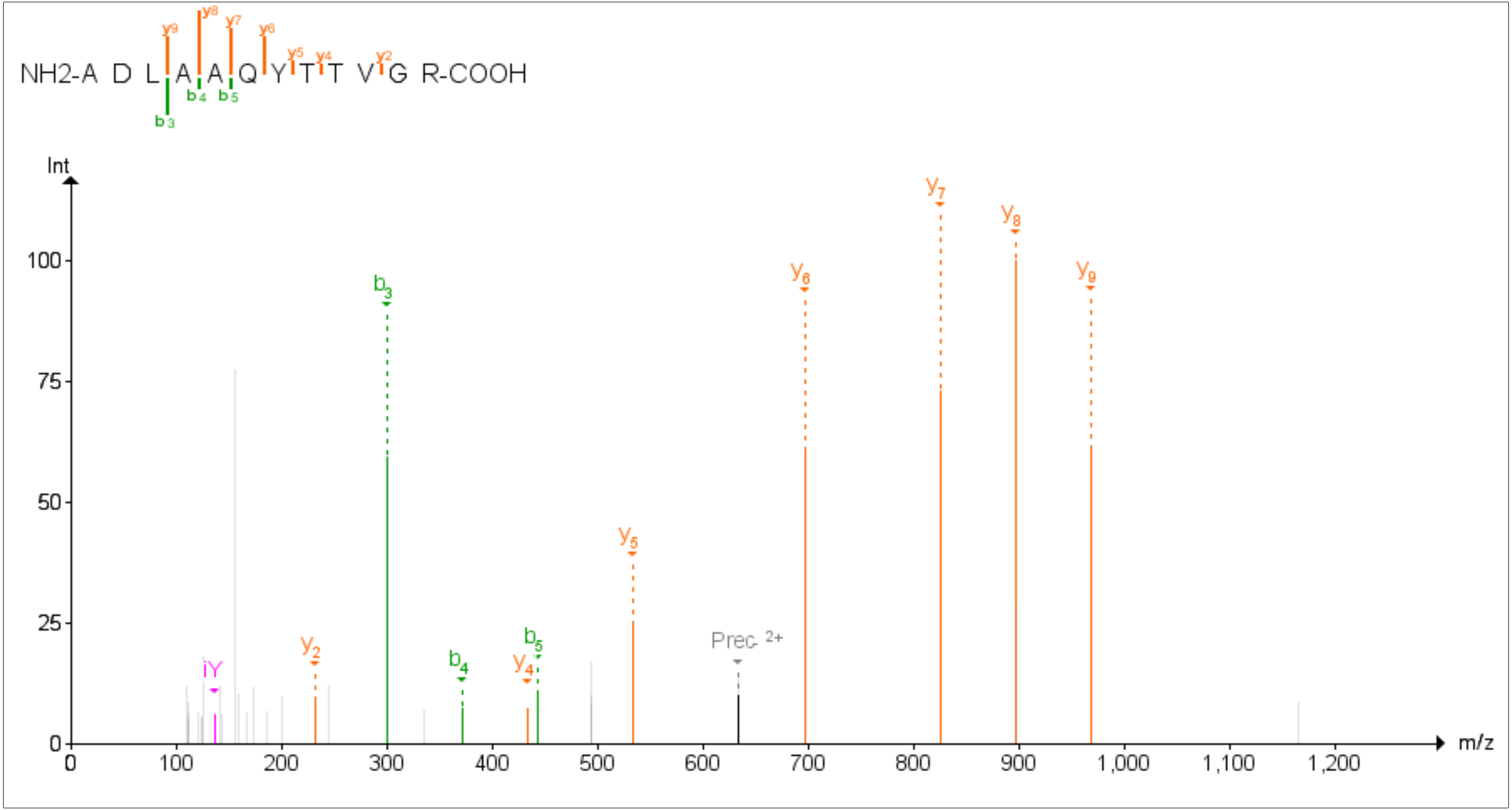

NH2-A D L A A Q Y T T V G R-COOH
y9
y8
y7
y6
y5
y4
y2
b3
b4
b5
Int
m/z
100
75
50
25
0
0
100
200
300
400
500
600
700
800
900
1,000
1,100
1,200
iY
y2
b3
b4
y4
b5
y5
Prec 2+
y6
y7
y8
y9

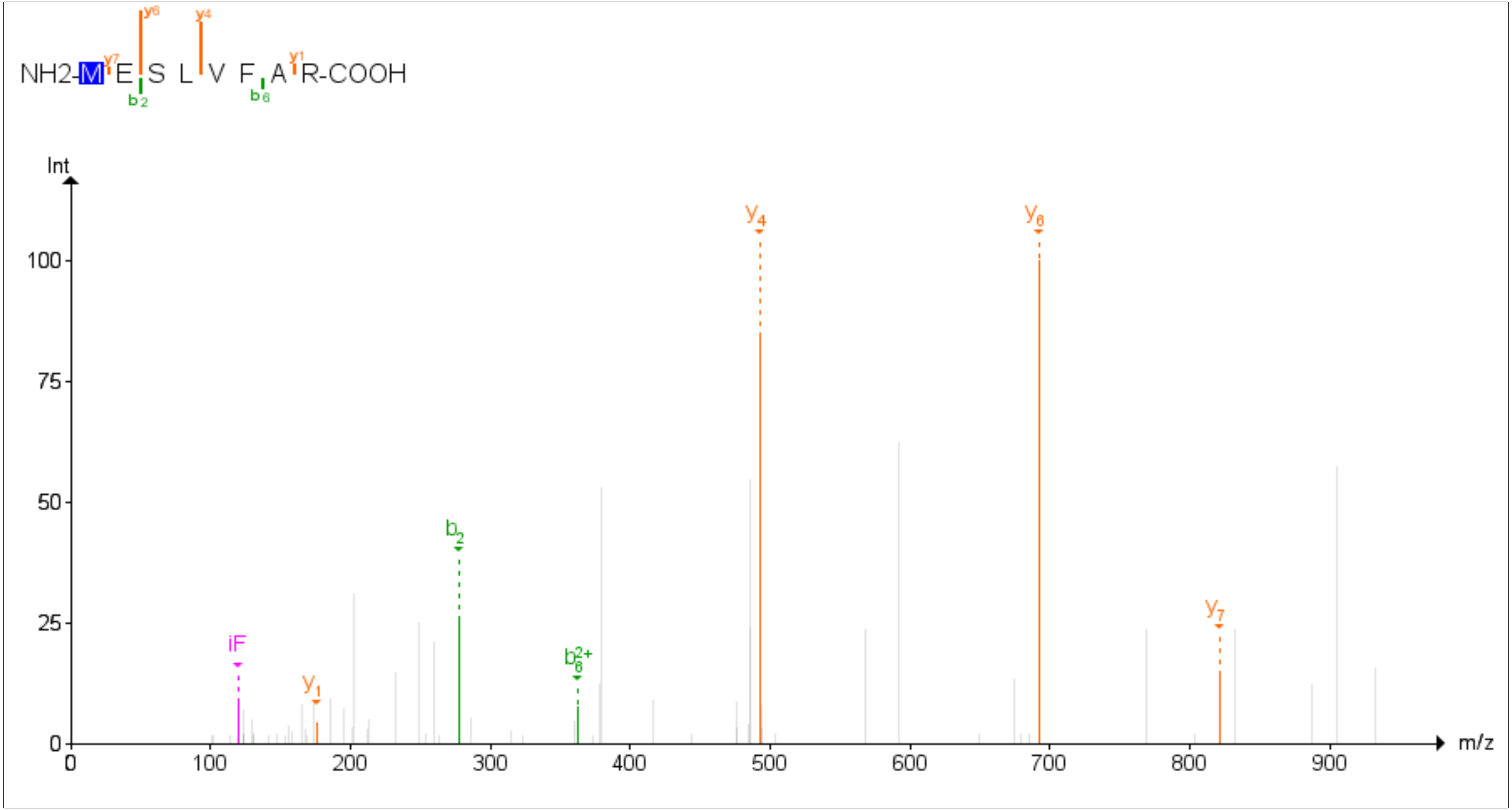

NH2-M E S L V F A R-COOH
y7
y6
y4
y1
b2
b6
Int
100
75
50
25
0
y4
y6
b2
iF
y1
b6 2+
y7
0
100
200
300
400
500
600
700
800
900
m/z

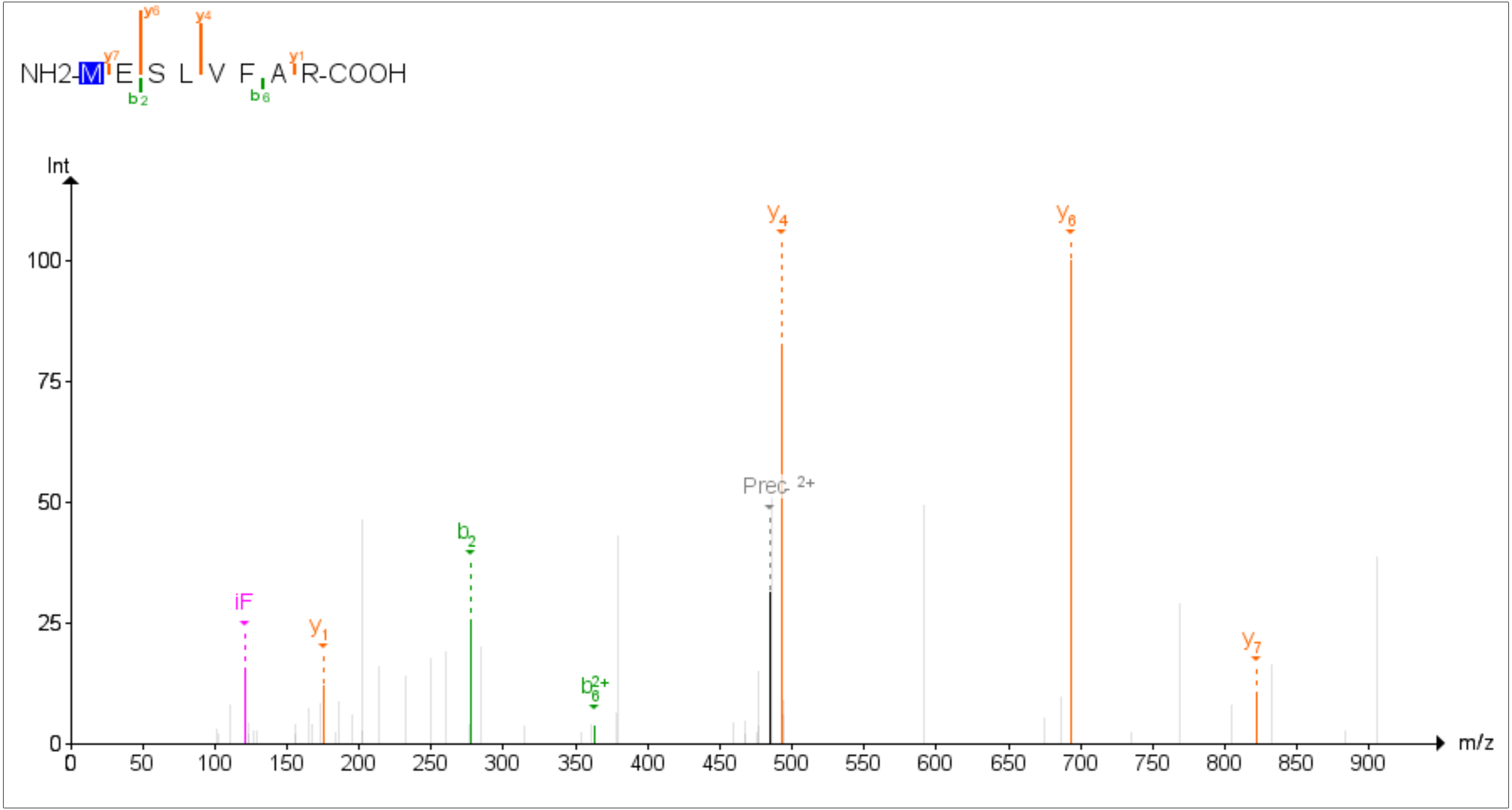
NH2-M E S L V F A R-COOH
y7
y6
y4
y1
b2
b6
Int
100
75
50
25
0
y4
y6
Prec 2+
b2
iF
y1
b6 2+
y7
0
50
100
150
200
250
300
350
400
450
500
550
600
650
700
750
800
850
900
m/z

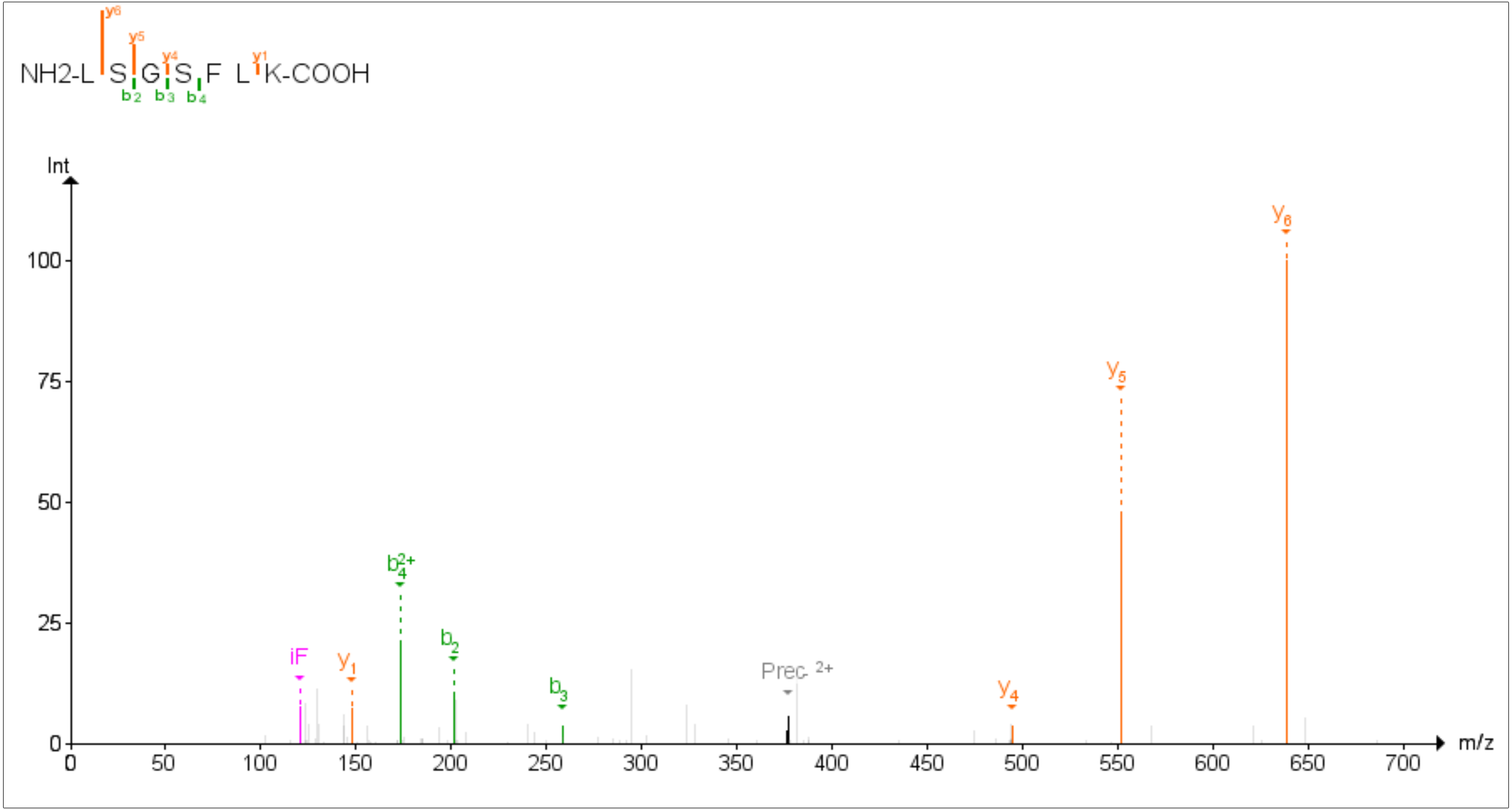

NH2-L S G S F L K-COOH
y6
y5
y4
y1
b2
b3
b4
Int
m/z
0
25
50
75
100
0
50
100
150
200
250
300
350
400
450
500
550
600
650
700
iF
y1
b4 2+
b2
b3
Prec- 2+
y4
y5
y6

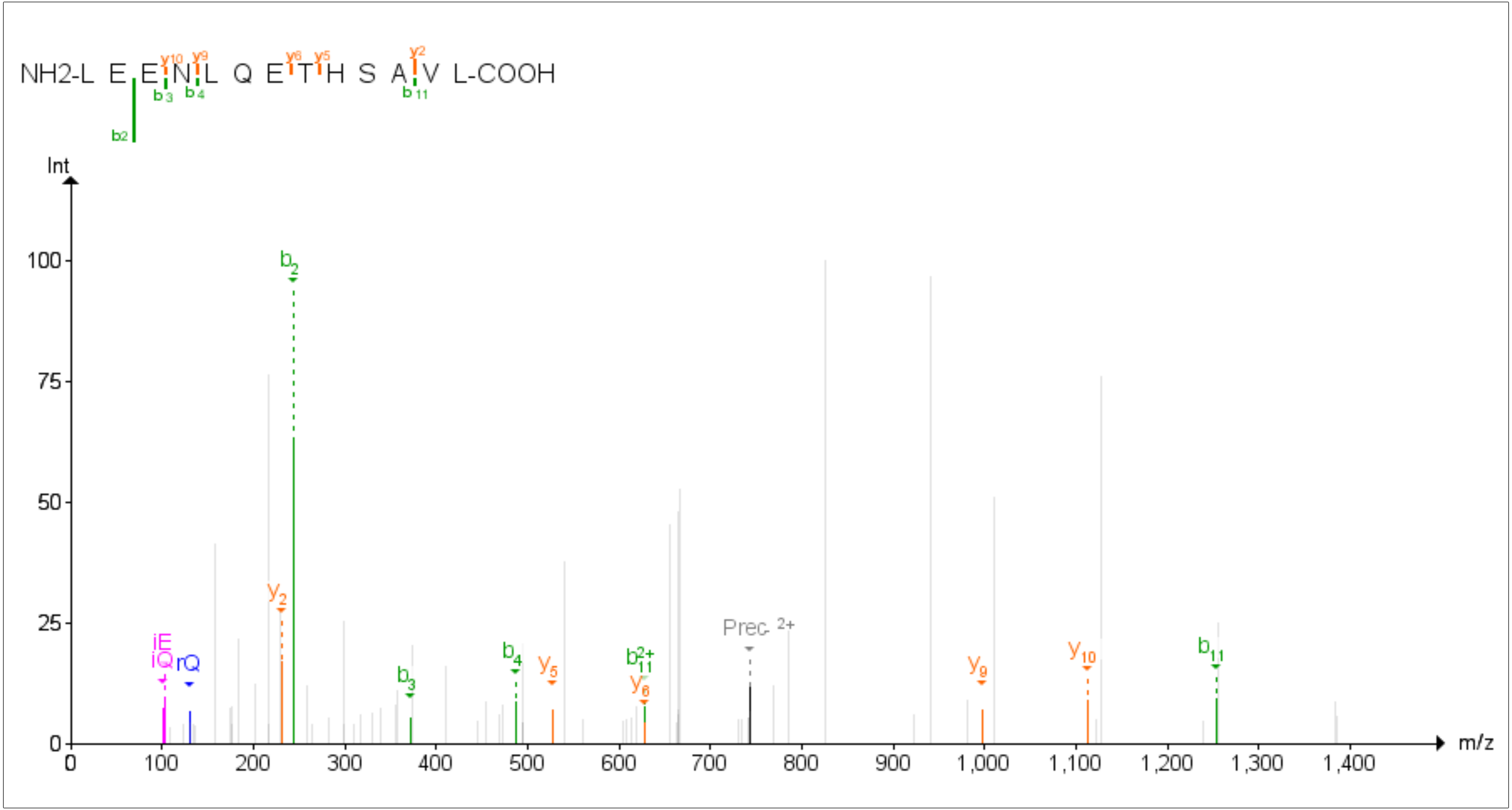

NH2-L E E N L Q E T H S A V L-COOH
y10 y9 y6 y5 y2
b2 b3 b4 b11
Int
100
75
50
25
0
b2
y2
iE
iQ
rQ
b3
b4
y5
b11 2+
y6
Prec. 2+
y9
y10
b11
0 100 200 300 400 500 600 700 800 900 1,000 1,100 1,200 1,300 1,400
m/z

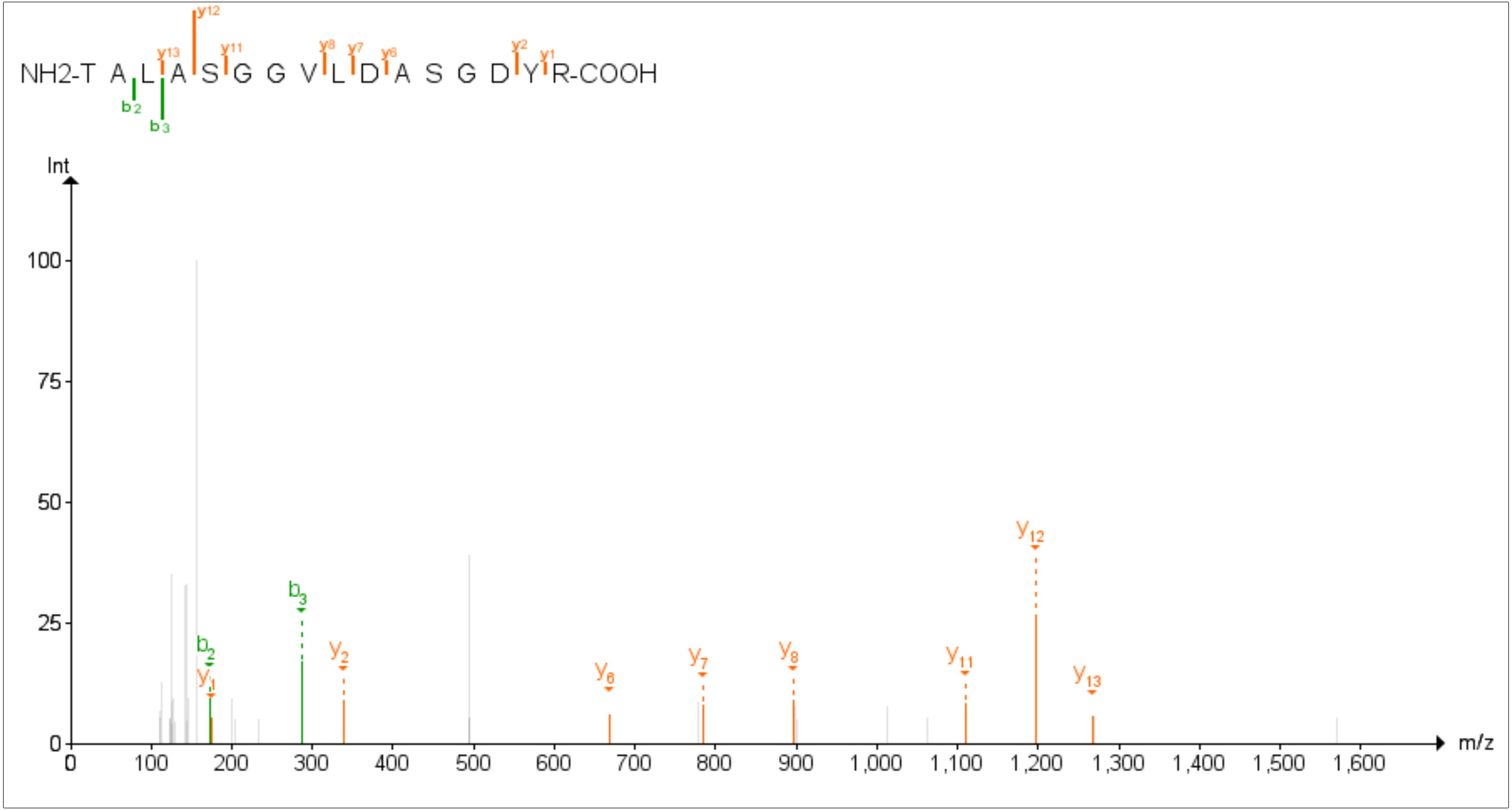

NH2-T A L A S G G V L D A S G D Y R-COOH
y12 y13 y11 y8 y7 y6 y2 y1
b2 b3
Int
100
75
50
25
0
m/z
0 100 200 300 400 500 600 700 800 900 1,000 1,100 1,200 1,300 1,400 1,500 1,600
b2 y1 b3 y2 y6 y7 y8 y11 y12 y13

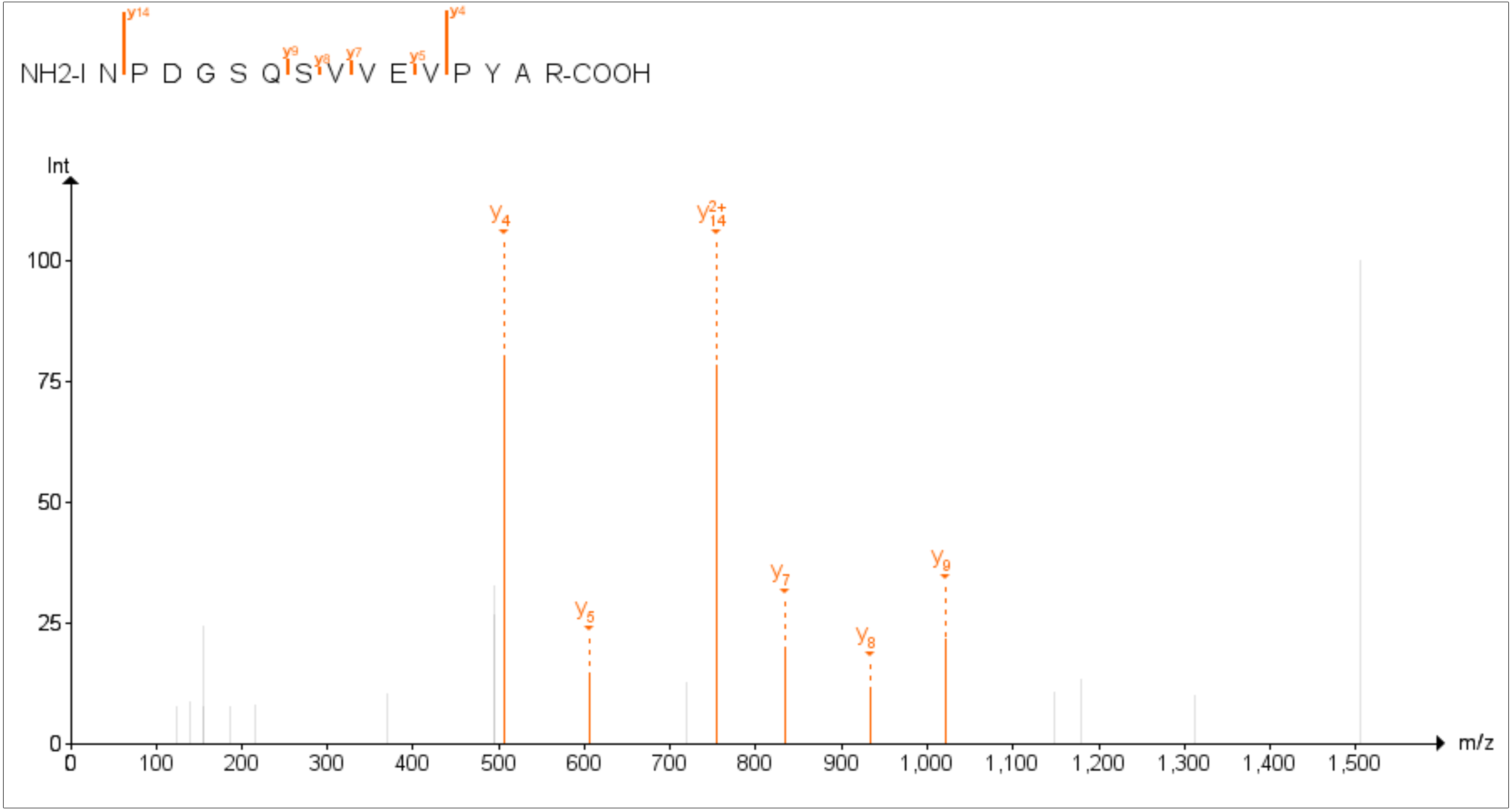
NH2-I N P D G S Q S V V E V P Y A R-COOH
y14
y9
y8
y7
y5
y4
Int
m/z
0
25
50
75
100
0
100
200
300
400
500
600
700
800
900
1,000
1,100
1,200
1,300
1,400
1,500
y4
y14 2+
y5
y7
y8
y9

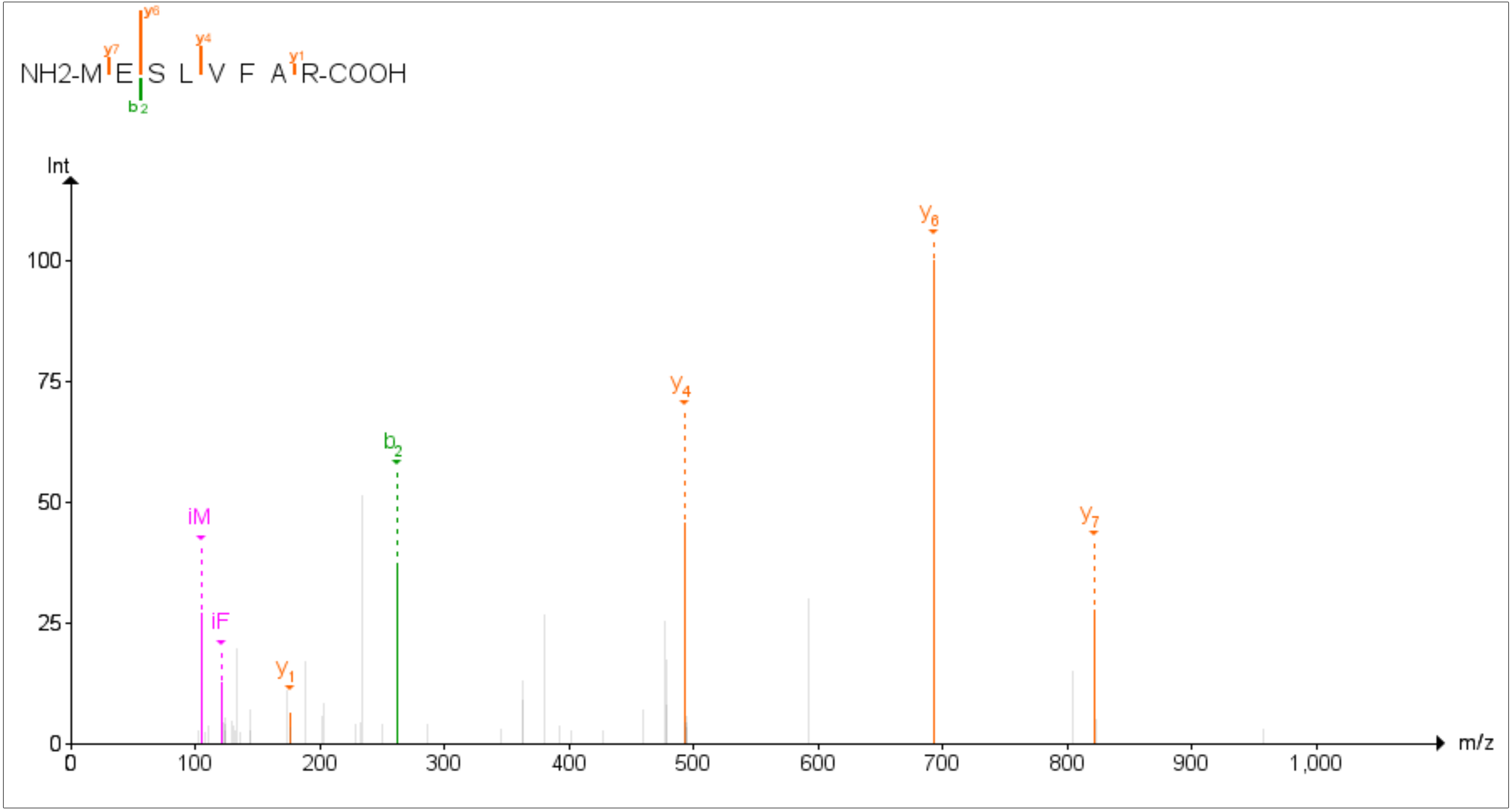

NH2-M E S L V F A R-COOH
y7
y6
y4
y1
b2
Int
100
75
50
25
0
iM
iF
y1
b2
y4
y6
y7
0
100
200
300
400
500
600
700
800
900
1,000
m/z

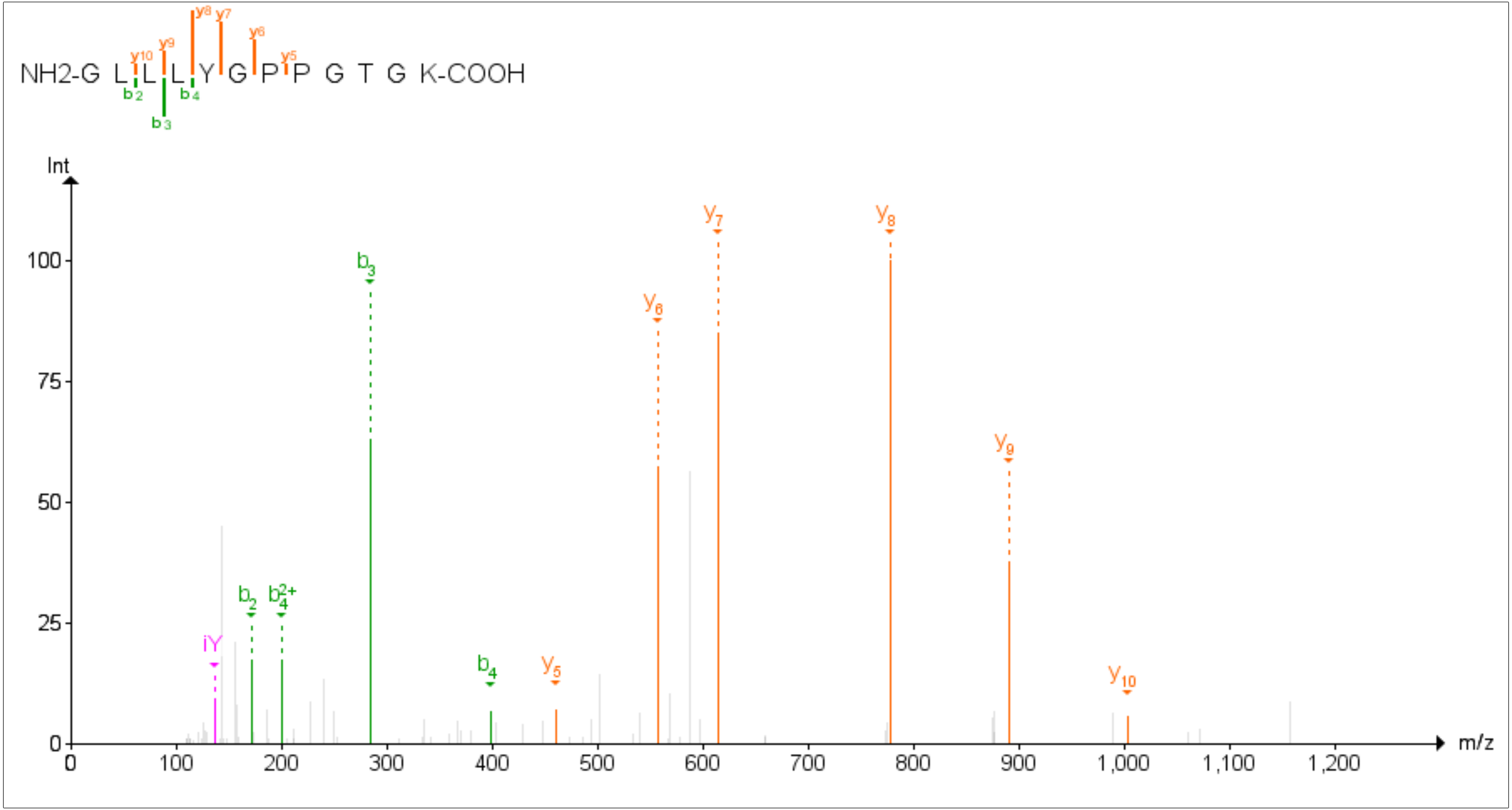

NH2-G L L L Y G P P G T G K-COOH
y10 y9 y8 y7 y6 y5
b2 b3 b4
Int
m/z
0
25
50
75
100
0
100
200
300
400
500
600
700
800
900
1,000
1,100
1,200
iY
b2
b4 2+
b3
b4
y5
y6
y7
y8
y9
y10

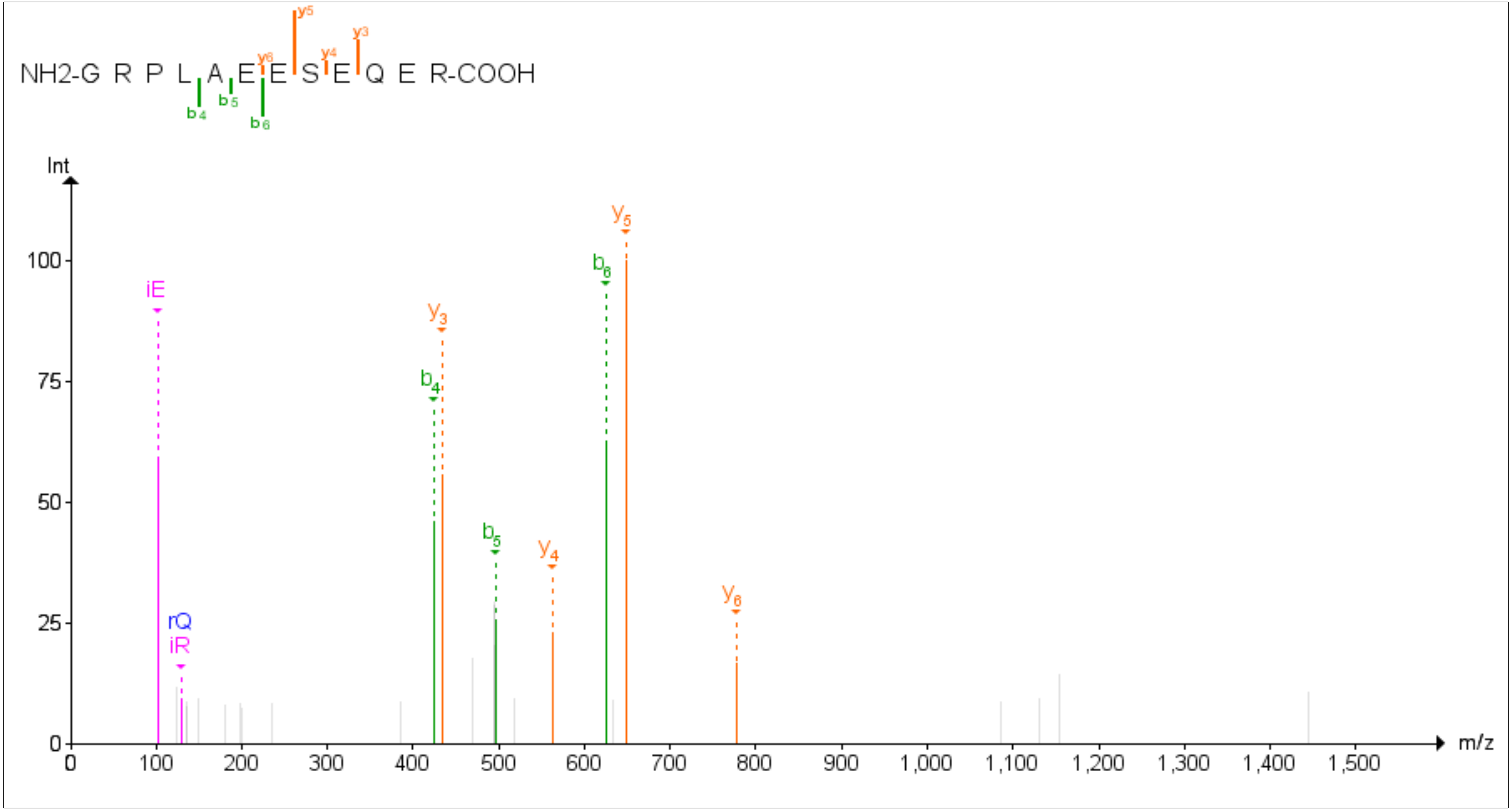

NH2-G R P L A E E S E Q E R-COOH
b4
b5
b6
y6
y5
y4
y3
Int
100
75
50
25
0
iE
rQ
iR
b4
y3
b5
y4
b6
y5
y6
0
100
200
300
400
500
600
700
800
900
1,000
1,100
1,200
1,300
1,400
1,500
m/z

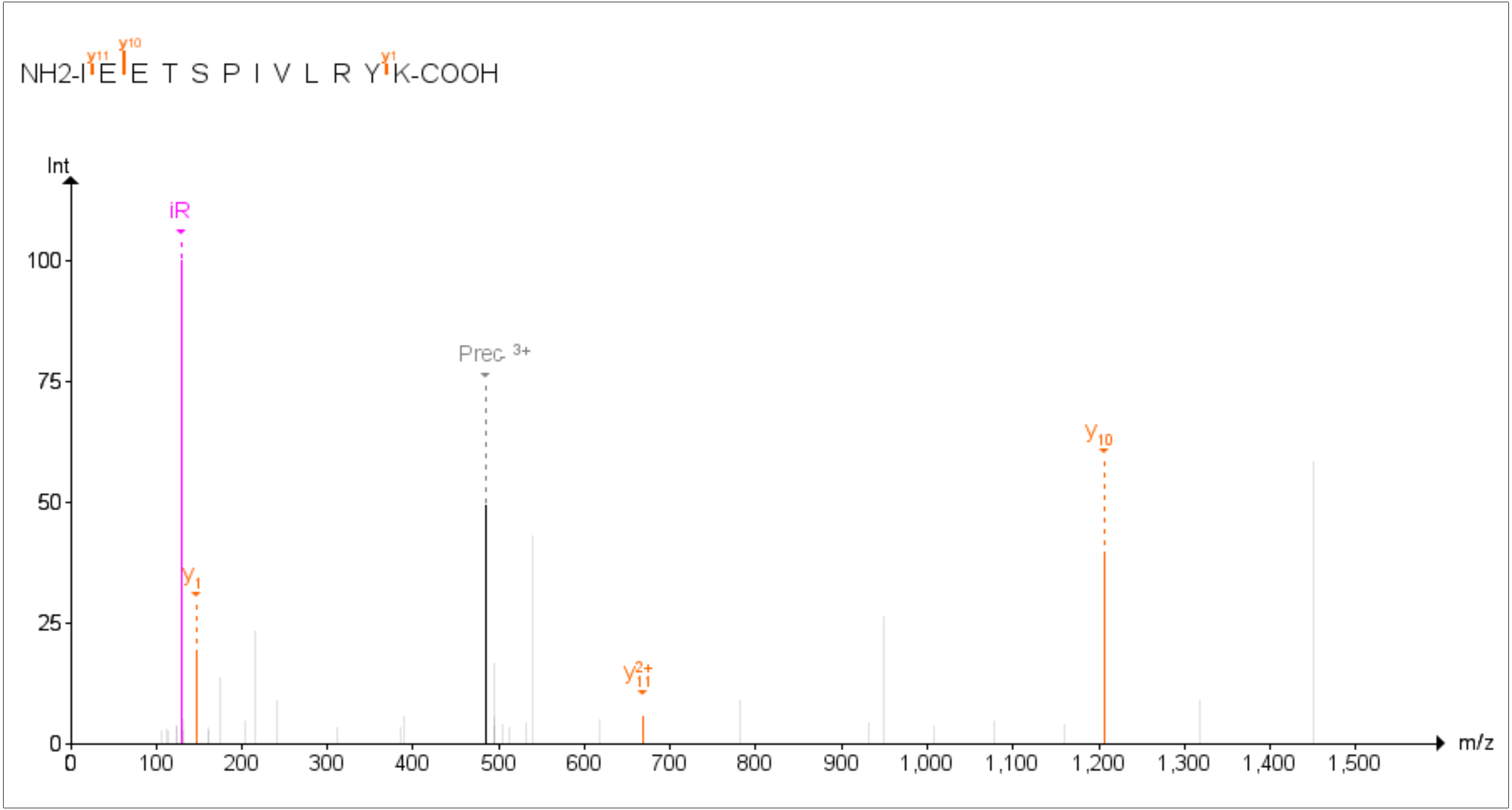

y11
y10
y1
NH2-I E E T S P I V L R Y K-COOH
Int
iR
Prec 3+
y10
y1
y11 2+
100
75
50
25
0
0
100
200
300
400
500
600
700
800
900
1,000
1,100
1,200
1,300
1,400
1,500
m/z

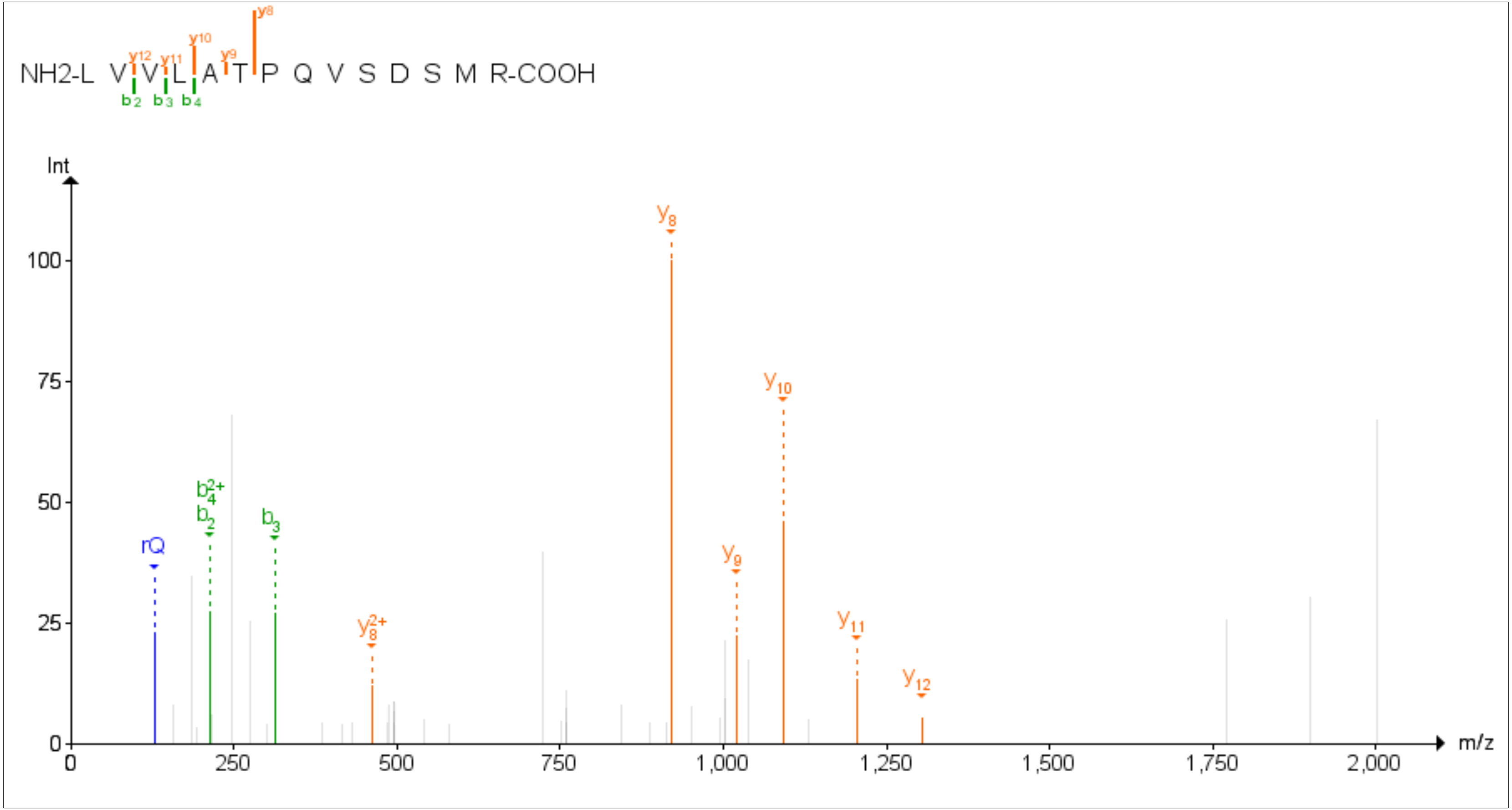
NH2-L V V L A T P Q V S D S M R-COOH
y12 y11 y10 y9 y8
b2 b3 b4
Int
m/z
rQ
b4 2+
b2
b3
y8 2+
y8
y9
y10
y11
y12
0
25
50
75
100
250
500
750
1,000
1,250
1,500
1,750
2,000

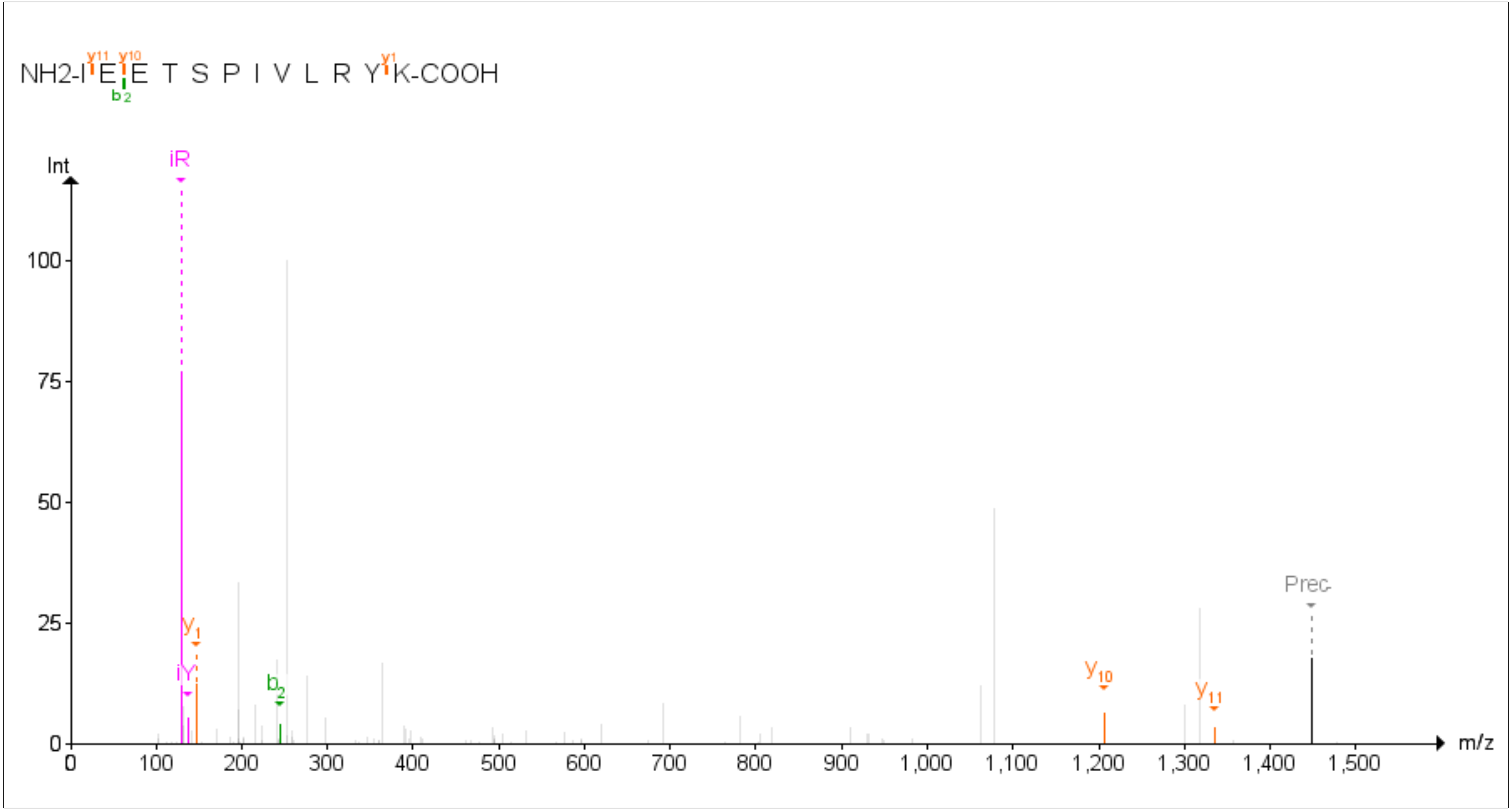

NH2-I E E T S P I V L R Y K-COOH
y11
y10
y1
b2
Int
iR
100
75
50
25
0
y1
iY
b2
y10
y11
Prec
0
100
200
300
400
500
600
700
800
900
1,000
1,100
1,200
1,300
1,400
1,500
m/z

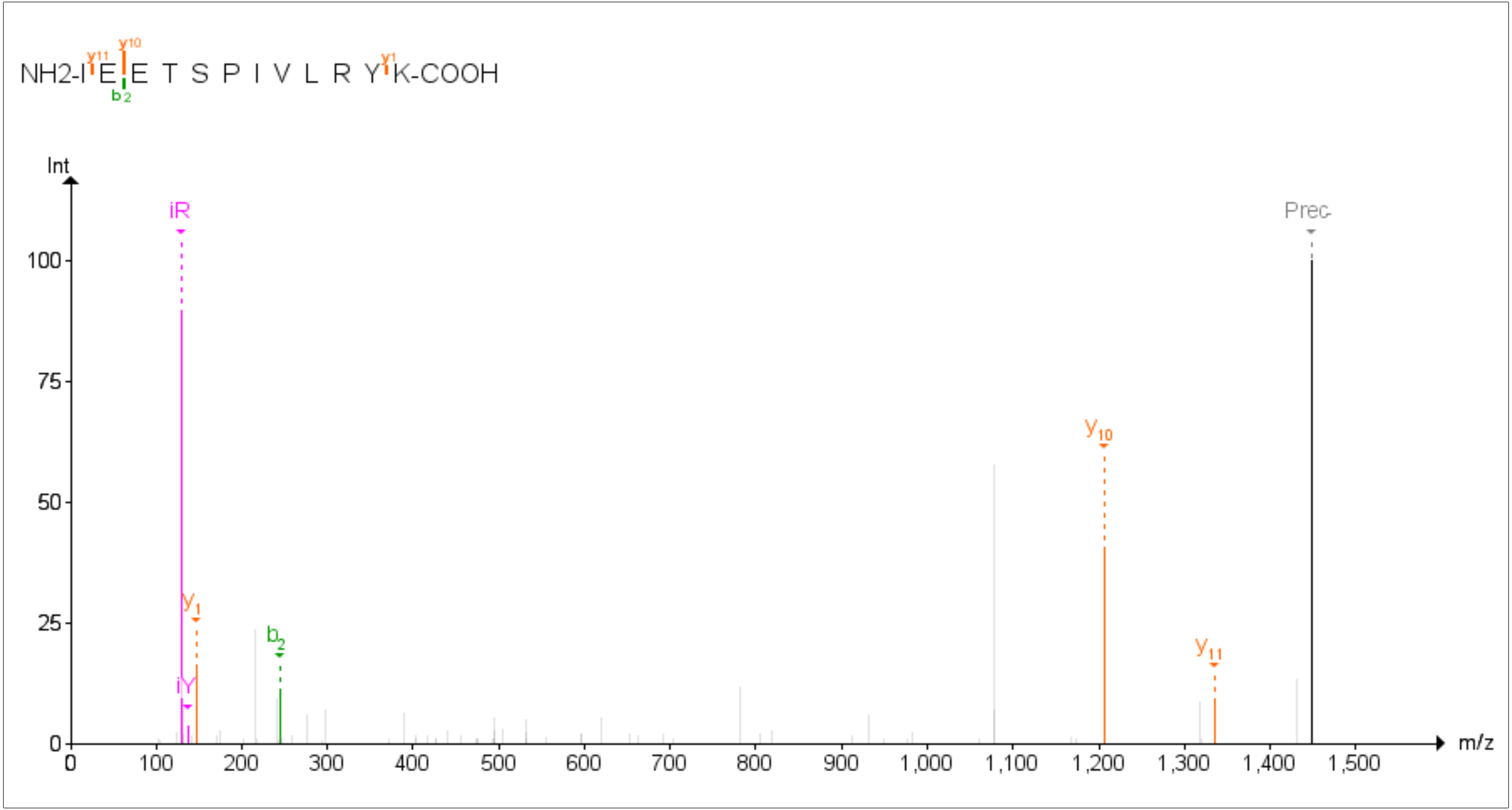

NH2-I E E T S P I V L R Y K-COOH
y11
y10
b2
y1
Int
iR
Prec
100
75
50
25
0
y10
y11
y1
b2
iY
0
100
200
300
400
500
600
700
800
900
1,000
1,100
1,200
1,300
1,400
1,500
m/z

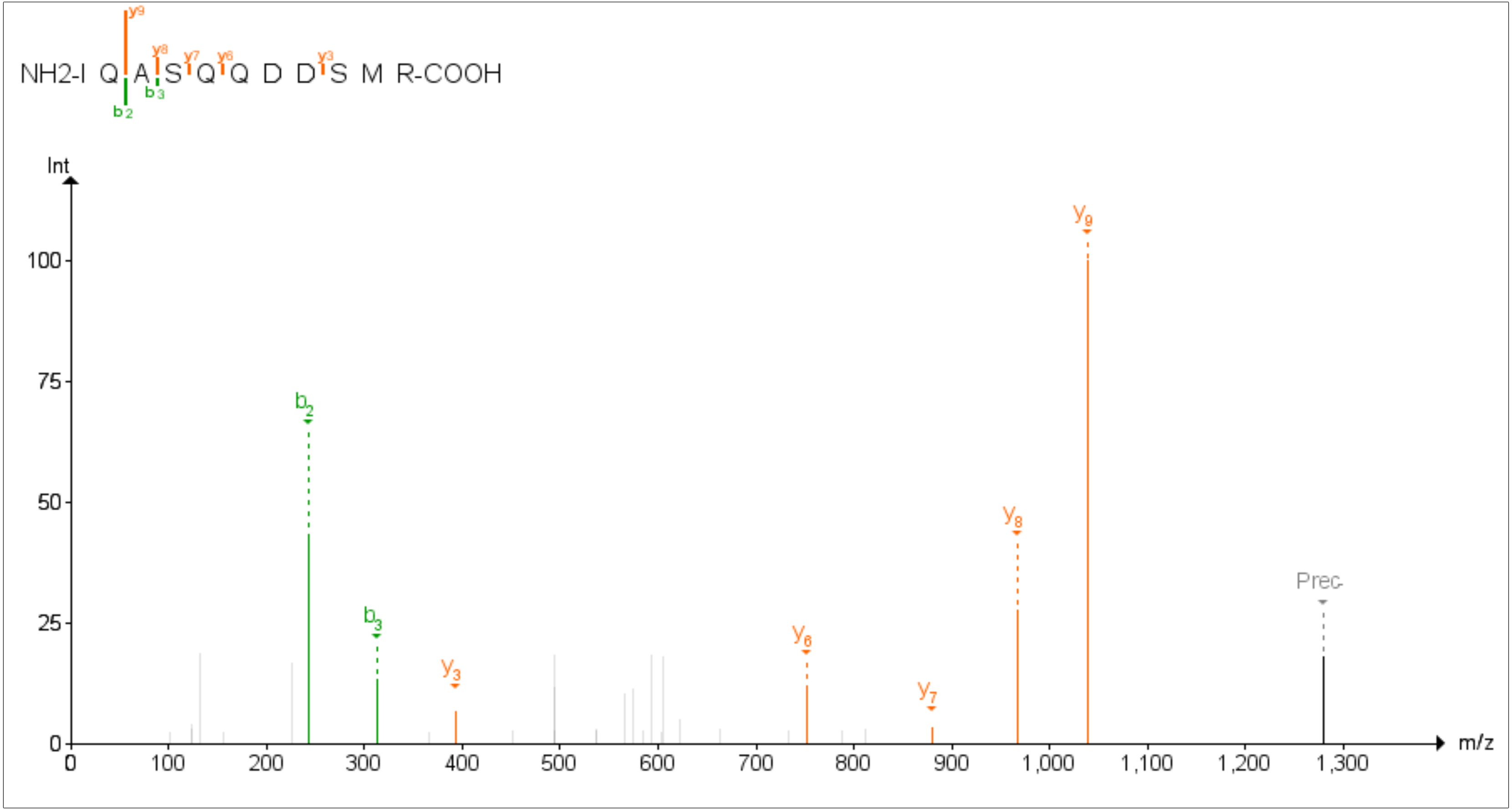

NH2-I Q A S Q Q D D S M R-COOH
y9
y8
y7
y6
y3
b2
b3
Int
100
75
50
25
0
b2
b3
y3
y6
y7
y8
y9
Prec
0
100
200
300
400
500
600
700
800
900
1,000
1,100
1,200
1,300
m/z

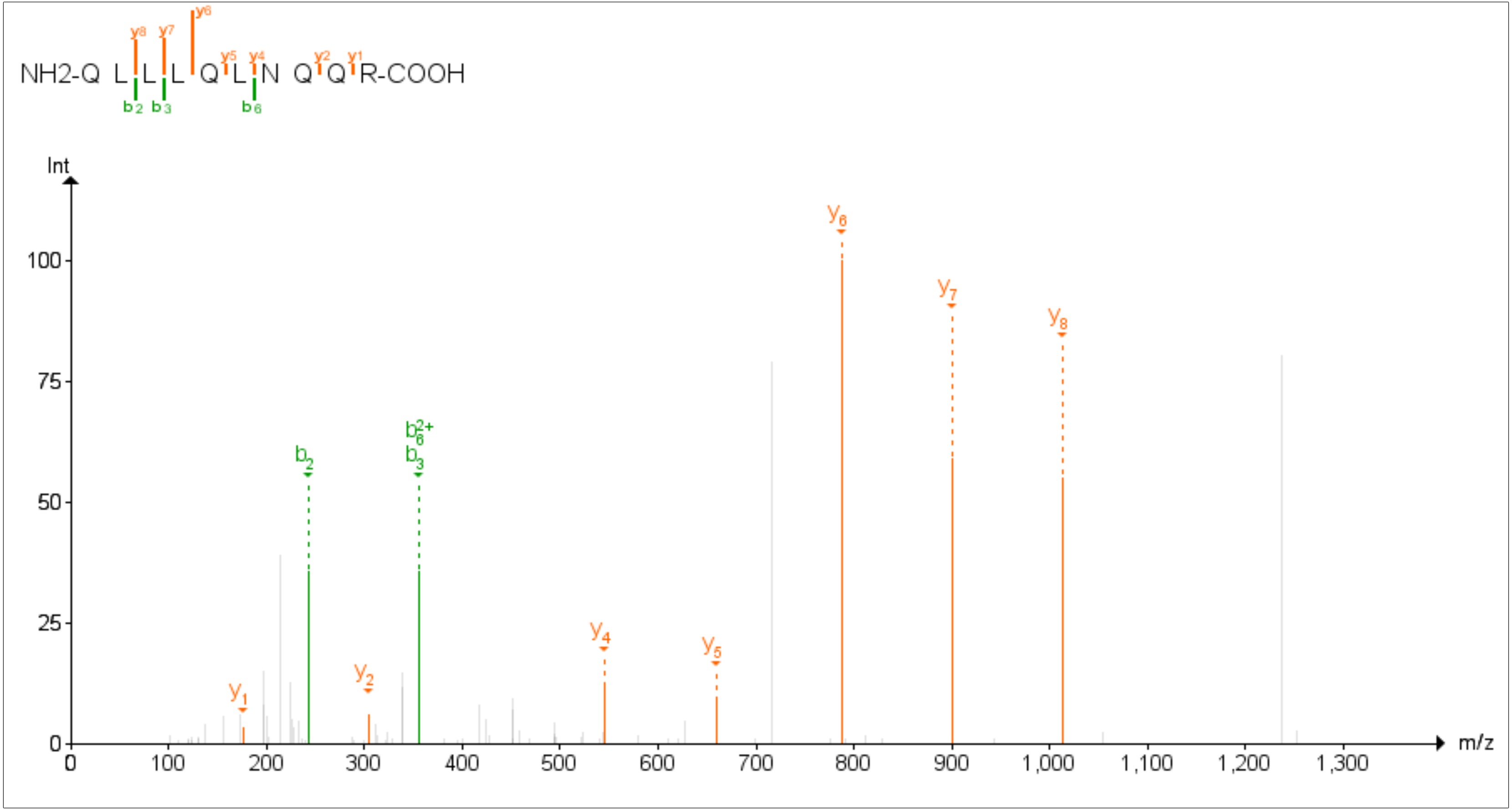

NH2-Q L L L Q L N Q Q R-COOH
y8 y7 y6 y5 y4 y2 y1
b2 b3 b6
Int
m/z
y1 y2 y4 y5 y6 y7 y8
b2 b3 b_6^{2+}
0 25 50 75 100
0 100 200 300 400 500 600 700 800 900 1,000 1,100 1,200 1,300

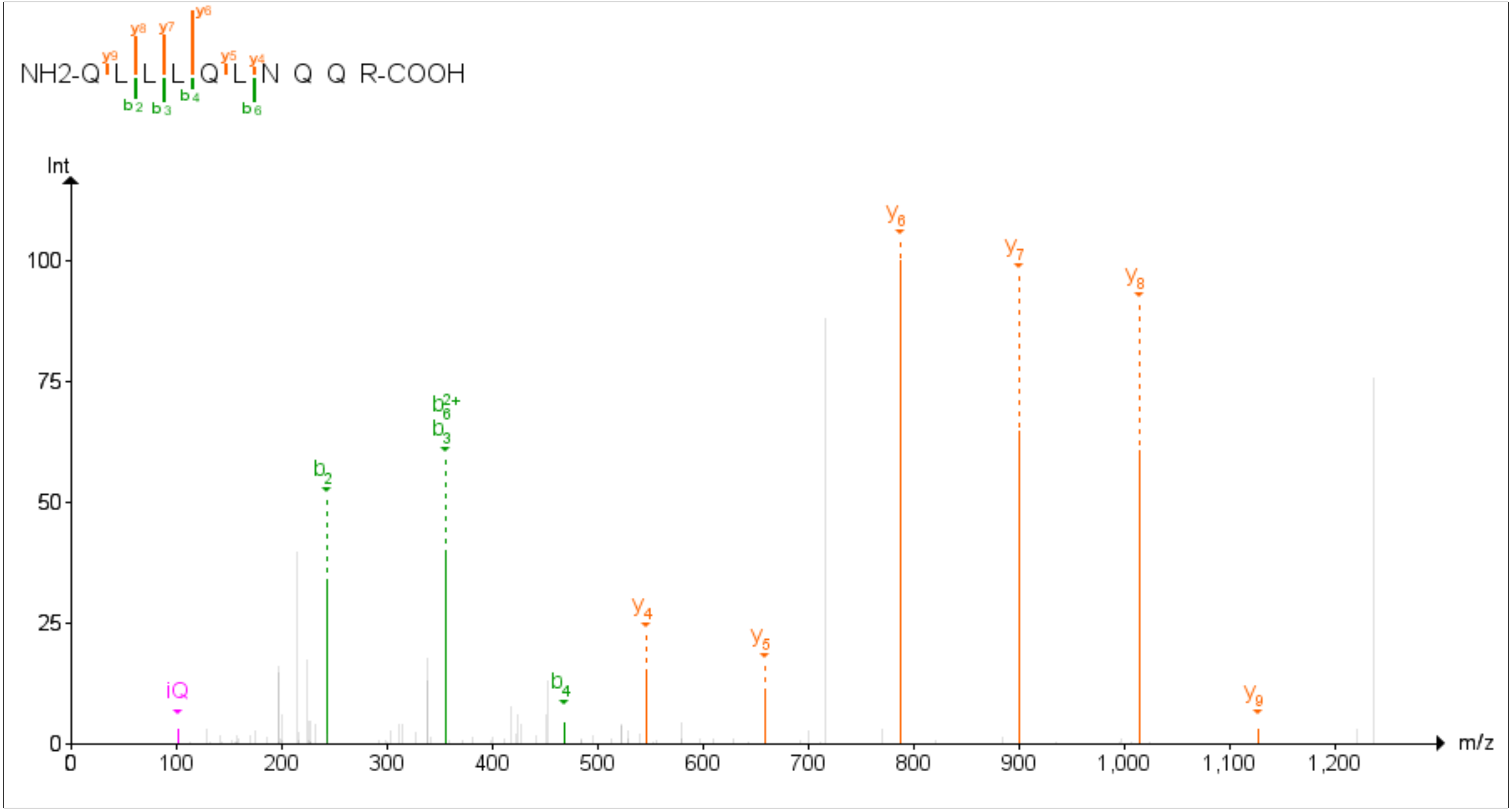
NH2-Q L L L Q L N Q Q R-COOH
y9 y8 y7 y6 y5 y4
b2 b3 b4 b6
Int
m/z
y6
y7
y8
y9
y4
y5
b2
b3
b6 2+
b4
iQ
0
25
50
75
100
0
100
200
300
400
500
600
700
800
900
1,000
1,100
1,200

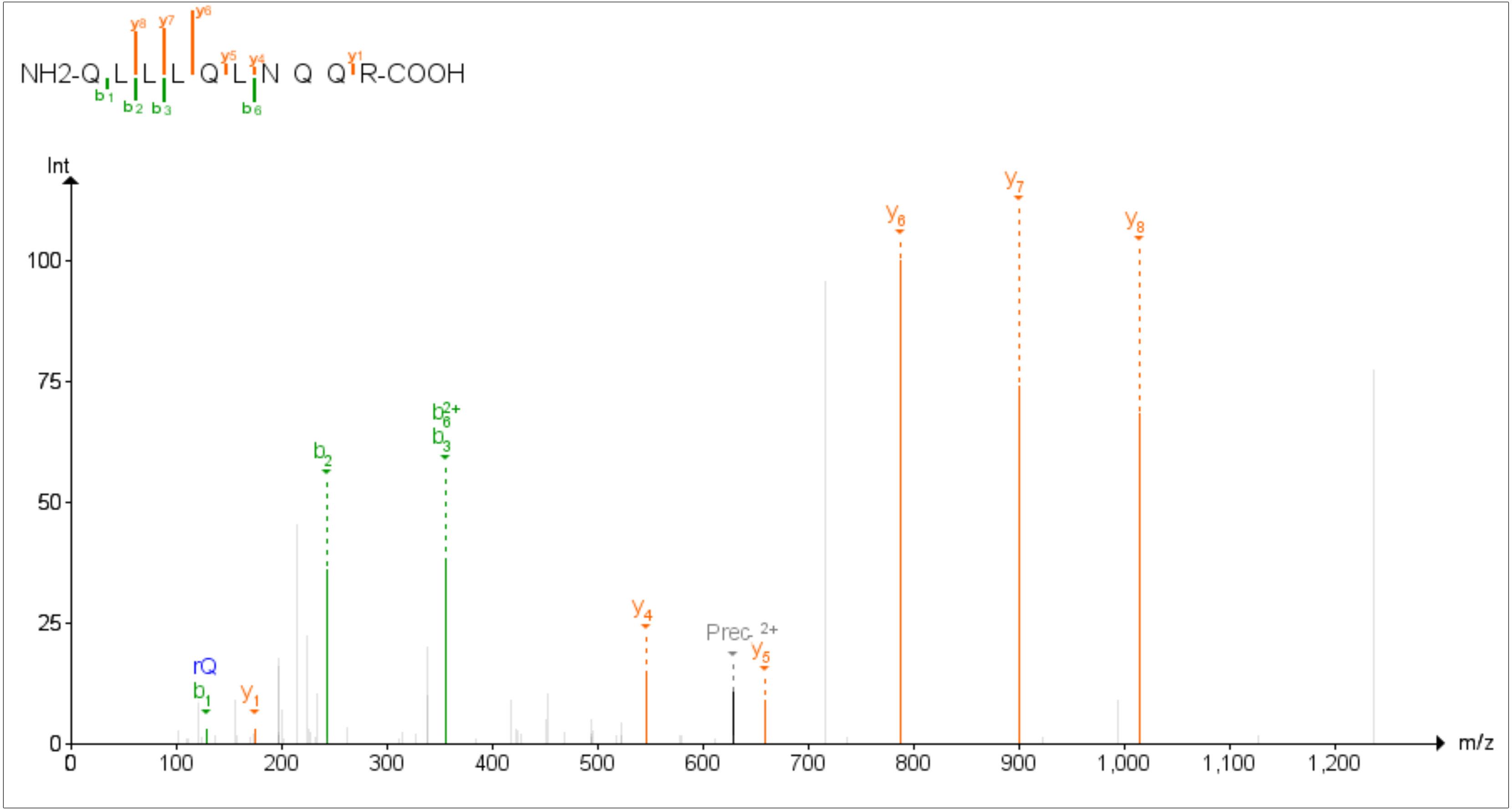

NH2-Q L L L Q L N Q Q R-COOH
b1 b2 b3 b6
y8 y7 y6 y5 y4 y1
Int
m/z
0
25
50
75
100
0
100
200
300
400
500
600
700
800
900
1,000
1,100
1,200
rQ
b1
y1
b2
b6 2+
b3
y4
Prec 2+
y5
y6
y7
y8

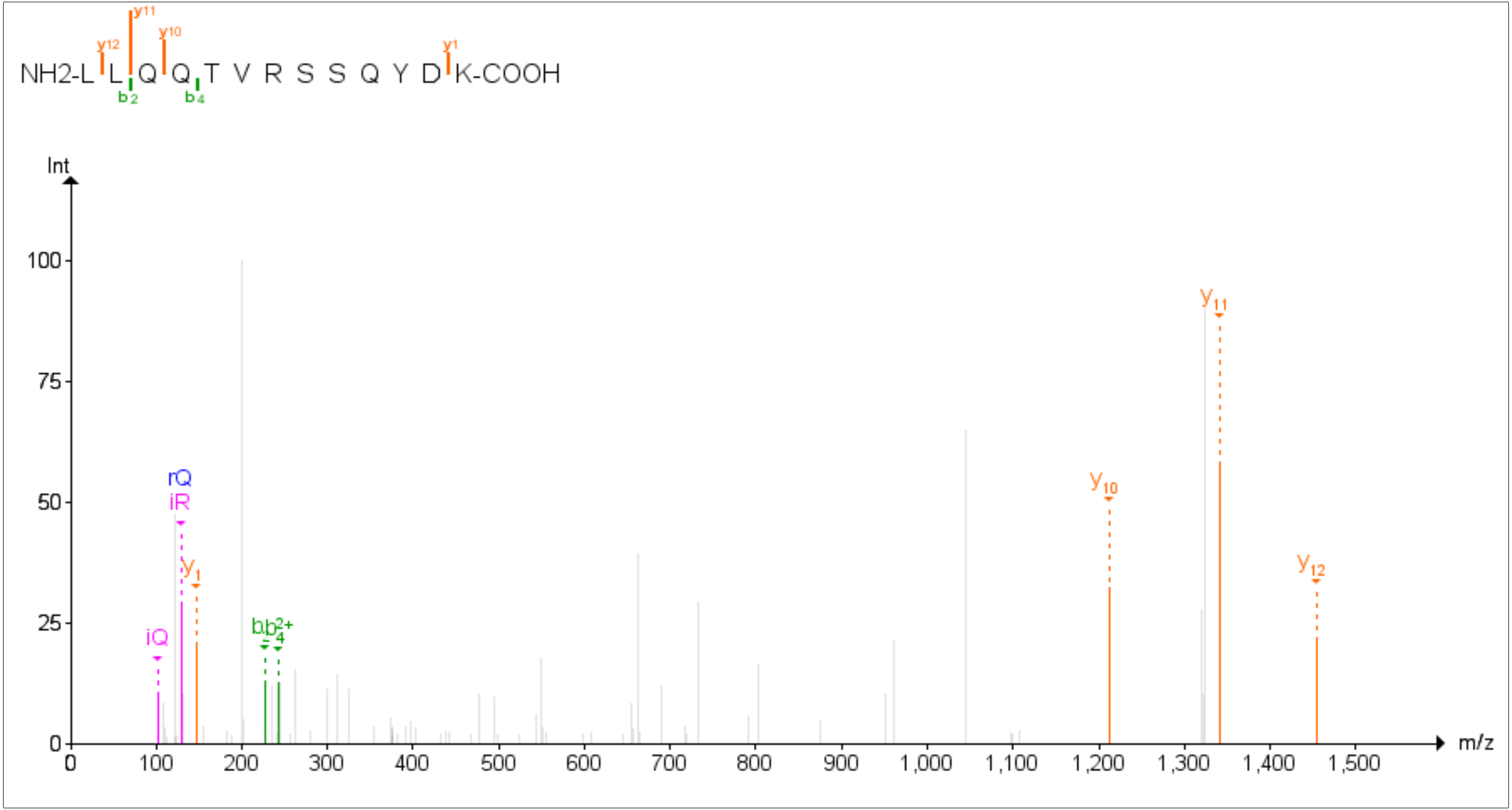

NH2-L L Q Q T V R S S Q Y D K-COOH
y12 y11 y10 y1
b2 b4
Int
100
75
50
25
0
rQ
iR
iQ
y1
b b4 2+
y10
y11
y12
0 100 200 300 400 500 600 700 800 900 1,000 1,100 1,200 1,300 1,400 1,500
m/z

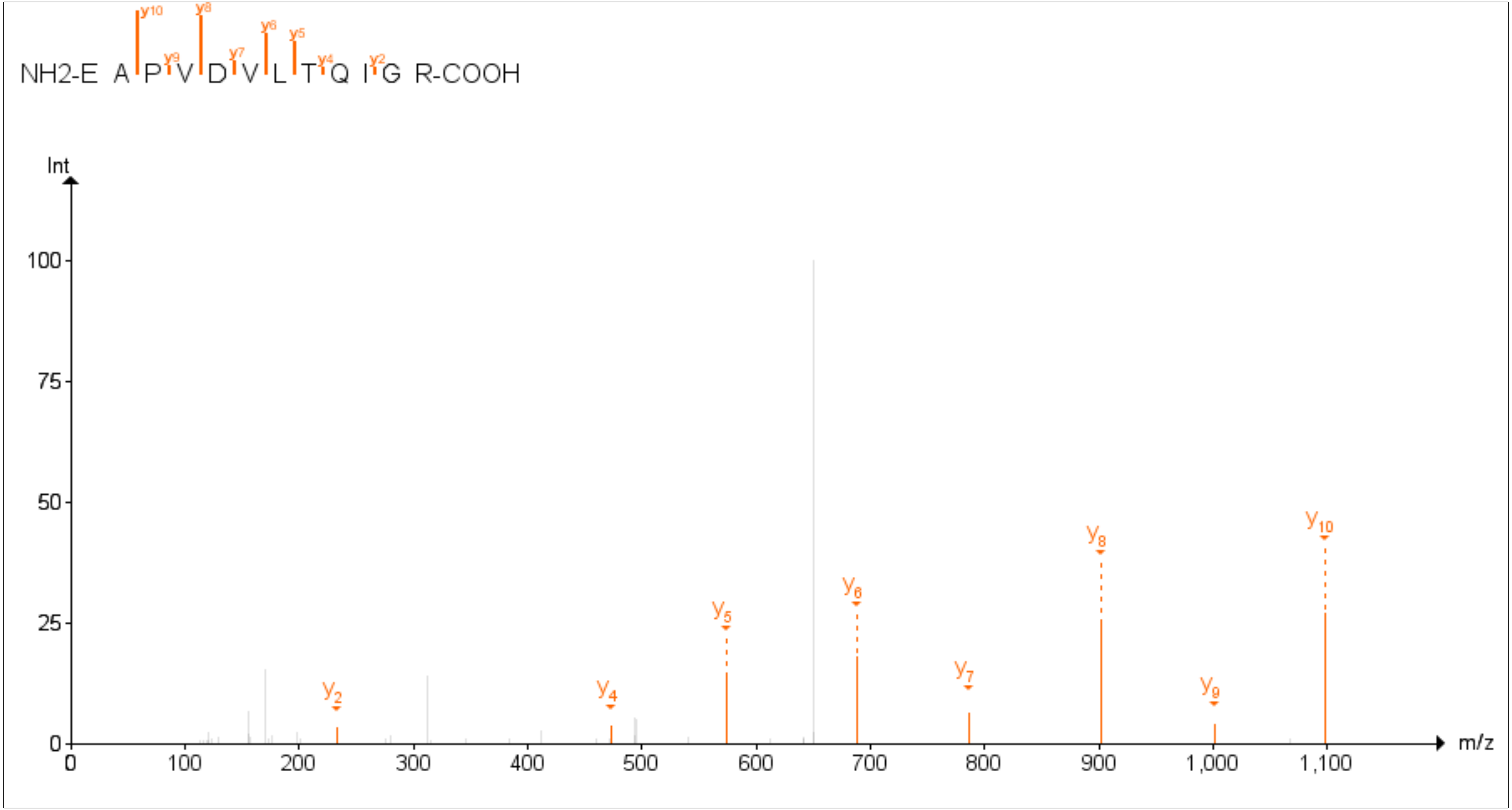

NH2-E A P V D V L T Q I G R-COOH
y10
y9
y8
y7
y6
y5
y4
y2
Int
100
75
50
25
0
m/z
0
100
200
300
400
500
600
700
800
900
1,000
1,100
y_2
y_4
y_5
y_6
y_7
y_8
y_9
y_{10}

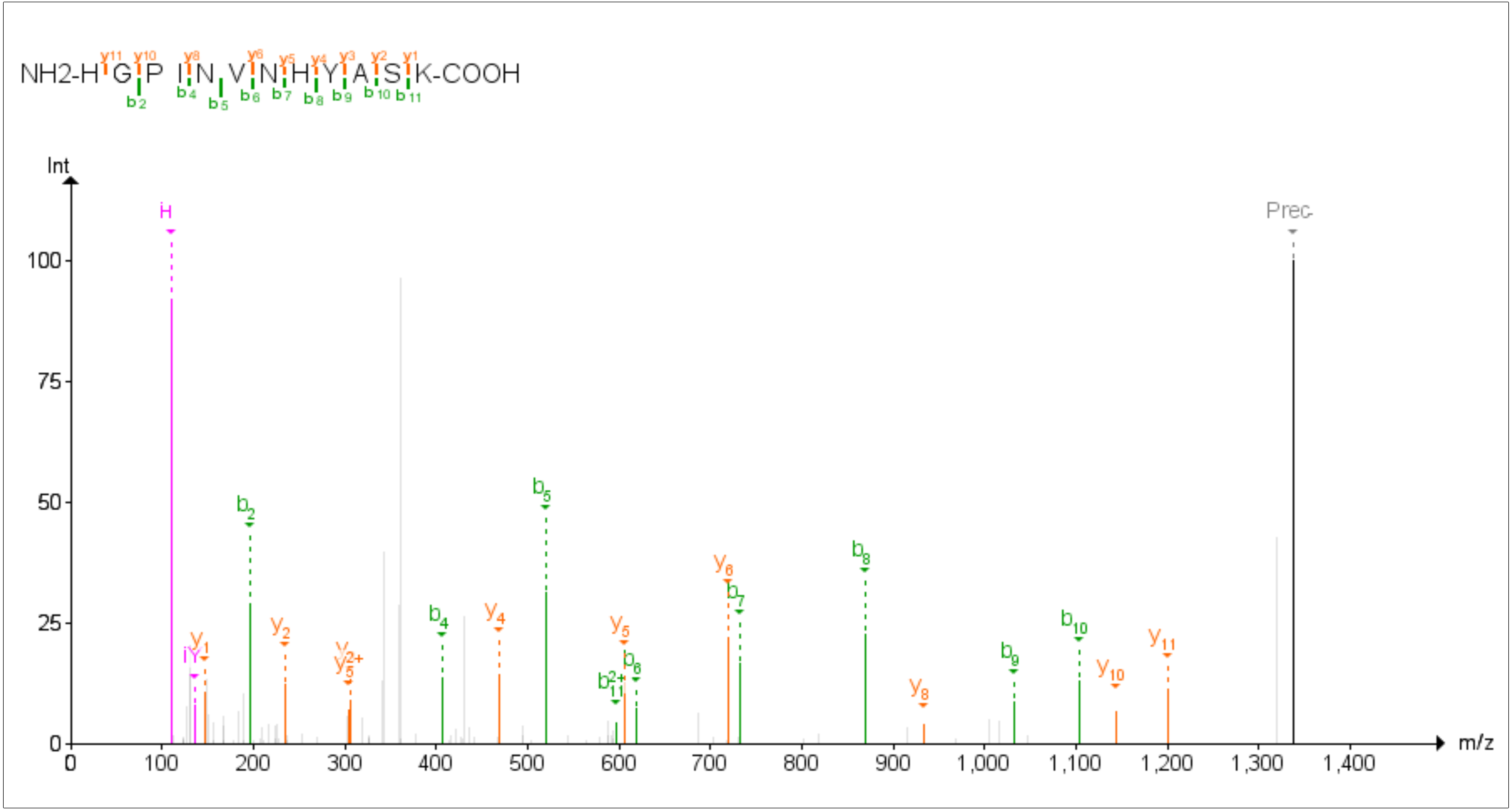
NH2-H G P I N V N H Y A S K-COOH
y11 y10 y8 y6 y5 y4 y3 y2 y1
b2 b4 b5 b6 b7 b8 b9 b10 b11
Int
iH
Prec
100
75
50
25
0
iY
y1
b2
y2
y5
y5 2+
b4
y4
b5
b11 2+
y5
b6
y6
b7
b8
y8
b9
b10
y10
y11
0
100
200
300
400
500
600
700
800
900
1,000
1,100
1,200
1,300
1,400
m/z

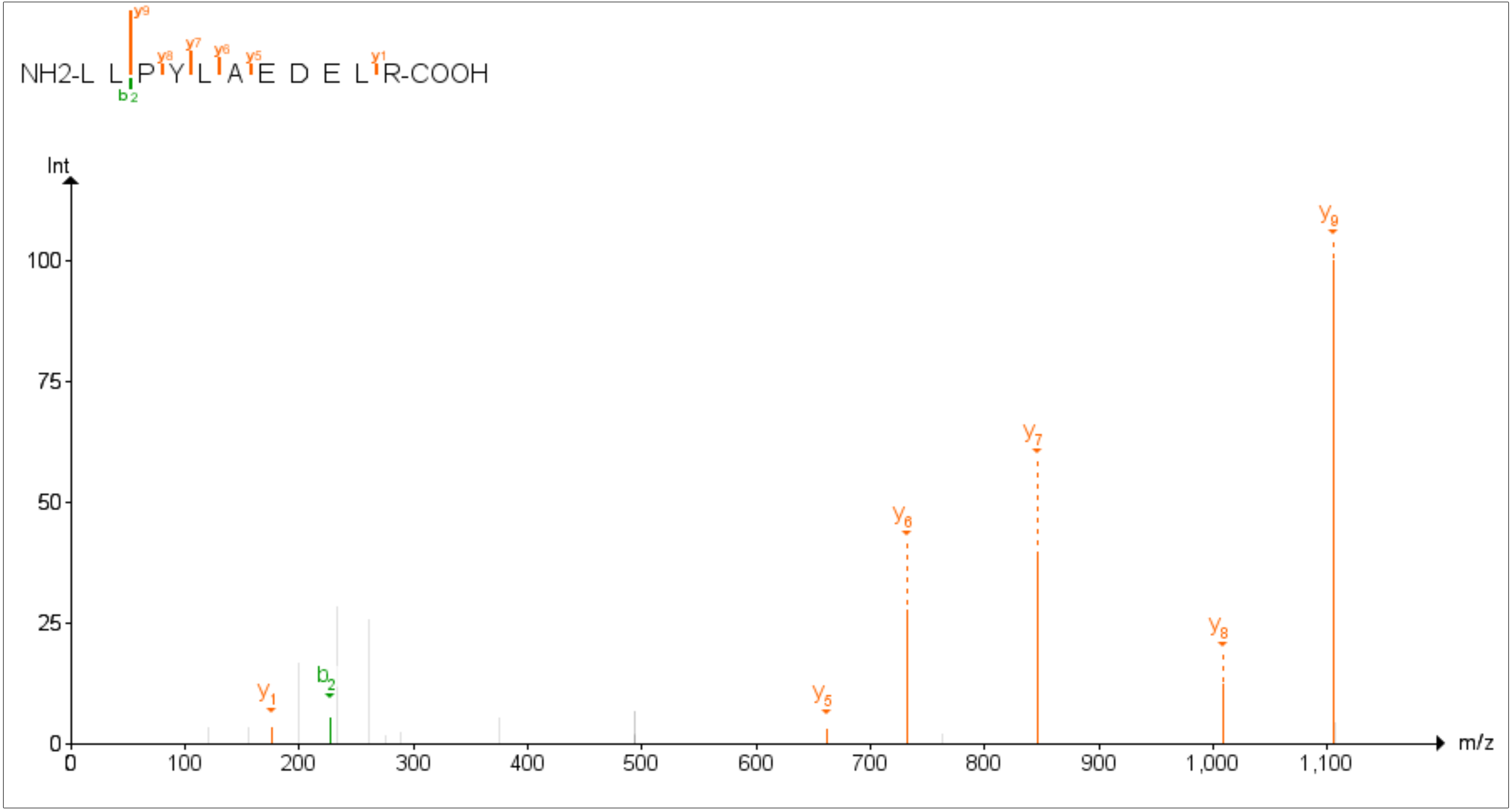
NH2-L L P Y L A E D E L R-COOH
y9
y8
y7
y6
y5
y1
b2
Int
100
75
50
25
0
m/z
0
100
200
300
400
500
600
700
800
900
1,000
1,100
y1
b2
y5
y6
y7
y8
y9

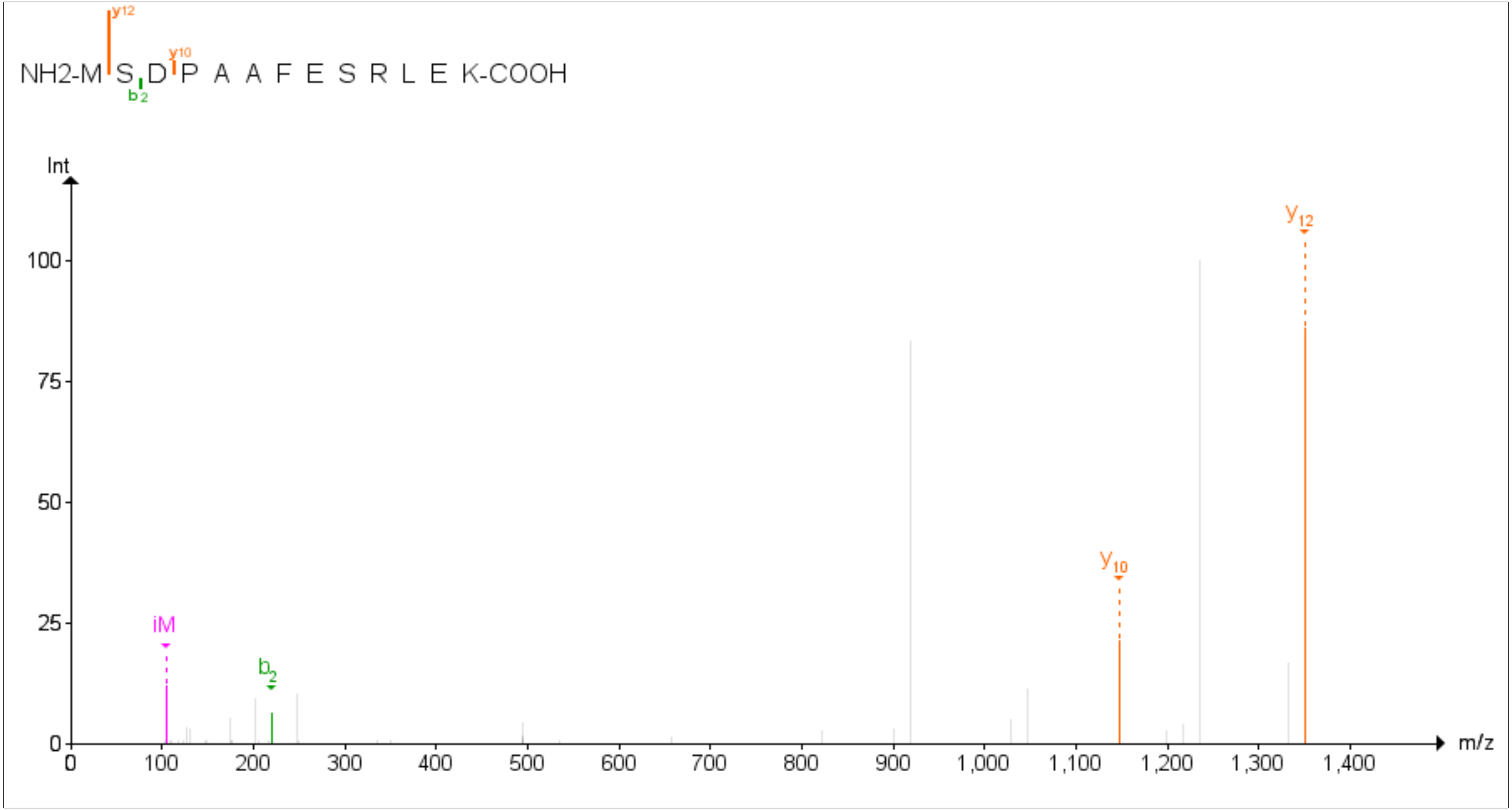

NH2-M S D P A A F E S R L E K-COOH
y12
y10
b2
Int
100
75
50
25
0
iM
b2
y10
y12
m/z
0
100
200
300
400
500
600
700
800
900
1,000
1,100
1,200
1,300
1,400

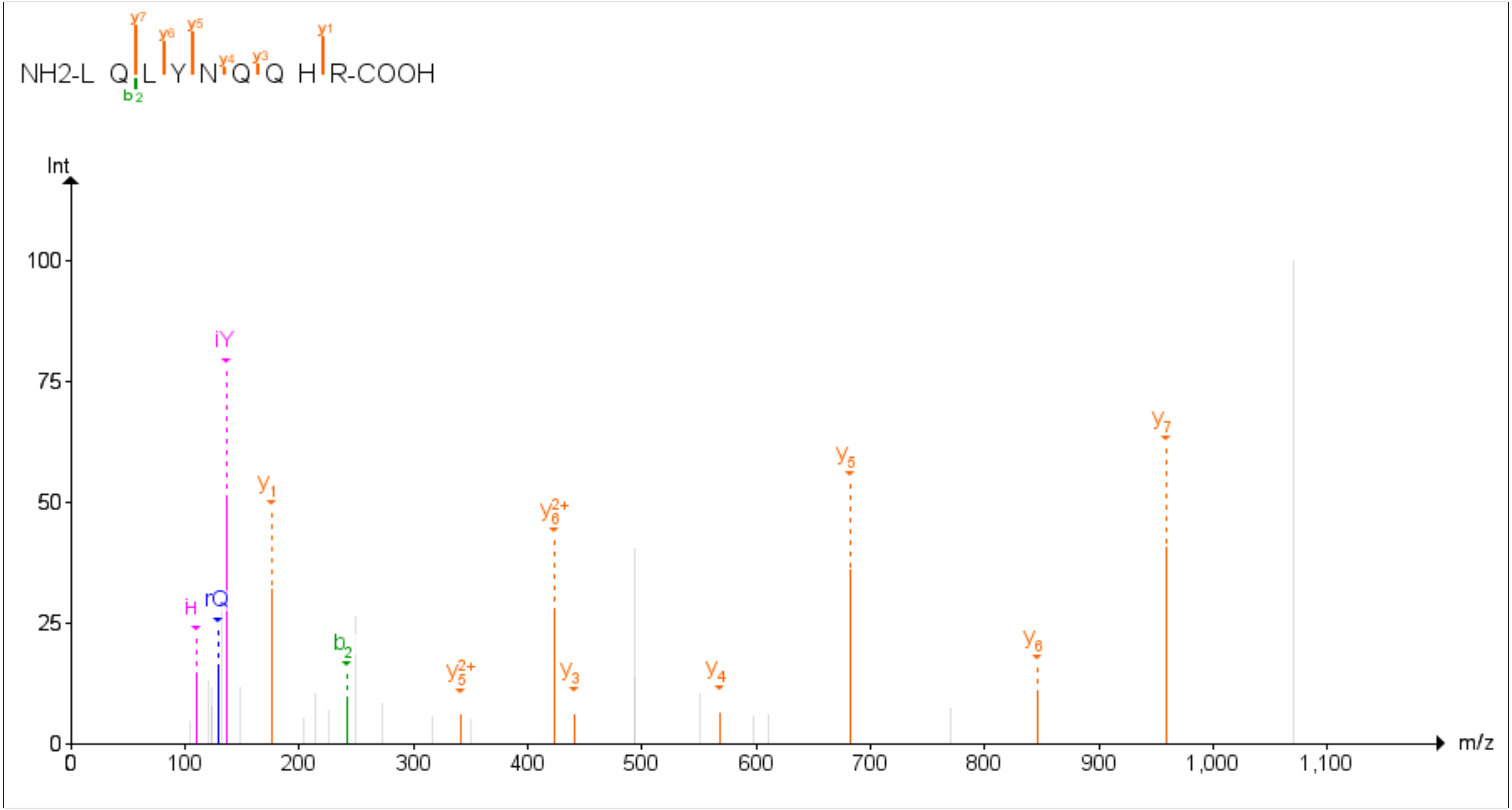

y7
y6
y5
y4
y3
y1
NH2-L Q L Y N Q Q H R-COOH
b2
Int
100
75
50
25
0
iY
y1
y6 2+
y5
y7
iH
rQ
b2
y5 2+
y3
y4
y6
0
100
200
300
400
500
600
700
800
900
1,000
1,100
m/z

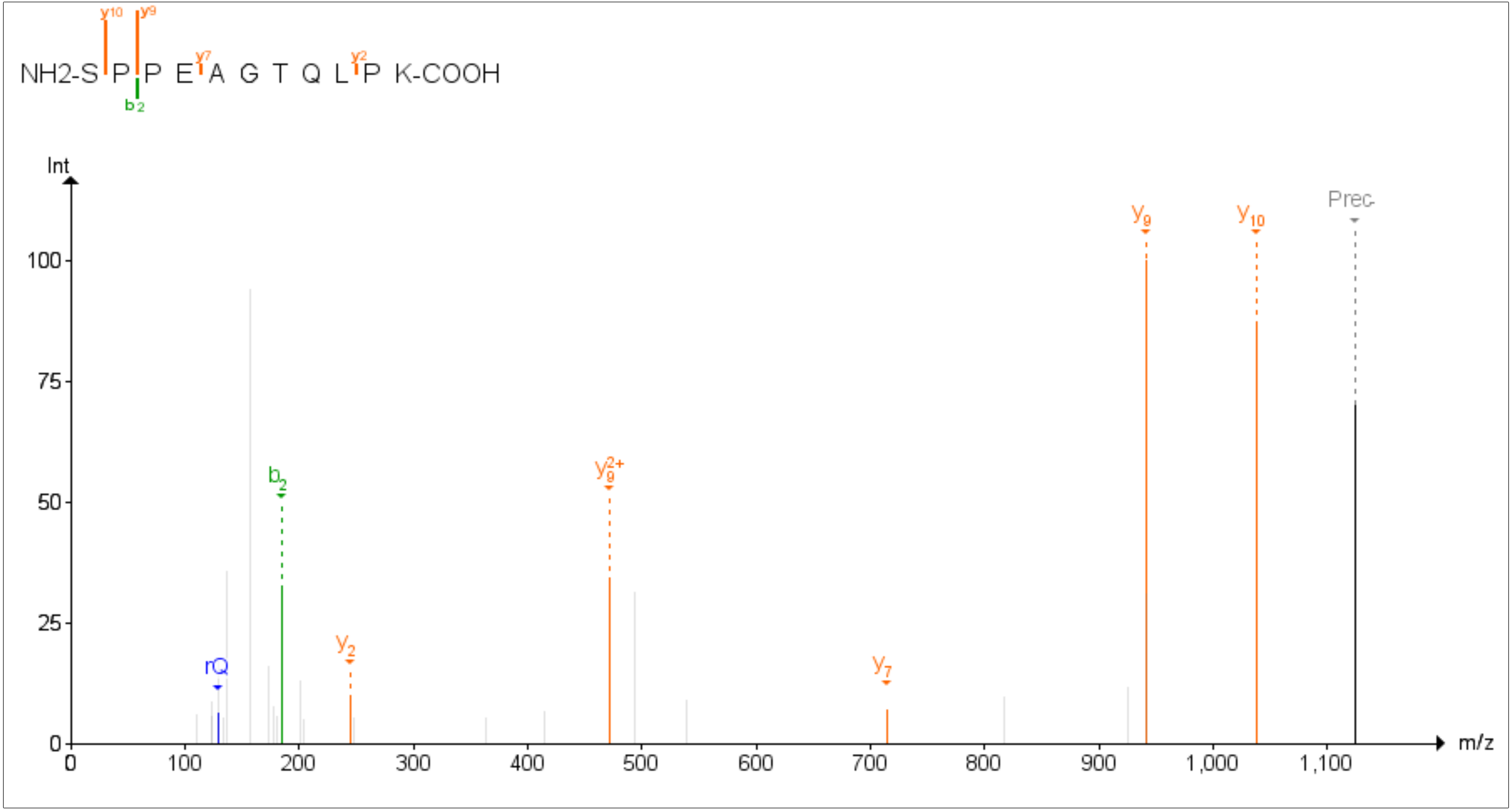

NH2-S P P E A G T Q L P K-COOH
y10
y9
y7
y2
b 2
Int
100
75
50
25
0
m/z
0
100
200
300
400
500
600
700
800
900
1,000
1,100
Prec
y9
y10
y9 2+
b2
y2
rQ
y7

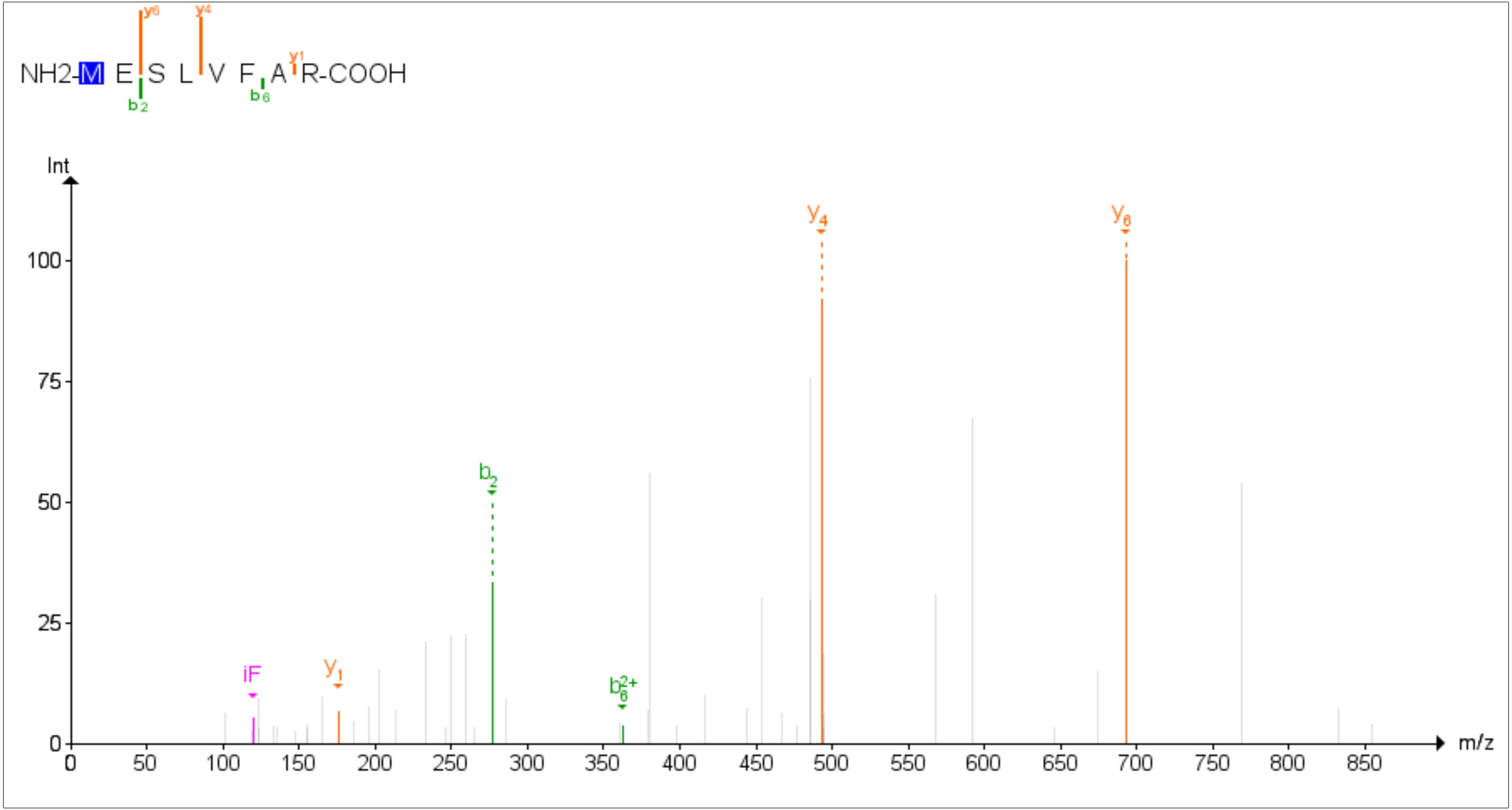

NH2-M E S L V F A R-COOH
y6
y4
y1
b2
b6
Int
100
75
50
25
0
y4
y6
b2
iF
y1
b6 2+
0
50
100
150
200
250
300
350
400
450
500
550
600
650
700
750
800
850
m/z

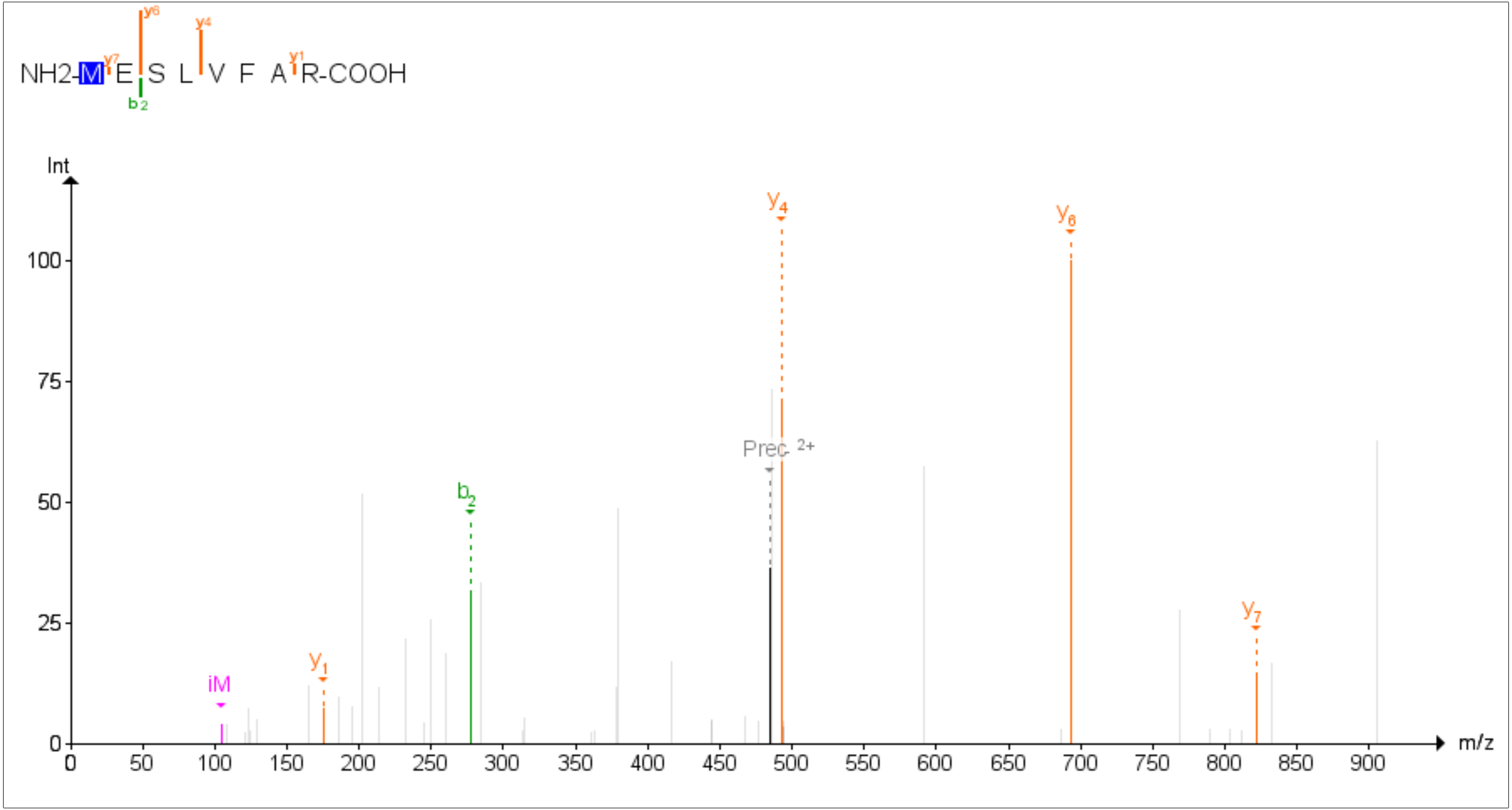

NH2-M E S L V F A R-COOH
y8
y4
y7
y1
b2
Int
100
75
50
25
0
y4
y6
Prec 2+
b2
y7
y1
iM
0 50 100 150 200 250 300 350 400 450 500 550 600 650 700 750 800 850 900
m/z

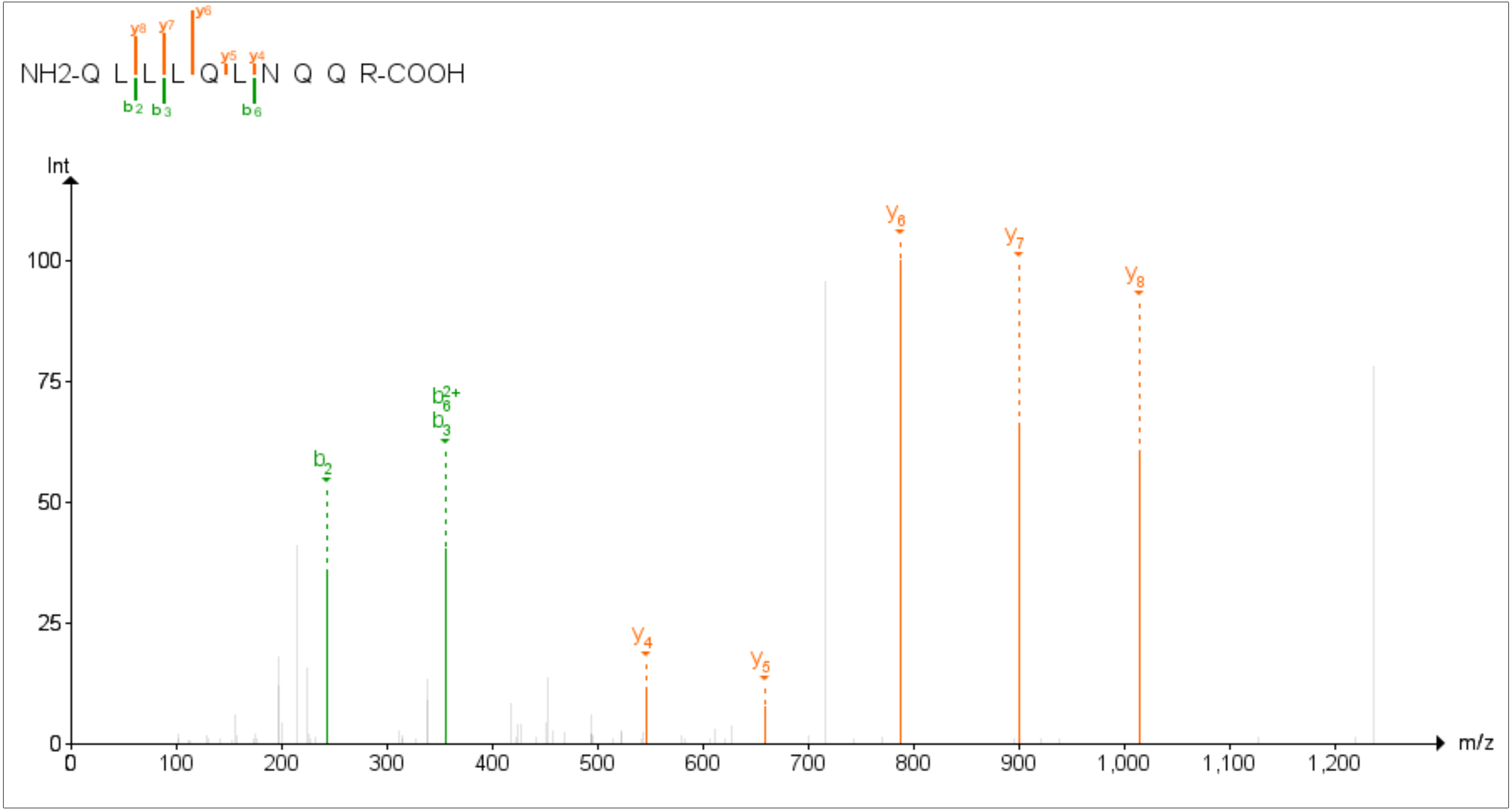
NH2-Q L L L Q L N Q Q R-COOH
y8 y7 y6 y5 y4
b2 b3 b6
Int
100
75
50
25
0
b2
b6 2+
b3
y4
y5
y6
y7
y8
0 100 200 300 400 500 600 700 800 900 1,000 1,100 1,200
m/z

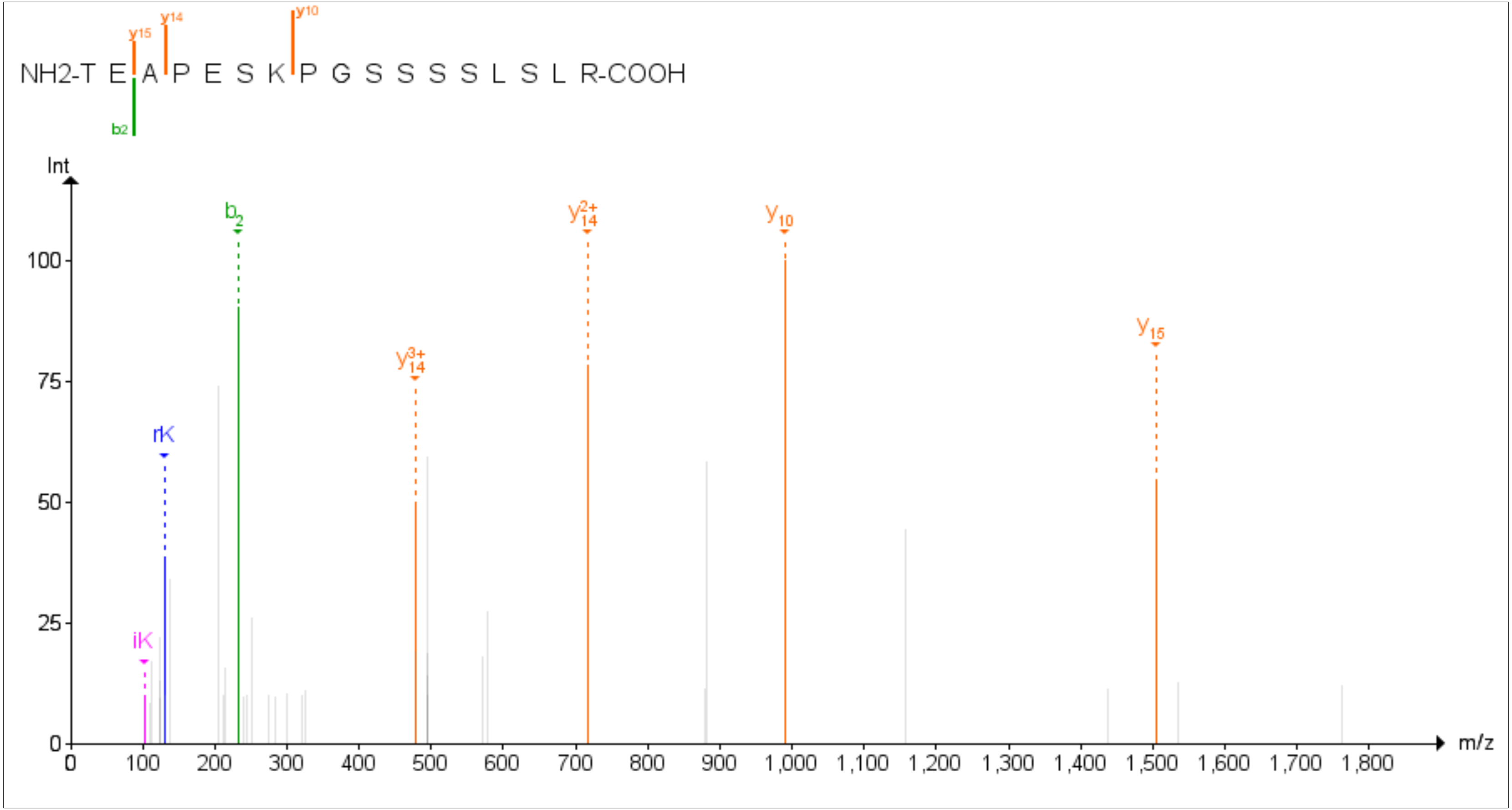

NH2-T E A P E S K P G S S S S L S L R-COOH
y15
y14
y10
b2
Int
100
75
50
25
0
b2
y14 2+
y10
y14 3+
y15
rK
iK
m/z
0 100 200 300 400 500 600 700 800 900 1,000 1,100 1,200 1,300 1,400 1,500 1,600 1,700 1,800

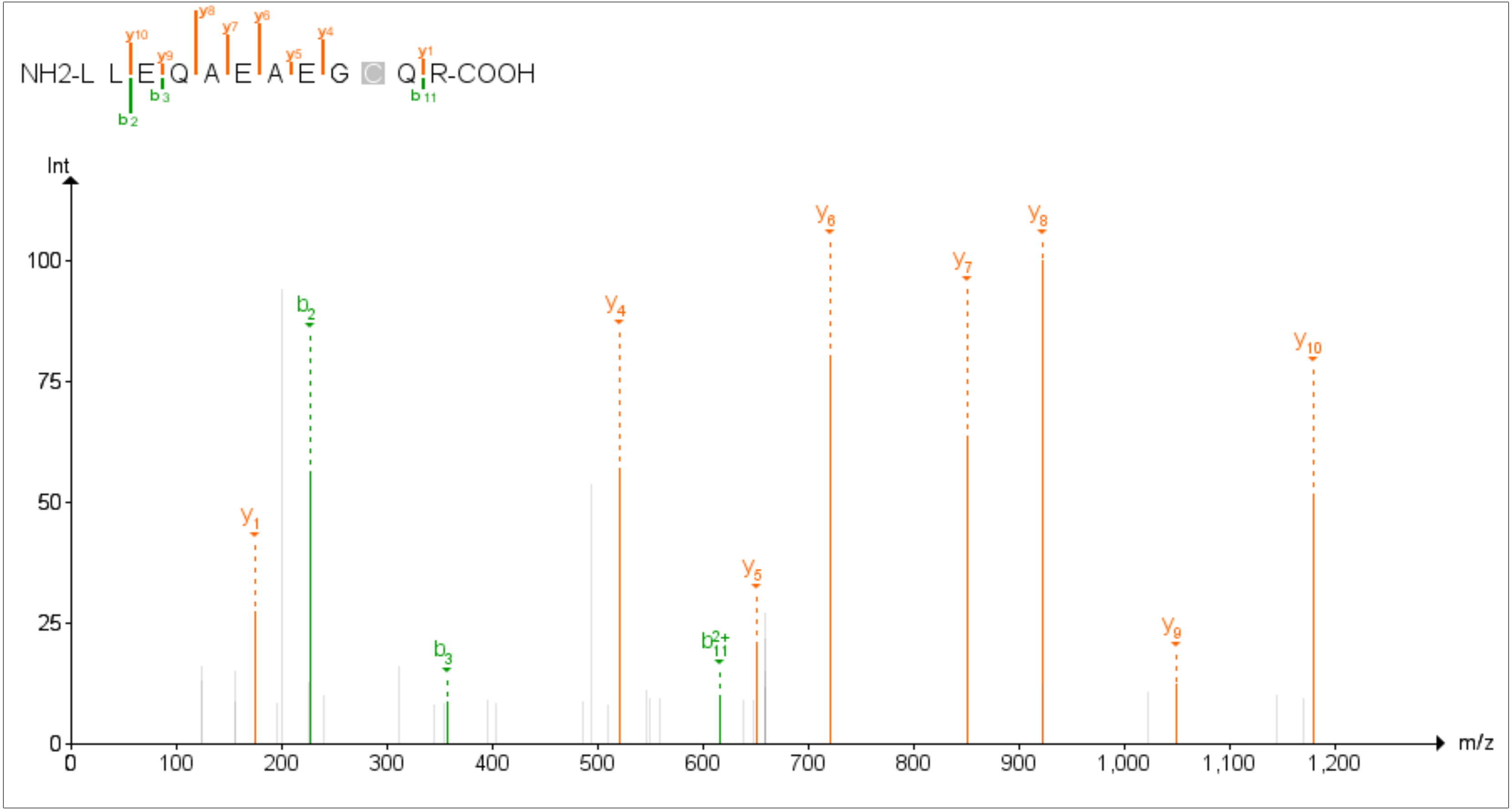
NH2-L L E Q A E A E G C Q R-COOH
y10 y9 y8 y7 y6 y5 y4 y1
b2 b3 b11
Int
100
75
50
25
0
m/z
0 100 200 300 400 500 600 700 800 900 1,000 1,100 1,200
y1 b2 b3 y4 b11 2+ y5 y6 y7 y8 y9 y10

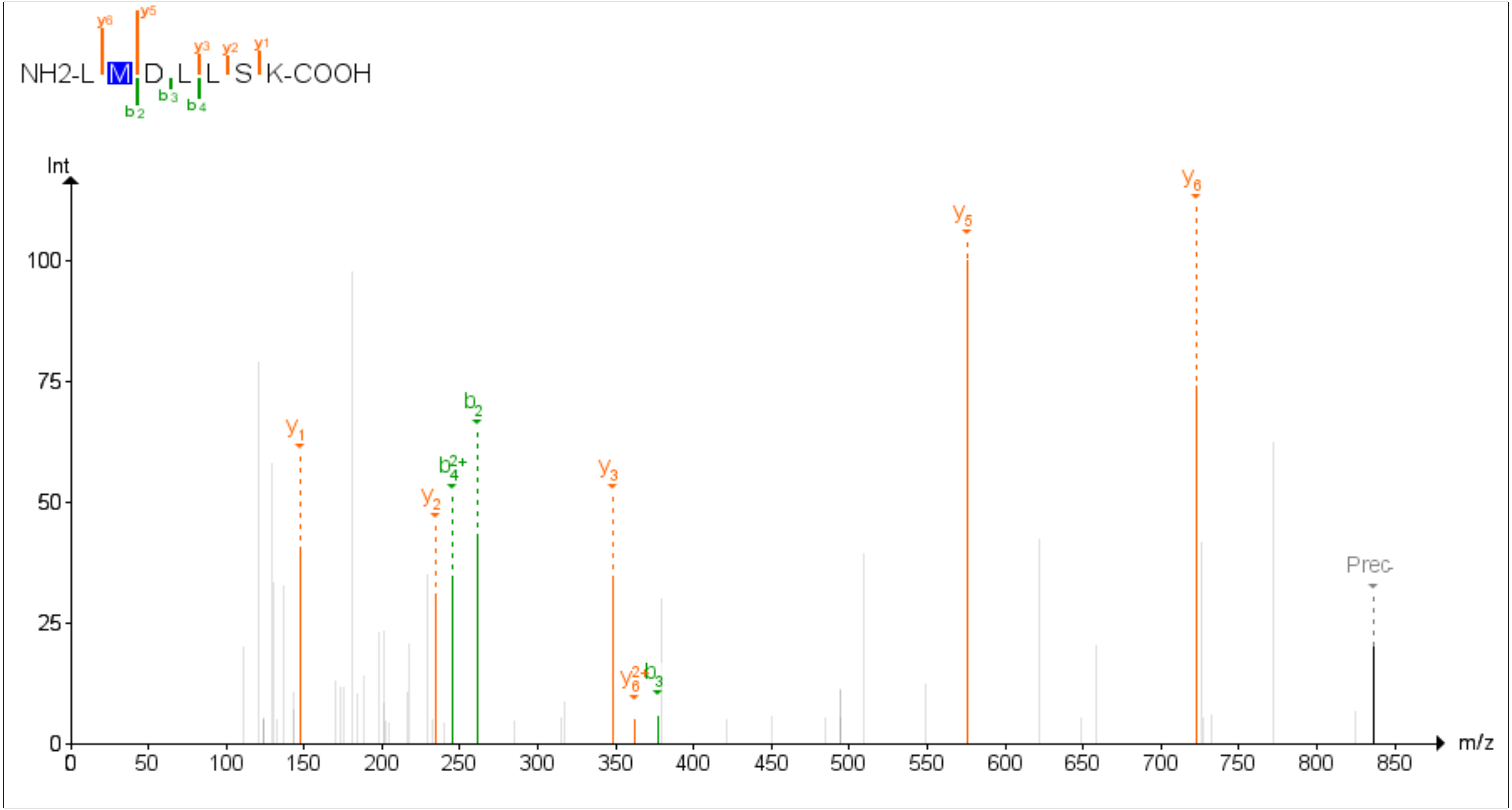

NH2-L M D L L S K-COOH
y6
y5
y3
y2
y1
b2
b3
b4
Int
100
75
50
25
0
y1
b2
b4 2+
y2
y3
y6 2+
b3
y5
y6
Prec
0
50
100
150
200
250
300
350
400
450
500
550
600
650
700
750
800
850
m/z

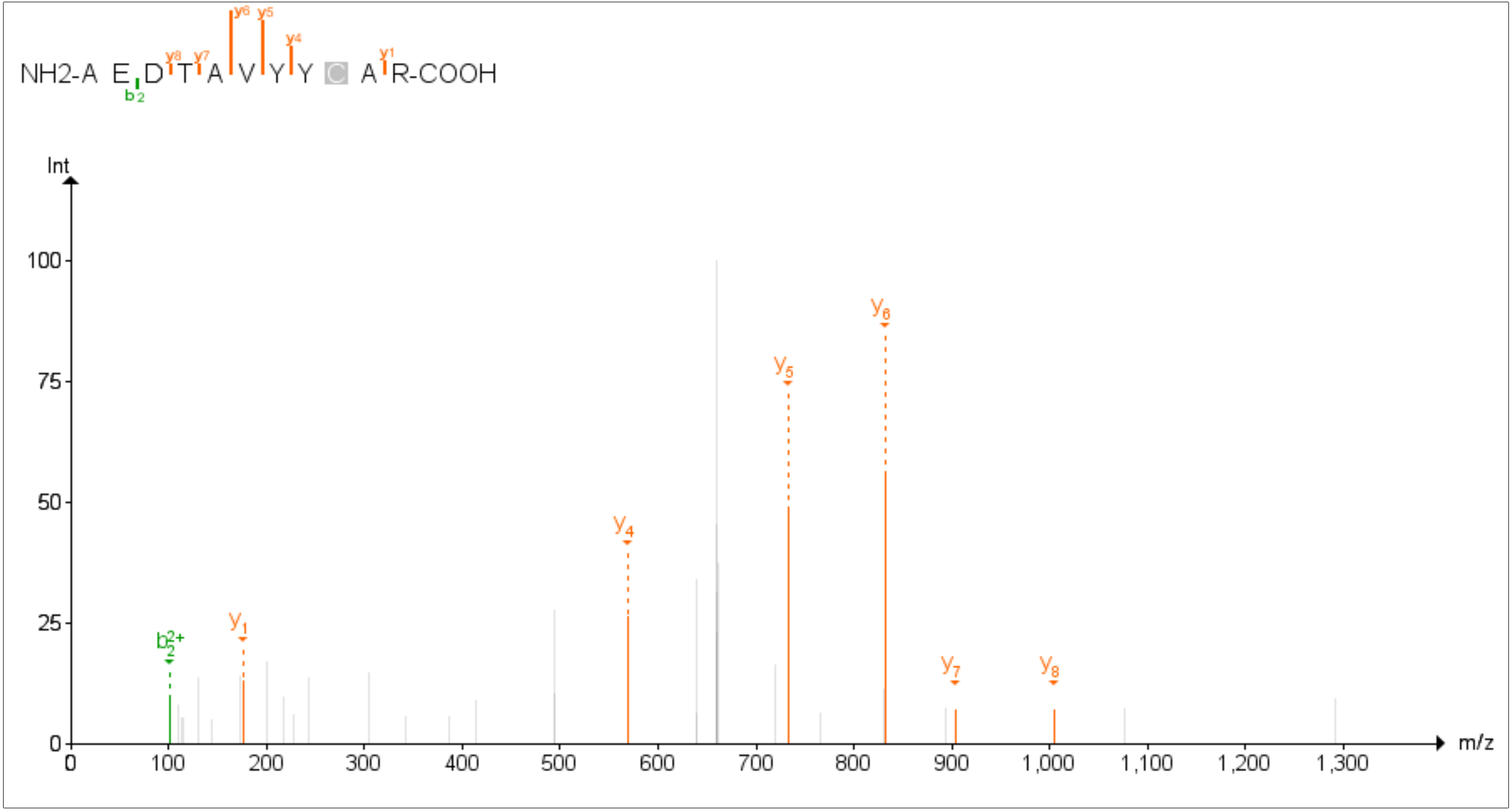
NH2-A E D T A V Y Y C A R-COOH
b2
y8
y7
y6
y5
y4
y1
Int
100
75
50
25
0
m/z
0
100
200
300
400
500
600
700
800
900
1,000
1,100
1,200
1,300
b2 2+
y1
y4
y5
y6
y7
y8

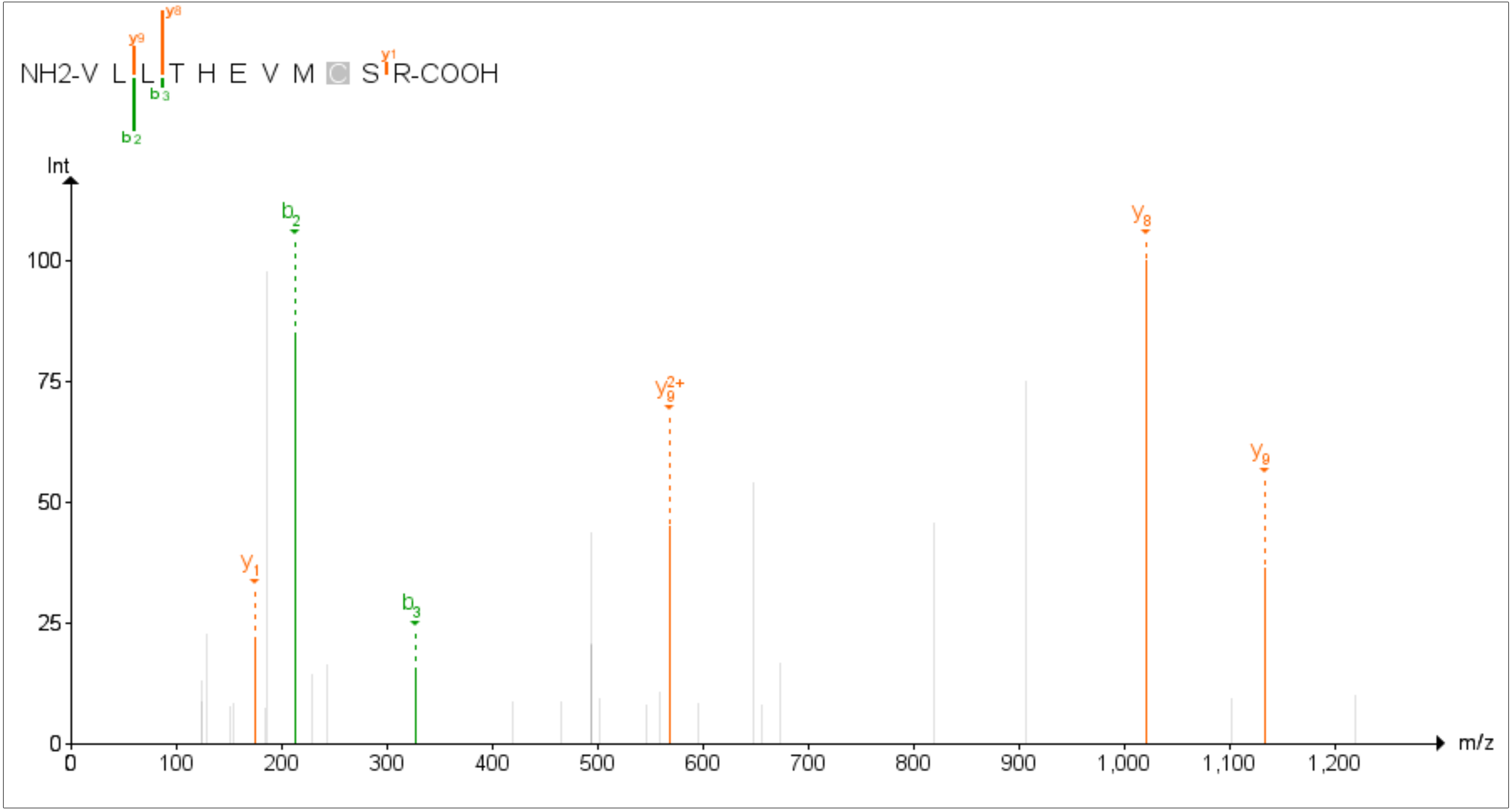

NH2-V L L T H E V M C S R-COOH
y9
y8
y1
b2
b3
Int
100
75
50
25
0
m/z
0
100
200
300
400
500
600
700
800
900
1,000
1,100
1,200
b_2
y_8
y_9^{2+}
y_9
y_1
b_3

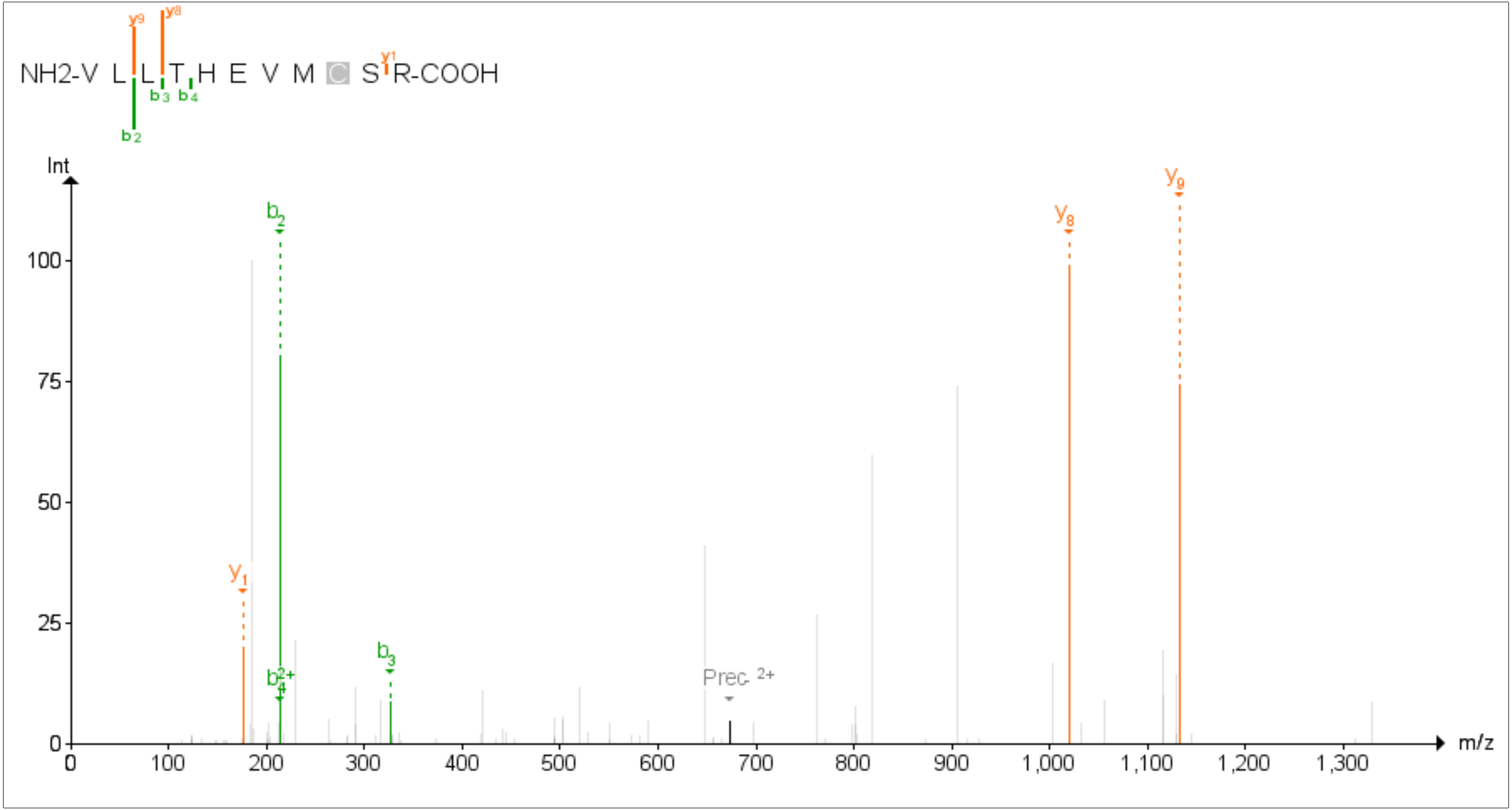

NH2-V L L T H E V M C S R-COOH
y9
y8
y1
b2
b3
b4
Int
100
75
50
25
0
b2
y8
y9
y1
b4 2+
b3
Prec. 2+
m/z
0
100
200
300
400
500
600
700
800
900
1,000
1,100
1,200
1,300

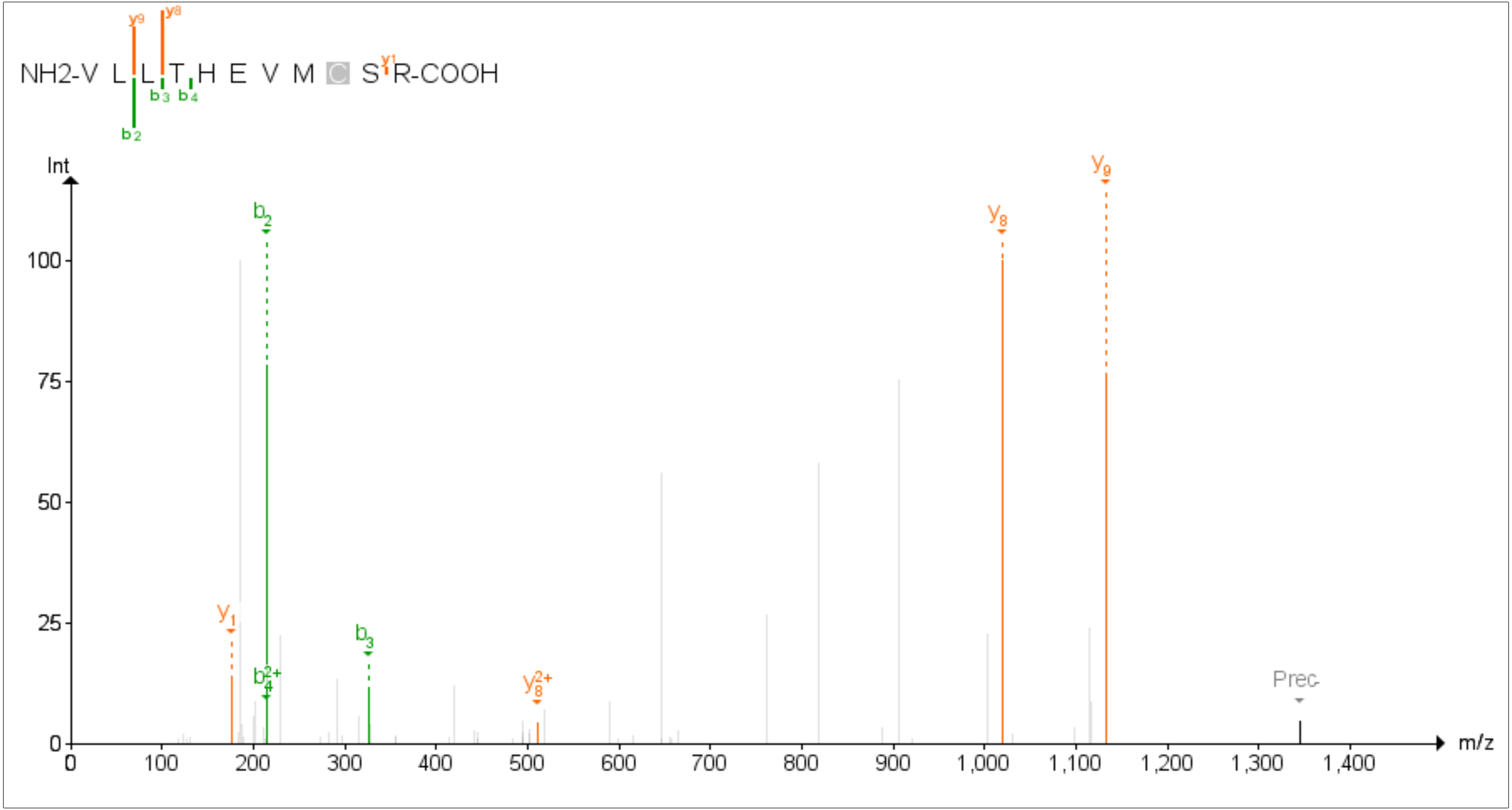

NH2-V L L T H E V M C S R-COOH
y9
y8
y1
b2
b3
b4
Int
100
75
50
25
0
m/z
0
100
200
300
400
500
600
700
800
900
1,000
1,100
1,200
1,300
1,400
y1
b2
b4 2+
b3
y8 2+
y8
y9
Prec

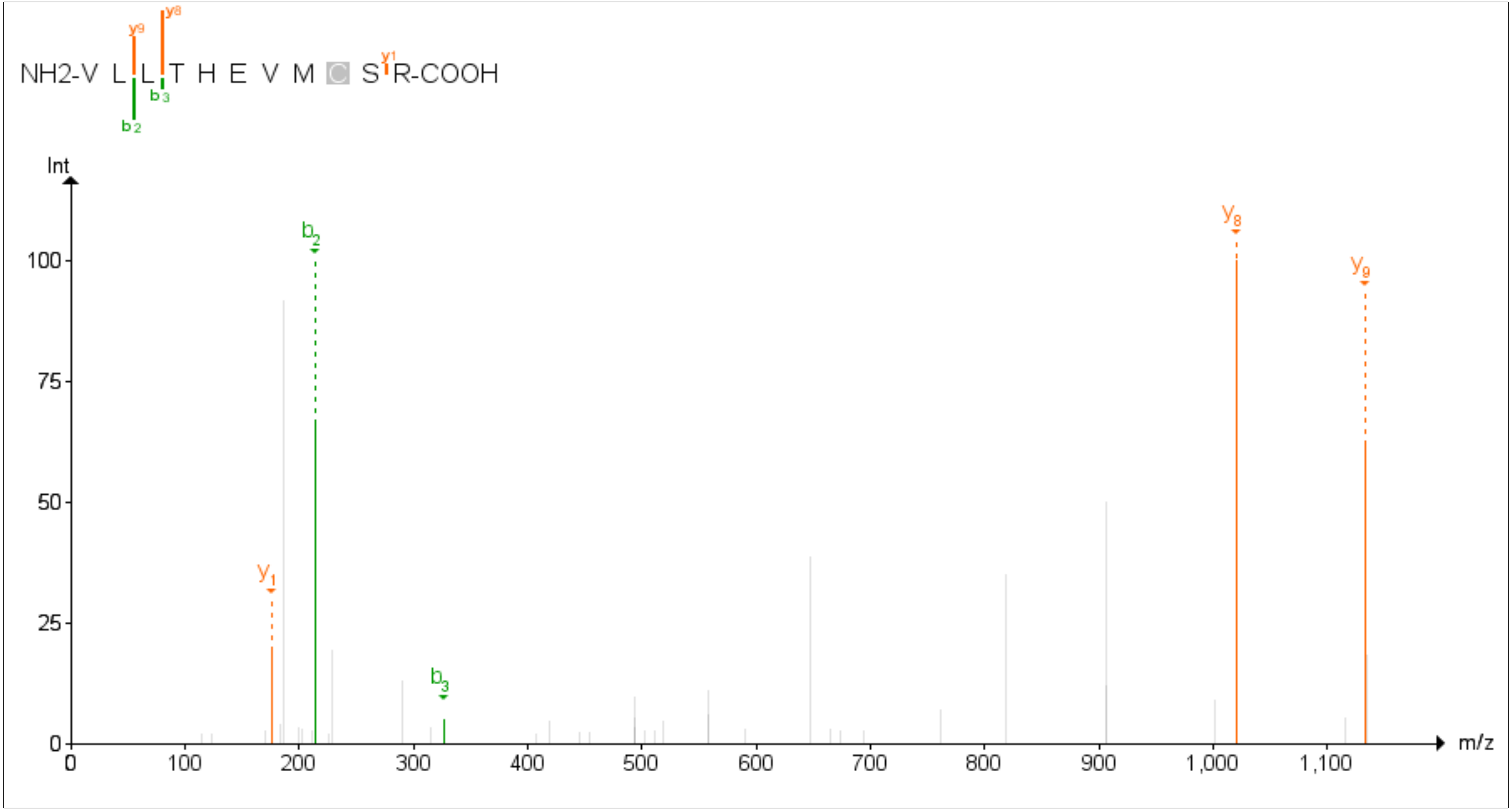

NH2-V L L T H E V M C S R-COOH
y9
y8
y1
b2
b3
Int
100
75
50
25
0
y8
y9
y1
b2
b3
0
100
200
300
400
500
600
700
800
900
1,000
1,100
m/z

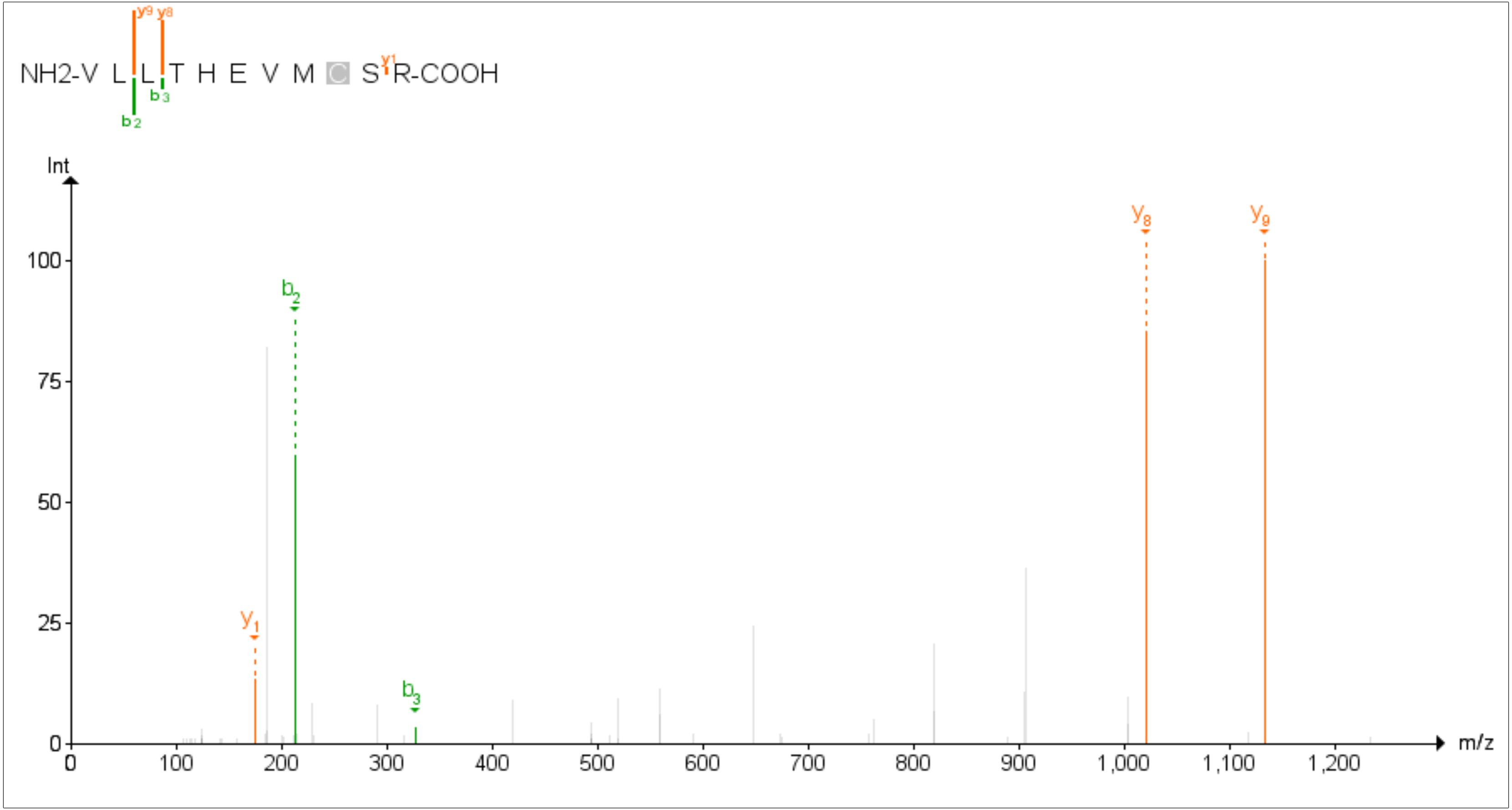

NH2-V L L T H E V M C S R-COOH
y9
y8
y1
b2
b3
Int
100
75
50
25
0
y8
y9
b2
y1
b3
0
100
200
300
400
500
600
700
800
900
1,000
1,100
1,200
m/z

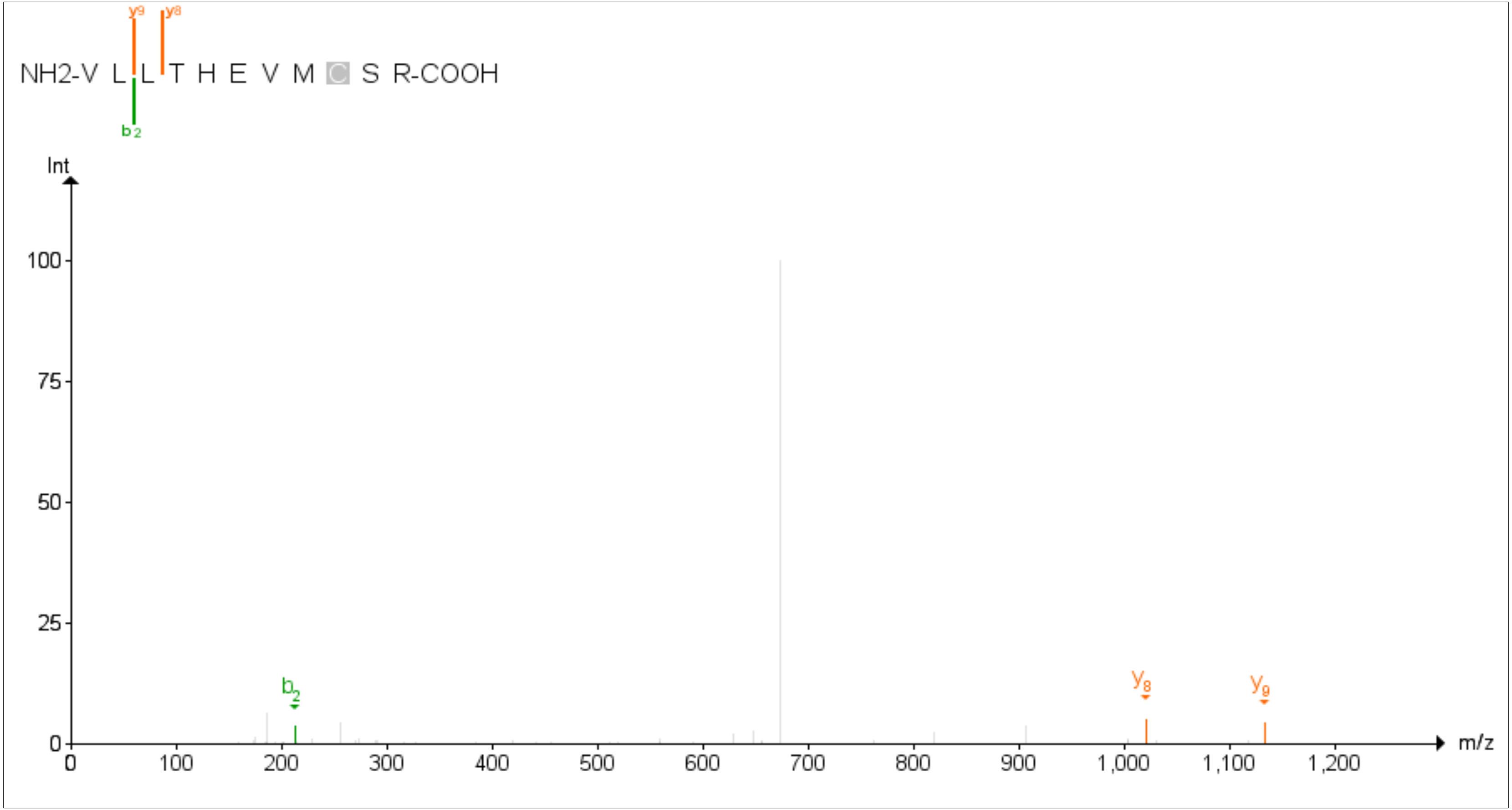
NH2-V L L T H E V M C S R-COOH
y9
y8
b2
Int
100
75
50
25
0
b2
y8
y9
0
100
200
300
400
500
600
700
800
900
1,000
1,100
1,200
m/z

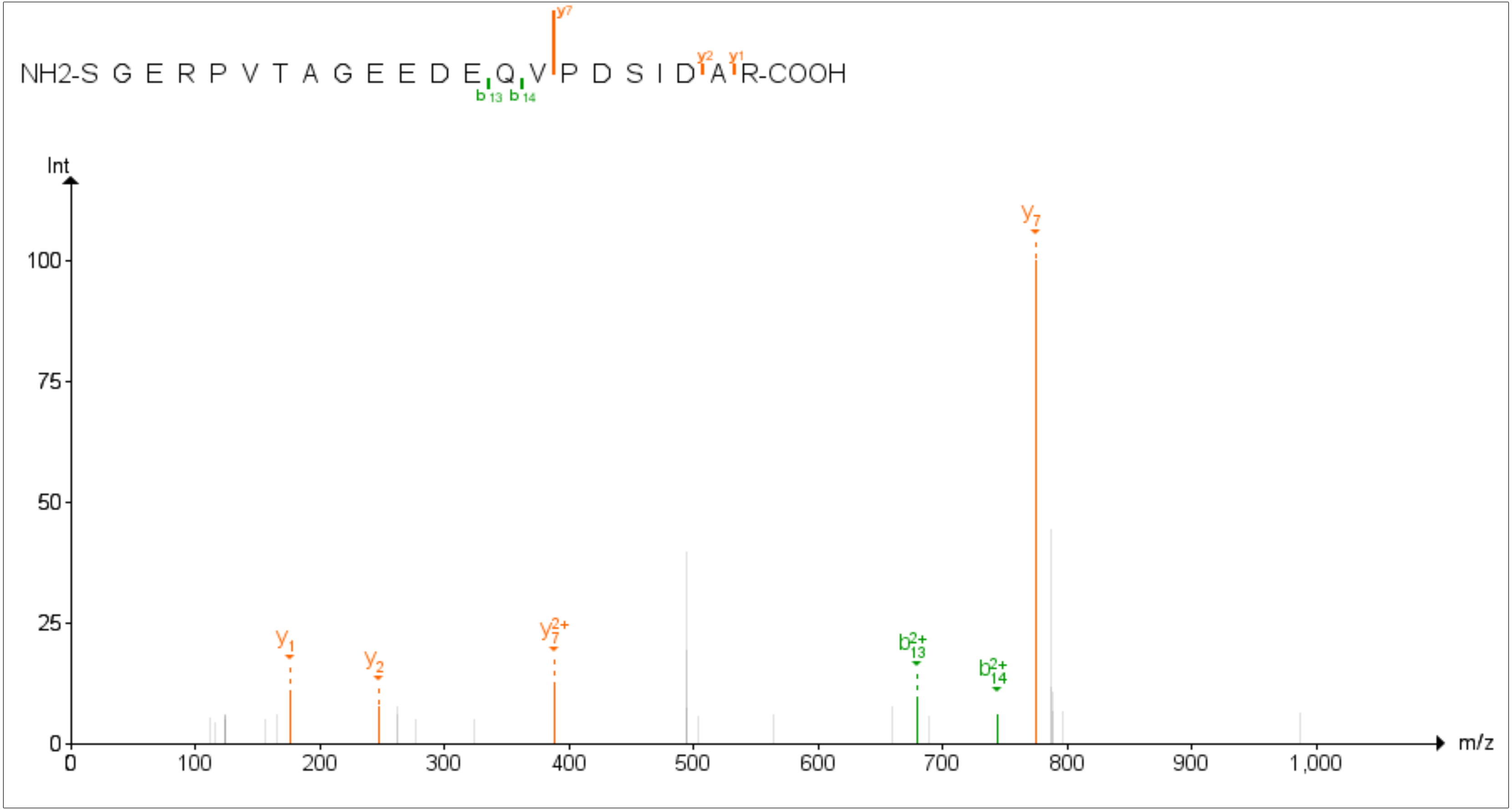

NH2-S G E R P V T A G E E D E Q V P D S I D A R-COOH
y7
y2
y1
b13
b14
Int
100
75
50
25
0
y7
y1
y2
y7 2+
b13 2+
b14 2+
0
100
200
300
400
500
600
700
800
900
1,000
m/z

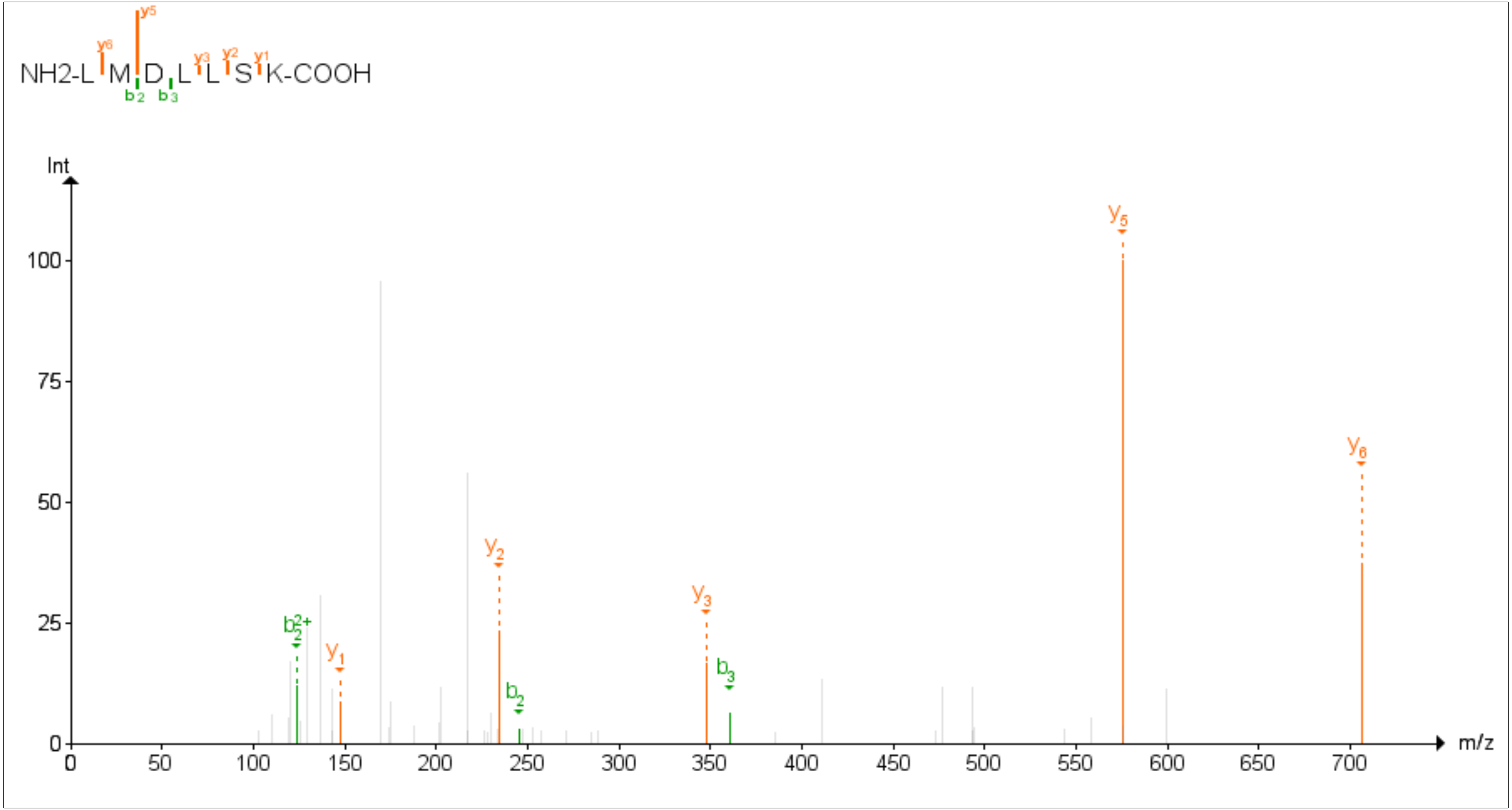

NH2-L M D L L S K-COOH
y6
y5
y3
y2
y1
b2
b3
Int
100
75
50
25
0
y5
y6
y2
y3
b2 2+
y1
b2
b3
0
50
100
150
200
250
300
350
400
450
500
550
600
650
700
m/z

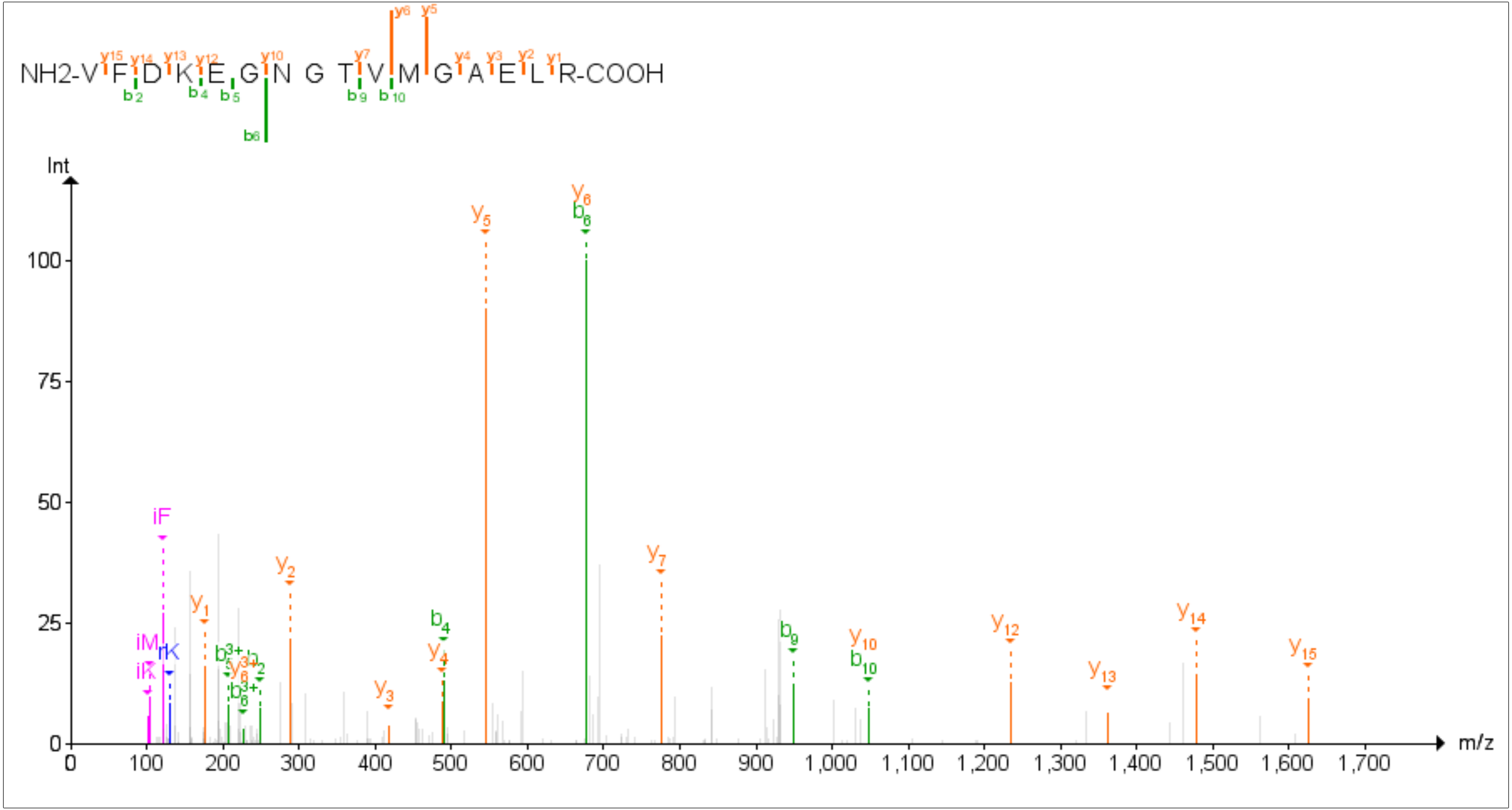

NH2-VFDKEGINGTIVMGAELR-COOH
y15 y14 y13 y12 y10 y7 y6 y5 y4 y3 y2 y1
b2 b4 b5 b6 b9 b10
Int
m/z
0
25
50
75
100
0 100 200 300 400 500 600 700 800 900 1,000 1,100 1,200 1,300 1,400 1,500 1,600 1,700
iF
iM
iK
rK
y1
y2
y3
y4
y5
y6
b6
y7
b4
b9
y10
b10
y12
y13
y14
y15

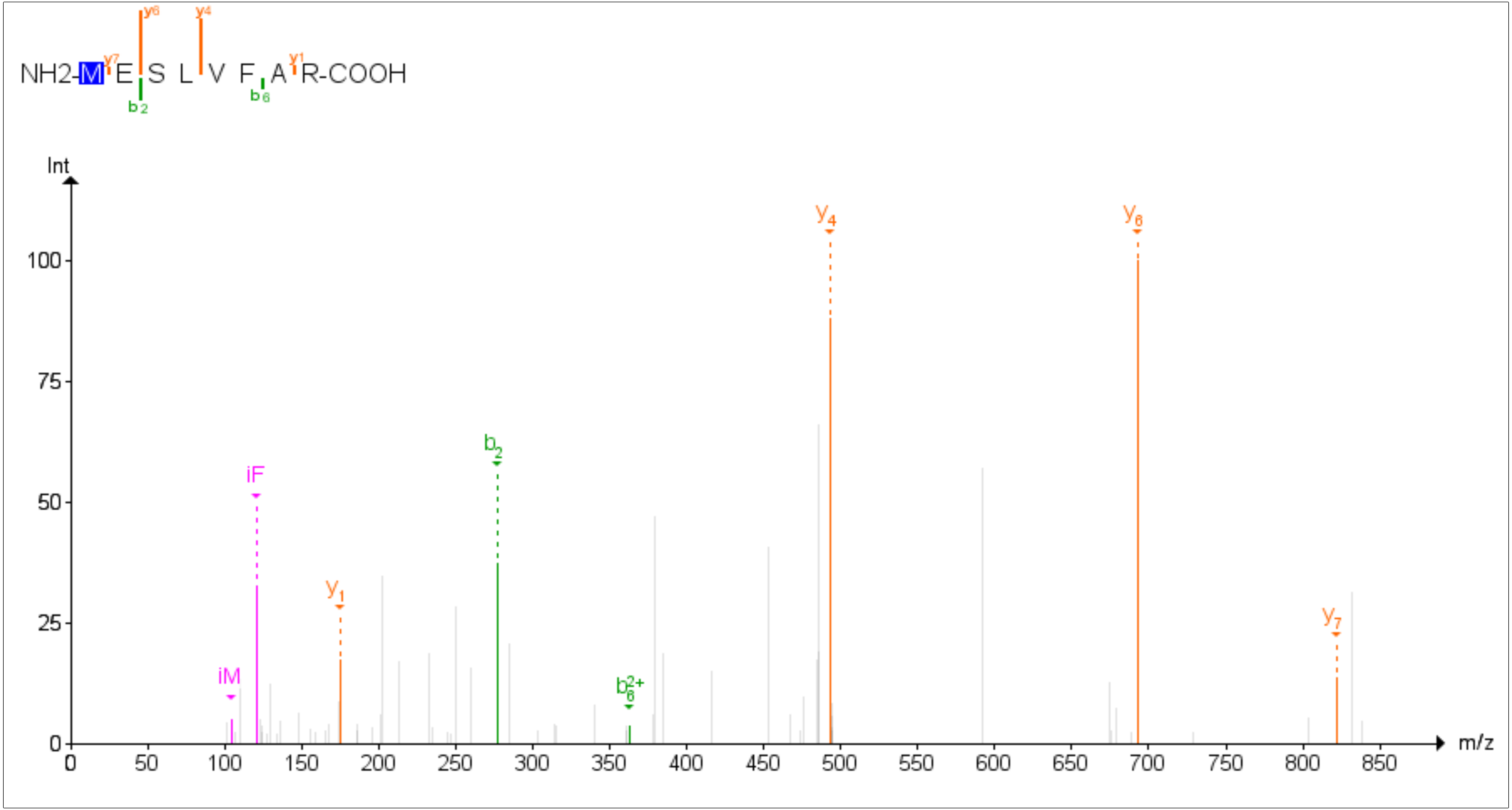

NH2-M E S L V F A R-COOH
y7
y6
y4
y1
b2
b6
Int
100
75
50
25
0
y4
y6
b2
iF
y1
iM
b6 2+
y7
0
50
100
150
200
250
300
350
400
450
500
550
600
650
700
750
800
850
m/z

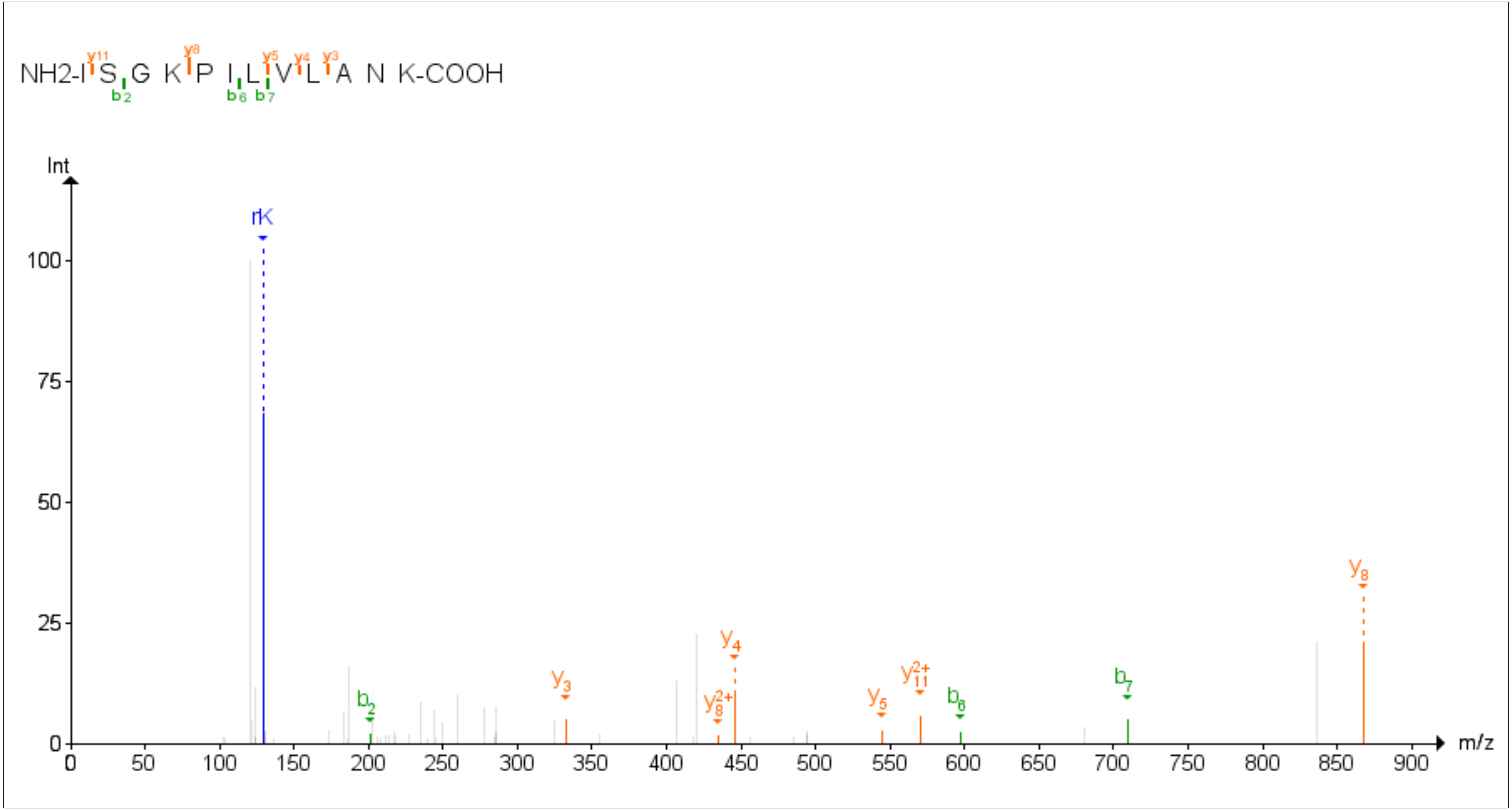

NH2-I S G K P I L V L A N K-COOH
y11
y8
y5
y4
y3
b2
b6
b7
Int
rK
100
75
50
25
0
y8
y4
y3
y8 2+
y5
y11 2+
b2
b6
b7
0
50
100
150
200
250
300
350
400
450
500
550
600
650
700
750
800
850
900
m/z

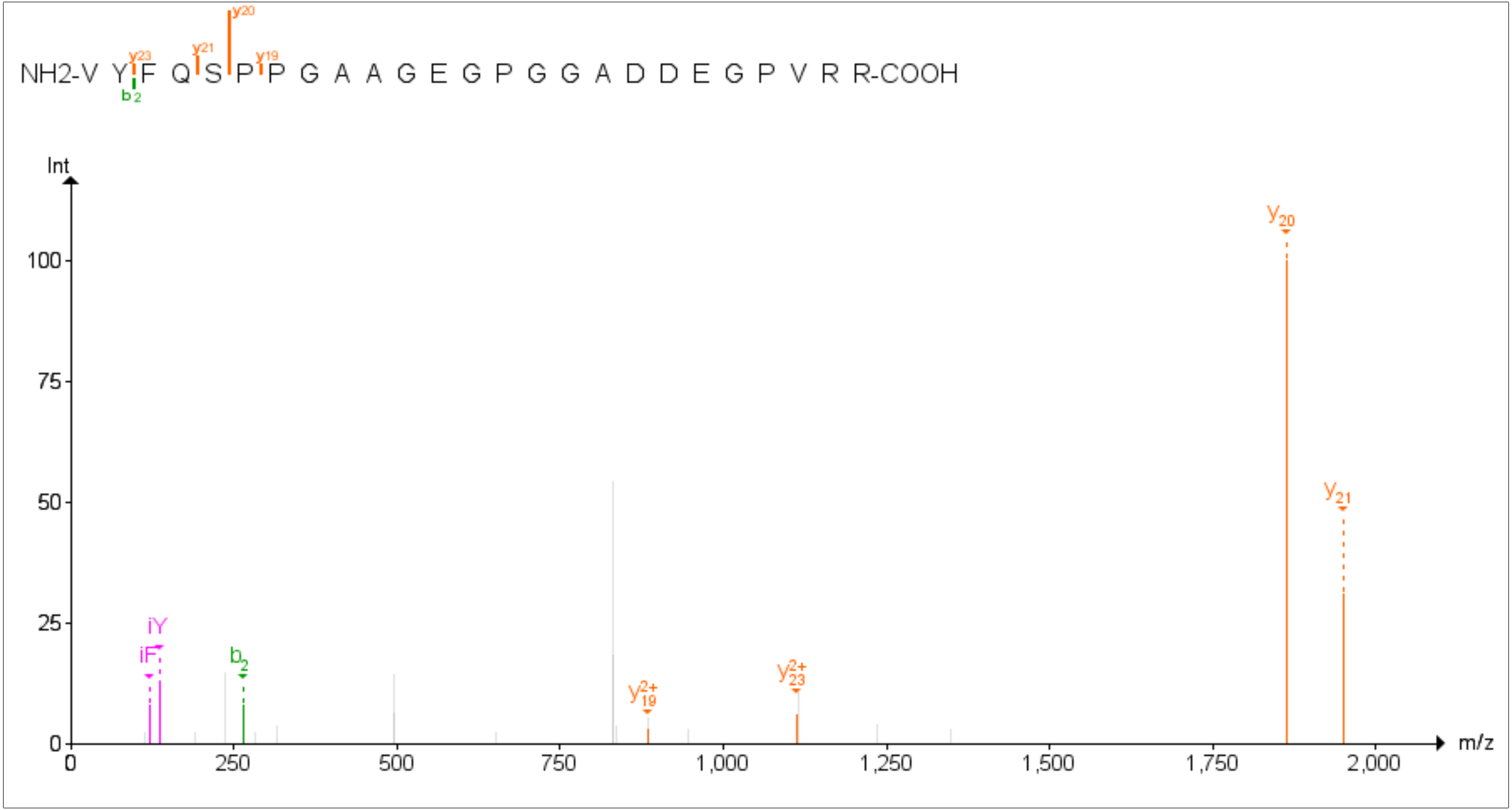

NH2-V Y F Q S P P G A A G E G P G G A D D E G P V R R-COOH
y23
y21
y20
y19
b2
Int
100
75
50
25
0
iY
iF
b2
y19 2+
y23 2+
y20
y21
0
250
500
750
1,000
1,250
1,500
1,750
2,000
m/z

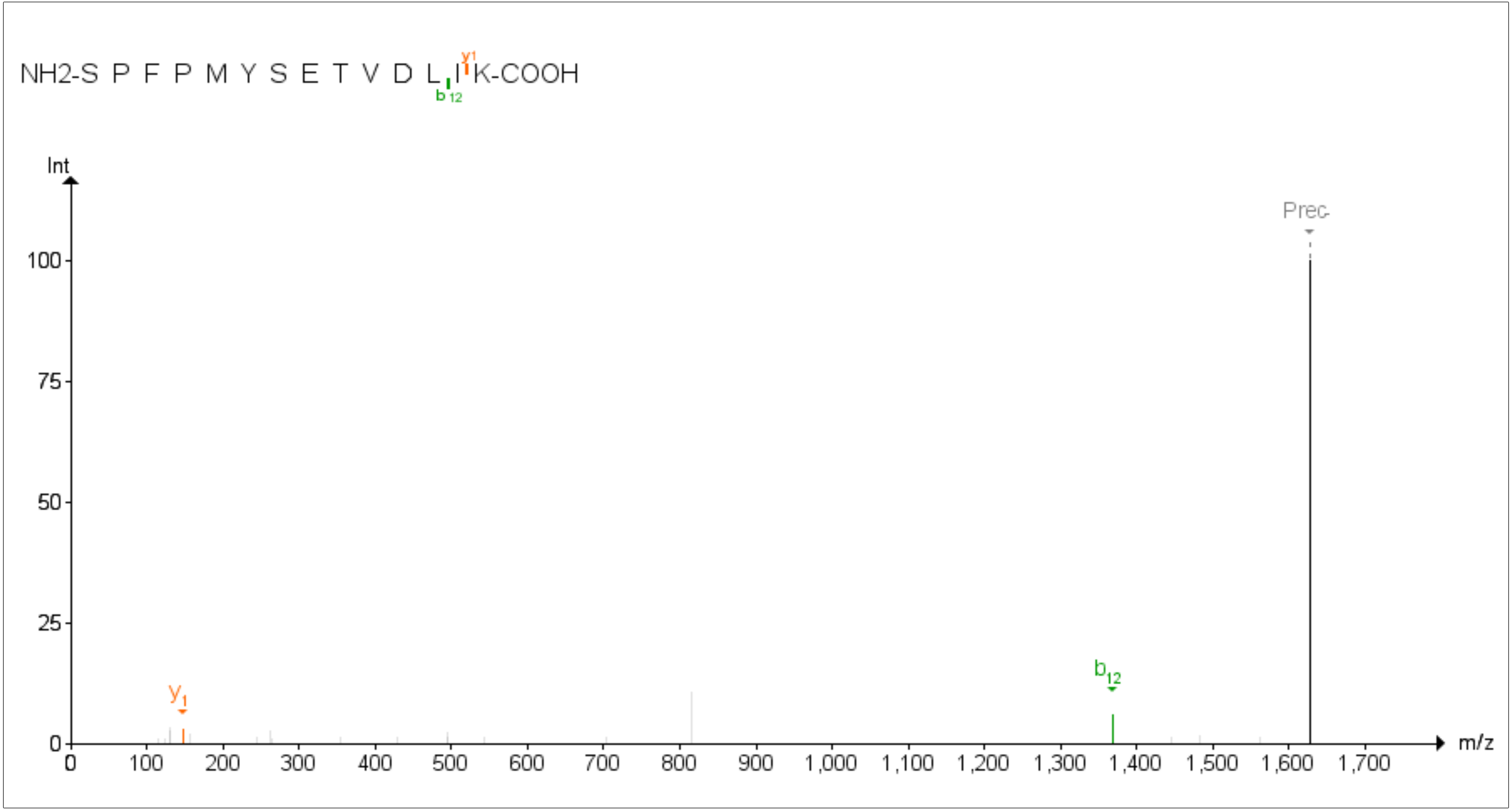

NH2-S P F P M Y S E T V D L I K-COOH
y1
b12
Int
Prec
100
75
50
25
0
y1
b12
0
100
200
300
400
500
600
700
800
900
1,000
1,100
1,200
1,300
1,400
1,500
1,600
1,700
m/z

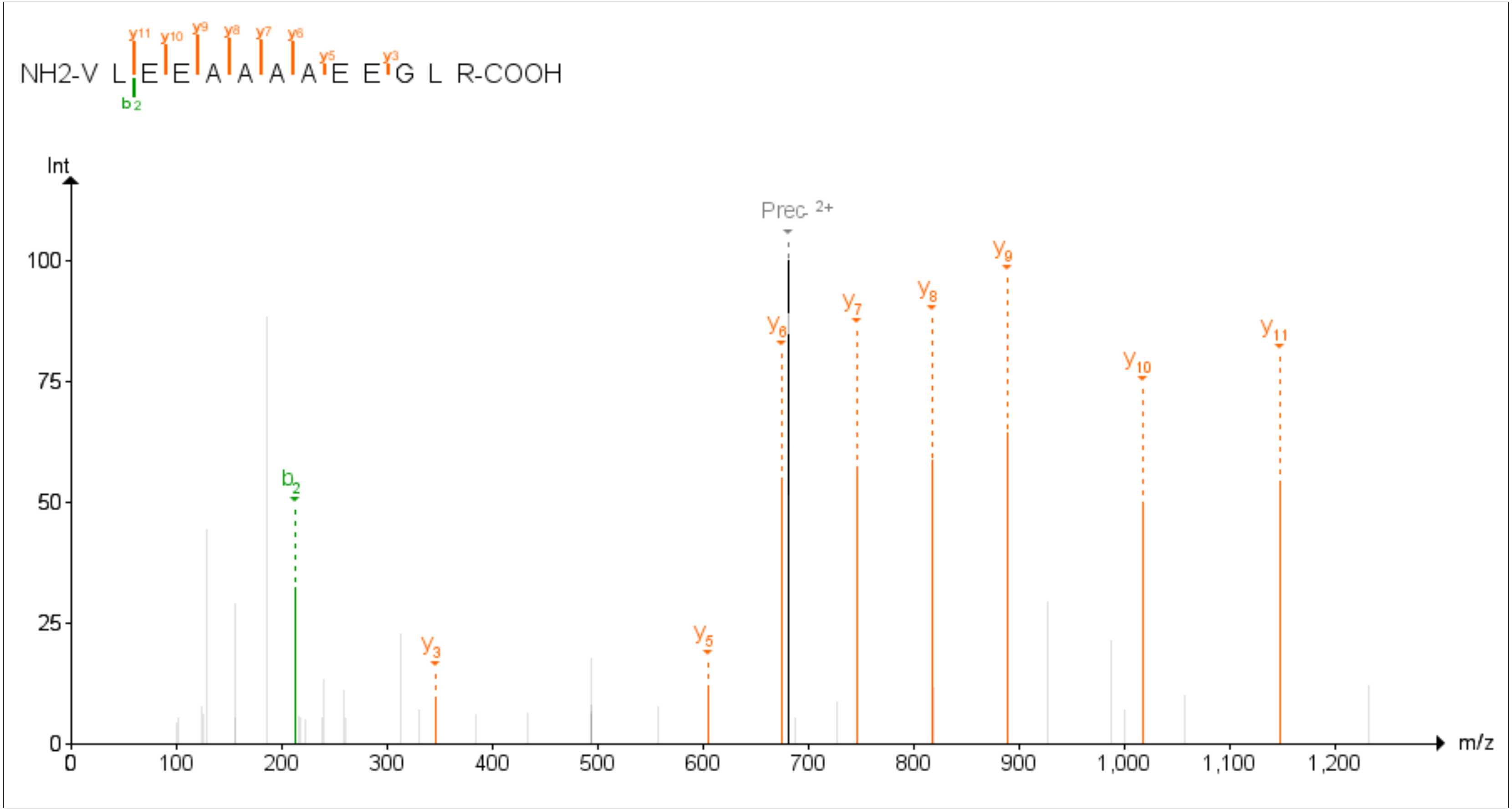

NH2-V L E E A A A A E E G L R-COOH
y11 y10 y9 y8 y7 y6 y5 y3
b2
Int
Prec 2+
y9
y8
y7
y6
y11
y10
b2
y3
y5
100
75
50
25
0
0 100 200 300 400 500 600 700 800 900 1,000 1,100 1,200
m/z

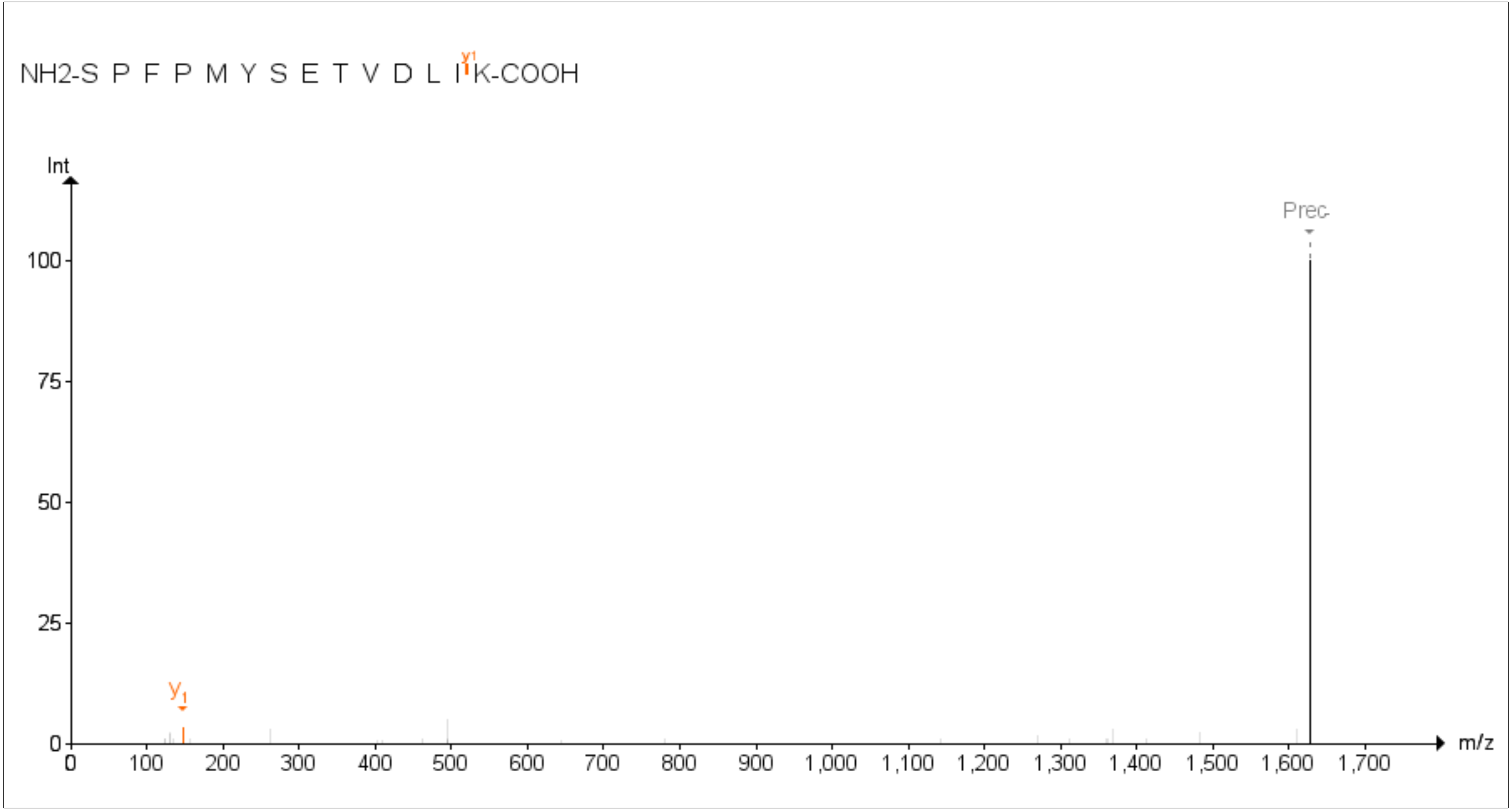

NH2-S P F P M Y S E T V D L I K-COOH
y1
Int
Prec-
100
75
50
25
0
y1
0
100
200
300
400
500
600
700
800
900
1,000
1,100
1,200
1,300
1,400
1,500
1,600
1,700
m/z

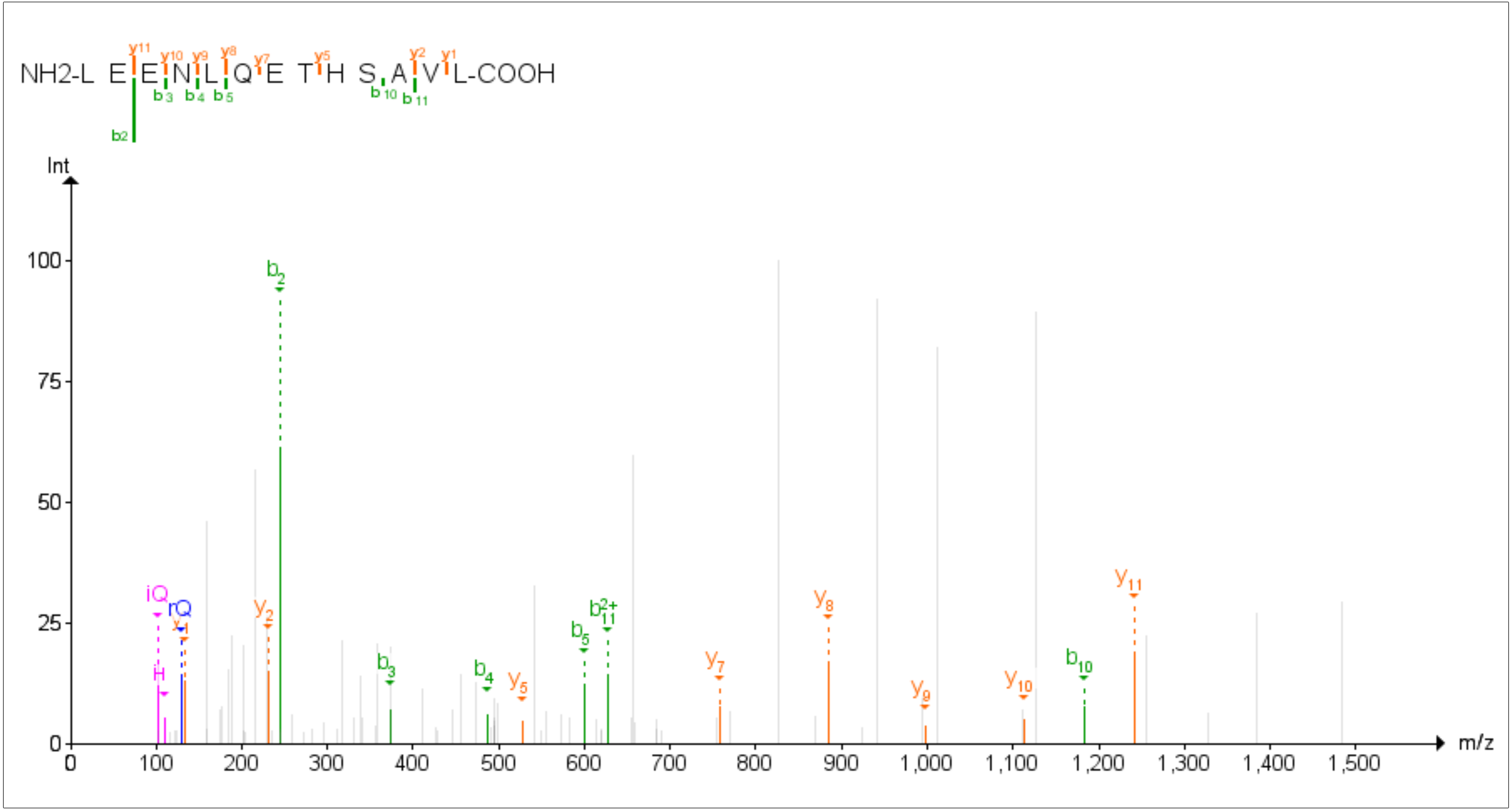

NH2-L E E N L Q E T H S A V L-COOH
y11 y10 y9 y8 y7 y5 y2 y1
b2 b3 b4 b5 b10 b11
Int
100
75
50
25
0
iQ
rQ
H
y1
y2
b2
b3
b4
y5
b5
b11 2+
y7
y8
y9
y10
b10
y11
0 100 200 300 400 500 600 700 800 900 1,000 1,100 1,200 1,300 1,400 1,500
m/z

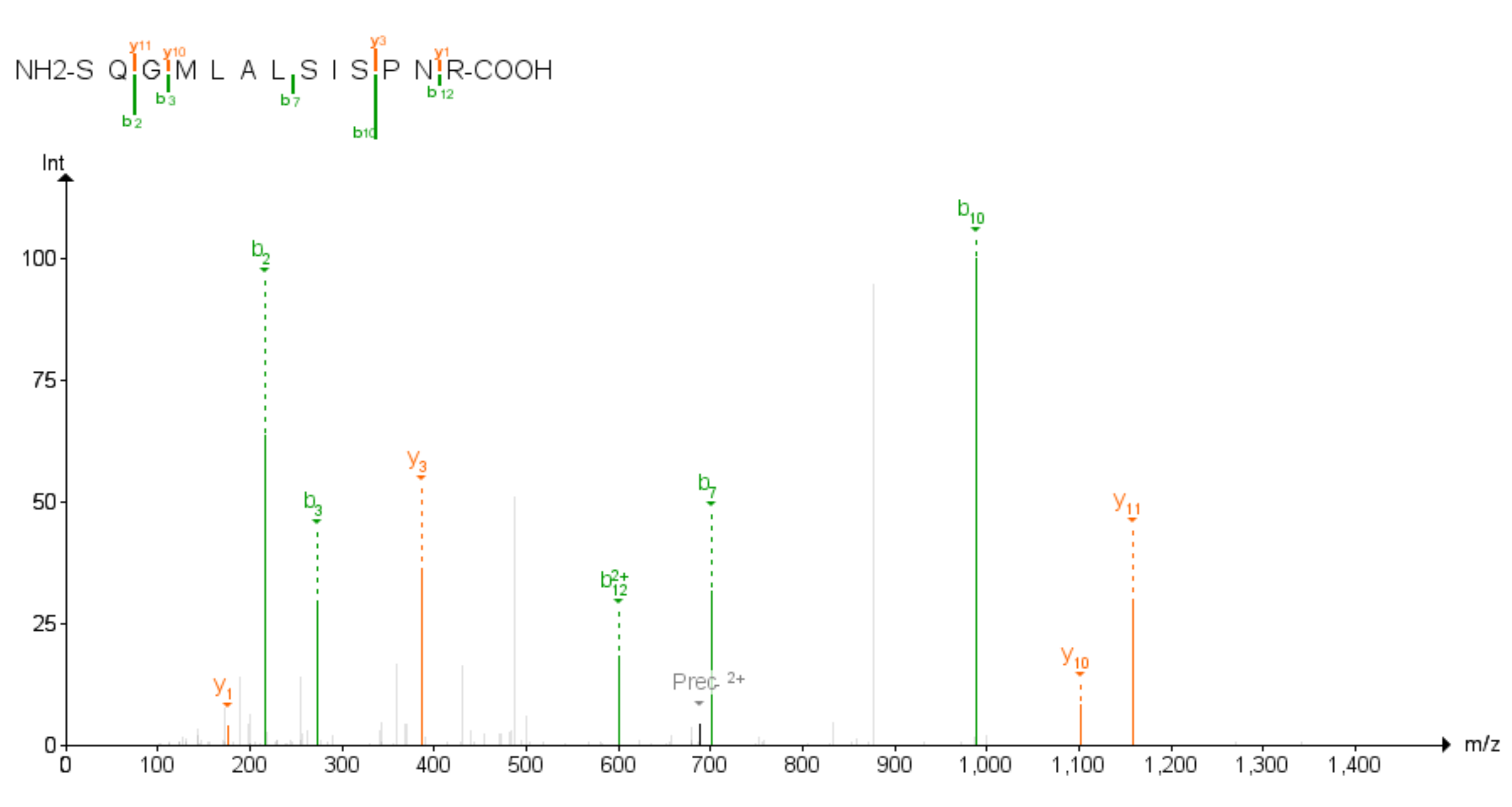
NH2-S Q G M L A L S I S P N R-COOH
y11 y10 y3 y1
b2 b3 b7 b10 b12
Int
100
75
50
25
0
b2
b3
y3
y1
b12 2+
Prec. 2+
b7
b10
y10
y11
0 100 200 300 400 500 600 700 800 900 1,000 1,100 1,200 1,300 1,400
m/z

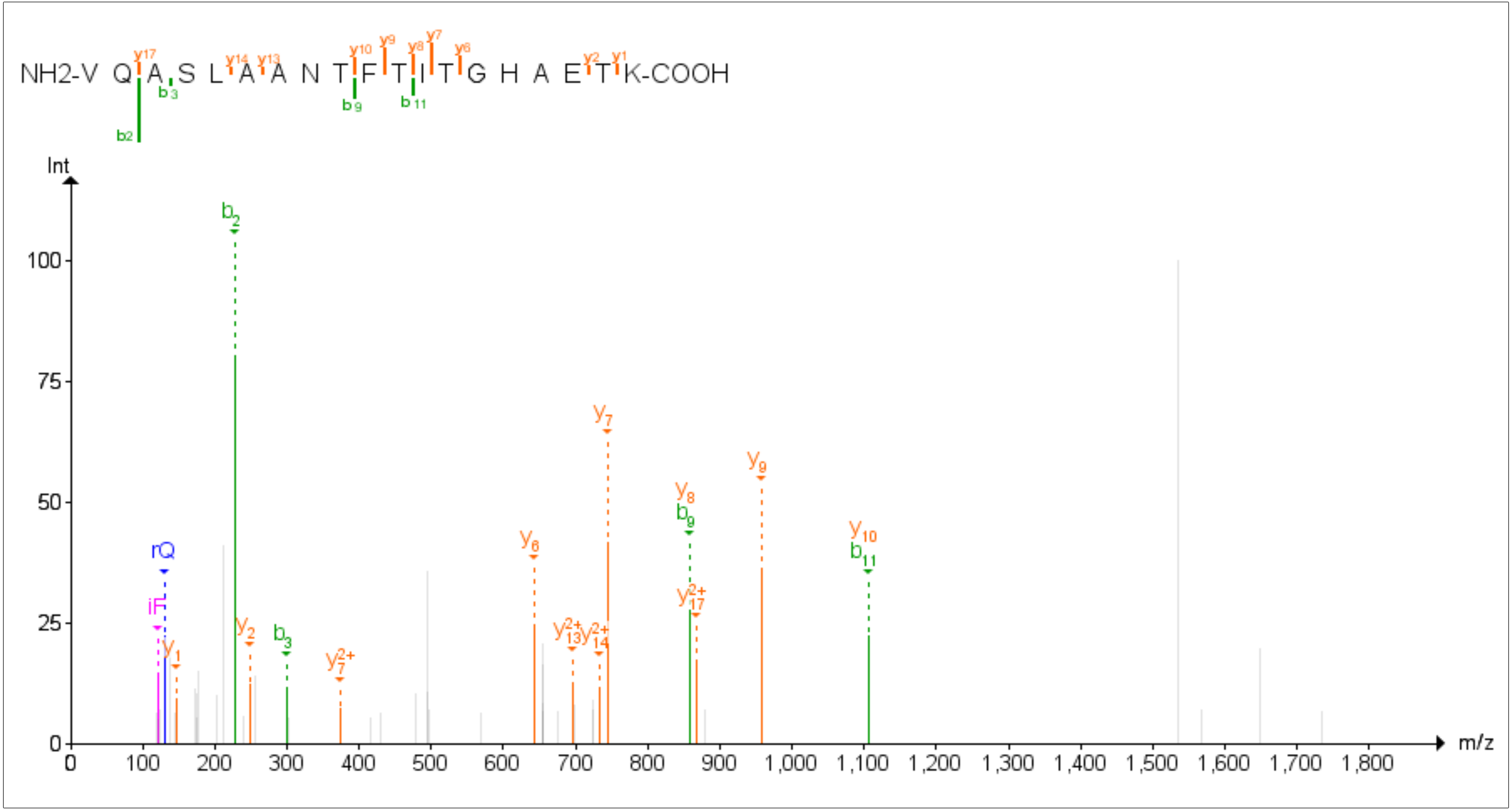

NH2-V Q A S L A A N T F T I T G H A E T K-COOH
y17
y14
y13
y10
y9
y8
y7
y6
y2
y1
b2
b3
b9
b11
Int
100
75
50
25
0
m/z
0
100
200
300
400
500
600
700
800
900
1,000
1,100
1,200
1,300
1,400
1,500
1,600
1,700
1,800
b2
rQ
iF
y1
y2
b3
y7 2+
y6
y13 2+
y14 2+
y7
y8
b9
y17 2+
y9
y10
b11

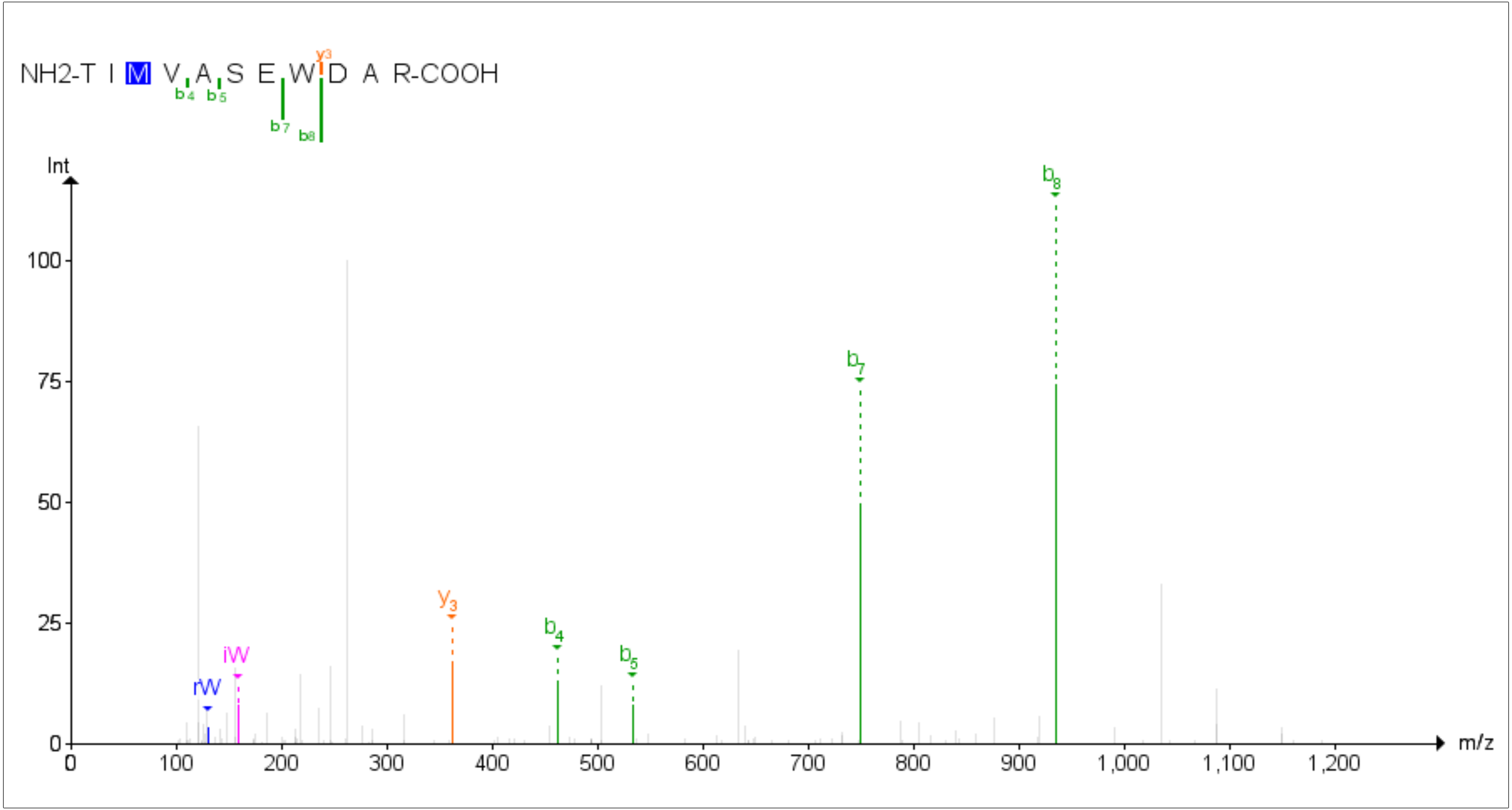

NH2-T I M V A S E W D A R-COOH
y3
b4
b5
b7
b8
Int
100
75
50
25
0
b8
b7
y3
b4
b5
iW
rW
0
100
200
300
400
500
600
700
800
900
1,000
1,100
1,200
m/z

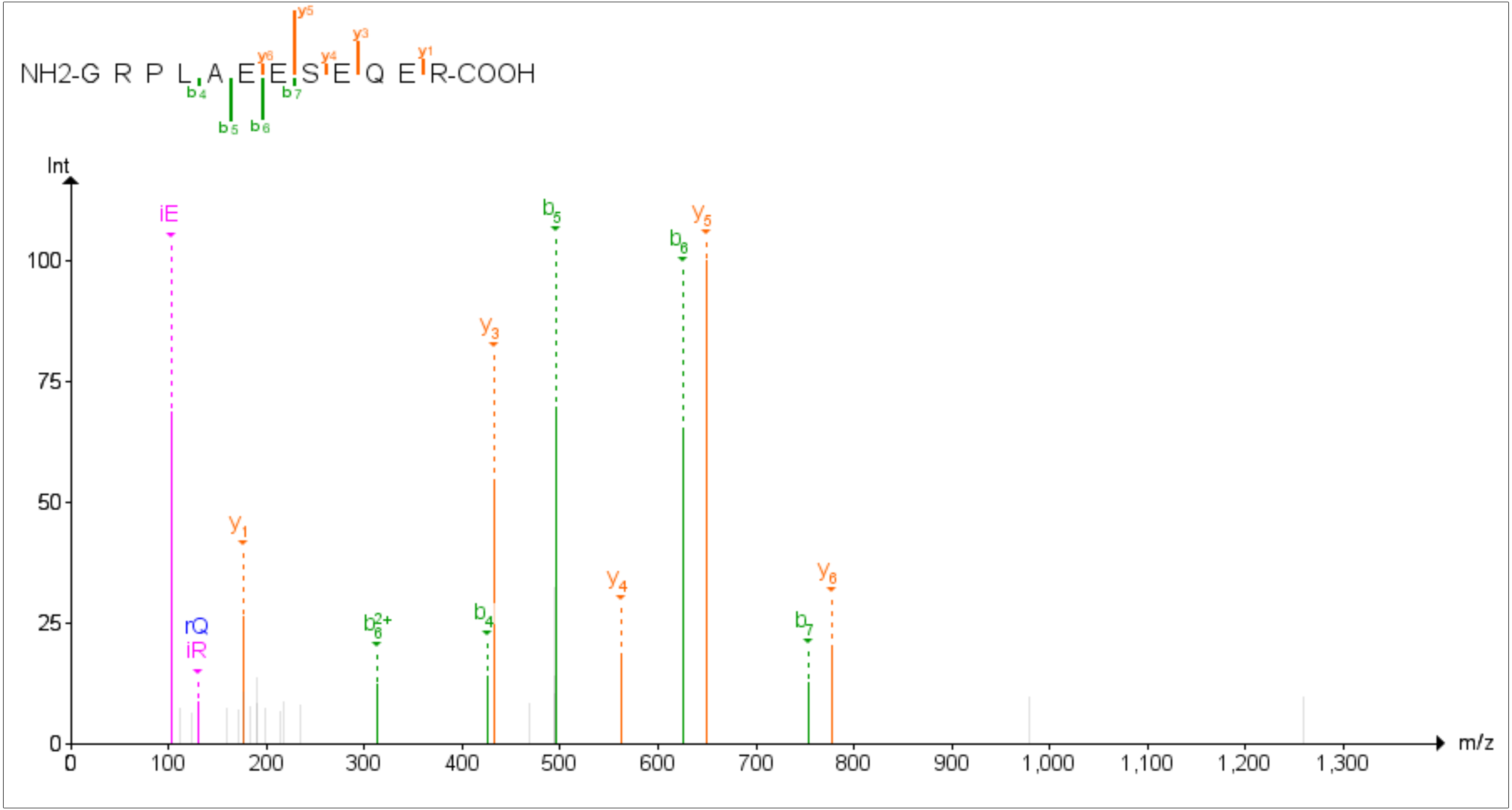

NH2-G R P L A E E S E Q E R-COOH
y5
y3
y6
y4
y1
b4
b5
b6
b7
Int
iE
b5
y5
b6
y3
y1
y4
y6
rQ
iR
b6 2+
b4
b7
m/z
0
25
50
75
100
0
100
200
300
400
500
600
700
800
900
1,000
1,100
1,200
1,300

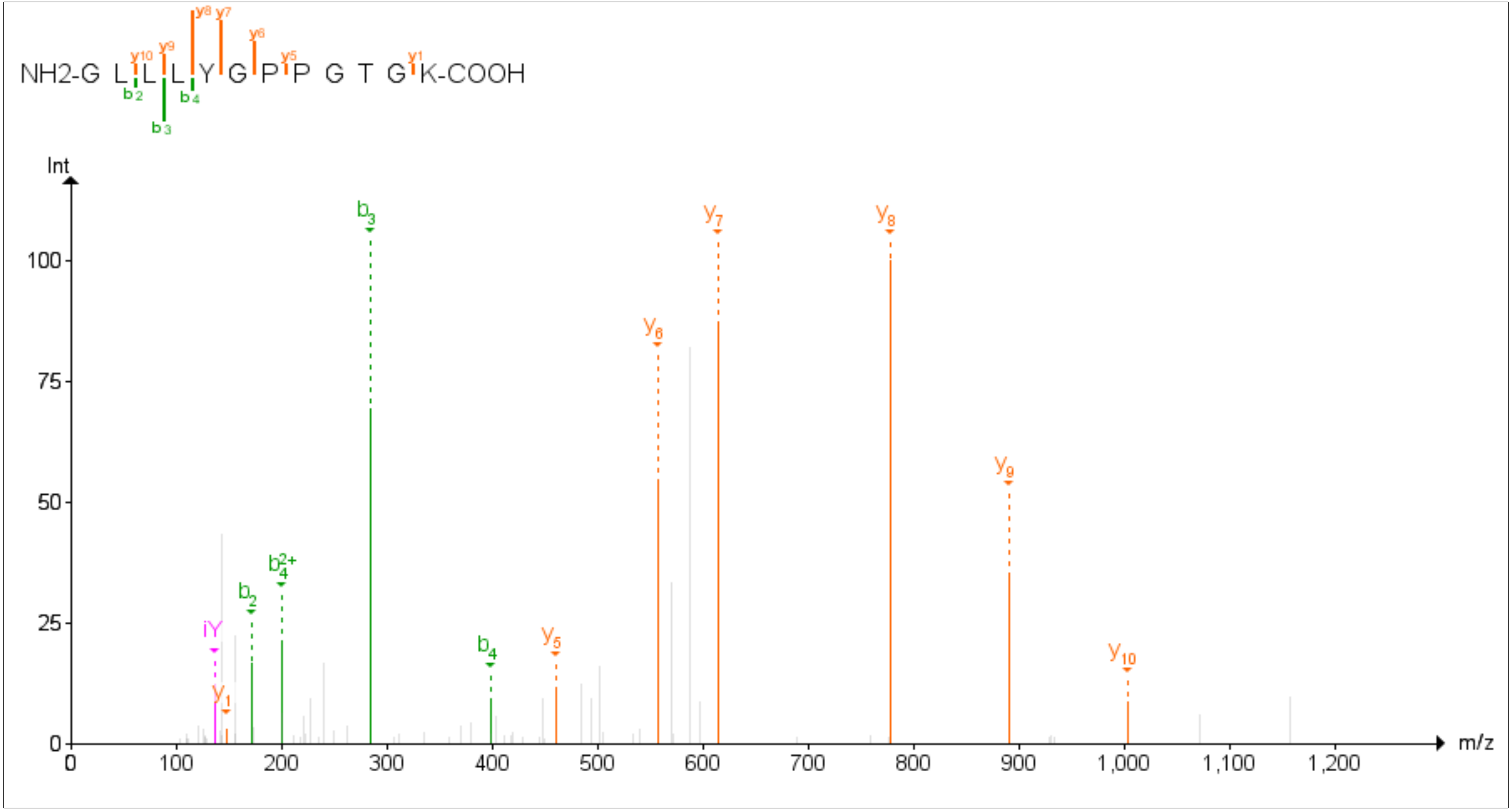

NH2-G L L L Y G P P G T G K-COOH
y10 y9 y8 y7 y6 y5 y1
b2 b3 b4
Int
100
75
50
25
0
b3
y7
y8
y6
y9
b4 2+
b2
iY
y1
b4
y5
y10
0 100 200 300 400 500 600 700 800 900 1,000 1,100 1,200
m/z

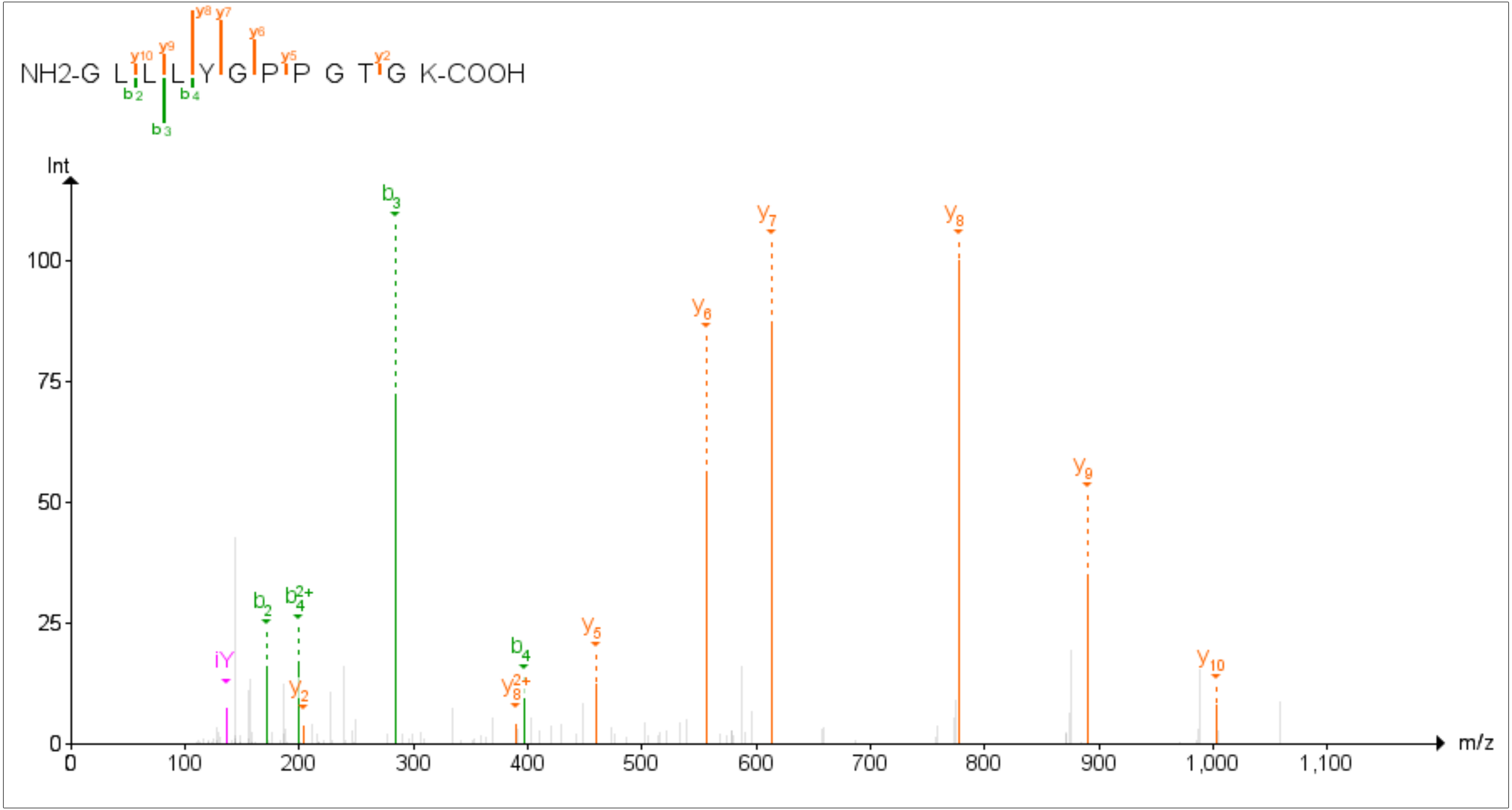

NH2-G L L L Y G P P G T G K-COOH
y10 y9 y8 y7 y6 y5 y2
b2 b3 b4
Int
m/z
b3
y7
y8
y6
y9
b2
b4 2+
iY
y2
b4
y8 2+
y5
y10

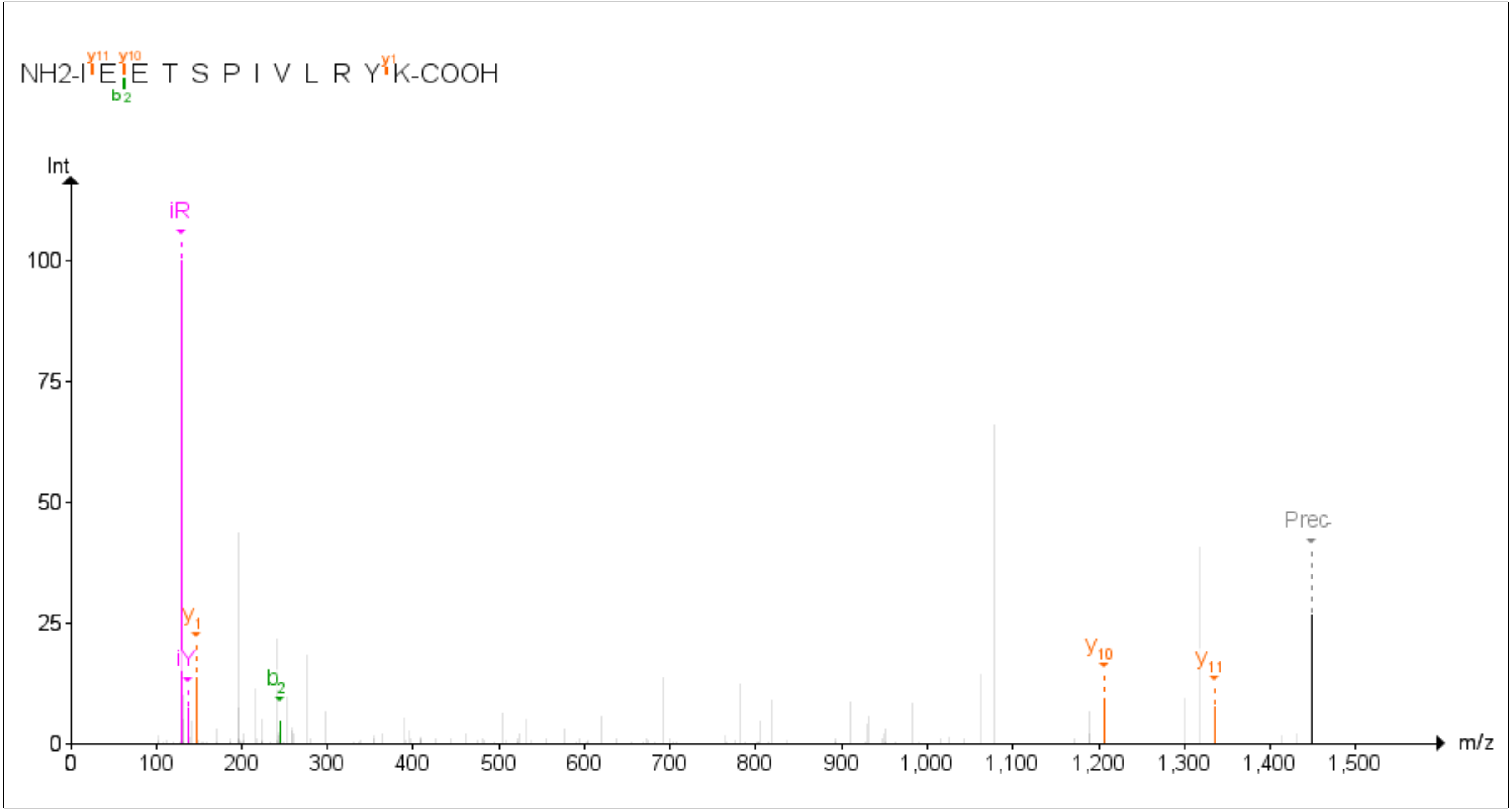

NH2-I E E T S P I V L R Y K-COOH
y11
y10
b2
y1
Int
iR
100
75
50
25
0
y1
iY
b2
y10
y11
Prec
m/z
0
100
200
300
400
500
600
700
800
900
1,000
1,100
1,200
1,300
1,400
1,500

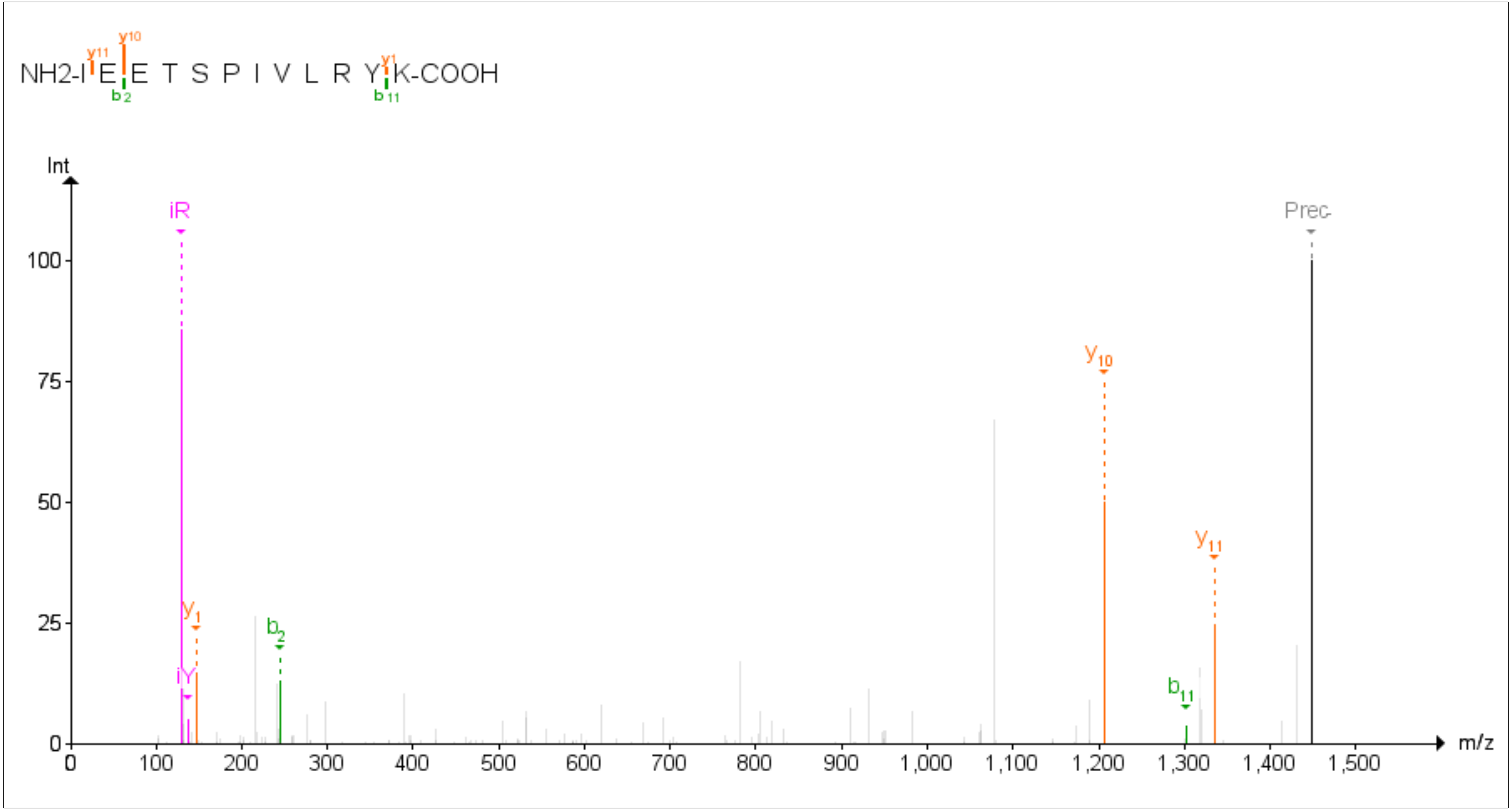

NH2-I E E T S P I V L R Y K-COOH
y11
y10
y1
b2
b11
Int
iR
Prec
y10
y11
y1
b2
iY
b11
100
75
50
25
0
0
100
200
300
400
500
600
700
800
900
1,000
1,100
1,200
1,300
1,400
1,500
m/z

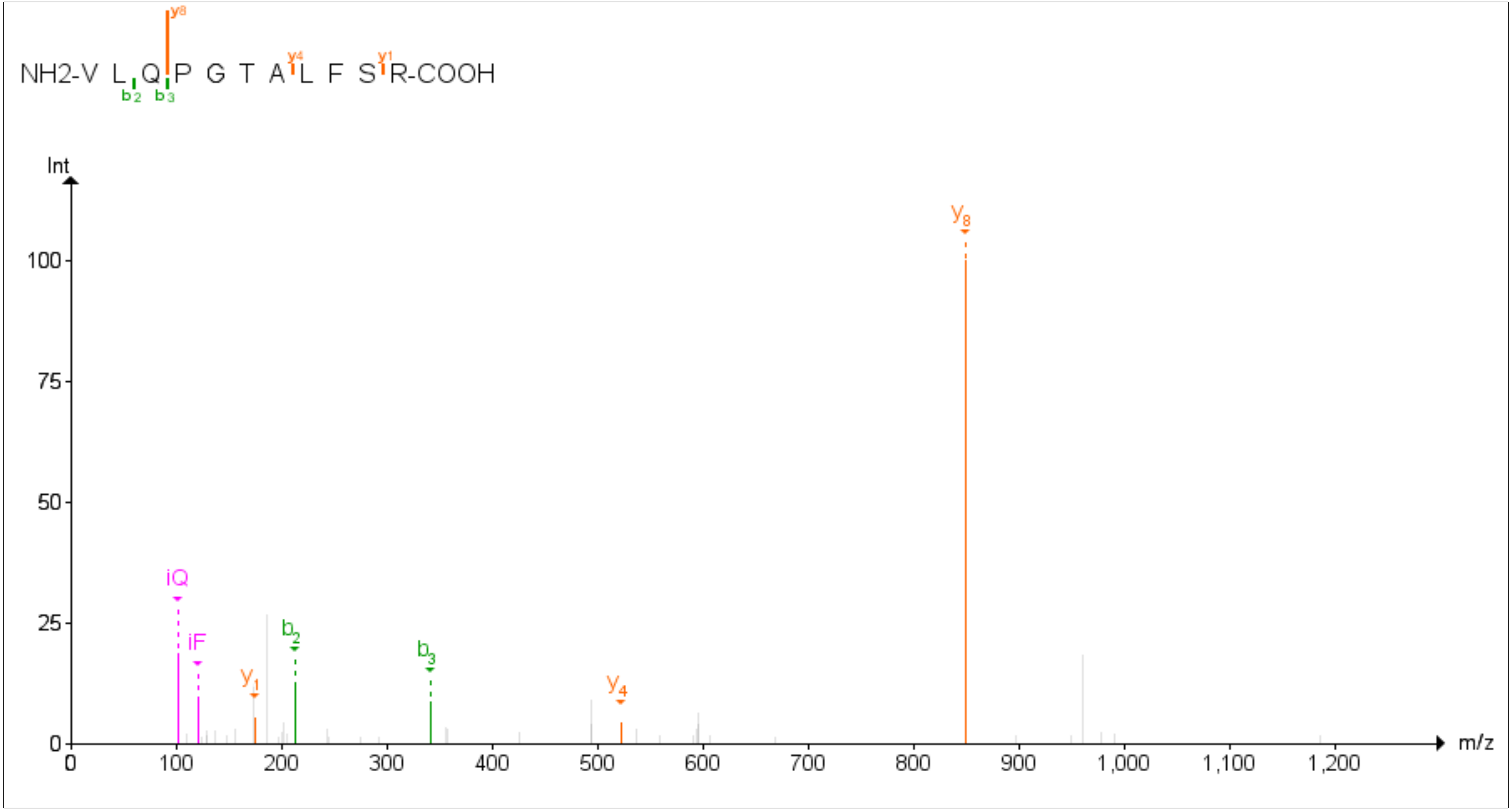

NH2-V L Q P G T A L F S R-COOH
y8
y4
y1
b2
b3
Int
100
75
50
25
0
iQ
iF
y1
b2
b3
y4
y8
0
100
200
300
400
500
600
700
800
900
1,000
1,100
1,200
m/z

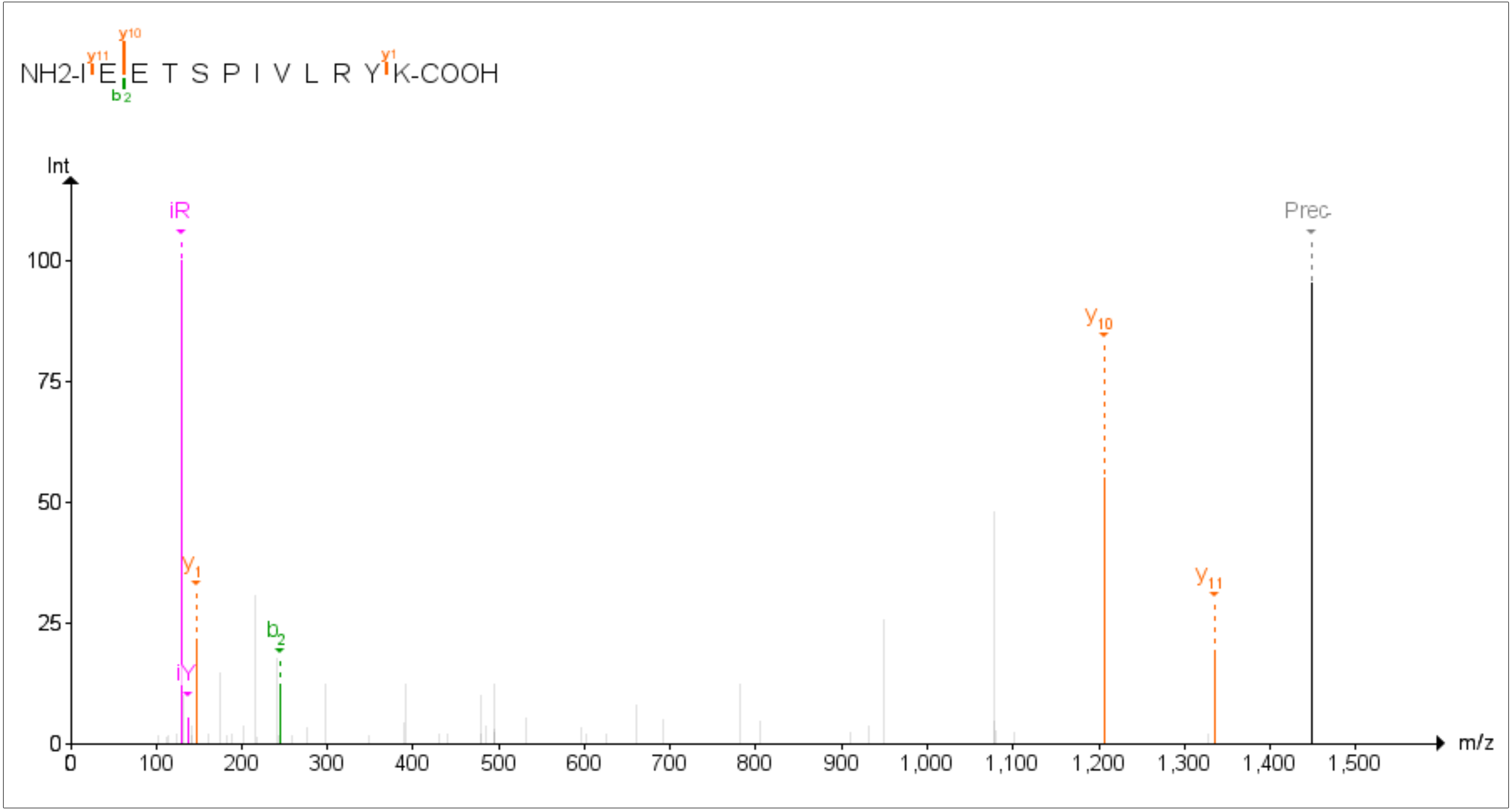

NH2-I E E T S P I V L R Y K-COOH
y10
y11
y1
b2
Int
iR
Prec.
y10
y11
y1
b2
iY
100
75
50
25
0
m/z
0
100
200
300
400
500
600
700
800
900
1,000
1,100
1,200
1,300
1,400
1,500

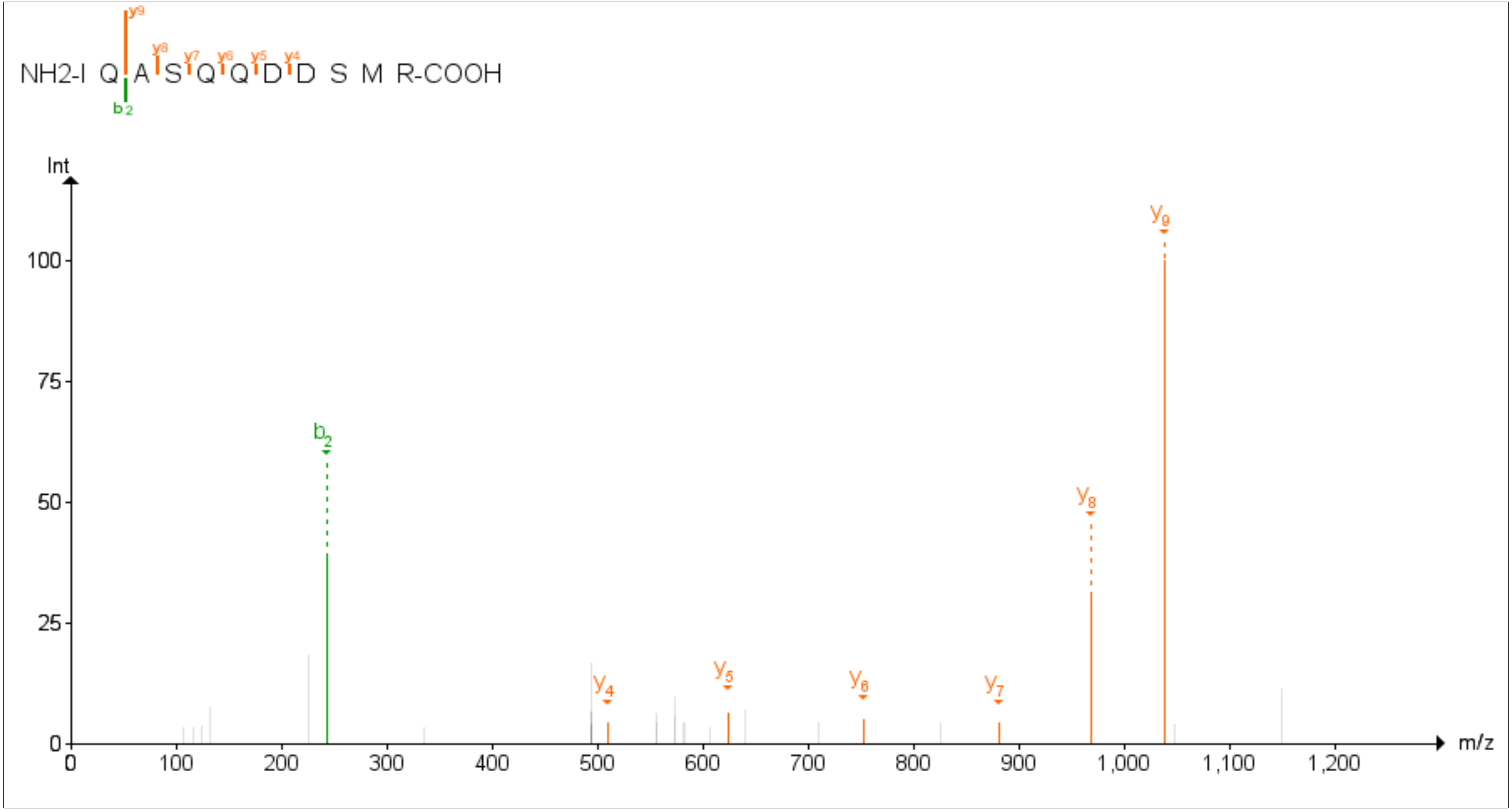

NH2-I Q A S Q Q D D S M R-COOH
y9
y8
y7
y6
y5
y4
b2
Int
m/z

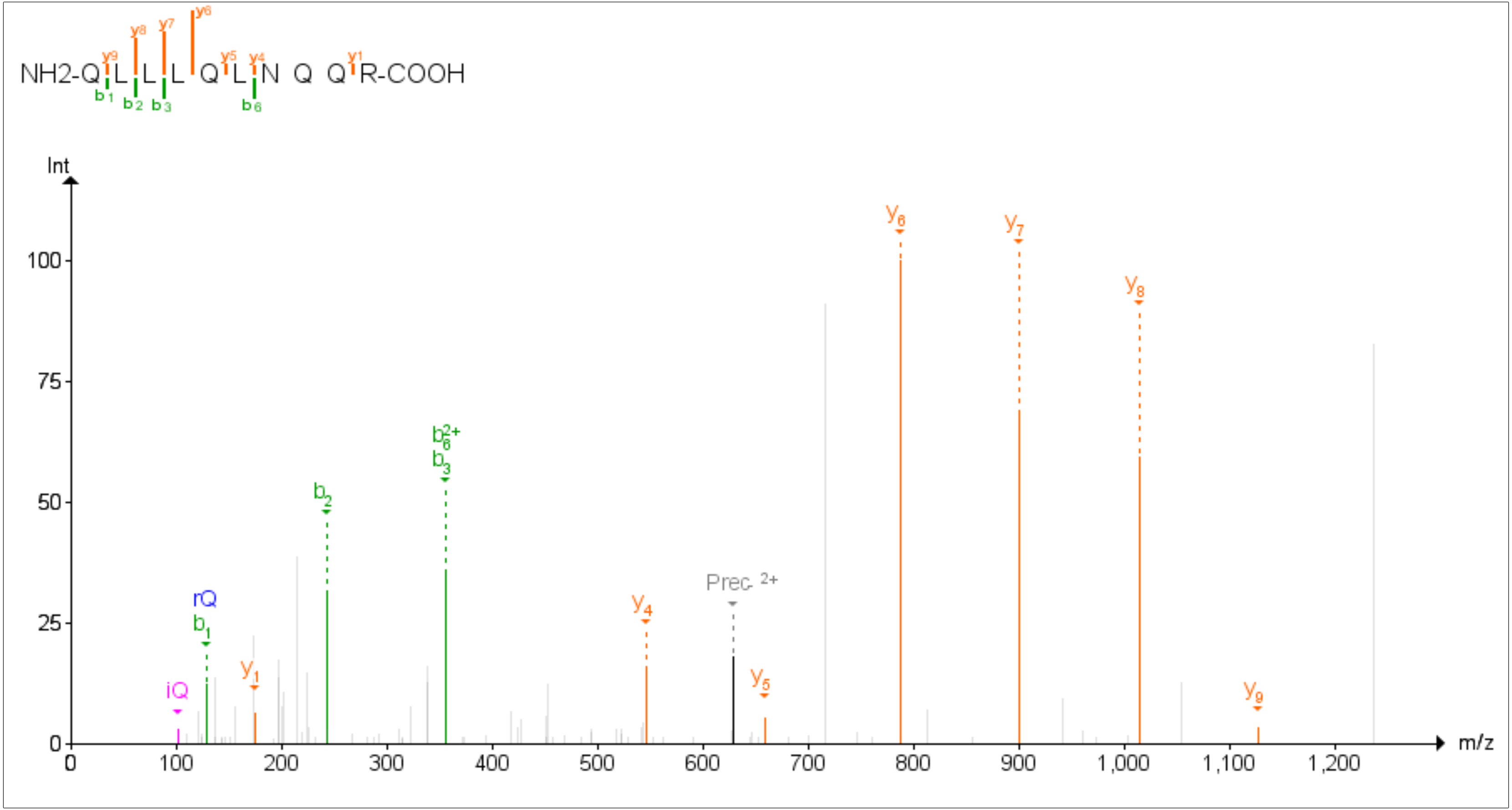

NH2-Q L L L Q L N Q Q R-COOH
y9 y8 y7 y6 y5 y4 y1
b1 b2 b3 b6
Int
100
75
50
25
0
y6
y7
y8
b6 2+
b3
b2
rQ
b1
iQ
y1
y4
Prec 2+
y5
y9
0 100 200 300 400 500 600 700 800 900 1,000 1,100 1,200
m/z

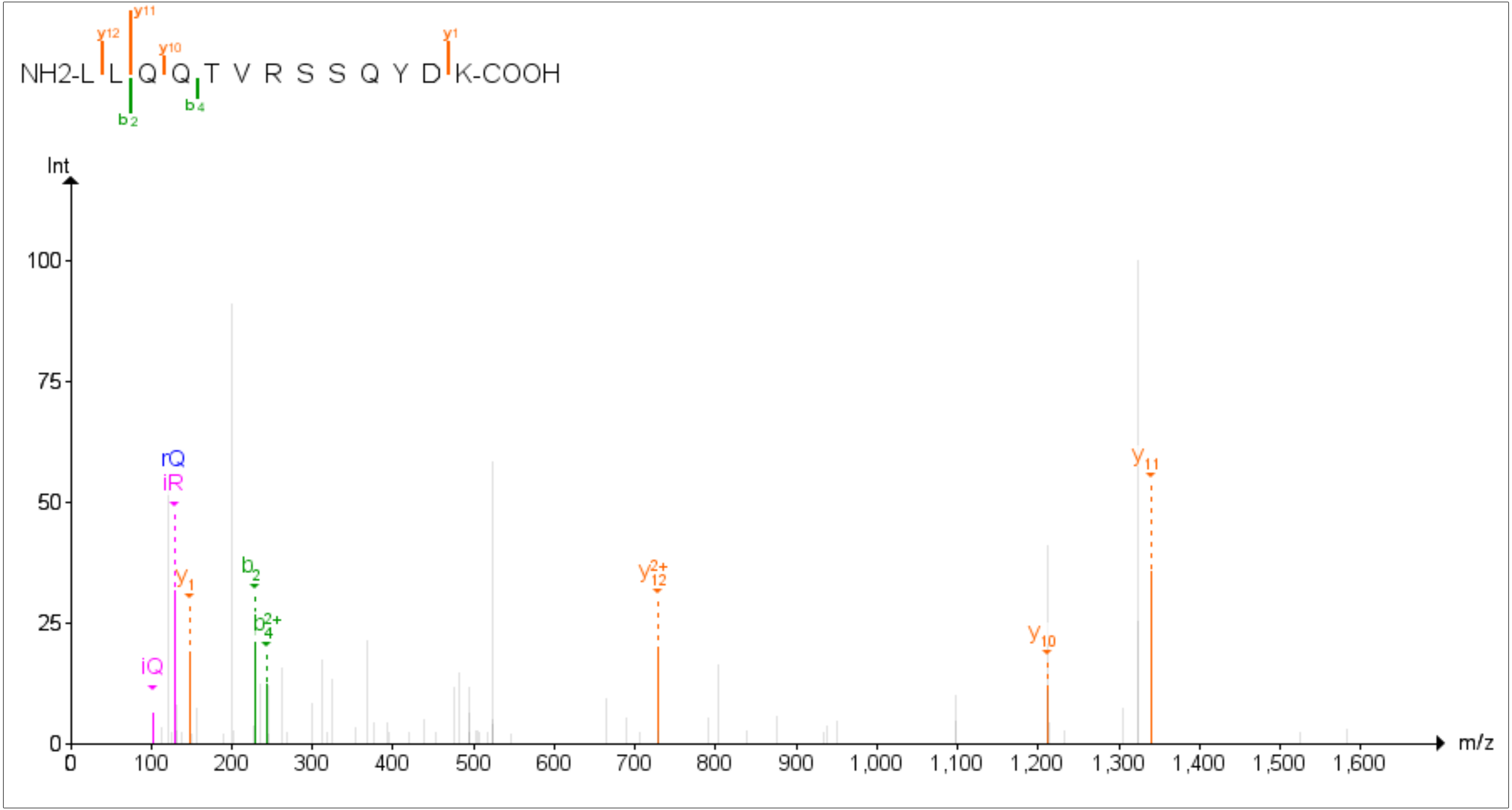

NH2-L L Q Q T V R S S Q Y D K-COOH
y12
y11
y10
y1
b2
b4
Int
m/z
rQ
iR
iQ
y1
b2
b4 2+
y12 2+
y10
y11
0
25
50
75
100
0
100
200
300
400
500
600
700
800
900
1,000
1,100
1,200
1,300
1,400
1,500
1,600

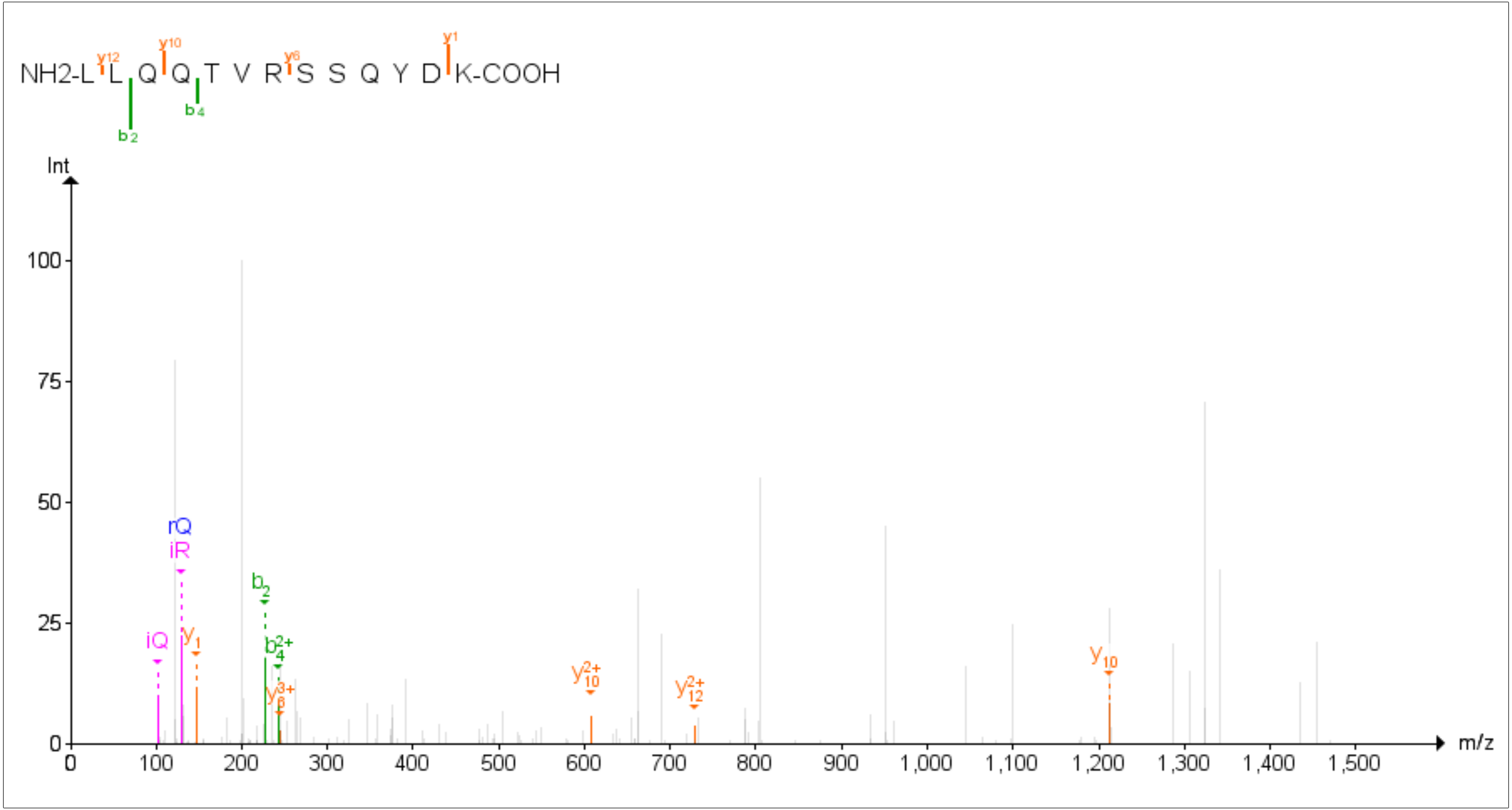

NH2-L L Q Q T V R S S Q Y D K-COOH
y12
y10
y6
y1
b2
b4
Int
m/z
rQ
iR
iQ
y1
b2
b4 2+
y6 3+
y10 2+
y12 2+
y10

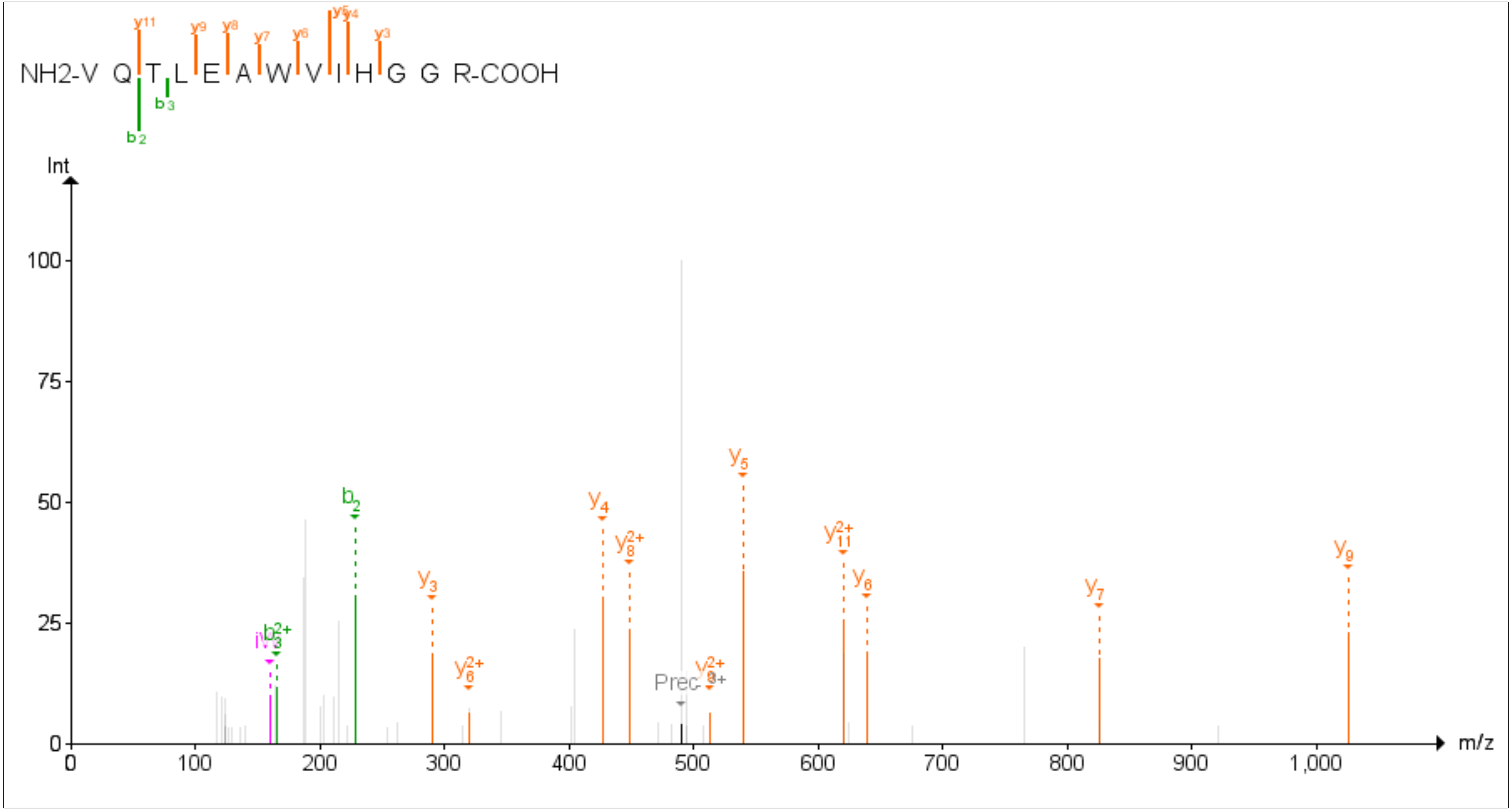

NH2-V Q T L E A W V I H G G R-COOH
y11 y9 y8 y7 y6 y5 y4 y3
b2 b3
Int
100
75
50
25
0
m/z
0 100 200 300 400 500 600 700 800 900 1,000
b2
y3
y4
y5
y7
y9
Prec 3+

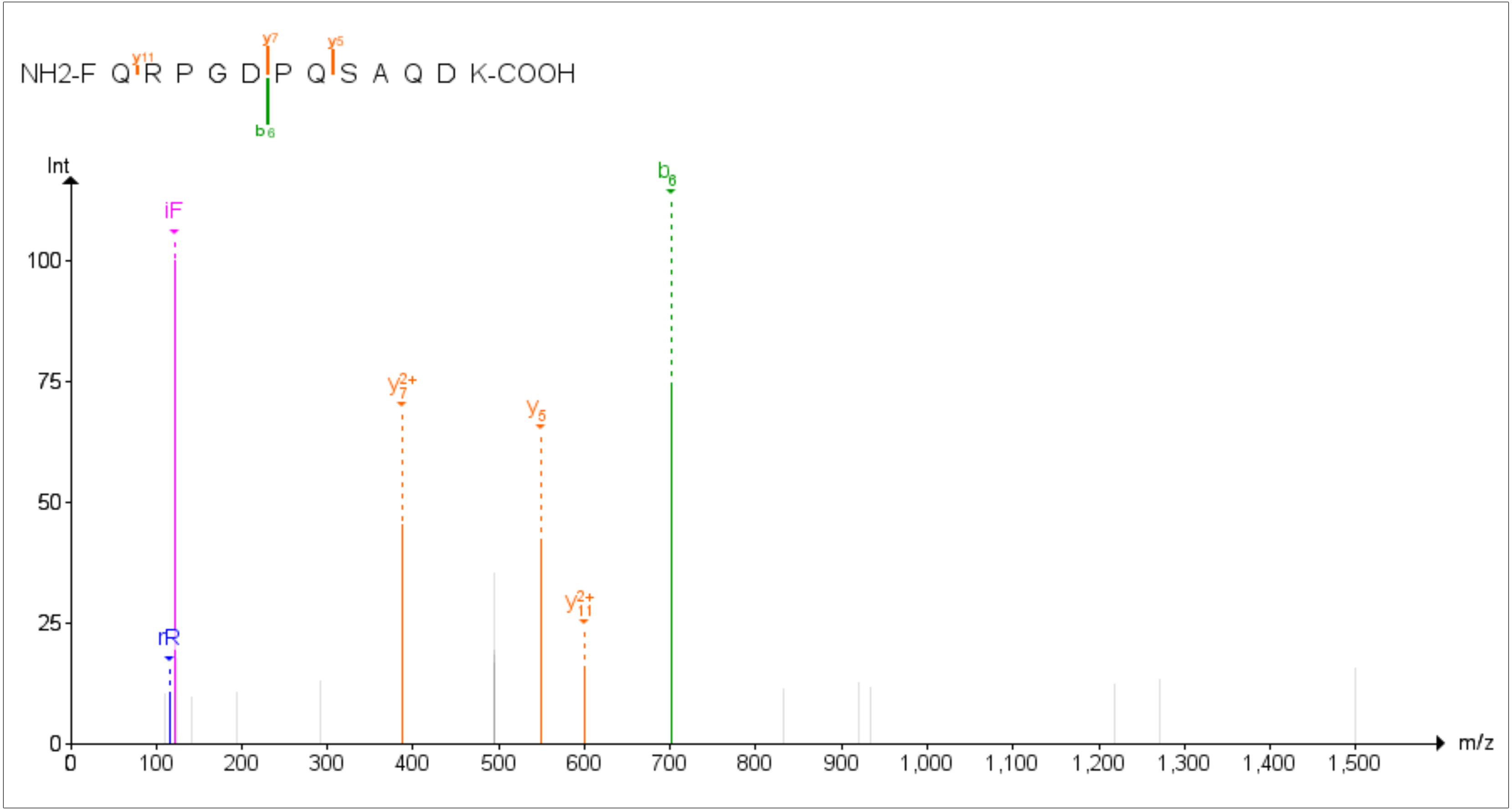

NH2-F Q R P G D P Q S A Q D K-COOH
y11
y7
y5
b6
Int
iF
b6
y7 2+
y5
y11 2+
rR
100
75
50
25
0
0
100
200
300
400
500
600
700
800
900
1,000
1,100
1,200
1,300
1,400
1,500
m/z

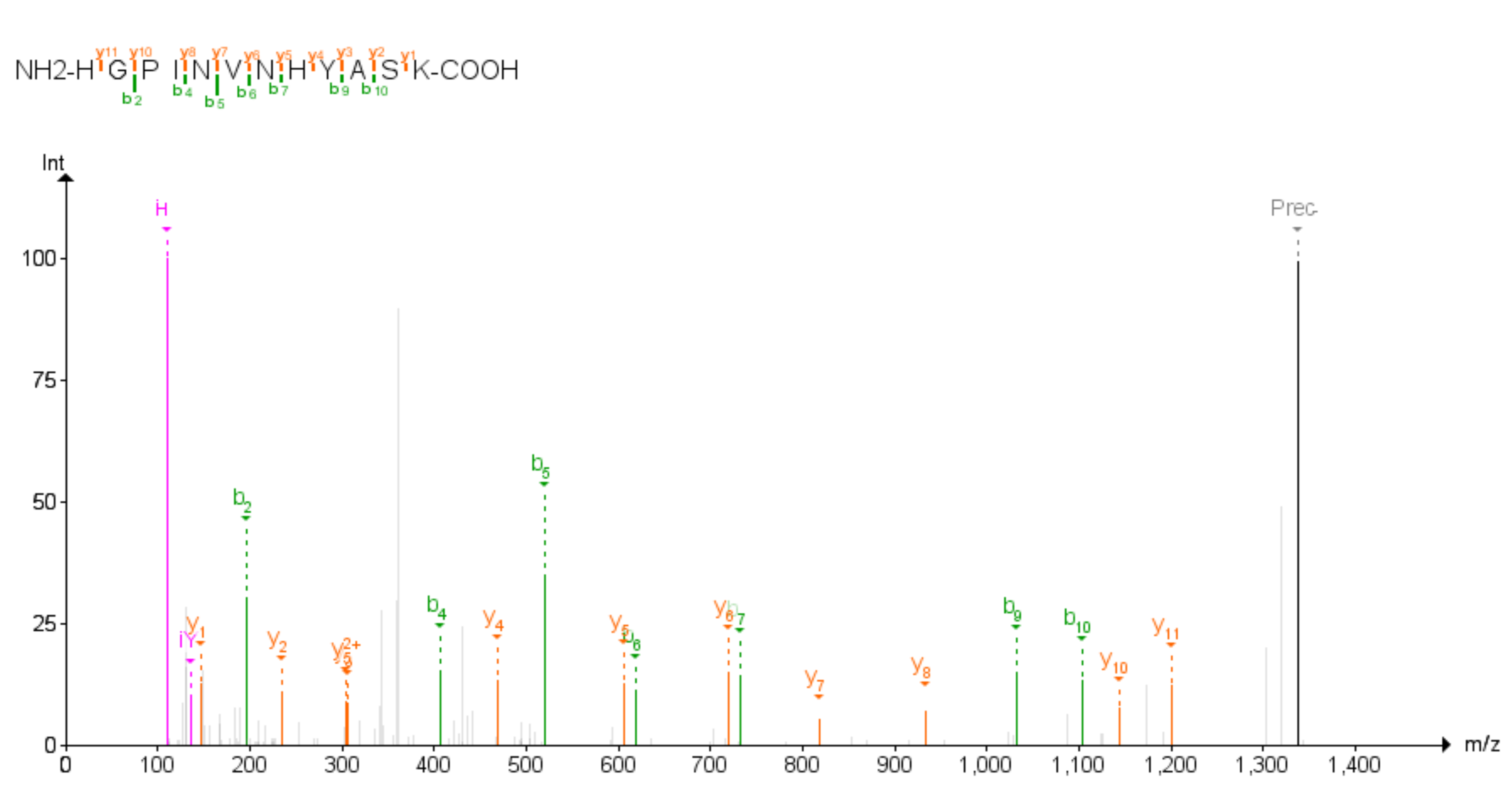

NH2-H G P I N V N H Y A S K-COOH
y11 y10 y8 y7 y6 y5 y4 y3 y2 y1
b2 b4 b5 b6 b7 b9 b10
Int
Prec
m/z

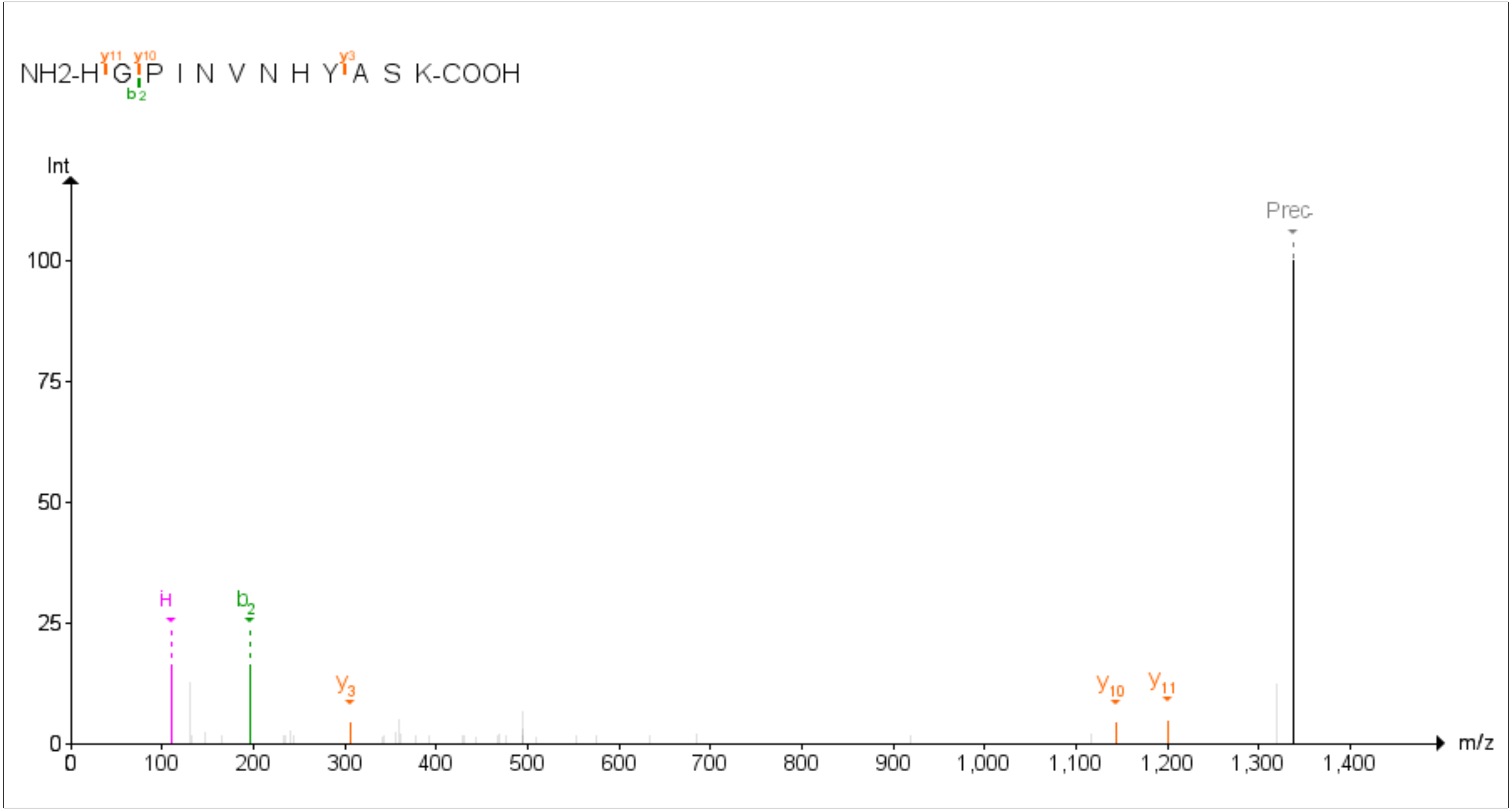

NH2-H G P I N V N H Y A S K-COOH
y11
y10
y3
b2
Int
Prec
m/z
100
75
50
25
0
H
b2
y3
y10
y11
0
100
200
300
400
500
600
700
800
900
1,000
1,100
1,200
1,300
1,400

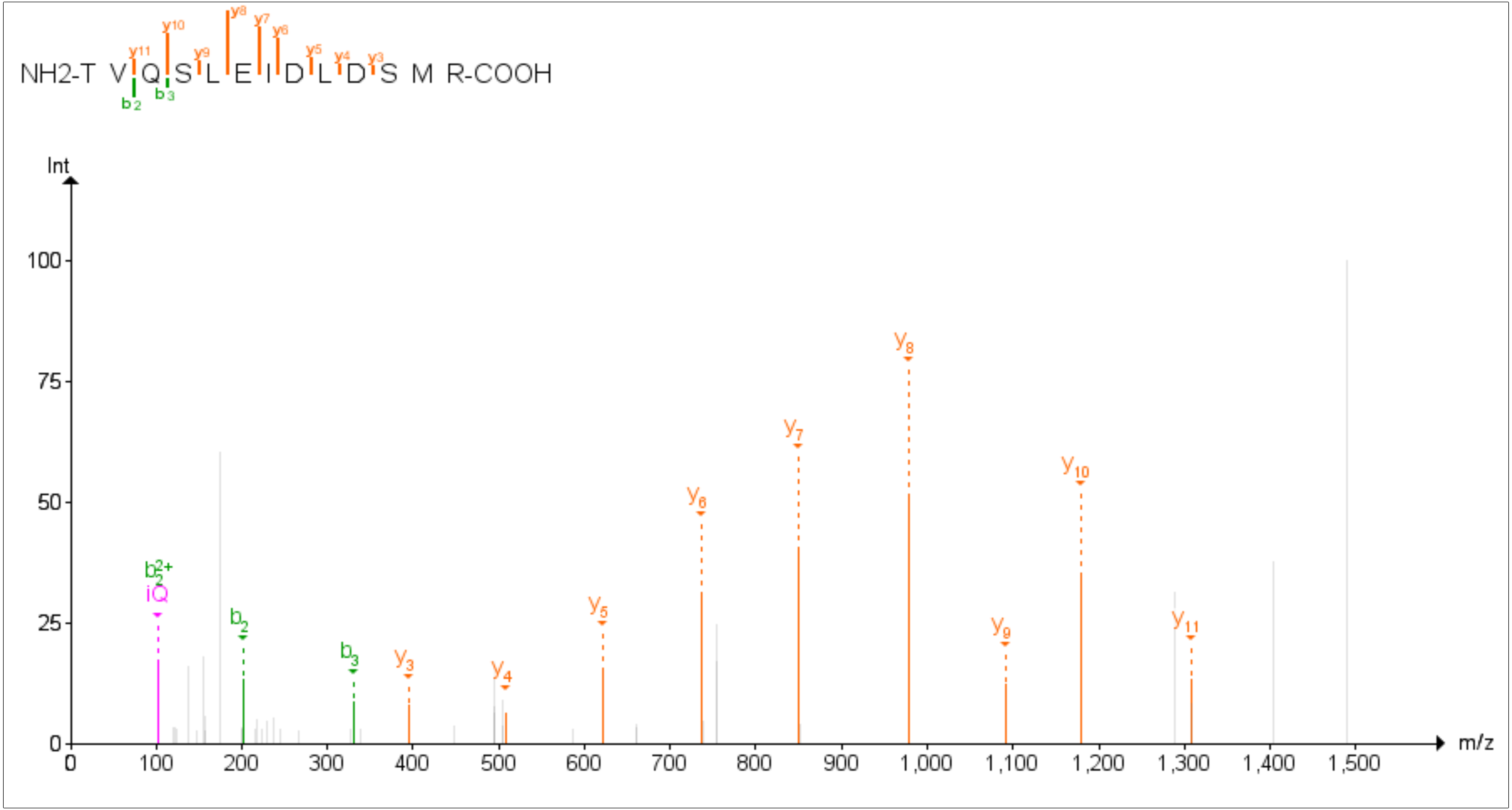

NH2-T V Q S L E I D L D S M R-COOH
y11 y10 y9 y8 y7 y6 y5 y4 y3
b2 b3
Int
100
75
50
25
0
m/z
0 100 200 300 400 500 600 700 800 900 1,000 1,100 1,200 1,300 1,400 1,500
b2 2+
iQ
b2
b3
y3
y4
y5
y6
y7
y8
y9
y10
y11

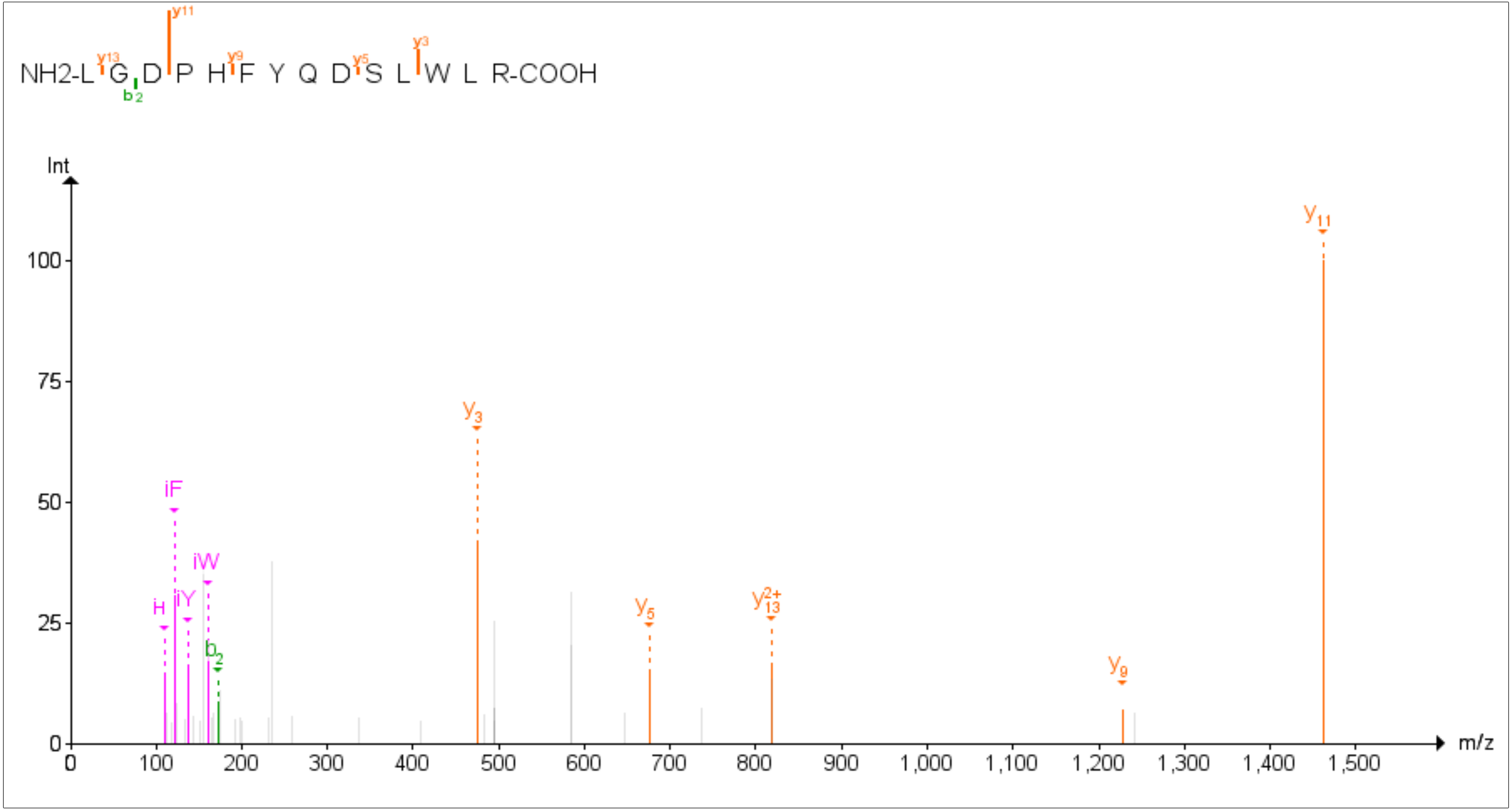
NH2-L G D P H F Y Q D S L W L R-COOH
y11
y13
y9
y5
y3
b2
Int
100
75
50
25
0
iF
iW
iH
iY
b2
y3
y5
y13 2+
y9
y11
m/z
0
100
200
300
400
500
600
700
800
900
1,000
1,100
1,200
1,300
1,400
1,500

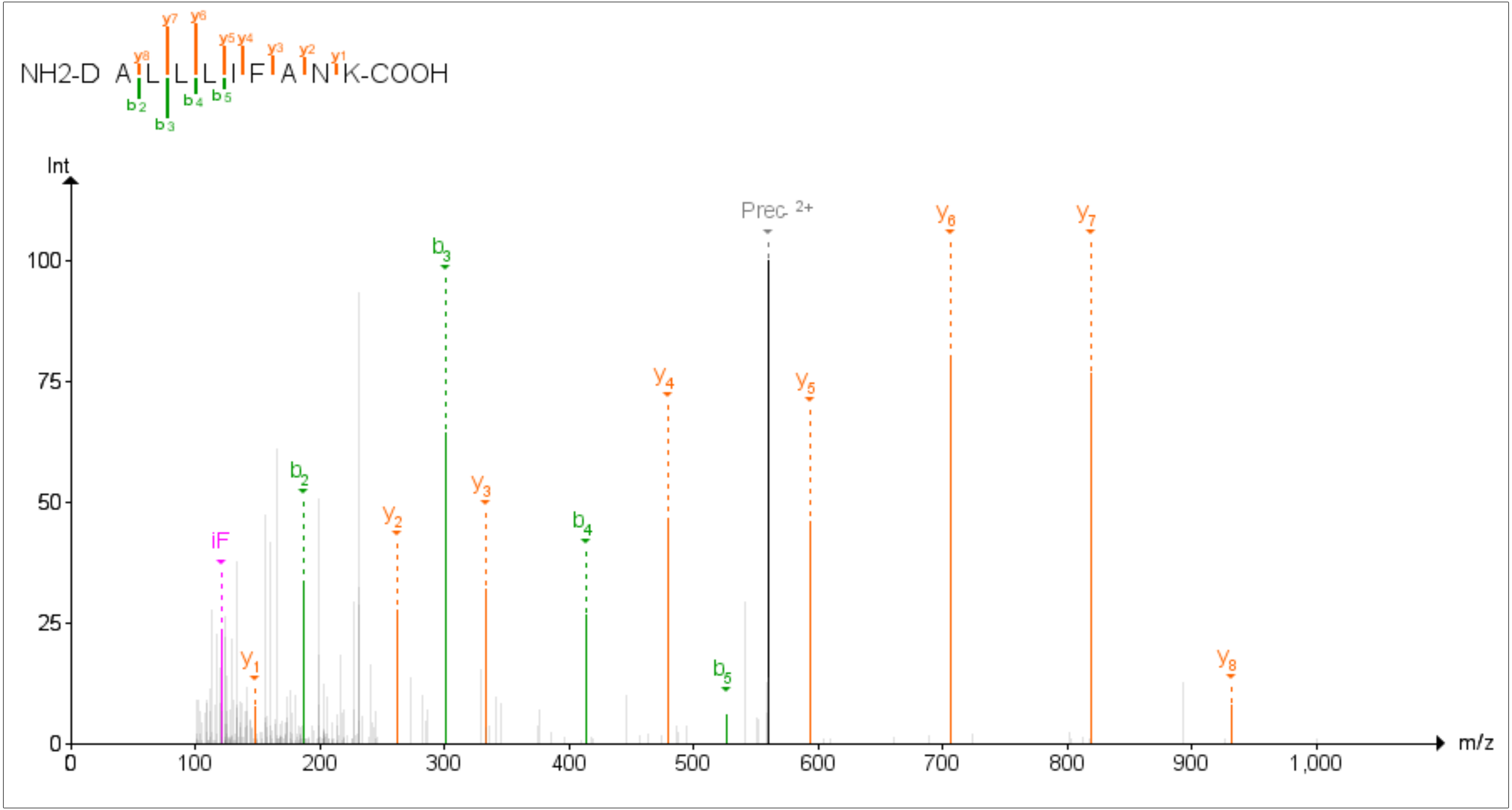

NH2-D A L L L I F A N K-COOH
y8 y7 y6 y5 y4 y3 y2 y1
b2 b3 b4 b5
Int
m/z
Prec 2+
y6
y7
b3
y4
y5
b2
y3
y2
b4
iF
y1
b5
y8
0
25
50
75
100
0
100
200
300
400
500
600
700
800
900
1,000

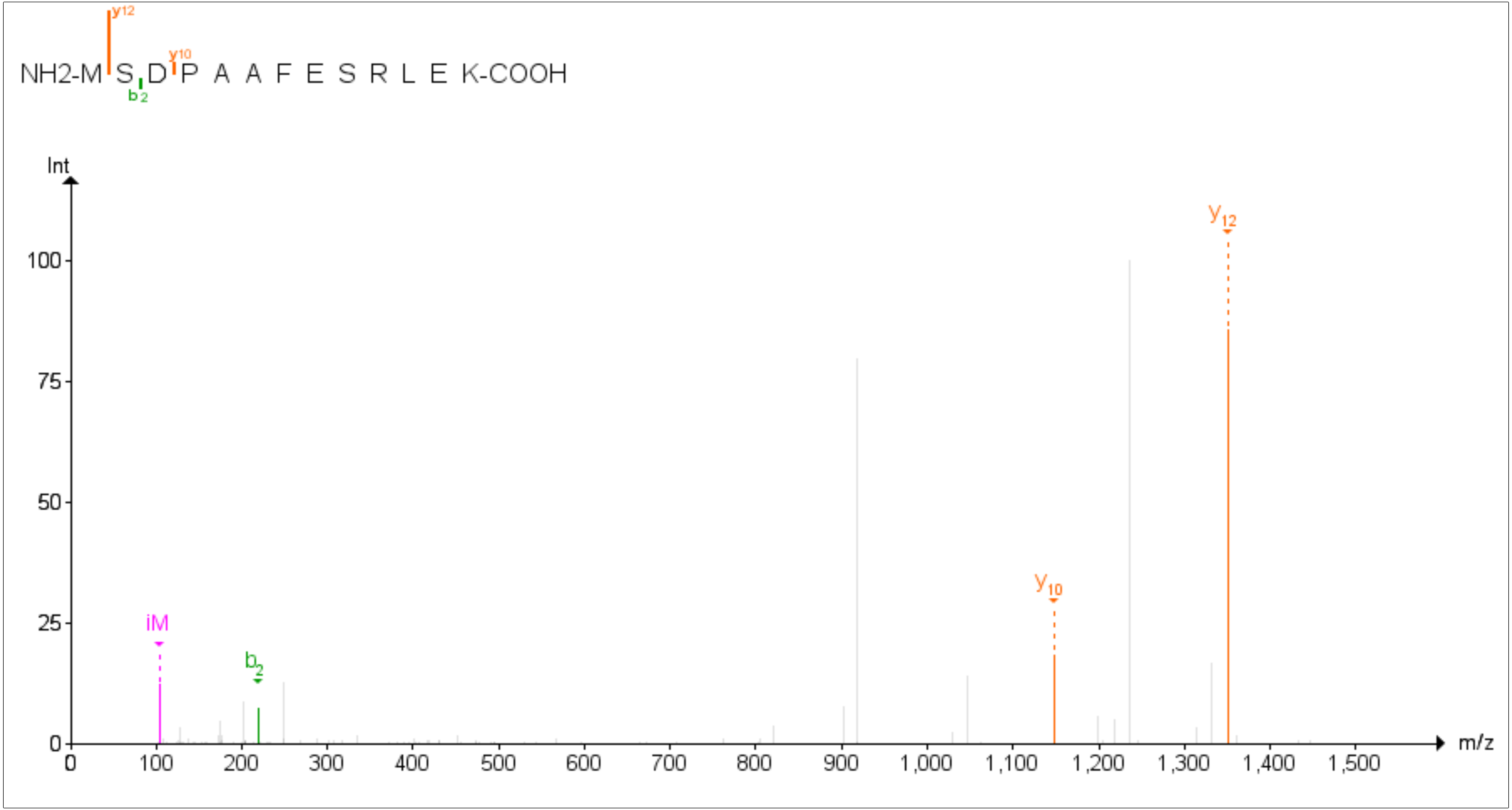

NH2-M S D P A A F E S R L E K-COOH
y12
y10
b2
Int
100
75
50
25
0
iM
b2
y10
y12
0
100
200
300
400
500
600
700
800
900
1,000
1,100
1,200
1,300
1,400
1,500
m/z

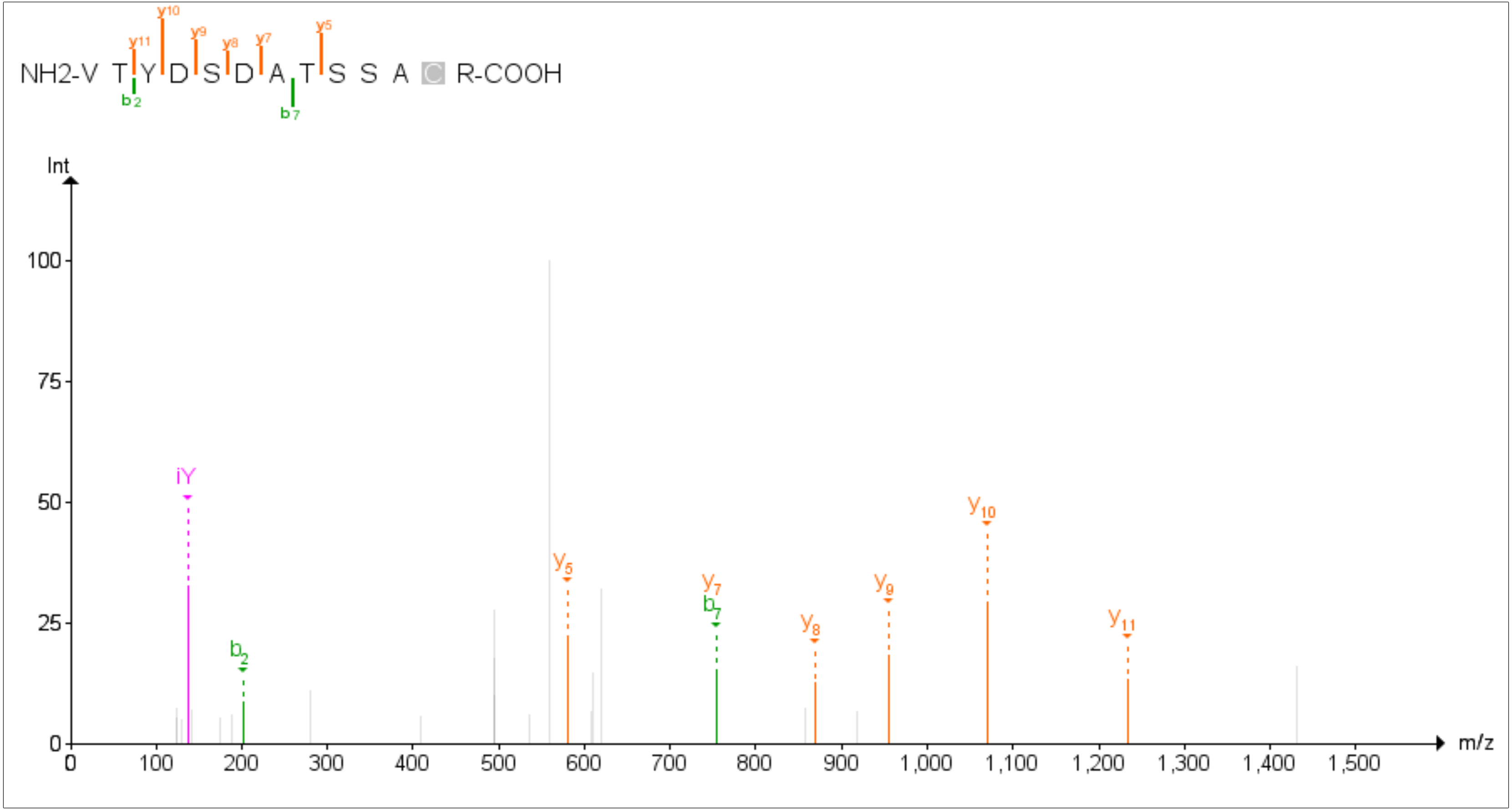

NH2-V T Y D S D A T S S A C R-COOH
y10 y11 y9 y8 y7 y5
b2 b7
Int
m/z
iY
y5
y7
b7
y8
y9
y10
y11
b2

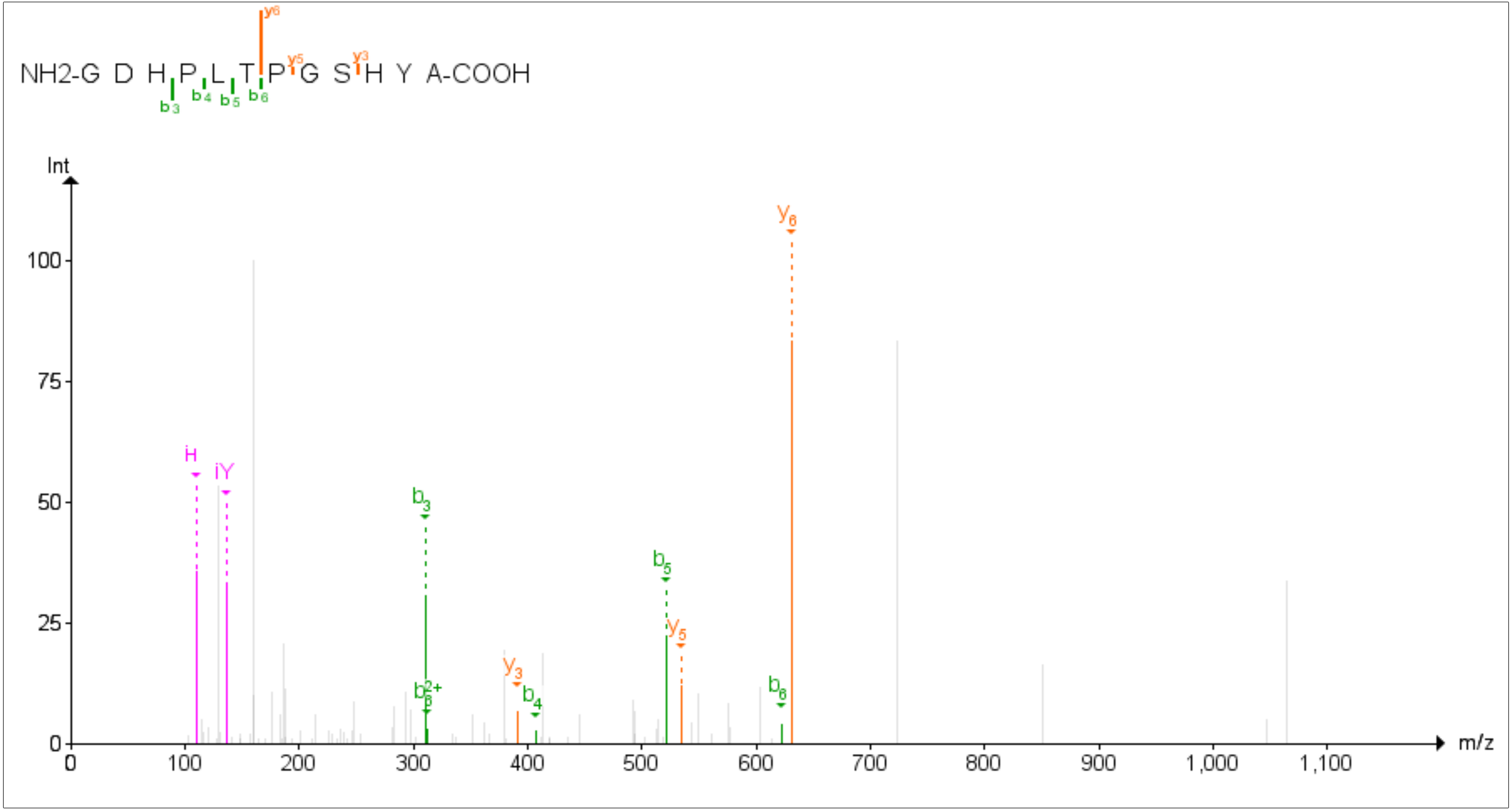

NH2-G D H P L T P G S H Y A-COOH
y6
y5
y3
b3
b4
b5
b6
Int
100
75
50
25
0
iH
iY
b3
b3 2+
y3
b4
b5
y5
b6
y6
0
100
200
300
400
500
600
700
800
900
1,000
1,100
m/z

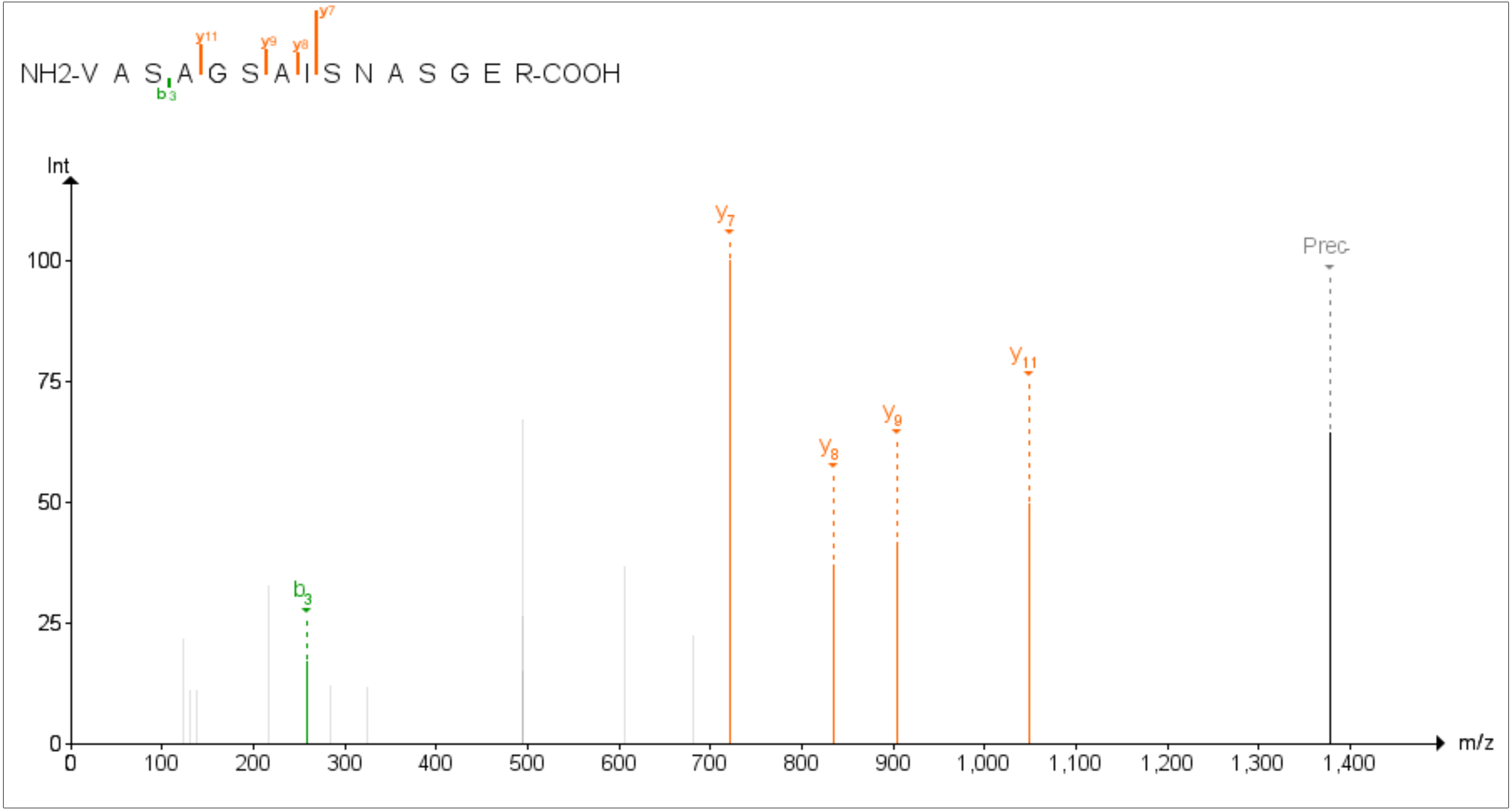

NH2-V A S A G S A I S N A S G E R-COOH
y11
y9
y8
y7
b3
Int
100
75
50
25
0
y7
y8
y9
y11
b3
Prec
m/z
0
100
200
300
400
500
600
700
800
900
1,000
1,100
1,200
1,300
1,400

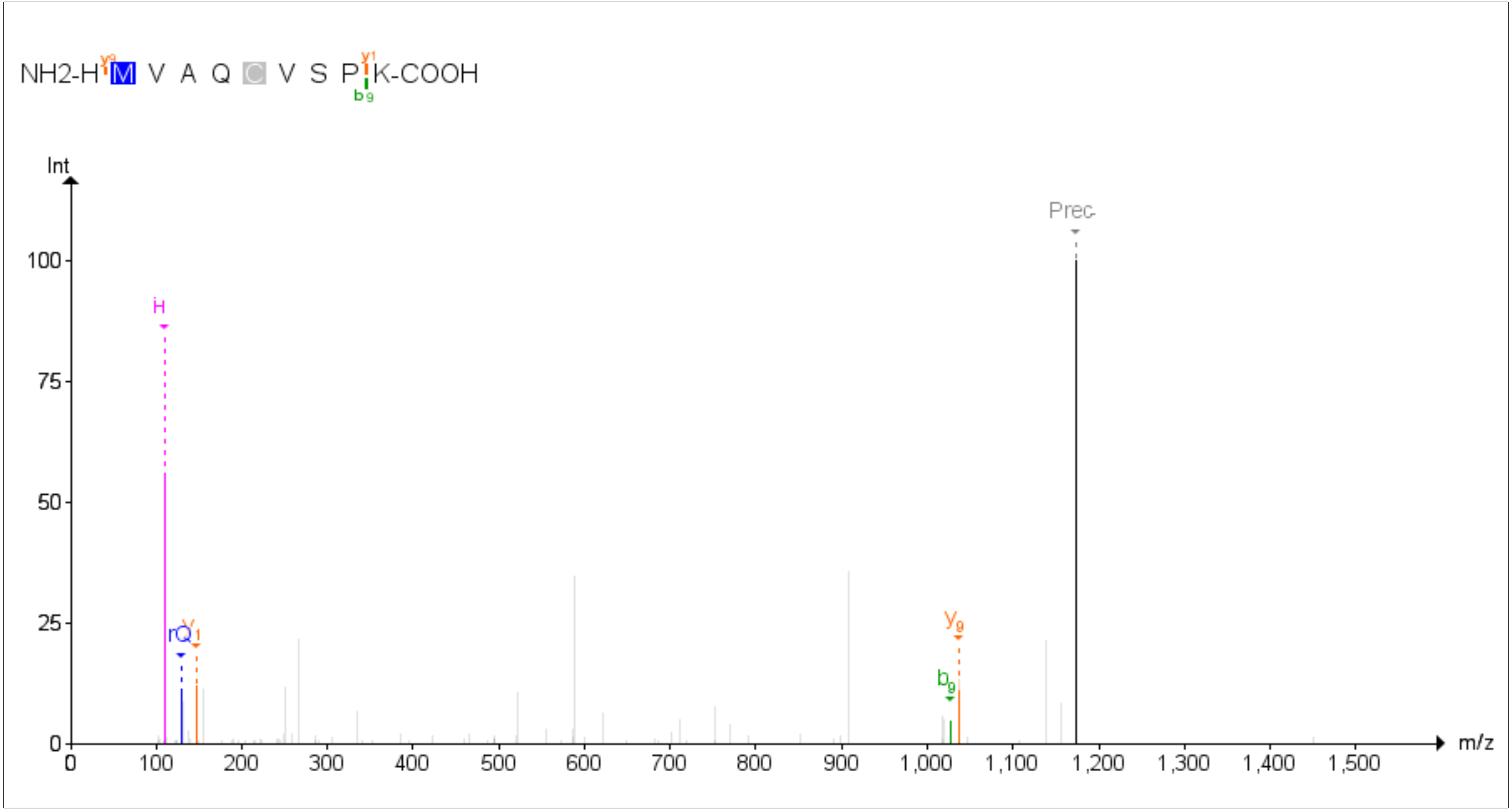

NH2-H M V A Q C V S P K-COOH
y9
y1
b9
Int
m/z
Prec.
H
rQ
y1
y9
b9
100
75
50
25
0
0
100
200
300
400
500
600
700
800
900
1,000
1,100
1,200
1,300
1,400
1,500

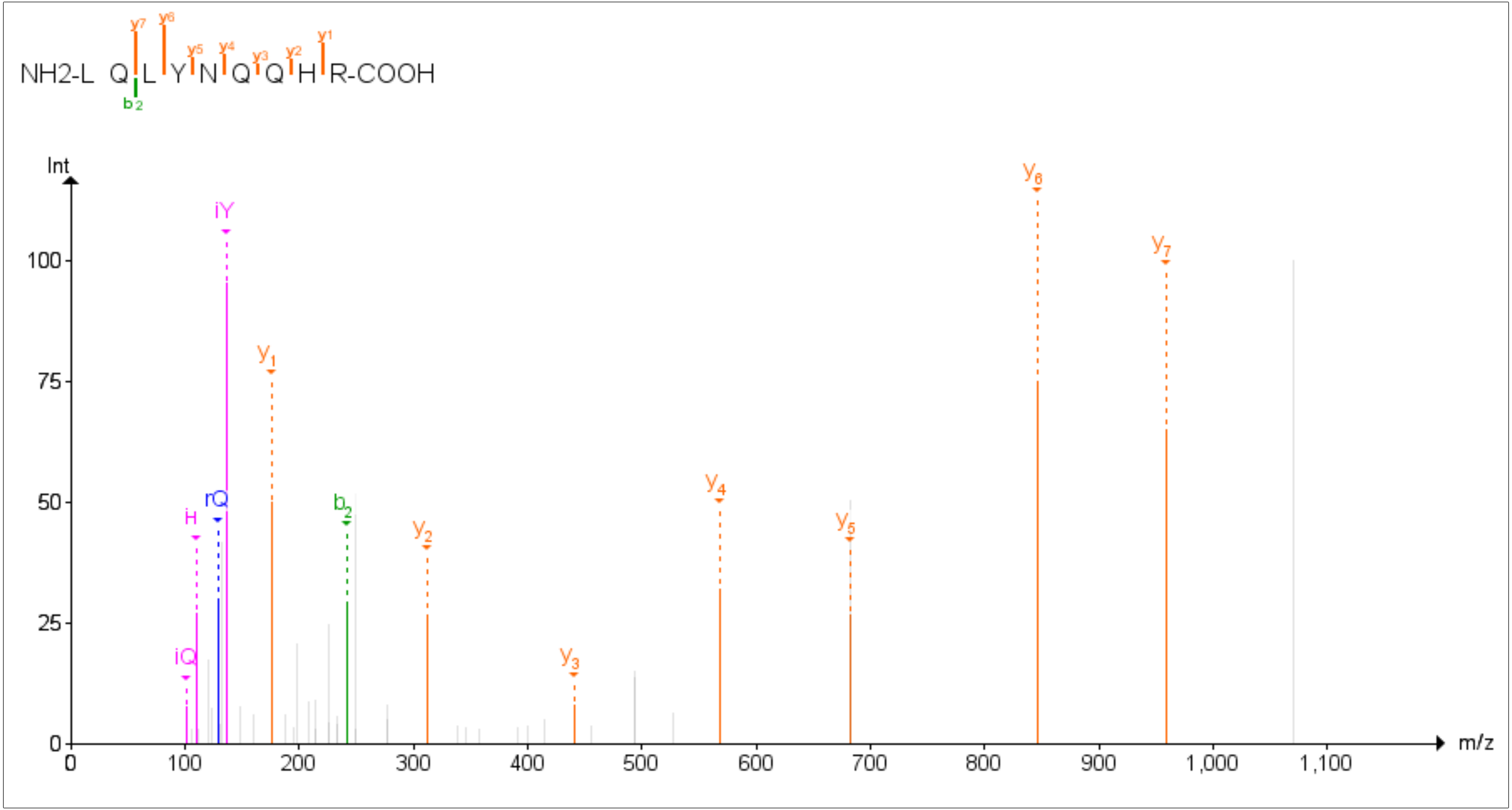

NH2-L Q L Y N Q Q H R-COOH
y7
y6
y5
y4
y3
y2
y1
b2
Int
iY
y1
rQ
H
b2
y2
iQ
y3
y4
y5
y6
y7
m/z
0
25
50
75
100
0
100
200
300
400
500
600
700
800
900
1,000
1,100

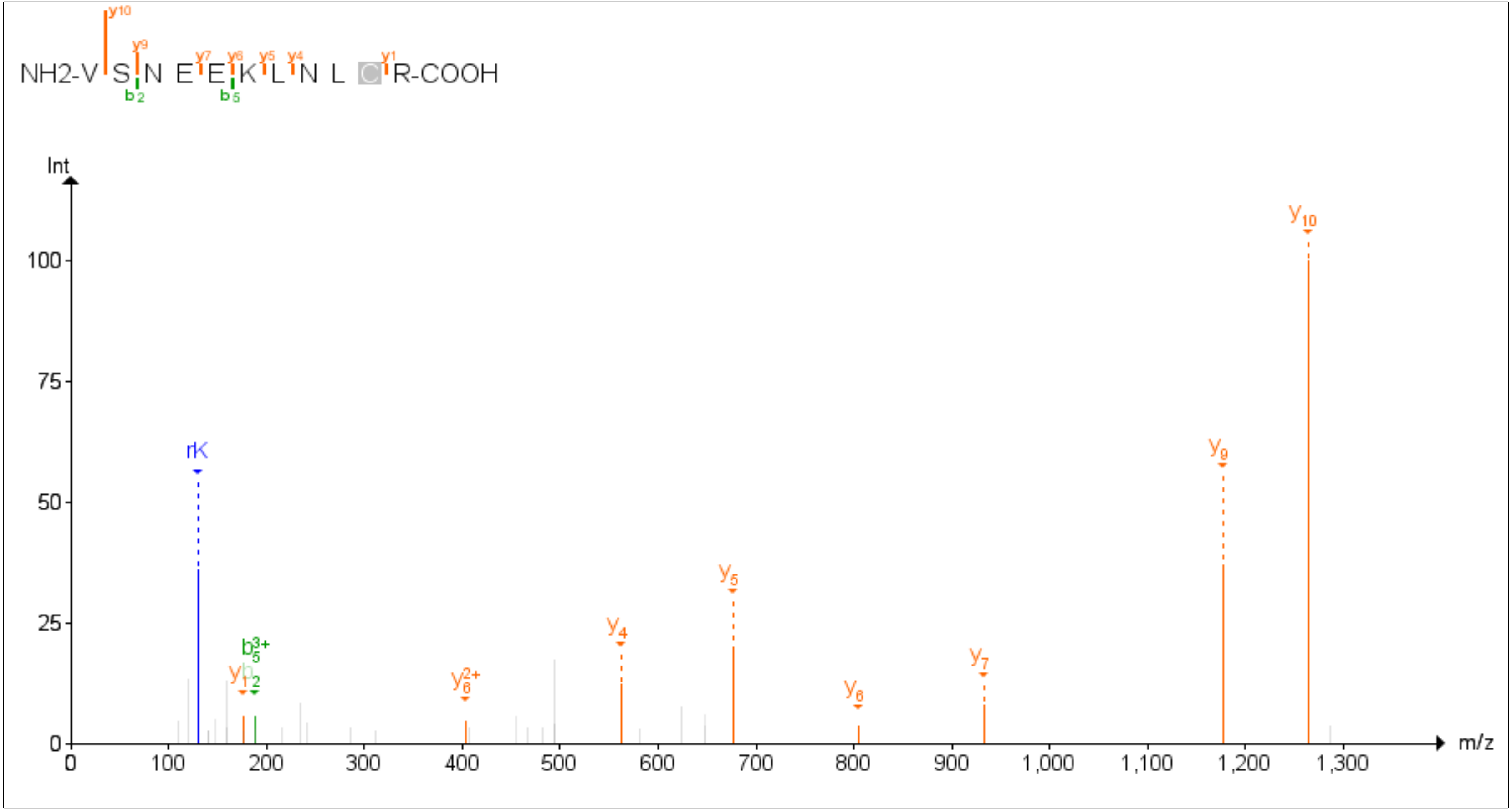

NH2-V S N E E K L N L C R-COOH
y10
y9
y7
y6
y5
y4
y1
b2
b5
Int
100
75
50
25
0
rK
b_5^{3+}
y_1
b_2
y_6^{2+}
y_4
y_5
y_6
y_7
y_9
y_{10}
0
100
200
300
400
500
600
700
800
900
1,000
1,100
1,200
1,300
m/z

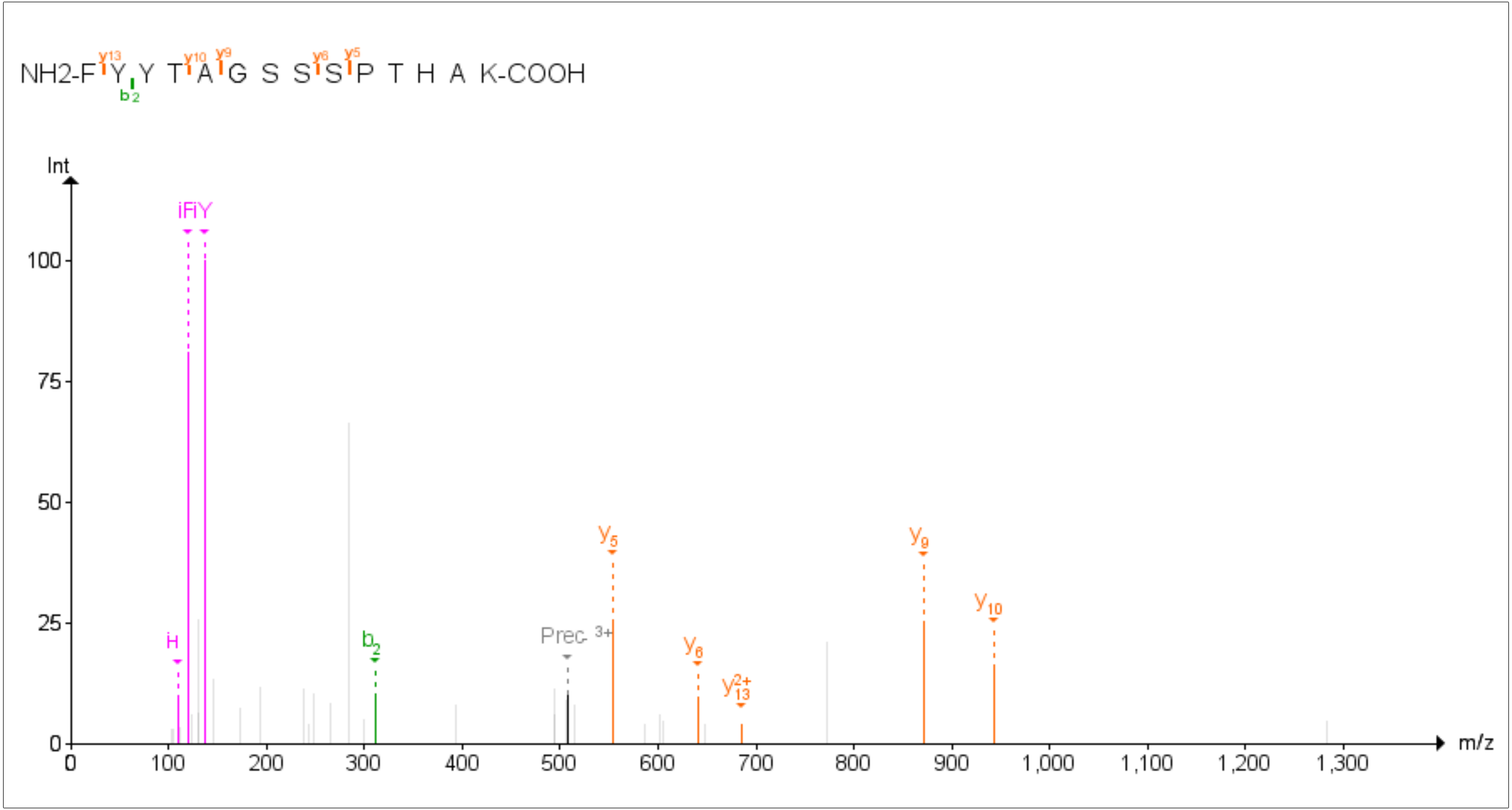
NH2-F Y Y T A G S S S P T H A K-COOH
y13
y10
y9
y6
y5
b2
Int
iF
iY
iH
b2
Prec 3+
y5
y6
y13 2+
y9
y10
0
25
50
75
100
0
100
200
300
400
500
600
700
800
900
1,000
1,100
1,200
1,300
m/z

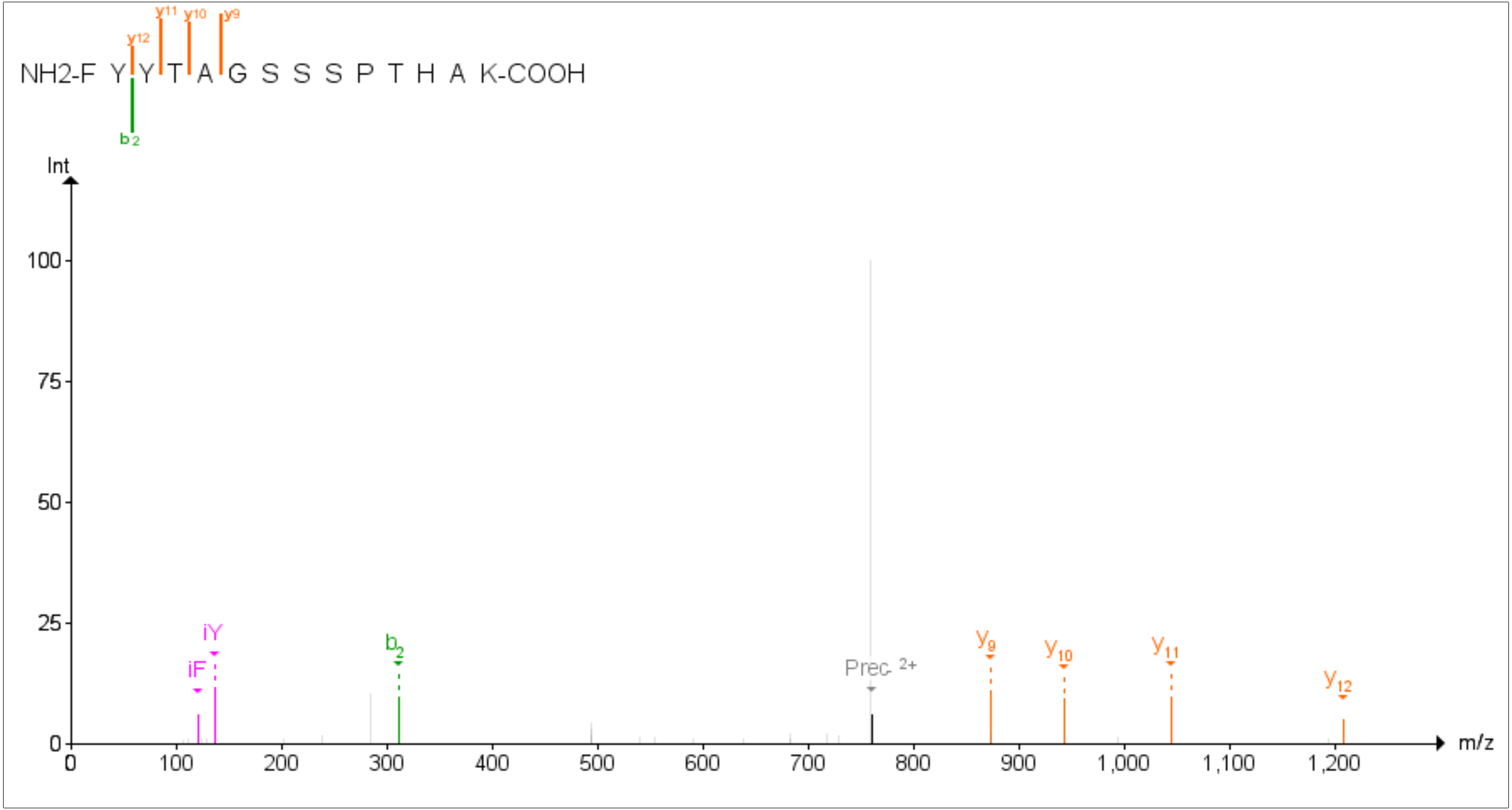

NH2-F Y Y T A G S S S P T H A K-COOH
y12
y11
y10
y9
b2
Int
100
75
50
25
0
iY
iF
b2
Prec 2+
y9
y10
y11
y12
0
100
200
300
400
500
600
700
800
900
1,000
1,100
1,200
m/z

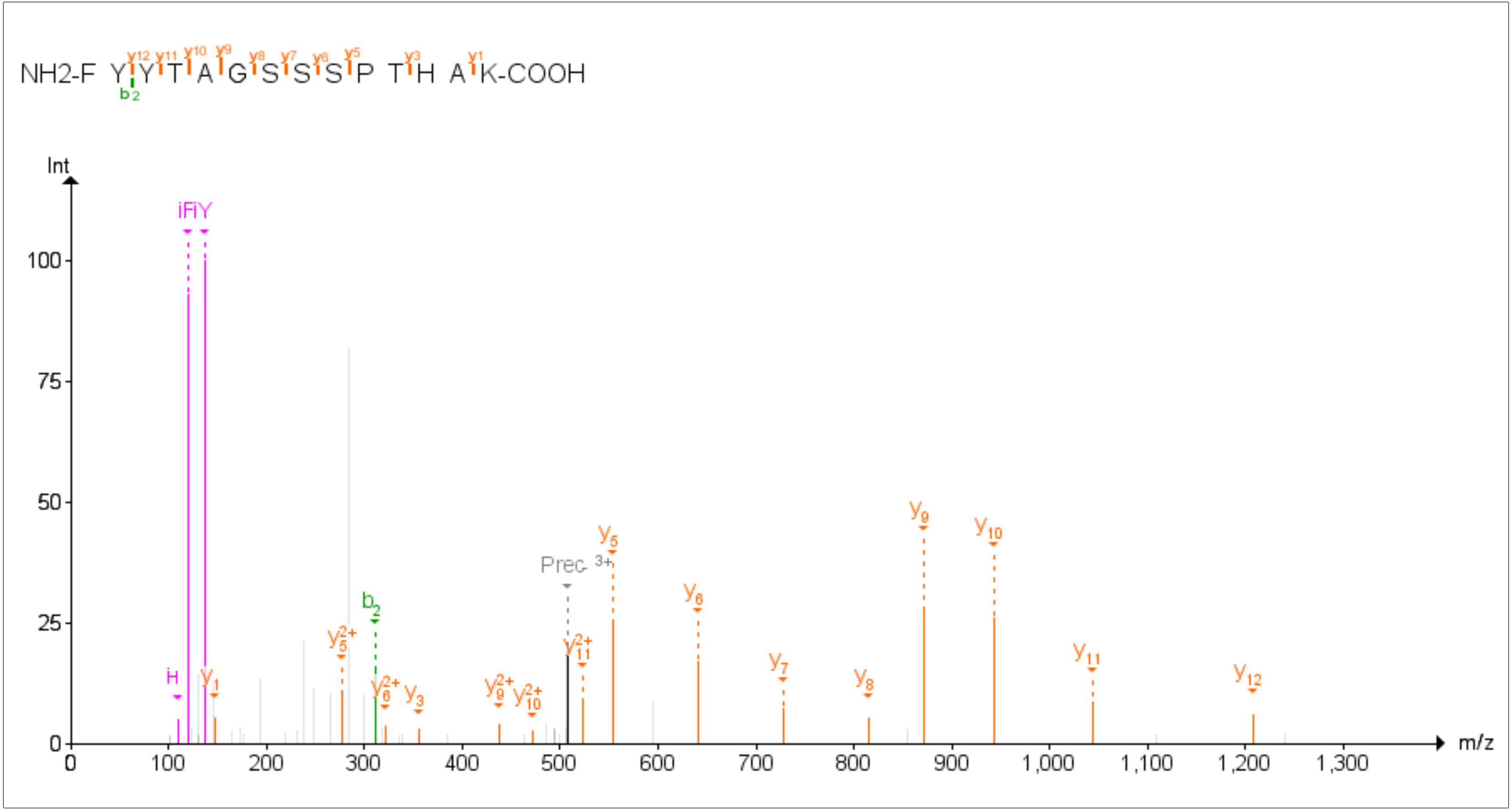

NH2-F Y Y T A G S S S P T H A K-COOH
y12 y11 y10 y9 y8 y7 y6 y5 y3 y1
b2
Int
m/z
iFiY
H
y1
y5 2+
b2
y6 2+
y3
y9 2+
y10 2+
Prec 3+
y11 2+
y5
y6
y7
y8
y9
y10
y11
y12
0
25
50
75
100
0
100
200
300
400
500
600
700
800
900
1,000
1,100
1,200
1,300

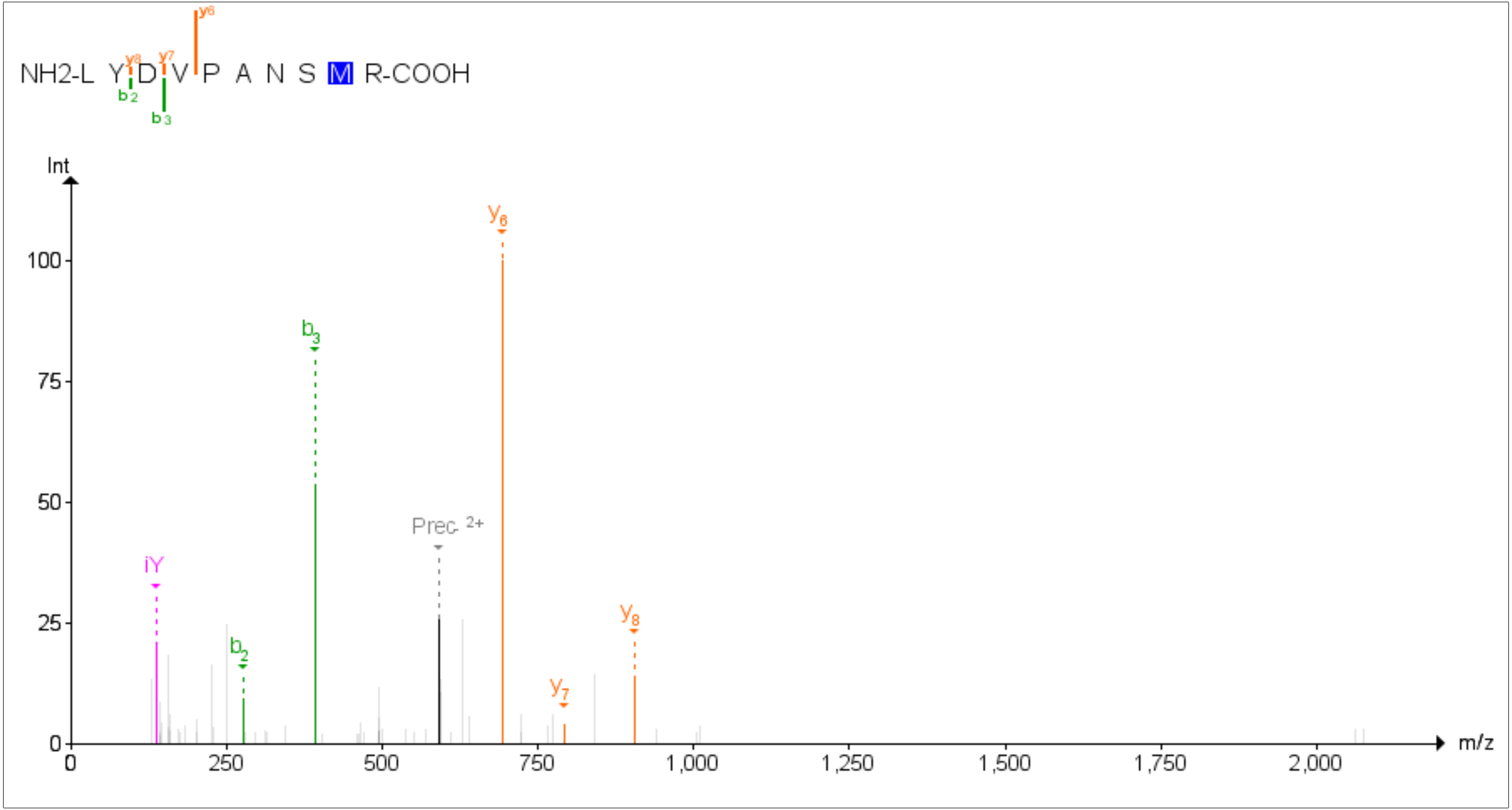

NH2-L Y D V P A N S M R-COOH
y8
y7
y6
b2
b3
Int
100
75
50
25
0
iY
b2
b3
Prec 2+
y6
y7
y8
0
250
500
750
1,000
1,250
1,500
1,750
2,000
m/z

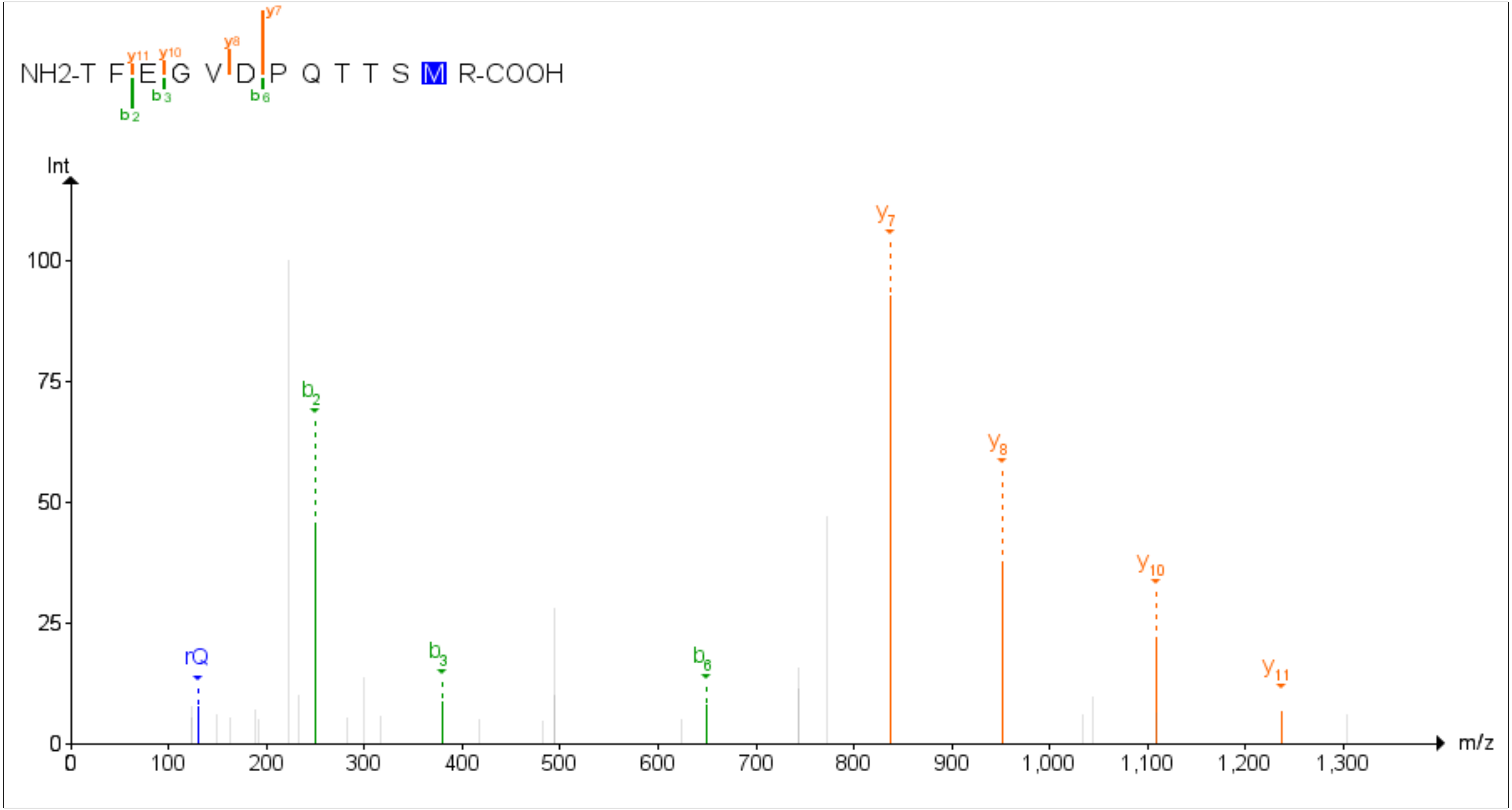

NH2-T F E G V D P Q T T S M R-COOH
y11 y10 y8 y7
b2 b3 b6
Int
100
75
50
25
0
rQ
b2
b3
b6
y7
y8
y10
y11
0 100 200 300 400 500 600 700 800 900 1,000 1,100 1,200 1,300
m/z

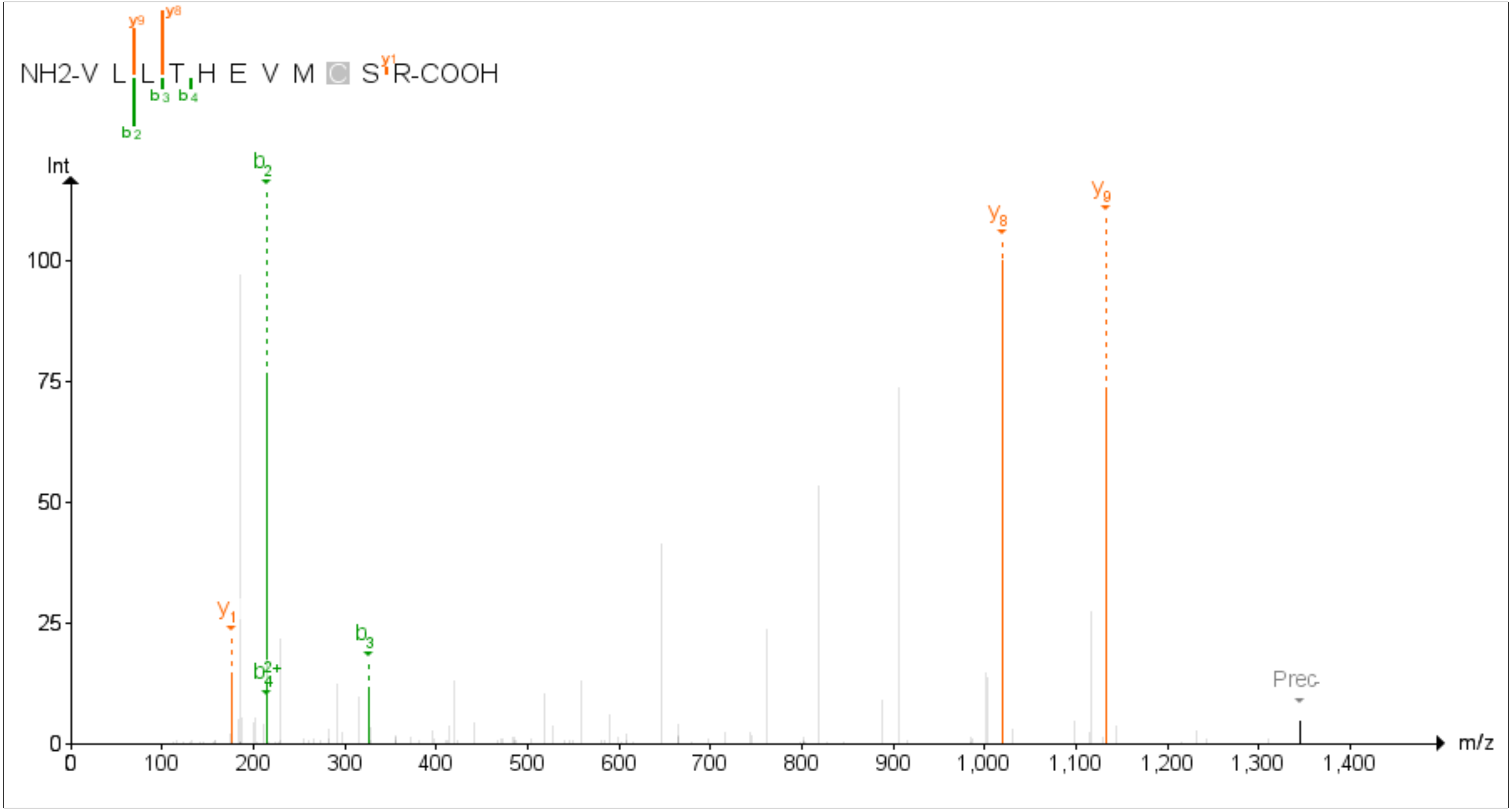

NH2-V L L T H E V M C S R-COOH
y9
y8
y1
b2
b3
b4
Int
100
75
50
25
0
b2
y8
y9
y1
b3
b4 2+
Prec
m/z
0
100
200
300
400
500
600
700
800
900
1,000
1,100
1,200
1,300
1,400

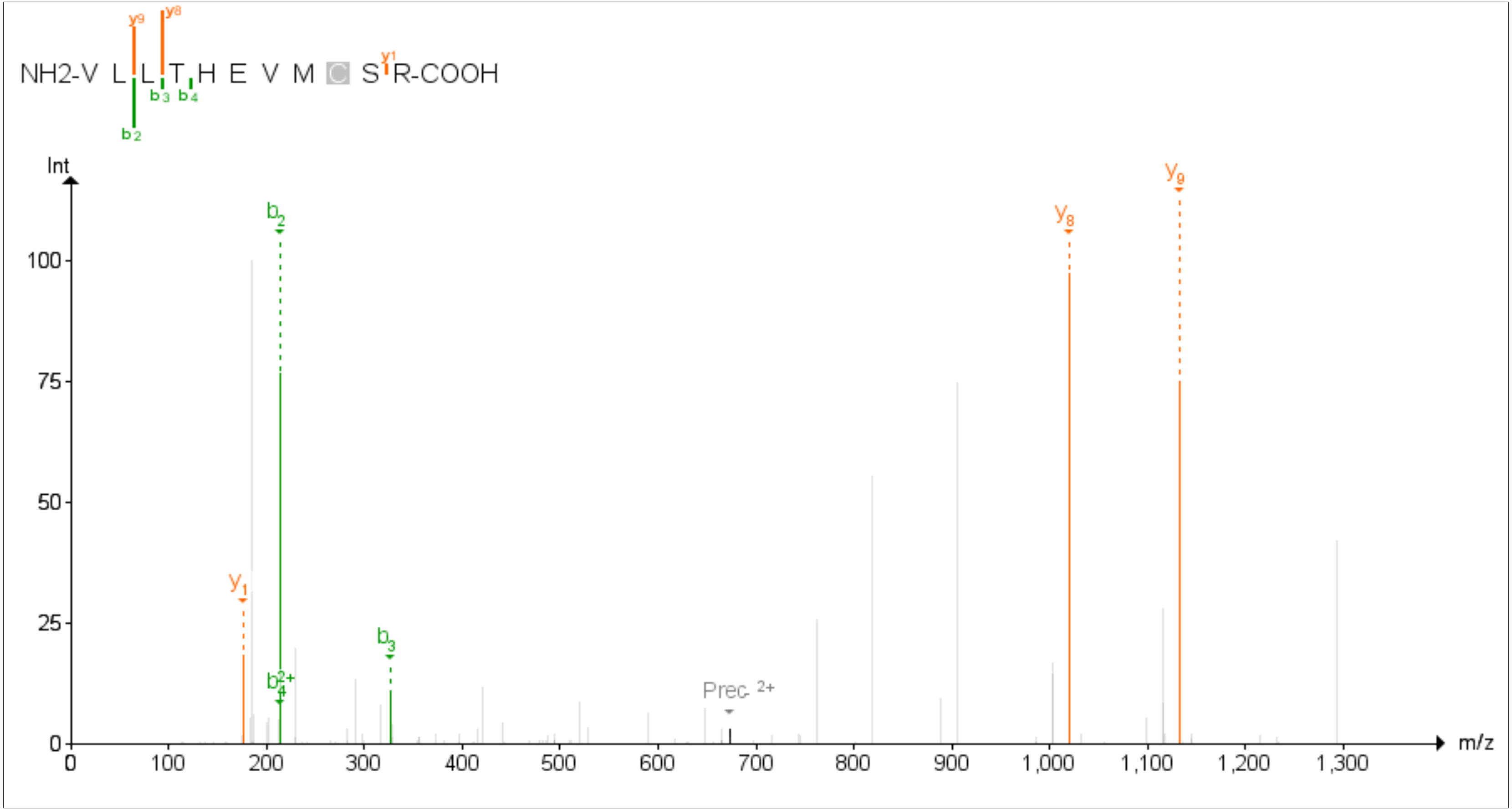

NH2-V L L T H E V M C S R-COOH
y9
y8
y1
b2
b3
b4
Int
100
75
50
25
0
b2
y8
y9
y1
b4 2+
b3
Prec. 2+
m/z
0
100
200
300
400
500
600
700
800
900
1,000
1,100
1,200
1,300

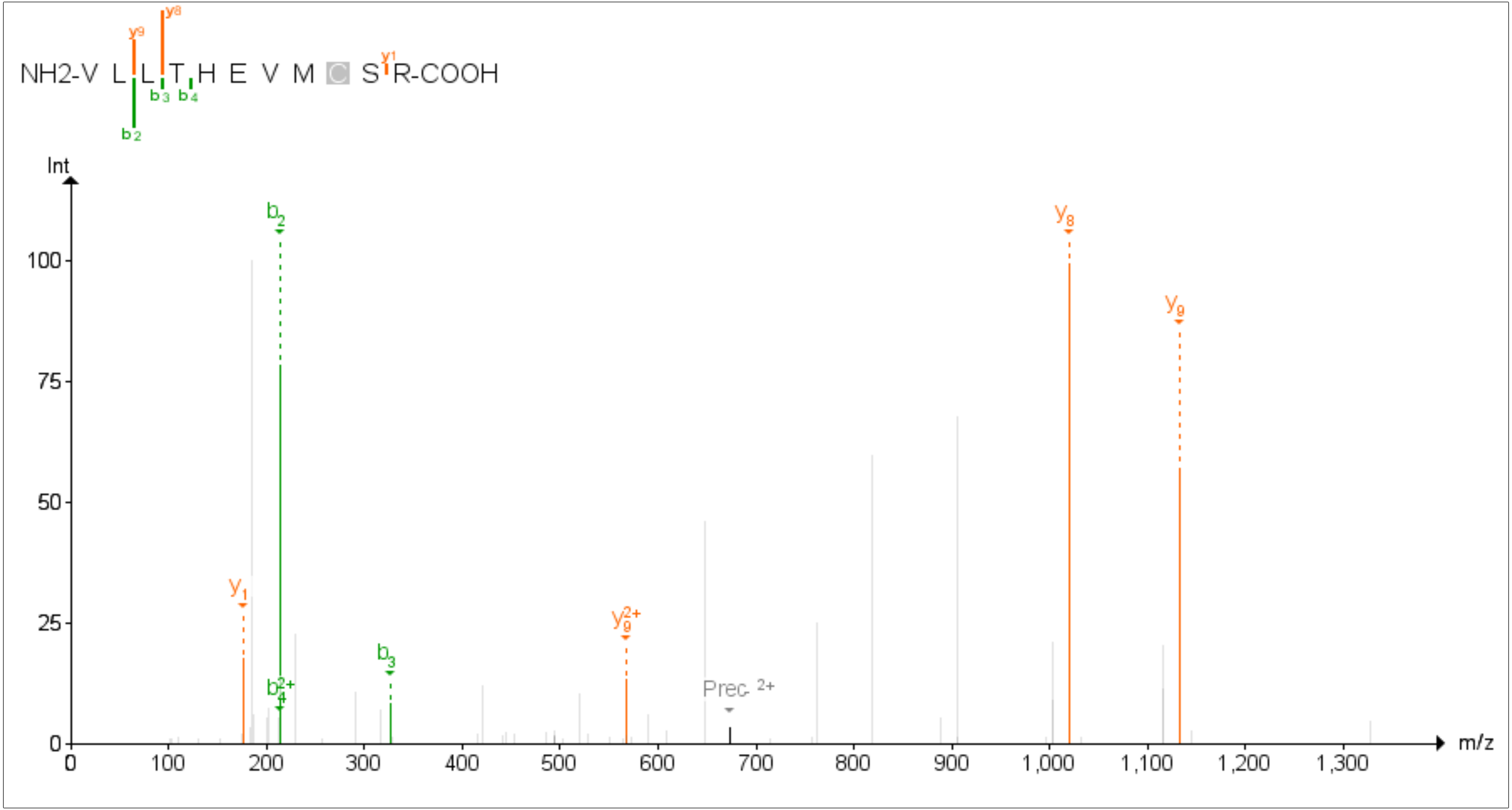
NH2-V L L T H E V M C S R-COOH
y9
y8
y1
b2
b3
b4
Int
100
75
50
25
0
b2
y8
y9
y1
b4 2+
b3
y9 2+
Prec. 2+
0
100
200
300
400
500
600
700
800
900
1,000
1,100
1,200
1,300
m/z

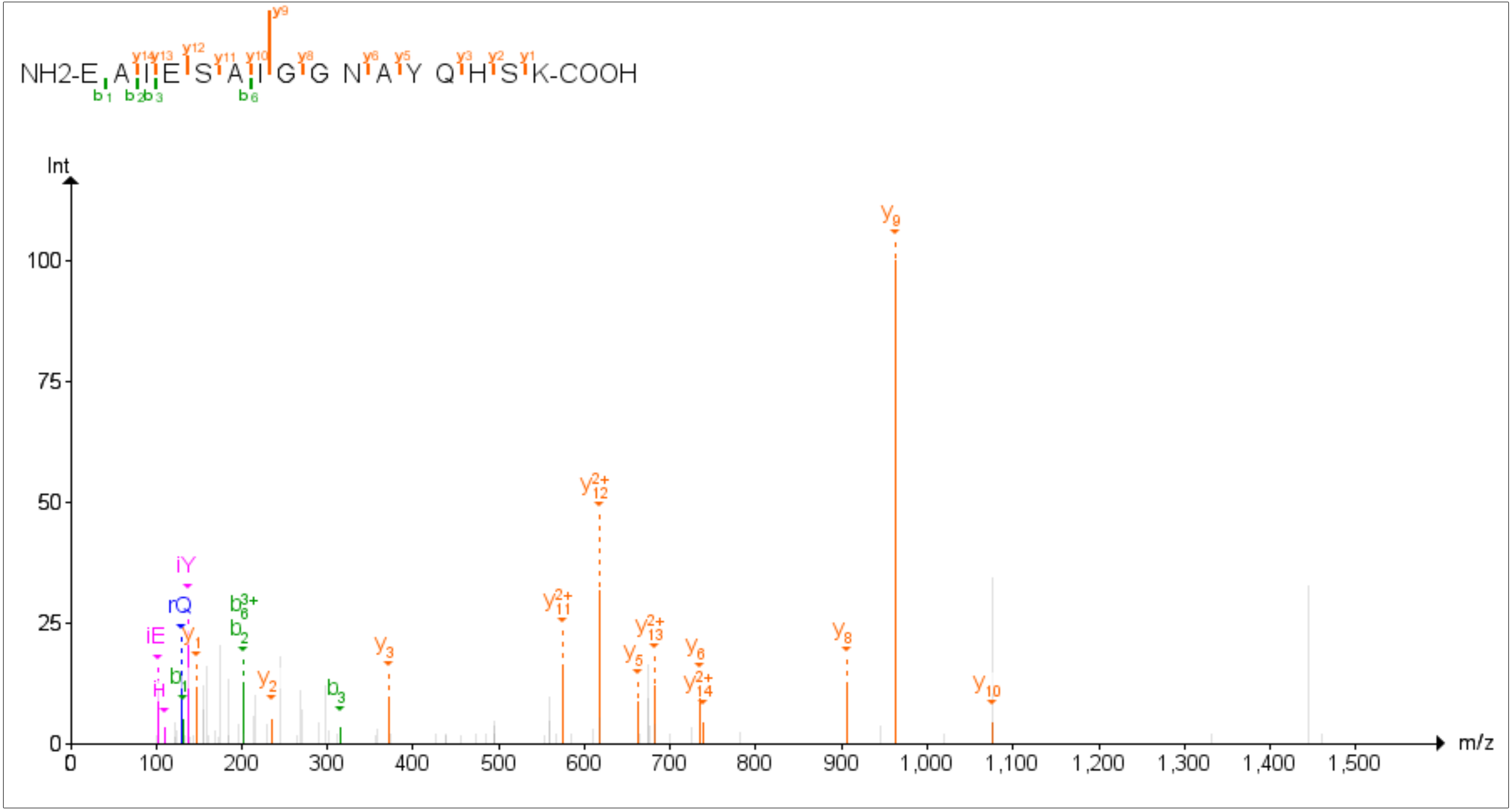

NH2-E A I I E S A I I G G N A Y Q H S K-COOH
Int
m/z
y9
y12 2+
y11 2+
y13 2+
y5
y6
y14 2+
y8
y10
y3
y2
y1
b6 3+
b2
b3
b1
iY
rQ
iE
iH

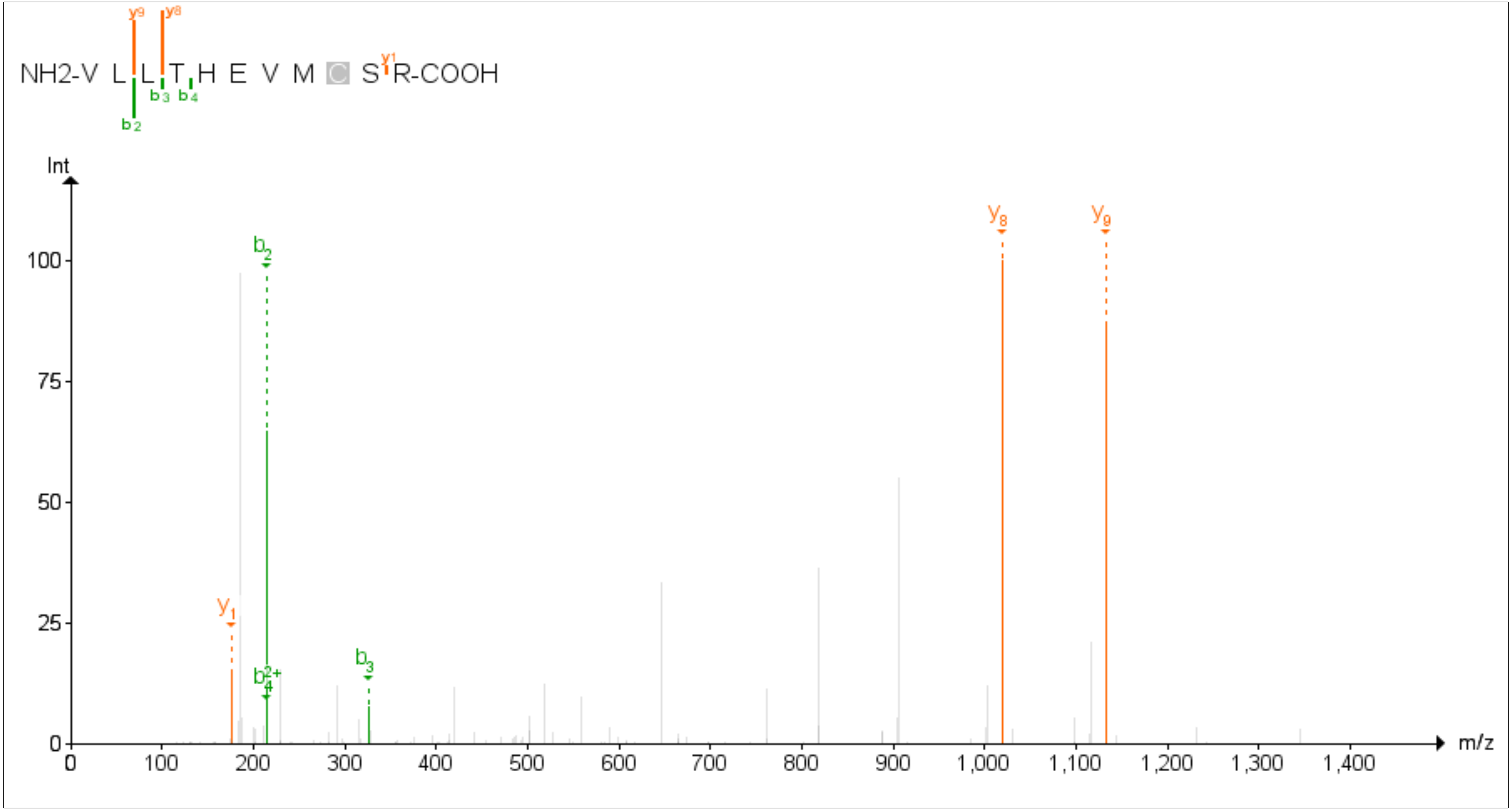

NH2-V L L T H E V M C S R-COOH
y9
y8
y1
b2
b3
b4
Int
100
75
50
25
0
b2
y8
y9
y1
b_4^{2+}
b3
0
100
200
300
400
500
600
700
800
900
1,000
1,100
1,200
1,300
1,400
m/z

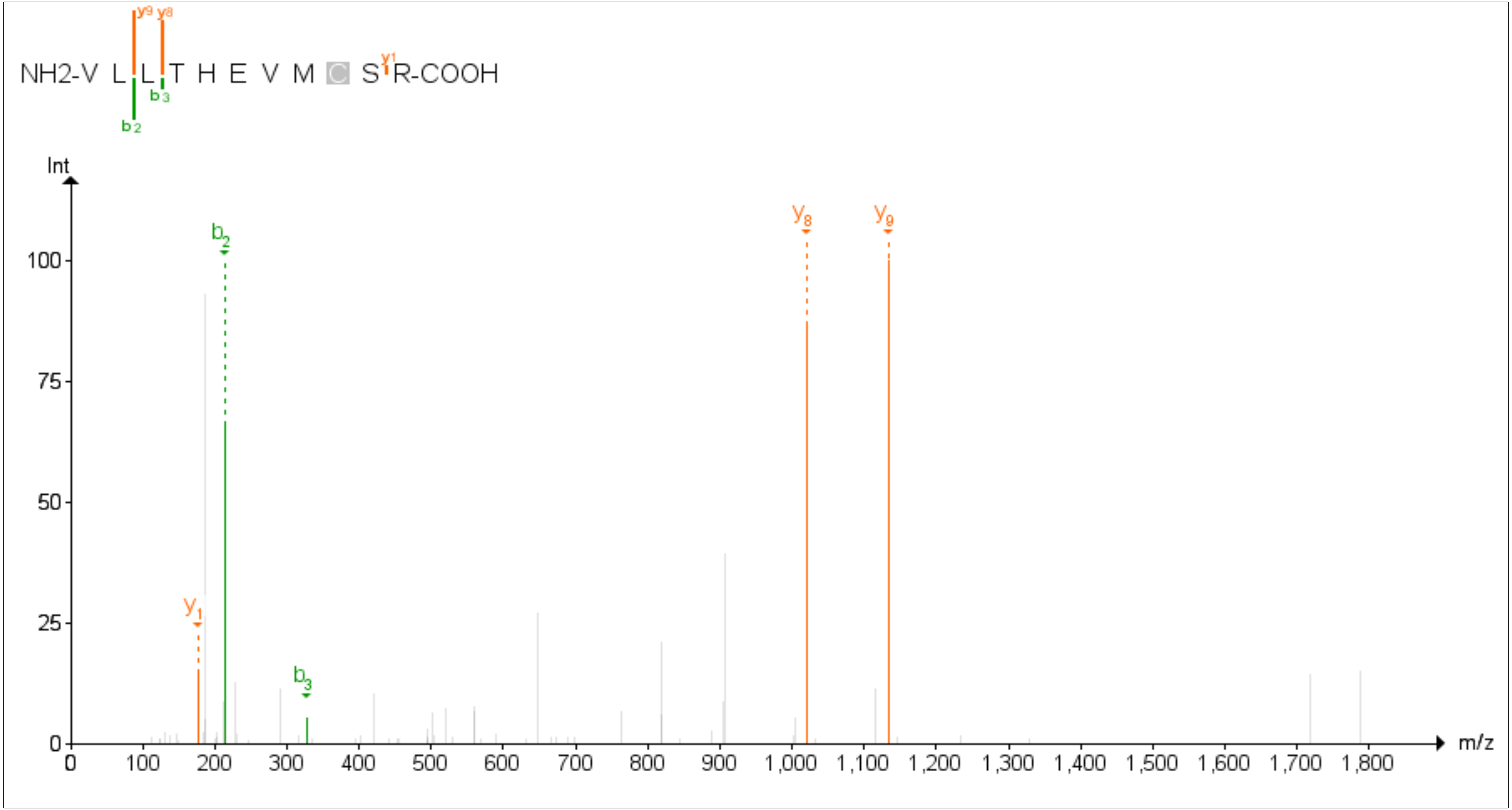

NH2-V L L T H E V M C S R-COOH
y9 y8 y1
b2 b3
Int
100
75
50
25
0
m/z
0 100 200 300 400 500 600 700 800 900 1,000 1,100 1,200 1,300 1,400 1,500 1,600 1,700 1,800
y1
b2
b3
y8
y9

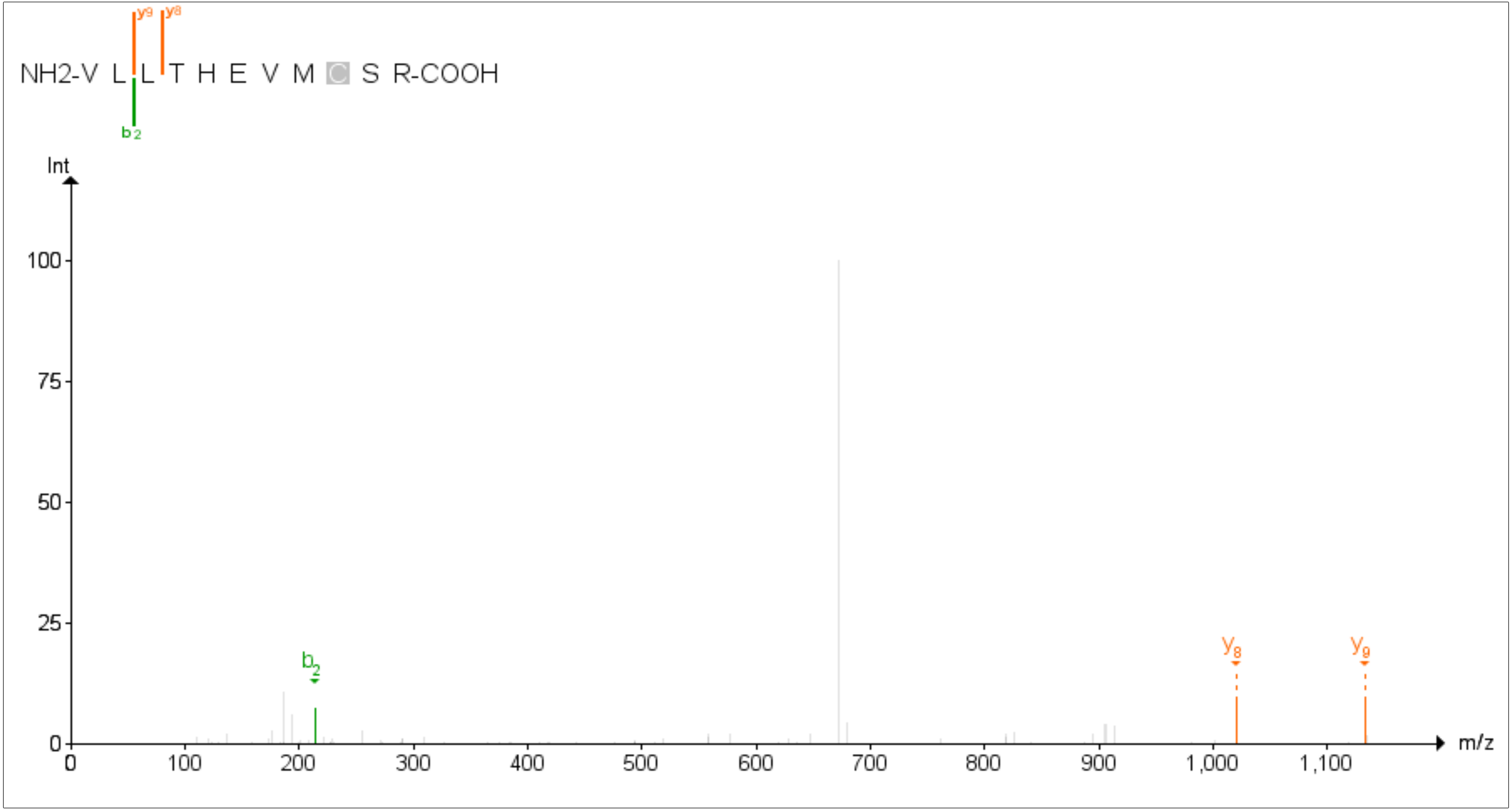

NH2-V L L T H E V M C S R-COOH
y9
y8
b2
Int
m/z
100
75
50
25
0
0
100
200
300
400
500
600
700
800
900
1,000
1,100
b2
y8
y9

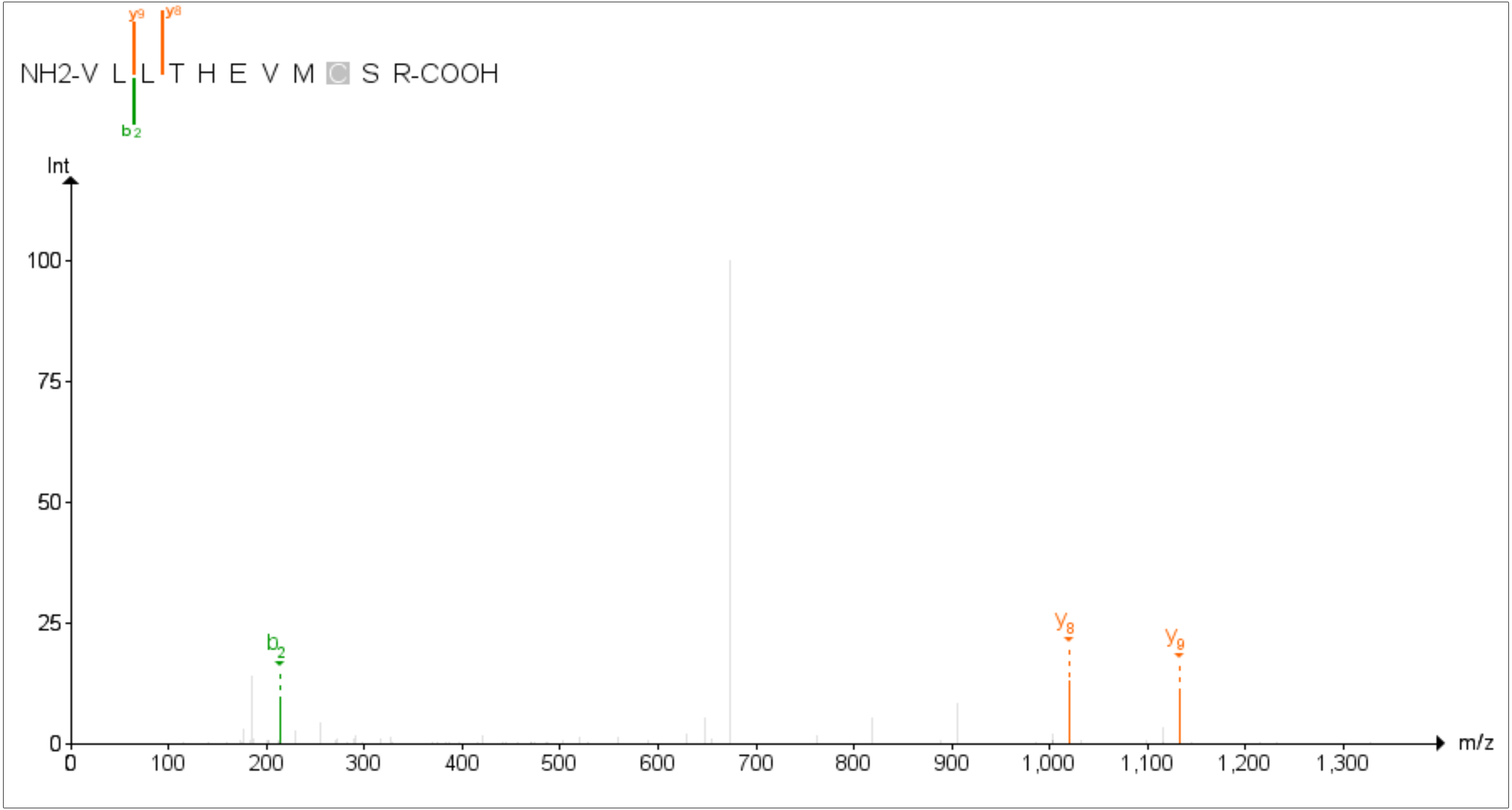

NH2-V L L T H E V M C S R-COOH
y9
y8
b2
Int
100
75
50
25
0
m/z
0
100
200
300
400
500
600
700
800
900
1,000
1,100
1,200
1,300
b2
y8
y9

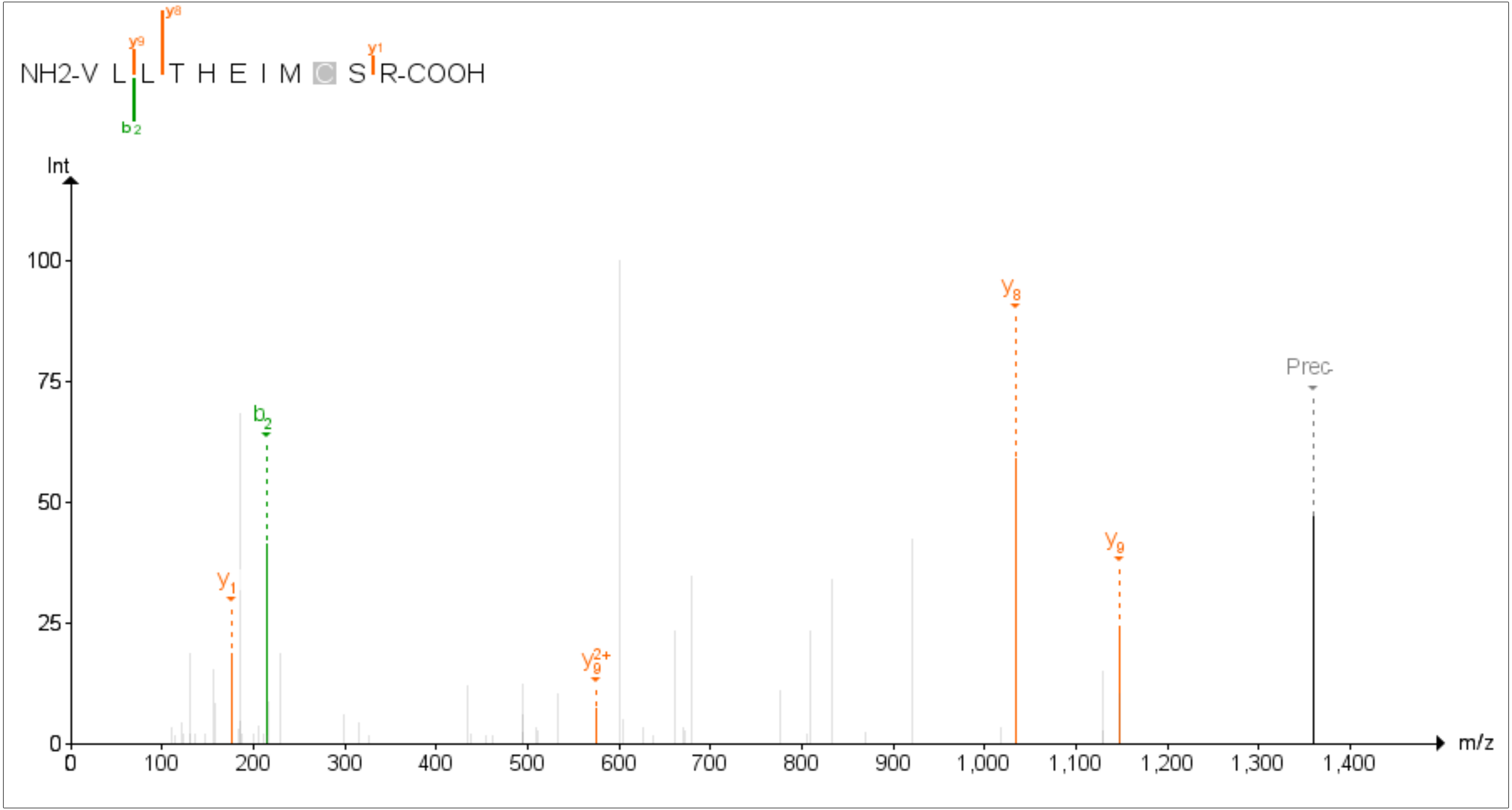

NH2-V L L T H E I M C S R-COOH
y8
y9
y1
b2
Int
100
75
50
25
0
y8
Prec.
b2
y9
y1
y9 2+
m/z
0
100
200
300
400
500
600
700
800
900
1,000
1,100
1,200
1,300
1,400

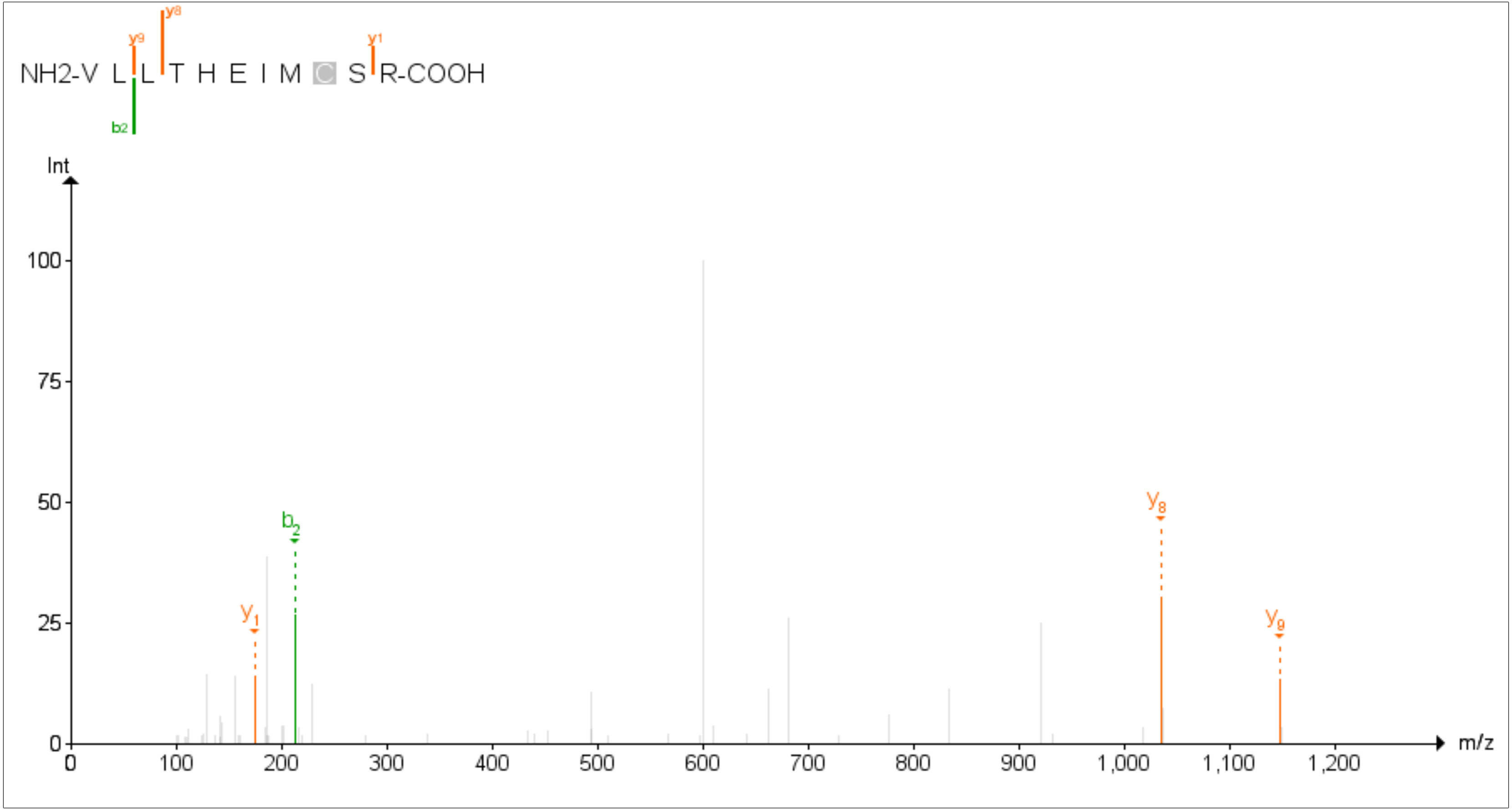

NH2-V L L T H E I M C S R-COOH
y8
y9
y1
b2
Int
100
75
50
25
0
m/z
0
100
200
300
400
500
600
700
800
900
1,000
1,100
1,200
y1
b2
y8
y9

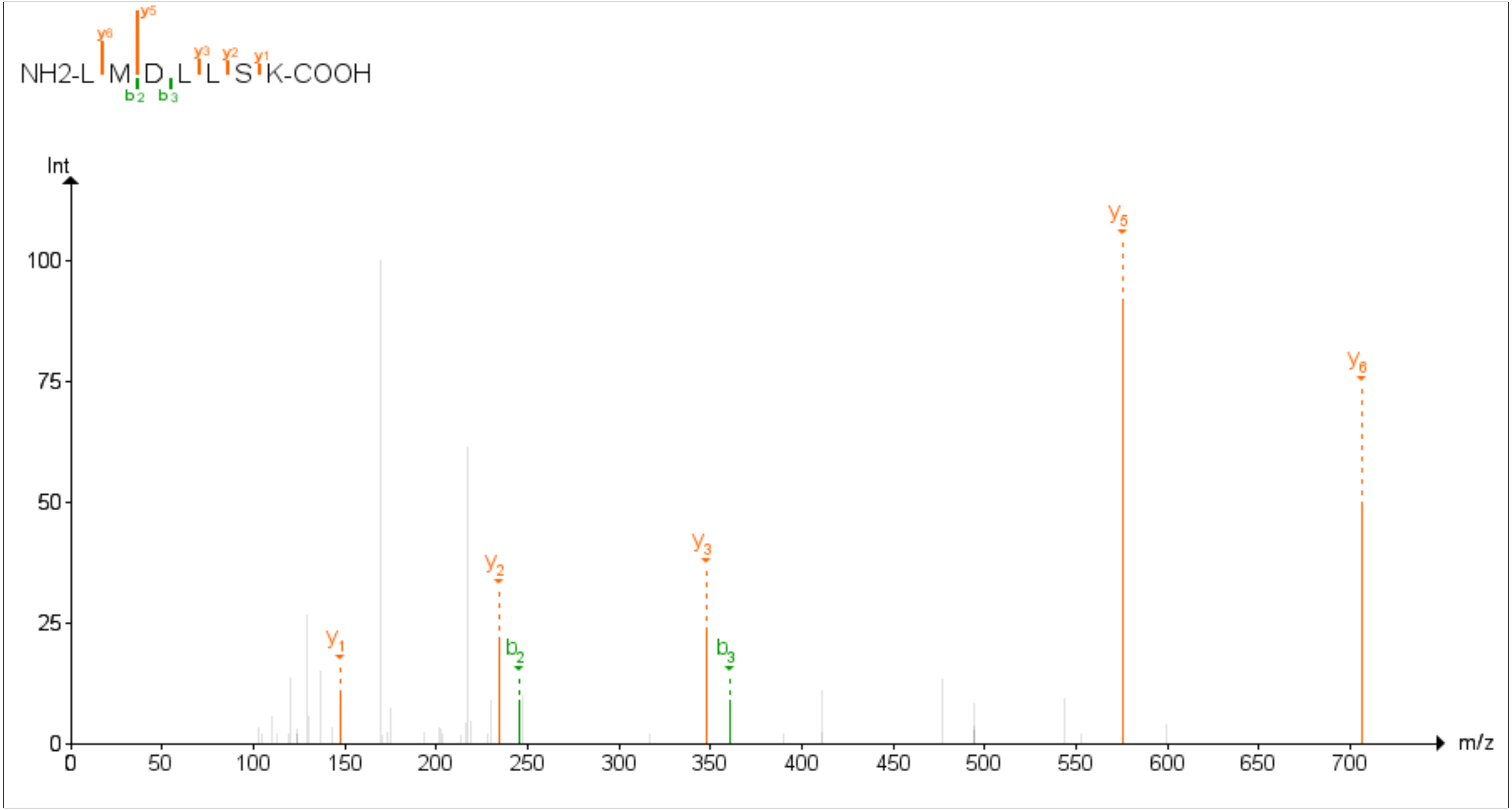

NH2-L M D L L S K-COOH
y6 y5 y3 y2 y1
b2 b3
Int
100
75
50
25
0
0 50 100 150 200 250 300 350 400 450 500 550 600 650 700
m/z
y1 y2 b2 y3 b3 y5 y6

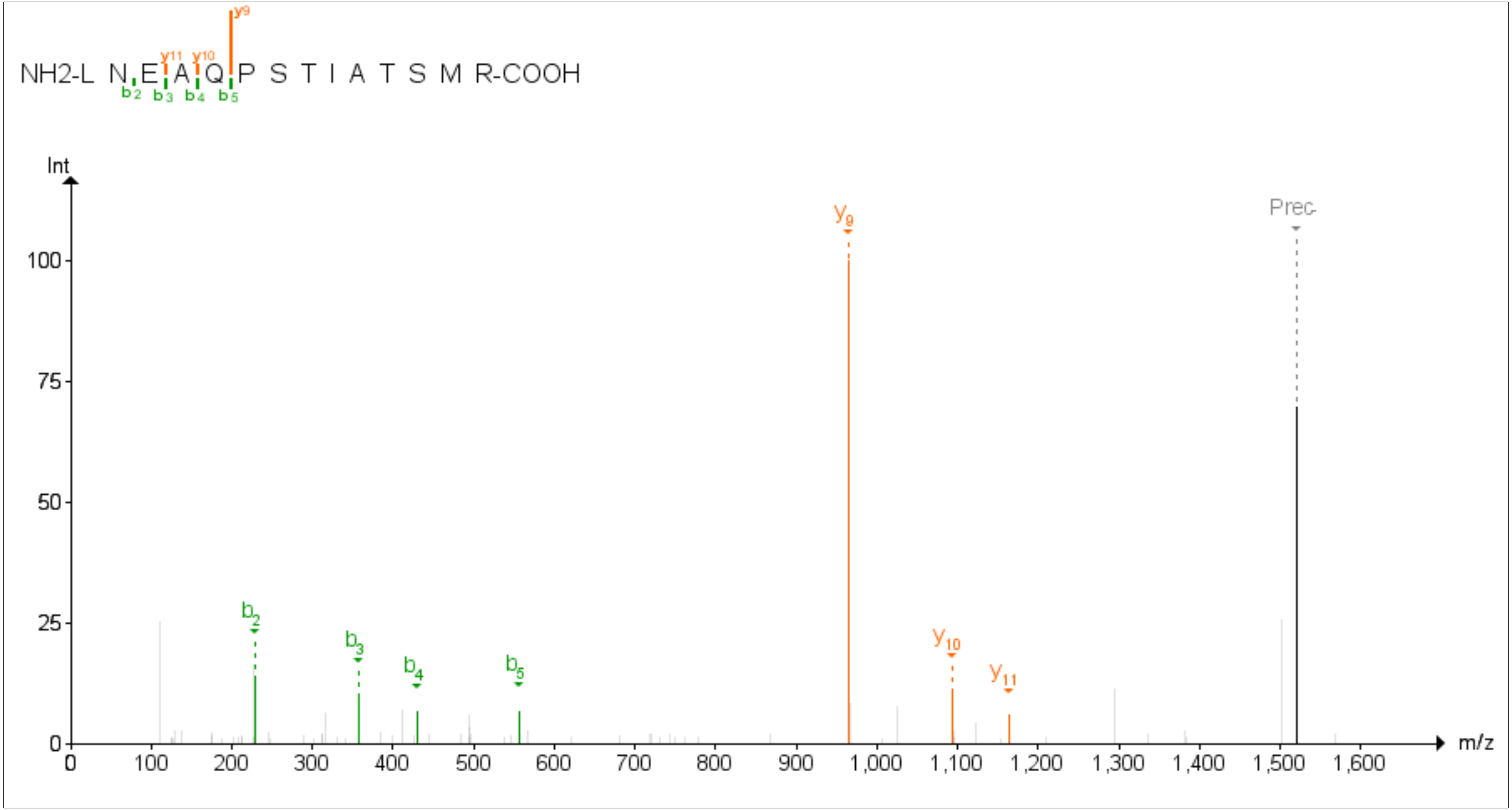

NH2-L N E A Q P S T I A T S M R-COOH
y9
y11
y10
b2
b3
b4
b5
Int
100
75
50
25
0
Prec
y9
y10
y11
b2
b3
b4
b5
0
100
200
300
400
500
600
700
800
900
1,000
1,100
1,200
1,300
1,400
1,500
1,600
m/z

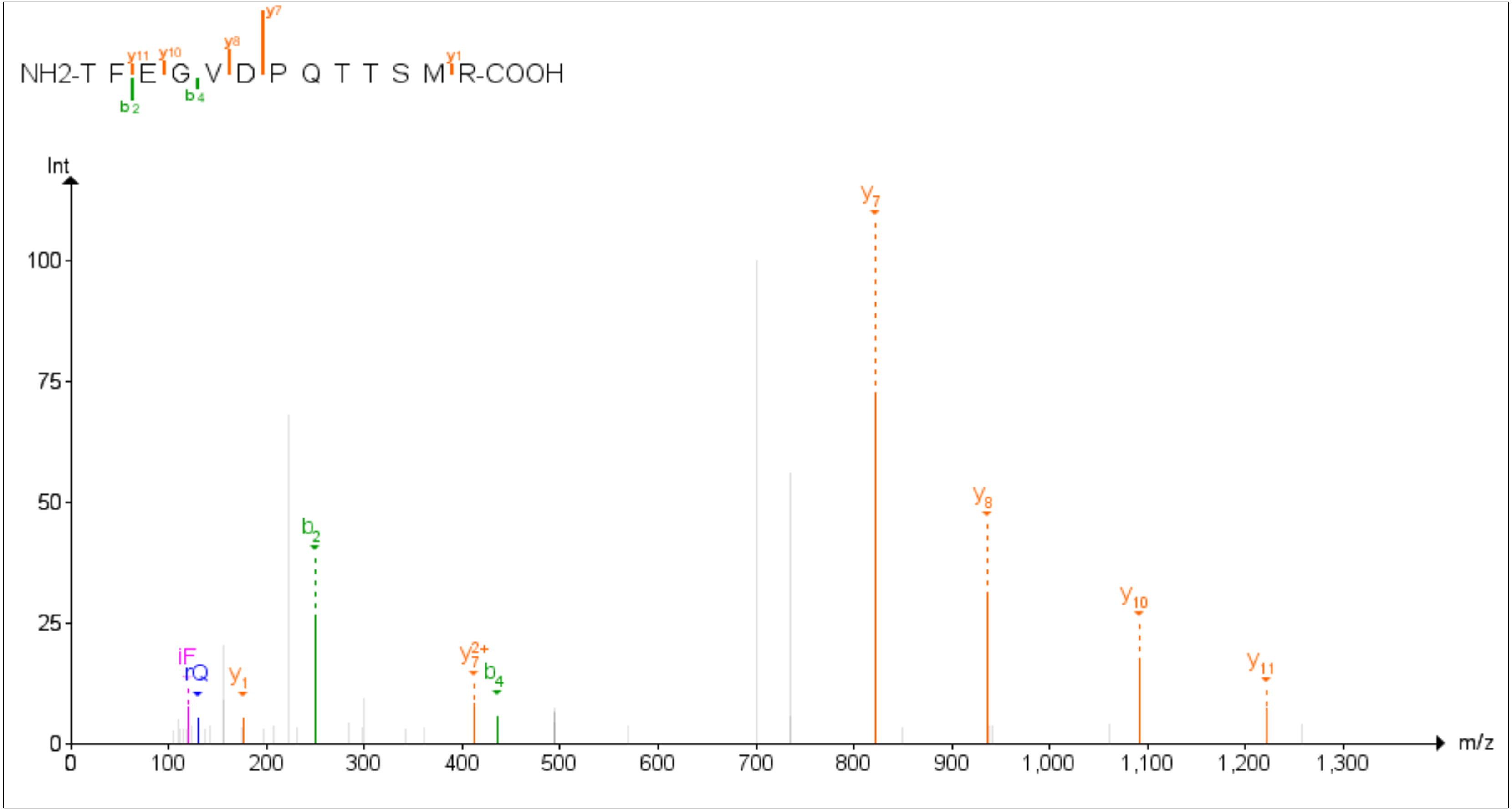
NH2-T F E G V D P Q T T S M R-COOH
y11
y10
y8
y7
y1
b2
b4
Int
100
75
50
25
0
m/z
0
100
200
300
400
500
600
700
800
900
1,000
1,100
1,200
1,300
y7
y8
y10
y11
b2
y7 2+
b4
y1
iF
rQ

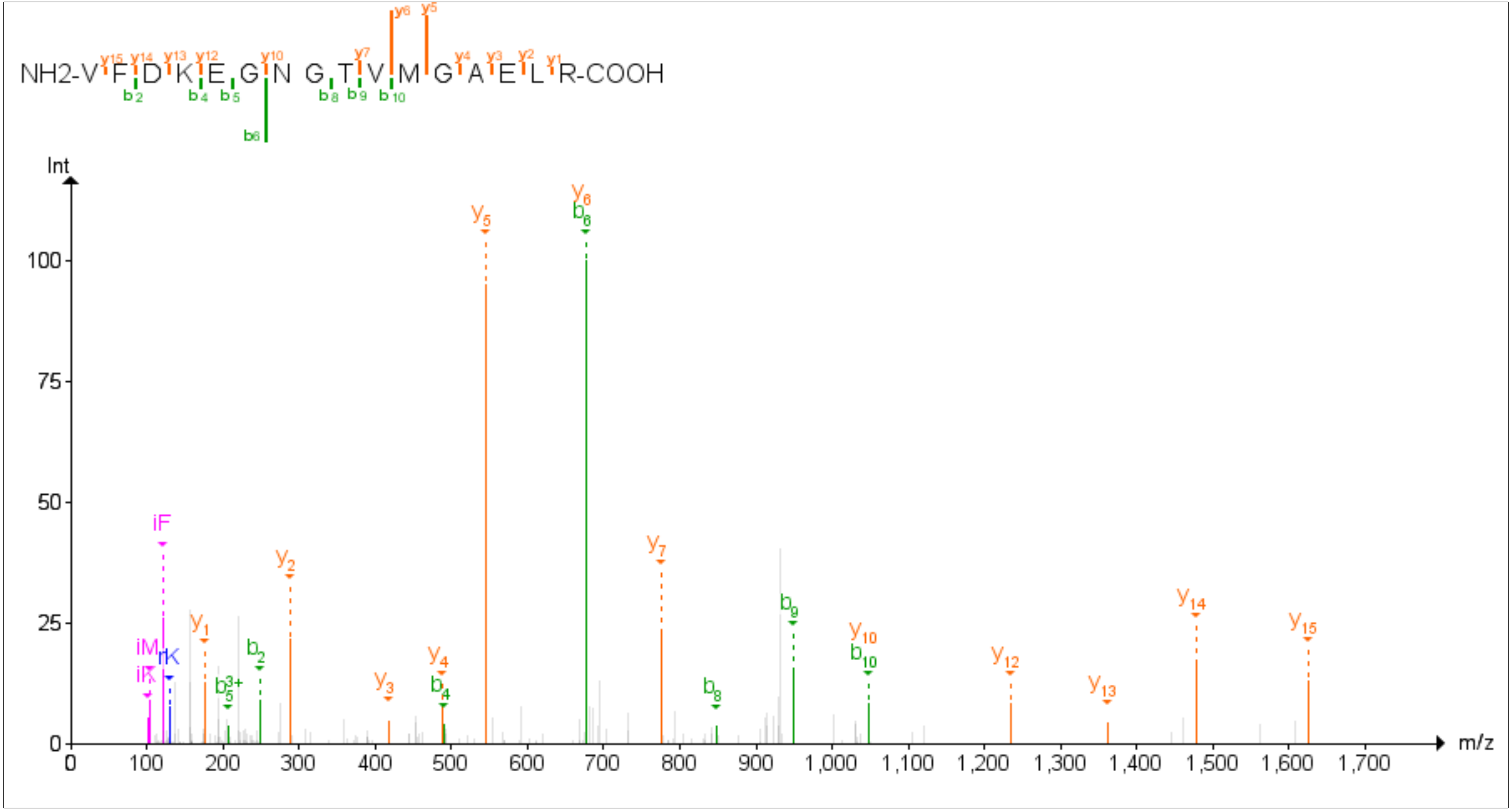
NH2-VFDKEGINGTVMGAELR-COOH
y15 y14 y13 y12 y10 y7 y6 y5 y4 y3 y2 y1
b2 b4 b5 b6 b8 b9 b10
Int
m/z
0
25
50
75
100
0
100
200
300
400
500
600
700
800
900
1,000
1,100
1,200
1,300
1,400
1,500
1,600
1,700
iF
iM
iK
rK
y1
b2
y2
y3
y4
b4
y5
y6
b6
y7
b8
b9
y10
b10
y12
y13
y14
y15

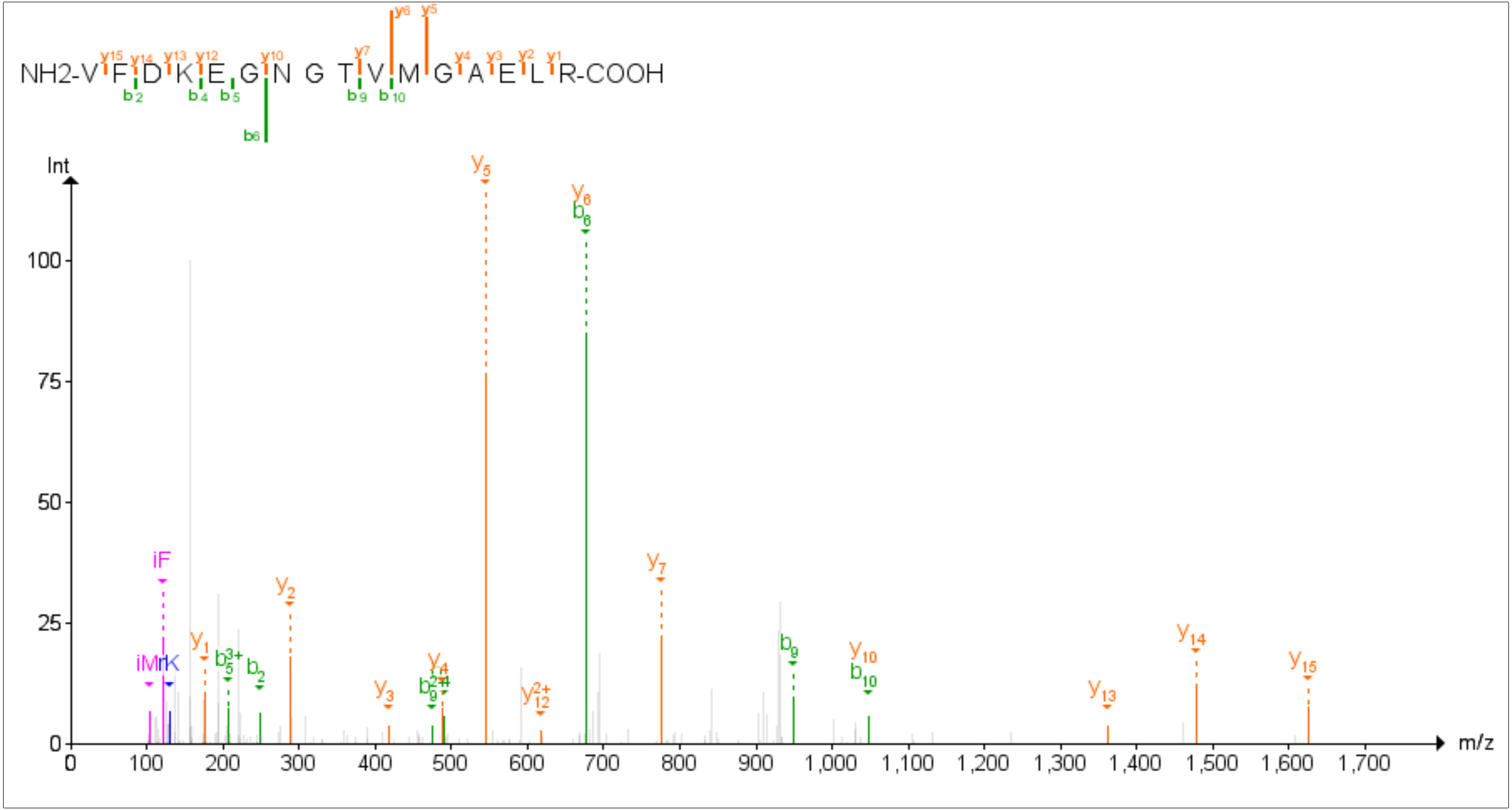

NH2-V F D K E G N G T V M G A E L R-COOH
y15 y14 y13 y12 y10 y7 y6 y5 y4 y3 y2 y1
b2 b4 b5 b6 b9 b10
Int
100
75
50
25
0
m/z
0 100 200 300 400 500 600 700 800 900 1,000 1,100 1,200 1,300 1,400 1,500 1,600 1,700
y5
y6
b6
iF
y2
y7
y1
iM
iK
b5 3+
b2
y3
b9 2+
y4
y12 2+
b9
y10
b10
y13
y14
y15

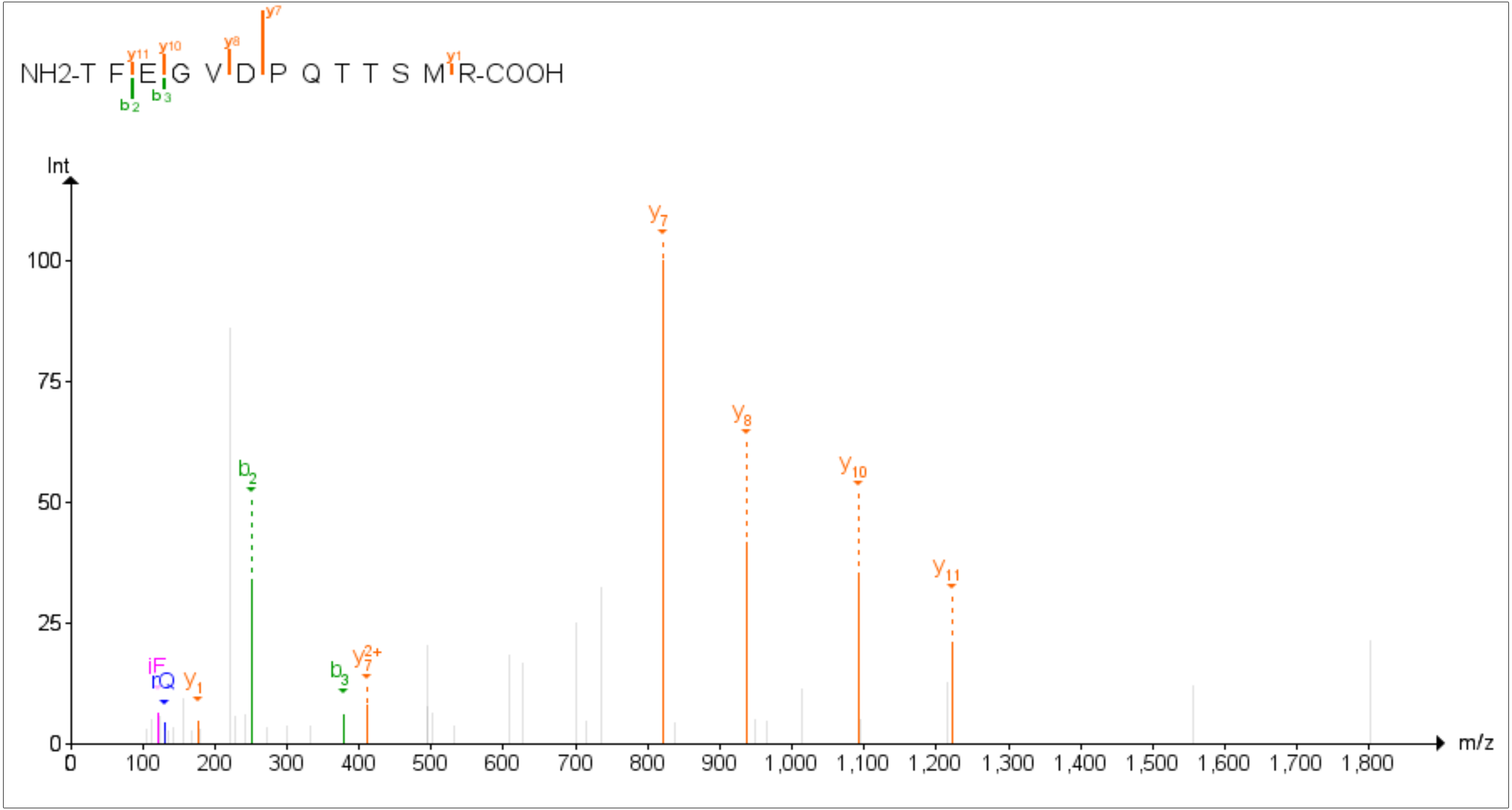

NH2-T F E G V D P Q T T S M R-COOH
y11 y10 y8 y7 y1
b2 b3
Int
100
75
50
25
0
y7
y8
y10
y11
b2
b3
y_7^{2+}
y1
iF
rQ
0 100 200 300 400 500 600 700 800 900 1,000 1,100 1,200 1,300 1,400 1,500 1,600 1,700 1,800
m/z

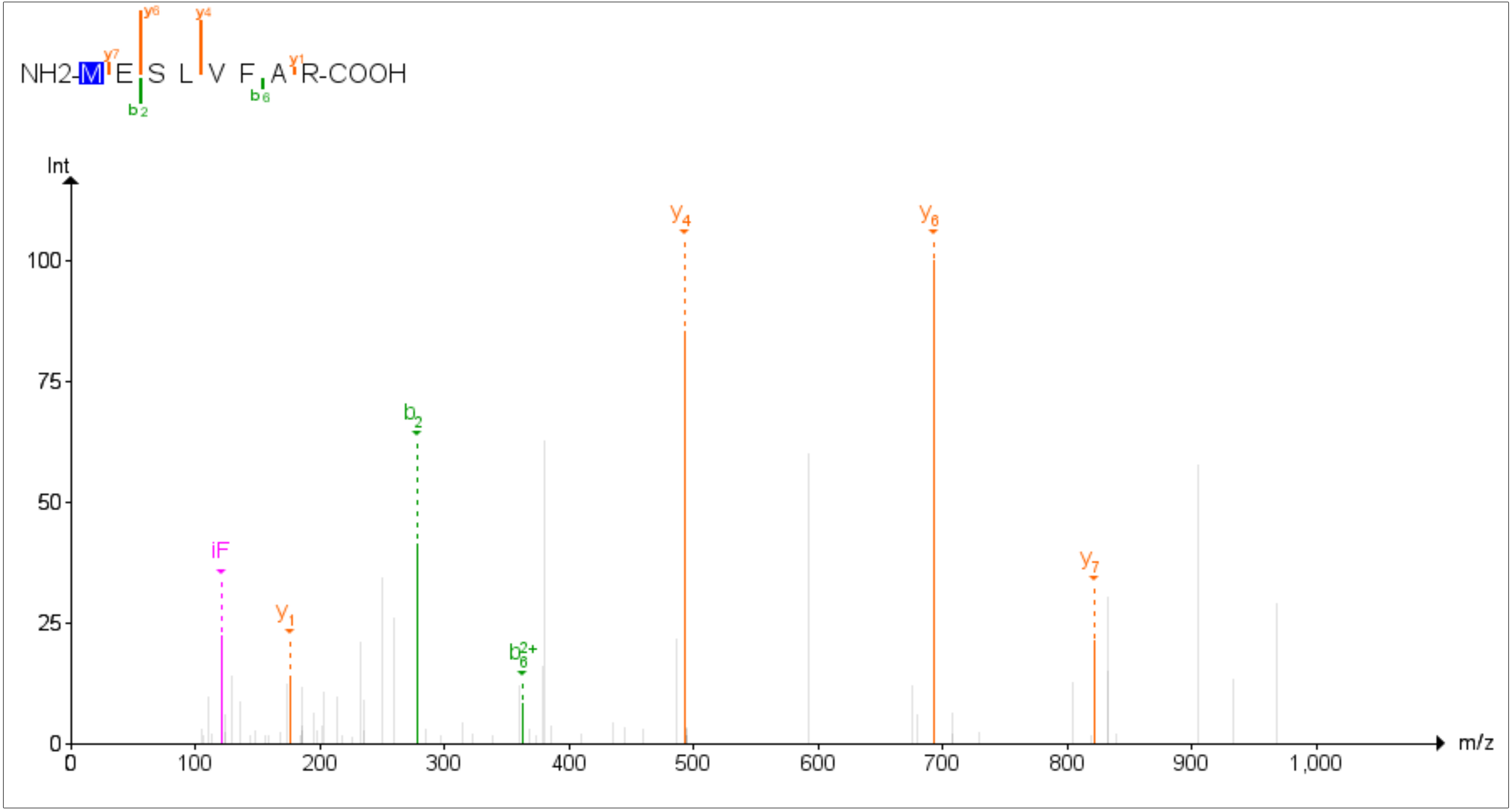

NH2-M E S L V F A R-COOH
y7
y6
y4
y1
b2
b6
Int
100
75
50
25
0
y4
y6
b2
iF
y1
b_6^{2+}
y7
0
100
200
300
400
500
600
700
800
900
1,000
m/z

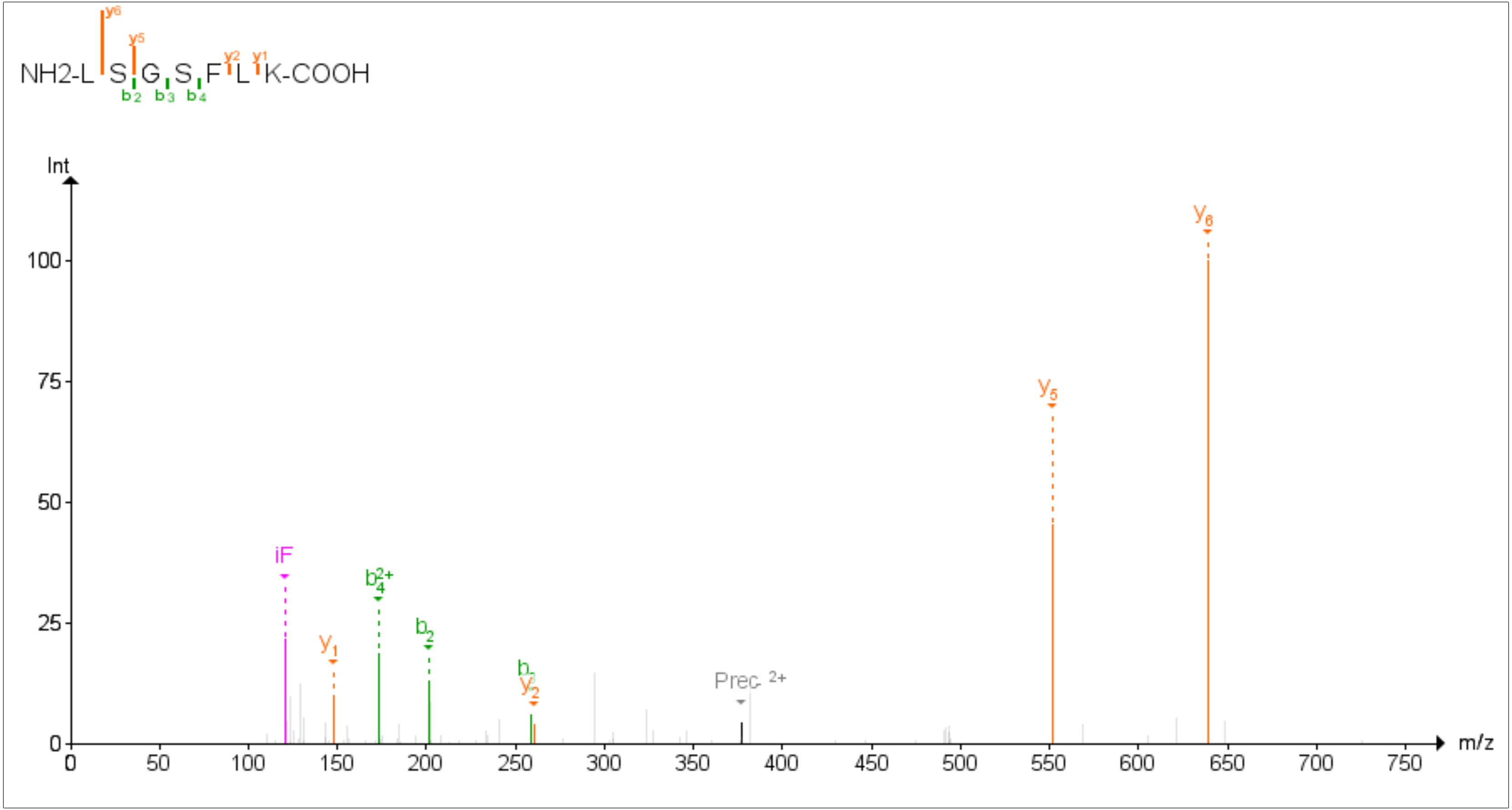
NH2-L S G S F L K-COOH
y6
y5
y2
y1
b2
b3
b4
Int
m/z
y6
y5
iF
b4 2+
b2
y1
b3
y2
Prec 2+
0
25
50
75
100
0
50
100
150
200
250
300
350
400
450
500
550
600
650
700
750

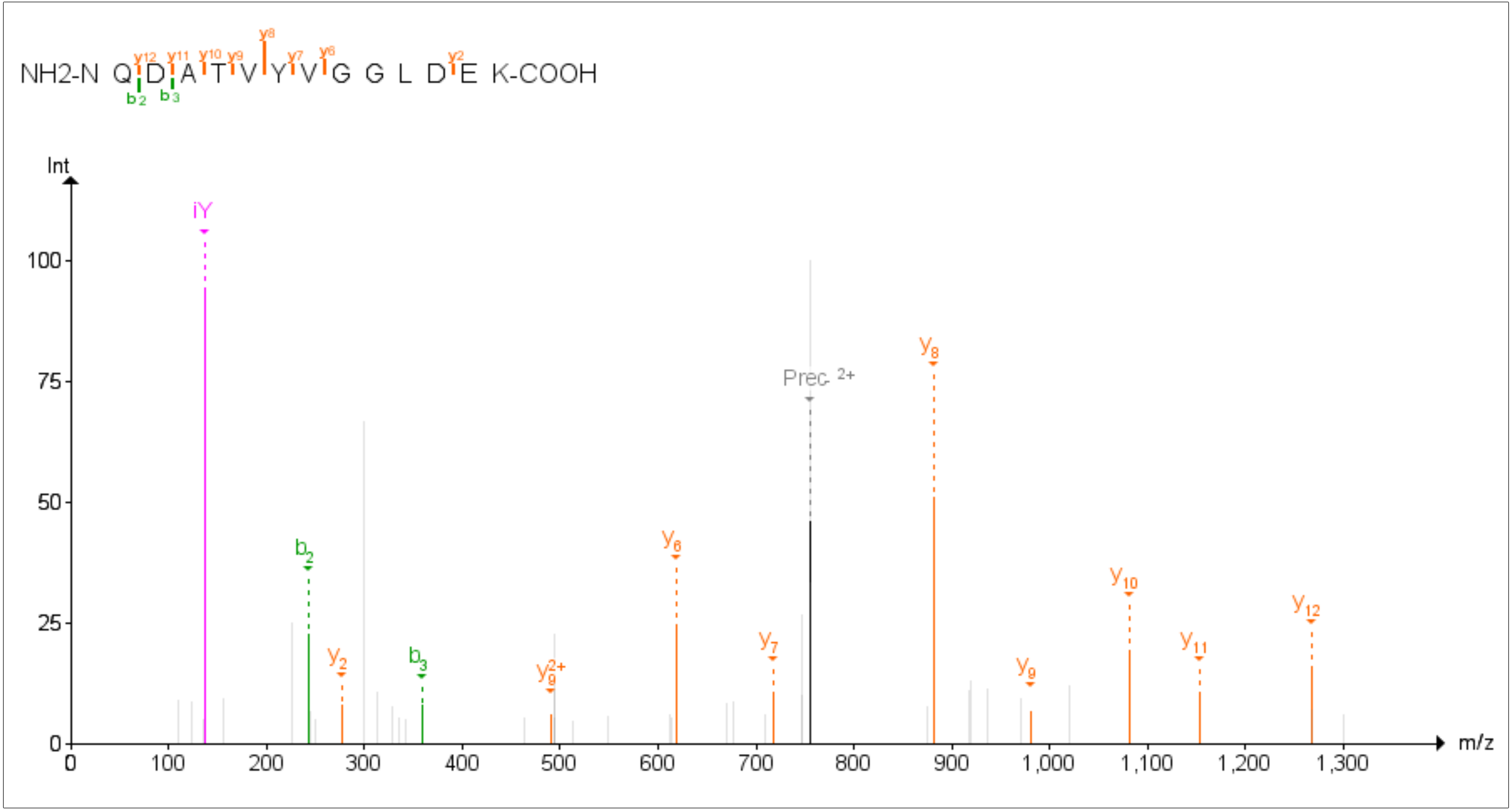

NH2-N Q D A T V Y V G G L D E K-COOH
y12 y11 y10 y9 y8 y7 y6 y2
b2 b3
Int
100
75
50
25
0
iY
b2
y2
b3
y9 2+
y6
y7
Prec. 2+
y8
y9
y10
y11
y12
0 100 200 300 400 500 600 700 800 900 1,000 1,100 1,200 1,300
m/z

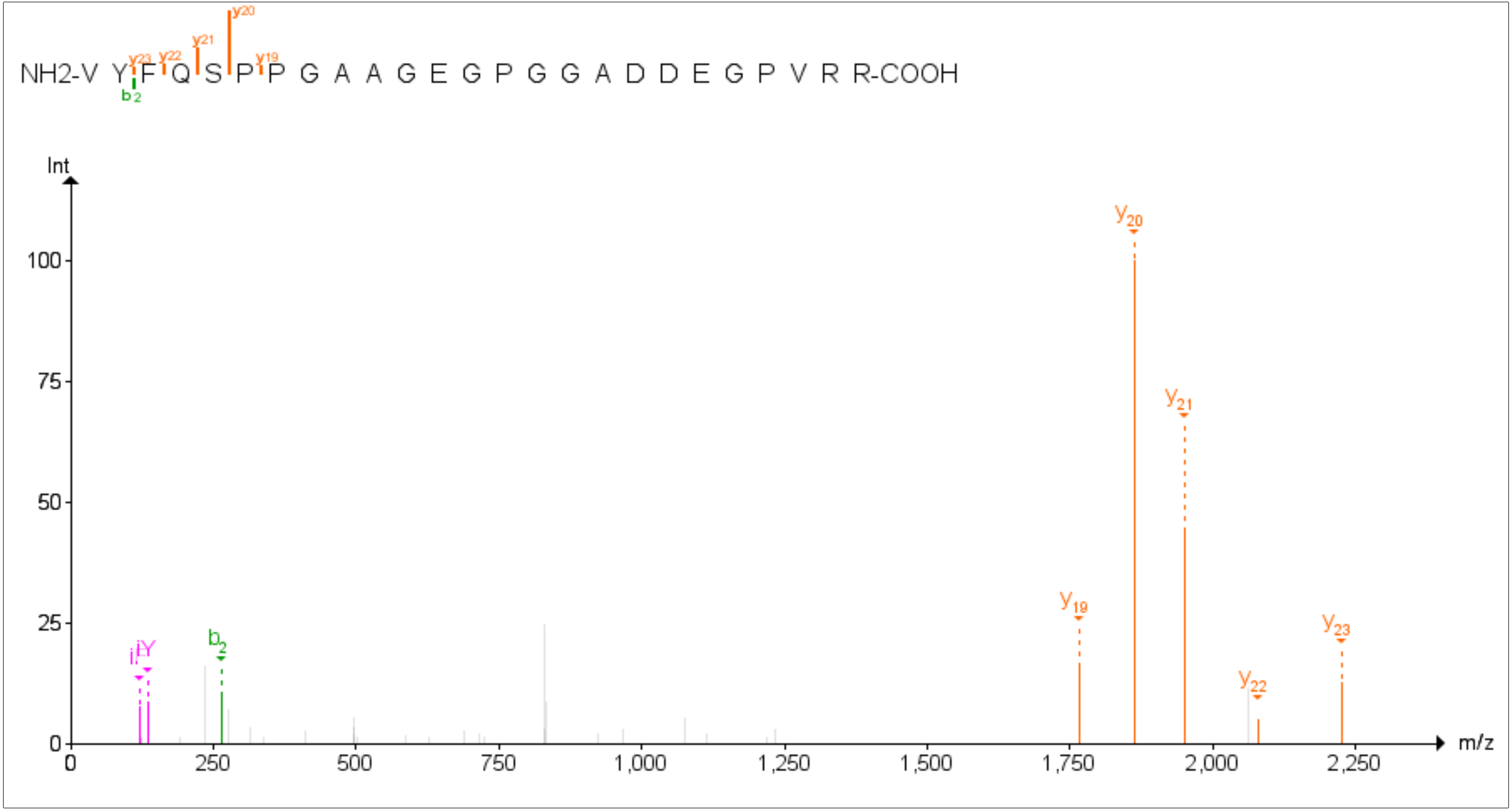

NH2-V Y F Q S P P G A A G E G P G G A D D E G P V R R-COOH
y20
y21
y23
y22
y19
b2
Int
100
75
50
25
0
m/z
0
250
500
750
1,000
1,250
1,500
1,750
2,000
2,250
y20
y21
y19
y23
y22
b2
iY

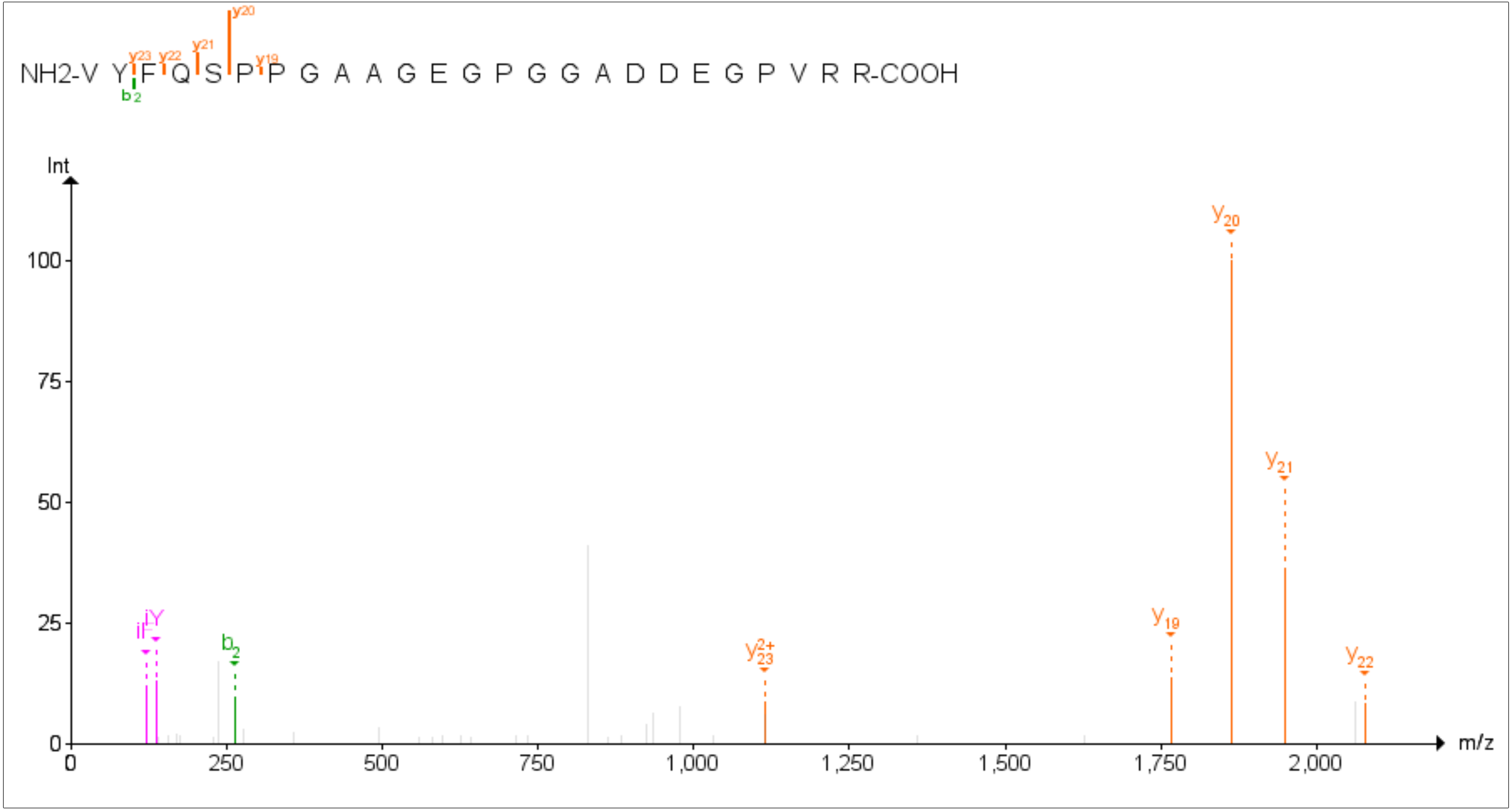

NH2-V Y F Q S P P G A A G E G P G G A D D E G P V R R-COOH
y20
y23
y22
y21
y19
b2
Int
100
75
50
25
0
m/z
0
250
500
750
1,000
1,250
1,500
1,750
2,000
iF
iY
b2
y23 2+
y19
y20
y21
y22

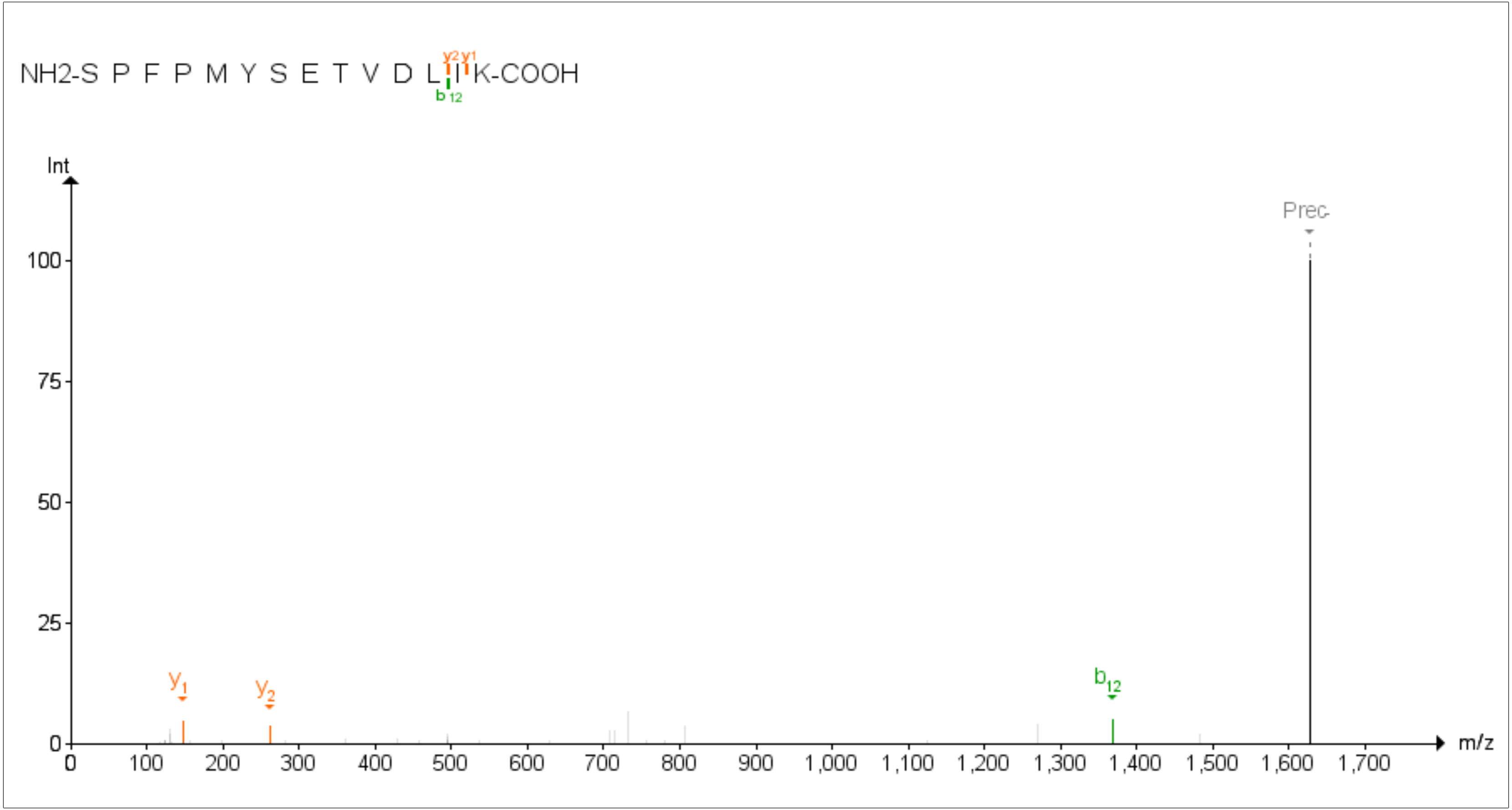

NH2-S P F P M Y S E T V D L I K-COOH
y2 y1
b 12
Int
100
75
50
25
0
Prec
y1
y2
b12
0 100 200 300 400 500 600 700 800 900 1,000 1,100 1,200 1,300 1,400 1,500 1,600 1,700
m/z

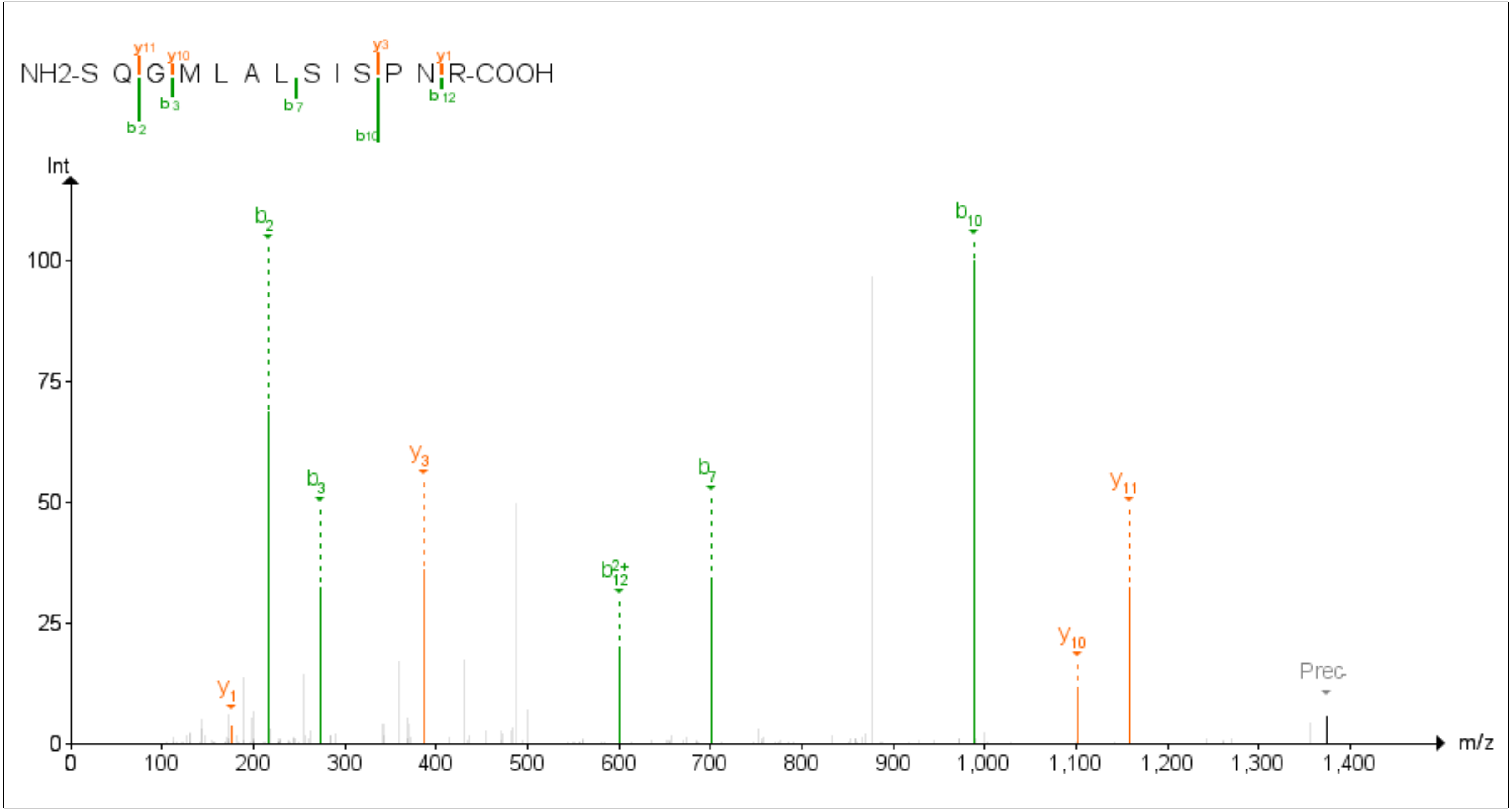
NH2-S Q G M L A L S I S P N R-COOH
y11
y10
y3
y1
b2
b3
b7
b10
b12
Int
100
75
50
25
0
b2
b10
b3
y3
b7
y11
b12 2+
y10
y1
Prec
m/z
0
100
200
300
400
500
600
700
800
900
1,000
1,100
1,200
1,300
1,400

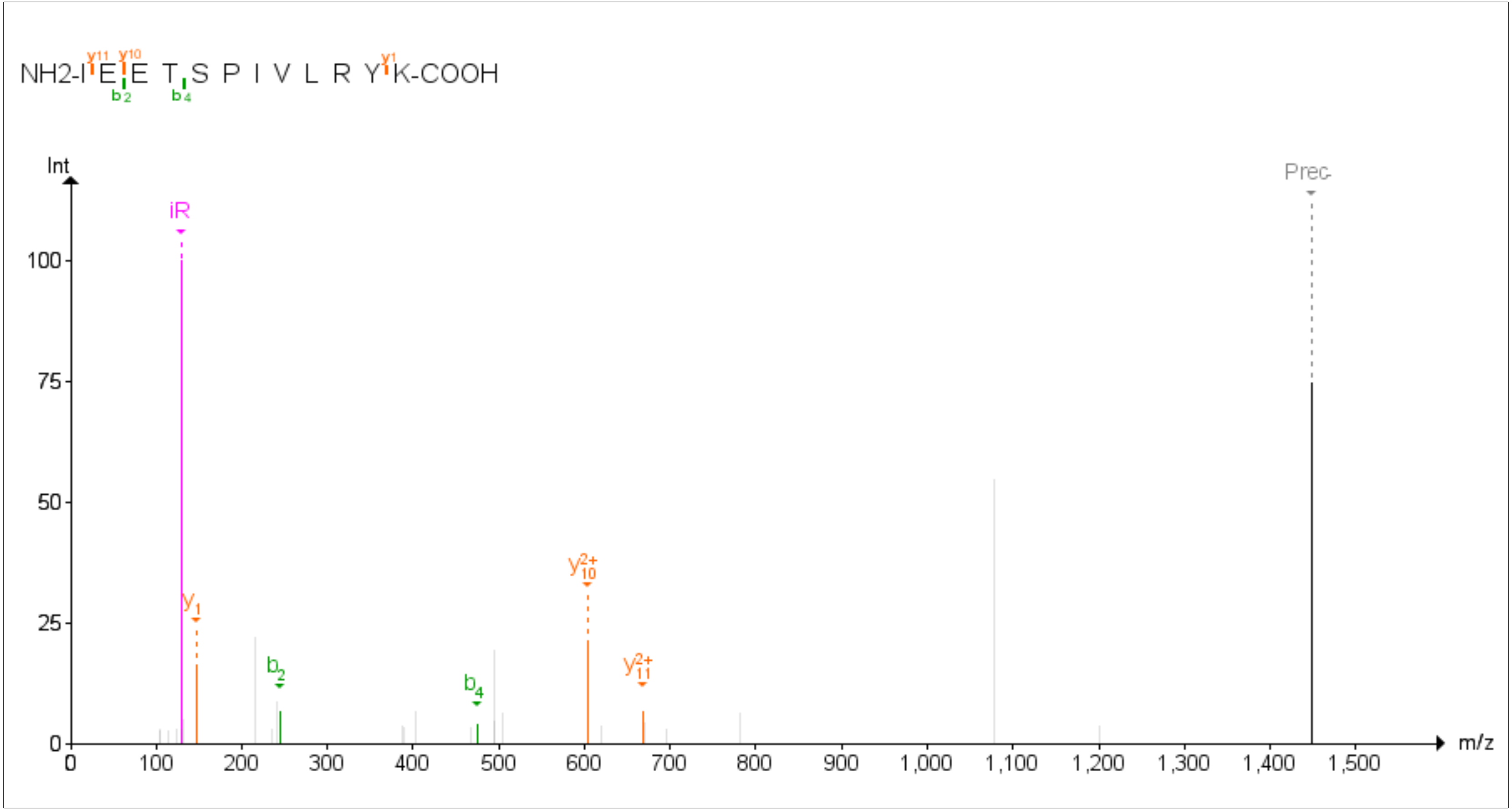

NH2-I E E T S P I V L R Y K-COOH
y11 y10 y1
b2 b4
Int
iR
Prec
y1
b2
b4
y_{10}^{2+}
y_{11}^{2+}
0
25
50
75
100
0
100
200
300
400
500
600
700
800
900
1,000
1,100
1,200
1,300
1,400
1,500
m/z

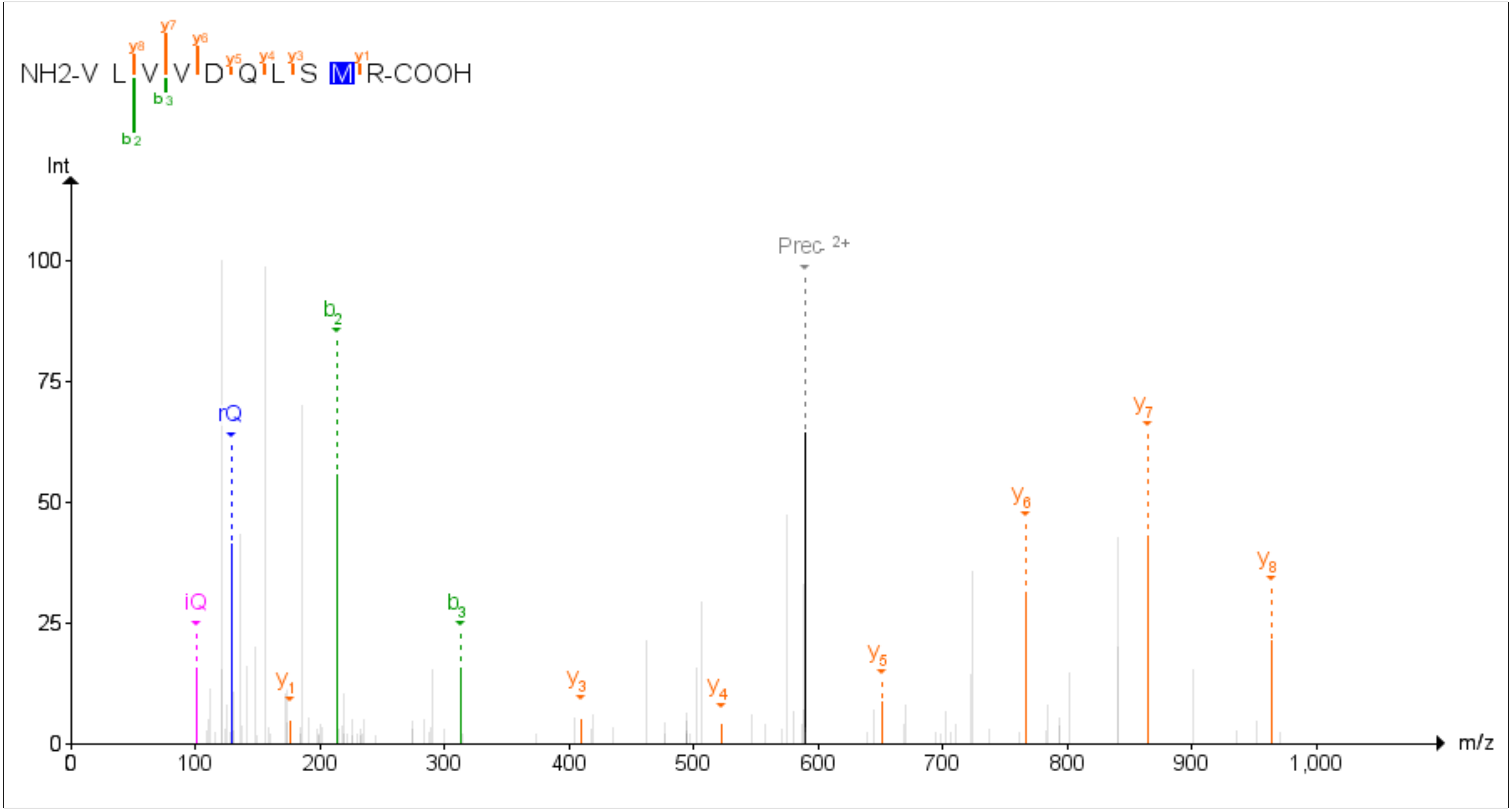

NH2-V L V V D Q L S M R-COOH
y8
y7
y6
y5
y4
y3
y1
b2
b3
Int
100
75
50
25
0
Prec 2+
b2
rQ
iQ
b3
y1
y3
y4
y5
y6
y7
y8
0
100
200
300
400
500
600
700
800
900
1,000
m/z

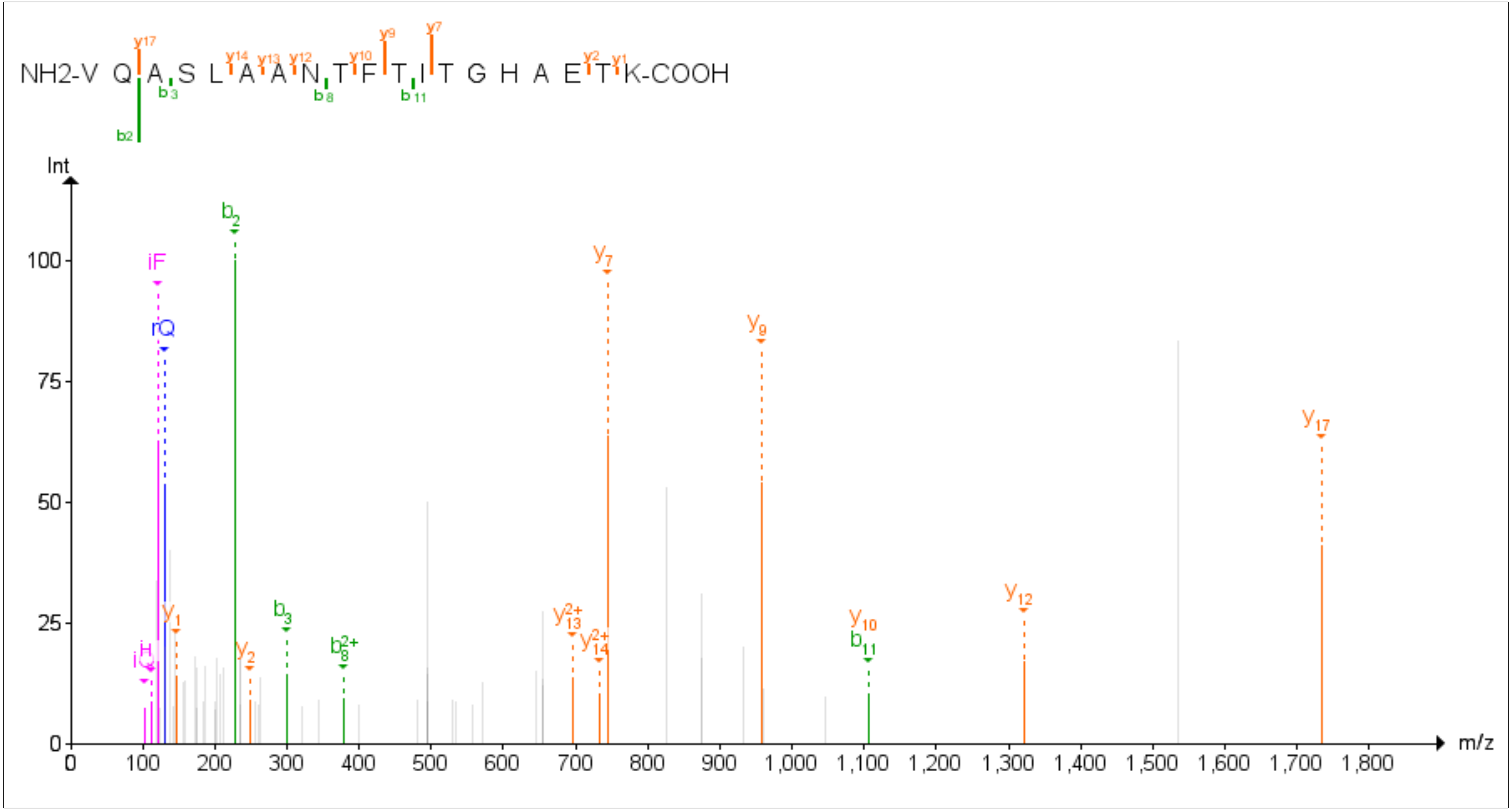

NH2-V Q A S L A A N T F T I T G H A E T K-COOH
y17 y14 y13 y12 y10 y9 y7 y2 y1
b2 b3 b8 b11
Int
m/z
iF
rQ
iH
iQ
b2
y7
y9
y17
y1
y2
b3
b8 2+
y13 2+
y14 2+
y10
b11
y12
0
25
50
75
100
0
100
200
300
400
500
600
700
800
900
1,000
1,100
1,200
1,300
1,400
1,500
1,600
1,700
1,800

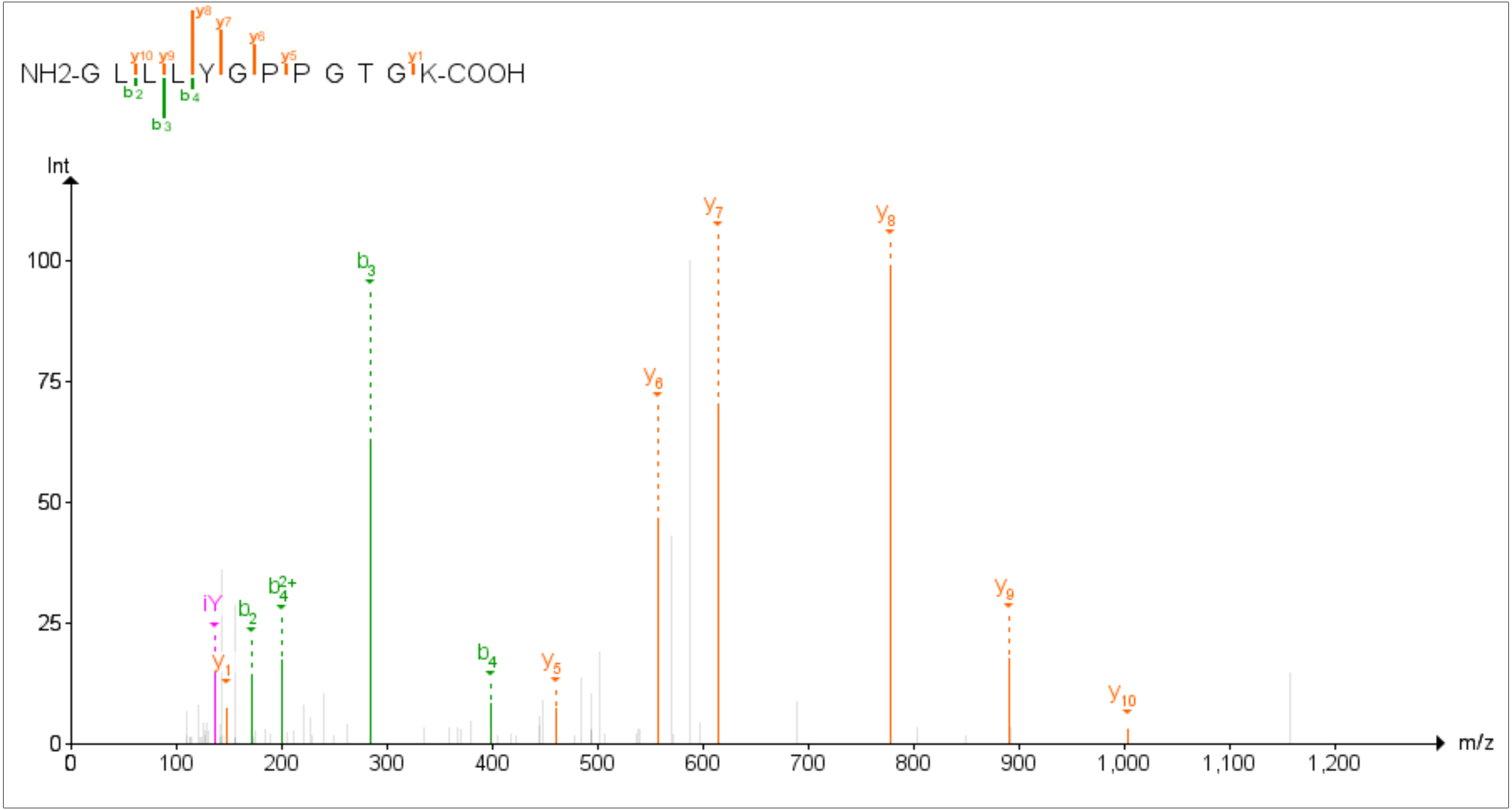

NH2-G L L L Y G P P G T G K-COOH
y10 y9 y8 y7 y6 y5 y1
b2 b3 b4
Int
m/z
iY
y1 b2 b4 2+ b3 b4 y5 y6 y7 y8 y9 y10
0
25
50
75
100
0 100 200 300 400 500 600 700 800 900 1,000 1,100 1,200

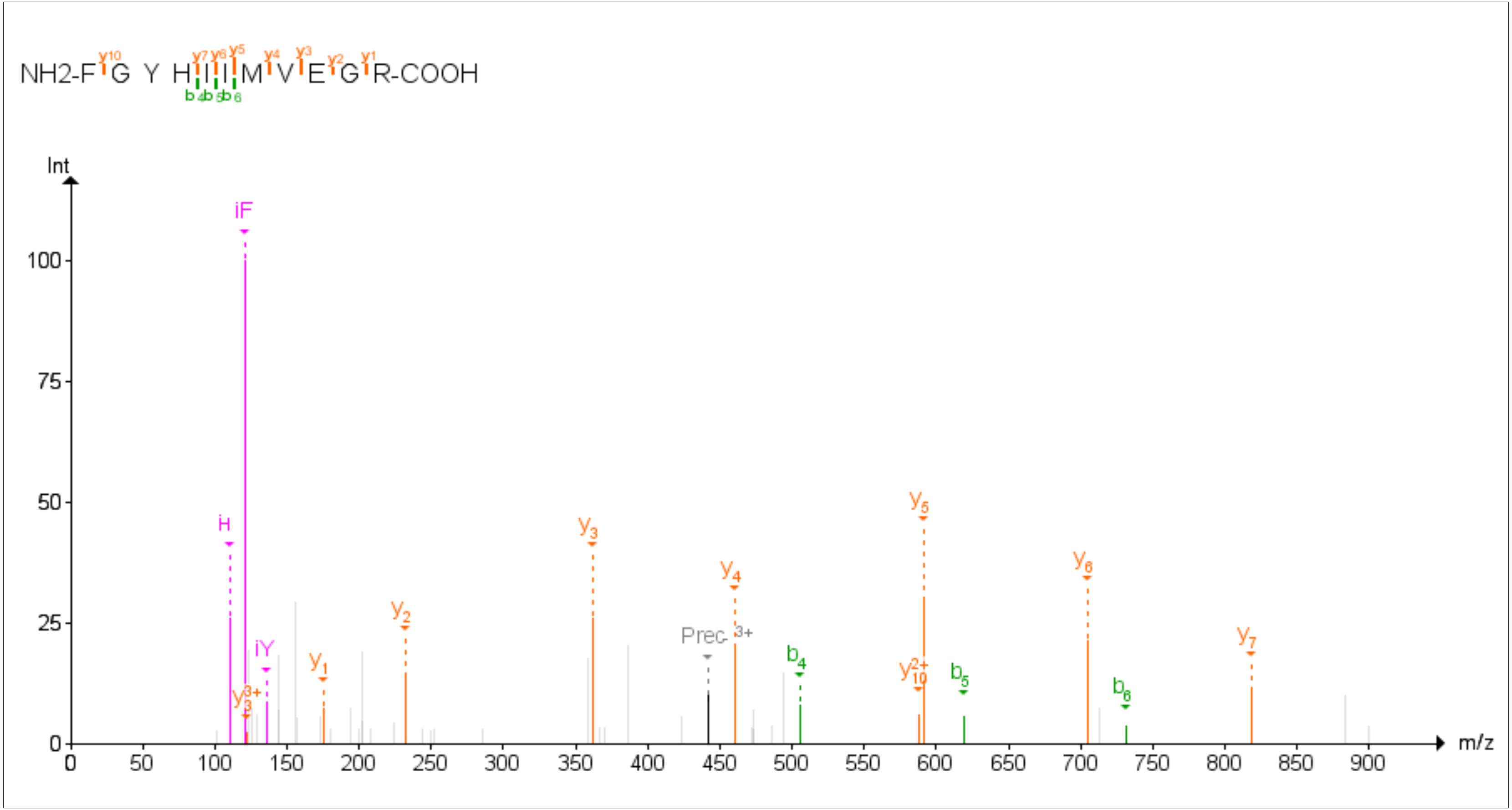

NH2-F G Y H I I I M V E G R-COOH
y10 y7 y6 y5 y4 y3 y2 y1
b4 b5 b6
Int
100
75
50
25
0
iF
iH
iY
y3 3+
y1
y2
y3
Prec 3+
y4
b4
y5
y10 2+
b5
y6
b6
y7
0 50 100 150 200 250 300 350 400 450 500 550 600 650 700 750 800 850 900
m/z

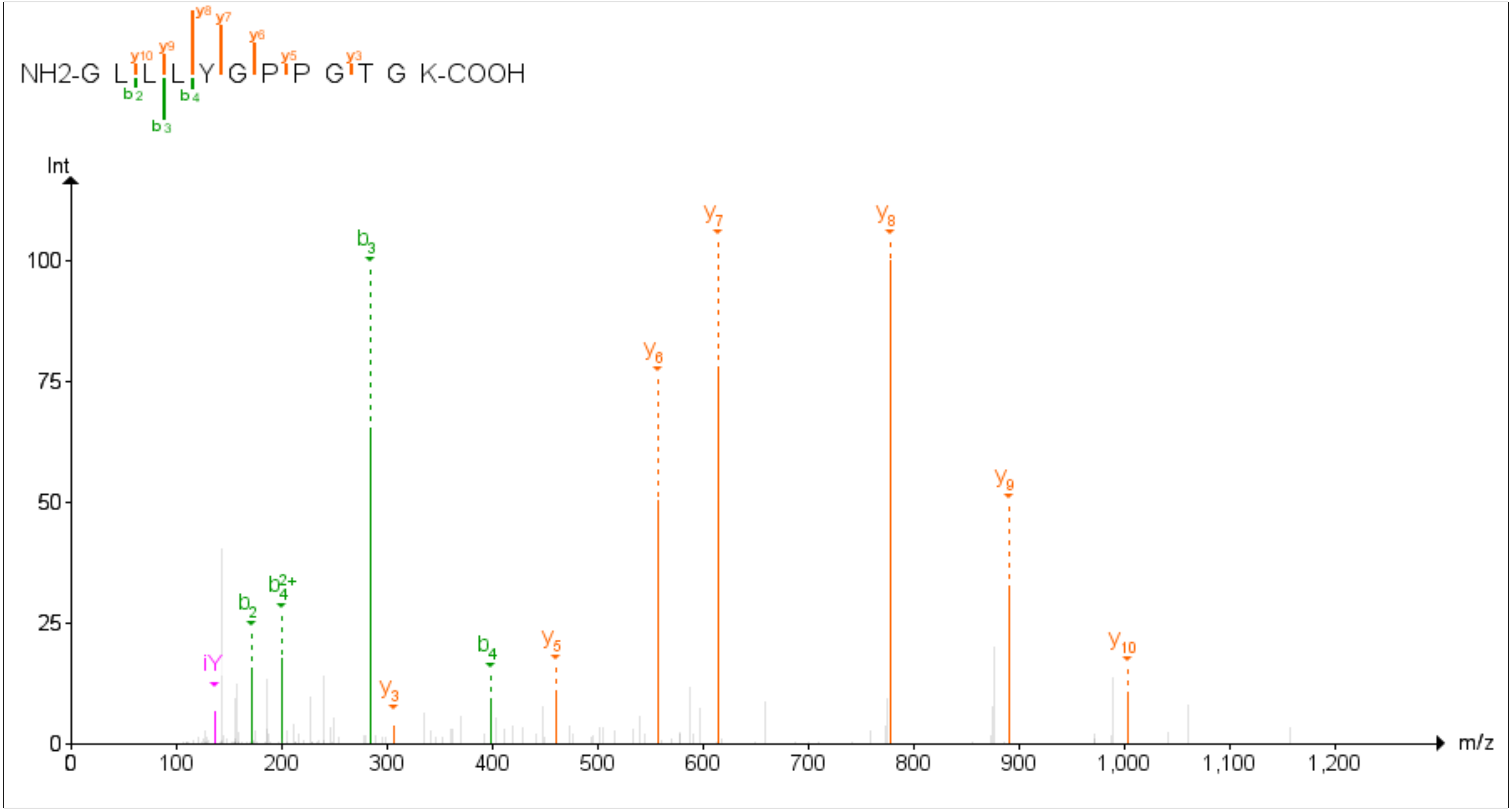

NH2-G L L L Y G P P G T G K-COOH
y10 y9 y8 y7 y6 y5 y3
b2 b3 b4
Int
m/z
b3
y7
y8
y6
y9
b2
b4 2+
b4
y5
y3
y10
iY

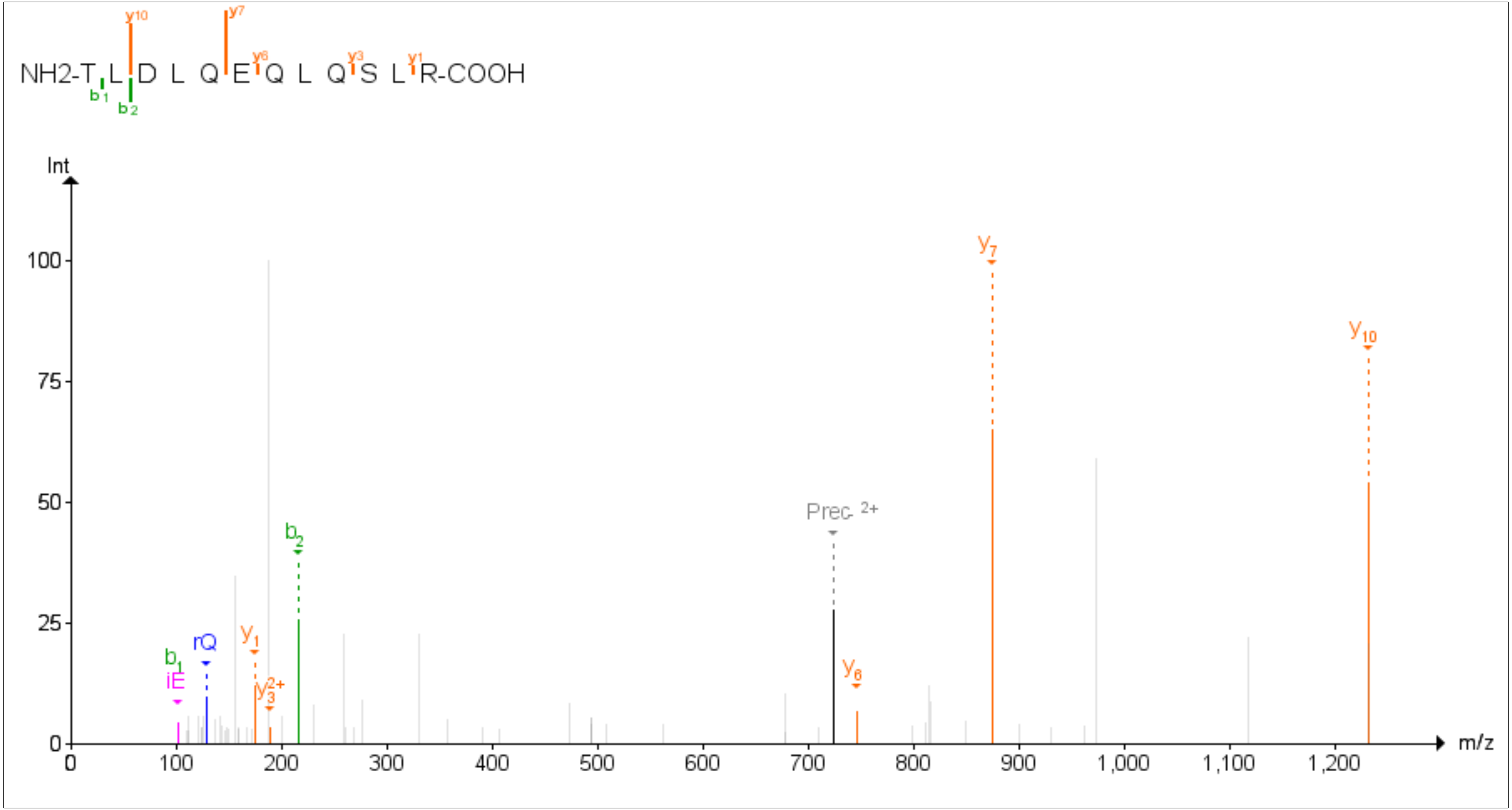
NH2-T L D L Q E Q L Q S L R-COOH
y10
y7
y6
y3
y1
b1
b2
Int
100
75
50
25
0
m/z
0
100
200
300
400
500
600
700
800
900
1,000
1,100
1,200
y7
y10
Prec- 2+
b2
b1
iE
rQ
y1
y3 2+
y6

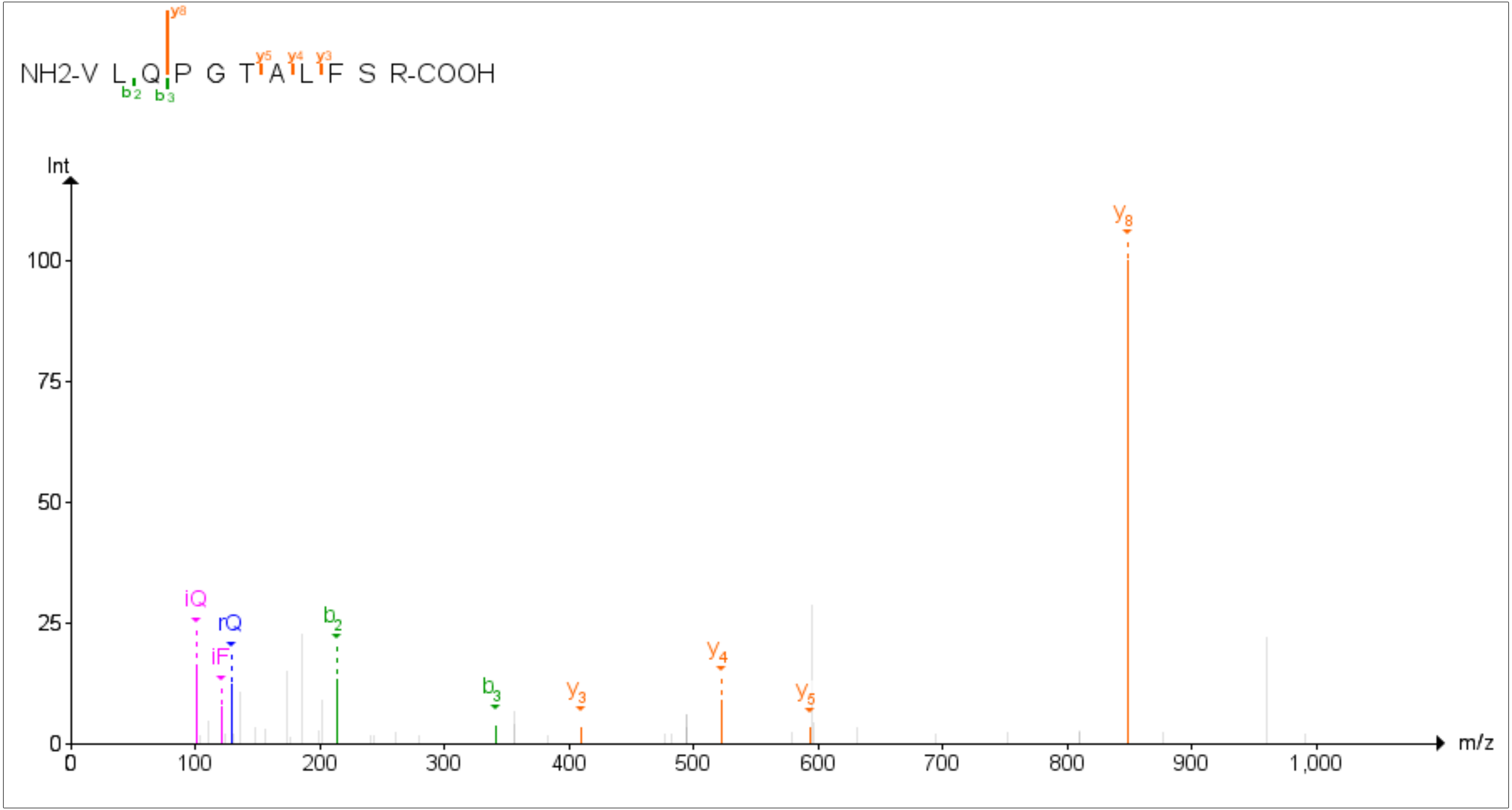

NH2-V L Q P G T A L F S R-COOH
y8
y5 y4 y3
b2 b3
Int
100
75
50
25
0
iQ
rQ
iF
b2
b3
y3
y4
y5
y8
0 100 200 300 400 500 600 700 800 900 1,000
m/z

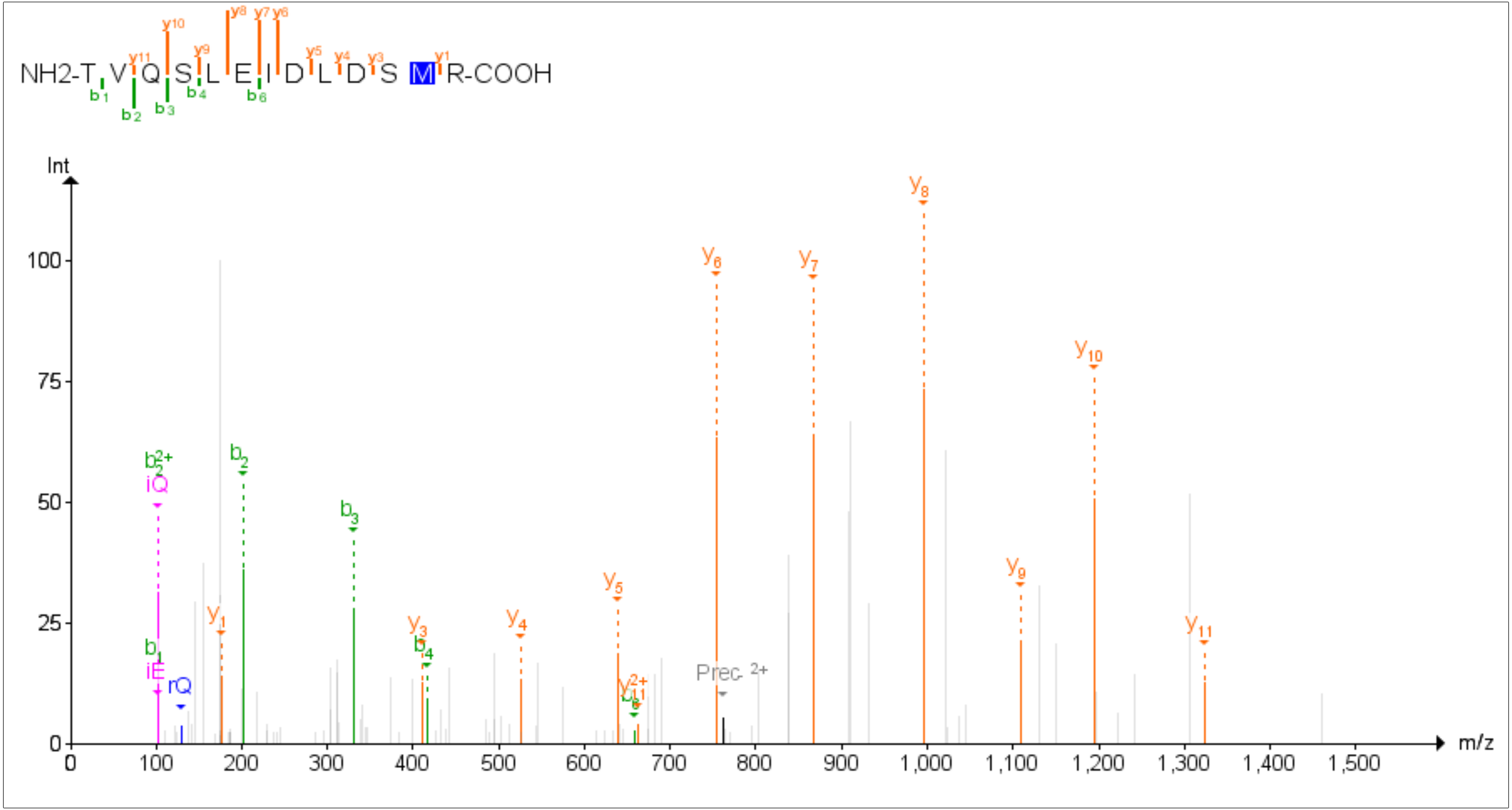

NH2-T V Q S L E I D L D S M R-COOH
Int
m/z
y1 y3 y4 y5 y6 y7 y8 y9 y10 y11
b1 b2 b3 b4 b6
b2 2+
iQ
iE
rQ
Prec 2+
y11 2+

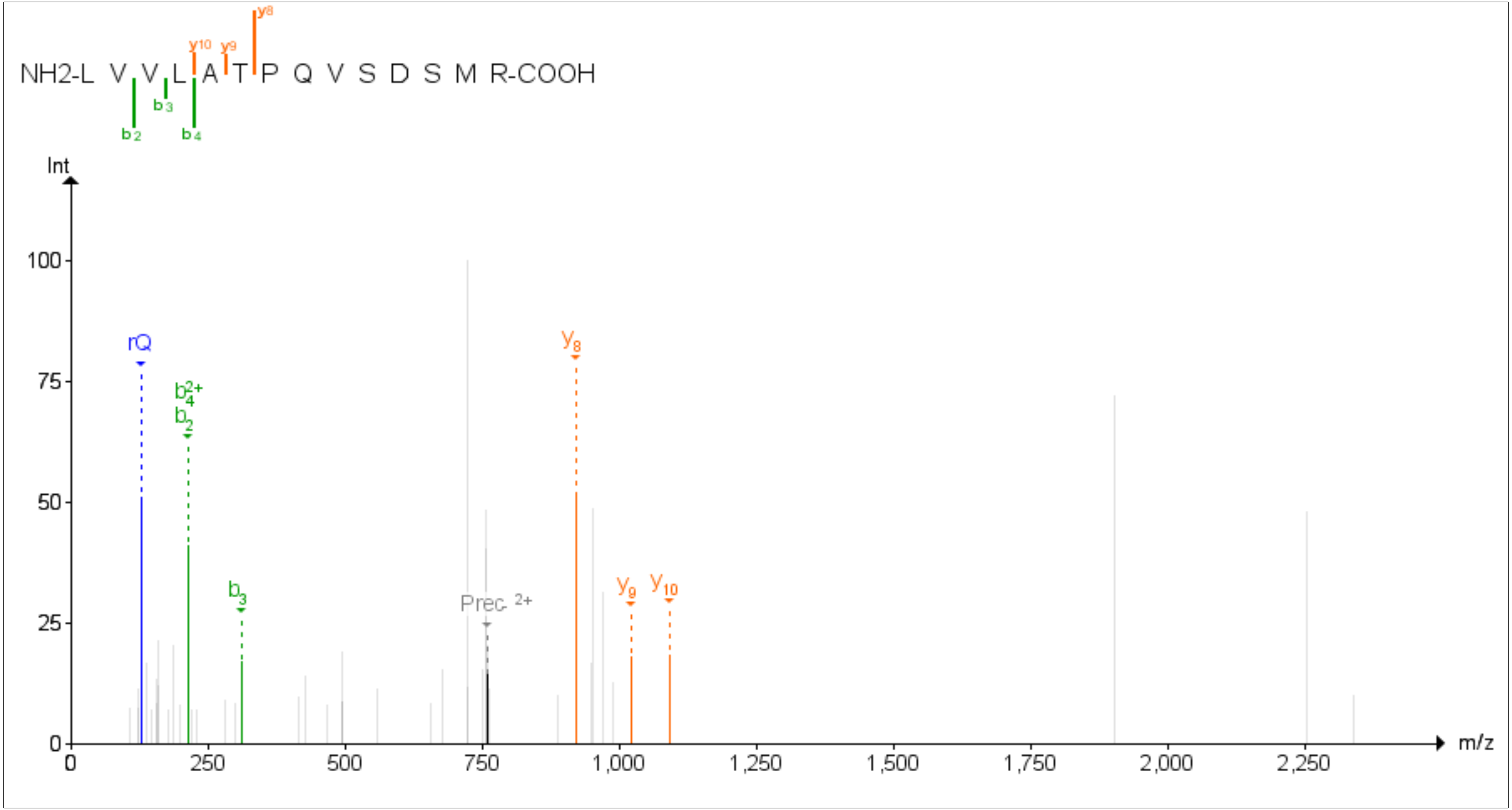

NH2-L V V L A T P Q V S D S M R-COOH
y10
y9
y8
b2
b3
b4
Int
100
75
50
25
0
rQ
b4 2+
b2
b3
Prec 2+
y8
y9
y10
0
250
500
750
1,000
1,250
1,500
1,750
2,000
2,250
m/z

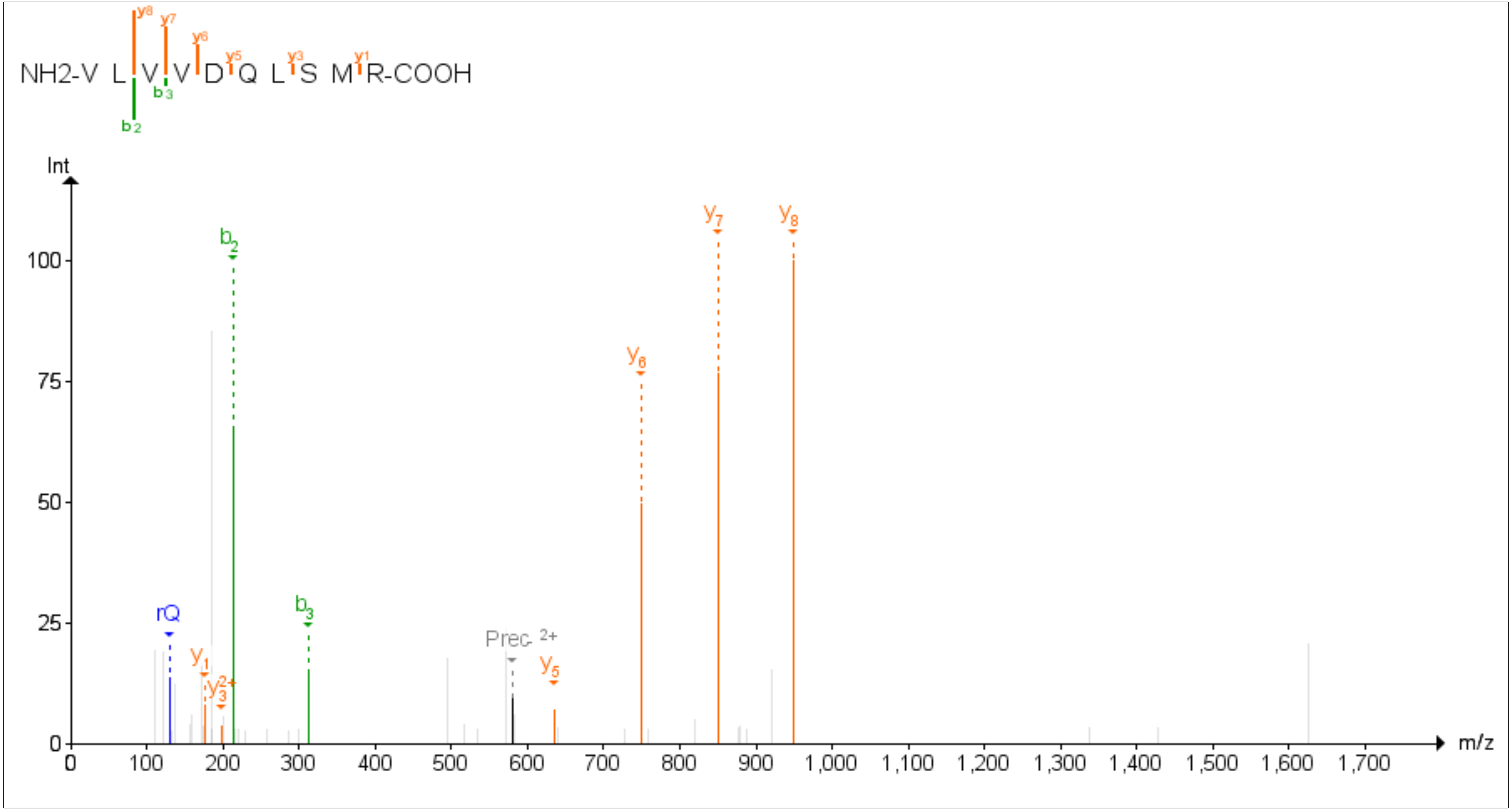

NH2-V L V V D Q L S M R-COOH
y8 y7 y6 y5 y3 y1
b2 b3
Int
100
75
50
25
0
m/z
0 100 200 300 400 500 600 700 800 900 1,000 1,100 1,200 1,300 1,400 1,500 1,600 1,700
b2
y7
y8
y6
rQ
b3
Prec- 2+
y1
y3 2+
y5

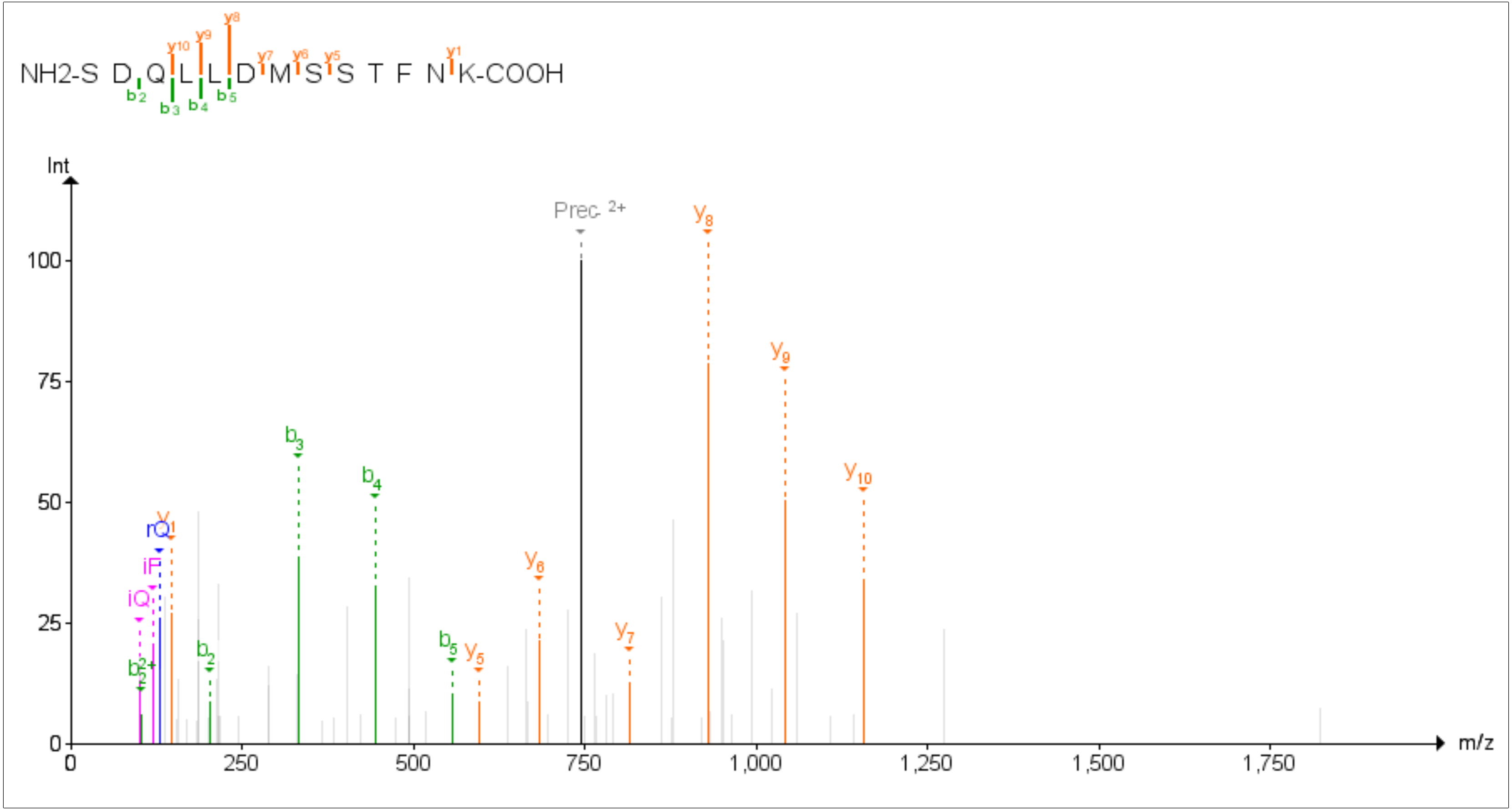

NH2-S D Q L L D M S S T F N K-COOH
y10 y9 y8 y7 y6 y5 y1
b2 b3 b4 b5
Int
Prec 2+
y8
y9
y10
b3
b4
b5
y5
y6
y7
b2
b2 2+
rQ
y1
iF
iQ
100
75
50
25
0
0
250
500
750
1,000
1,250
1,500
1,750
m/z

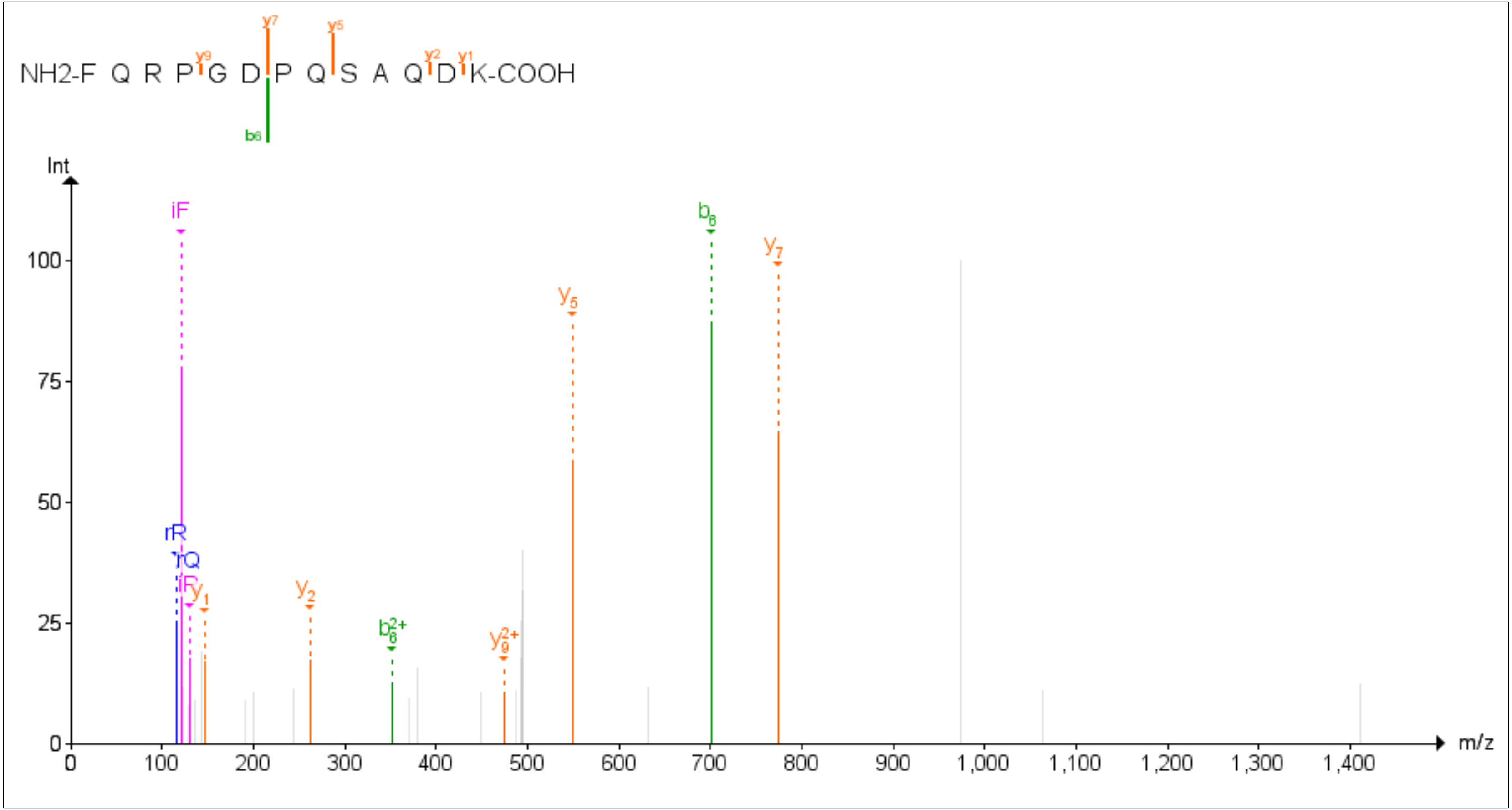

NH2-F Q R P G D P Q S A Q D K-COOH
y7
y5
y9
y2
y1
b6
Int
iF
b6
y7
y5
rR
rQ
iR
y1
y2
b6 2+
y9 2+
100
75
50
25
0
0
100
200
300
400
500
600
700
800
900
1,000
1,100
1,200
1,300
1,400
m/z

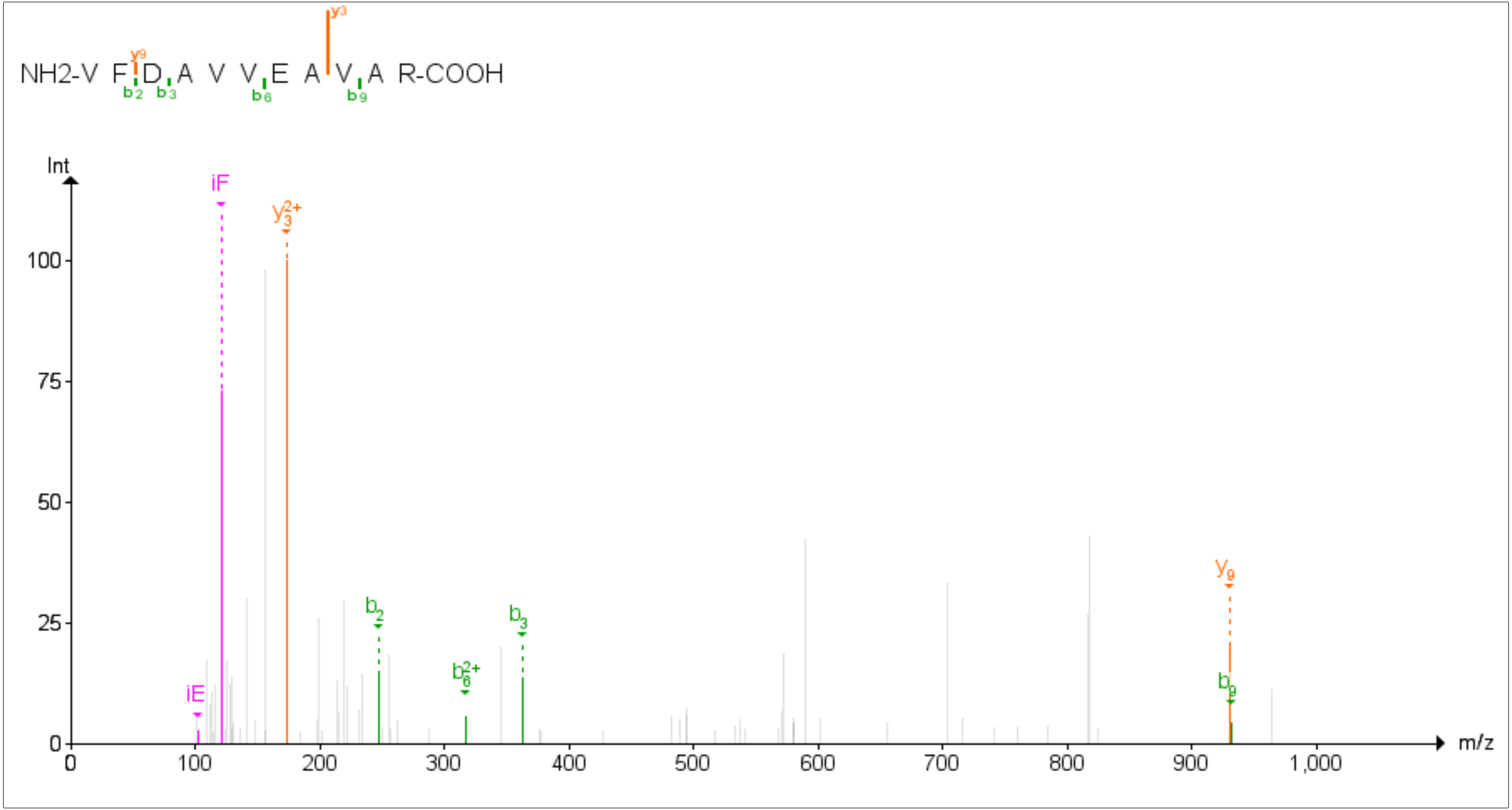

NH2-V F D A V V E A V A R-COOH
y9
y3
b2
b3
b6
b9
Int
iF
y_3^{2+}
iE
b_2
b_6^{2+}
b_3
y_9
b_9
100
75
50
25
0
0
100
200
300
400
500
600
700
800
900
1,000
m/z

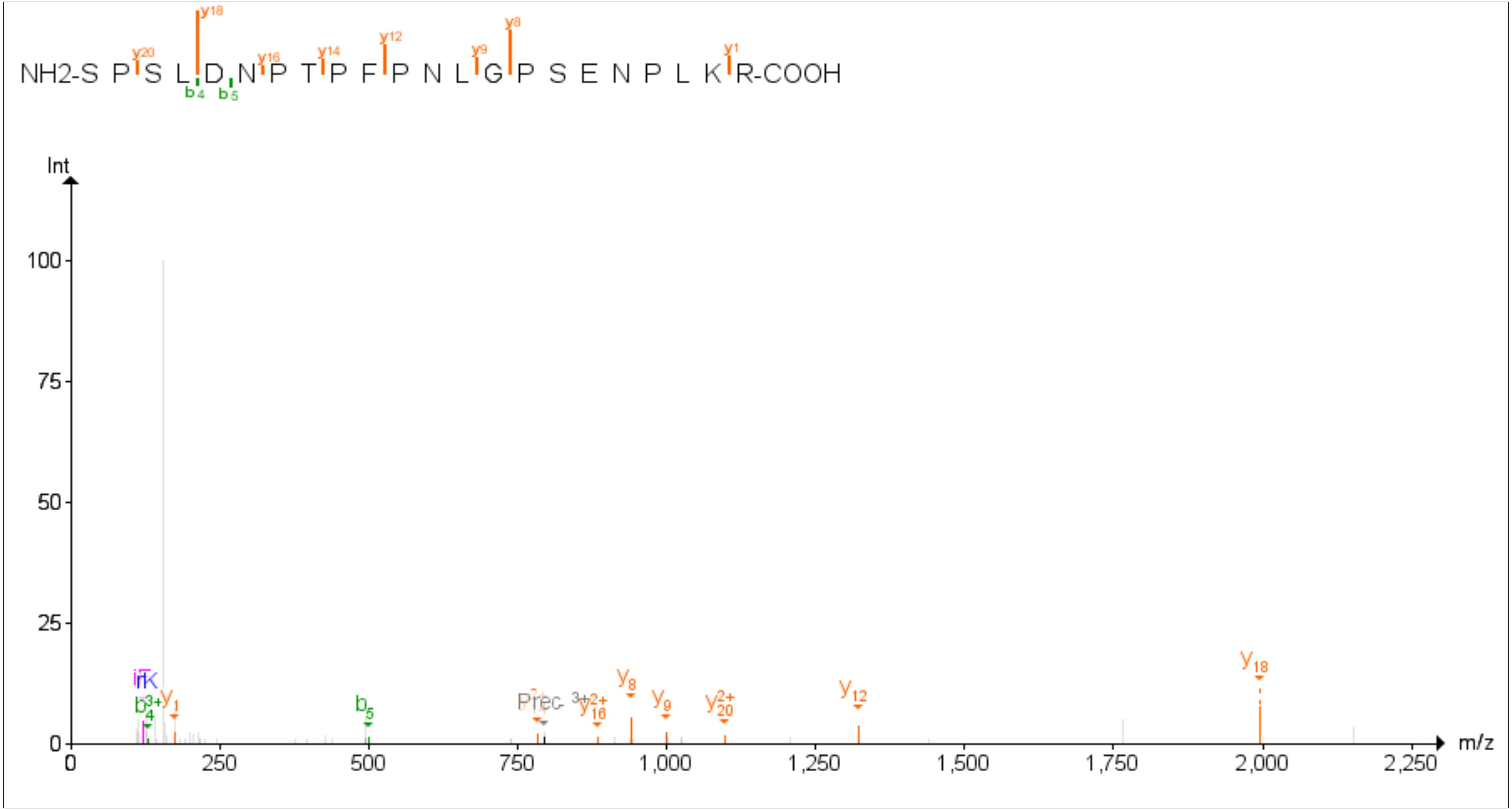

NH2-S P S L D N P T P F P N L G P S E N P L K R-COOH
y20
y18
y16
y14
y12
y9
y8
y1
b4
b5
Int
100
75
50
25
0
m/z
0
250
500
750
1,000
1,250
1,500
1,750
2,000
2,250
y1
y8
y9
y12
y18
b5
Prec 3+

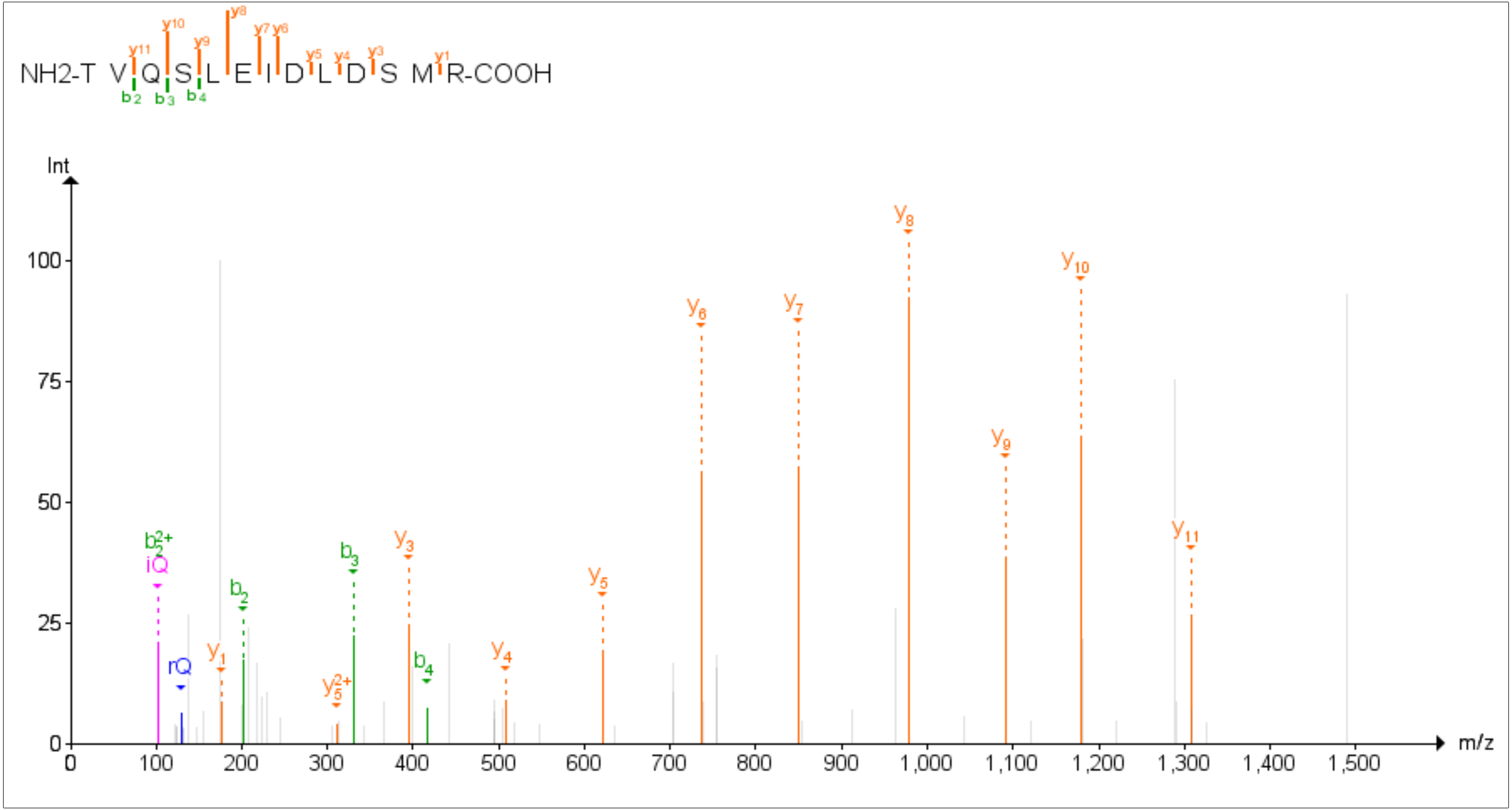

NH2-T V Q S L E I D L D S M R-COOH
y11 y10 y9 y8 y7 y6 y5 y4 y3 y1
b2 b3 b4
Int
100
75
50
25
0
m/z
0 100 200 300 400 500 600 700 800 900 1,000 1,100 1,200 1,300 1,400 1,500
y8
y10
y6
y7
y9
y11
b2 2+
iQ
rQ
y1
b2
b3
y3
y5 2+
b4
y4
y5

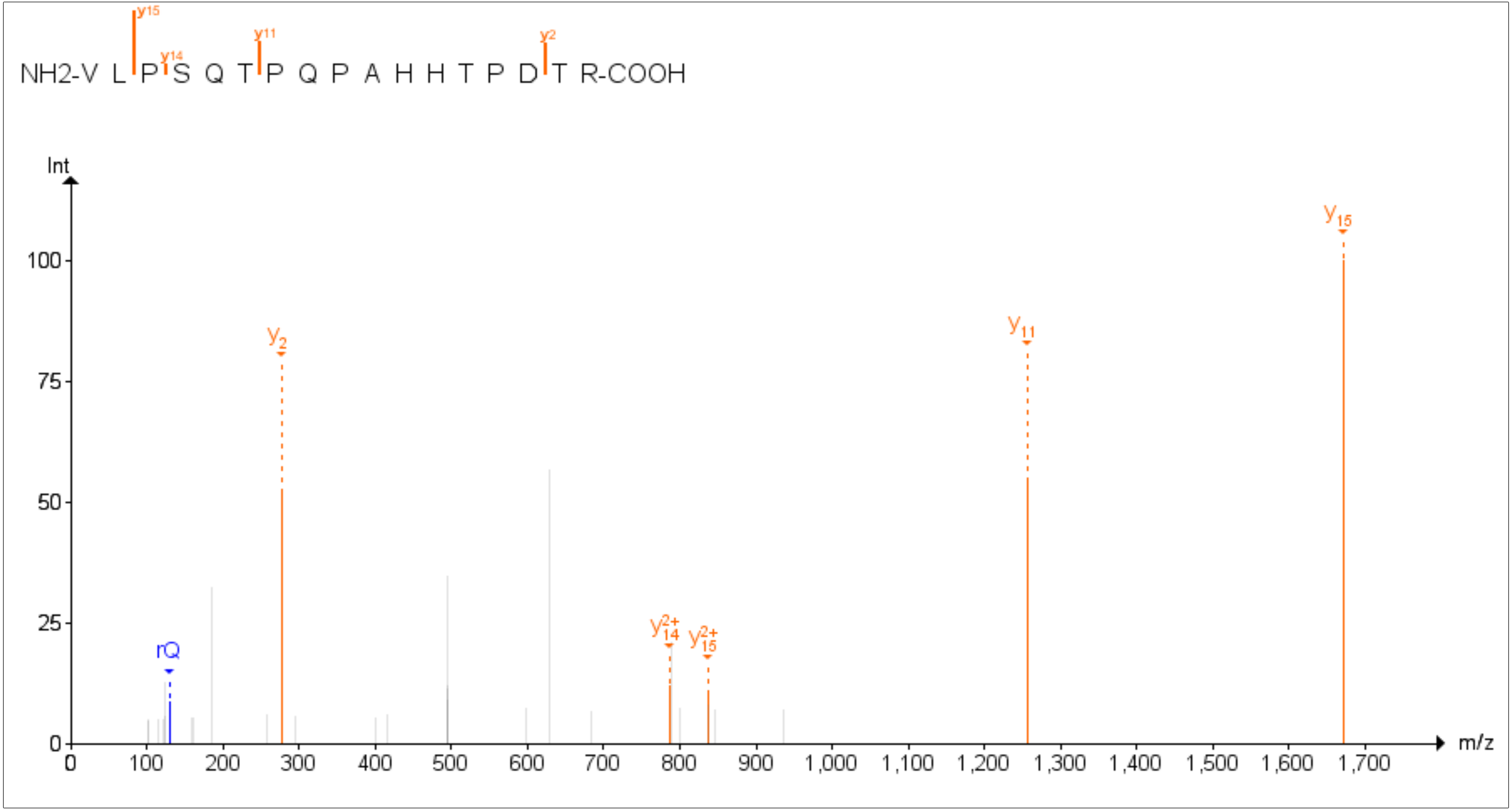

NH2-V L P S Q T P Q P A H H T P D T R-COOH
y15
y14
y11
y2
Int
100
75
50
25
0
rQ
y2
y2+14
y2+15
y11
y15
0
100
200
300
400
500
600
700
800
900
1,000
1,100
1,200
1,300
1,400
1,500
1,600
1,700
m/z

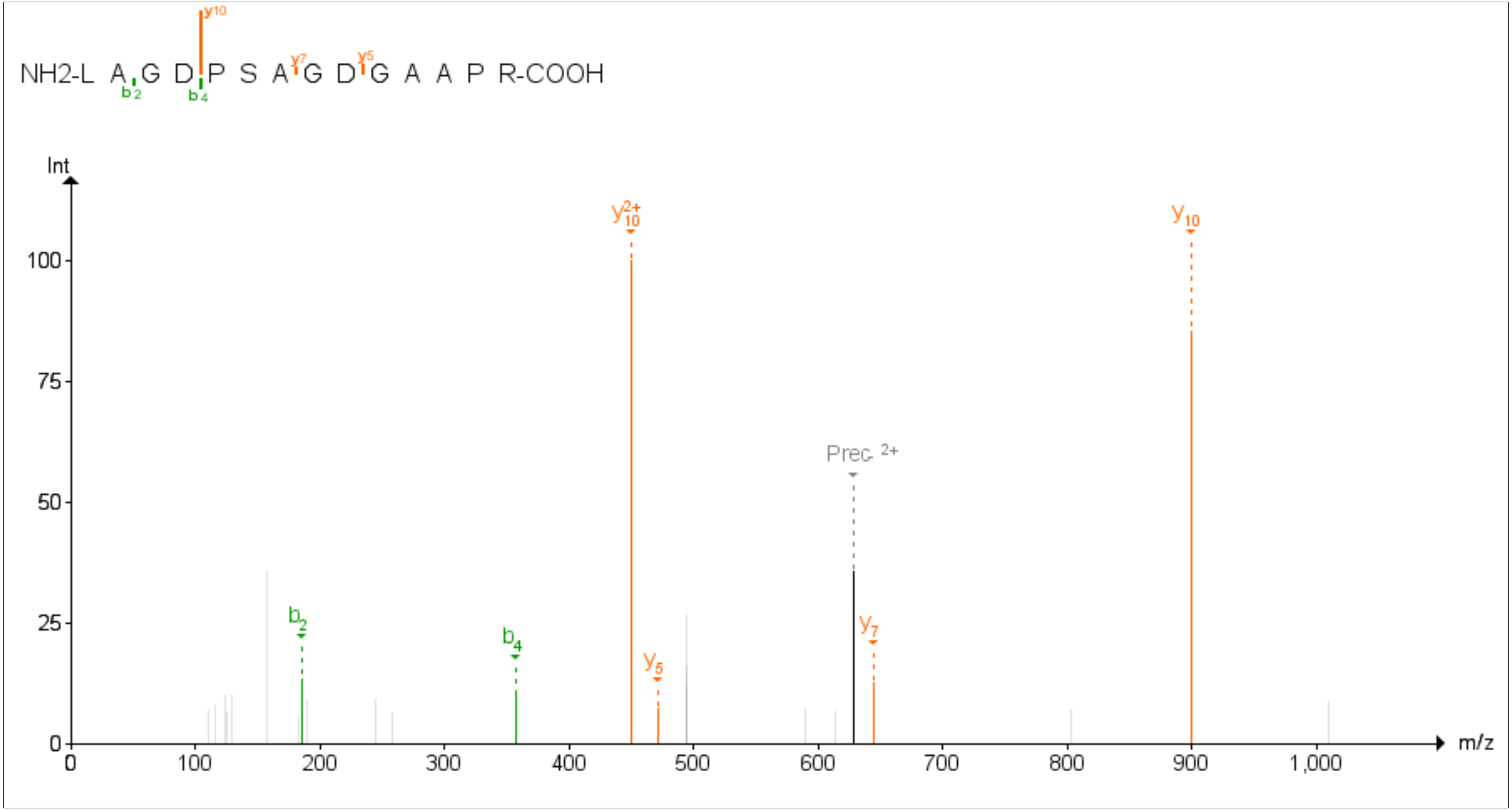

NH2-L A G D P S A G D G A A P R-COOH
y10
y7
y5
b2
b4
Int
100
75
50
25
0
y10 2+
y10
Prec 2+
b2
b4
y5
y7
0
100
200
300
400
500
600
700
800
900
1,000
m/z

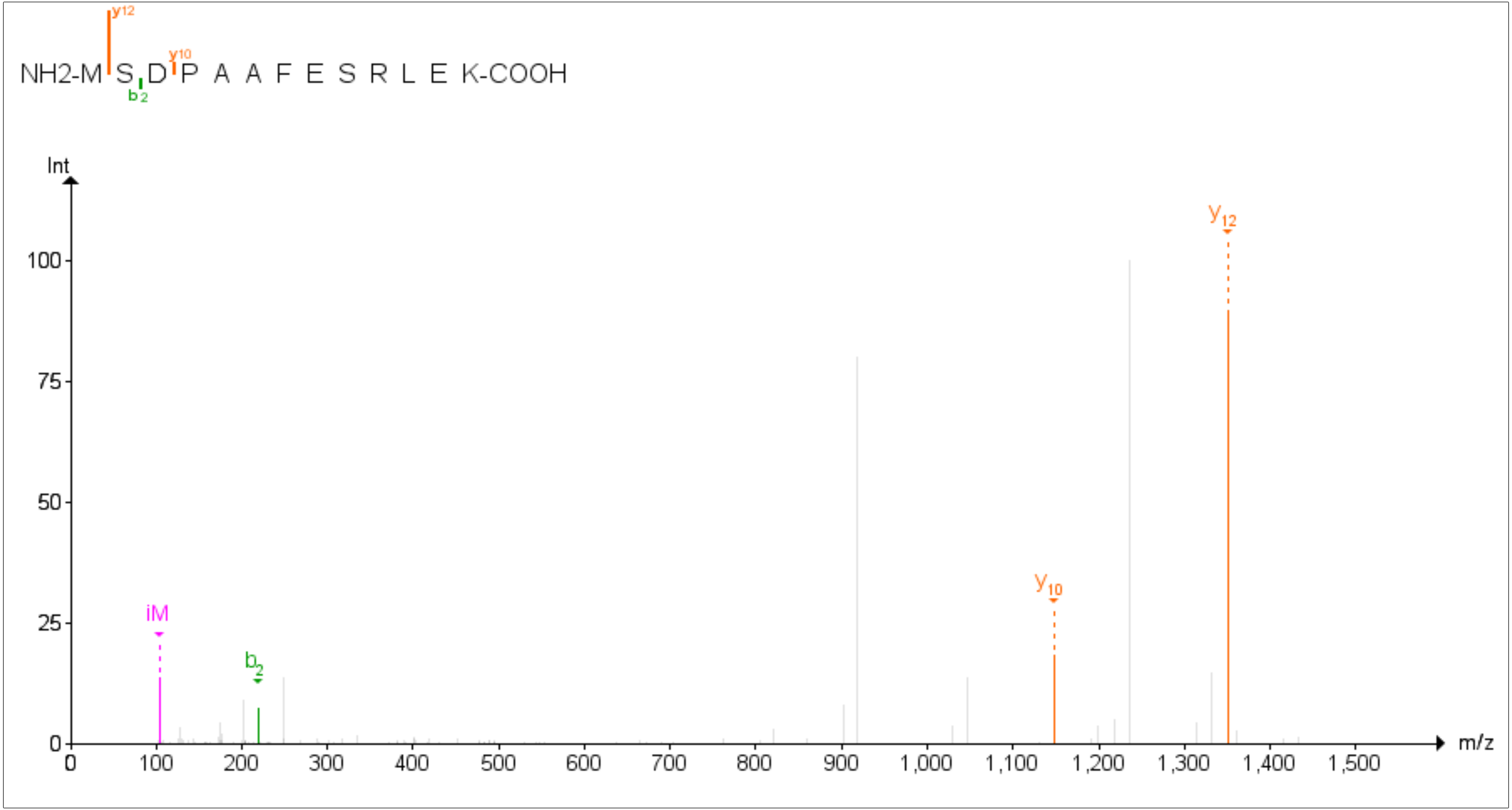
NH2-M S D P A A F E S R L E K-COOH
y12
y10
b2
Int
m/z
0
25
50
75
100
0
100
200
300
400
500
600
700
800
900
1,000
1,100
1,200
1,300
1,400
1,500
iM
b2
y10
y12

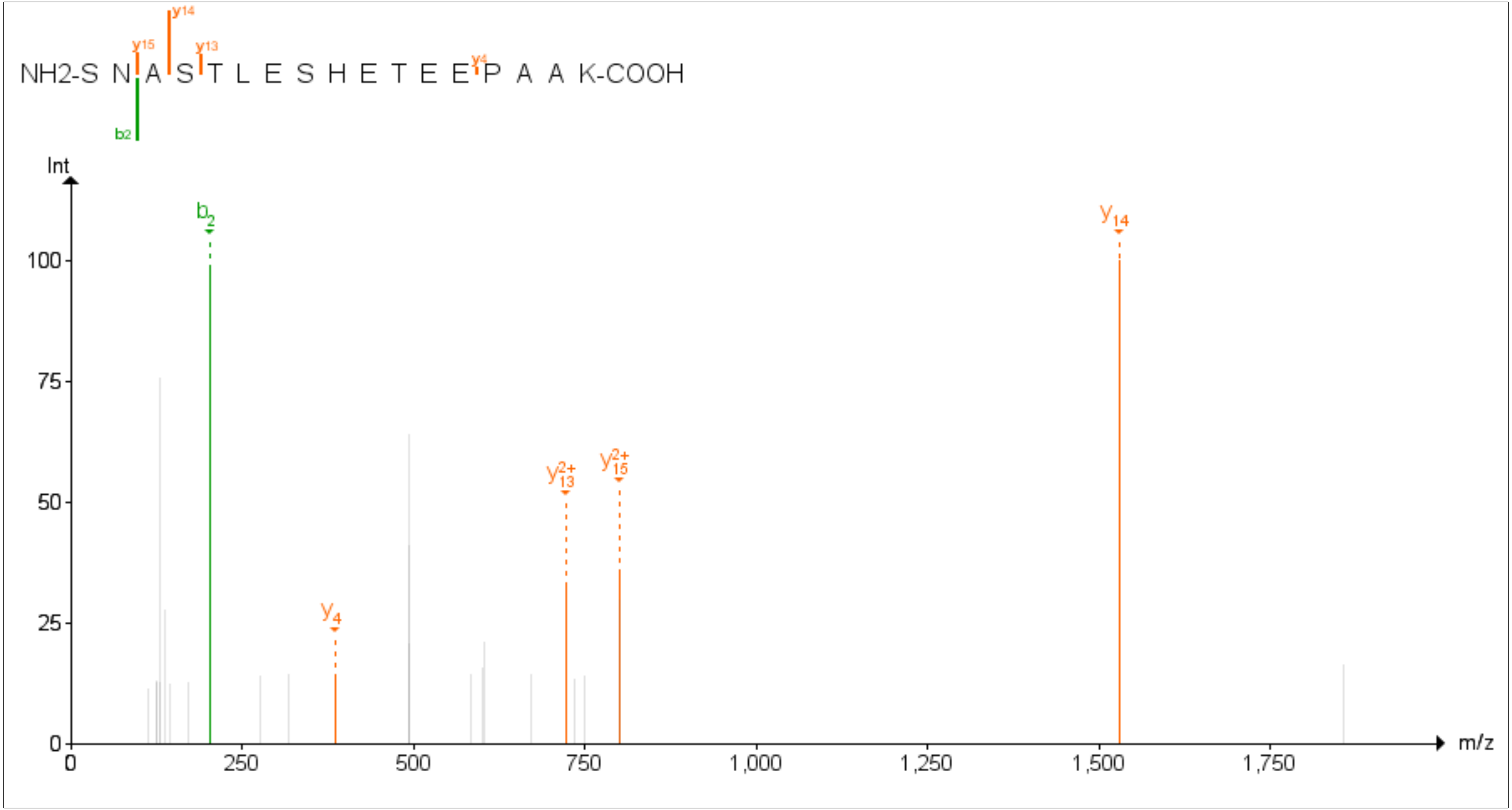

NH2-S N A S T L E S H E T E E P A A K-COOH
y15
y14
y13
y4
b2
Int
m/z
100
75
50
25
0
250
500
750
1,000
1,250
1,500
1,750
b_2
y_{14}
y_4
y_{13}^{2+}
y_{15}^{2+}

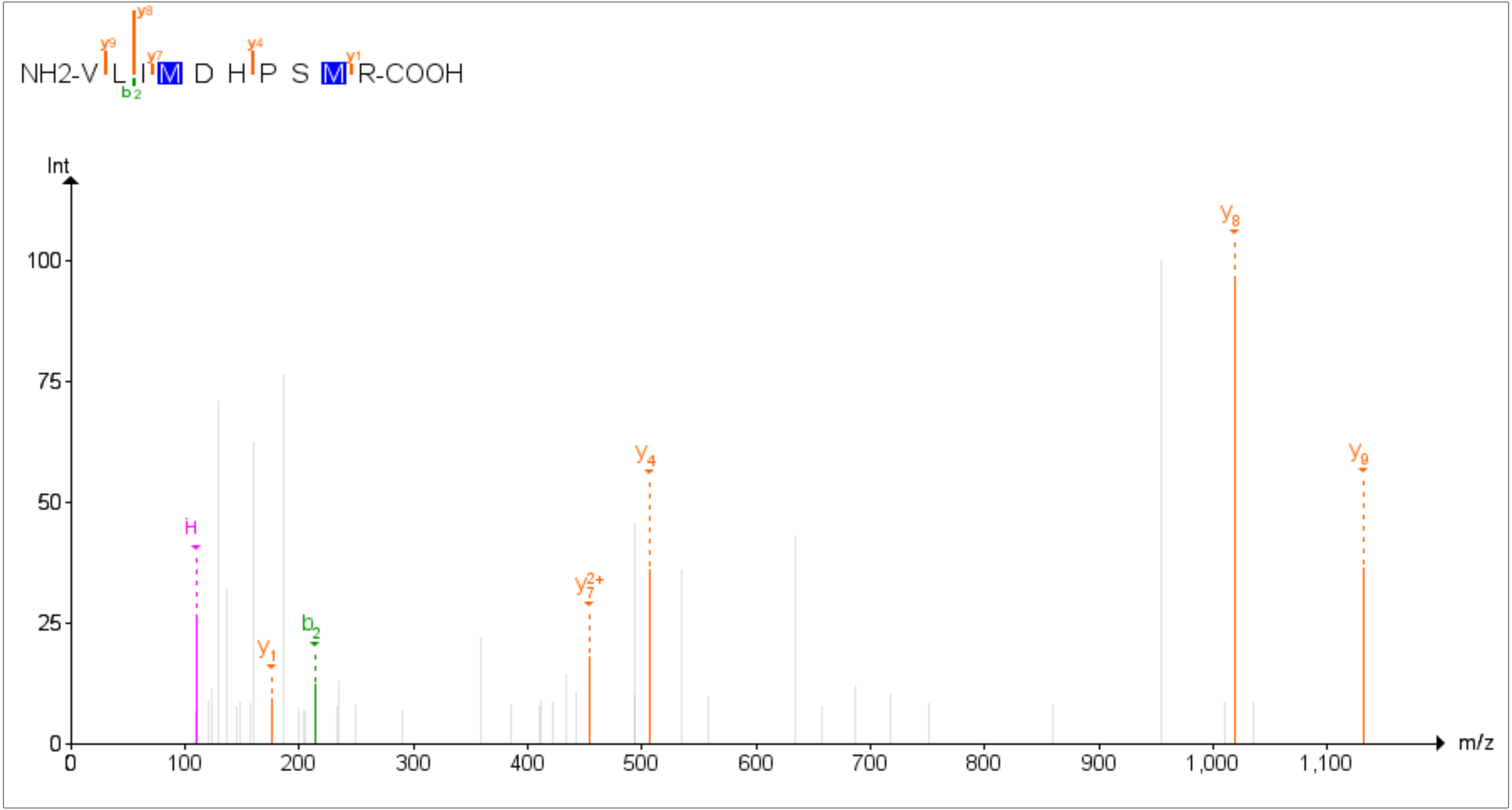

NH2-V L I M D H P S M R-COOH
y9
y8
y7
y4
y1
b2
Int
100
75
50
25
0
H
y1
b2
y7 2+
y4
y8
y9
0
100
200
300
400
500
600
700
800
900
1,000
1,100
m/z

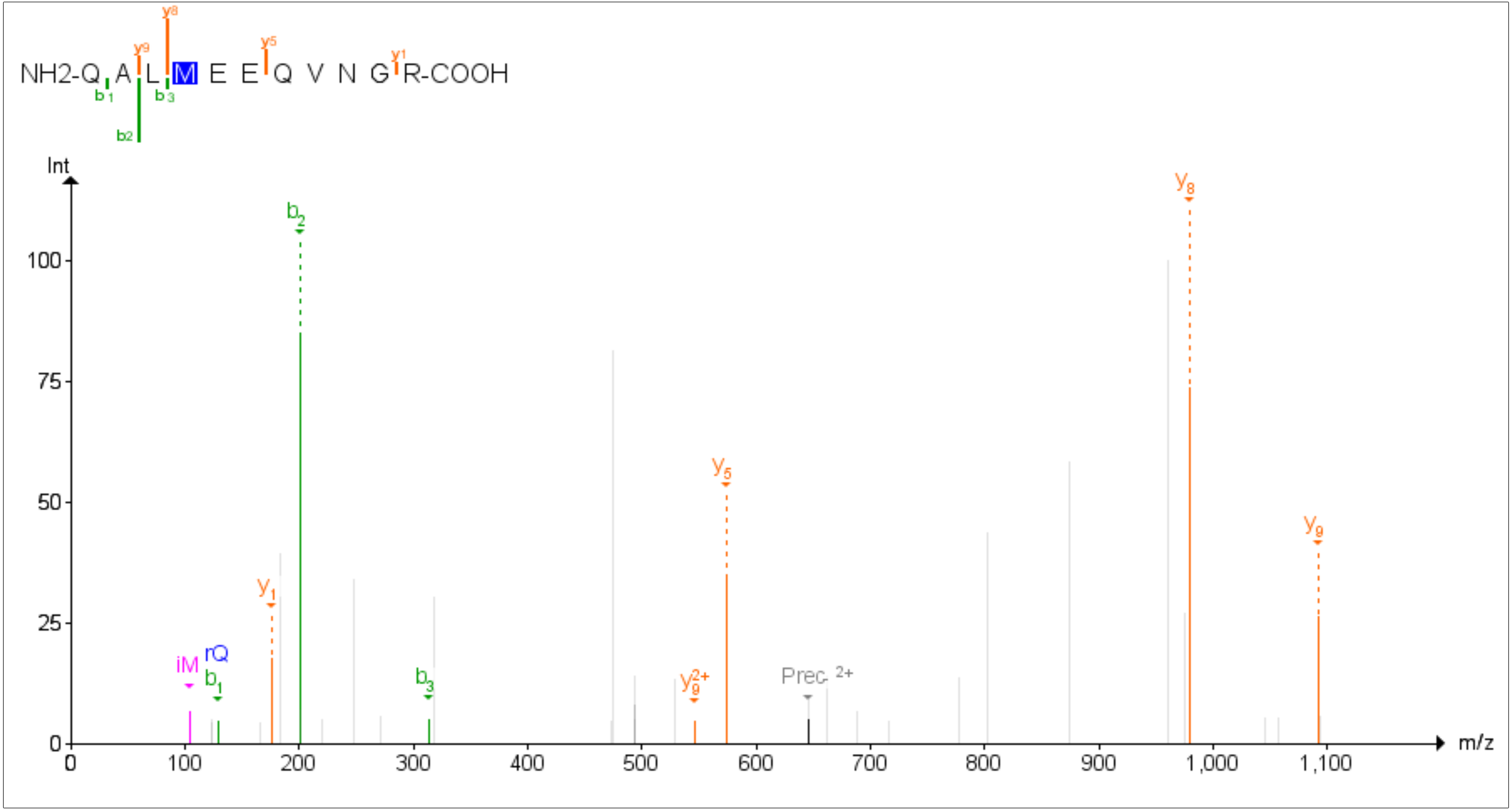

NH2-Q A L M E E Q V N G R-COOH
b1 b2 b3 y9 y8 y5 y1
Int
m/z
b2
y8
y5
y9
y1
iM
rQ
b1
b3
y9 2+
Prec 2+
100
75
50
25
0
0
100
200
300
400
500
600
700
800
900
1,000
1,100

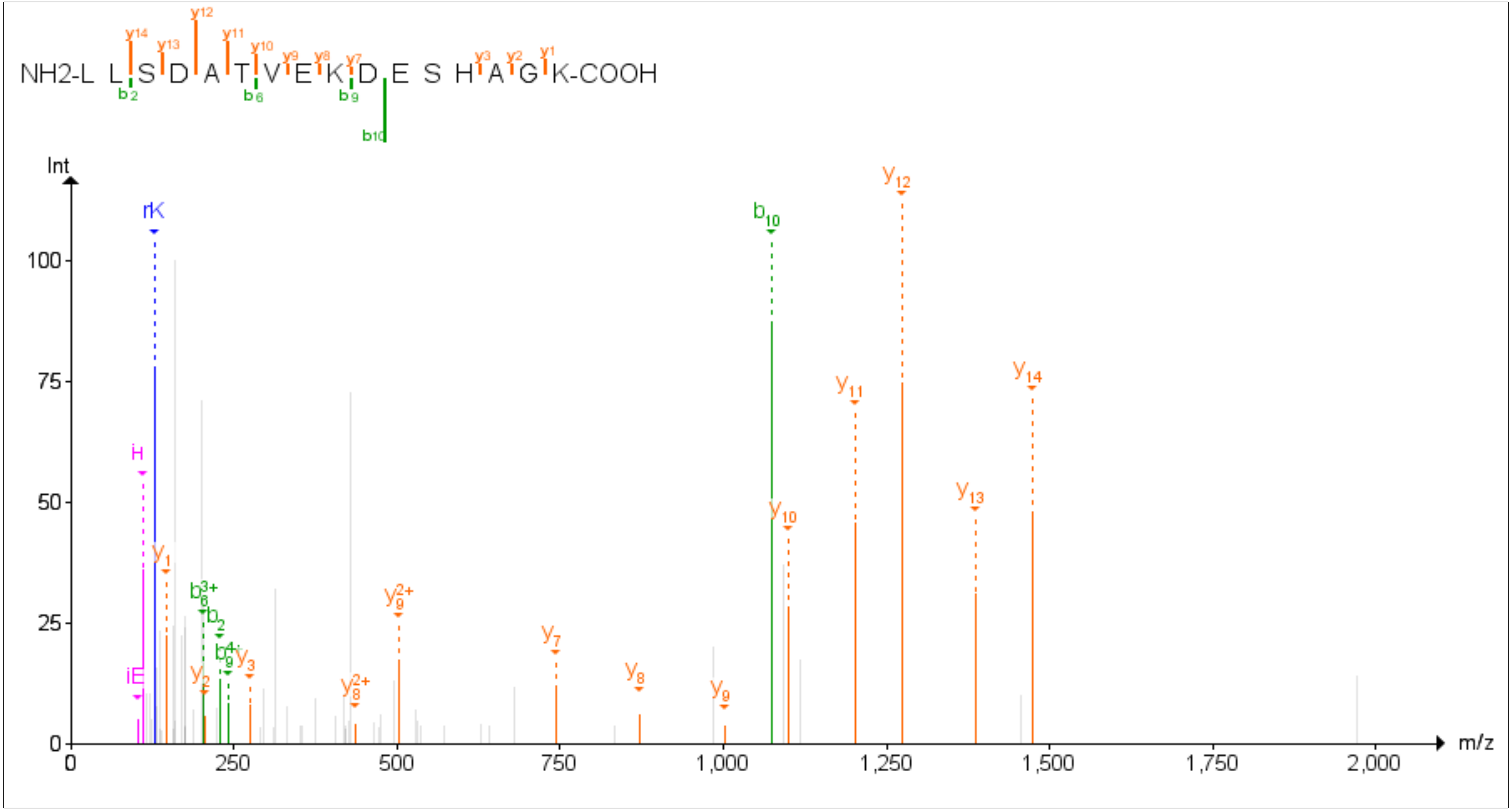
NH2-L L S D A T V E K D E S H A G K-COOH
y14
y13
y12
y11
y10
y9
y8
y7
y3
y2
y1
b2
b6
b9
b10
Int
m/z
0
25
50
75
100
250
500
750
1,000
1,250
1,500
1,750
2,000
rK
iH
iE
y1
y2
y3
b2
y8 2+
y9 2+
y7
y8
y9
b10
y10
y11
y12
y13
y14

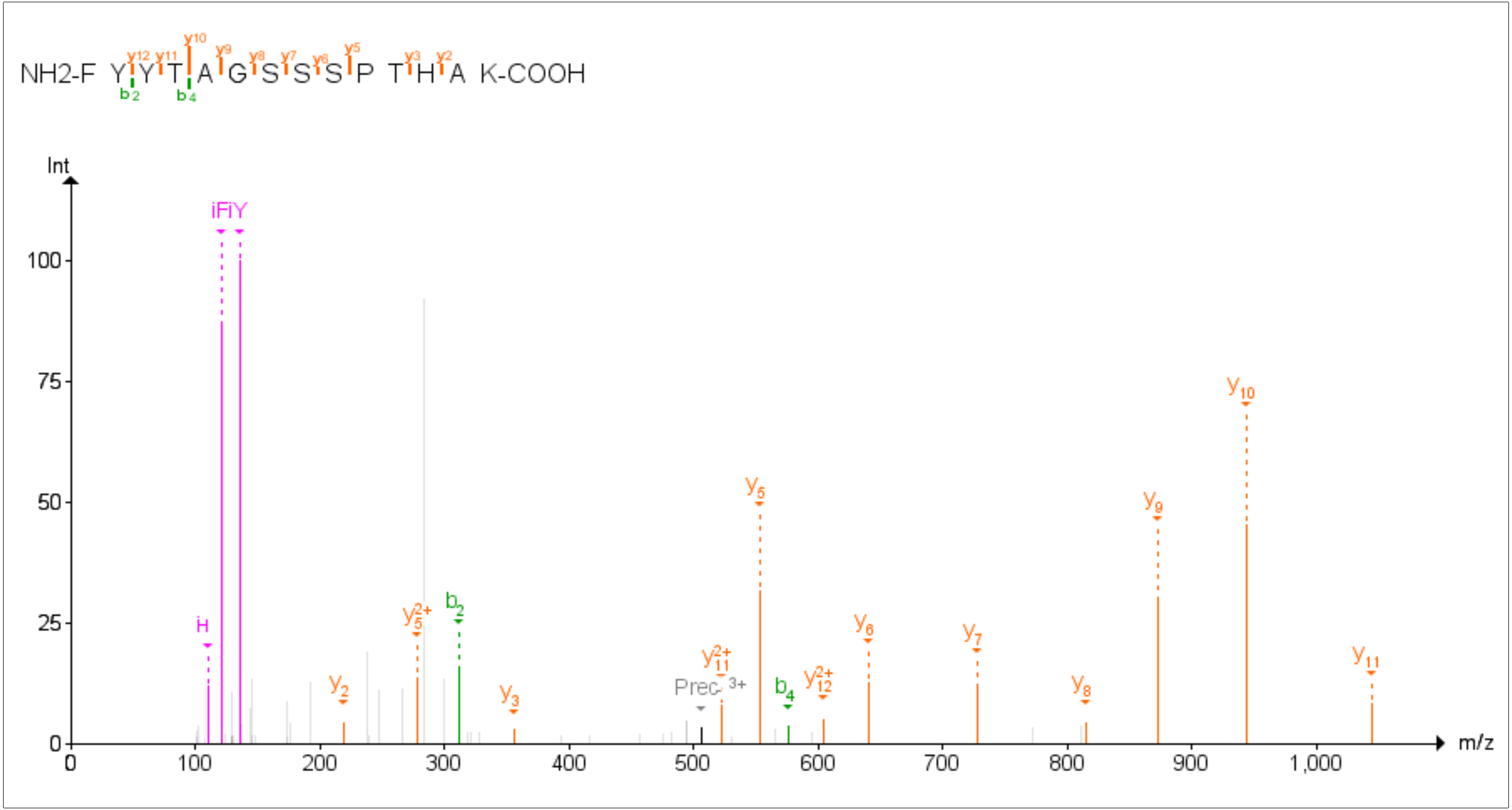

NH2-F Y Y T A G S S S P T H A K-COOH
y12 y11 y10 y9 y8 y7 y6 y5 y3 y2
b2 b4
Int
100
75
50
25
0
iFiY
H
y2
y5 2+
b2
y3
Prec- 3+
y11 2+
y5
b4
y12 2+
y6
y7
y8
y9
y10
y11
0
100
200
300
400
500
600
700
800
900
1,000
m/z

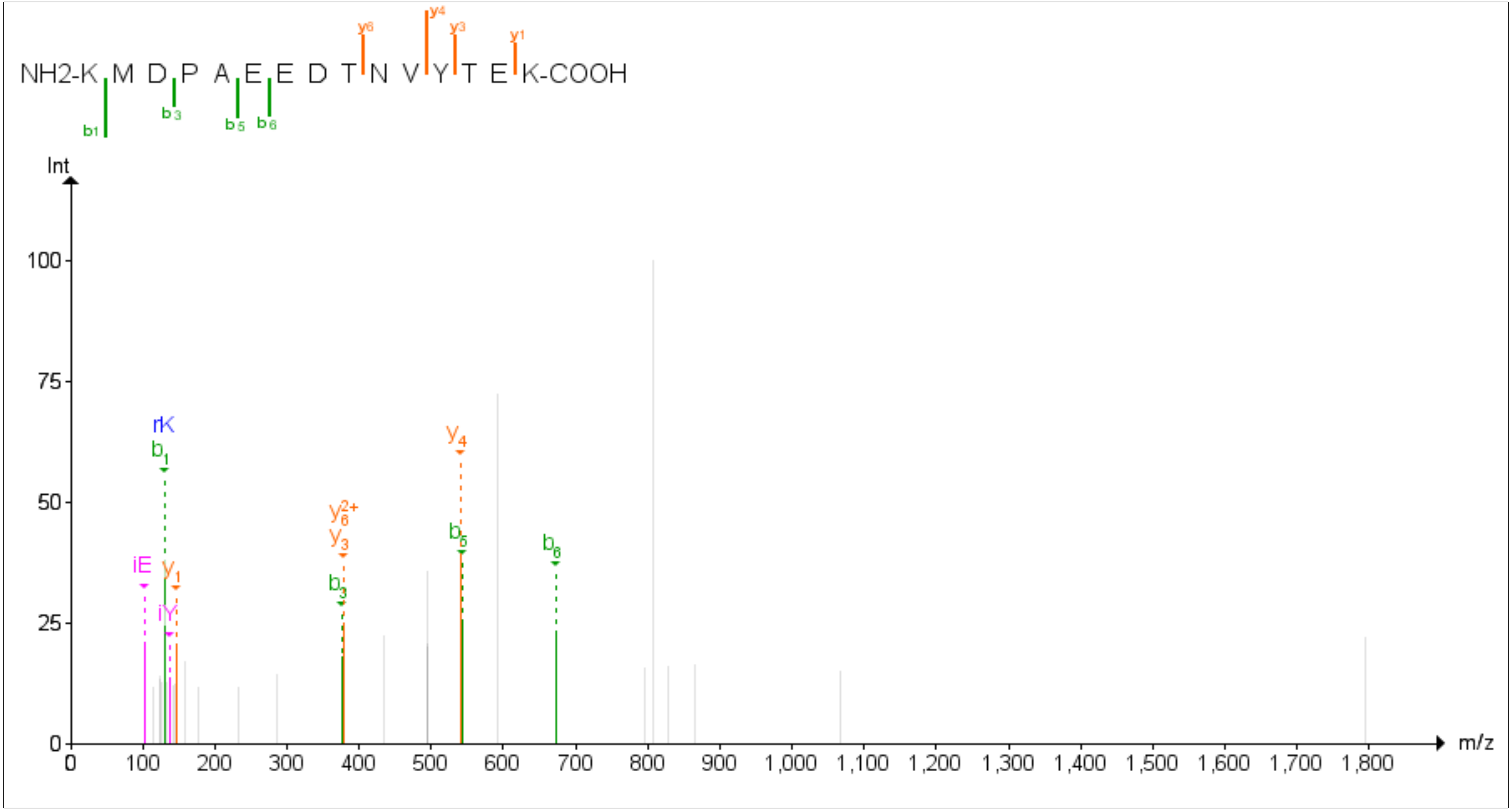

NH2-K M D P A E E D T N V Y T E K-COOH
b1
b3
b5
b6
y6
y4
y3
y1
Int
100
75
50
25
0
rK
b1
y4
y6 2+
y3
b5
b6
iE
y1
b3
iY
m/z
0
100
200
300
400
500
600
700
800
900
1,000
1,100
1,200
1,300
1,400
1,500
1,600
1,700
1,800

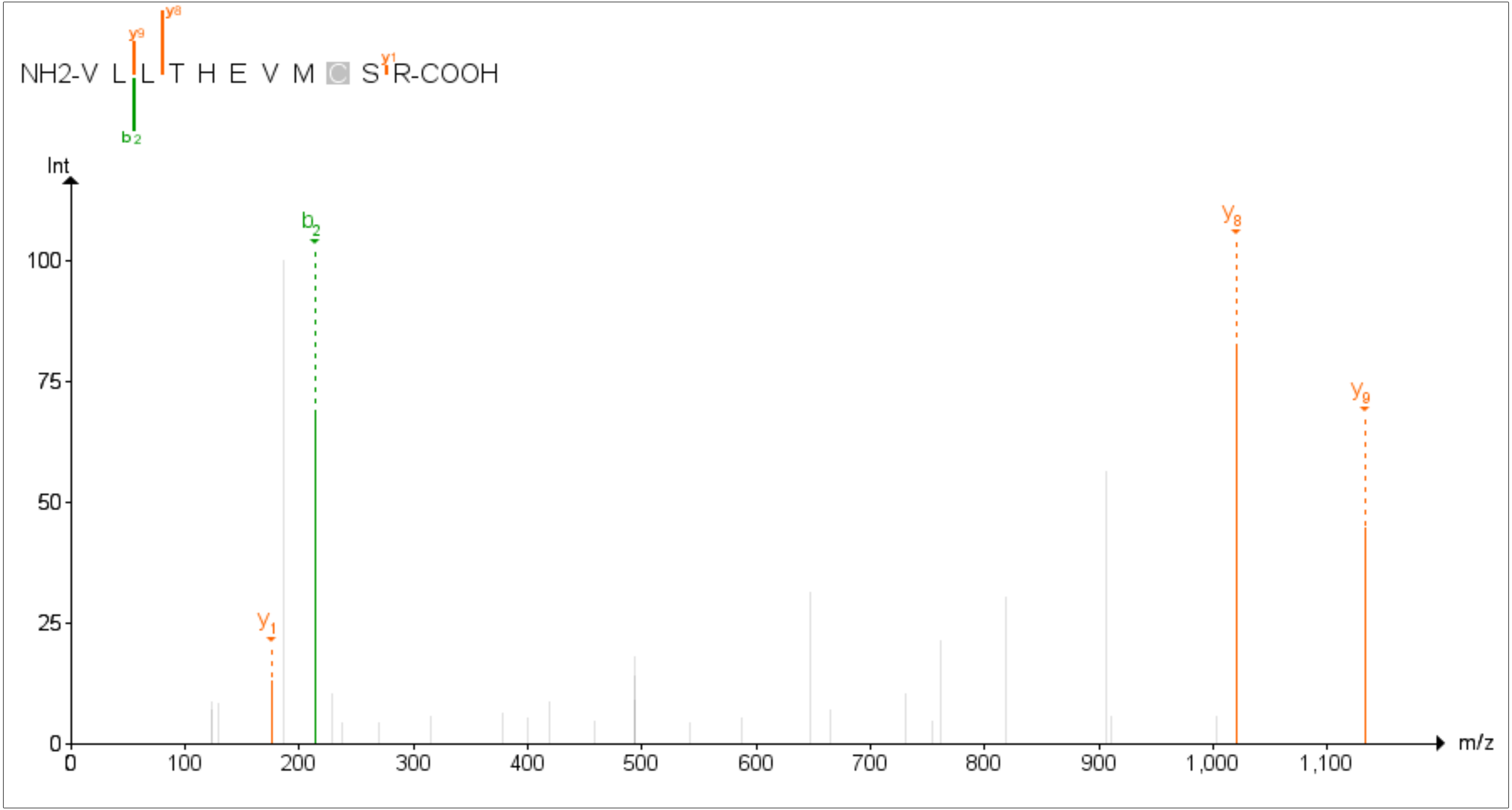

NH2-V L L T H E V M C S R-COOH
y9
y8
y1
b2
Int
100
75
50
25
0
b2
y8
y9
y1
0
100
200
300
400
500
600
700
800
900
1,000
1,100
m/z

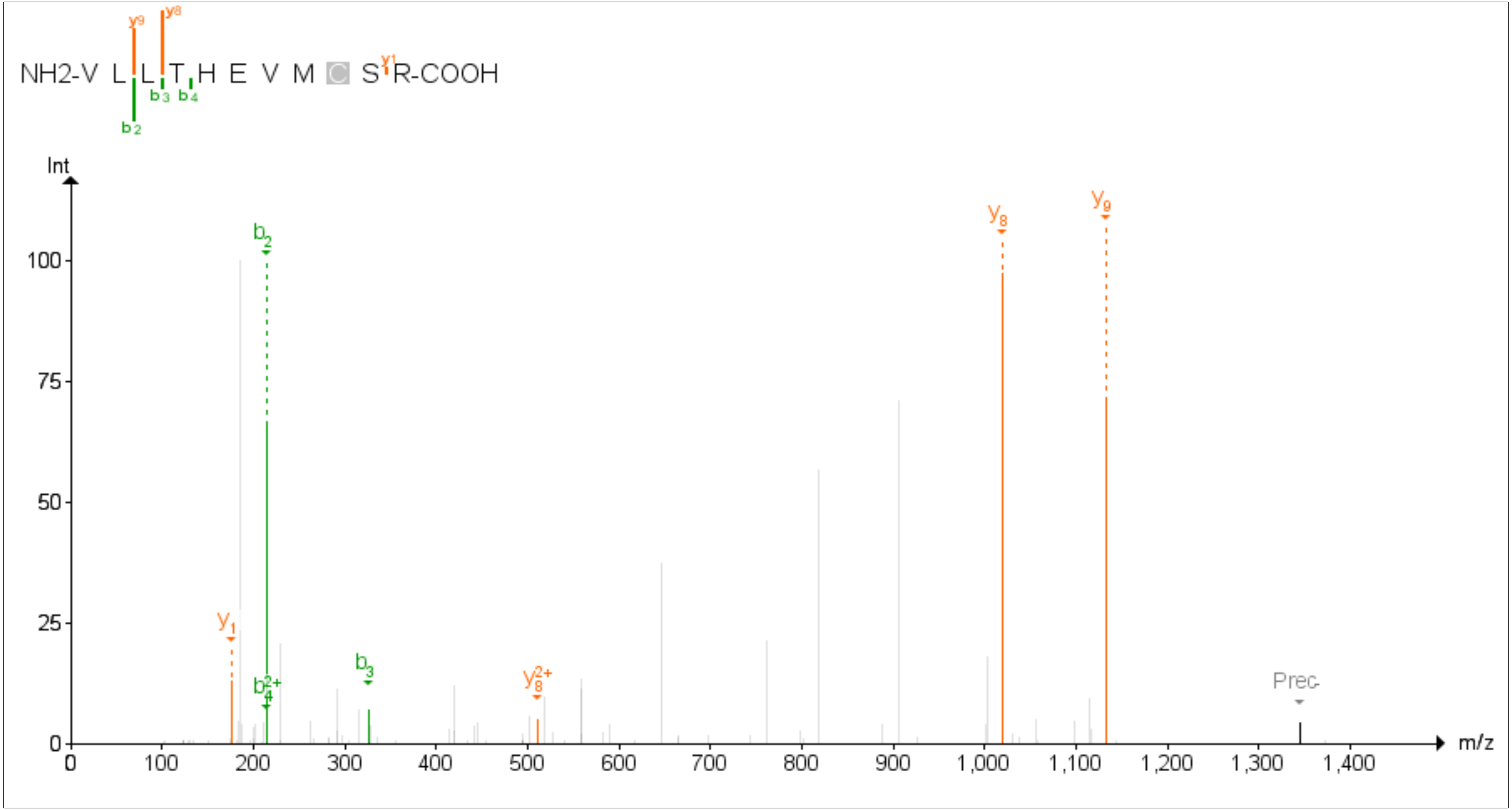

NH2-V L L T H E V M C S R-COOH
y9
y8
y1
b2
b3
b4
Int
100
75
50
25
0
b2
y8
y9
y1
b3
b4 2+
y8 2+
Prec
m/z
0
100
200
300
400
500
600
700
800
900
1,000
1,100
1,200
1,300
1,400

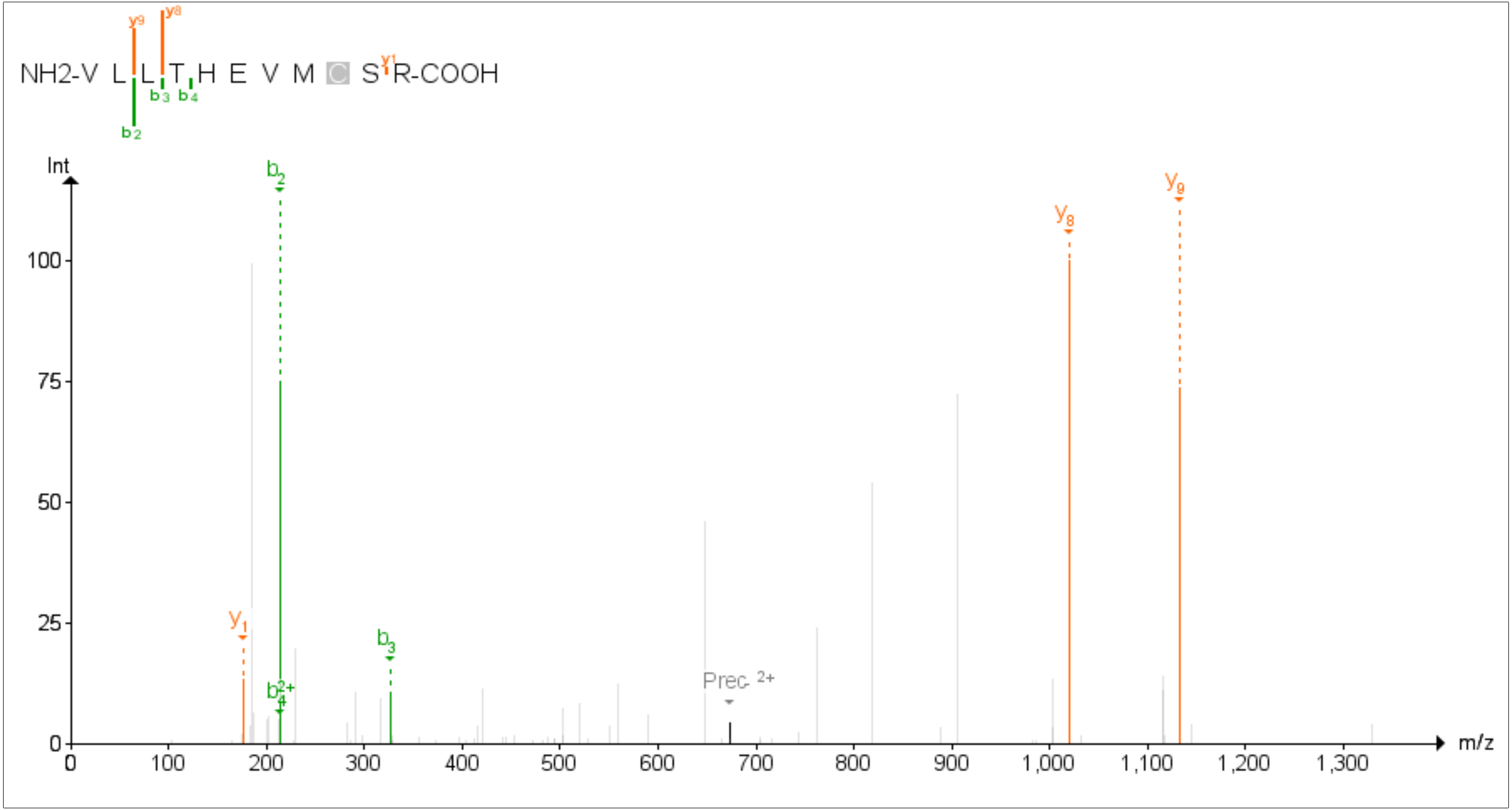

NH2-V L L T H E V M C S R-COOH
y9 y8 y1
b2 b3 b4
Int
100
75
50
25
0
b2
y8
y9
y1
b3
b4 2+
Prec 2+
0 100 200 300 400 500 600 700 800 900 1,000 1,100 1,200 1,300
m/z

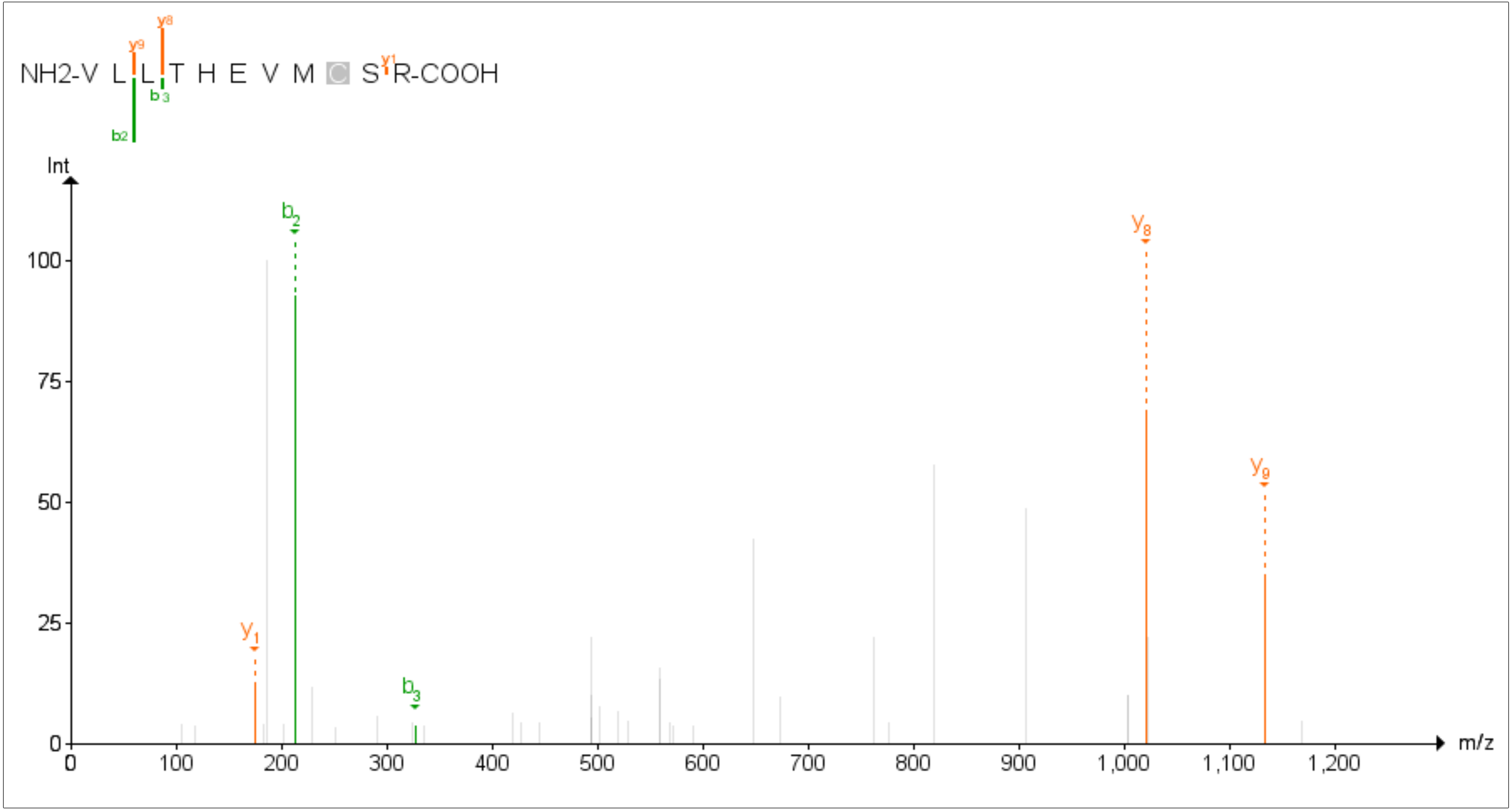

NH2-V L L T H E V M C S R-COOH
y9
y8
y1
b2
b3
Int
100
75
50
25
0
b2
b3
y1
y8
y9
0
100
200
300
400
500
600
700
800
900
1,000
1,100
1,200
m/z

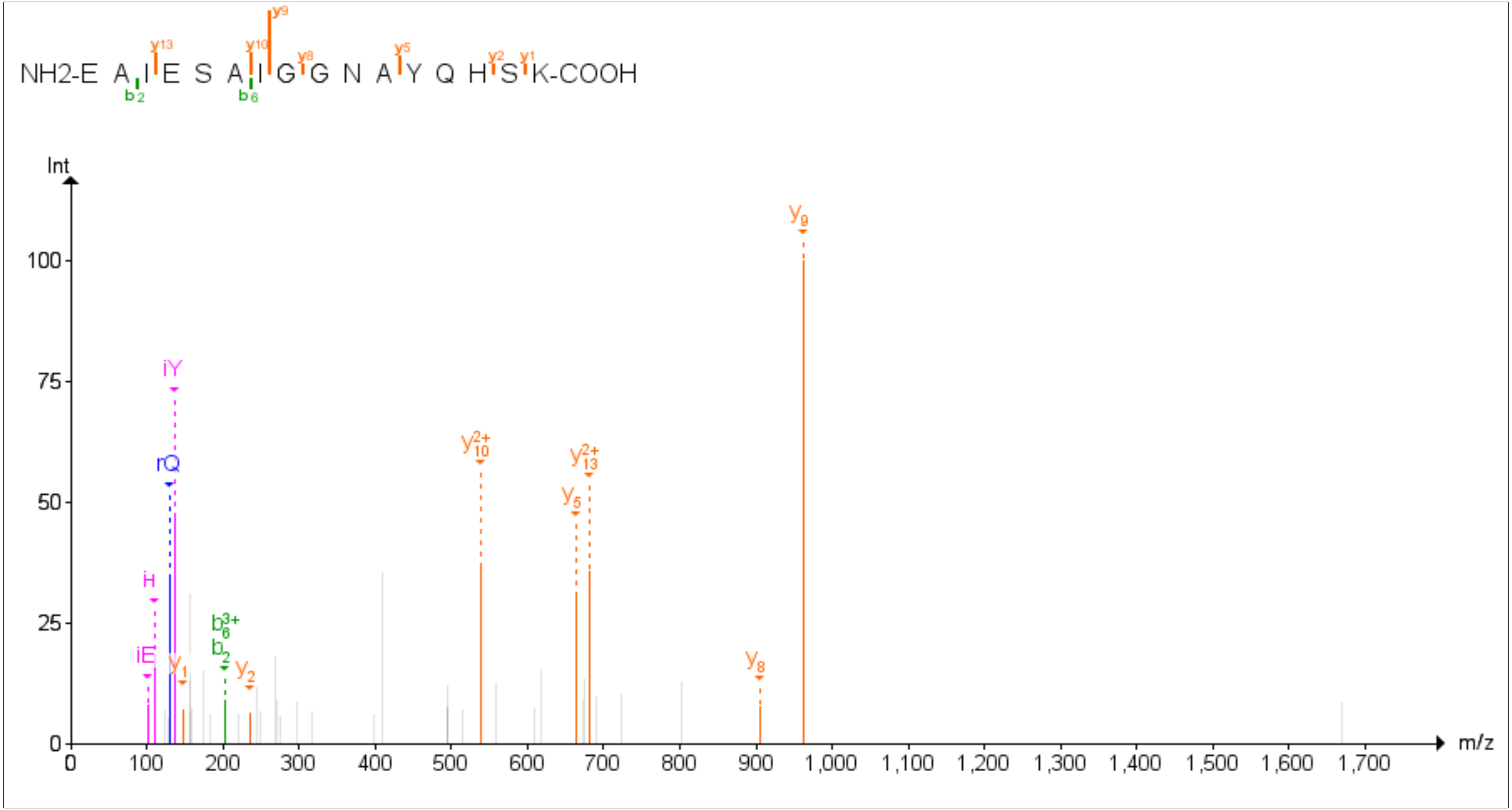

NH2-E A I E S A I G G N A Y Q H S K-COOH
y13 y10 y9 y8 y5 y2 y1
b2 b6
Int
100
75
50
25
0
iY
rQ
iH
iE
y1
b_6^{3+}
b_2
y_2
y_{10}^{2+}
y_{13}^{2+}
y_5
y_8
y_9
0 100 200 300 400 500 600 700 800 900 1,000 1,100 1,200 1,300 1,400 1,500 1,600 1,700
m/z

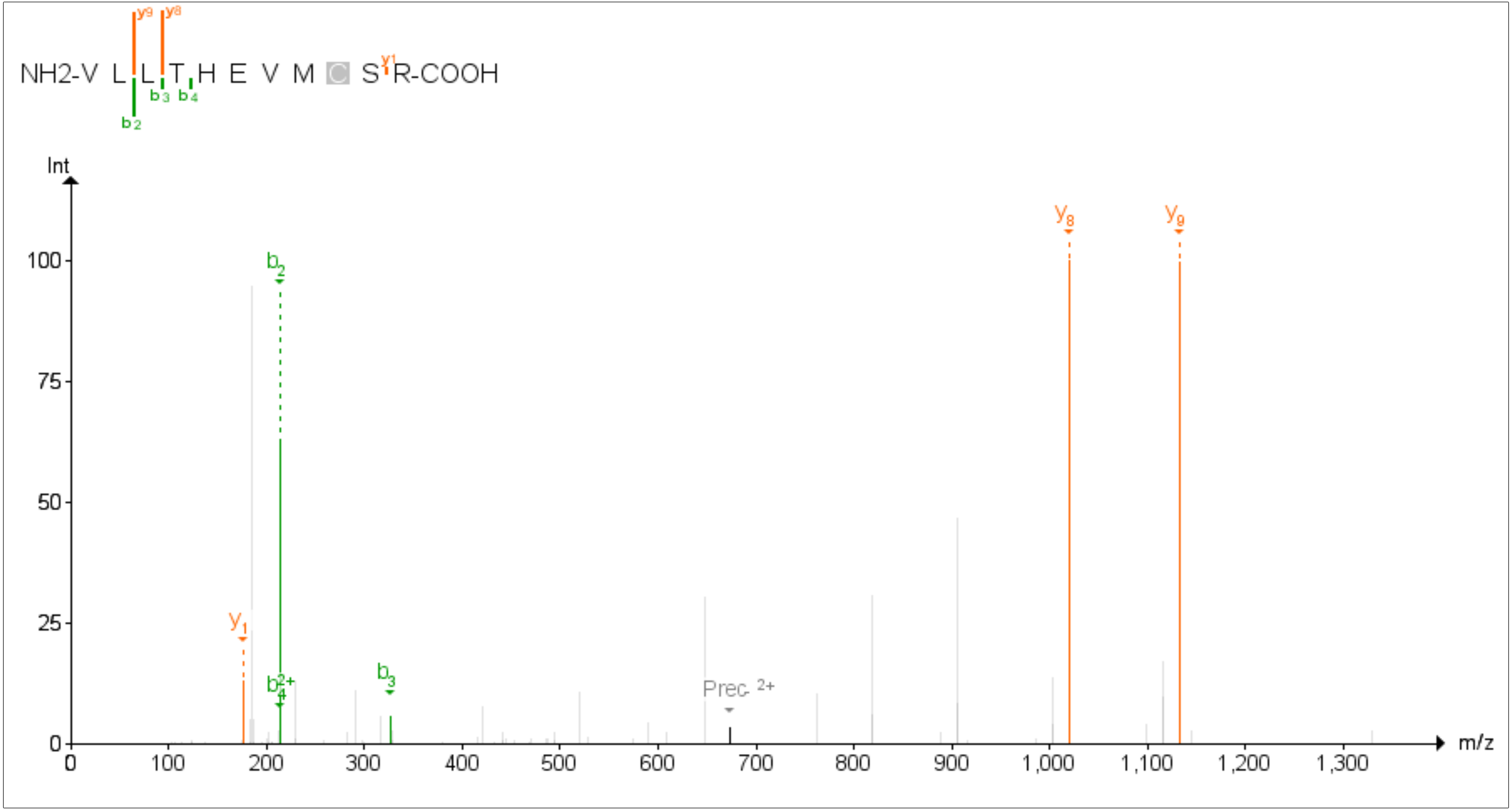

NH2-V L L T H E V M C S R-COOH
y9
y8
y1
b2
b3
b4
Int
100
75
50
25
0
m/z
0
100
200
300
400
500
600
700
800
900
1,000
1,100
1,200
1,300
y8
y9
b2
y1
b4 2+
b3
Prec. 2+

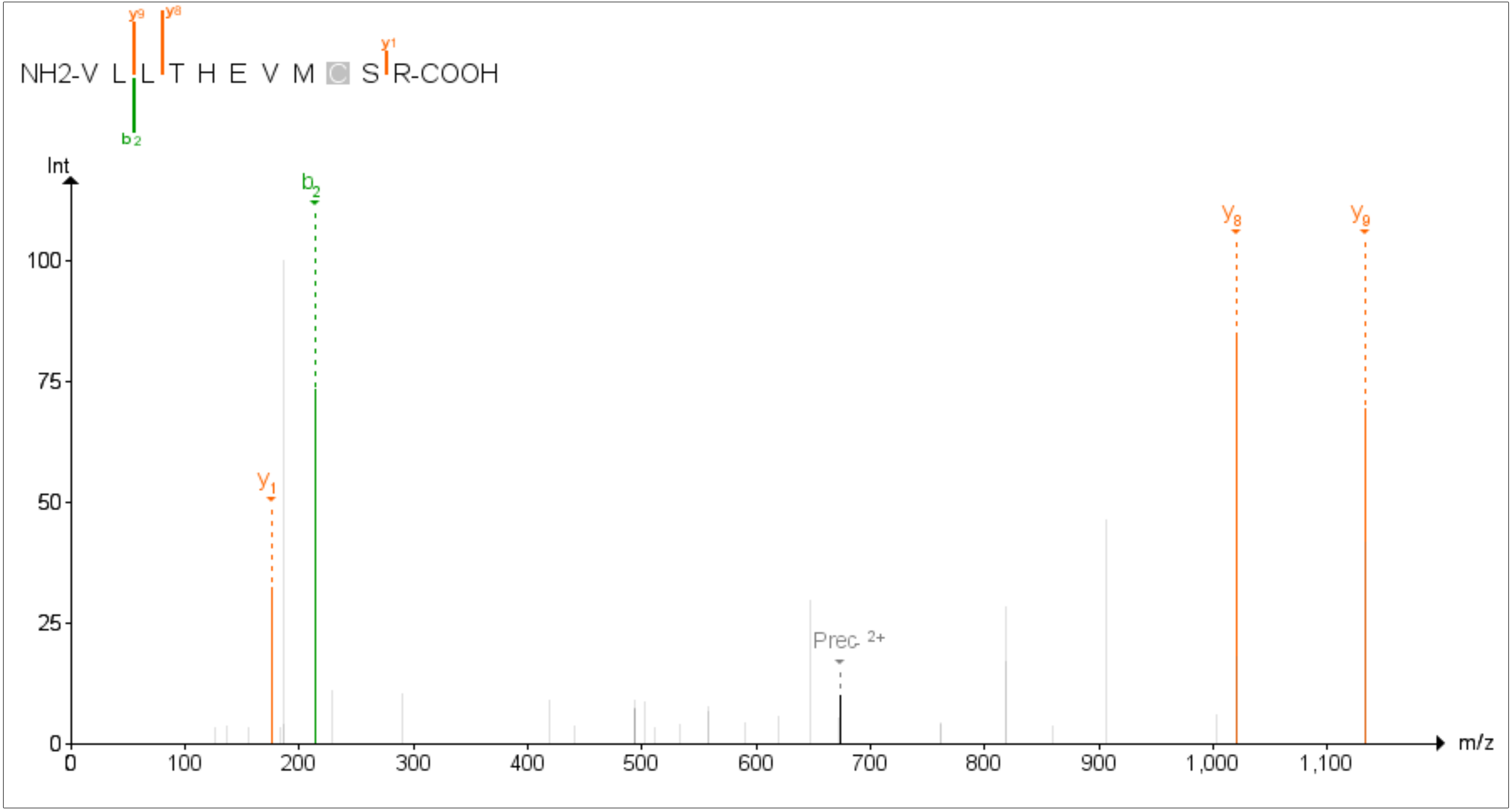

NH2-V L L T H E V M C S R-COOH
y9
y8
y1
b2
Int
100
75
50
25
0
b2
y8
y9
y1
Prec. 2+
m/z
0
100
200
300
400
500
600
700
800
900
1,000
1,100

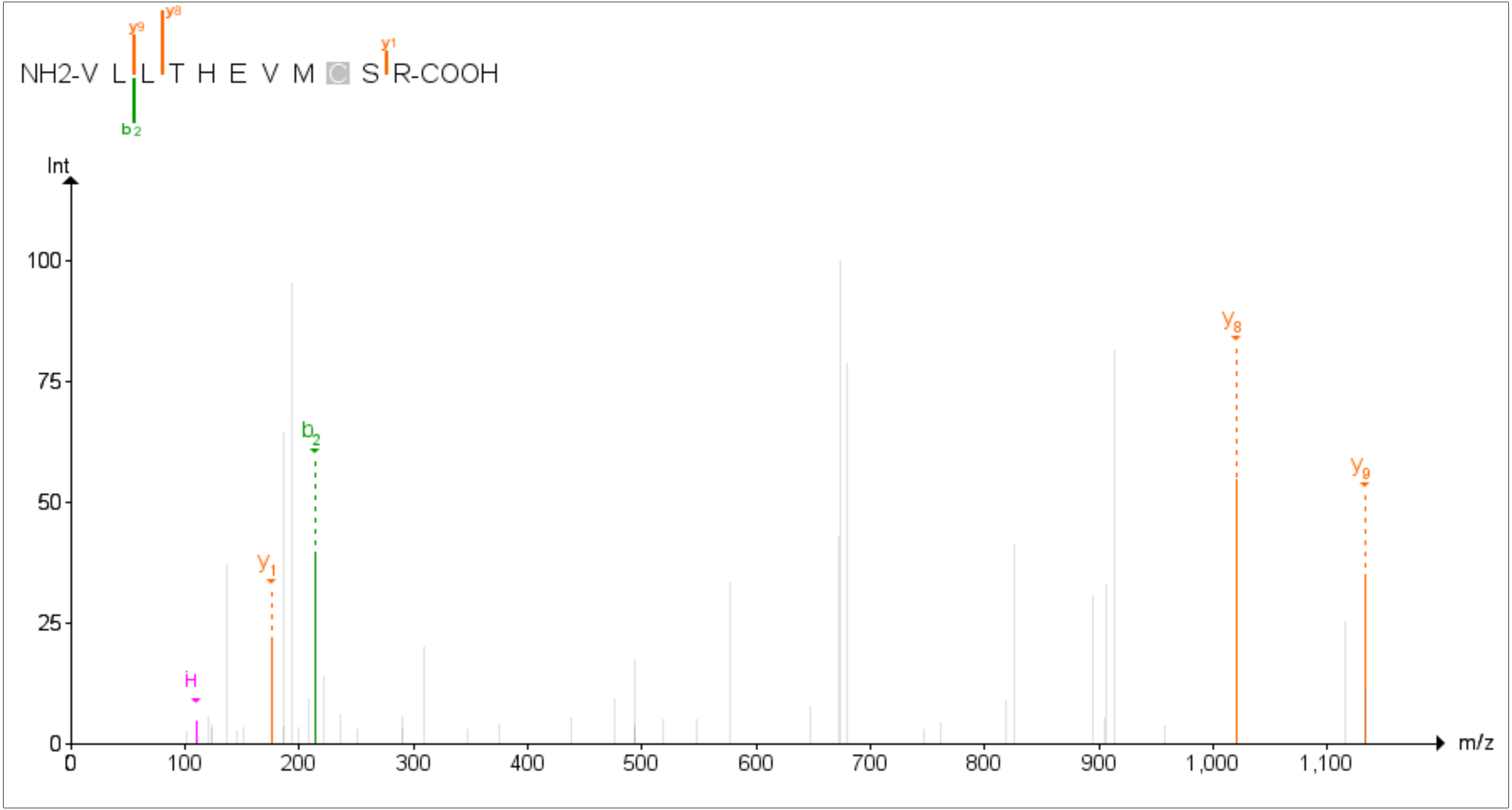
NH2-V L L T H E V M C S R-COOH
y9
y8
y1
b2
Int
100
75
50
25
0
y8
y9
b2
y1
H
0
100
200
300
400
500
600
700
800
900
1,000
1,100
m/z

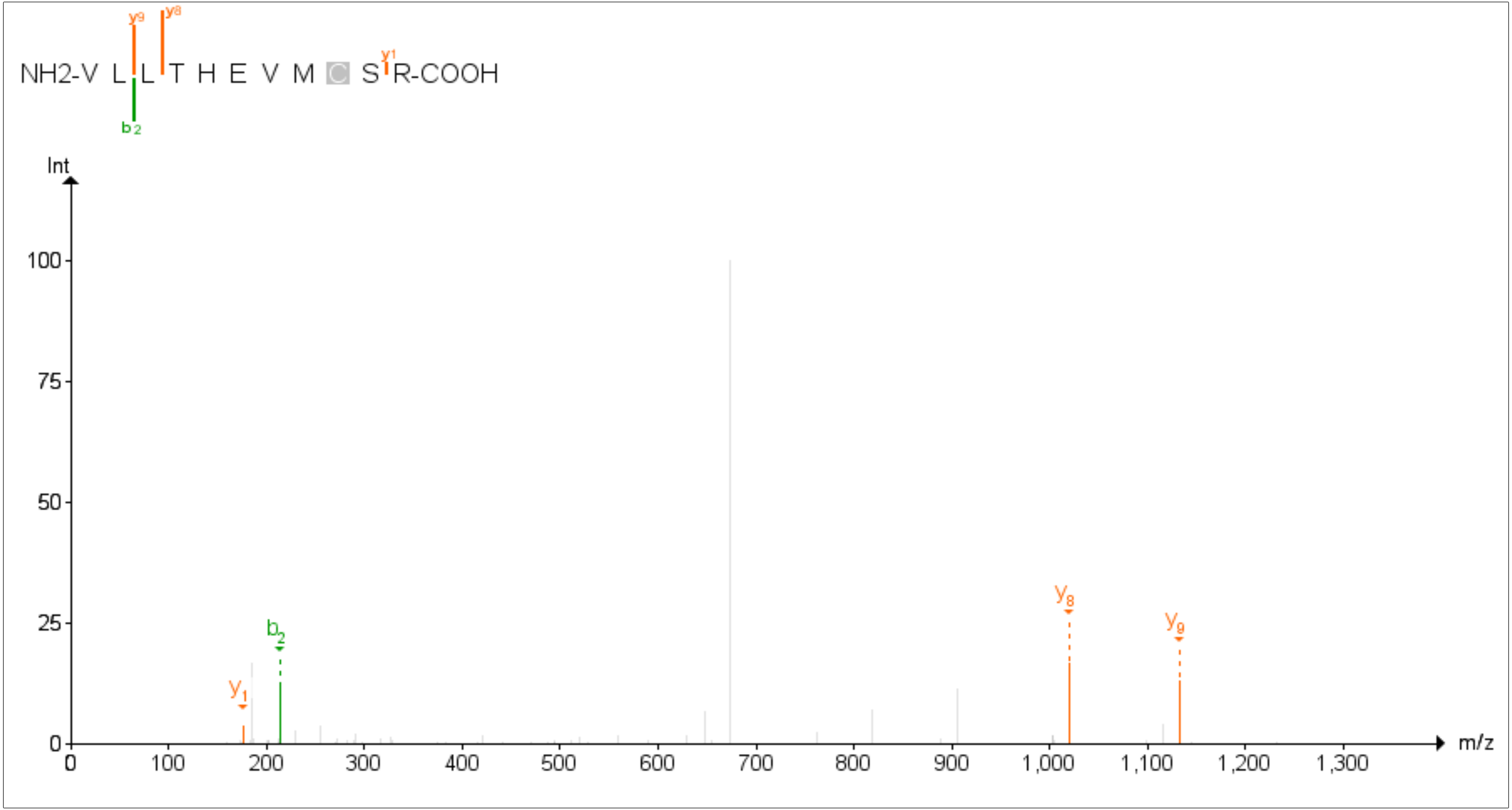

NH2-V L L T H E V M C S R-COOH
y9
y8
y1
b2
Int
100
75
50
25
0
m/z
0
100
200
300
400
500
600
700
800
900
1,000
1,100
1,200
1,300
y1
b2
y8
y9

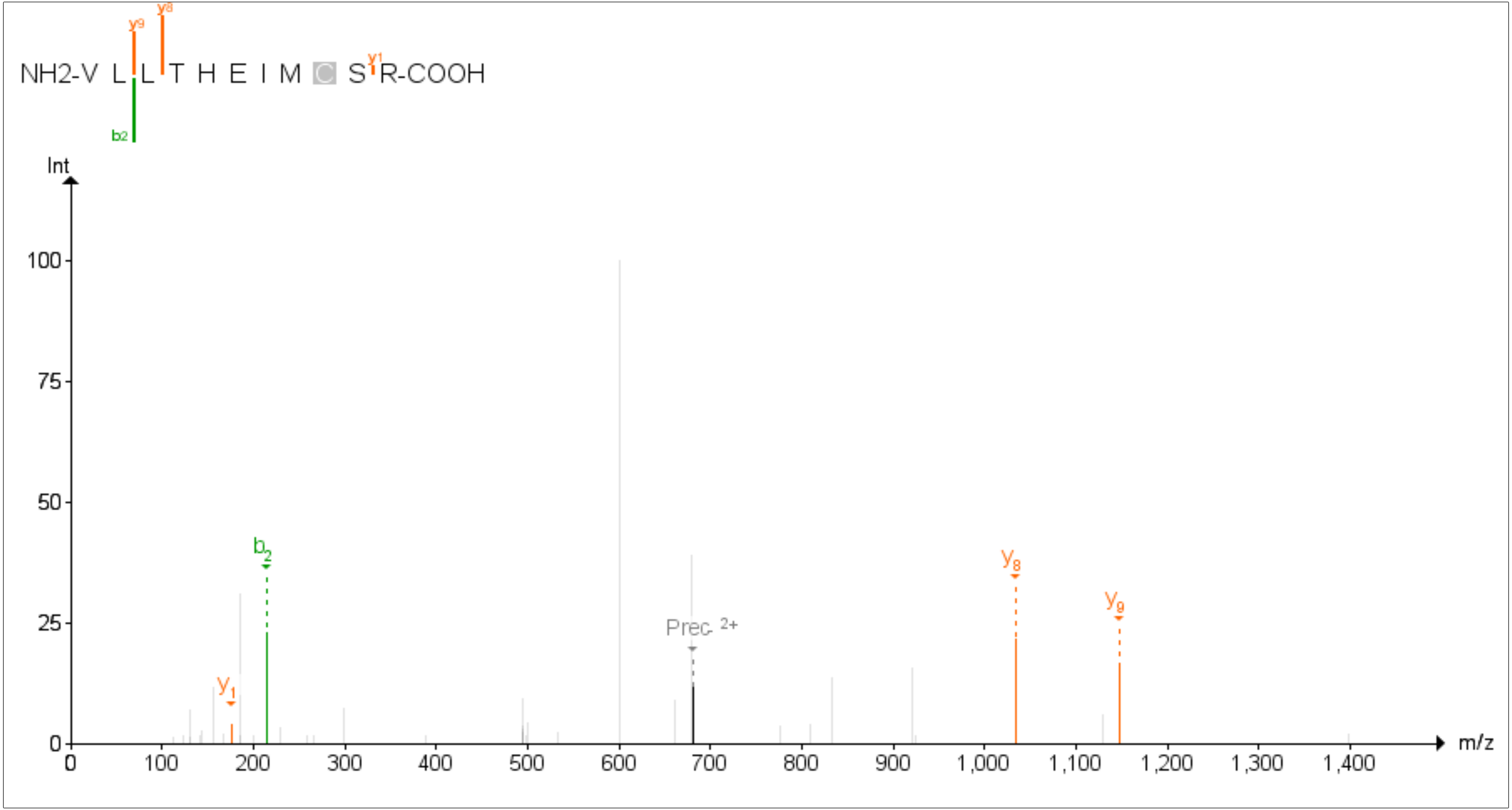

NH2-V L L T H E I M C S R-COOH
y9
y8
y1
b2
Int
100
75
50
25
0
b2
y1
Prec 2+
y8
y9
0
100
200
300
400
500
600
700
800
900
1,000
1,100
1,200
1,300
1,400
m/z

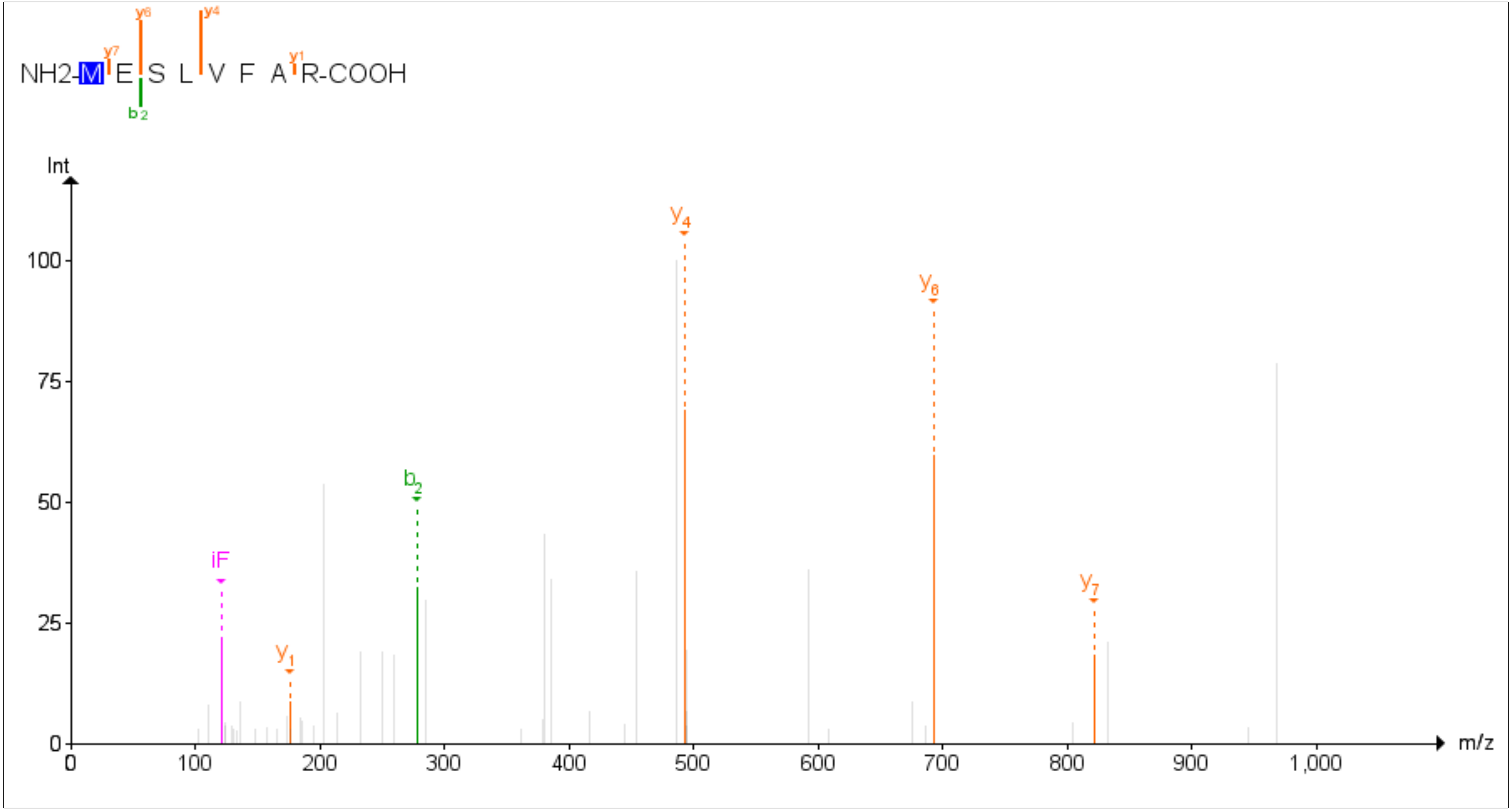

NH2-M E S L V F A R-COOH
y7
y6
y4
y1
b2
Int
100
75
50
25
0
y4
y6
y7
b2
iF
y1
0
100
200
300
400
500
600
700
800
900
1,000
m/z

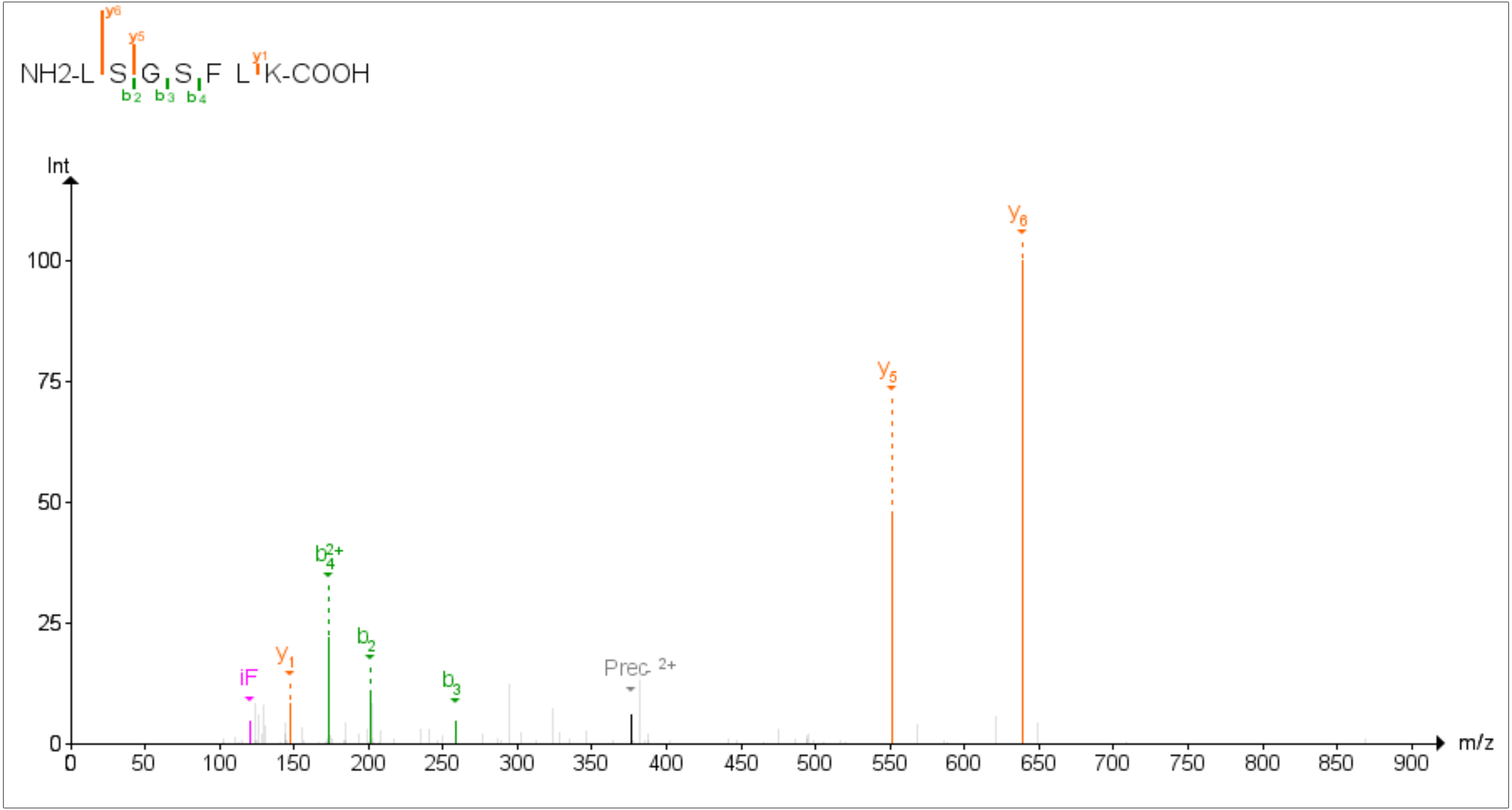

NH2-L S G S F L K-COOH
y6
y5
y1
b2
b3
b4
Int
100
75
50
25
0
iF
y1
b4 2+
b2
b3
Prec 2+
y5
y6
0 50 100 150 200 250 300 350 400 450 500 550 600 650 700 750 800 850 900
m/z

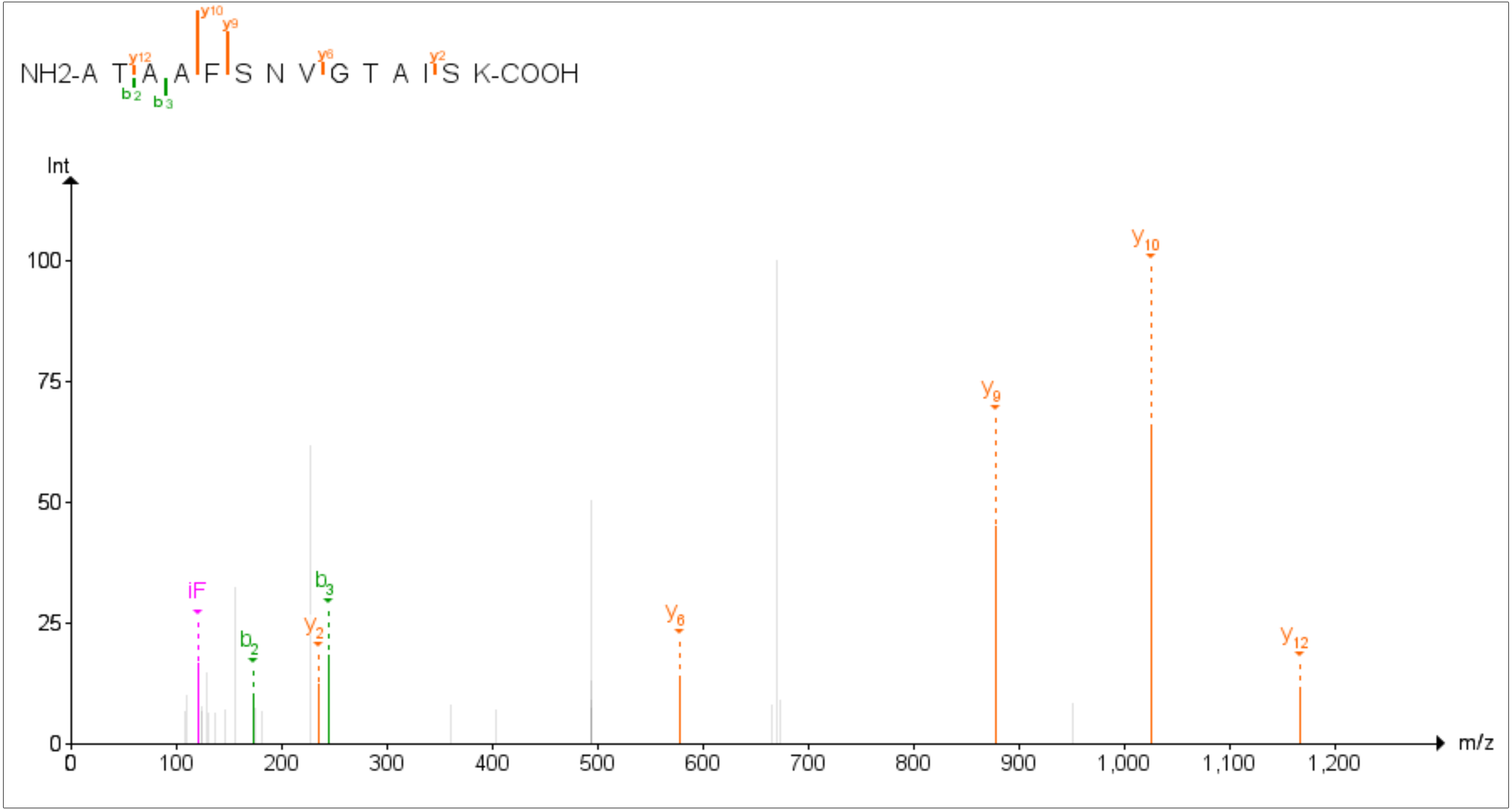

NH2-A T A A F S N V G T A I S K-COOH
y10
y9
y12
y6
y2
b2
b3
Int
100
75
50
25
0
iF
b2
y2
b3
y6
y9
y10
y12
0
100
200
300
400
500
600
700
800
900
1,000
1,100
1,200
m/z

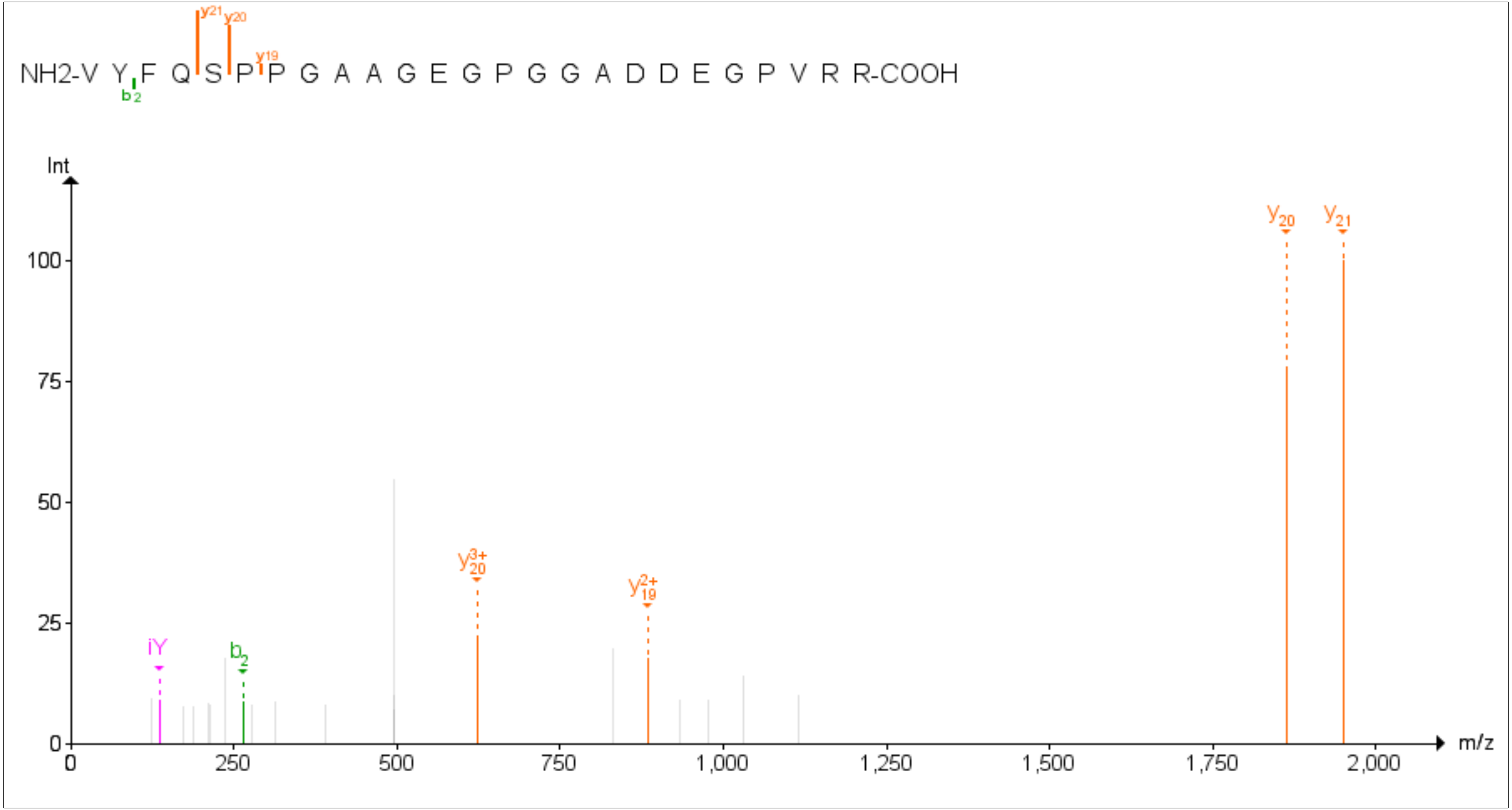

NH2-V Y F Q S P P G A A G E G P G G A D D E G P V R R-COOH
b2
y21
y20
y19
Int
m/z
iY
b2
y20 3+
y19 2+
y20
y21
0
25
50
75
100
0
250
500
750
1,000
1,250
1,500
1,750
2,000

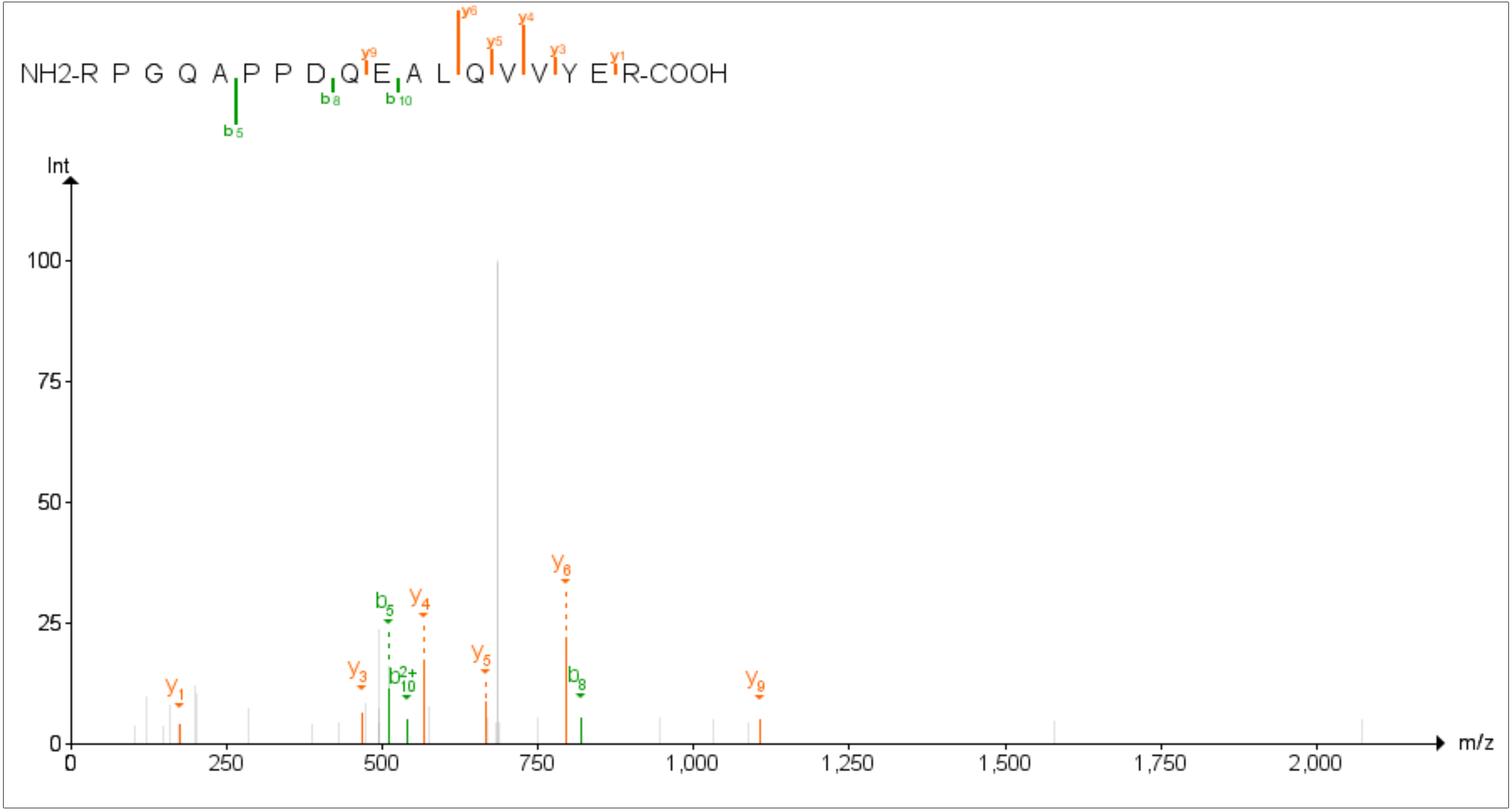

NH2-R P G Q A P P D Q E A L Q V V Y E R-COOH
y6
y4
y5
y9
y3
y1
b5
b8
b10
Int
100
75
50
25
0
0
250
500
750
1,000
1,250
1,500
1,750
2,000
m/z
y1
y3
b5
b10 2+
y4
y5
y6
b8
y9

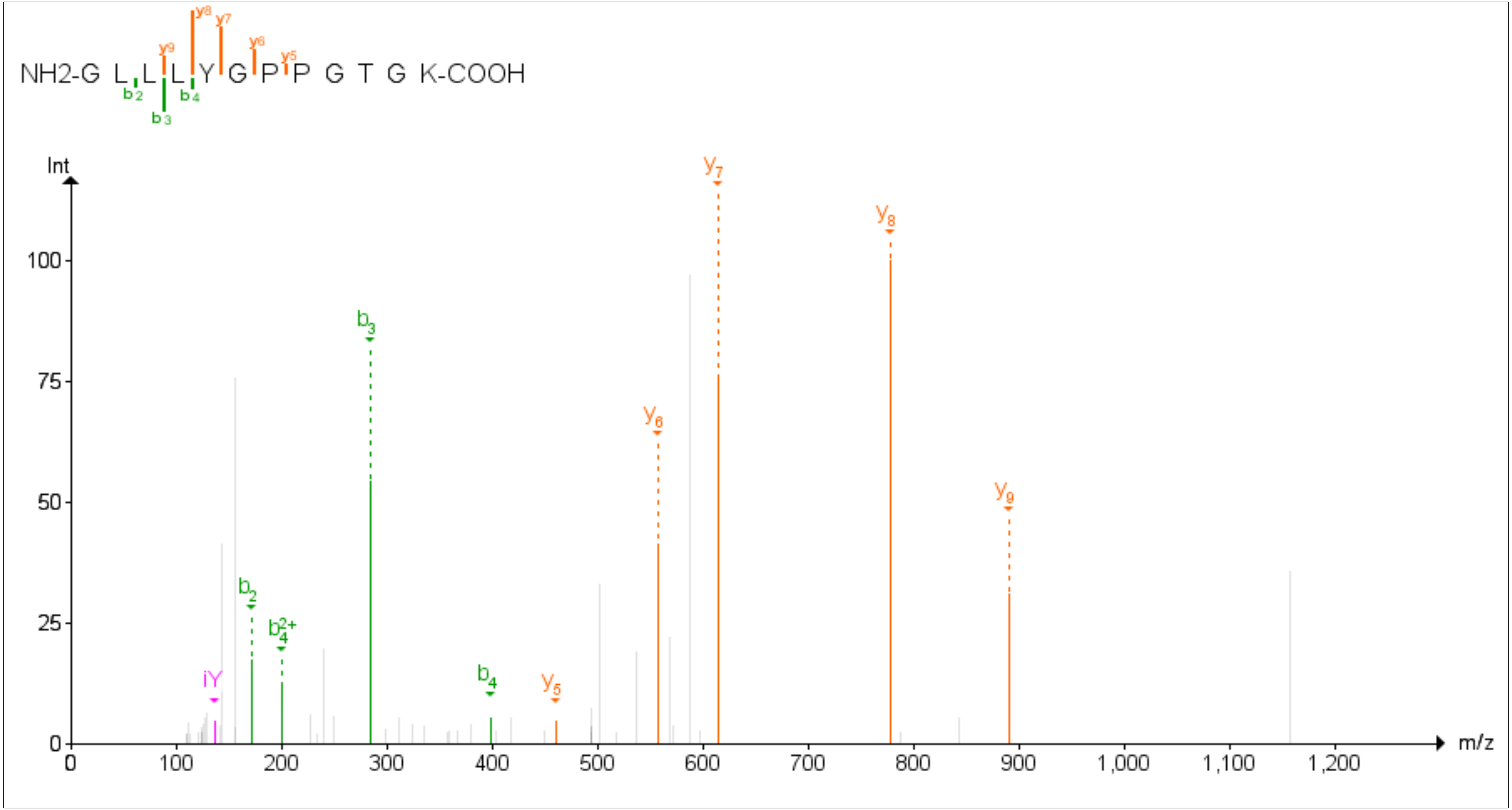

NH2-G L L L Y G P P G T G K-COOH
b2 b3 b4
y9 y8 y7 y6 y5
Int
0
25
50
75
100
m/z
0
100
200
300
400
500
600
700
800
900
1,000
1,100
1,200
iY
b2
b_4^{2+}
b3
b4
y5
y6
y7
y8
y9

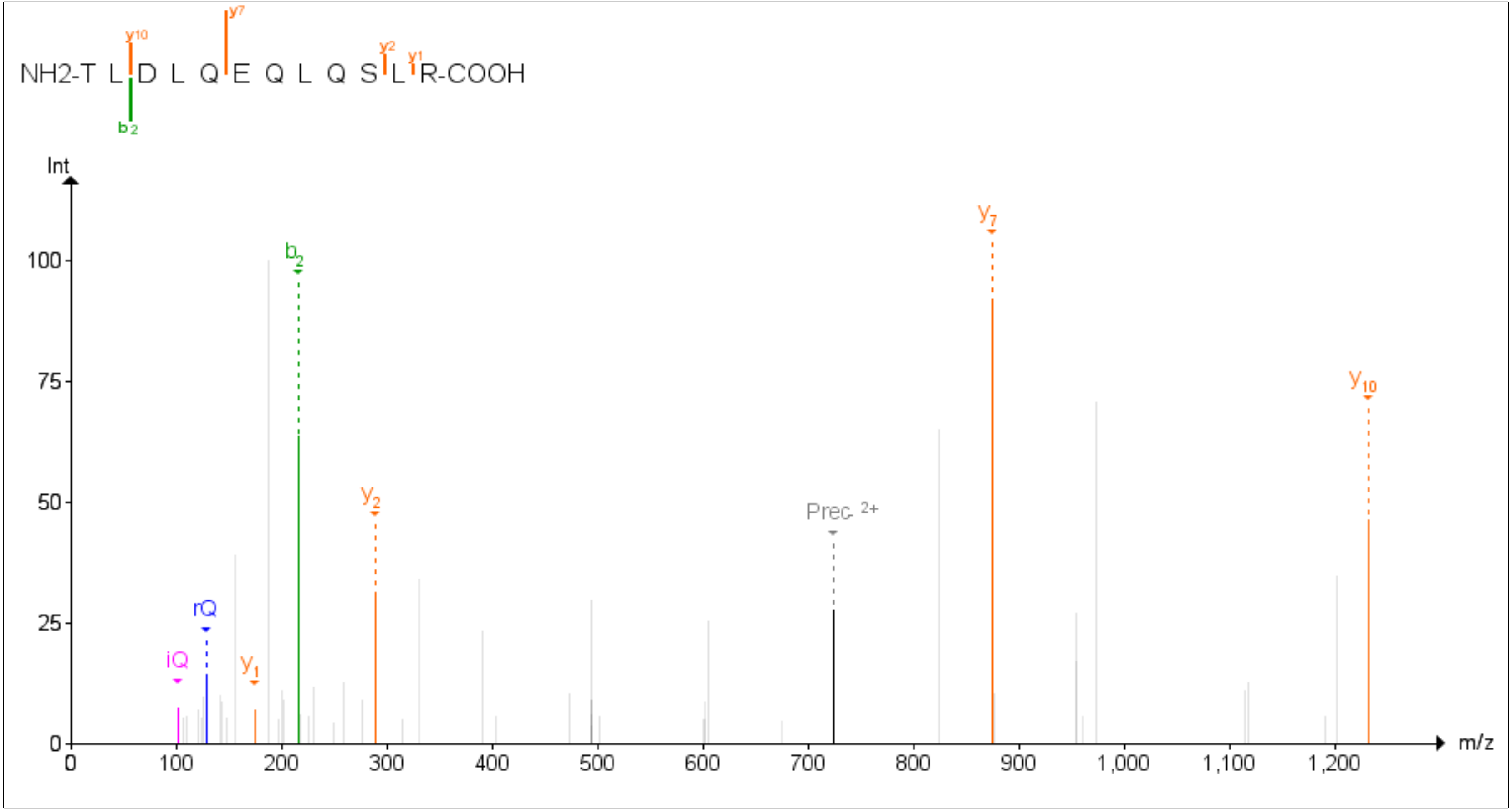
NH2-T L D L Q E Q L Q S L R-COOH
y10
y7
y2
y1
b2
Int
100
75
50
25
0
b2
y7
y10
y2
Prec- 2+
rQ
iQ
y1
0
100
200
300
400
500
600
700
800
900
1,000
1,100
1,200
m/z

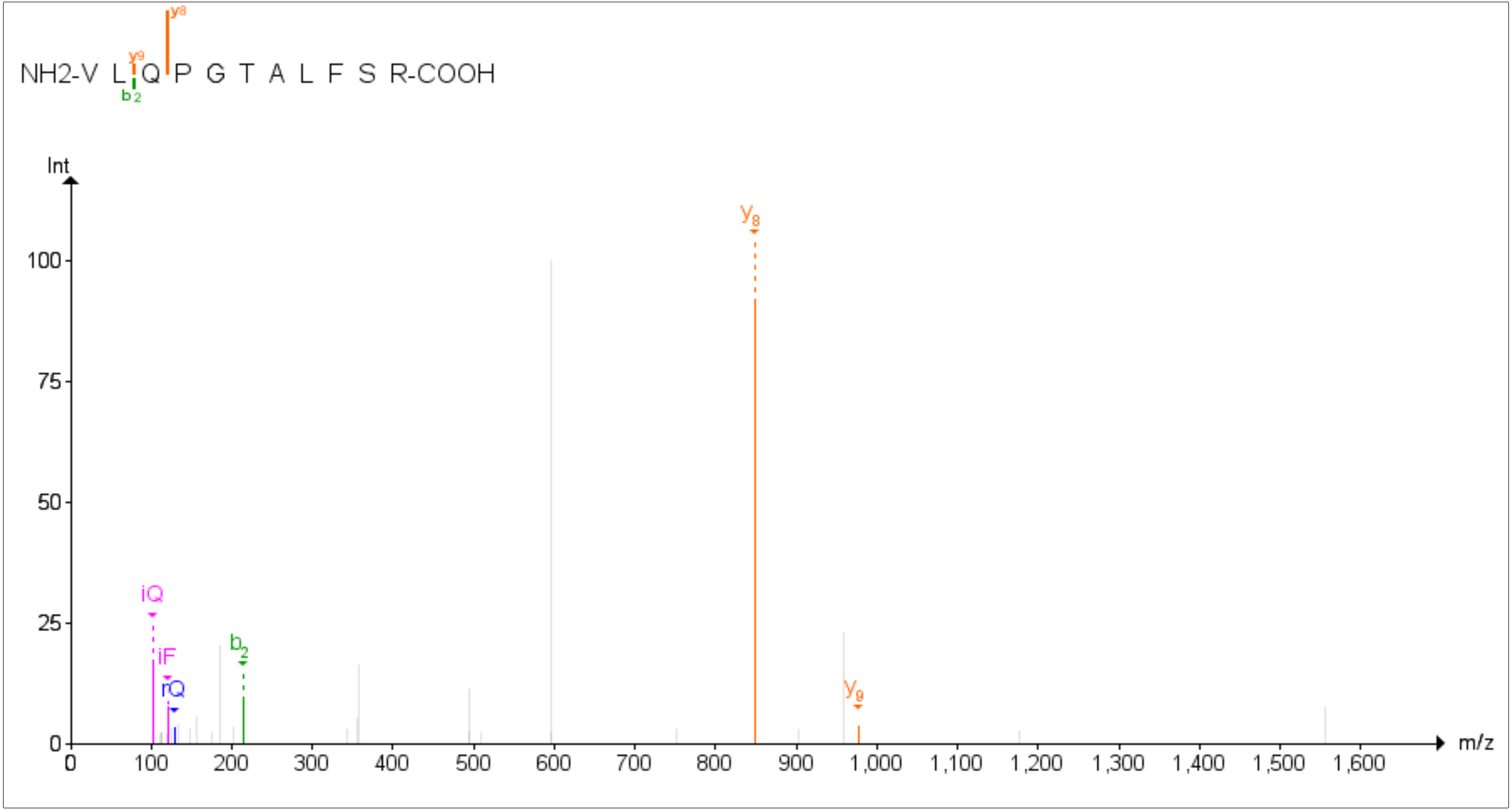

NH2-V L Q P G T A L F S R-COOH
y8
y9
b2
Int
100
75
50
25
0
y8
y9
iQ
iF
rQ
b2
0
100
200
300
400
500
600
700
800
900
1,000
1,100
1,200
1,300
1,400
1,500
1,600
m/z

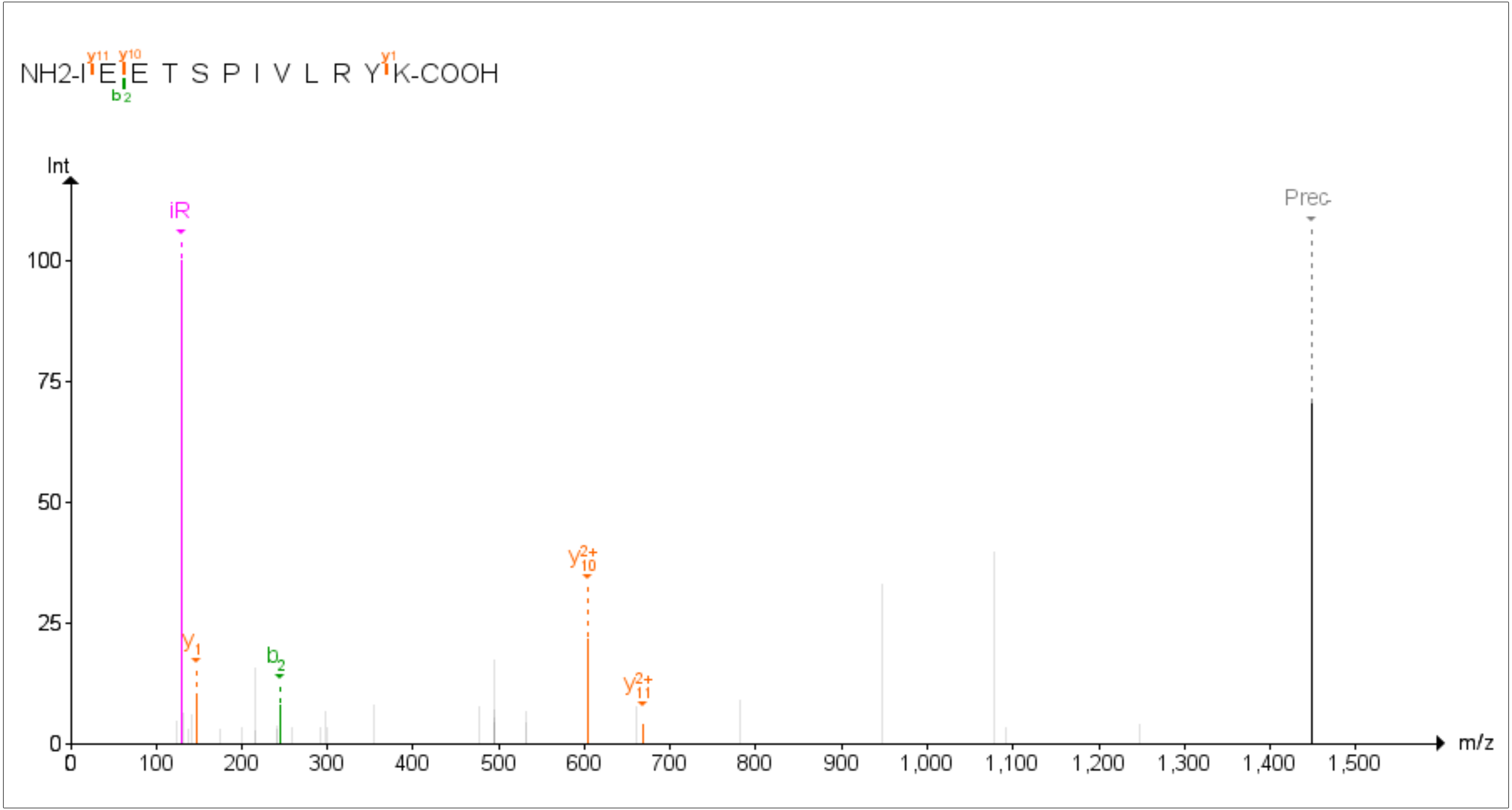

NH2-I E E T S P I V L R Y K-COOH
y11
y10
y1
b2
Int
iR
Prec
100
75
50
25
0
y1
b2
y10 2+
y11 2+
0
100
200
300
400
500
600
700
800
900
1,000
1,100
1,200
1,300
1,400
1,500
m/z

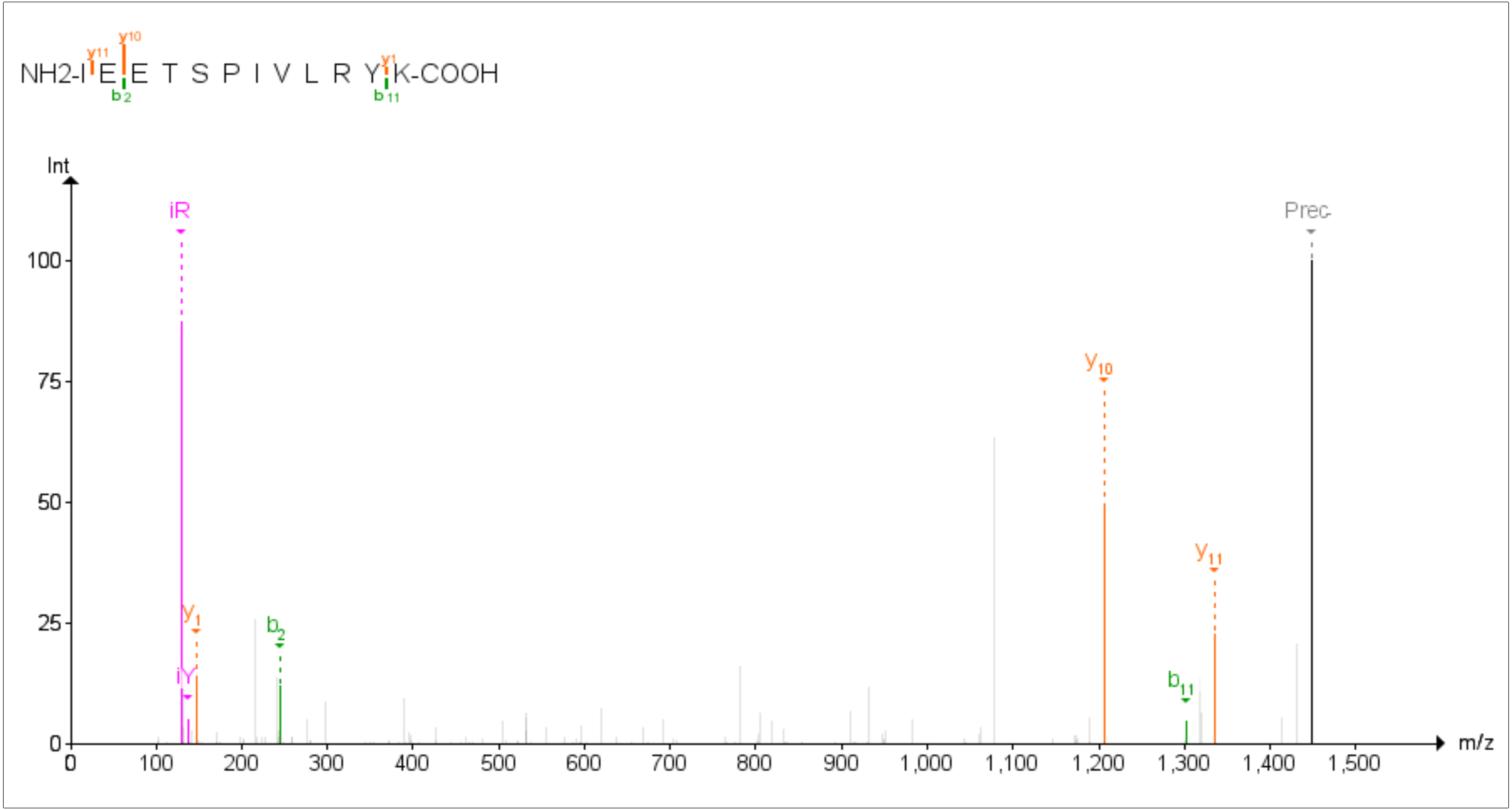

NH2-I E E T S P I V L R Y K-COOH
y11
y10
y1
b2
b11
Int
iR
Prec
y10
y11
y1
b2
iY
b11
100
75
50
25
0
0
100
200
300
400
500
600
700
800
900
1,000
1,100
1,200
1,300
1,400
1,500
m/z

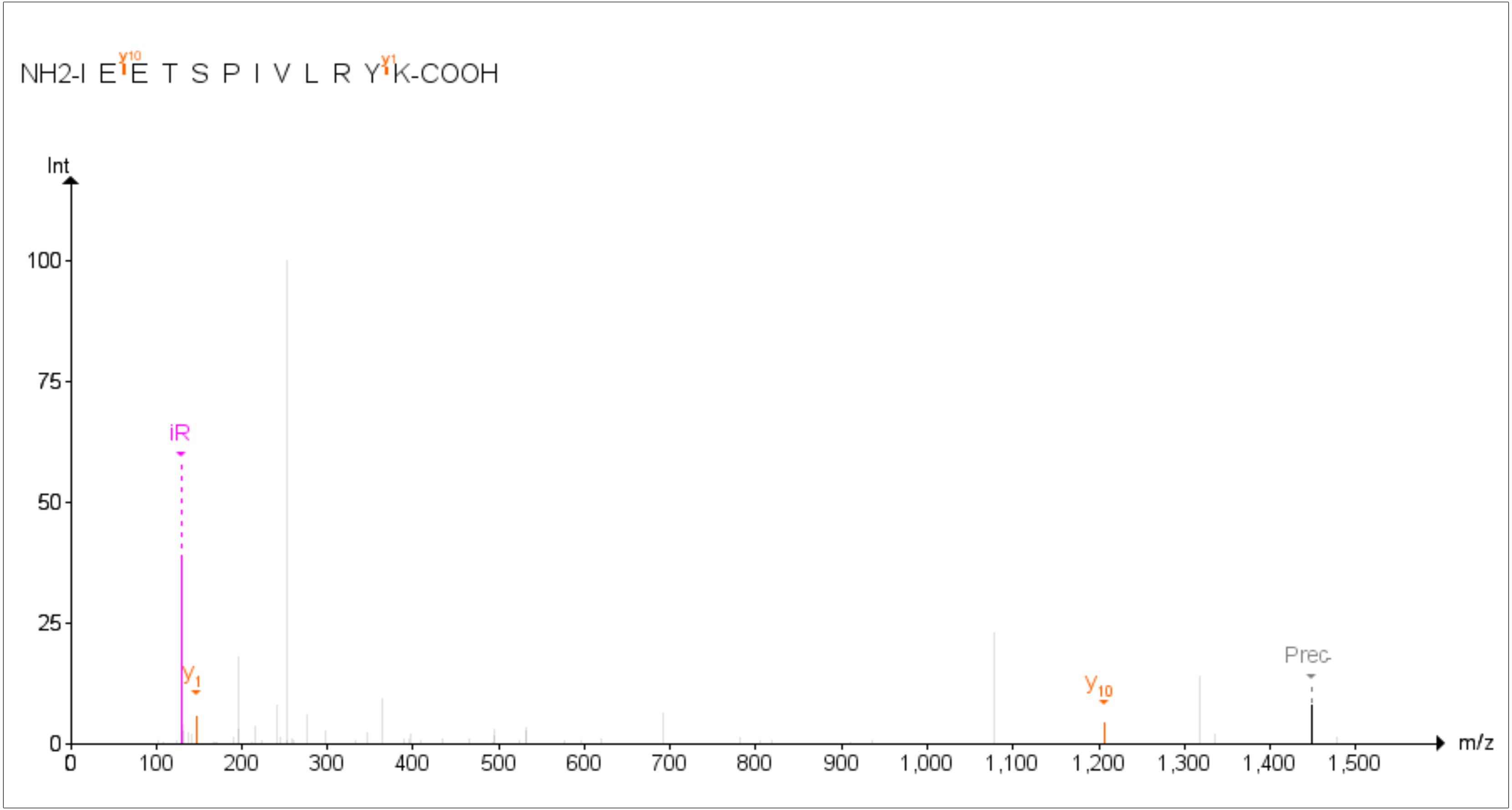

NH2-I E E T S P I V L R Y K-COOH
y10
y1
Int
100
75
50
25
0
iR
y1
y10
Prec-
0
100
200
300
400
500
600
700
800
900
1,000
1,100
1,200
1,300
1,400
1,500
m/z

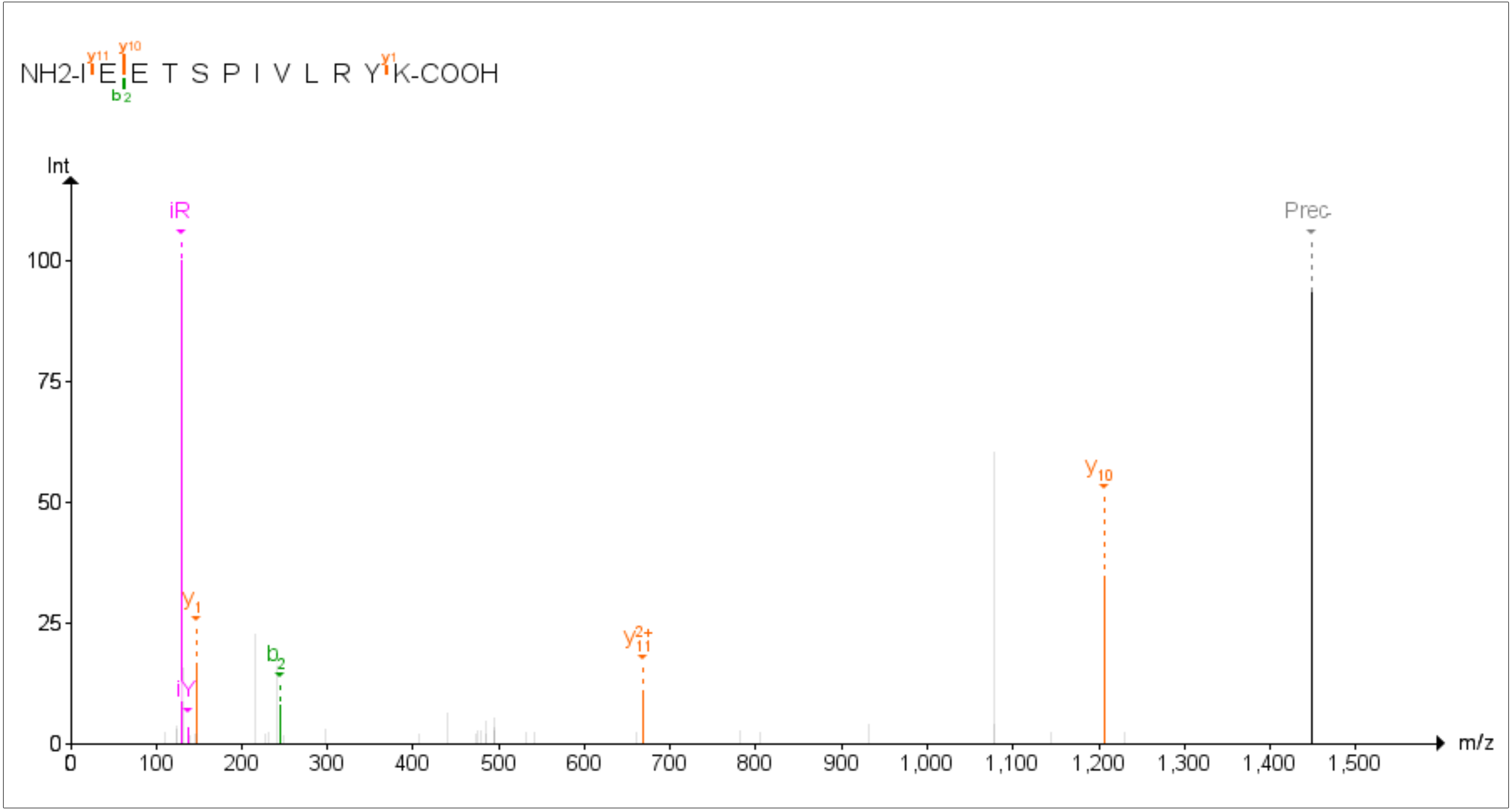

NH2-I E E T S P I V L R Y K-COOH
y11
y10
y1
b2
Int
iR
Prec
100
75
50
25
0
y1
iY
b2
y11 2+
y10
0
100
200
300
400
500
600
700
800
900
1,000
1,100
1,200
1,300
1,400
1,500
m/z

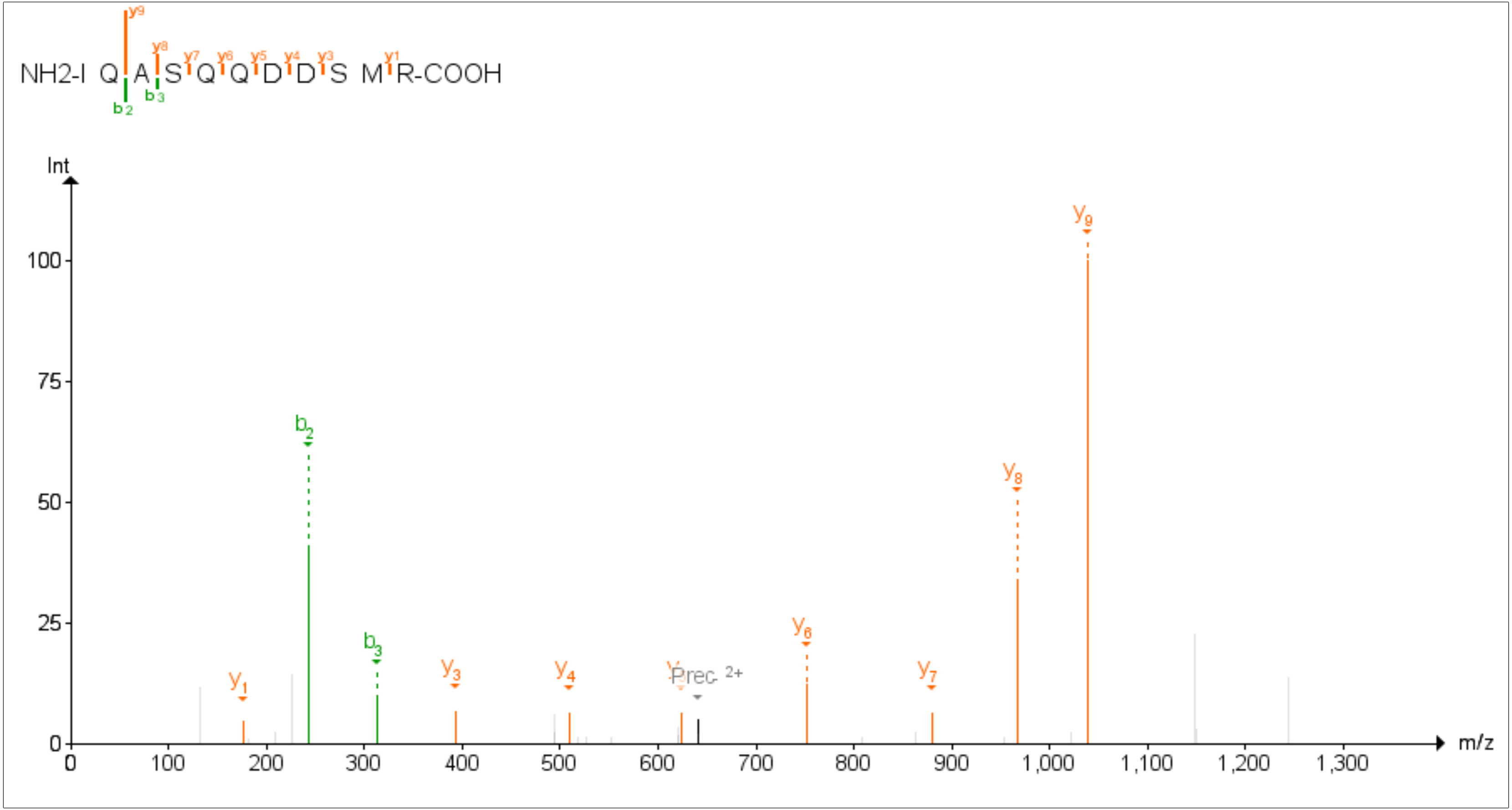

NH2-I Q A S Q Q D D S M R-COOH
y9
y8
y7
y6
y5
y4
y3
y1
b2
b3
Int
100
75
50
25
0
b2
b3
y1
y3
y4
Prec 2+
y6
y7
y8
y9
m/z
0
100
200
300
400
500
600
700
800
900
1,000
1,100
1,200
1,300

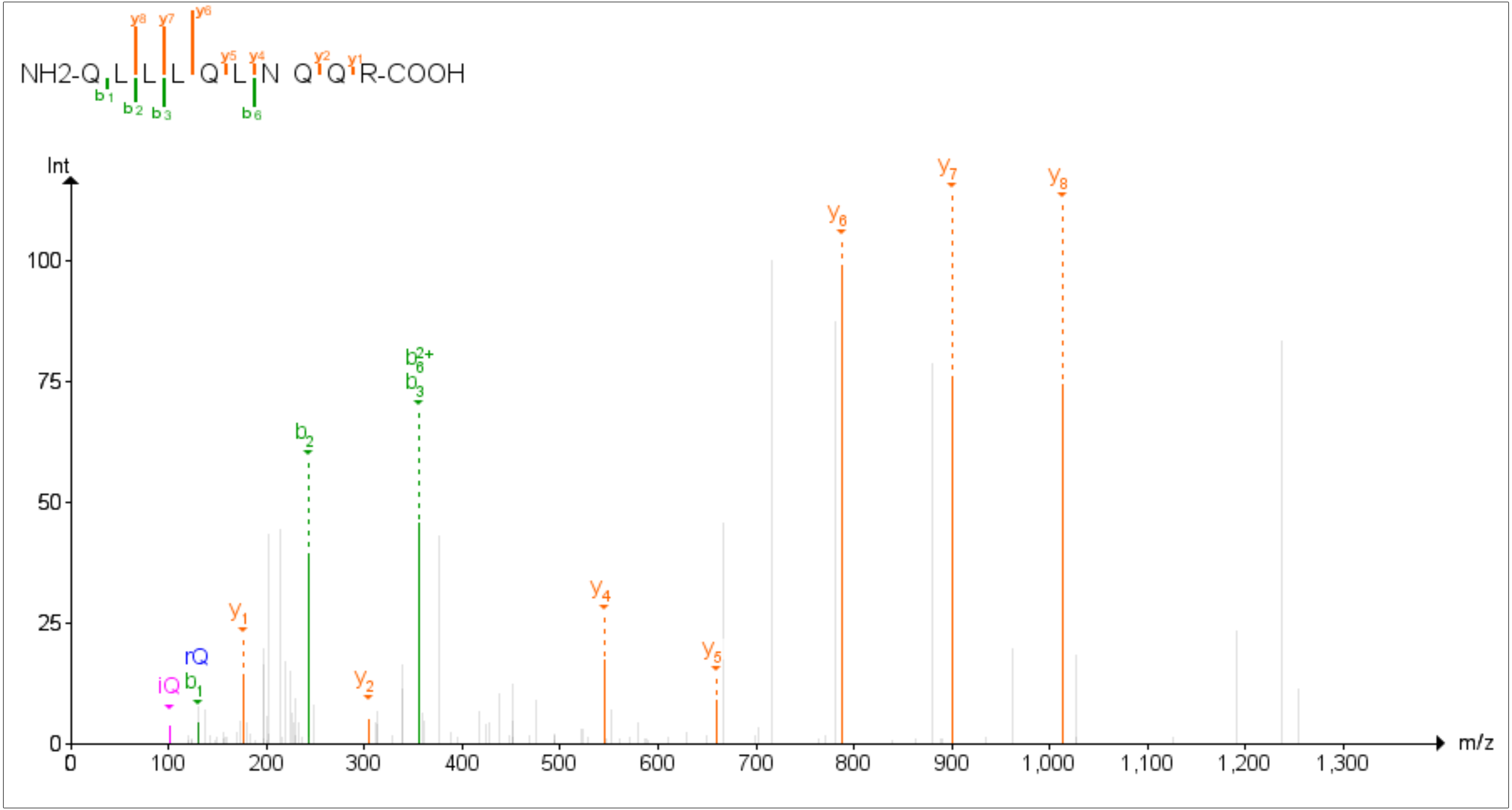

NH2-Q L L L Q L N Q Q R-COOH
y8 y7 y6 y5 y4 y2 y1
b1 b2 b3 b6
Int
100
75
50
25
0
iQ
rQ
b1
y1
b2
y2
b6 2+
b3
y4
y5
y6
y7
y8
0 100 200 300 400 500 600 700 800 900 1,000 1,100 1,200 1,300
m/z

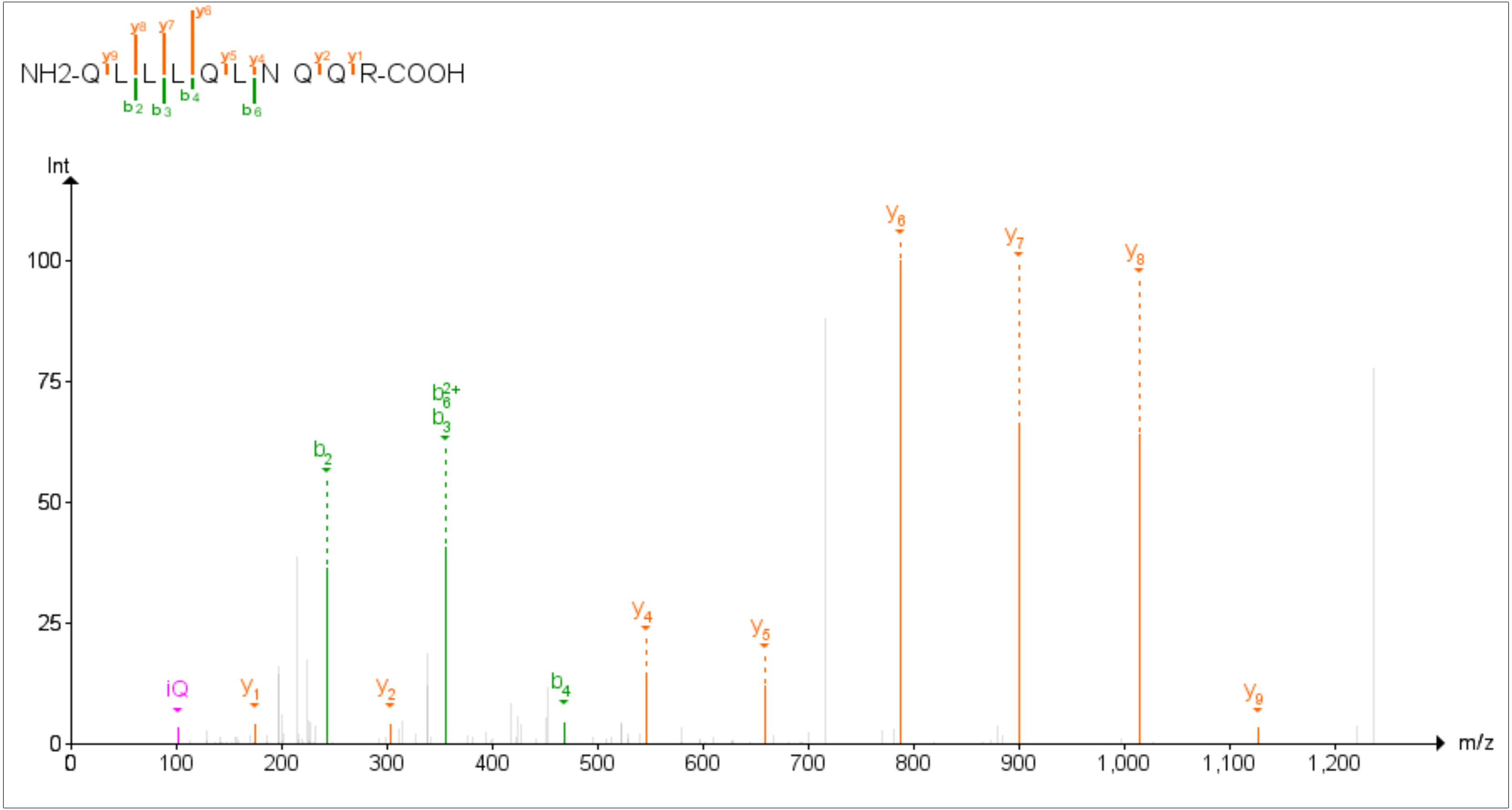
NH2-Q L L L Q L N Q Q R-COOH
y9 y8 y7 y6 y5 y4 y2 y1
b2 b3 b4 b6
Int
m/z
0 25 50 75 100
0 100 200 300 400 500 600 700 800 900 1,000 1,100 1,200
iQ
y1
y2
b2
b_6^{2+}
b_3
b4
y4
y5
y6
y7
y8
y9

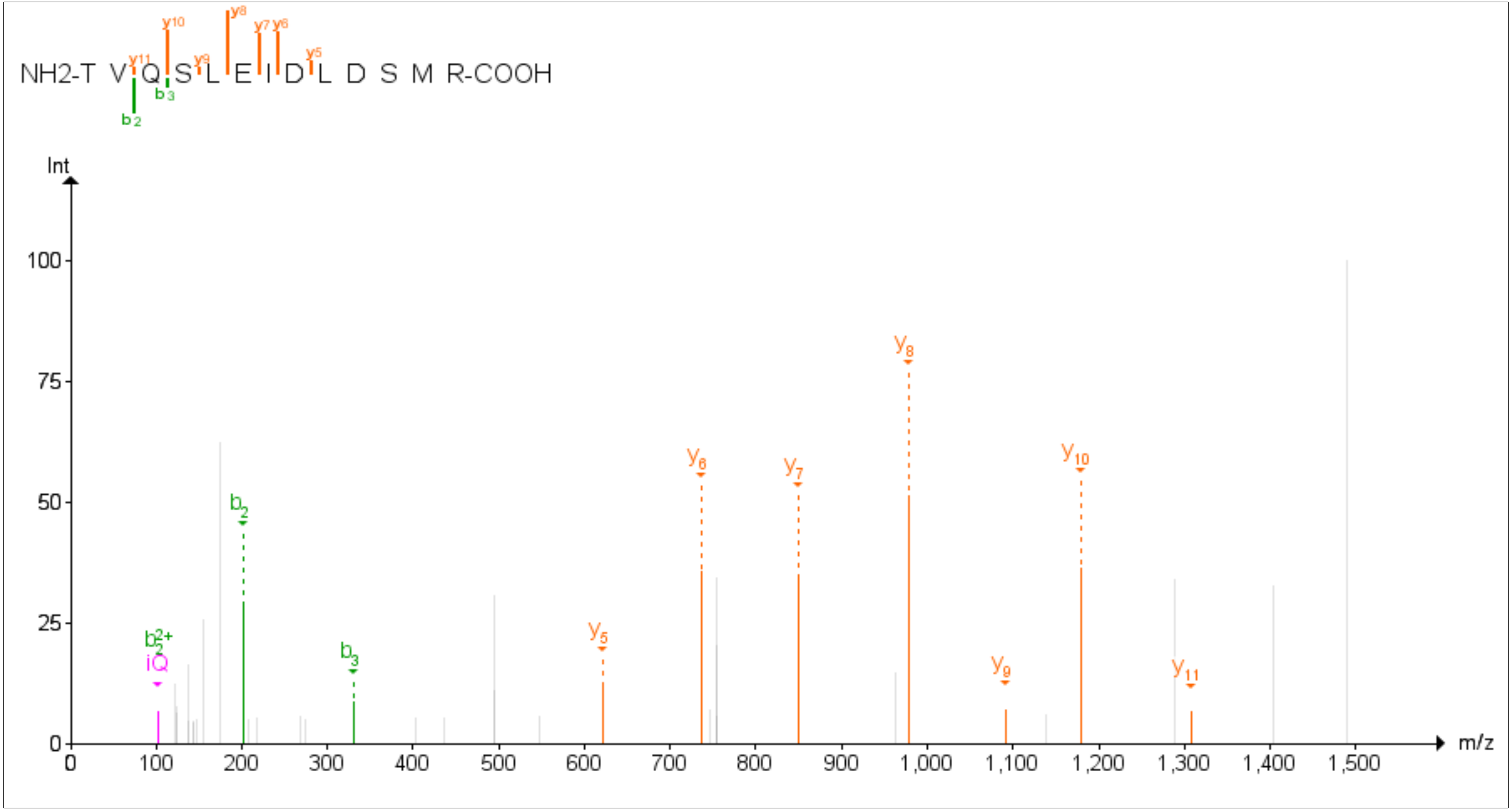

NH2-T V Q S L E I D L D S M R-COOH
y10
y8
y7
y6
y11
y9
y5
b3
b2
Int
100
75
50
25
0
m/z
0
100
200
300
400
500
600
700
800
900
1,000
1,100
1,200
1,300
1,400
1,500
b2
b2 2+
iQ
b3
y5
y6
y7
y8
y9
y10
y11

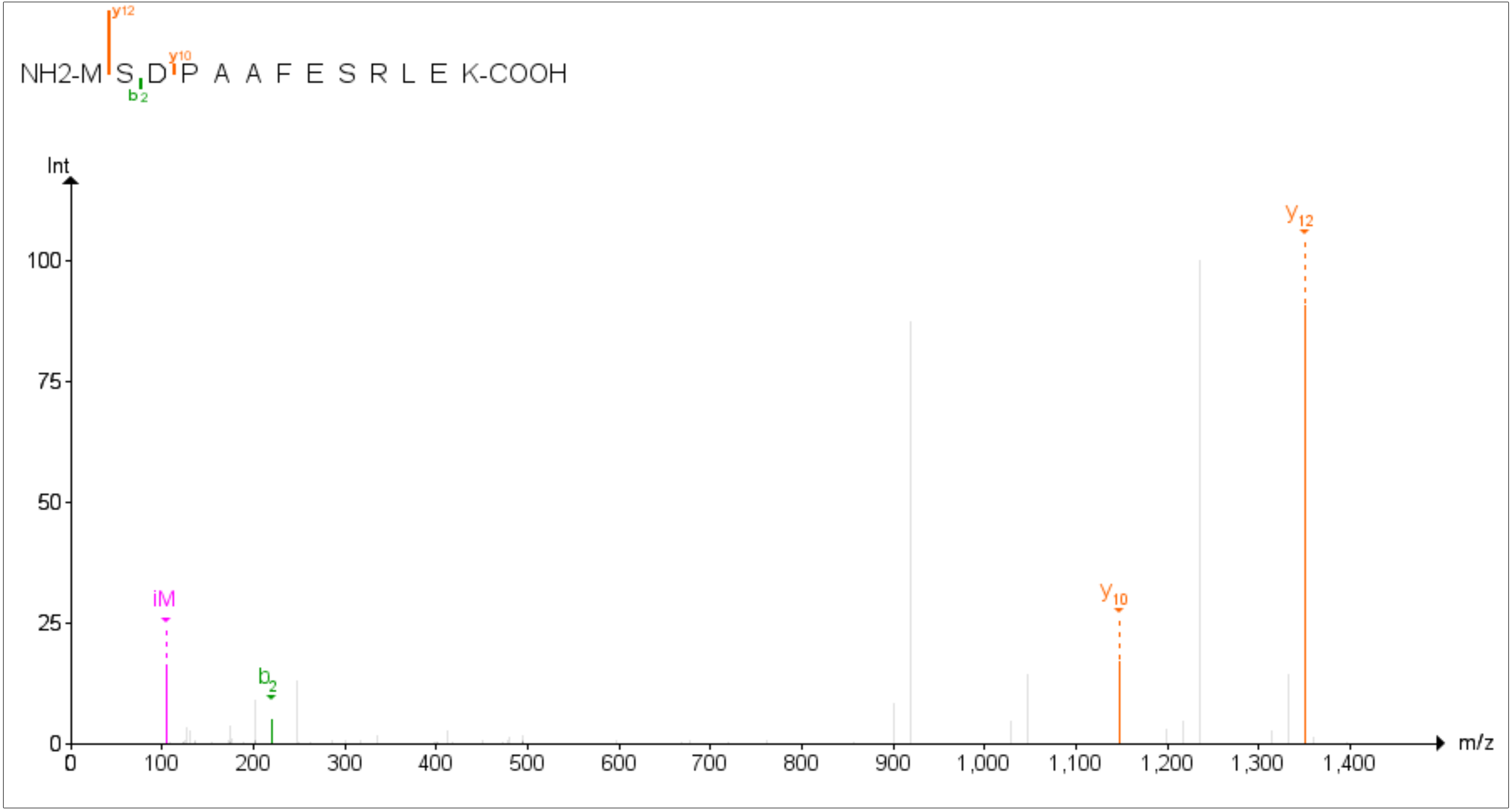

y12
y10
NH2-M S D P A A F E S R L E K-COOH
b2
Int
100
75
50
25
0
y12
y10
iM
b2
0
100
200
300
400
500
600
700
800
900
1,000
1,100
1,200
1,300
1,400
m/z

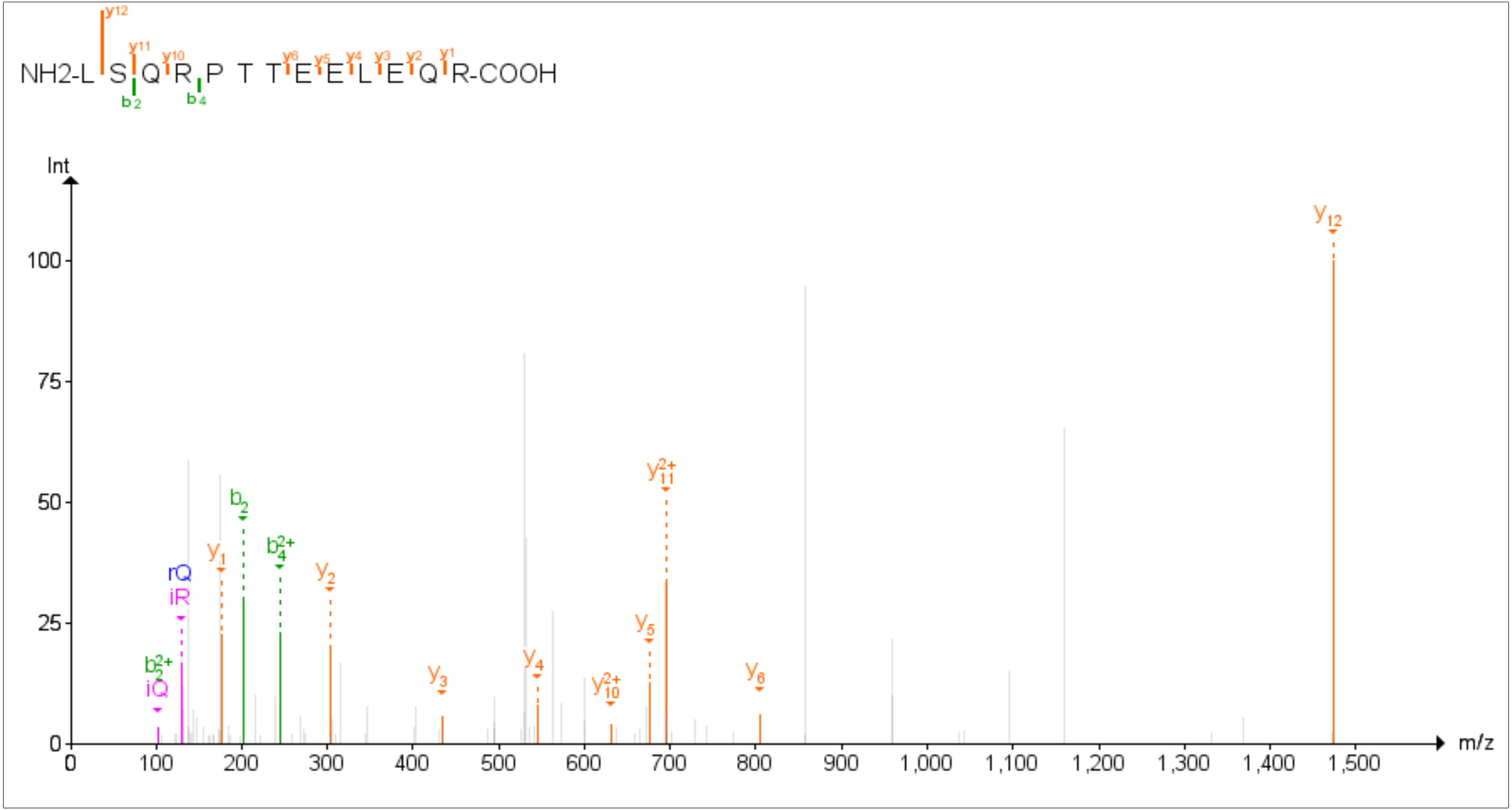

NH2-L S Q R P T T E E L E Q R-COOH
y12 y11 y10 y6 y5 y4 y3 y2 y1
b2 b4
Int
m/z
0
25
50
75
100
0 100 200 300 400 500 600 700 800 900 1,000 1,100 1,200 1,300 1,400 1,500
y12
y11 2+
b2
y1
b4 2+
rQ
iR
y2
y5
y4
y3
y10 2+
y6
b2 2+
iQ

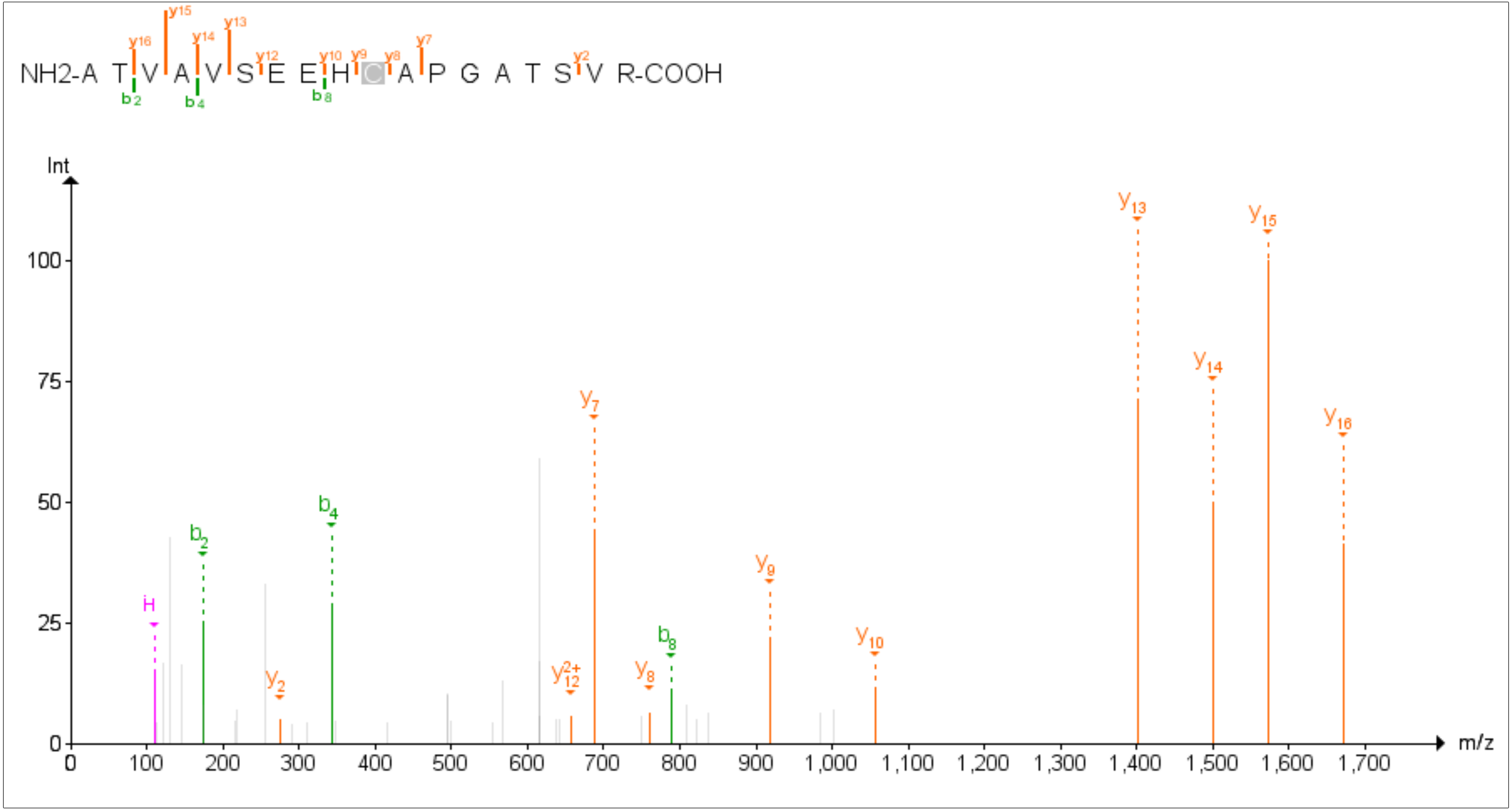

NH2-A T V A V S E E H C A P G A T S V R-COOH
y16 y15 y14 y13 y12 y10 y9 y8 y7 y2
b2 b4 b8
Int
100
75
50
25
0
0 100 200 300 400 500 600 700 800 900 1,000 1,100 1,200 1,300 1,400 1,500 1,600 1,700
m/z
y13 y15 y14 y16 y7 b4 b2 y9 H y10 b8 y8 y2

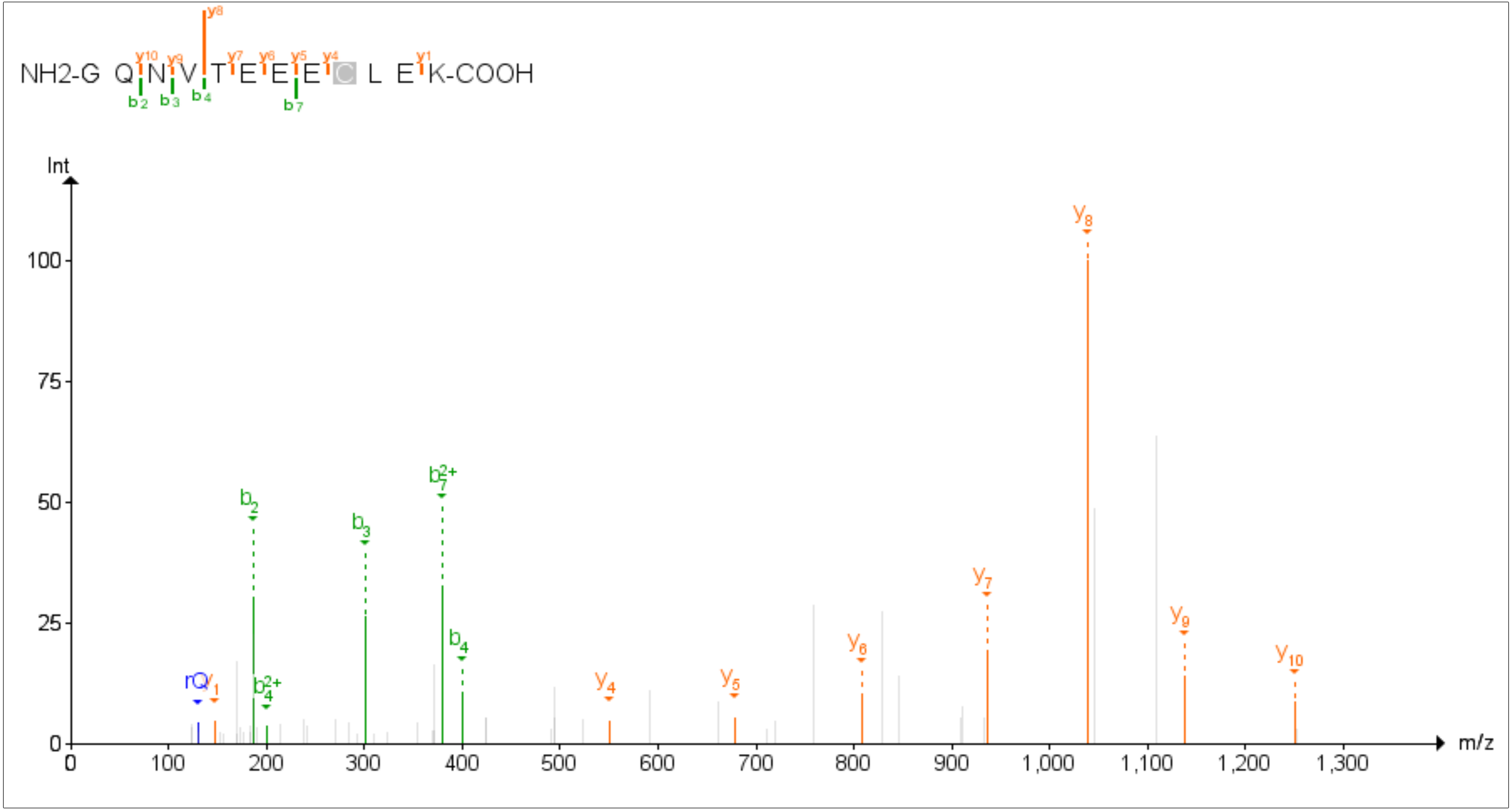

NH2-G Q N V T E E E C L E K-COOH
y10 y9 y8 y7 y6 y5 y4 y1
b2 b3 b4 b7
Int
100
75
50
25
0
0 100 200 300 400 500 600 700 800 900 1,000 1,100 1,200 1,300
m/z
rQ
y1
b2
b4 2+
b3
b7 2+
b4
y4
y5
y6
y7
y8
y9
y10

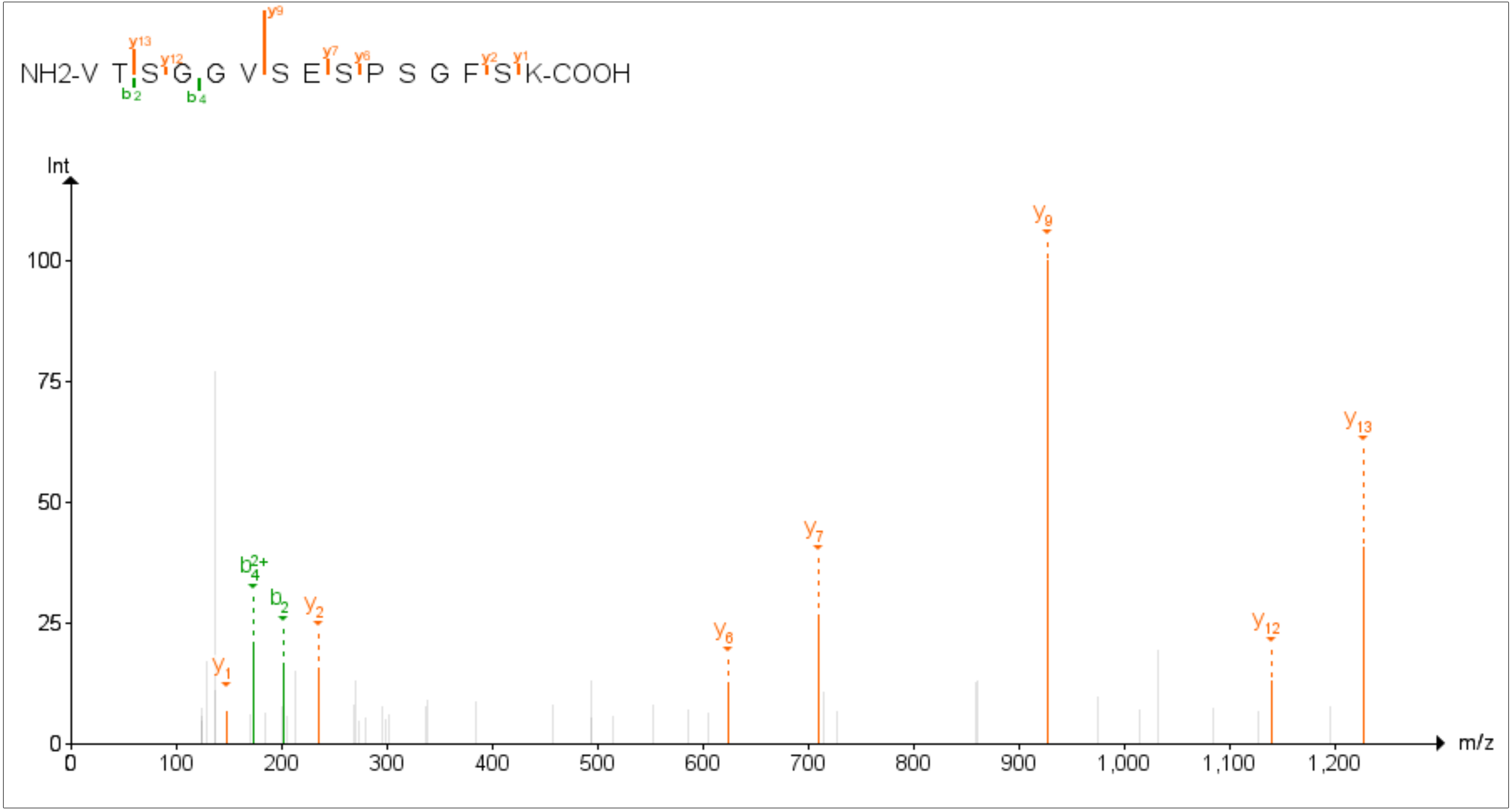

NH2-V T S G G V S E S P S G F S K-COOH
b2
b4
y13
y12
y9
y7
y6
y2
y1
Int
m/z
0
25
50
75
100
0
100
200
300
400
500
600
700
800
900
1,000
1,100
1,200

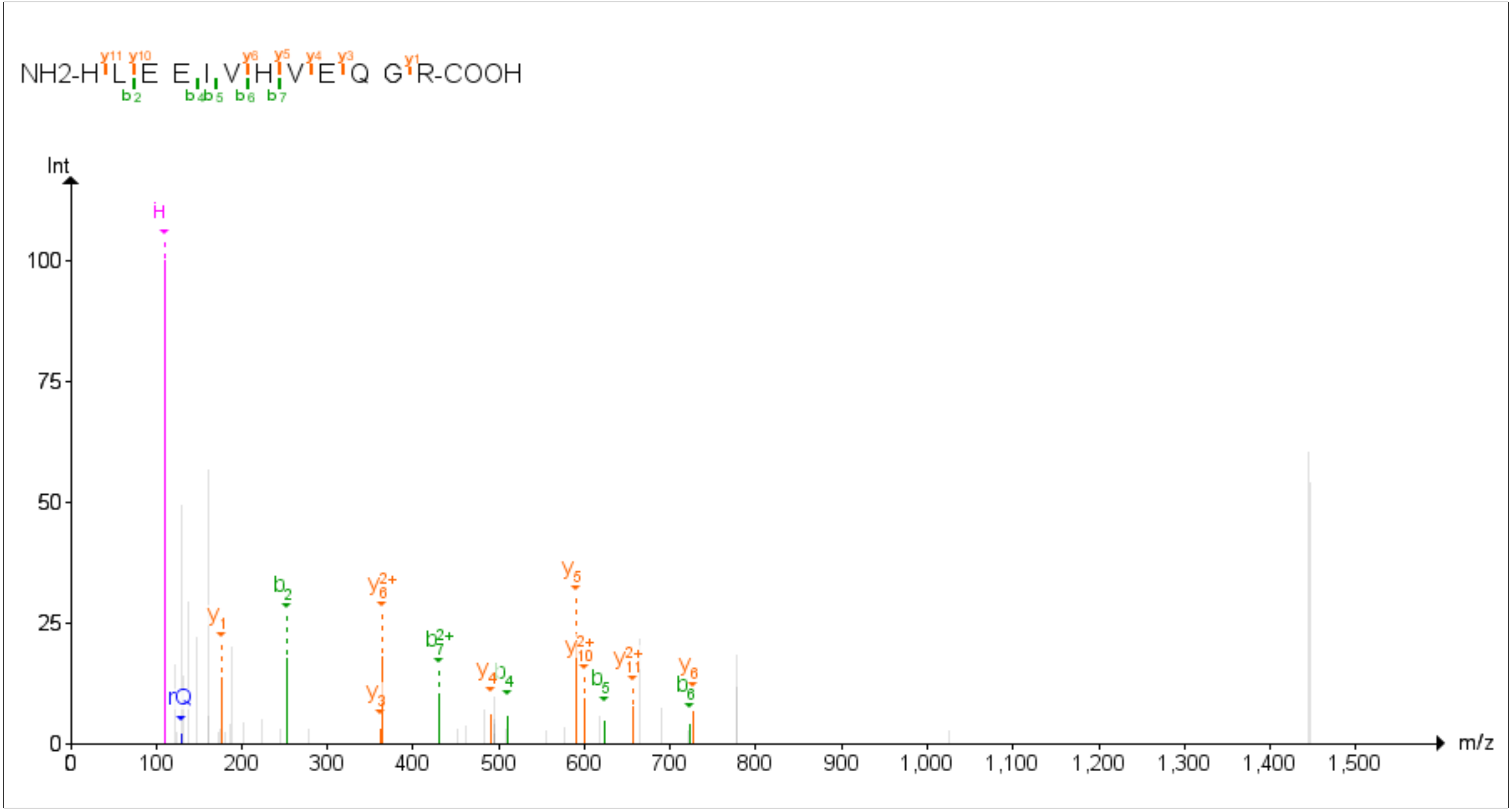

NH2-H L E E I V H V E Q G R-COOH
y11 y10 y6 y5 y4 y3 y1
b2 b4 b5 b6 b7
Int
m/z
H
rQ
y1
b2
y6 2+
b7 2+
y3
y4
b4
y5
y10 2+
b5
y11 2+
y6
b6
0
25
50
75
100
0
100
200
300
400
500
600
700
800
900
1,000
1,100
1,200
1,300
1,400
1,500

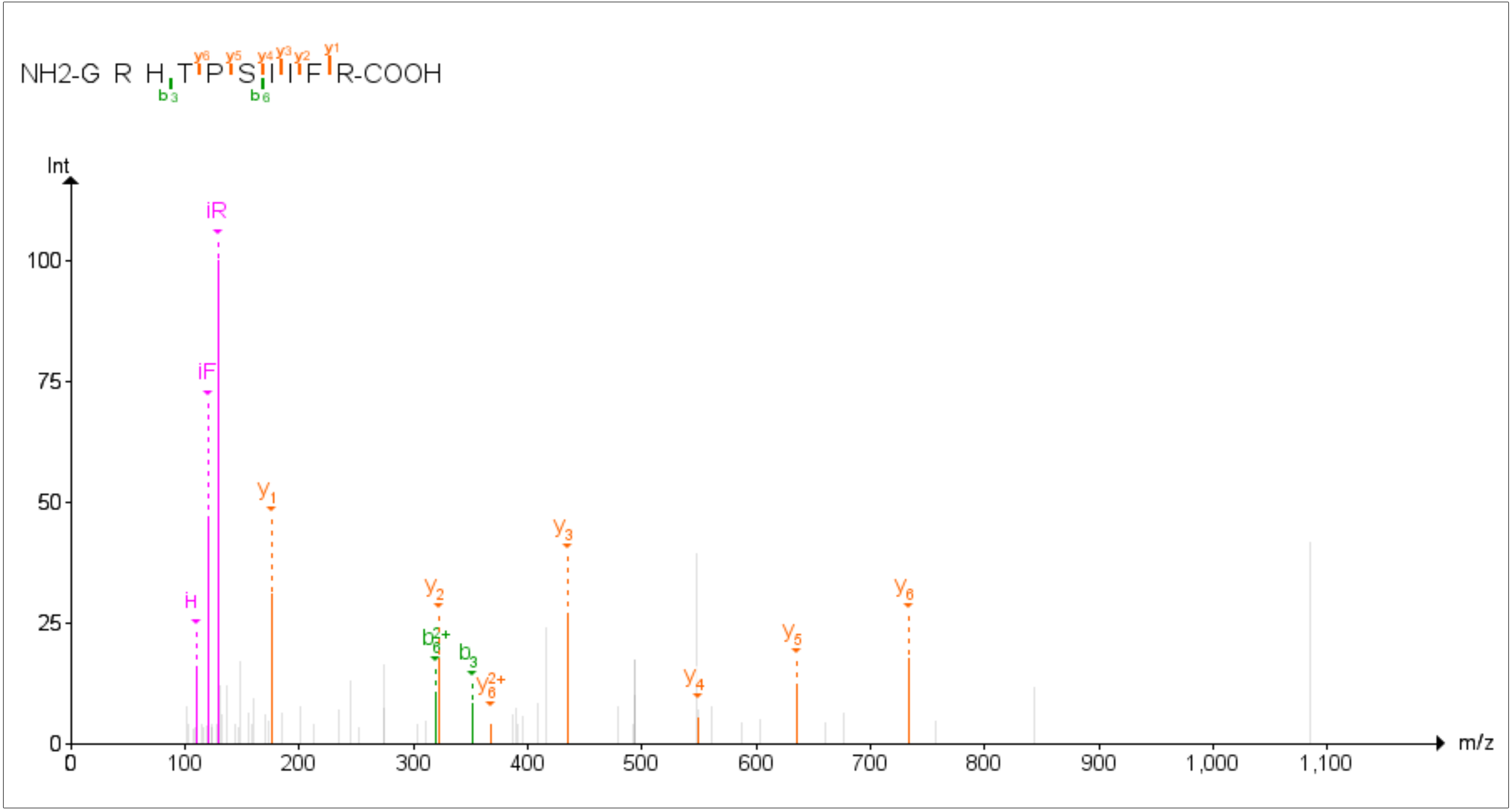
NH2-G R H T P S I I F R-COOH
y6 y5 y4 y3 y2 y1
b3 b6
Int
100
75
50
25
0
iR
iF
iH
y1
y2
y3
y4
y5
y6
b6 2+
b3
y6 2+
0
100
200
300
400
500
600
700
800
900
1,000
1,100
m/z

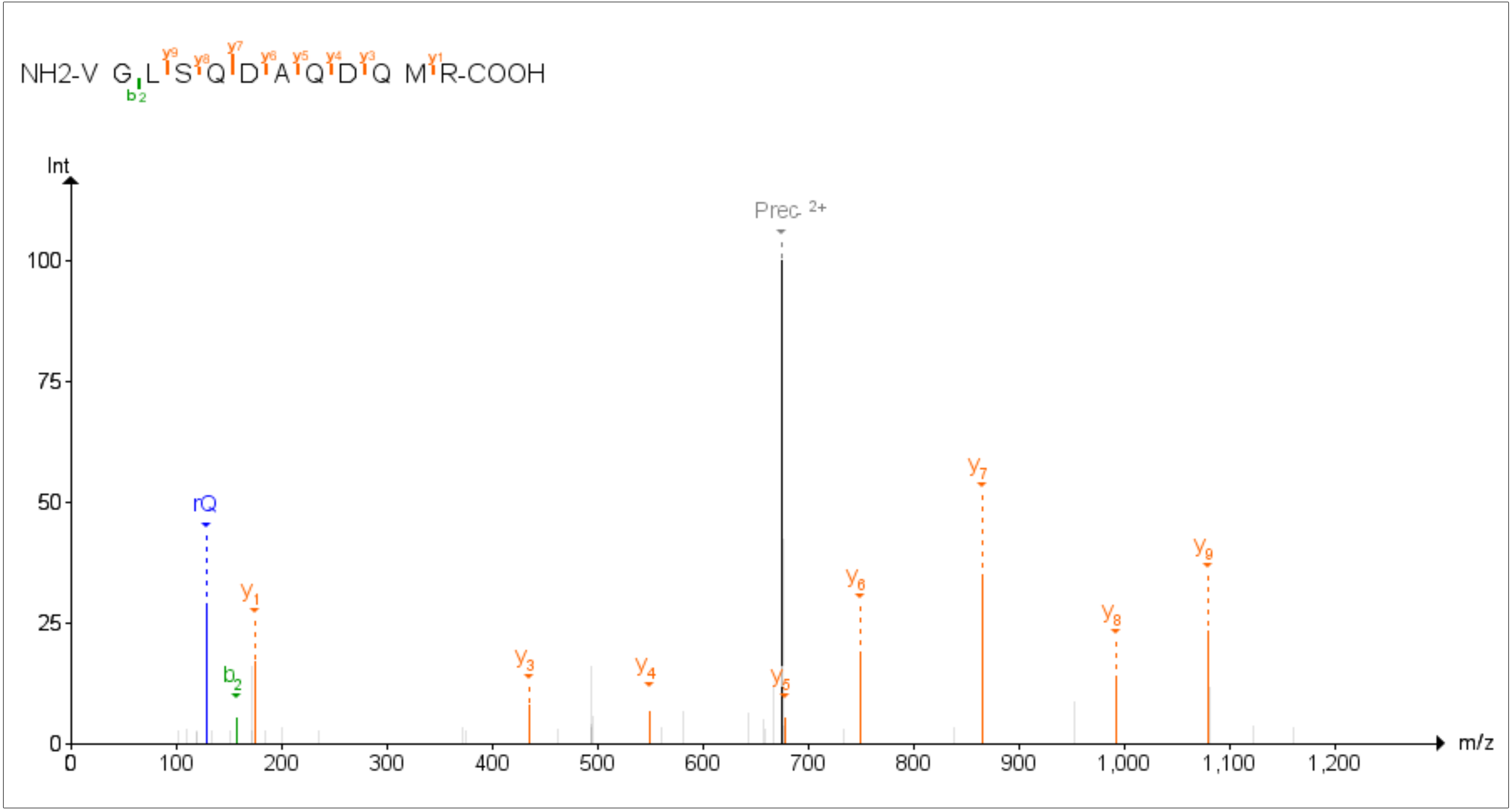

NH2-V G L S Q D A Q D Q M R-COOH
y9 y8 y7 y6 y5 y4 y3 y1
b2
Int
Prec- 2+
100
75
50
25
0
rQ
y1
b2
y3
y4
y5
y6
y7
y8
y9
0
100
200
300
400
500
600
700
800
900
1,000
1,100
1,200
m/z

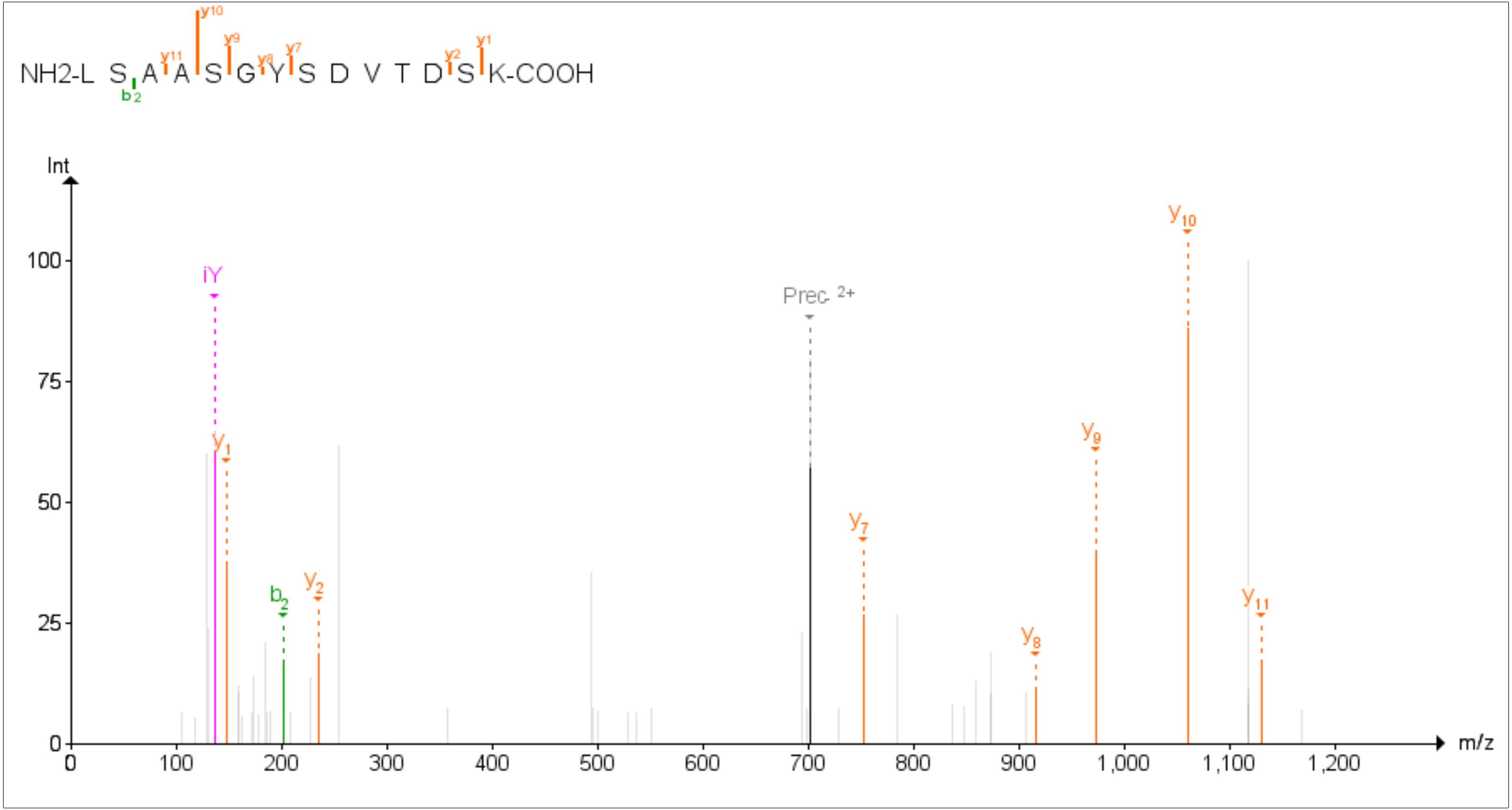

NH2-L S A A S G Y S D V T D S K-COOH
y10
y9
y11
y8
y7
y2
y1
b2
Int
100
75
50
25
0
iY
y1
b2
y2
Prec. 2+
y7
y8
y9
y10
y11
0
100
200
300
400
500
600
700
800
900
1,000
1,100
1,200
m/z

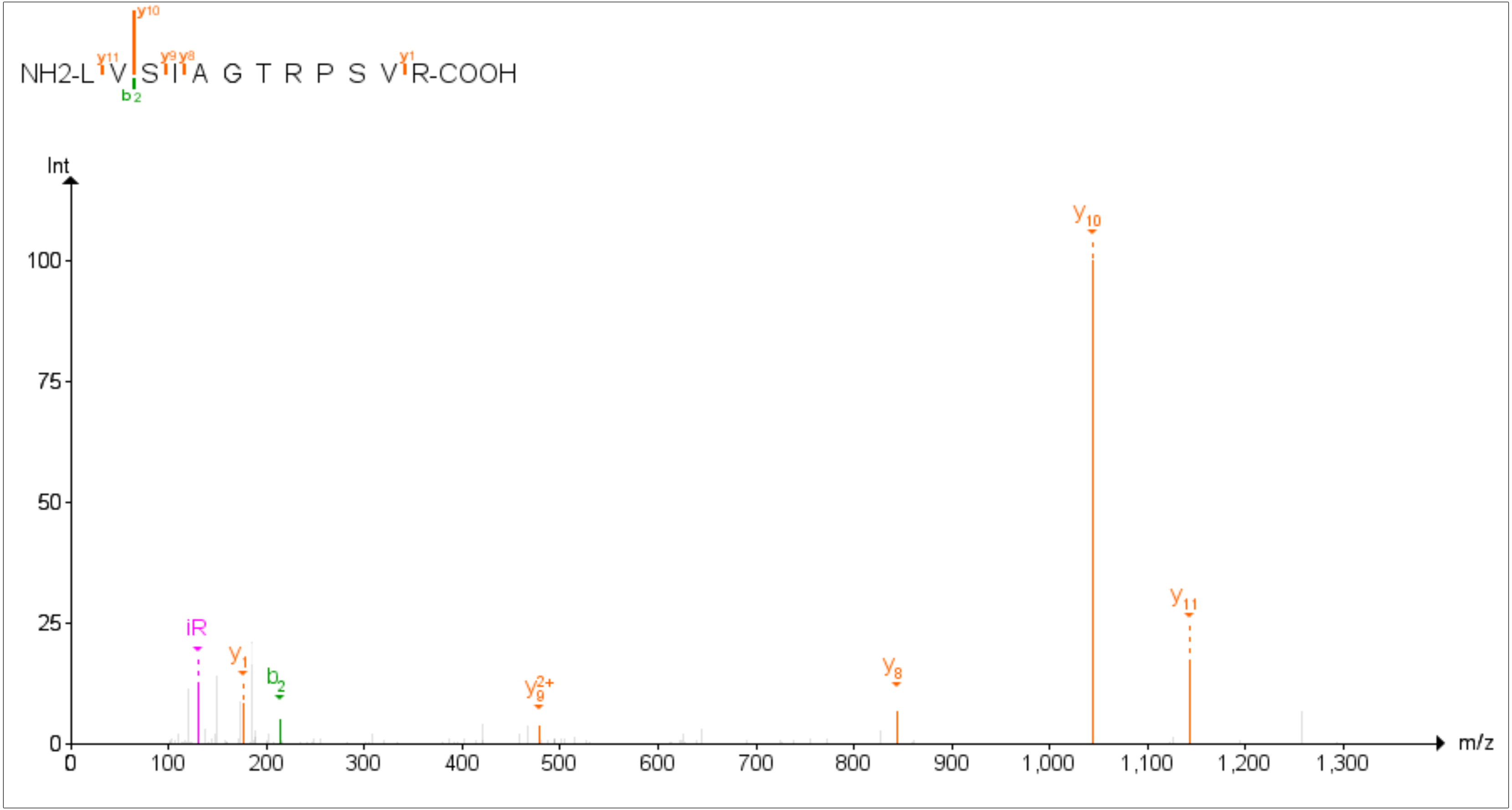

NH2-L V S I A G T R P S V R-COOH
y10
y11
y9
y8
y1
b2
Int
iR
y1
b2
y9 2+
y8
y10
y11
m/z
0
25
50
75
100
0
100
200
300
400
500
600
700
800
900
1,000
1,100
1,200
1,300

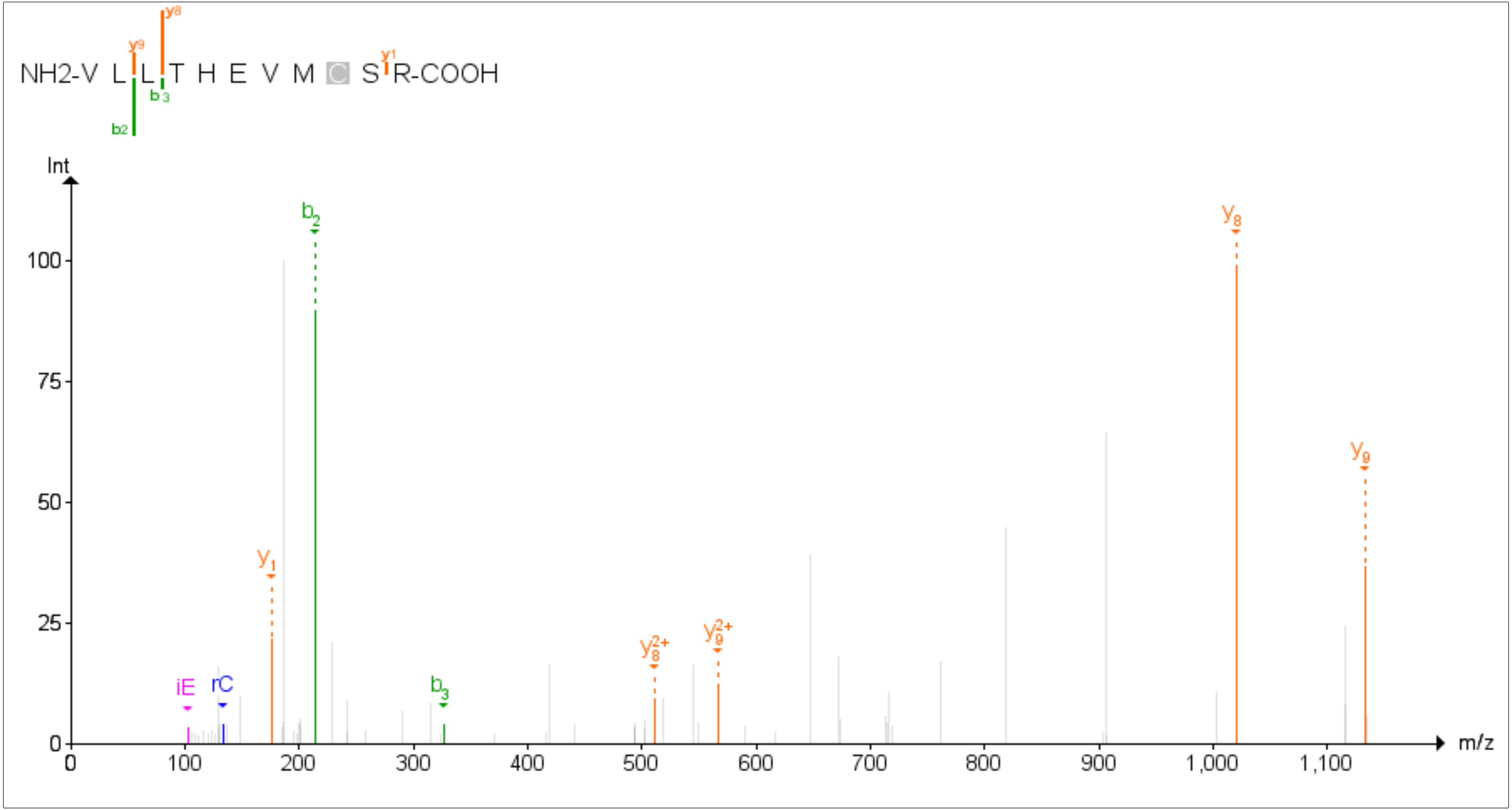

NH2-V L L T H E V M C S R-COOH
y9
y8
y1
b2
b3
Int
100
75
50
25
0
b2
y8
y9
y1
y8 2+
y9 2+
iE
rC
b3
0
100
200
300
400
500
600
700
800
900
1,000
1,100
m/z

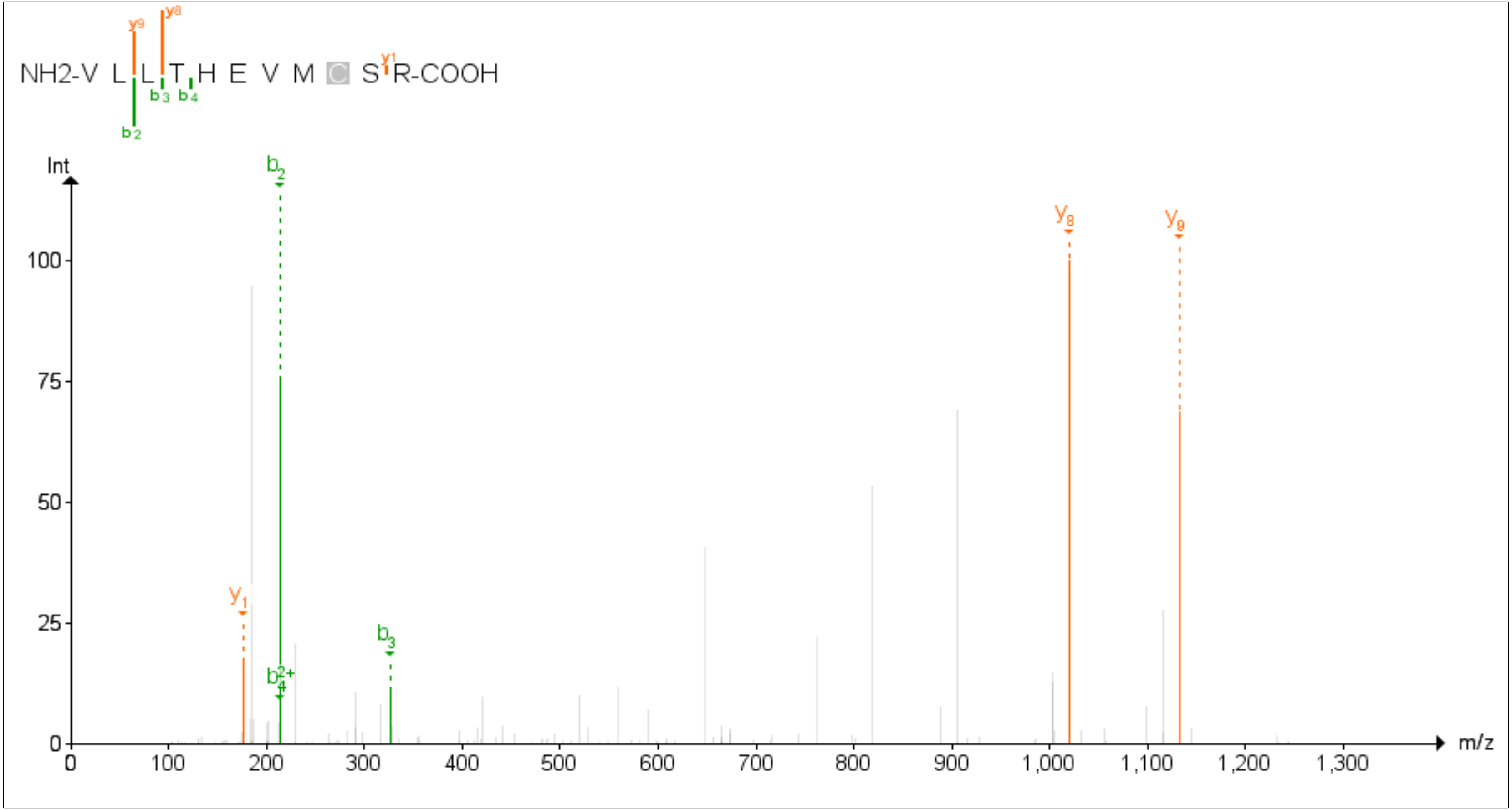

NH2-V L L T H E V M C S R-COOH
y9
y8
y1
b2
b3
b4
Int
100
75
50
25
0
b2
y8
y9
y1
b4 2+
b3
0
100
200
300
400
500
600
700
800
900
1,000
1,100
1,200
1,300
m/z

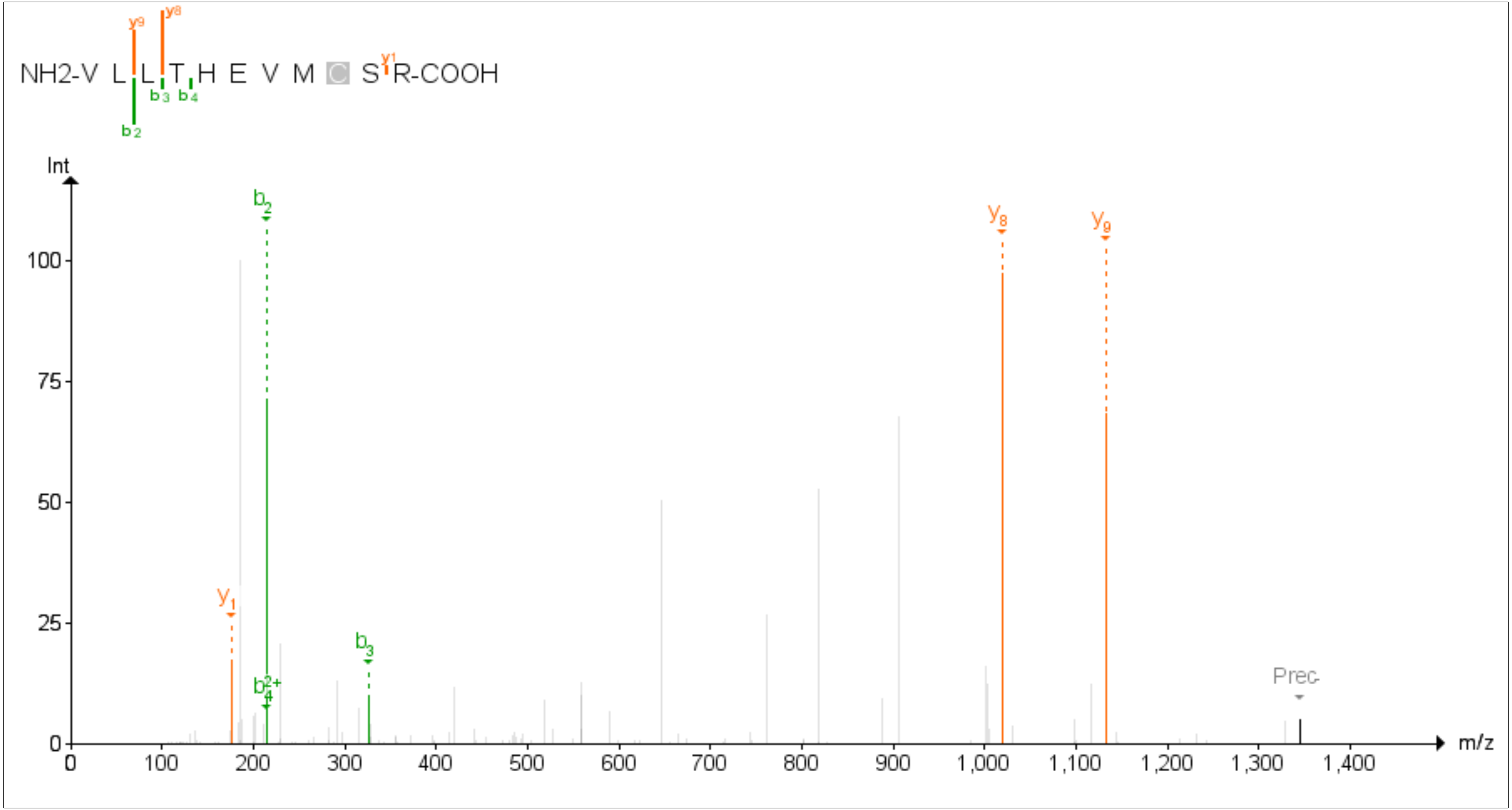
NH2-V L L T H E V M C S R-COOH
y9
y8
y1
b2
b3
b4
Int
100
75
50
25
0
b2
y8
y9
y1
b3
b4 2+
Prec.
0
100
200
300
400
500
600
700
800
900
1,000
1,100
1,200
1,300
1,400
m/z

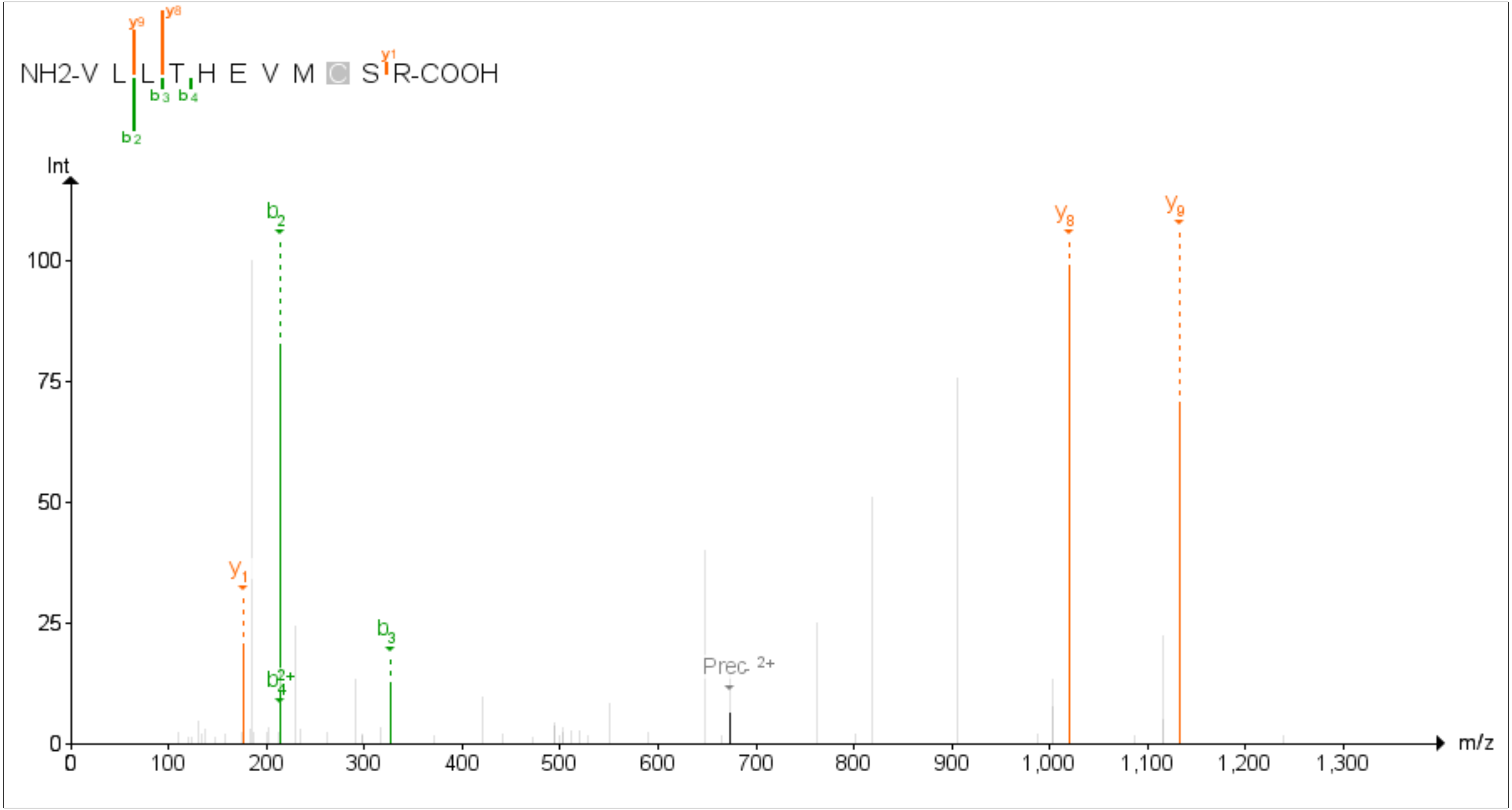

NH2-V L L T H E V M C S R-COOH
y9
y8
y1
b2
b3
b4
Int
100
75
50
25
0
b2
y8
y9
y1
b3
b4 2+
Prec 2+
0
100
200
300
400
500
600
700
800
900
1,000
1,100
1,200
1,300
m/z

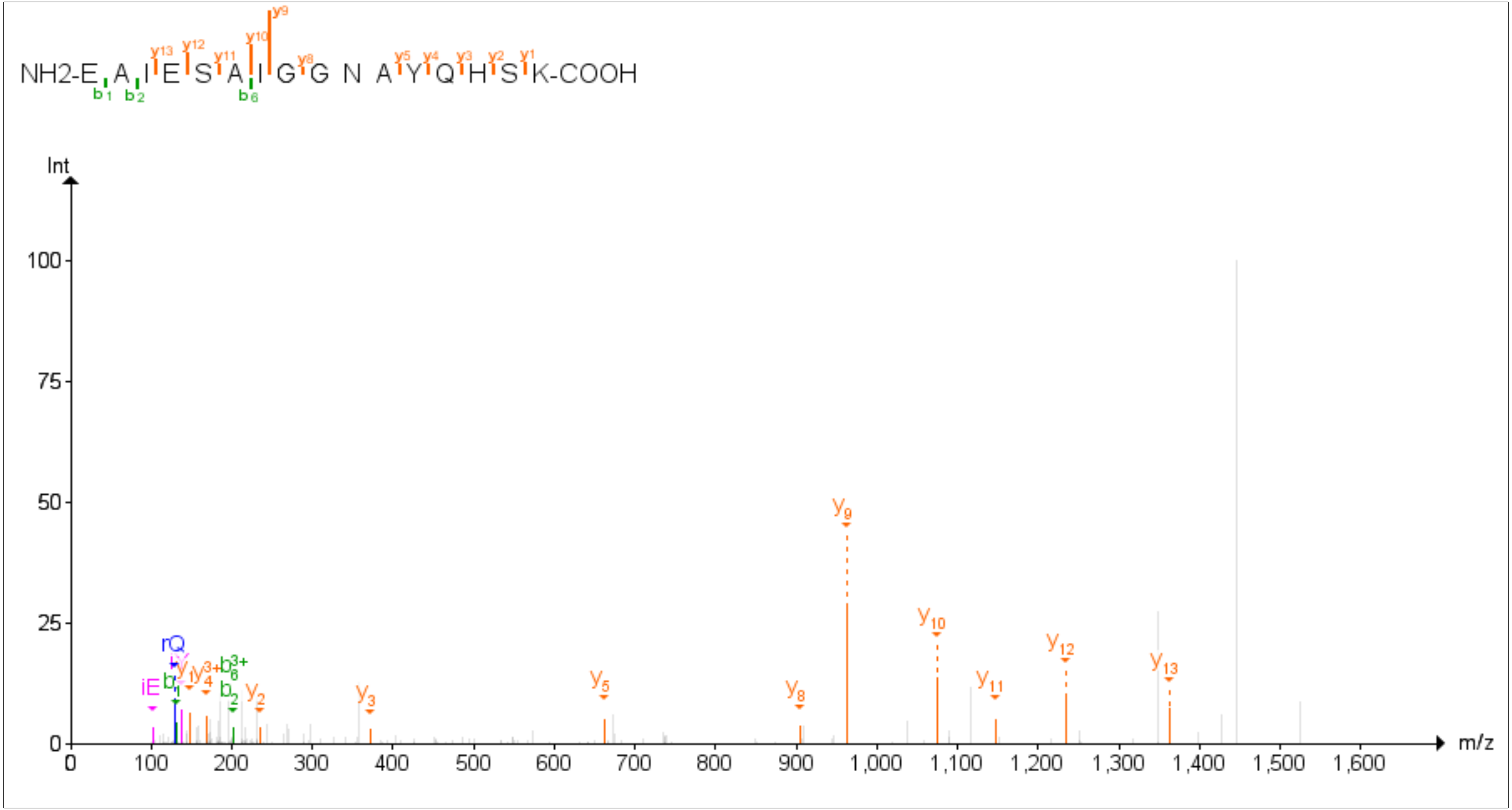

NH2-E A I E S A I G G N A Y Q H S K-COOH
b1 b2 b6
y13 y12 y11 y10 y9 y8 y5 y4 y3 y2 y1
Int
100
75
50
25
0
y9
y10
y12
y11
y13
y5
y8
y3
y2
y4
b2
b6 3+
y3+
rQ
iE
b1
y1
0 100 200 300 400 500 600 700 800 900 1,000 1,100 1,200 1,300 1,400 1,500 1,600
m/z

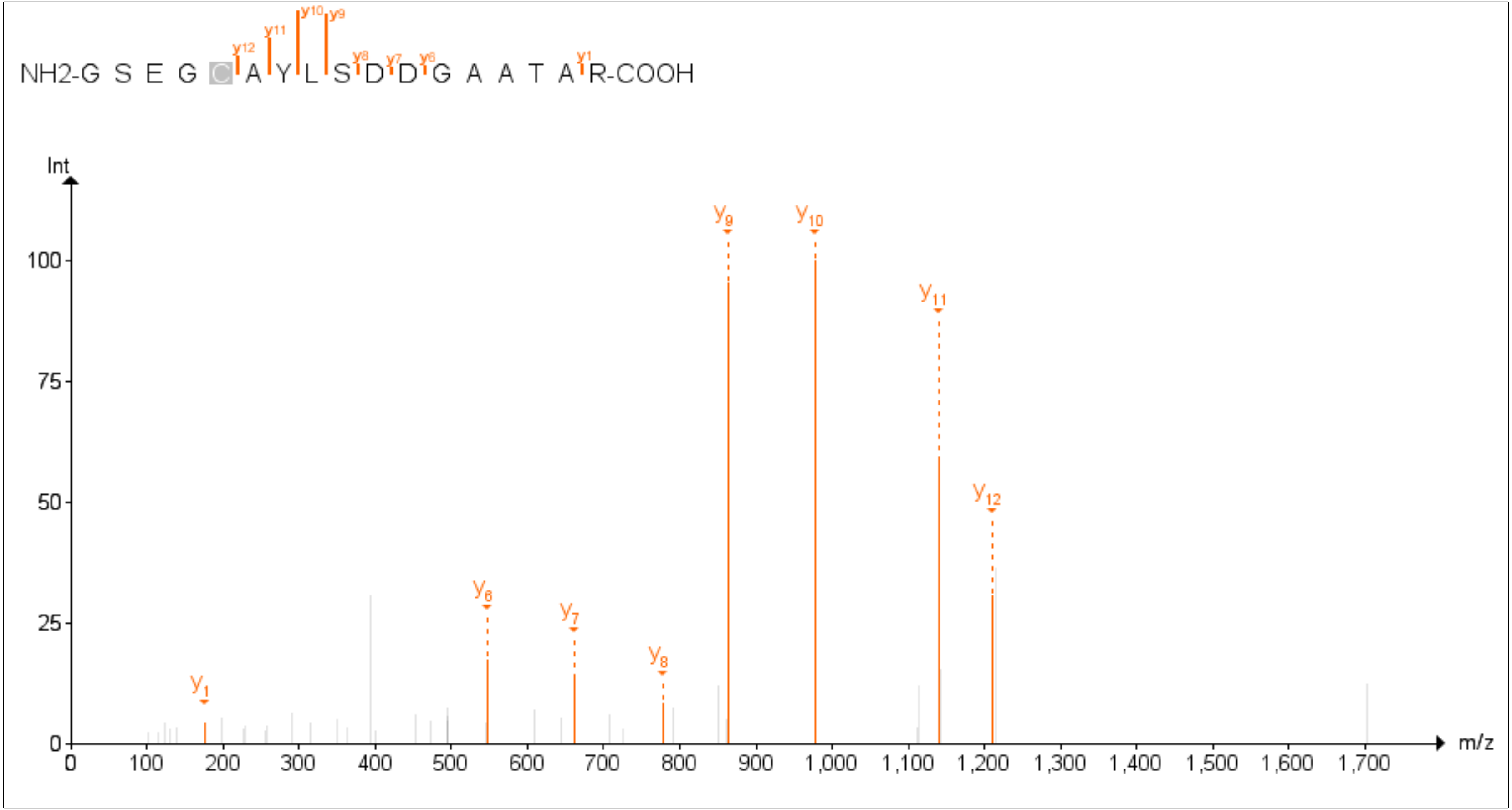

NH2-G S E G C A Y L S D D G A A T A R-COOH
y12 y11 y10 y9 y8 y7 y6 y1
Int
m/z
0
25
50
75
100
0 100 200 300 400 500 600 700 800 900 1,000 1,100 1,200 1,300 1,400 1,500 1,600 1,700
y1
y6
y7
y8
y9
y10
y11
y12

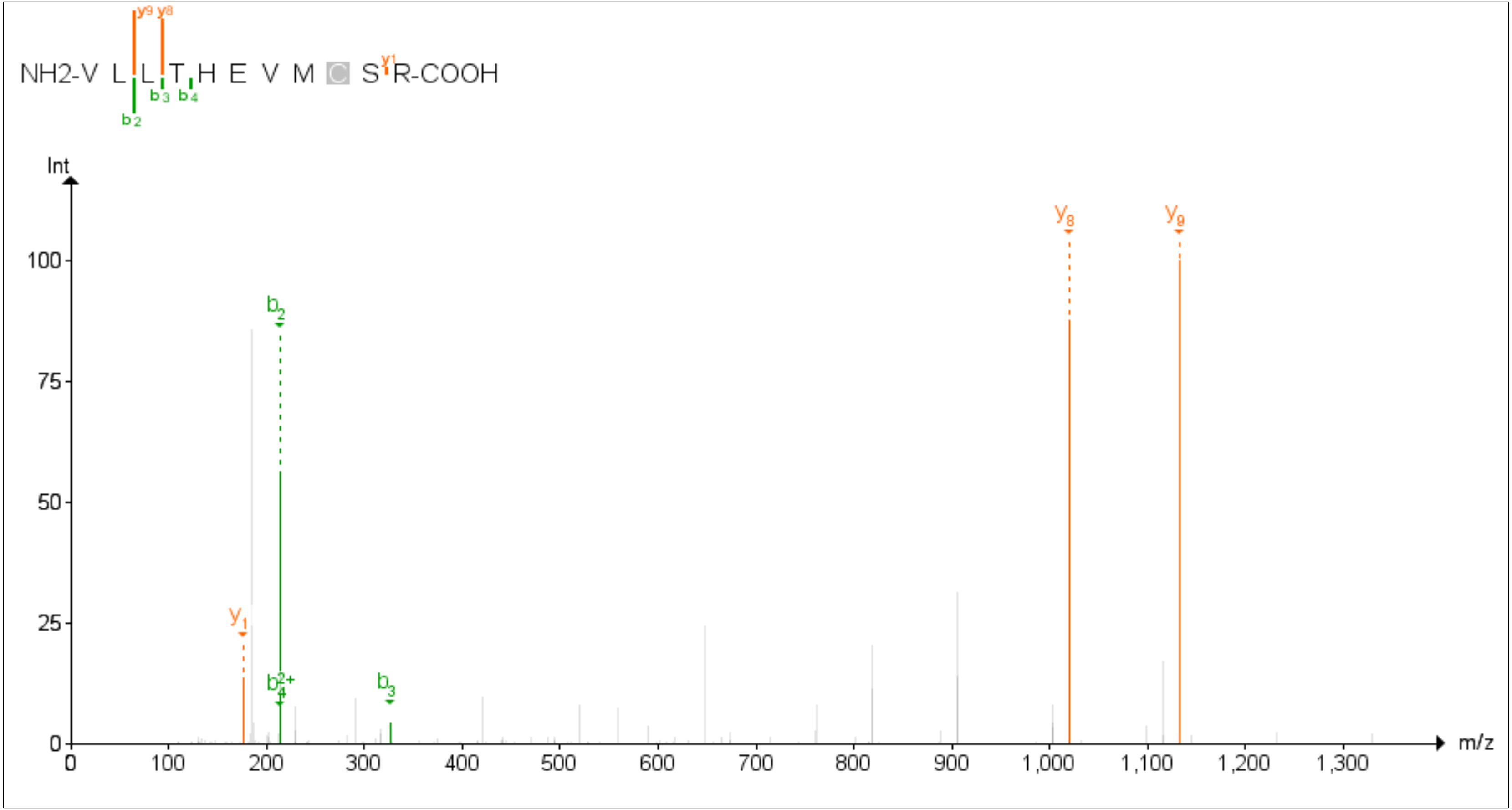
NH2-V L L T H E V M C S R-COOH
y9 y8 y1
b2 b3 b4
Int
100
75
50
25
0
y8
y9
b2
y1
b4 2+
b3
0 100 200 300 400 500 600 700 800 900 1,000 1,100 1,200 1,300
m/z

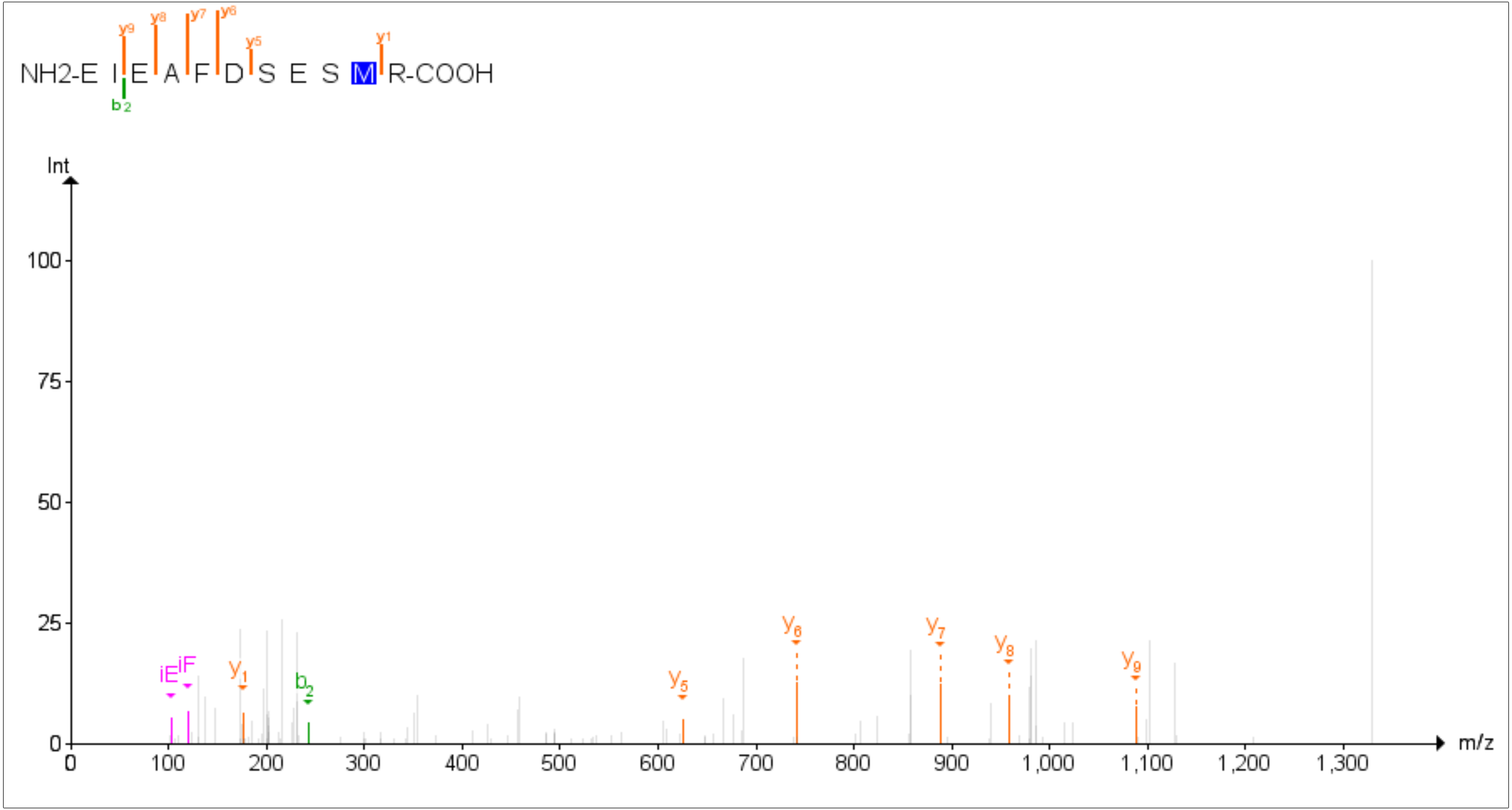

NH2-E I E A F D S E S M R-COOH
y9
y8
y7
y6
y5
y1
b2
Int
100
75
50
25
0
iE
iF
y1
b2
y5
y6
y7
y8
y9
0
100
200
300
400
500
600
700
800
900
1,000
1,100
1,200
1,300
m/z

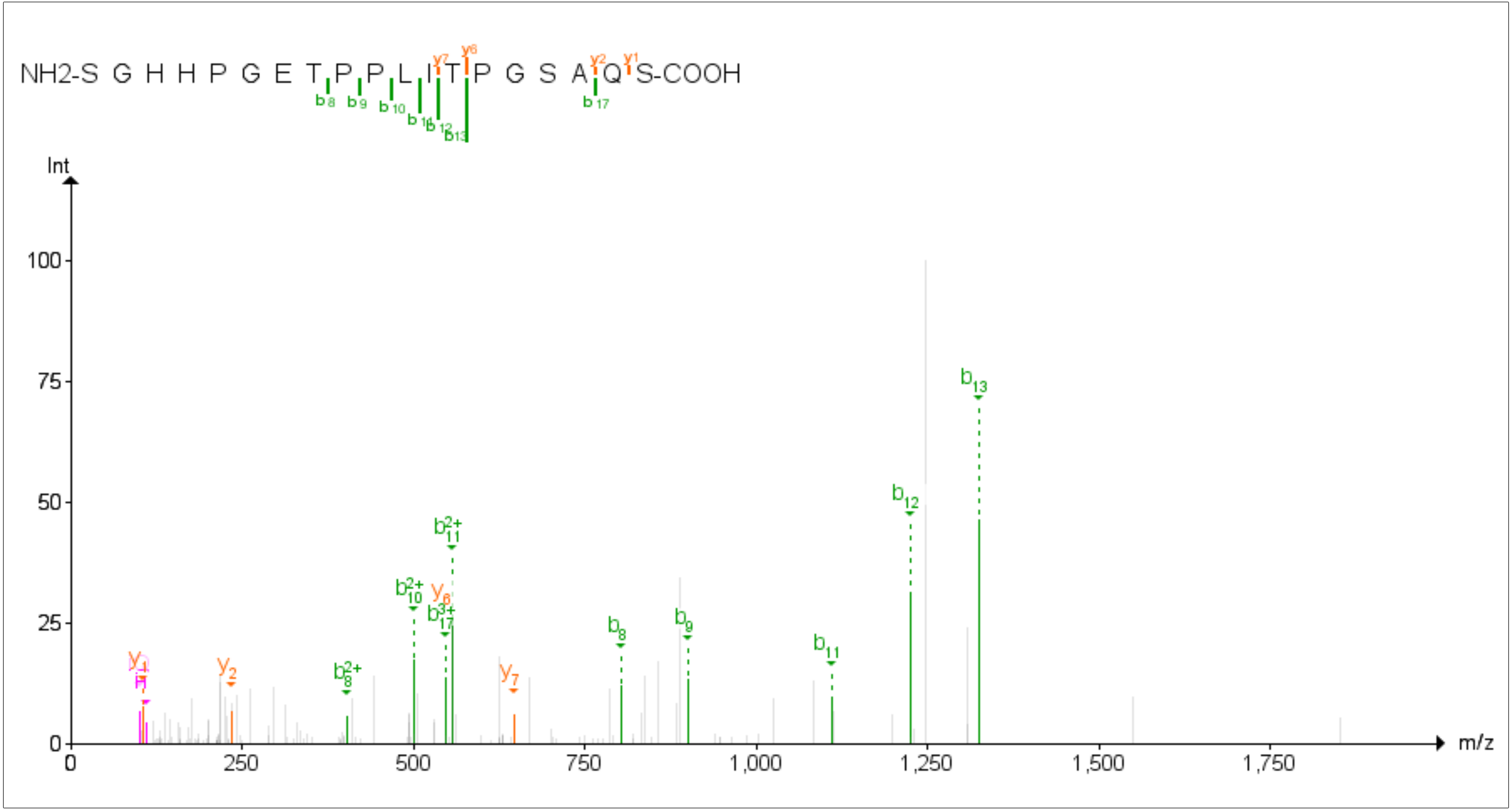

NH2-S G H H P G E T P P L I T P G S A Q S-COOH
Int
m/z
100
75
50
25
0
250
500
750
1,000
1,250
1,500
1,750
b13
b12
b11
b9
b8
b11 2+
b10 2+
b17 3+
b8 2+
y6
y7
y2
y1

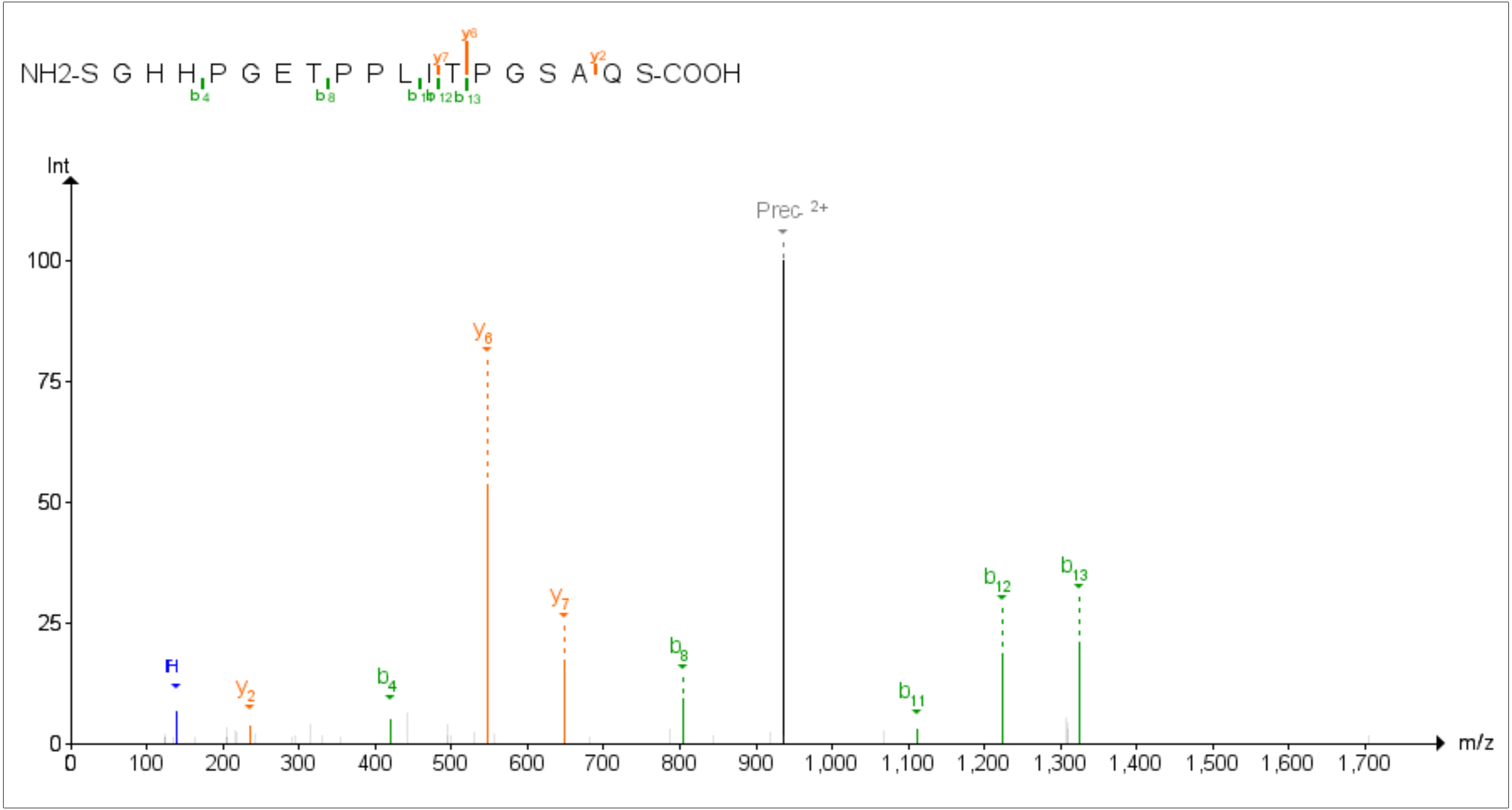
NH2-S G H H P G E T P P L I T P G S A Q S-COOH
b4
b8
b12
b13
y7
y6
y2
Int
100
75
50
25
0
Prec 2+
y6
y7
y2
b4
b8
b11
b12
b13
m/z
0
100
200
300
400
500
600
700
800
900
1,000
1,100
1,200
1,300
1,400
1,500
1,600
1,700

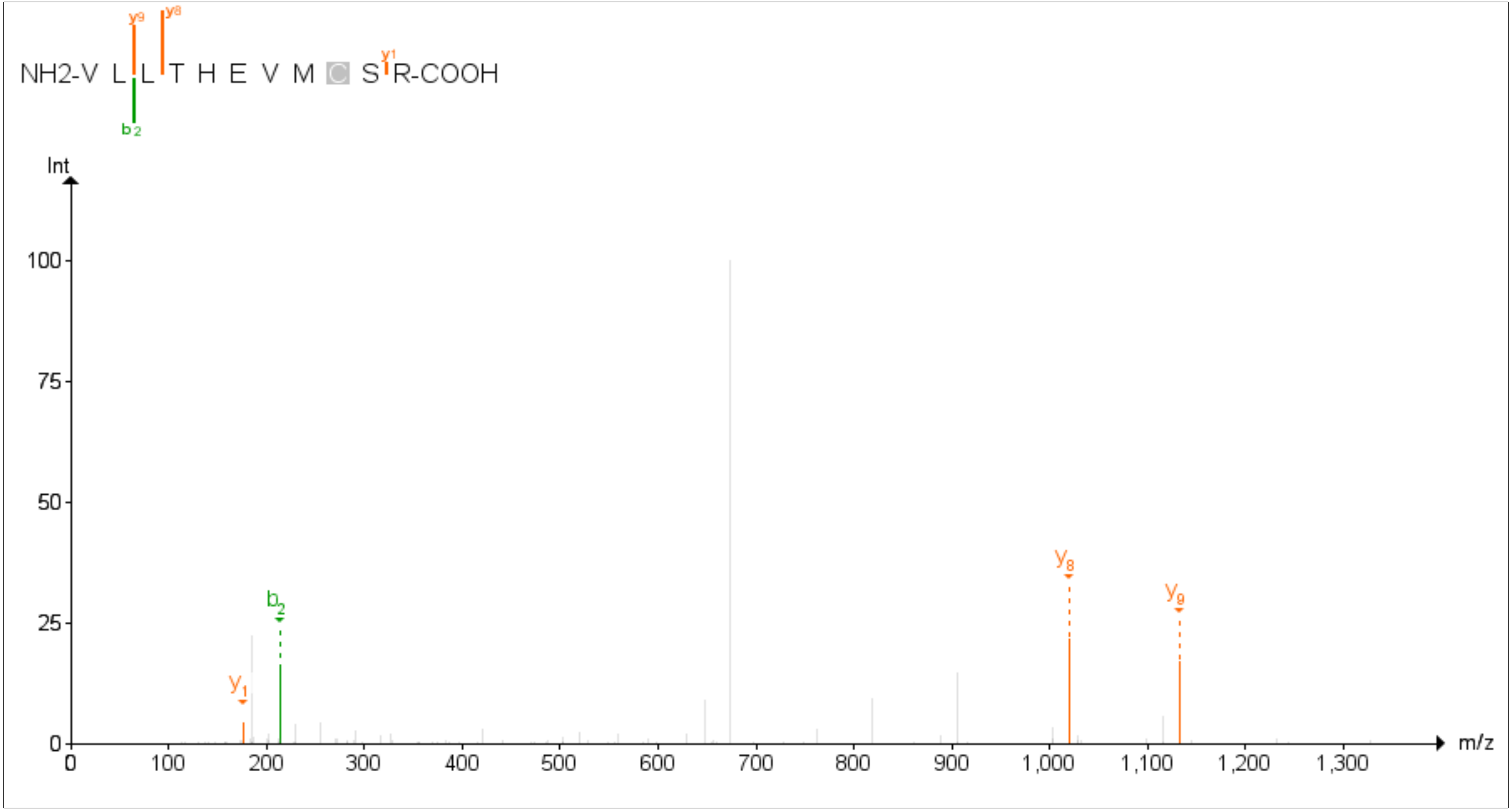

NH2-V L L T H E V M C S R-COOH
y9
y8
y1
b2
Int
100
75
50
25
0
m/z
0
100
200
300
400
500
600
700
800
900
1,000
1,100
1,200
1,300

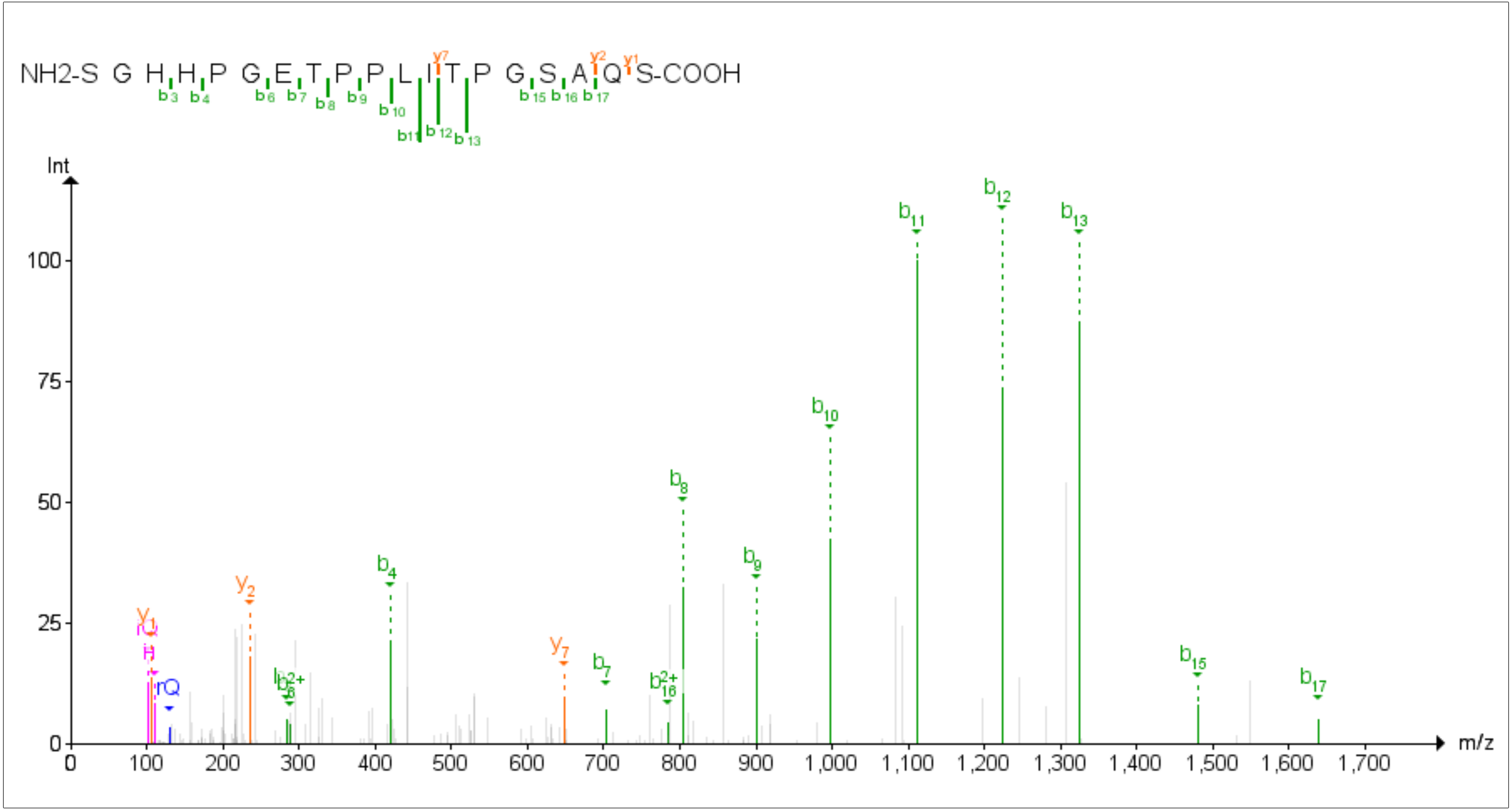

NH2-S G H H P G E T P P L I T P G S A Q S-COOH
y7 y2 y1
b3 b4 b6 b7 b8 b9 b10 b11 b12 b13 b15 b16 b17
Int
100
75
50
25
0
m/z
0 100 200 300 400 500 600 700 800 900 1,000 1,100 1,200 1,300 1,400 1,500 1,600 1,700

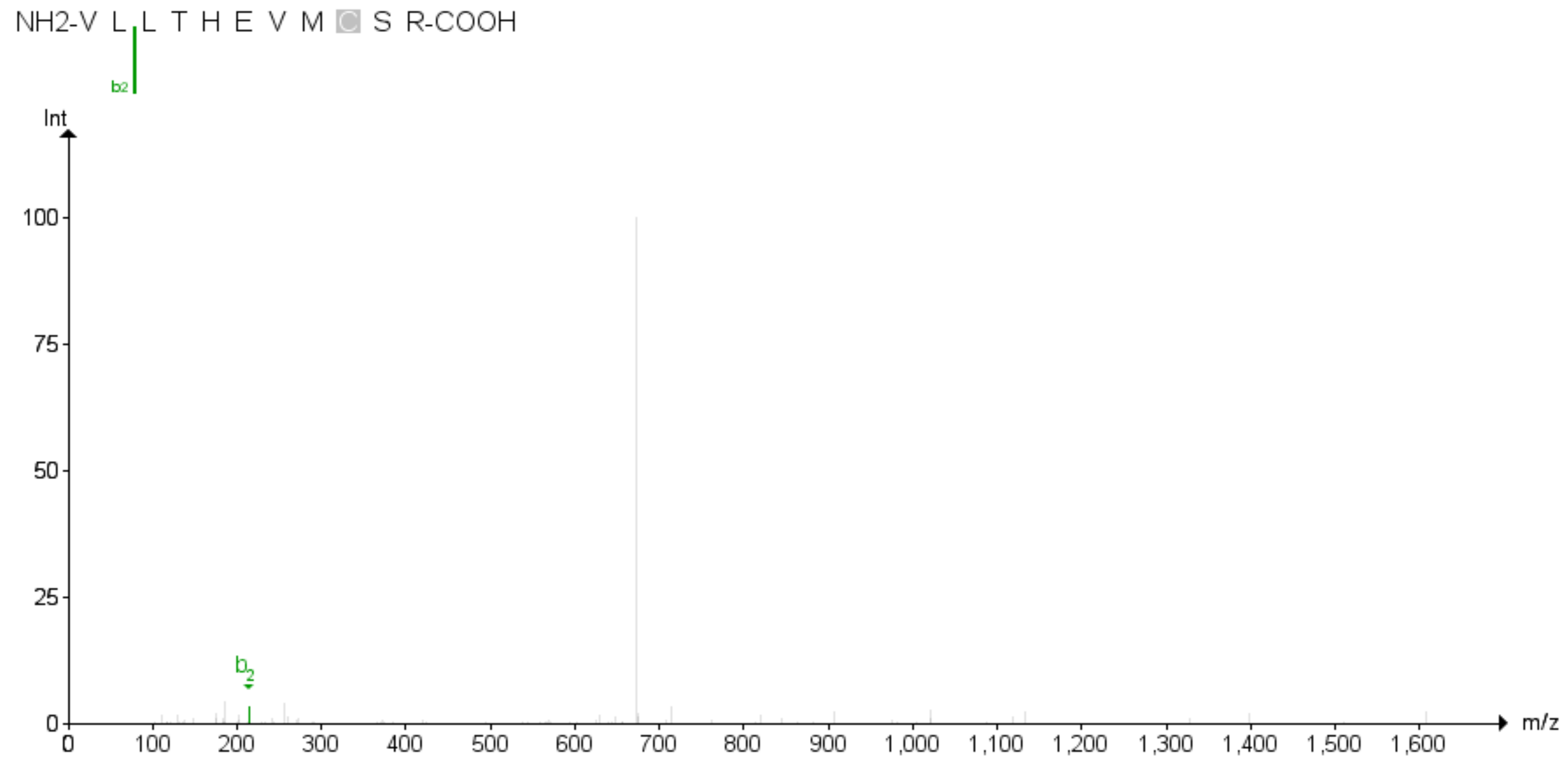

NH2-V L L T H E V M C S R-COOH
b2
Int
100
75
50
25
0
b2
0
100
200
300
400
500
600
700
800
900
1,000
1,100
1,200
1,300
1,400
1,500
1,600
m/z

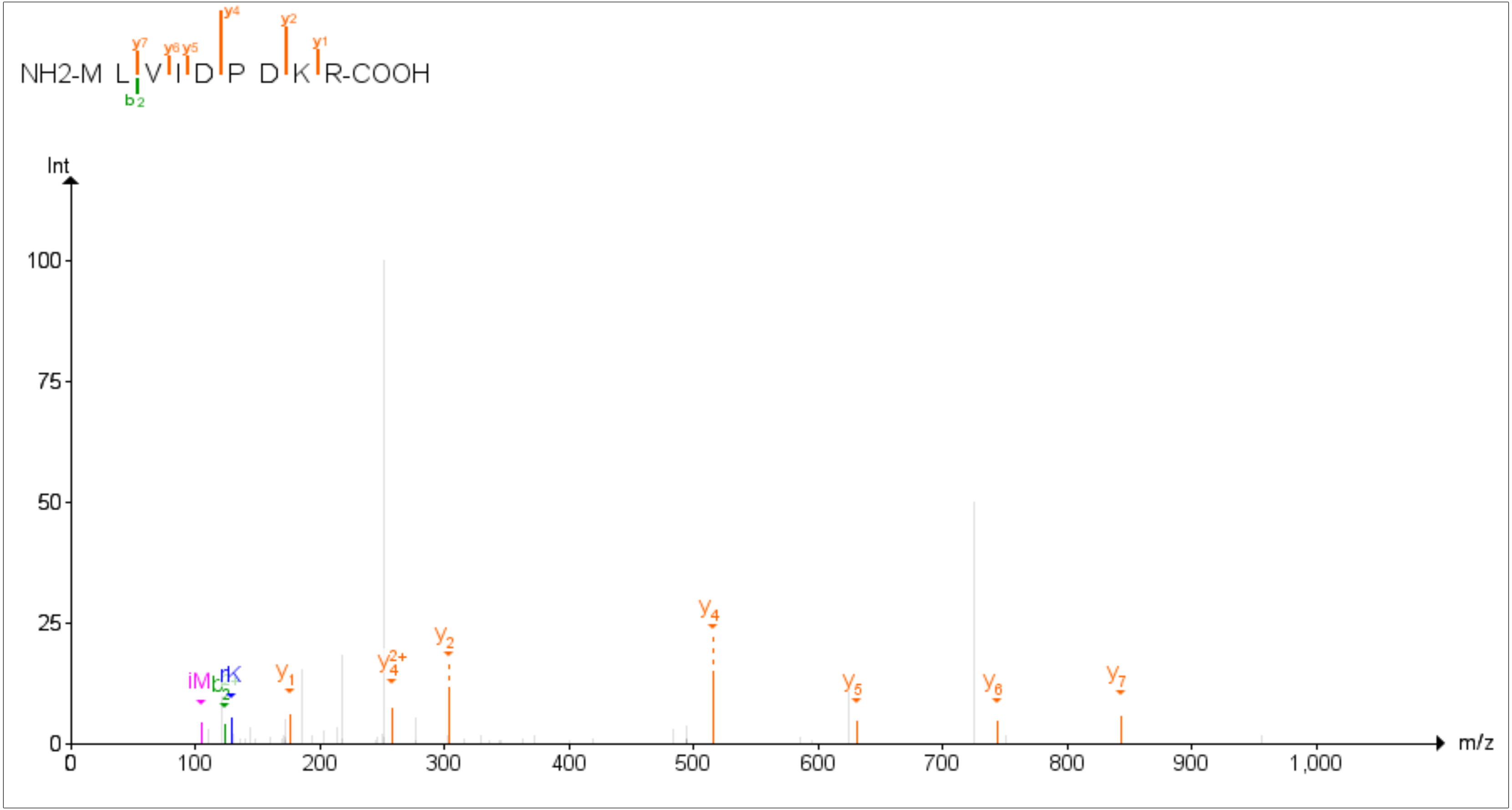

NH2-M L V I D P D K R-COOH
y7
y6
y5
y4
y2
y1
b2
Int
100
75
50
25
0
m/z
0
100
200
300
400
500
600
700
800
900
1,000
iM
rK
y1
y4 2+
y2
y4
y5
y6
y7

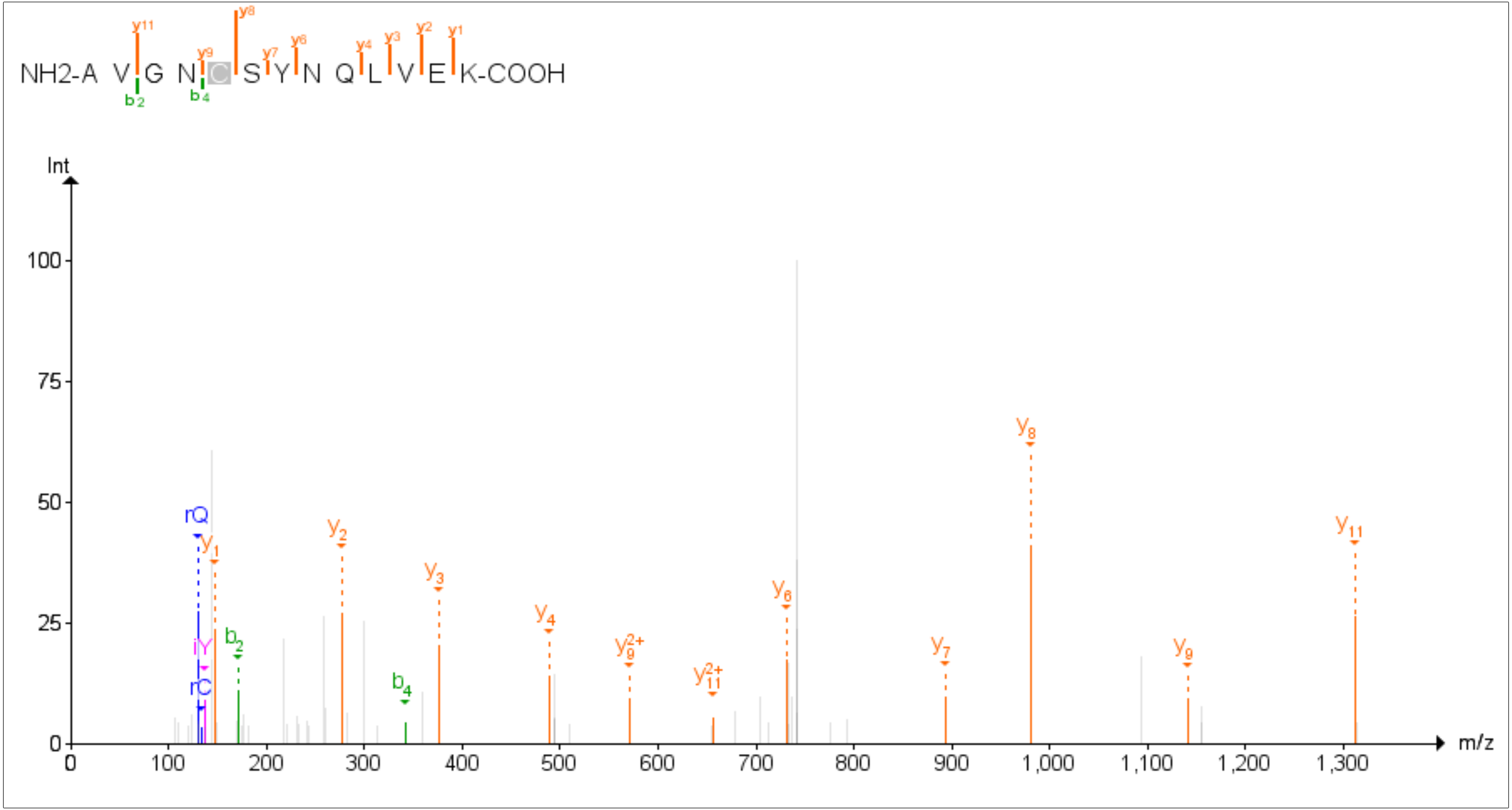

NH2-A V G N C S Y N Q L V E K-COOH
y11 y9 y8 y7 y6 y4 y3 y2 y1
b2 b4
Int
100
75
50
25
0
rQ
y1
iY
rC
b2
y2
b4
y3
y4
y9 2+
y11 2+
y6
y7
y8
y9
y11
0 100 200 300 400 500 600 700 800 900 1,000 1,100 1,200 1,300
m/z

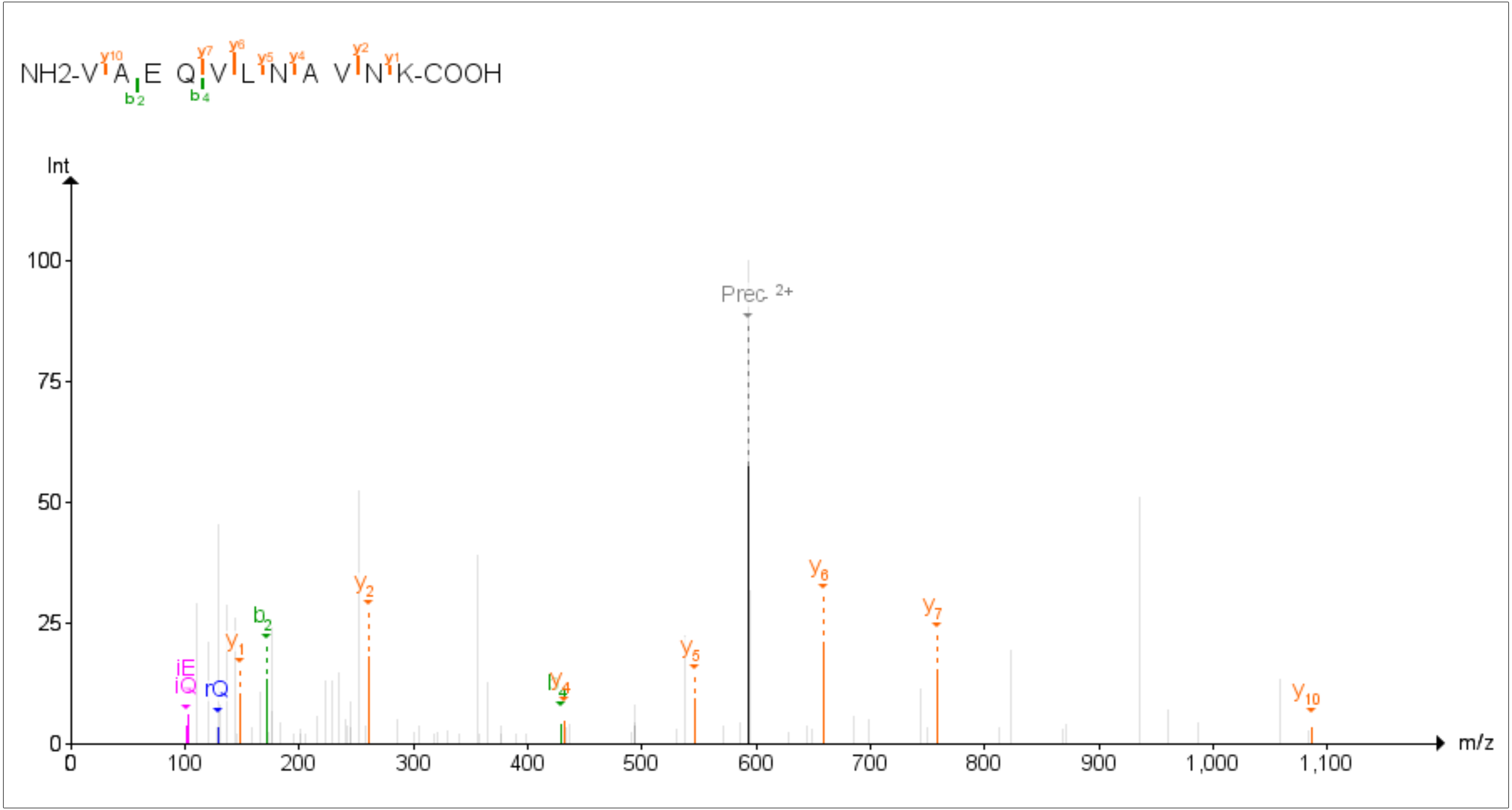

NH2-V A E Q V L N A V N K-COOH
y10 y7 y6 y5 y4 y2 y1
b2 b4
Int
100
75
50
25
0
Prec 2+
y2
b2
y1
iE
iQ
rQ
y4
y5
y6
y7
y10
0 100 200 300 400 500 600 700 800 900 1,000 1,100
m/z

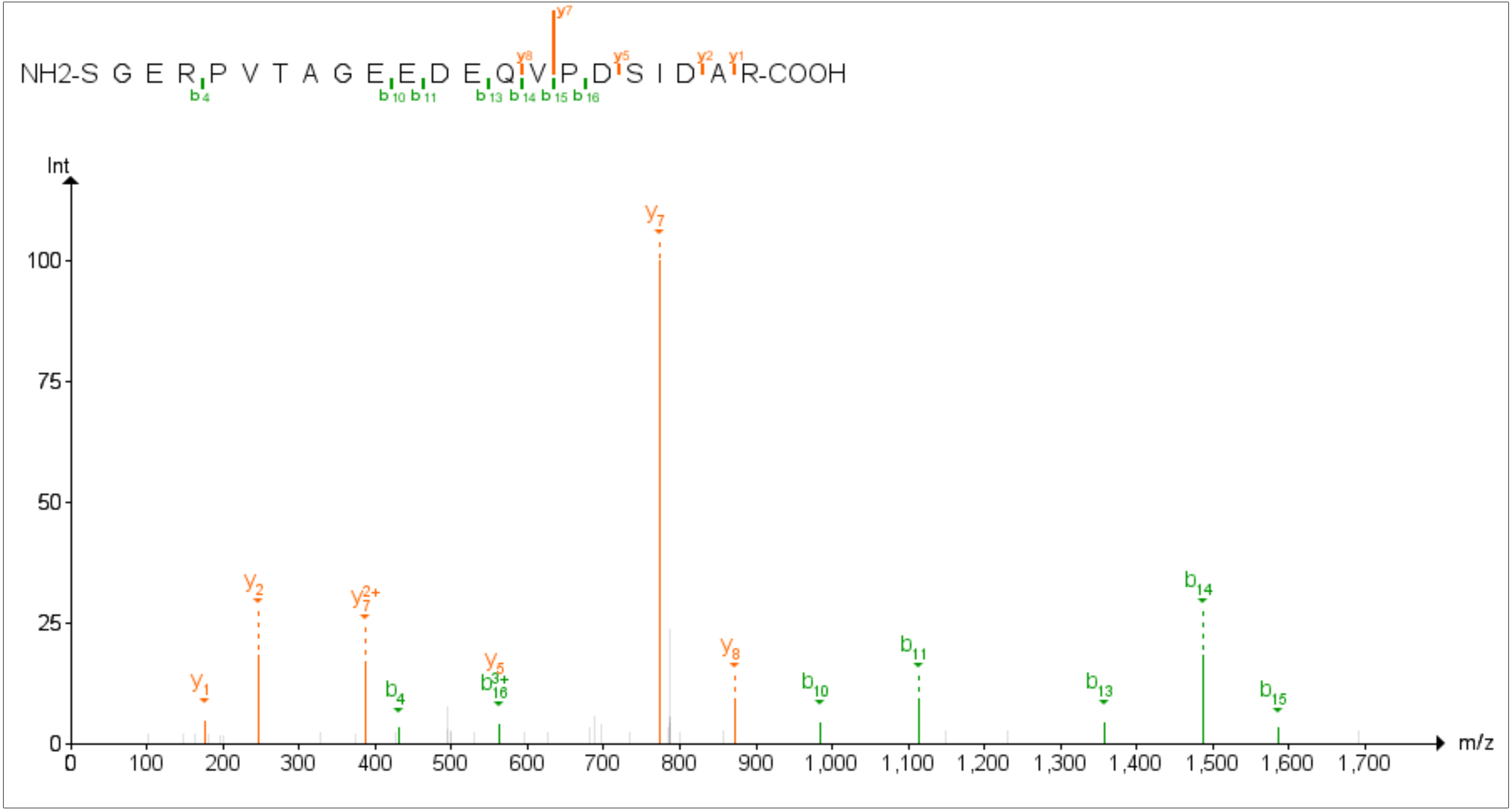
NH2-S G E R P V T A G E E D E Q V P D S I D A R-COOH
y7
y8
y5
y2
y1
b4
b10
b11
b13
b14
b15
b16
Int
100
75
50
25
0
y7
y2
y7 2+
y1
b4
y5
b16 3+
y8
b10
b11
b13
b14
b15
0
100
200
300
400
500
600
700
800
900
1,000
1,100
1,200
1,300
1,400
1,500
1,600
1,700
m/z

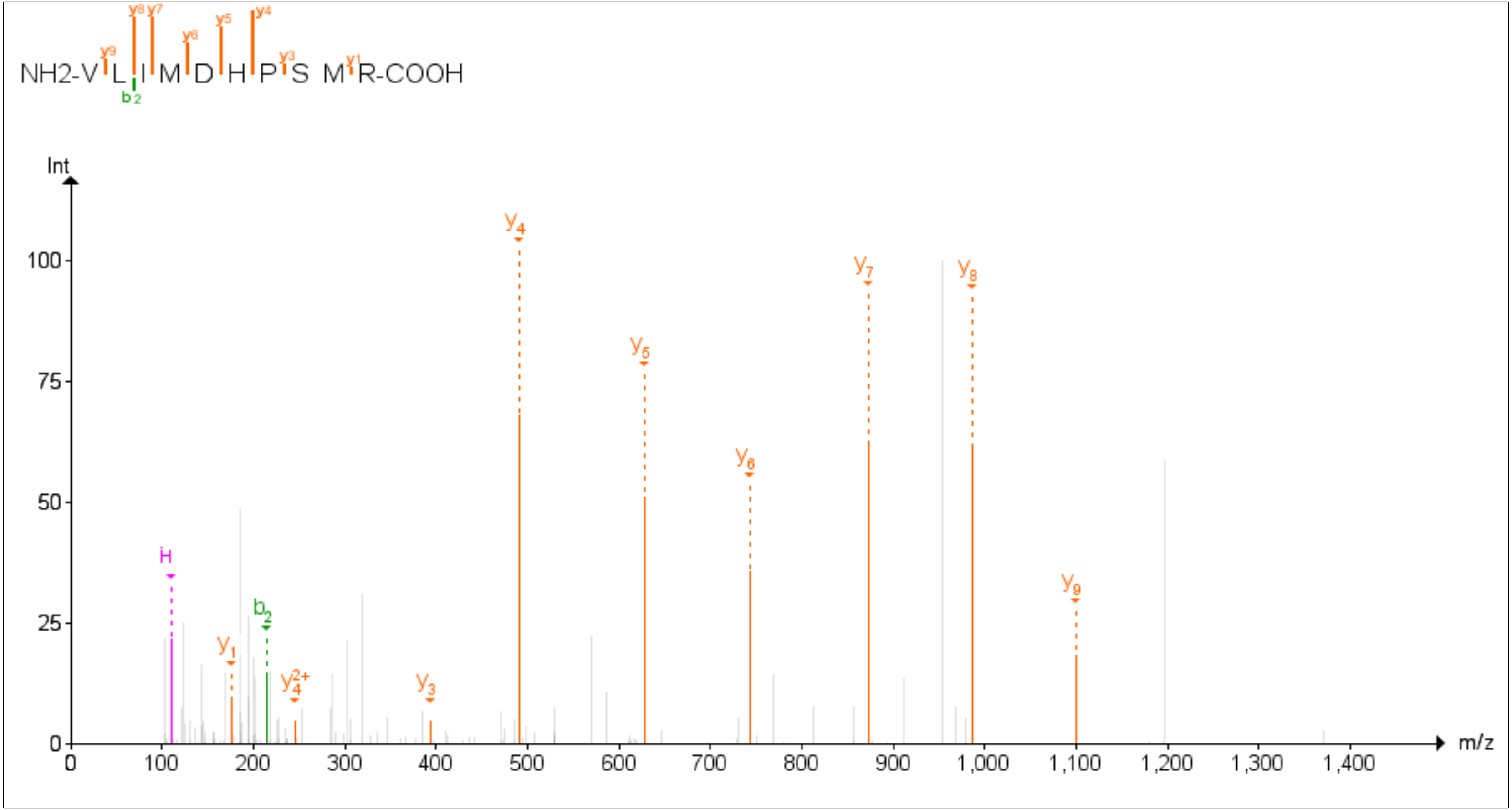

NH2-V L I M D H P S M R-COOH
y9 y8 y7 y6 y5 y4 y3 y1
b2
Int
m/z
0
25
50
75
100
100
200
300
400
500
600
700
800
900
1,000
1,100
1,200
1,300
1,400
y4
y5
y6
y7
y8
y9
H
b2
y1
y_4^{2+}
y3

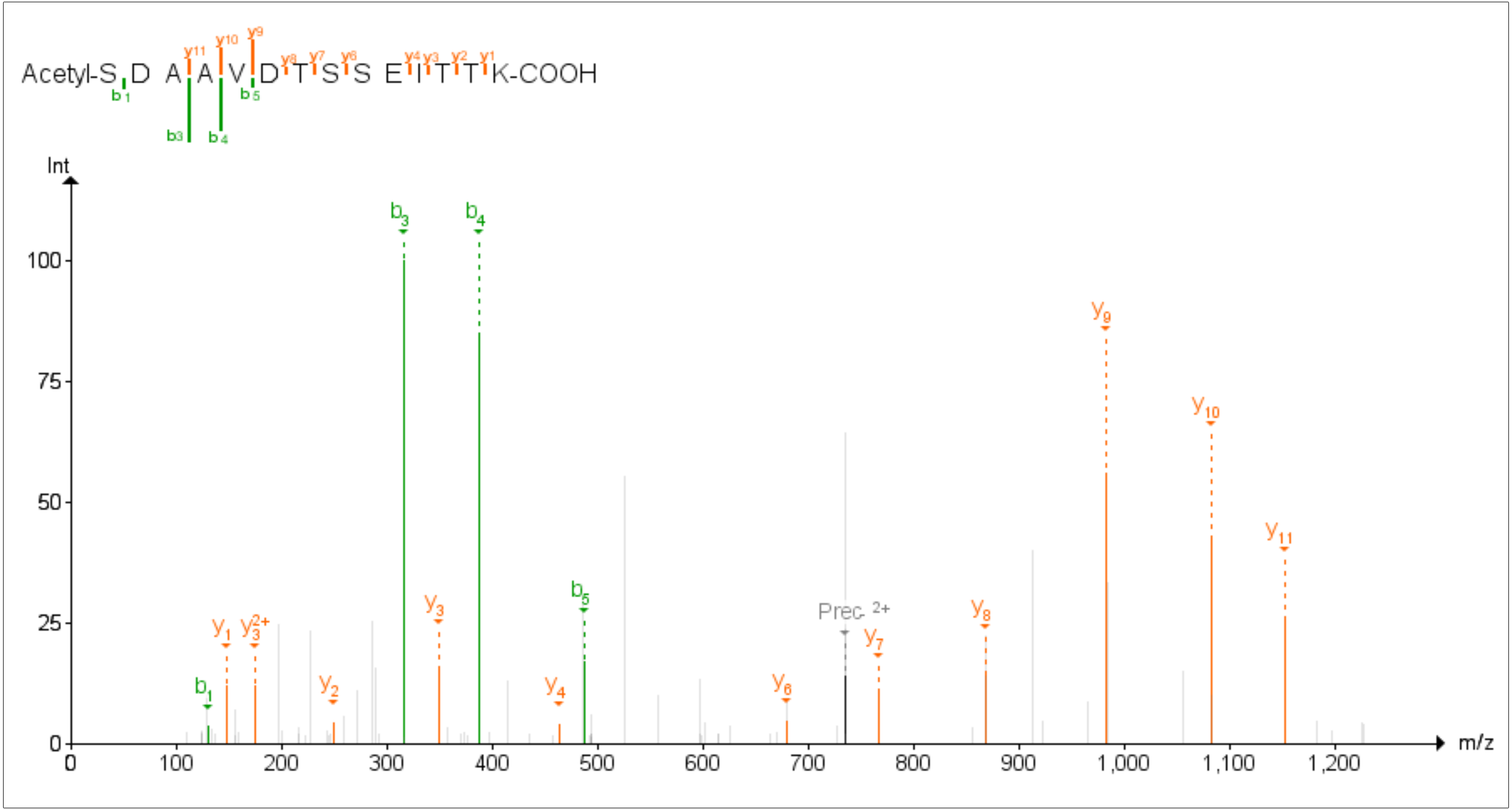

Acetyl-S D A A V D T S S E I T T K-COOH
b1 b3 b4 b5
y11 y10 y9 y8 y7 y6 y4 y3 y2 y1
Int
100
75
50
25
0
m/z
0 100 200 300 400 500 600 700 800 900 1,000 1,100 1,200
b3 b4 y9 y10 y11 b5 y3 Prec 2+ y8 y7 y1 y3 2+ y2 y4 y6 b1

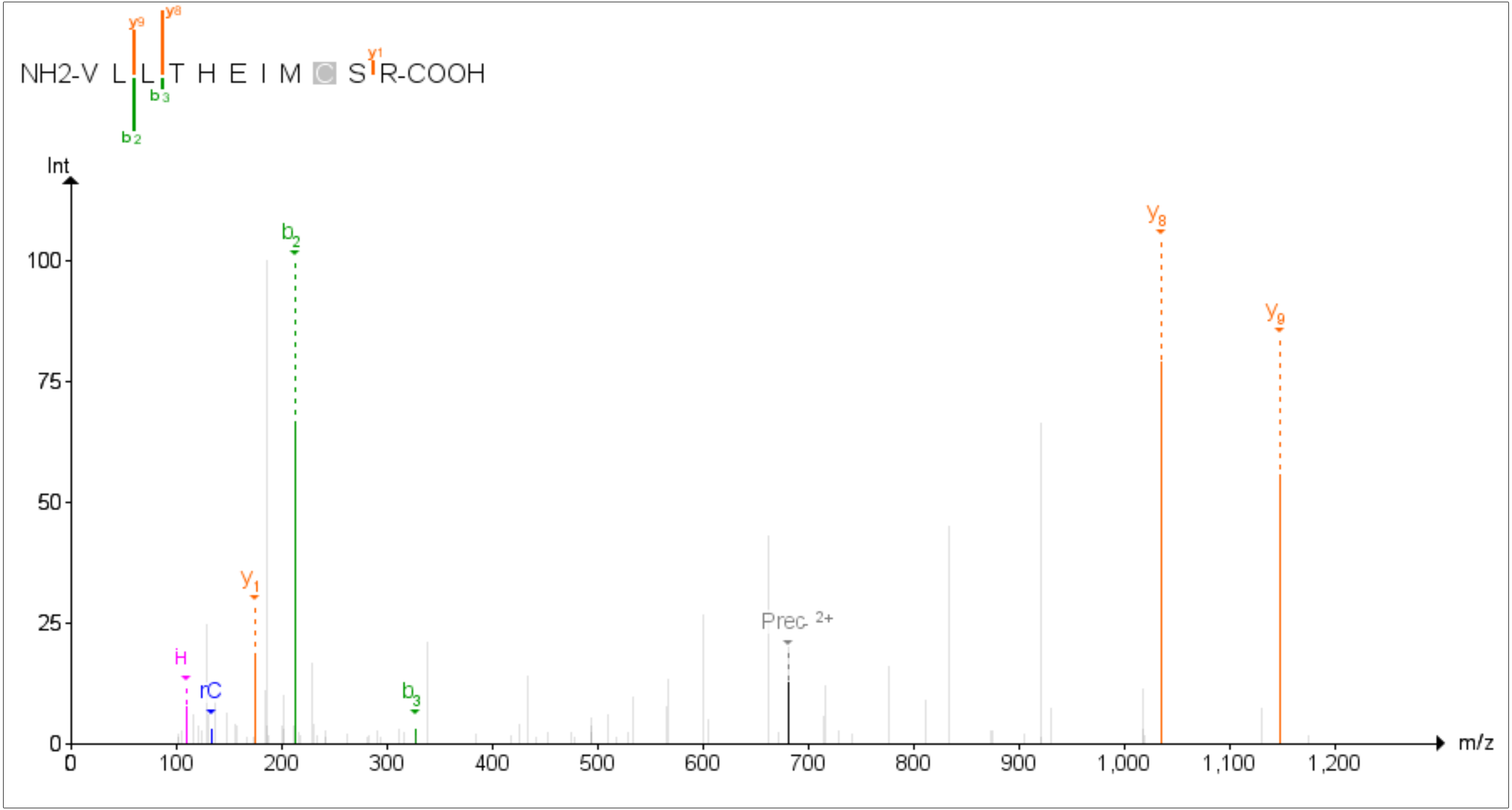

NH2-V L L T H E I M C S R-COOH
y9
y8
y1
b2
b3
Int
100
75
50
25
0
b2
y8
y9
y1
H
rC
b3
Prec 2+
0
100
200
300
400
500
600
700
800
900
1,000
1,100
1,200
m/z

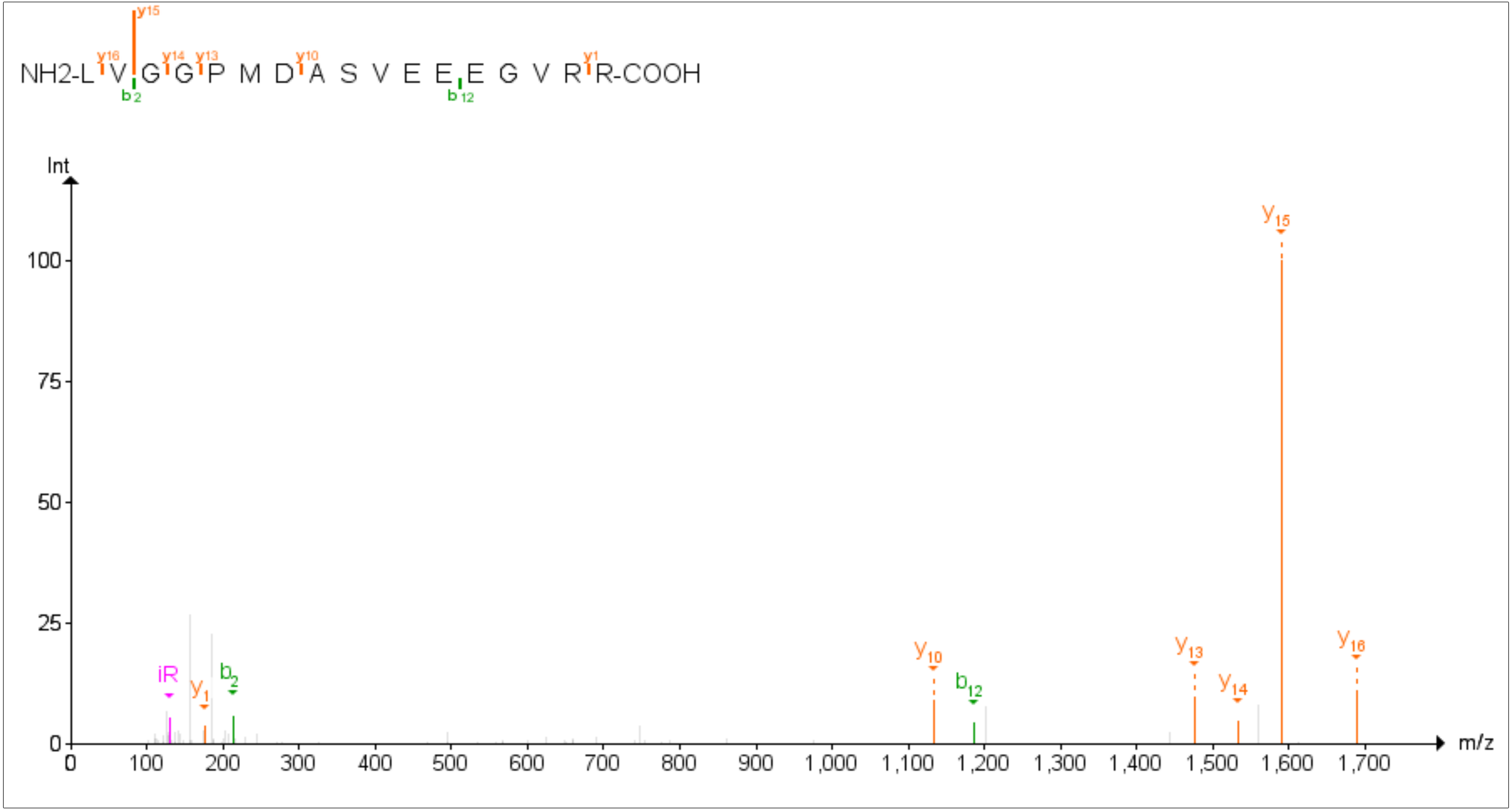

NH2-L V G G P M D A S V E E E G V R R-COOH
y15
y16
y14
y13
y10
y1
b2
b12
Int
100
75
50
25
0
y15
y10
y13
y14
y16
b12
iR
y1
b2
0
100
200
300
400
500
600
700
800
900
1,000
1,100
1,200
1,300
1,400
1,500
1,600
1,700
m/z

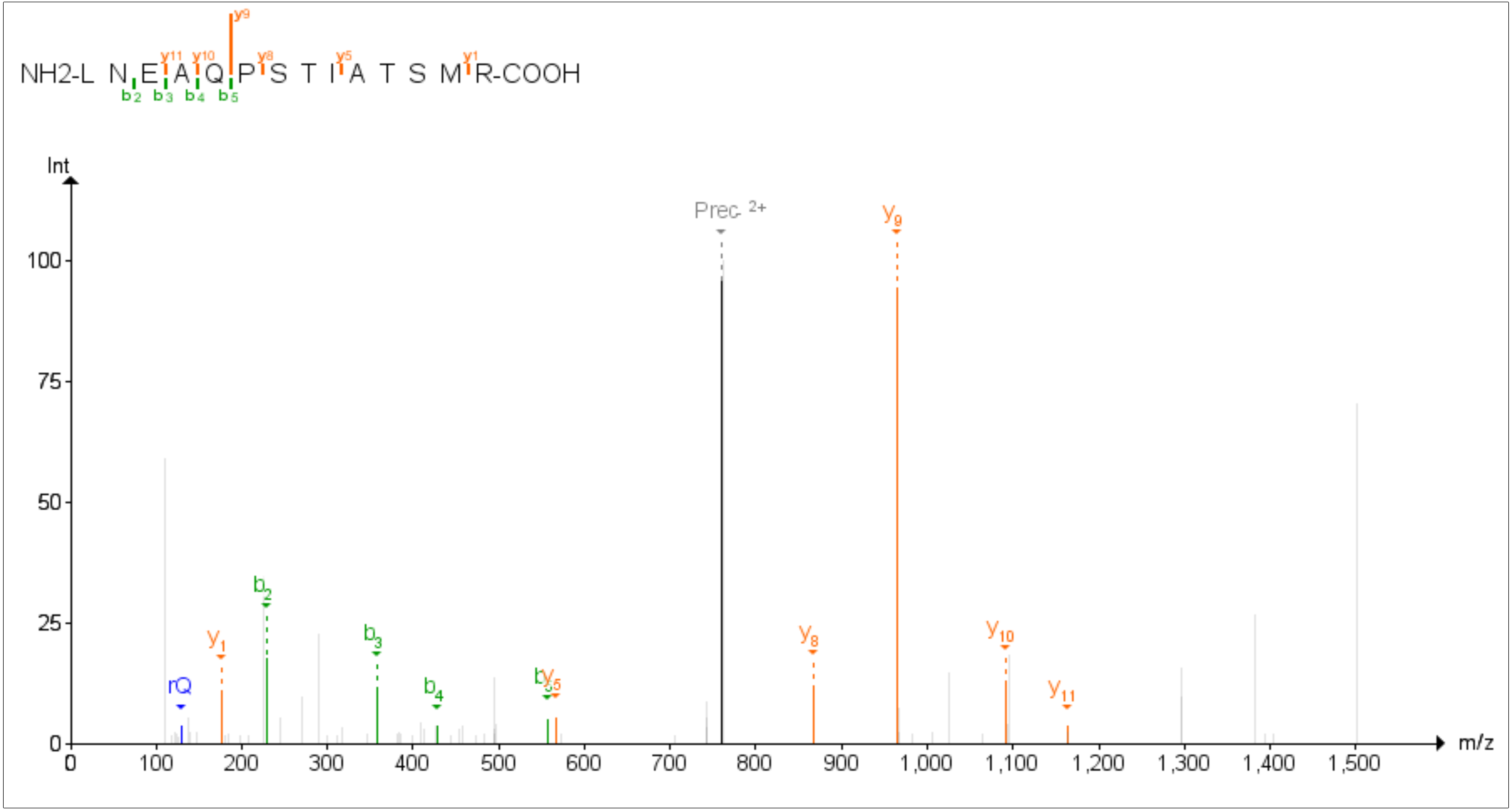

NH2-L N E A Q P S T I A T S M R-COOH
y9
y11
y10
y8
y5
y1
b2
b3
b4
b5
Int
100
75
50
25
0
Prec 2+
y9
y8
y10
y11
b2
b3
b4
b5
y5
y1
rQ
0
100
200
300
400
500
600
700
800
900
1,000
1,100
1,200
1,300
1,400
1,500
m/z

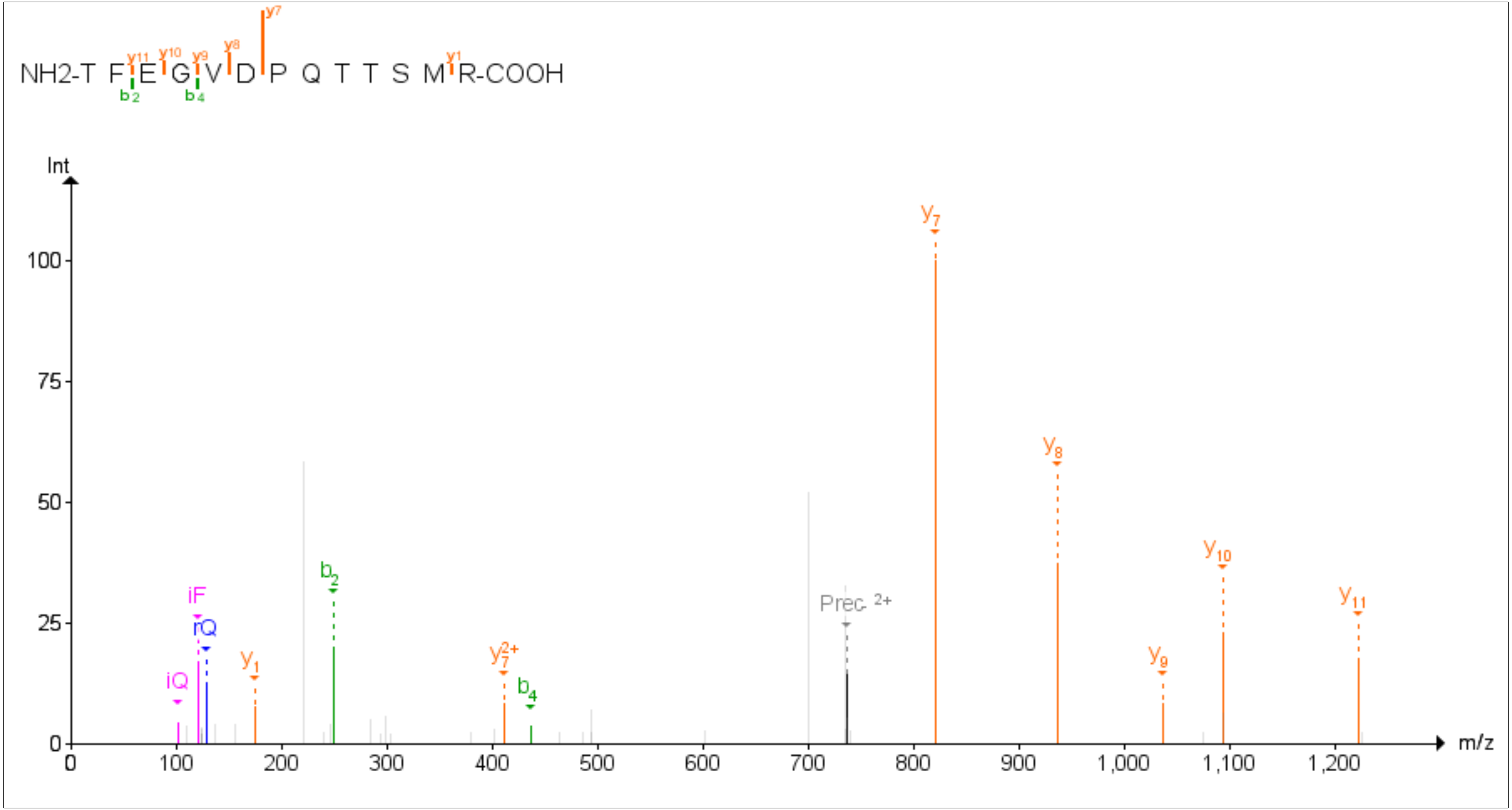

NH2-T F E G V D P Q T T S M R-COOH
y11 y10 y9 y8 y7 y1
b2 b4
Int
100
75
50
25
0
m/z
0 100 200 300 400 500 600 700 800 900 1,000 1,100 1,200
y7
y8
y10
y11
y9
Prec 2+
b2
iF
rQ
iQ
y1
y7 2+
b4

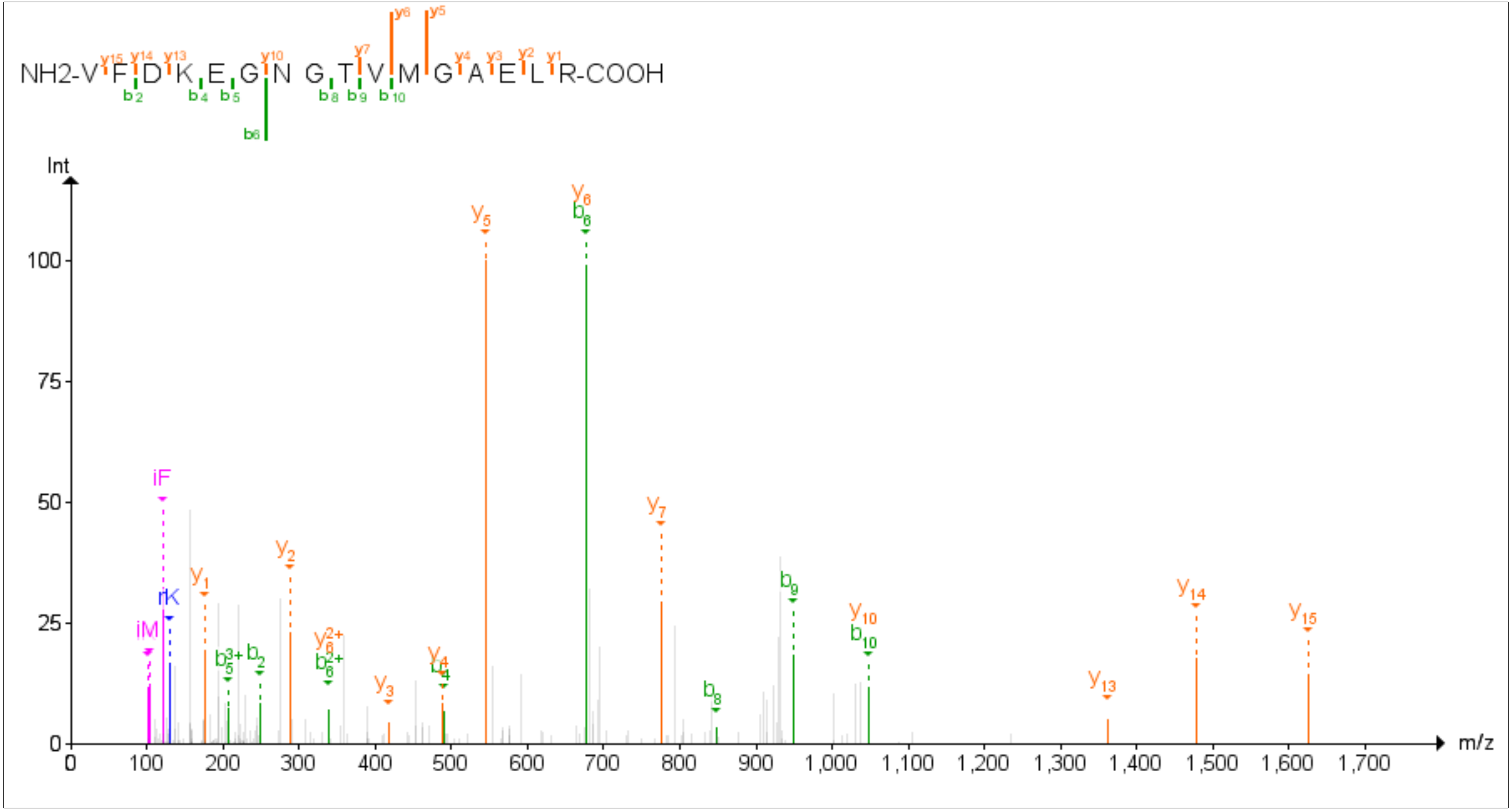

NH2-V F D K E G N G T V M G A E L R-COOH
y15 y14 y13 y10 y7 y6 y5 y4 y3 y2 y1
b2 b4 b5 b6 b8 b9 b10
Int
m/z
iF
iM
rK
y1
y2
y3
y4
y5
y6
y7
y10
y13
y14
y15
b2
b6
b8
b9
b10
0
25
50
75
100
0
100
200
300
400
500
600
700
800
900
1,000
1,100
1,200
1,300
1,400
1,500
1,600
1,700

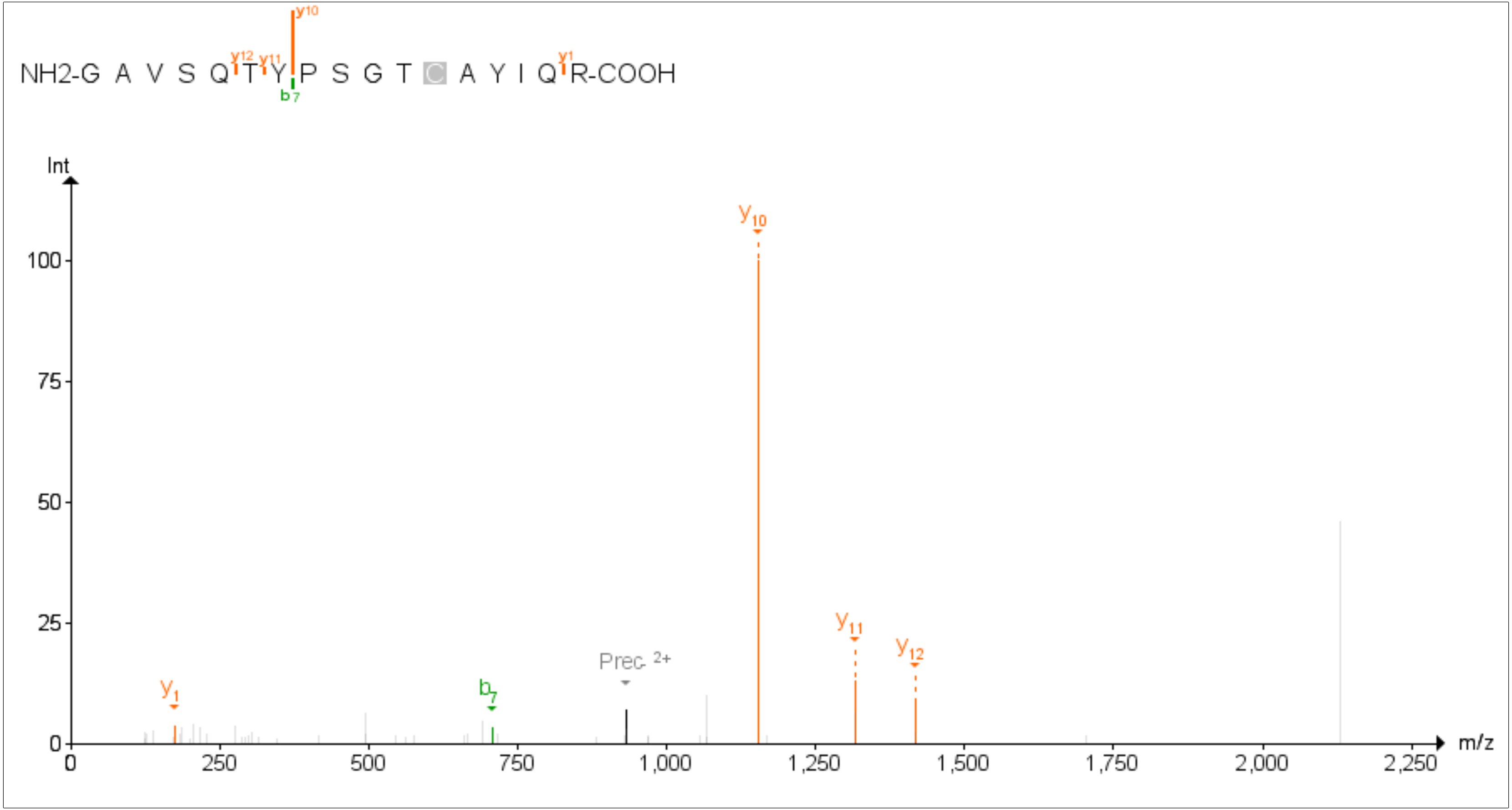
NH2-G A V S Q T Y P S G T C A Y I Q R-COOH
y10
y12
y11
y1
b7
Int
100
75
50
25
0
y10
y11
y12
y1
b7
Prec 2+
0
250
500
750
1,000
1,250
1,500
1,750
2,000
2,250
m/z

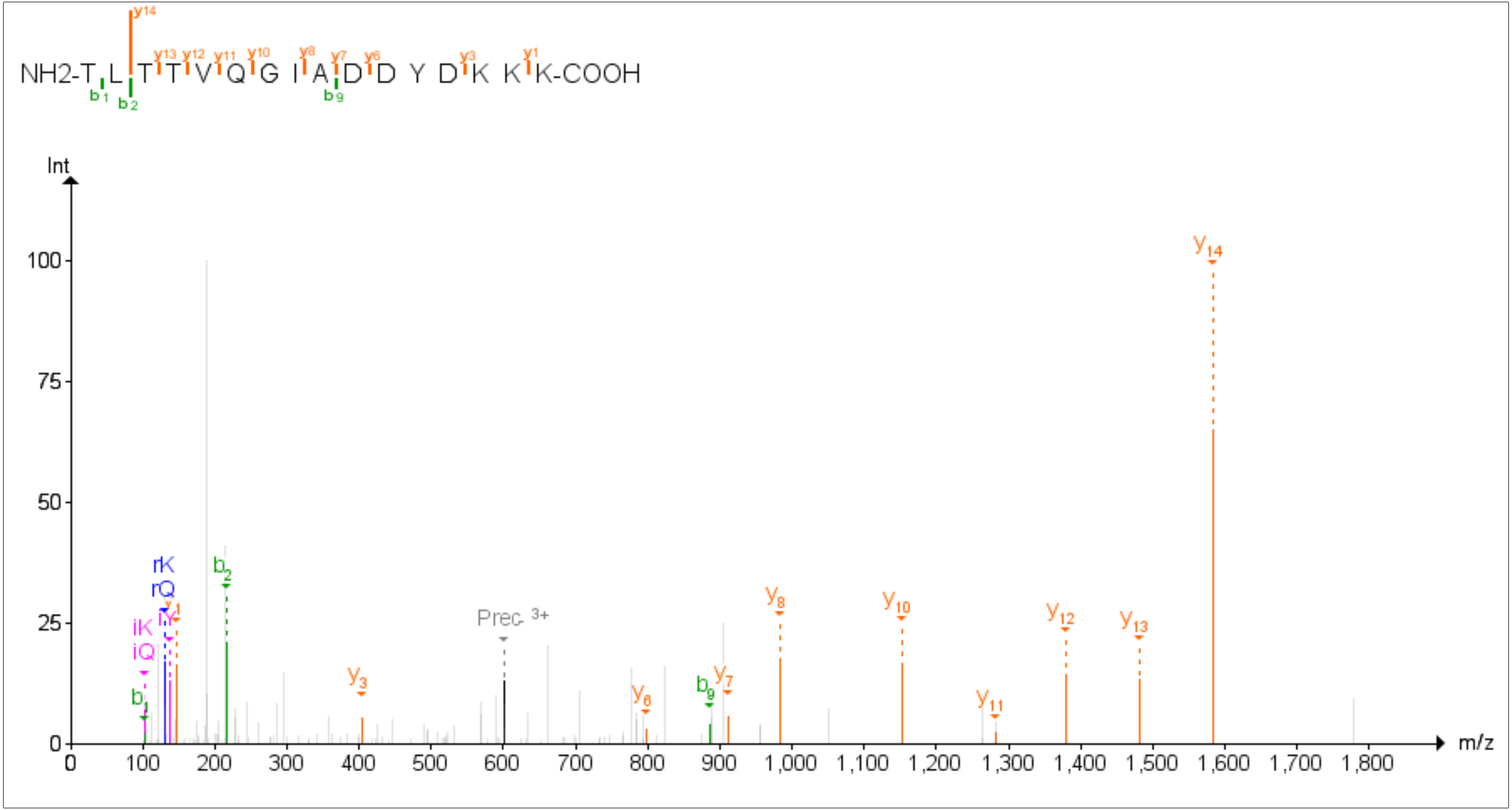

NH2-T L T T V Q G I A D D Y D K K K-COOH
y14 y13 y12 y11 y10 y8 y7 y6 y3 y1
b1 b2 b9
Int
100
75
50
25
0
m/z
0 100 200 300 400 500 600 700 800 900 1,000 1,100 1,200 1,300 1,400 1,500 1,600 1,700 1,800
y14
rK
rQ
iK
iQ
b2
y1
b1
y3
Prec 3+
y6
b9
y7
y8
y10
y11
y12
y13

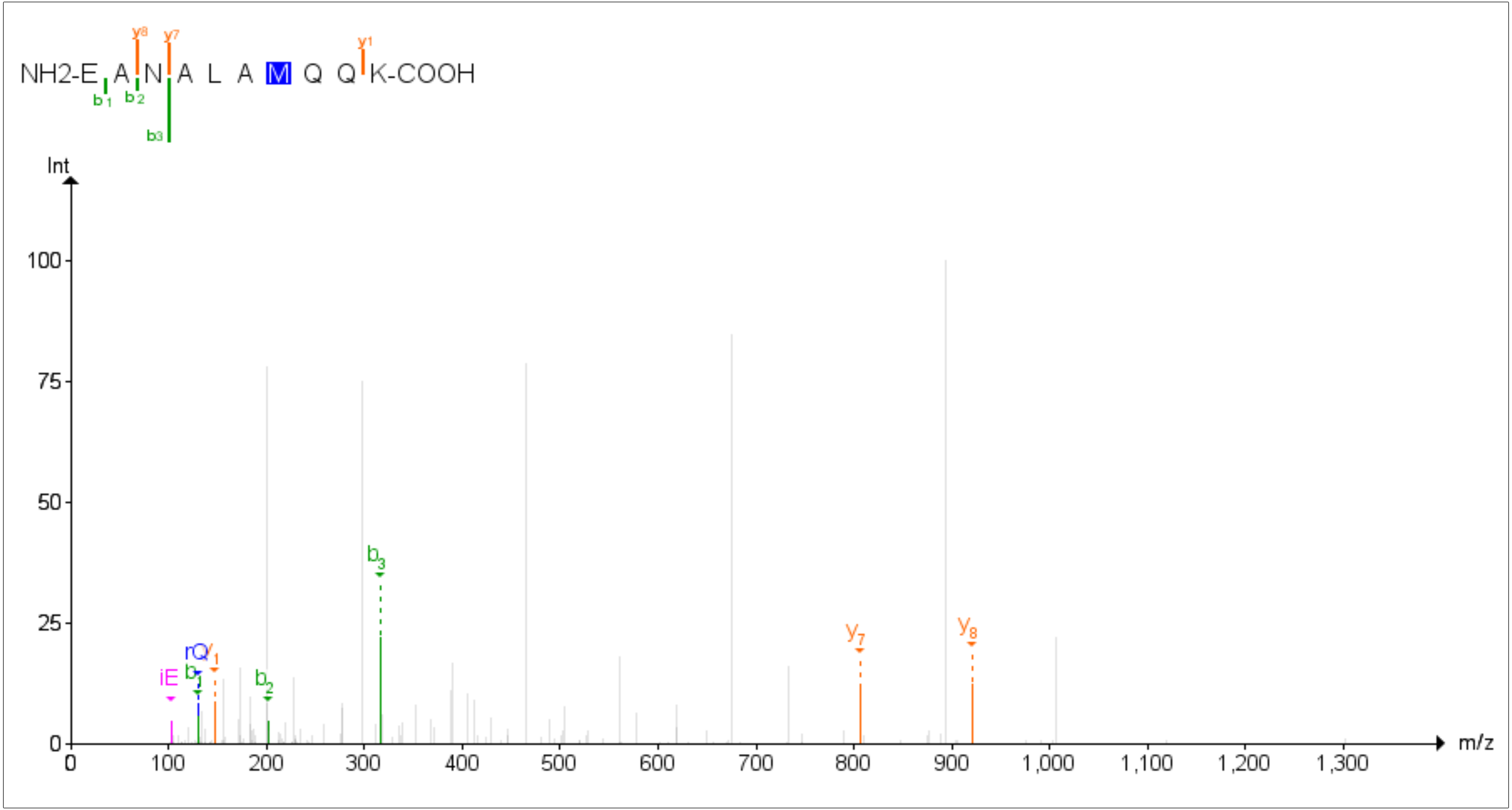

NH2-E A N A L A M Q Q K-COOH
y8
y7
y1
b 1
b 2
b3
Int
100
75
50
25
0
b3
y7
y8
iE
rQ
b1
y1
b2
0
100
200
300
400
500
600
700
800
900
1,000
1,100
1,200
1,300
m/z

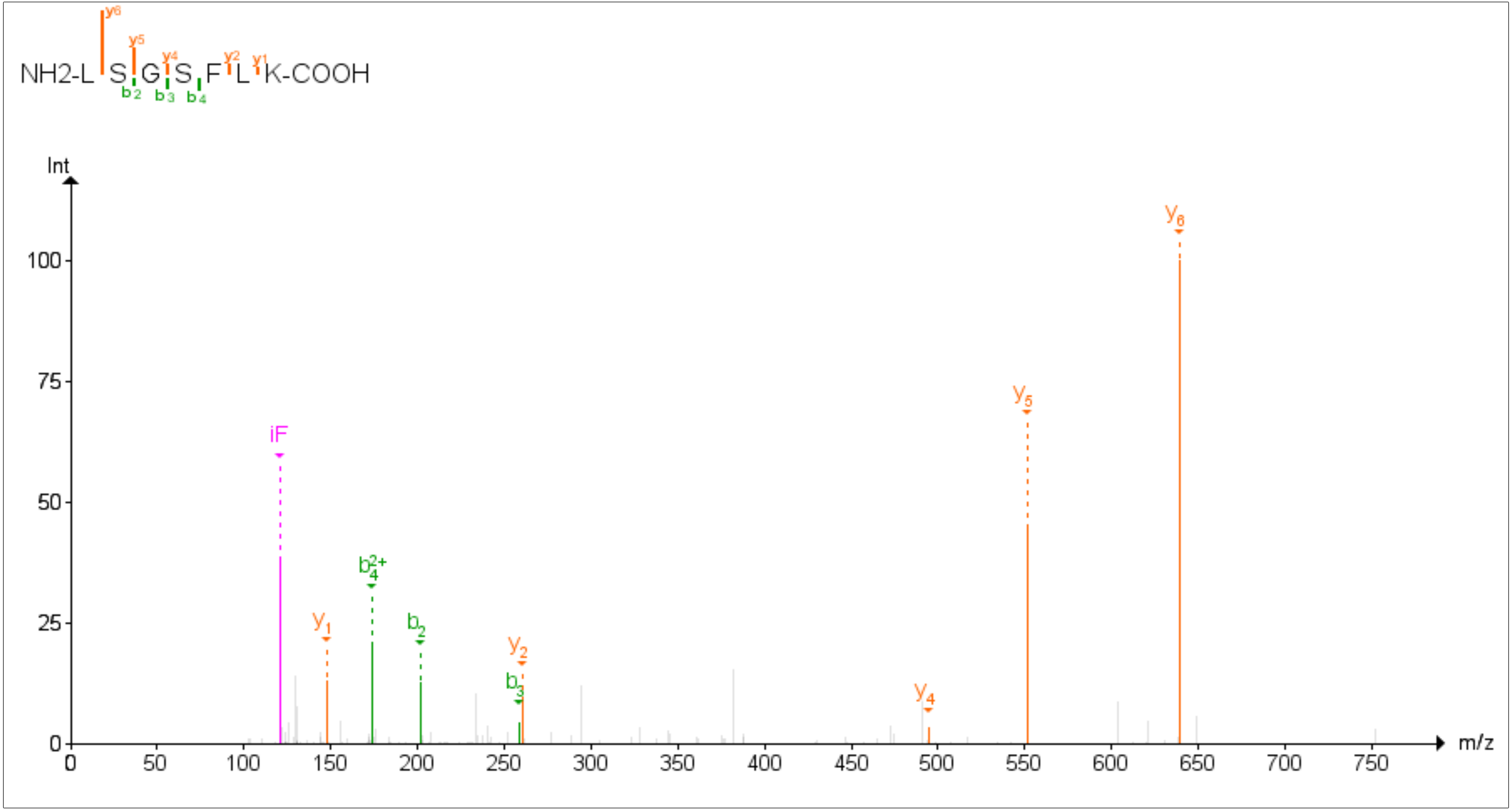

NH2-L S G S F L K-COOH
y6 y5 y4 y2 y1
b2 b3 b4
Int
100
75
50
25
0
iF
b_4^{2+}
y_1
b_2
y_2
b_3
y_4
y_5
y_6
0 50 100 150 200 250 300 350 400 450 500 550 600 650 700 750
m/z

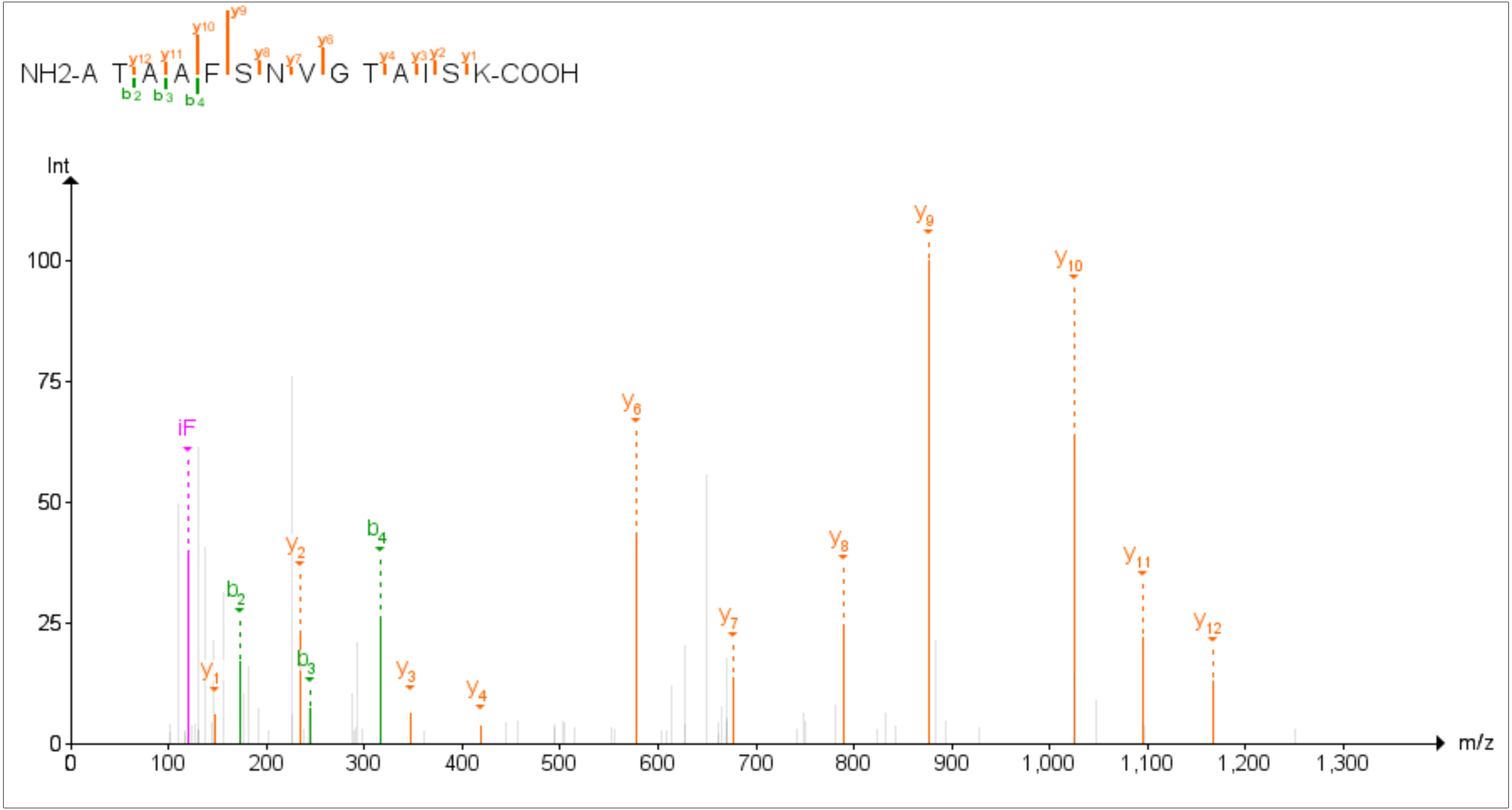
NH2-A T A A F S N V G T A I S K-COOH
y12 y11 y10 y9 y8 y7 y6 y4 y3 y2 y1
b2 b3 b4
Int
100
75
50
25
0
iF
y1
b2
y2
b3
b4
y3
y4
y6
y7
y8
y9
y10
y11
y12
0 100 200 300 400 500 600 700 800 900 1,000 1,100 1,200 1,300
m/z

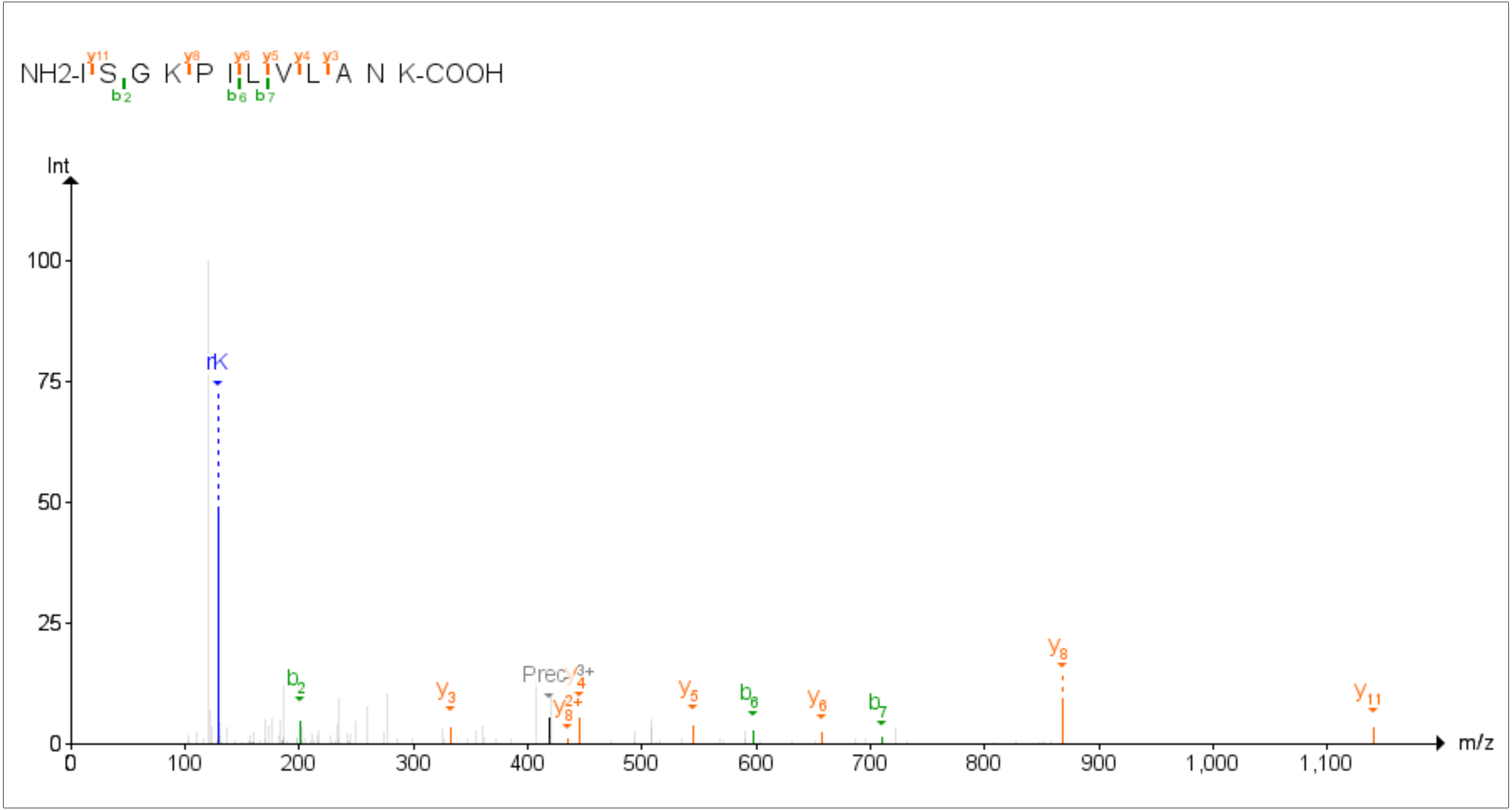

NH2-I S G K P I L V L A N K-COOH
y11 y8 y6 y5 y4 y3
b2 b6 b7
Int
100
75
50
25
0
rK
b2
y3
Prec3+
y4
y8 2+
y5
b6
y6
b7
y8
y11
0
100
200
300
400
500
600
700
800
900
1,000
1,100
m/z

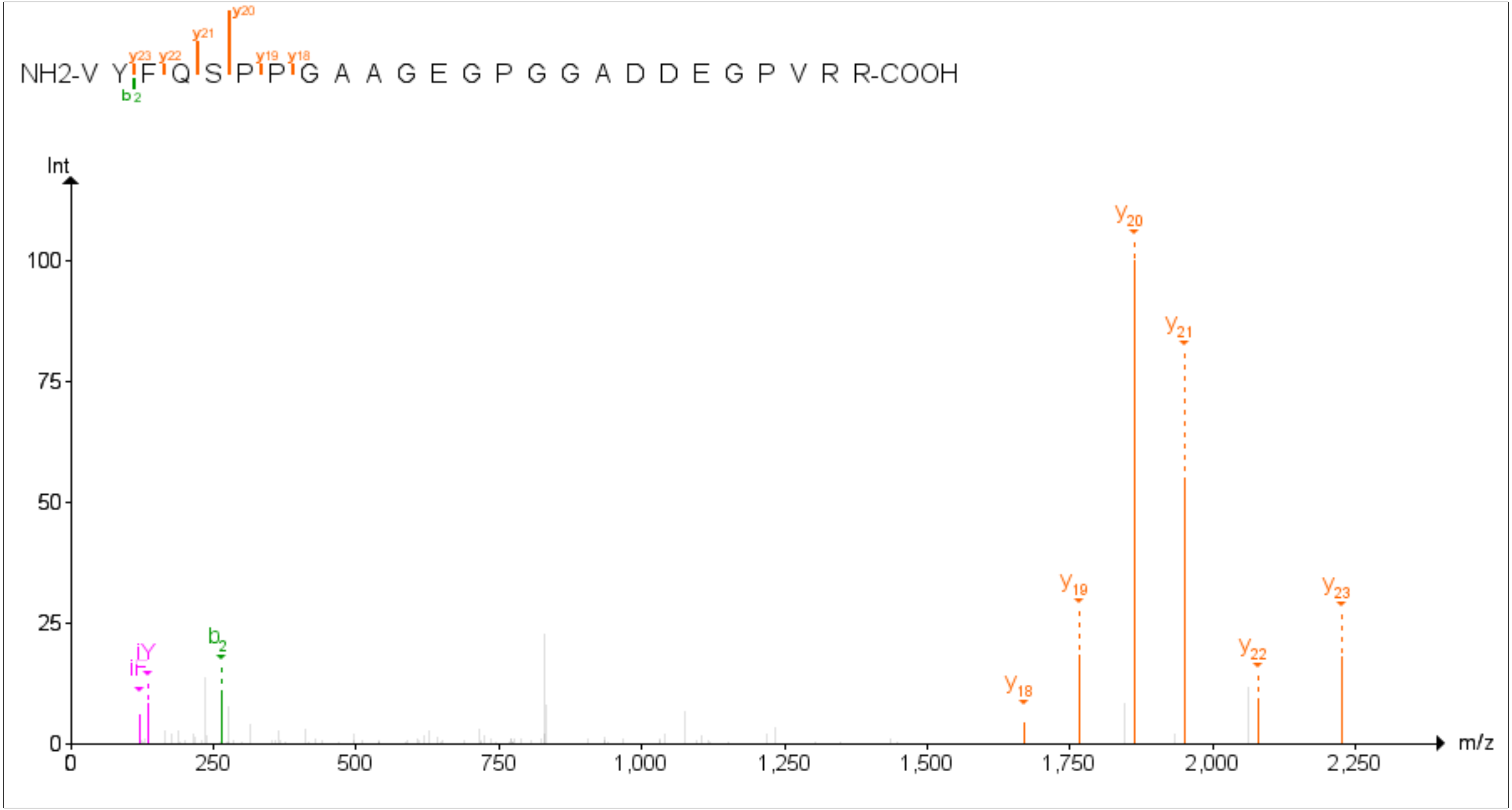

NH2-V Y F Q S P P G A A G E G P G G A D D E G P V R R-COOH
y23 y22 y21 y20 y19 y18
b2
Int
100
75
50
25
0
iY
iF
b2
y18
y19
y20
y21
y22
y23
0
250
500
750
1,000
1,250
1,500
1,750
2,000
2,250
m/z

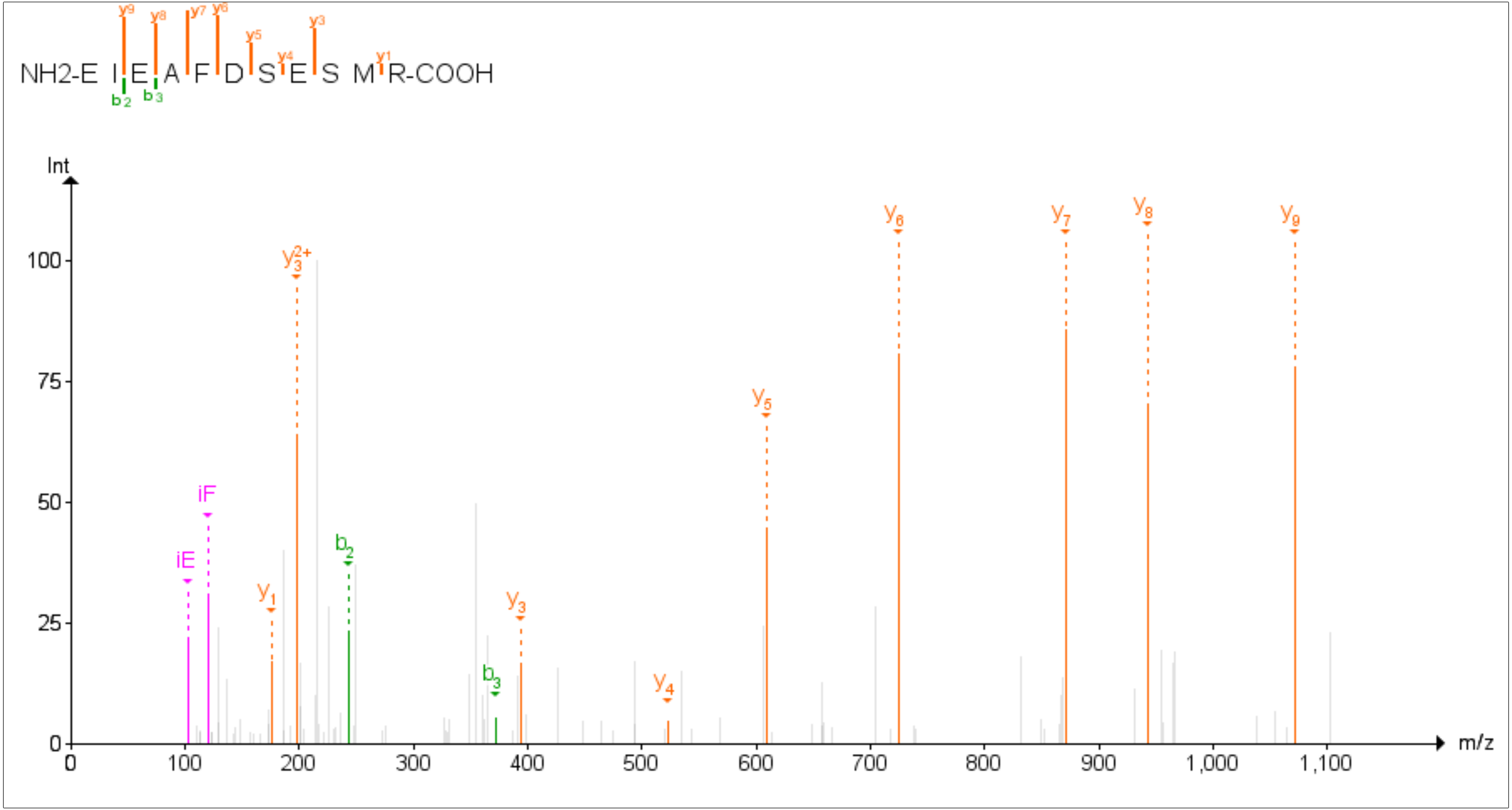
NH2-E I E A F D S E S M R-COOH
y9 y8 y7 y6 y5 y4 y3 y1
b2 b3
Int
100
75
50
25
0
m/z
0 100 200 300 400 500 600 700 800 900 1,000 1,100
iE iF y1 y3^2+ b2 b3 y3 y4 y5 y6 y7 y8 y9

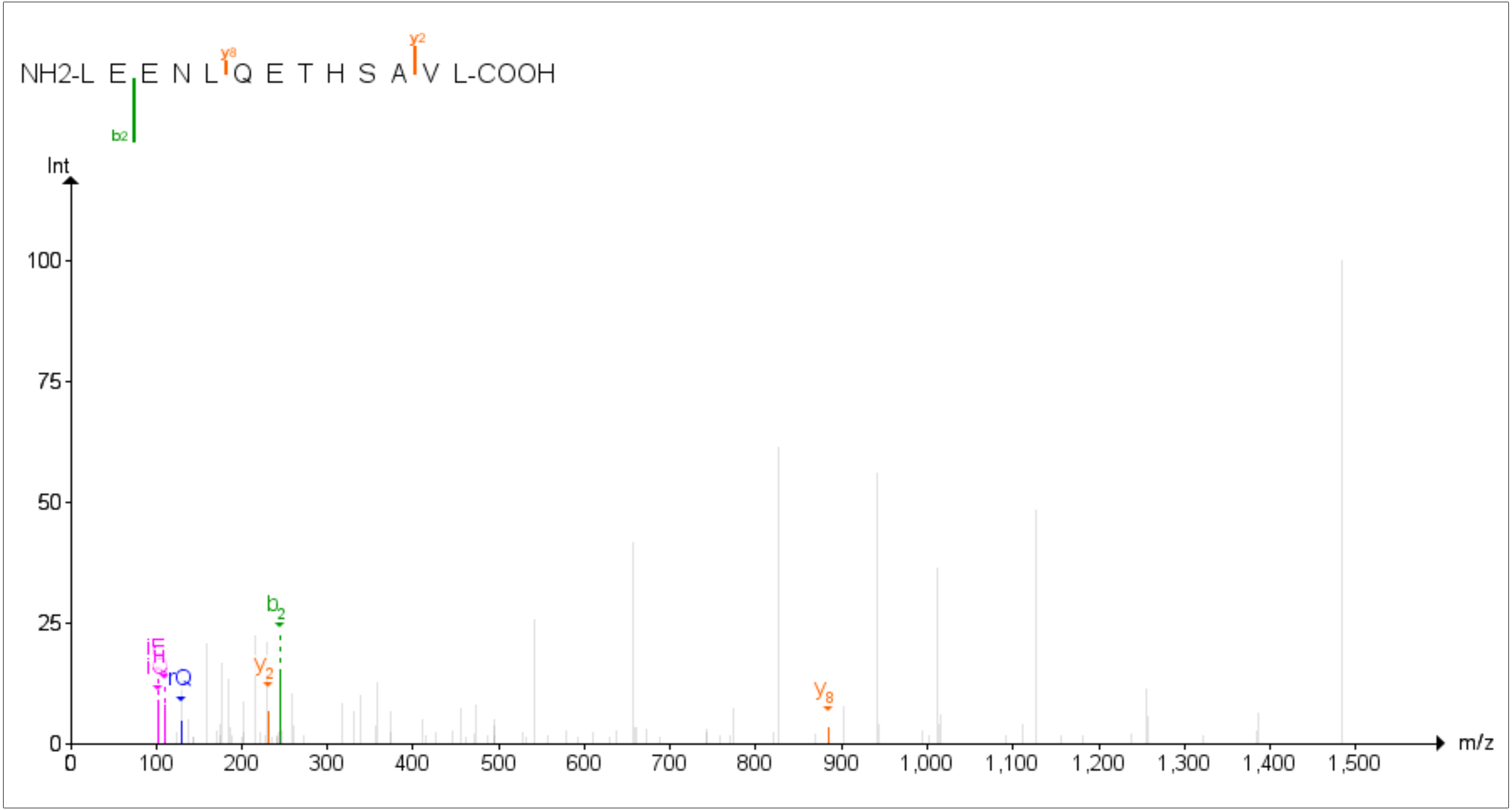

NH2-L E E N L Q E T H S A V L-COOH
b2
y8
y2
Int
100
75
50
25
0
m/z
0
100
200
300
400
500
600
700
800
900
1,000
1,100
1,200
1,300
1,400
1,500
b2
y2
y8
rQ

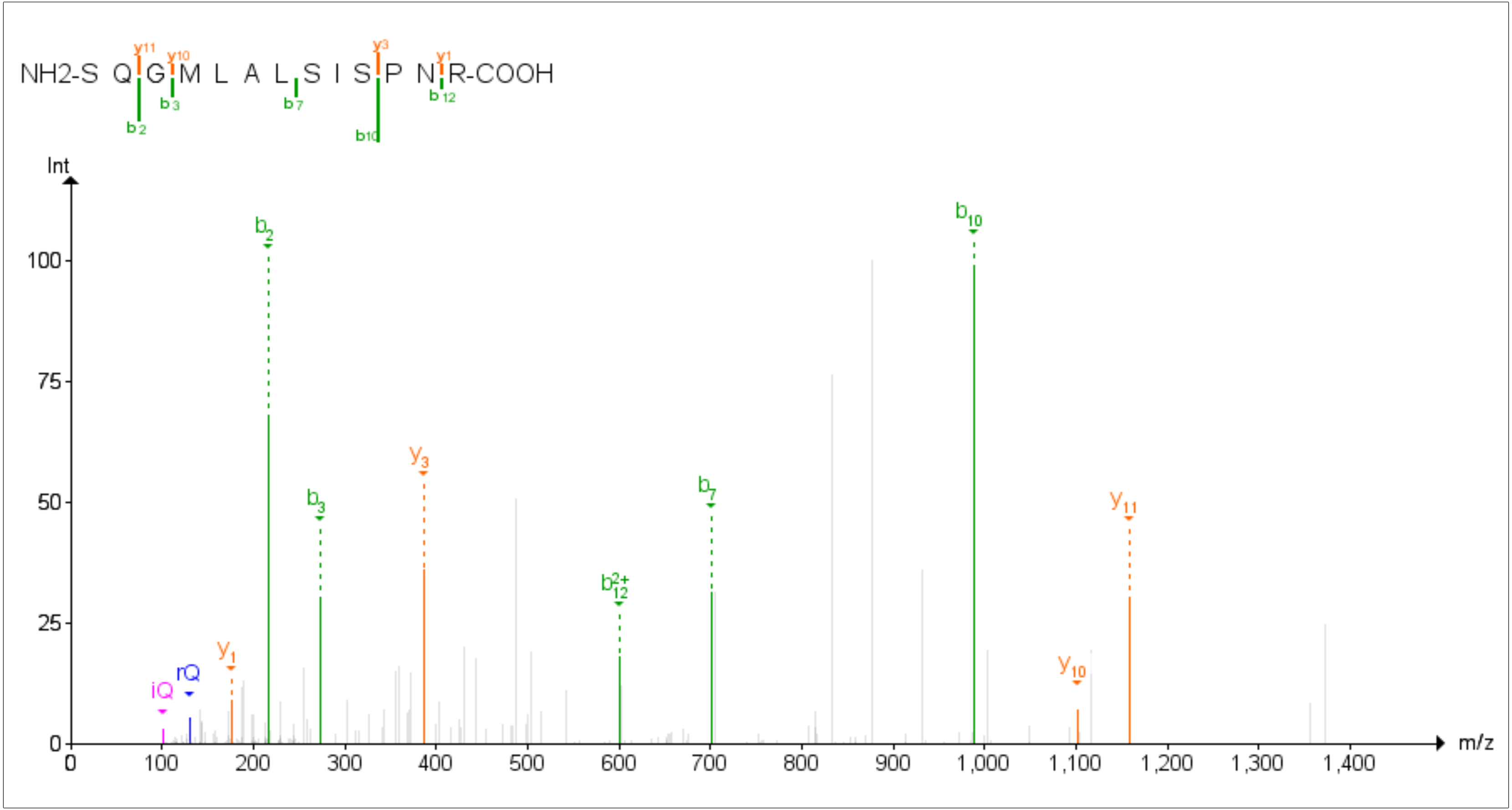

NH2-S Q G M L A L S I S P N R-COOH
y11
y10
y3
y1
b2
b3
b7
b10
b12
Int
m/z
b2
b10
y3
b3
b7
b12 2+
y11
y10
y1
rQ
iQ
0
25
50
75
100
0
100
200
300
400
500
600
700
800
900
1,000
1,100
1,200
1,300
1,400

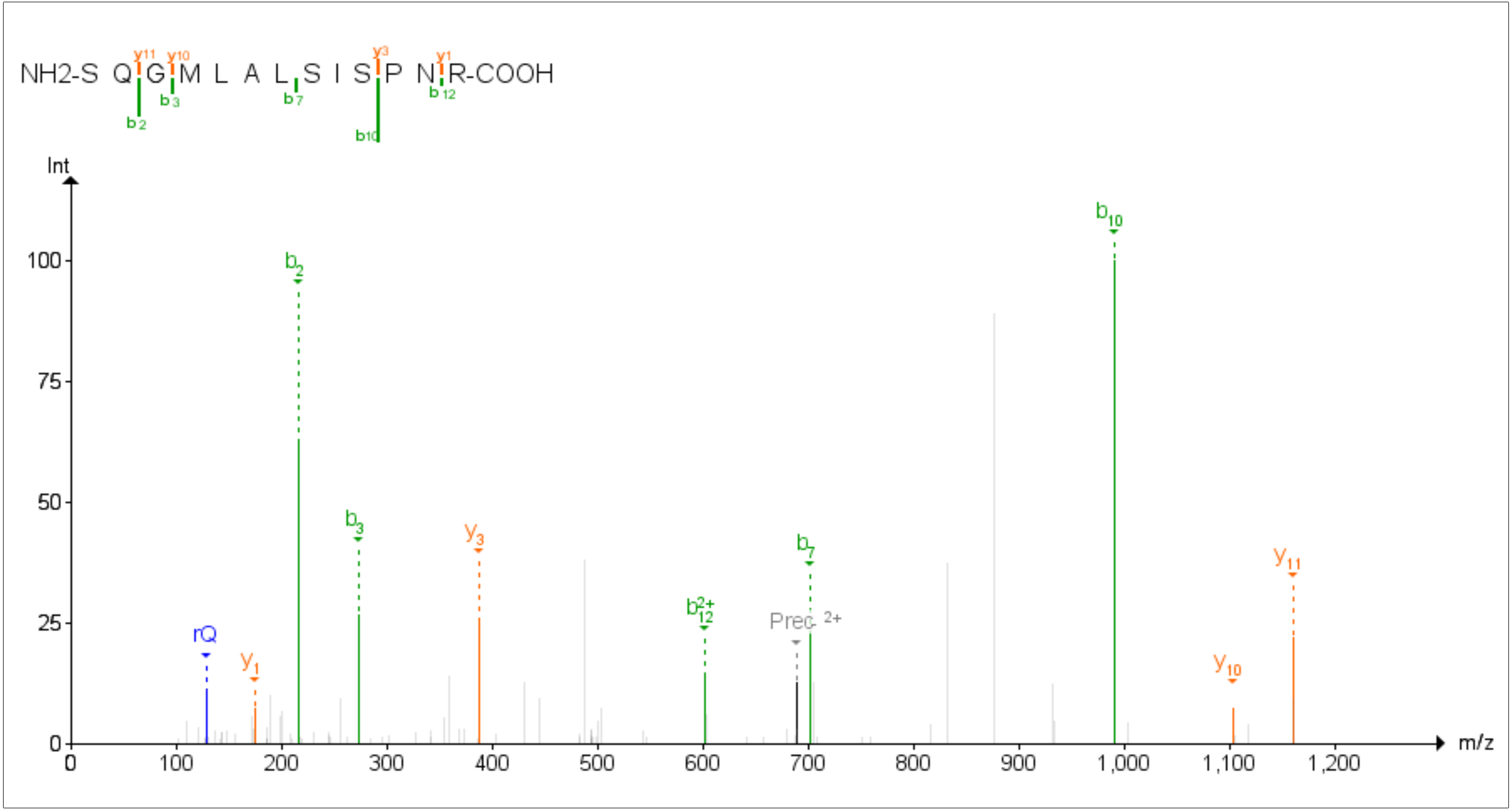

NH2-S Q G M L A L S I S P N R-COOH
y11 y10 y3 y1
b2 b3 b7 b10 b12
Int
100
75
50
25
0
b2
b3
y3
b10
rQ
y1
b12 2+
Prec 2+
b7
y10
y11
0 100 200 300 400 500 600 700 800 900 1,000 1,100 1,200
m/z

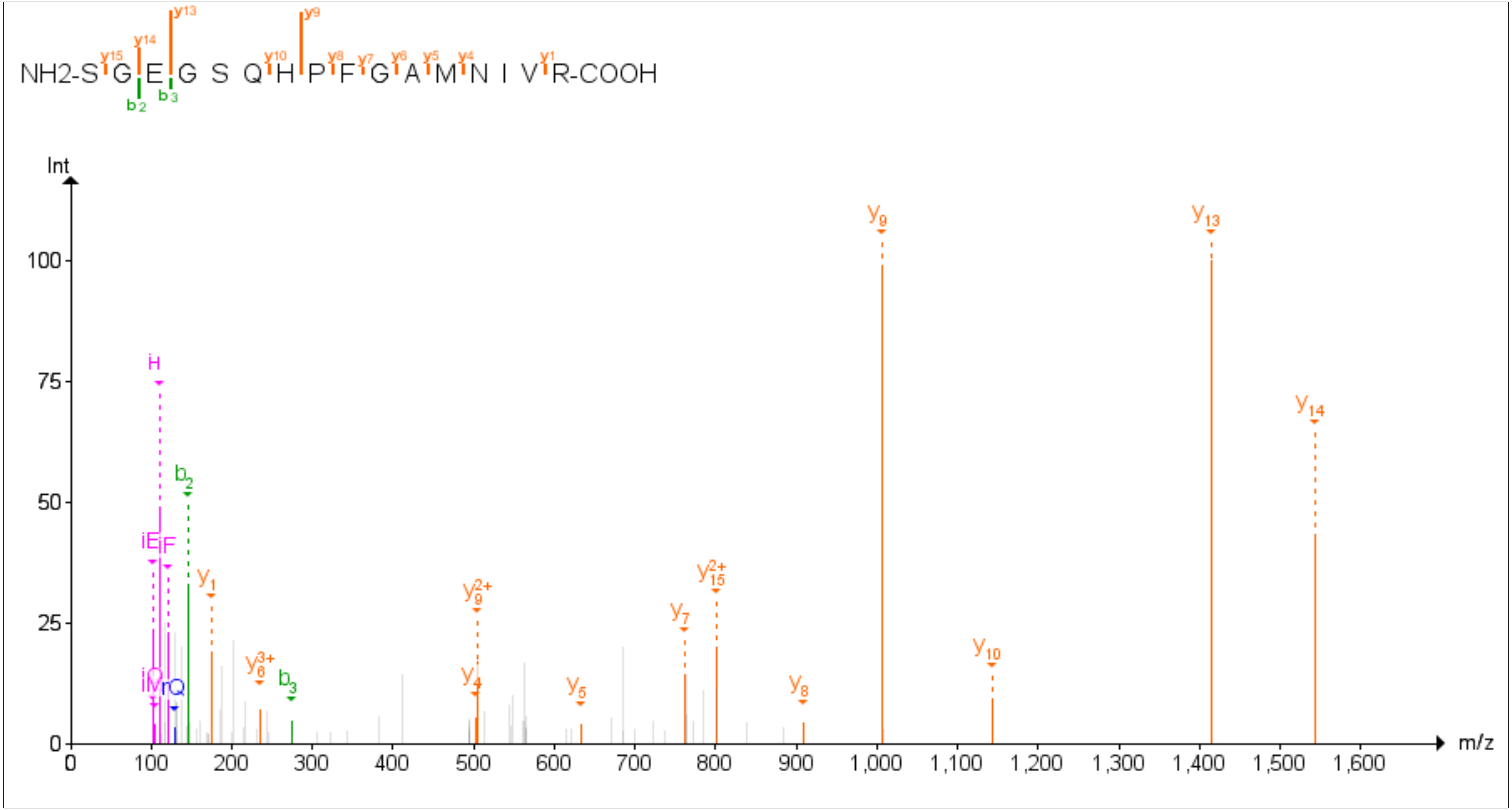

NH2-S G E G S Q H P F G A M N I V R-COOH
y15 y14 y13 y10 y9 y8 y7 y6 y5 y4 y1
b2 b3
Int
m/z
0
25
50
75
100
0
100
200
300
400
500
600
700
800
900
1,000
1,100
1,200
1,300
1,400
1,500
1,600
iH
b2
iE
iF
y1
iQ
iM
rQ
y6 3+
b3
y9 2+
y4
y5
y7
y15 2+
y8
y9
y10
y13
y14

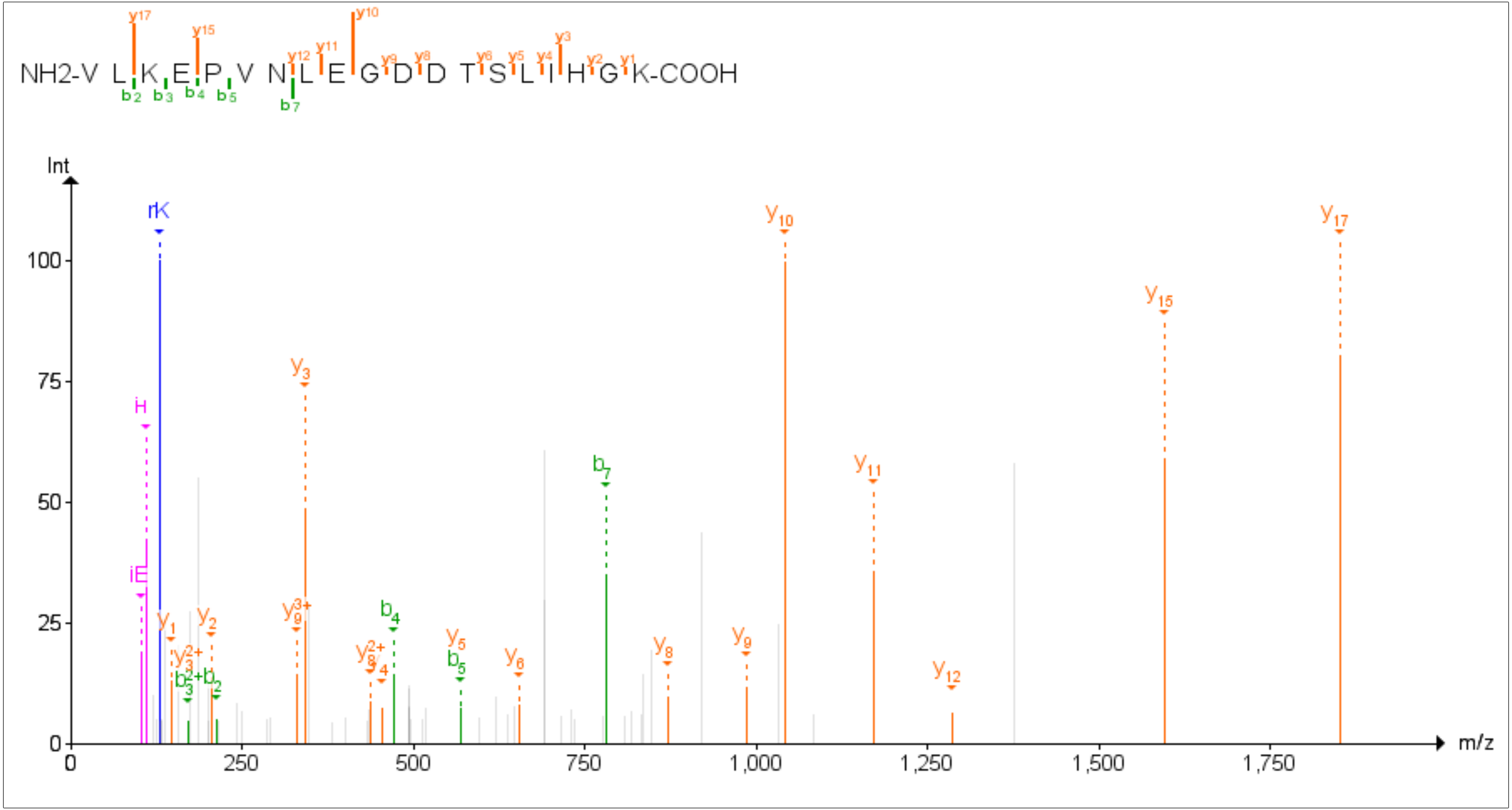

NH2-V L K E P V N L E G D D T S L I H G K-COOH
y17 y15 y12 y11 y10 y9 y8 y6 y5 y4 y3 y2 y1
b2 b3 b4 b5 b7
Int
m/z
0
25
50
75
100
250
500
750
1,000
1,250
1,500
1,750
rK
iH
iE
y1
y2
y3
y9 3+
y3 2+
b3 2+
b2
y8 2+
y4
b4
y5
b5
y6
b7
y8
y9
y10
y11
y12
y15
y17

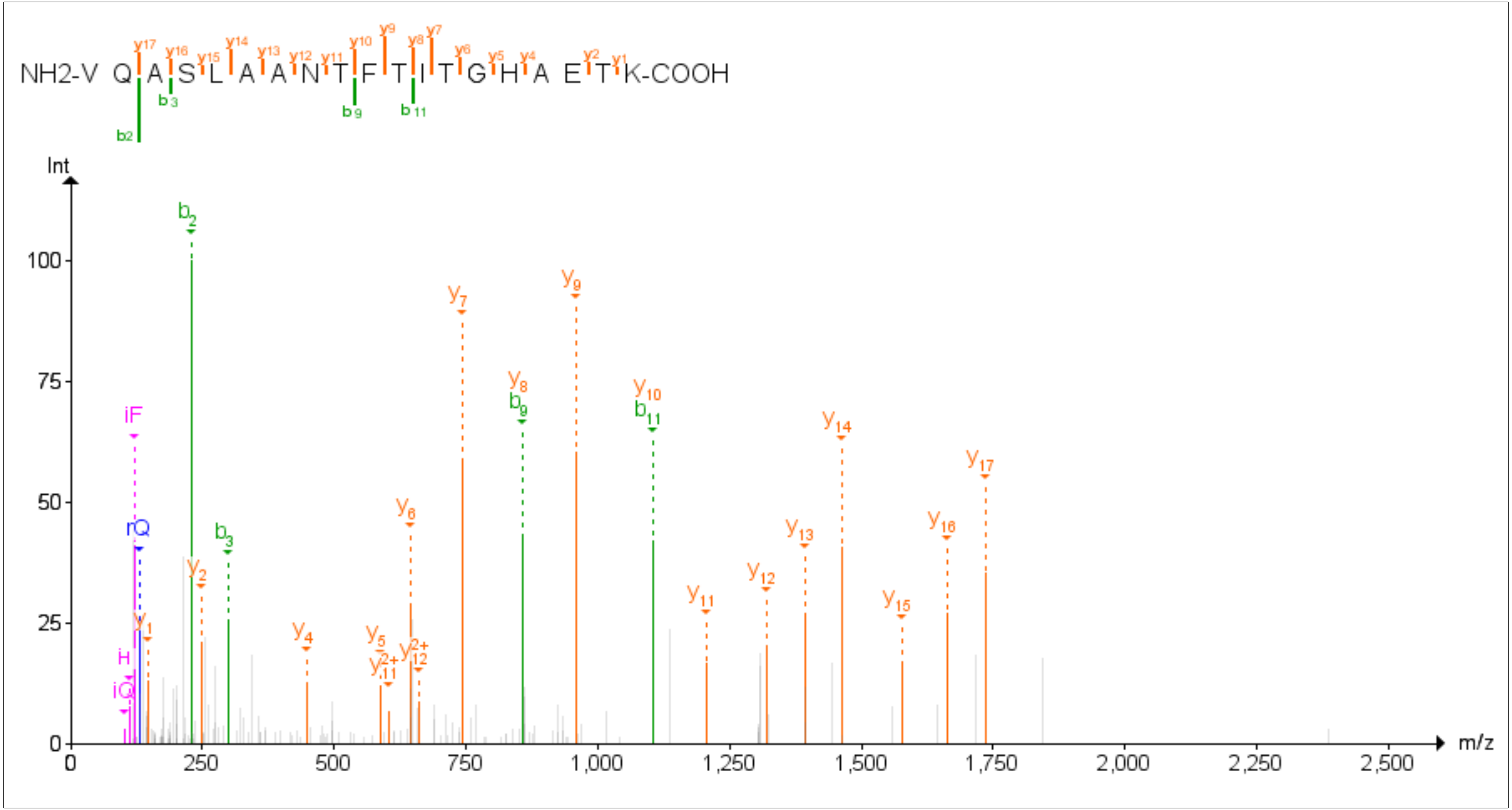

NH2-V Q A S L A A N T F T I T G H A E T K-COOH
Int
m/z
b2
b3
b9
b11
y1
y2
y4
y5
y6
y7
y8
y9
y10
y11
y12
y13
y14
y15
y16
y17
y11 2+
y12 2+
iF
rQ
iH
iQ
0
25
50
75
100
0
250
500
750
1,000
1,250
1,500
1,750
2,000
2,250
2,500

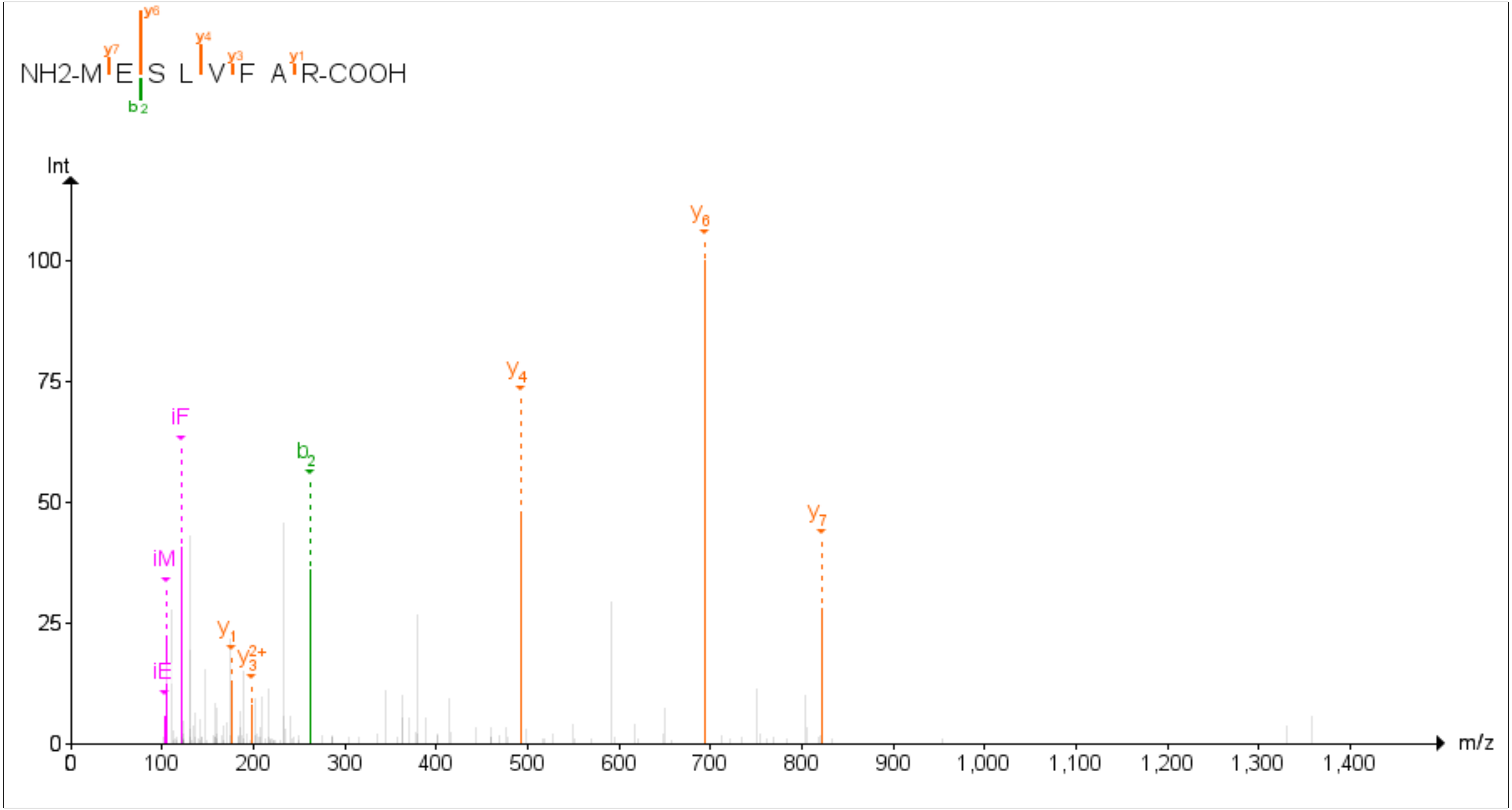

NH2-M E S L V F A R-COOH
y7
y6
y4
y3
y1
b2
Int
100
75
50
25
0
iF
iM
iE
b2
y1
y3 2+
y4
y6
y7
0
100
200
300
400
500
600
700
800
900
1,000
1,100
1,200
1,300
1,400
m/z

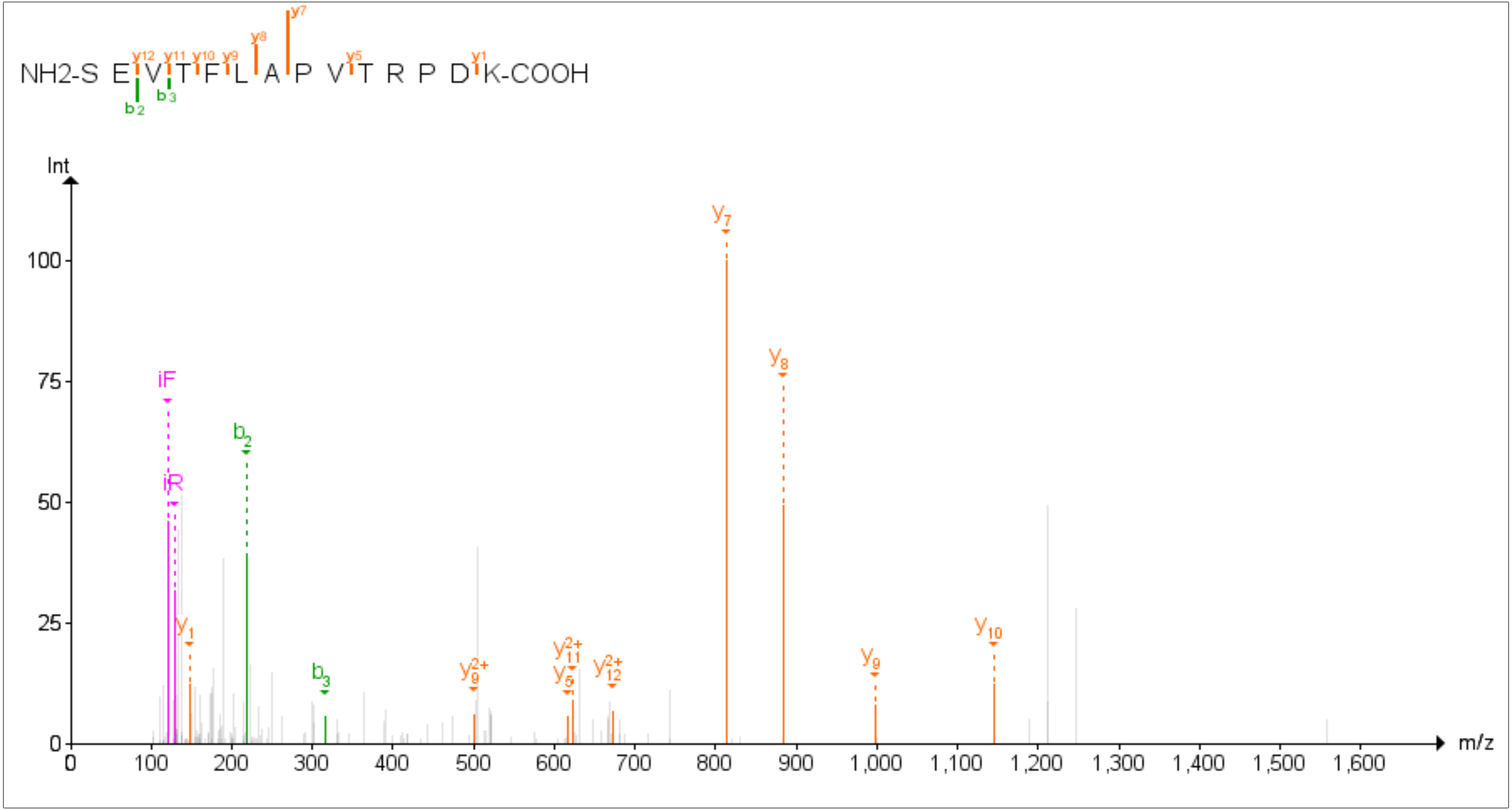
NH2-S E V T F L A P V T R P D K-COOH
y12 y11 y10 y9 y8 y7 y5 y1
b2 b3
Int
m/z
y7
y8
iF
b2
iR
y1
b3
y9 2+
y11 2+
y5
y12 2+
y9
y10
0
25
50
75
100
0
100
200
300
400
500
600
700
800
900
1,000
1,100
1,200
1,300
1,400
1,500
1,600

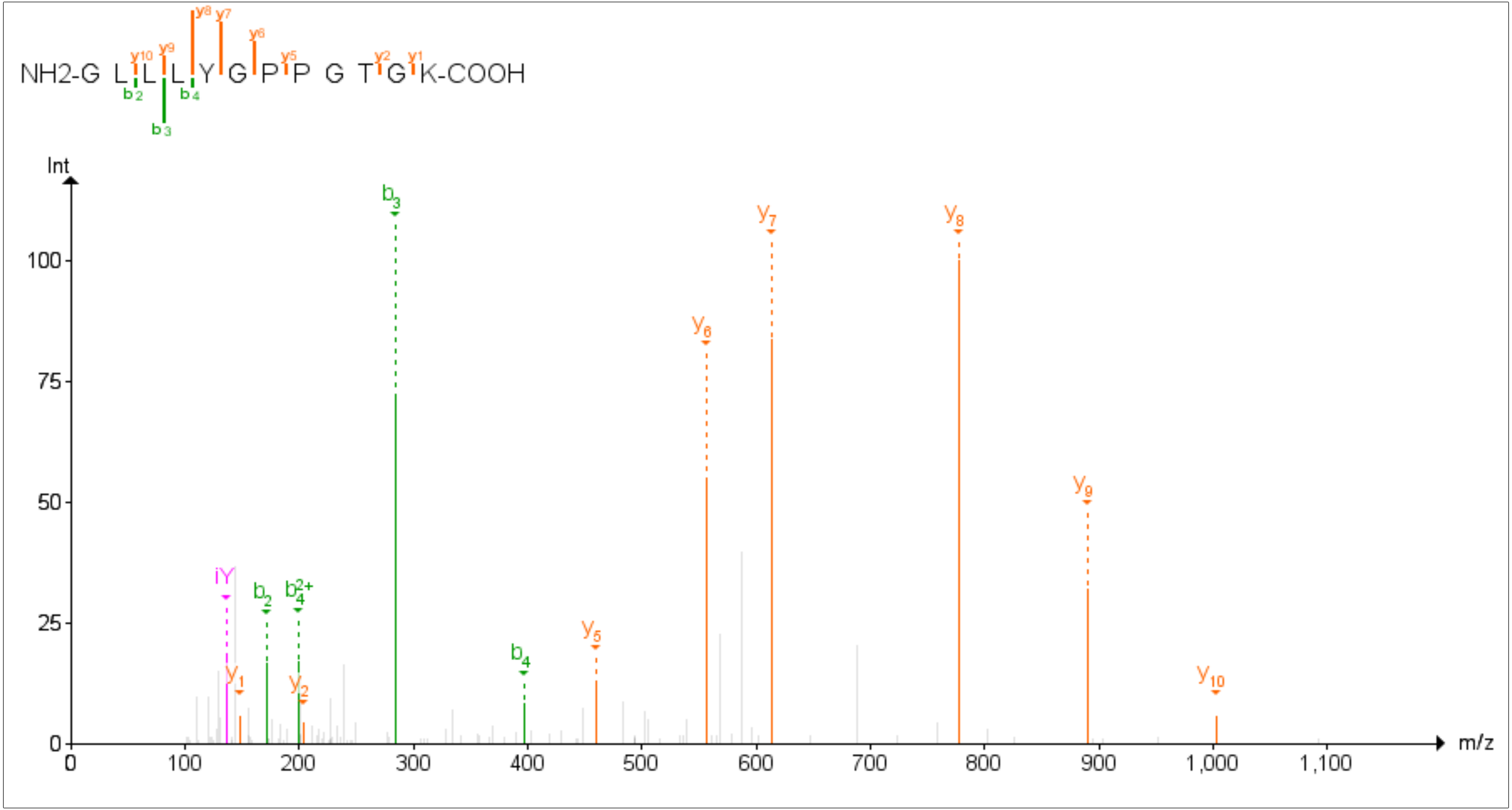

NH2-G L L L Y G P P G T G K-COOH
y10 y9 y8 y7 y6 y5 y2 y1
b2 b3 b4
Int
100
75
50
25
0
b3
y7
y8
y6
y9
iY
b2
b_4^{2+}
y1
y2
b4
y5
y10
0
100
200
300
400
500
600
700
800
900
1,000
1,100
m/z

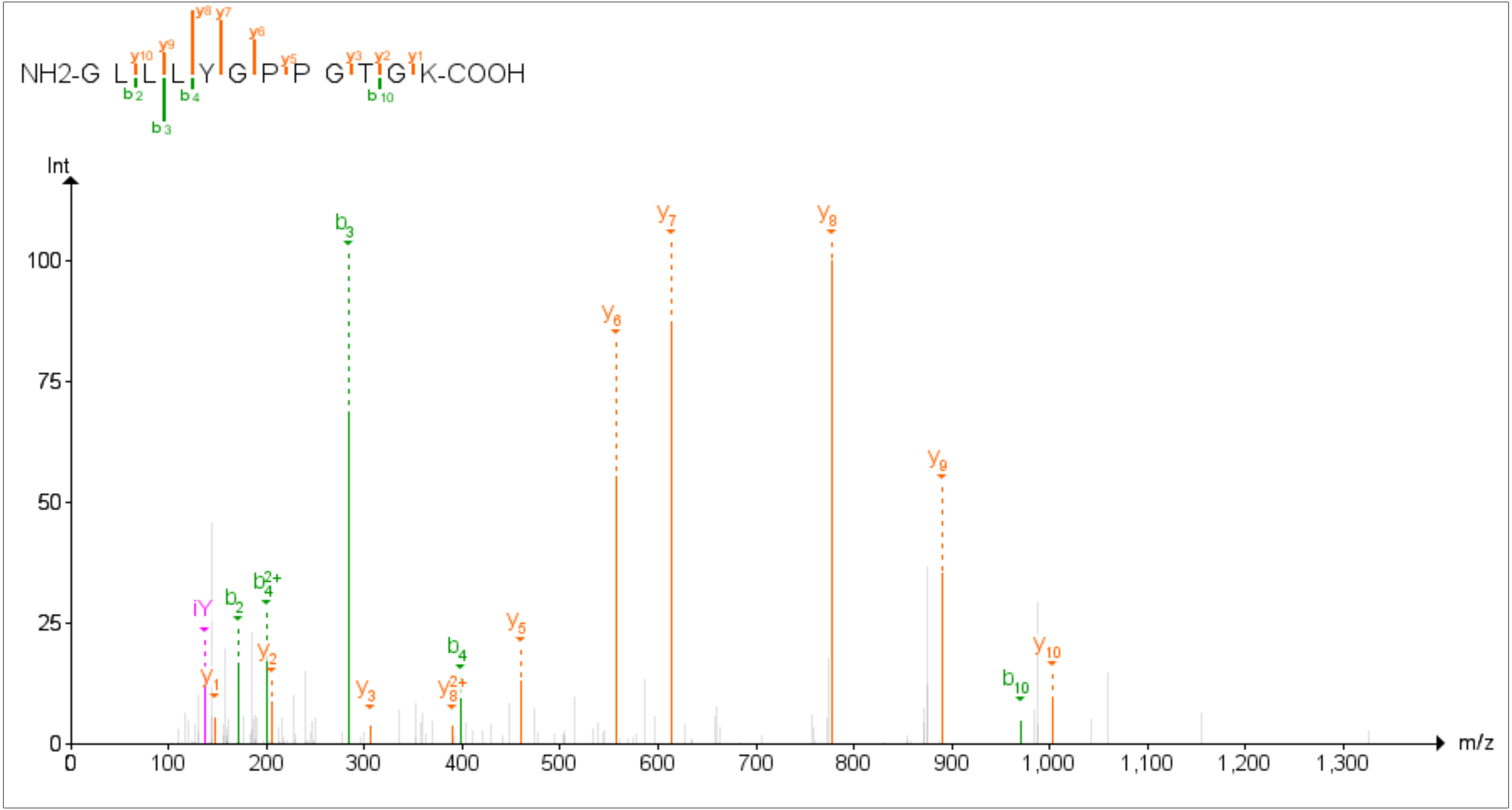
NH2-G L L L Y G P P G T G K-COOH
y10 y9 y8 y7 y6 y5 y3 y2 y1
b2 b3 b4 b10
Int
m/z
b3
y7
y8
y6
y9
iY
b2
b_4^{2+}
y2
y1
y3
y_8^{2+}
b4
y5
b10
y10
0
25
50
75
100
0
100
200
300
400
500
600
700
800
900
1,000
1,100
1,200
1,300

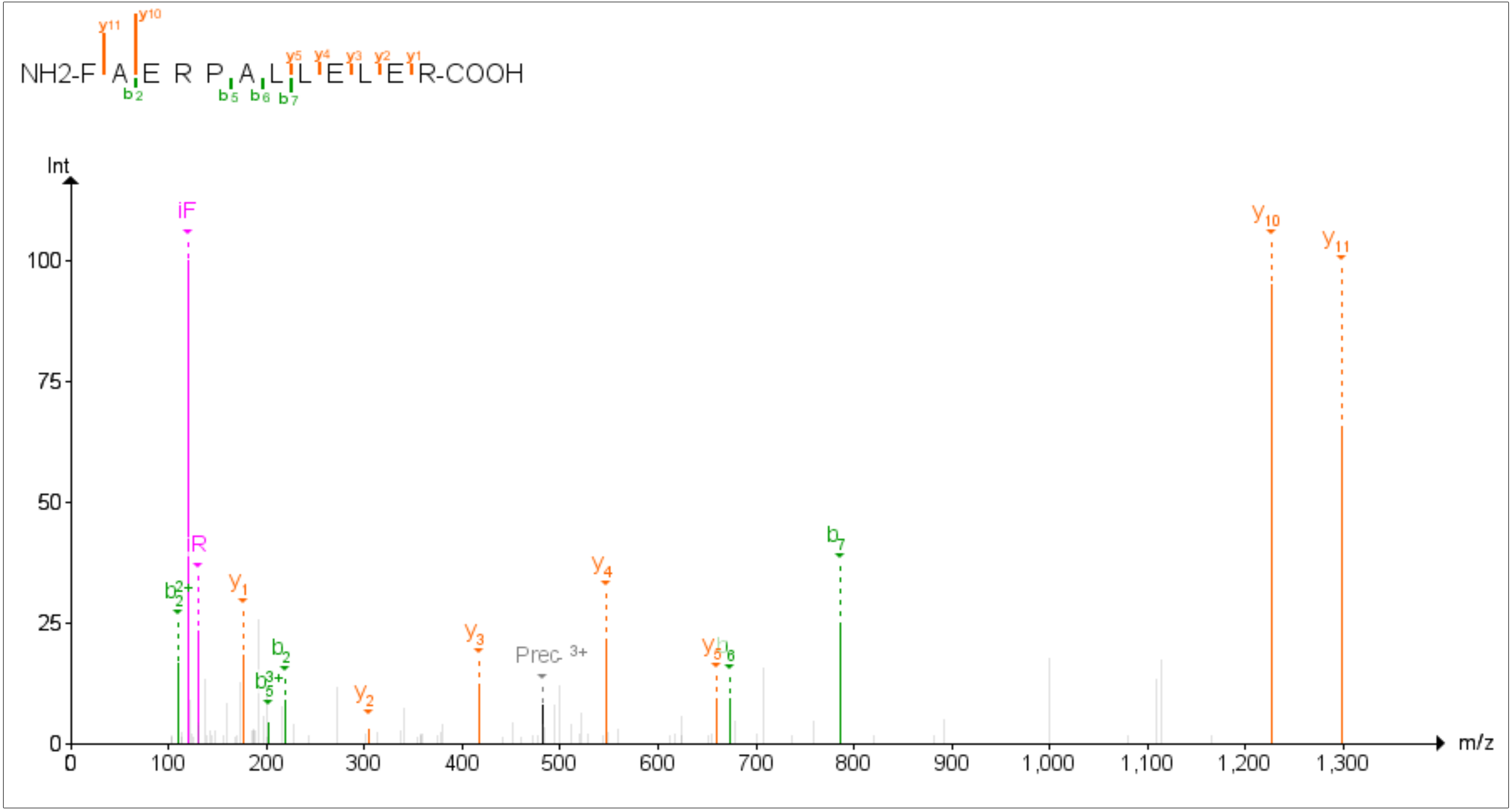

NH2-F A E R P A L L E L E R-COOH
y11
y10
y5
y4
y3
y2
y1
b2
b5
b6
b7
Int
iF
iR
y10
y11
y1
y2
y3
y4
y5
b7
b2
Prec. 3+
0
25
50
75
100
0
100
200
300
400
500
600
700
800
900
1,000
1,100
1,200
1,300
m/z

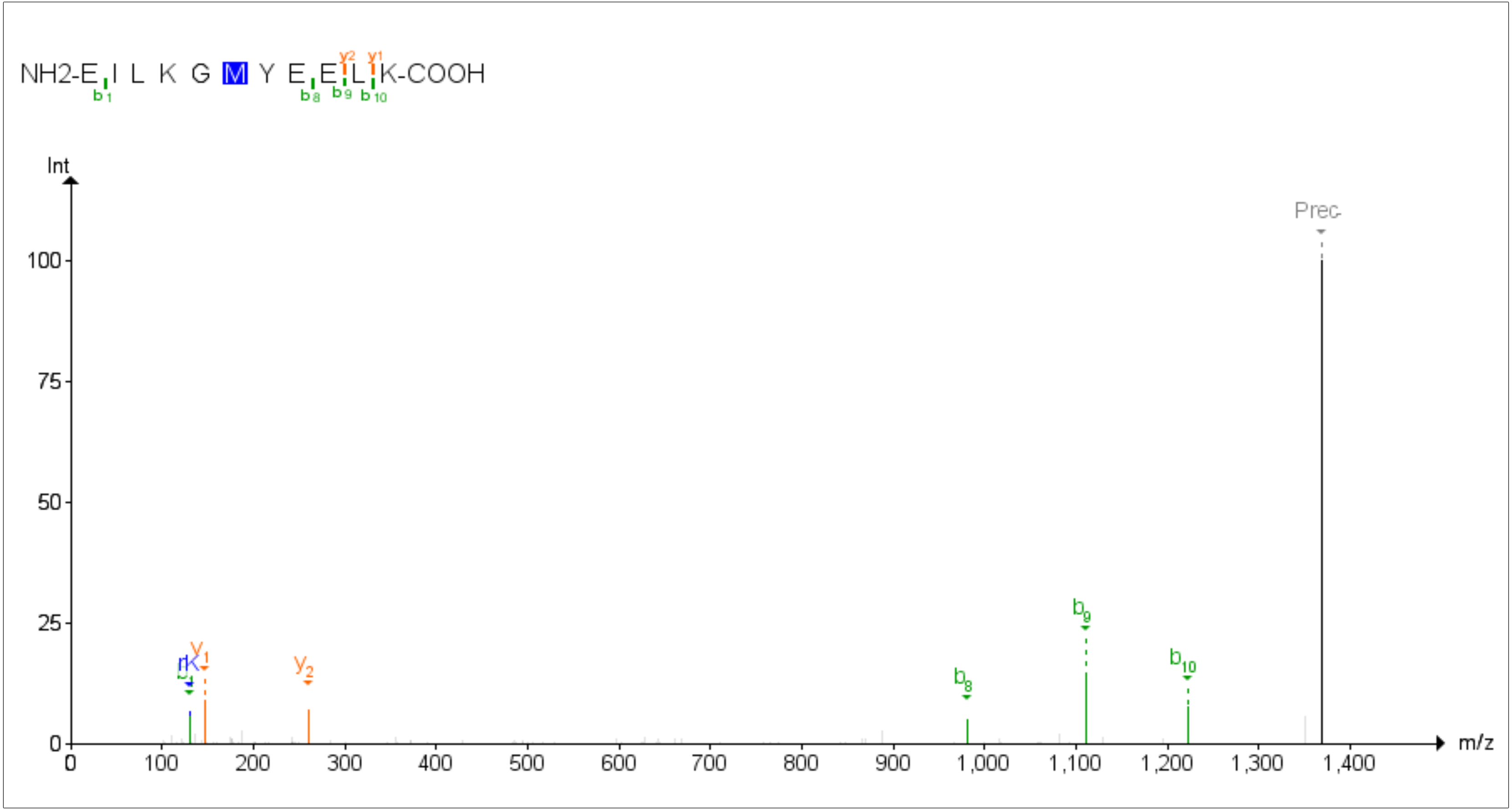

NH2-E I L K G M Y E E L K-COOH
b1
b8
b9
b10
y2
y1
Int
100
75
50
25
0
Prec
b9
b10
b8
y1
y2
b1
m/z
0
100
200
300
400
500
600
700
800
900
1,000
1,100
1,200
1,300
1,400

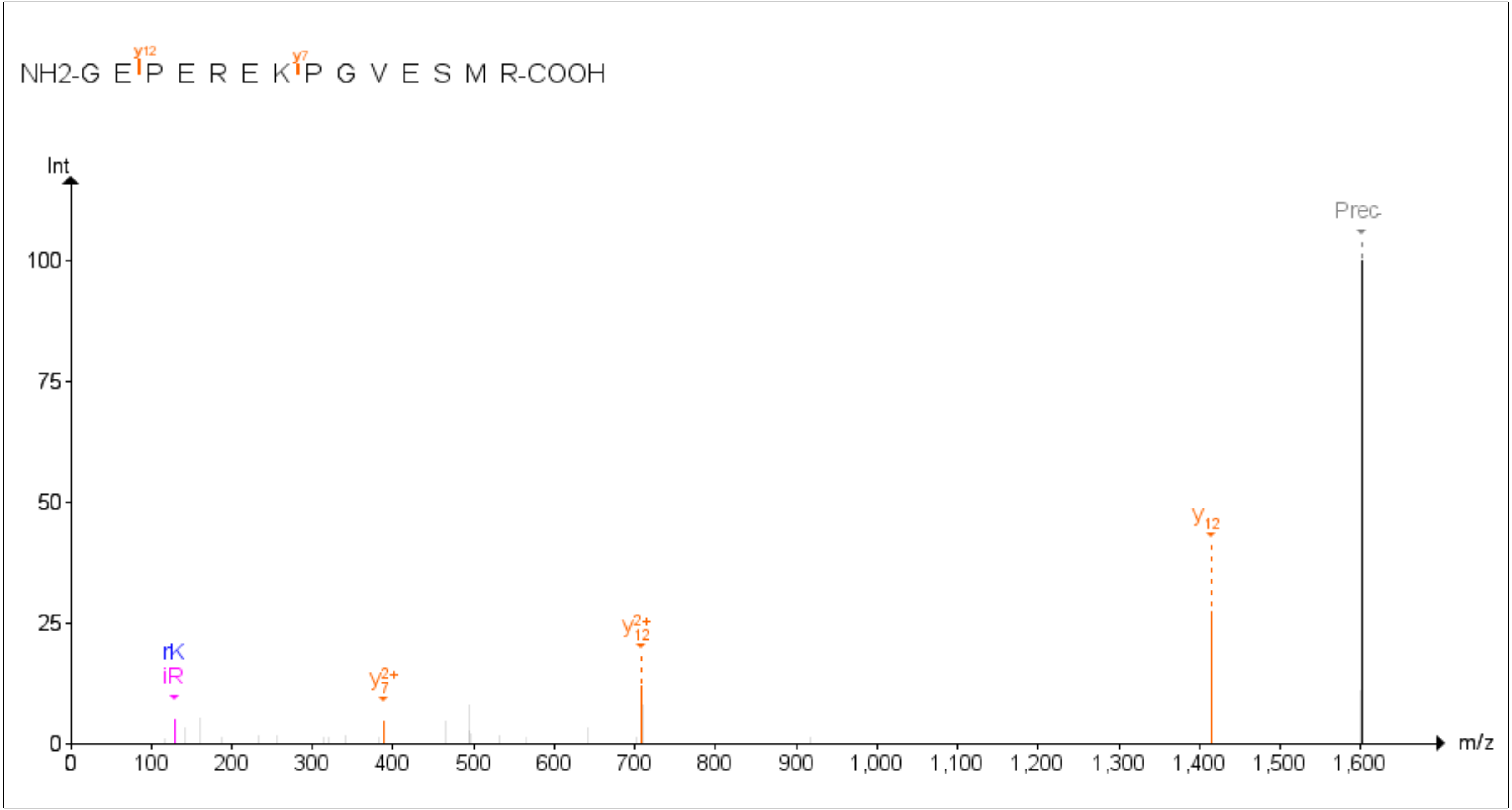

NH2-G E P E R E K P G V E S M R-COOH
y12
y7
Int
100
75
50
25
0
Prec
y12
y12 2+
y7 2+
rK
iR
0
100
200
300
400
500
600
700
800
900
1,000
1,100
1,200
1,300
1,400
1,500
1,600
m/z

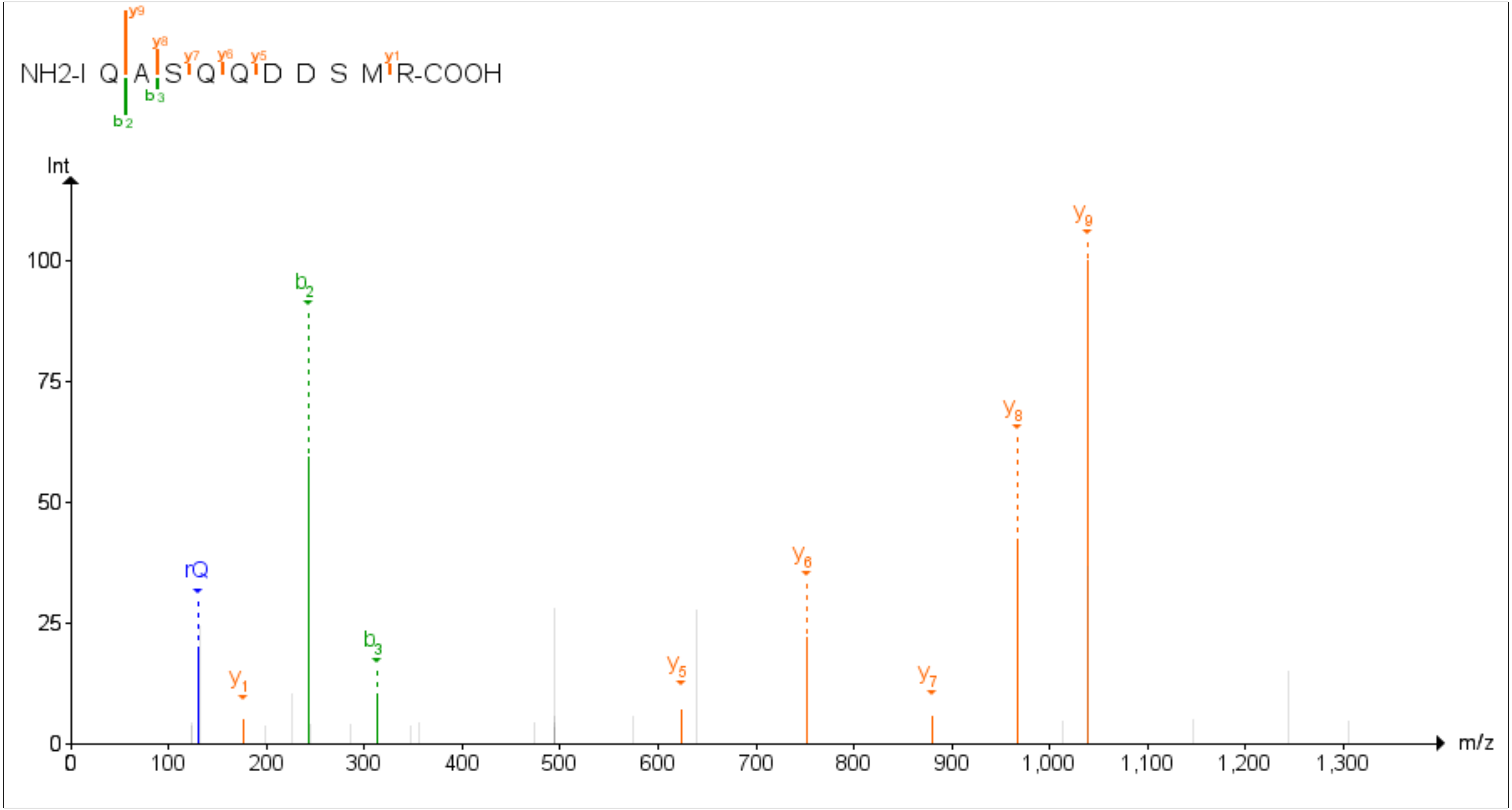

NH2-I Q A S Q Q D D S M R-COOH
y9
y8
y7
y6
y5
y1
b 2
b 3
Int
100
75
50
25
0
b2
b3
rQ
y1
y5
y6
y7
y8
y9
0
100
200
300
400
500
600
700
800
900
1,000
1,100
1,200
1,300
m/z

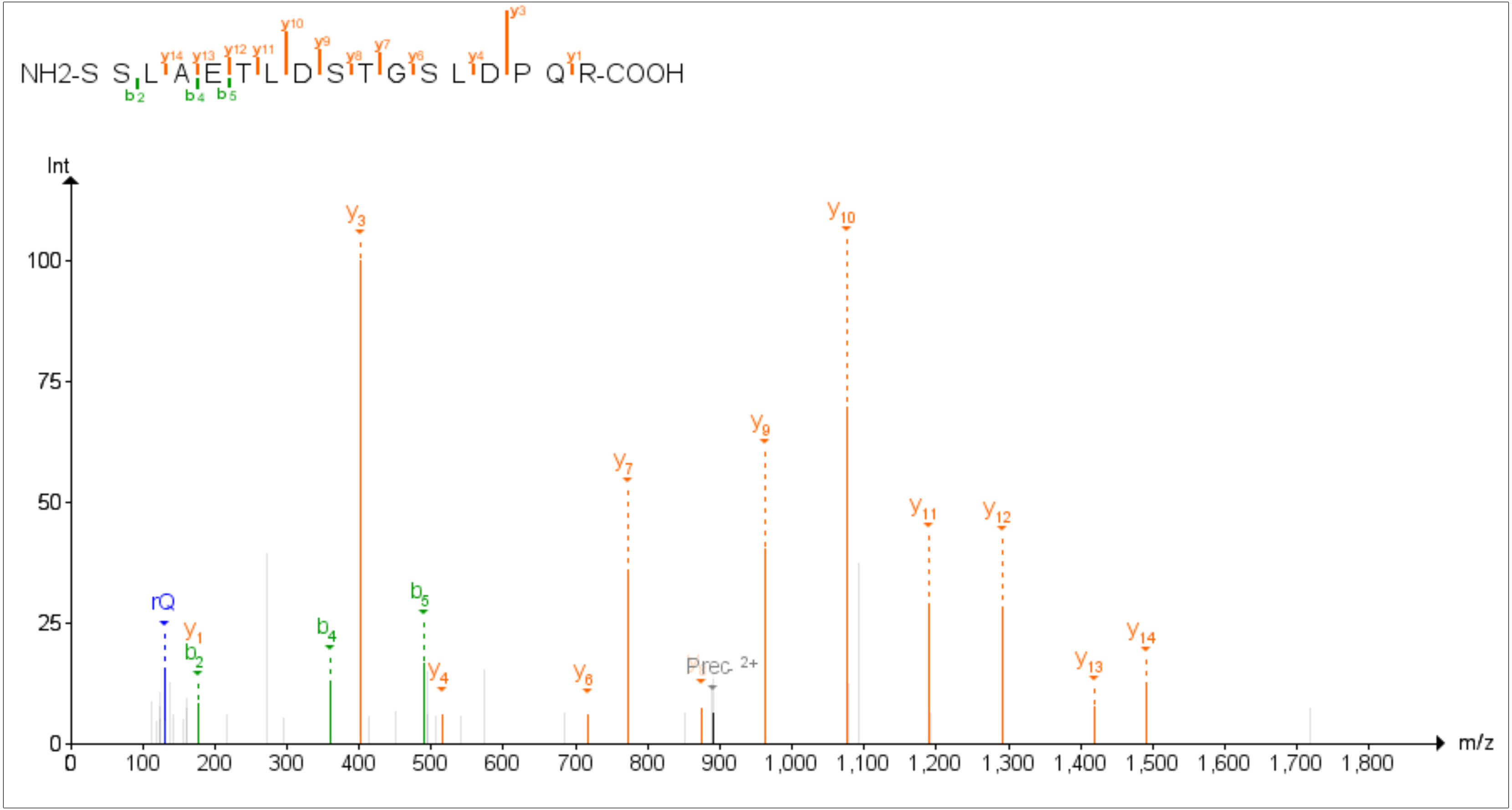

NH2-S S L A E T L D S T G S L D P Q R-COOH
y14 y13 y12 y11 y10 y9 y8 y7 y6 y4 y3 y1
b2 b4 b5
Int
m/z
0 25 50 75 100
0 100 200 300 400 500 600 700 800 900 1,000 1,100 1,200 1,300 1,400 1,500 1,600 1,700 1,800
rQ
y1
b2
b4
y3
b5
y4
y6
y7
Prec 2+
y9
y10
y11
y12
y13
y14

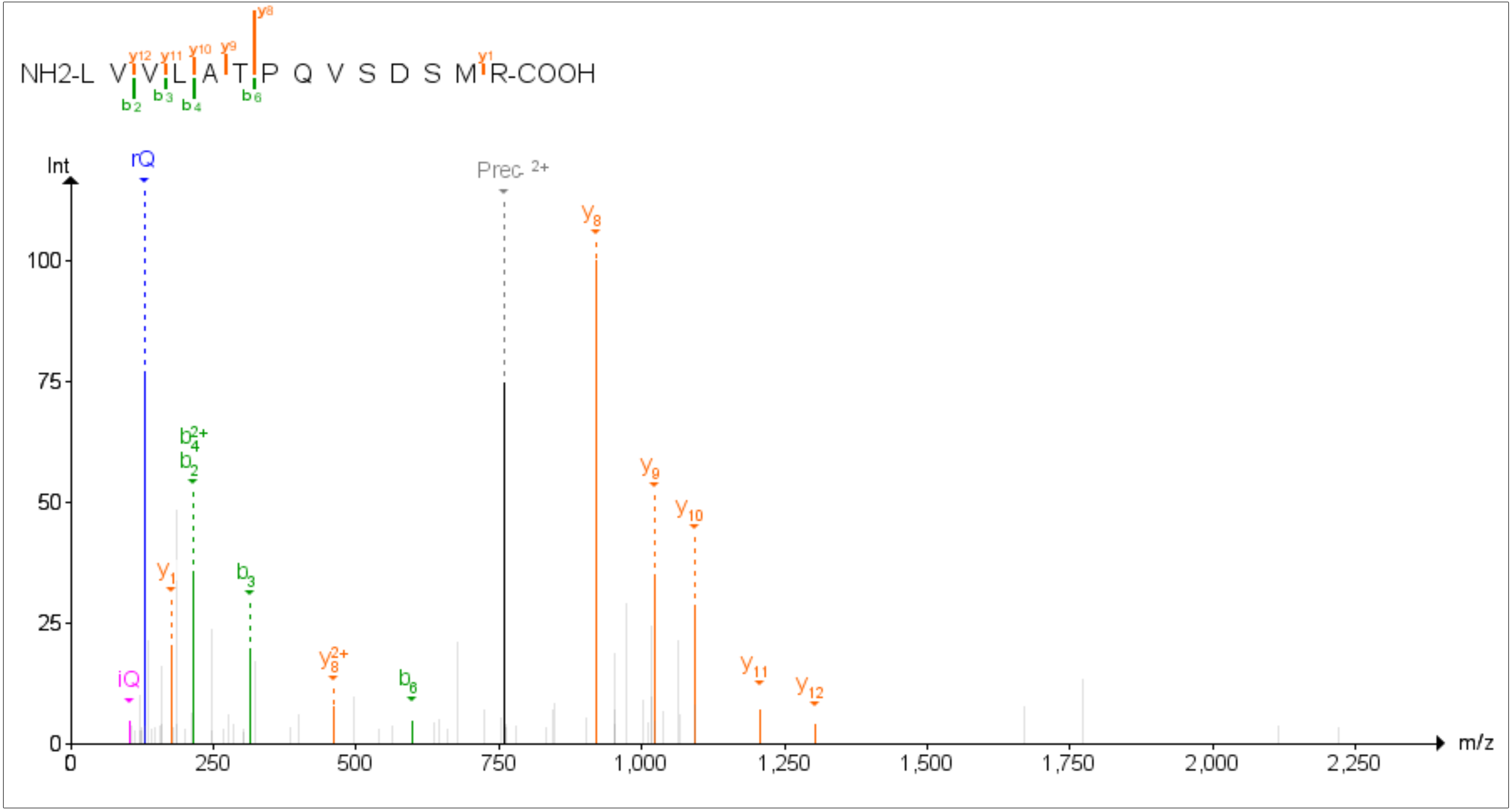

NH2-L V V L A T P Q V S D S M R-COOH
y12 y11 y10 y9 y8 y1
b2 b3 b4 b6
Int
rQ
Prec 2+
y8
100
75
50
25
0
b4 2+
b2
y9
y10
y1
b3
y8 2+
b6
iQ
y11
y12
0
250
500
750
1,000
1,250
1,500
1,750
2,000
2,250
m/z

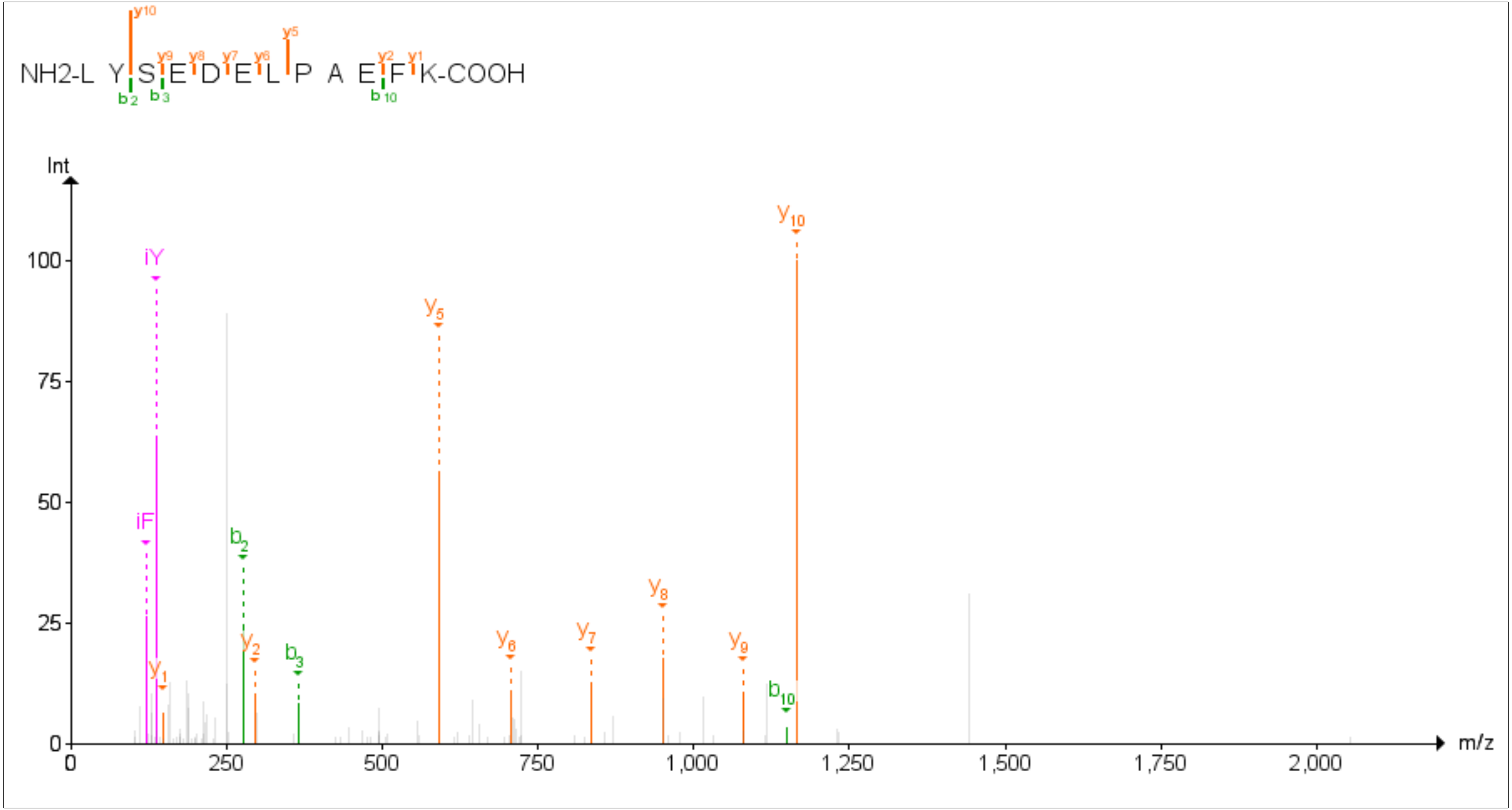

NH2-L Y S E D E L P A E F K-COOH
y10
y9
y8
y7
y6
y5
y2
y1
b2
b3
b10
Int
iY
iF
100
75
50
25
0
m/z
250
500
750
1,000
1,250
1,500
1,750
2,000

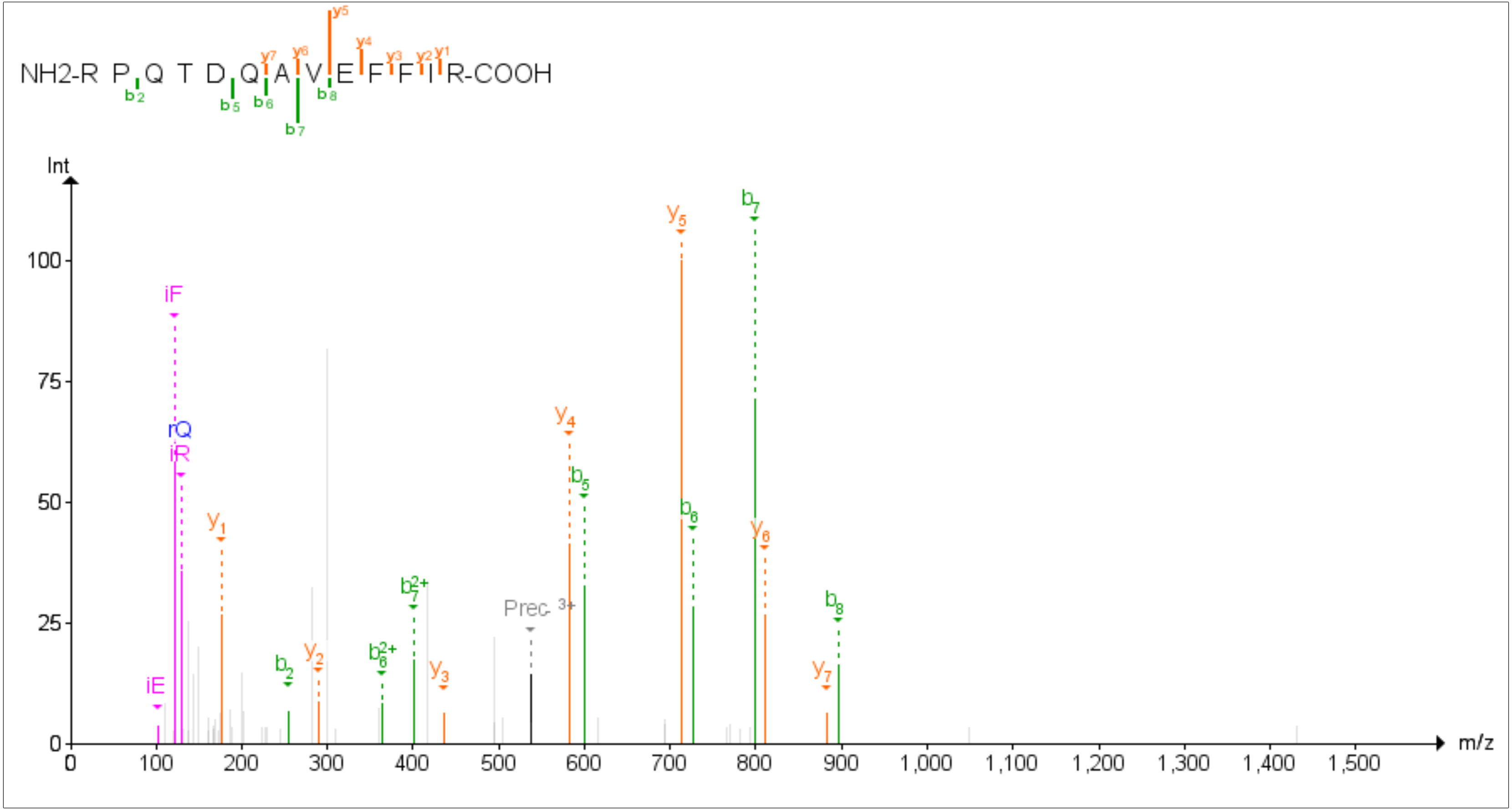
NH2-R P Q T D Q A V E F F I R-COOH
b2 b5 b6 b7 b8
y7 y6 y5 y4 y3 y2 y1
Int
m/z
0
25
50
75
100
0 100 200 300 400 500 600 700 800 900 1,000 1,100 1,200 1,300 1,400 1,500
iF
rQ
iR
iE
y1
b2
y2
b6 2+
b7 2+
y3
Prec 3+
y4
b5
y5
b6
b7
y6
y7
b8

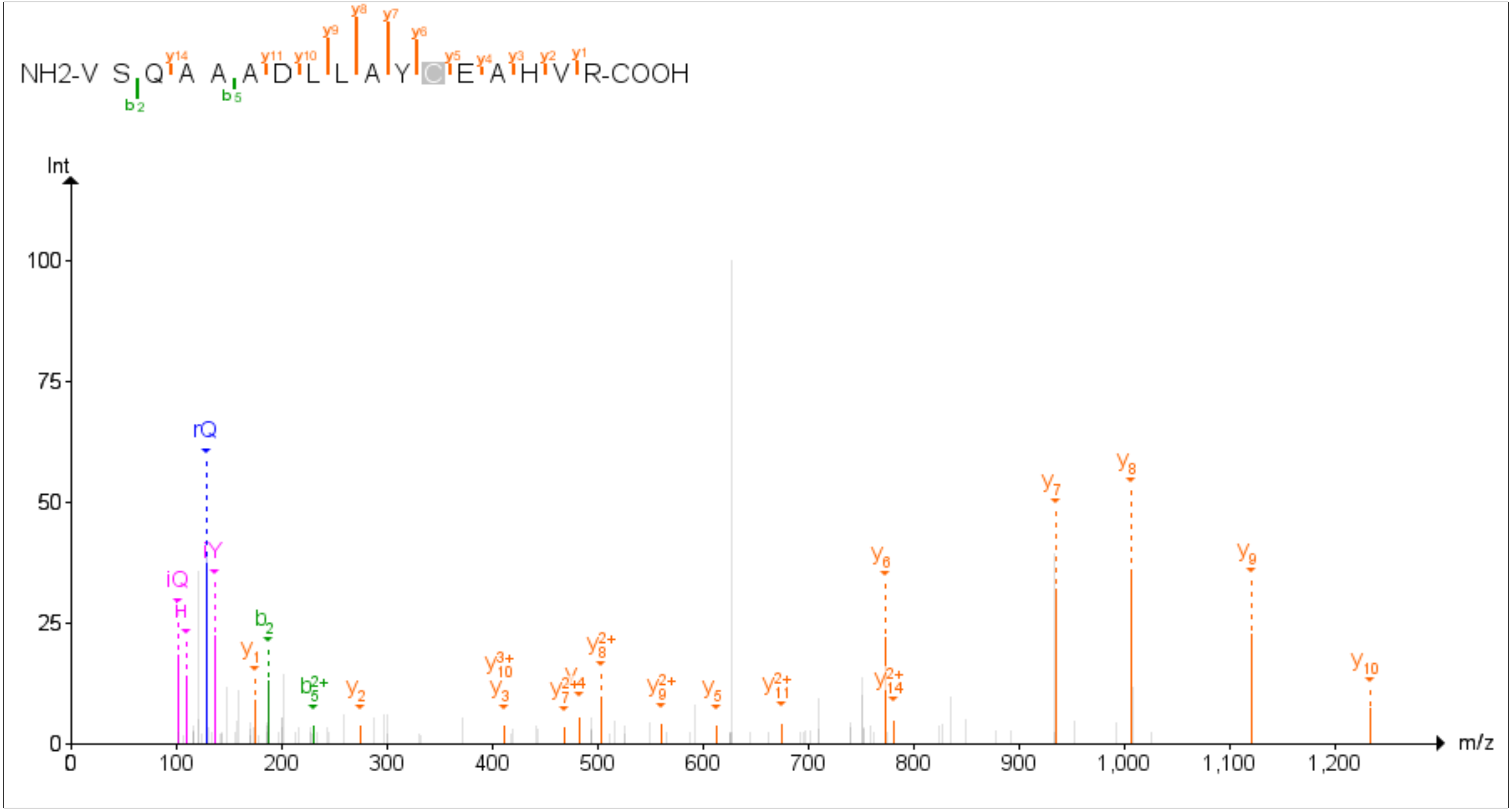

NH2-V S Q A A A D L L A Y C E A H V R-COOH
Int
100
75
50
25
0
m/z
0
100
200
300
400
500
600
700
800
900
1,000
1,100
1,200
rQ
iQ
iY
H
b2
b5 2+
y1
y2
y3
y10 3+
y7 2+
y8 2+
y9 2+
y5
y11 2+
y6
y14 2+
y7
y8
y9
y10

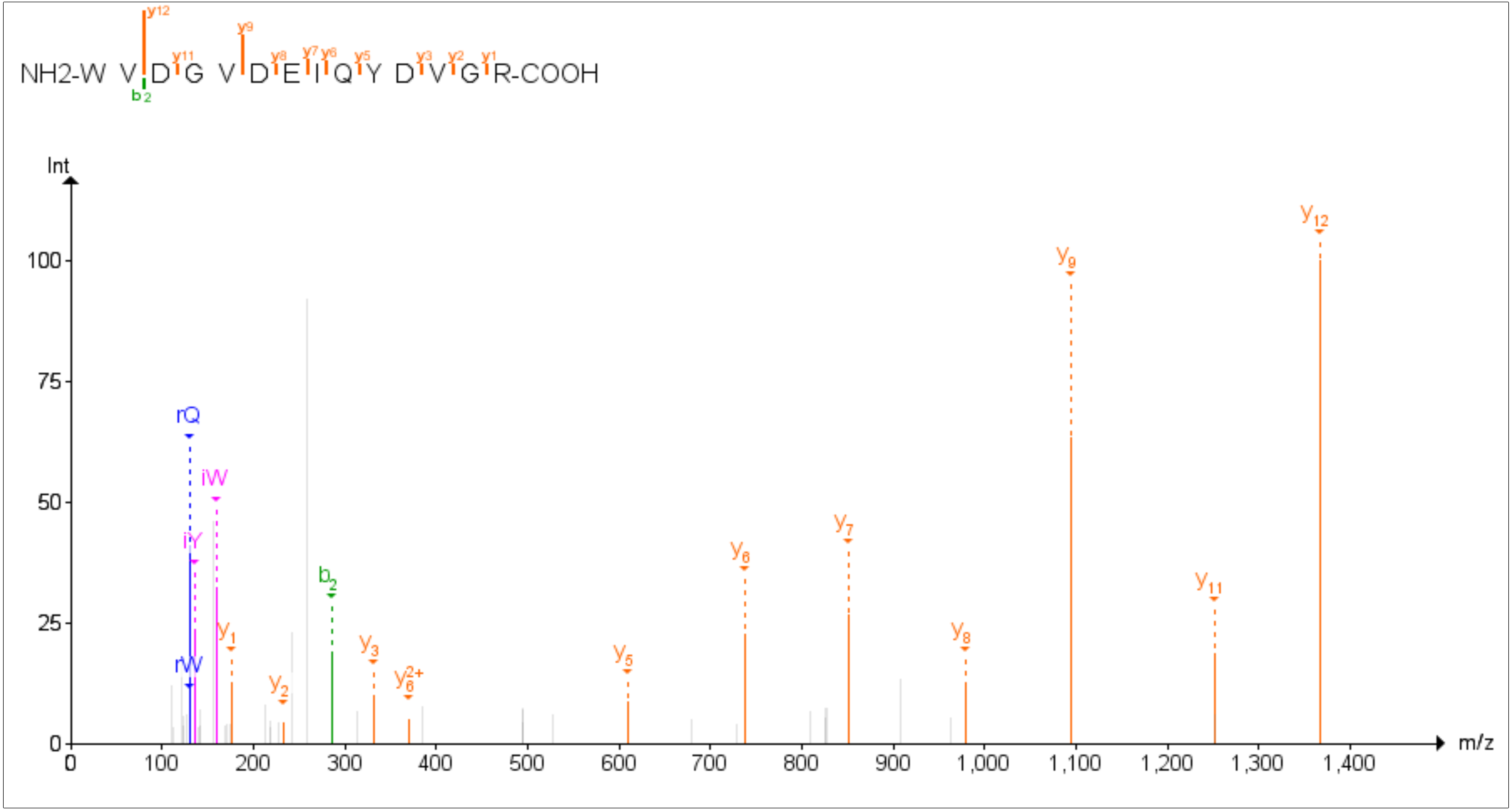

NH2-W V D G V D E I Q Y D V G R-COOH
y12
y11
y9
y8
y7
y6
y5
y3
y2
y1
b2
Int
m/z
0
25
50
75
100
0
100
200
300
400
500
600
700
800
900
1,000
1,100
1,200
1,300
1,400
rQ
iW
iY
rW
y1
y2
b2
y3
y6 2+
y5
y6
y7
y8
y9
y11
y12

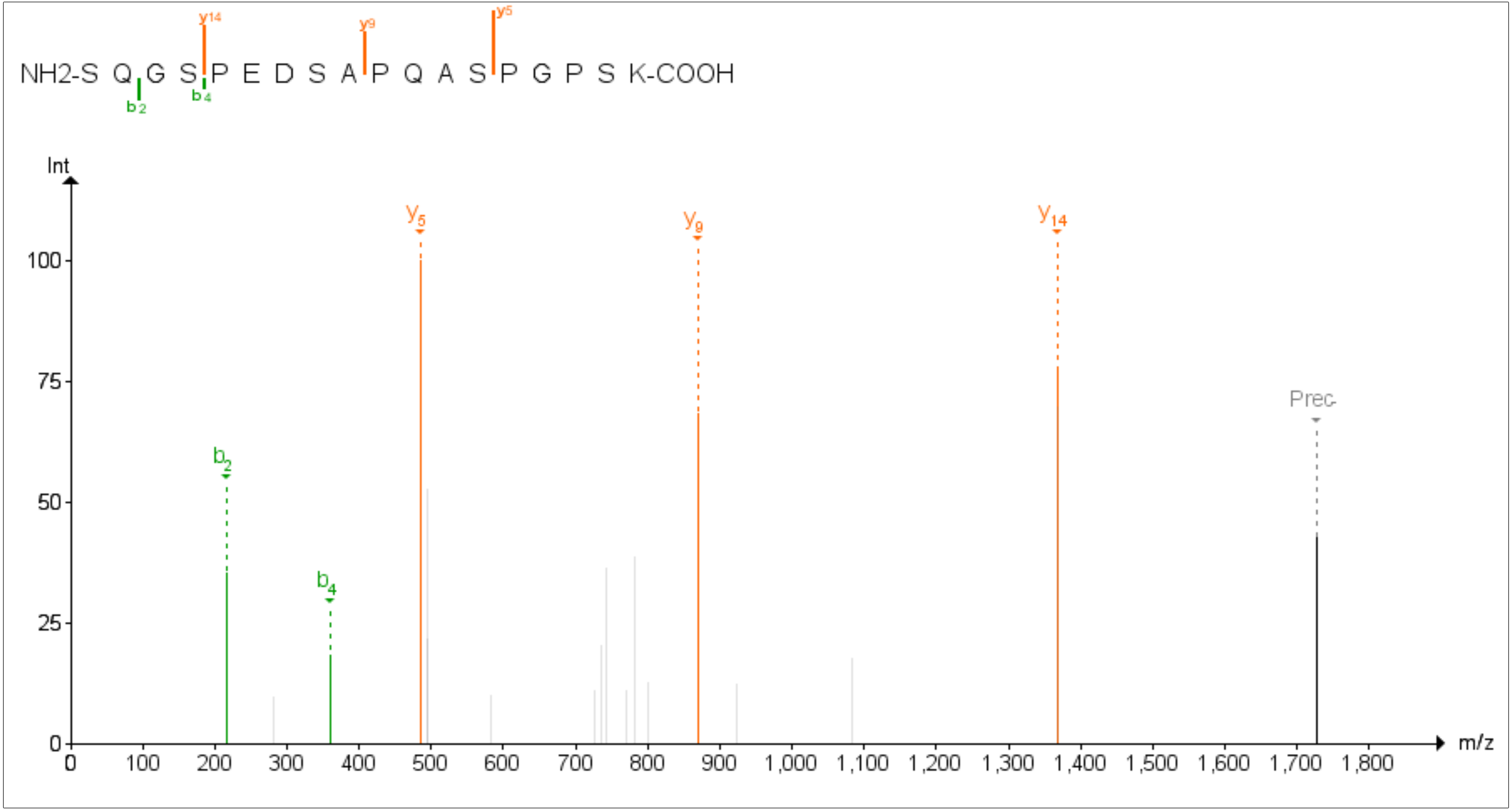

NH2-S Q G S P E D S A P Q A S P G P S K-COOH
y14
y9
y5
b2
b4
Int
100
75
50
25
0
y5
y9
y14
b2
b4
Prec
0
100
200
300
400
500
600
700
800
900
1,000
1,100
1,200
1,300
1,400
1,500
1,600
1,700
1,800
m/z

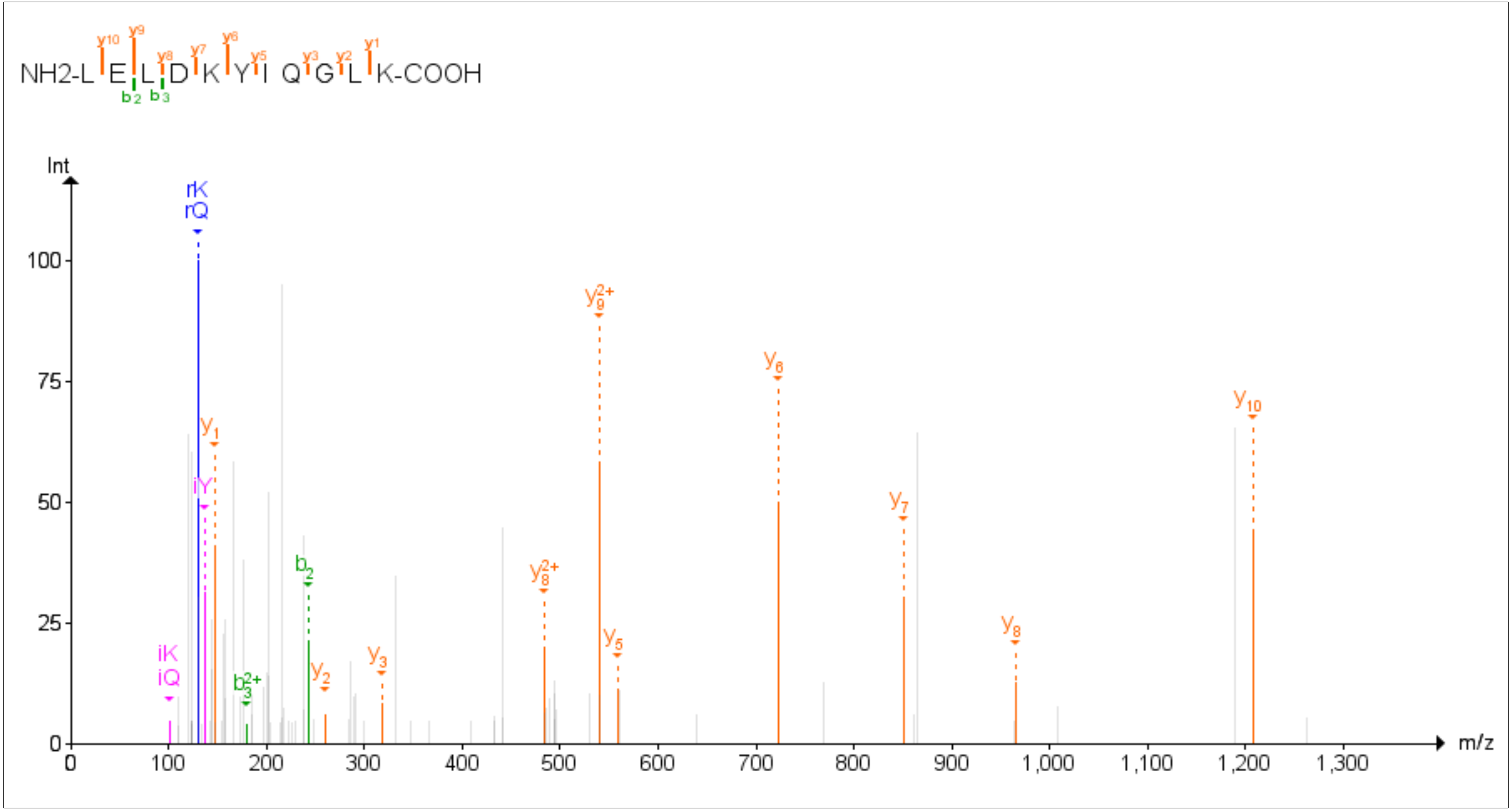

NH2-L E L D K Y I Q G L K-COOH
y10 y9 y8 y7 y6 y5 y3 y2 y1
b2 b3
Int
rK
rQ
100
75
50
25
0
y1
iY
iK
iQ
b_3^{2+}
b_2
y_2
y_3
y_8^{2+}
y_9^{2+}
y_5
y_6
y_7
y_8
y_{10}
0 100 200 300 400 500 600 700 800 900 1,000 1,100 1,200 1,300
m/z

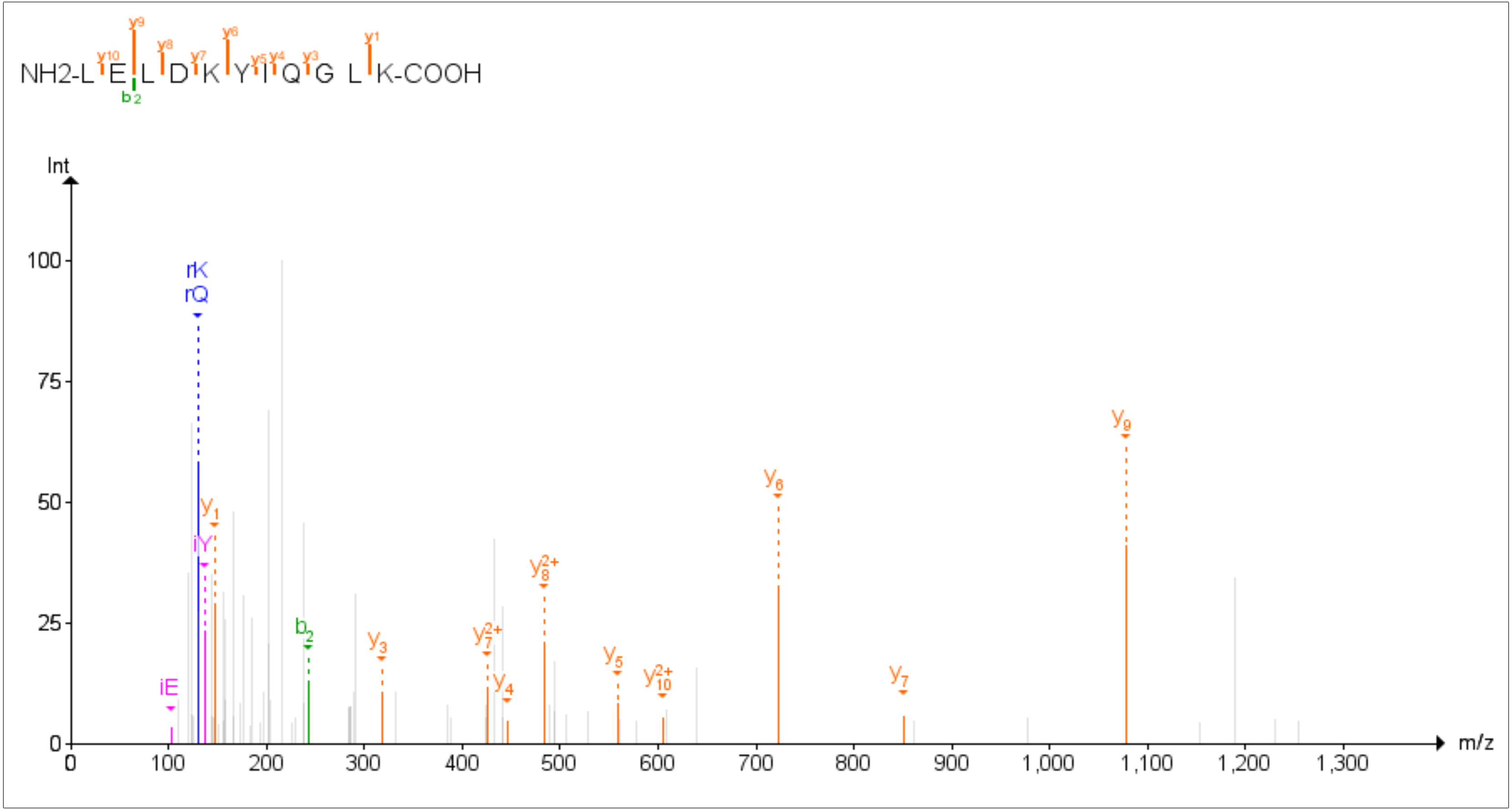

NH2-L E L D K Y I Q G L K-COOH
y10 y9 y8 y7 y6 y5 y4 y3 y1
b2
Int
m/z
rK
rQ
iY
iE
y1
b2
y3
y7 2+
y4
y8 2+
y5
y10 2+
y6
y7
y9
0
25
50
75
100
0
100
200
300
400
500
600
700
800
900
1,000
1,100
1,200
1,300

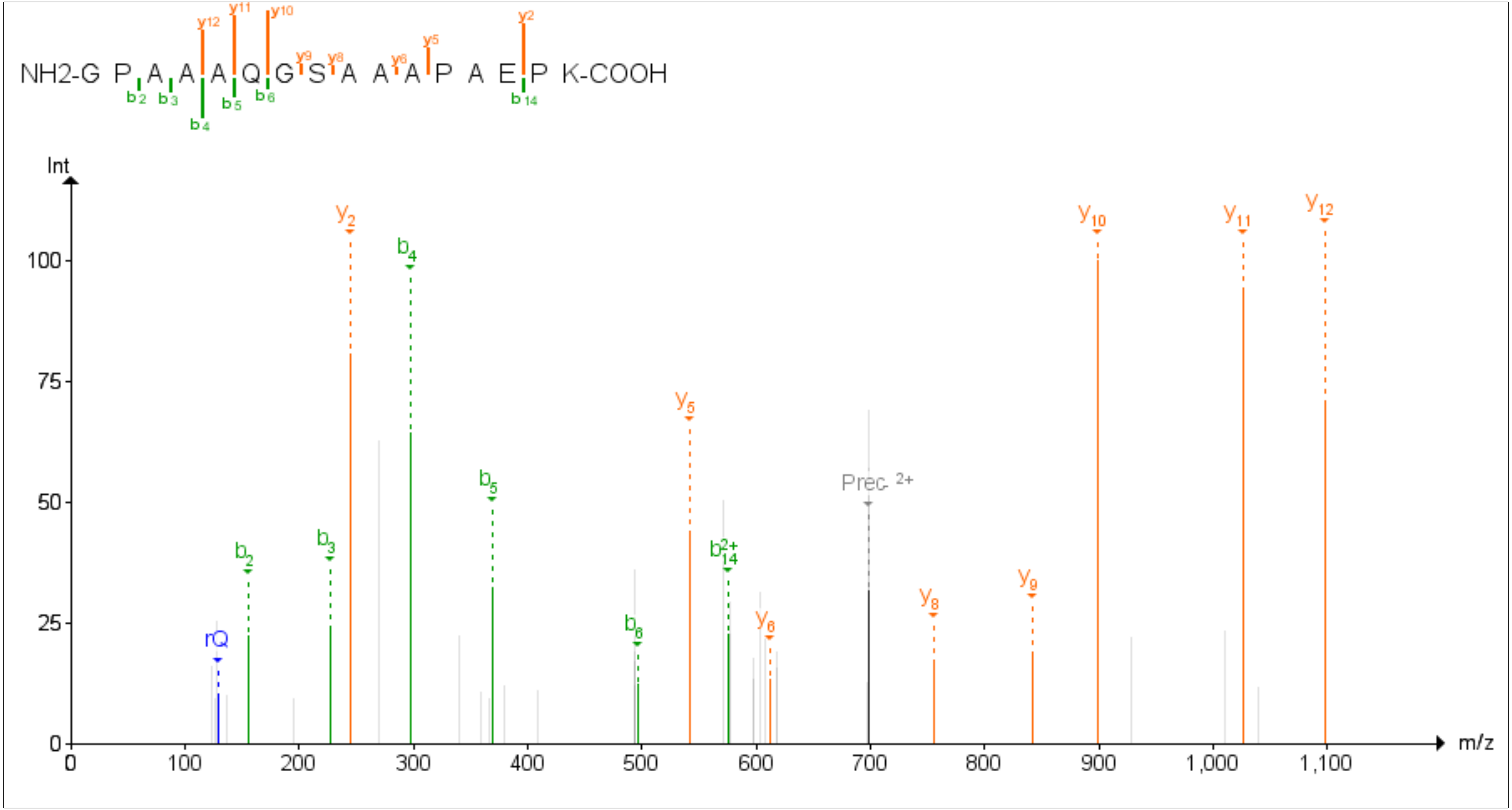

NH2-G P A A A Q G S A A A P A E P K-COOH
y12 y11 y10 y9 y8 y6 y5 y2
b2 b3 b4 b5 b6 b14
Int
m/z
0
25
50
75
100
0 100 200 300 400 500 600 700 800 900 1,000 1,100
rQ
b2
b3
y2
b4
b5
b6
y5
b14 2+
y6
Prec. 2+
y8
y9
y10
y11
y12

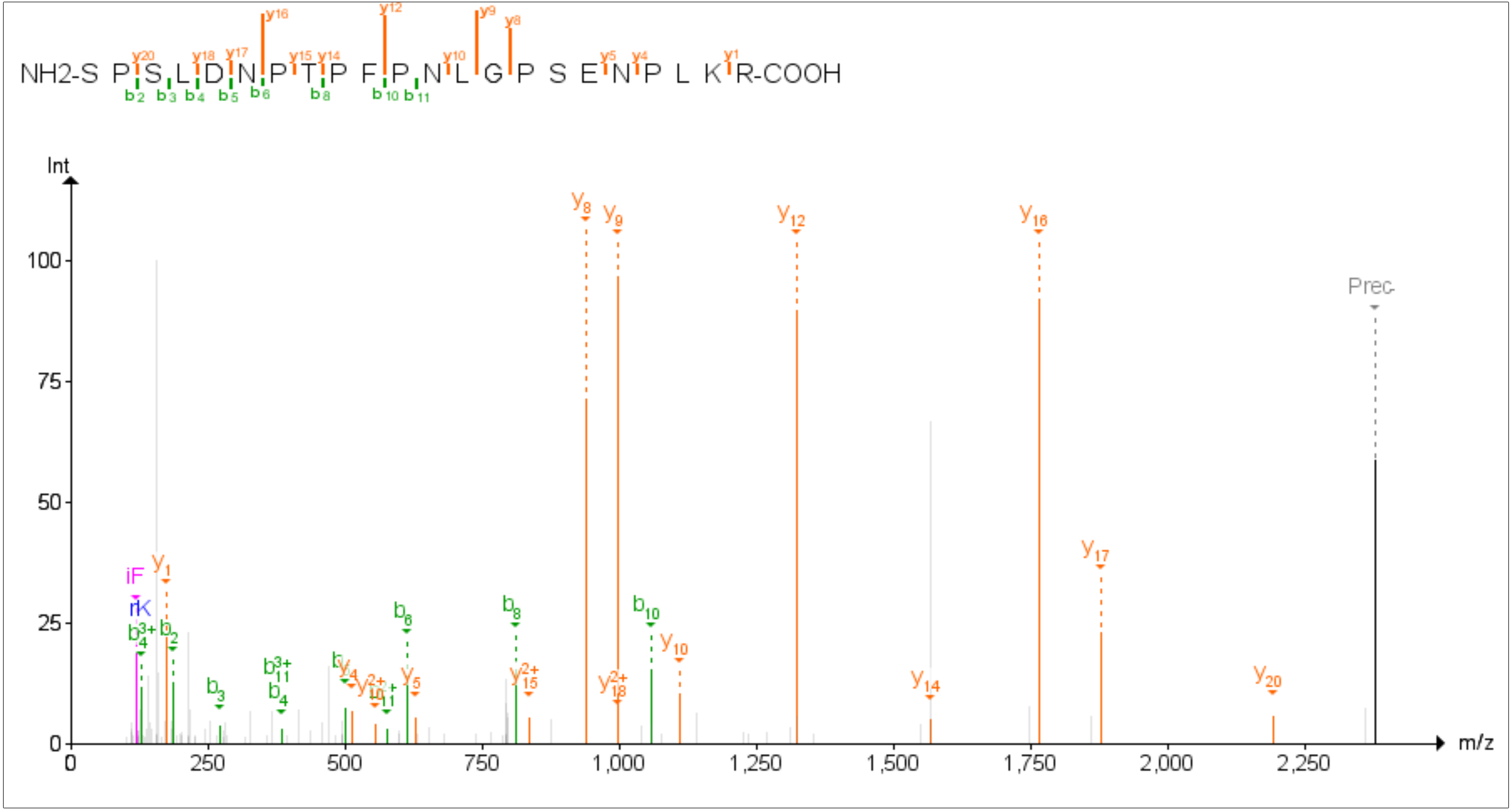

NH2-S P S L D N P T P F P N L G P S E N P L K R-COOH
y20 y18 y17 y16 y15 y14 y12 y10 y9 y8 y5 y4 y1
b2 b3 b4 b5 b6 b8 b10 b11
Int
100
75
50
25
0
m/z
0 250 500 750 1,000 1,250 1,500 1,750 2,000 2,250
y8 y9 y12 y16 Prec
y17
iF y1
rK
b6 b8 b10
y10
y14 y20

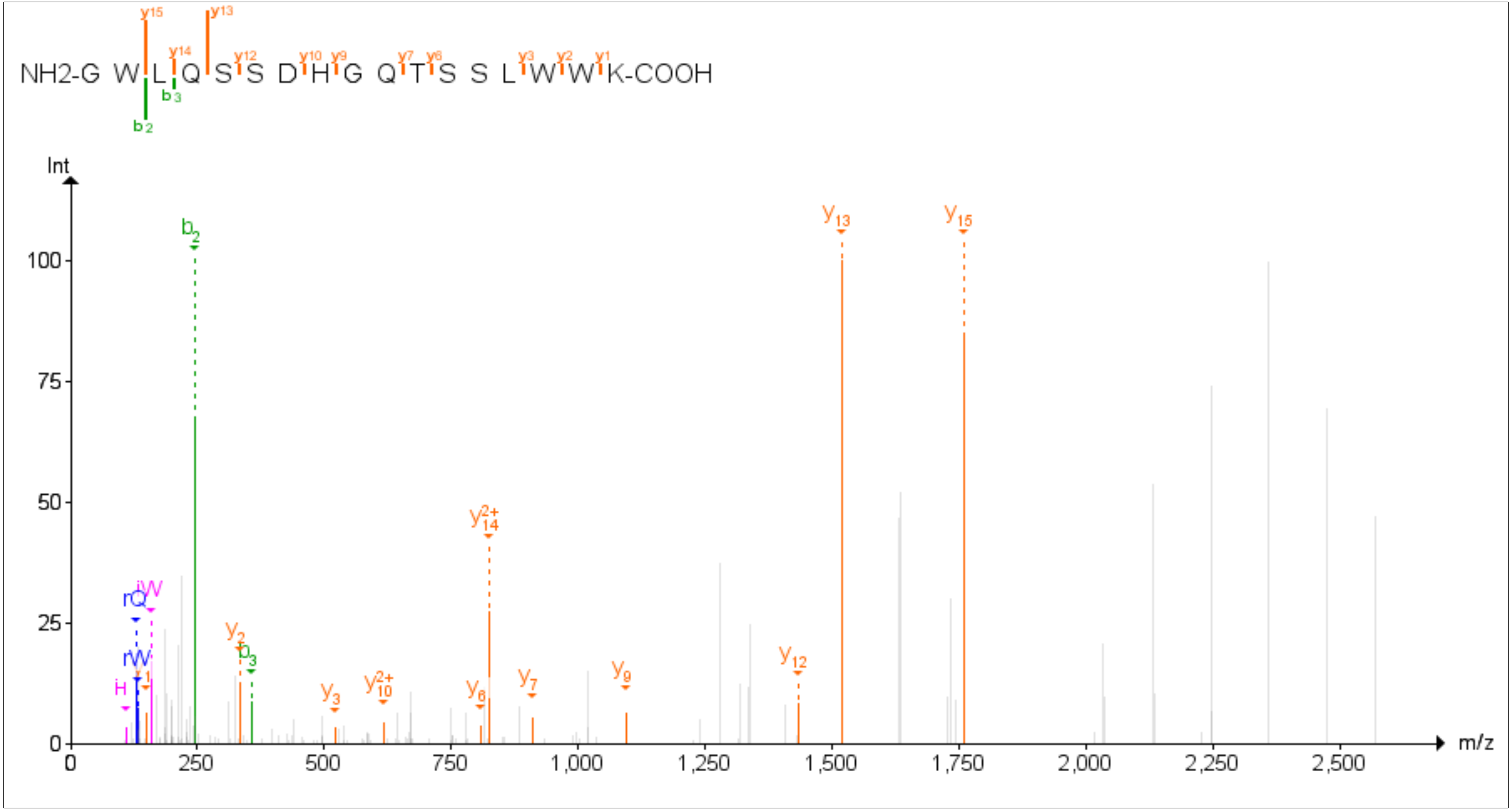

NH2-G W L Q S S D H G Q T S S L W W K-COOH
y15
y13
y14
y12
y10
y9
y7
y6
y3
y2
y1
b2
b3
Int
m/z
0
25
50
75
100
250
500
750
1,000
1,250
1,500
1,750
2,000
2,250
2,500
rQ
rW
iW
iH

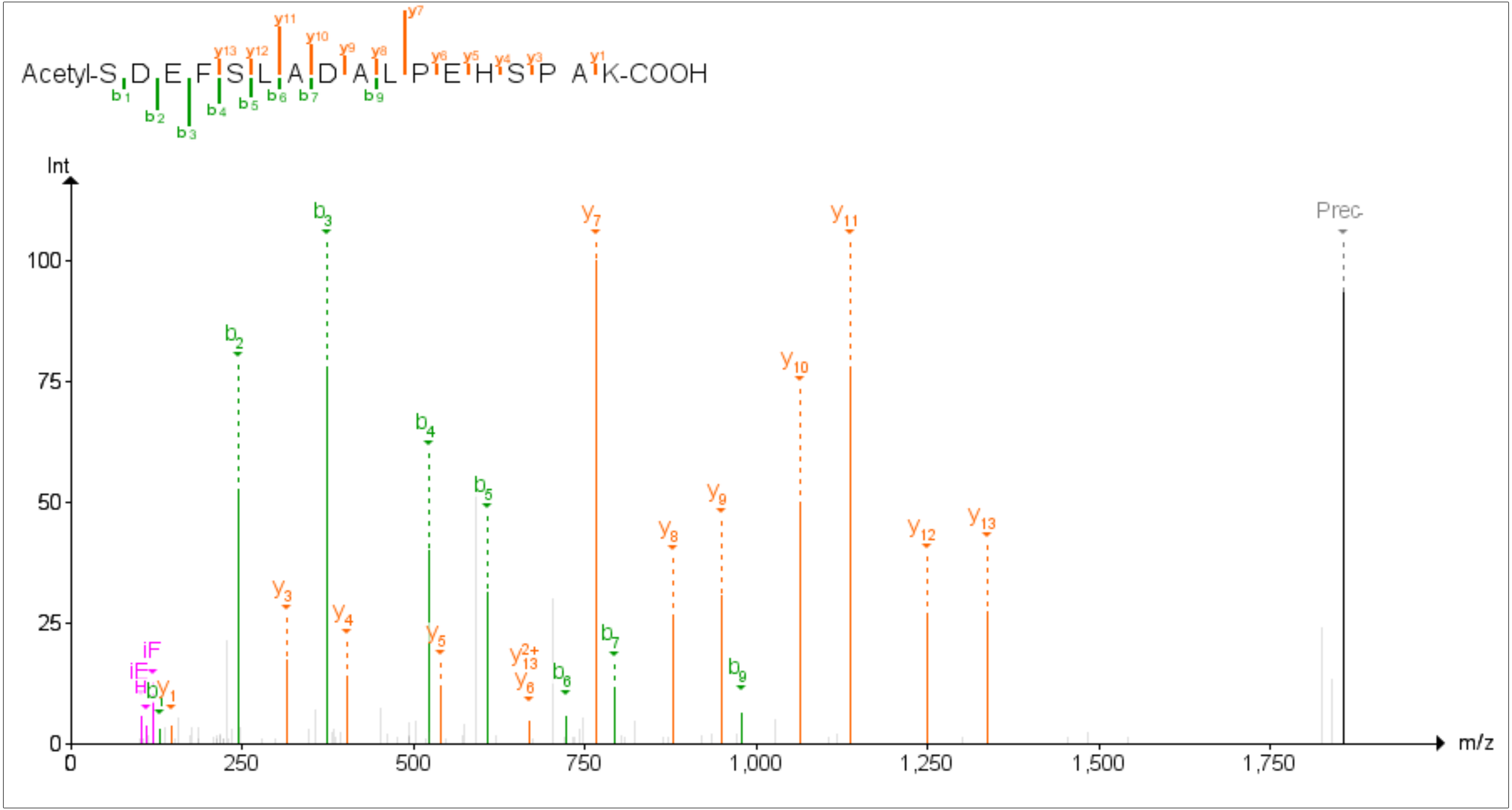

Acetyl-S D E F S L A D A L P E H S P A K-COOH
b1 b2 b3 b4 b5 b6 b7 b9
y13 y12 y11 y10 y9 y8 y7 y6 y5 y4 y3 y1
Int
100
75
50
25
0
0
250
500
750
1,000
1,250
1,500
1,750
m/z
Prec.
b2
b3
b4
b5
b6
b7
b9
b1
y1
y3
y4
y5
y6
y7
y8
y9
y10
y11
y12
y13
y13 2+
iF
iF
H

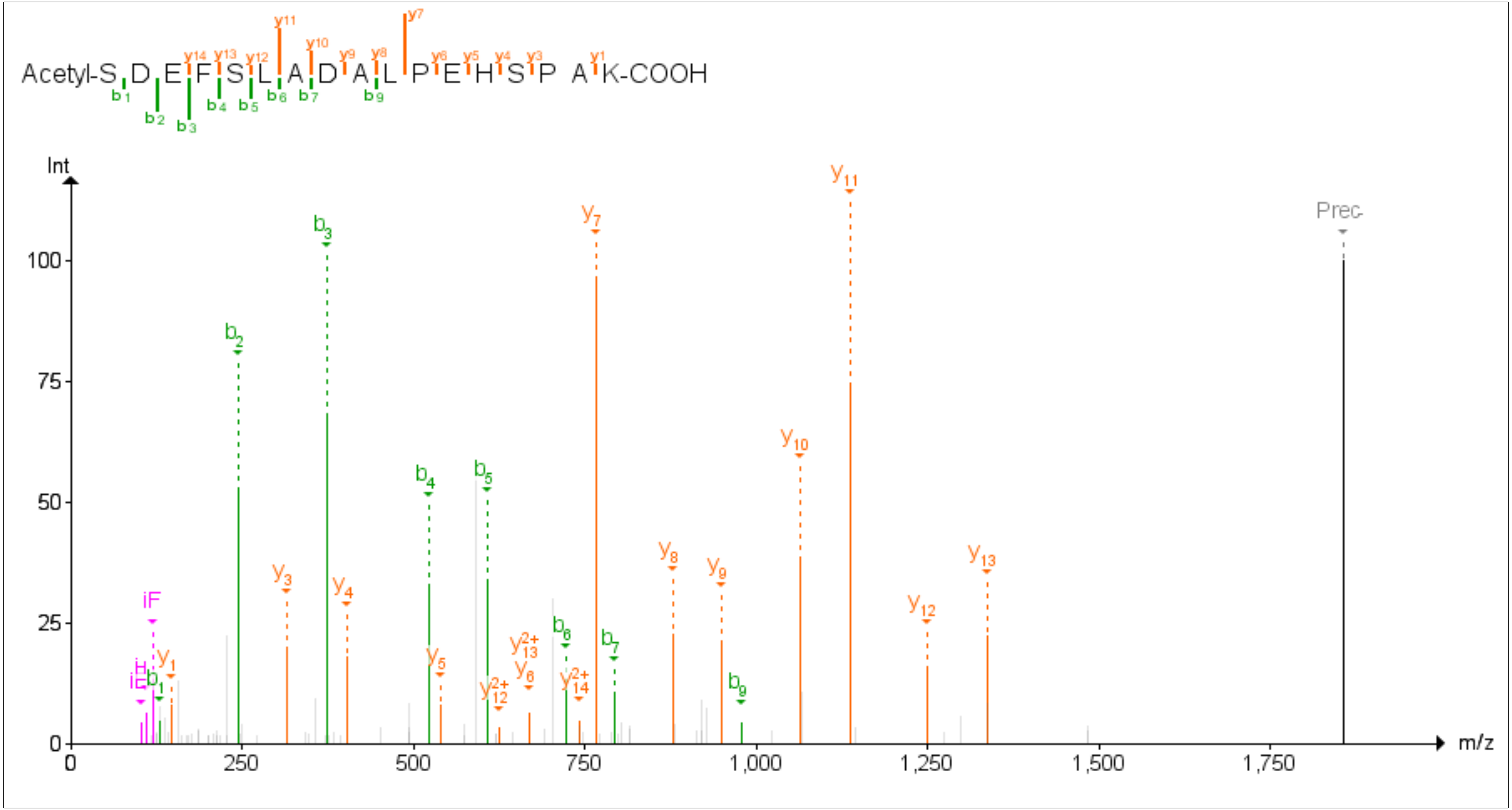

Acetyl-S D E F S L A D A L P E H S P A K-COOH
y14 y13 y12 y11 y10 y9 y8 y7 y6 y5 y4 y3 y1
b1 b2 b3 b4 b5 b6 b7 b9
Int
100
75
50
25
0
m/z
0
250
500
750
1,000
1,250
1,500
1,750
Prec
y11
y7
b3
b2
y10
b4
b5
y8
y13
y9
y3
y4
y12
iF
y1
iH
iE
b1
y5
y2+13
y6
y2+12
b6
b7
y2+14
b9

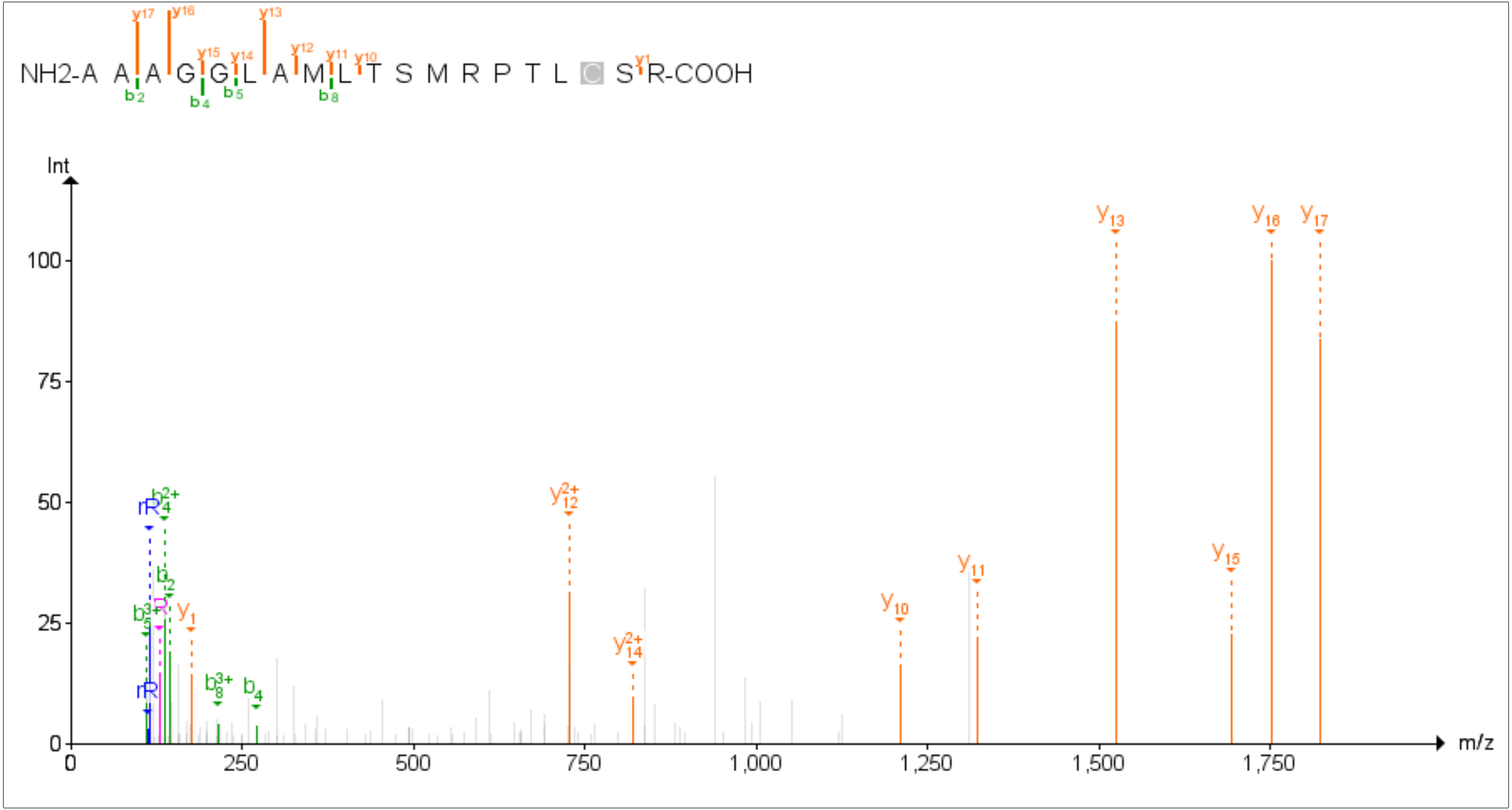

NH2-A A A G G L A M L T S M R P T L C S R-COOH
y17 y16 y15 y14 y13 y12 y11 y10 y1
b2 b4 b5 b8
Int
100
75
50
25
0
0 250 500 750 1,000 1,250 1,500 1,750
m/z
y13 y16 y17
y15 y11 y10
y12 2+
y14 2+
y1
rR
b4 2+
b2
b5 3+
b8 3+
b4

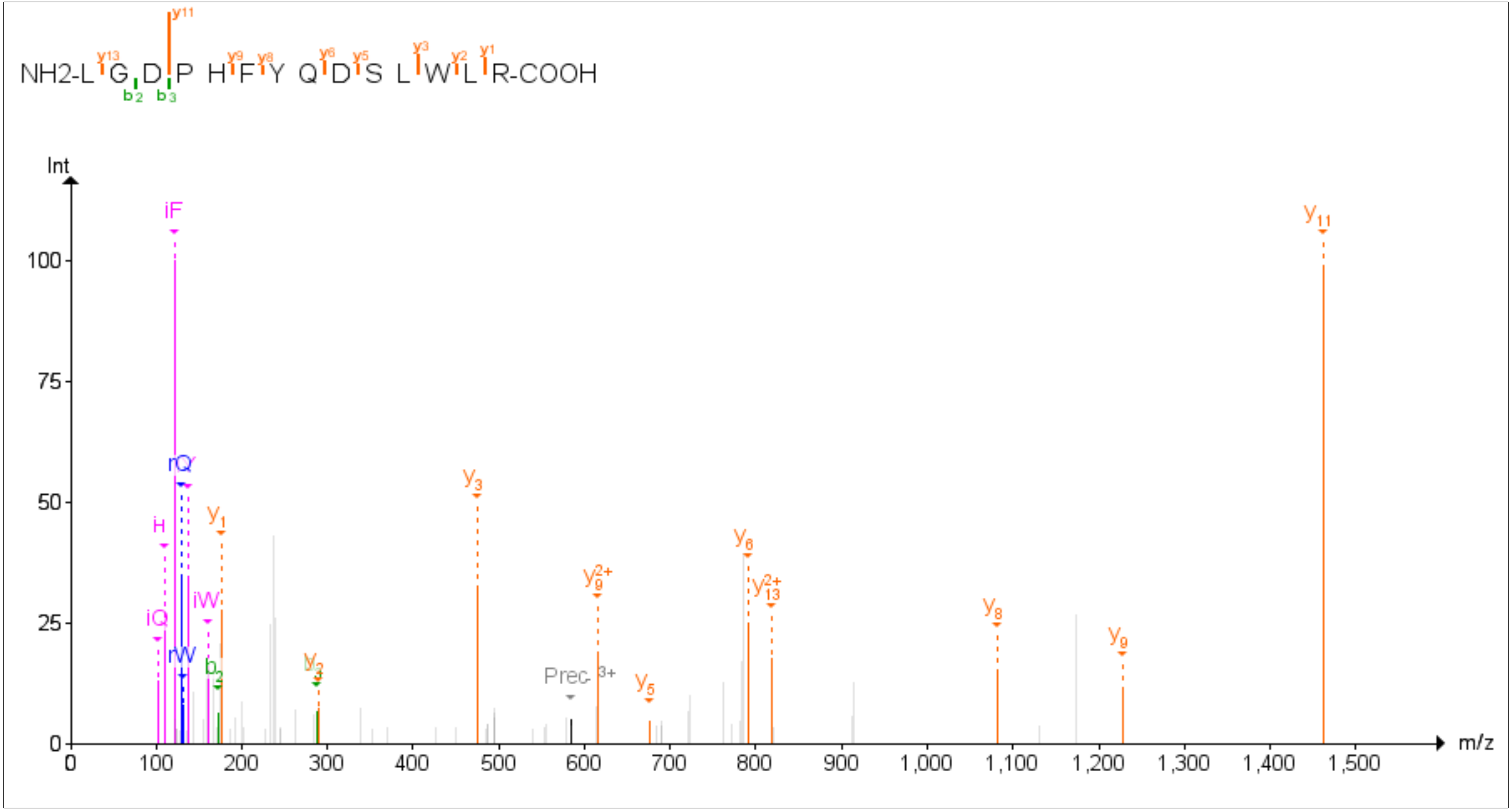

NH2-L G D P H F Y Q D S L W L R-COOH
y13 y11 y9 y8 y6 y5 y3 y2 y1
b2 b3
Int
m/z
iF
y11
y3
y1
iH
rQ
iW
iQ
rW
b2
y3
Prec 3+
y9 2+
y5
y6
y13 2+
y8
y9
0
25
50
75
100
0
100
200
300
400
500
600
700
800
900
1,000
1,100
1,200
1,300
1,400
1,500

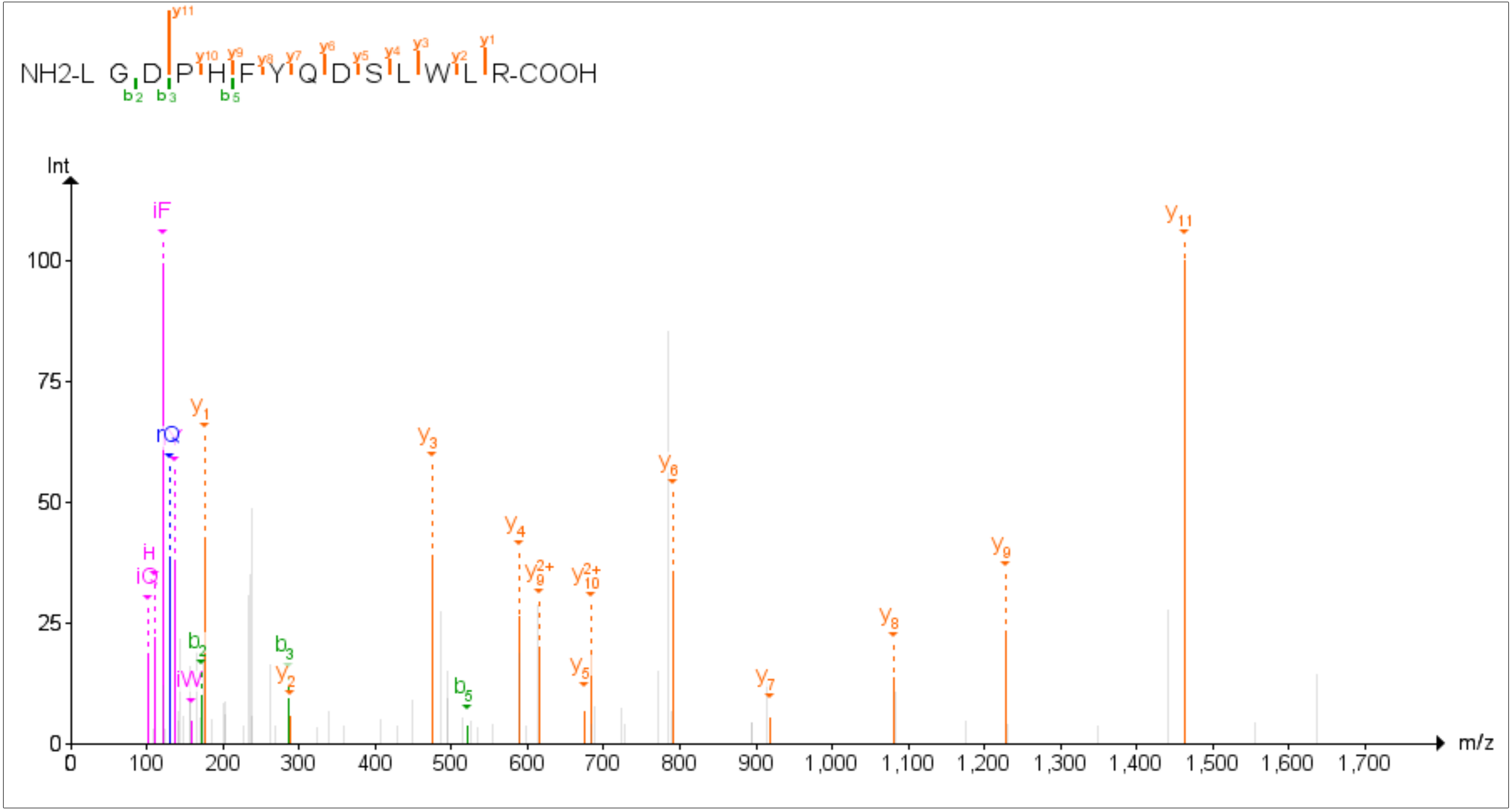

NH2-L G D P H F Y Q D S L W L R-COOH
y11 y10 y9 y8 y7 y6 y5 y4 y3 y2 y1
b2 b3 b5
Int
100
75
50
25
0
iF
rQ
y1
iH
iQ
b2
iW
b3
y2
y3
b5
y4
y9 2+
y10 2+
y5
y6
y7
y8
y9
y11
0
100
200
300
400
500
600
700
800
900
1,000
1,100
1,200
1,300
1,400
1,500
1,600
1,700
m/z

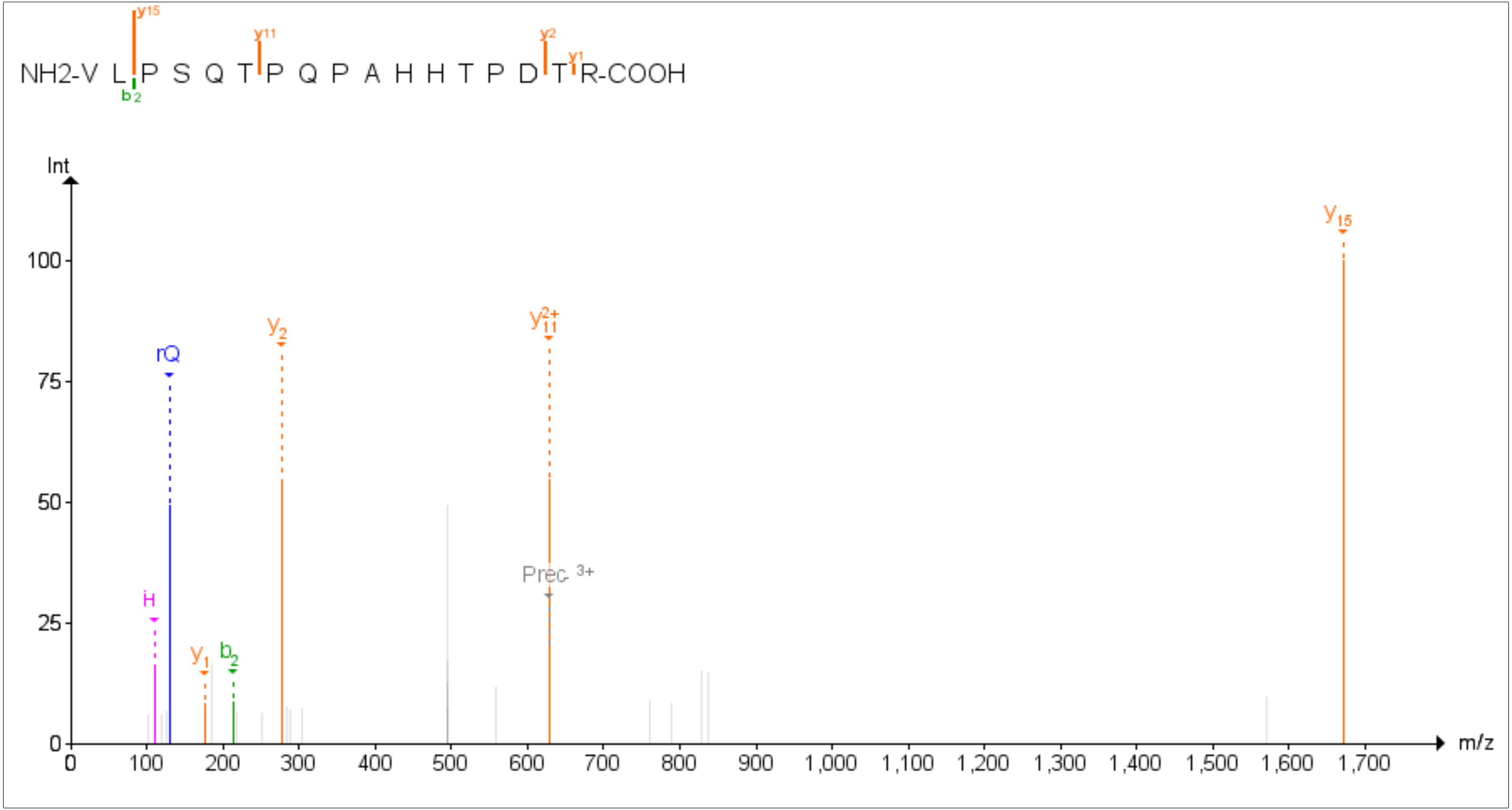

NH2-V L P S Q T P Q P A H H T P D T R-COOH
y15
y11
y2
y1
b2
Int
100
75
50
25
0
y15
y2
rQ
y11 2+
Prec- 3+
H
y1
b2
0
100
200
300
400
500
600
700
800
900
1,000
1,100
1,200
1,300
1,400
1,500
1,600
1,700
m/z

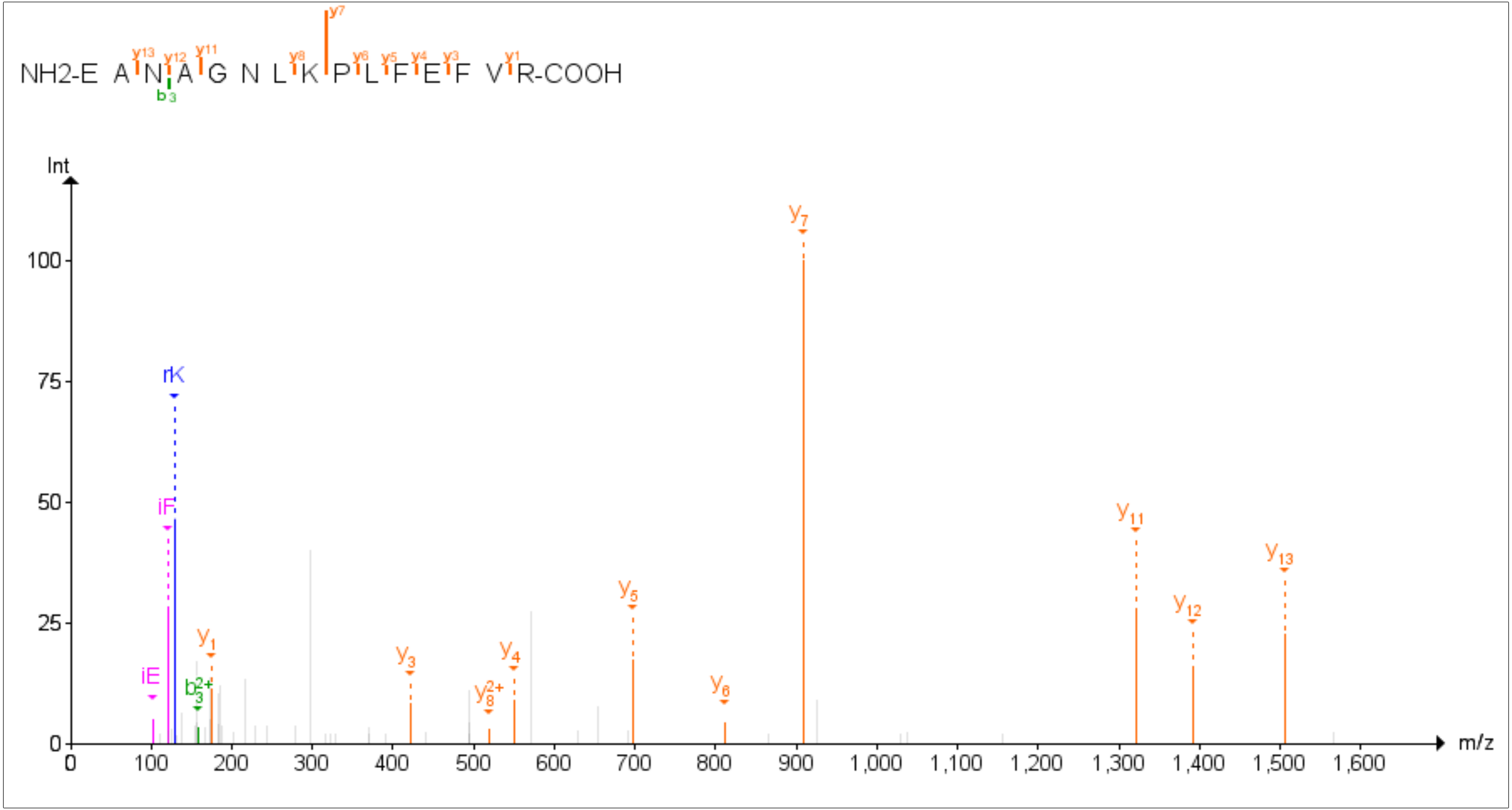

NH2-E A N A G N L K P L F E F V R-COOH
y13 y12 y11 y8 y7 y6 y5 y4 y3 y1
b3
Int
100
75
50
25
0
rK
iF
iE
b3 2+
y1
y3
y8 2+
y4
y5
y6
y7
y11
y12
y13
0
100
200
300
400
500
600
700
800
900
1,000
1,100
1,200
1,300
1,400
1,500
1,600
m/z

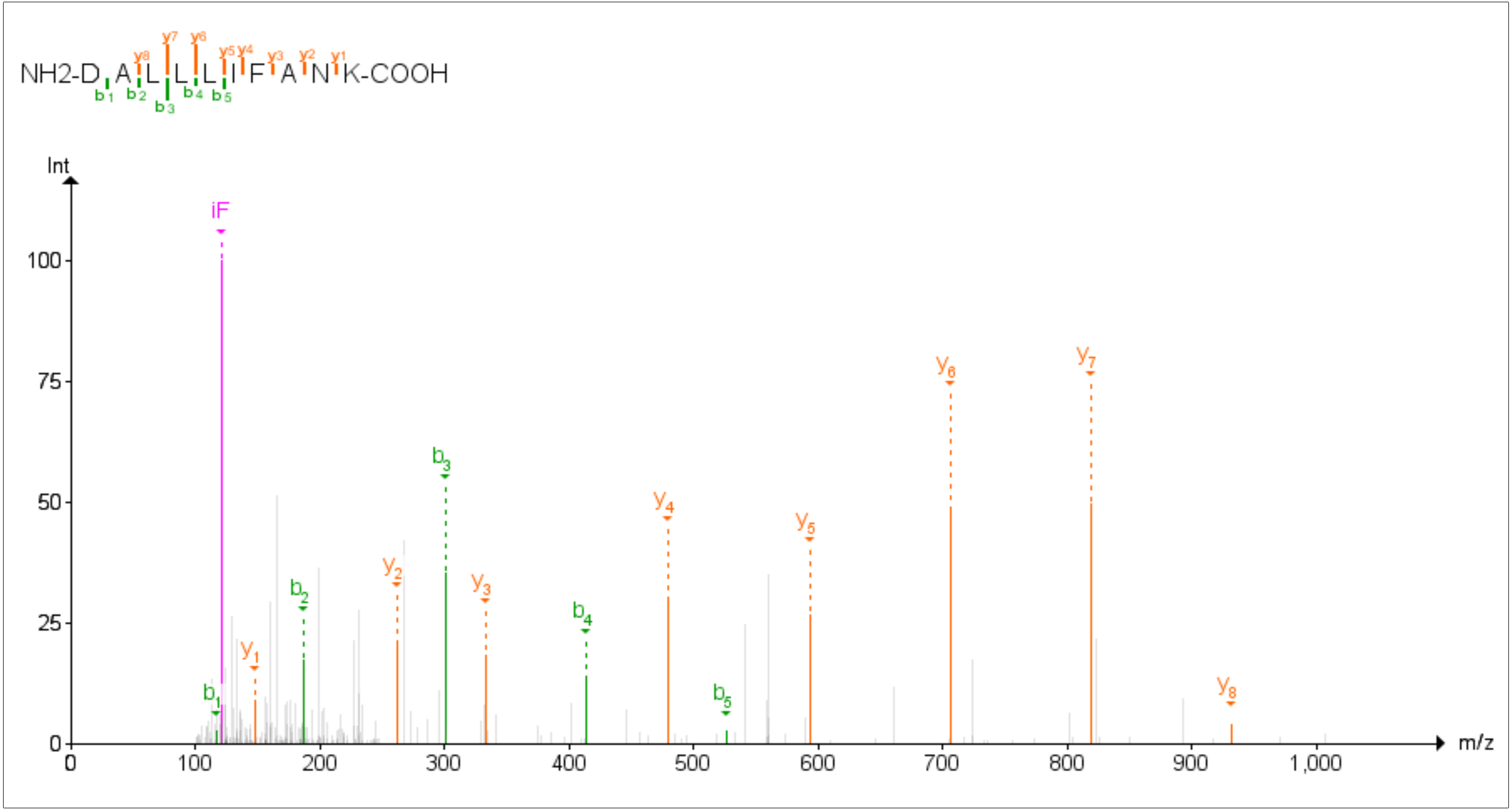

NH2-D A L L L I F A N K-COOH
y7 y6 y8 y5 y4 y3 y2 y1
b1 b2 b3 b4 b5
Int
iF
100
75
50
25
0
b1
y1
b2
y2
b3
y3
b4
y4
b5
y5
y6
y7
y8
0
100
200
300
400
500
600
700
800
900
1,000
m/z

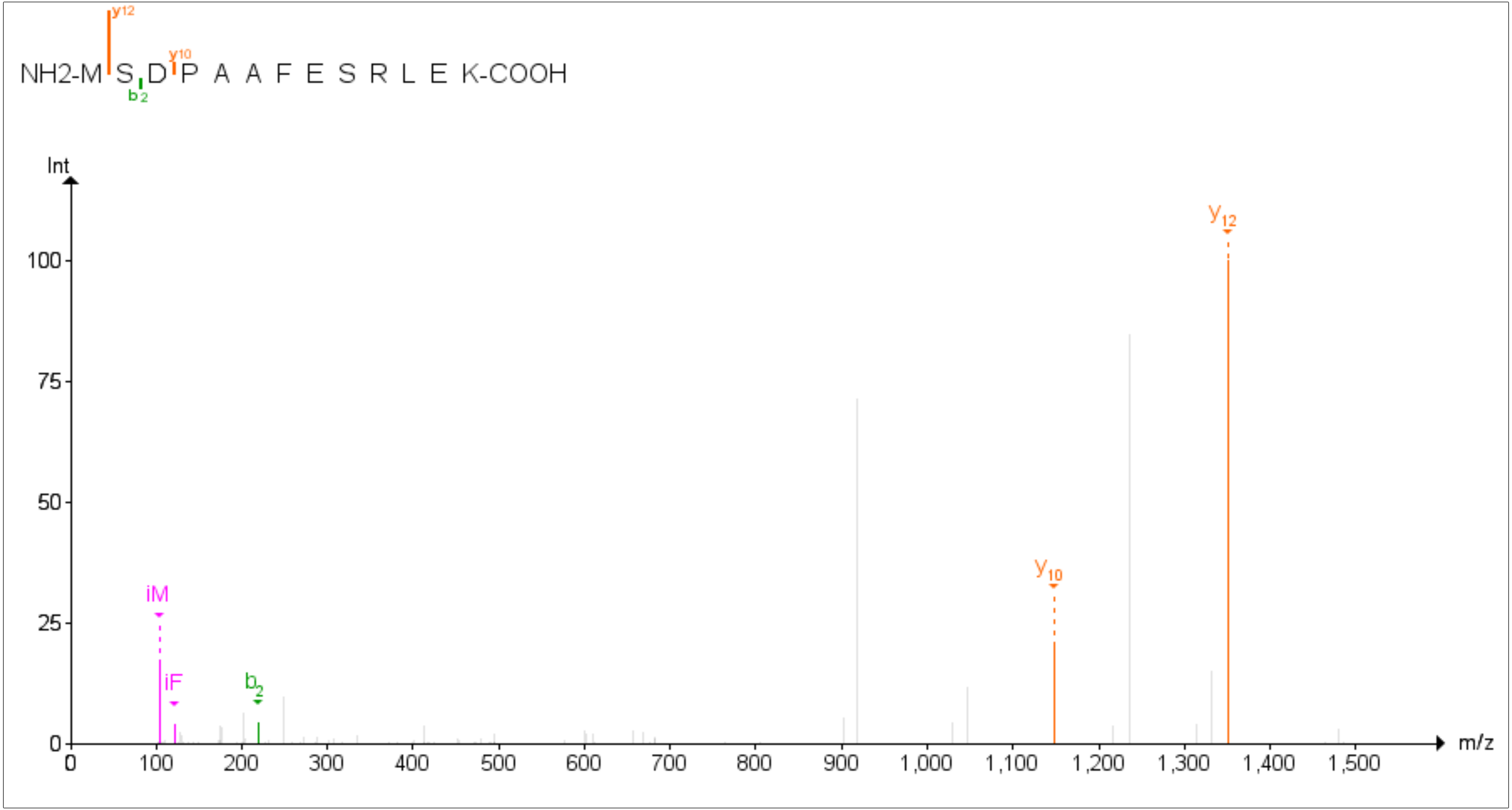

y12
y10
NH2-M S D P A A F E S R L E K-COOH
b2
Int
100
75
50
25
0
y12
y10
iM
iF
b2
0
100
200
300
400
500
600
700
800
900
1,000
1,100
1,200
1,300
1,400
1,500
m/z

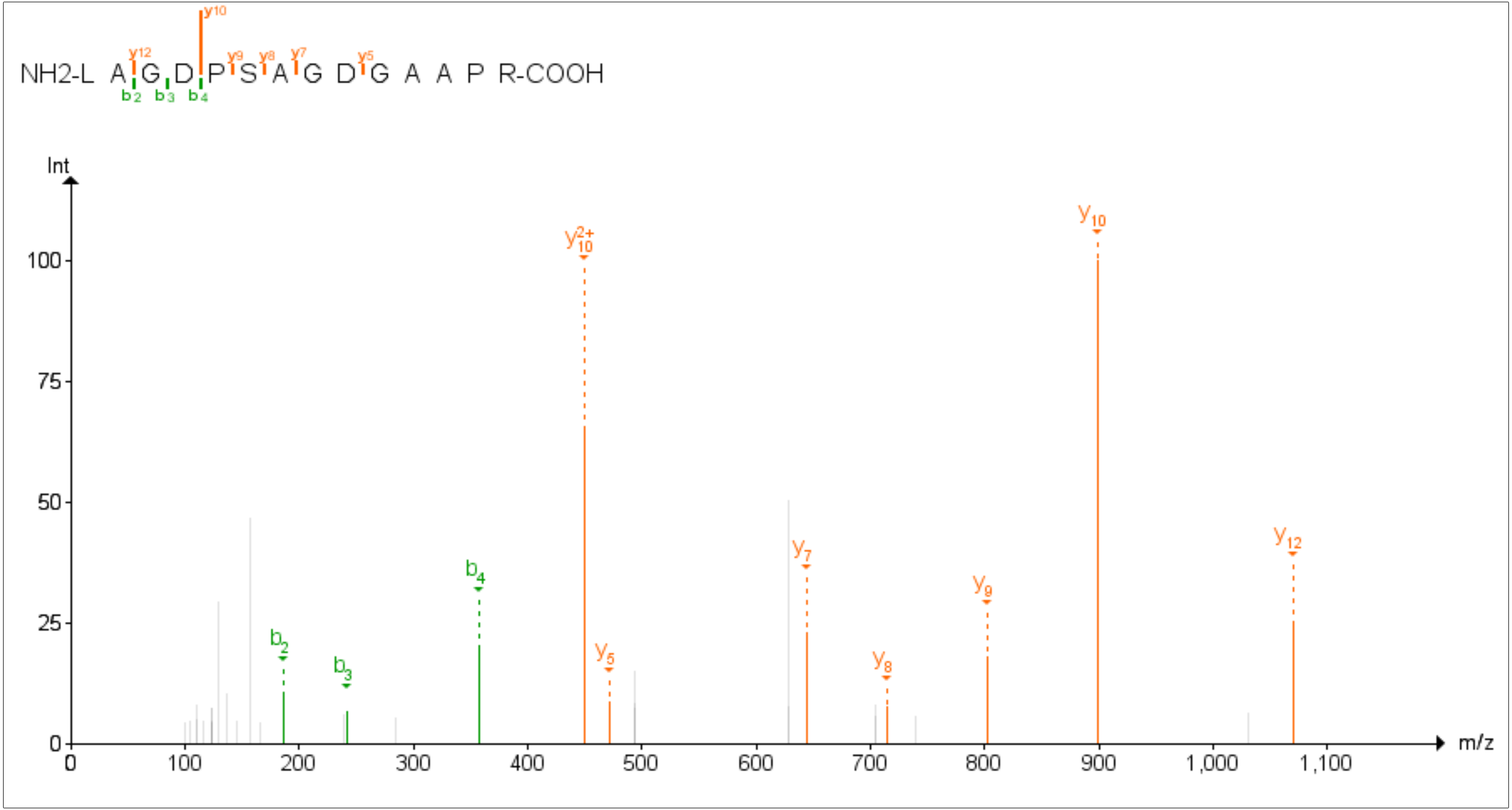

NH2-L A G D P S A G D G A A P R-COOH
y12 y10 y9 y8 y7 y5
b2 b3 b4
Int
m/z
0 25 50 75 100
0 100 200 300 400 500 600 700 800 900 1,000 1,100
b2 b3 b4 y10 2+ y5 y7 y8 y9 y10 y12

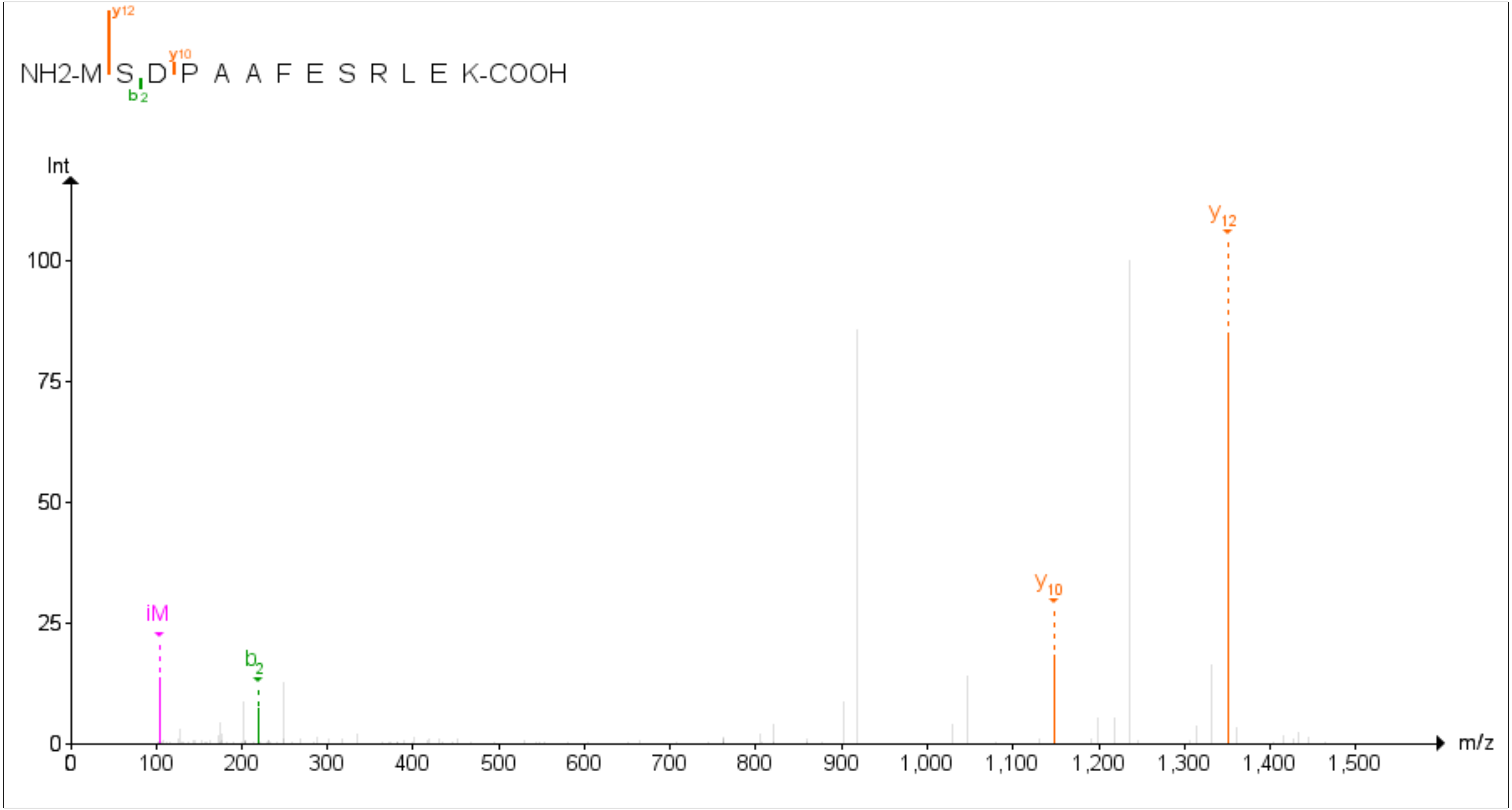
NH2-M S D P A A F E S R L E K-COOH
y12
y10
b2
Int
100
75
50
25
0
iM
b2
y10
y12
m/z
0
100
200
300
400
500
600
700
800
900
1,000
1,100
1,200
1,300
1,400
1,500

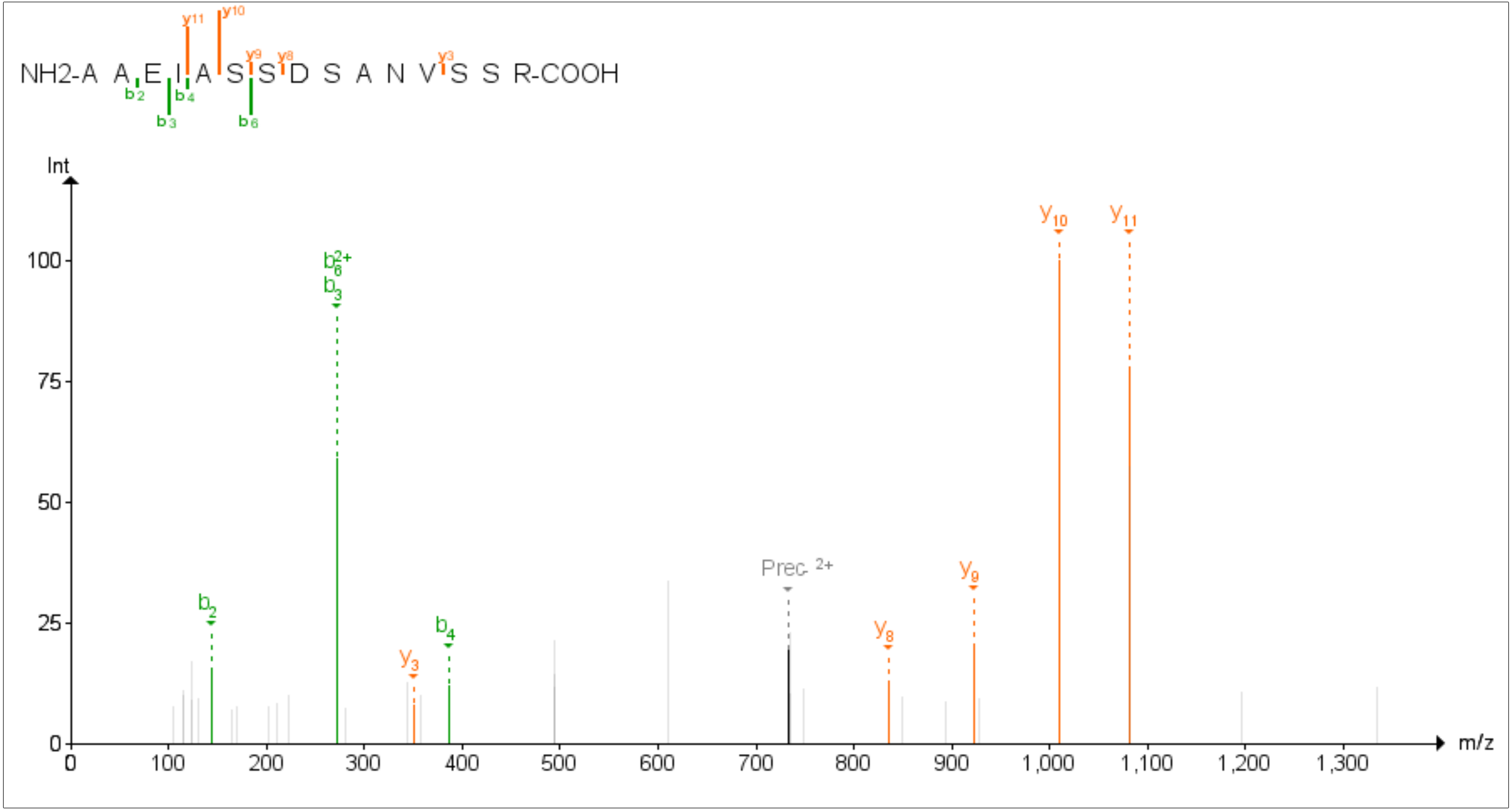

NH2-A A E I A S S D S A N V S S R-COOH
y11 y10 y9 y8 y3
b2 b3 b4 b6
Int
100
75
50
25
0
b6 2+
b3
b2
y3
b4
Prec 2+
y8
y9
y10
y11
0 100 200 300 400 500 600 700 800 900 1,000 1,100 1,200 1,300
m/z

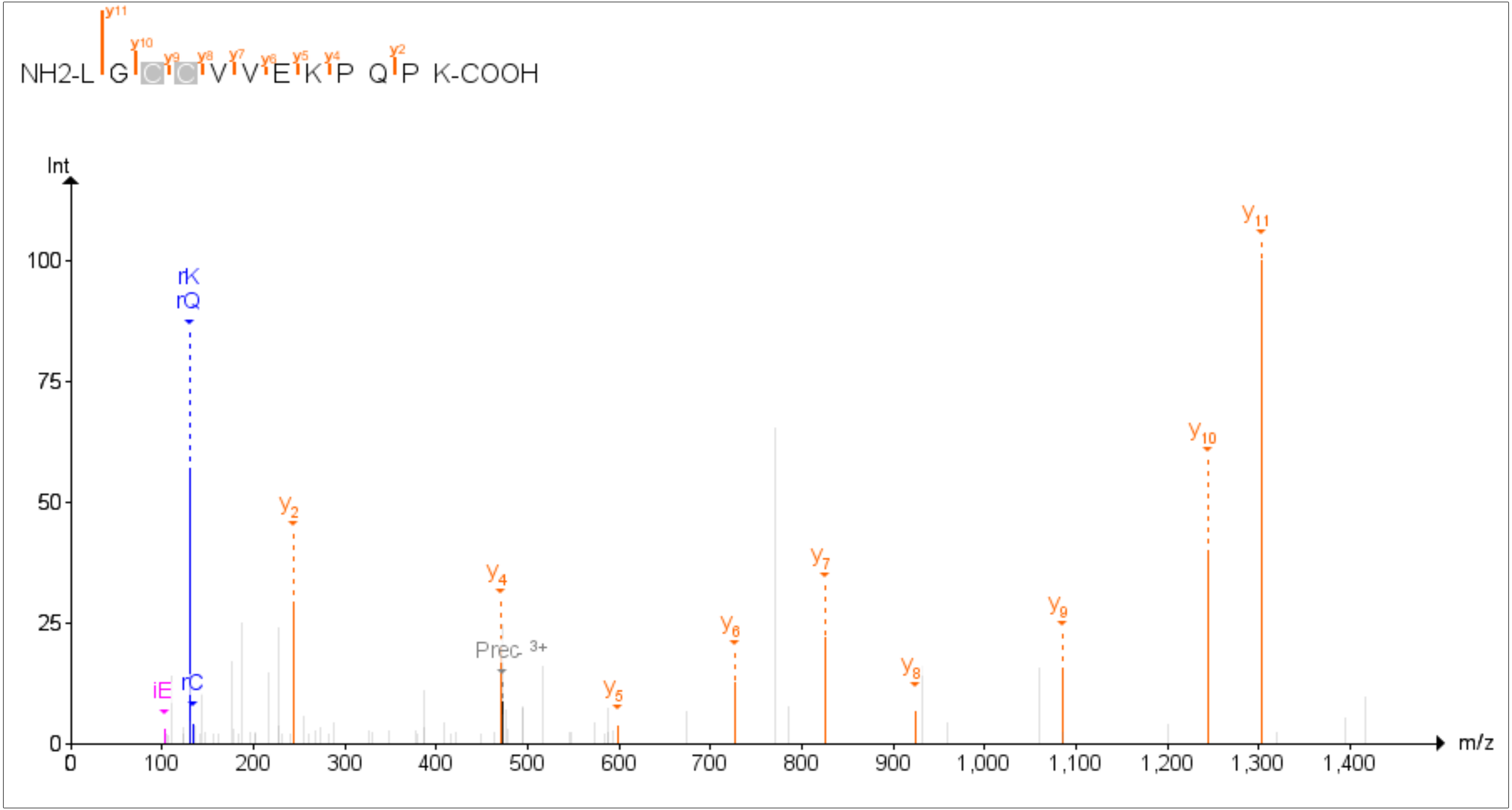

y11
y10
y9
y8
y7
y6
y5
y4
y2
NH2-L G C C V V E K P Q P K-COOH
Int
100
75
50
25
0
rK
rQ
y2
y4
Prec 3+
iE
rC
y5
y6
y7
y8
y9
y10
y11
0
100
200
300
400
500
600
700
800
900
1,000
1,100
1,200
1,300
1,400
m/z

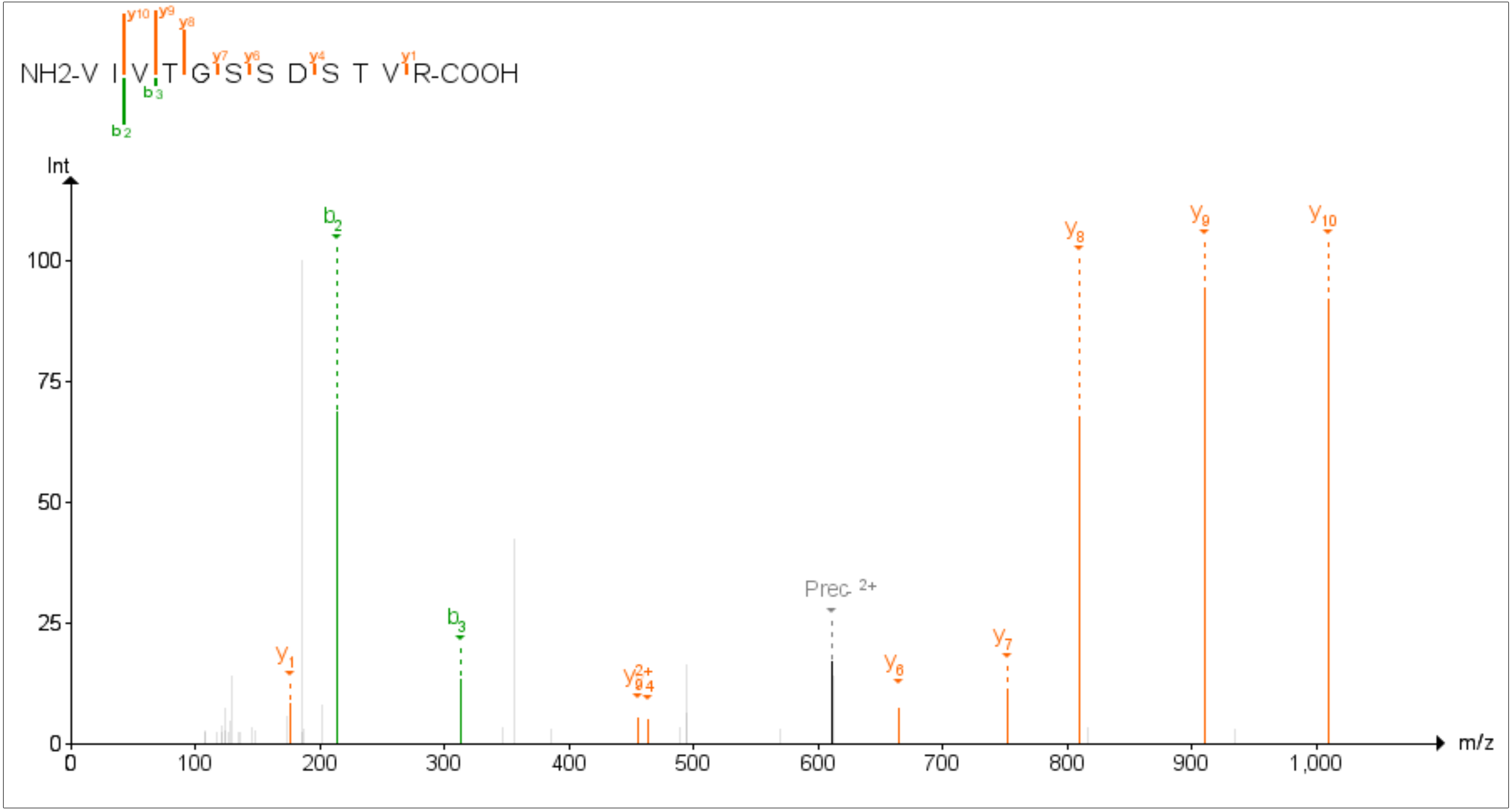
NH2-V I V T G S S D S T V R-COOH
y10 y9 y8 y7 y6 y4 y1
b2 b3
Int
m/z
Prec 2+
y9 2+
y4
y1
b2
b3
y6
y7
y8
y9
y10
0
25
50
75
100
0
100
200
300
400
500
600
700
800
900
1,000

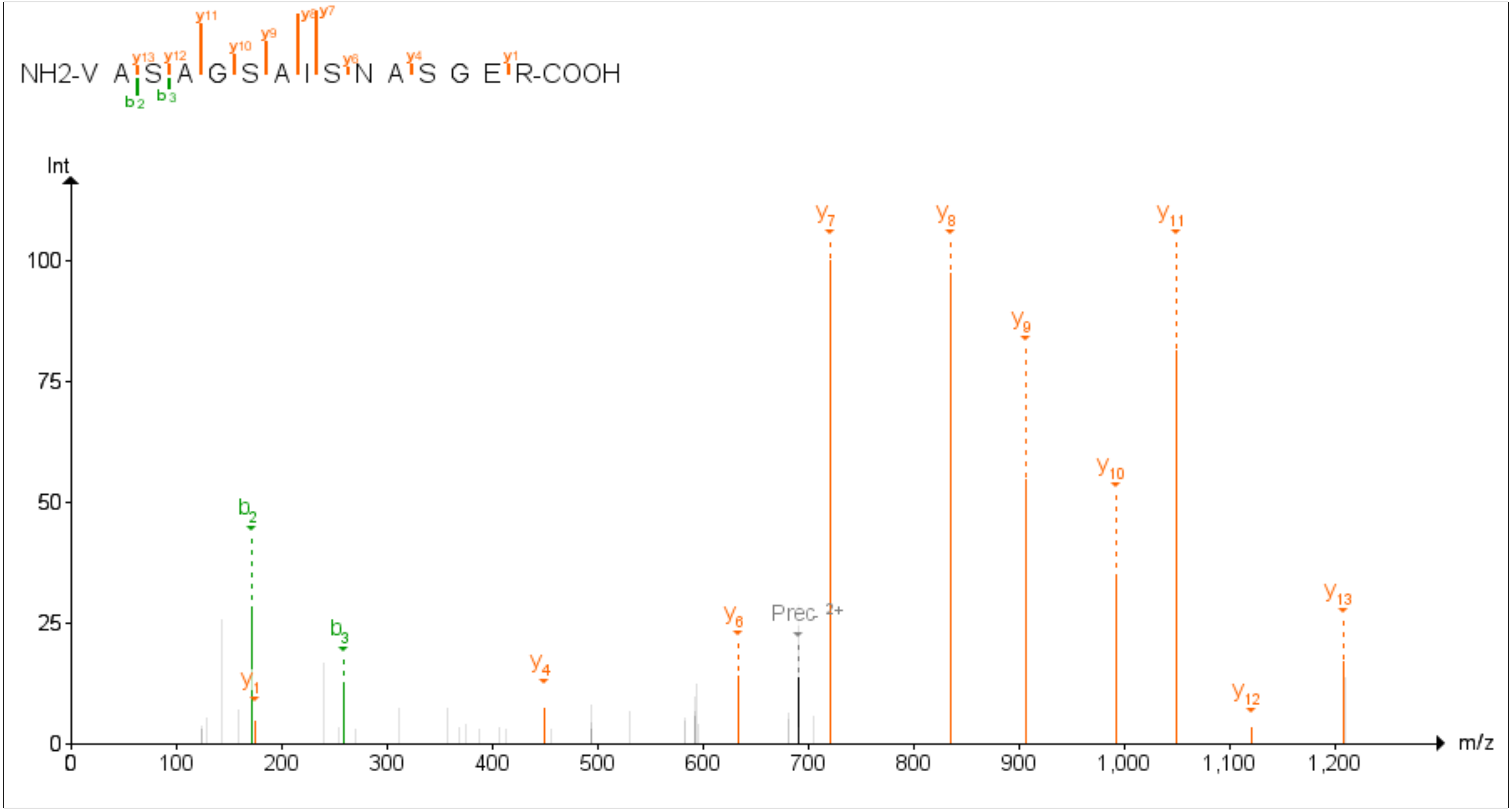

NH2-V A S A G S A I S N A S G E R-COOH
y11 y13 y12 y10 y9 y8 y7 y6 y4 y1
b2 b3
Int
100
75
50
25
0
0 100 200 300 400 500 600 700 800 900 1,000 1,100 1,200
m/z
y7 y8 y11 y9 y10 b2 b3 y1 y4 y6 Prec 2+ y12 y13

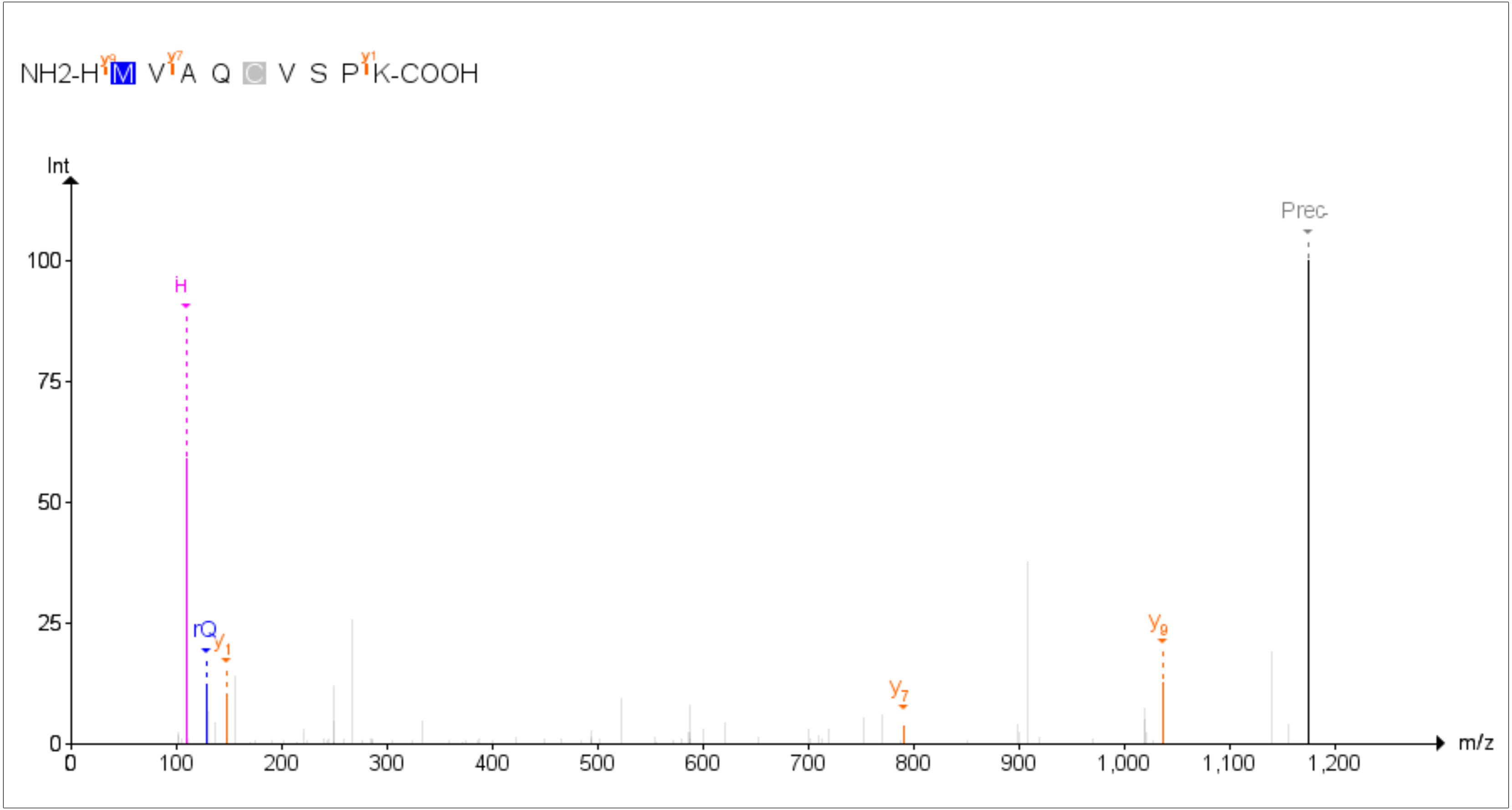

NH2-H M V A Q C V S P K-COOH
y9
y7
y1
Int
100
75
50
25
0
Prec
H
rQ
y1
y7
y9
0
100
200
300
400
500
600
700
800
900
1,000
1,100
1,200
m/z

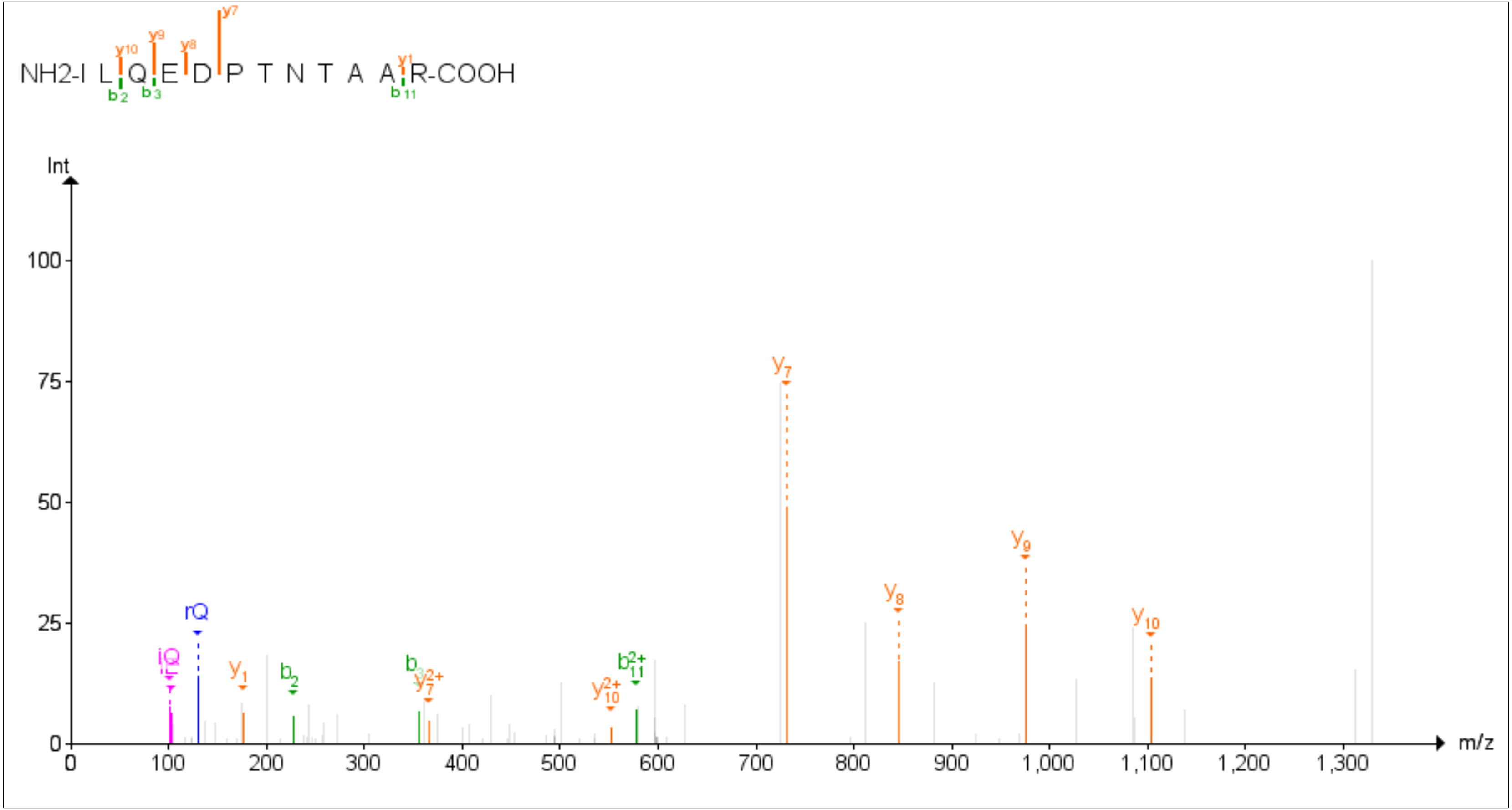
NH2-I L Q E D P T N T A A R-COOH
y10 y9 y8 y7 y1
b2 b3 b11
Int
m/z
0
25
50
75
100
0
100
200
300
400
500
600
700
800
900
1,000
1,100
1,200
1,300
iQ
rQ
y1
b2
b3
y7 2+
y10 2+
b11 2+
y7
y8
y9
y10

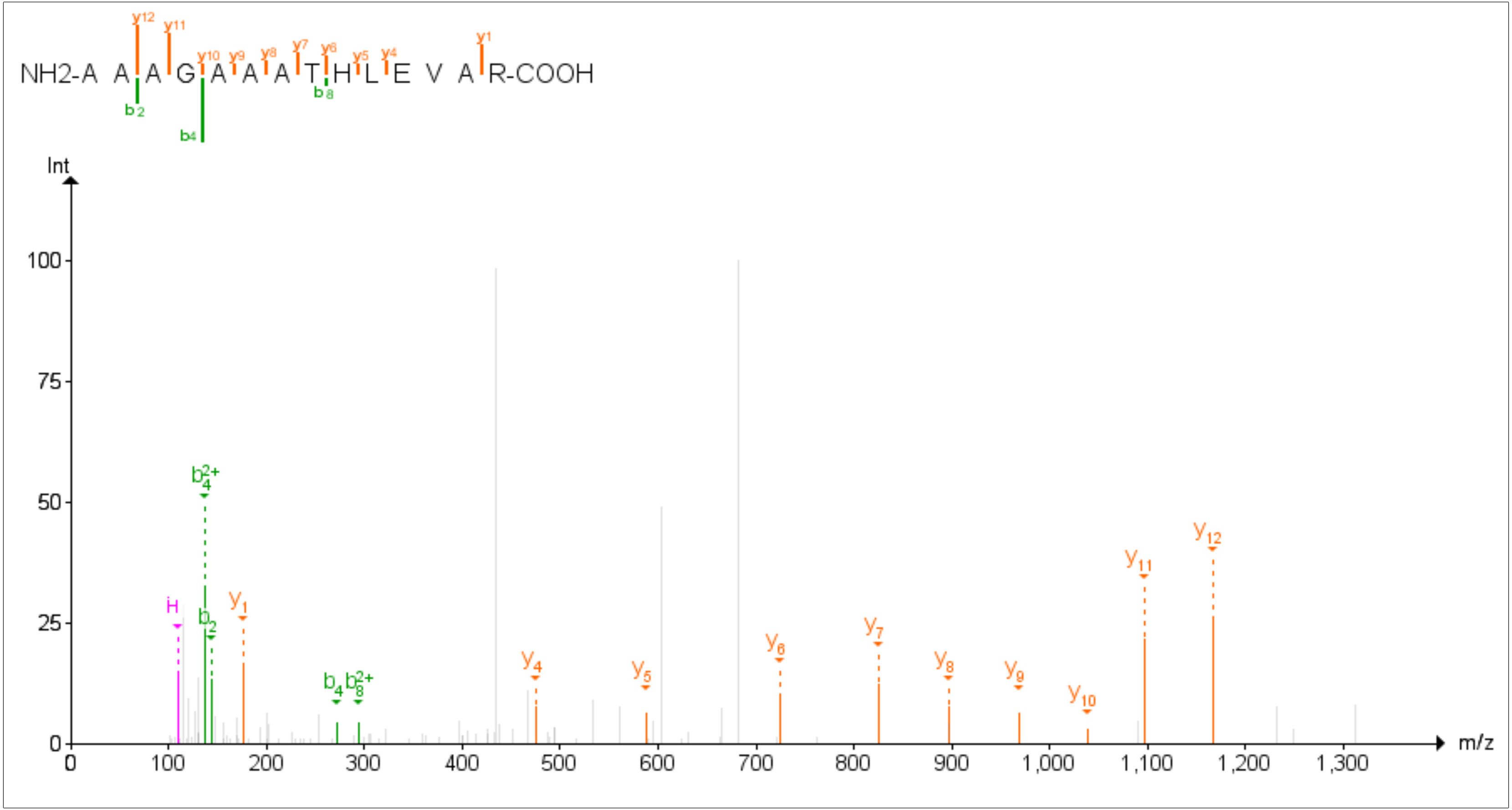

NH2-A A A G A A A T H L E V A R-COOH
y12 y11 y10 y9 y8 y7 y6 y5 y4 y1
b2 b4 b8
Int
100
75
50
25
0
m/z
0 100 200 300 400 500 600 700 800 900 1,000 1,100 1,200 1,300
H
b2
b4 2+
y1
b4
b8 2+
y4
y5
y6
y7
y8
y9
y10
y11
y12

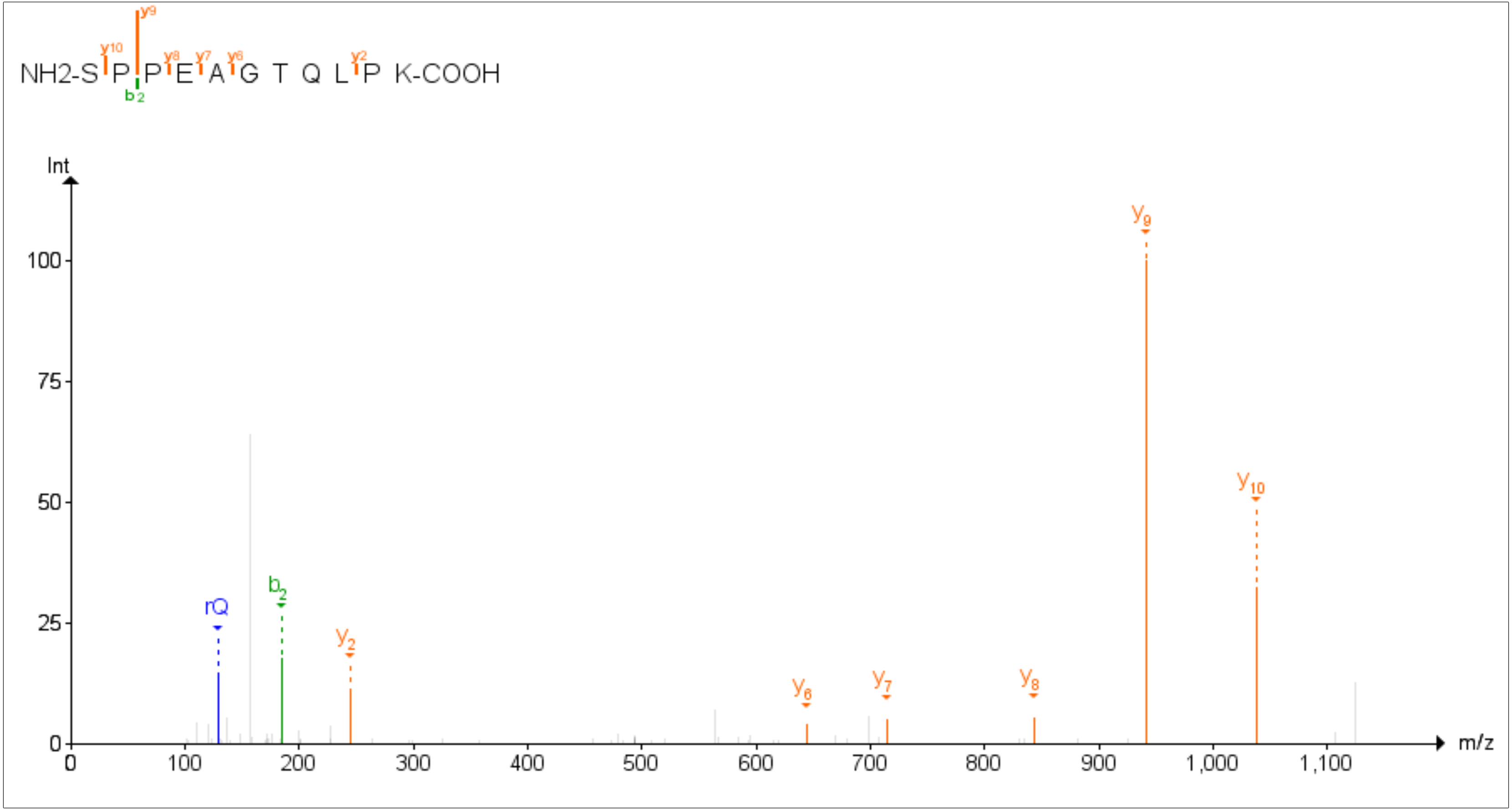

NH2-S P P E A G T Q L P K-COOH
y10 y9 y8 y7 y6 y2
b2
Int
100
75
50
25
0
rQ
b2
y2
y6
y7
y8
y9
y10
0 100 200 300 400 500 600 700 800 900 1,000 1,100
m/z

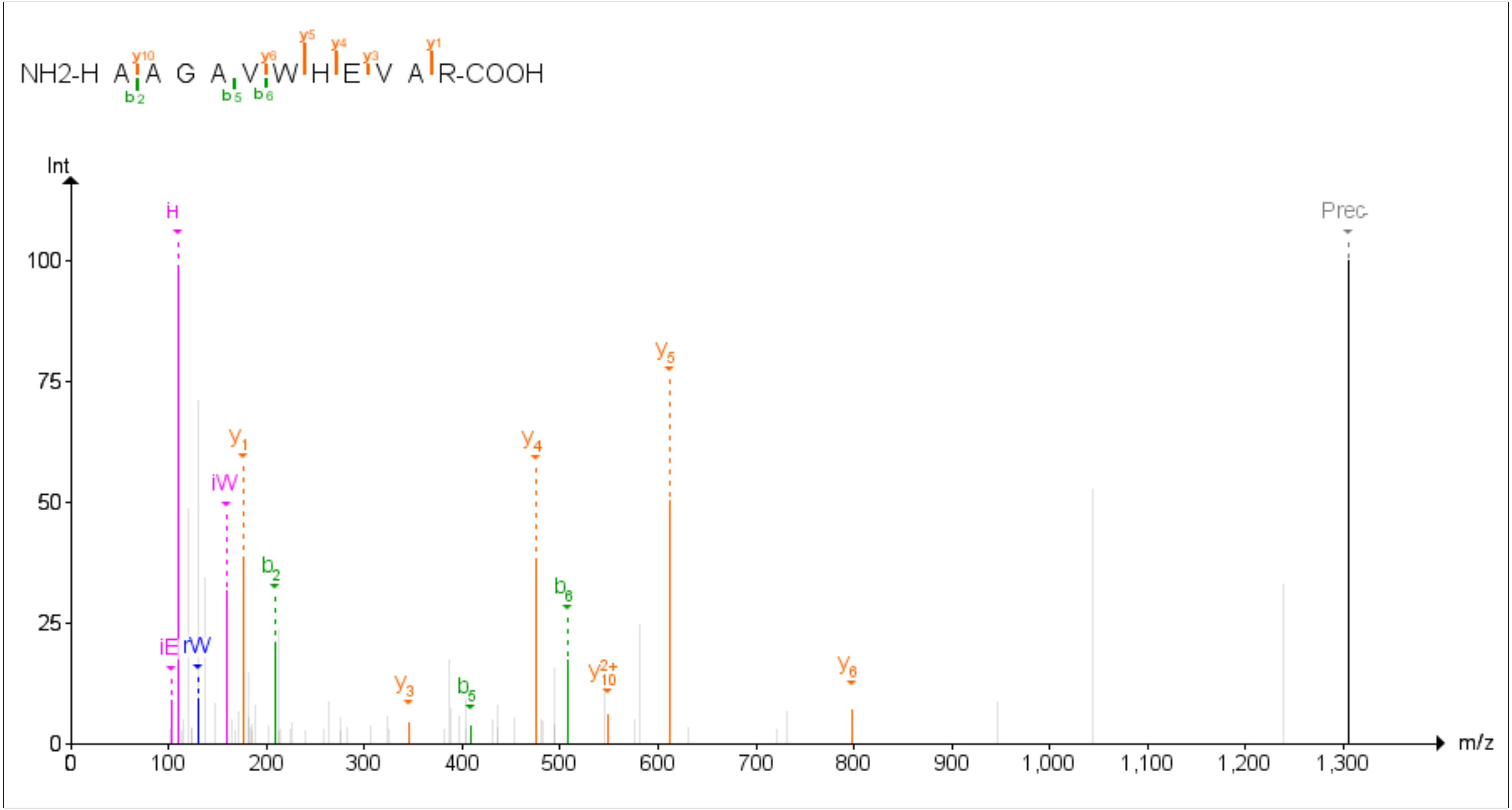

NH2-H A A G A V W H E V A R-COOH
y10
y6
y5
y4
y3
y1
b2
b5
b6
Int
100
75
50
25
0
iH
iW
iE
rW
y1
y4
y5
b2
b6
y3
b5
y10 2+
y6
Prec.
0
100
200
300
400
500
600
700
800
900
1,000
1,100
1,200
1,300
m/z

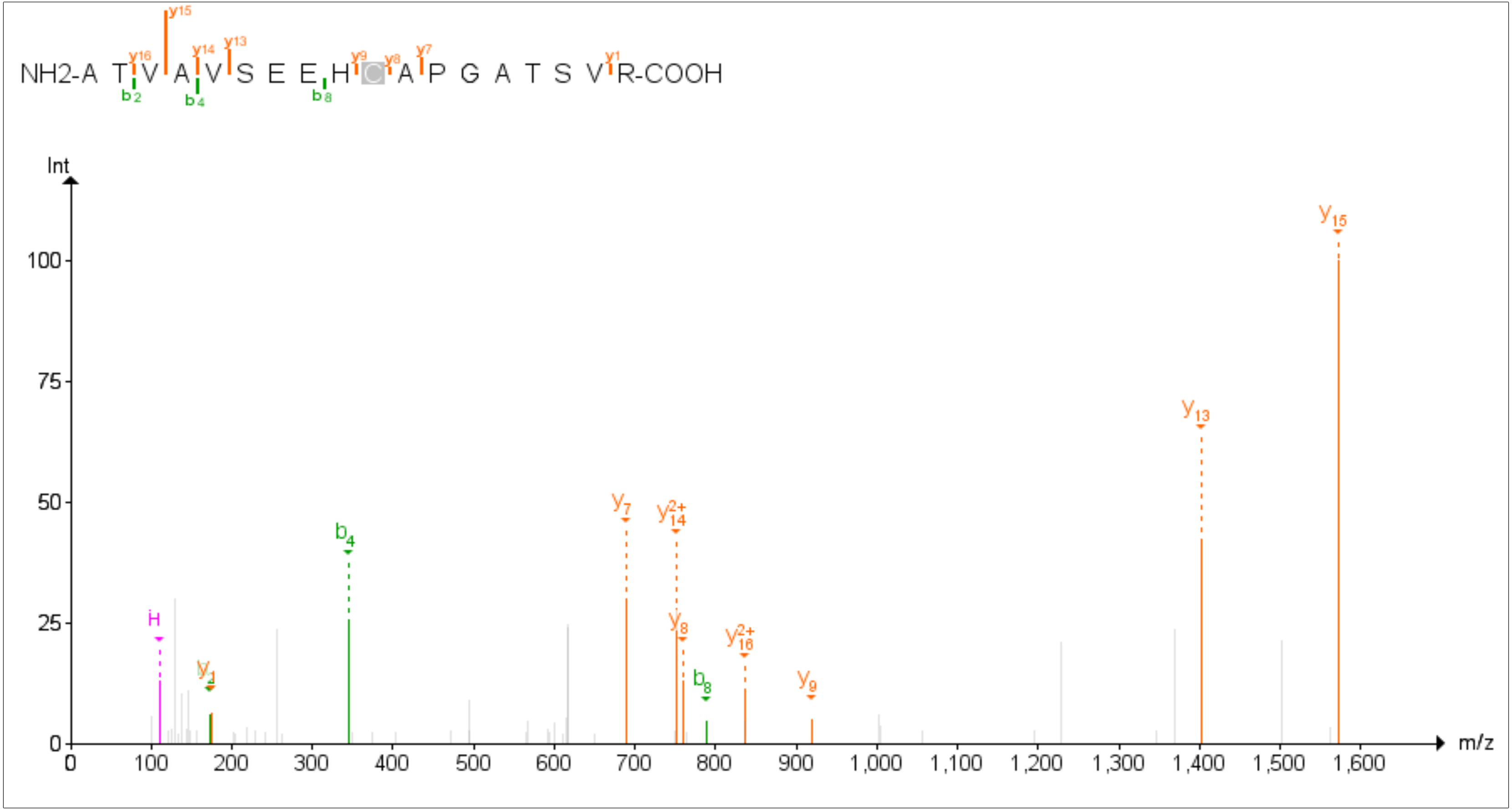

NH2-A T V A V S E E H C A P G A T S V R-COOH
Int
m/z

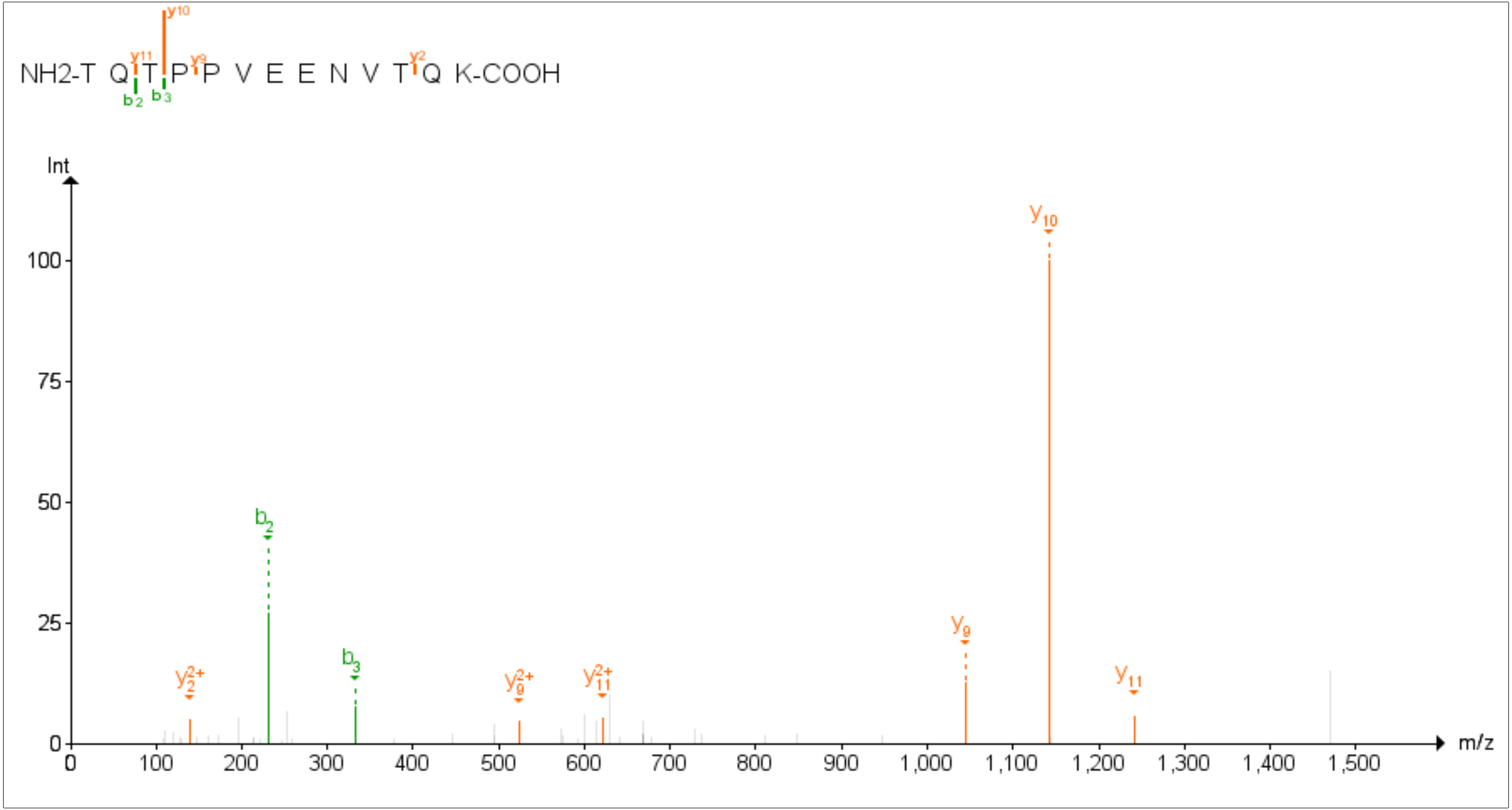

NH2-T Q T P P V E E N V T Q K-COOH
y10
y11
y9
y2
b2
b3
Int
100
75
50
25
0
m/z
0
100
200
300
400
500
600
700
800
900
1,000
1,100
1,200
1,300
1,400
1,500
y10
b2
b3
y2 2+
y9 2+
y11 2+
y9
y11

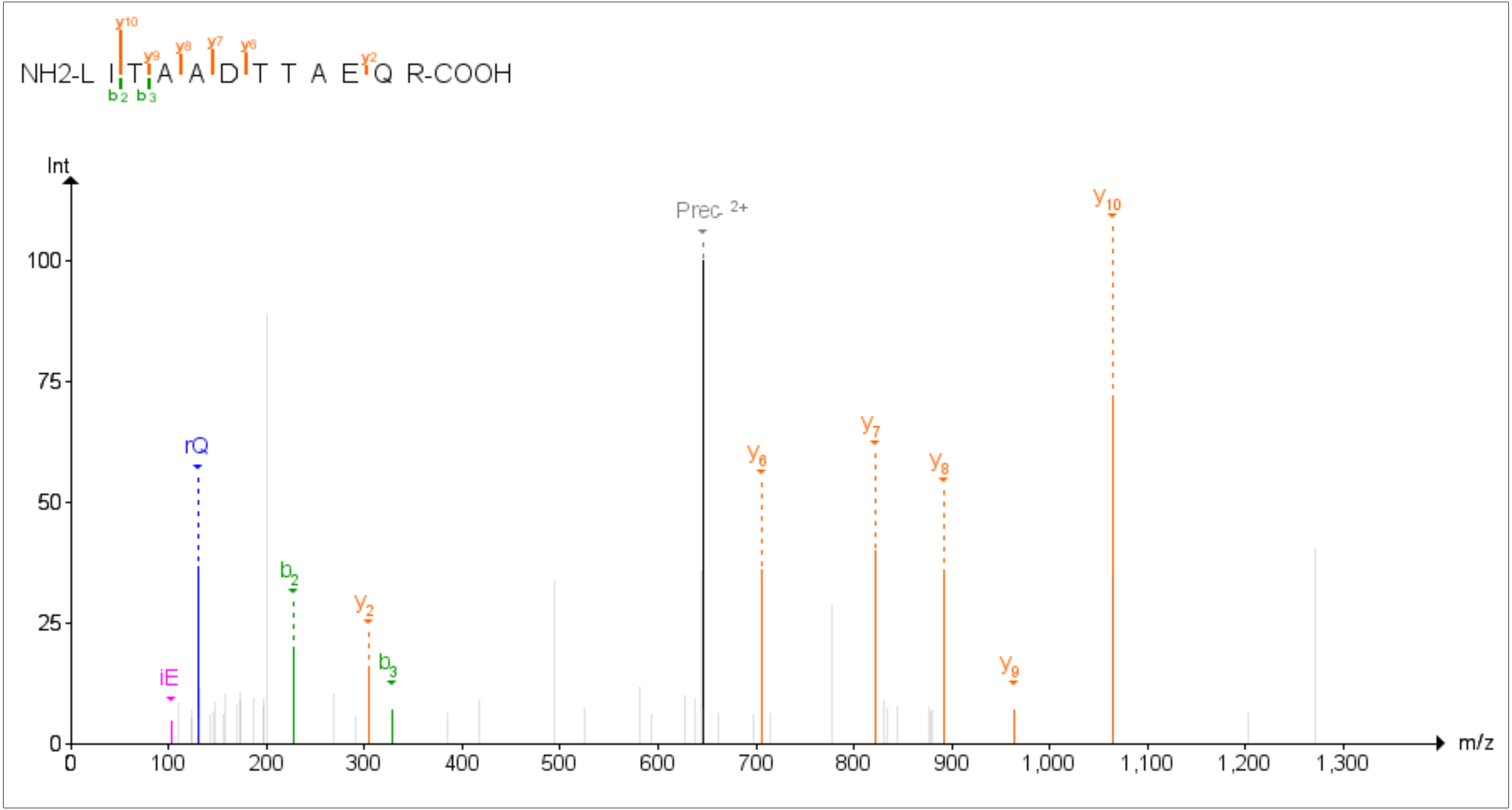

NH2-L I T A A D T T A E Q R-COOH
y10
y9
y8
y7
y6
y2
b 2
b 3
Int
100
75
50
25
0
Prec 2+
y10
rQ
y6
y7
y8
b2
y2
b3
iE
y9
0
100
200
300
400
500
600
700
800
900
1,000
1,100
1,200
1,300
m/z

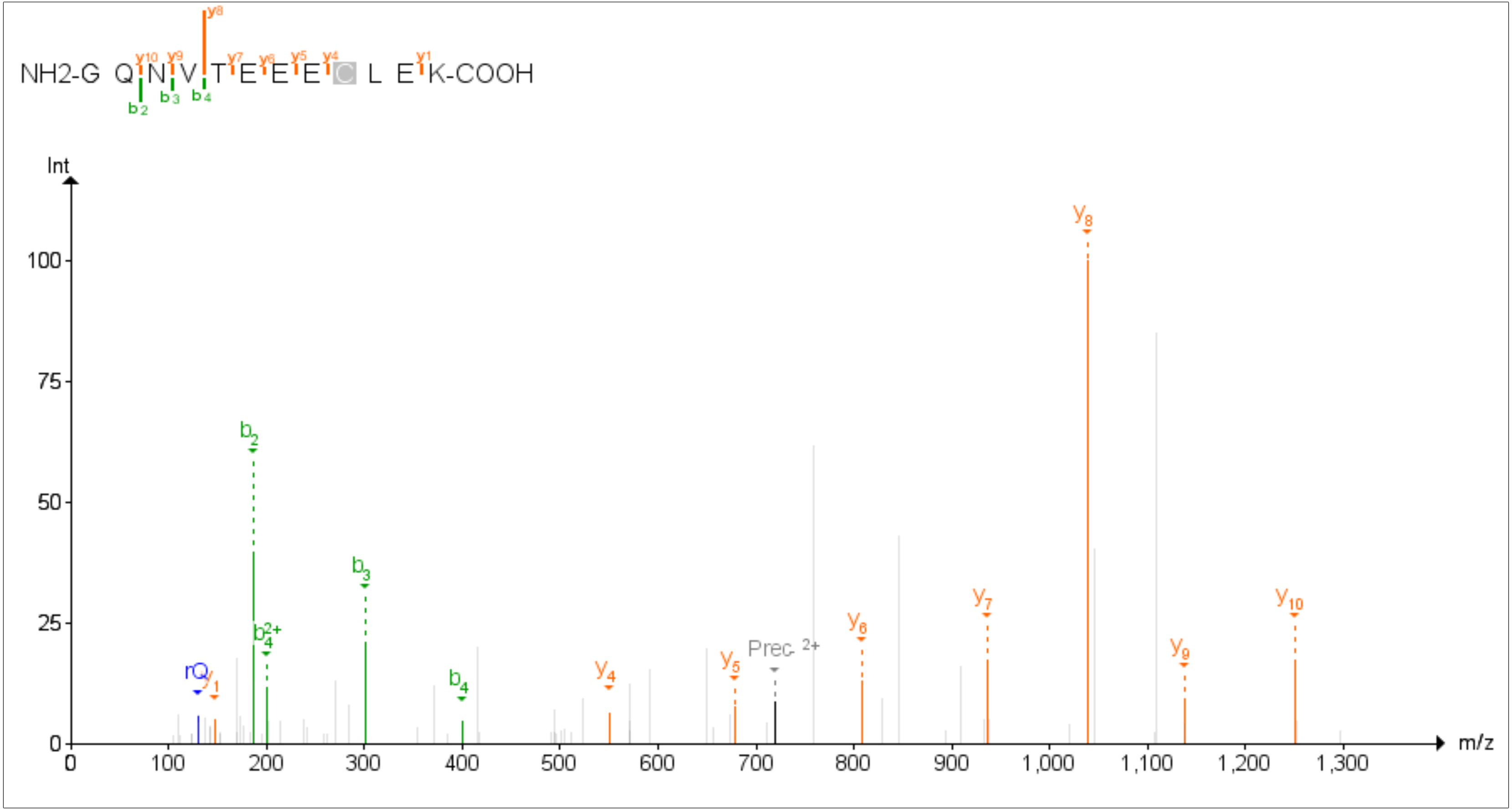
NH2-G Q N V T E E E C L E K-COOH
y10 y9 y8 y7 y6 y5 y4 y1
b2 b3 b4
Int
m/z
y8
y7
y10
y6
y9
y5
Prec 2+
y4
b2
b3
b4 2+
b4
rQ
y1
0
25
50
75
100
0
100
200
300
400
500
600
700
800
900
1,000
1,100
1,200
1,300

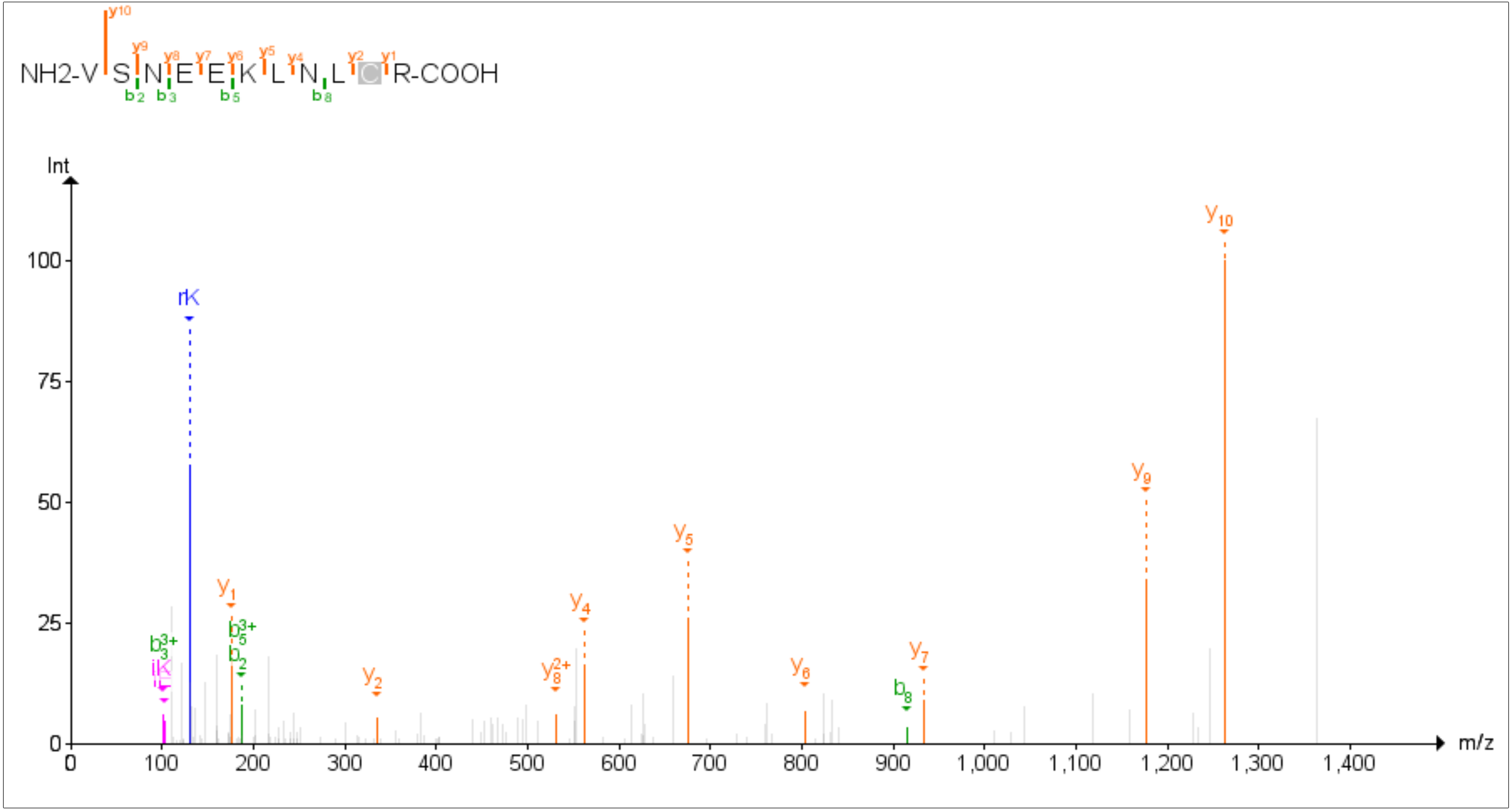

NH2-VSNEEKLNLCR-COOH
Int
m/z
rK
iK
y10
y9
y5
y4
y1
y2
y8 2+
y6
y7
b3 3+
b5 3+
b2
b8

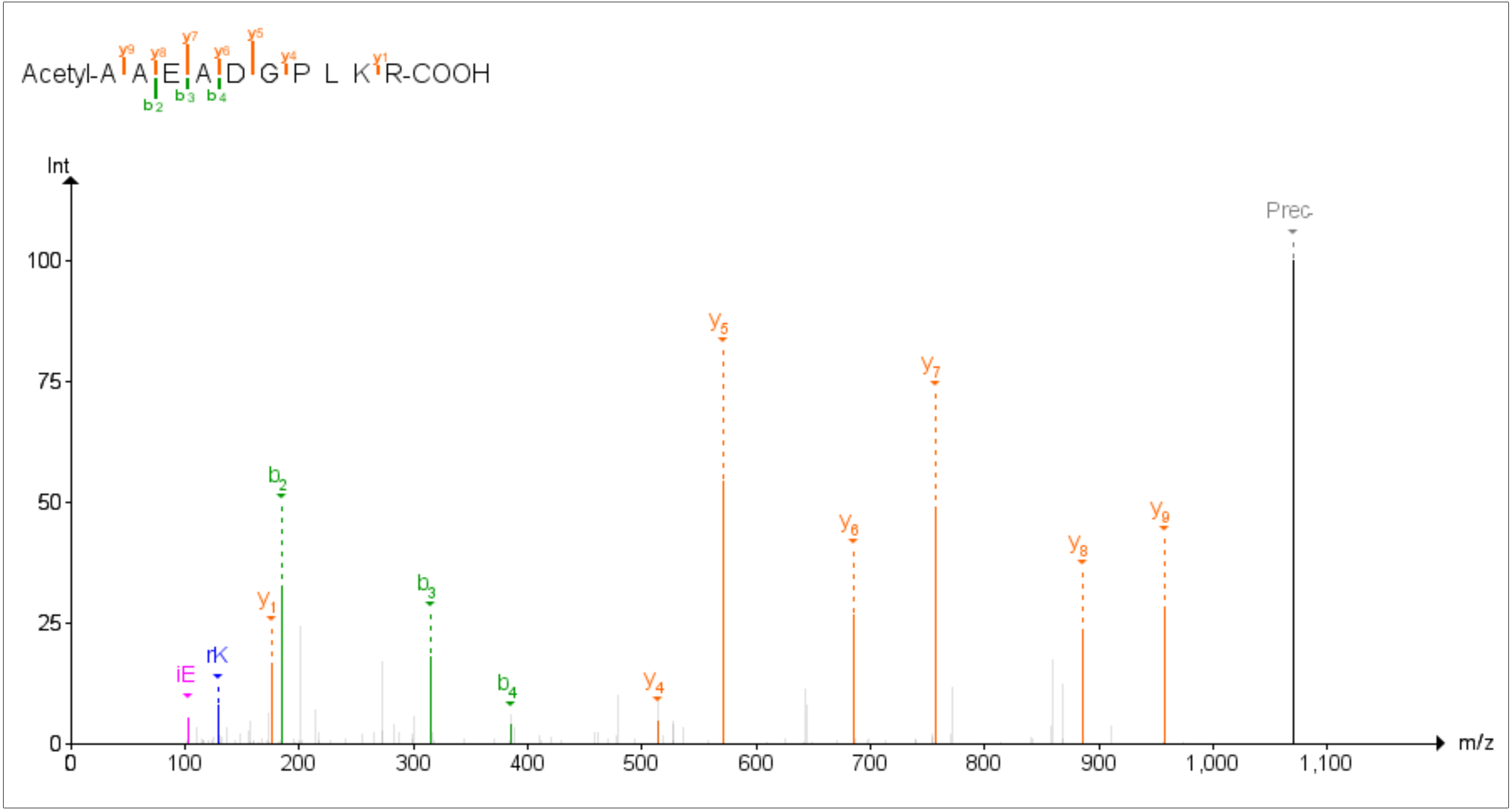
Acetyl-A A E A D G P L K R-COOH
y9 y8 y7 y6 y5 y4 y1
b2 b3 b4
Int
m/z
Prec
y5 y7 y6 y9 y8 b2 b3 y1 b4 y4 rK iE
0 25 50 75 100
0 100 200 300 400 500 600 700 800 900 1,000 1,100

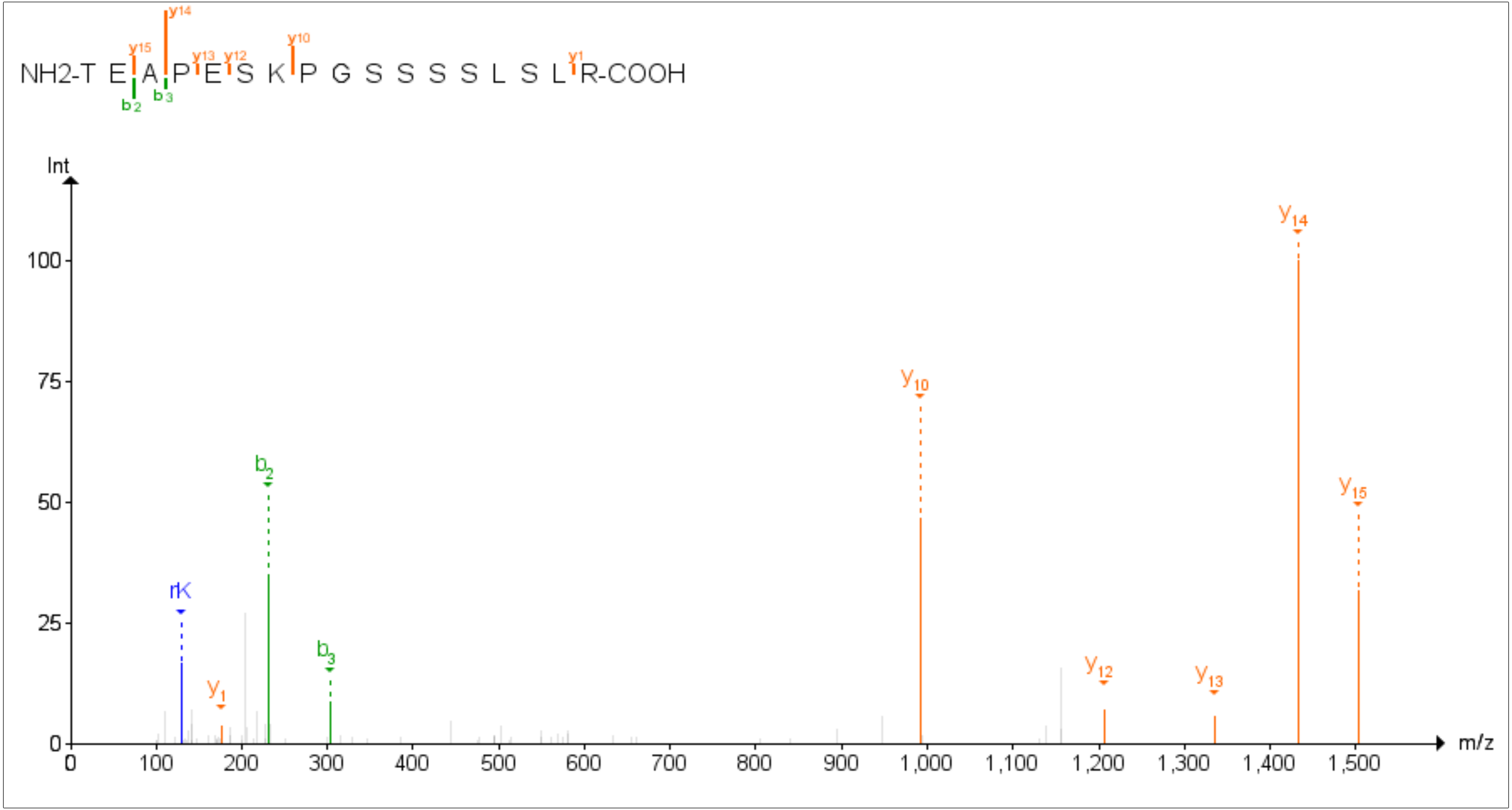

NH2-T E A P E S K P G S S S S L S L R-COOH
y14
y15
y13
y12
y10
y1
b2
b3
Int
m/z
y14
y10
b2
y15
rK
b3
y1
y12
y13

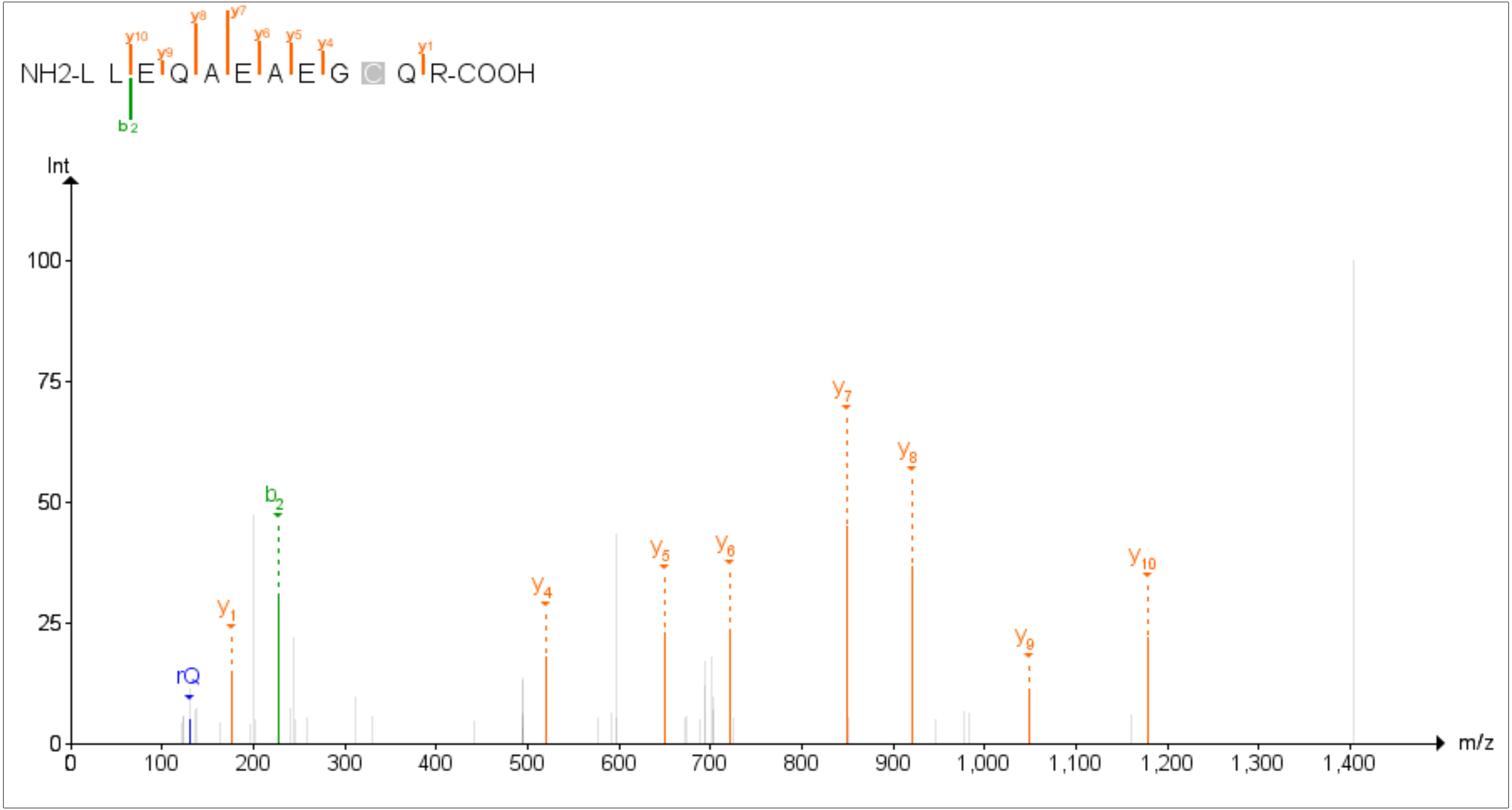
NH2-L L E Q A E A E G C Q R-COOH
y10 y9 y8 y7 y6 y5 y4 y1
b2
Int
100
75
50
25
0
m/z
0 100 200 300 400 500 600 700 800 900 1,000 1,100 1,200 1,300 1,400
rQ
y1
b2
y4
y5
y6
y7
y8
y9
y10

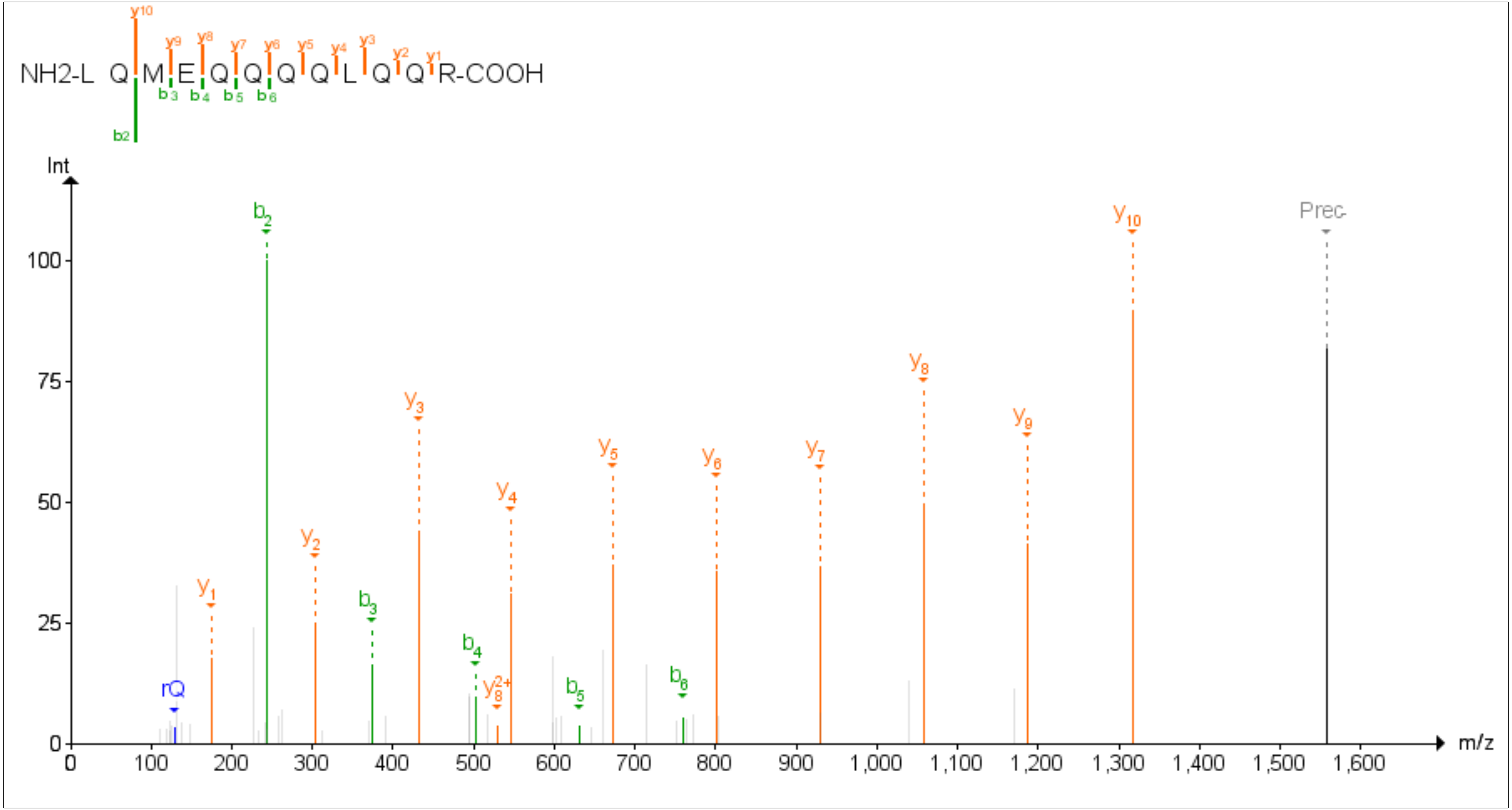

NH2-L Q M E Q Q Q Q L Q Q R-COOH
y10 y9 y8 y7 y6 y5 y4 y3 y2 y1
b2 b3 b4 b5 b6
Int
m/z
0 25 50 75 100
0 100 200 300 400 500 600 700 800 900 1,000 1,100 1,200 1,300 1,400 1,500 1,600
rQ
y1 y2 y3 y4 y5 y6 y7 y8 y9 y10
b2 b3 b4 b5 b6
y8 2+
Prec

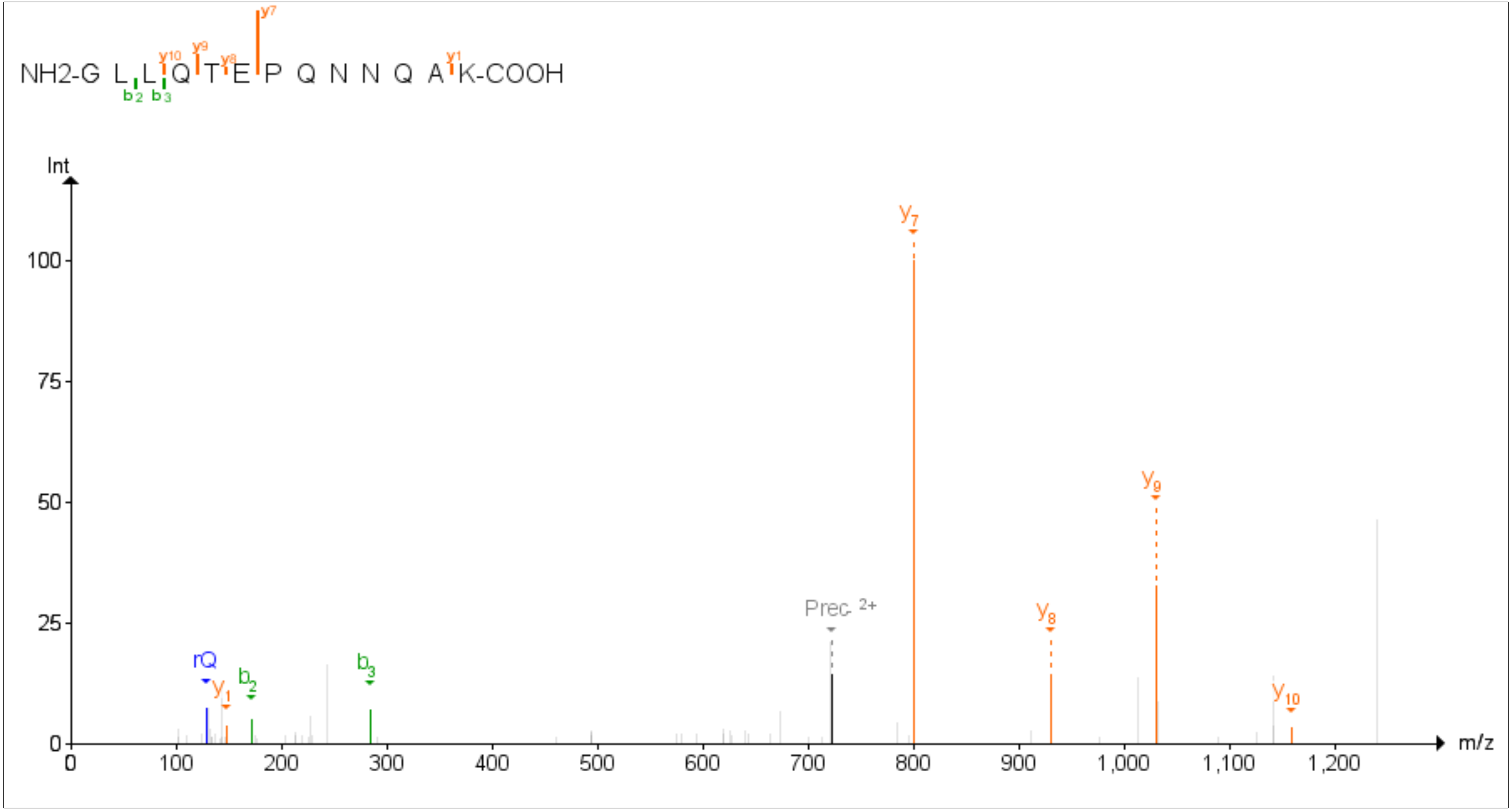

NH2-G L L Q T E P Q N N Q A K-COOH
y7
y10
y9
y8
y1
b2
b3
Int
100
75
50
25
0
y7
y9
y8
y10
Prec 2+
rQ
y1
b2
b3
m/z
0
100
200
300
400
500
600
700
800
900
1,000
1,100
1,200

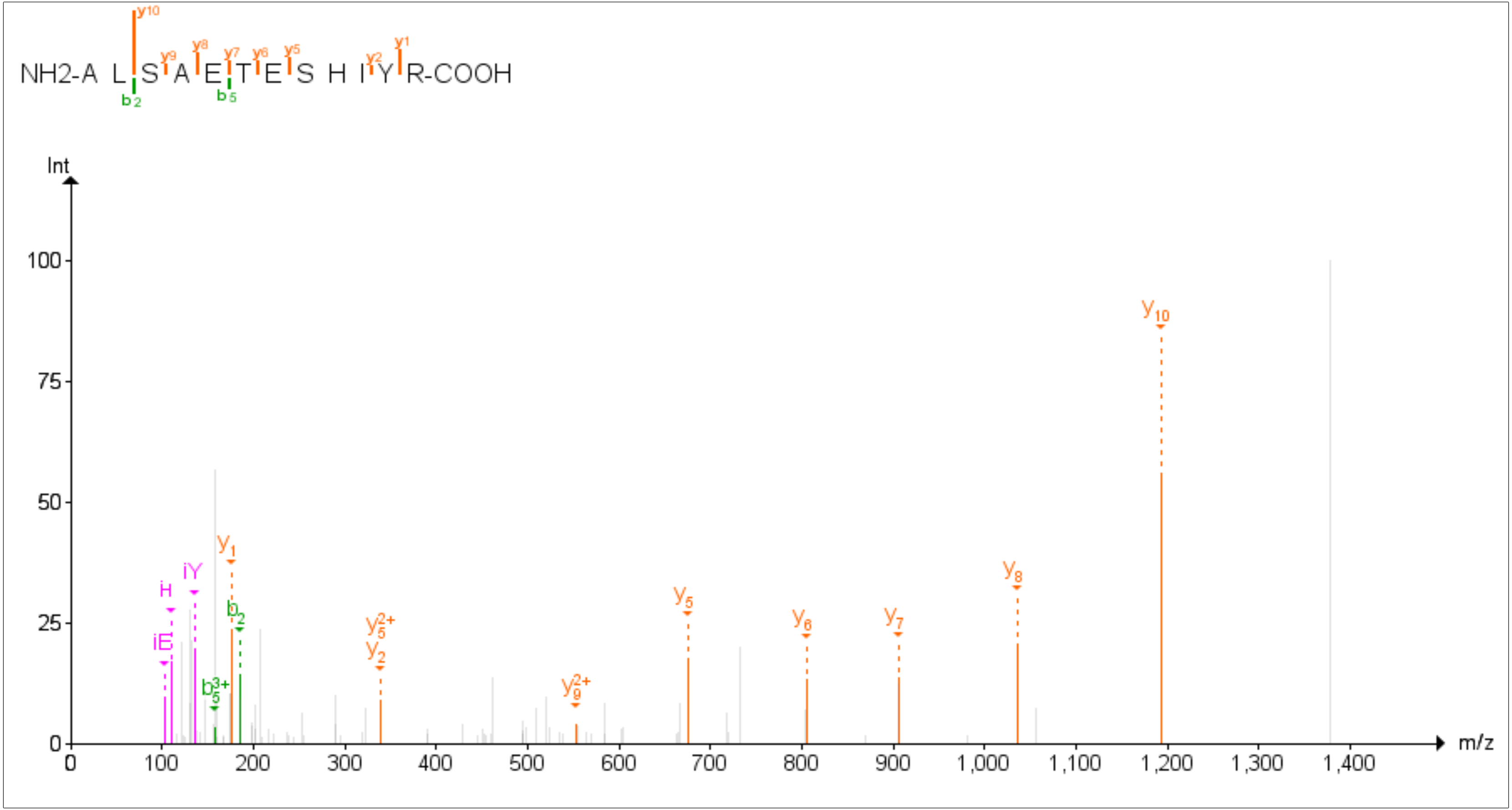

NH2-A L S A E T E S H I Y R-COOH
y10
y9
y8
y7
y6
y5
y2
y1
b2
b5
Int
100
75
50
25
0
y10
y8
y7
y6
y5
y1
iY
iH
b2
y5 2+
y2
iE
b5 3+
y9 2+
m/z
0
100
200
300
400
500
600
700
800
900
1,000
1,100
1,200
1,300
1,400

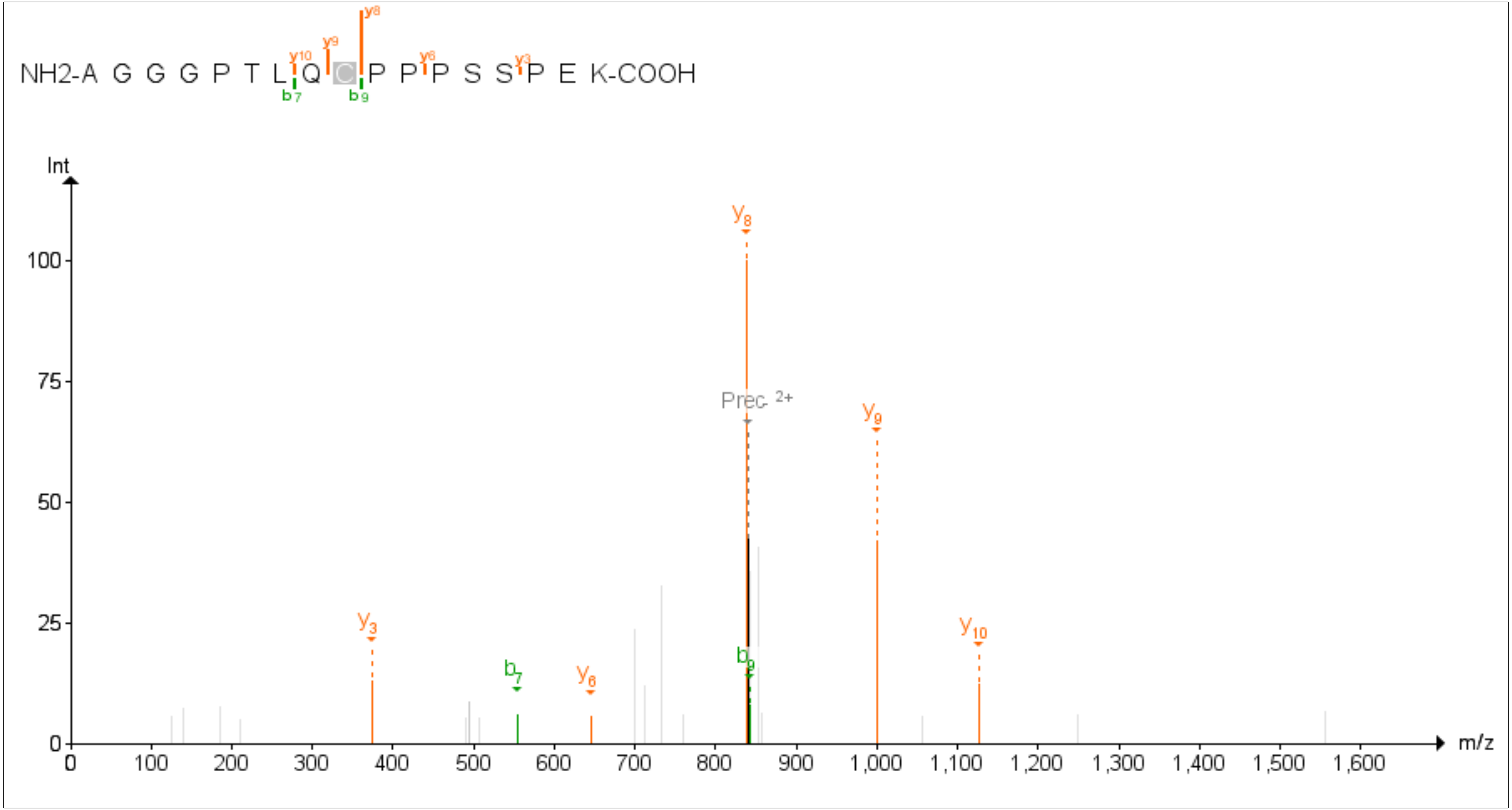

NH2-A G G G P T L Q C P P P S S P E K-COOH
y10 y9 y8 y6 y3
b7 b9
Int
100
75
50
25
0
Prec 2+
y8
y9
y3
b7
y6
b9
y10
0 100 200 300 400 500 600 700 800 900 1,000 1,100 1,200 1,300 1,400 1,500 1,600
m/z

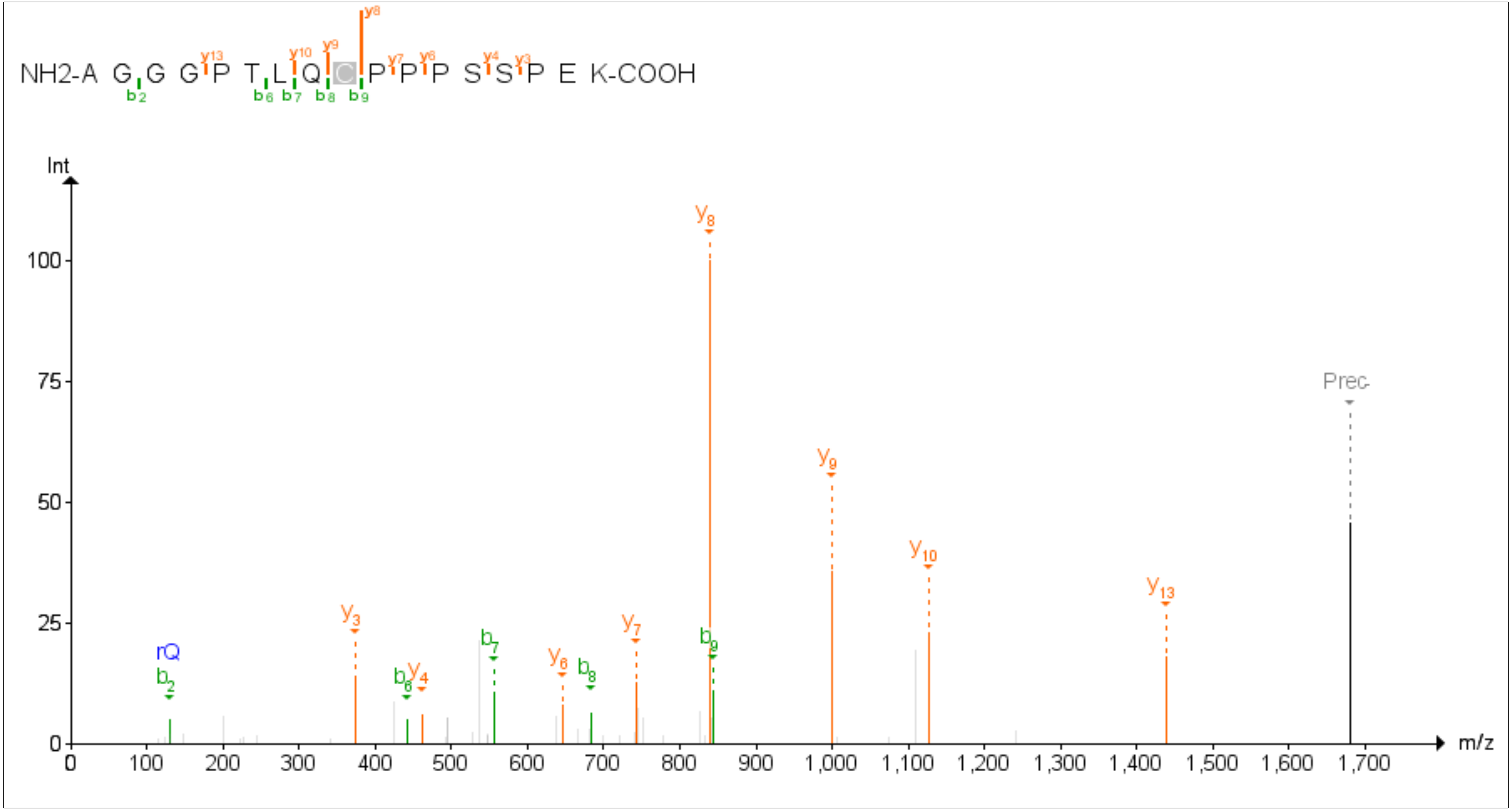

NH2-A G G G P T L Q C P P P S S P E K-COOH
y13 y10 y9 y8 y7 y6 y4 y3
b2 b6 b7 b8 b9
Int
100
75
50
25
0
y8
y9
y10
y13
y3
y7
y6
y4
b9
b8
b7
b6
b2
rQ
Prec.
m/z
0
100
200
300
400
500
600
700
800
900
1,000
1,100
1,200
1,300
1,400
1,500
1,600
1,700

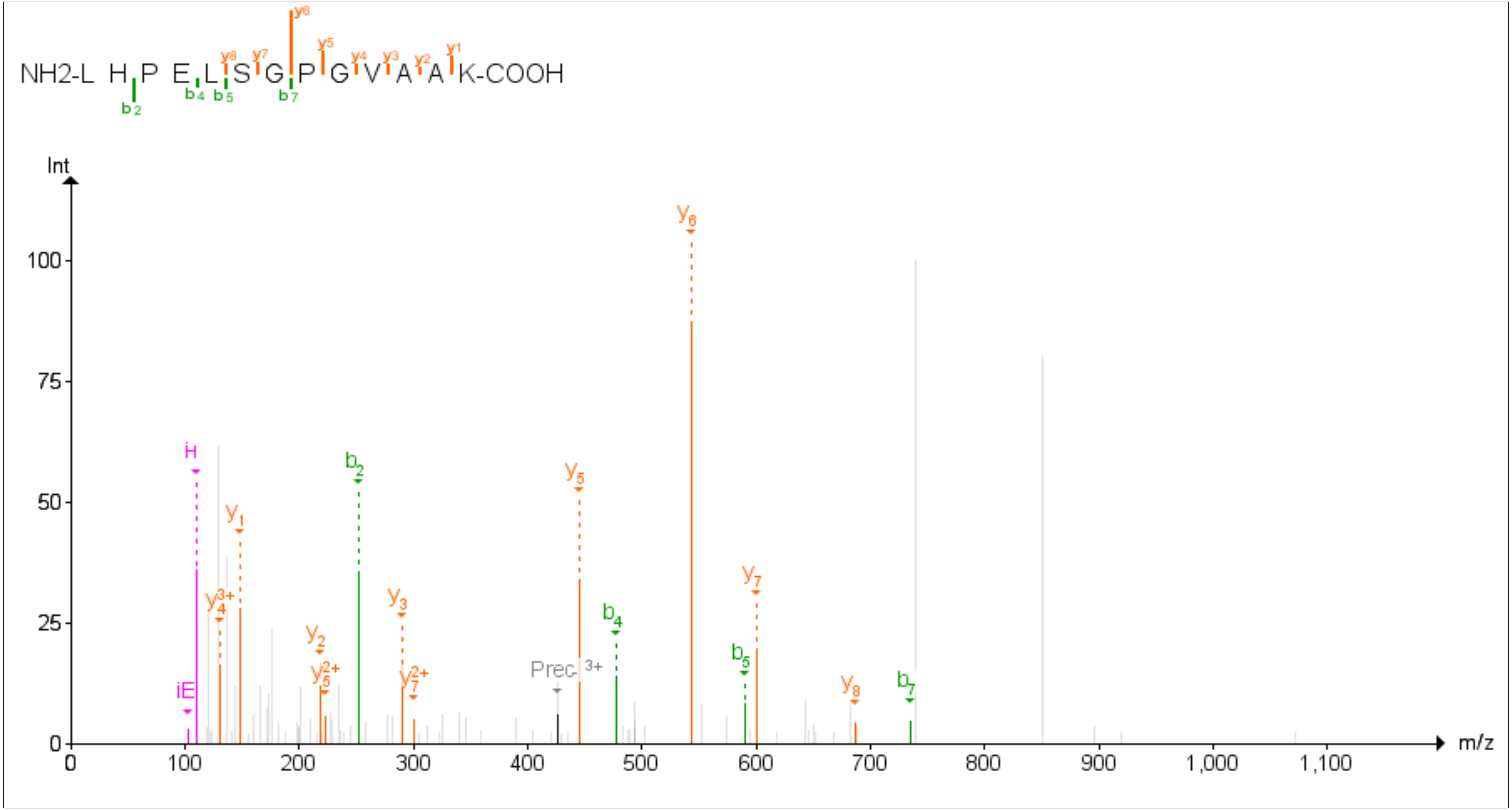

NH2-L H P E L S G P G V A A K-COOH
y8 y7 y6 y5 y4 y3 y2 y1
b2 b4 b5 b7
Int
m/z
100
75
50
25
0
0 100 200 300 400 500 600 700 800 900 1,000 1,100
iH
iE
y1
y4 3+
b2
y2
y5 2+
y3
y7 2+
Prec 3+
y5
b4
y6
b5
y7
y8
b7

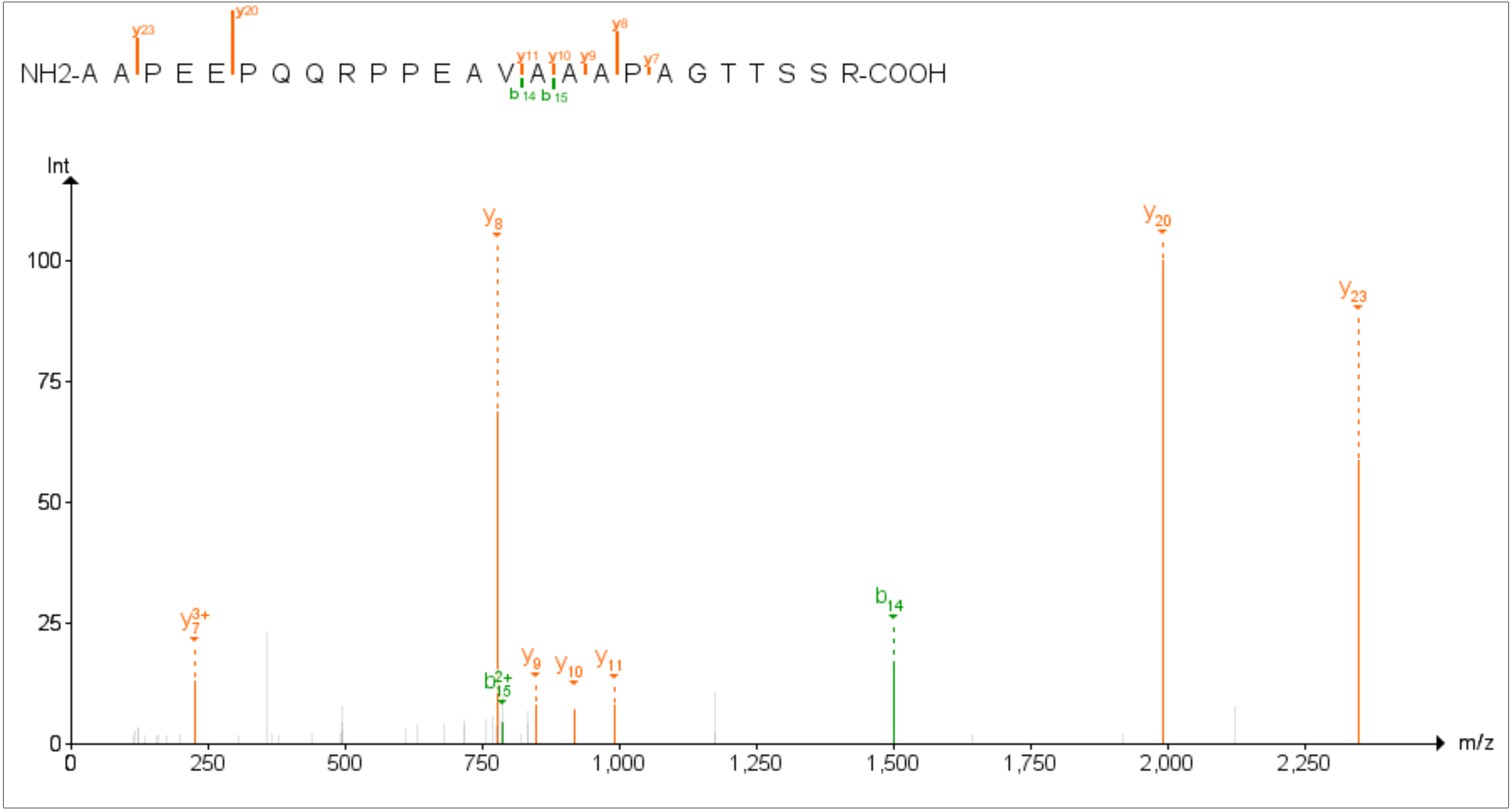
NH2-A A P E E P Q Q R P P E A V A A A P A G T T S S R-COOH
y23
y20
y11
y10
y9
y8
y7
b 14
b 15
Int
100
75
50
25
0
y_8
y_{20}
y_{23}
y_7^{3+}
b_{15}^{2+}
y_9
y_{10}
y_{11}
b_{14}
0
250
500
750
1,000
1,250
1,500
1,750
2,000
2,250
m/z

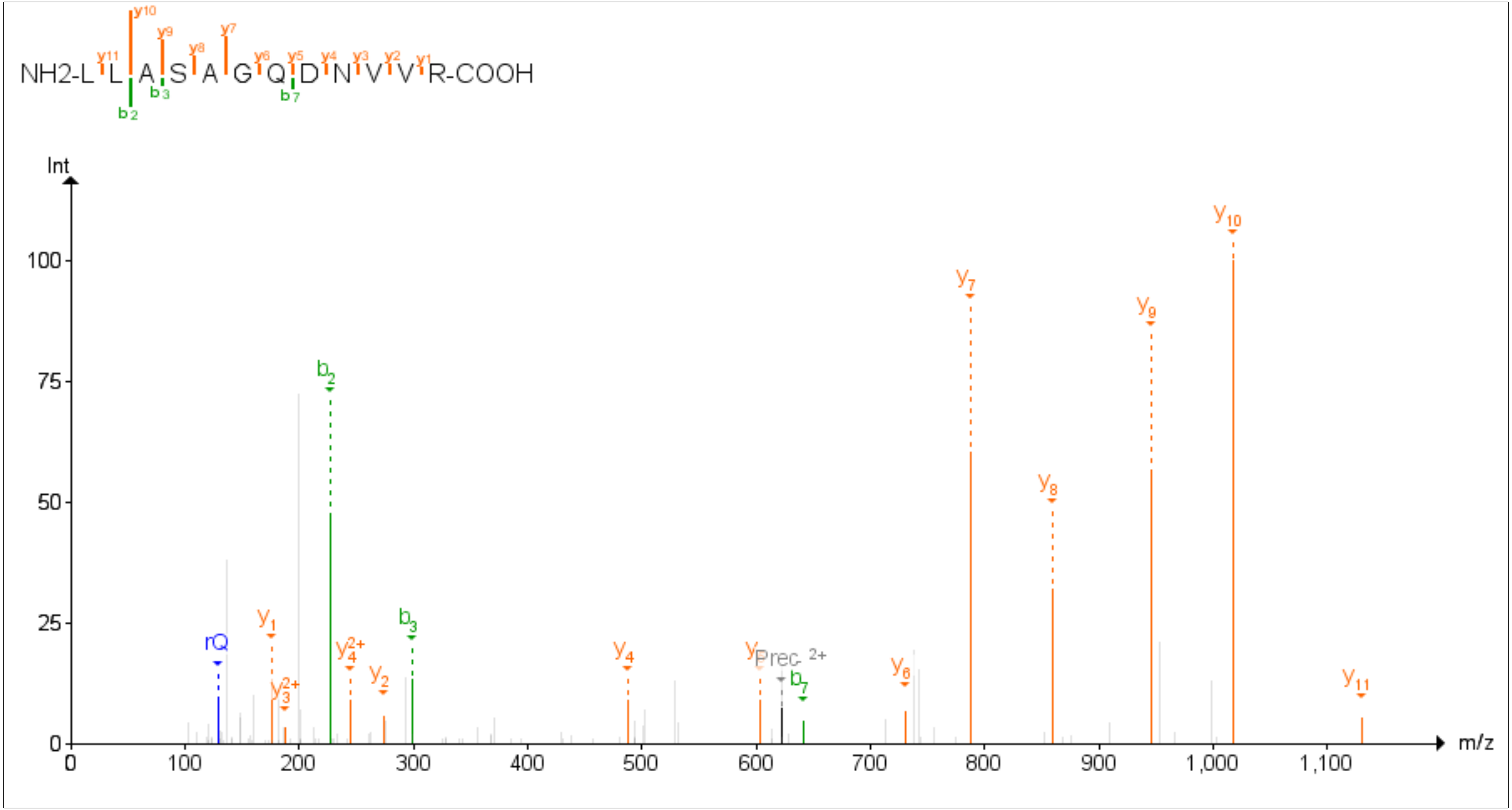

NH2-L L A S A G Q D N V V R-COOH
y11 y10 y9 y8 y7 y6 y5 y4 y3 y2 y1
b2 b3 b7
Int
100
75
50
25
0
m/z
0
100
200
300
400
500
600
700
800
900
1,000
1,100
rQ
y_1
y_3^{2+}
b_2
y_4^{2+}
y_2
b_3
y_4
Prec 2+
b_7
y_6
y_7
y_8
y_9
y_{10}
y_{11}

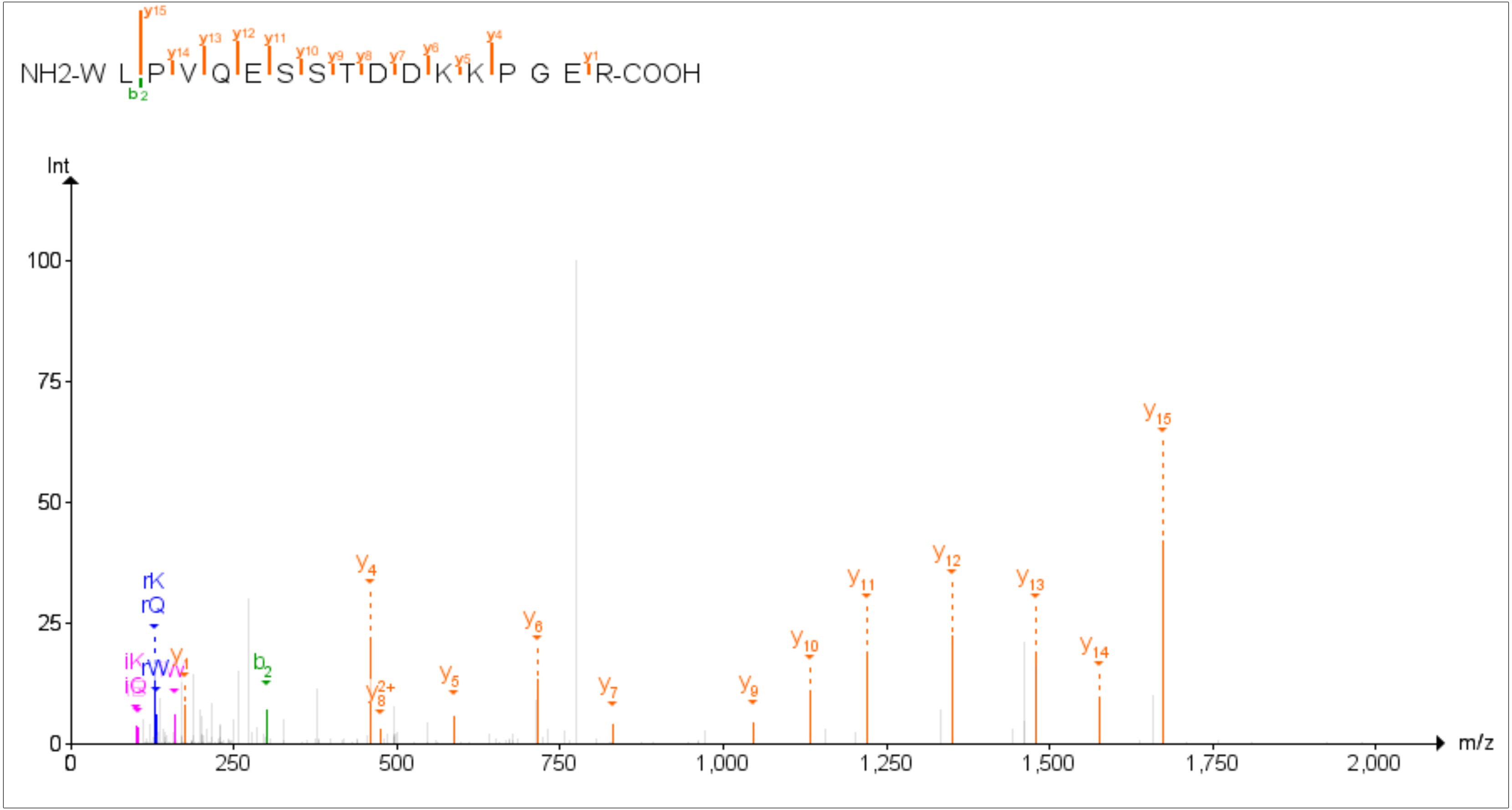

NH2-W L P V Q E S S T D D K K P G E R-COOH
y15
y14
y13
y12
y11
y10
y9
y8
y7
y6
y5
y4
y1
b2
Int
100
75
50
25
0
m/z
0
250
500
750
1,000
1,250
1,500
1,750
2,000
rK
rQ
iK
rW
iQ
y1
b2
y4
y8 2+
y5
y6
y7
y9
y10
y11
y12
y13
y14
y15

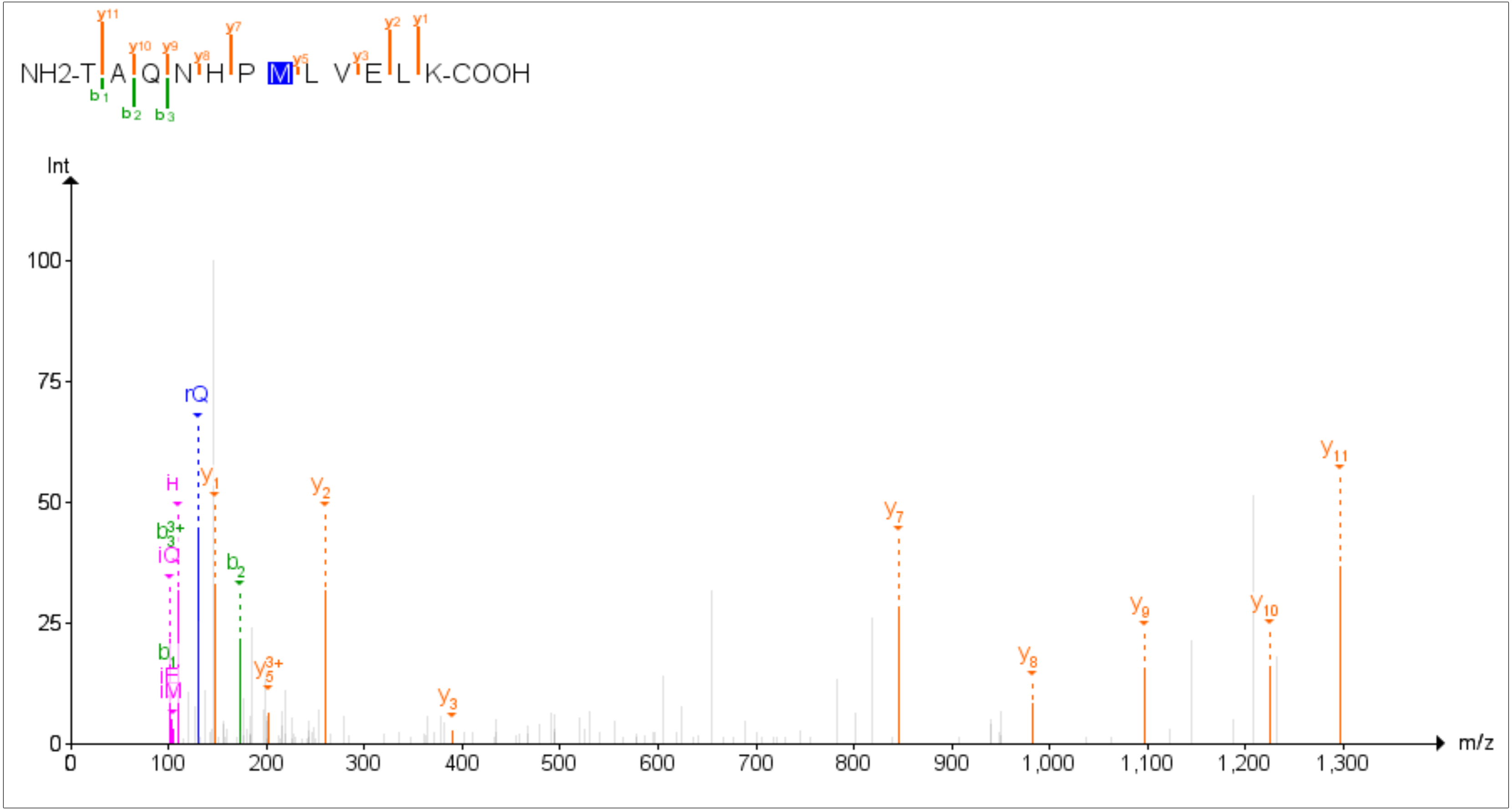

NH2-T A Q N H P M L V E L K-COOH
y11 y10 y9 y8 y7 y5 y3 y2 y1
b1 b2 b3
Int
100
75
50
25
0
rQ
iH
y1
y2
b3 3+
iQ
b2
b1
iE
iM
y5 3+
y3
y7
y8
y9
y10
y11
0 100 200 300 400 500 600 700 800 900 1,000 1,100 1,200 1,300
m/z

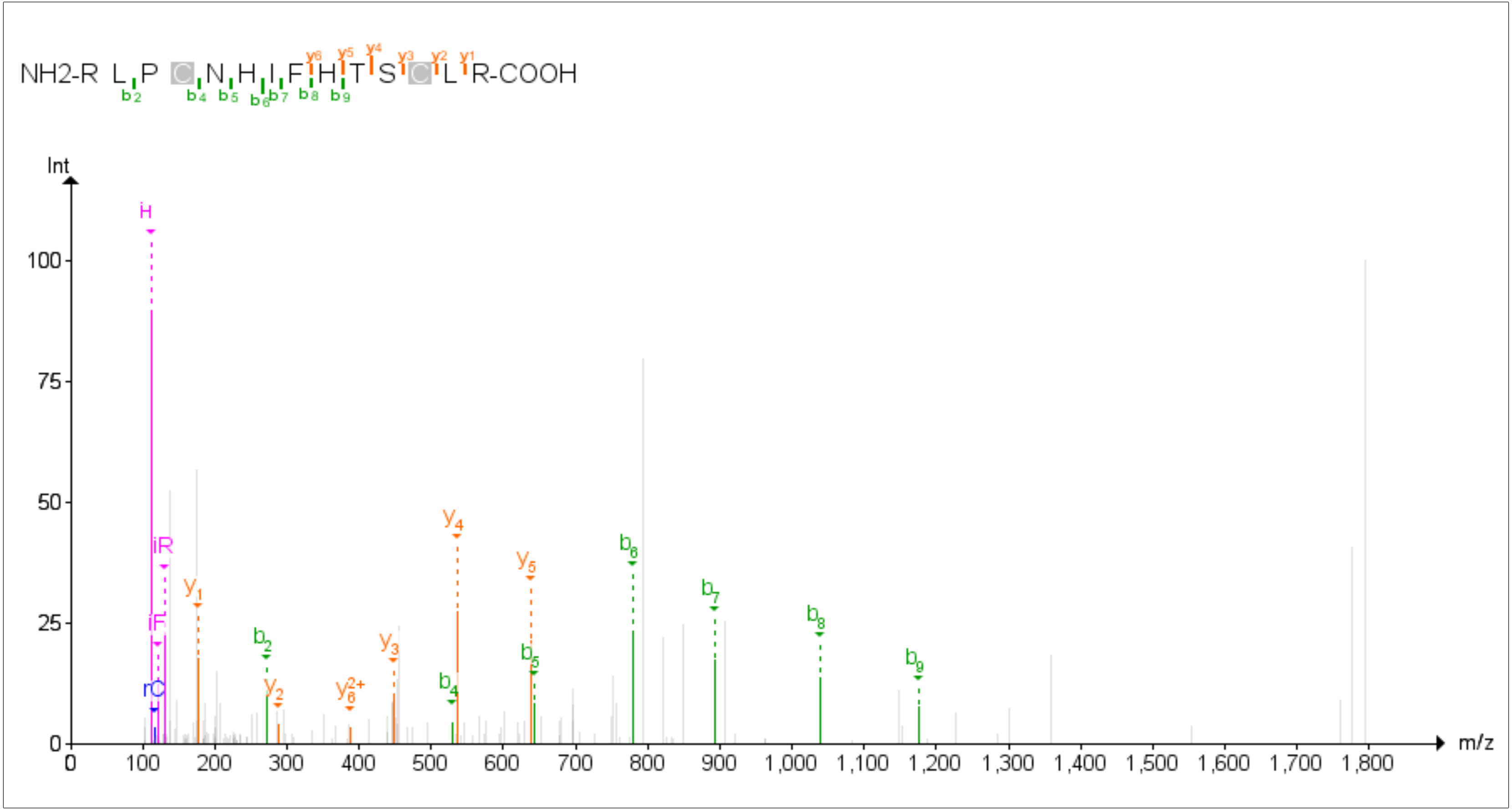

NH2-R L P C N H I F H T S C L R-COOH
y6 y5 y4 y3 y2 y1
b2 b4 b5 b6 b7 b8 b9
Int
m/z
iH
iR
iF
rC
y1
y2
y3
y4
y5
y6 2+
b2
b4
b5
b6
b7
b8
b9
0
25
50
75
100
0
100
200
300
400
500
600
700
800
900
1,000
1,100
1,200
1,300
1,400
1,500
1,600
1,700
1,800

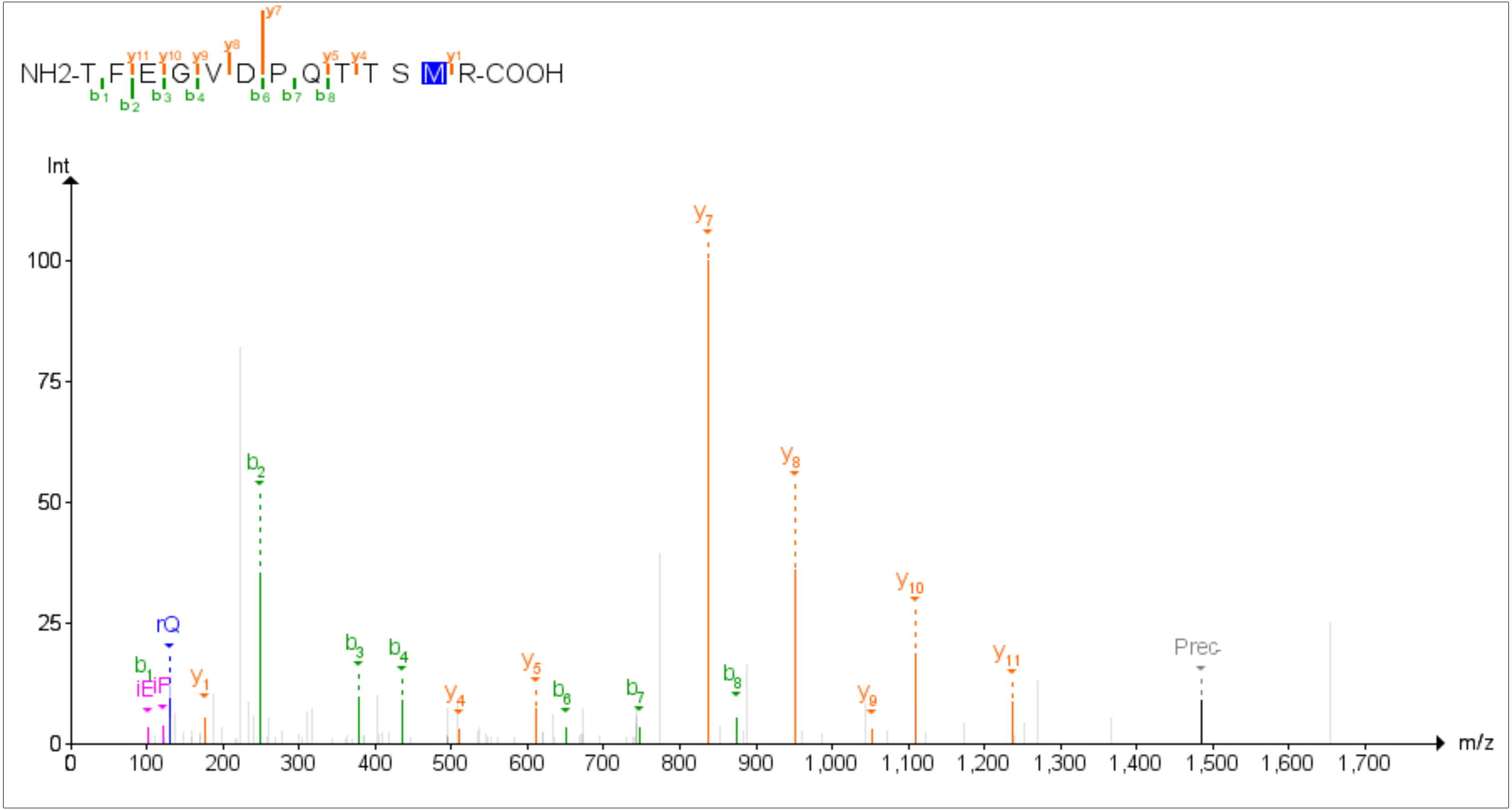
NH2-T F E G V D P Q T T S M R-COOH
y11 y10 y9 y8 y7 y5 y4 y1
b1 b2 b3 b4 b6 b7 b8
Int
100
75
50
25
0
m/z
0 100 200 300 400 500 600 700 800 900 1,000 1,100 1,200 1,300 1,400 1,500 1,600 1,700
y7
y8
y10
y11
y9
y5
y4
y1
b2
b3
b4
b6
b7
b8
b1
rQ
iE
iP
Prec

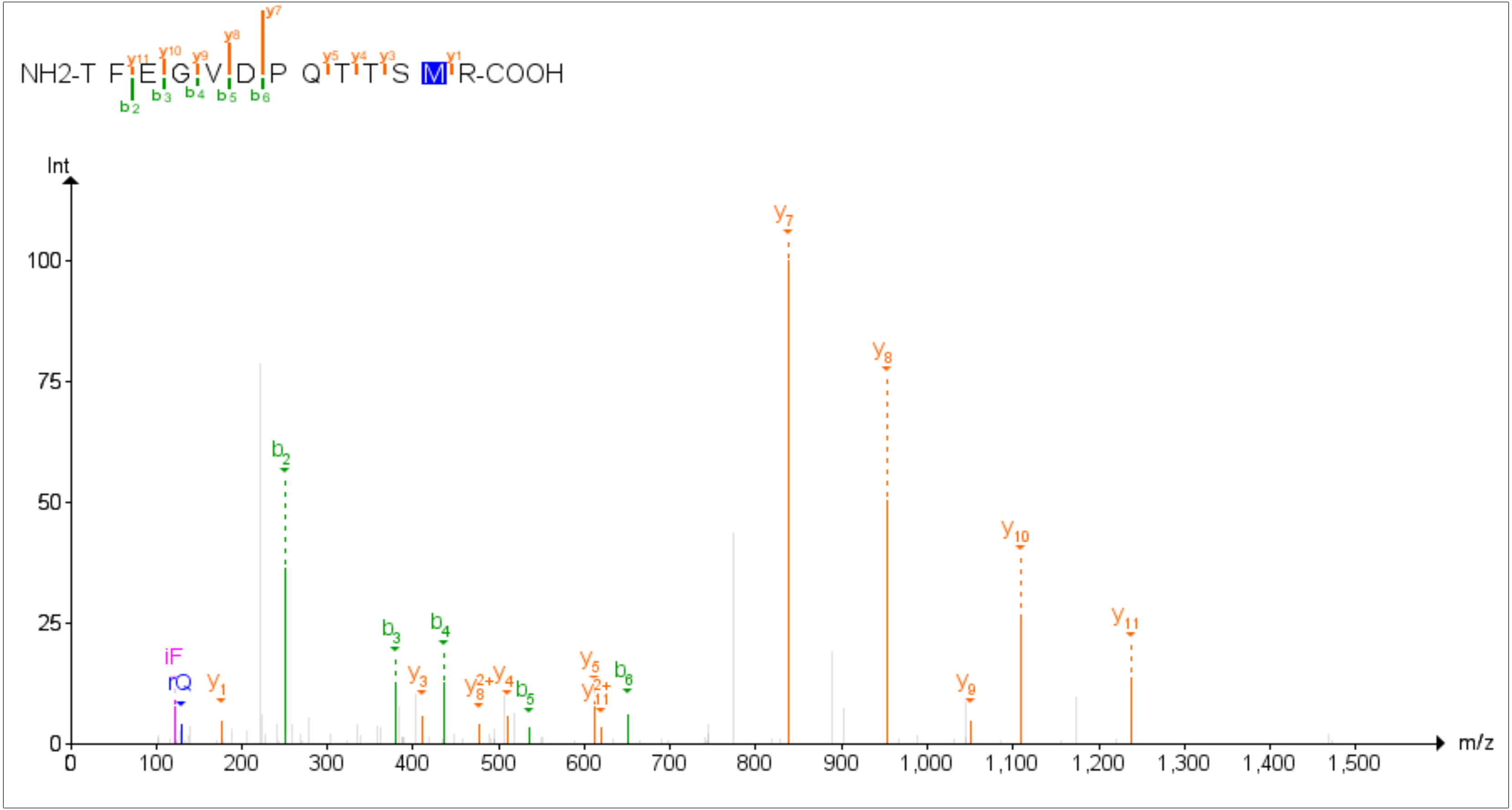
NH2-T F E G V D P Q T T T S M R-COOH
y11 y10 y9 y8 y7 y5 y4 y3 y1
b2 b3 b4 b5 b6
Int
100
75
50
25
0
y7
y8
b2
y10
y11
b3
b4
iF
rQ
y1
y3
y8 2+
y4
b5
y5
y11 2+
b6
y9
0 100 200 300 400 500 600 700 800 900 1,000 1,100 1,200 1,300 1,400 1,500
m/z

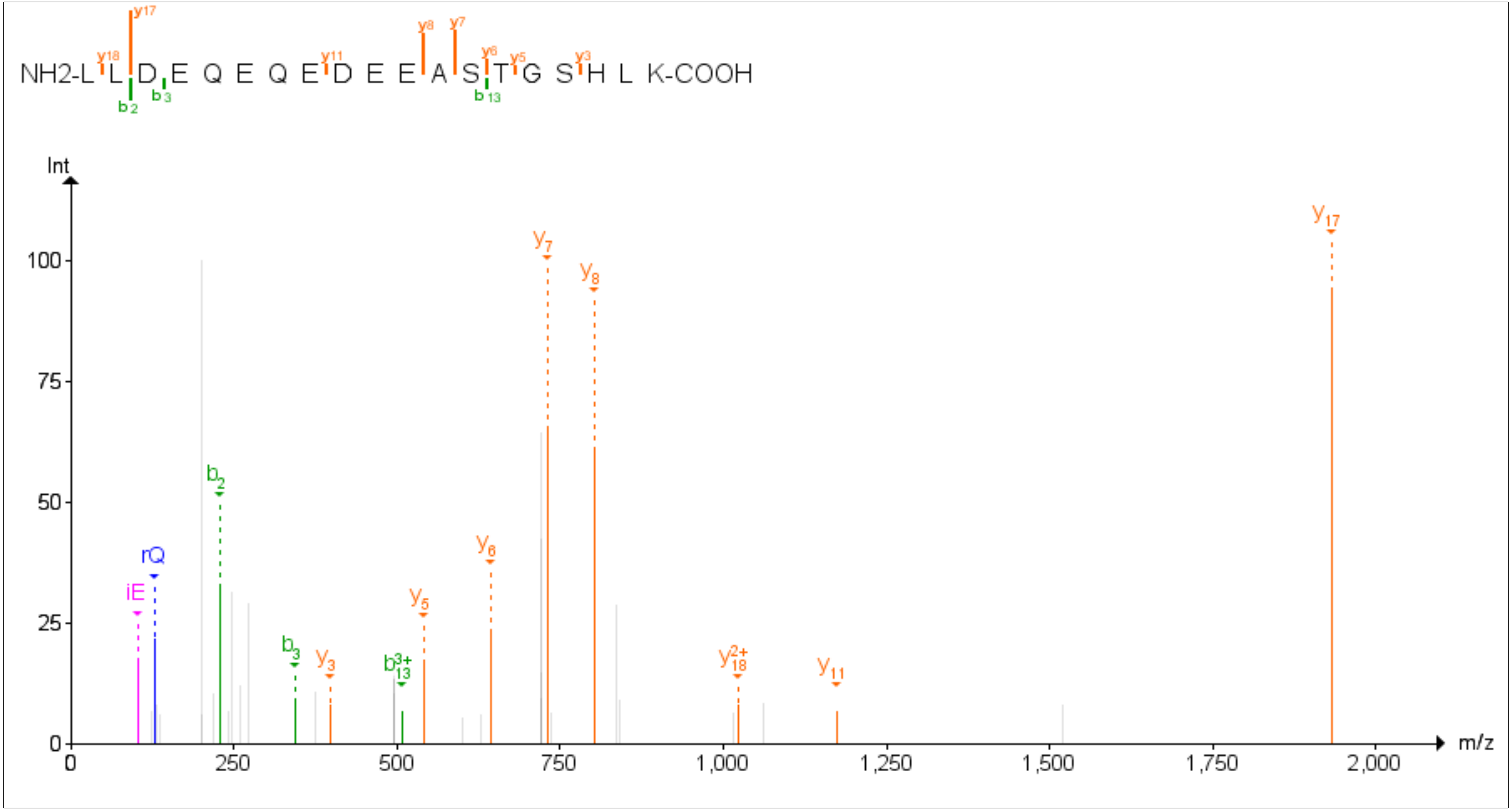

NH2-L L D E Q E Q E D E E A S T G S H L K-COOH
y17
y18
y11
y8
y7
y6
y5
y3
b2
b3
b13
Int
100
75
50
25
0
m/z
0
250
500
750
1,000
1,250
1,500
1,750
2,000
y17
y7
y8
b2
y6
rQ
iE
y5
b3
y3
b13 3+
y18 2+
y11

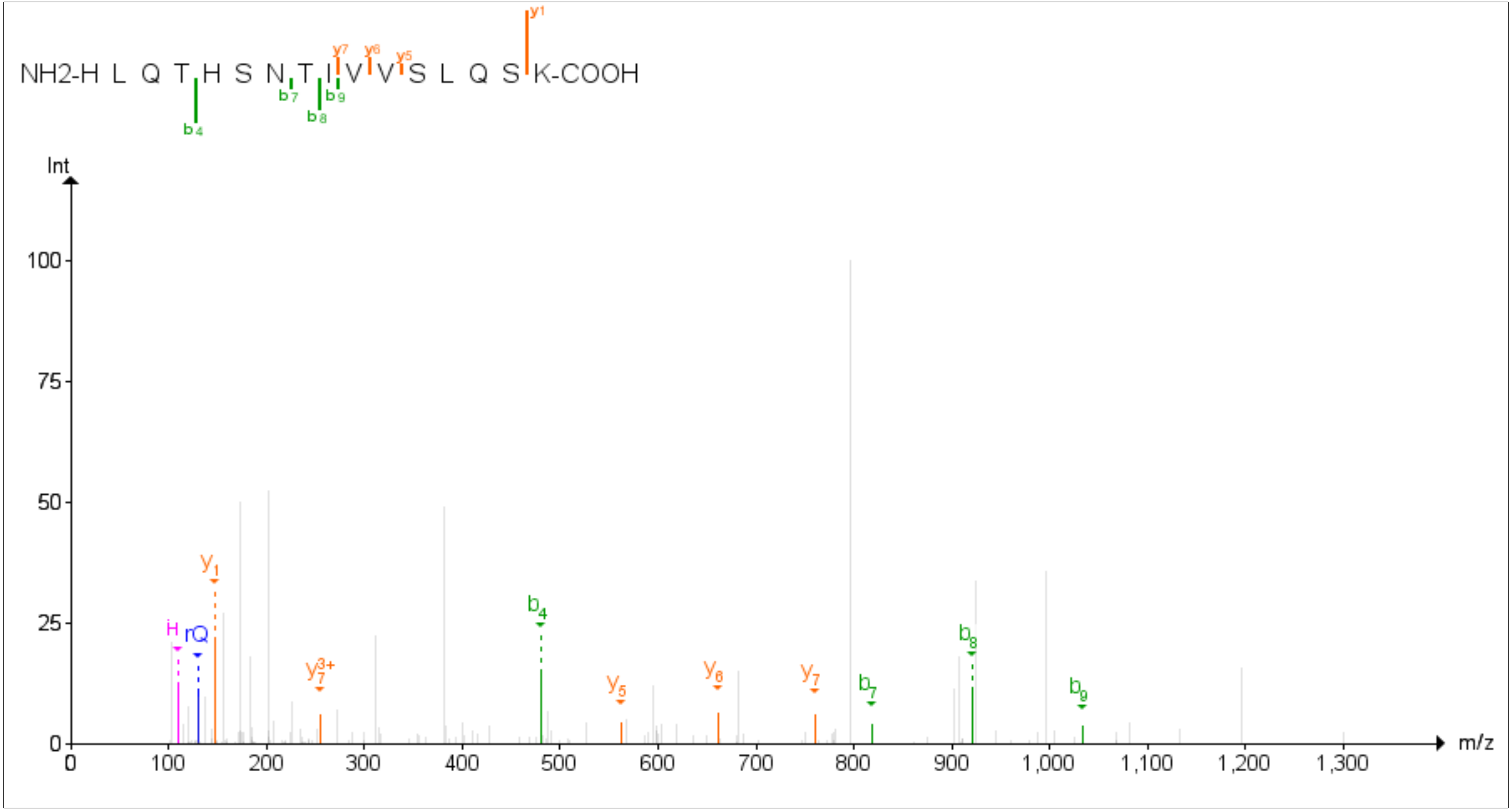

NH2-H L Q T H S N T I V V S L Q S K-COOH
y1
y7
y6
y5
b4
b7
b8
b9
Int
100
75
50
25
0
m/z
0
100
200
300
400
500
600
700
800
900
1,000
1,100
1,200
1,300
iH
rQ
y1
y7 3+
b4
y5
y6
y7
b7
b8
b9

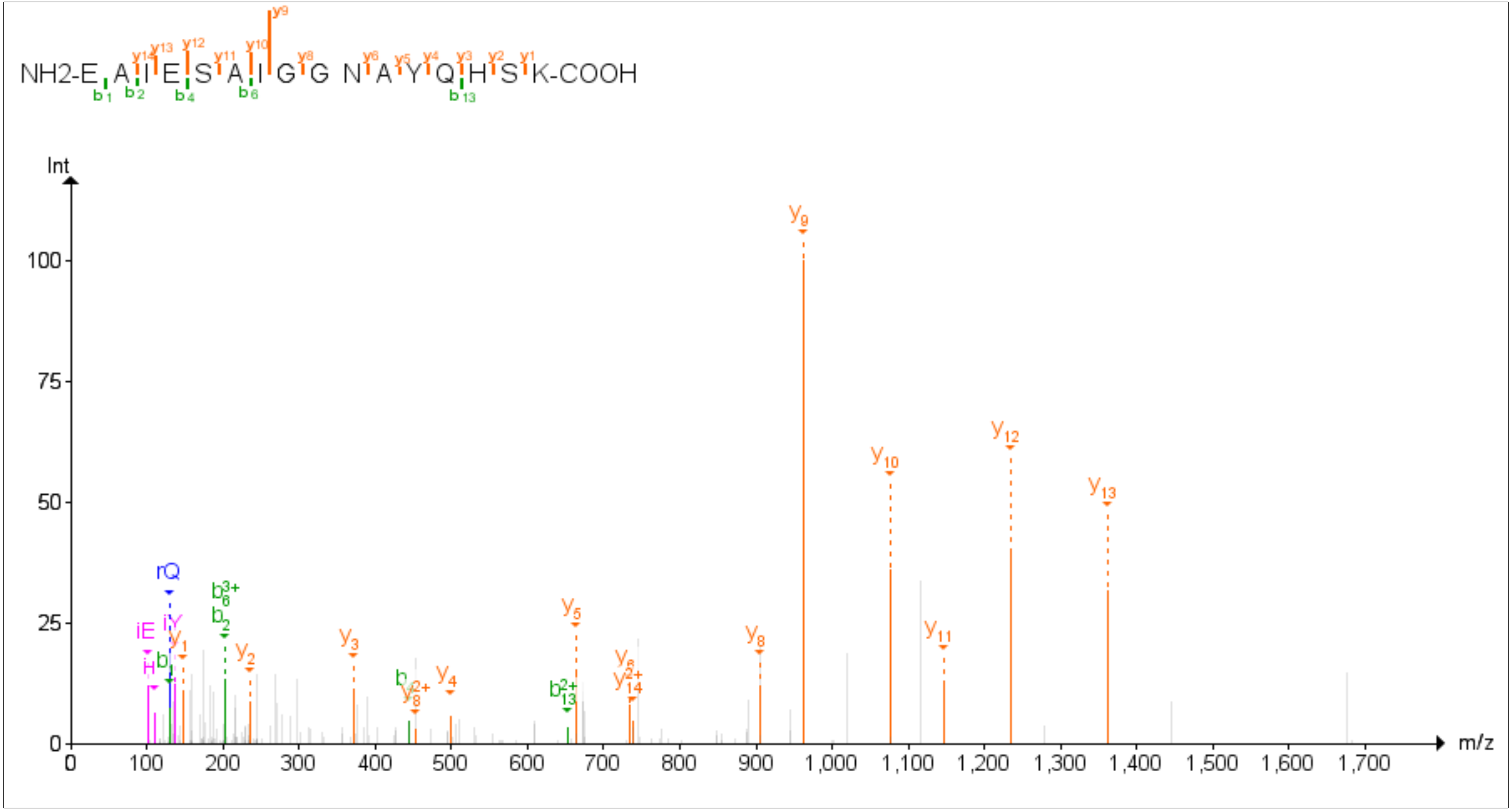

NH2-E A I E S A I G G N A Y Q H S K-COOH
b1 b2 b4 b6 b13
y14 y13 y12 y11 y10 y9 y8 y6 y5 y4 y3 y2 y1
Int
100
75
50
25
0
m/z
0 100 200 300 400 500 600 700 800 900 1,000 1,100 1,200 1,300 1,400 1,500 1,600 1,700
y9
y10
y12
y13
y11
y8
y5
y3
y2
y1
y4
rQ
iE
iY
H
b6 3+
b2
b13 2+
y8 2+
y14 2+

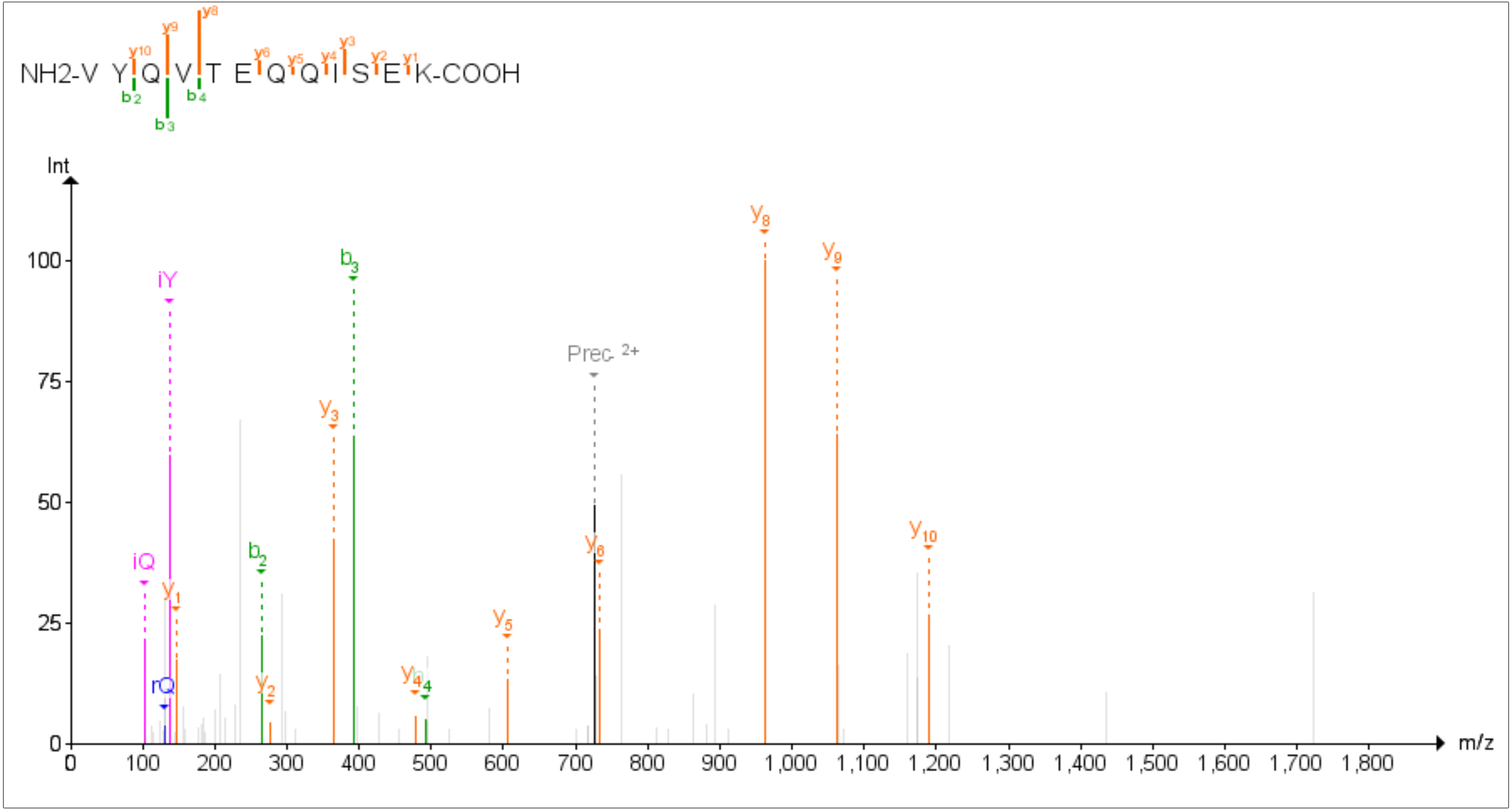

NH2-V Y Q V T E Q Q I S E K-COOH
y10 y9 y8 y6 y5 y4 y3 y2 y1
b2 b3 b4
Int
m/z
0 25 50 75 100
0 100 200 300 400 500 600 700 800 900 1,000 1,100 1,200 1,300 1,400 1,500 1,600 1,700 1,800
iY
iQ
rQ
y1
b2
y2
y3
b3
y4
b4
y5
Prec 2+
y6
y8
y9
y10

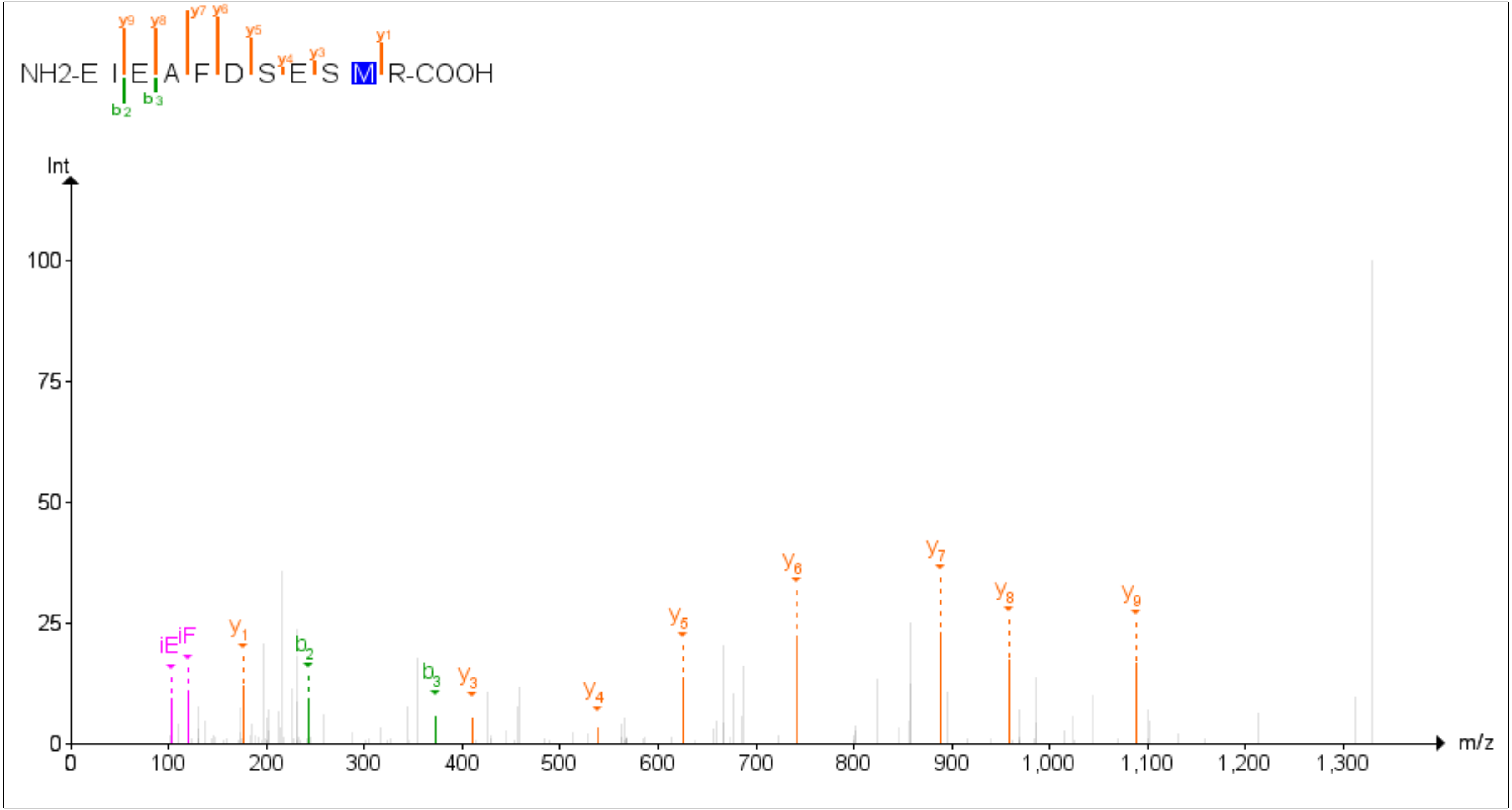

NH2-E I E A F D S E S M R-COOH
y9 y8 y7 y6 y5 y4 y3 y1
b2 b3
Int
100
75
50
25
0
m/z
0 100 200 300 400 500 600 700 800 900 1,000 1,100 1,200 1,300
iE iF
y_1 b_2 b_3 y_3 y_4 y_5 y_6 y_7 y_8 y_9

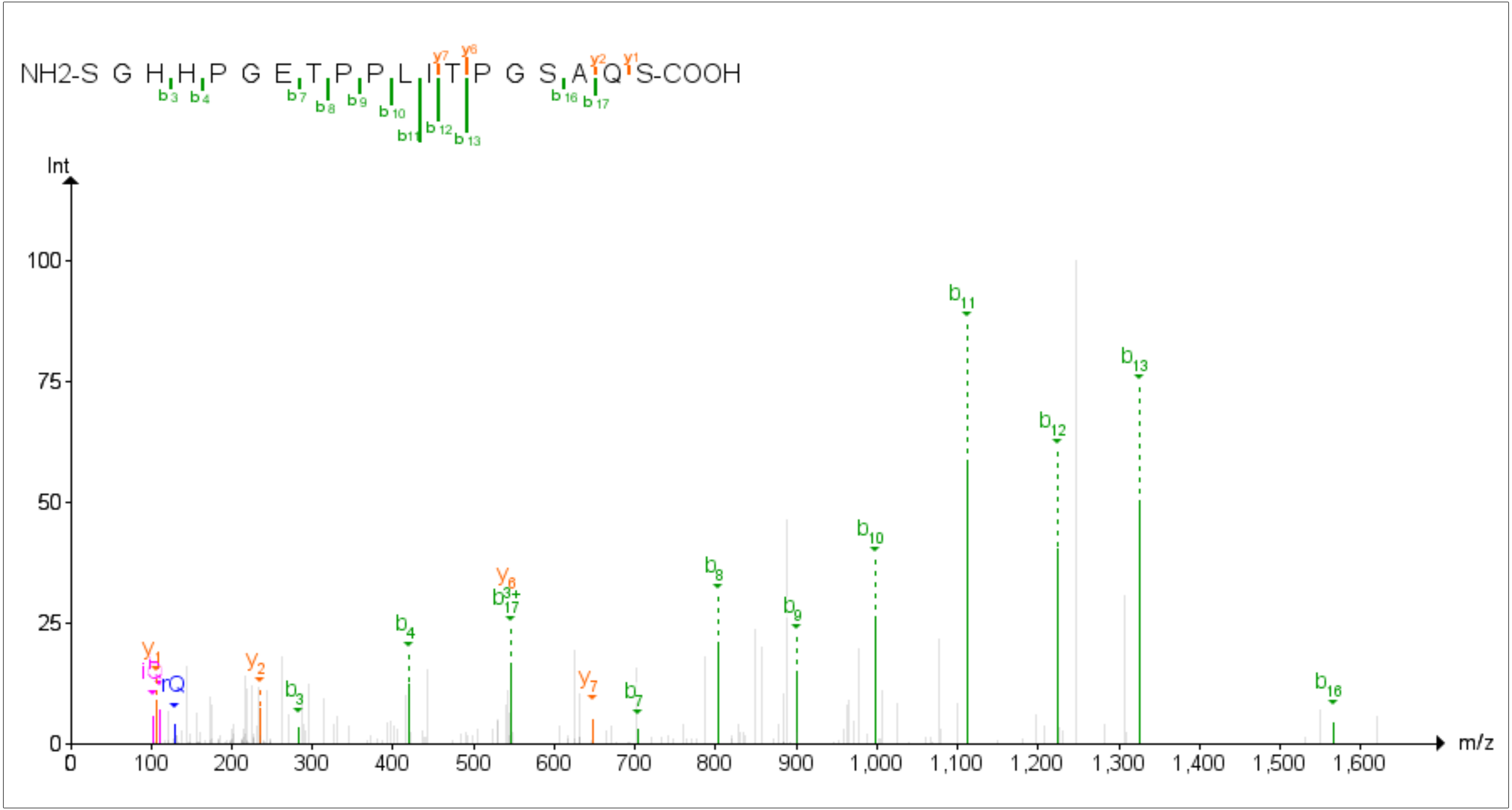

NH2-S G H H P G E T P P L I T P G S A Q S-COOH
b3 b4 b7 b8 b9 b10 b11 b12 b13 b16 b17
y7 y6 y2 y1
Int
m/z
0
25
50
75
100
0 100 200 300 400 500 600 700 800 900 1,000 1,100 1,200 1,300 1,400 1,500 1,600
y1
iQ
rQ
y2
b3
b4
y6
b17 3+
y7
b7
b8
b9
b10
b11
b12
b13
b16

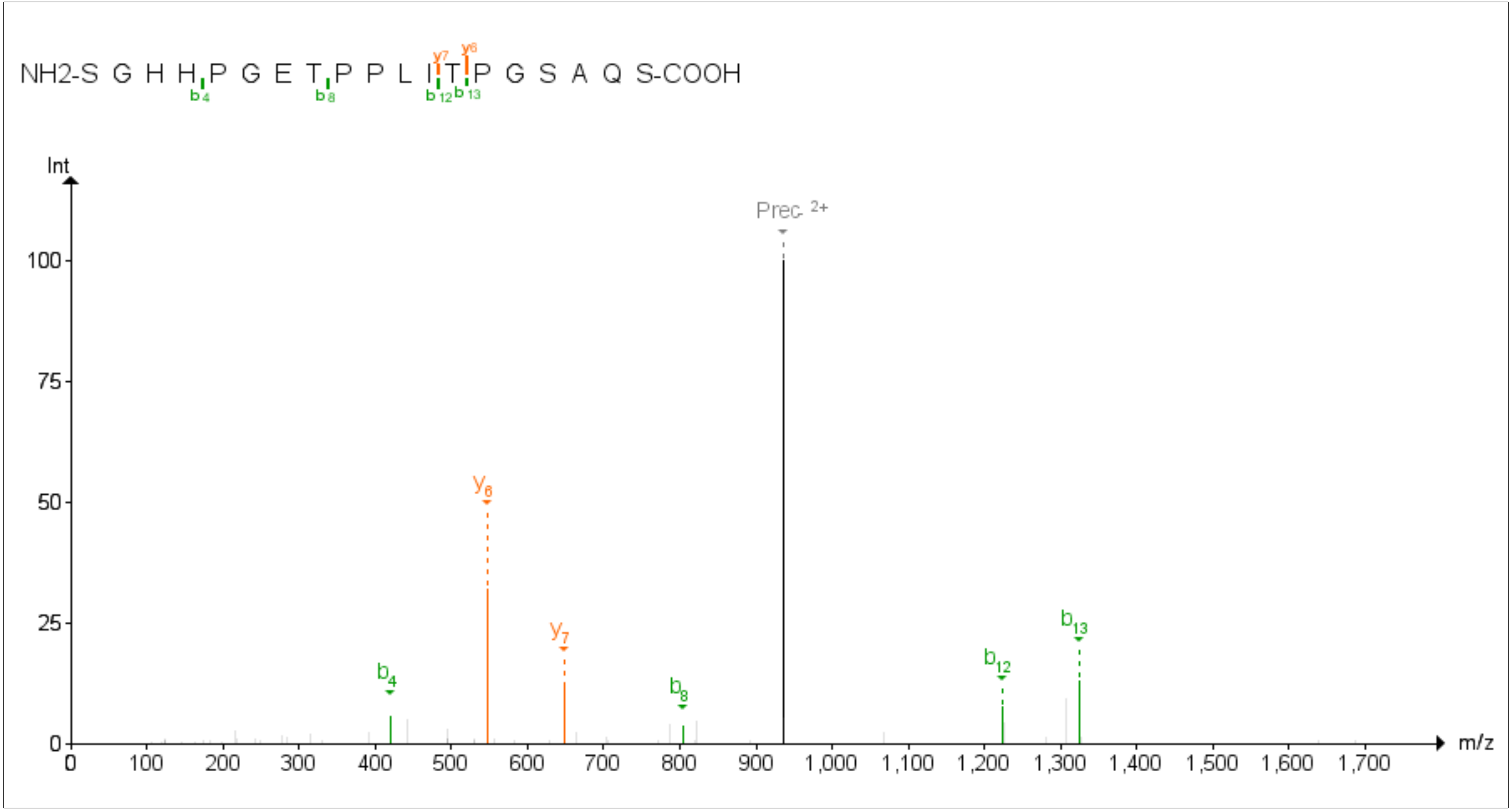
NH2-S G H H P G E T P P L I T P G S A Q S-COOH
b4
b8
b12
b13
y7
y6
Int
Prec. 2+
100
75
50
25
0
y6
y7
b4
b8
b12
b13
0
100
200
300
400
500
600
700
800
900
1,000
1,100
1,200
1,300
1,400
1,500
1,600
1,700
m/z

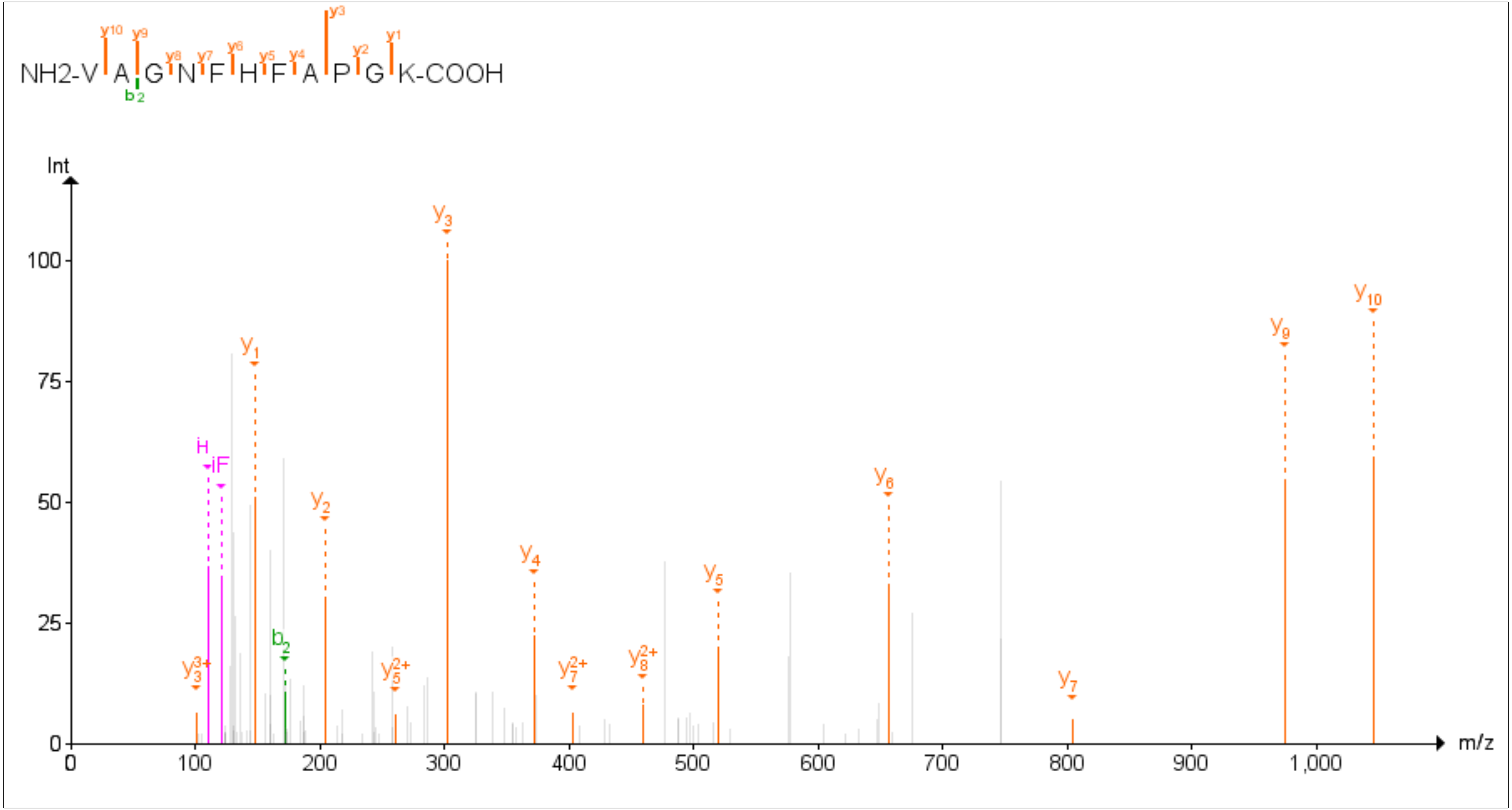

NH2-V A G N F H F A P G K-COOH
y10 y9 y8 y7 y6 y5 y4 y3 y2 y1
b2
Int
100
75
50
25
0
m/z
0
100
200
300
400
500
600
700
800
900
1,000
y3
y10
y9
y1
H
iF
y6
y2
y4
y5
b2
y3 3+
y5 2+
y7 2+
y8 2+
y7

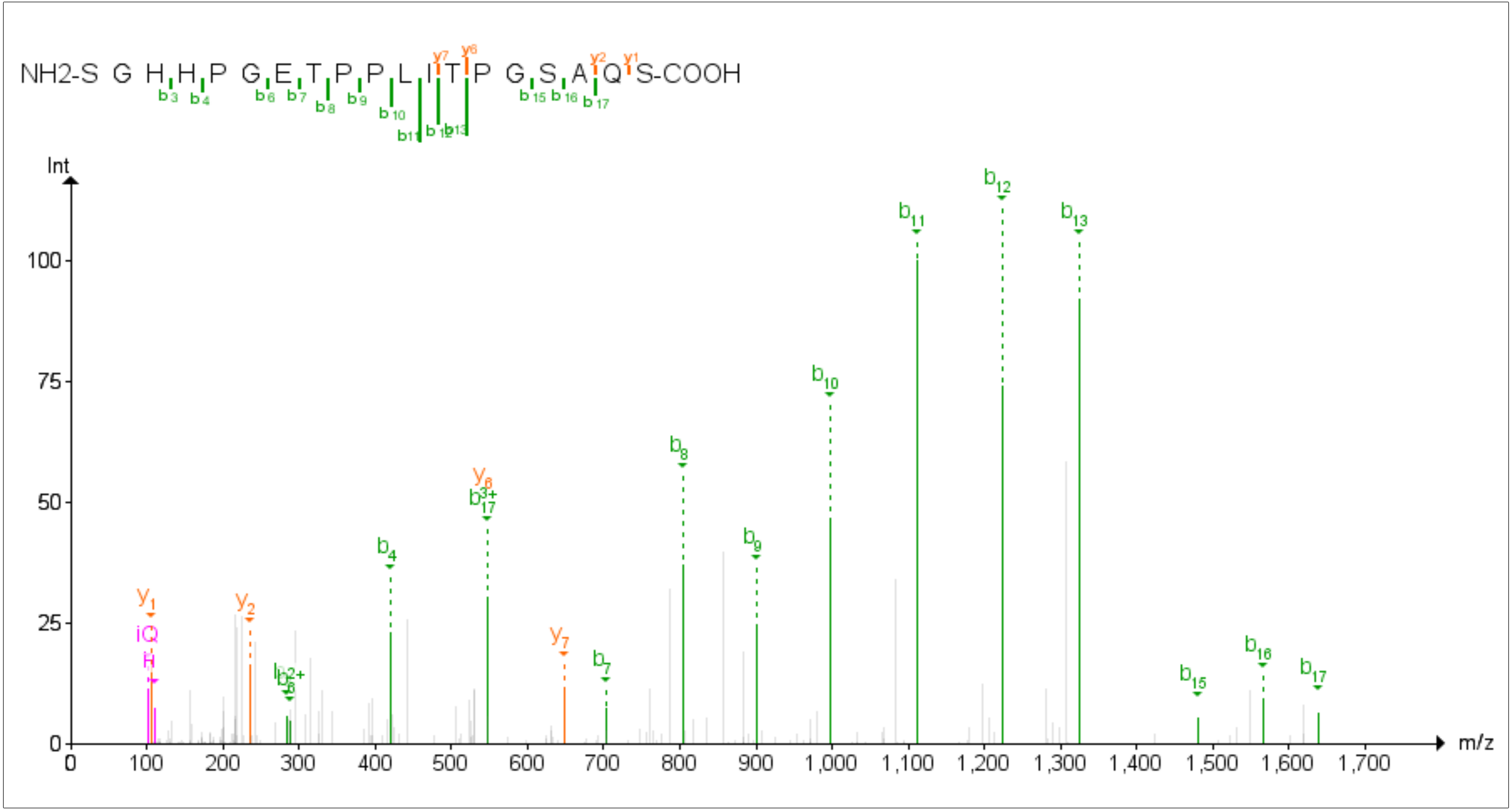

NH2-S G H H P G E T P P L I T P G S A Q S-COOH
Int
m/z
0
25
50
75
100
100
200
300
400
500
600
700
800
900
1,000
1,100
1,200
1,300
1,400
1,500
1,600
1,700

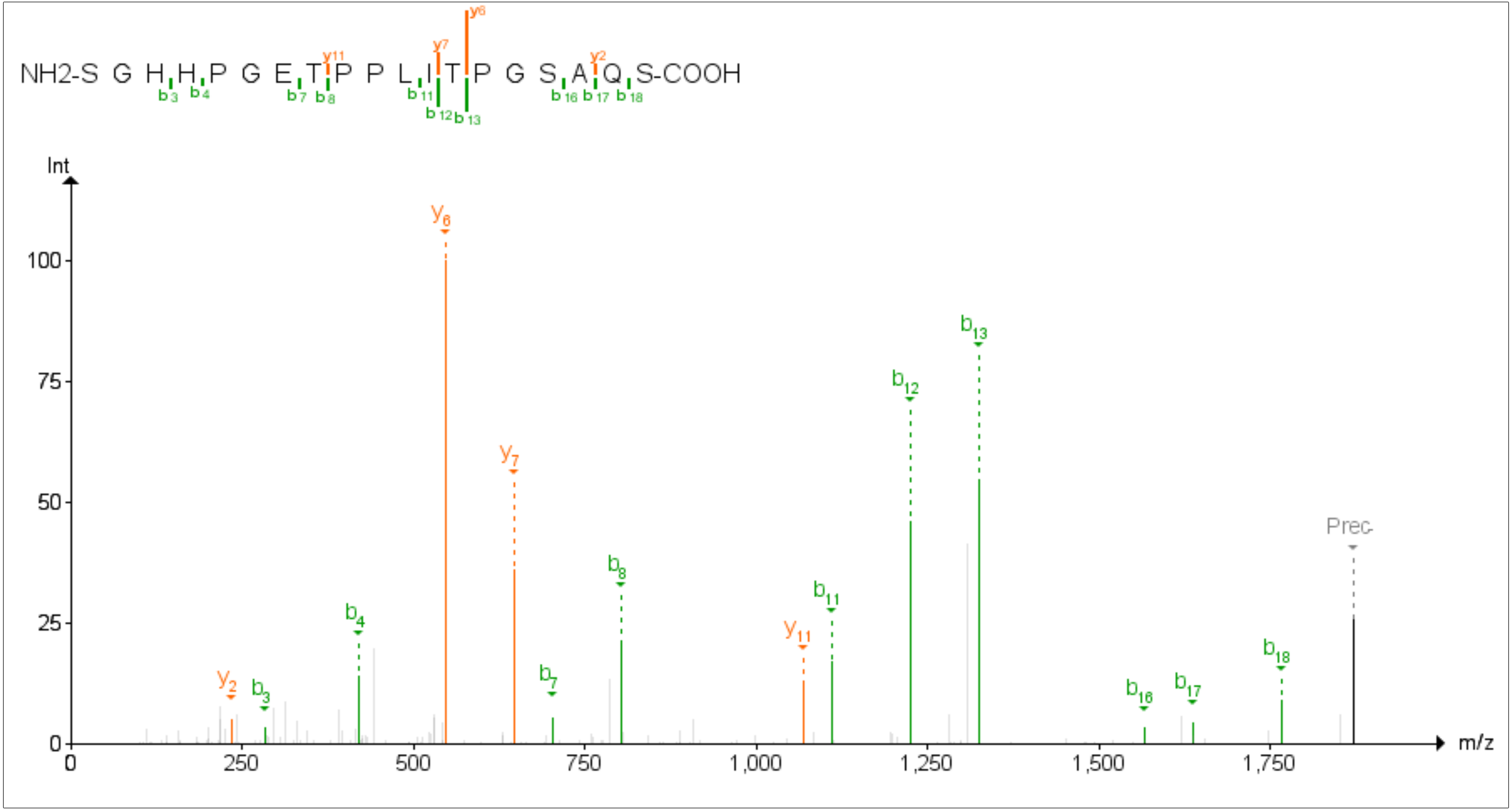

NH2-S G H H P G E T P P L I T P G S A Q S-COOH
y11 y7 y6 y2
b3 b4 b7 b8 b11 b12 b13 b16 b17 b18
Int
100
75
50
25
0
y6
y7
b13
b12
b8
b11
y11
b4
y2
b3
b7
b16
b17
b18
Prec
0
250
500
750
1,000
1,250
1,500
1,750
m/z

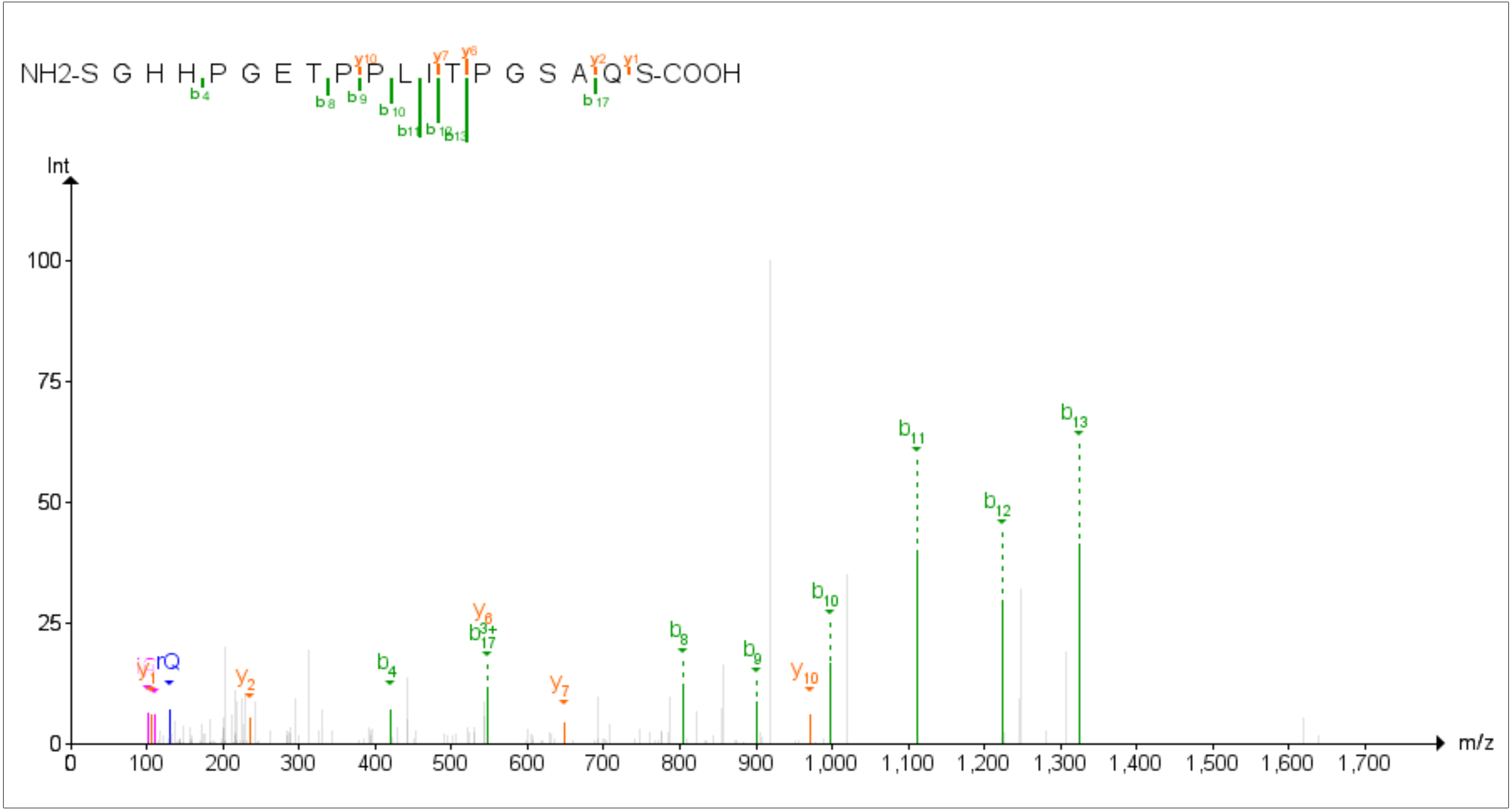
NH2-S G H H P G E T P P L I T P G S A Q S-COOH
y10 y7 y6 y2 y1
b4 b8 b9 b10 b11 b12 b13 b17
Int
m/z
0
25
50
75
100
0
100
200
300
400
500
600
700
800
900
1,000
1,100
1,200
1,300
1,400
1,500
1,600
1,700
y1
rQ
y2
b4
y6
b17 3+
y7
b8
b9
y10
b10
b11
b12
b13

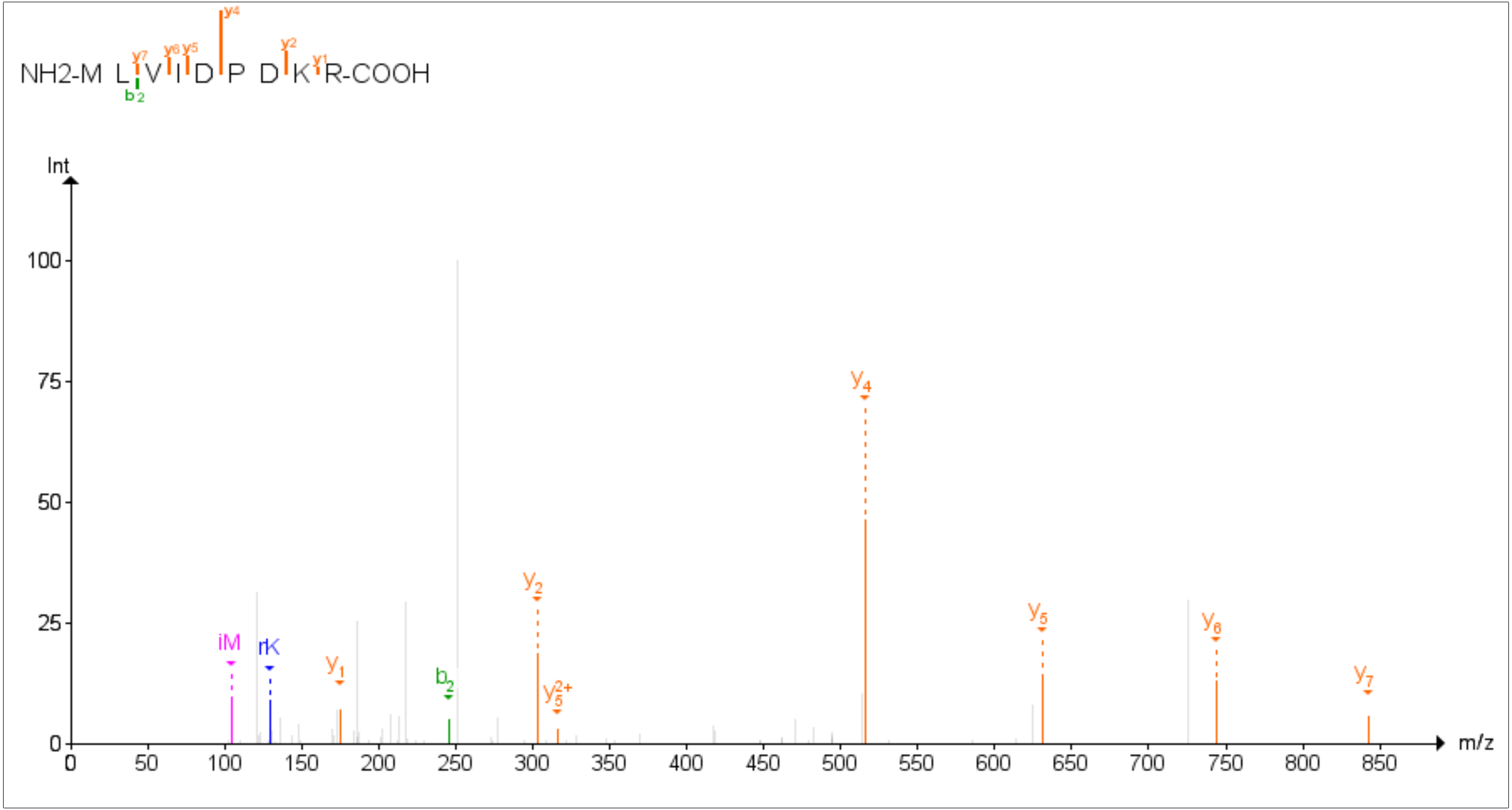

NH2-M L V I D P D K R-COOH
y7 y6 y5 y4 y2 y1
b2
Int
100
75
50
25
0
m/z
0 50 100 150 200 250 300 350 400 450 500 550 600 650 700 750 800 850
iM
rK
y1
b2
y2
y5 2+
y4
y5
y6
y7

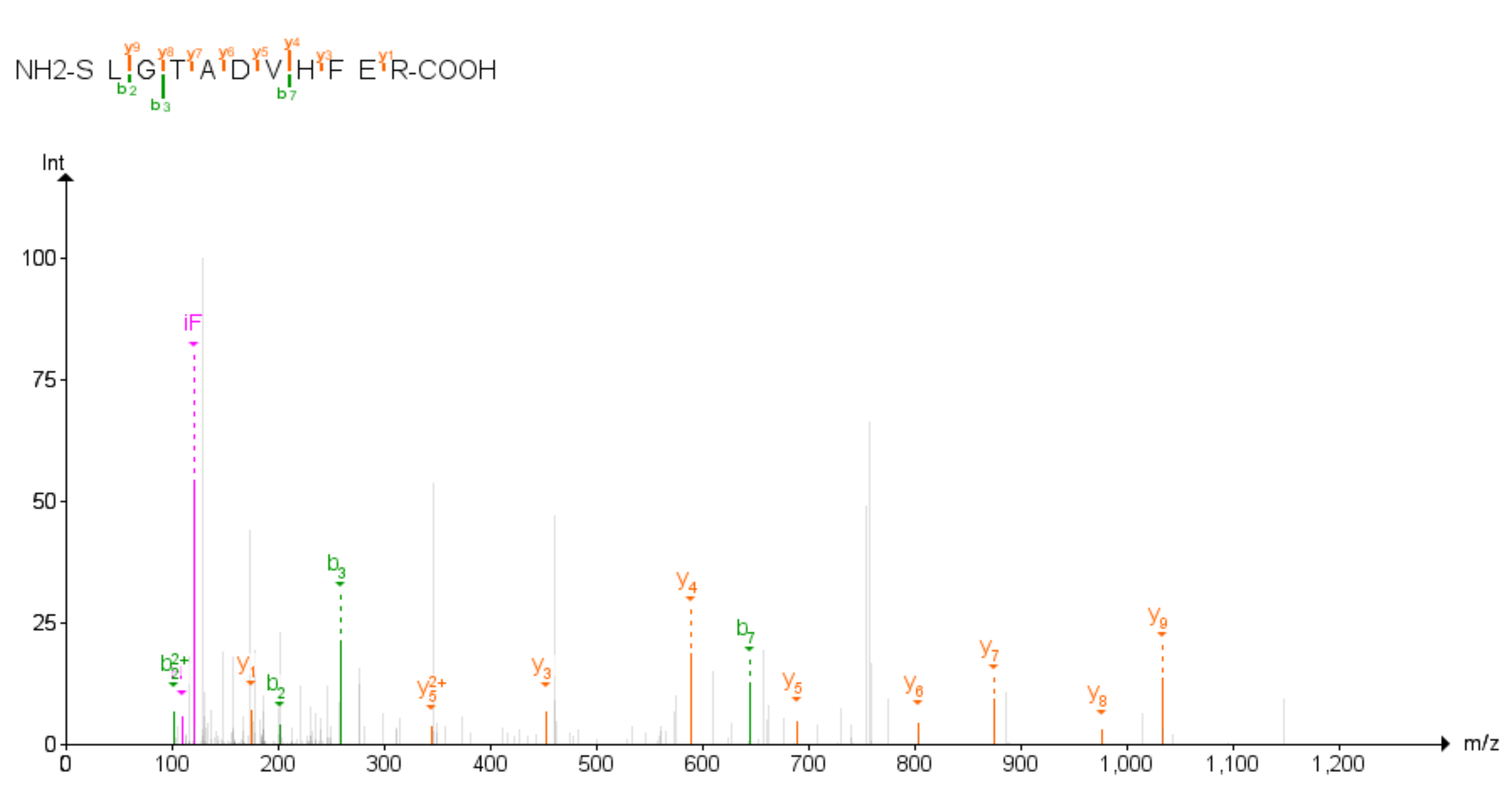

NH2-S L G T A D V H F E R-COOH
y9 y8 y7 y6 y5 y4 y3 y1
b2 b3 b7
Int
100
75
50
25
0
iF
b3
y4
b7
y9
y7
y1
b2
y3
y5
y6
y8
m/z
0 100 200 300 400 500 600 700 800 900 1,000 1,100 1,200

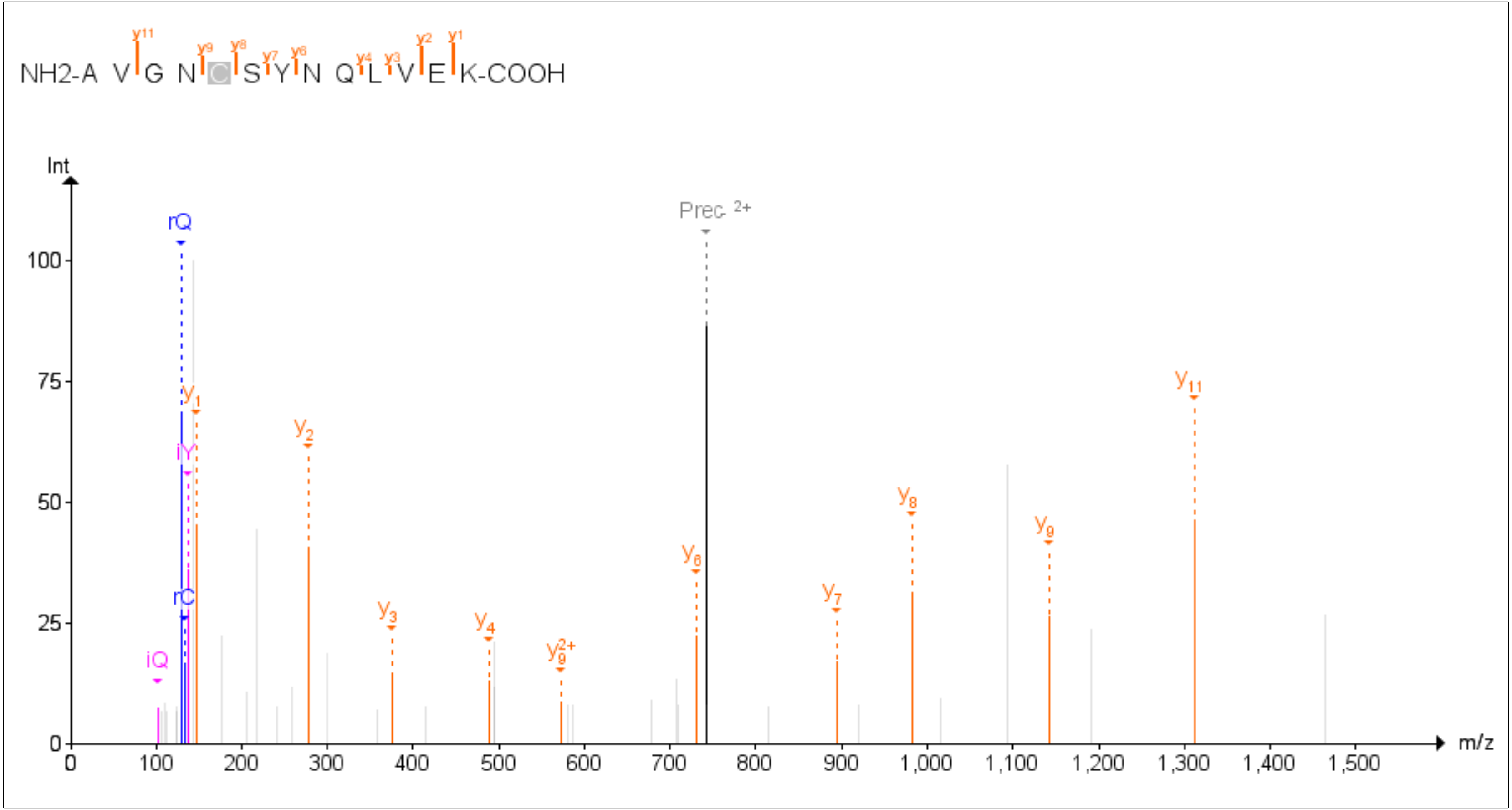

NH2-A V G N C S Y N Q L V E K-COOH
y11 y9 y8 y7 y6 y4 y3 y2 y1
Int
100
75
50
25
0
rQ
Prec 2+
y1
y2
iY
y11
y8
y9
y6
y7
rC
y3
y4
iQ
y9 2+
0 100 200 300 400 500 600 700 800 900 1,000 1,100 1,200 1,300 1,400 1,500
m/z

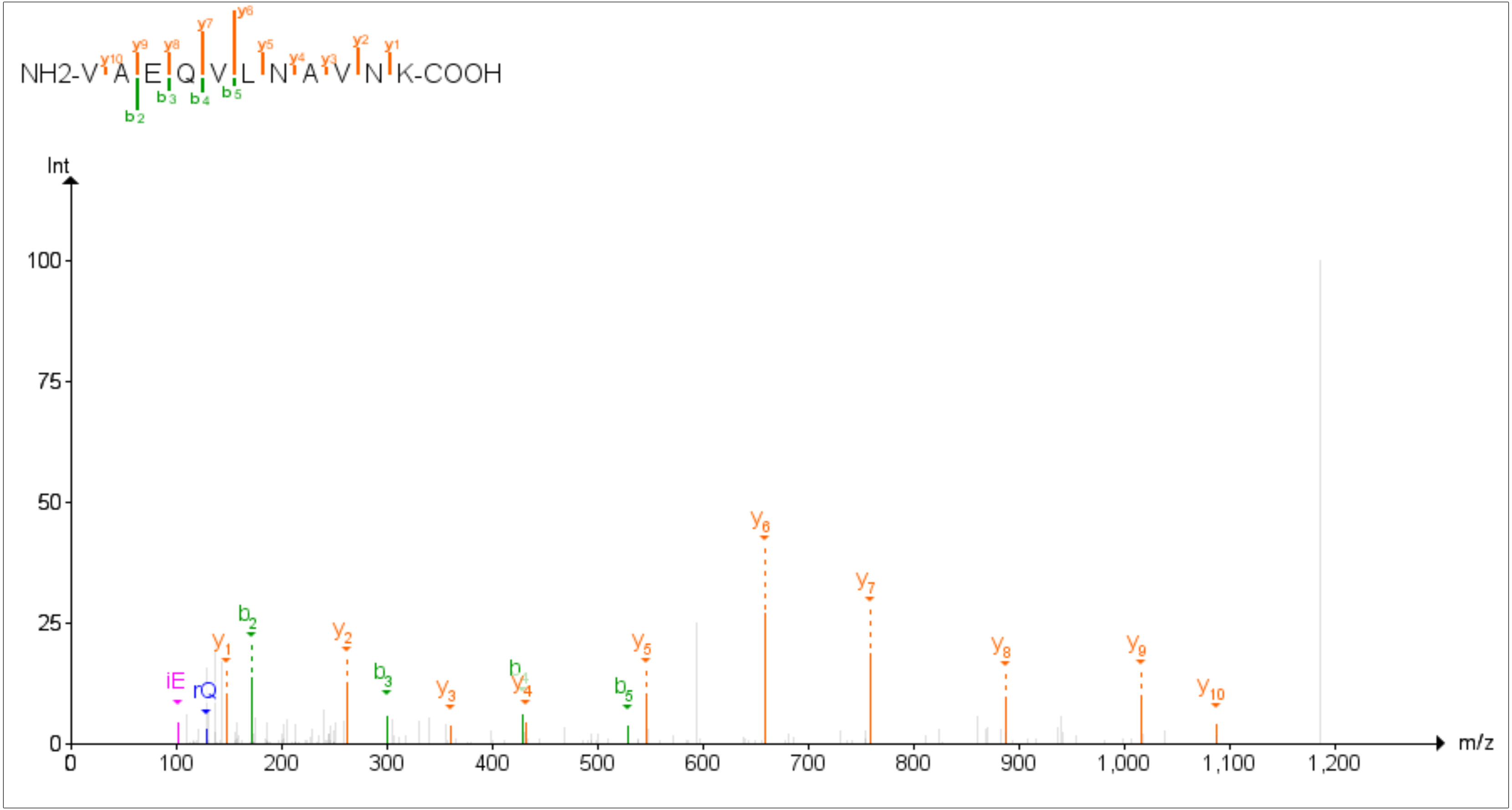

NH2-V A E Q V L N A V N K-COOH
y10 y9 y8 y7 y6 y5 y4 y3 y2 y1
b2 b3 b4 b5
Int
100
75
50
25
0
m/z
0 100 200 300 400 500 600 700 800 900 1,000 1,100 1,200
iE
rQ
y1
b2
y2
b3
y3
b4
y4
b5
y5
y6
y7
y8
y9
y10

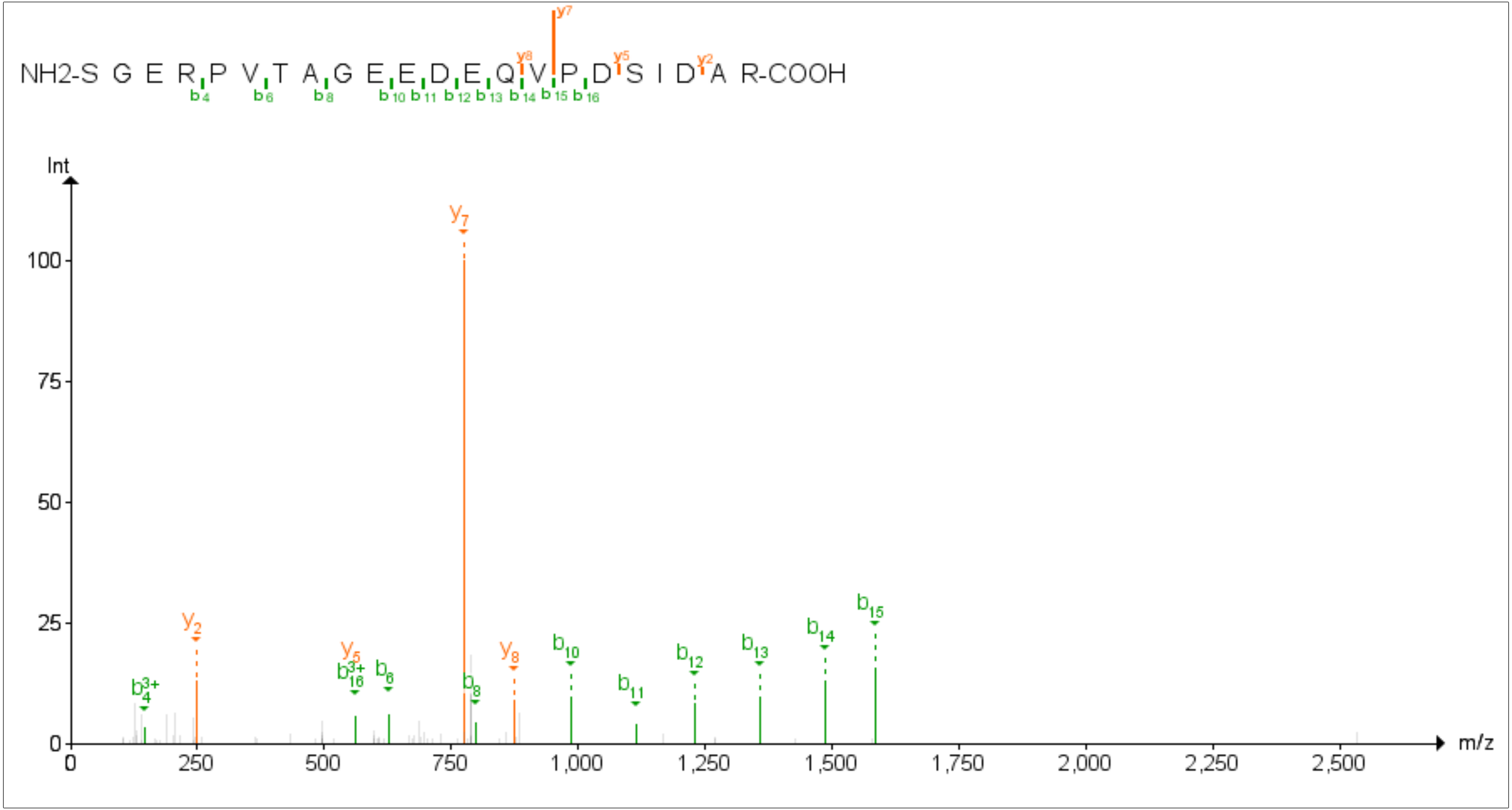

NH2-S G E R P V T A G E E D E Q V P D S I D A R-COOH
b4 b6 b8 b10 b11 b12 b13 b14 b15 b16
y7 y8 y5 y2
Int
100
75
50
25
0
m/z
0
250
500
750
1,000
1,250
1,500
1,750
2,000
2,250
2,500

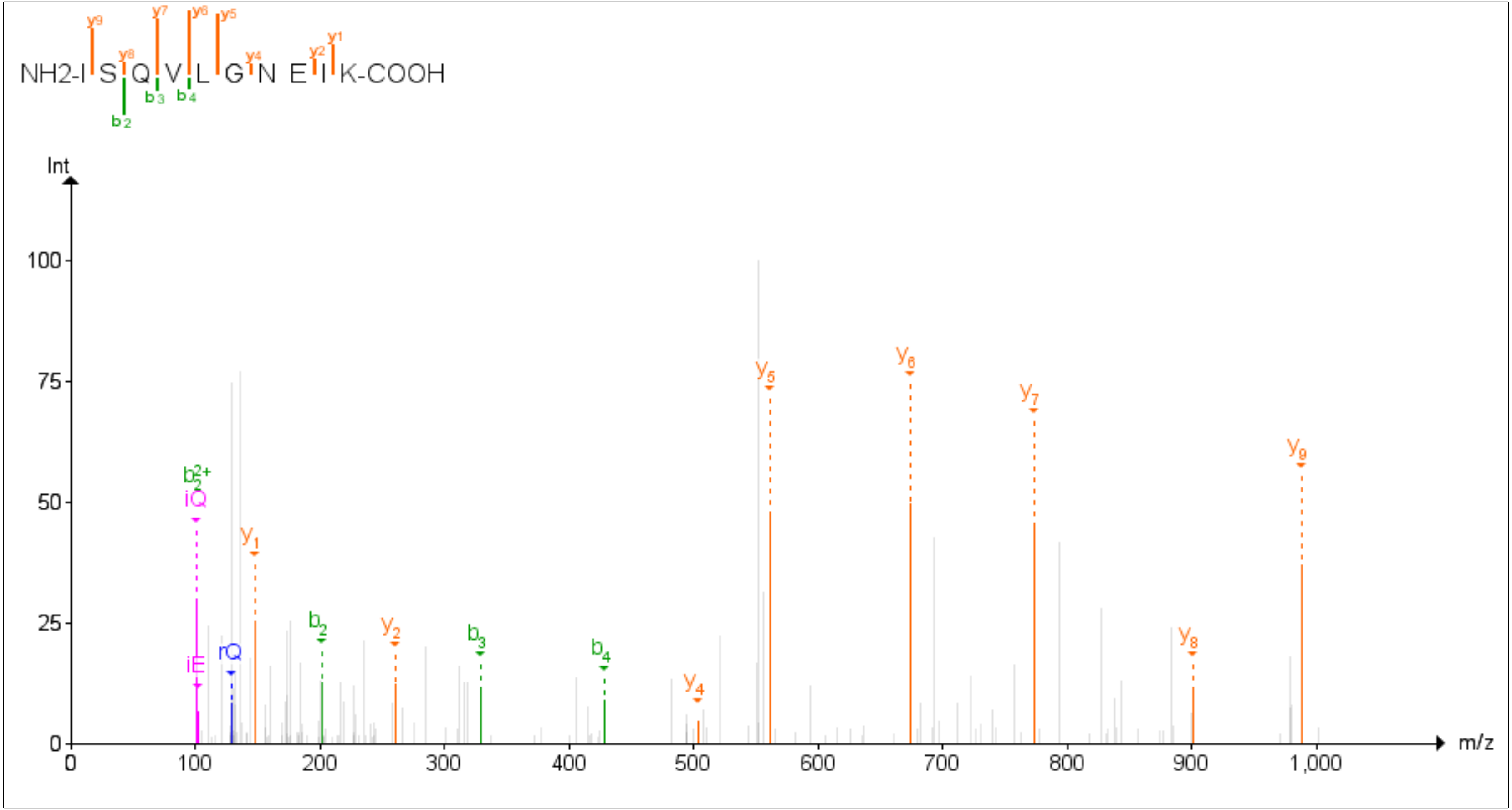

NH2-I S Q V L G N E I K-COOH
y9 y8 y7 y6 y5 y4 y2 y1
b2 b3 b4
Int
100
75
50
25
0
m/z
0 100 200 300 400 500 600 700 800 900 1,000
b2 2+
iQ
iE
rQ
y1 y2 y4 y5 y6 y7 y8 y9
b2 b3 b4

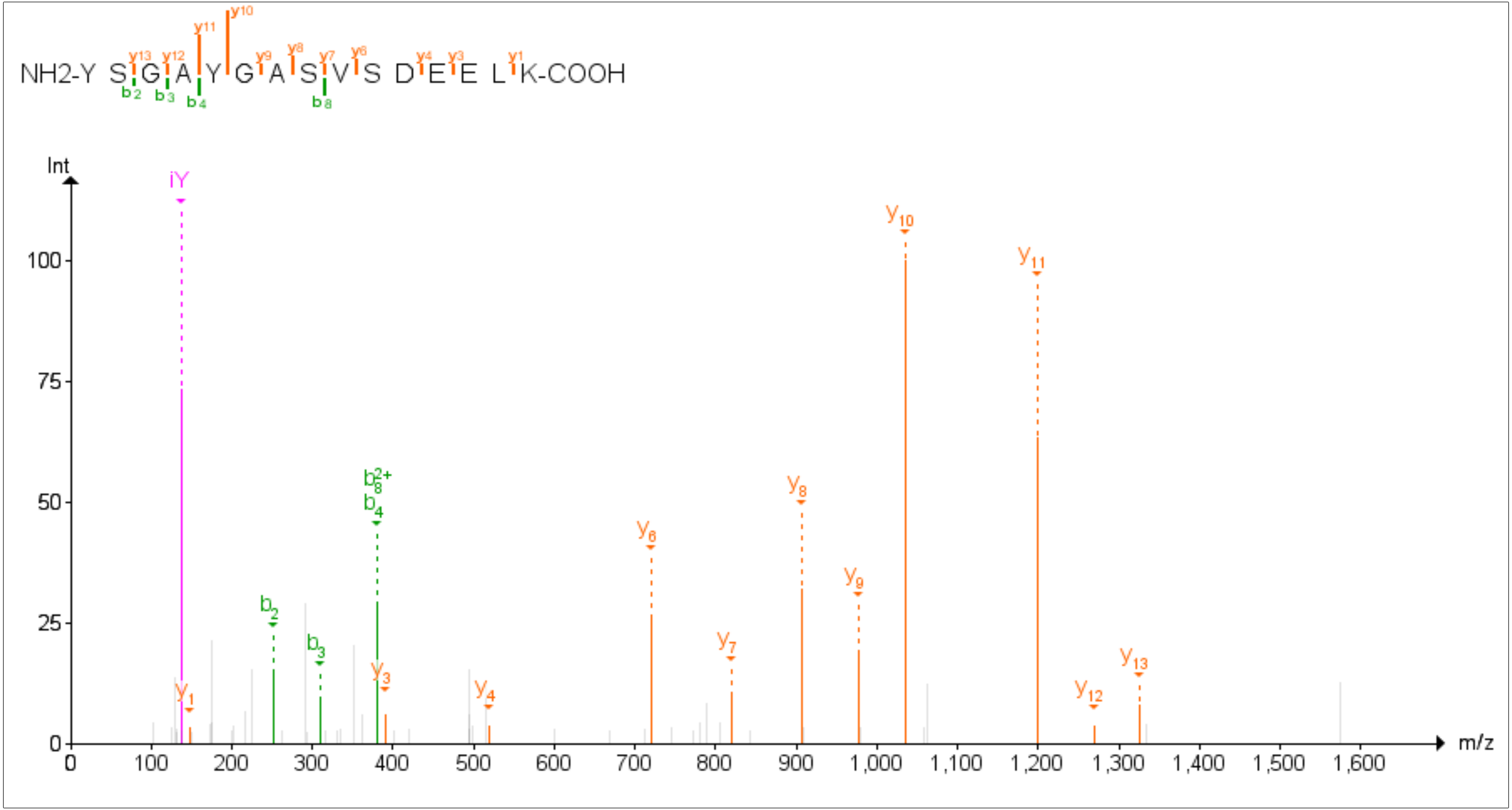

NH2-Y S G A Y G A S V S D E E L K-COOH
y13 y12 y11 y10 y9 y8 y7 y6 y4 y3 y1
b2 b3 b4 b8
Int
iY
y_{10}
y_{11}
b_8^{2+}
b_4
y_8
y_6
y_9
b_2
b_3
y_7
y_3
y_{13}
y_1
y_4
y_{12}
100
75
50
25
0
0 100 200 300 400 500 600 700 800 900 1,000 1,100 1,200 1,300 1,400 1,500 1,600
m/z

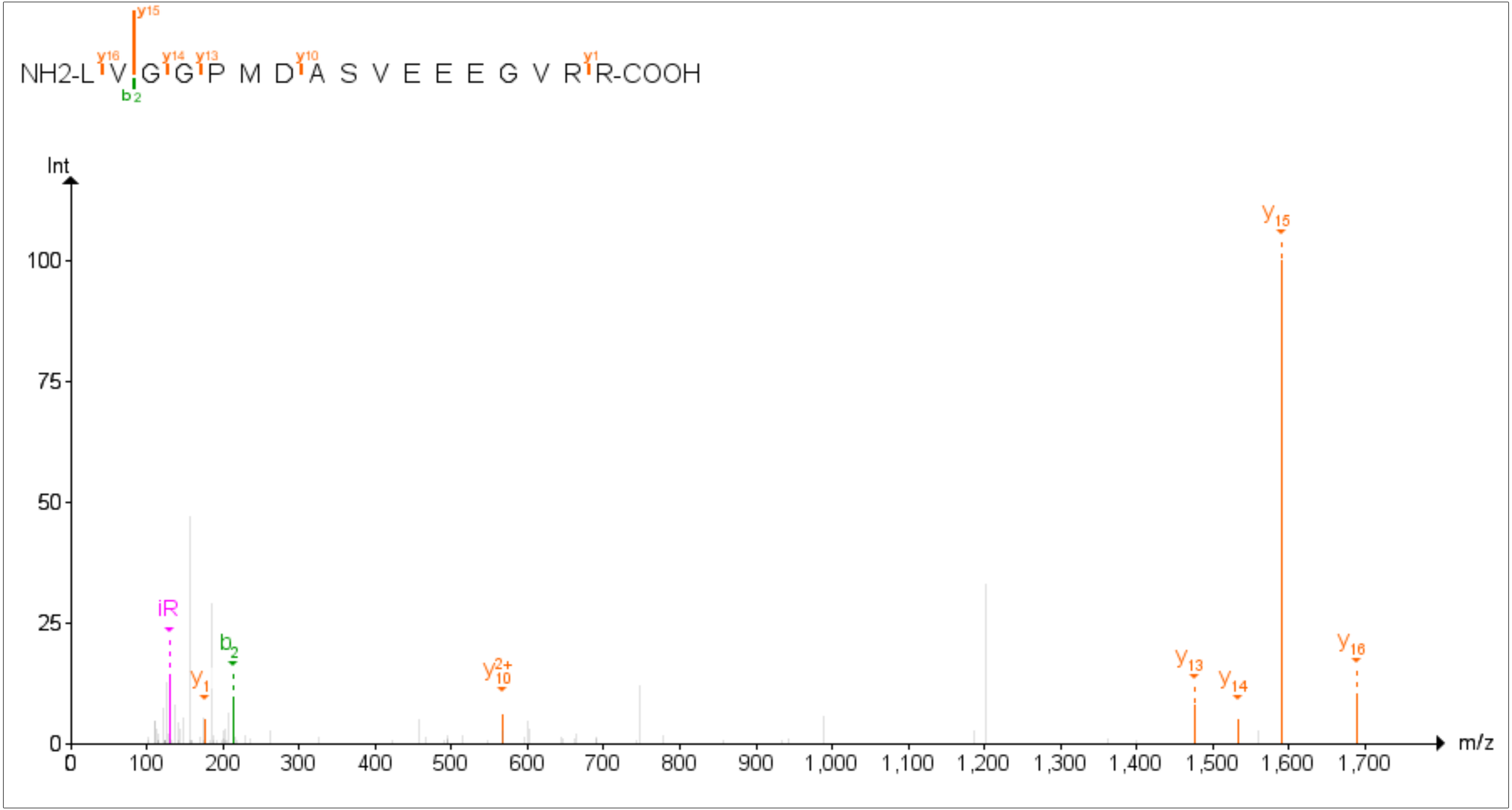
NH2-L V G G P M D A S V E E E G V R R-COOH
y15
y16
y14
y13
y10
y1
b2
Int
100
75
50
25
0
iR
y1
b2
y10 2+
y13
y14
y15
y16
0
100
200
300
400
500
600
700
800
900
1,000
1,100
1,200
1,300
1,400
1,500
1,600
1,700
m/z

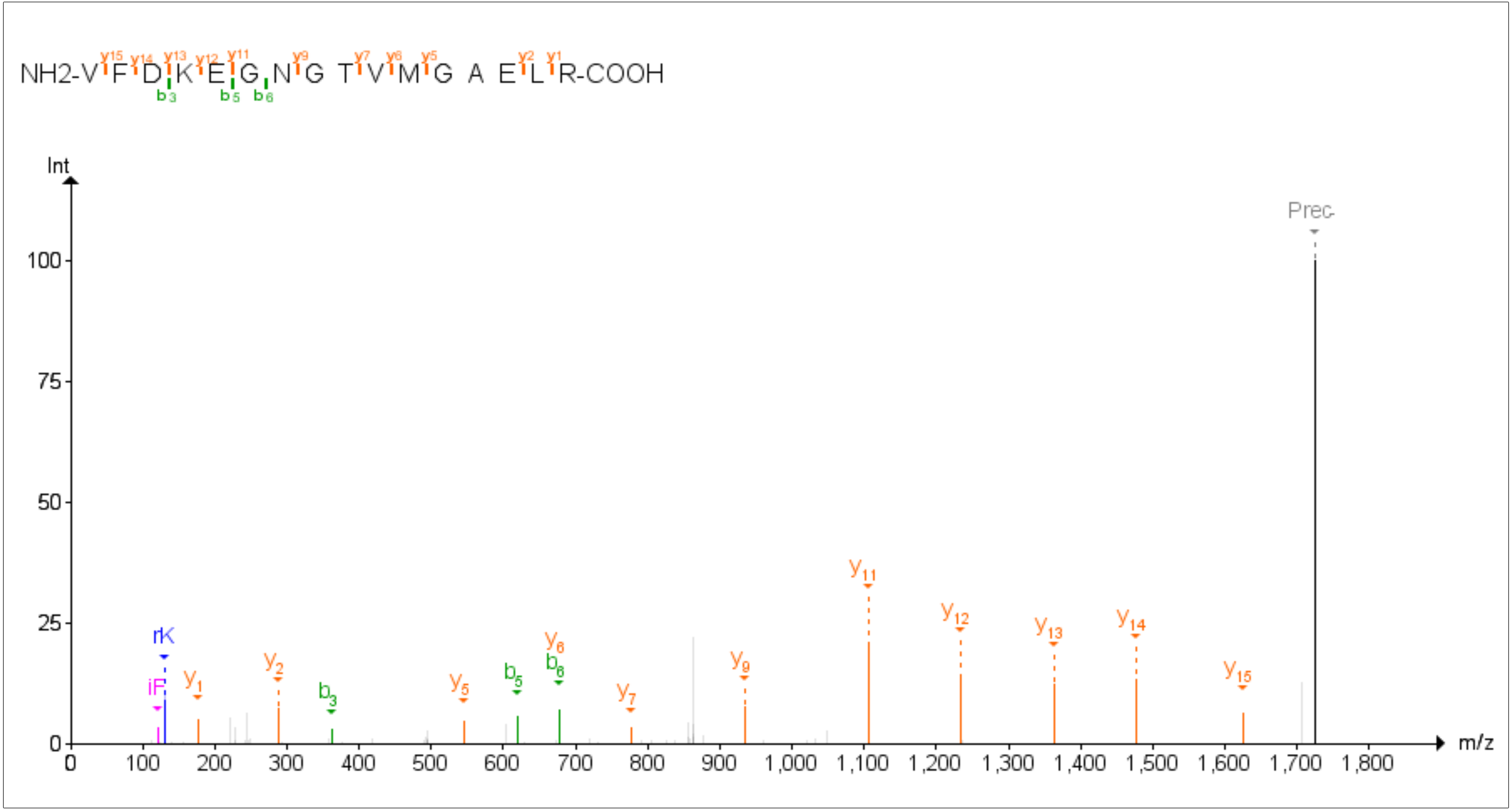
NH2-V F D K E G N G T V M G A E L R-COOH
y15 y14 y13 y12 y11 y9 y7 y6 y5 y2 y1
b3 b5 b6
Int
Prec
rK
iF
y1
y2
b3
y5
b5
y6
b6
y7
y9
y11
y12
y13
y14
y15
0
25
50
75
100
0 100 200 300 400 500 600 700 800 900 1,000 1,100 1,200 1,300 1,400 1,500 1,600 1,700 1,800
m/z

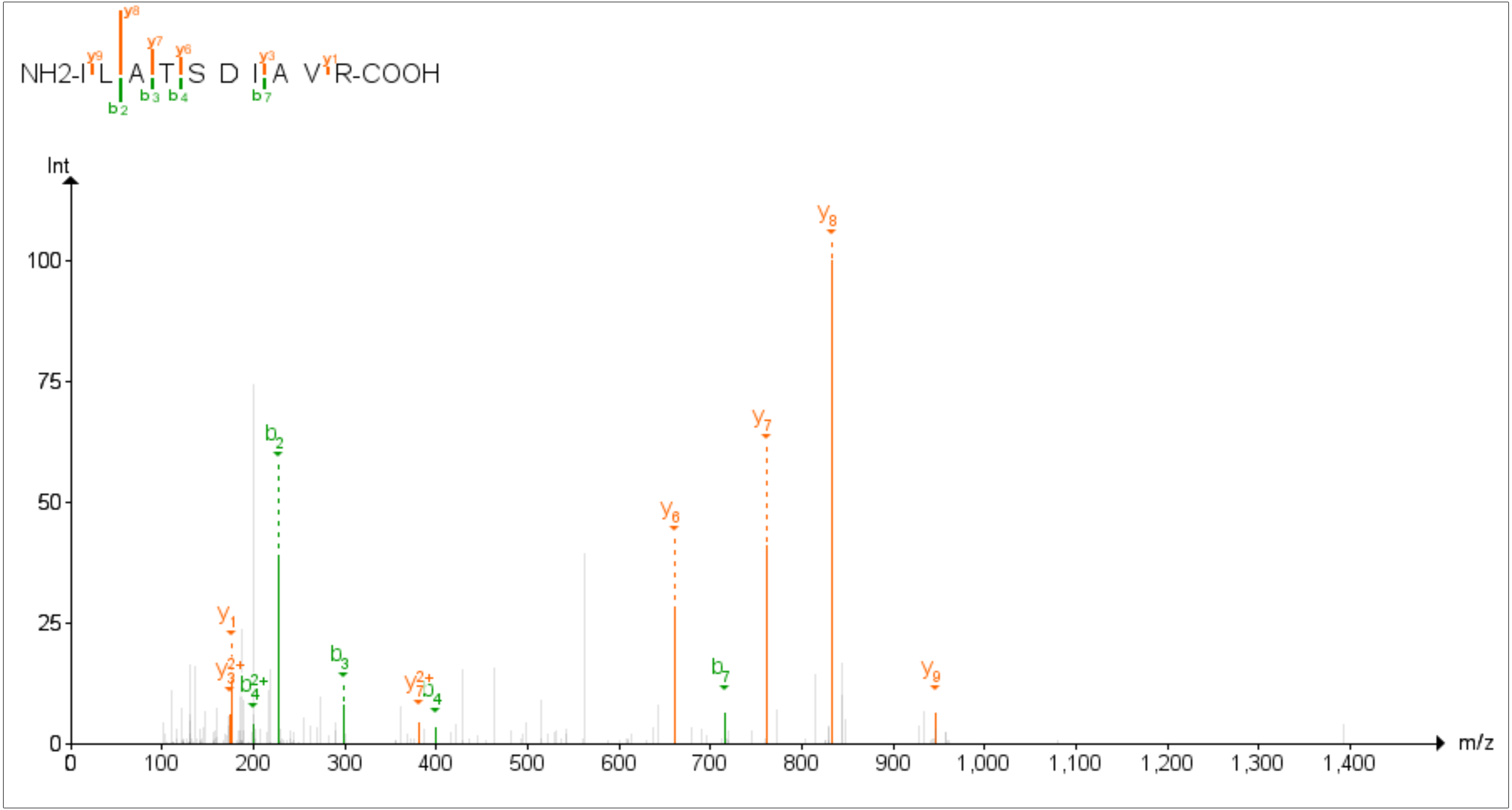

NH2-I L A T S D I A V R-COOH
y9 y8 y7 y6 y3 y1
b2 b3 b4 b7
Int
100
75
50
25
0
y8
y7
y6
b2
y1
y3 2+
b4 2+
b3
y7 2+
b4
b7
y9
0 100 200 300 400 500 600 700 800 900 1,000 1,100 1,200 1,300 1,400
m/z

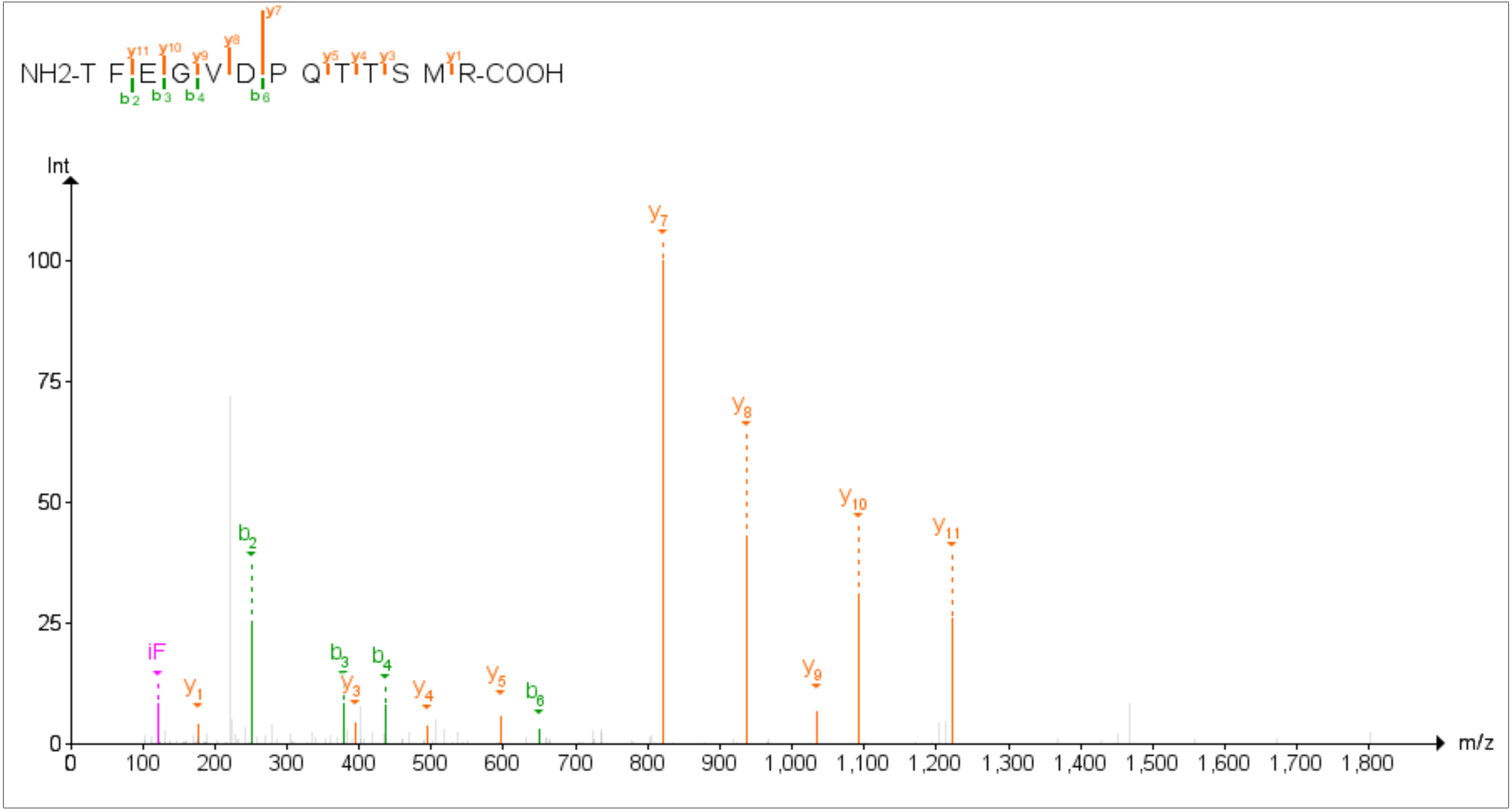

NH2-T F E G V D P Q T T S M R-COOH
y11 y10 y9 y8 y7 y5 y4 y3 y1
b2 b3 b4 b6
Int
100
75
50
25
0
m/z
0 100 200 300 400 500 600 700 800 900 1,000 1,100 1,200 1,300 1,400 1,500 1,600 1,700 1,800
y7
y8
y10
y11
b2
iF
y1
b3
y3
b4
y4
y5
b6
y9

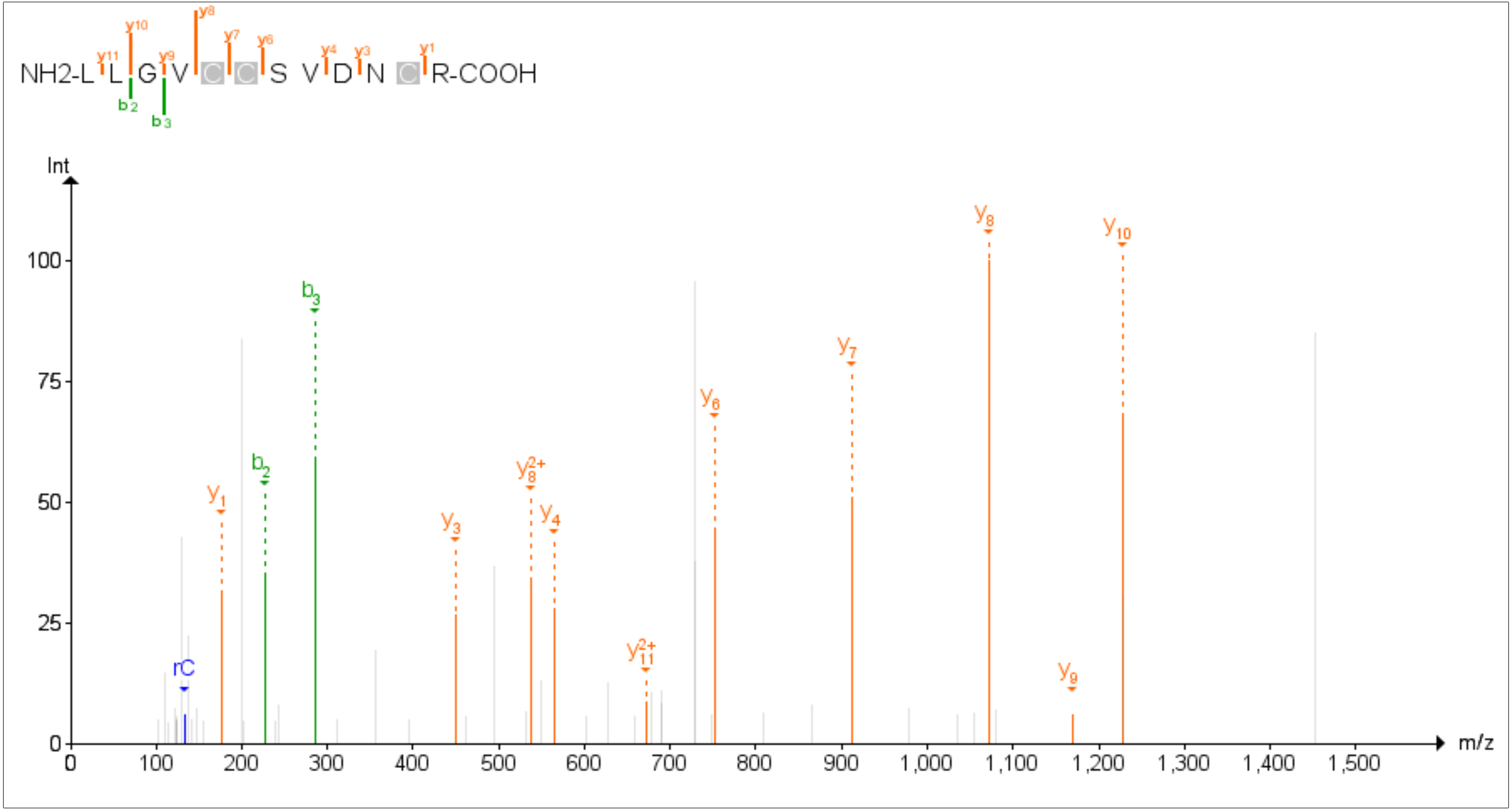

NH2-L L G V C C S V D N C R-COOH
y11 y10 y9 y8 y7 y6 y4 y3 y1
b2 b3
Int
100
75
50
25
0
m/z
0 100 200 300 400 500 600 700 800 900 1,000 1,100 1,200 1,300 1,400 1,500
rC
y1
b2
b3
y3
y8 2+
y4
y11 2+
y6
y7
y8
y9
y10

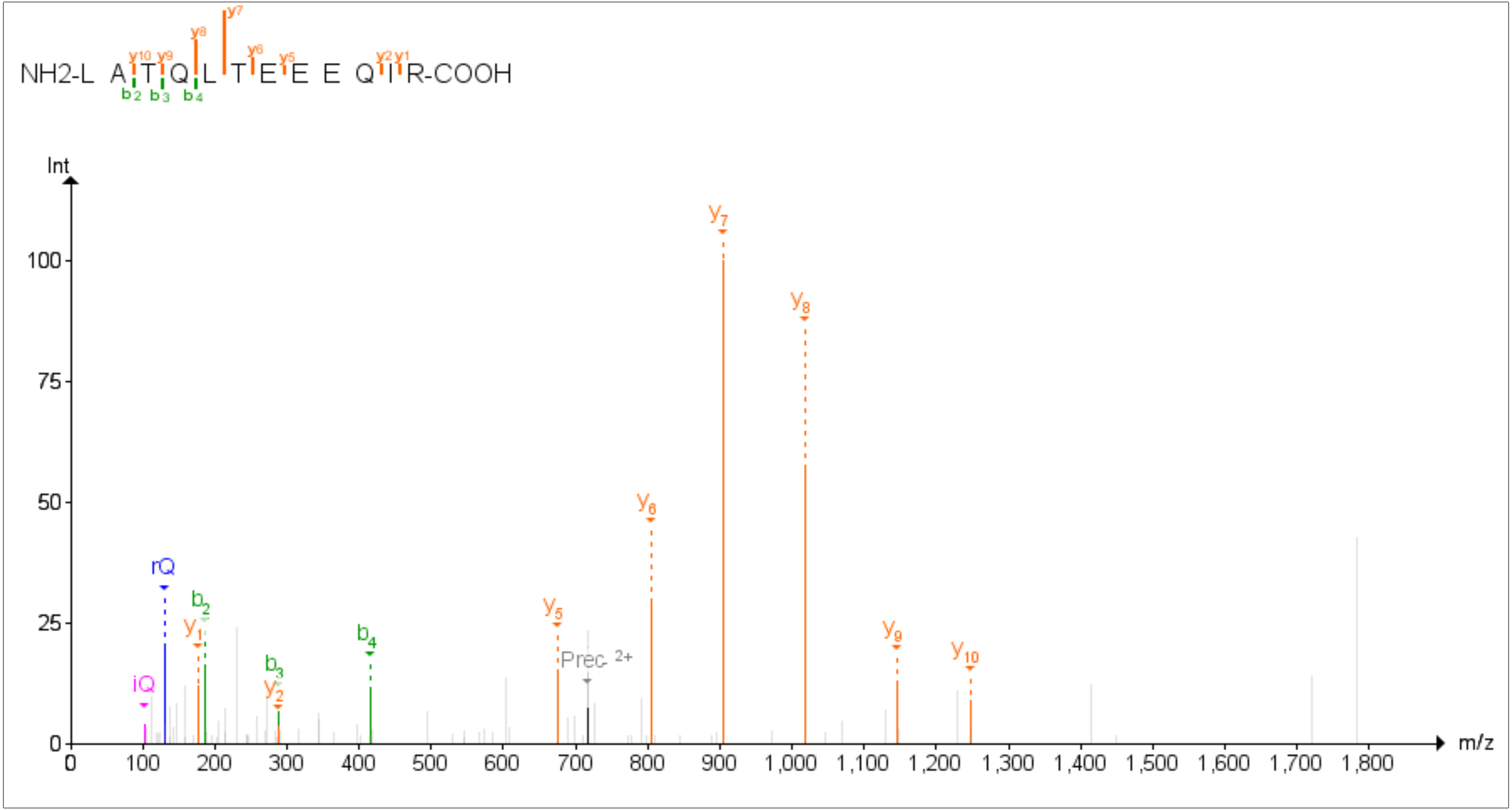

NH2-L A T Q L T E E E Q I R-COOH
y10 y9 y8 y7 y6 y5 y2 y1
b2 b3 b4
Int
100
75
50
25
0
rQ
iQ
b2
b3
b4
y1
y2
y5
y6
y7
y8
y9
y10
Prec 2+
0 100 200 300 400 500 600 700 800 900 1,000 1,100 1,200 1,300 1,400 1,500 1,600 1,700 1,800
m/z

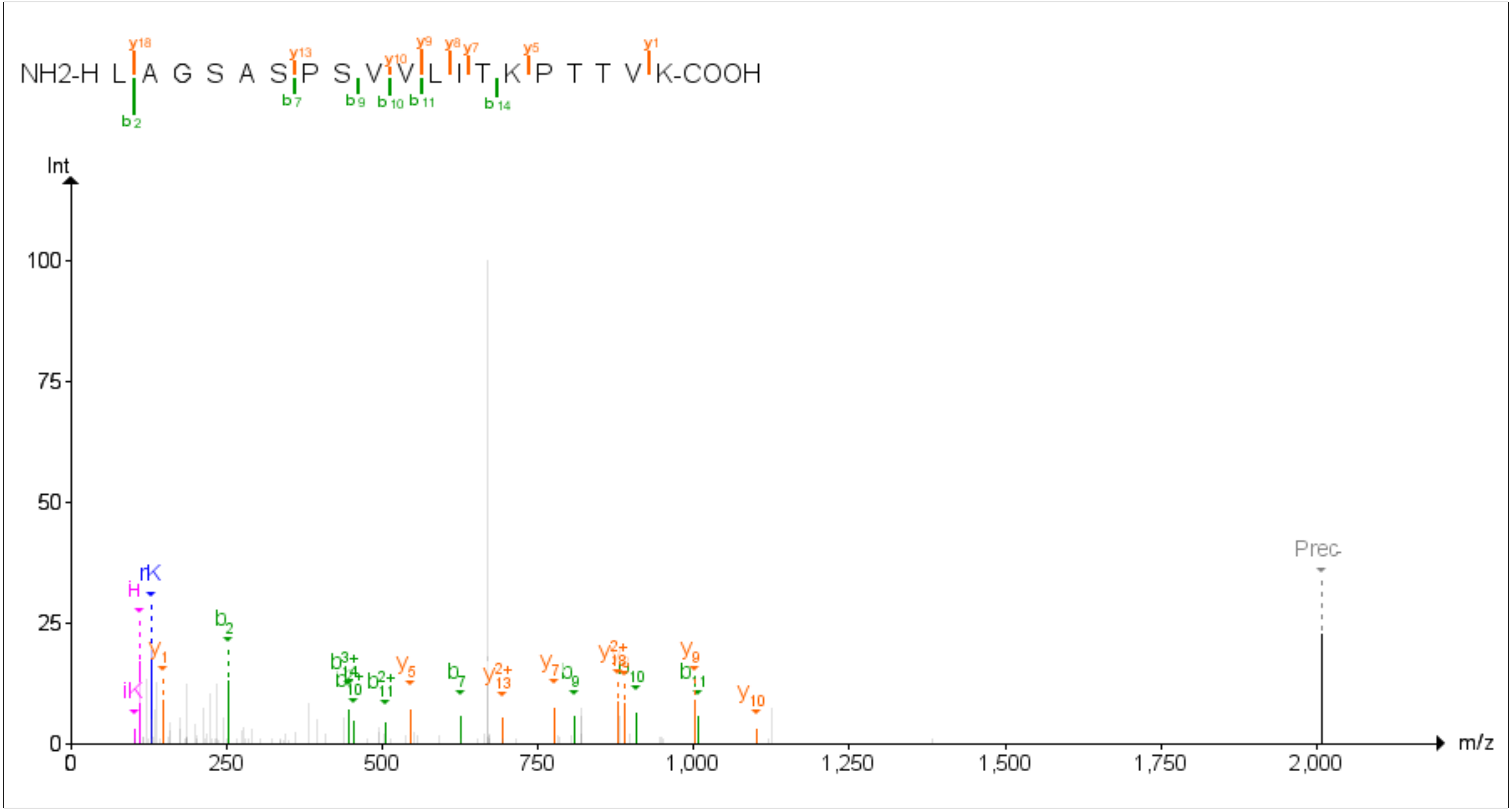

NH2-H L A G S A S P S V V L I T K P T T V K-COOH
y18 y13 y10 y9 y8 y7 y5 y1
b2 b7 b9 b10 b11 b14
Int
100
75
50
25
0
0
250
500
750
1,000
1,250
1,500
1,750
2,000
m/z
Prec
rK
iH
iK
y1
b2
y5
b7
y7
b9
b10
y9
b11
y10

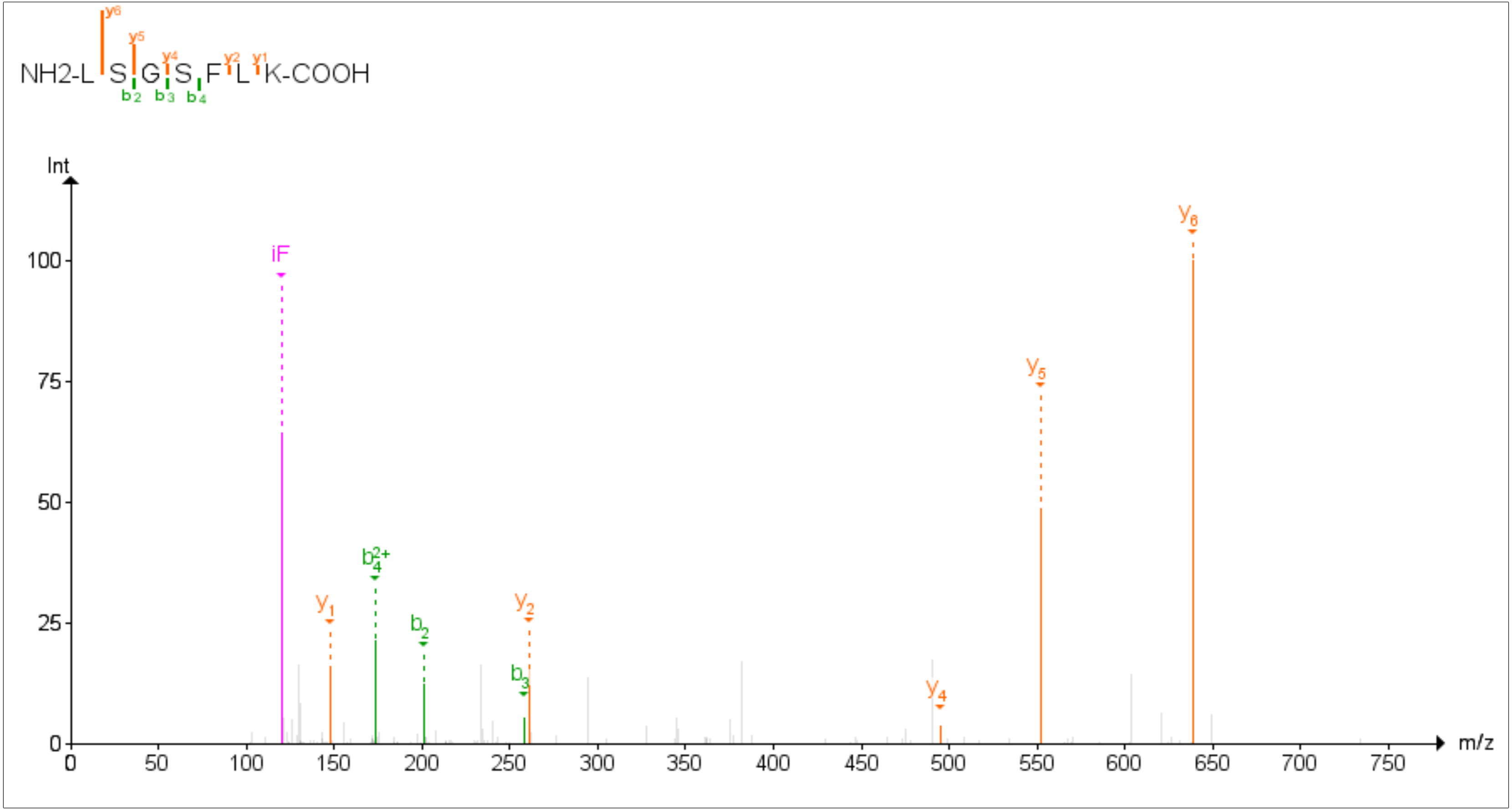

NH2-L S G S F L K-COOH
y6
y5
y4
y2
y1
b2
b3
b4
Int
100
75
50
25
0
iF
y6
y5
y4
y1
y2
b4 2+
b2
b3
0
50
100
150
200
250
300
350
400
450
500
550
600
650
700
750
m/z

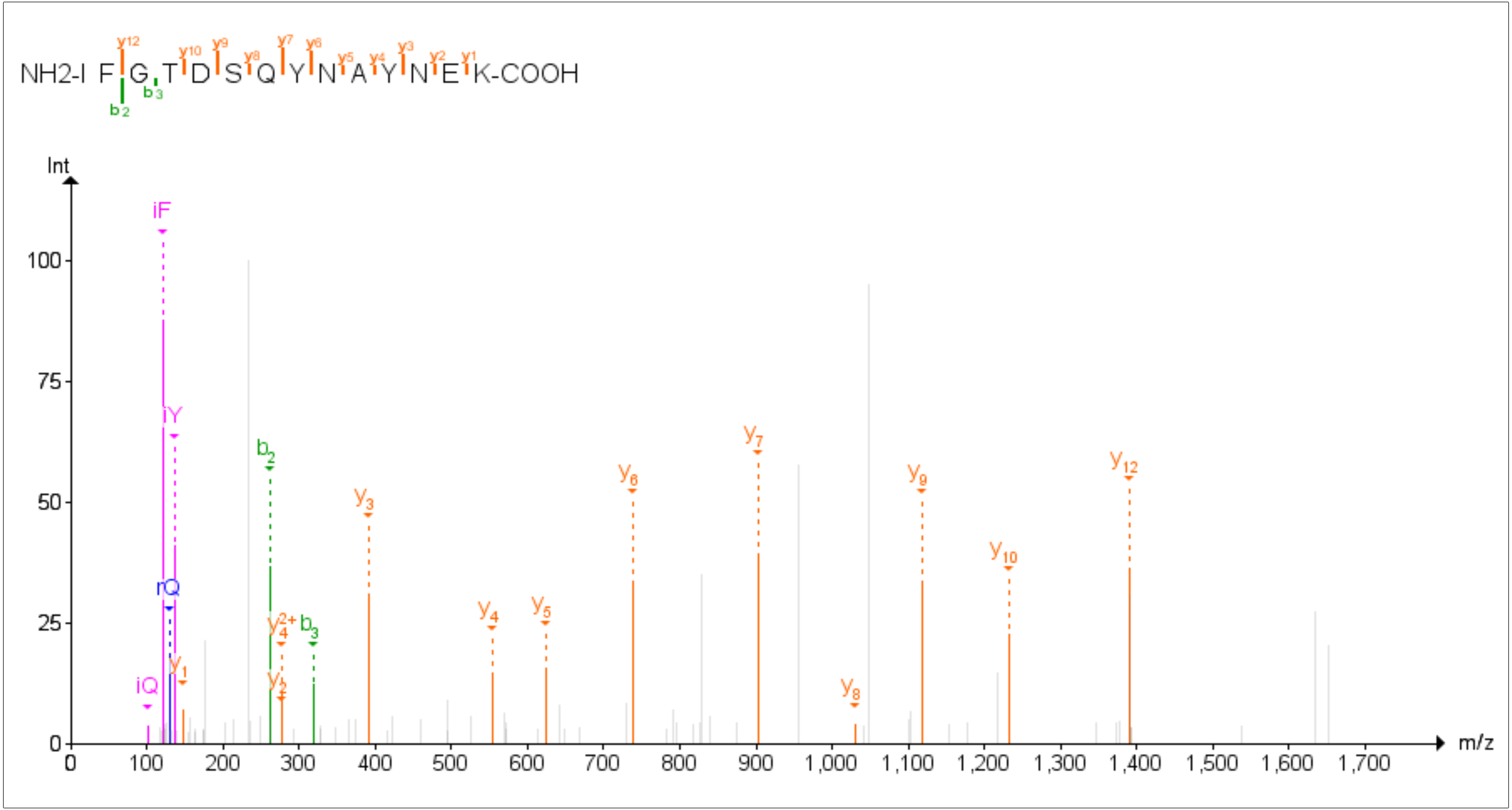

NH2-I F G T D S Q Y N A Y N E K-COOH
y12 y10 y9 y8 y7 y6 y5 y4 y3 y2 y1
b2 b3
Int
m/z
iF
iY
iQ
rQ
b2
b3
y1
y2
y3
y4
y5
y6
y7
y8
y9
y10
y12
0
25
50
75
100
0
100
200
300
400
500
600
700
800
900
1,000
1,100
1,200
1,300
1,400
1,500
1,600
1,700

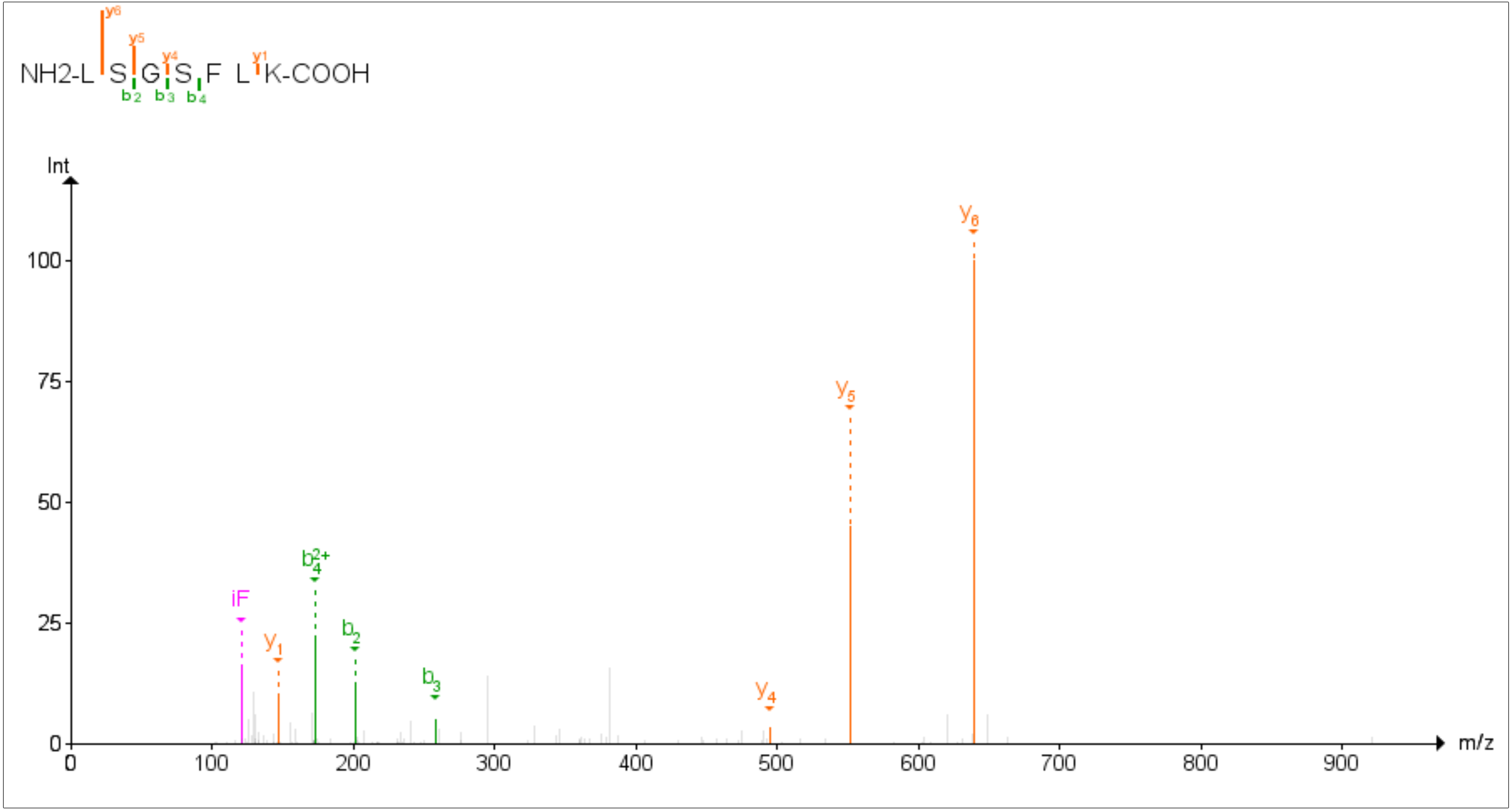
NH2-L S G S F L K-COOH
y6
y5
y4
y1
b2
b3
b4
Int
100
75
50
25
0
m/z
0
100
200
300
400
500
600
700
800
900
y6
y5
iF
y1
b4 2+
b2
b3
y4

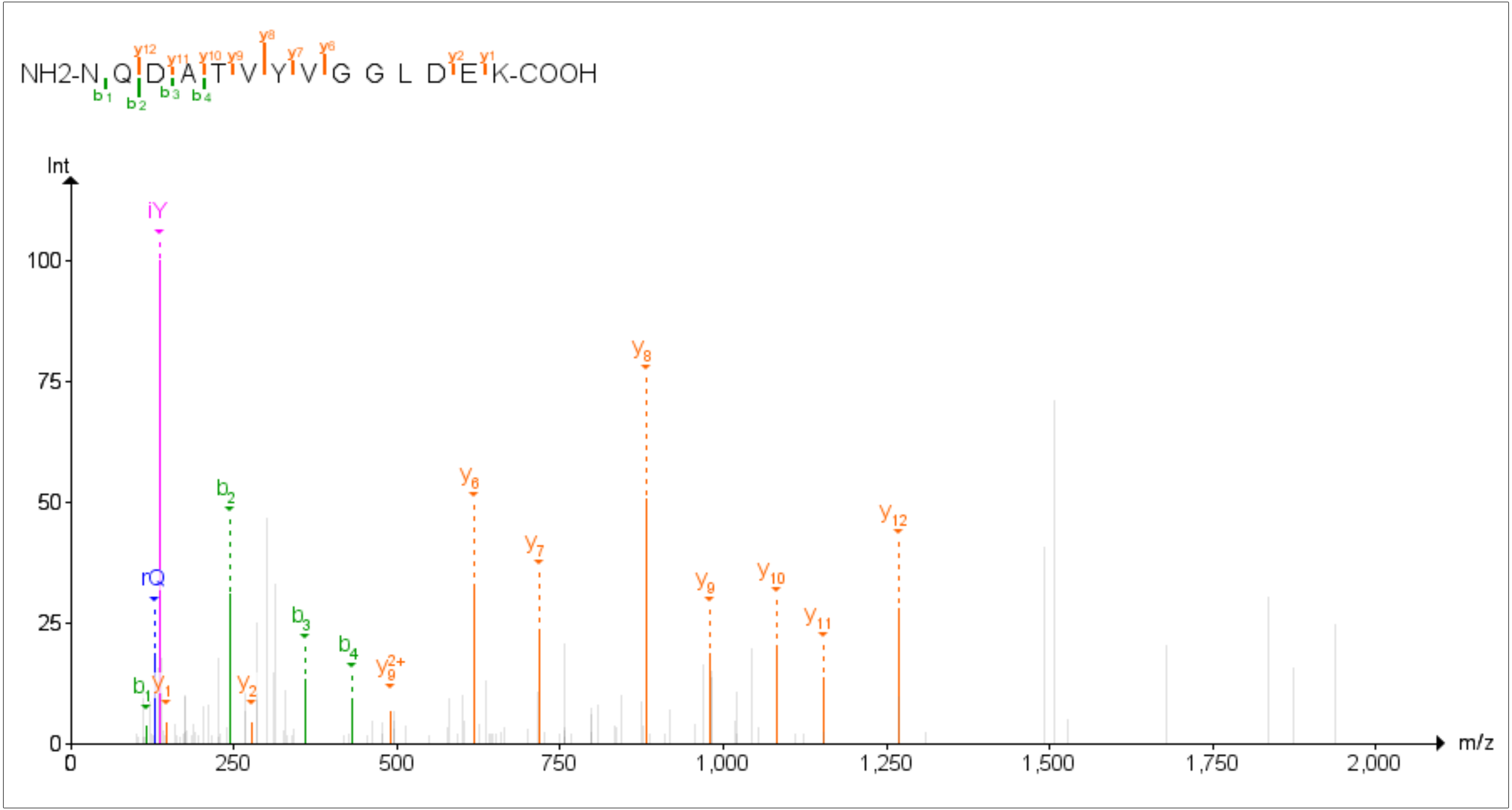
NH2-N Q D A T V Y V G G L D E K-COOH
y12 y11 y10 y9 y8 y7 y6 y2 y1
b1 b2 b3 b4
Int
iY
rQ
b1
y1
b2
y2
b3
b4
y9 2+
y6
y7
y8
y9
y10
y11
y12
0
25
50
75
100
0
250
500
750
1,000
1,250
1,500
1,750
2,000
m/z

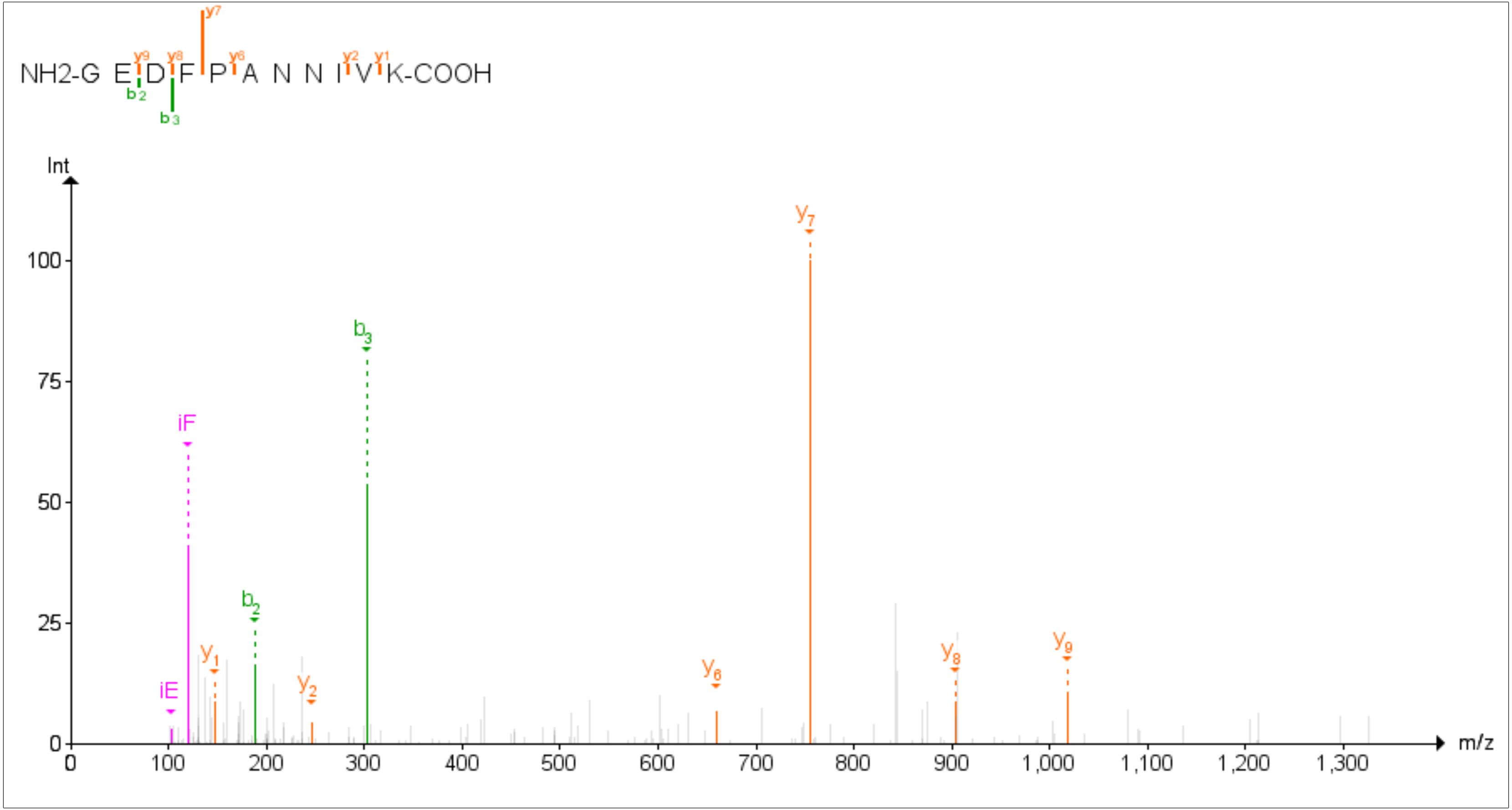

NH2-G E D F P A N N I V K-COOH
y9
y8
y7
y6
y2
y1
b2
b3
Int
100
75
50
25
0
iF
iE
y1
b2
y2
b3
y6
y7
y8
y9
0
100
200
300
400
500
600
700
800
900
1,000
1,100
1,200
1,300
m/z

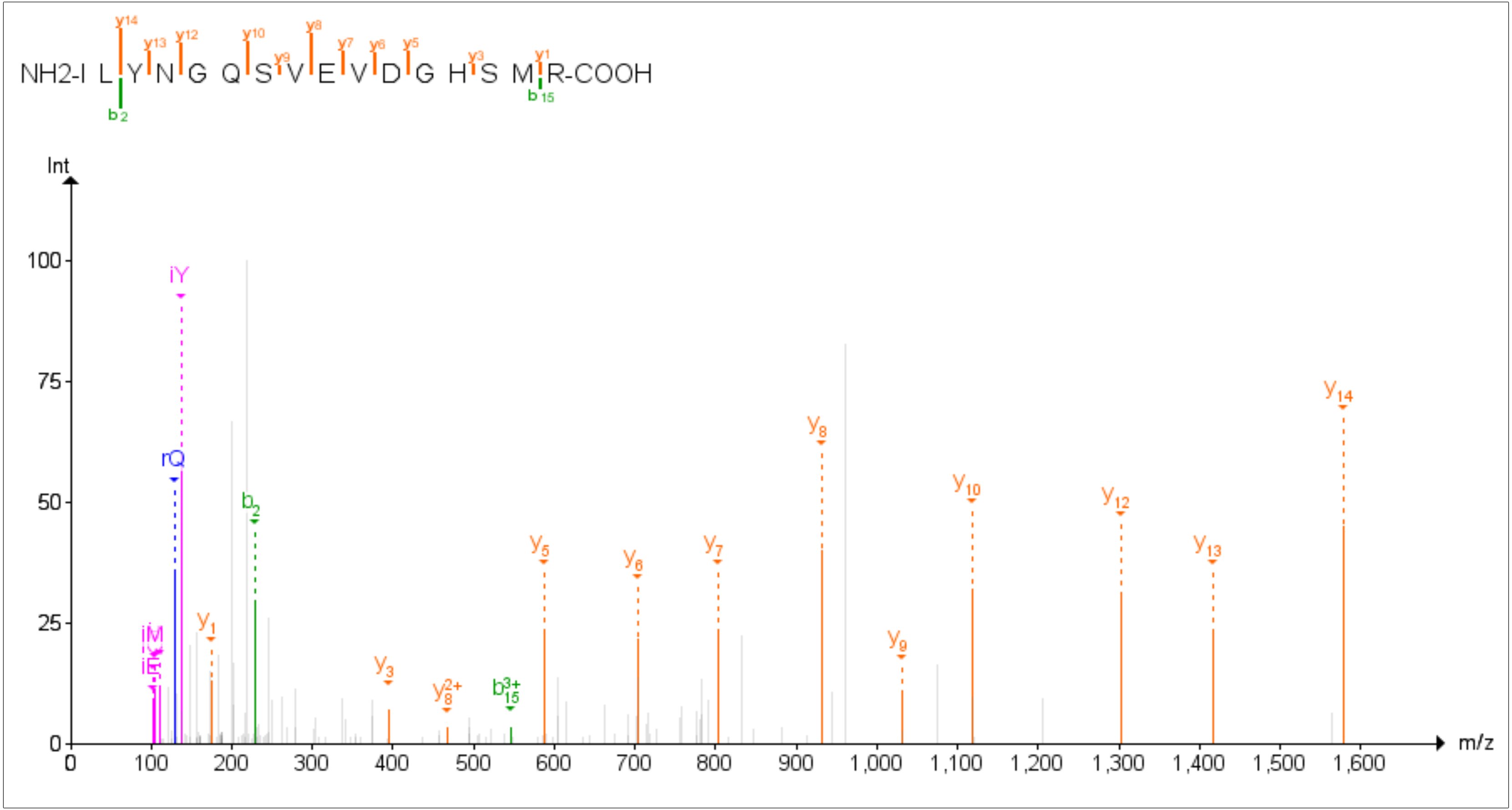

NH2-I L Y N G Q S V E V D G H S M R-COOH
y14 y13 y12 y10 y9 y8 y7 y6 y5 y3 y1
b2 b15
Int
100
75
50
25
0
iY
rQ
b2
iM
iF
y1
y3
y2+8
b3+15
y5
y6
y7
y8
y9
y10
y12
y13
y14
0 100 200 300 400 500 600 700 800 900 1,000 1,100 1,200 1,300 1,400 1,500 1,600
m/z

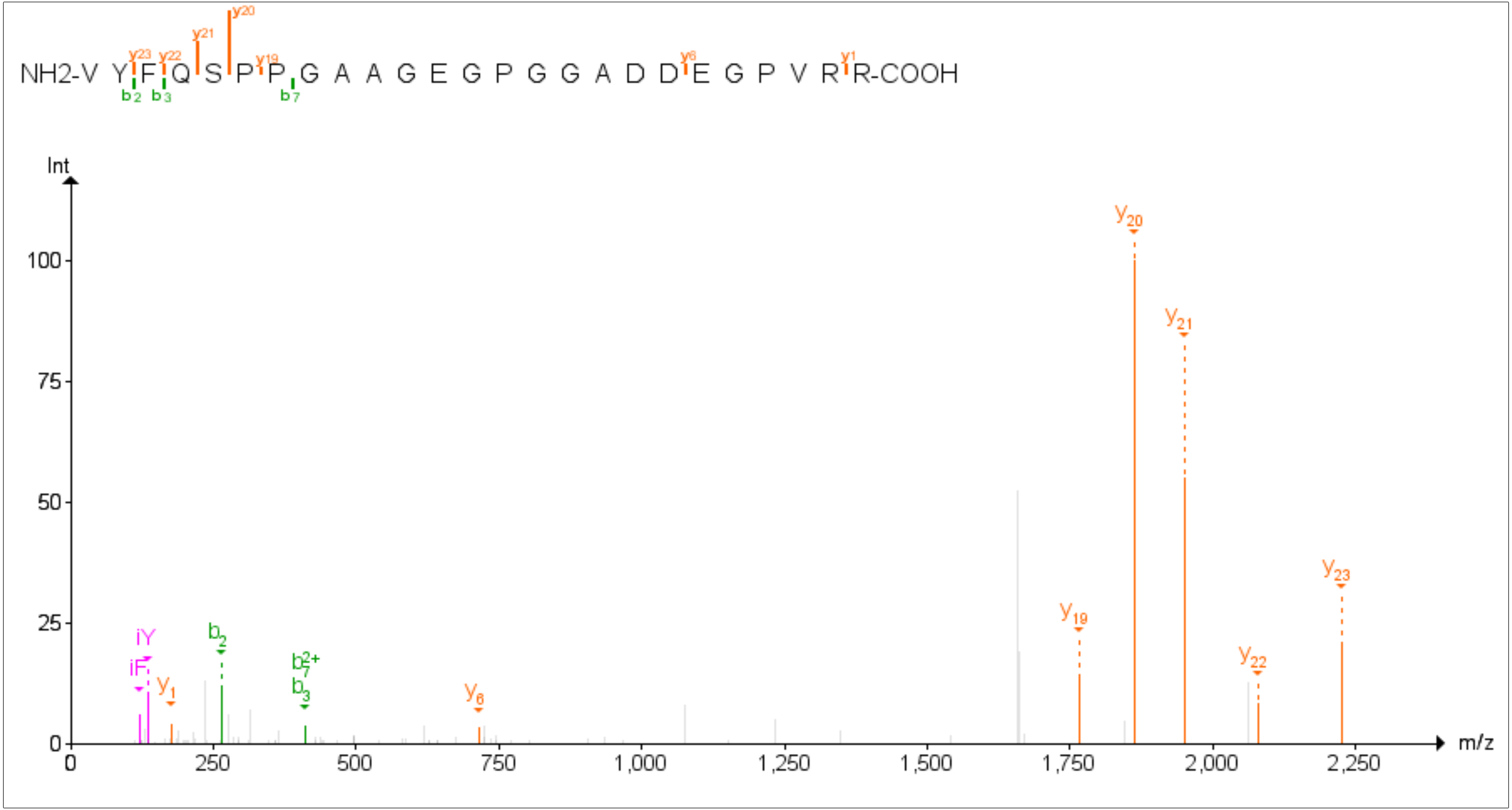
NH2-V Y F Q S P P G A A G E G P G G A D D E G P V R R-COOH
y23 y22 y21 y20 y19 y6 y1
b2 b3 b7
Int
100
75
50
25
0
m/z
0
250
500
750
1,000
1,250
1,500
1,750
2,000
2,250
iY
iF
y1
b2
b7 2+
b3
y6
y19
y20
y21
y22
y23

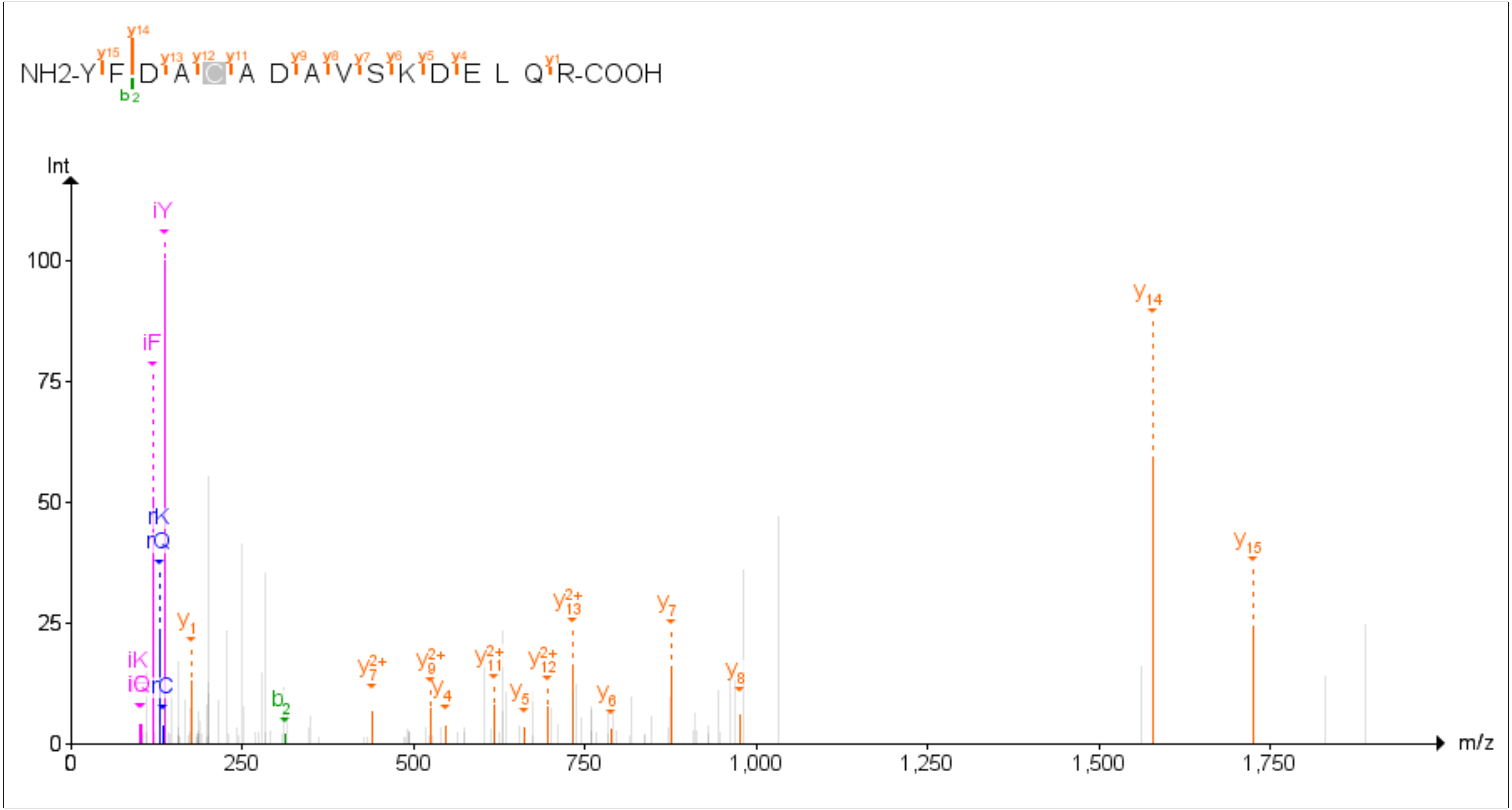
NH2-Y F D A C A D A V S K D E L Q R-COOH
y15 y14 y13 y12 y11 y9 y8 y7 y6 y5 y4 y1
b2
Int
100
75
50
25
0
iY
iF
rK
rQ
iK
iQ
rC
y1
b2
y7 2+
y9 2+
y4
y11 2+
y5
y12 2+
y13 2+
y6
y7
y8
y14
y15
0
250
500
750
1,000
1,250
1,500
1,750
m/z

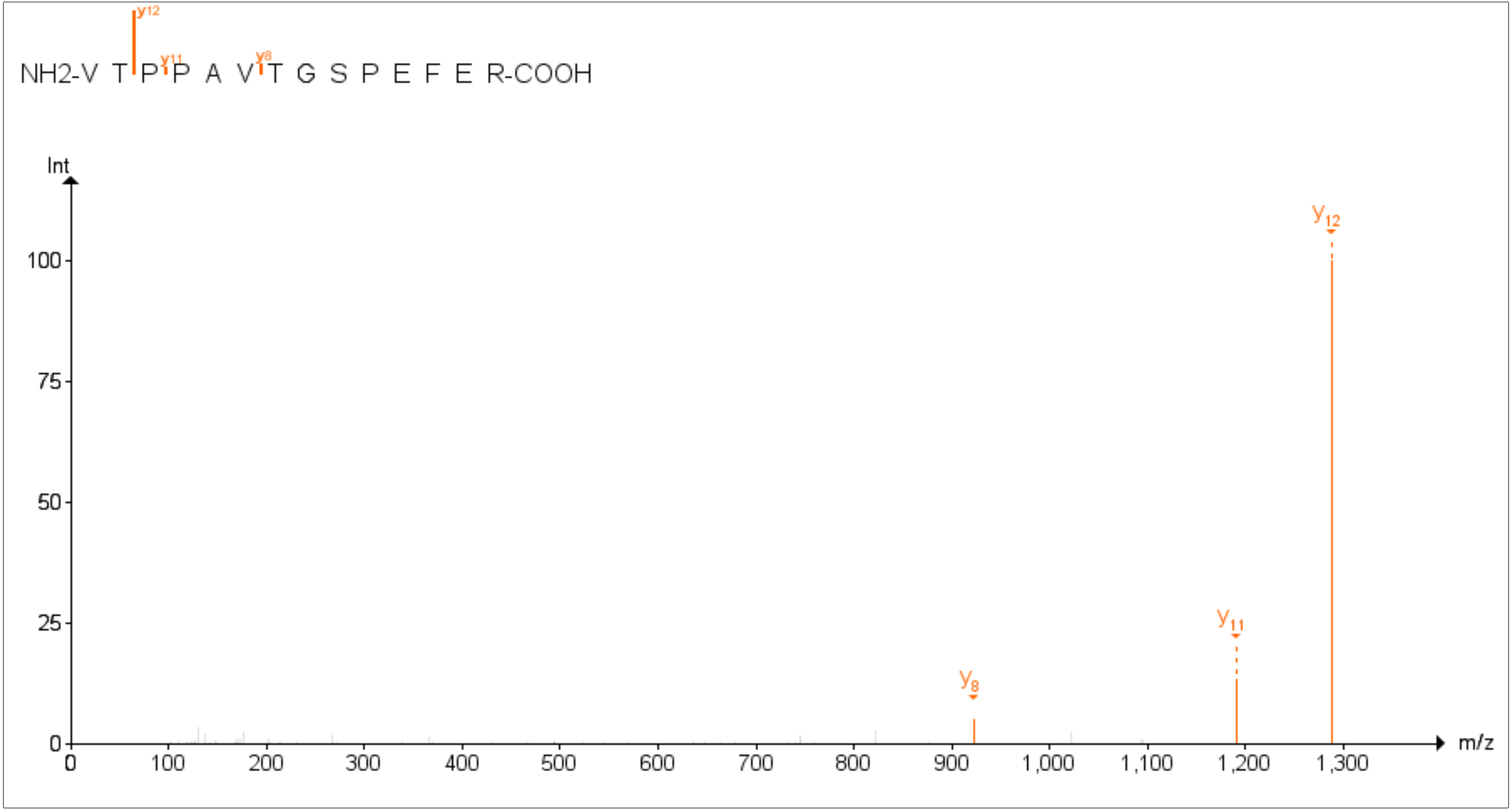

y12
y11
y8
NH2-V T P P A V T G S P E F E R-COOH
Int
100
75
50
25
0
0
100
200
300
400
500
600
700
800
900
1,000
1,100
1,200
1,300
m/z
y12
y11
y8

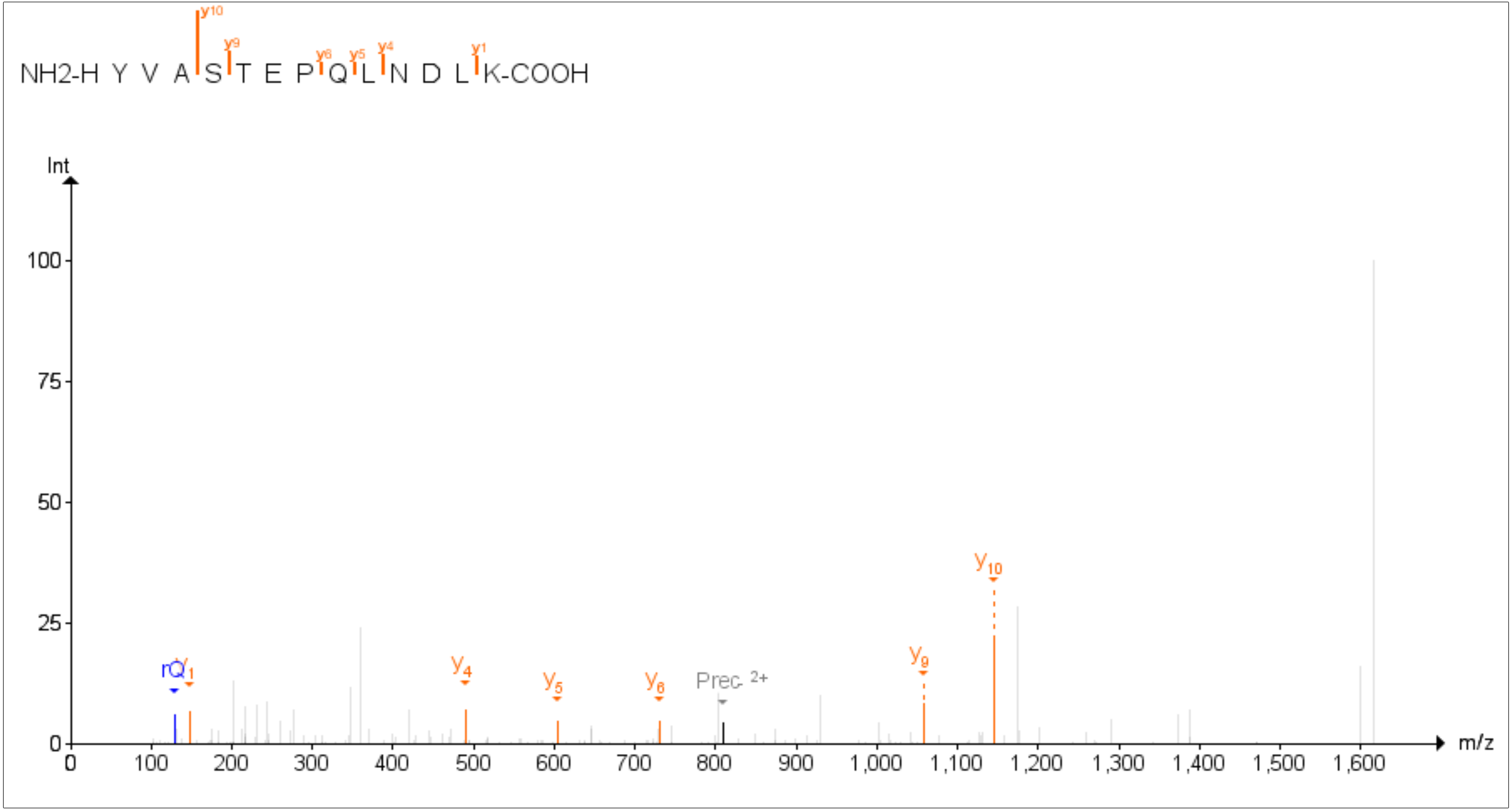

NH2-H Y V A S T E P Q L N D L K-COOH
y10
y9
y6
y5
y4
y1
Int
100
75
50
25
0
rQ
y1
y4
y5
y6
Prec 2+
y9
y10
m/z
0
100
200
300
400
500
600
700
800
900
1,000
1,100
1,200
1,300
1,400
1,500
1,600

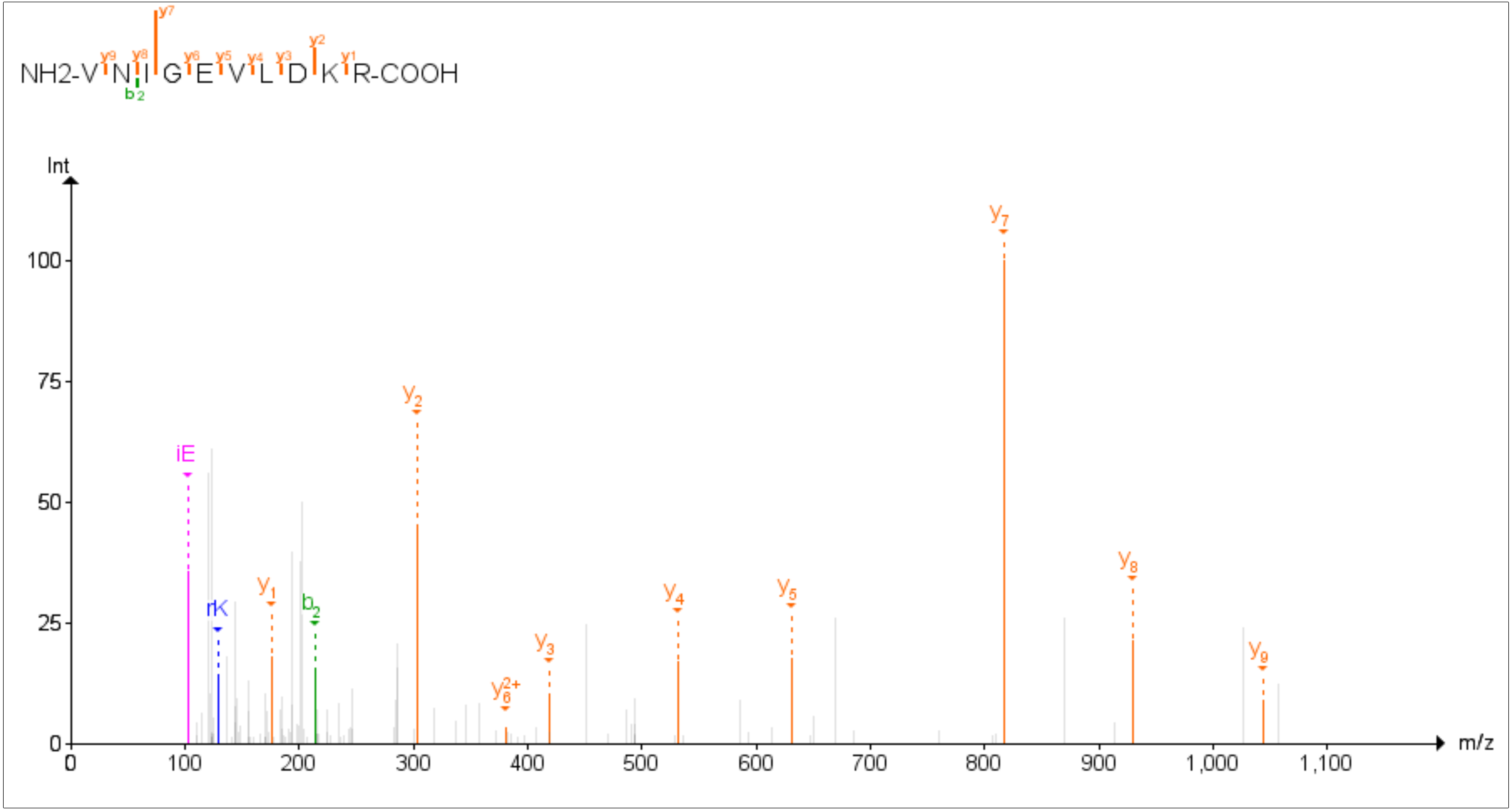
NH2-V N I G E V L D K R-COOH
y9 y8 y7 y6 y5 y4 y3 y2 y1
b2
Int
100
75
50
25
0
m/z
0 100 200 300 400 500 600 700 800 900 1,000 1,100
iE
rK
y1
b2
y2
y6 2+
y3
y4
y5
y7
y8
y9

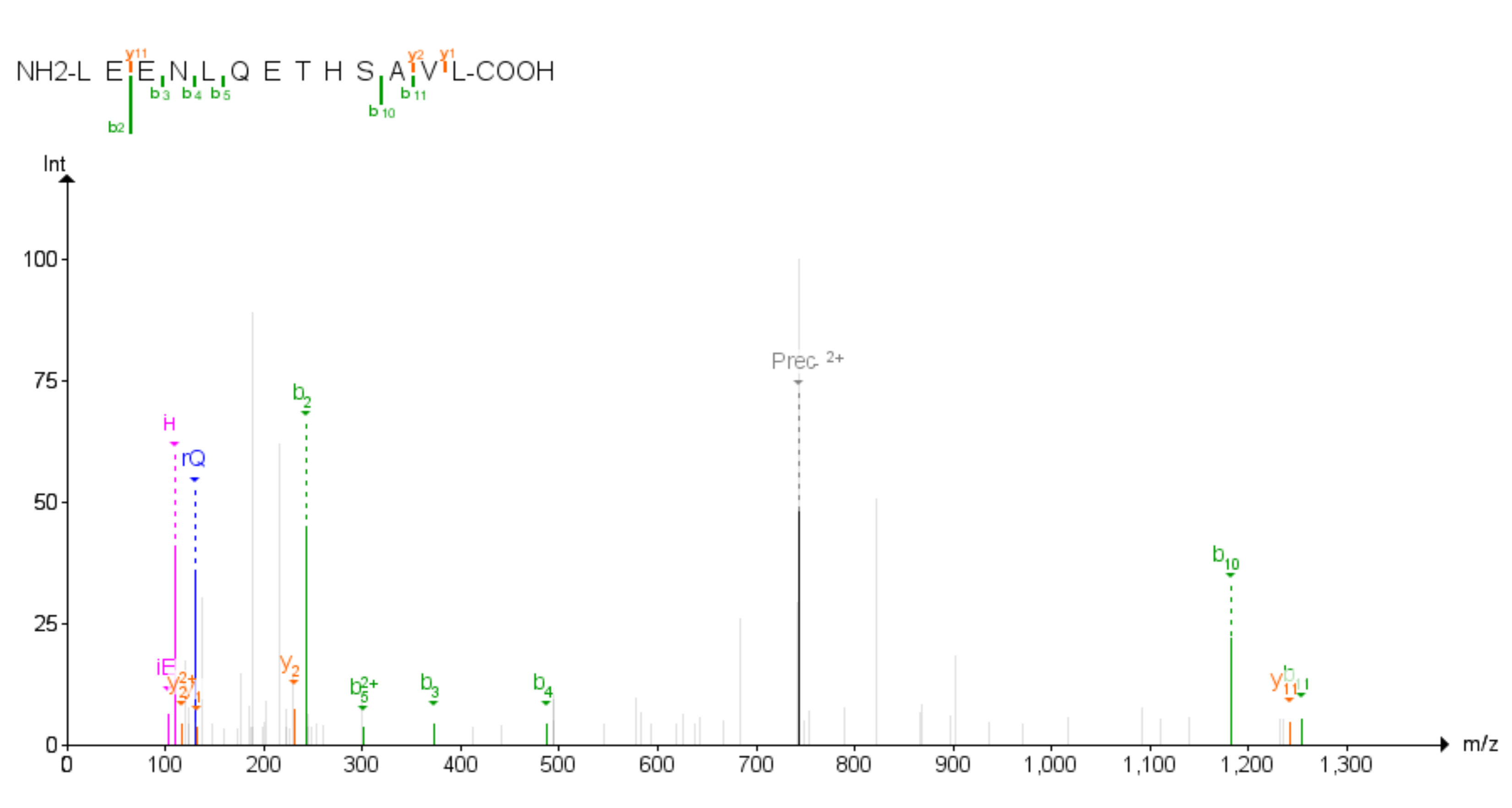

NH2-L E E N L Q E T H S A V L-COOH
y11
y2
y1
b2
b3
b4
b5
b10
b11
Int
100
75
50
25
0
Prec 2+
b2
iH
rQ
b10
iE
y2
y2 2+
y1
b5 2+
b3
b4
y11
b11
m/z
0
100
200
300
400
500
600
700
800
900
1,000
1,100
1,200
1,300

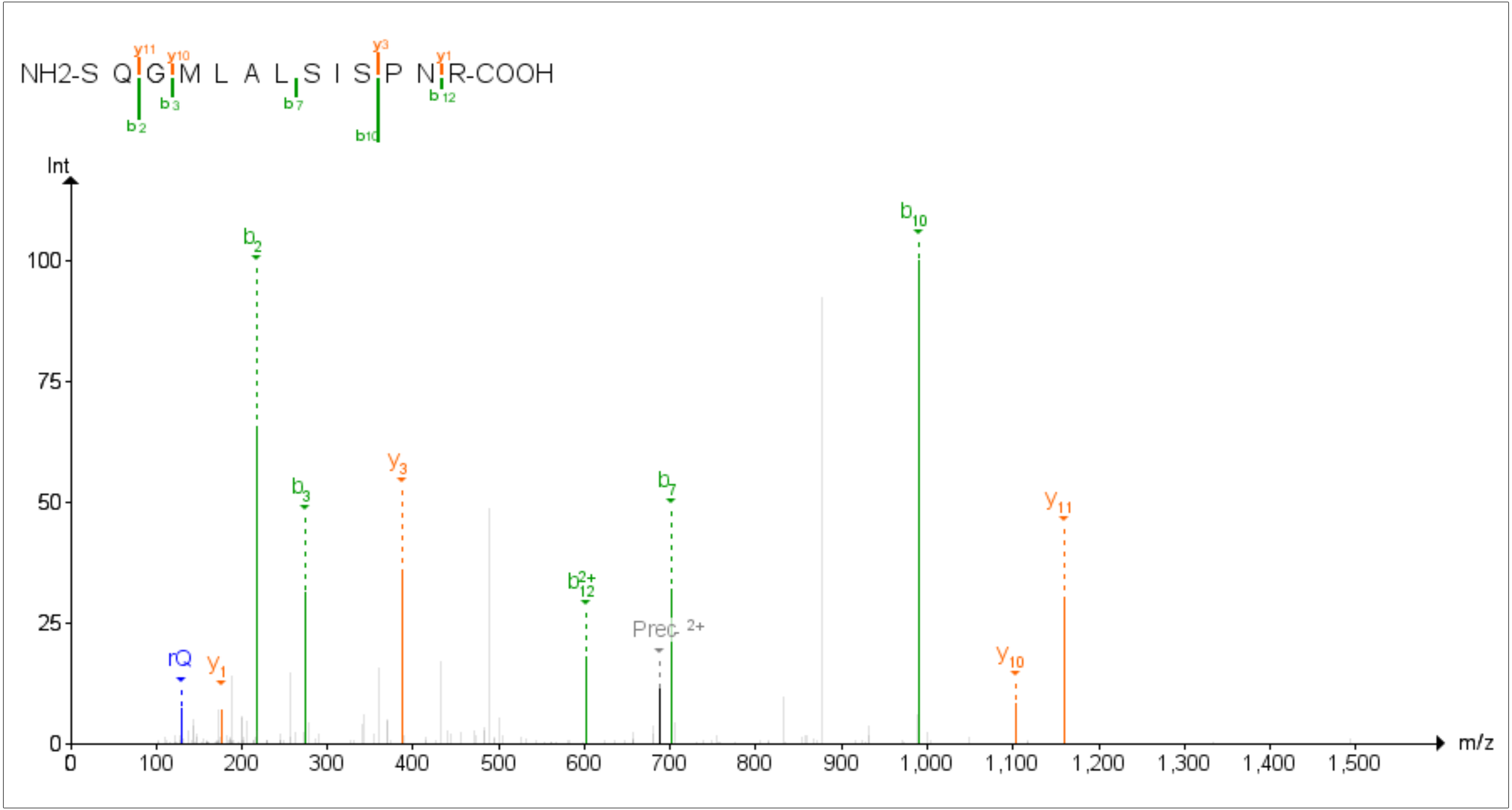

NH2-S Q G M L A L S I S P N R-COOH
y11
y10
y3
y1
b2
b3
b7
b10
b12
Int
100
75
50
25
0
b2
b10
b3
y3
b7
y11
b12 2+
Prec 2+
rQ
y1
y10
0
100
200
300
400
500
600
700
800
900
1,000
1,100
1,200
1,300
1,400
1,500
m/z

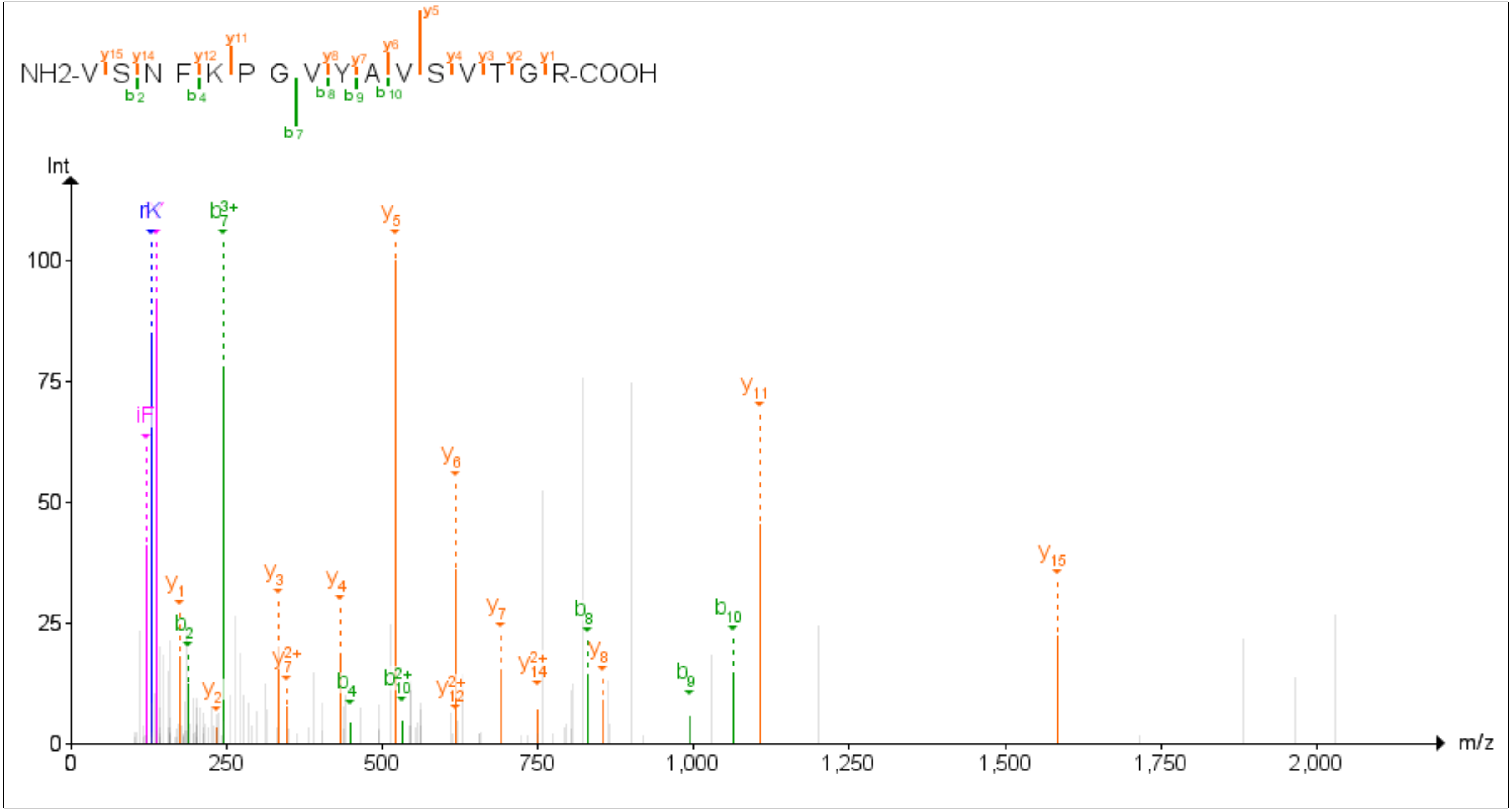
NH2-VSNFKPGVYAVSVTGR-COOH
y15 y14 y12 y11 y8 y7 y6 y5 y4 y3 y2 y1
b2 b4 b7 b8 b9 b10
Int
m/z
0
25
50
75
100
0
250
500
750
1,000
1,250
1,500
1,750
2,000
iF
rK
b_7^{3+}
y_5
y_{11}
y_6
y_{15}
y_1
b_2
y_2
y_3
y_7^{2+}
y_4
b_4
b_{10}^{2+}
y_{12}^{2+}
y_7
y_{14}^{2+}
b_8
y_8
b_9
b_{10}

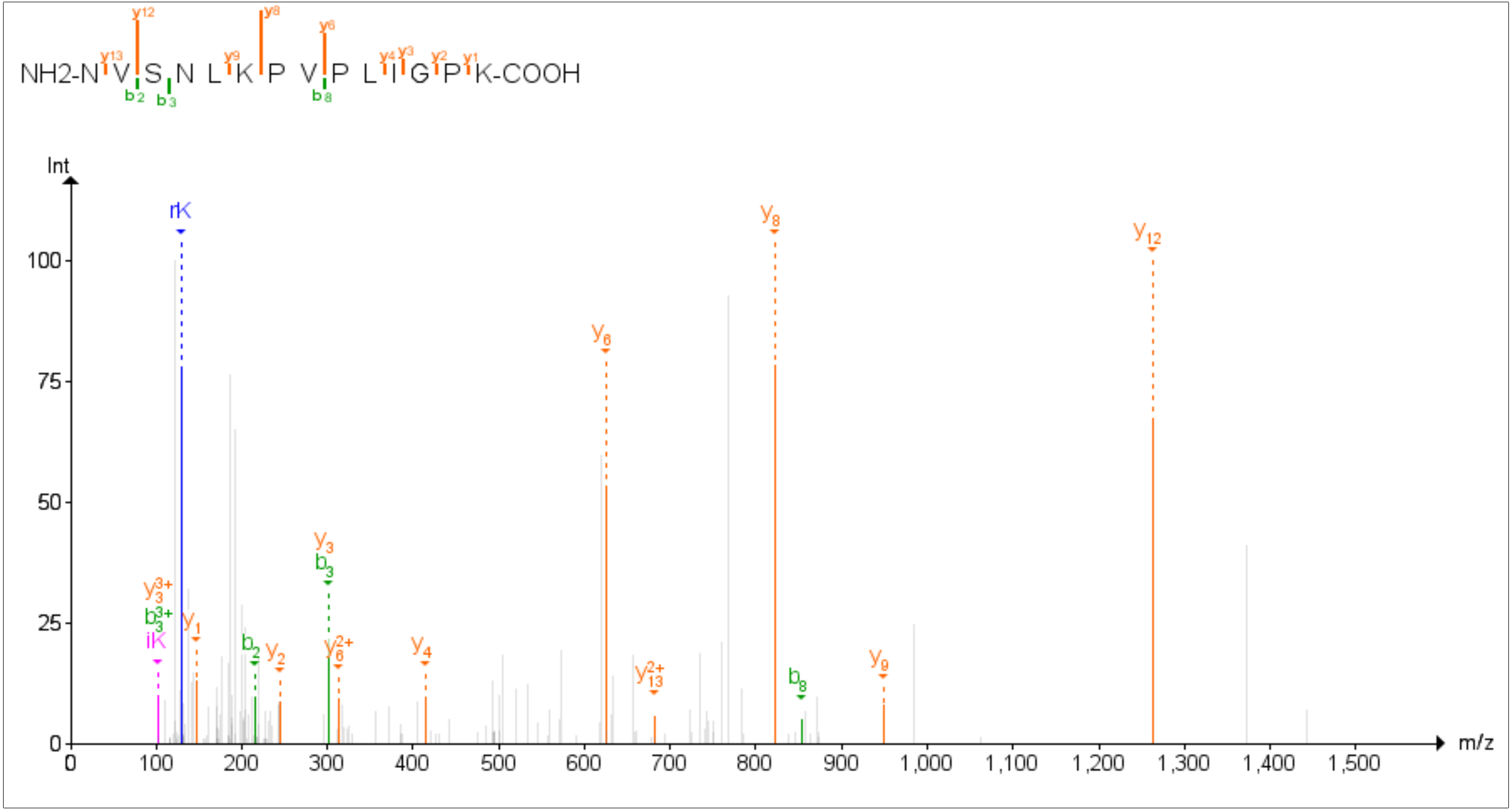
NH2-N V S N L K P V P L I G P K-COOH
y13 y12 y9 y8 y6 y4 y3 y2 y1
b2 b3 b8
Int
100
75
50
25
0
rK
y8
y12
y6
y3
b3
y3 3+
b3 3+
iK
y1
b2
y2
y6 2+
y4
y13 2+
b8
y9
0 100 200 300 400 500 600 700 800 900 1,000 1,100 1,200 1,300 1,400 1,500
m/z

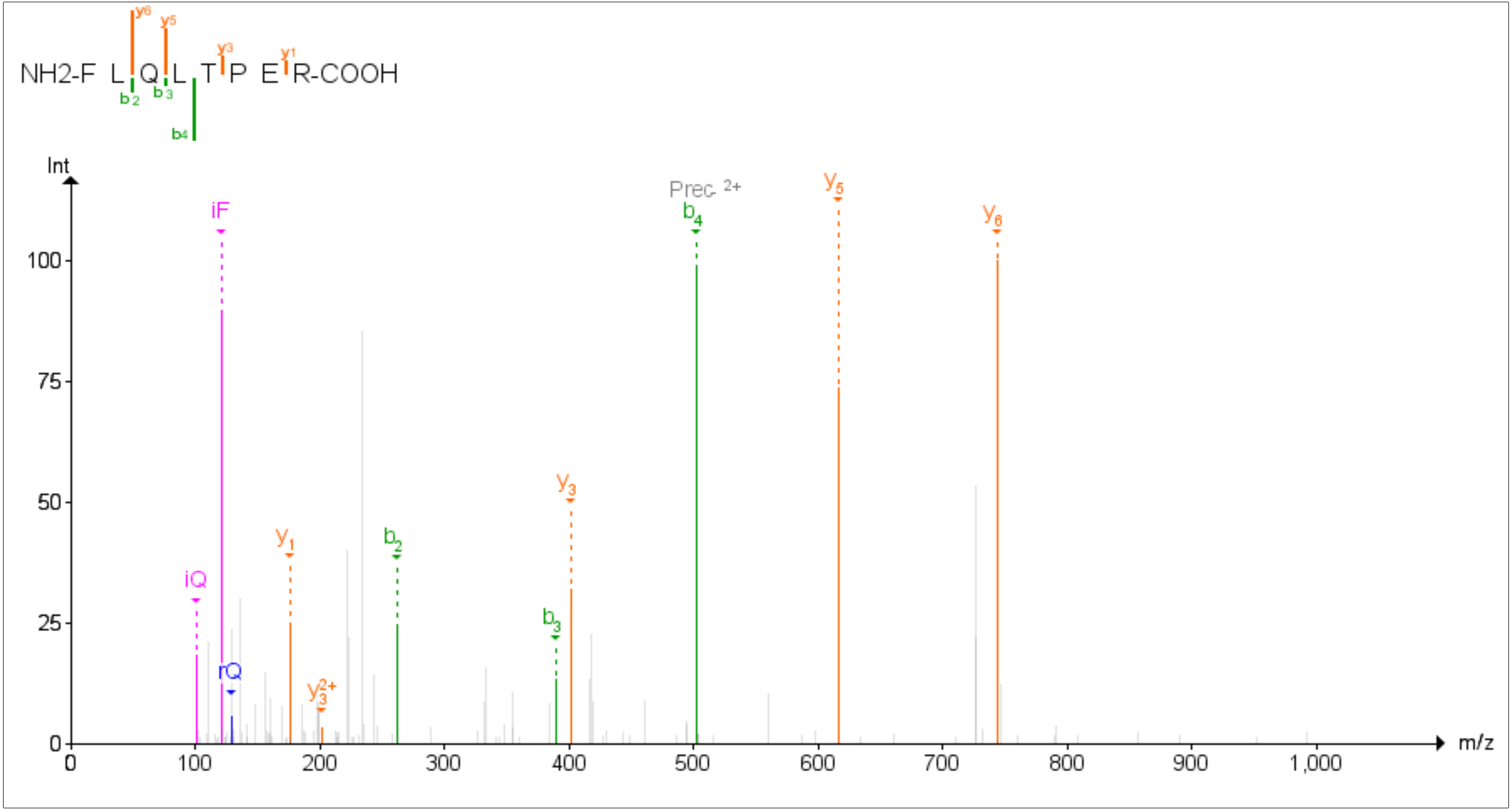

NH2-F L Q L T P E R-COOH
y6
y5
y3
y1
b2
b3
b4
Int
100
75
50
25
0
iF
iQ
rQ
y1
y3 2+
b2
b3
y3
Prec 2+
b4
y5
y6
0
100
200
300
400
500
600
700
800
900
1,000
m/z

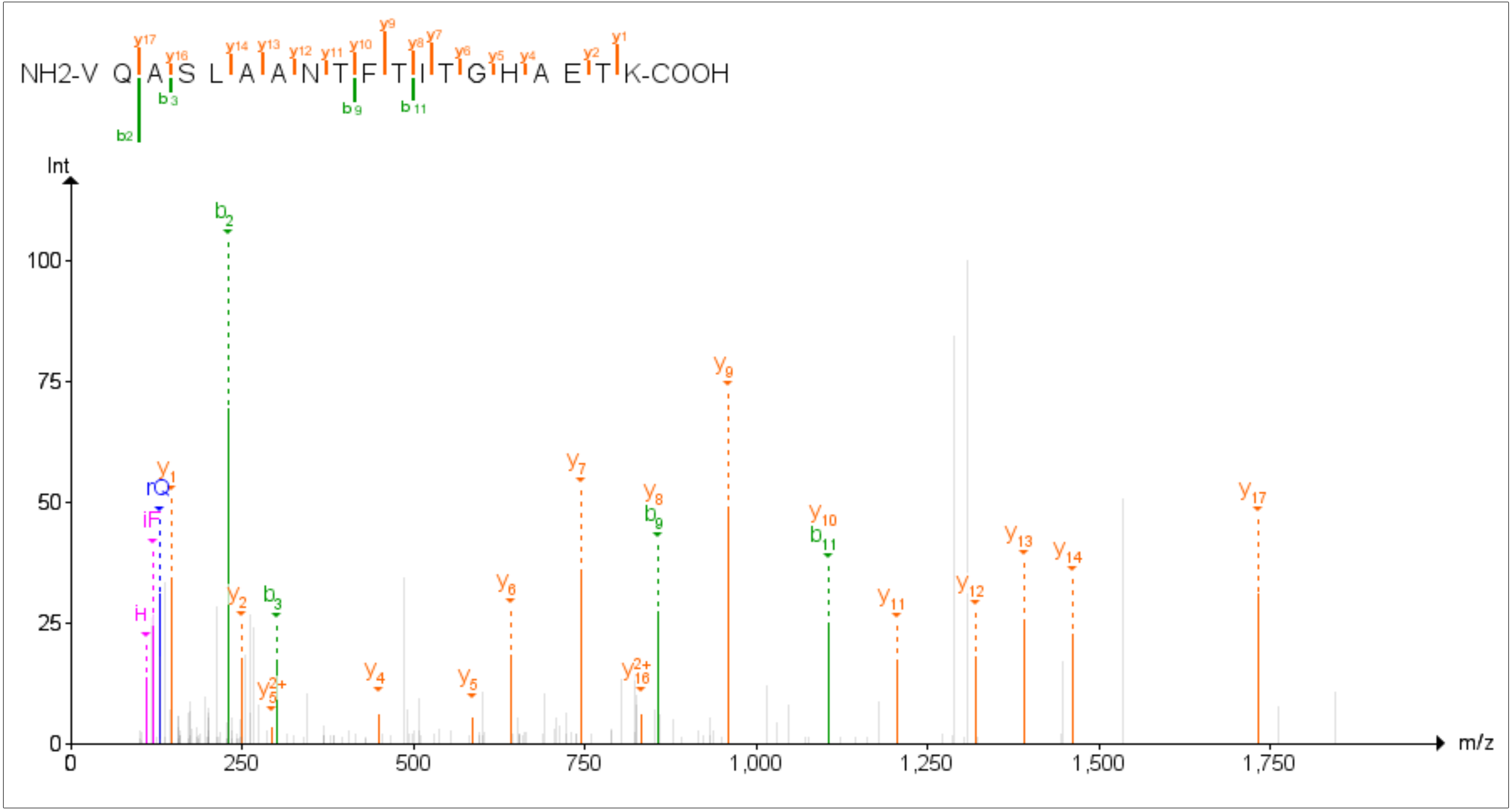

NH2-V Q A S L A A N T F T I T G H A E T K-COOH
y17 y16 y14 y13 y12 y11 y10 y9 y8 y7 y6 y5 y4 y2 y1
b2 b3 b9 b11
Int
100
75
50
25
0
0
250
500
750
1,000
1,250
1,500
1,750
m/z
b2
y9
y1
rQ
iF
iH
y7
y8
b9
y10
b11
y17
y13
y14
y2
b3
y6
y11
y12
y5 2+
y4
y5
y16 2+

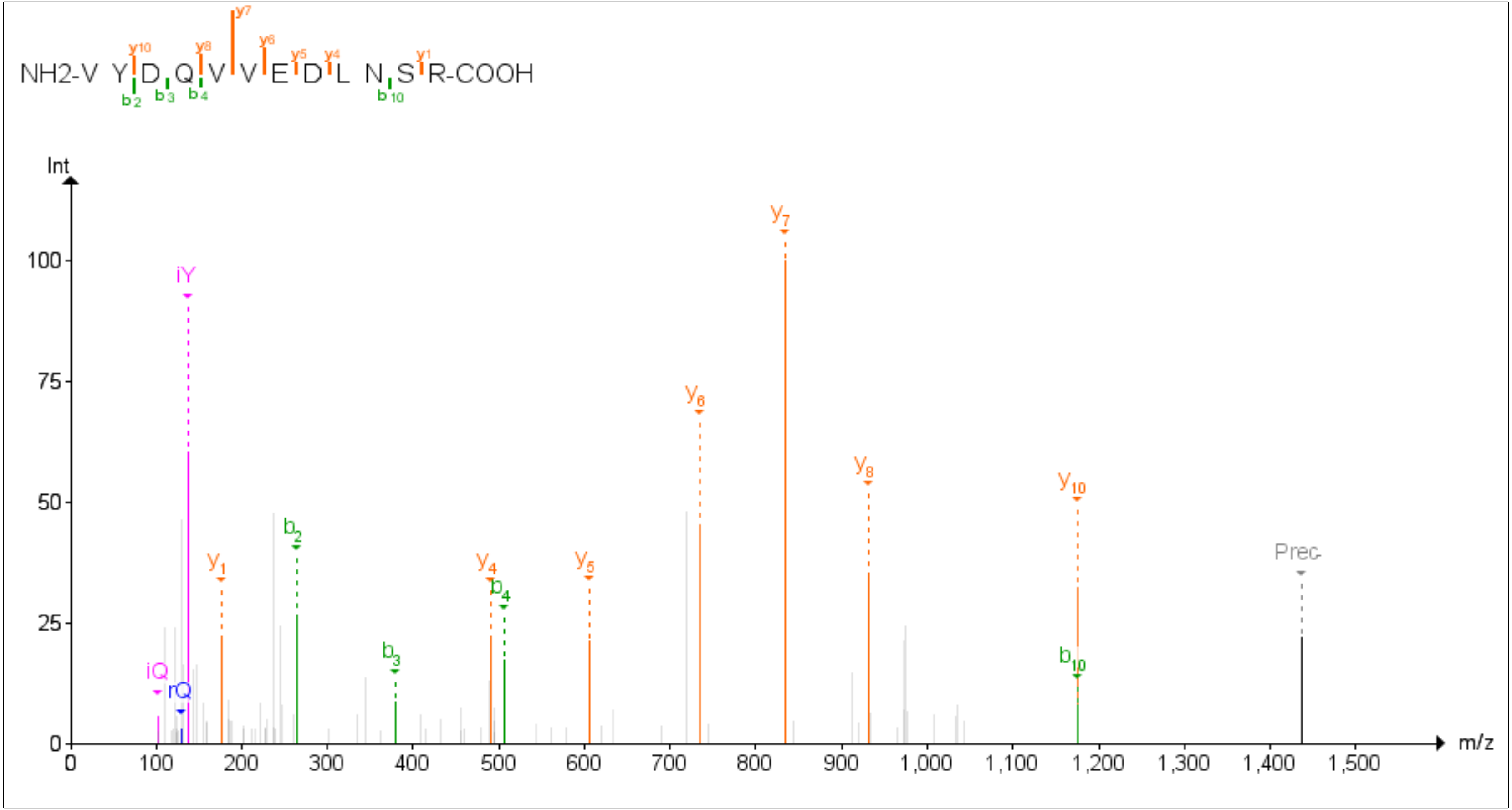

NH2-V Y D Q V V E D L N S R-COOH
y10 y8 y7 y6 y5 y4 y1
b2 b3 b4 b10
Int
100
75
50
25
0
iY
iQ
rQ
y1
b2
b3
y4
b4
y5
y6
y7
y8
y10
b10
Prec
0
100
200
300
400
500
600
700
800
900
1,000
1,100
1,200
1,300
1,400
1,500
m/z

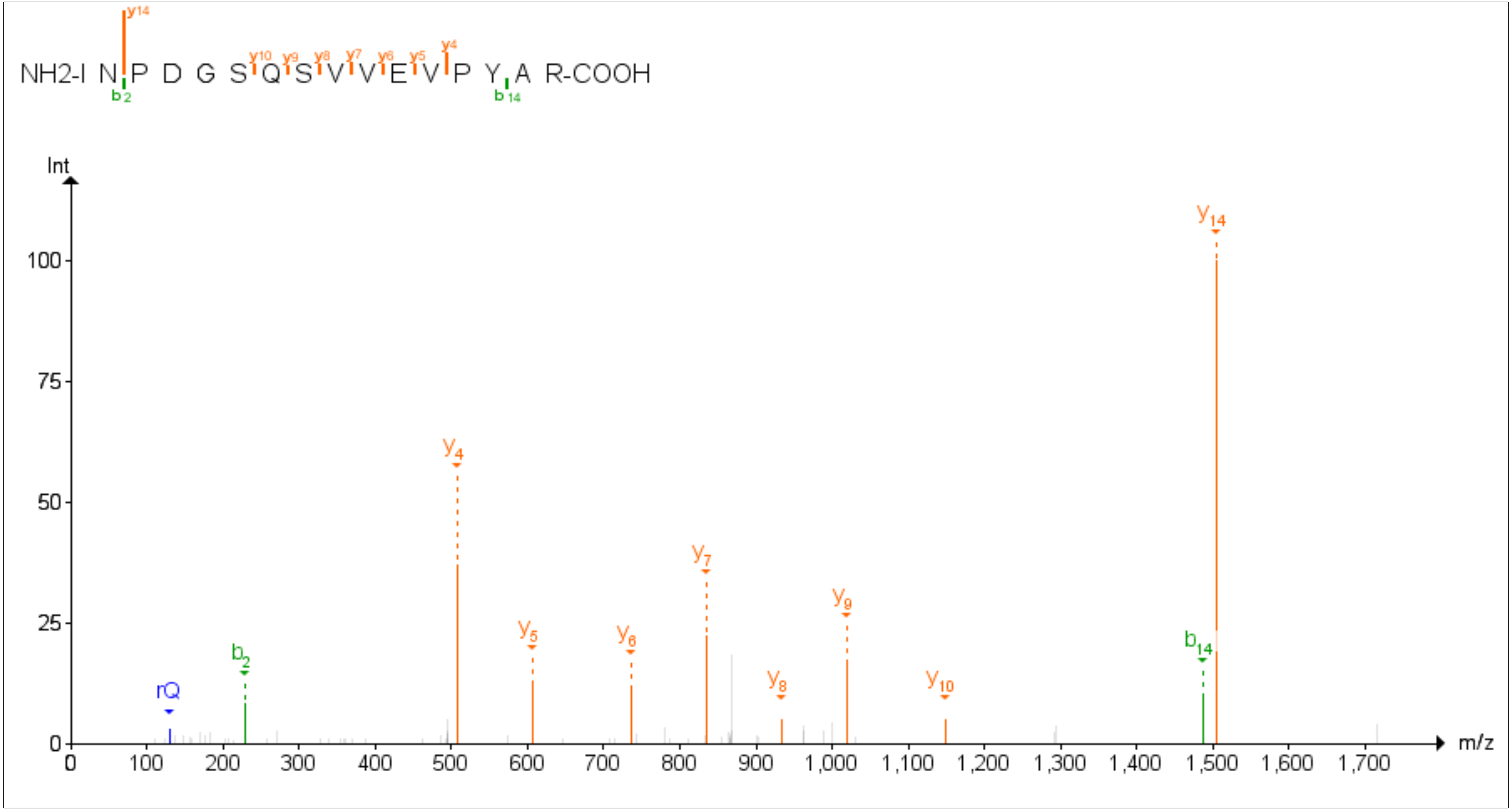

NH2-I N P D G S Q S V V E V P Y A R-COOH
Int
m/z
y14
y4
y5
y6
y7
y8
y9
y10
b2
b14
rQ

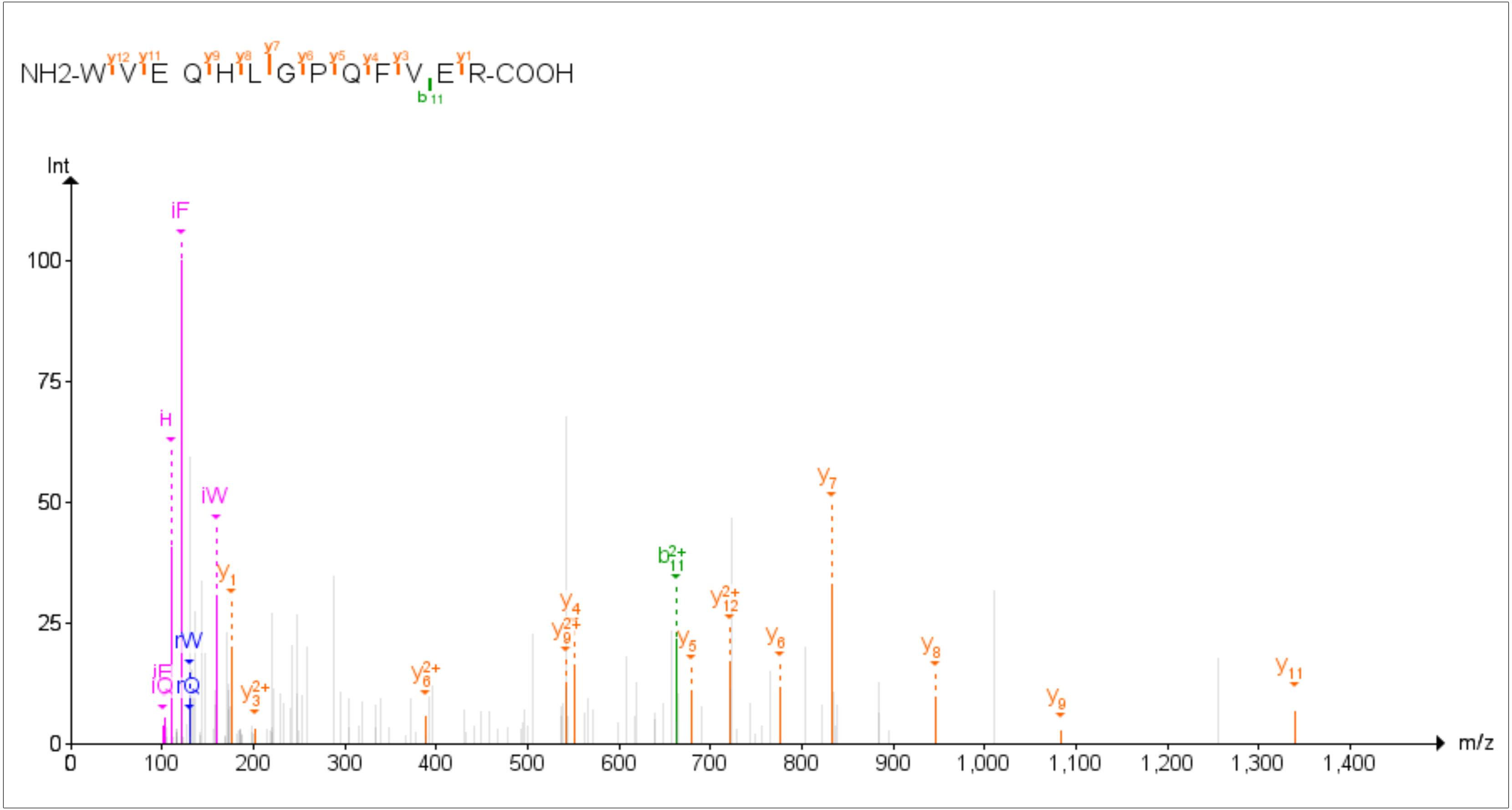

NH2-W V E Q H L G P Q F V E R-COOH
y12 y11 y9 y8 y7 y6 y5 y4 y3 y1
b11
Int
100
75
50
25
0
iF
iH
iW
y1
rW
iE
iQ
rQ
y3 2+
y6 2+
y4
y9 2+
b11 2+
y5
y12 2+
y6
y7
y8
y9
y11
0
100
200
300
400
500
600
700
800
900
1,000
1,100
1,200
1,300
1,400
m/z

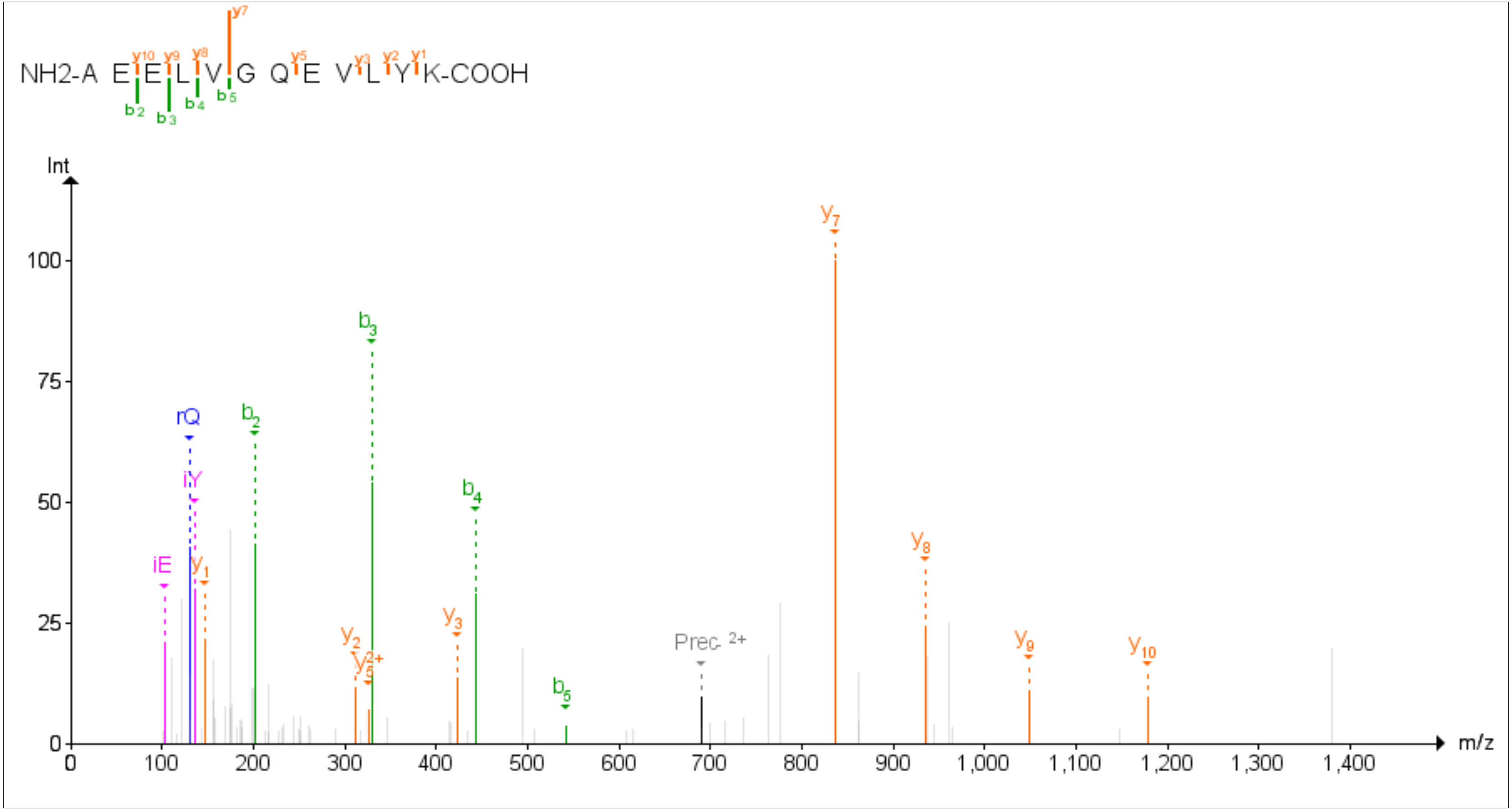

NH2-A E E L V G Q E V L Y K-COOH
y10 y9 y8 y7 y5 y3 y2 y1
b2 b3 b4 b5
Int
m/z
rQ
iY
iE
y1
b2
b3
b4
b5
y2
y5 2+
y3
Prec 2+
y7
y8
y9
y10

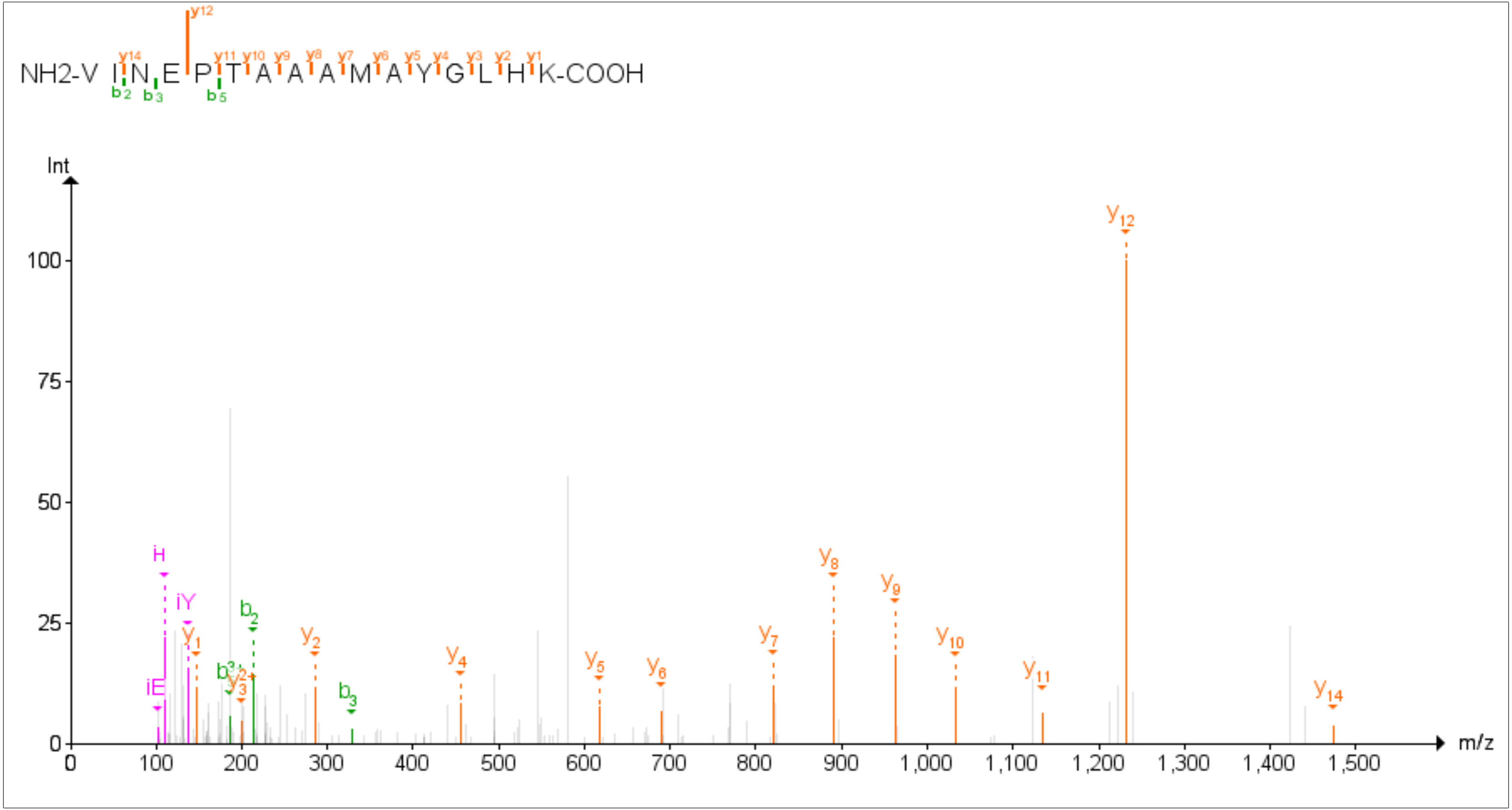

NH2-V I N E P T A A A M A Y G L H K-COOH
y14 y12 y11 y10 y9 y8 y7 y6 y5 y4 y3 y2 y1
b2 b3 b5
Int
100
75
50
25
0
iH
iY
iE
b2
b3
y1
y2
y3
y4
y5
y6
y7
y8
y9
y10
y11
y12
y14
0 100 200 300 400 500 600 700 800 900 1,000 1,100 1,200 1,300 1,400 1,500
m/z

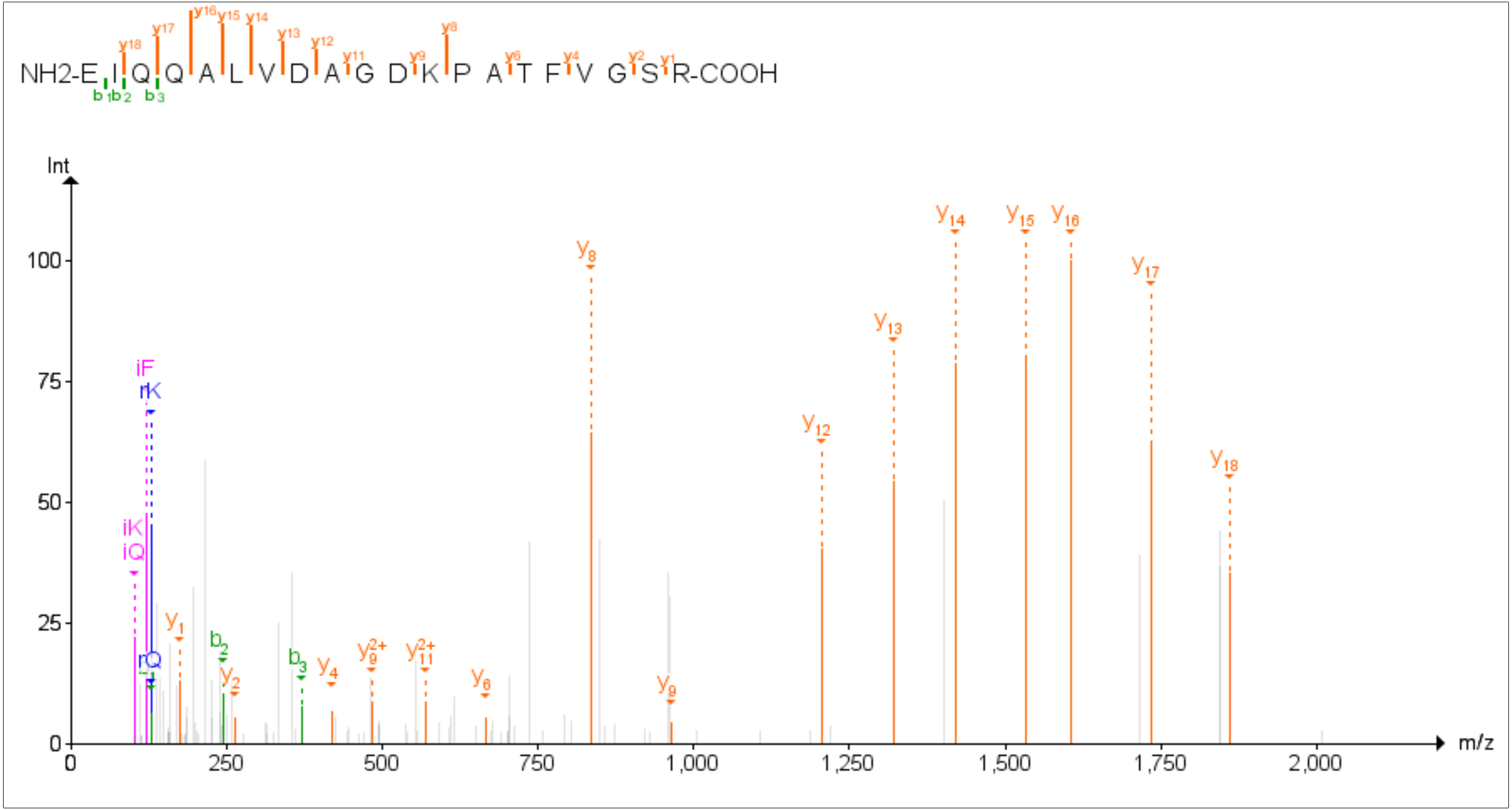

NH2-E I Q Q A L V D A G D K P A T F V G S R-COOH
b1 b2 b3
y18 y17 y16 y15 y14 y13 y12 y11 y9 y8 y6 y4 y2 y1
Int
100
75
50
25
0
iF
rK
iK
iQ
rQ
y1
b2
y2
b3
y4
y9 2+
y11 2+
y6
y8
y9
y12
y13
y14
y15
y16
y17
y18
0
250
500
750
1,000
1,250
1,500
1,750
2,000
m/z

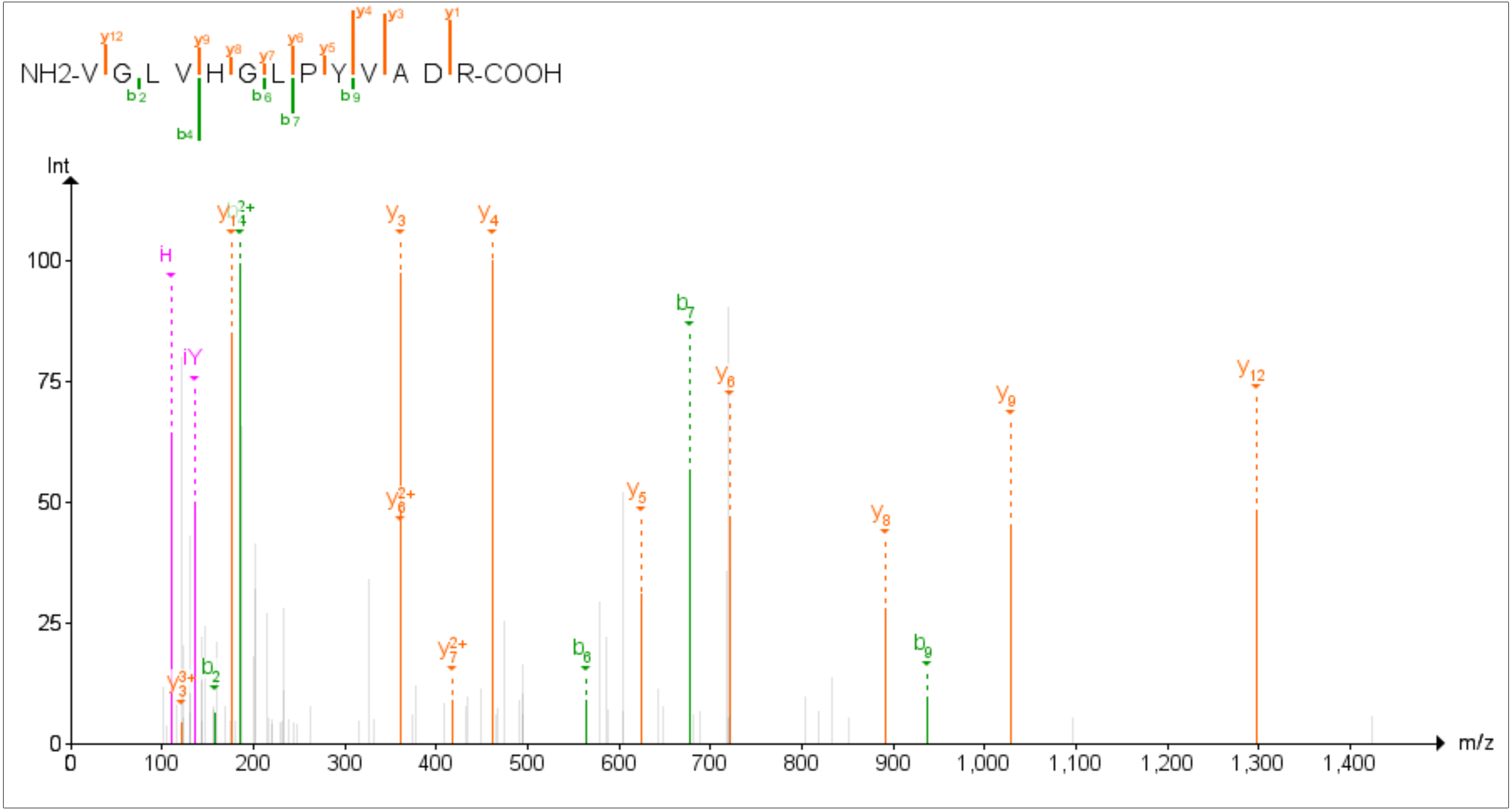

NH2-V G L V H G L P Y V A D R-COOH
y12 y9 y8 y7 y6 y5 y4 y3 y1
b2 b4 b6 b7 b9
Int
m/z
iH
iY
y3 y14 b2+
y4
b7
y6
y12
y9
y6 2+
y5
y8
y7 2+
b6
b9
y3 3+
b2
0 25 50 75 100
0 100 200 300 400 500 600 700 800 900 1,000 1,100 1,200 1,300 1,400

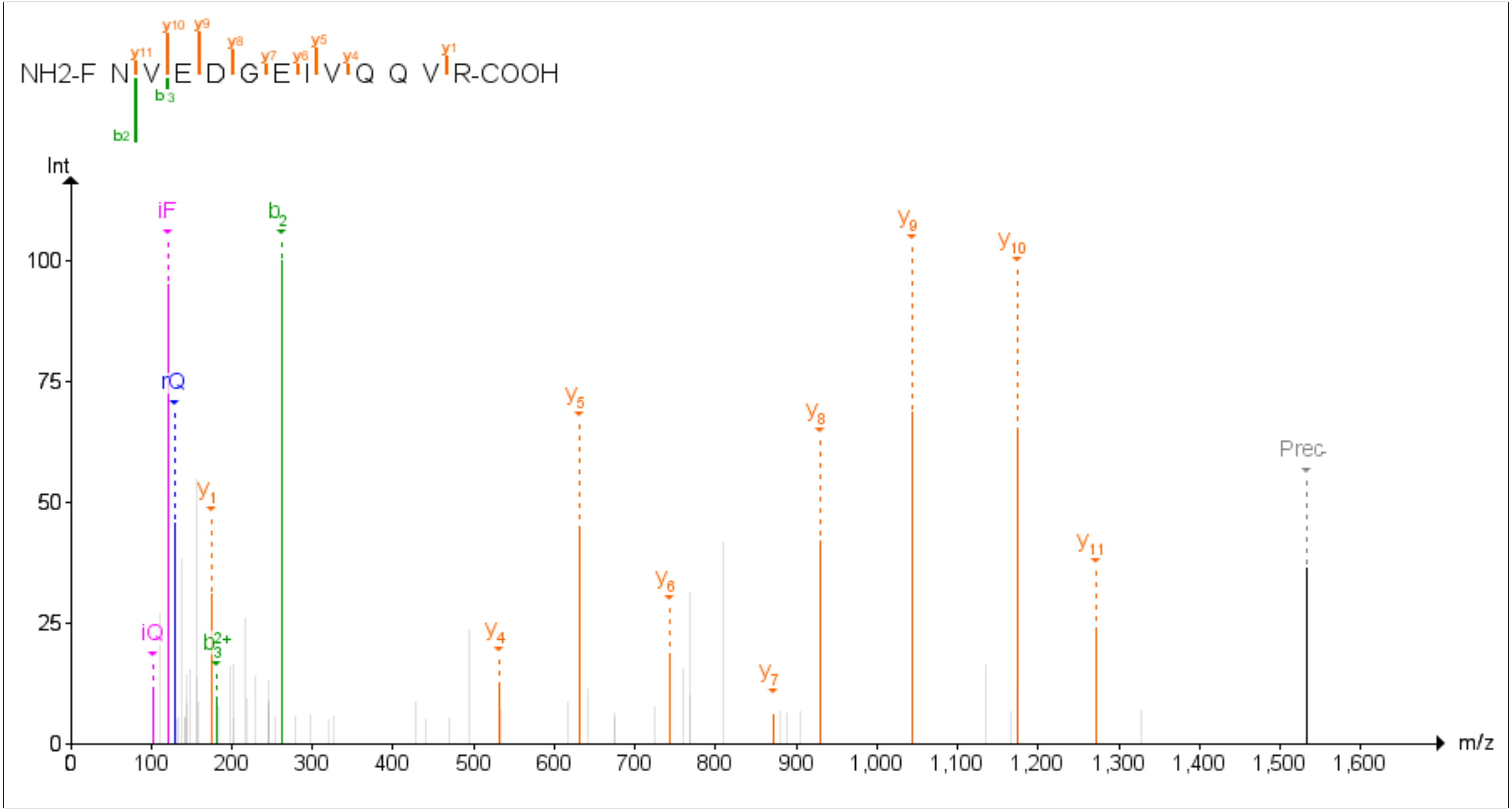

NH2-F N V E D G E I V Q Q V R-COOH
y11 y10 y9 y8 y7 y6 y5 y4 y1
b2 b3
Int
iF
b2
rQ
y1
iQ
b3 2+
y4
y5
y6
y7
y8
y9
y10
y11
Prec
m/z
0
25
50
75
100
0 100 200 300 400 500 600 700 800 900 1,000 1,100 1,200 1,300 1,400 1,500 1,600

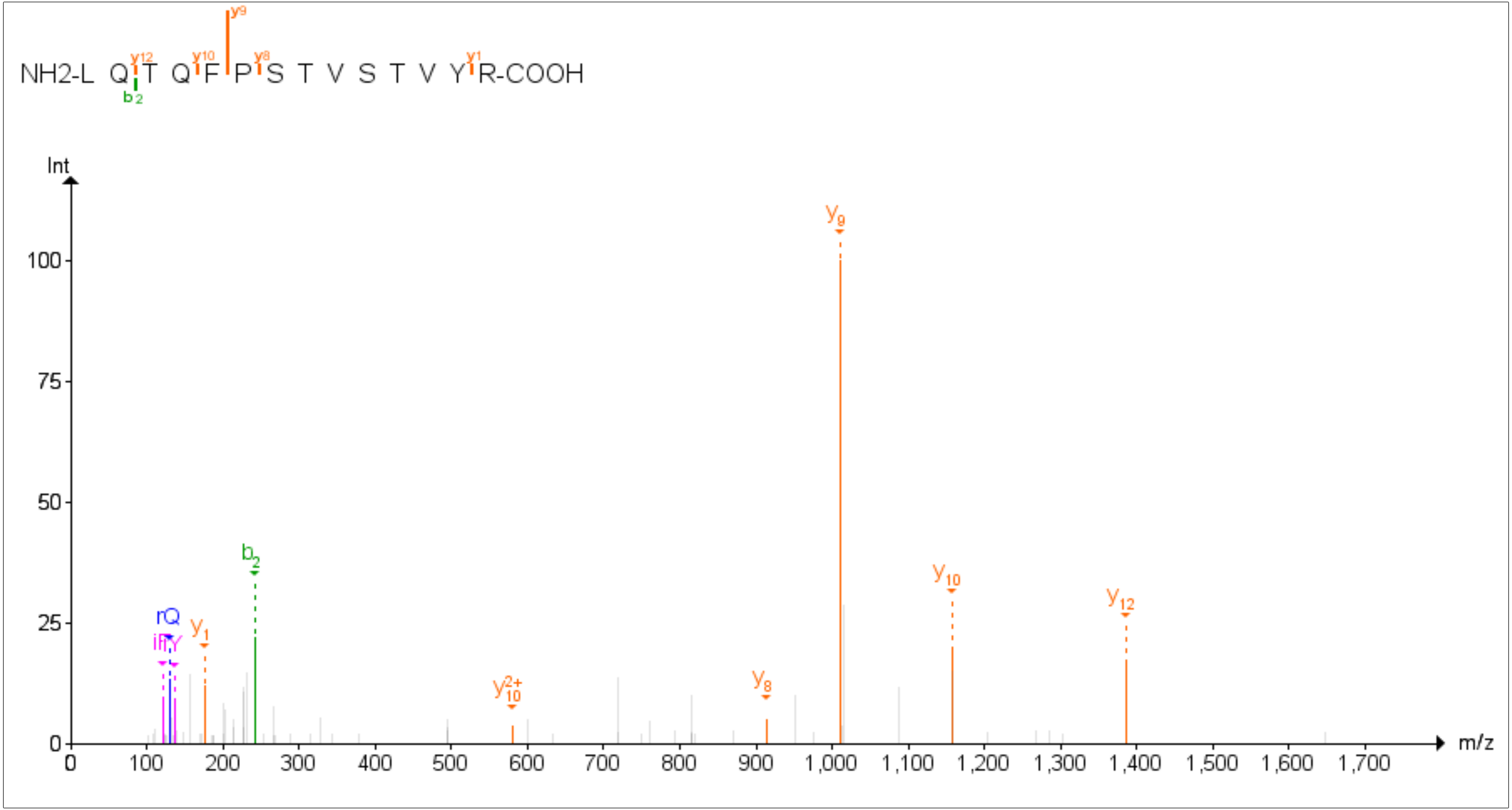
NH2-L Q T Q F P S T V S T V Y R-COOH
y12
y10
y9
y8
y1
b2
Int
100
75
50
25
0
y9
y10
y12
b2
rQ
imY
y1
y_{10}^{2+}
y8
0
100
200
300
400
500
600
700
800
900
1,000
1,100
1,200
1,300
1,400
1,500
1,600
1,700
m/z

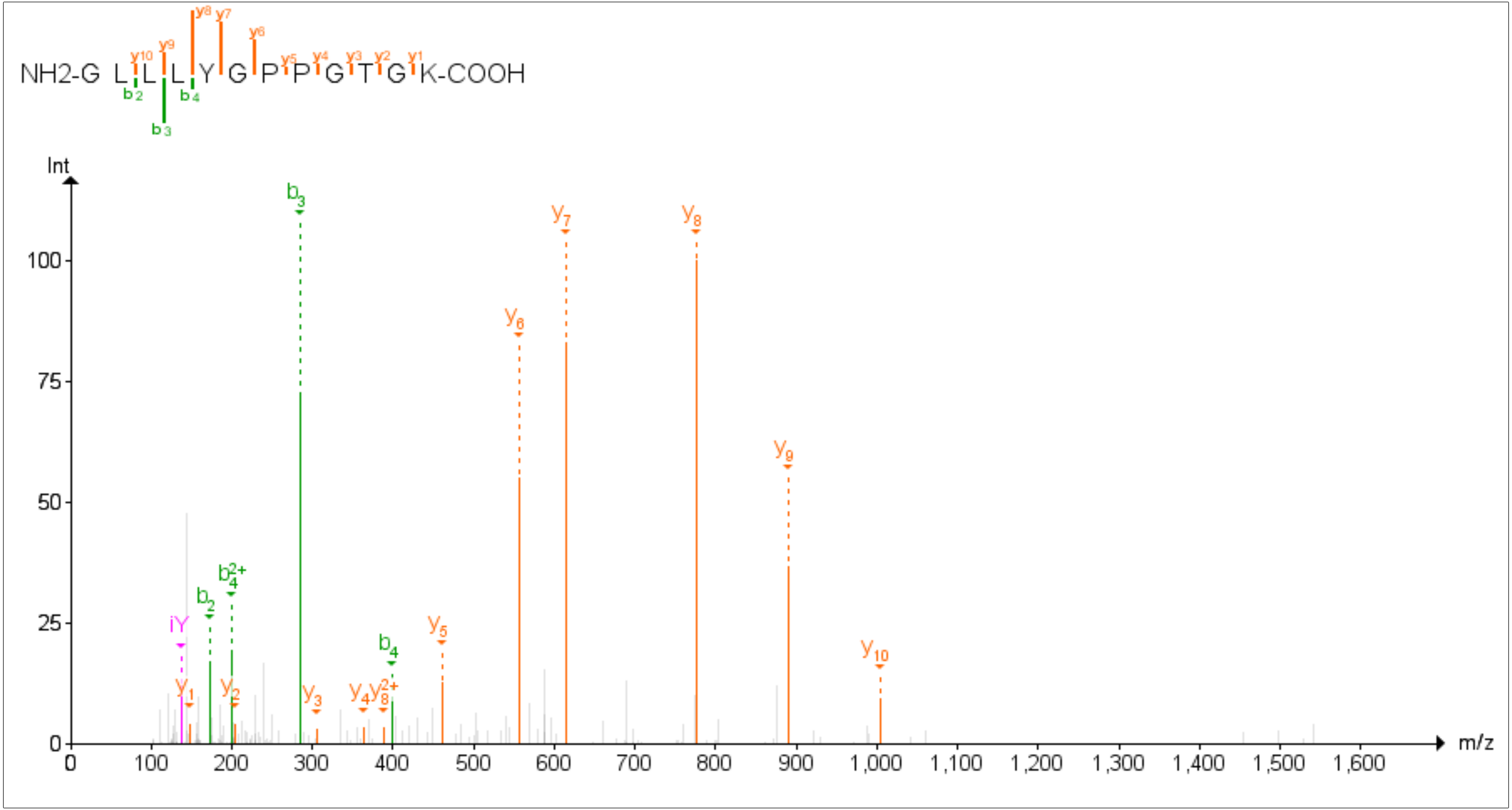

NH2-G L L L Y G P P G T G K-COOH
y10 y9 y8 y7 y6 y5 y4 y3 y2 y1
b2 b3 b4
Int
100
75
50
25
0
b3
y7
y8
y6
y9
y10
y5
b4
b4 2+
b2
iY
y1
y2
y3
y4
y8 2+
0 100 200 300 400 500 600 700 800 900 1,000 1,100 1,200 1,300 1,400 1,500 1,600
m/z

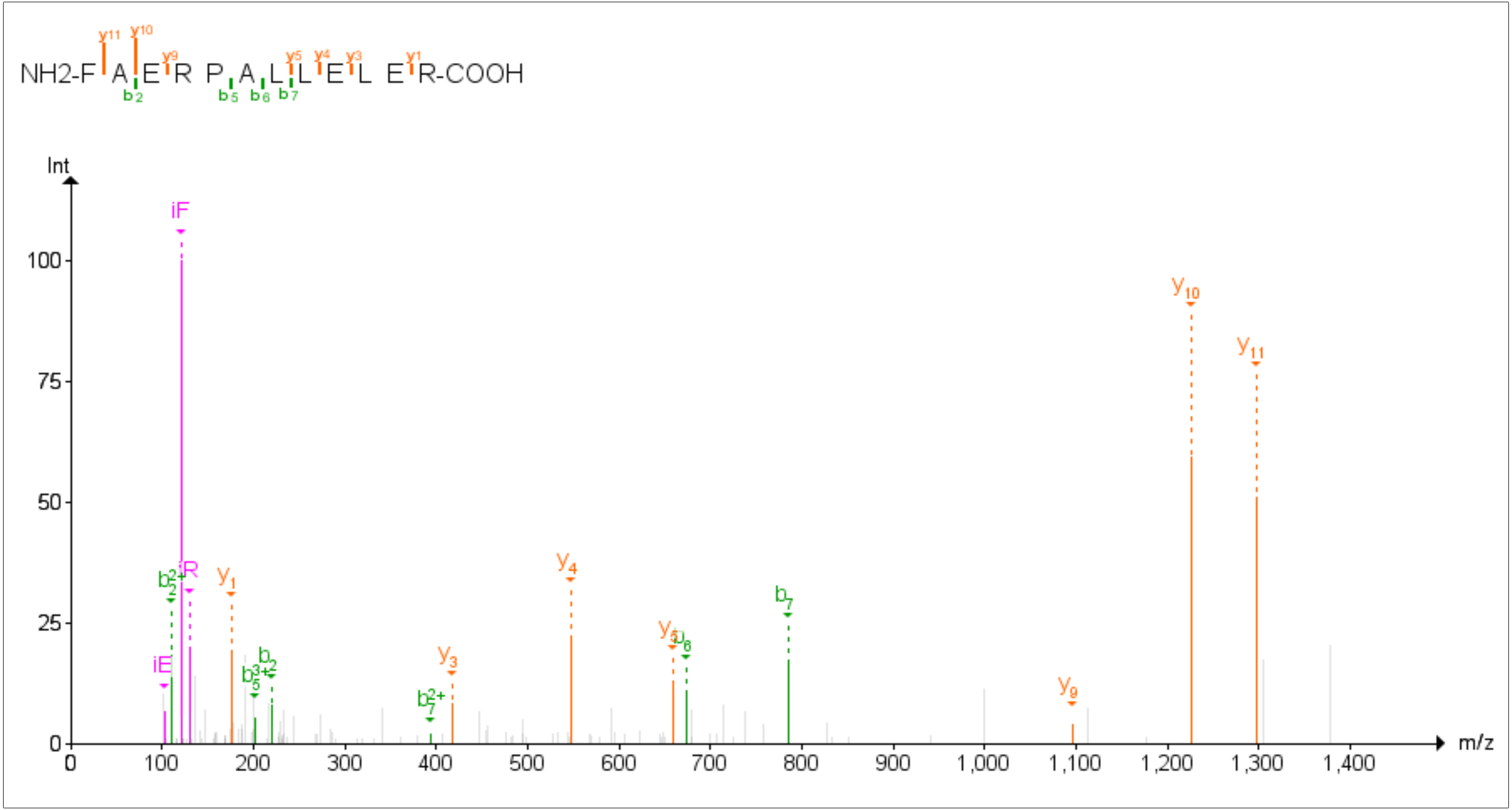

NH2-F A E R P A L L L E L E R-COOH
y11 y10 y9 y5 y4 y3 y1
b2 b5 b6 b7
Int
iF
y10
y11
b2 2+
iR
y1
y4
b7
y5
b6
iE
b2
b5 3+
y3
b7 2+
y9
m/z
0
25
50
75
100
0 100 200 300 400 500 600 700 800 900 1,000 1,100 1,200 1,300 1,400

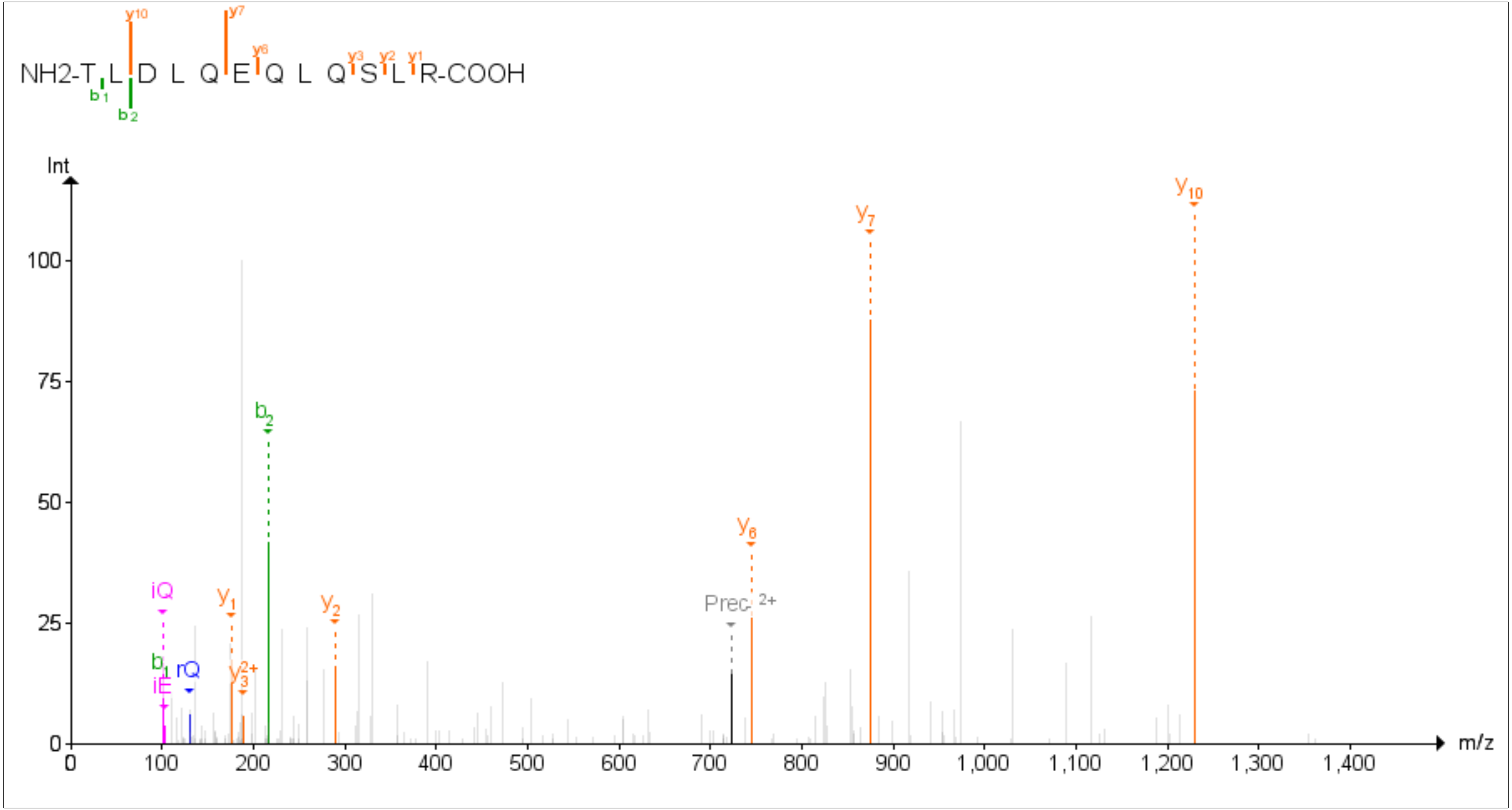

NH2-T L D L Q E Q L Q S L R-COOH
y10
y7
y6
y3
y2
y1
b1
b2
Int
100
75
50
25
0
m/z
0
100
200
300
400
500
600
700
800
900
1,000
1,100
1,200
1,300
1,400
y10
y7
b2
y6
iQ
y1
y2
Prec 2+
b1
rQ
iE
y3 2+

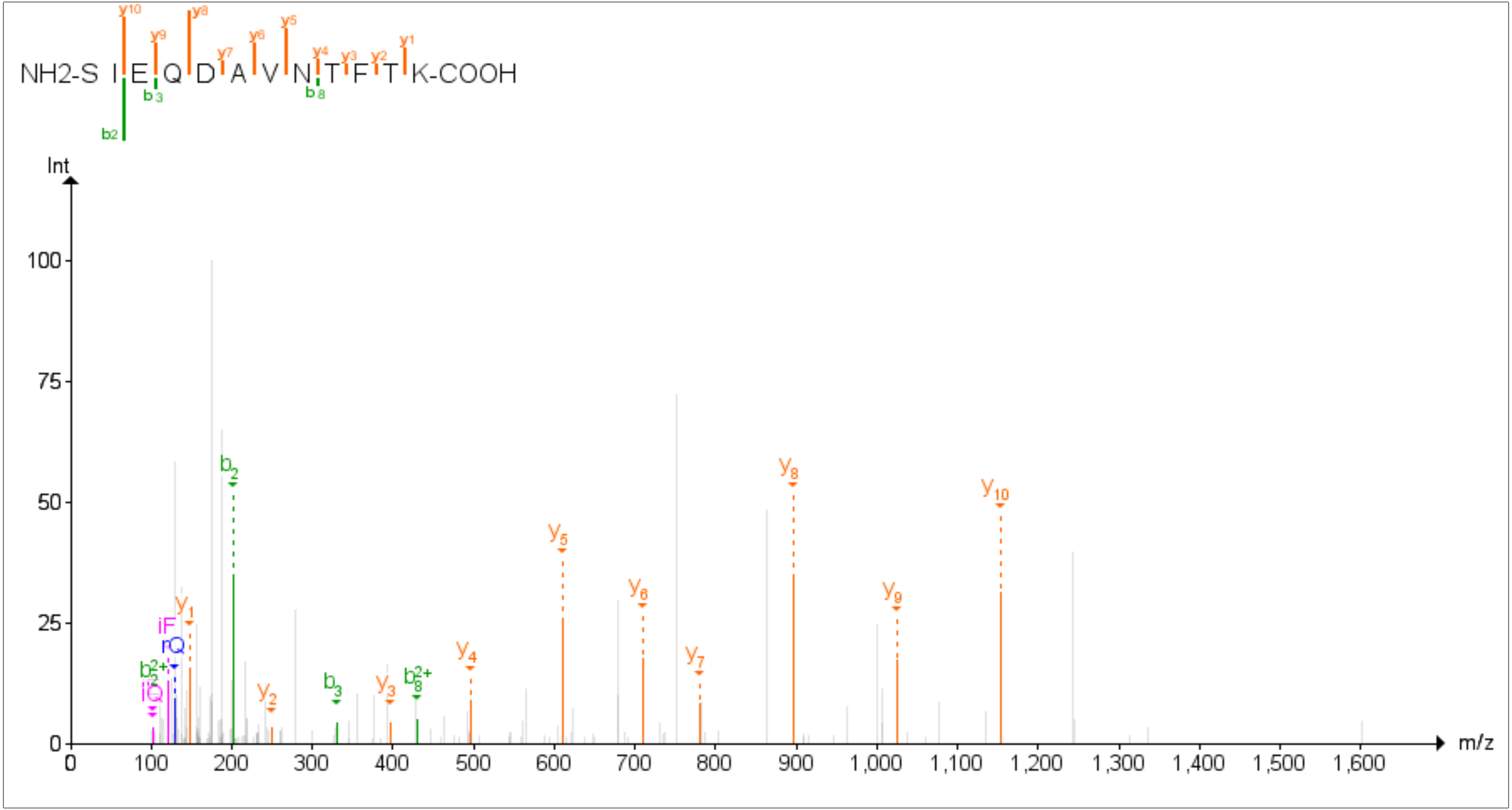

NH2-S I E Q D A V N T F T K-COOH
y10
y9
y8
y7
y6
y5
y4
y3
y2
y1
b2
b3
b8
Int
m/z
0
25
50
75
100
0
100
200
300
400
500
600
700
800
900
1,000
1,100
1,200
1,300
1,400
1,500
1,600
b2
y1
iF
rQ
b2 2+
iQ
y2
b3
y3
b8 2+
y4
y5
y6
y7
y8
y9
y10

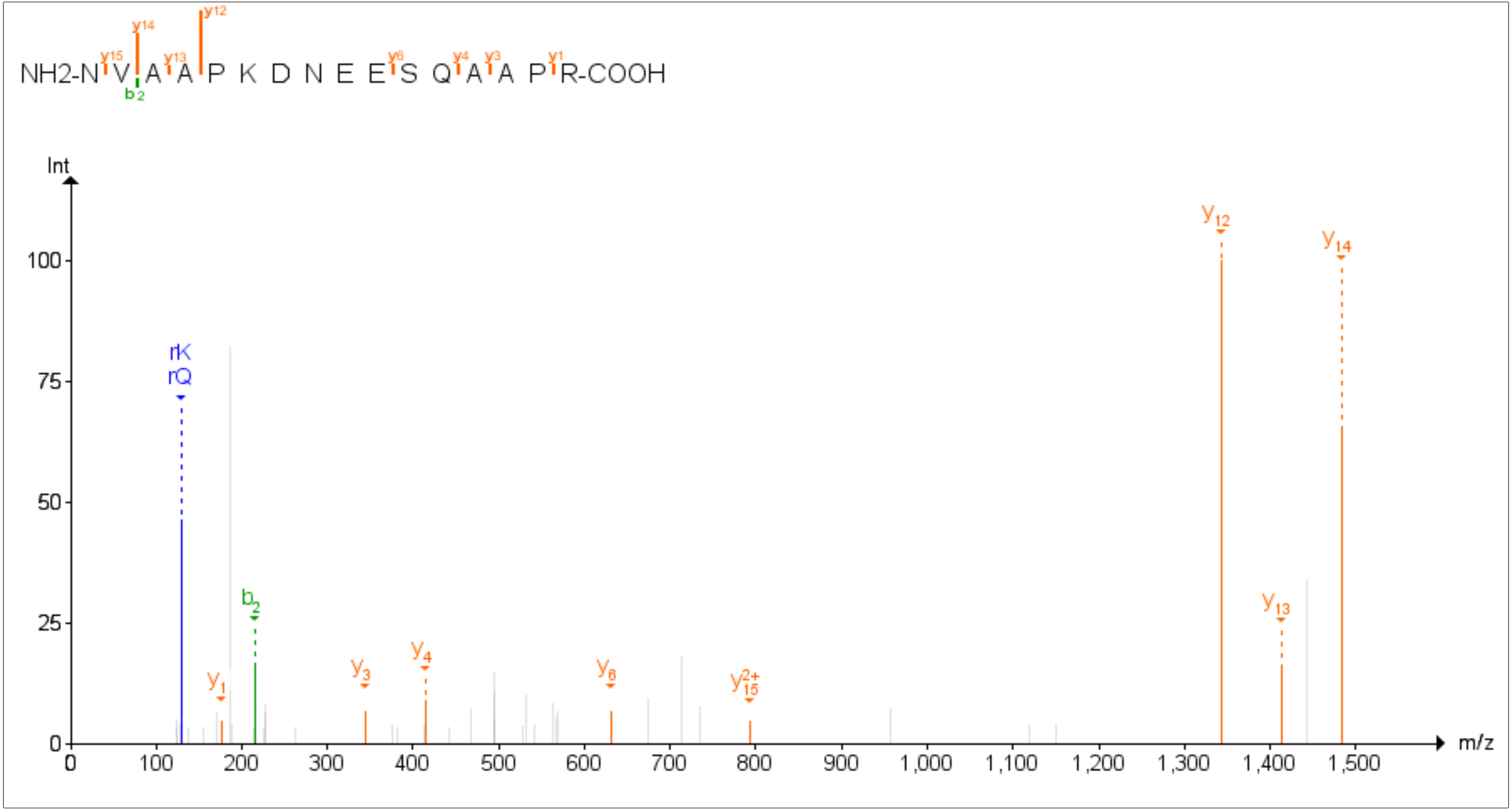

NH2-N V A A P K D N E E S Q A A P R-COOH
y15 y14 y13 y12 y6 y4 y3 y1
b2
Int
100
75
50
25
0
rK
rQ
b2
y1
y3
y4
y6
y15 2+
y12
y14
y13
0 100 200 300 400 500 600 700 800 900 1,000 1,100 1,200 1,300 1,400 1,500
m/z

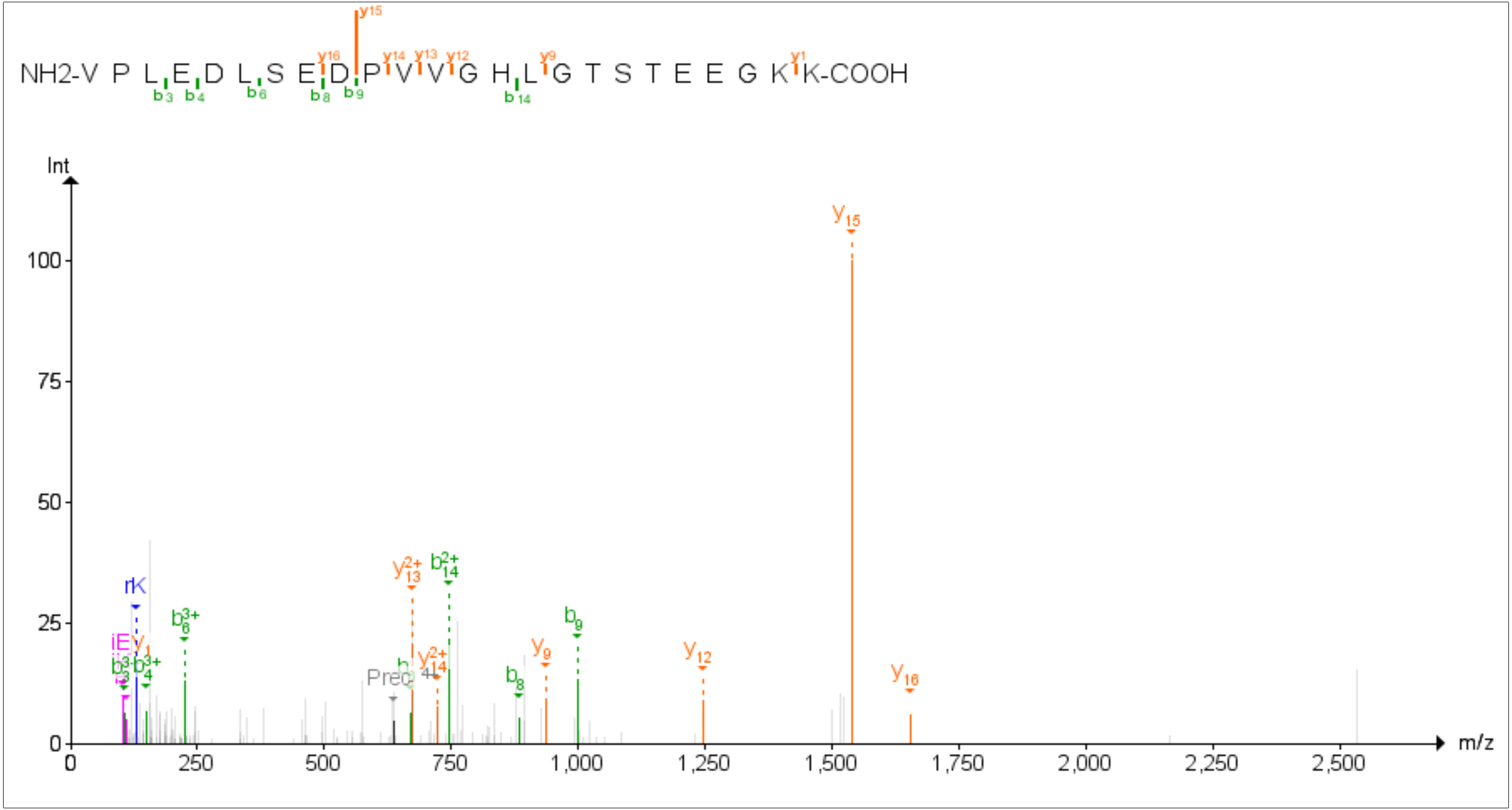

NH2-V P L E D L S E D P V V G H L G T S T E E G K K-COOH
Int
m/z
0
25
50
75
100
250
500
750
1,000
1,250
1,500
1,750
2,000
2,250
2,500
rK
iE
Prec

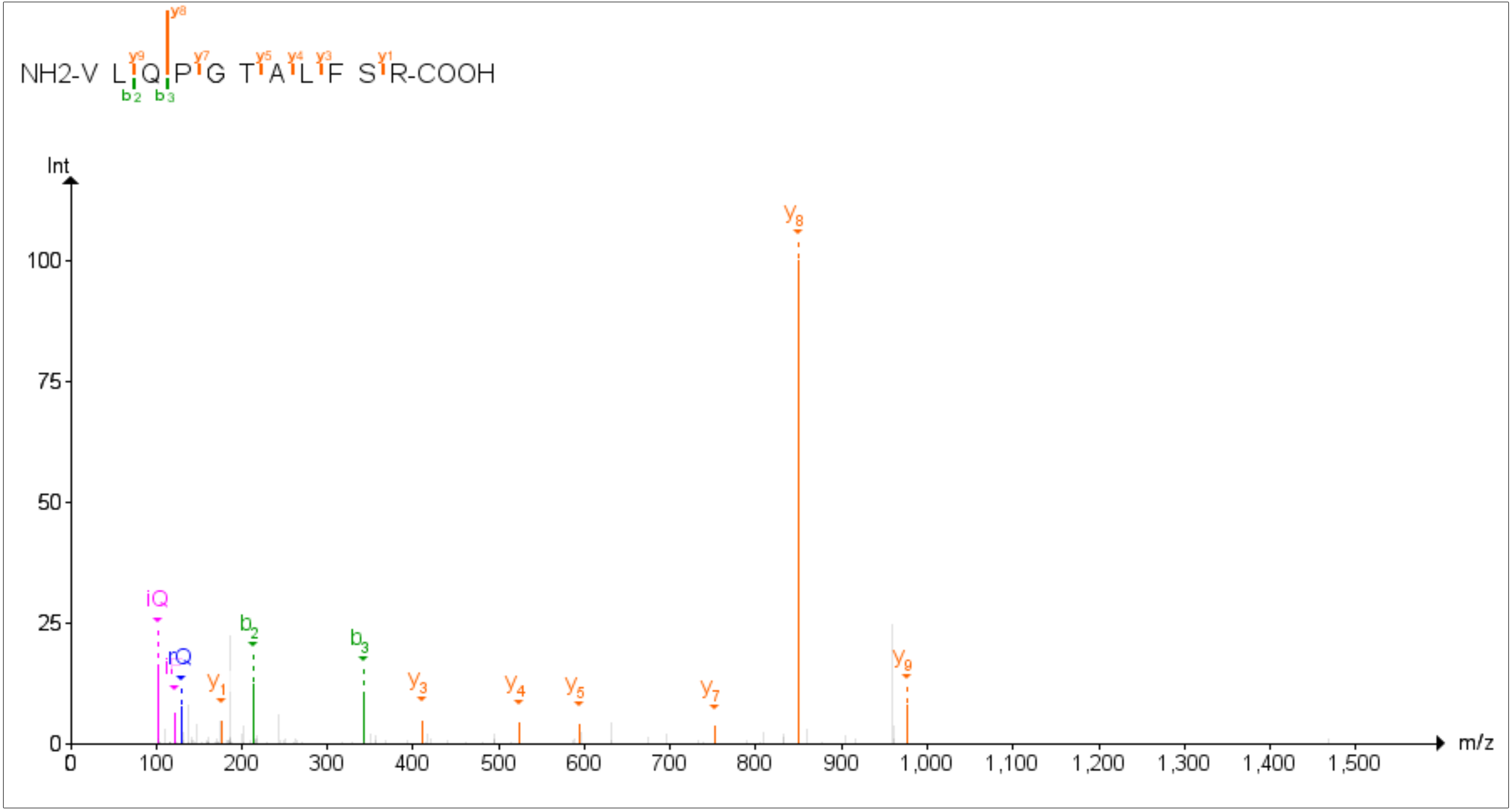

NH2-V L Q P G T A L F S R-COOH
y8
y9
y7
y5
y4
y3
y1
b2
b3
Int
100
75
50
25
0
iQ
rQ
b2
b3
y1
y3
y4
y5
y7
y8
y9
0
100
200
300
400
500
600
700
800
900
1,000
1,100
1,200
1,300
1,400
1,500
m/z

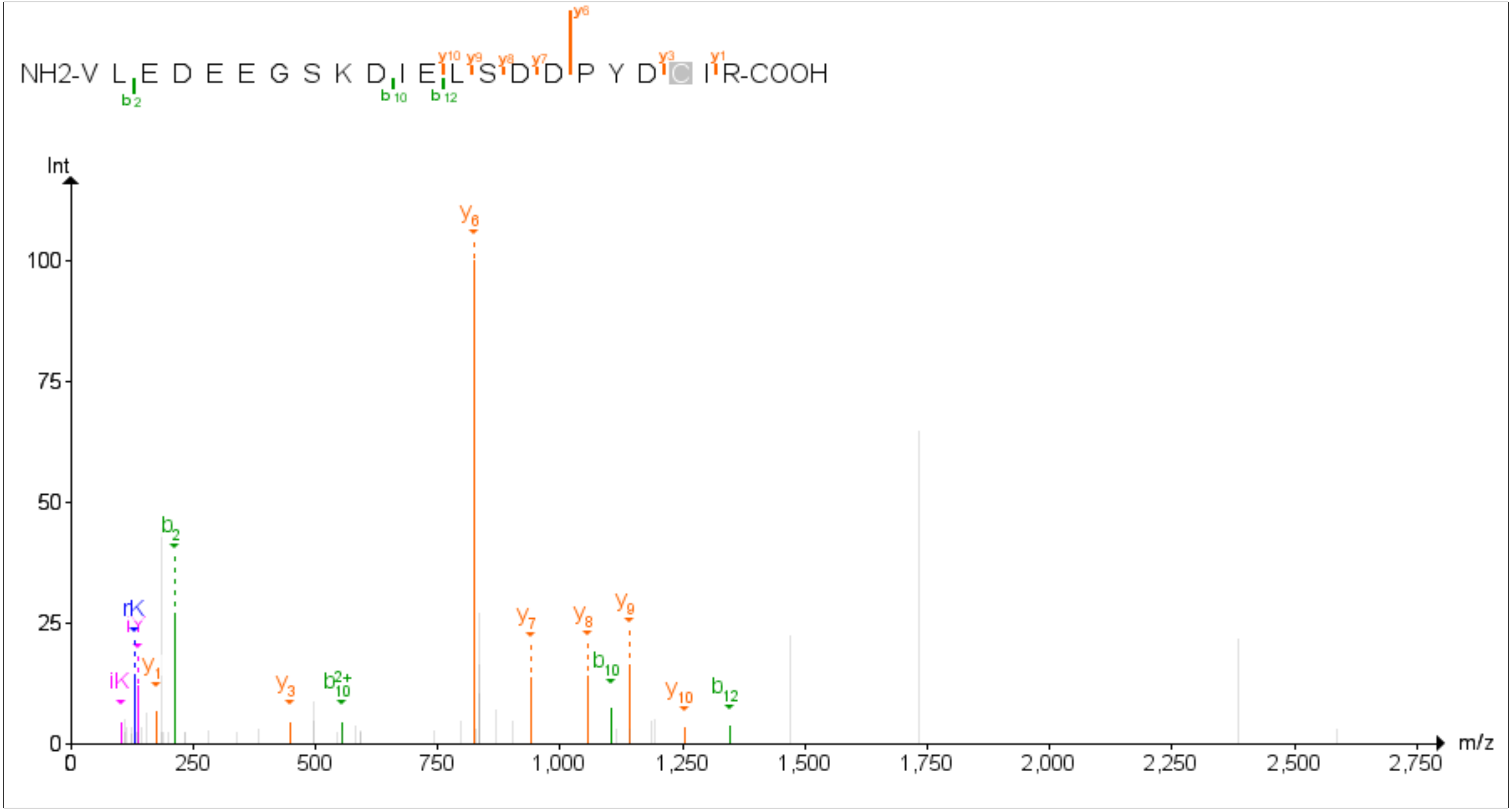

NH2-V L E D E E G S K D I E L S D D P Y D C I R-COOH
b2
b10
b12
y10
y9
y8
y7
y6
y3
y1
Int
100
75
50
25
0
y6
b2
rK
iY
iK
y1
y3
b10 2+
y7
y8
y9
b10
y10
b12
0
250
500
750
1,000
1,250
1,500
1,750
2,000
2,250
2,500
2,750
m/z

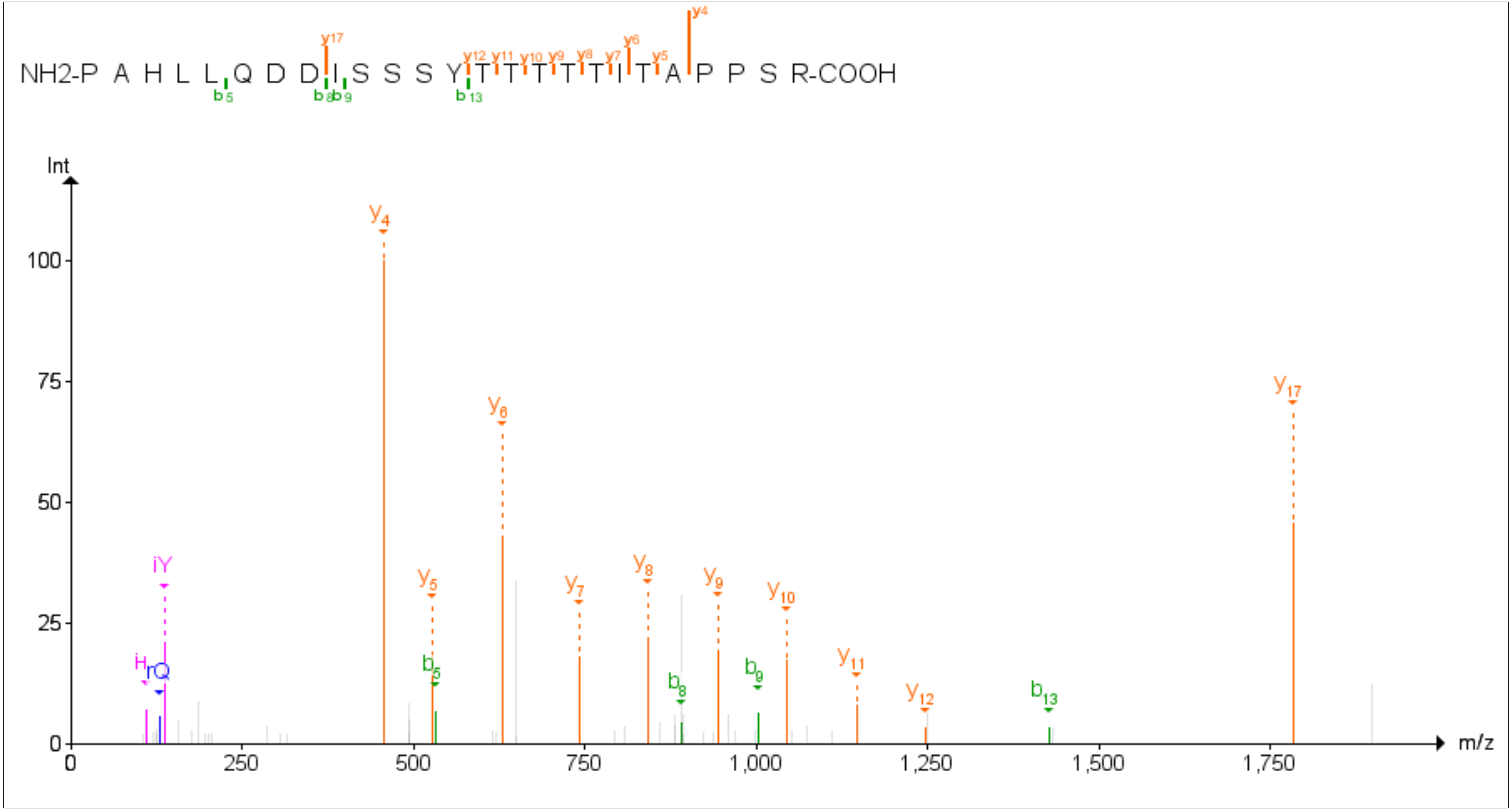

NH2-P A H L L Q D D I S S S Y T T T T T I T A P P S R-COOH
y4 y6 y17 y12 y11 y10 y9 y8 y7 y5
b5 b8 b9 b13
Int
100
75
50
25
0
y4
y6
y17
iY
y5
y7
y8
y9
y10
y11
y12
iH
rQ
b5
b8
b9
b13
0
250
500
750
1,000
1,250
1,500
1,750
m/z

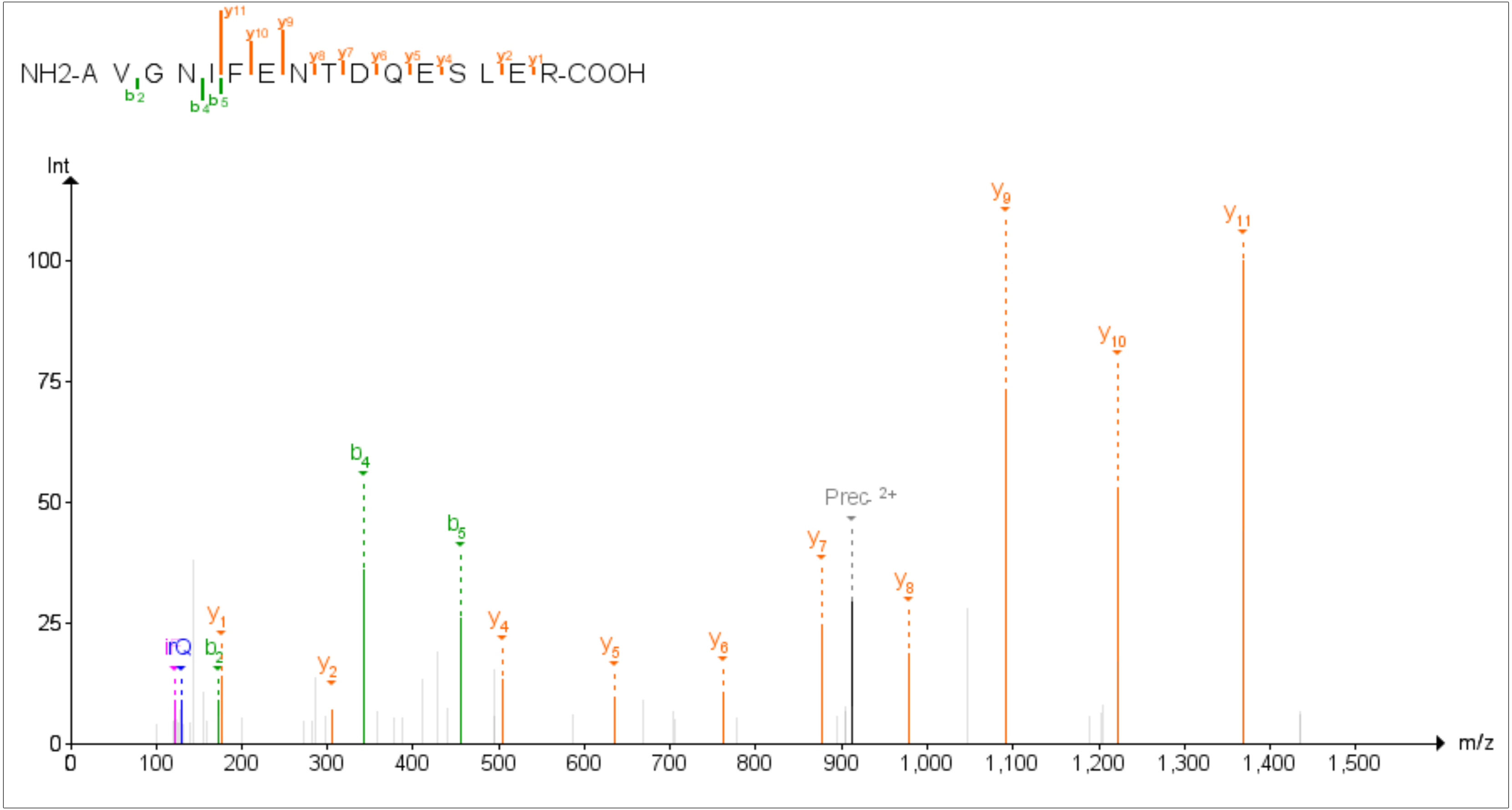

NH2-A V G N I F E N T D Q E S L E R-COOH
Int
m/z
Prec. 2+
irQ

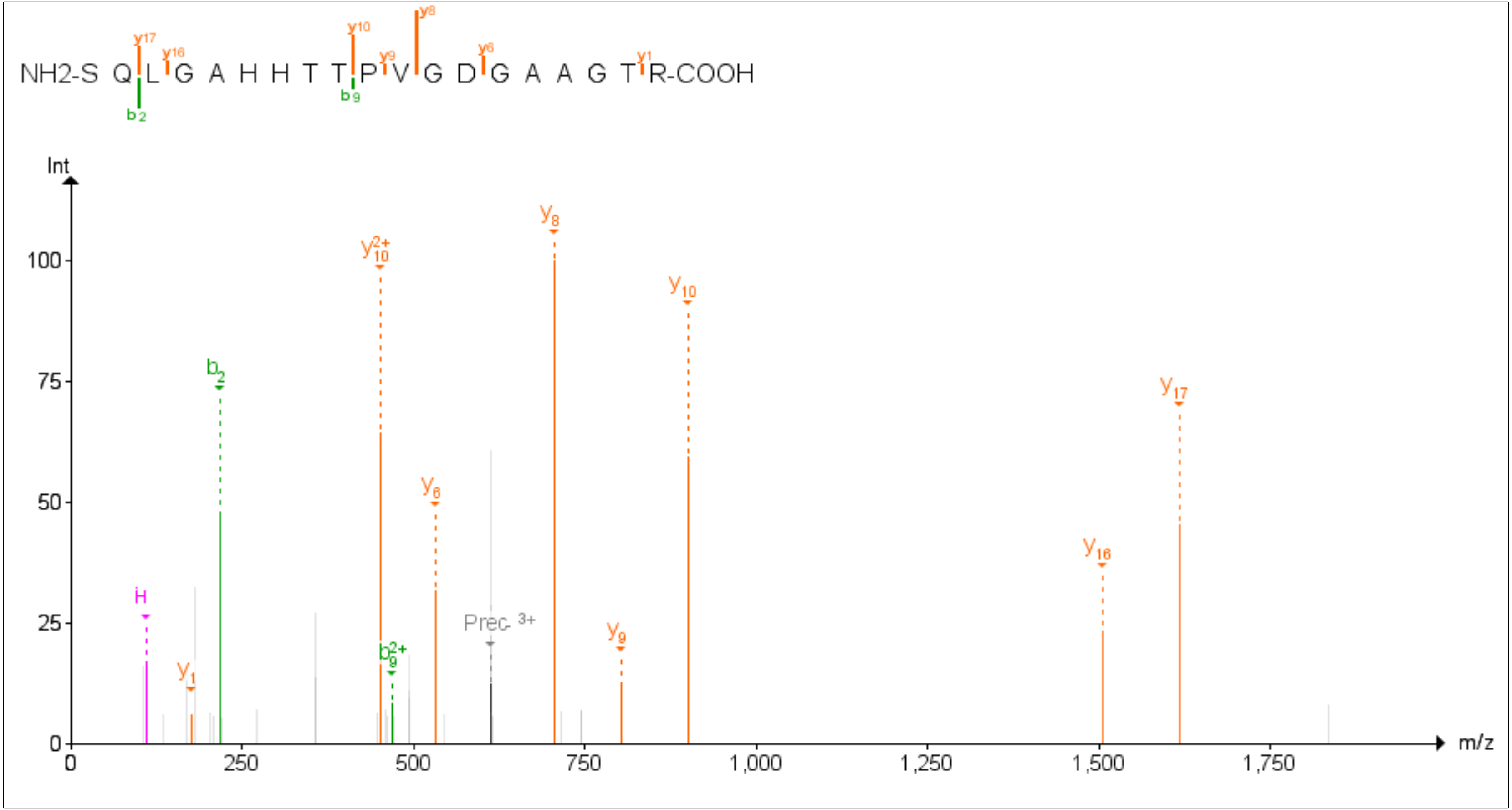
NH2-S Q L G A H H T T P V G D G A A G T R-COOH
y17
y16
y10
y9
y8
y6
y1
b2
b9
Int
m/z
Prec. 3+
y8
y10 2+
y10
b2
y6
y17
y16
y9
y1
b9 2+
0
25
50
75
100
250
500
750
1,000
1,250
1,500
1,750

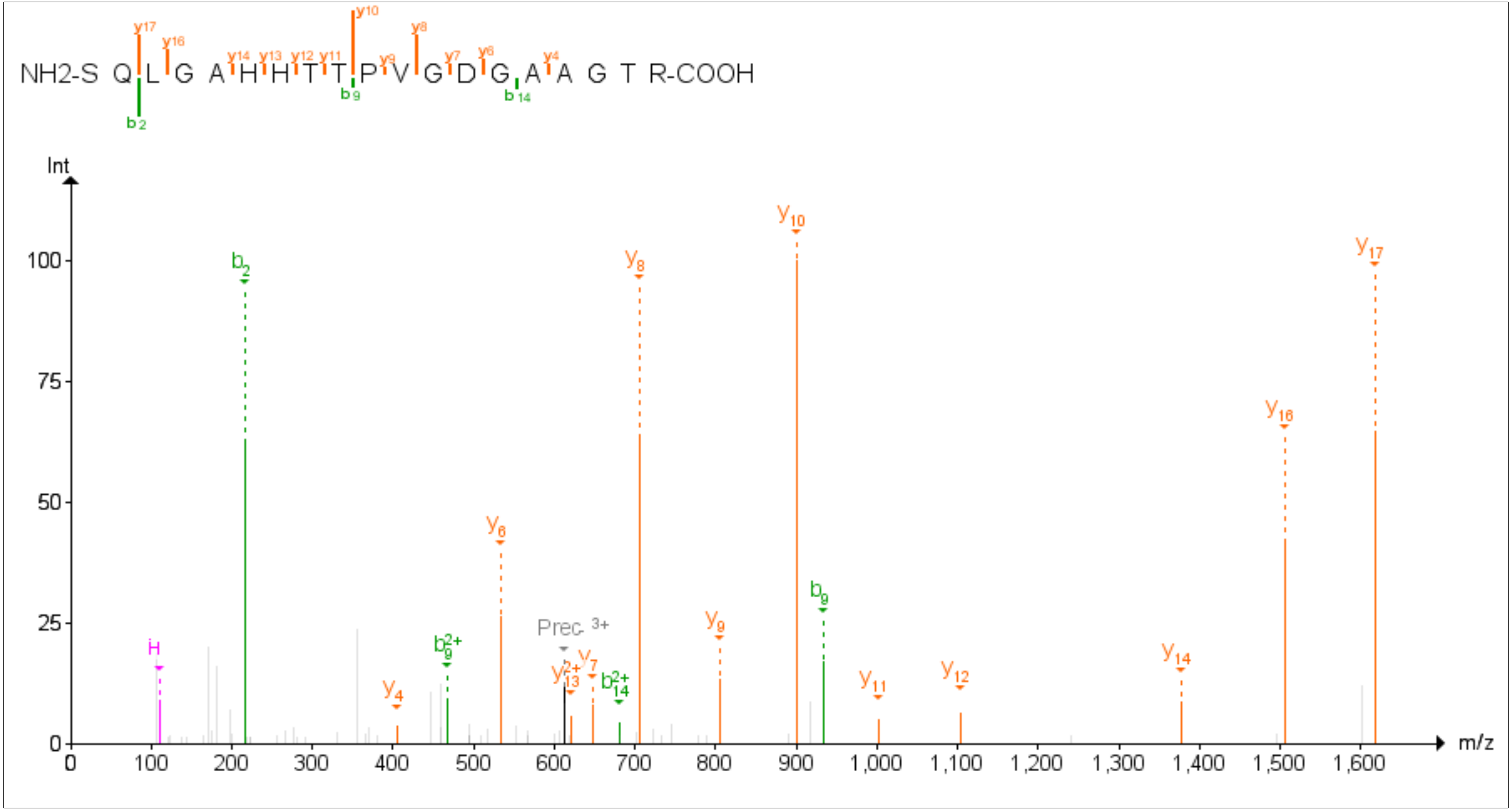

NH2-S Q L G A H H T T T P V G D G A A G T R-COOH
y17 y16 y14 y13 y12 y11 y10 y9 y8 y7 y6 y4
b2 b9 b14
Int
100
75
50
25
0
b2
y10
y8
y17
y16
y6
b9
y9
Prec 3+
H
b9 2+
y4
y13 2+
y7
b14 2+
y11
y12
y14
0 100 200 300 400 500 600 700 800 900 1,000 1,100 1,200 1,300 1,400 1,500 1,600
m/z

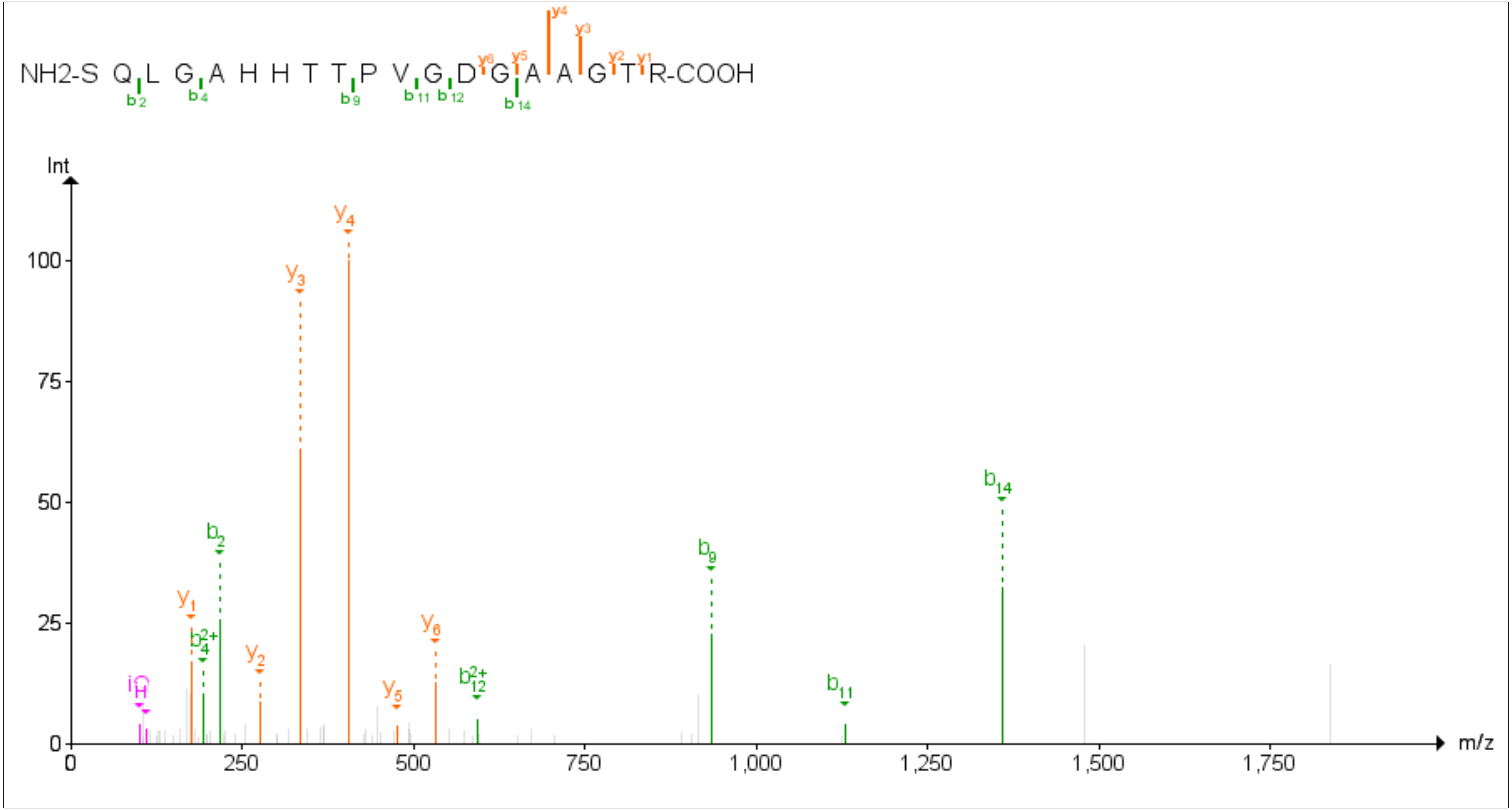

NH2-S Q L G A H H T T P V G D G A A G T R-COOH
Int
100
75
50
25
0
m/z
0
250
500
750
1,000
1,250
1,500
1,750

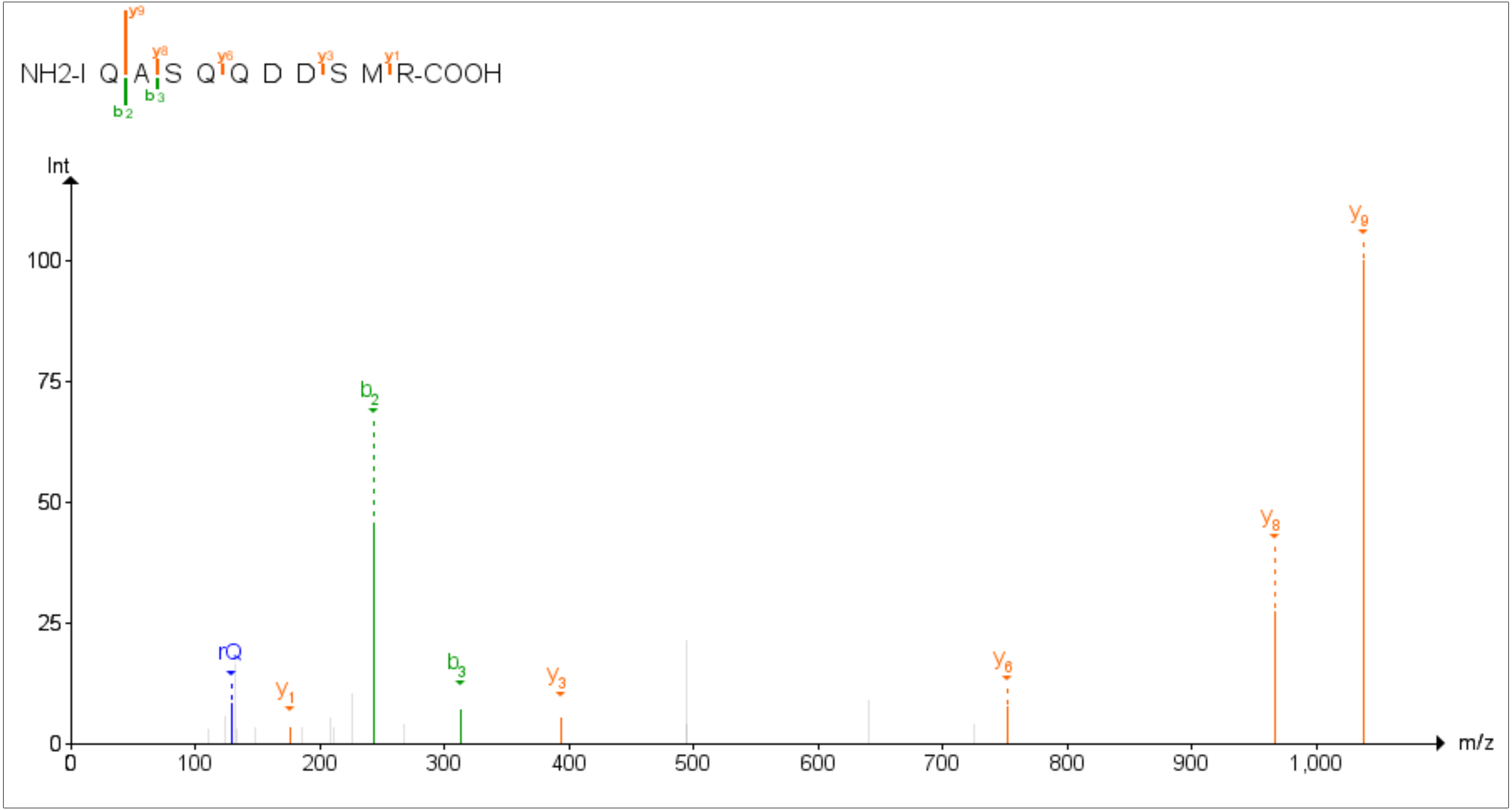

NH2-I Q A S Q Q D D S M R-COOH
y9
y8
y6
y3
y1
b2
b3
Int
100
75
50
25
0
rQ
m/z
0
100
200
300
400
500
600
700
800
900
1,000

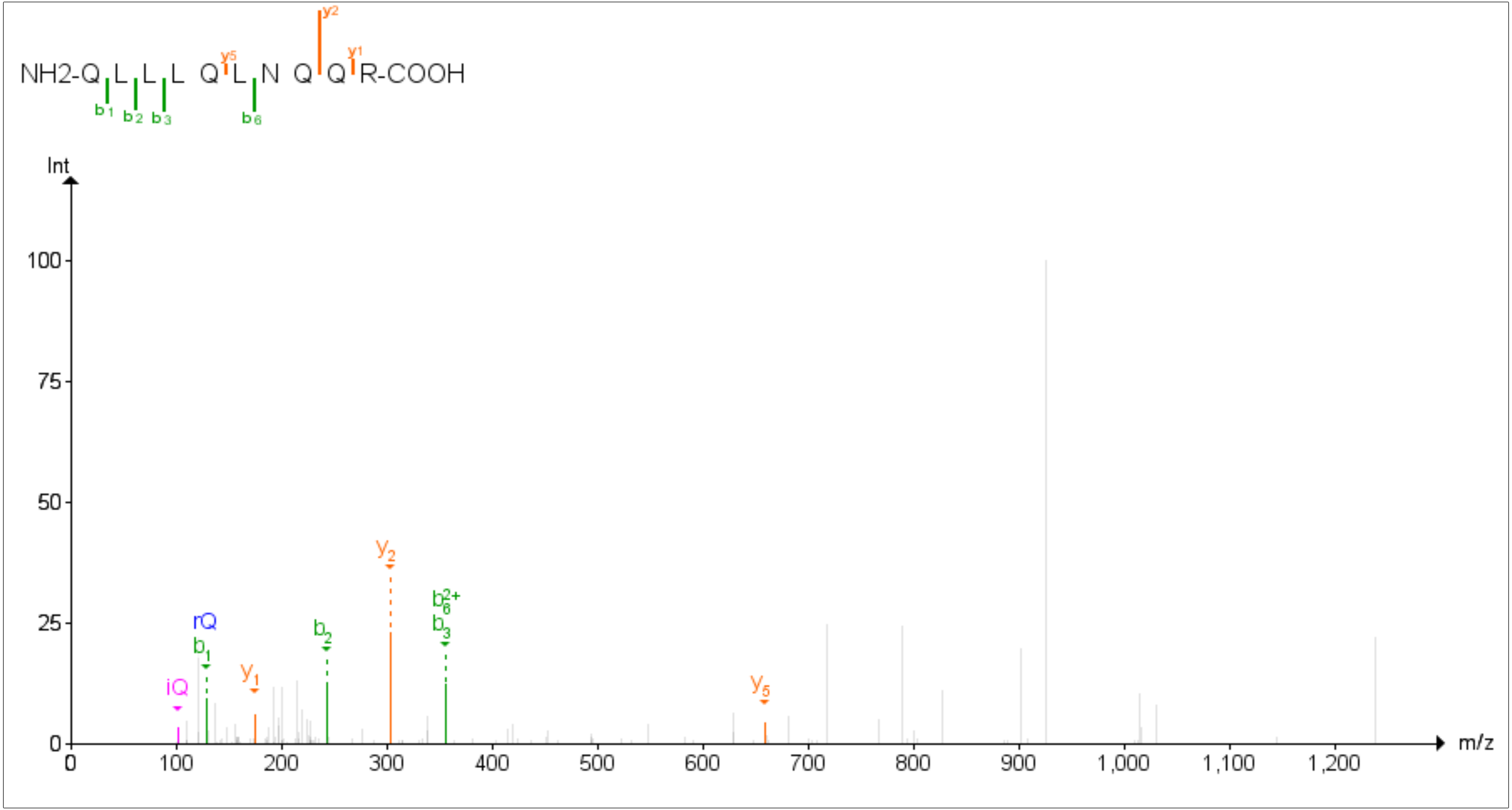

NH2-Q L L L Q L N Q Q R-COOH
y2
y5
y1
b 1
b 2
b 3
b 6
Int
100
75
50
25
0
y2
b6 2+
b3
rQ
b1
b2
iQ
y1
y5
0
100
200
300
400
500
600
700
800
900
1,000
1,100
1,200
m/z

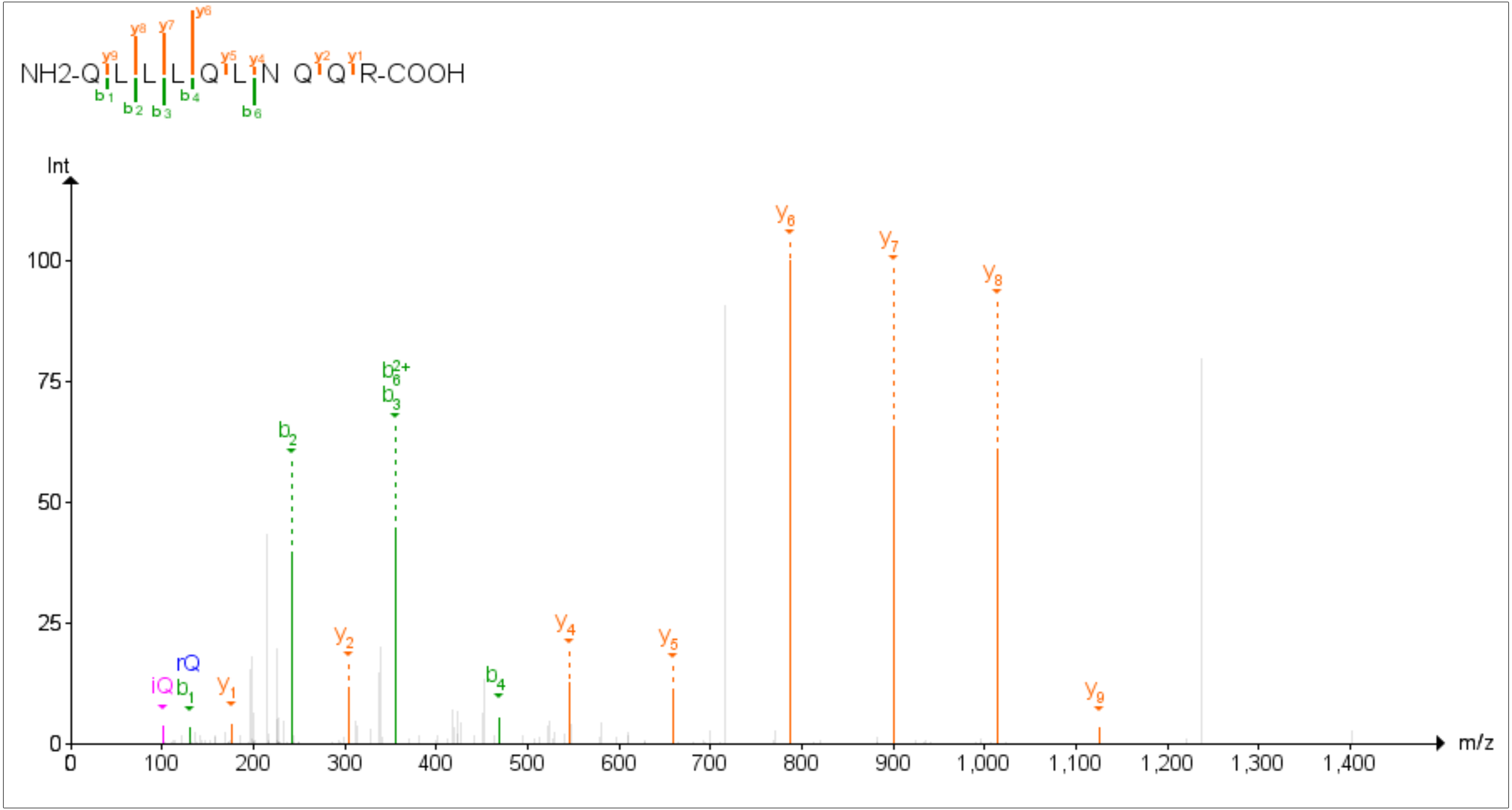

NH2-Q L L L L Q L N Q Q R-COOH
y9 y8 y7 y6 y5 y4 y2 y1
b1 b2 b3 b4 b6
Int
100
75
50
25
0
m/z
0 100 200 300 400 500 600 700 800 900 1,000 1,100 1,200 1,300 1,400
y6 y7 y8 y9 y5 y4 y2 y1
b2 b3 b6 2+ b4 b1
rQ
iQ

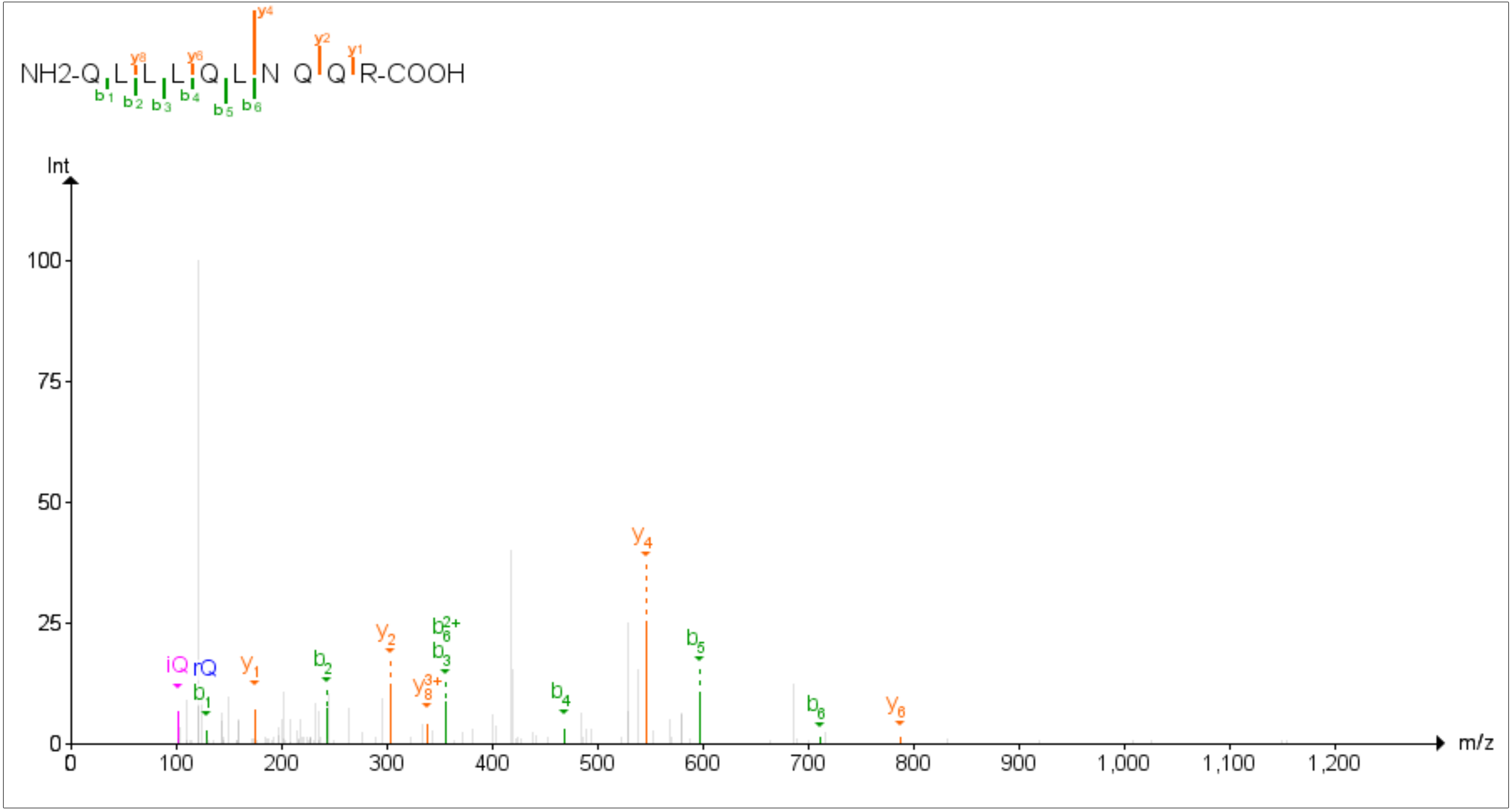

NH2-Q L L L Q L N Q Q R-COOH
y8 y6 y4 y2 y1
b1 b2 b3 b4 b5 b6
Int
100
75
50
25
0
iQ rQ
b1
y1
b2
y2
y8 3+
b6 2+
b3
b4
y4
b5
b6
y6
0 100 200 300 400 500 600 700 800 900 1,000 1,100 1,200
m/z

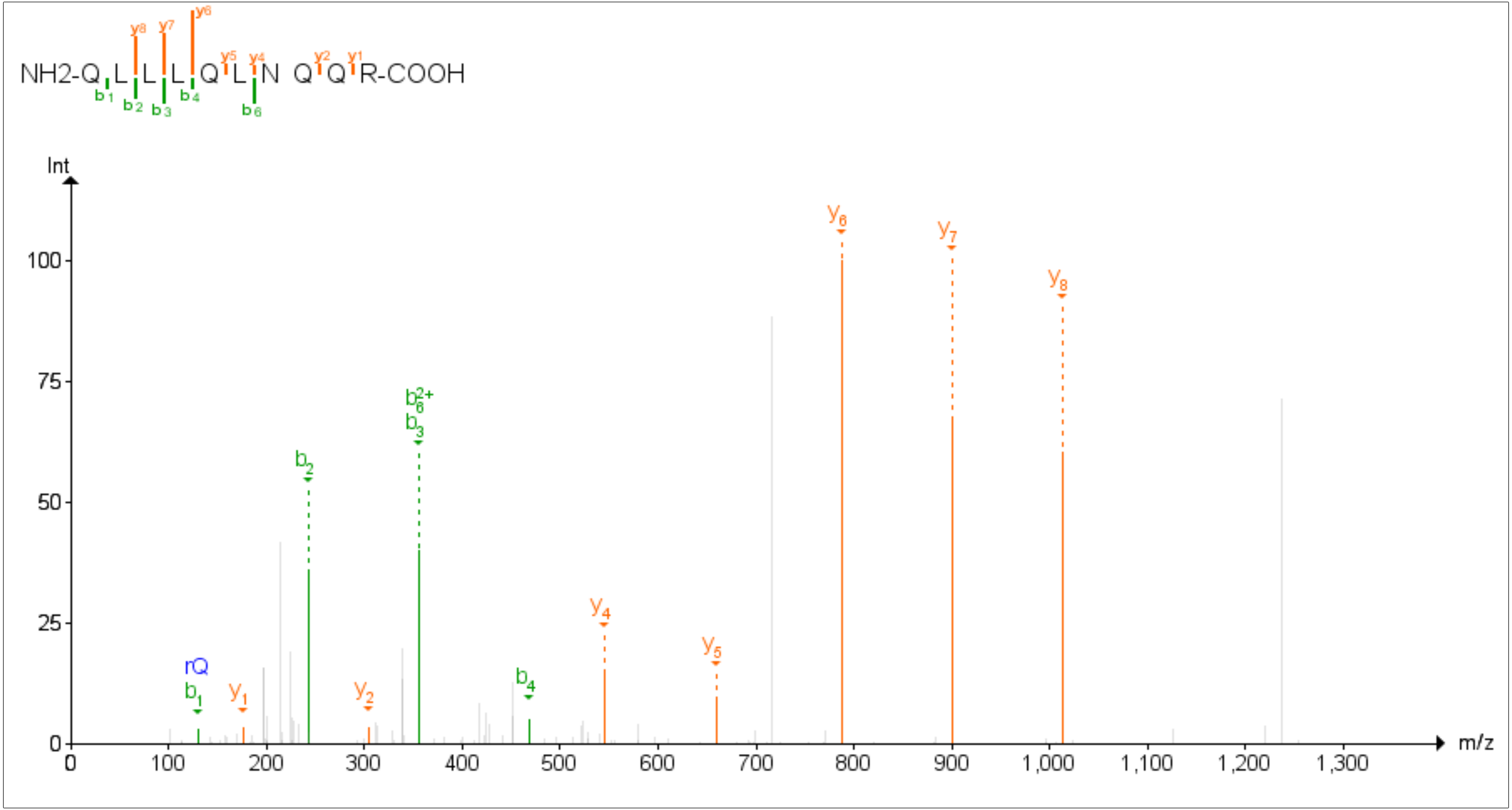

NH2-Q L L L Q L N Q Q R-COOH
y8 y7 y6 y5 y4 y2 y1
b1 b2 b3 b4 b6
Int
100
75
50
25
0
rQ
b_1
y_1
b_2
y_2
b_6^{2+}
b_3
b_4
y_4
y_5
y_6
y_7
y_8
0
100
200
300
400
500
600
700
800
900
1,000
1,100
1,200
1,300
m/z

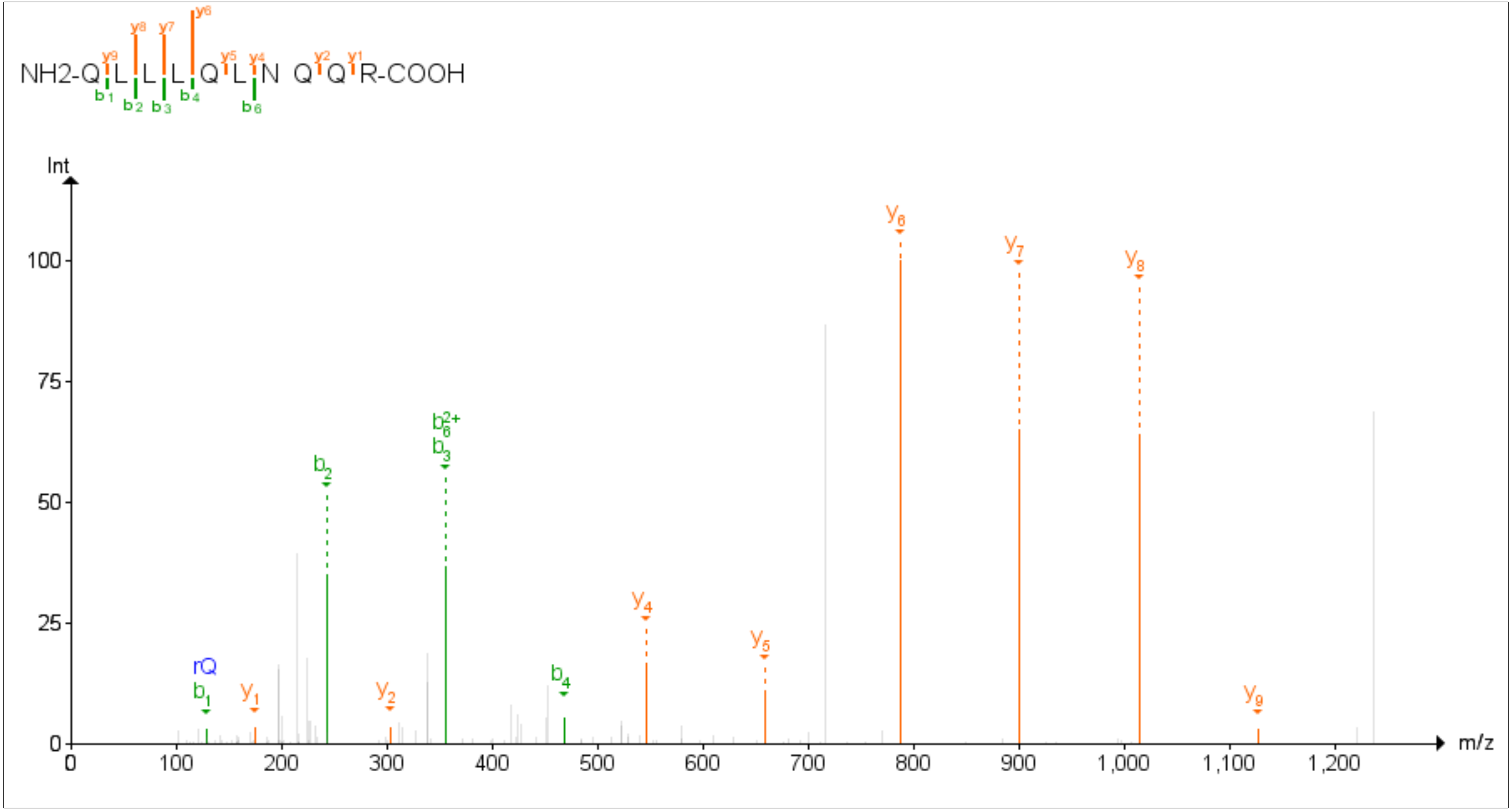

NH2-Q L L L Q L N Q Q R-COOH
y9 y8 y7 y6 y5 y4 y2 y1
b1 b2 b3 b4 b6
Int
100
75
50
25
0
m/z
0 100 200 300 400 500 600 700 800 900 1,000 1,100 1,200
rQ
b1
y1
b2
y2
b6 2+
b3
b4
y4
y5
y6
y7
y8
y9

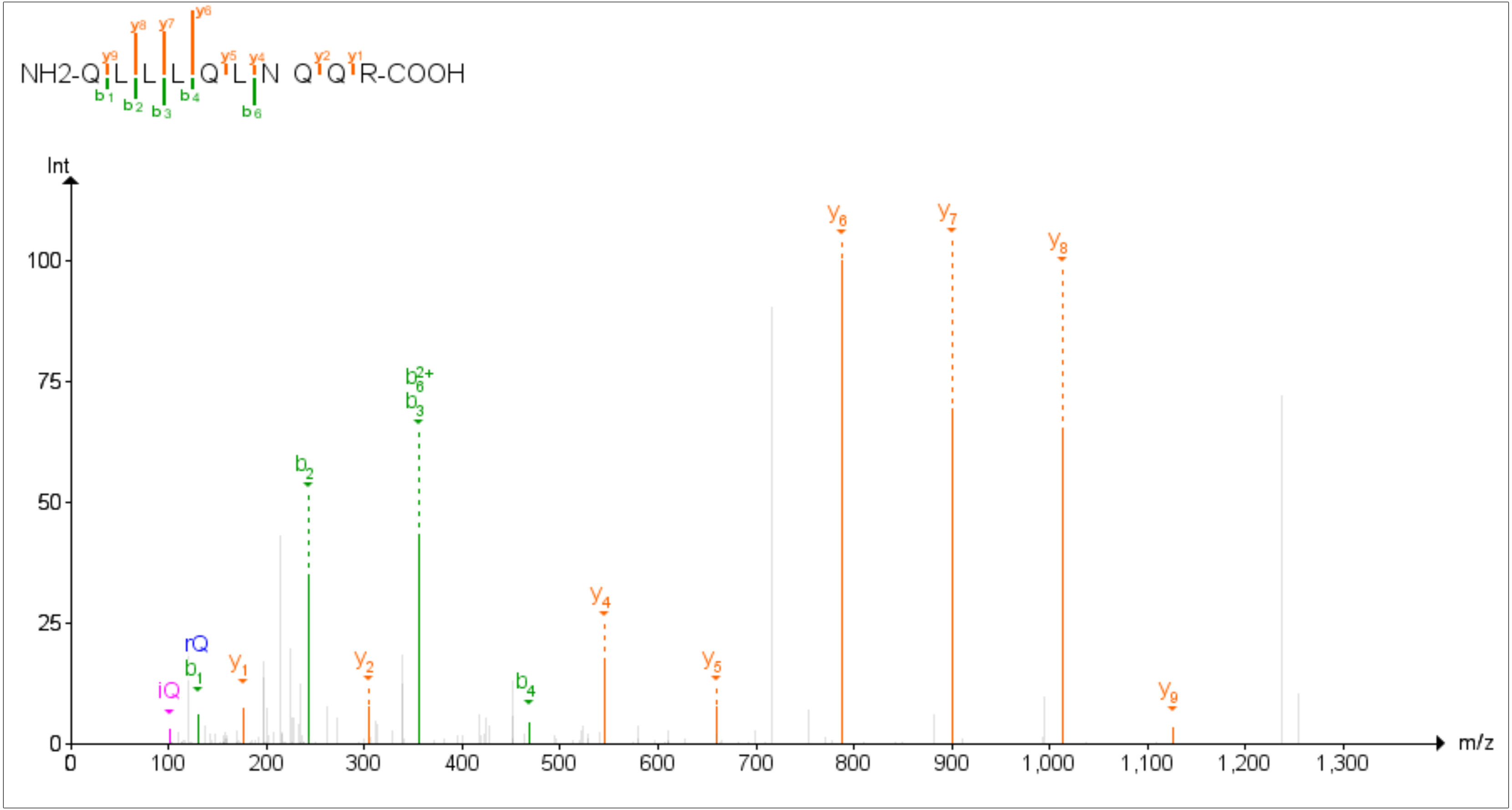
NH2-QLLLLQLNQQR-COOH
y9 y8 y7 y6 y5 y4 y2 y1
b1 b2 b3 b4 b6
Int
100
75
50
25
0
m/z
0 100 200 300 400 500 600 700 800 900 1,000 1,100 1,200 1,300
iQ
rQ
b1
y1
b2
y2
b6 2+
b3
b4
y4
y5
y6
y7
y8
y9

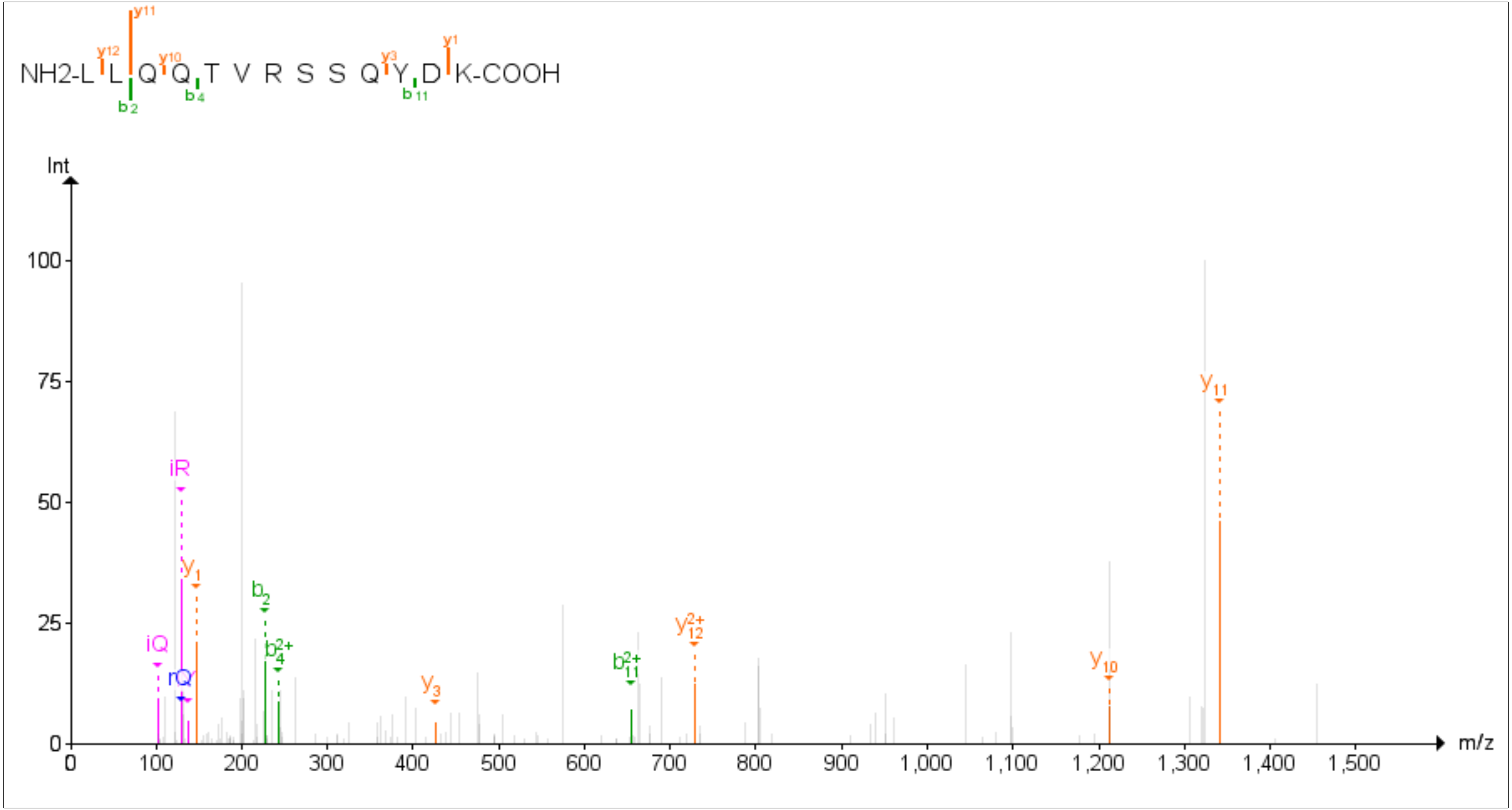

NH2-L L Q Q T V R S S Q Y D K-COOH
y12 y11 y10 y3 y1
b2 b4 b11
Int
100
75
50
25
0
iR
y1
b2
iQ
b4 2+
rQ
y3
b11 2+
y12 2+
y10
y11
0 100 200 300 400 500 600 700 800 900 1,000 1,100 1,200 1,300 1,400 1,500
m/z

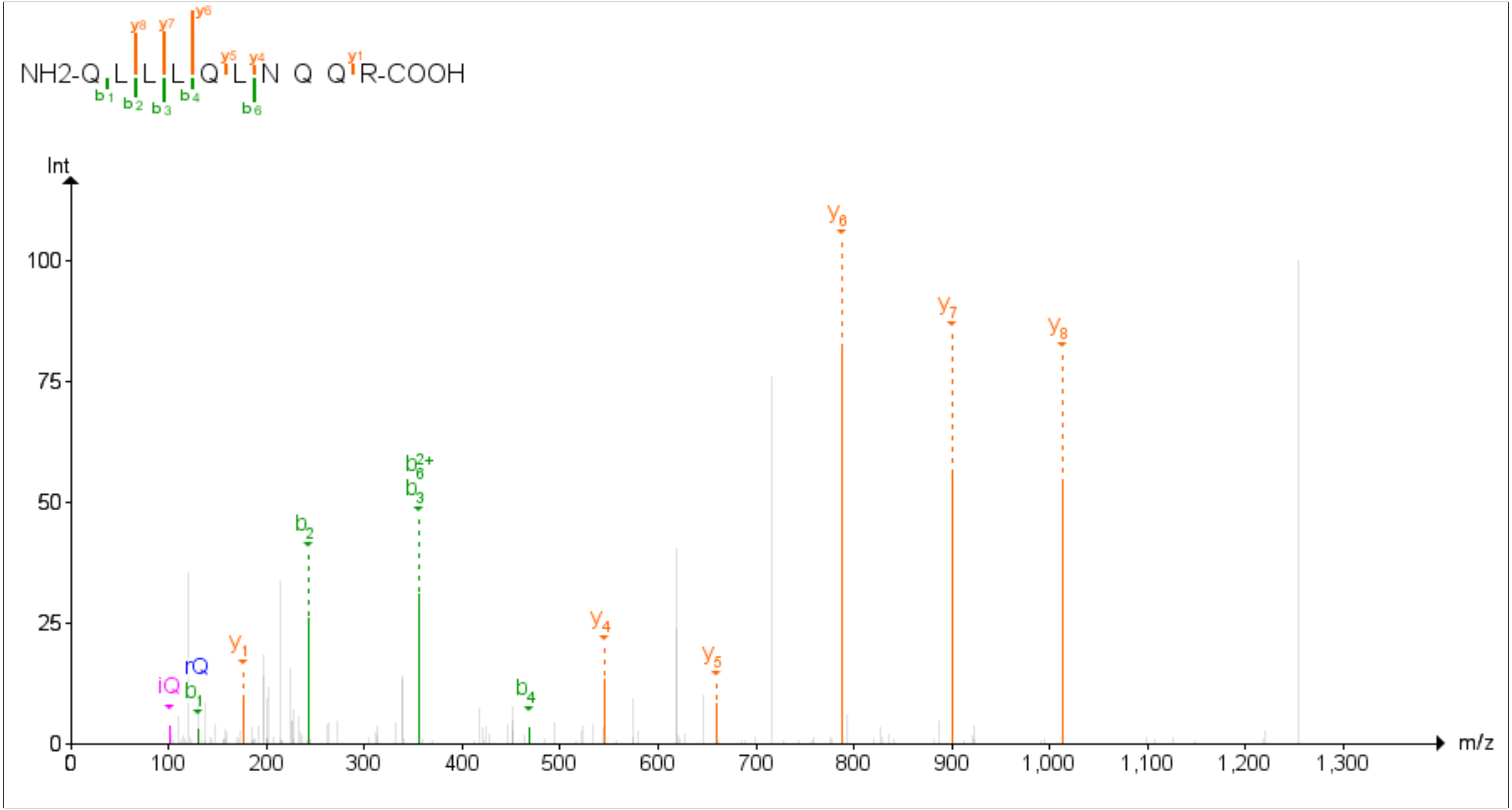

NH2-Q L L L L Q L N Q Q R-COOH
y8 y7 y6 y5 y4 y1
b1 b2 b3 b4 b6
Int
m/z
0
25
50
75
100
0 100 200 300 400 500 600 700 800 900 1,000 1,100 1,200 1,300
iQ
rQ
b1
y1
b2
b6 2+
b3
b4
y4
y5
y6
y7
y8

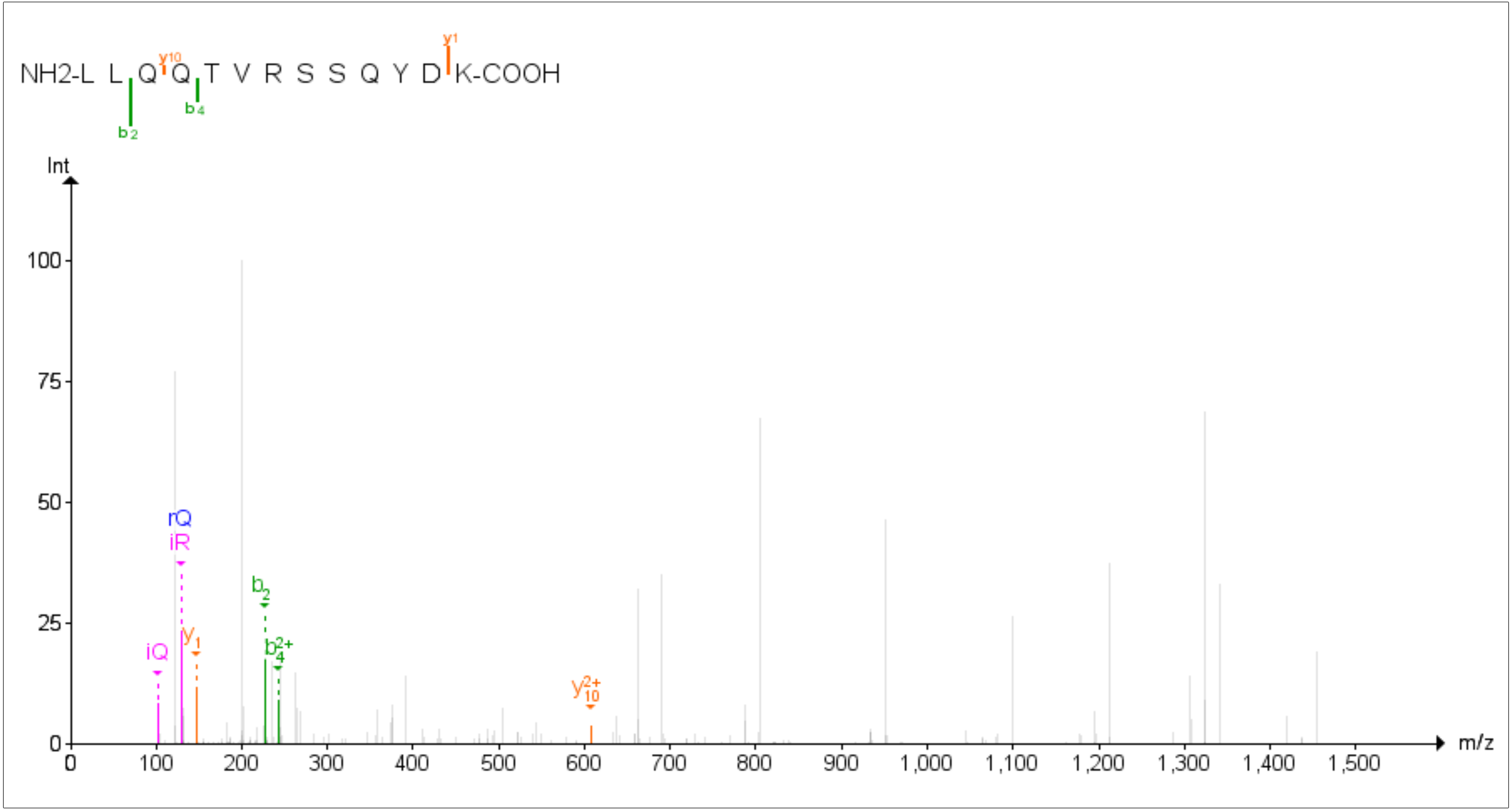

NH2-L L Q Q T V R S S Q Y D K-COOH
y10
y1
b2
b4
Int
100
75
50
25
0
rQ
iR
iQ
y1
b2
b4 2+
y10 2+
0
100
200
300
400
500
600
700
800
900
1,000
1,100
1,200
1,300
1,400
1,500
m/z

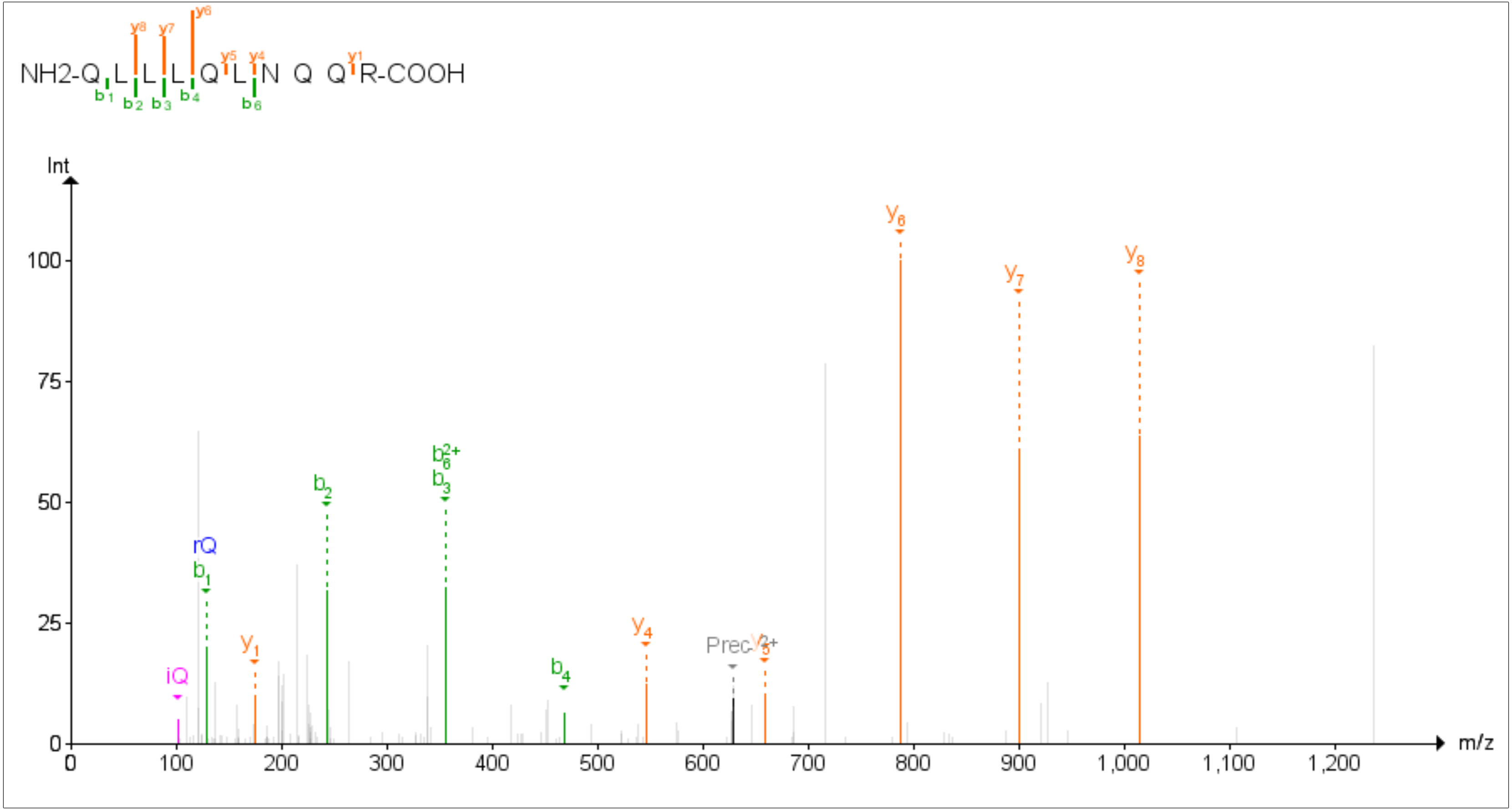

NH2-Q L L L Q L N Q Q R-COOH
y8 y7 y6 y5 y4 y1
b1 b2 b3 b4 b6
Int
m/z
y6
y7
y8
b6 2+
b3
b2
rQ
b1
y1
iQ
b4
y4
Prec 2+
y3

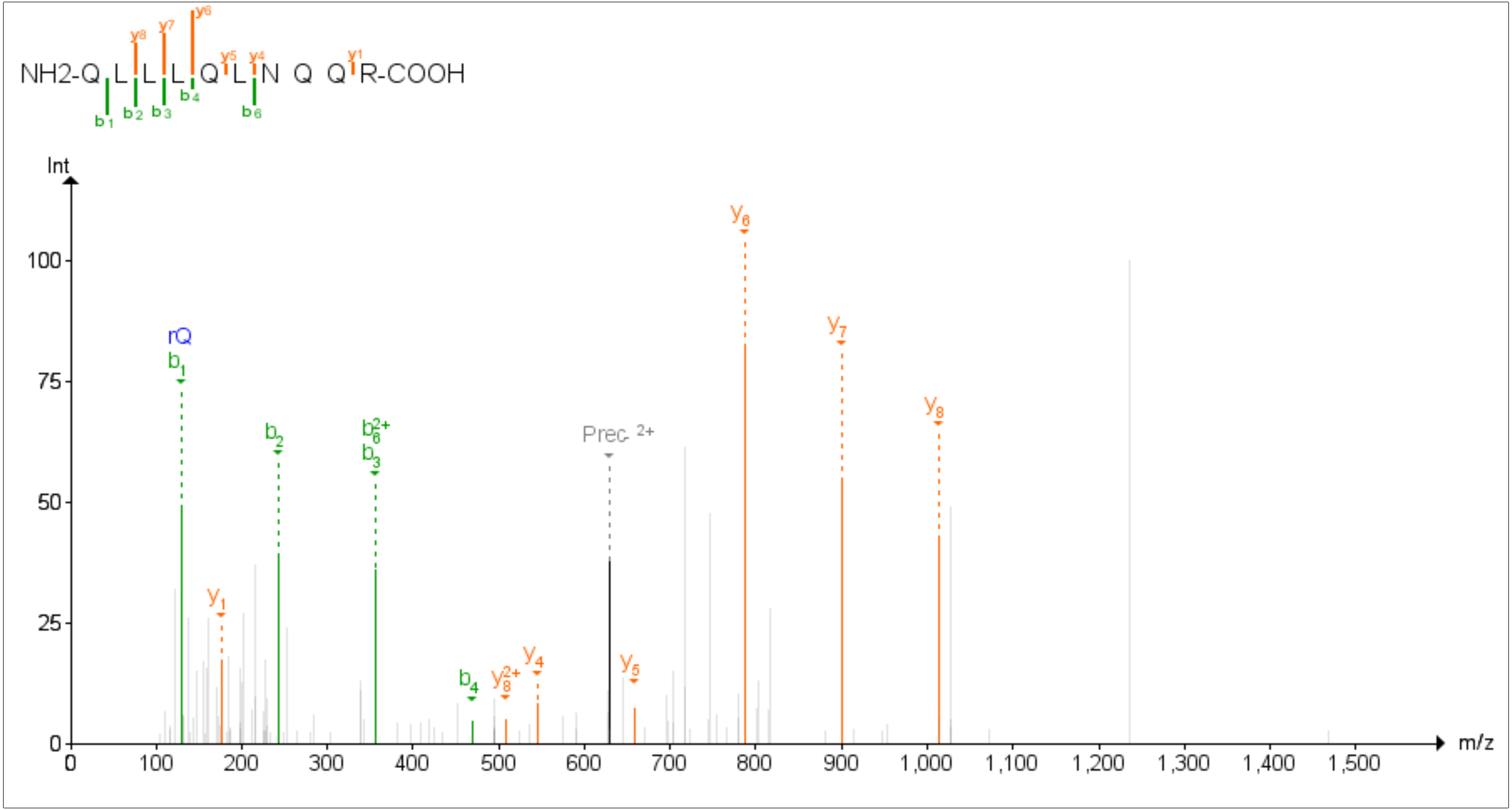

NH2-Q L L L Q L N Q Q R-COOH
y8 y7 y6 y5 y4 y1
b1 b2 b3 b4 b6
Int
m/z
rQ
b1
b2
b6 2+
b3
Prec 2+
y1
b4
y8 2+
y4
y5
y6
y7
y8
0
25
50
75
100
0 100 200 300 400 500 600 700 800 900 1,000 1,100 1,200 1,300 1,400 1,500

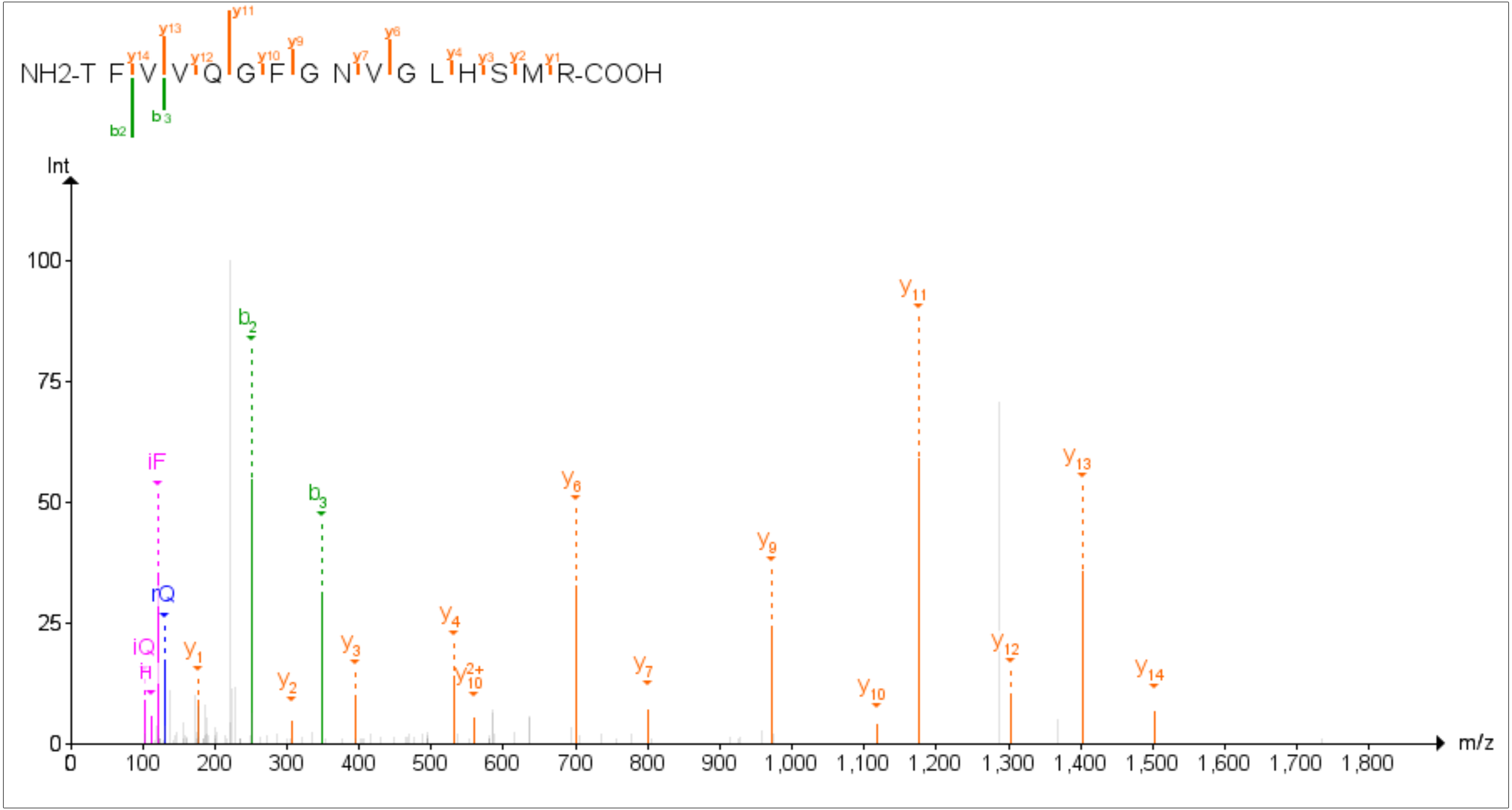
NH2-T F V V Q G F G N V G L H S M R-COOH
y14 y13 y12 y11 y10 y9 y8 y7 y4 y3 y2 y1
b2 b3
Int
m/z
100
75
50
25
0
0 100 200 300 400 500 600 700 800 900 1,000 1,100 1,200 1,300 1,400 1,500 1,600 1,700 1,800
iF
iQ
iH
rQ
y1
y2
y3
y4
y8
y7
y9
y10
y11
y12
y13
y14
b2
b3

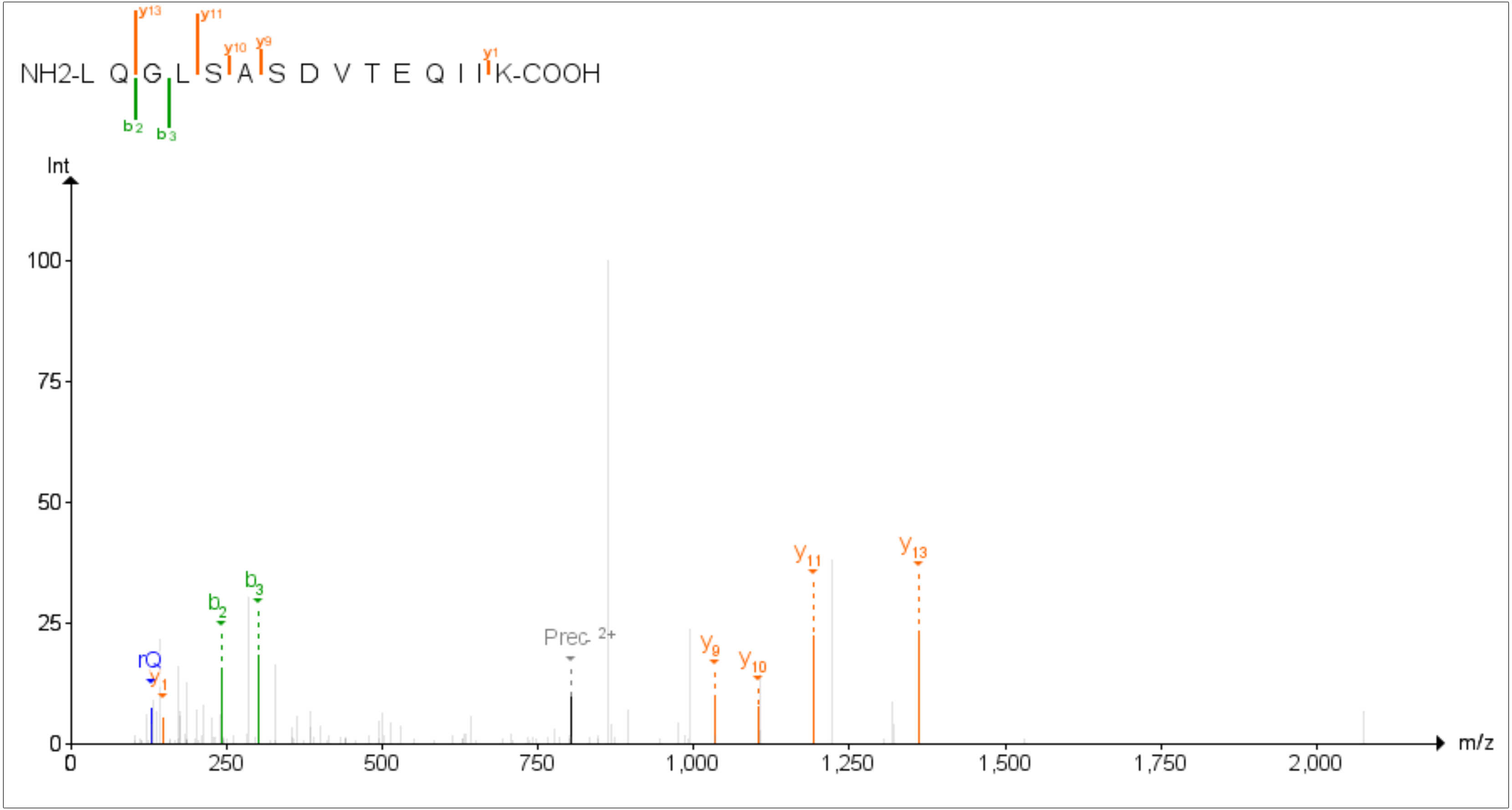

NH2-L Q G L S A S D V T E Q I I K-COOH
y13
y11
y10
y9
y1
b2
b3
Int
100
75
50
25
0
rQ
y1
b2
b3
Prec 2+
y9
y10
y11
y13
0
250
500
750
1,000
1,250
1,500
1,750
2,000
m/z

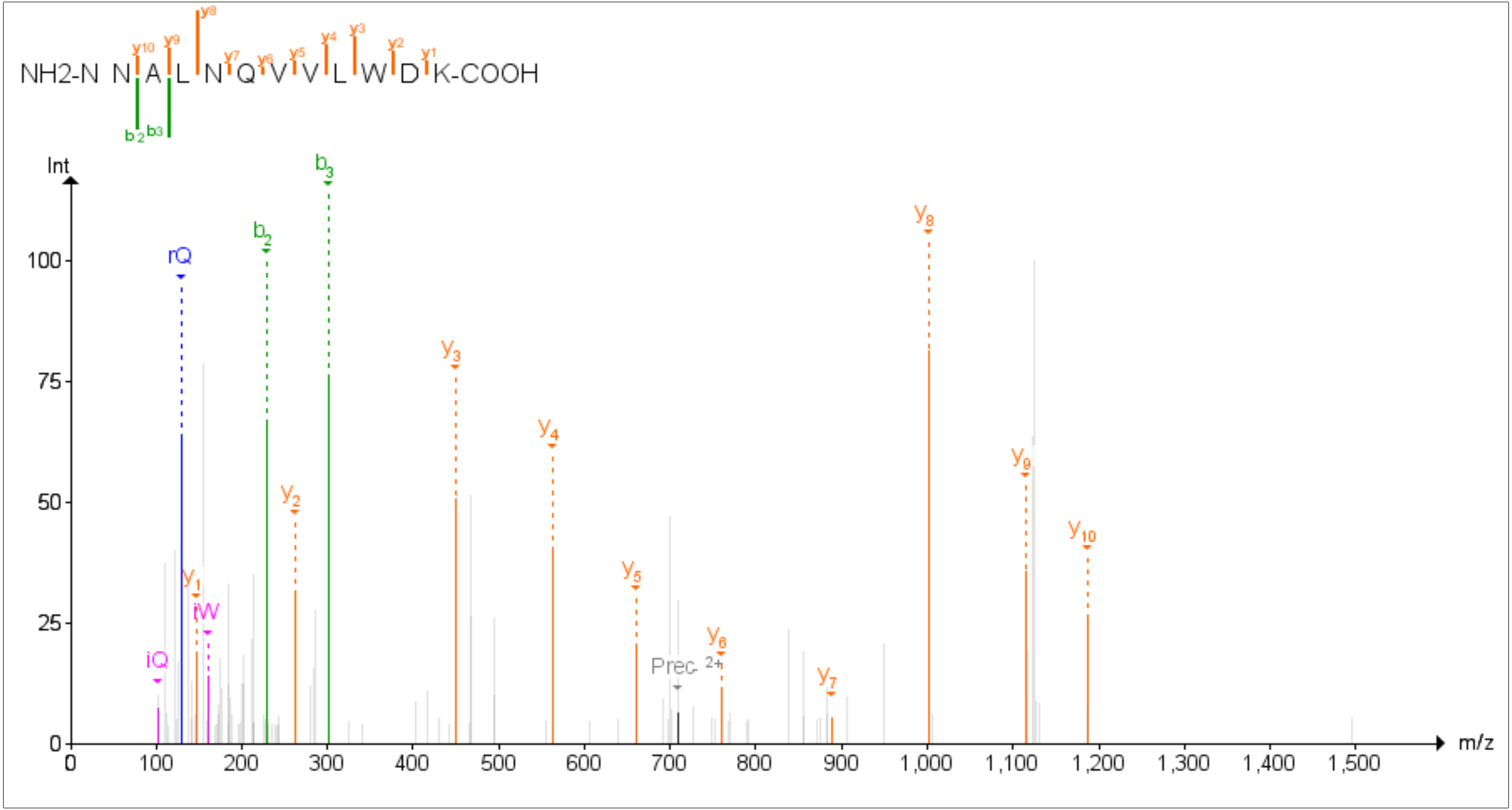

NH2-N N A L N Q V V L W D K-COOH
y10 y9 y8 y7 y6 y5 y4 y3 y2 y1
b2 b3
Int
m/z
rQ
b2
b3
y1
y2
y3
y4
y5
y6
y7
y8
y9
y10
iQ
iW
Prec 2+
0
25
50
75
100
0
100
200
300
400
500
600
700
800
900
1,000
1,100
1,200
1,300
1,400
1,500

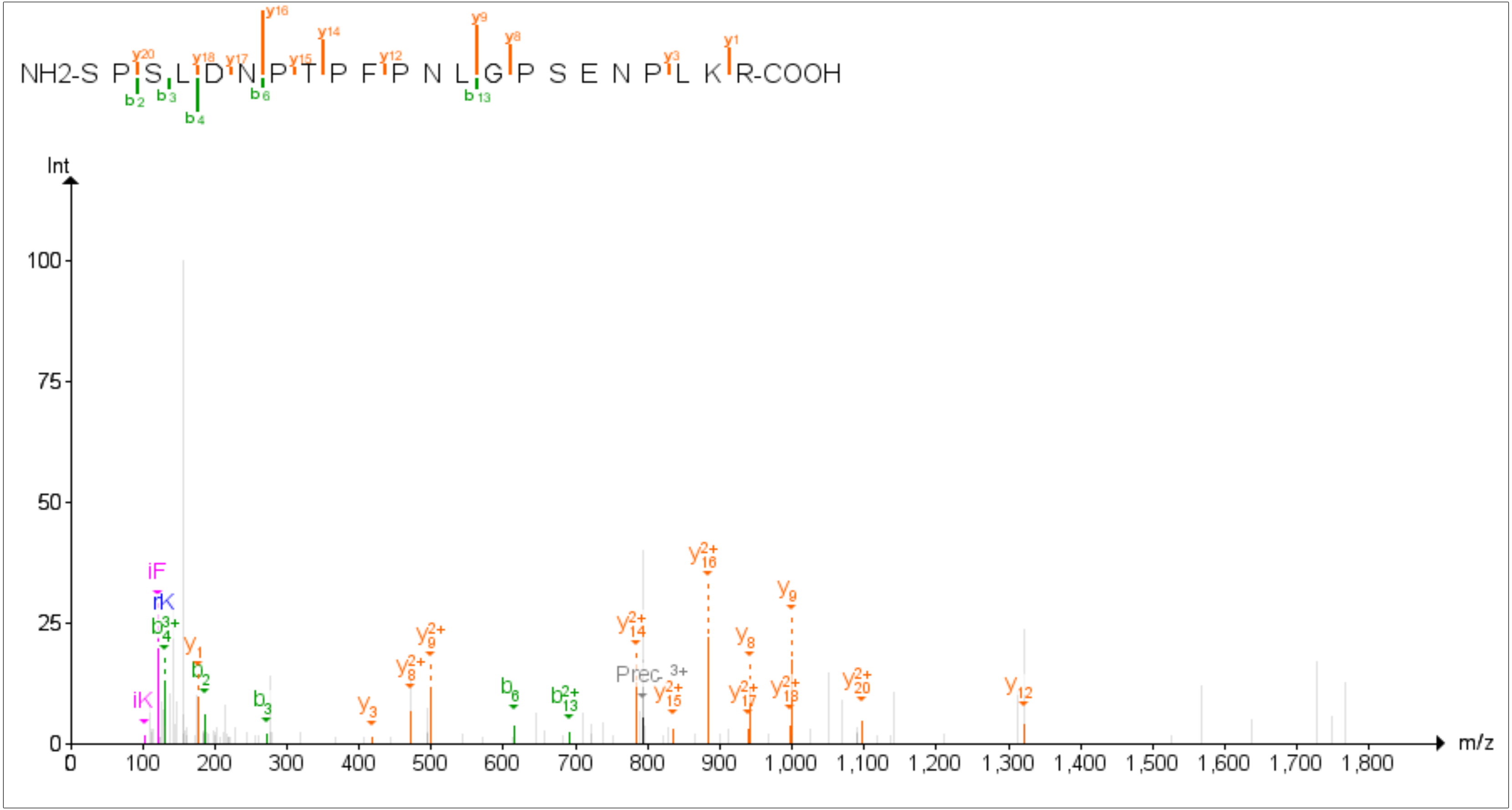

NH2-S P S L D N P T P F P N L G P S E N P L K R-COOH
Int
m/z
Prec 3+
iF
iK

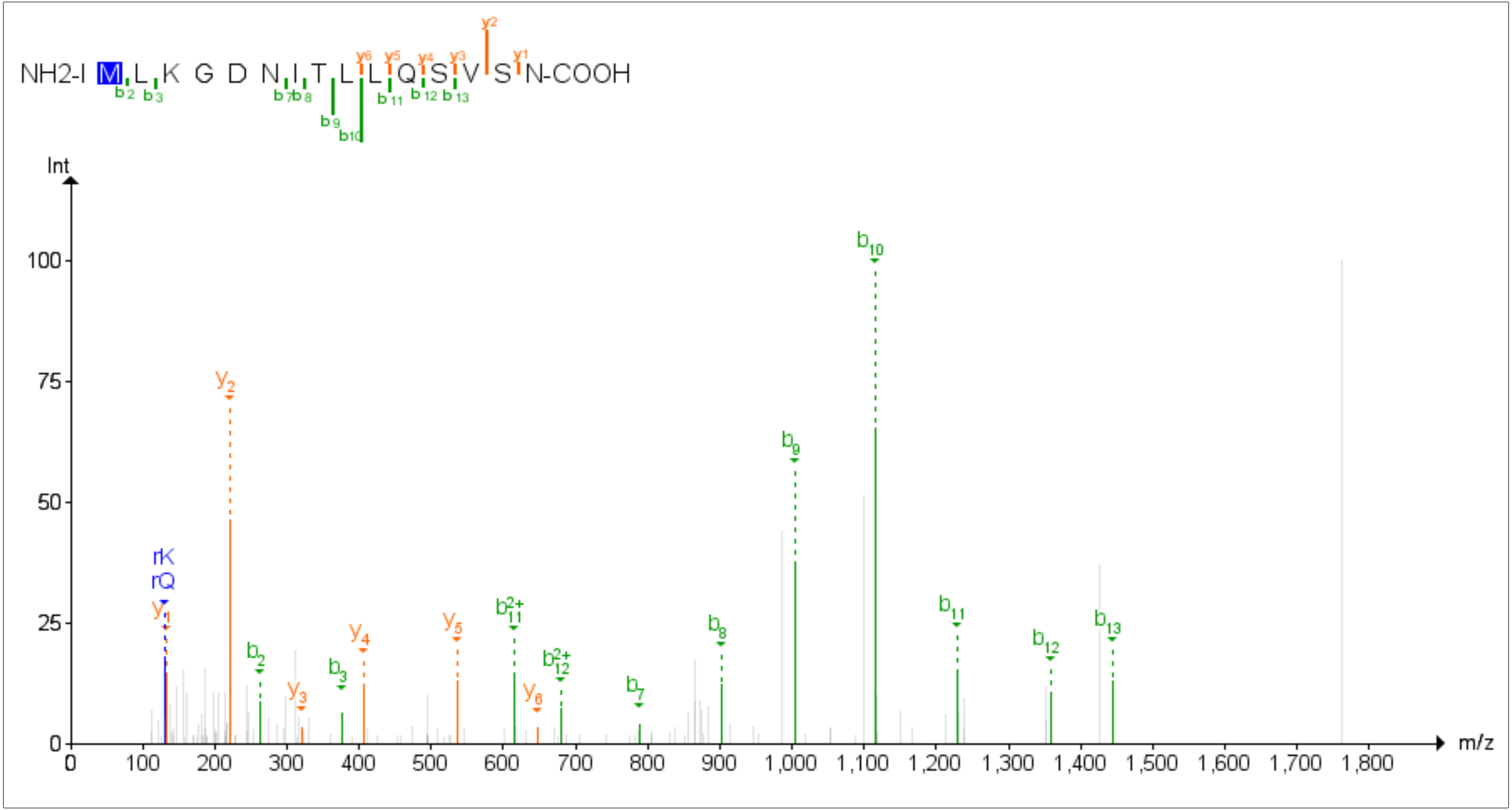

NH2-I M L K G D N I T L L Q S V S N-COOH
Int
m/z
y2
y1
y3
y4
y5
y6
b2
b3
b7
b8
b9
b10
b11
b12
b13
rK
rQ
b2+11
b2+12

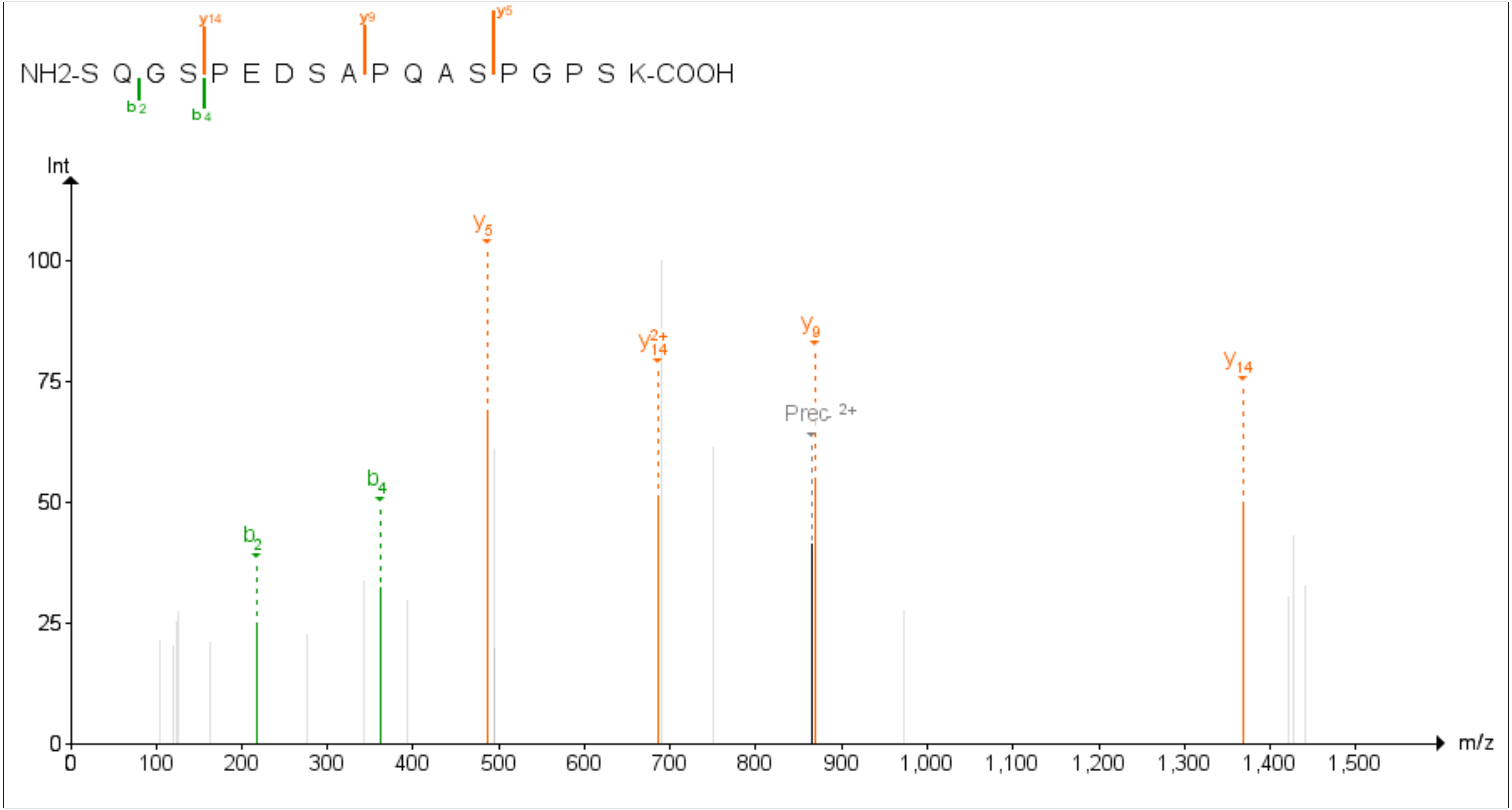

NH2-S Q G S P E D S A P Q A S P G P S K-COOH
y14
y9
y5
b2
b4
Int
m/z
y5
y14 2+
y9
Prec 2+
y14
b2
b4
0
25
50
75
100
0
100
200
300
400
500
600
700
800
900
1,000
1,100
1,200
1,300
1,400
1,500

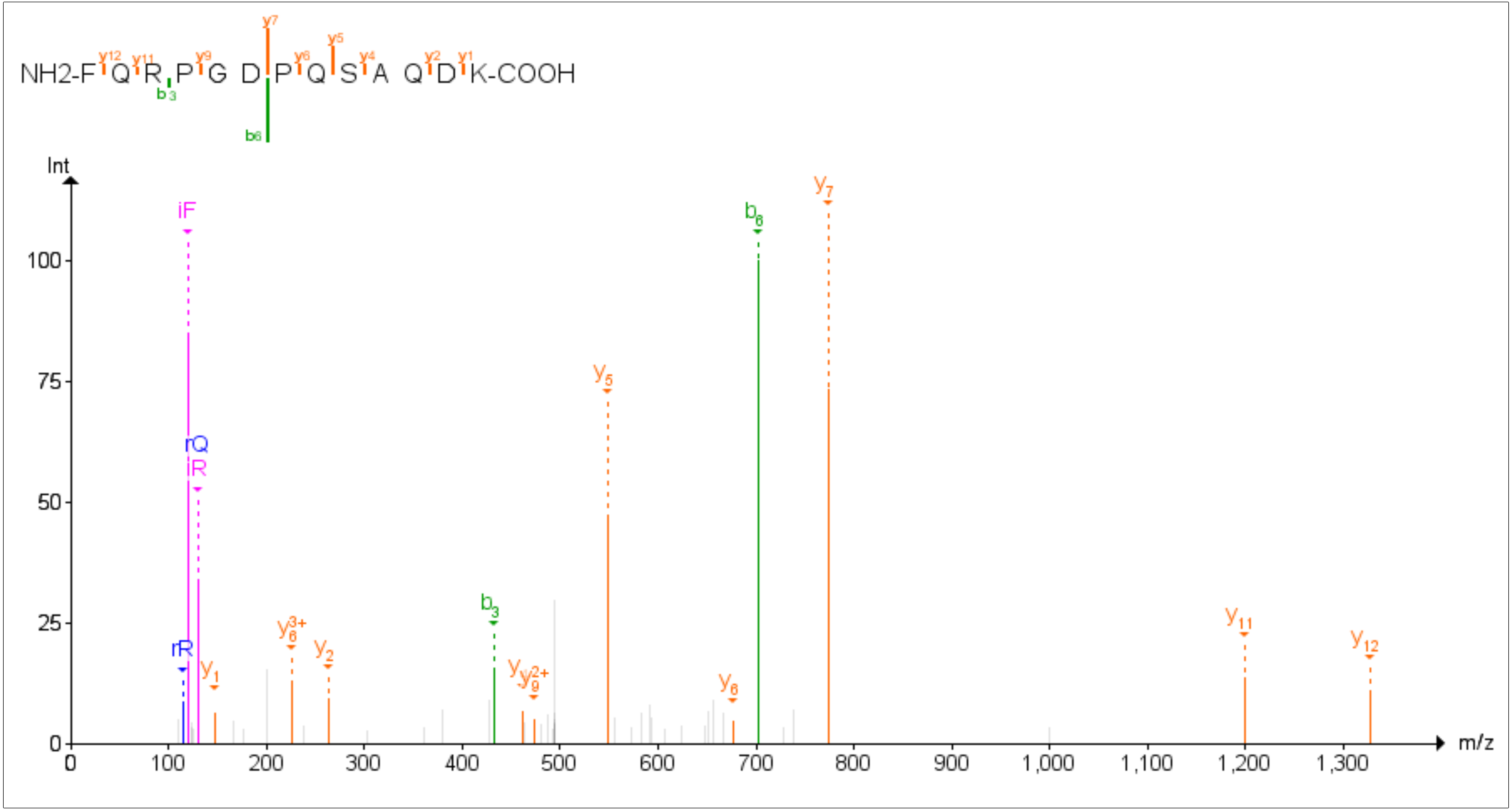

NH2-F Q R P G D P Q S A Q D K-COOH
y12 y11 y9 y7 y6 y5 y4 y2 y1
b3 b6
Int
100
75
50
25
0
iF
rQ
iR
rR
y1
y6 3+
y2
b3
y y9 2+
y5
y6
b6
y7
y11
y12
0 100 200 300 400 500 600 700 800 900 1,000 1,100 1,200 1,300
m/z

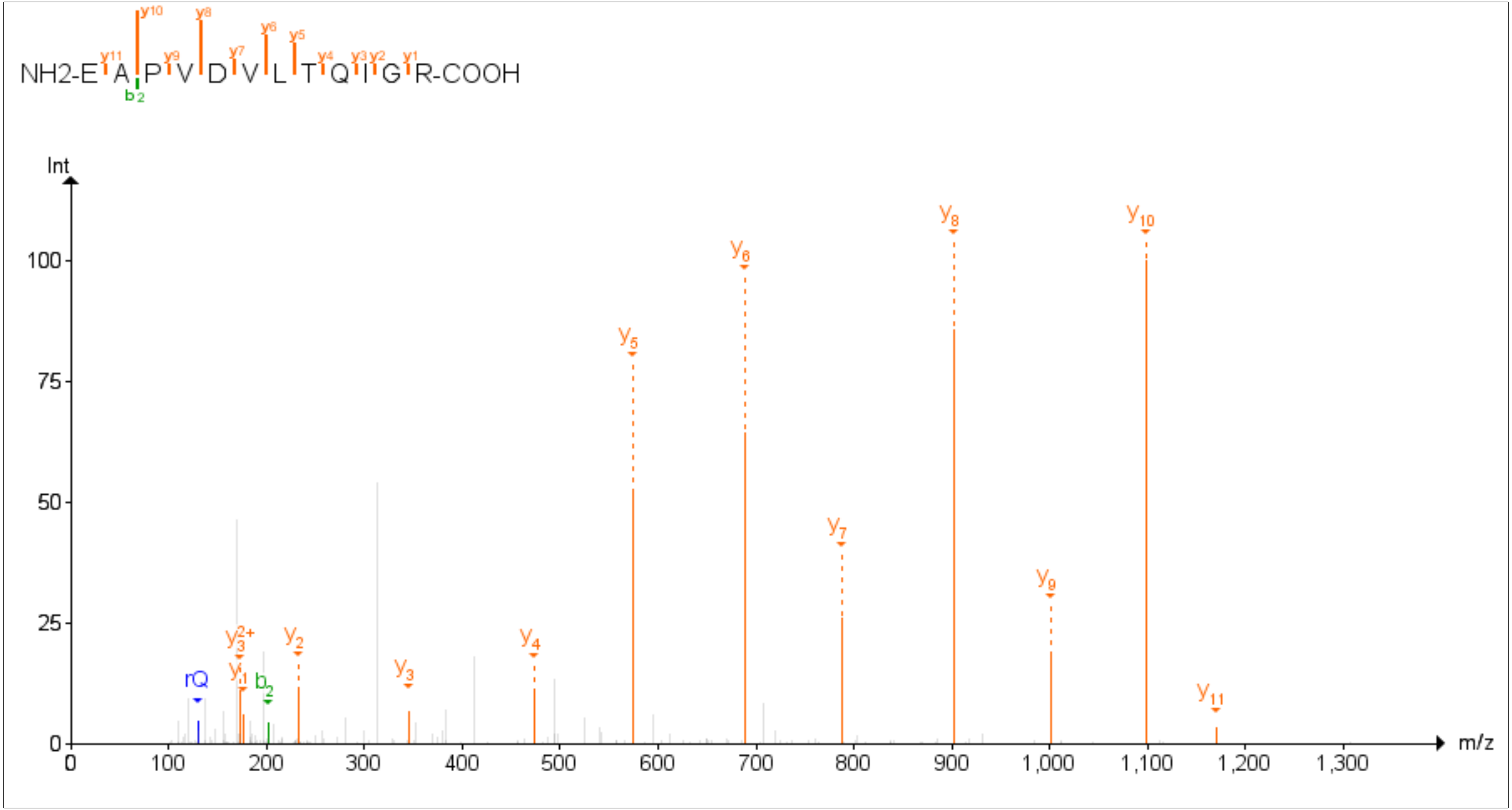
NH2-E A P V D V L T Q I G R-COOH
y11 y10 y9 y8 y7 y6 y5 y4 y3 y2 y1
b2
Int
100
75
50
25
0
m/z
0 100 200 300 400 500 600 700 800 900 1,000 1,100 1,200 1,300
rQ
y1
y3 2+
b2
y2
y3
y4
y5
y6
y7
y8
y9
y10
y11

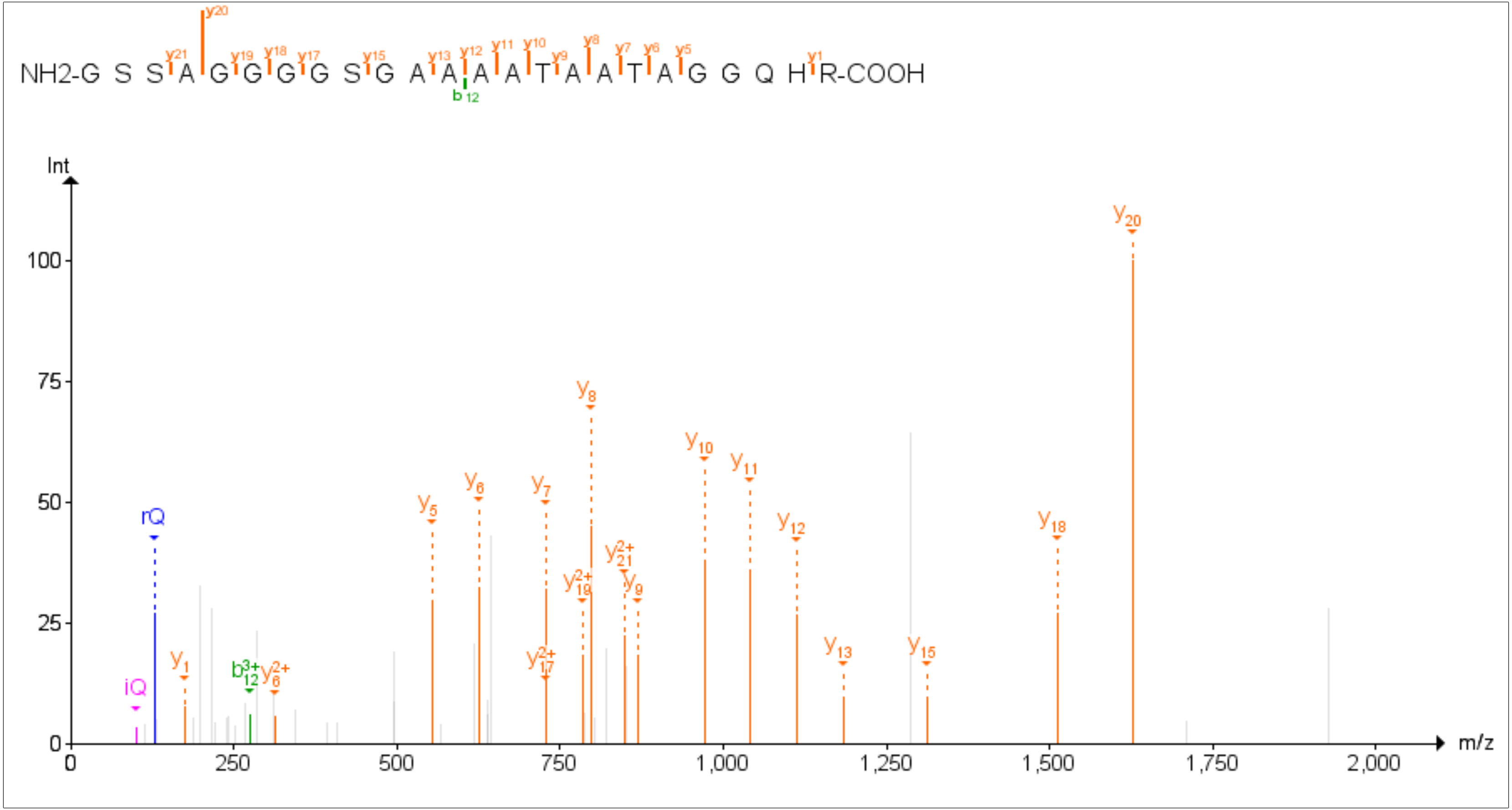
NH2-G S S A G G G G S G A A A A T A A T A G G Q H R-COOH
y21 y20 y19 y18 y17 y15 y13 y12 y11 y10 y9 y8 y7 y6 y5 y1
b 12
Int
m/z
0
25
50
75
100
250
500
750
1,000
1,250
1,500
1,750
2,000
iQ
rQ
y_1
b_{12}^{3+}
y_6^{2+}
y_5
y_6
y_7
y_{17}^{2+}
y_{19}^{2+}
y_8
y_{21}^{2+}
y_9
y_{10}
y_{11}
y_{12}
y_{13}
y_{15}
y_{18}
y_{20}

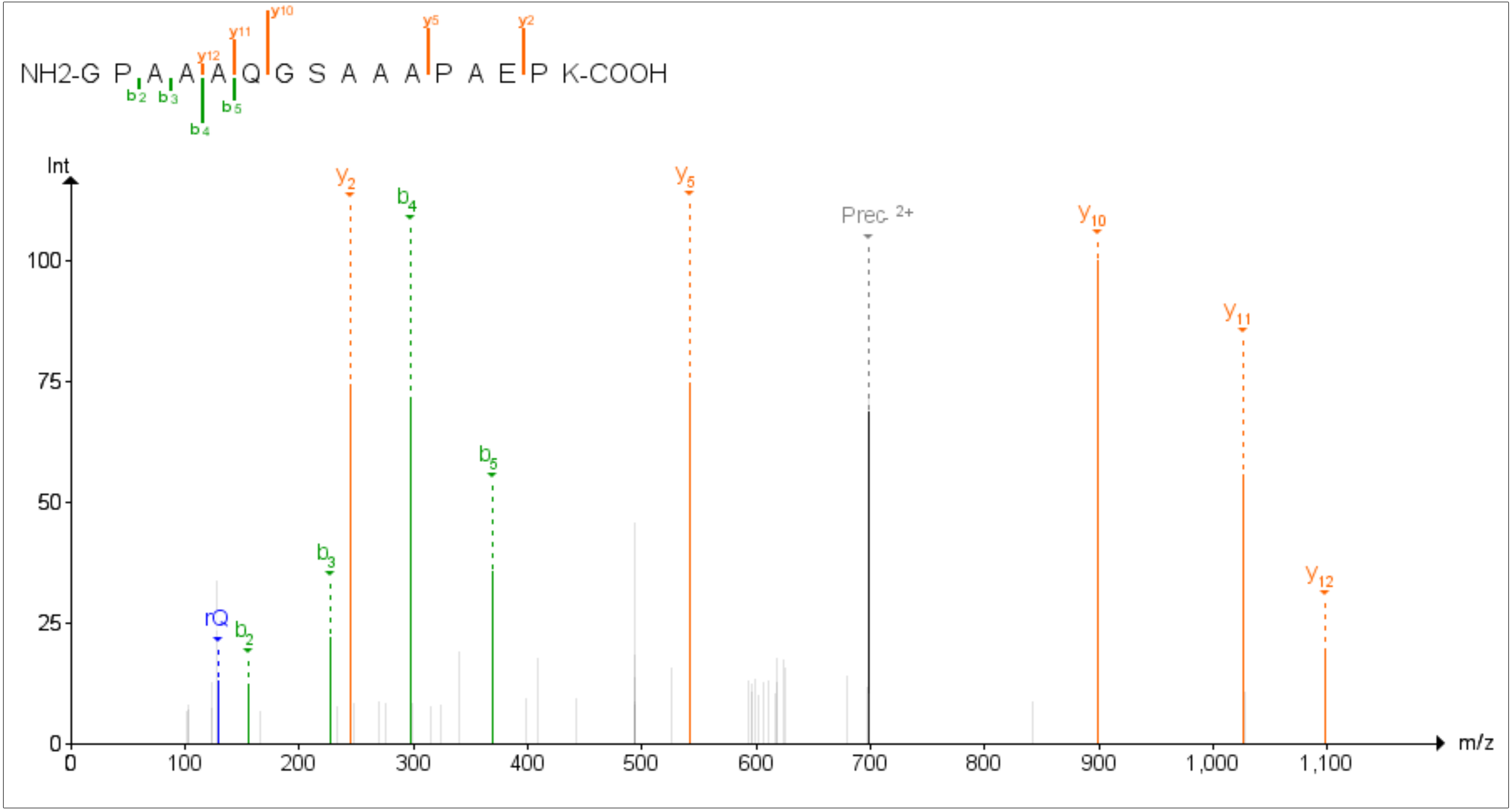

NH2-G P A A A Q G S A A A P A E P K-COOH
y10
y11
y12
y5
y2
b2
b3
b4
b5
Int
m/z
y2
b4
y5
Prec 2+
y10
y11
b5
b3
y12
rQ
b2
0
25
50
75
100
0
100
200
300
400
500
600
700
800
900
1,000
1,100

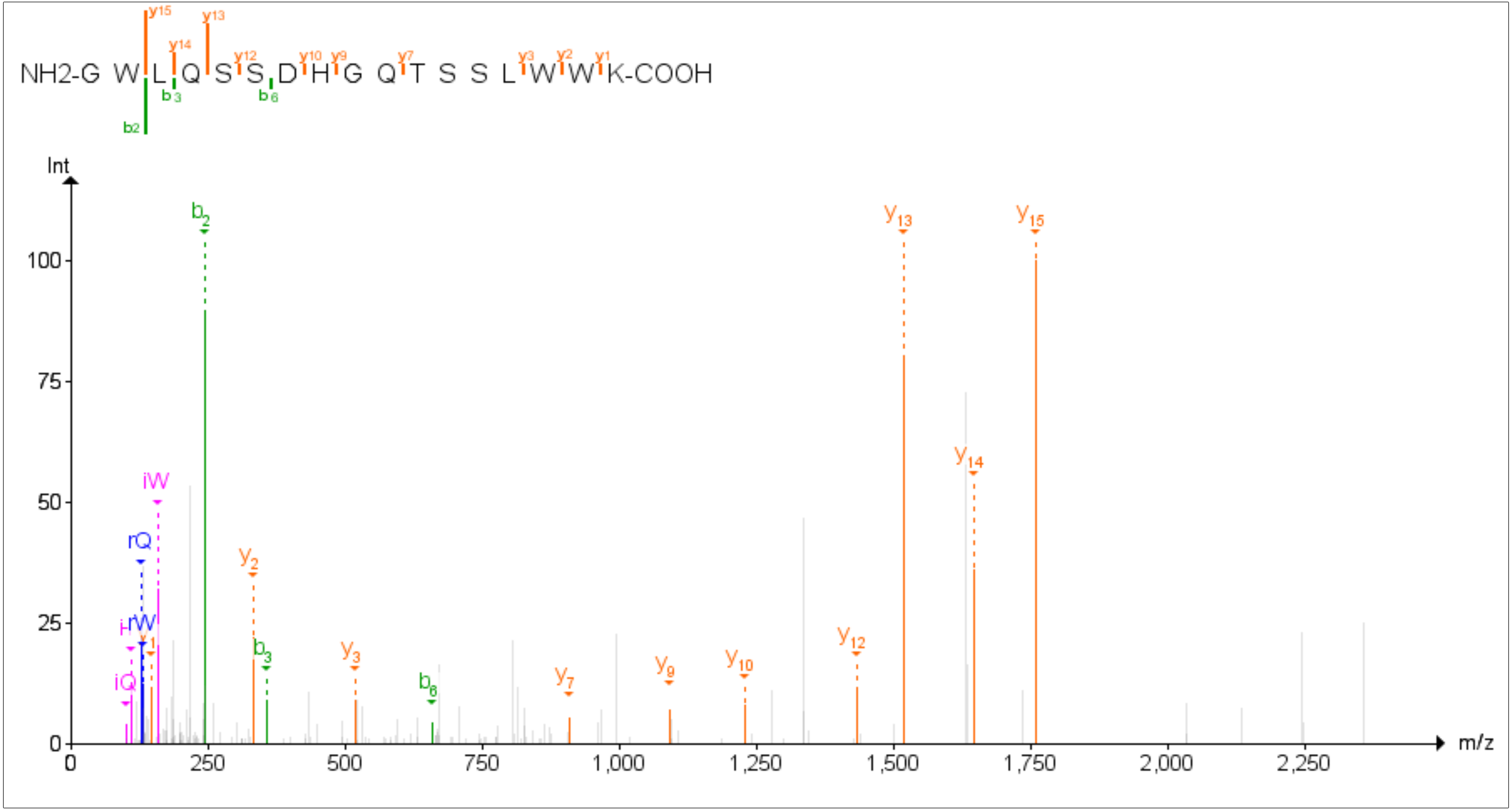

NH2-G W L Q S S D H G Q T S S L W W K-COOH
y15 y14 y13 y12 y10 y9 y7 y3 y2 y1
b2 b3 b6
Int
100
75
50
25
0
b2
y13
y15
y14
iW
rQ
iQ
rW
y2
b3
y3
b6
y7
y9
y10
y12
0 250 500 750 1,000 1,250 1,500 1,750 2,000 2,250
m/z

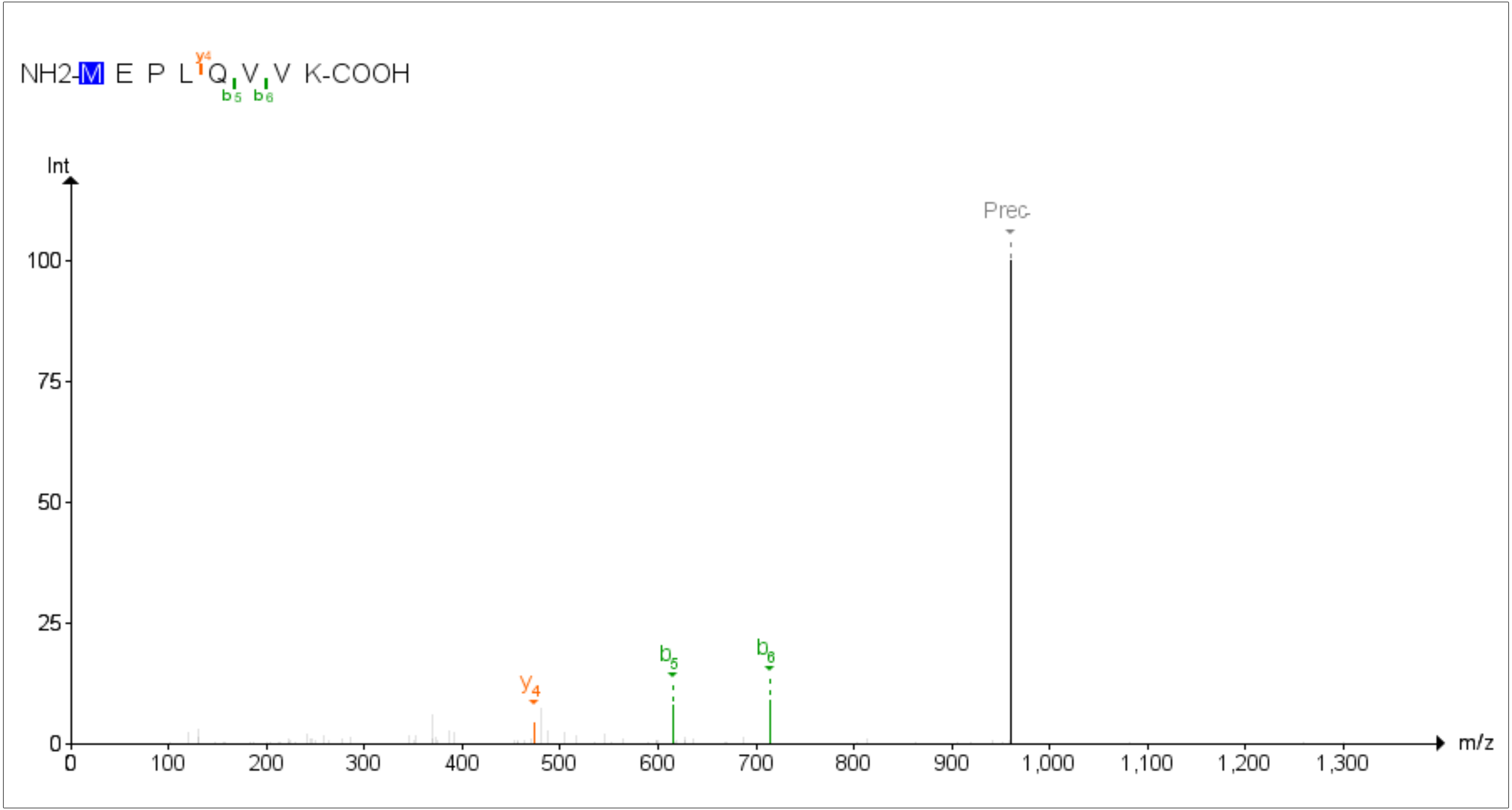

NH2-M E P L Q V V K-COOH
y4
b5
b6
Int
100
75
50
25
0
Prec
y4
b5
b6
0
100
200
300
400
500
600
700
800
900
1,000
1,100
1,200
1,300
m/z

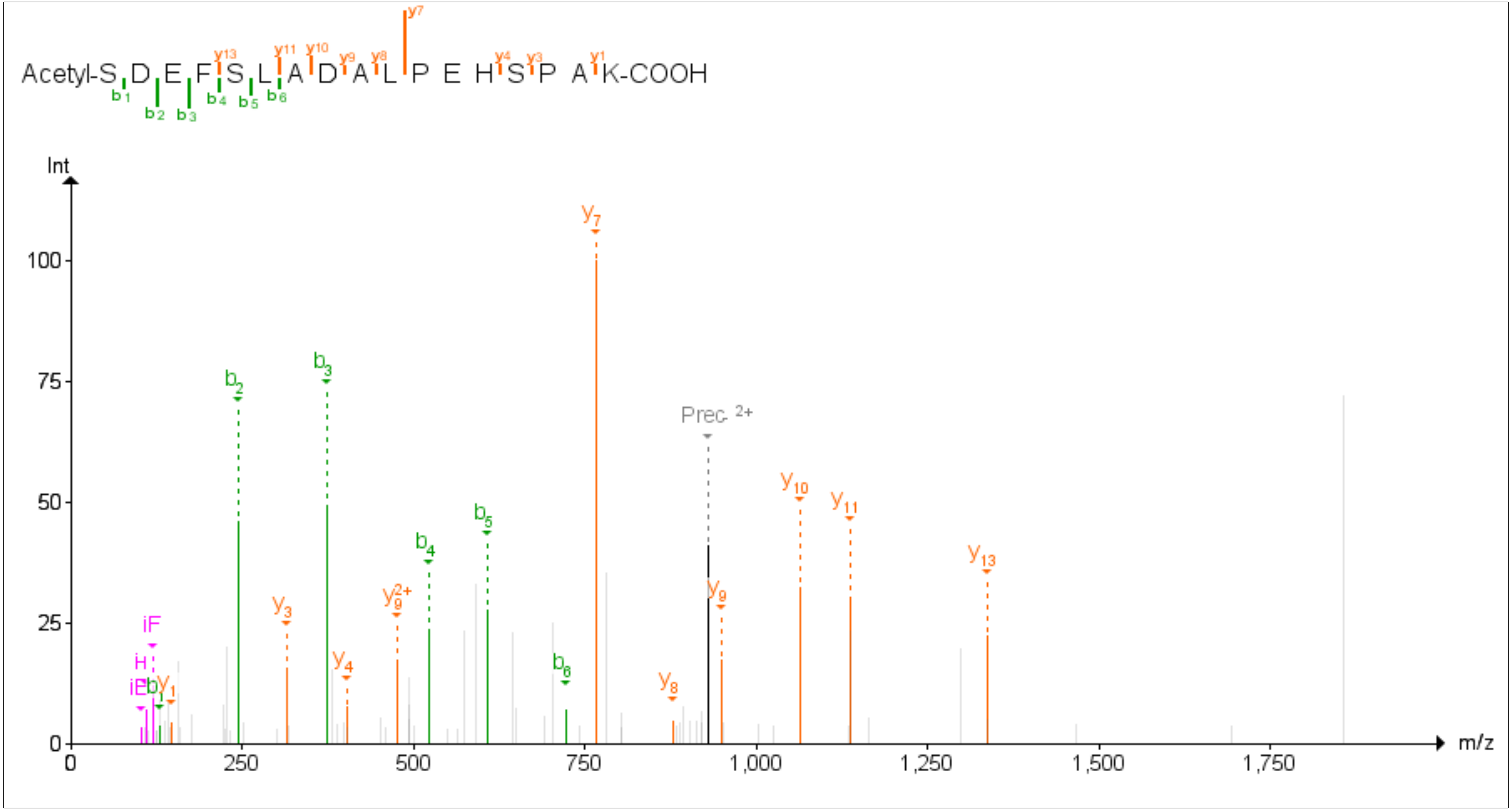
Acetyl-S D E F S L A D A L P E H S P A K-COOH
b1 b2 b3 b4 b5 b6
y13 y11 y10 y9 y8 y7 y4 y3 y1
Int
100
75
50
25
0
m/z
0
250
500
750
1,000
1,250
1,500
1,750
y7
b3
b2
Prec 2+
y10
y11
b5
b4
y13
y9 2+
y9
y3
iF
y4
iH
b6
y8
iE b y1

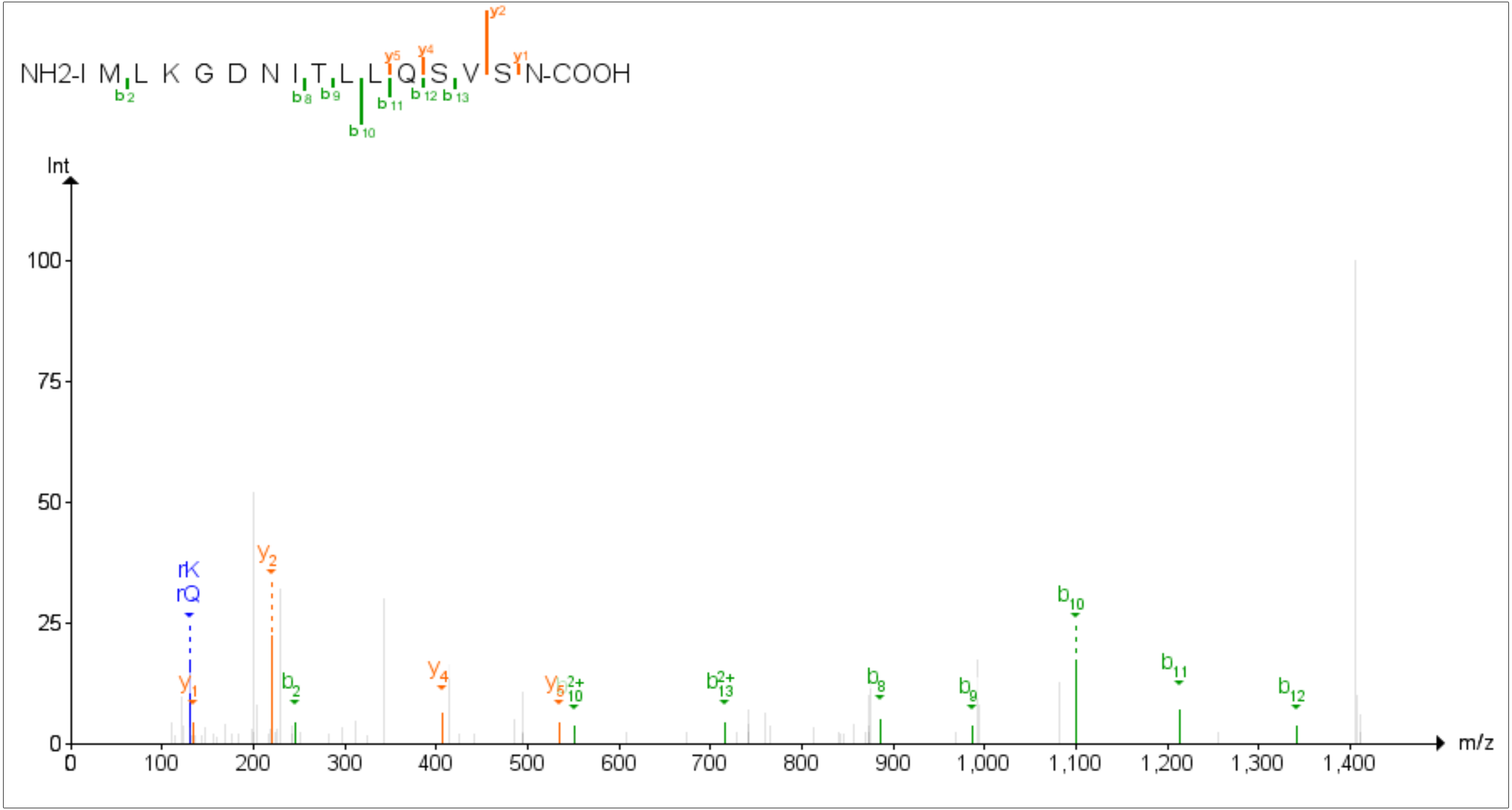

NH2-I M L K G D N I T L L Q S V S N-COOH
b2
b8
b9
b10
b11
b12
b13
y5
y4
y2
y1
Int
100
75
50
25
0
rK
rQ
y2
y1
b2
y4
y5
b10 2+
b13 2+
b8
b9
b10
b11
b12
0
100
200
300
400
500
600
700
800
900
1,000
1,100
1,200
1,300
1,400
m/z

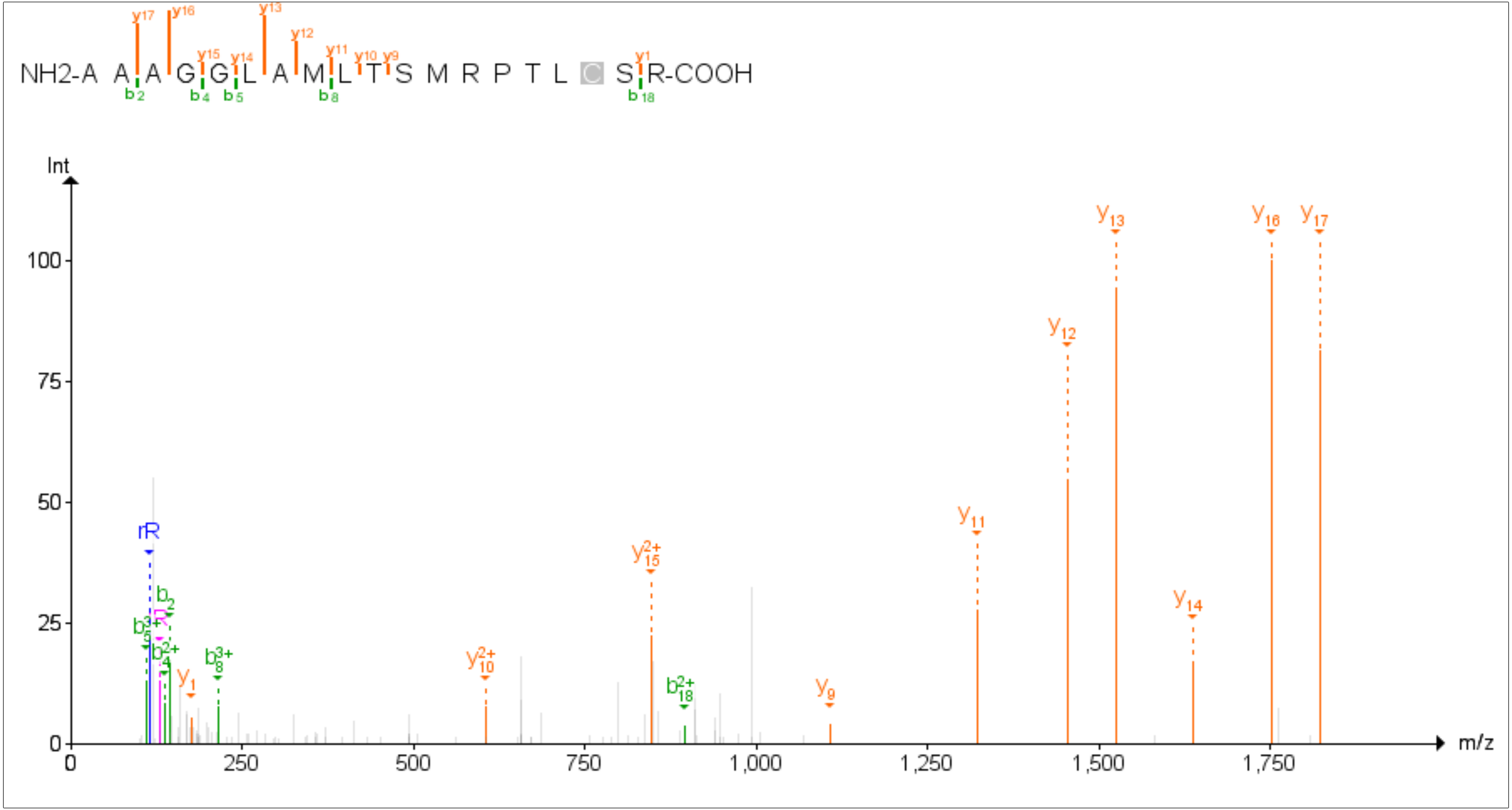
NH2-A A A G G L A M L T S M R P T L C S R-COOH
y17 y16 y15 y14 y13 y12 y11 y10 y9 y1
b2 b4 b5 b8 b18
Int
100
75
50
25
0
m/z
0
250
500
750
1,000
1,250
1,500
1,750
y13
y16
y17
y12
y11
y14
y15 2+
y10 2+
b18 2+
y9
rR
b2
b5 3+
b4 2+
b8 3+
y1

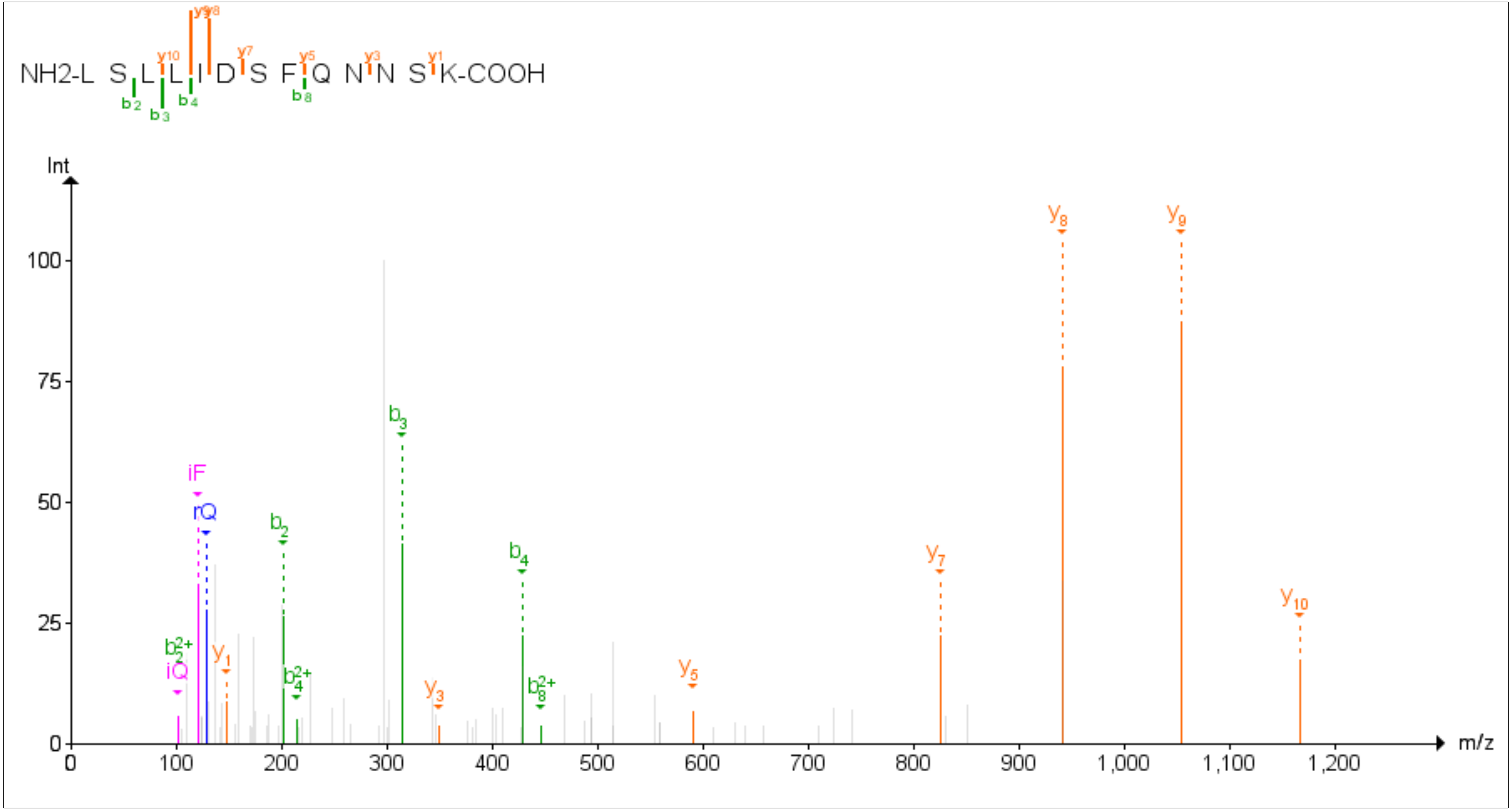

NH2-L S L L I D S F Q N N S K-COOH
b2 b3 b4 b8
y10 y9 y8 y7 y5 y3 y1
Int
m/z
iF
rQ
iQ
b2
b3
b4
b2 2+
b4 2+
b8 2+
y1
y3
y5
y7
y8
y9
y10
0
25
50
75
100
0
100
200
300
400
500
600
700
800
900
1,000
1,100
1,200

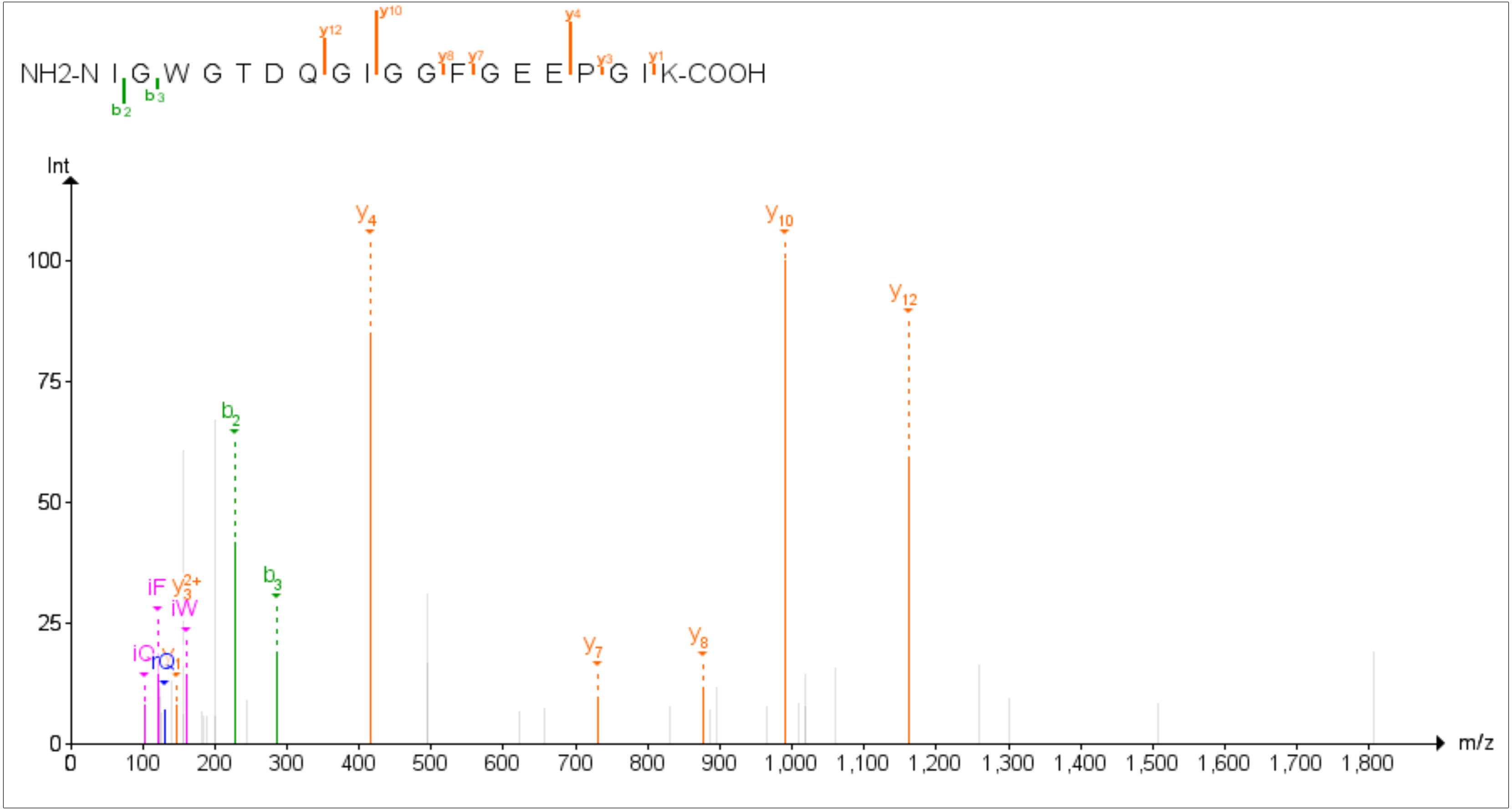

NH2-N I G W G T D Q G I G G F G E E P G I K-COOH
b2
b3
y12
y10
y8
y7
y4
y3
y1
Int
m/z
y4
y10
y12
b2
b3
iF
y3 2+
iW
iQ
rQ
y1
y7
y8
0
25
50
75
100
0
100
200
300
400
500
600
700
800
900
1,000
1,100
1,200
1,300
1,400
1,500
1,600
1,700
1,800

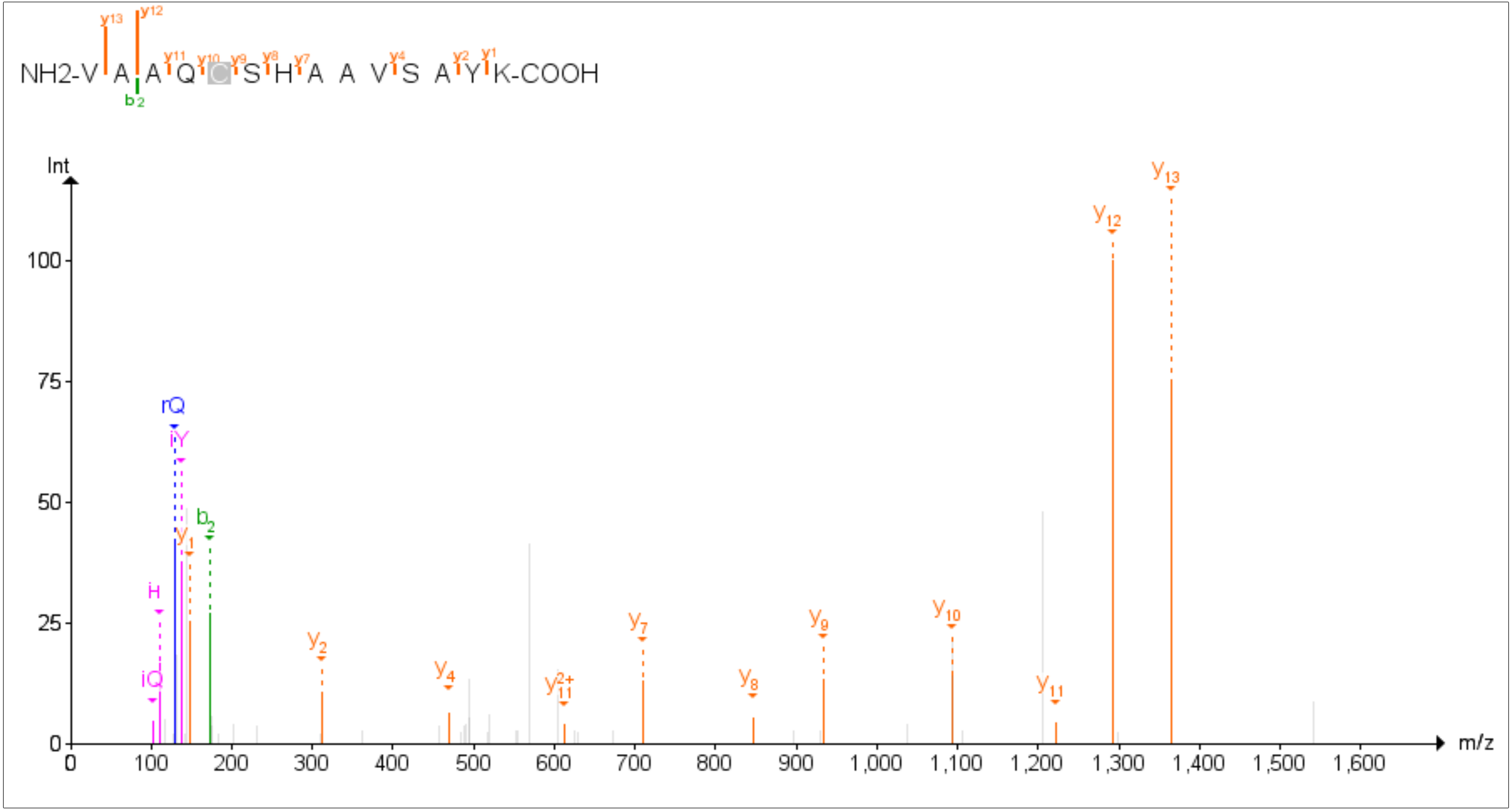

NH2-V A A Q C S H A A V S A Y K-COOH
y13
y12
y11
y10
y9
y8
y7
y4
y2
y1
b2
Int
100
75
50
25
0
rQ
iY
iH
iQ
y1
b2
y2
y4
y11 2+
y7
y8
y9
y10
y11
y12
y13
m/z
0
100
200
300
400
500
600
700
800
900
1,000
1,100
1,200
1,300
1,400
1,500
1,600

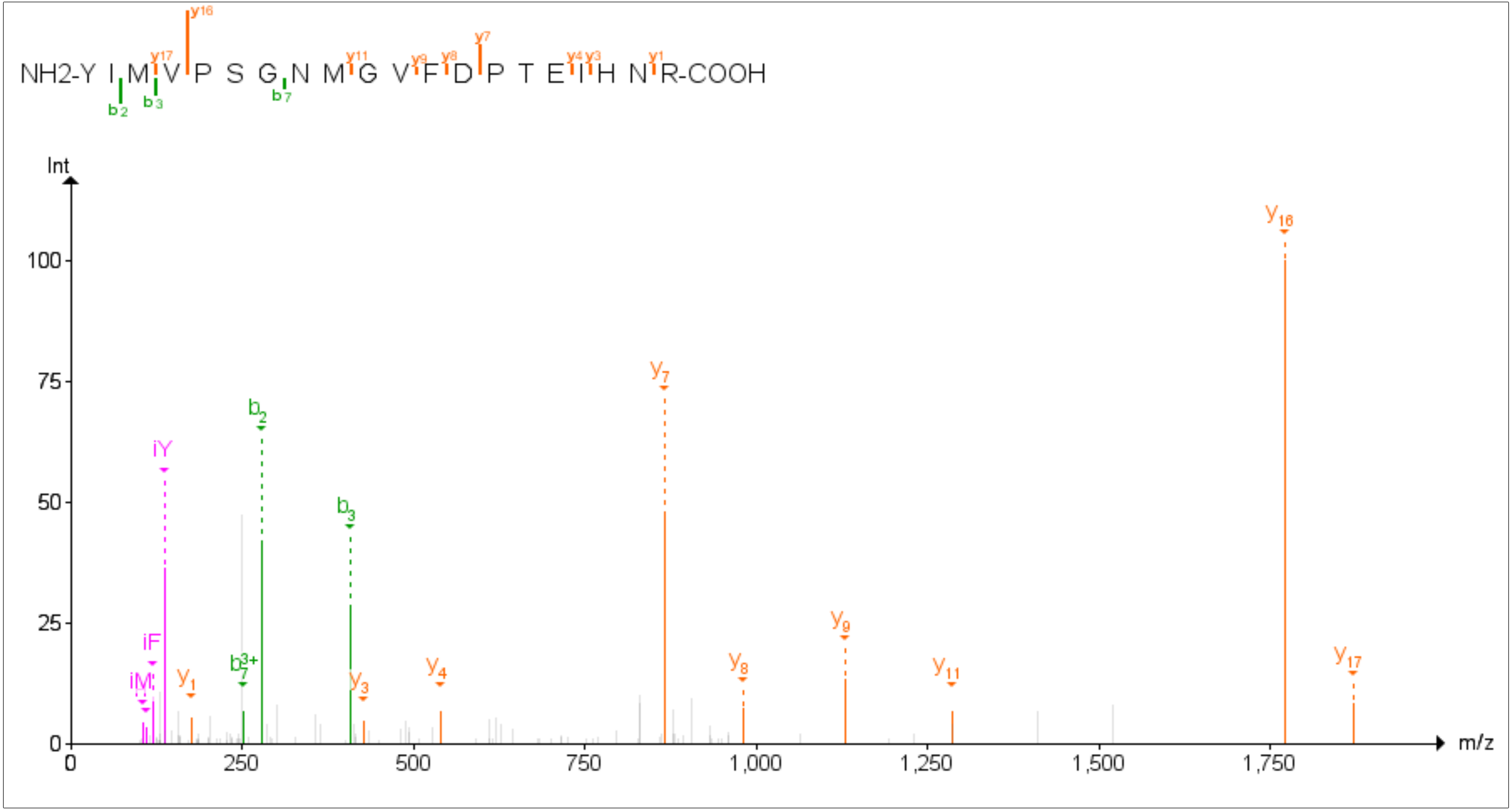
NH2-Y I M V P S G N M G V F D P T E I H N R-COOH
y16
y17
y11
y9
y8
y7
y4
y3
y1
b2
b3
b7
Int
m/z
iY
iF
iM
b2
b3
b7 3+
y1
y3
y4
y7
y8
y9
y11
y16
y17
0
25
50
75
100
0
250
500
750
1,000
1,250
1,500
1,750

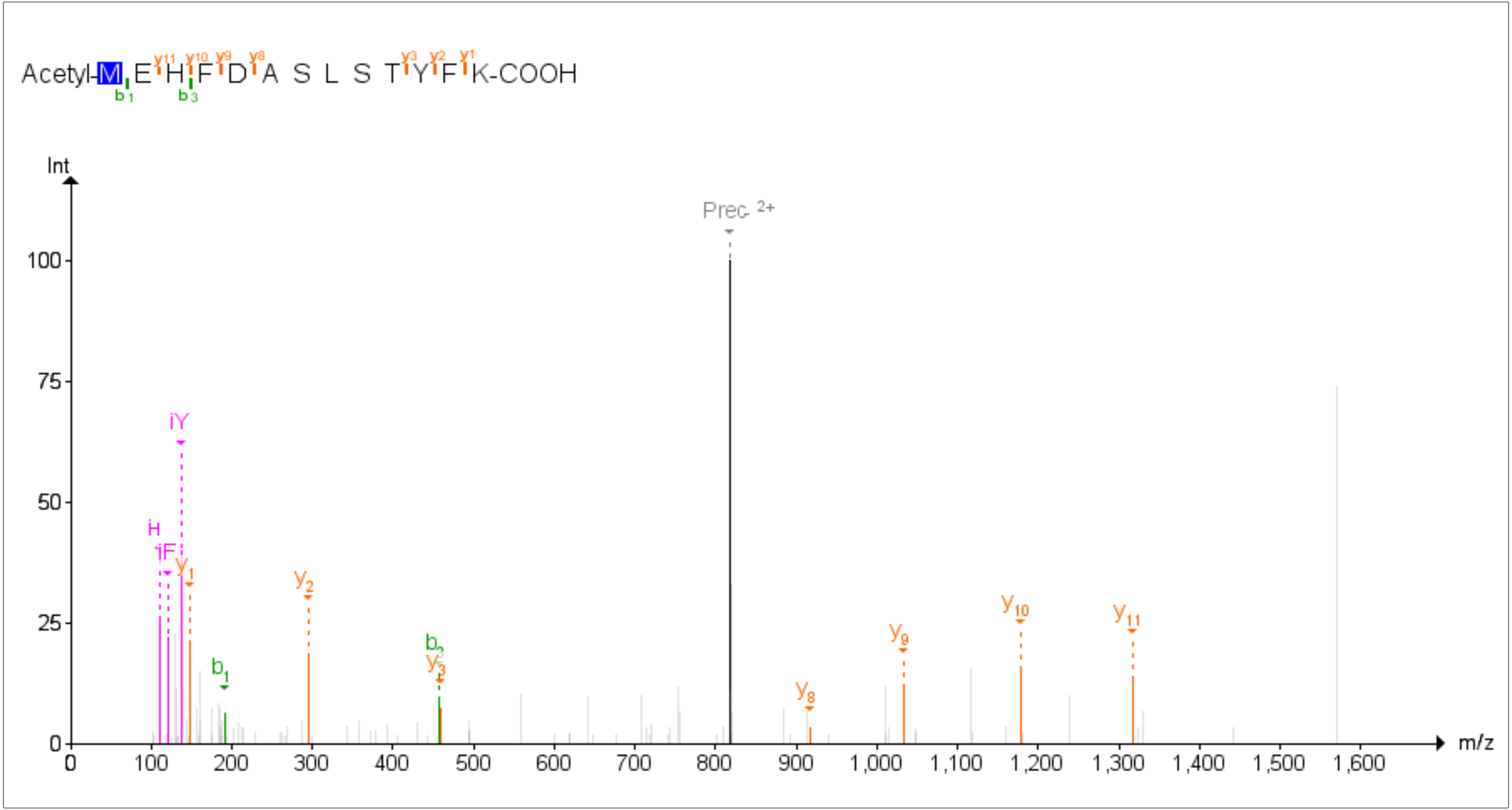

Acetyl-M E H F D A S L S T Y F K-COOH
b1
b3
y11 y10 y9 y8 y3 y2 y1
Int
Prec. 2+
iY
iH
iF
y1
y2
b1
b3
y3
y8
y9
y10
y11
0
25
50
75
100
0
100
200
300
400
500
600
700
800
900
1,000
1,100
1,200
1,300
1,400
1,500
1,600
m/z

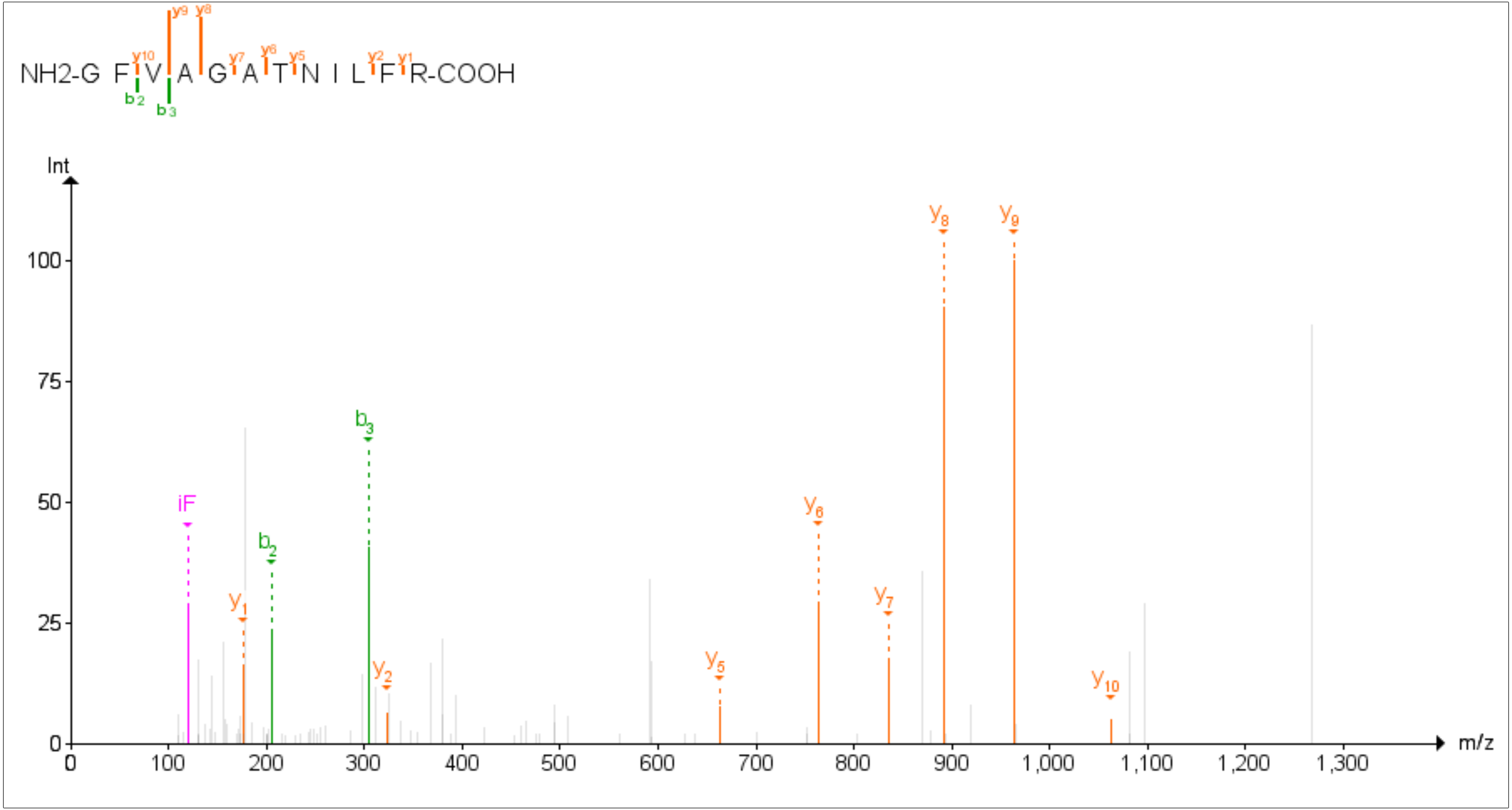
NH2-G F V A G A T N I L F R-COOH
y9 y8 y10 y7 y6 y5 y2 y1
b2 b3
Int
m/z
iF
y1 b2 b3 y2 y5 y6 y7 y8 y9 y10
0 25 50 75 100
0 100 200 300 400 500 600 700 800 900 1,000 1,100 1,200 1,300

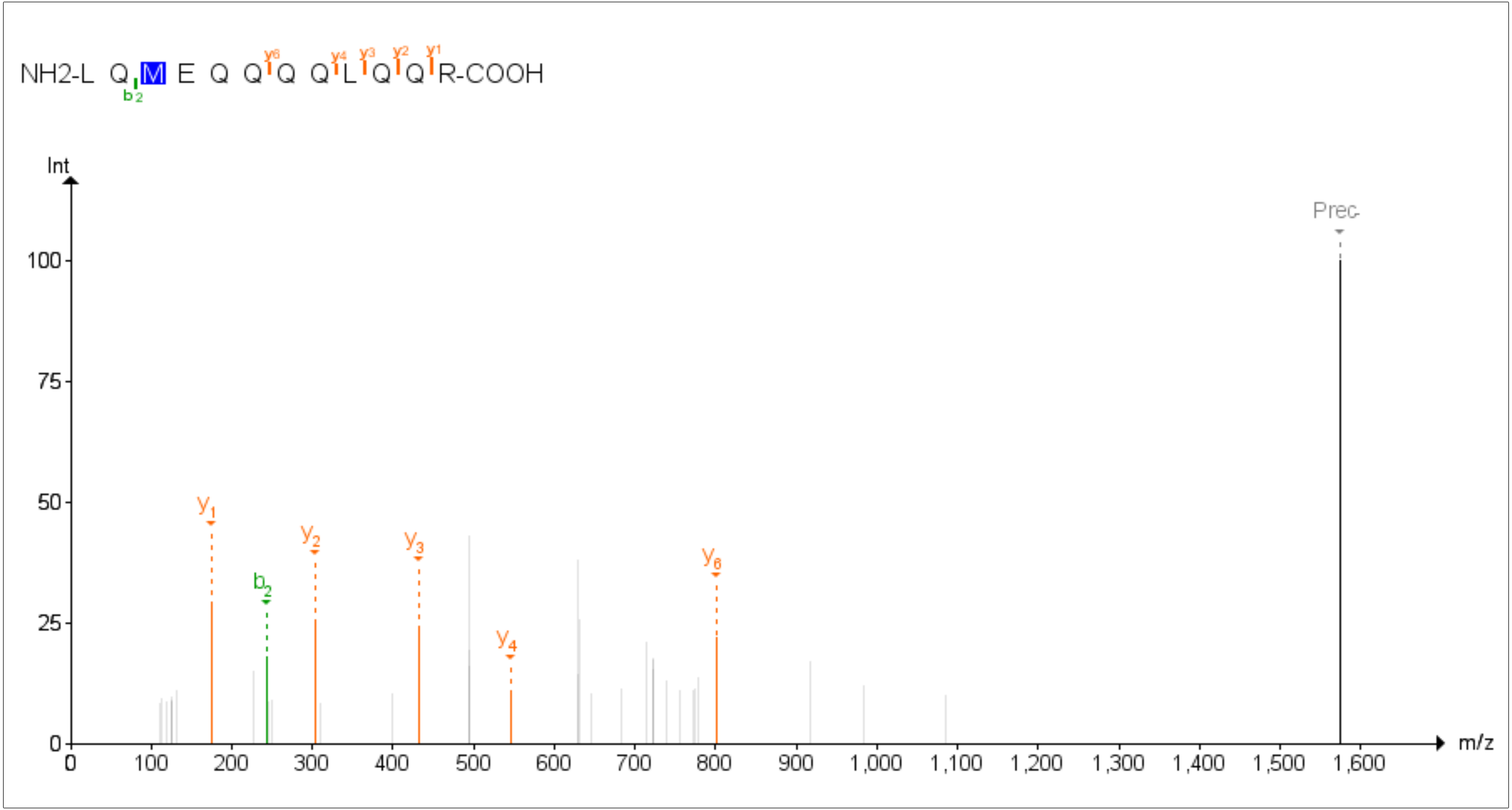
NH2-L Q M E Q Q Q Q L Q Q R-COOH
b2
y6
y4
y3
y2
y1
Int
Prec
100
75
50
25
0
y1
b2
y2
y3
y4
y6
0
100
200
300
400
500
600
700
800
900
1,000
1,100
1,200
1,300
1,400
1,500
1,600
m/z

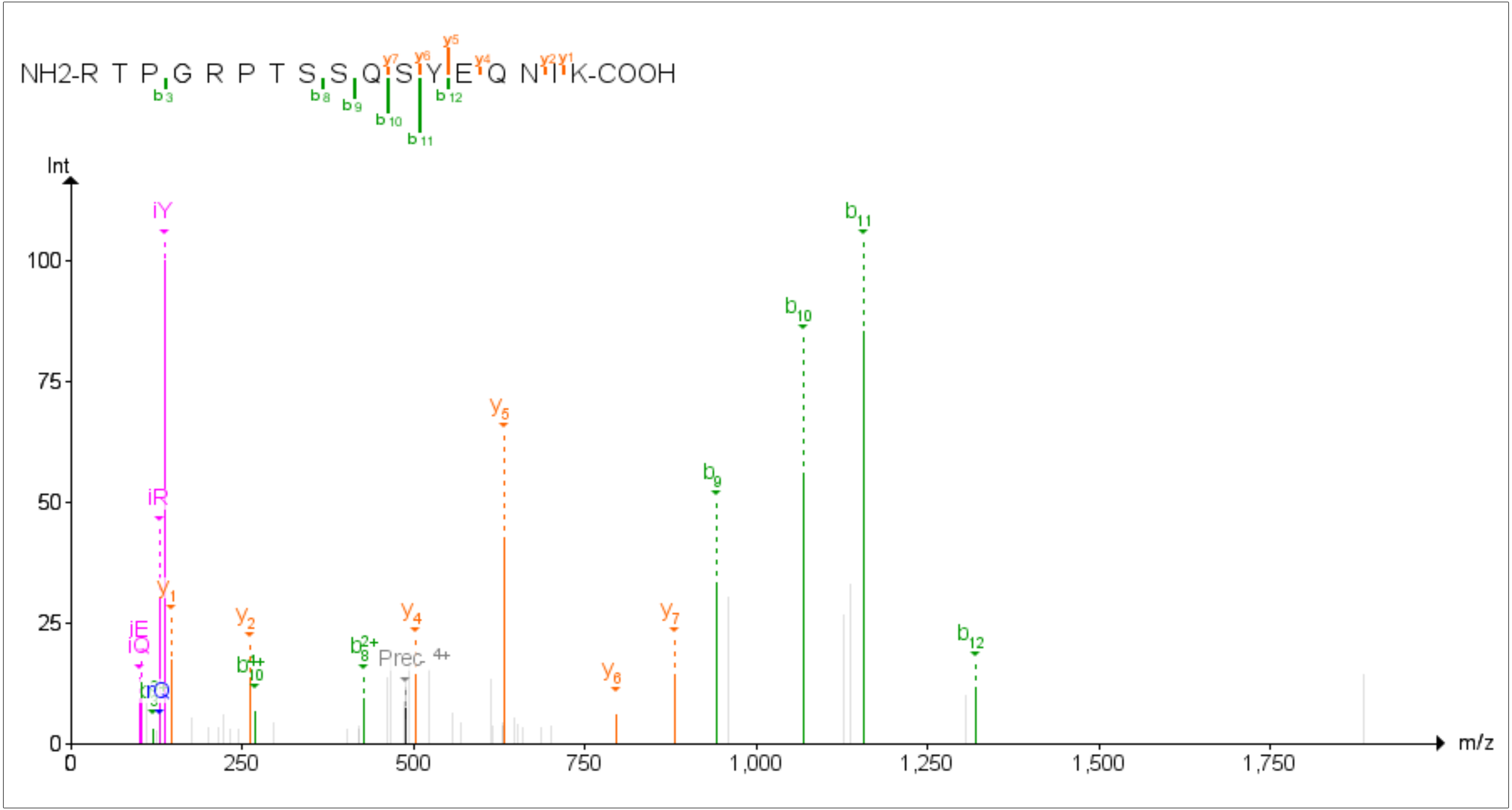
NH2-R T P G R P T S S Q S Y E Q N I K-COOH
Int
m/z
0
25
50
75
100
0
250
500
750
1,000
1,250
1,500
1,750
iY
iR
iE
iQ
b11
b10
b9
b12
y5
y1
y2
y4
y7
y6
Prec 4+

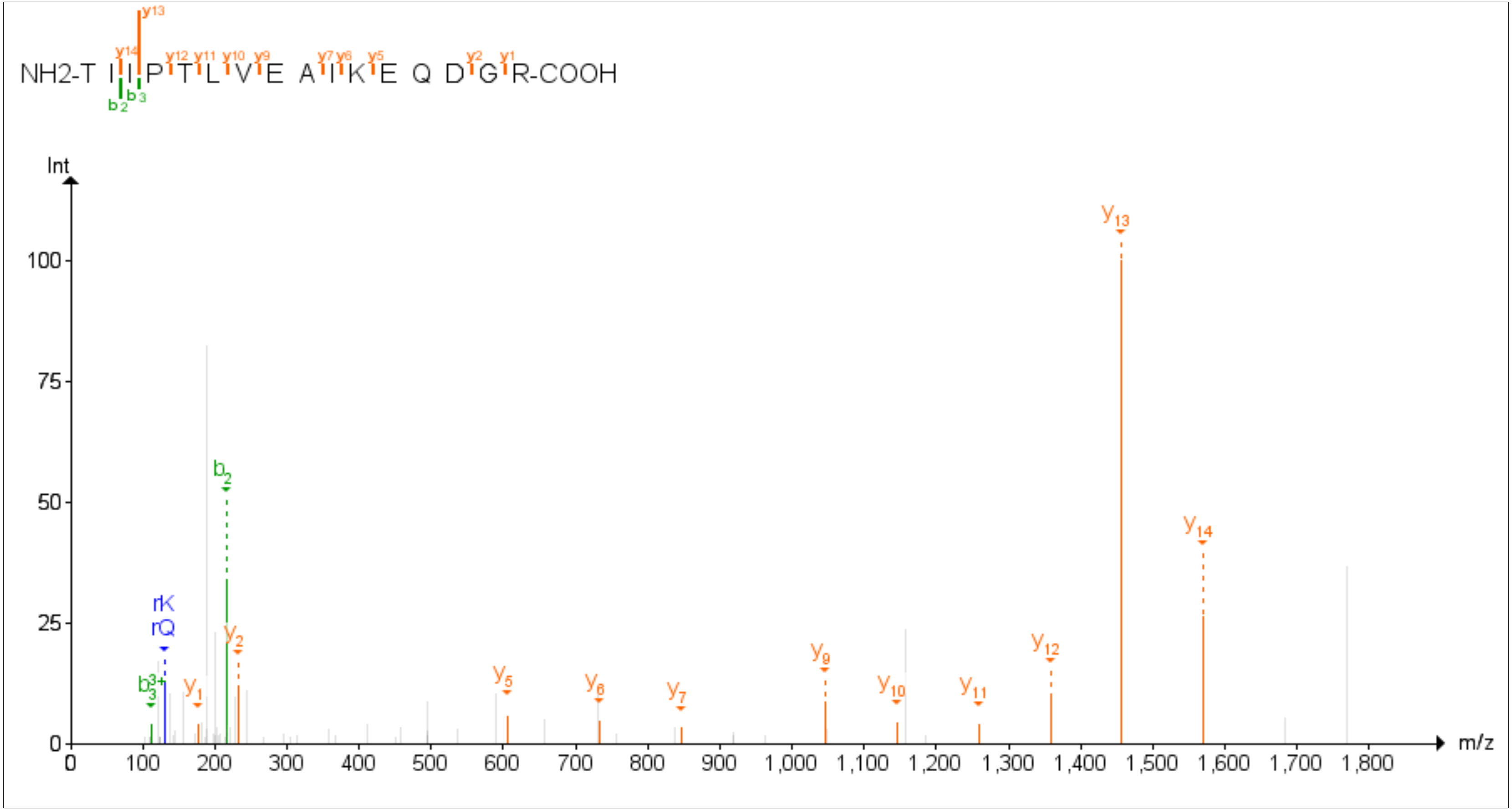

NH2-T I I P T L V E A I K E Q D G R-COOH
y13
y14
y12
y11
y10
y9
y7
y6
y5
y2
y1
b2
b3
Int
100
75
50
25
0
m/z
0
100
200
300
400
500
600
700
800
900
1,000
1,100
1,200
1,300
1,400
1,500
1,600
1,700
1,800
rK
rQ

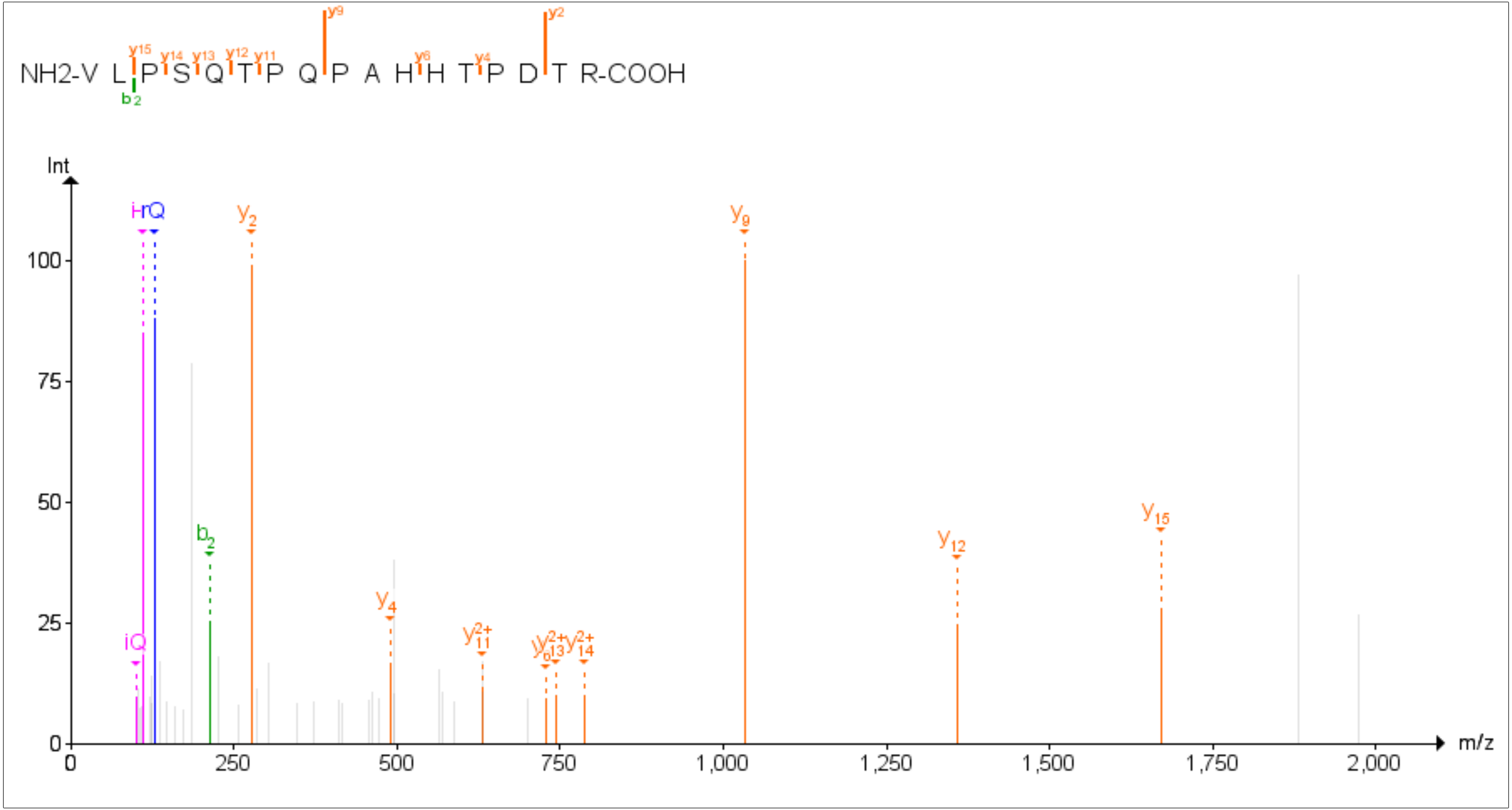

NH2-V L P S Q T P Q P A H H T P D T R-COOH
y15 y14 y13 y12 y11 y9 y6 y4 y2
b2
Int
m/z
iH rQ
y2
y9
b2
y4
iQ
y15
y12
0
25
50
75
100
0
250
500
750
1,000
1,250
1,500
1,750
2,000

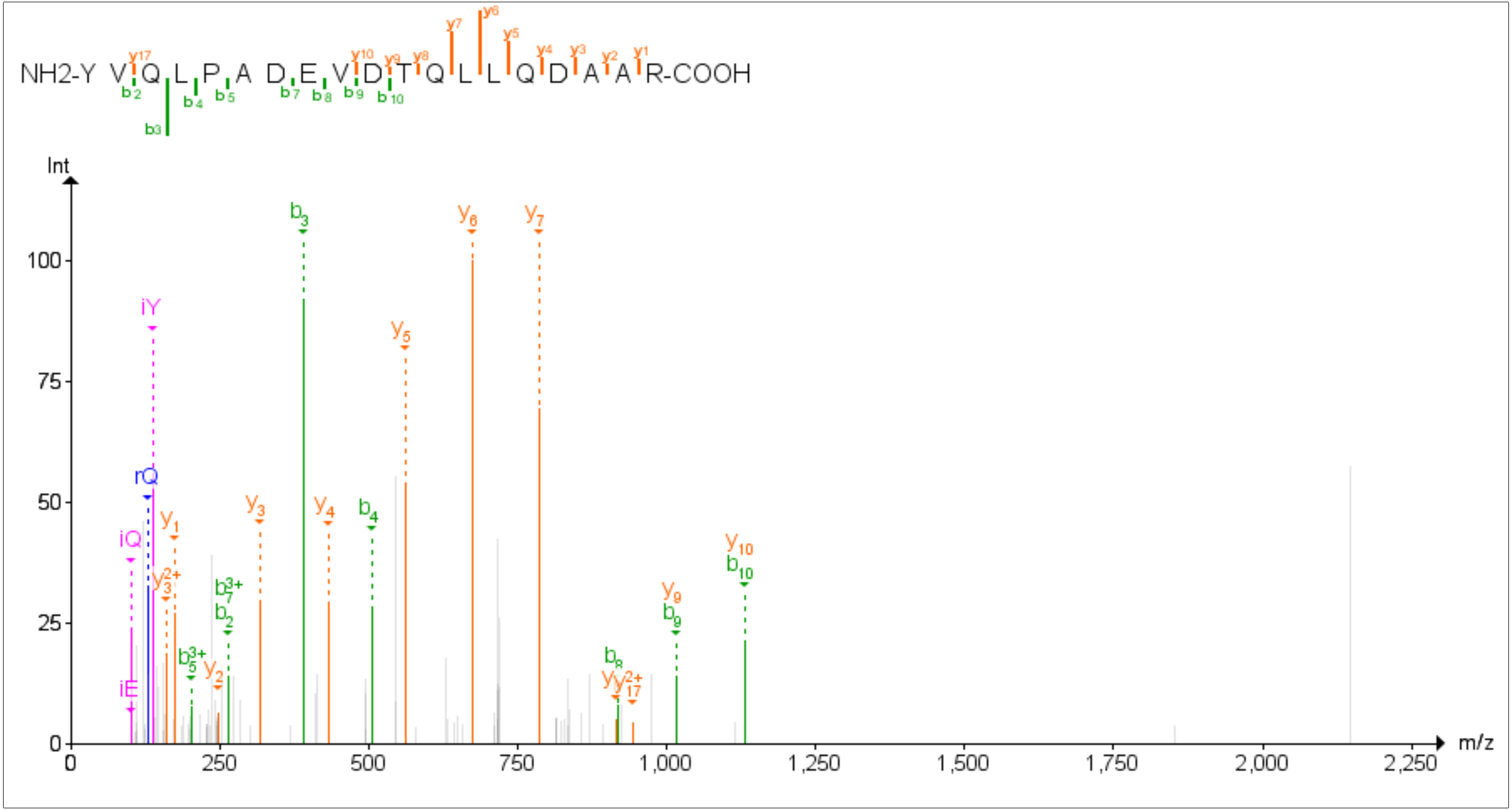

NH2-Y V Q L P A D E V D T Q L L Q D A A R-COOH
y17 y10 y9 y8 y7 y6 y5 y4 y3 y2 y1
b2 b3 b4 b5 b7 b8 b9 b10
Int
m/z
0
25
50
75
100
250
500
750
1,000
1,250
1,500
1,750
2,000
2,250
iY
rQ
iQ
iE

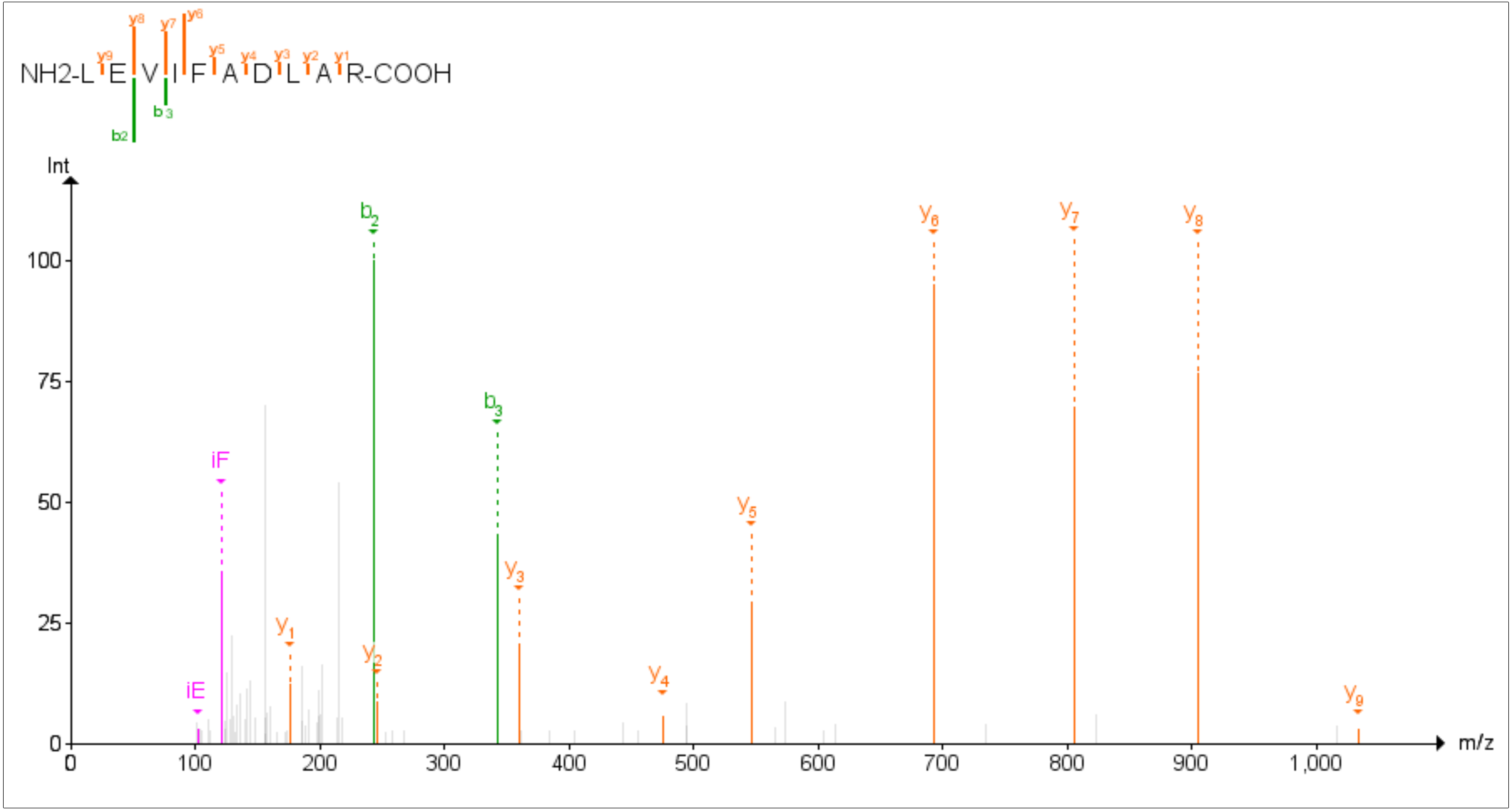

NH2-L E V I F A D L A R-COOH
y9 y8 y7 y6 y5 y4 y3 y2 y1
b2 b3
Int
100
75
50
25
0
m/z
0 100 200 300 400 500 600 700 800 900 1,000
b2
b3
y6
y7
y8
iF
iE
y1
y2
y3
y4
y5
y9

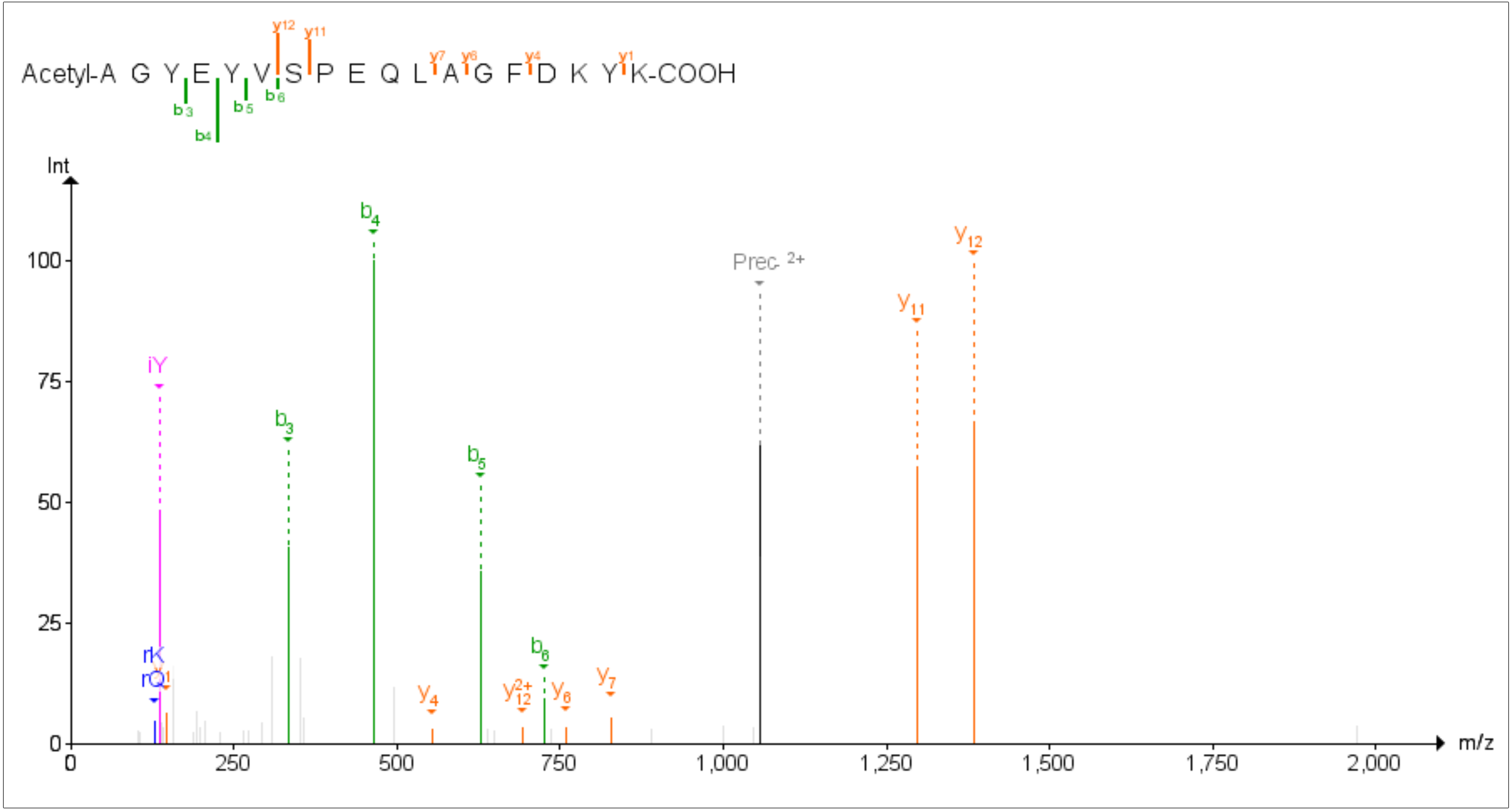

Acetyl-A G Y E Y V S P E Q L A G F D K Y K-COOH
y12
y11
y7
y6
y4
y1
b3
b4
b5
b6
Int
100
75
50
25
0
iY
rK
rQ
b4
b3
b5
b6
y4
y12 2+
y6
y7
Prec 2+
y11
y12
m/z
0
250
500
750
1,000
1,250
1,500
1,750
2,000

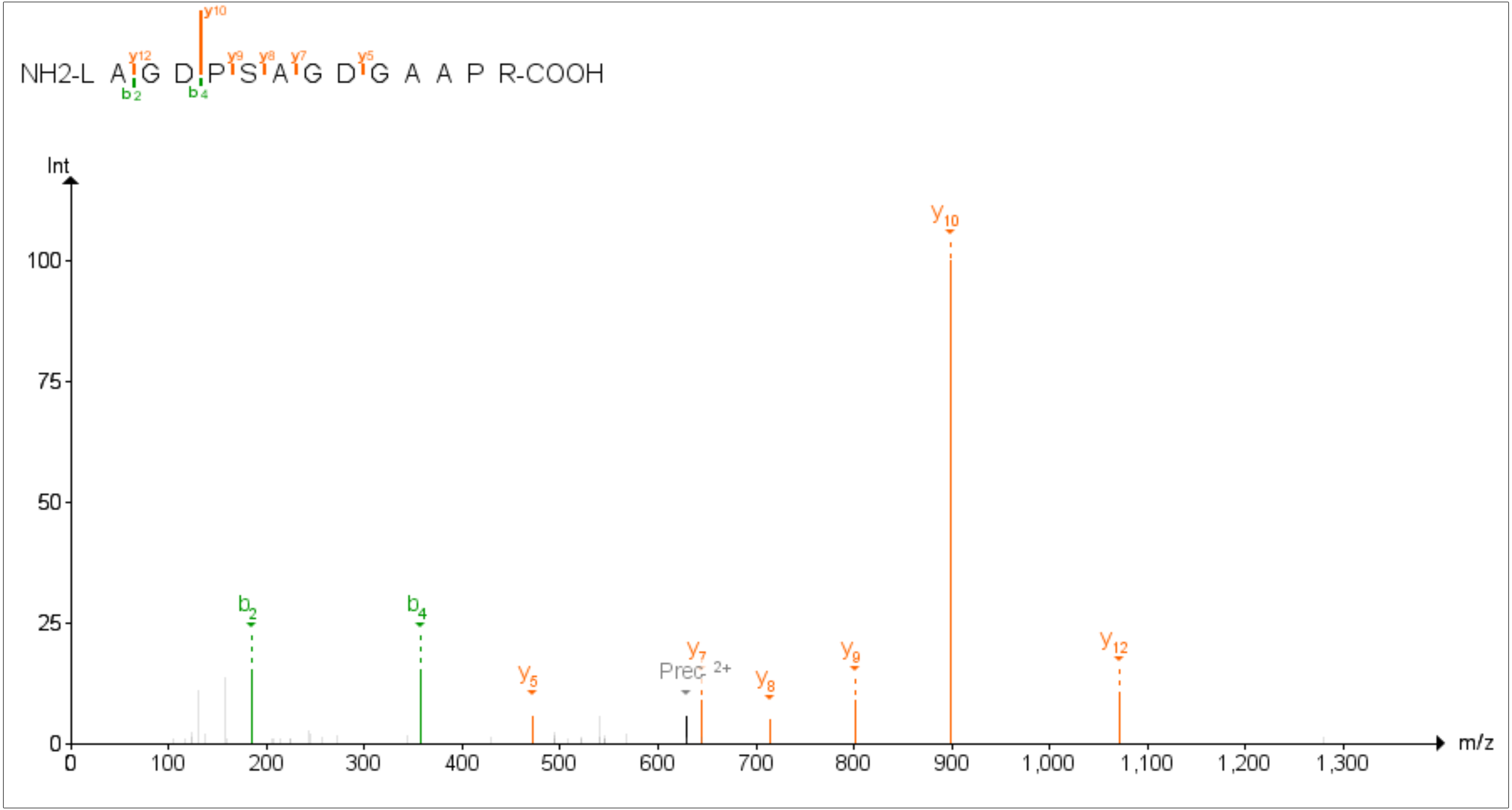

NH2-L A G D P S A G D G A A P R-COOH
y12 y10 y9 y8 y7 y5
b2 b4
Int
100
75
50
25
0
b2
b4
y5
Prec 2+
y7
y8
y9
y10
y12
0 100 200 300 400 500 600 700 800 900 1,000 1,100 1,200 1,300
m/z

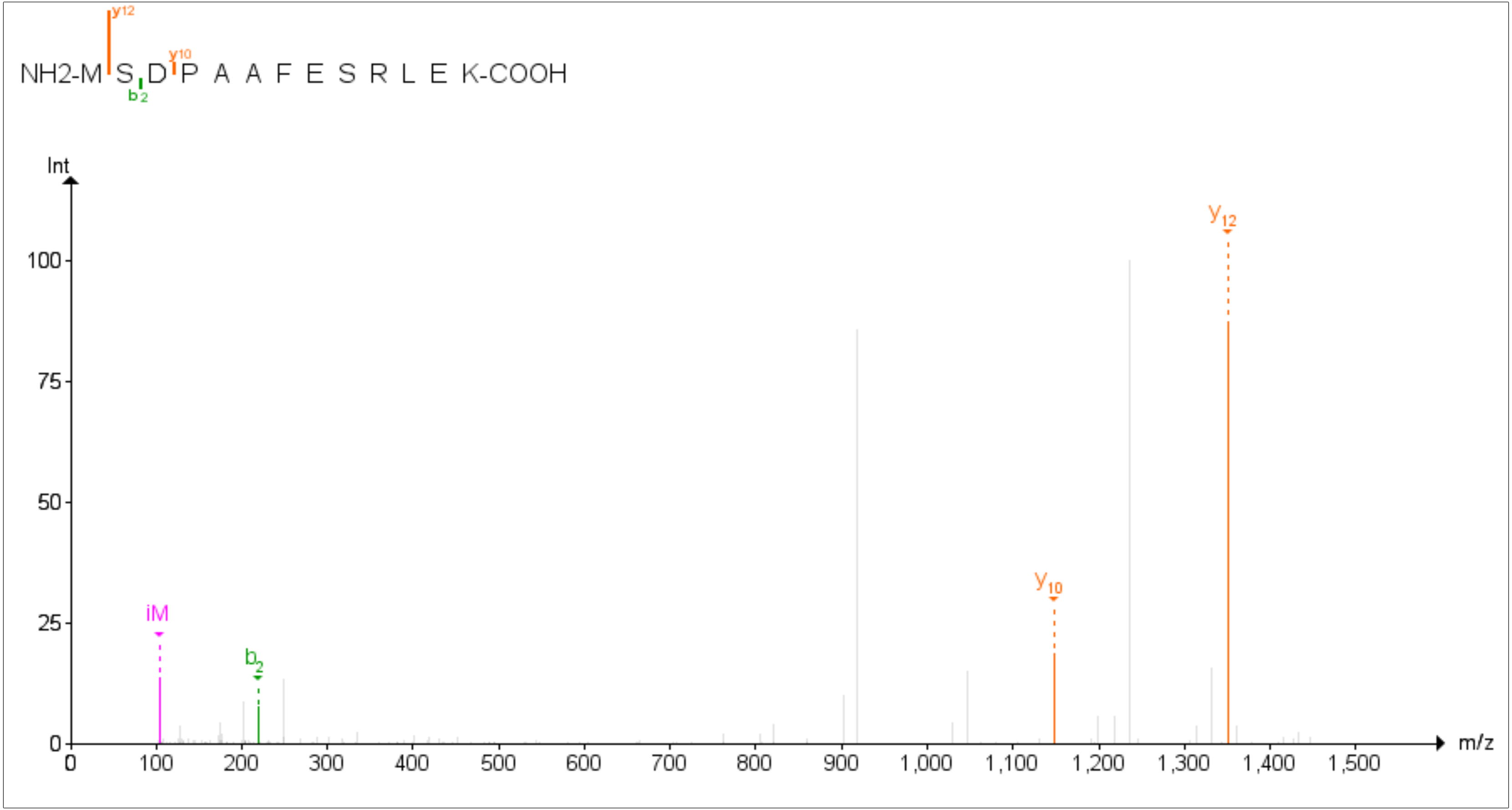

NH2-M S D P A A F E S R L E K-COOH
y12
y10
b2
Int
100
75
50
25
0
iM
b2
y10
y12
0
100
200
300
400
500
600
700
800
900
1,000
1,100
1,200
1,300
1,400
1,500
m/z

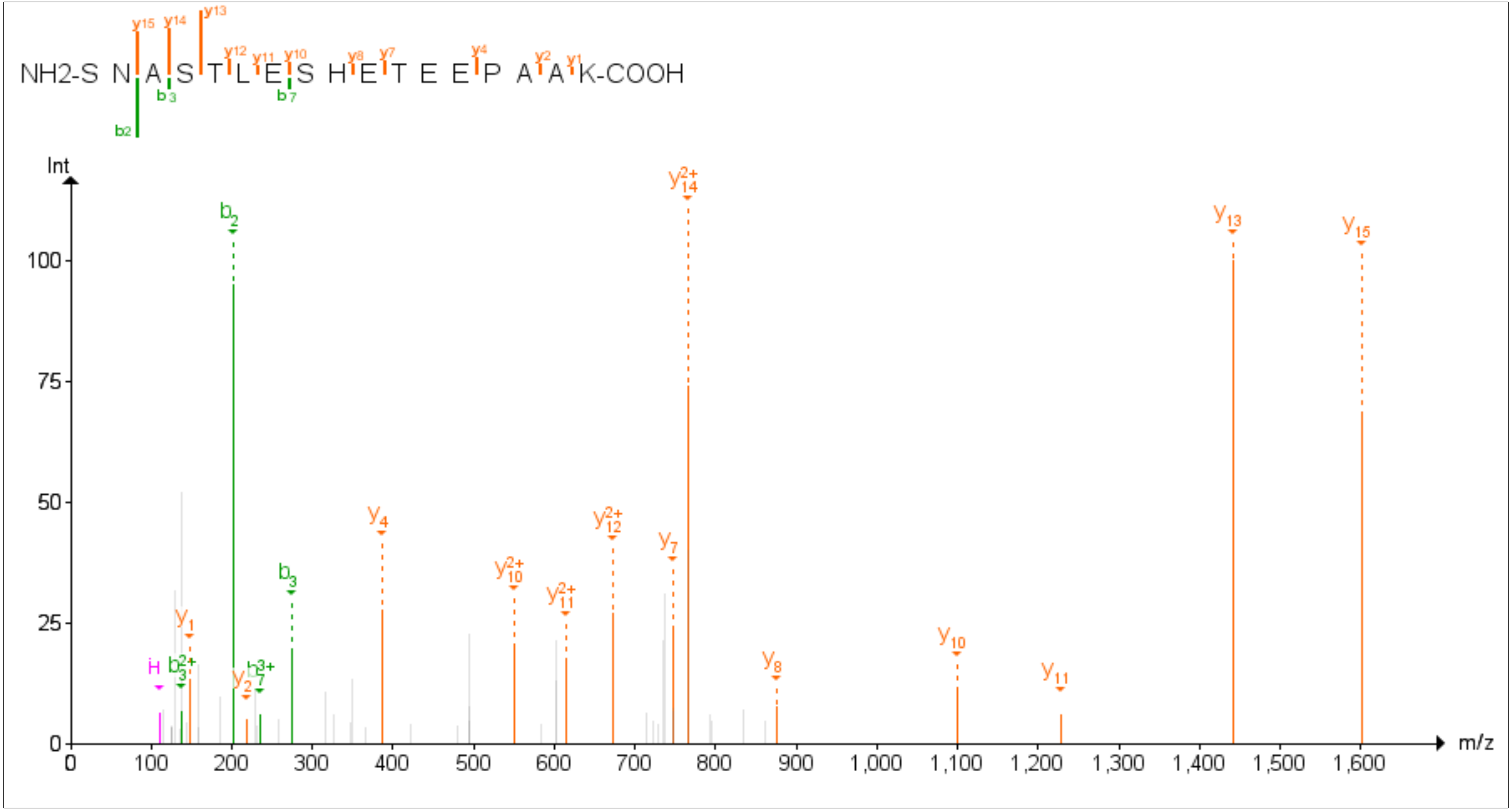
NH2-S N A S T L E S H E T E E P A A K-COOH
y15 y14 y13 y12 y11 y10 y8 y7 y4 y2 y1
b2 b3 b7
Int
m/z
0
25
50
75
100
0 100 200 300 400 500 600 700 800 900 1,000 1,100 1,200 1,300 1,400 1,500 1,600
b2
b3
y4
y14 2+
y13
y15
y12 2+
y7
y10 2+
y11 2+
y1
y2
y8
y10
y11
H

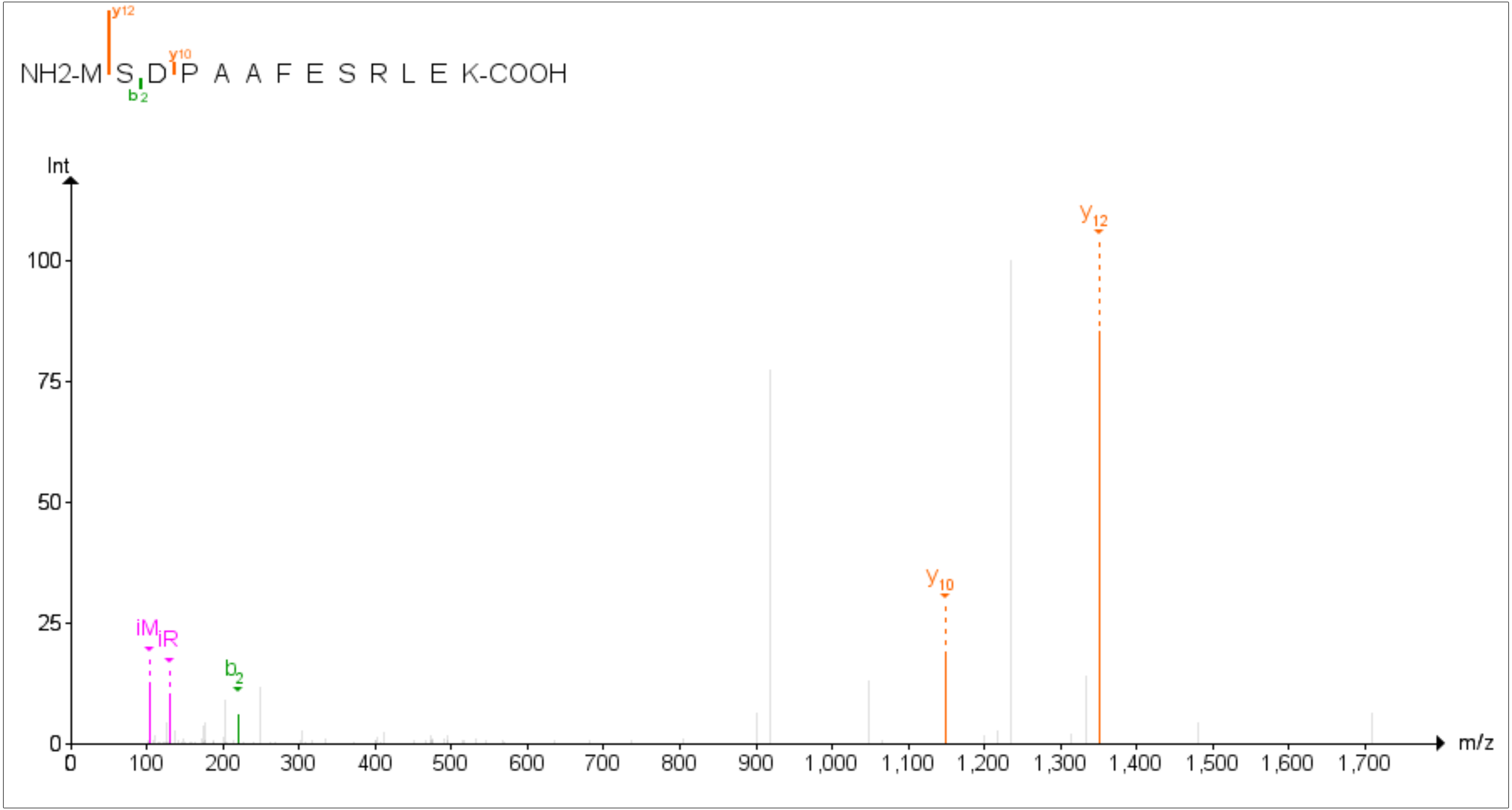

NH2-M S D P A A F E S R L E K-COOH
y12
y10
b2
Int
100
75
50
25
0
y12
y10
iM
iR
b2
0
100
200
300
400
500
600
700
800
900
1,000
1,100
1,200
1,300
1,400
1,500
1,600
1,700
m/z

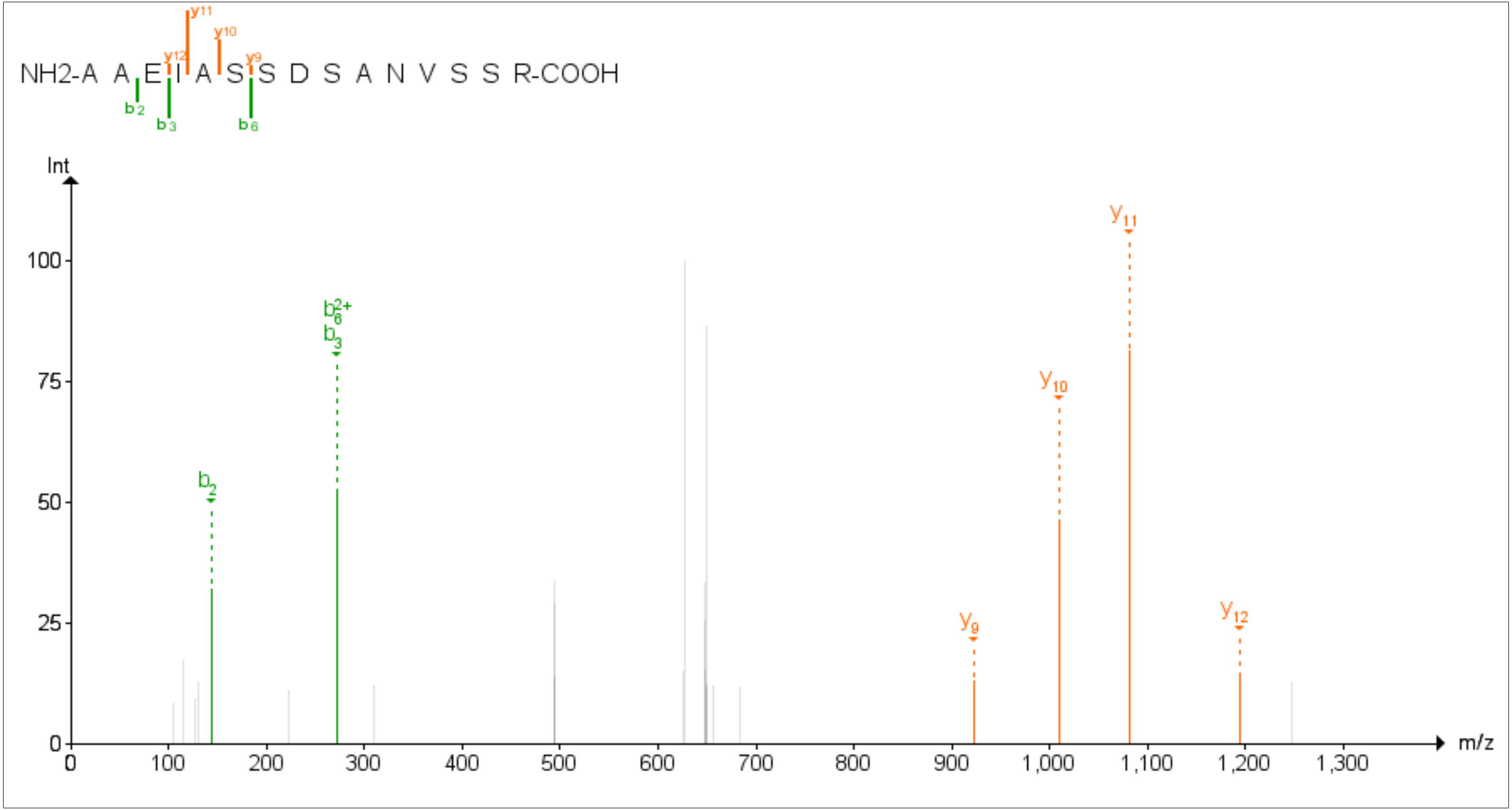

NH2-A A E I A S S D S A N V S S R-COOH
y11
y10
y12
y9
b2
b3
b6
Int
100
75
50
25
0
b_2
b_6^{2+}
b_3
y_{11}
y_{10}
y_9
y_{12}
0
100
200
300
400
500
600
700
800
900
1,000
1,100
1,200
1,300
m/z

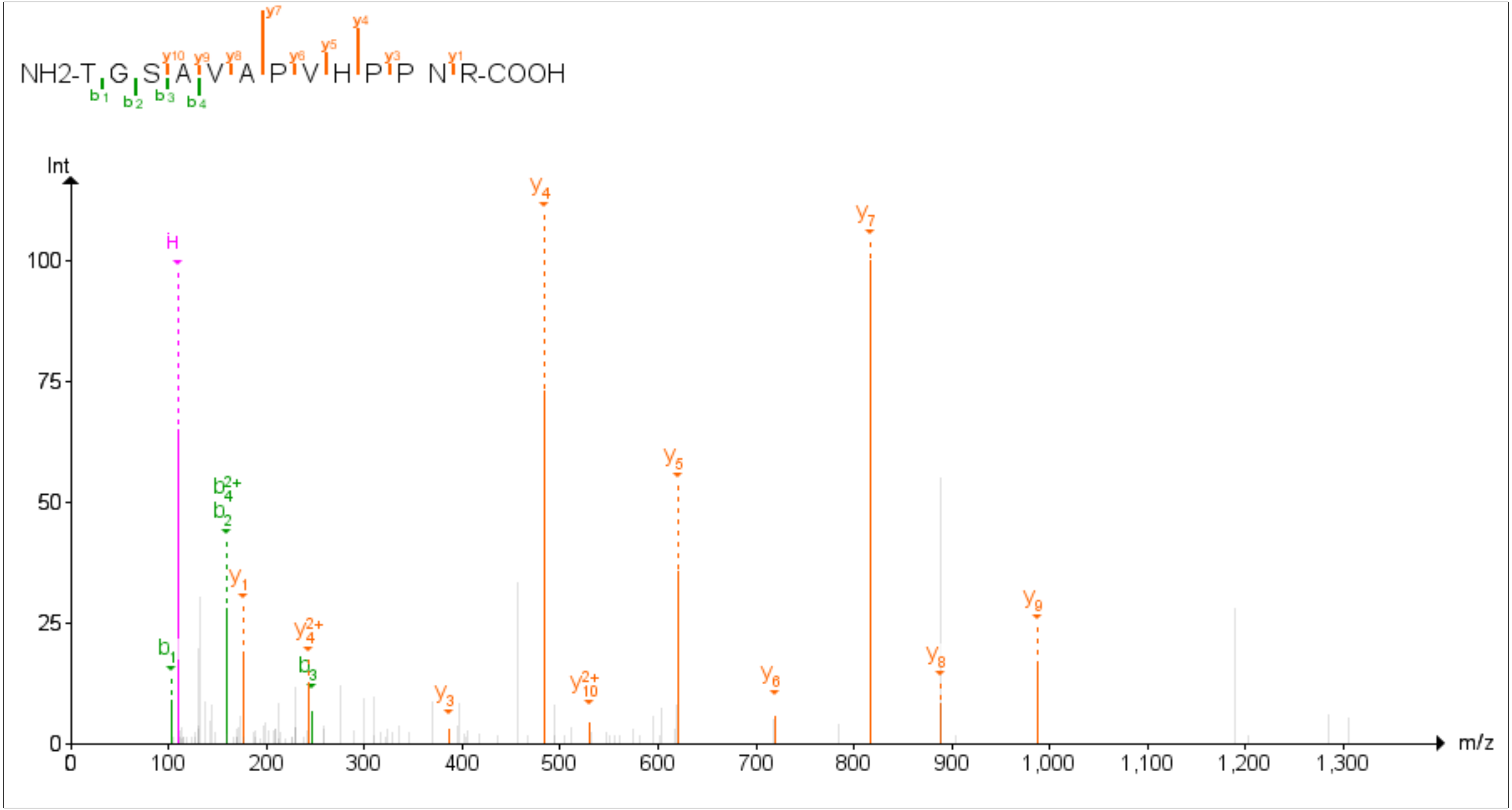

NH2-T G S A V A P V H P P N R-COOH
b1 b2 b3 b4
y10 y9 y8 y7 y6 y5 y4 y3 y1
Int
100
75
50
25
0
m/z
0 100 200 300 400 500 600 700 800 900 1,000 1,100 1,200 1,300
H
y4
y7
y5
b4 2+
b2
y1
b1
y4 2+
b3
y3
y10 2+
y6
y8
y9

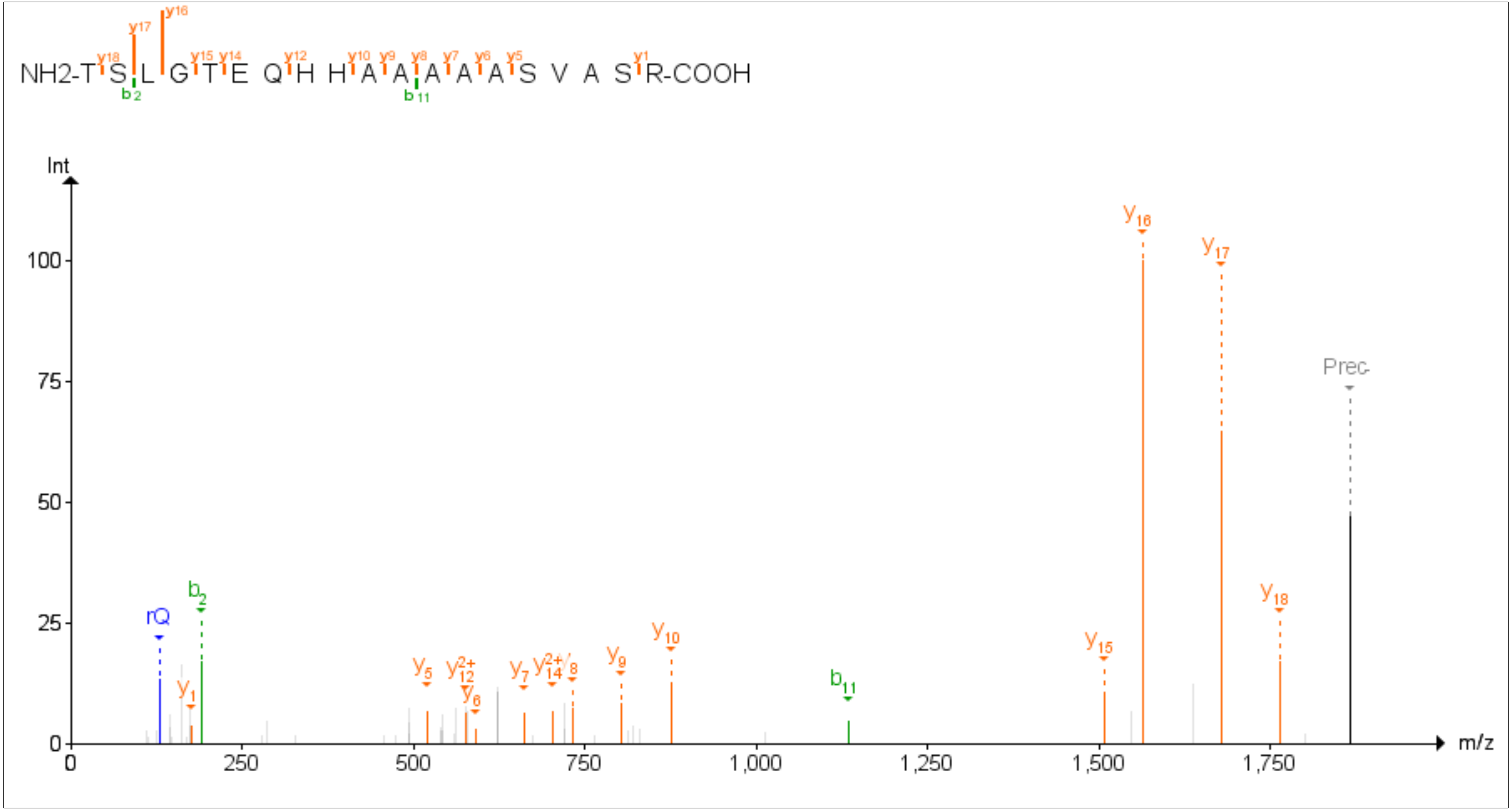

NH2-T S L G T E Q H H A A A A A S V A S R-COOH
y18 y17 y16 y15 y14 y12 y10 y9 y8 y7 y6 y5 y1
b2 b11
Int
100
75
50
25
0
0 250 500 750 1,000 1,250 1,500 1,750
m/z
rQ
b2
y1
y5
y12 2+
y6
y7
y14 2+
y8
y9
y10
b11
y15
y16
y17
y18
Prec.

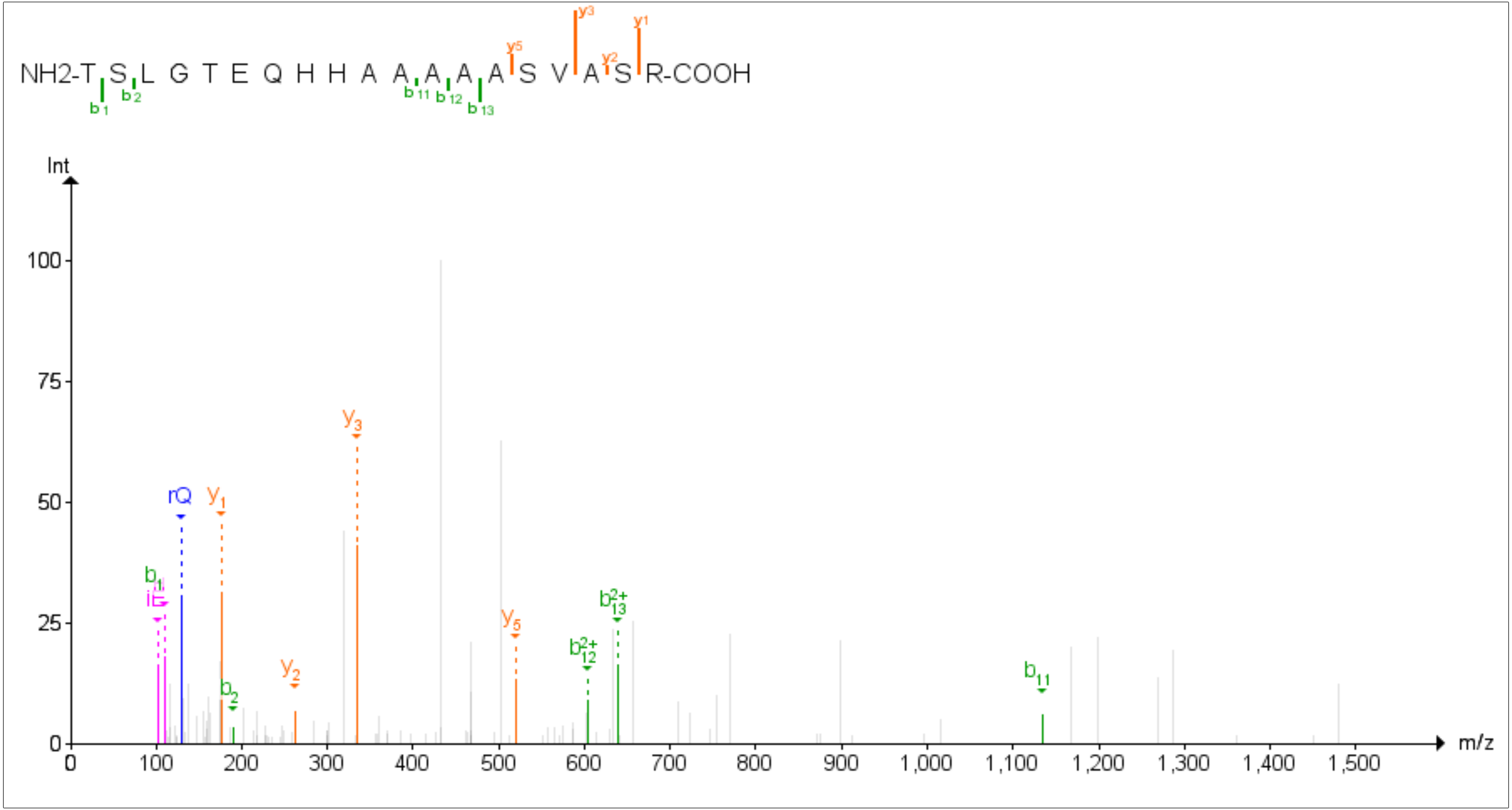

NH2-T S L G T E Q H H A A A A A S V A S R-COOH
b1 b2 b11 b12 b13
y5 y3 y2 y1
Int
100
75
50
25
0
rQ
y1
y3
b1
y2
b2
y5
b12 2+
b13 2+
b11
0 100 200 300 400 500 600 700 800 900 1,000 1,100 1,200 1,300 1,400 1,500
m/z

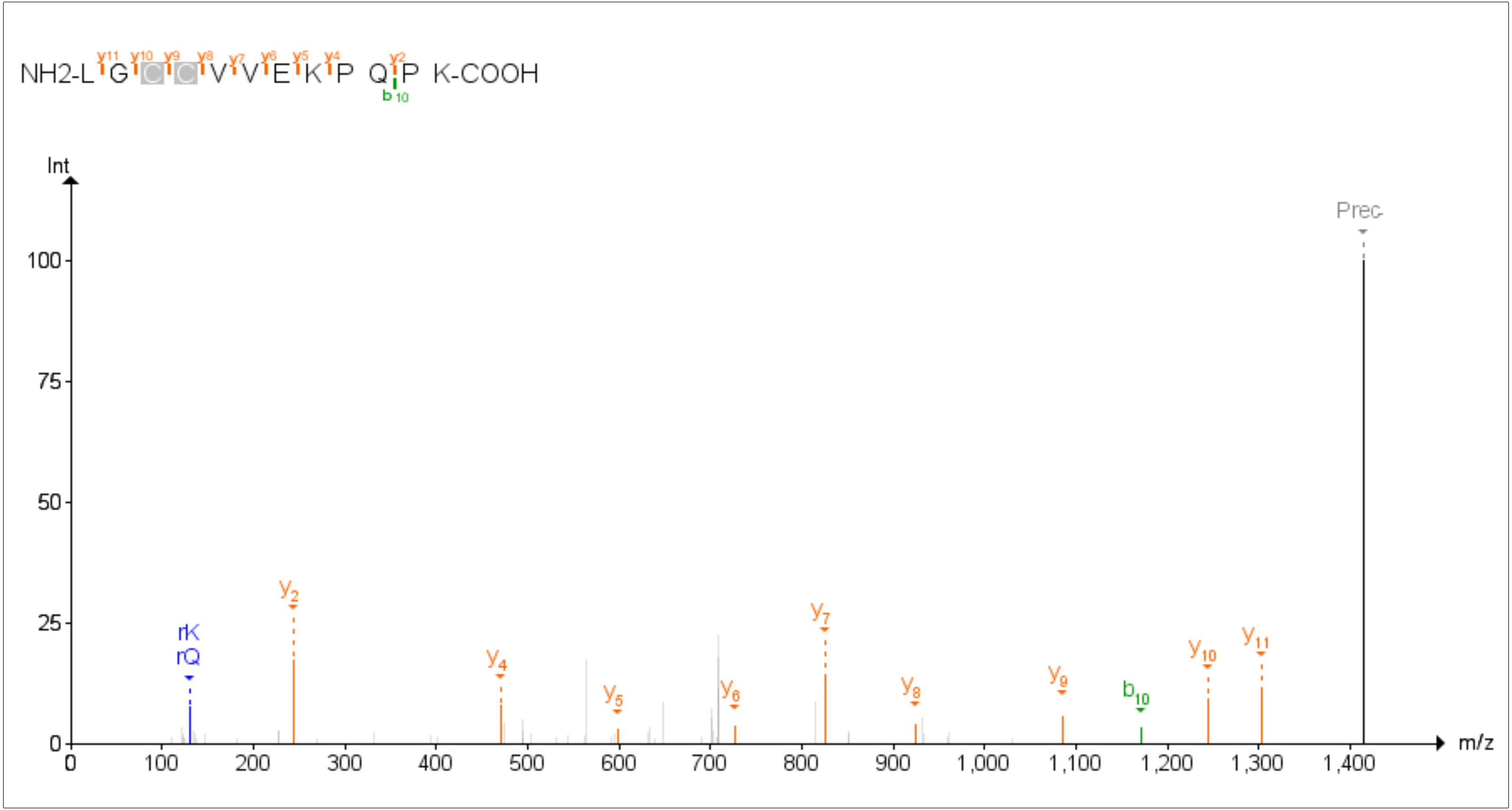

NH2-L G C C V V E K P Q P K-COOH
y11 y10 y9 y8 y7 y6 y5 y4 y2
b10
Int
100
75
50
25
0
Prec.
rK
rQ
y2
y4
y5
y6
y7
y8
y9
b10
y10
y11
0
100
200
300
400
500
600
700
800
900
1,000
1,100
1,200
1,300
1,400
m/z

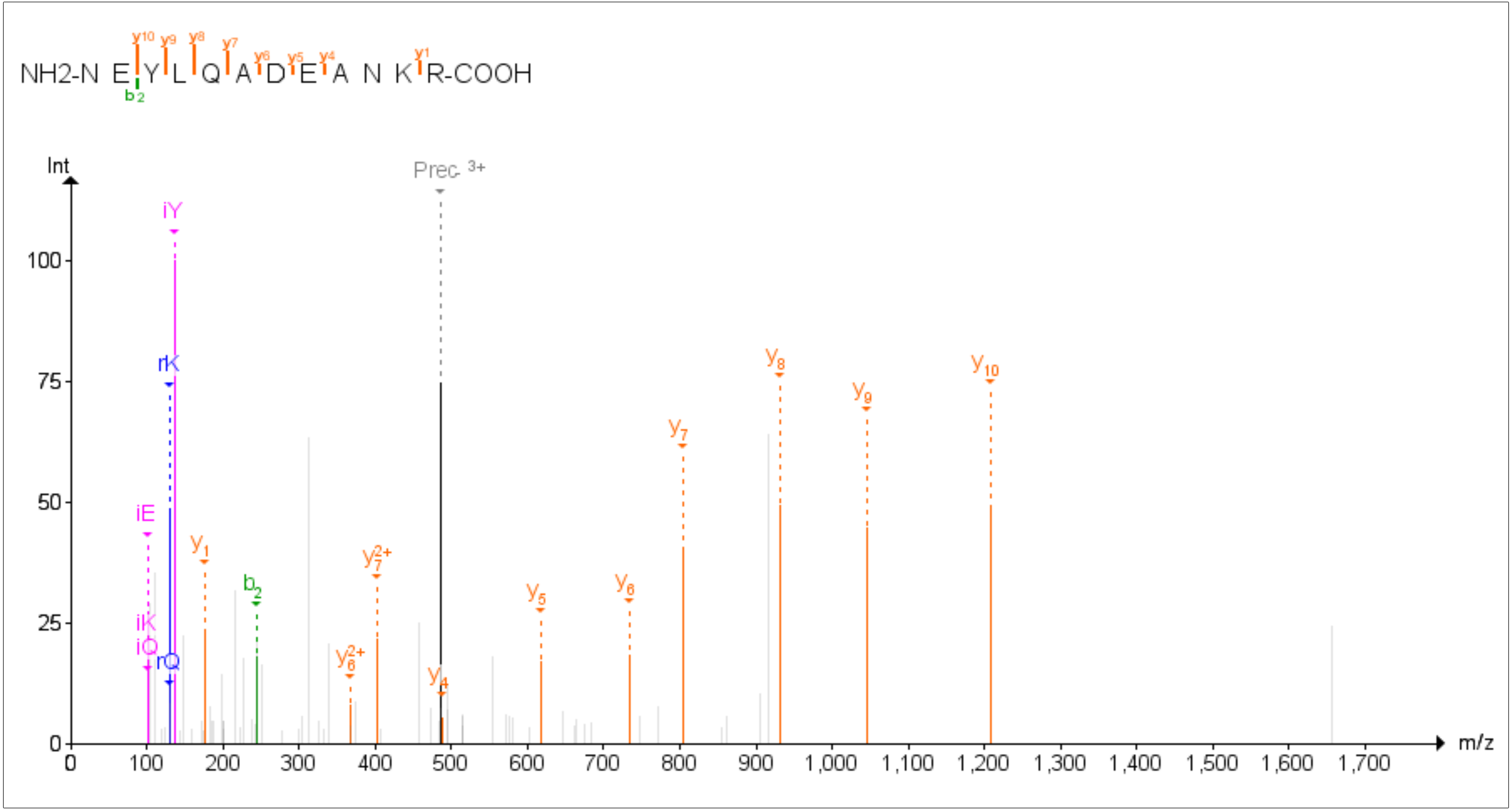

NH2-N E Y L Q A D E A N K R-COOH
y10 y9 y8 y7 y6 y5 y4 y1
b2
Int
Prec 3+
iY
rK
iE
iK
iQ
rQ
y1
b2
y7 2+
y6 2+
y4
y5
y6
y7
y8
y9
y10
m/z
0
25
50
75
100
0 100 200 300 400 500 600 700 800 900 1,000 1,100 1,200 1,300 1,400 1,500 1,600 1,700

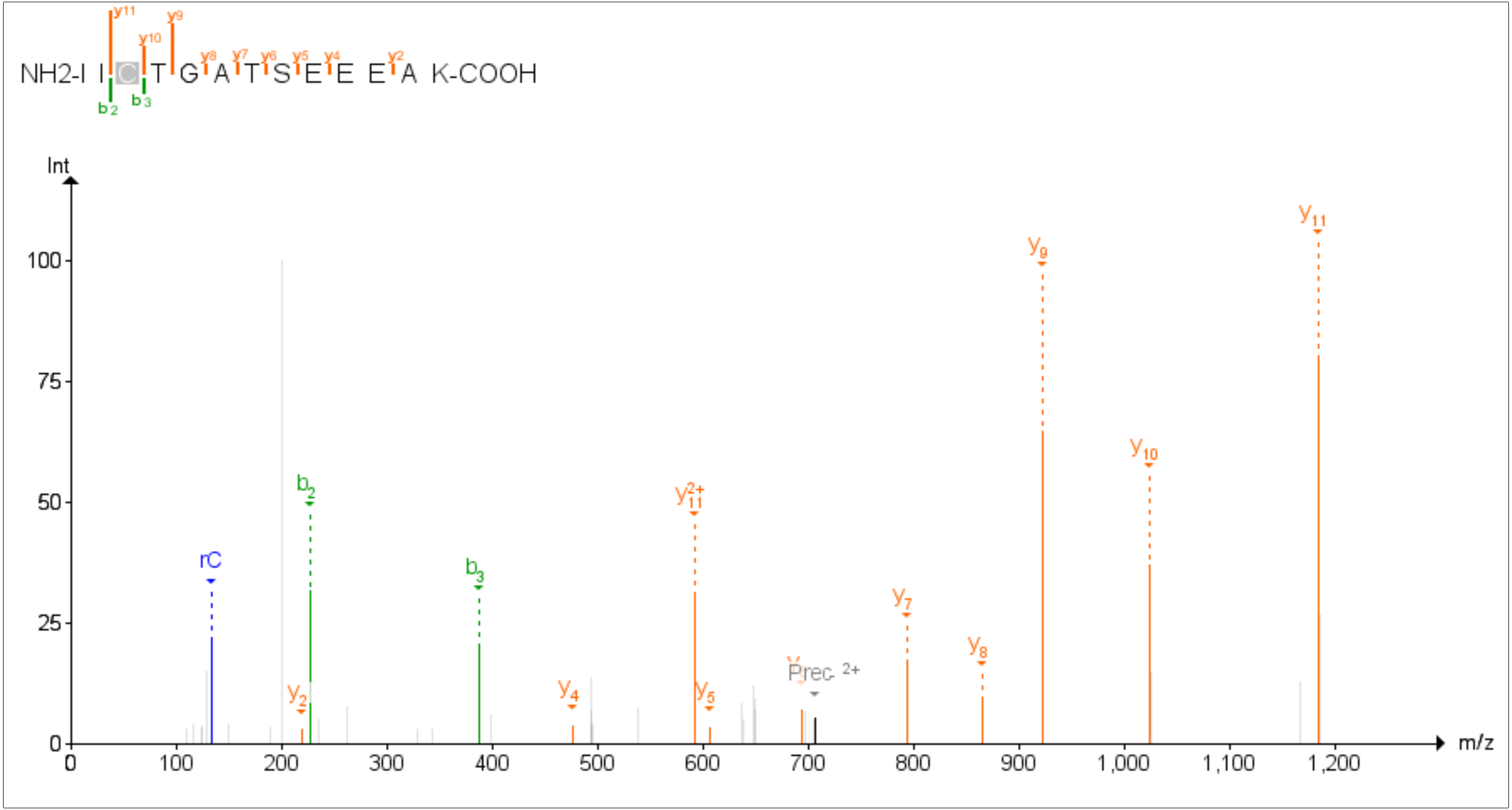

NH2-I I C T G A T S E E E A K-COOH
y11
y10
y9
y8
y7
y6
y5
y4
y2
b 2
b 3
Int
100
75
50
25
0
rC
b2
y2
b3
y4
y11 2+
y5
Prec- 2+
y7
y8
y9
y10
y11
0
100
200
300
400
500
600
700
800
900
1,000
1,100
1,200
m/z

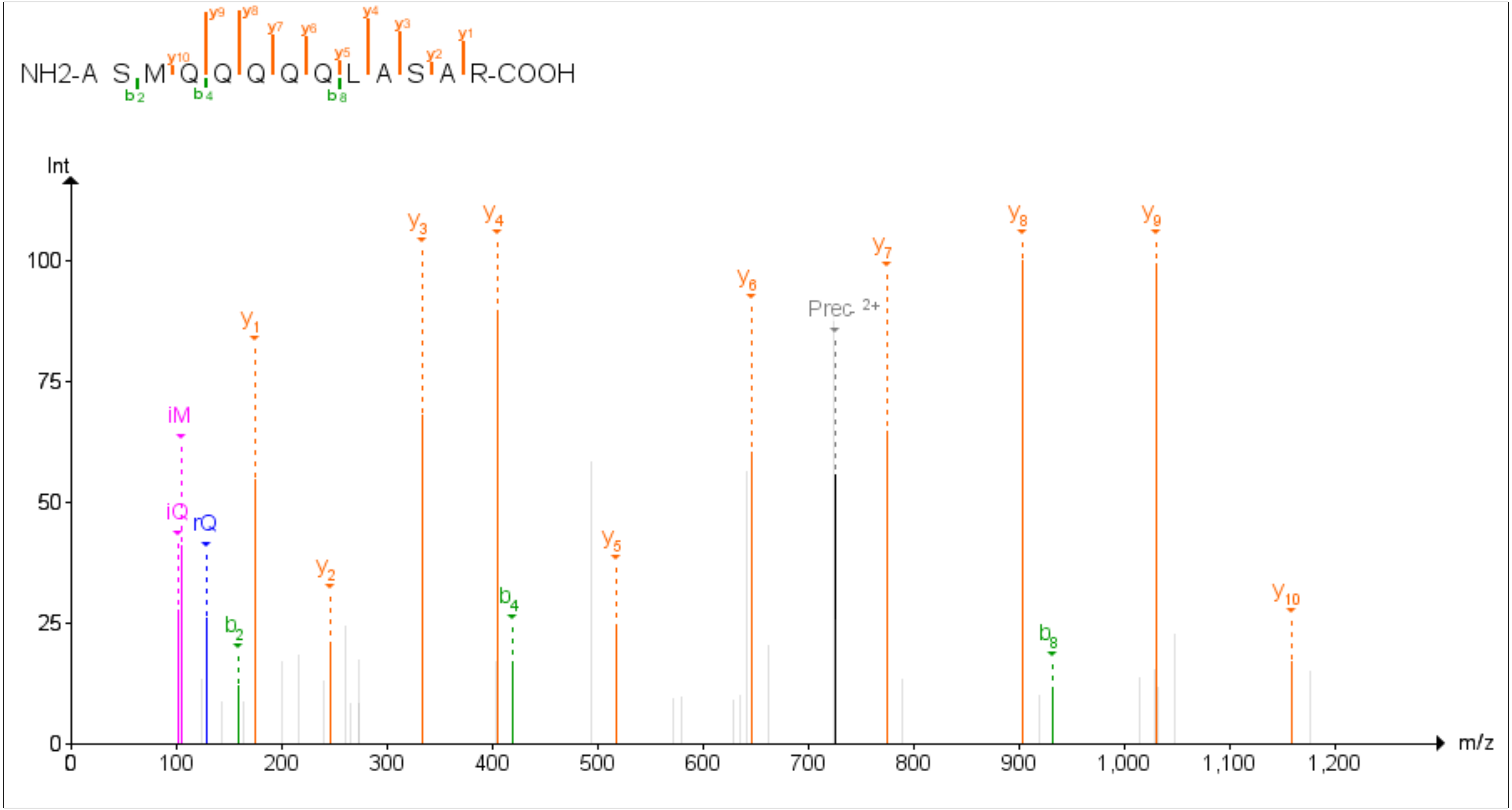

NH2-A S M Q Q Q Q Q L A S A R-COOH
y10 y9 y8 y7 y6 y5 y4 y3 y2 y1
b2 b4 b8
Int
100
75
50
25
0
y3
y4
y8
y9
y7
y6
Prec. 2+
y1
iM
iQ
rQ
y5
y2
b4
b2
y10
b8
0
100
200
300
400
500
600
700
800
900
1,000
1,100
1,200
m/z

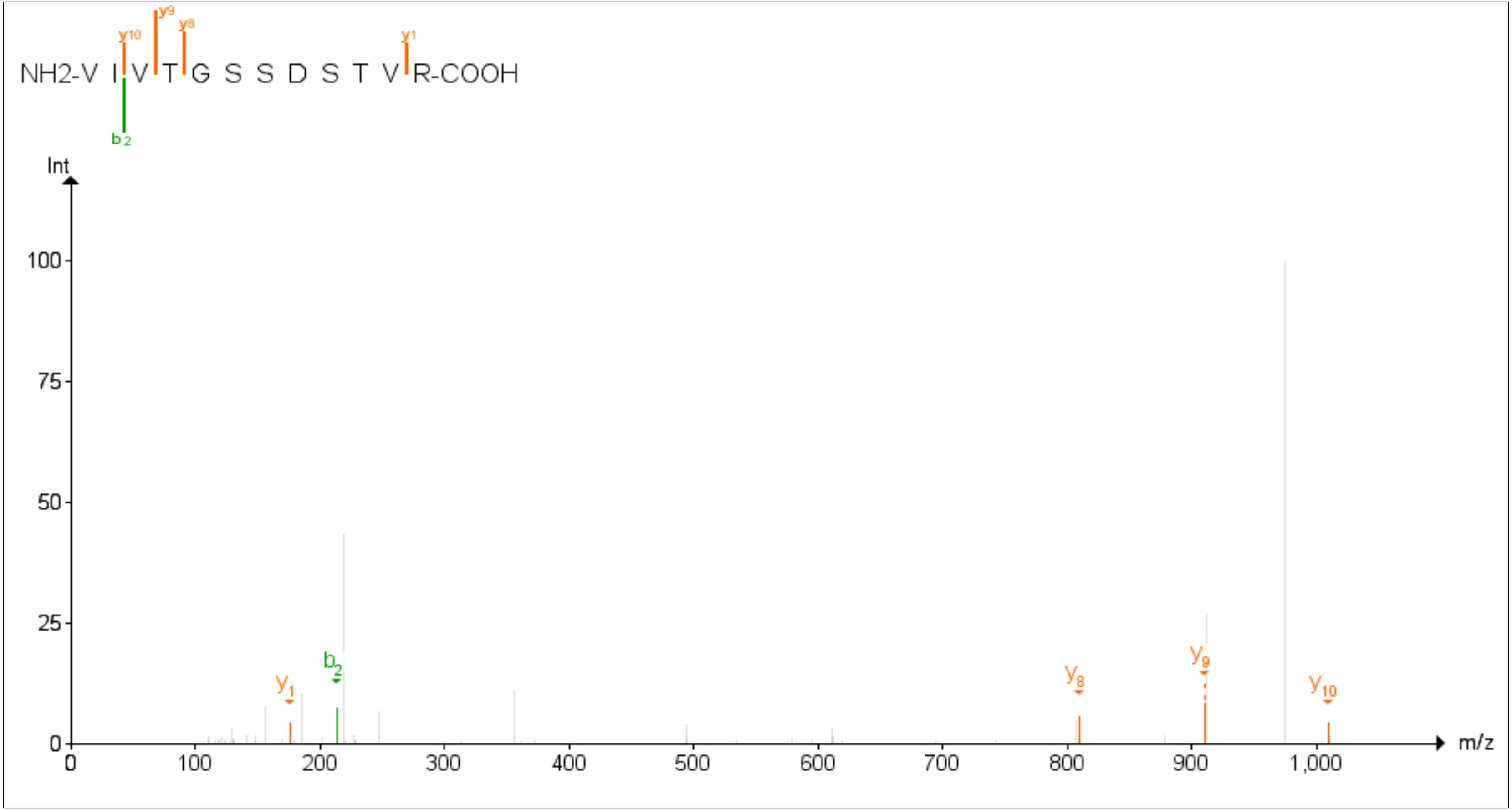
NH2-V I V T G S S D S T V R-COOH
y10
y9
y8
y1
b2
Int
100
75
50
25
0
y1
b2
y8
y9
y10
0
100
200
300
400
500
600
700
800
900
1,000
m/z

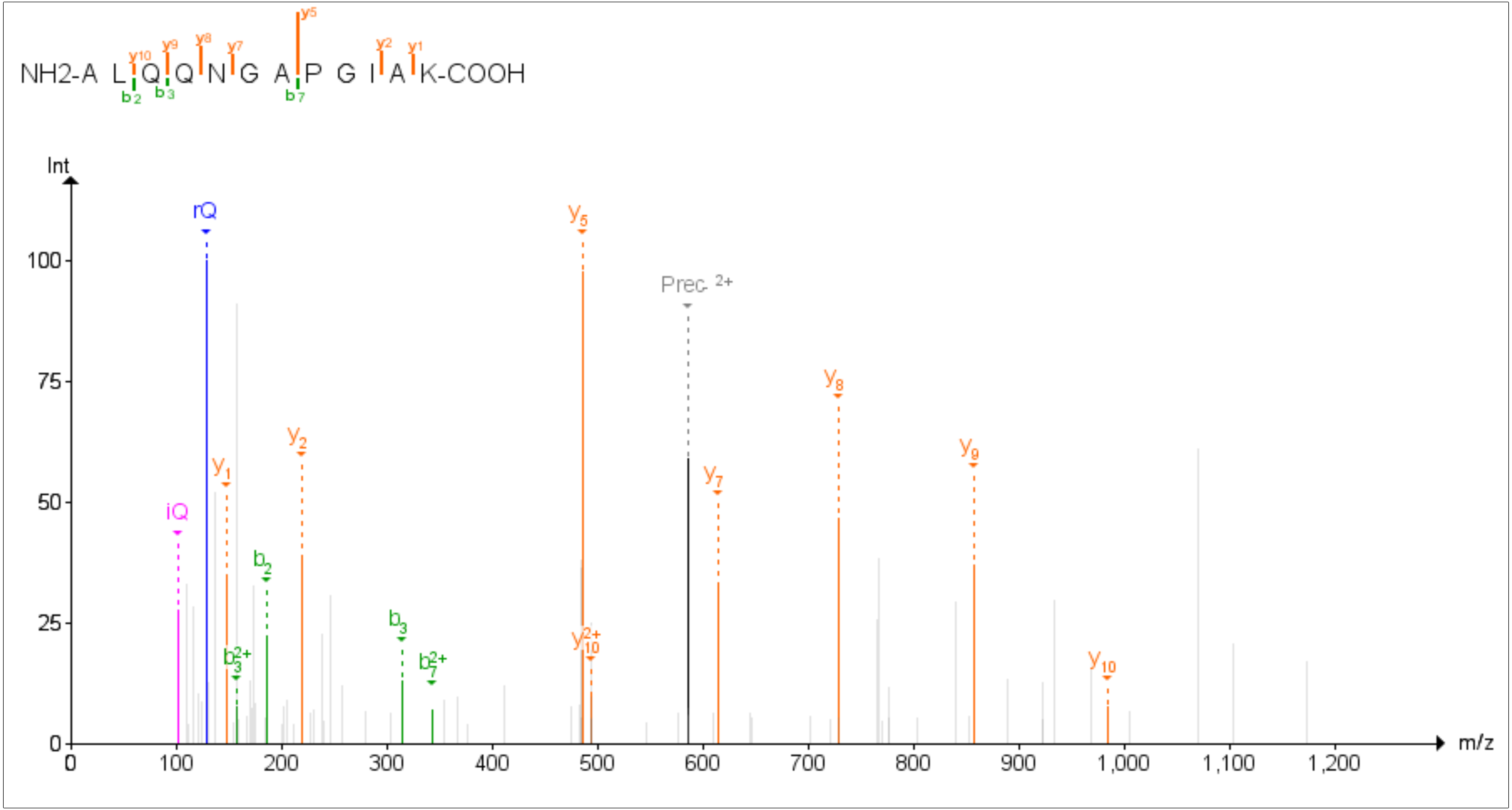

NH2-A L Q Q N G A P G I A K-COOH
y10 y9 y8 y7 y5 y2 y1
b2 b3 b7
Int
m/z
rQ
iQ
y1
y2
b2
b3
b3 2+
b7 2+
y5
y10 2+
Prec 2+
y7
y8
y9
y10
0
25
50
75
100
0
100
200
300
400
500
600
700
800
900
1,000
1,100
1,200

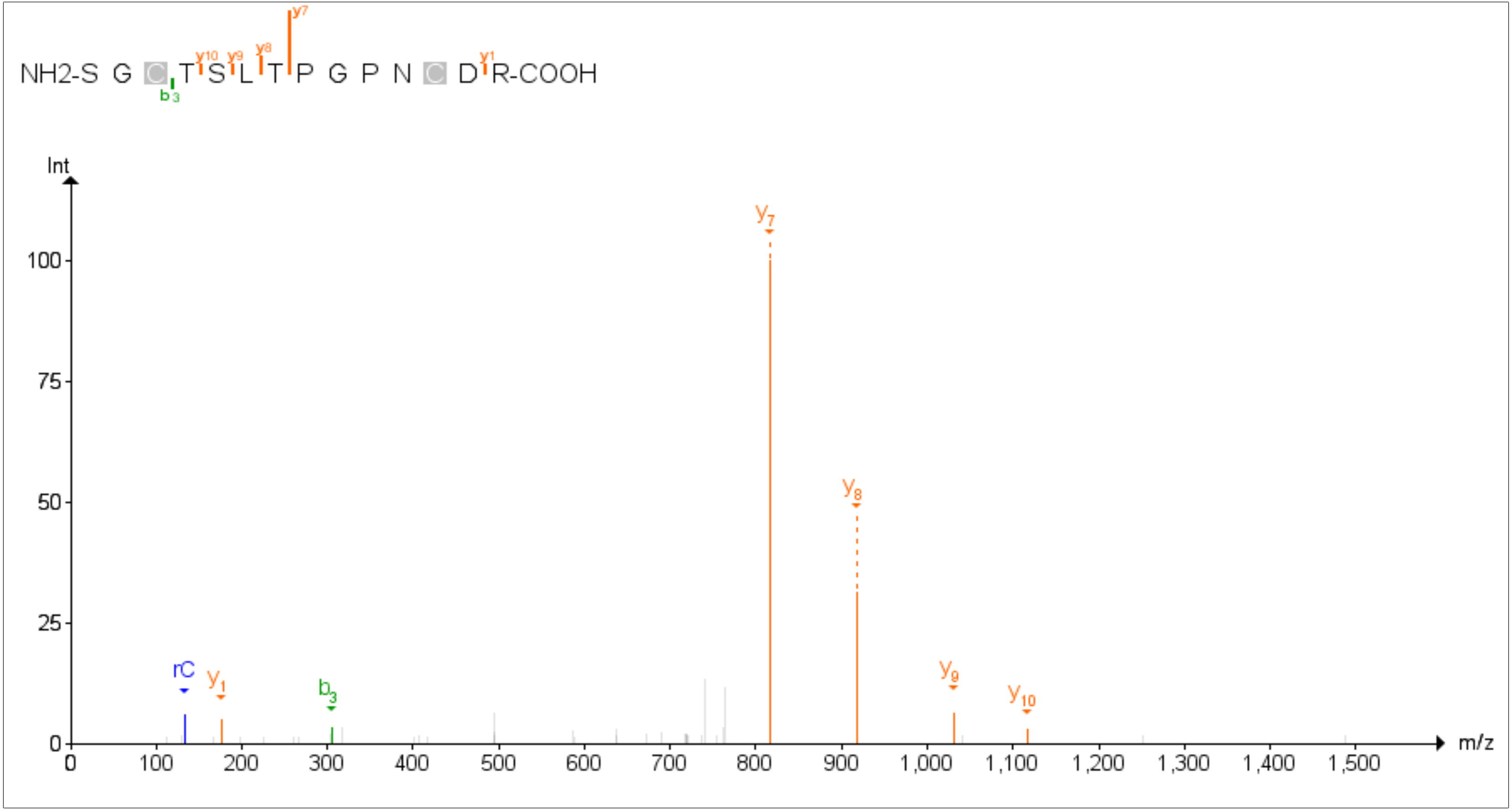

NH2-S G C T S L T P G P N C D R-COOH
y7
y10
y9
y8
y1
b3
Int
100
75
50
25
0
y7
y8
rC
y1
b3
y9
y10
0
100
200
300
400
500
600
700
800
900
1,000
1,100
1,200
1,300
1,400
1,500
m/z
