# Supplementary material for: Surface Proteomics of Cancer-Derived Extracellular Vesicles Reveals Universal Heat Shock Proteins and Cancer Signatures
Source: Mol Cell Proteomics. 2026 Aug 22;25(10):101649. doi: 10.1016/j.mcpro.2026.101649 (PMC13634791; doi:10.1016/j.mcpro.2026.101649)
Supplement: Supplementary file 3 [file mmc4.pdf]

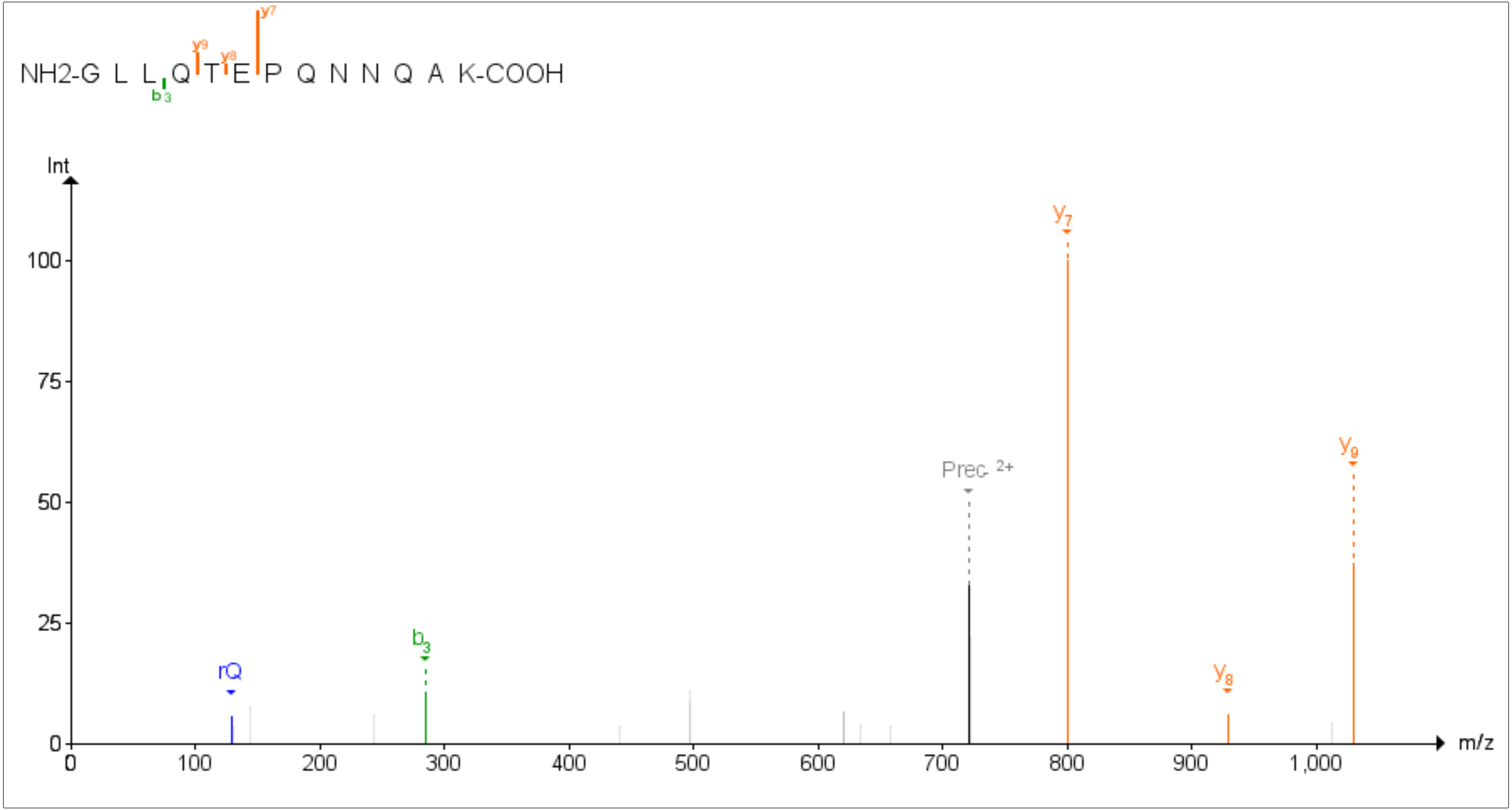

NH2-G L L Q T E P Q N N Q A K-COOH
y9
y8
y7
b3
Int
100
75
50
25
0
y7
Prec 2+
y9
y8
b3
rQ
0
100
200
300
400
500
600
700
800
900
1,000
m/z

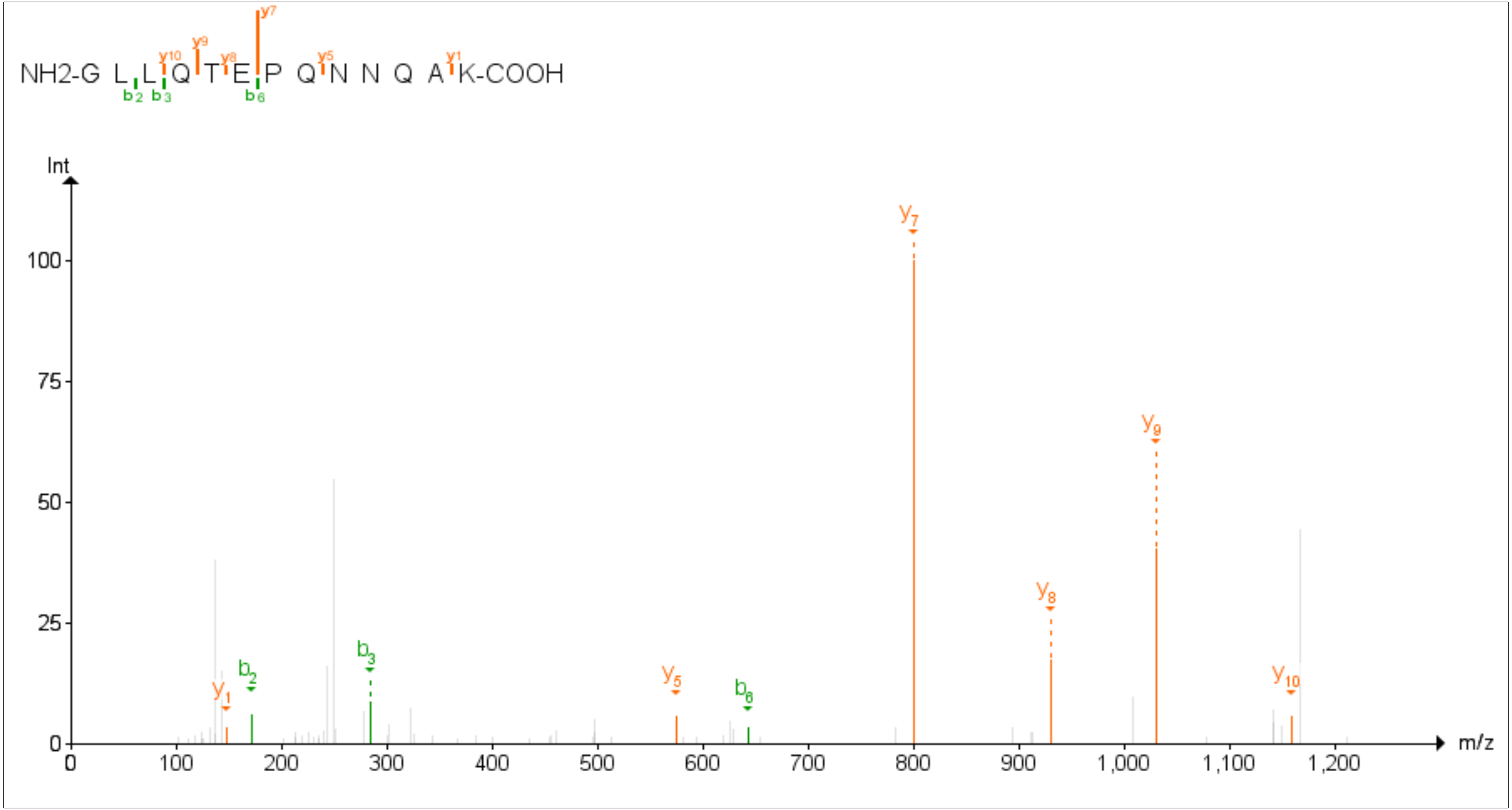
NH2-G L L Q T E P Q N N Q A K-COOH
y7
y9
y10
y8
y5
y1
b2
b3
b6
Int
100
75
50
25
0
m/z
0
100
200
300
400
500
600
700
800
900
1,000
1,100
1,200
y7
y9
y8
y10
y5
b6
b3
b2
y1

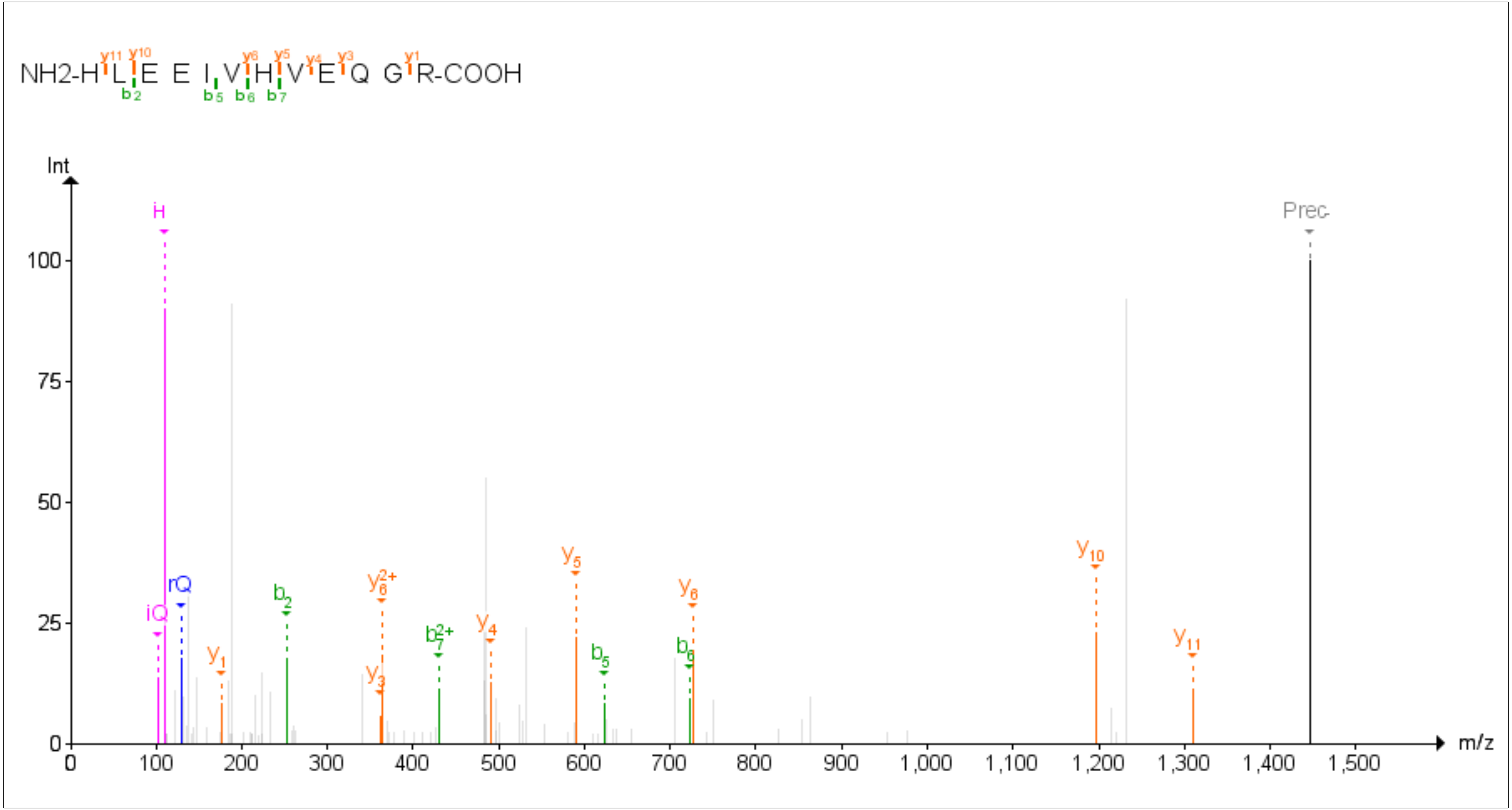

NH2-H L E E I V H V E Q G R-COOH
y11 y10 y6 y5 y4 y3 y1
b2 b5 b6 b7
Int
100
75
50
25
0
H
Prec.
rQ
iQ
y1
b2
y6 2+
y3
b7 2+
y4
y5
b5
y6
b6
y10
y11
0
100
200
300
400
500
600
700
800
900
1,000
1,100
1,200
1,300
1,400
1,500
m/z

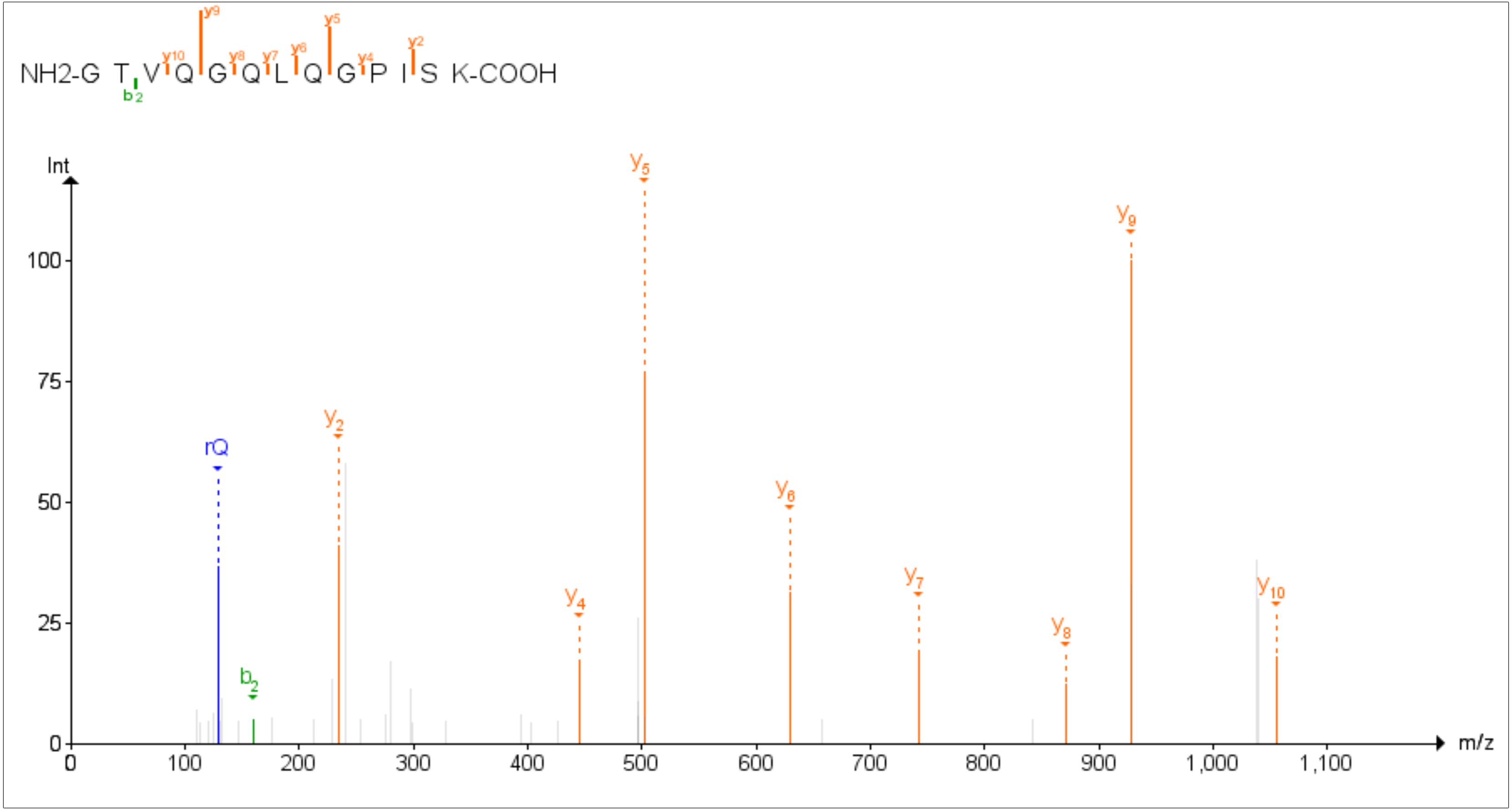

NH2-G T V Q G Q L Q G P I S K-COOH
y9 y10 y8 y7 y6 y5 y4 y2 b2
Int
100
75
50
25
0
y5
y9
y2
rQ
y6
y7
y4
y8
y10
b2
0 100 200 300 400 500 600 700 800 900 1,000 1,100
m/z

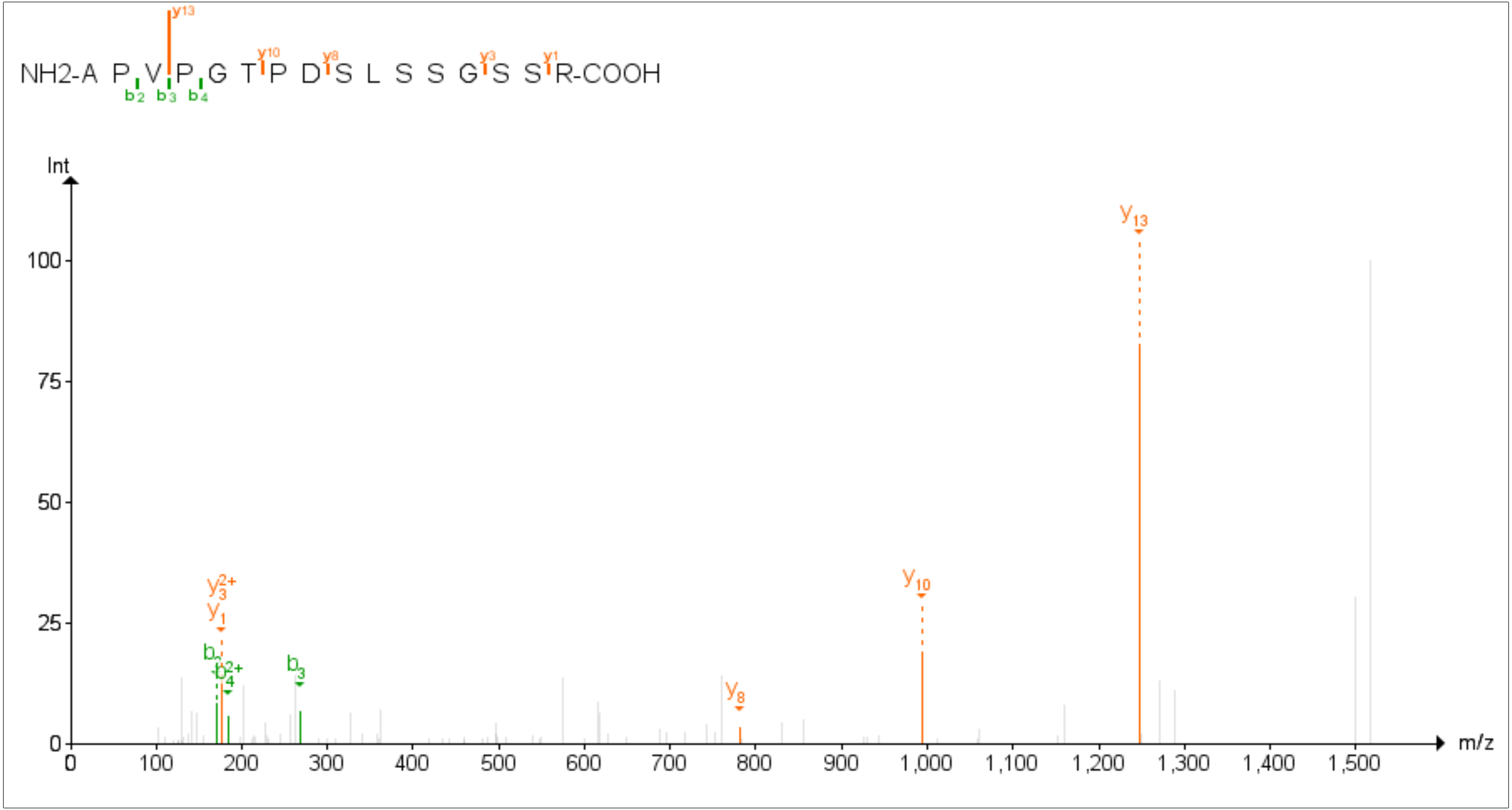

NH2-A P V P G T P D S L S S G S S R-COOH
y13
y10
y8
y3
y1
b2
b3
b4
Int
100
75
50
25
0
m/z
0
100
200
300
400
500
600
700
800
900
1,000
1,100
1,200
1,300
1,400
1,500
y13
y10
y8
y3 2+
y1
b3
b4 2+

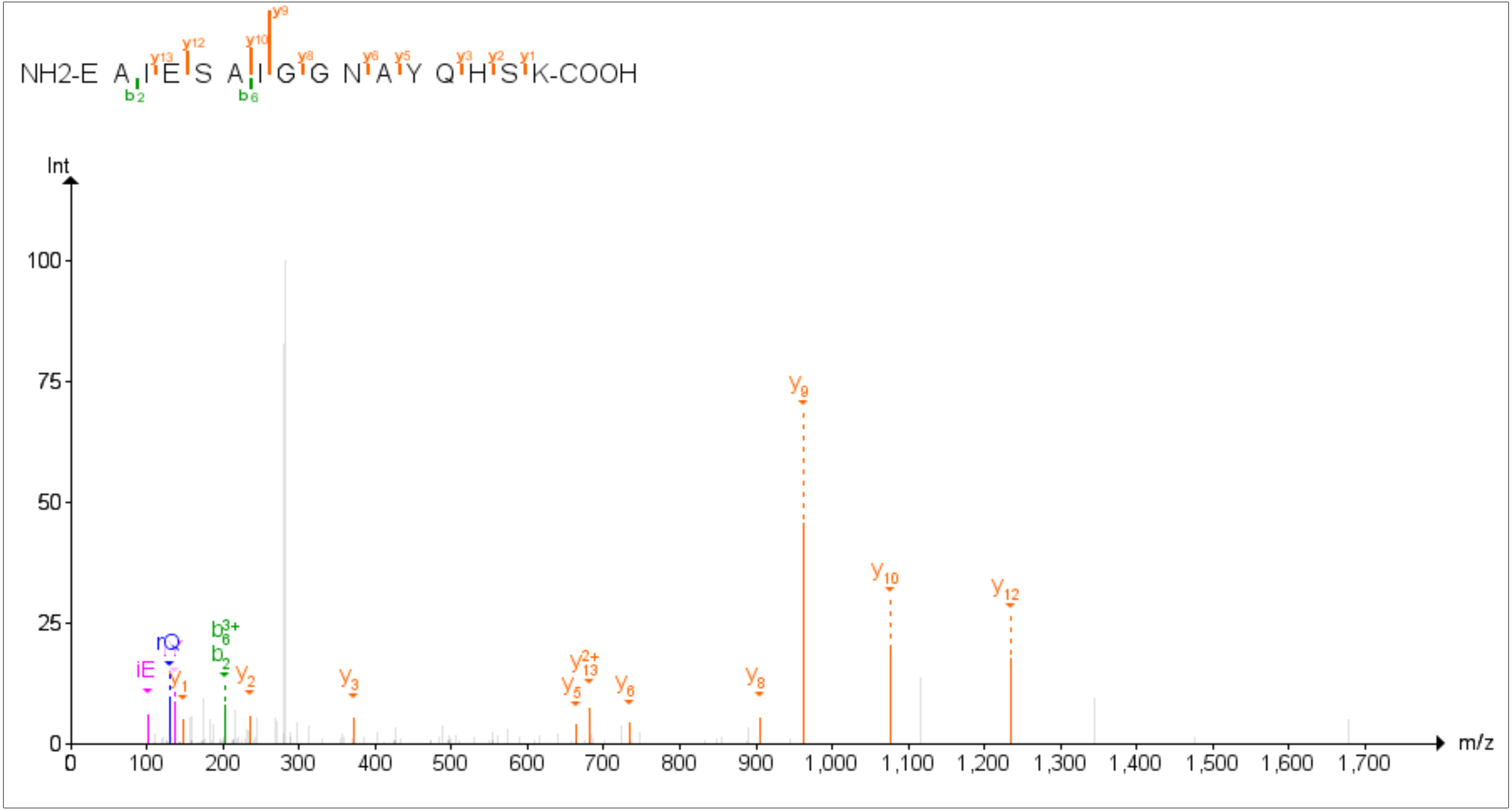

NH2-E A I E S A I G G N A Y Q H S K-COOH
y13 y12 y10 y9 y8 y6 y5 y3 y2 y1
b2 b6
Int
m/z
y9
y10
y12
iE
rQ
y1
b6 3+
b2
y2
y3
y13 2+
y5
y6
y8

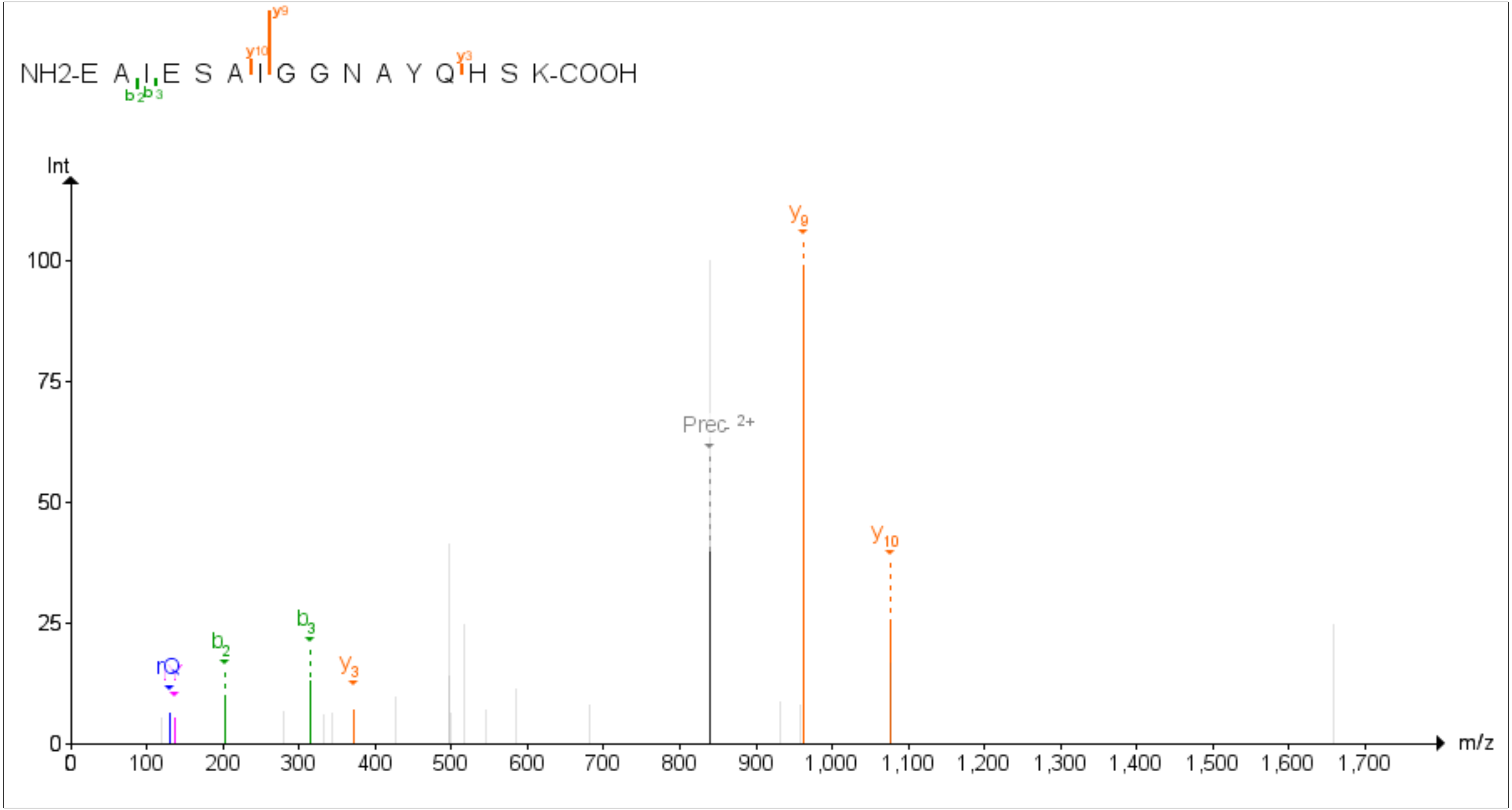

NH2-E A I E S A I G G N A Y Q H S K-COOH
b2 b3
y10 y9 y3
Int
100
75
50
25
0
Prec 2+
y9
y10
b2
b3
y3
m/z
0
100
200
300
400
500
600
700
800
900
1,000
1,100
1,200
1,300
1,400
1,500
1,600
1,700

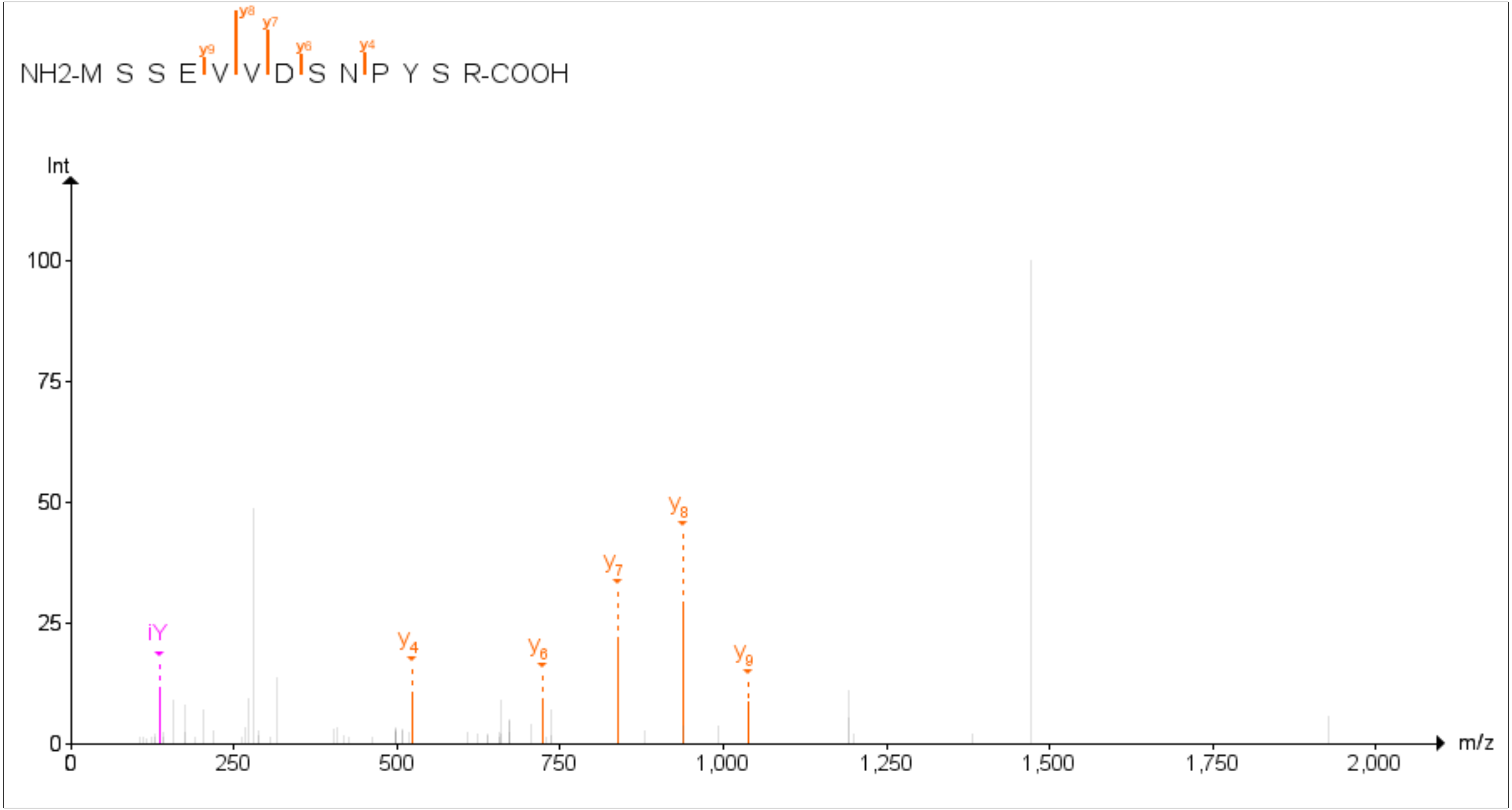

NH2-M S S E V V D S N P Y S R-COOH
y9 y8 y7 y6 y4
Int
100
75
50
25
0
iY
y4
y6
y7
y8
y9
0
250
500
750
1,000
1,250
1,500
1,750
2,000
m/z

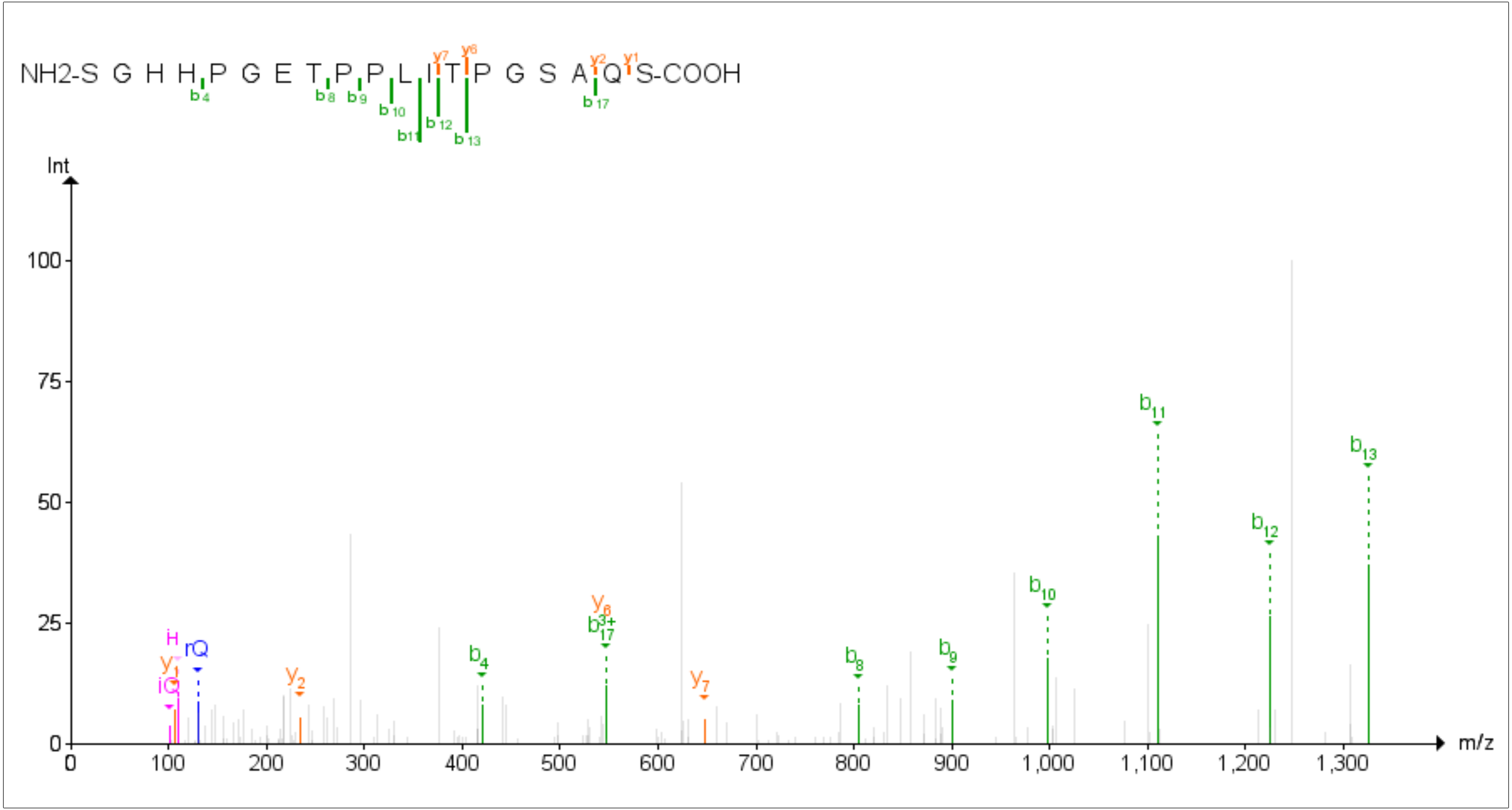
NH2-S G H H P G E T P P L I T P G S A Q S-COOH
b4
b8
b9
b10
b11
b12
b13
b17
y7
y6
y2
y1
Int
100
75
50
25
0
m/z
0
100
200
300
400
500
600
700
800
900
1,000
1,100
1,200
1,300
iH
rQ
y1
iQ
y2
b4
y6
b17 3+
y7
b8
b9
b10
b11
b12
b13

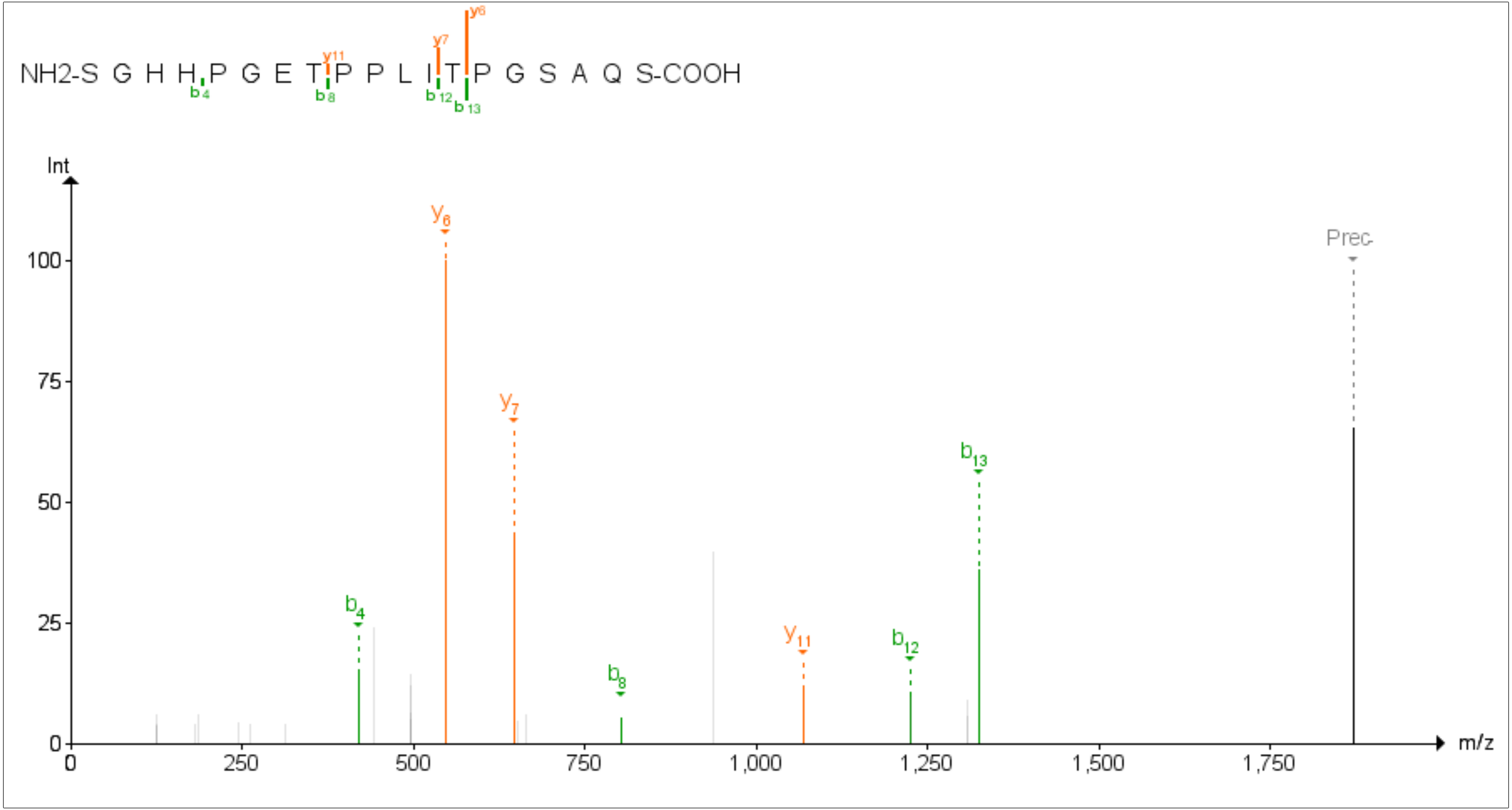

NH2-S G H H P G E T P P L I T P G S A Q S-COOH
b4
b8
b12
b13
y11
y7
y6
Int
m/z
Prec.
0
25
50
75
100
0
250
500
750
1,000
1,250
1,500
1,750

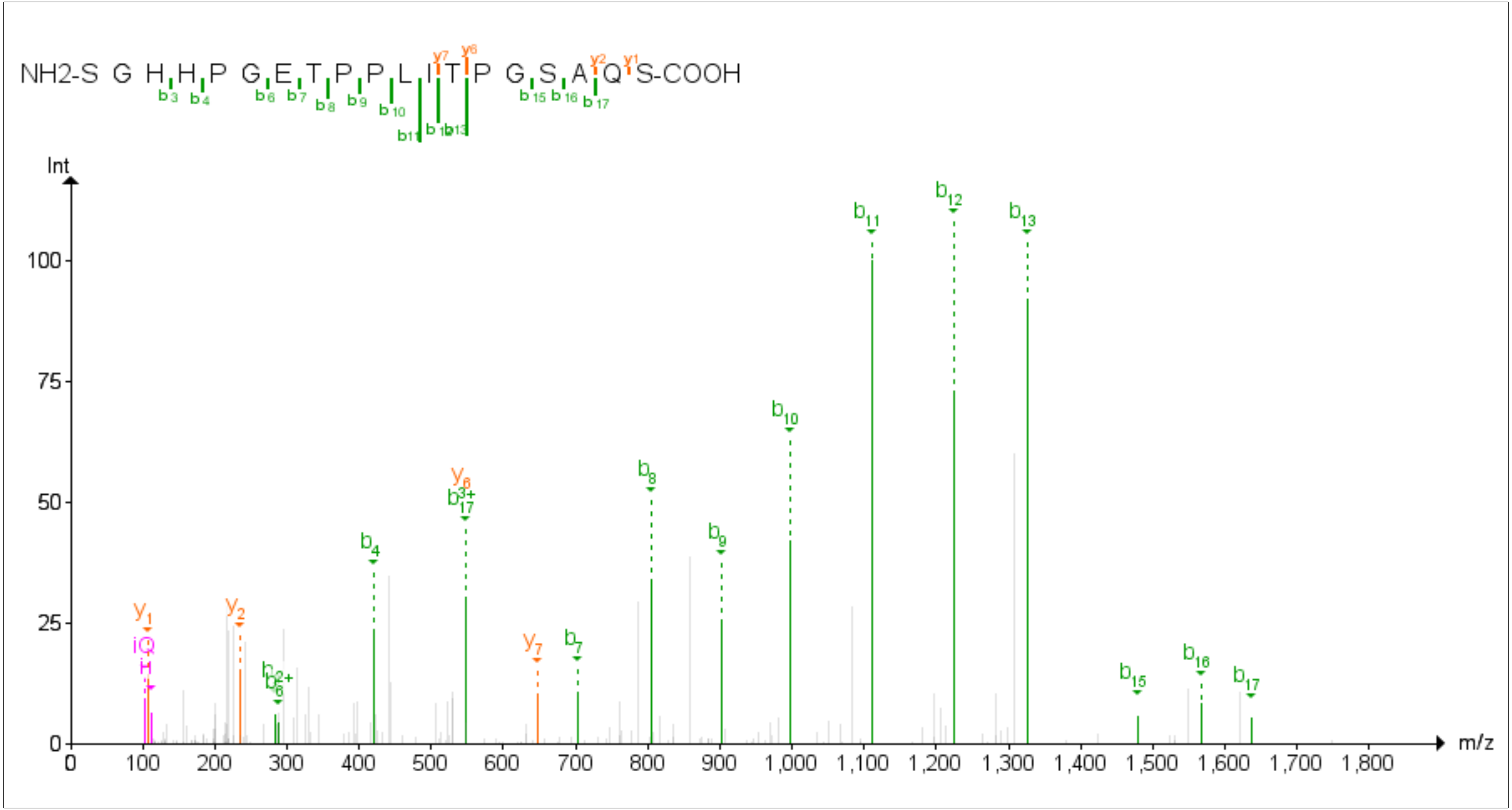

NH2-S G H H P G E T P P L I T P G S A Q S-COOH
Int
m/z
b3 b4 b6 b7 b8 b9 b10 b11 b12 b13 b15 b16 b17
y1 y2 y6 y7
iQ
H

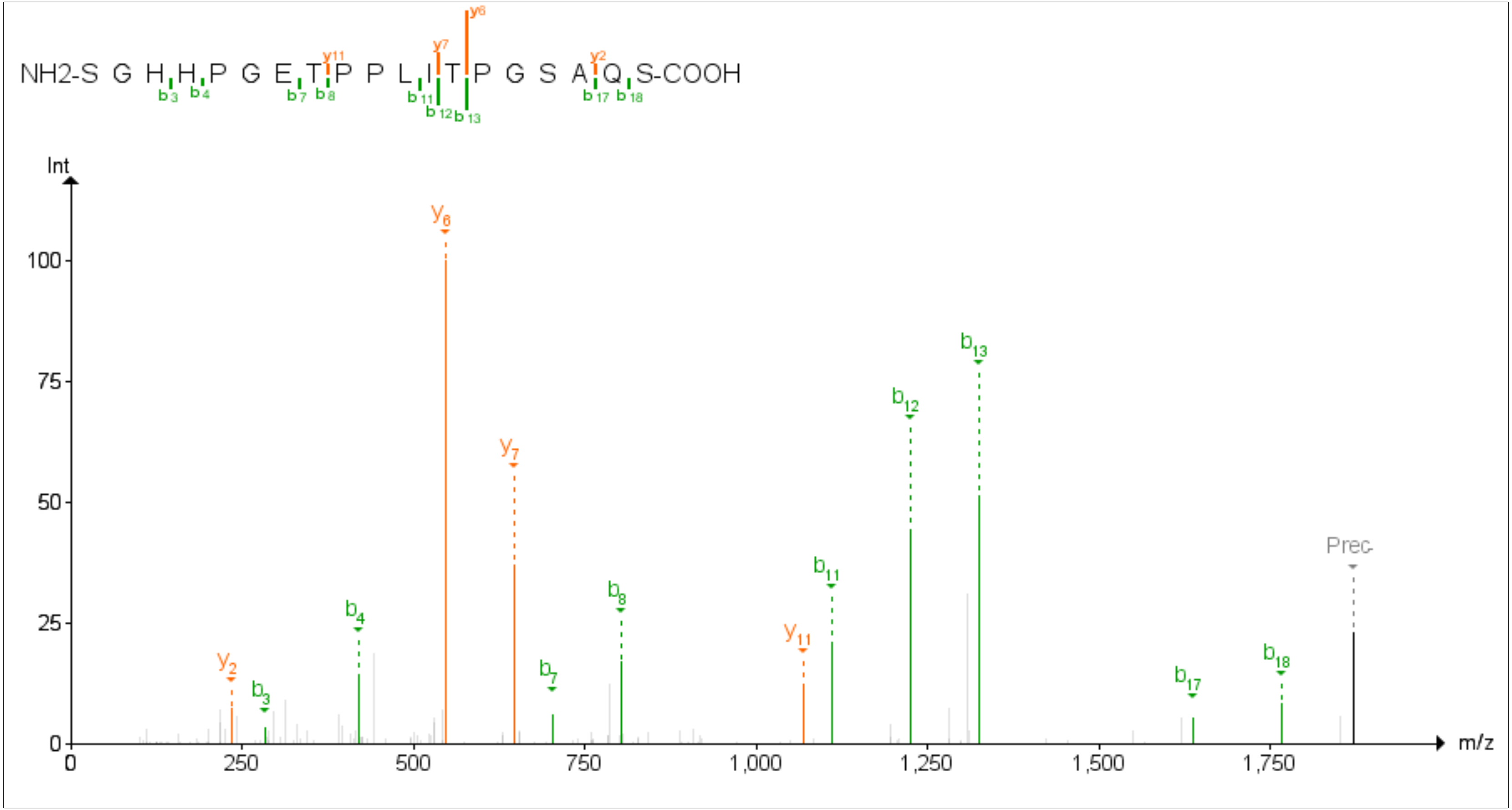

NH2-S G H H P G E T P P L I T P G S A Q S-COOH
y11 y7 y6 y2
b3 b4 b7 b8 b11 b12 b13 b17 b18
Int
100
75
50
25
0
y6
y7
y2
b3
b4
b7
b8
y11
b11
b12
b13
b17
b18
Prec
0
250
500
750
1,000
1,250
1,500
1,750
m/z

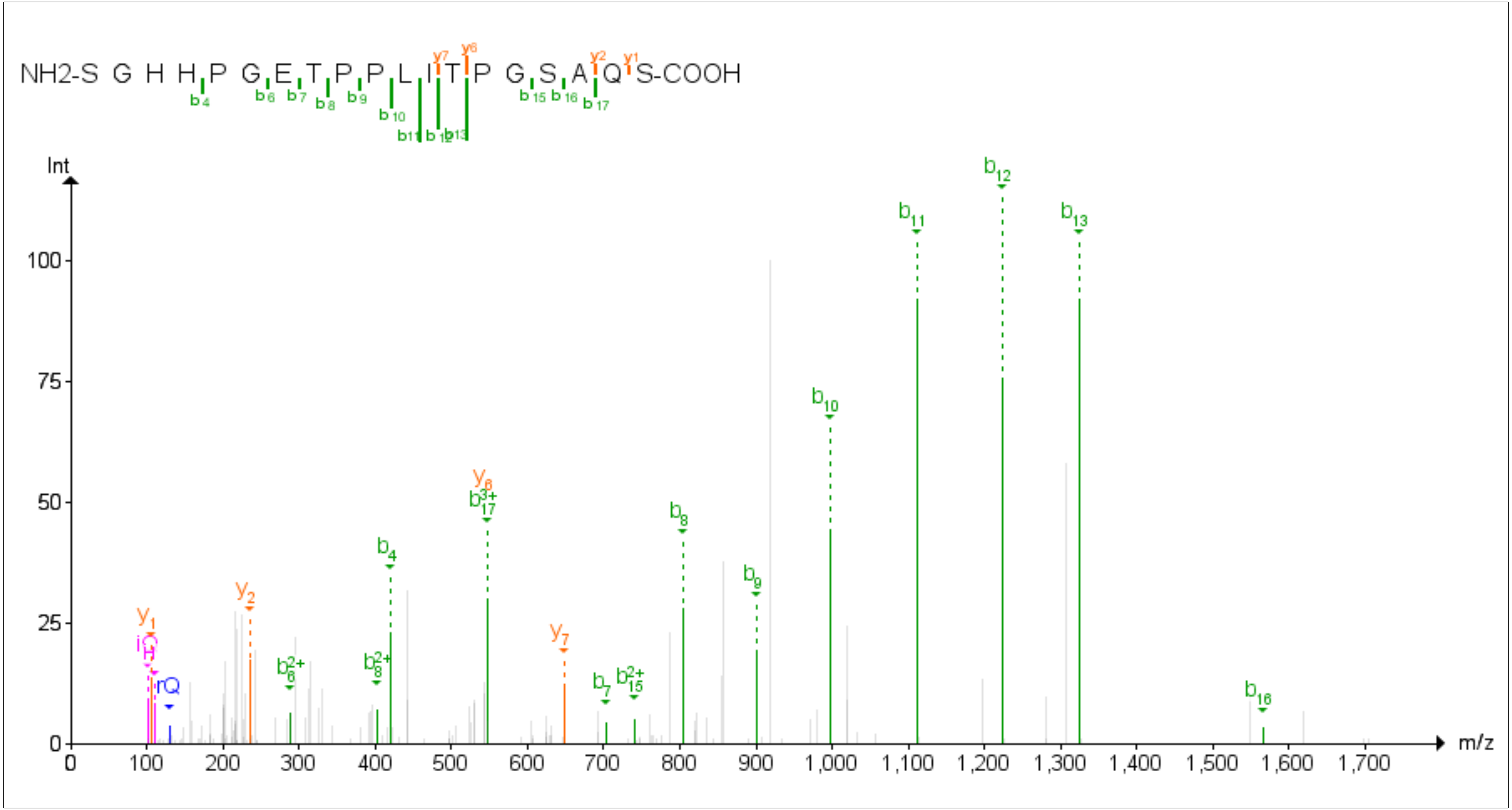

NH2-S G H H P G E T P P L I T P G S A Q S-COOH
b4 b6 b7 b8 b9 b10 b11 b12 b13 b15 b16 b17
y7 y6 y2 y1
Int
100
75
50
25
0
m/z
0 100 200 300 400 500 600 700 800 900 1,000 1,100 1,200 1,300 1,400 1,500 1,600 1,700
y1
iQ
iH
rQ
y2
b6 2+
b8 2+
b4
y6
b17 3+
y7
b7
b15 2+
b8
b9
b10
b11
b12
b13
b16

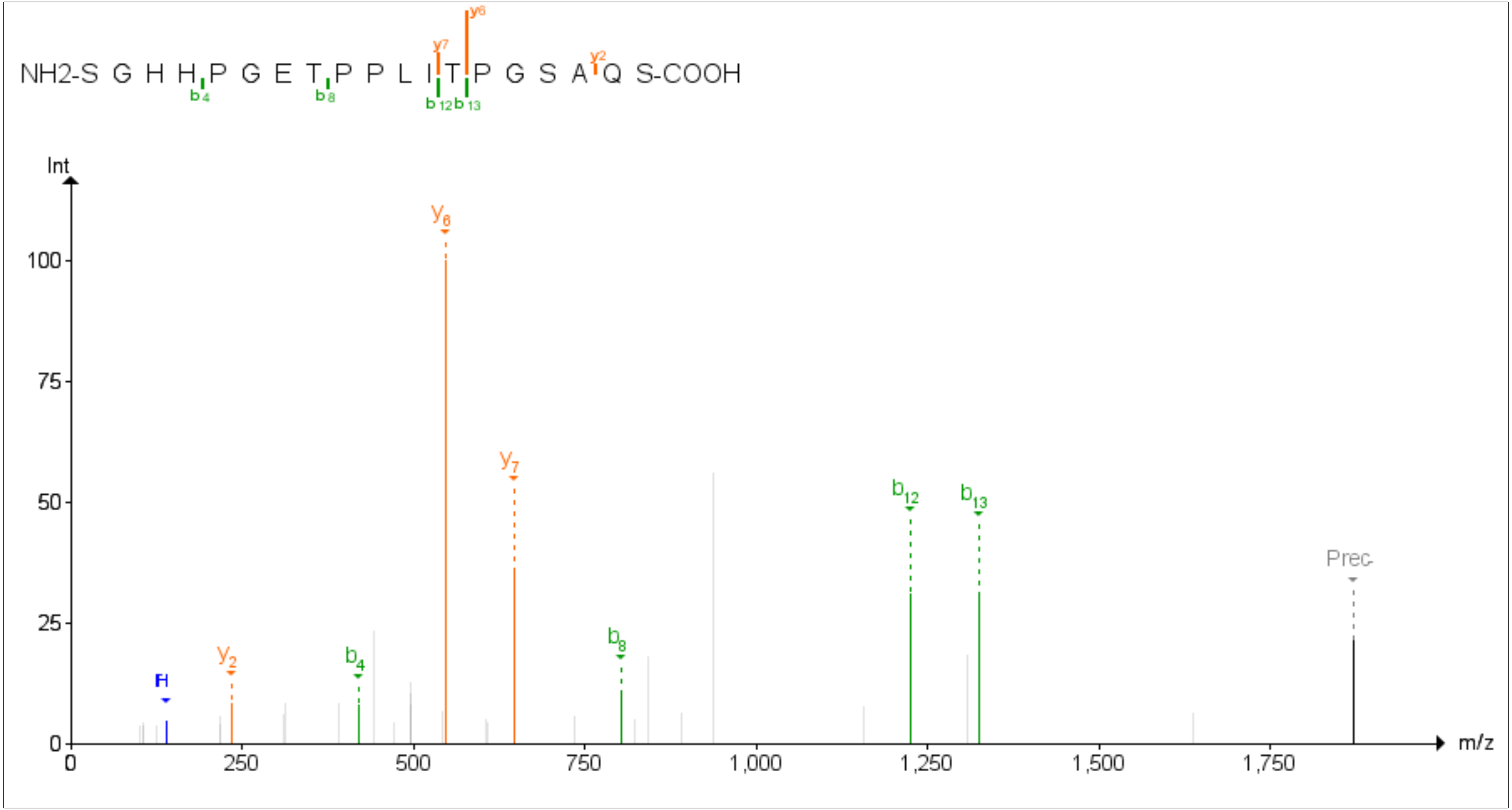

NH2-S G H H P G E T P P L I T P G S A Q S-COOH
b4
b8
b12
b13
y7
y6
y2
Int
100
75
50
25
0
m/z
0
250
500
750
1,000
1,250
1,500
1,750
H
y2
b4
y6
y7
b8
b12
b13
Prec-

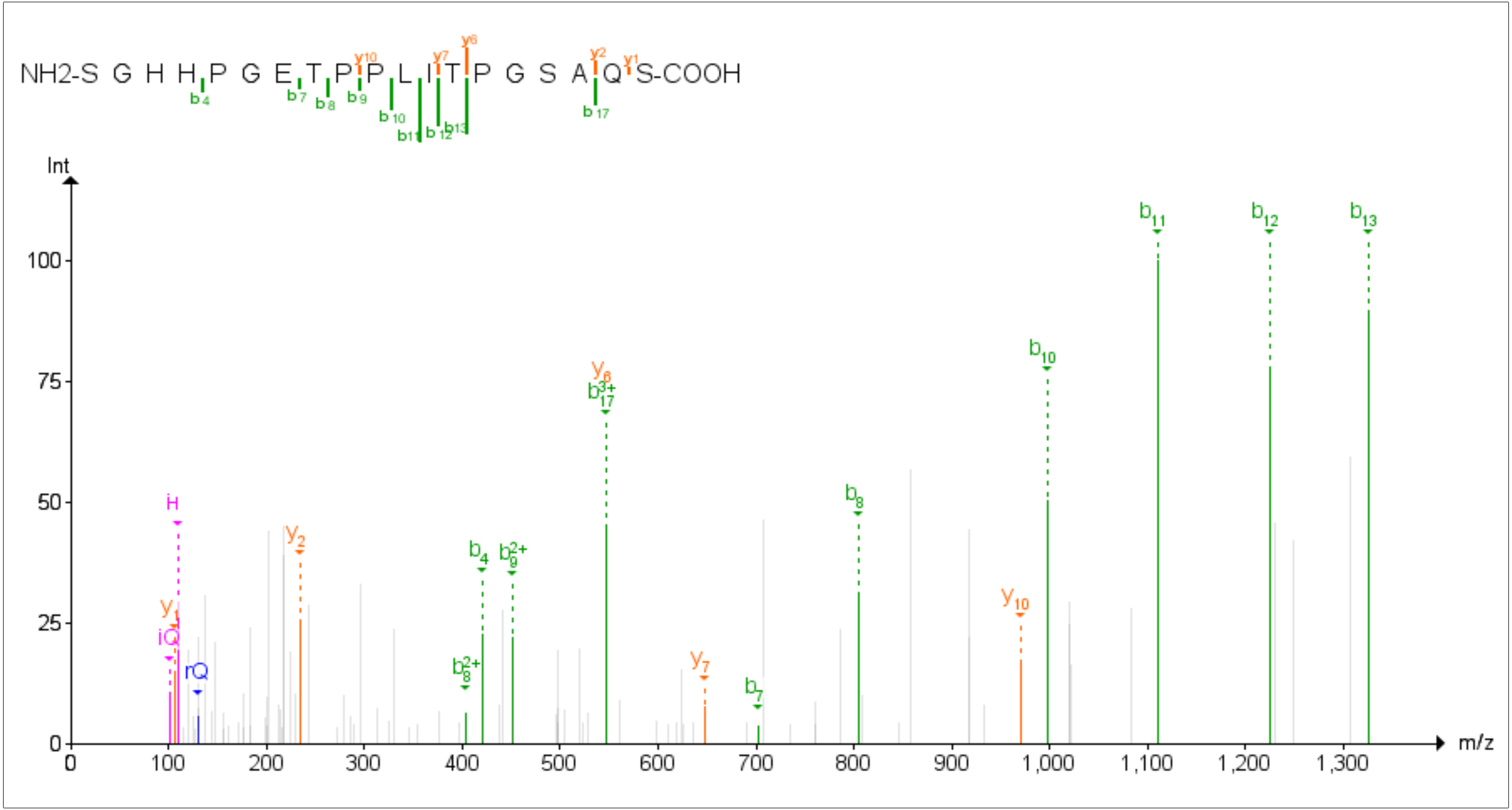

NH2-S G H H P G E T P P L I T P G S A Q S-COOH
Int
m/z
b11
b12
b13
b10
y6
b17 3+
b8
b4
b9 2+
y2
y1
iH
iQ
rQ
b8 2+
y7
b7
y10

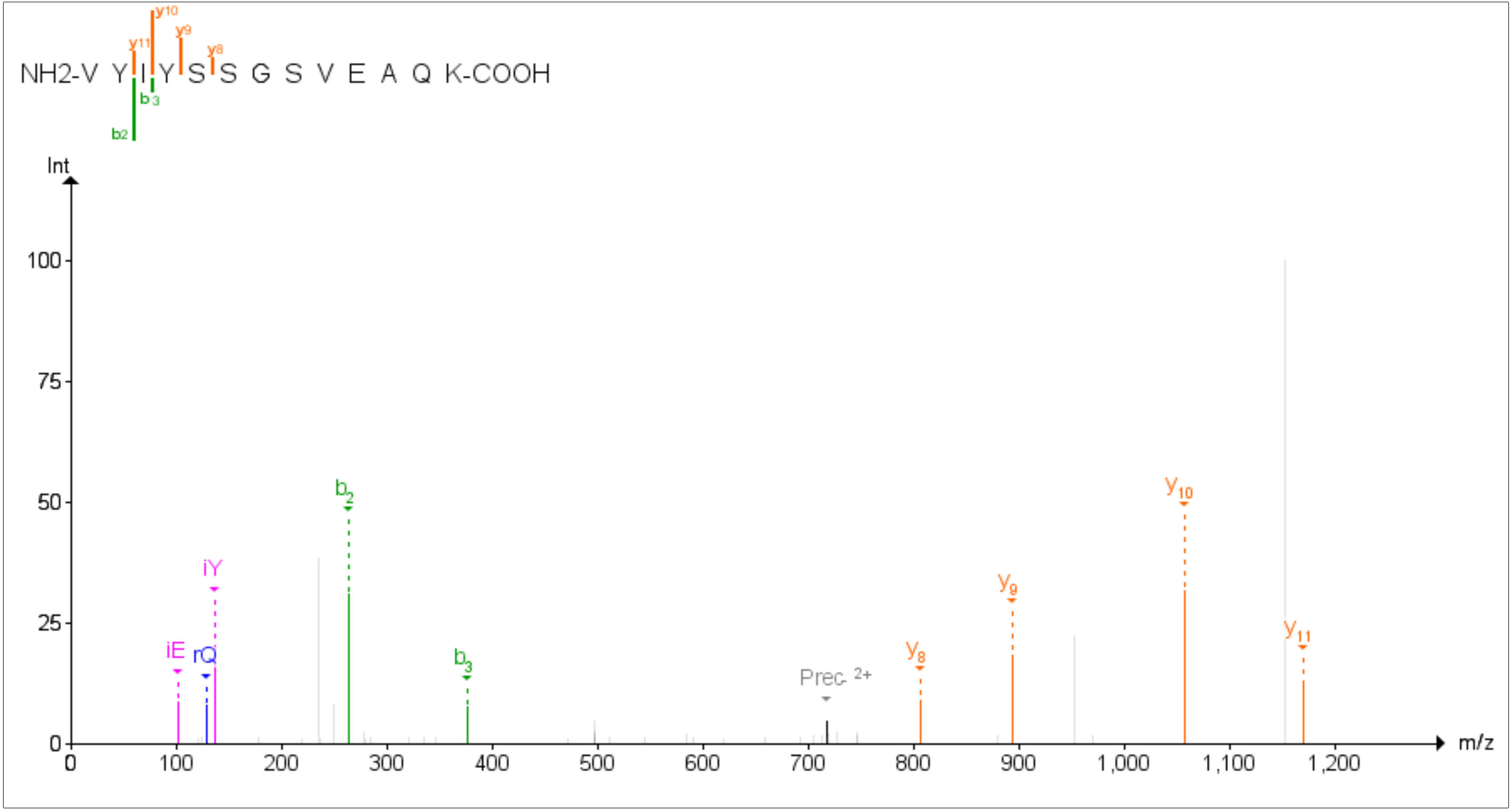

NH2-V Y I Y S S G S V E A Q K-COOH
y10
y9
y11
y8
b3
b2
Int
100
75
50
25
0
b2
iY
iE
rQ
b3
Prec 2+
y8
y9
y10
y11
0
100
200
300
400
500
600
700
800
900
1,000
1,100
1,200
m/z

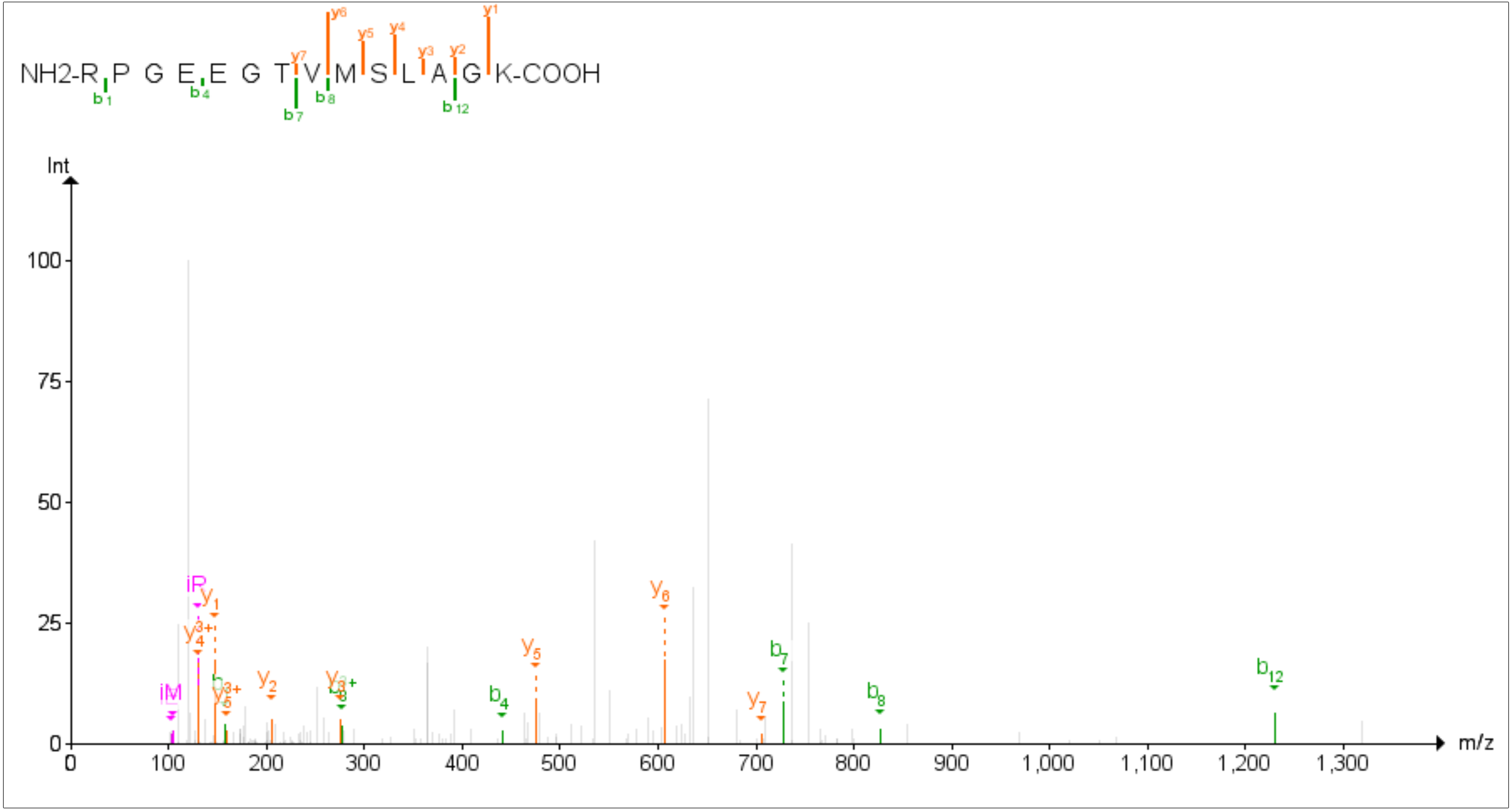
NH2-R P G E E G T V M S L A G K-COOH
b1 b4 b7 b8 b12
y7 y6 y5 y4 y3 y2 y1
Int
100
75
50
25
0
iP
y1
y4 3+
iM
y2
y6
y5
y7
b7
b4
b8
b12
0 100 200 300 400 500 600 700 800 900 1,000 1,100 1,200 1,300
m/z

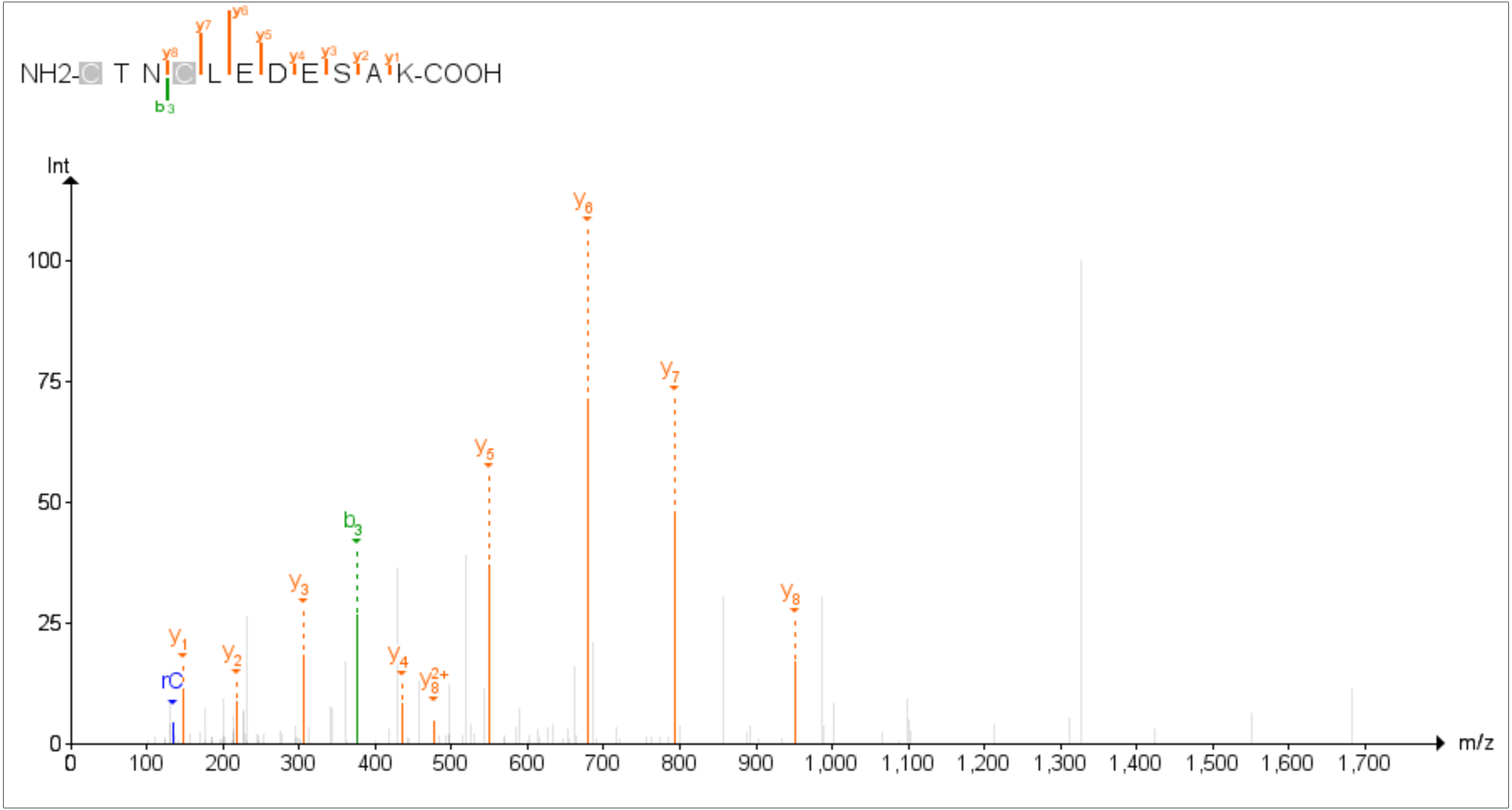

NH2-C T N C L E D E S A K-COOH
y8 y7 y6 y5 y4 y3 y2 y1
b3
Int
m/z
y6
y7
y5
b3
y3
y8
y1
y2
y4
rC
y8 2+

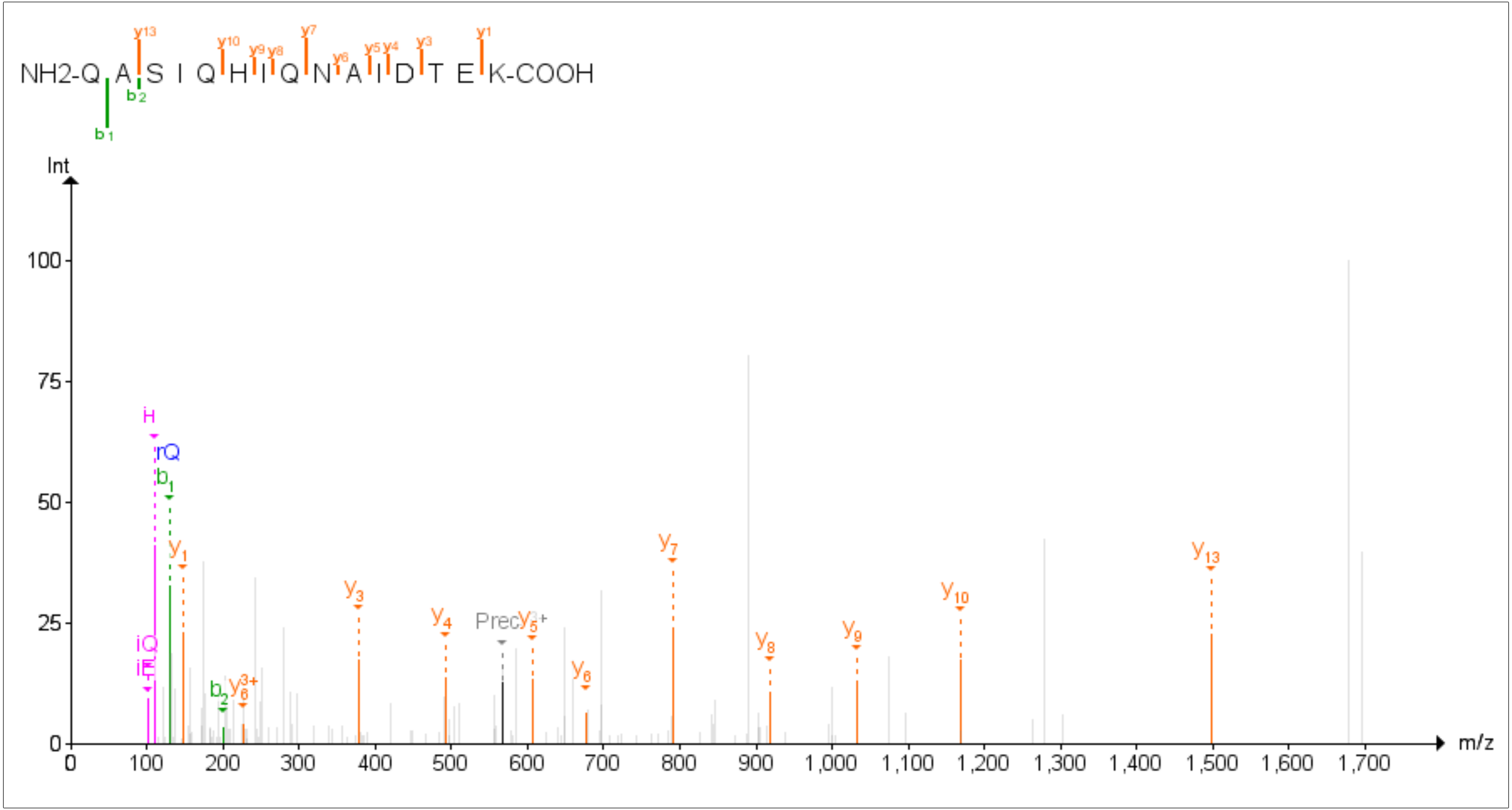

NH2-Q A S I Q H I Q N A I D T E K-COOH
y13 y10 y9 y8 y7 y6 y5 y4 y3 y1
b1 b2
Int
m/z
0
25
50
75
100
0 100 200 300 400 500 600 700 800 900 1,000 1,100 1,200 1,300 1,400 1,500 1,600 1,700
iH
rQ
iQ
iE
y1
y3
y4
y5
y6
y7
y8
y9
y10
y13
Prec

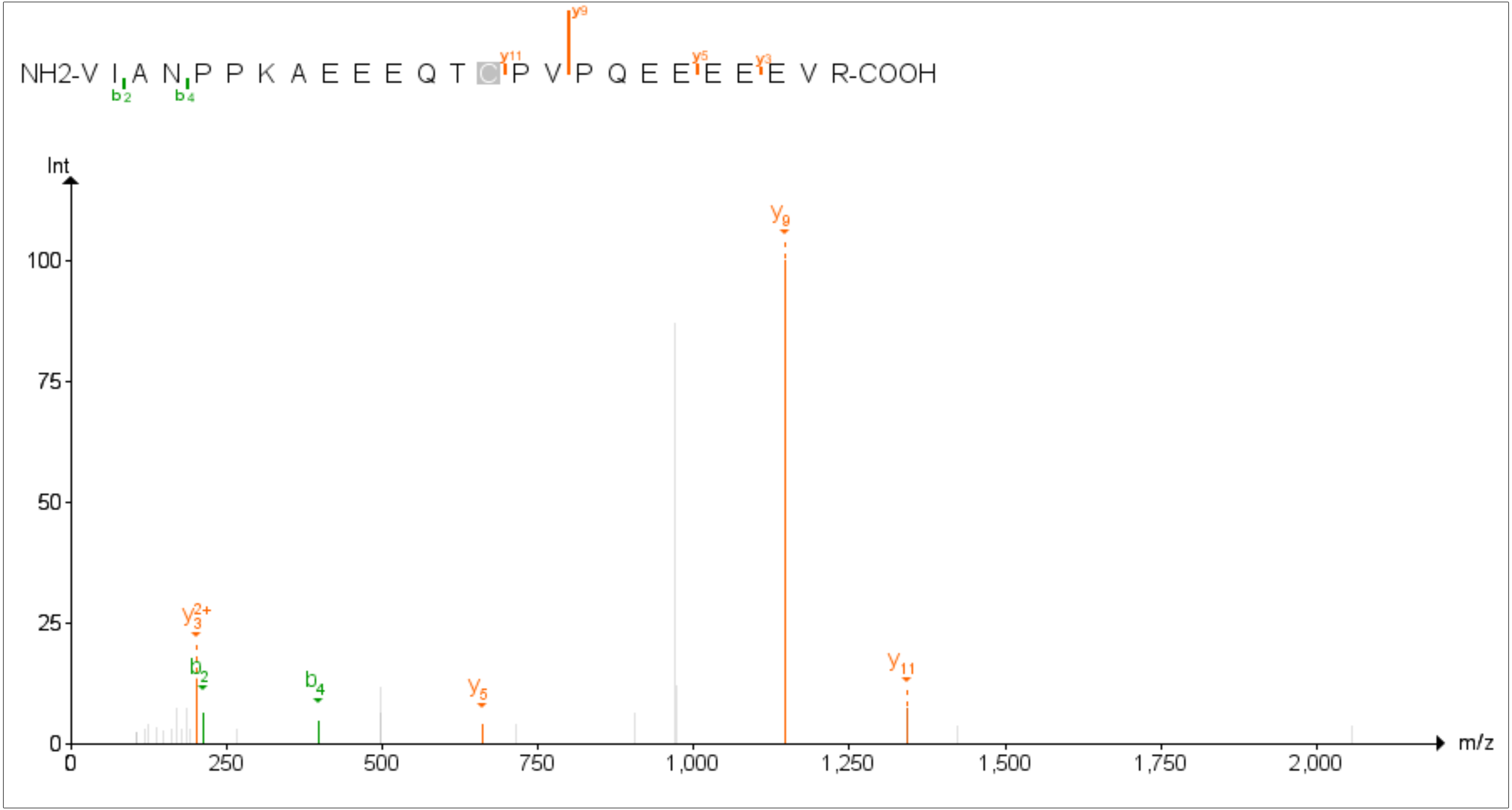
NH2-V I A N P P K A E E E Q T C P V P Q E E E E E V R-COOH
b2
b4
y11
y9
y5
y3
Int
100
75
50
25
0
y9
y3 2+
b2
b4
y5
y11
0
250
500
750
1,000
1,250
1,500
1,750
2,000
m/z

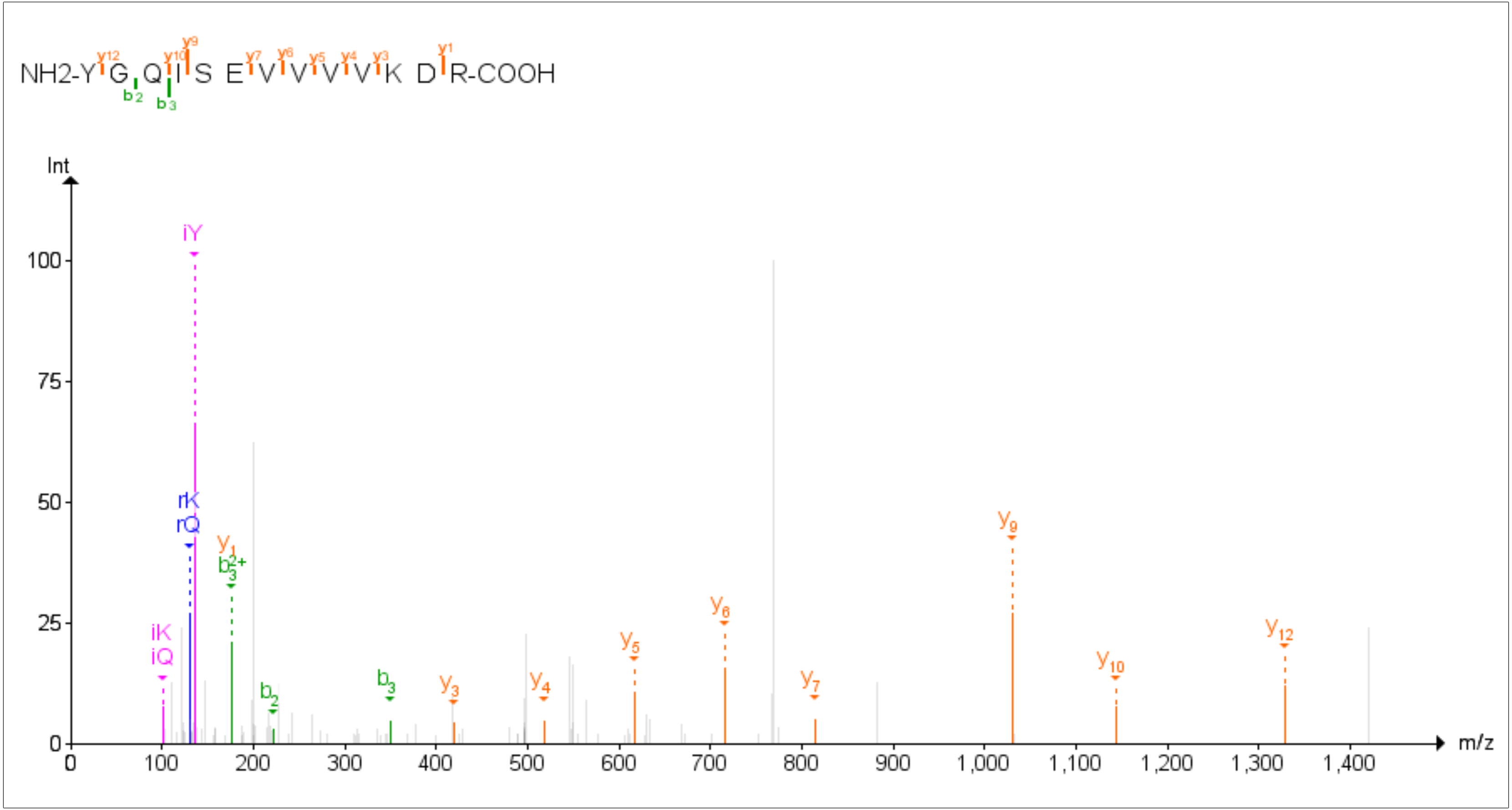
NH2-Y G Q I S E V V V V K D R-COOH
y12 y10 y9 y7 y6 y5 y4 y3 y1
b2 b3
Int
m/z
iY
rK
rQ
iK
iQ
y1
b3 2+
b2
b3
y3
y4
y5
y6
y7
y9
y10
y12
0
25
50
75
100
0
100
200
300
400
500
600
700
800
900
1,000
1,100
1,200
1,300
1,400

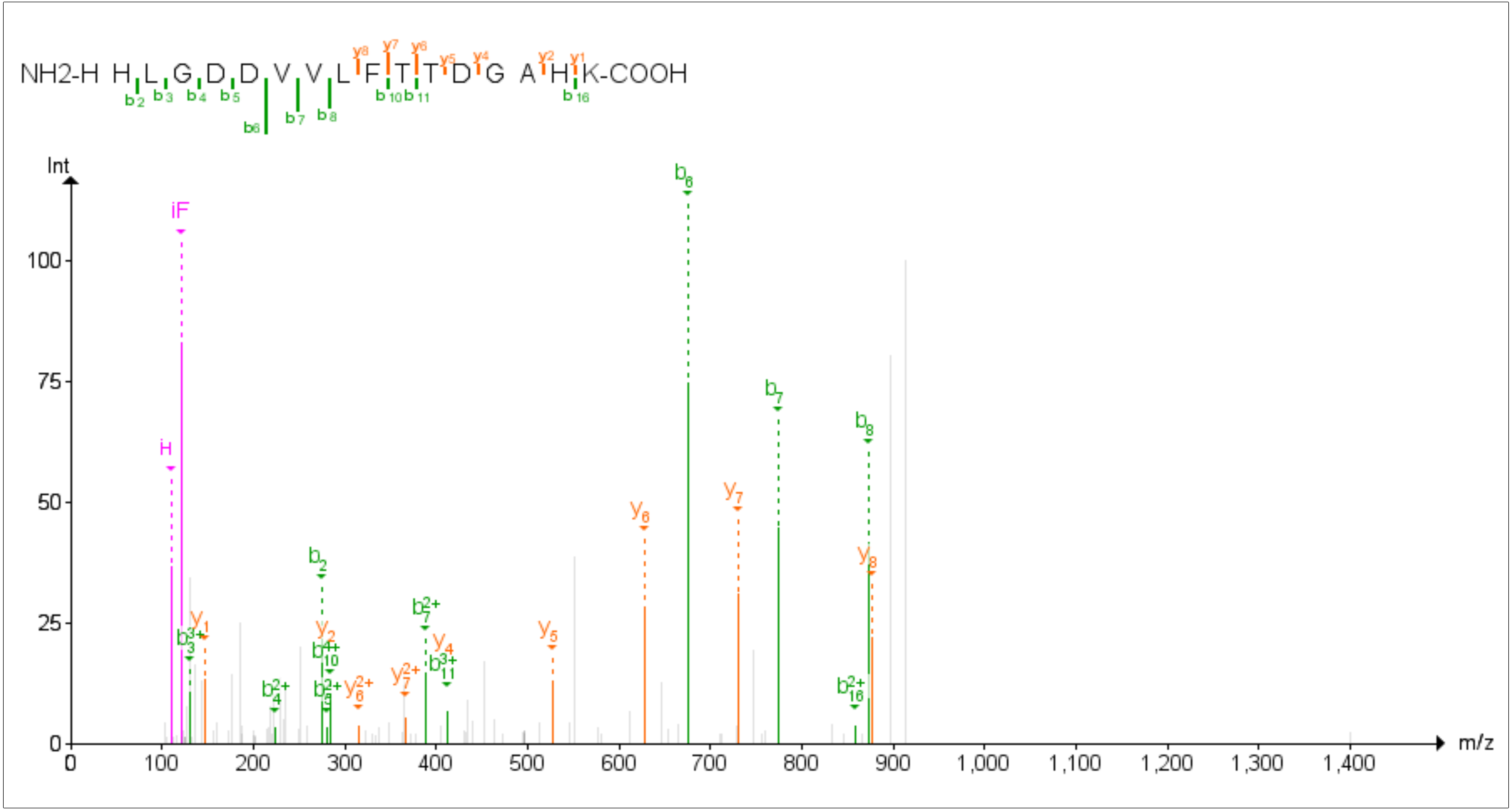

NH2-H H L G D D V V L F T T D G A H K-COOH
b2 b3 b4 b5 b6 b7 b8 b10 b11 b16
y8 y7 y6 y5 y4 y2 y1
Int
m/z
0
25
50
75
100
0
100
200
300
400
500
600
700
800
900
1,000
1,100
1,200
1,300
1,400
iF
iH
b6
b7
b8
y8
y6
y7
y5
y4
y2
y1
b2
b3 3+
b4 2+
b10 4+
b5 2+
y6 2+
y7 2+
b7 2+
b11 3+
b16 2+

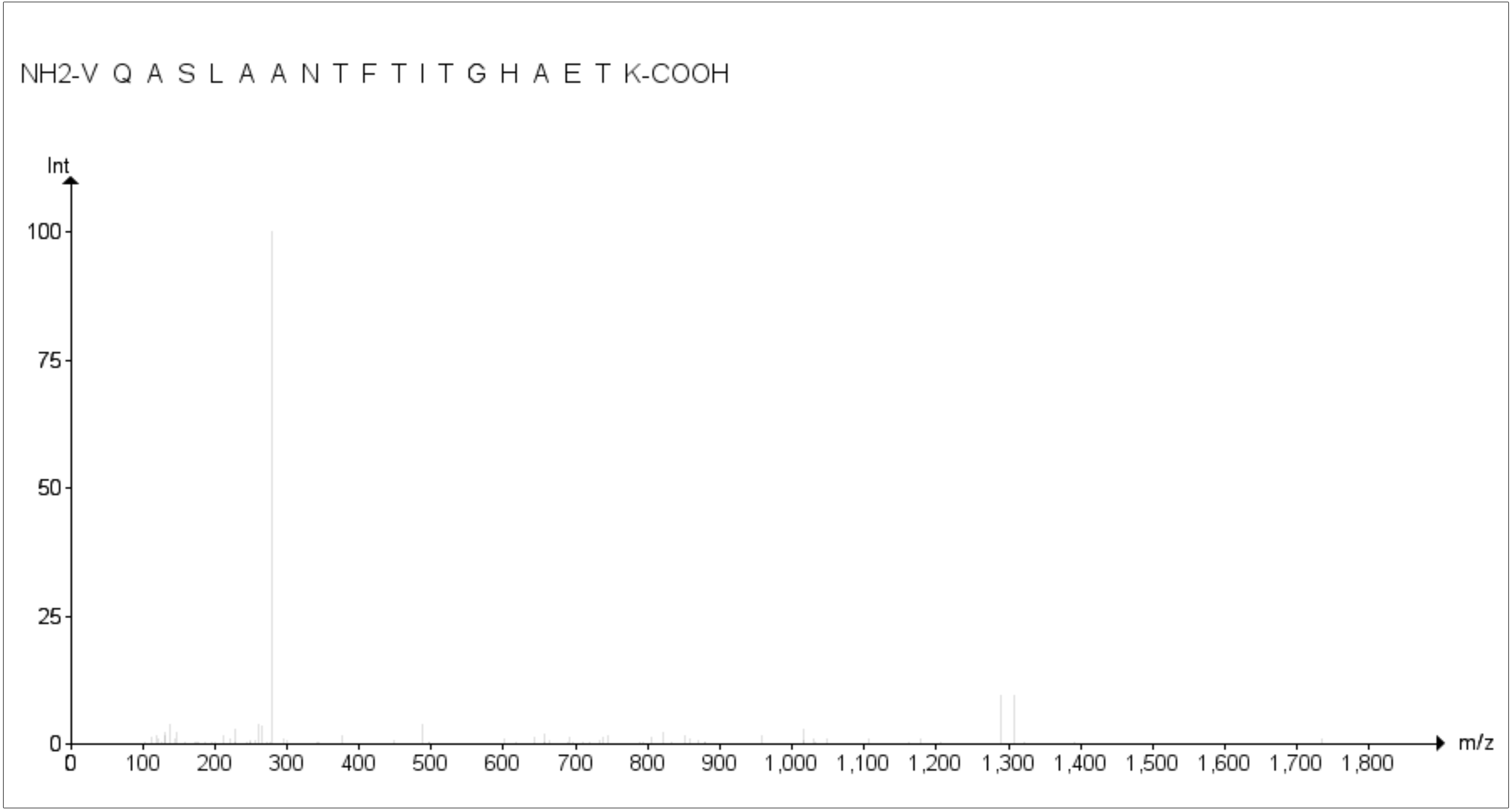
NH2-V Q A S L A A N T F T I T G H A E T K-COOH
Int
100
75
50
25
0
m/z
0
100
200
300
400
500
600
700
800
900
1,000
1,100
1,200
1,300
1,400
1,500
1,600
1,700
1,800

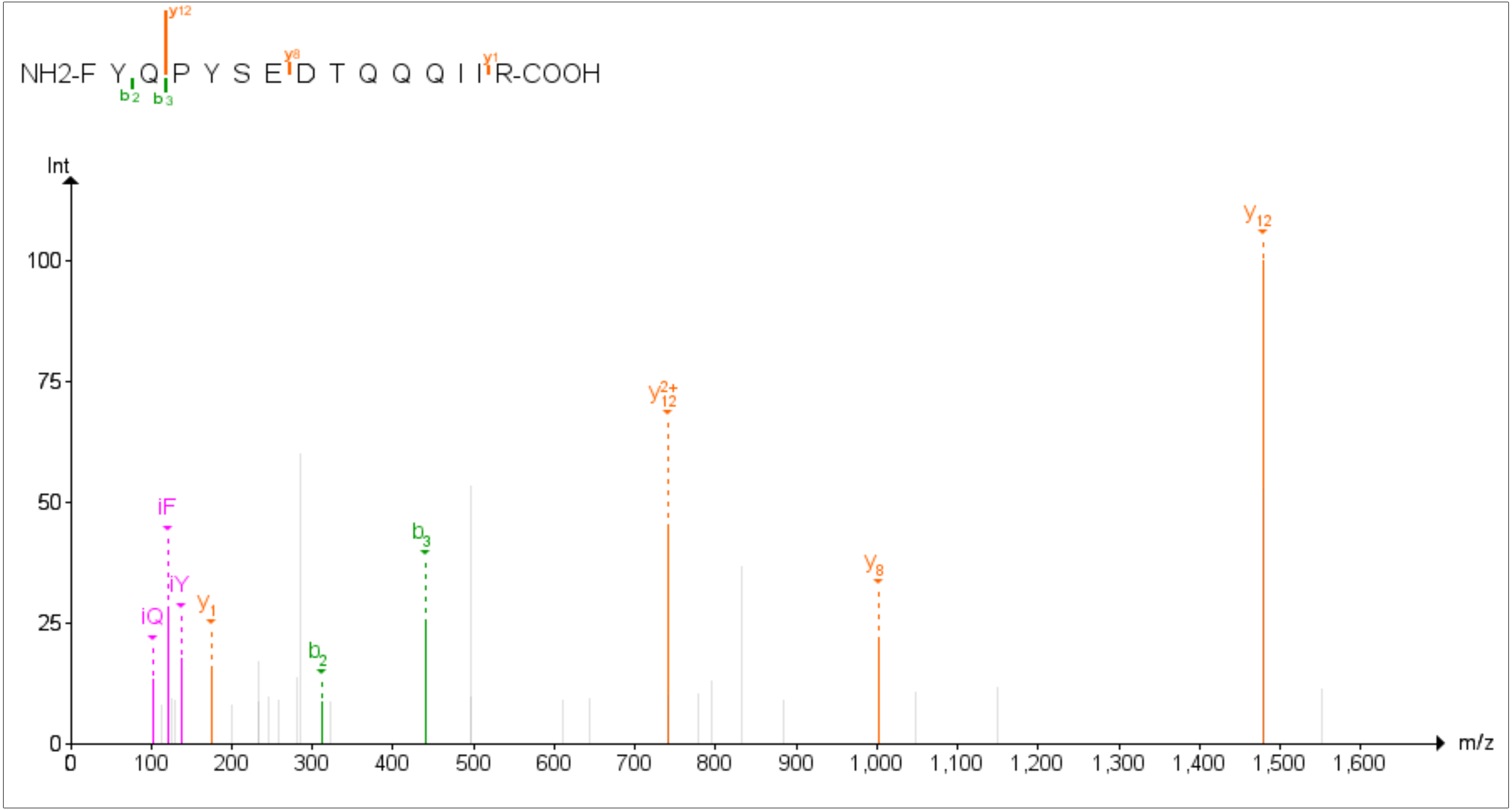

NH2-F Y Q P Y S E D T Q Q Q I I R-COOH
y12
y8
y1
b2
b3
Int
100
75
50
25
0
iF
iY
iQ
y1
b2
b3
y_{12}^{2+}
y8
y12
0
100
200
300
400
500
600
700
800
900
1,000
1,100
1,200
1,300
1,400
1,500
1,600
m/z

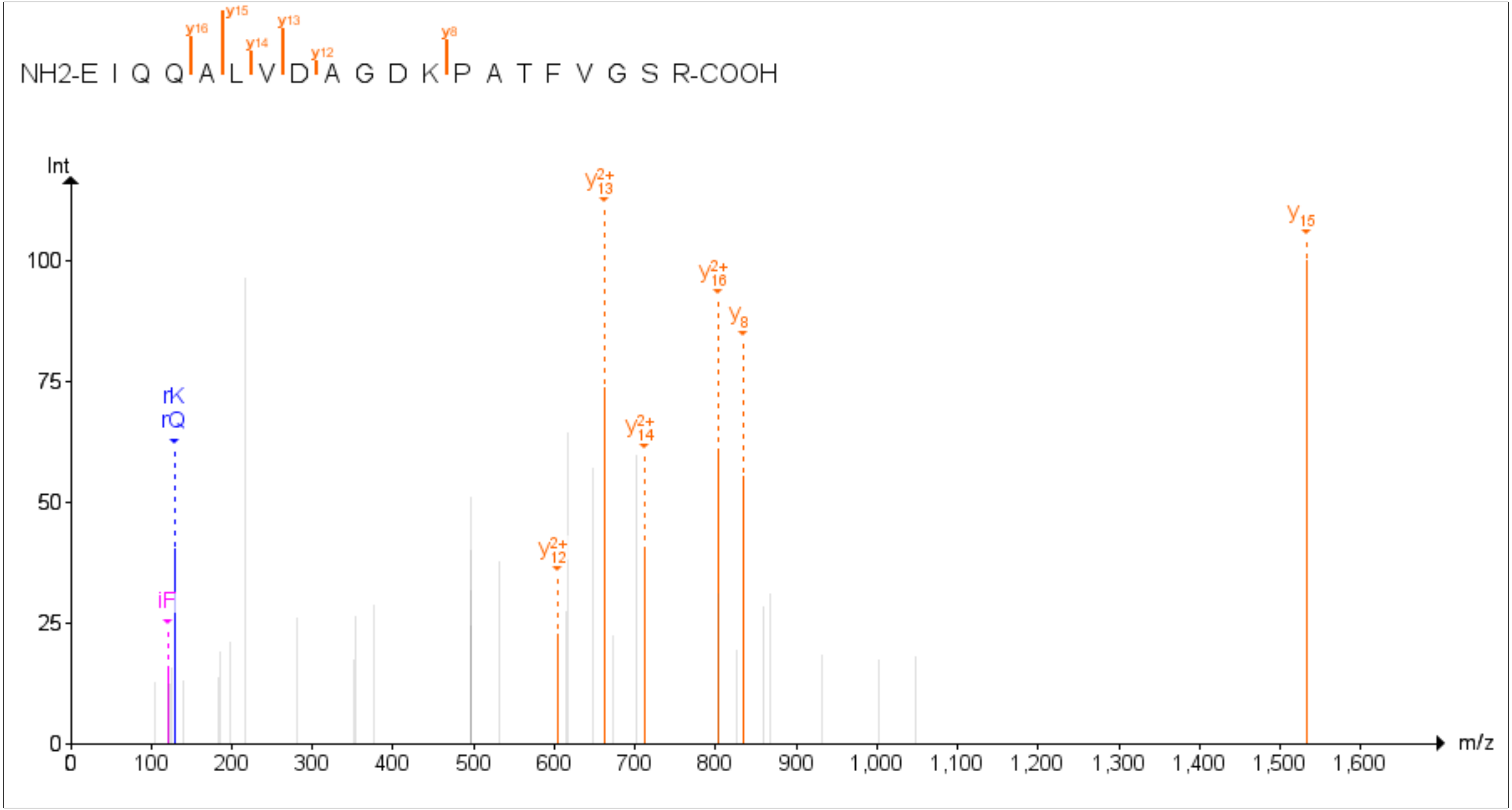

NH2-E I Q Q A L V D A G D K P A T F V G S R-COOH
y16
y15
y14
y13
y12
y8
Int
m/z
rK
rQ
iF
y_{13}^{2+}
y_{15}
y_{16}^{2+}
y_8
y_{14}^{2+}
y_{12}^{2+}

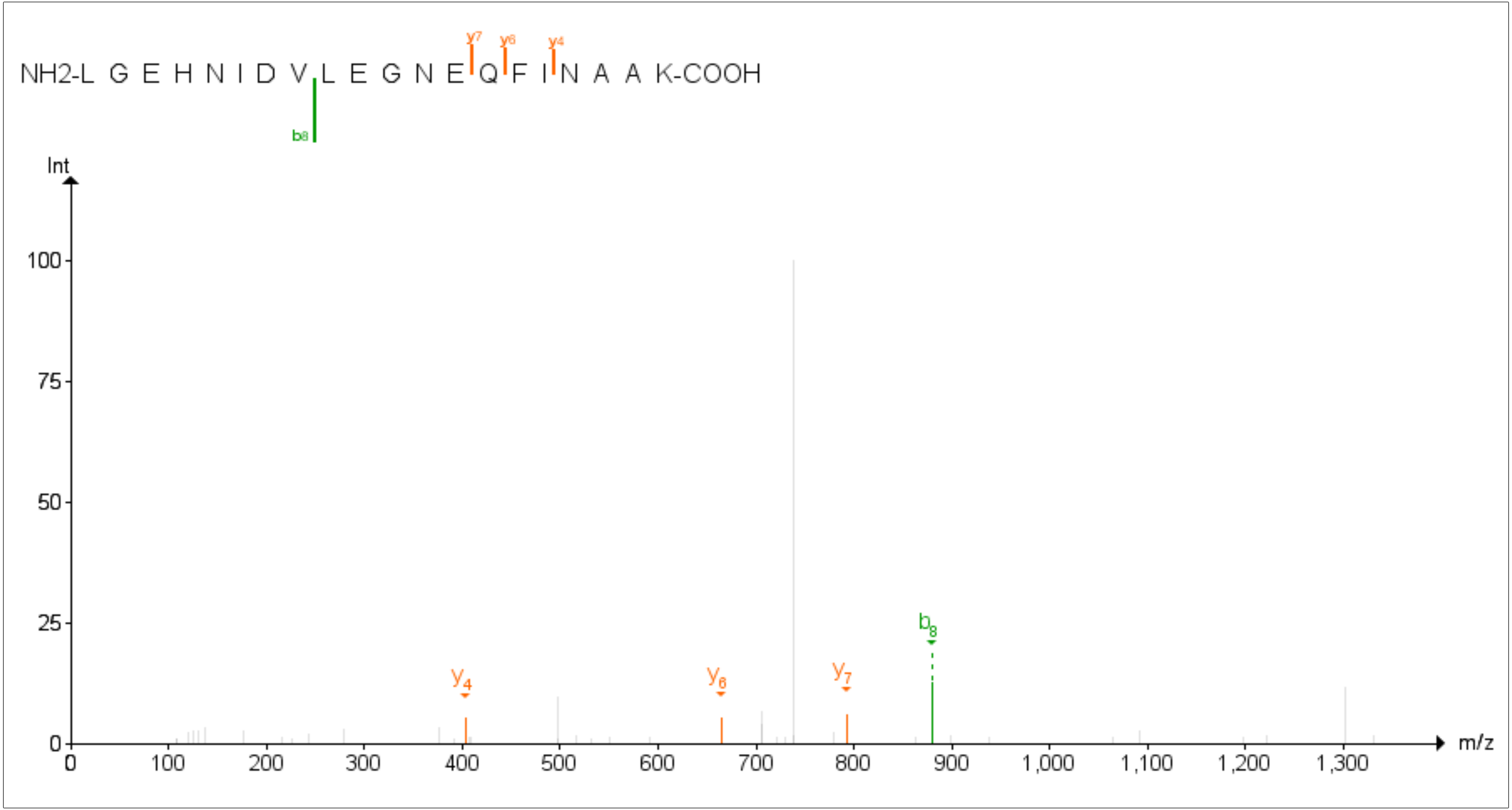

NH2-L G E H N I D V L E G N E Q F I N A A K-COOH
y7
y6
y4
b8
Int
100
75
50
25
0
m/z
0
100
200
300
400
500
600
700
800
900
1,000
1,100
1,200
1,300
y4
y6
y7
b8

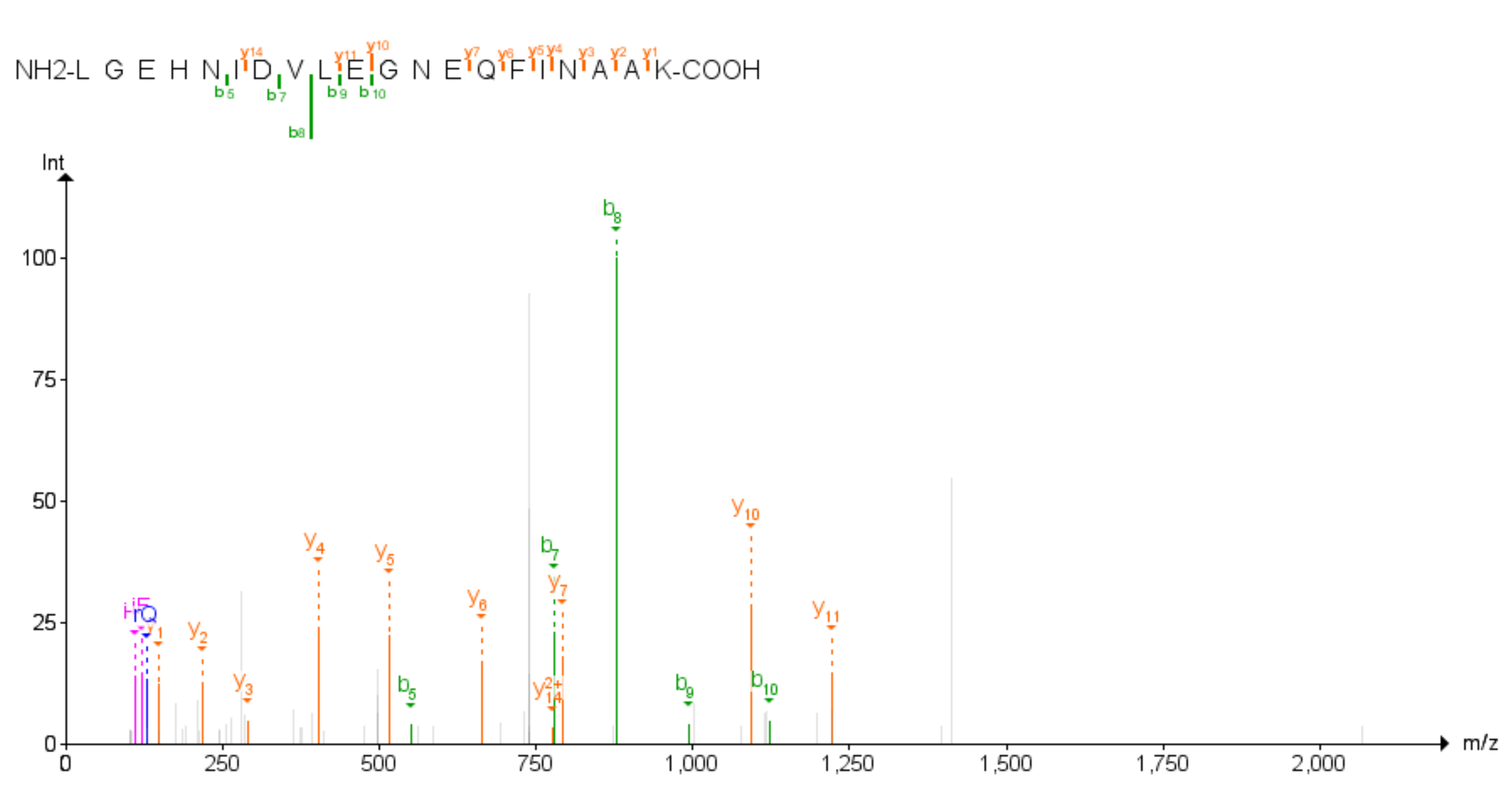
NH2-L G E H N I D V L E G N E Q F I N A A K-COOH
y14 y11 y10 y7 y6 y5 y4 y3 y2 y1
b5 b7 b8 b9 b10
Int
100
75
50
25
0
0 250 500 750 1,000 1,250 1,500 1,750 2,000
m/z
b8
y10
y4
y5
b7
y7
y6
y11
y1
y2
y3
b5
b9
b10
rQ

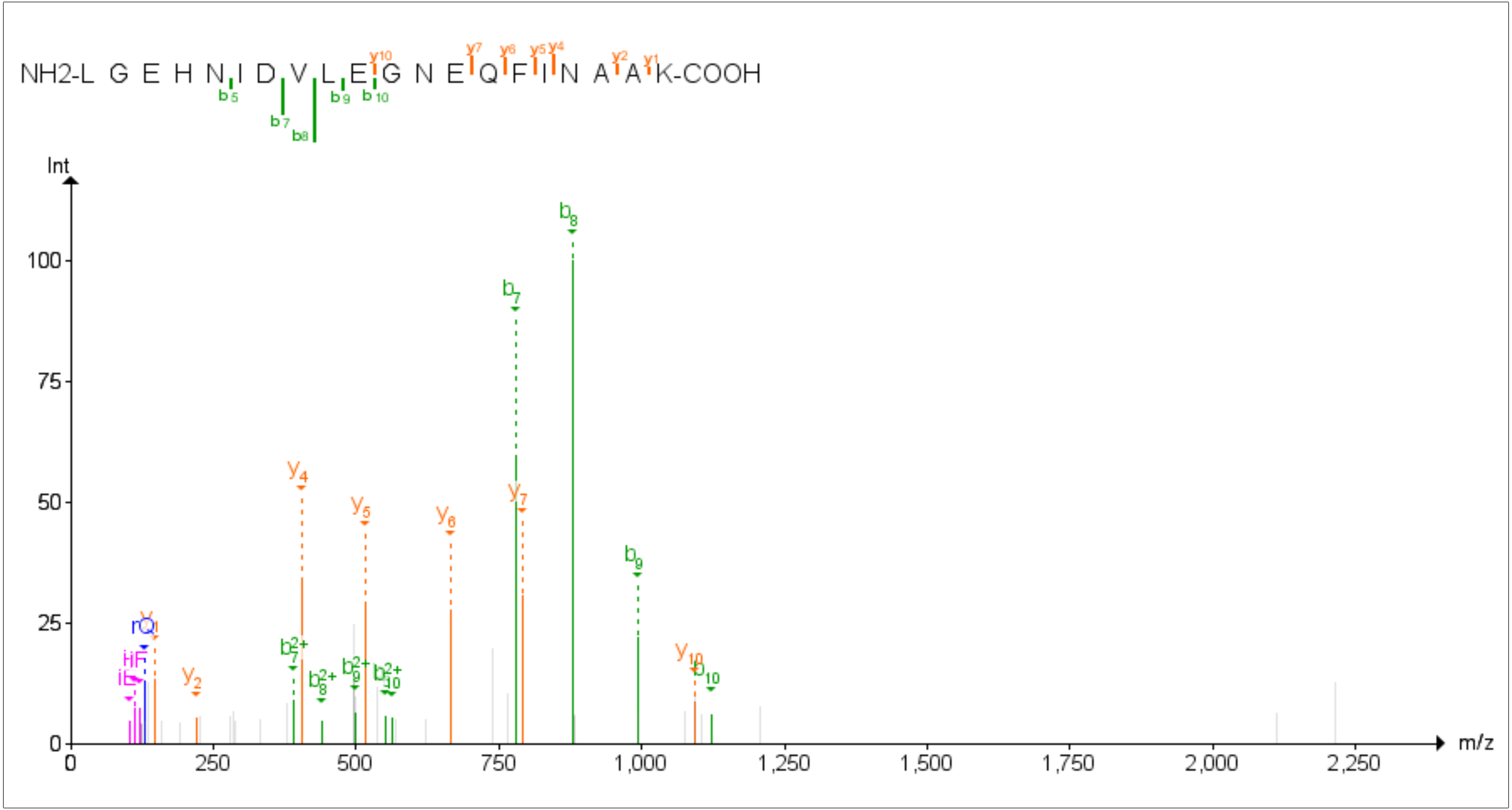

NH2-L G E H N I D V L E G N E Q F I N A A K-COOH
b5 b7 b8 b9 b10
y10 y7 y6 y5 y4 y2 y1
Int
100
75
50
25
0
m/z
0
250
500
750
1,000
1,250
1,500
1,750
2,000
2,250
b8
b7
y4
y5
y6
y7
b9
y10
b10
y2
b7 2+
b8 2+
b9 2+
b10 2+
rQ
iF
iL

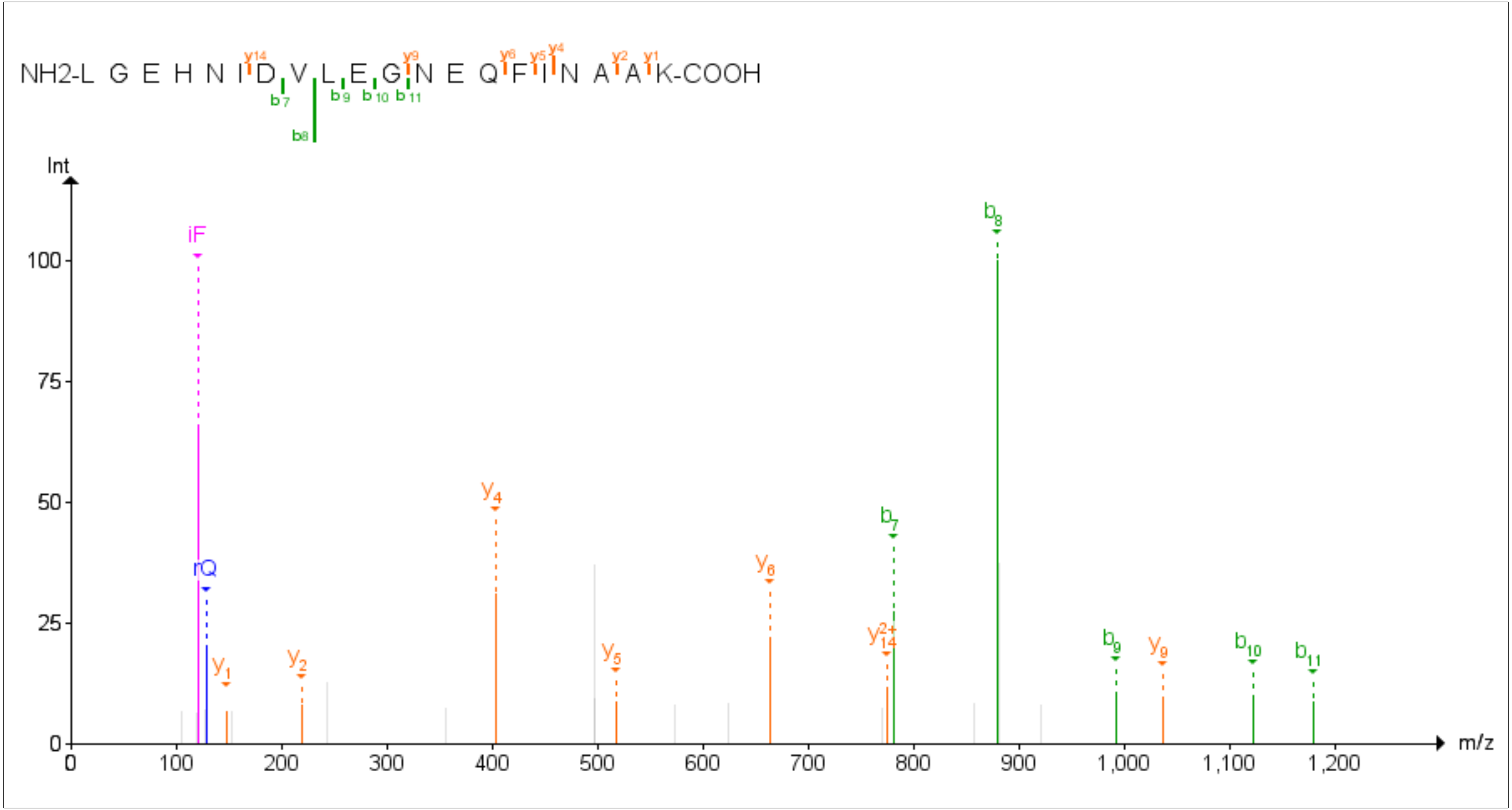

NH2-L G E H N I D V L E G N E Q F I N A A K-COOH
y14 y9 y6 y5 y4 y2 y1
b7 b8 b9 b10 b11
Int
100
75
50
25
0
iF
rQ
y1
y2
y4
y5
y6
y14 2+
b7
b8
b9
y9
b10
b11
0 100 200 300 400 500 600 700 800 900 1,000 1,100 1,200
m/z

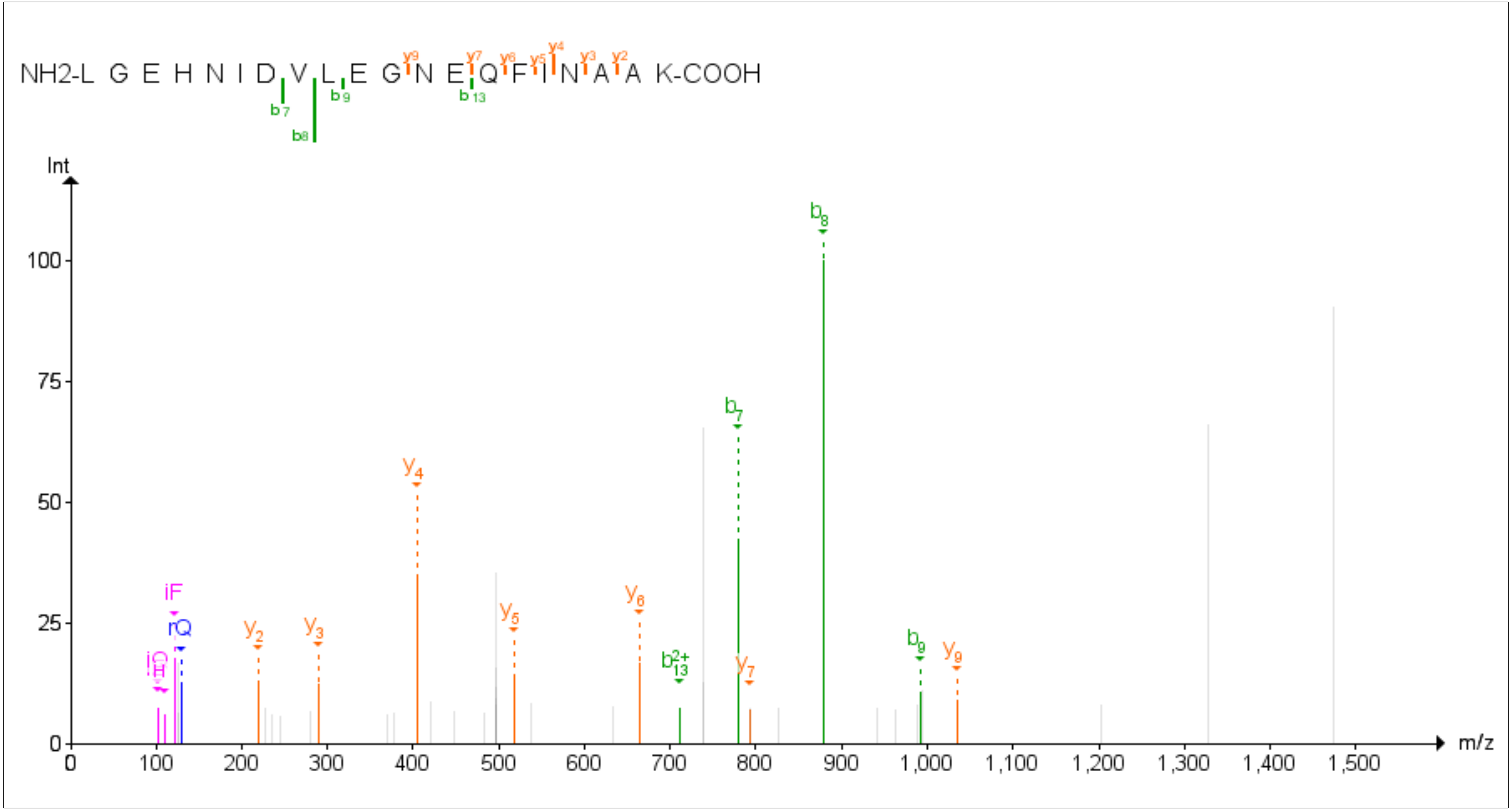

NH2-L G E H N I D V L E G N E Q F I N A A K-COOH
y9 y7 y6 y5 y4 y3 y2
b7 b8 b9 b13
Int
100
75
50
25
0
iF
rQ
iQ
iH
y2 y3 y4 y5 y6 y7 y9
b2+13 b7 b8 b9
m/z
0 100 200 300 400 500 600 700 800 900 1,000 1,100 1,200 1,300 1,400 1,500

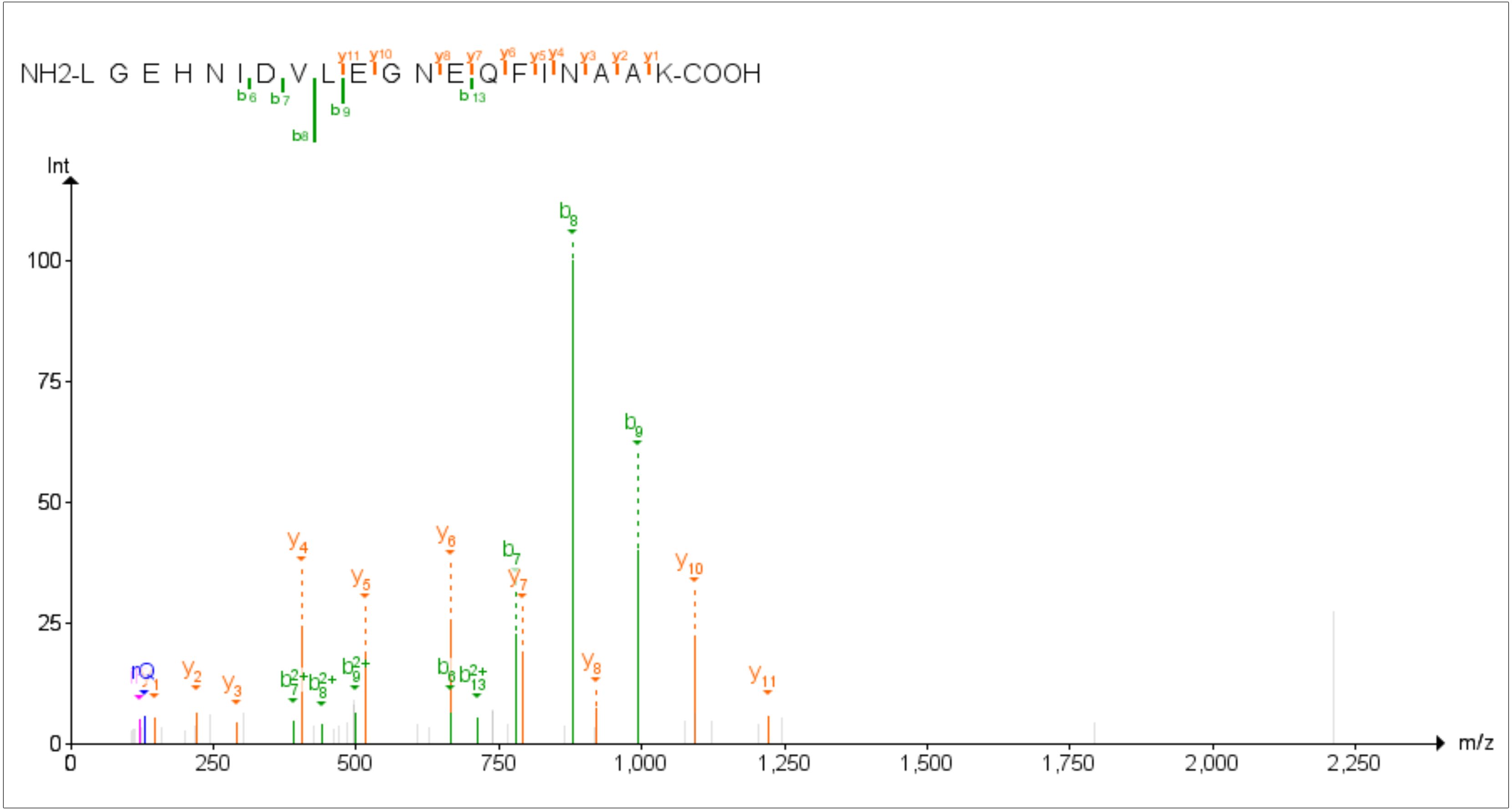

NH2-L G E H N I D V L E G N E Q F I N A A K-COOH
Int
m/z
b8
b9
y4
y6
b7
y7
y5
y10
y8
y11
y2
y3
b7 2+
b8 2+
b9 2+
b6
b13 2+

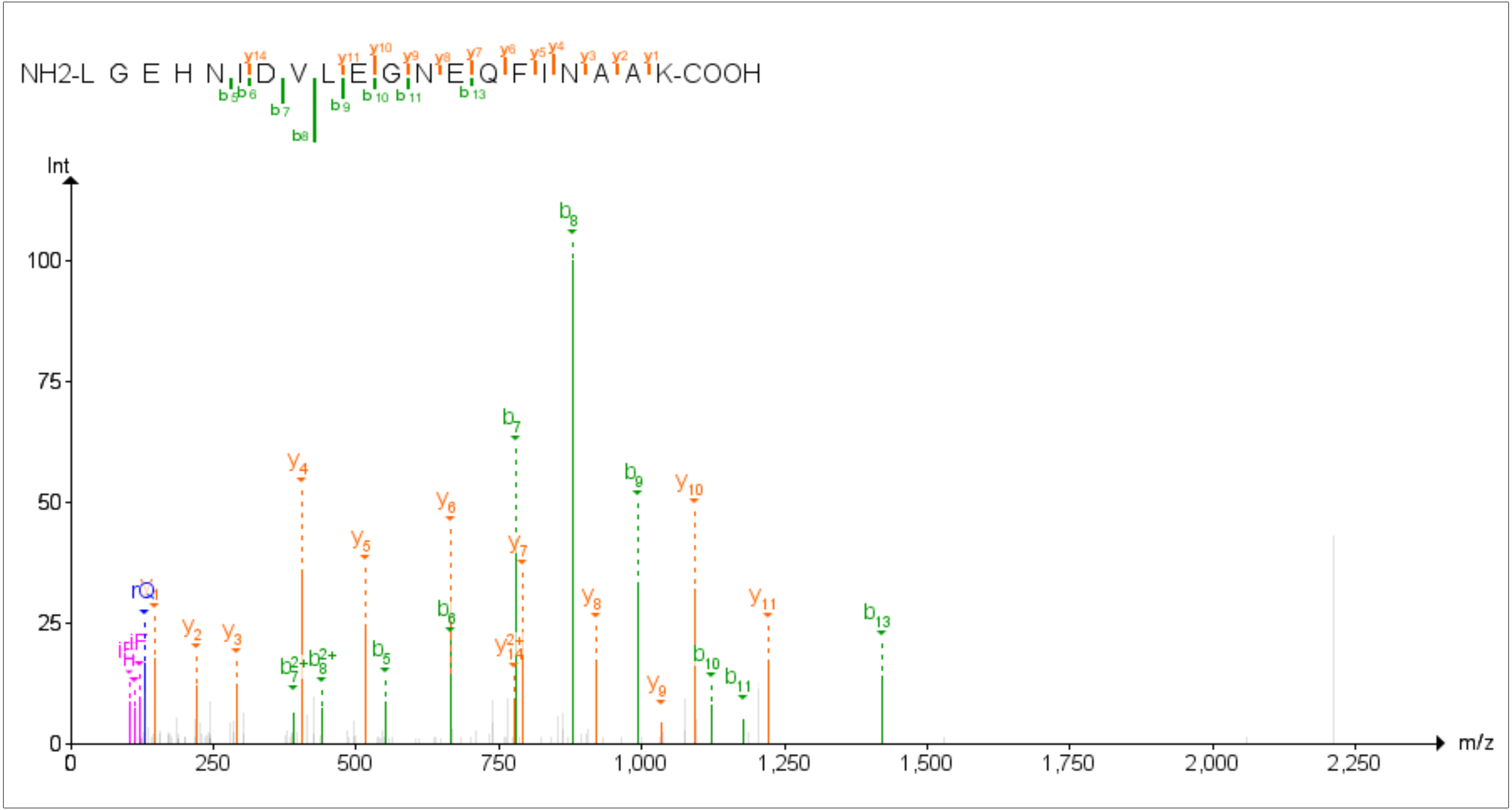

NH2-L G E H N I D V L E G N E Q F I N A A K-COOH
Int
100
75
50
25
0
250
500
750
1,000
1,250
1,500
1,750
2,000
2,250
m/z

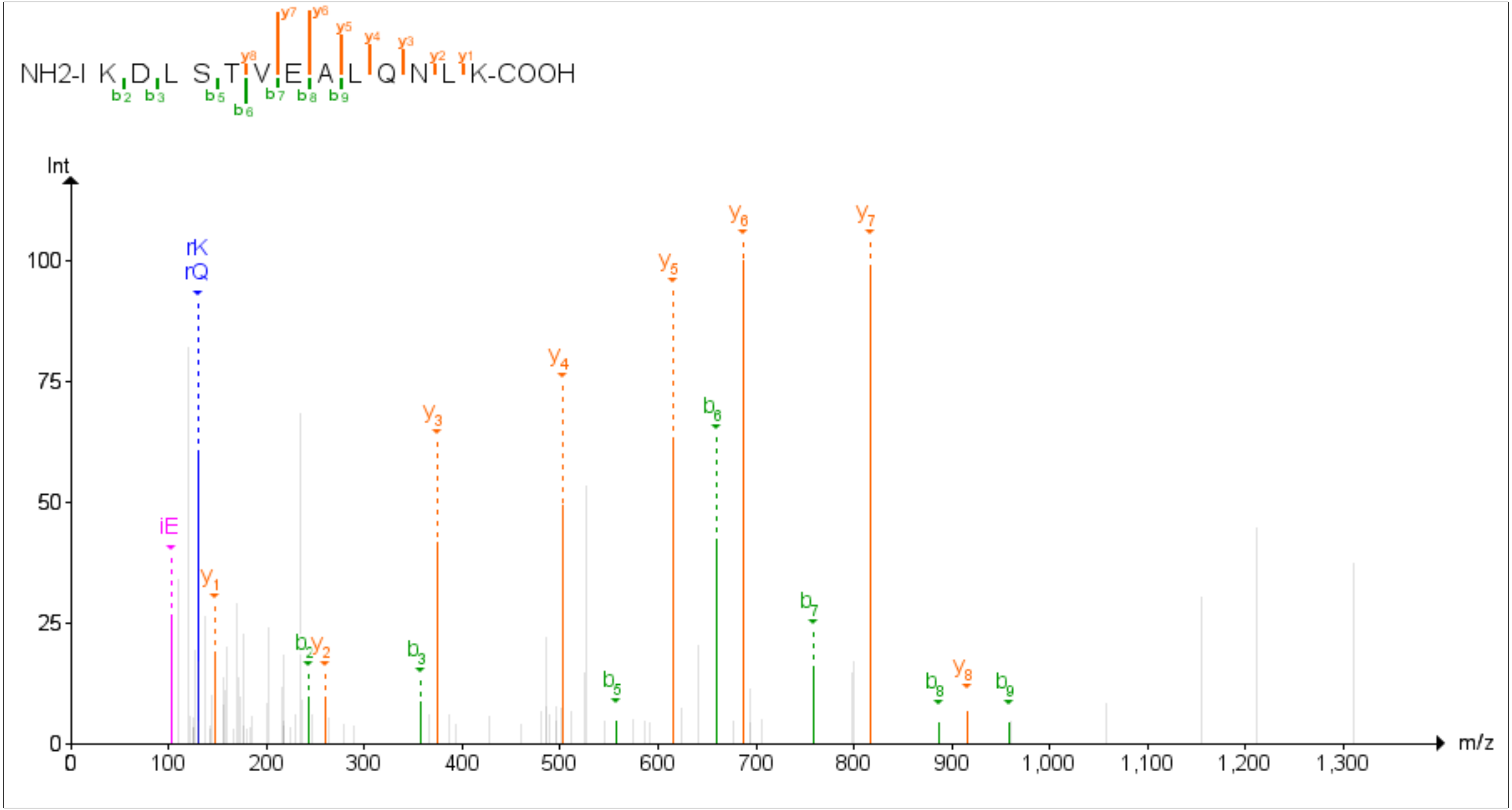

NH2-I K D L S T V E A L Q N L K-COOH
y8 y7 y6 y5 y4 y3 y2 y1
b2 b3 b5 b6 b7 b8 b9
Int
m/z
rK
rQ
iE
y1 y2 y3 y4 y5 y6 y7 y8
b2 b3 b5 b6 b7 b8 b9
0 25 50 75 100
0 100 200 300 400 500 600 700 800 900 1,000 1,100 1,200 1,300

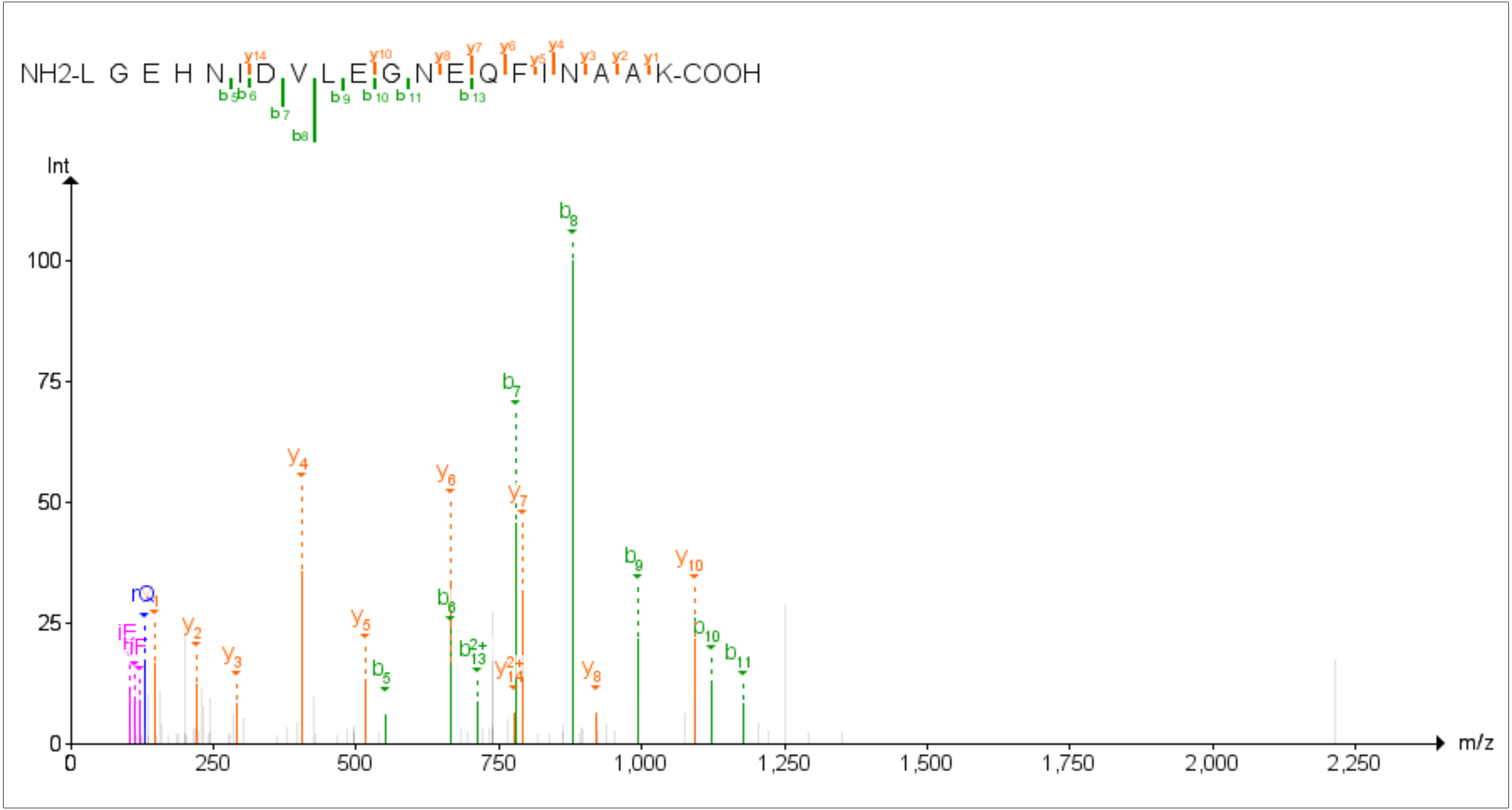

NH2-L G E H N I D V L E G N E Q F I N A A K-COOH
Int
m/z

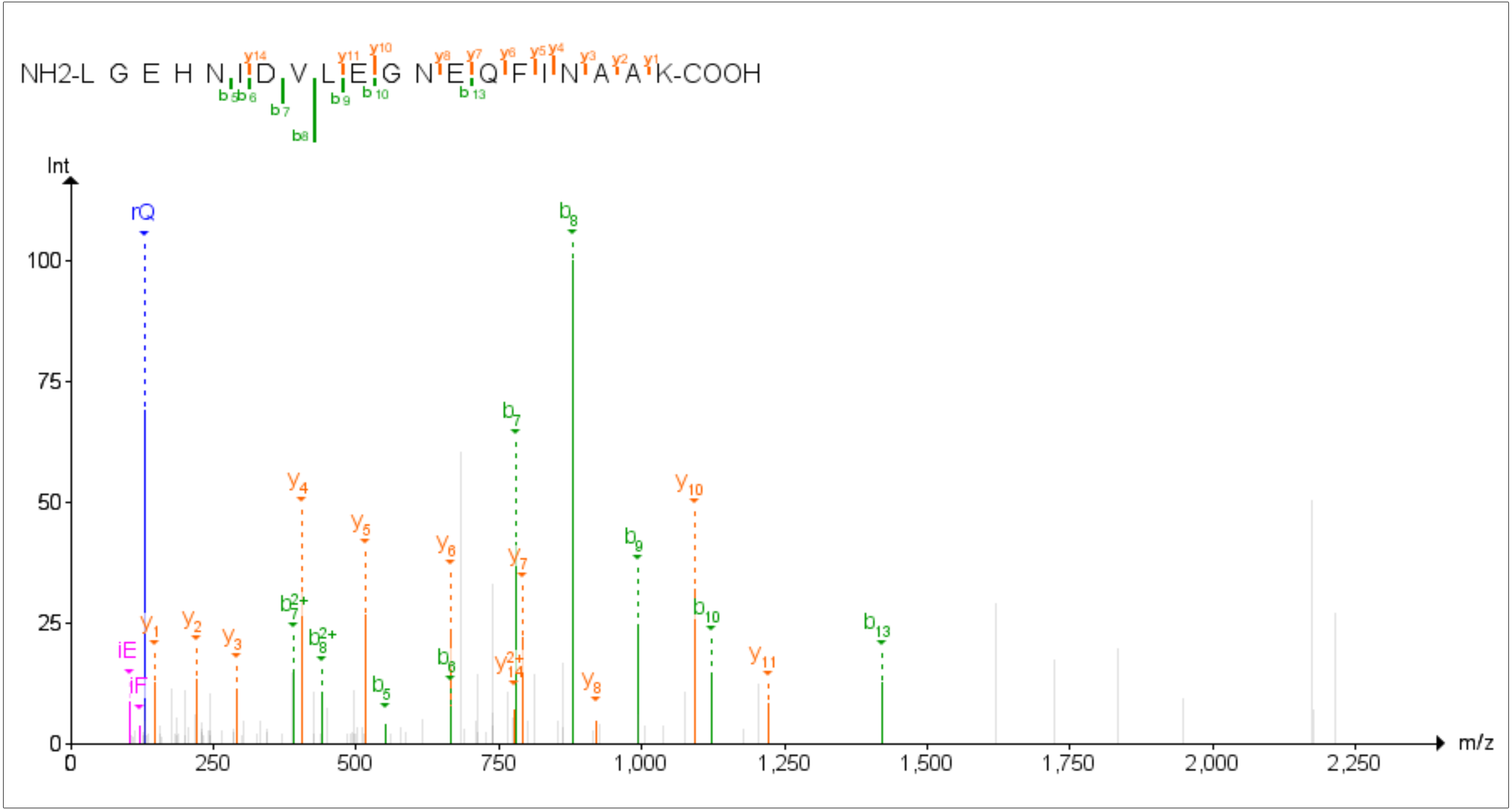
NH2-L G E H N I D V L E G N E Q F I N A A K-COOH
Int
m/z
rQ
iE
iF
0
25
50
75
100
250
500
750
1,000
1,250
1,500
1,750
2,000
2,250

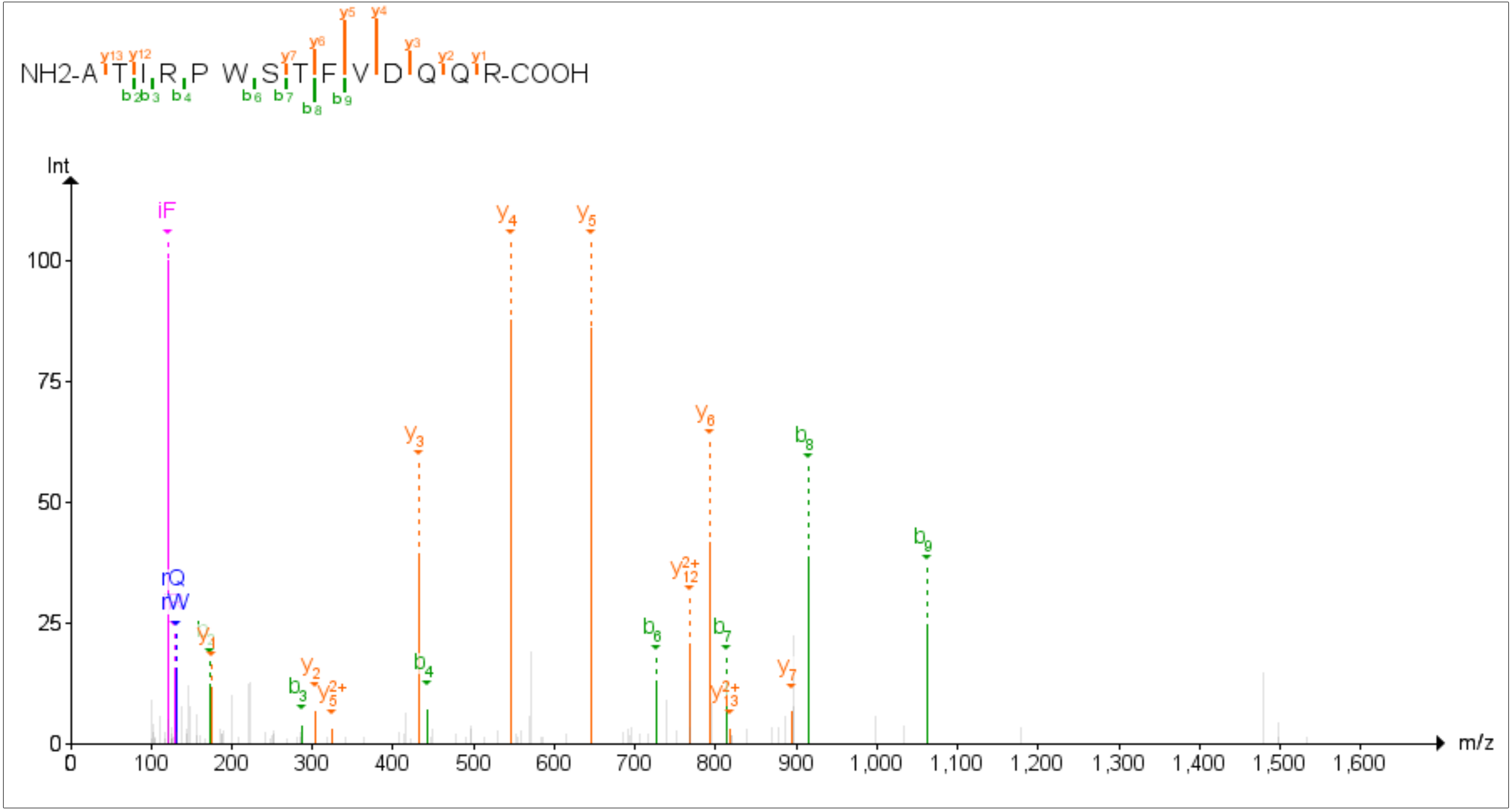

NH2-ATIIRPWSTFVDQQR-COOH
y13 y12 y7 y6 y5 y4 y3 y2 y1
b2 b3 b4 b6 b7 b8 b9
Int
100
75
50
25
0
iF
y4
y5
y6
y3
b8
b9
rQ
rW
y1
y2
b3
y5 2+
b4
b6
y12 2+
b7
y13 2+
y7
0 100 200 300 400 500 600 700 800 900 1,000 1,100 1,200 1,300 1,400 1,500 1,600
m/z

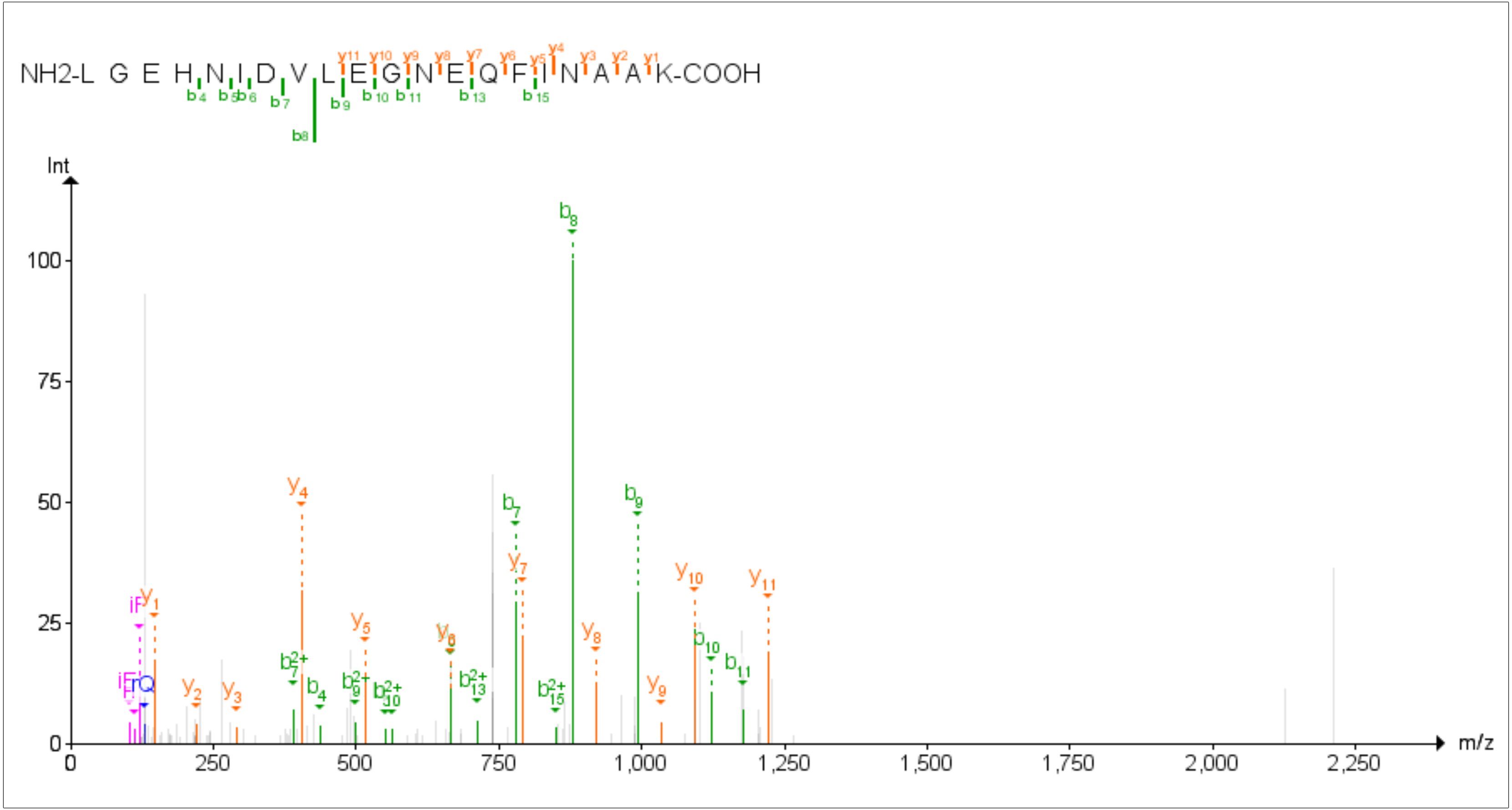
NH2-L G E H N I D V L E G N E Q F I N A A K-COOH
Int
100
75
50
25
0
0
250
500
750
1,000
1,250
1,500
1,750
2,000
2,250
m/z

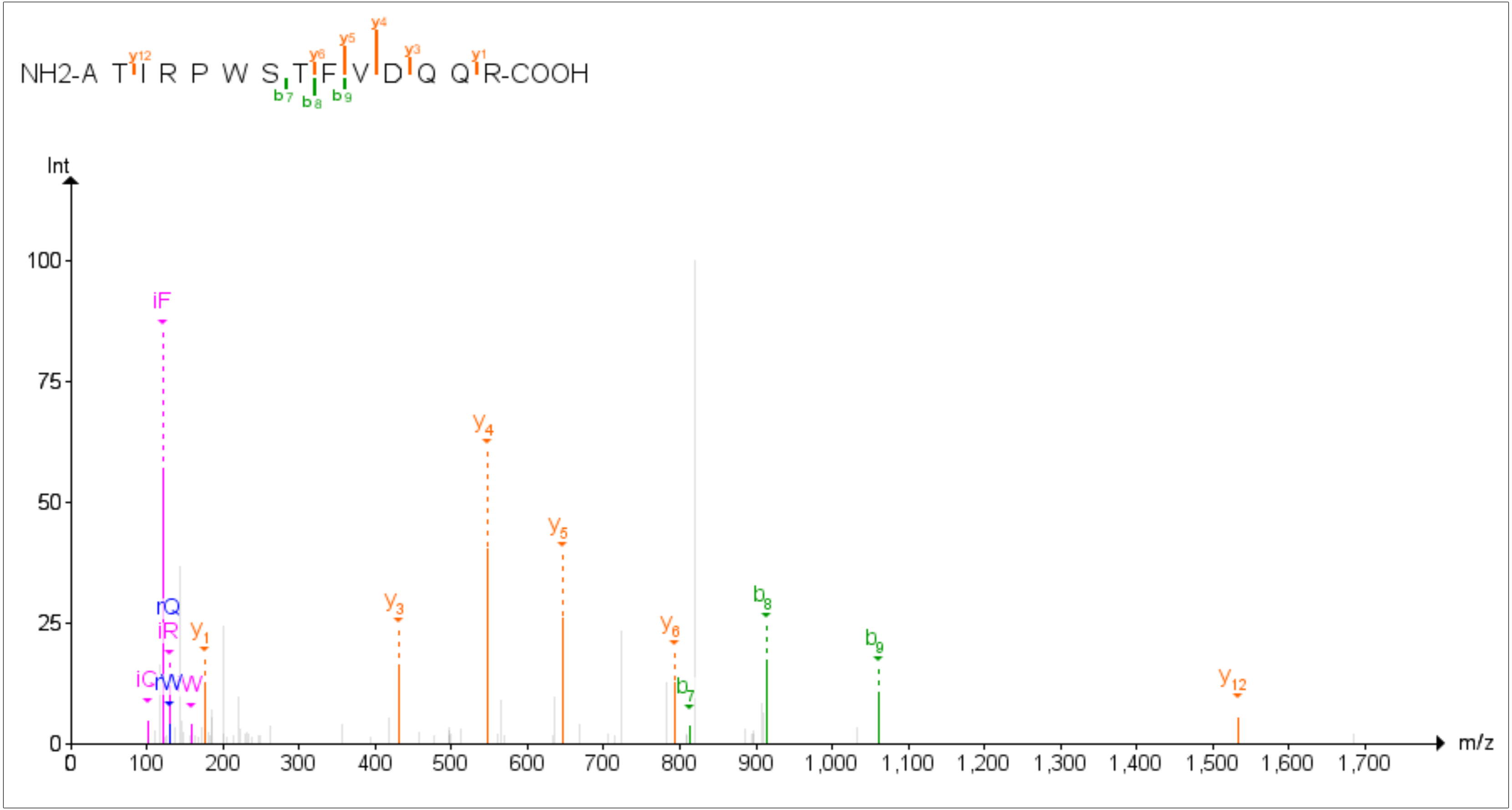

NH2-A T I R P W S T F V D Q Q R-COOH
y12
y6
y5
y4
y3
y1
b7
b8
b9
Int
100
75
50
25
0
iF
y4
y5
y3
rQ
iR
y1
iC
rW
W
y6
b8
b7
b9
y12
0
100
200
300
400
500
600
700
800
900
1,000
1,100
1,200
1,300
1,400
1,500
1,600
1,700
m/z

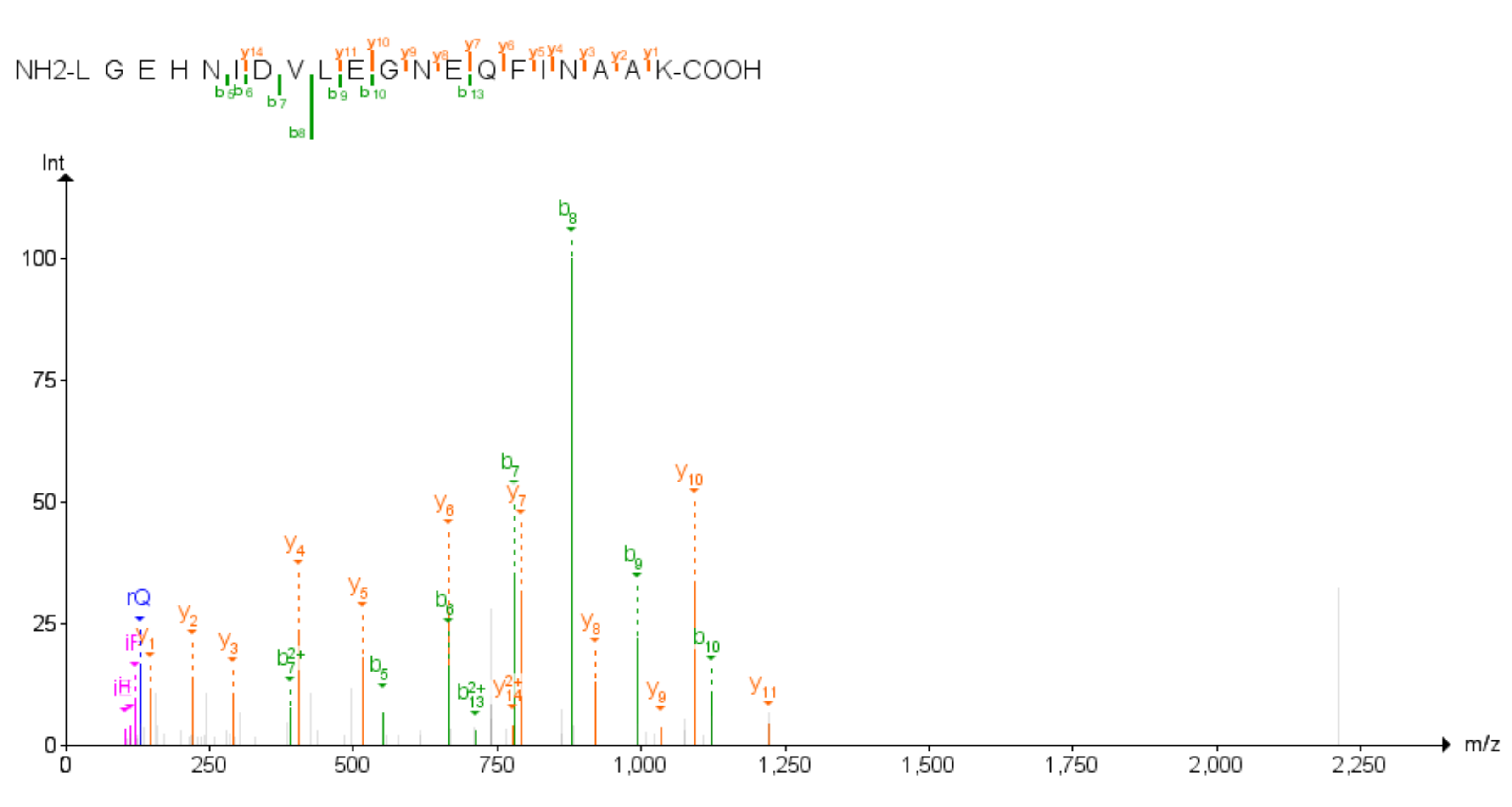

NH2-L G E H N I D V L E G N E Q F I N A A K-COOH
Int
100
75
50
25
0
m/z
0
250
500
750
1,000
1,250
1,500
1,750
2,000
2,250

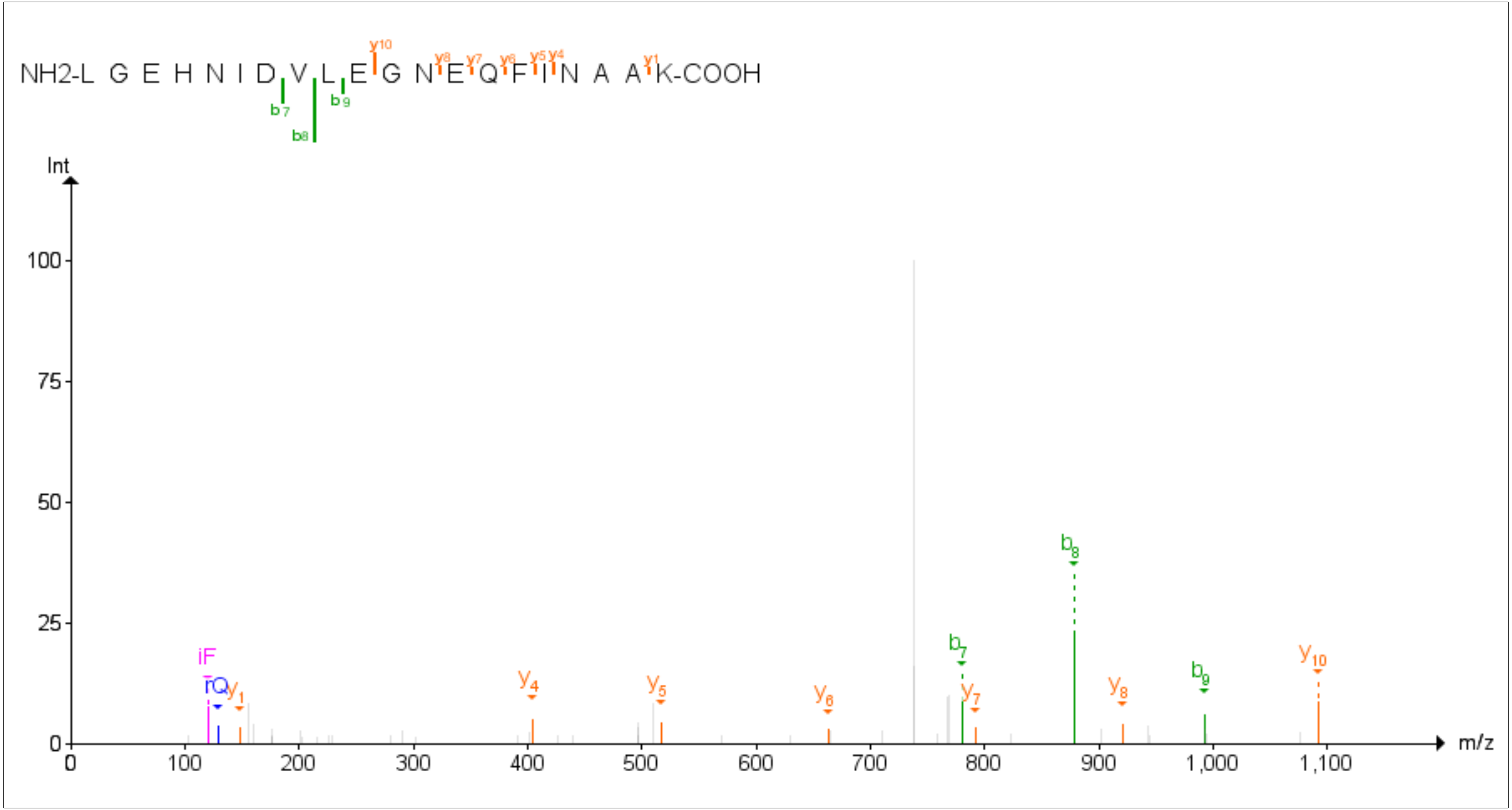

NH2-L G E H N I D V L E G N E Q F I N A A K-COOH
b7
b8
b9
y10
y8
y7
y6
y5
y4
y1
Int
100
75
50
25
0
iF
rQ
y1
y4
y5
y6
b7
y7
b8
y8
b9
y10
0
100
200
300
400
500
600
700
800
900
1,000
1,100
m/z

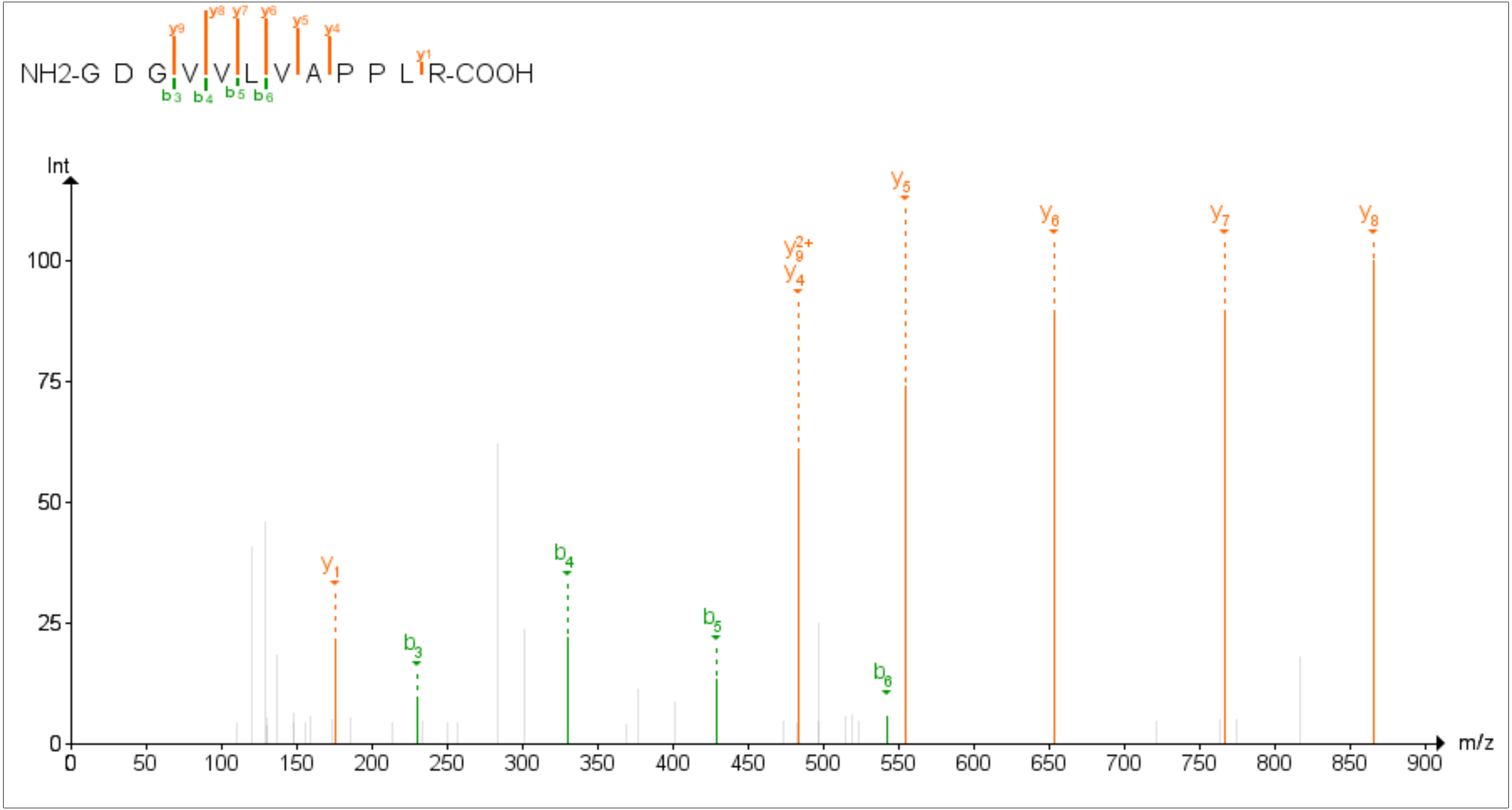
NH2-G D G V V L V A P P L R-COOH
y9 y8 y7 y6 y5 y4 y1
b3 b4 b5 b6
Int
m/z
y1
b3
b4
b5
y9 2+
y4
b6
y5
y6
y7
y8

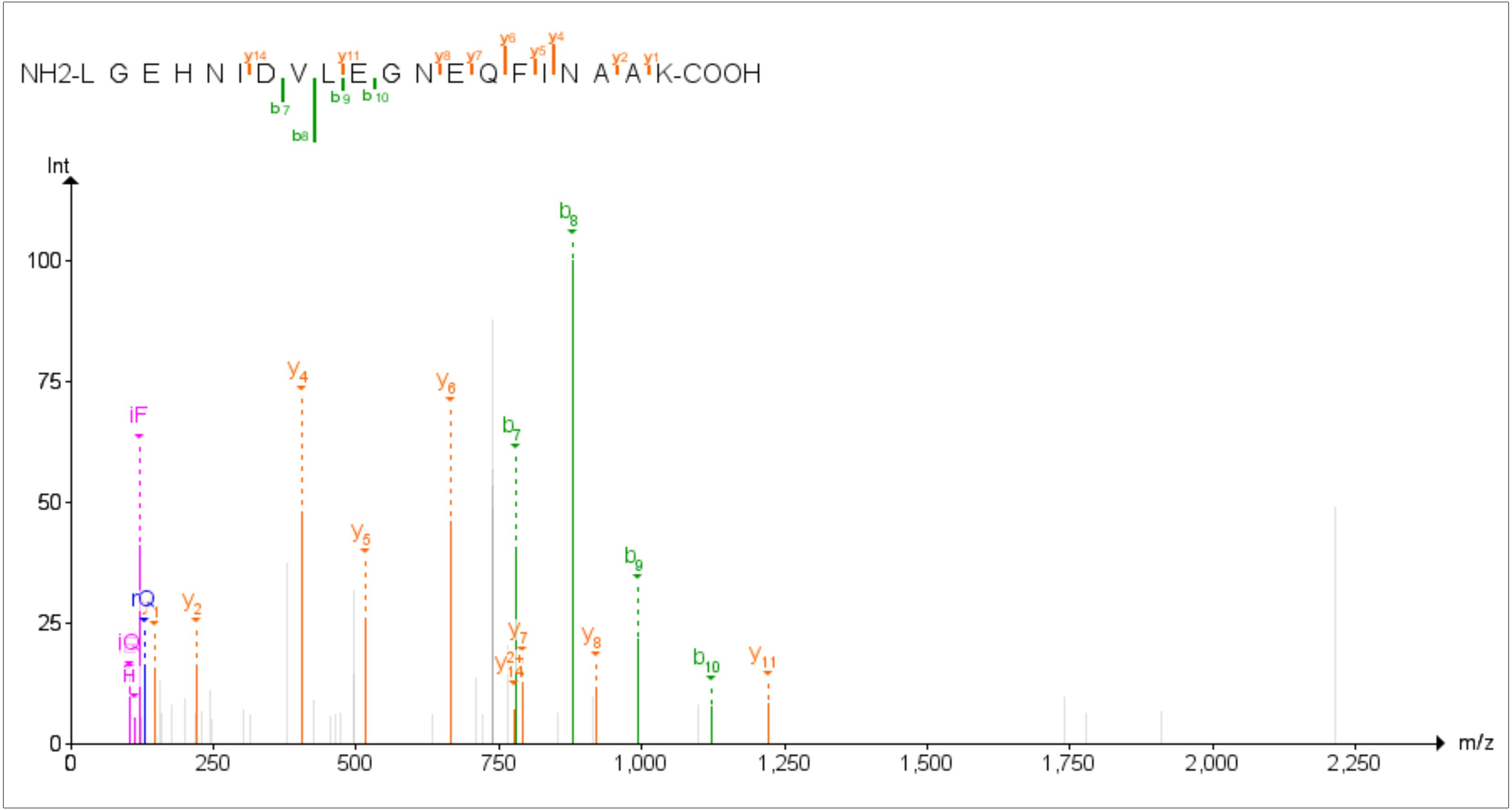

NH2-L G E H N I D V L E G N E Q F I N A A K-COOH
y14
y11
y8
y7
y6
y5
y4
y2
y1
b7
b8
b9
b10
Int
m/z
0
25
50
75
100
250
500
750
1,000
1,250
1,500
1,750
2,000
2,250
iF
rQ
iQ
y1
y2
y4
y5
y6
b7
y7
y14 2+
b8
y8
b9
b10
y11

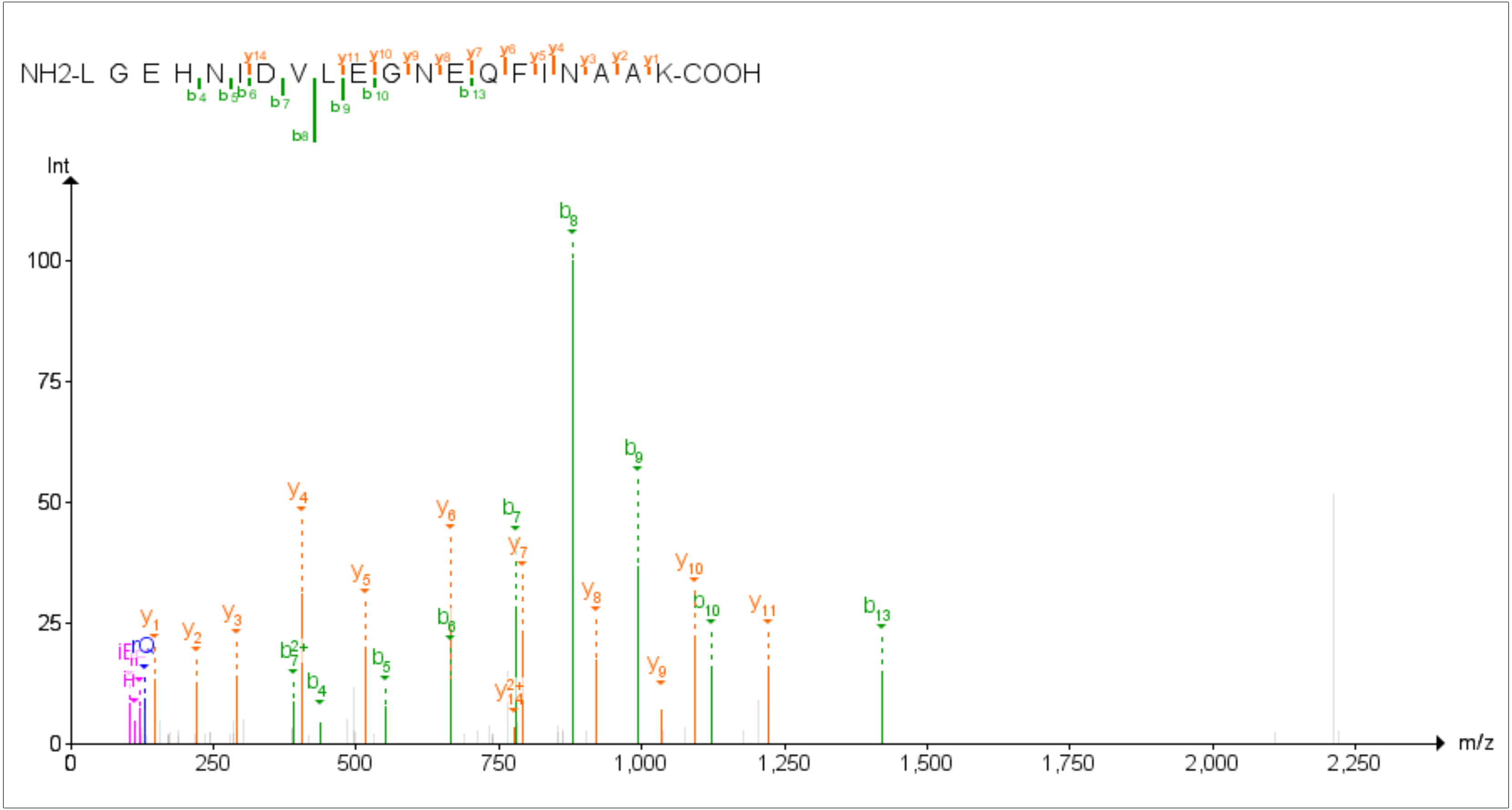

NH2-L G E H N I D V L E G N E Q F I N A A K-COOH
Int
m/z
0
25
50
75
100
250
500
750
1,000
1,250
1,500
1,750
2,000
2,250
b8
b9
b7
y4
y6
y7
y5
y10
y8
b10
y11
b13
y9
y1
y2
y3
b6
b5
b4
y14 2+
b7 2+

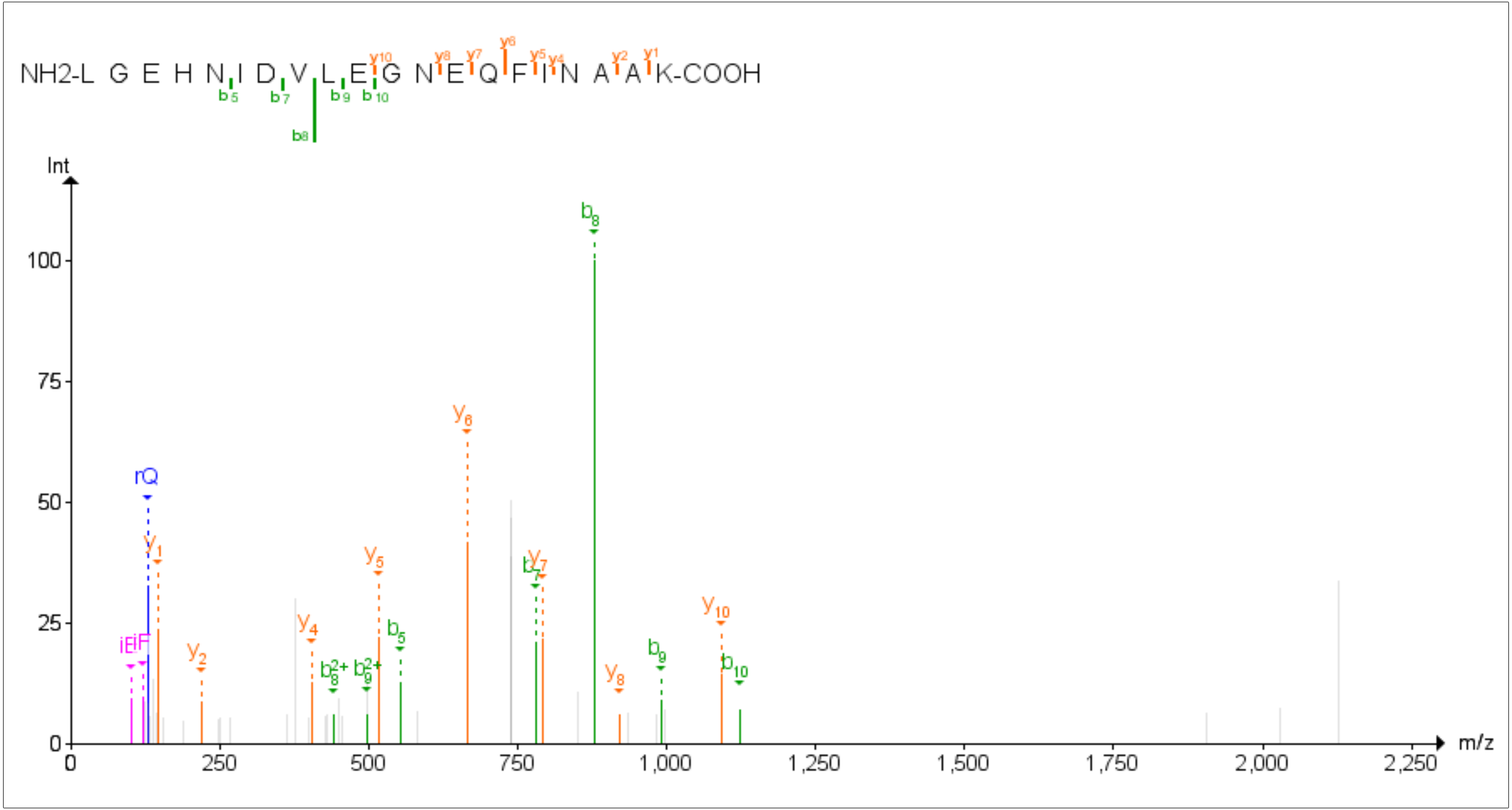
NH2-L G E H N I D V L E G N E Q F I N A A K-COOH
Int
m/z
b8
y6
rQ
y1
y5
b7
y7
y4
y2
iEiF
b5
b8 2+
b9 2+
y10
b9
b10
y8
0
25
50
75
100
250
500
750
1,000
1,250
1,500
1,750
2,000
2,250

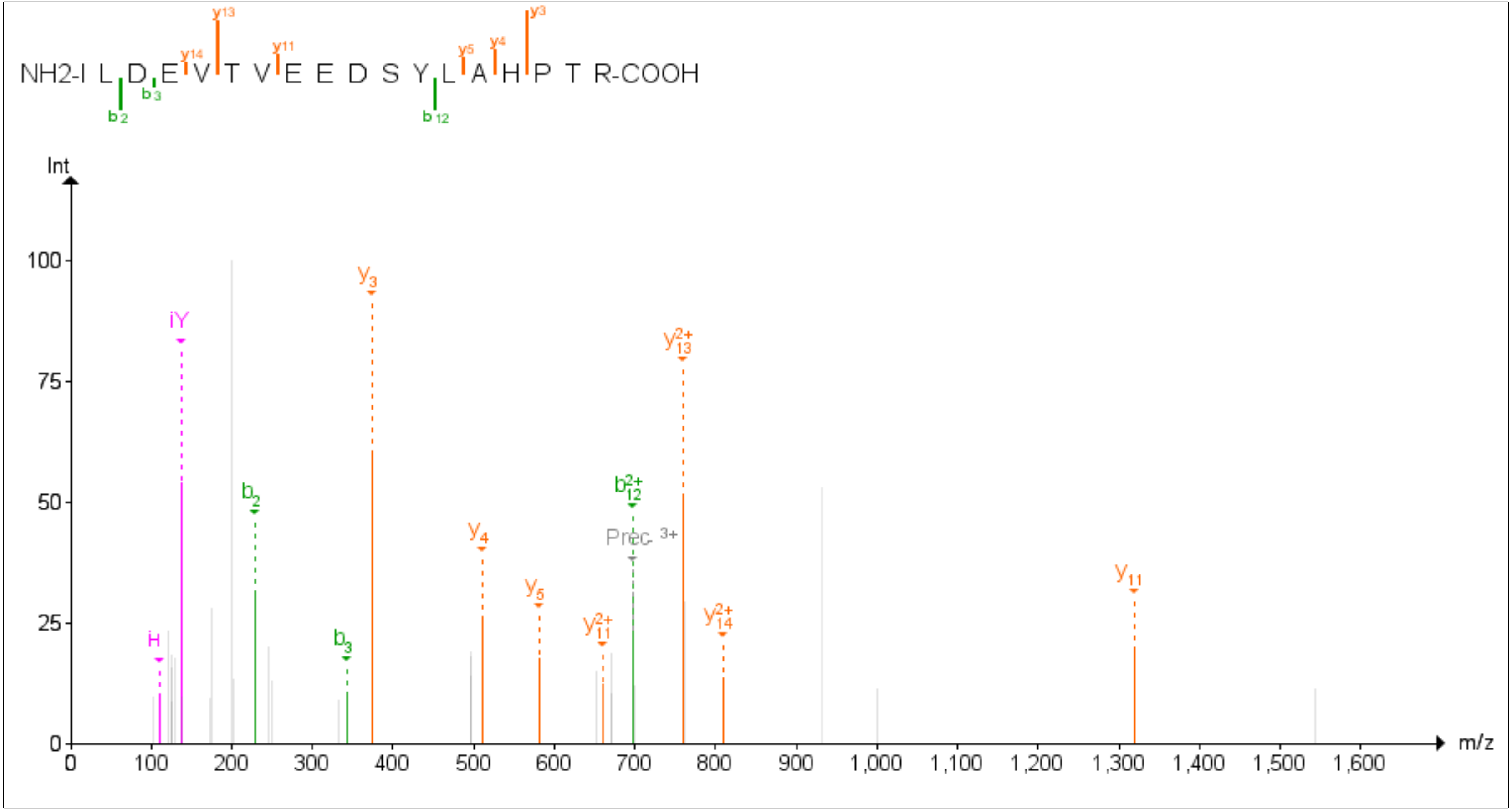

NH2-I L D E V T V E E D S Y L A H P T R-COOH
Int
m/z
iY
iH
b2
b3
y3
y4
y5
y13 2+
b12 2+
Prec. 3+
y11 2+
y14 2+
y11

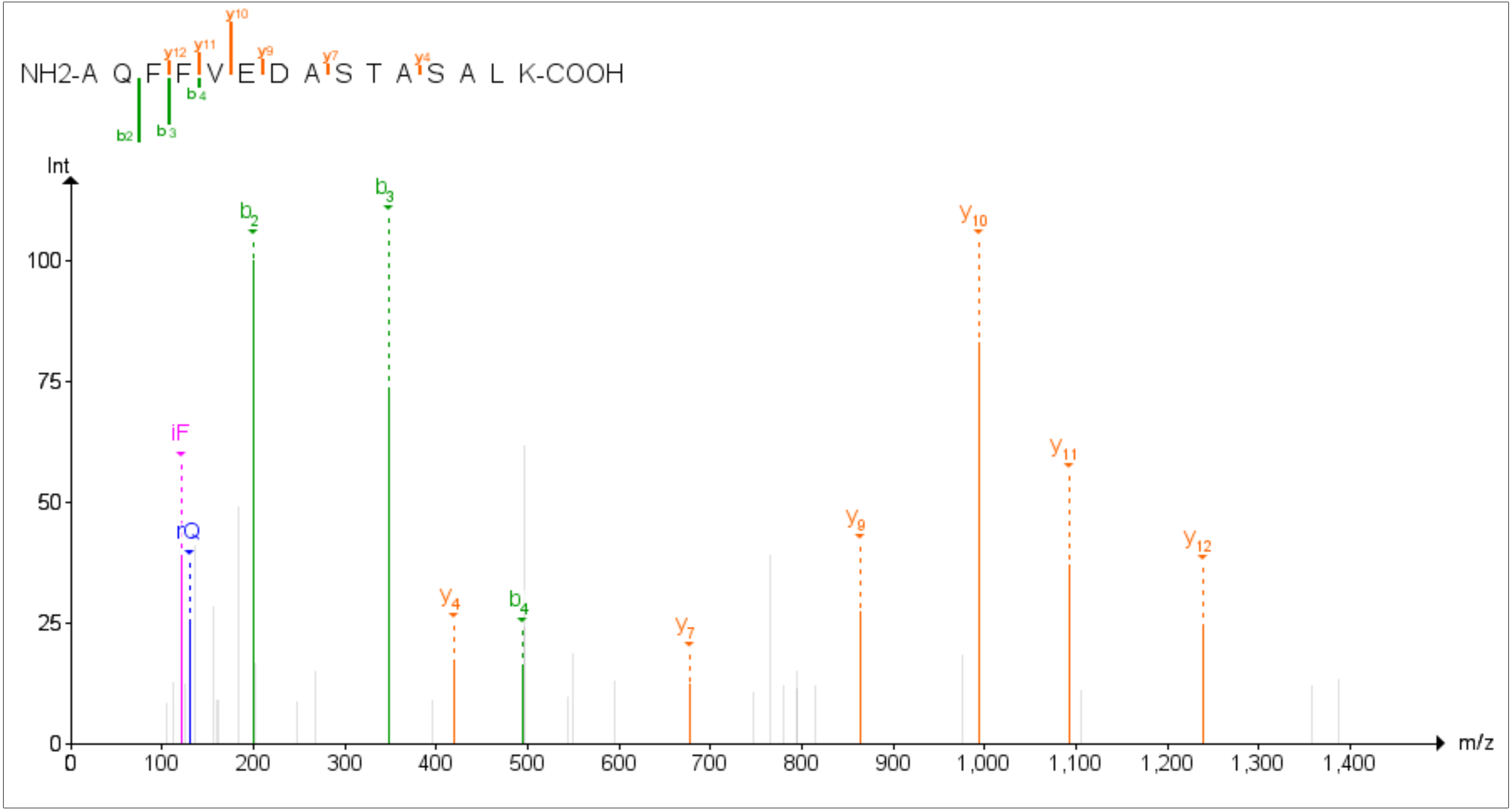
NH2-A Q F F V E D A S T A S A L K-COOH
y10 y12 y11 y9 y7 y4
b2 b3 b4
Int
iF
rQ
b2
b3
y4
b4
y7
y9
y10
y11
y12
0 25 50 75 100
0 100 200 300 400 500 600 700 800 900 1,000 1,100 1,200 1,300 1,400
m/z

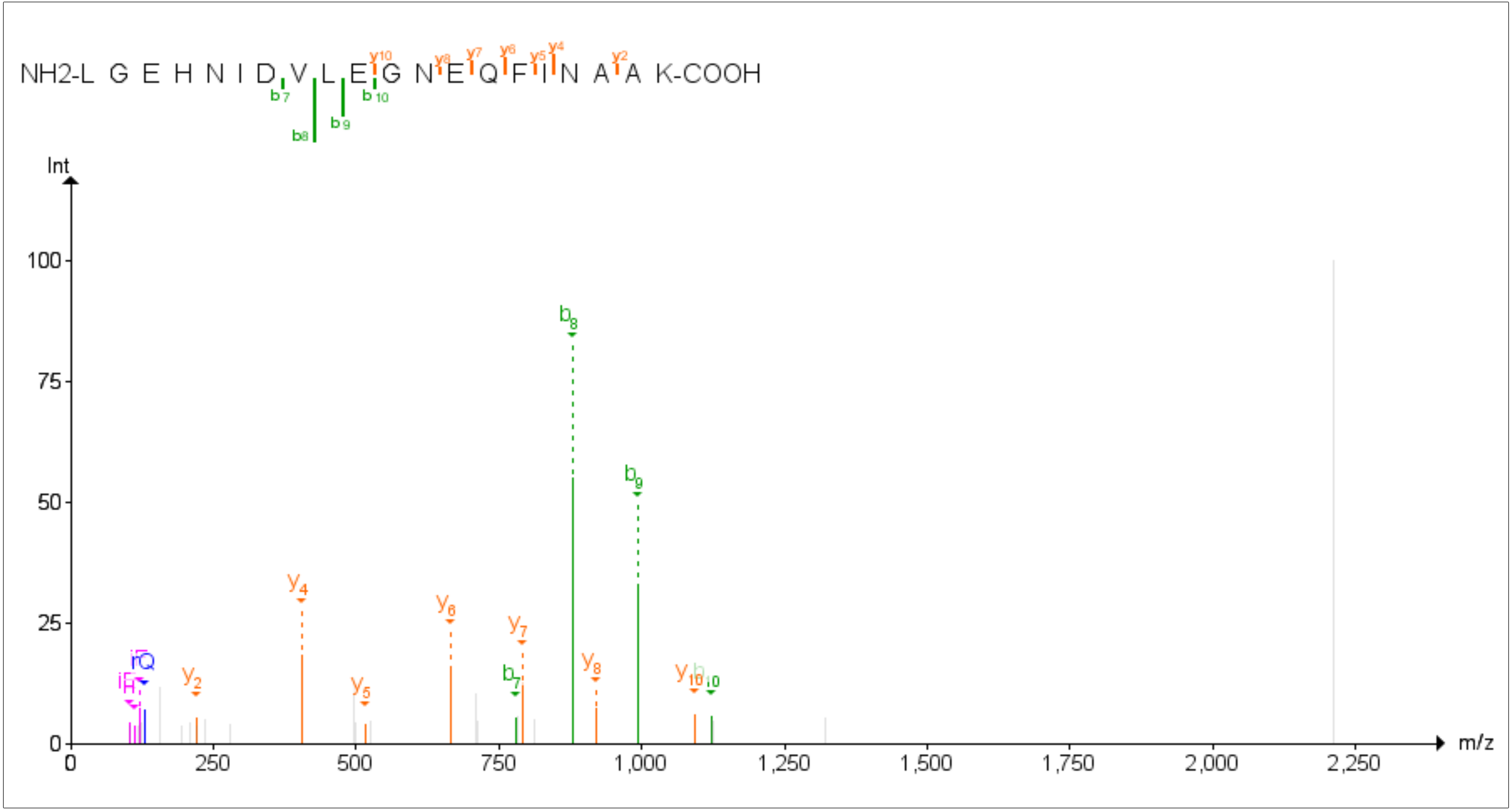

NH2-L G E H N I D V L E G N E Q F I N A A K-COOH
b7
b8
b9
b10
y10
y8
y7
y6
y5
y4
y2
Int
100
75
50
25
0
m/z
0
250
500
750
1,000
1,250
1,500
1,750
2,000
2,250
b8
b9
y4
y6
y7
b7
y8
y2
y5
y10
iQ

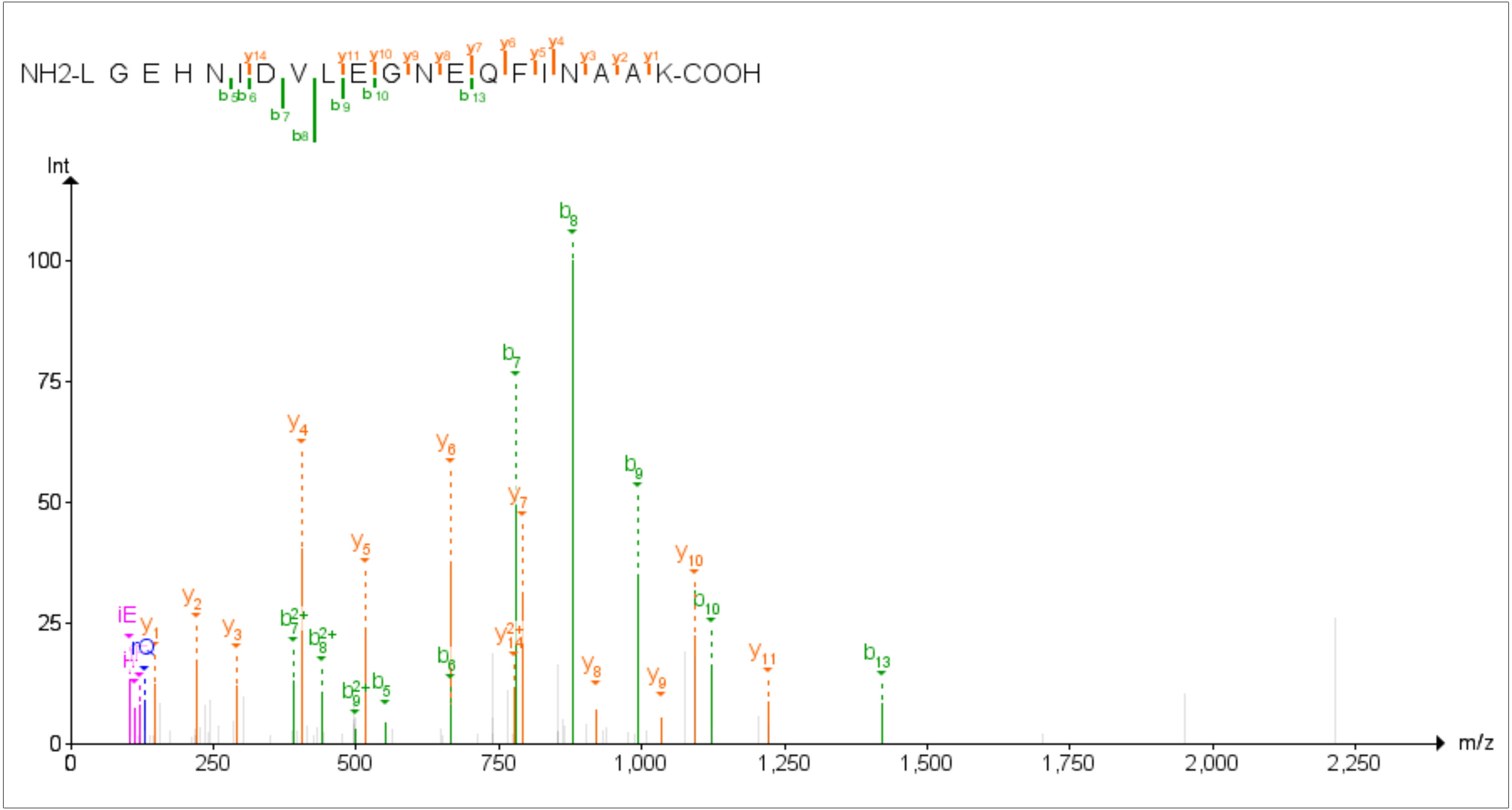
NH2-L G E H N I D V L E G N E Q F I N A A K-COOH
Int
m/z
b8
b7
b9
y4
y6
y7
y5
y10
b10
y2
y3
y1
iE
rQ
y8
y9
y11
b13
b6
b5
0
25
50
75
100
250
500
750
1,000
1,250
1,500
1,750
2,000
2,250

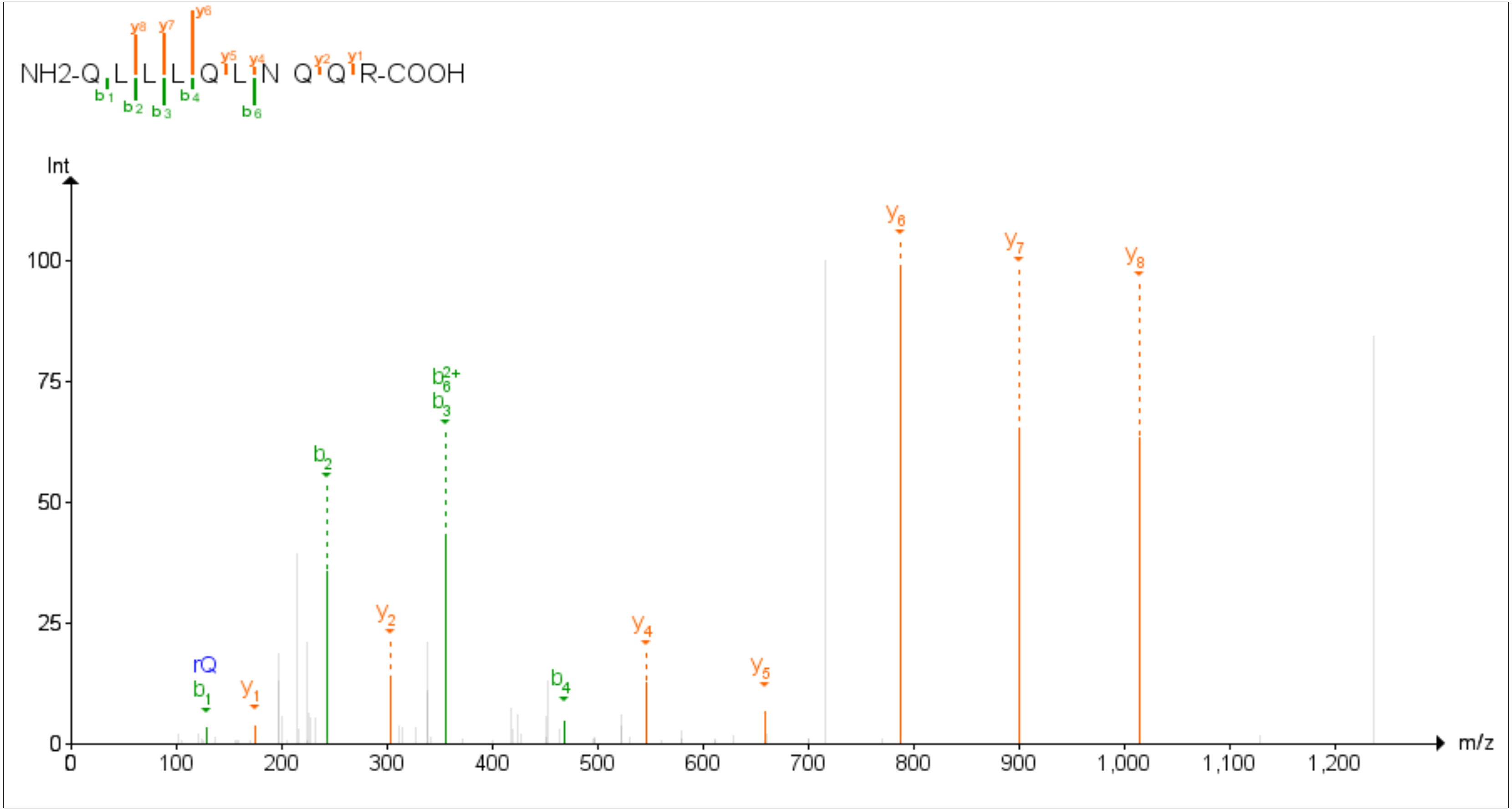

NH2-QLLLLQLN QQR-COOH
Int
m/z
y6
y7
y8
b2
b6 2+
b3
y2
y4
y5
b4
rQ
b1
y1

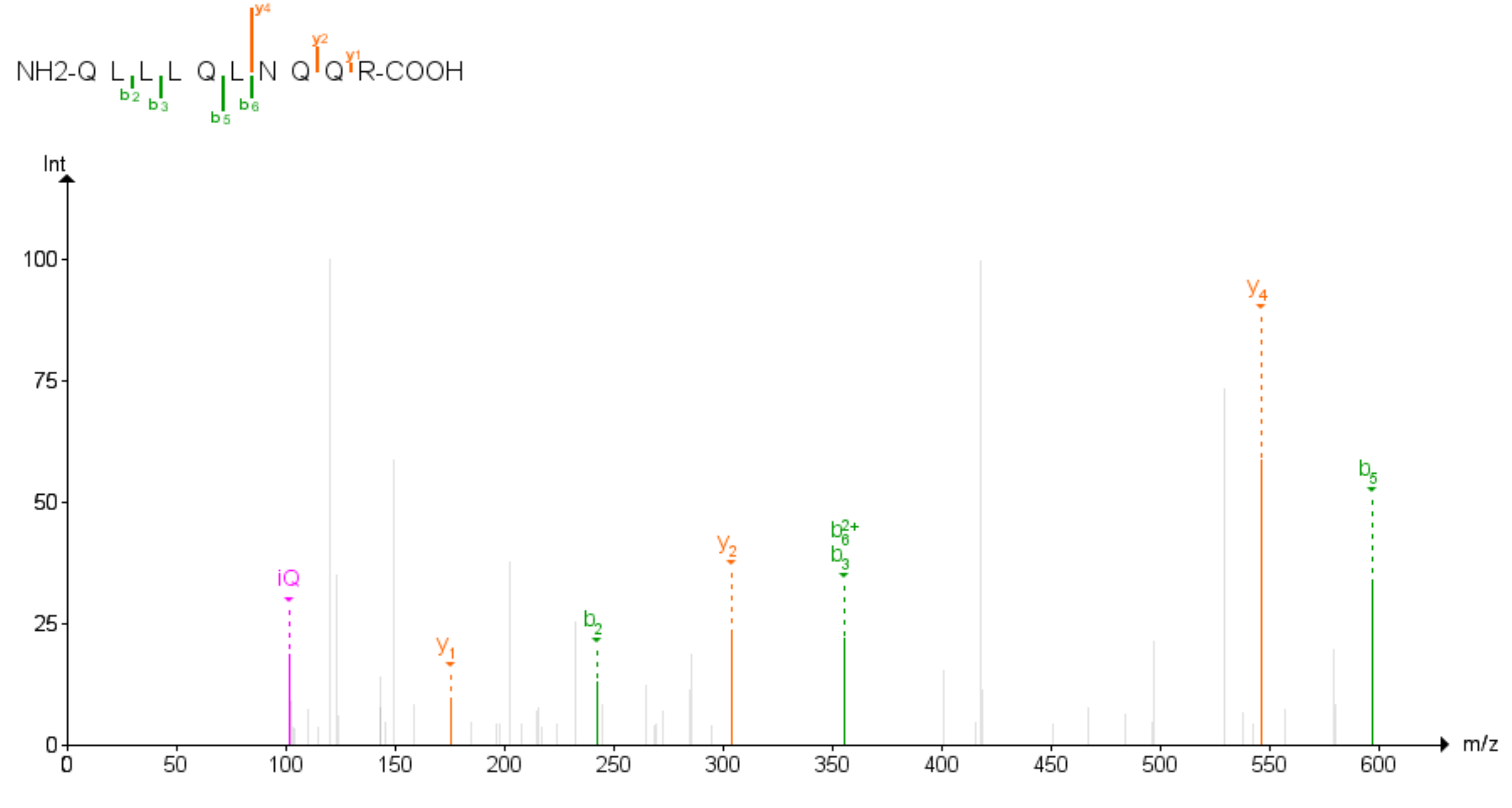

NH2-Q L L L Q L N Q Q R-COOH
y4
y2
y1
b2
b3
b5
b6
Int
100
75
50
25
0
y4
b5
b6 2+
b3
y2
b2
y1
iQ
0
50
100
150
200
250
300
350
400
450
500
550
600
m/z

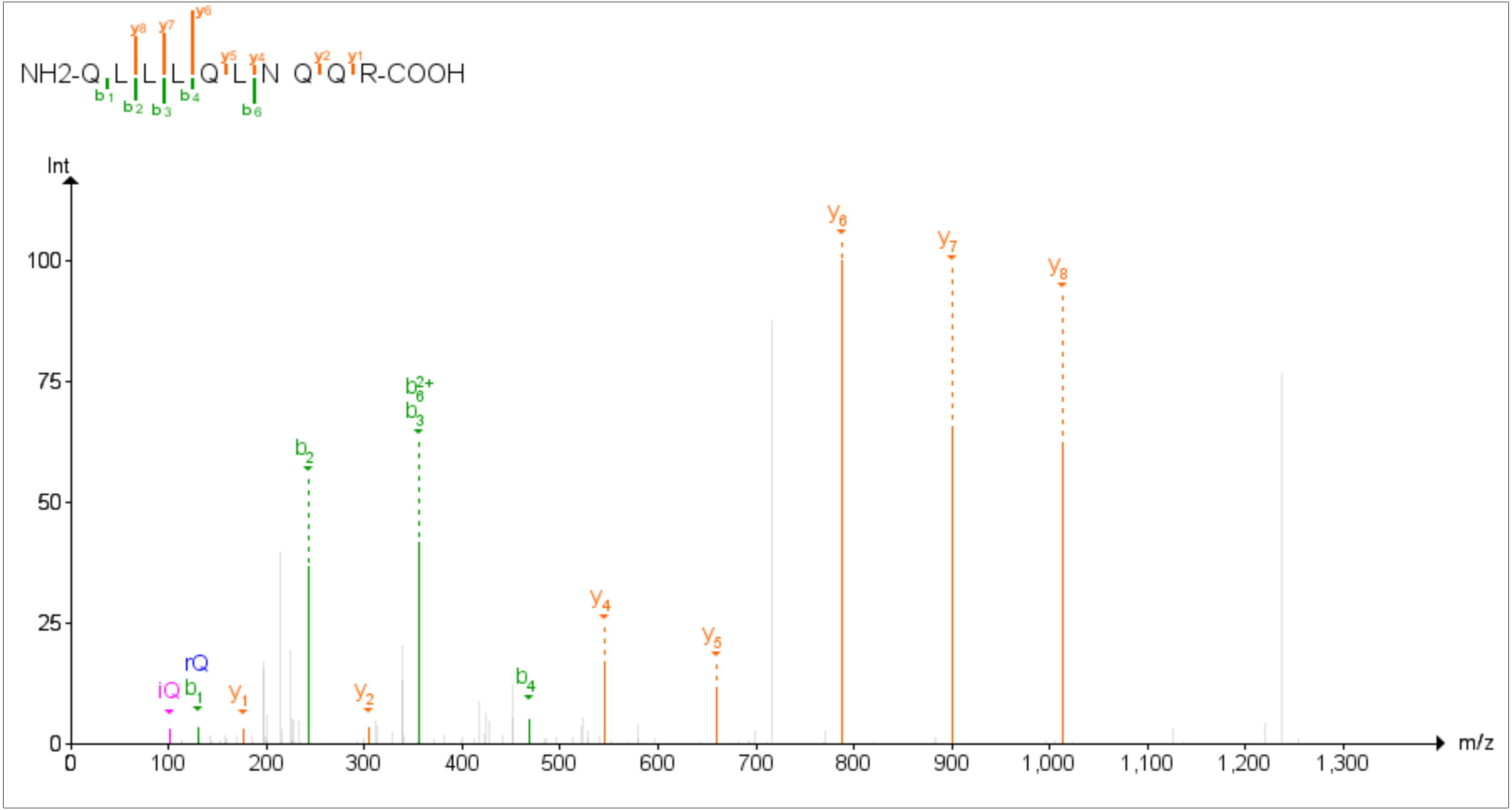

NH2-Q L L L L Q L N Q Q R-COOH
Int
m/z
iQ
rQ

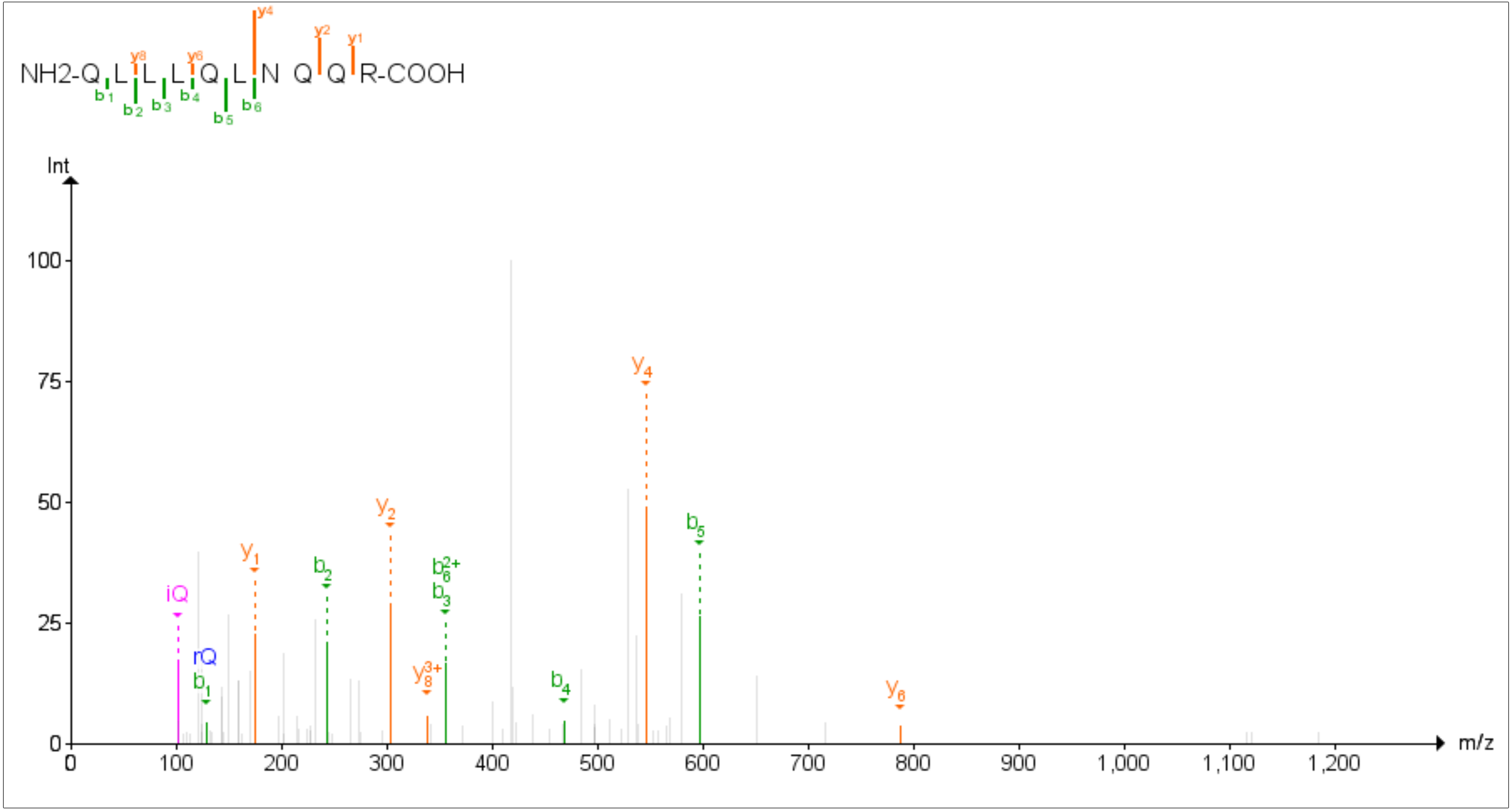

NH2-Q L L L Q L N Q Q R-COOH
y8 y6 y4 y2 y1
b1 b2 b3 b4 b5 b6
Int
100
75
50
25
0
iQ
rQ
b1
y1
b2
y2
b6 2+
b3
y8 3+
b4
y4
b5
y6
0
100
200
300
400
500
600
700
800
900
1,000
1,100
1,200
m/z

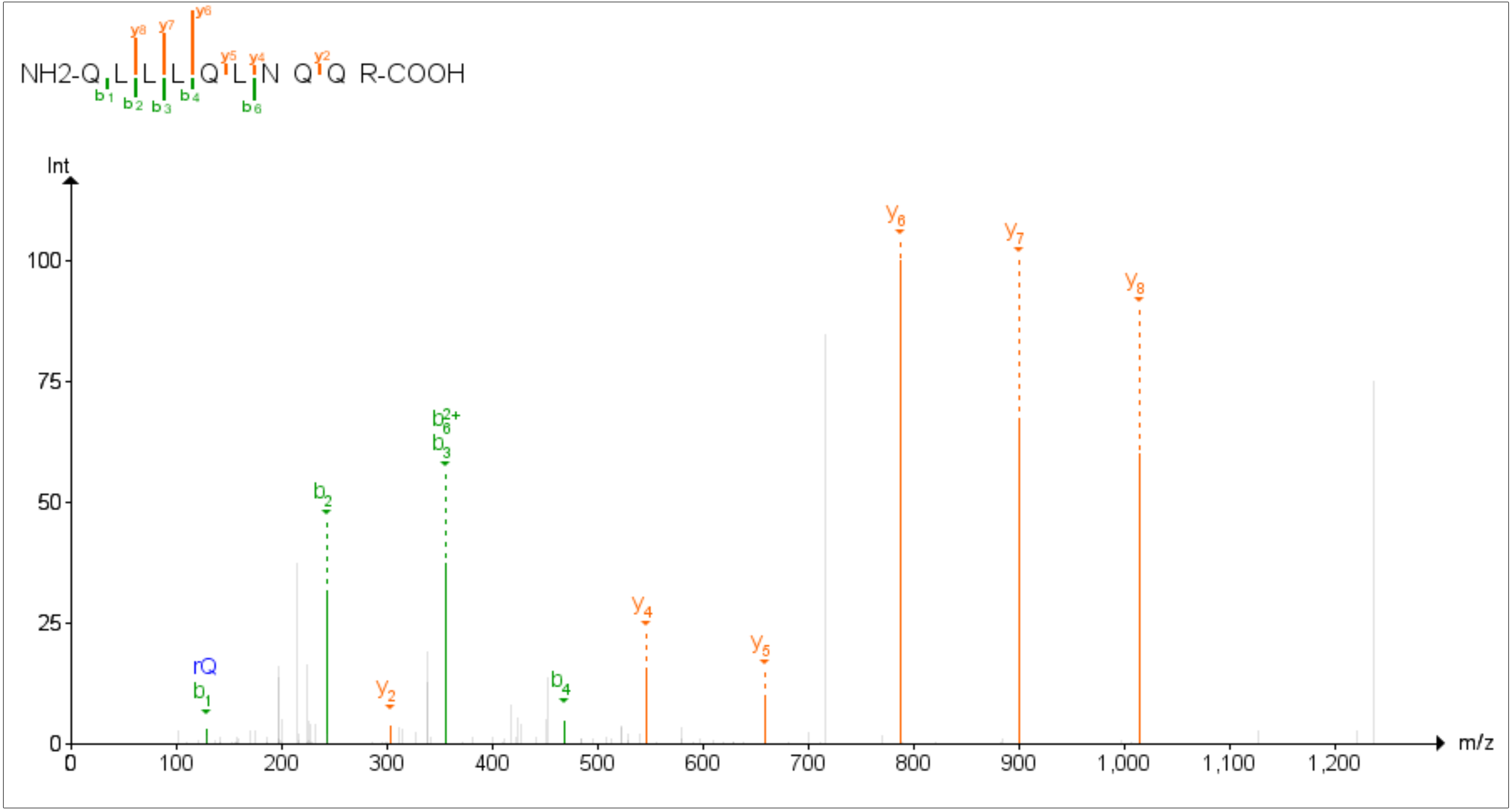

NH2-Q L L L L Q L N Q Q R-COOH
b1 b2 b3 b4 b6
y8 y7 y6 y5 y4 y2
Int
m/z
rQ
b1
b2
b6 2+
b3
y2
b4
y4
y5
y6
y7
y8

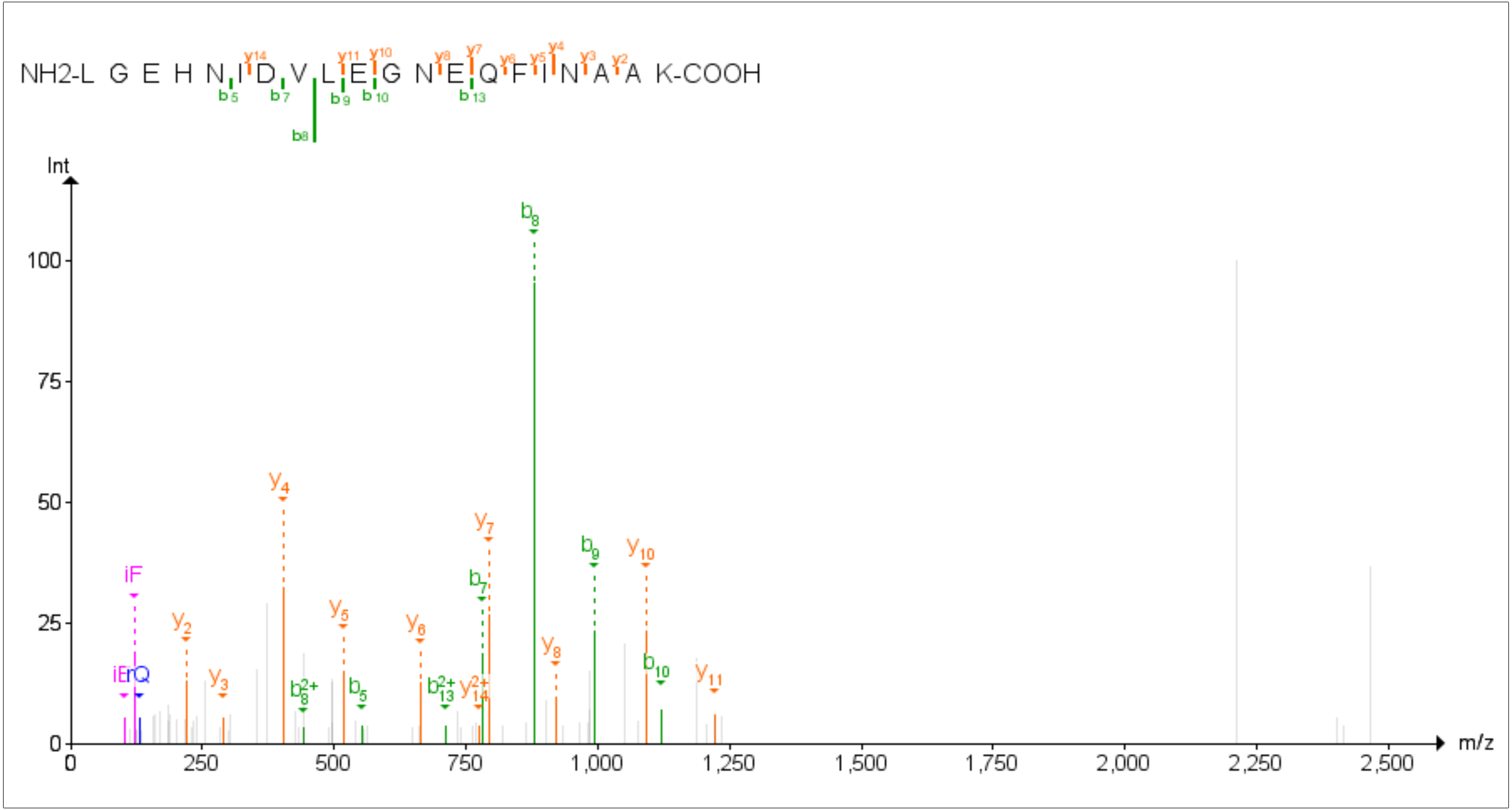
NH2-L G E H N I D V L E G N E Q F I N A A K-COOH
y14 y11 y10 y8 y7 y6 y5 y4 y3 y2
b5 b7 b8 b9 b10 b13
Int
m/z
0 25 50 75 100
0 250 500 750 1,000 1,250 1,500 1,750 2,000 2,250 2,500
b8
y4
y7
b9
y10
b7
iF
y2
y5
y6
y8
b10
y11
iE
rQ
y3
b8 2+
b5
b13 2+
y14 2+

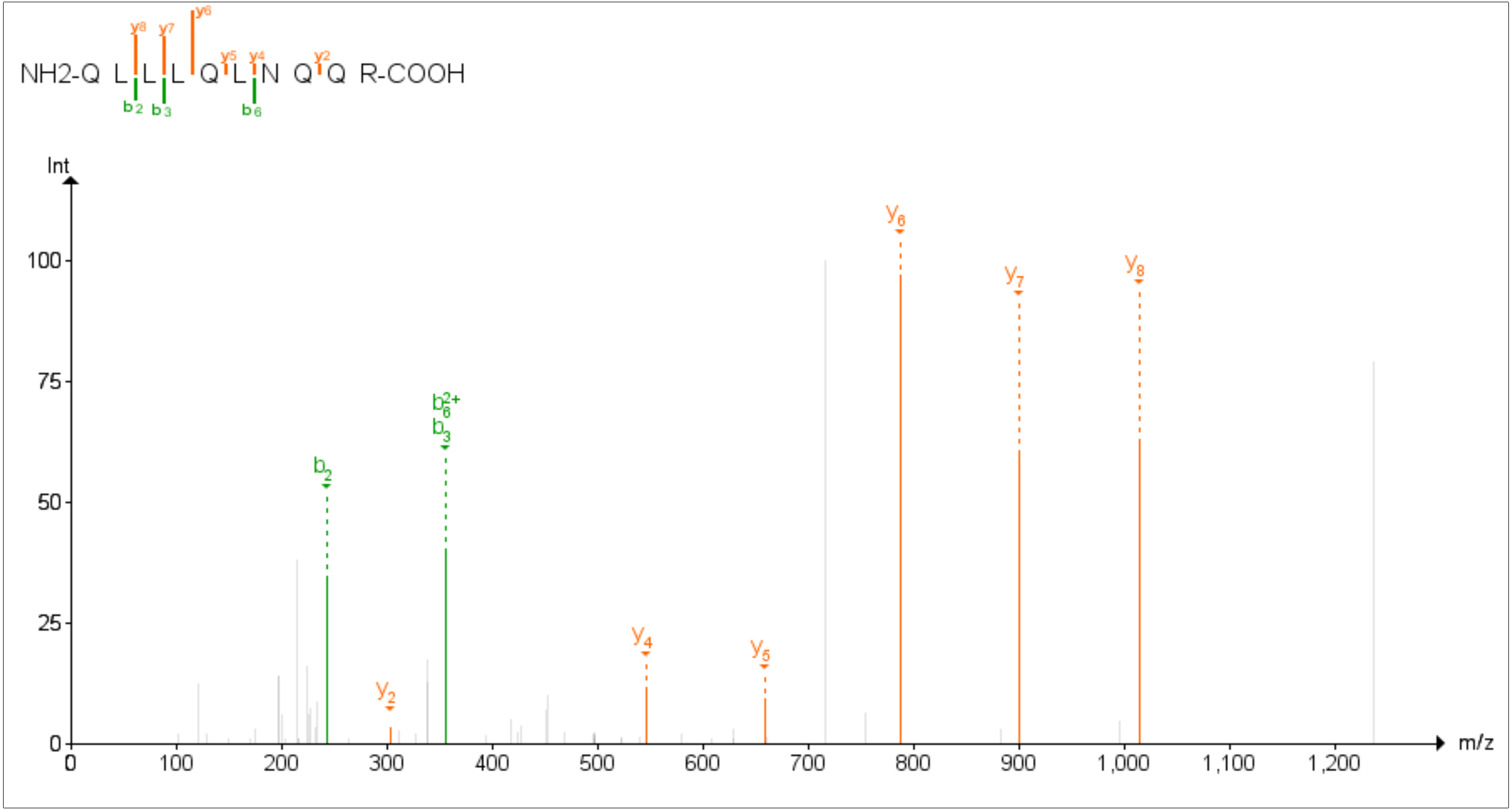

NH2-Q L L L Q L N Q Q R-COOH
y8
y7
y6
y5
y4
y2
b2
b3
b6
Int
100
75
50
25
0
m/z
0
100
200
300
400
500
600
700
800
900
1,000
1,100
1,200
b2
b6 2+
b3
y2
y4
y5
y6
y7
y8

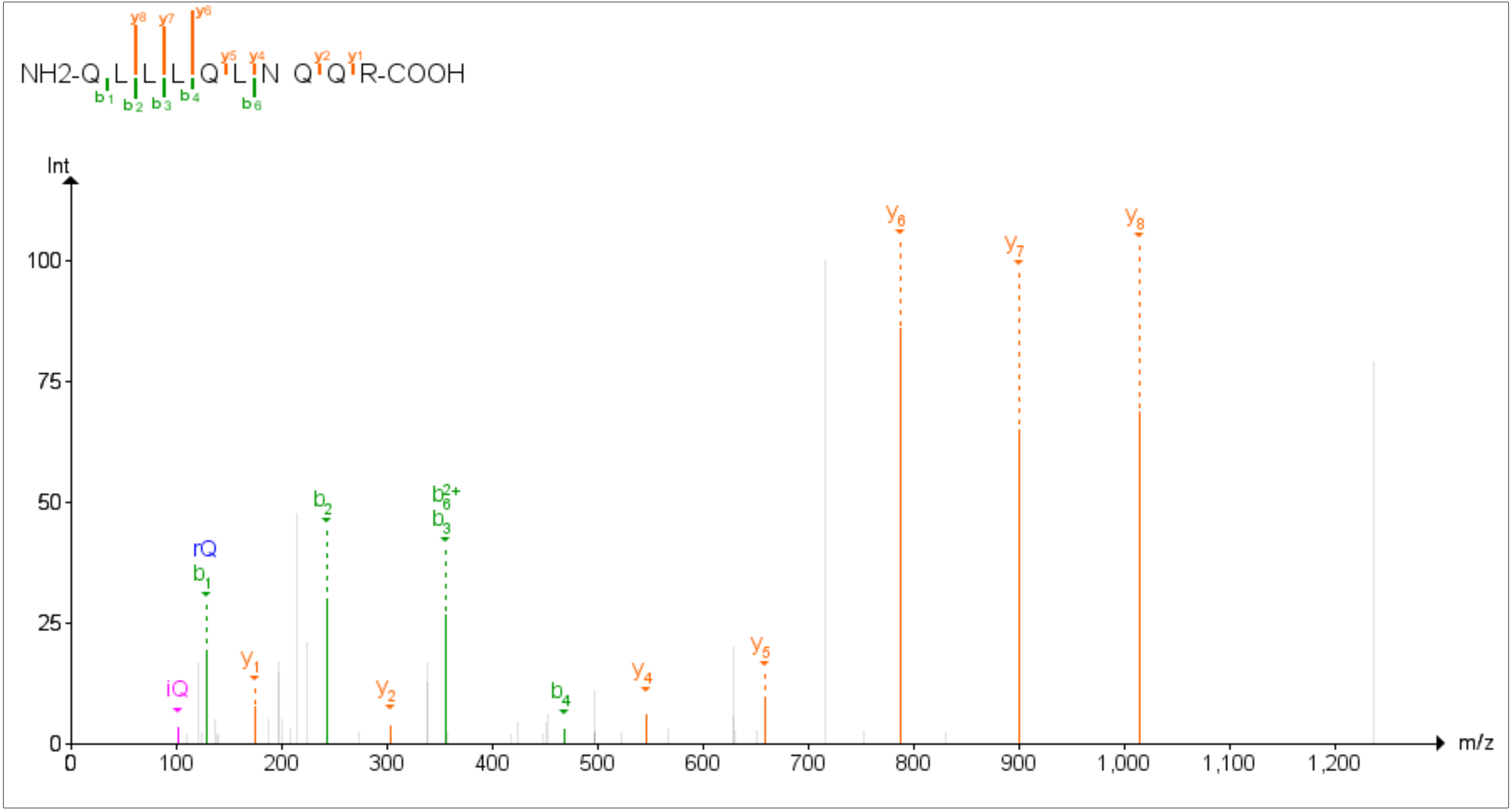

NH2-Q L L L Q L N Q Q R-COOH
y8 y7 y6 y5 y4 y2 y1
b1 b2 b3 b4 b6
Int
100
75
50
25
0
rQ
b1
iQ
y1
b2
y2
b6 2+
b3
b4
y4
y5
y6
y7
y8
0
100
200
300
400
500
600
700
800
900
1,000
1,100
1,200
m/z

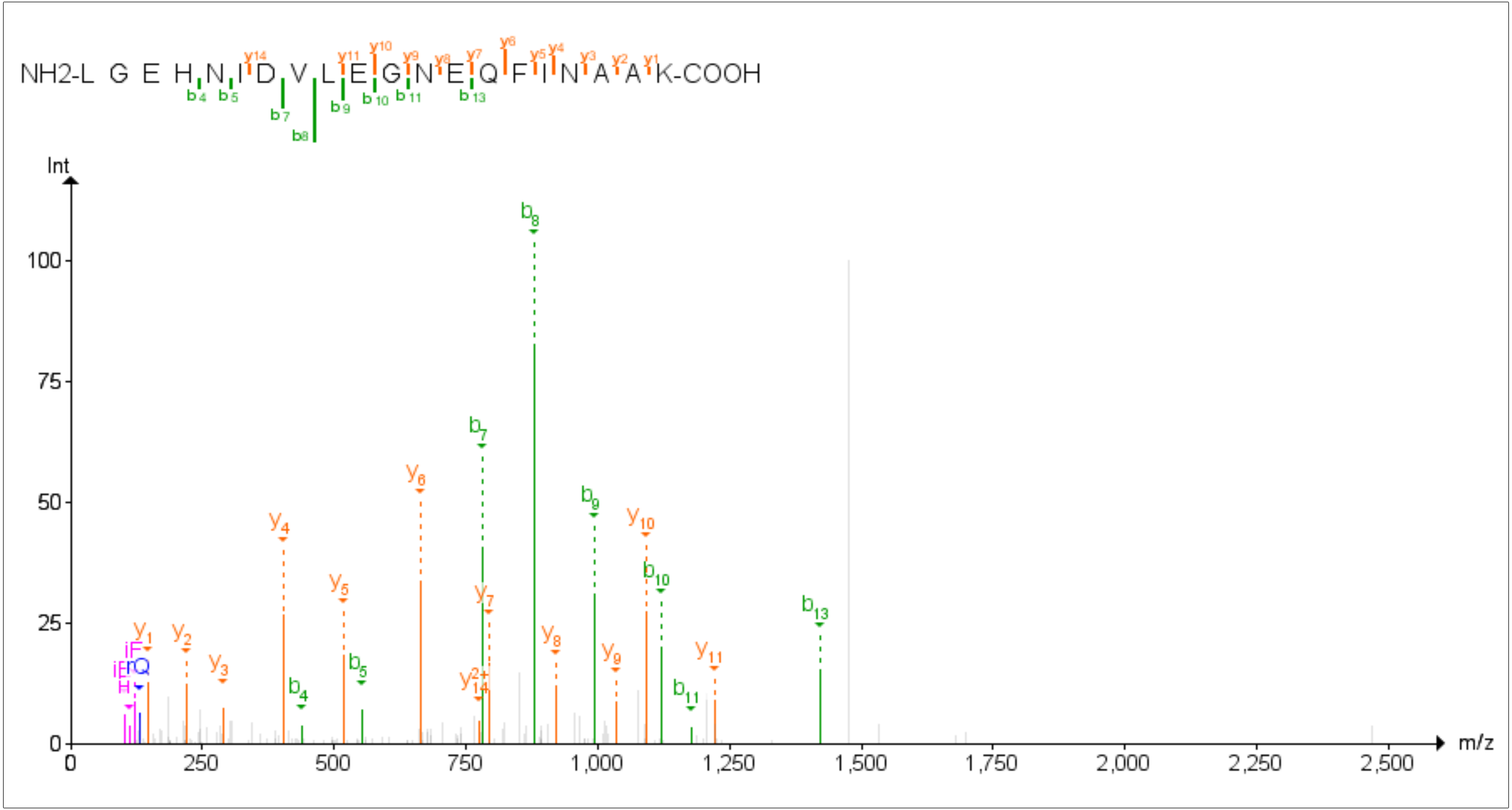
NH2-L G E H N I D V L E G N E Q F I N A A K-COOH
y14 y11 y10 y9 y8 y7 y6 y5 y4 y3 y2 y1
b4 b5 b7 b8 b9 b10 b11 b13
Int
100
75
50
25
0
0 250 500 750 1,000 1,250 1,500 1,750 2,000 2,250 2,500
m/z
b8 b7 y6 b9 y10 y4 b10 y5 y7 b13 b5 y8 y9 y11 y1 y2 y3 b4 b11 y14 2+
iF iE rQ H

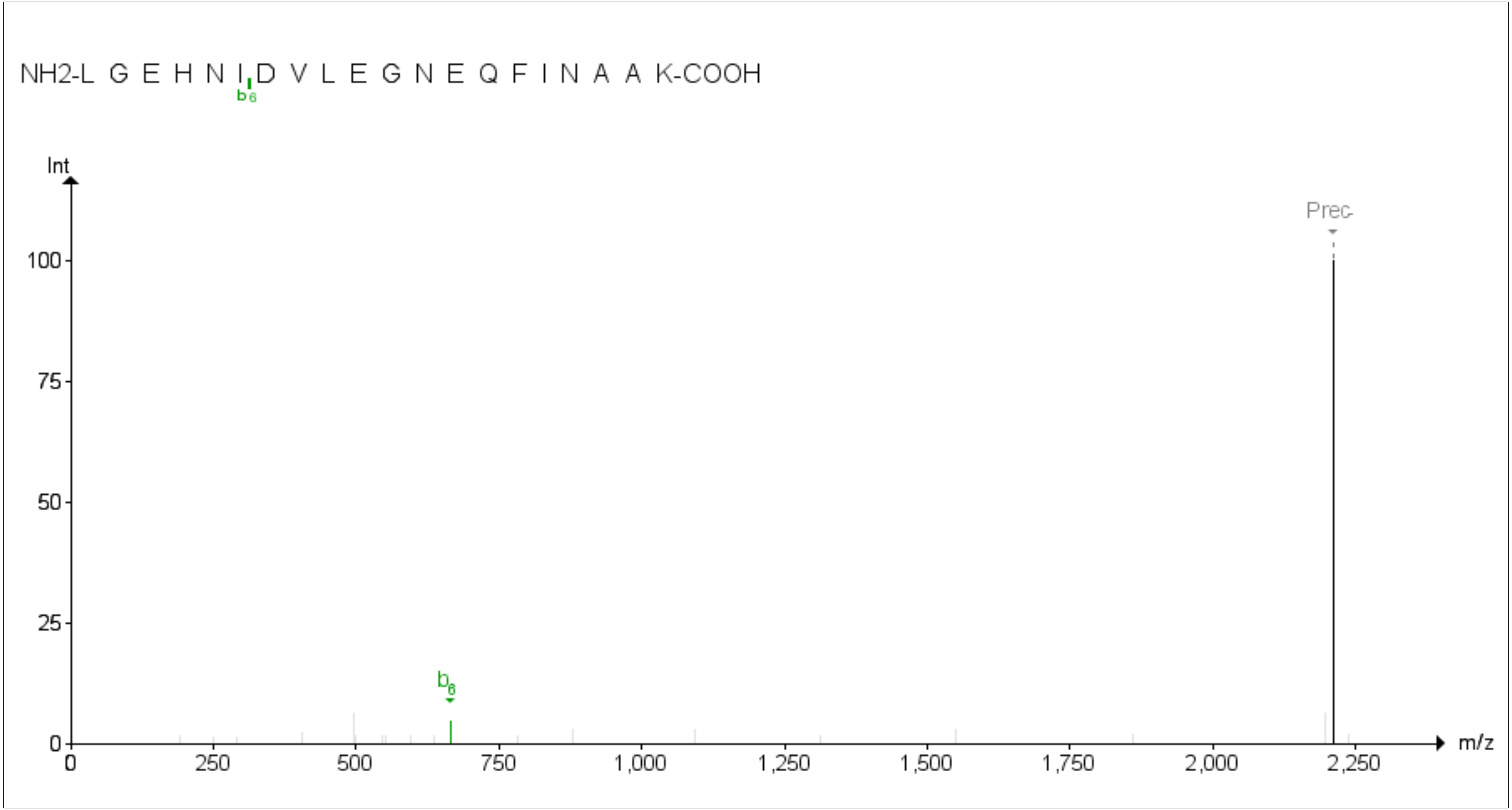

NH2-L G E H N I D V L E G N E Q F I N A A K-COOH
b6
Int
Prec
b6
100
75
50
25
0
0
250
500
750
1,000
1,250
1,500
1,750
2,000
2,250
m/z

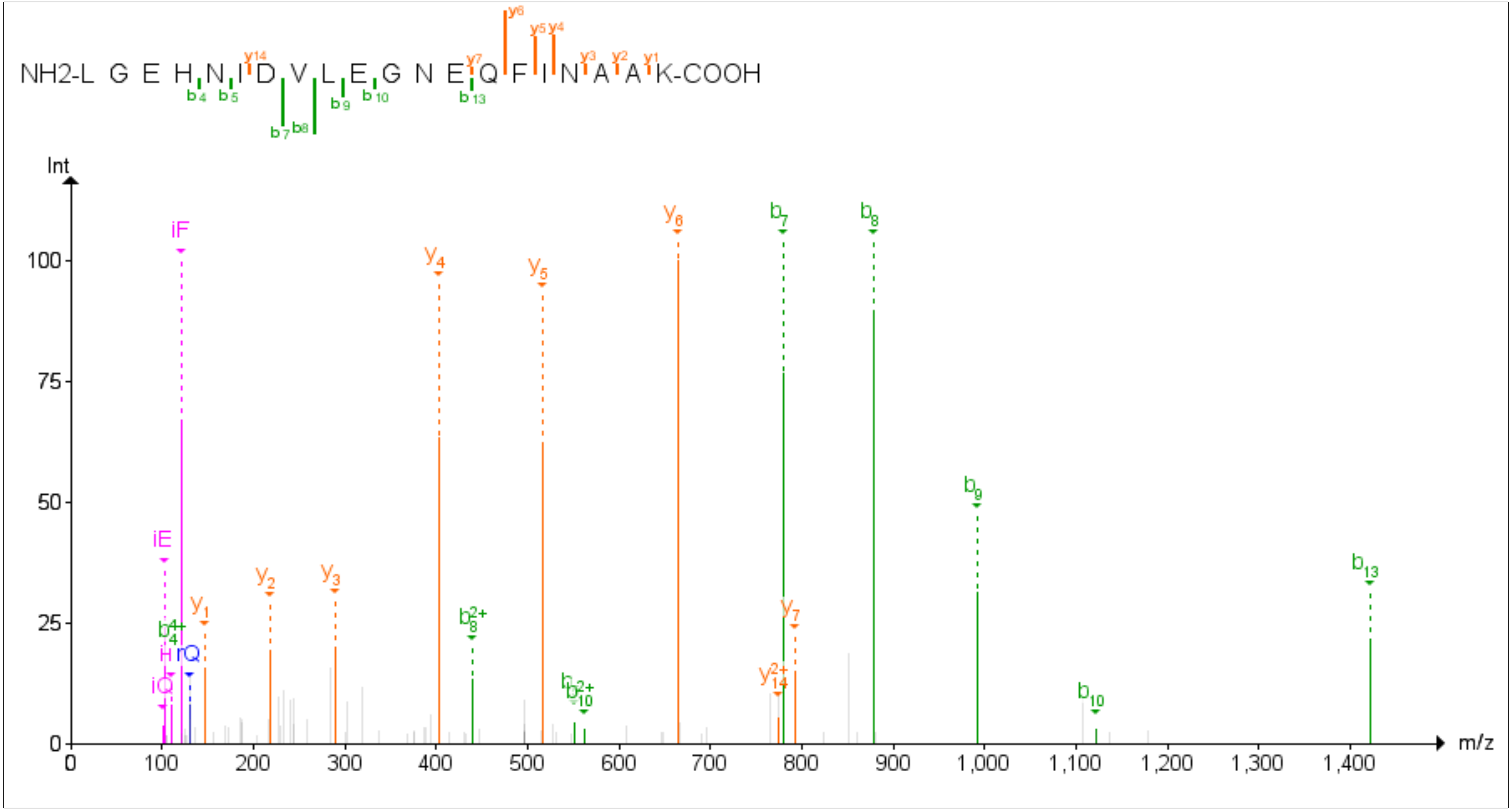

NH2-L G E H N I D V L E G N E Q F I N A A K-COOH
b4 b5 b7 b8 b9 b10 b13
y14 y7 y6 y5 y4 y3 y2 y1
Int
m/z
0
25
50
75
100
0
100
200
300
400
500
600
700
800
900
1,000
1,100
1,200
1,300
1,400
iF
iE
iQ
H
rQ

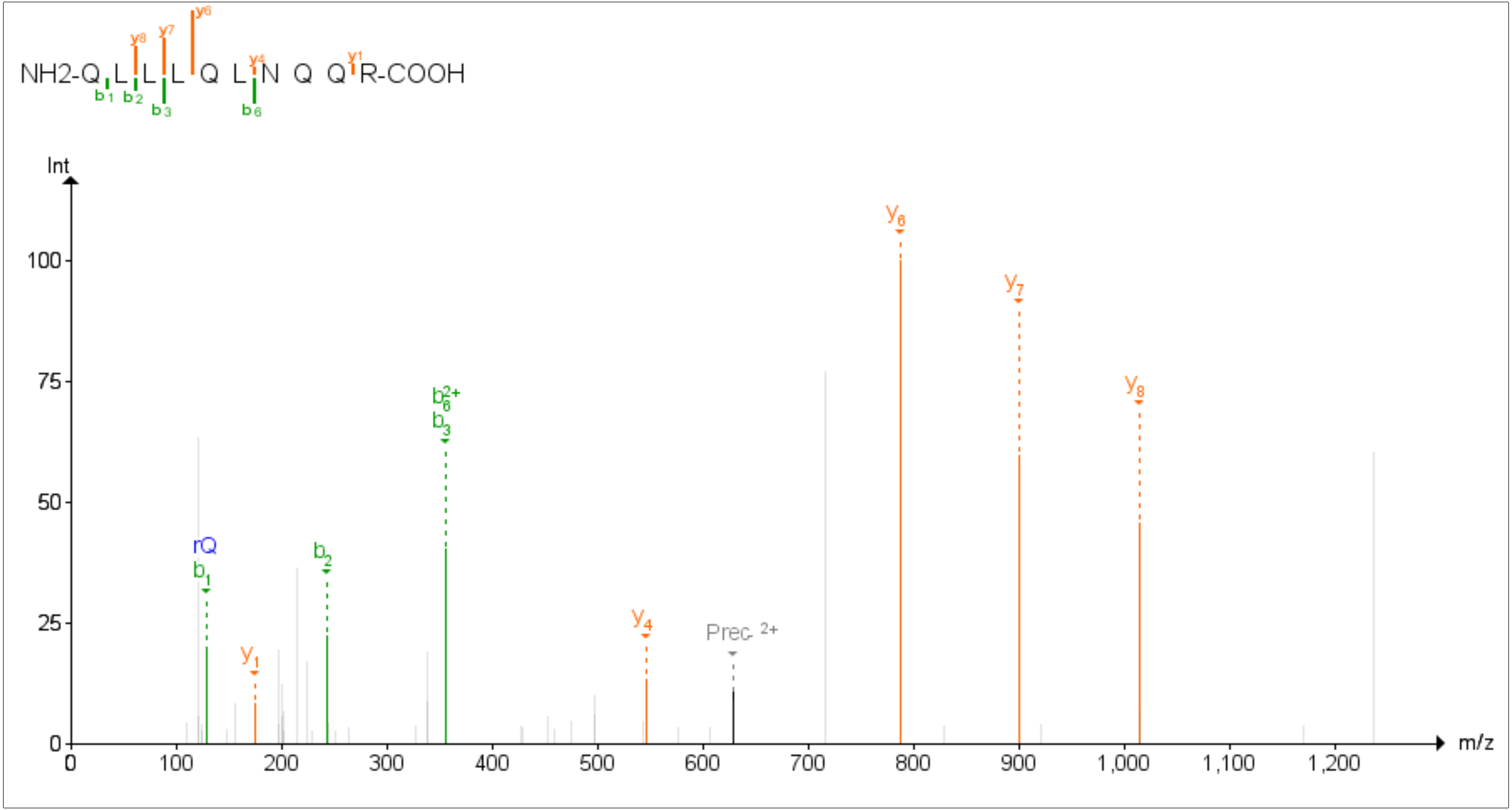

NH2-Q L L L Q L N Q Q R-COOH
y8 y7 y6 y4 y1
b1 b2 b3 b6
Int
m/z
y6
y7
y8
b6 2+
b3
rQ
b1
b2
y1
y4
Prec 2+
0
25
50
75
100
0
100
200
300
400
500
600
700
800
900
1,000
1,100
1,200

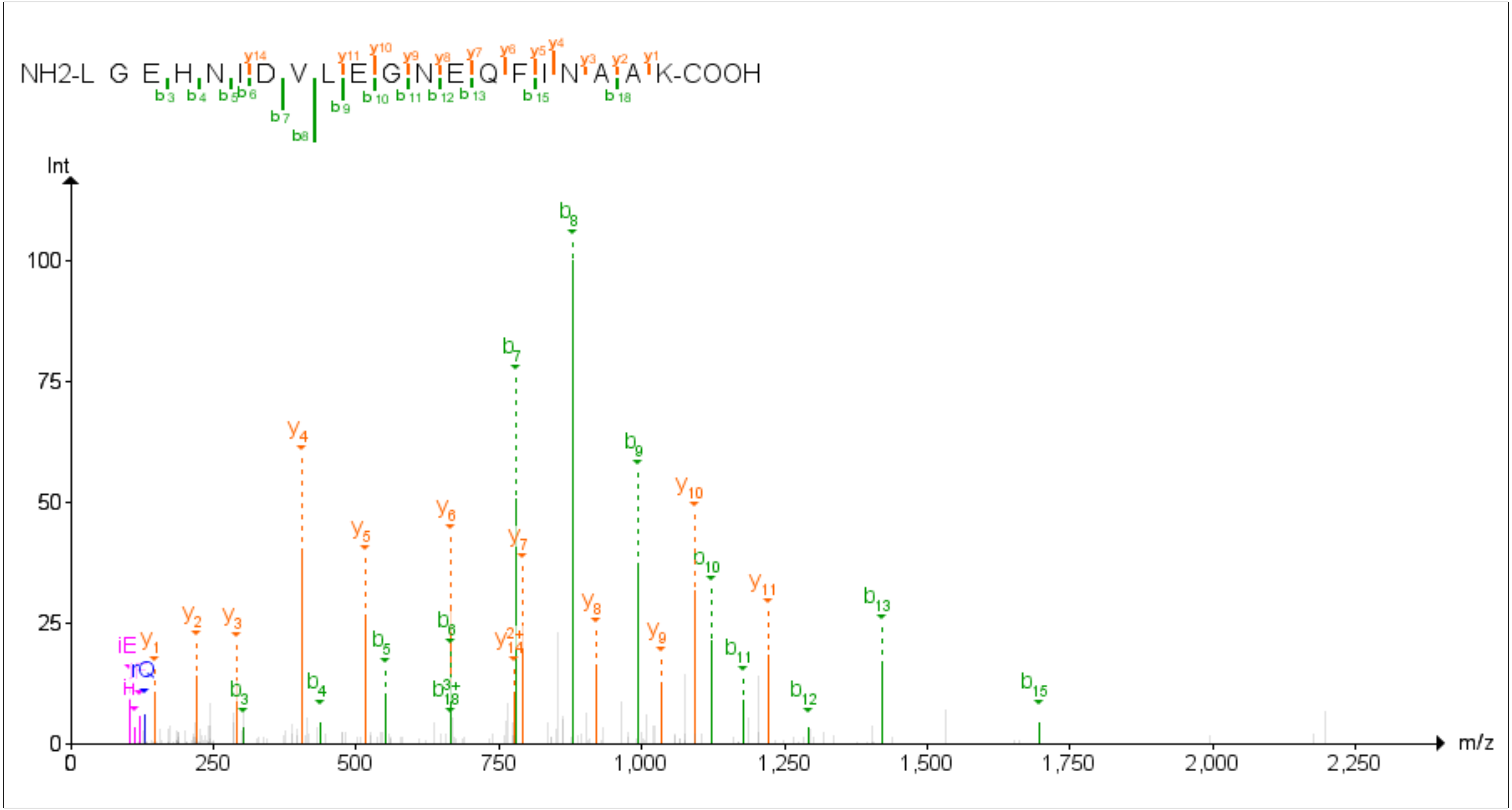
NH2-L G E H N I D V L E G N E Q F I N A A K-COOH
Int
m/z
0
25
50
75
100
250
500
750
1,000
1,250
1,500
1,750
2,000
2,250

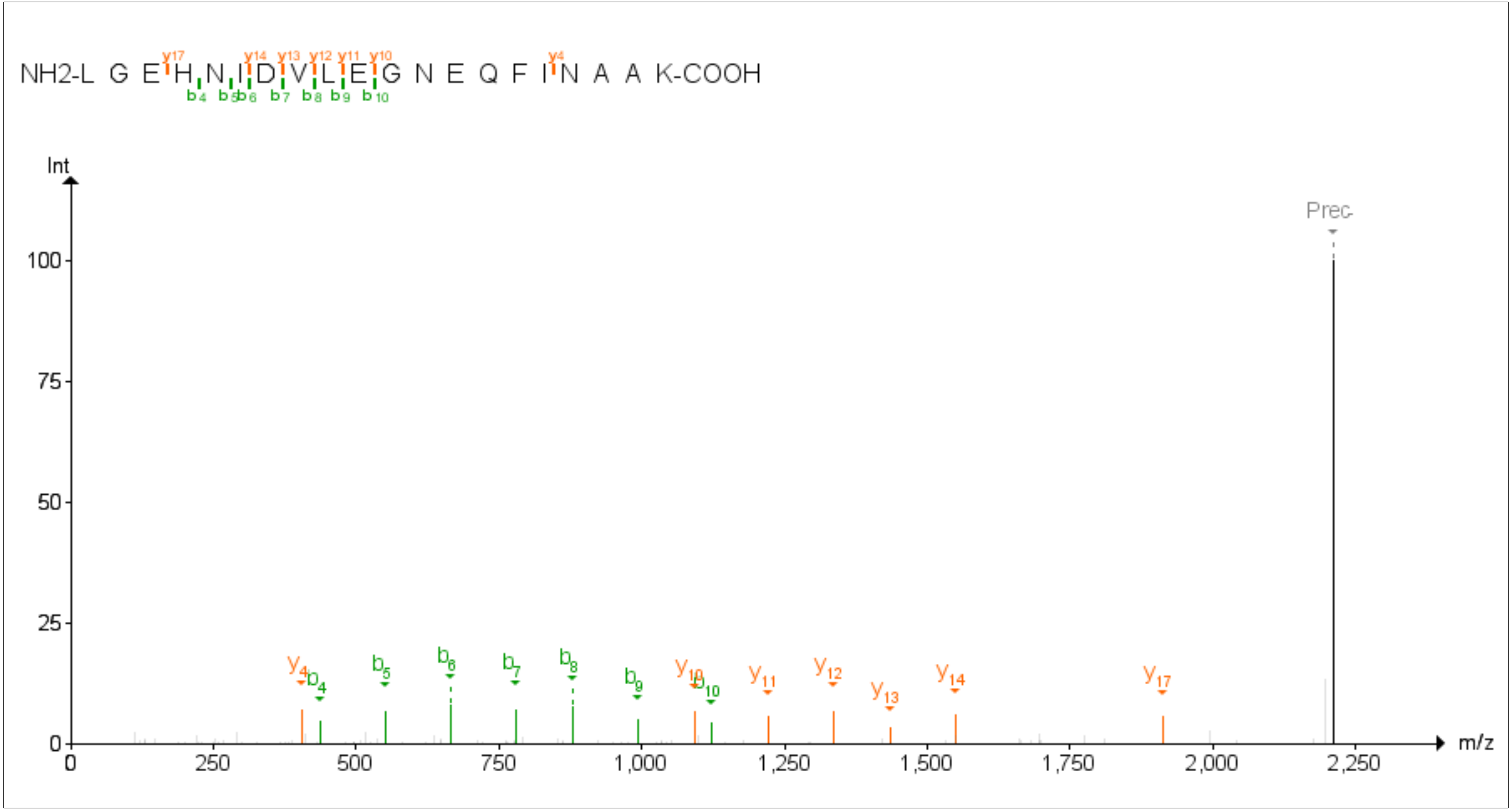
NH2-L G E H N I D V L E G N E Q F I N A A K-COOH
y17 y14 y13 y12 y11 y10 y4
b4 b5 b6 b7 b8 b9 b10
Int
Prec
100
75
50
25
0
m/z
0
250
500
750
1,000
1,250
1,500
1,750
2,000
2,250

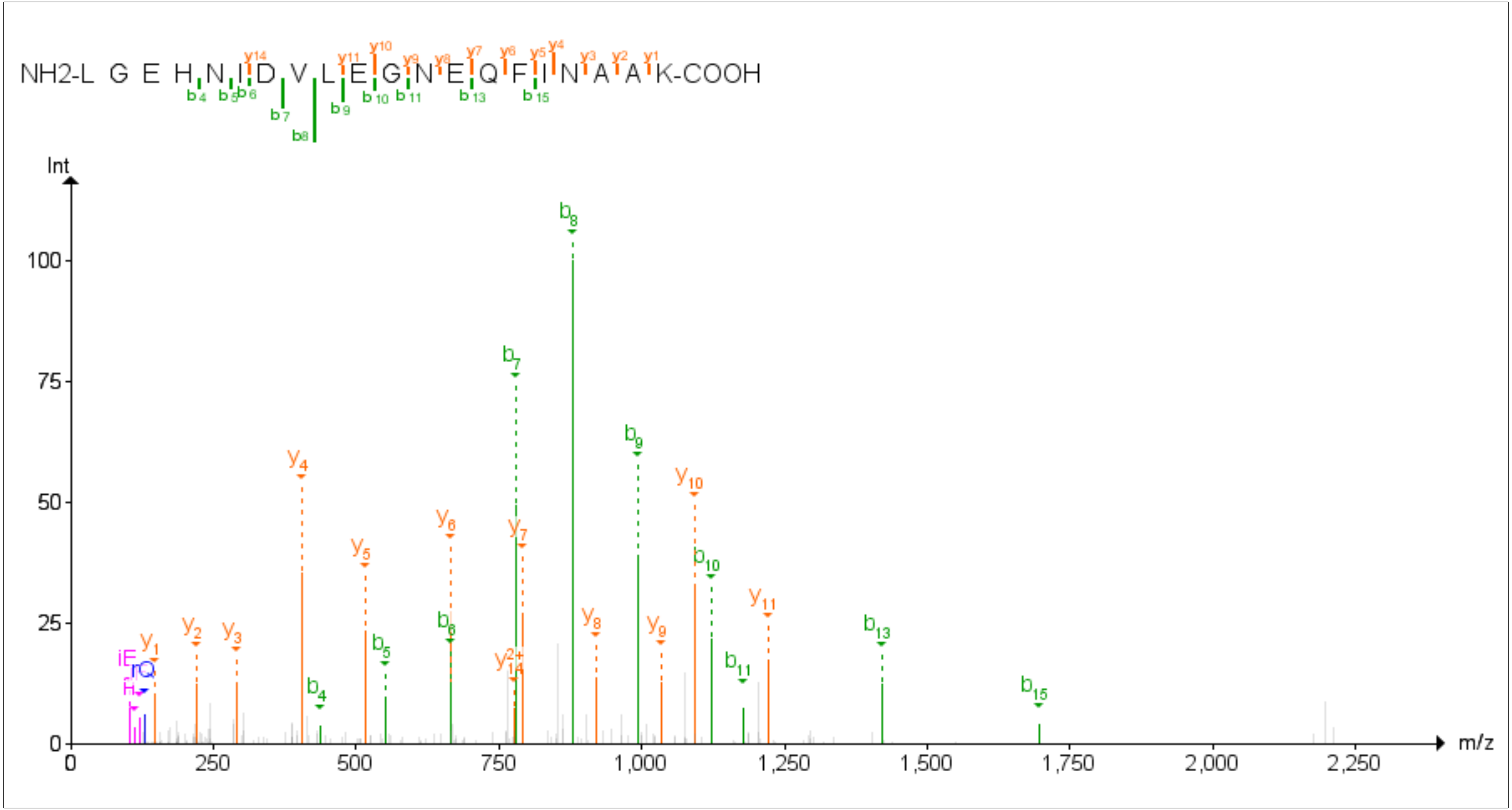

NH2-L G E H N I D V L E G N E Q F I N A A K-COOH
Int
m/z
b8
b7
b9
y4
y10
y6
y7
y5
b10
y11
b6
b13
y8
y9
b5
y1
y2
y3
b4
b11
b15
iE
rQ

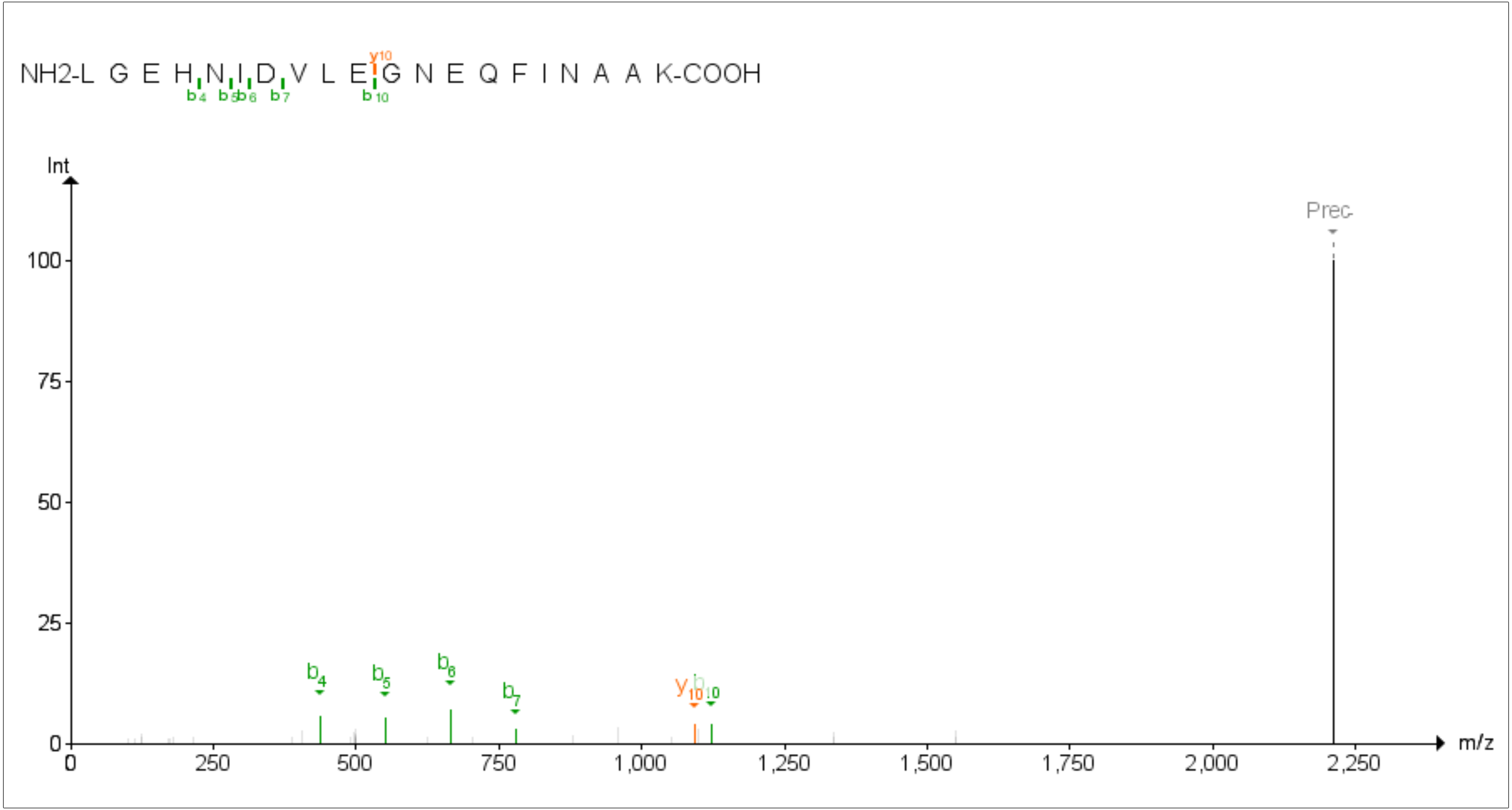

y10
NH2-L G E H N I D V L E G N E Q F I N A A K-COOH
b4 b5 b6 b7 b10
Int
Prec
100
75
50
25
0
b4
b5
b6
b7
y10
b10
0
250
500
750
1,000
1,250
1,500
1,750
2,000
2,250
m/z

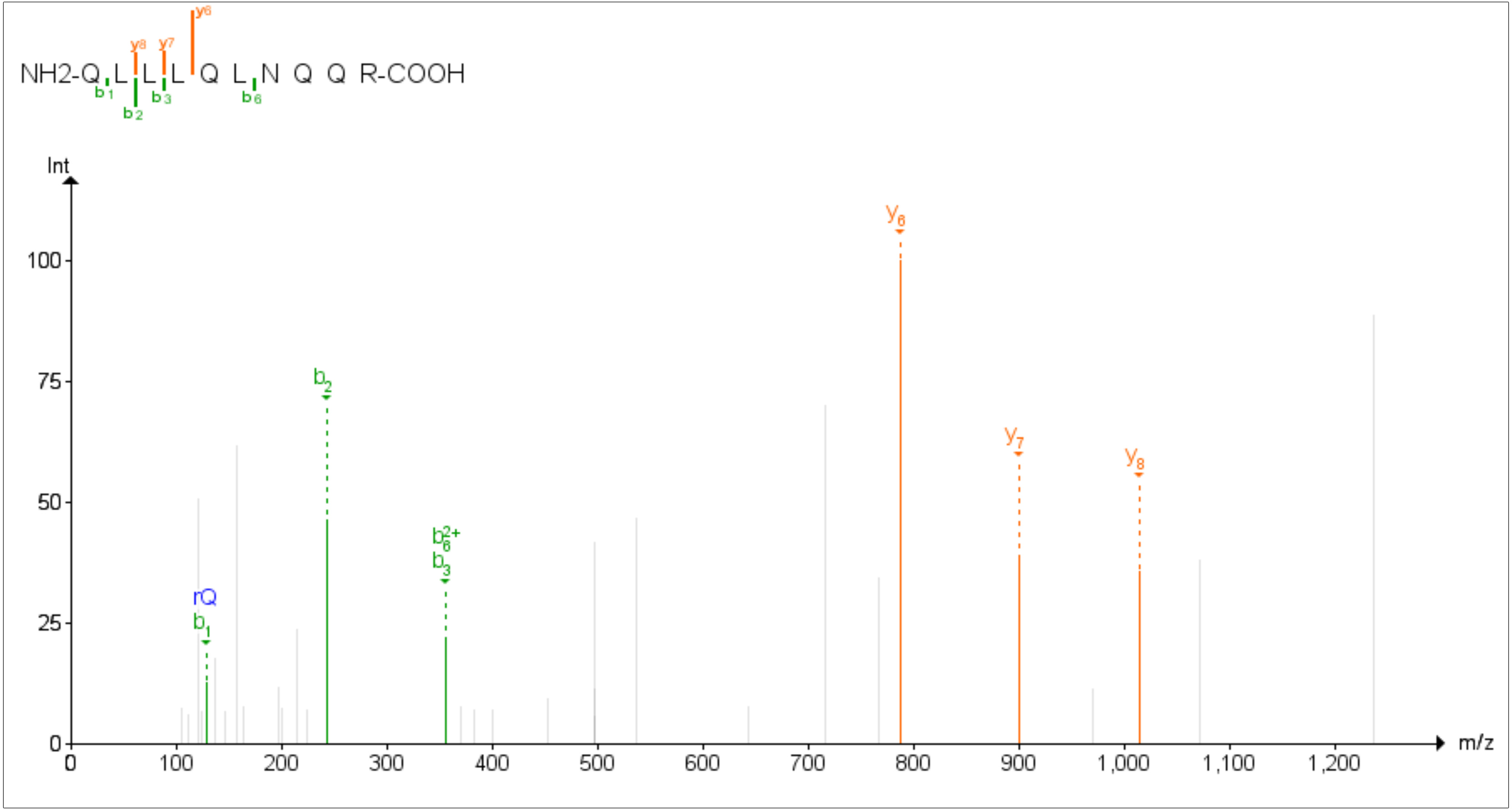
NH2-Q L L L Q L N Q Q R-COOH
y8 y7 y6
b1 b2 b3 b6
Int
m/z
y6
y7
y8
b2
b3
rQ
b1
0
25
50
75
100
0
100
200
300
400
500
600
700
800
900
1,000
1,100
1,200

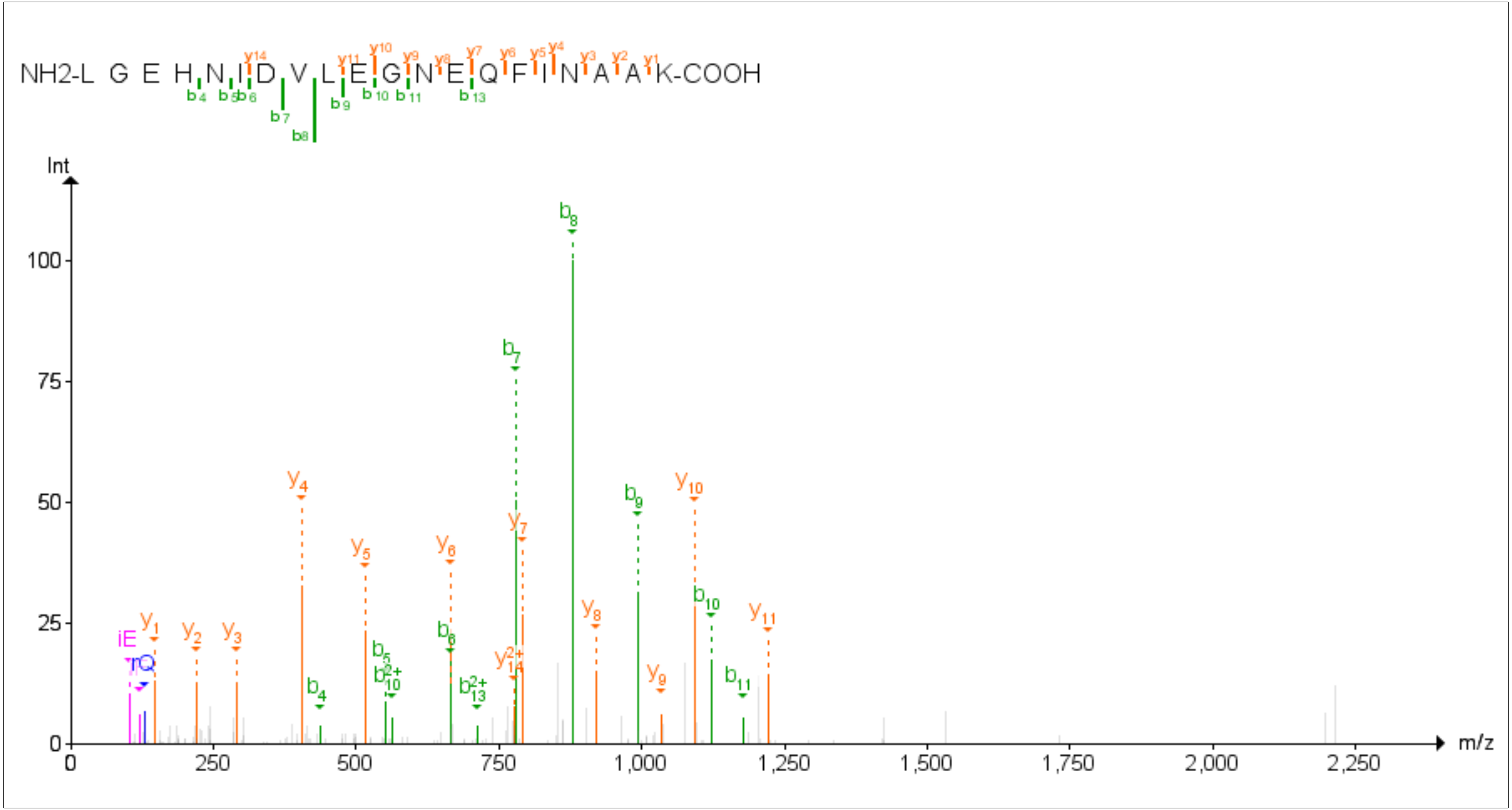

NH2-L G E H N I D V L E G N E Q F I N A A K-COOH
Int
m/z
0
25
50
75
100
250
500
750
1,000
1,250
1,500
1,750
2,000
2,250

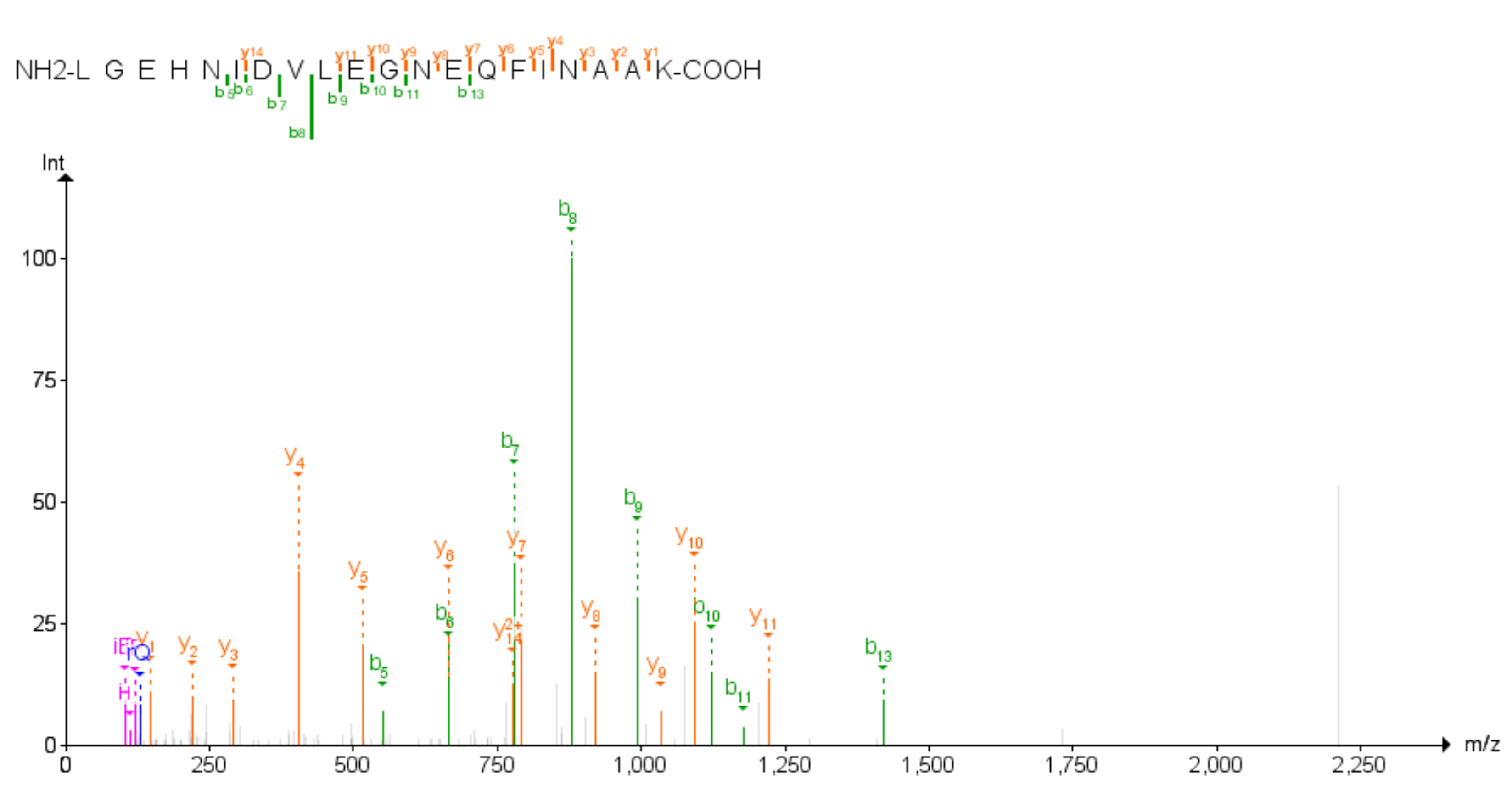

NH2-L G E H N I D V L E G N E Q F I N A A K-COOH
Int
m/z
0
25
50
75
100
250
500
750
1,000
1,250
1,500
1,750
2,000
2,250

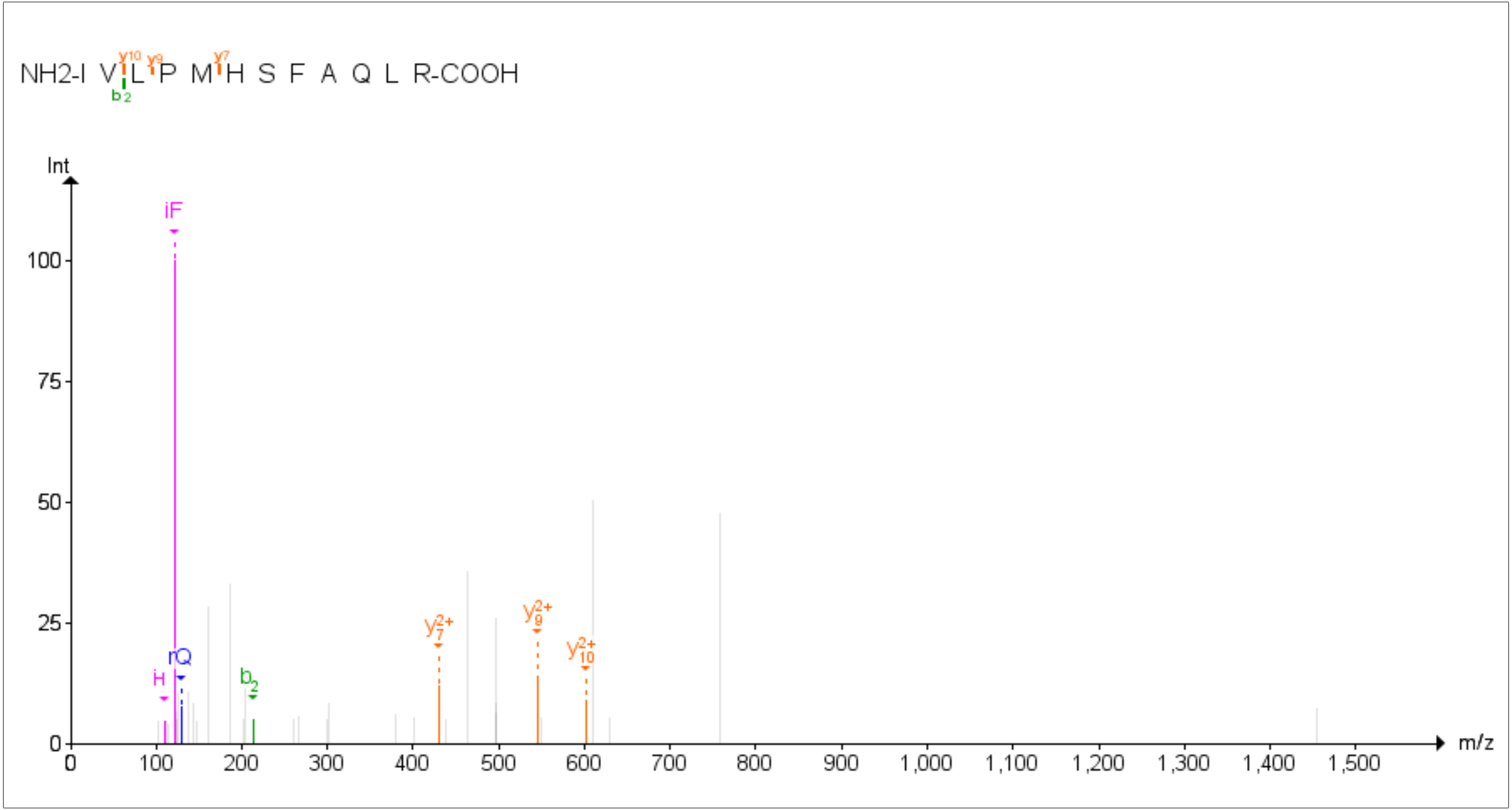

NH2-I V L P M H S F A Q L R-COOH
y10
y9
y7
b2
Int
iF
100
75
50
25
0
H
rQ
b2
y7 2+
y9 2+
y10 2+
0
100
200
300
400
500
600
700
800
900
1,000
1,100
1,200
1,300
1,400
1,500
m/z

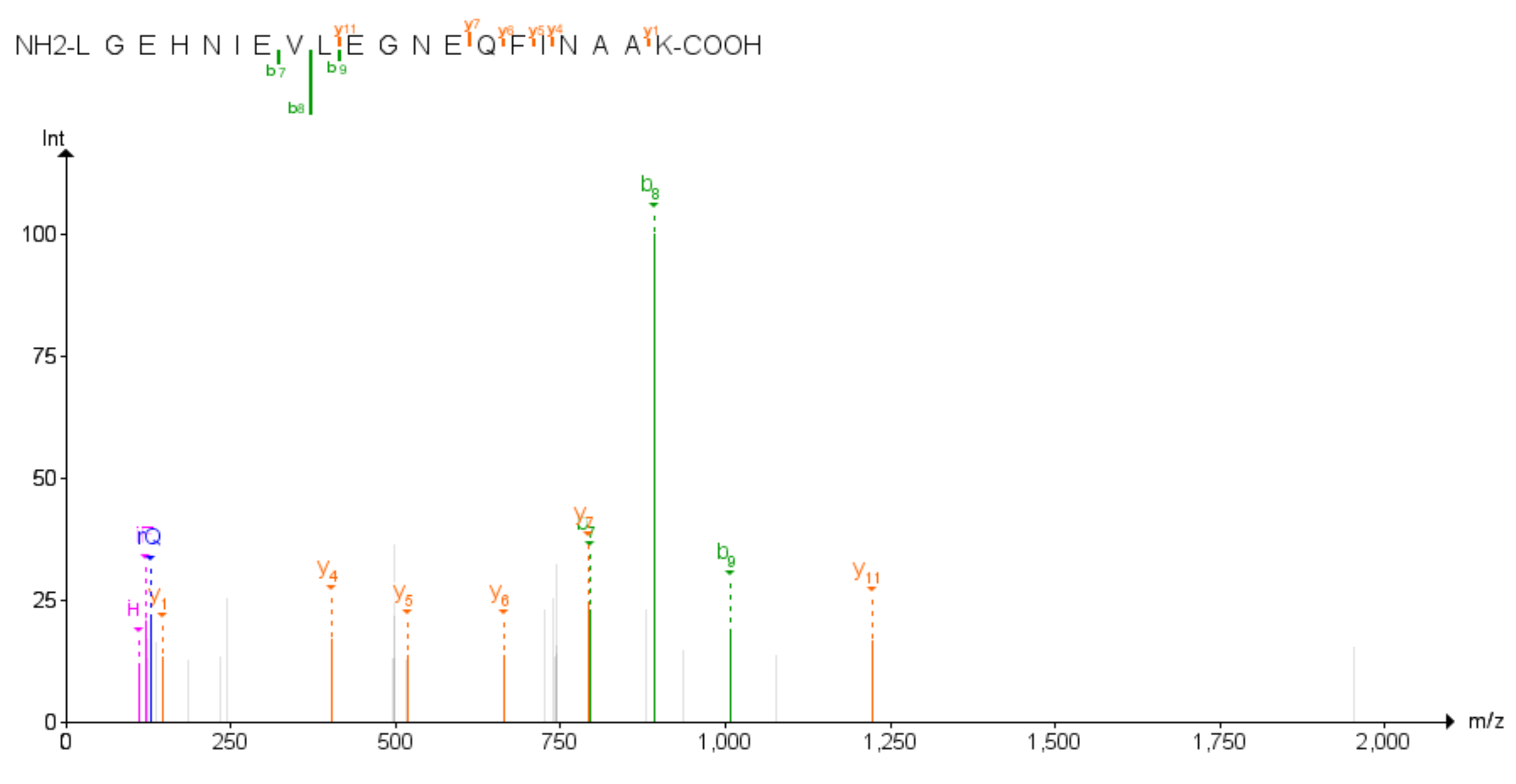

NH2-L G E H N I E V L E G N E Q F I N A A K-COOH
b7
b8
b9
y11
y7
y6
y5
y4
y1
Int
100
75
50
25
0
m/z
0
250
500
750
1,000
1,250
1,500
1,750
2,000
b8
y7
b7
b9
y11
y4
y5
y6
y1
rQ
H

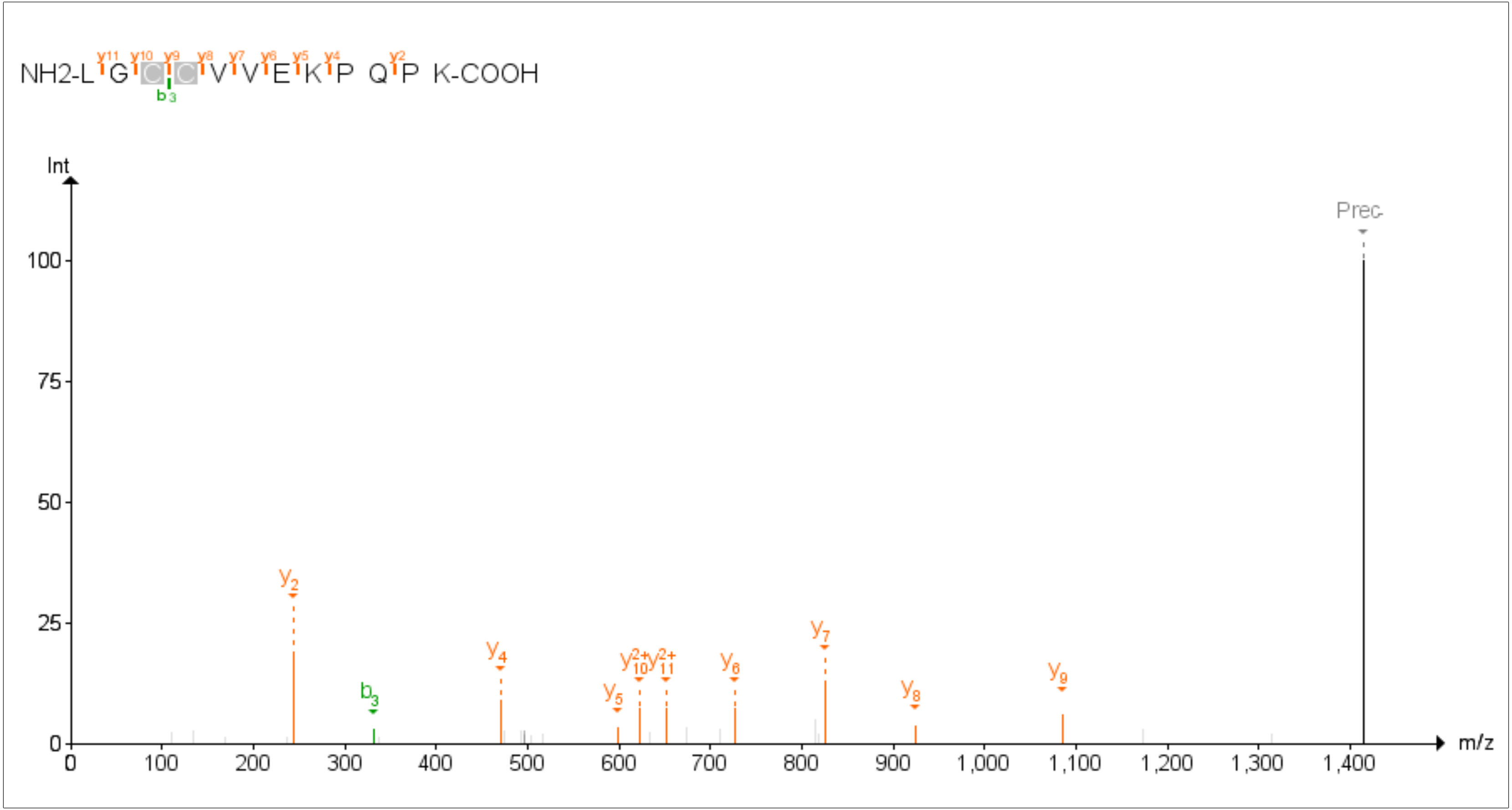

NH2-L G C C V V E K P Q P K-COOH
y11 y10 y9 y8 y7 y6 y5 y4 y2
b3
Int
m/z
Prec
y2
b3
y4
y5
y10 2+
y11 2+
y6
y7
y8
y9
0
25
50
75
100
0
100
200
300
400
500
600
700
800
900
1,000
1,100
1,200
1,300
1,400

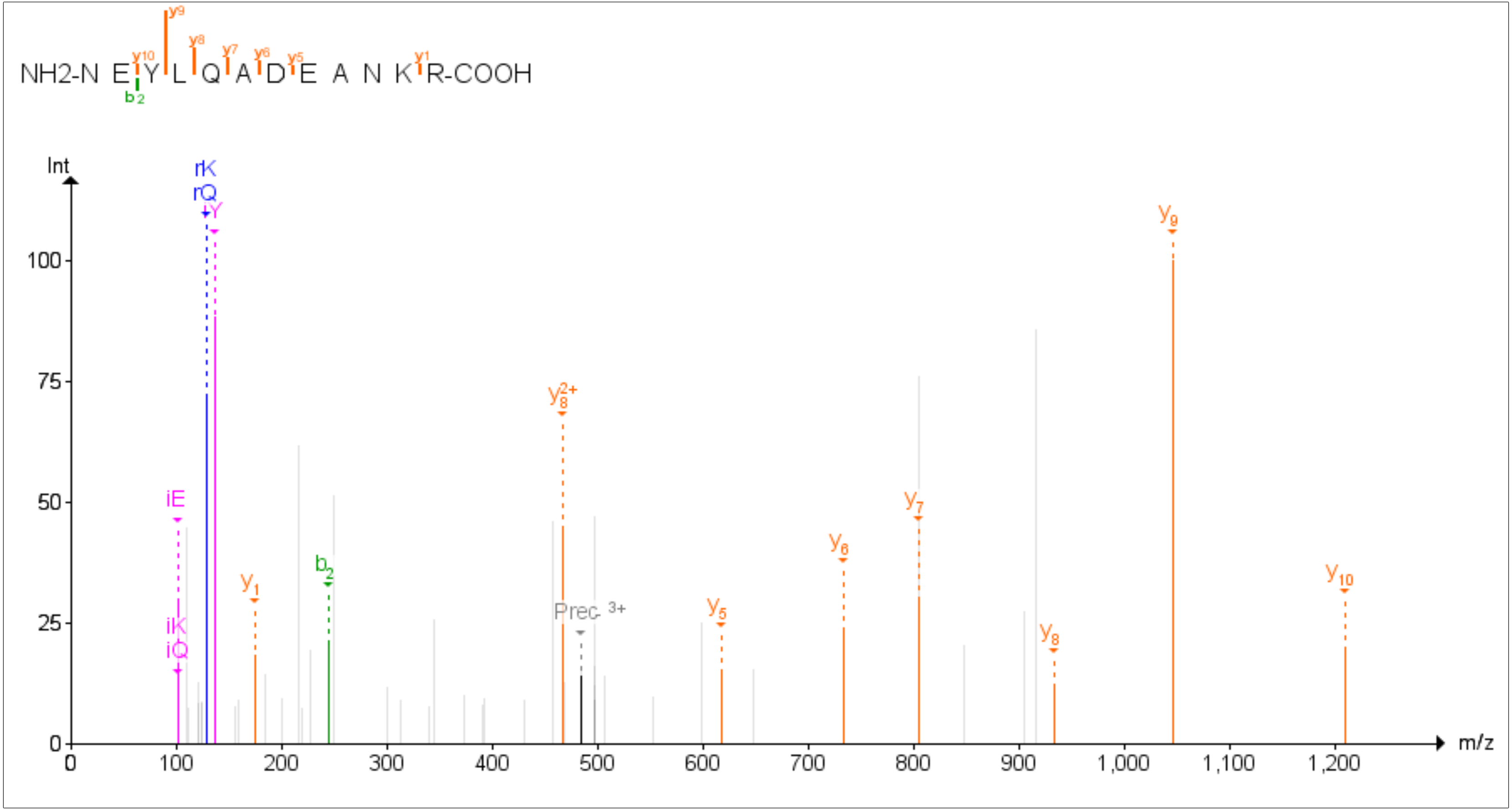
NH2-N E Y L Q A D E A N K R-COOH
y9
y10
y8
y7
y6
y5
y1
b2
Int
rK
rQ
iY
iE
iK
iQ
y1
b2
y8 2+
Prec 3+
y5
y6
y7
y8
y9
y10
m/z
0
25
50
75
100
0
100
200
300
400
500
600
700
800
900
1,000
1,100
1,200

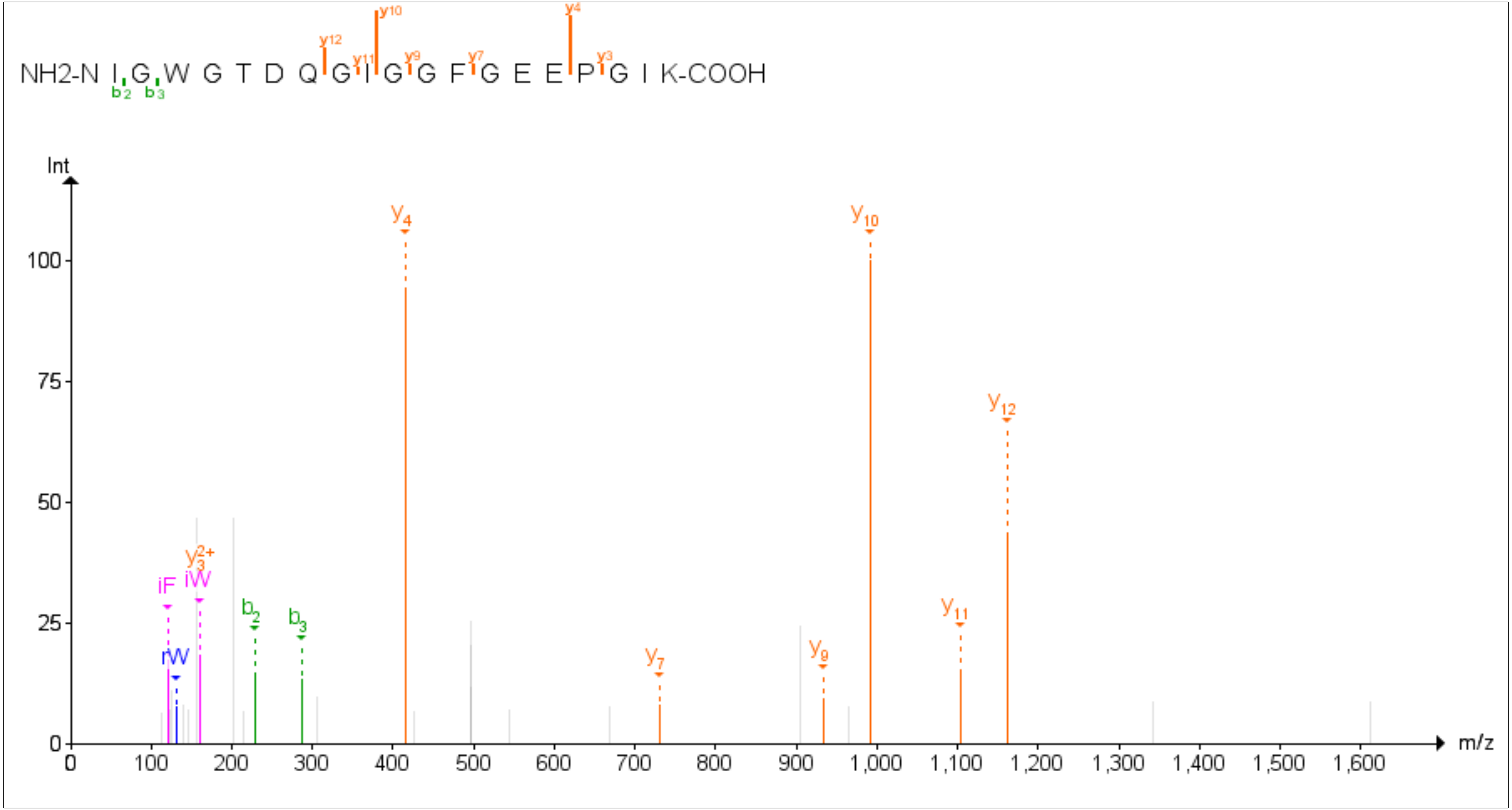

NH2-N I G W G T D Q G I G G F G E E P G I K-COOH
b2 b3
y12 y11 y10 y9 y7 y4 y3
Int
100
75
50
25
0
y4
y10
y12
y11
y9
y7
b2
b3
iF
iW
rW
y_3^{2+}
0 100 200 300 400 500 600 700 800 900 1,000 1,100 1,200 1,300 1,400 1,500 1,600
m/z

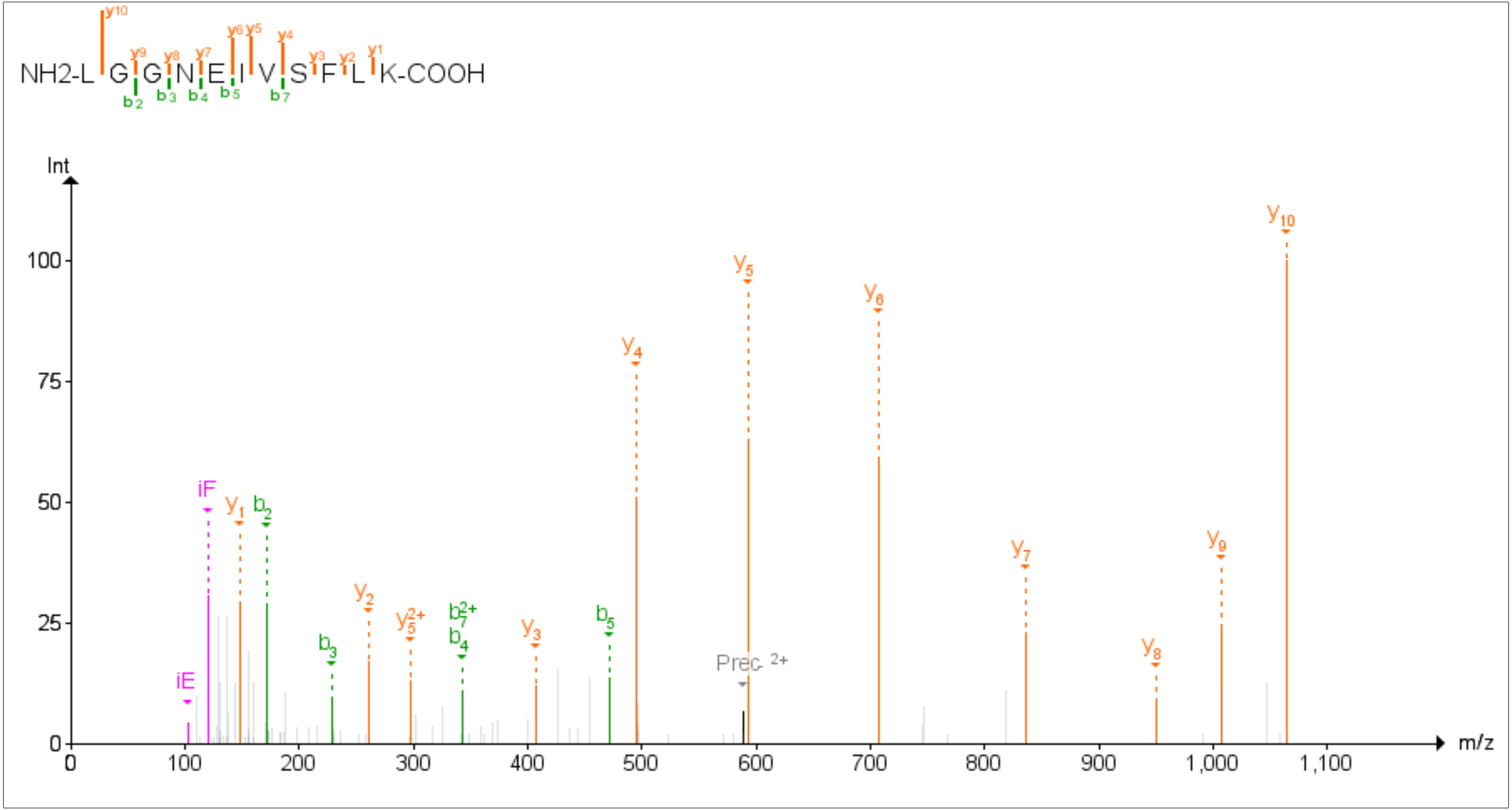

NH2-L G G N E I V S F L K-COOH
y10
y9
y8
y7
y6
y5
y4
y3
y2
y1
b2
b3
b4
b5
b7
Int
100
75
50
25
0
m/z
0
100
200
300
400
500
600
700
800
900
1,000
1,100
iE
iF
y1
b2
b3
y2
y5 2+
b7 2+
b4
y3
b5
y4
Prec 2+
y5
y6
y7
y8
y9
y10

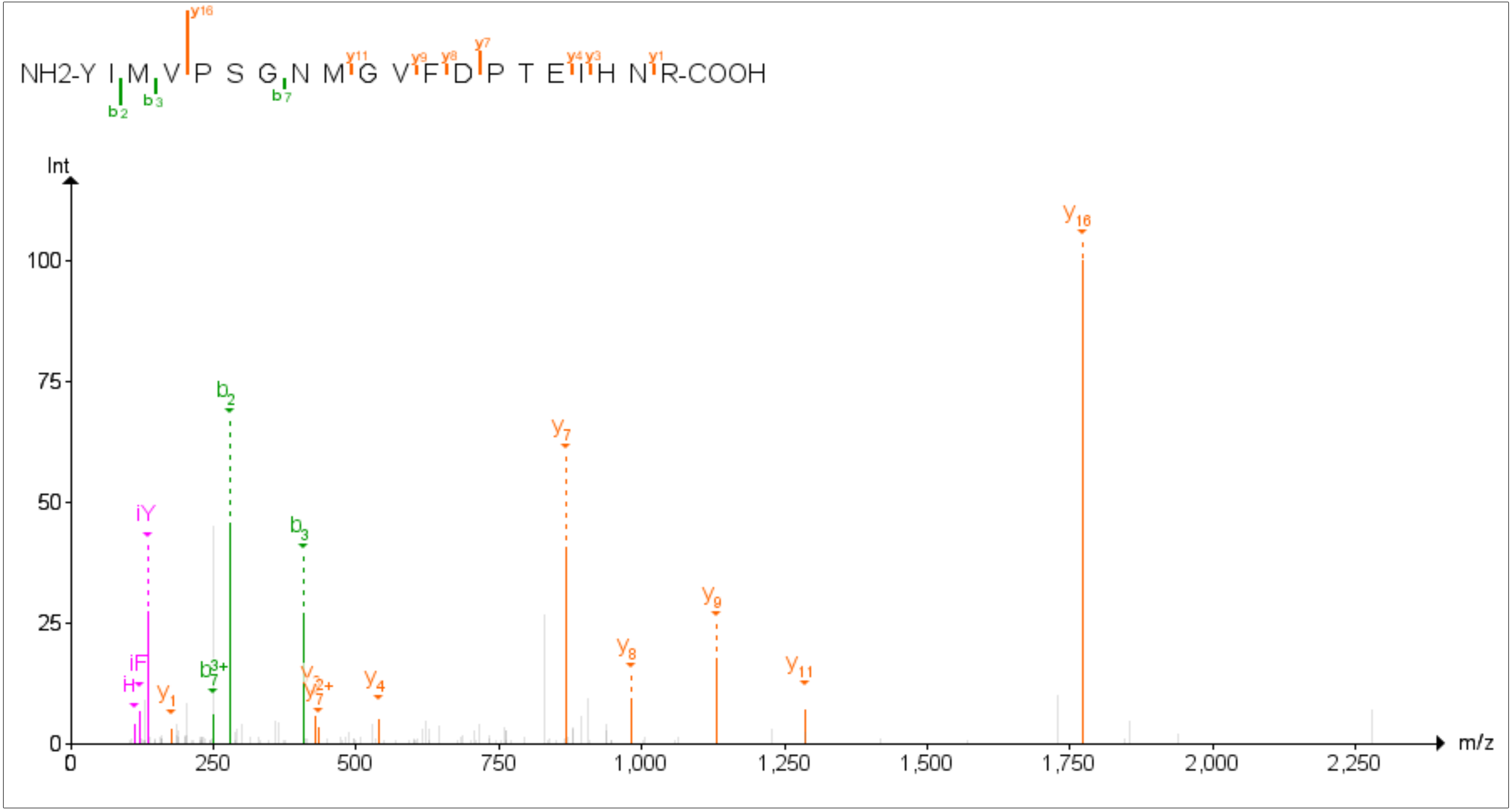

NH2-Y I M V P S G N M G V F D P T E I H N R-COOH
y16
y11
y9
y8
y7
y4
y3
y1
b2
b3
b7
Int
100
75
50
25
0
m/z
250
500
750
1,000
1,250
1,500
1,750
2,000
2,250
iY
iF
iH
y1
b7 3+
y7 2+
y4
b2
b3
y7
y8
y9
y11
y16

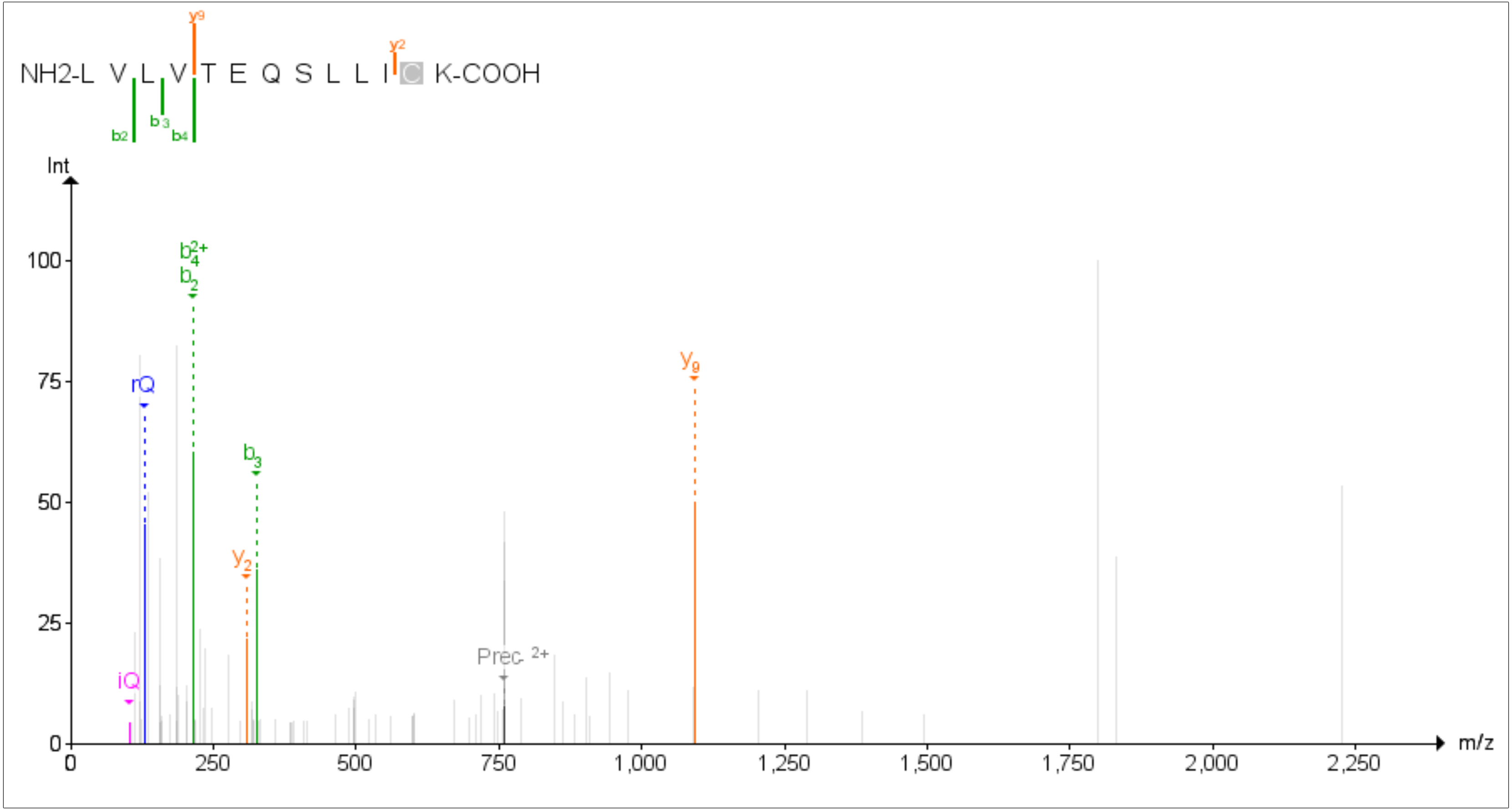

NH2-L V L V T E Q S L L I C K-COOH
y9
y2
b2
b3
b4
Int
m/z
100
75
50
25
0
250
500
750
1,000
1,250
1,500
1,750
2,000
2,250
b4 2+
b2
rQ
b3
y2
y9
iQ
Prec 2+

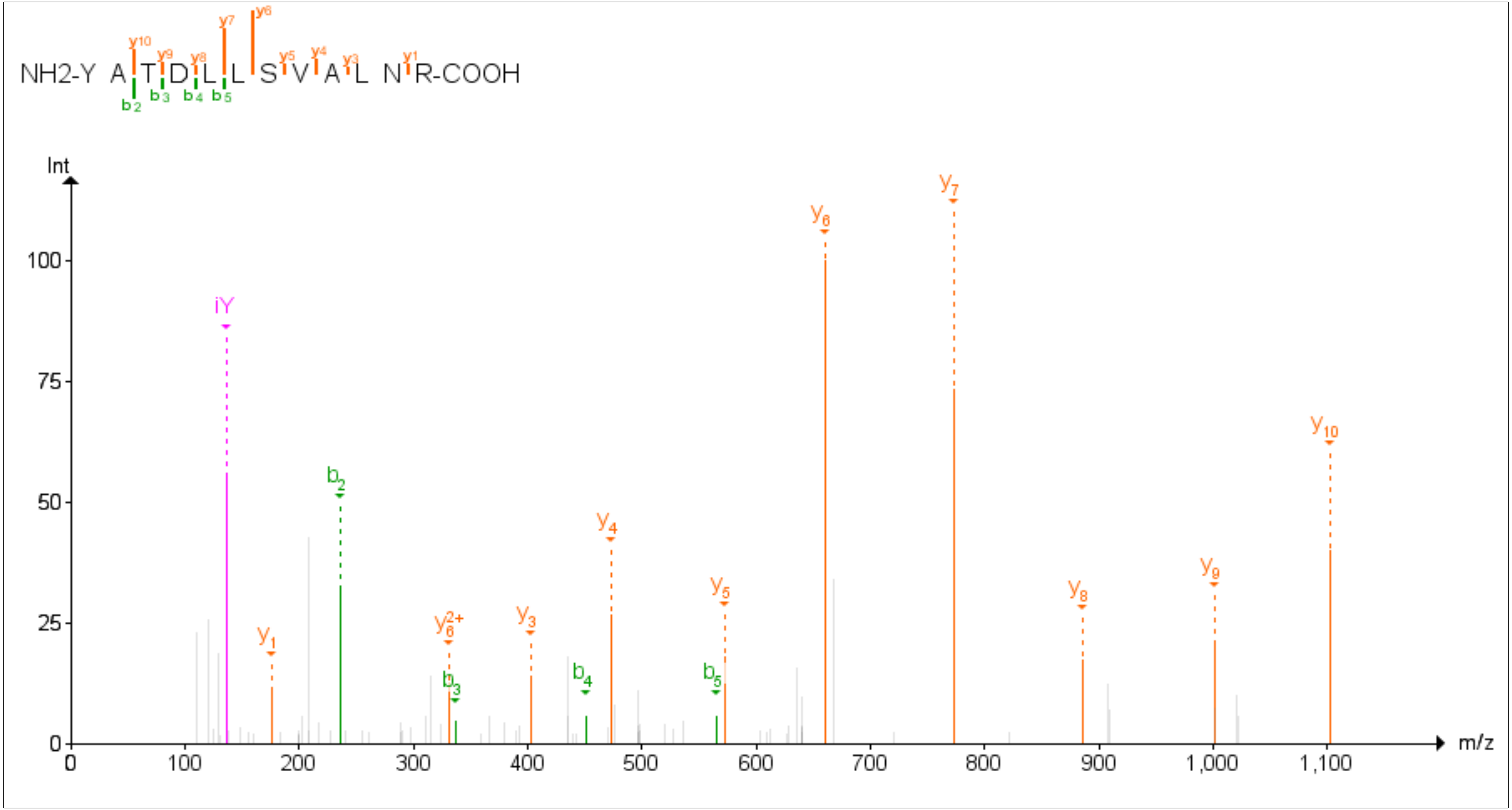

NH2-Y A T D L L S V A L N R-COOH
y10 y9 y8 y7 y6 y5 y4 y3 y1
b2 b3 b4 b5
Int
100
75
50
25
0
iY
b2
y1
y6 2+
b3
y3
b4
y4
b5
y5
y6
y7
y8
y9
y10
0 100 200 300 400 500 600 700 800 900 1,000 1,100
m/z

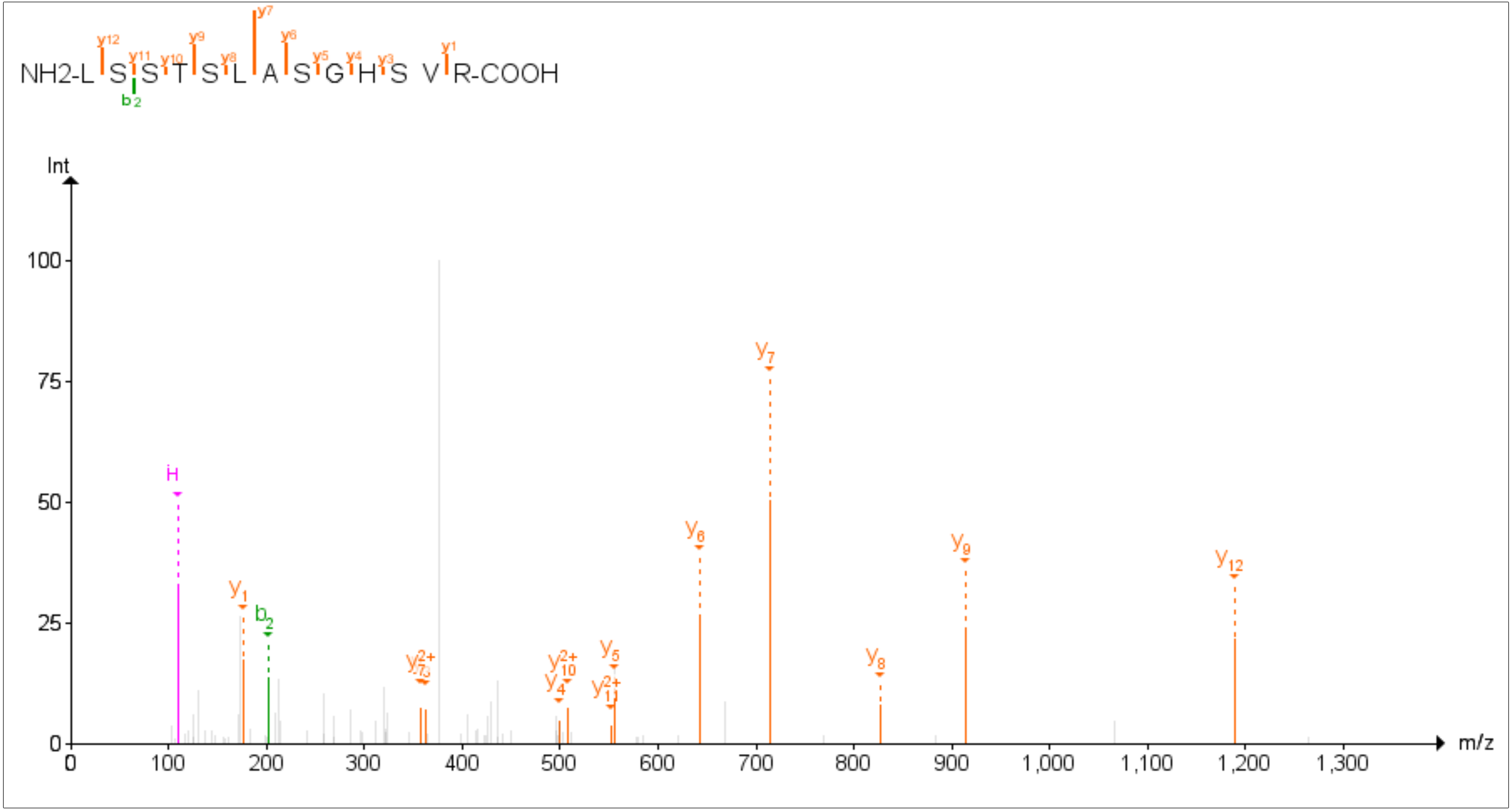
NH2-L S S T S L A S G H S V R-COOH
y12 y11 y10 y9 y8 y7 y6 y5 y4 y3 y1
b2
Int
100
75
50
25
0
m/z
0 100 200 300 400 500 600 700 800 900 1,000 1,100 1,200 1,300
H
y1
b2
y7 2+
y3
y10 2+
y4
y5
y11 2+
y6
y7
y8
y9
y12

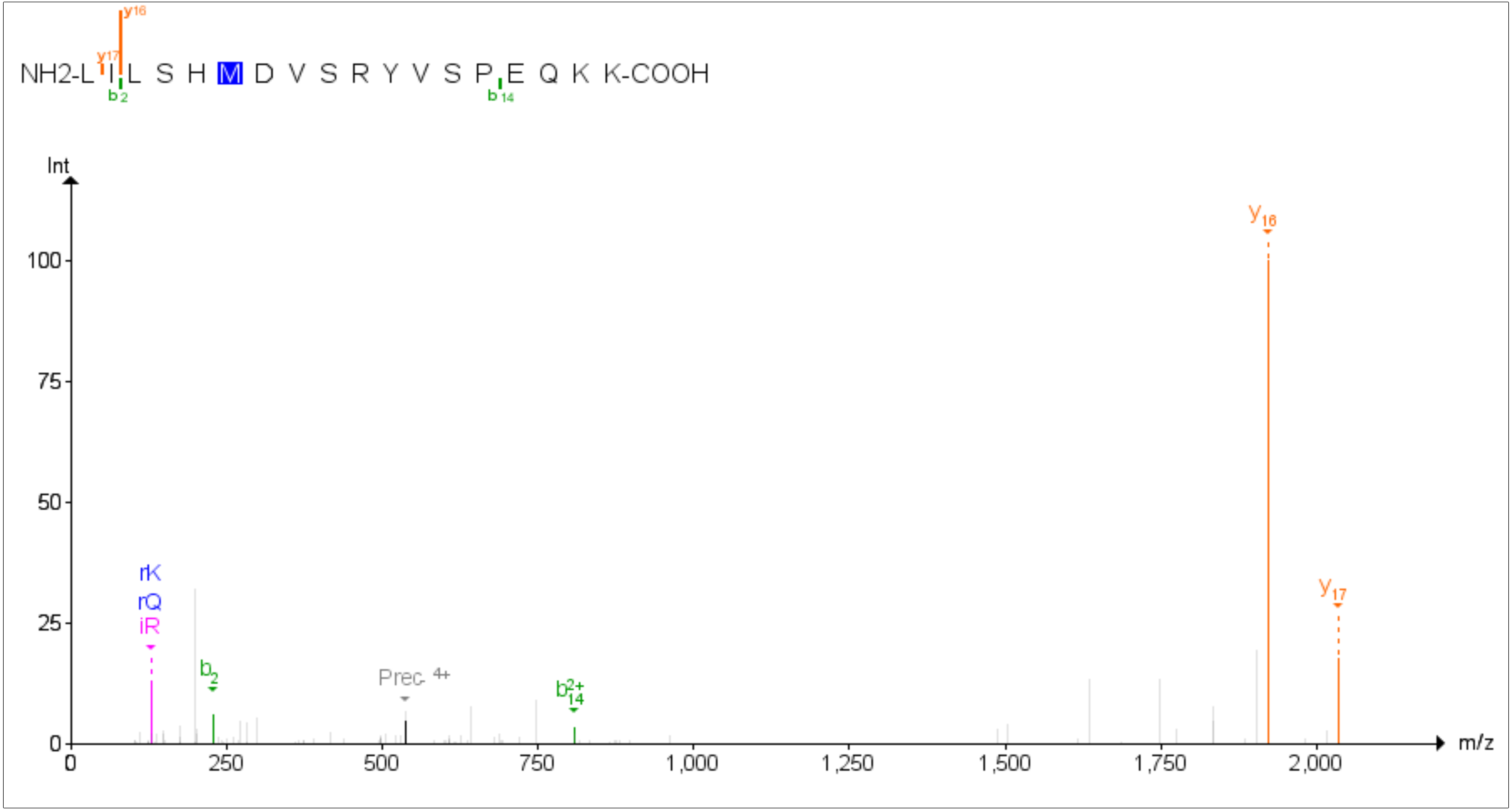

y16
y17
NH2-L I L S H M D V S R Y V S P E Q K K-COOH
b 2
b 14
Int
100
75
50
25
0
rK
rQ
iR
b2
Prec 4+
b14 2+
y16
y17
0
250
500
750
1,000
1,250
1,500
1,750
2,000
m/z

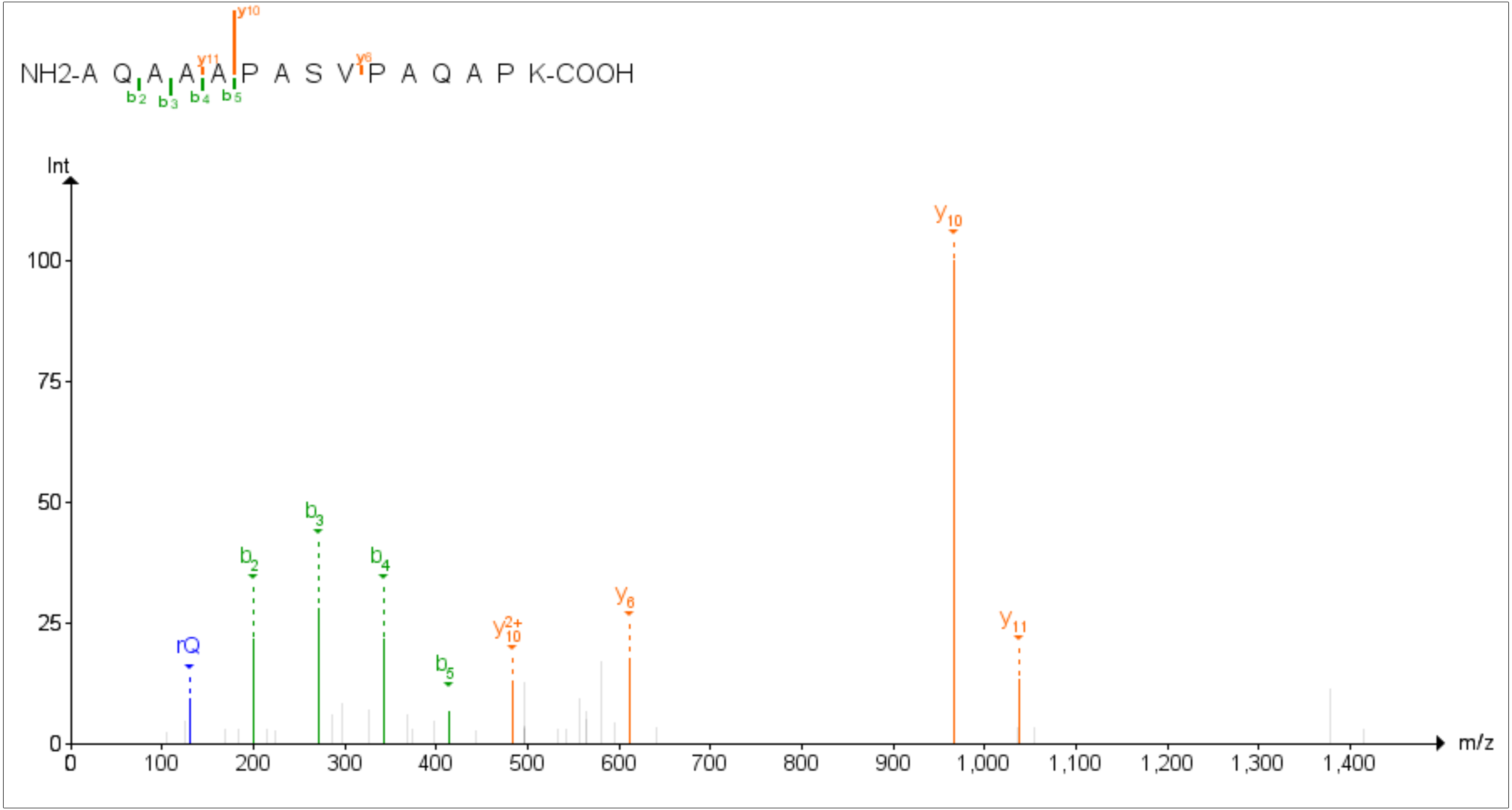

NH2-A Q A A A P A S V P A Q A P K-COOH
y10
y11
y6
b2
b3
b4
b5
Int
100
75
50
25
0
m/z
0
100
200
300
400
500
600
700
800
900
1,000
1,100
1,200
1,300
1,400
rQ
b2
b3
b4
b5
y10 2+
y6
y10
y11

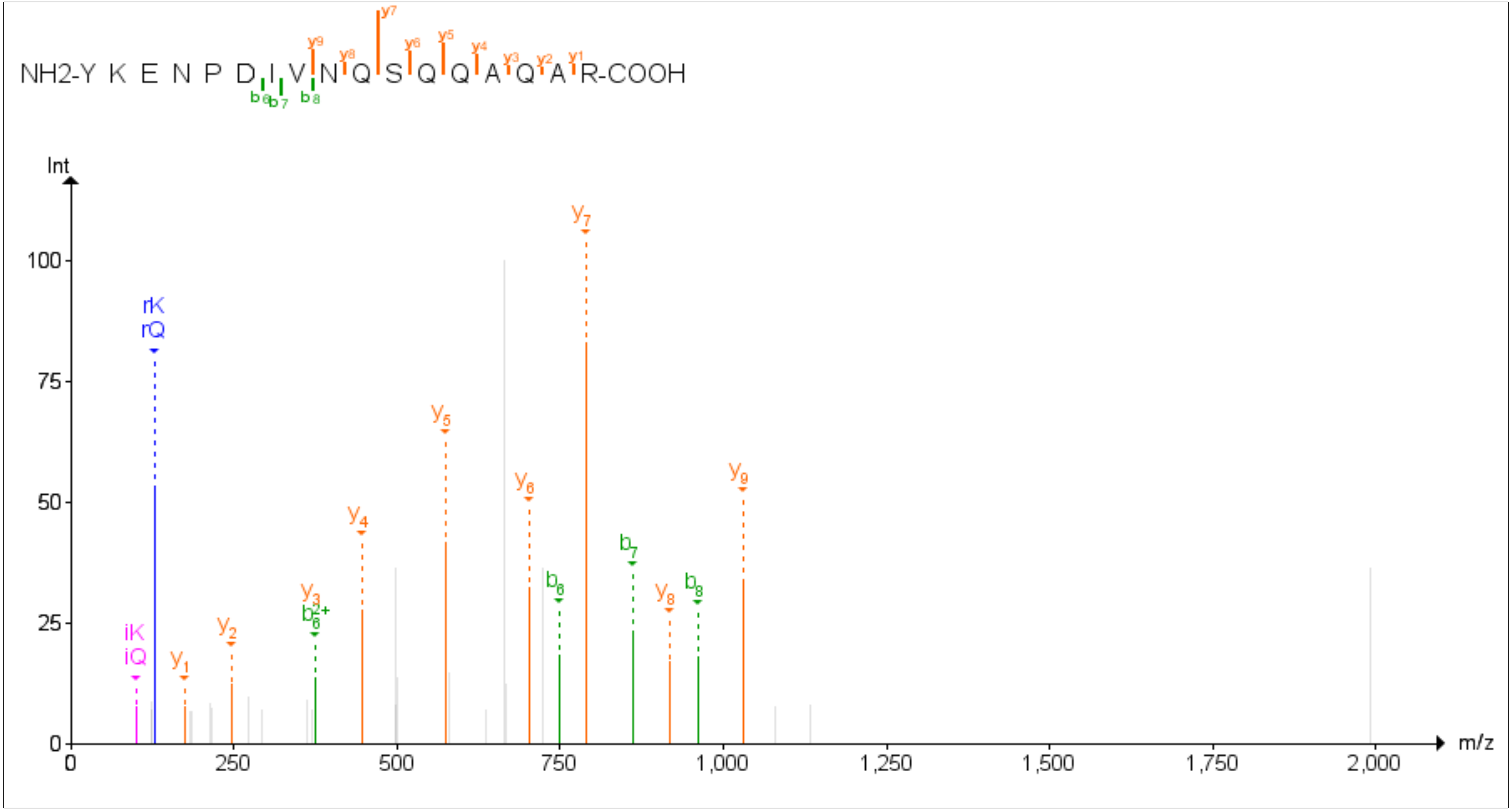

NH2-Y K E N P D I V N Q S Q Q A Q A R-COOH
y9 y8 y7 y6 y5 y4 y3 y2 y1
b6 b7 b8
Int
100
75
50
25
0
rK
rQ
iK
iQ
y1 y2 y3 y4 y5 y6 y7 y8 y9
b6 b7 b8
b6 2+
0
250
500
750
1,000
1,250
1,500
1,750
2,000
m/z

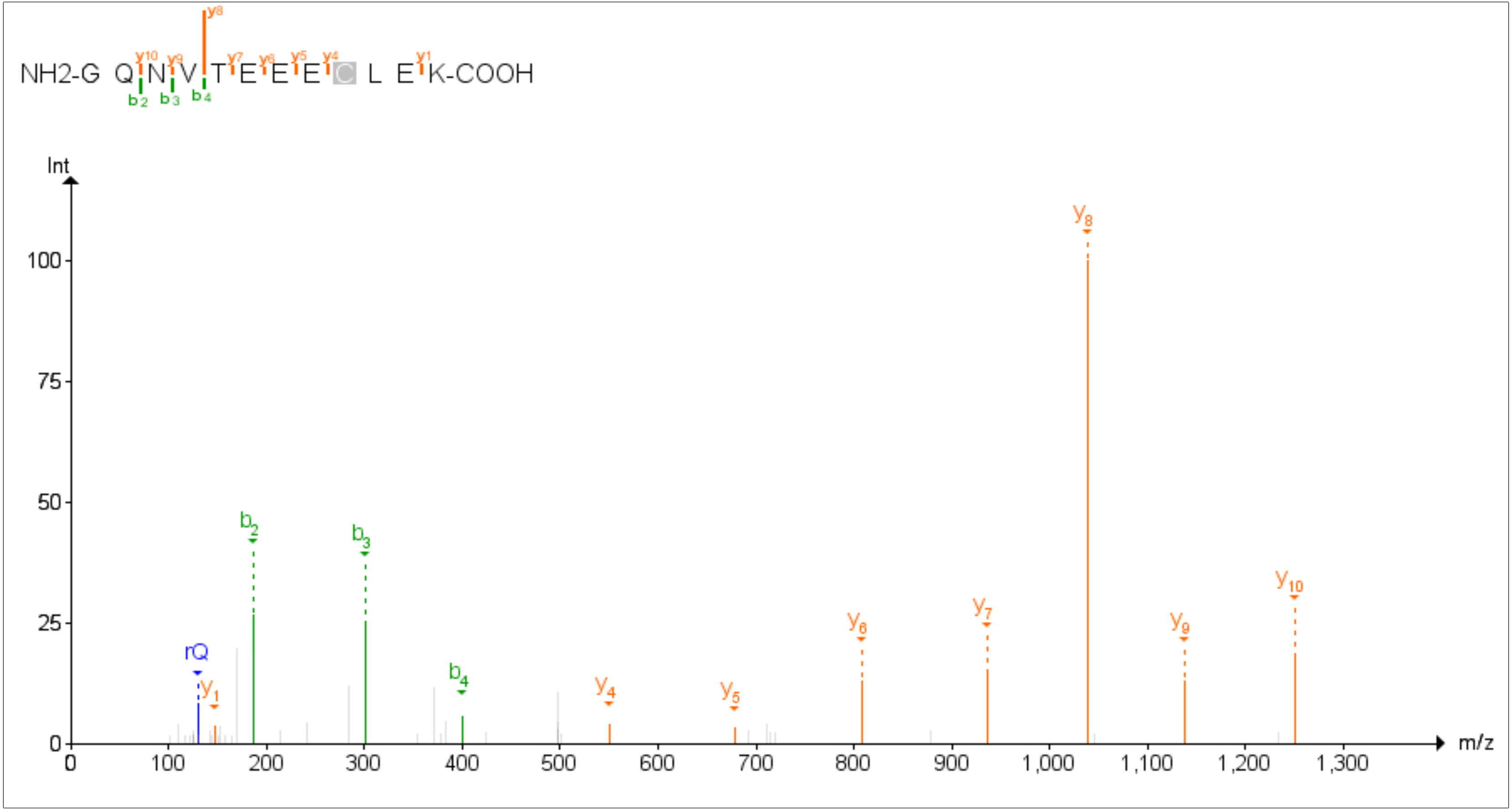

NH2-G Q N V T E E E C L E K-COOH
y10 y9 y8 y7 y6 y5 y4 y1
b2 b3 b4
Int
100
75
50
25
0
rQ
y1
b2
b3
b4
y4
y5
y6
y7
y8
y9
y10
0
100
200
300
400
500
600
700
800
900
1,000
1,100
1,200
1,300
m/z

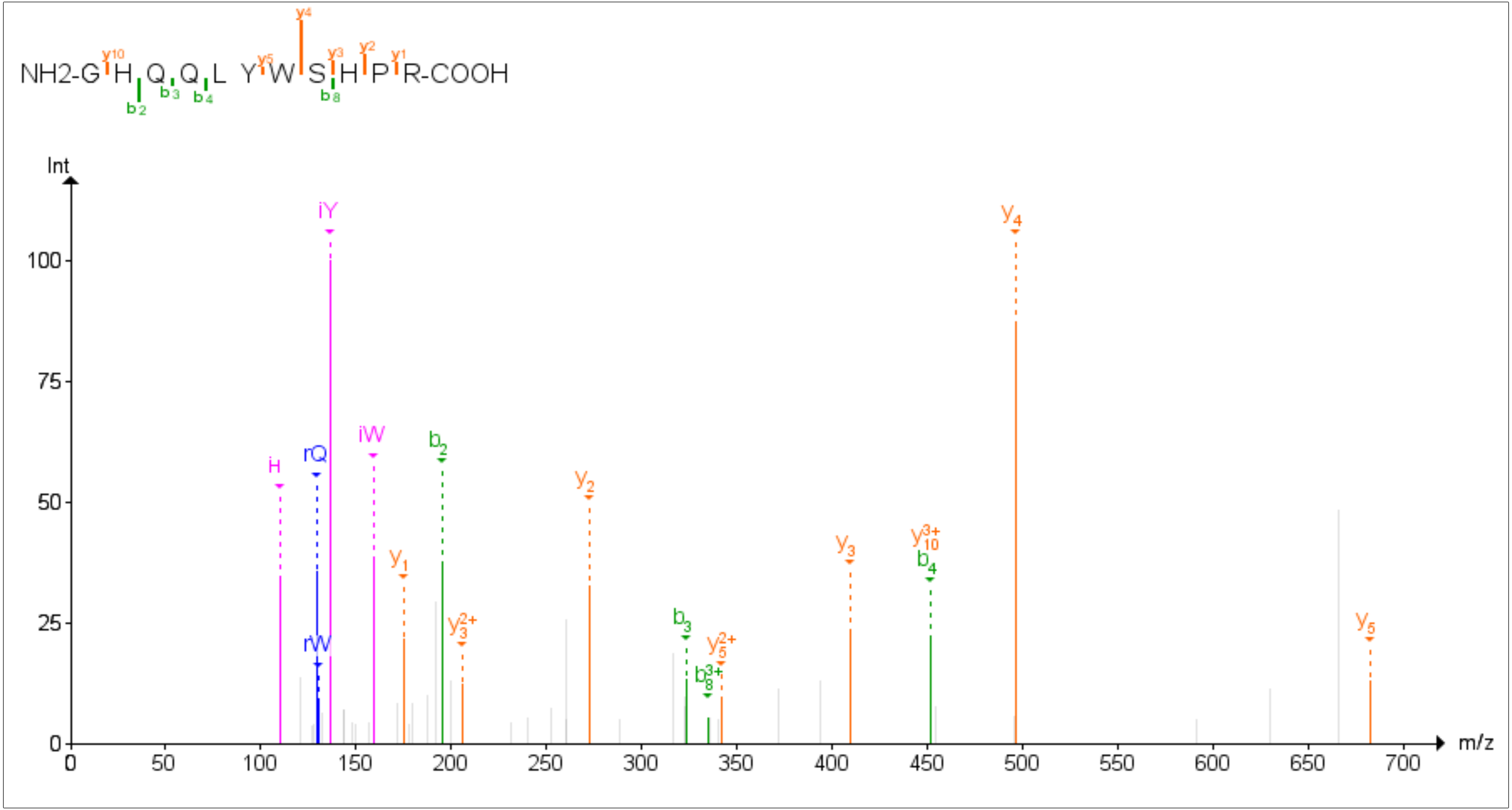

NH2-G H Q Q L Y W S H P R-COOH
y10 y5 y4 y3 y2 y1
b2 b3 b4 b8
Int
100
75
50
25
0
iY
y4
iW
iH
rQ
b2
y2
y1
y3
y10 3+
b4
y3 2+
rW
b3
y5 2+
b8 3+
y5
0 50 100 150 200 250 300 350 400 450 500 550 600 650 700
m/z

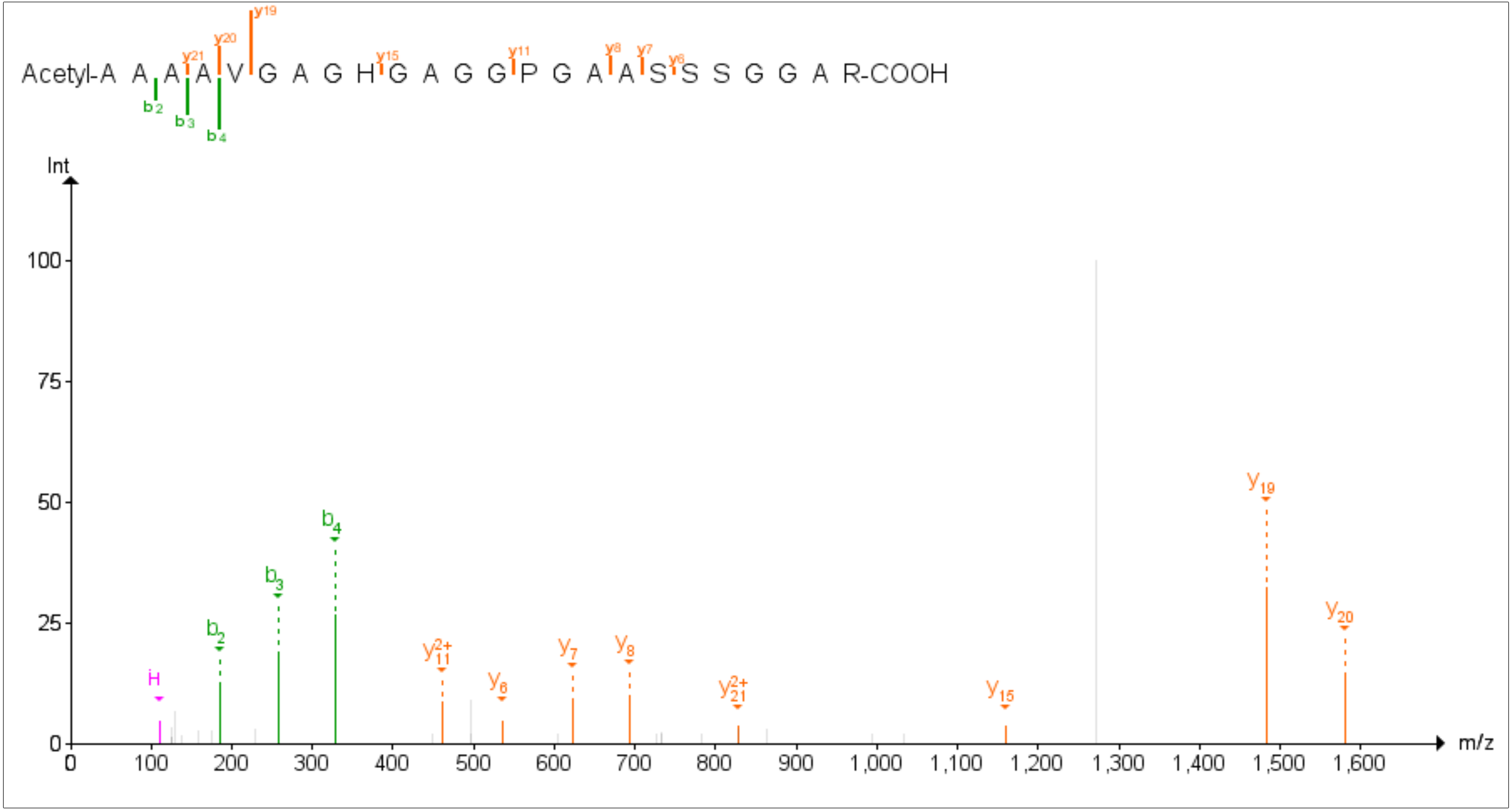

Acetyl-A A A A V G A G H G A G G P G A A S S S G G A R-COOH
y19
y20
y21
y15
y11
y8
y7
y6
b2
b3
b4
Int
100
75
50
25
0
m/z
0
100
200
300
400
500
600
700
800
900
1,000
1,100
1,200
1,300
1,400
1,500
1,600
H
b2
b3
b4
y11 2+
y6
y7
y8
y21 2+
y15
y19
y20

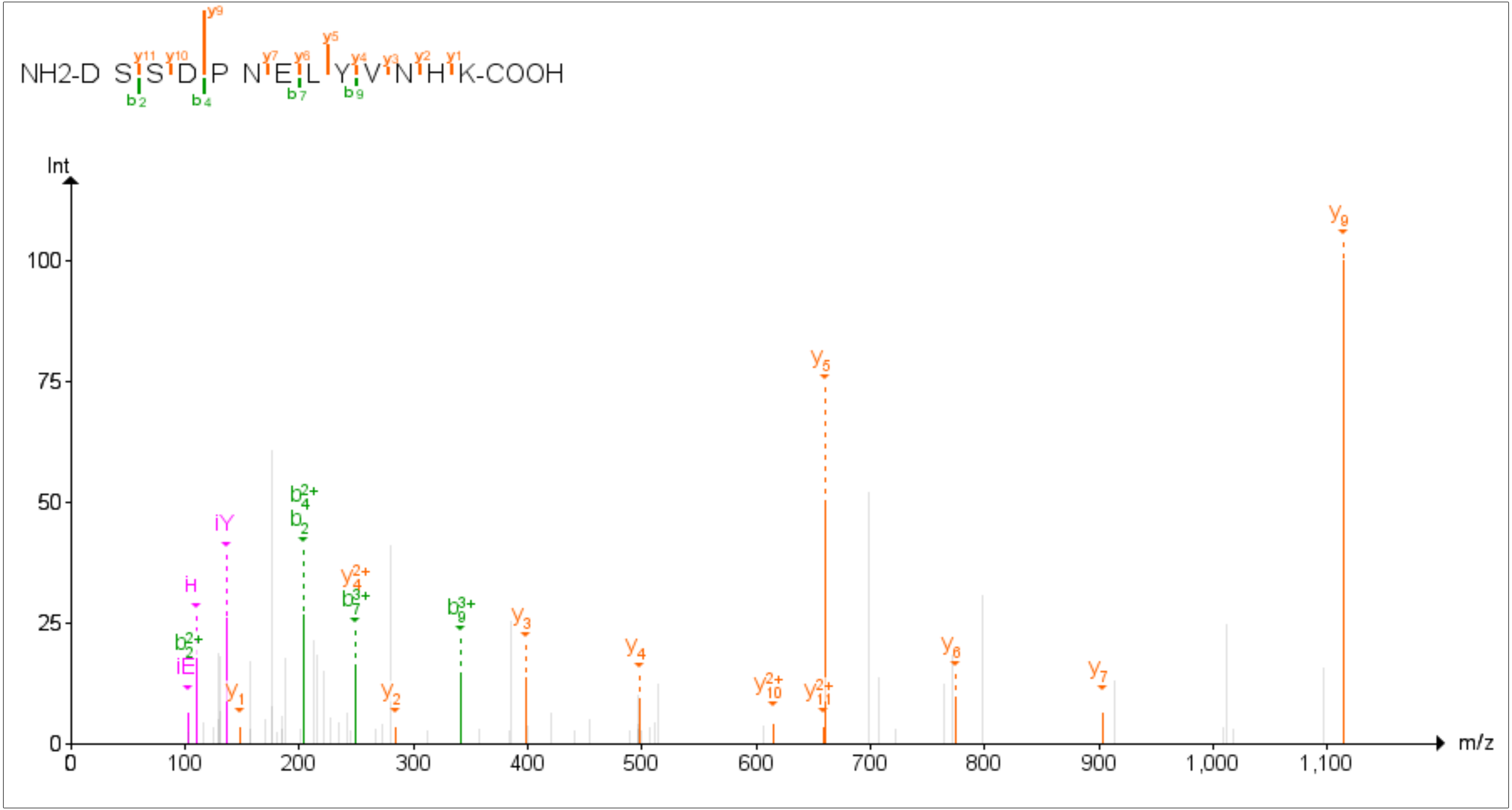

NH2-D S S D P N E L Y V N H K-COOH
y11 y10 y9 y7 y6 y5 y4 y3 y2 y1
b2 b4 b7 b9
Int
m/z
0
25
50
75
100
0
100
200
300
400
500
600
700
800
900
1,000
1,100
iE
iH
iY
y9
y5
y3
y4
y6
y7
y1
y2

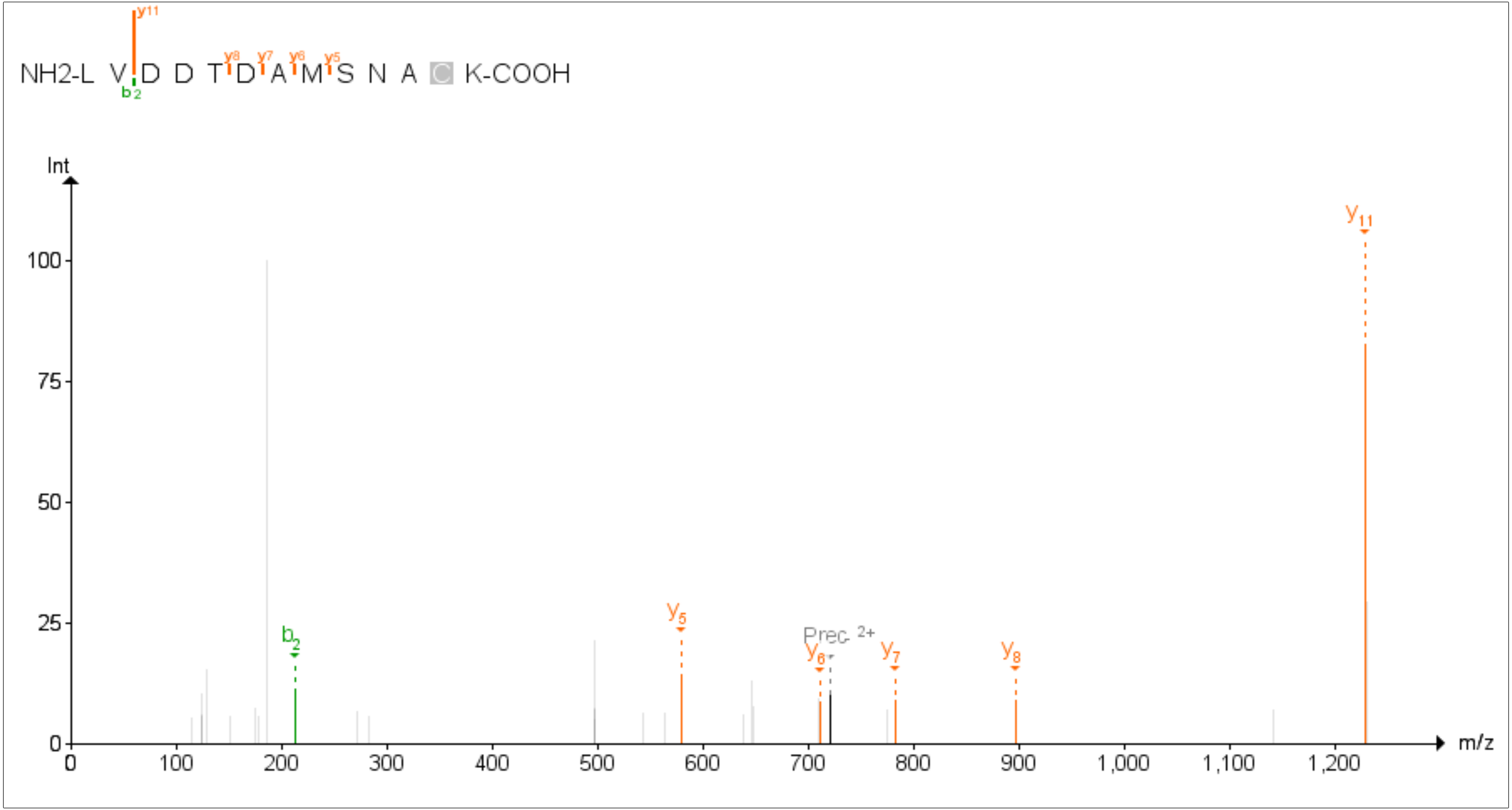

NH2-L V D D T D A M S N A C K-COOH
y11
y8
y7
y6
y5
b2
Int
100
75
50
25
0
y11
y5
b2
Prec. 2+
y6
y7
y8
0
100
200
300
400
500
600
700
800
900
1,000
1,100
1,200
m/z

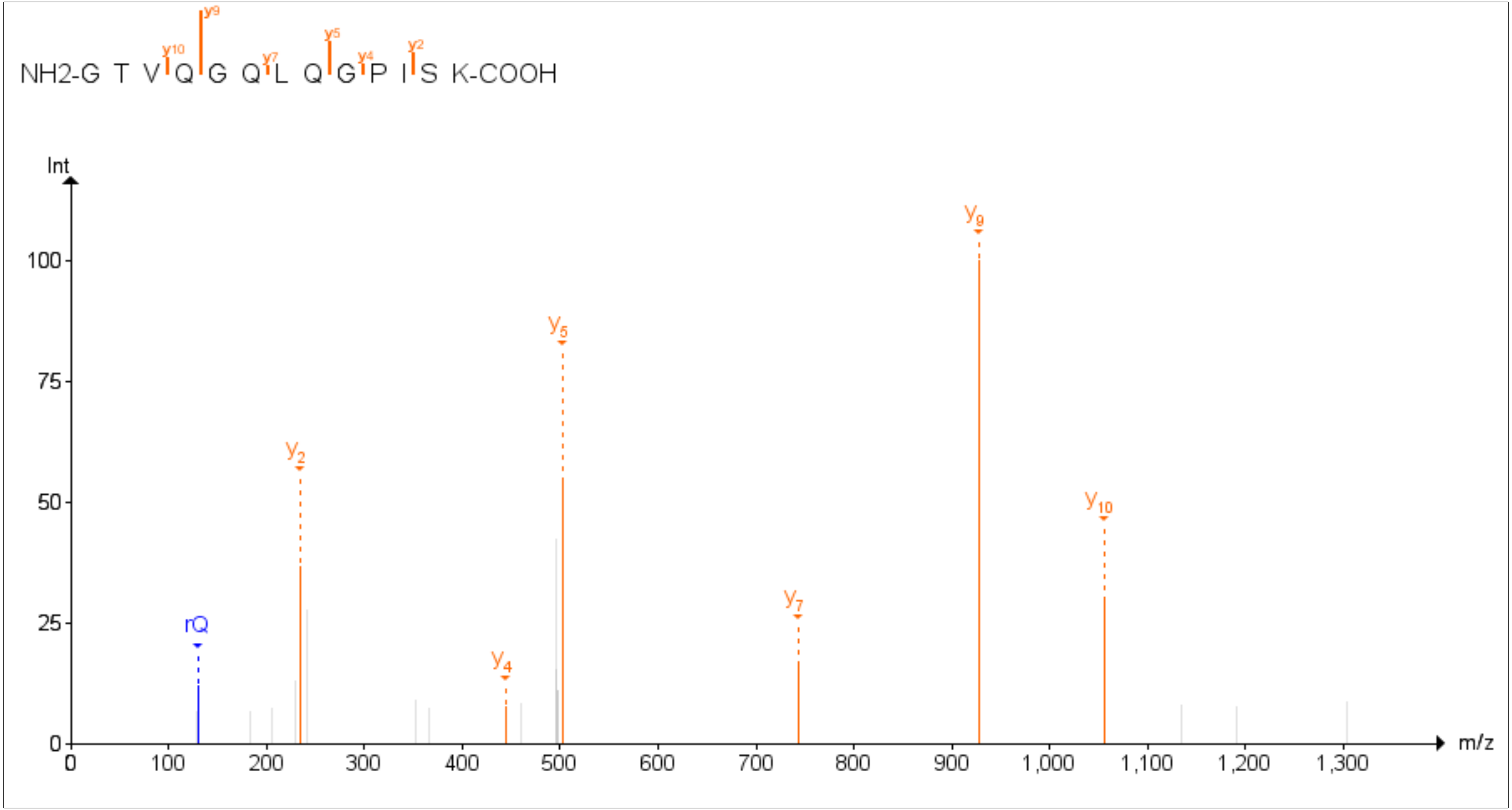
NH2-G T V Q G Q L Q G P I S K-COOH
y10 y9 y7 y5 y4 y2
Int
100
75
50
25
0
rQ
y2
y4
y5
y7
y9
y10
0 100 200 300 400 500 600 700 800 900 1,000 1,100 1,200 1,300
m/z

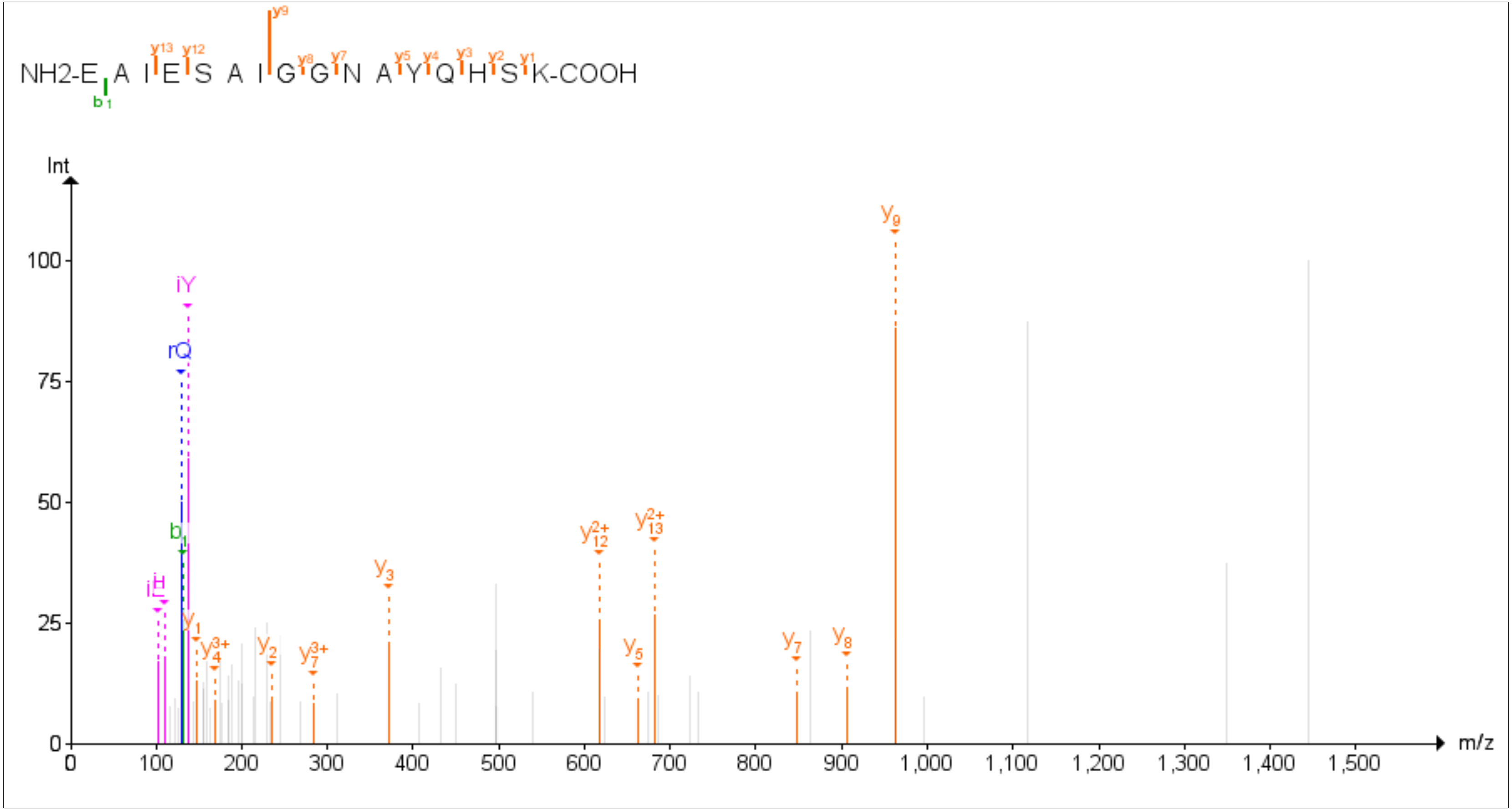

NH2-E A I E S A I G G N A Y Q H S K-COOH
b1
y13 y12 y9 y8 y7 y5 y4 y3 y2 y1
Int
m/z
iY
rQ
b1
iH
iL
y1
y4 3+
y2
y7 3+
y3
y12 2+
y13 2+
y5
y7
y8
y9

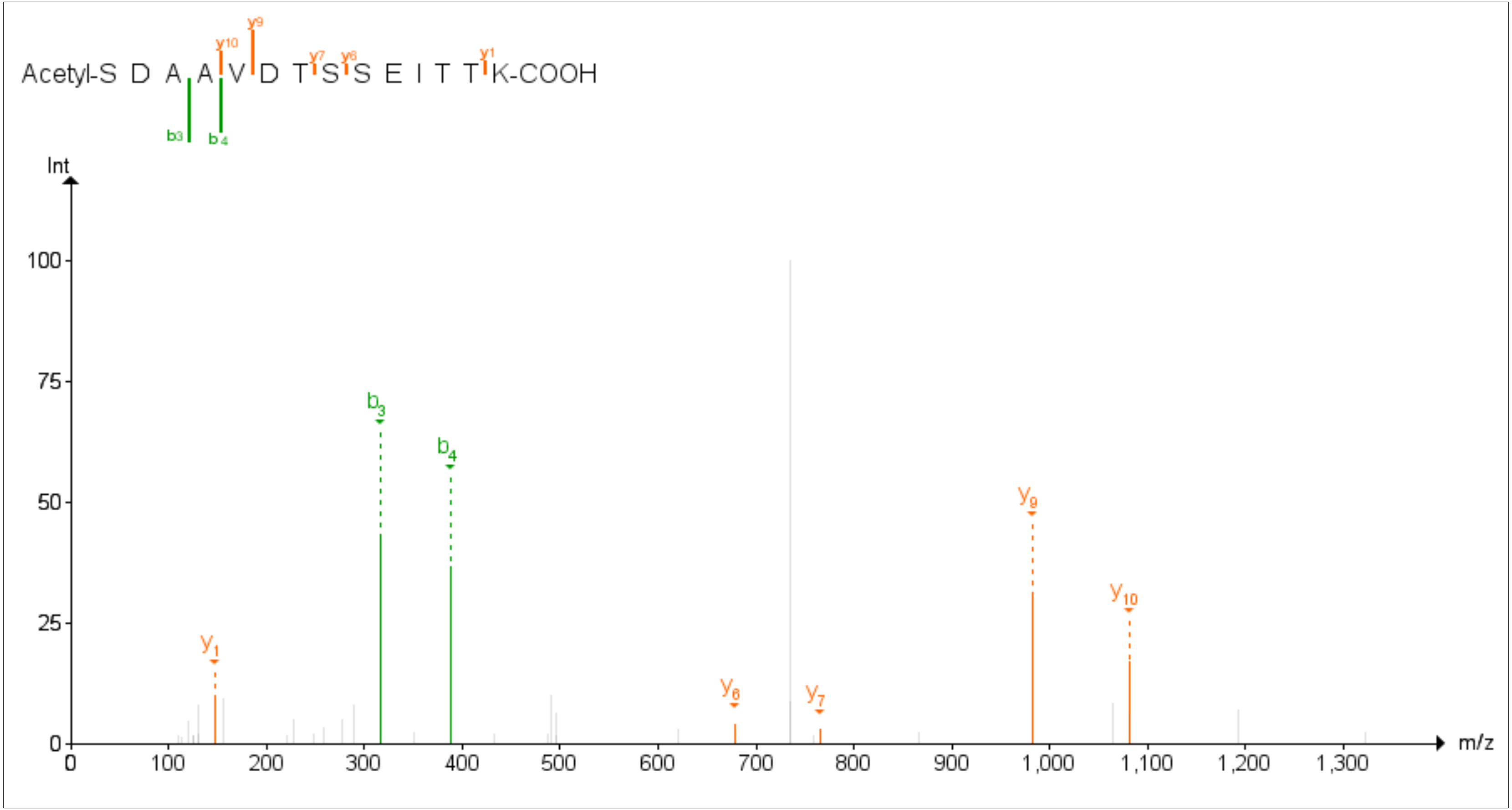

Acetyl-S D A A V D T S S E I T T K-COOH
y10
y9
y7
y6
y1
b3
b4
Int
100
75
50
25
0
b3
b4
y1
y6
y7
y9
y10
0
100
200
300
400
500
600
700
800
900
1,000
1,100
1,200
1,300
m/z

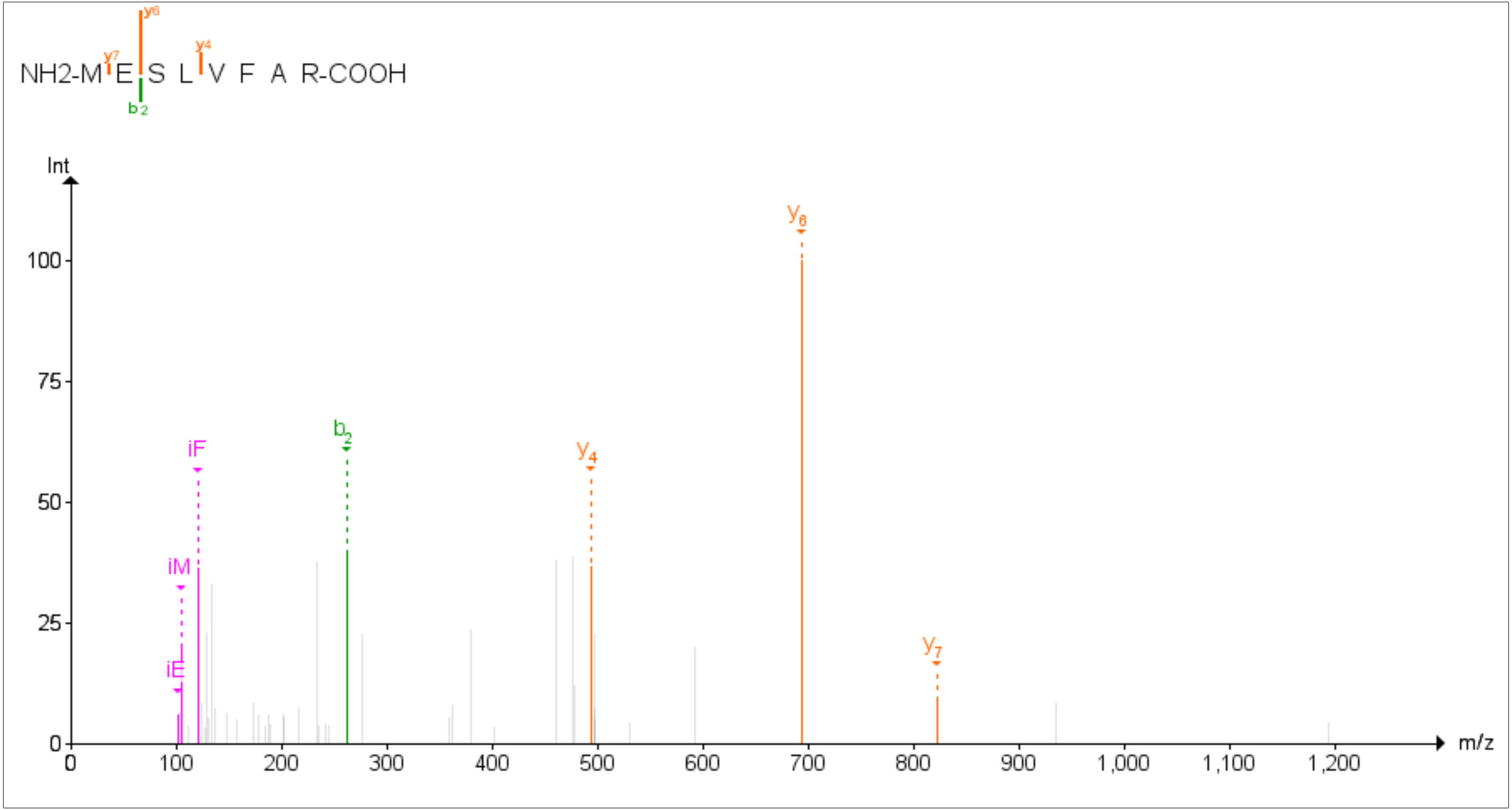

NH2-M E S L V F A R-COOH
y7
y6
y4
b2
Int
100
75
50
25
0
iF
iM
iE
b2
y4
y6
y7
0
100
200
300
400
500
600
700
800
900
1,000
1,100
1,200
m/z

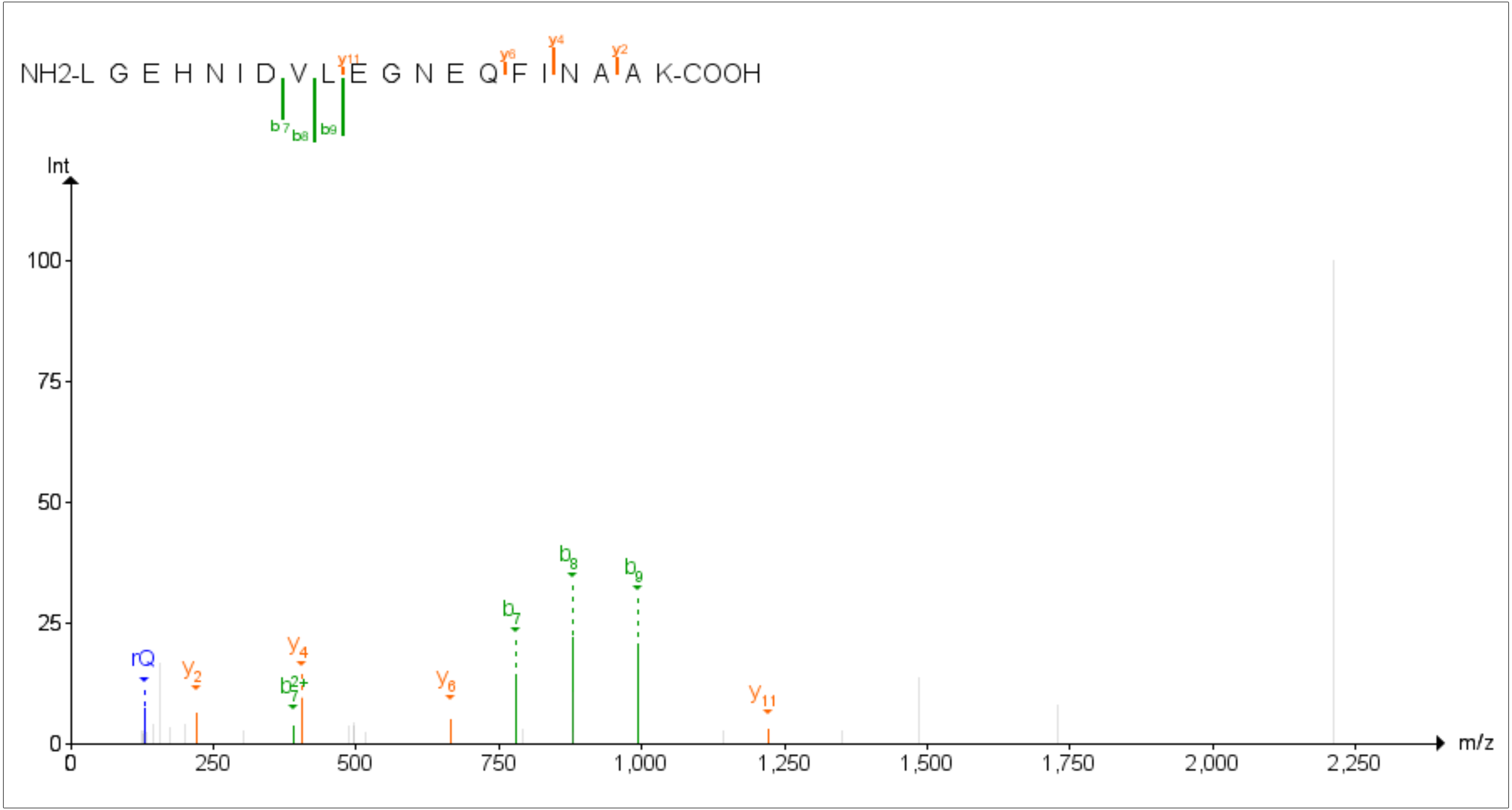

NH2-L G E H N I D V L E G N E Q F I N A A K-COOH
y11 y6 y4 y2
b7 b8 b9
Int
100
75
50
25
0
rQ
y2
y4
b7 2+
y6
b7
b8
b9
y11
0 250 500 750 1,000 1,250 1,500 1,750 2,000 2,250
m/z

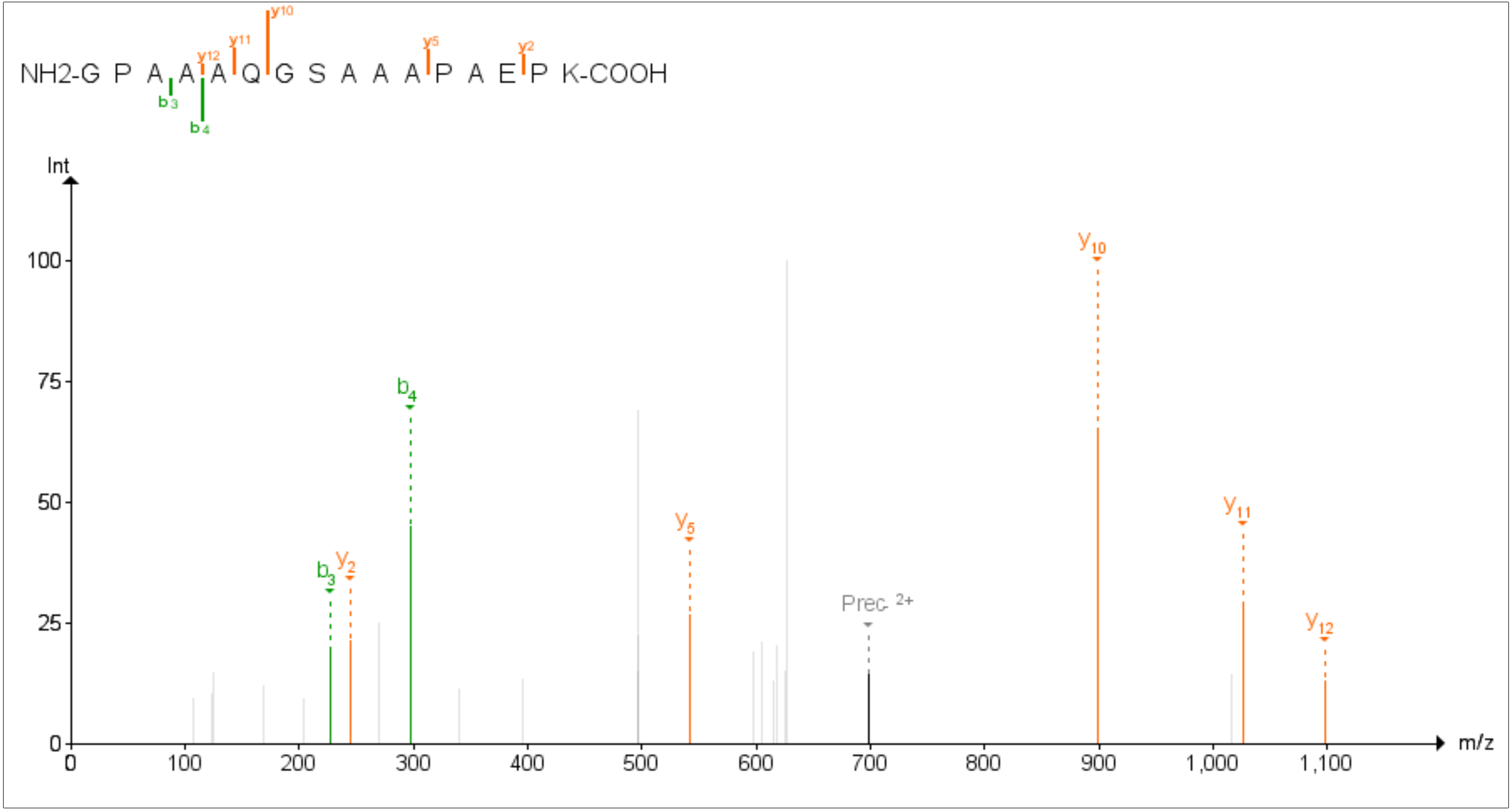

NH2-G P A A A Q G S A A A P A E P K-COOH
y10
y11
y12
y5
y2
b3
b4
Int
100
75
50
25
0
b4
b3
y2
y5
y10
y11
y12
Prec. 2+
0
100
200
300
400
500
600
700
800
900
1,000
1,100
m/z

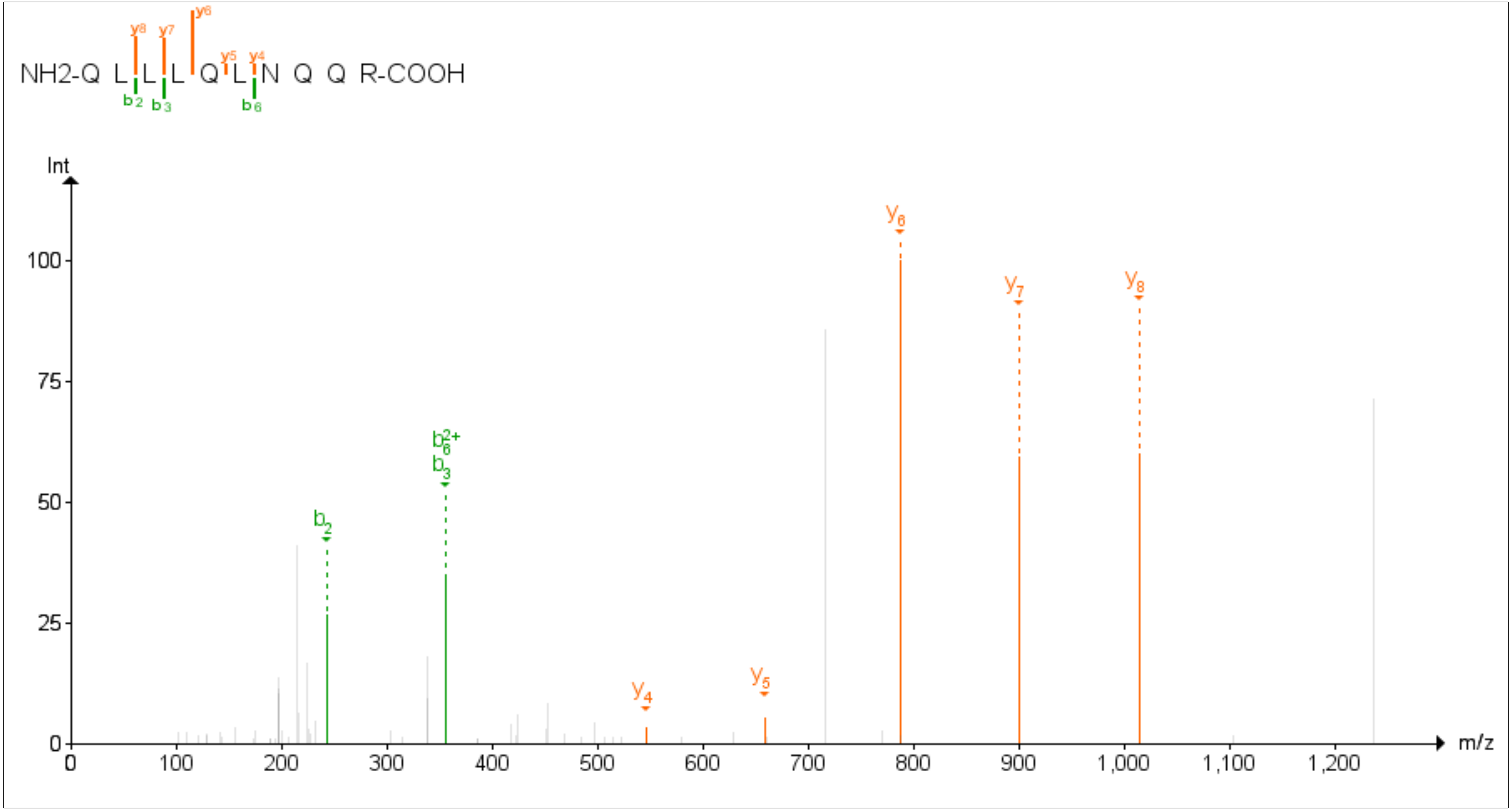

NH2-Q L L L Q L N Q Q R-COOH
y8 y7 y6 y5 y4
b2 b3 b6
Int
m/z
y6
y7
y8
b6 2+
b3
b2
y4
y5

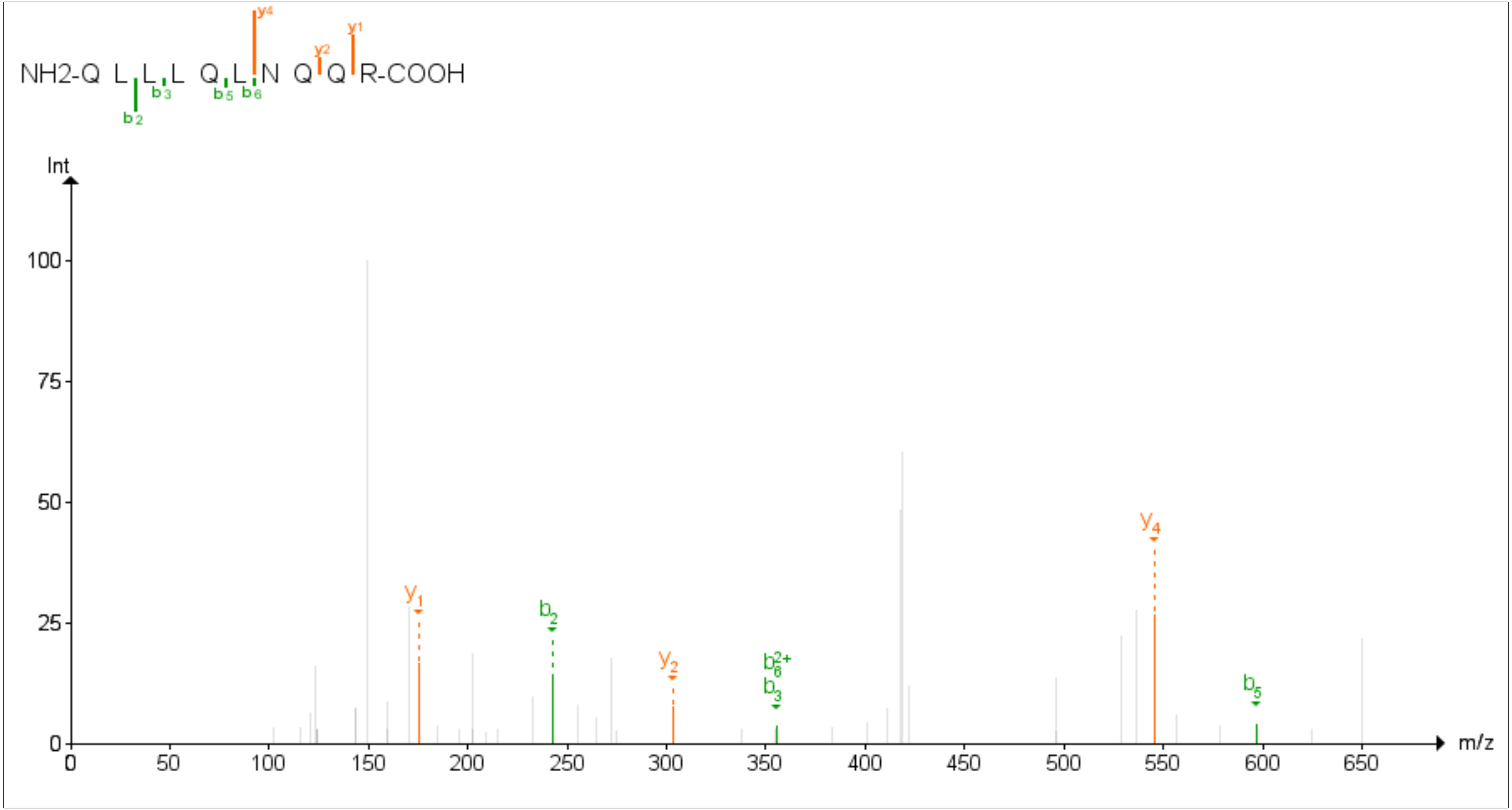
NH2-Q L L L Q L N Q Q R-COOH
y4
y1
y2
b2
b3
b5
b6
Int
100
75
50
25
0
m/z
0
50
100
150
200
250
300
350
400
450
500
550
600
650
y1
b2
y2
b6 2+
b3
y4
b5

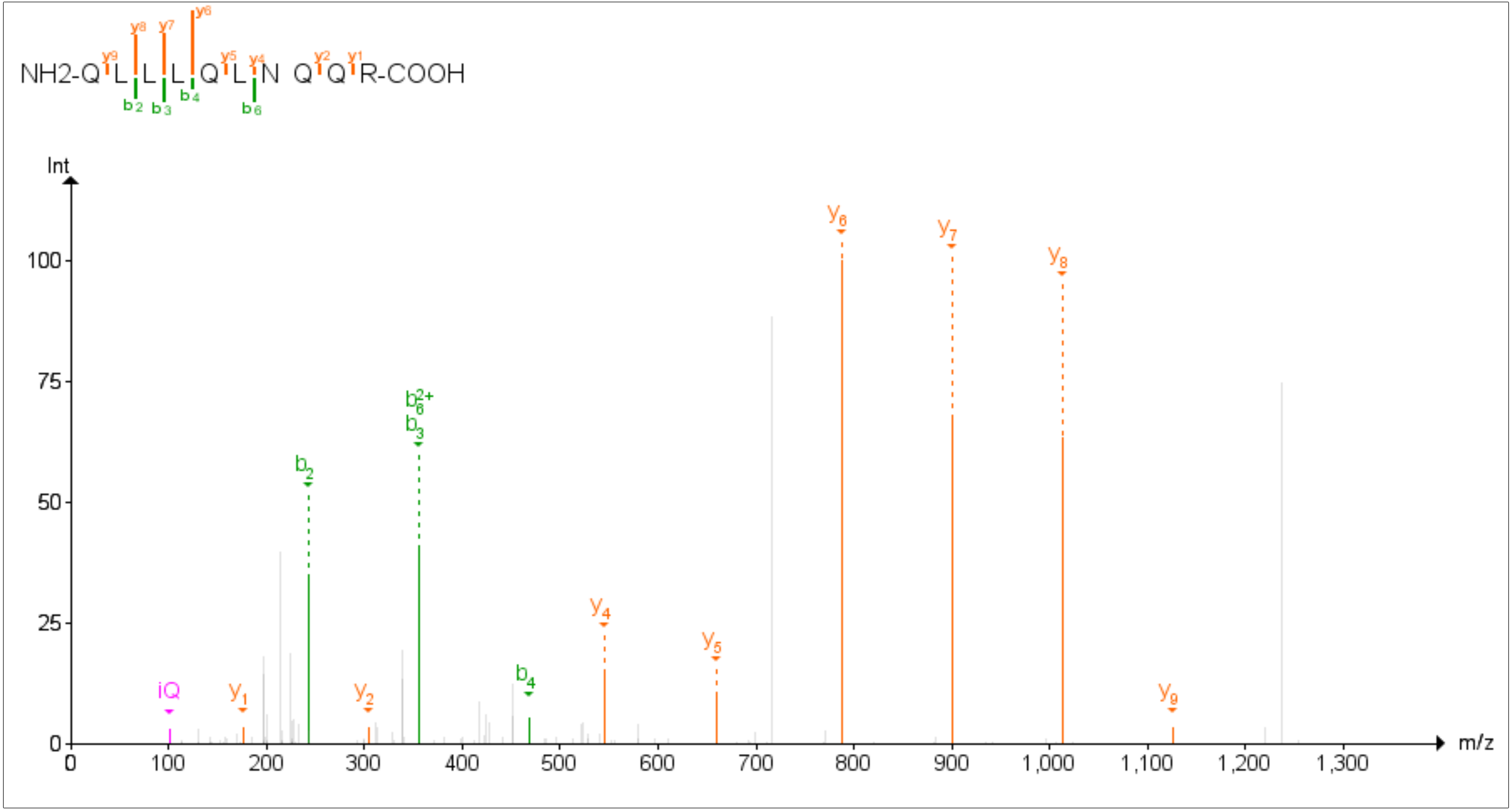

NH2-Q L L L Q L N Q Q R-COOH
y9 y8 y7 y6 y5 y4 y2 y1
b2 b3 b4 b6
Int
100
75
50
25
0
iQ
y1
y2
b2
b6 2+
b3
b4
y4
y5
y6
y7
y8
y9
0 100 200 300 400 500 600 700 800 900 1,000 1,100 1,200 1,300
m/z

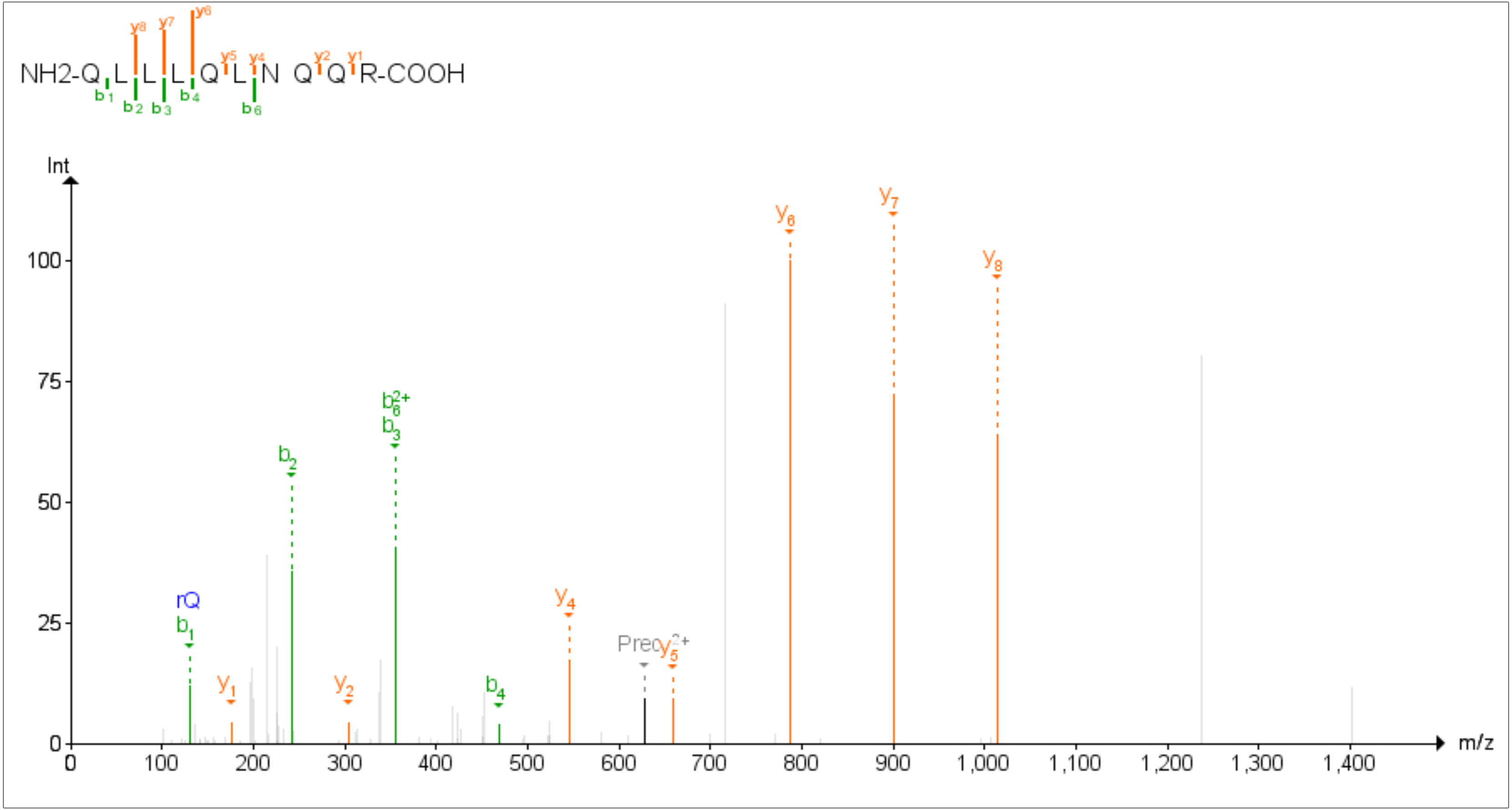

NH2-Q L L L Q L N Q Q R-COOH
y8 y7 y6 y5 y4 y2 y1
b1 b2 b3 b4 b6
Int
m/z
rQ
b1
y1
b2
y2
b6 2+
b3
b4
y4
Prec
y5 2+
y6
y7
y8
0
25
50
75
100
0
100
200
300
400
500
600
700
800
900
1,000
1,100
1,200
1,300
1,400

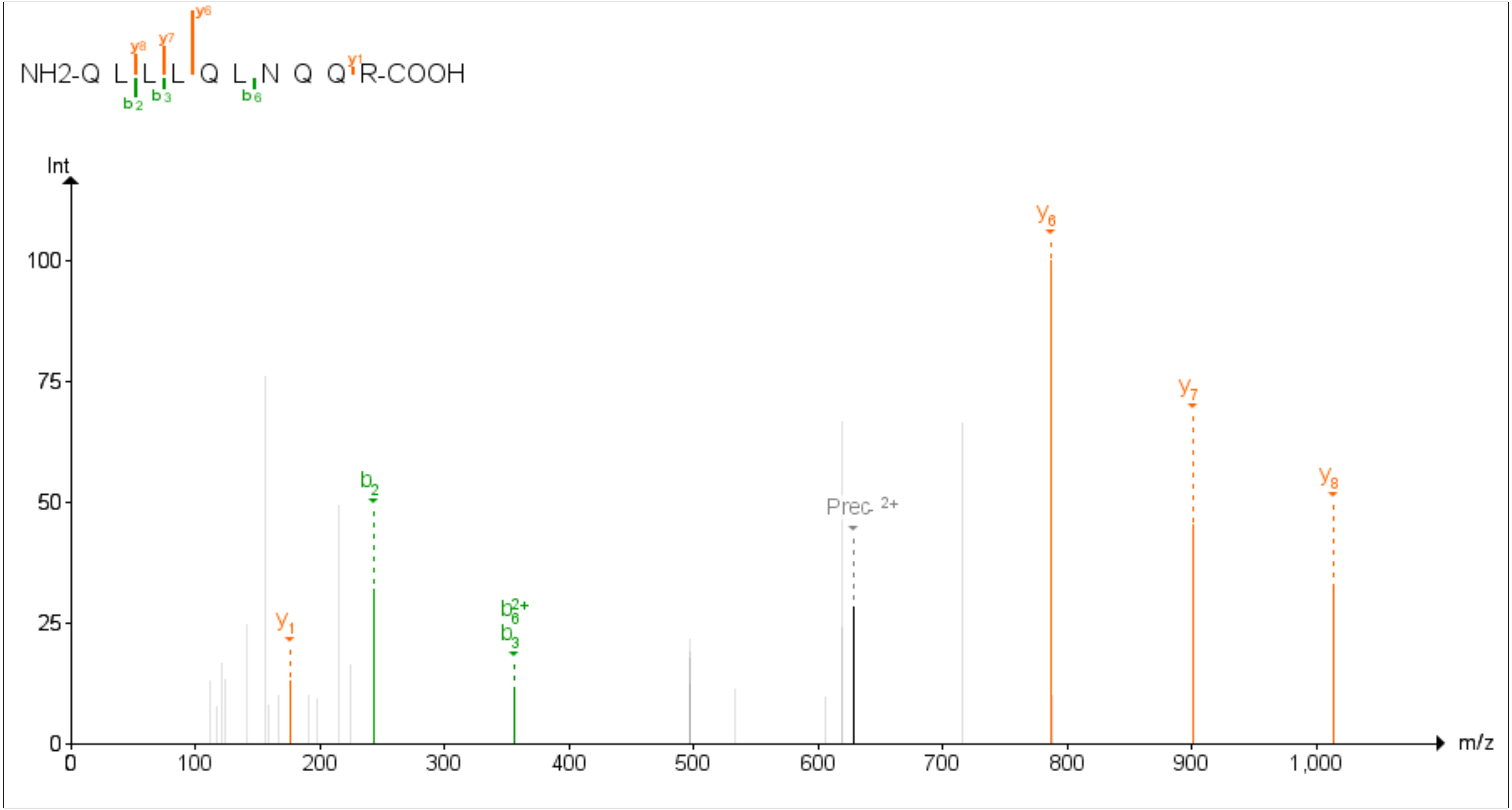

NH2-Q L L L Q L N Q Q R-COOH
y8 y7 y6 y1
b2 b3 b6
Int
100
75
50
25
0
y6
y7
y8
b2
Prec. 2+
y1
b6 2+
b3
0
100
200
300
400
500
600
700
800
900
1,000
m/z

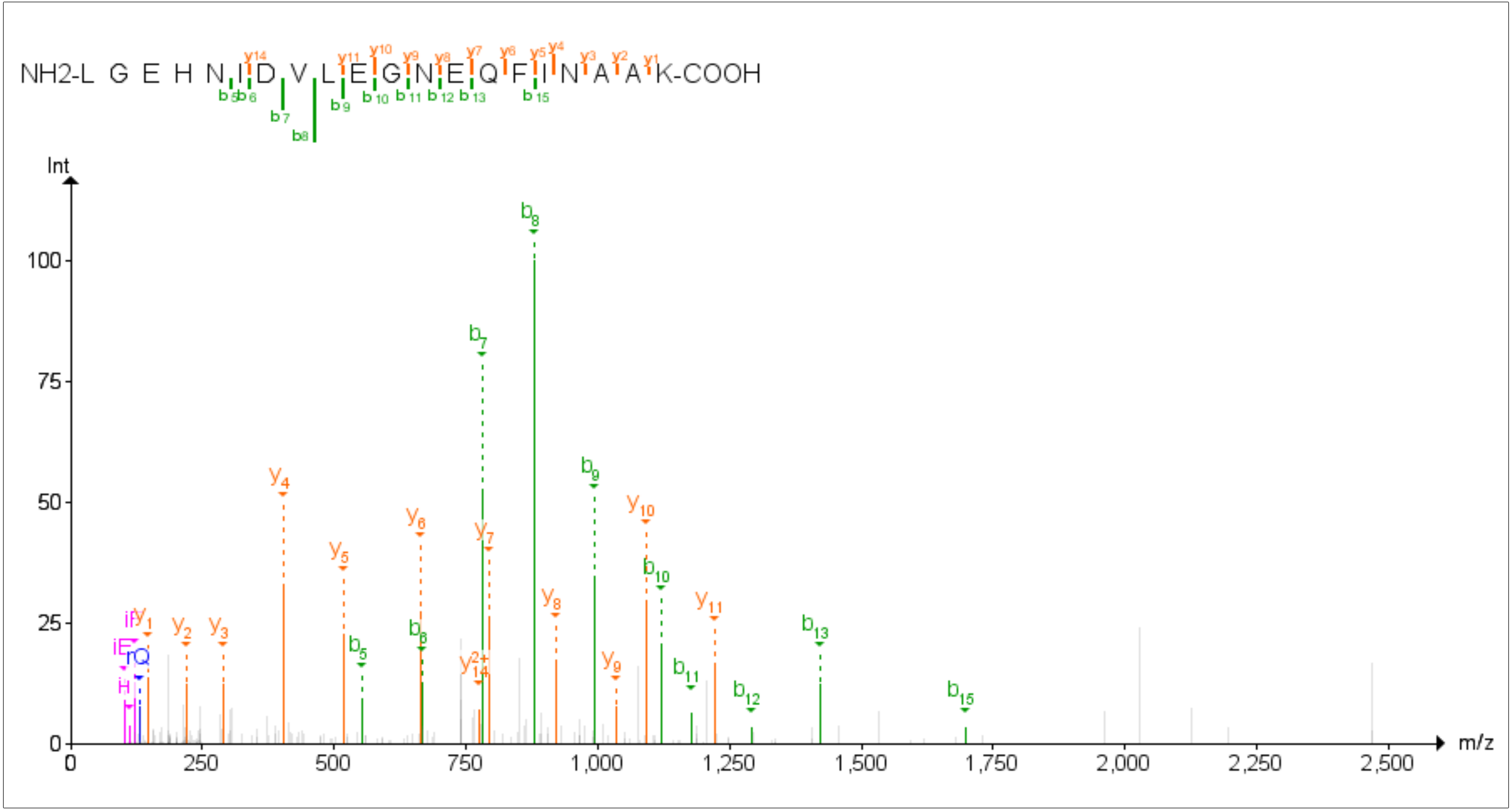
NH2-L G E H N I D V L E G N E Q F I N A A K-COOH
Int
m/z
0
25
50
75
100
250
500
750
1,000
1,250
1,500
1,750
2,000
2,250
2,500

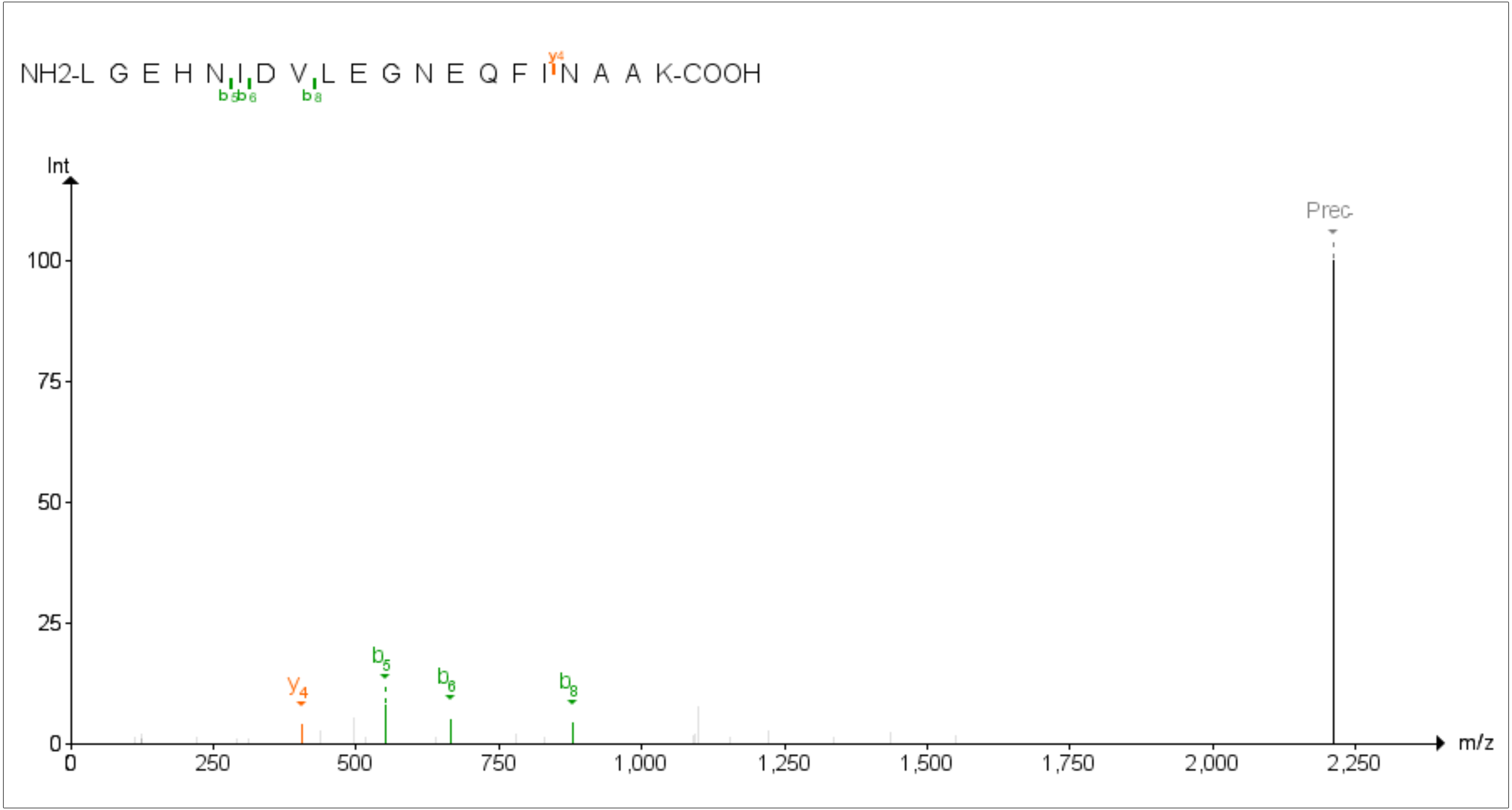

NH2-L G E H N I D V L E G N E Q F I N A A K-COOH
b5 b6 b8 y4
Int
100
75
50
25
0
Prec
y4
b5
b6
b8
0 250 500 750 1,000 1,250 1,500 1,750 2,000 2,250
m/z

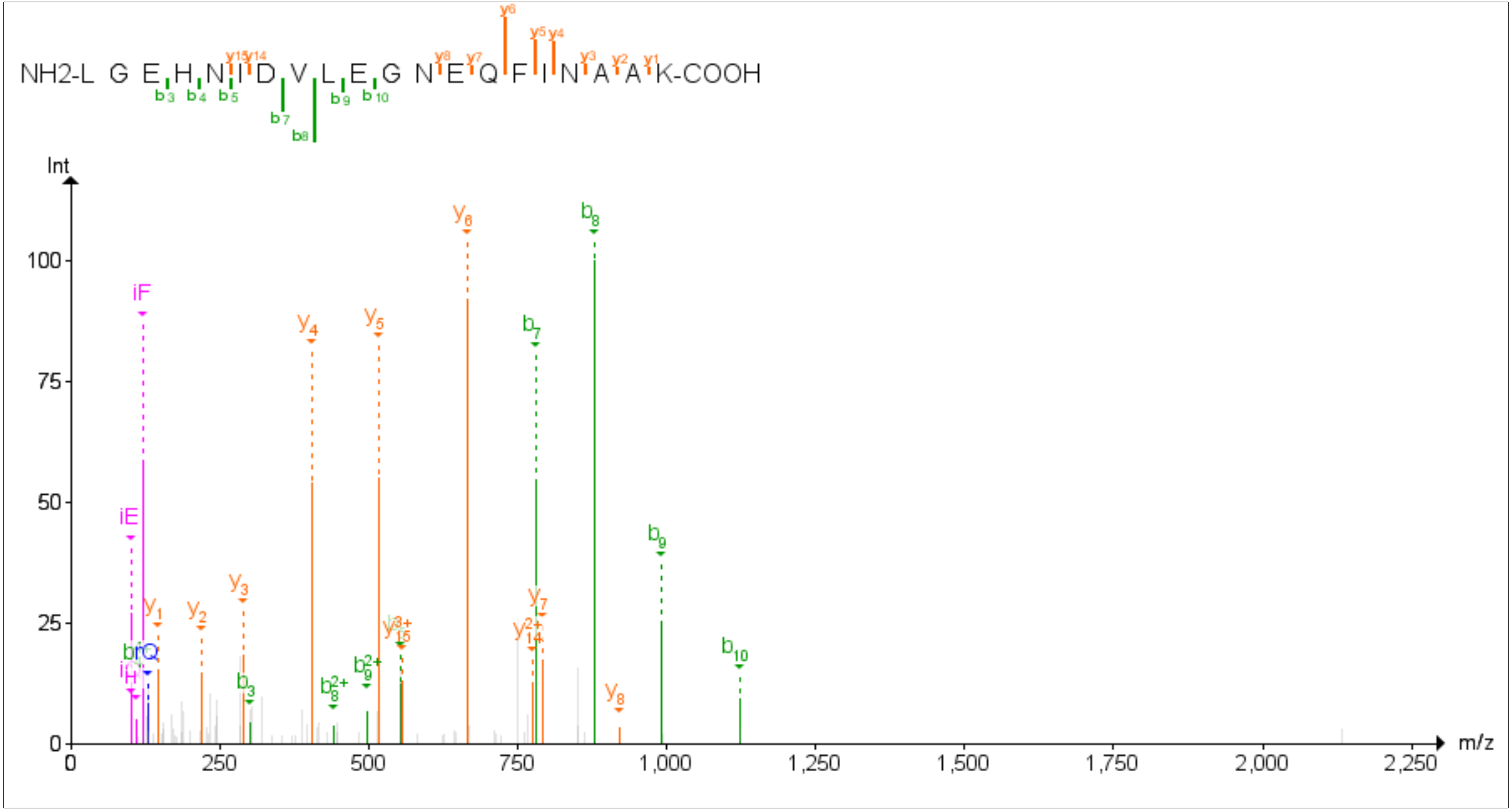
NH2-L G E H N I D V L E G N E Q F I N A A K-COOH
Int
m/z
0
25
50
75
100
250
500
750
1,000
1,250
1,500
1,750
2,000
2,250

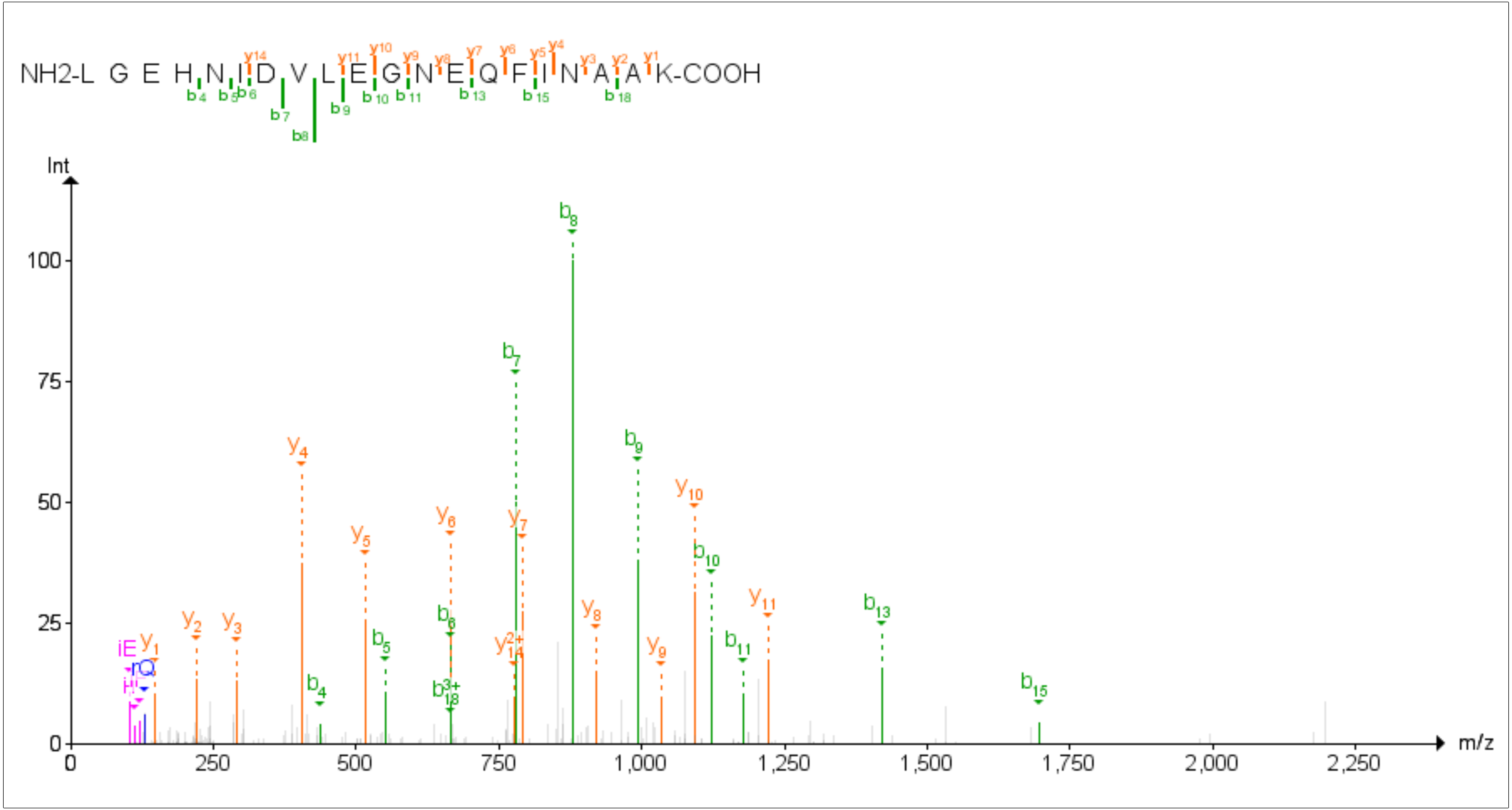

NH2-L G E H N I D V L E G N E Q F I N A A K-COOH
y14 y11 y10 y9 y8 y7 y6 y5 y4 y3 y2 y1
b4 b5 b6 b7 b8 b9 b10 b11 b13 b15 b18
Int
m/z
0
25
50
75
100
250
500
750
1,000
1,250
1,500
1,750
2,000
2,250

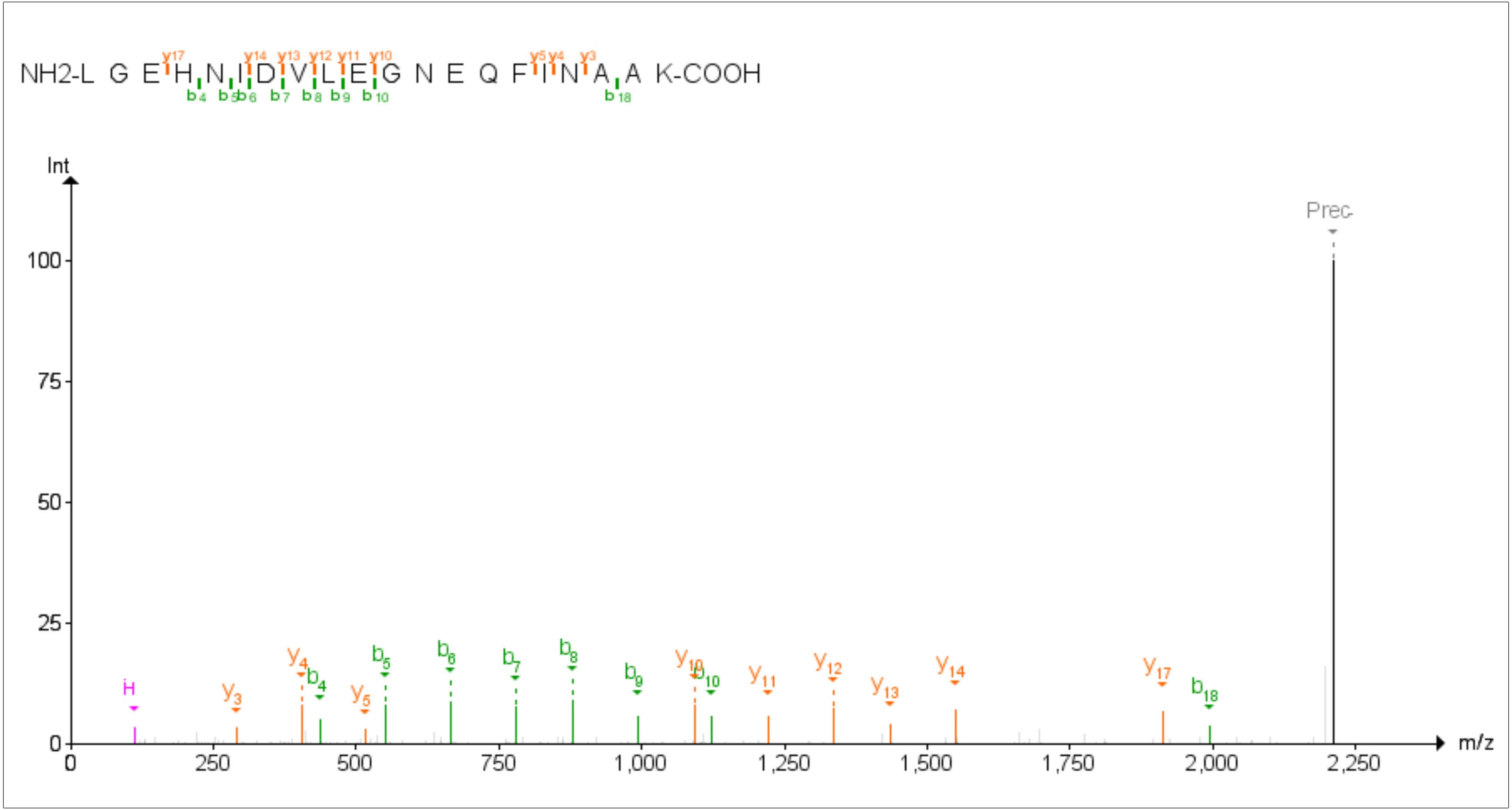
NH2-L G E H N I D V L E G N E Q F I N A A K-COOH
y17 y14 y13 y12 y11 y10 y5 y4 y3
b4 b5 b6 b7 b8 b9 b10 b18
Int
Prec.
m/z
0
25
50
75
100
0
250
500
750
1,000
1,250
1,500
1,750
2,000
2,250
H
y3 y4 b4 y5 b5 b6 b7 b8 b9 y10 b10 y11 y12 y13 y14 y17 b18

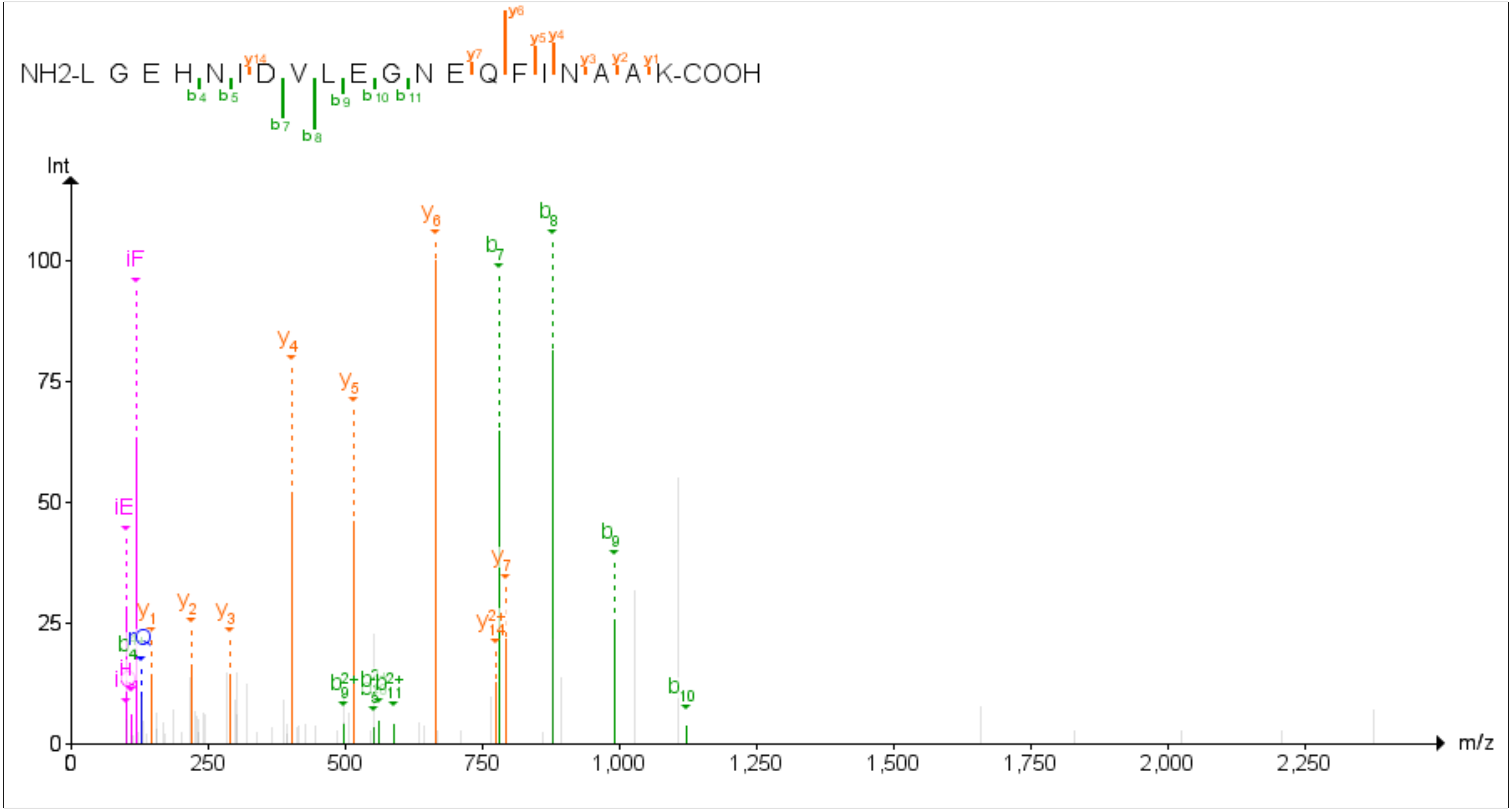

NH2-L G E H N I D V L E G N E Q F I N A A K-COOH
y14 y7 y6 y5 y4 y3 y2 y1
b4 b5 b7 b8 b9 b10 b11
Int
100
75
50
25
0
iF
iE
y1
y2
y3
y4
y5
y6
y7
b7
b8
b9
b10
m/z
0
250
500
750
1,000
1,250
1,500
1,750
2,000
2,250

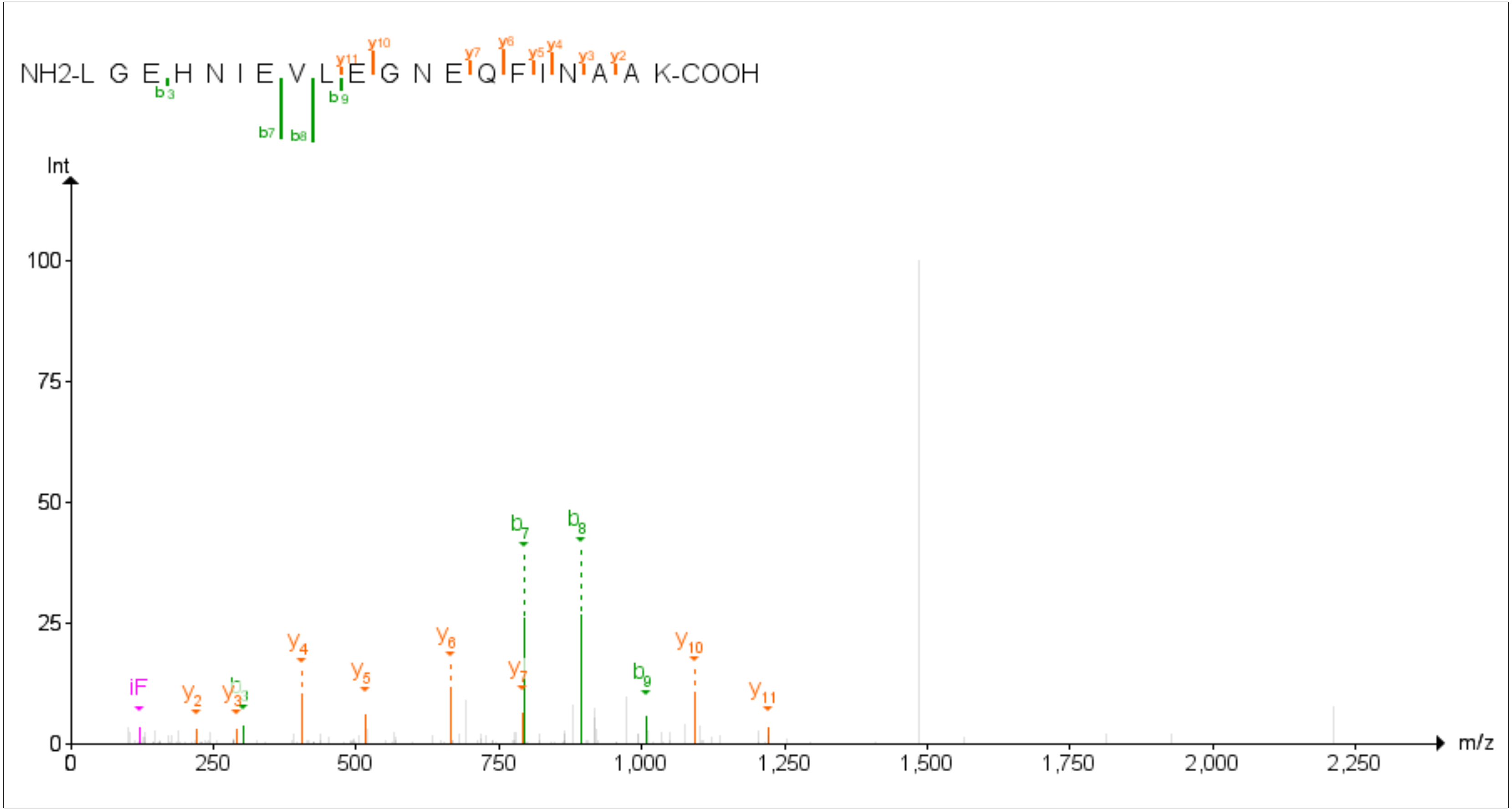
NH2-L G E H N I E V L I E G N E Q F I N A A K-COOH
b3
b7
b8
b9
y11
y10
y7
y6
y5
y4
y3
y2
Int
100
75
50
25
0
iF
y2
y3
b3
y4
y5
y6
y7
b7
b8
b9
y10
y11
0
250
500
750
1,000
1,250
1,500
1,750
2,000
2,250
m/z

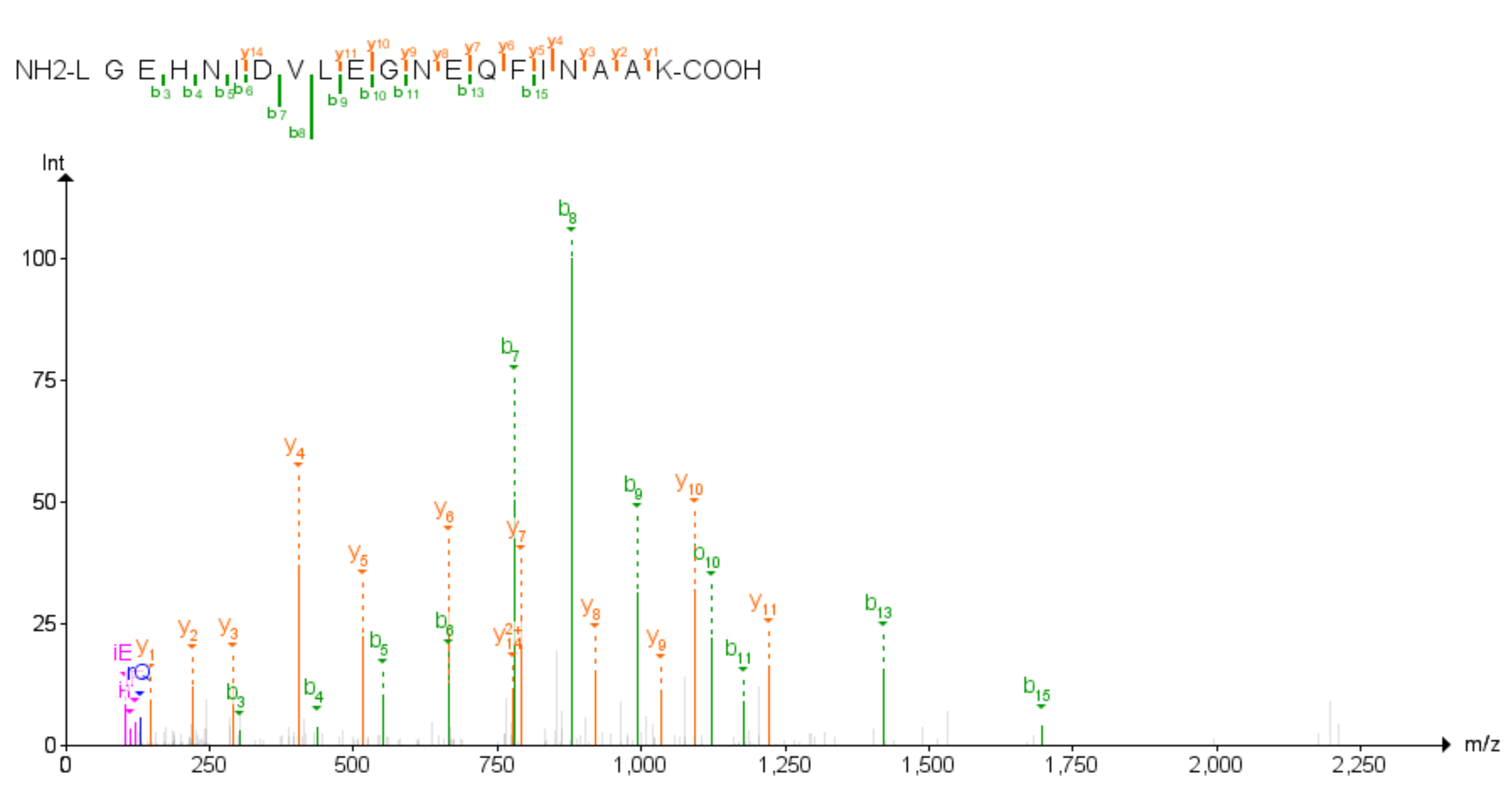

NH2-L G E H N I D V L E G N E Q F I N A A K-COOH
Int
m/z
0
25
50
75
100
0
250
500
750
1,000
1,250
1,500
1,750
2,000
2,250

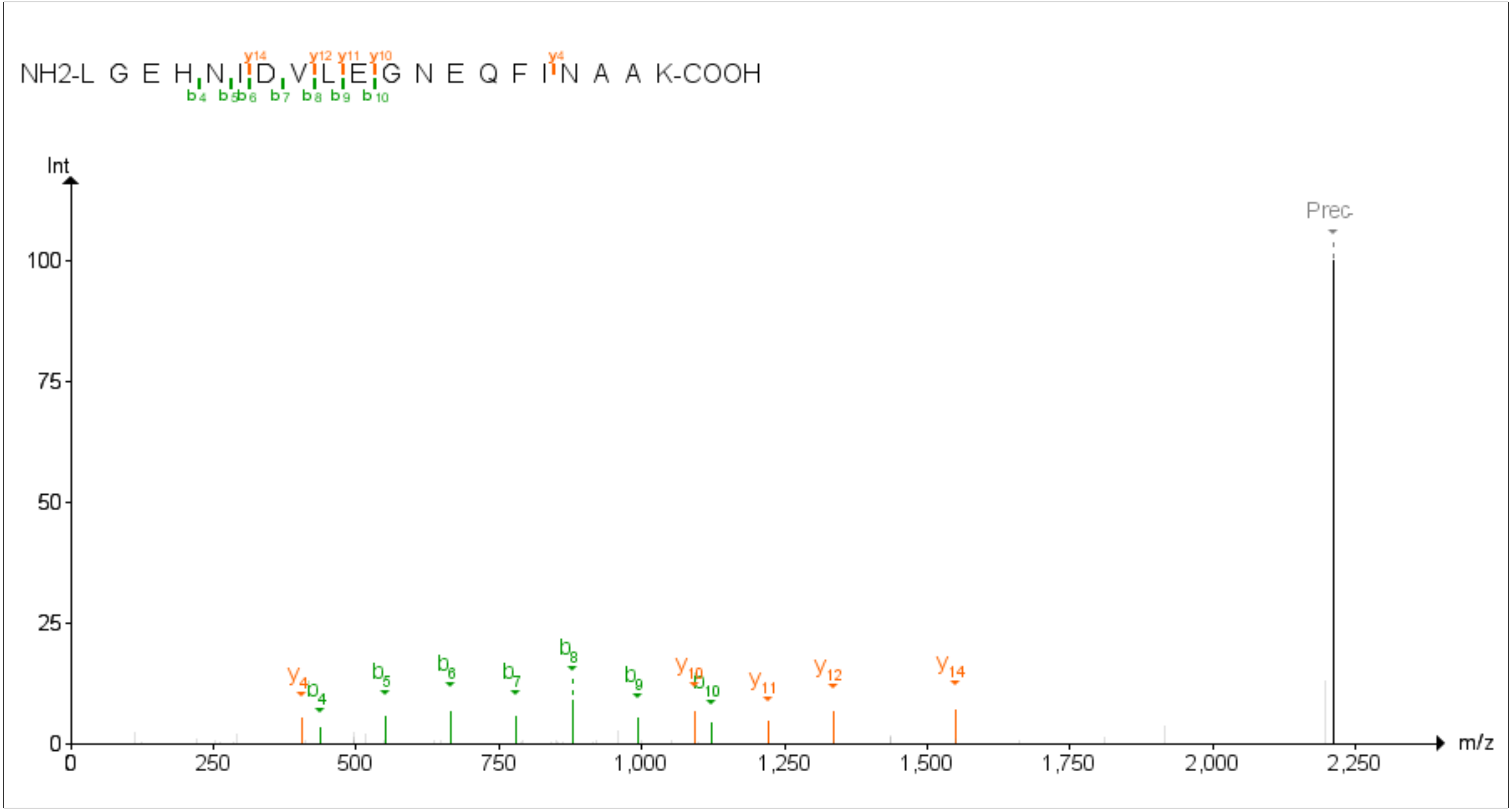

NH2-L G E H N I D V L E G N E Q F I N A A K-COOH
y14 y12 y11 y10 y4
b4 b5 b6 b7 b8 b9 b10
Int
100
75
50
25
0
Prec
y4 b4 b5 b6 b7 b8 b9 y10 b10 y11 y12 y14
0 250 500 750 1,000 1,250 1,500 1,750 2,000 2,250
m/z

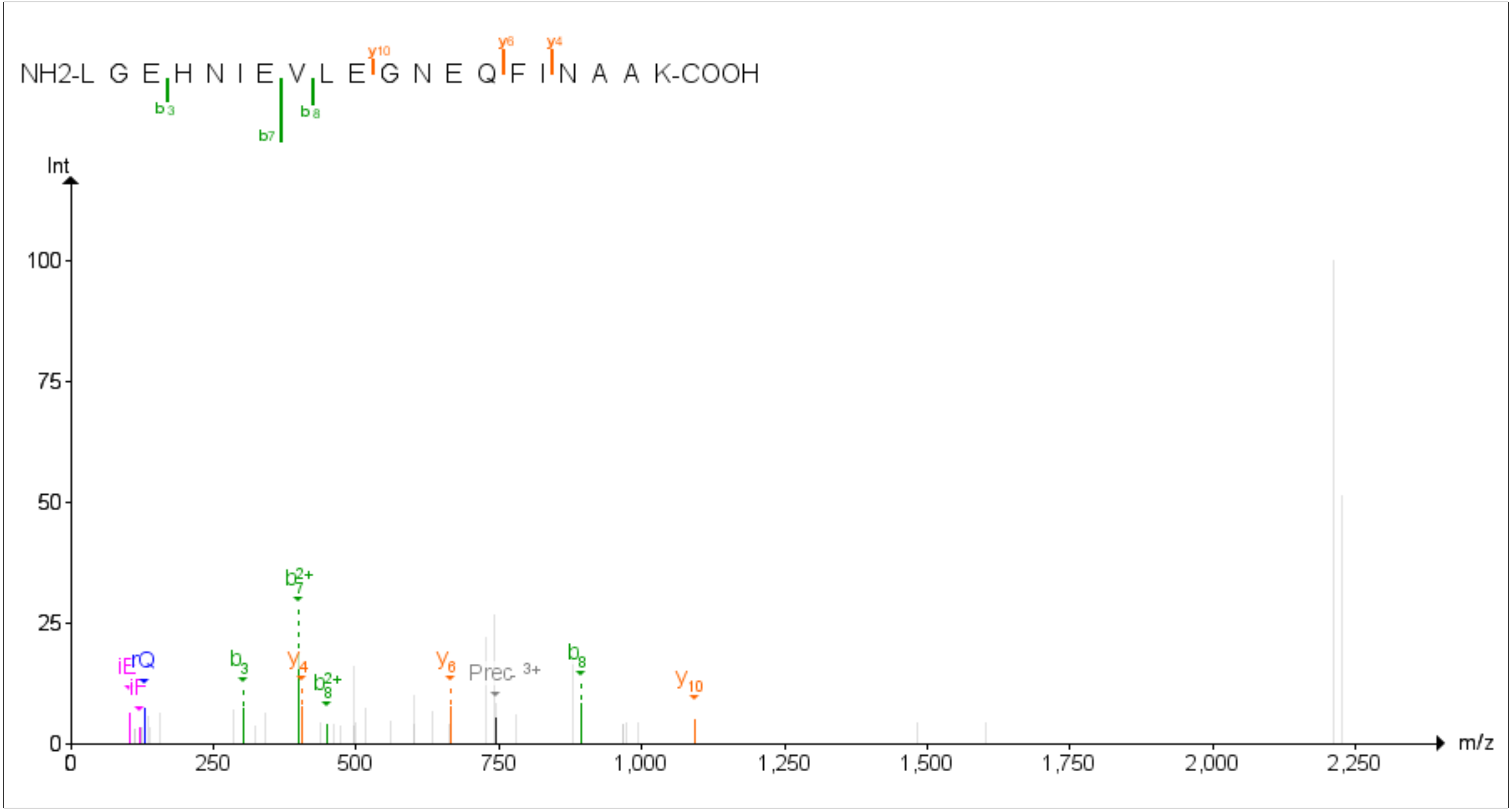
NH2-L G E H N I E V L E G N E Q F I N A A K-COOH
y10
y6
y4
b3
b7
b8
Int
100
75
50
25
0
m/z
0
250
500
750
1,000
1,250
1,500
1,750
2,000
2,250
Prec 3+

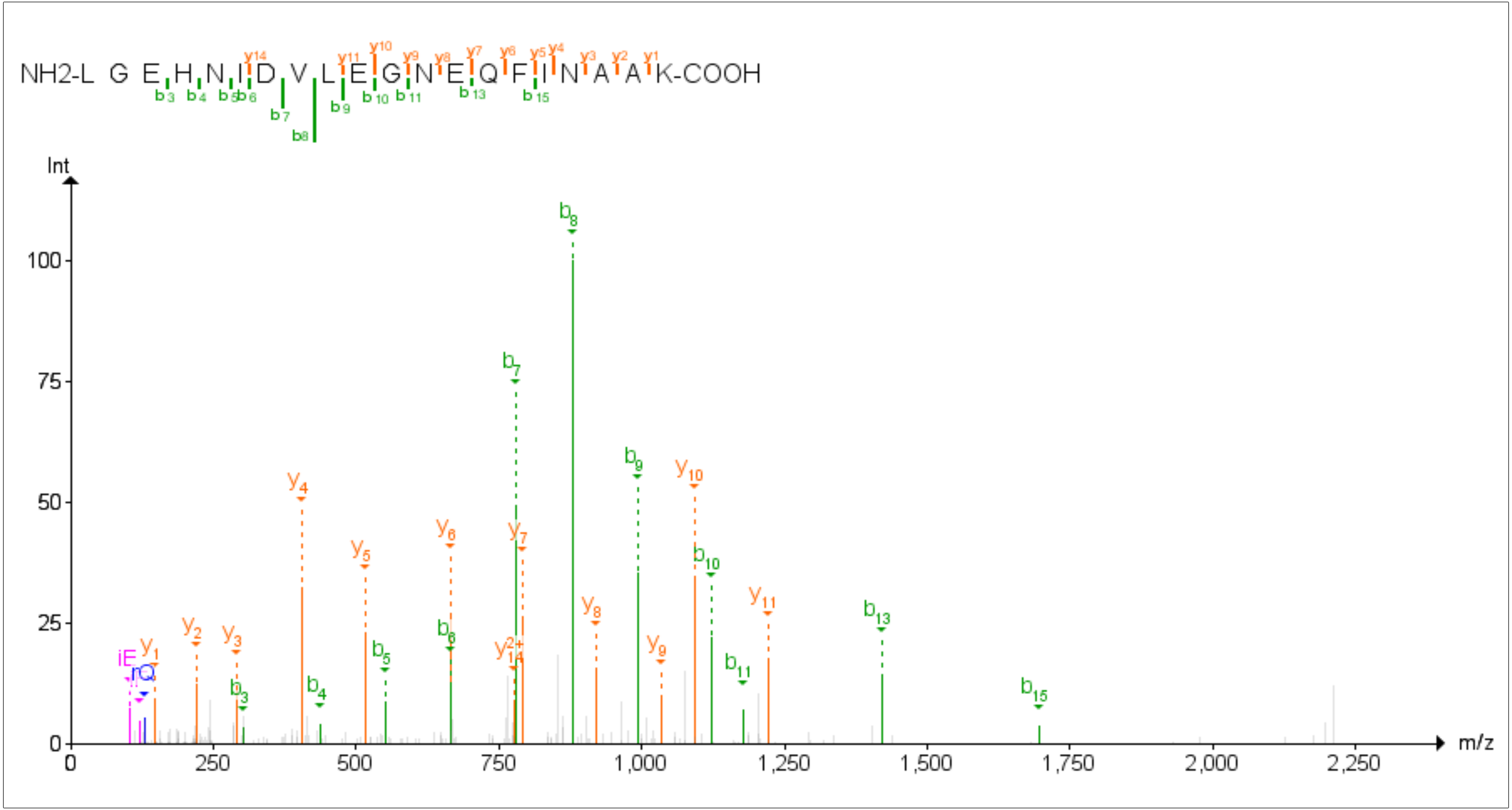
NH2-L G E H N I D V L E G N E Q F I N A A K-COOH
Int
m/z
b8
b7
b9
y10
b10
y4
y6
y7
y5
y11
b13
y8
y9
b6
b5
y2
y3
y1
iE
rQ
y14 2+
b3
b4
b11
b15
0
25
50
75
100
0
250
500
750
1,000
1,250
1,500
1,750
2,000
2,250

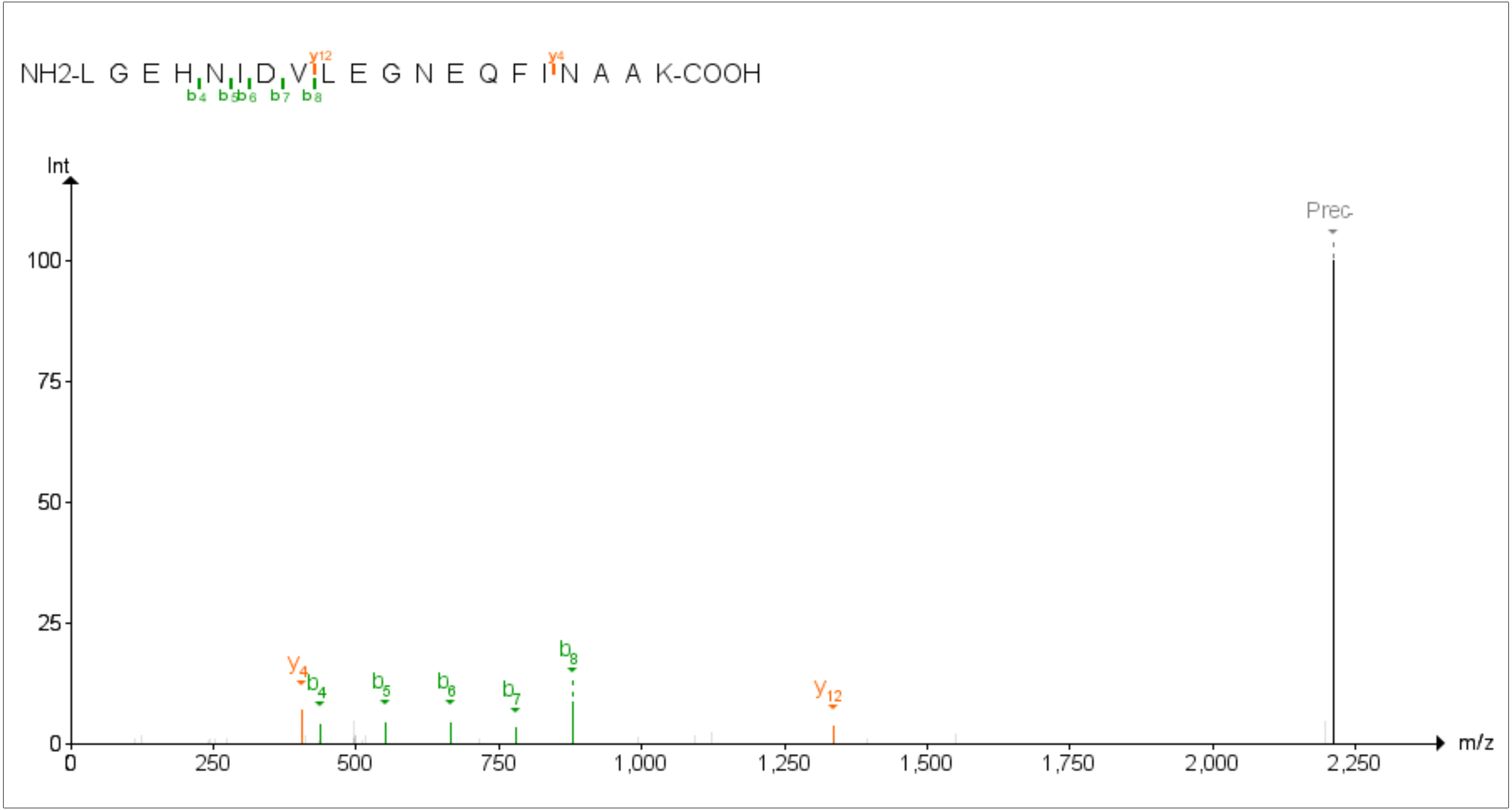

NH2-L G E H N I D V L E G N E Q F I N A A K-COOH
y12
y4
b4 b5 b6 b7 b8
Int
100
75
50
25
0
Prec
y4
b4
b5
b6
b7
b8
y12
m/z
0
250
500
750
1,000
1,250
1,500
1,750
2,000
2,250

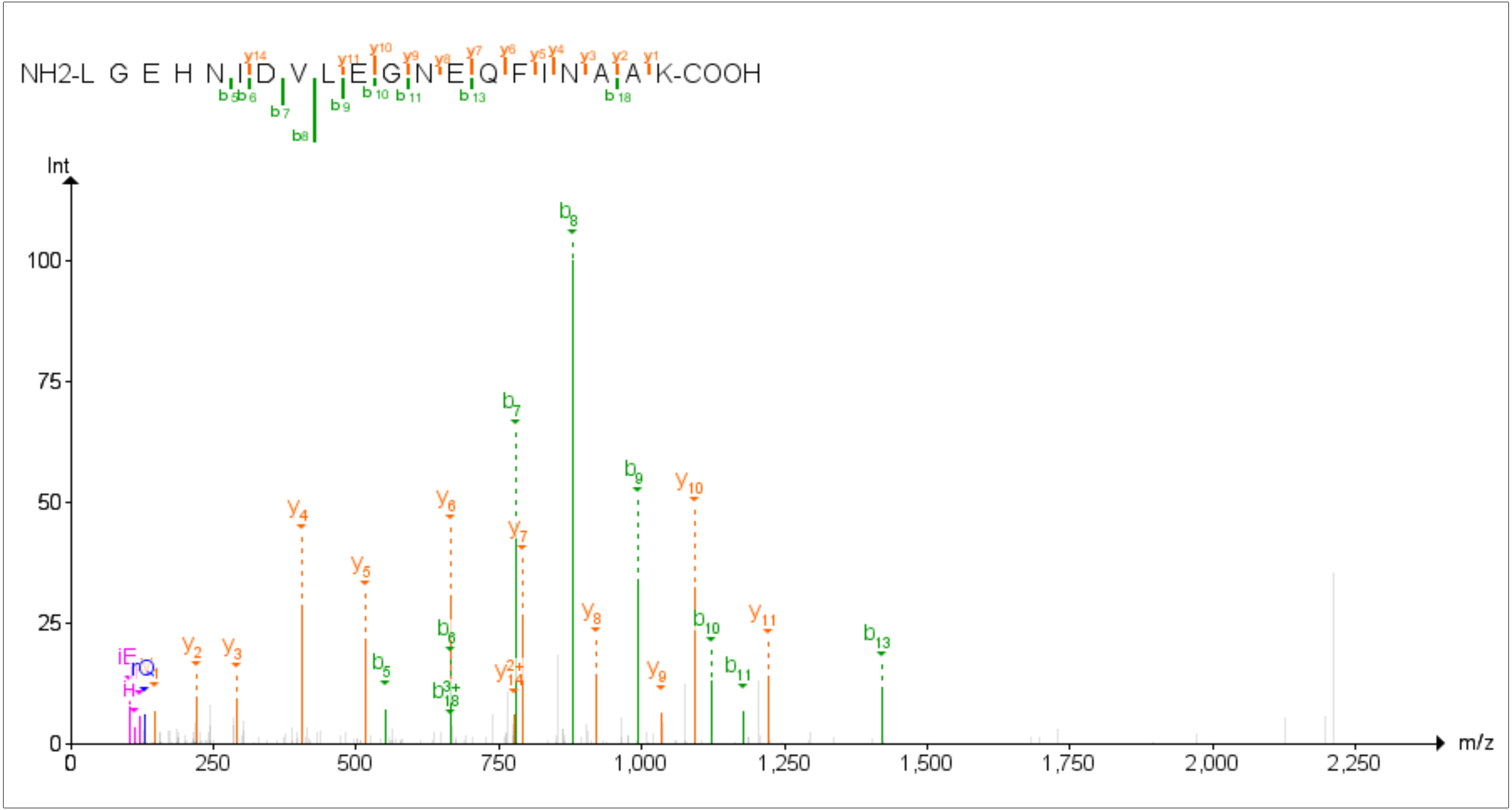

NH2-L G E H N I D V L E G N E Q F I N A A K-COOH
Int
m/z
b8
b7
b9
y10
y4
y6
y7
y5
y8
y9
y11
b10
b11
b13
b5
b6
y2
y3

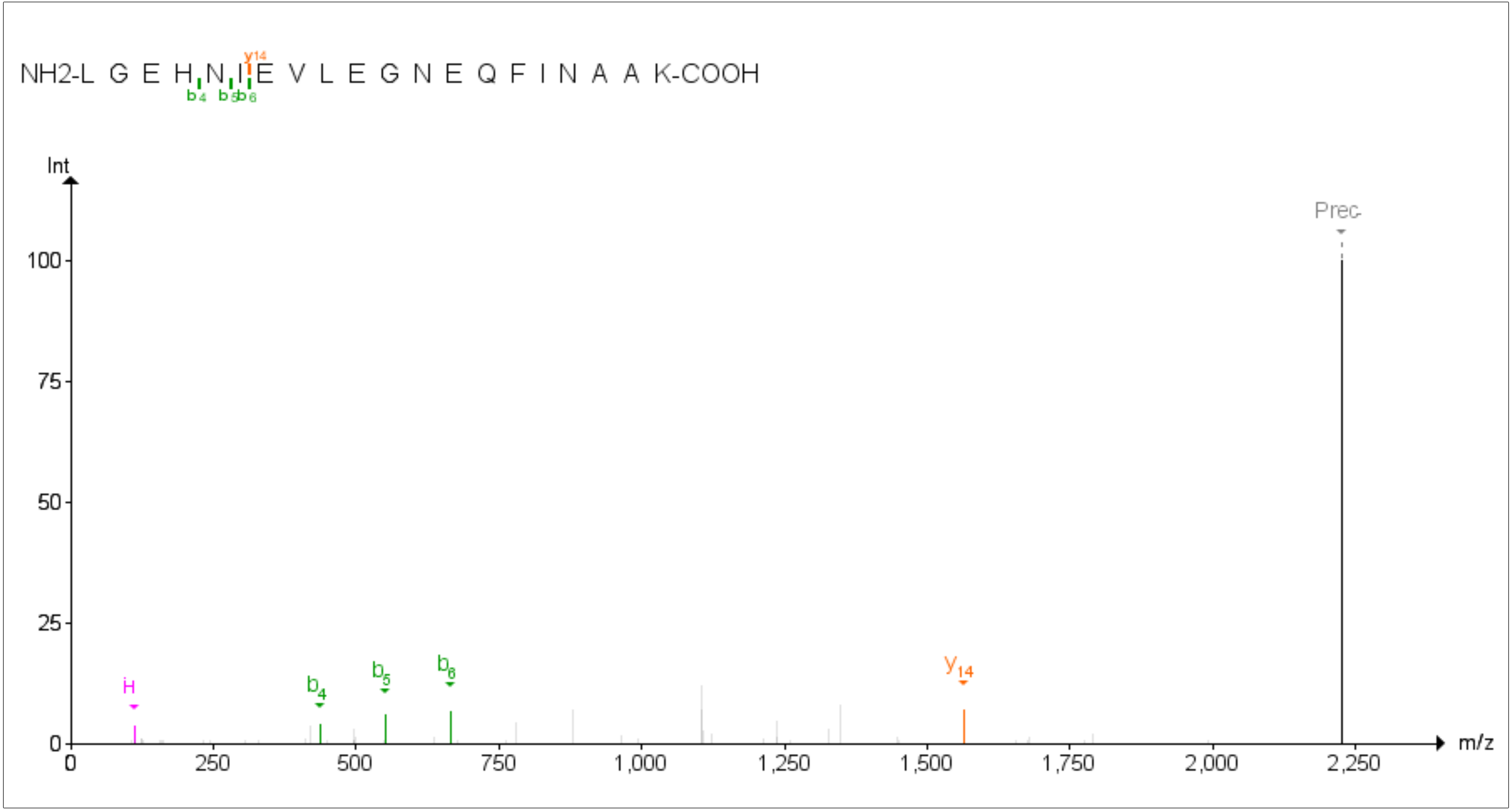
NH2-L G E H N I E V L E G N E Q F I N A A K-COOH
y14
b4 b5 b6
Int
100
75
50
25
0
Prec.
H
b4
b5
b6
y14
0
250
500
750
1,000
1,250
1,500
1,750
2,000
2,250
m/z

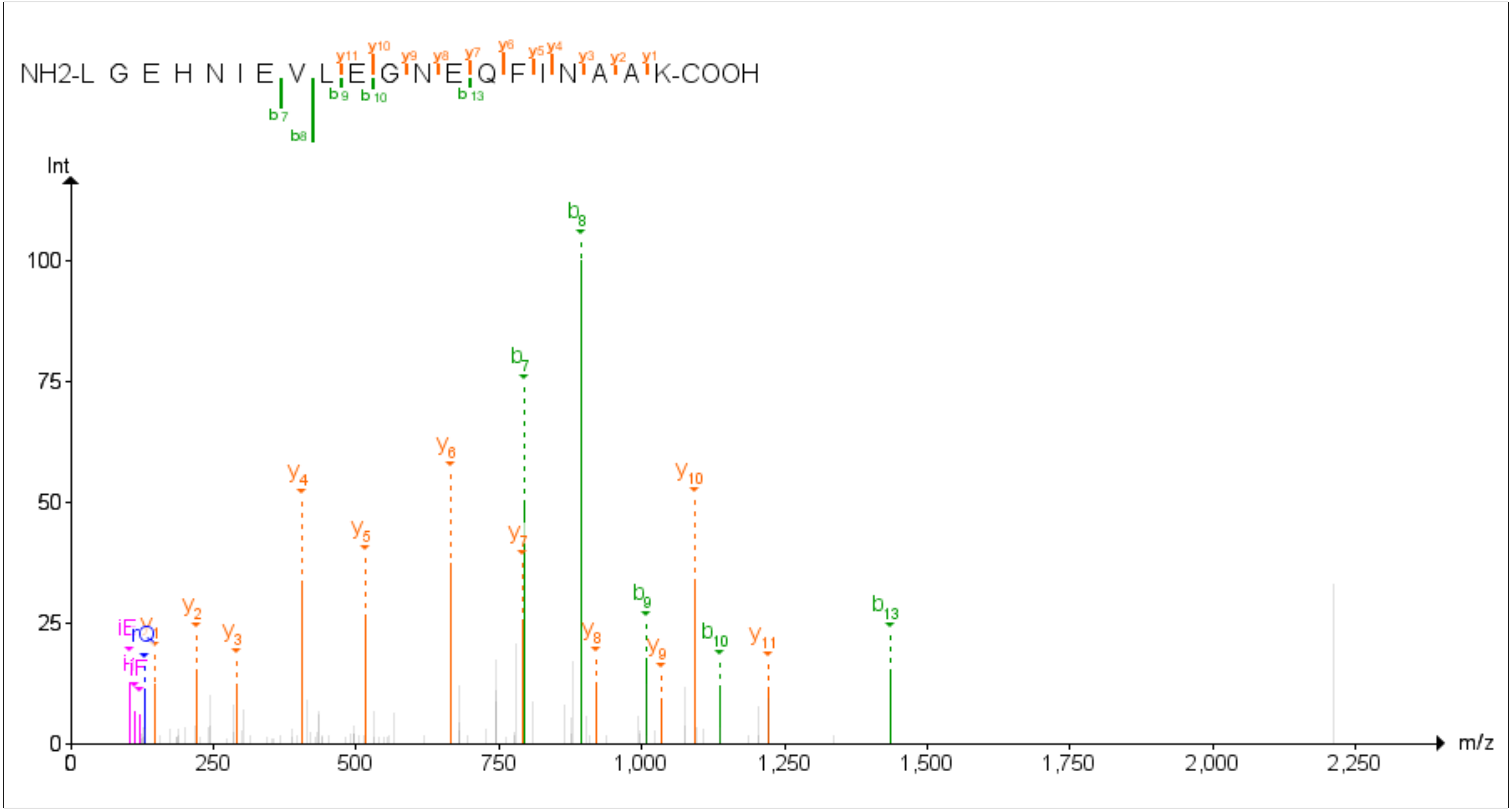

NH2-L G E H N I E V L E G N E Q F I N A A K-COOH
y11 y10 y9 y8 y7 y6 y5 y4 y3 y2 y1
b7 b8 b9 b10 b13
Int
100
75
50
25
0
m/z
0
250
500
750
1,000
1,250
1,500
1,750
2,000
2,250
b8
b7
y6
y4
y10
y5
y7
y2
y3
b9
b13
y8
y9
b10
y11

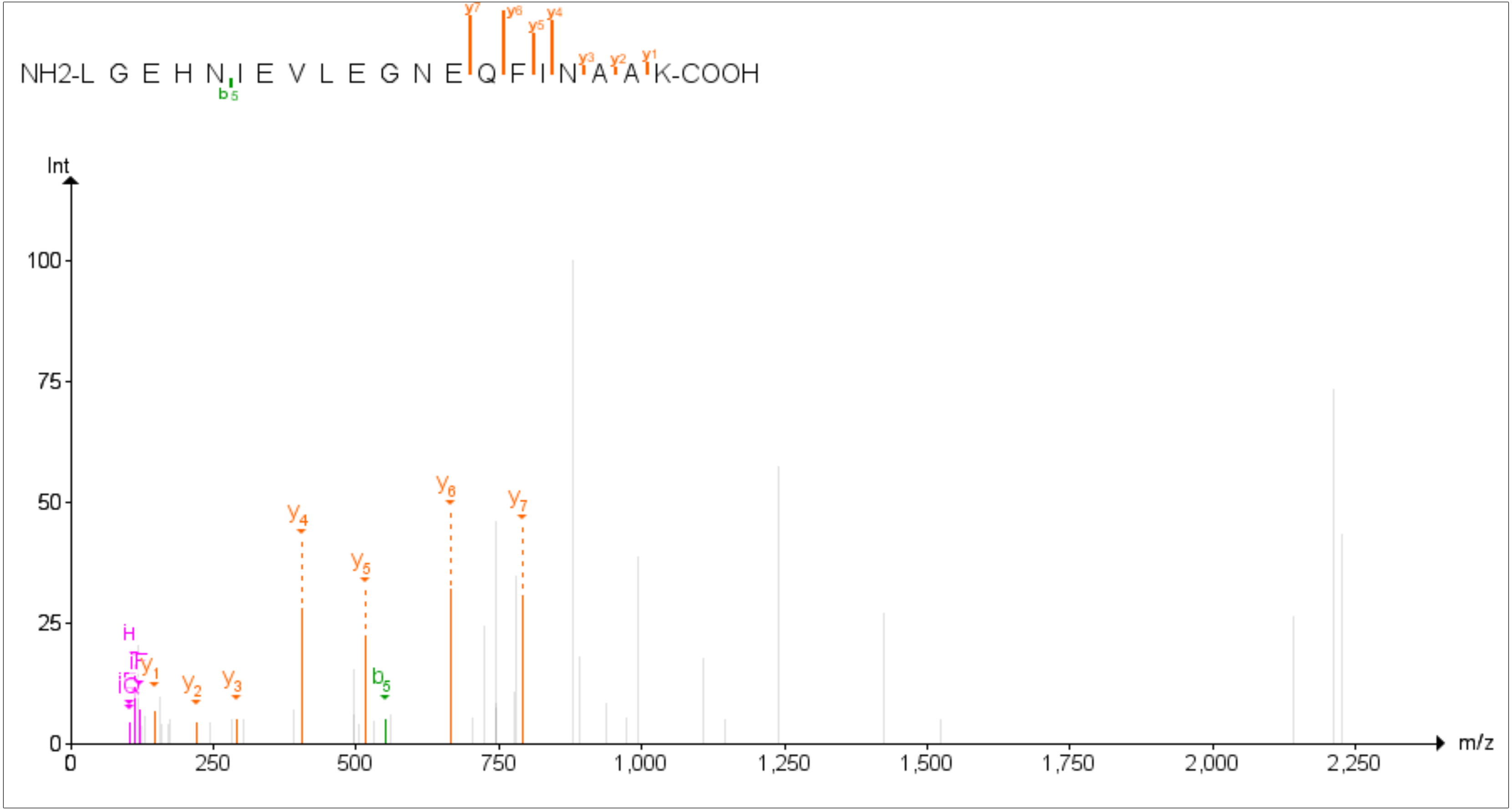

NH2-L G E H N I E V L E G N E Q F I N A A K-COOH
b5
y7 y6 y5 y4 y3 y2 y1
Int
100
75
50
25
0
m/z
0
250
500
750
1,000
1,250
1,500
1,750
2,000
2,250
y1
y2
y3
y4
y5
y6
y7
b5
iH
iF
iQ

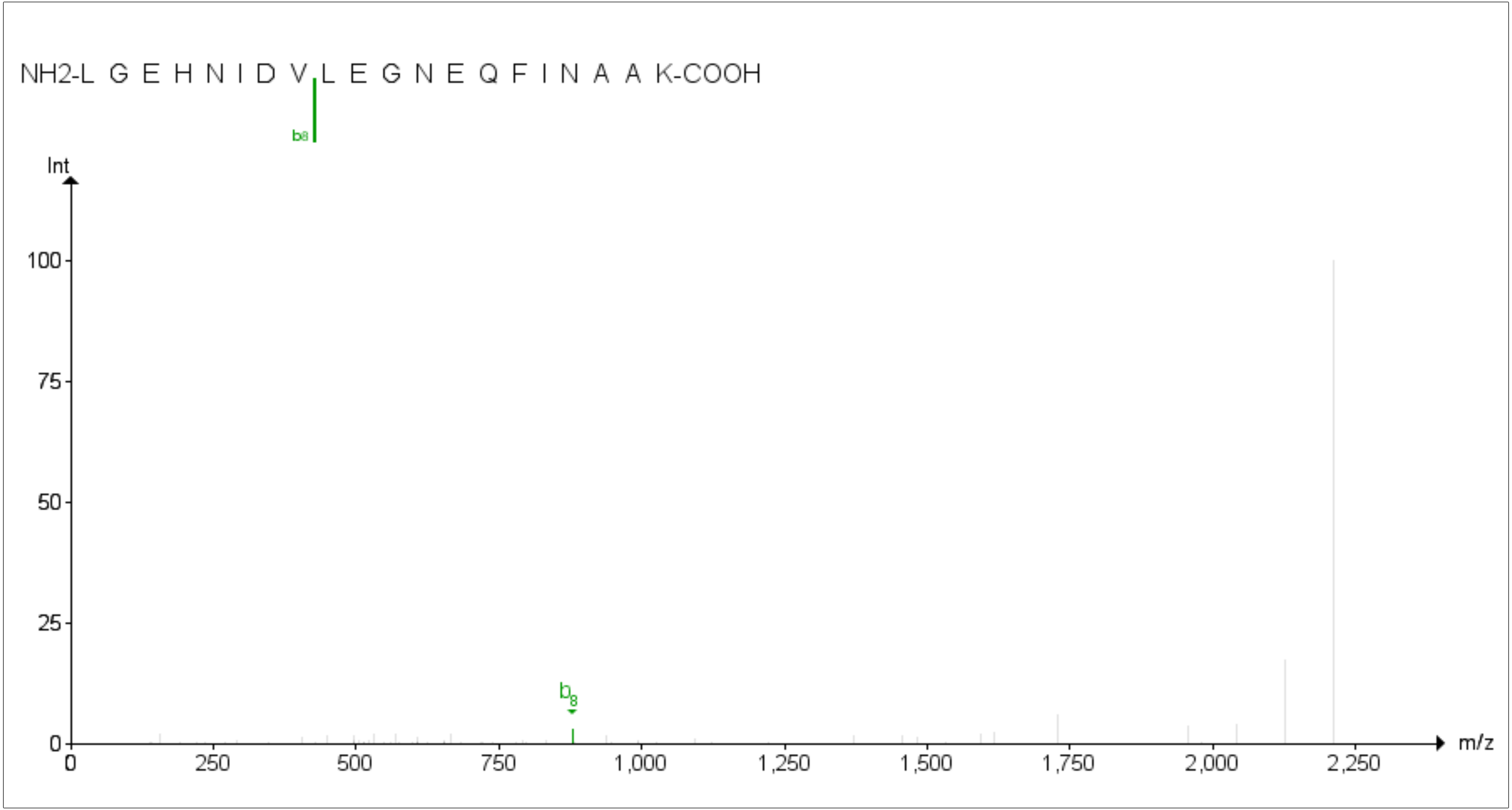
NH2-L G E H N I D V L E G N E Q F I N A A K-COOH
b8
Int
100
75
50
25
0
b8
0
250
500
750
1,000
1,250
1,500
1,750
2,000
2,250
m/z

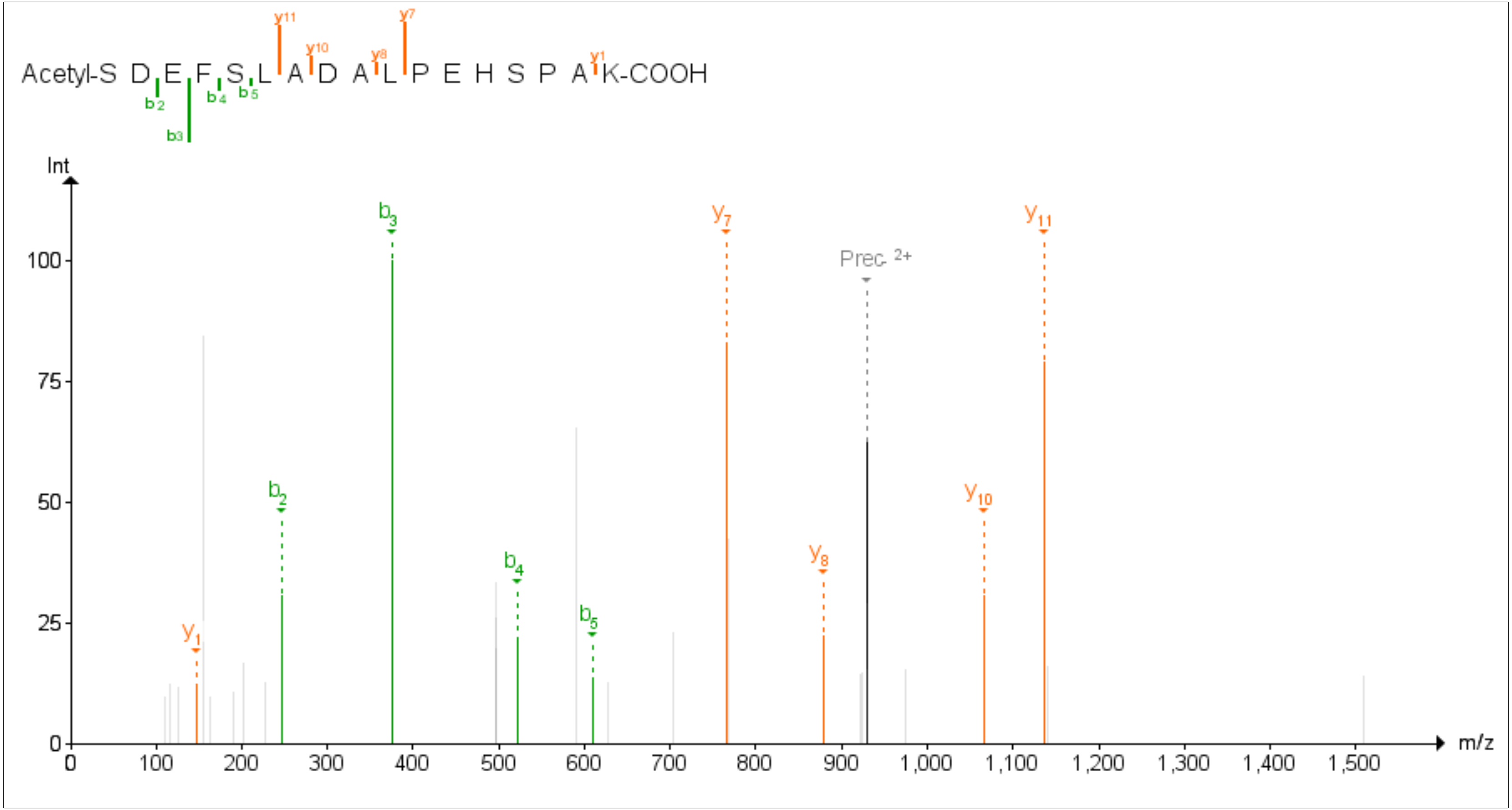

Acetyl-S D E F S L A D A L P E H S P A K-COOH
y11 y10 y8 y7 y1
b2 b3 b4 b5
Int
100
75
50
25
0
b3
y7
y11
Prec 2+
b2
y10
b4
y8
b5
y1
0 100 200 300 400 500 600 700 800 900 1,000 1,100 1,200 1,300 1,400 1,500
m/z

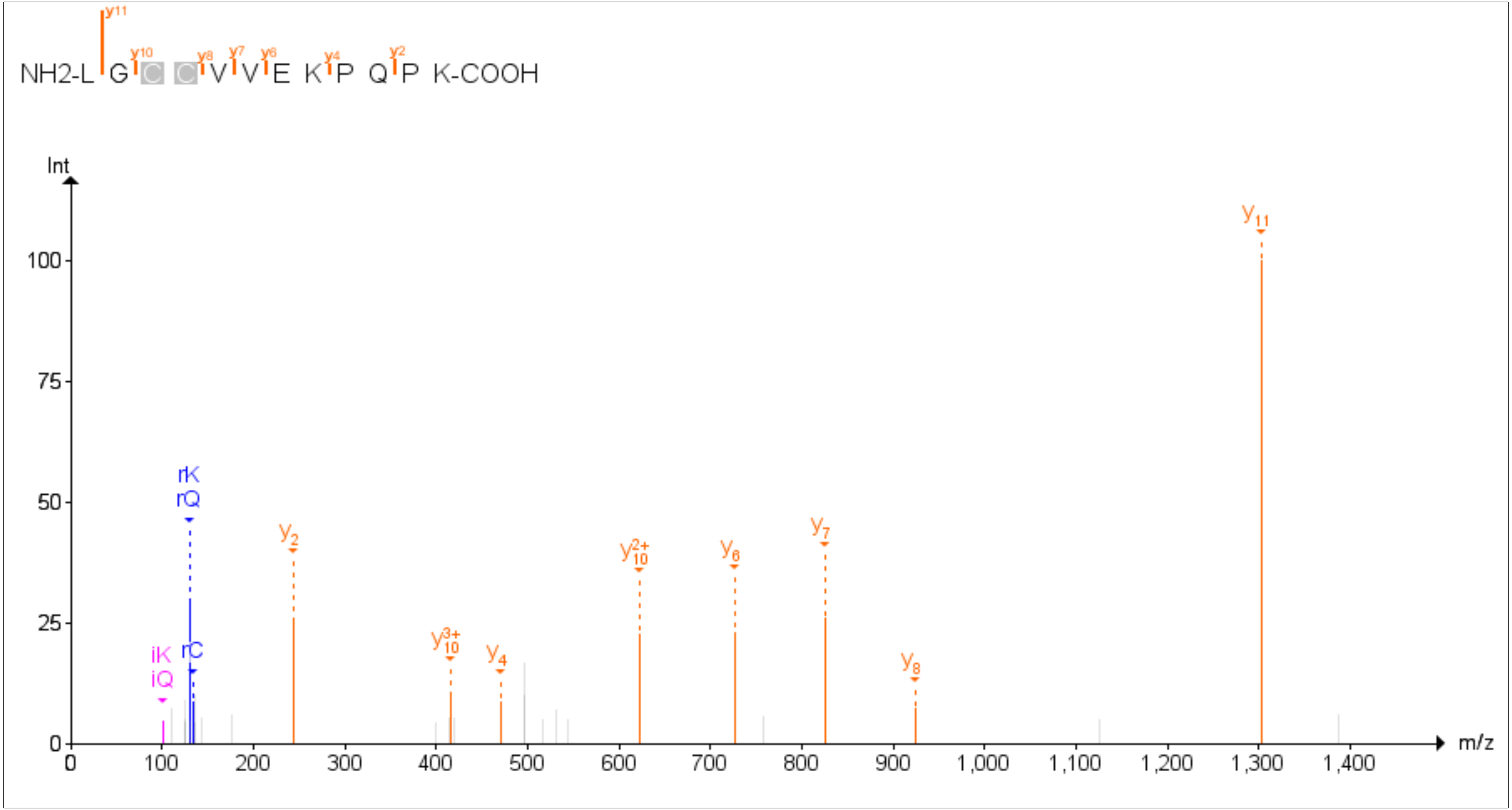

NH2-L G C C V V E K P Q P K-COOH
y11
y10
y8
y7
y6
y4
y2
Int
100
75
50
25
0
rK
rQ
rC
iK
iQ
y11
y2
y10 2+
y10 3+
y4
y6
y7
y8
m/z
0
100
200
300
400
500
600
700
800
900
1,000
1,100
1,200
1,300
1,400

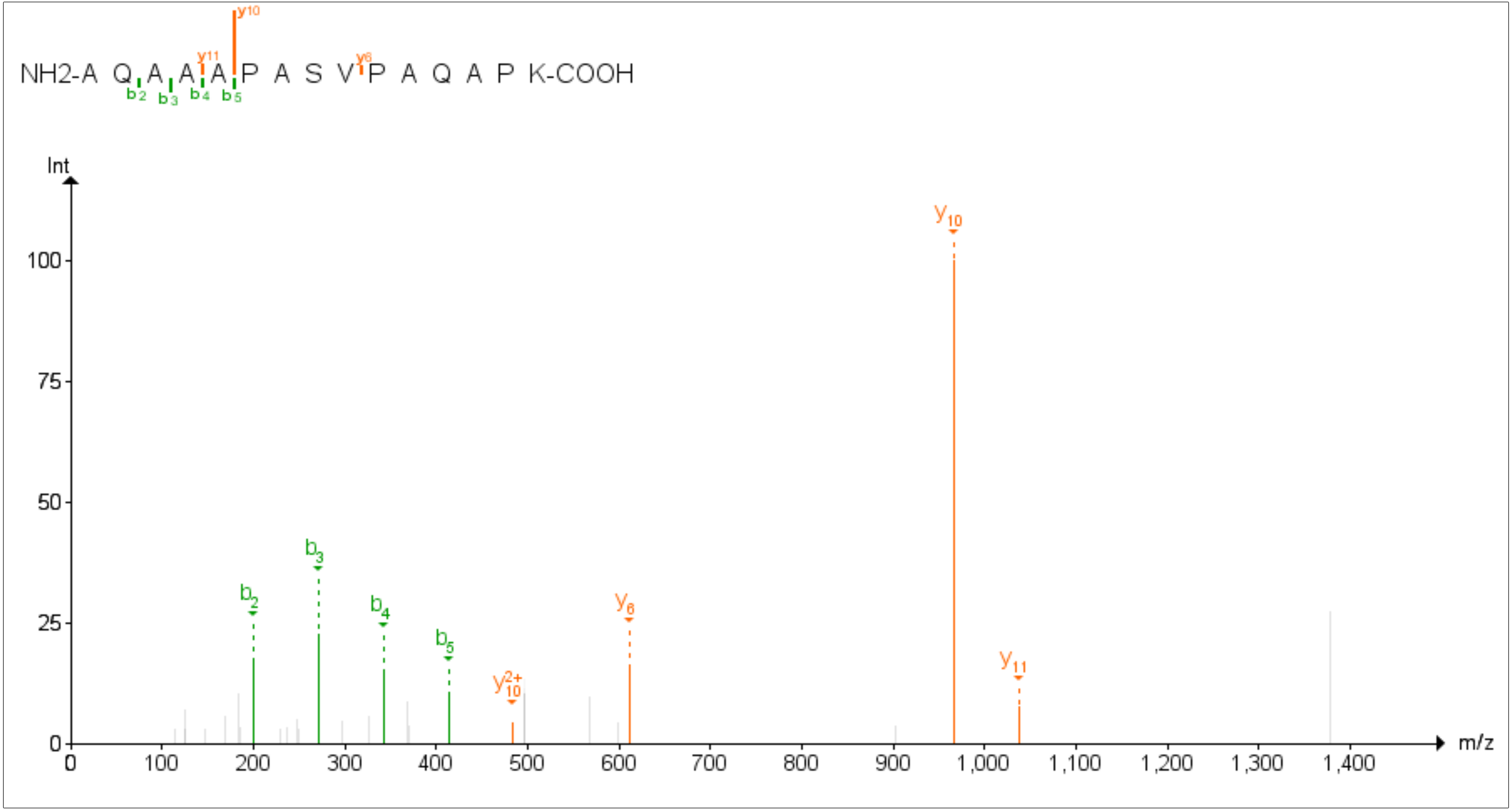

NH2-A Q A A A P A S V P A Q A P K-COOH
Int
m/z
b2
b3
b4
b5
y6
y10
y11

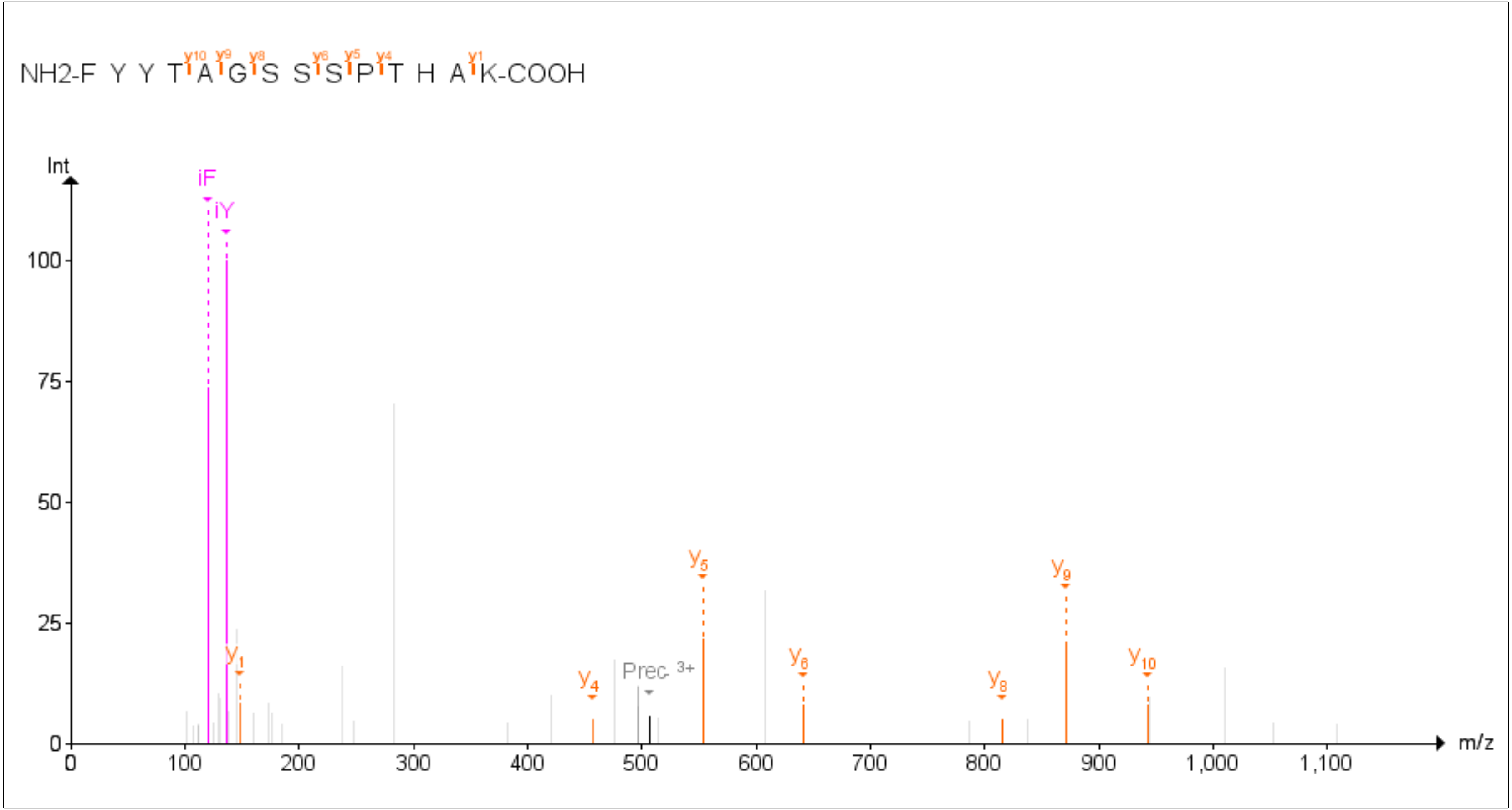

NH2-F Y Y T A G S S S P T H A K-COOH
y10 y9 y8 y6 y5 y4 y1
Int
iF
iY
y1
y4
Prec 3+
y5
y6
y8
y9
y10
m/z
0
25
50
75
100
0
100
200
300
400
500
600
700
800
900
1,000
1,100

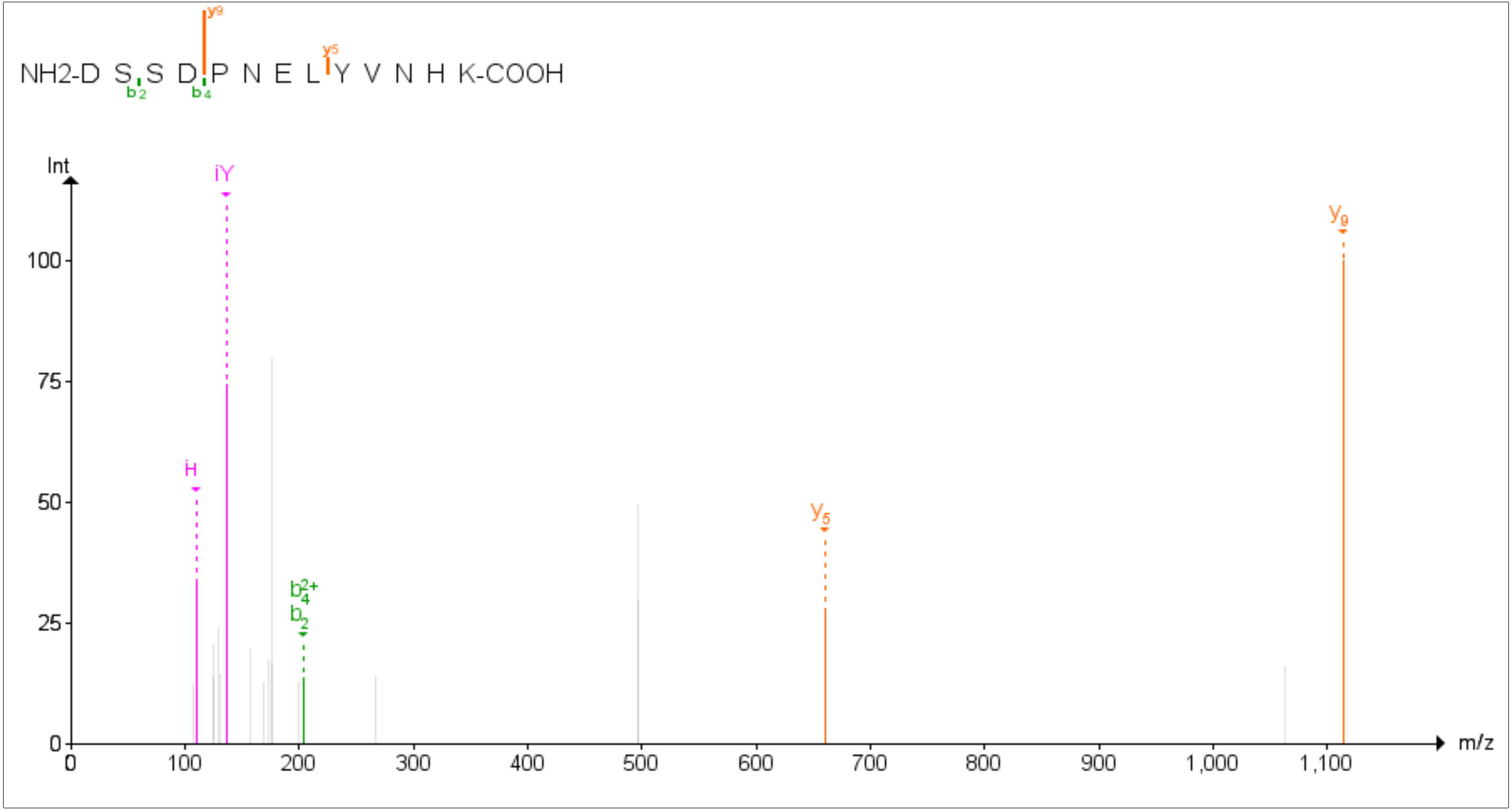

NH2-D S S D P N E L Y V N H K-COOH
y9
y5
b2
b4
Int
iY
iH
y9
y5
b4 2+
b2
100
75
50
25
0
0
100
200
300
400
500
600
700
800
900
1,000
1,100
m/z

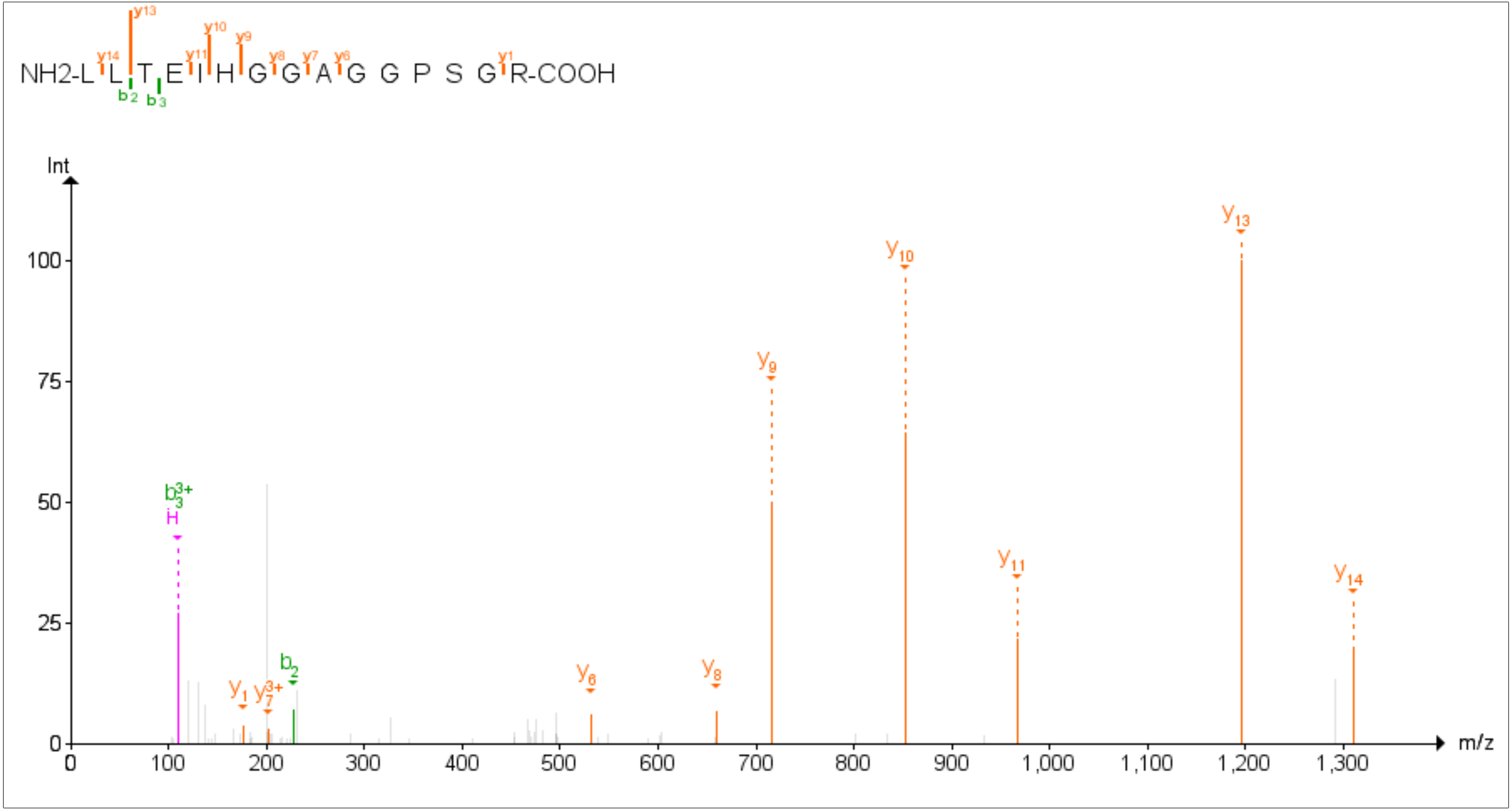

NH2-L L T E I H G G A G G P S G R-COOH
y14 y13 y11 y10 y9 y8 y7 y6 y1
b2 b3
Int
m/z
b3 3+
H
y1
y7 3+
b2
y6
y8
y9
y10
y11
y13
y14
0
25
50
75
100
0
100
200
300
400
500
600
700
800
900
1,000
1,100
1,200
1,300

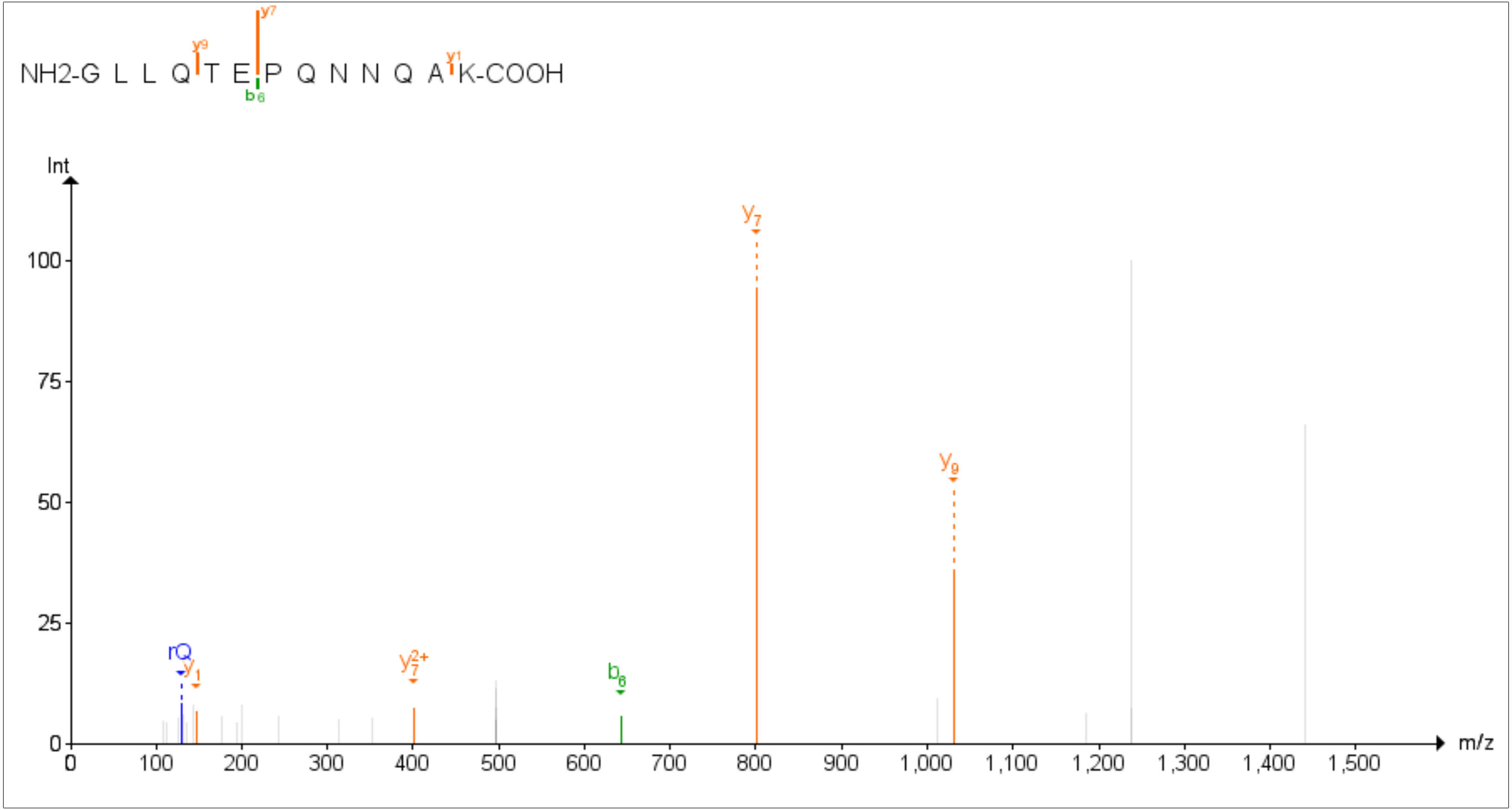

NH2-G L L Q T E P Q N N Q A K-COOH
y9
y7
y1
b6
Int
100
75
50
25
0
y7
y9
rQ
y1
y7 2+
b6
0
100
200
300
400
500
600
700
800
900
1,000
1,100
1,200
1,300
1,400
1,500
m/z

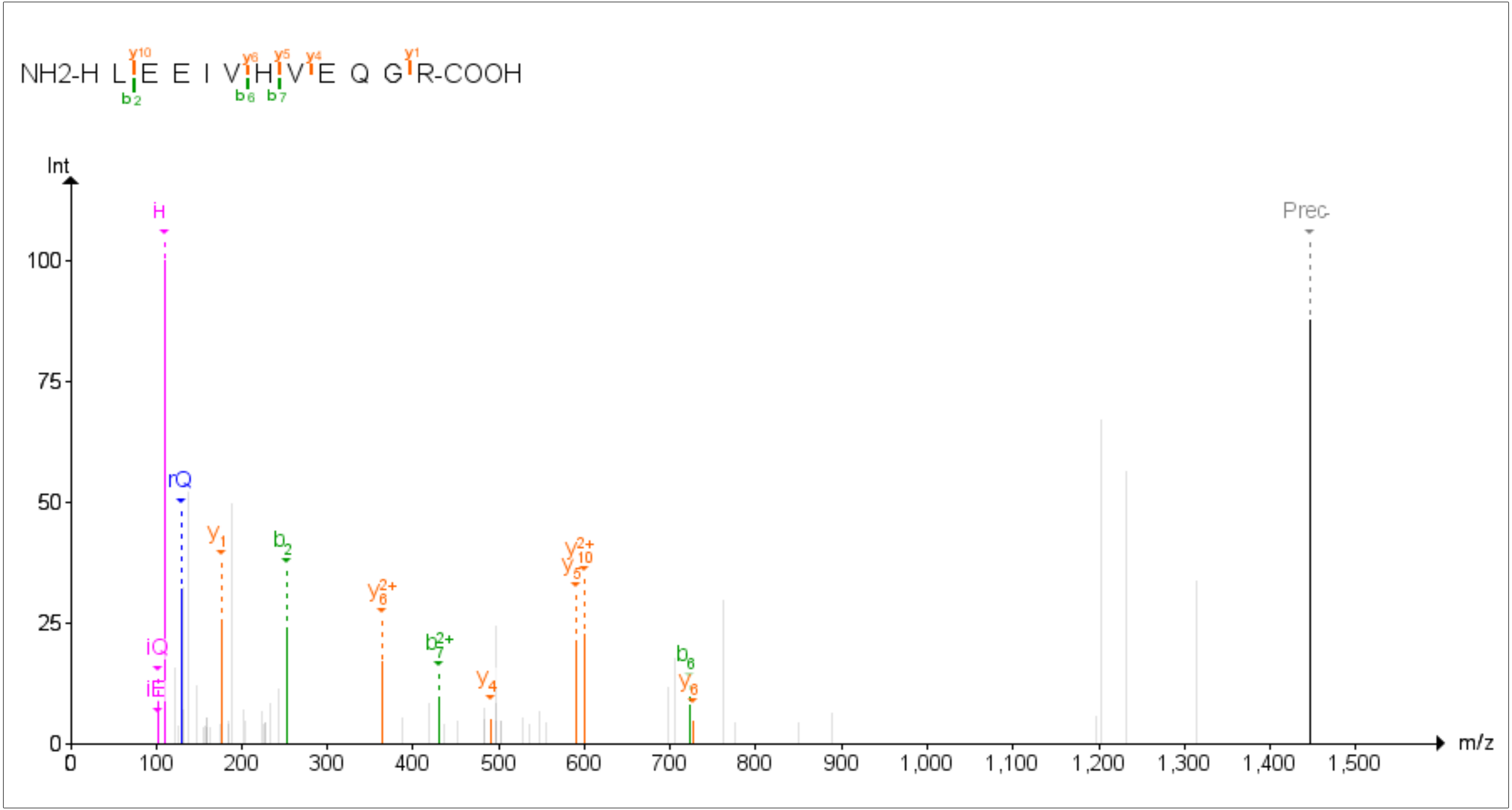

NH2-H L E E I V H V E Q G R-COOH
y10 y6 y5 y4 y1
b2 b6 b7
Int
m/z
H
rQ
iQ
Prec
y1
b2
b6
y4
y6
0
25
50
75
100
0
100
200
300
400
500
600
700
800
900
1,000
1,100
1,200
1,300
1,400
1,500

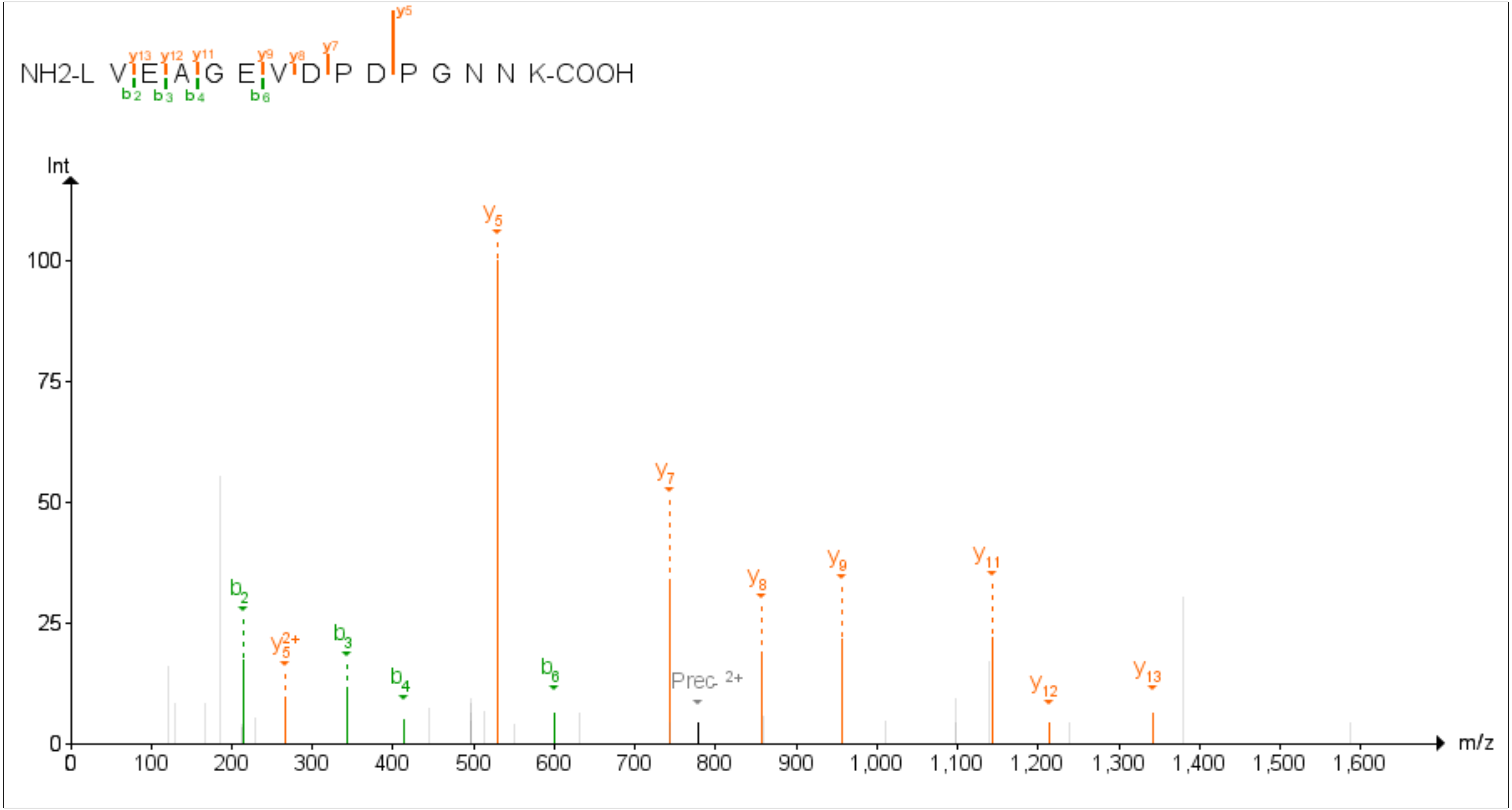

NH2-L V E A G E V D P D P G N N K-COOH
y13 y12 y11 y9 y8 y7 y5
b2 b3 b4 b6
Int
100
75
50
25
0
y5
y7
y8
y9
y11
y12
y13
b2
b3
b4
b6
y5 2+
Prec. 2+
0 100 200 300 400 500 600 700 800 900 1,000 1,100 1,200 1,300 1,400 1,500 1,600
m/z

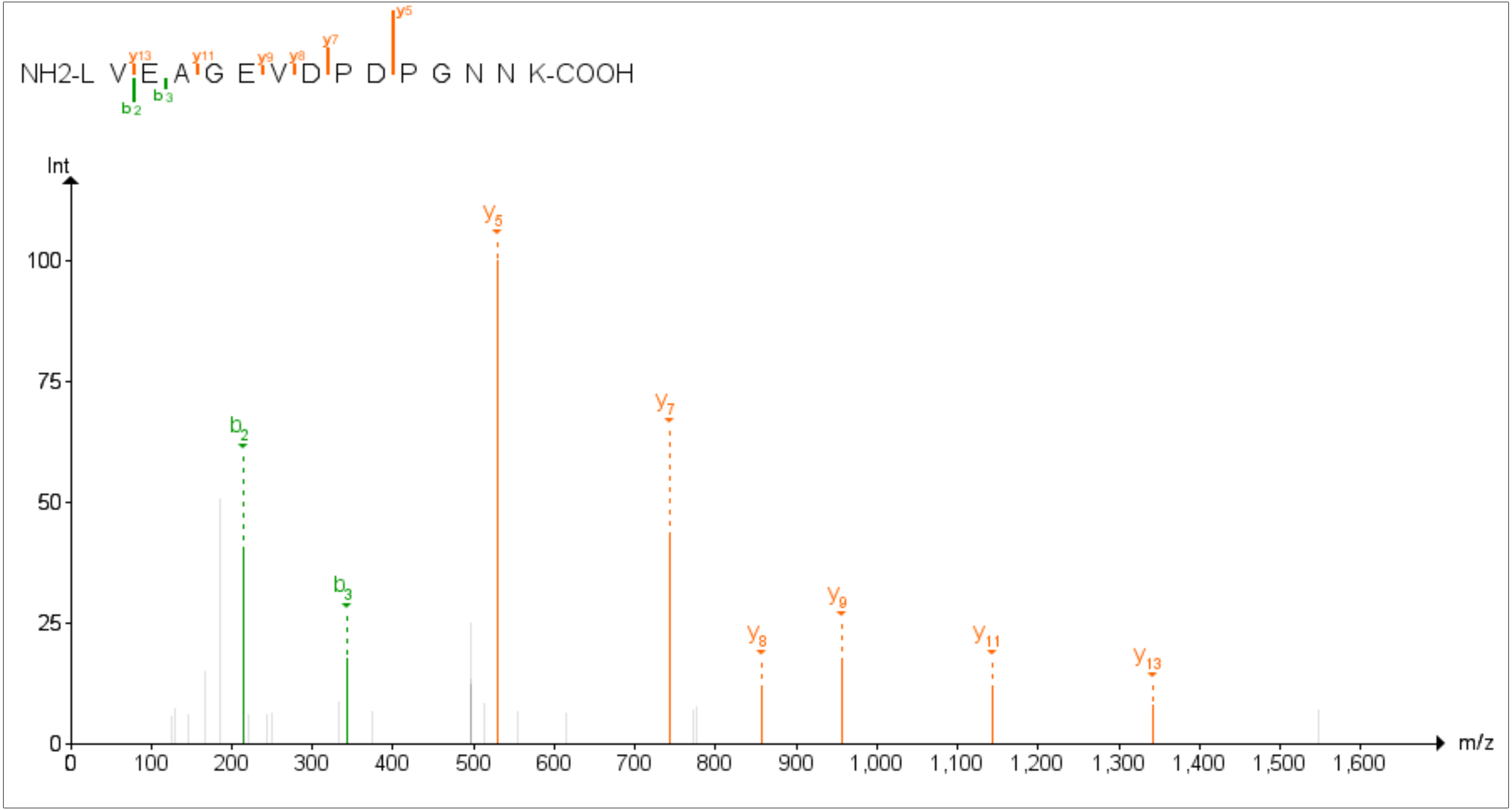
NH2-L V E A G E V D P D P G N N K-COOH
y13 y11 y9 y8 y7 y5
b2 b3
Int
m/z
0
25
50
75
100
0 100 200 300 400 500 600 700 800 900 1,000 1,100 1,200 1,300 1,400 1,500 1,600
b2
b3
y5
y7
y8
y9
y11
y13

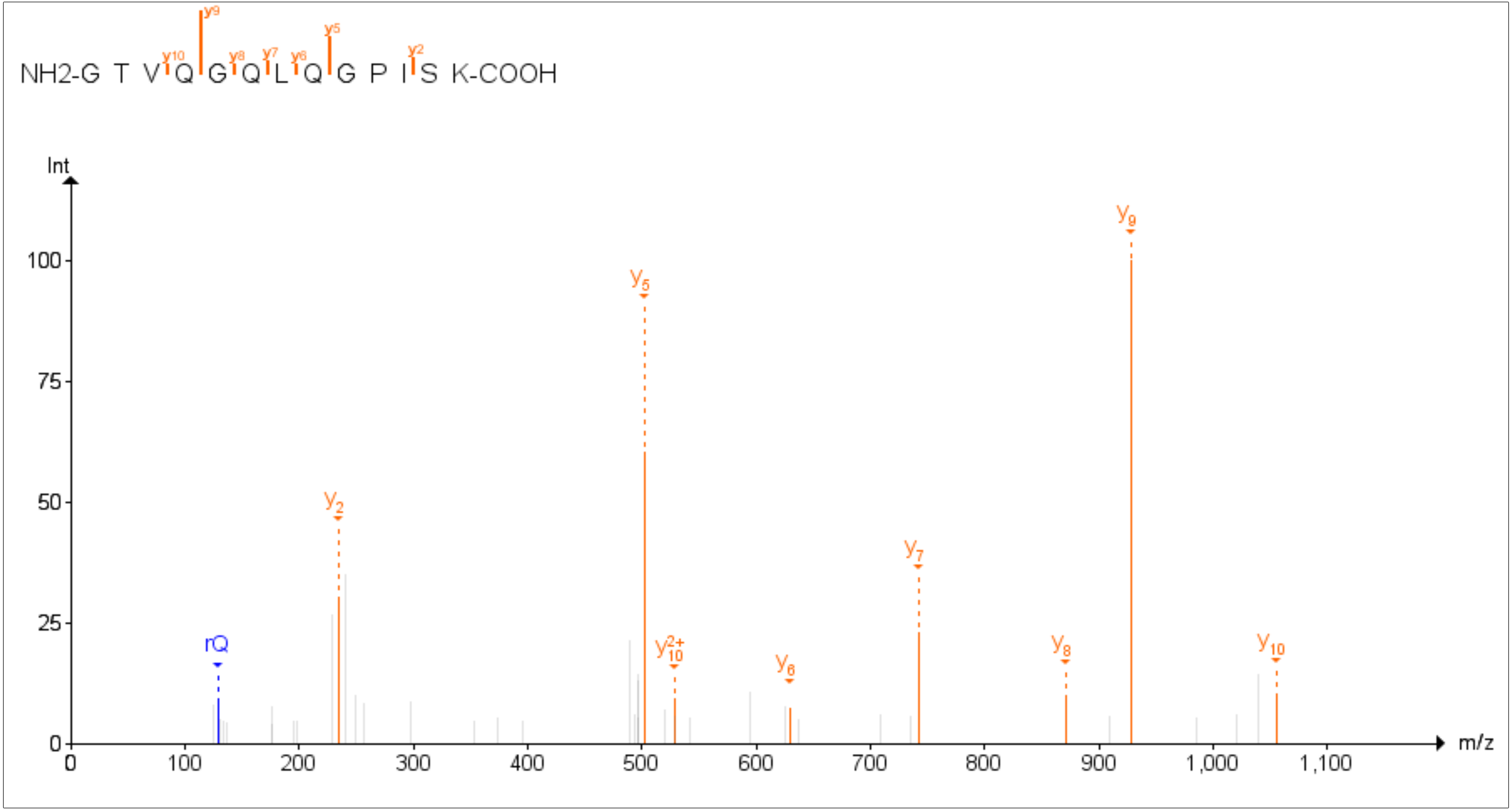
NH2-G T V Q G Q L Q G P I S K-COOH
y10 y9 y8 y7 y6 y5 y2
Int
100
75
50
25
0
rQ
y_2
y_5
y_{10}^{2+}
y_6
y_7
y_8
y_9
y_{10}
0 100 200 300 400 500 600 700 800 900 1,000 1,100
m/z

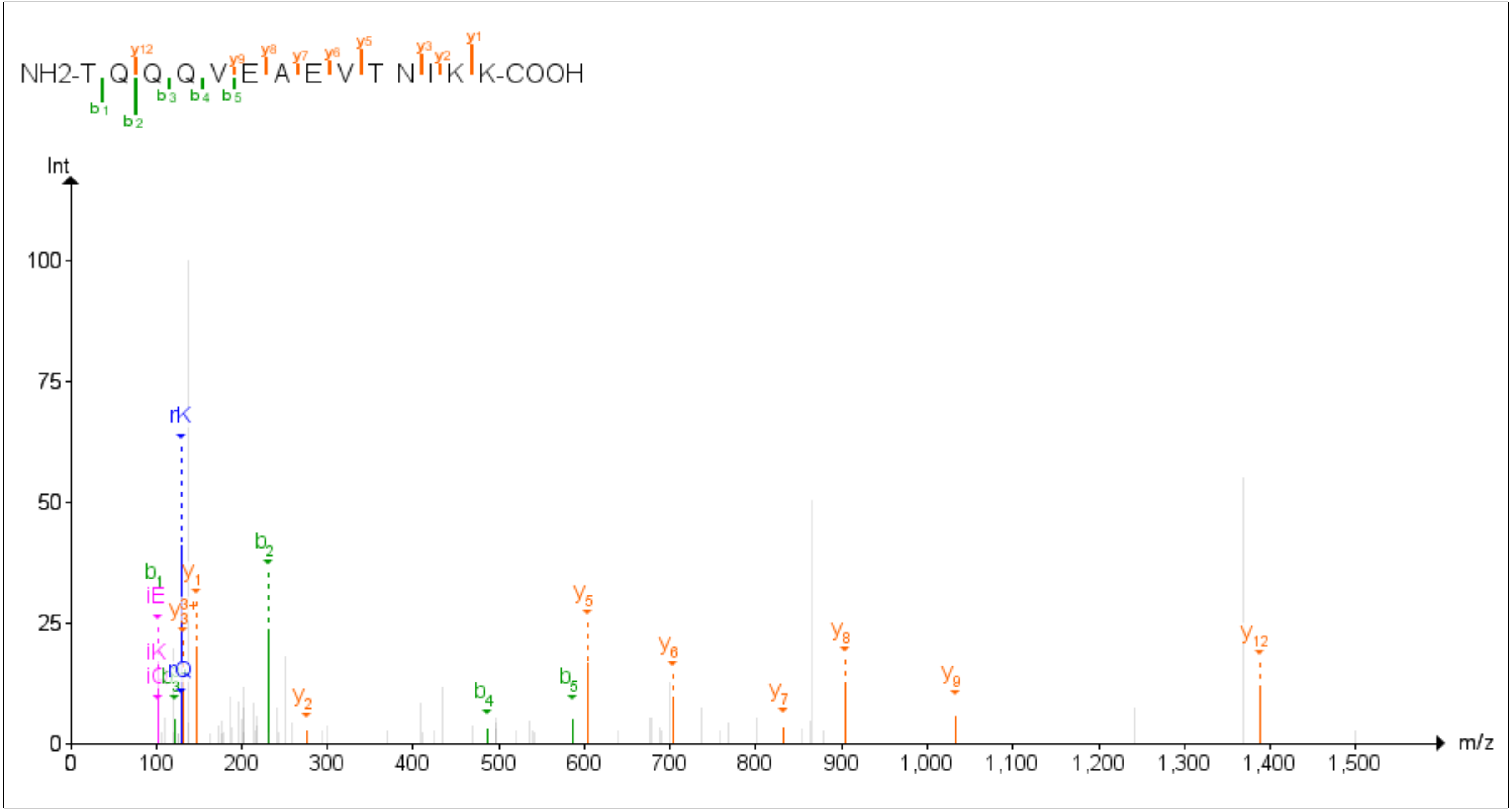

NH2-T Q Q Q V E A E V T N I K K-COOH
Int
m/z
rK
rQ
b1
b2
b3
b4
b5
y1
y2
y5
y6
y7
y8
y9
y12
iE
iK

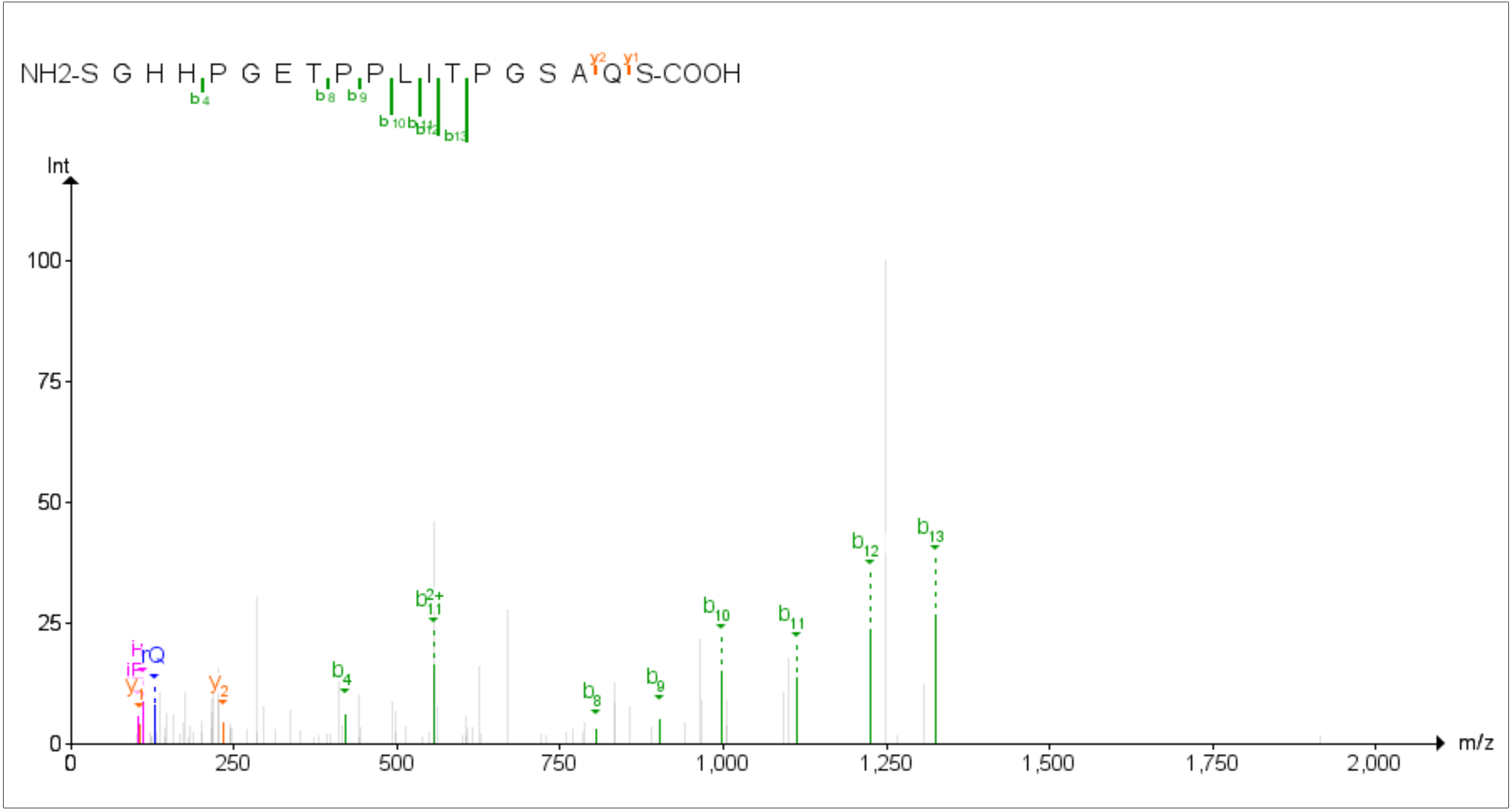
NH2-S G H H P G E T P P L I T P G S A Q S-COOH
b4 b8 b9 b10 b11 b12 b13
y2 y1
Int
100
75
50
25
0
0 250 500 750 1,000 1,250 1,500 1,750 2,000
m/z
y1 iF iH rQ y2 b4 b11 2+ b8 b9 b10 b11 b12 b13

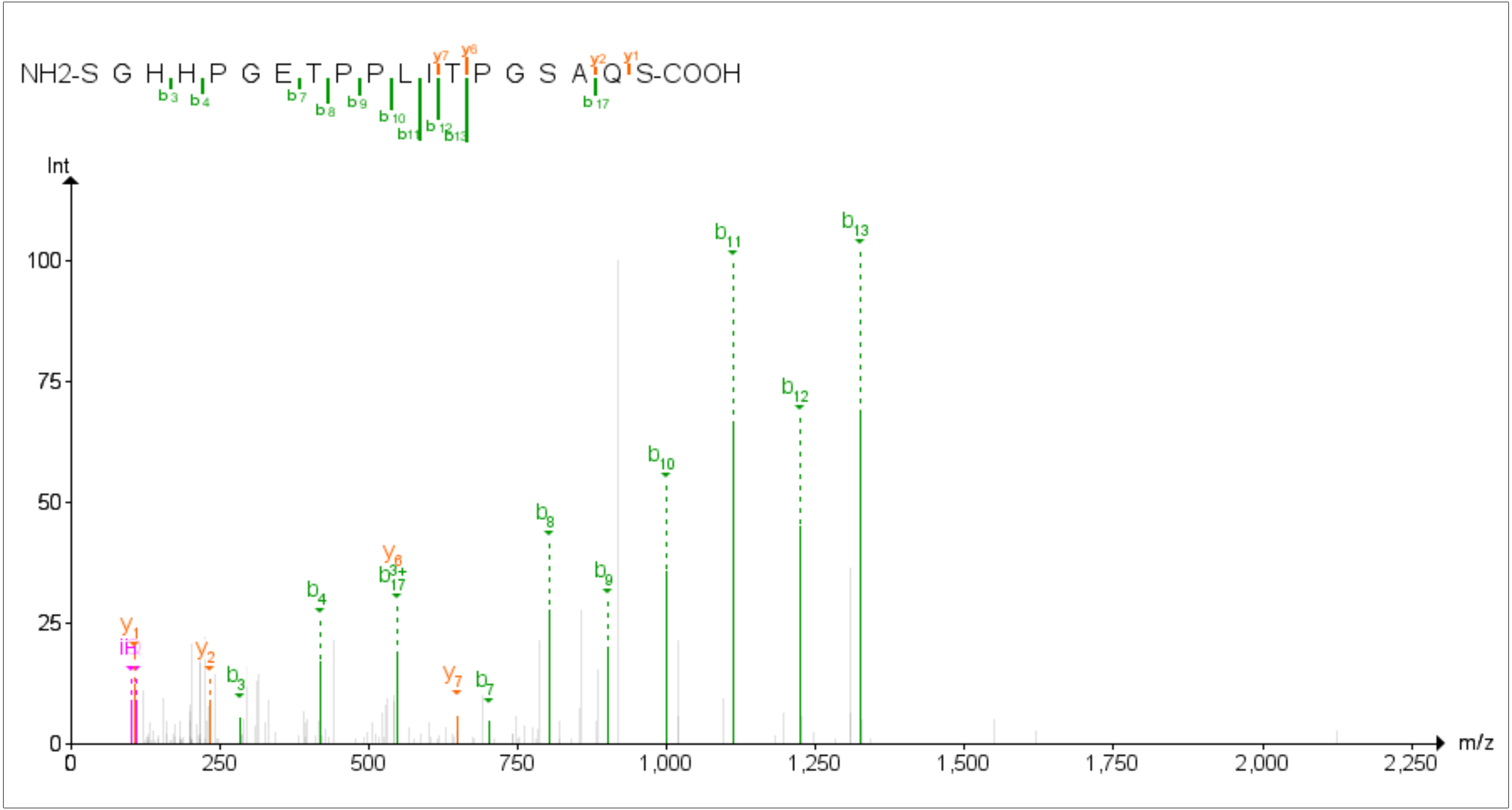

NH2-S G H H P G E T P P L I T P G S A Q S-COOH
Int
m/z
b3
b4
b7
b8
b9
b10
b11
b12
b13
b17
y1
y2
y6
y7
0
25
50
75
100
0
250
500
750
1,000
1,250
1,500
1,750
2,000
2,250

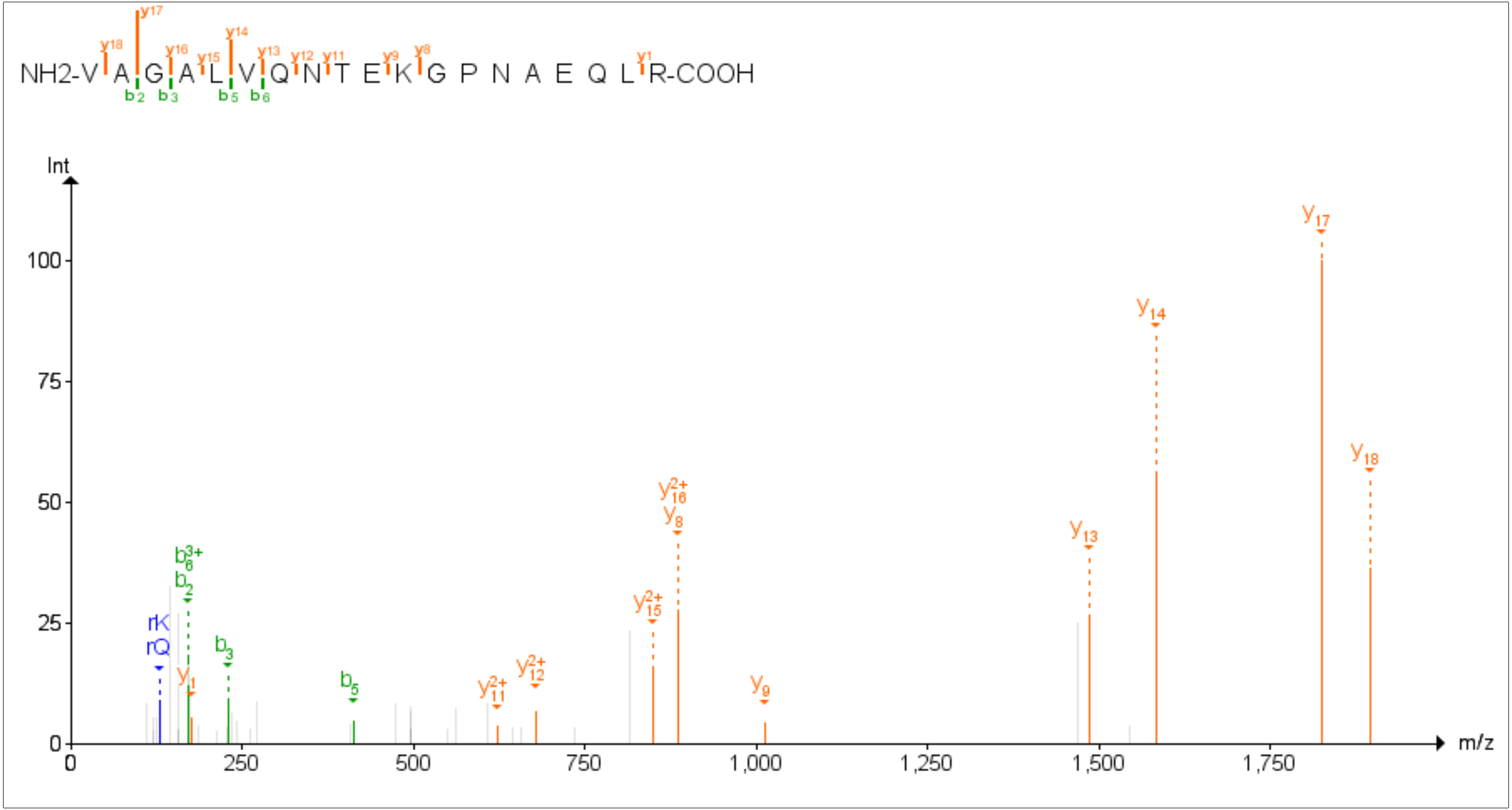
NH2-V A G A L V Q N T E K G P N A E Q L R-COOH
y18 y17 y16 y15 y14 y13 y12 y11 y9 y8 y1
b2 b3 b5 b6
Int
100
75
50
25
0
250
500
750
1,000
1,250
1,500
1,750
m/z
y17
y14
y18
y13
y16 2+
y8
y15 2+
y12 2+
y11 2+
y9
b6 3+
b2
rK
rQ
b3
y1
b5

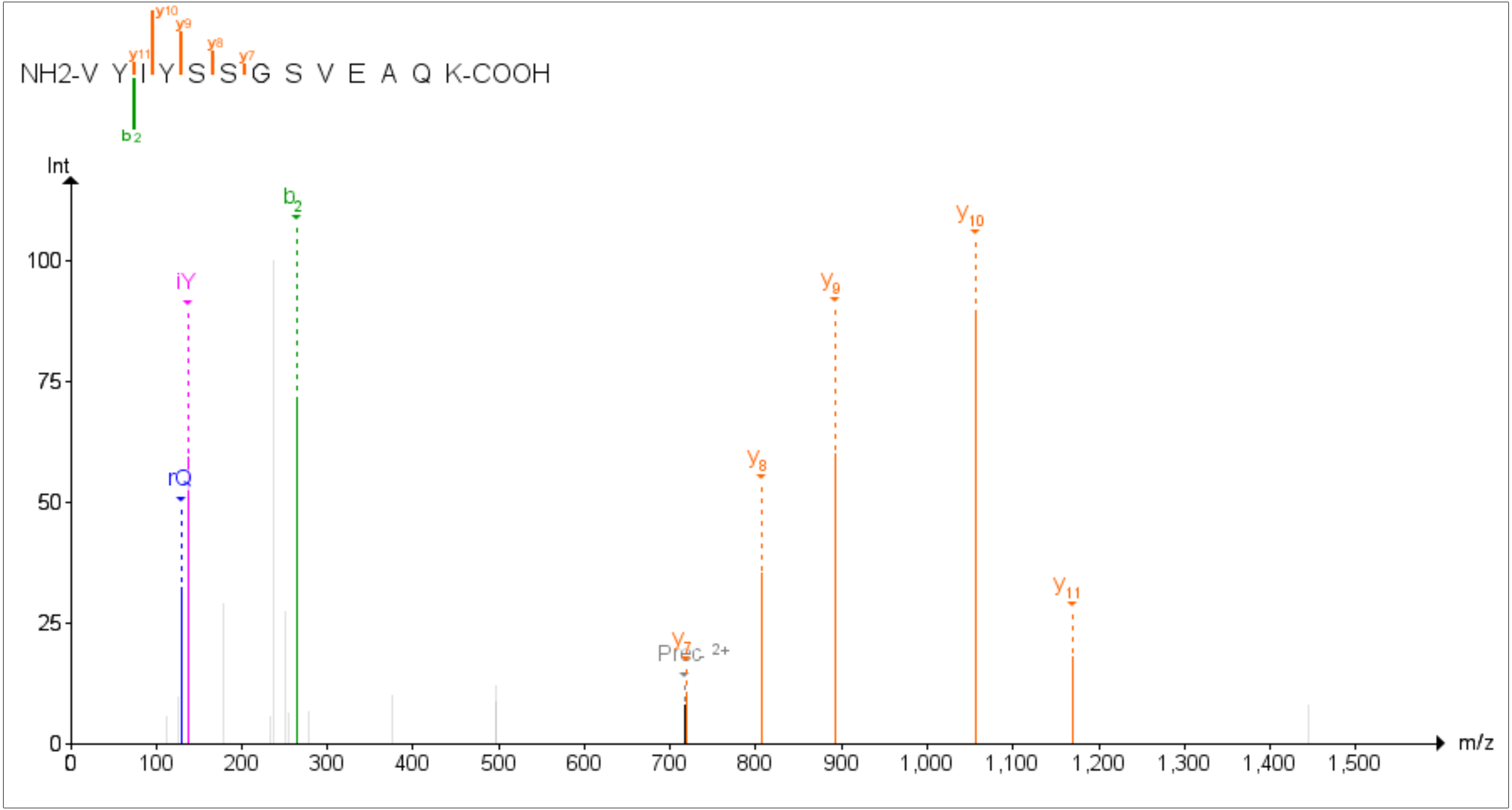

NH2-V Y I Y S S G S V E A Q K-COOH
y10
y9
y11
y8
y7
b2
Int
100
75
50
25
0
iY
rQ
b2
y10
y9
y8
y7
Prec 2+
y11
0
100
200
300
400
500
600
700
800
900
1,000
1,100
1,200
1,300
1,400
1,500
m/z

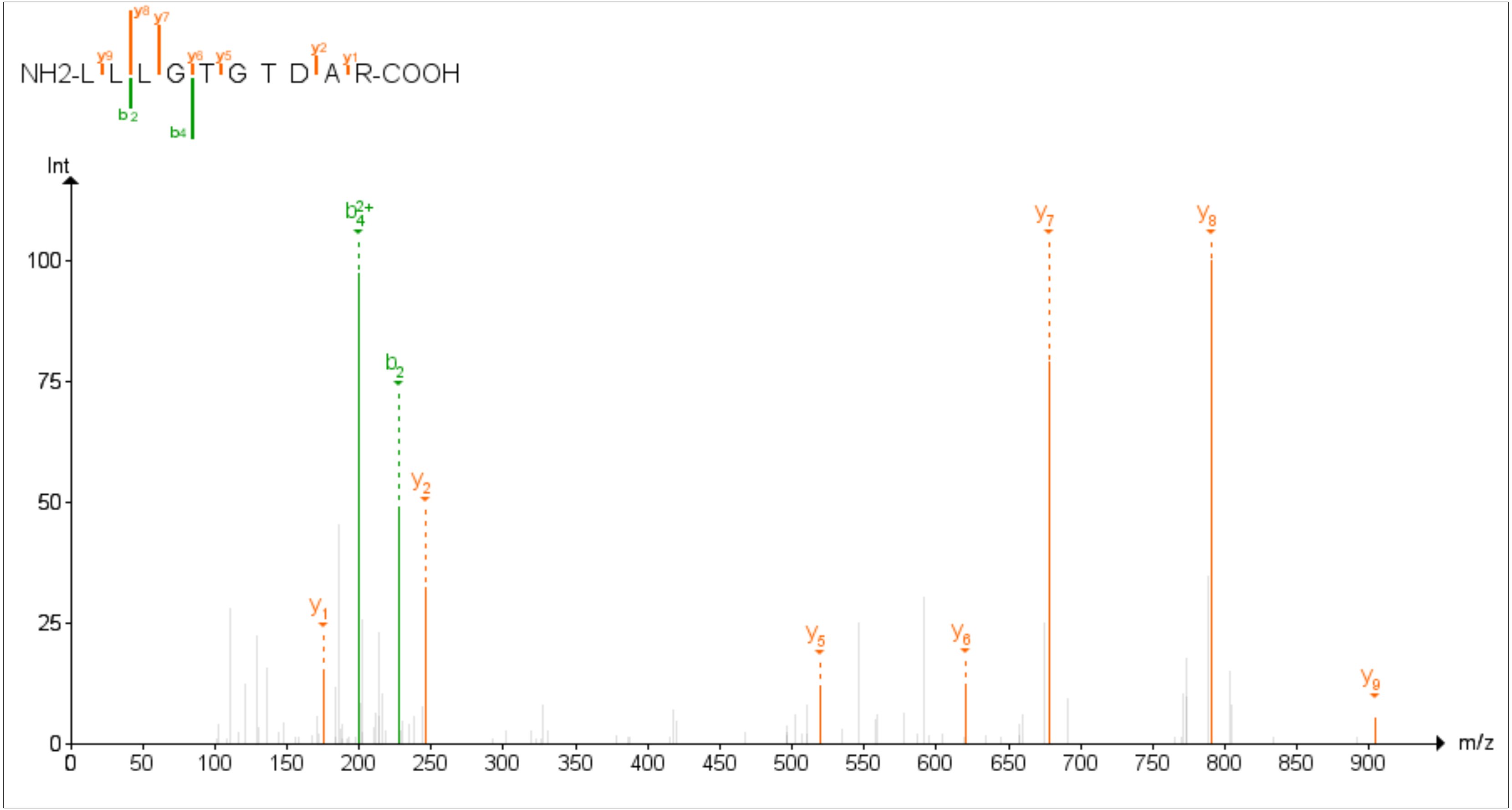

NH2-L L L G T G T D A R-COOH
y9
y8
y7
y6
y5
y2
y1
b2
b4
Int
m/z
b_4^{2+}
b_2
y_1
y_2
y_5
y_6
y_7
y_8
y_9
0
25
50
75
100
0 50 100 150 200 250 300 350 400 450 500 550 600 650 700 750 800 850 900

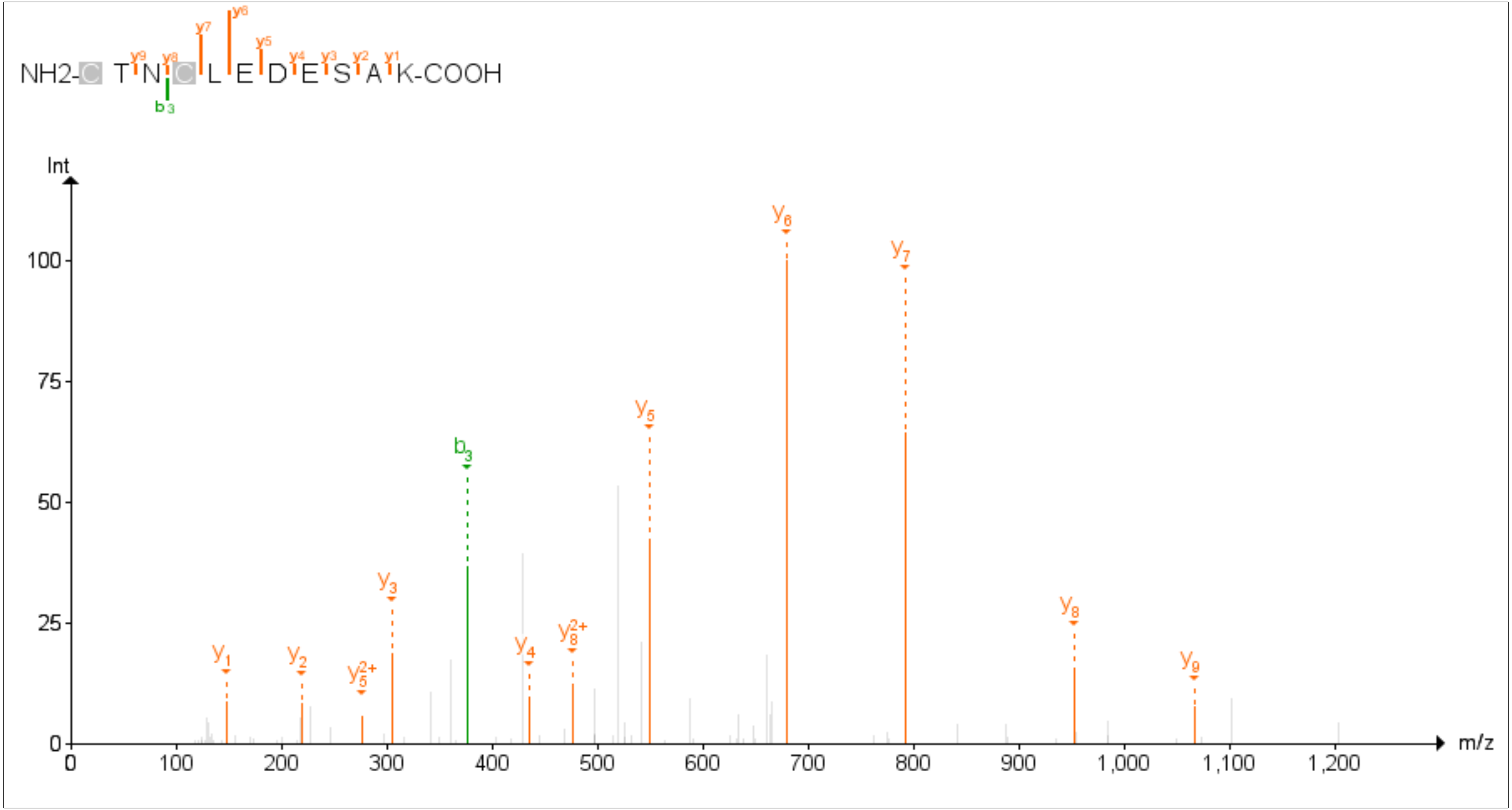

NH2-C T N C L E D E S A K-COOH
y9 y8 y7 y6 y5 y4 y3 y2 y1
b3
Int
100
75
50
25
0
m/z
0 100 200 300 400 500 600 700 800 900 1,000 1,100 1,200
y1 y2 y5 2+ y3 b3 y4 y8 2+ y5 y6 y7 y8 y9

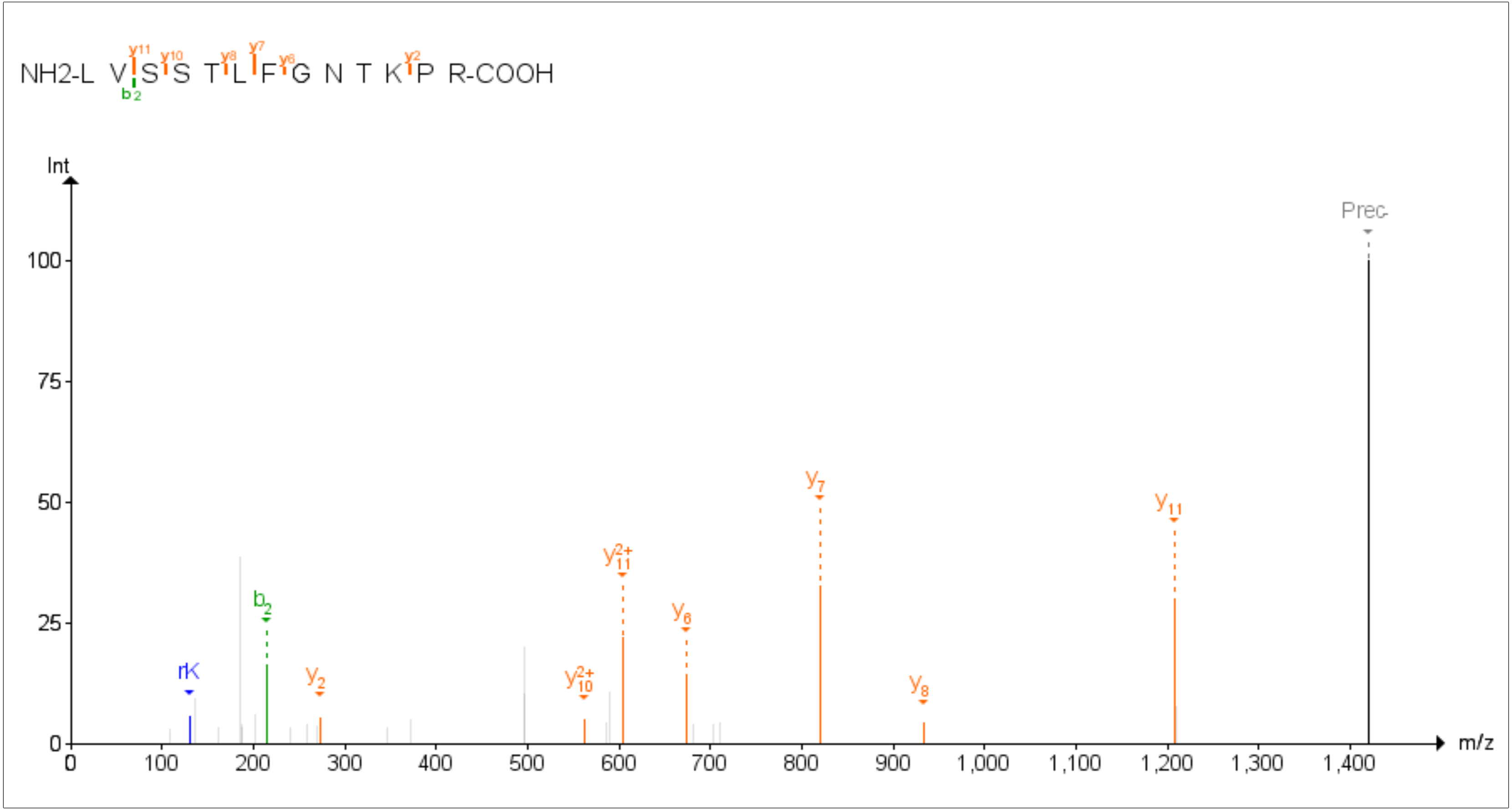
NH2-L V S S T L F G N T K P R-COOH
y11
y10
y8
y7
y6
y2
b2
Int
Prec.
rK
b2
y2
y10 2+
y11 2+
y6
y7
y8
y11
m/z

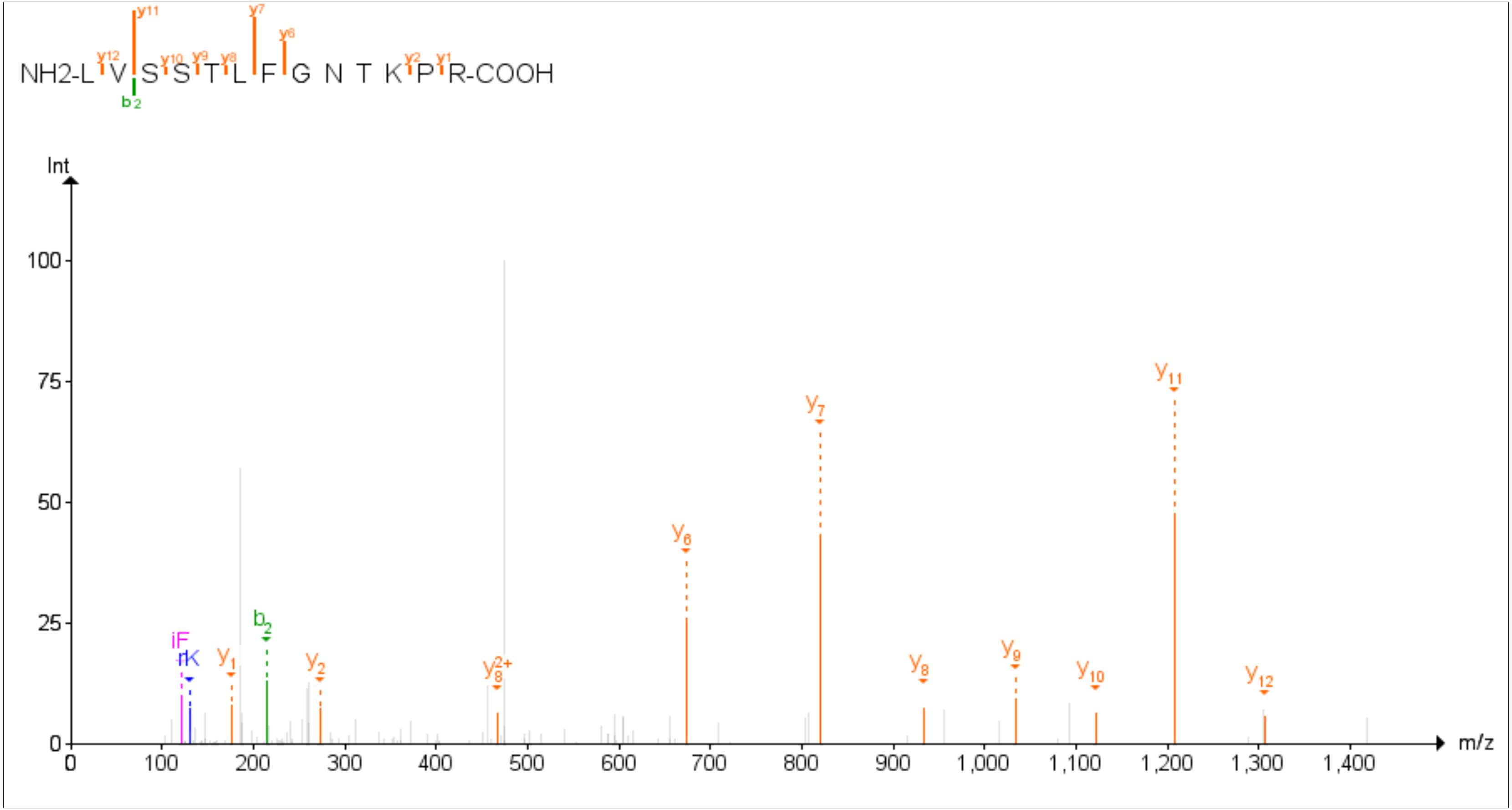

NH2-L V S S T L F G N T K P R-COOH
y11 y7 y6 y12 y10 y9 y8 y2 y1
b2
Int
100
75
50
25
0
y11
y7
y6
b2
iF
rK
y1
y2
y8 2+
y8
y9
y10
y12
0 100 200 300 400 500 600 700 800 900 1,000 1,100 1,200 1,300 1,400
m/z

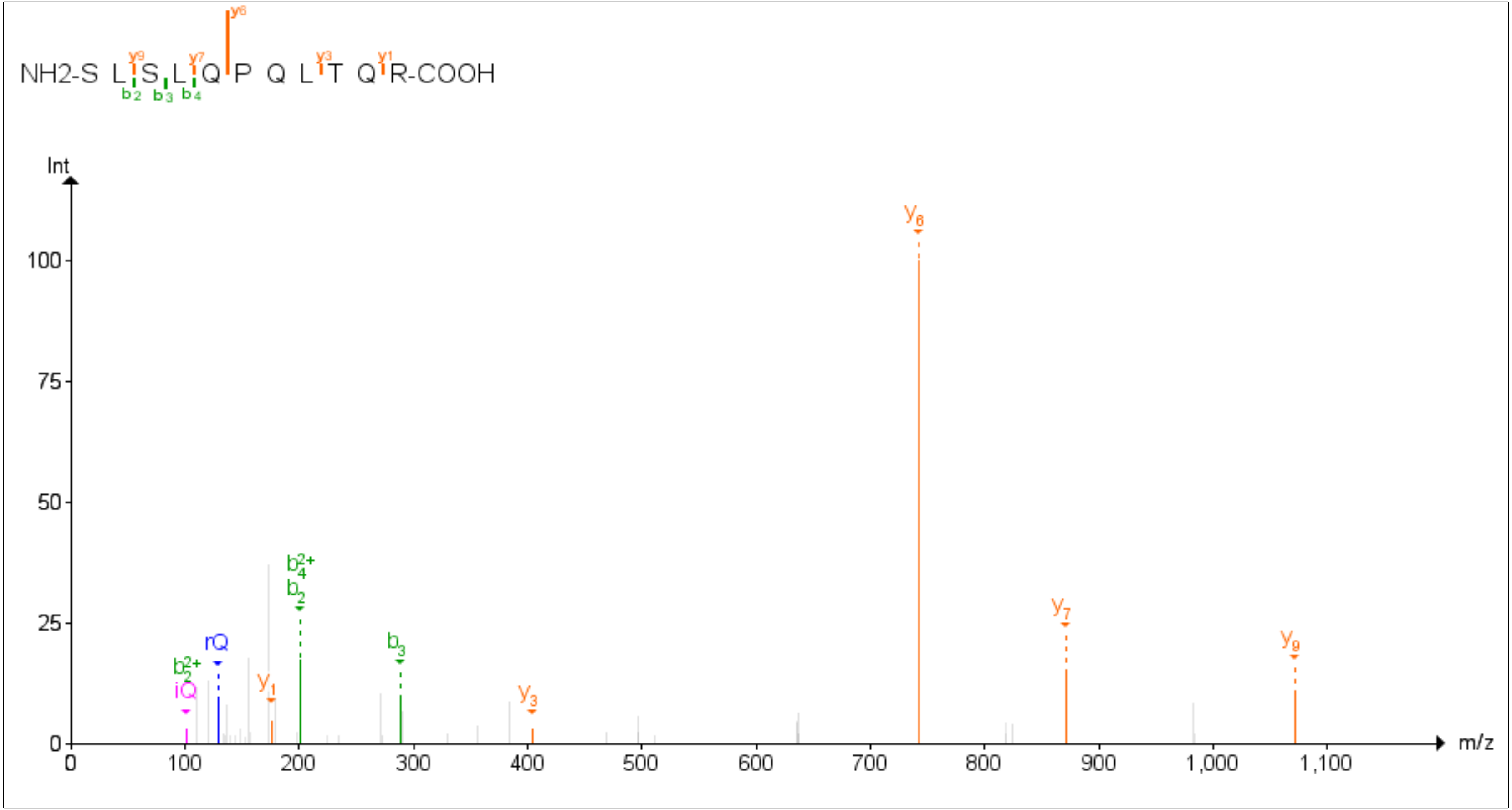

NH2-S L S L Q P Q L T Q R-COOH
y9 y7 y6 y3 y1
b2 b3 b4
Int
100
75
50
25
0
y6
y7
y9
b_4^{2+}
b_2
b_3
rQ
b_2^{2+}
iQ
y_1
y_3
0 100 200 300 400 500 600 700 800 900 1,000 1,100
m/z

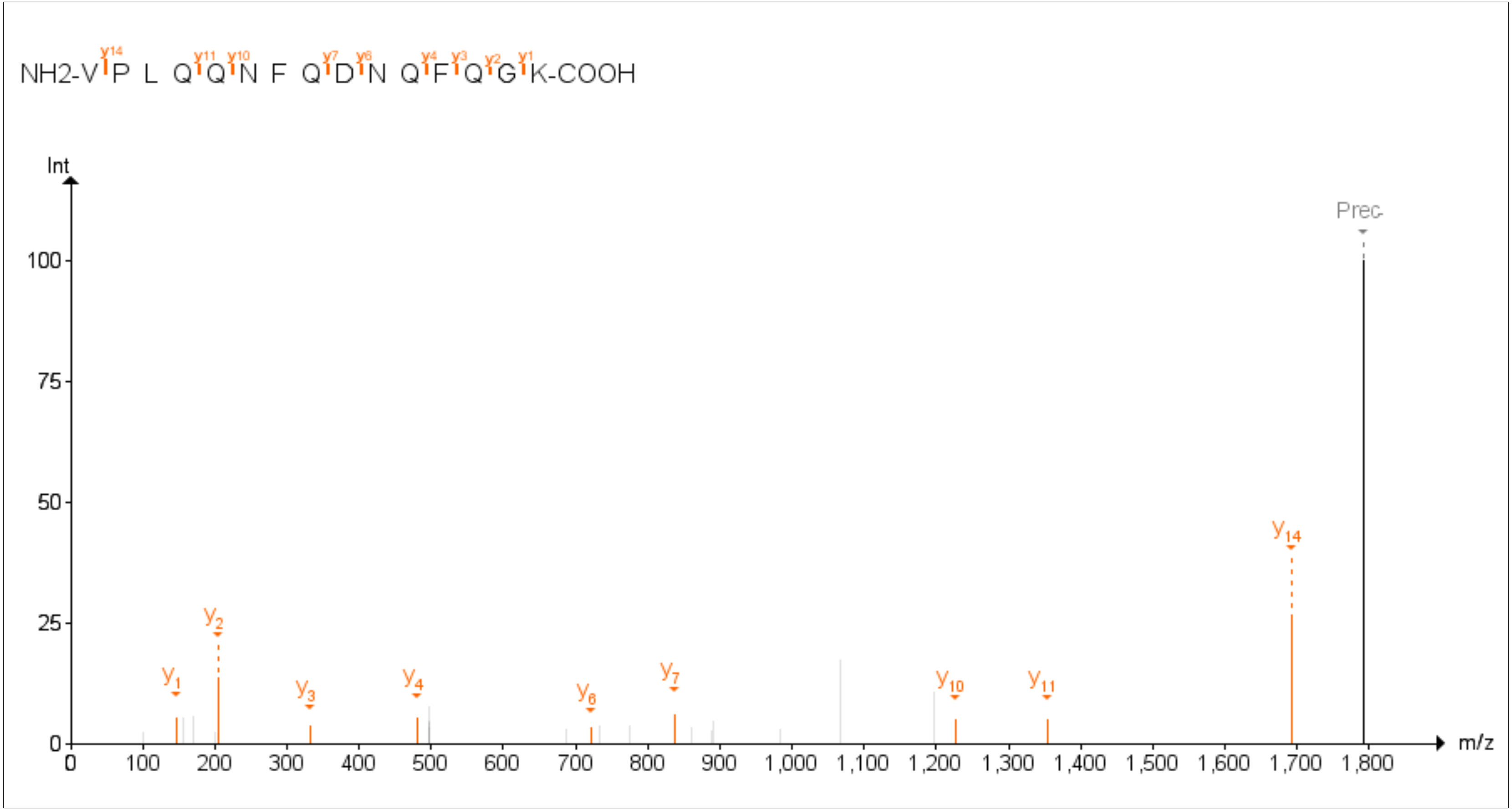

NH2-V P L Q Q N F Q D N Q F Q G K-COOH
y14 y11 y10 y7 y6 y4 y3 y2 y1
Int
100
75
50
25
0
Prec
y14
y2
y1
y3
y4
y6
y7
y10
y11
0 100 200 300 400 500 600 700 800 900 1,000 1,100 1,200 1,300 1,400 1,500 1,600 1,700 1,800
m/z

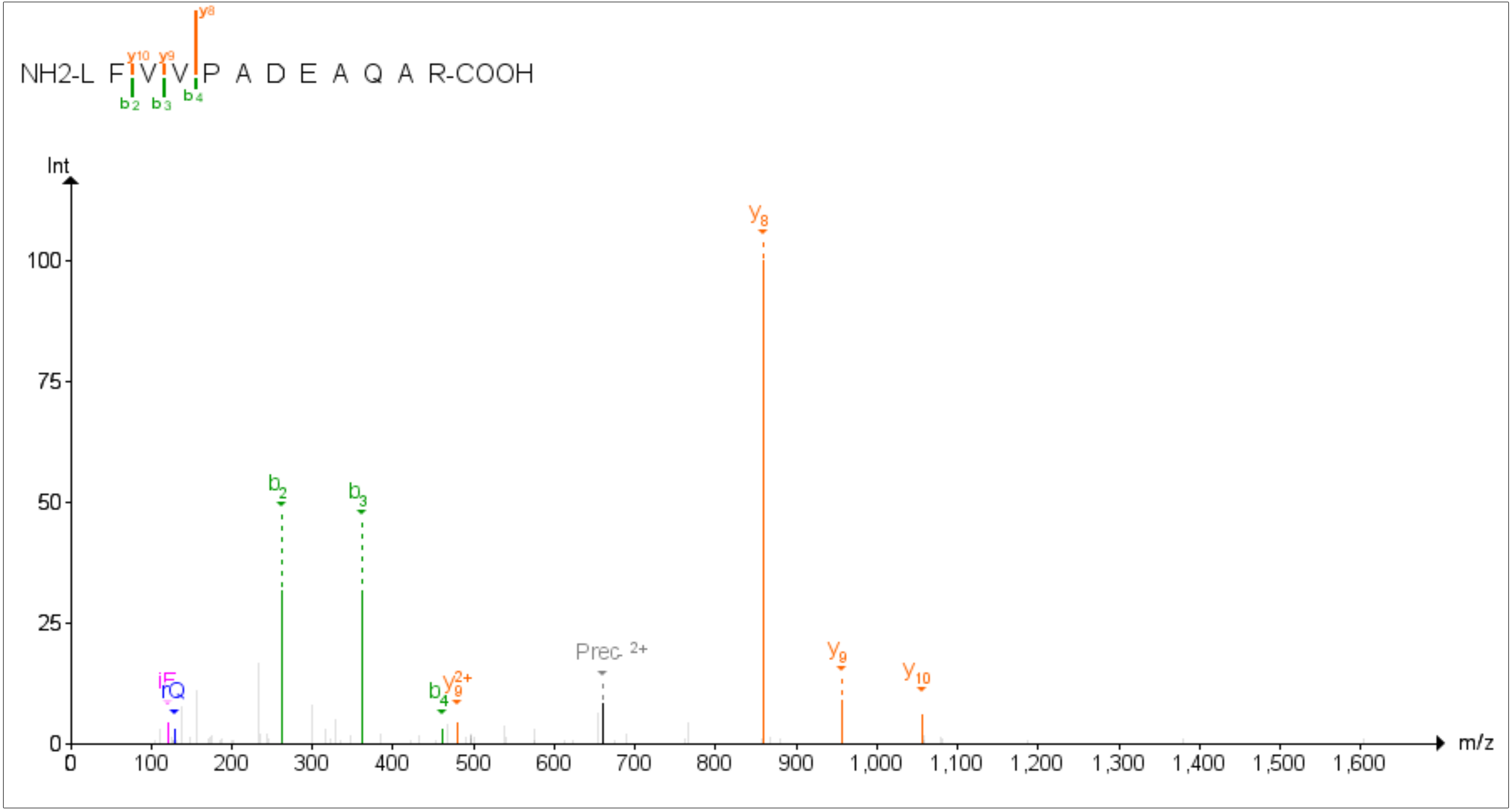

NH2-L F V V P A D E A Q A R-COOH
y10 y9 y8
b2 b3 b4
Int
m/z
y8
b2
b3
Prec 2+
y9
y10
b4
y9 2+
iF
rQ

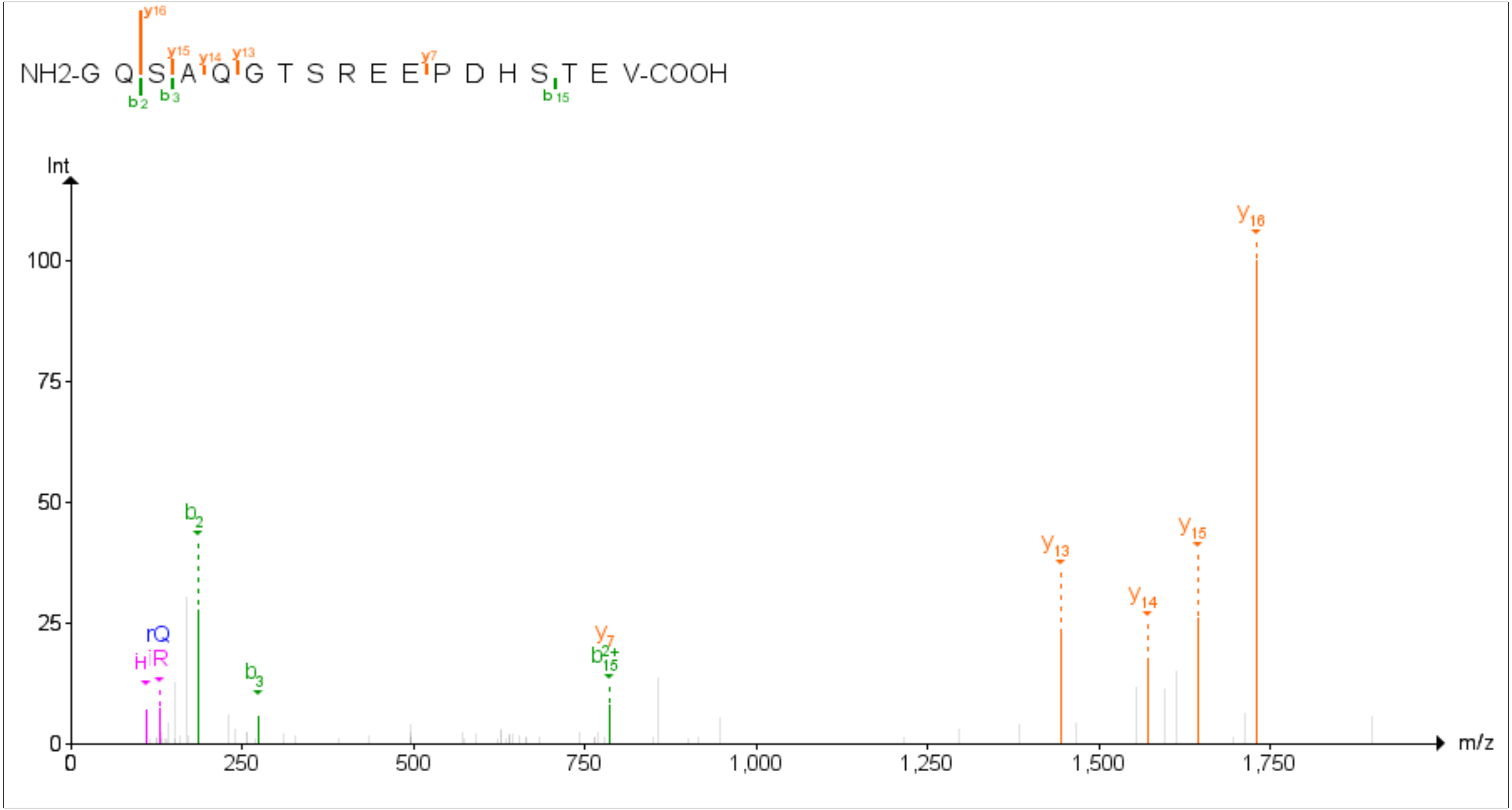

NH2-G Q S A Q G T S R E E P D H S T E V-COOH
y16
y15
y14
y13
y7
b2
b3
b15
Int
100
75
50
25
0
m/z
0
250
500
750
1,000
1,250
1,500
1,750
y16
b2
y13
y15
y14
rQ
H
iR
b3
y7
b15 2+

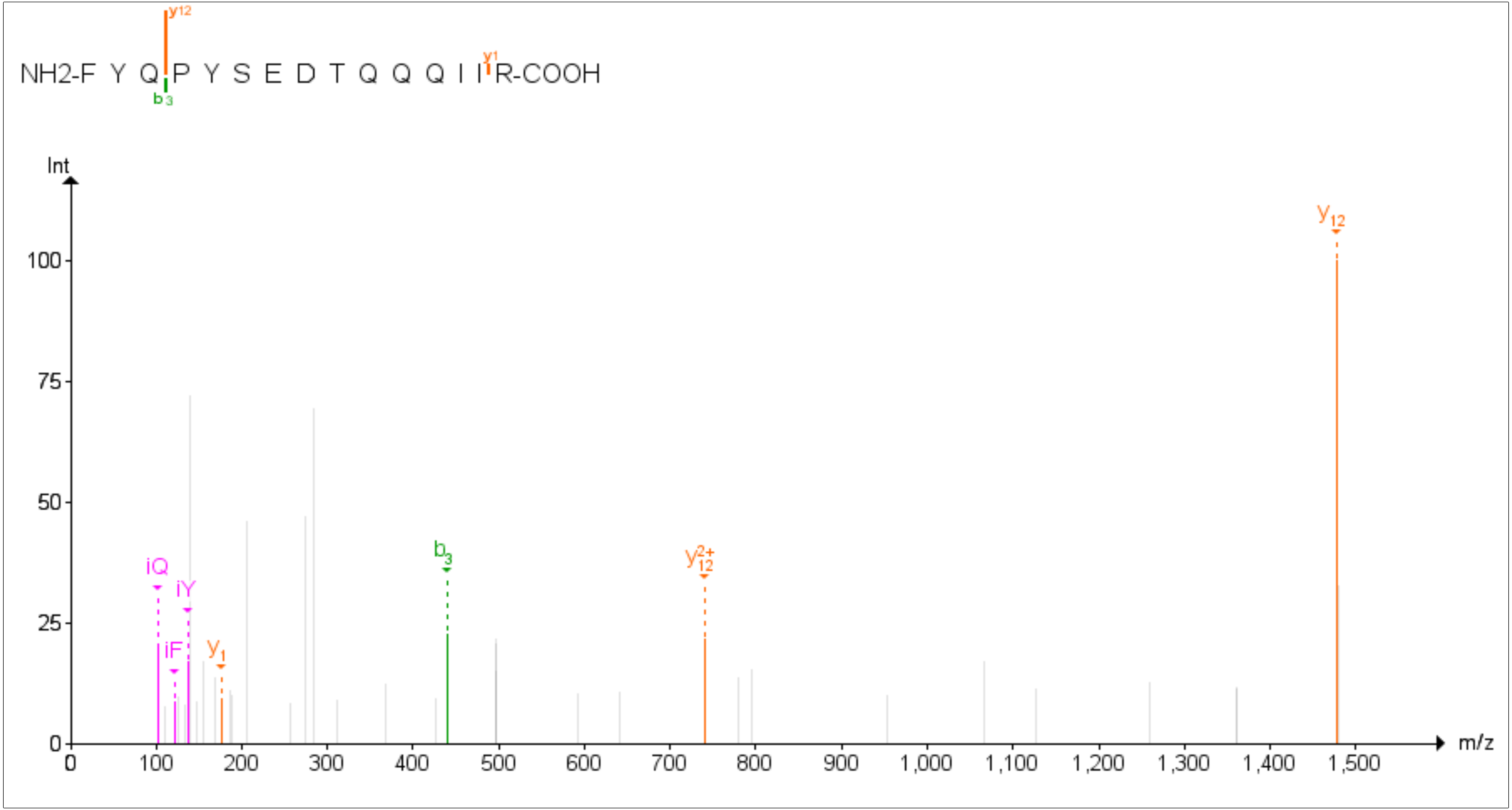

NH2-F Y Q P Y S E D T Q Q Q I I R-COOH
y12
b3
y1
Int
100
75
50
25
0
m/z
0
100
200
300
400
500
600
700
800
900
1,000
1,100
1,200
1,300
1,400
1,500
y12
iQ
iY
iF
y1
b3
y12 2+

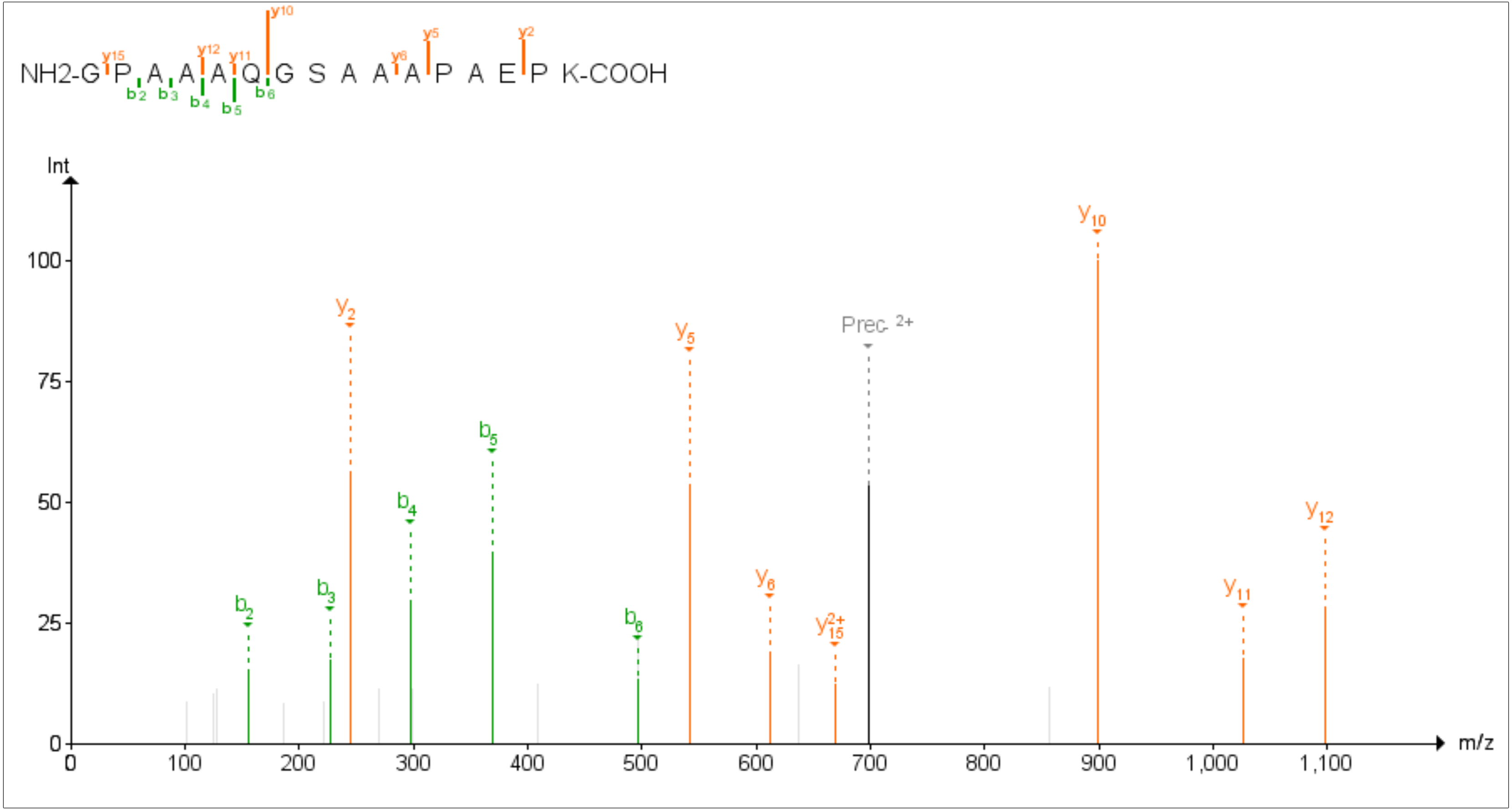

NH2-G P A A A Q G S A A A P A E P K-COOH
y15 y12 y11 y10 y6 y5 y2
b2 b3 b4 b5 b6
Int
m/z
Prec. 2+
y2 y5 y6 y10 y11 y12 y15 2+
b2 b3 b4 b5 b6
0 25 50 75 100
0 100 200 300 400 500 600 700 800 900 1,000 1,100

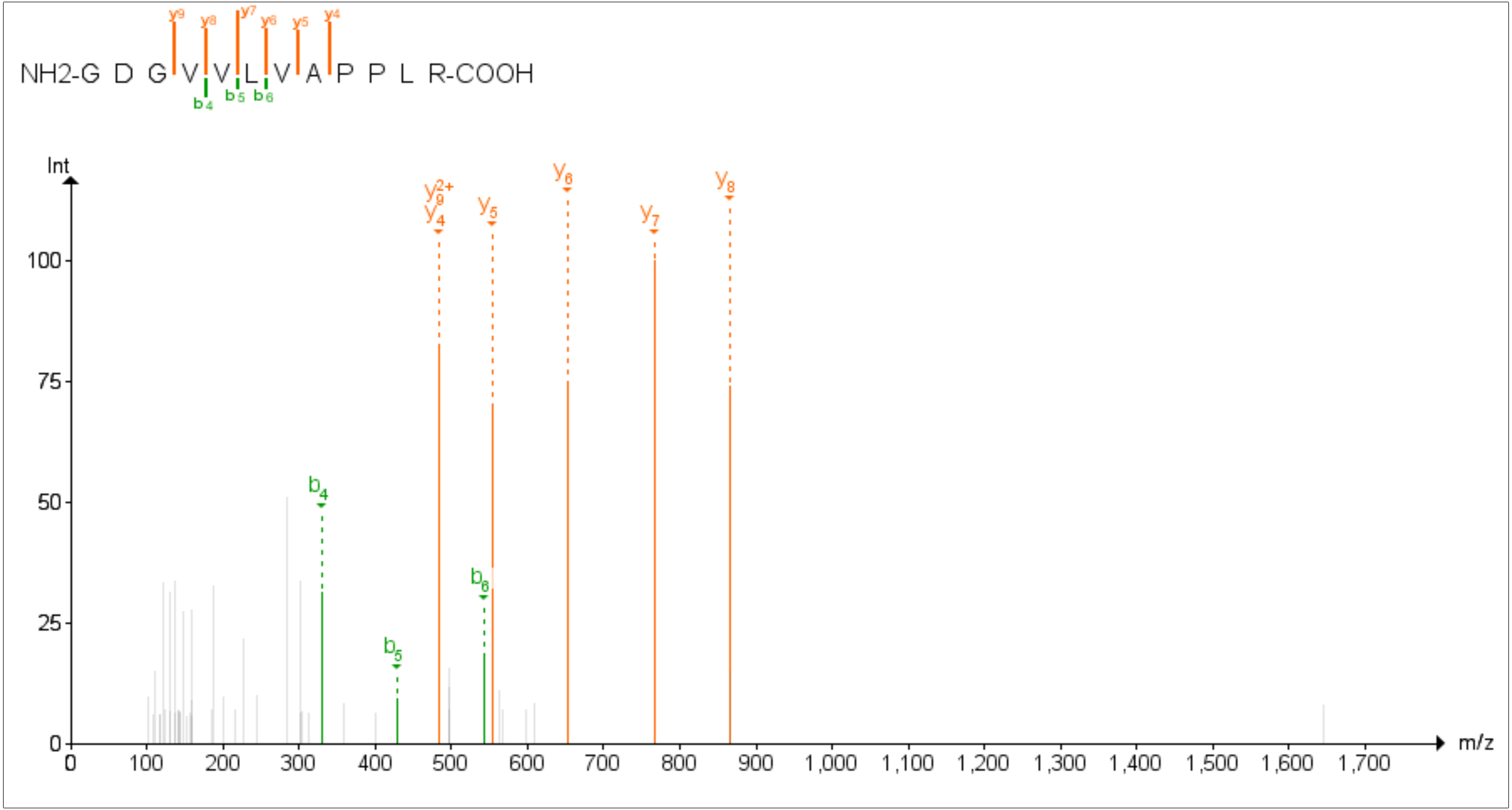

NH2-G D G V V L V A P P L R-COOH
y9 y8 y7 y6 y5 y4
b4 b5 b6
Int
100
75
50
25
0
m/z
0 100 200 300 400 500 600 700 800 900 1,000 1,100 1,200 1,300 1,400 1,500 1,600 1,700
y9 2+
y4
y5
y6
y7
y8
b4
b5
b6

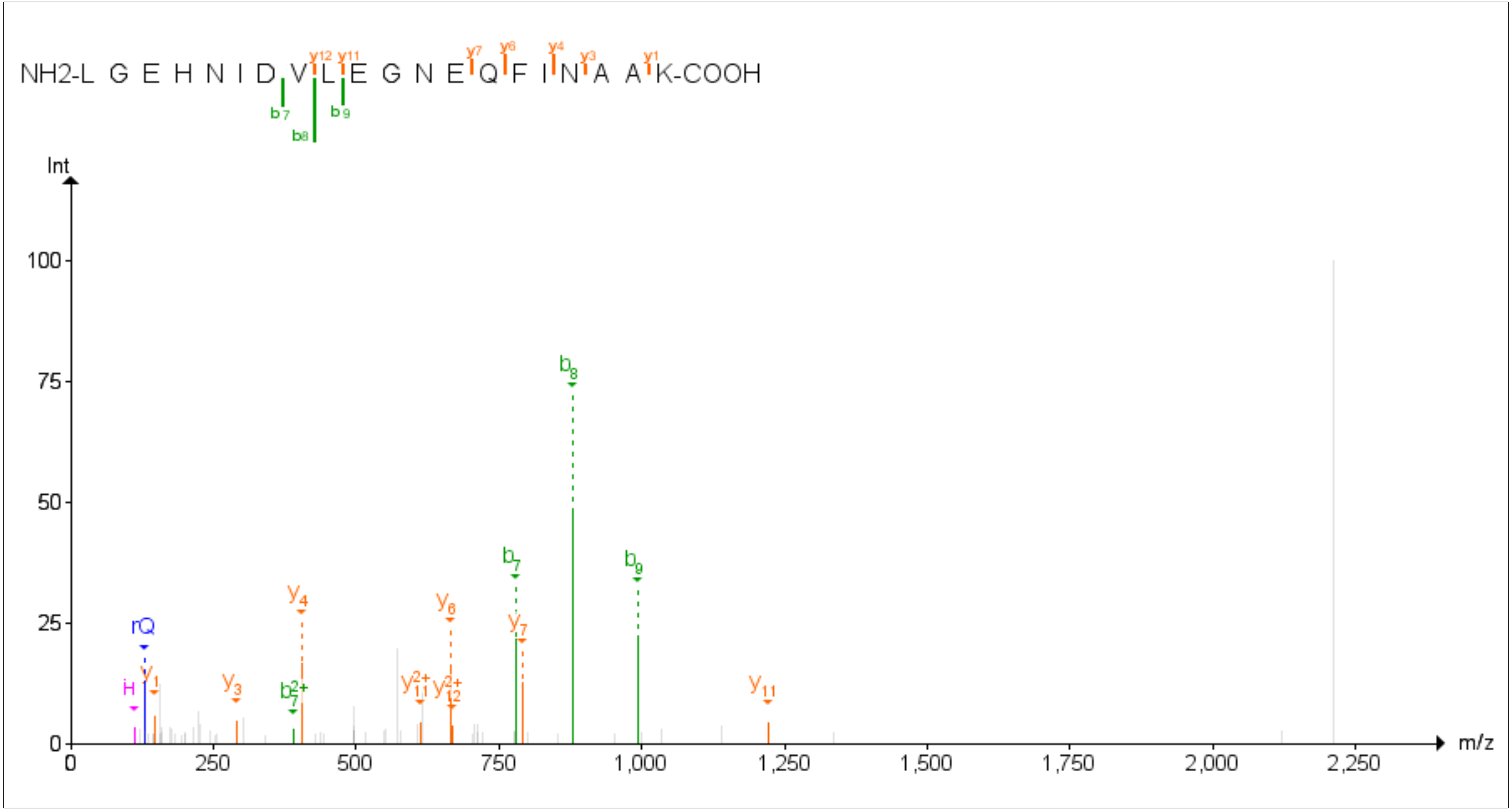
NH2-L G E H N I D V L E G N E Q F I N A A K-COOH
y12 y11 y7 y6 y4 y3 y1
b7 b8 b9
Int
m/z
0 25 50 75 100
0 250 500 750 1,000 1,250 1,500 1,750 2,000 2,250
b8 b7 b9 y4 y6 y7 rQ y1 iH y3 b7 2+ y11 2+ y12 2+ y11

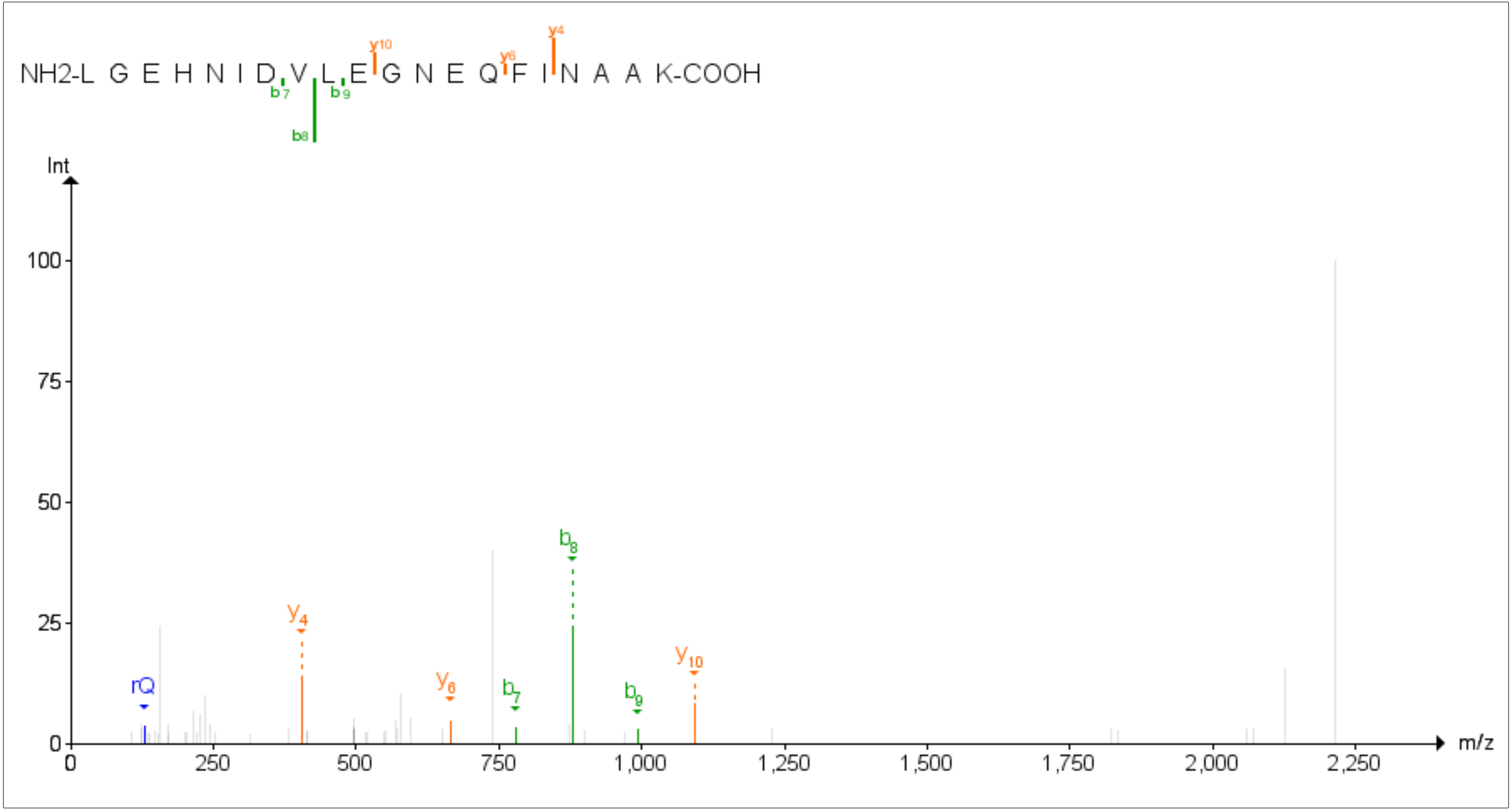

NH2-L G E H N I D V L E G N E Q F I N A A K-COOH
y10
y6
y4
b7
b9
b8
Int
100
75
50
25
0
m/z
0
250
500
750
1,000
1,250
1,500
1,750
2,000
2,250
rQ
y4
y6
b7
b8
b9
y10

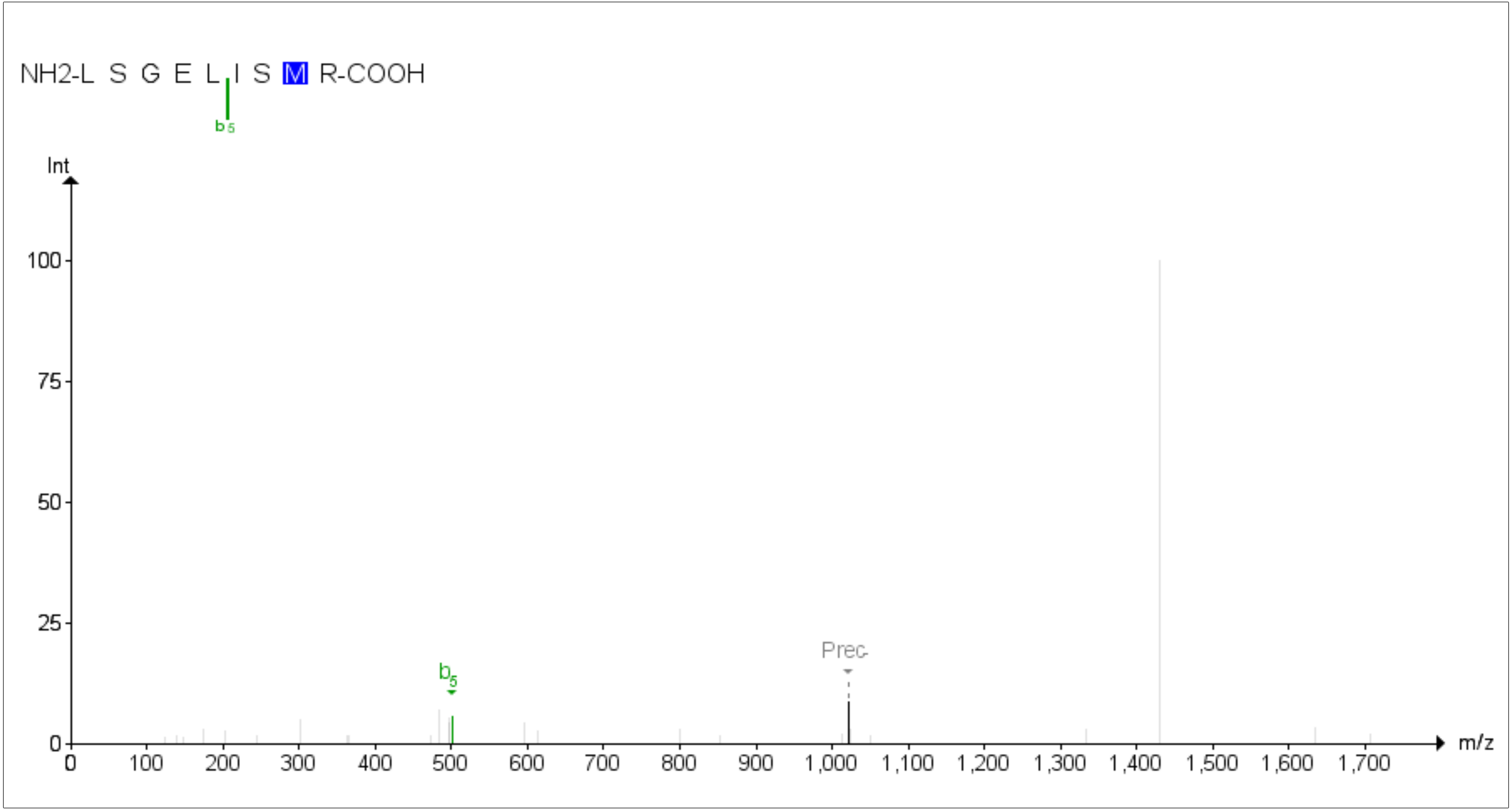

NH2-L S G E L I S M R-COOH
b5
Int
100
75
50
25
0
b5
Prec
0
100
200
300
400
500
600
700
800
900
1,000
1,100
1,200
1,300
1,400
1,500
1,600
1,700
m/z

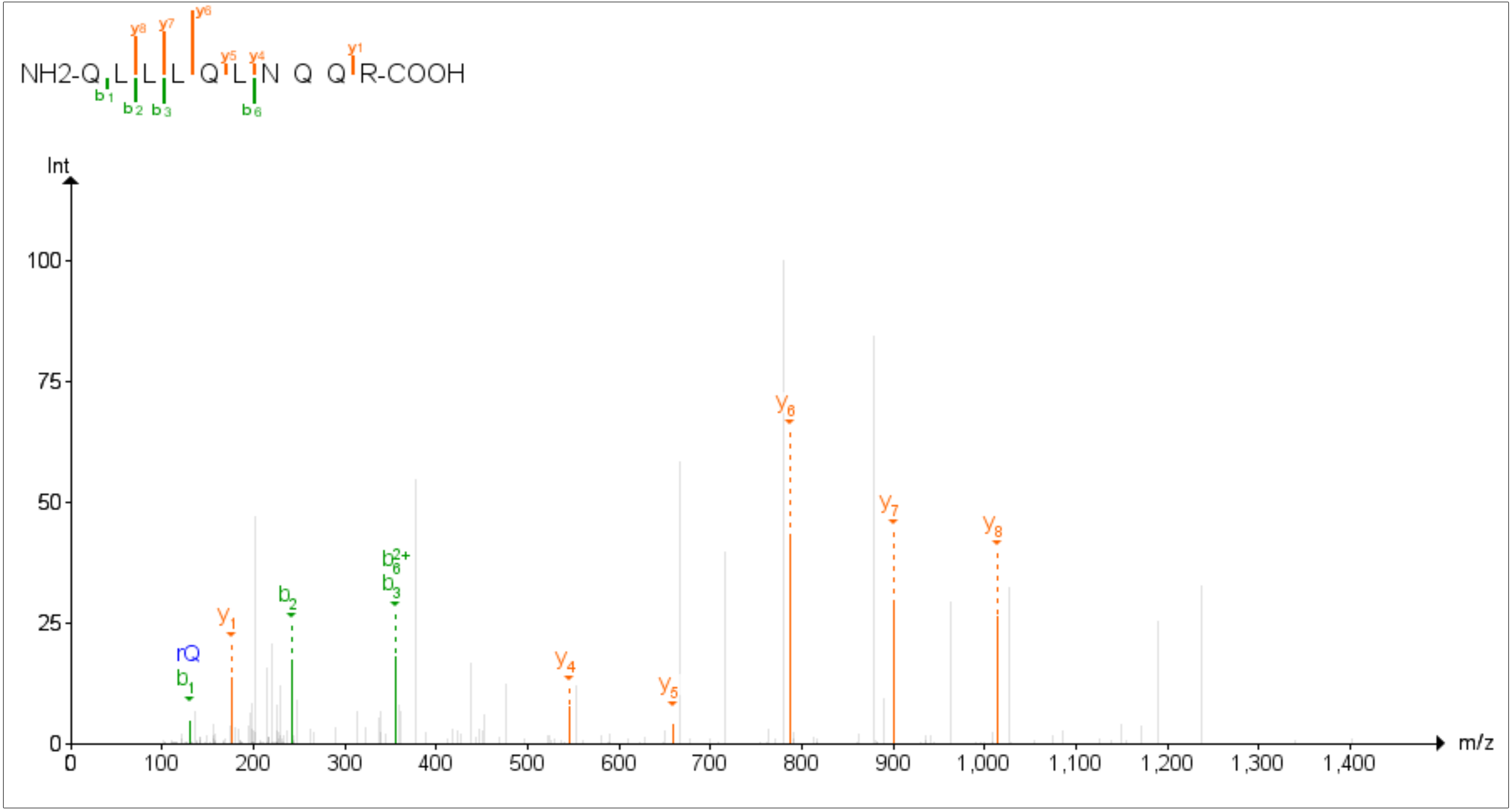
NH2-Q L L L Q L N Q Q R-COOH
y8
y7
y6
y5
y4
y1
b1
b2
b3
b6
Int
m/z
rQ
b1
y1
b2
b3
y4
y5
y6
y7
y8

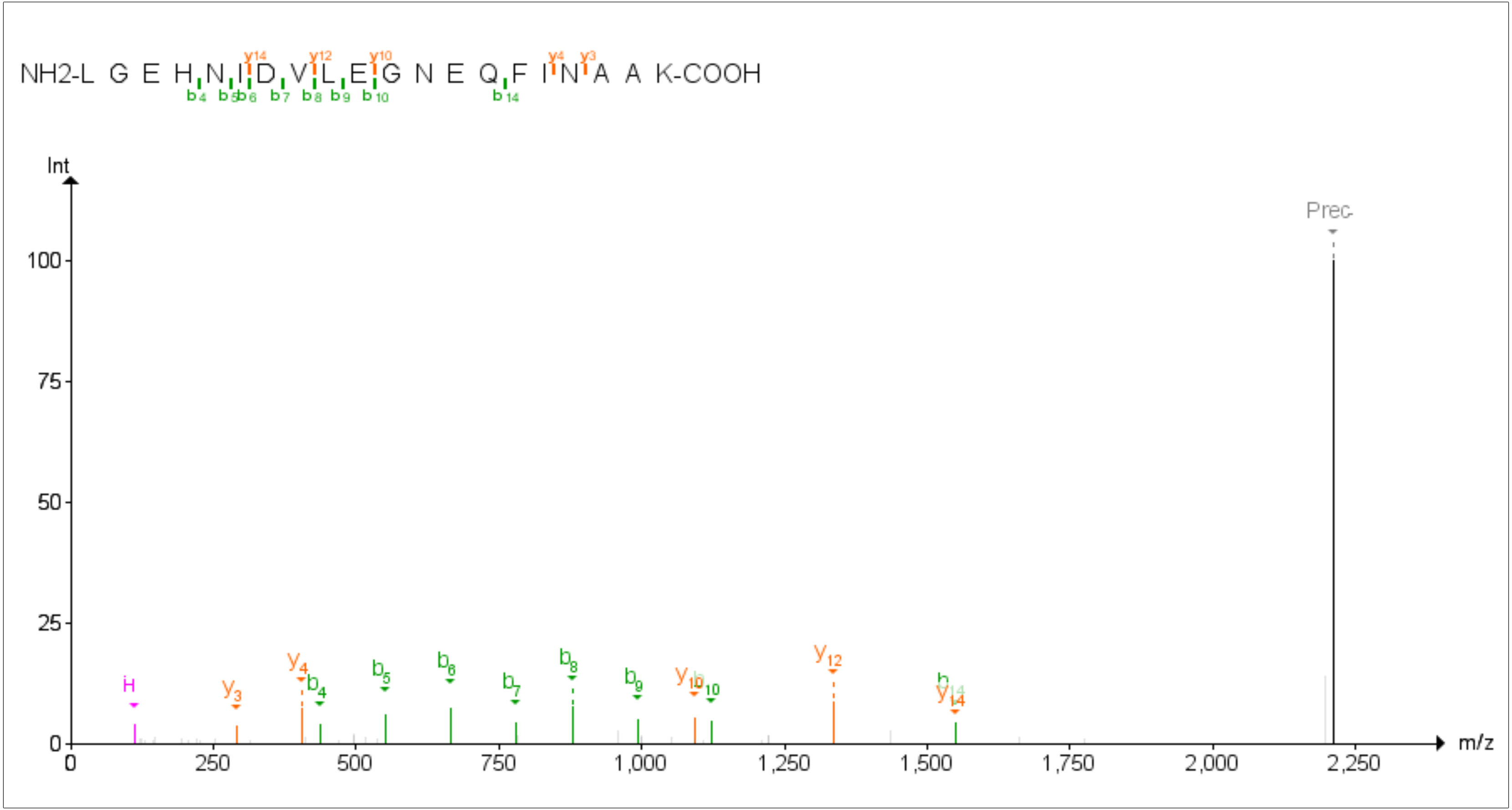

NH2-L G E H N I D V L E G N E Q F I N A A K-COOH
y14 y12 y10 y4 y3
b4 b5 b6 b7 b8 b9 b10 b14
Int
100
75
50
25
0
Prec
H
y3 y4 b4 b5 b6 b7 b8 b9 y10 b10 y12 y14
0
250
500
750
1,000
1,250
1,500
1,750
2,000
2,250
m/z

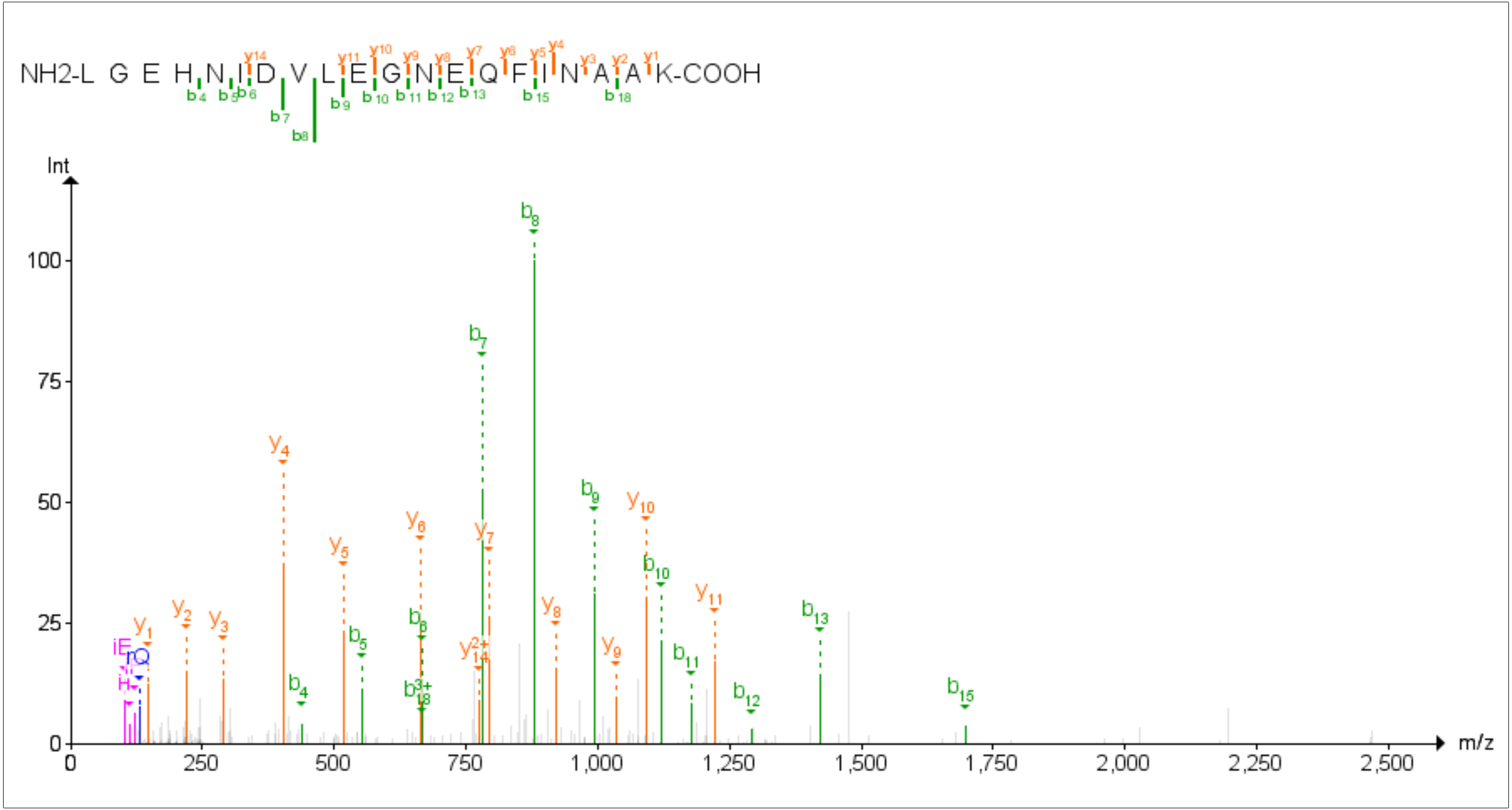

NH2-L G E H N I D V L E G N E Q F I N A A K-COOH
Int
m/z
0
25
50
75
100
0
250
500
750
1,000
1,250
1,500
1,750
2,000
2,250
2,500

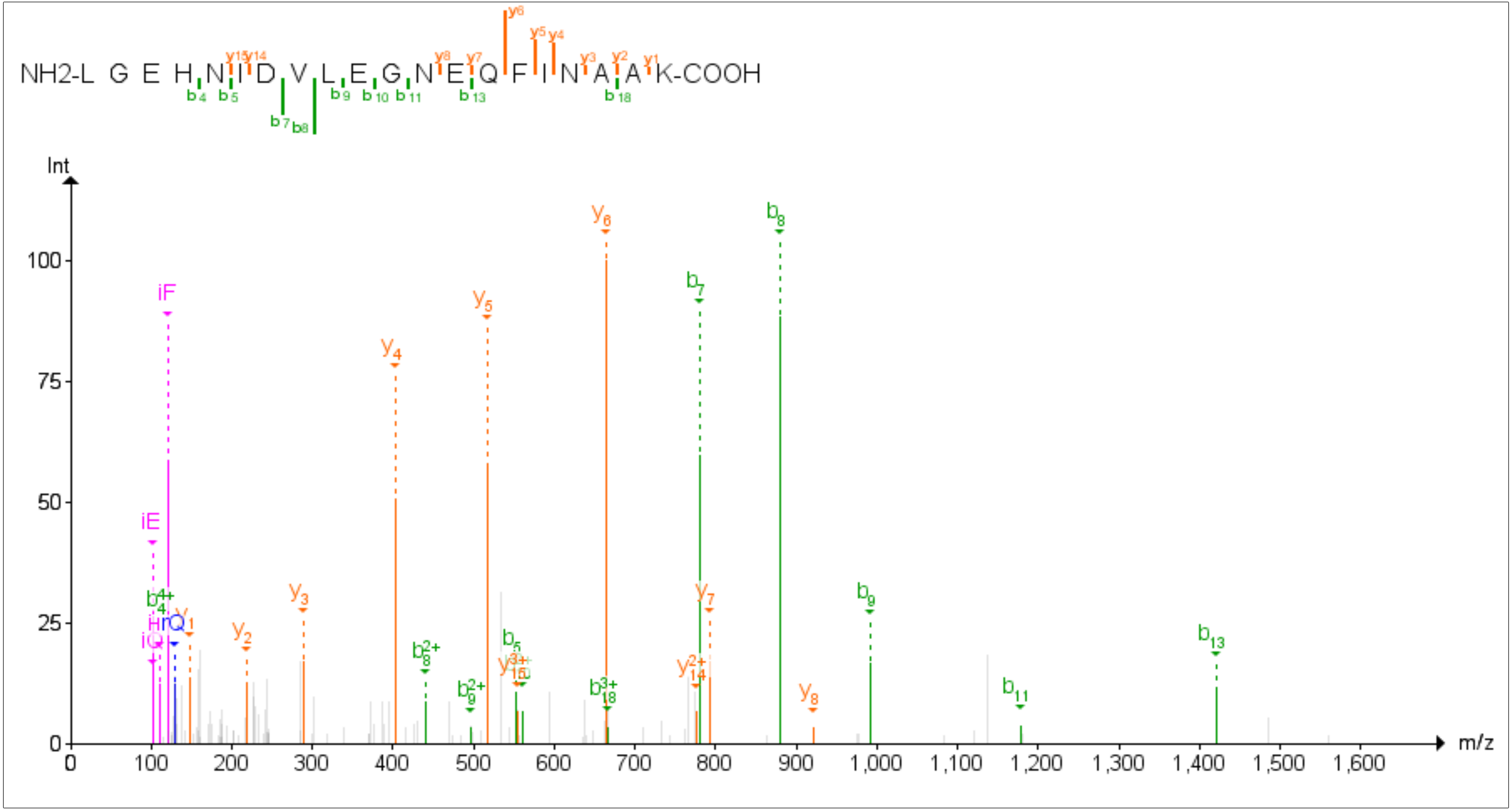

NH2-L G E H N I D V L E G N E Q F I N A A K-COOH
Int
m/z
0
25
50
75
100
0
100
200
300
400
500
600
700
800
900
1,000
1,100
1,200
1,300
1,400
1,500
1,600

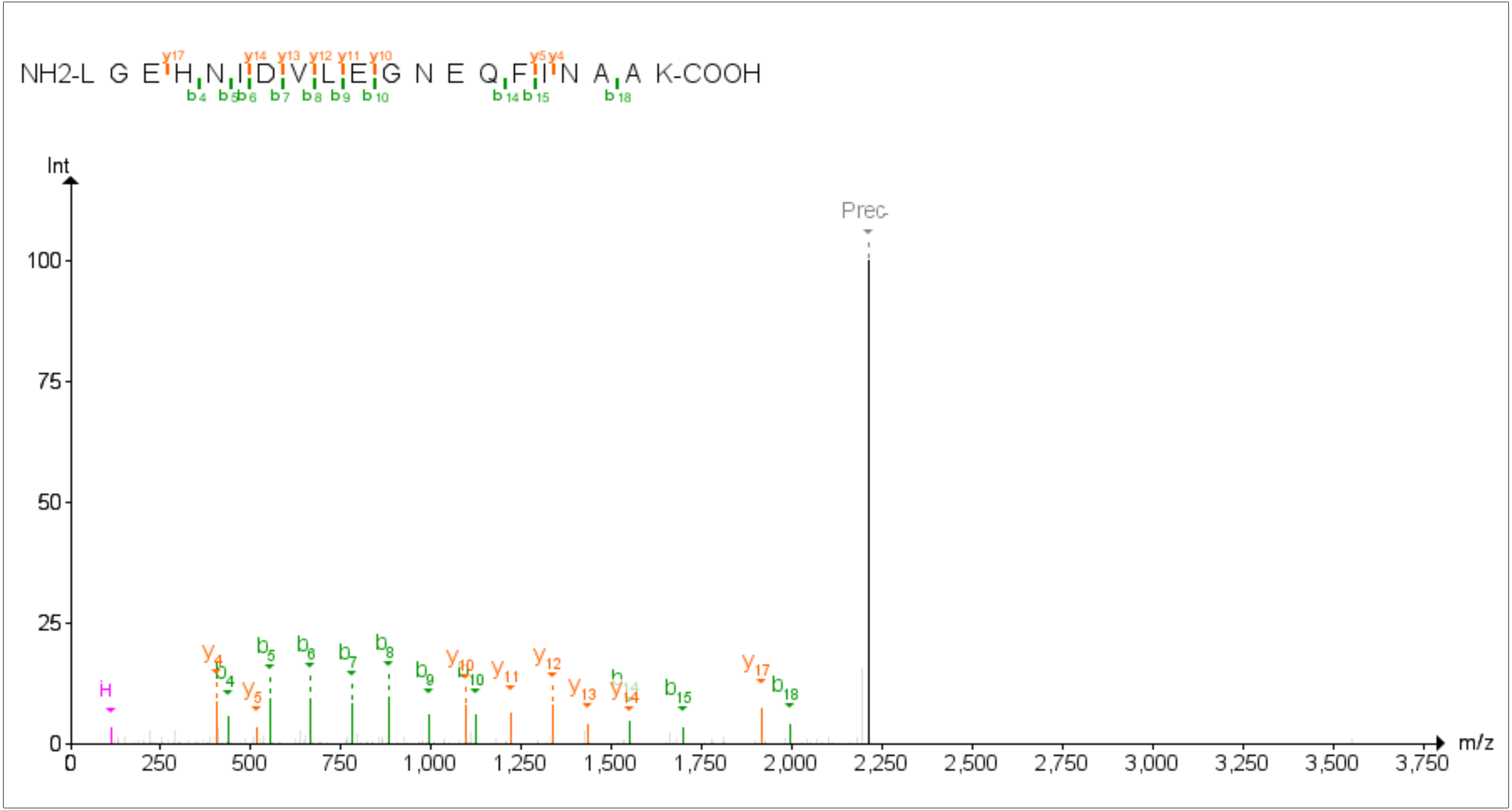

NH2-L G E H N I D V L E G N E Q F I N A A K-COOH
y17 y14 y13 y12 y11 y10 y5 y4
b4 b5 b6 b7 b8 b9 b10 b14 b15 b18
Int
Prec
100
75
50
25
0
H
y4 b4 y5 b5 b6 b7 b8 b9 y10 b10 y11 y12 y13 y14 b15 y17 b18
0
250
500
750
1,000
1,250
1,500
1,750
2,000
2,250
2,500
2,750
3,000
3,250
3,500
3,750
m/z

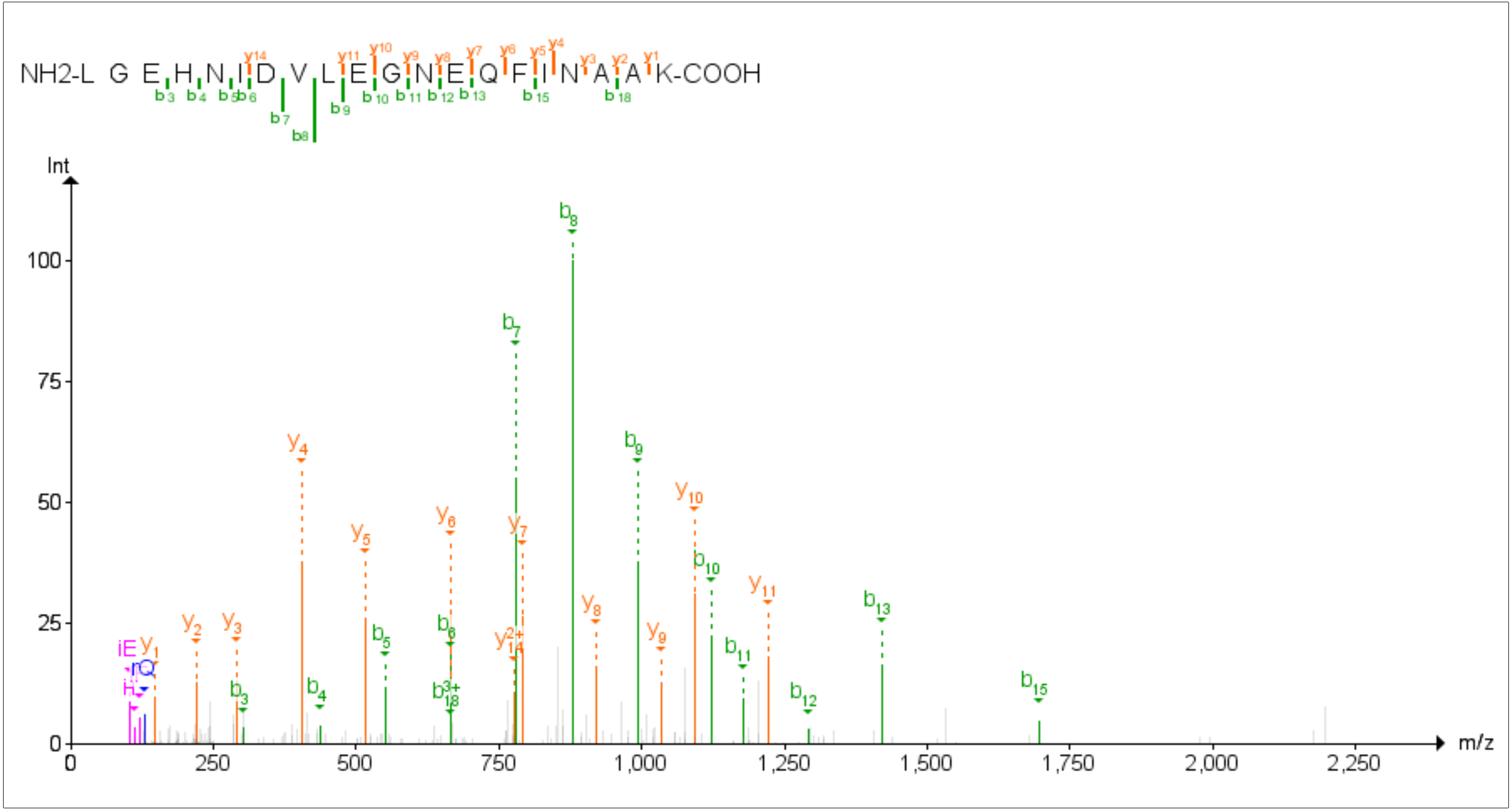
NH2-L G E H N I D V L E G N E Q F I N A A K-COOH
y14 y11 y10 y9 y8 y7 y6 y5 y4 y3 y2 y1
b3 b4 b5 b6 b7 b8 b9 b10 b11 b12 b13 b15 b18
Int
100
75
50
25
0
m/z
0
250
500
750
1,000
1,250
1,500
1,750
2,000
2,250
b8
b7
b9
y4
y10
y6
y7
y5
b10
y11
b13
y8
y9
b6
b5
y14 2+
b11
y2
y3
b3+ 18
b15
iE
y1
nQ
iH
b3
b4
b12

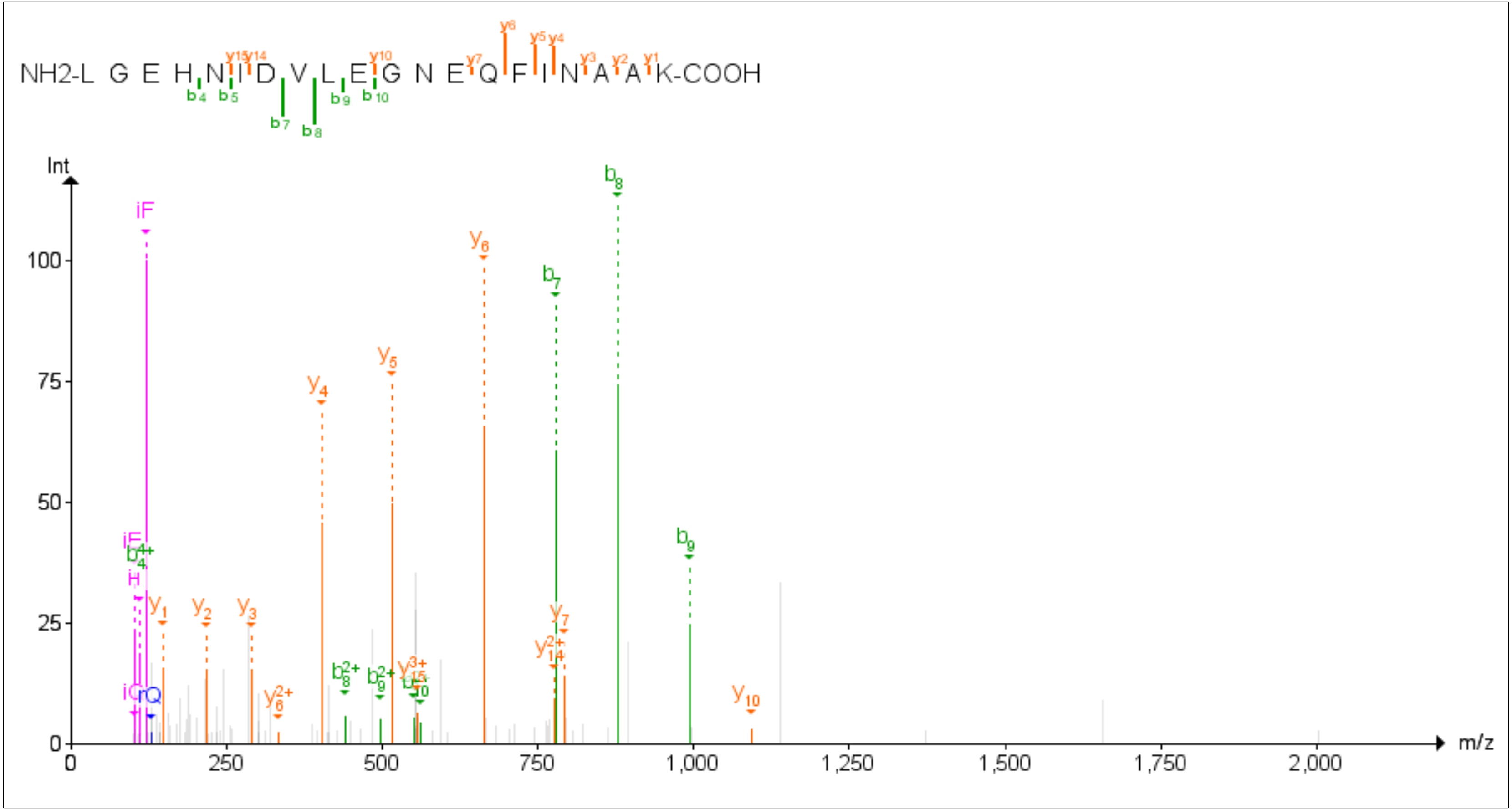

NH2-L G E H N I D V L E G N E Q F I N A A K-COOH
b4 b5 b7 b8 b9 b10
y15 y14 y10 y7 y6 y5 y4 y3 y2 y1
Int
m/z
0
25
50
75
100
250
500
750
1,000
1,250
1,500
1,750
2,000
iF
b8
y6
b7
y5
y4
b9
y1
y2
y3
y7
y10
rQ

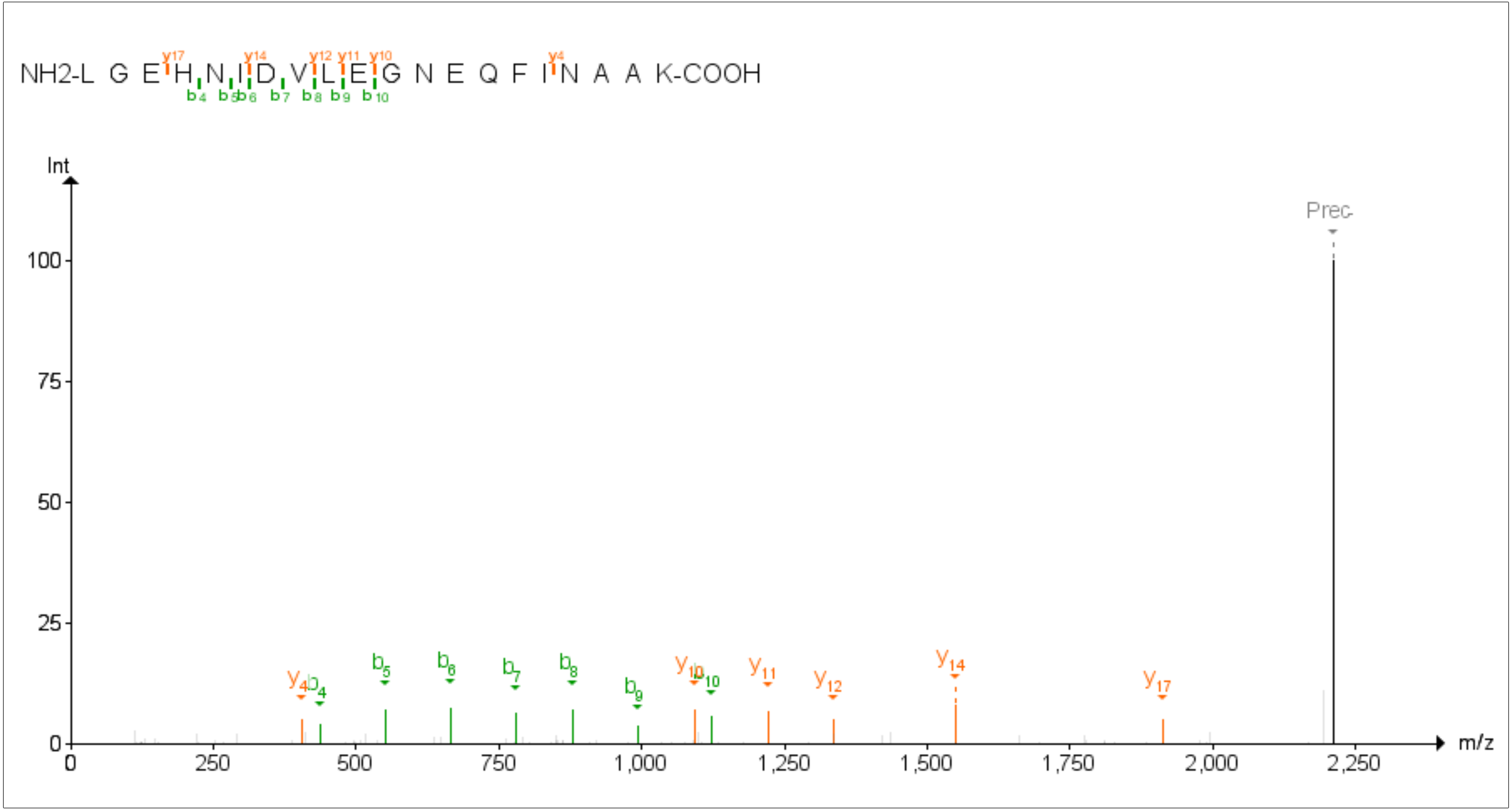

NH2-L G E H N I D V L E G N E Q F I N A A K-COOH
y17 y14 y12 y11 y10 y4
b4 b5 b6 b7 b8 b9 b10
Int
100
75
50
25
0
Prec
y4 b4 b5 b6 b7 b8 b9 y10 b10 y11 y12 y14 y17
0 250 500 750 1,000 1,250 1,500 1,750 2,000 2,250
m/z

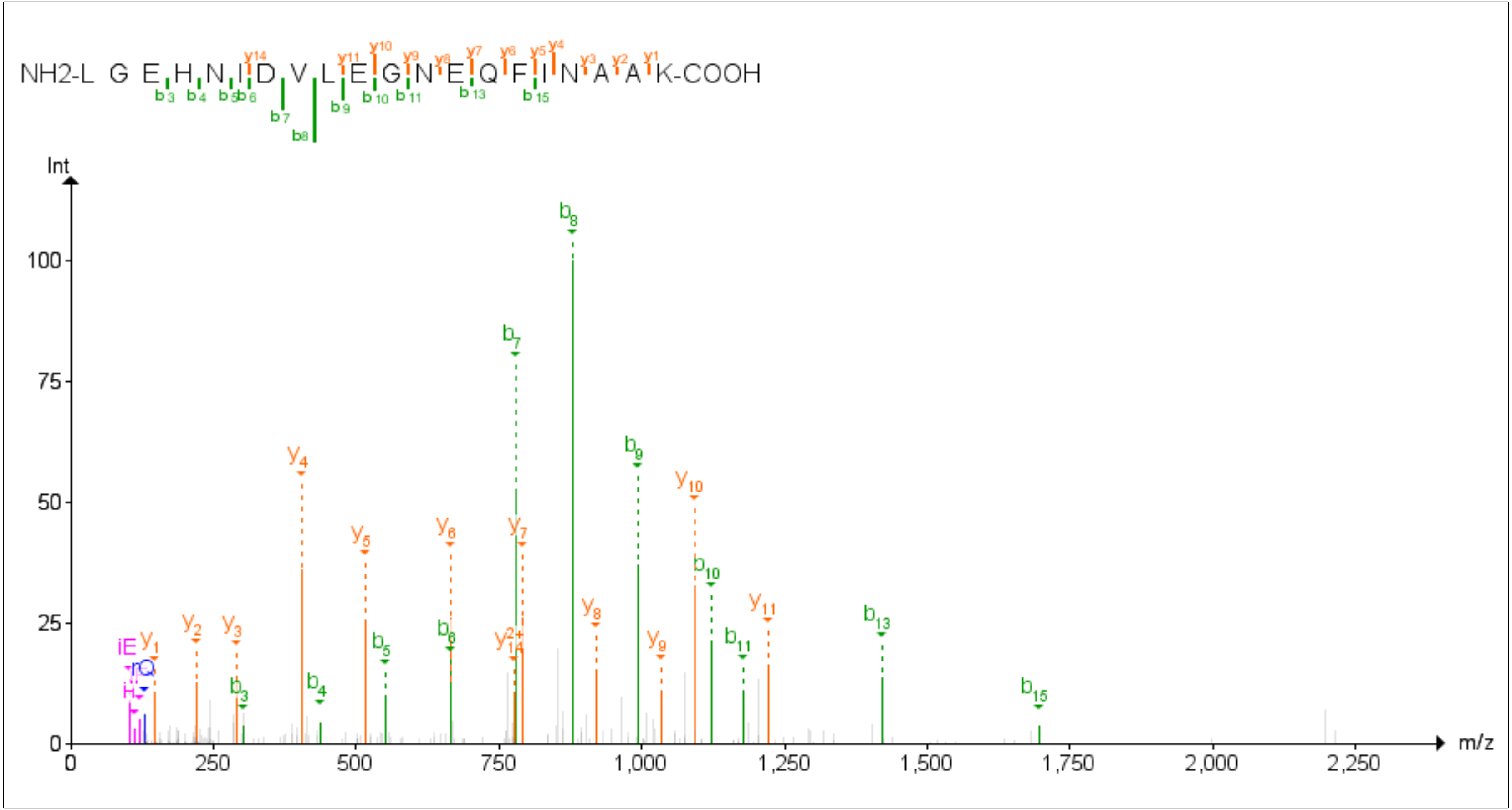

NH2-L G E H N I D V L E G N E Q F I N A A K-COOH
Int
m/z
b8
b7
b9
y4
y10
y5
y6
y7
b10
y11
b13
y8
y9
b11
b6
b5
y14 2+
y1
y2
y3
b3
b4
b15
iE
nQ
H

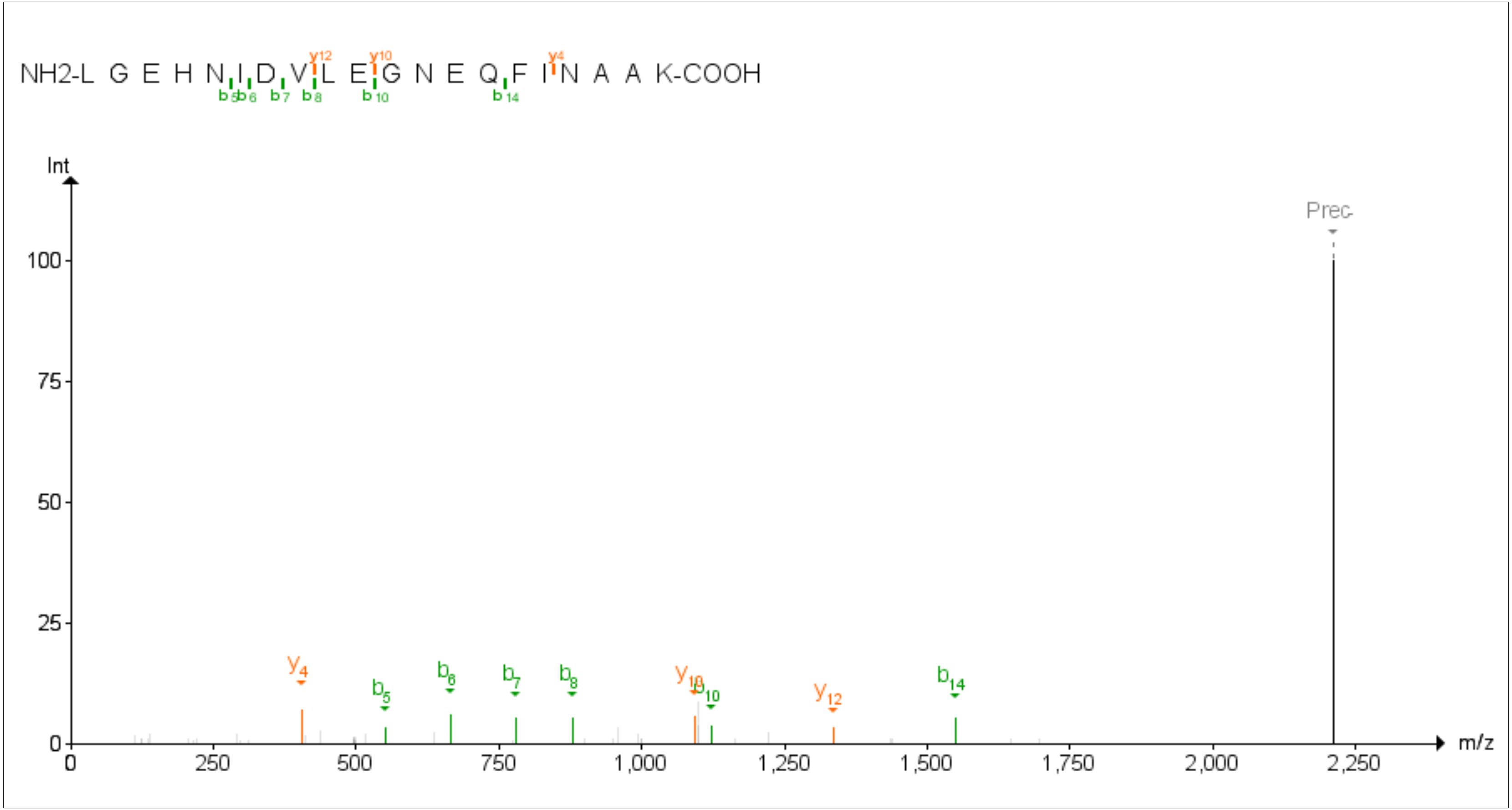

NH2-L G E H N I D V L E G N E Q F I N A A K-COOH
Int
m/z
Prec.
y4
b5
b6
b7
b8
y10
b10
y12
b14

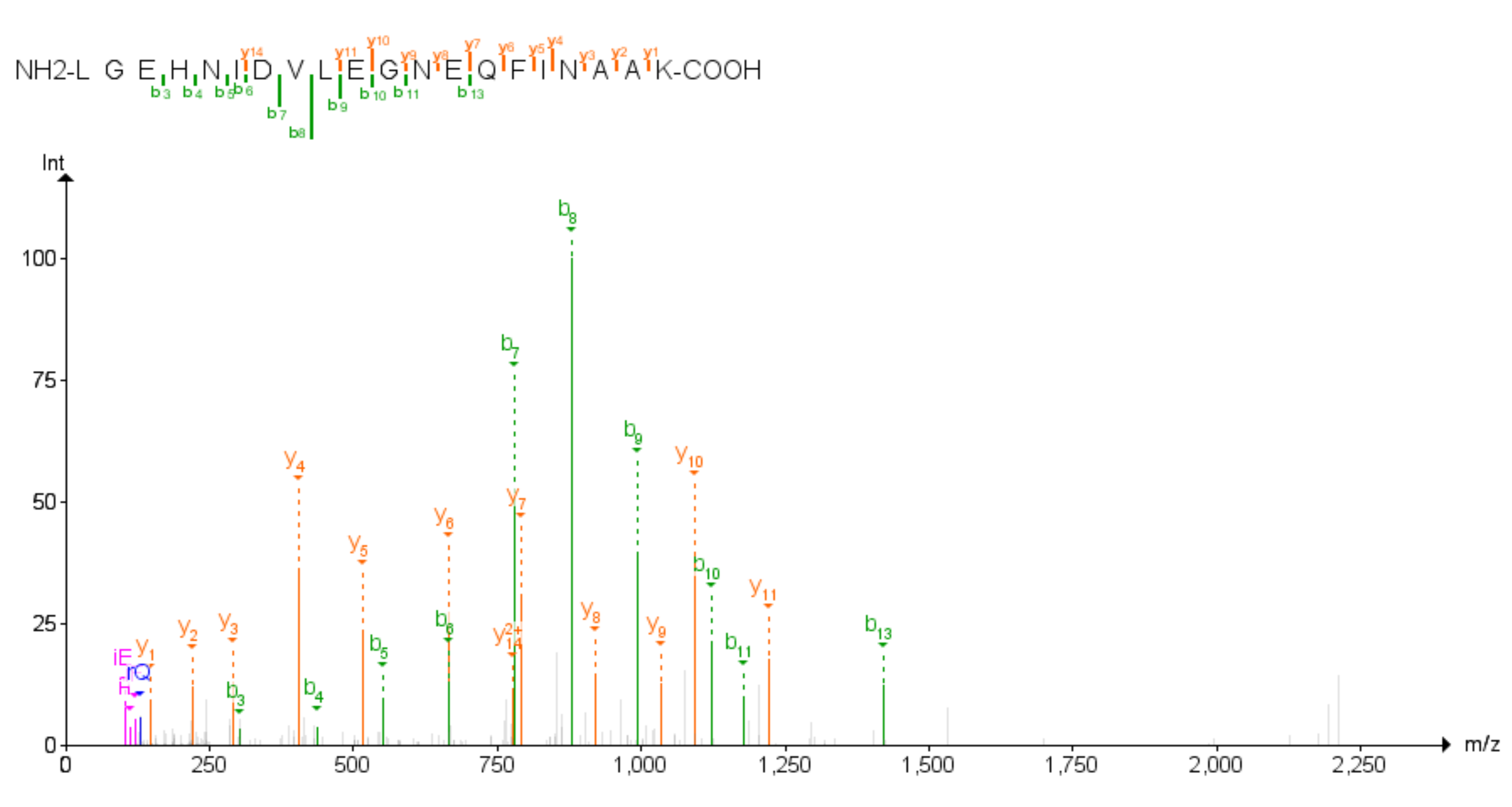

NH2-L G E H N I D V L E G N E Q F I N A A K-COOH
Int
m/z
100
75
50
25
0
250
500
750
1,000
1,250
1,500
1,750
2,000
2,250

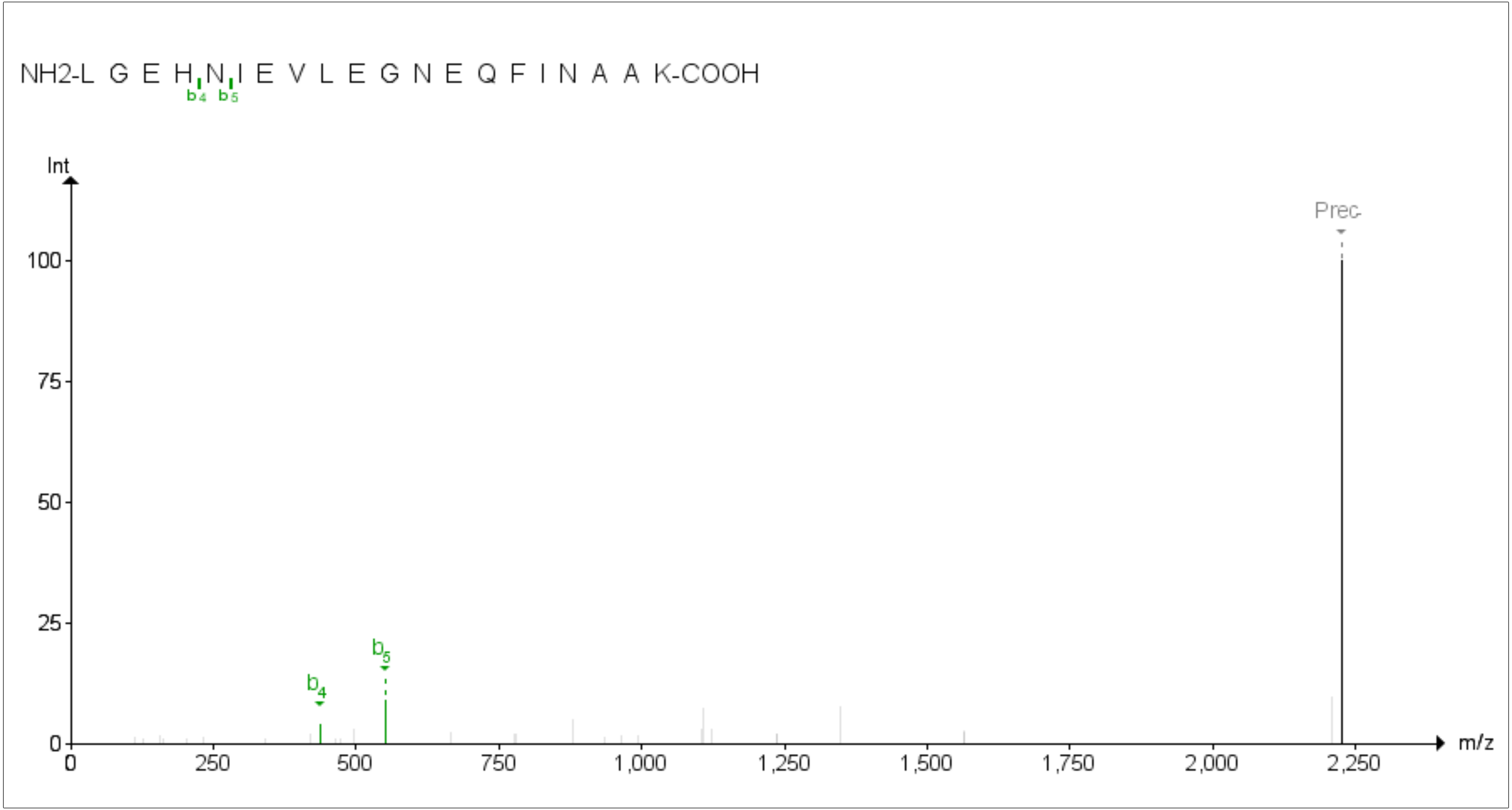

NH2-L G E H N I E V L E G N E Q F I N A A K-COOH
b4
b5
Int
Prec
100
75
50
25
0
b4
b5
0
250
500
750
1,000
1,250
1,500
1,750
2,000
2,250
m/z

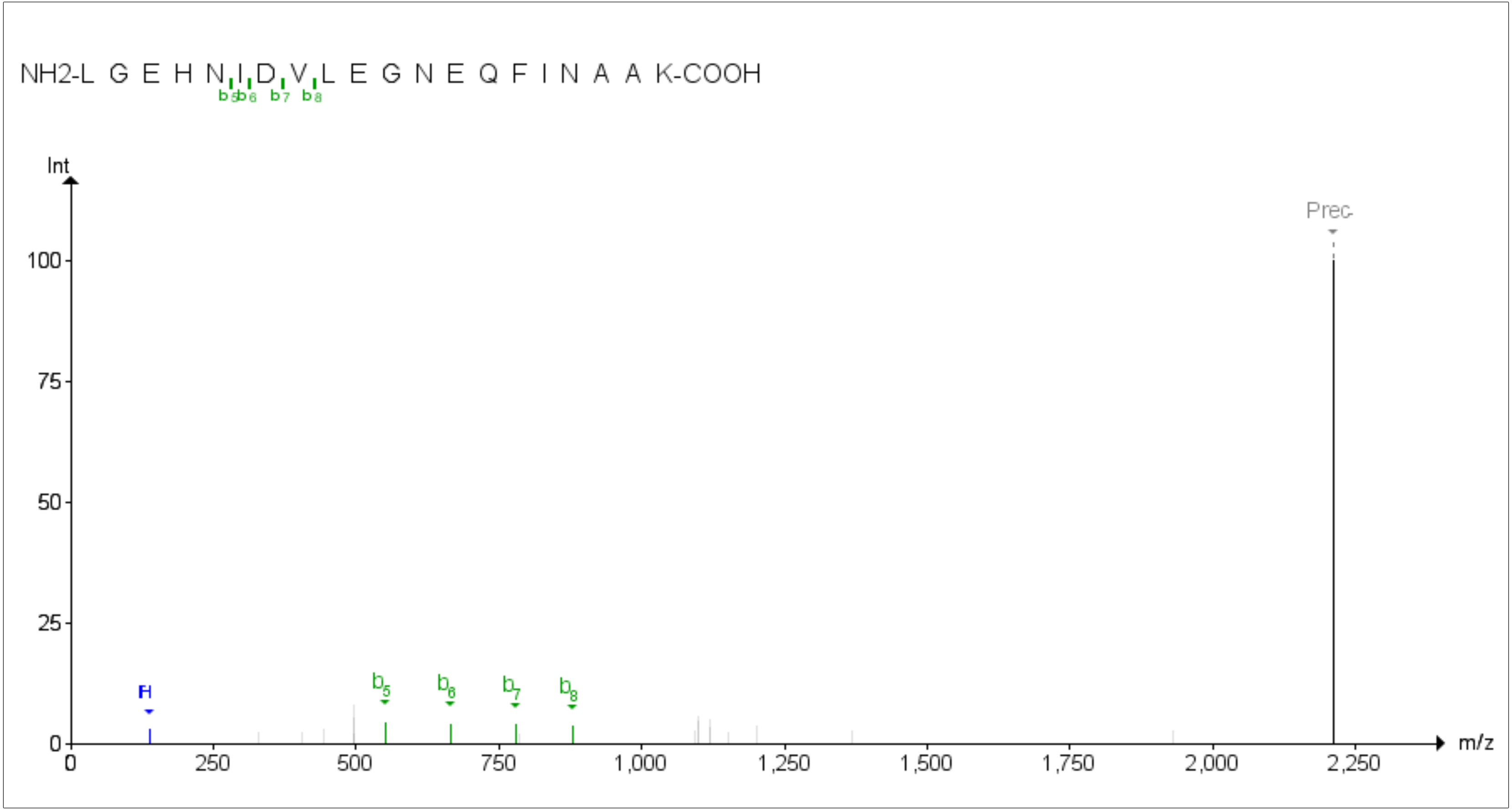

NH2-L G E H N I D V L E G N E Q F I N A A K-COOH
b5 b6 b7 b8
Int
100
75
50
25
0
Prec
H
b5
b6
b7
b8
0
250
500
750
1,000
1,250
1,500
1,750
2,000
2,250
m/z

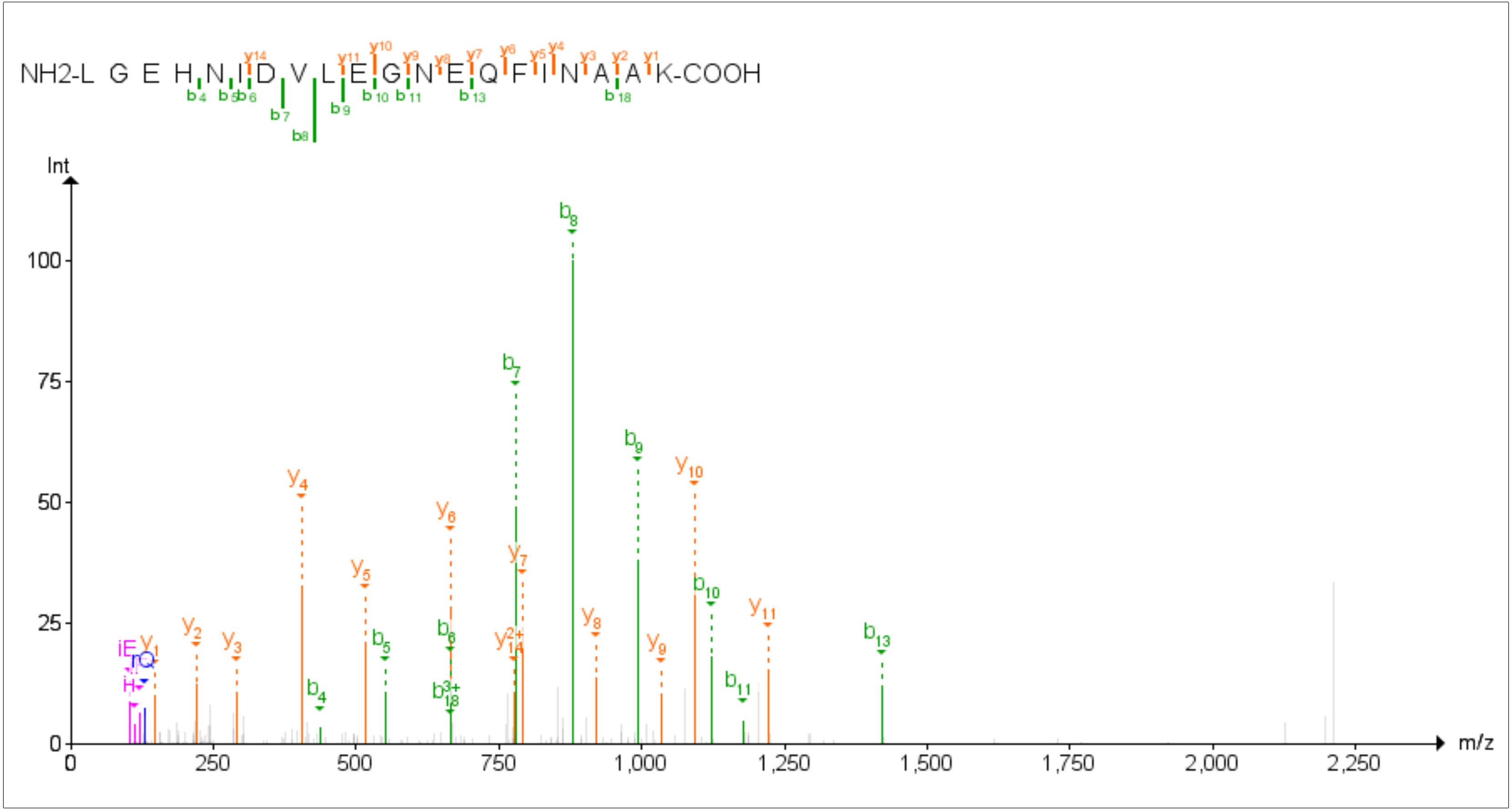
NH2-L G E H N I D V L E G N E Q F I N A A K-COOH
Int
m/z
0
25
50
75
100
250
500
750
1,000
1,250
1,500
1,750
2,000
2,250

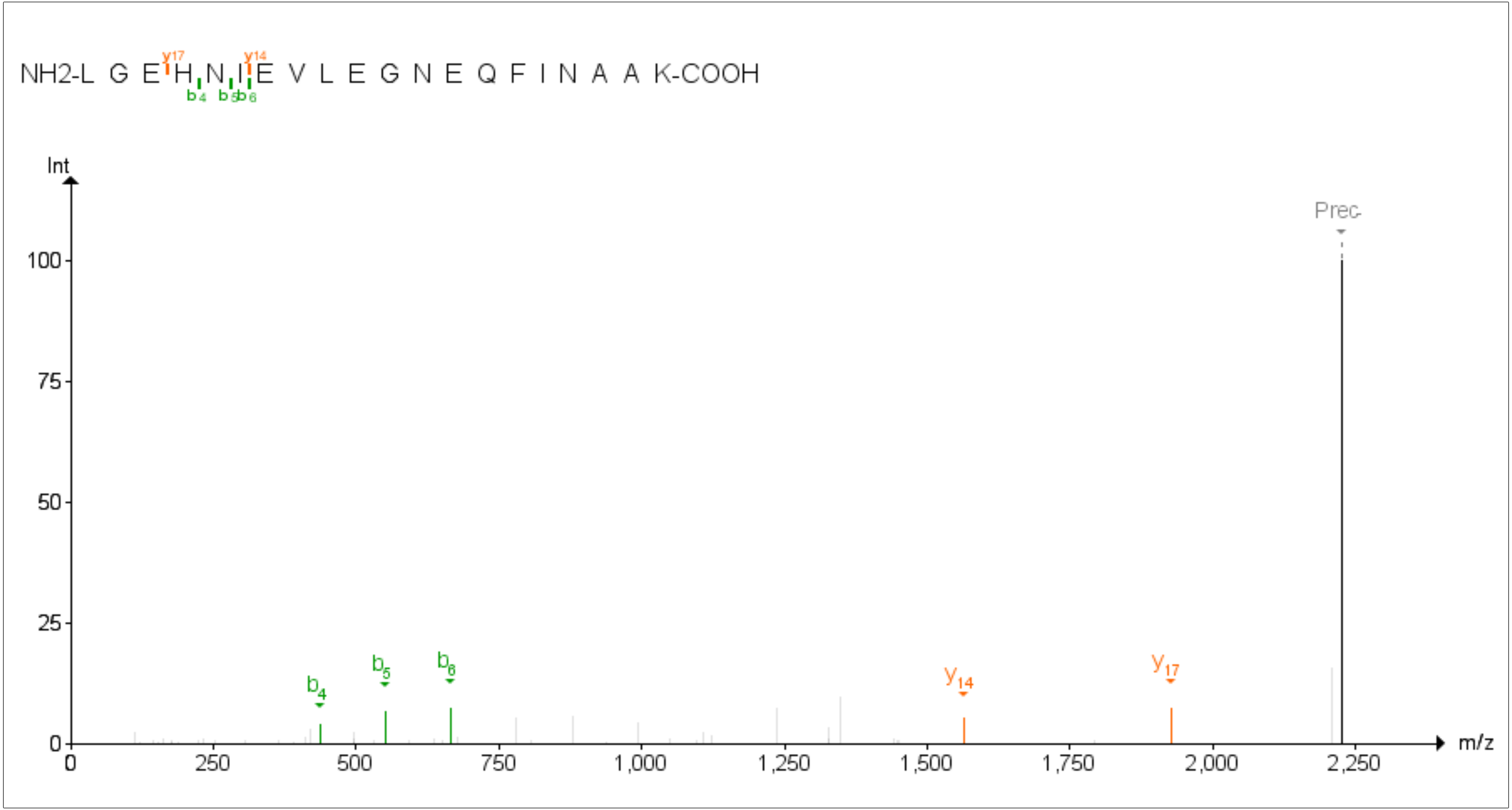

NH2-L G E H N I E V L E G N E Q F I N A A K-COOH
y17
y14
b4
b5
b6
Int
100
75
50
25
0
Prec.
b4
b5
b6
y14
y17
0
250
500
750
1,000
1,250
1,500
1,750
2,000
2,250
m/z

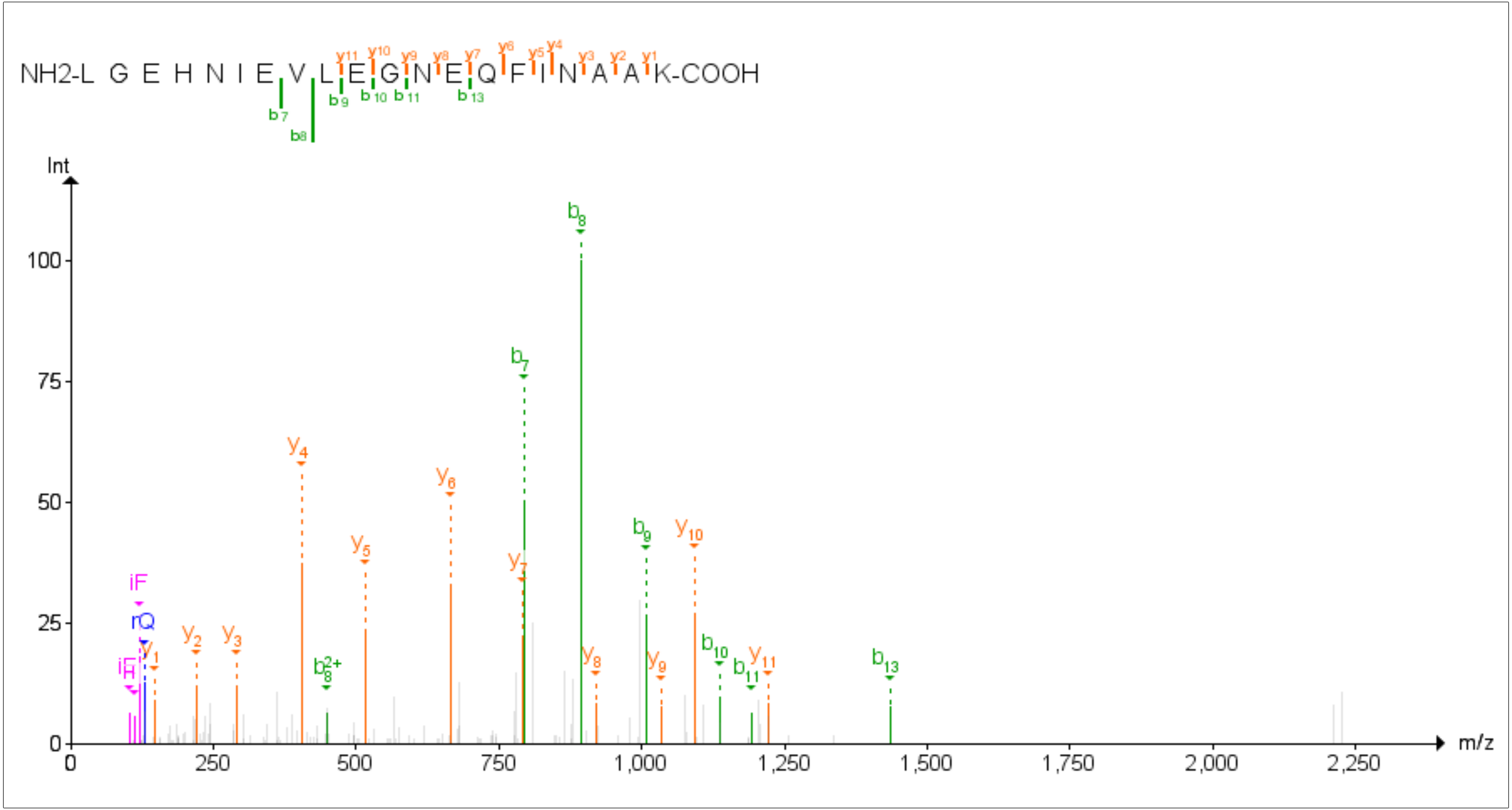

NH2-L G E H N I E V L E G N E Q F I N A A K-COOH
Int
m/z
b8
b7
y4
y6
y5
y7
b9
y10
iF
rQ
y2
y3
y1
iH
b8 2+
y8
y9
b10
b11
y11
b13

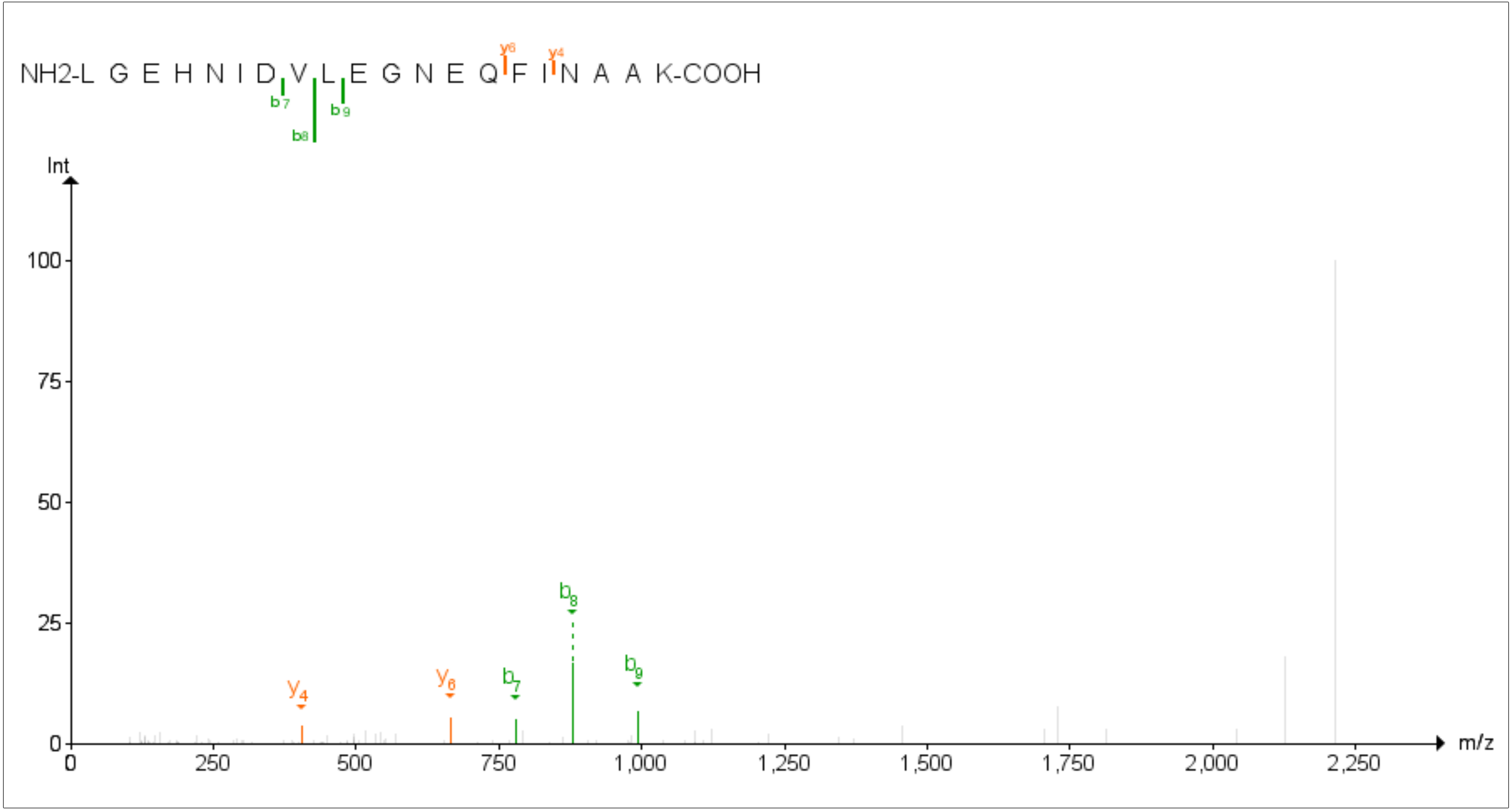

NH2-L G E H N I D V L E G N E Q F I N A A K-COOH
y6
y4
b7
b8
b9
Int
100
75
50
25
0
m/z
0
250
500
750
1,000
1,250
1,500
1,750
2,000
2,250
y4
y6
b7
b8
b9

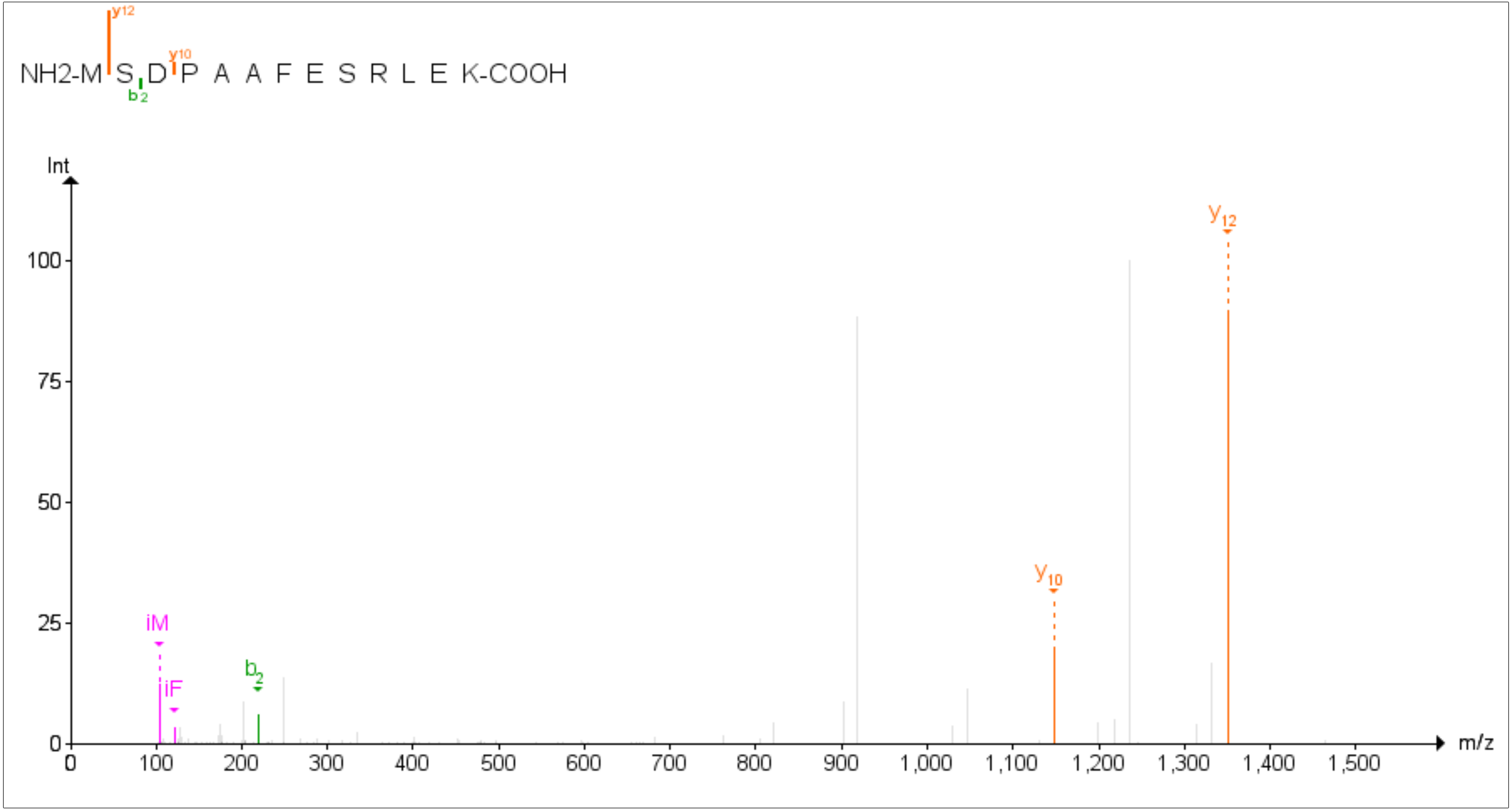
NH2-M S D P A A F E S R L E K-COOH
y12
y10
b2
Int
100
75
50
25
0
iM
iF
b2
y10
y12
0
100
200
300
400
500
600
700
800
900
1,000
1,100
1,200
1,300
1,400
1,500
m/z

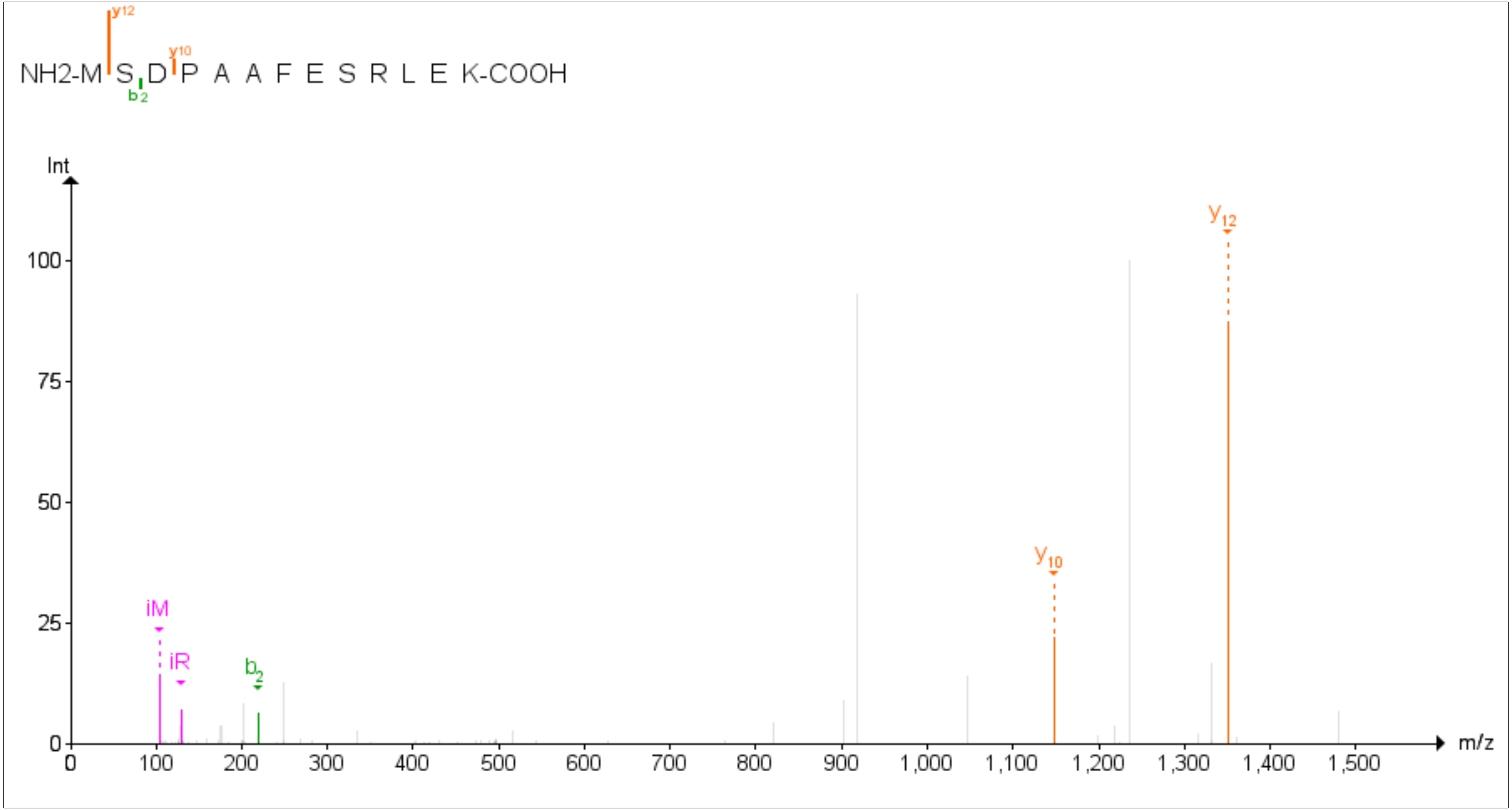

NH2-M S D P A A F E S R L E K-COOH
y12
y10
b2
Int
100
75
50
25
0
iM
iR
b2
y10
y12
0
100
200
300
400
500
600
700
800
900
1,000
1,100
1,200
1,300
1,400
1,500
m/z

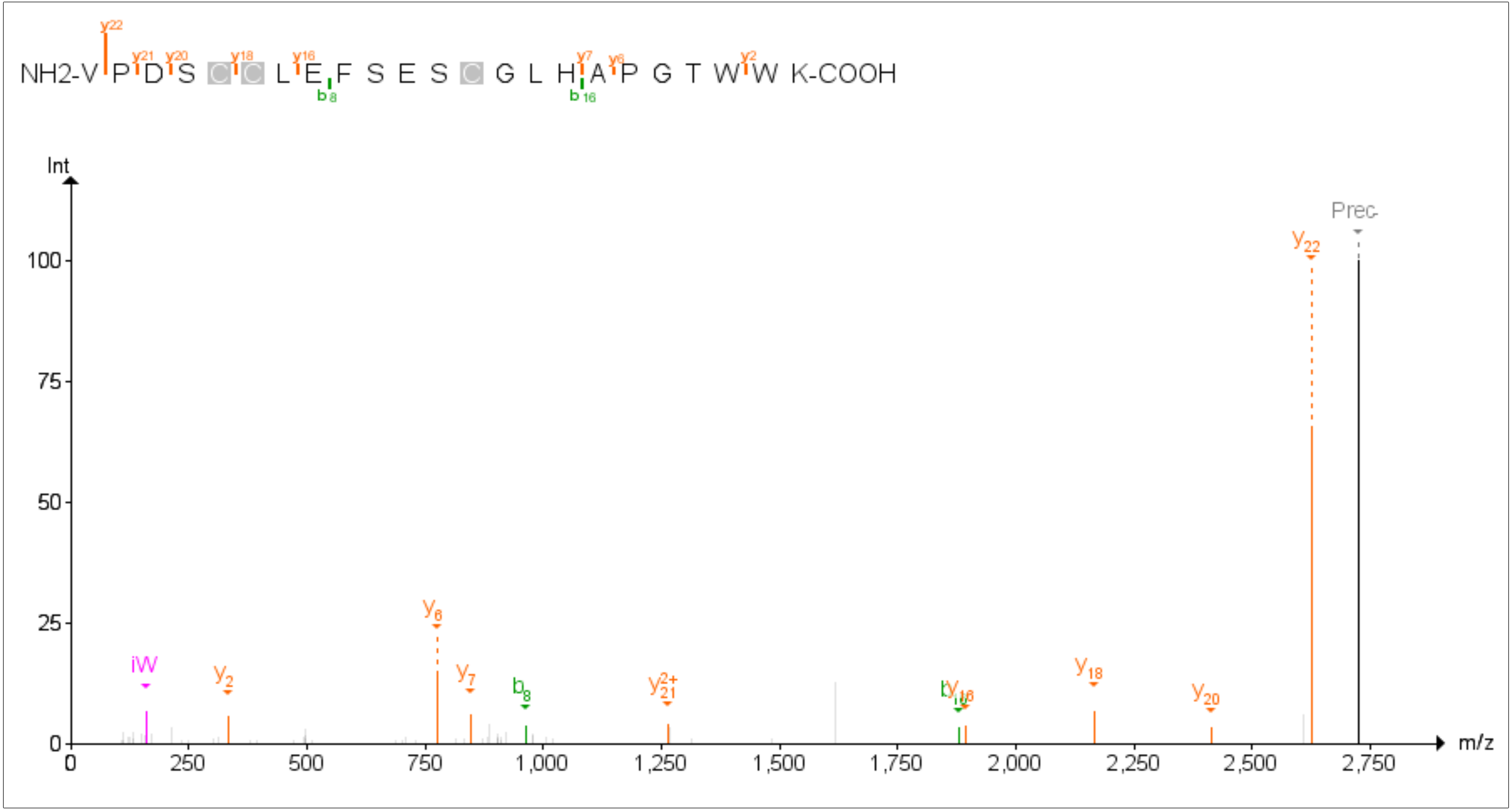

NH2-V P D S C C L E F S E S C G L H A P G T W W K-COOH
y22
y21
y20
y18
y16
y7
y6
y2
b8
b16
Int
100
75
50
25
0
Prec.
y22
y6
iW
y2
y7
b8
y21 2+
y18
y20
0
250
500
750
1,000
1,250
1,500
1,750
2,000
2,250
2,500
2,750
m/z

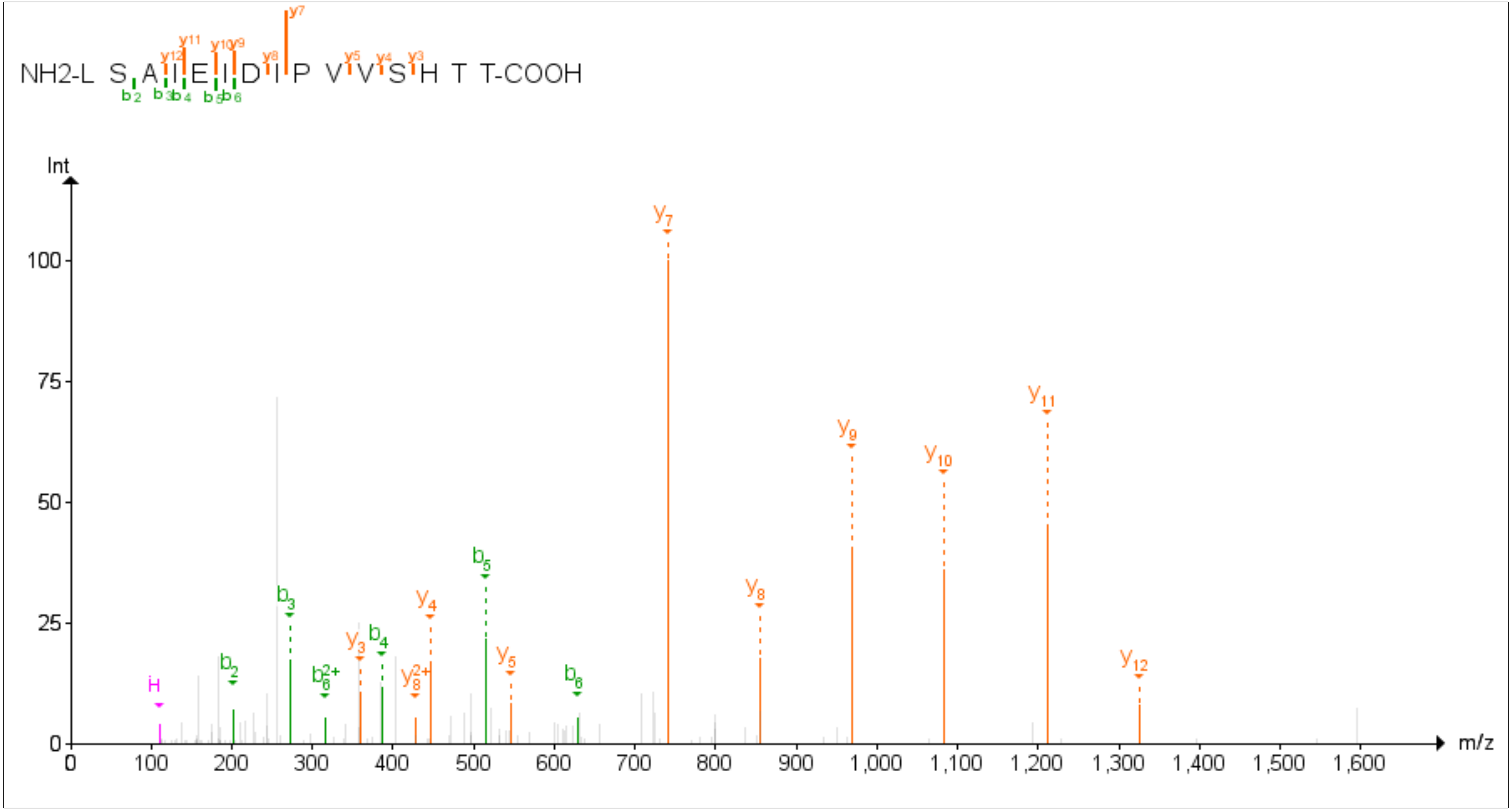
NH2-L S A I E I D I P V V S H T T-COOH
Int
m/z
y7
y11
y9
y10
y8
y4
y12
b5
y3
y4
b3
b4
y5
y3
b6
b2
y8 2+
b6 2+
H

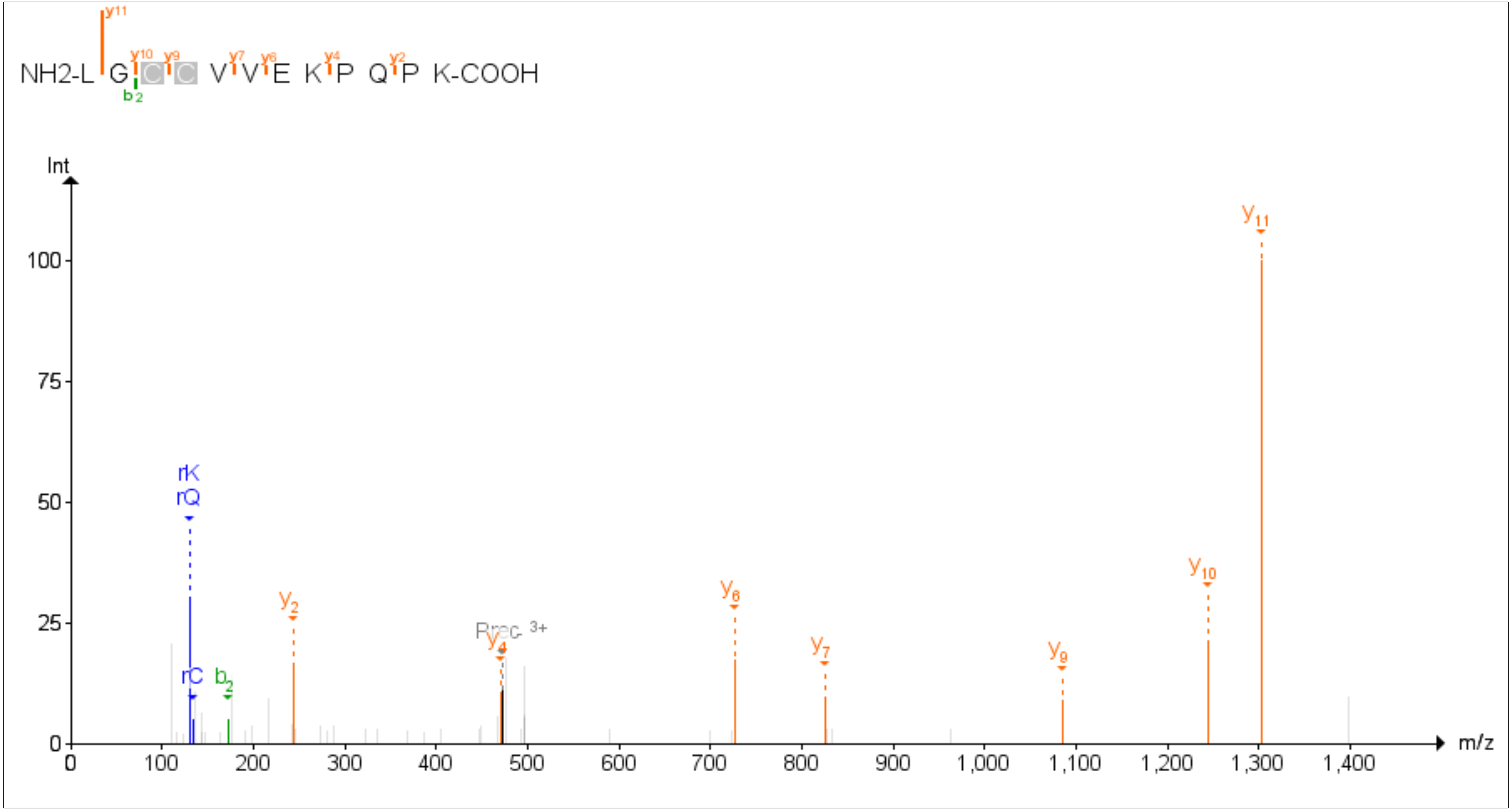

NH2-L G C C V V E K P Q P K-COOH
y11
y10
y9
y7
y6
y4
y2
b2
Int
m/z
rK
rQ
rC
Prec 3+

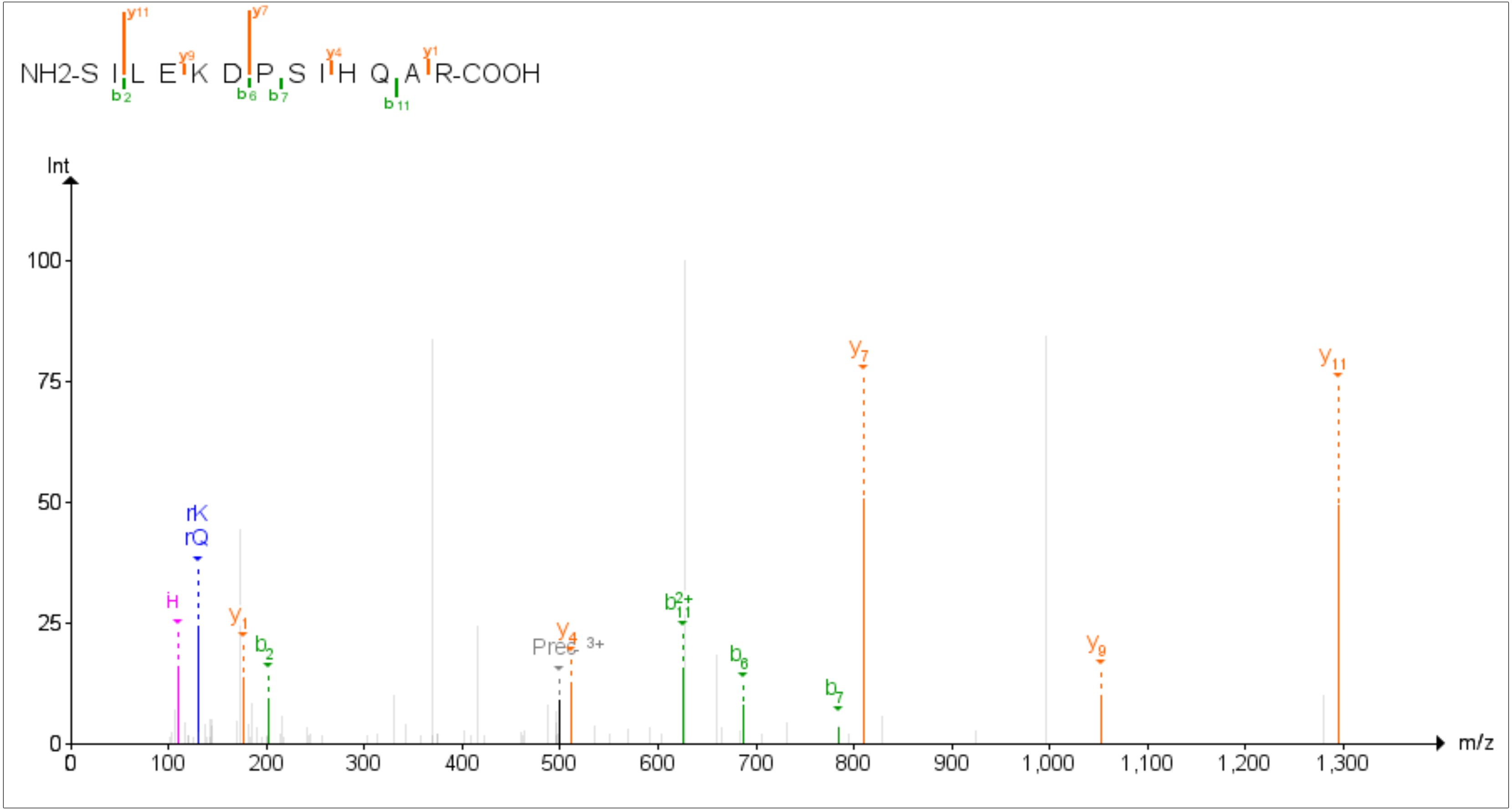

NH2-S I L E K D P S I H Q A R-COOH
y11
y9
y7
y4
y1
b2
b6
b7
b11
Int
m/z
100
75
50
25
0
0
100
200
300
400
500
600
700
800
900
1,000
1,100
1,200
1,300
rK
rQ
H
y1
b2
Prec 3+
y4
b11 2+
b6
b7
y7
y9
y11

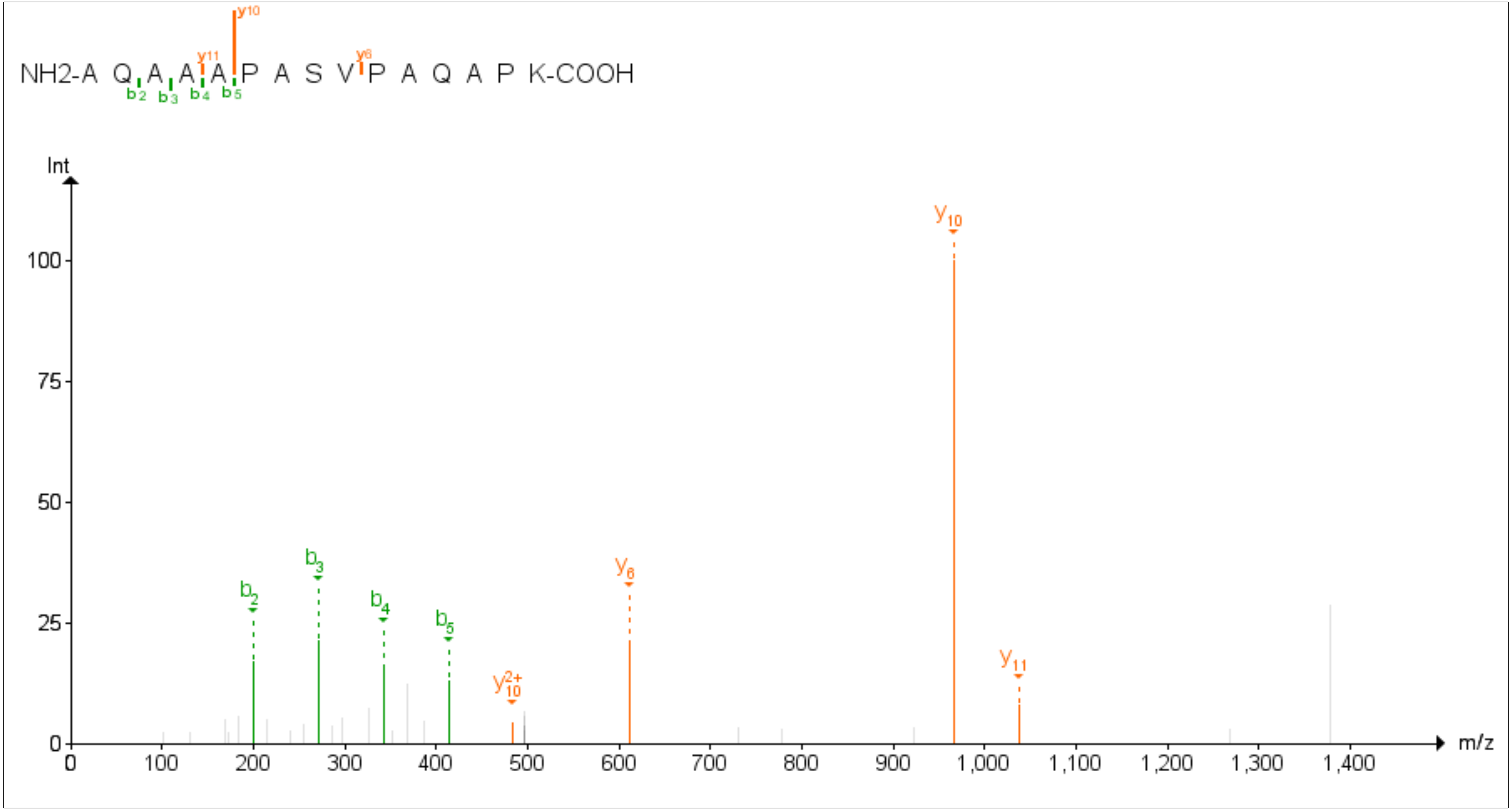

NH2-A Q A A A P A S V P A Q A P K-COOH
y10
y11
y6
b2
b3
b4
b5
Int
100
75
50
25
0
m/z
0
100
200
300
400
500
600
700
800
900
1,000
1,100
1,200
1,300
1,400
y10
b2
b3
b4
b5
y10 2+
y6
y11

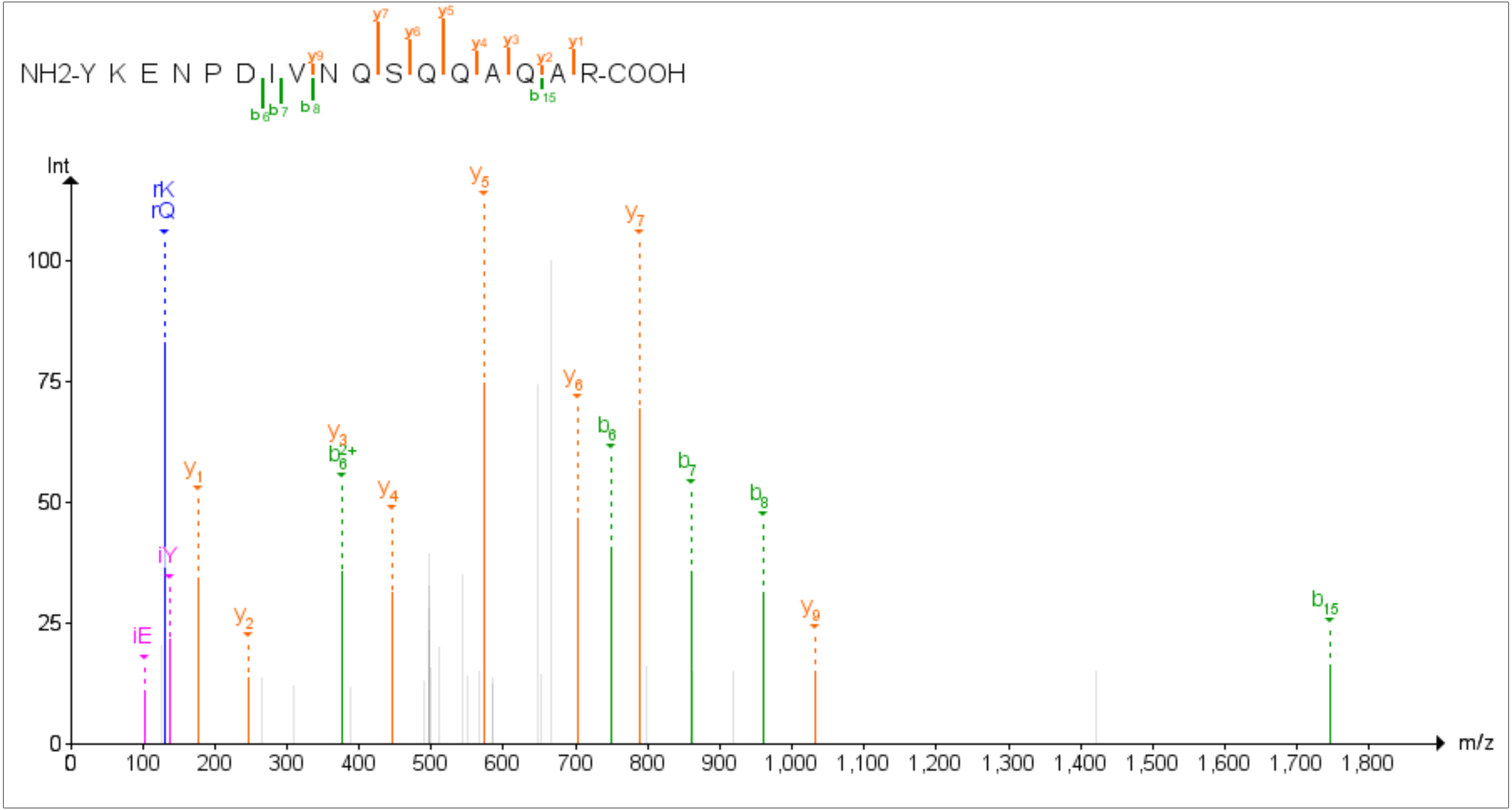
NH2-Y K E N P D I V N Q S Q Q A Q A R-COOH
Int
m/z
rK
rQ
iY
iE
y1
y2
y3
y4
y5
y6
y7
y9
b6 2+
b6
b7
b8
b15

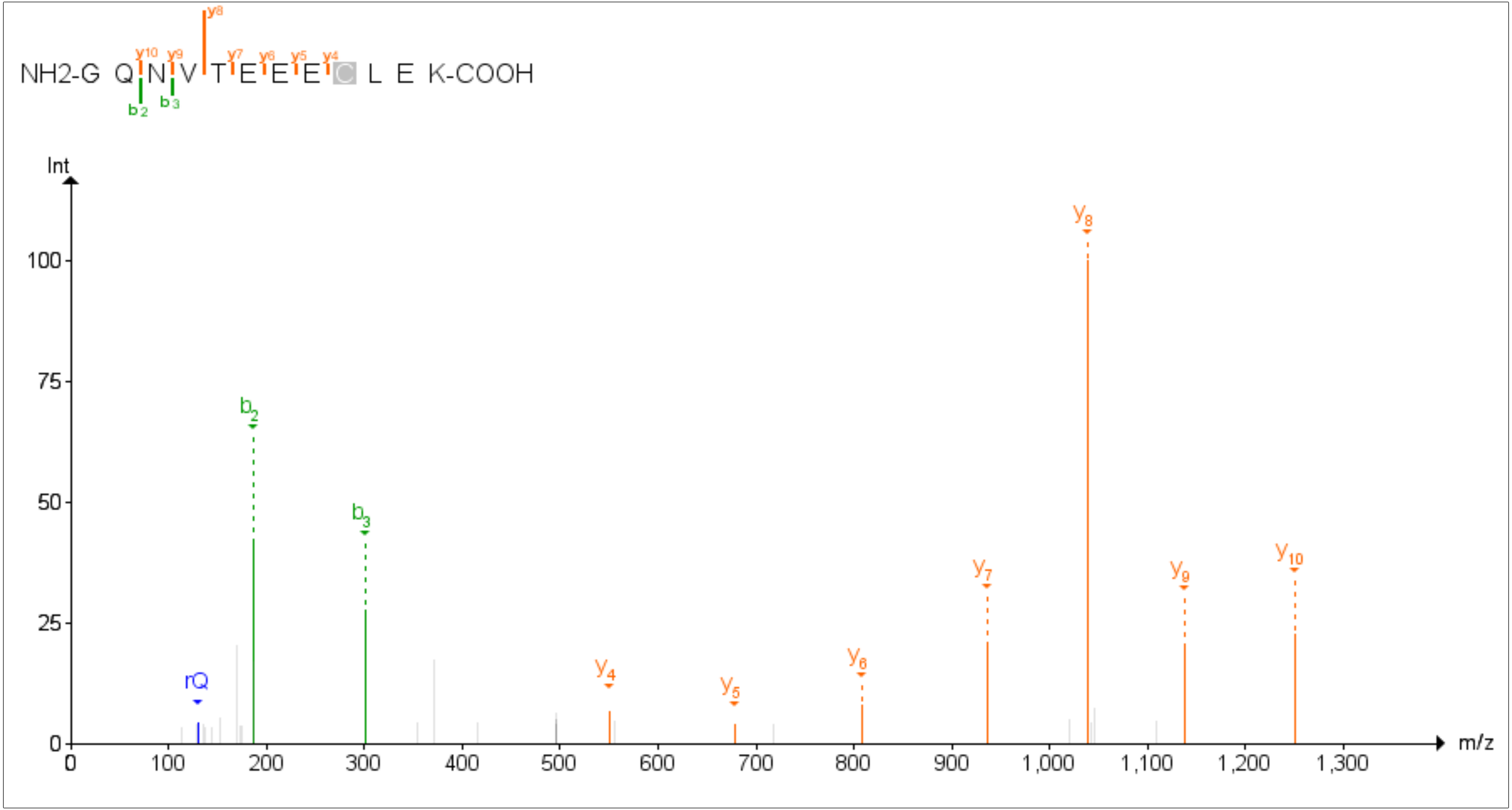

NH2-G Q N V T E E E C L E K-COOH
y10 y9 y8 y7 y6 y5 y4
b2 b3
Int
100
75
50
25
0
rQ
b2
b3
y4
y5
y6
y7
y8
y9
y10
0 100 200 300 400 500 600 700 800 900 1,000 1,100 1,200 1,300
m/z

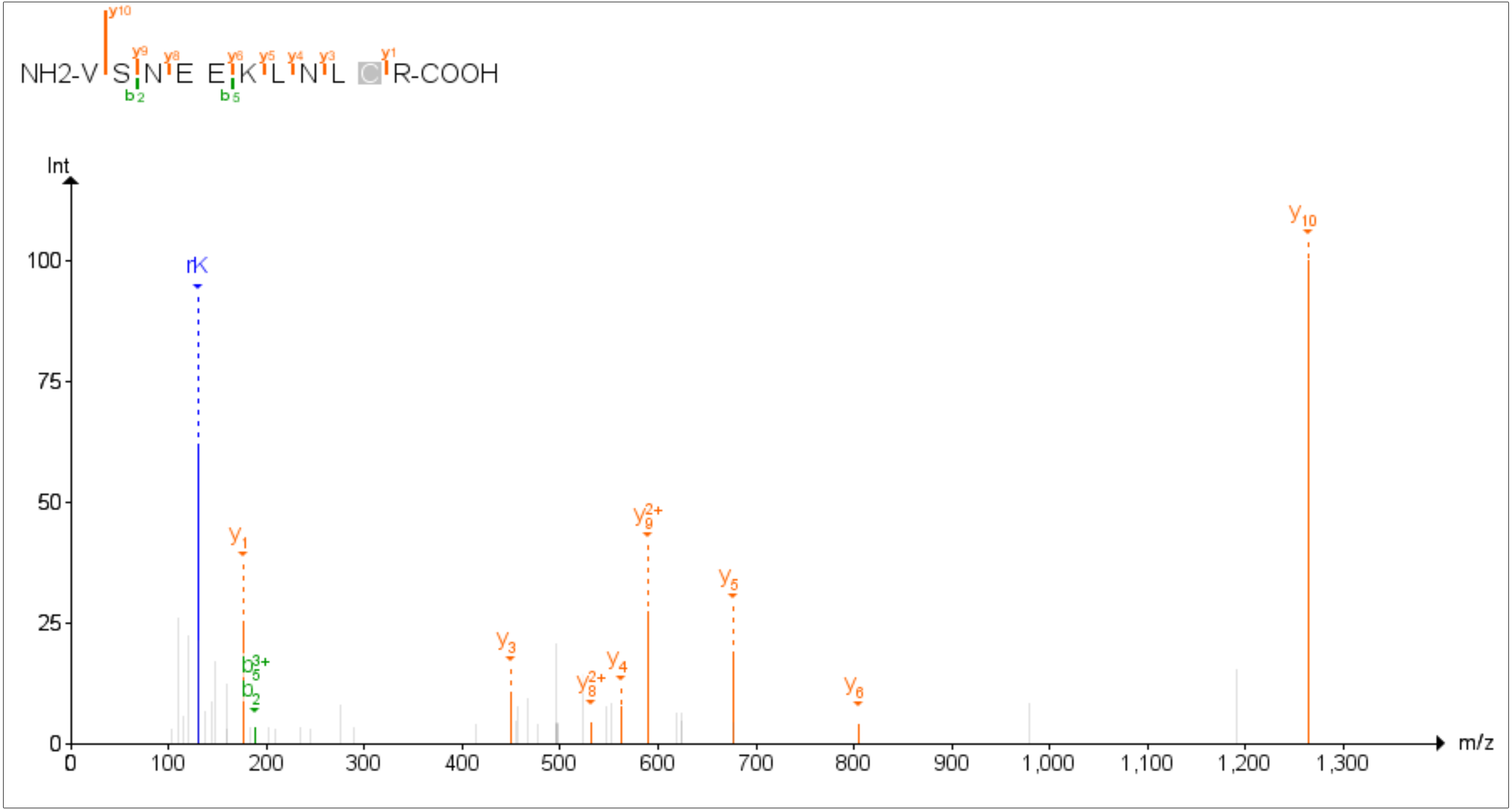

NH2-V S N E E K L N L C R-COOH
y10
y9
y8
y6
y5
y4
y3
y1
b2
b5
Int
m/z
rK
y10
y1
b5 3+
b2
y3
y8 2+
y4
y9 2+
y5
y6

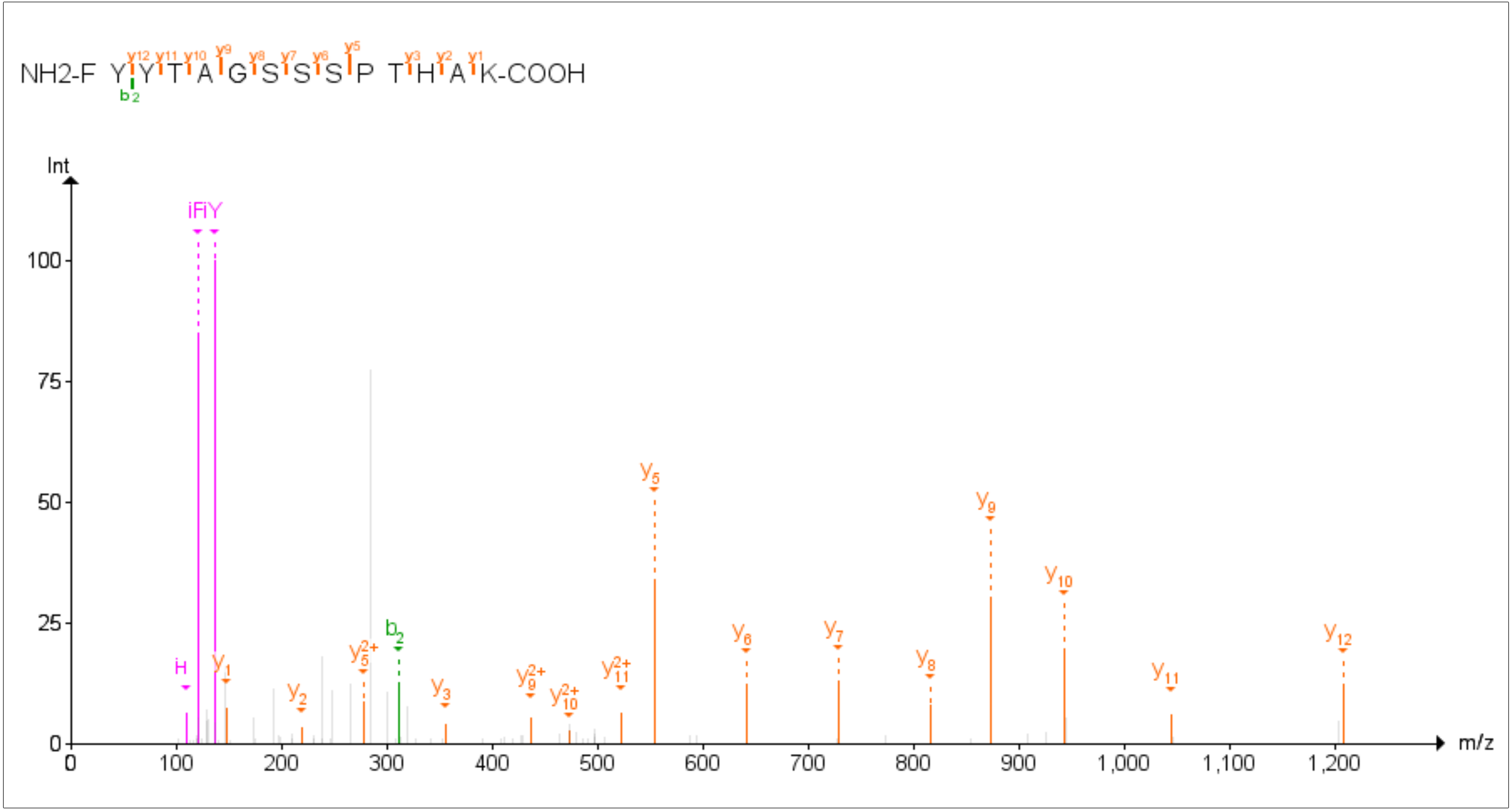

NH2-F Y Y T A G S S S P T H A K-COOH
y12 y11 y10 y9 y8 y7 y6 y5 y3 y2 y1
b2
Int
100
75
50
25
0
iFiY
iH
y1
y2
y5 2+
b2
y3
y9 2+
y10 2+
y11 2+
y5
y6
y7
y8
y9
y10
y11
y12
0
100
200
300
400
500
600
700
800
900
1,000
1,100
1,200
m/z

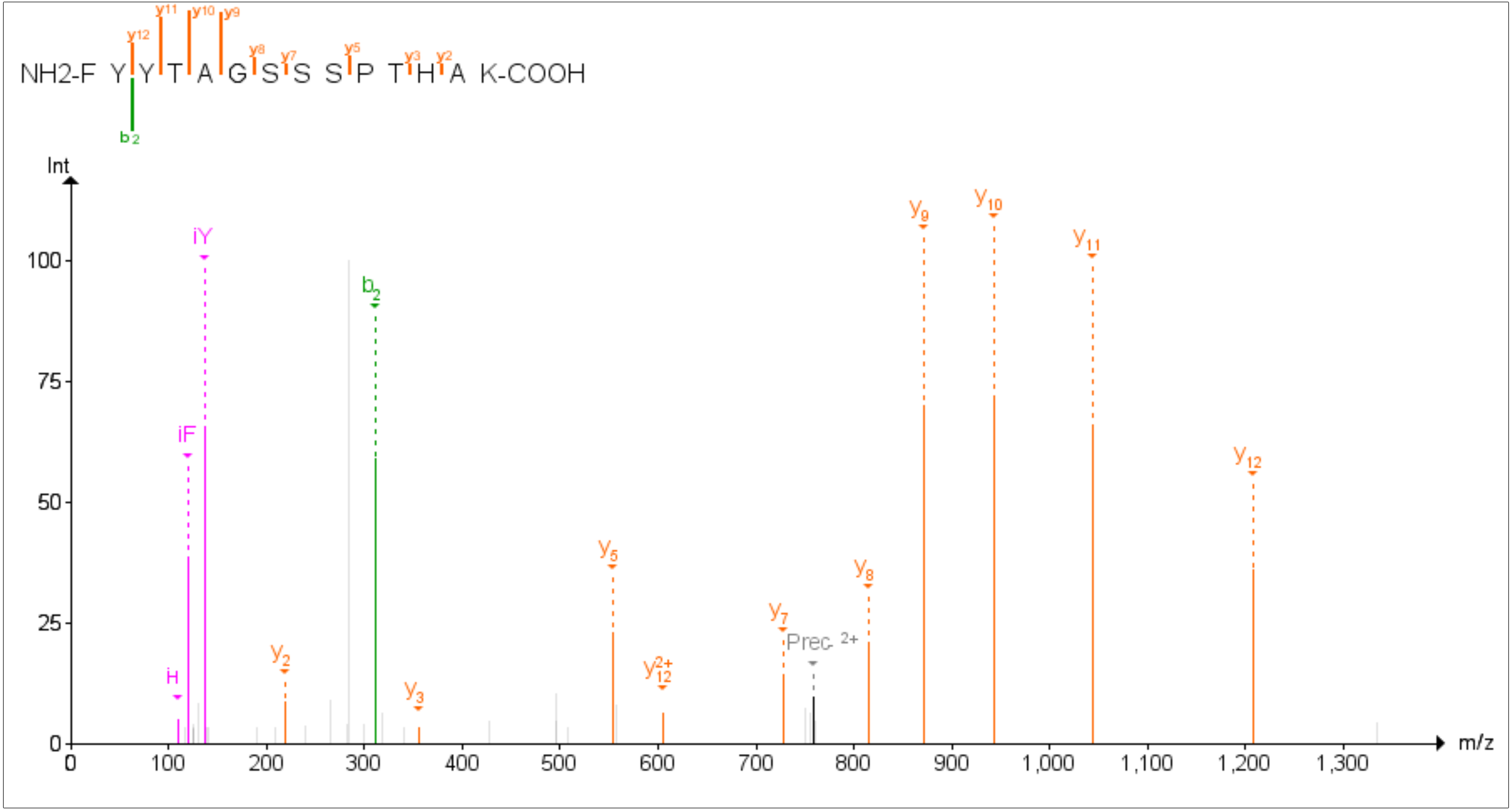

NH2-F Y Y T A G S S S P T H A K-COOH
y12
y11
y10
y9
y8
y7
y5
y3
y2
b2
Int
100
75
50
25
0
iY
iF
iH
b2
y2
y3
y5
y12 2+
y7
Prec 2+
y8
y9
y10
y11
y12
0
100
200
300
400
500
600
700
800
900
1,000
1,100
1,200
1,300
m/z

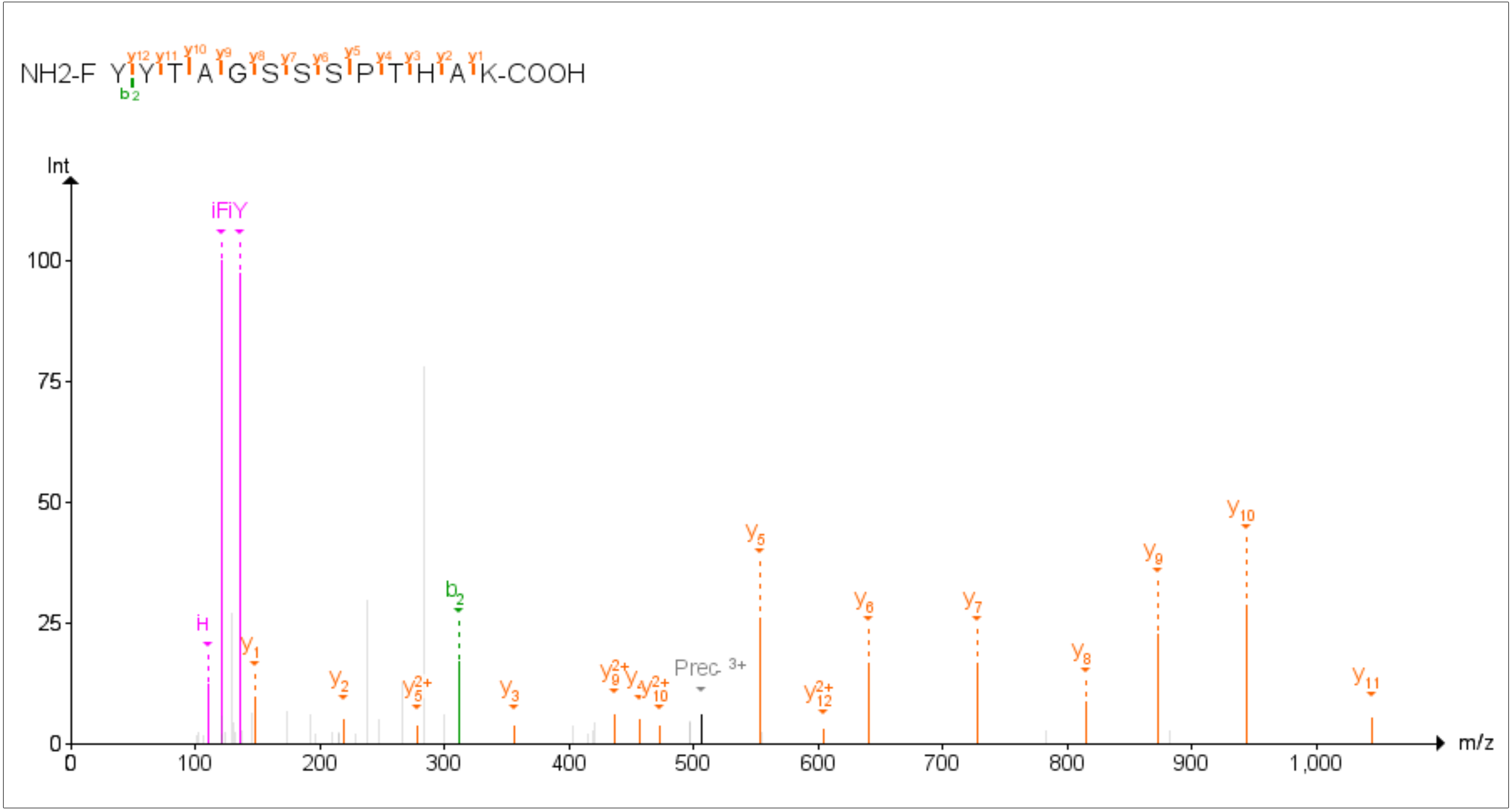

NH2-F Y Y T A G S S S P T H A K-COOH
Int
m/z
iFiY
iH
Prec 3+

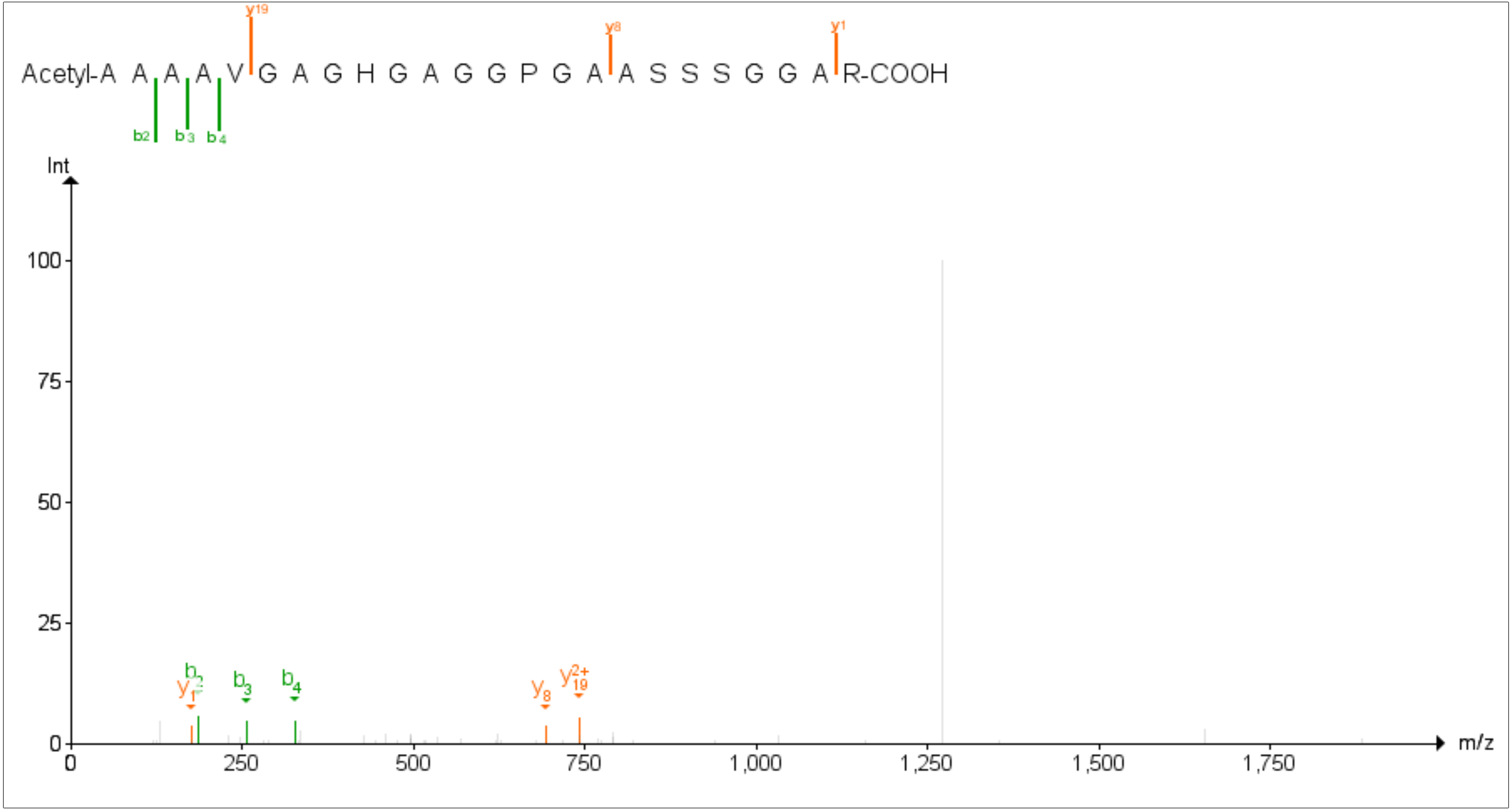

Acetyl-A A A A V G A G H G A G G P G A A S S S G G A R-COOH
y19
y8
y1
b2
b3
b4
Int
100
75
50
25
0
m/z
0
250
500
750
1,000
1,250
1,500
1,750

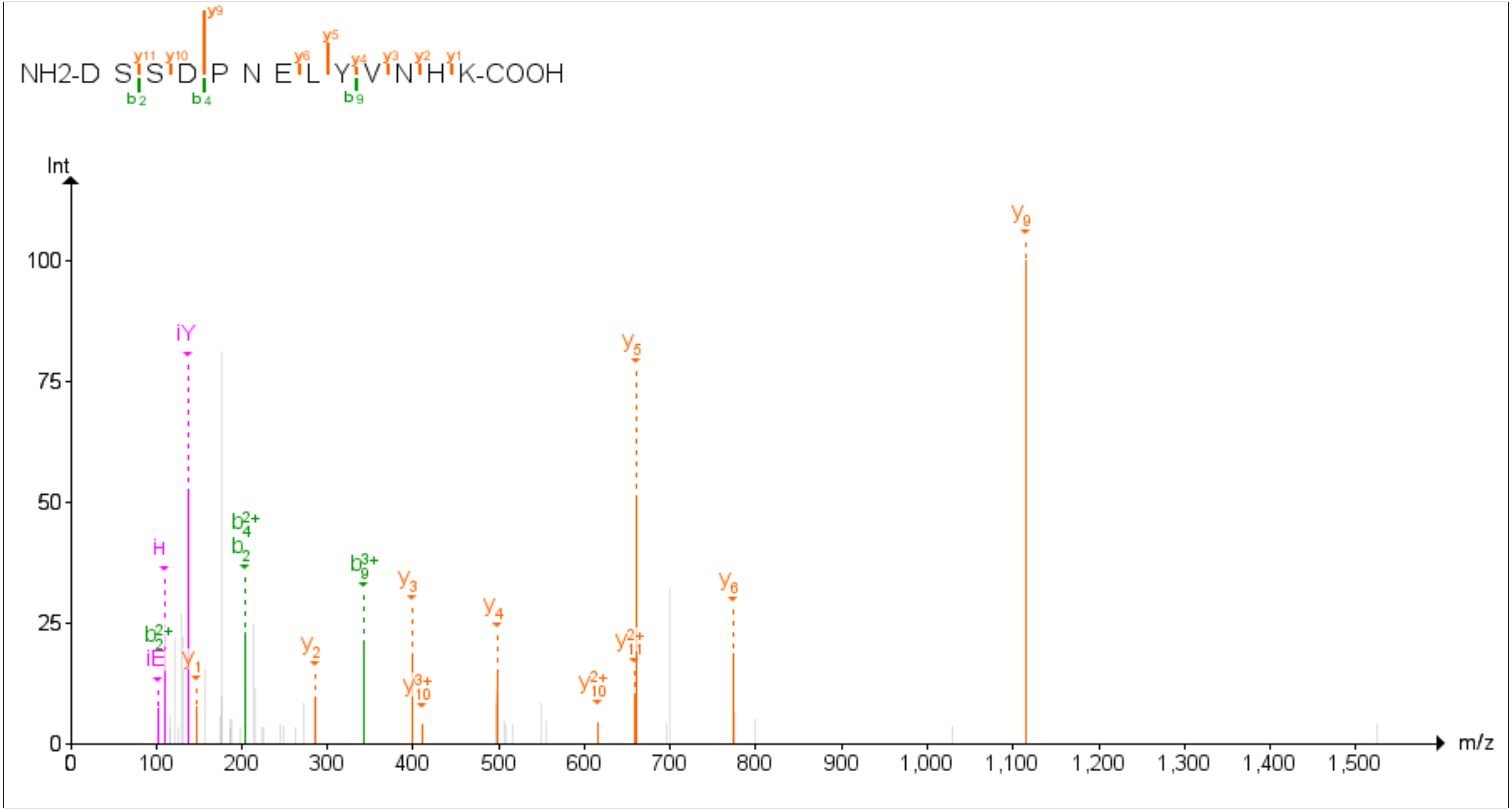

NH2-D S S D P N E L Y V N H K-COOH
y11 y10 y9 y6 y5 y4 y3 y2 y1
b2 b4 b9
Int
m/z
y9
y5
iY
iH
b4 2+
b2
b9 3+
y3
y6
y4
b2 2+
iE
y1
y2
y10 3+
y11 2+
y10 2+
0 25 50 75 100
0 100 200 300 400 500 600 700 800 900 1,000 1,100 1,200 1,300 1,400 1,500

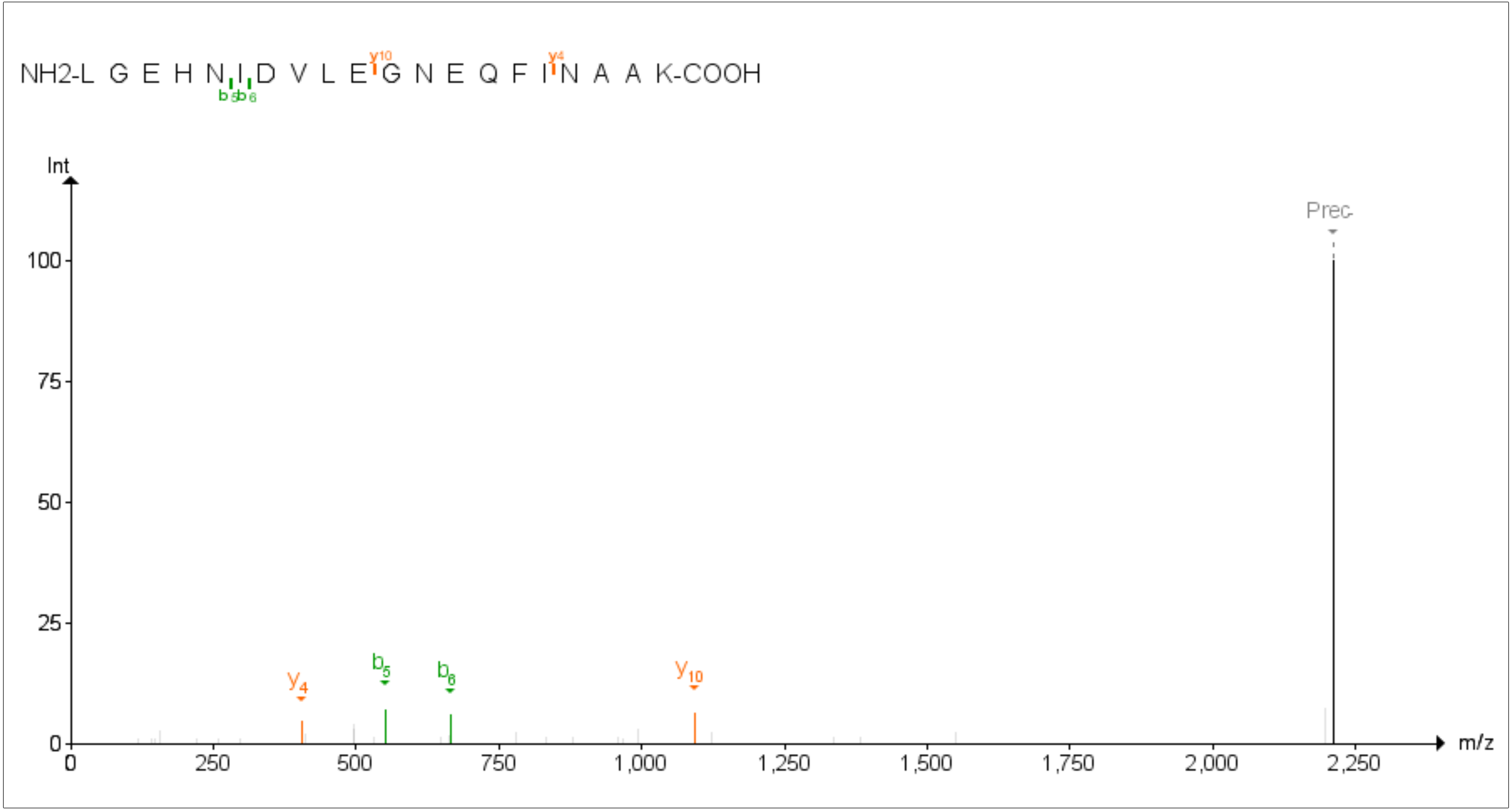

NH2-L G E H N I D V L E G N E Q F I N A A K-COOH
y10
y4
b5
b6
Int
100
75
50
25
0
Prec
y4
b5
b6
y10
m/z
0
250
500
750
1,000
1,250
1,500
1,750
2,000
2,250

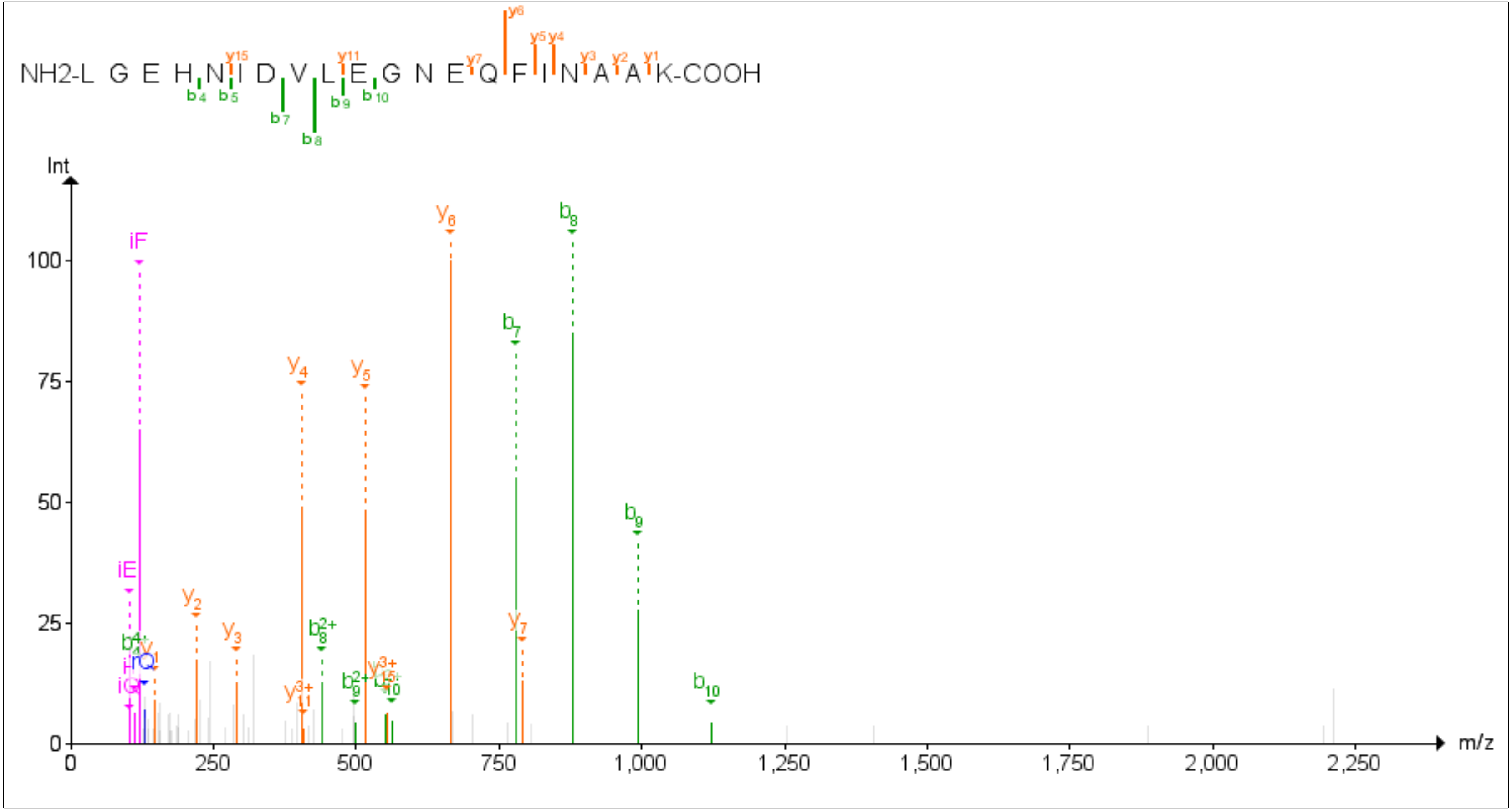
NH2-L G E H N I D V L E G N E Q F I N A A K-COOH
y15 y11 y7 y6 y5 y4 y3 y2 y1
b4 b5 b7 b8 b9 b10
Int
m/z
y6
b8
iF
b7
y4
y5
b9
iE
y2
y3
y7
b10
0
25
50
75
100
0
250
500
750
1,000
1,250
1,500
1,750
2,000
2,250

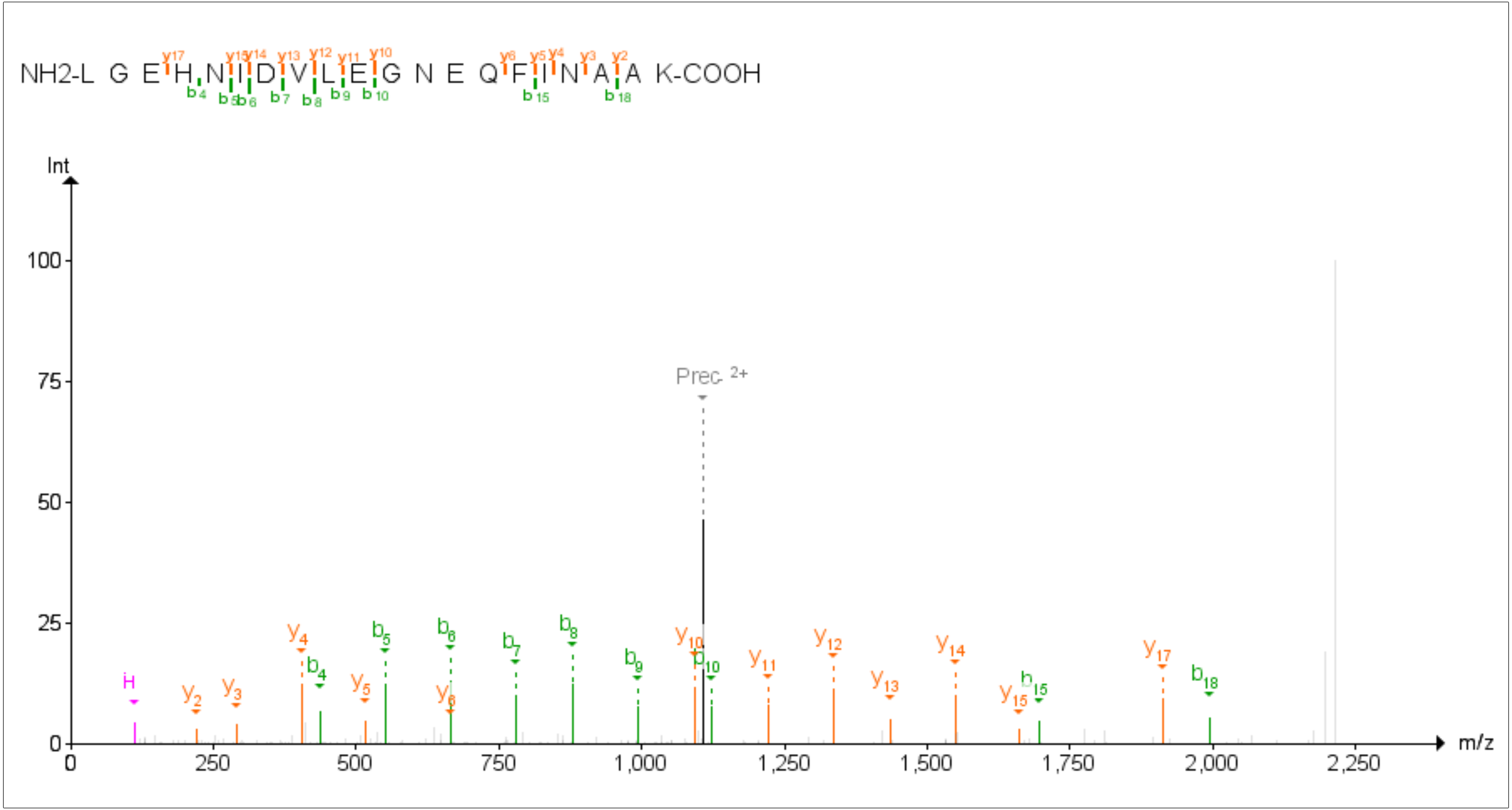

NH2-L G E H N I I D V L E G N E Q F I I N A A K-COOH
Int
Prec 2+
m/z

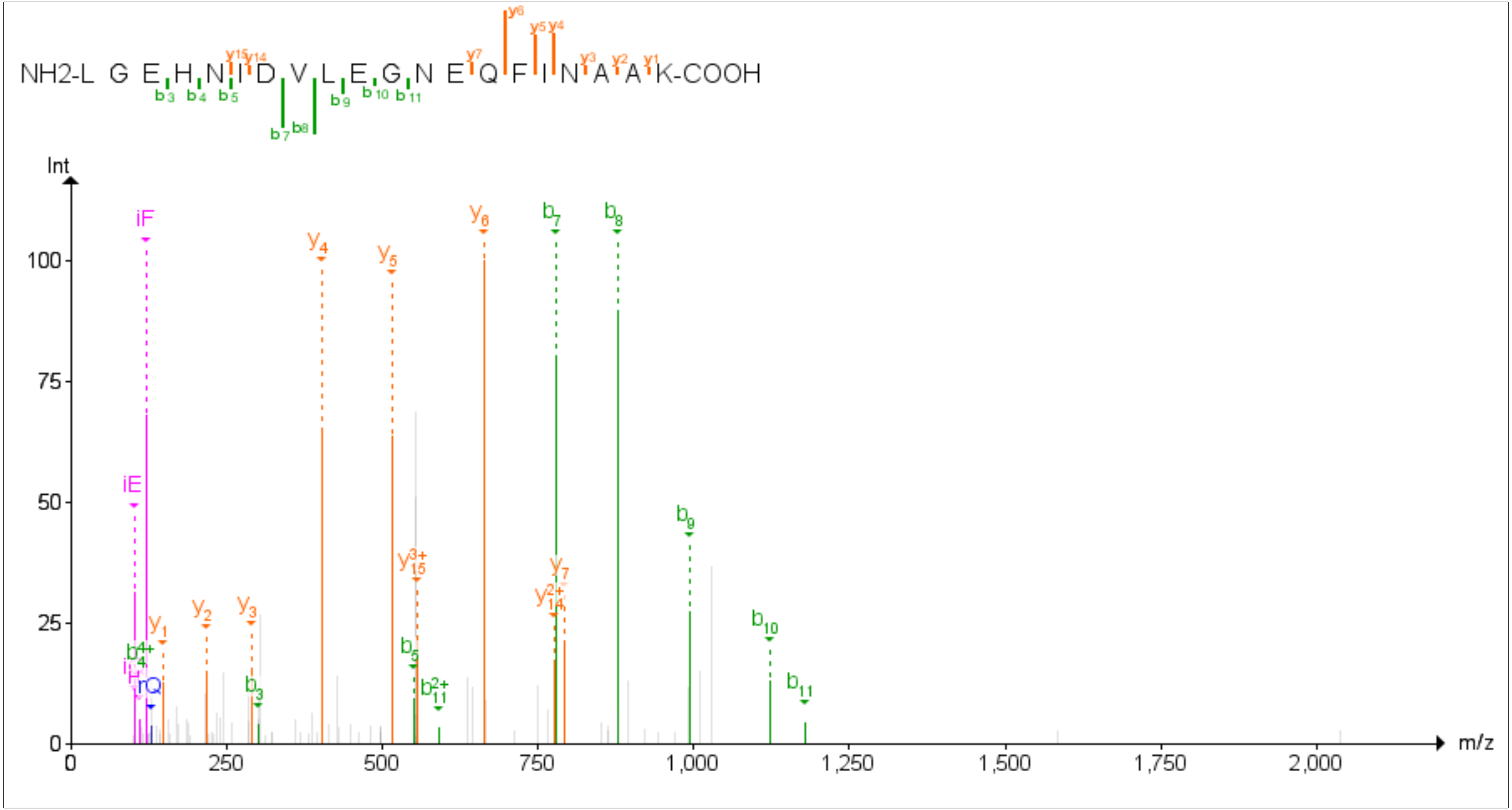
NH2-L G E H N I D V L E G N E Q F I N A A K-COOH
Int
m/z
0
25
50
75
100
0
250
500
750
1,000
1,250
1,500
1,750
2,000
iF
iE
iH
rQ
y1
y2
y3
y4
y5
y6
y7
y15 3+
y14 2+
b4 4+
b3
b5
b7
b8
b9
b10
b11
b11 2+

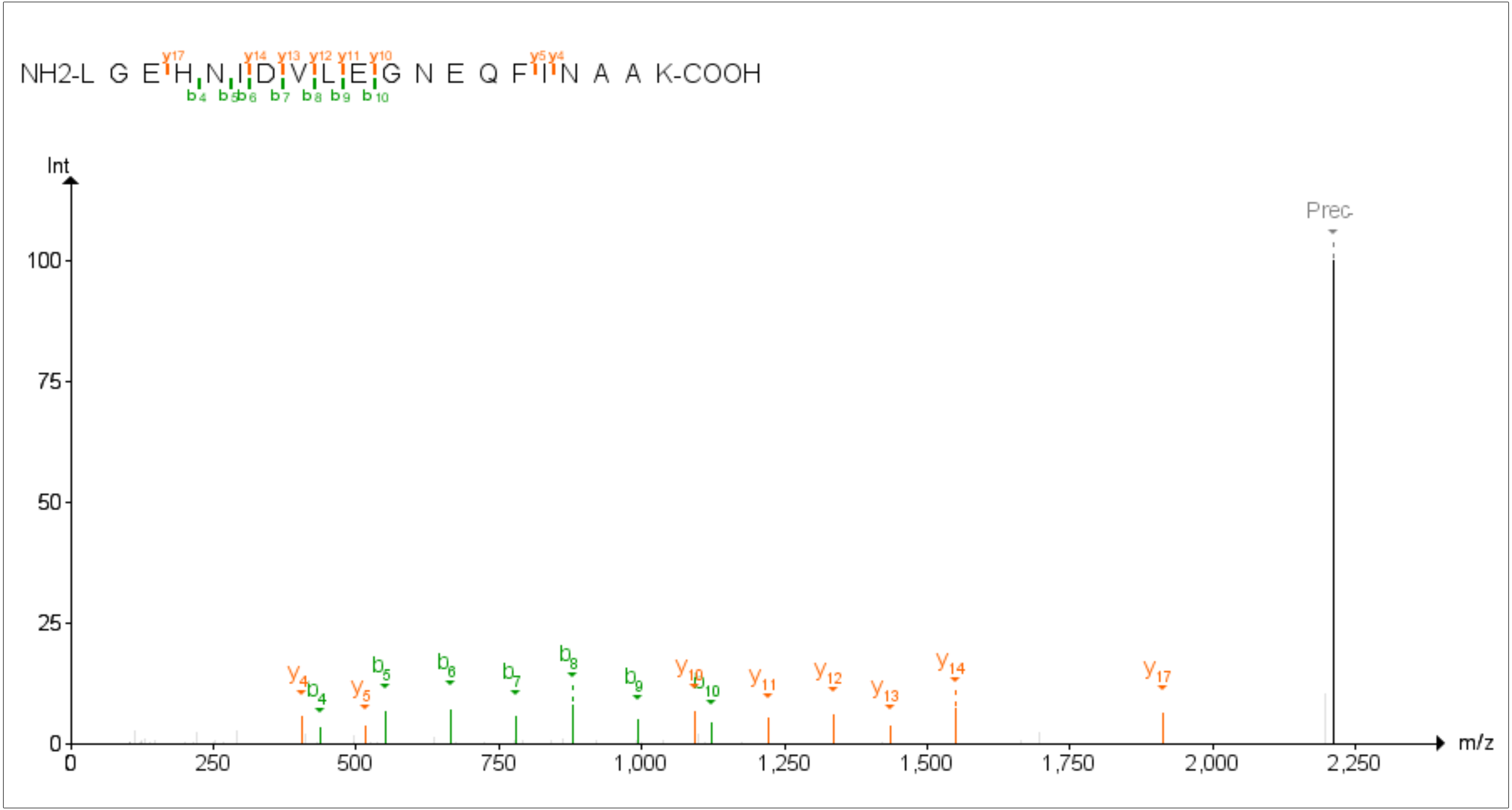

NH2-L G E H N I D V L E G N E Q F I N A A K-COOH
y17 y14 y13 y12 y11 y10 y5 y4
b4 b5 b6 b7 b8 b9 b10
Int
Prec
100
75
50
25
0
y4 b4 y5 b5 b6 b7 b8 b9 y10 b10 y11 y12 y13 y14 y17
0
250
500
750
1,000
1,250
1,500
1,750
2,000
2,250
m/z

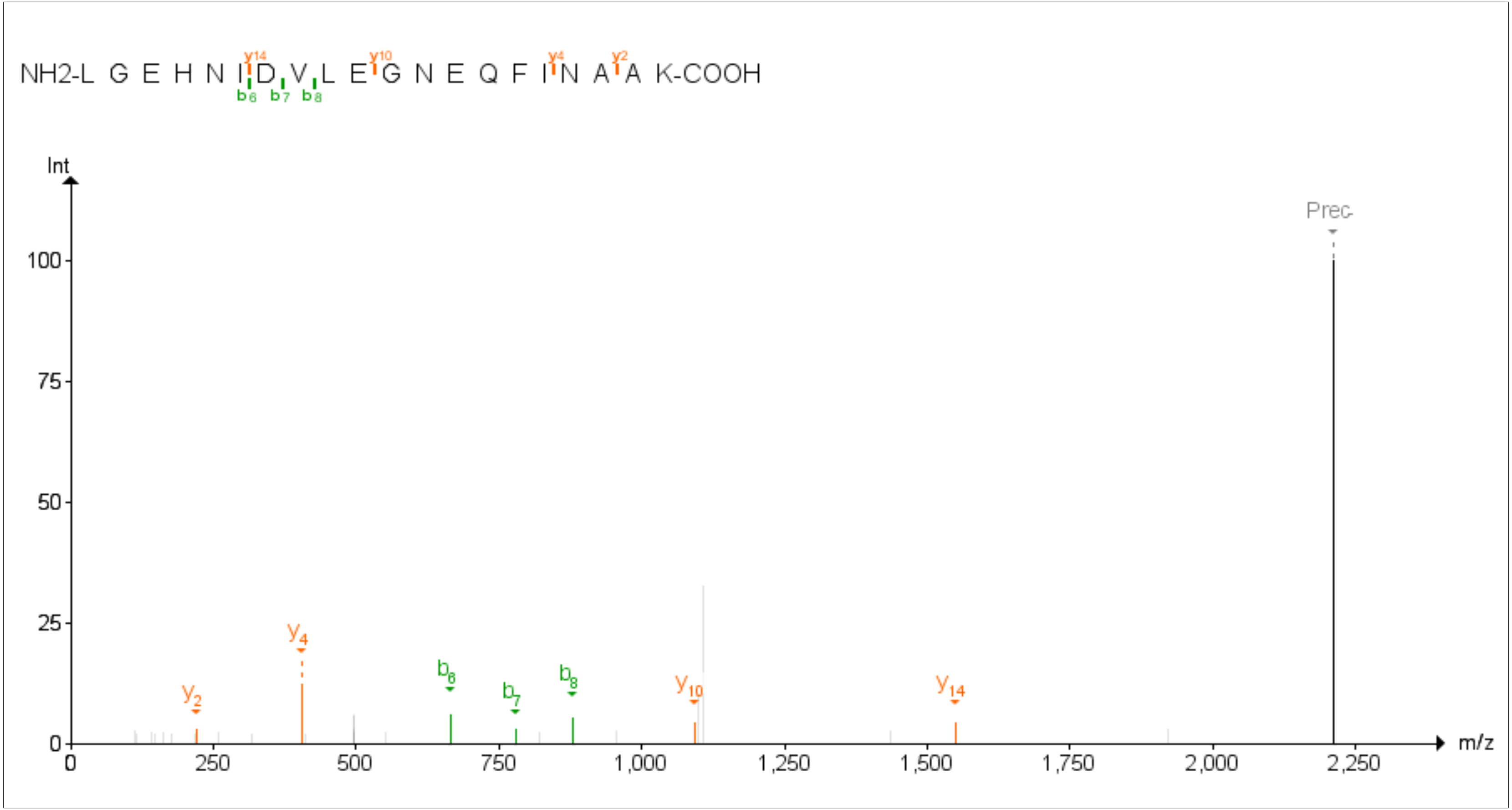

NH2-L G E H N I D V L E G N E Q F I N A A K-COOH
y14 y10 y4 y2
b6 b7 b8
Int
Prec
100
75
50
25
0
y4
y2
b6
b7
b8
y10
y14
0
250
500
750
1,000
1,250
1,500
1,750
2,000
2,250
m/z

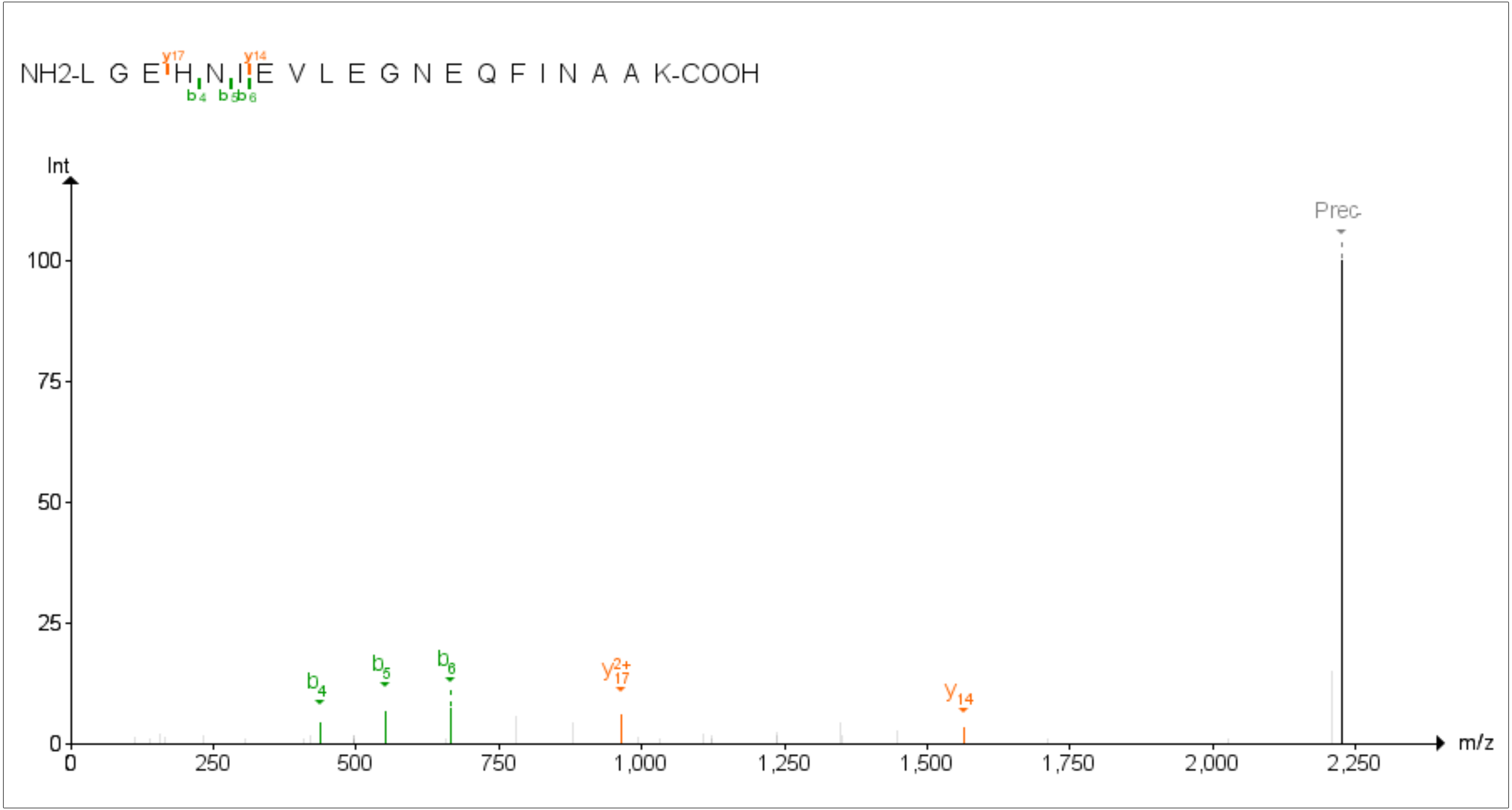
NH2-L G E H N I E V L E G N E Q F I N A A K-COOH
y17
y14
b4
b5
b6
Int
Prec.
100
75
50
25
0
b4
b5
b6
y17 2+
y14
m/z
0
250
500
750
1,000
1,250
1,500
1,750
2,000
2,250

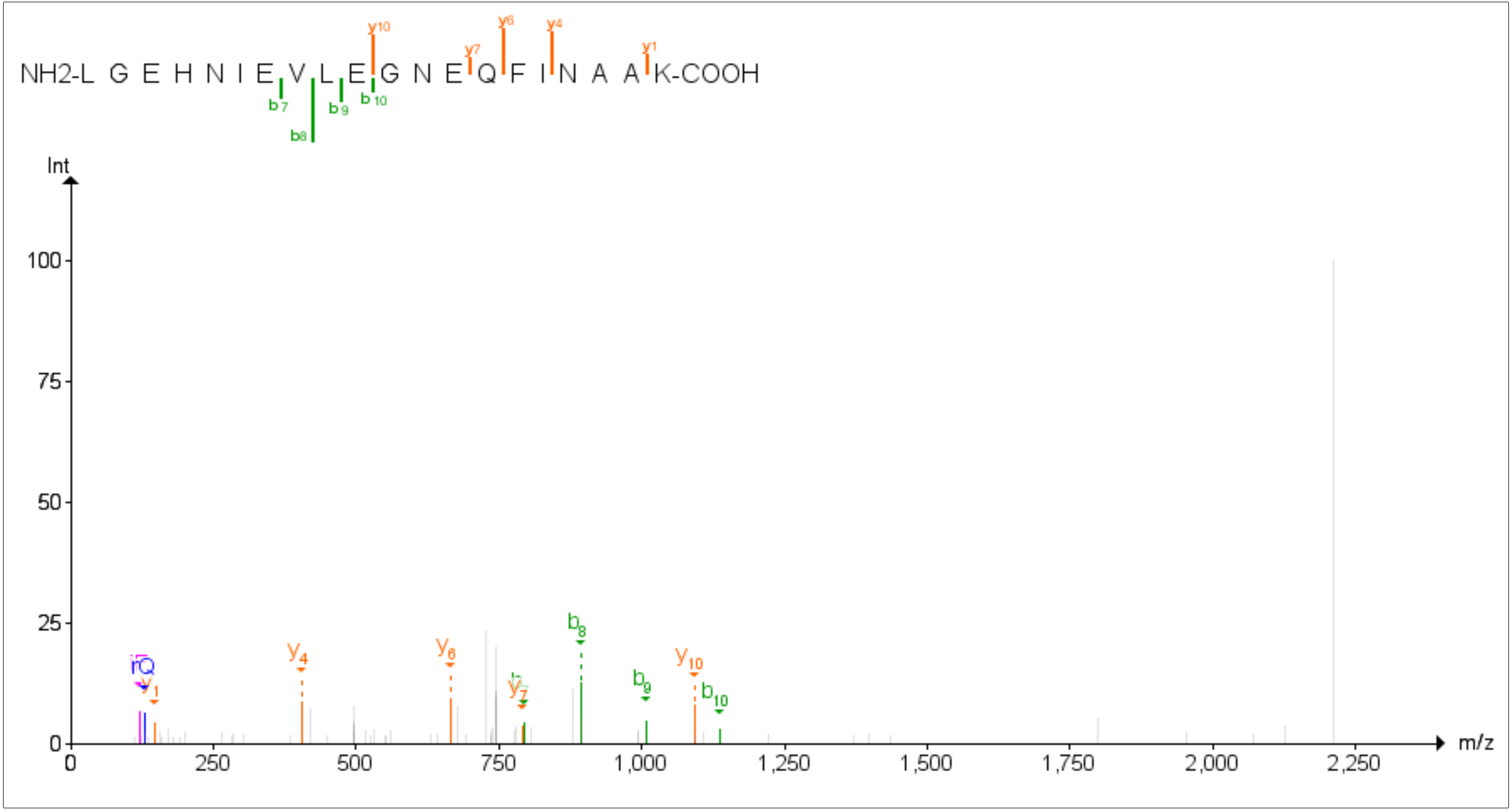

NH2-L G E H N I E V L E G N E Q F I N A A K-COOH
y10
y7
y6
y4
y1
b7
b8
b9
b10
Int
100
75
50
25
0
y4
y6
b8
b9
y10
b10
y7
y1
m/z
0
250
500
750
1,000
1,250
1,500
1,750
2,000
2,250

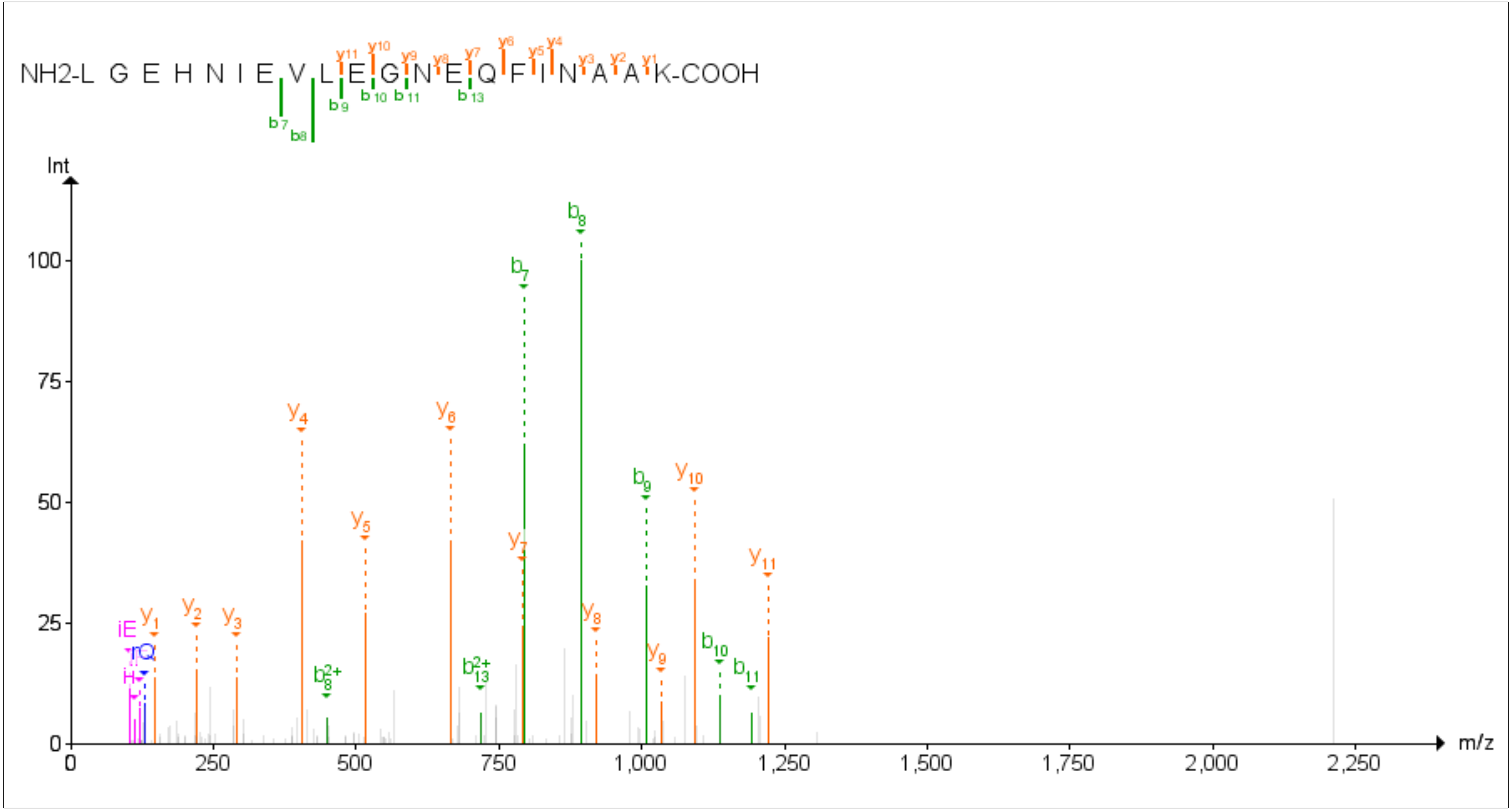
NH2-L G E H N I E V L E G N E Q F I N A A K-COOH
Int
m/z
0
25
50
75
100
250
500
750
1,000
1,250
1,500
1,750
2,000
2,250
b7
b8
b9
b10
b11
b13
y1
y2
y3
y4
y5
y6
y7
y8
y9
y10
y11
iE
nQ
H

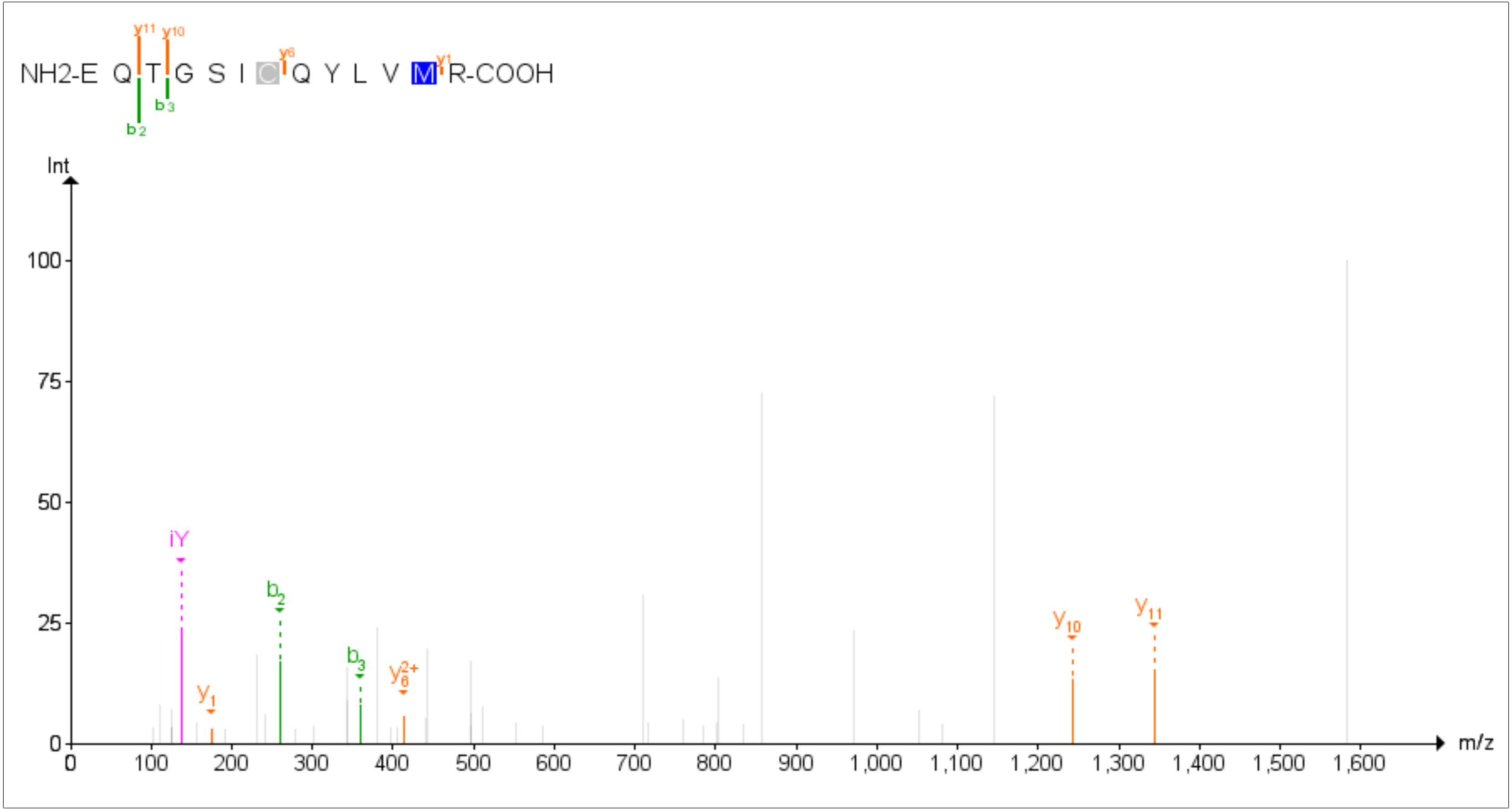

NH2-E Q T G S I C Q Y L V M R-COOH
y11
y10
y6
y1
b2
b3
Int
100
75
50
25
0
iY
b2
b3
y1
y6 2+
y10
y11
0
100
200
300
400
500
600
700
800
900
1,000
1,100
1,200
1,300
1,400
1,500
1,600
m/z

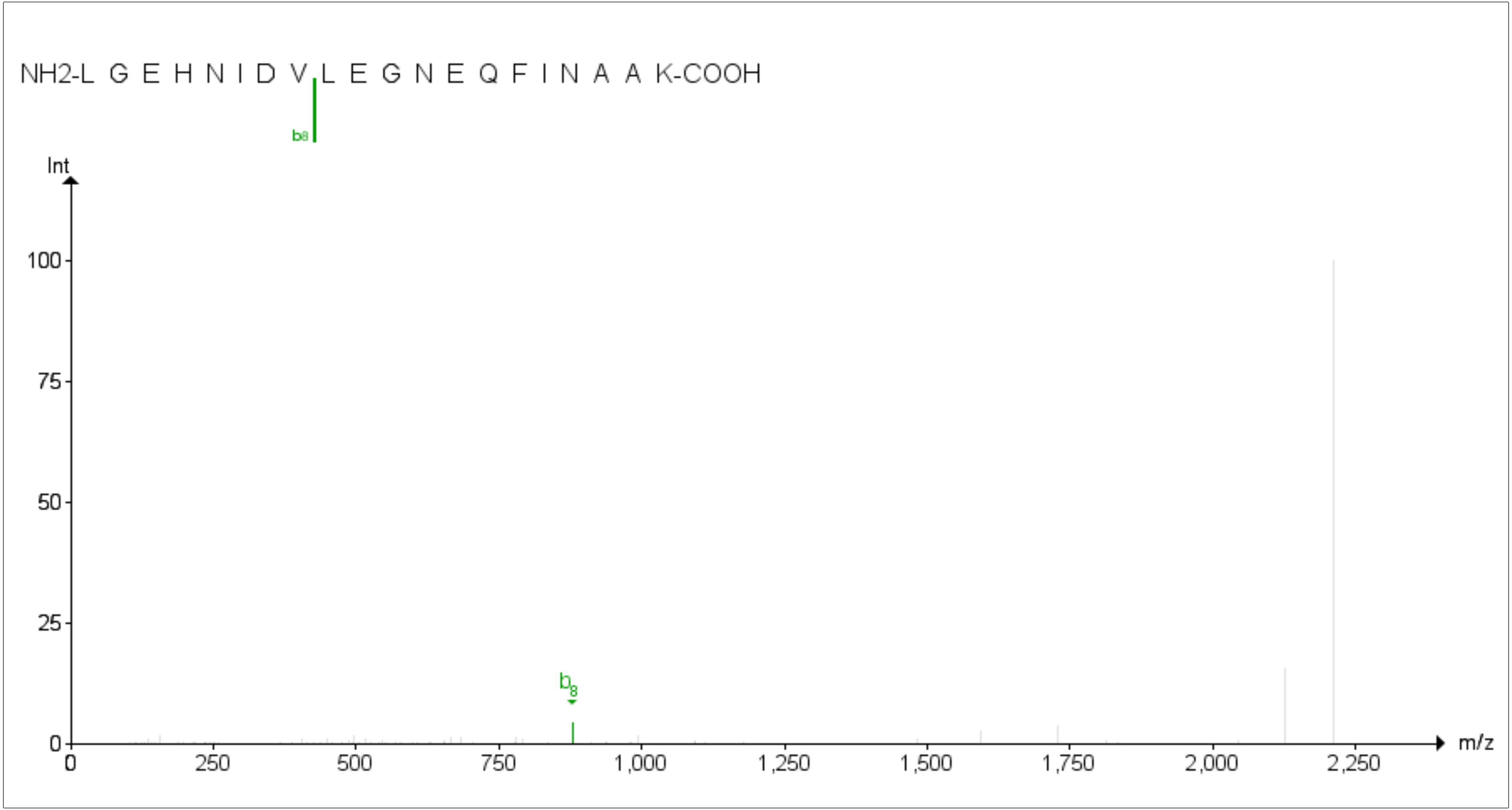
NH2-L G E H N I D V L E G N E Q F I N A A K-COOH
b8
Int
100
75
50
25
0
b8
0
250
500
750
1,000
1,250
1,500
1,750
2,000
2,250
m/z

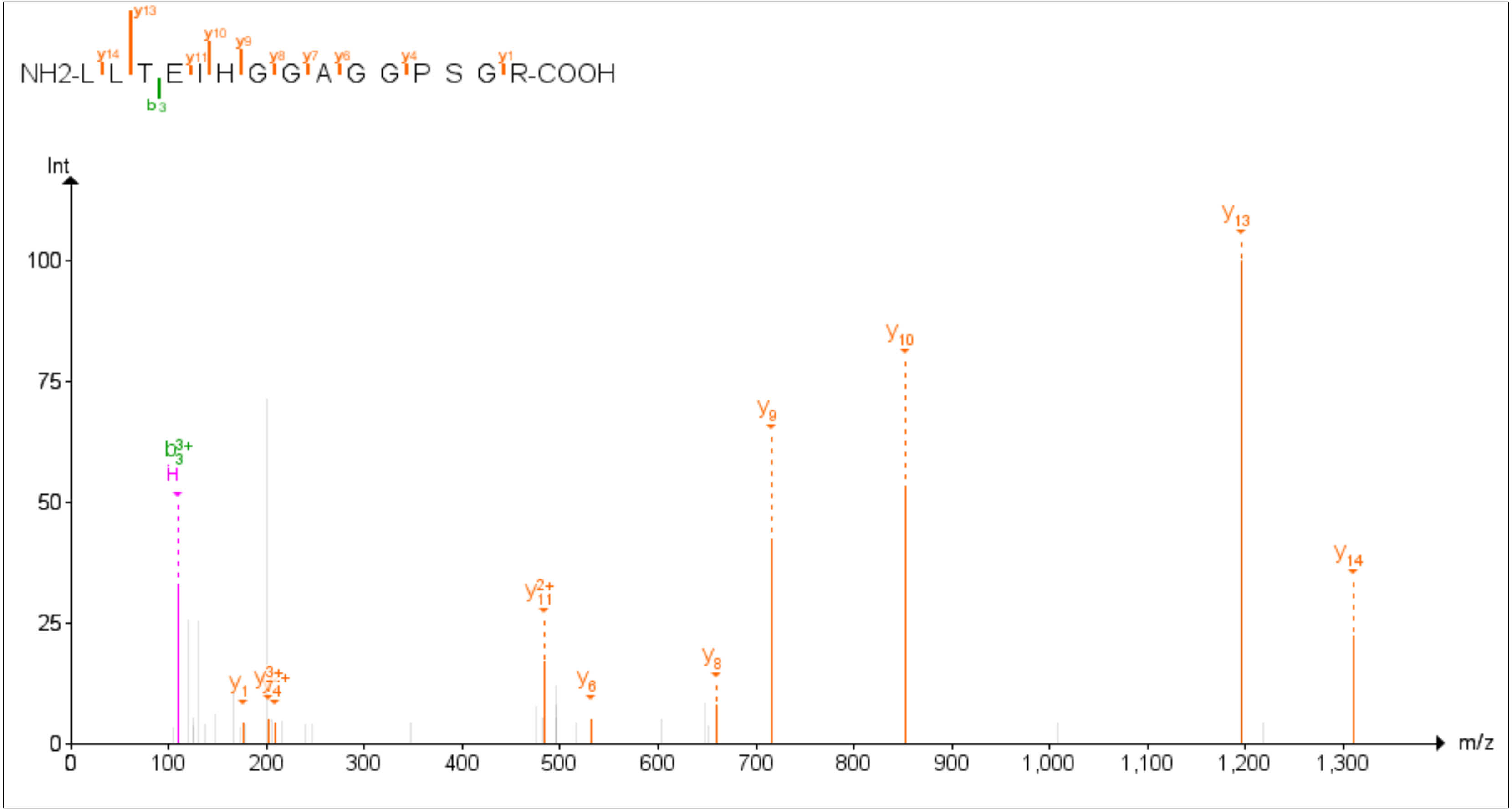

NH2-L L T E I H G G A G G P S G R-COOH
y14
y13
y11
y10
y9
y8
y7
y6
y4
y1
b3
Int
100
75
50
25
0
m/z
0
100
200
300
400
500
600
700
800
900
1,000
1,100
1,200
1,300
b3 3+
H
y1
y7 3+
y4 2+
y11 2+
y6
y8
y9
y10
y13
y14

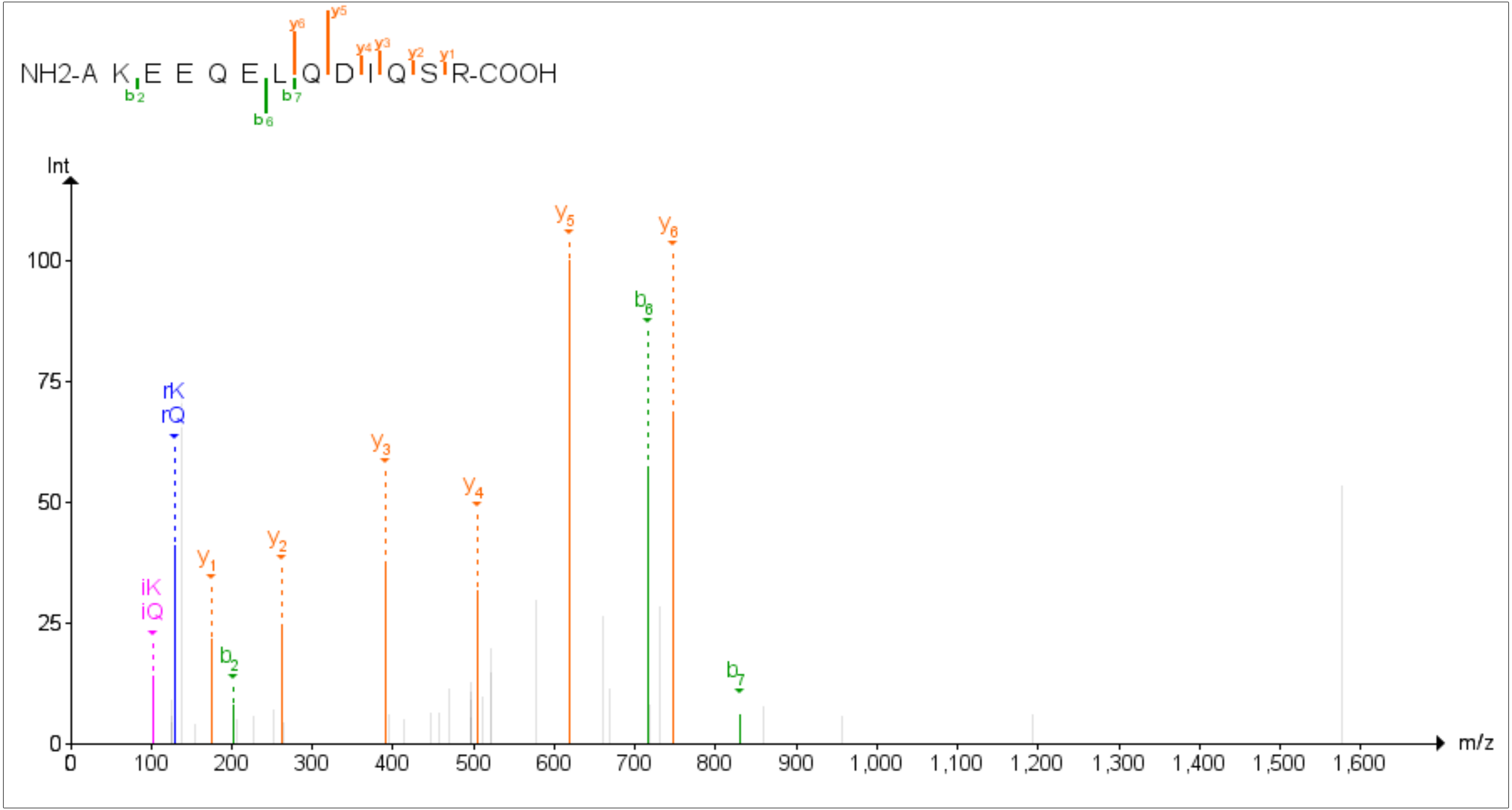

NH2-A K E E Q E L Q D I Q S R-COOH
b2
b6
b7
y6
y5
y4
y3
y2
y1
Int
100
75
50
25
0
iK
iQ
rK
rQ
y1
b2
y2
y3
y4
y5
b6
y6
b7
0
100
200
300
400
500
600
700
800
900
1,000
1,100
1,200
1,300
1,400
1,500
1,600
m/z

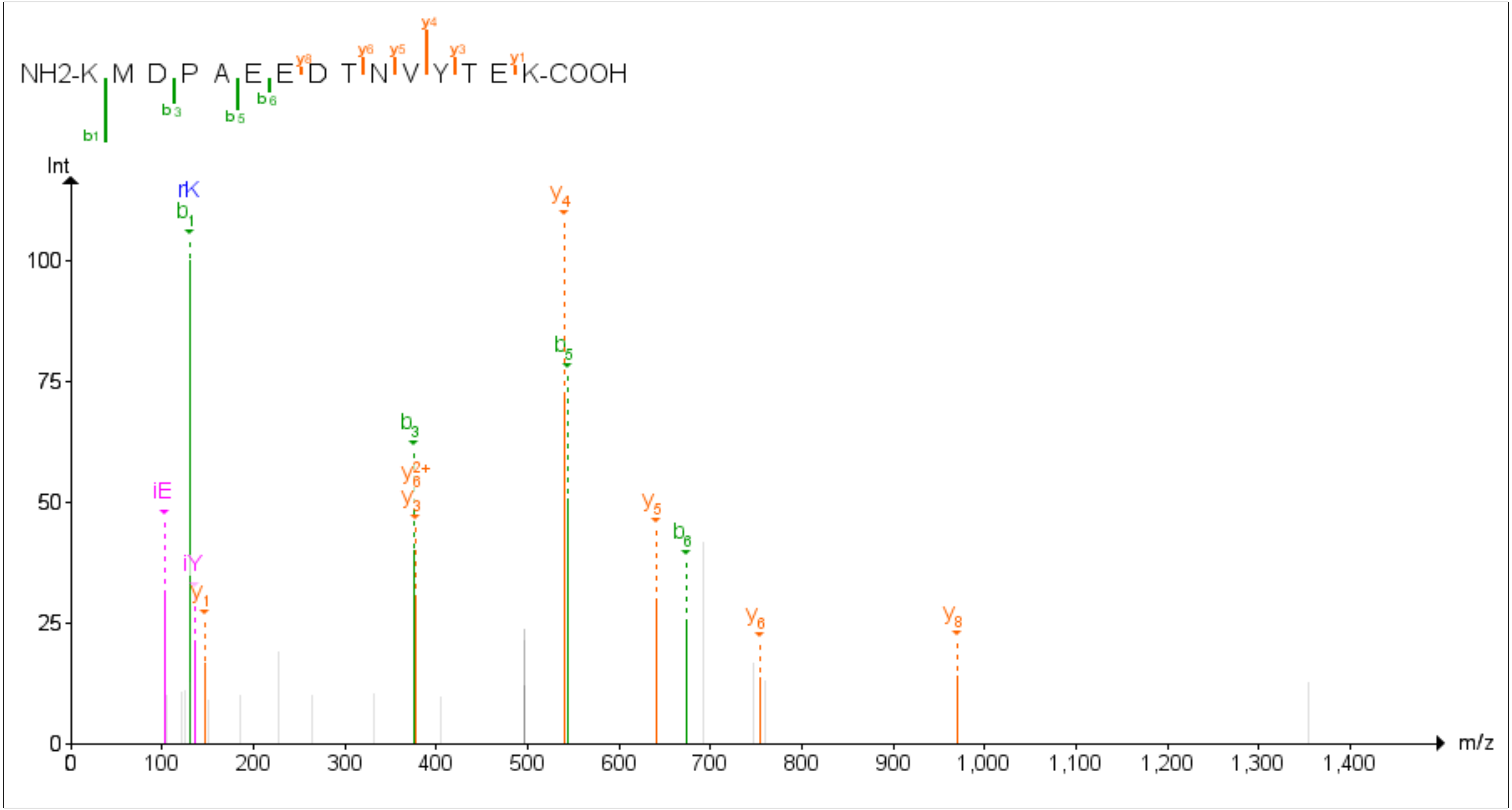
NH2-K M D P A E E D T N V Y T E K-COOH
b1 b3 b5 b6
y8 y6 y5 y4 y3 y1
Int
m/z
rK
b1
iE
iY
y1
b3
y6 2+
y3
y4
b5
y5
b6
y6
y8
0
25
50
75
100
0
100
200
300
400
500
600
700
800
900
1,000
1,100
1,200
1,300
1,400

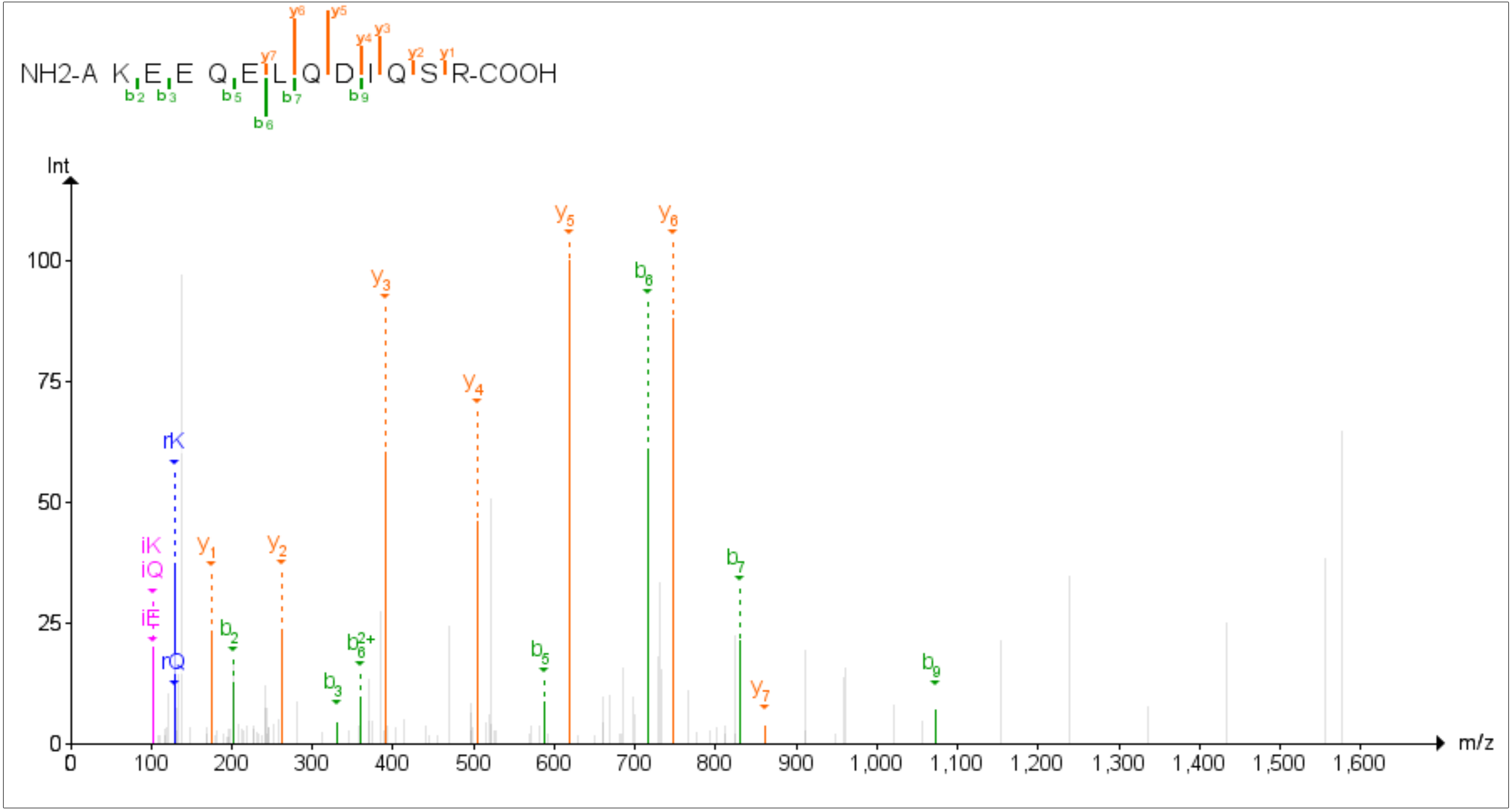

NH2-A K E E Q E L Q D I Q S R-COOH
b2 b3 b5 b6 b7 b9
y7 y6 y5 y4 y3 y2 y1
Int
100
75
50
25
0
rK
iK
iQ
iE
rQ
y1 y2 y3 y4 y5 y6 y7
b2 b3 b6 2+ b5 b6 b7 b9
0 100 200 300 400 500 600 700 800 900 1,000 1,100 1,200 1,300 1,400 1,500 1,600
m/z

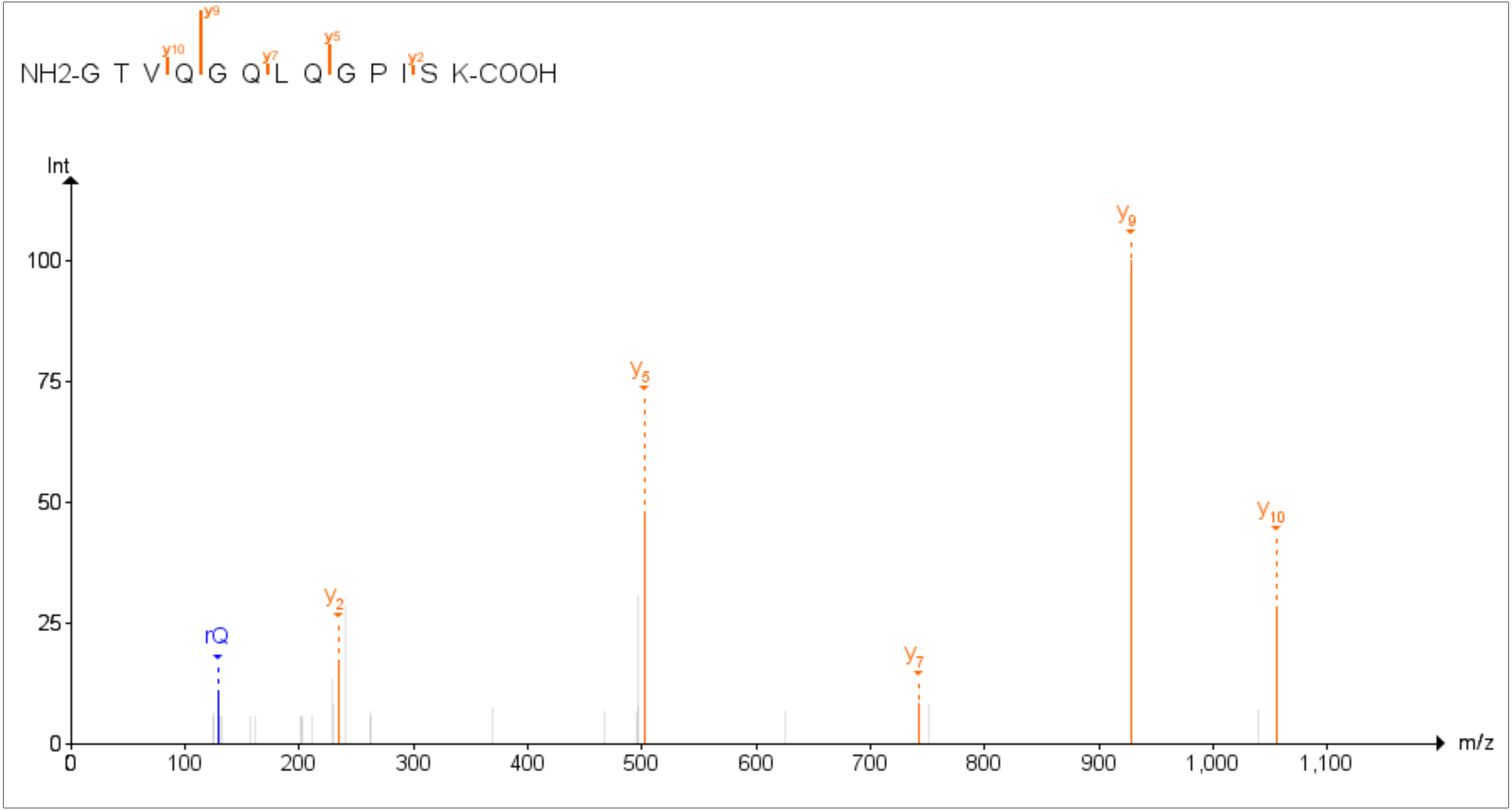

NH2-G T V Q G Q L Q G P I S K-COOH
y10
y9
y7
y5
y2
Int
100
75
50
25
0
rQ
y2
y5
y7
y9
y10
0
100
200
300
400
500
600
700
800
900
1,000
1,100
m/z

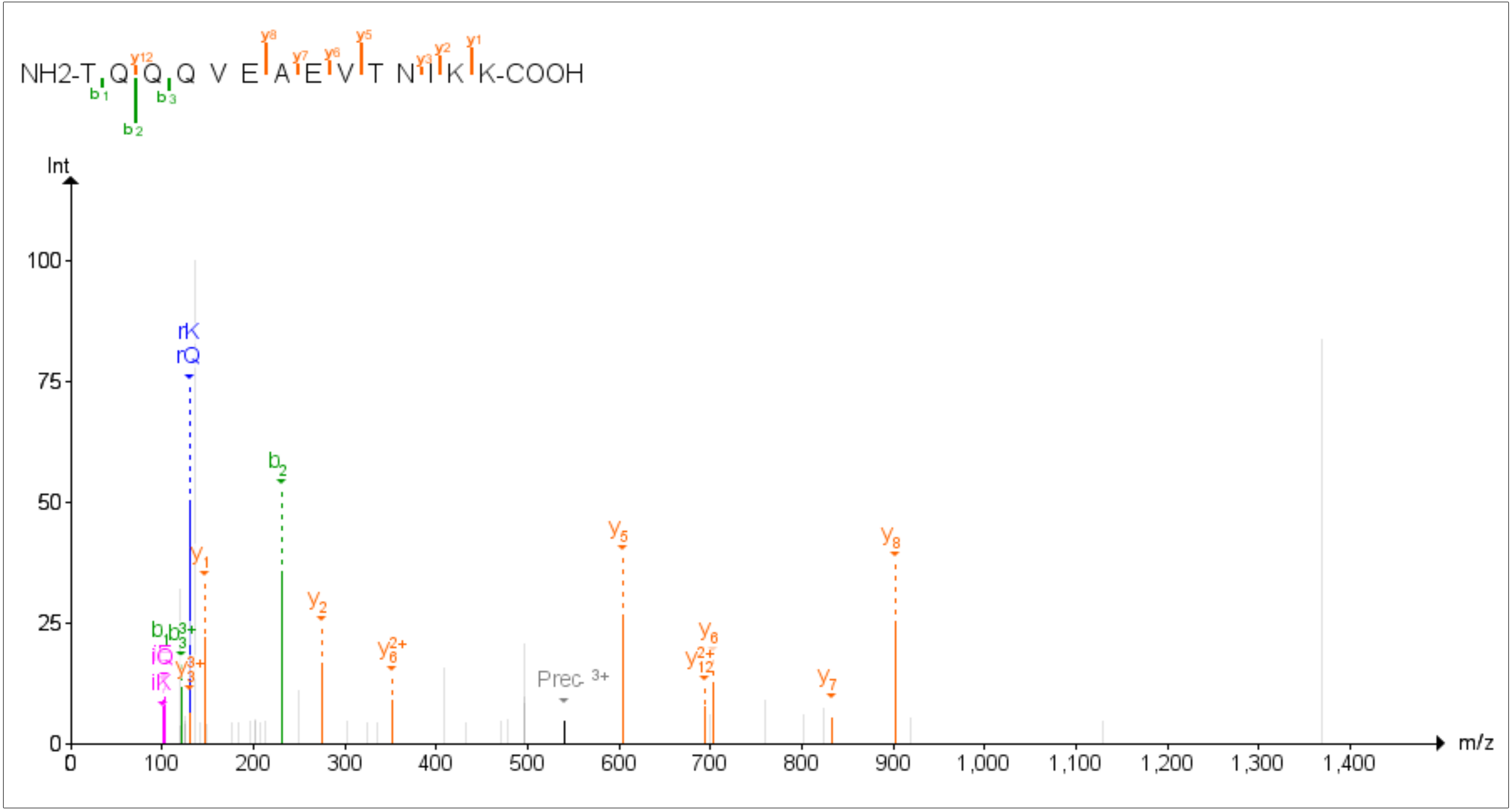
NH2-T Q Q Q V E A E V T N I K K-COOH
b1
b2
b3
y12
y8
y7
y6
y5
y3
y2
y1
Int
rK
rQ
b2
y1
b1
b3
3+
iQ
iK
y3
3+
y2
y6
2+
Prec 3+
y5
y6
y12
2+
y7
y8
m/z
0
25
50
75
100
0
100
200
300
400
500
600
700
800
900
1,000
1,100
1,200
1,300
1,400

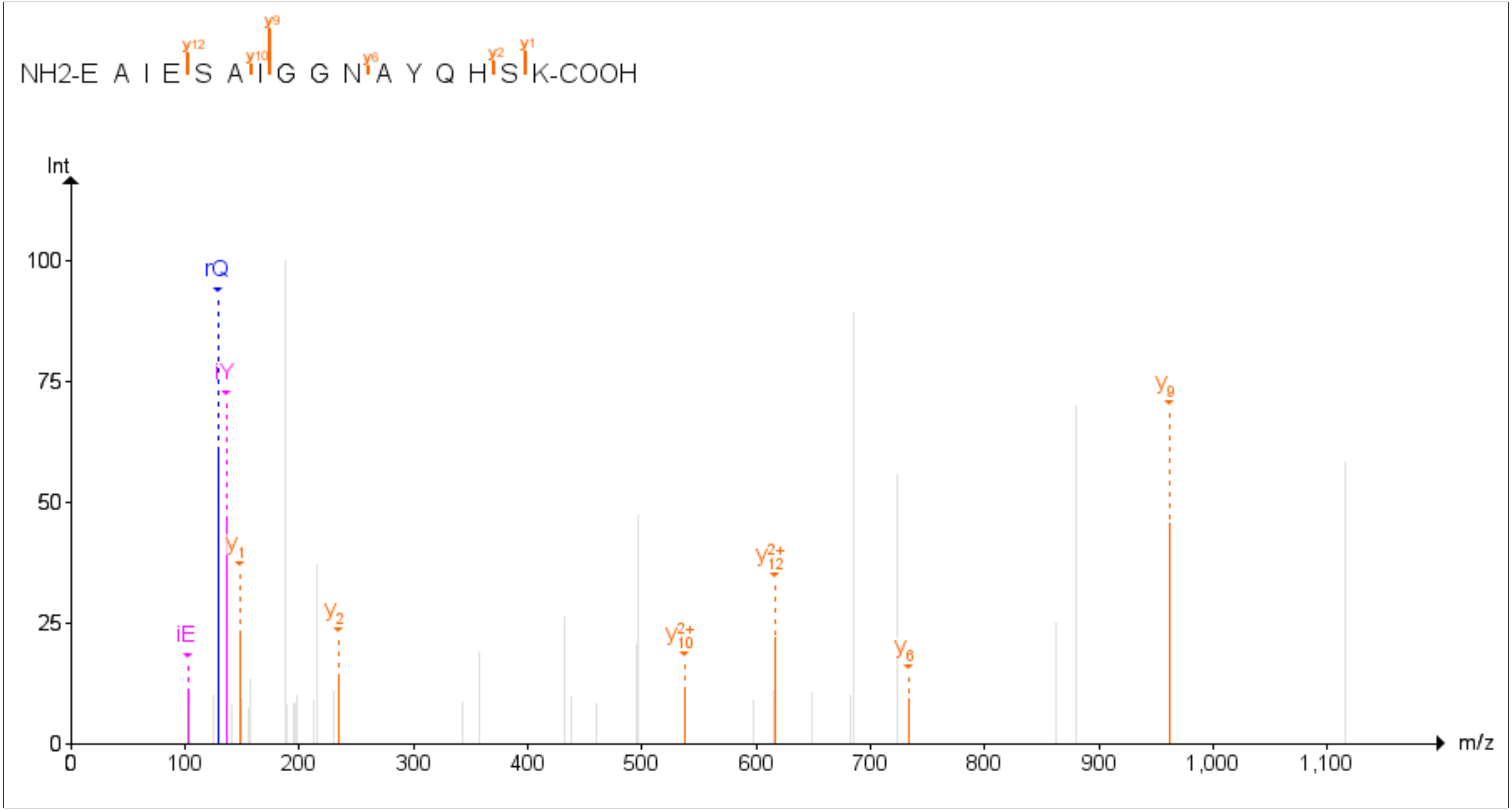

NH2-E A I E S A I G G N A Y Q H S K-COOH
y12
y10
y9
y6
y2
y1
Int
100
75
50
25
0
rQ
iY
iE
y1
y2
y10 2+
y12 2+
y6
y9
0
100
200
300
400
500
600
700
800
900
1,000
1,100
m/z

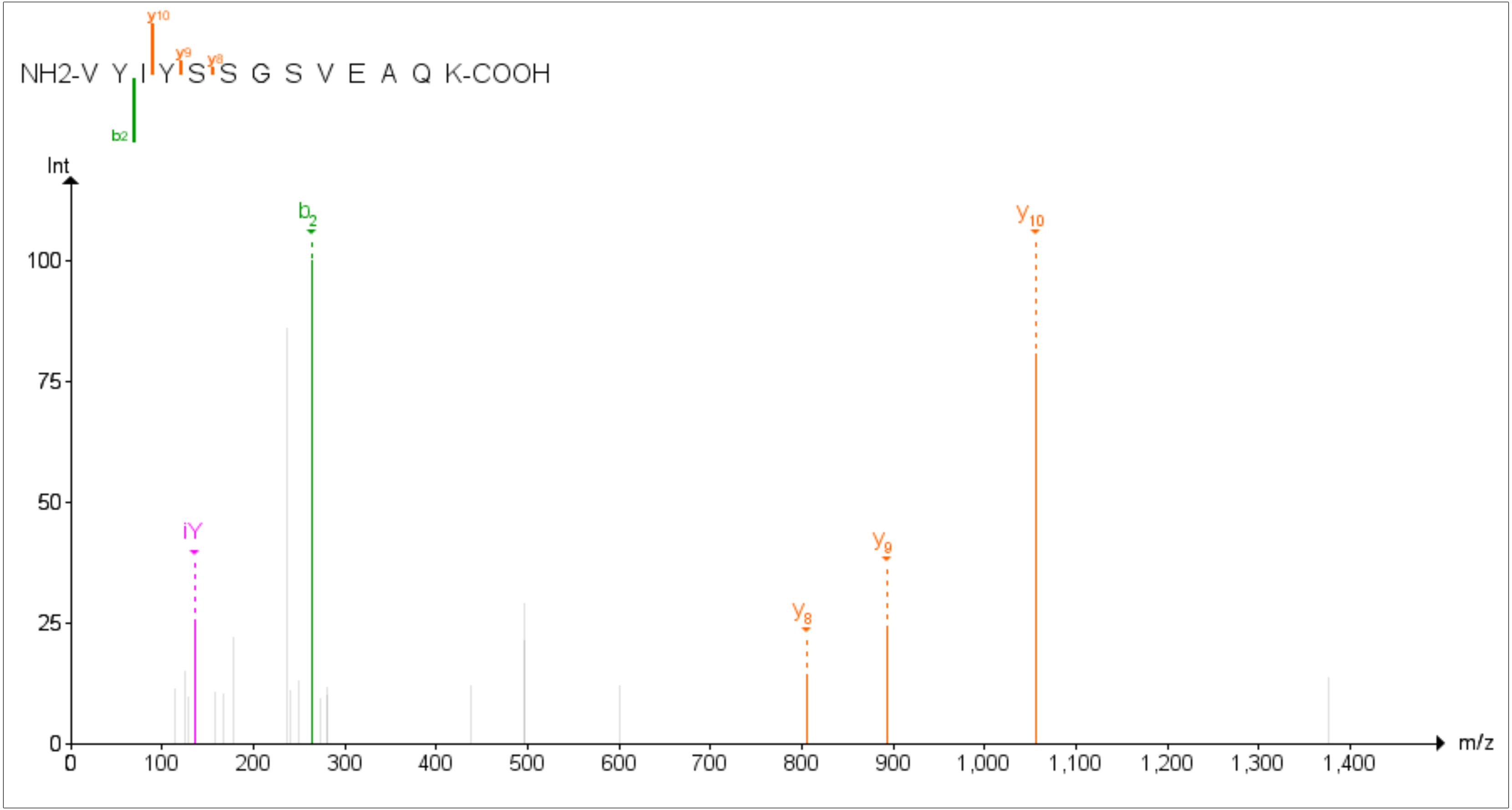

NH2-V Y I Y S S G S V E A Q K-COOH
y10
y9
y8
b2
Int
b2
y10
iY
y9
y8
100
75
50
25
0
0
100
200
300
400
500
600
700
800
900
1,000
1,100
1,200
1,300
1,400
m/z

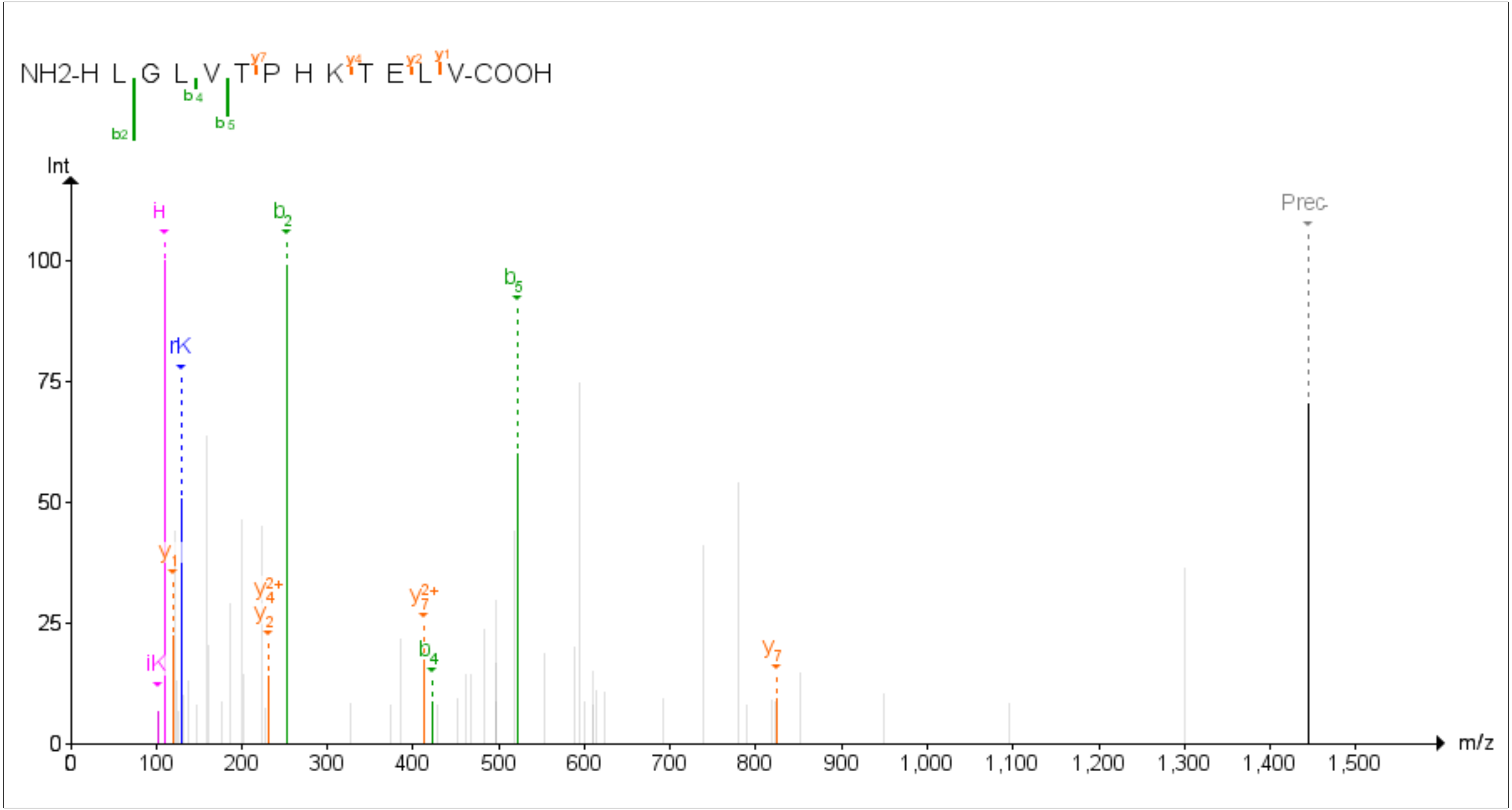

NH2-H L G L V T P H K T E L V-COOH
b2
b4
b5
y7
y4
y2
y1
Int
m/z
iH
b2
Prec
b5
rK
y1
y4 2+
y2
y7 2+
b4
iK
y7
0
25
50
75
100
0
100
200
300
400
500
600
700
800
900
1,000
1,100
1,200
1,300
1,400
1,500

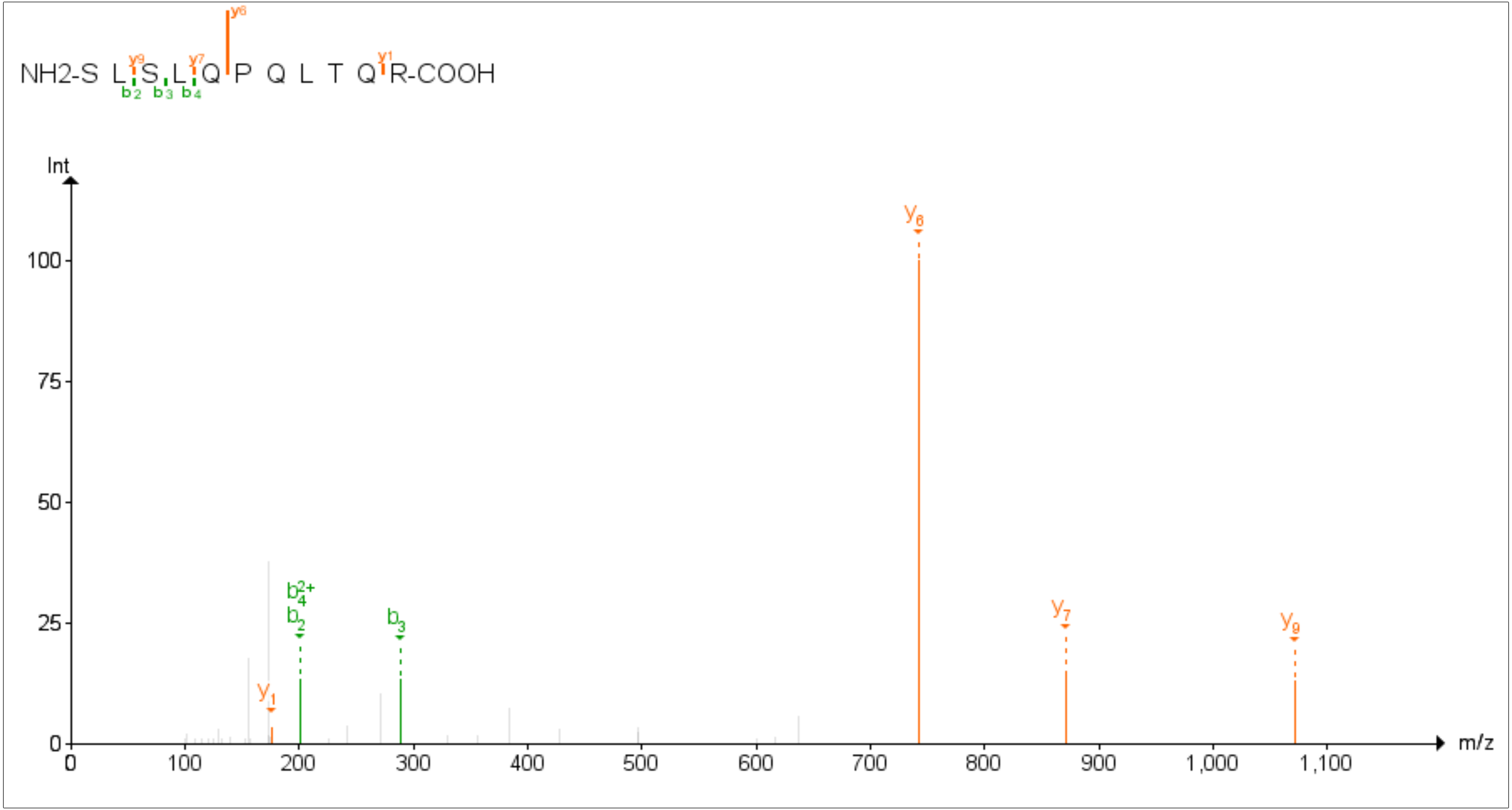

NH2-S L S L Q P Q L T Q R-COOH
y9 y7 y6 y1
b2 b3 b4
Int
100
75
50
25
0
y6
y7
y9
y1
b4 2+
b2
b3
0 100 200 300 400 500 600 700 800 900 1,000 1,100
m/z

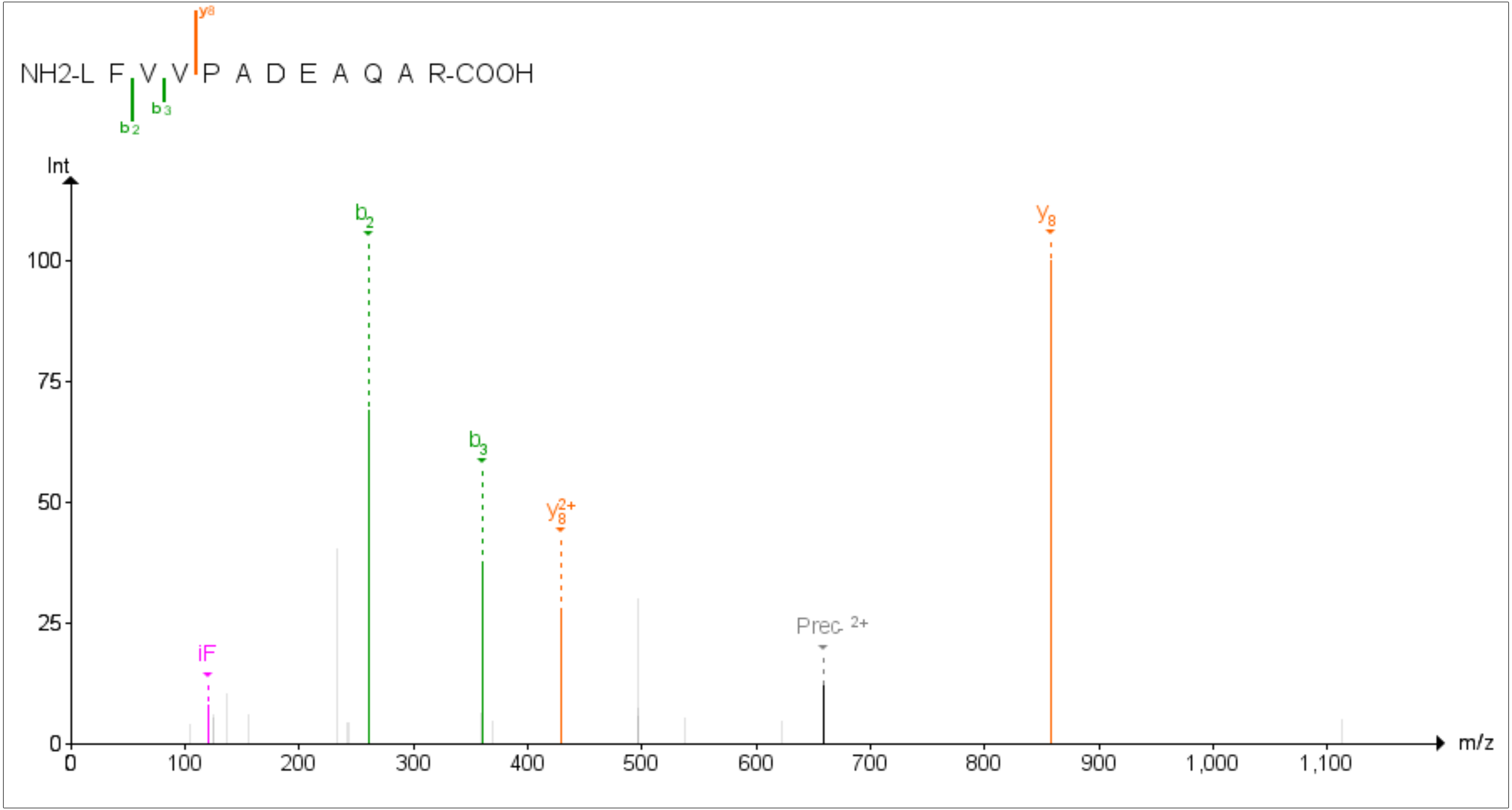

NH2-L F V V P A D E A Q A R-COOH
y8
b2
b3
Int
m/z
b2
b3
y8
y8 2+
iF
Prec- 2+
0
25
50
75
100
0
100
200
300
400
500
600
700
800
900
1,000
1,100

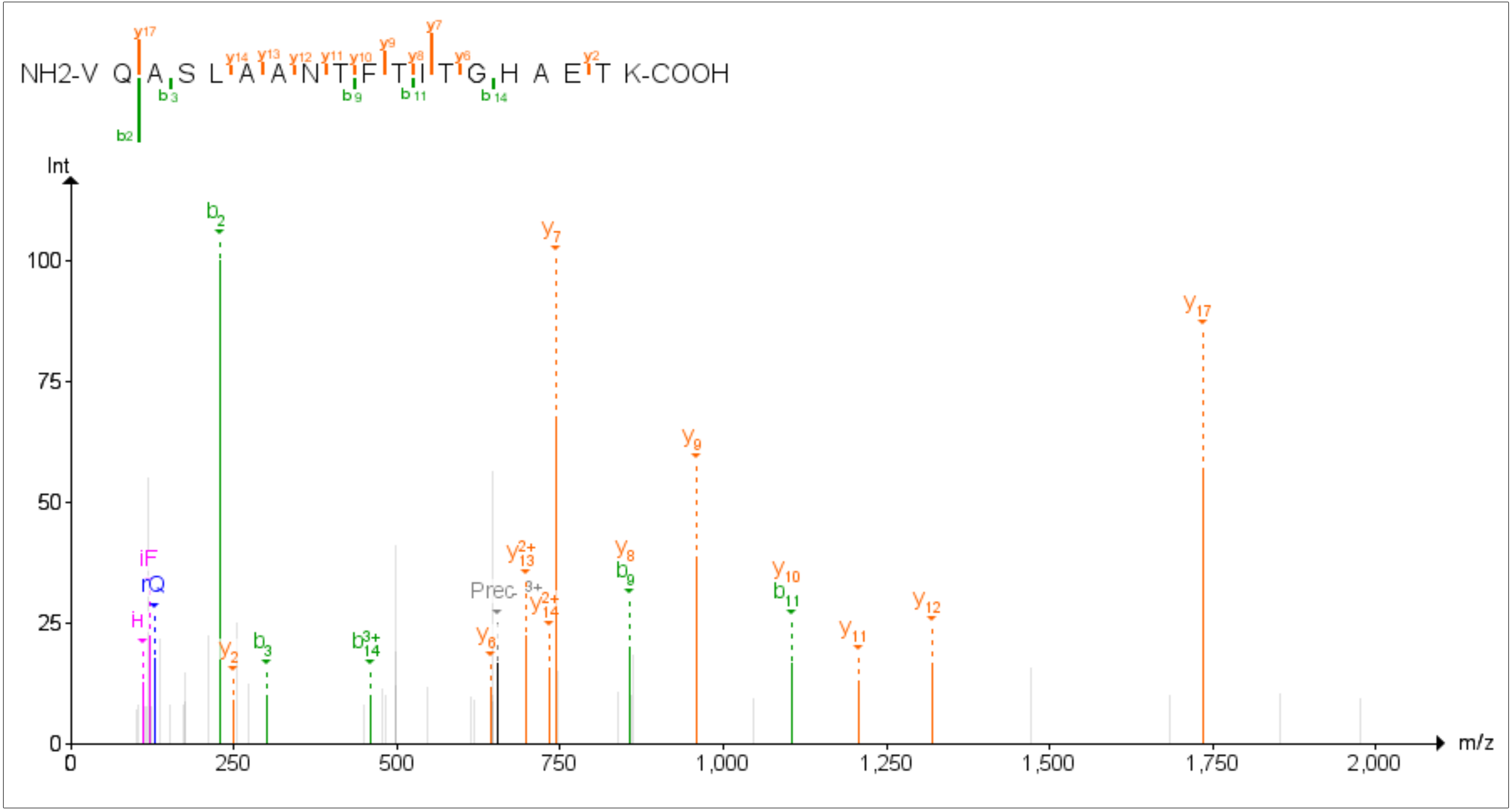

NH2-V Q A S L A A N T F T I T G H A E T K-COOH
Int
m/z
0
25
50
75
100
250
500
750
1,000
1,250
1,500
1,750
2,000
Prec
iF
rQ
iH

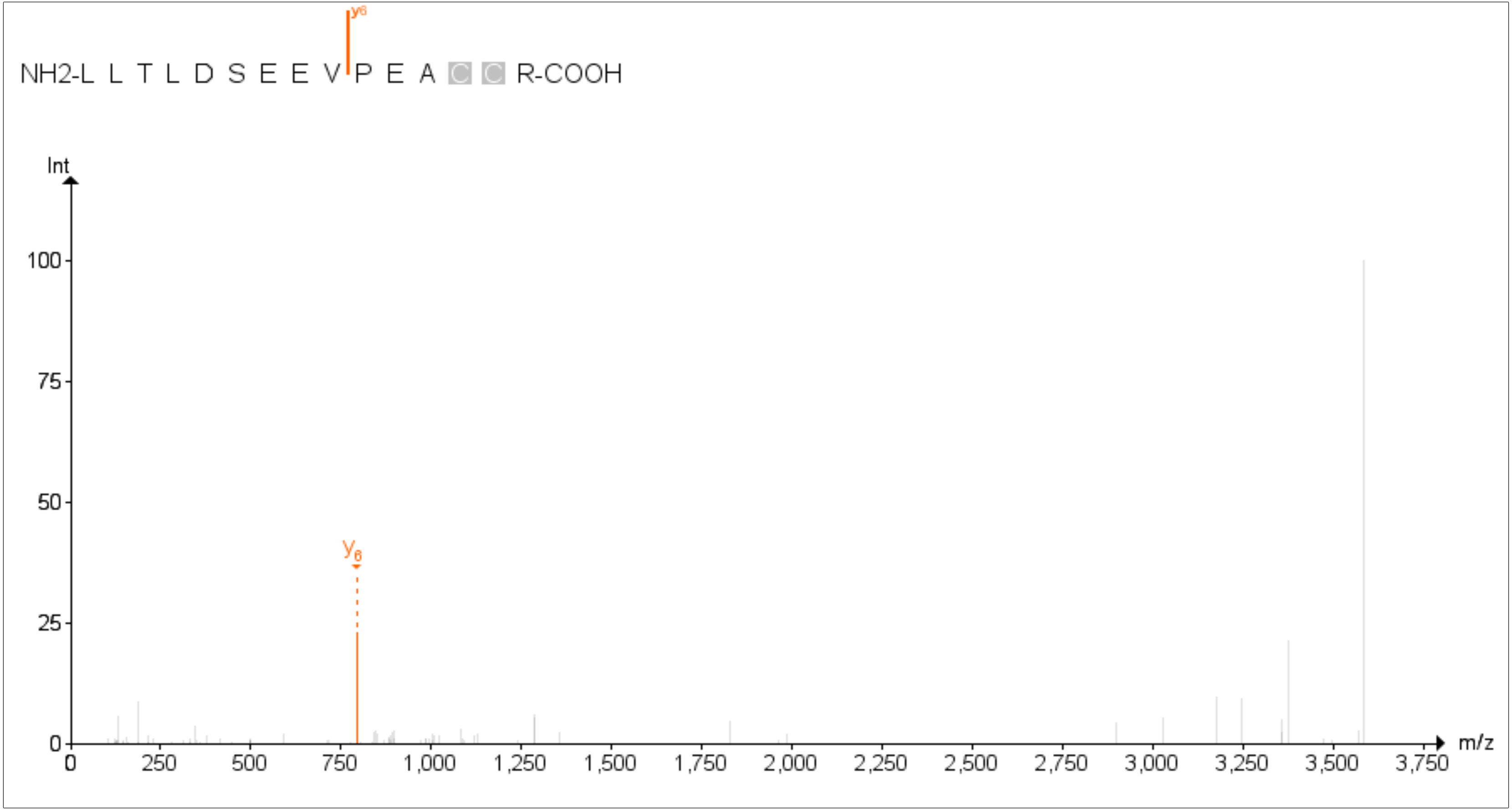

y6
NH2-L L T L D S E E V P E A C C R-COOH
Int
100
75
50
25
0
y6
0
250
500
750
1,000
1,250
1,500
1,750
2,000
2,250
2,500
2,750
3,000
3,250
3,500
3,750
m/z

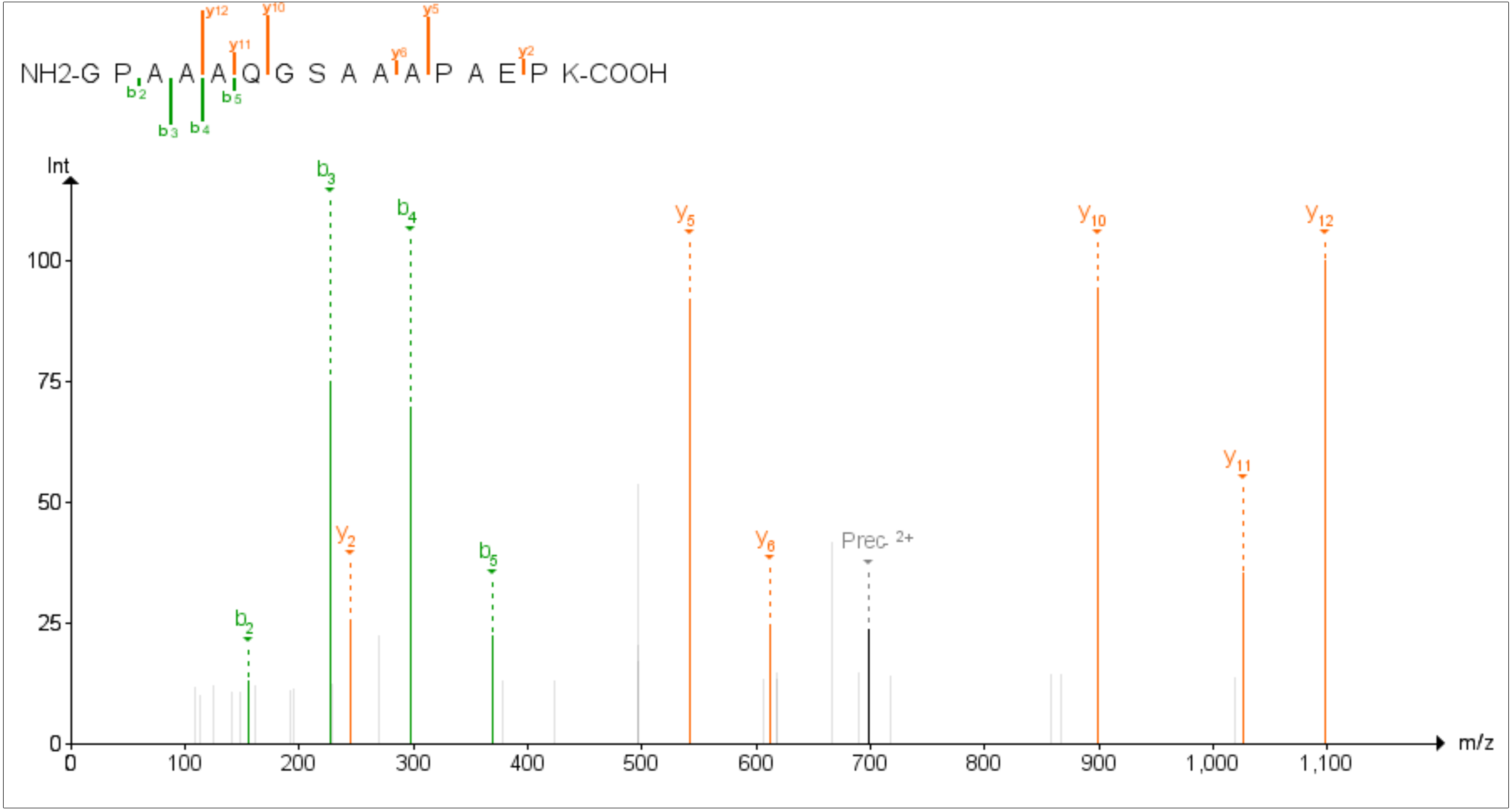

NH2-G P A A A Q G S A A A P A E P K-COOH
y12 y11 y10 y6 y5 y2
b2 b3 b4 b5
Int
m/z
Prec. 2+

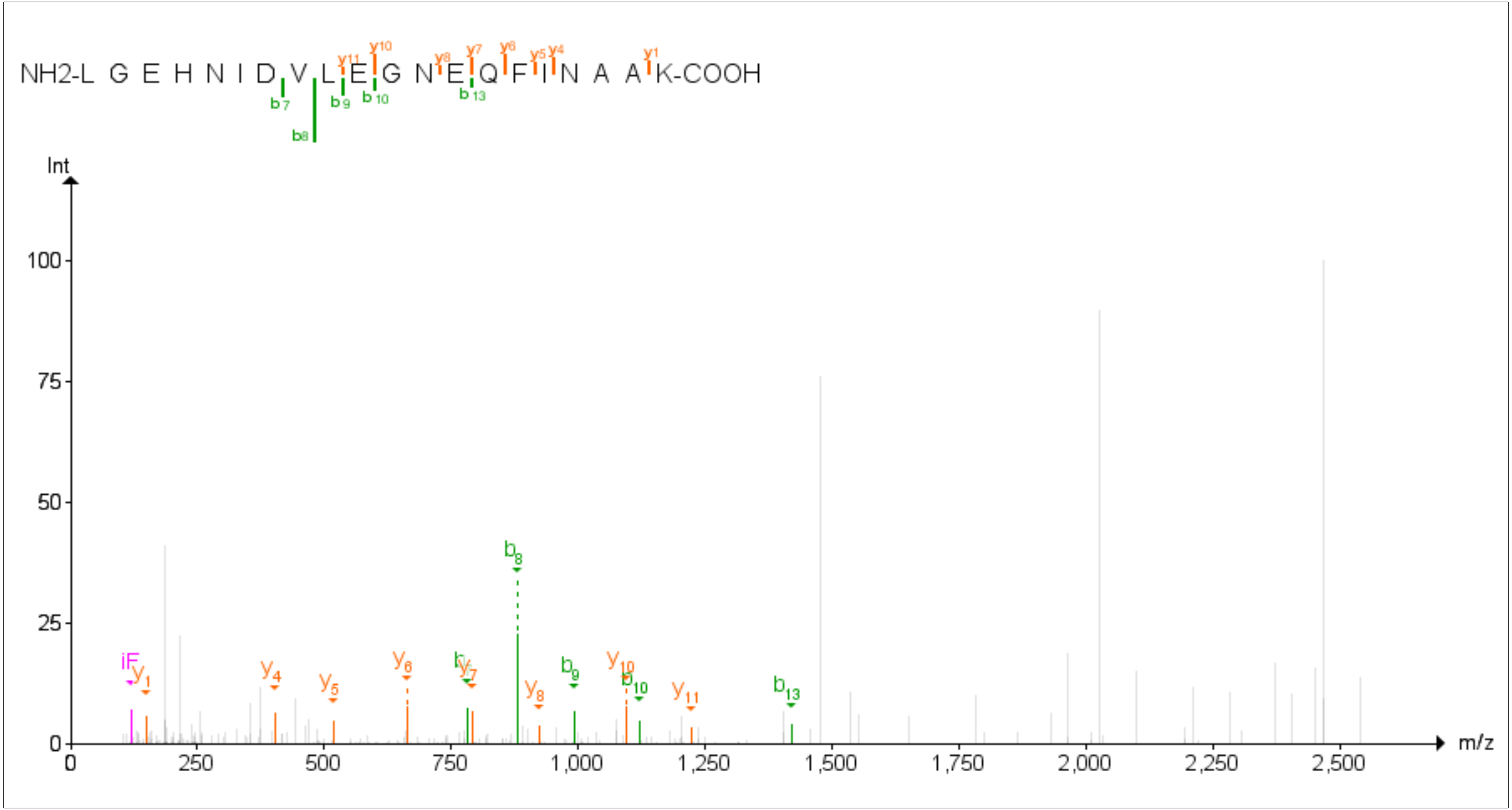

NH2-L G E H N I D V L E G N E Q F I N A A K-COOH
Int
m/z

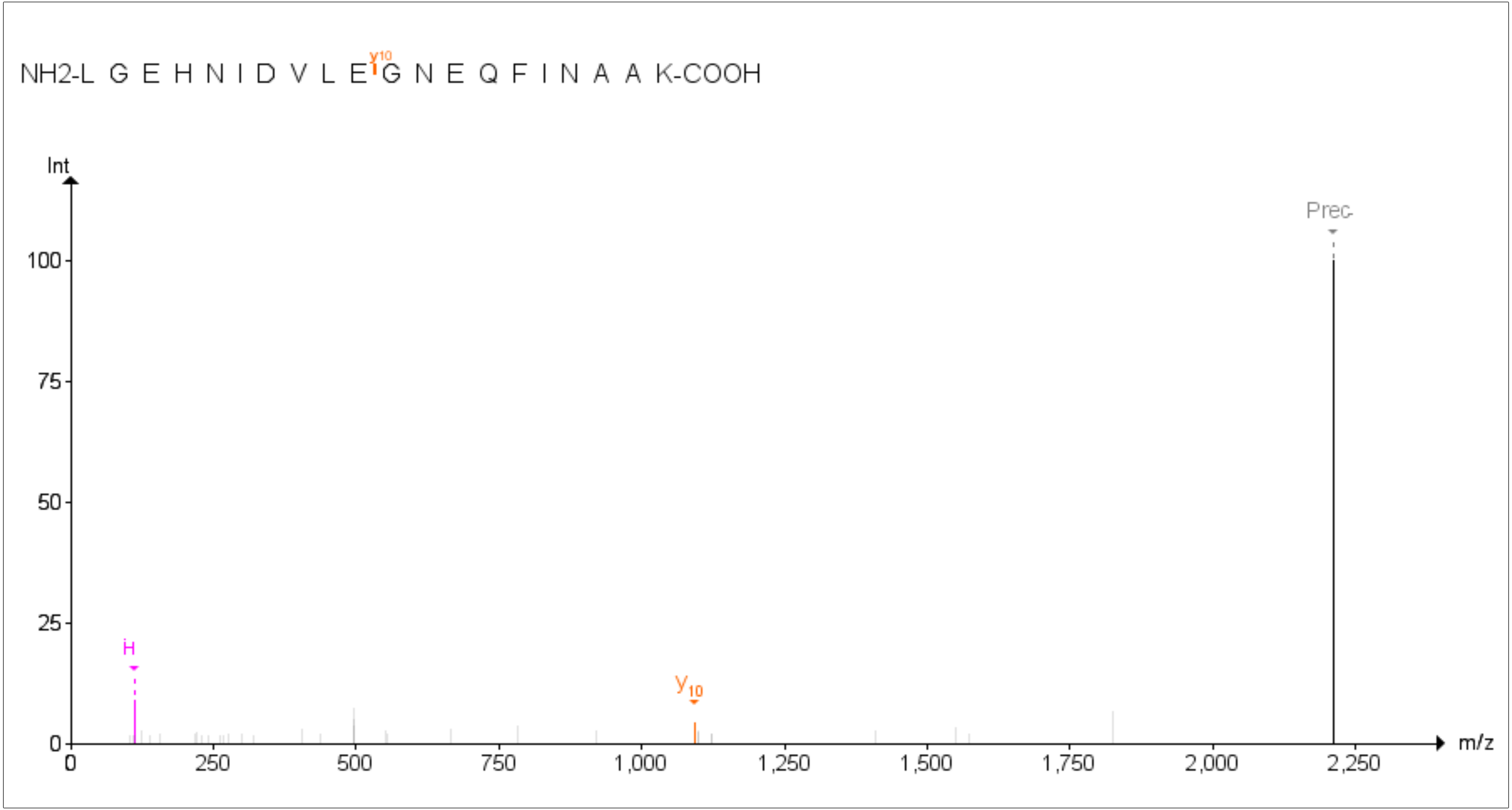

NH2-L G E H N I D V L E G N E Q F I N A A K-COOH
y10
Int
100
75
50
25
0
Prec
H
y10
0
250
500
750
1,000
1,250
1,500
1,750
2,000
2,250
m/z

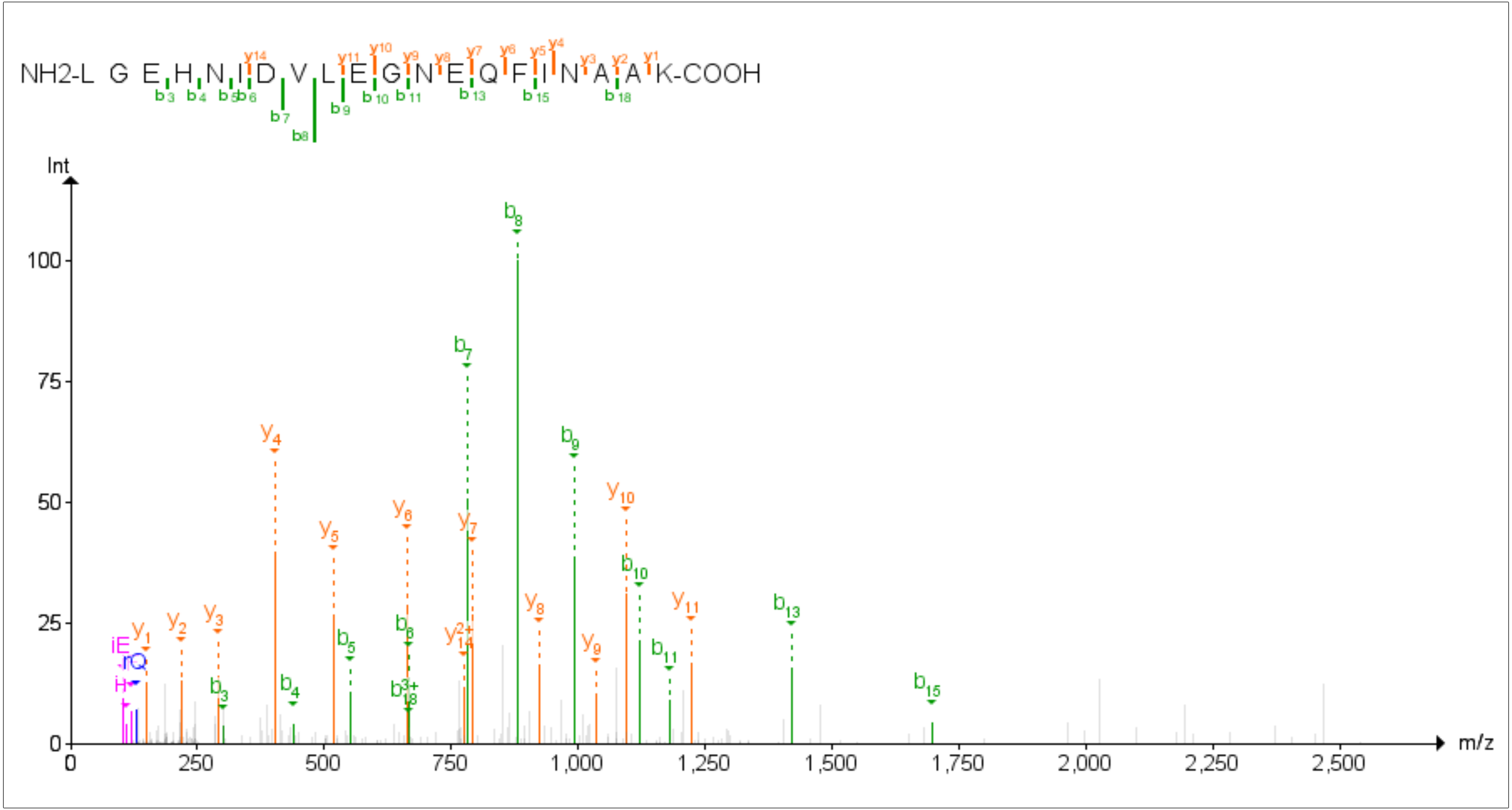
NH2-L G E H N I D V L E G N E Q F I N A A K-COOH
Int
m/z
0
25
50
75
100
250
500
750
1,000
1,250
1,500
1,750
2,000
2,250
2,500

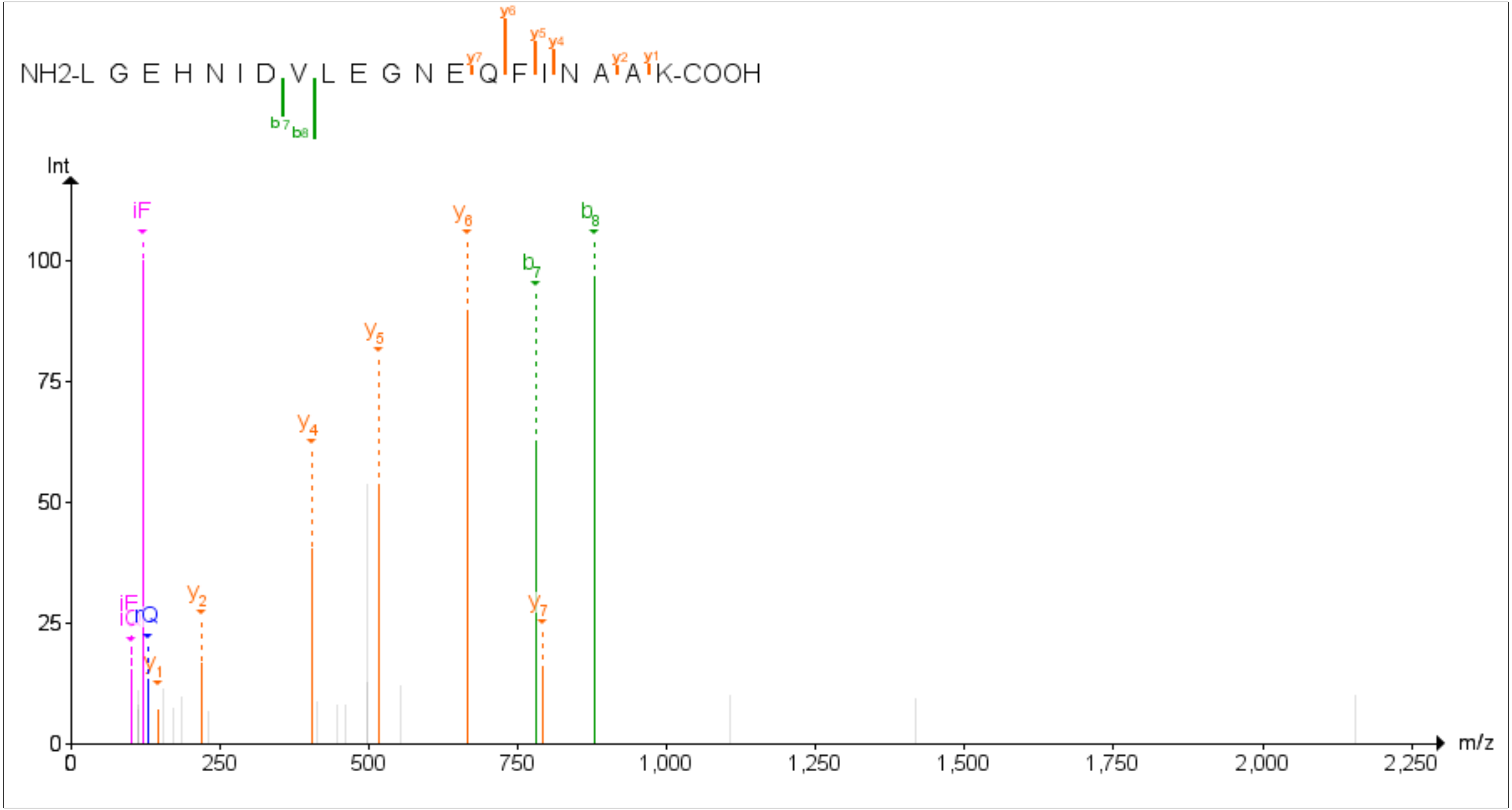
NH2-L G E H N I D V L E G N E Q F I N A A K-COOH
y8
y7
y5
y4
y2
y1
b7
b8
Int
iF
y6
b8
b7
y5
y4
iF
iQ
rQ
y2
y1
y7
100
75
50
25
0
250
500
750
1,000
1,250
1,500
1,750
2,000
2,250
m/z

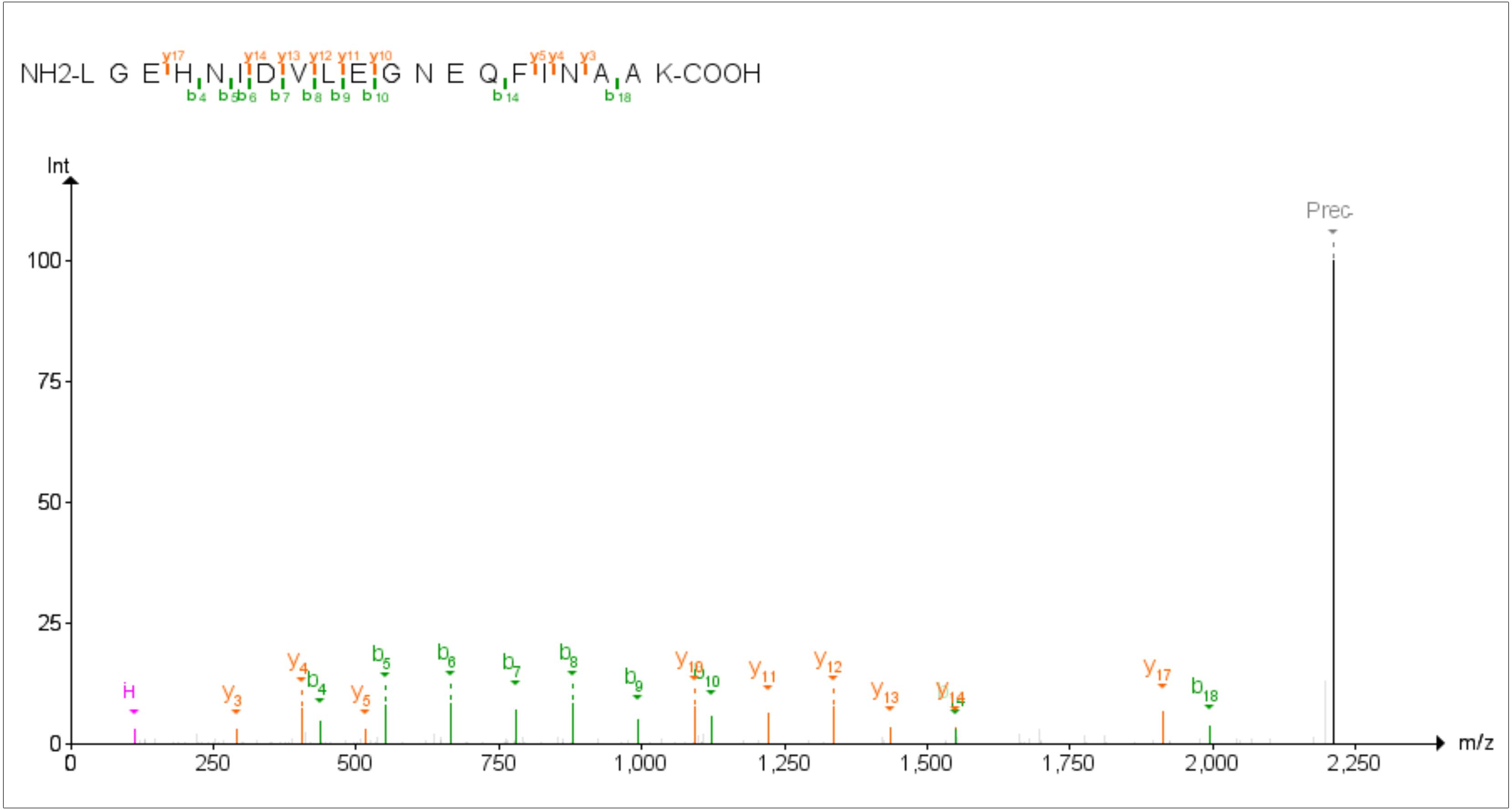

NH2-L G E H N I D V L E G N E Q F I N A A K-COOH
y17 y14 y13 y12 y11 y10 y5 y4 y3
b4 b5 b6 b7 b8 b9 b10 b14 b18
Int
Prec
100
75
50
25
0
H
y3 y4 b4 y5 b5 b6 b7 b8 b9 y10 b10 y11 y12 y13 y14 y17 b18
0 250 500 750 1,000 1,250 1,500 1,750 2,000 2,250
m/z

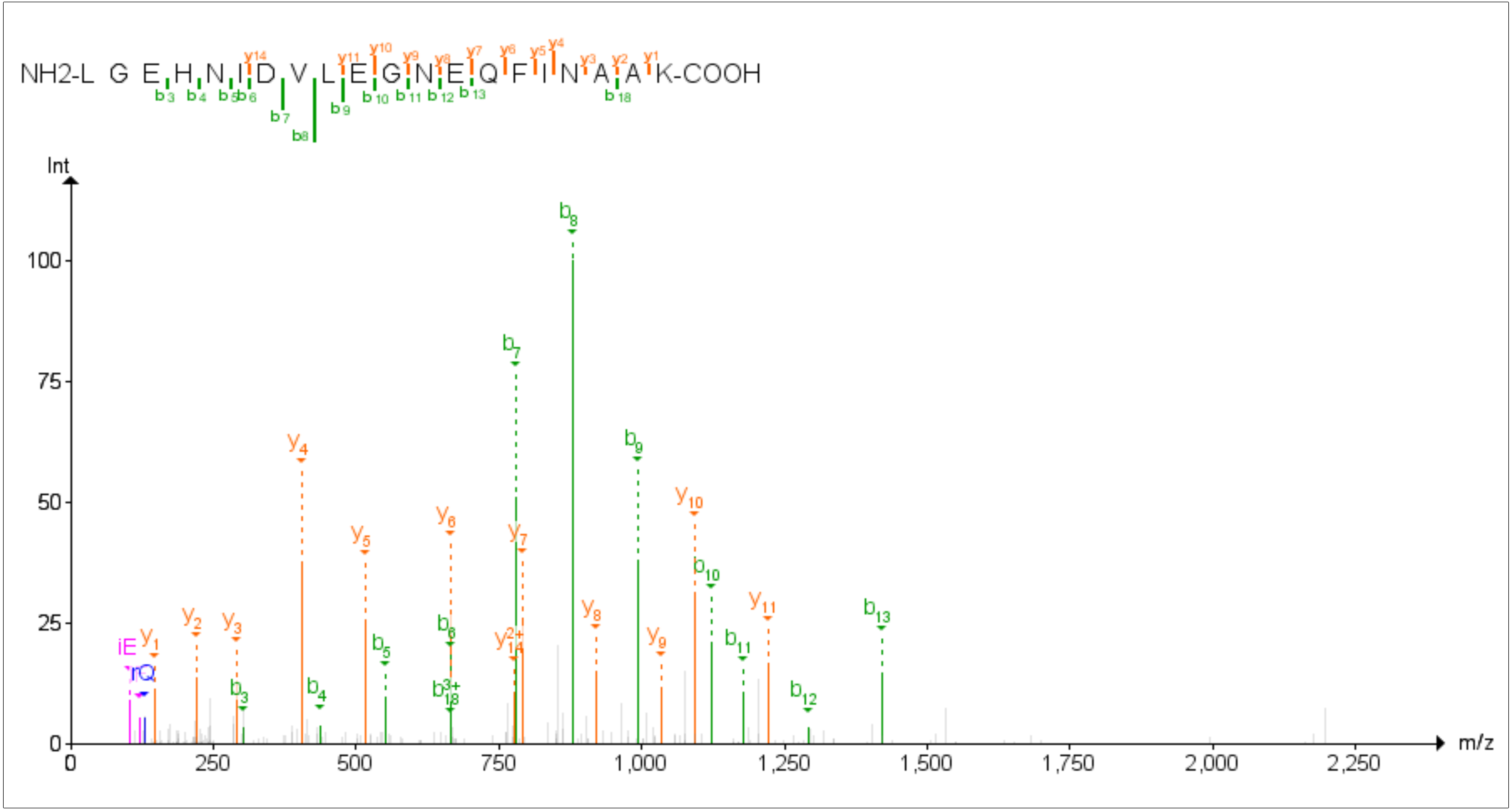

NH2-L G E H N I D V L E G N E Q F I N A A K-COOH
Int
m/z
0
25
50
75
100
250
500
750
1,000
1,250
1,500
1,750
2,000
2,250

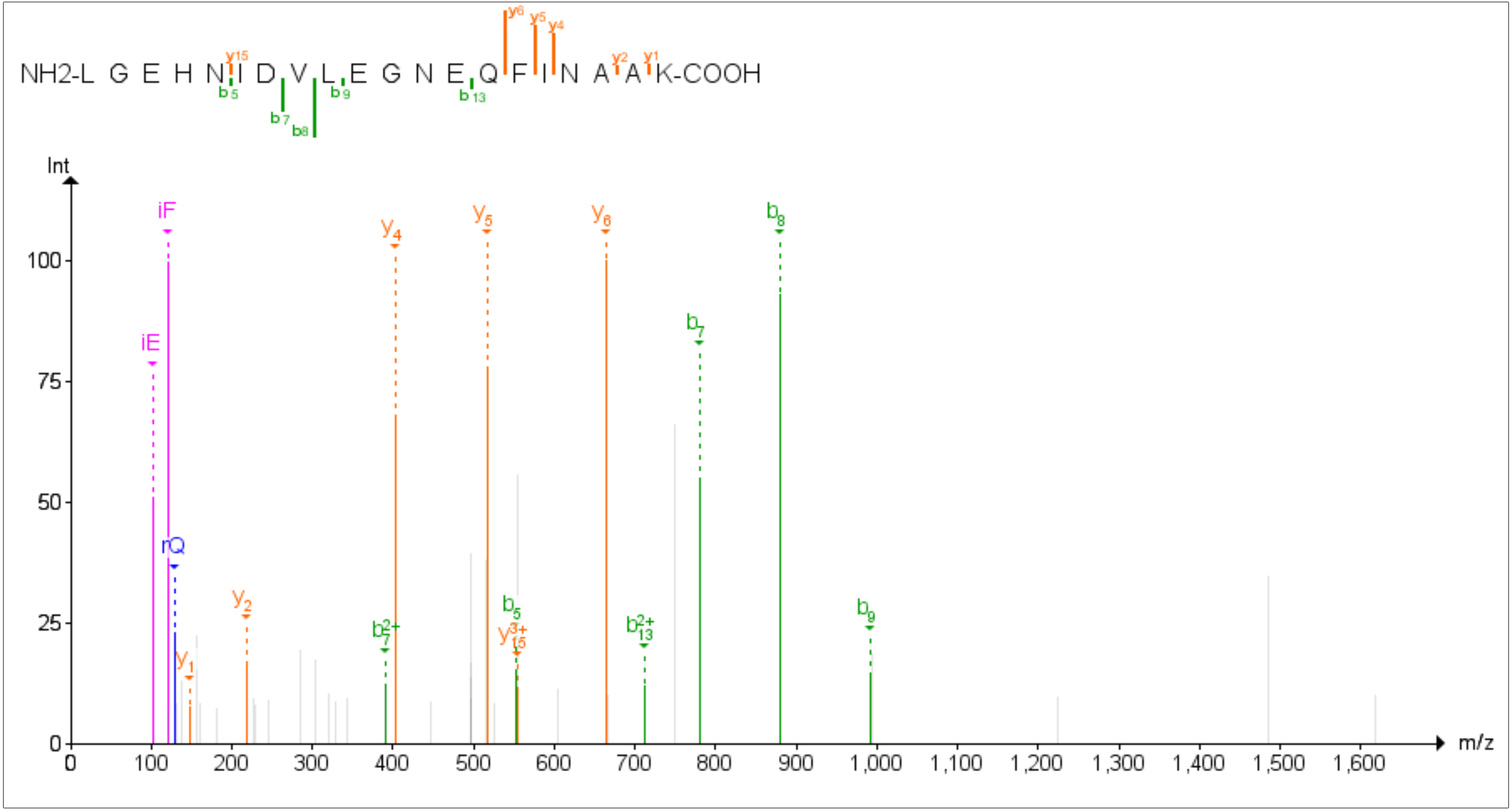

NH2-L G E H N I D V L E G N E Q F I N A A K-COOH
y15 y6 y5 y4 y2 y1
b5 b7 b8 b9 b13
Int
m/z
iF
iE
rQ
y1
y2
y4
y5
y6
b8
b7
b5
b9

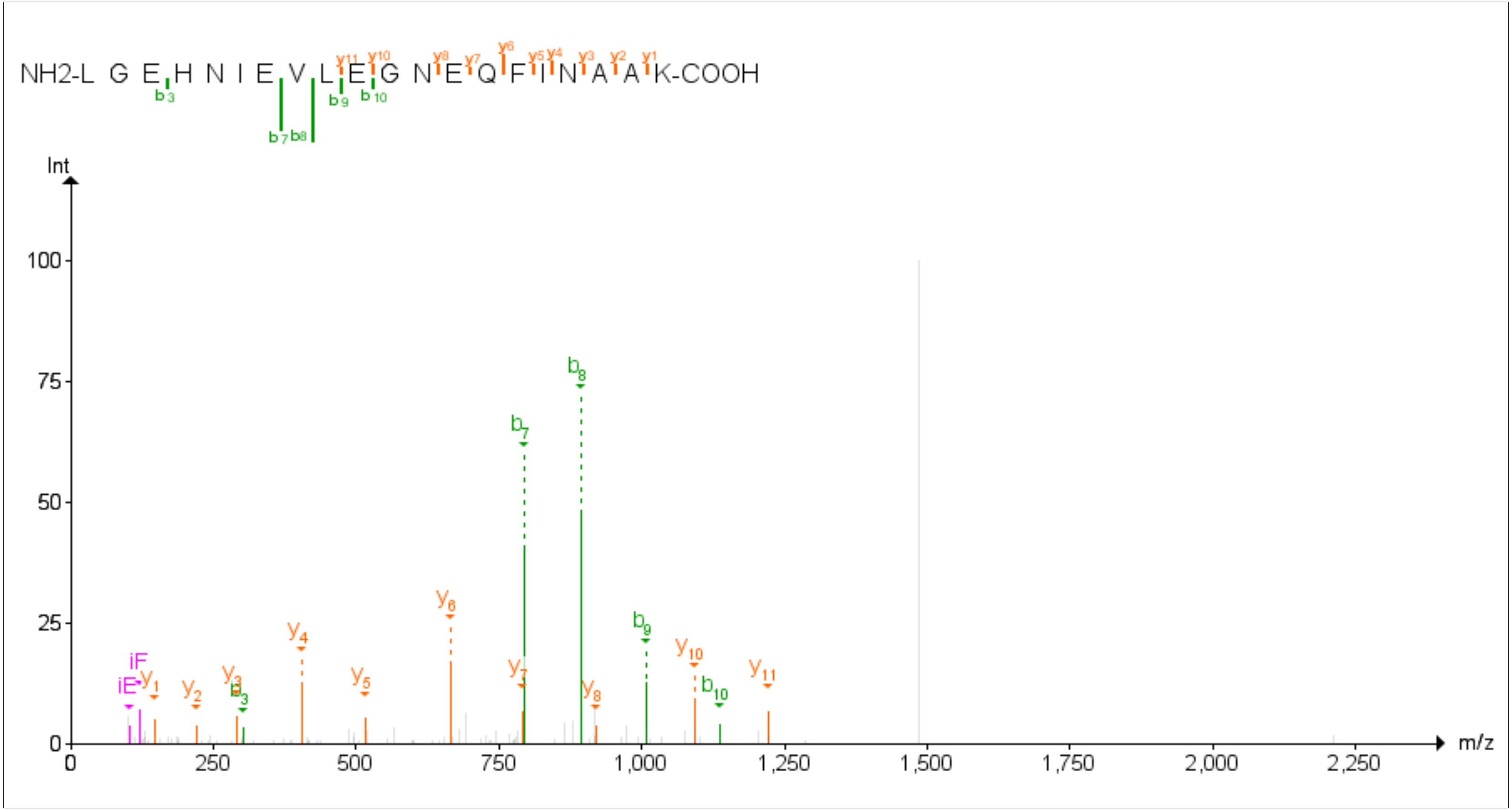

NH2-L G E H N I E V L I E G N E Q F I N A A K-COOH
b3 b7 b8 b9 b10
y11 y10 y8 y7 y6 y5 y4 y3 y2 y1
Int
100
75
50
25
0
m/z
0
250
500
750
1,000
1,250
1,500
1,750
2,000
2,250
iF
iE
y1
y2
y3
b3
y4
y5
y6
y7
b7
b8
y8
b9
y10
b10
y11

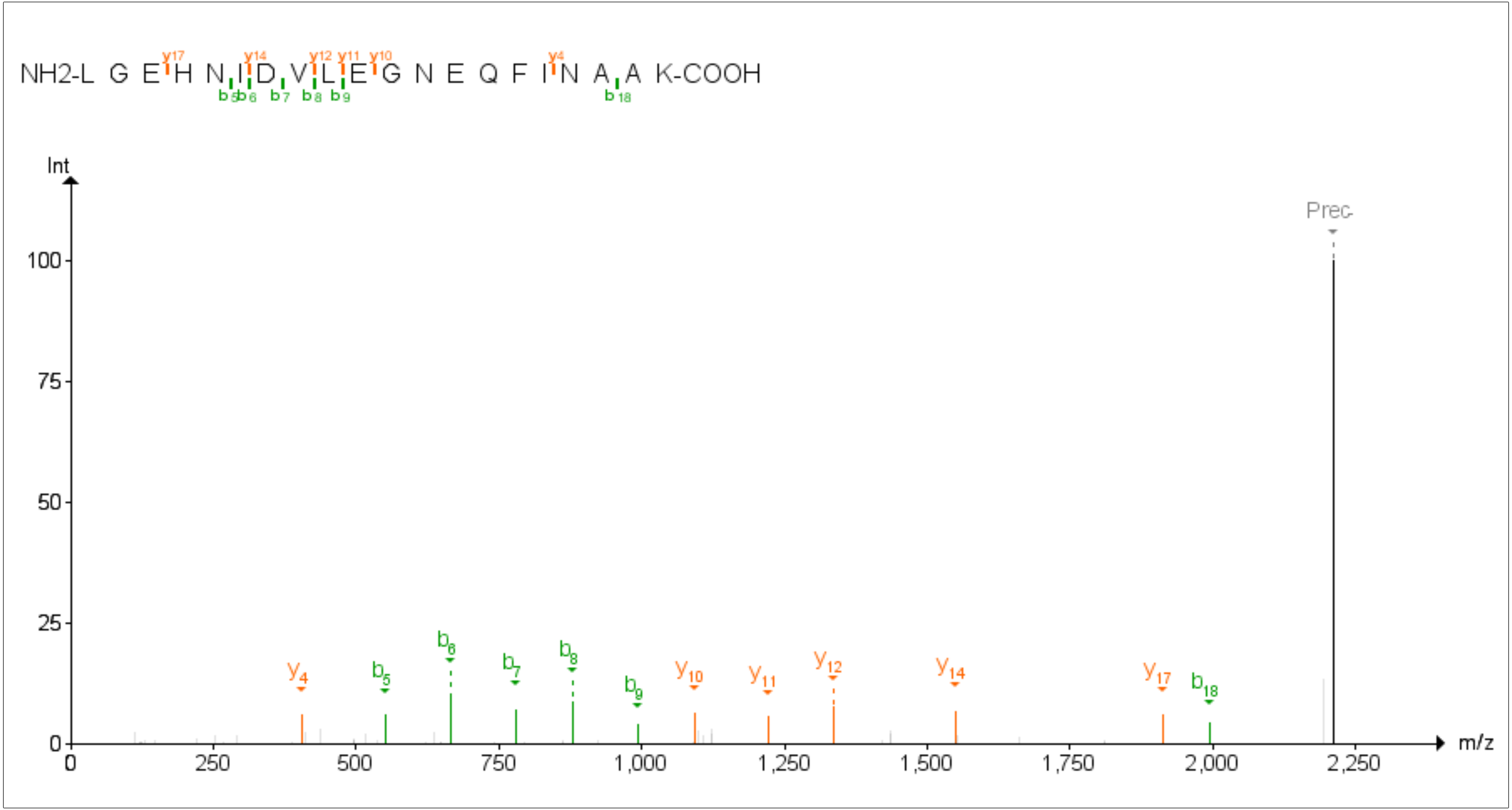

NH2-L G E H N I D V L E G N E Q F I N A A K-COOH
y17
y14
y12
y11
y10
y4
b5
b6
b7
b8
b9
b18
Int
Prec.
100
75
50
25
0
250
500
750
1,000
1,250
1,500
1,750
2,000
2,250
m/z

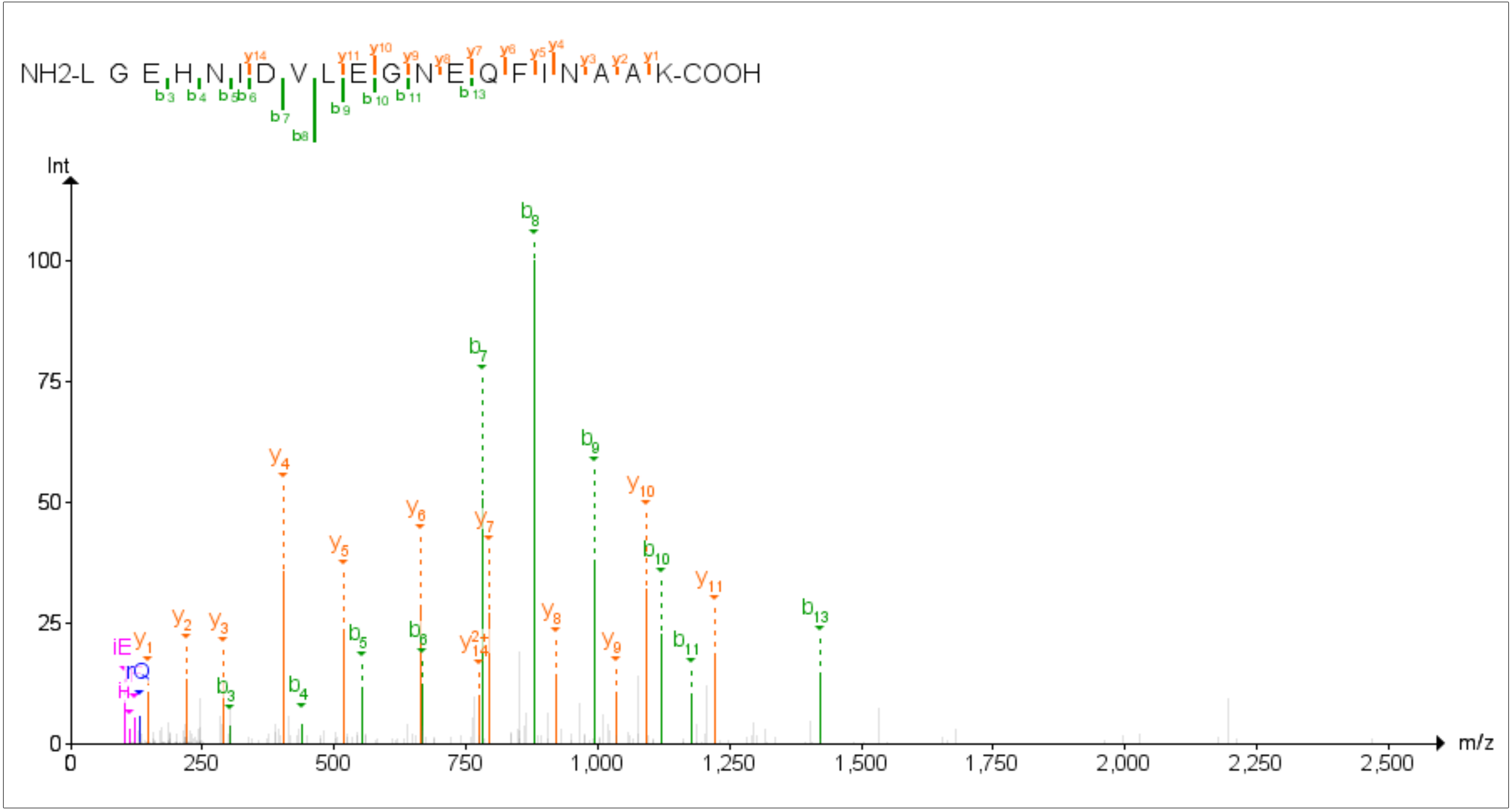

NH2-L G E H N I D V L E G N E Q F I N A A K-COOH
y14 y11 y10 y9 y8 y7 y6 y5 y4 y3 y2 y1
b3 b4 b5 b6 b7 b8 b9 b10 b11 b13
Int
m/z
0
25
50
75
100
250
500
750
1,000
1,250
1,500
1,750
2,000
2,250
2,500
iE
rQ
H

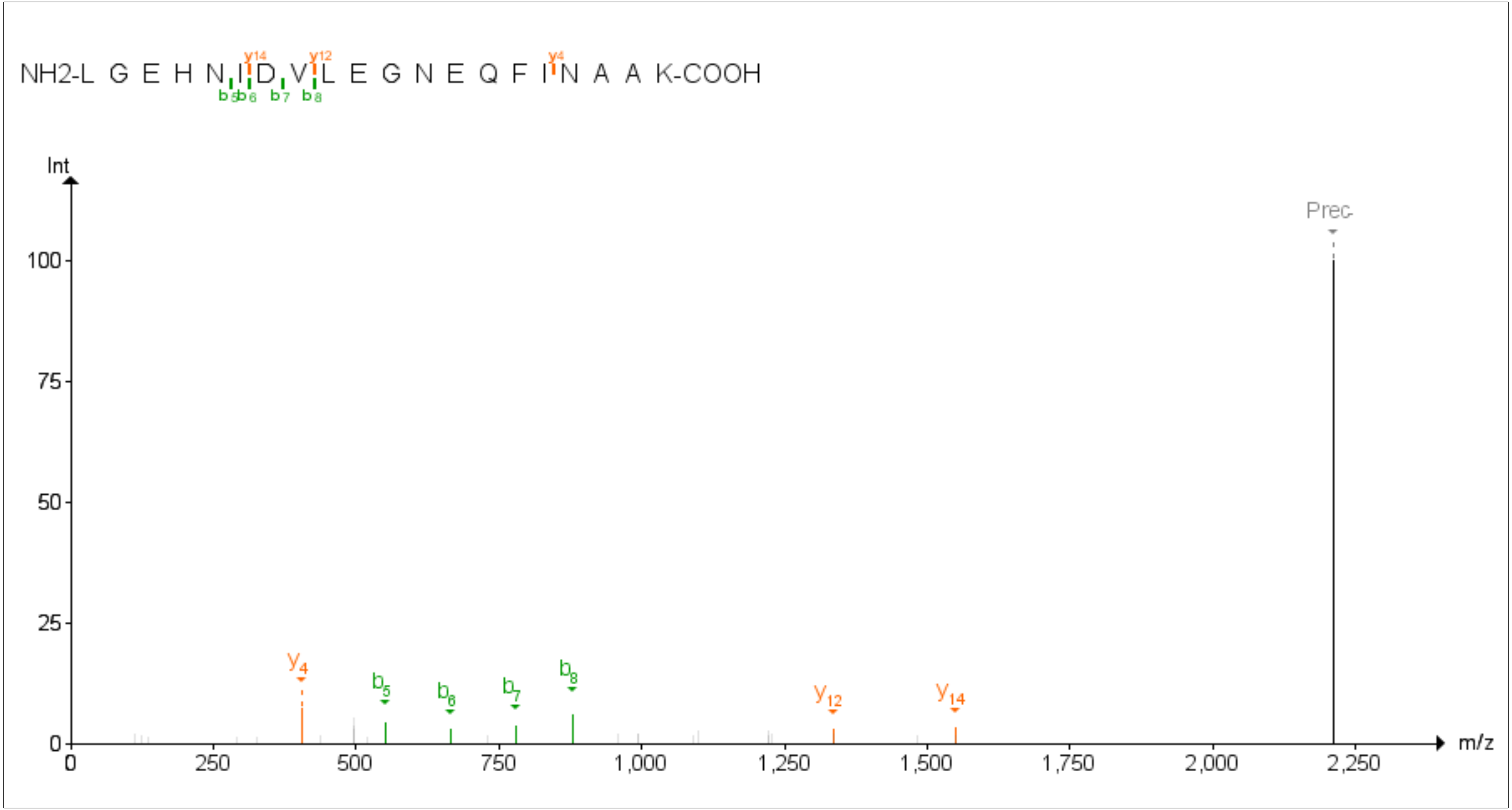
NH2-L G E H N I D V L E G N E Q F I N A A K-COOH
y14
y12
y4
b5
b6
b7
b8
Int
100
75
50
25
0
Prec
y4
b5
b6
b7
b8
y12
y14
0
250
500
750
1,000
1,250
1,500
1,750
2,000
2,250
m/z

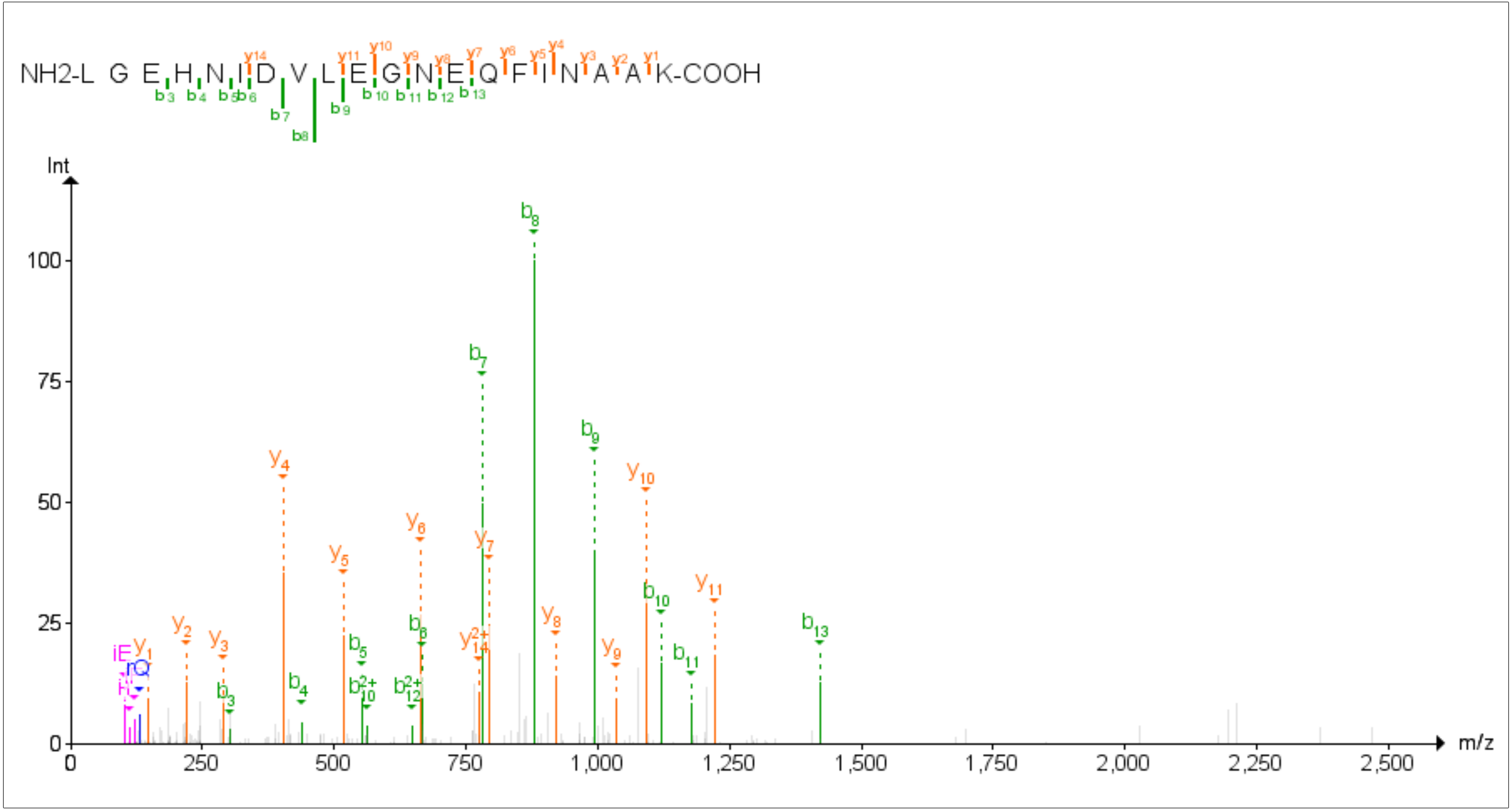
NH2-L G E H N I D V L E G N E Q F I N A A K-COOH
Int
m/z
0
25
50
75
100
250
500
750
1,000
1,250
1,500
1,750
2,000
2,250
2,500

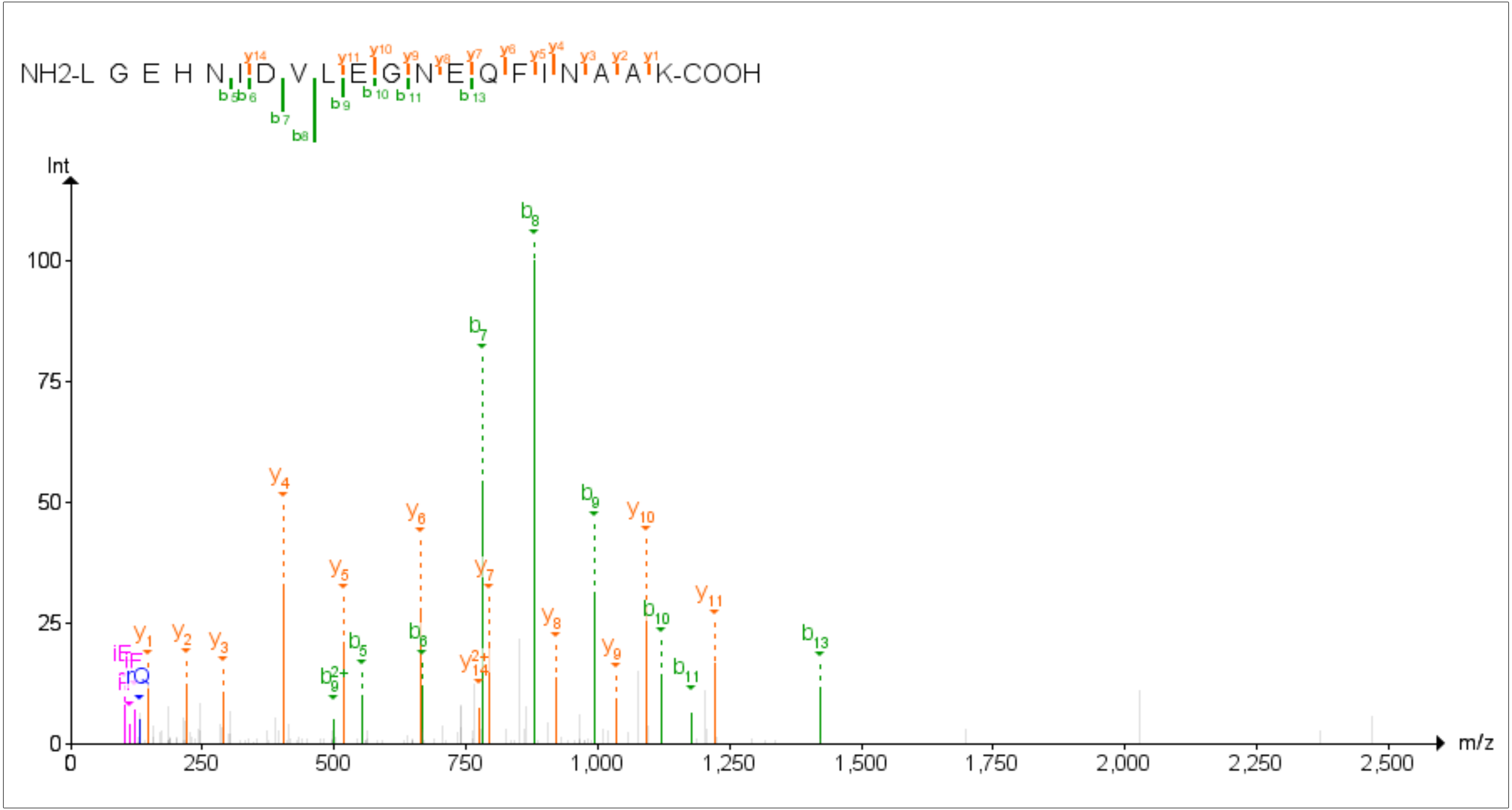

NH2-L G E H N I D V L E G N E Q F I N A A K-COOH
Int
m/z
0
25
50
75
100
250
500
750
1,000
1,250
1,500
1,750
2,000
2,250
2,500

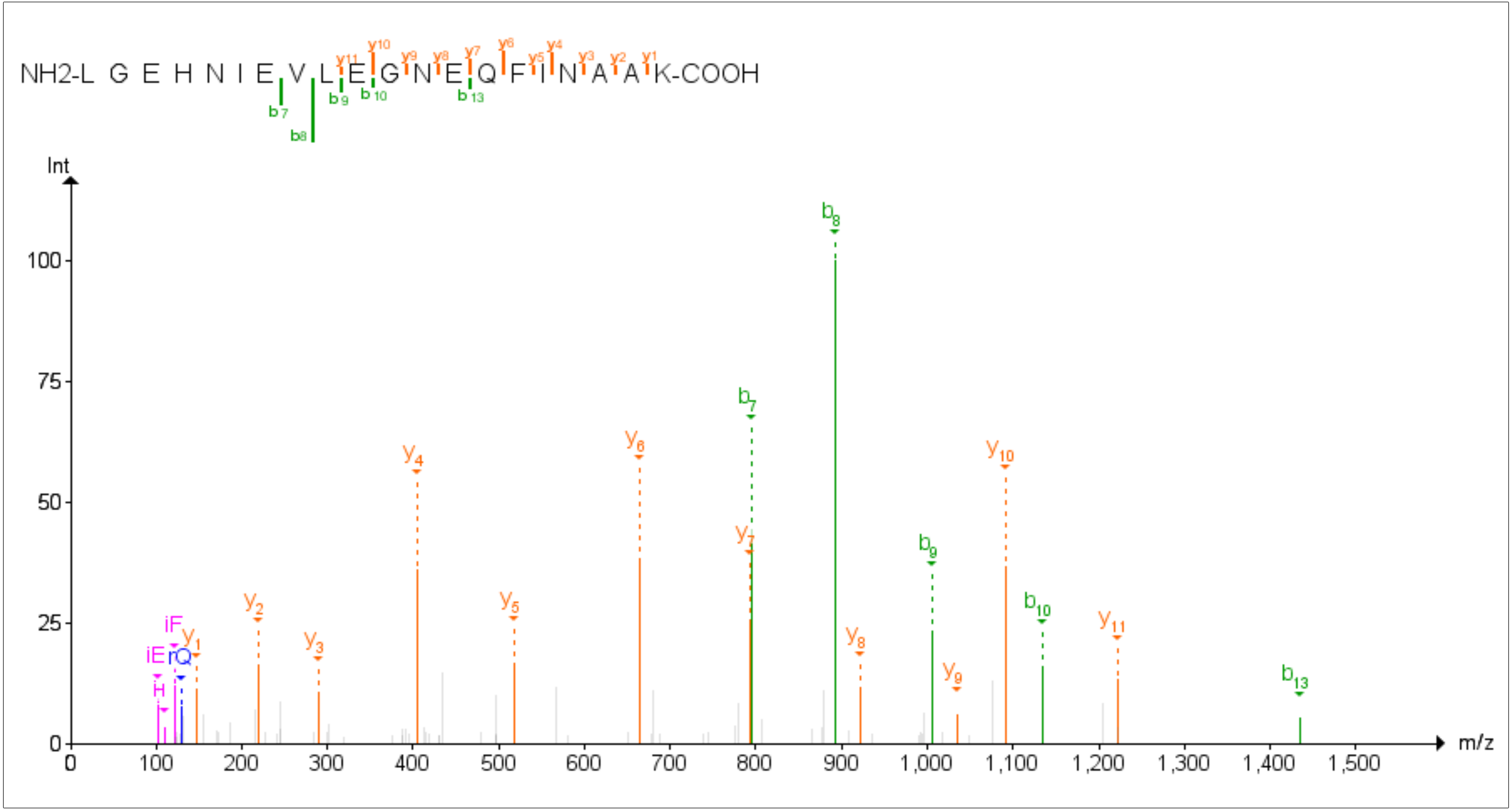

NH2-L G E H N I E V L E G N E Q F I N A A K-COOH
Int
m/z

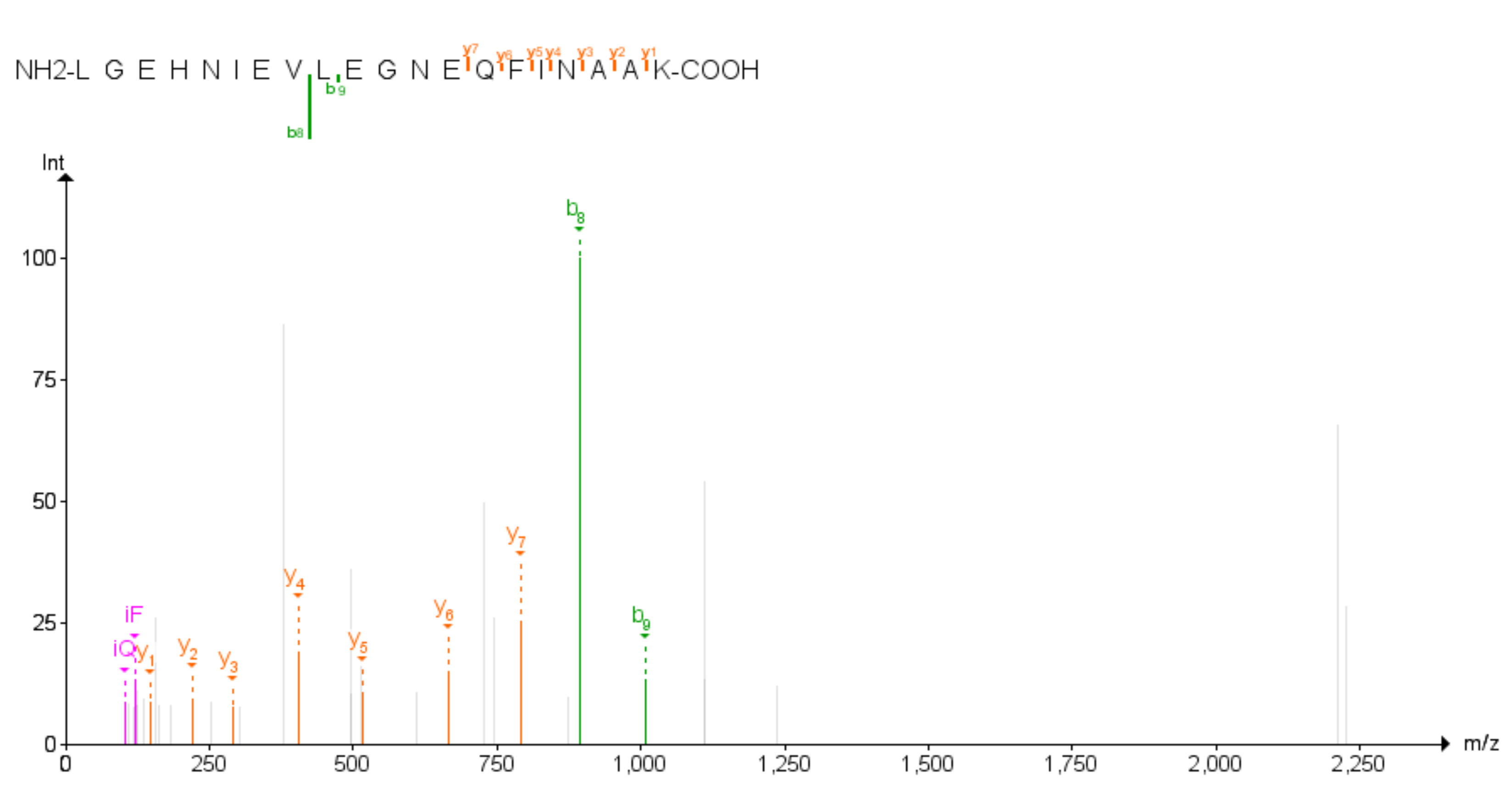

NH2-L G E H N I E V L E G N E Q F I N A A K-COOH
b8
b9
y7
y6
y5
y4
y3
y2
y1
Int
m/z
0
25
50
75
100
0
250
500
750
1,000
1,250
1,500
1,750
2,000
2,250
iF
iQ
y_1
y_2
y_3
y_4
y_5
y_6
y_7
b_8
b_9

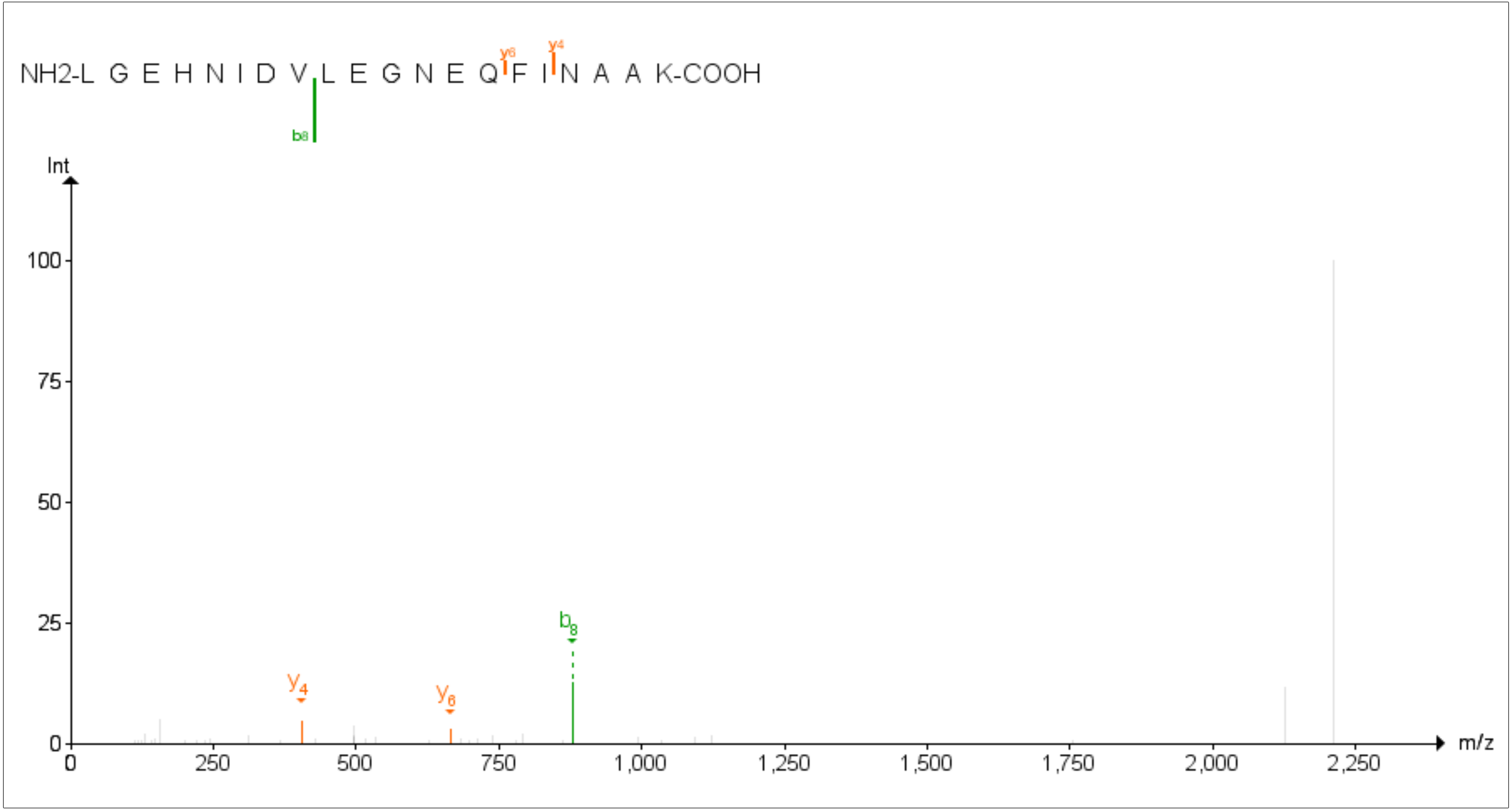

NH2-L G E H N I D V L E G N E Q F I N A A K-COOH
y6
y4
b8
Int
100
75
50
25
0
m/z
0
250
500
750
1,000
1,250
1,500
1,750
2,000
2,250
y4
y6
b8

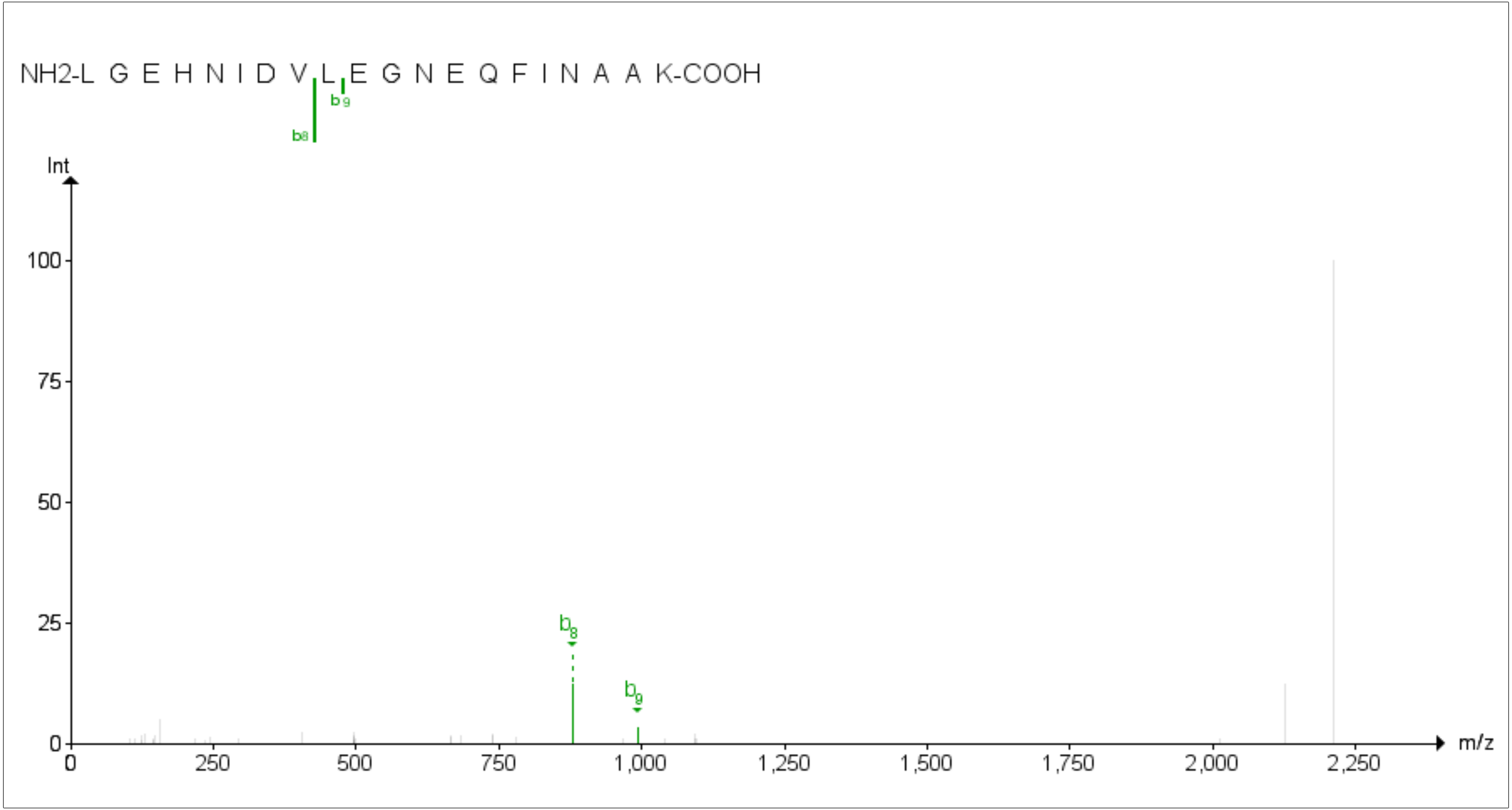

NH2-L G E H N I D V L E G N E Q F I N A A K-COOH
b8
b9
Int
100
75
50
25
0
b8
b9
0
250
500
750
1,000
1,250
1,500
1,750
2,000
2,250
m/z

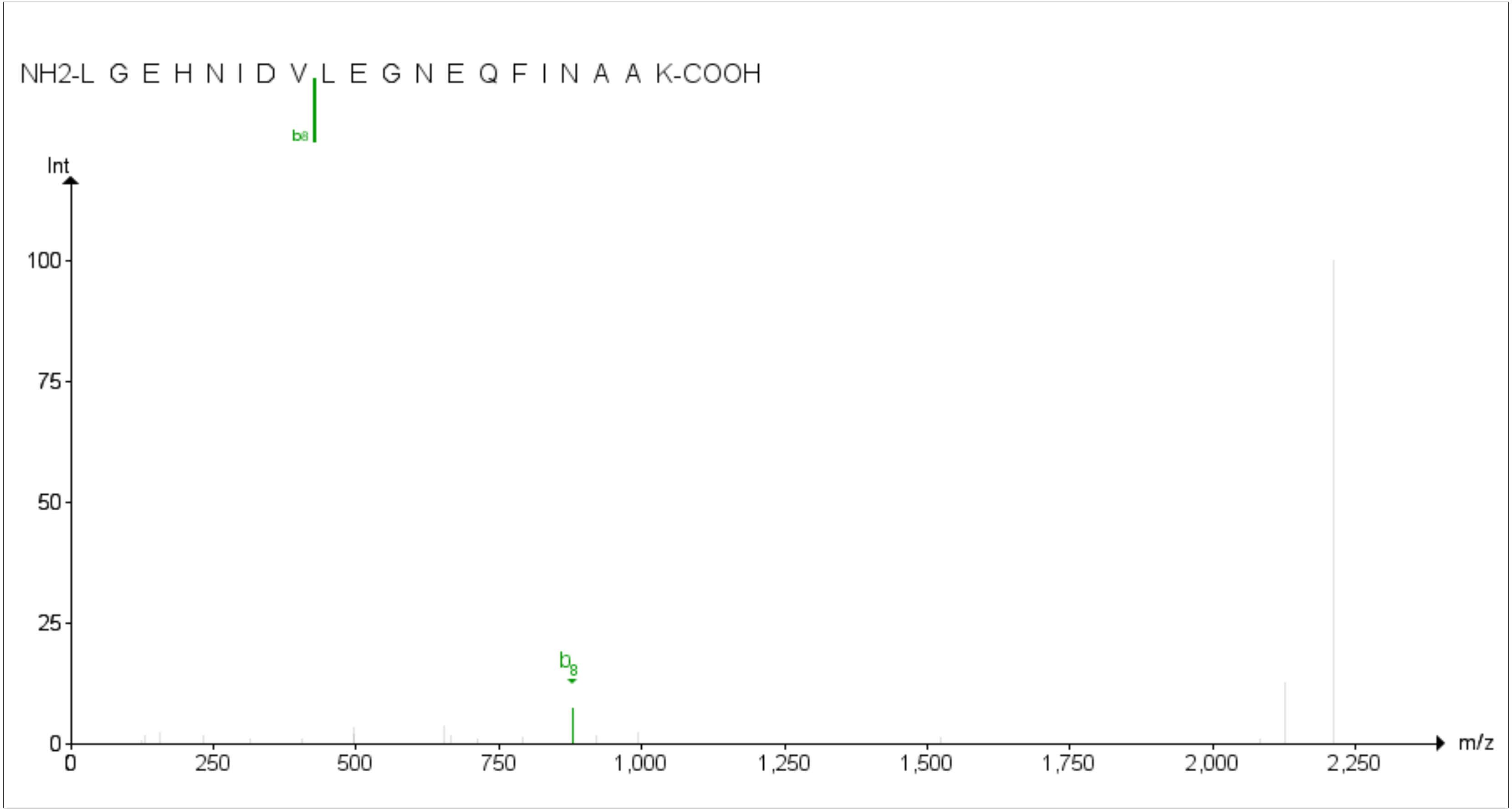
NH2-L G E H N I D V L E G N E Q F I N A A K-COOH
b8
Int
100
75
50
25
0
b8
0
250
500
750
1,000
1,250
1,500
1,750
2,000
2,250
m/z

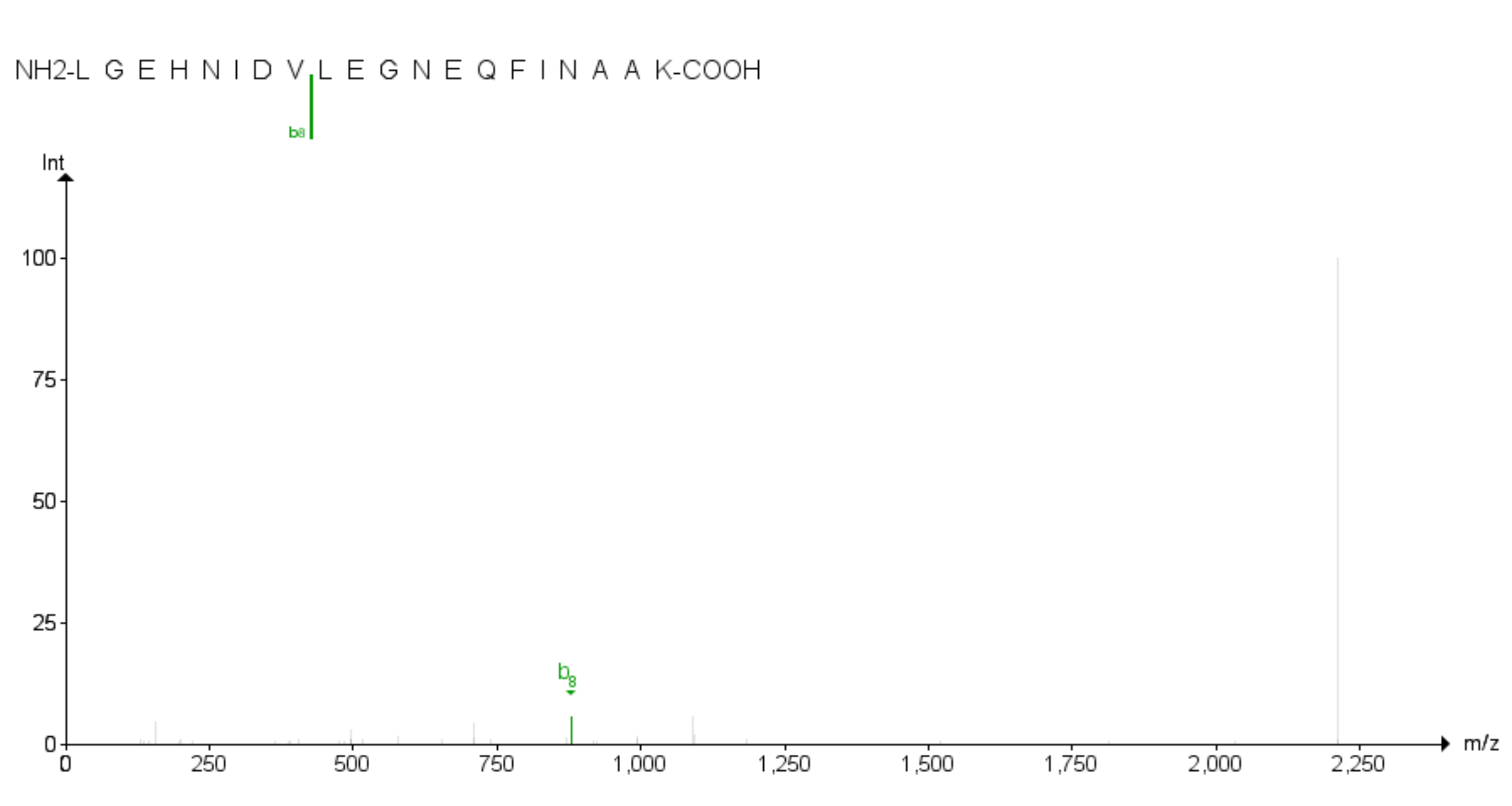

NH2-L G E H N I D V L E G N E Q F I N A A K-COOH
b8
Int
100
75
50
25
0
b8
0
250
500
750
1,000
1,250
1,500
1,750
2,000
2,250
m/z

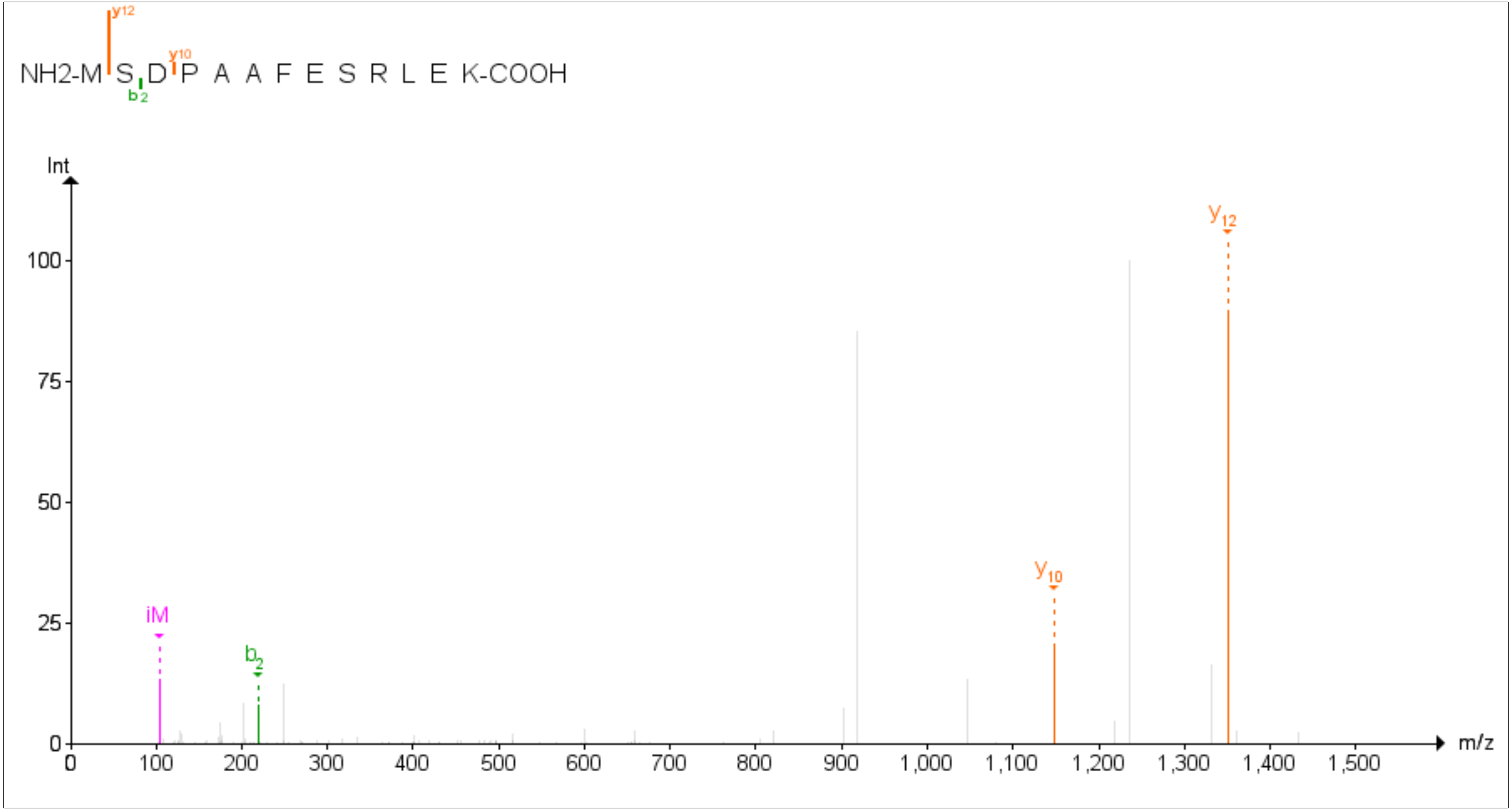

y12
y10
NH2-M S D P A A F E S R L E K-COOH
b2
Int
100
75
50
25
0
y12
y10
iM
b2
0
100
200
300
400
500
600
700
800
900
1,000
1,100
1,200
1,300
1,400
1,500
m/z

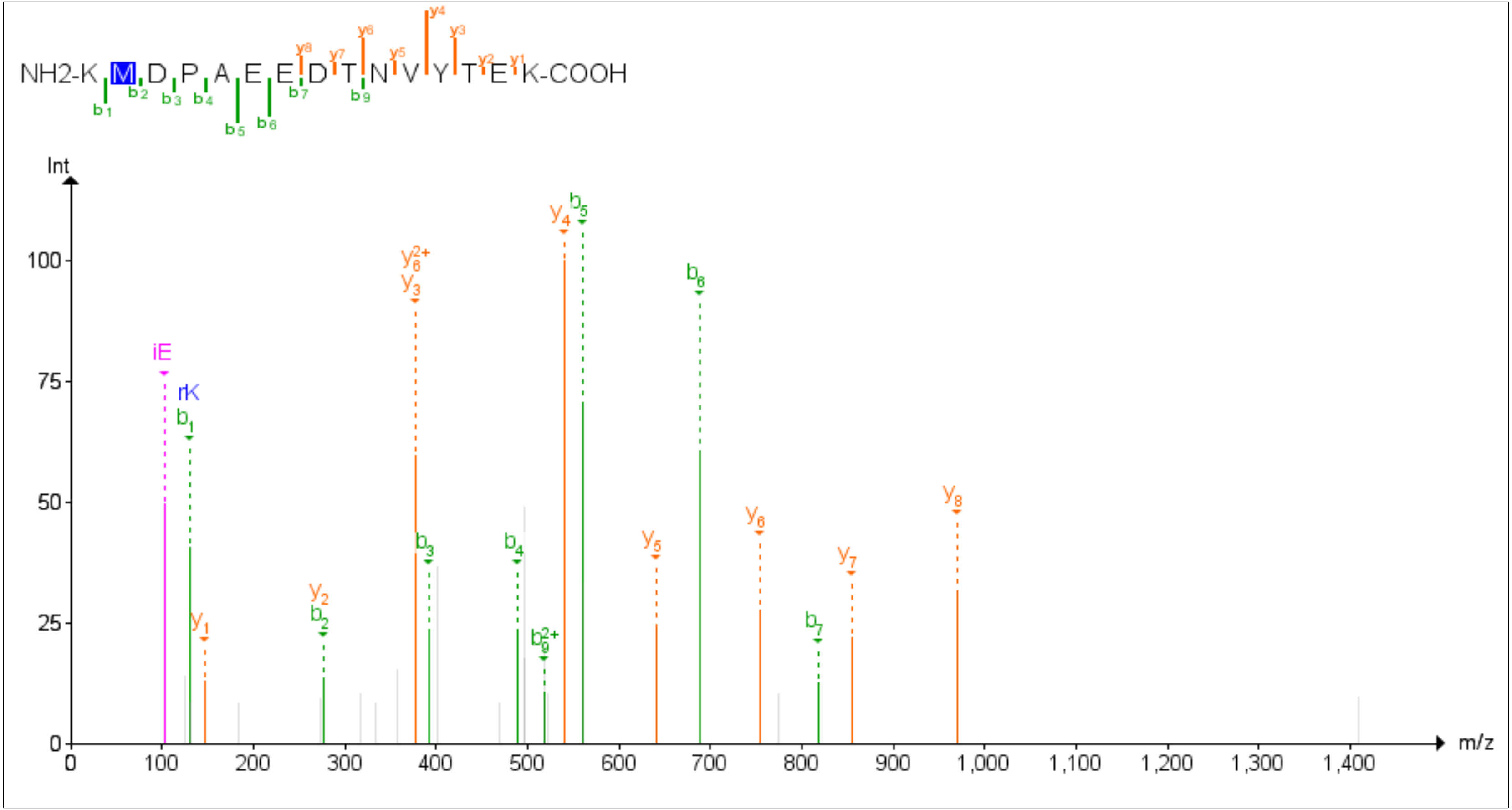

NH2-K M D P A E E D T N V Y T E K-COOH
b1 b2 b3 b4 b5 b6 b7 b9
y8 y7 y6 y5 y4 y3 y2 y1
Int
m/z
iE
rK
b_1
y_1
y_2
b_2
y_6^{2+}
y_3
b_3
b_4
b_9^{2+}
y_4
b_5
b_6
y_5
y_6
b_7
y_7
y_8
0 25 50 75 100
0 100 200 300 400 500 600 700 800 900 1,000 1,100 1,200 1,300 1,400

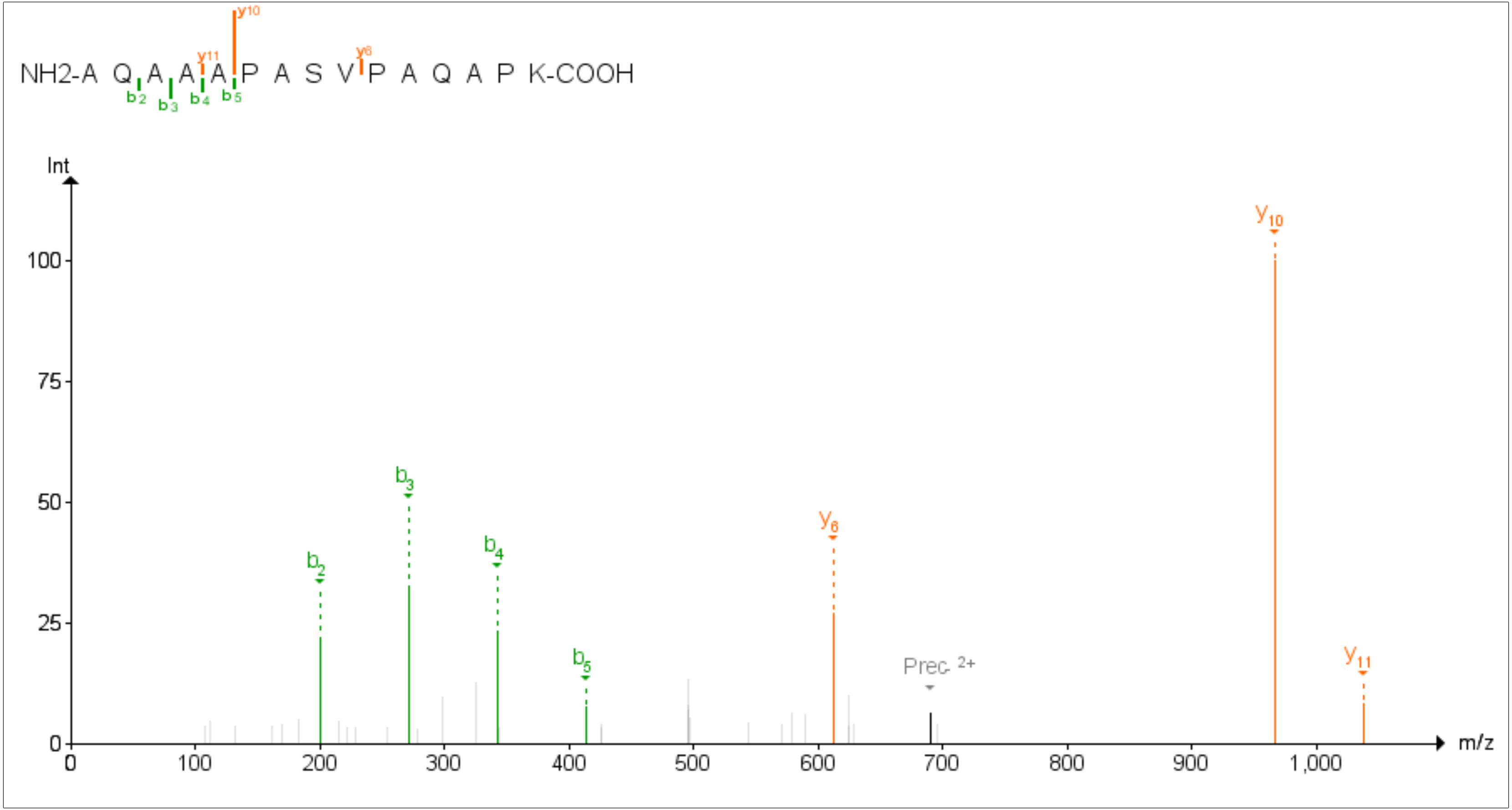

NH2-A Q A A A P A S V P A Q A P K-COOH
y10
y11
y6
b2
b3
b4
b5
Int
100
75
50
25
0
m/z
0
100
200
300
400
500
600
700
800
900
1,000
y10
b3
y6
b4
b2
b5
Prec 2+
y11

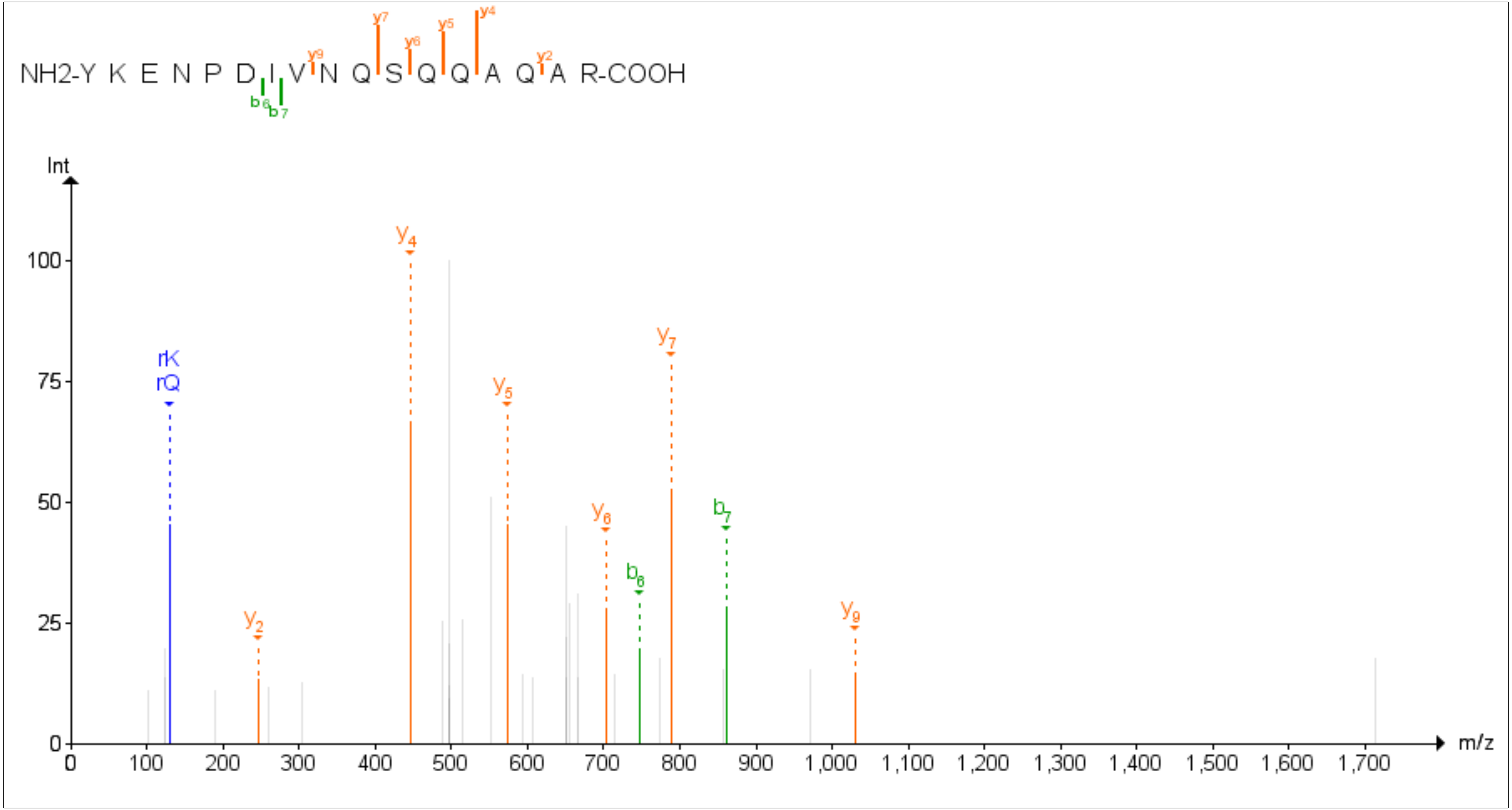

NH2-Y K E N P D I V N Q S Q Q A Q A R-COOH
y7
y6
y5
y4
y9
y2
b6
b7
Int
100
75
50
25
0
rK
rQ
y4
y5
y7
y6
b6
b7
y2
y9
0
100
200
300
400
500
600
700
800
900
1,000
1,100
1,200
1,300
1,400
1,500
1,600
1,700
m/z

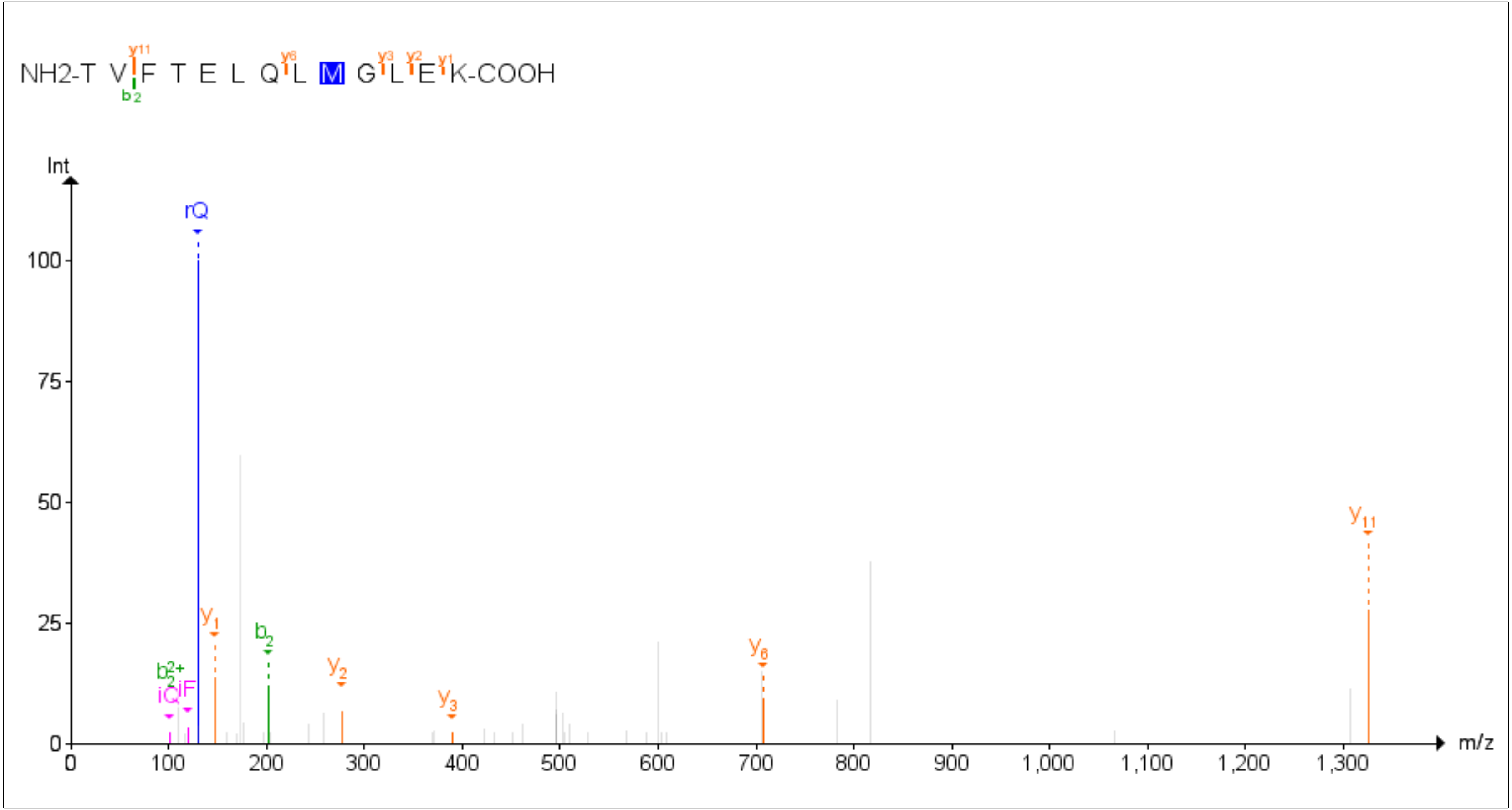

NH2-T V F T E L Q L M G L E K-COOH
y11
b2
y6
y3
y2
y1
Int
m/z
rQ
y1
b2
b2 2+
iQ
iF
y2
y3
y6
y11
0
25
50
75
100
0
100
200
300
400
500
600
700
800
900
1,000
1,100
1,200
1,300

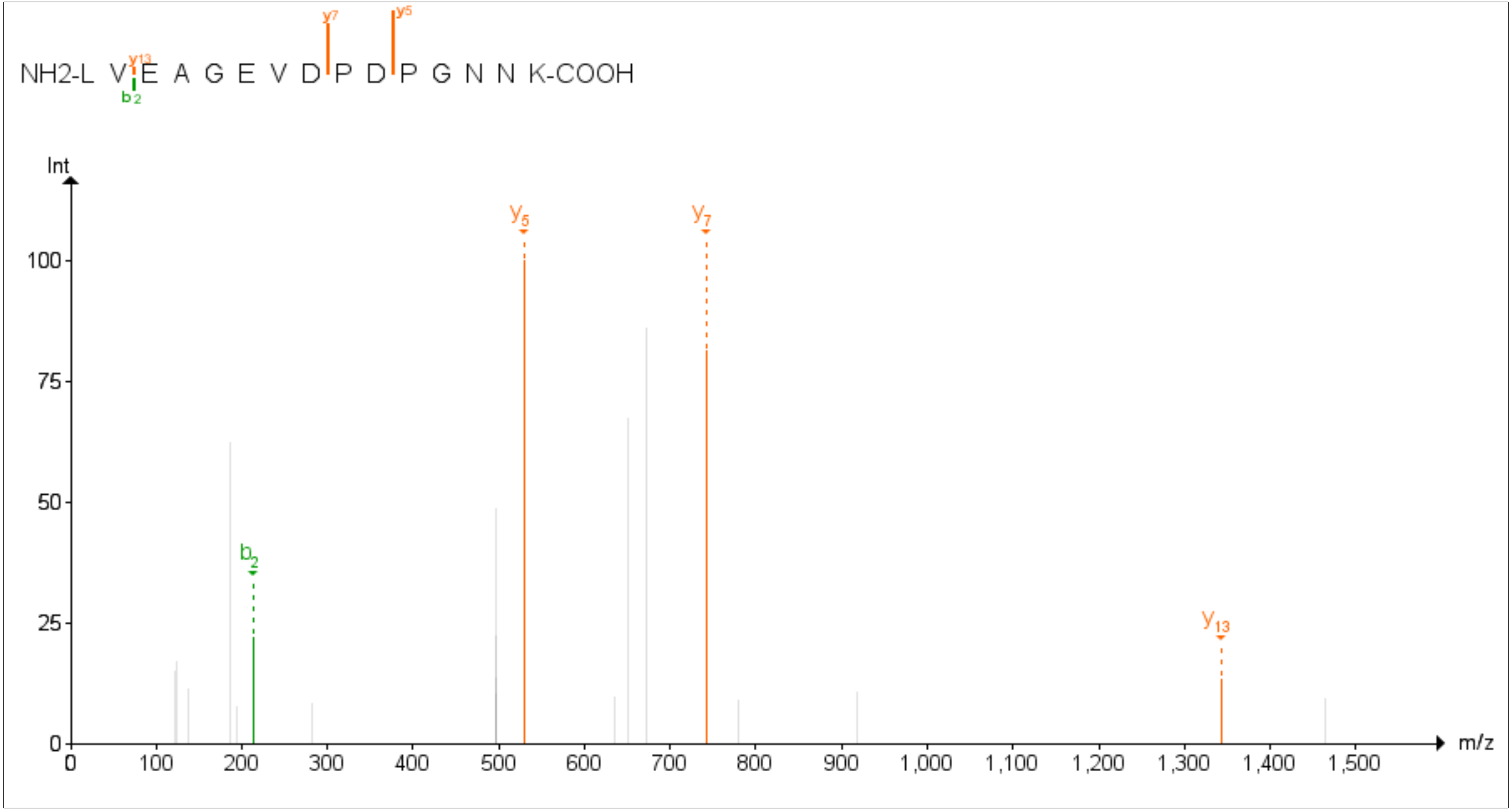

NH2-L V E A G E V D P D P G N N K-COOH
y7
y5
y13
b2
Int
100
75
50
25
0
y5
y7
b2
y13
0
100
200
300
400
500
600
700
800
900
1,000
1,100
1,200
1,300
1,400
1,500
m/z

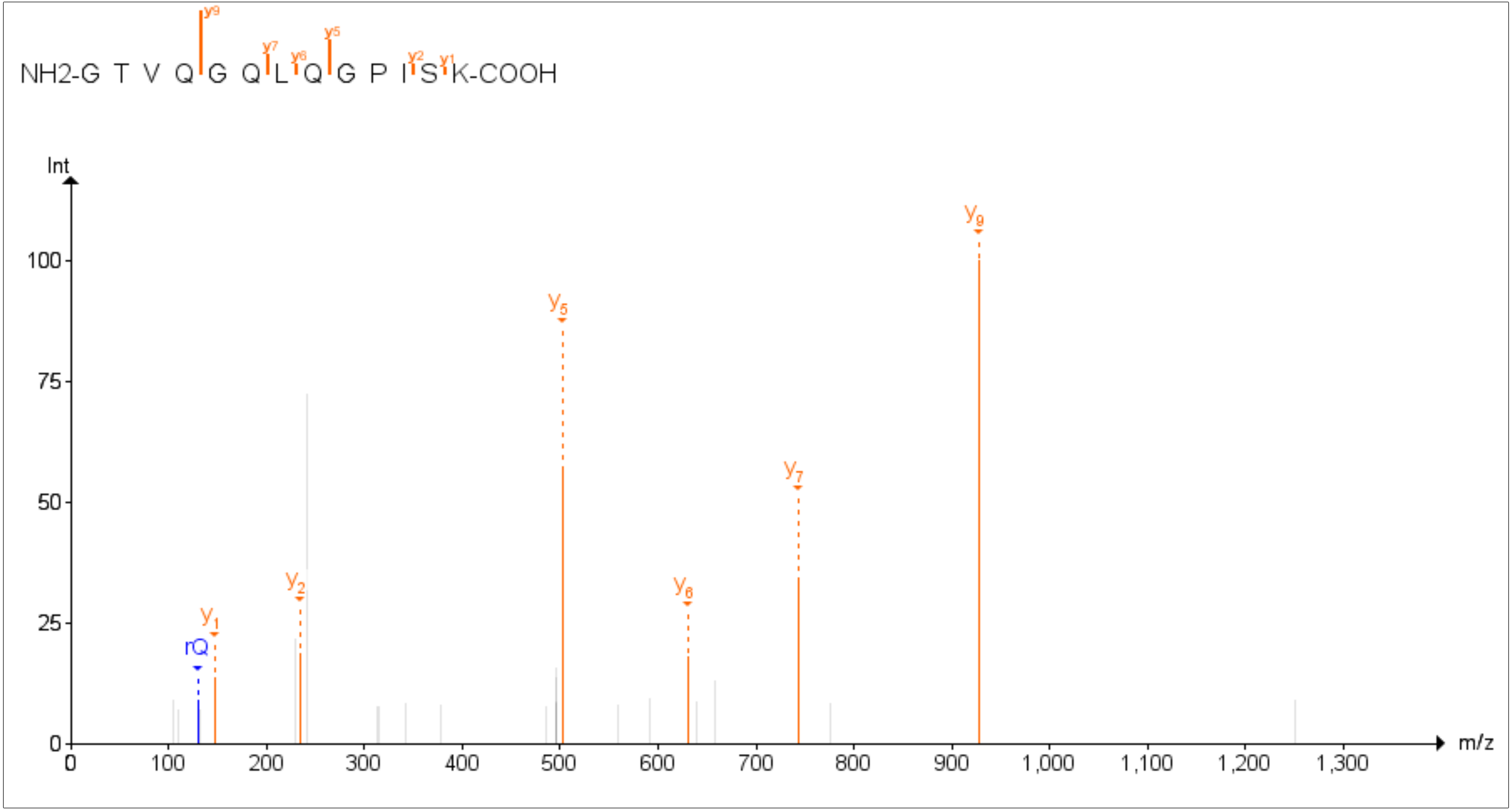

NH2-G T V Q G Q L Q G P I S K-COOH
y9
y7
y6
y5
y2
y1
Int
100
75
50
25
0
y9
y5
y7
y6
y2
y1
rQ
0
100
200
300
400
500
600
700
800
900
1,000
1,100
1,200
1,300
m/z

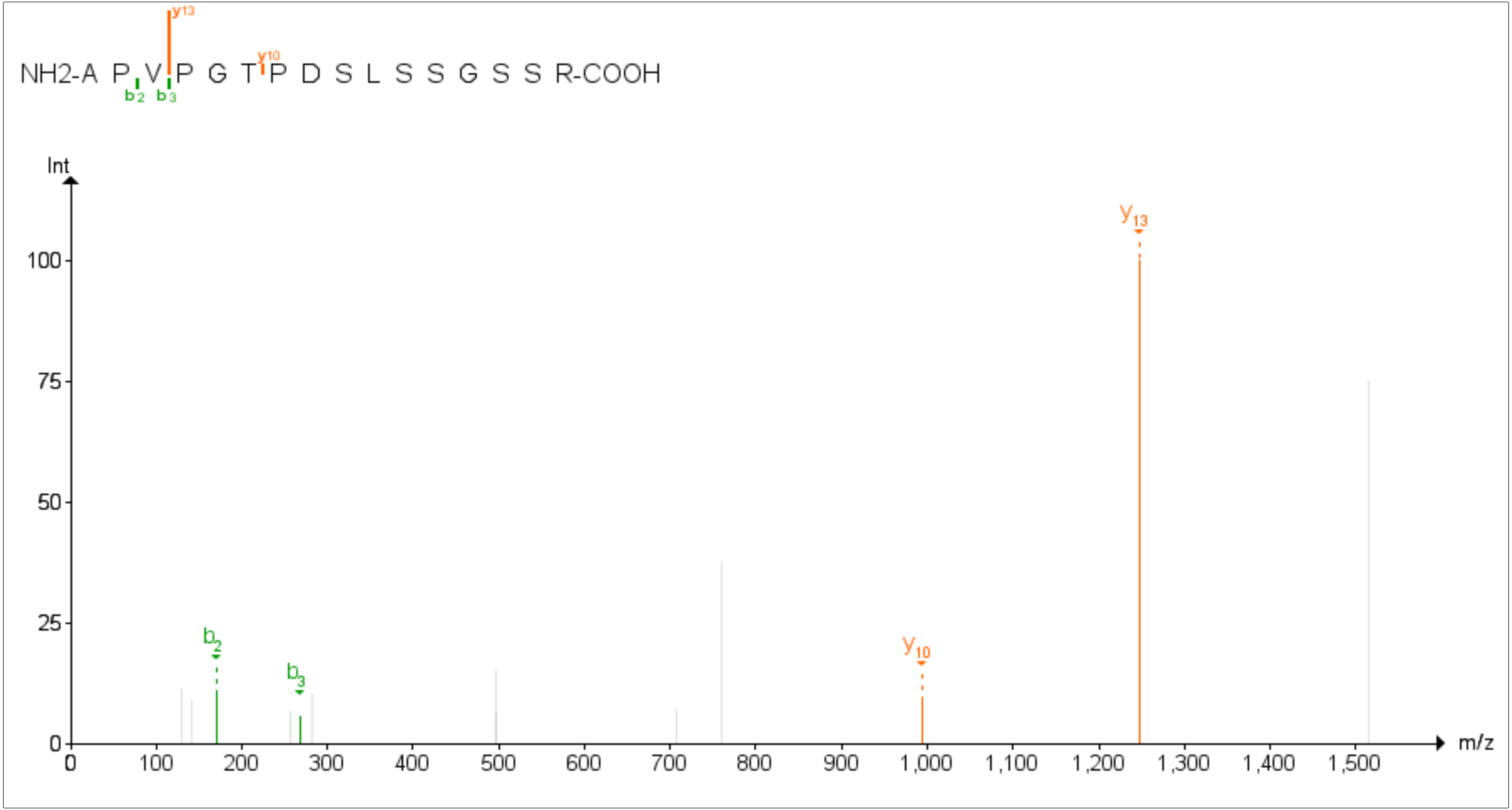

NH2-A P V P G T P D S L S S G S S R-COOH
y13
y10
b2
b3
Int
100
75
50
25
0
m/z
0
100
200
300
400
500
600
700
800
900
1,000
1,100
1,200
1,300
1,400
1,500

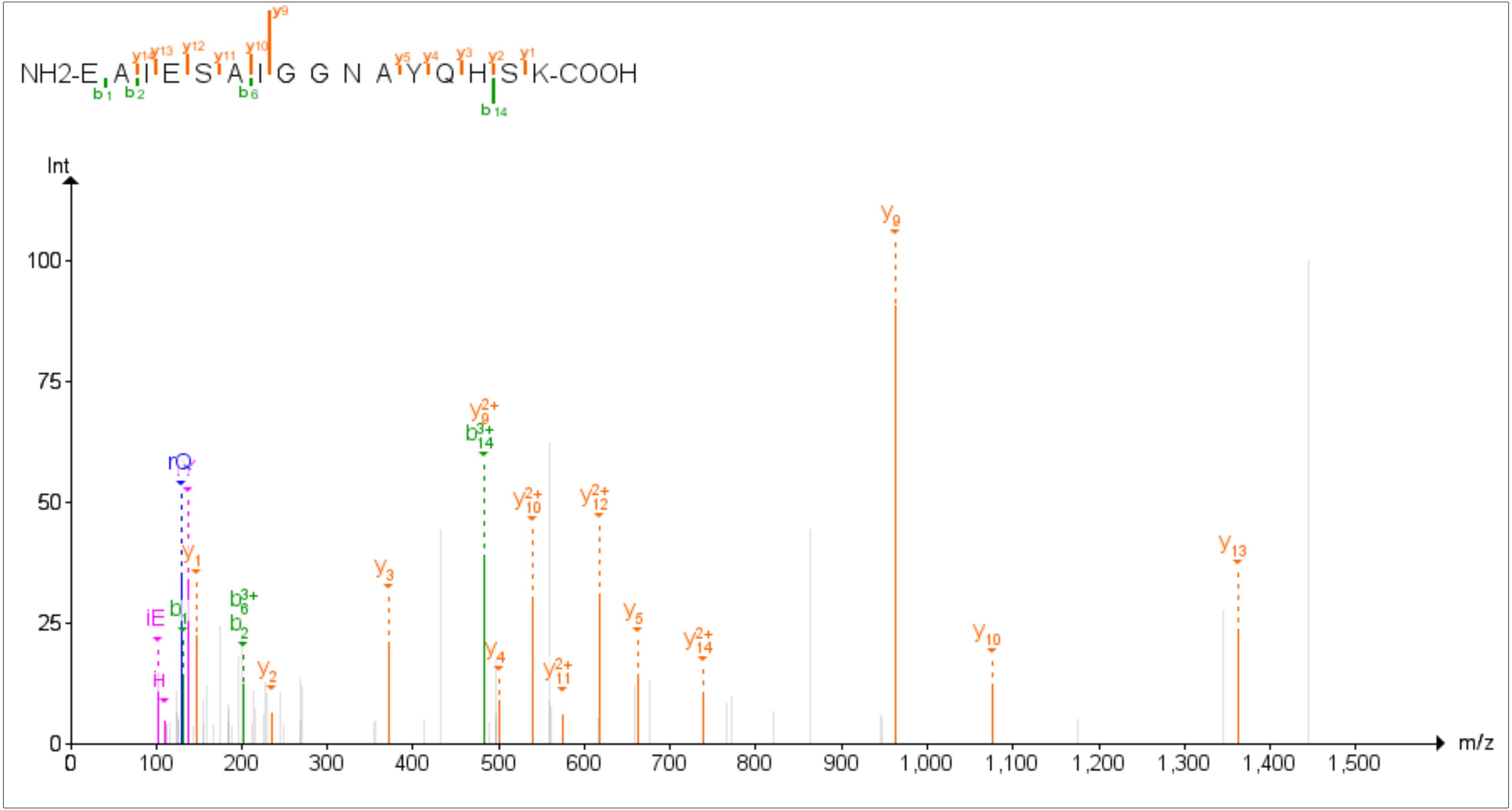

NH2-E A I E S A I G G N A Y Q H S K-COOH
Int
m/z
y9
y13
y10
y12
y14
y5
y1
y3
y2
y4
y11
rQ
iE
iH
b1
b2
b6
b14

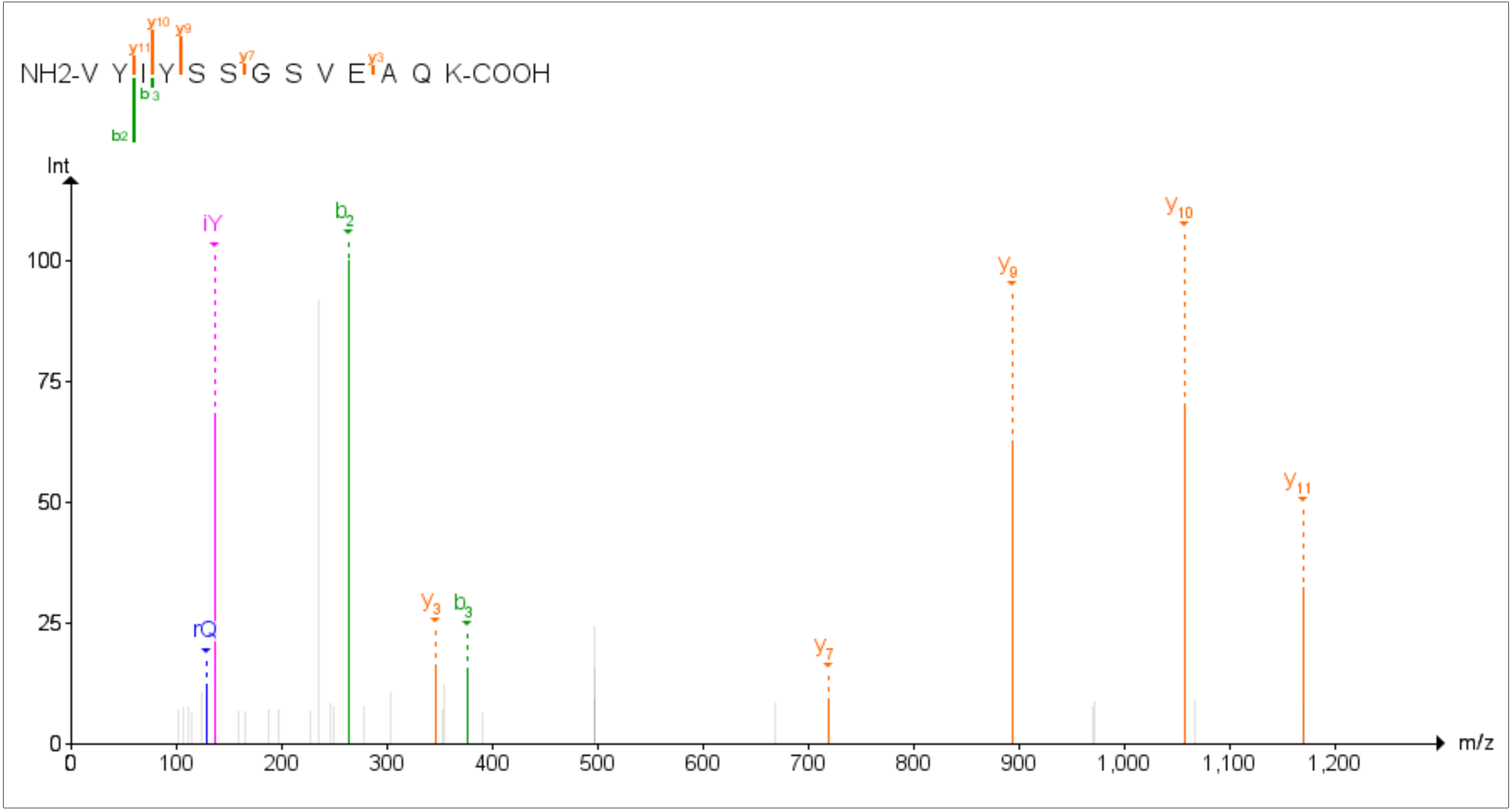

NH2-V Y I Y S S G S V E A Q K-COOH
y10
y9
y11
y7
y3
b3
b2
Int
iY
b2
y10
y9
y11
y3
b3
rQ
y7
100
75
50
25
0
0
100
200
300
400
500
600
700
800
900
1,000
1,100
1,200
m/z

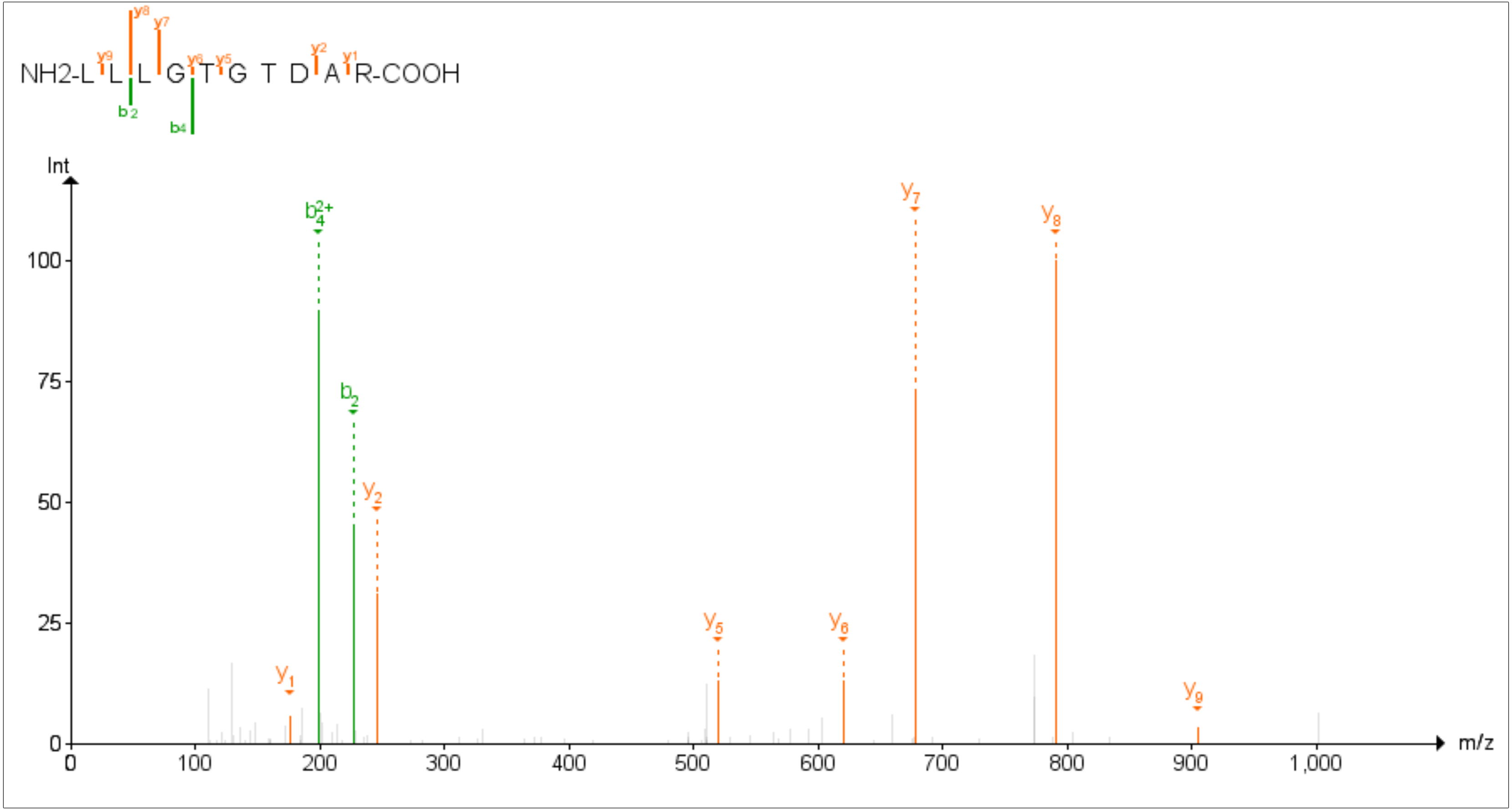
NH2-L L L G T G T D A R-COOH
y9
y8
y7
y6
y5
y2
y1
b2
b4
Int
m/z
b_4^{2+}
b_2
y_1
y_2
y_5
y_6
y_7
y_8
y_9
0
25
50
75
100
0
100
200
300
400
500
600
700
800
900
1,000

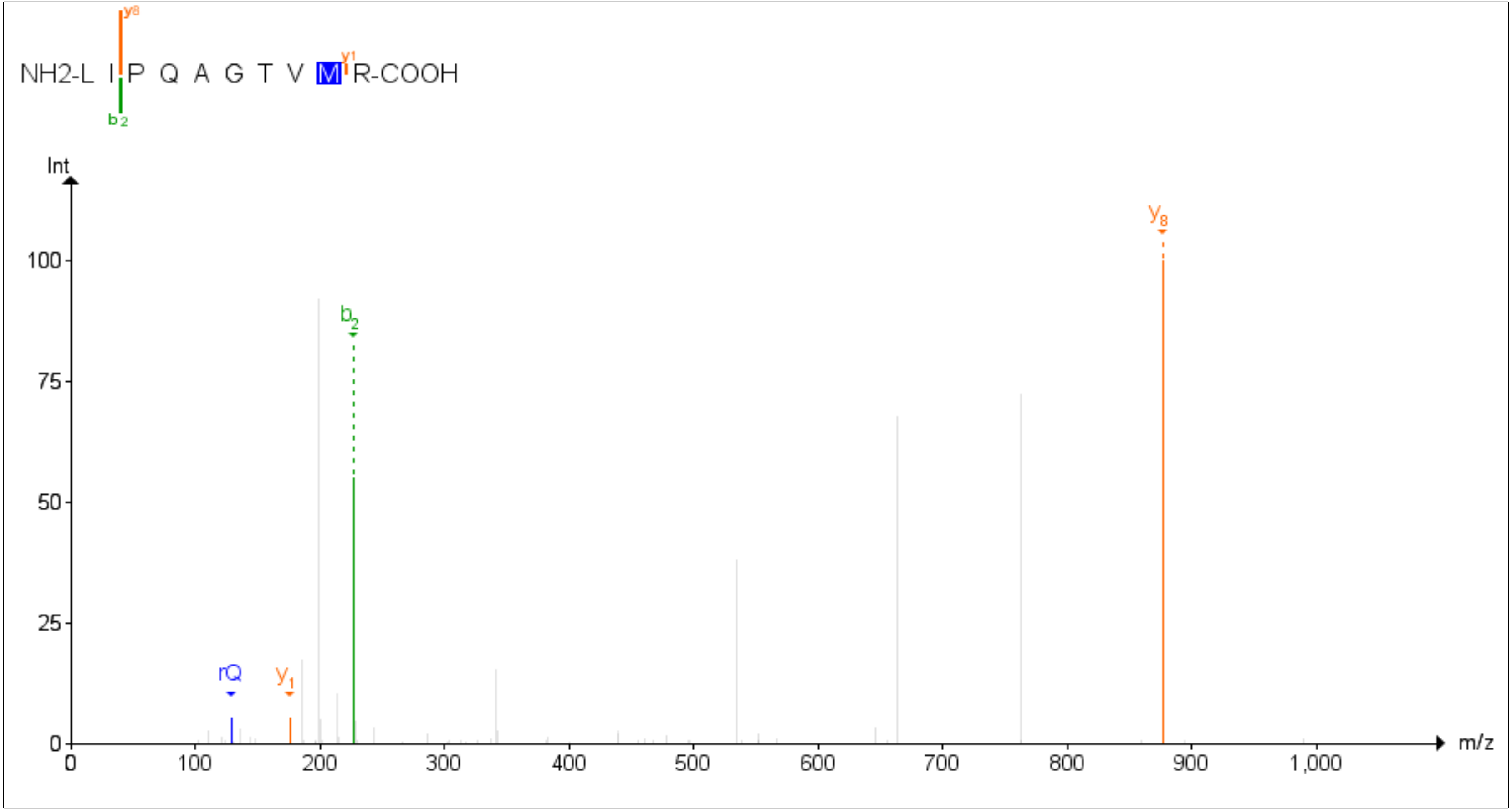

NH2-L I P Q A G T V M R-COOH
y8
y1
b2
Int
100
75
50
25
0
rQ
y1
b2
y8
0
100
200
300
400
500
600
700
800
900
1,000
m/z

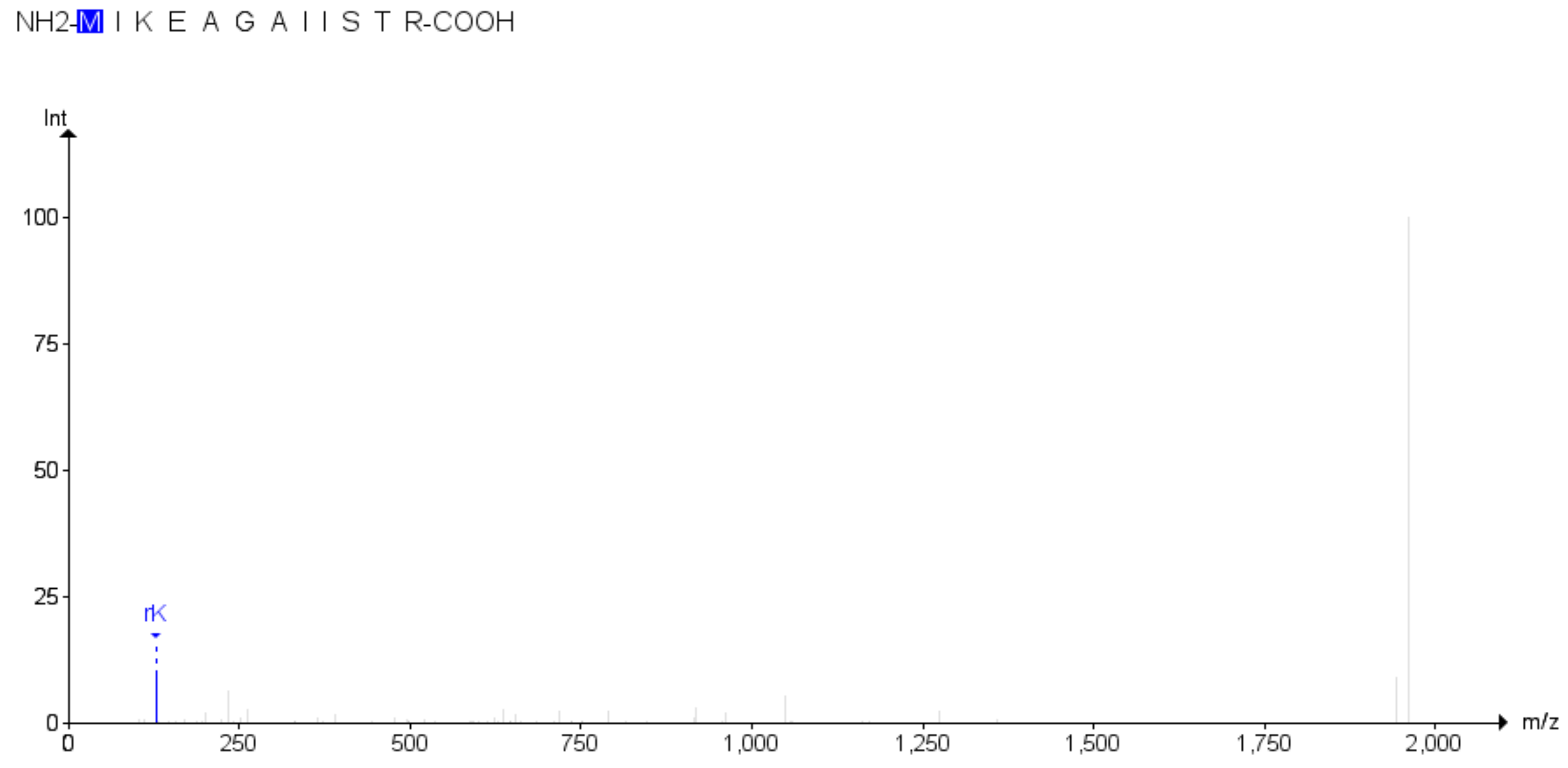

NH2-M I K E A G A I I S T R-COOH
Int
100
75
50
25
0
rK
0
250
500
750
1,000
1,250
1,500
1,750
2,000
m/z

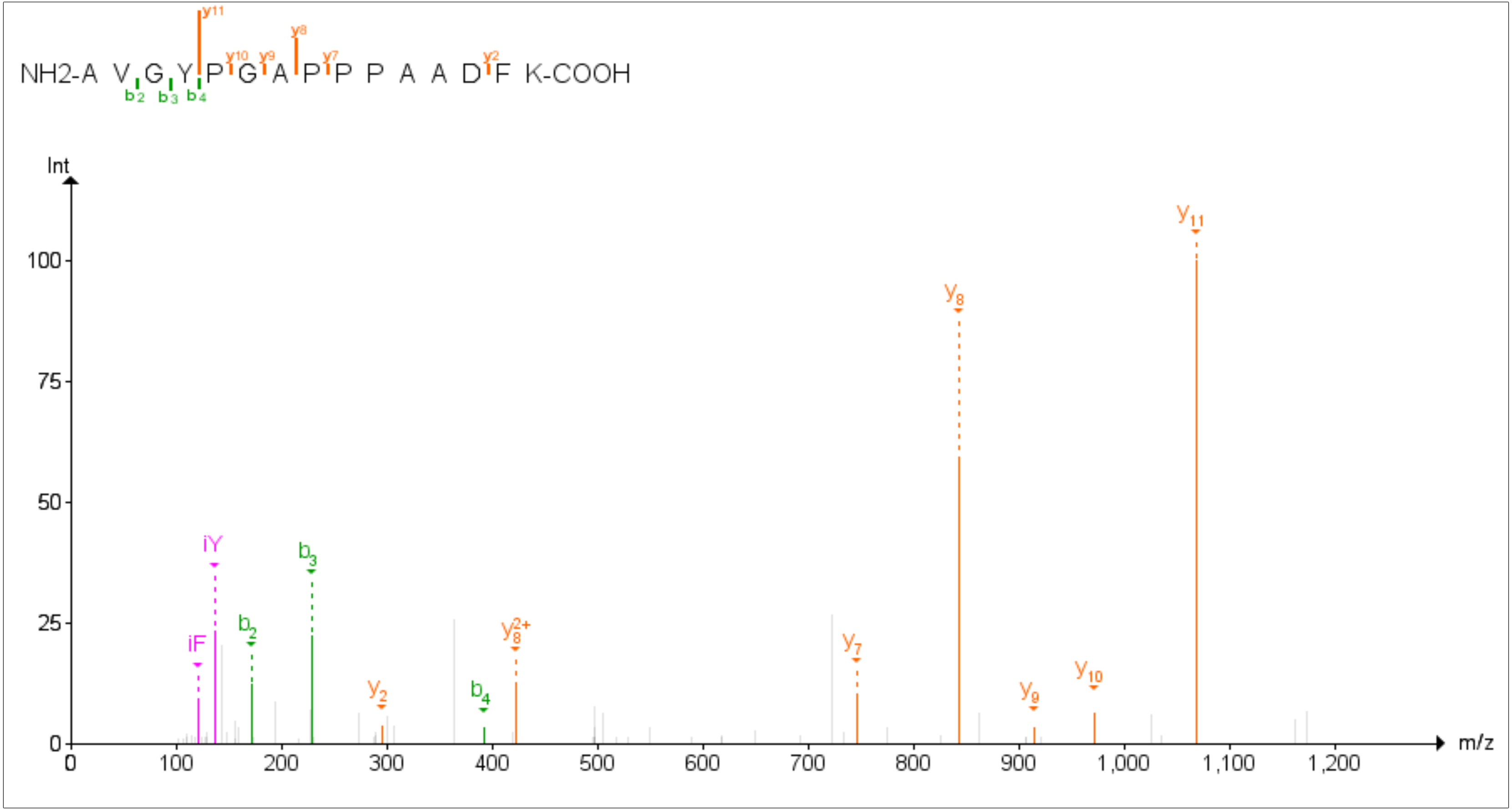

NH2-A V G Y P G A P P P A A D F K-COOH
y11
y10
y9
y8
y7
y2
b2
b3
b4
Int
100
75
50
25
0
iY
iF
b2
b3
y2
b4
y8 2+
y7
y8
y9
y10
y11
0
100
200
300
400
500
600
700
800
900
1,000
1,100
1,200
m/z

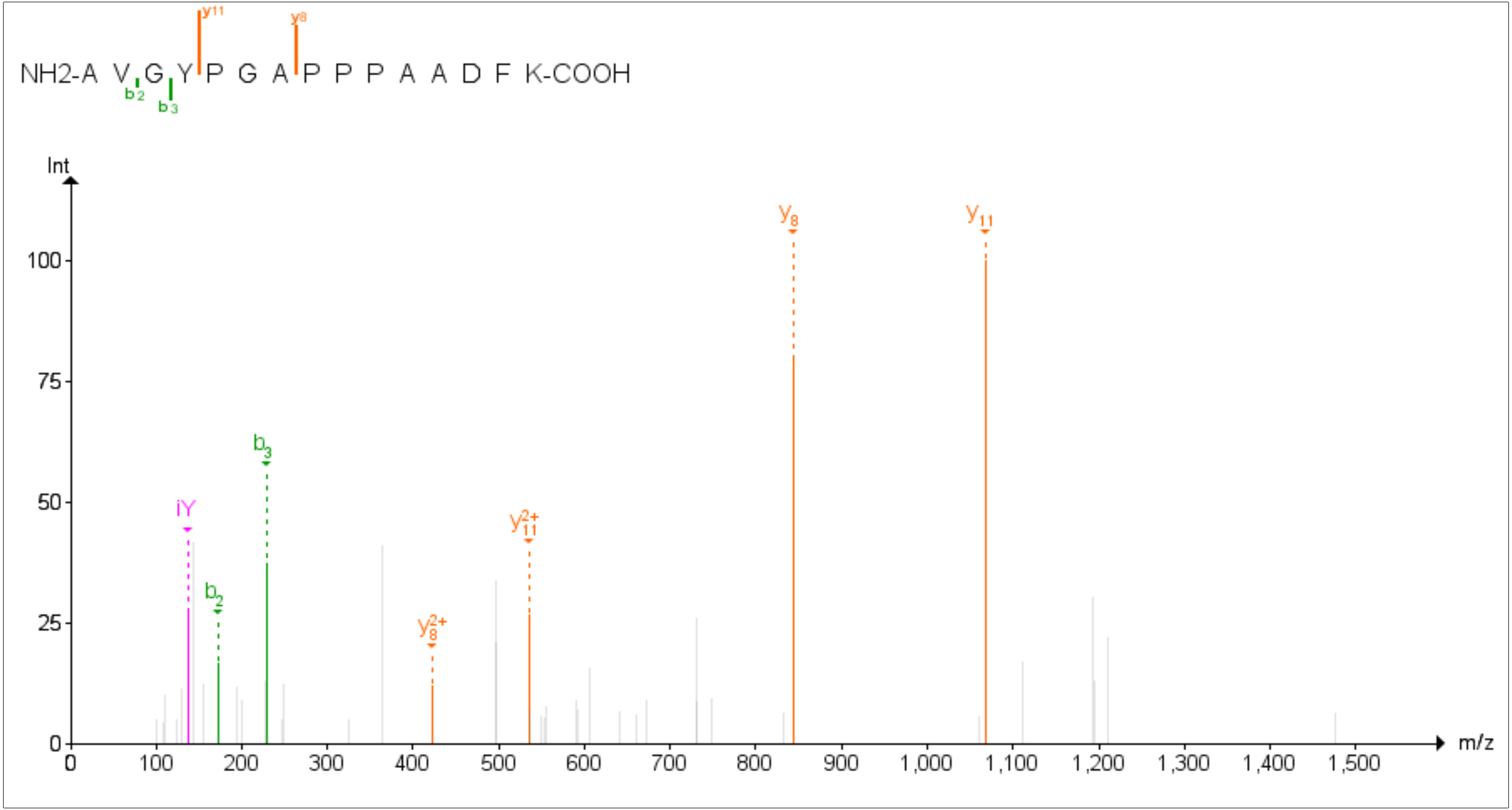

NH2-A V G Y P G A P P P A A D F K-COOH
y11
y8
b2
b3
Int
100
75
50
25
0
m/z
0
100
200
300
400
500
600
700
800
900
1,000
1,100
1,200
1,300
1,400
1,500
iY
b_2
b_3
y_8^{2+}
y_{11}^{2+}
y_8
y_{11}

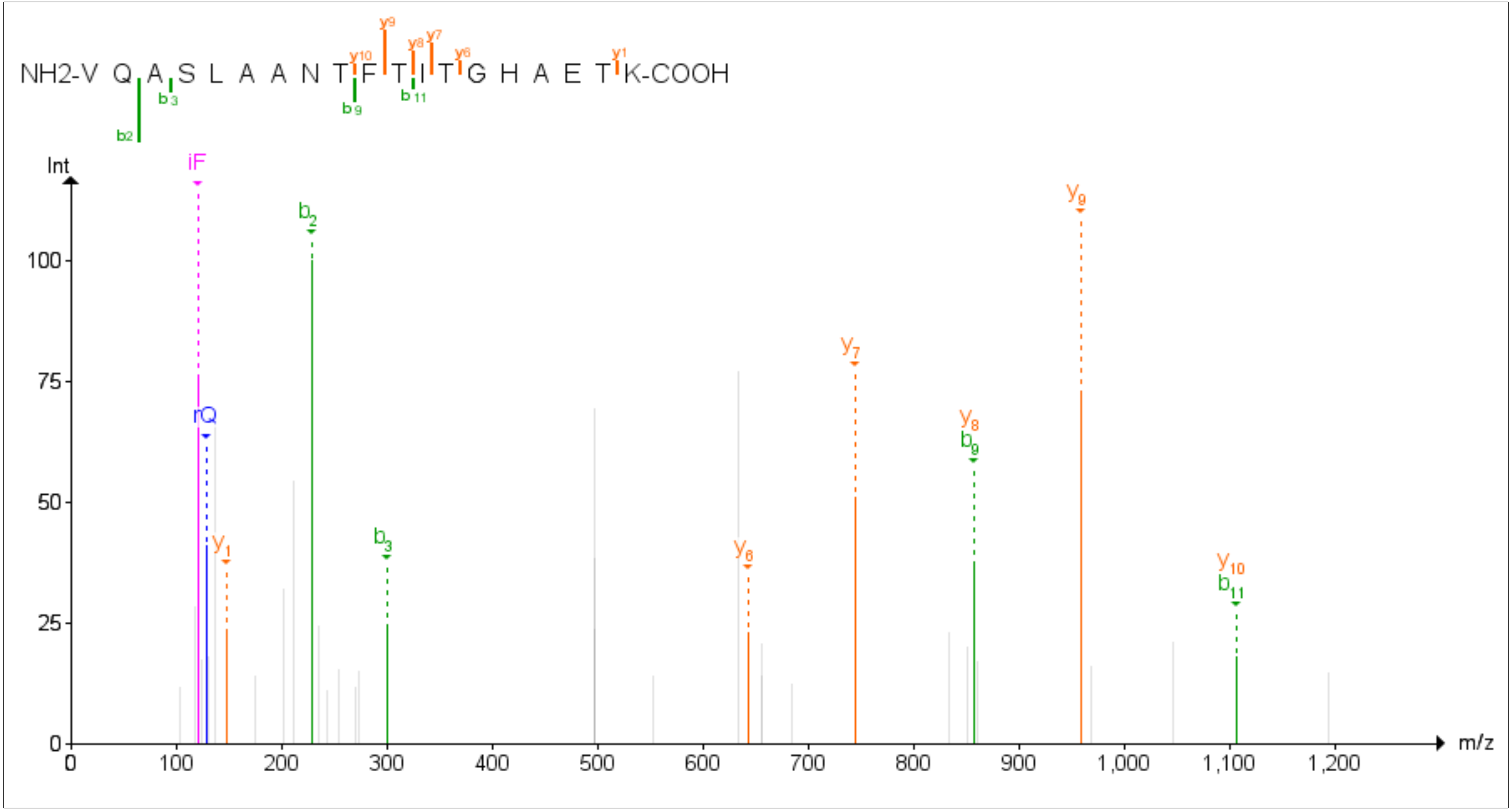
NH2-V Q A S L A A N T F T I T G H A E T K-COOH
Int
m/z
iF
rQ
b2
b3
b9
b11
y1
y6
y7
y8
y9
y10

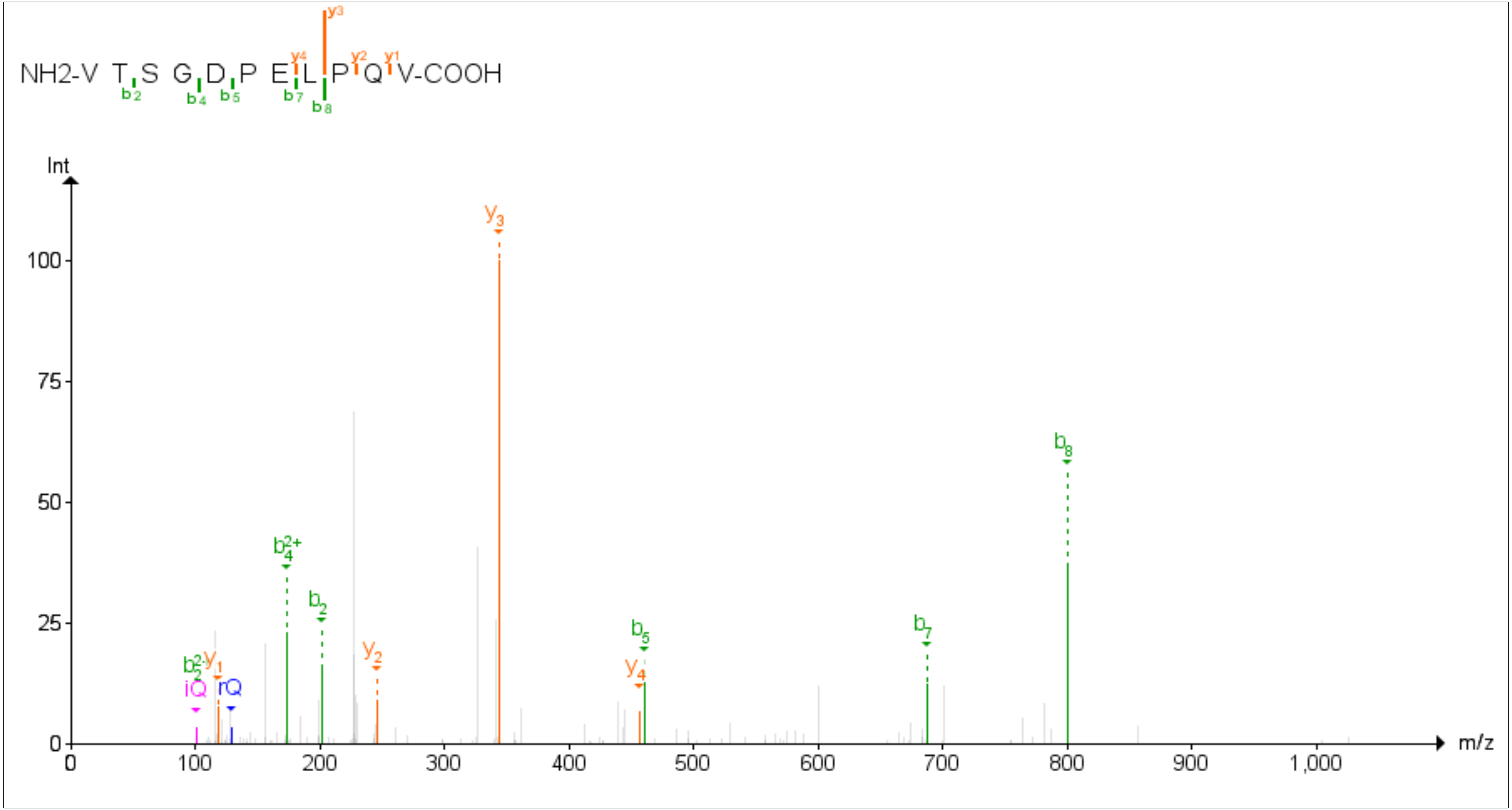
NH2-V T S G D P E L P Q V-COOH
b2 b4 b5 b7 b8
y4 y3 y2 y1
Int
m/z
y3
b8
b4 2+
b2
y2
b5
y4
b7
b2 2+
y1
iQ
rQ
0
25
50
75
100
0
100
200
300
400
500
600
700
800
900
1,000

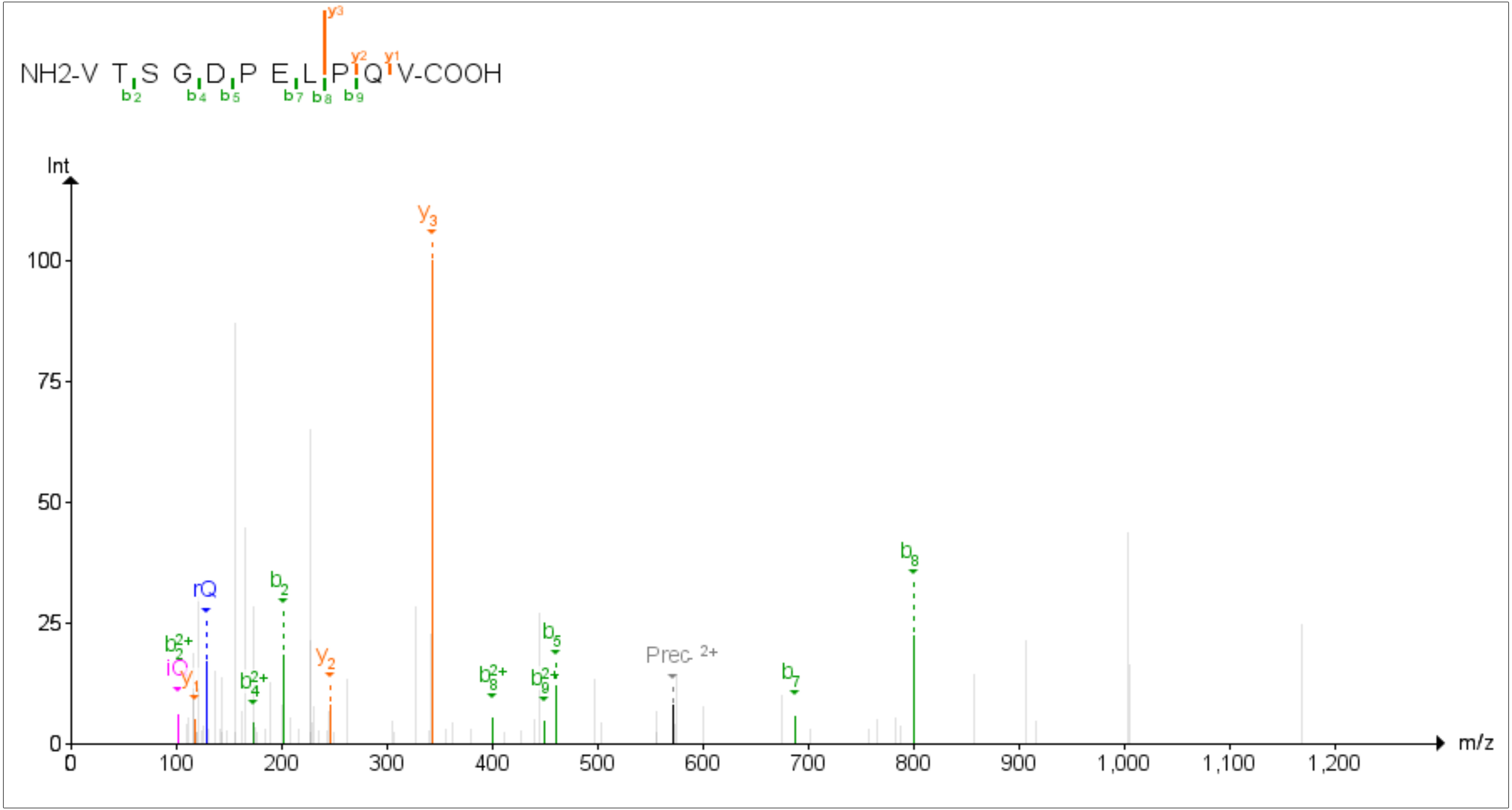

NH2-V T S G D P E L P Q V-COOH
b2 b4 b5 b7 b8 b9
y3 y2 y1
Int
100
75
50
25
0
y3
b8
rQ
b2
b2 2+
iQ
y1
b4 2+
y2
b8 2+
b9 2+
b5
Prec. 2+
b7
0
100
200
300
400
500
600
700
800
900
1,000
1,100
1,200
m/z

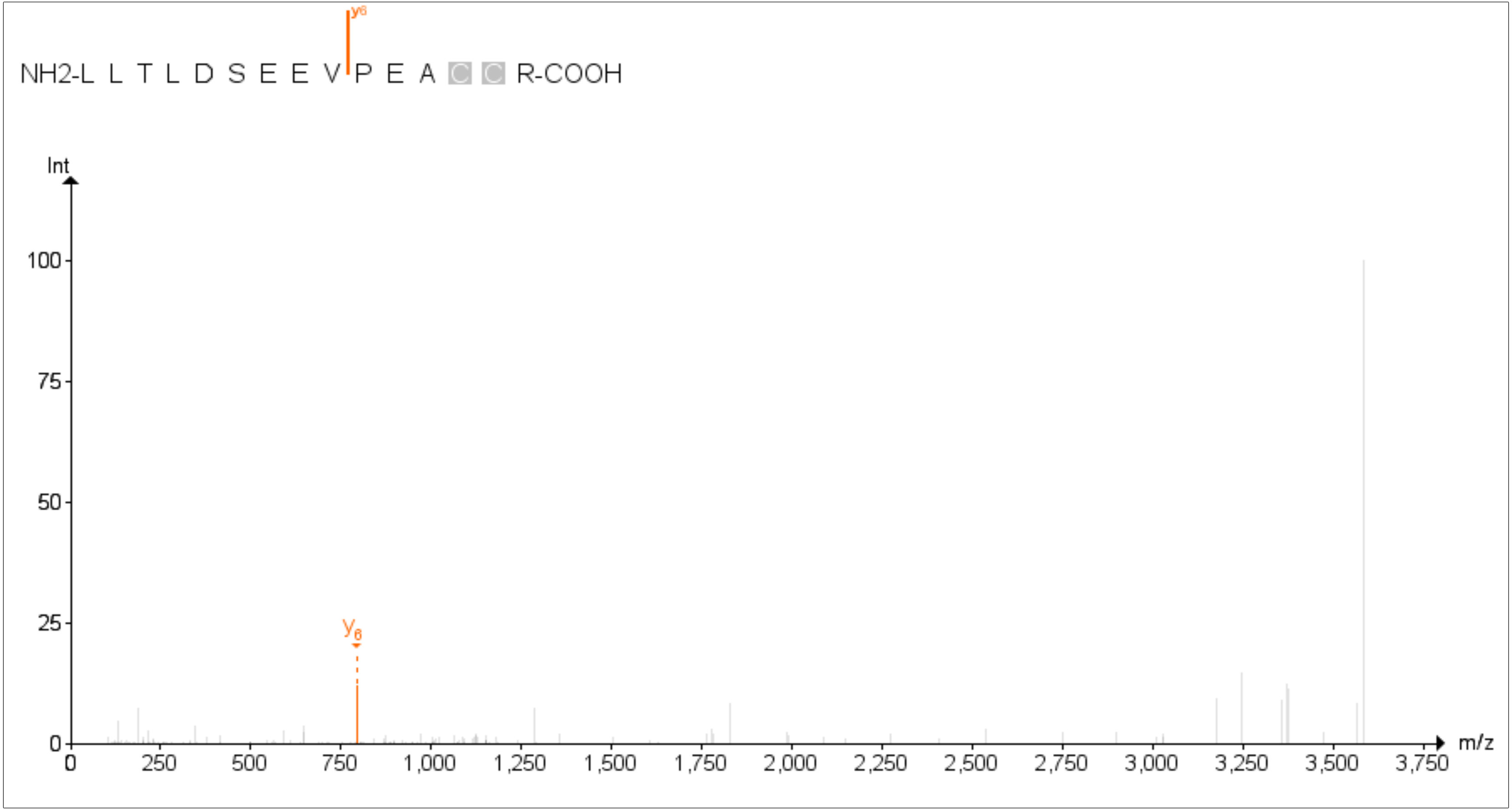

y6
NH2-L L T L D S E E V P E A C C R-COOH
Int
100
75
50
25
0
y6
0
250
500
750
1,000
1,250
1,500
1,750
2,000
2,250
2,500
2,750
3,000
3,250
3,500
3,750
m/z

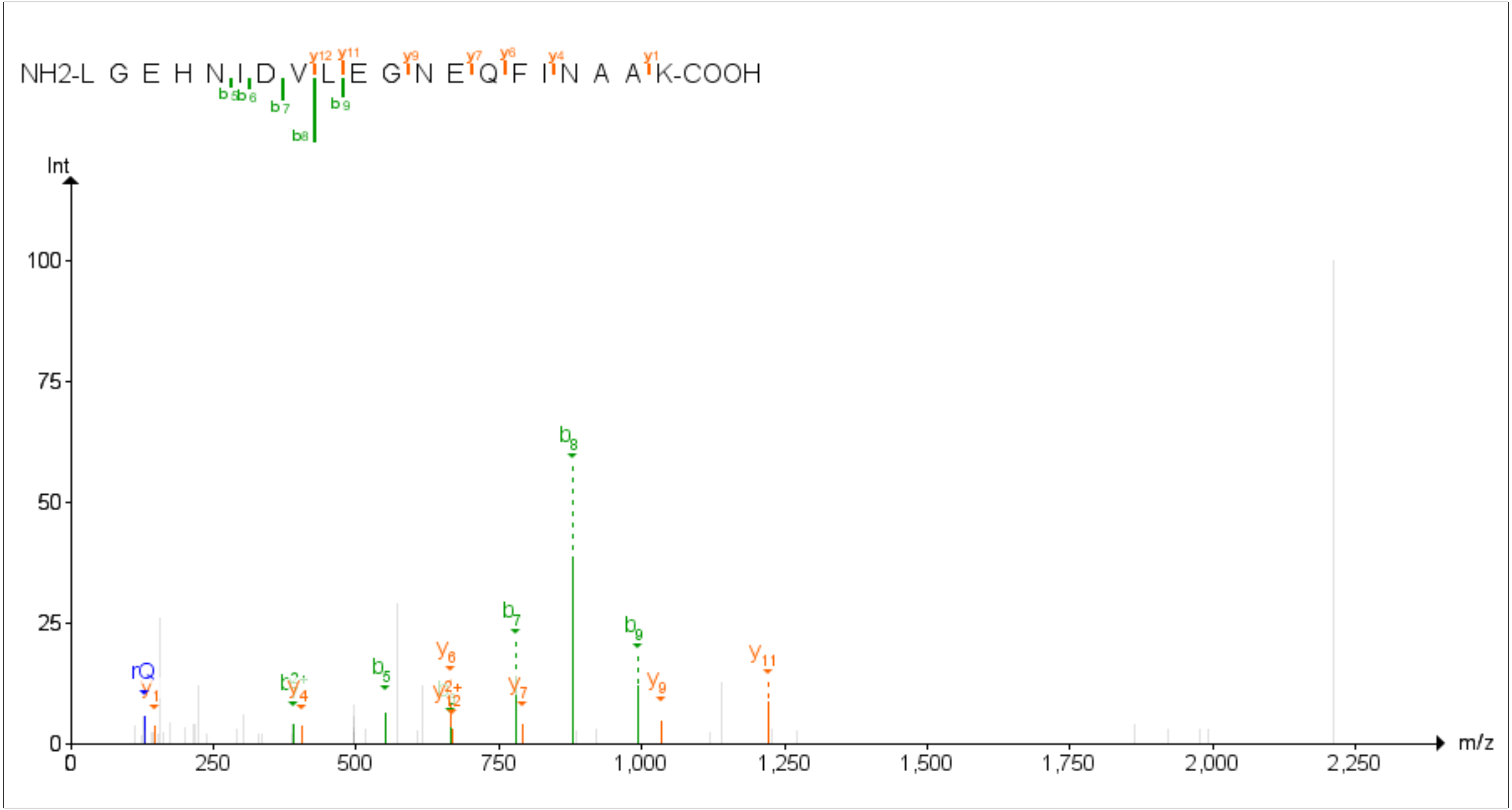
NH2-L G E H N I D V L E G N E Q F I N A A K-COOH
Int
m/z
0
25
50
75
100
250
500
750
1,000
1,250
1,500
1,750
2,000
2,250
b8
b7
b9
b5
y6
y7
y9
y11
y1
y4
rQ

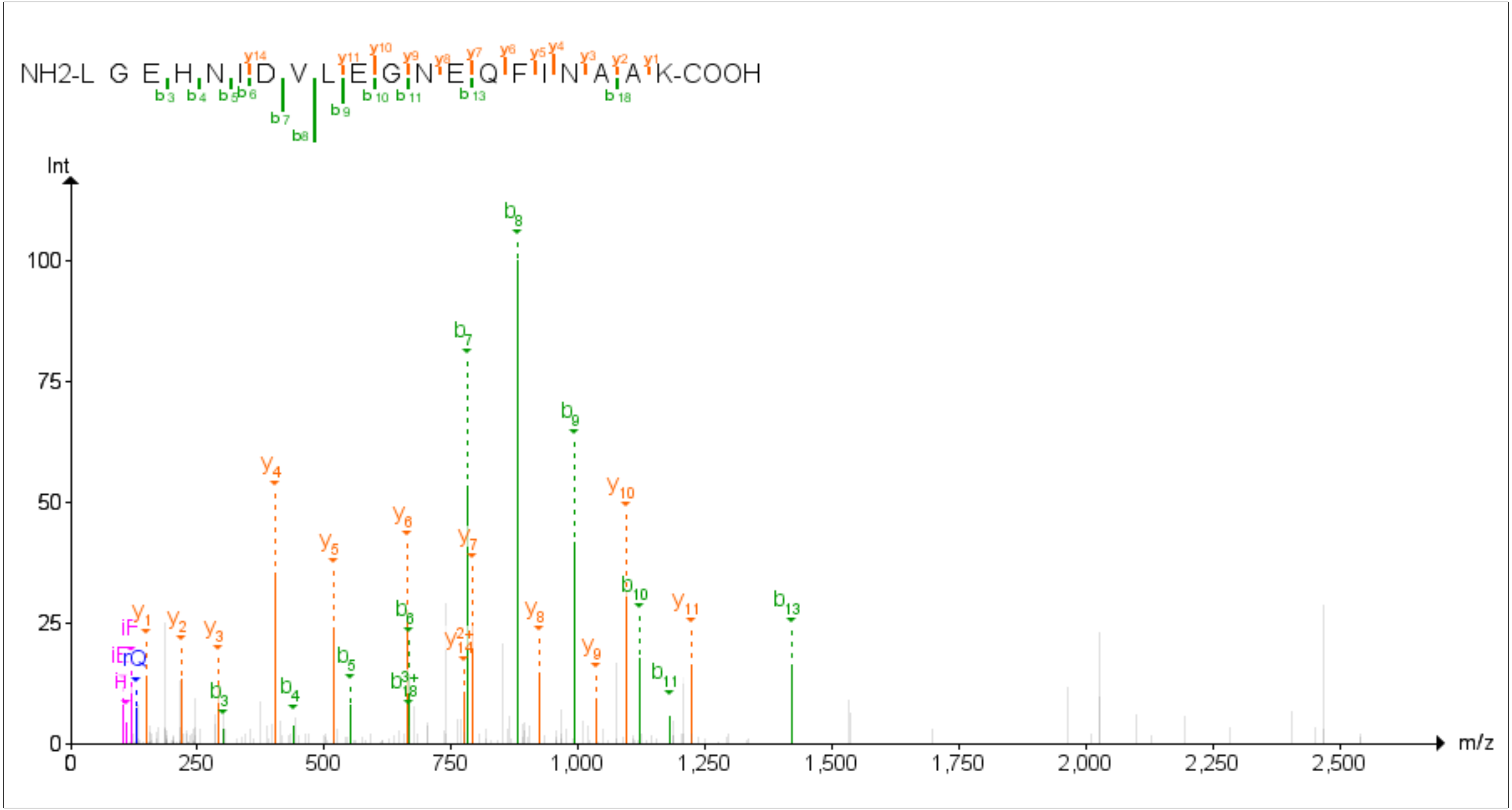
NH2-L G E H N I D V L E G N E Q F I N A A K-COOH
Int
m/z
0
25
50
75
100
250
500
750
1,000
1,250
1,500
1,750
2,000
2,250
2,500

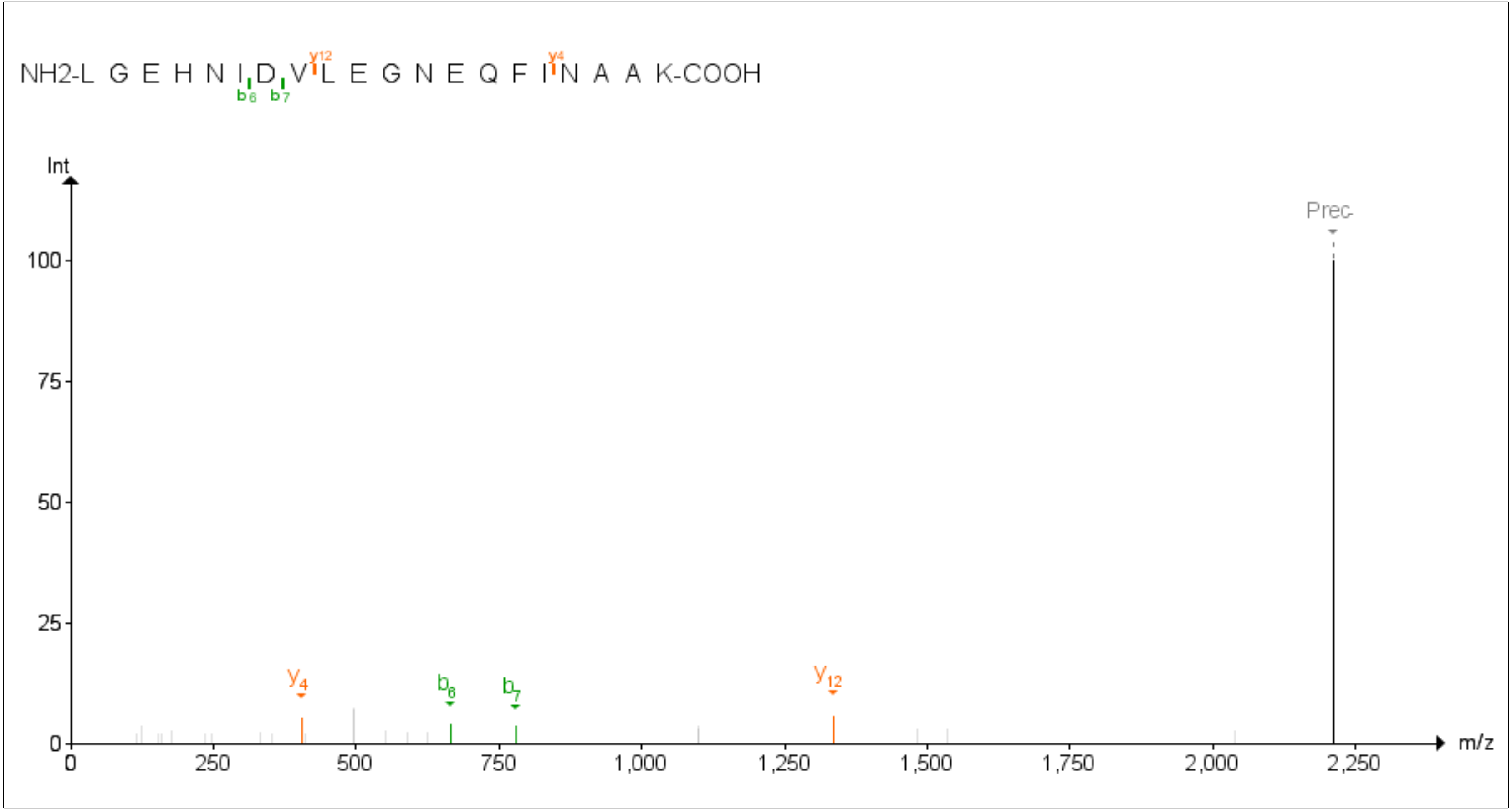

NH2-L G E H N I D V L E G N E Q F I N A A K-COOH
y12
y4
b6
b7
Int
100
75
50
25
0
Prec
y4
b6
b7
y12
0
250
500
750
1,000
1,250
1,500
1,750
2,000
2,250
m/z

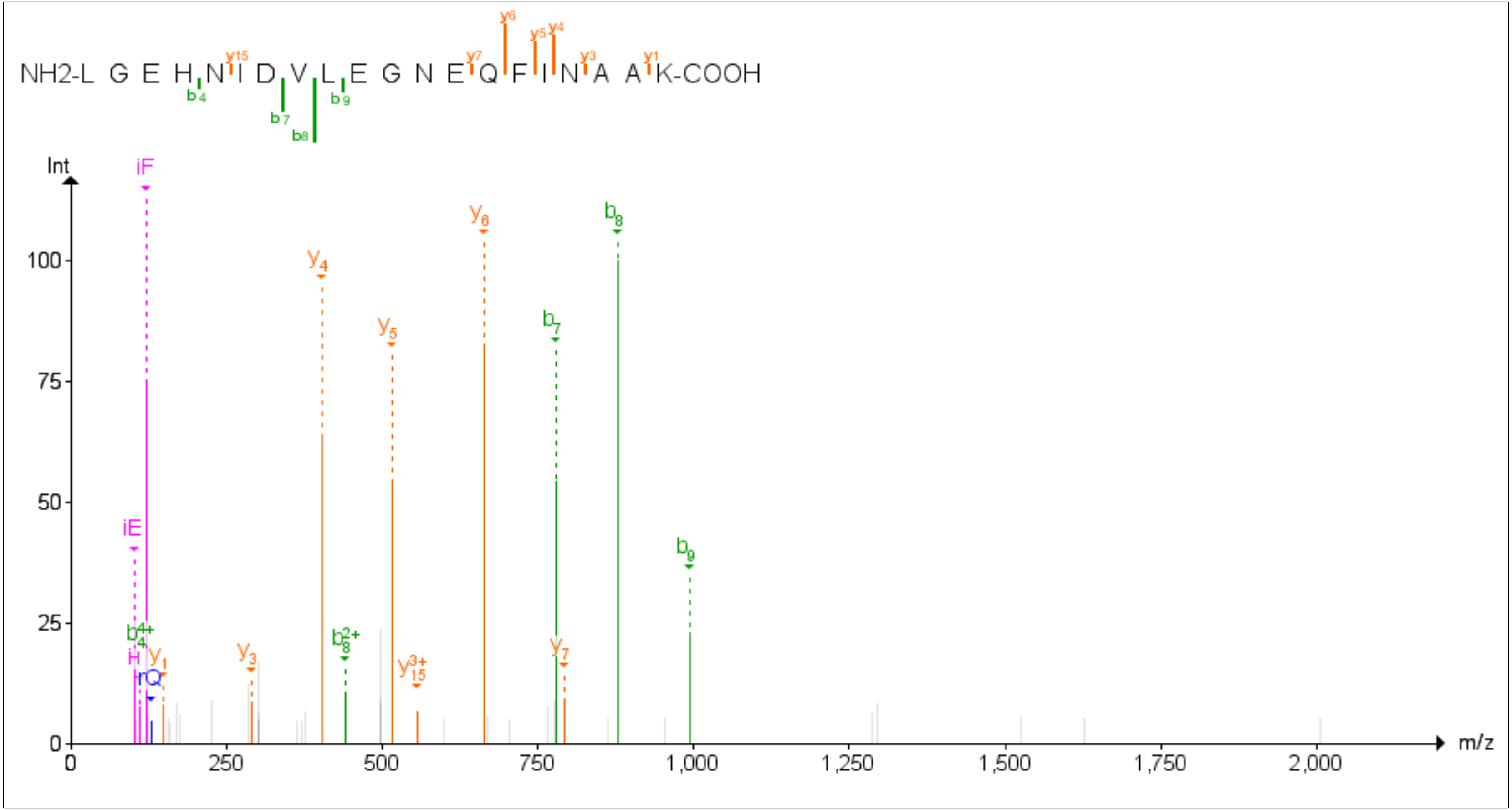

NH2-L G E H N I D V L E G N E Q F I N A A K-COOH
y15 y7 y6 y5 y4 y3 y1
b4 b7 b8 b9
Int
m/z
iF
iE
iH
iQ
b_4^{4+}
y_1
y_3
y_4
y_5
y_6
y_7
b_7
b_8
b_9
b_8^{2+}
y_{15}^{3+}
0
25
50
75
100
0
250
500
750
1,000
1,250
1,500
1,750
2,000

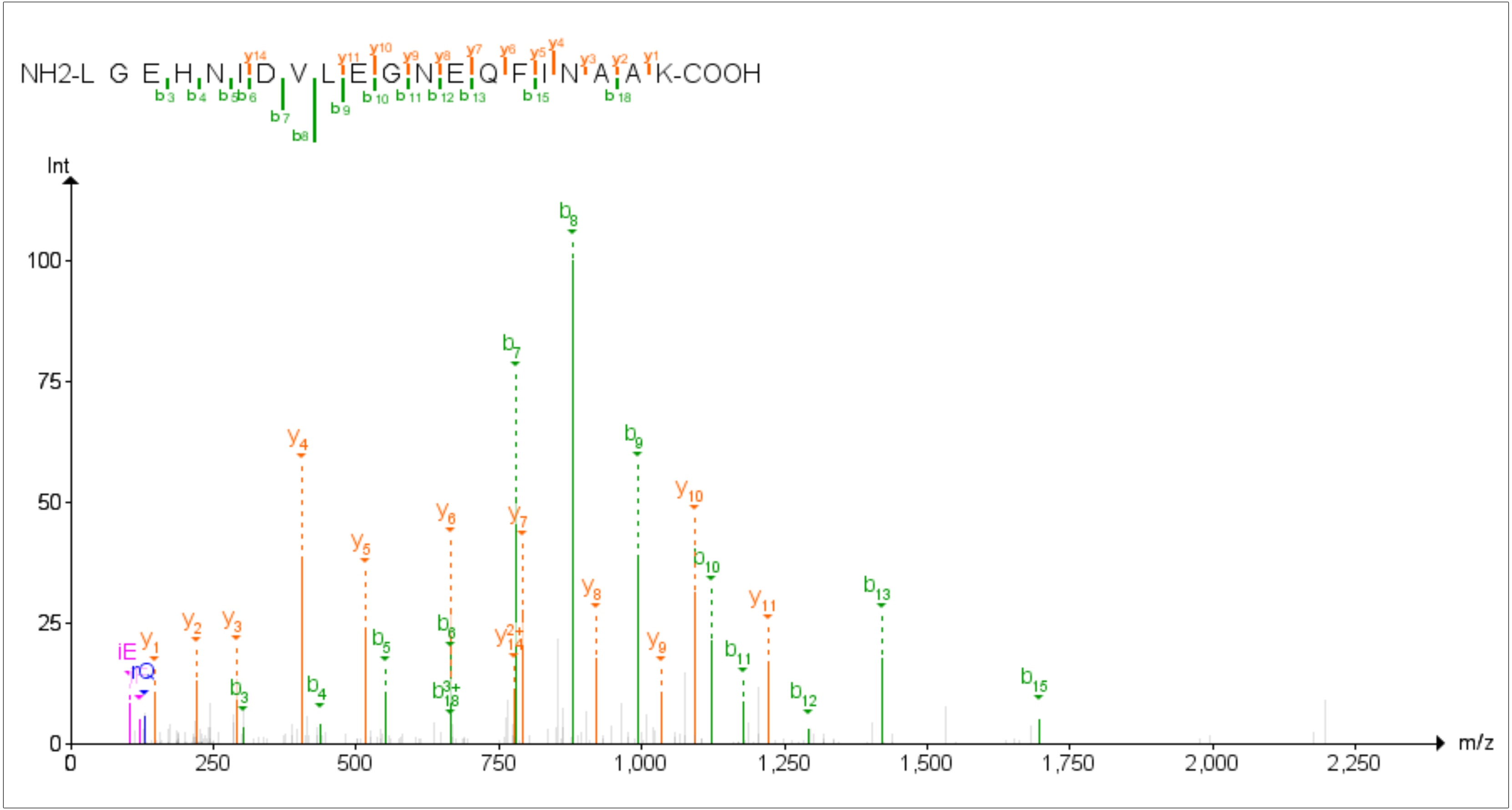

NH2-L G E H N I D V L E G N E Q F I N A A K-COOH
Int
m/z
0
25
50
75
100
250
500
750
1,000
1,250
1,500
1,750
2,000
2,250

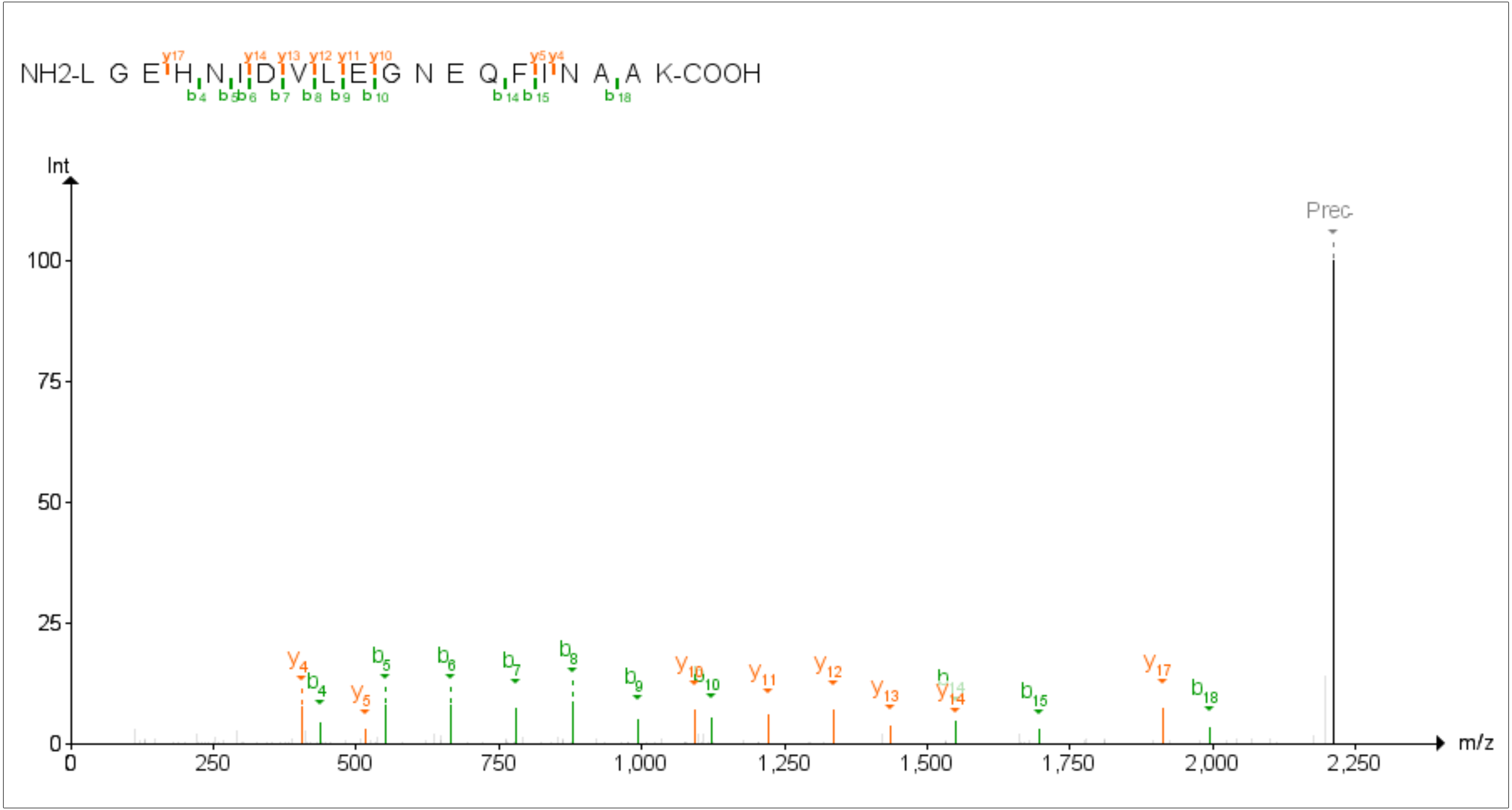

NH2-L G E H N I D V L E G N E Q F I N A A K-COOH
Int
Prec
100
75
50
25
0
250
500
750
1,000
1,250
1,500
1,750
2,000
2,250
m/z

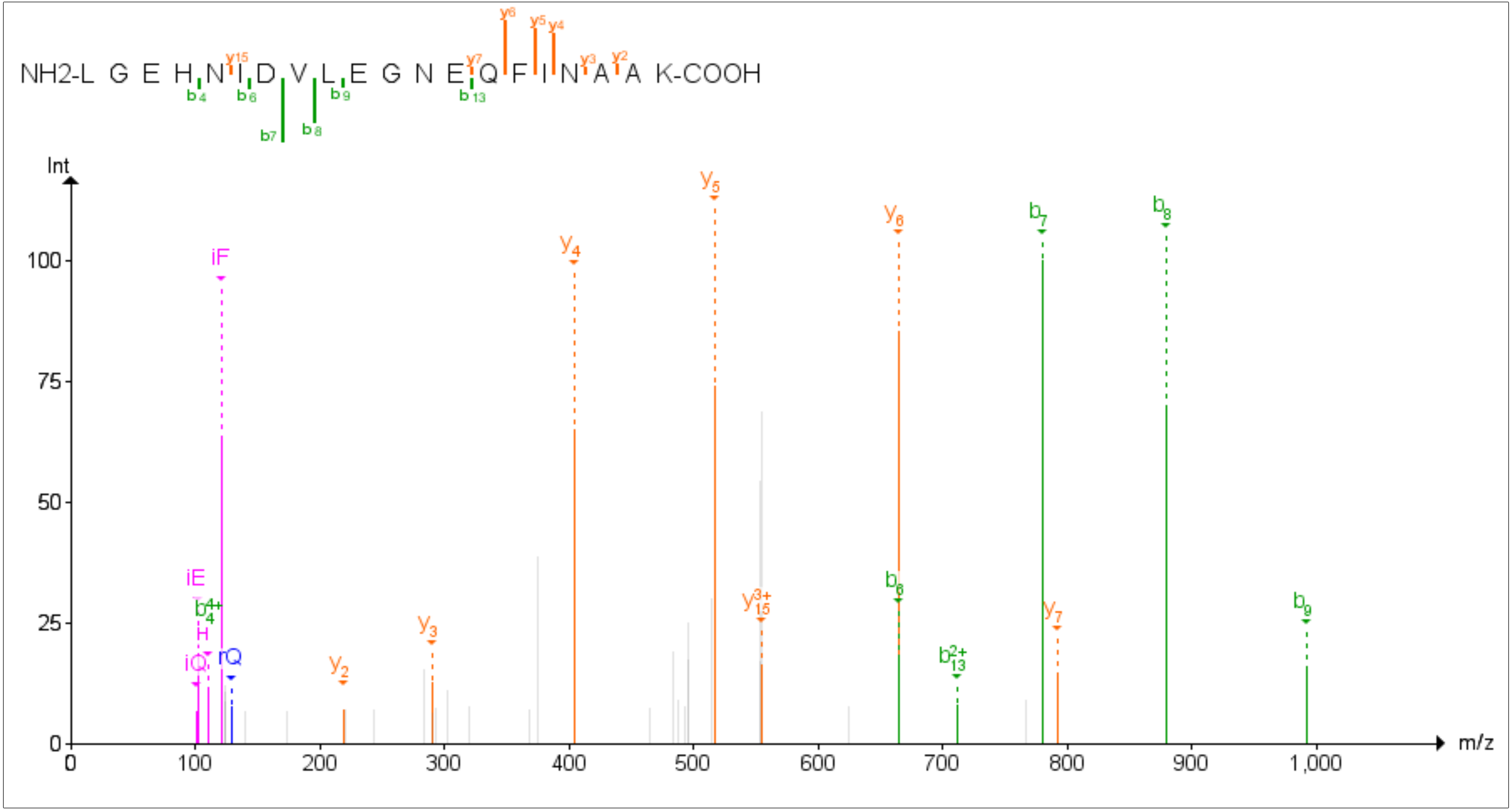
NH2-L G E H N I D V L E G N E Q F I N A A K-COOH
b4 b6 b7 b8 b9 b13
y15 y7 y6 y5 y4 y3 y2
Int
m/z
iF
iE
iQ
H
rQ
b_4^{4+}
y_2
y_3
y_4
y_5
y_6
y_7
y_{15}^{3+}
b_6
b_7
b_8
b_9
b_{13}^{2+}
0
25
50
75
100
0
100
200
300
400
500
600
700
800
900
1,000

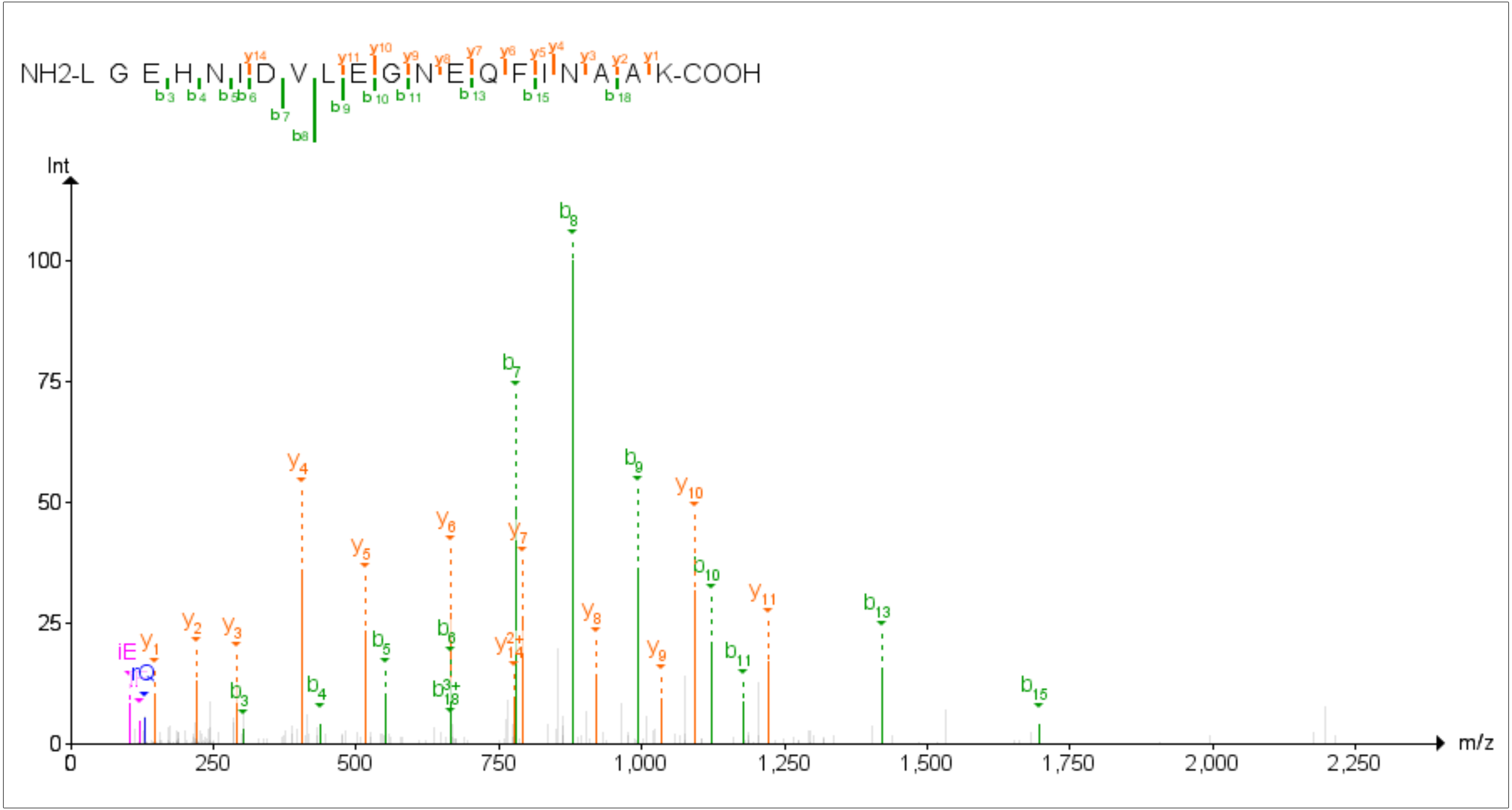

NH2-L G E H N I D V L E G N E Q F I N A A K-COOH
y14 y11 y10 y9 y8 y7 y6 y5 y4 y3 y2 y1
b3 b4 b5 b6 b7 b8 b9 b10 b11 b13 b15 b18
Int
100
75
50
25
0
m/z
0
250
500
750
1,000
1,250
1,500
1,750
2,000
2,250
b8
b7
b9
y10
y4
y6
y7
y5
b10
y11
b13
y8
y9
b11
b15
y1
y2
y3
iE
nQ
b3
b4
b5
b6
b3+ 18
y2+ 14

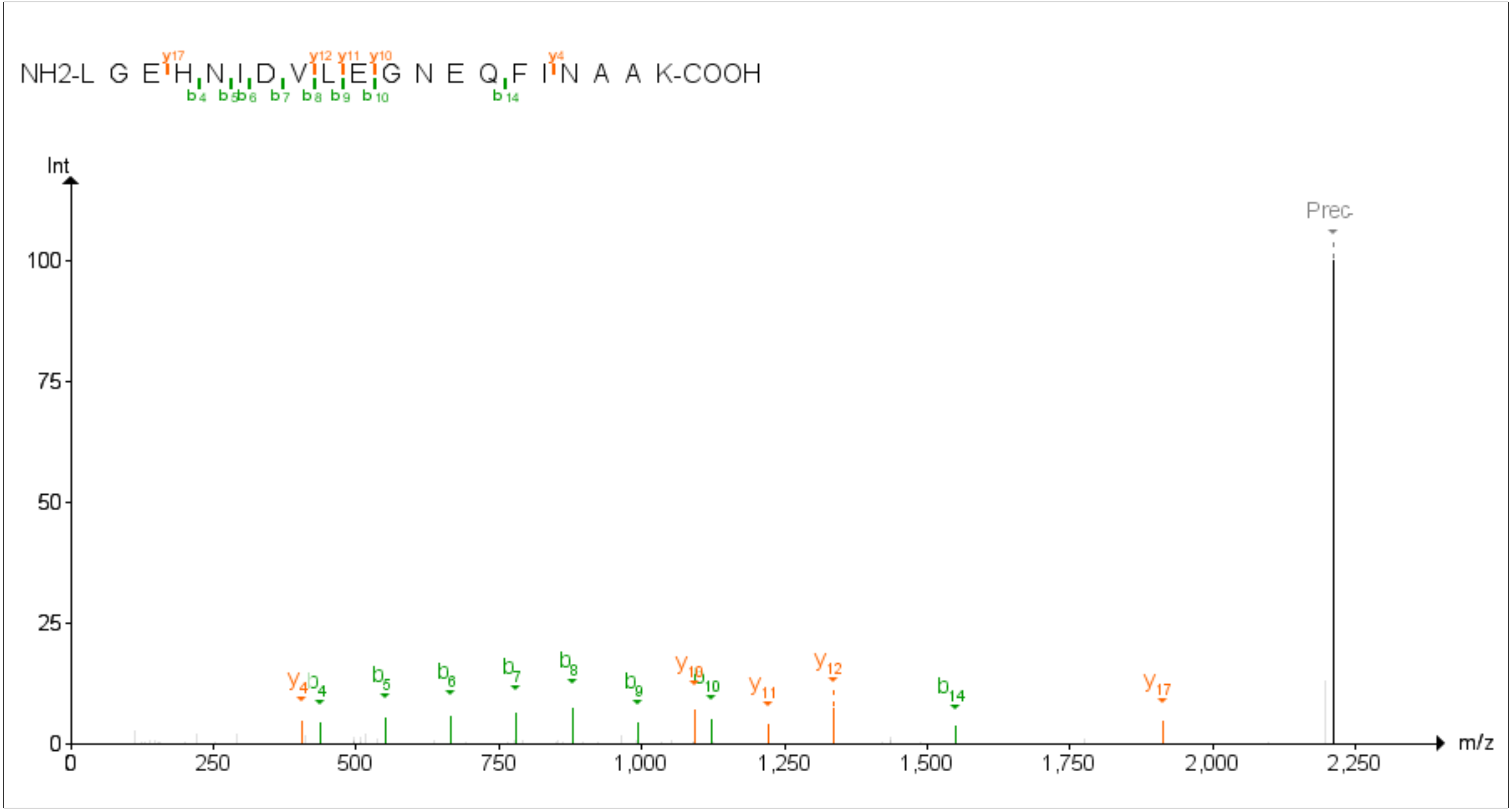

NH2-L G E H N I D V L E G N E Q F I N A A K-COOH
y17 y12 y11 y10 y4
b4 b5 b6 b7 b8 b9 b10 b14
Int
100
75
50
25
0
Prec
y4 b4 b5 b6 b7 b8 b9 y10 b10 y11 y12 b14 y17
0 250 500 750 1,000 1,250 1,500 1,750 2,000 2,250
m/z

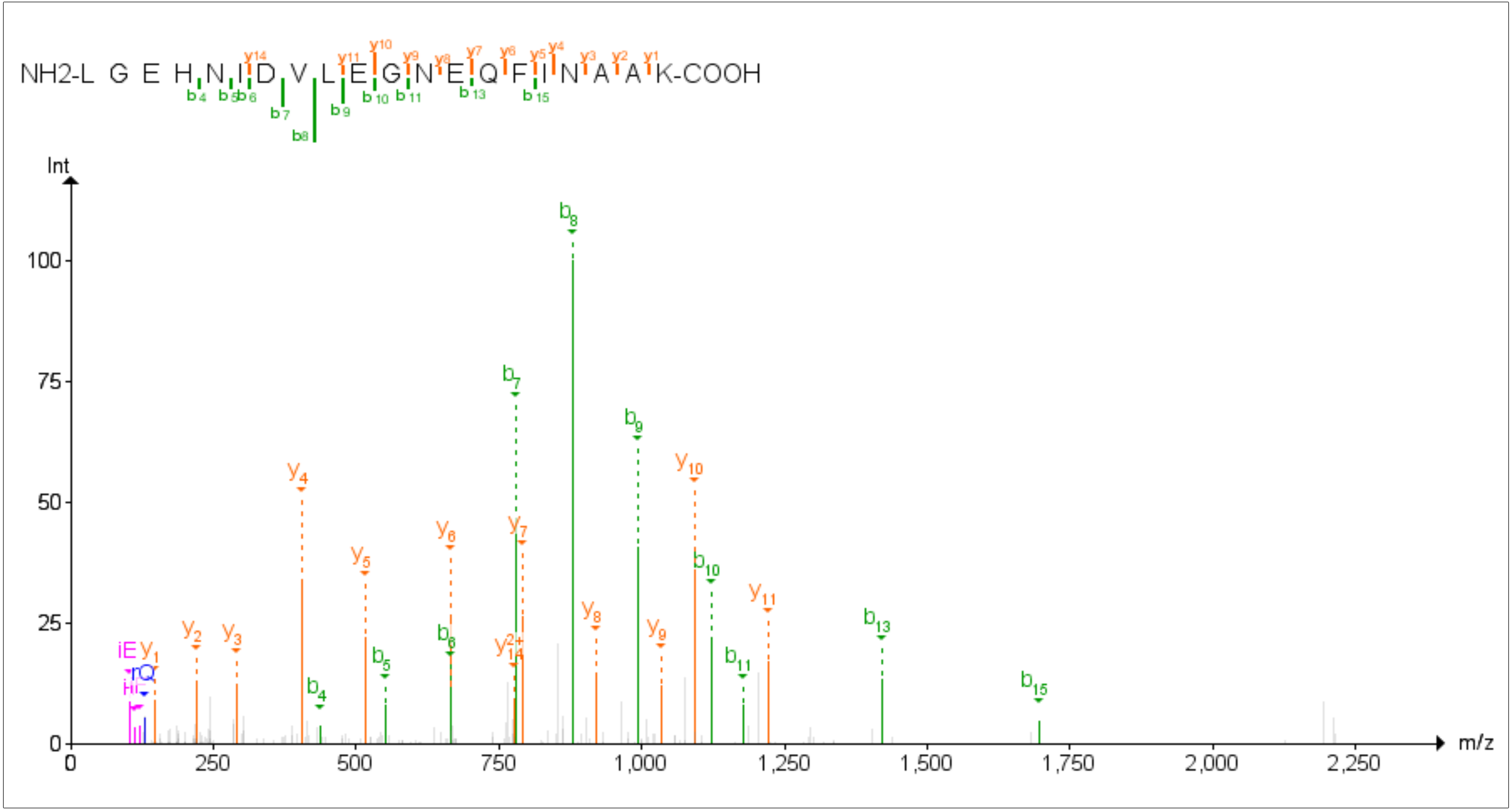
NH2-L G E H N I D V L E G N E Q F I N A A K-COOH
Int
m/z
0
25
50
75
100
250
500
750
1,000
1,250
1,500
1,750
2,000
2,250

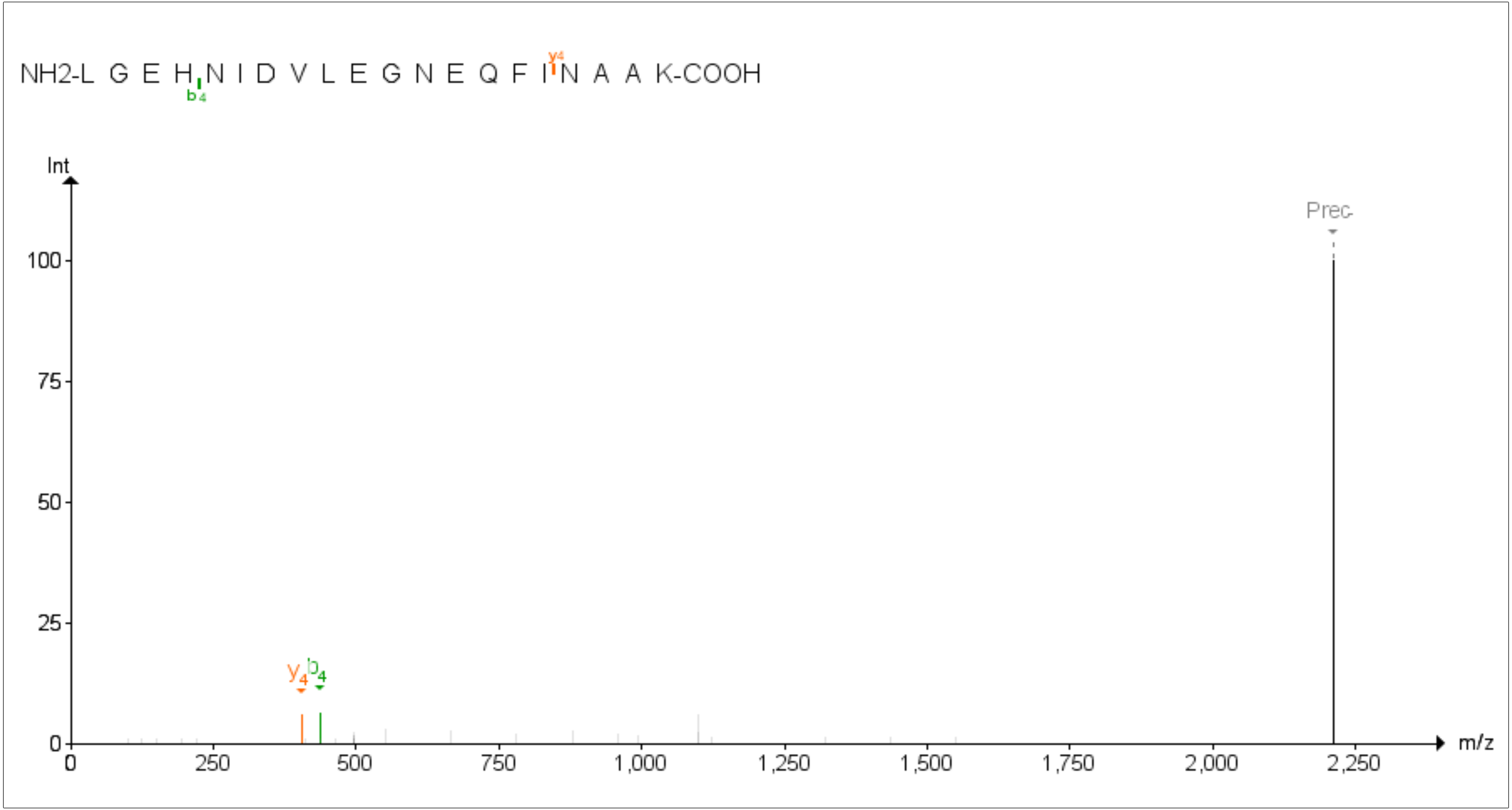

NH2-L G E H N I D V L E G N E Q F I N A A K-COOH
b4
y4
Int
100
75
50
25
0
Prec
m/z
0
250
500
750
1,000
1,250
1,500
1,750
2,000
2,250

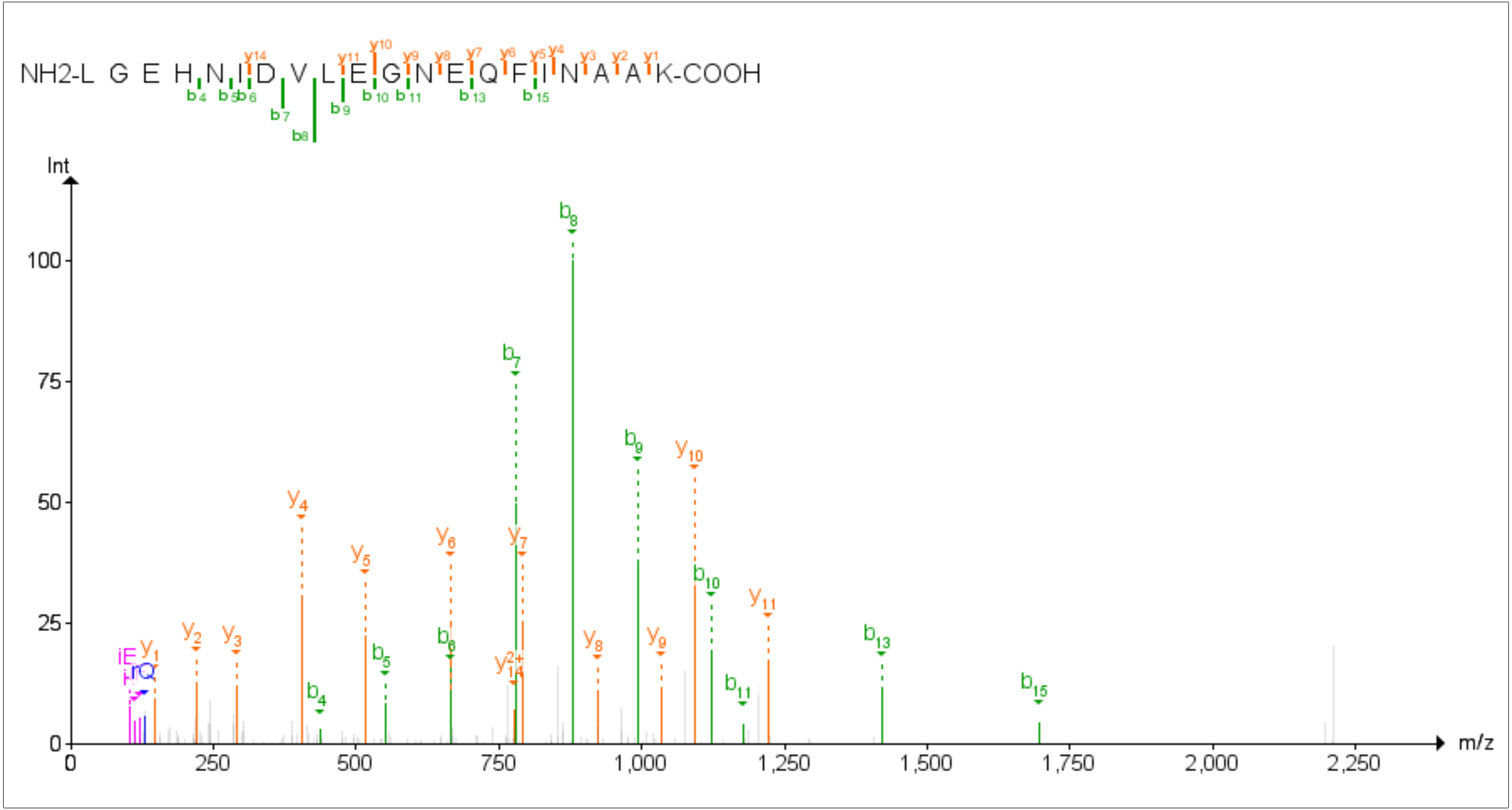

NH2-L G E H N I D V L E G N E Q F I N A A K-COOH
y14 y11 y10 y9 y8 y7 y6 y5 y4 y3 y2 y1
b4 b5 b6 b7 b8 b9 b10 b11 b13 b15
Int
m/z
0
25
50
75
100
250
500
750
1,000
1,250
1,500
1,750
2,000
2,250

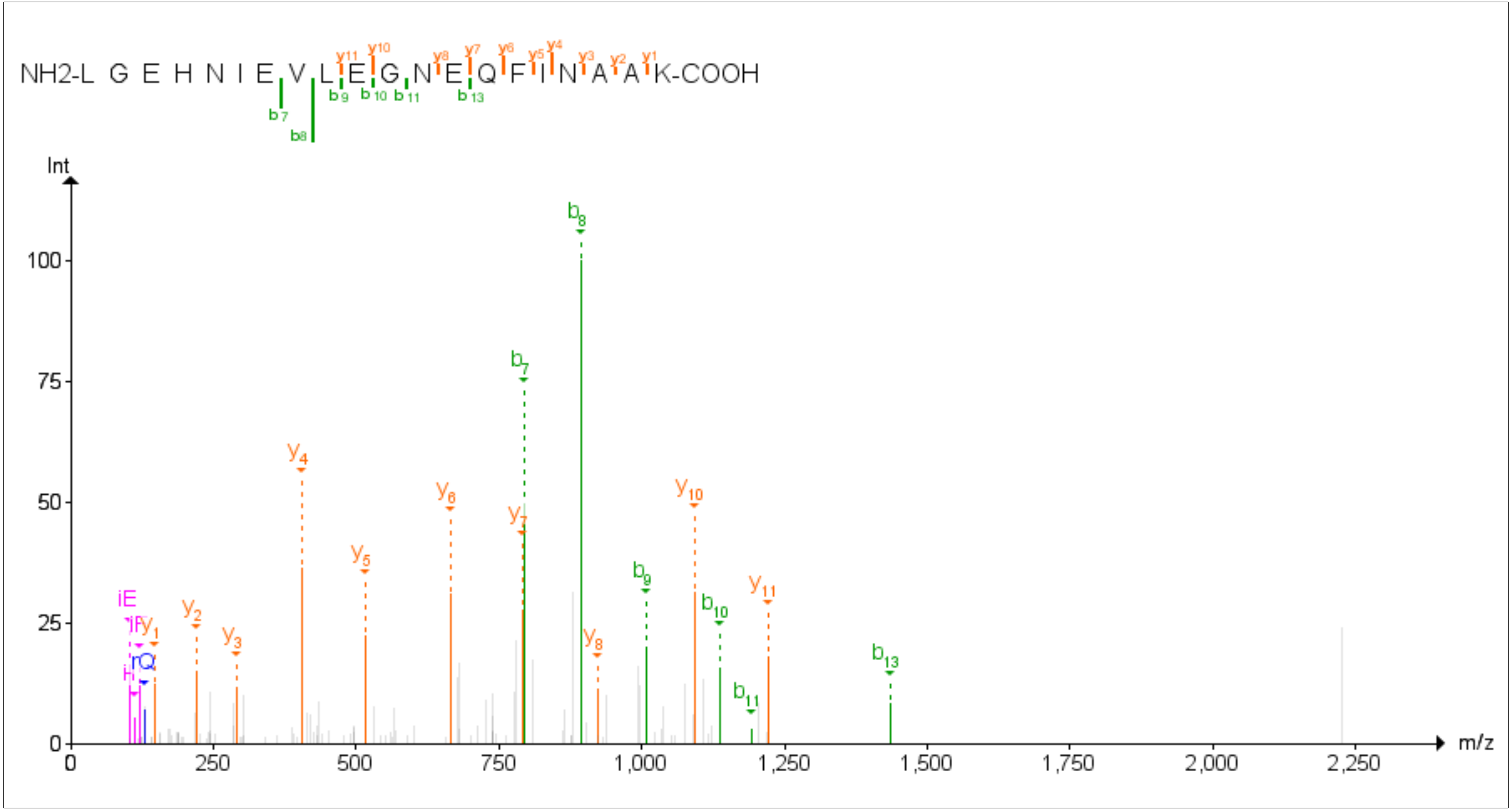
NH2-L G E H N I E V L E G N E Q F I N A A K-COOH
y11 y10 y8 y7 y6 y5 y4 y3 y2 y1
b7 b8 b9 b10 b11 b13
Int
100
75
50
25
0
m/z
0
250
500
750
1,000
1,250
1,500
1,750
2,000
2,250
b8
b7
y4
y6
y7
y10
y5
b9
b10
y11
iE
y2
y1
y3
y8
rQ
b11
b13

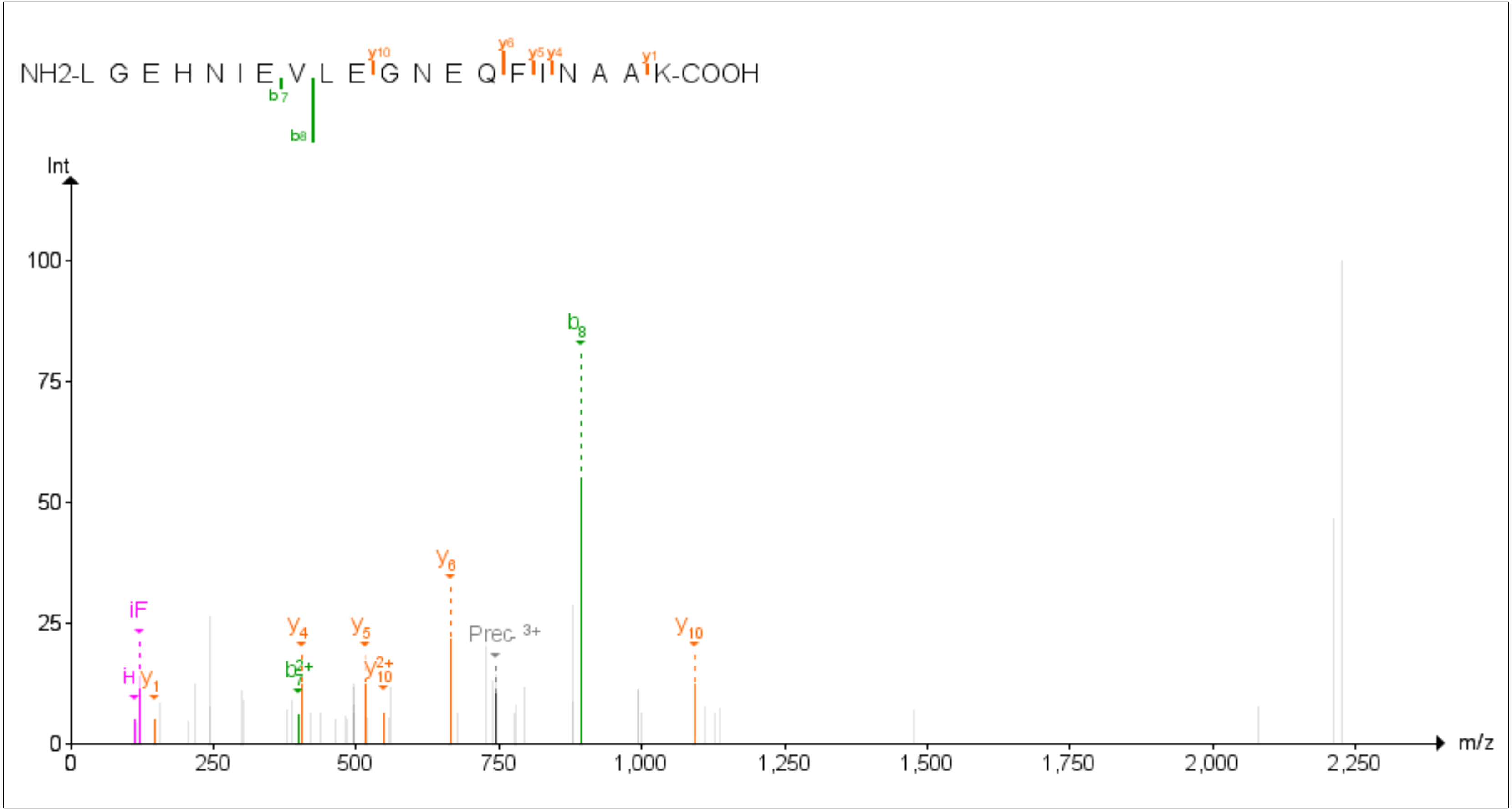

NH2-L G E H N I E V L E G N E Q F I N A A K-COOH
b7
b8
y10
y6
y5
y4
y1
Int
100
75
50
25
0
b8
y6
iF
y4
y5
Prec 3+
y10
H
y1
b7 2+
y10 2+
0
250
500
750
1,000
1,250
1,500
1,750
2,000
2,250
m/z

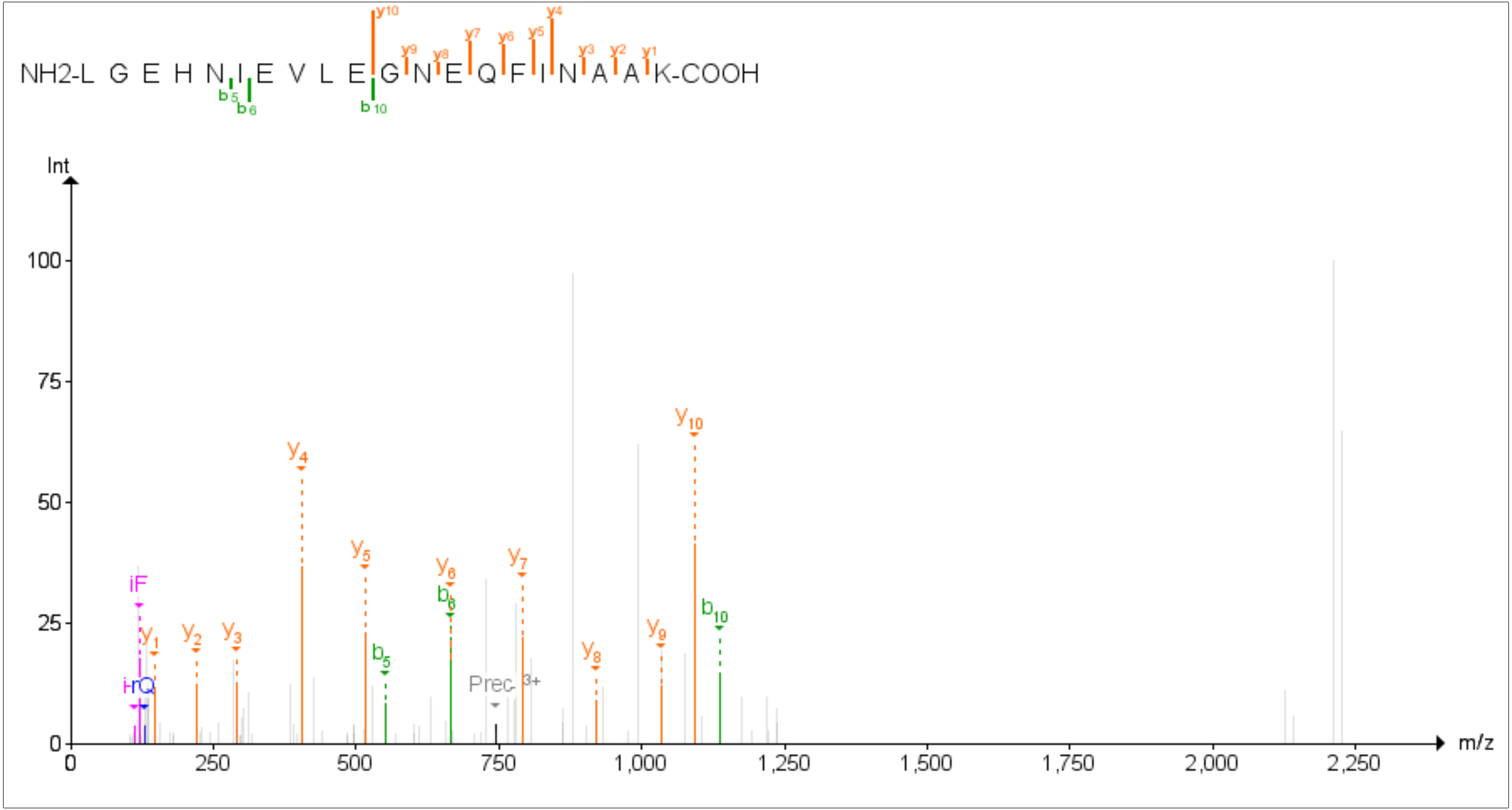

NH2-L G E H N I E V L E G N E Q F I N A A K-COOH
y10 y9 y8 y7 y6 y5 y4 y3 y2 y1
b5 b6 b10
Int
100
75
50
25
0
m/z
0
250
500
750
1,000
1,250
1,500
1,750
2,000
2,250
iF
iQ
y1 y2 y3 y4 y5 y6 y7 y8 y9 y10
b5 b6 b10
Prec 3+

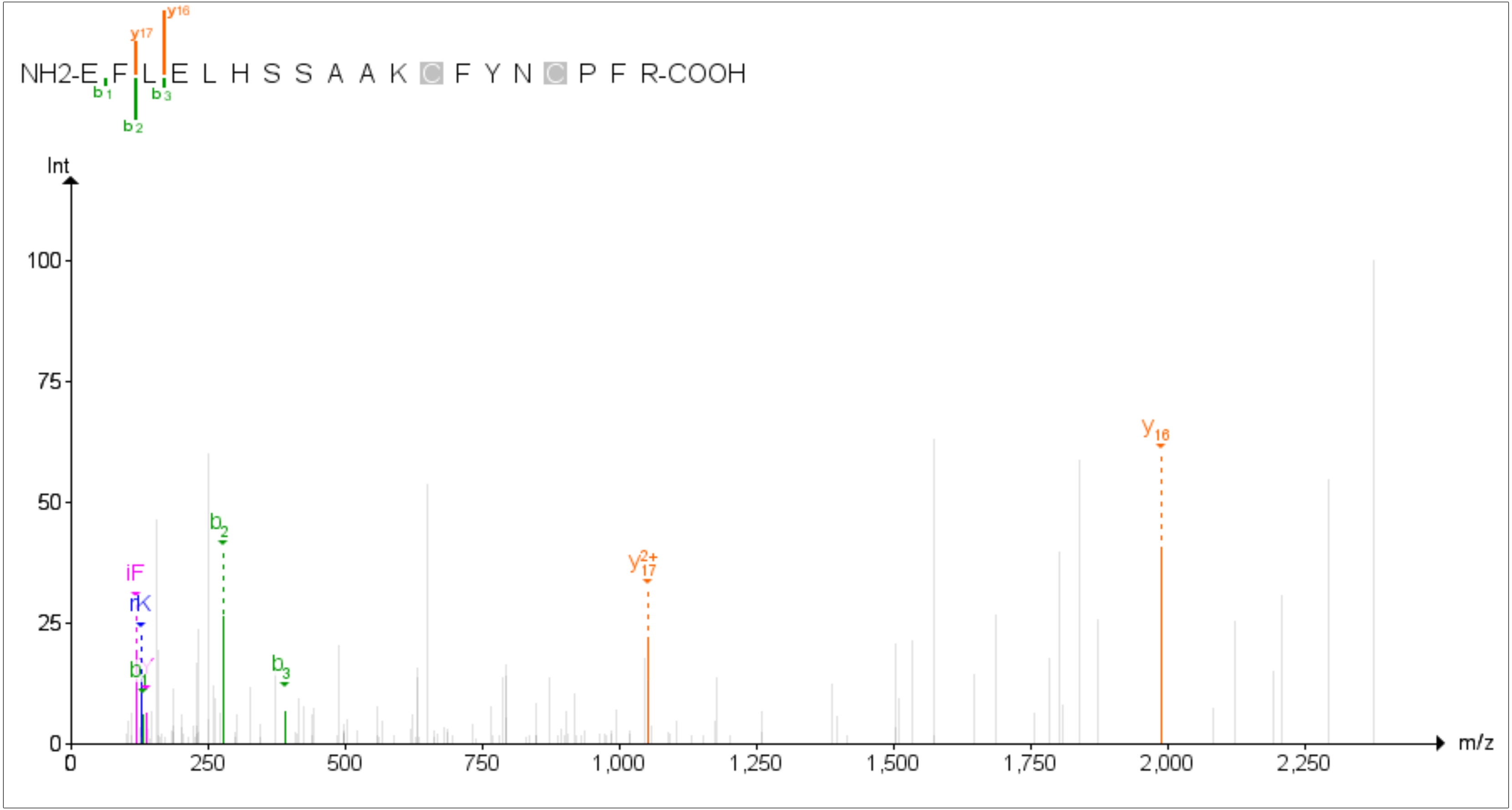

NH2-E F L E L H S S A A K C F Y N C P F R-COOH
y17
y16
b1
b2
b3
Int
100
75
50
25
0
iF
rK
b2
b3
y16
m/z
0
250
500
750
1,000
1,250
1,500
1,750
2,000
2,250

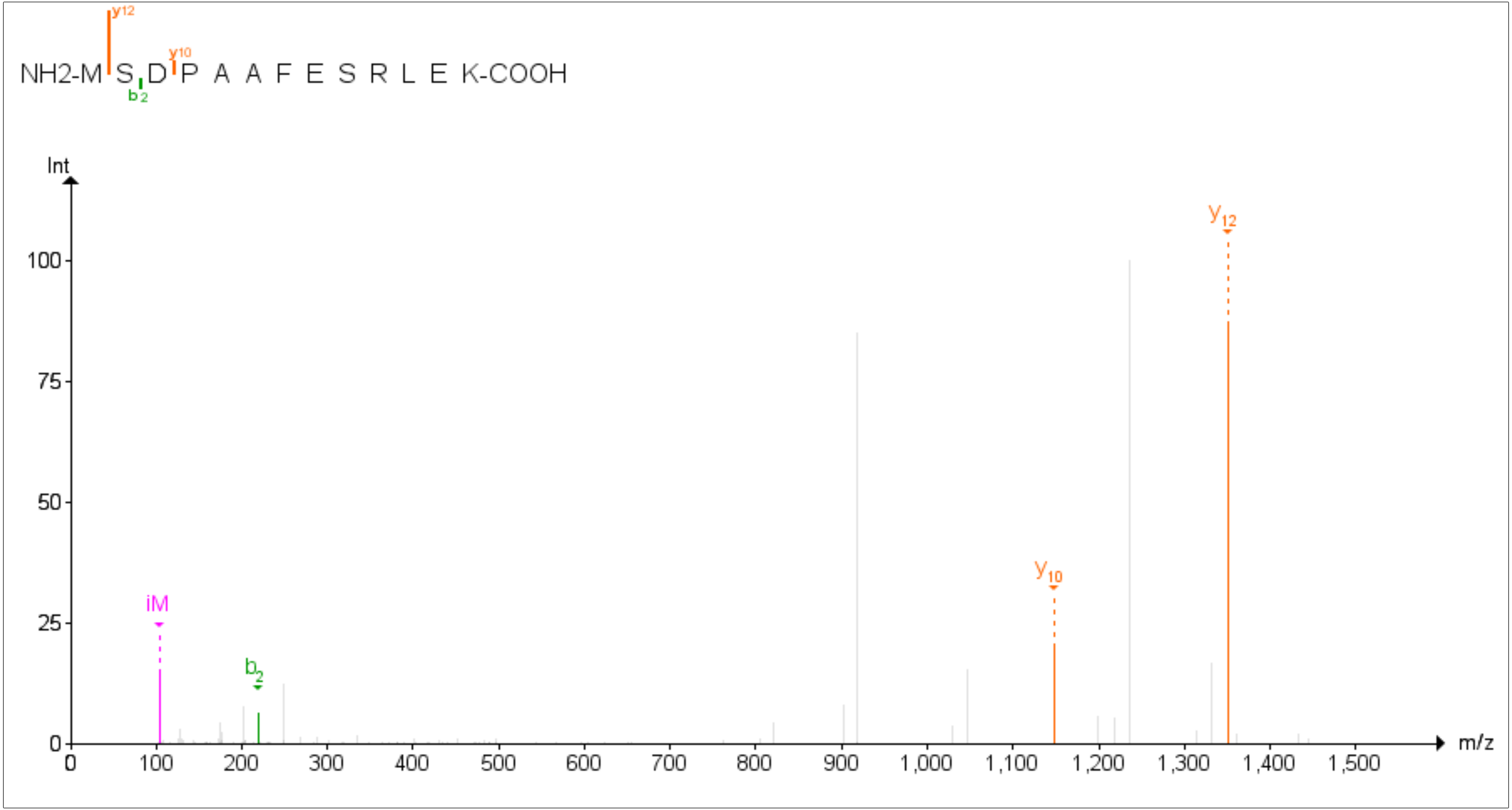

NH2-M S D P A A F E S R L E K-COOH
y12
y10
b2
Int
100
75
50
25
0
iM
b2
y10
y12
m/z
0
100
200
300
400
500
600
700
800
900
1,000
1,100
1,200
1,300
1,400
1,500

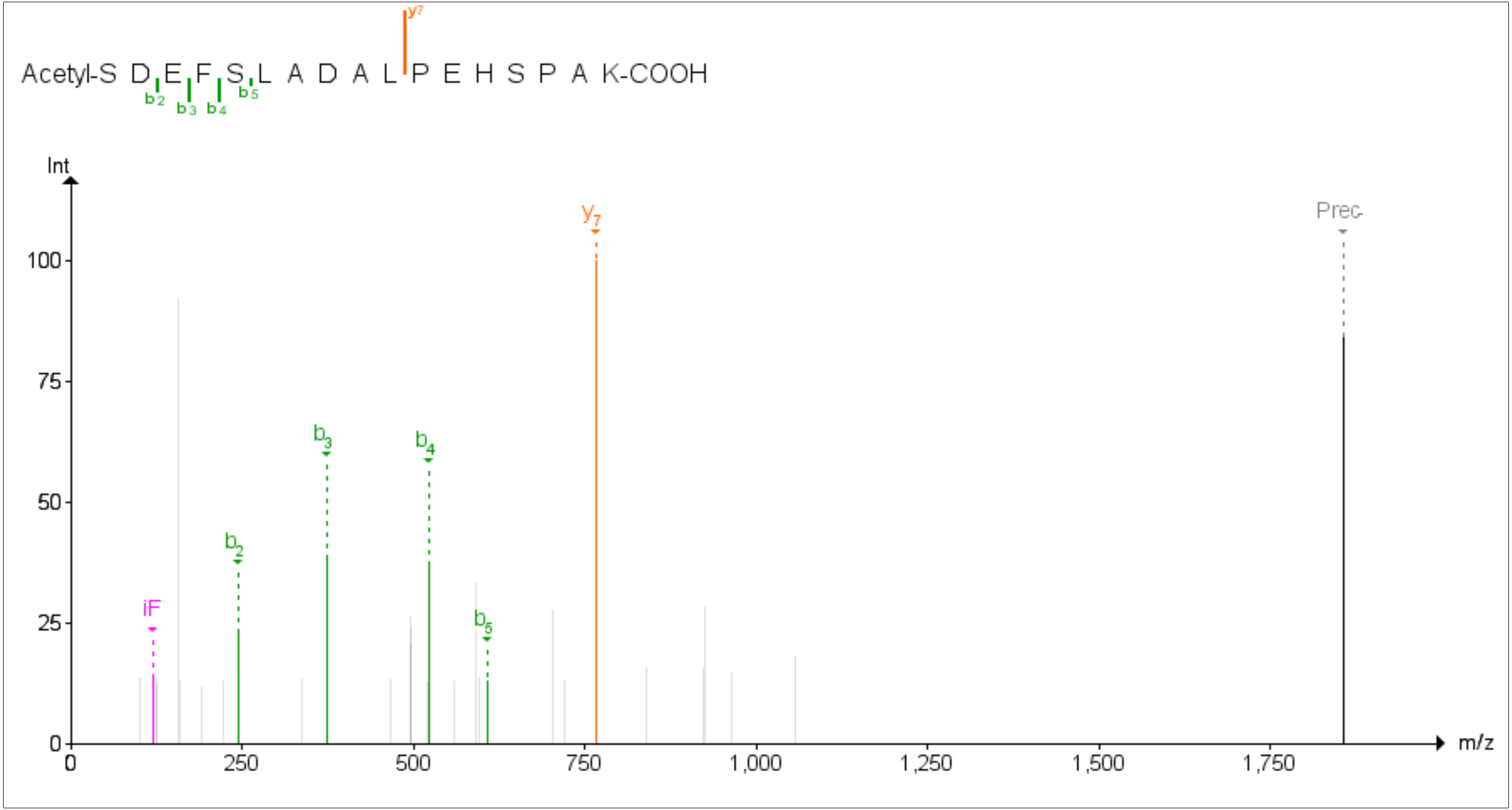

Acetyl-S D E F S L A D A L P E H S P A K-COOH
b2 b3 b4 b5 y7
Int
100
75
50
25
0
iF
b2
b3
b4
b5
y7
Prec
0 250 500 750 1,000 1,250 1,500 1,750
m/z

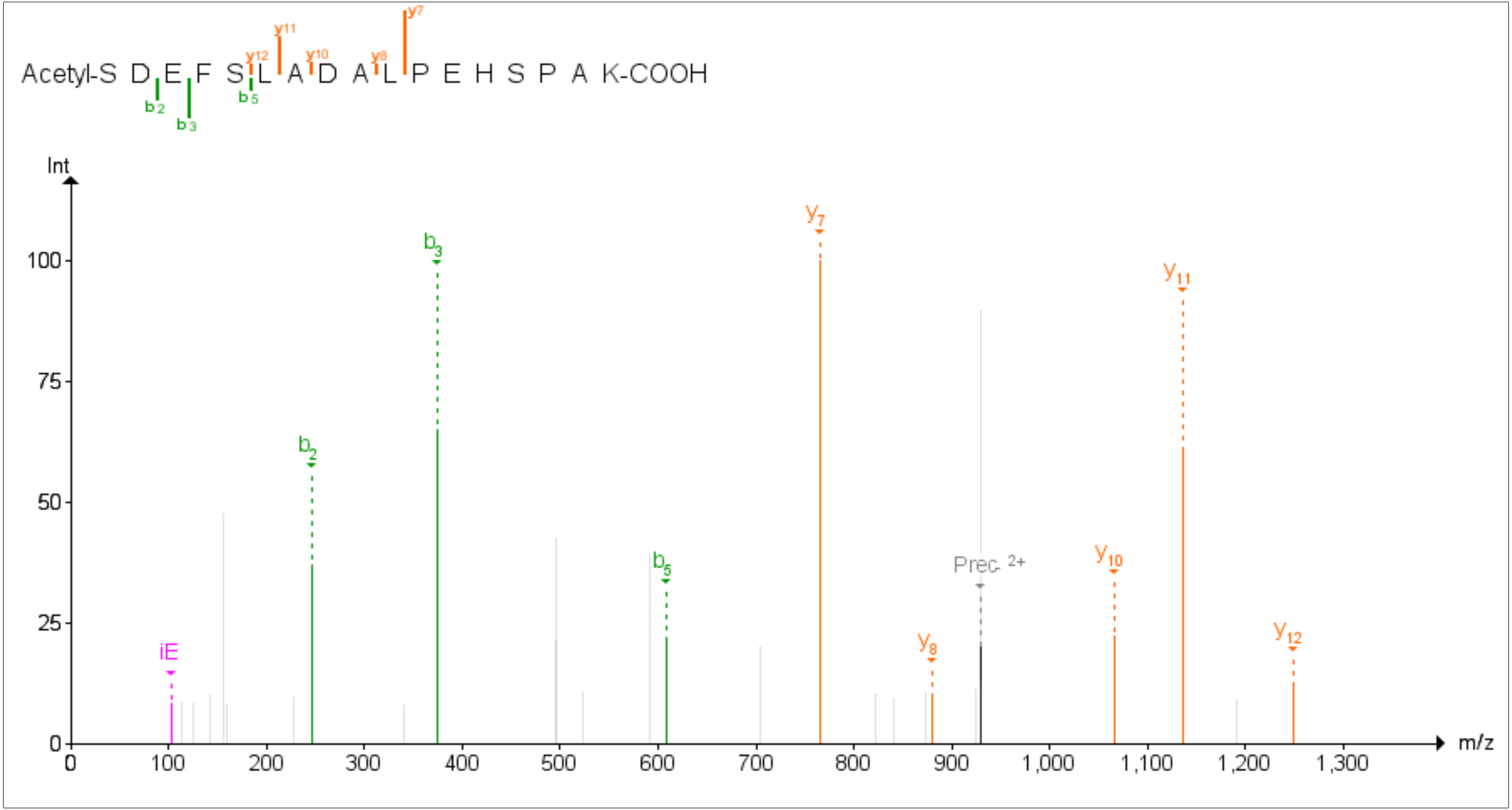

Acetyl-S D E F S L A D A L P E H S P A K-COOH
y7
y11
y12
y10
y8
b2
b3
b5
Int
m/z
0
25
50
75
100
0
100
200
300
400
500
600
700
800
900
1,000
1,100
1,200
1,300
iE
Prec 2+

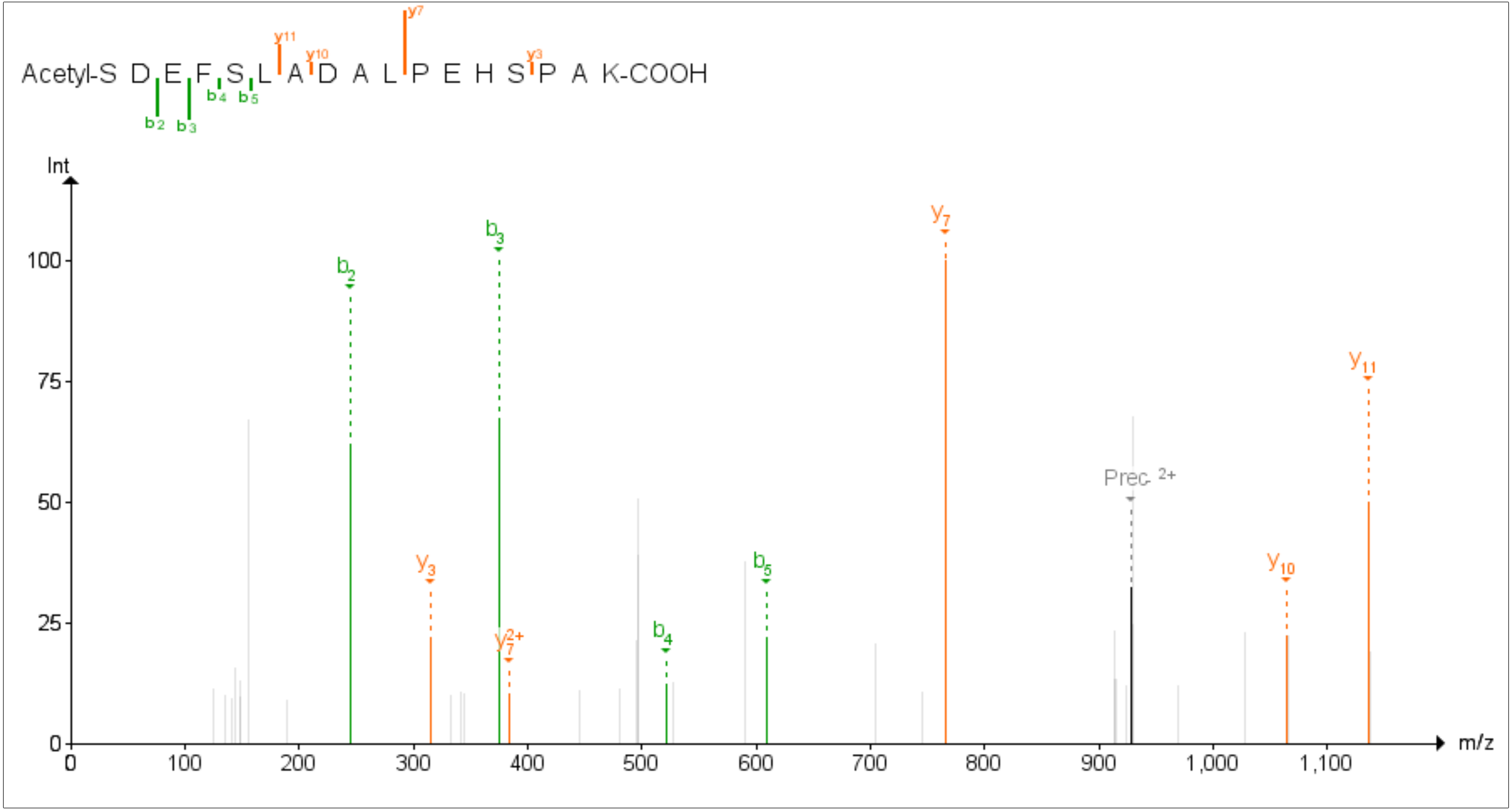

Acetyl-S D E F S L A D A L P E H S P A K-COOH
b2 b3 b4 b5
y11 y10 y7 y3
Int
100
75
50
25
0
0 100 200 300 400 500 600 700 800 900 1,000 1,100
m/z
b2 b3 y3 y7 2+ b4 b5 y7 Prec. 2+ y10 y11

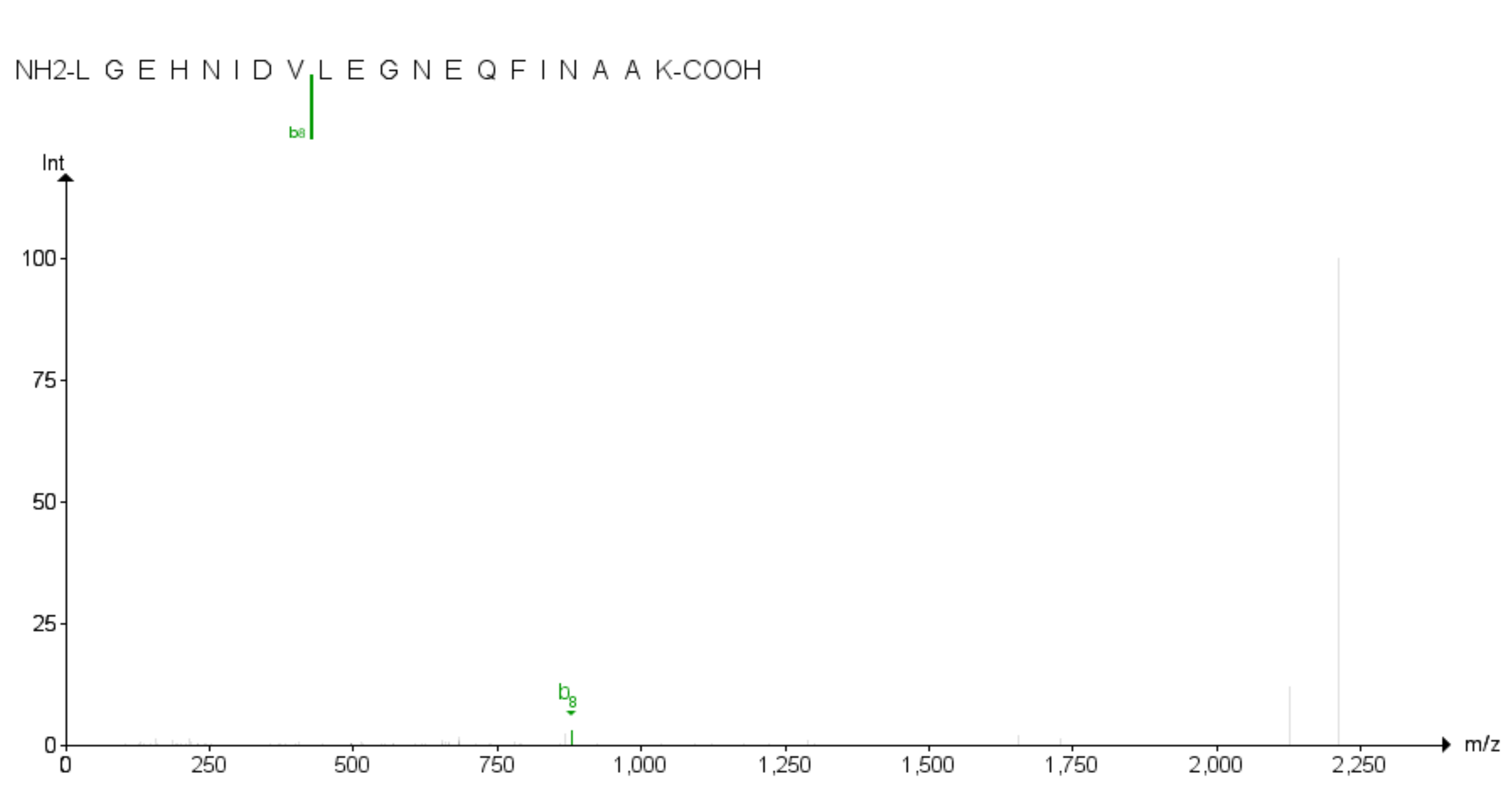

NH2-L G E H N I D V L E G N E Q F I N A A K-COOH
b8
Int
100
75
50
25
0
b8
0
250
500
750
1,000
1,250
1,500
1,750
2,000
2,250
m/z

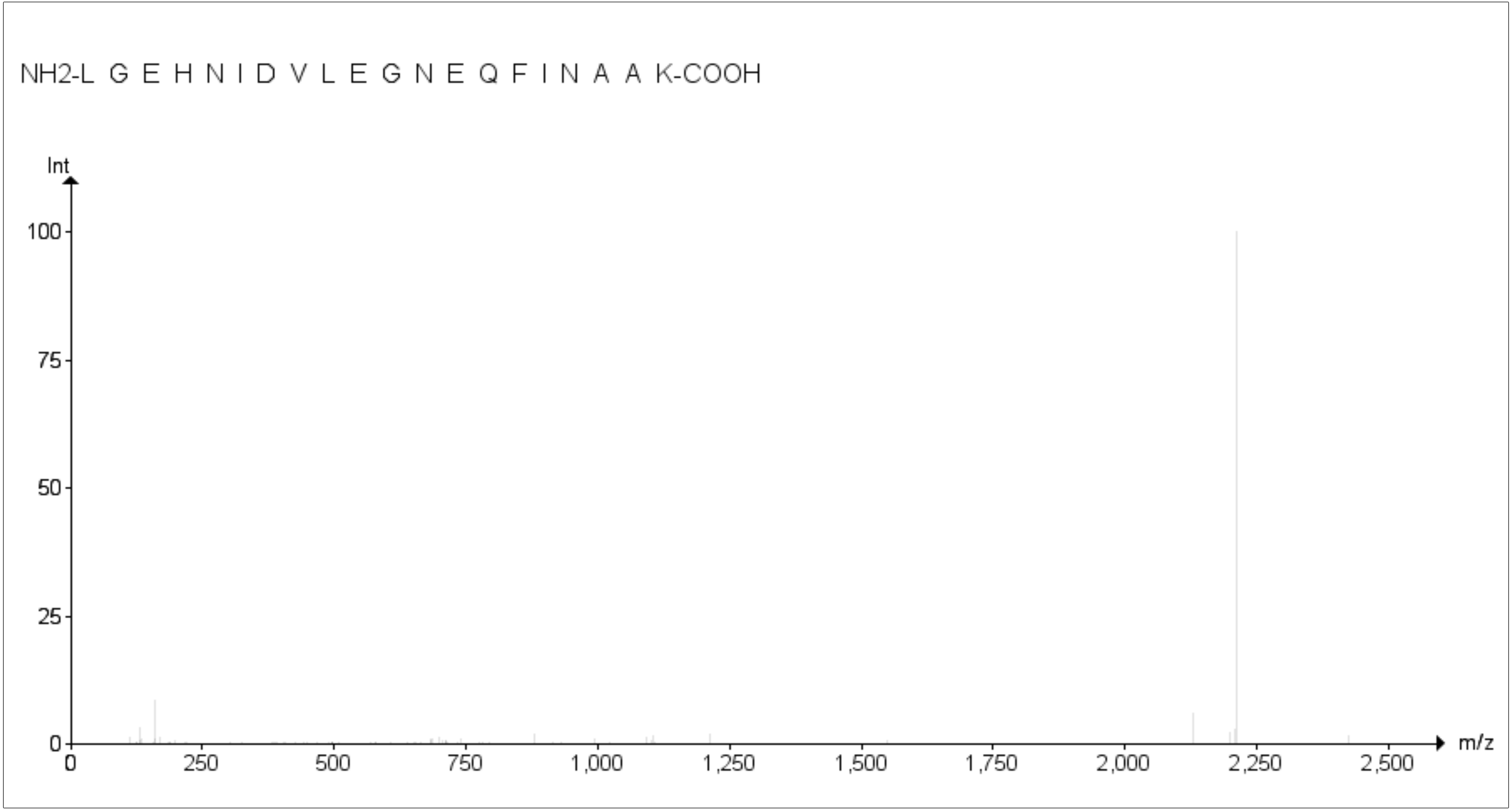
NH2-L G E H N I D V L E G N E Q F I N A A K-COOH
Int
100
75
50
25
0
m/z
0
250
500
750
1,000
1,250
1,500
1,750
2,000
2,250
2,500

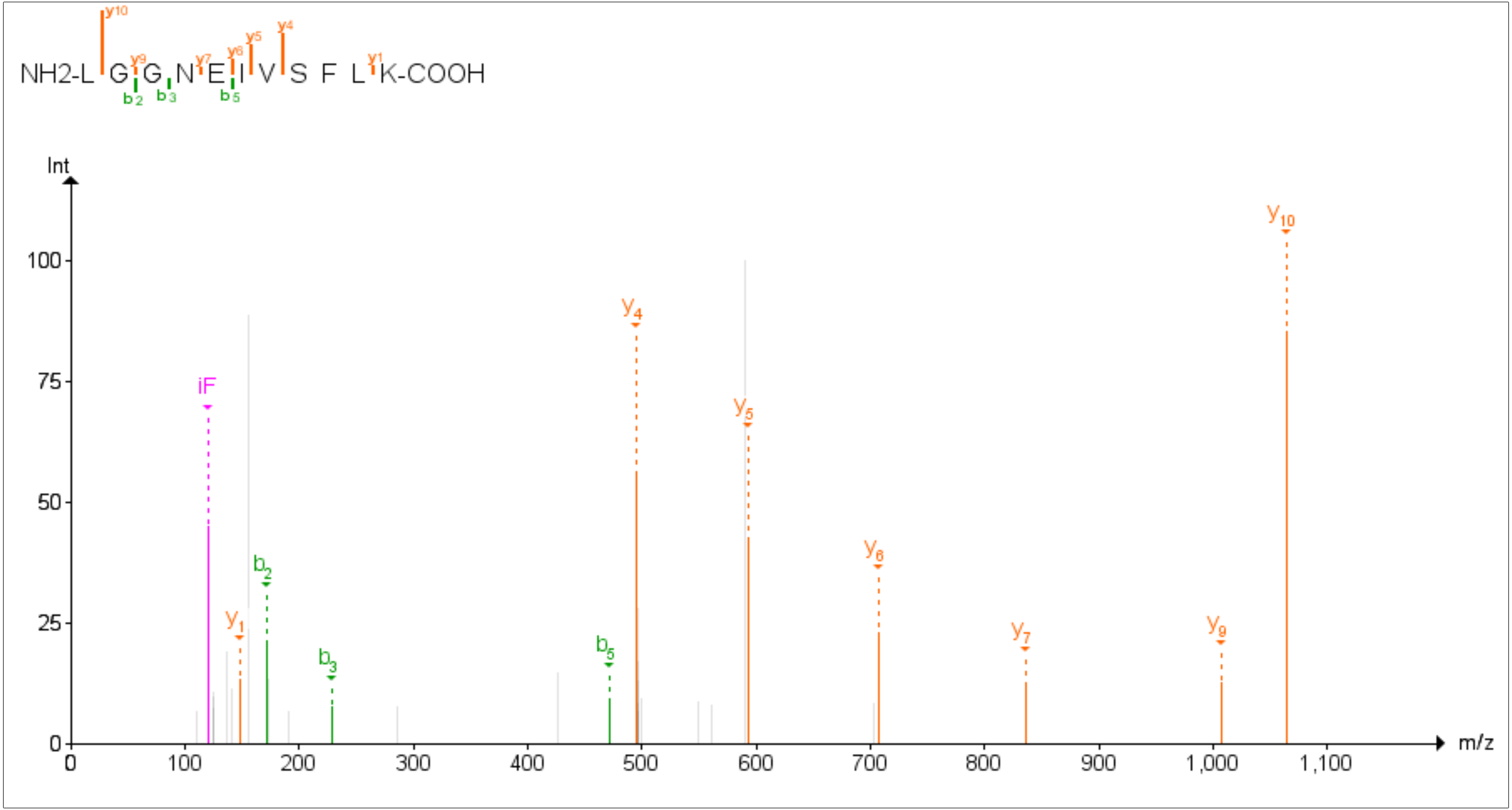

NH2-L G G N E I V S F L K-COOH
y10
y9
y7
y6
y5
y4
y1
b2
b3
b5
Int
100
75
50
25
0
iF
y1
b2
b3
b5
y4
y5
y6
y7
y9
y10
0
100
200
300
400
500
600
700
800
900
1,000
1,100
m/z

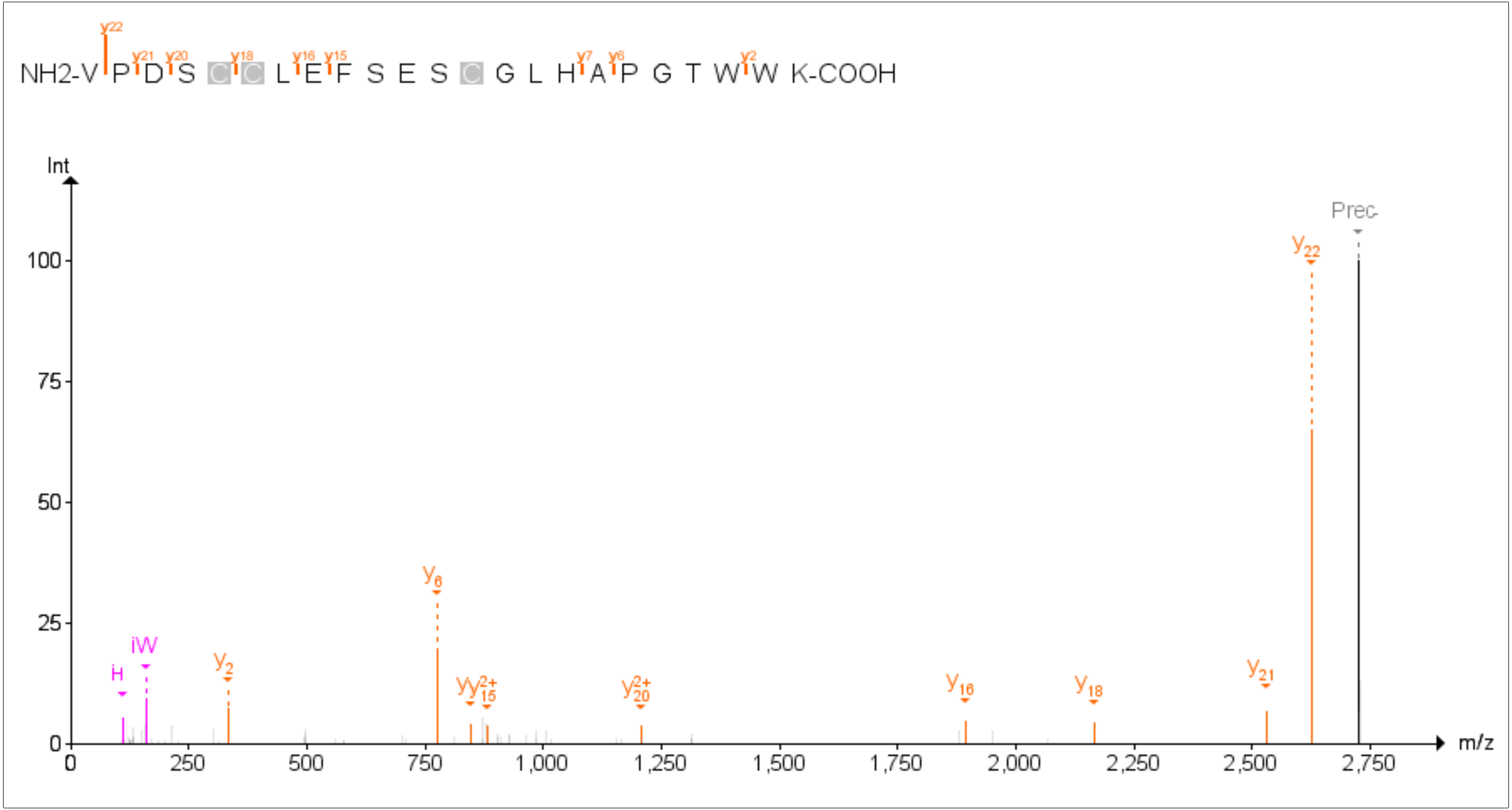

NH2-V P D S C C L E F S E S C G L H A P G T W W K-COOH
y22 y21 y20 y18 y16 y15 y7 y6 y2
Int
100
75
50
25
0
Prec.
y22
y6
iW
H
y2
yy15 2+
y20 2+
y16
y18
y21
m/z
0
250
500
750
1,000
1,250
1,500
1,750
2,000
2,250
2,500
2,750

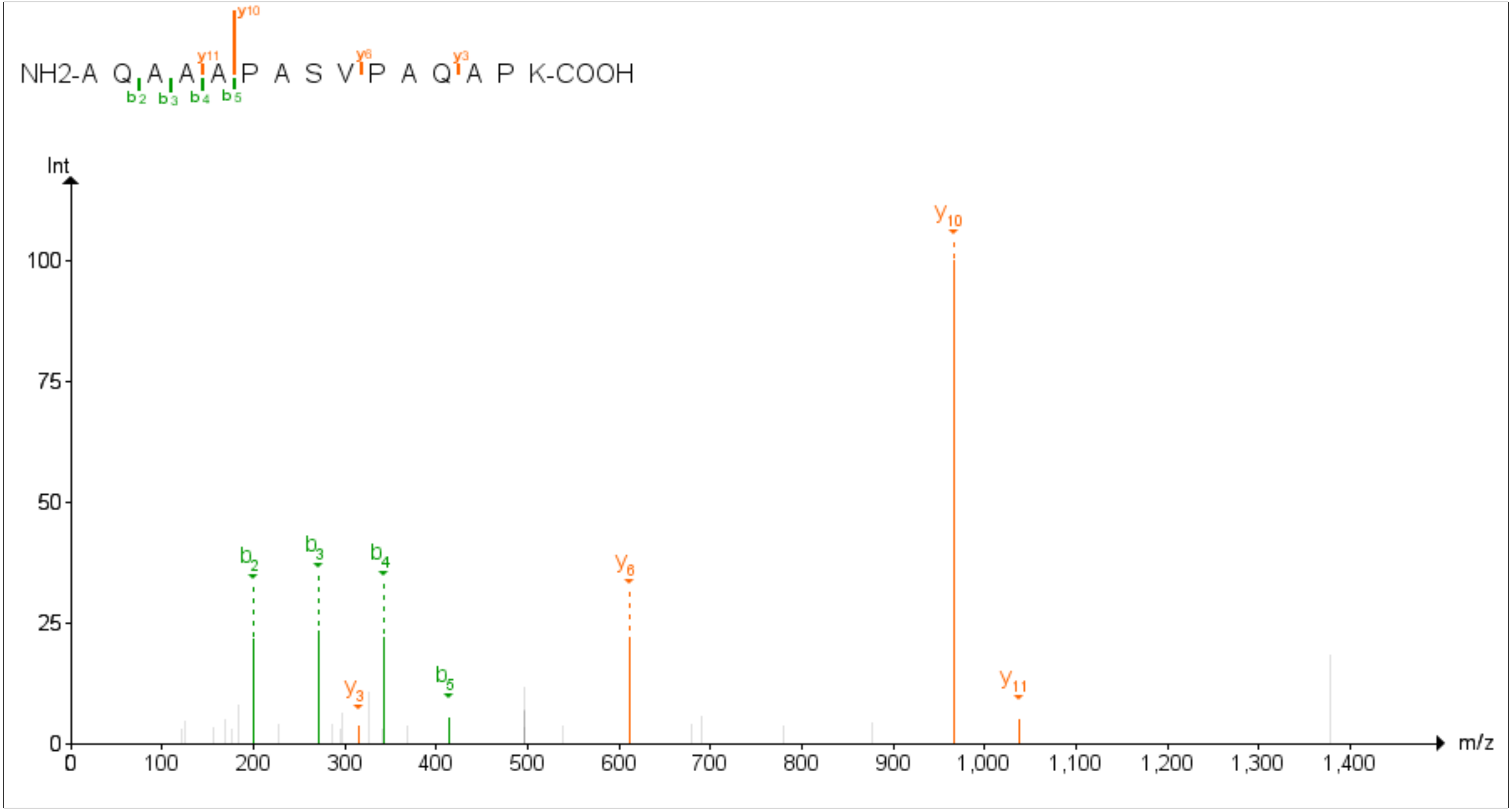

NH2-A Q A A A P A S V P A Q A P K-COOH
y10
y11
y6
y3
b2
b3
b4
b5
Int
100
75
50
25
0
m/z
0
100
200
300
400
500
600
700
800
900
1,000
1,100
1,200
1,300
1,400

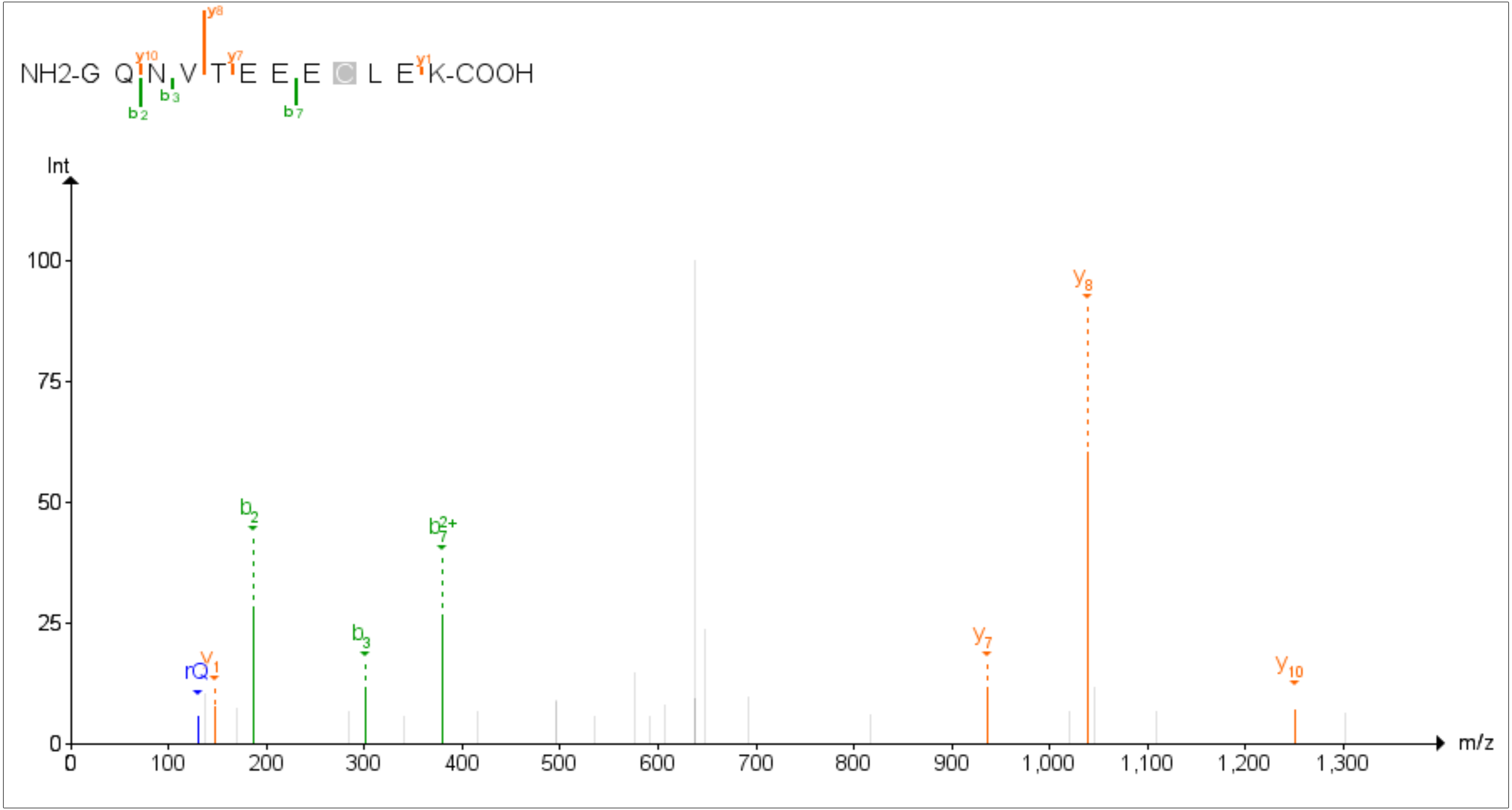

NH2-G Q N V T E E E C L E K-COOH
y10 y8 y7 y1
b2 b3 b7
Int
100
75
50
25
0
b2
b7 2+
b3
rQ
y1
y7
y8
y10
0 100 200 300 400 500 600 700 800 900 1,000 1,100 1,200 1,300
m/z

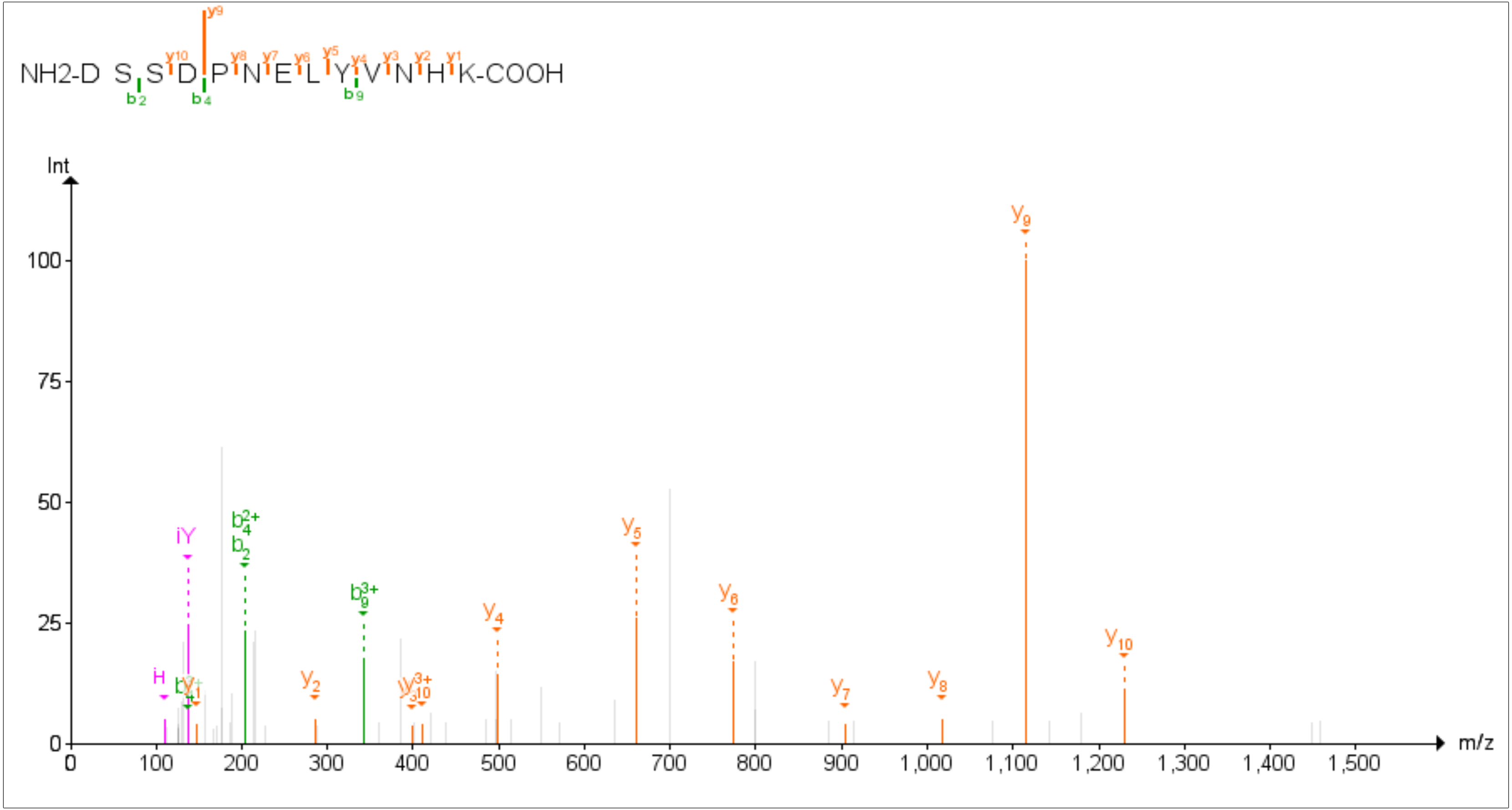
NH2-D S S D P N E L Y V N H K-COOH
Int
m/z
0
25
50
75
100
0
100
200
300
400
500
600
700
800
900
1,000
1,100
1,200
1,300
1,400
1,500
y9
y5
y6
y4
y10
y7
y8
y2
iY
b4 2+
b2
b9 3+

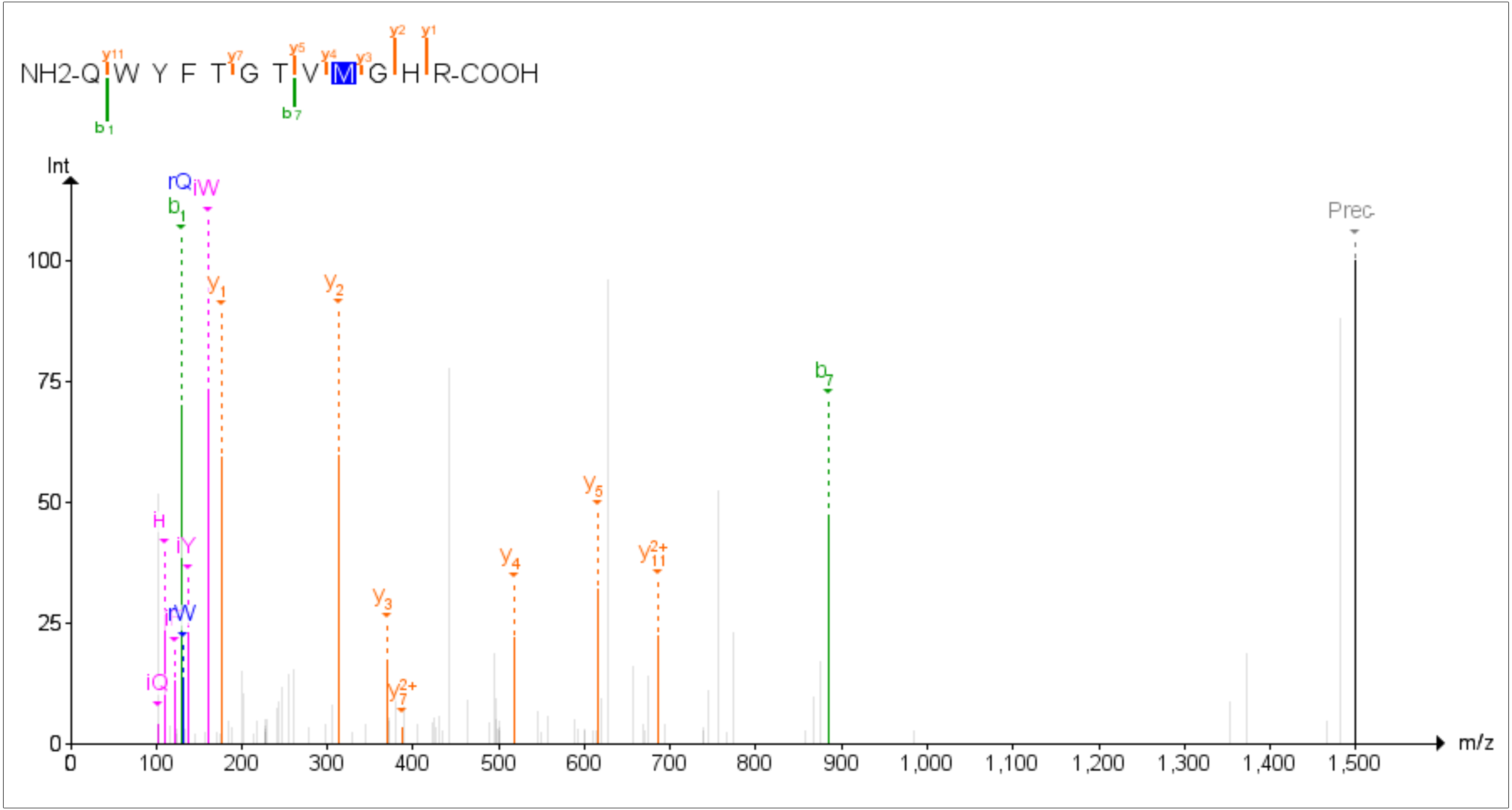

NH2-Q W Y F T G T V M G H R-COOH
y11
y7
y5
y4
y3
y2
y1
b1
b7
Int
m/z
rQ
iW
b1
y1
y2
b7
Prec
iH
iY
rW
iQ
y3
y4
y5
y_{11}^{2+}
y_7^{2+}
0
25
50
75
100
0
100
200
300
400
500
600
700
800
900
1,000
1,100
1,200
1,300
1,400
1,500

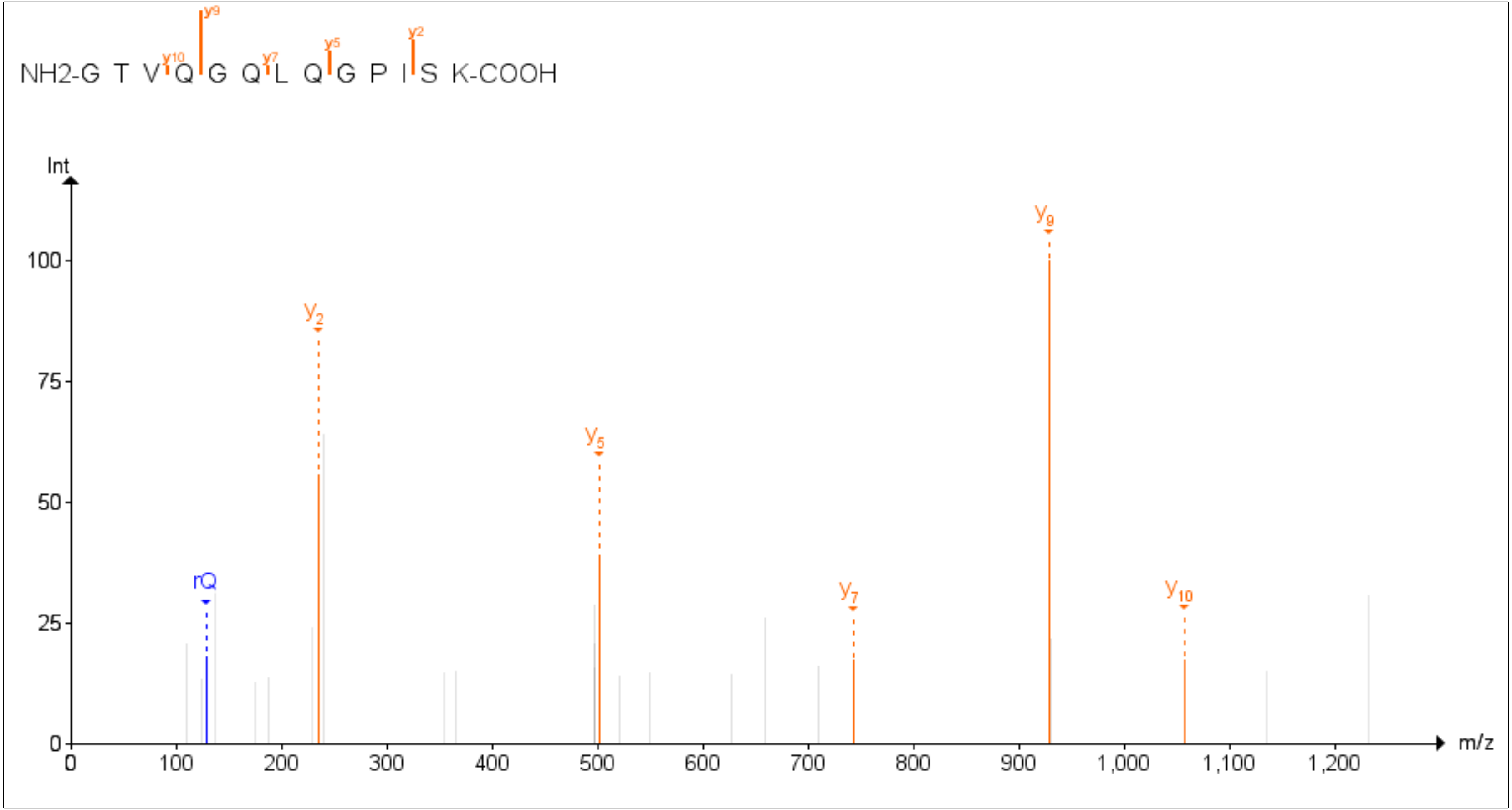
NH2-G T V Q G Q L Q G P I S K-COOH
y10 y9 y7 y5 y2
Int
m/z
rQ
y2
y5
y7
y9
y10
0
25
50
75
100
0
100
200
300
400
500
600
700
800
900
1,000
1,100
1,200

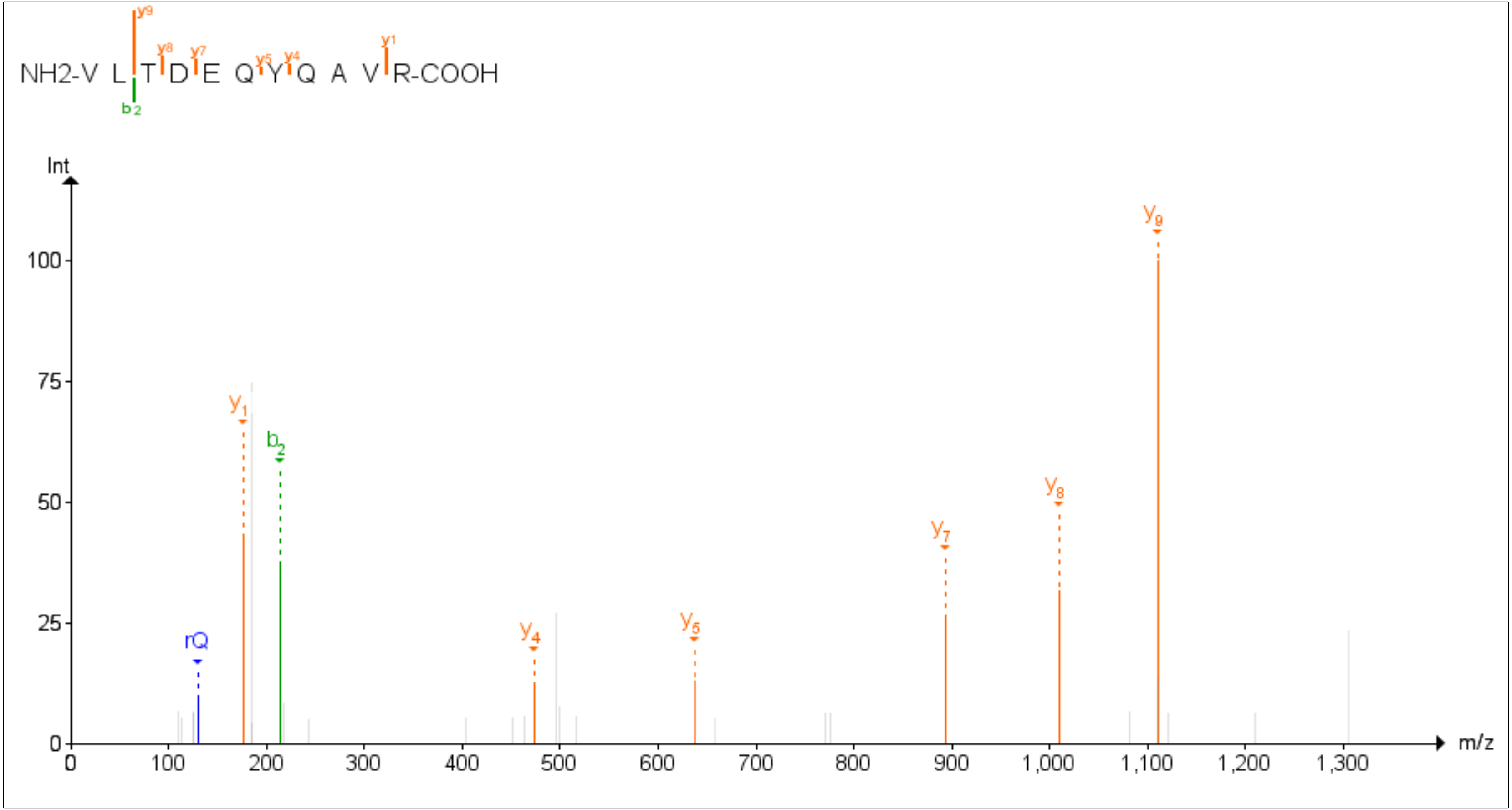

NH2-V L T D E Q Y Y Q A V R-COOH
y9
y8
y7
y5
y4
y1
b2
Int
100
75
50
25
0
y9
y1
b2
y8
y7
y4
y5
rQ
0
100
200
300
400
500
600
700
800
900
1,000
1,100
1,200
1,300
m/z

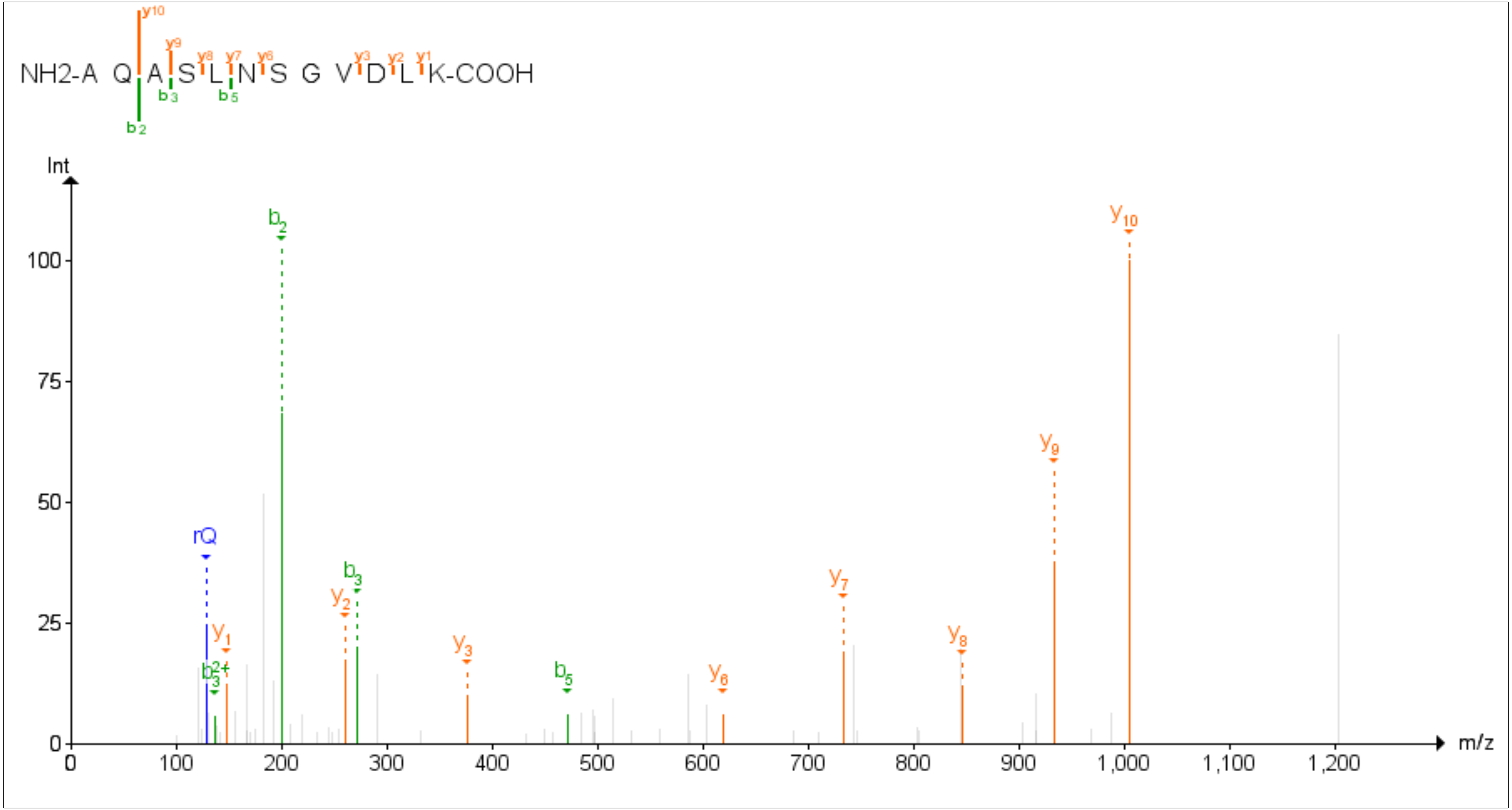

NH2-A Q A S L N S G V D L K-COOH
y10 y9 y8 y7 y6 y3 y2 y1
b2 b3 b5
Int
100
75
50
25
0
b2
y10
y9
rQ
b3
y2
y7
y1
y8
b3 2+
y3
b5
y6
0 100 200 300 400 500 600 700 800 900 1,000 1,100 1,200
m/z

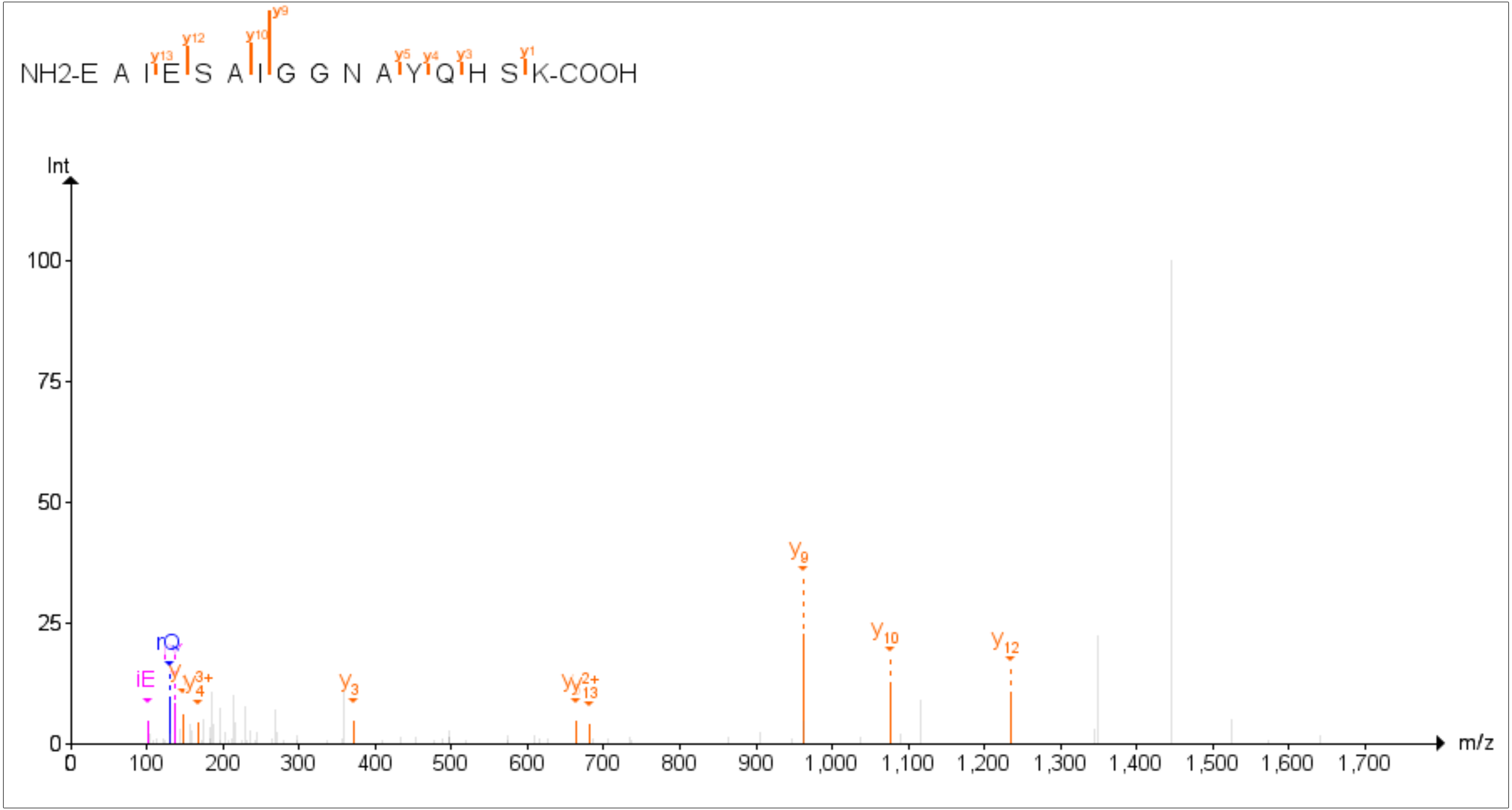
NH2-E A I E S A I G G N A Y Q H S K-COOH
y13 y12 y10 y9 y5 y4 y3 y1
Int
100
75
50
25
0
m/z
0 100 200 300 400 500 600 700 800 900 1,000 1,100 1,200 1,300 1,400 1,500 1,600 1,700
rQ
iE
y4 3+
y3
y13 2+
y9
y10
y12

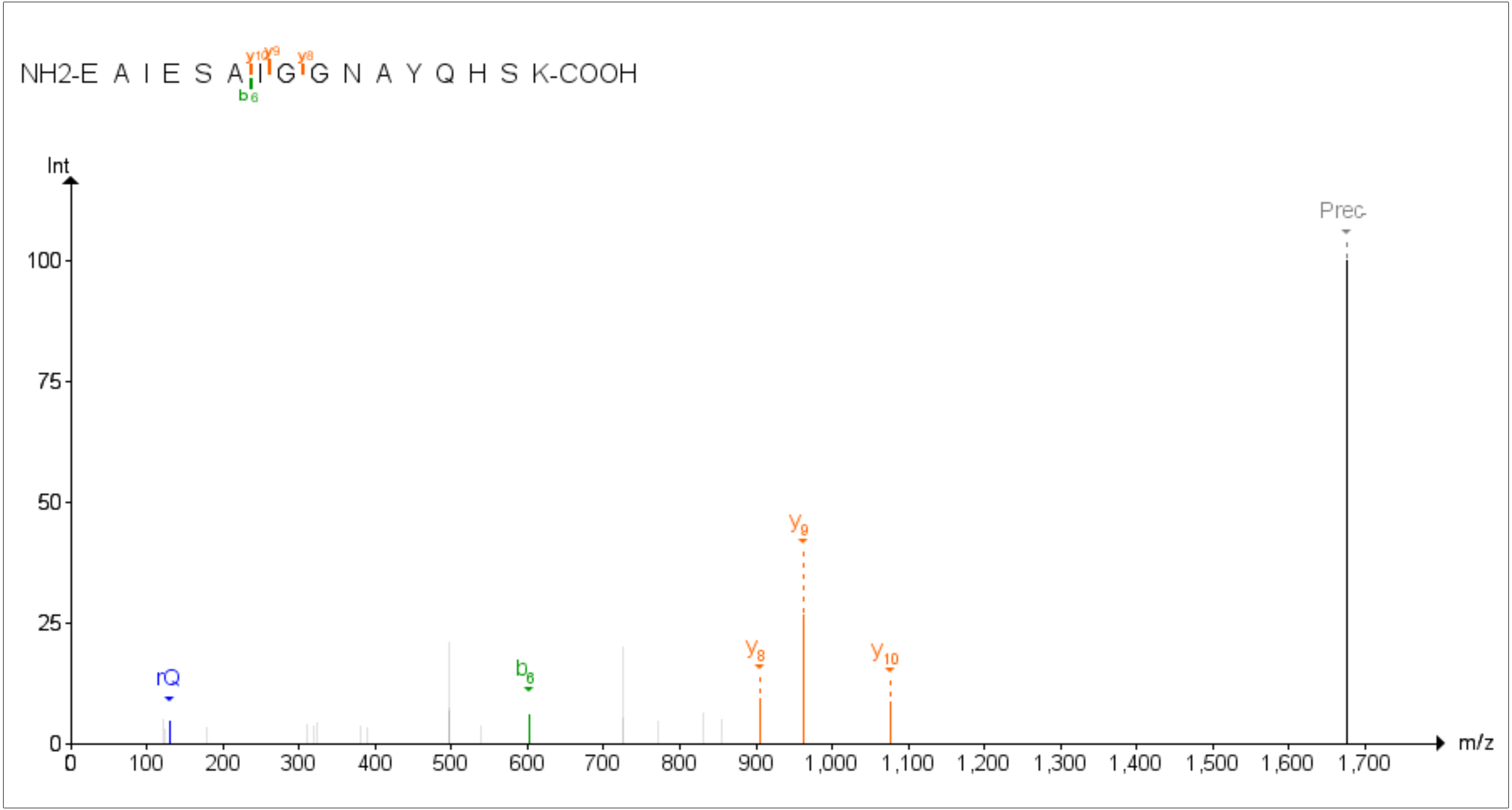

NH2-E A I E S A I G G N A Y Q H S K-COOH
y10
y9
y8
b6
Int
Prec
y9
y8
y10
b6
rQ
100
75
50
25
0
0
100
200
300
400
500
600
700
800
900
1,000
1,100
1,200
1,300
1,400
1,500
1,600
1,700
m/z

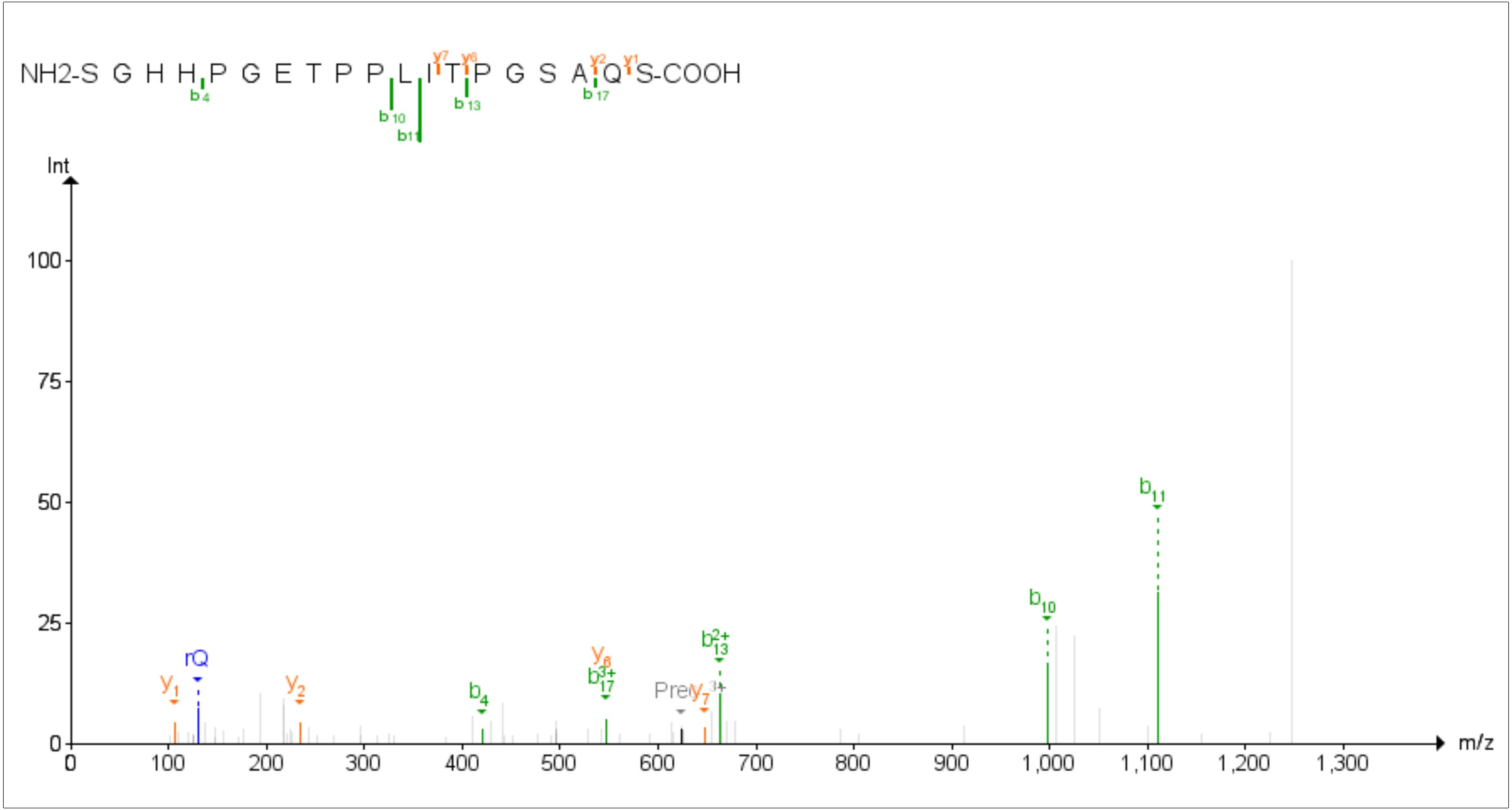
NH2-S G H H P G E T P P L I T P G S A Q S-COOH
y7 y6 y2 y1
b4 b10 b11 b13 b17
Int
100
75
50
25
0
rQ
y1
y2
b4
y6
b17 3+
Prec 3+
y7
b13 2+
b10
b11
0 100 200 300 400 500 600 700 800 900 1,000 1,100 1,200 1,300
m/z

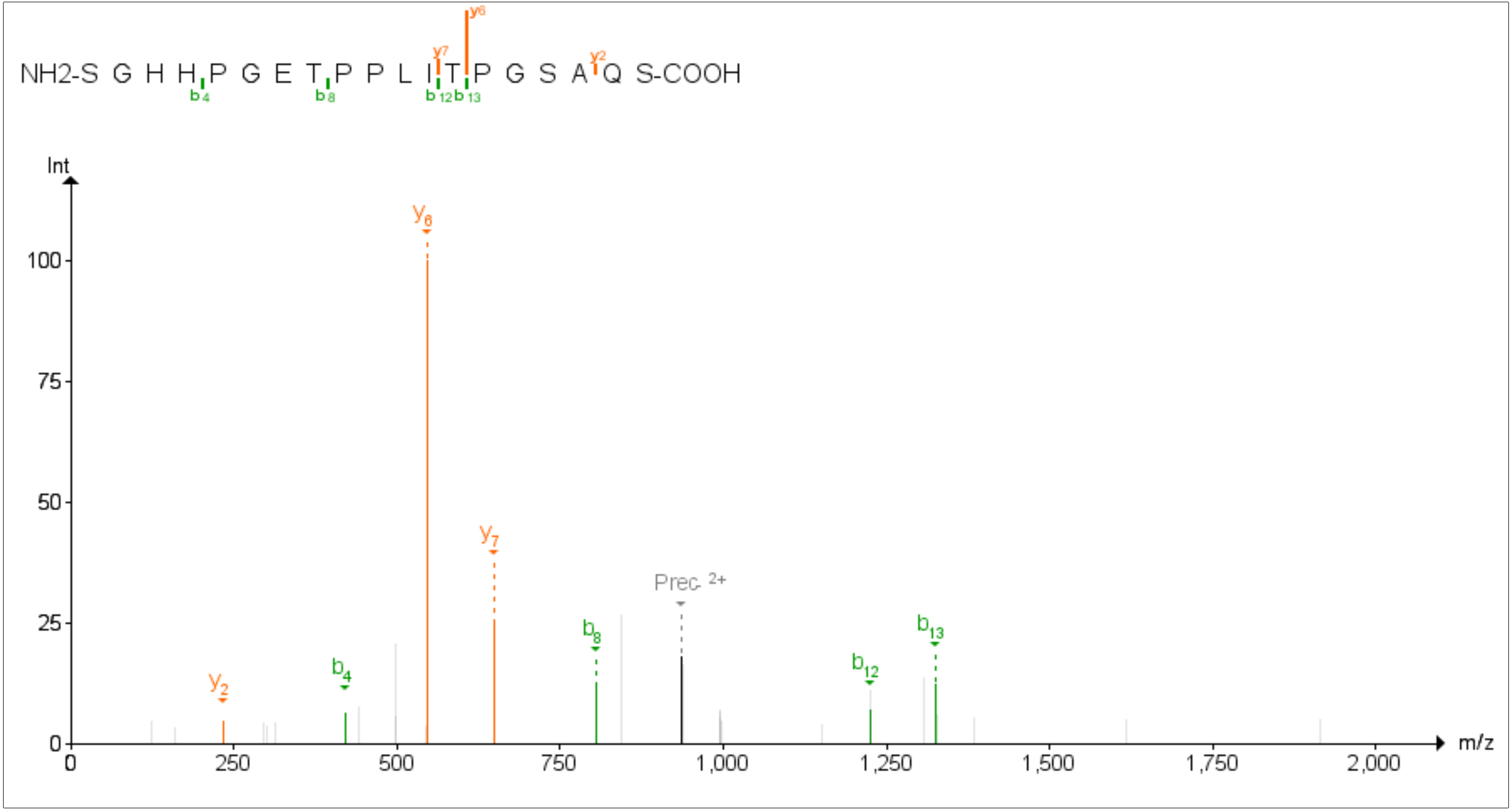

NH2-S G H H P G E T P P L I T P G S A Q S-COOH
b4 b8 b12 b13
y6 y7 y2
Int
100
75
50
25
0
y6
y7
Prec 2+
y2
b4
b8
b12
b13
0 250 500 750 1,000 1,250 1,500 1,750 2,000
m/z

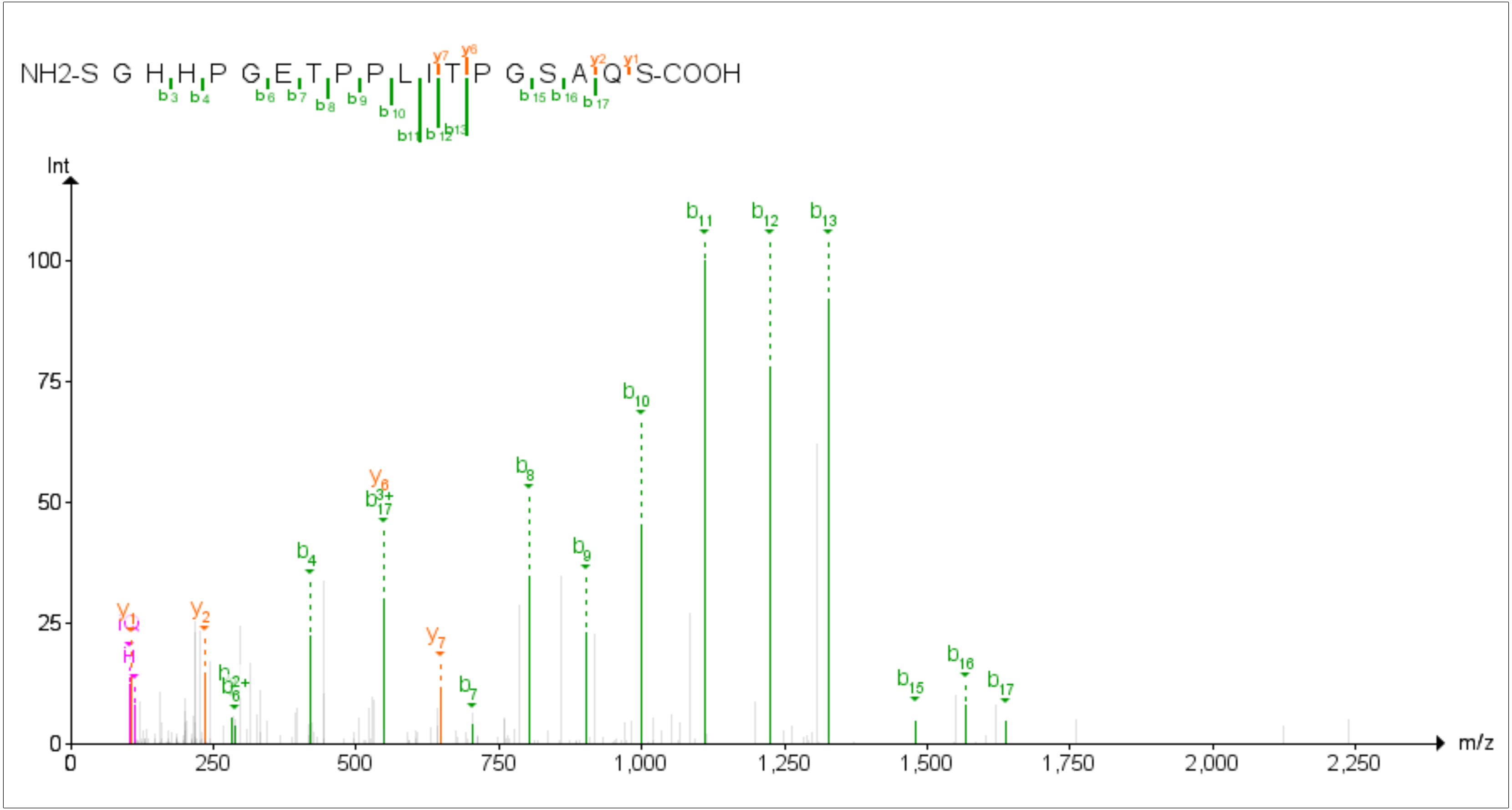

NH2-S G H H P G E T P P L I T P G S A Q S-COOH
b3 b4 b6 b7 b8 b9 b10 b11 b12 b13 b15 b16 b17
y7 y6 y2 y1
Int
m/z
0
25
50
75
100
250
500
750
1,000
1,250
1,500
1,750
2,000
2,250

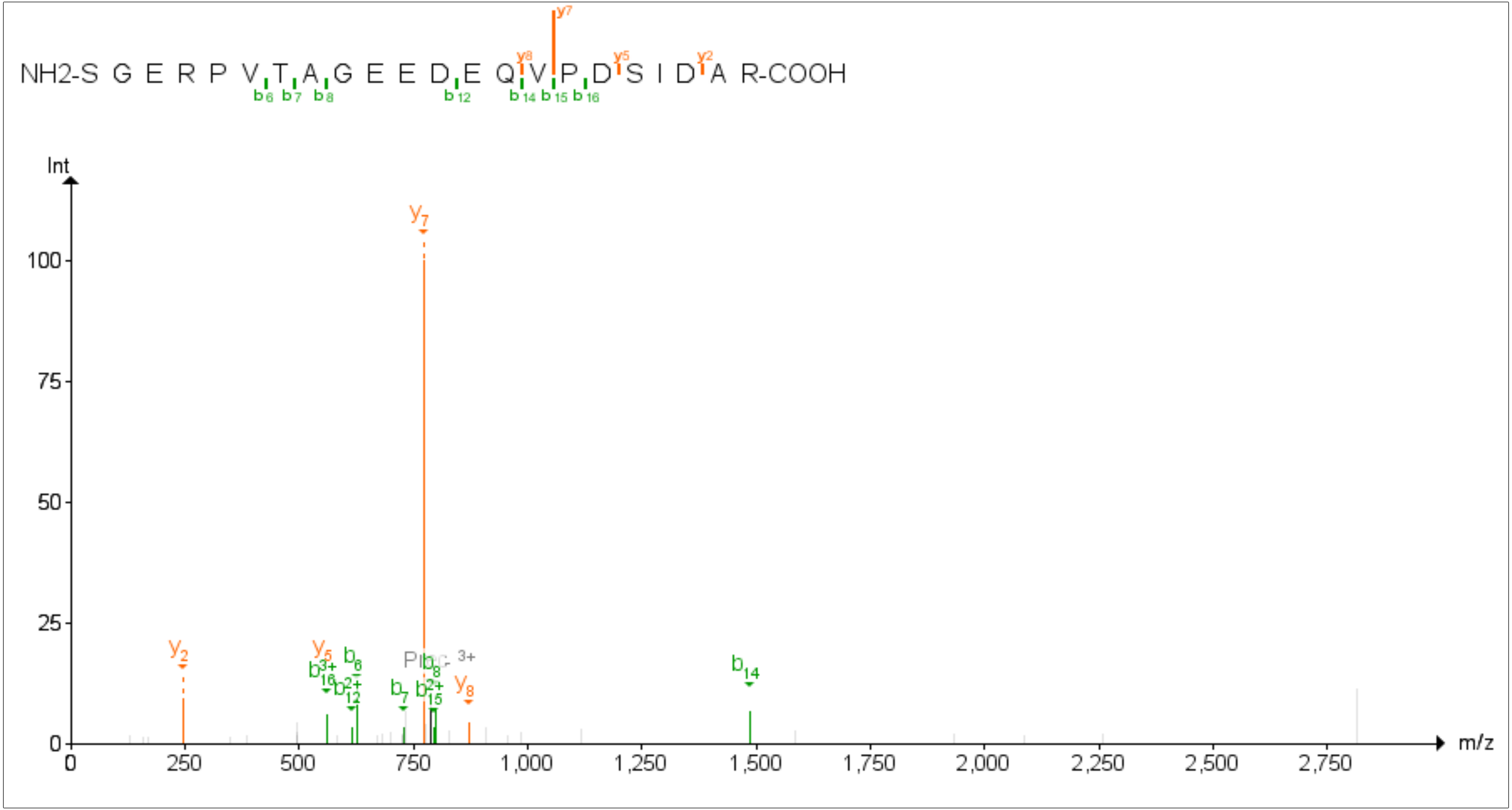
NH2-S G E R P V T A G E E D E Q V P D S I D A R-COOH
y7
y8
y5
y2
b6
b7
b8
b12
b14
b15
b16
Int
100
75
50
25
0
y7
y2
y5
b6
b16 3+
b12 2+
b7
b8
b15 2+
y8
b14
Prec 3+
0
250
500
750
1,000
1,250
1,500
1,750
2,000
2,250
2,500
2,750
m/z

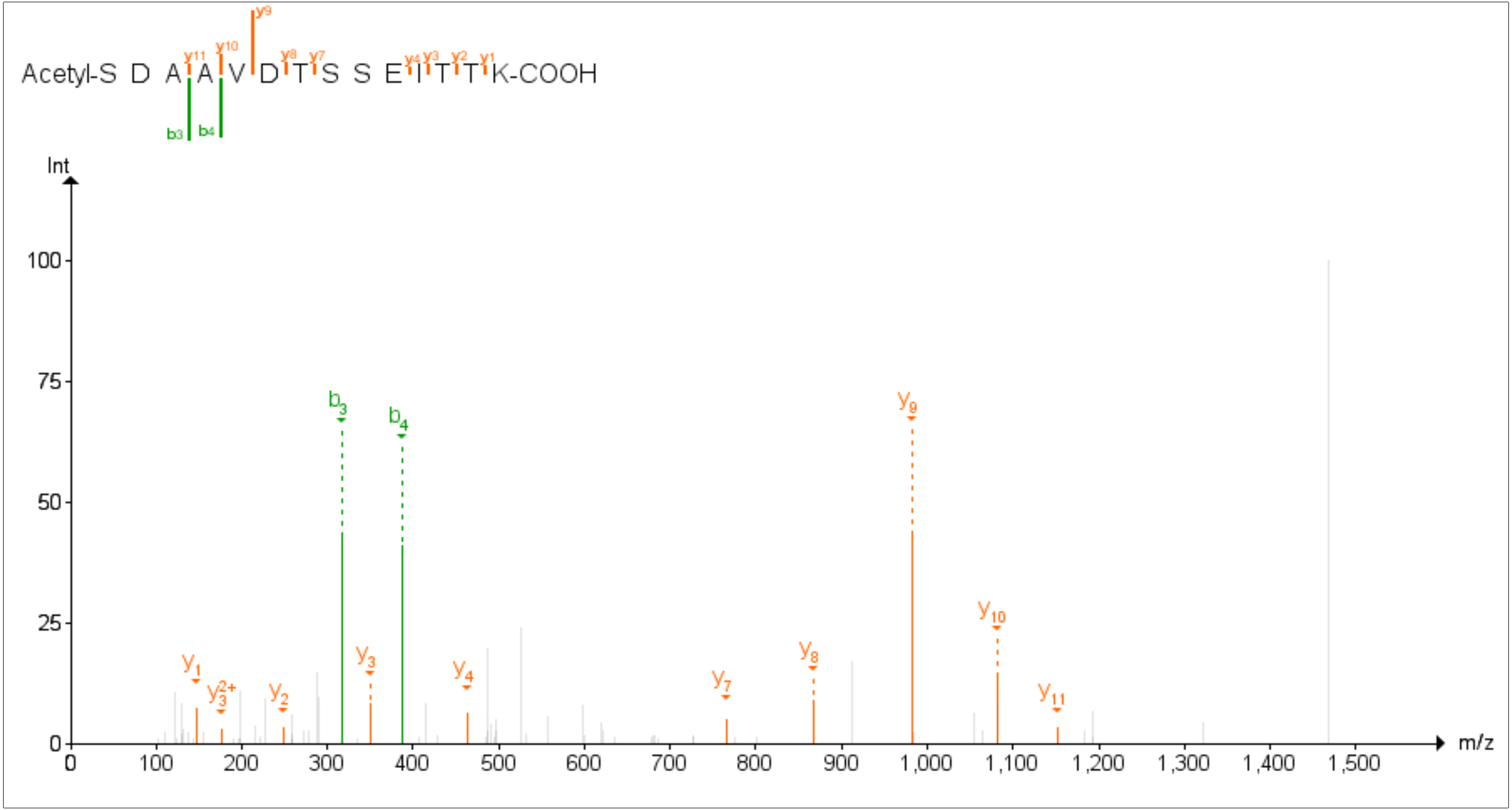

Acetyl-S D A A V D T S S E I T T K-COOH
y9 y11 y10 y8 y7 y4 y3 y2 y1
b3 b4
Int
m/z

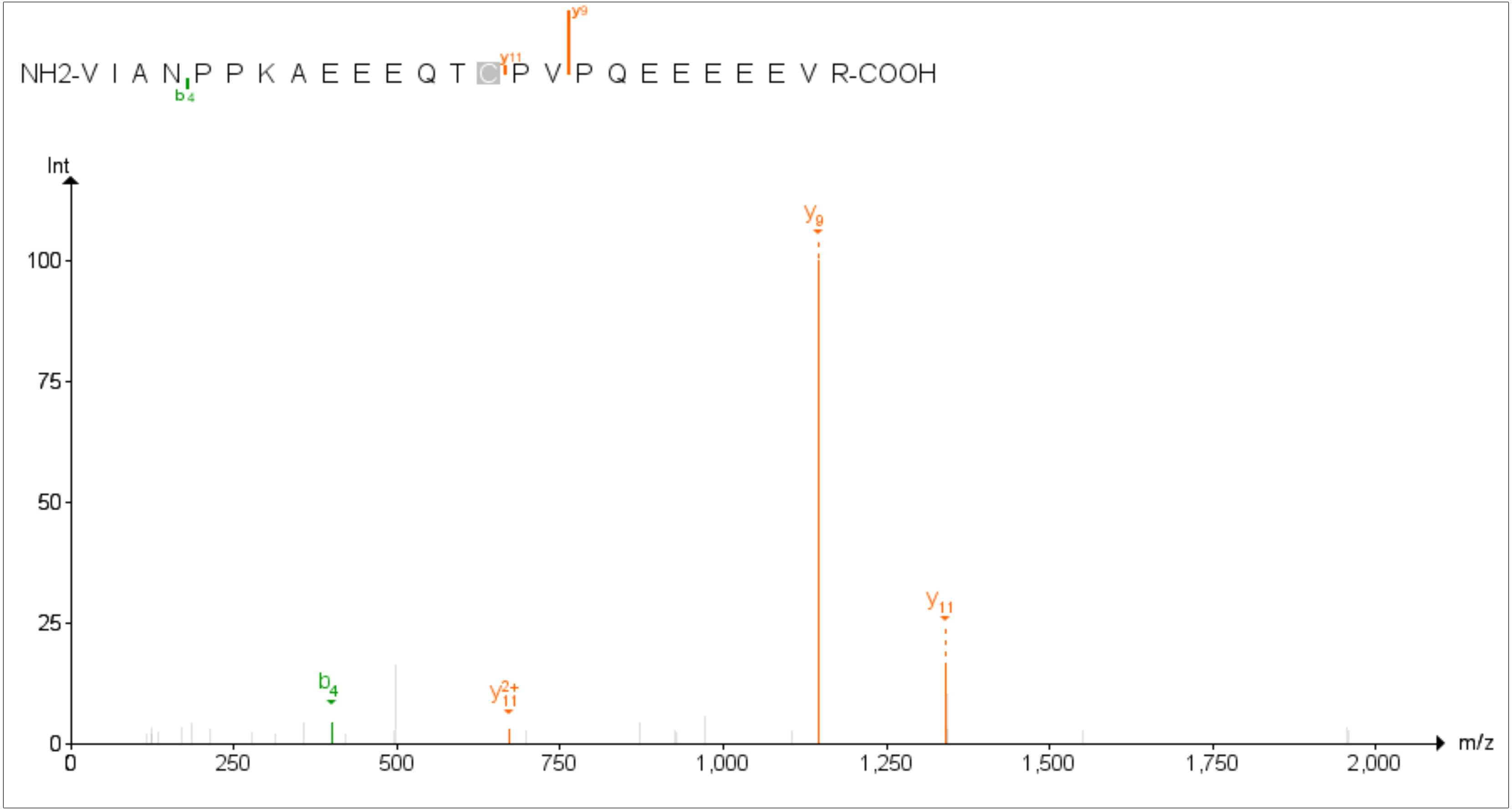
NH2-V I A N P P K A E E E Q T C P V P Q E E E E E V R-COOH
b4
y11
y9
Int
100
75
50
25
0
y9
y11
b4
y11 2+
0
250
500
750
1,000
1,250
1,500
1,750
2,000
m/z

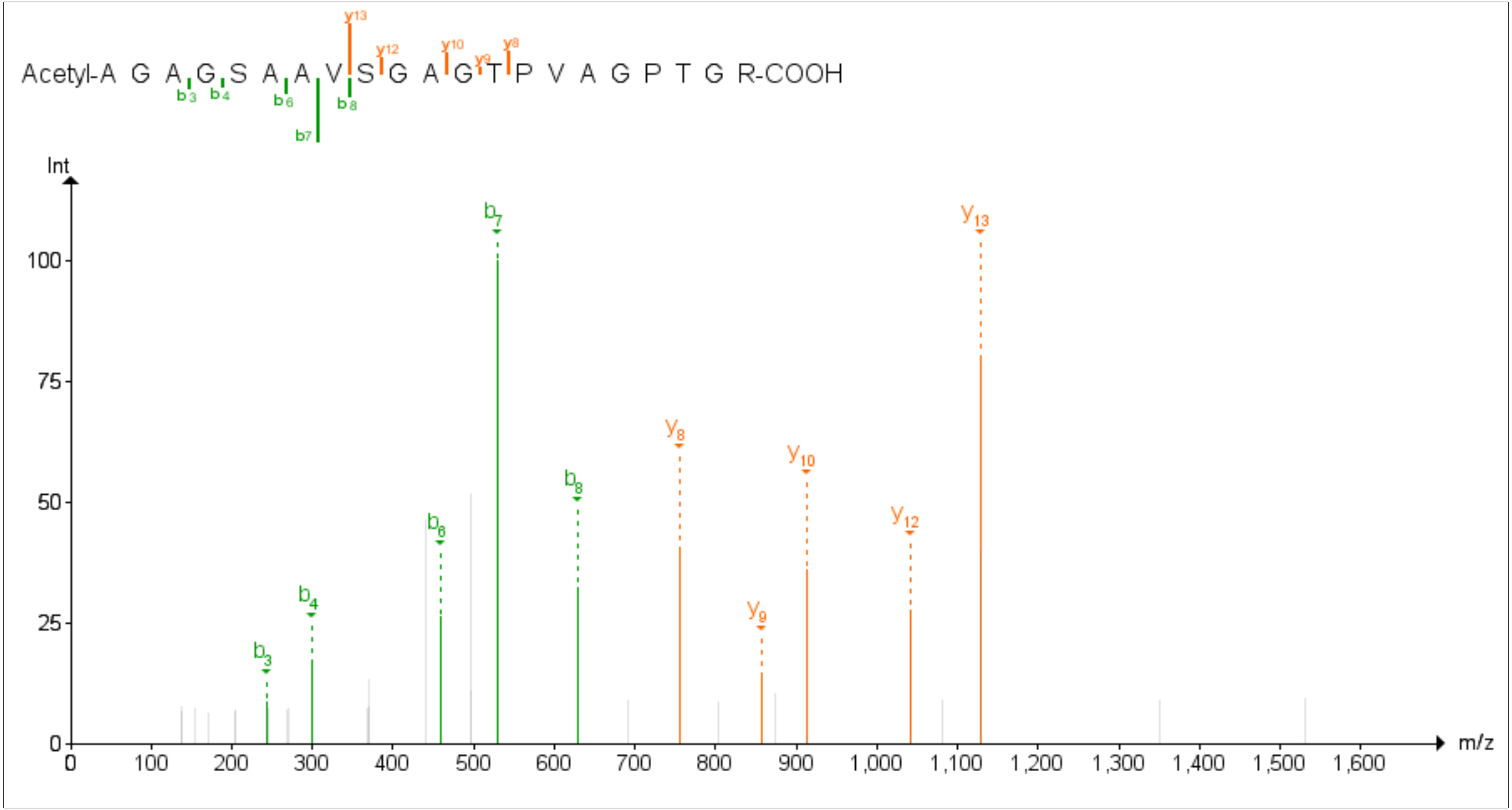

Acetyl-A G A G S A A V S G A G T P V A G P T G R-COOH
b3
b4
b6
b7
b8
y13
y12
y10
y9
y8
Int
100
75
50
25
0
m/z
0
100
200
300
400
500
600
700
800
900
1,000
1,100
1,200
1,300
1,400
1,500
1,600

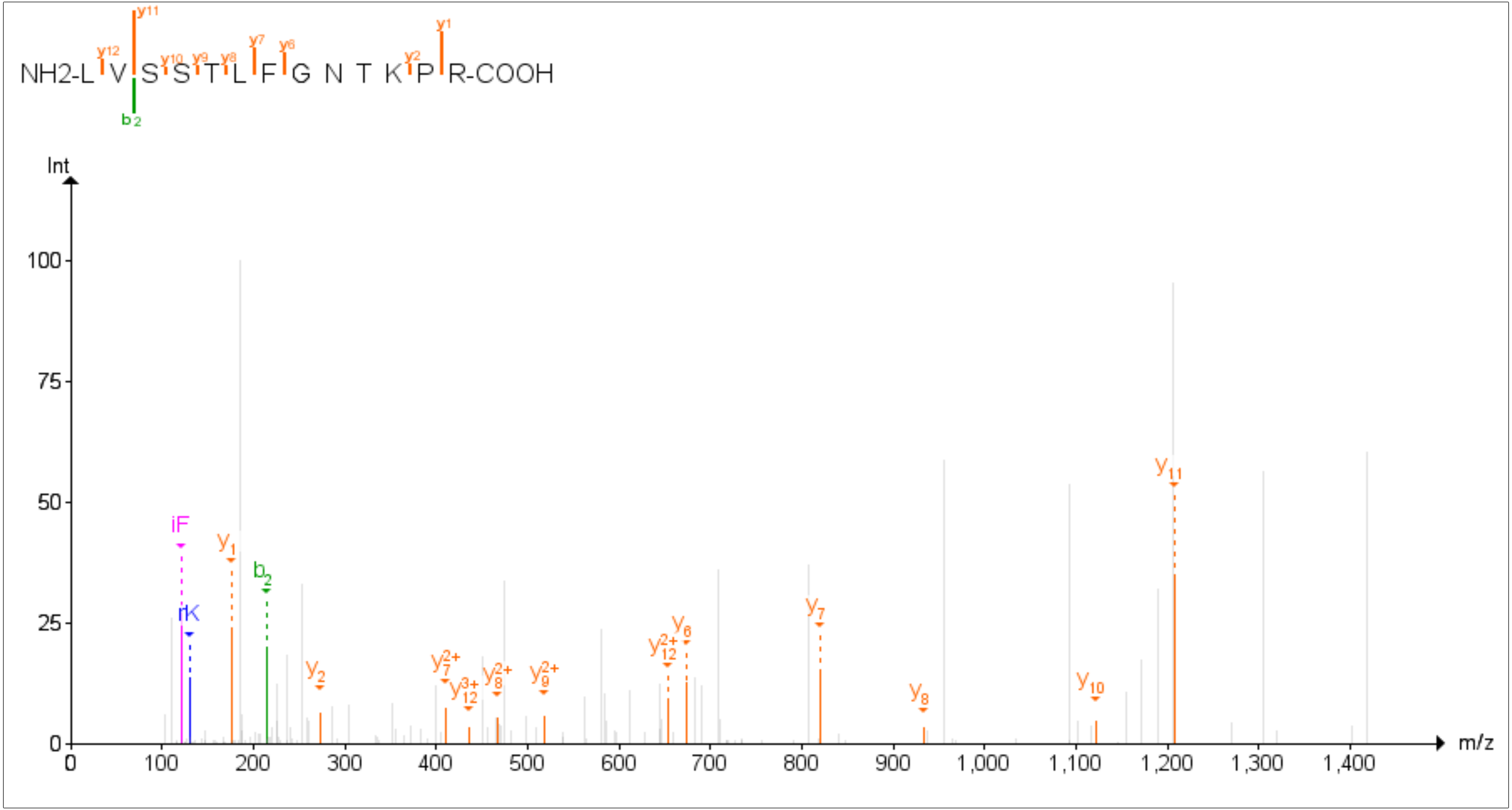

NH2-L V S S T L F G N T K P R-COOH
y12 y11 y10 y9 y8 y7 y6 y2 y1
b2
Int
100
75
50
25
0
iF
rK
y1
b2
y2
y7 2+
y12 3+
y8 2+
y9 2+
y12 2+
y6
y7
y8
y10
y11
0 100 200 300 400 500 600 700 800 900 1,000 1,100 1,200 1,300 1,400
m/z

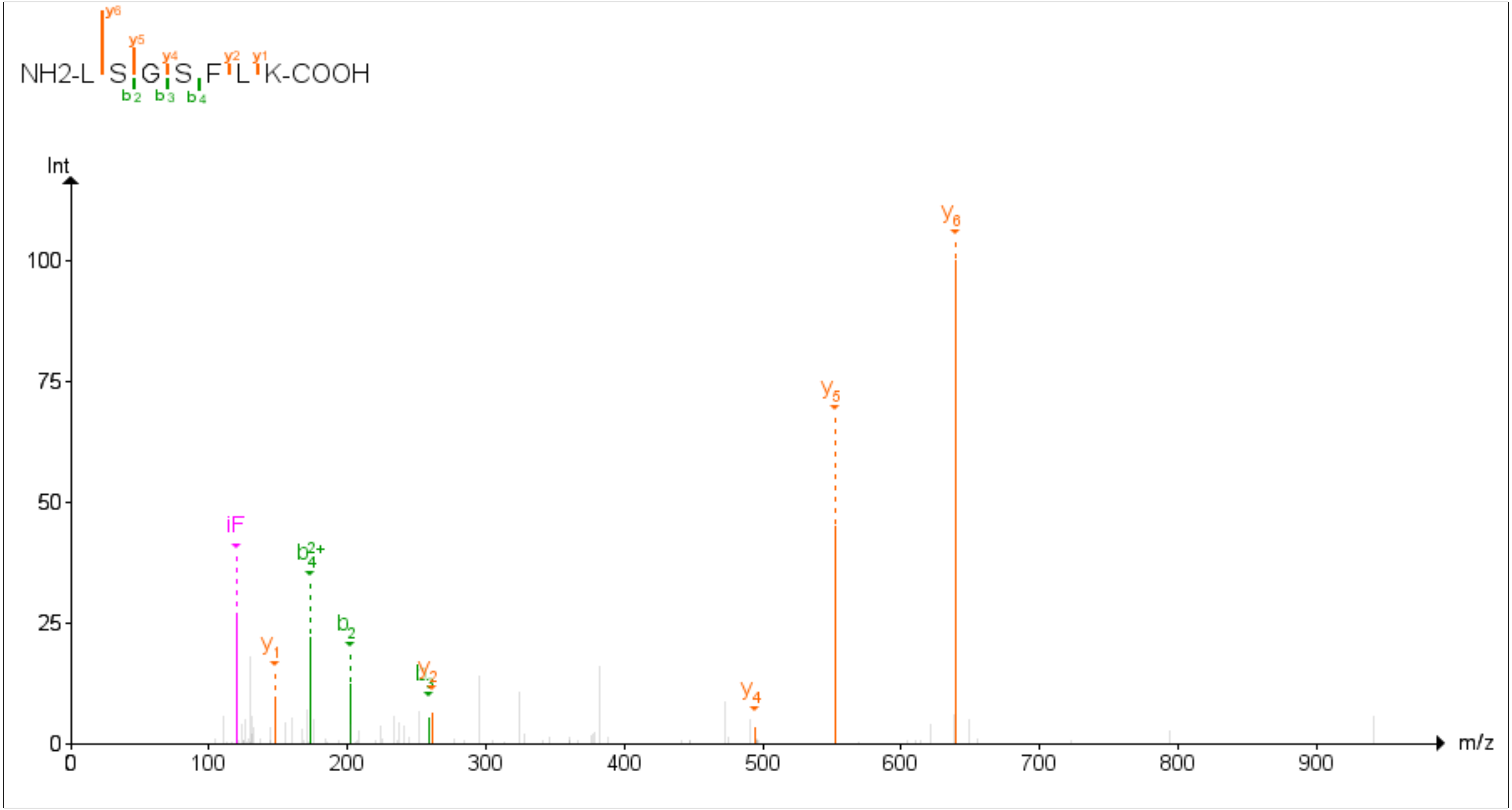
NH2-L S G S F L K-COOH
y6
y5
y4
y2
y1
b2
b3
b4
Int
100
75
50
25
0
iF
b_4^{2+}
b_2
y_1
y_2
b_3
y_4
y_5
y_6
m/z
0
100
200
300
400
500
600
700
800
900

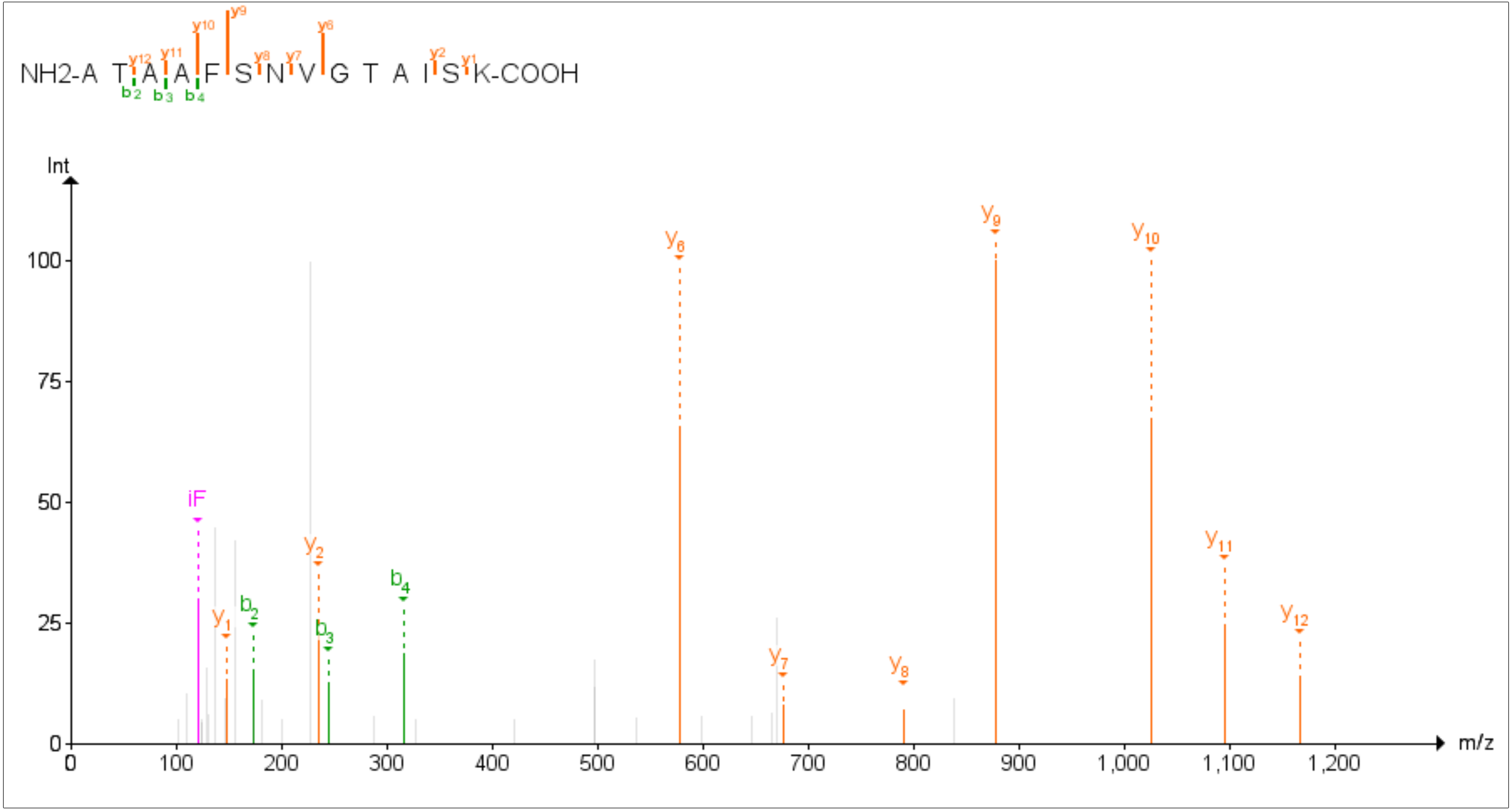

NH2-A T A A F S N V G T A I S K-COOH
y12 y11 y10 y9 y8 y7 y6 y2 y1
b2 b3 b4
Int
m/z
iF

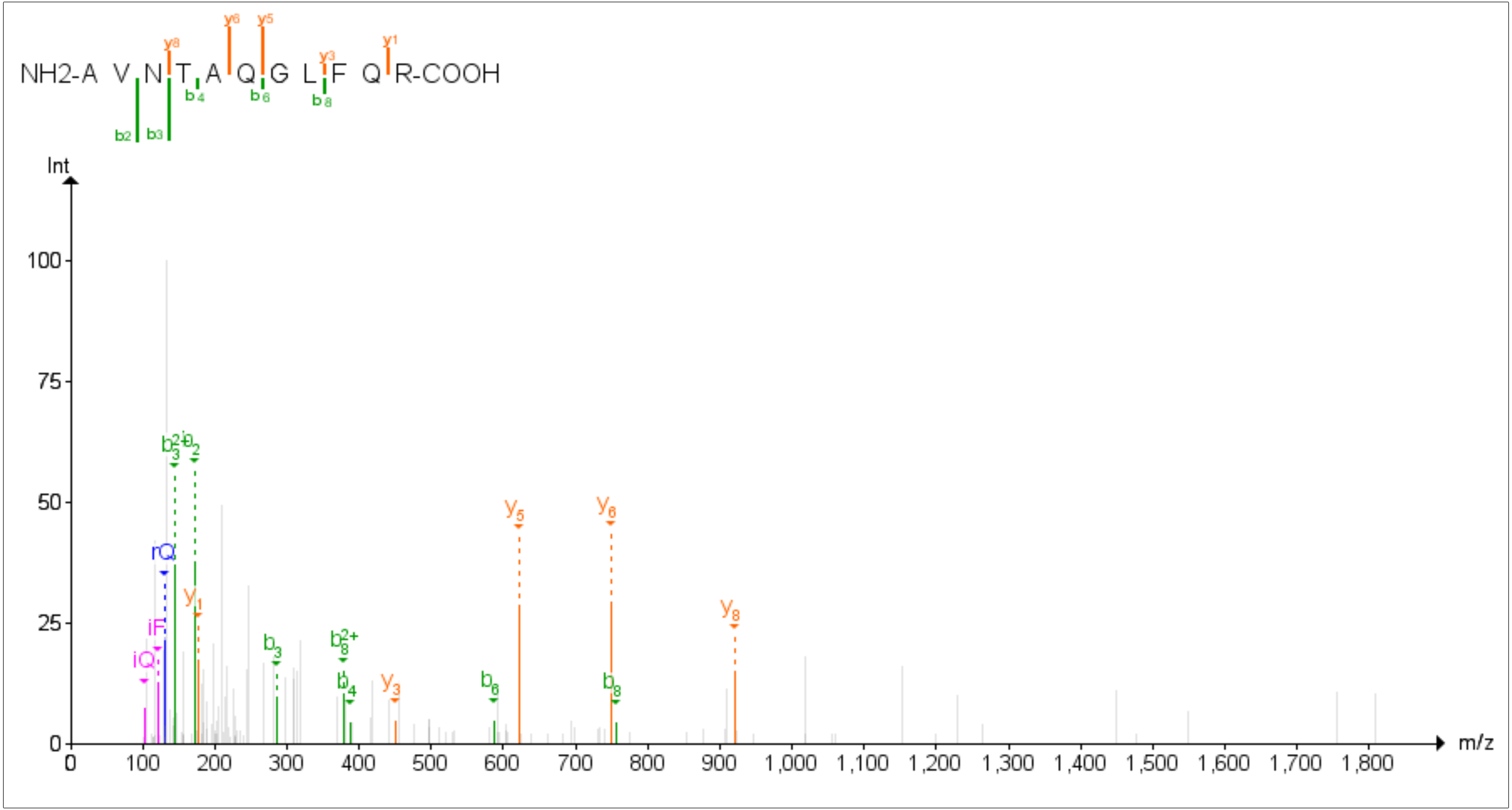

NH2-A V N T A Q G L F Q R-COOH
y8 y6 y5 y3 y1
b2 b3 b4 b6 b8
Int
m/z
0
25
50
75
100
0 100 200 300 400 500 600 700 800 900 1,000 1,100 1,200 1,300 1,400 1,500 1,600 1,700 1,800
b3 2+
b2
rQ
y1
iF
iQ
b3
b8 2+
b4
y3
b6
y5
y6
b8
y8

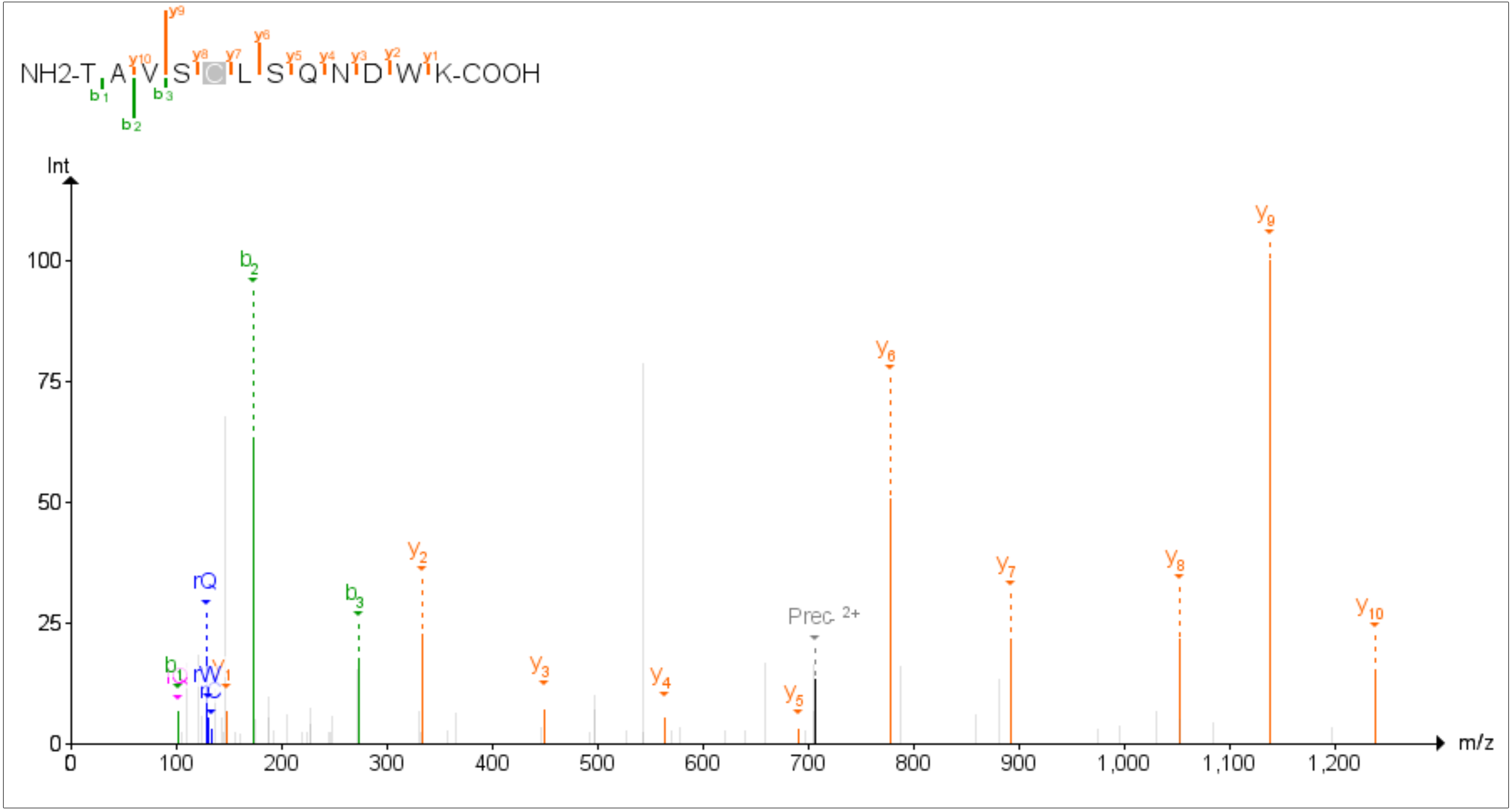

NH2-T A V S C L S Q N D W K-COOH
y9 y10 y8 y7 y6 y5 y4 y3 y2 y1
b1 b2 b3
Int
m/z
Prec 2+
rQ
rW

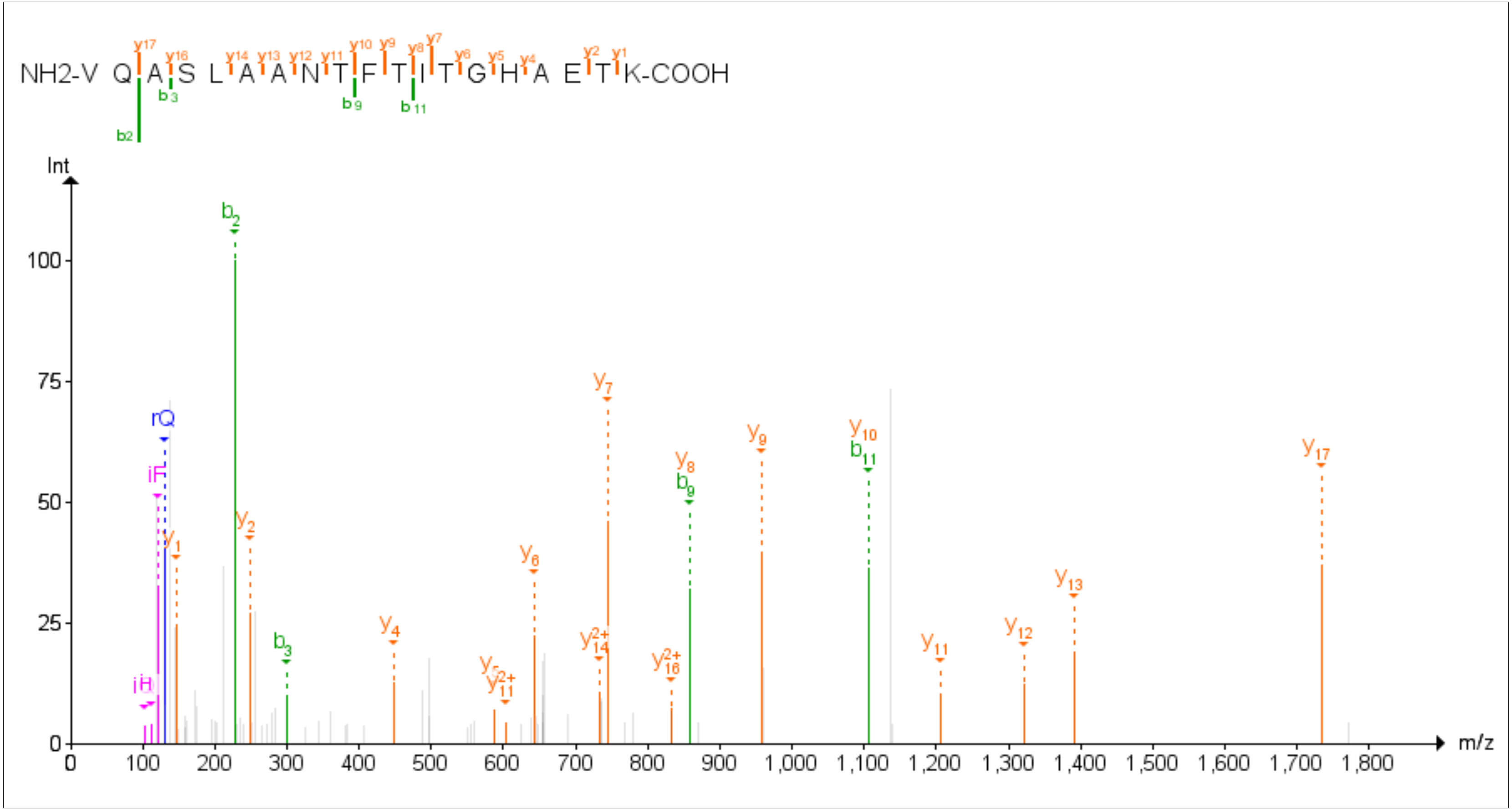

NH2-V Q A S L A A N T F T I T G H A E T K-COOH
Int
m/z
b2
b3
b9
b11
y1
y2
y4
y5
y6
y7
y8
y9
y10
y11
y12
y13
y14
y16
y17
rQ
iF
iH

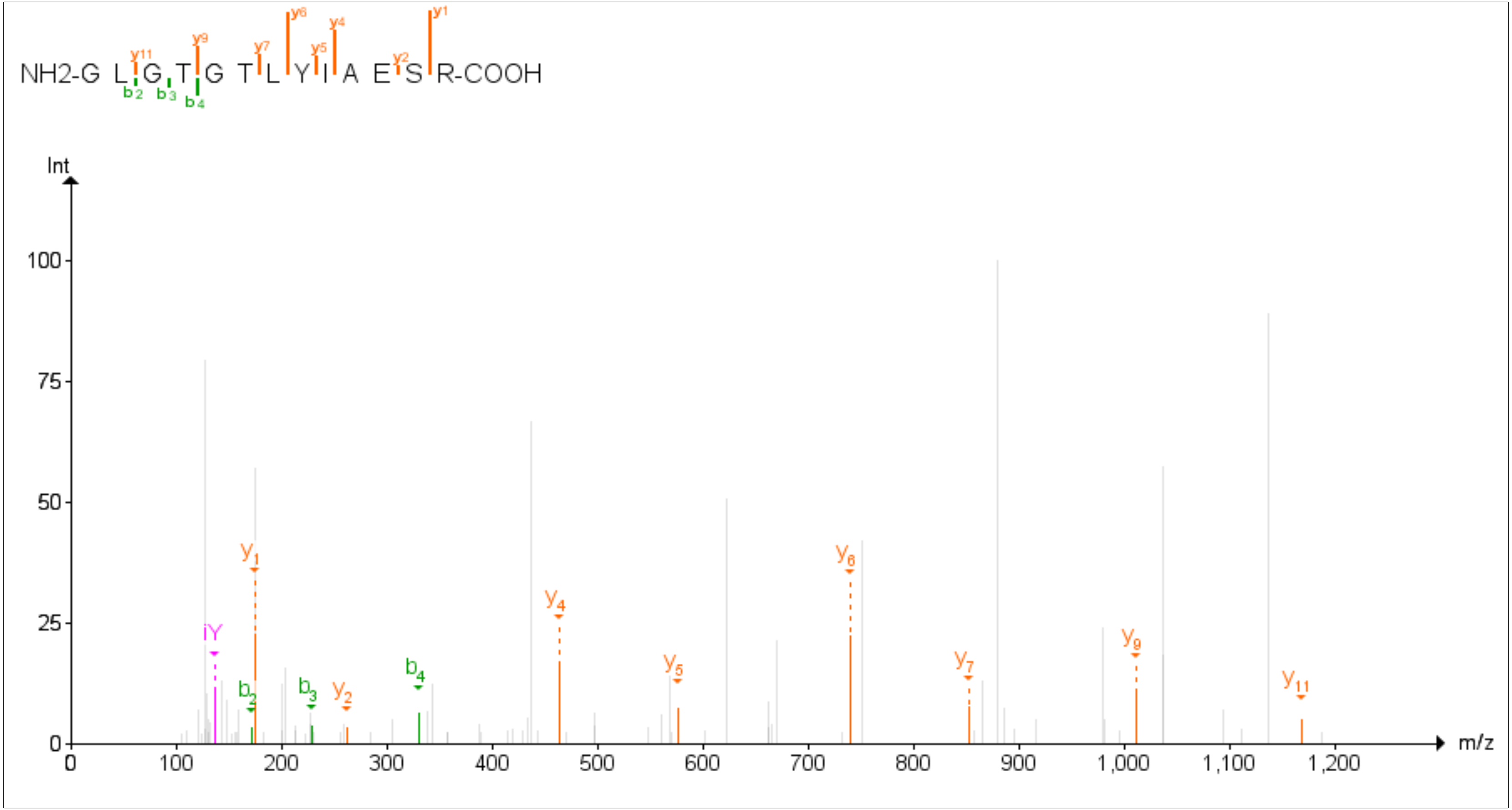
NH2-G L G T G T L Y I A E S R-COOH
y11 y9 y7 y6 y5 y4 y2 y1
b2 b3 b4
Int
100
75
50
25
0
iY
y1
b2
b3
y2
b4
y4
y5
y6
y7
y9
y11
m/z
0
100
200
300
400
500
600
700
800
900
1,000
1,100
1,200

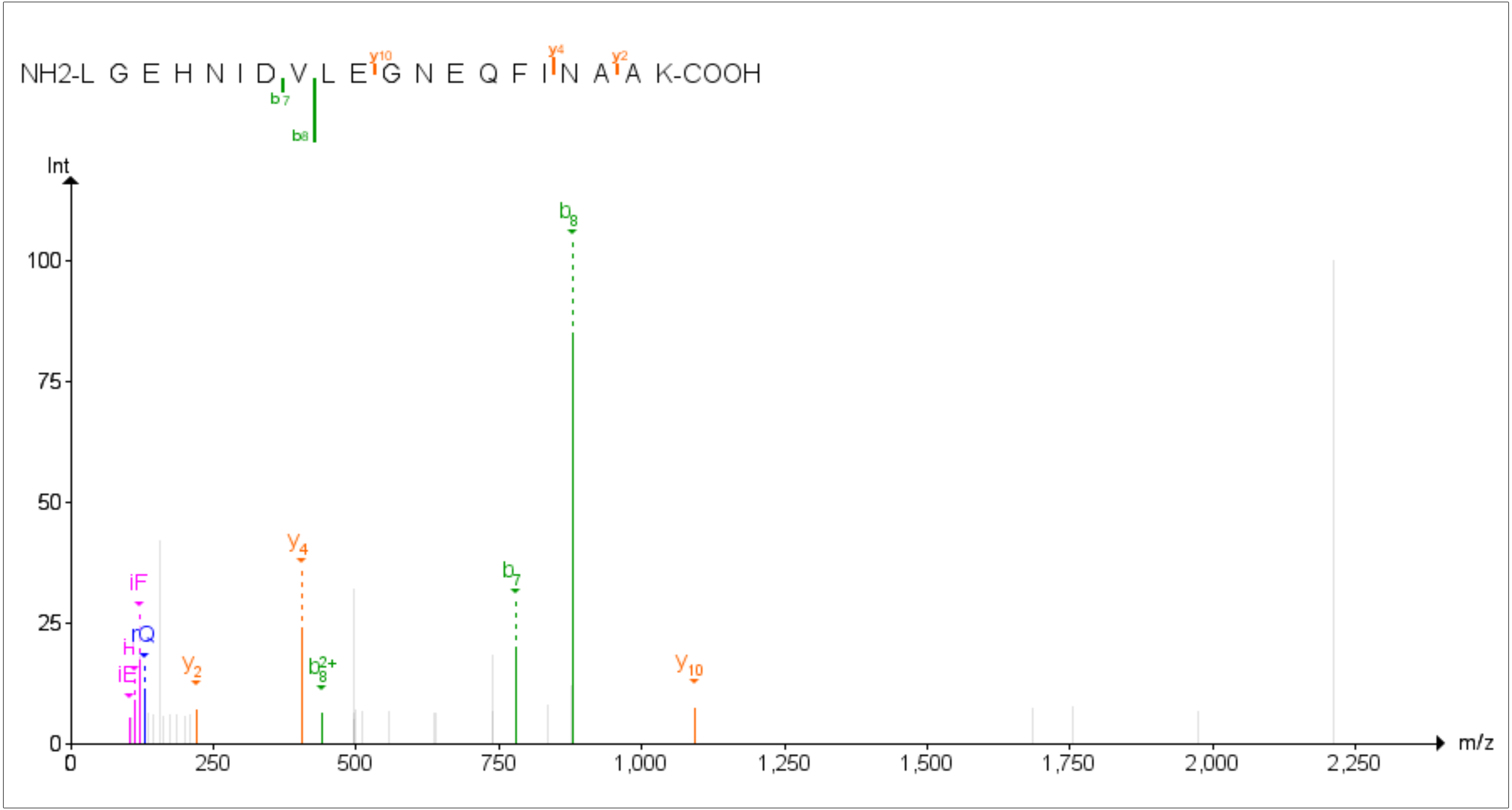

NH2-L G E H N I D V L E G N E Q F I N A A K-COOH
b7
b8
y10
y4
y2
Int
m/z
100
75
50
25
0
0
250
500
750
1,000
1,250
1,500
1,750
2,000
2,250
b8
y4
iF
b7
rQ
iH
y2
b8 2+
iE
y10

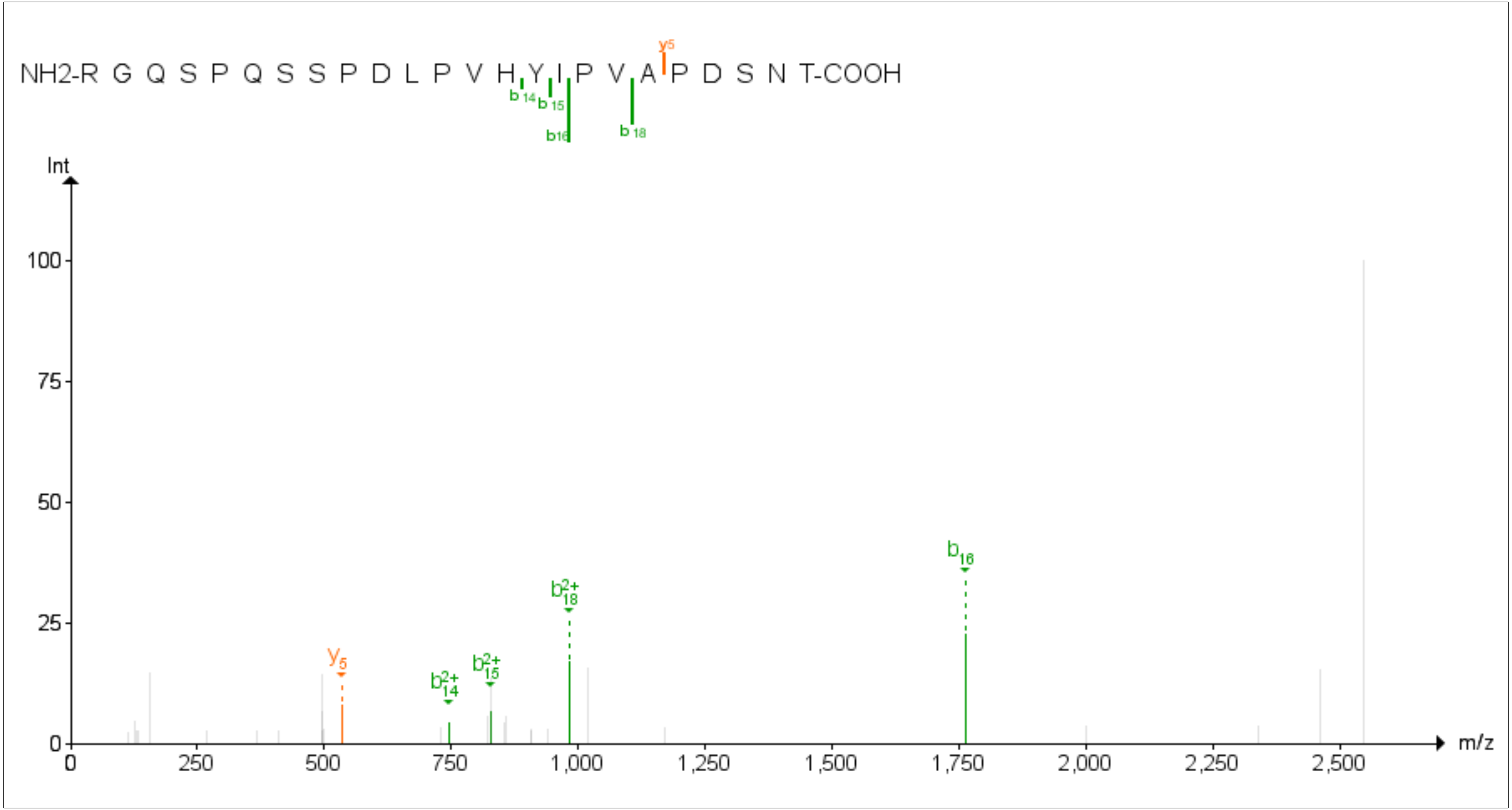
NH2-R G Q S P Q S S P D L P V H Y I P V A P D S N T-COOH
y5
b 14
b 15
b16
b 18
Int
100
75
50
25
0
m/z
0
250
500
750
1,000
1,250
1,500
1,750
2,000
2,250
2,500
y5
b2+ 14
b2+ 15
b2+ 18
b16

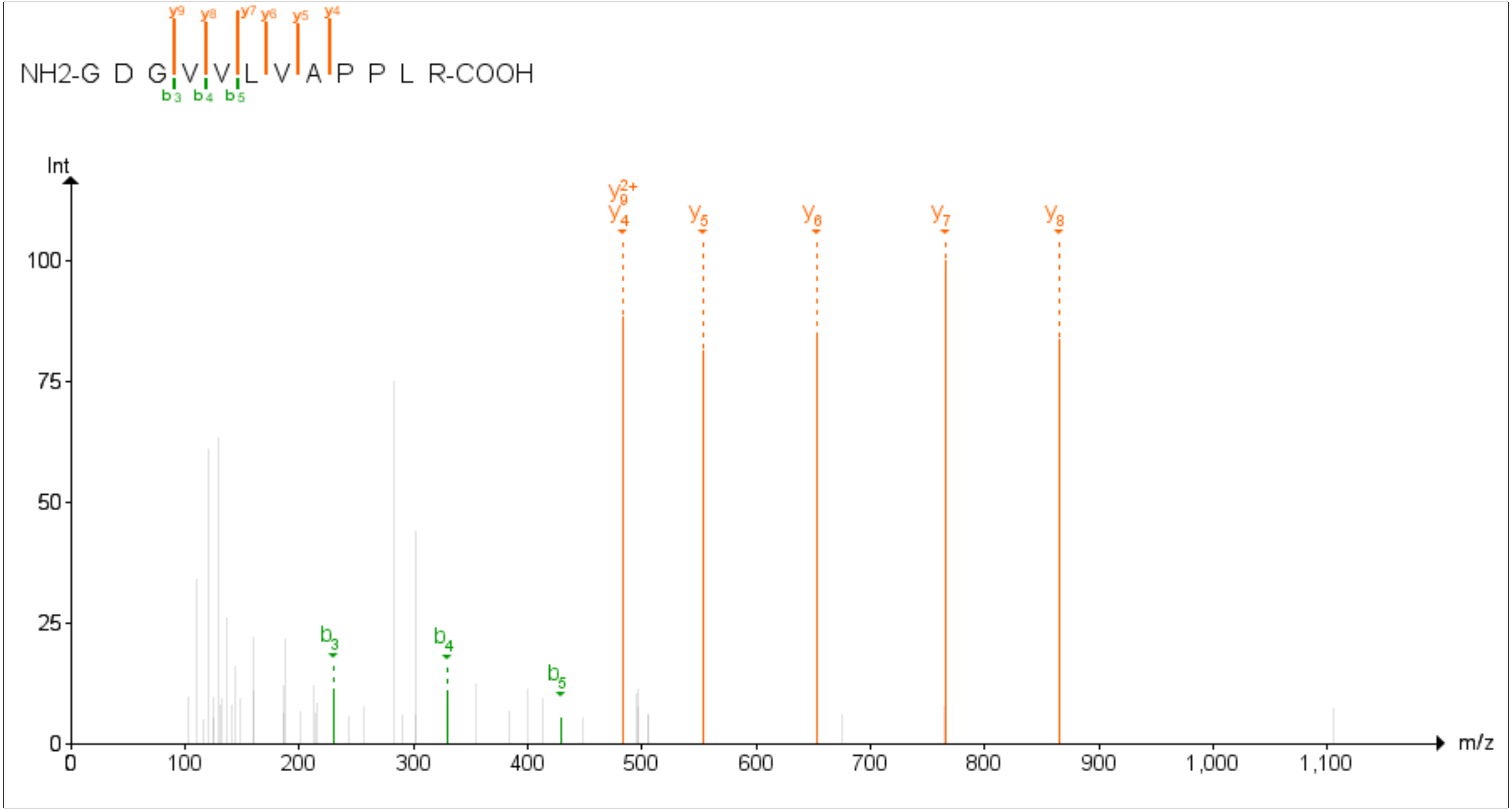

NH2-G D G V V L V A P P L R-COOH
y9 y8 y7 y6 y5 y4
b3 b4 b5
Int
100
75
50
25
0
m/z
0 100 200 300 400 500 600 700 800 900 1,000 1,100
y9 2+
y4
y5
y6
y7
y8
b3
b4
b5

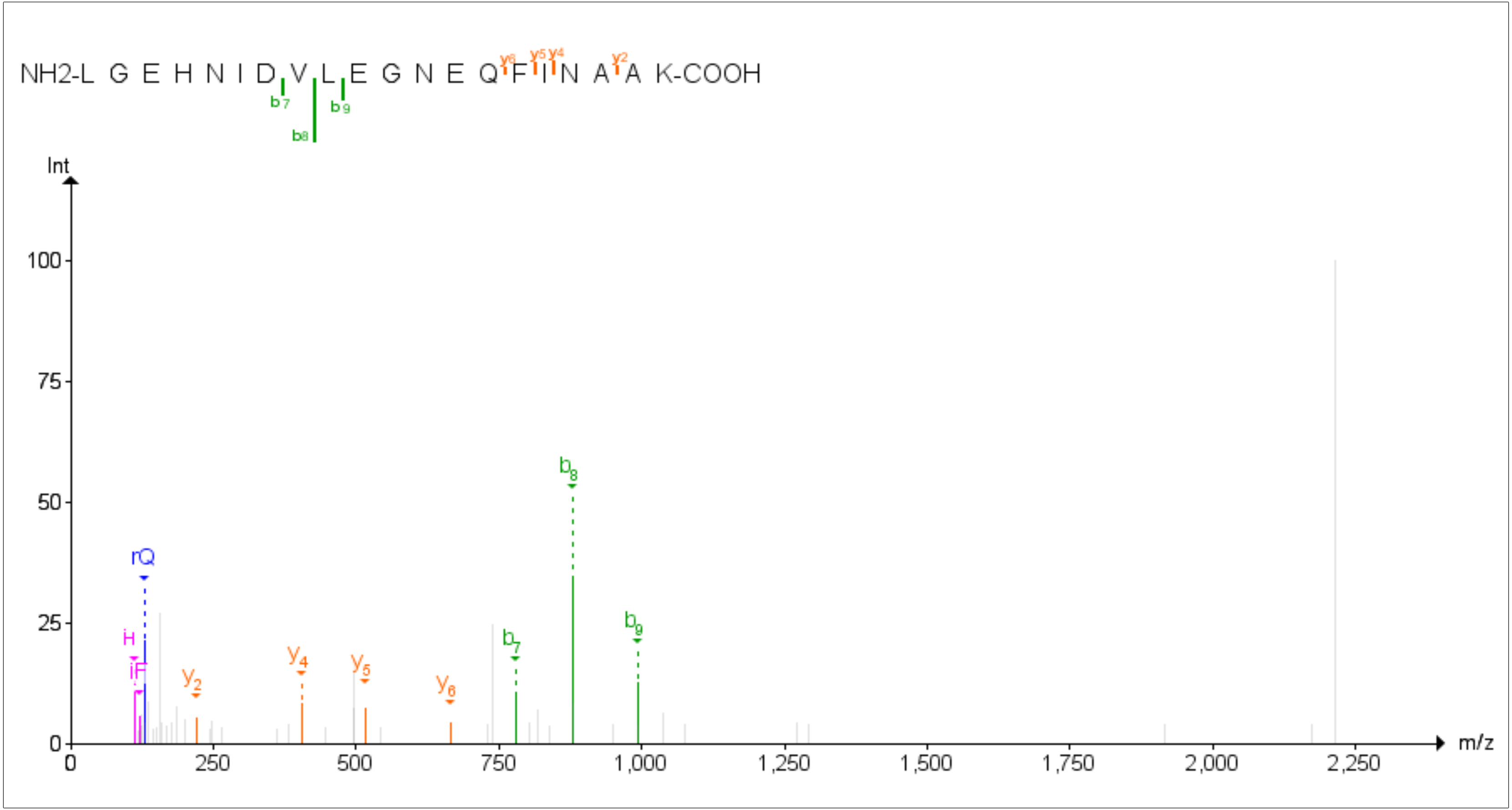
NH2-L G E H N I D V L E G N E Q F I N A A K-COOH
b7
b8
b9
y6
y5
y4
y2
Int
100
75
50
25
0
rQ
H
iF
y2
y4
y5
y6
b7
b8
b9
0
250
500
750
1,000
1,250
1,500
1,750
2,000
2,250
m/z

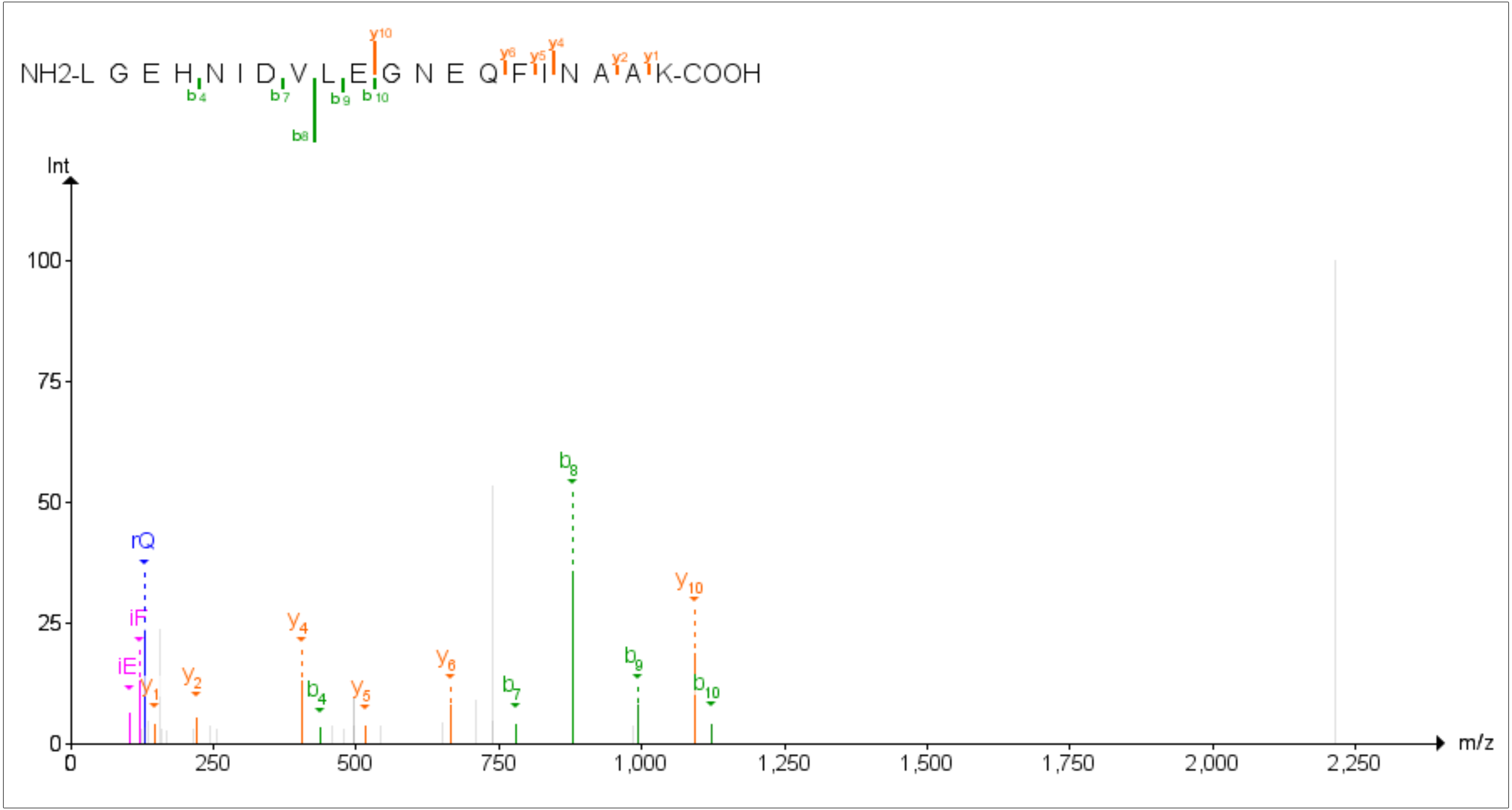
NH2-L G E H N I D V L E G N E Q F I N A A K-COOH
b4 b7 b8 b9 b10
y10 y6 y5 y4 y2 y1
Int
100
75
50
25
0
rQ
iF
iE
y1
y2
y4
b4
y5
y6
b7
b8
b9
y10
b10
0
250
500
750
1,000
1,250
1,500
1,750
2,000
2,250
m/z

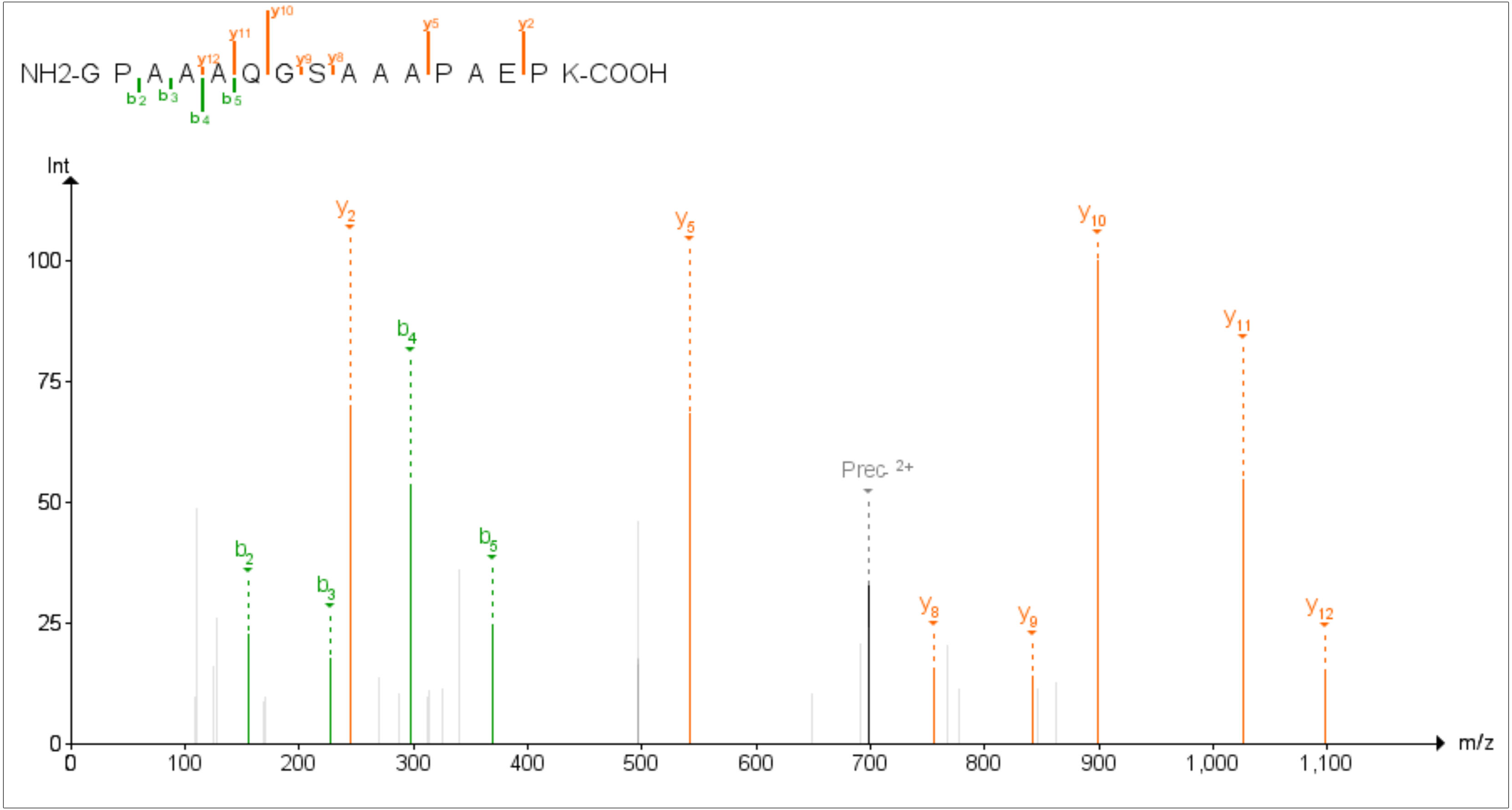

NH2-G P A A A Q G S A A A P A E P K-COOH
y10 y11 y12 y9 y8 y5 y2
b2 b3 b4 b5
Int
m/z
y2
y5
y10
y11
y12
y8
y9
b2
b3
b4
b5
Prec 2+
0
25
50
75
100
0
100
200
300
400
500
600
700
800
900
1,000
1,100

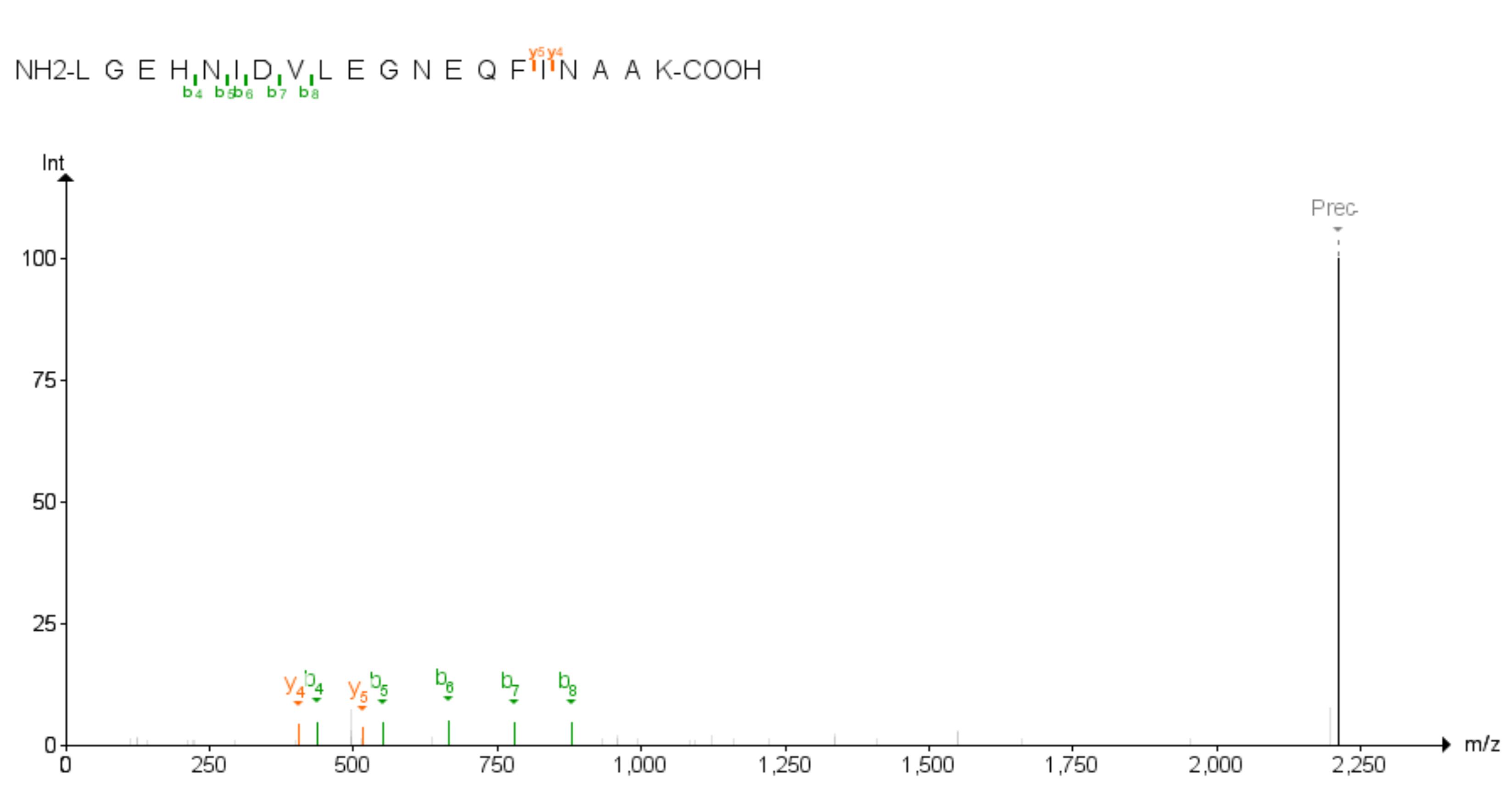

NH2-L G E H N I D V L E G N E Q F I N A A K-COOH
b4 b5 b6 b7 b8
y5 y4
Int
100
75
50
25
0
Prec
y4 b4 y5 b5 b6 b7 b8
0 250 500 750 1,000 1,250 1,500 1,750 2,000 2,250
m/z

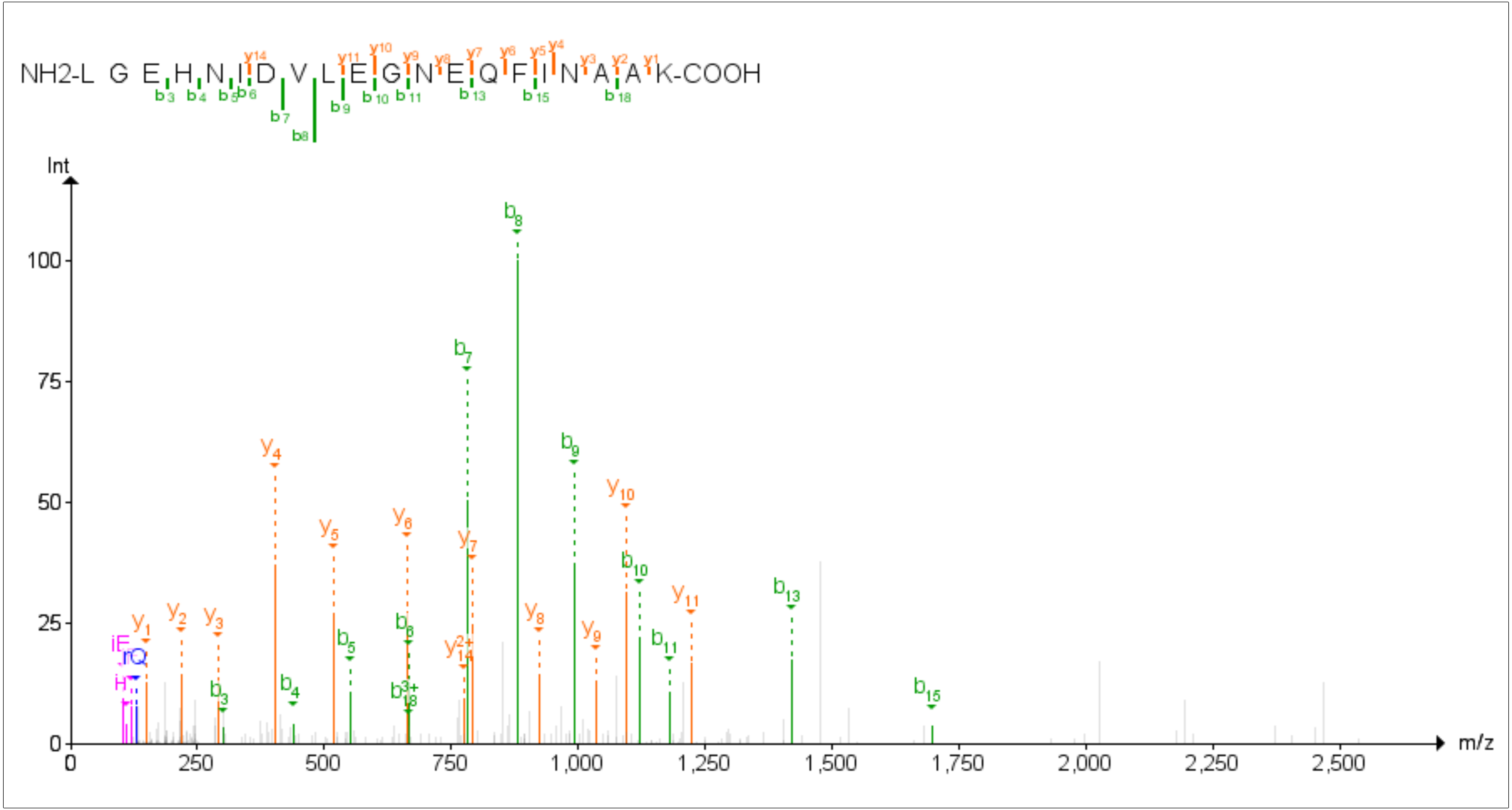

NH2-L G E H N I D V L E G N E Q F I N A A K-COOH
Int
m/z
0
25
50
75
100
250
500
750
1,000
1,250
1,500
1,750
2,000
2,250
2,500

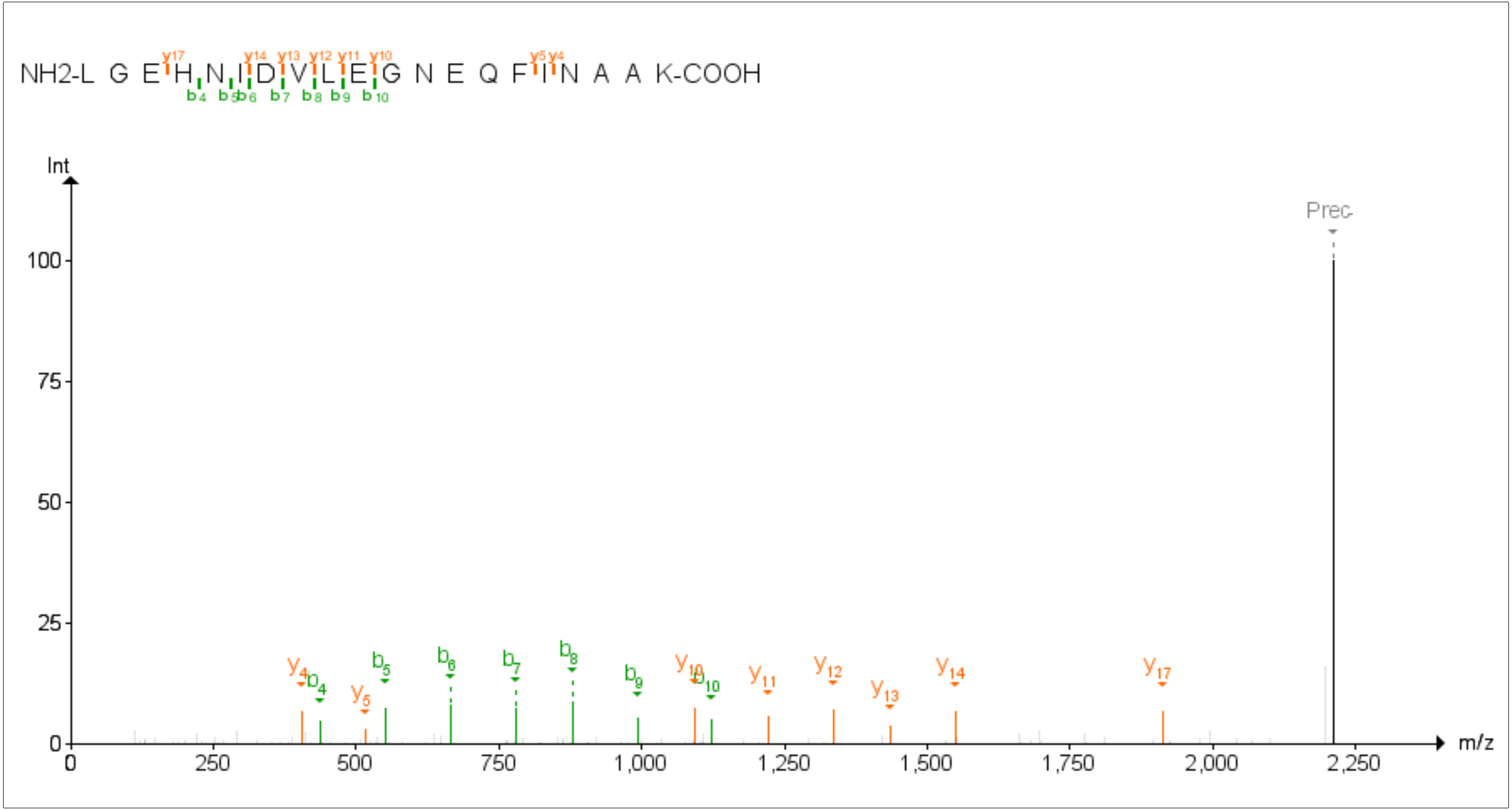

NH2-L G E H N I D V L E G N E Q F I N A A K-COOH
Int
Prec
m/z

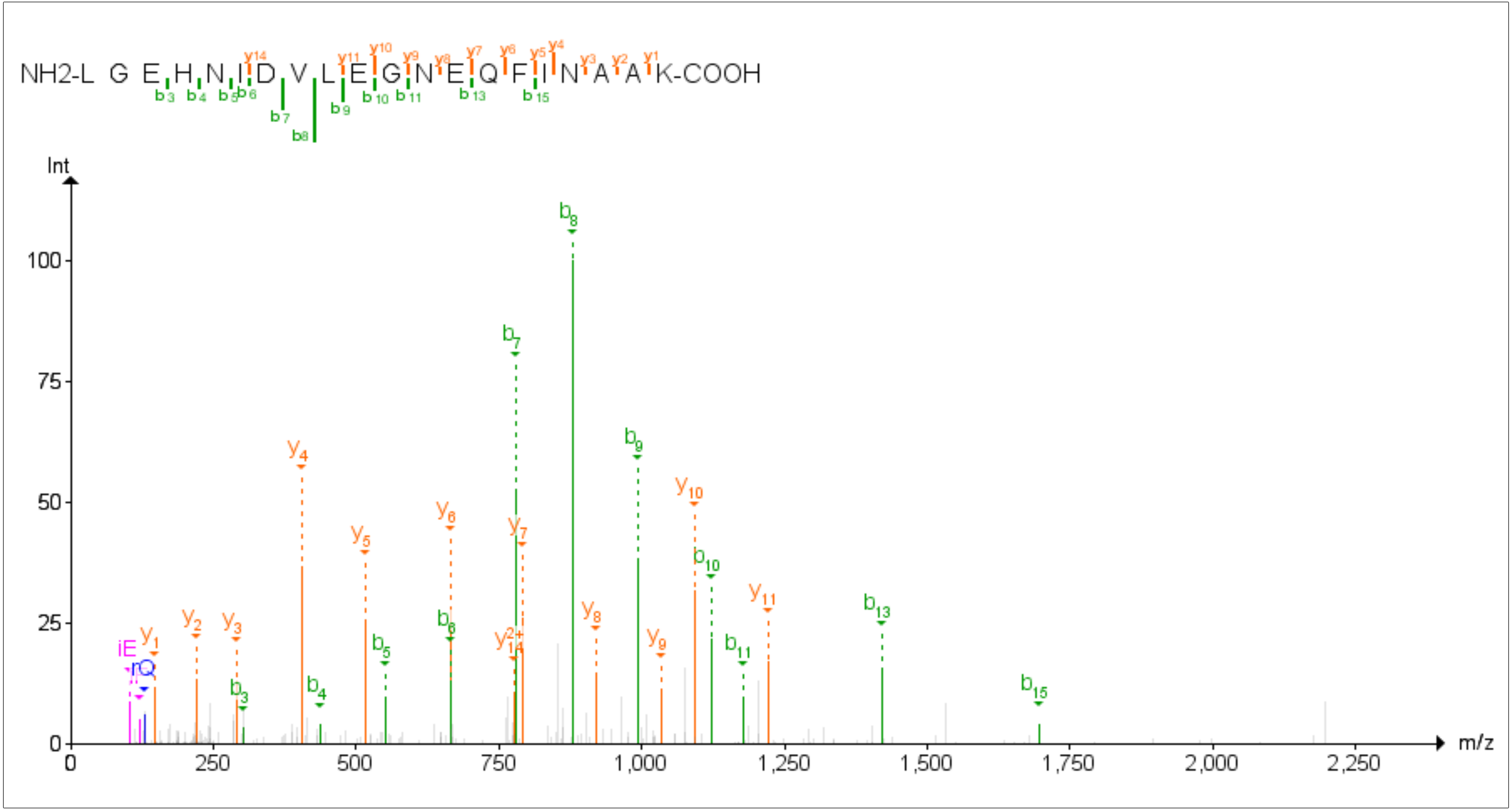

NH2-L G E H N I D V L E G N E Q F I N A A K-COOH
Int
m/z
0
25
50
75
100
250
500
750
1,000
1,250
1,500
1,750
2,000
2,250

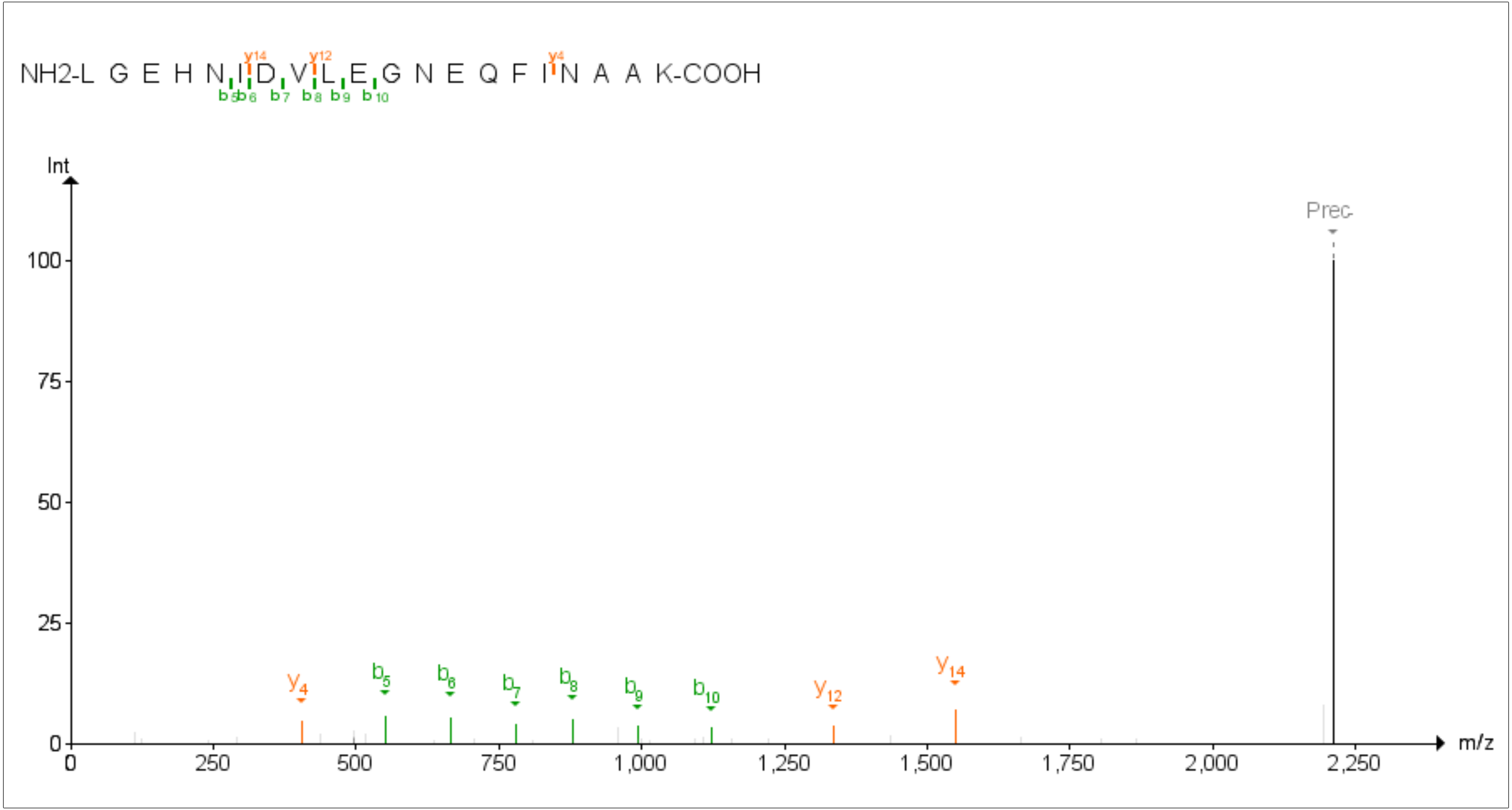

NH2-L G E H N I D V L E G N E Q F I N A A K-COOH
y14 y12 y4
b5 b6 b7 b8 b9 b10
Int
100
75
50
25
0
Prec
y4 b5 b6 b7 b8 b9 b10 y12 y14
0 250 500 750 1,000 1,250 1,500 1,750 2,000 2,250
m/z

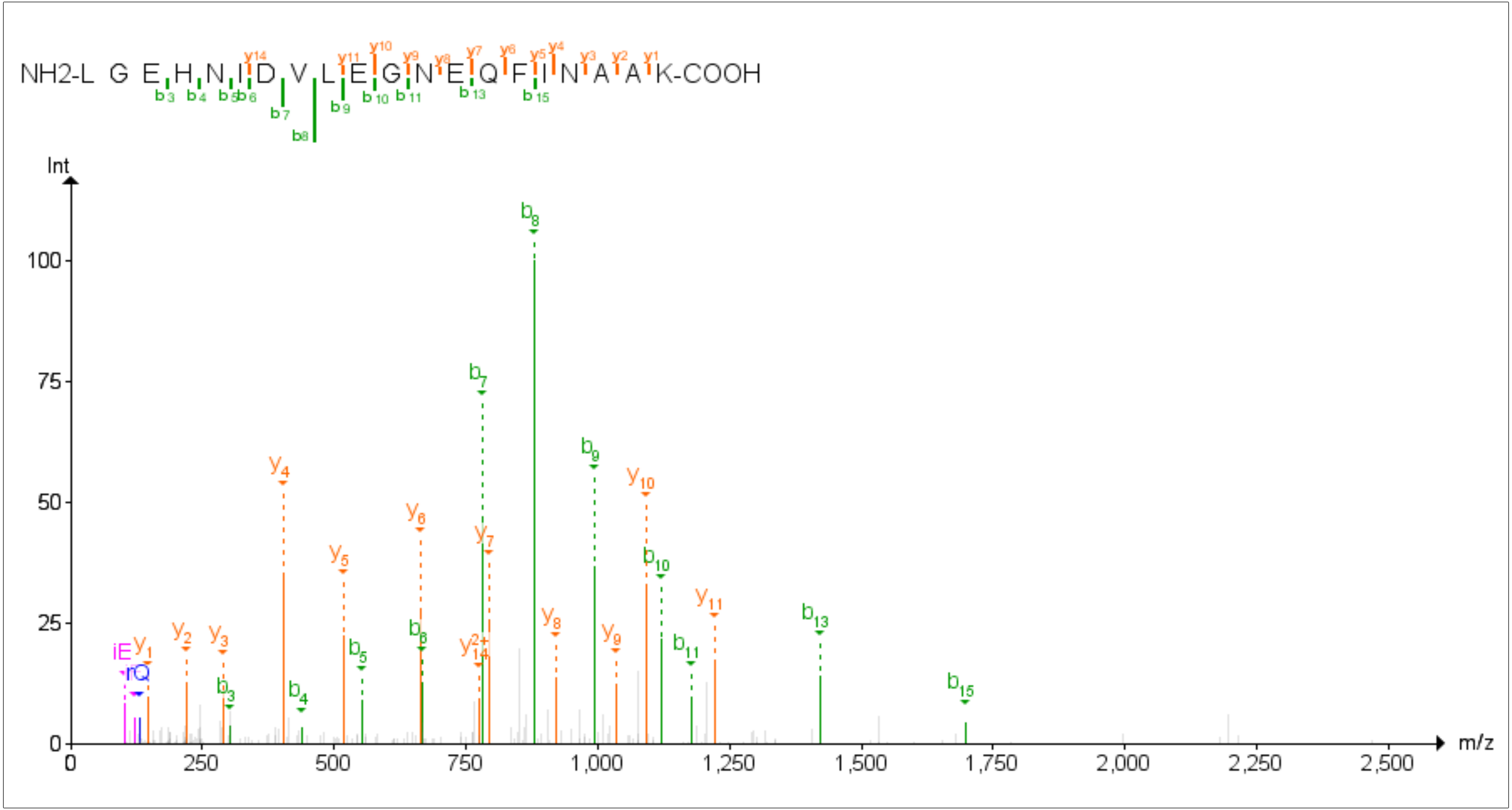

NH2-L G E H N I D V L E G N E Q F I N A A K-COOH
Int
m/z

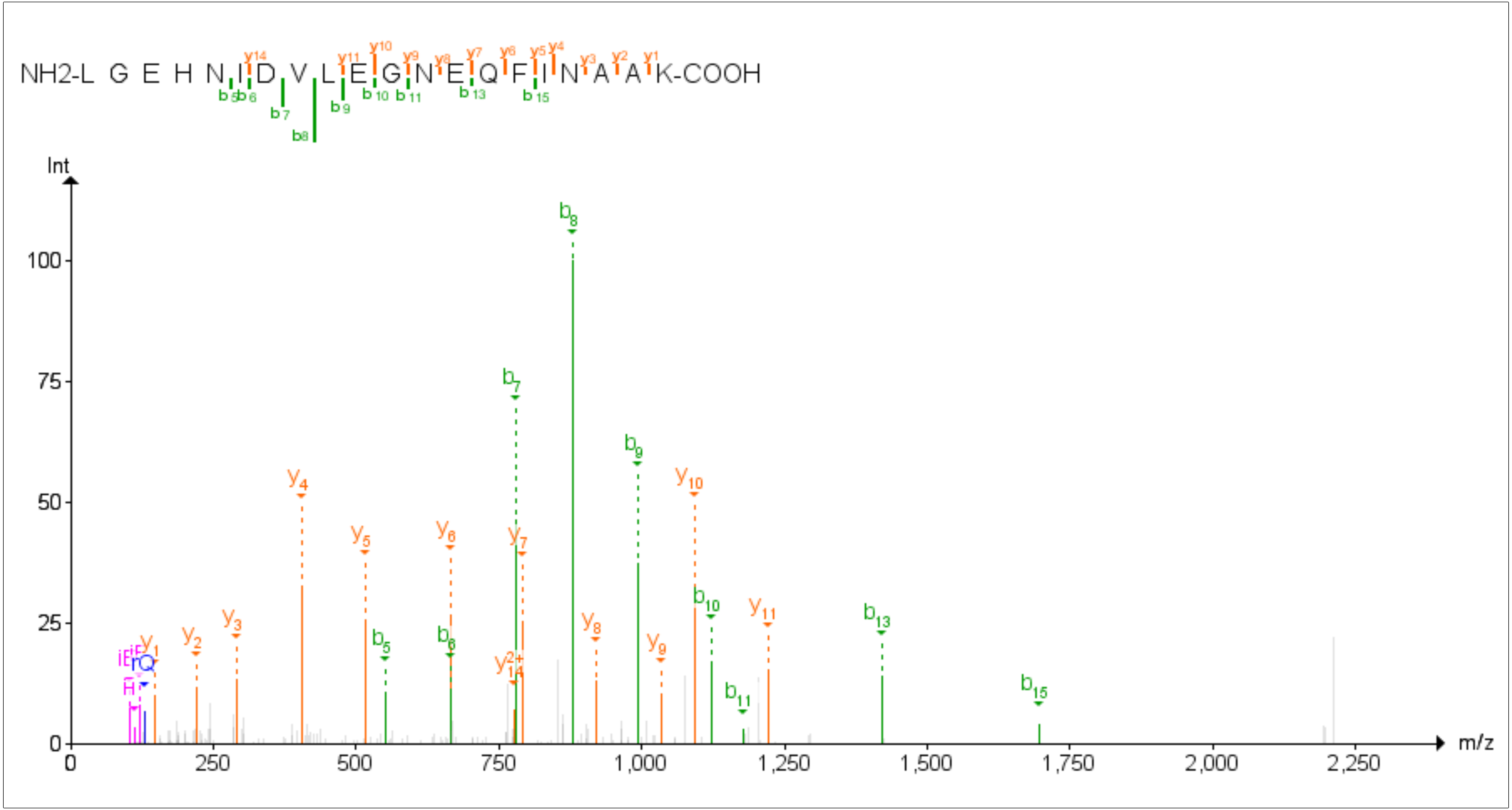

NH2-L G E H N I D V L E G N E Q F I N A A K-COOH
Int
m/z
0
25
50
75
100
250
500
750
1,000
1,250
1,500
1,750
2,000
2,250

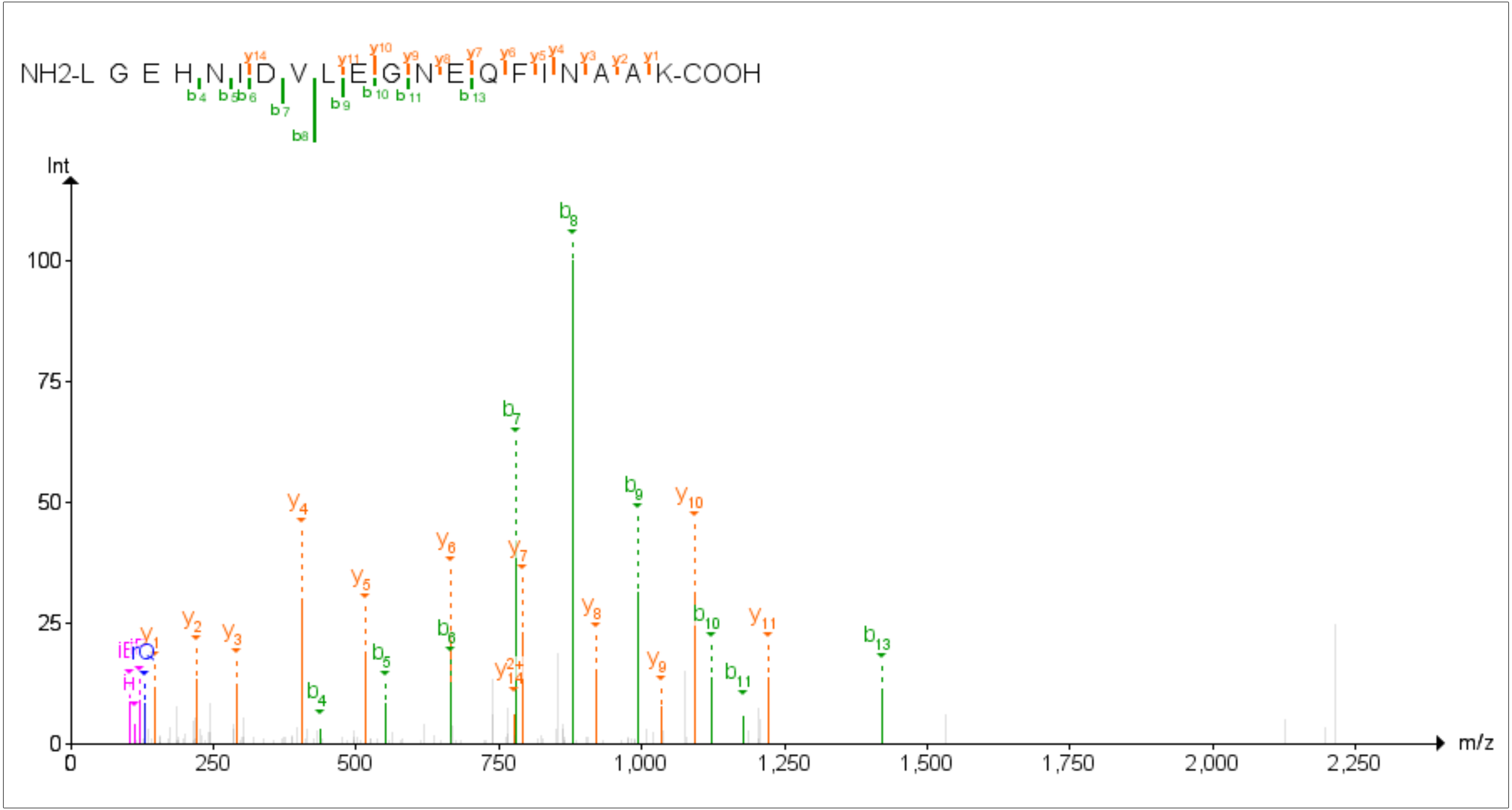

NH2-L G E H N I D V L E G N E Q F I N A A K-COOH
Int
m/z
0
25
50
75
100
250
500
750
1,000
1,250
1,500
1,750
2,000
2,250

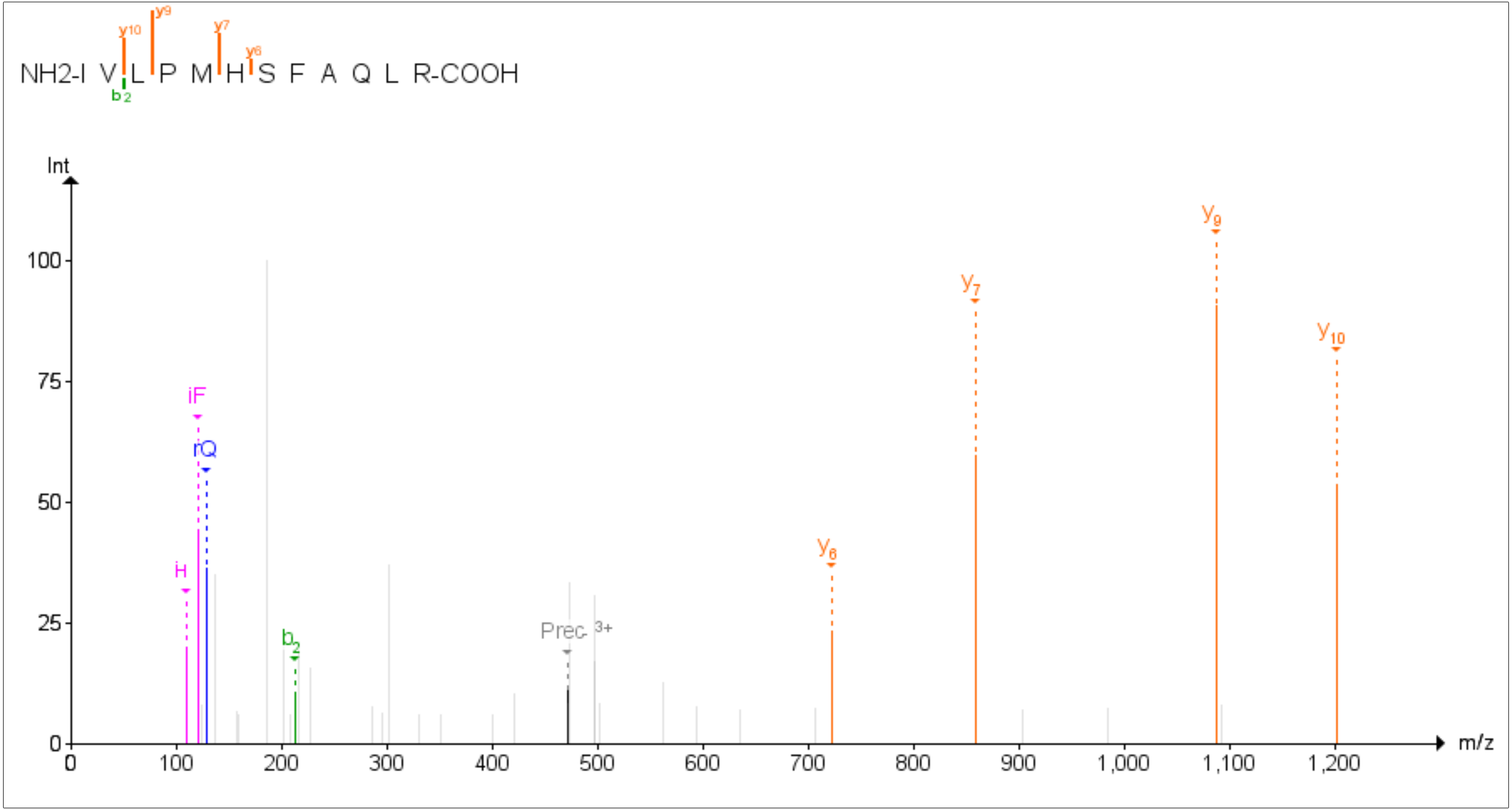

NH2-I V L P M H S F A Q L R-COOH
y10
y9
y7
y6
b2
Int
100
75
50
25
0
iF
rQ
iH
b2
Prec- 3+
y6
y7
y9
y10
0
100
200
300
400
500
600
700
800
900
1,000
1,100
1,200
m/z

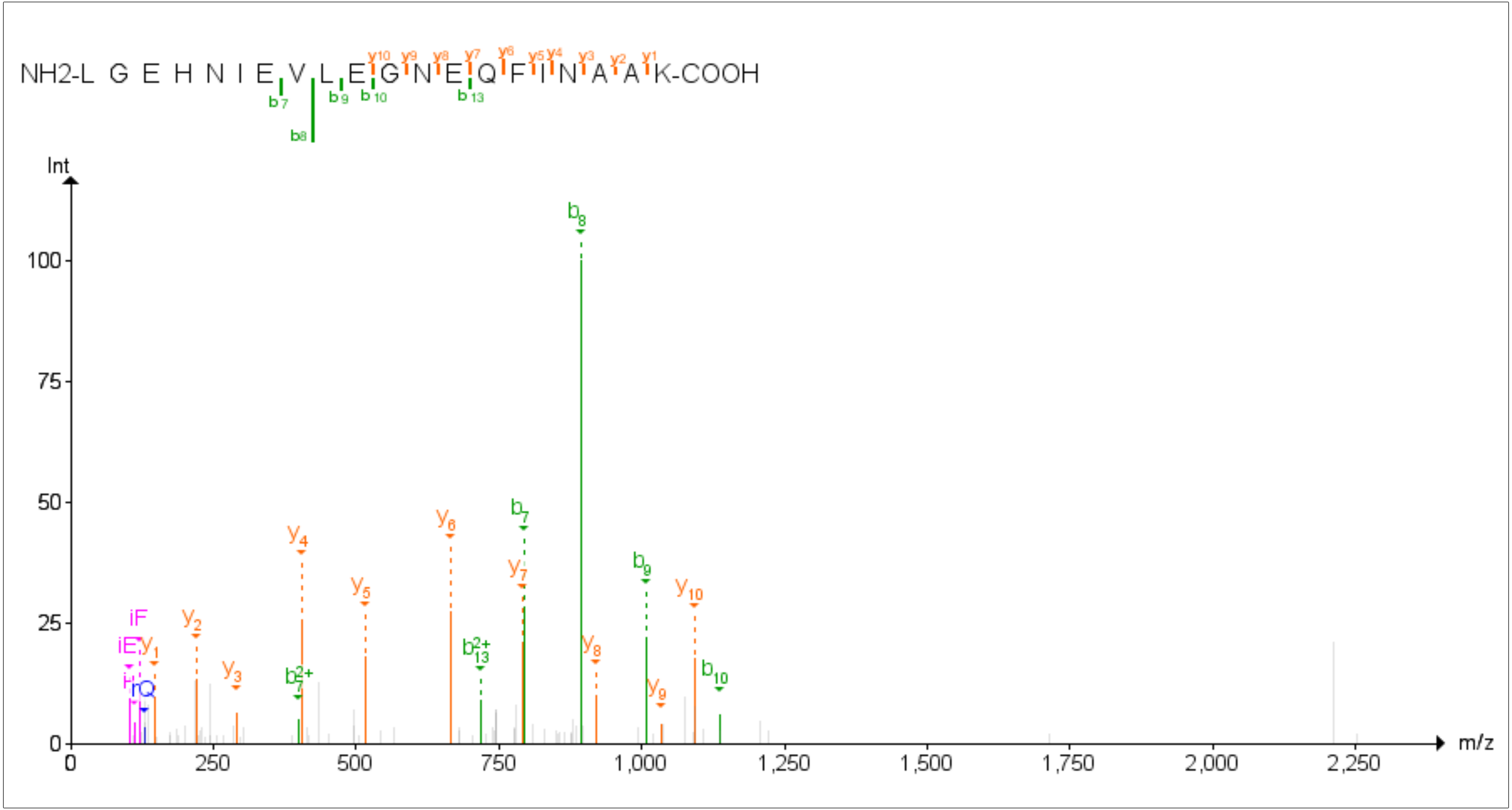
NH2-L G E H N I E V L E G N E Q F I N A A K-COOH
y10 y9 y8 y7 y6 y5 y4 y3 y2 y1
b7 b8 b9 b10 b13
Int
100
75
50
25
0
0
250
500
750
1,000
1,250
1,500
1,750
2,000
2,250
m/z
b8
b7
y6
y4
y7
b9
y5
y10
iF
y2
iE
y1
b13 2+
y8
y3
b7 2+
b10
y9
iQ

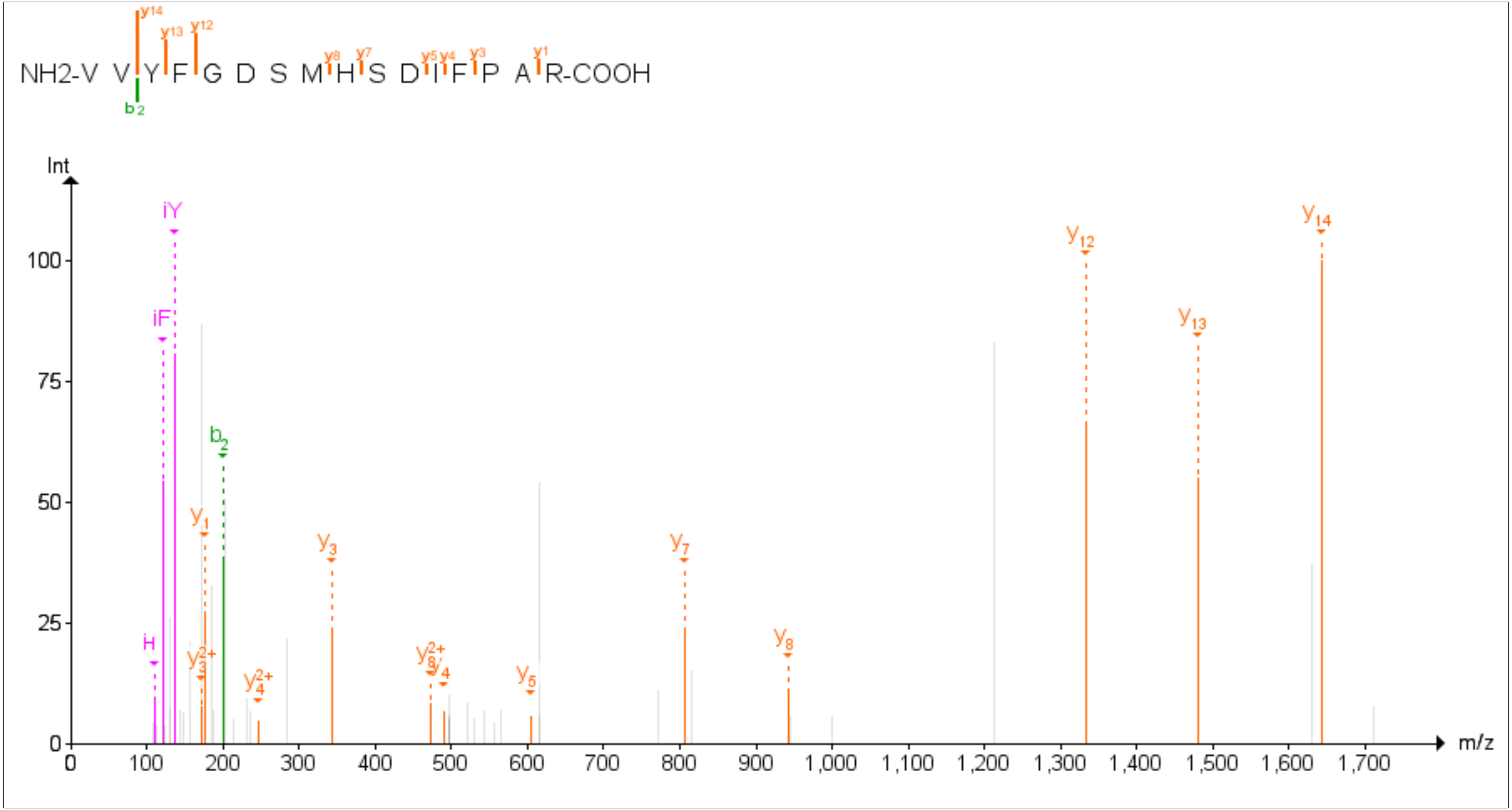

NH2-V V Y F G D S M H S D I F P A R-COOH
y14
y13
y12
y8
y7
y5
y4
y3
y1
b 2
Int
m/z
iY
iF
iH
b2
y1
y_3^{2+}
y_4^{2+}
y3
y_8^{2+}
y4
y5
y7
y8
y12
y13
y14
0
25
50
75
100
0
100
200
300
400
500
600
700
800
900
1,000
1,100
1,200
1,300
1,400
1,500
1,600
1,700

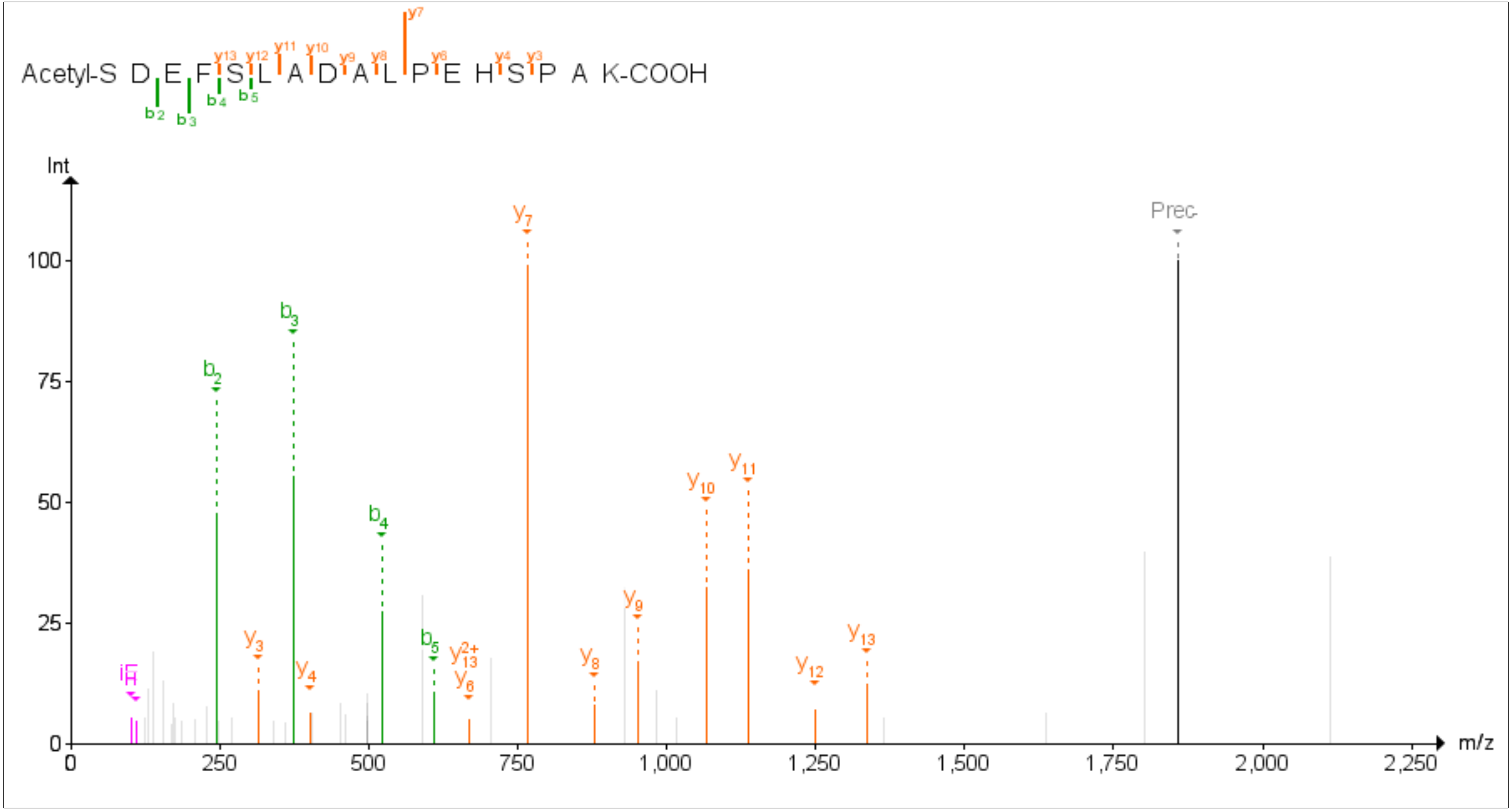

Acetyl-S D E F S L A D A L P E H S P A K-COOH
Int
m/z
Prec.

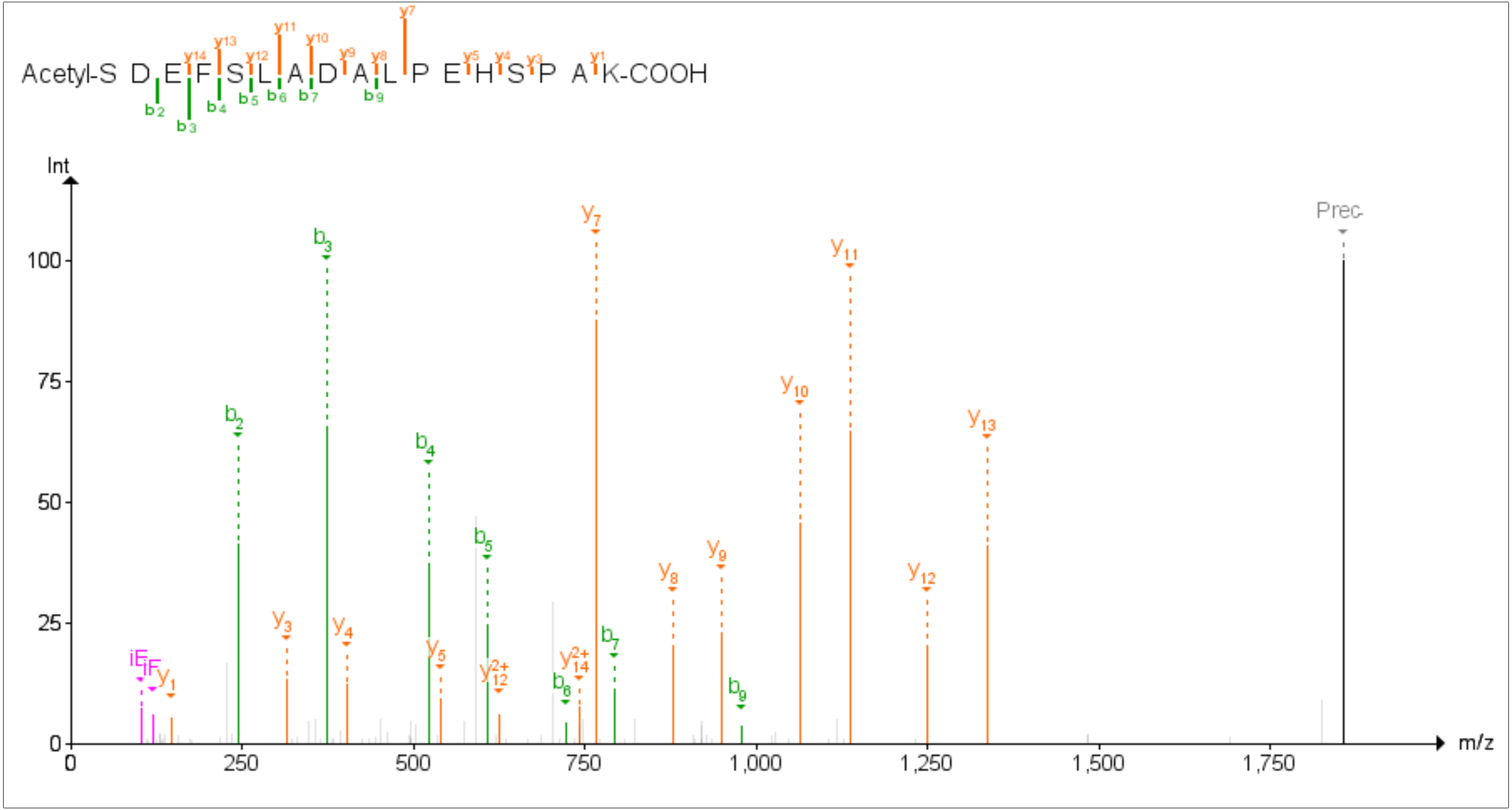

Acetyl-S D E F S L A D A L P E H S P A K-COOH
Int
m/z
Prec

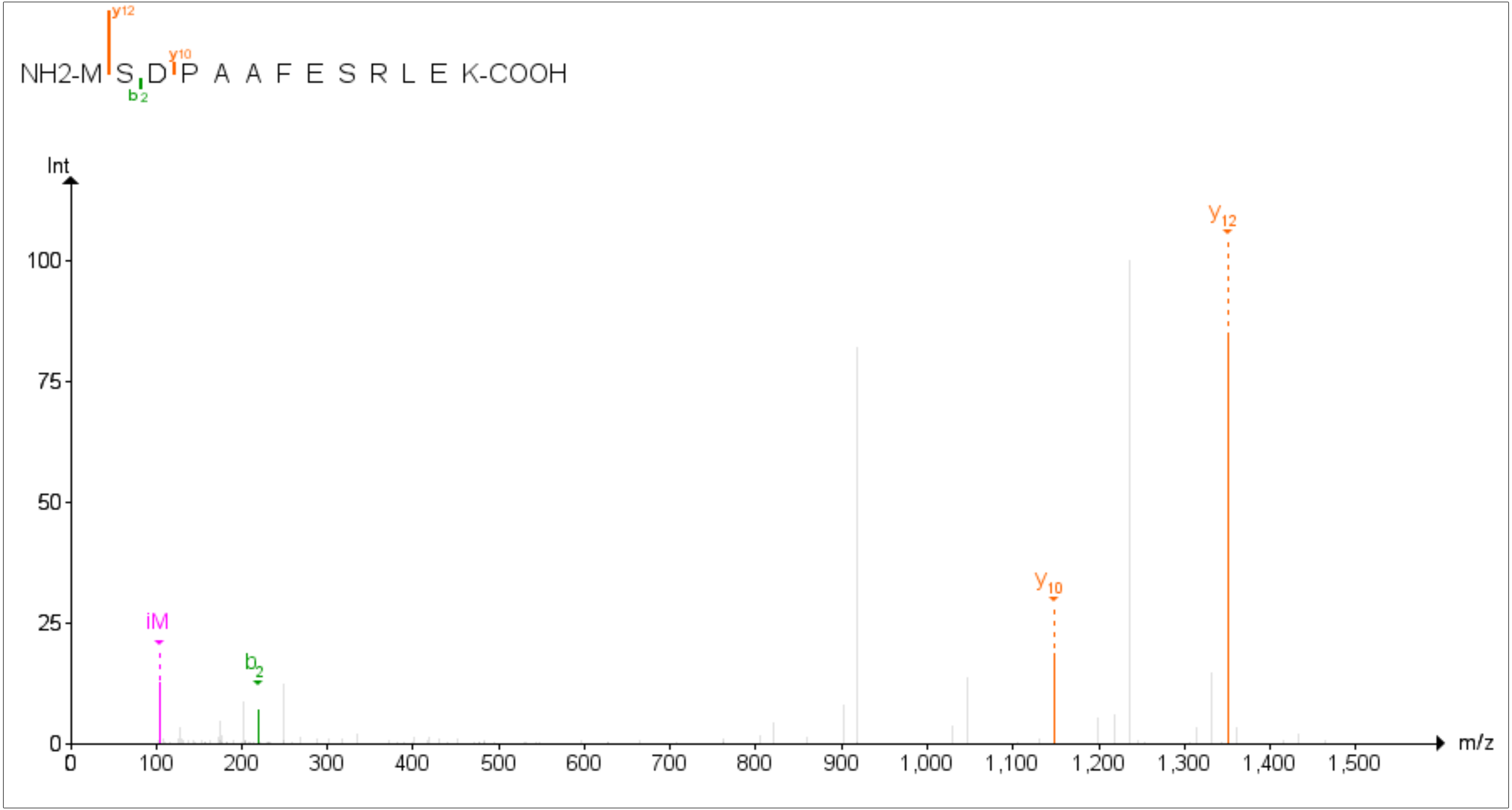

NH2-M S D P A A F E S R L E K-COOH
y12
y10
b2
Int
100
75
50
25
0
iM
b2
y10
y12
0
100
200
300
400
500
600
700
800
900
1,000
1,100
1,200
1,300
1,400
1,500
m/z

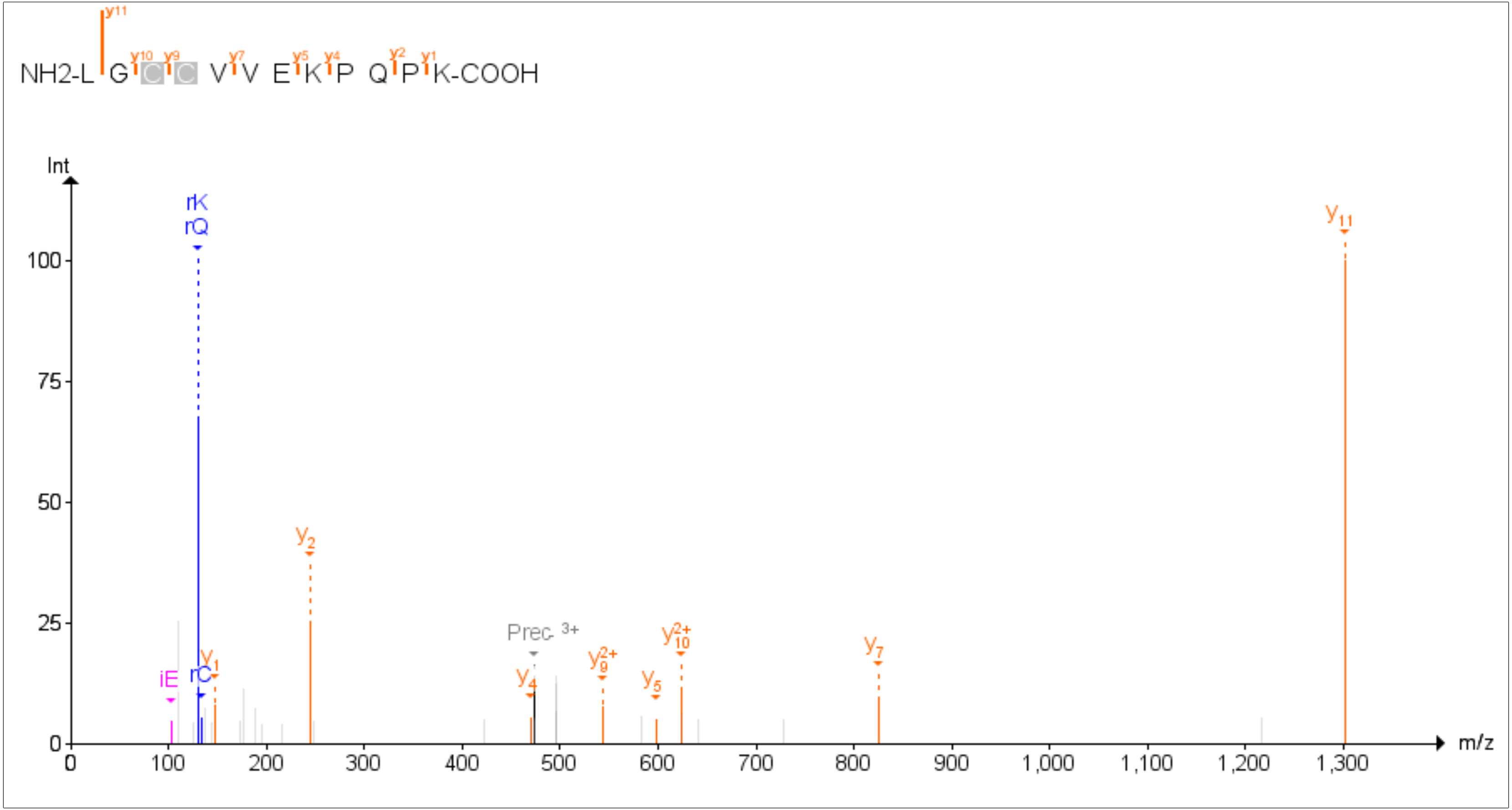
NH2-L G C C V V E K P Q P K-COOH
y11
y10
y9
y7
y5
y4
y2
y1
Int
100
75
50
25
0
rK
rQ
y11
y2
Prec 3+
y2+ 10
y2+ 9
y7
y5
y4
y1
iE
rC
m/z
0
100
200
300
400
500
600
700
800
900
1,000
1,100
1,200
1,300

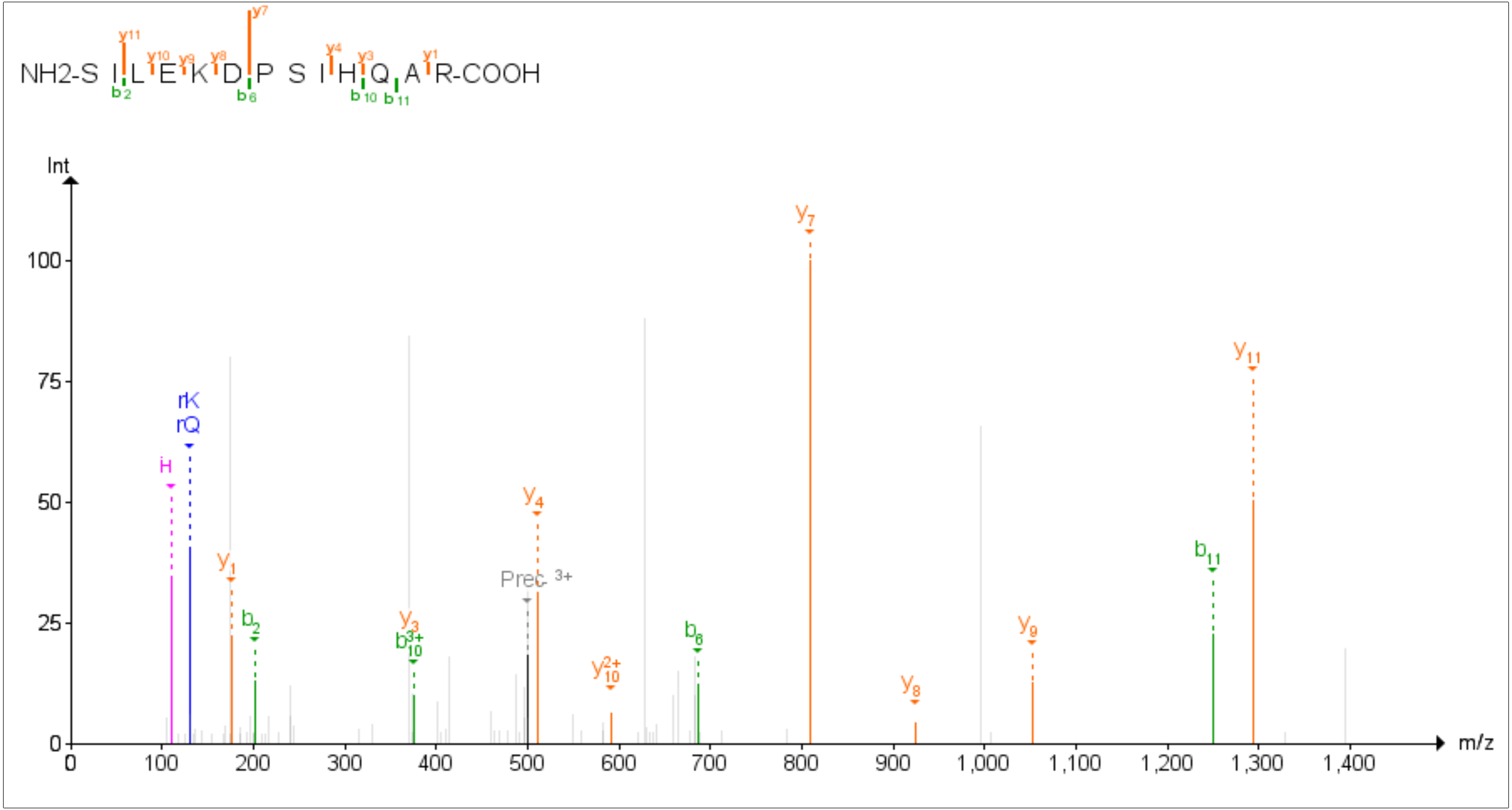
NH2-S I L E K D P S I H Q A R-COOH
Int
m/z
y7
y11
y4
y1
b11
y3
b10 3+
y9
b6
y10 2+
y8
b2
rK
rQ
H
Prec 3+
100
75
50
25
0
0
100
200
300
400
500
600
700
800
900
1,000
1,100
1,200
1,300
1,400

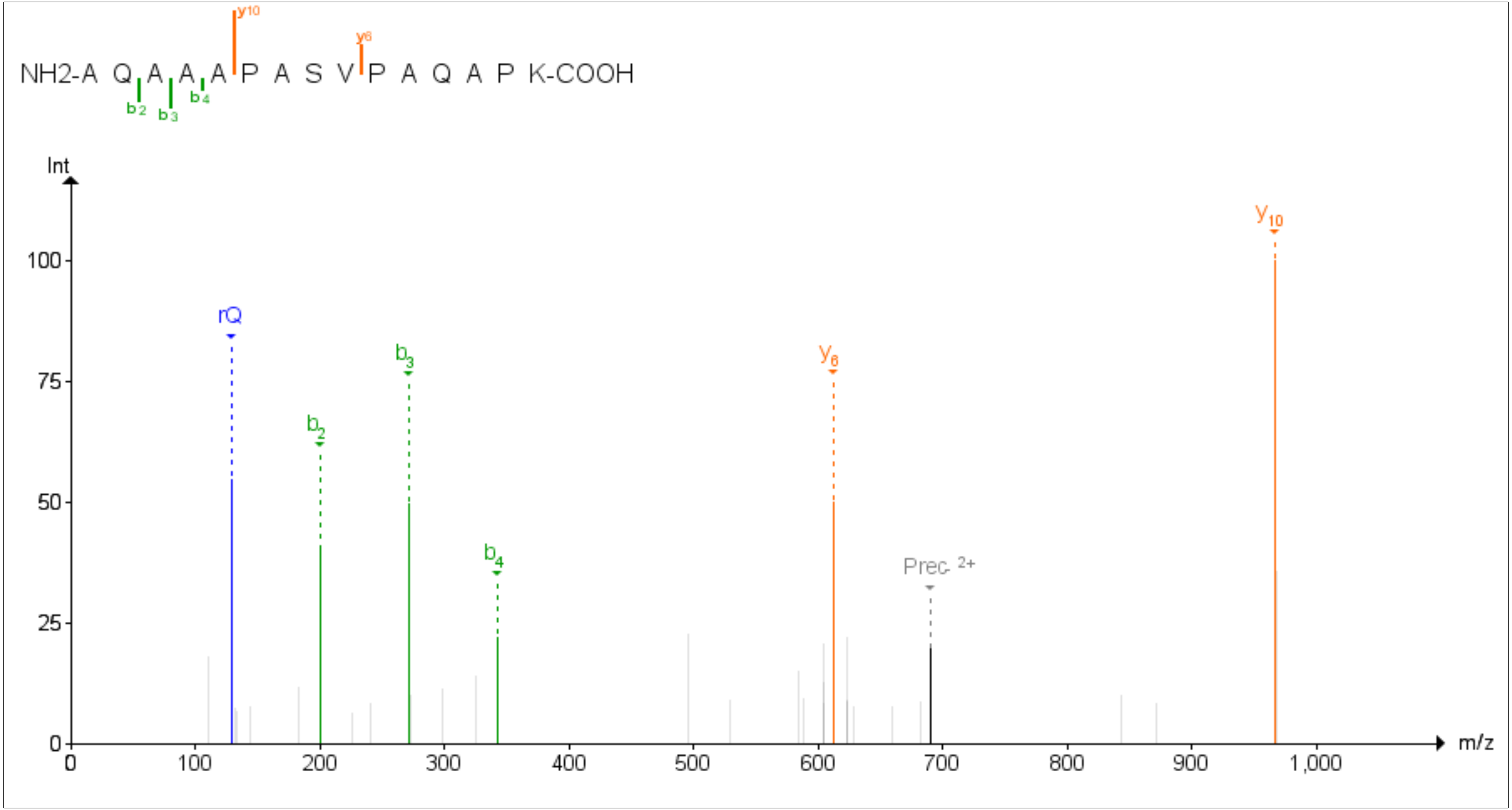

NH2-A Q A A A P A S V P A Q A P K-COOH
y10
y6
b2
b3
b4
Int
m/z
rQ
b2
b3
b4
y6
y10
Prec 2+
0
25
50
75
100
0
100
200
300
400
500
600
700
800
900
1,000

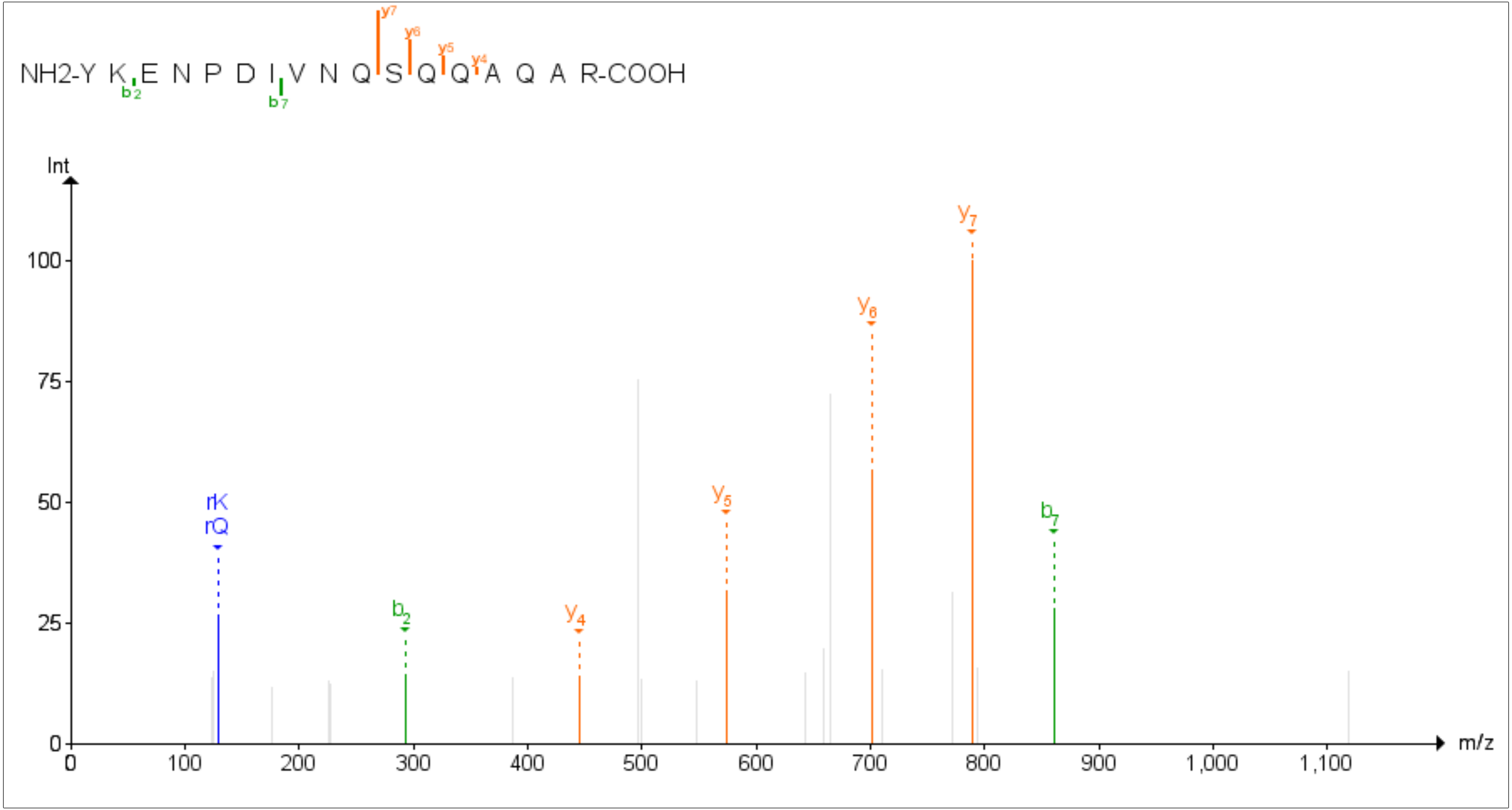

NH2-Y K E N P D I V N Q S Q Q A Q A R-COOH
b2
b7
y7
y6
y5
y4
Int
m/z
rK
rQ
b2
y4
y5
y6
y7
b7
0
25
50
75
100
0
100
200
300
400
500
600
700
800
900
1,000
1,100

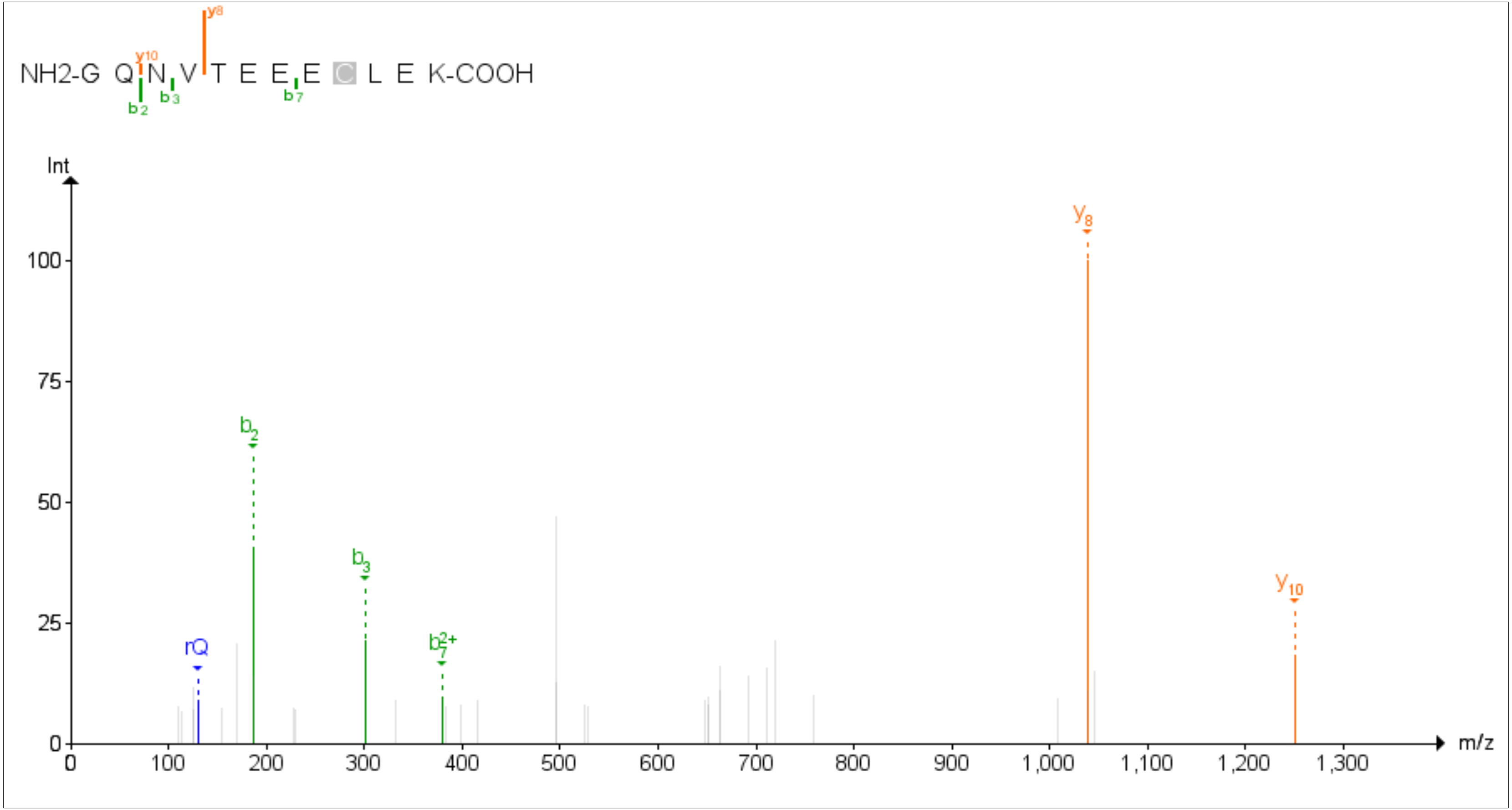

NH2-G Q N V T E E E C L E K-COOH
y8
y10
b2
b3
b7
Int
100
75
50
25
0
y8
b2
b3
rQ
b7 2+
y10
0
100
200
300
400
500
600
700
800
900
1,000
1,100
1,200
1,300
m/z

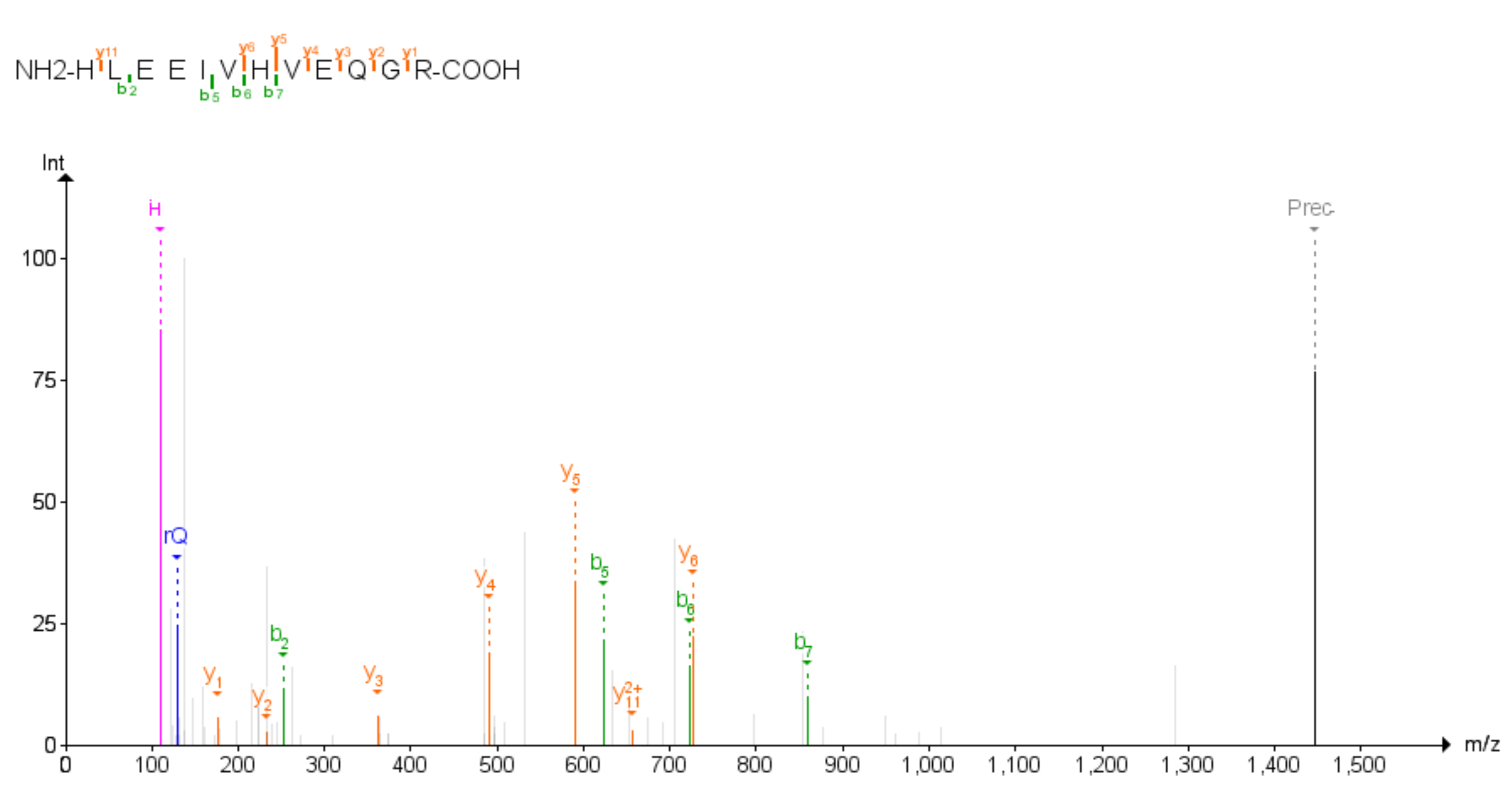

NH2-H L E E I V H V E Q G R-COOH
y11 y6 y5 y4 y3 y2 y1
b2 b5 b6 b7
Int
H
Prec
rQ
y1
y2
b2
y3
y4
y5
b5
y6
b6
y11 2+
b7
100
75
50
25
0
0
100
200
300
400
500
600
700
800
900
1,000
1,100
1,200
1,300
1,400
1,500
m/z

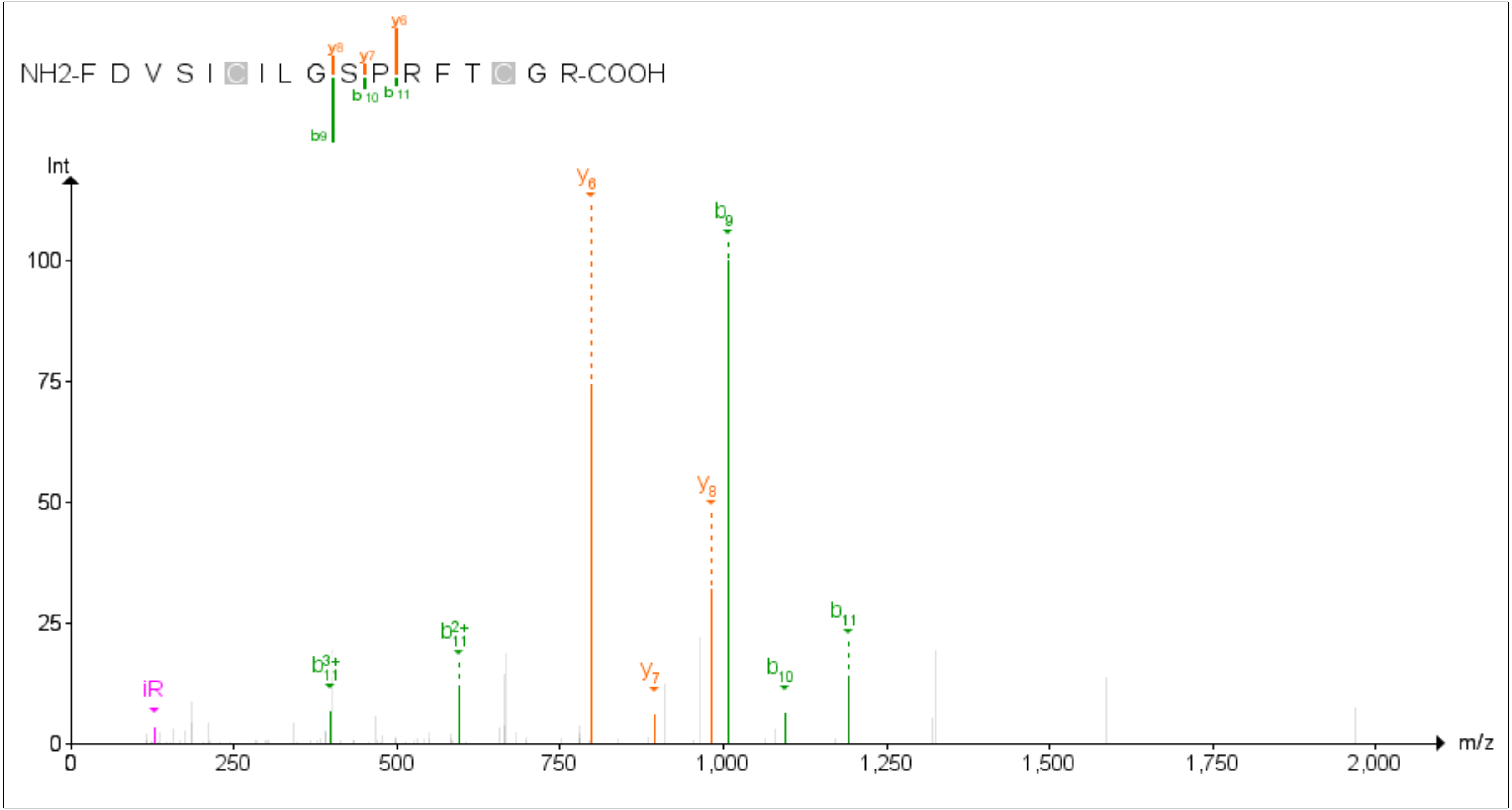

NH2-F D V S I C I L G S P R F T C G R-COOH
y8
y7
y6
b9
b 10
b 11
Int
100
75
50
25
0
iR
b11 3+
b11 2+
y6
y7
y8
b9
b10
b11
0
250
500
750
1,000
1,250
1,500
1,750
2,000
m/z

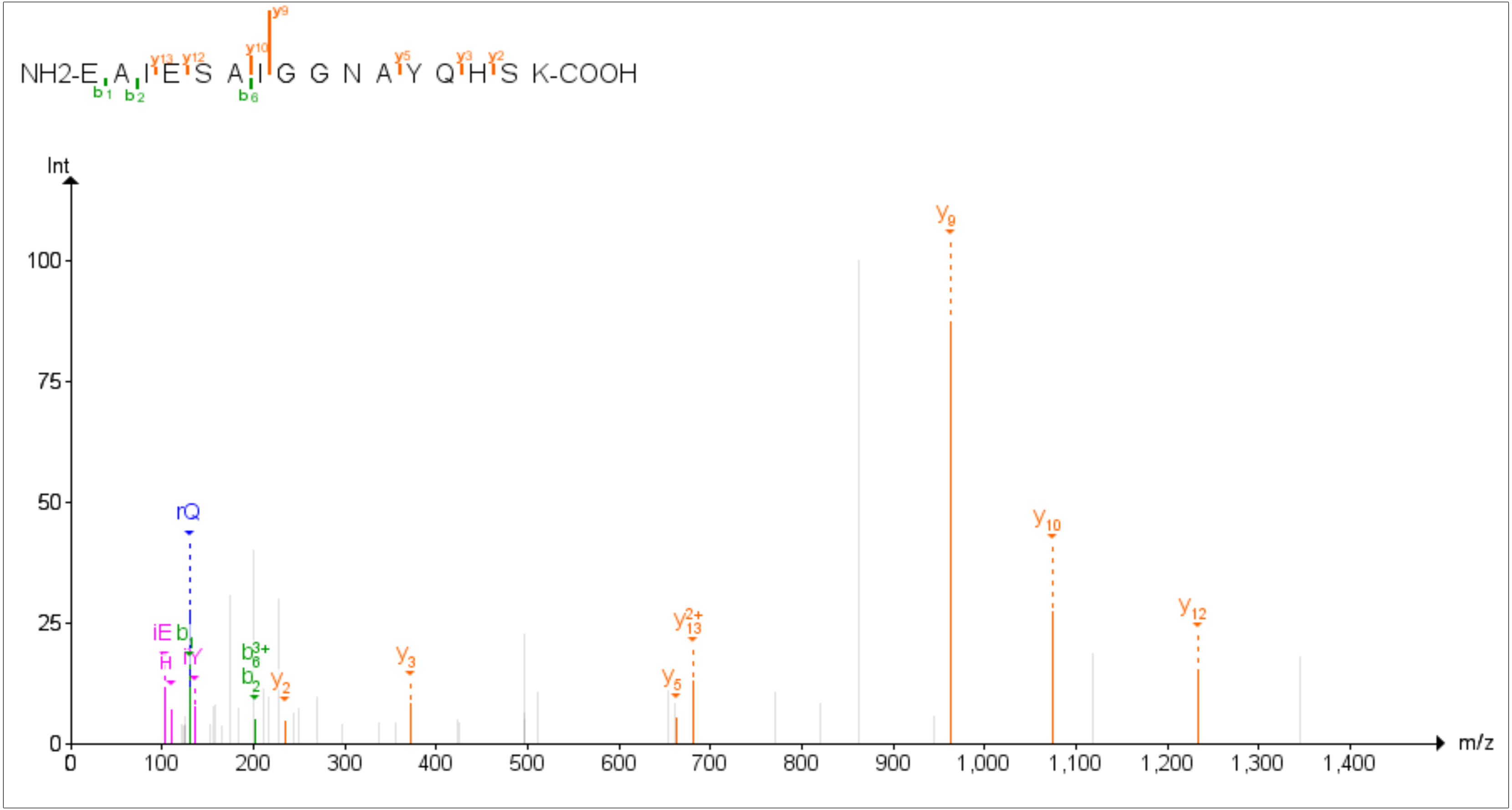

NH2-E A I E S A I G G N A Y Q H S K-COOH
b1 b2 b6
y13 y12 y10 y9 y5 y3 y2
Int
m/z
y9
y10
y12
y13 2+
y5
y3
y2
b6 3+
b2
rQ
iE
b1
iH
iY

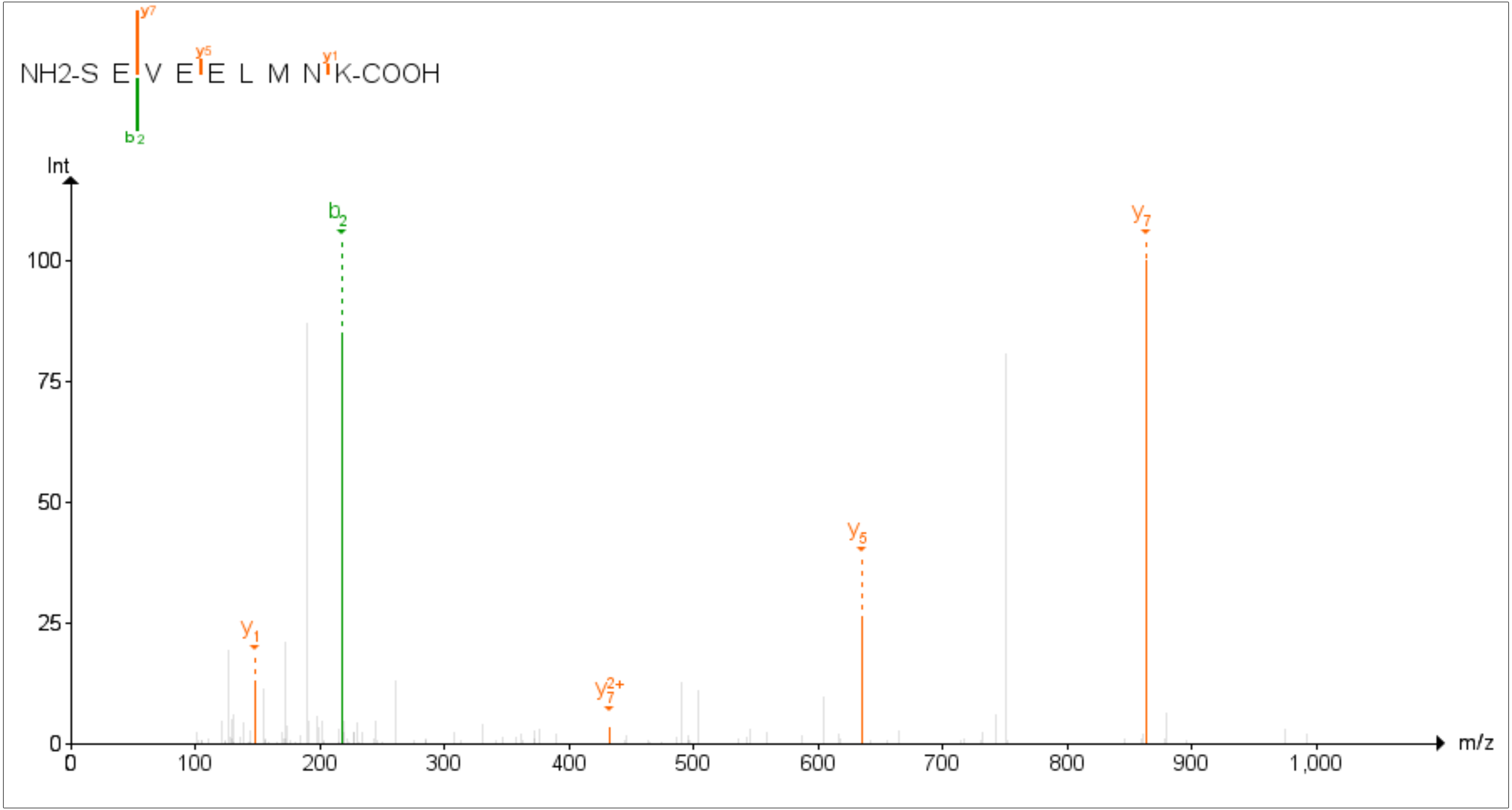

NH2-S E V E E L M N K-COOH
y7
y5
y1
b2
Int
100
75
50
25
0
b2
y7
y5
y1
y7 2+
m/z
0
100
200
300
400
500
600
700
800
900
1,000

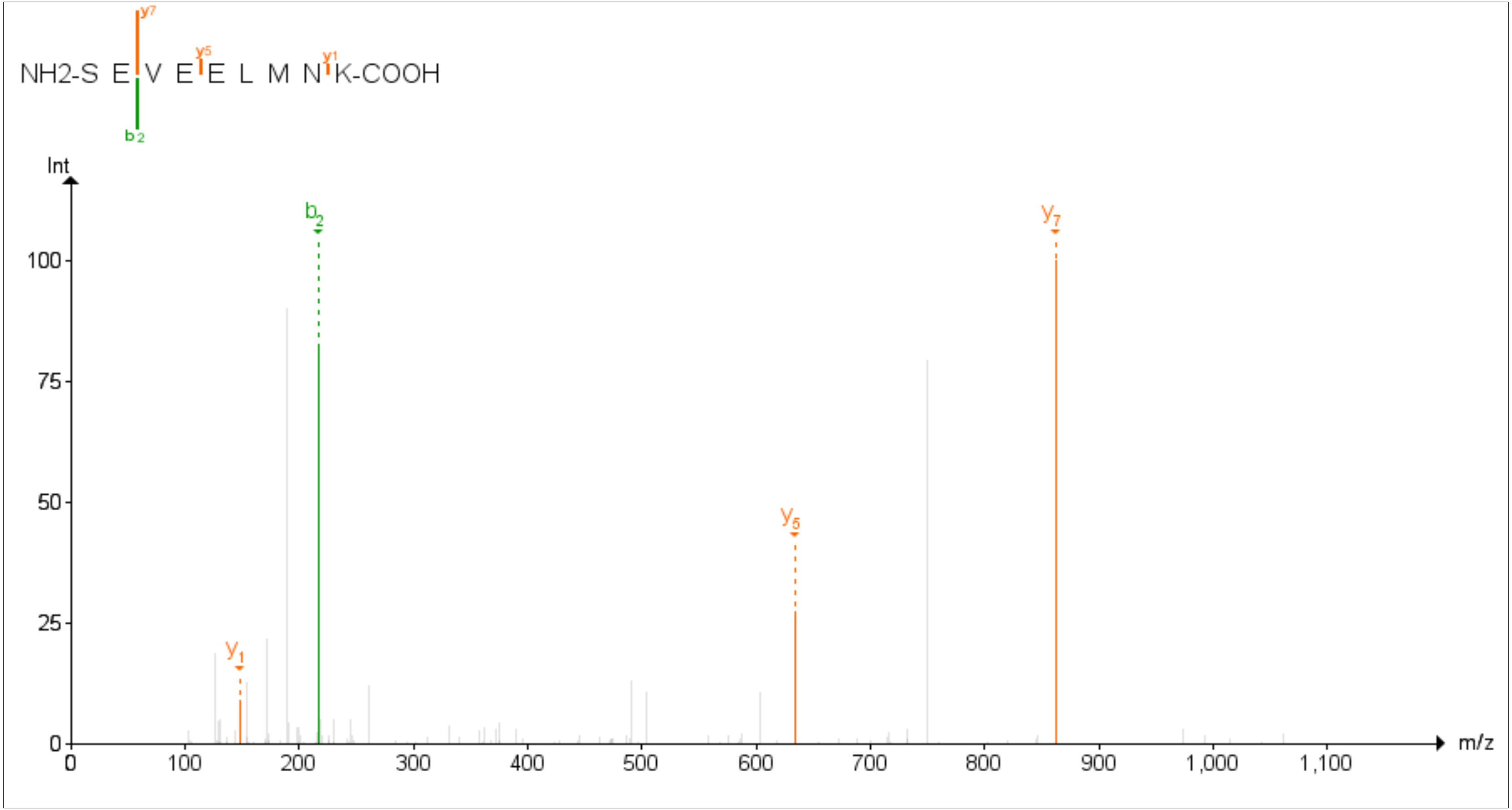

NH2-S E V E E L M N K-COOH
y7
y5
y1
b2
Int
100
75
50
25
0
b2
y7
y5
y1
0
100
200
300
400
500
600
700
800
900
1,000
1,100
m/z

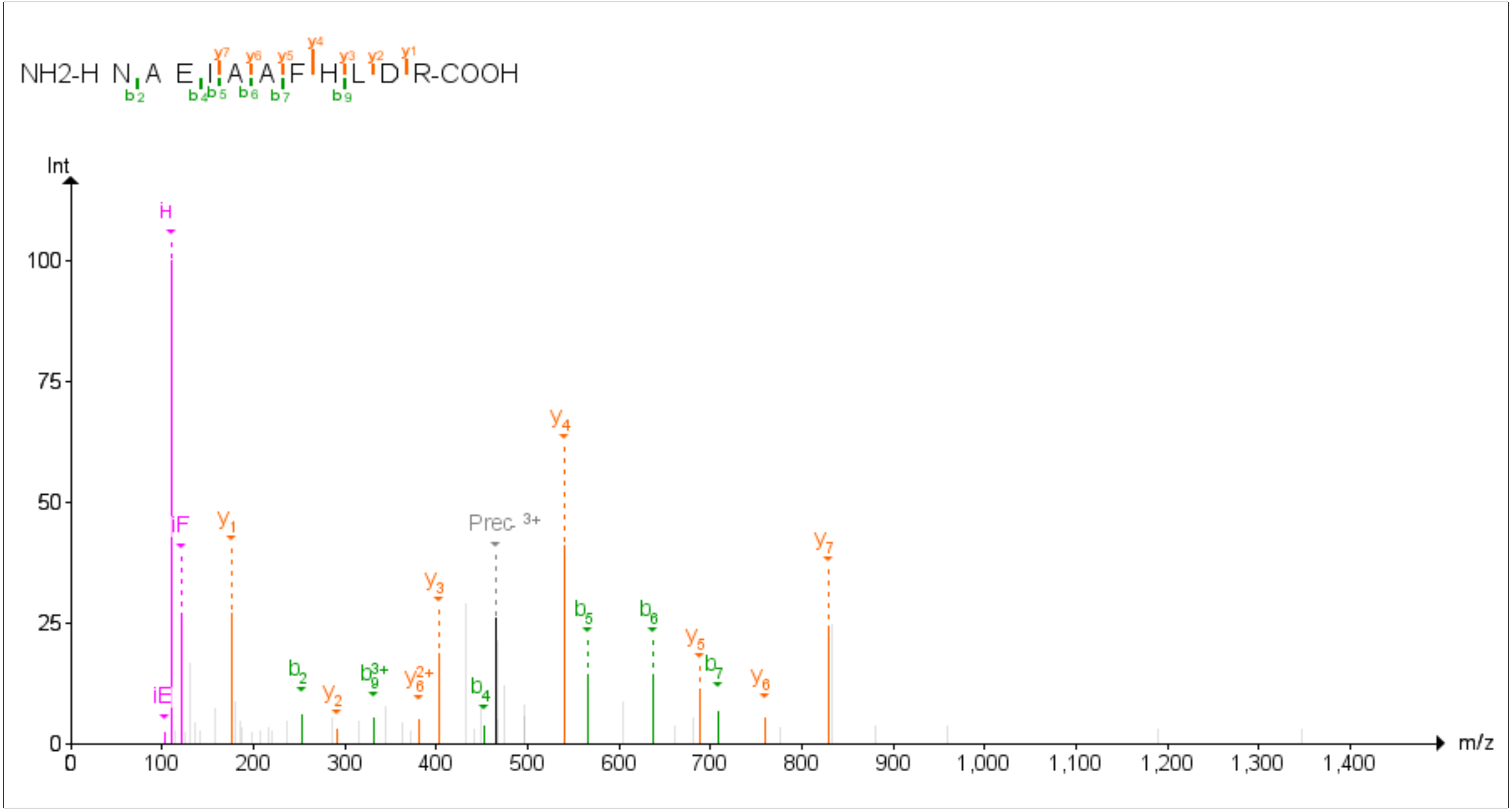

NH2-H N A E I A A F H L D R-COOH
y7 y6 y5 y4 y3 y2 y1
b2 b4 b5 b6 b7 b9
Int
iH
iF
iE
y1
b2
y2
b9 3+
y6 2+
y3
b4
Prec 3+
y4
b5
b6
y5
b7
y6
y7
0
25
50
75
100
0
100
200
300
400
500
600
700
800
900
1,000
1,100
1,200
1,300
1,400
m/z

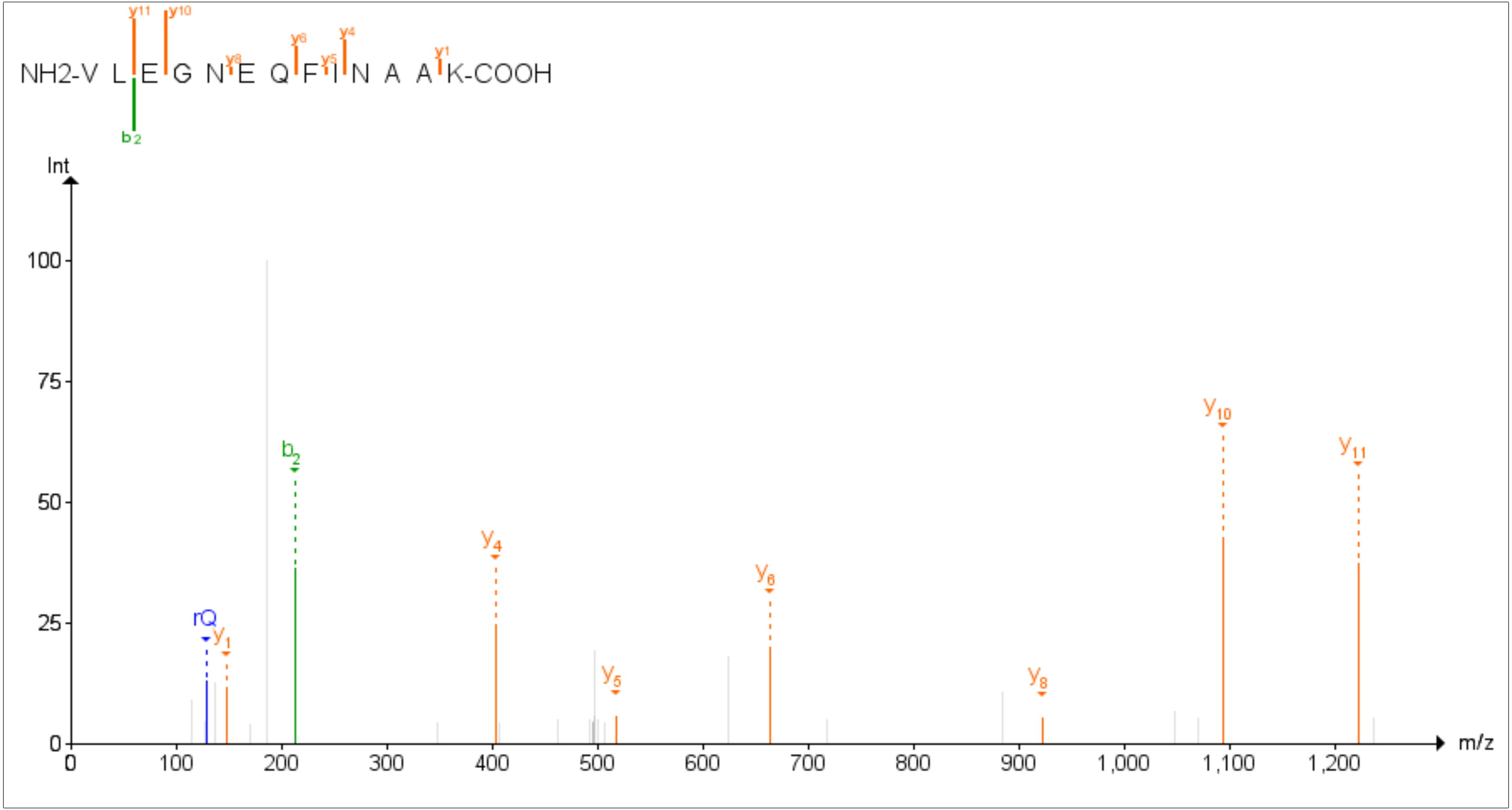

NH2-V L E G N E Q F I N A A K-COOH
y11
y10
y8
y6
y5
y4
y1
b2
Int
100
75
50
25
0
rQ
y1
b2
y4
y5
y6
y8
y10
y11
0
100
200
300
400
500
600
700
800
900
1,000
1,100
1,200
m/z

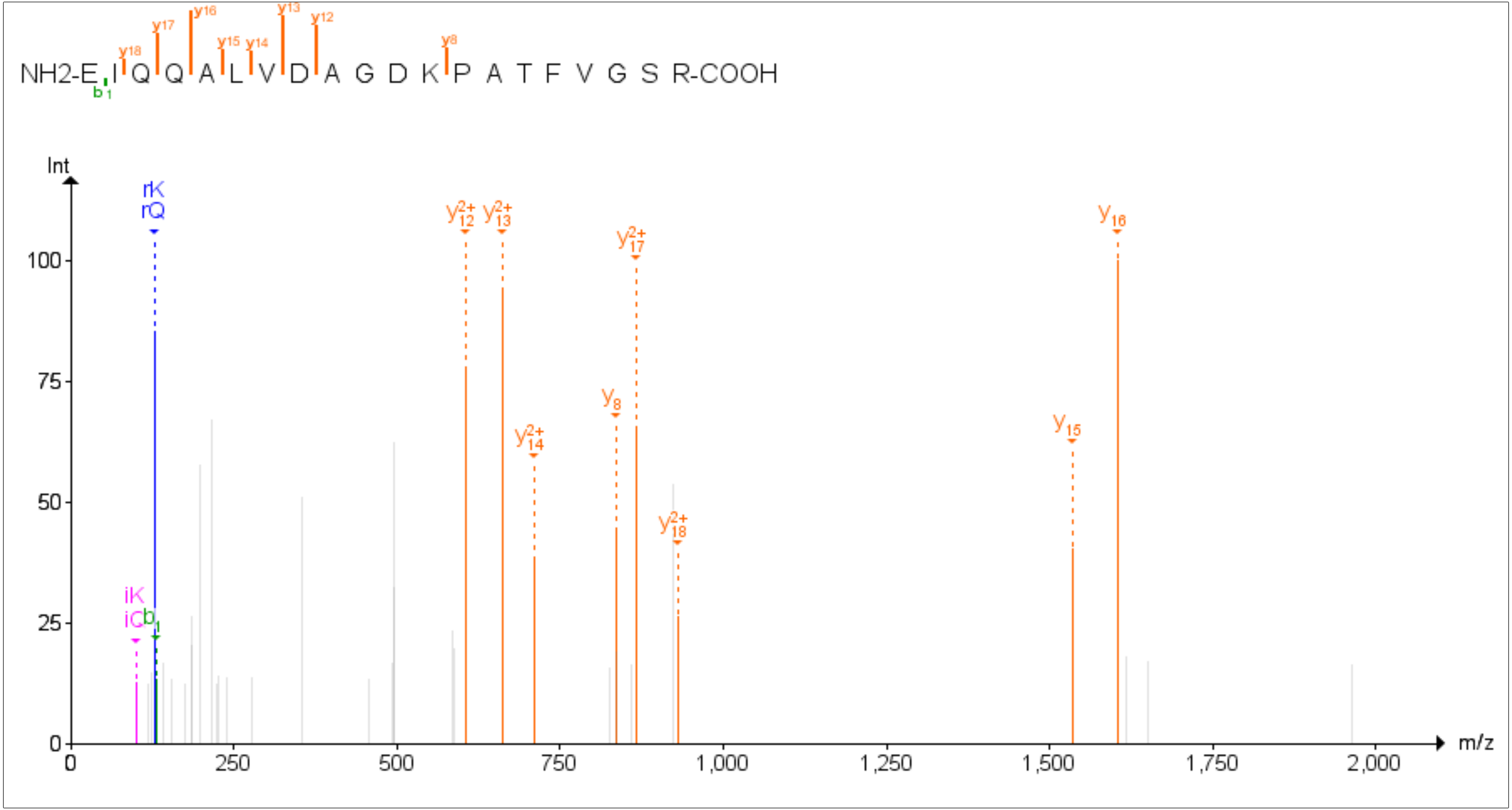

NH2-E I Q Q A L V D A G D K P A T F V G S R-COOH
b1
y18
y17
y16
y15
y14
y13
y12
y8
Int
m/z
rK
rQ
iK
iQ
b1
y12 2+
y13 2+
y14 2+
y17 2+
y18 2+
y8
y15
y16
0
25
50
75
100
0
250
500
750
1,000
1,250
1,500
1,750
2,000

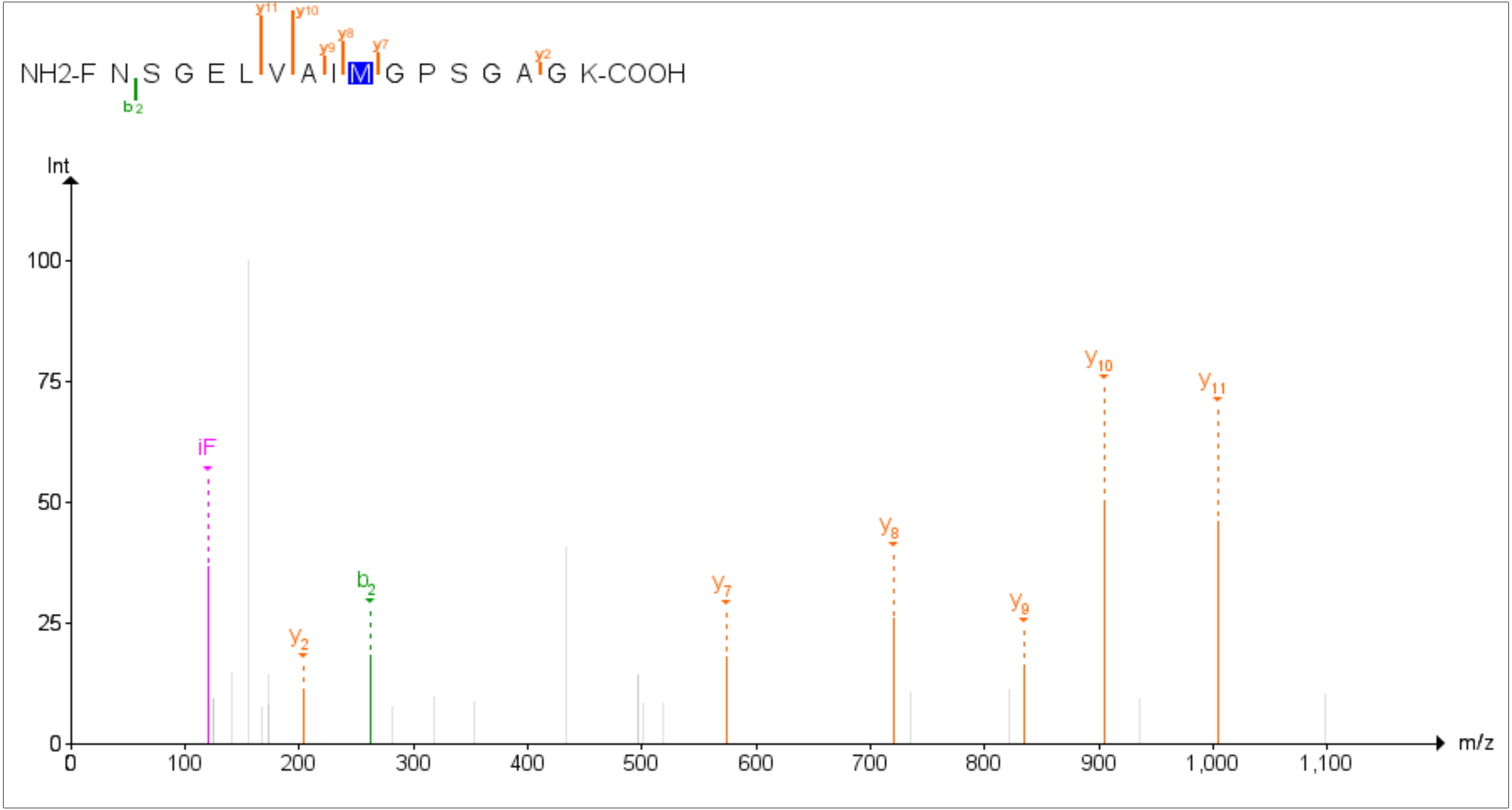

NH2-F N S G E L V A I M G P S G A G K-COOH
b2
y11
y10
y9
y8
y7
y2
Int
100
75
50
25
0
iF
y2
b2
y7
y8
y9
y10
y11
0
100
200
300
400
500
600
700
800
900
1,000
1,100
m/z

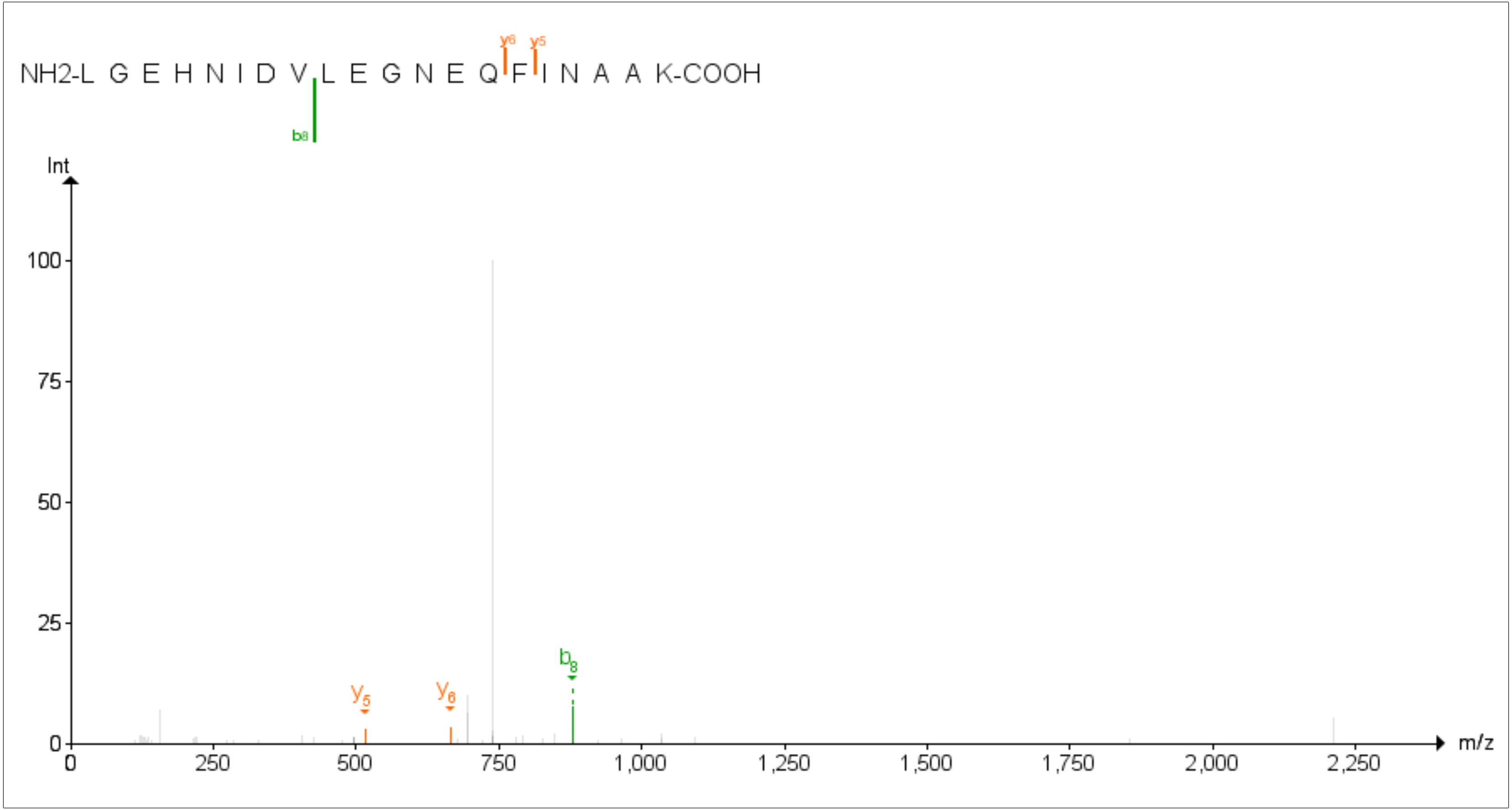
NH2-L G E H N I D V L E G N E Q F I N A A K-COOH
y6
y5
b8
Int
100
75
50
25
0
y5
y6
b8
0
250
500
750
1,000
1,250
1,500
1,750
2,000
2,250
m/z

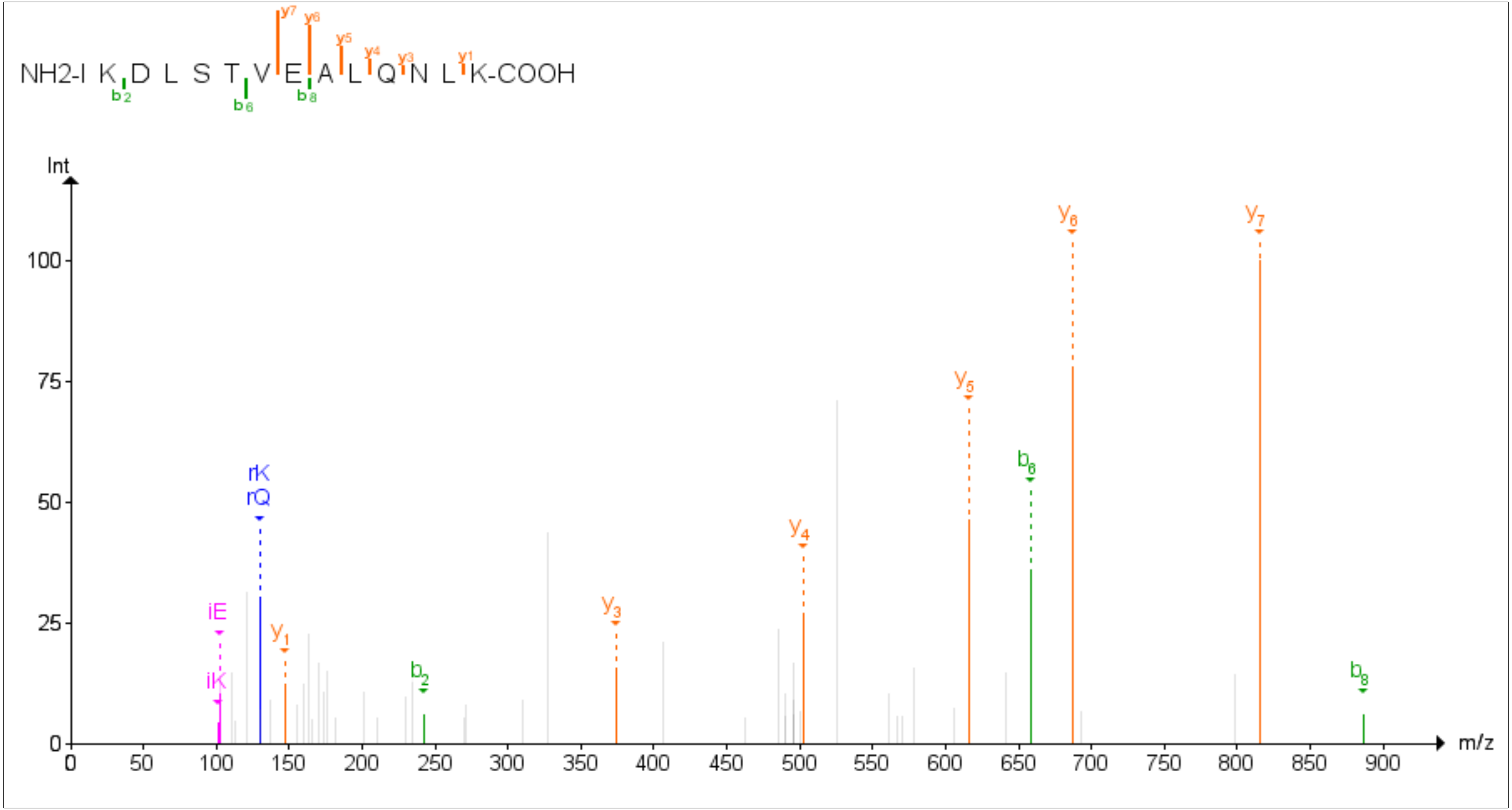

NH2-I K D L S T V E A L Q N L K-COOH
y7 y6 y5 y4 y3 y1
b2 b6 b8
Int
m/z
y6
y7
y5
b6
rK
rQ
y4
y3
iE
y1
b2
iK
b8
0
25
50
75
100
0 50 100 150 200 250 300 350 400 450 500 550 600 650 700 750 800 850 900

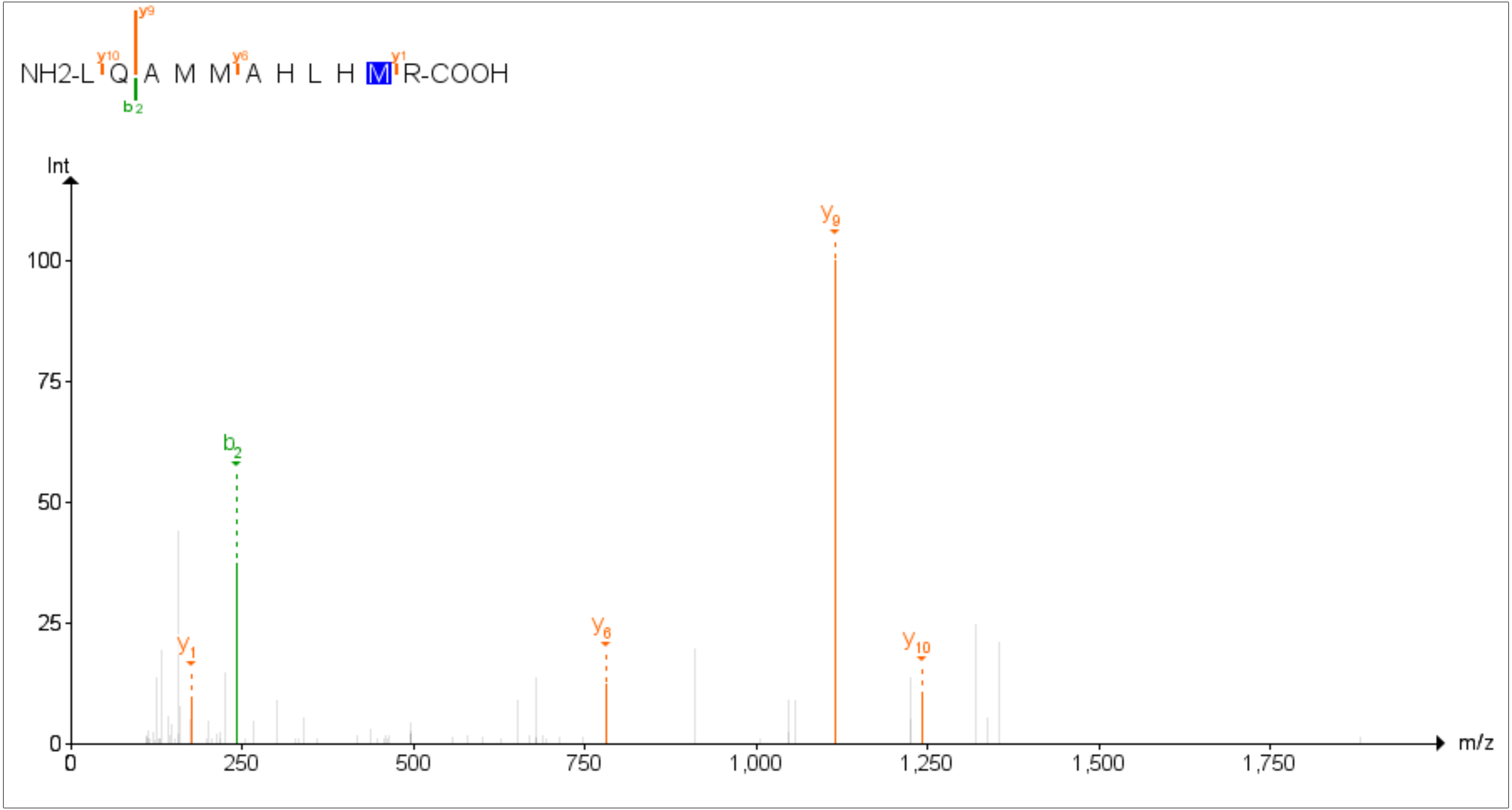

NH2-L Q A M M A H L H M R-COOH
y9
y10
y6
y1
b2
Int
100
75
50
25
0
y9
b2
y1
y6
y10
0
250
500
750
1,000
1,250
1,500
1,750
m/z

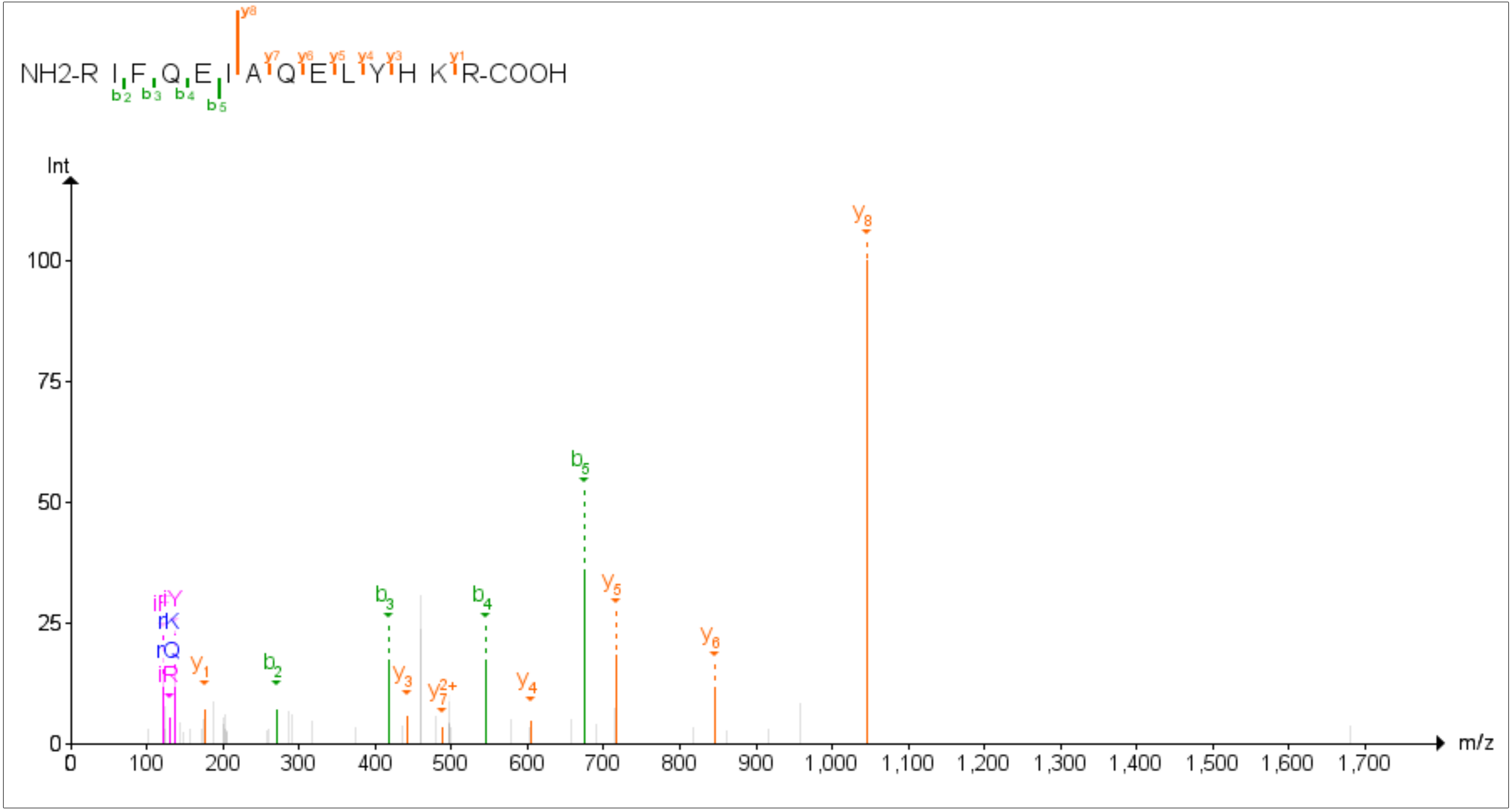

NH2-R I F Q E I A Q E L Y H K R-COOH
b2 b3 b4 b5
y8 y7 y6 y5 y4 y3 y1
Int
m/z
0
25
50
75
100
0
100
200
300
400
500
600
700
800
900
1,000
1,100
1,200
1,300
1,400
1,500
1,600
1,700
y8
b5
y5
y6
b3
b4
y1
b2
y3
y7 2+
y4
iY
rK
rQ
iR

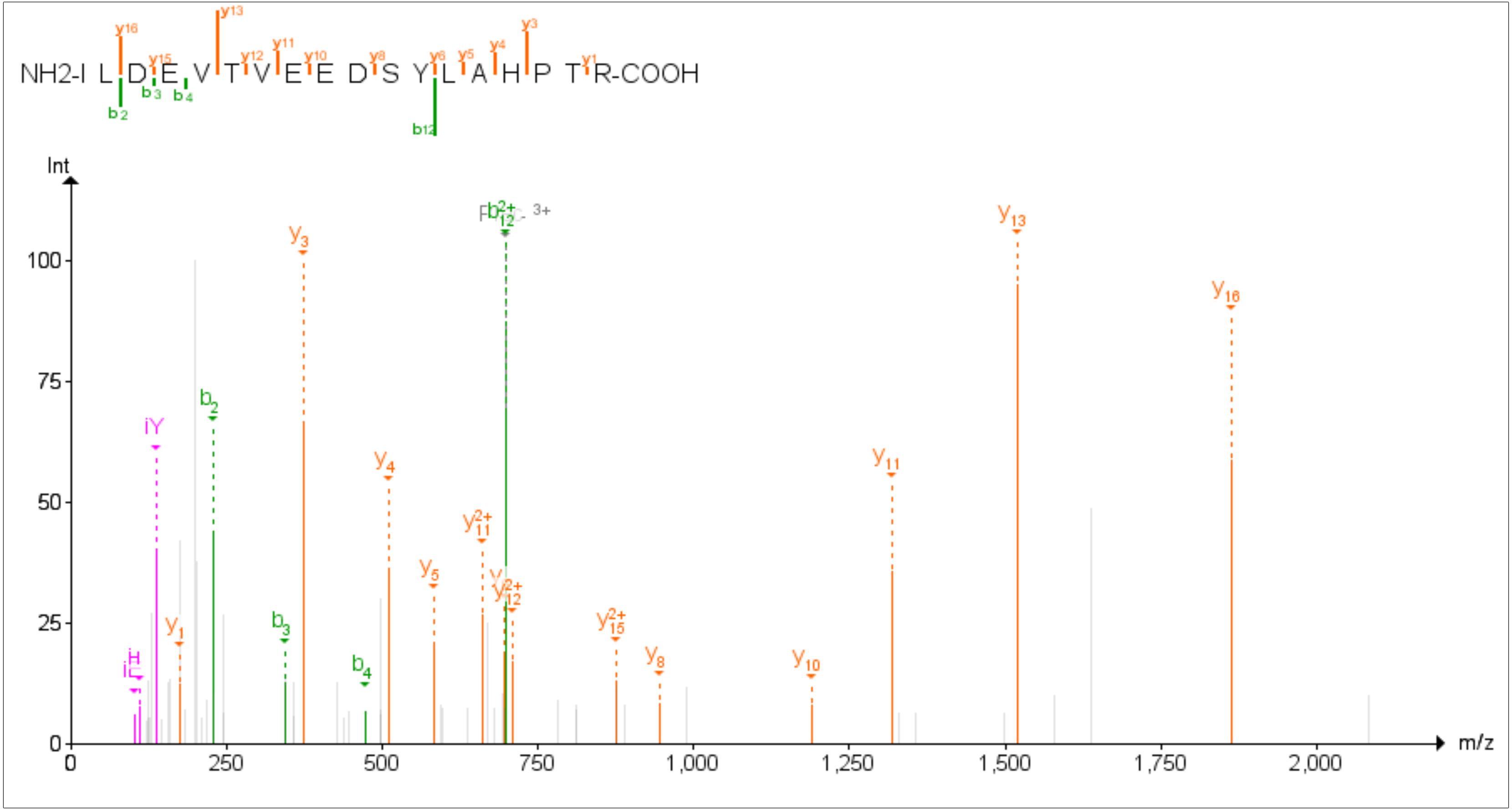

NH2-I L D E V T V E E D S Y L A H P T R-COOH
Int
m/z
0
25
50
75
100
250
500
750
1,000
1,250
1,500
1,750
2,000
iY
b2
y3
b2+ 12
y13
y16
y4
y2+ 11
y11
y5
y2+ 12
y1
b3
b4
y2+ 15
y8
y10
iH
iL

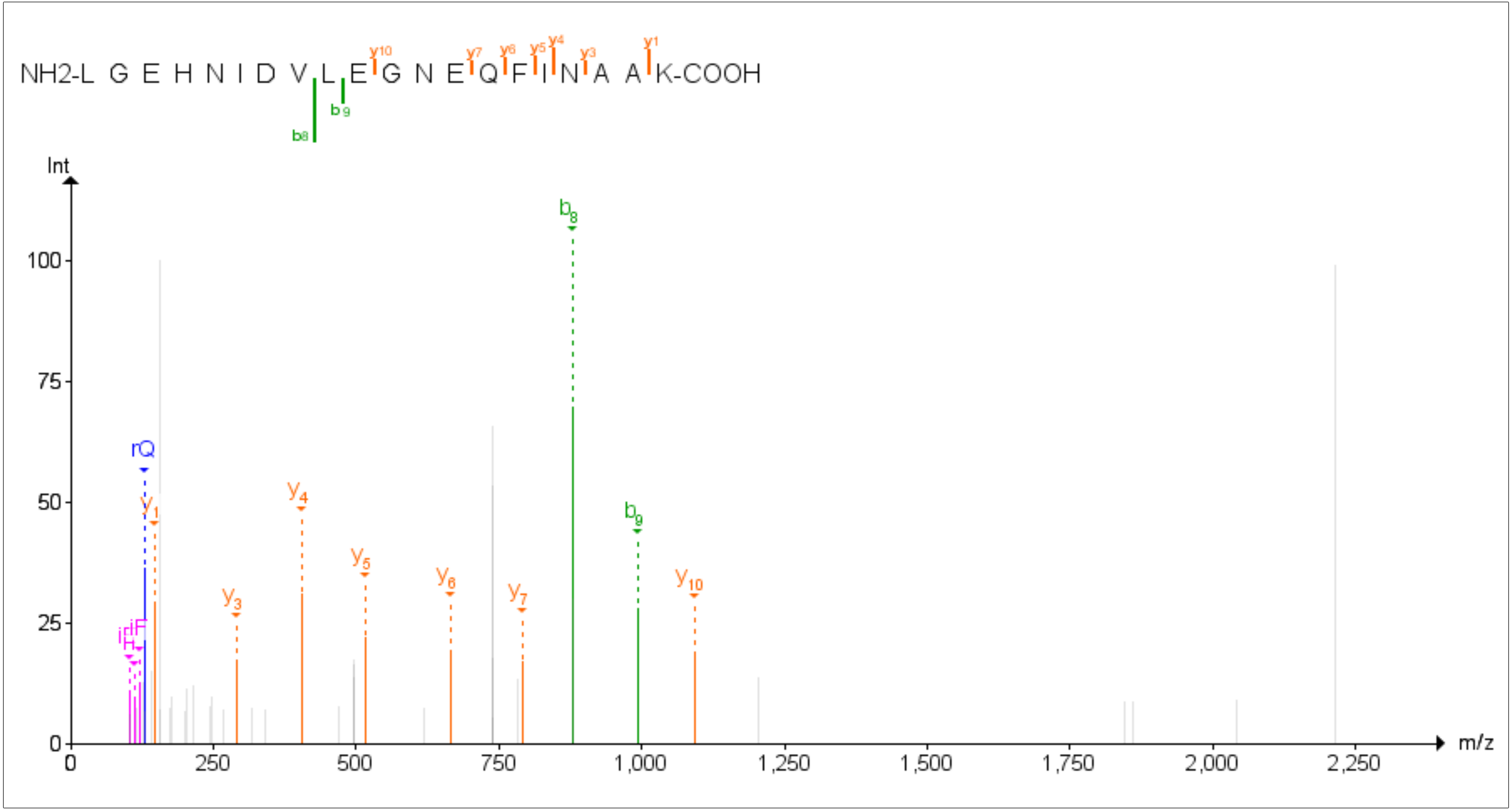

NH2-L G E H N I D V L E G N E Q F I N A A K-COOH
b8
b9
y10
y7
y6
y5
y4
y3
y1
Int
m/z
0
25
50
75
100
250
500
750
1,000
1,250
1,500
1,750
2,000
2,250
rQ
iH
iF

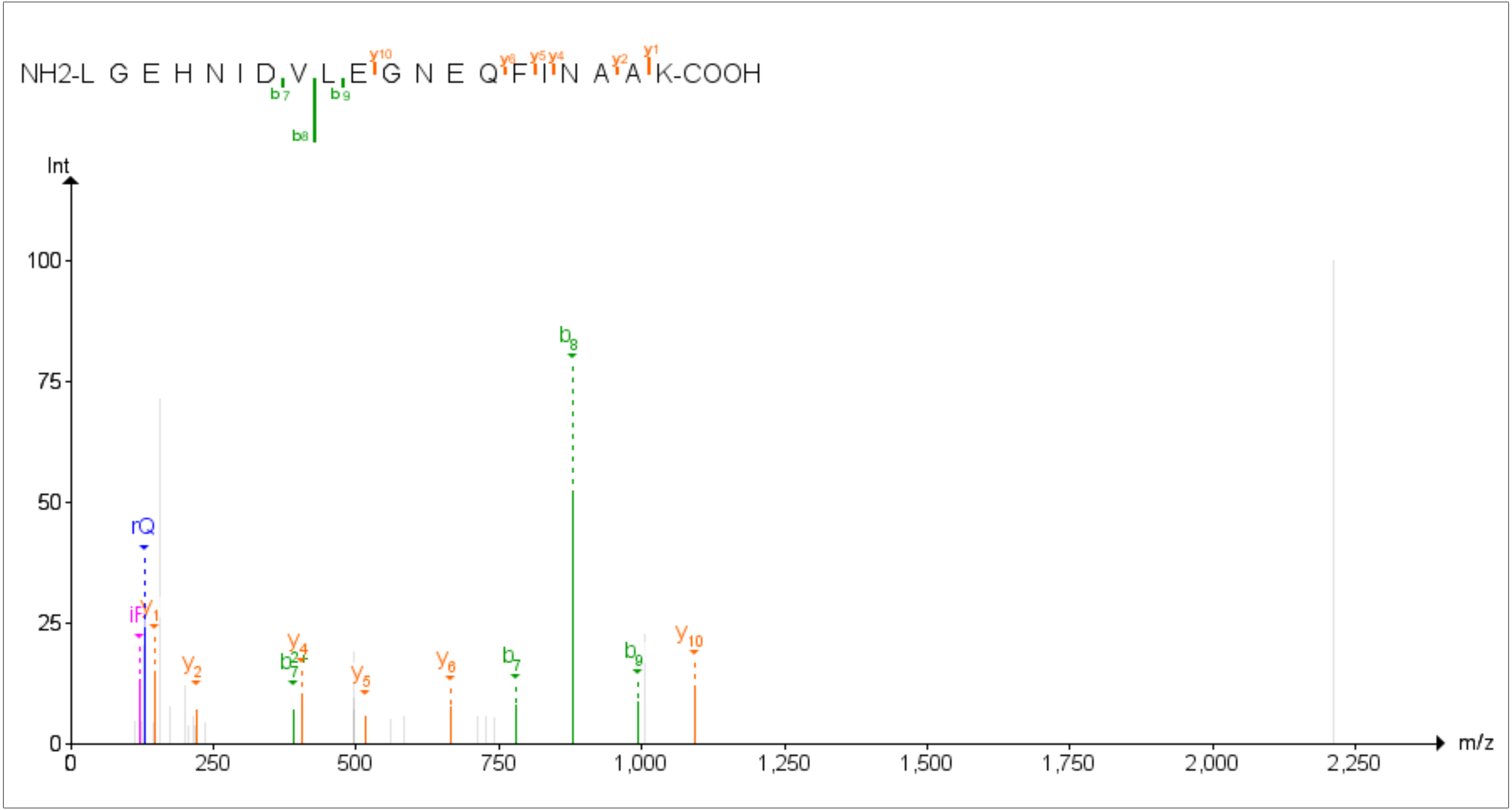
NH2-L G E H N I D V L E G N E Q F I N A A K-COOH
b7
b8
b9
y10
y6
y5
y4
y2
y1
Int
100
75
50
25
0
rQ
iF
b8
b7
b9
y1
y2
y4
y5
y6
y10
0
250
500
750
1,000
1,250
1,500
1,750
2,000
2,250
m/z

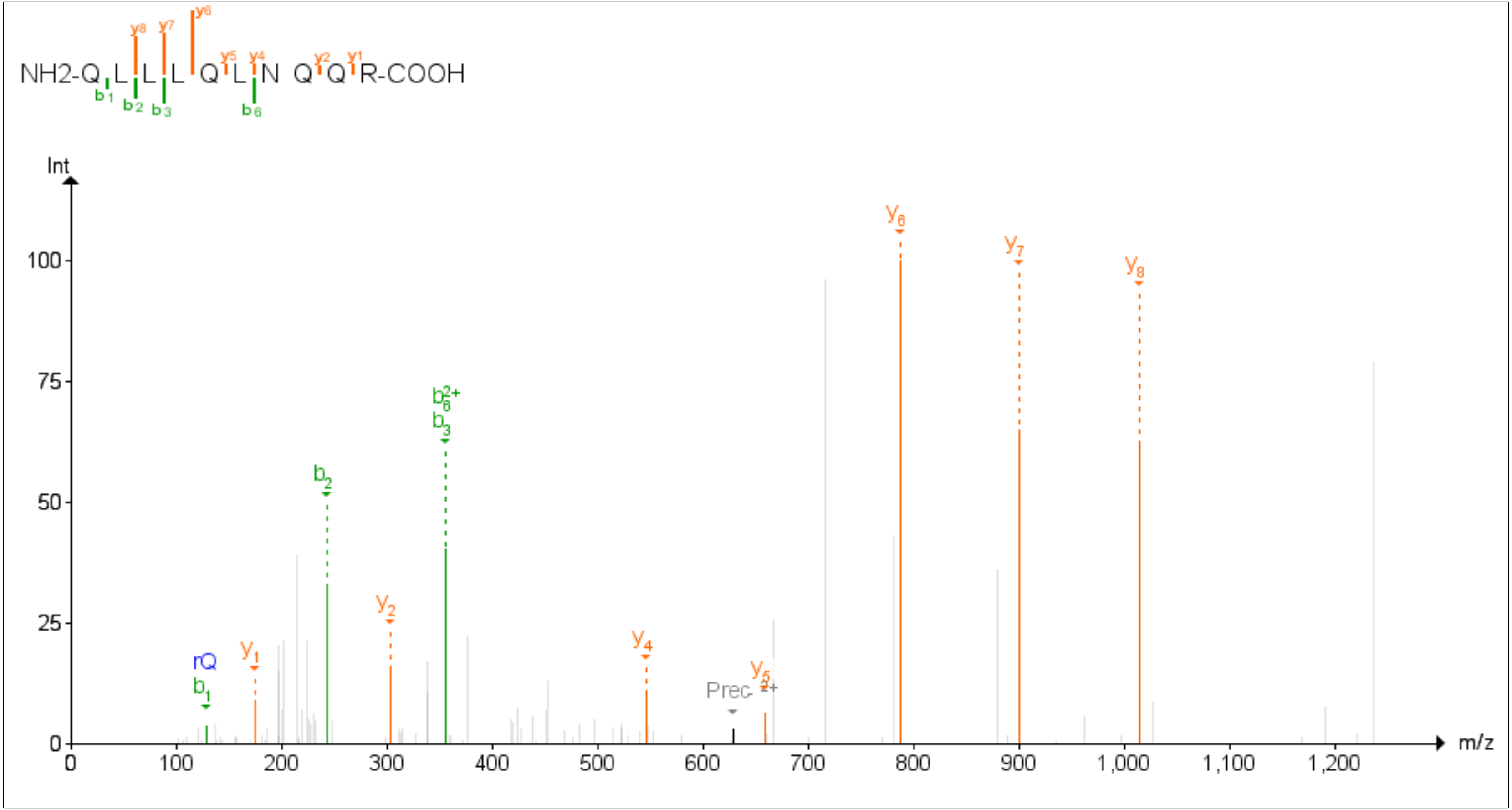

NH2-QLLLLQLNQQR-COOH
Int
m/z

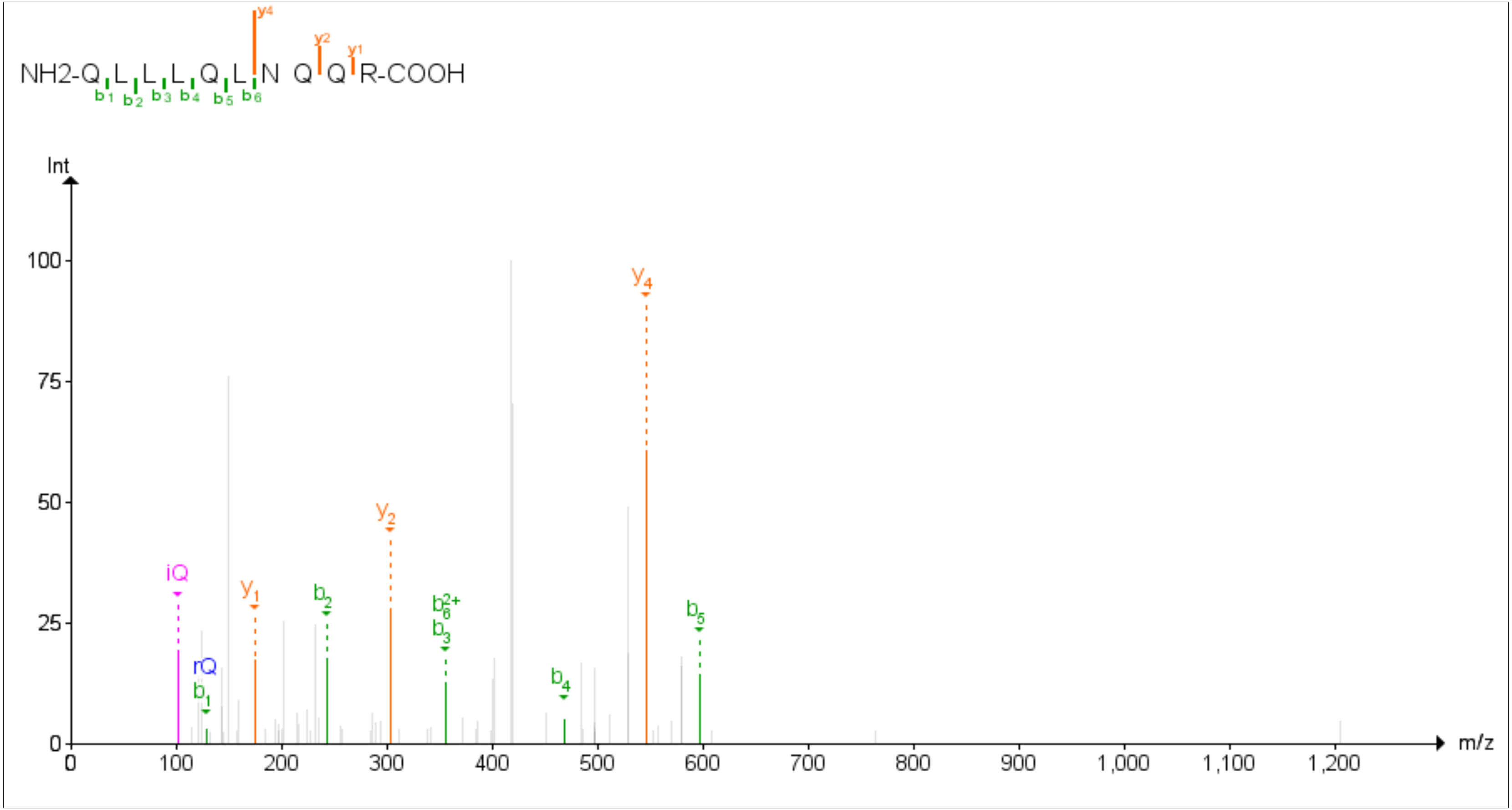
NH2-Q L L L Q L N Q Q R-COOH
b1 b2 b3 b4 b5 b6
y4 y2 y1
Int
100
75
50
25
0
m/z
0
100
200
300
400
500
600
700
800
900
1,000
1,100
1,200
iQ
rQ
b1
y1
b2
y2
b6 2+
b3
b4
y4
b5

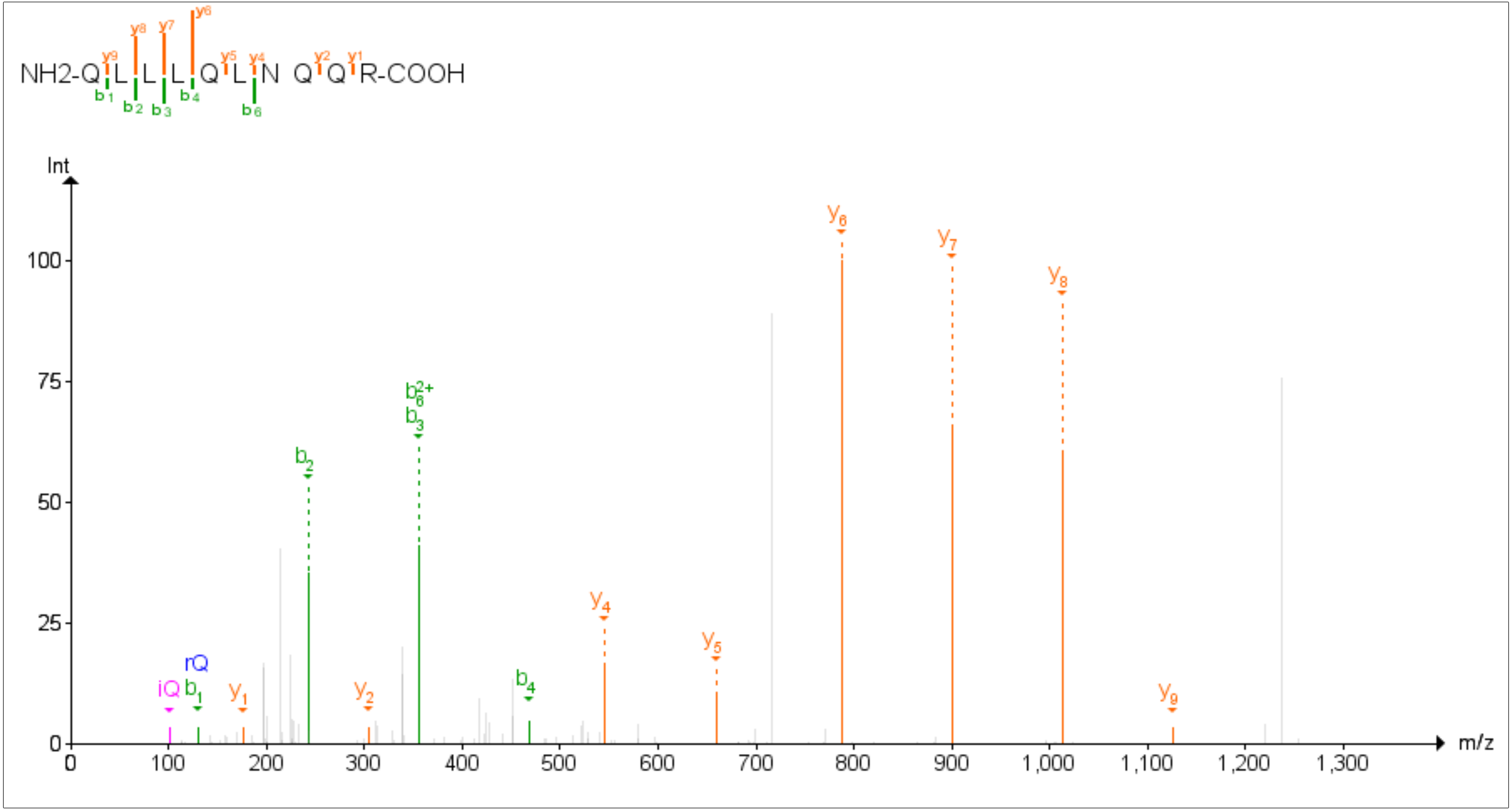

NH2-Q L L L L Q L N Q Q R-COOH
Int
m/z
y6
y7
y8
y4
y5
b2
b3
b4
b1
y1
y2
y9
iQ
rQ

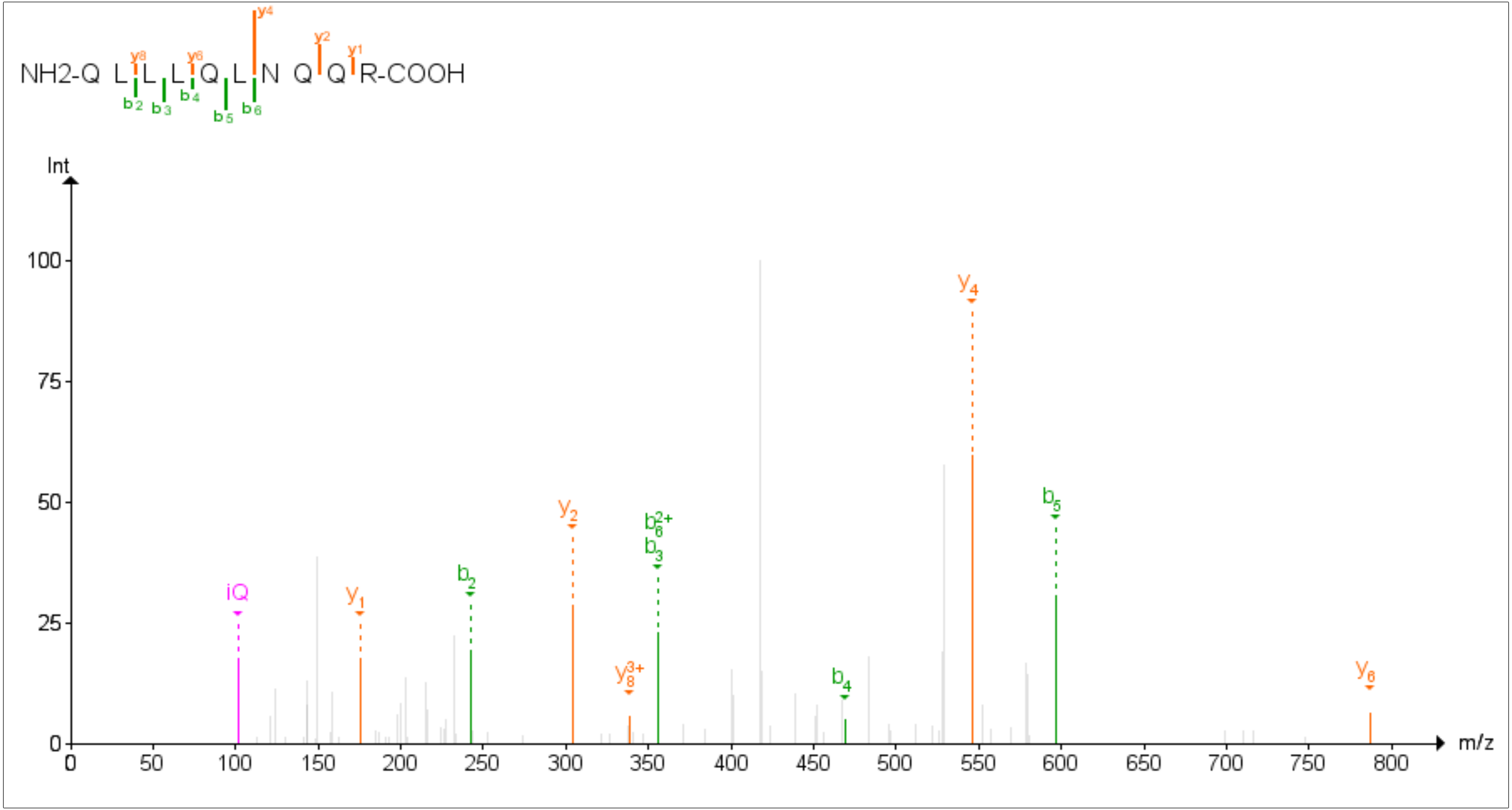

NH2-Q L L L Q L N Q Q R-COOH
y8 y6 y4 y2 y1
b2 b3 b4 b5 b6
Int
100
75
50
25
0
iQ
y1
b2
y2
y8 3+
b6 2+
b3
b4
y4
b5
y6
0 50 100 150 200 250 300 350 400 450 500 550 600 650 700 750 800
m/z

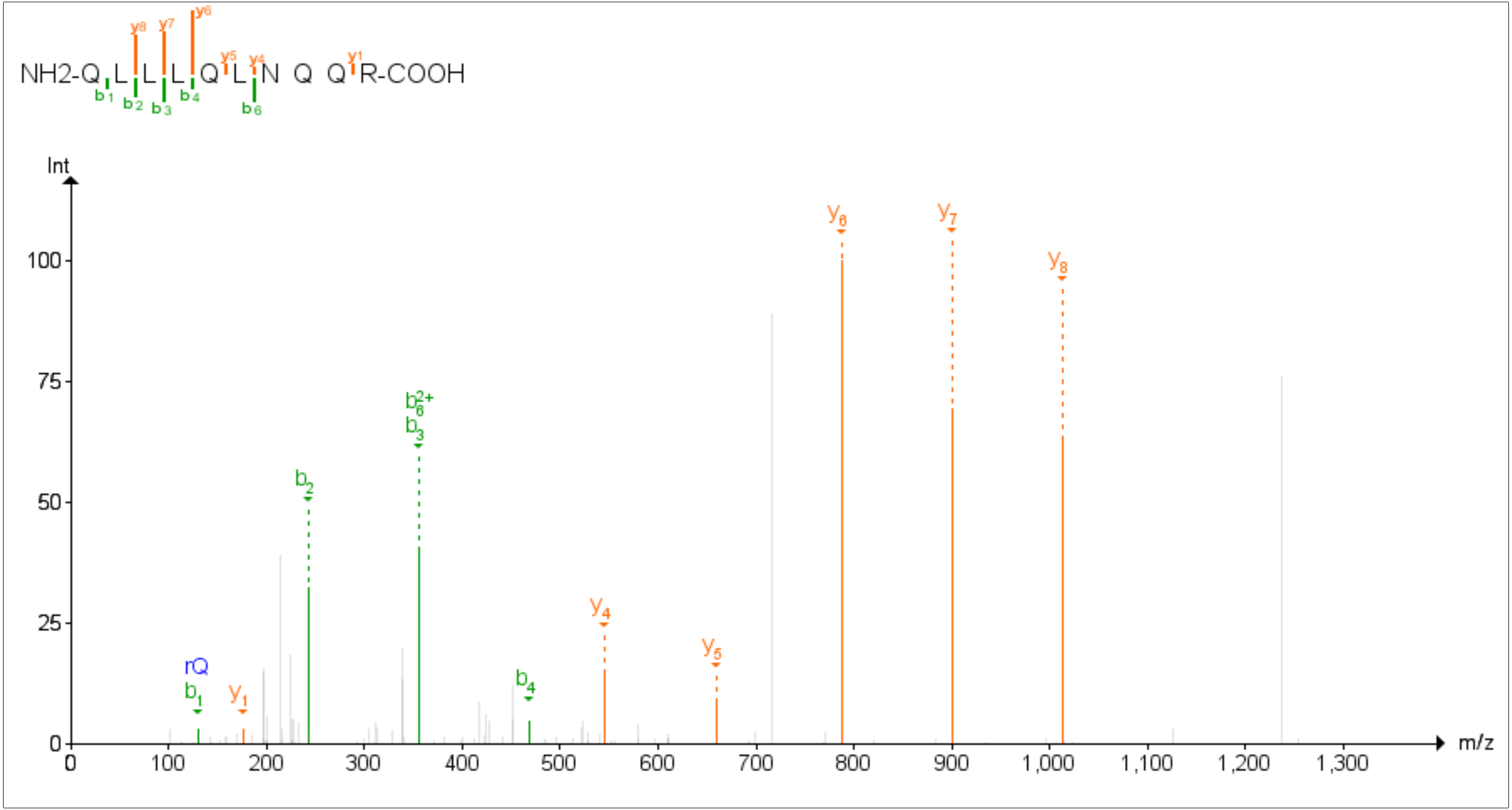

NH2-Q L L L Q L N Q Q R-COOH
y8 y7 y6 y5 y4 y1
b1 b2 b3 b4 b6
Int
100
75
50
25
0
m/z
0
100
200
300
400
500
600
700
800
900
1,000
1,100
1,200
1,300
rQ
b_1
y_1
b_2
b_6^{2+}
b_3
b_4
y_4
y_5
y_6
y_7
y_8

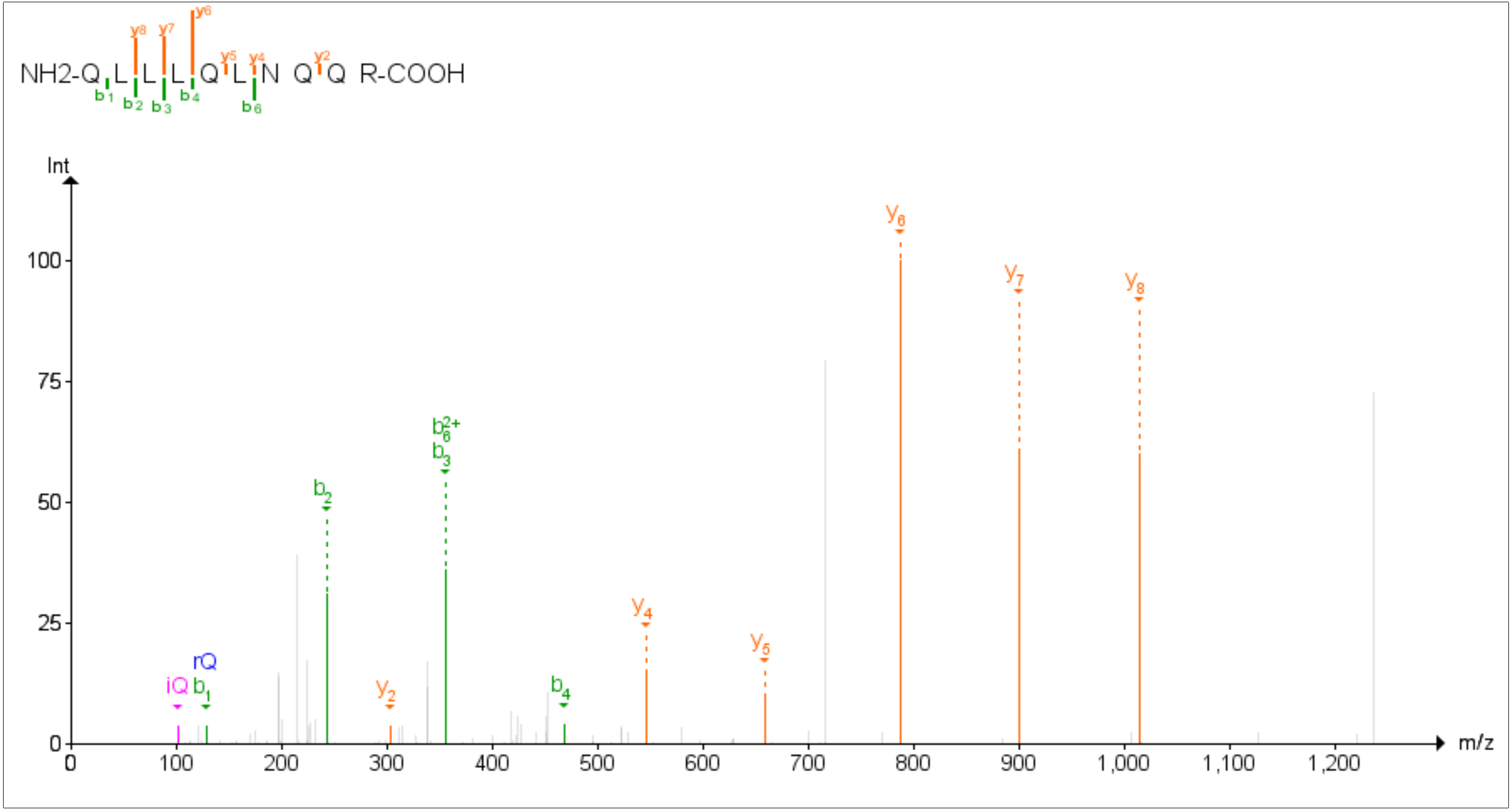

NH2-Q L L L Q L N Q Q R-COOH
y8 y7 y6 y5 y4 y2
b1 b2 b3 b4 b6
Int
m/z
y6
y7
y8
b6 2+
b3
b2
y4
y5
y2
b4
iQ
rQ
b1
0
25
50
75
100
0
100
200
300
400
500
600
700
800
900
1,000
1,100
1,200

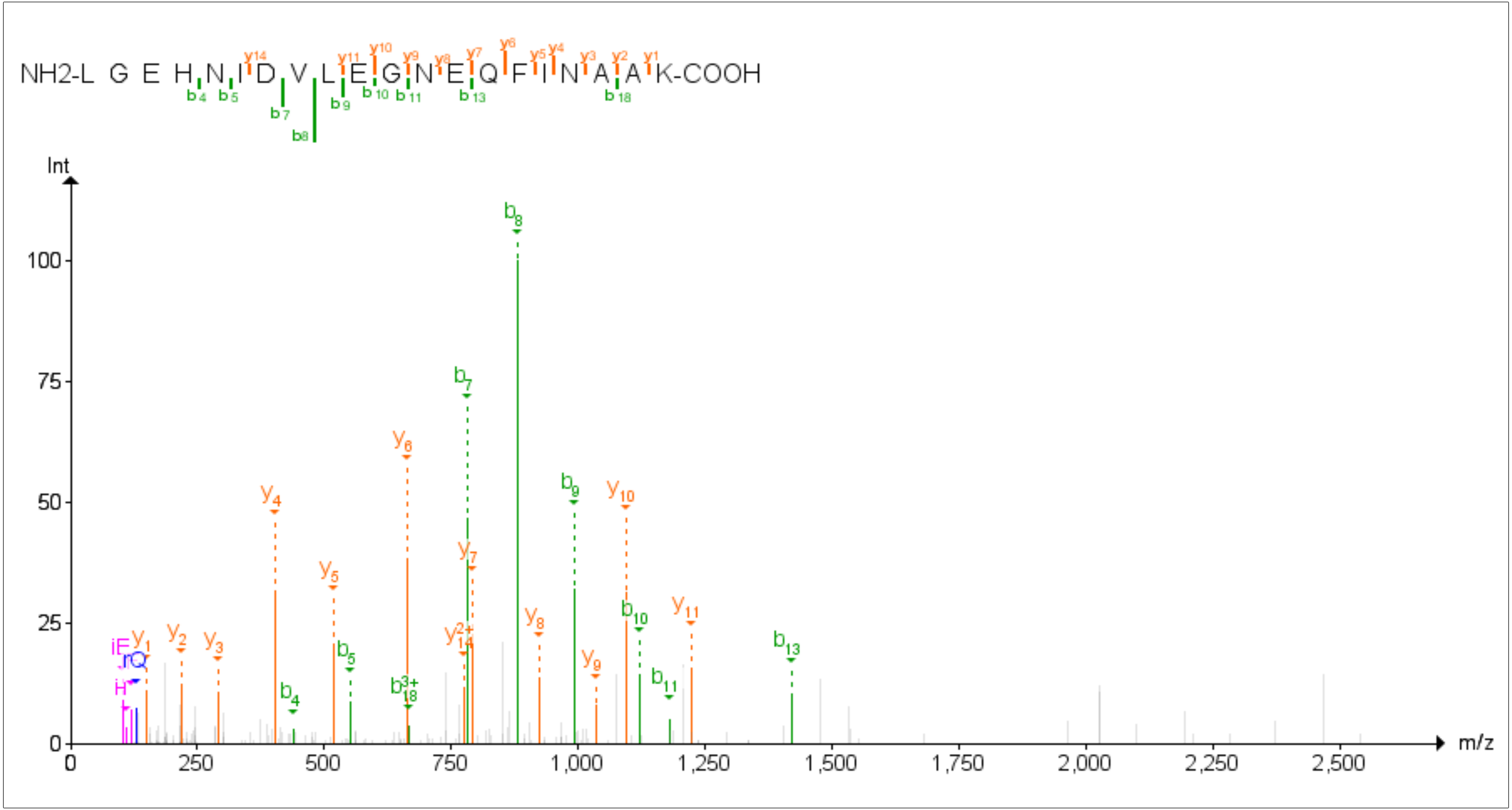

NH2-L G E H N I D V L E G N E Q F I N A A K-COOH
Int
m/z
0
25
50
75
100
250
500
750
1,000
1,250
1,500
1,750
2,000
2,250
2,500

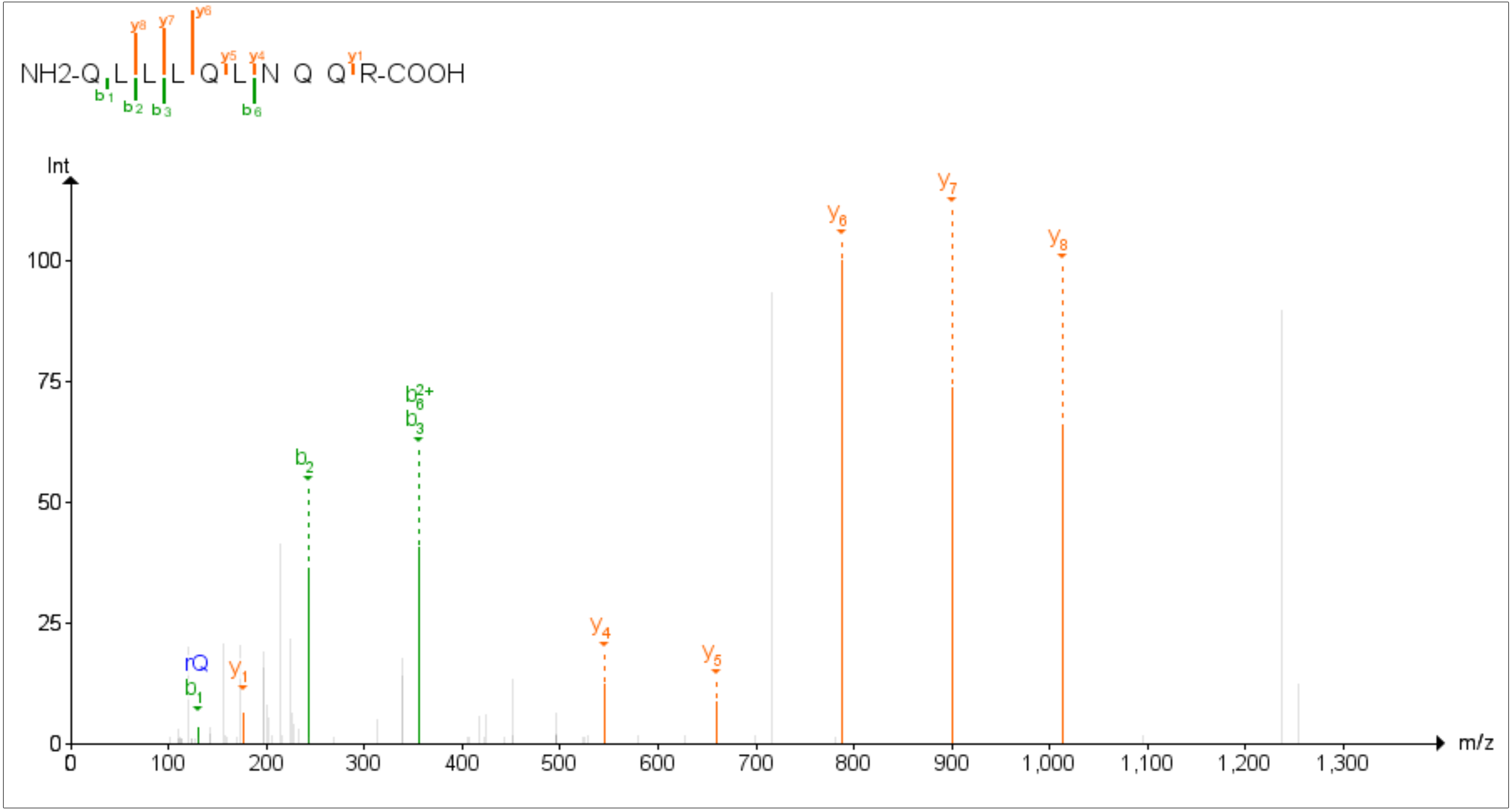

NH2-Q L L L Q L N Q Q R-COOH
y8 y7 y6 y5 y4 y1
b1 b2 b3 b6
Int
m/z
y6
y7
y8
b2
b3
y4
y5
rQ
b1
y1

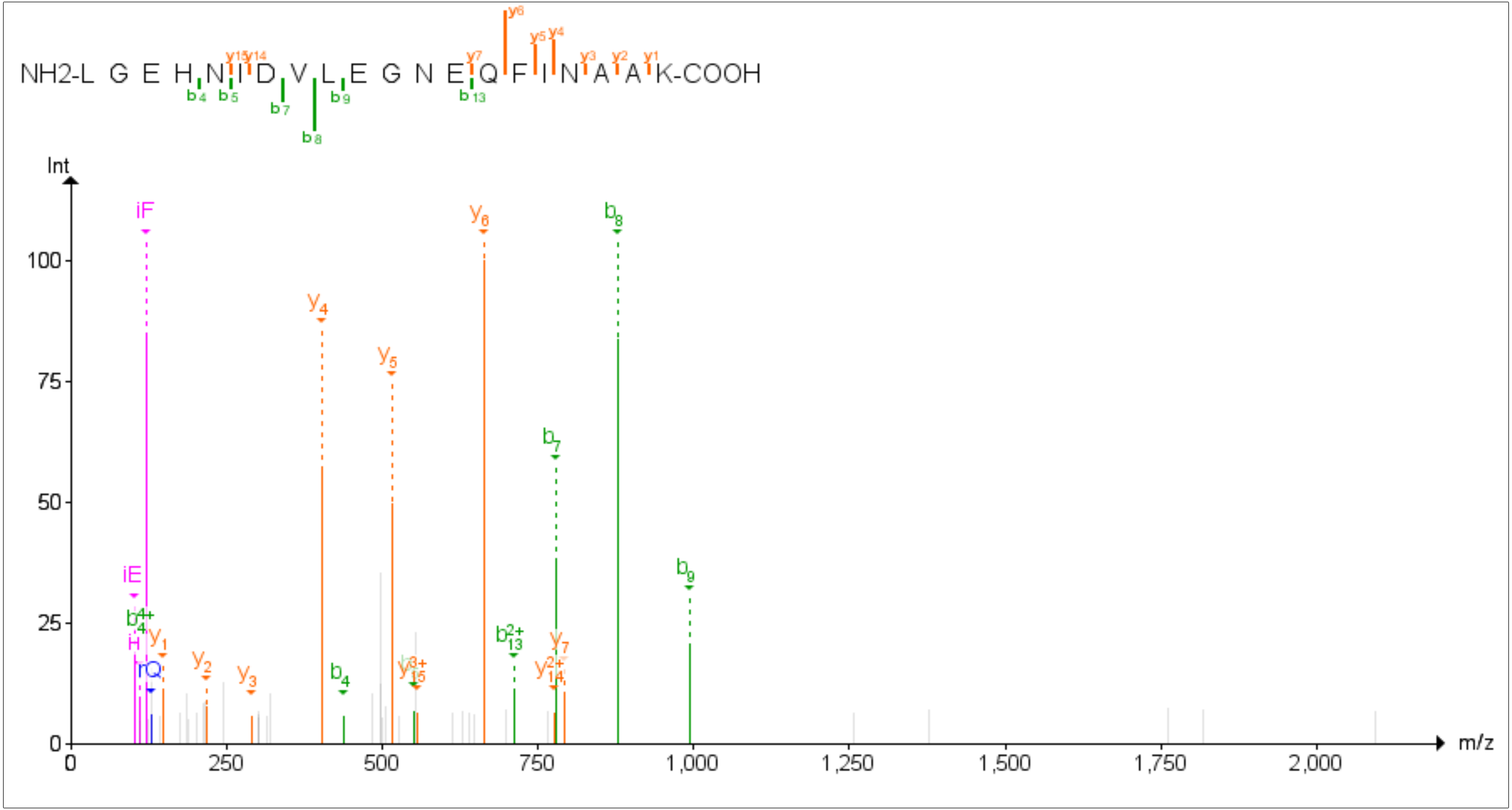
NH2-L G E H N I D V L E G N E Q F I N A A K-COOH
Int
m/z
0
25
50
75
100
250
500
750
1,000
1,250
1,500
1,750
2,000
iF
iE
y6
b8
y4
y5
b7
b9
y1
y2
y3
b4
rQ
y7

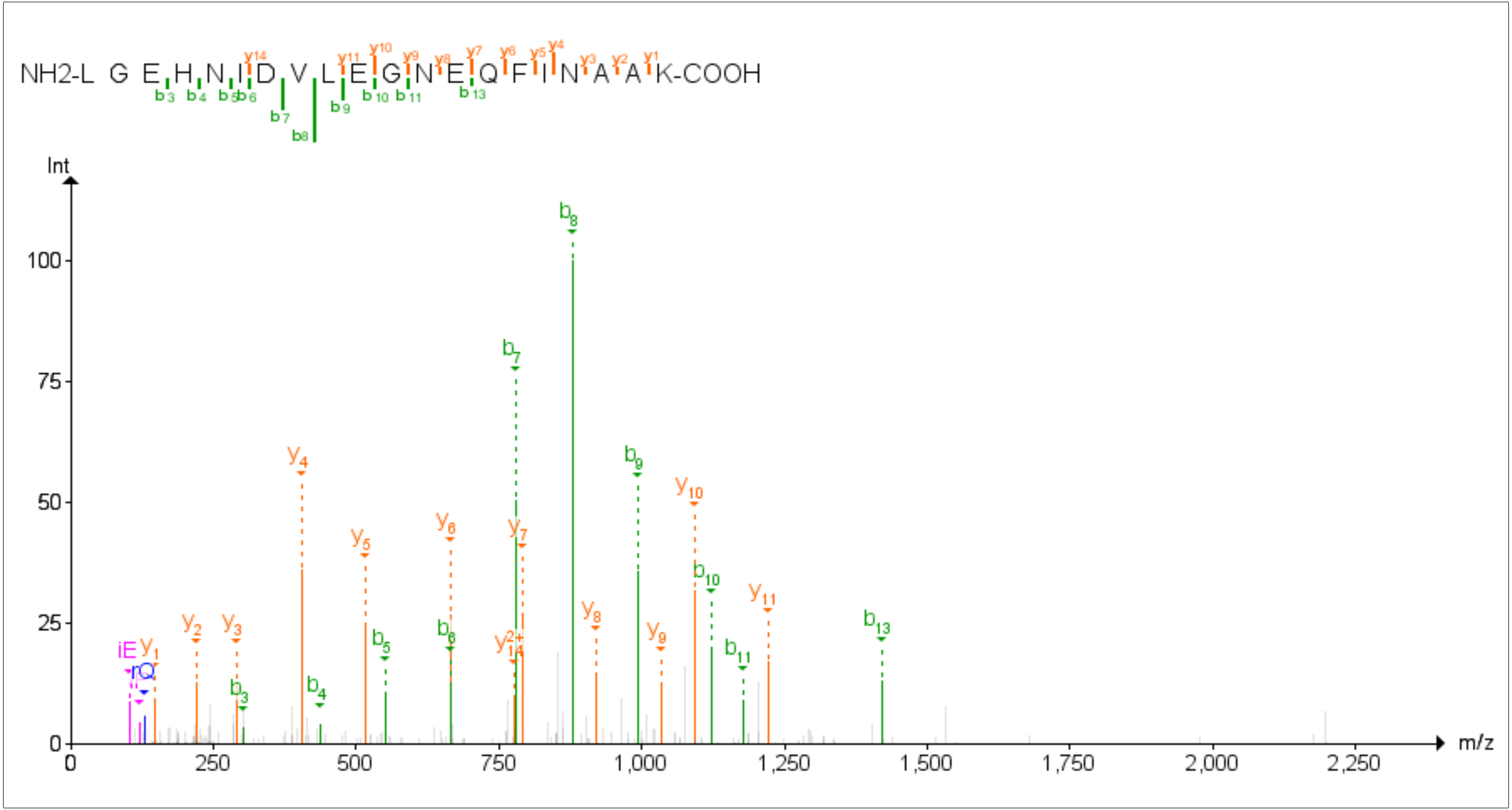

NH2-L G E H N I D V L E G N E Q F I N A A K-COOH
Int
m/z
b8
b7
b9
y10
y4
y5
y6
y7
b10
y11
y8
y9
b13
b11
b5
b6
y2
y3
iE
y1
nQ
b3
b4
0
25
50
75
100
250
500
750
1,000
1,250
1,500
1,750
2,000
2,250

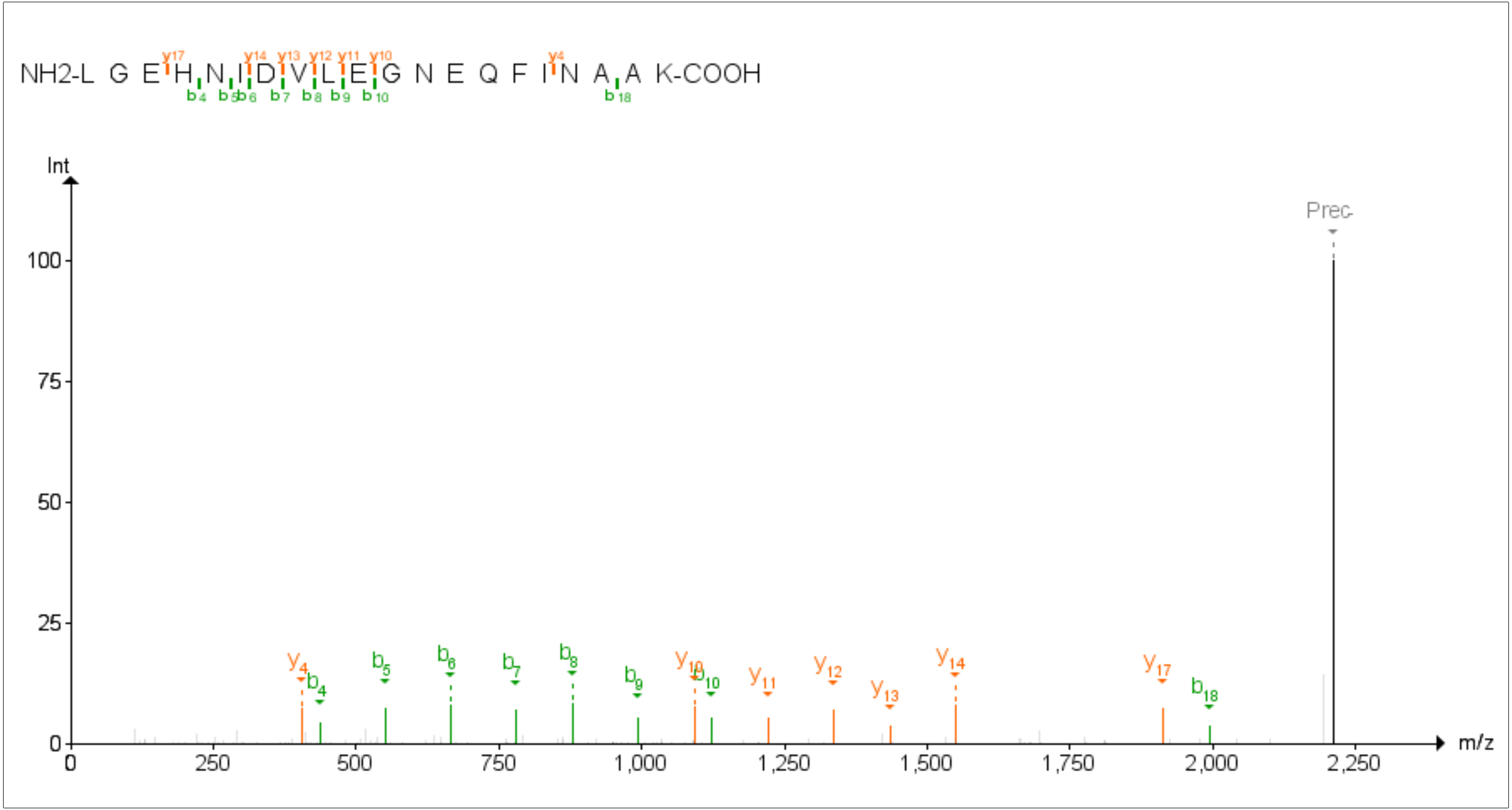

NH2-L G E H N I D V L E G N E Q F I N A A K-COOH
y17 y14 y13 y12 y11 y10 y4
b4 b5 b6 b7 b8 b9 b10 b18
Int
Prec.
100
75
50
25
0
y4 b4 b5 b6 b7 b8 b9 y10 b10 y11 y12 y13 y14 y17 b18
0
250
500
750
1,000
1,250
1,500
1,750
2,000
2,250
m/z

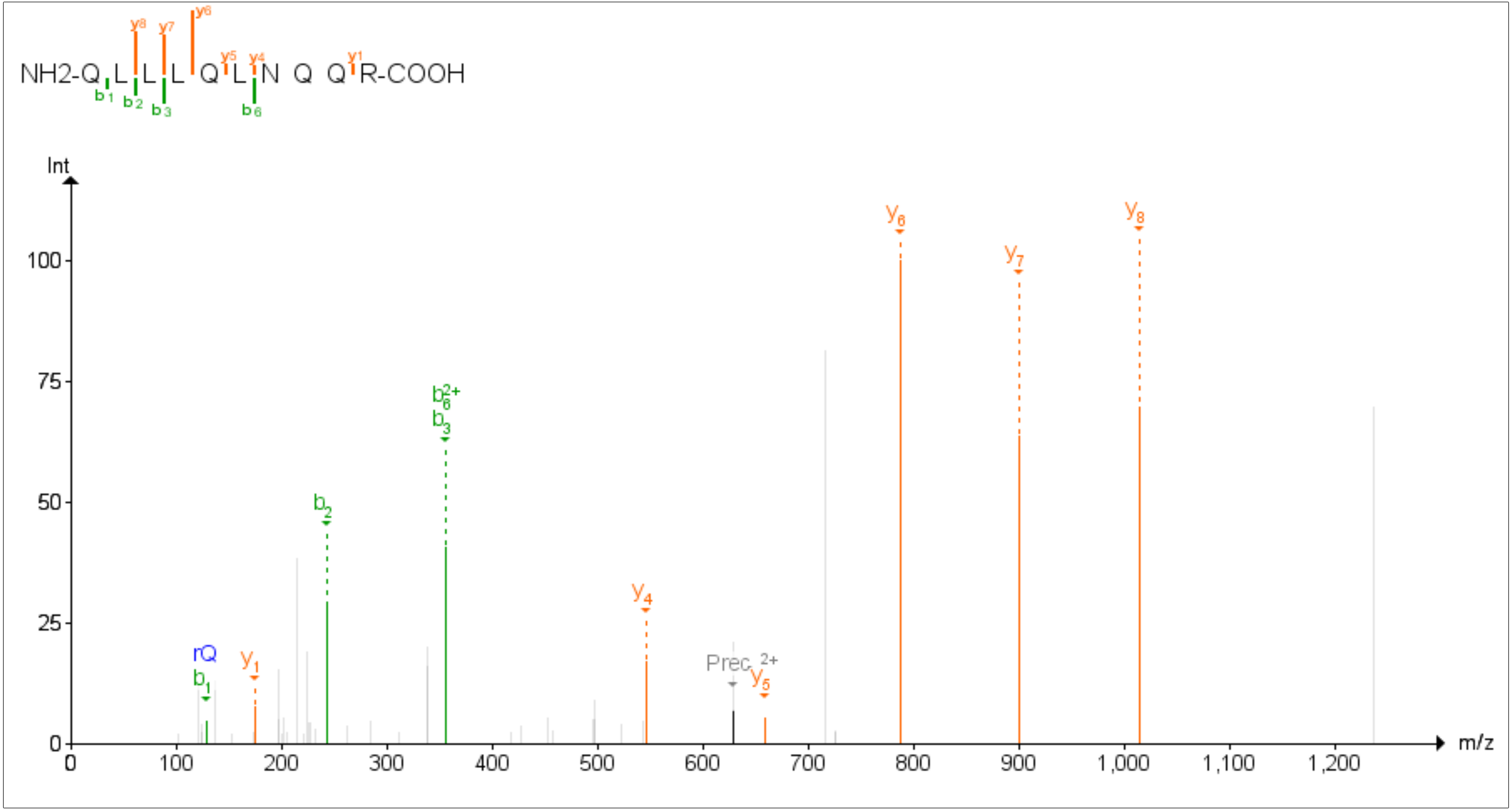
NH2-Q L L L Q L N Q Q R-COOH
y8 y7 y6 y5 y4 y1
b1 b2 b3 b6
Int
m/z
y6
y7
y8
b6 2+
b3
b2
y4
rQ
b1
y1
Prec 2+
y5
0
25
50
75
100
0
100
200
300
400
500
600
700
800
900
1,000
1,100
1,200

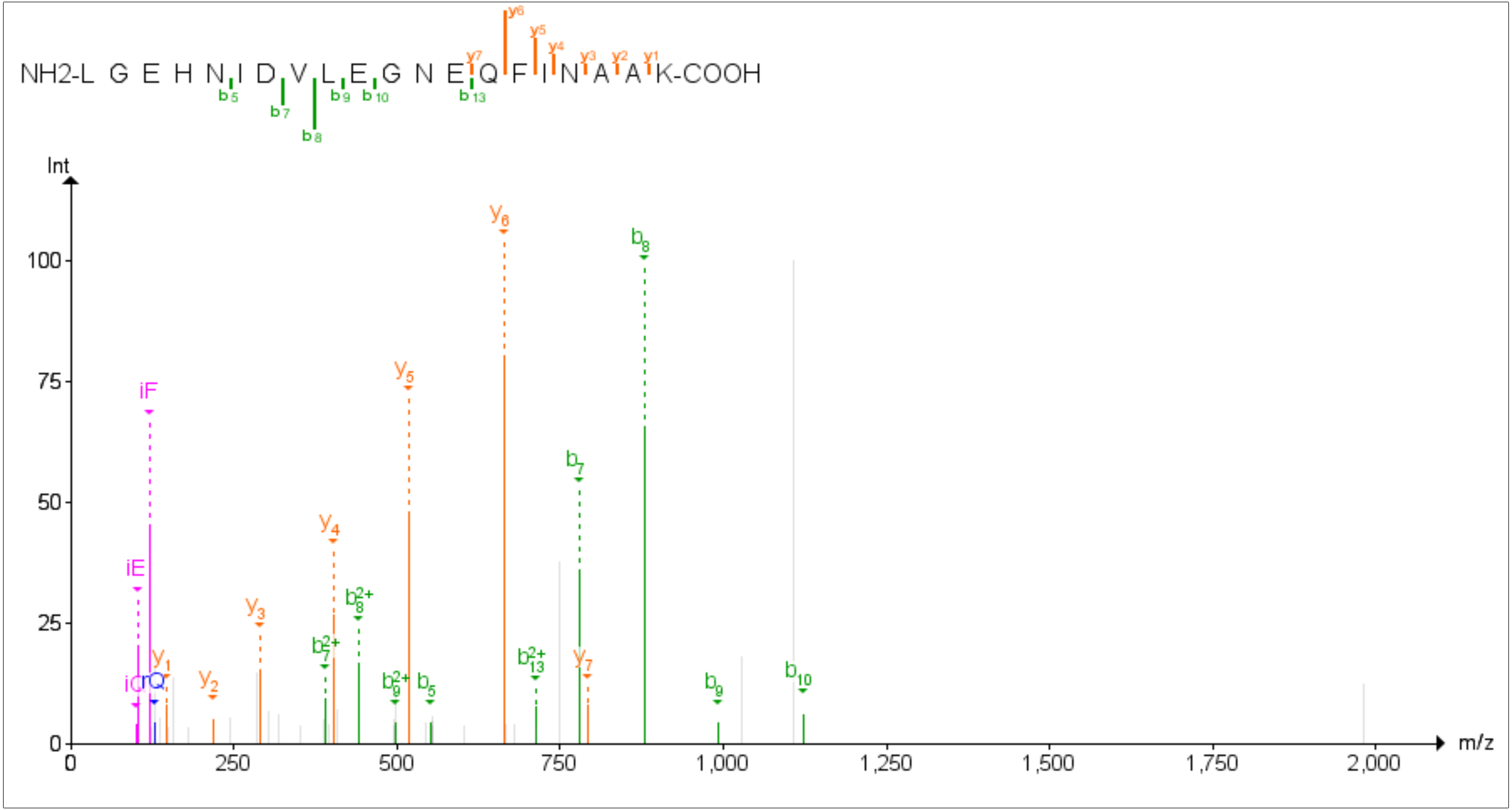

NH2-L G E H N I D V L E G N E Q F I N A A K-COOH
b5 b7 b8 b9 b10 b13
y7 y6 y5 y4 y3 y2 y1
Int
m/z
0
25
50
75
100
250
500
750
1,000
1,250
1,500
1,750
2,000
iF
iE
iQ
rQ
y1 y2 y3 y4 y5 y6 y7
b7 2+ b8 2+ b9 2+ b5 b13 2+ b7 b8 b9 b10

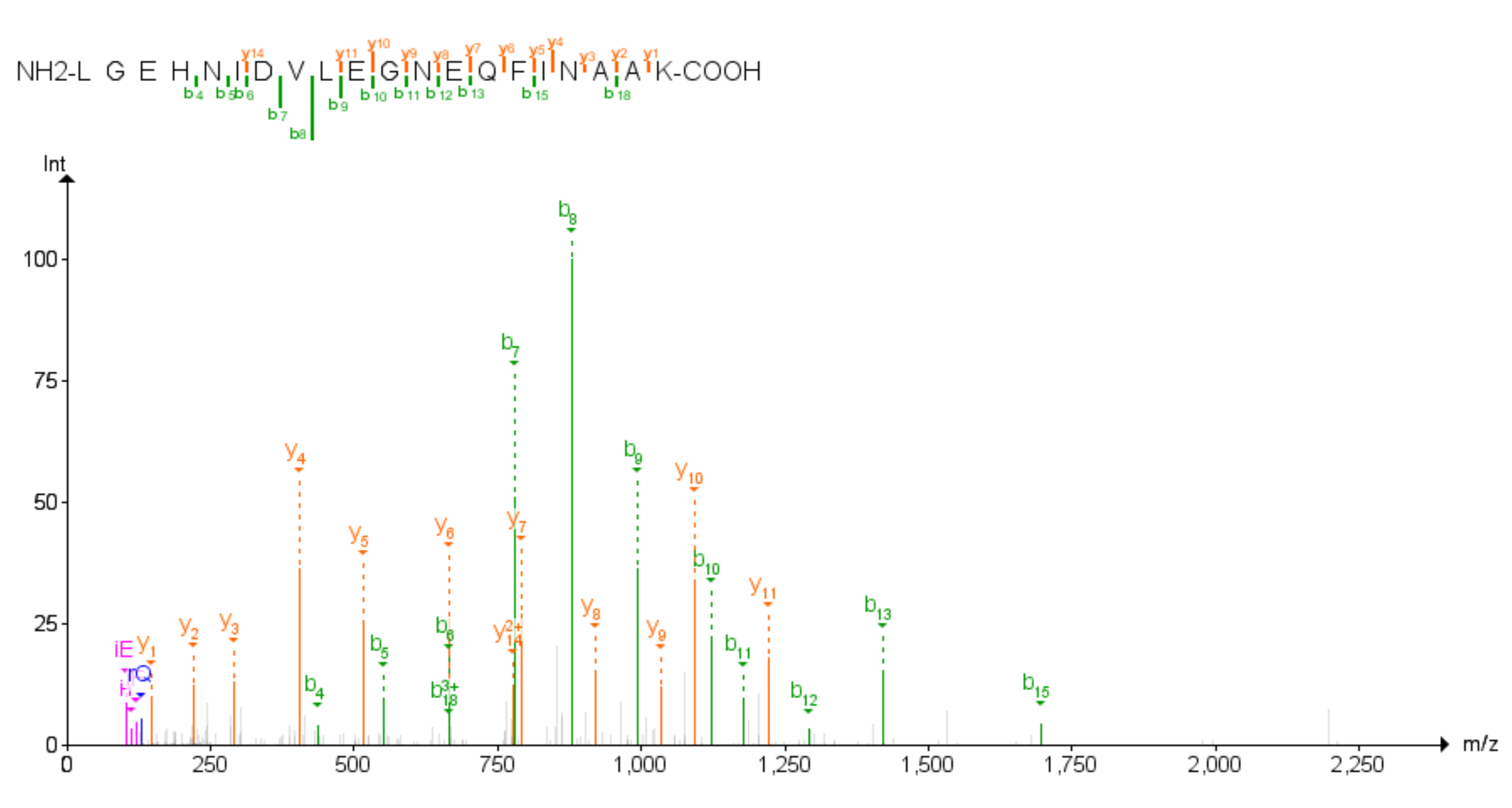

NH2-L G E H N I D V L E G N E Q F I N A A K-COOH
Int
m/z

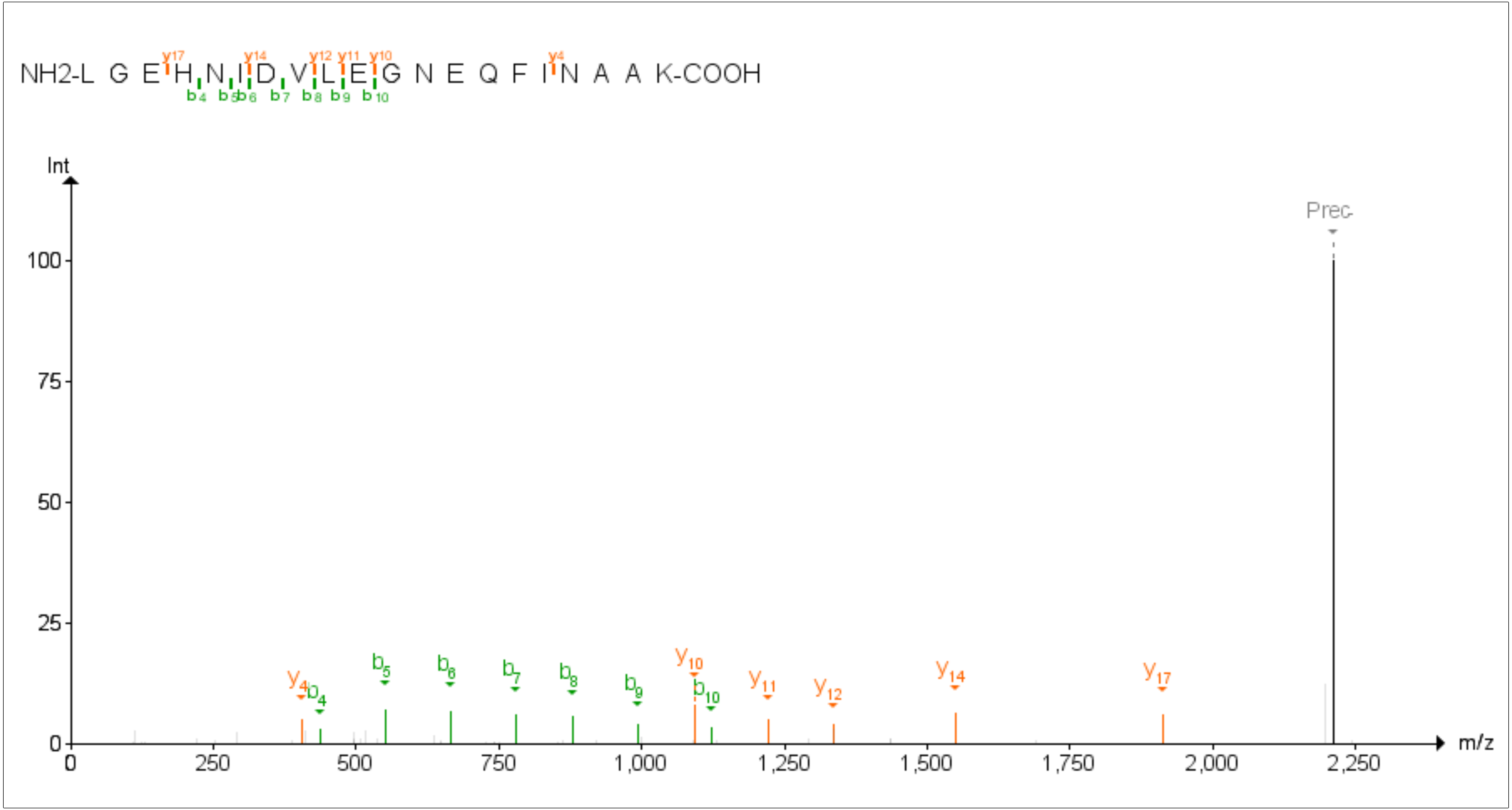
NH2-L G E H N I D V L E G N E Q F I N A A K-COOH
y17 y14 y12 y11 y10 y4
b4 b5 b6 b7 b8 b9 b10
Int
Prec
100
75
50
25
0
m/z
0
250
500
750
1,000
1,250
1,500
1,750
2,000
2,250

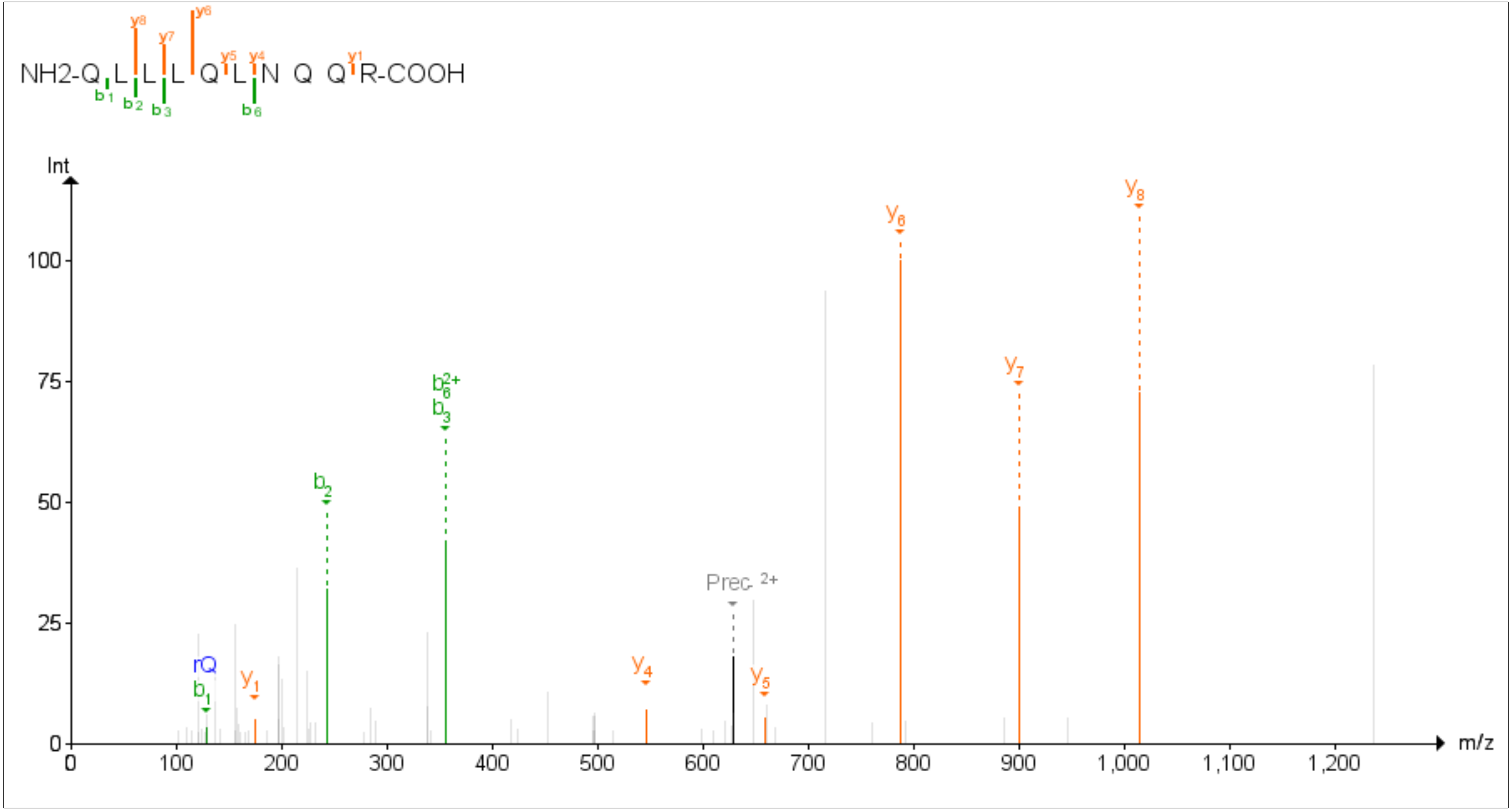

NH2-Q L L L Q L N Q Q R-COOH
b1 b2 b3 b6
y8 y7 y6 y5 y4 y1
Int
100
75
50
25
0
m/z
0 100 200 300 400 500 600 700 800 900 1,000 1,100 1,200
rQ
b1
y1
b2
b6 2+
b3
Prec 2+
y4
y5
y6
y7
y8

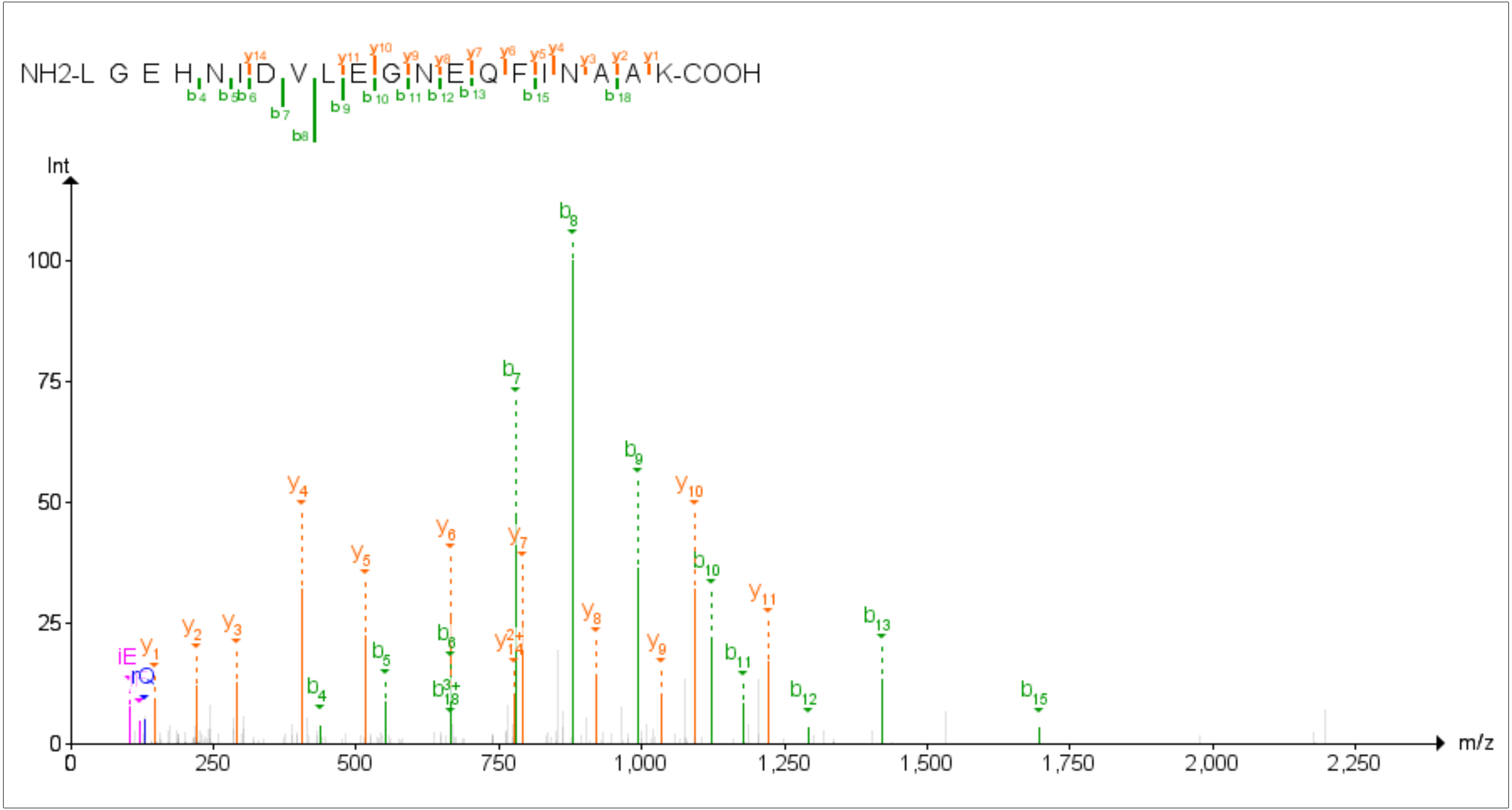
NH2-L G E H N I D V L E G N E Q F I N A A K-COOH
Int
m/z
0
25
50
75
100
250
500
750
1,000
1,250
1,500
1,750
2,000
2,250
iE
rQ

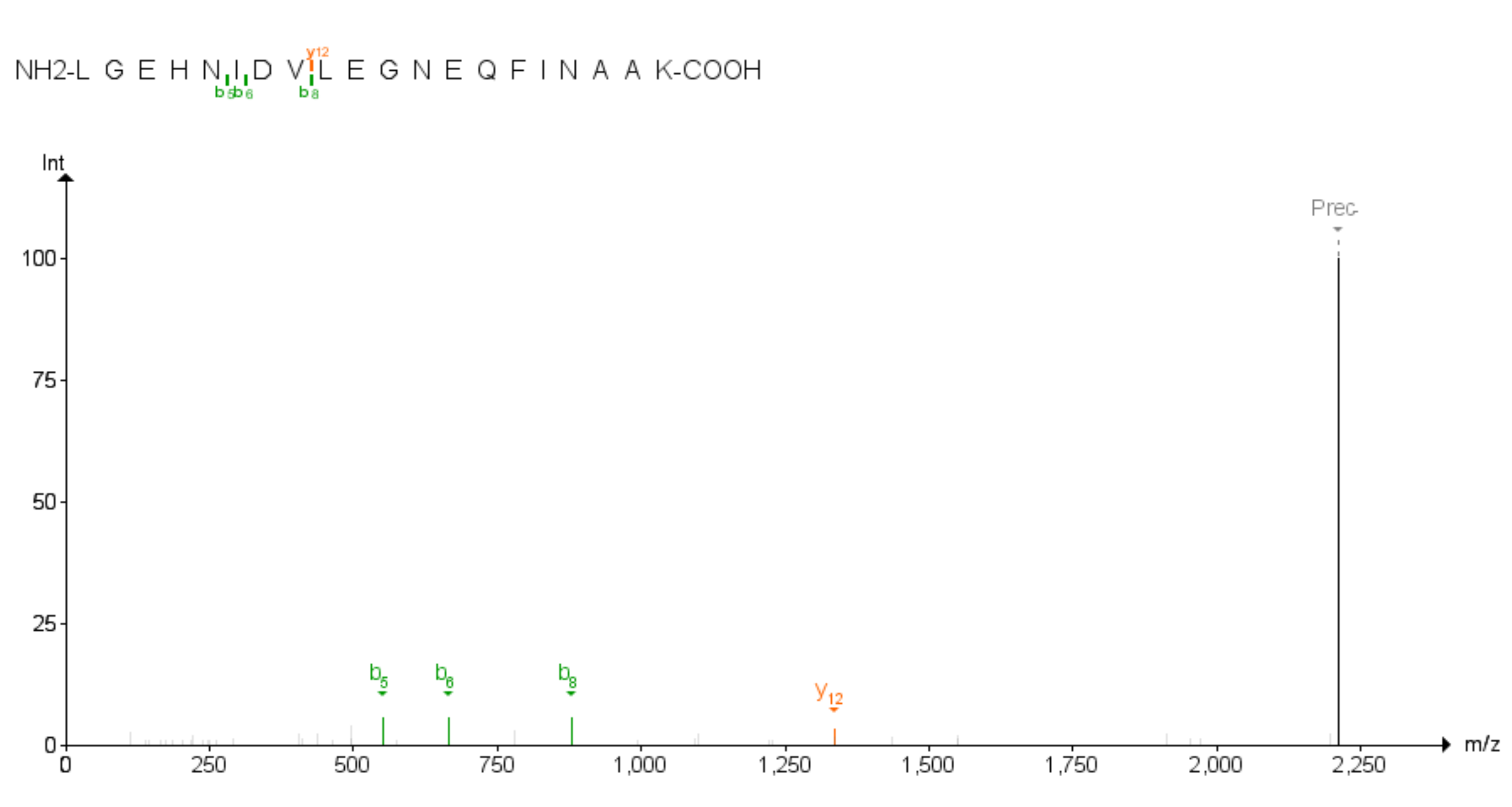
NH2-L G E H N I D V I L E G N E Q F I N A A K-COOH
y12
b5 b6 b8
Int
Prec.
100
75
50
25
0
b5
b6
b8
y12
0
250
500
750
1,000
1,250
1,500
1,750
2,000
2,250
m/z

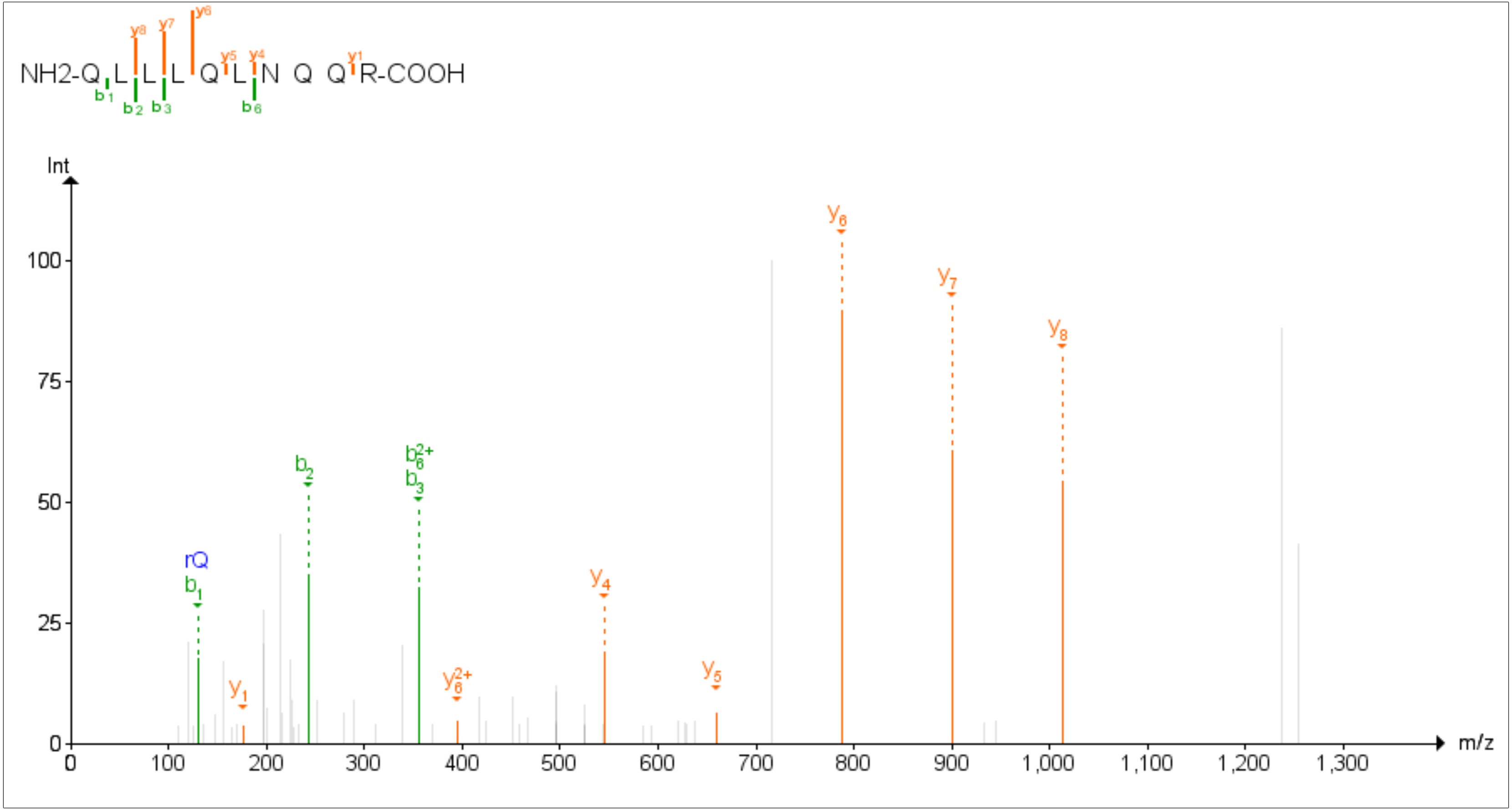

NH2-Q L L L L Q L N Q Q R-COOH
y8 y7 y6 y5 y4 y1
b1 b2 b3 b6
Int
100
75
50
25
0
rQ
b1
y1
b2
b6 2+
b3
y6 2+
y4
y5
y6
y7
y8
0 100 200 300 400 500 600 700 800 900 1,000 1,100 1,200 1,300
m/z

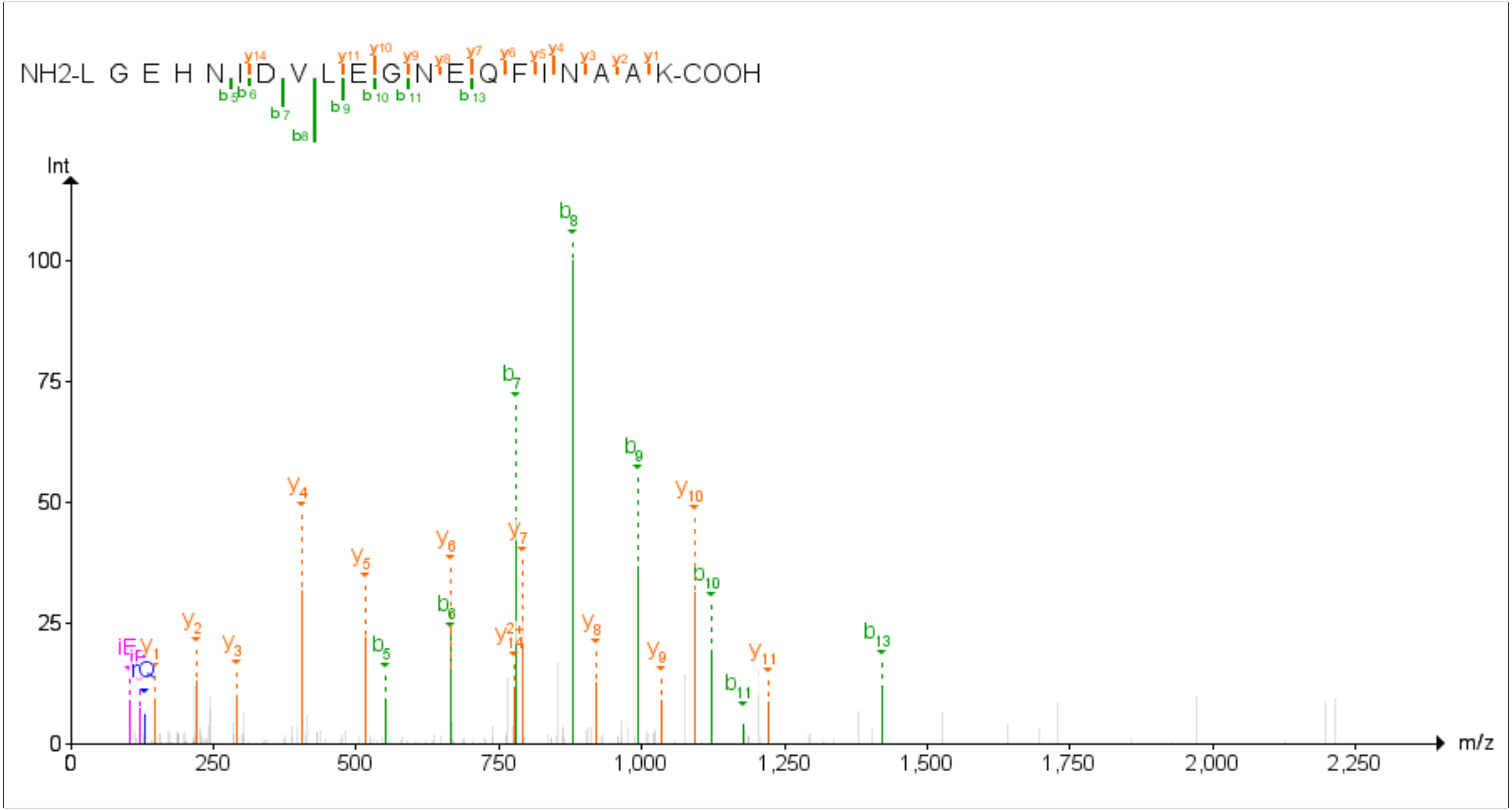
NH2-L G E H N I D V L E G N E Q F I N A A K-COOH
Int
m/z
0
25
50
75
100
250
500
750
1,000
1,250
1,500
1,750
2,000
2,250

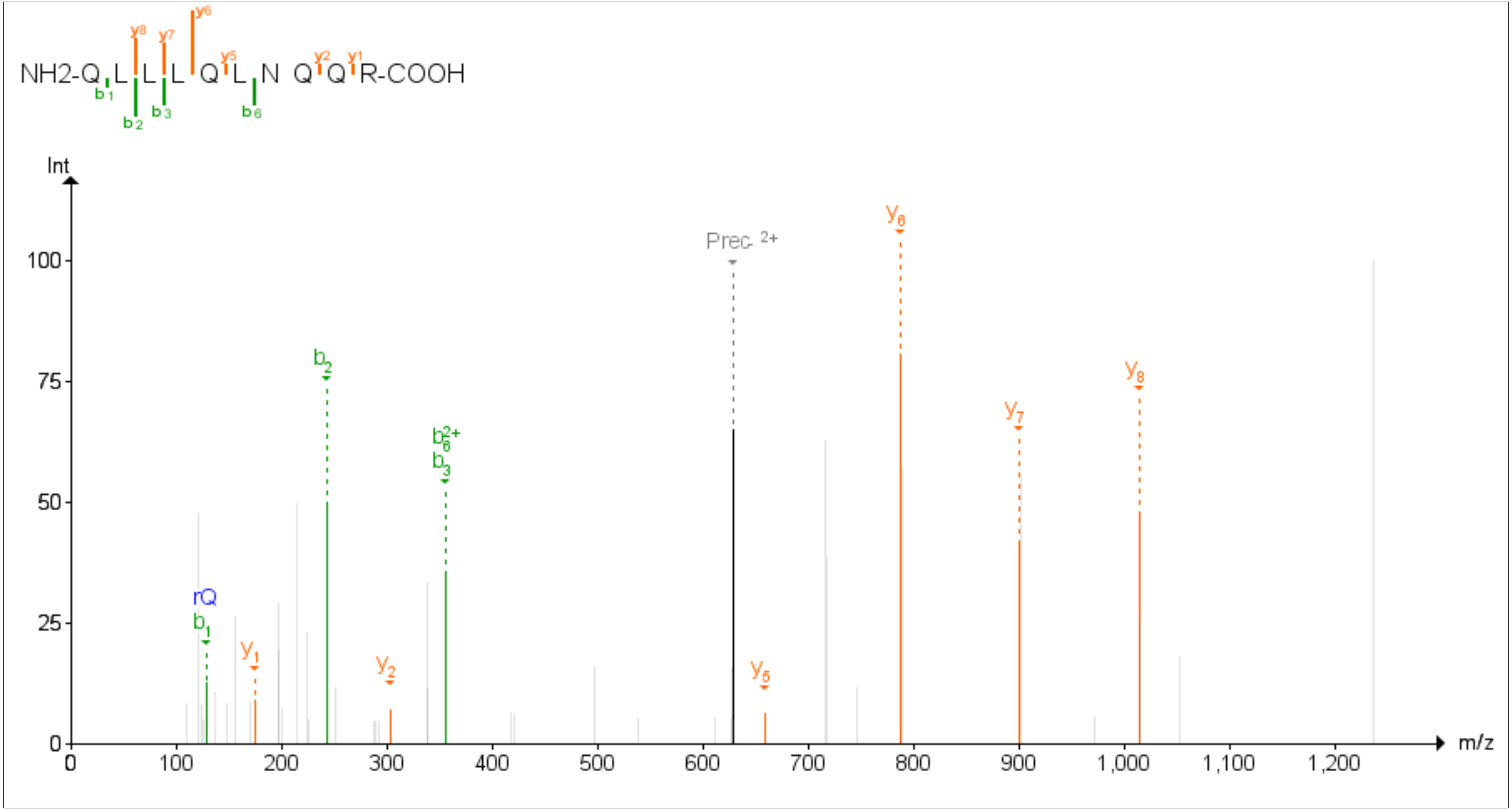

NH2-Q L L L Q L N Q Q R-COOH
y8 y7 y6 y5 y2 y1
b1 b2 b3 b6
Int
m/z
Prec 2+
y6
b2
b6 2+
b3
y8
y7
rQ
b1
y1
y2
y5
0
25
50
75
100
0
100
200
300
400
500
600
700
800
900
1,000
1,100
1,200

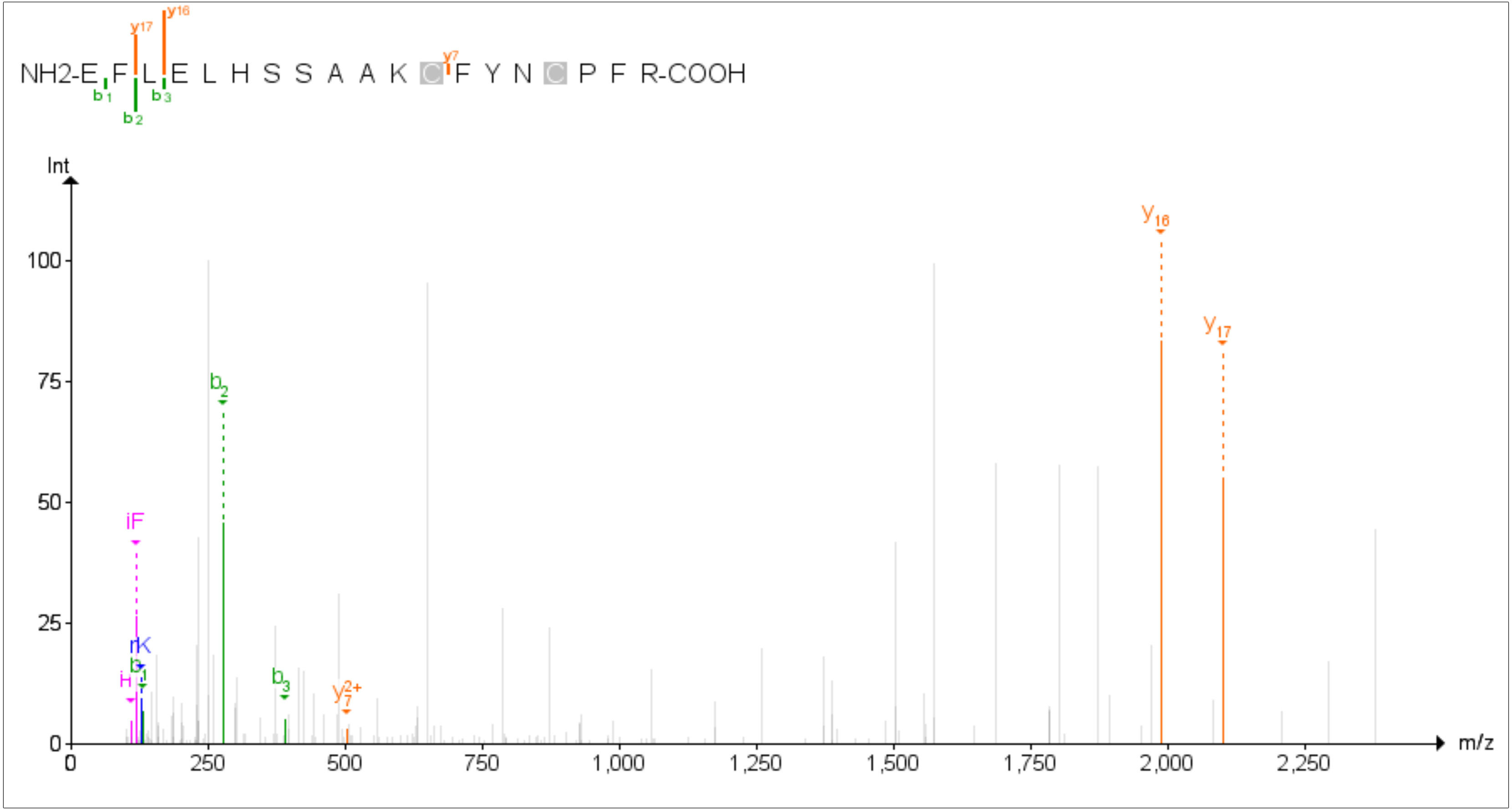
NH2-E F L E L H S S A A K C F Y N C P F R-COOH
b1
b2
b3
y17
y16
y7
Int
100
75
50
25
0
0
250
500
750
1,000
1,250
1,500
1,750
2,000
2,250
m/z
y16
y17
b2
iF
rK
b1
iH
b3
y7 2+

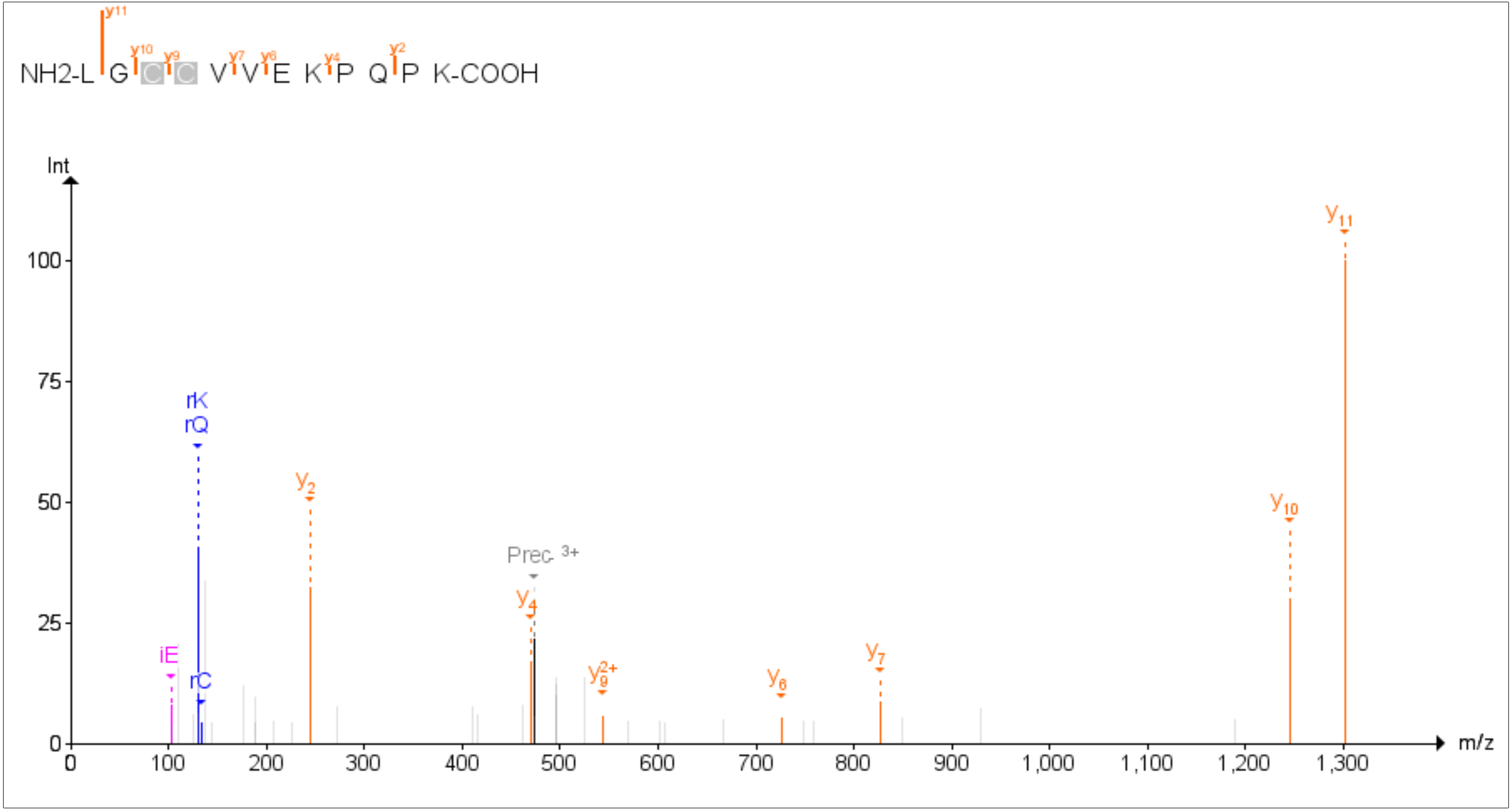

NH2-L G C C V V E K P Q P K-COOH
y11
y10
y9
y7
y6
y4
y2
Int
m/z
rK
rQ
rC
iE
Prec- 3+
y2
y4
y9 2+
y6
y7
y10
y11

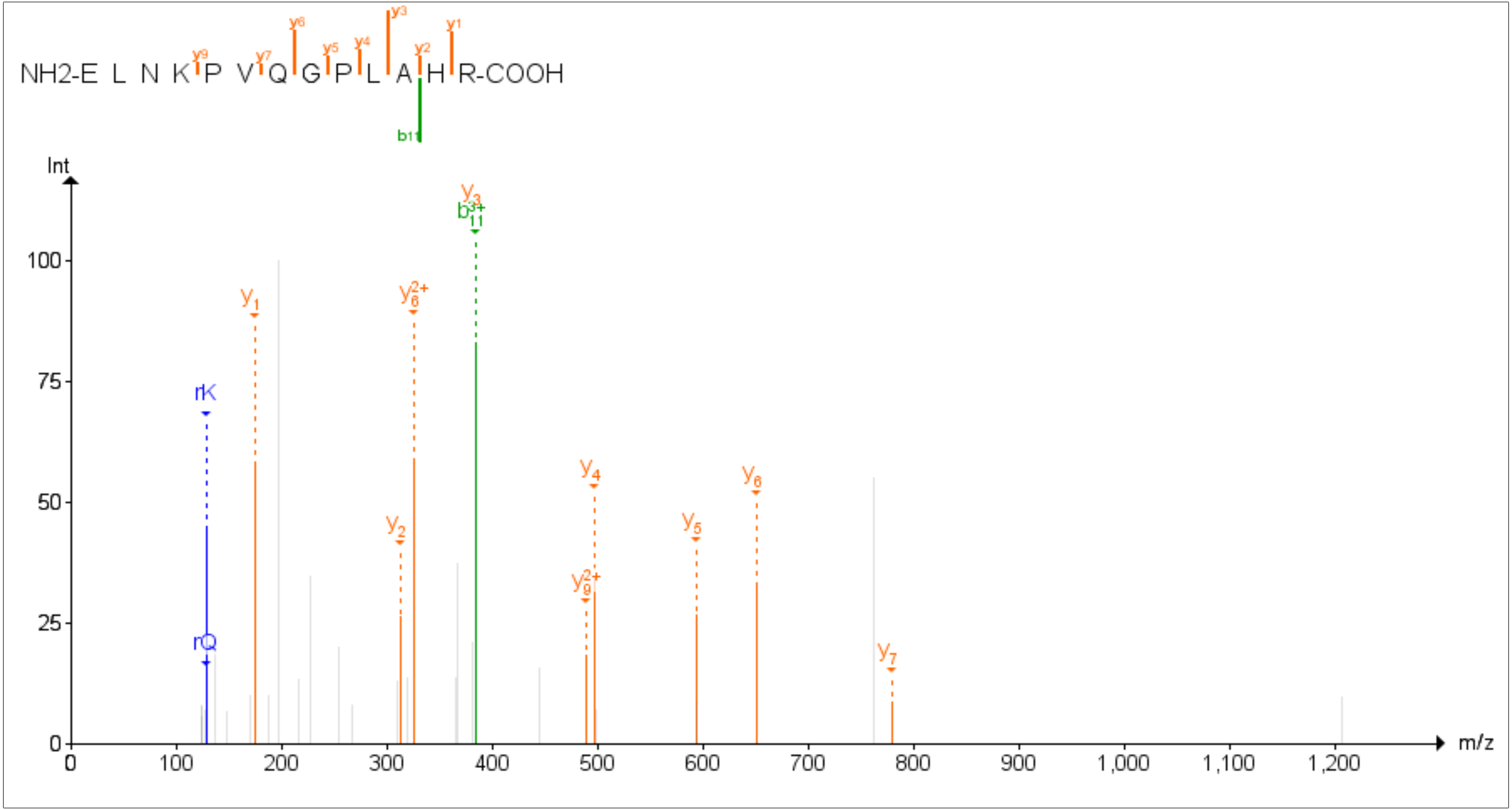

NH2-E L N K P V Q G P L A H R-COOH
y9
y7
y6
y5
y4
y3
y2
y1
b11
Int
100
75
50
25
0
rK
rQ
y1
y6 2+
y3
b11 3+
y2
y4
y9 2+
y5
y6
y7
0
100
200
300
400
500
600
700
800
900
1,000
1,100
1,200
m/z

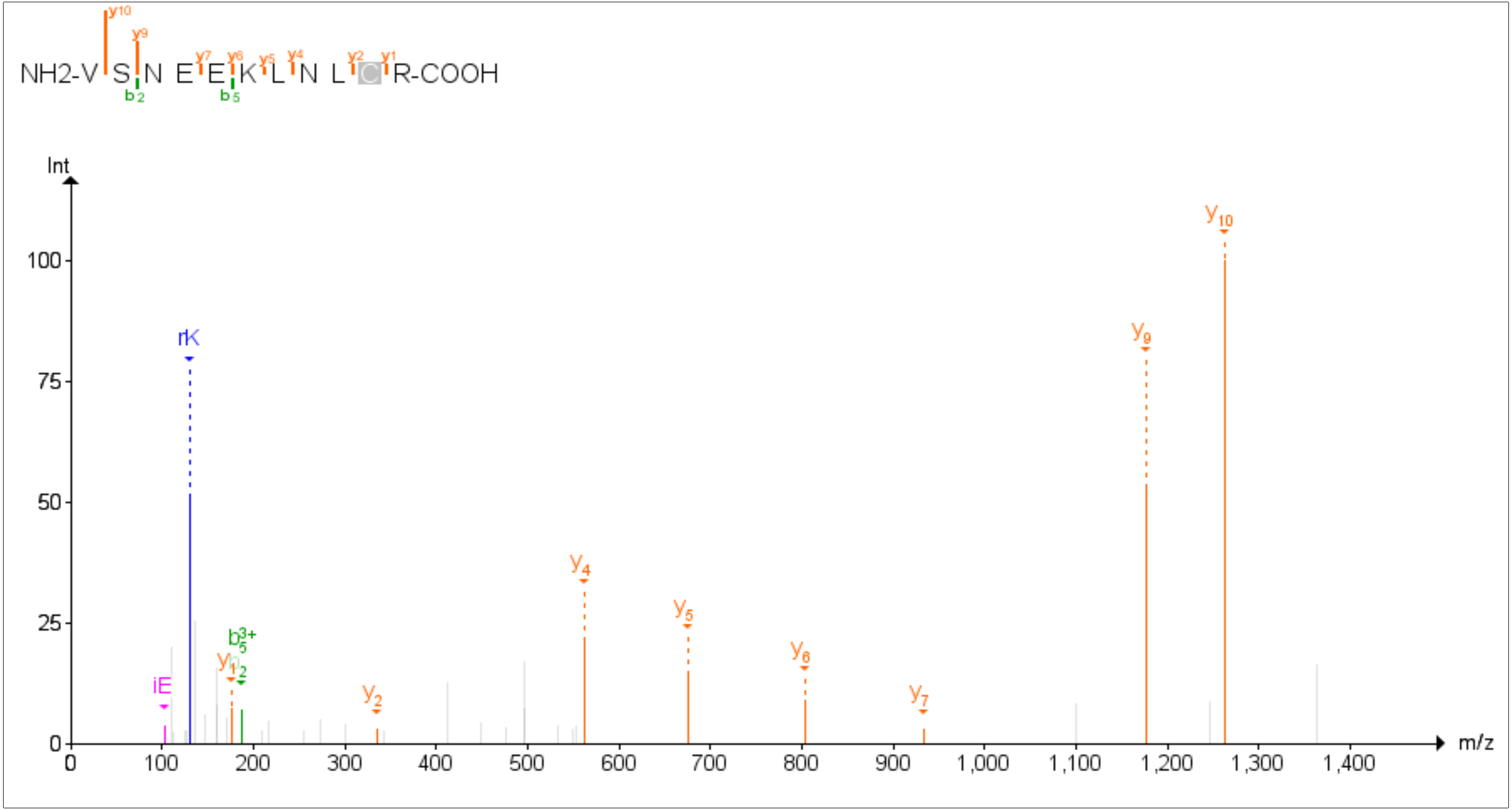

NH2-V S N E E K L N L C R-COOH
y10
y9
y7
y6
y5
y4
y2
y1
b2
b5
Int
rK
iE
y1
b2
b5 3+
y2
y4
y5
y6
y7
y9
y10
m/z
0
25
50
75
100
0
100
200
300
400
500
600
700
800
900
1,000
1,100
1,200
1,300
1,400

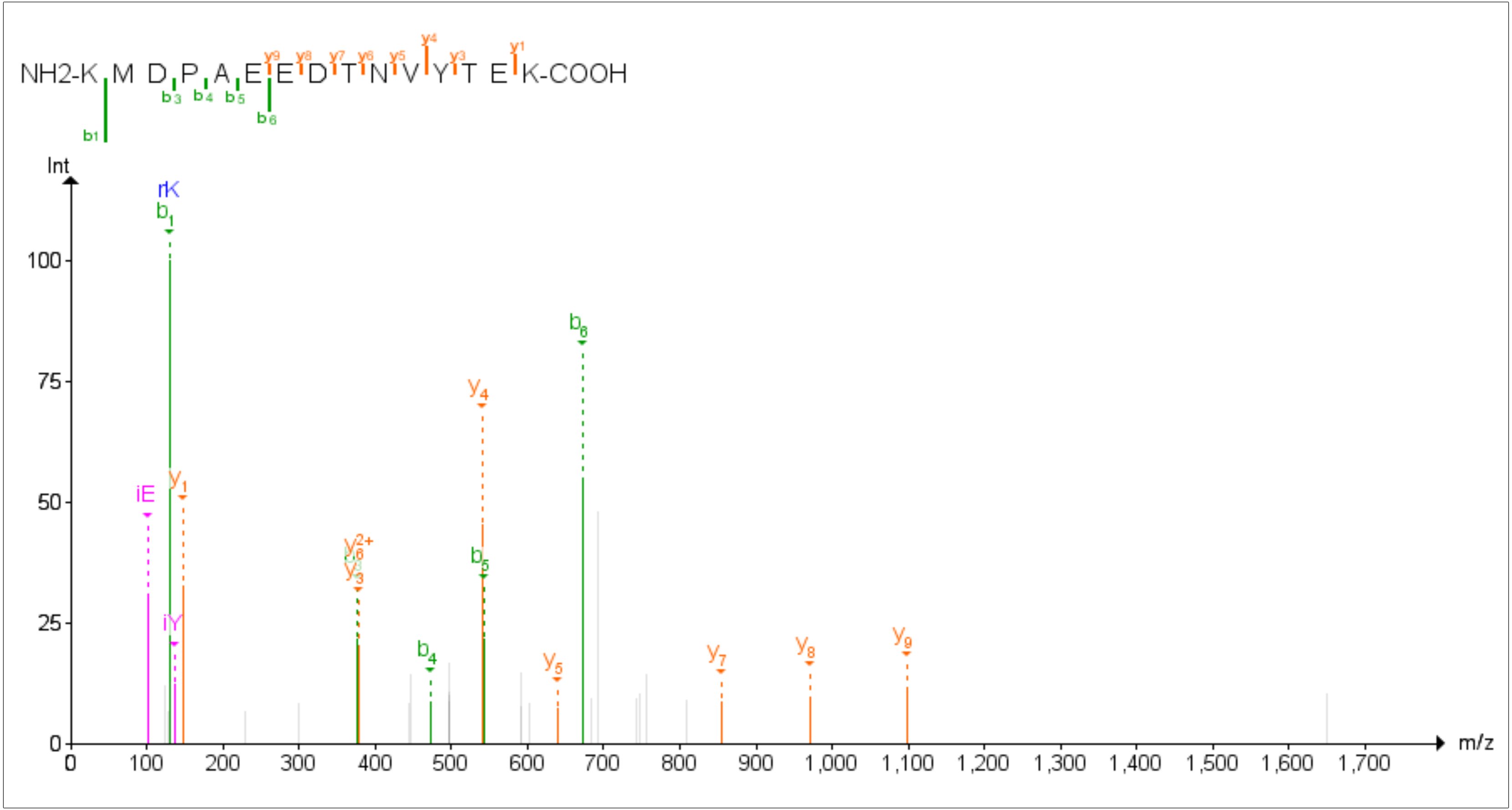

NH2-K M D P A E E D T N V Y T E K-COOH
b1
b3
b4
b5
b6
y9
y8
y7
y6
y5
y4
y3
y1
Int
rK
b1
iE
y1
iY
y6 2+
y3
b4
b5
y4
b6
y5
y7
y8
y9
100
75
50
25
0
0
100
200
300
400
500
600
700
800
900
1,000
1,100
1,200
1,300
1,400
1,500
1,600
1,700
m/z

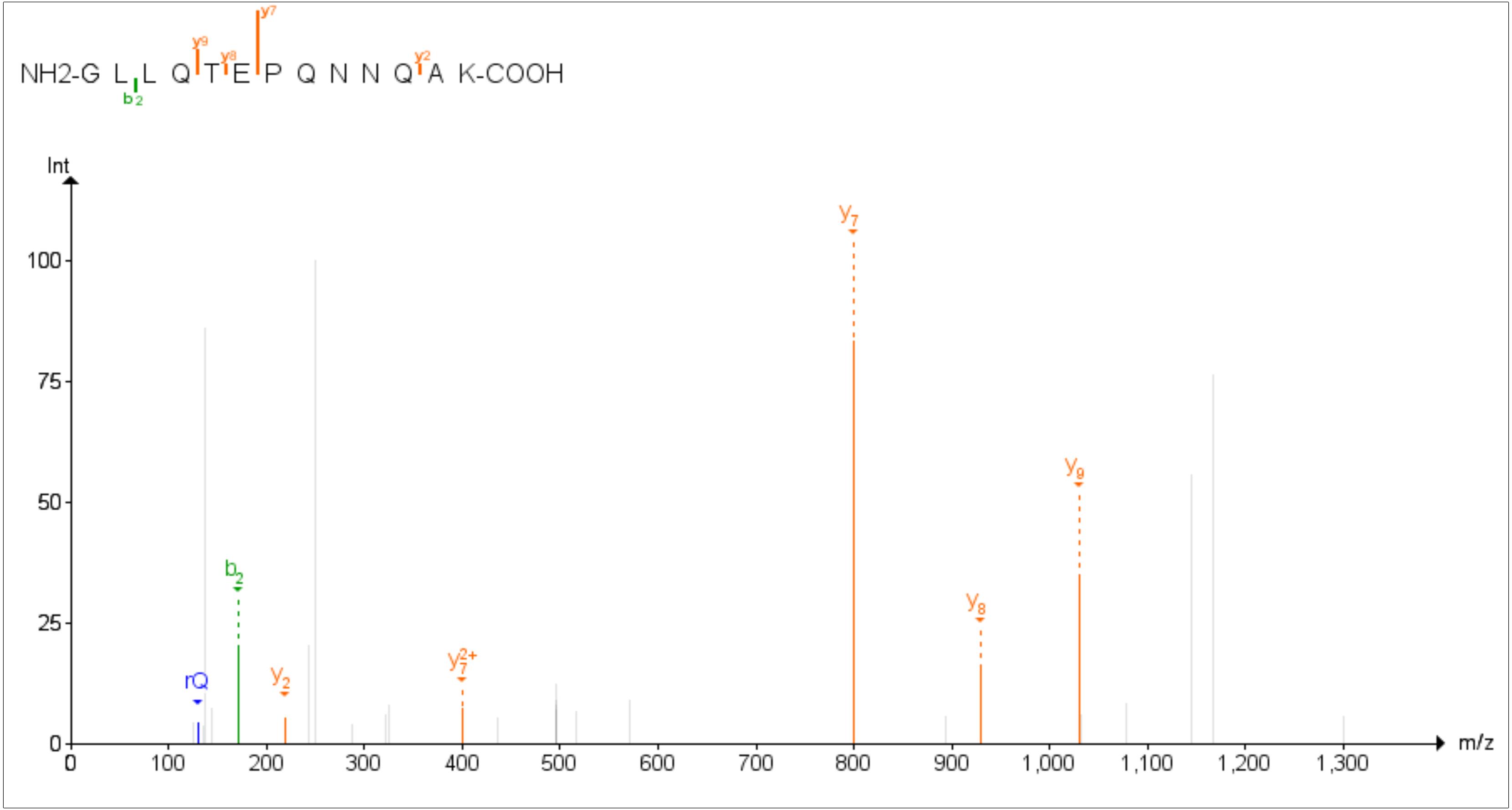
NH2-G L L Q T E P Q N N Q A K-COOH
b2
y9
y8
y7
y2
Int
m/z
y7
y9
y8
b2
rQ
y2
y7 2+
0
25
50
75
100
0
100
200
300
400
500
600
700
800
900
1,000
1,100
1,200
1,300

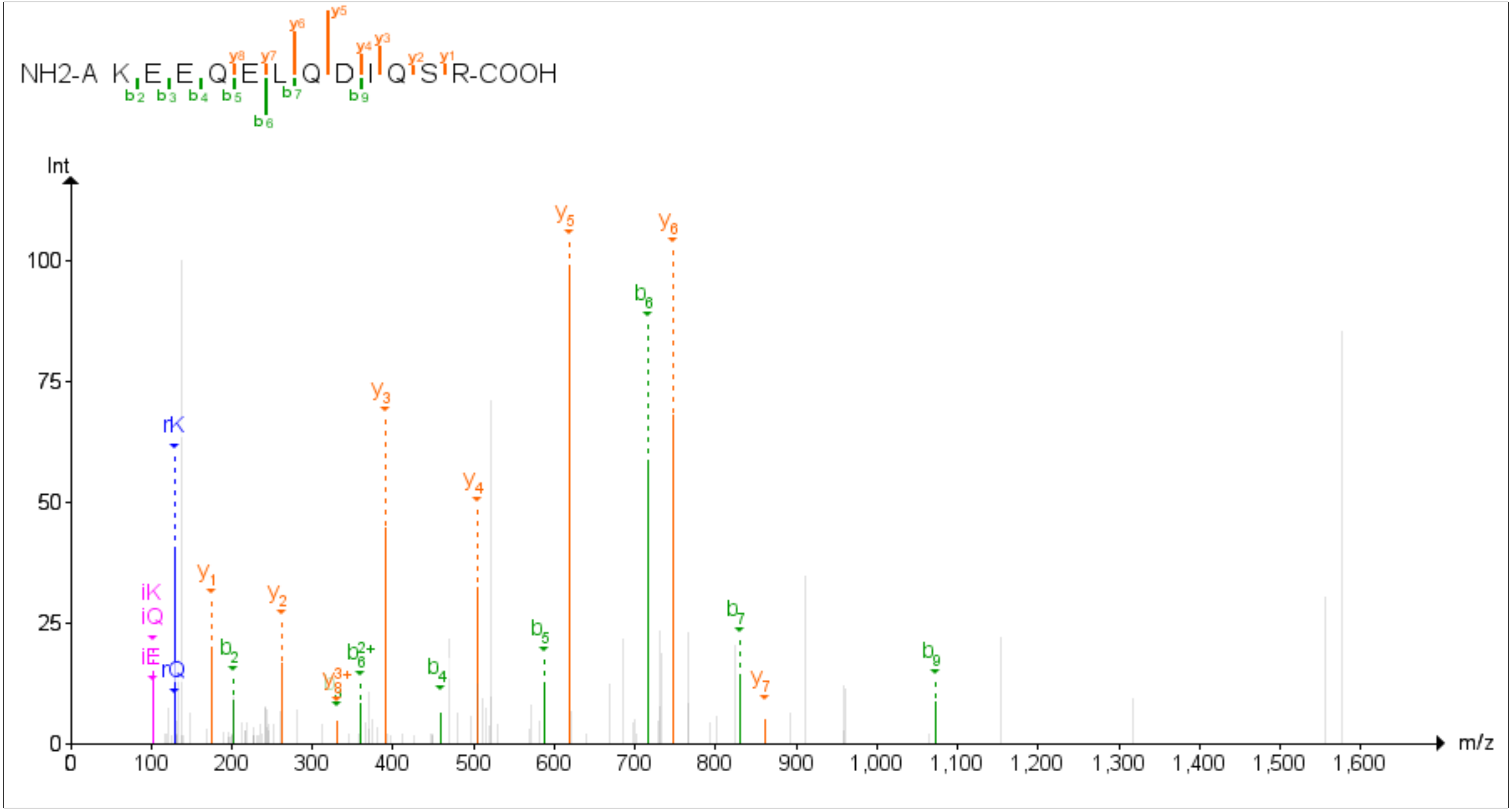
NH2-A K E E Q E L Q D I Q S R-COOH
b2 b3 b4 b5 b6 b7 b9
y8 y7 y6 y5 y4 y3 y2 y1
Int
100
75
50
25
0
m/z
0 100 200 300 400 500 600 700 800 900 1,000 1,100 1,200 1,300 1,400 1,500 1,600
y5 y6 b6 y3 rK y4 y1 y2 iK iQ iE rQ b2 y8 3+ b6 2+ b4 b5 b7 y7 b9

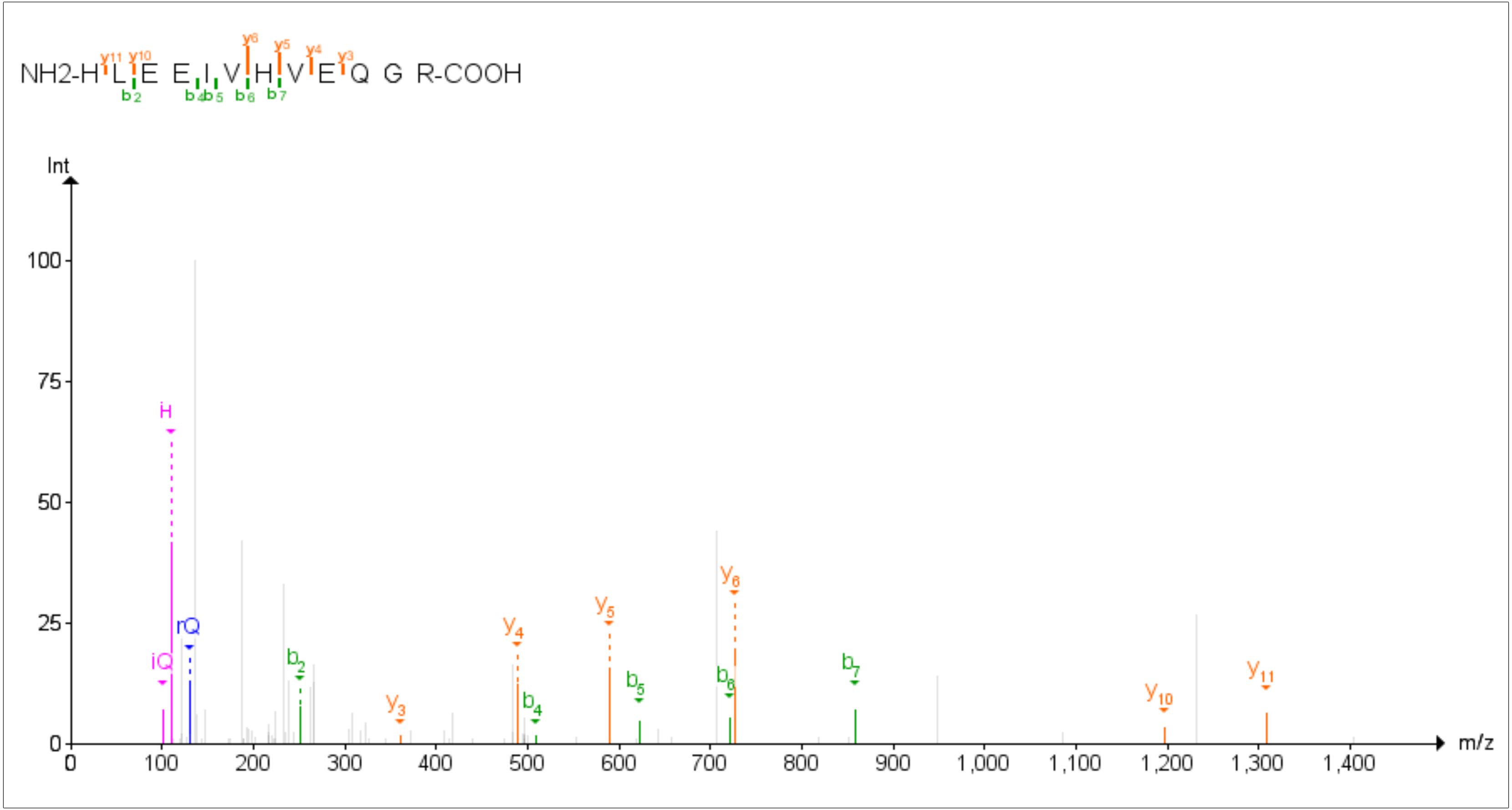

NH2-H L E E I V H V E Q G R-COOH
y11 y10 y6 y5 y4 y3
b2 b4 b5 b6 b7
Int
100
75
50
25
0
iH
rQ
iQ
b2
y3
y4
b4
y5
b5
y6
b6
b7
y10
y11
0 100 200 300 400 500 600 700 800 900 1,000 1,100 1,200 1,300 1,400
m/z

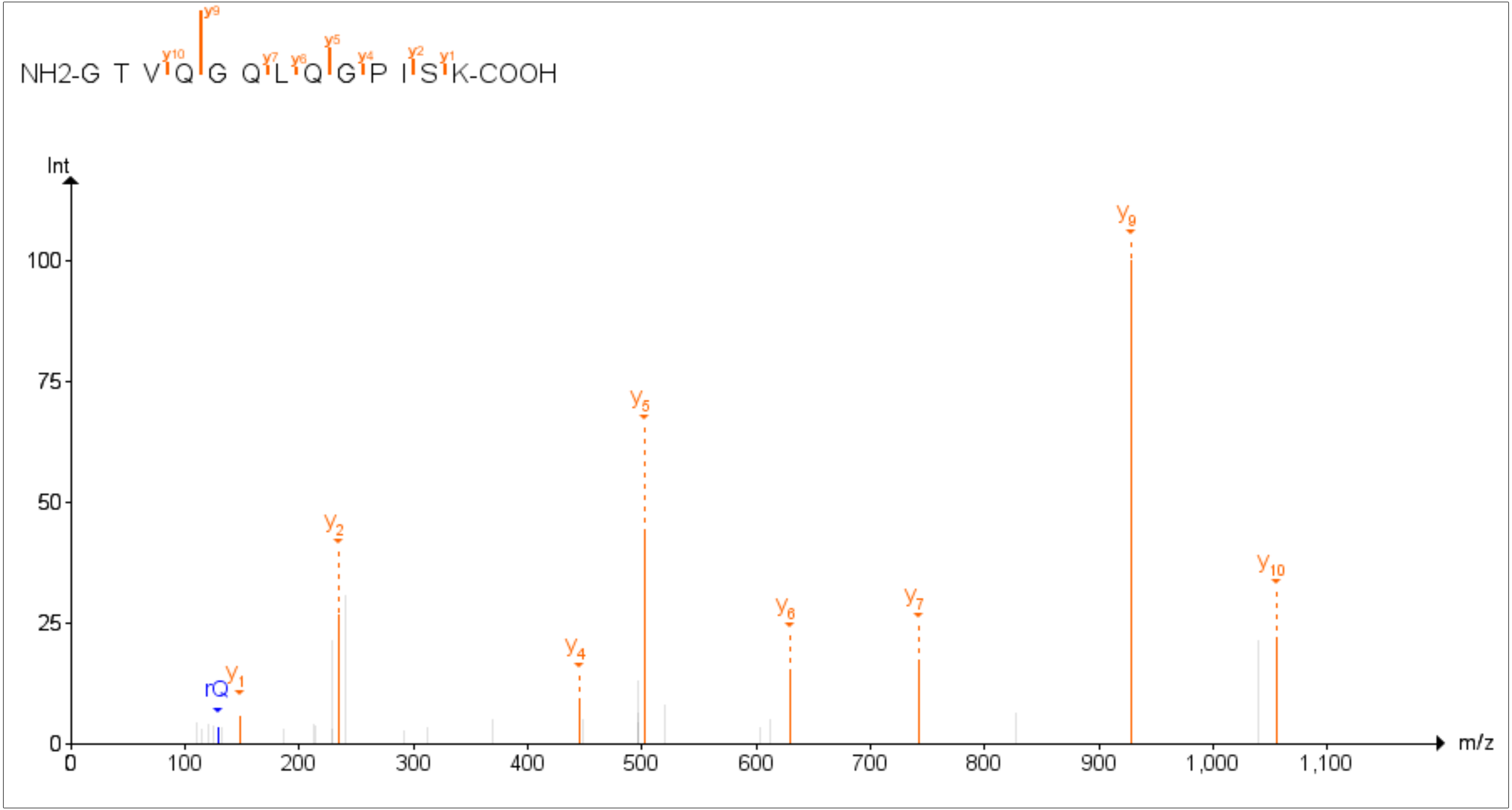

NH2-G T V Q G Q L Q G P I S K-COOH
y9
y10
y7
y6
y5
y4
y2
y1
Int
100
75
50
25
0
y9
y5
y2
y10
y6
y7
y4
y1
rQ
m/z
0
100
200
300
400
500
600
700
800
900
1,000
1,100

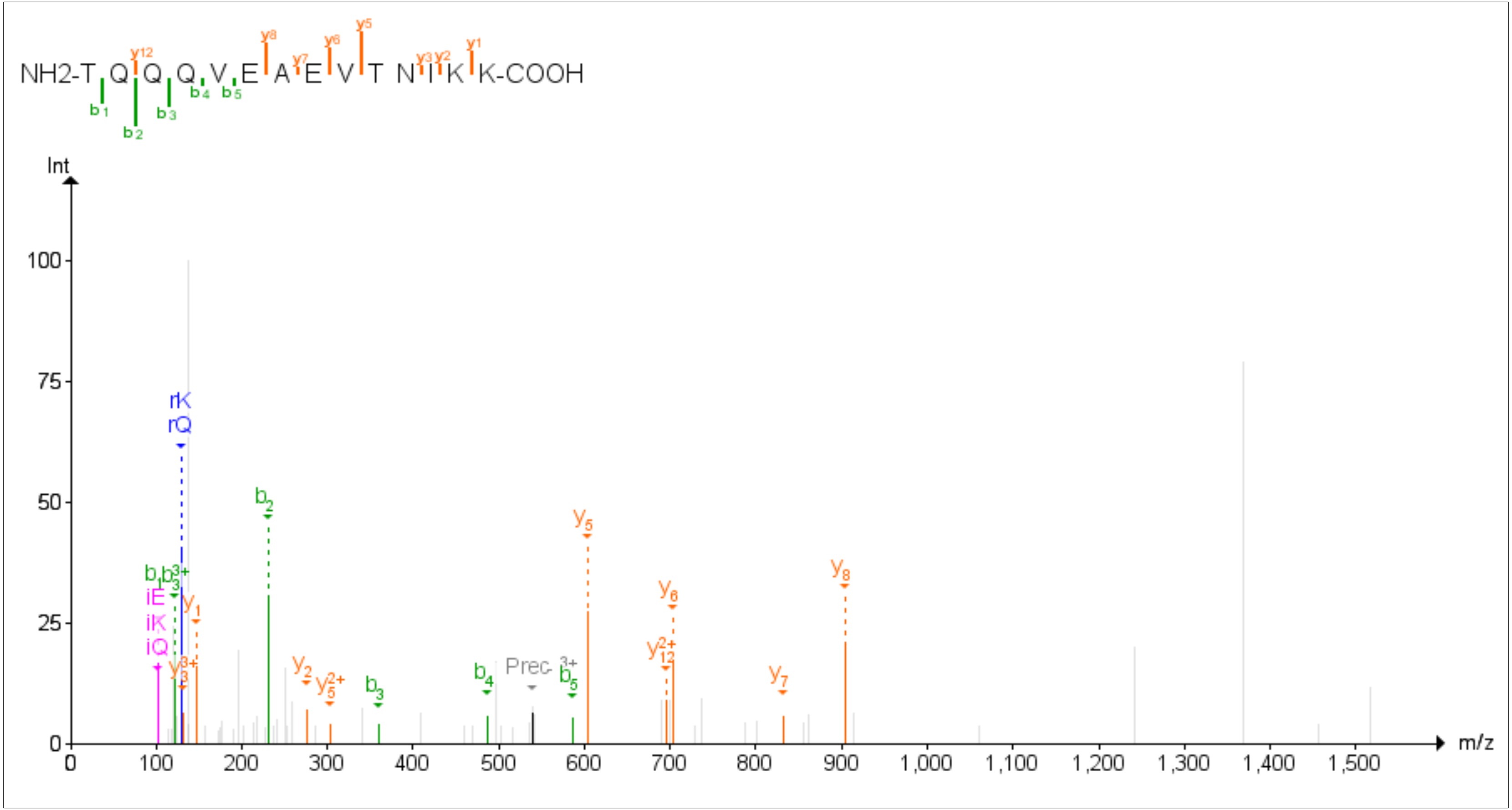

NH2-T Q Q Q V E A E V T N I K K-COOH
y12 y8 y7 y6 y5 y3 y2 y1
b1 b2 b3 b4 b5
Int
100
75
50
25
0
rK
rQ
b2
y5
b1
b3 3+
iE
iK
iQ
y1
y3 3+
y2
y5 2+
b3
b4
Prec 3+
b5
y6
y12 2+
y7
y8
0 100 200 300 400 500 600 700 800 900 1,000 1,100 1,200 1,300 1,400 1,500
m/z

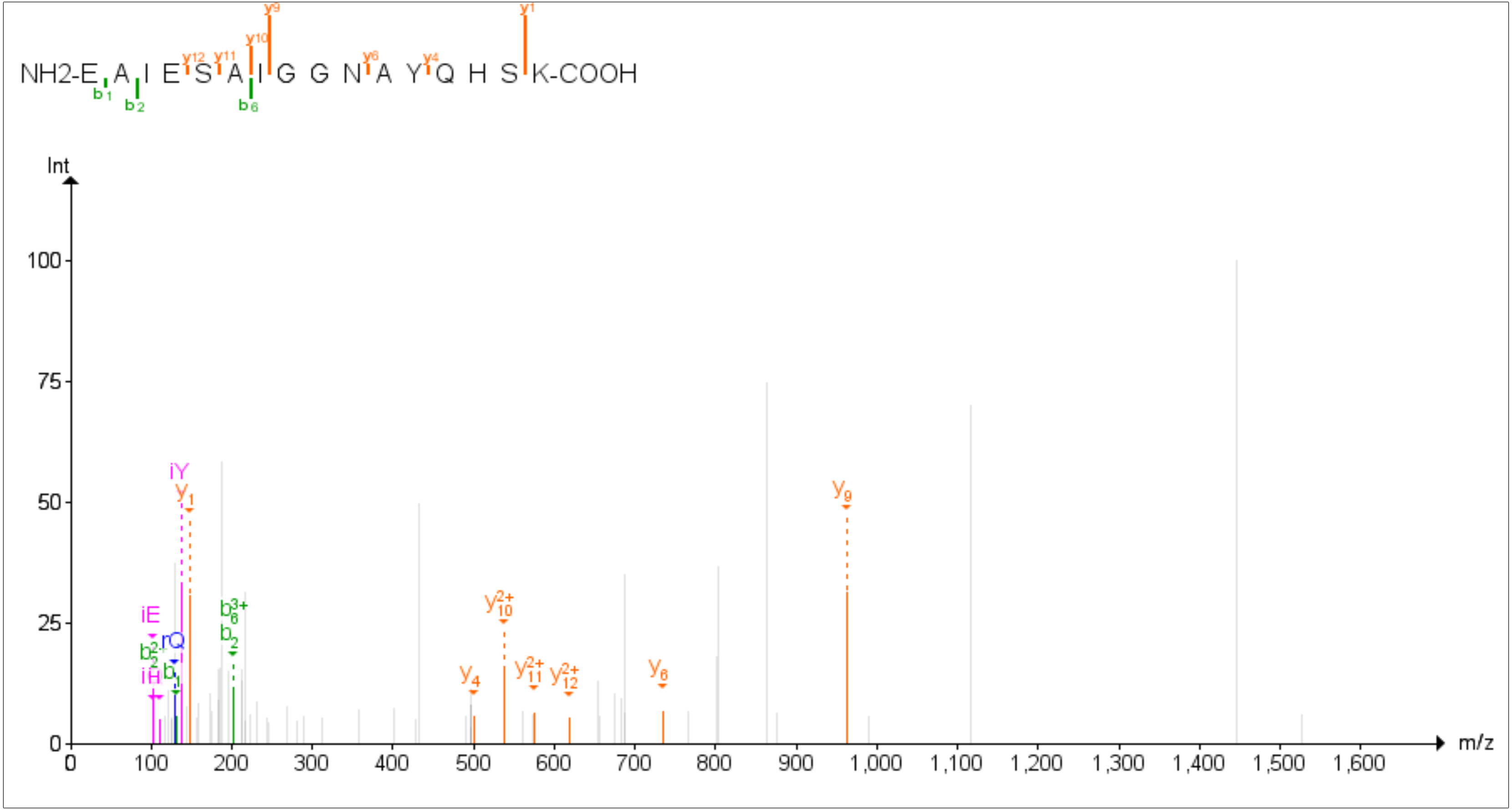

NH2-E A I E S A I G G N A Y Q H S K-COOH
b1
b2
b6
y12
y11
y10
y9
y6
y4
y1
Int
100
75
50
25
0
iY
y1
y9
iE
rQ
b2 2+
iH
b1
b6 3+
b2
y10 2+
y4
y11 2+
y12 2+
y6
m/z
0
100
200
300
400
500
600
700
800
900
1,000
1,100
1,200
1,300
1,400
1,500
1,600

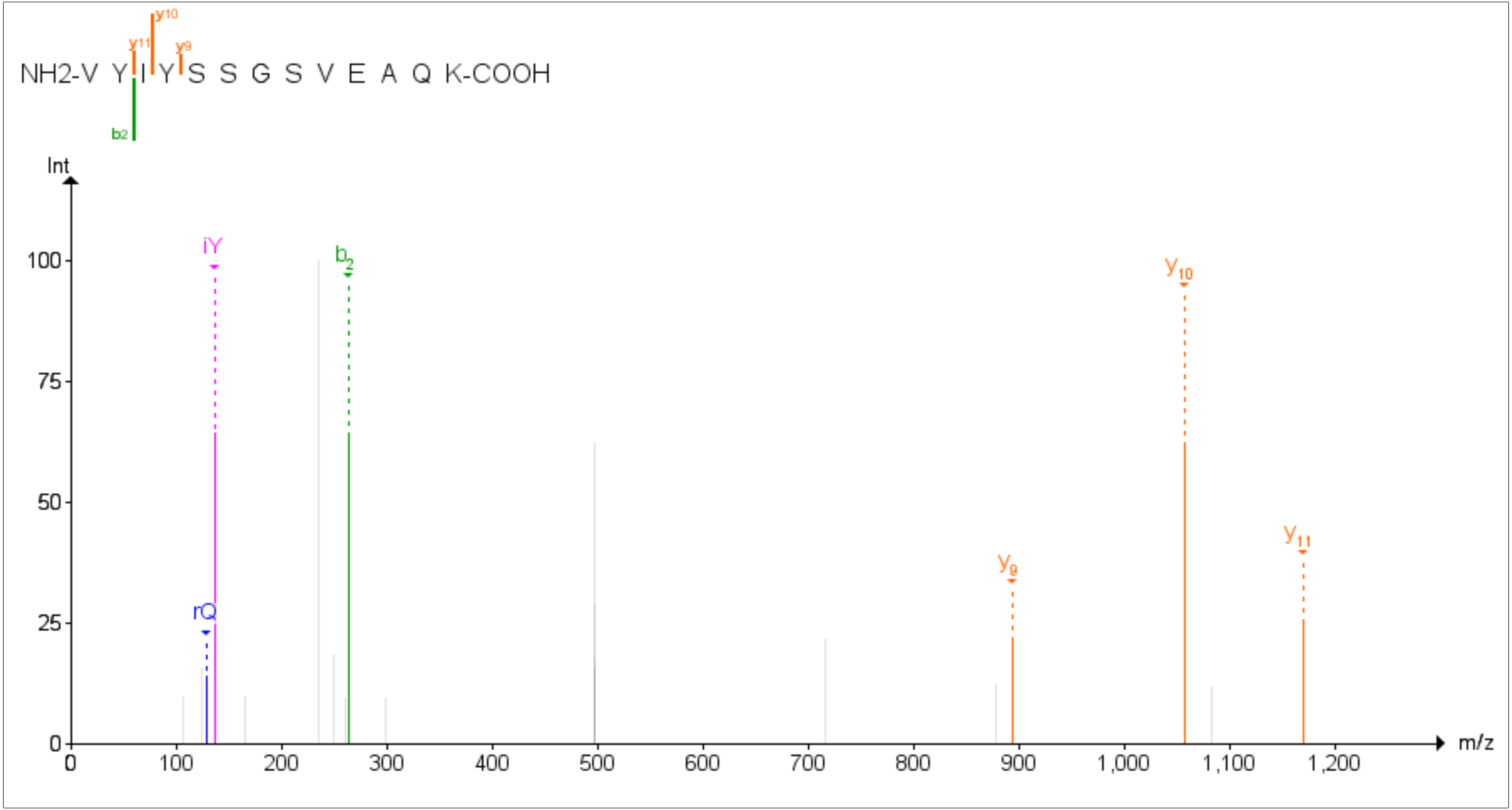
NH2-V Y I Y S S G S V E A Q K-COOH
y10
y11
y9
b2
Int
iY
b2
y10
y11
y9
rQ
100
75
50
25
0
0
100
200
300
400
500
600
700
800
900
1,000
1,100
1,200
m/z

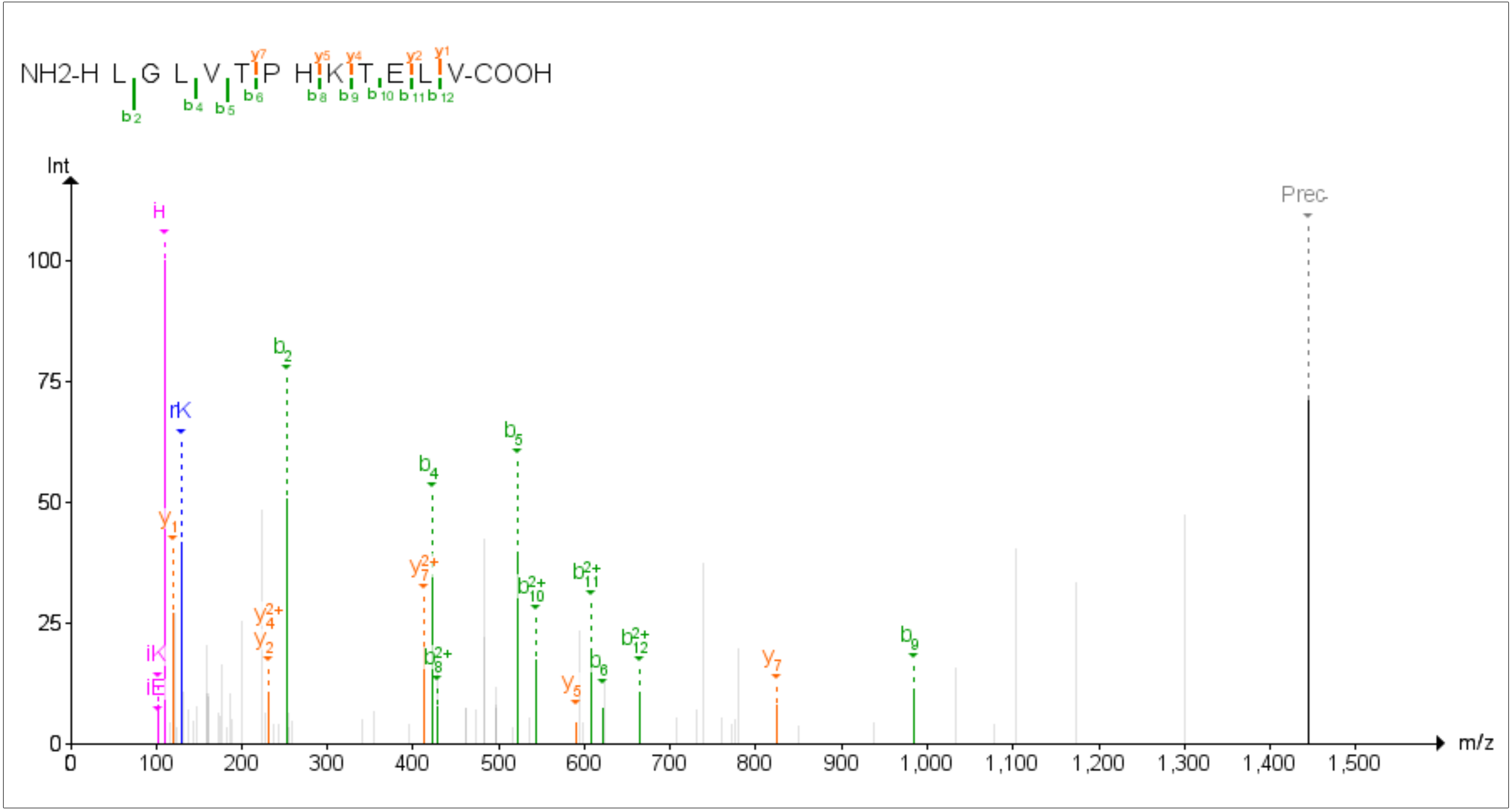

NH2-H L G L V T P H K T E L V-COOH
Int
m/z
Prec

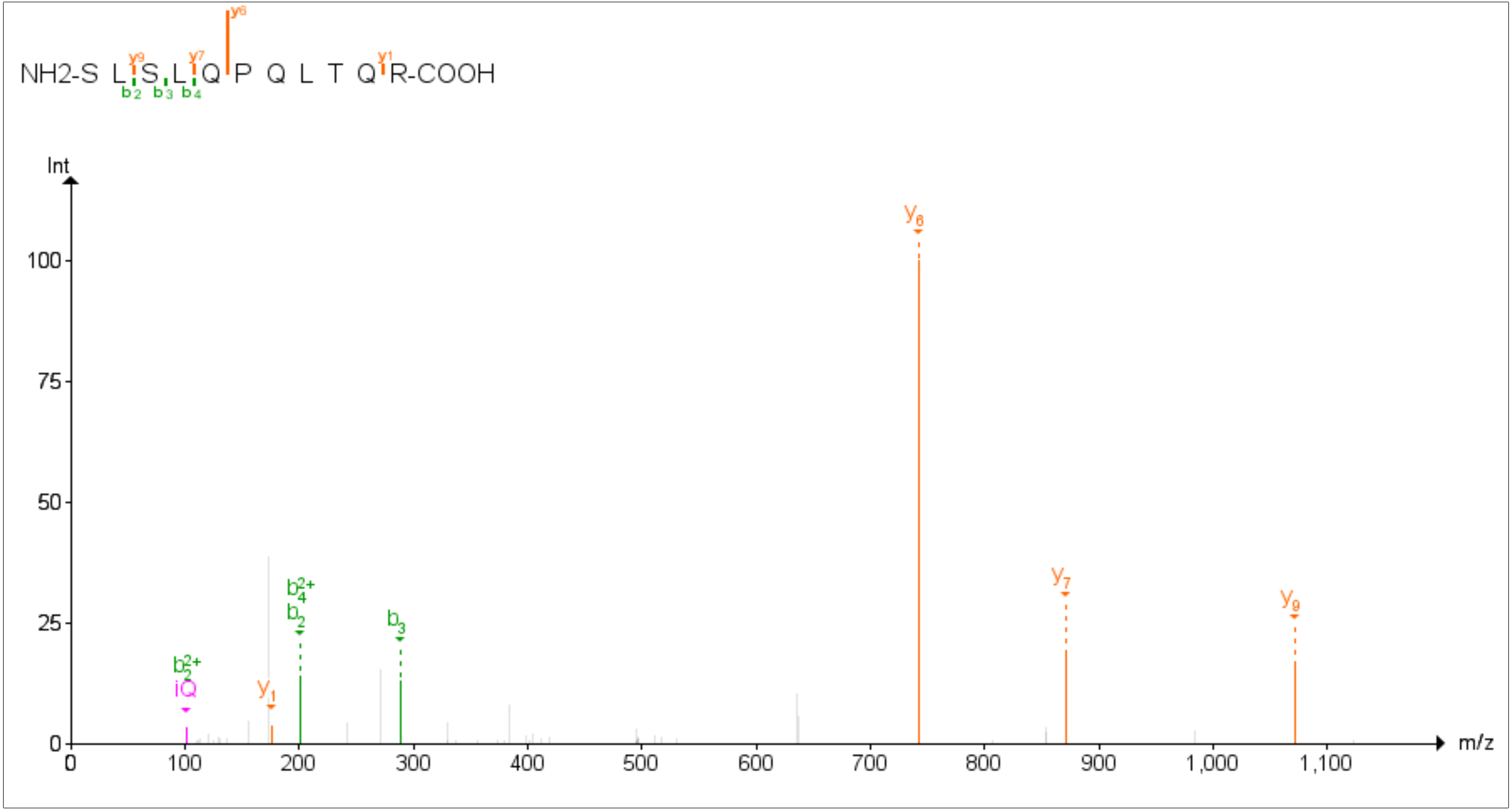
NH2-S L S L Q P Q L T Q R-COOH
y9 y7 y6 y1
b2 b3 b4
Int
100
75
50
25
0
y6
y7
y9
b4 2+
b2
b3
b2 2+
iQ
y1
0 100 200 300 400 500 600 700 800 900 1,000 1,100
m/z

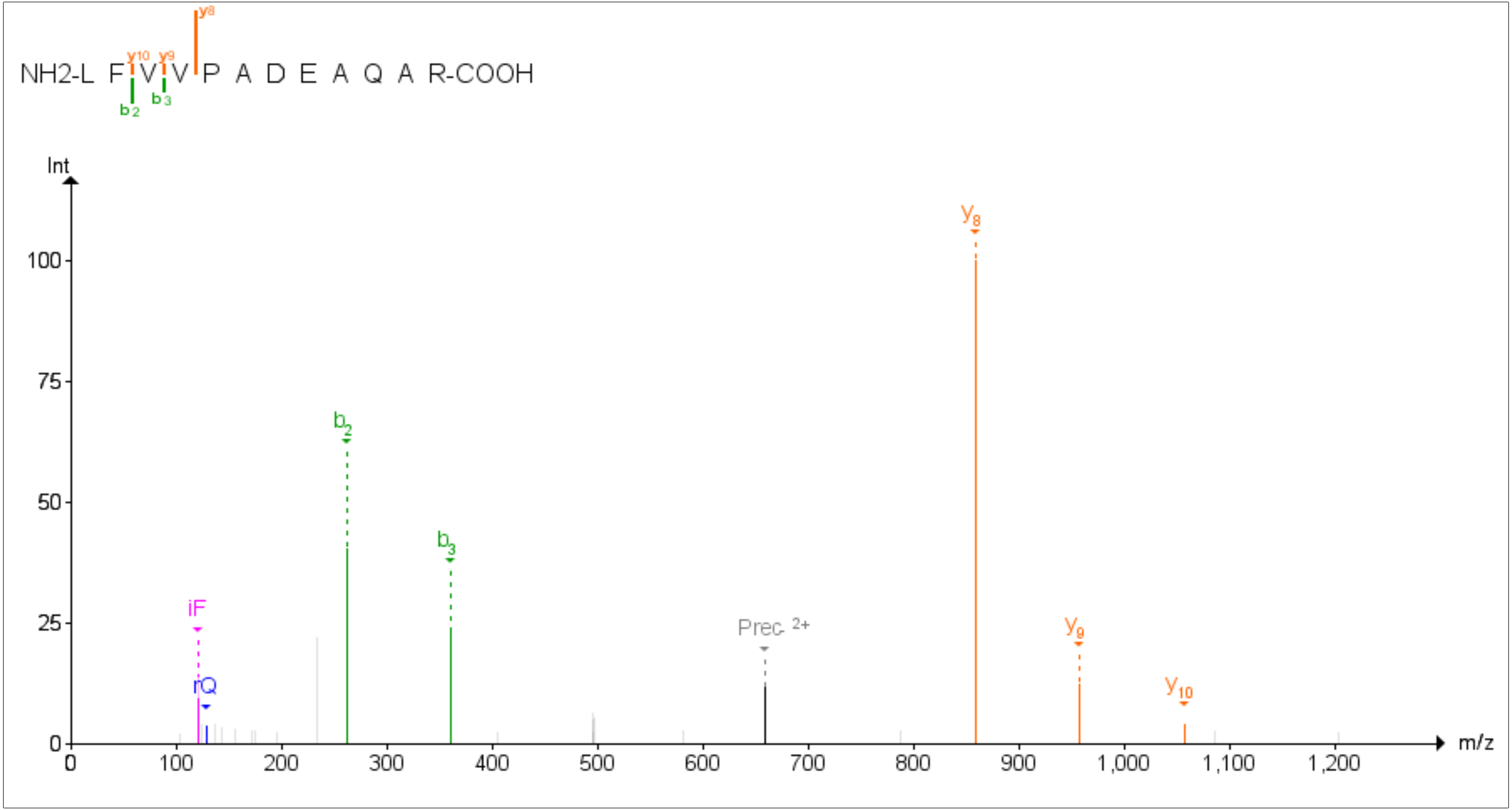

NH2-L F V V P A D E A Q A R-COOH
y10
y9
y8
b2
b3
Int
100
75
50
25
0
iF
rQ
b2
b3
Prec 2+
y8
y9
y10
0
100
200
300
400
500
600
700
800
900
1,000
1,100
1,200
m/z

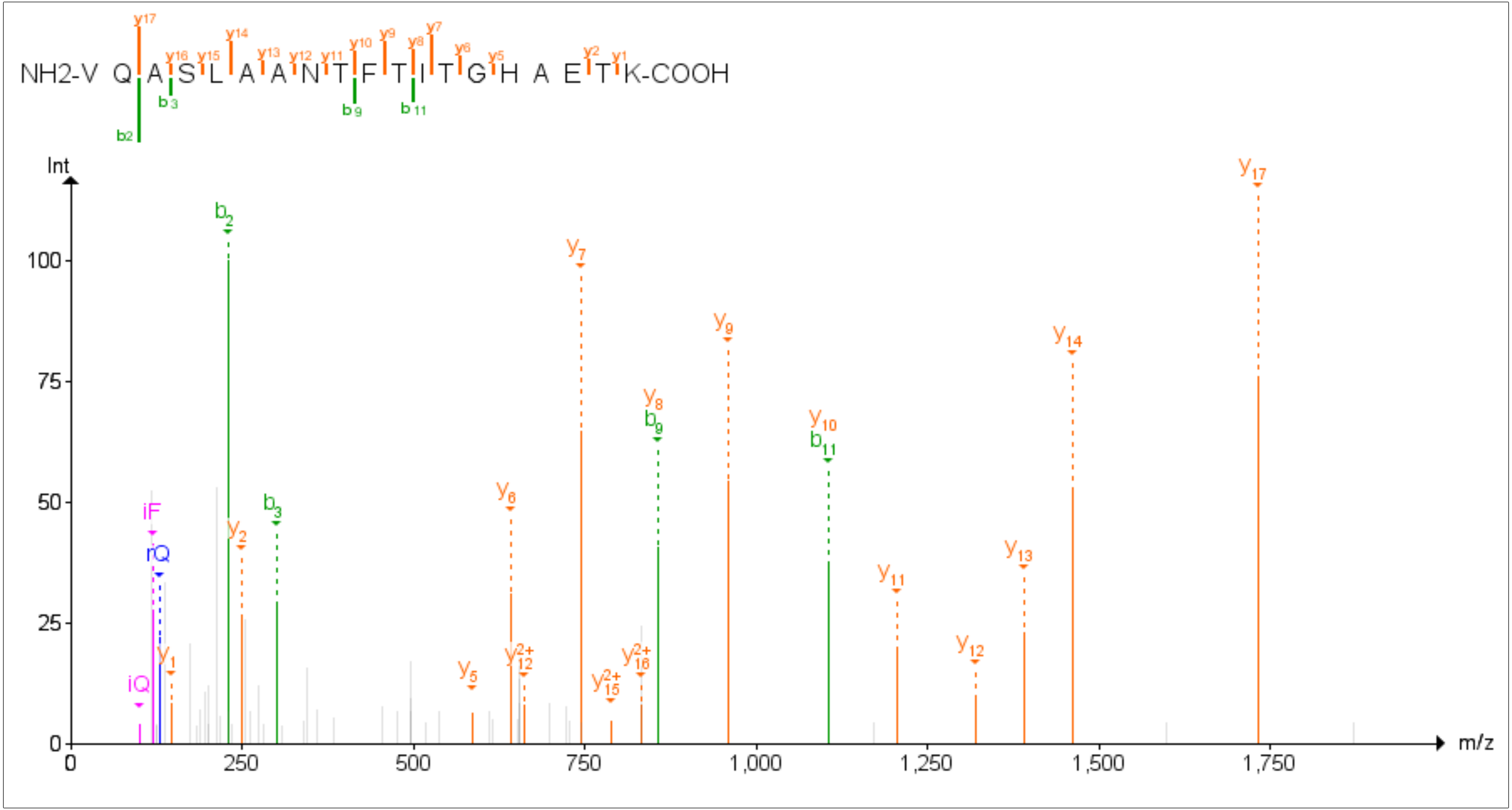

NH2-V Q A S L A A N T F T I T G H A E T K-COOH
Int
m/z
100
75
50
25
0
250
500
750
1,000
1,250
1,500
1,750

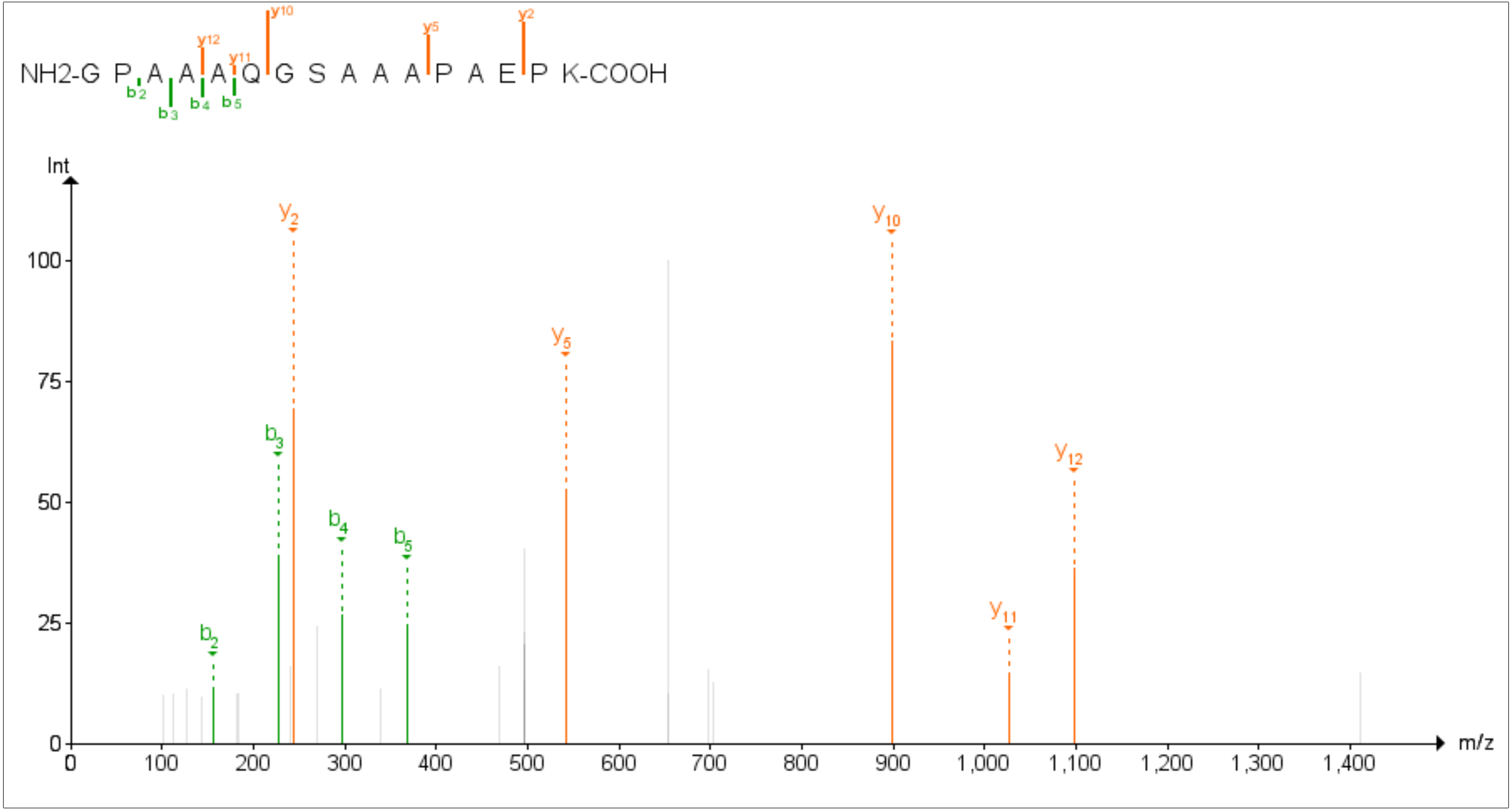

NH2-G P A A A Q G S A A A P A E P K-COOH
y10
y12
y11
y5
y2
b2
b3
b4
b5
Int
100
75
50
25
0
m/z
0
100
200
300
400
500
600
700
800
900
1,000
1,100
1,200
1,300
1,400
y2
y10
y5
b3
b4
b5
y12
y11
b2

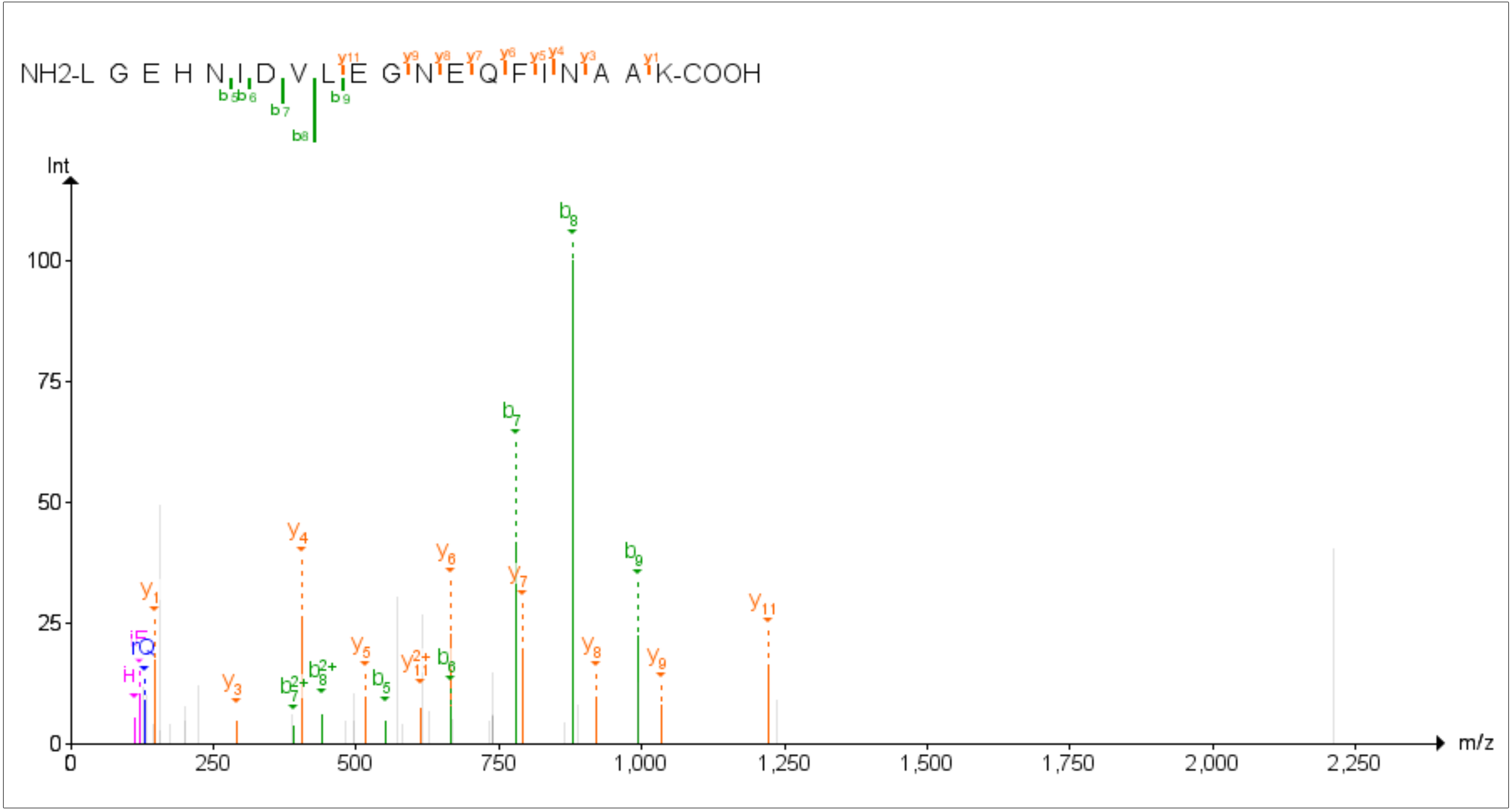
NH2-L G E H N I D V L E G N E Q F I N A A K-COOH
Int
m/z
0
25
50
75
100
250
500
750
1,000
1,250
1,500
1,750
2,000
2,250

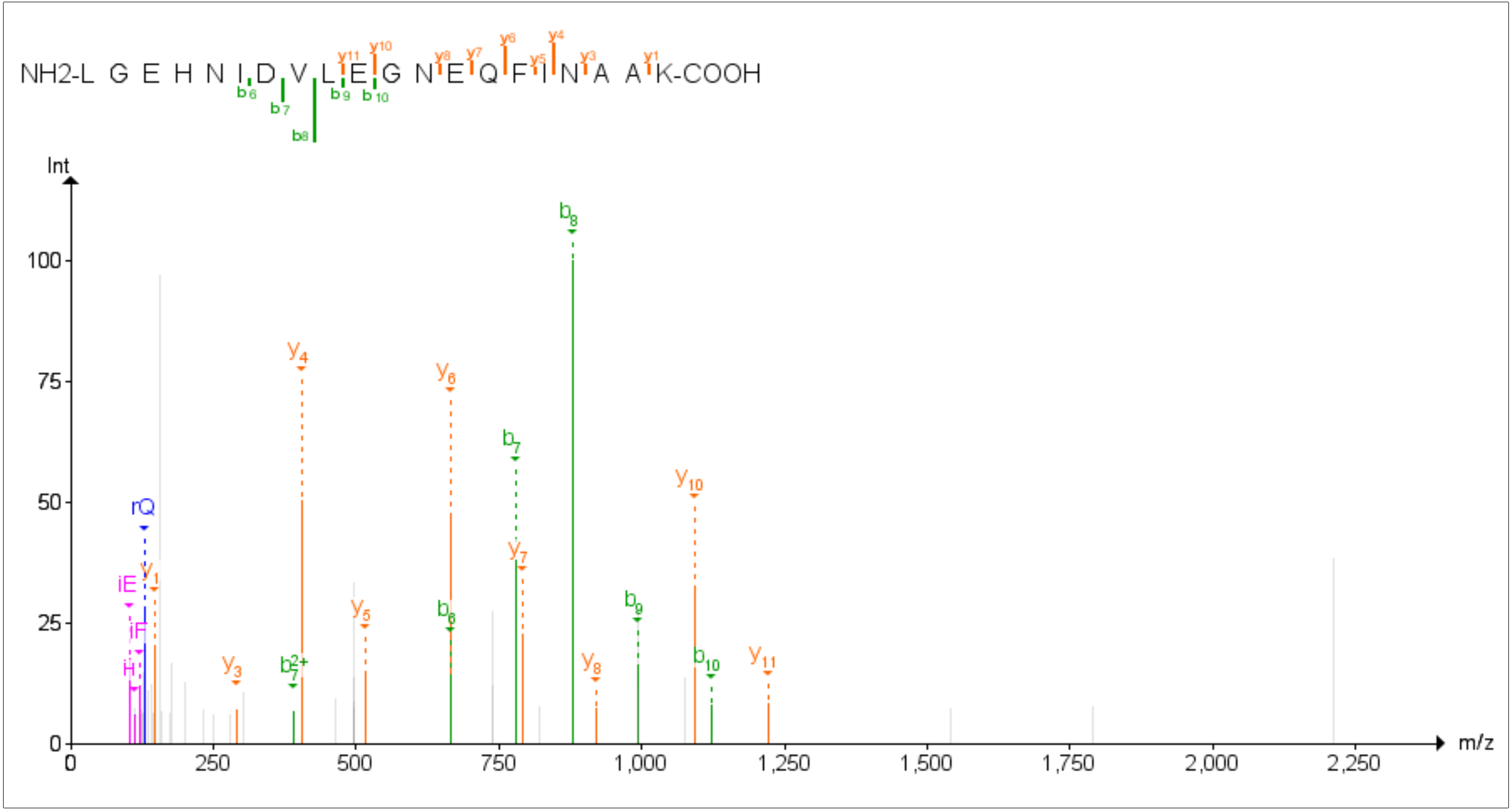

NH2-L G E H N I D V L E G N E Q F I N A A K-COOH
Int
100
75
50
25
0
m/z
0
250
500
750
1,000
1,250
1,500
1,750
2,000
2,250
b8
y4
y6
b7
y10
rQ
y7
iE
y1
y5
b6
b9
iF
iH
y3
b7 2+
y8
b10
y11

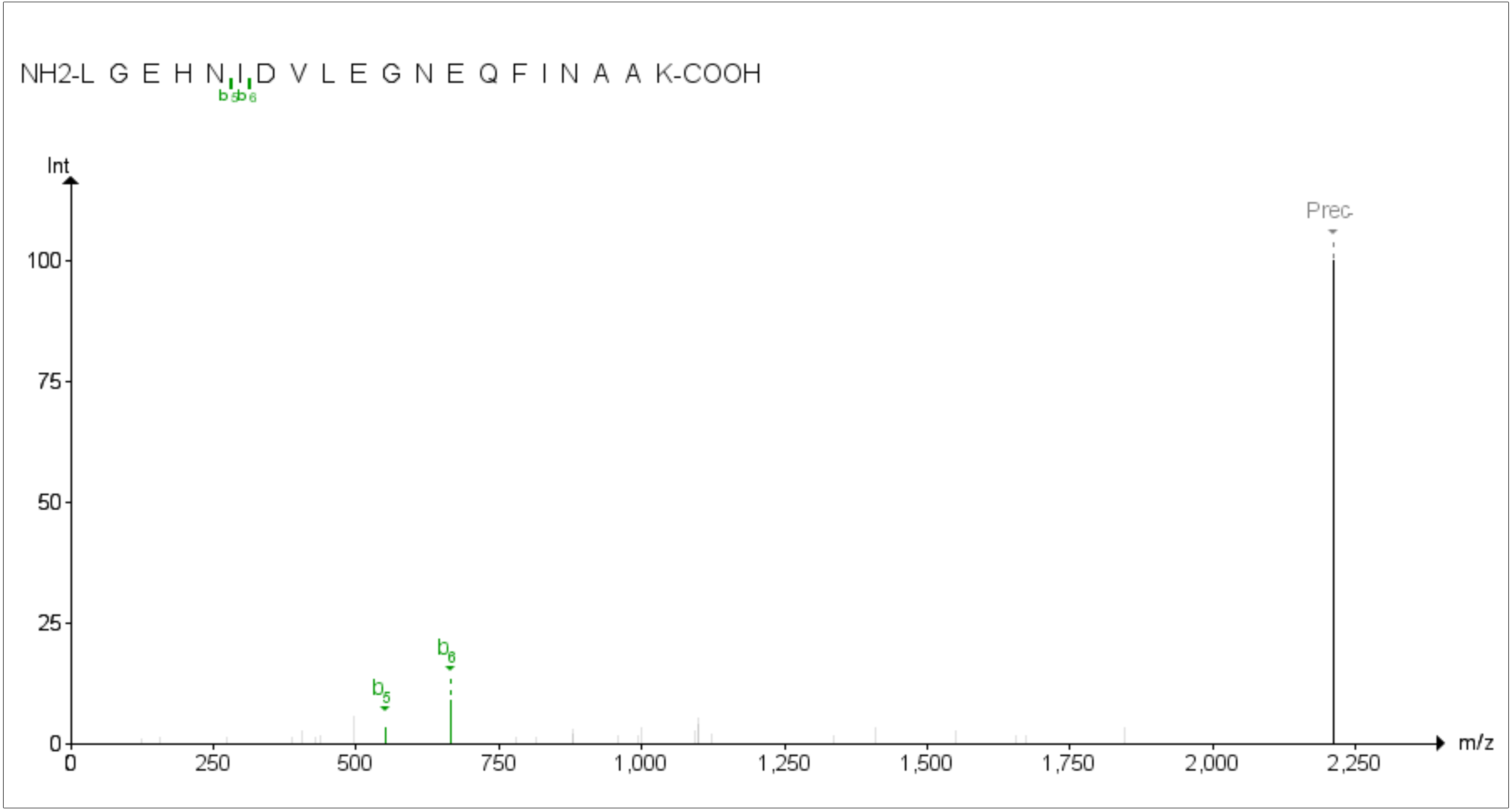
NH2-L G E H N I D V L E G N E Q F I N A A K-COOH
b5 b6
Int
100
75
50
25
0
Prec
b5
b6
0
250
500
750
1,000
1,250
1,500
1,750
2,000
2,250
m/z

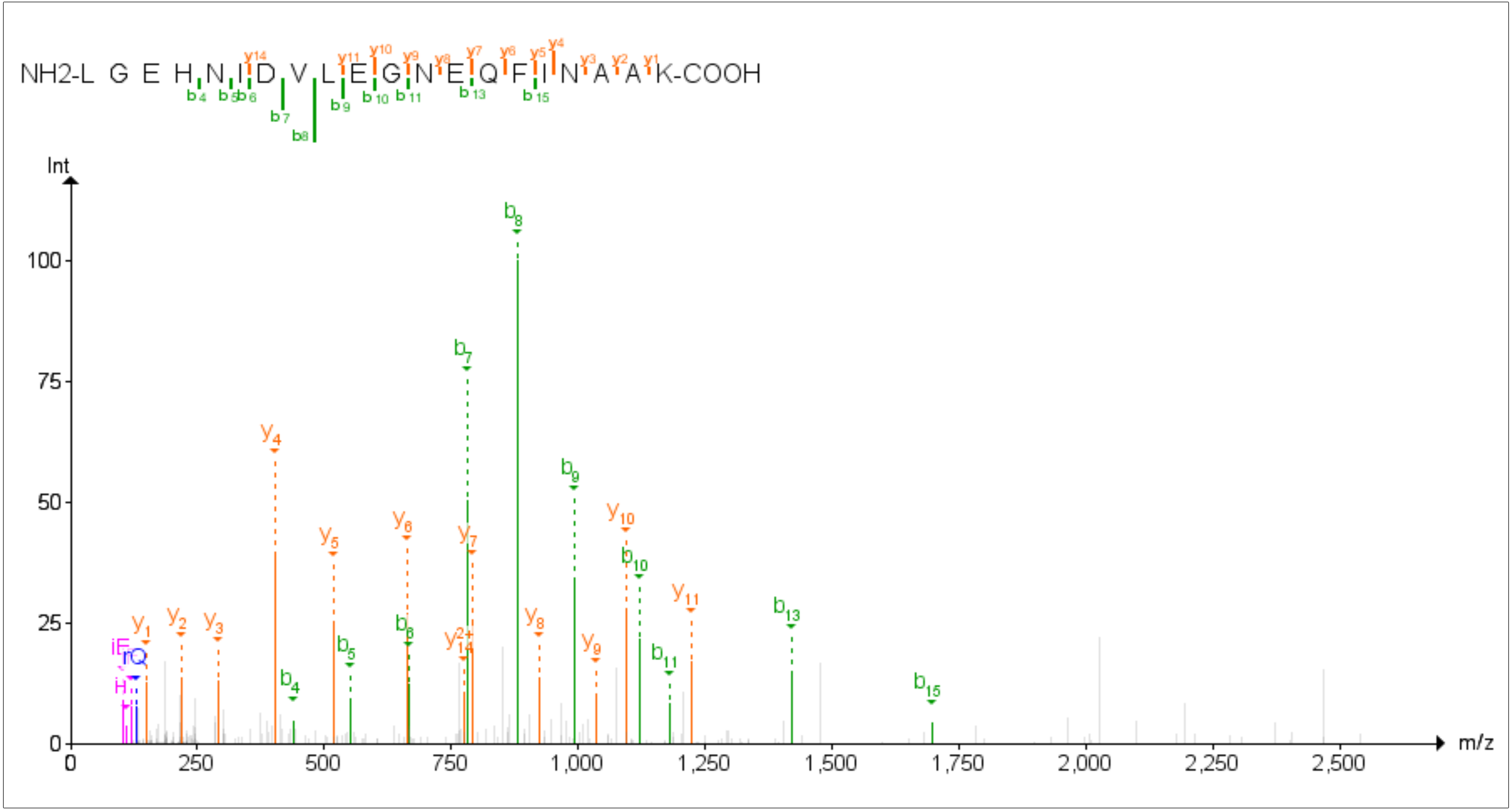

NH2-L G E H N I D V L E G N E Q F I N A A K-COOH
Int
m/z
0
25
50
75
100
250
500
750
1,000
1,250
1,500
1,750
2,000
2,250
2,500

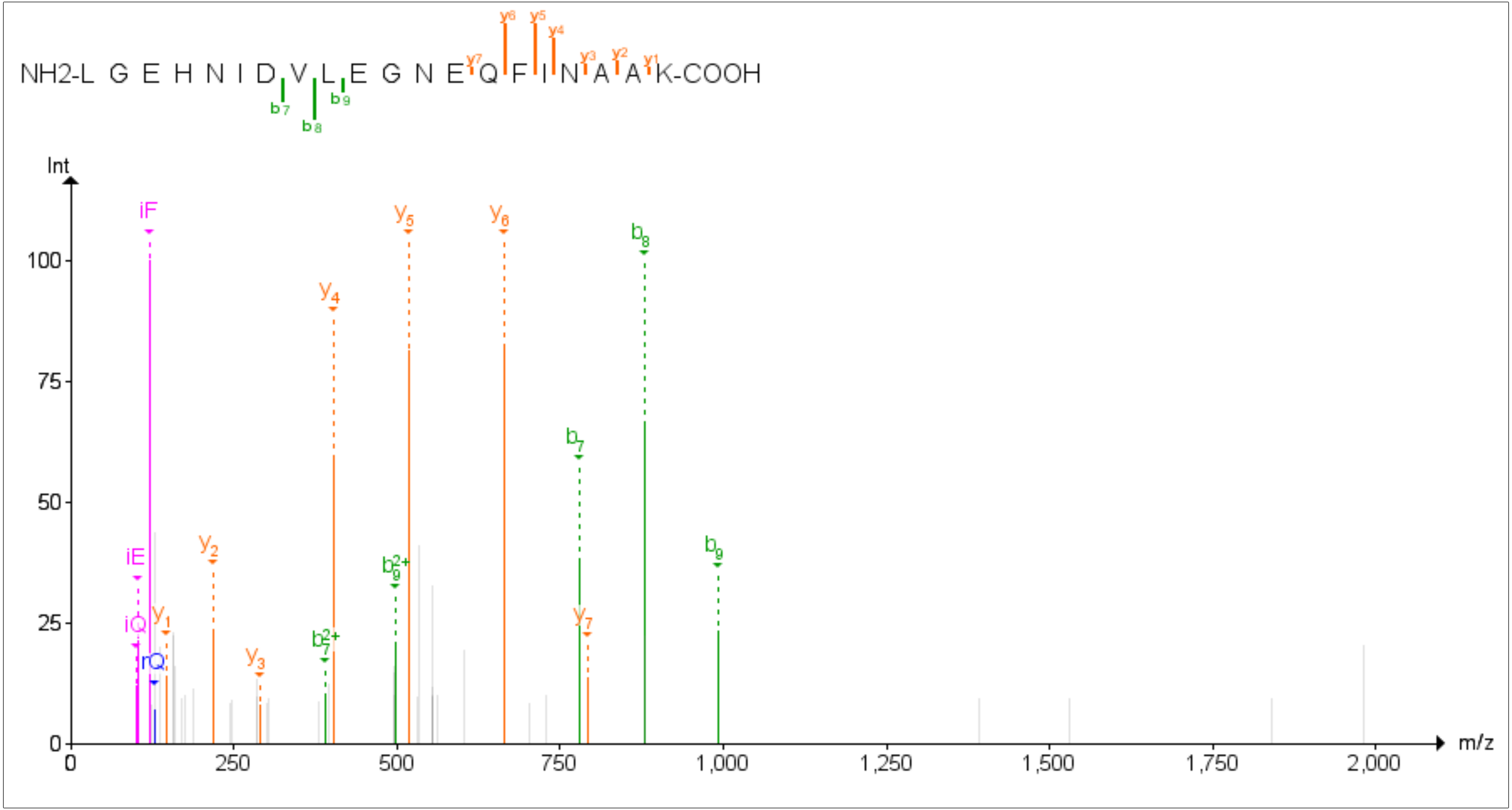
NH2-L G E H N I D V L E G N E Q F I N A A K-COOH
b7 b8 b9
y7 y6 y5 y4 y3 y2 y1
Int
m/z
0
25
50
75
100
250
500
750
1,000
1,250
1,500
1,750
2,000
iF
iE
iQ
rQ
y1
y2
y3
y4
y5
y6
y7
b7
b8
b9
b7 2+
b9 2+

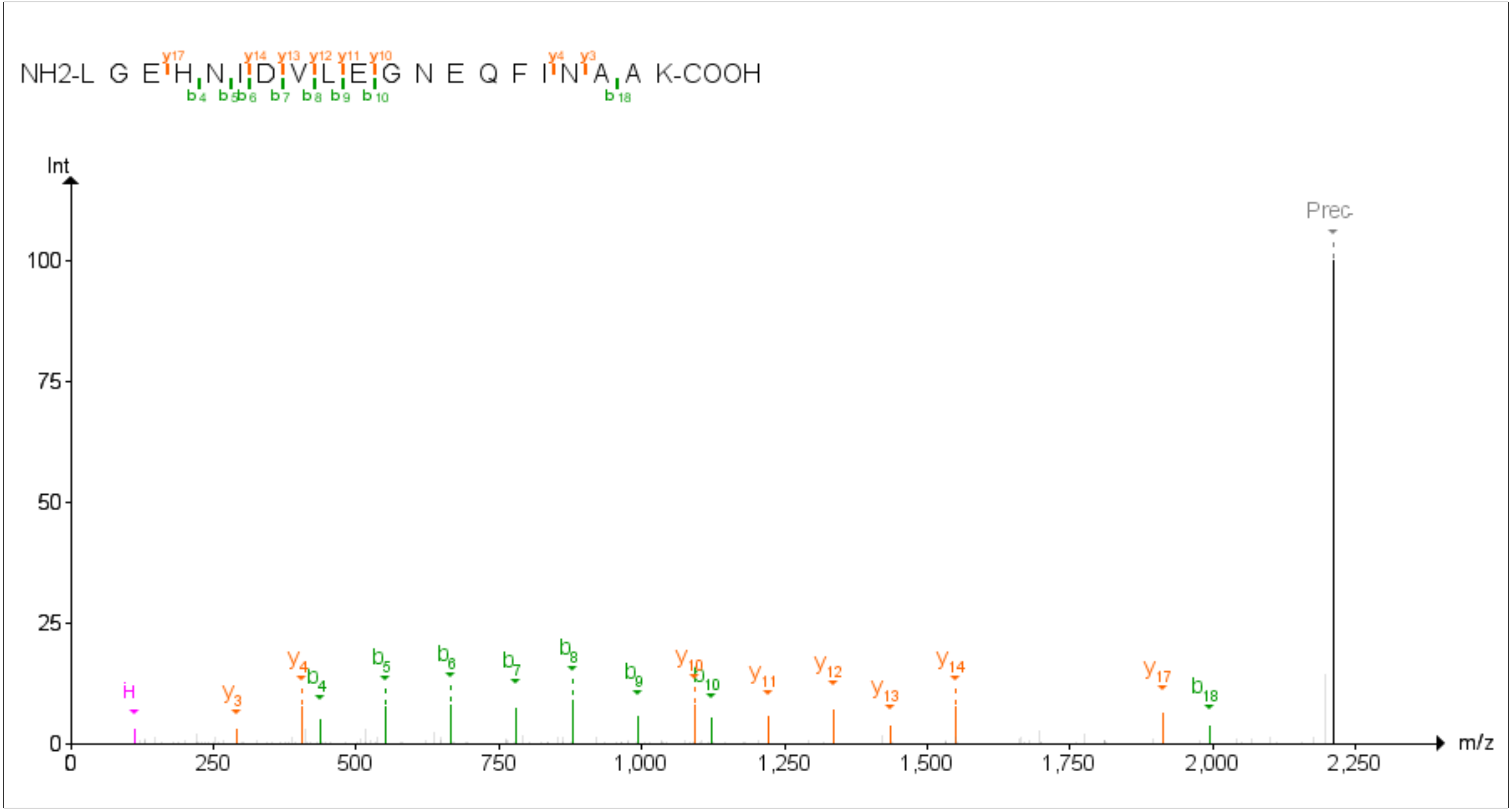

NH2-L G E H N I D V L E G N E Q F I N A A K-COOH
y17 y14 y13 y12 y11 y10 y4 y3
b4 b5 b6 b7 b8 b9 b10 b18
Int
Prec
100
75
50
25
0
H
y3 y4 b4 b5 b6 b7 b8 b9 y10 b10 y11 y12 y13 y14 y17 b18
0 250 500 750 1,000 1,250 1,500 1,750 2,000 2,250
m/z

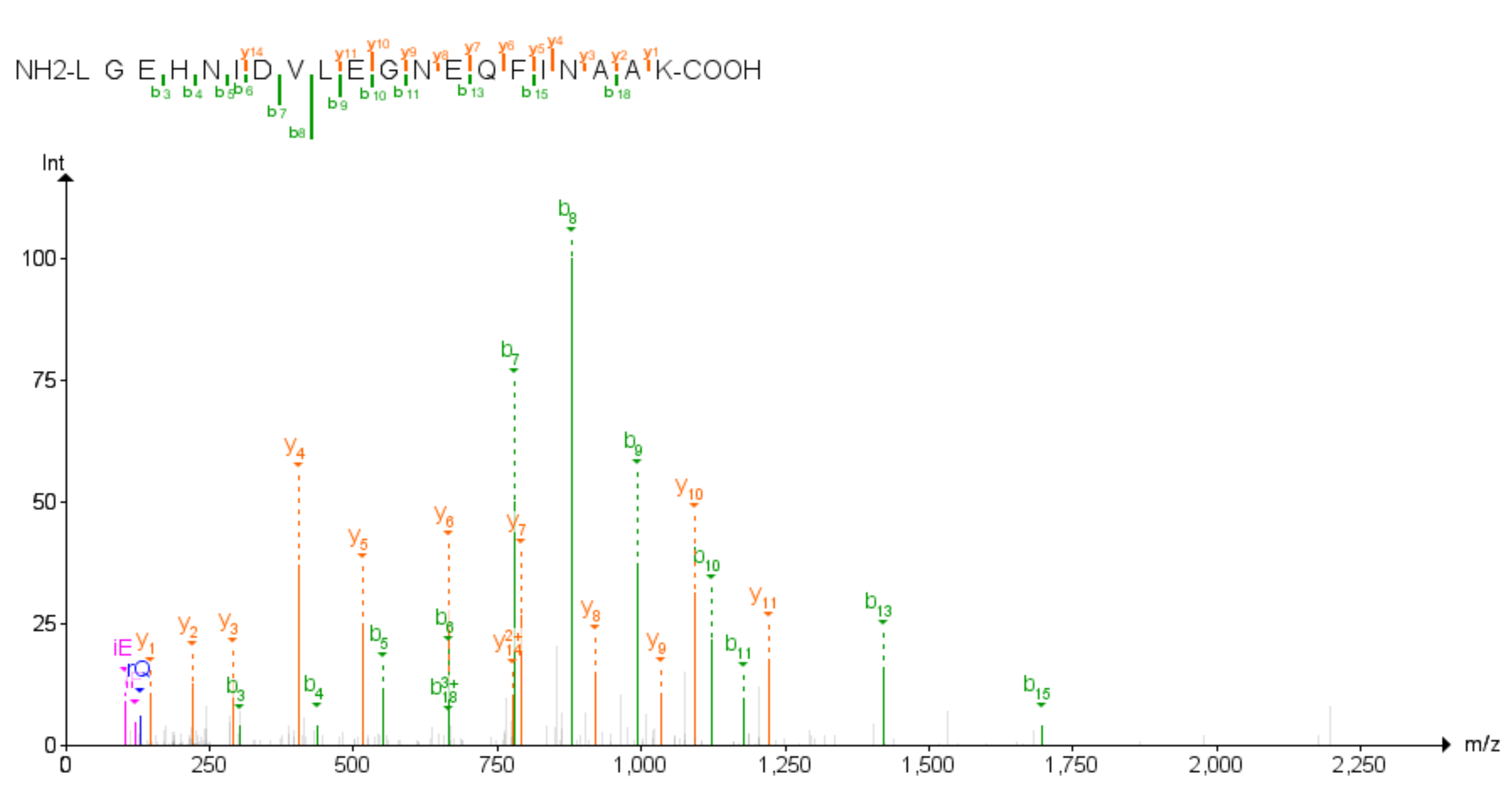

NH2-L G E H N I D V L E G N E Q F I N A A K-COOH
Int
m/z
0
25
50
75
100
0
250
500
750
1,000
1,250
1,500
1,750
2,000
2,250
b8
b7
b9
y10
b10
y11
b13
b11
b15
y4
y5
y6
y7
y8
y9
y1
y2
y3
b3
b4
b5
b6
iE

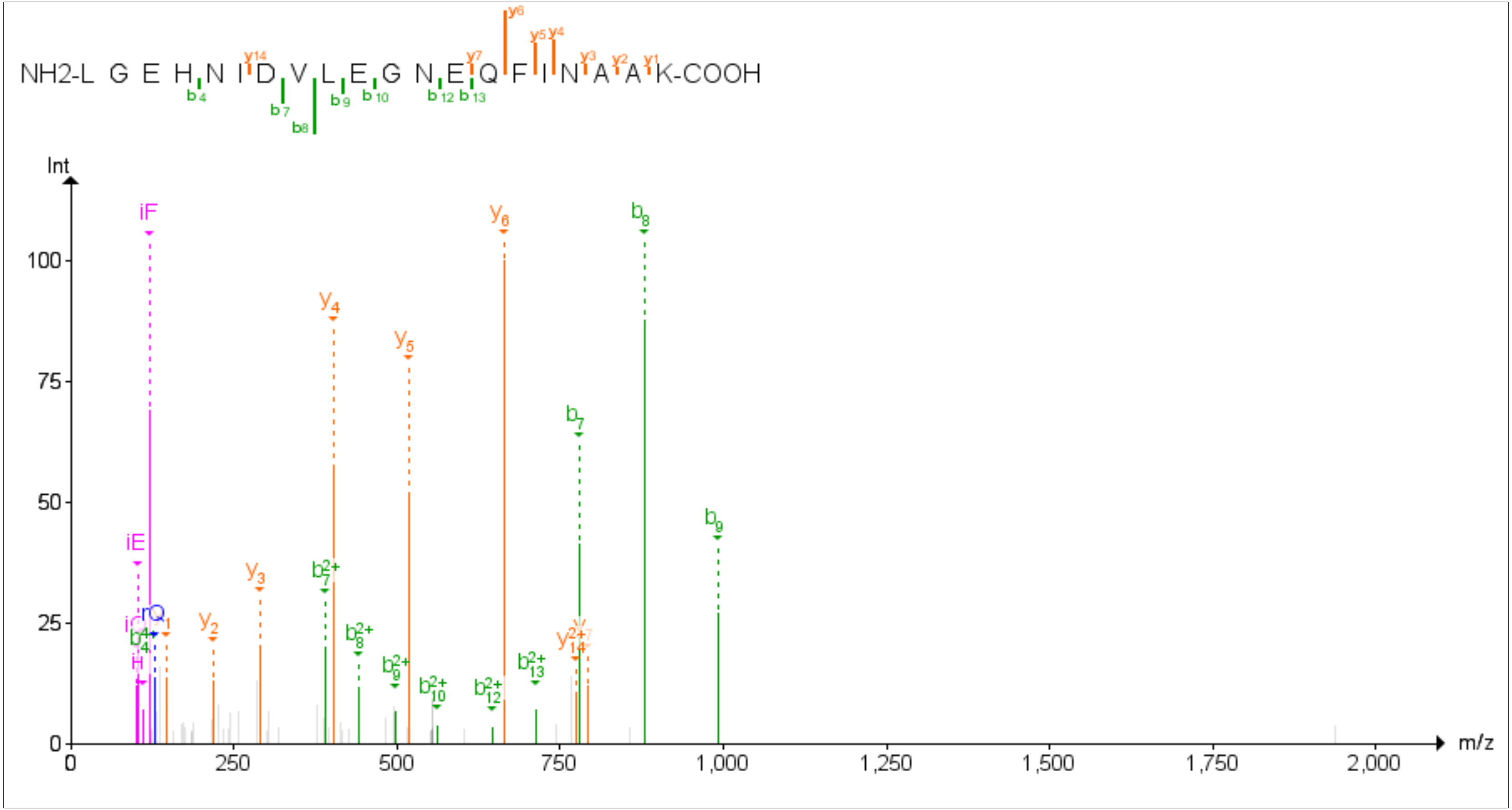

NH2-L G E H N I D V L E G N E Q F I N A A K-COOH
Int
m/z
0
25
50
75
100
250
500
750
1,000
1,250
1,500
1,750
2,000
iF
iE
y6
b8
y4
y5
b7
b9
y3
y2

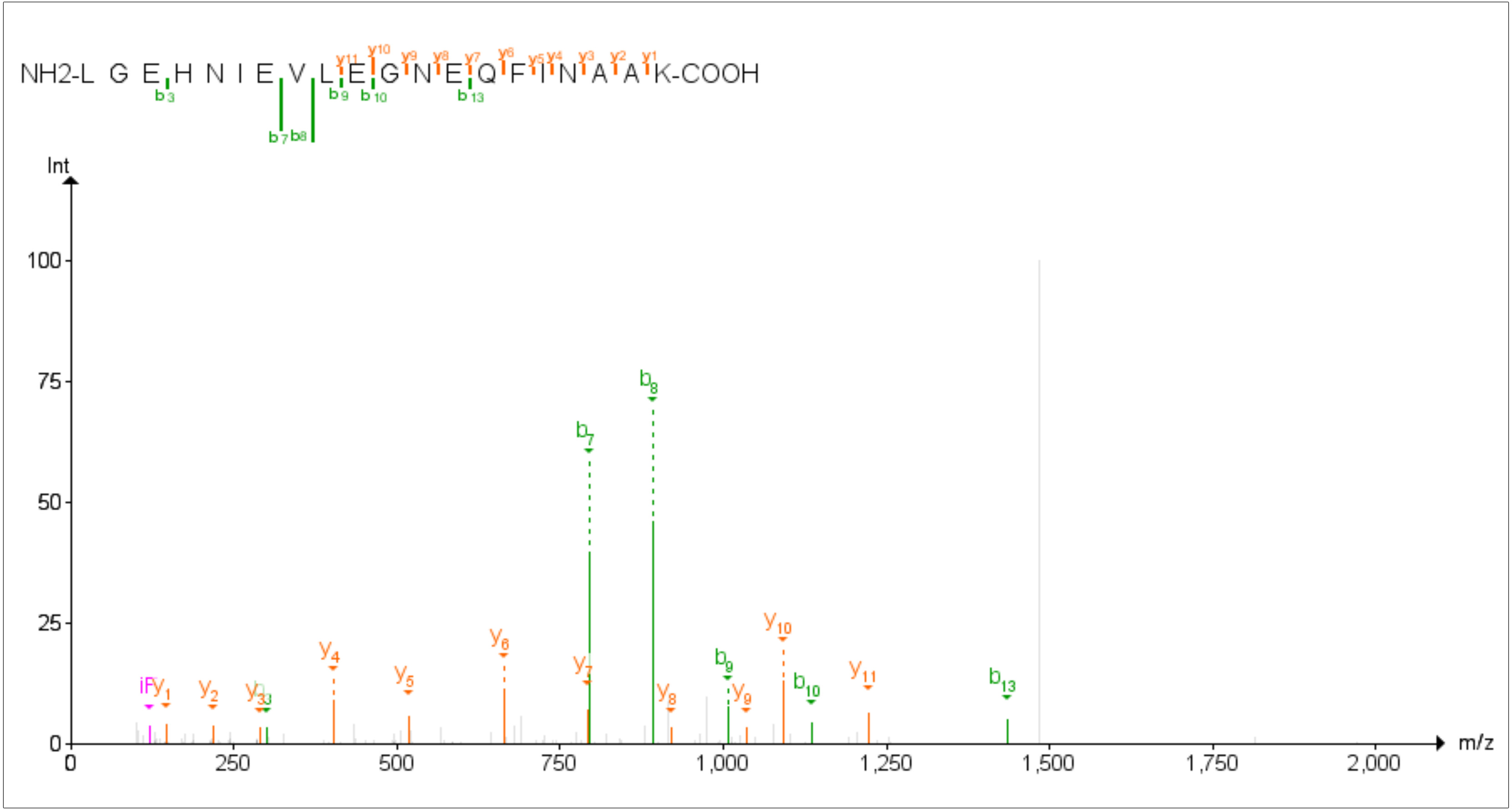
NH2-L G E H N I E V L E G N E Q F I N A A K-COOH
Int
m/z
100
75
50
25
0
0
250
500
750
1,000
1,250
1,500
1,750
2,000
b3 b7 b8 b9 b10 b13
y1 y2 y3 y4 y5 y6 y7 y8 y9 y10 y11

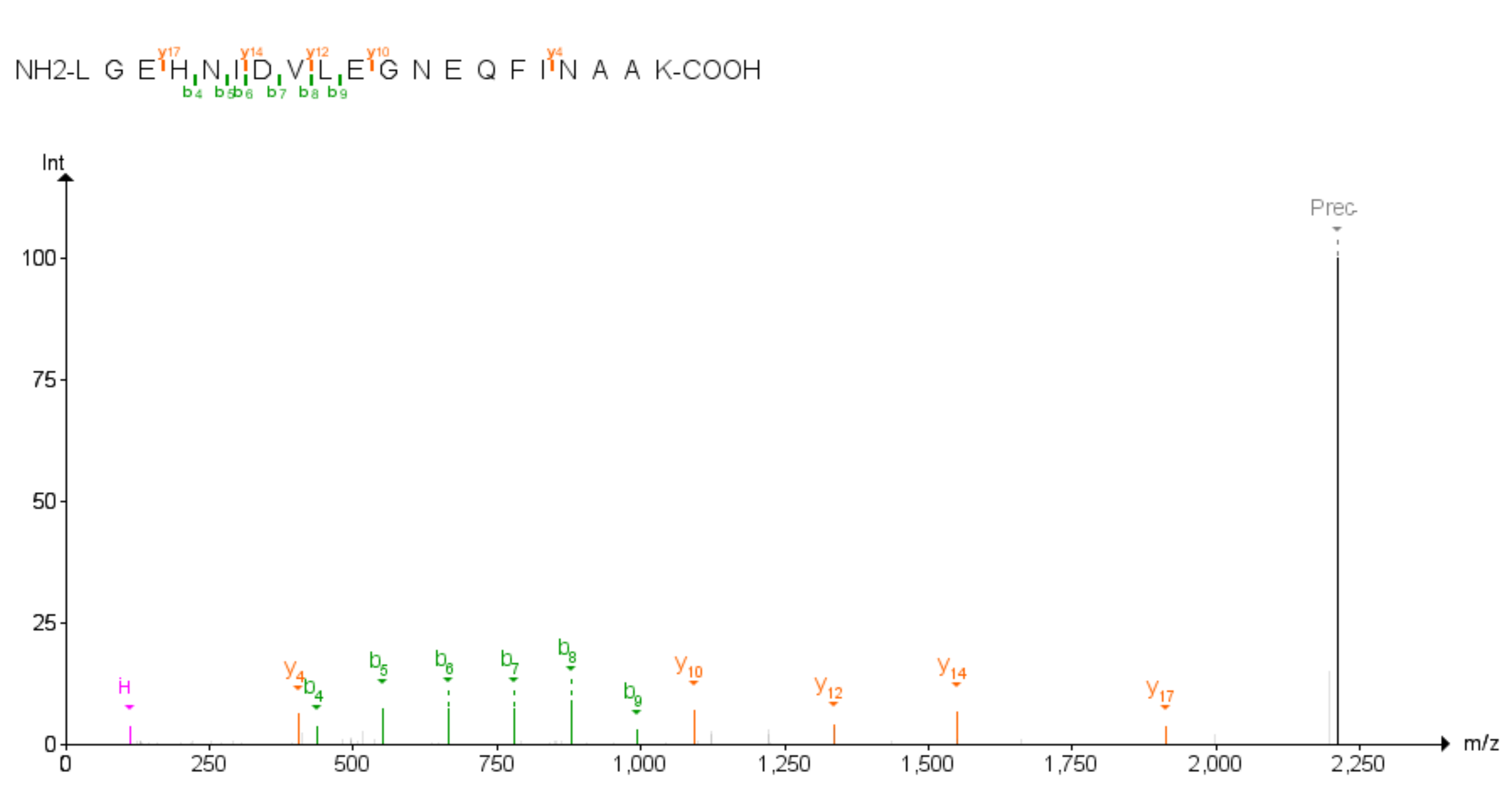

NH2-L G E H N I D V L E G N E Q F I N A A K-COOH
y17 y14 y12 y10 y4
b4 b5 b6 b7 b8 b9
Int
Prec
H
y4
b4
b5
b6
b7
b8
b9
y10
y12
y14
y17
m/z
0
25
50
75
100
0
250
500
750
1,000
1,250
1,500
1,750
2,000
2,250

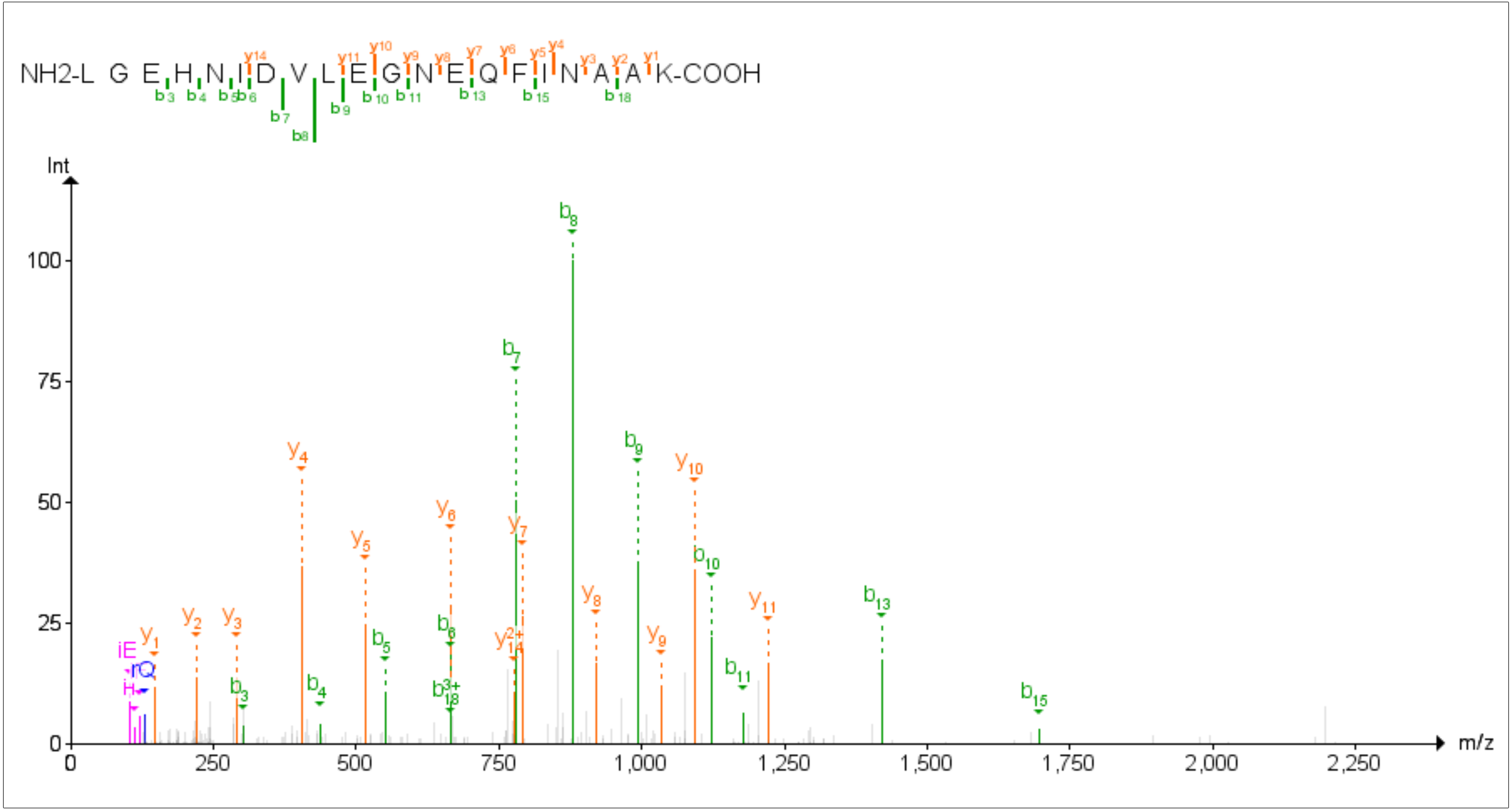
NH2-L G E H N I D V L E G N E Q F I N A A K-COOH
Int
m/z
0
25
50
75
100
250
500
750
1,000
1,250
1,500
1,750
2,000
2,250
b8
b7
b9
b10
b13
b15
y4
y5
y6
y7
y8
y9
y10
y11
y1
y2
y3
b3
b4
b5
b6
b11
iE
rQ
iH

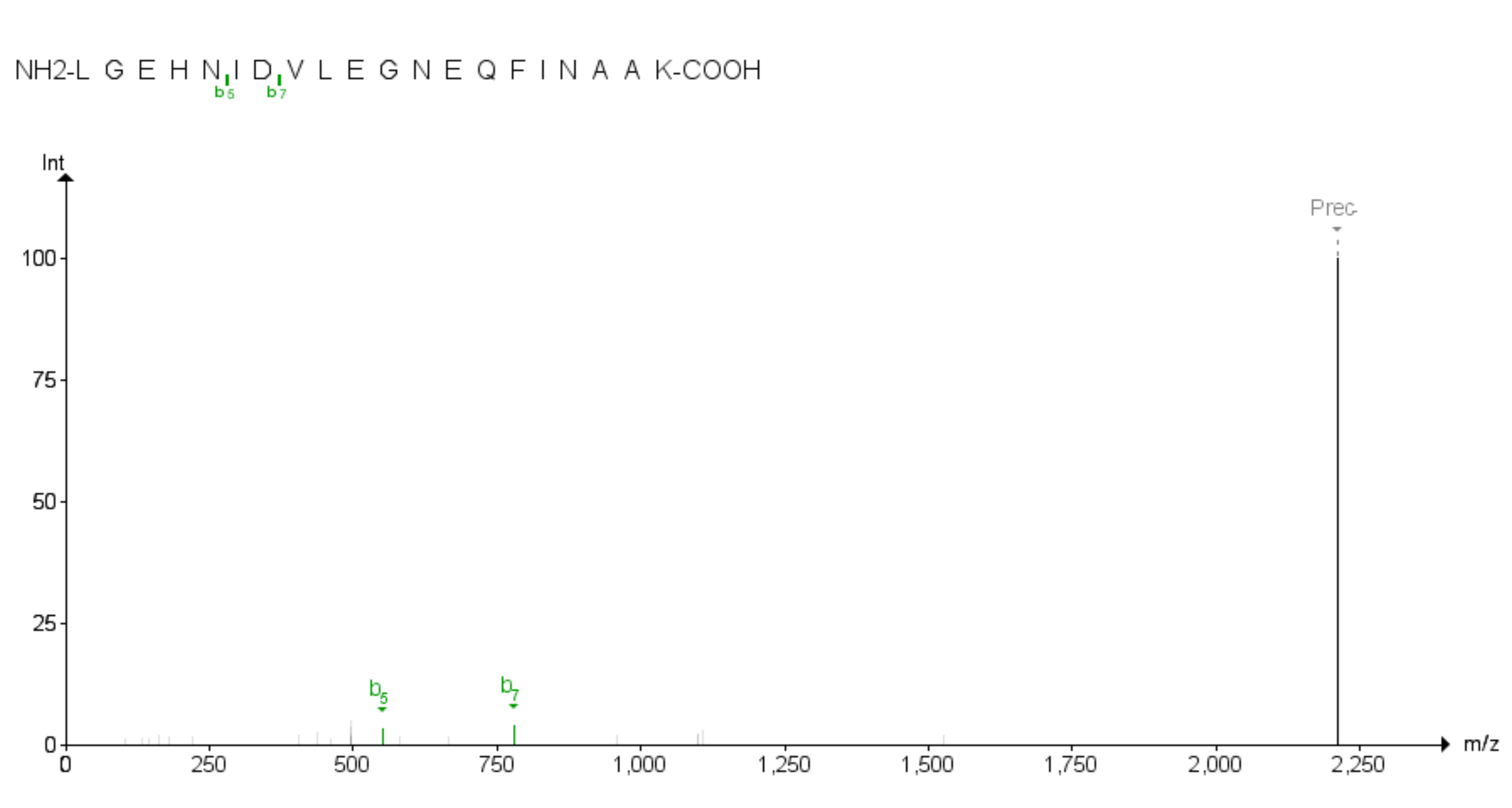
NH2-L G E H N I D V L E G N E Q F I N A A K-COOH
b5
b7
Int
100
75
50
25
0
Prec
b5
b7
0
250
500
750
1,000
1,250
1,500
1,750
2,000
2,250
m/z

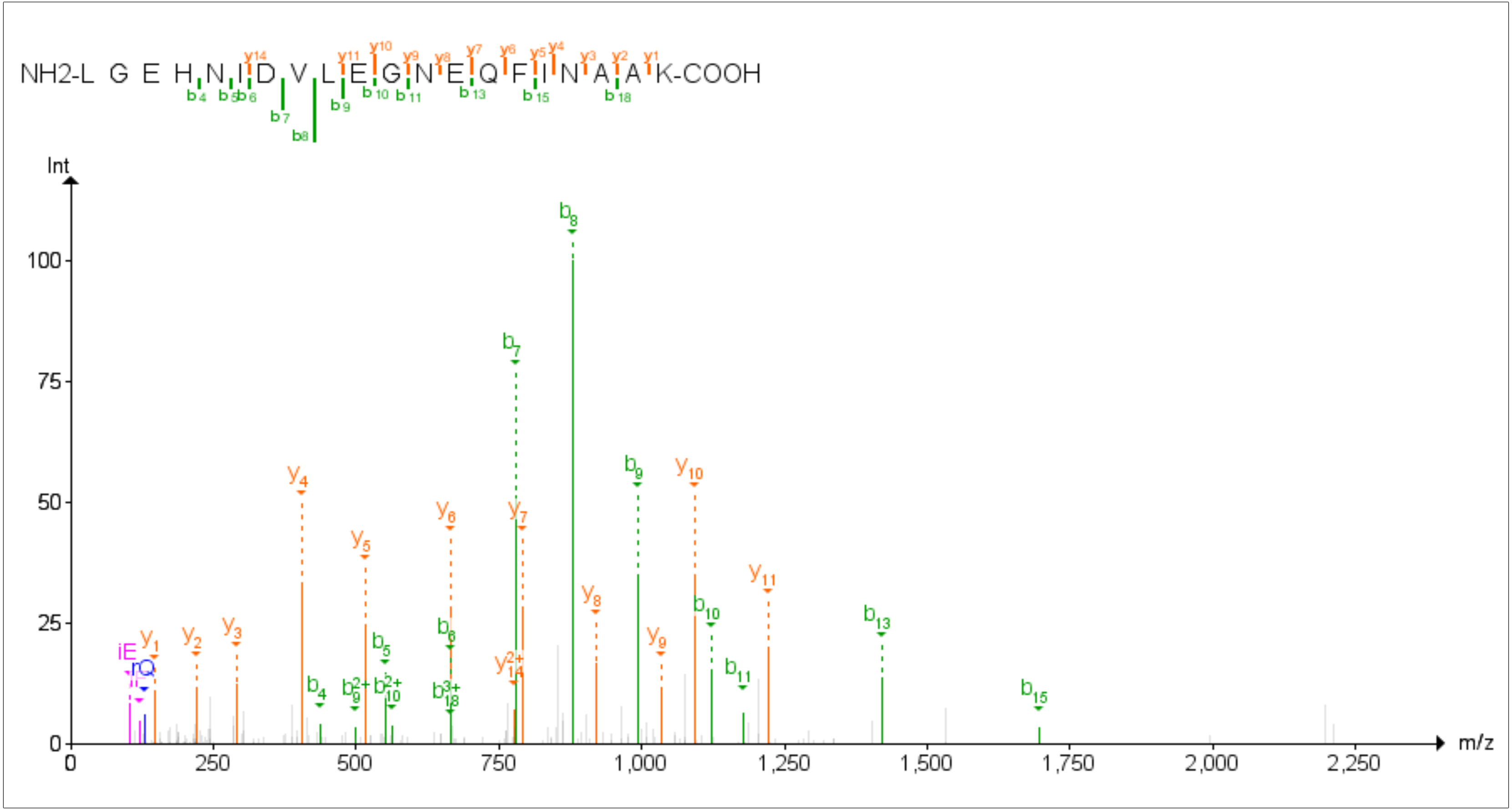
NH2-L G E H N I D V L E G N E Q F I N A A K-COOH
Int
m/z
0
25
50
75
100
250
500
750
1,000
1,250
1,500
1,750
2,000
2,250

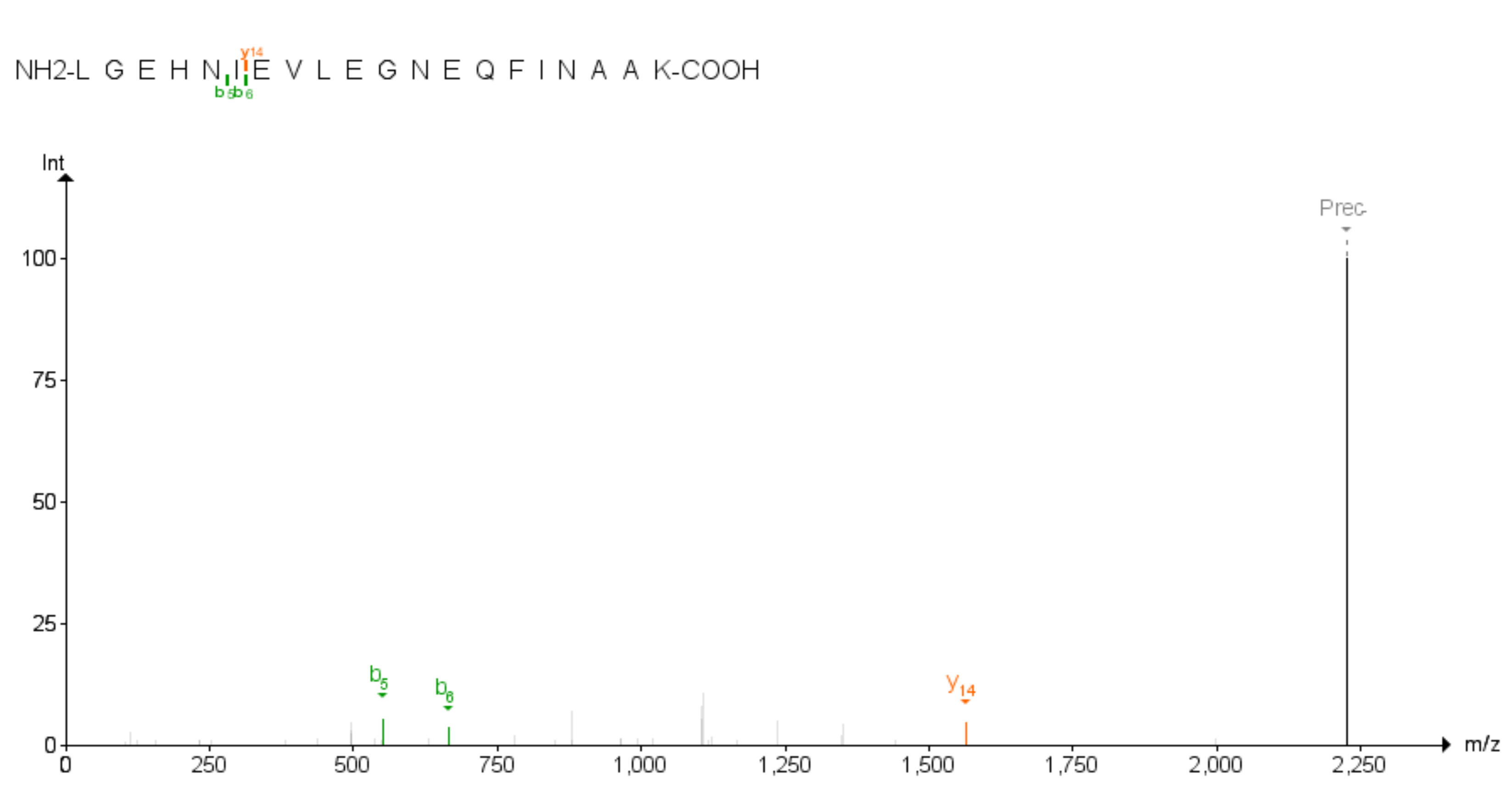

NH2-L G E H N I E V L E G N E Q F I N A A K-COOH
y14
b5
b6
Int
100
75
50
25
0
Prec.
b5
b6
y14
0
250
500
750
1,000
1,250
1,500
1,750
2,000
2,250
m/z

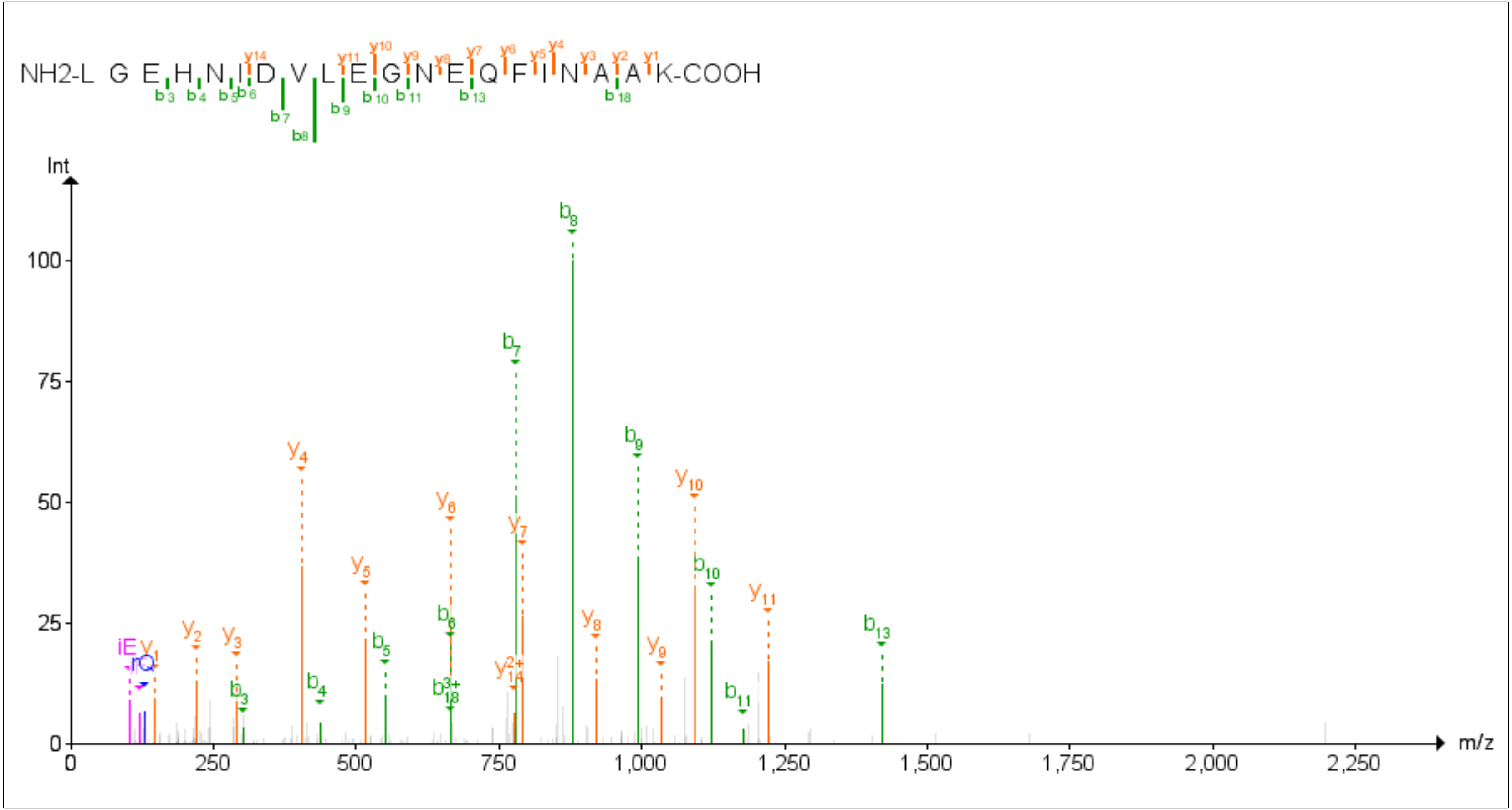

NH2-L G E H N I D V L E G N E Q F I N A A K-COOH
Int
m/z
0
25
50
75
100
250
500
750
1,000
1,250
1,500
1,750
2,000
2,250

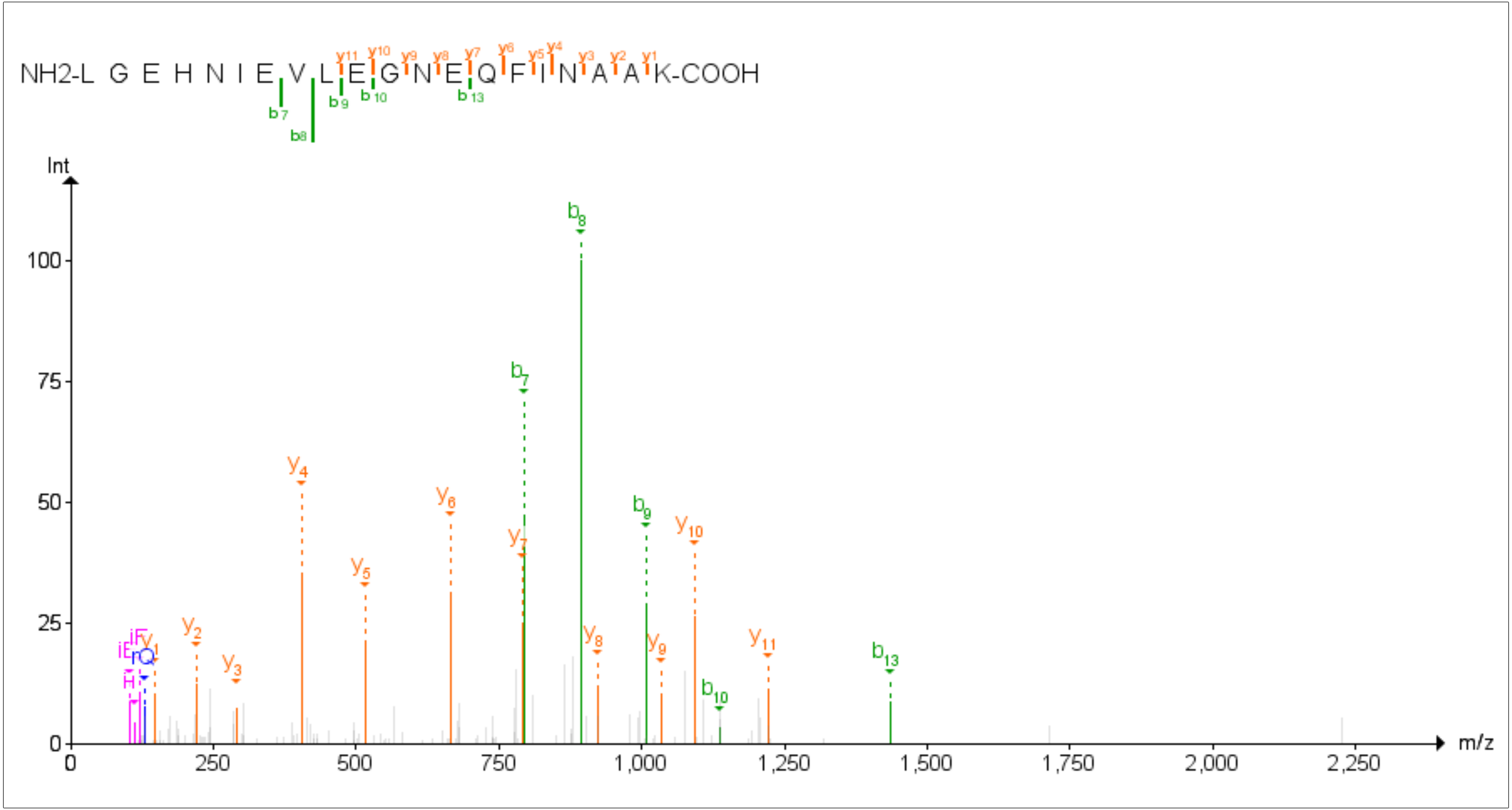

NH2-L G E H N I E V L E G N E Q F I N A A K-COOH
y11 y10 y9 y8 y7 y6 y5 y4 y3 y2 y1
b7 b8 b9 b10 b13
Int
100
75
50
25
0
m/z
0
250
500
750
1,000
1,250
1,500
1,750
2,000
2,250

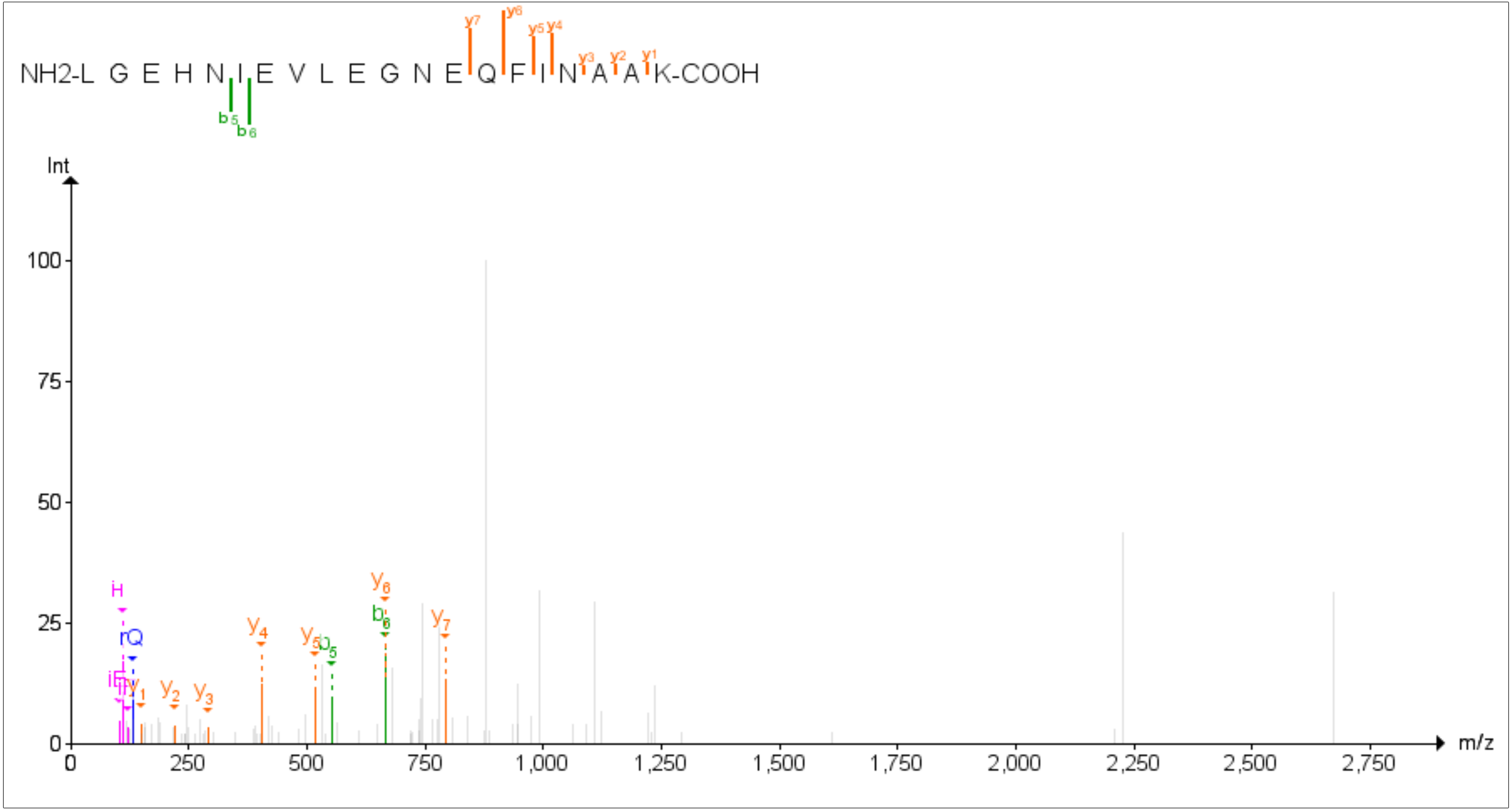

NH2-L G E H N I E V L E G N E Q F I N A A K-COOH
b5
b6
y7
y6
y5
y4
y3
y2
y1
Int
100
75
50
25
0
m/z
0
250
500
750
1,000
1,250
1,500
1,750
2,000
2,250
2,500
2,750
rQ
y4
y5
b5
y6
b6
y7
y1
y2
y3

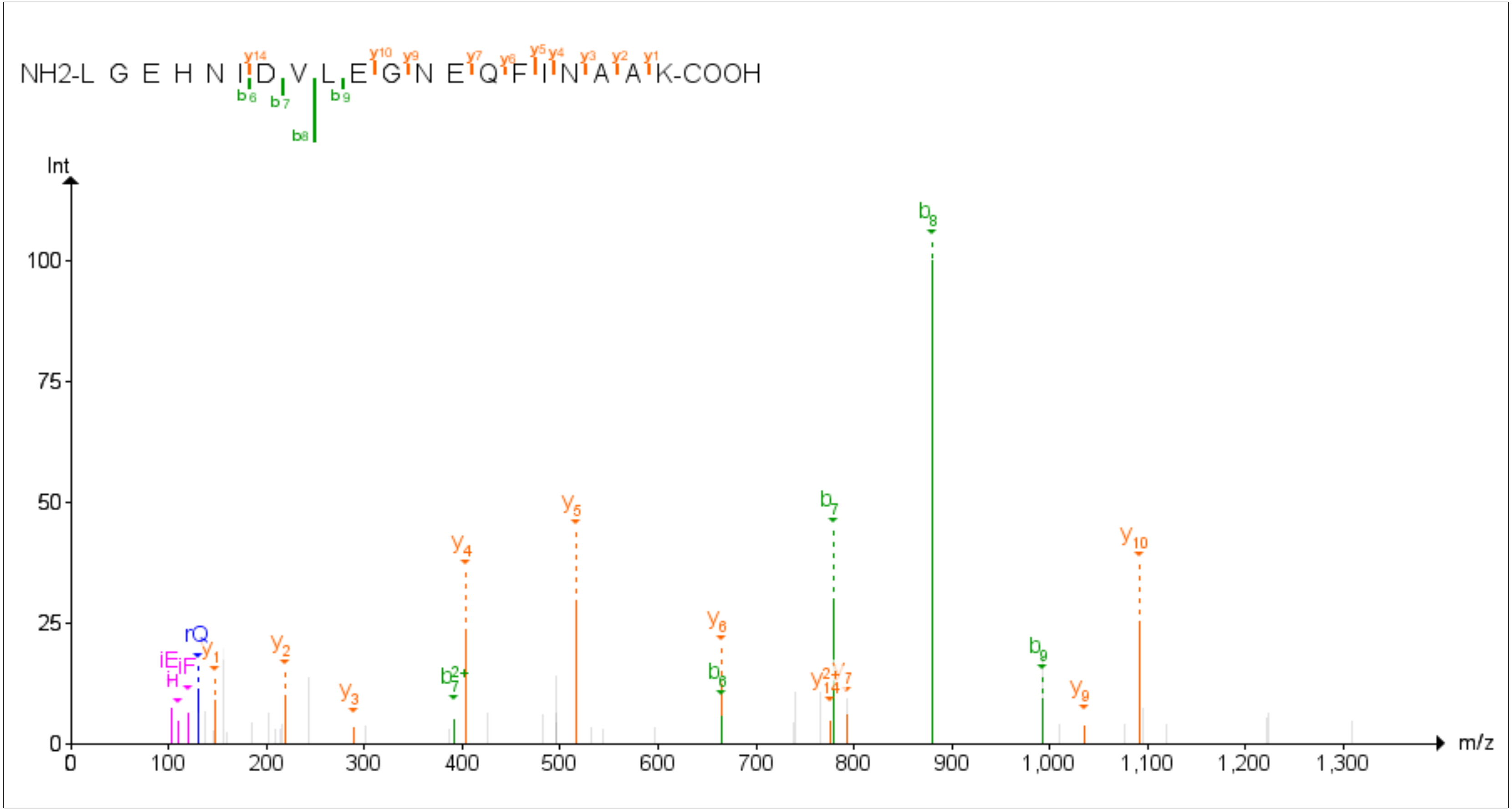

NH2-L G E H N I D V L E G N E Q F I N A A K-COOH
y14 y10 y9 y7 y6 y5 y4 y3 y2 y1
b6 b7 b8 b9
Int
m/z
0
25
50
75
100
0
100
200
300
400
500
600
700
800
900
1,000
1,100
1,200
1,300
b8
y5
y4
b7
y10
y6
b6
b9
y9
y3
y2
y1
rQ
iE
iF
H

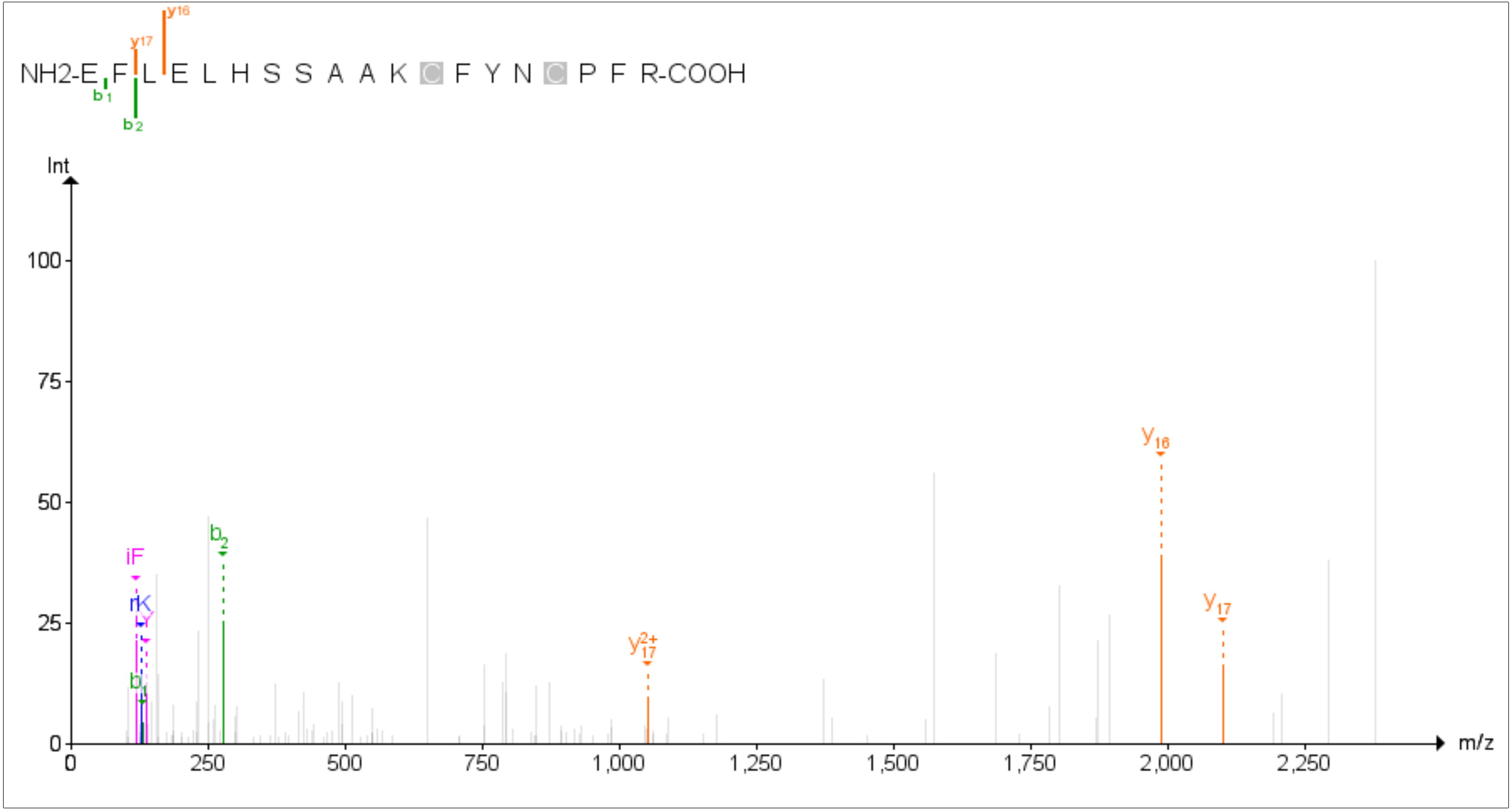

NH2-E F L E L H S S A A K C F Y N C P F R-COOH
y16
y17
b1
b2
Int
100
75
50
25
0
iF
rK
b2
b1
y17 2+
y16
y17
0
250
500
750
1,000
1,250
1,500
1,750
2,000
2,250
m/z

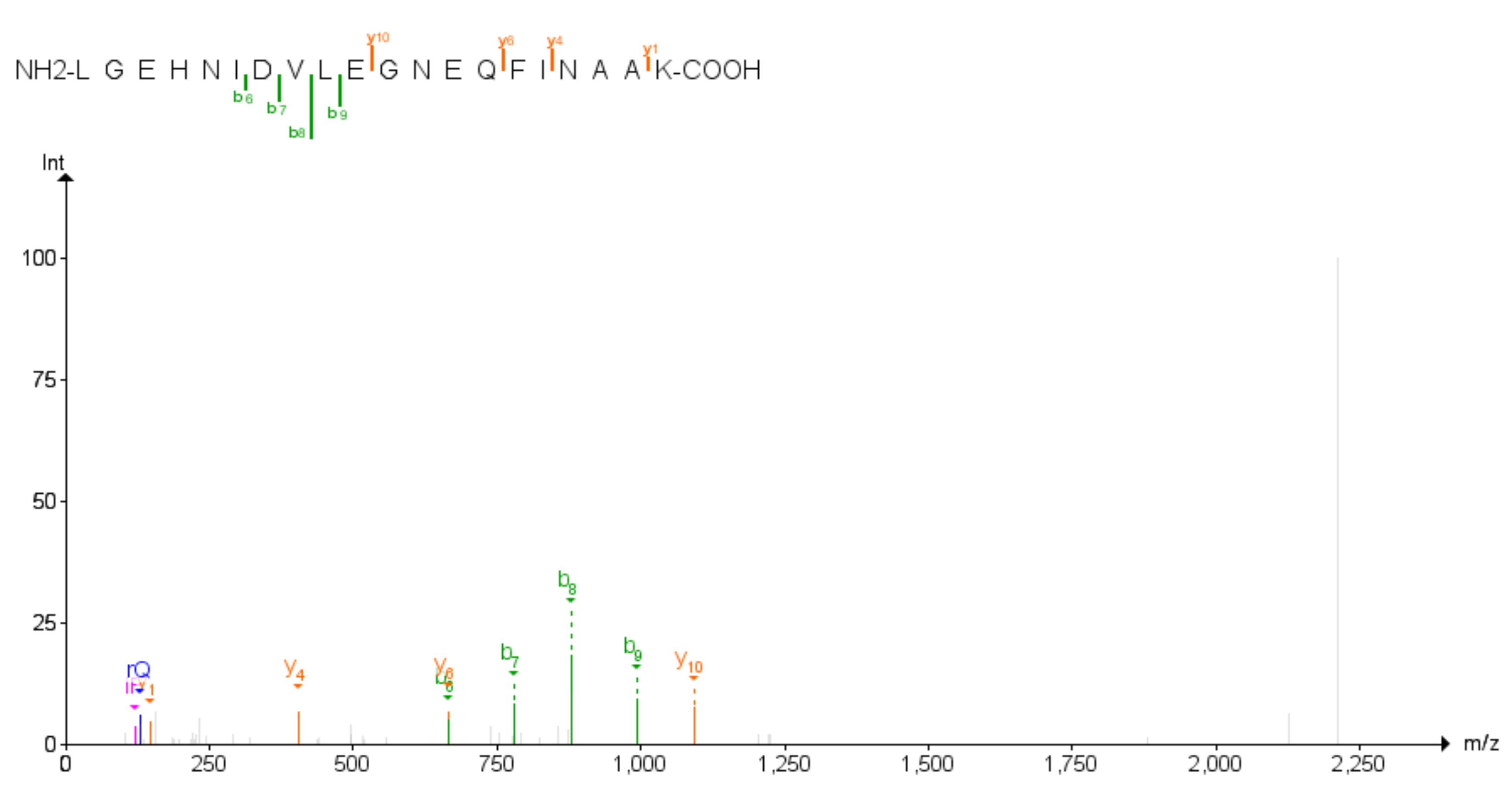
NH2-L G E H N I D V L E G N E Q F I N A A K-COOH
y10
y6
y4
y1
b6
b7
b8
b9
Int
100
75
50
25
0
b8
b7
b9
y10
y4
y6
b6
IQ
y1
m/z
0
250
500
750
1,000
1,250
1,500
1,750
2,000
2,250

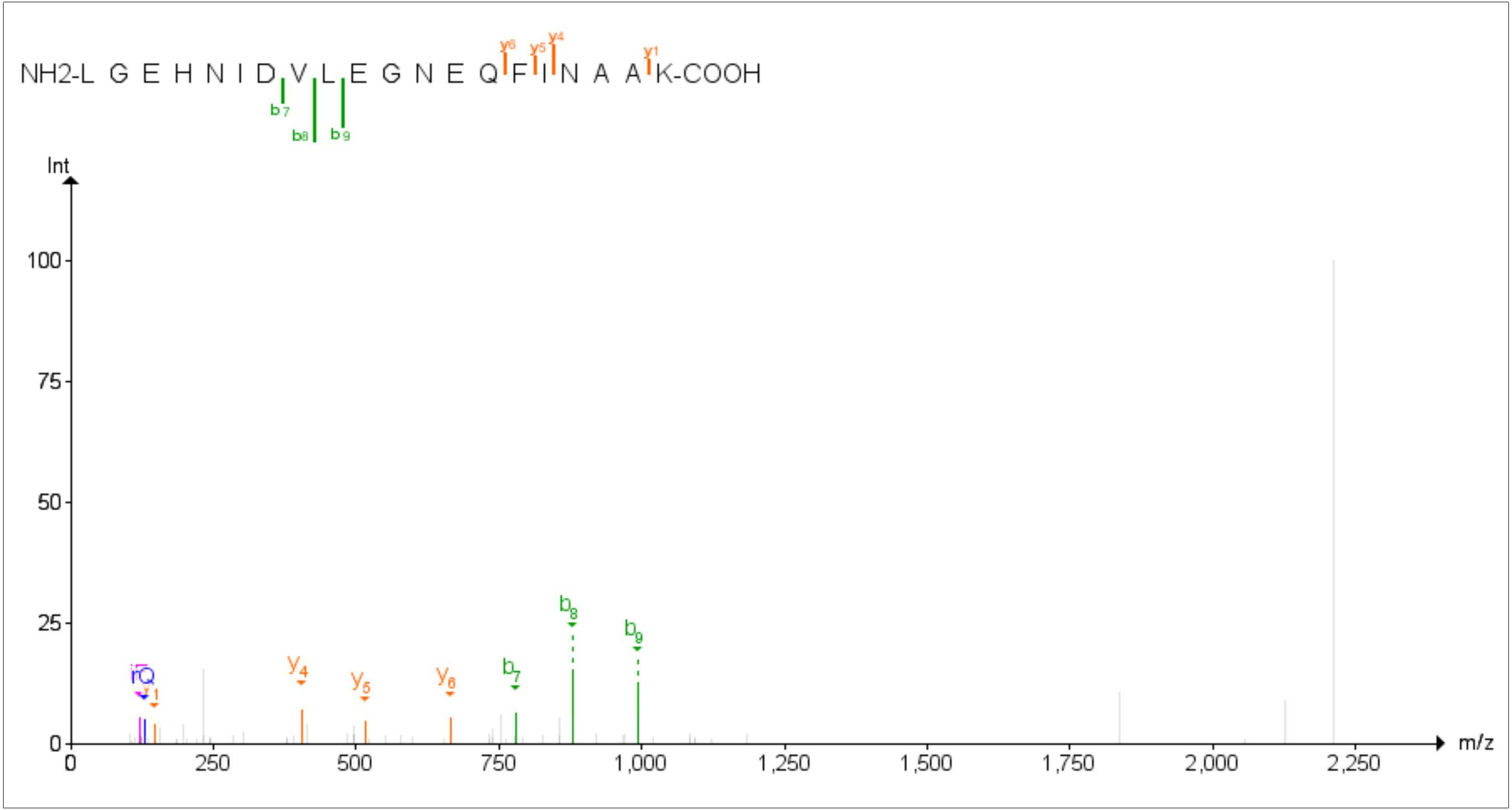

NH2-L G E H N I D V L E G N E Q F I N A A K-COOH
y6 y5 y4 y1
b7 b8 b9
Int
100
75
50
25
0
m/z
0 250 500 750 1,000 1,250 1,500 1,750 2,000 2,250
iQ
y1
y4
y5
y6
b7
b8
b9

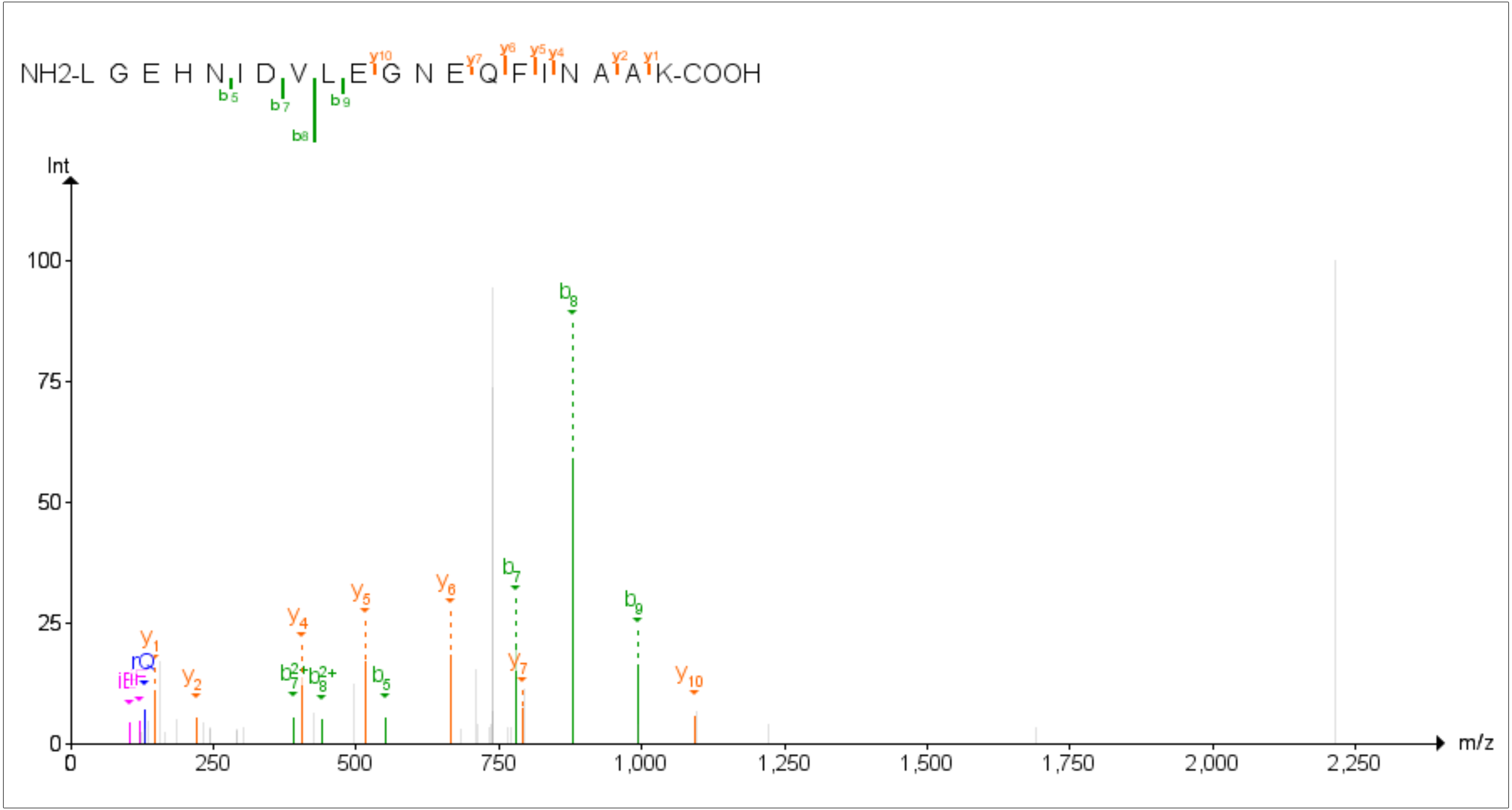

NH2-L G E H N I D V L E G N E Q F I N A A K-COOH
b5
b7
b8
b9
y10
y7
y6
y5
y4
y2
y1
Int
100
75
50
25
0
m/z
0
250
500
750
1,000
1,250
1,500
1,750
2,000
2,250

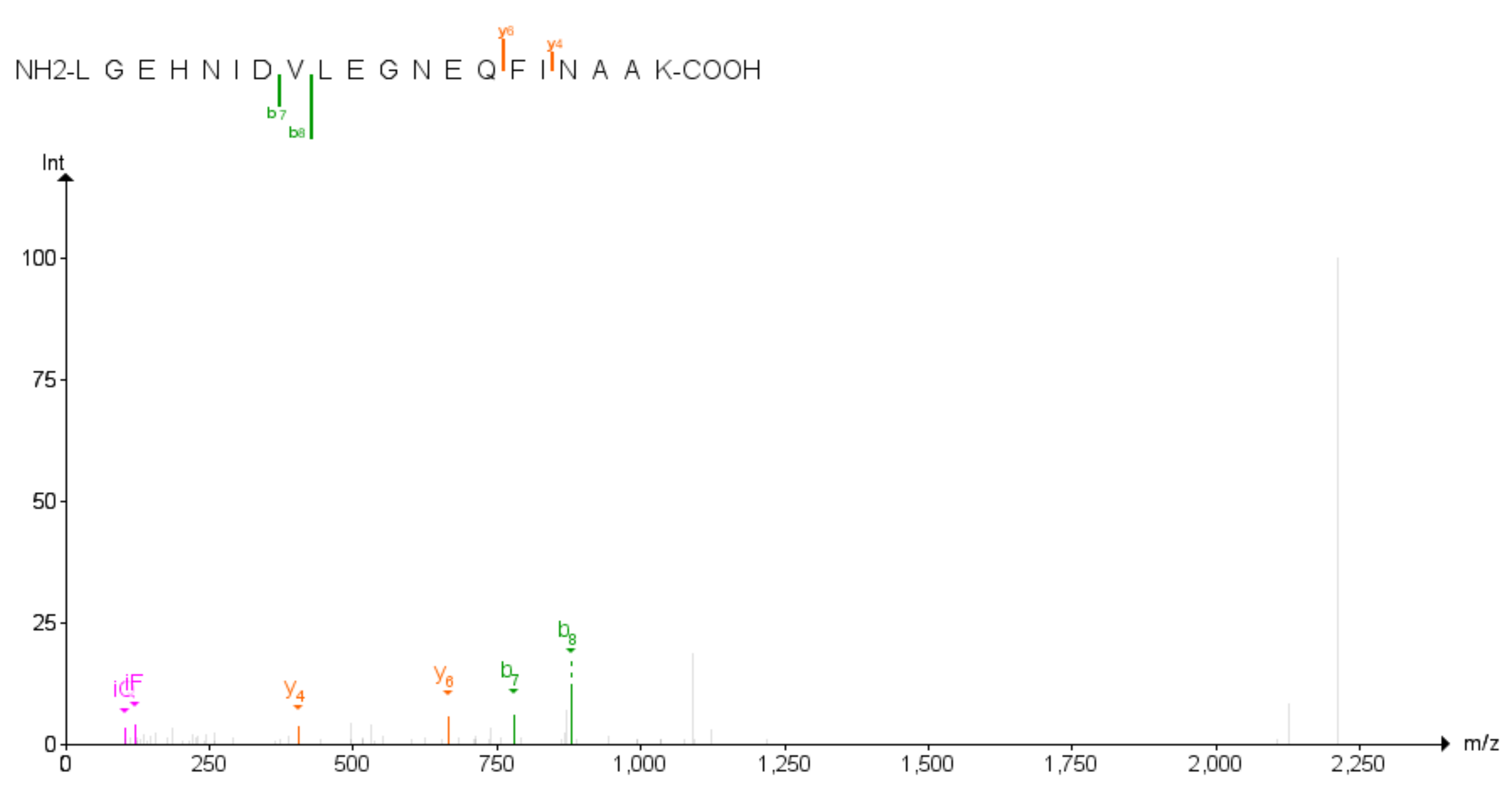

NH2-L G E H N I D V L E G N E Q F I N A A K-COOH
y6
y4
b7
b8
Int
100
75
50
25
0
0
250
500
750
1,000
1,250
1,500
1,750
2,000
2,250
m/z
y4
y6
b7
b8

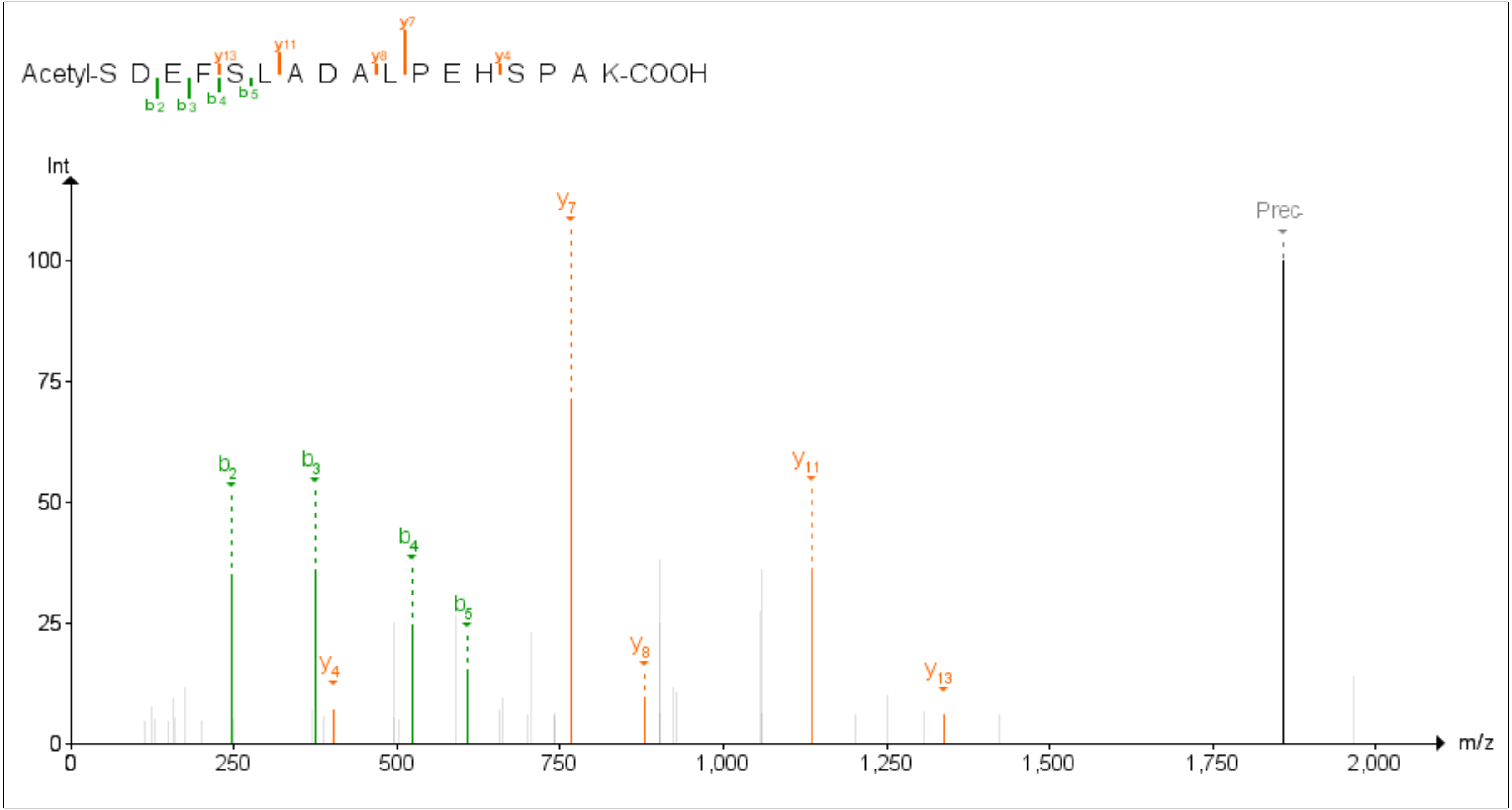
Acetyl-S D E F S L A D A L P E H S P A K-COOH
b2 b3 b4 b5
y13 y11 y8 y7 y4
Int
100
75
50
25
0
Prec
y7
b2
b3
b4
b5
y4
y8
y11
y13
0 250 500 750 1,000 1,250 1,500 1,750 2,000
m/z

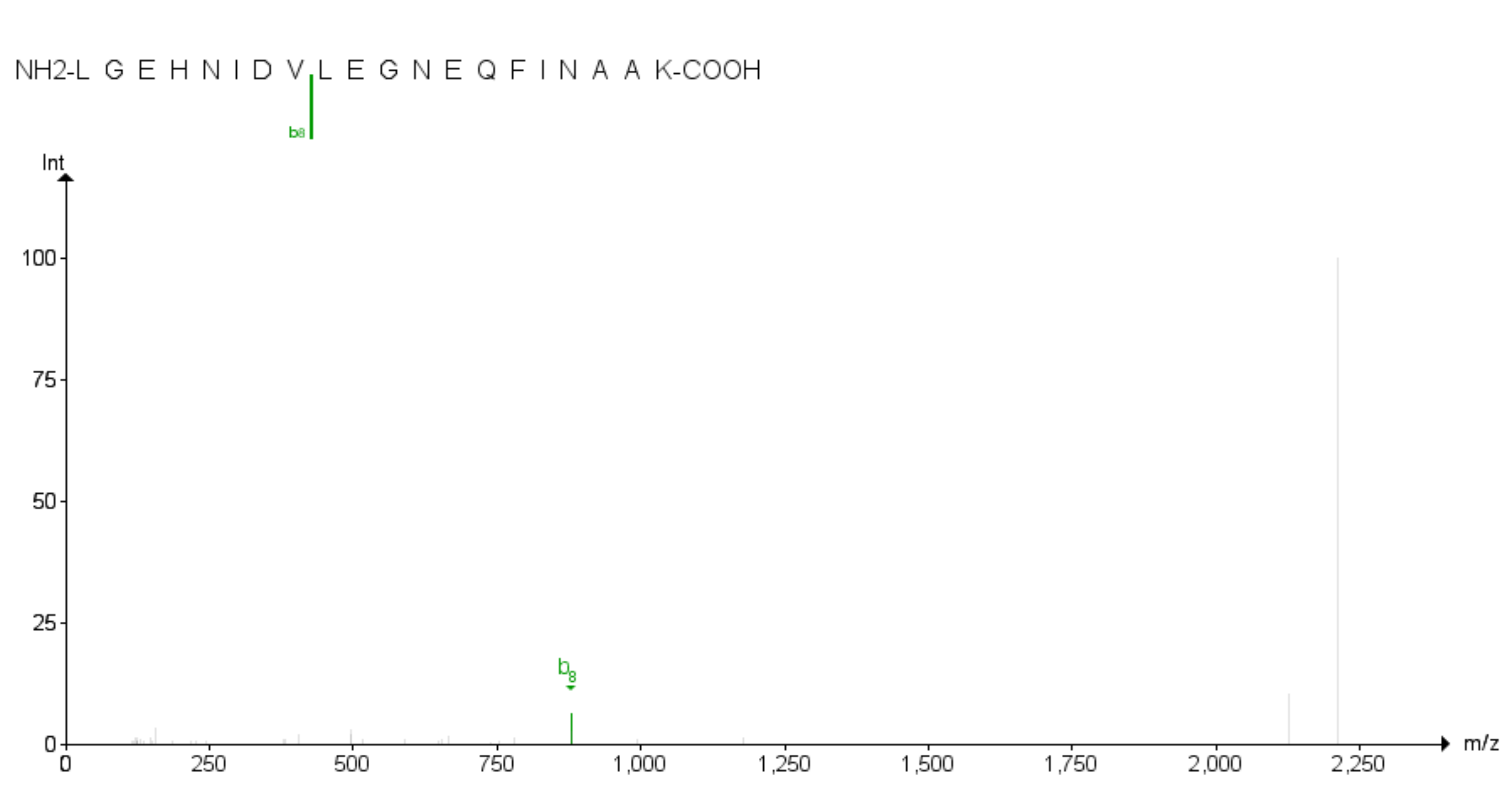
NH2-L G E H N I D V L E G N E Q F I N A A K-COOH
b8
Int
100
75
50
25
0
b8
m/z
0
250
500
750
1,000
1,250
1,500
1,750
2,000
2,250

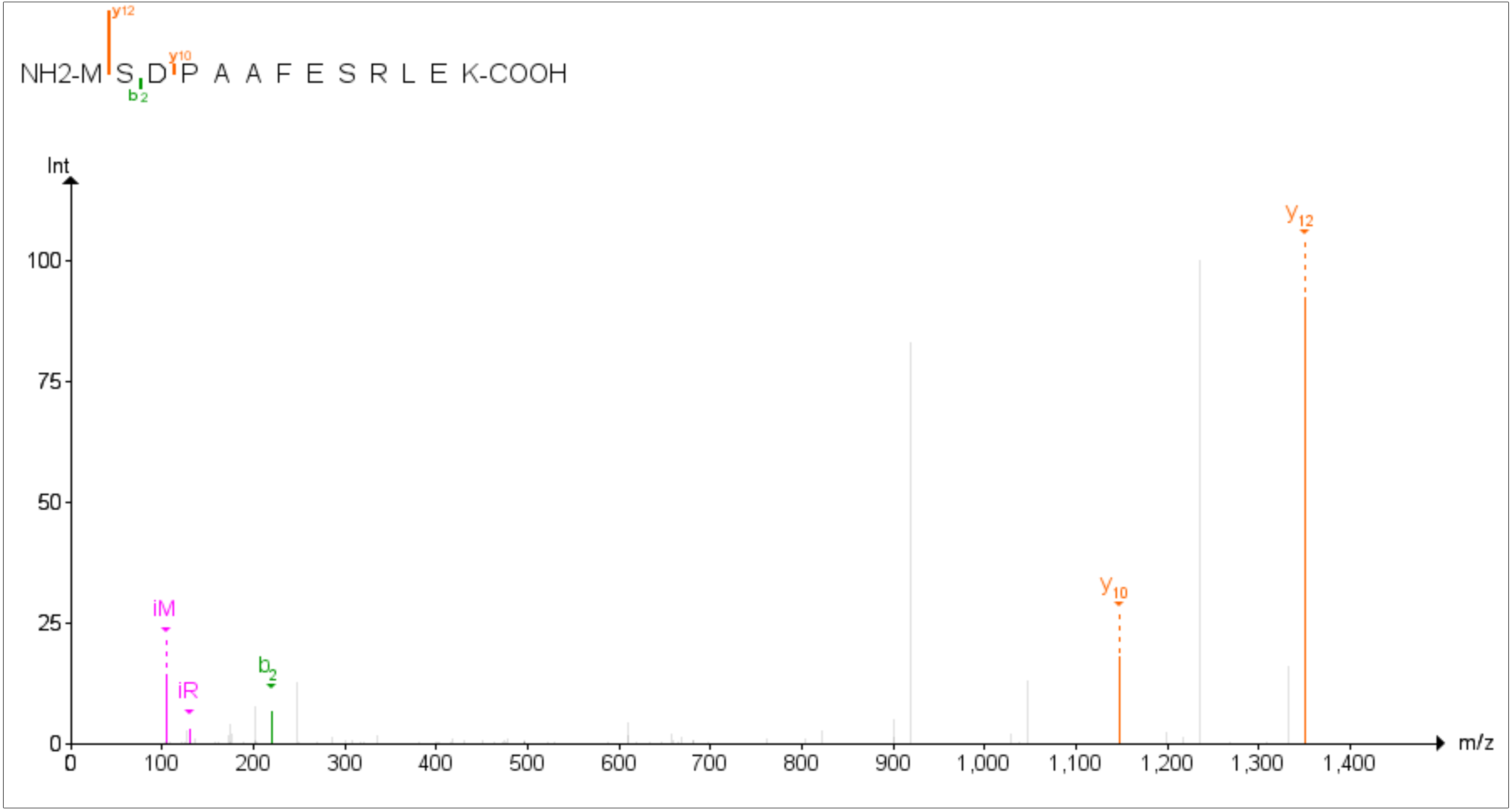

NH2-M S D P A A F E S R L E K-COOH
y12
y10
b2
Int
100
75
50
25
0
iM
iR
b2
y10
y12
0
100
200
300
400
500
600
700
800
900
1,000
1,100
1,200
1,300
1,400
m/z

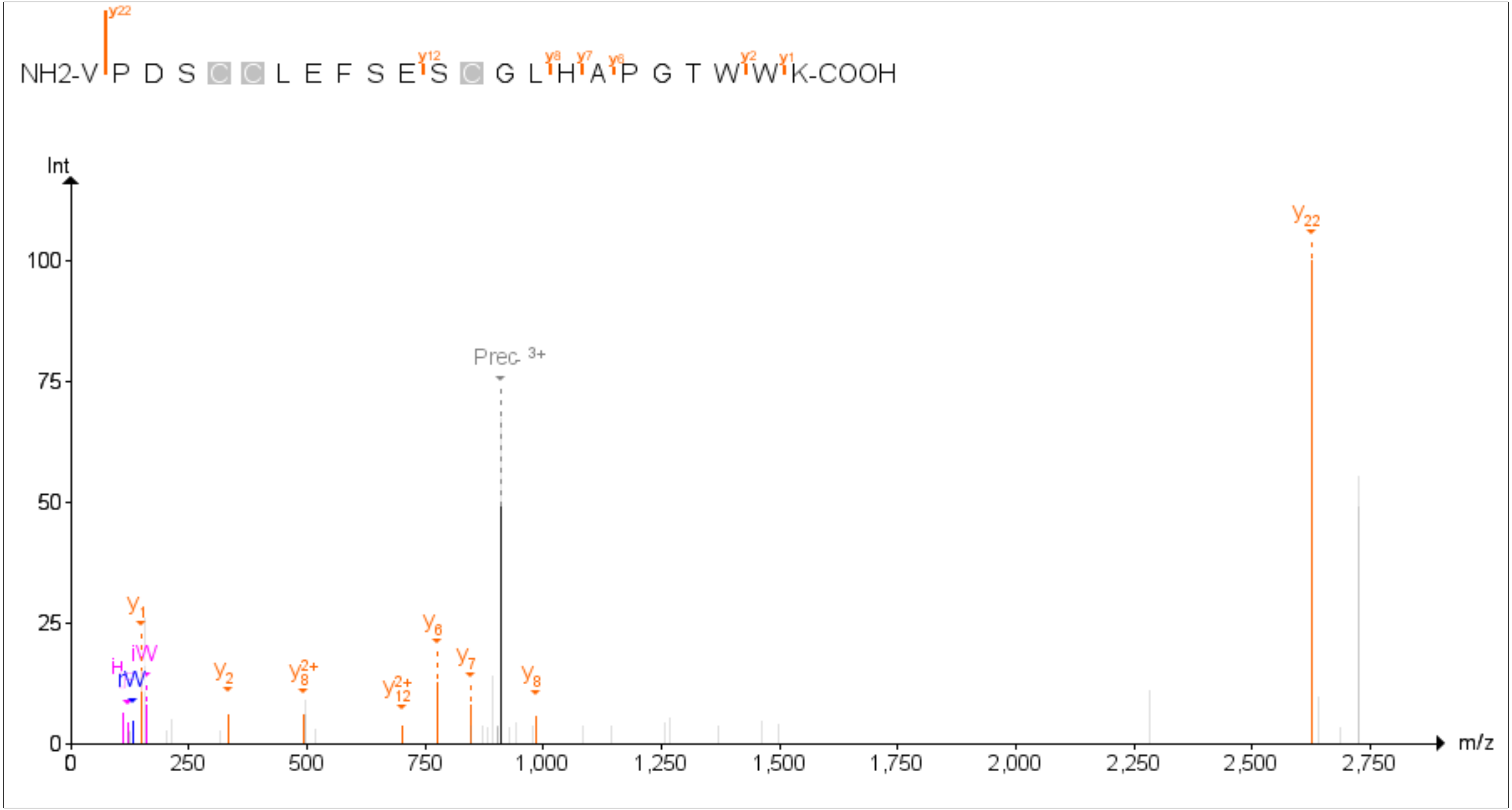

NH2-V P D S C C L E F S E S C G L H A P G T W W K-COOH
y22
y12
y8
y7
y6
y2
y1
Int
100
75
50
25
0
Prec. 3+
y22
y1
iW
iH
rW
y2
y8 2+
y12 2+
y6
y7
y8
0
250
500
750
1,000
1,250
1,500
1,750
2,000
2,250
2,500
2,750
m/z

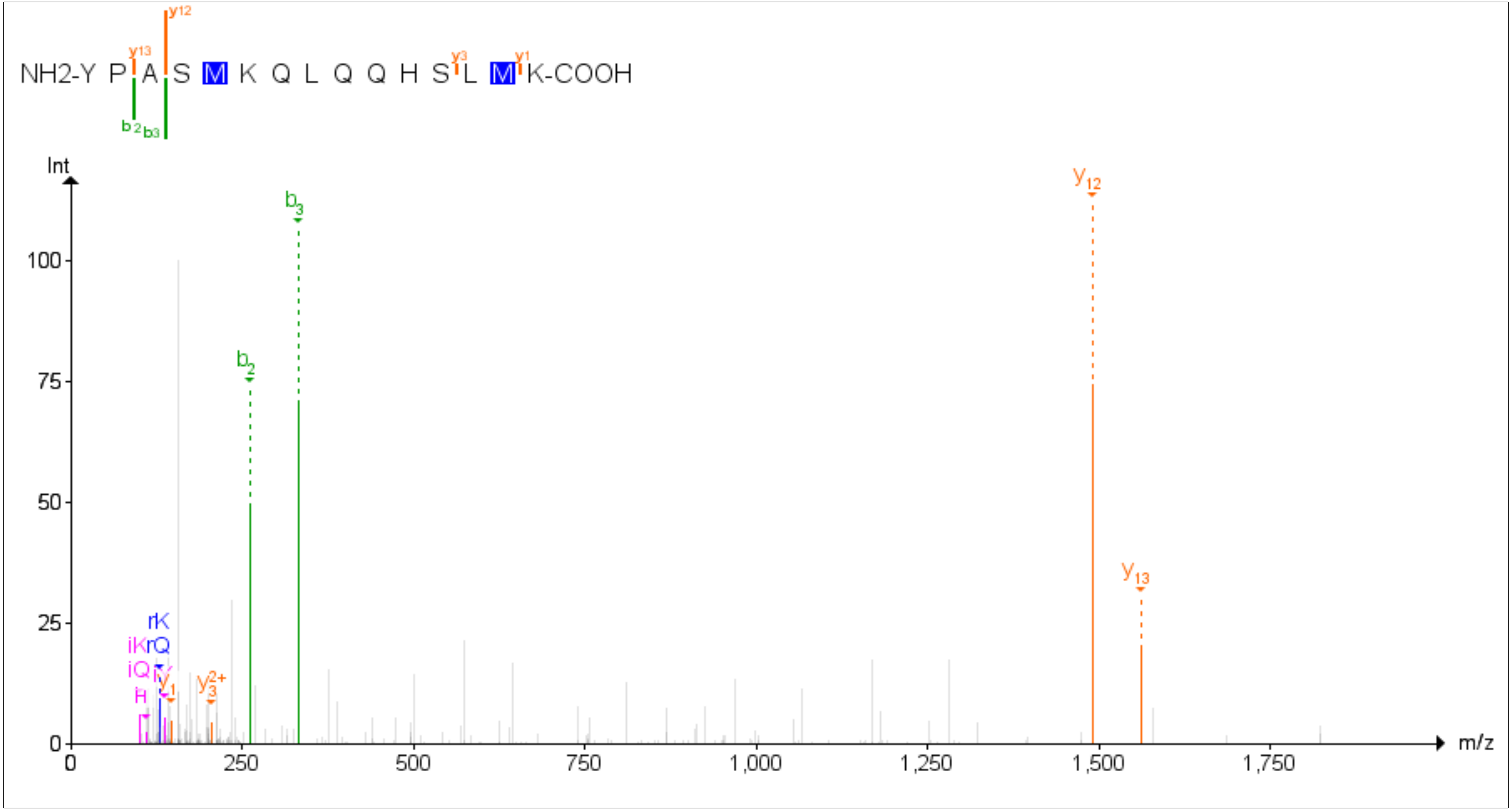

NH2-Y P A S M K Q L Q Q H S L M K-COOH
y12
y13
y3
y1
b2
b3
Int
100
75
50
25
0
b3
b2
y12
y13
rK
iK rQ
iQ
H
y1
y3 2+
0
250
500
750
1,000
1,250
1,500
1,750
m/z

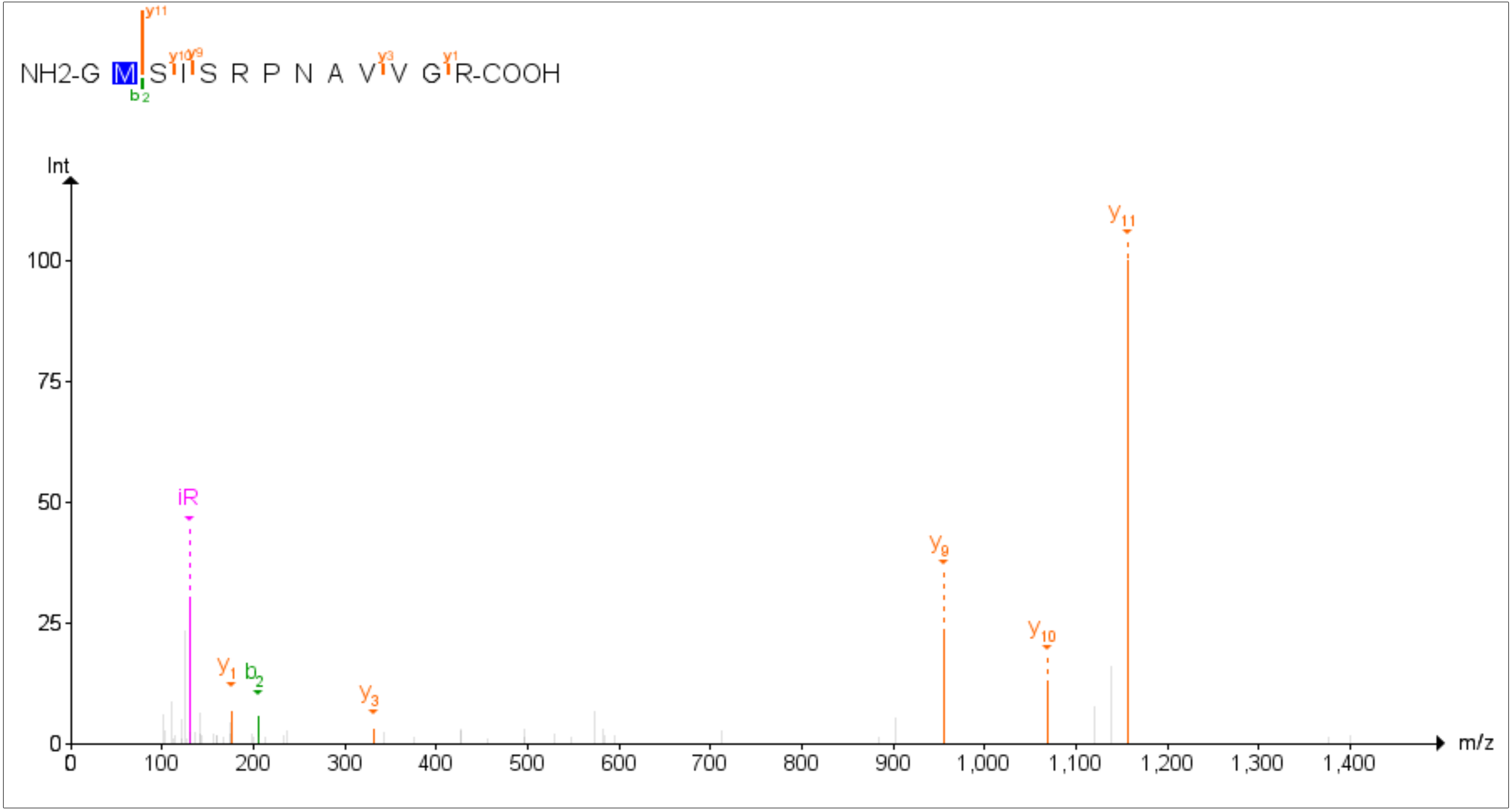
NH2-G M S I S R P N A V V G R-COOH
y11
y10
y9
y3
y1
b2
Int
100
75
50
25
0
iR
y1
b2
y3
y9
y10
y11
0
100
200
300
400
500
600
700
800
900
1,000
1,100
1,200
1,300
1,400
m/z

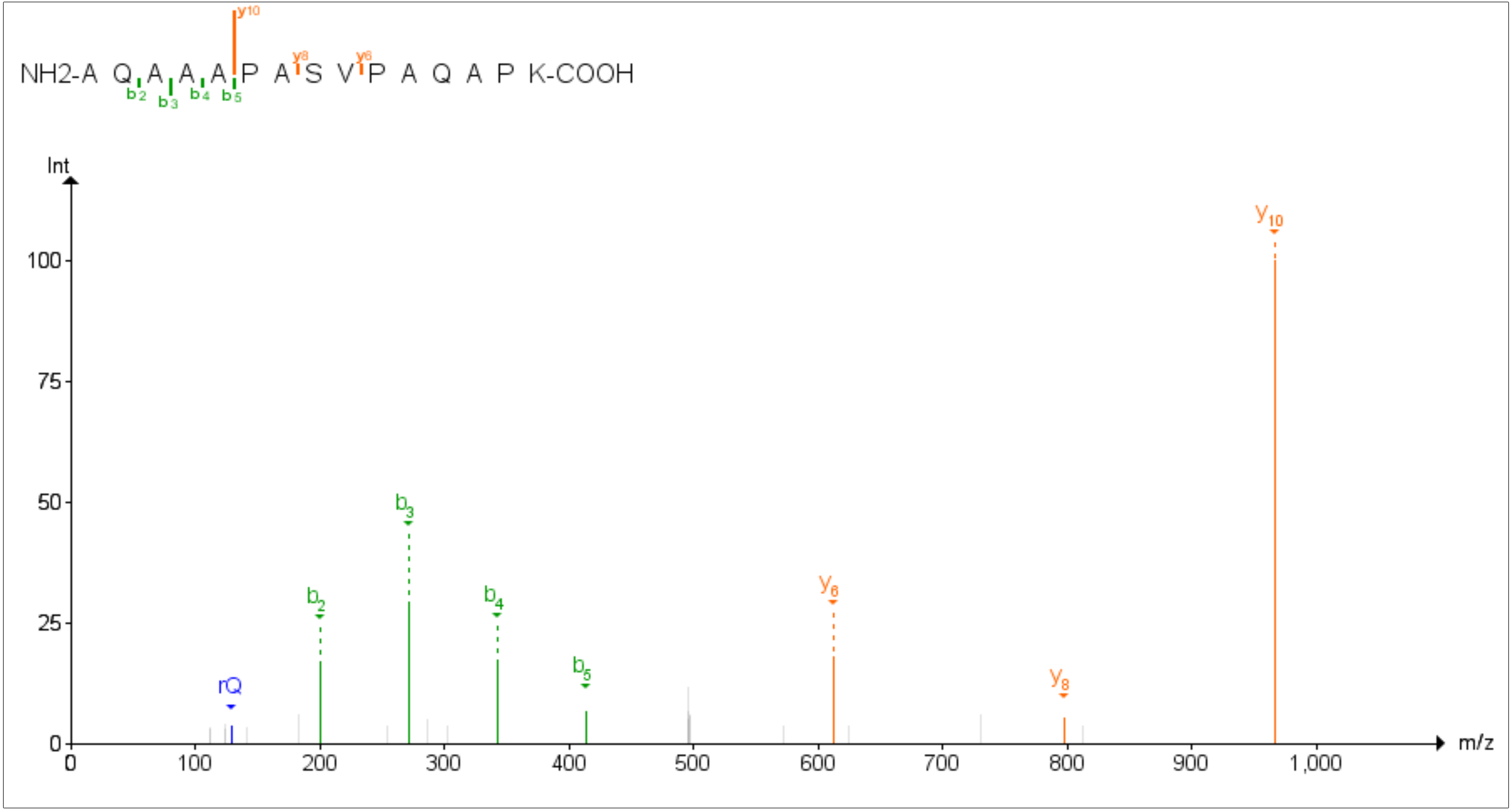

NH2-A Q A A A P A S V P A Q A P K-COOH
y10
y8
y6
b2
b3
b4
b5
Int
100
75
50
25
0
rQ
b2
b3
b4
b5
y6
y8
y10
0
100
200
300
400
500
600
700
800
900
1,000
m/z

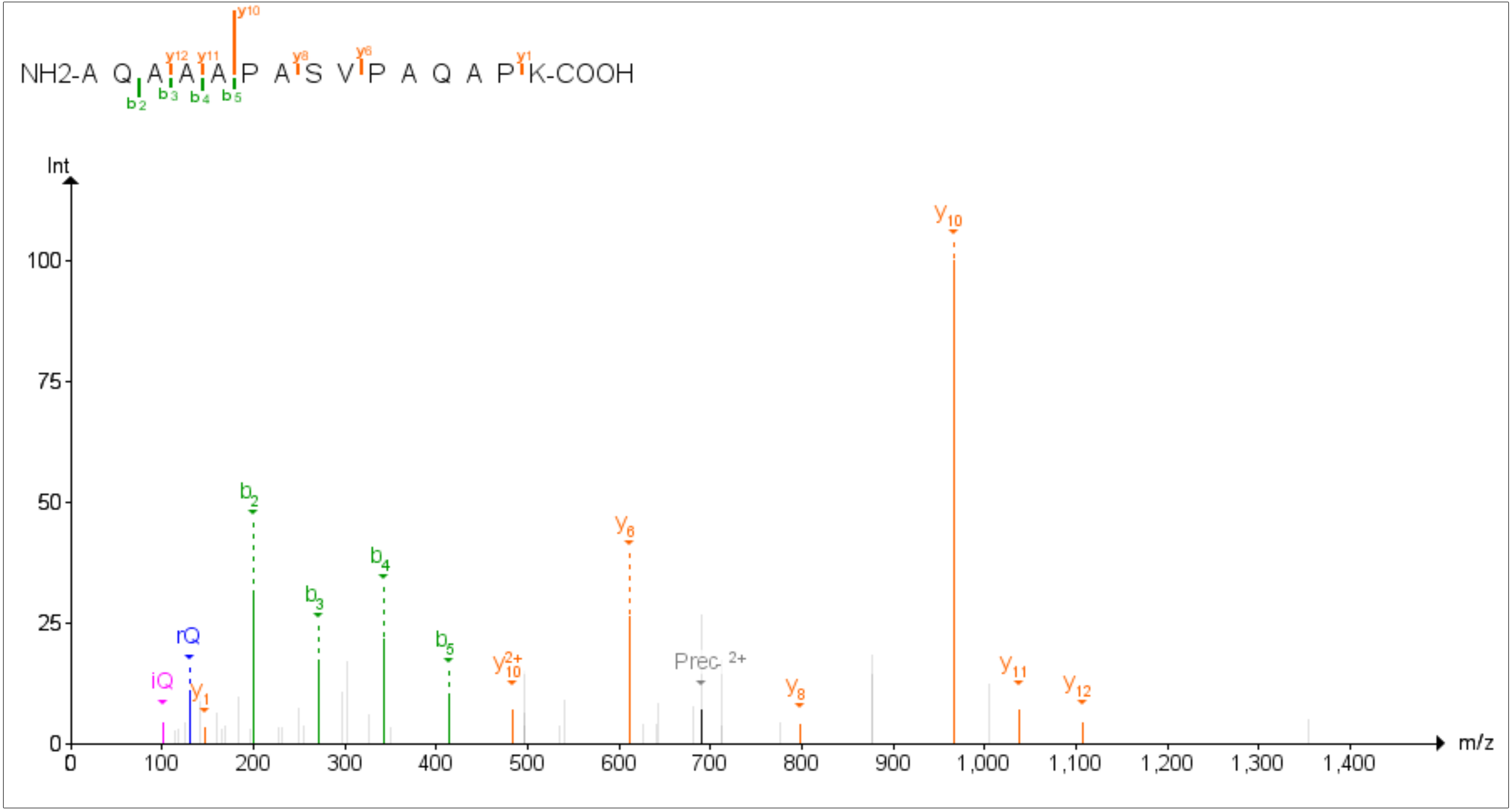

NH2-A Q A A A P A S V P A Q A P K-COOH
y10
y12
y11
y8
y6
y1
b2
b3
b4
b5
Int
100
75
50
25
0
y10
b2
y6
b4
b3
b5
rQ
iQ
y1
y10 2+
Prec- 2+
y8
y11
y12
0
100
200
300
400
500
600
700
800
900
1,000
1,100
1,200
1,300
1,400
m/z

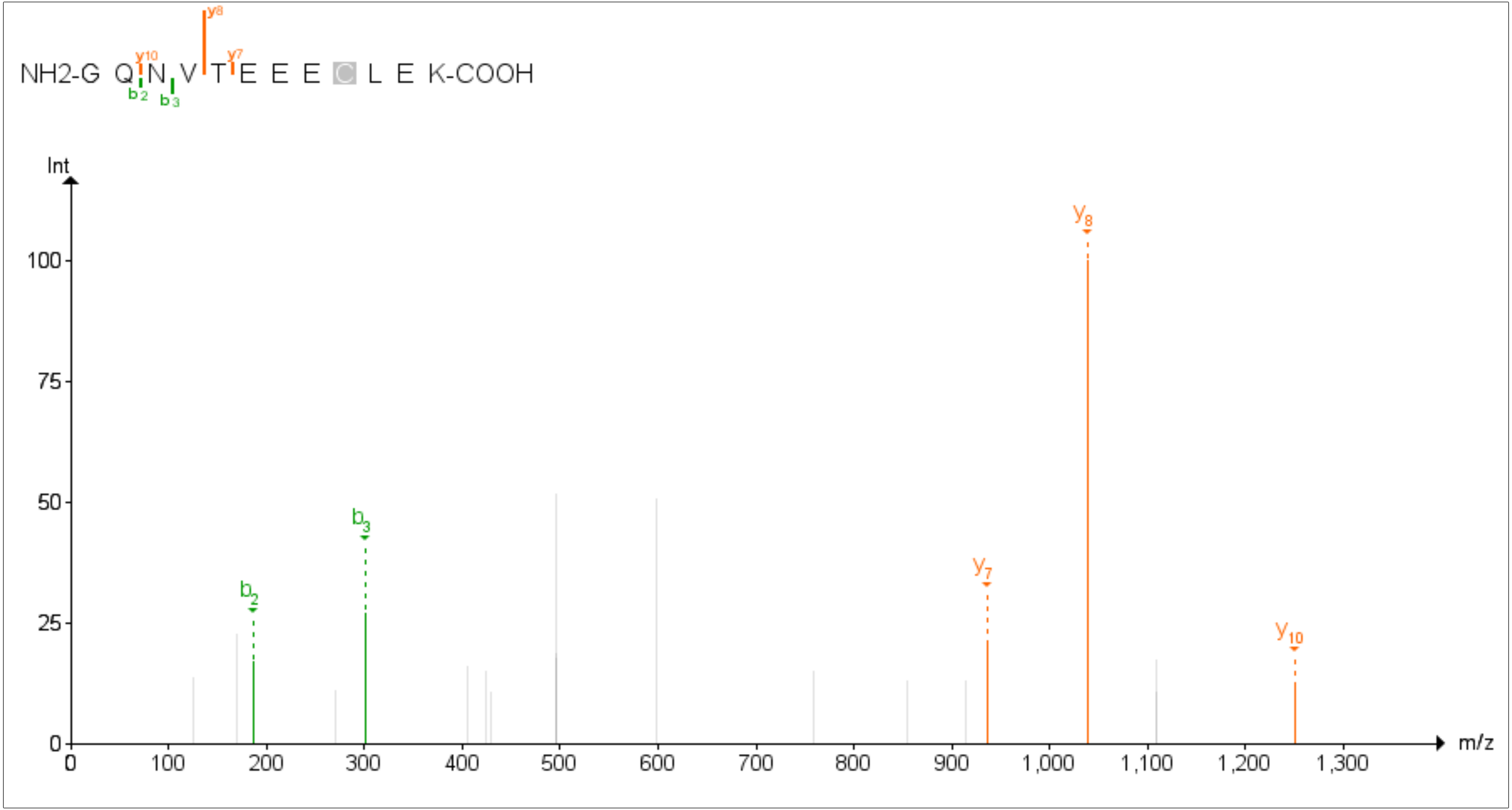

NH2-G Q N V T E E E C L E K-COOH
y10 y8 y7
b2 b3
Int
100
75
50
25
0
b2
b3
y7
y8
y10
0 100 200 300 400 500 600 700 800 900 1,000 1,100 1,200 1,300
m/z

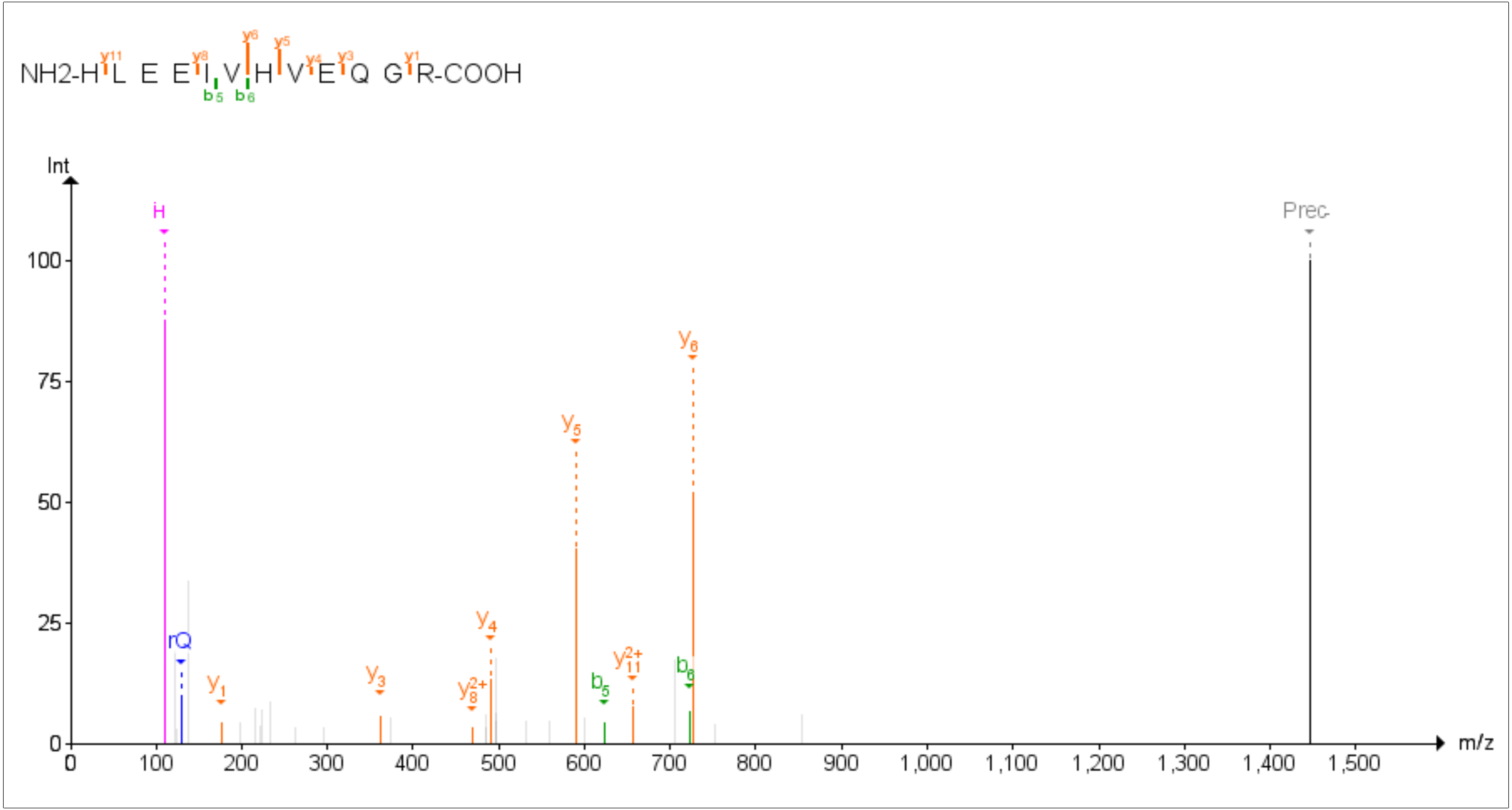

NH2-H L E E I V H V E Q G R-COOH
y11
y8
y6
y5
y4
y3
y1
b5
b6
Int
H
Prec
100
75
50
25
0
rQ
y1
y3
y4
y8 2+
y5
b5
y11 2+
b6
y6
0
100
200
300
400
500
600
700
800
900
1,000
1,100
1,200
1,300
1,400
1,500
m/z

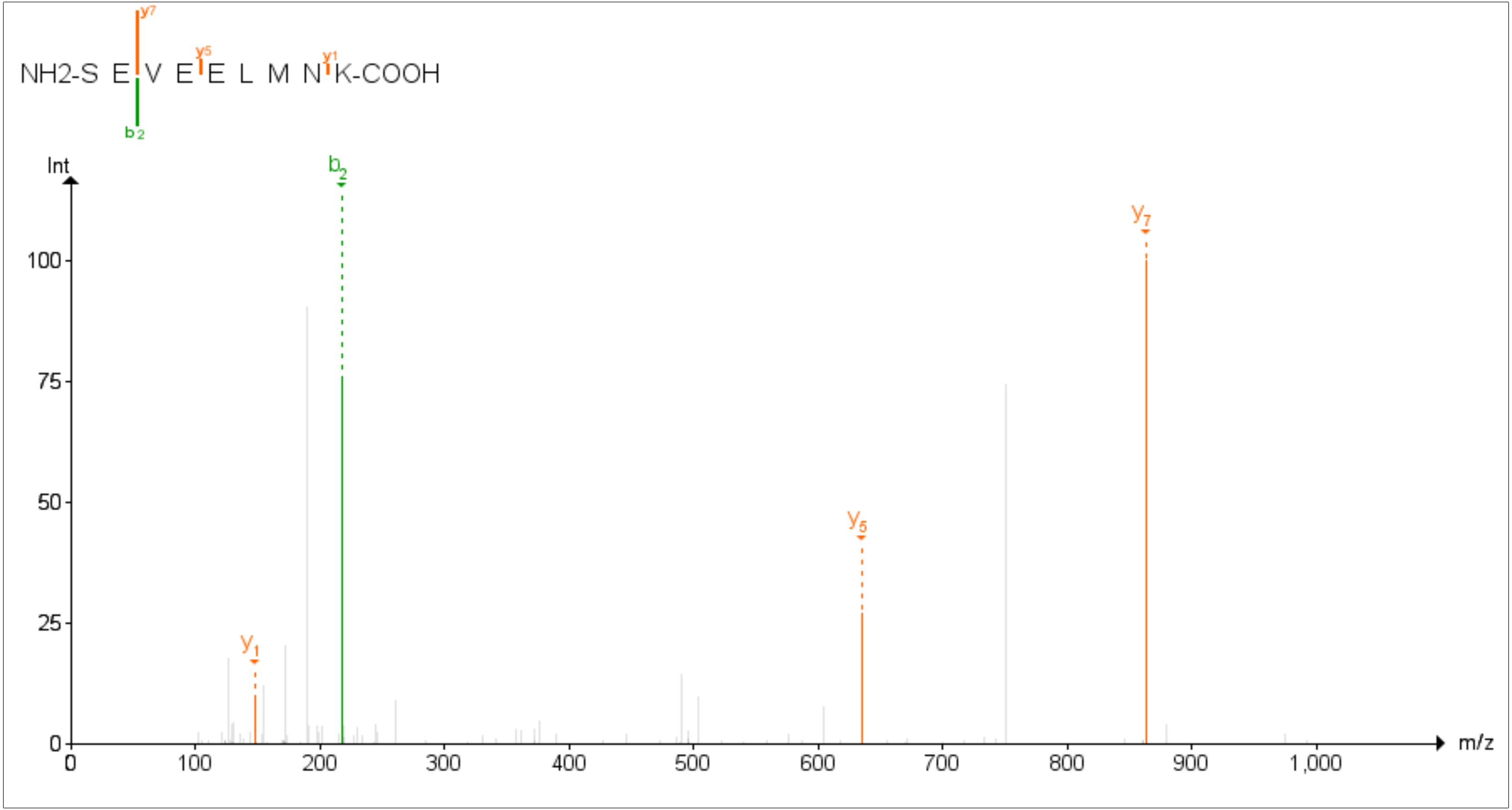
NH2-S E V E E L M N K-COOH
y7
y5
y1
b2
Int
100
75
50
25
0
m/z
0
100
200
300
400
500
600
700
800
900
1,000

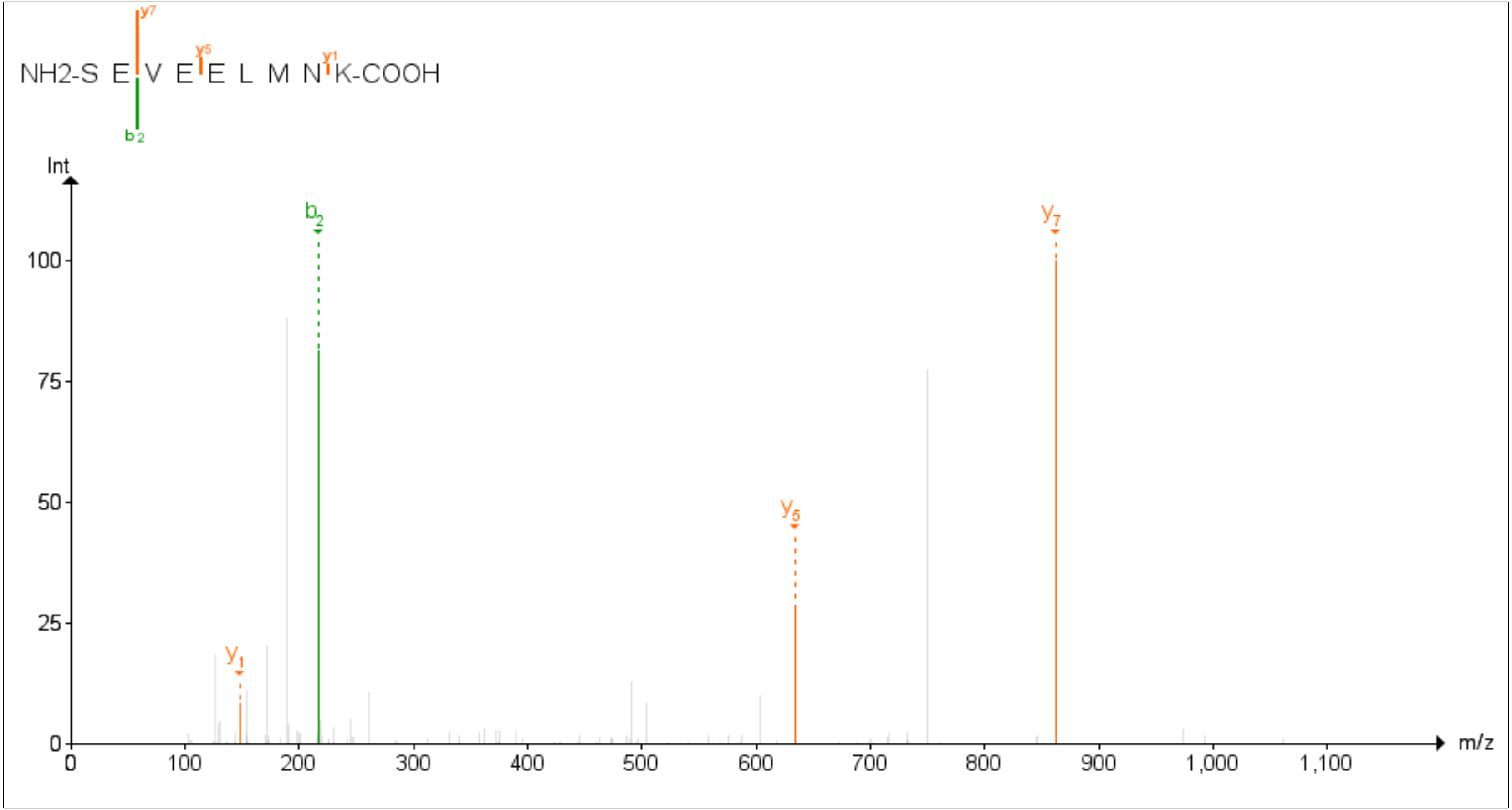

NH2-S E V E E L M N K-COOH
y7
y5
y1
b2
Int
m/z
0
25
50
75
100
0
100
200
300
400
500
600
700
800
900
1,000
1,100
b_2
y_7
y_5
y_1

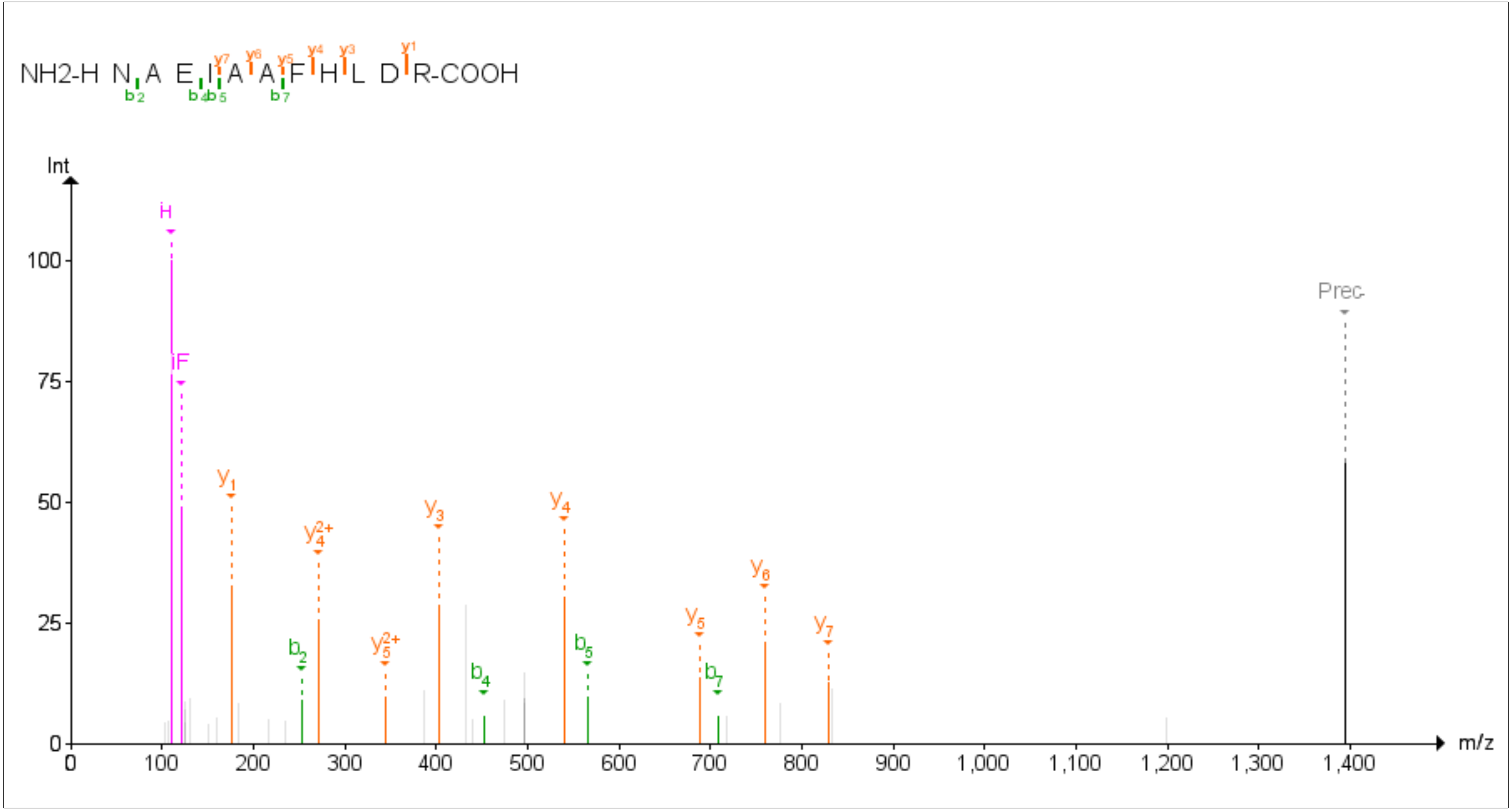

NH2-H N A E I A A F H L D R-COOH
b2 b4 b5 b7
y7 y6 y5 y4 y3 y1
Int
m/z
iH
iF
y1
y4 2+
y3
y4
y6
y5 2+
y5
y7
b2
b4
b5
b7
Prec
0
25
50
75
100
0
100
200
300
400
500
600
700
800
900
1,000
1,100
1,200
1,300
1,400

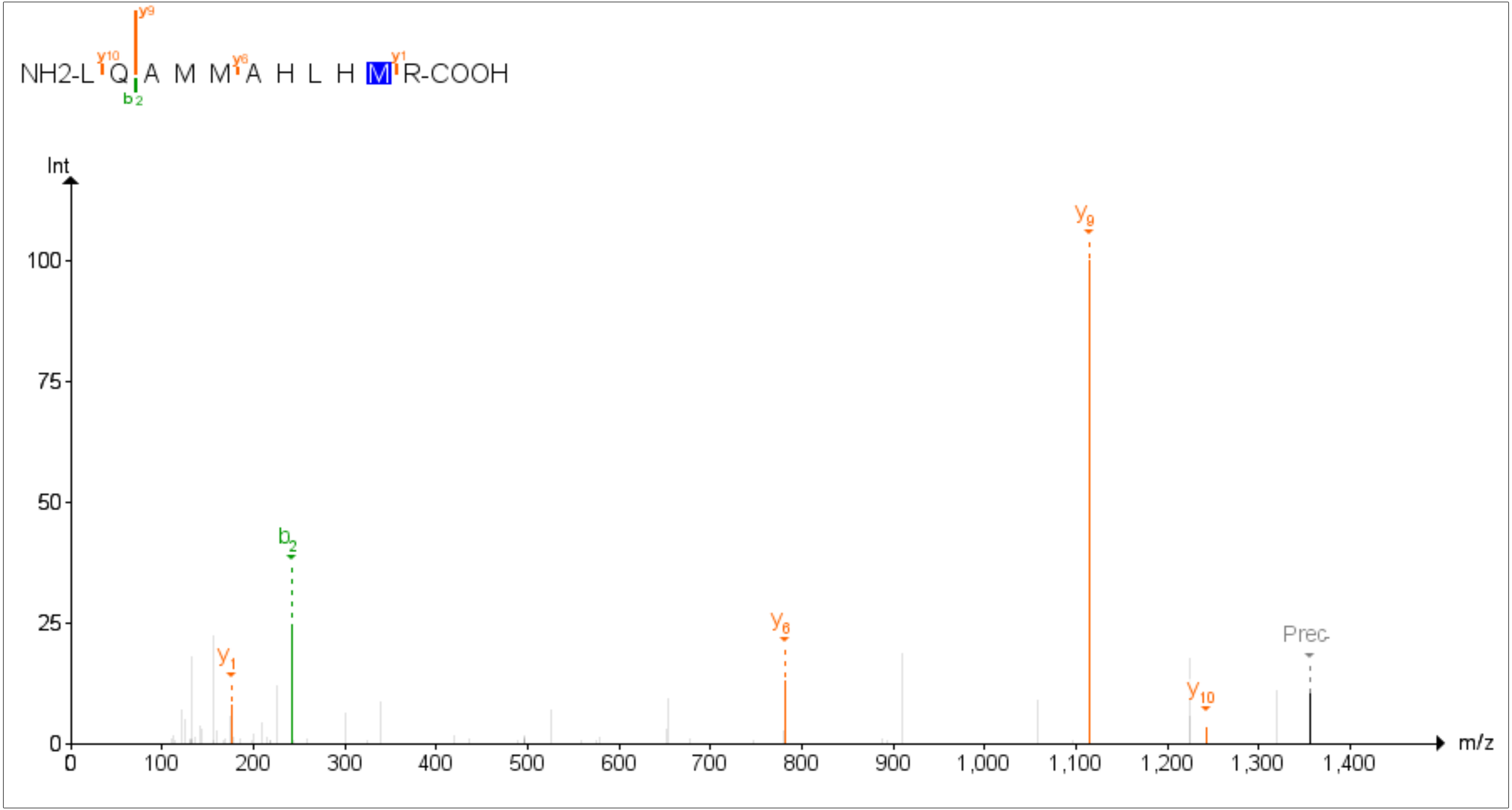

NH2-L Q A M M A H L H M R-COOH
y9
y10
y6
y1
b2
Int
100
75
50
25
0
y9
b2
y6
y1
Prec
y10
m/z
0
100
200
300
400
500
600
700
800
900
1,000
1,100
1,200
1,300
1,400

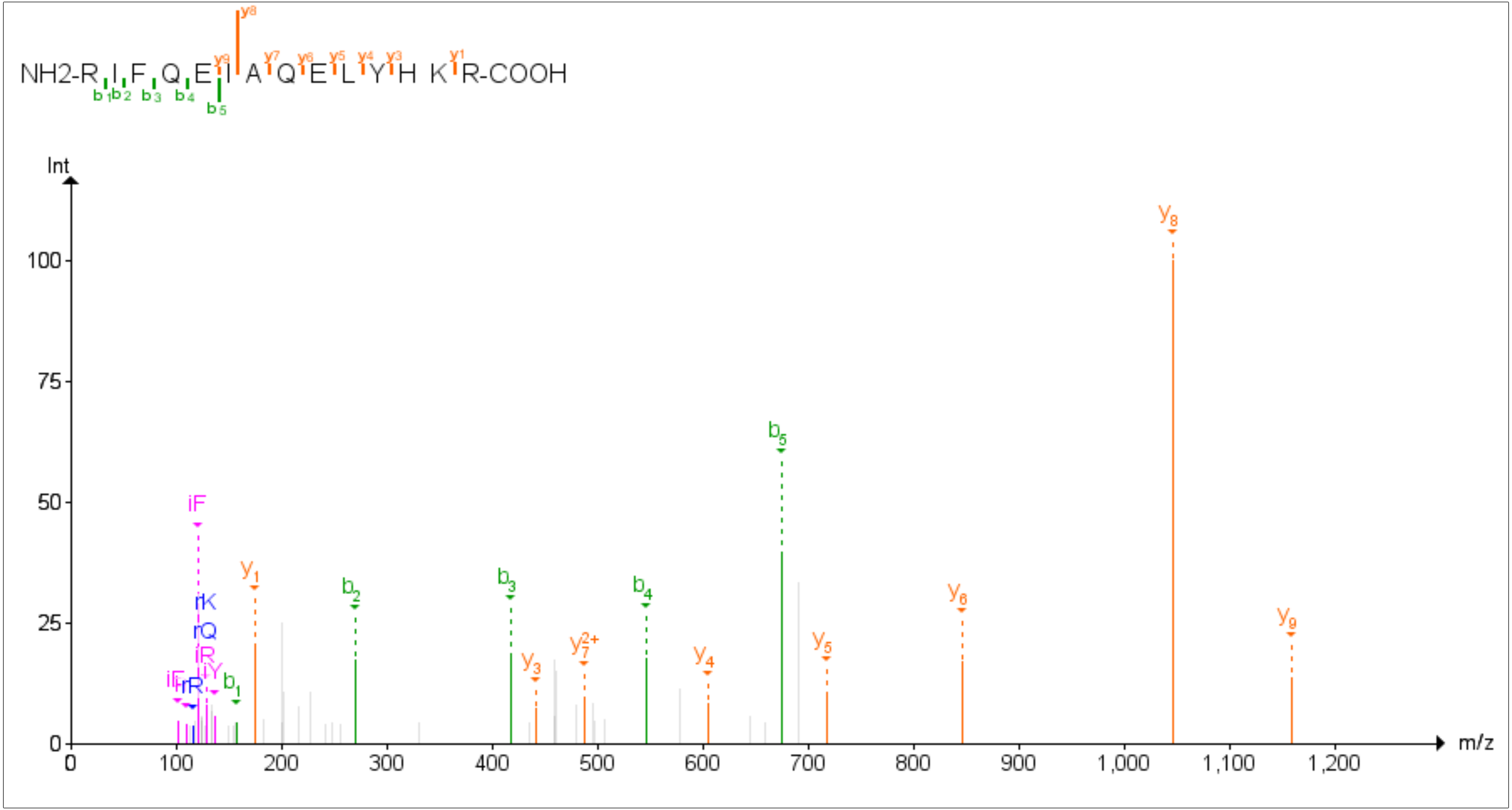

NH2-R I F Q E I A Q E L Y H K R-COOH
Int
m/z
iF
rK
rQ
iR
iY
rR
b1
b2
b3
b4
b5
y1
y3
y4
y5
y6
y7 2+
y8
y9

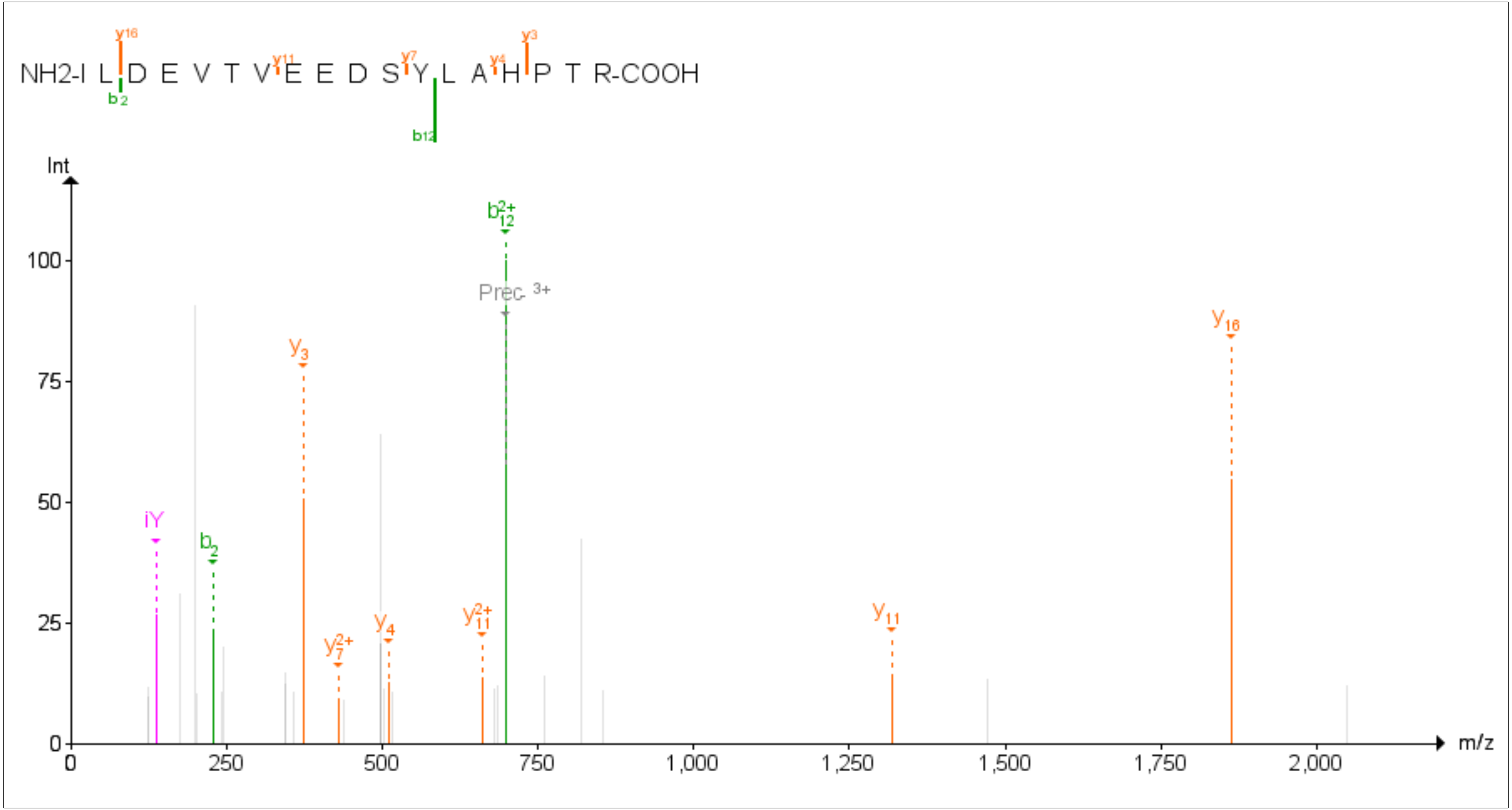

NH2-I L D E V T V E E D S Y L A H P T R-COOH
y16
y11
y7
y4
y3
b2
b12
Int
100
75
50
25
0
iY
b2
y3
y7 2+
y4
y11 2+
b12 2+
Prec 3+
y11
y16
0
250
500
750
1,000
1,250
1,500
1,750
2,000
m/z

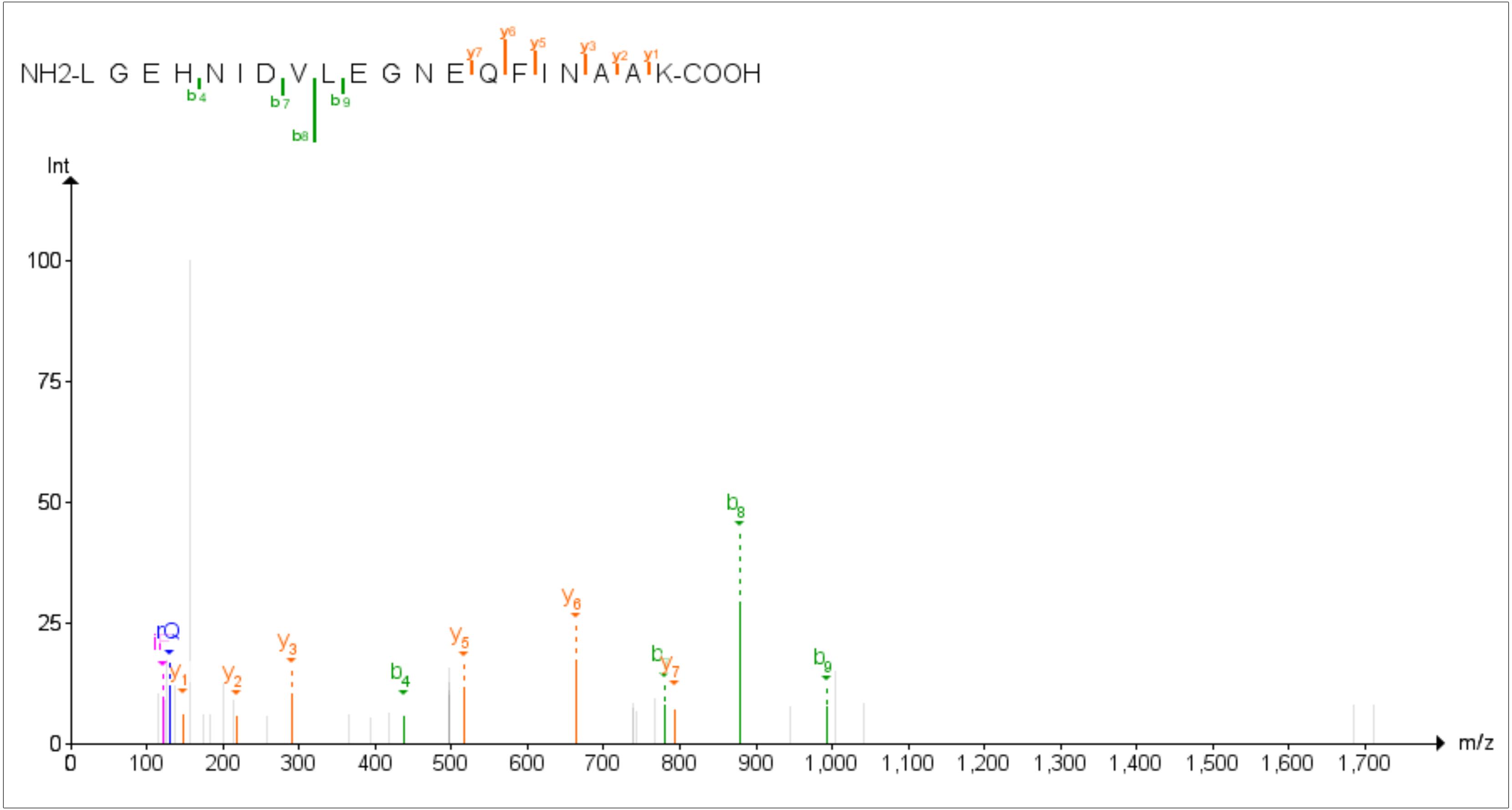

NH2-L G E H N I D V L E G N E Q F I N A A K-COOH
b4
b7
b8
b9
y7
y6
y5
y3
y2
y1
Int
100
75
50
25
0
iI
rQ
y1
y2
y3
b4
y5
y6
b7
y7
b8
b9
0
100
200
300
400
500
600
700
800
900
1,000
1,100
1,200
1,300
1,400
1,500
1,600
1,700
m/z

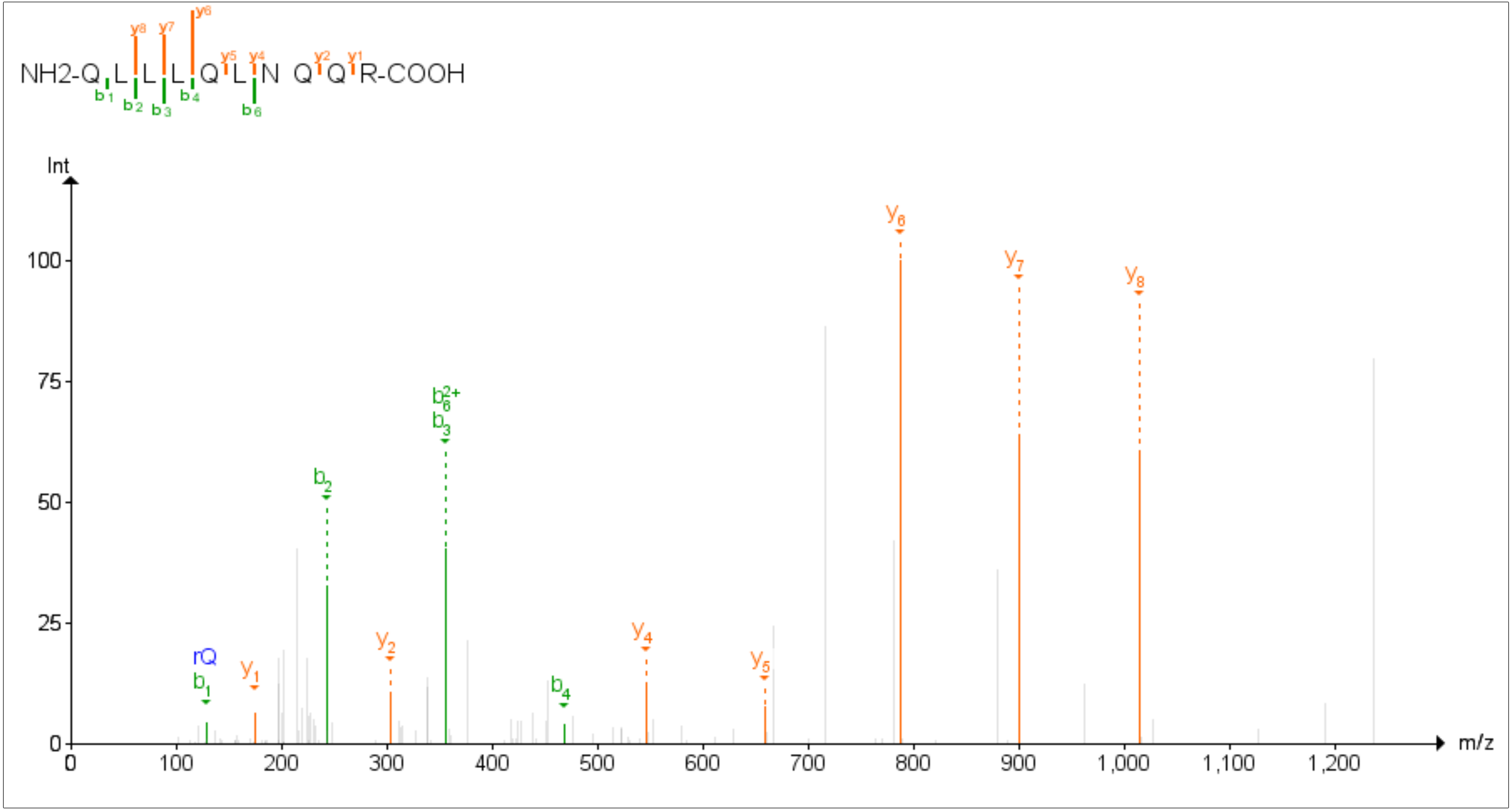

NH2-Q L L L Q L N Q Q R-COOH
b1 b2 b3 b4 b6
y8 y7 y6 y5 y4 y2 y1
Int
m/z
0
25
50
75
100
0
100
200
300
400
500
600
700
800
900
1,000
1,100
1,200
rQ
b1
y1
b2
y2
b6 2+
b3
b4
y4
y5
y6
y7
y8

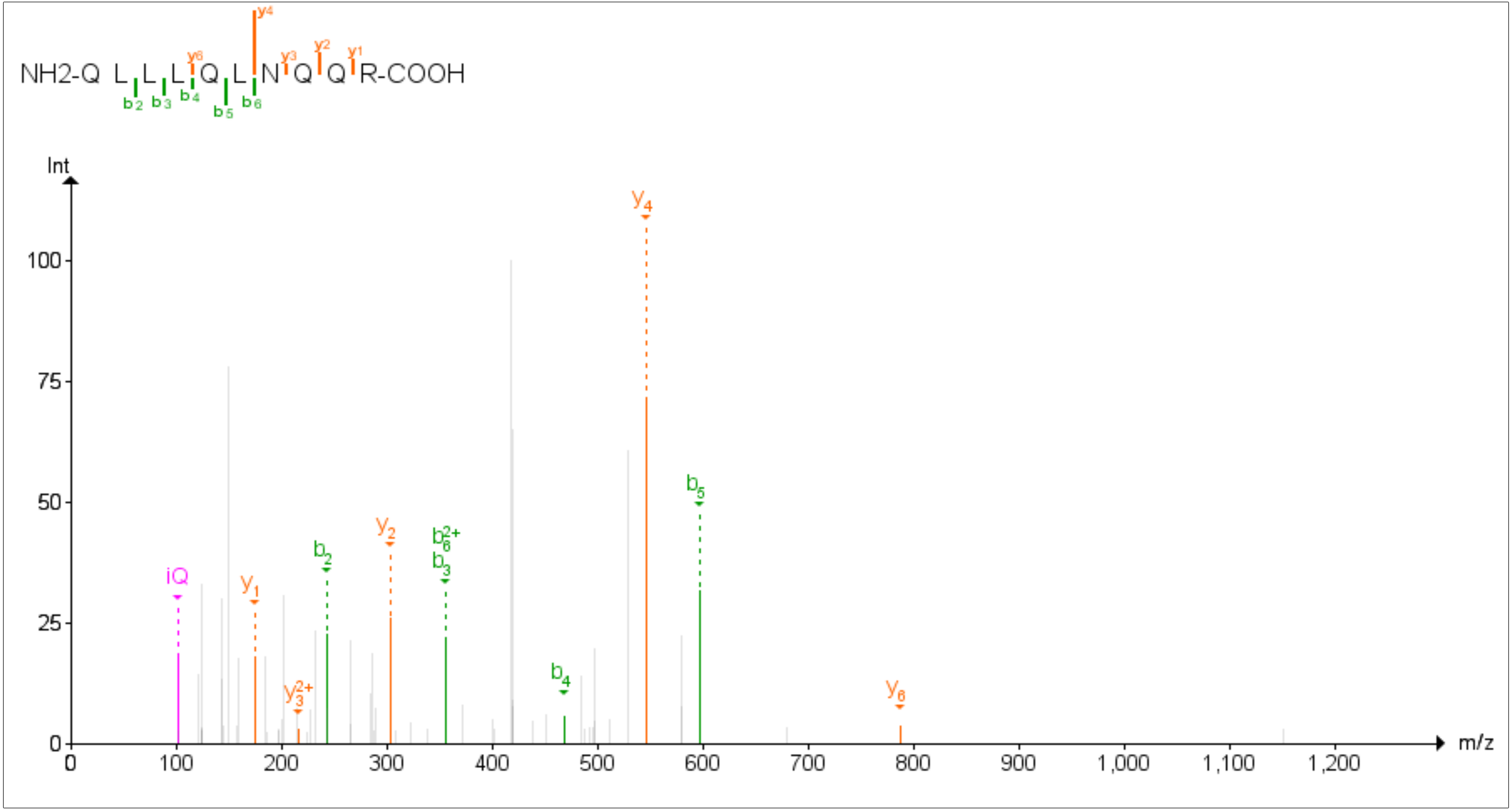

NH2-Q L L L Q L N Q Q R-COOH
y4
y6
y3
y2
y1
b2
b3
b4
b5
b6
Int
100
75
50
25
0
iQ
y_1
y_3^{2+}
b_2
y_2
b_6^{2+}
b_3
b_4
y_4
b_5
y_6
0
100
200
300
400
500
600
700
800
900
1,000
1,100
1,200
m/z

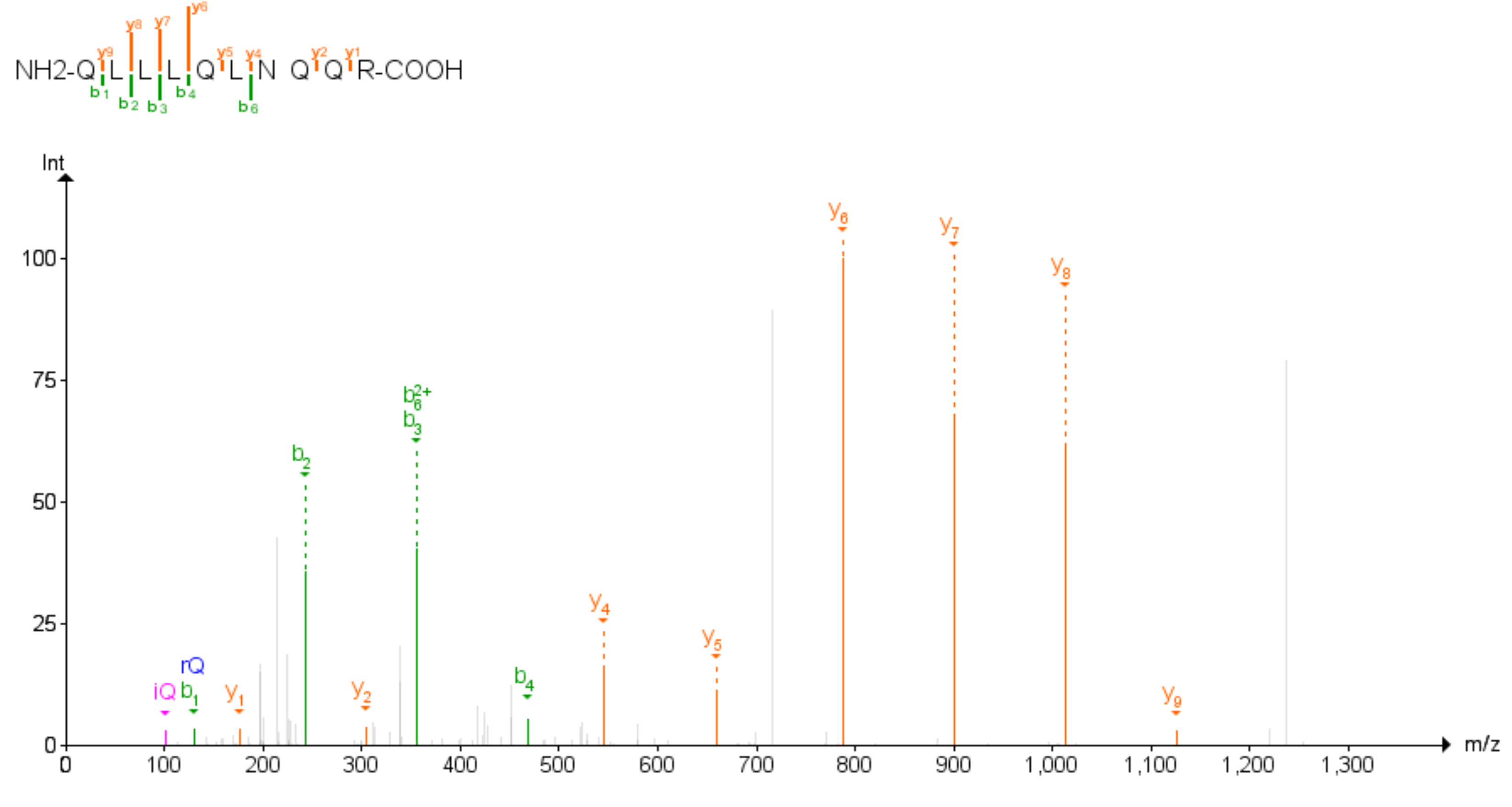
NH2-Q L L L Q L N Q Q R-COOH
y9 y8 y7 y6 y5 y4 y2 y1
b1 b2 b3 b4 b6
Int
100
75
50
25
0
y6
y7
y8
b6 2+
b3
b2
y4
y5
rQ
iQ
b1
y1
y2
b4
y9
0 100 200 300 400 500 600 700 800 900 1,000 1,100 1,200 1,300
m/z

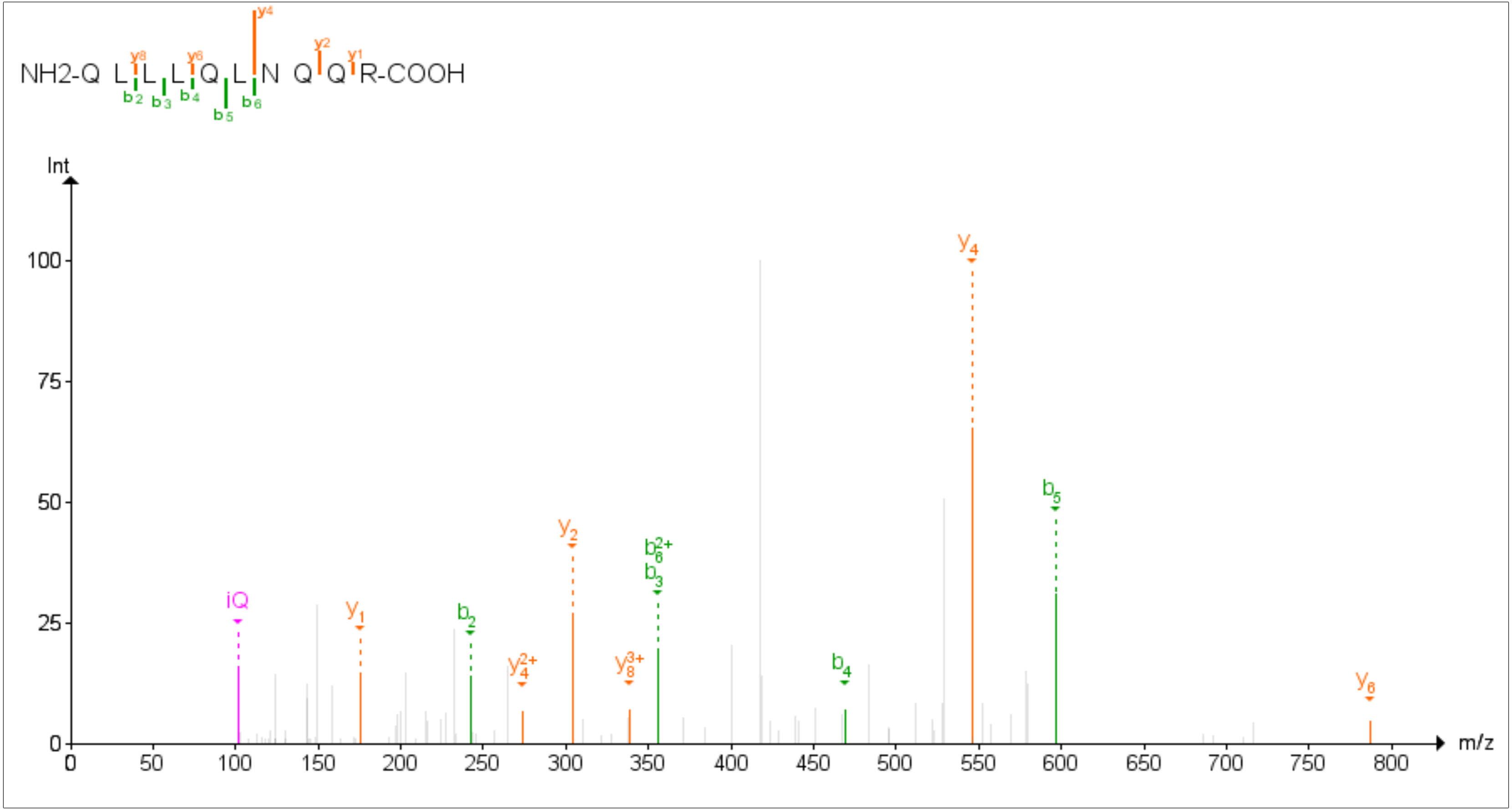

NH2-Q L L L Q L N Q Q R-COOH
y8 y6 y4 y2 y1
b2 b3 b4 b5 b6
Int
100
75
50
25
0
iQ
y1
b2
y4 2+
y2
y8 3+
b6 2+
b3
b4
y4
b5
y6
0 50 100 150 200 250 300 350 400 450 500 550 600 650 700 750 800
m/z

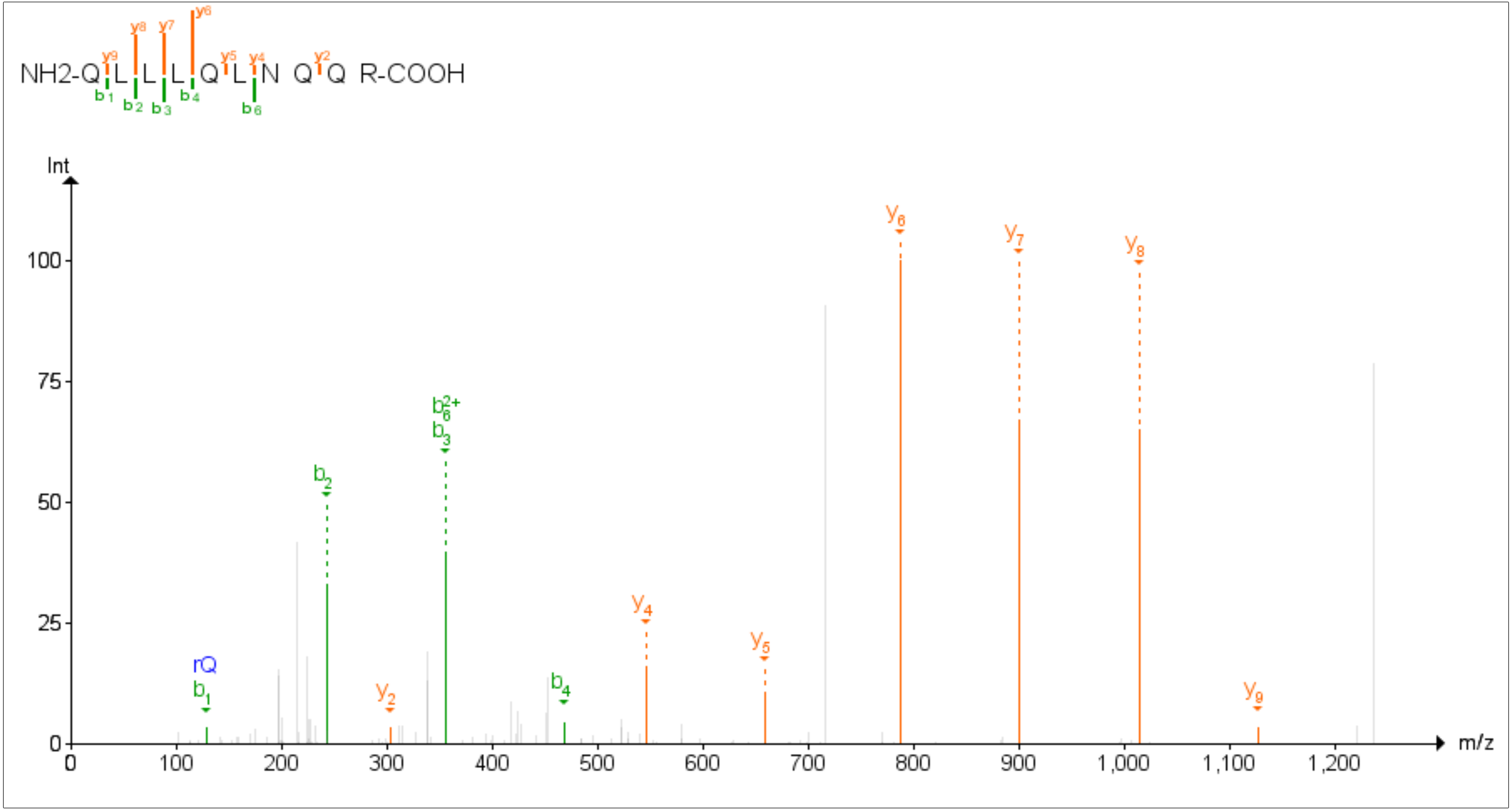
NH2-Q L L L Q L N Q Q R-COOH
y9 y8 y7 y6 y5 y4 y2
b1 b2 b3 b4 b6
Int
100
75
50
25
0
rQ
b1
b2
b6 2+
b3
y2
b4
y4
y5
y6
y7
y8
y9
0 100 200 300 400 500 600 700 800 900 1,000 1,100 1,200
m/z

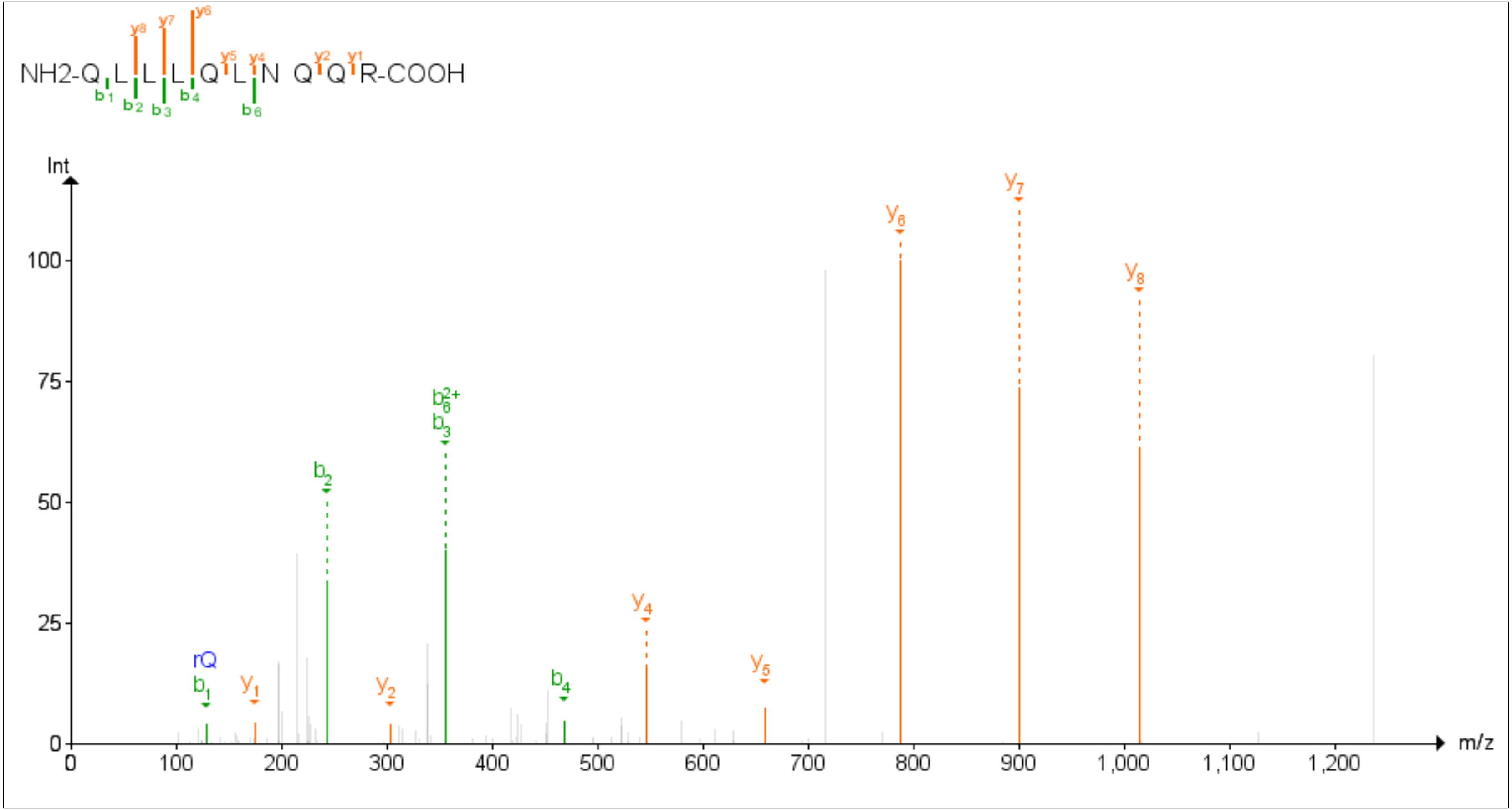

NH2-Q L L L Q L N Q Q R-COOH
y8 y7 y6 y5 y4 y2 y1
b1 b2 b3 b4 b6
Int
100
75
50
25
0
rQ
b1
y1
b2
y2
b6 2+
b3
b4
y4
y5
y6
y7
y8
0 100 200 300 400 500 600 700 800 900 1,000 1,100 1,200
m/z

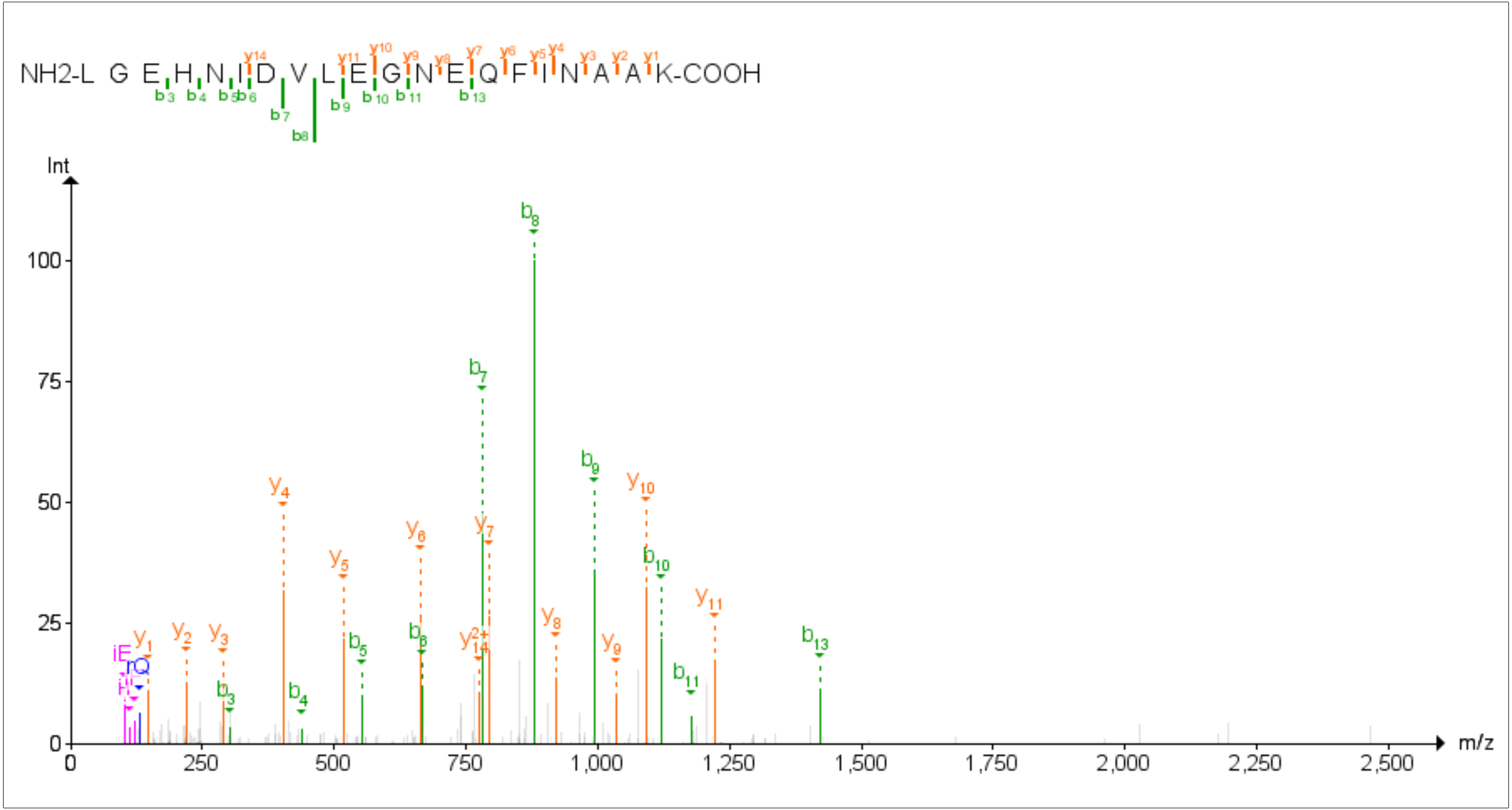
NH2-L G E H N I D V L E G N E Q F I N A A K-COOH
Int
m/z
0
25
50
75
100
250
500
750
1,000
1,250
1,500
1,750
2,000
2,250
2,500
b8
b7
b9
y10
y4
y6
y7
b10
y5
y11
y8
y9
b5
b6
b11
b13
y1
y2
y3
b3
b4
iE
rQ

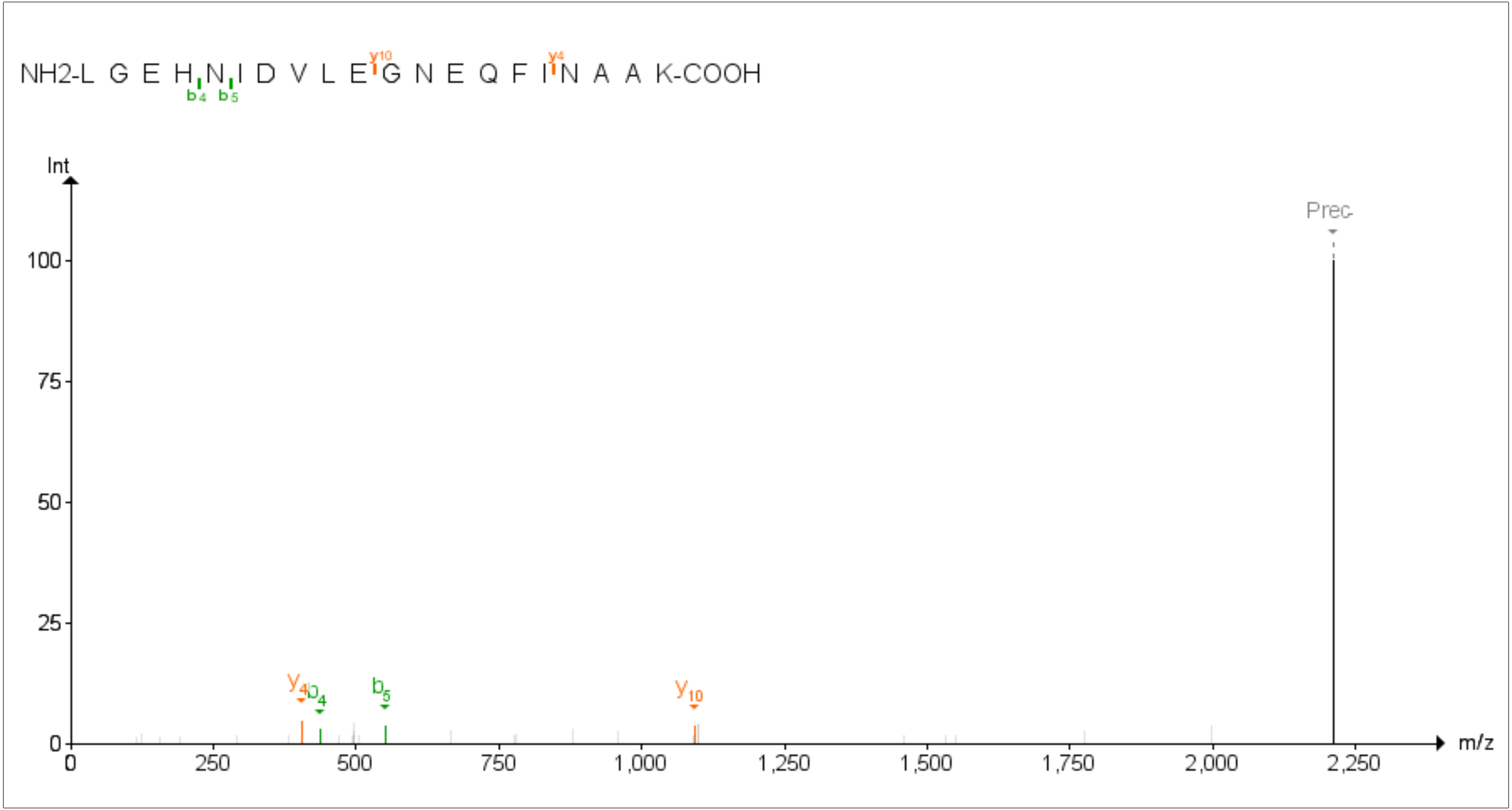

NH2-L G E H N I D V L E G N E Q F I N A A K-COOH
b4
b5
y10
y4
Int
100
75
50
25
0
Prec
y4
b4
b5
y10
0
250
500
750
1,000
1,250
1,500
1,750
2,000
2,250
m/z

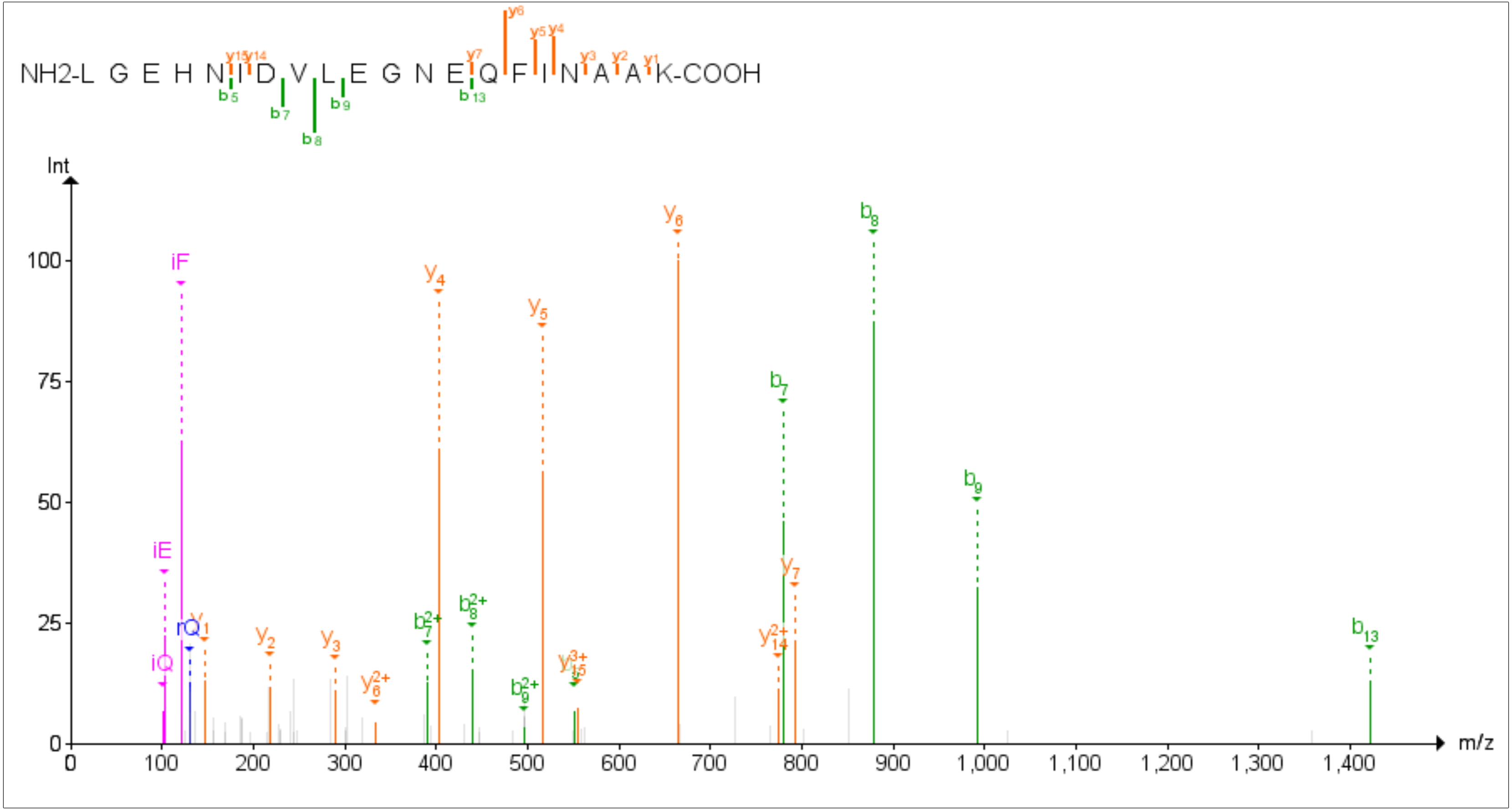

NH2-L G E H N I D V L E G N E Q F I N A A K-COOH
y15 y14 y7 y6 y5 y4 y3 y2 y1
b5 b7 b8 b9 b13
Int
m/z
0
25
50
75
100
0
100
200
300
400
500
600
700
800
900
1,000
1,100
1,200
1,300
1,400
iF
iE
iQ
rQ
y1
y2
y3
y_6^{2+}
b_7^{2+}
y4
b_8^{2+}
b_9^{2+}
y5
y_{15}^{3+}
y6
y_{14}^{2+}
b7
y7
b8
b9
b13

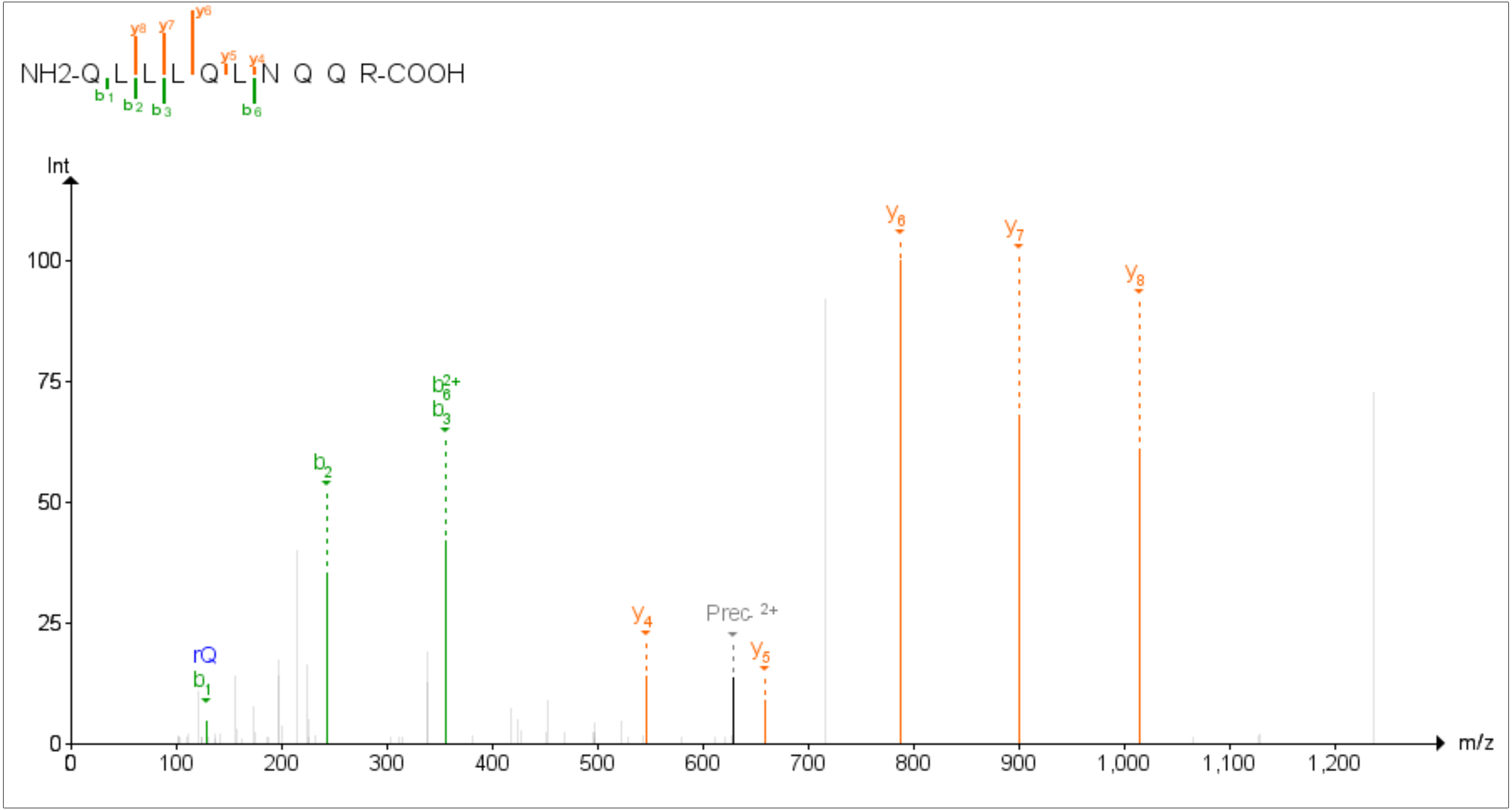

NH2-Q L L L Q L N Q Q R-COOH
y8 y7 y6 y5 y4
b1 b2 b3 b6
Int
100
75
50
25
0
y6
y7
y8
b6 2+
b3
b2
y4
Prec 2+
y5
rQ
b1
0 100 200 300 400 500 600 700 800 900 1,000 1,100 1,200
m/z

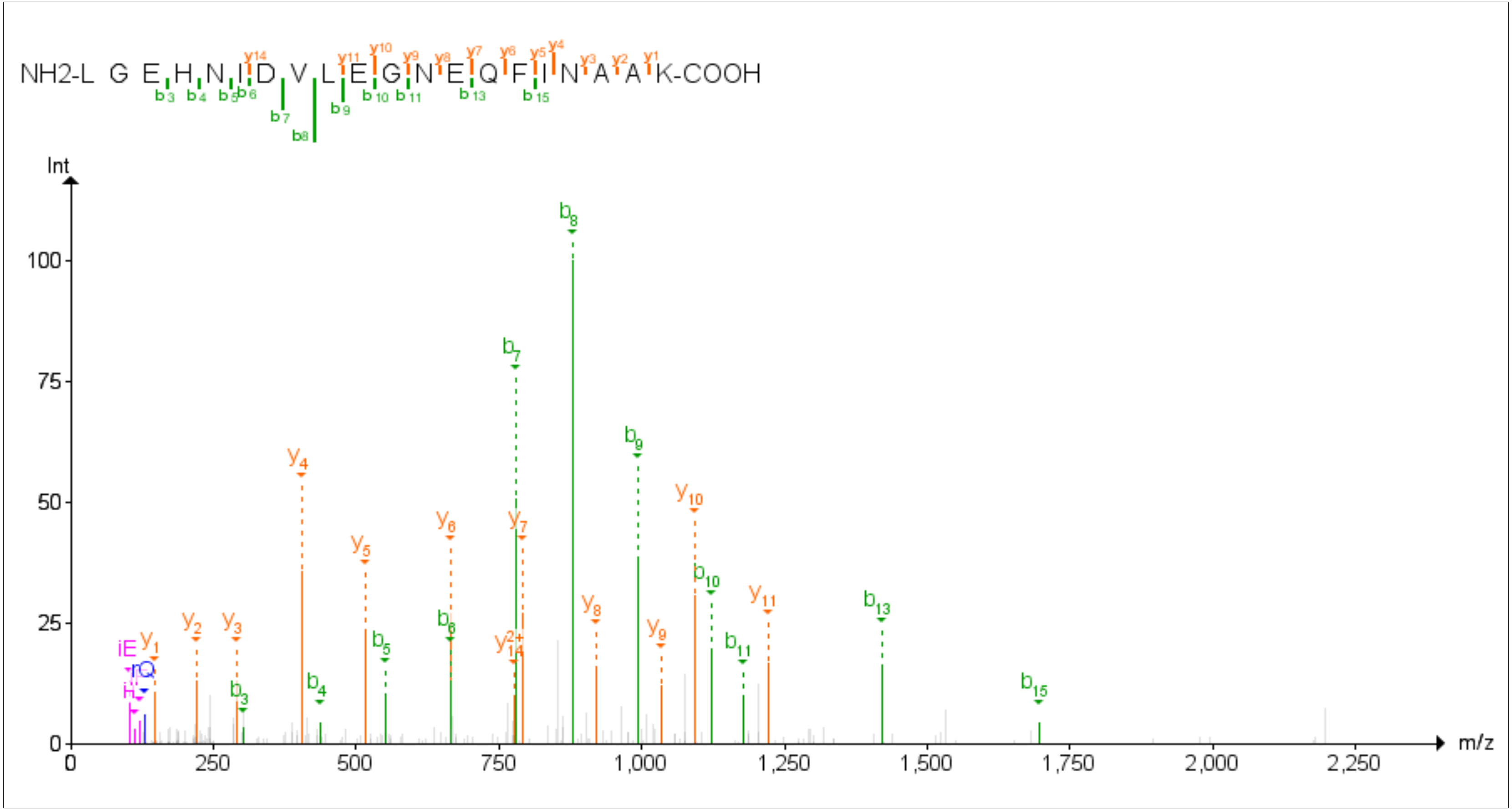
NH2-L G E H N I D V L E G N E Q F I N A A K-COOH
Int
m/z

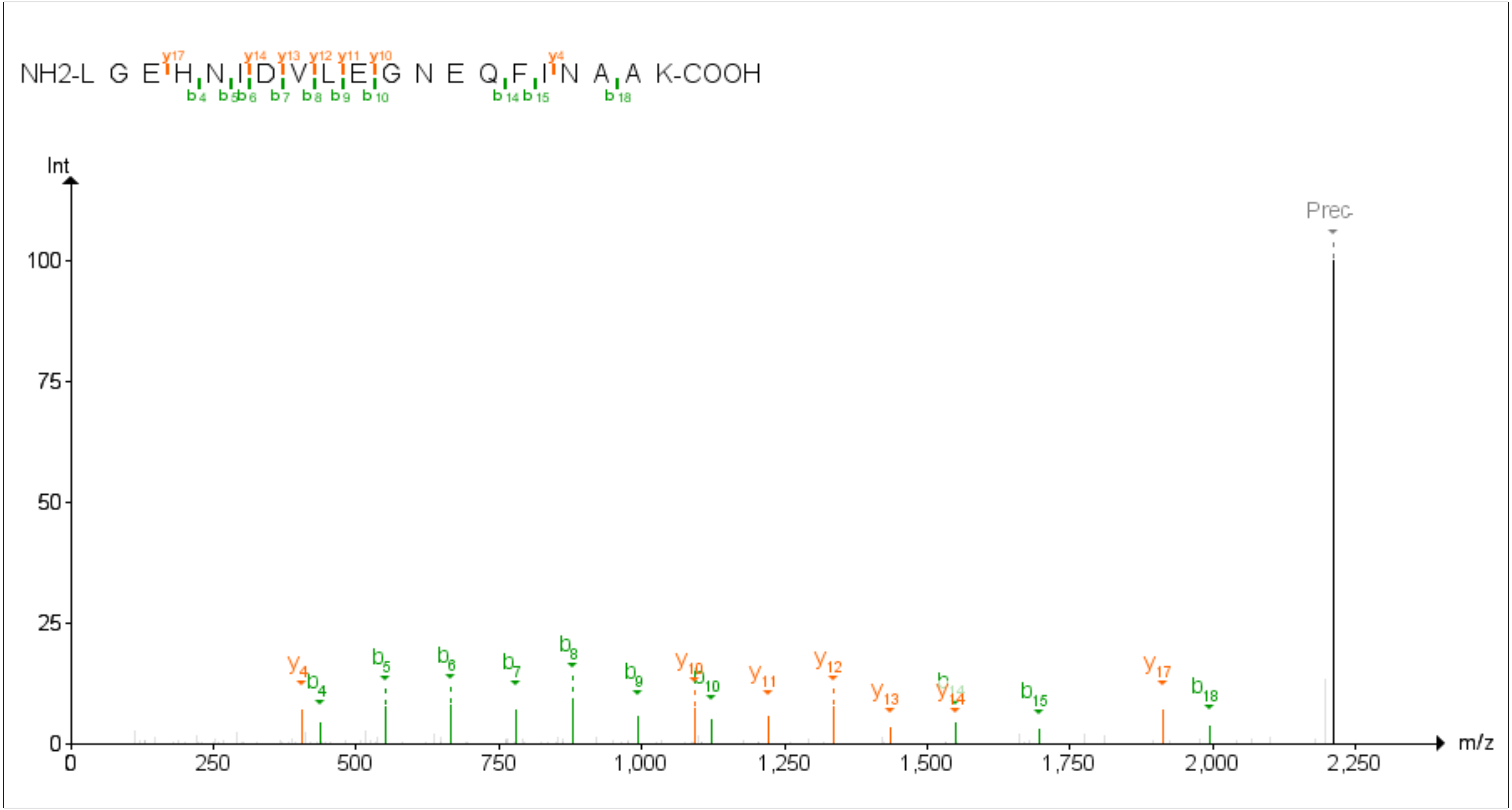

NH2-L G E H N I D V L E G N E Q F I N A A K-COOH
y17 y14 y13 y12 y11 y10 y4
b4 b5 b6 b7 b8 b9 b10 b14 b15 b18
Int
Prec
100
75
50
25
0
0 250 500 750 1,000 1,250 1,500 1,750 2,000 2,250
m/z

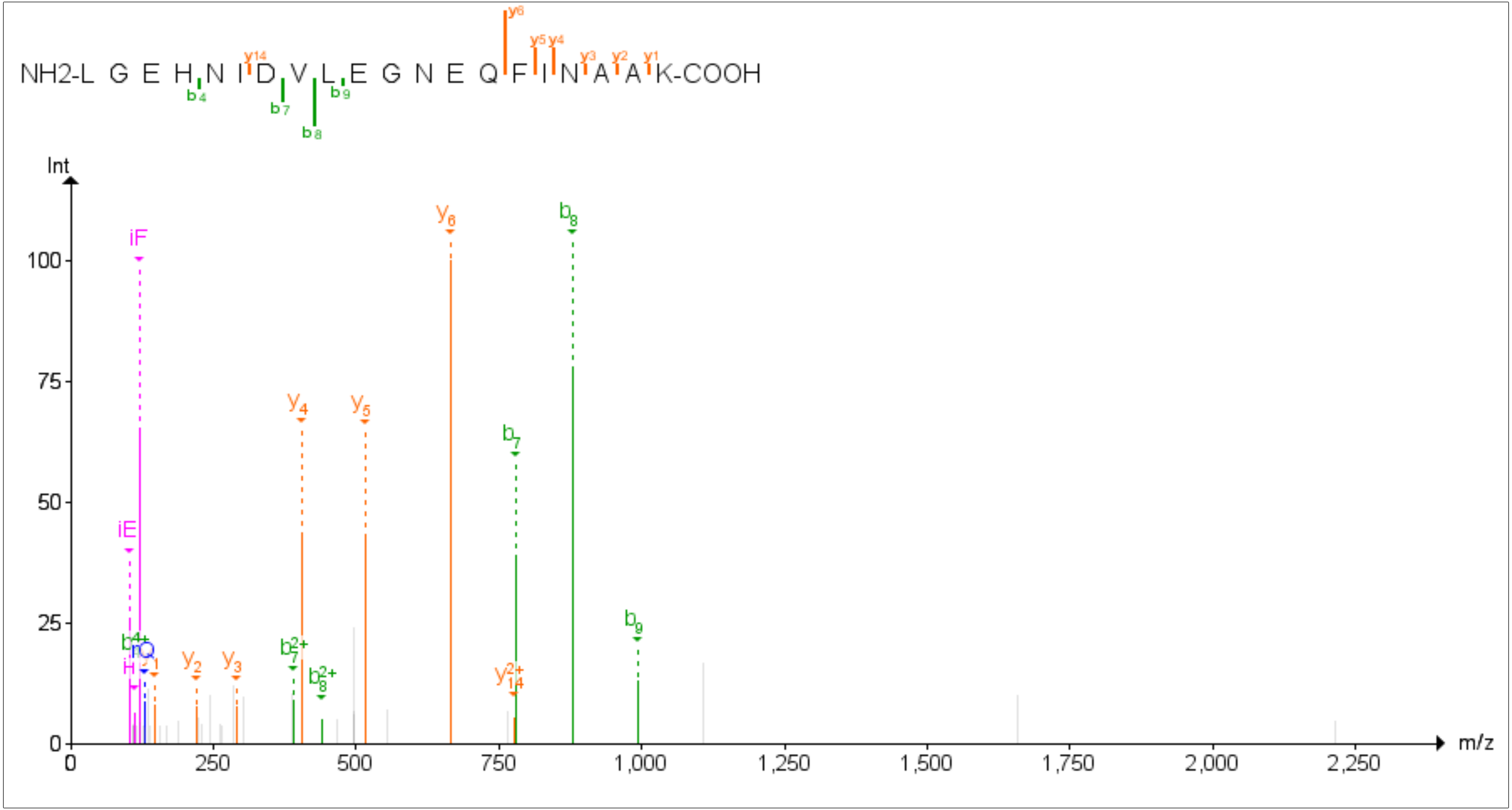

NH2-L G E H N I D V L E G N E Q F I N A A K-COOH
y14 y6 y5 y4 y3 y2 y1
b4 b7 b8 b9
Int
100
75
50
25
0
iF
iE
y6
b8
y4
y5
b7
b9
y2
y3
y14 2+
b7 2+
b8 2+
b4+
nQ
0
250
500
750
1,000
1,250
1,500
1,750
2,000
2,250
m/z

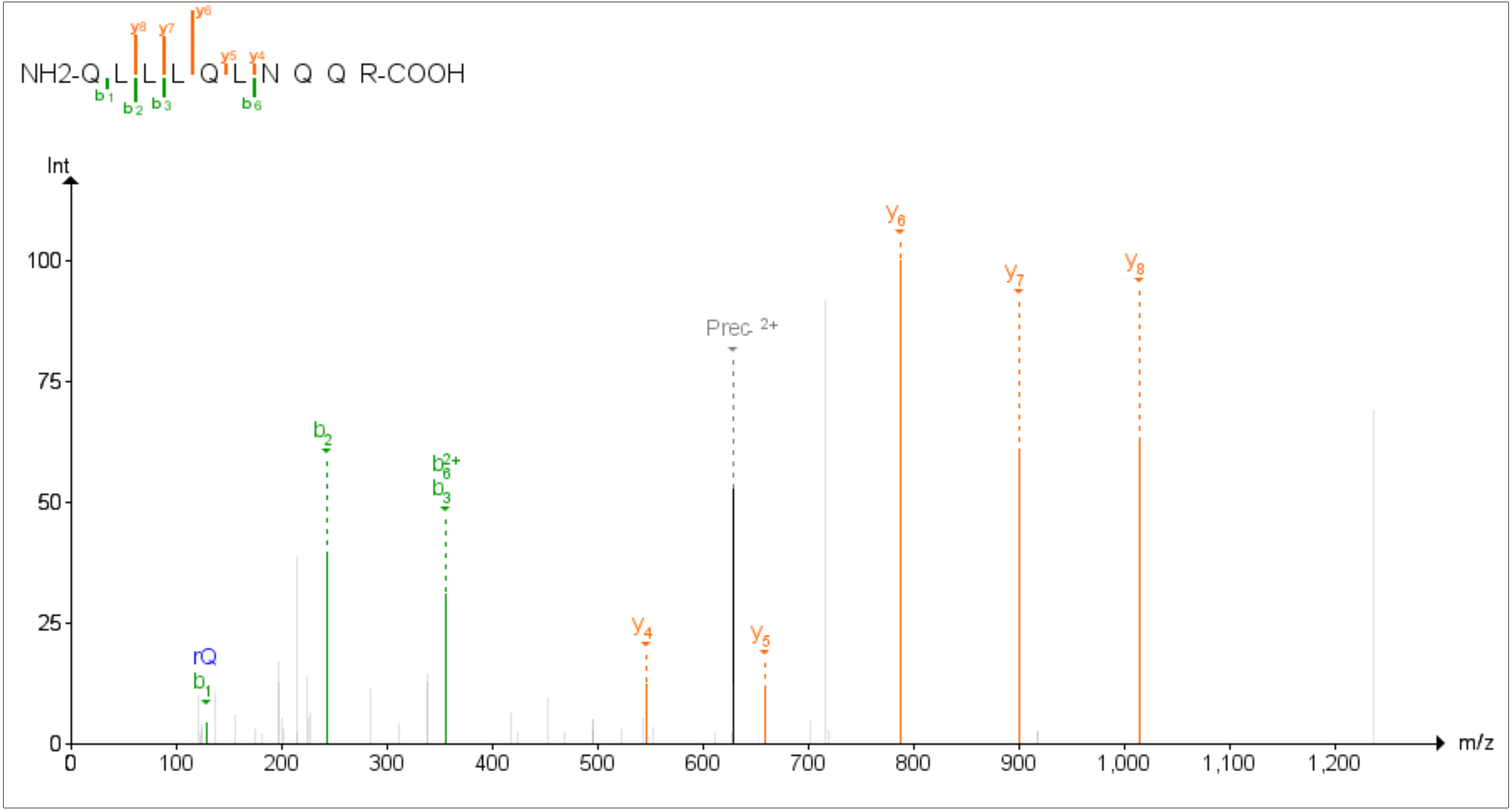

NH2-Q L L L Q L N Q Q R-COOH
y8 y7 y6 y5 y4
b1 b2 b3 b6
Int
100
75
50
25
0
rQ
b1
b2
b6 2+
b3
y4
y5
Prec 2+
y6
y7
y8
0 100 200 300 400 500 600 700 800 900 1,000 1,100 1,200
m/z

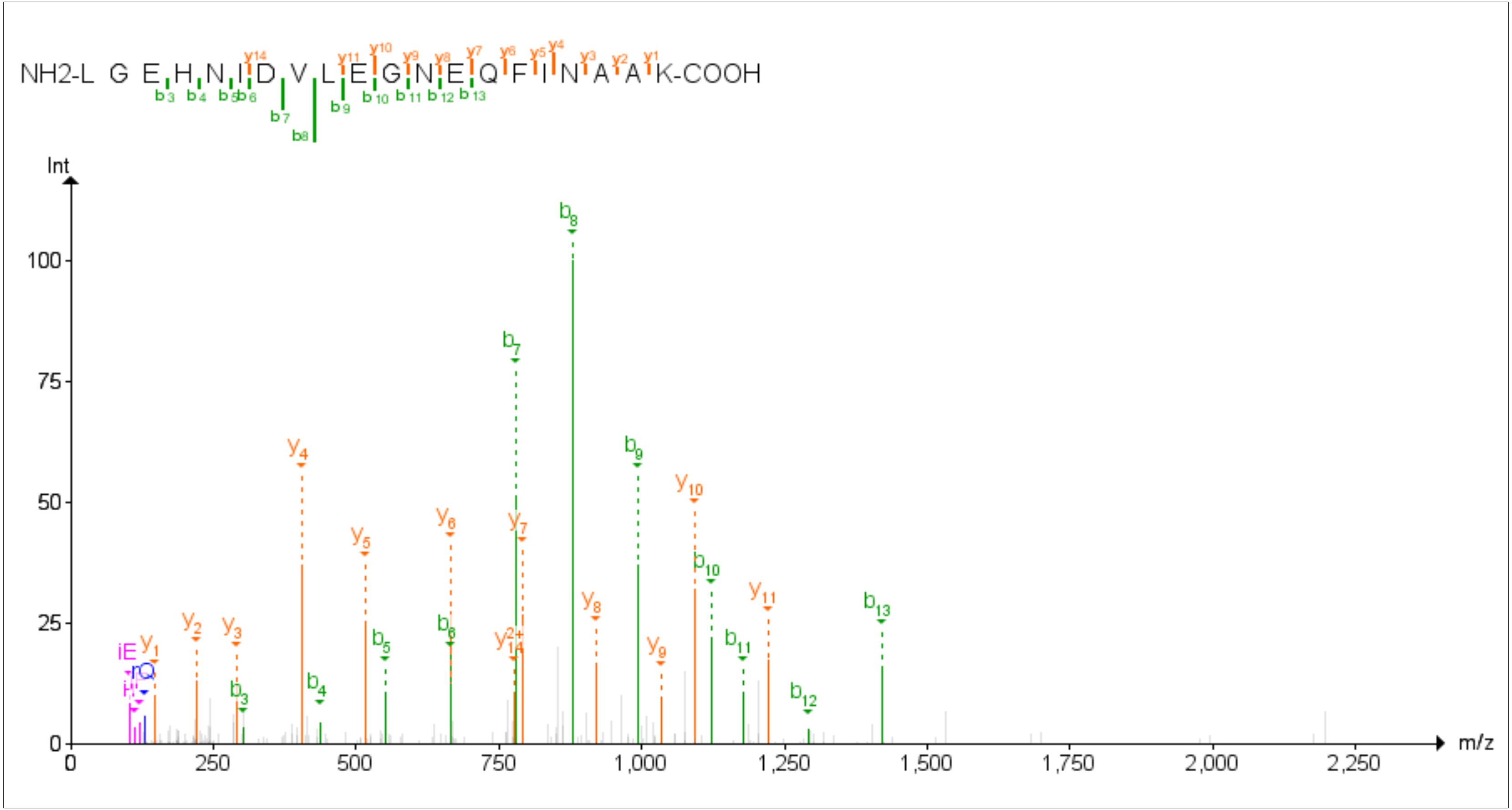

NH2-L G E H N I D V L E G N E Q F I N A A K-COOH
Int
m/z
0
25
50
75
100
250
500
750
1,000
1,250
1,500
1,750
2,000
2,250

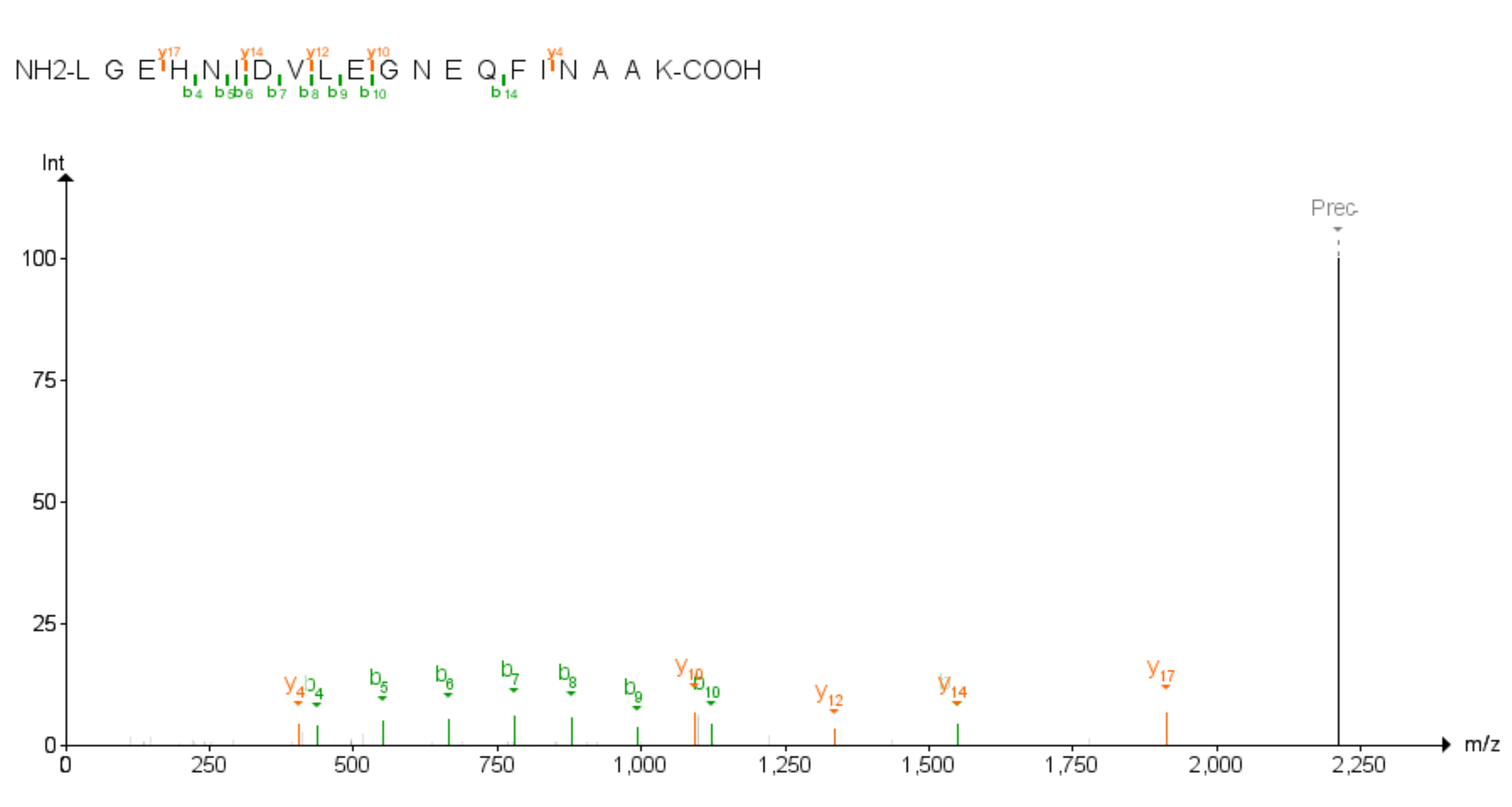
NH2-L G E H N I D V L E G N E Q F I N A A K-COOH
y17 y14 y12 y10 y4
b4 b5 b6 b7 b8 b9 b10 b14
Int
100
75
50
25
0
Prec.
y4 b4 b5 b6 b7 b8 b9 y10 b10 y12 y14 y17
0 250 500 750 1,000 1,250 1,500 1,750 2,000 2,250
m/z

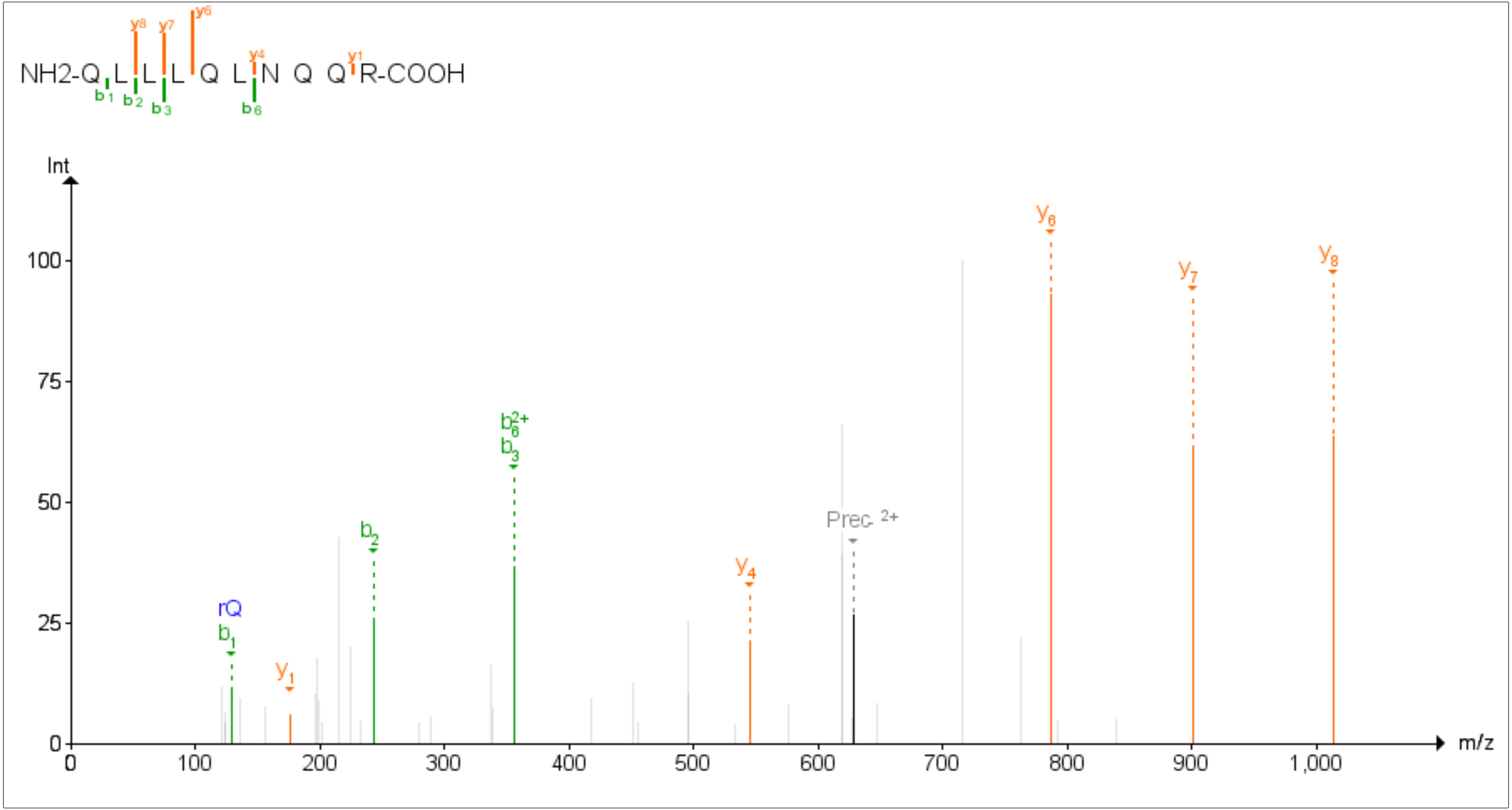

NH2-Q L L L Q L N Q Q R-COOH
y8 y7 y6 y4 y1
b1 b2 b3 b6
Int
100
75
50
25
0
y6
y7
y8
b6 2+
b3
b2
rQ
b1
y1
y4
Prec 2+
0 100 200 300 400 500 600 700 800 900 1,000
m/z

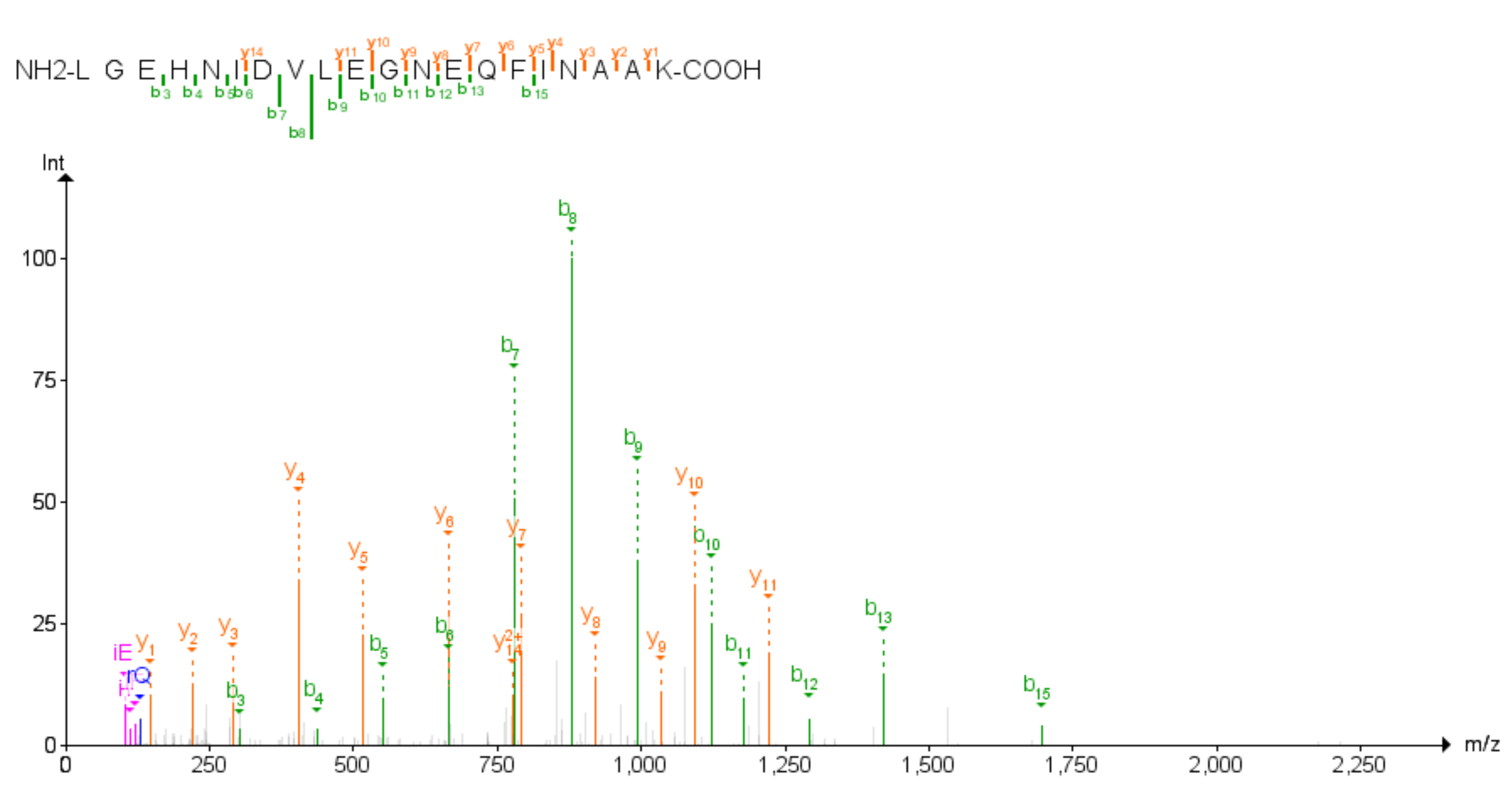
NH2-L G E H N I D V L E G N E Q F I N A A K-COOH
y14 y11 y10 y9 y8 y7 y6 y5 y4 y3 y2 y1
b3 b4 b5 b6 b7 b8 b9 b10 b11 b12 b13 b15
Int
100
75
50
25
0
m/z
0
250
500
750
1,000
1,250
1,500
1,750
2,000
2,250
b8
b7
b9
y4
y10
b10
y6
y7
y5
y11
b13
y8
b5
b6
y9
b11
iE
y1
y2
y3
rQ
b12
b15
b3
b4

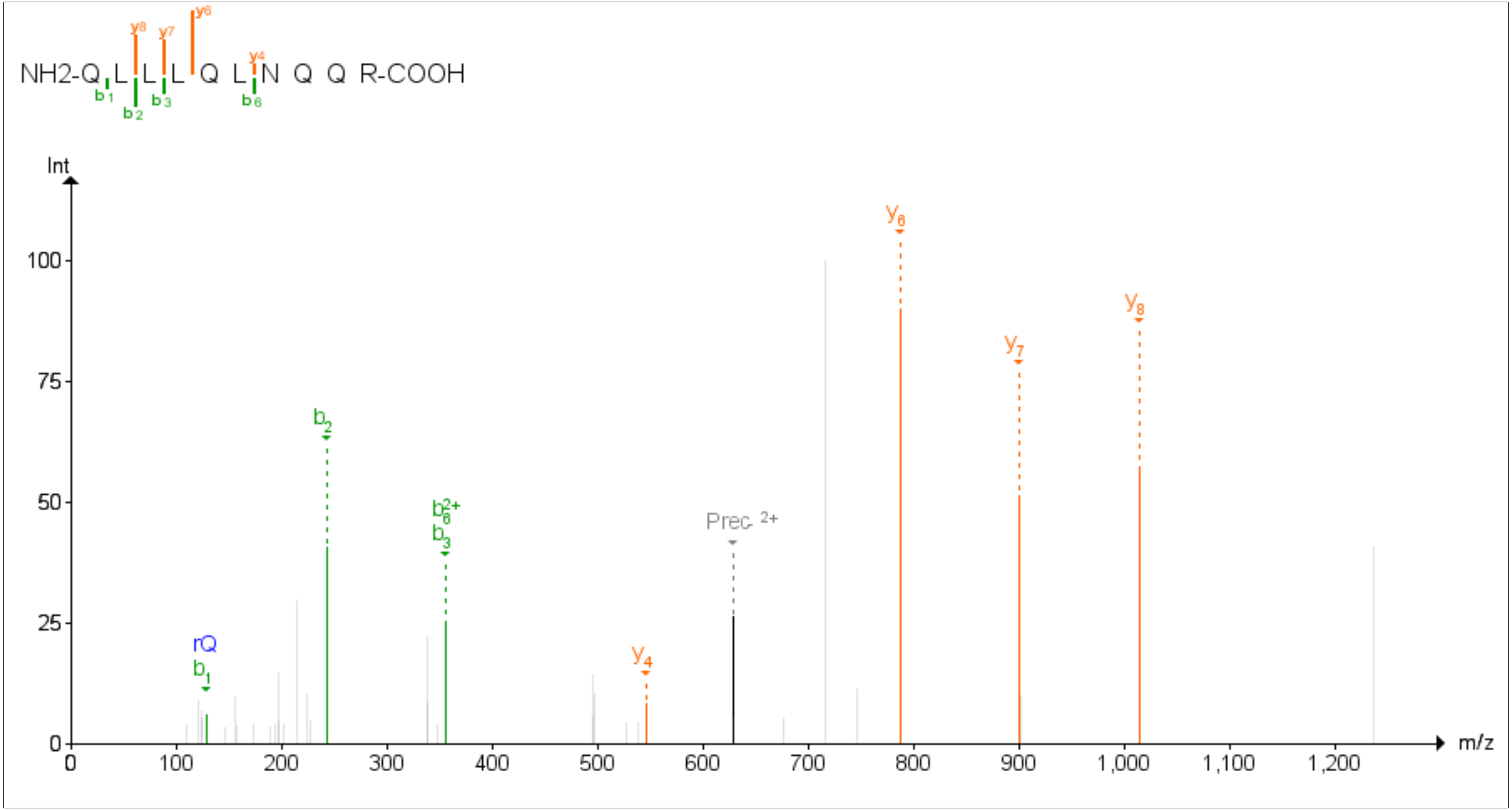

NH2-Q L L L Q L N Q Q R-COOH
y8
y7
y6
y4
b1
b2
b3
b6
Int
100
75
50
25
0
y6
y8
y7
b2
b6 2+
b3
Prec 2+
rQ
b1
y4
0
100
200
300
400
500
600
700
800
900
1,000
1,100
1,200
m/z

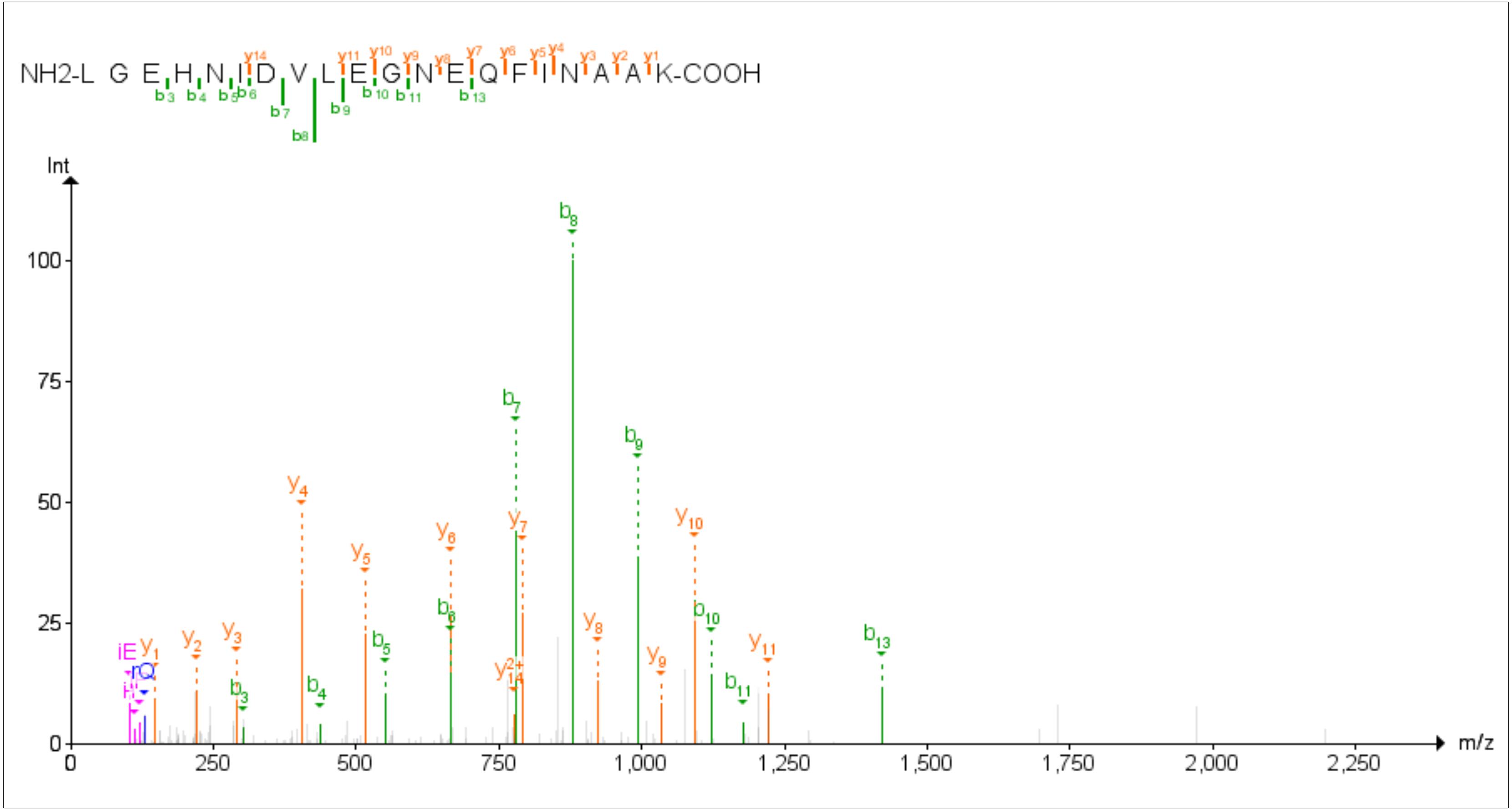
NH2-L G E H N I D V L E G N E Q F I N A A K-COOH
y14 y11 y10 y9 y8 y7 y6 y5 y4 y3 y2 y1
b3 b4 b5 b6 b7 b8 b9 b10 b11 b13
Int
m/z
0
25
50
75
100
250
500
750
1,000
1,250
1,500
1,750
2,000
2,250
b8
b7
b9
y4
y5
y6
y7
y10
b6
b10
b5
y8
y9
y11
b11
b13
b3
b4
y1
y2
y3
iE
nQ
iH
y14 2+

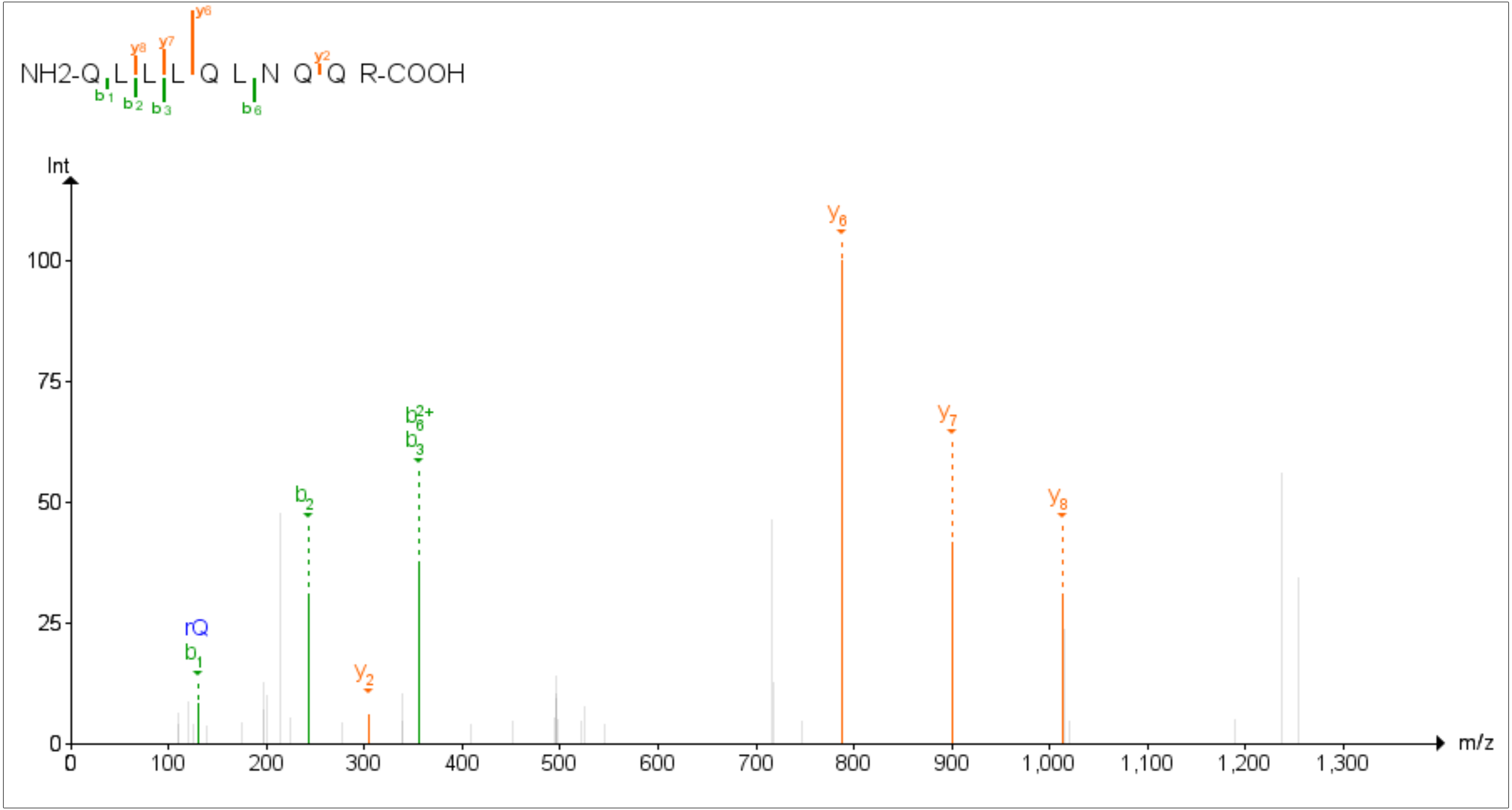

NH2-Q L L L Q L N Q Q R-COOH
y8
y7
y6
y2
b1
b2
b3
b6
Int
y6
y7
y8
y2
b2
b6 2+
b3
rQ
b1
m/z
0
25
50
75
100
0
100
200
300
400
500
600
700
800
900
1,000
1,100
1,200
1,300

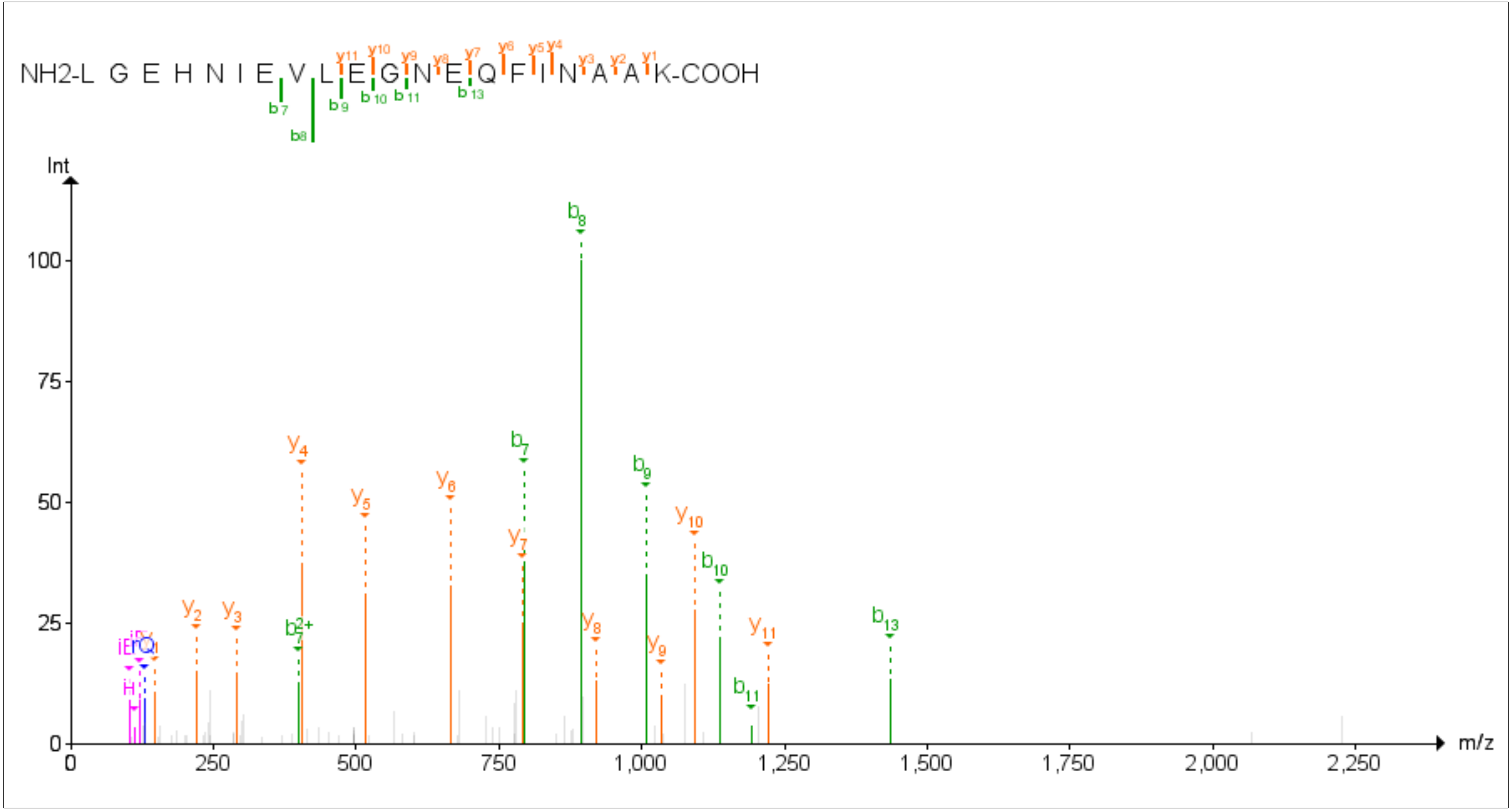

NH2-L G E H N I E V L E G N E Q F I N A A K-COOH
Int
m/z
b8
b7
y4
b9
y6
y5
y7
y10
b10
b13
y2
y3
y8
y9
y11
b11
0
25
50
75
100
0
250
500
750
1,000
1,250
1,500
1,750
2,000
2,250

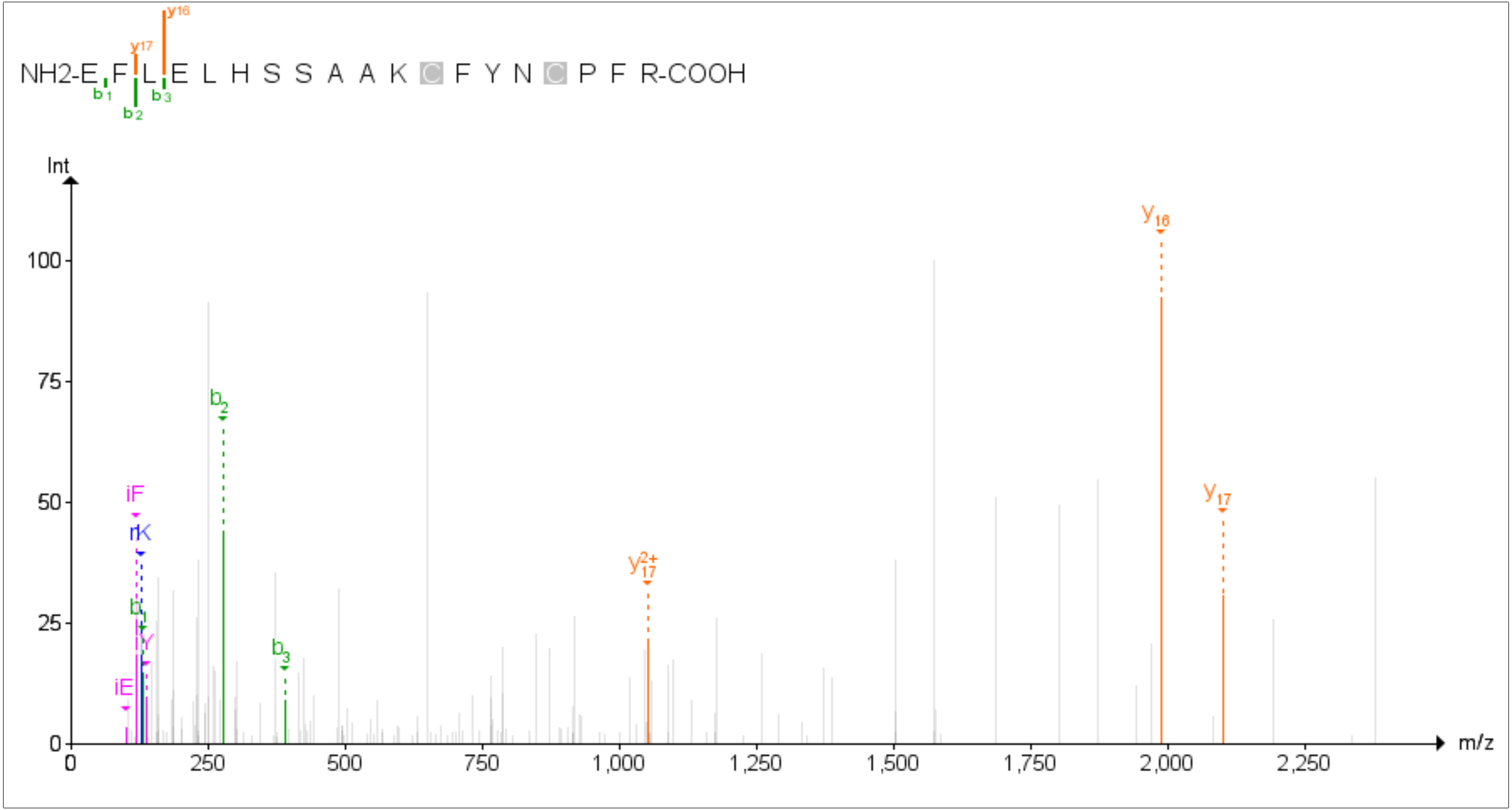
NH2-E F L E L H S S A A K C F Y N C P F R-COOH
b1 b2 b3 y17 y16
Int
m/z
0 25 50 75 100
0 250 500 750 1,000 1,250 1,500 1,750 2,000 2,250
y16 y17 y17 2+ b2 b3 b1 iF rK iE iY

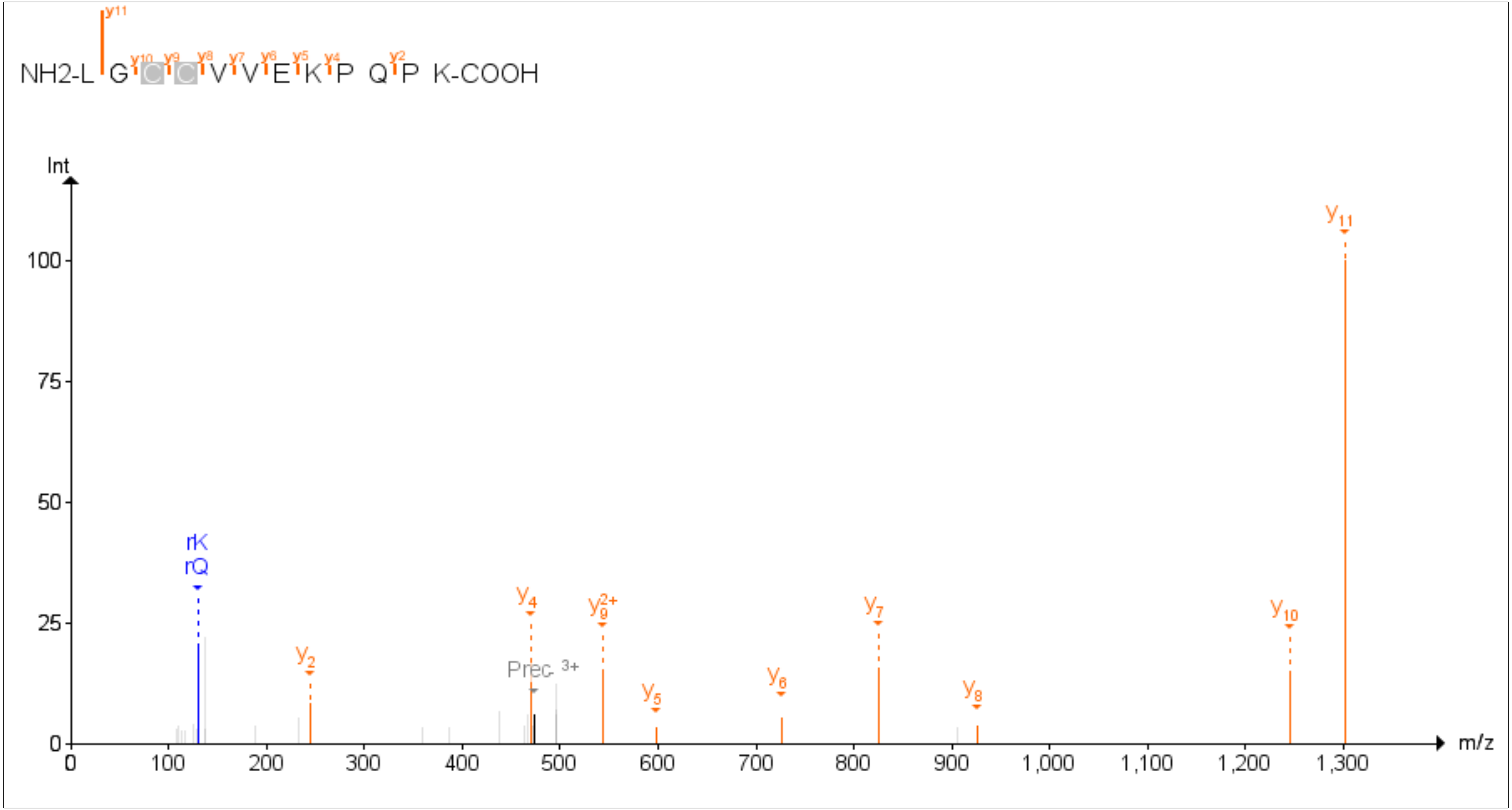

NH2-L G C C V V E K P Q P K-COOH
y11 y10 y9 y8 y7 y6 y5 y4 y2
Int
100
75
50
25
0
rK
rQ
y2
Prec 3+
y4
y9 2+
y5
y6
y7
y8
y10
y11
0 100 200 300 400 500 600 700 800 900 1,000 1,100 1,200 1,300
m/z

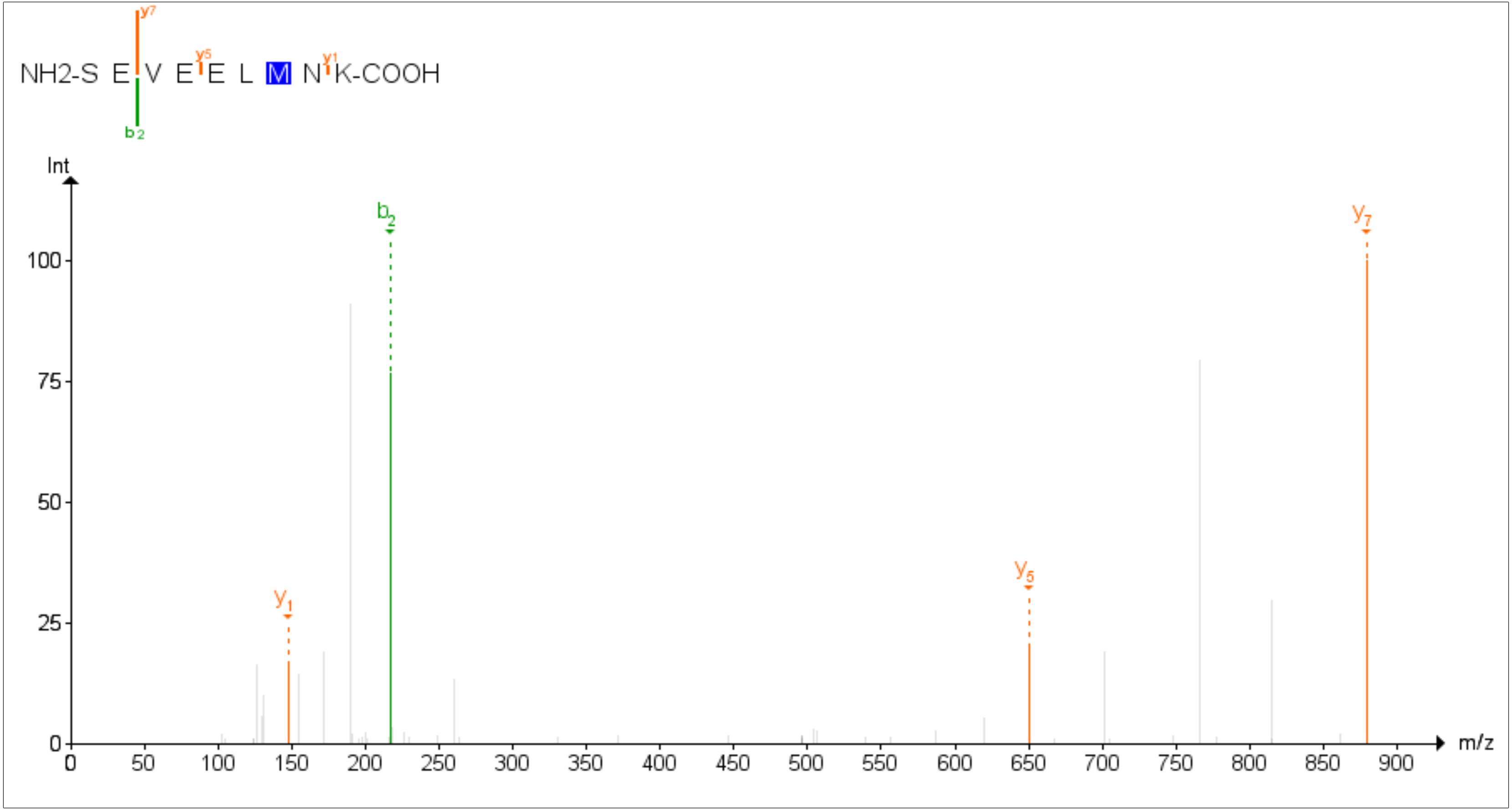

NH2-S E V E E L M N K-COOH
y7
y5
y1
b2
Int
100
75
50
25
0
m/z
0
50
100
150
200
250
300
350
400
450
500
550
600
650
700
750
800
850
900
b2
y7
y5
y1

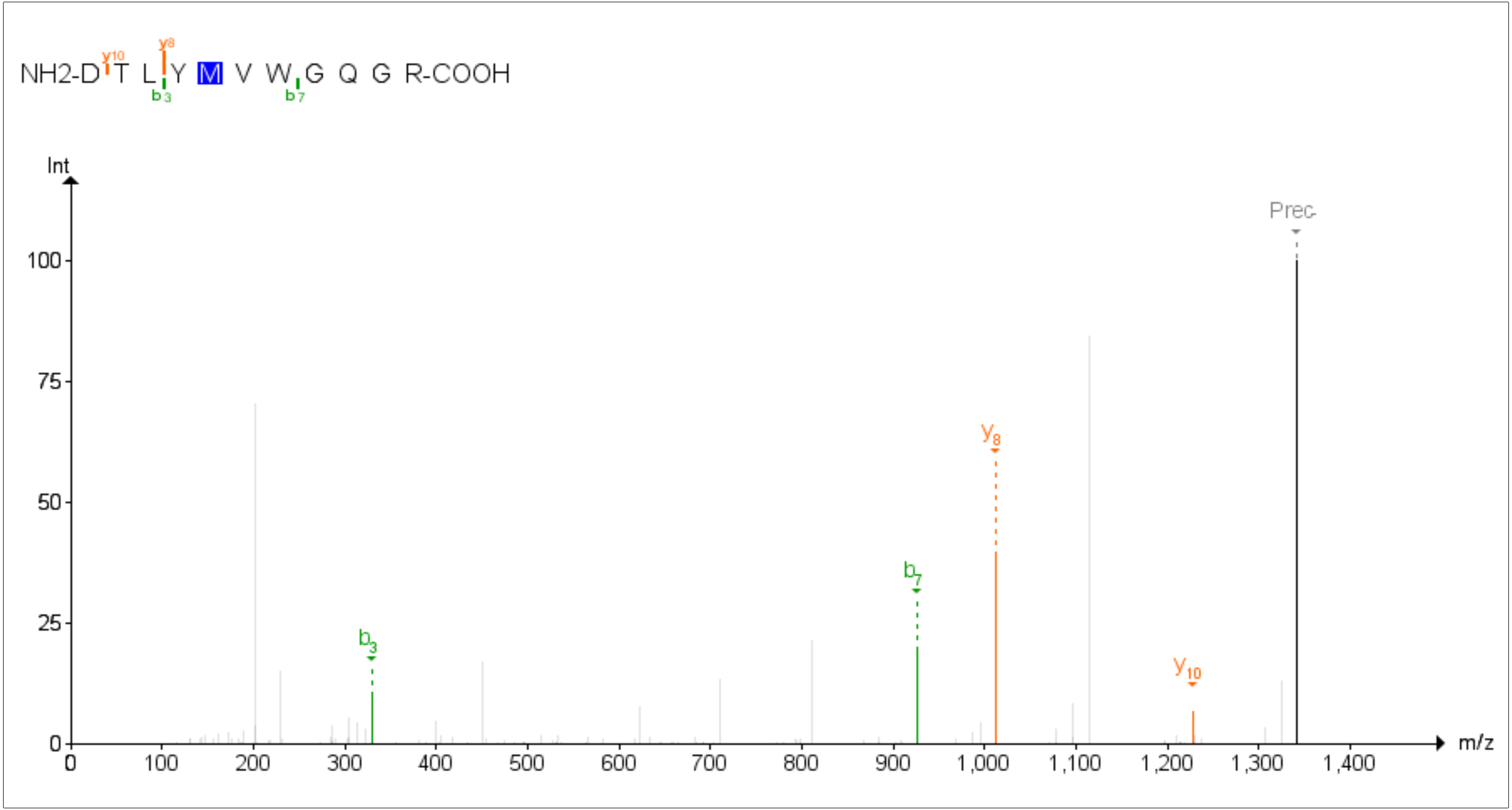

NH2-D T L Y M V W G Q G R-COOH
y10
y8
b3
b7
Int
Prec.
100
75
50
25
0
0
100
200
300
400
500
600
700
800
900
1,000
1,100
1,200
1,300
1,400
m/z

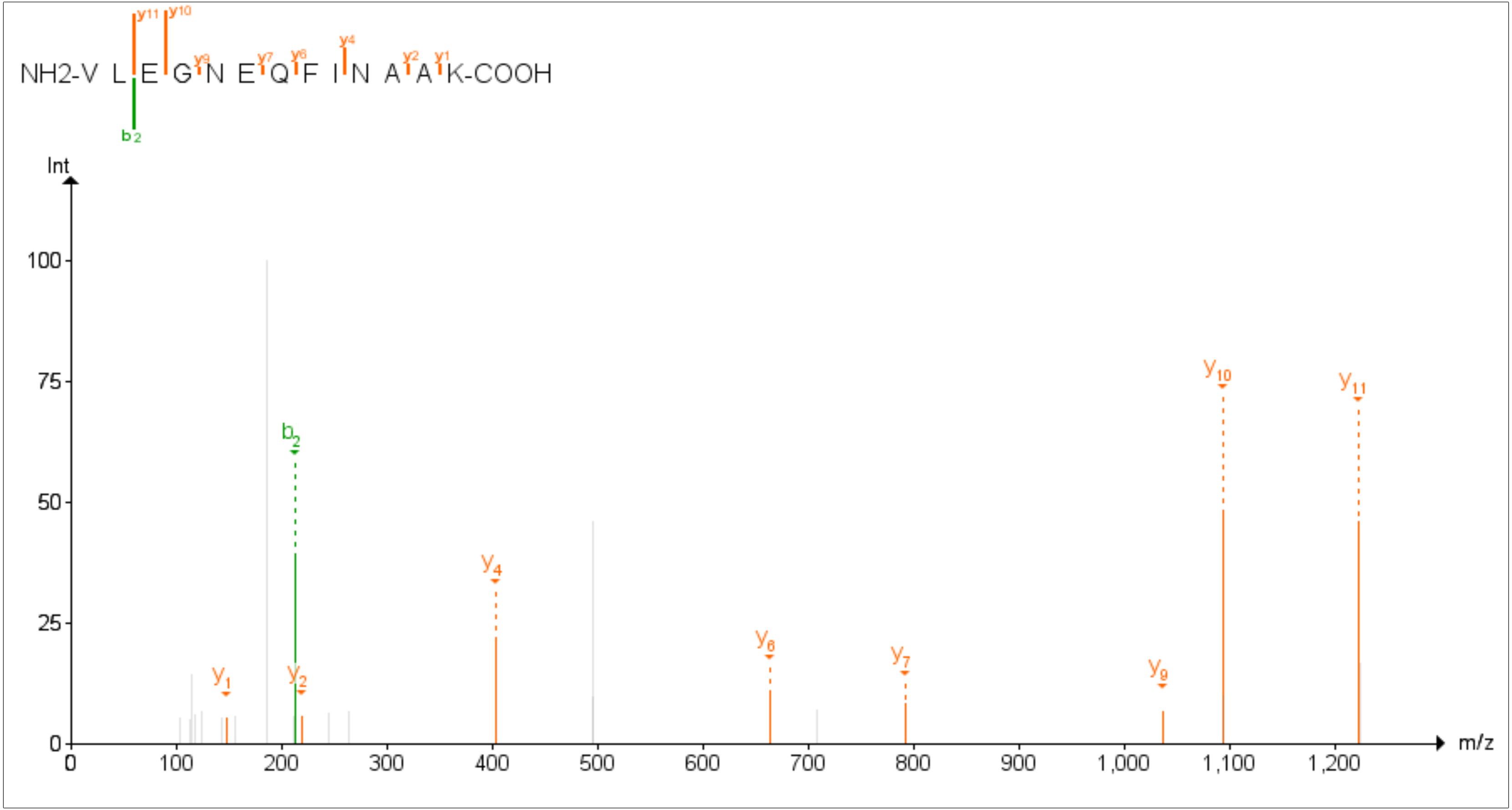

NH2-V L E G N E Q F I N A A K-COOH
y11
y10
y9
y7
y6
y4
y2
y1
b2
Int
100
75
50
25
0
m/z
0
100
200
300
400
500
600
700
800
900
1,000
1,100
1,200

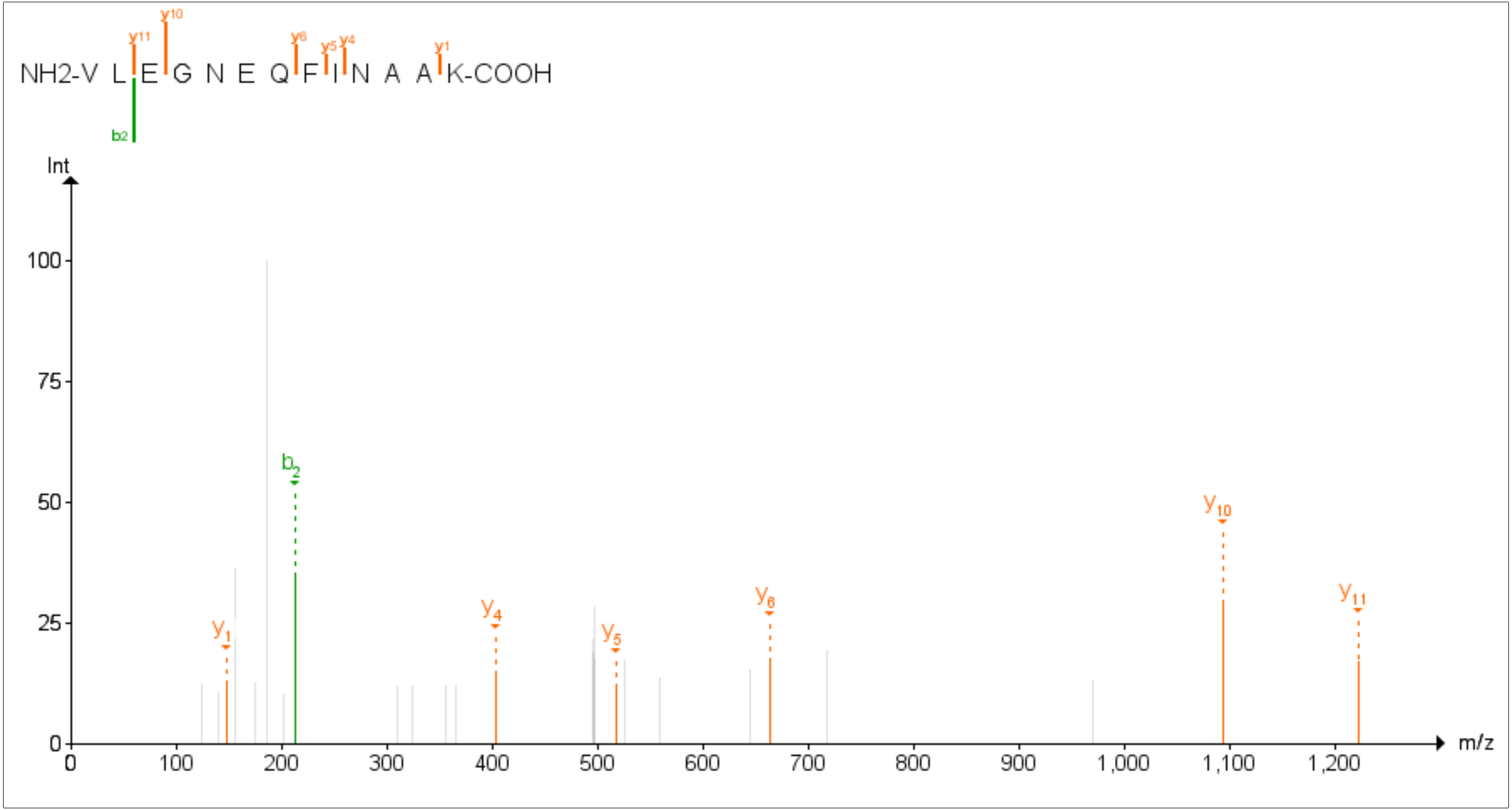
NH2-V L E G N E Q F I N A A K-COOH
y11
y10
y6
y5
y4
y1
b2
Int
100
75
50
25
0
m/z
0
100
200
300
400
500
600
700
800
900
1,000
1,100
1,200
b2
y1
y4
y5
y6
y10
y11

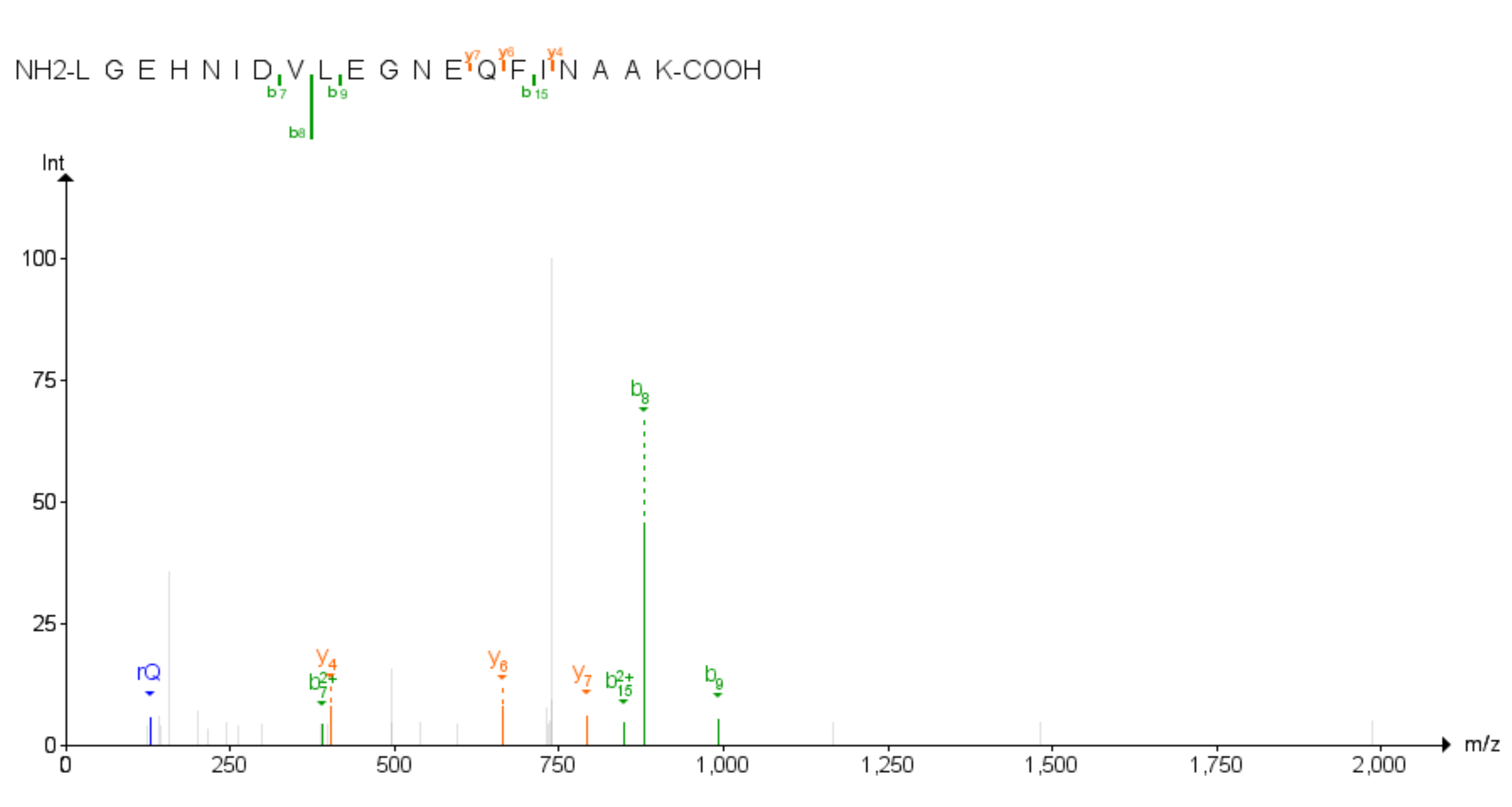

NH2-L G E H N I D V L E G N E Q F I N A A K-COOH
b7
b8
b9
b15
y7
y6
y4
Int
100
75
50
25
0
rQ
y4
b7 2+
y6
y7
b15 2+
b8
b9
0
250
500
750
1,000
1,250
1,500
1,750
2,000
m/z

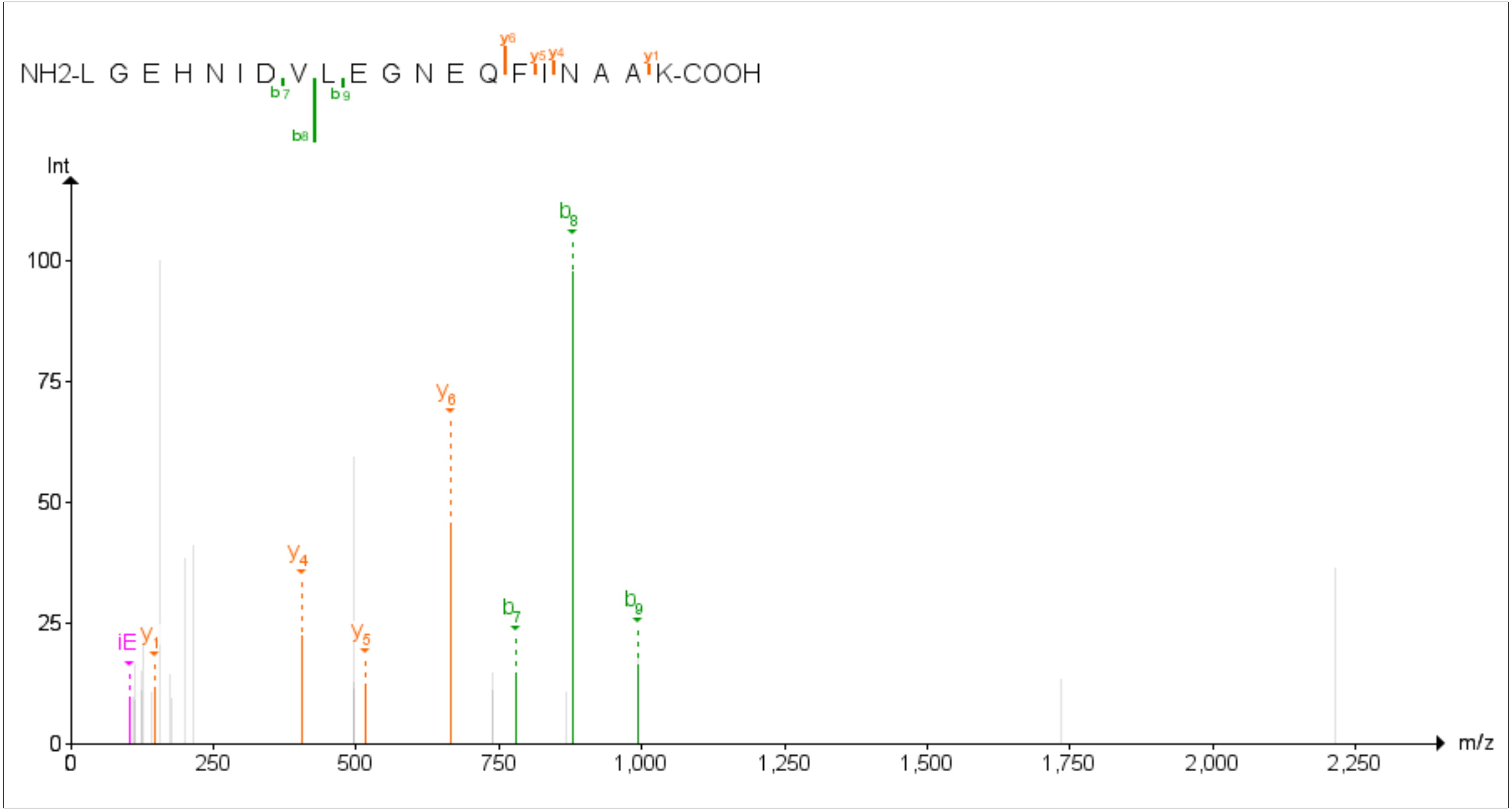
NH2-L G E H N I D V L E G N E Q F I N A A K-COOH
y6 y5 y4 y1
b7 b9 b8
Int
100
75
50
25
0
b8
y6
y4
y5
y1
iE
b7
b9
0 250 500 750 1,000 1,250 1,500 1,750 2,000 2,250
m/z

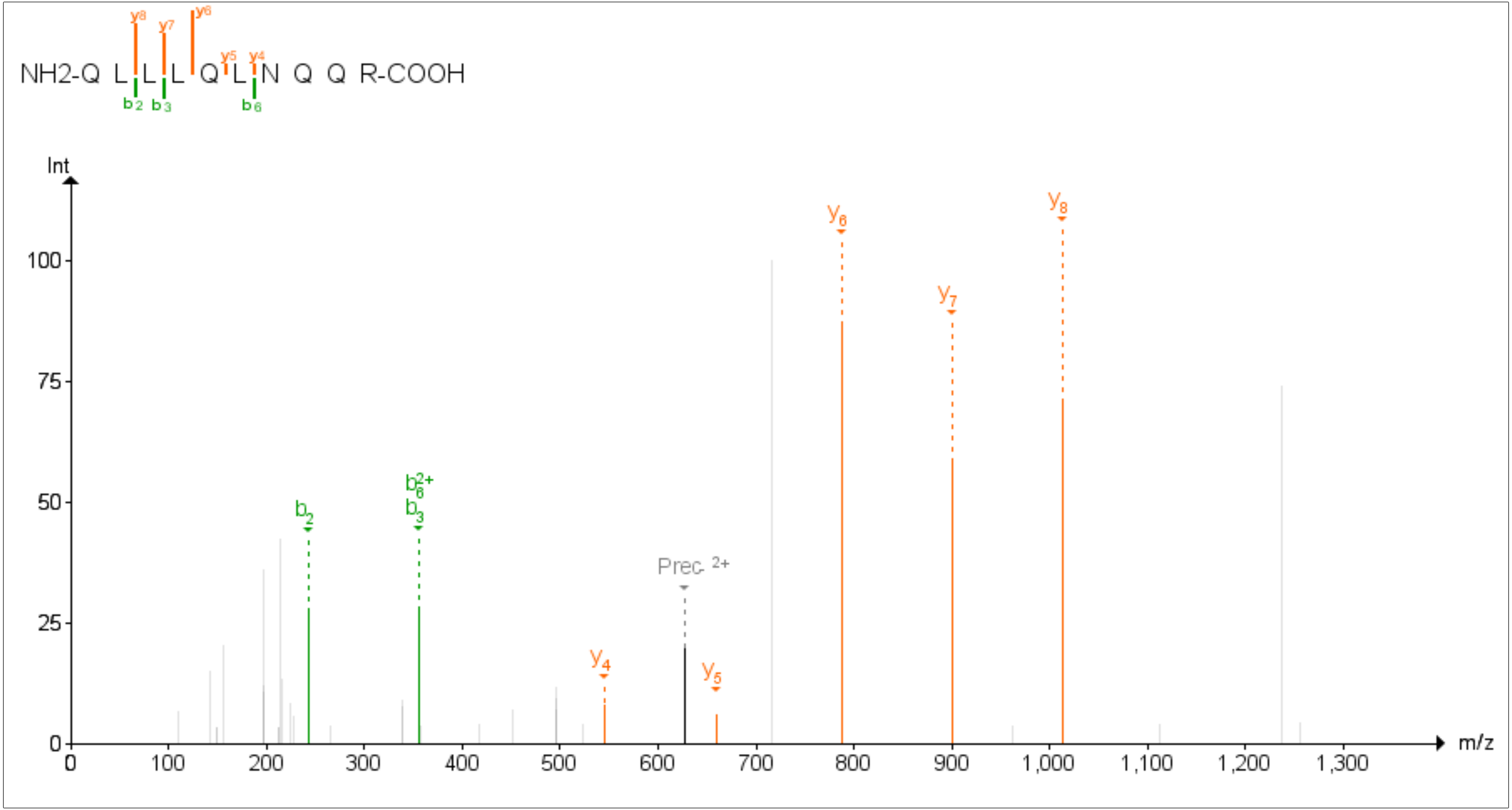

NH2-Q L L L Q L N Q Q R-COOH
y8
y7
y6
y5
y4
b2
b3
b6
Int
100
75
50
25
0
y6
y8
y7
b2
b6 2+
b3
Prec. 2+
y4
y5
0
100
200
300
400
500
600
700
800
900
1,000
1,100
1,200
1,300
m/z

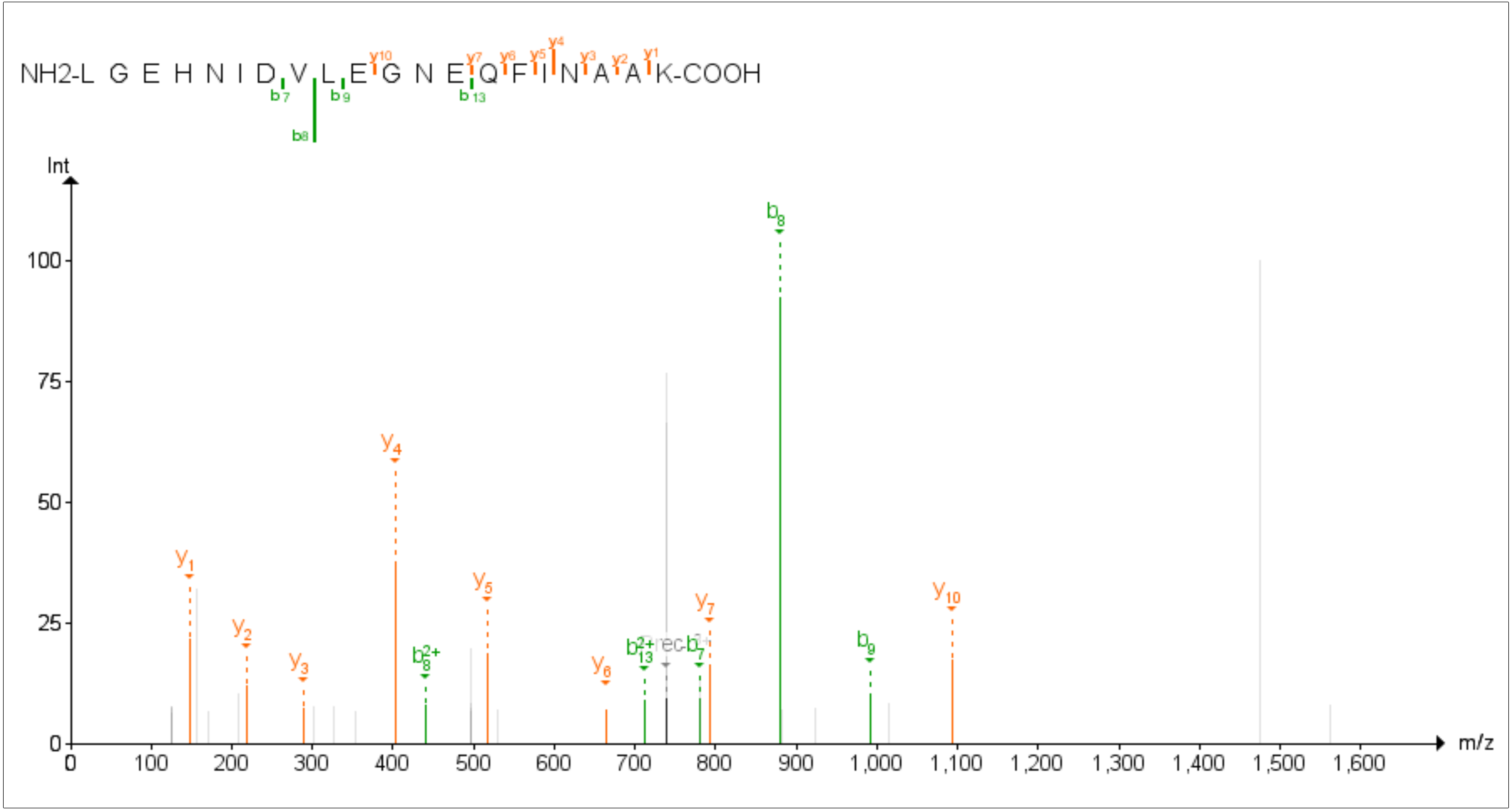

NH2-L G E H N I D V L E G N E Q F I N A A K-COOH
b7
b8
b9
b13
y10
y7
y6
y5
y4
y3
y2
y1
Int
m/z
0
25
50
75
100
0
100
200
300
400
500
600
700
800
900
1,000
1,100
1,200
1,300
1,400
1,500
1,600
y1
y2
y3
y4
b8 2+
y5
y6
b13 2+
Prec
b7
y7
b8
b9
y10

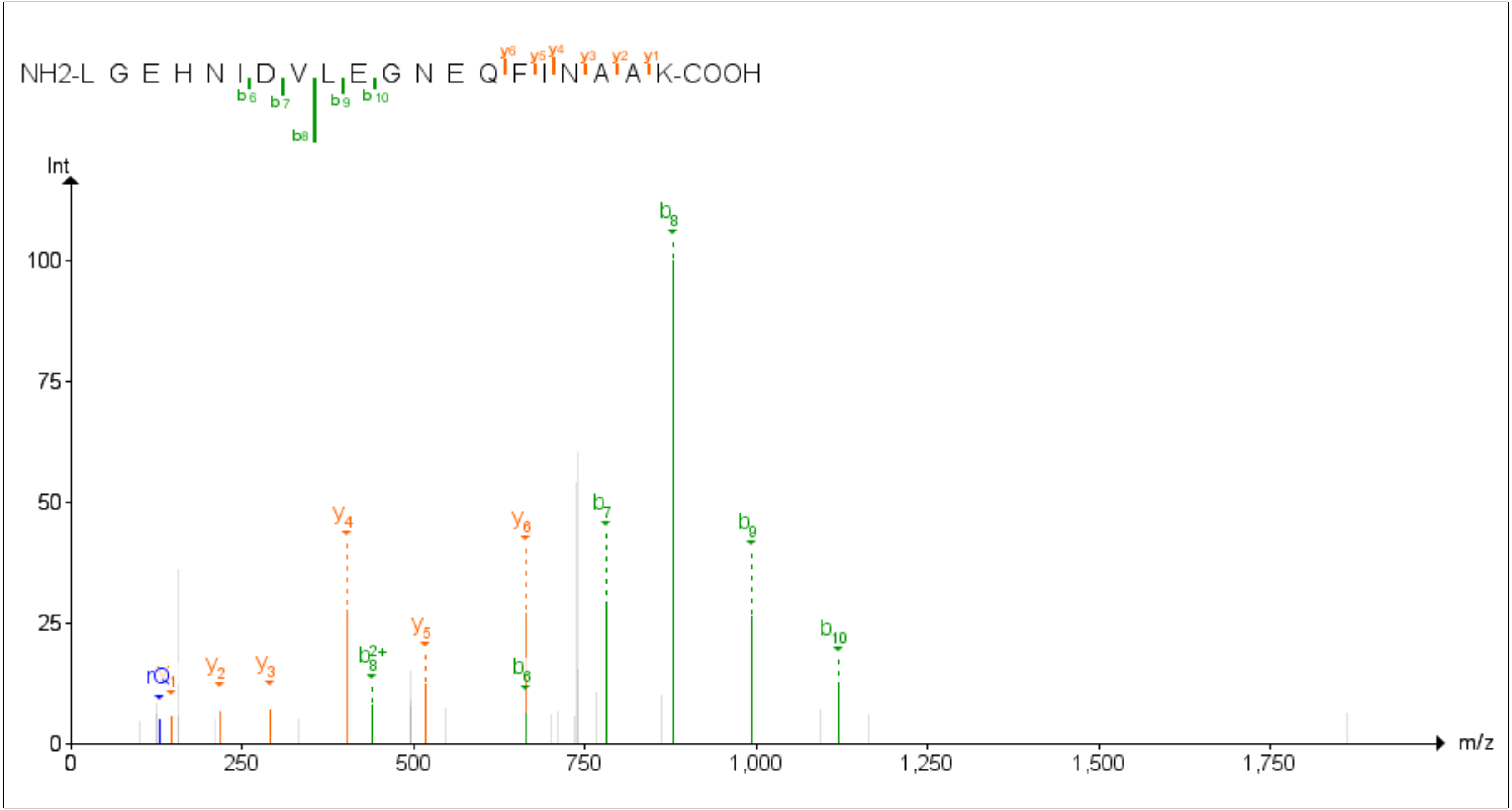

NH2-L G E H N I D V L E G N E Q F I N A A K-COOH
b6 b7 b8 b9 b10
y6 y5 y4 y3 y2 y1
Int
m/z
b8
b7
b9
b10
y4
y6
y5
y2
y3
b8 2+
b6
rQ
y1
100
75
50
25
0
250
500
750
1,000
1,250
1,500
1,750

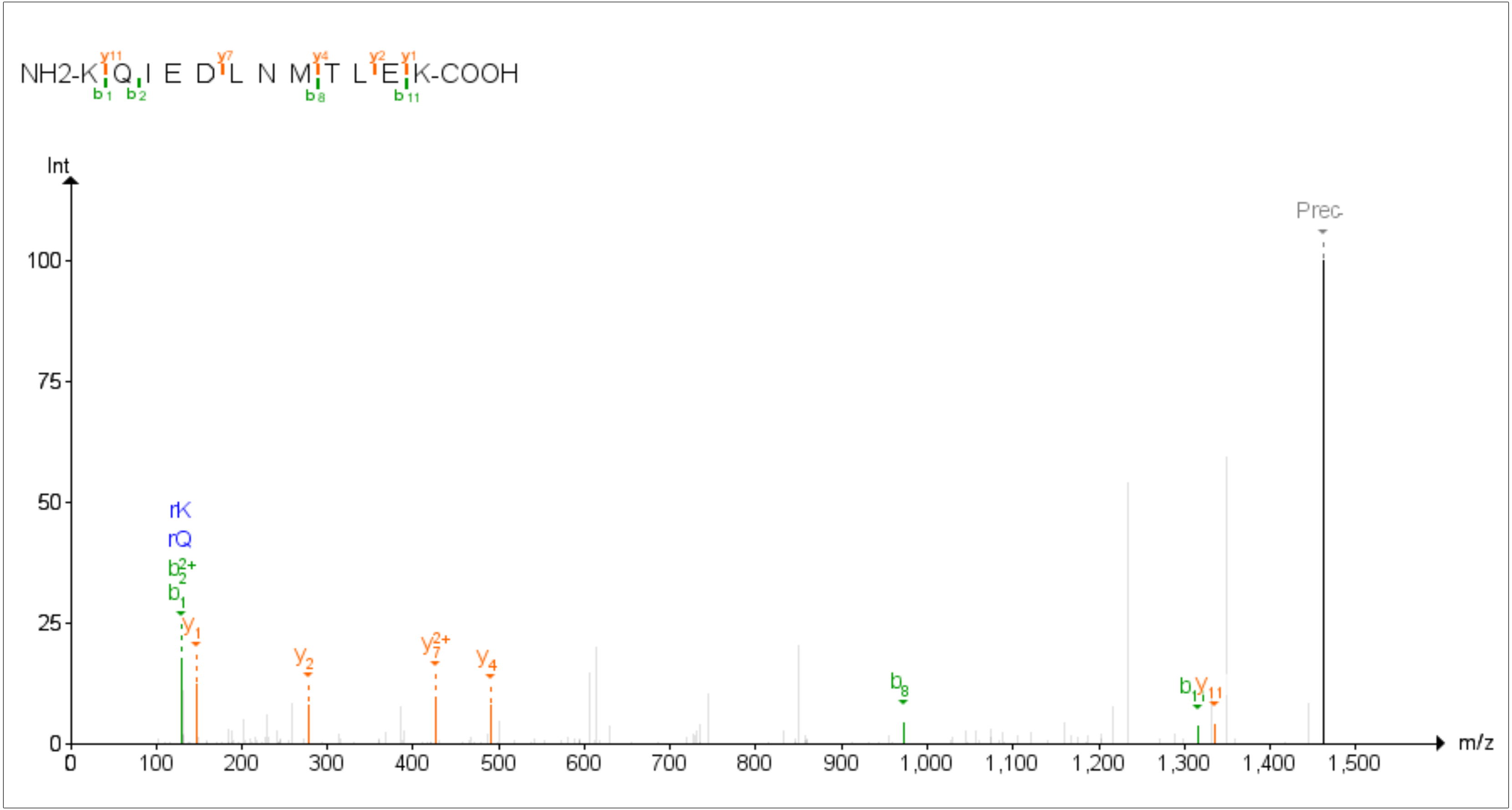

NH2-K Q I E D L N M T L E K-COOH
y11 y7 y4 y2 y1
b1 b2 b8 b11
Int
Prec.
rK
rQ
b_2^{2+}
b_1
y_1
y_2
y_7^{2+}
y_4
b_8
b_{11}
y_{11}
0
25
50
75
100
100
200
300
400
500
600
700
800
900
1,000
1,100
1,200
1,300
1,400
1,500
m/z

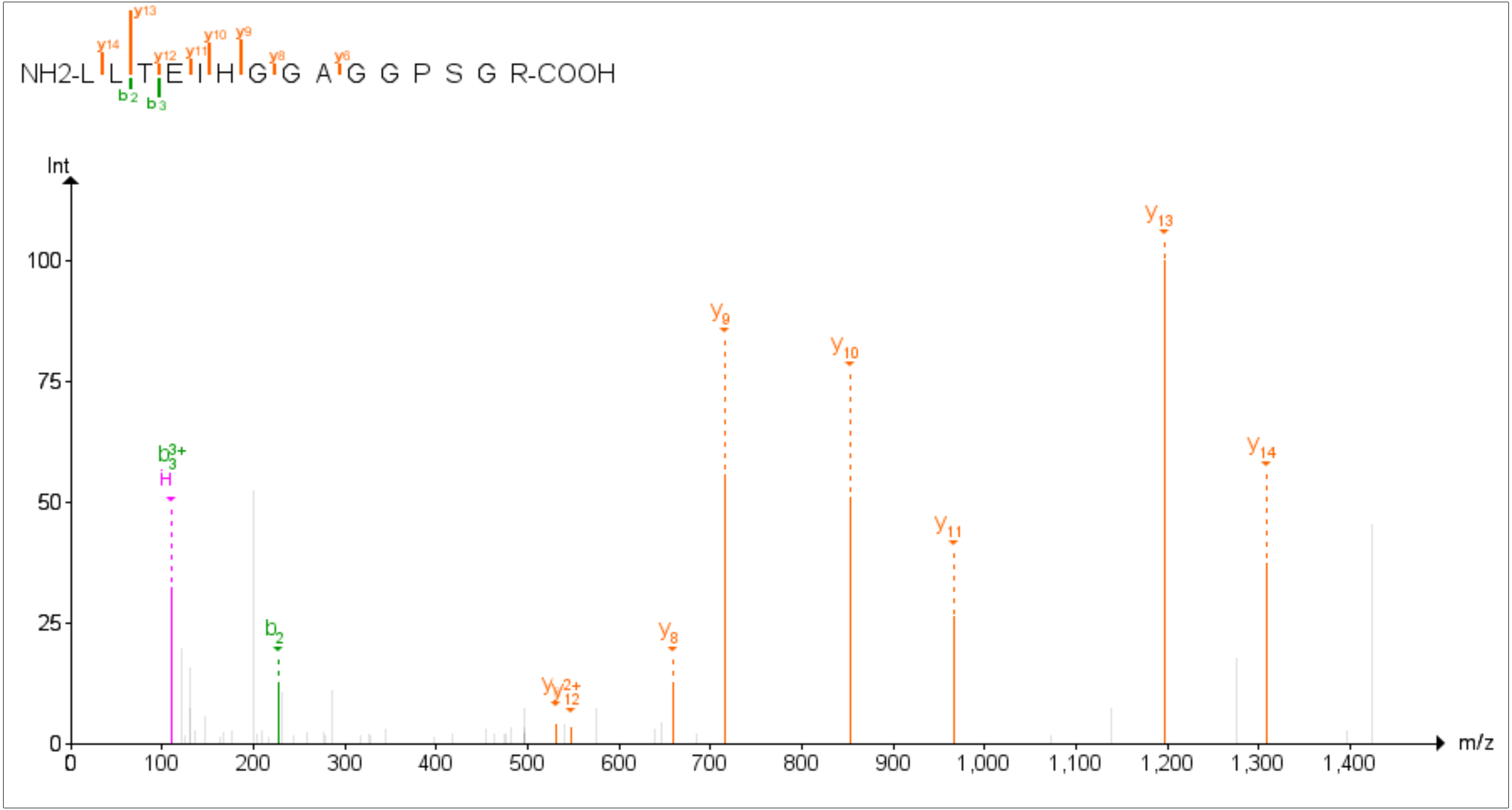

NH2-L L T E I H G G A G G P S G R-COOH
y14 y13 y12 y11 y10 y9 y8 y6
b2 b3
Int
m/z
0
25
50
75
100
0
100
200
300
400
500
600
700
800
900
1,000
1,100
1,200
1,300
1,400
b3 3+
H
b2
y12 2+
y8
y9
y10
y11
y13
y14

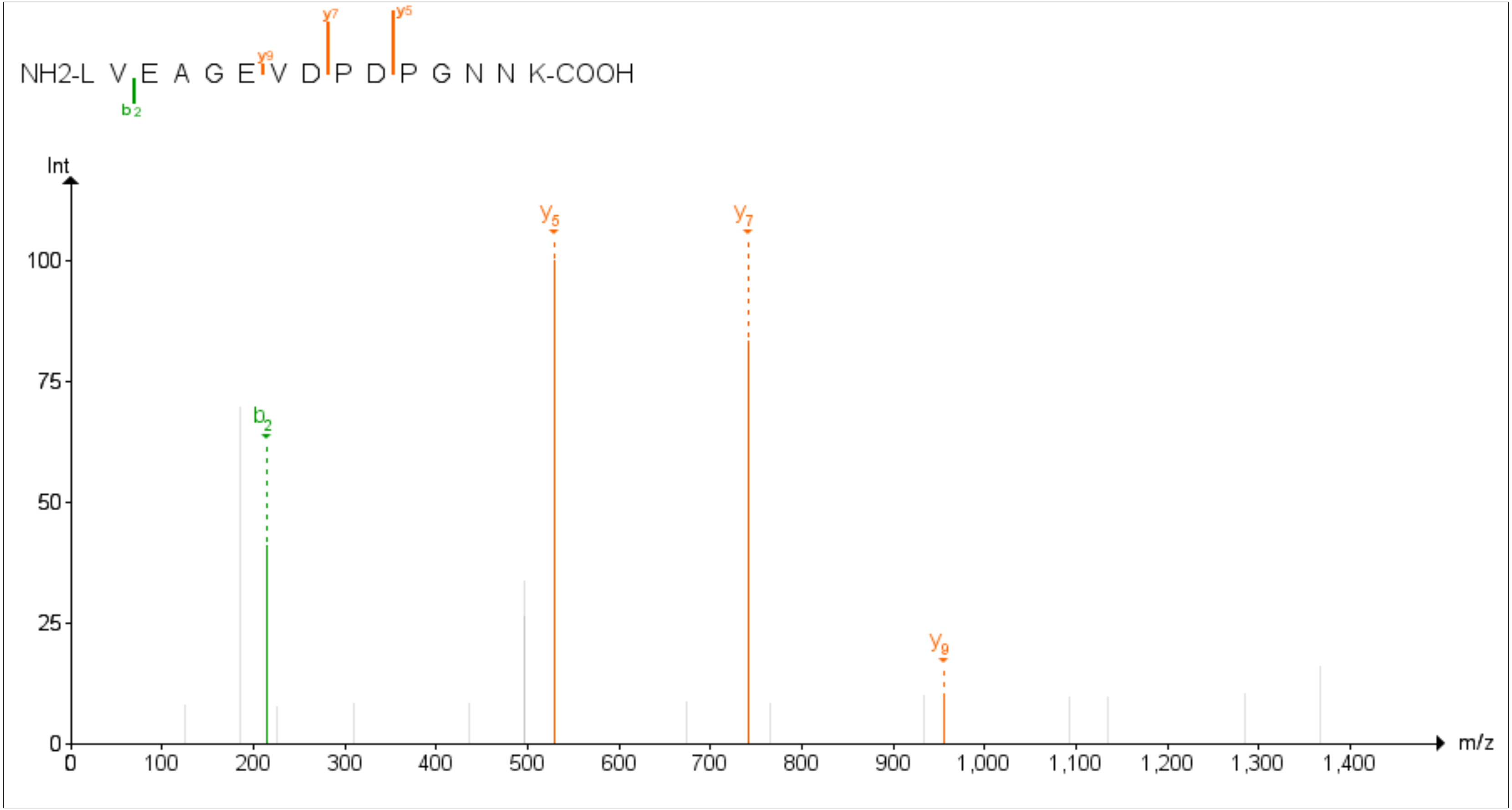

NH2-L V E A G E V D P D P G N N K-COOH
b2
y9
y7
y5
Int
m/z
0
25
50
75
100
0
100
200
300
400
500
600
700
800
900
1,000
1,100
1,200
1,300
1,400

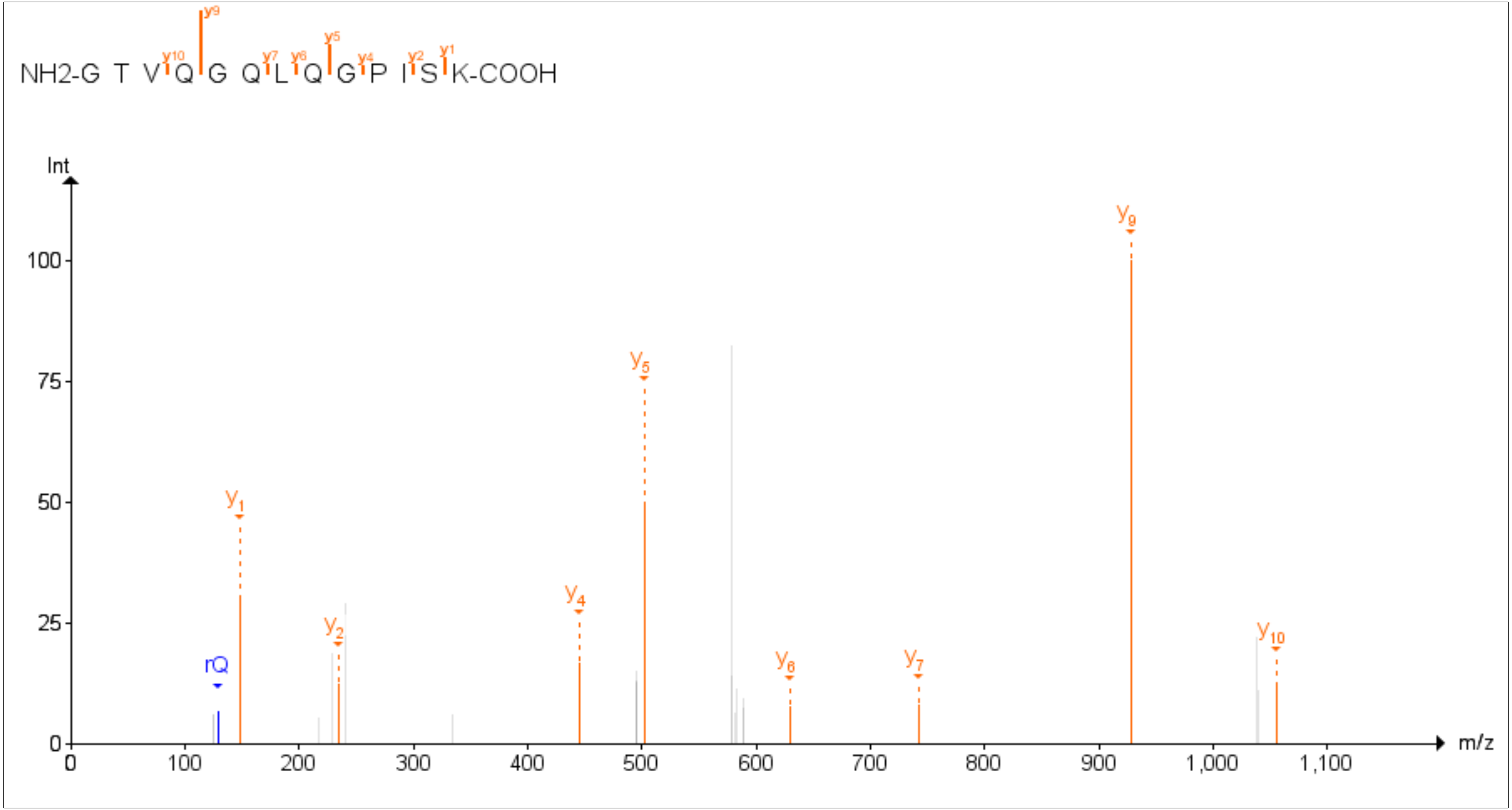

NH2-G T V Q G Q L Q G P I S K-COOH
y9 y10 y7 y6 y5 y4 y2 y1
Int
100
75
50
25
0
y1 y2 y4 y5 y6 y7 y9 y10
rQ
0 100 200 300 400 500 600 700 800 900 1,000 1,100
m/z

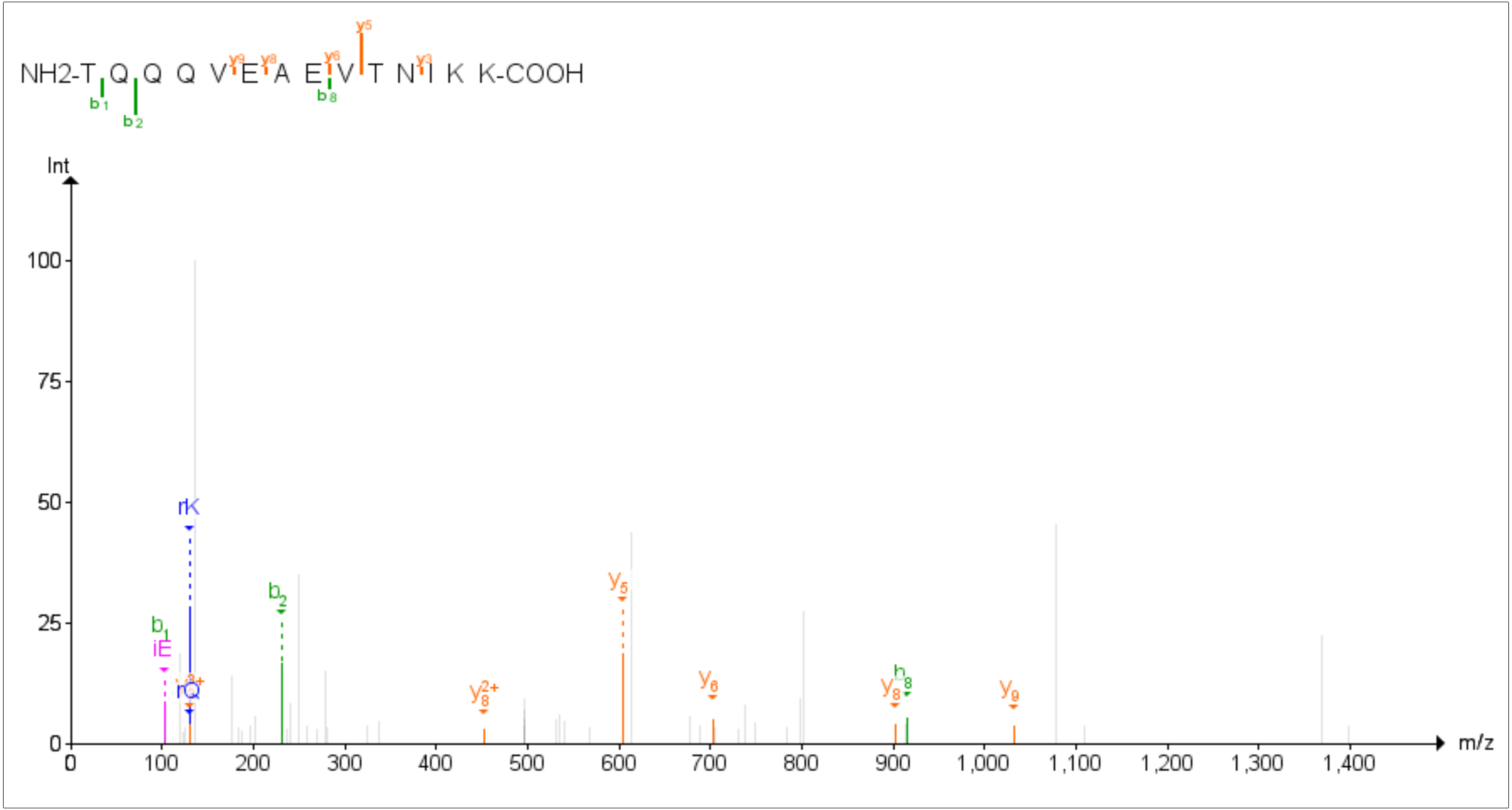

NH2-T Q Q Q V E A E V T N I K K-COOH
b1
b2
b8
y9
y8
y6
y5
y3
Int
100
75
50
25
0
m/z
0
100
200
300
400
500
600
700
800
900
1,000
1,100
1,200
1,300
1,400
rK
b2
y5
b1
iE
rQ
y8 2+
y6
b8
y8
y9

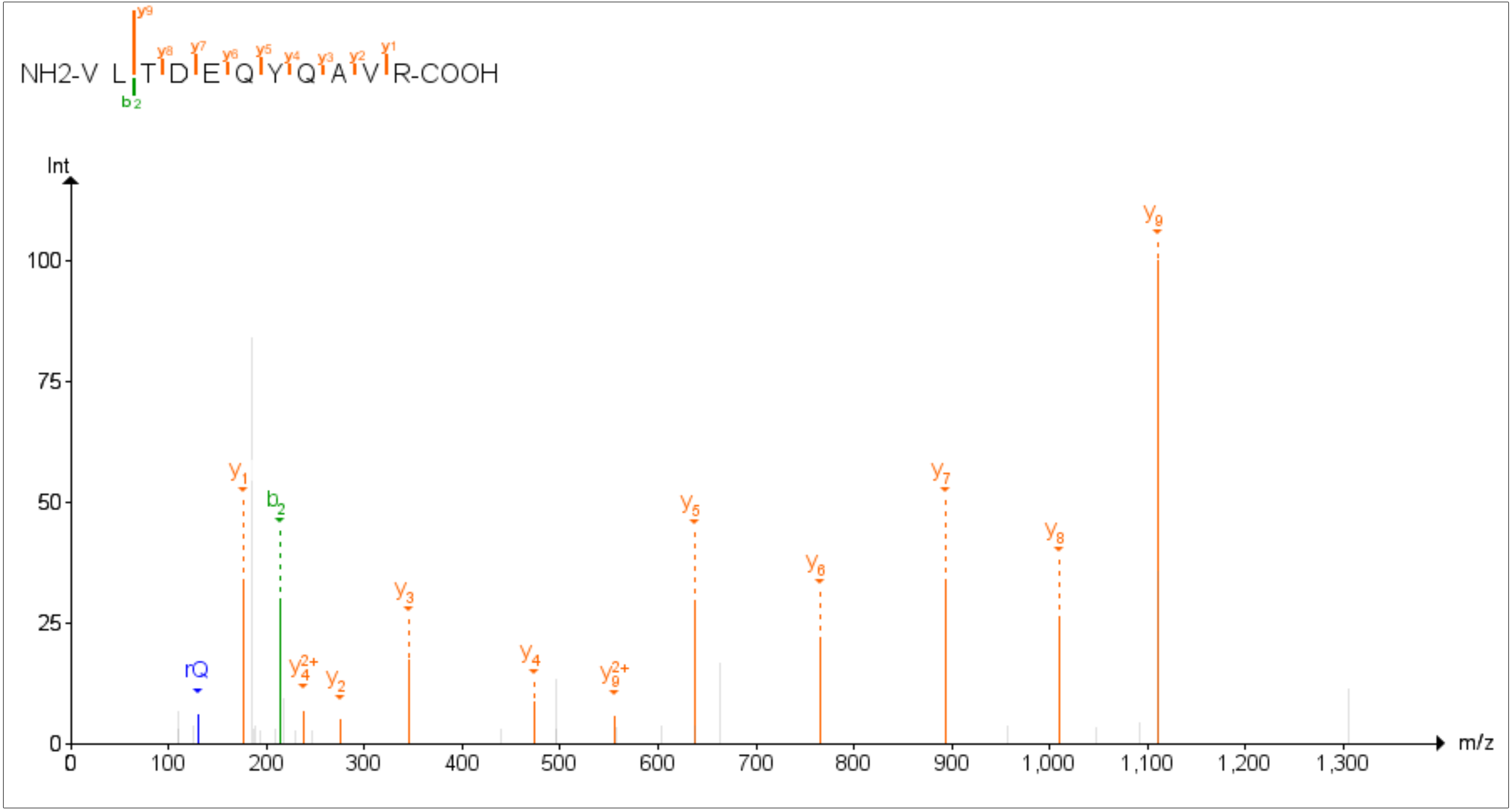

NH2-V L T D E Q Y Q A V R-COOH
y9 y8 y7 y6 y5 y4 y3 y2 y1
b2
Int
m/z
y9
y1
b2
y5
y7
y8
y6
y3
y4
rQ
y4 2+
y2
y9 2+
0
25
50
75
100
0
100
200
300
400
500
600
700
800
900
1,000
1,100
1,200
1,300

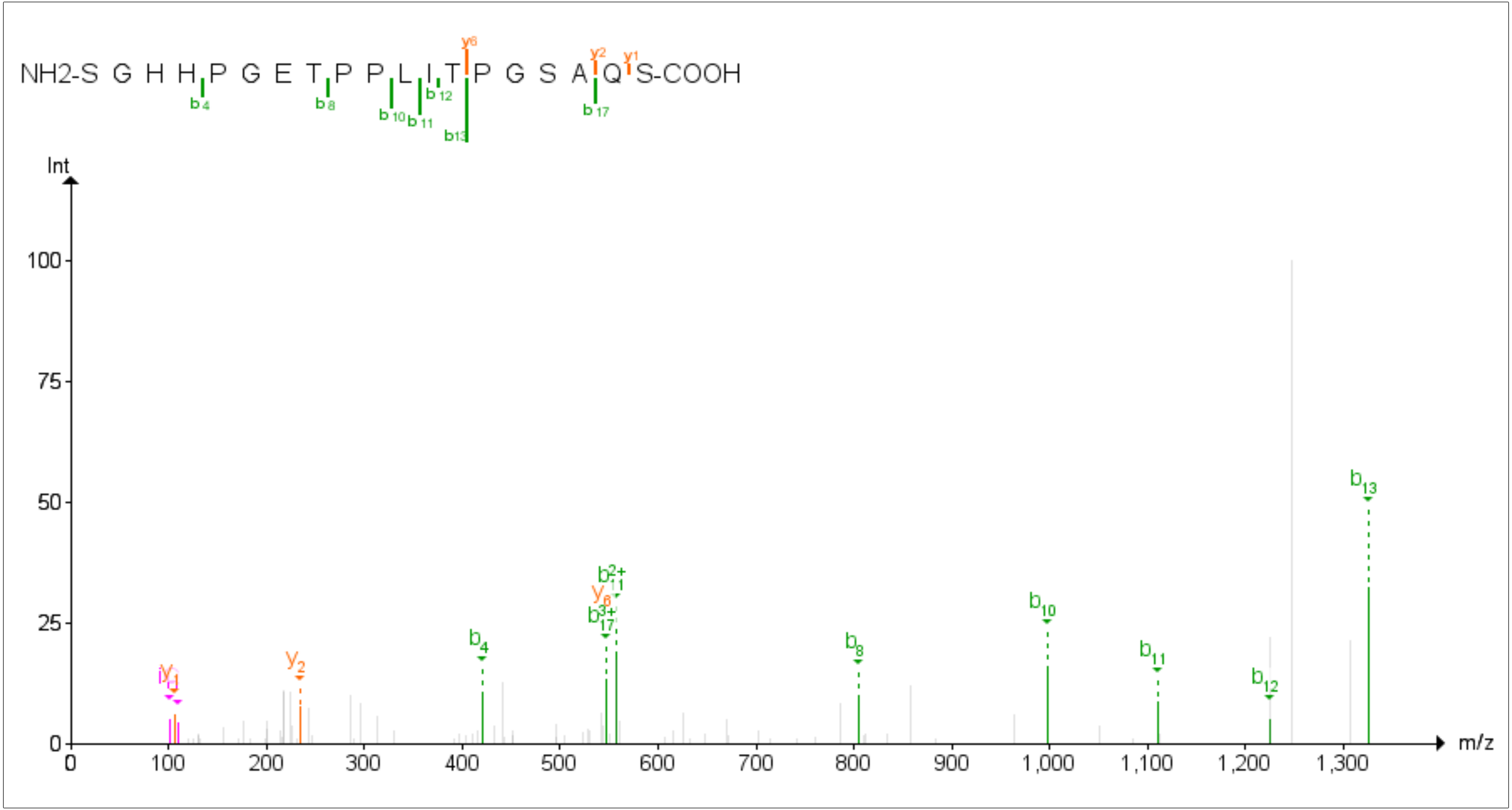

NH2-S G H H P G E T P P L I T P G S A Q S-COOH
b4
b8
b10
b11
b12
b13
b17
y6
y2
y1
Int
100
75
50
25
0
m/z
0
100
200
300
400
500
600
700
800
900
1,000
1,100
1,200
1,300

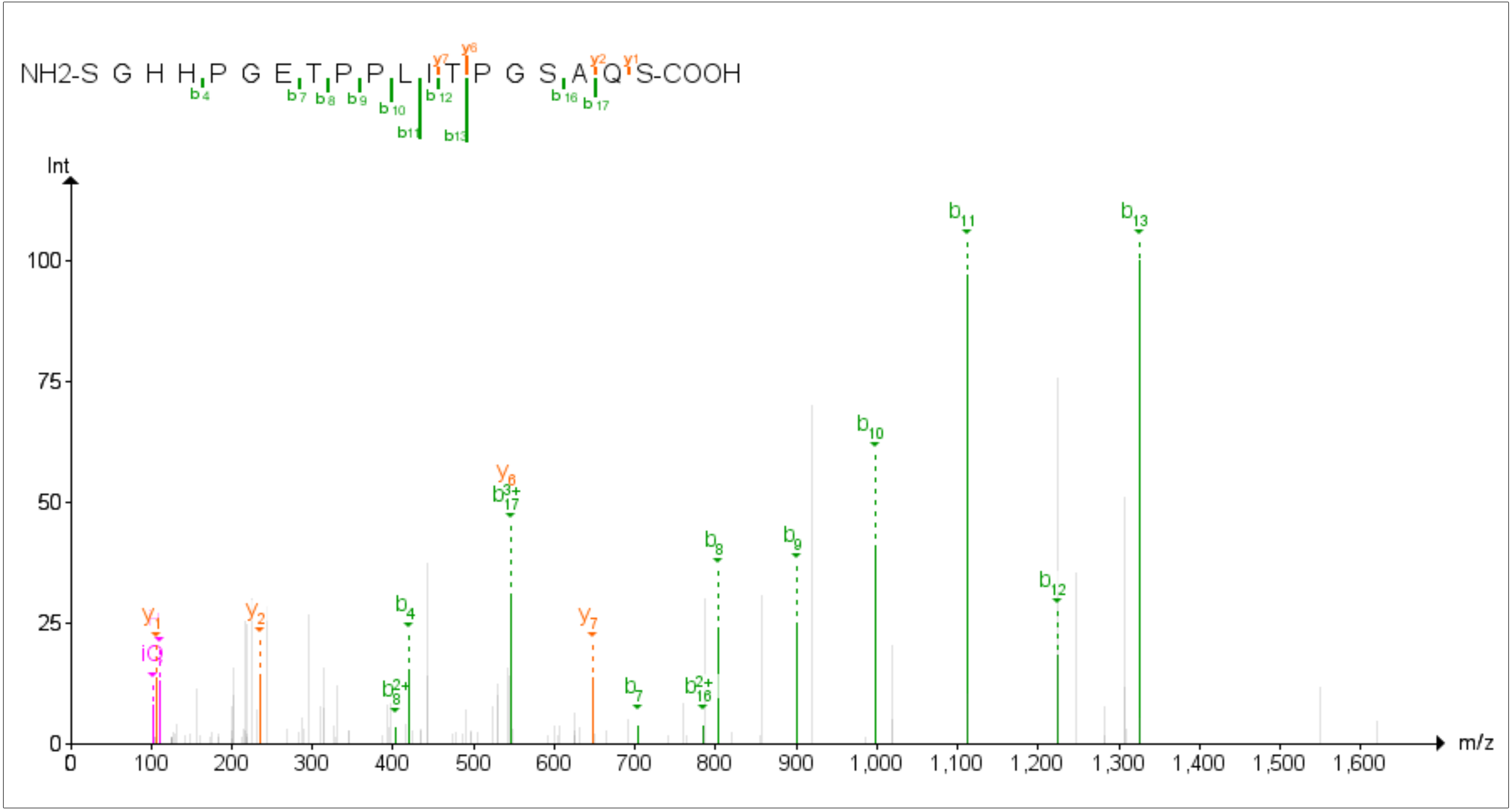

NH2-S G H H P G E T P P L I T P G S A Q S-COOH
Int
m/z

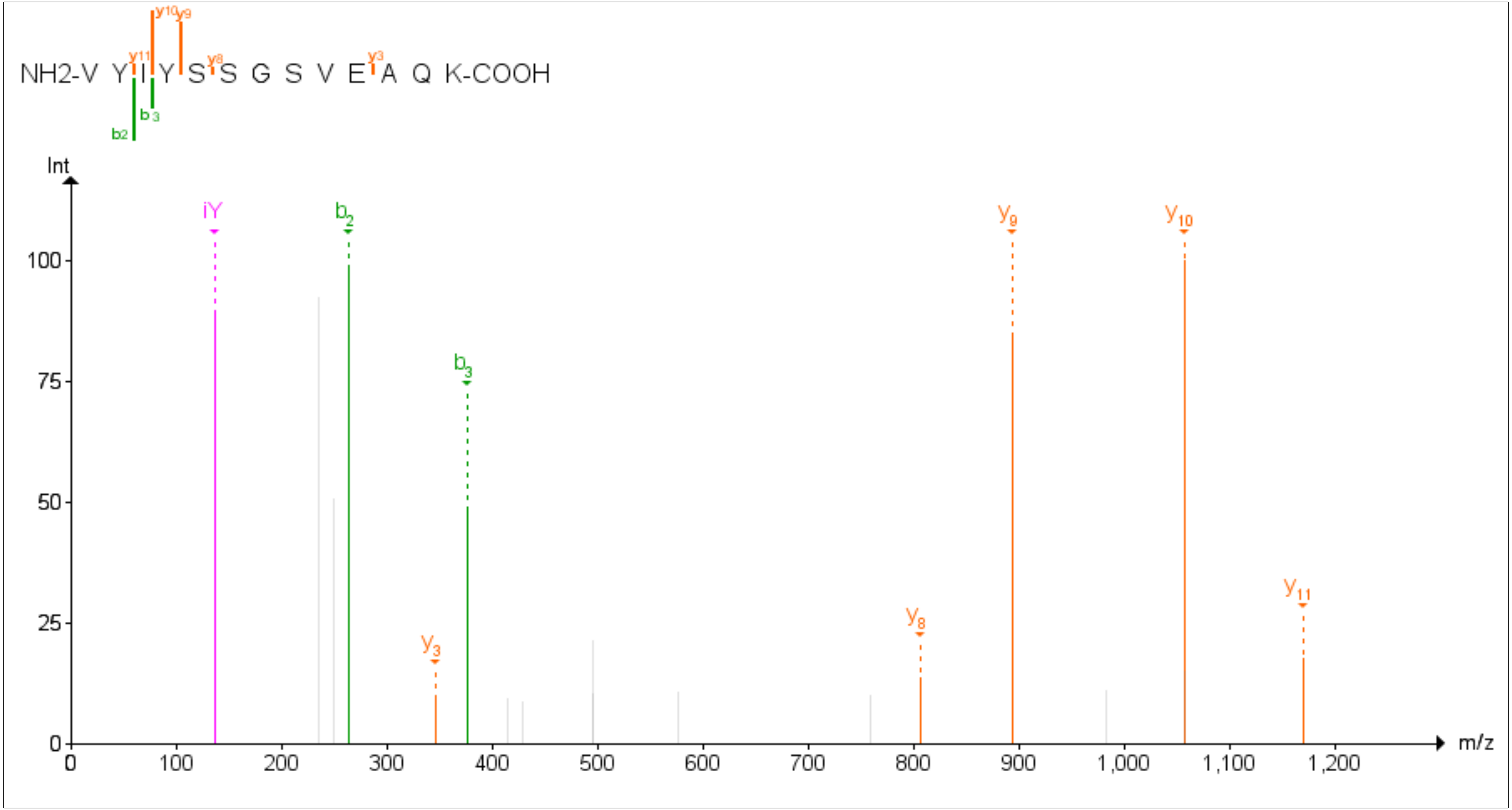

NH2-V Y I Y S S G S V E A Q K-COOH
y10 y9 y11 y8 y3
b2 b3
Int
iY
b2
b3
y3
y8
y9
y10
y11
100
75
50
25
0
0 100 200 300 400 500 600 700 800 900 1,000 1,100 1,200
m/z

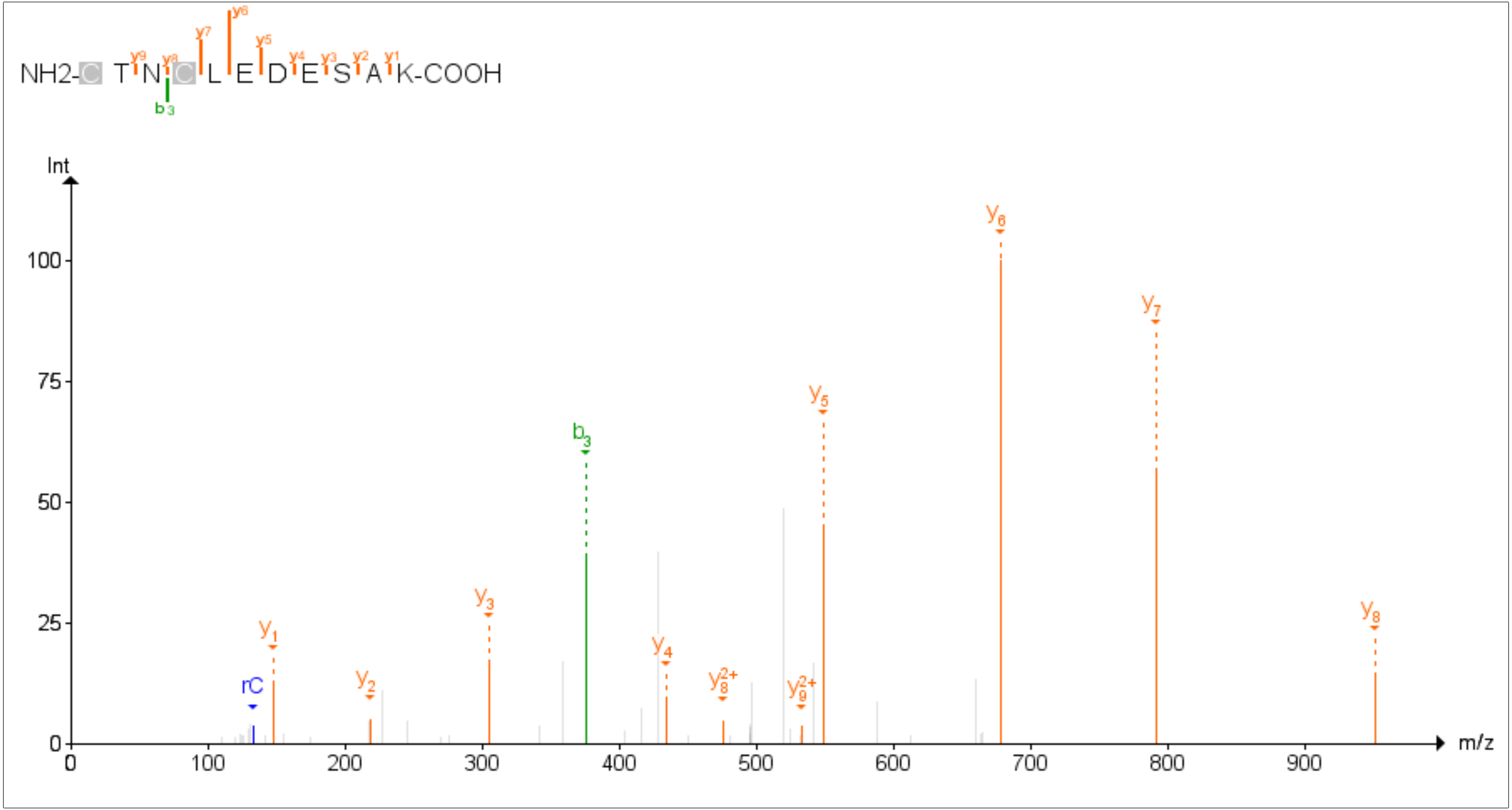

NH2-C T N C L E D E S A K-COOH
y9 y8 y7 y6 y5 y4 y3 y2 y1
b3
Int
m/z
100
75
50
25
0
0 100 200 300 400 500 600 700 800 900
y6
y7
y5
b3
y3
y1
rC
y2
y4
y8 2+
y9 2+
y8

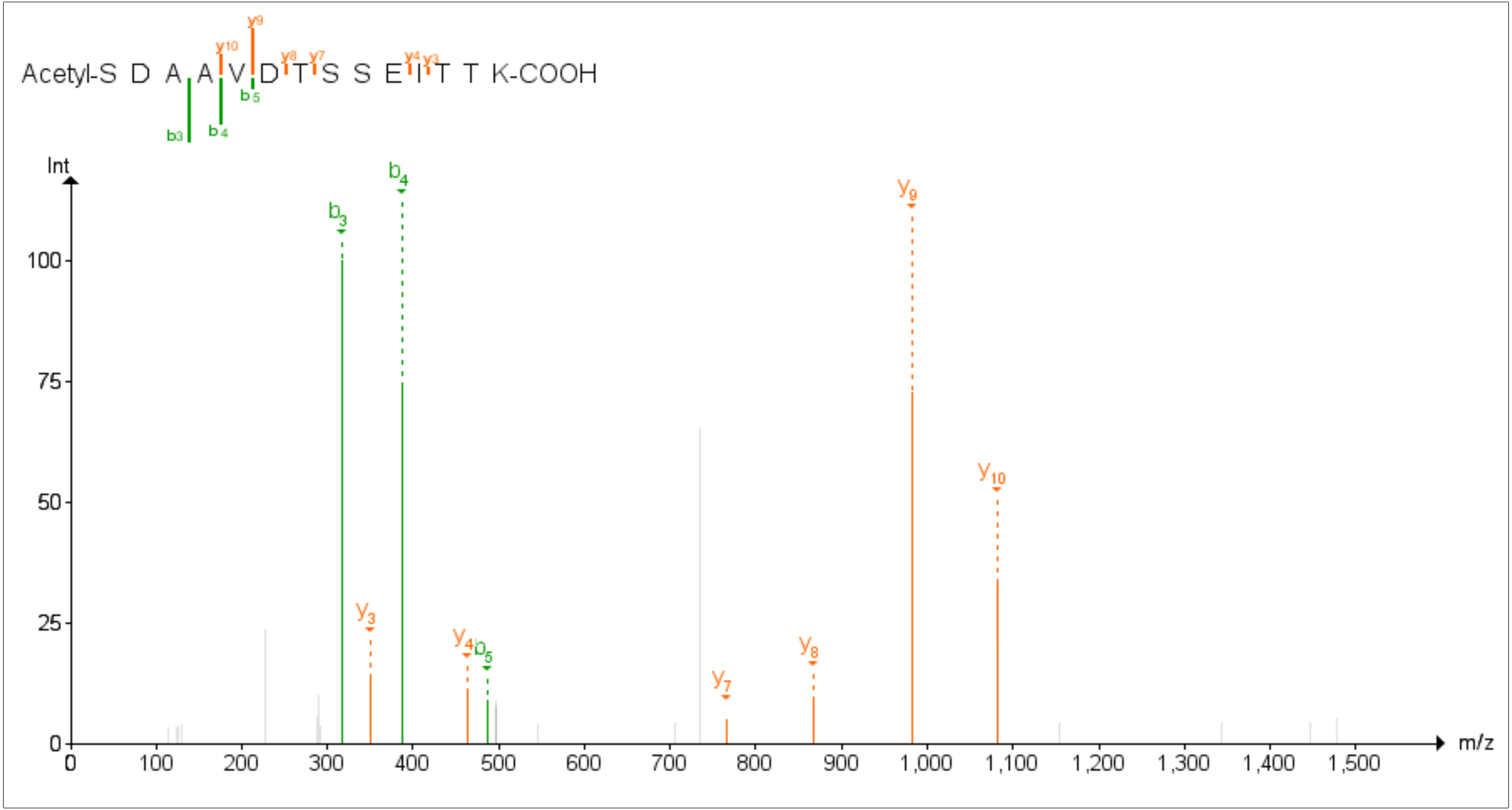

Acetyl-S D A A V D T S S E I T T K-COOH
y10 y9 y8 y7 y4 y3
b3 b4 b5
Int
100
75
50
25
0
b3 b4 y9 y10 y3 y4 b5 y7 y8
0 100 200 300 400 500 600 700 800 900 1,000 1,100 1,200 1,300 1,400 1,500
m/z

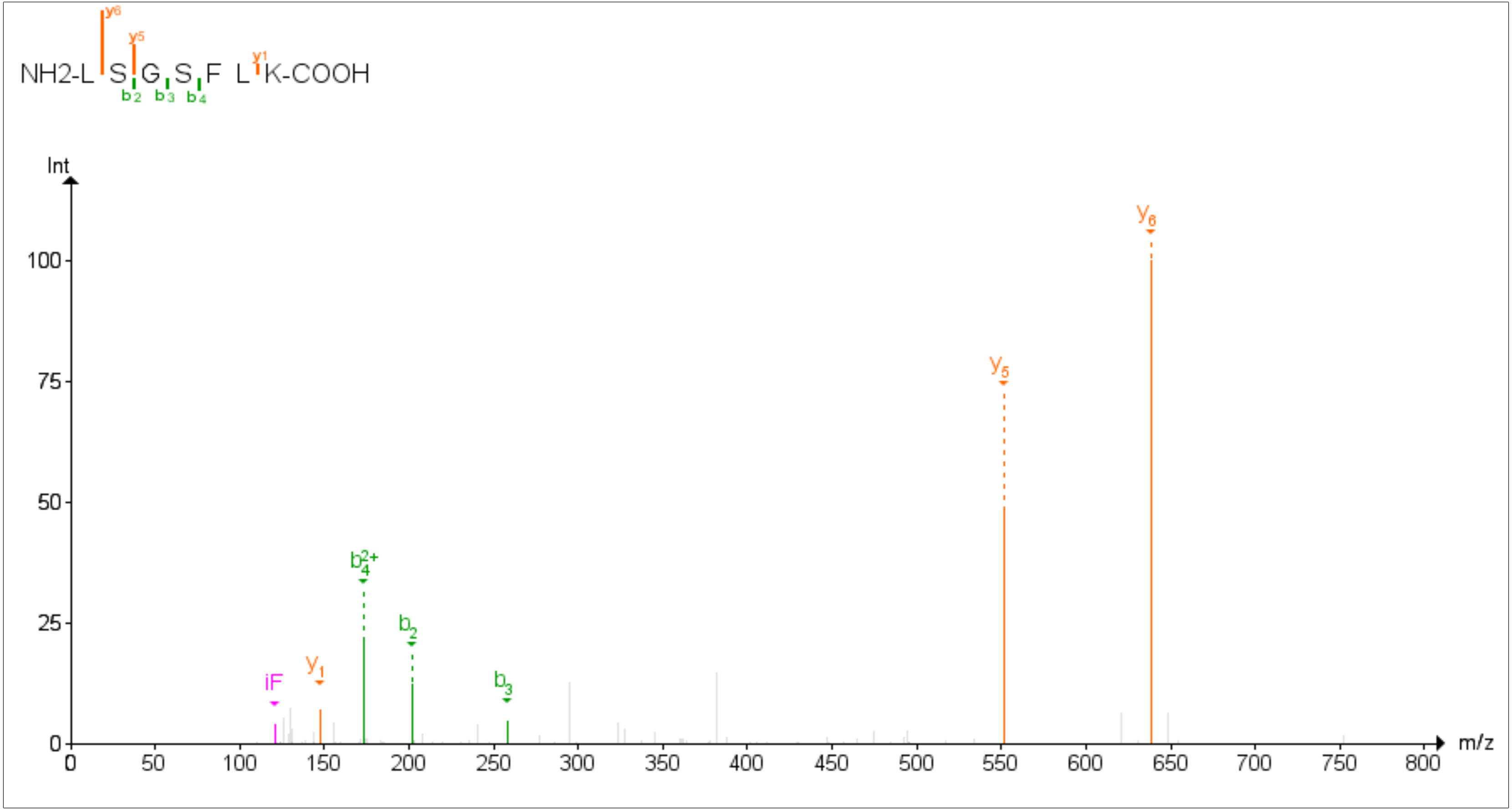
NH2-L S G S F L K-COOH
y6
y5
y1
b2
b3
b4
Int
100
75
50
25
0
y6
y5
b4 2+
b2
b3
y1
iF
0
50
100
150
200
250
300
350
400
450
500
550
600
650
700
750
800
m/z

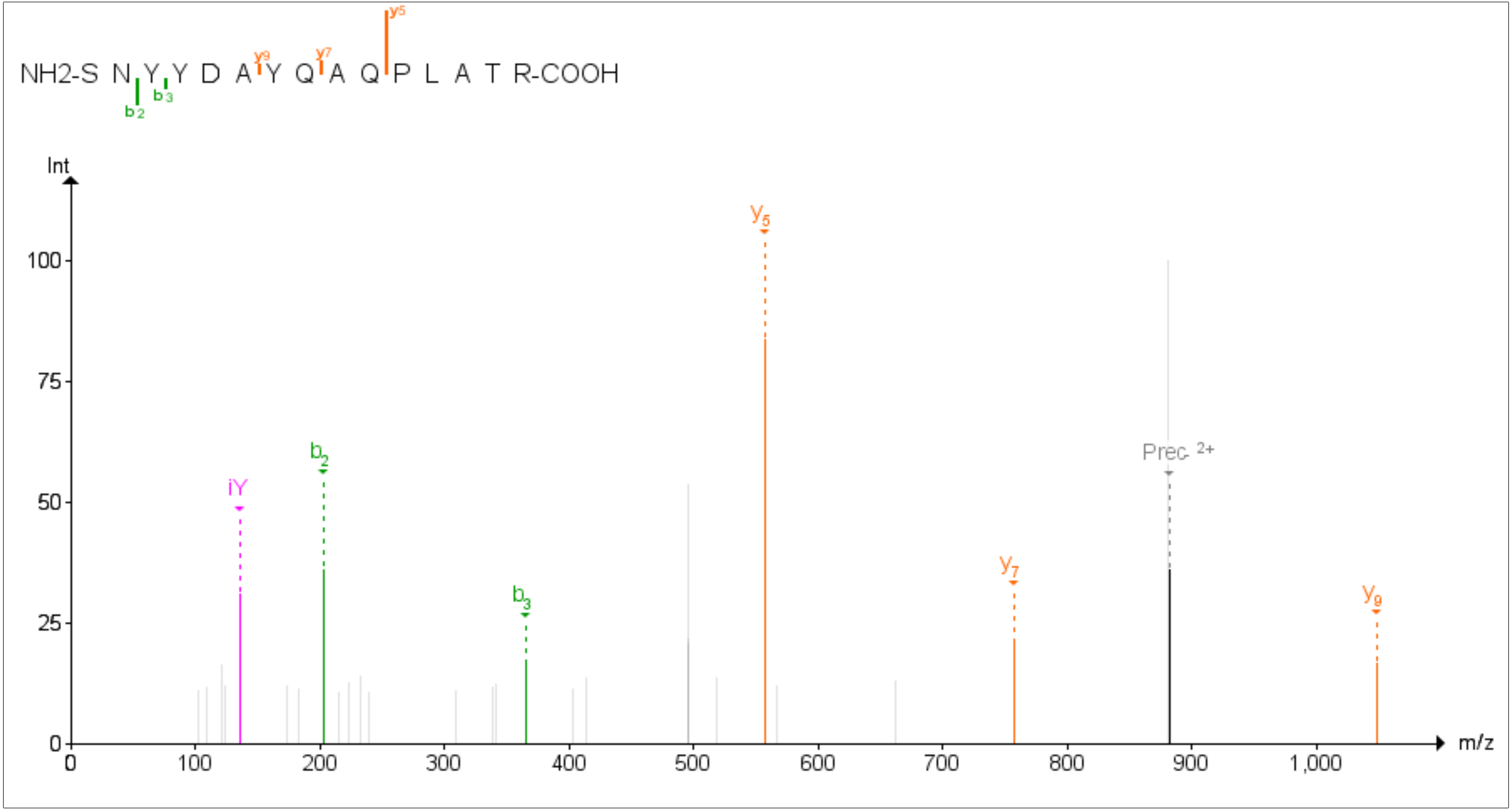

NH2-S N Y Y D A Y Q A Q P L A T R-COOH
b2
b3
y9
y7
y5
Int
100
75
50
25
0
iY
b2
b3
y5
y7
y9
Prec. 2+
0
100
200
300
400
500
600
700
800
900
1,000
m/z

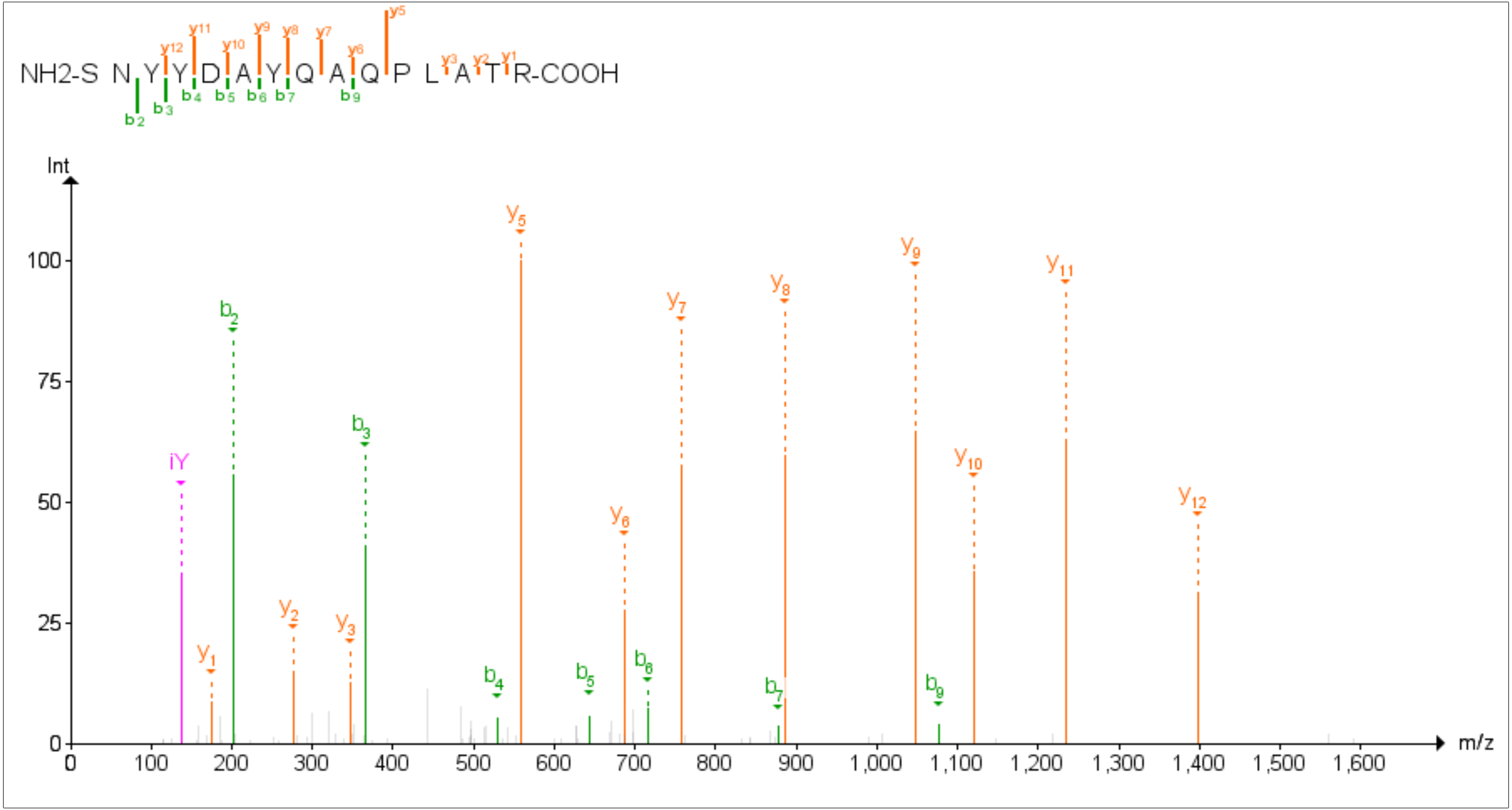

NH2-S N Y Y D A Y Q A Q P L A T R-COOH
Int
m/z
iY
b2
b3
b4
b5
b6
b7
b9
y1
y2
y3
y5
y6
y7
y8
y9
y10
y11
y12

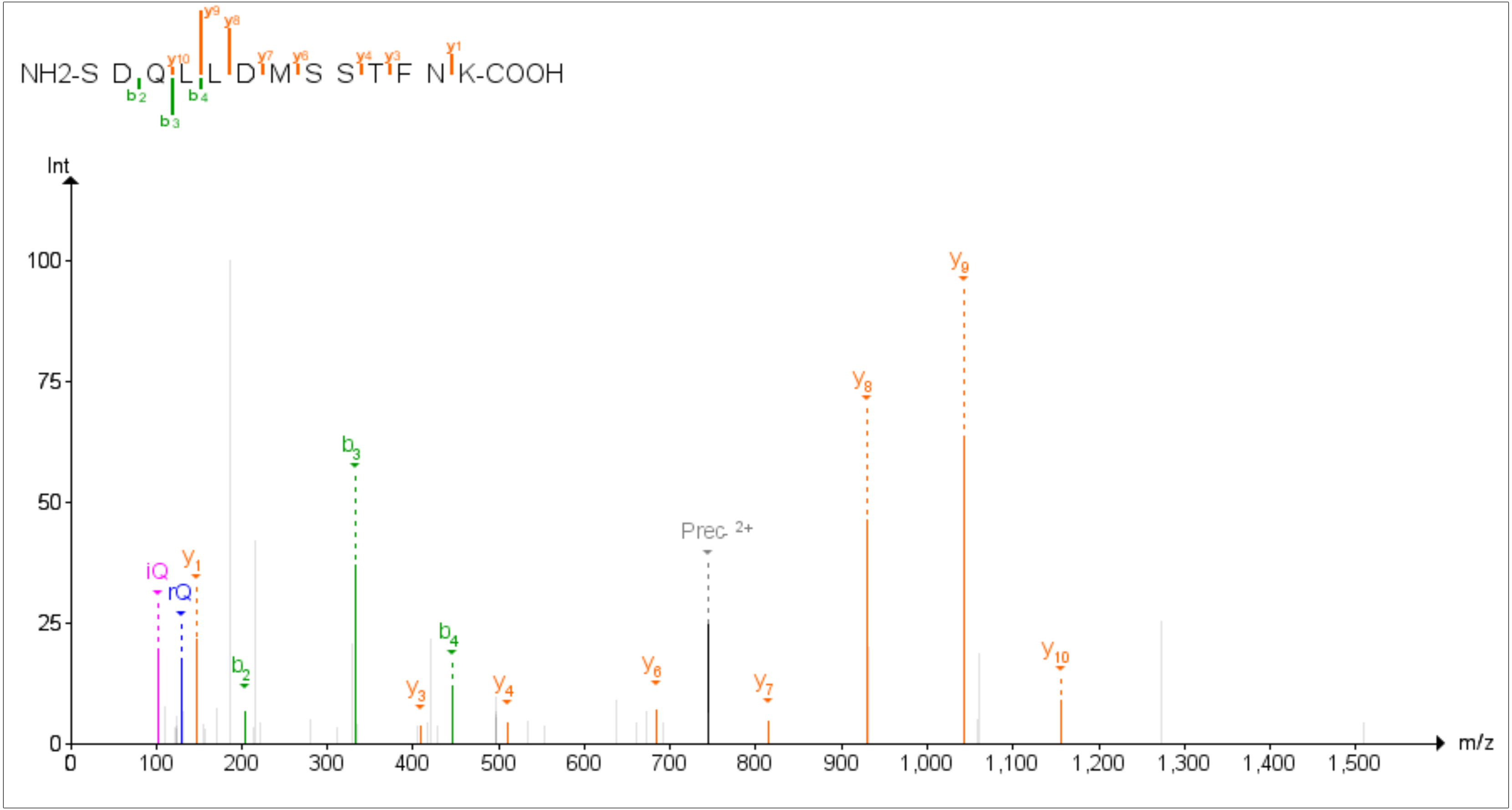

NH2-S D Q L L D M S S T F N K-COOH
y9 y8 y10 y7 y6 y4 y3 y1
b2 b3 b4
Int
100
75
50
25
0
iQ
rQ
y1
b2
b3
y3
b4
y4
y6
Prec 2+
y7
y8
y9
y10
0 100 200 300 400 500 600 700 800 900 1,000 1,100 1,200 1,300 1,400 1,500
m/z

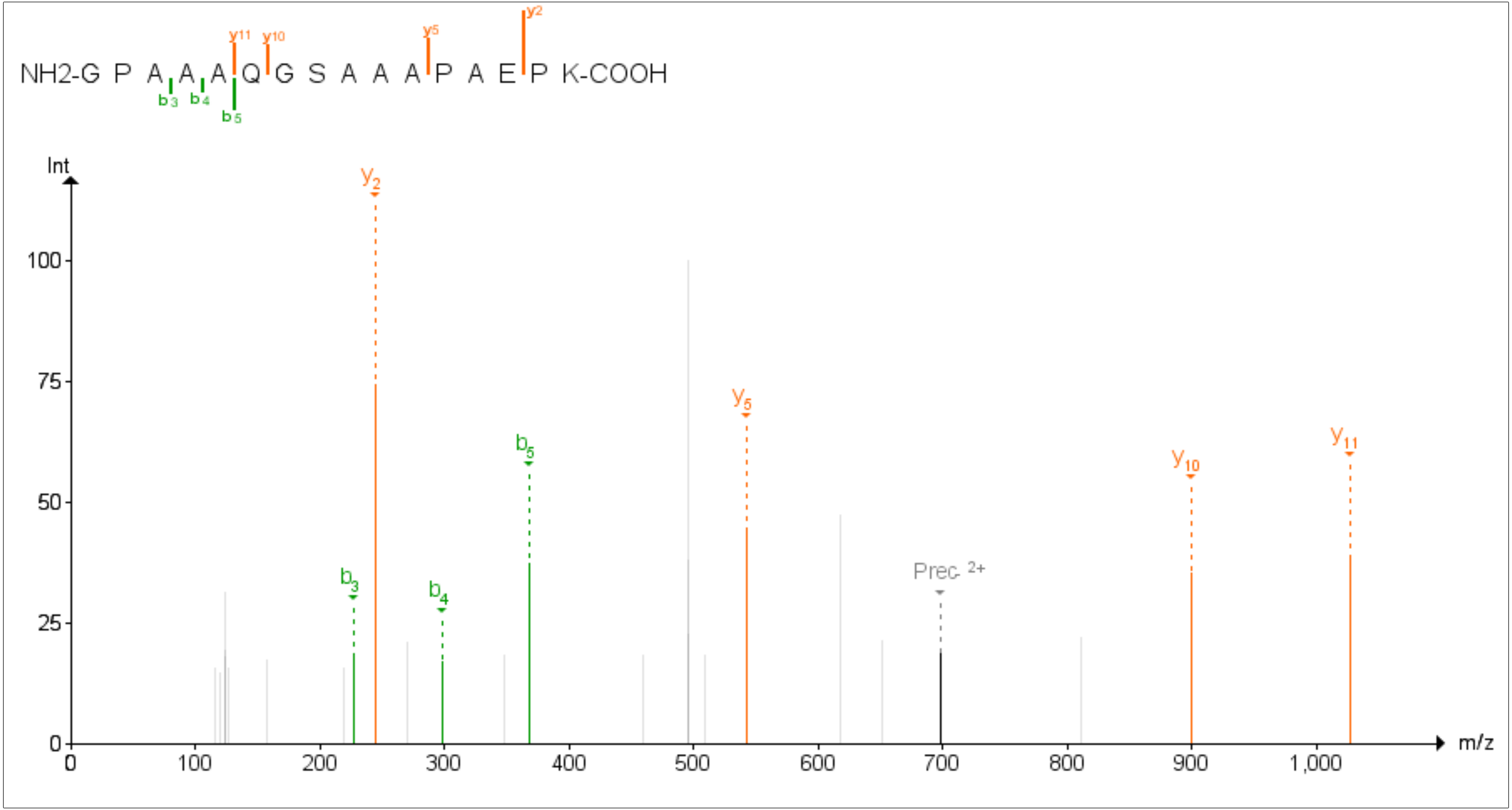

NH2-G P A A A Q G S A A A P A E P K-COOH
y11 y10 y5 y2
b3 b4 b5
Int
100
75
50
25
0
y2
y5
b5
b3
b4
Prec 2+
y10
y11
0 100 200 300 400 500 600 700 800 900 1,000
m/z

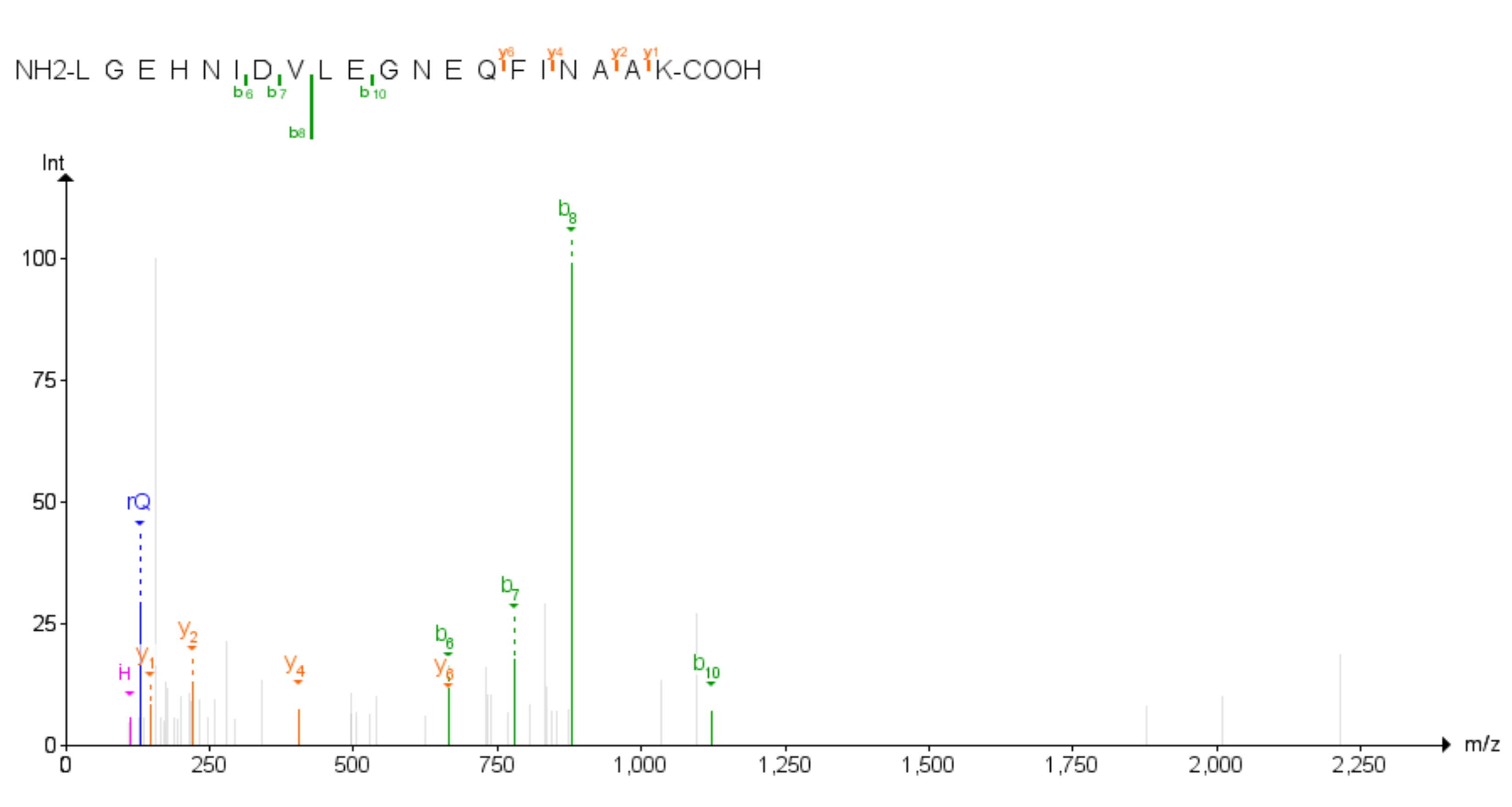

NH2-L G E H N I D V L E G N E Q F I N A A K-COOH
y6 y4 y2 y1
b6 b7 b10
b8
Int
100
75
50
25
0
m/z
0
250
500
750
1,000
1,250
1,500
1,750
2,000
2,250
b8
rQ
b7
y2
y1
iH
y4
b6
y6
b10

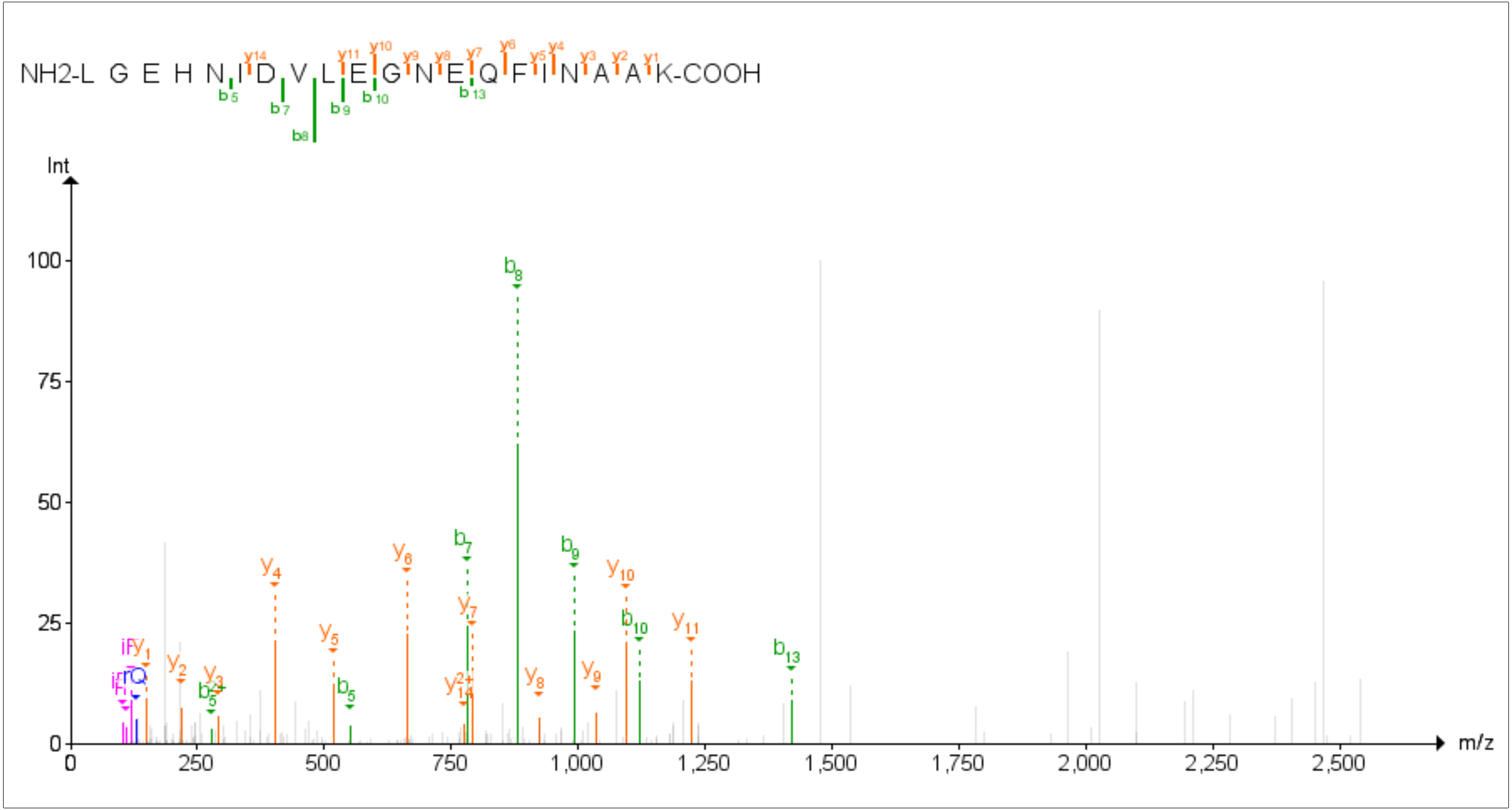
NH2-L G E H N I D V L E G N E Q F I N A A K-COOH
Int
m/z
0
25
50
75
100
250
500
750
1,000
1,250
1,500
1,750
2,000
2,250
2,500
b8
b7
b9
b10
b13
y4
y6
y10
y11
y5
b5
y1
y2
y3
y7
y8
y9
rQ

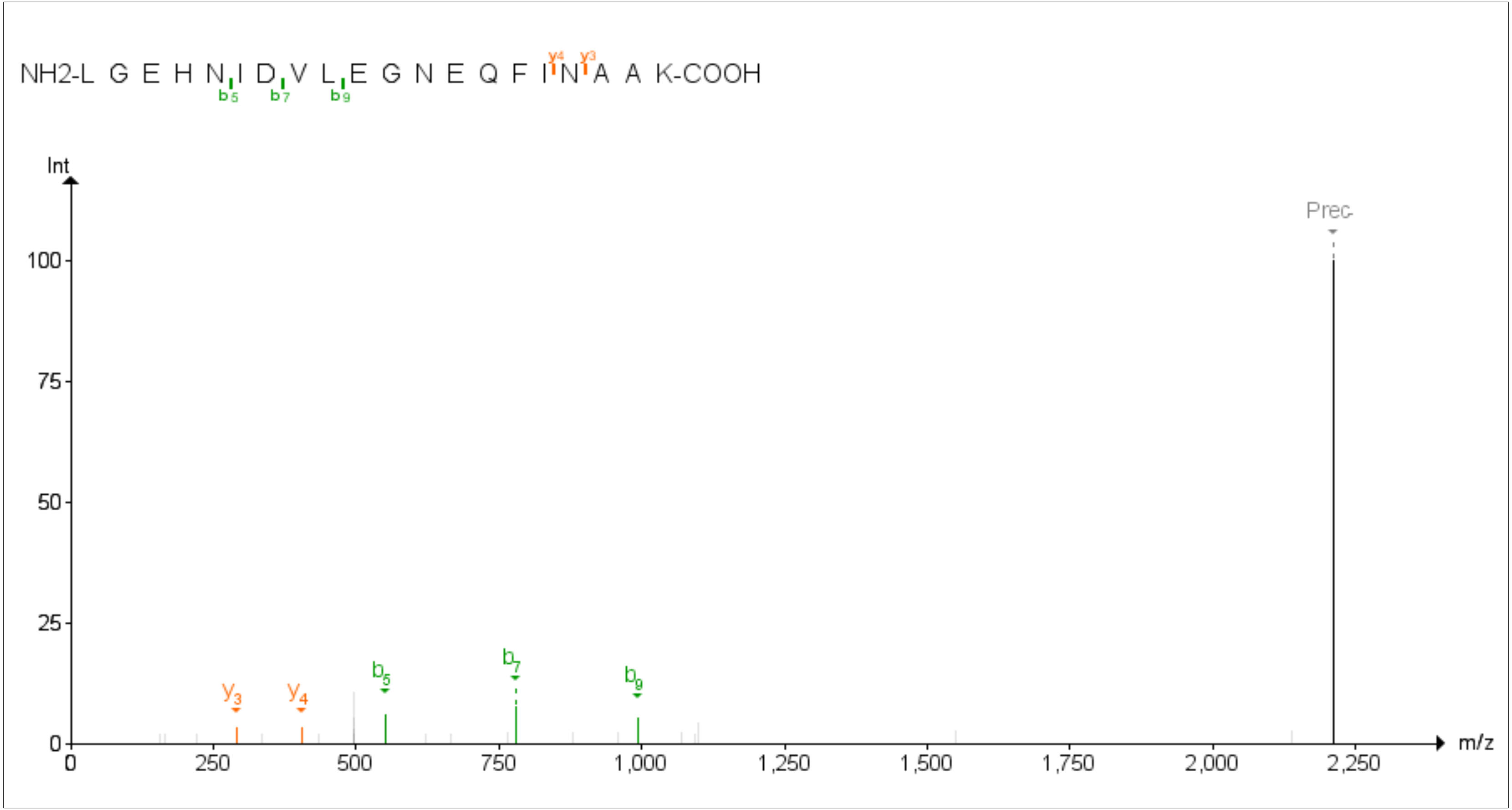
NH2-L G E H N I D V L E G N E Q F I N A A K-COOH
b5
b7
b9
y4
y3
Int
Prec
100
75
50
25
0
y3
y4
b5
b7
b9
0
250
500
750
1,000
1,250
1,500
1,750
2,000
2,250
m/z

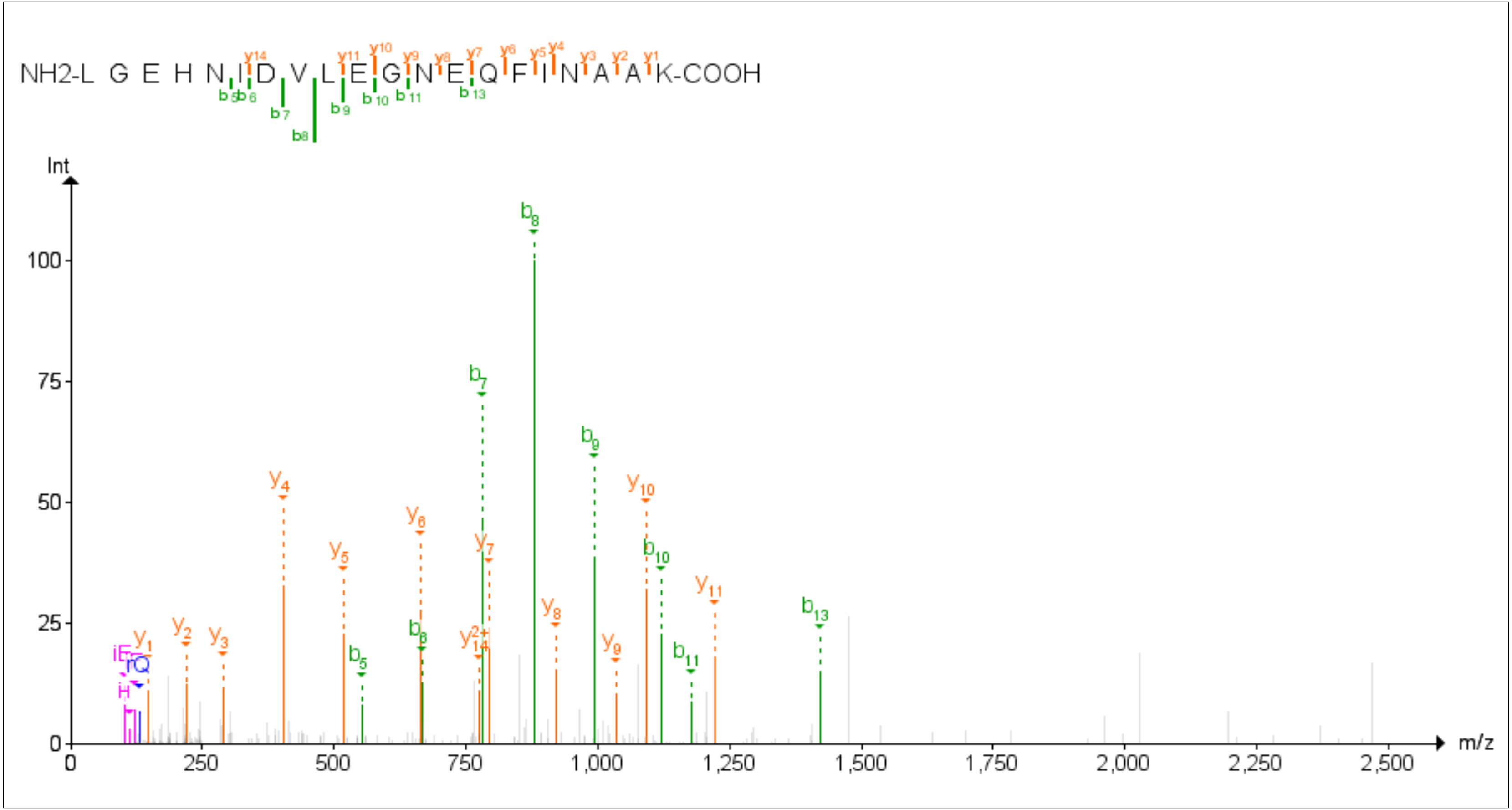

NH2-L G E H N I D V L E G N E Q F I N A A K-COOH
Int
m/z

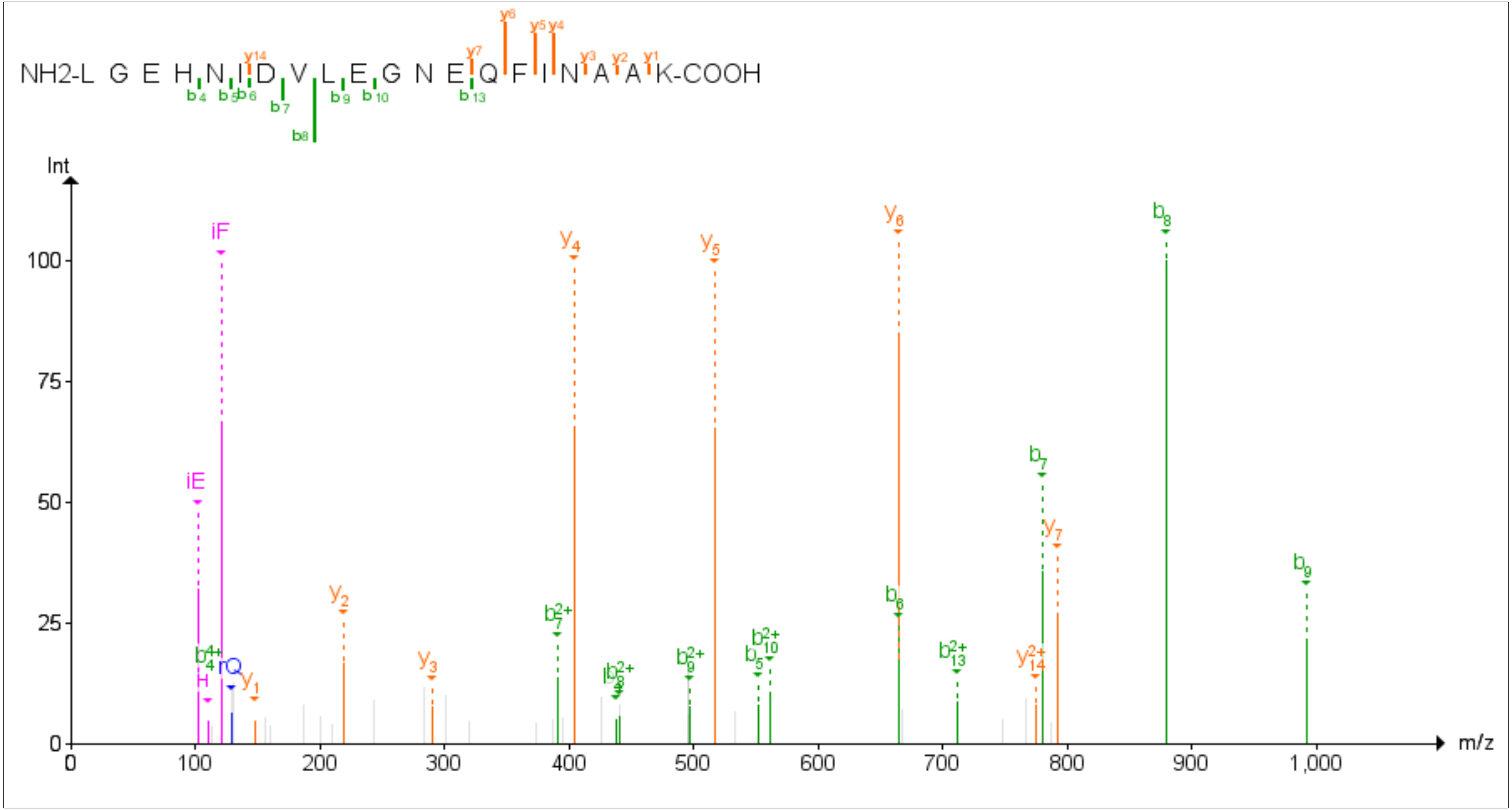

NH2-L G E H N I D V L E G N E Q F I N A A K-COOH
b4 b5 b6 b7 b8 b9 b10 b13
y14 y7 y6 y5 y4 y3 y2 y1
Int
m/z
0
25
50
75
100
200
300
400
500
600
700
800
900
1,000
iF
iE
H
rQ
y1
y2
y3
y4
y5
y6
y7
b8
b7
b9
b6
b5
b4 4+
b7 2+
b8 2+
b9 2+
b10 2+
b13 2+
y14 2+

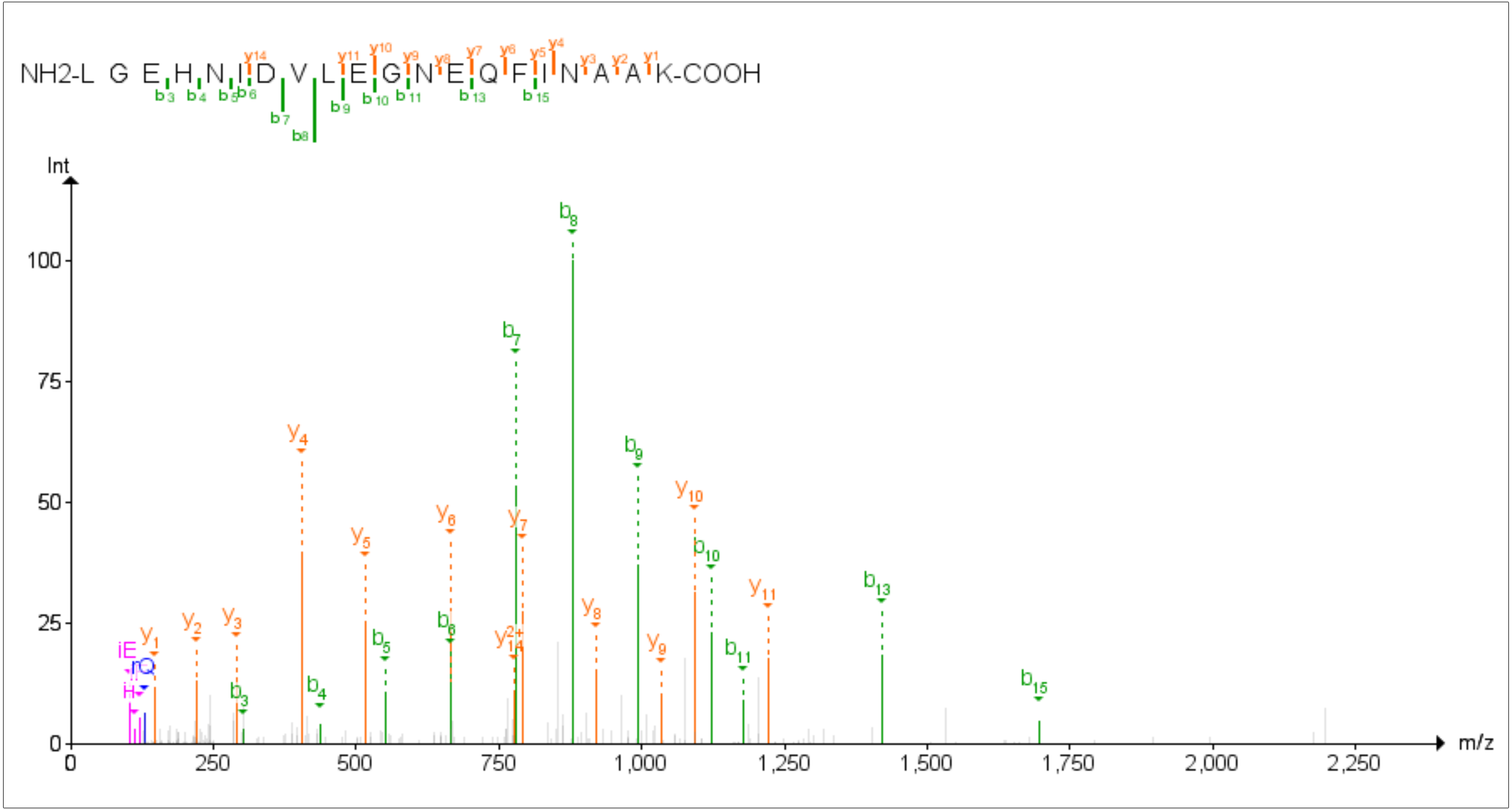
NH2-L G E H N I D V L E G N E Q F I N A A K-COOH
y14 y11 y10 y9 y8 y7 y6 y5 y4 y3 y2 y1
b3 b4 b5 b6 b7 b8 b9 b10 b11 b13 b15
Int
100
75
50
25
0
m/z
0
250
500
750
1,000
1,250
1,500
1,750
2,000
2,250
b8
b7
b9
y4
y10
b10
y5
y6
y7
y11
b13
y8
y9
b11
b15
y1
y2
y3
b3
b4
b5
b6
iE
iQ
iH

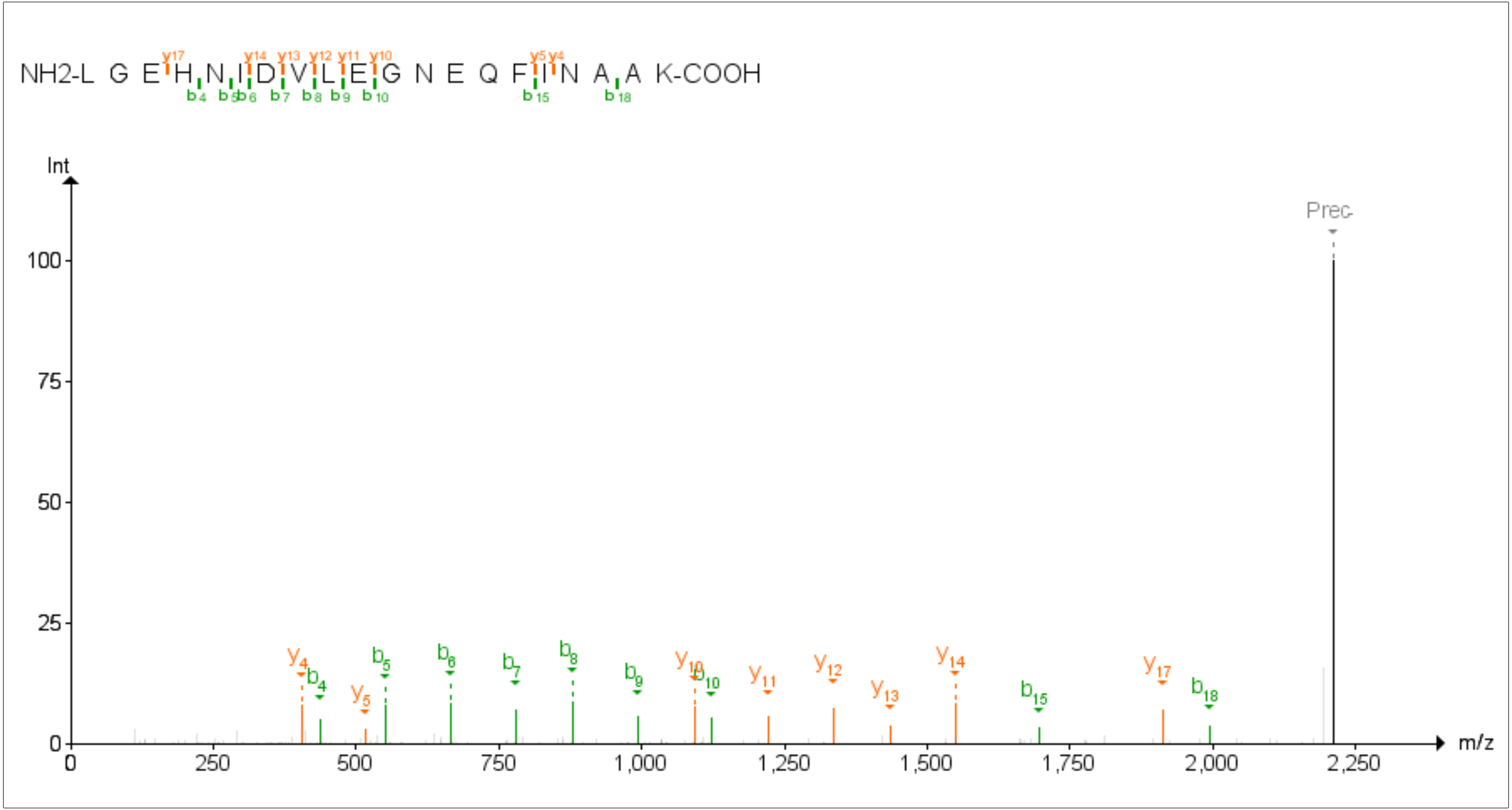

NH2-L G E H N I D V L E G N E Q F I N A A K-COOH
y17 y14 y13 y12 y11 y10 y5 y4
b4 b5 b6 b7 b8 b9 b10 b15 b18
Int
Prec
100
75
50
25
0
0
250
500
750
1,000
1,250
1,500
1,750
2,000
2,250
m/z
y4 b4 y5 b5 b6 b7 b8 b9 y10 b10 y11 y12 y13 y14 b15 y17 b18

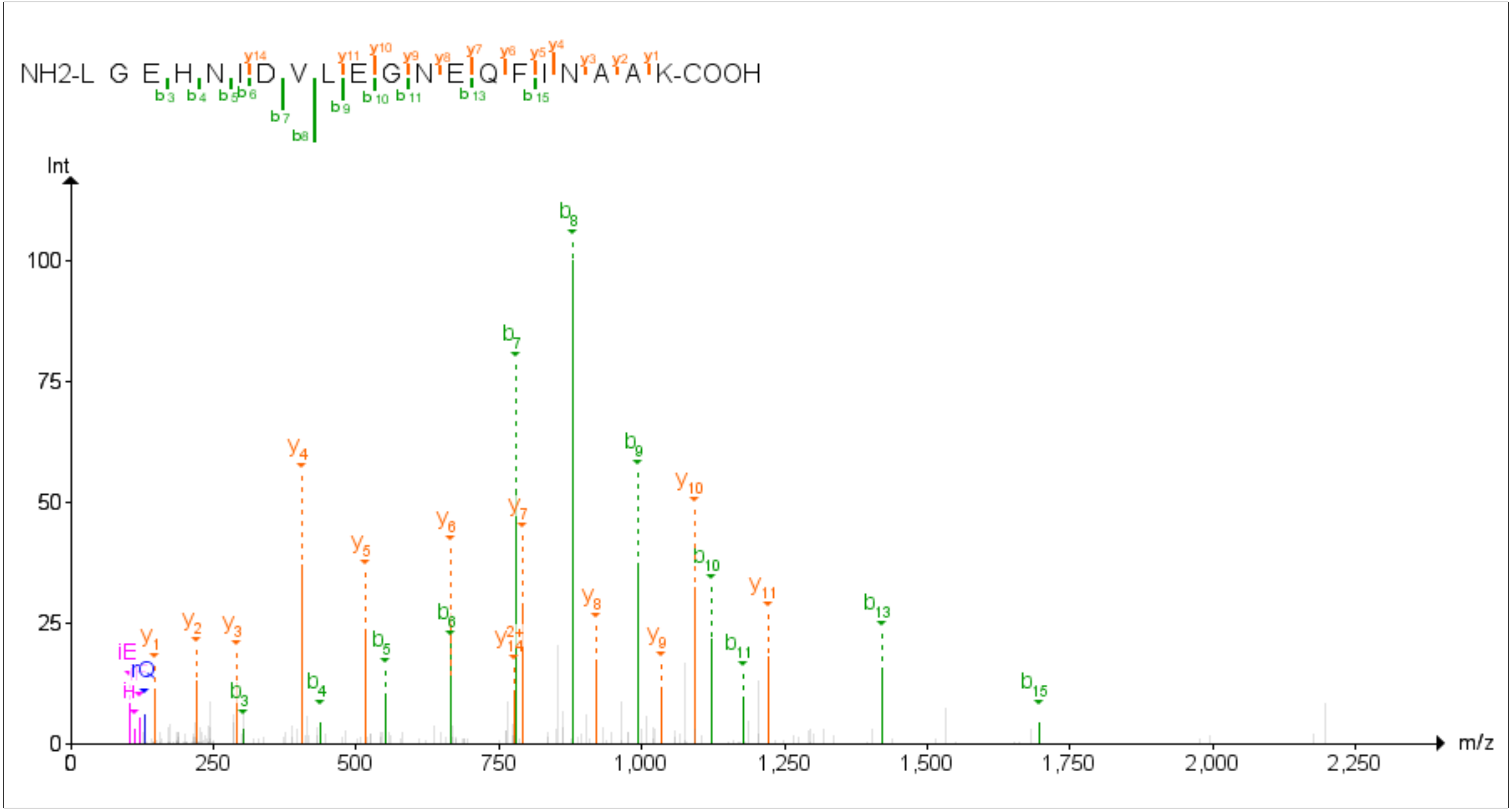

NH2-L G E H N I D V L E G N E Q F I N A A K-COOH
Int
m/z
0
25
50
75
100
250
500
750
1,000
1,250
1,500
1,750
2,000
2,250

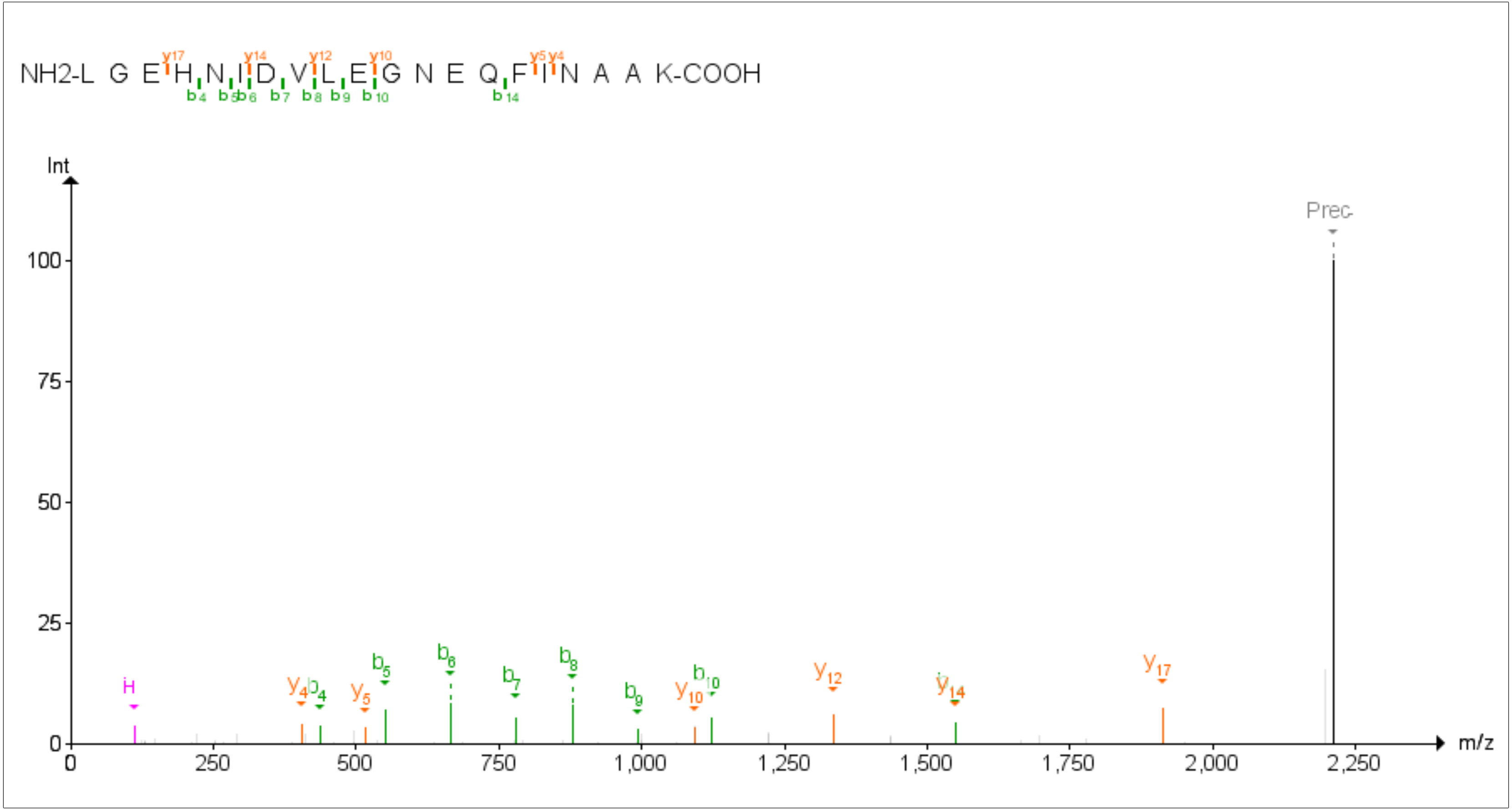

NH2-L G E H N I D V L E G N E Q F I N A A K-COOH
y17 y14 y12 y10 y5 y4
b4 b5 b6 b7 b8 b9 b10 b14
Int
Prec
100
75
50
25
0
H
y4 b4 y5 b5 b6 b7 b8 b9 y10 b10 y12 y14 y17
0 250 500 750 1,000 1,250 1,500 1,750 2,000 2,250
m/z

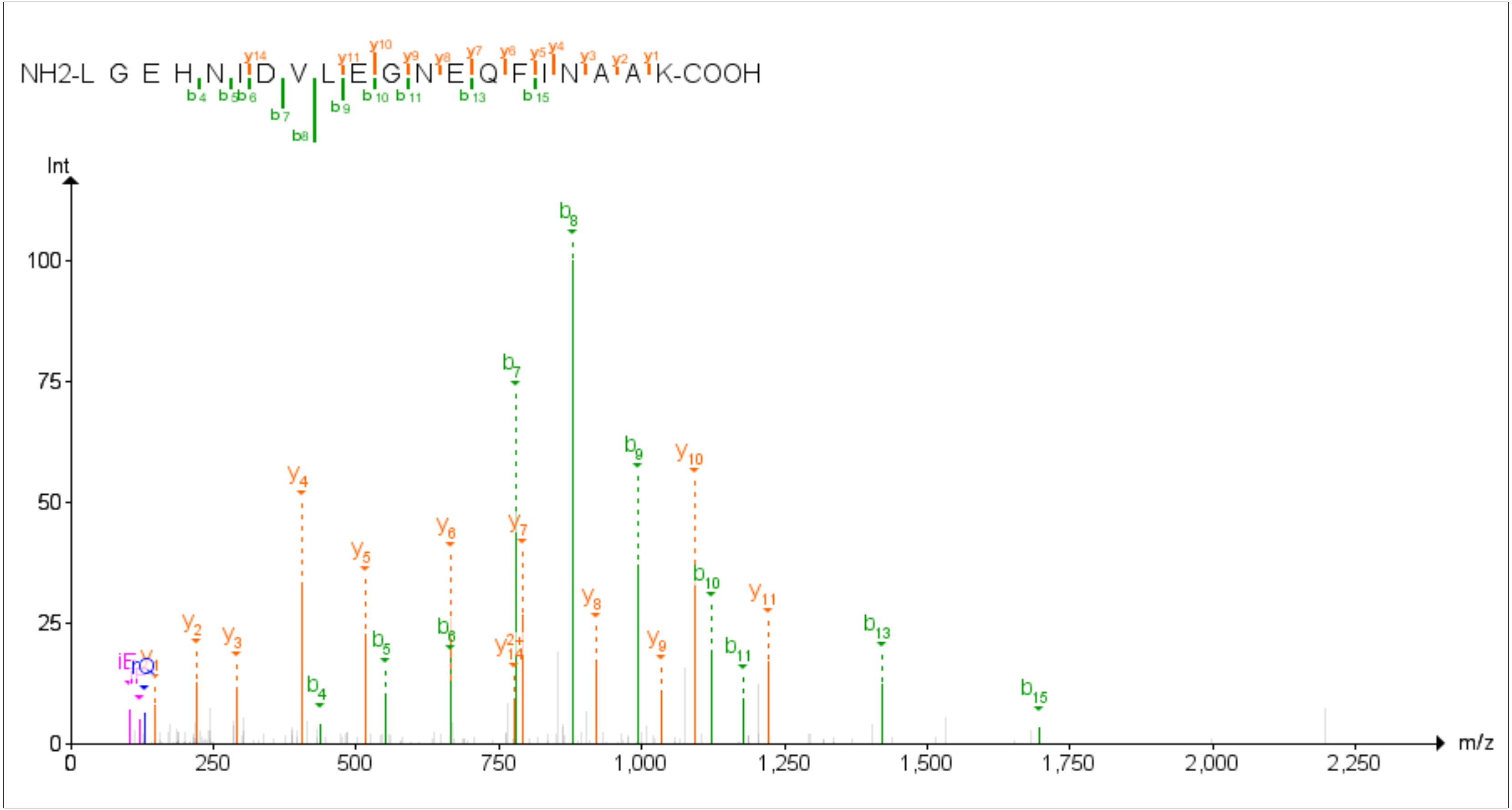

NH2-L G E H N I D V L E G N E Q F I N A A K-COOH
Int
m/z
b8
b7
b9
y10
y4
y6
y7
y5
b10
y8
y11
b13
y2
y3
b5
b6
y14 2+
y9
b11
b4
b15

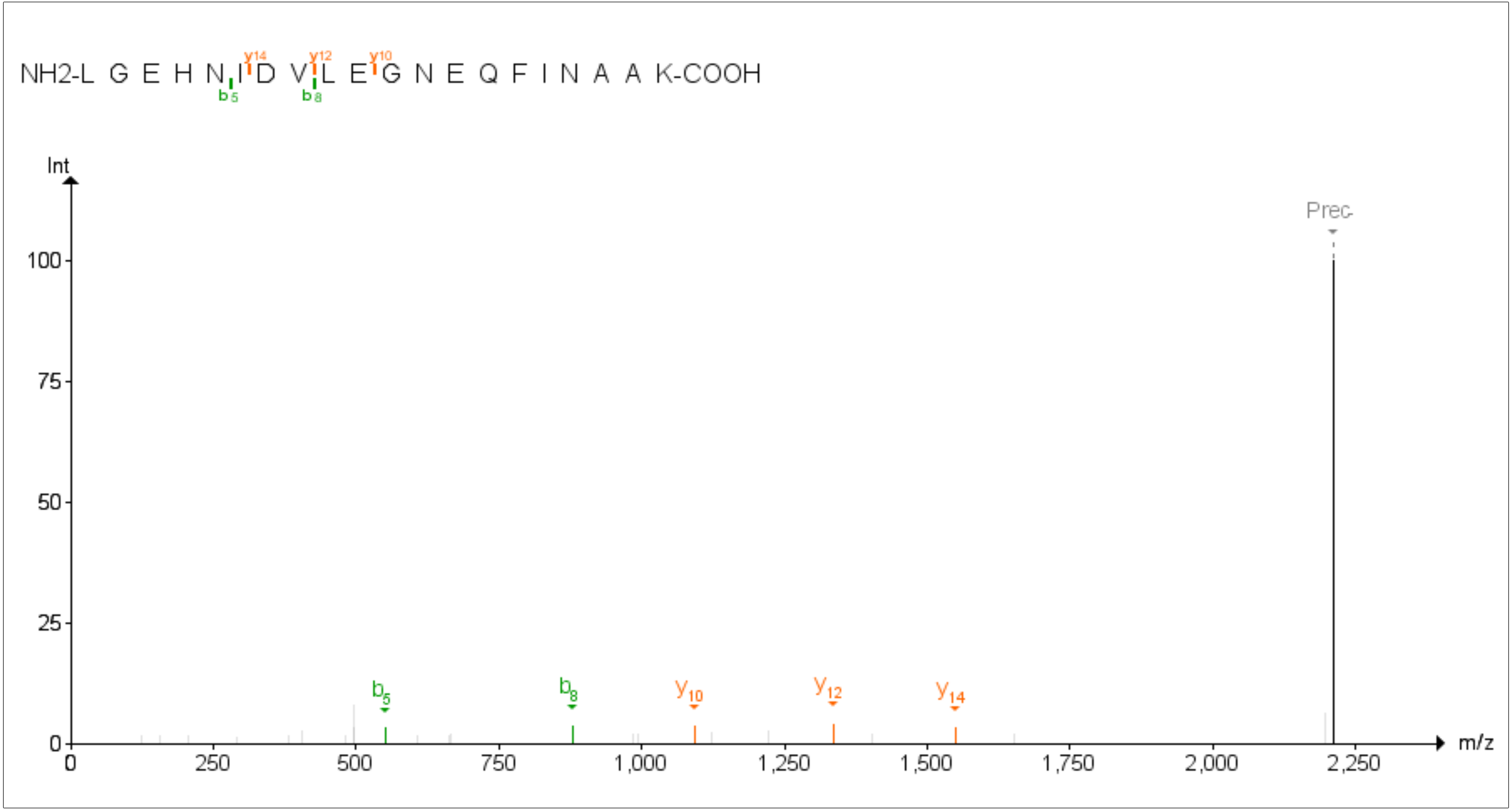

NH2-L G E H N I D V I L E G N E Q F I N A A K-COOH
y14
y12
y10
b5
b8
Int
Prec
100
75
50
25
0
b5
b8
y10
y12
y14
0
250
500
750
1,000
1,250
1,500
1,750
2,000
2,250
m/z

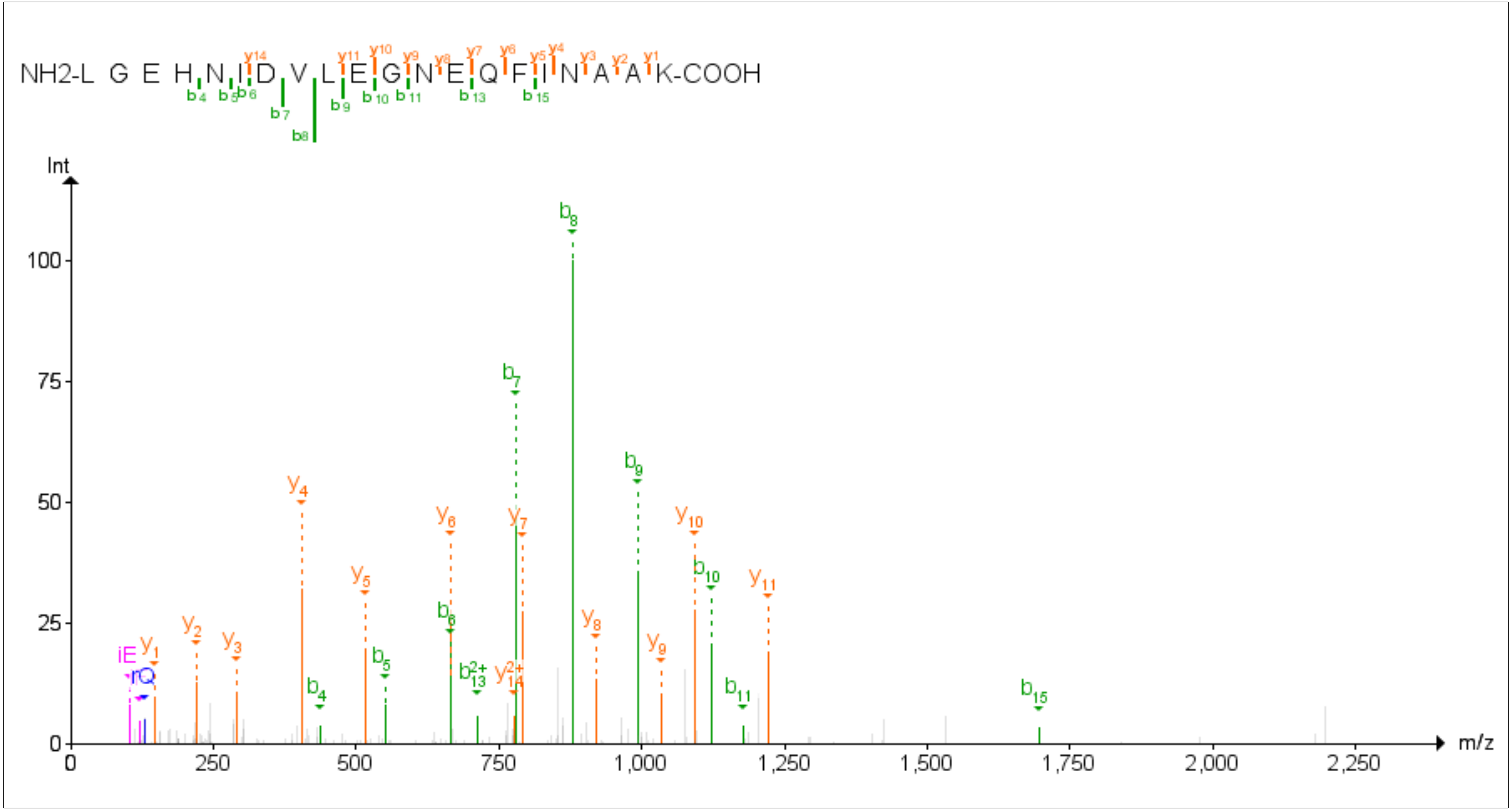

NH2-L G E H N I D V L E G N E Q F I N A A K-COOH
Int
m/z
b8
b7
b9
y4
y6
y7
y10
b10
y5
y11
b6
y8
y9
y2
y3
y1
iE
IQ
b4
b5
b11
b15
0
25
50
75
100
250
500
750
1,000
1,250
1,500
1,750
2,000
2,250

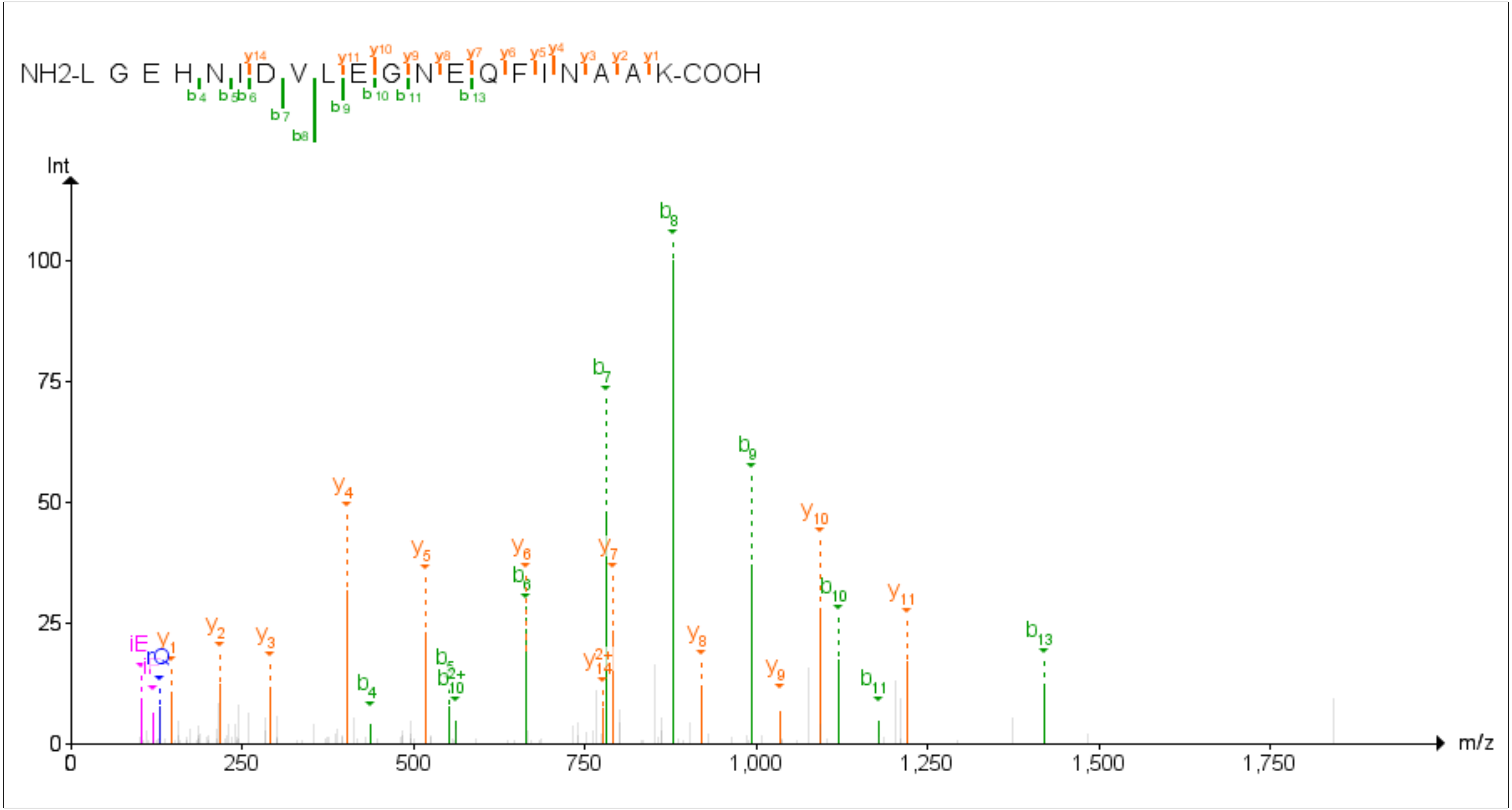

NH2-L G E H N I D V L E G N E Q F I N A A K-COOH
b4 b5 b6 b7 b8 b9 b10 b11 b13
y14 y11 y10 y9 y8 y7 y6 y5 y4 y3 y2 y1
Int
m/z
0
25
50
75
100
250
500
750
1,000
1,250
1,500
1,750
iE
iI
rQ

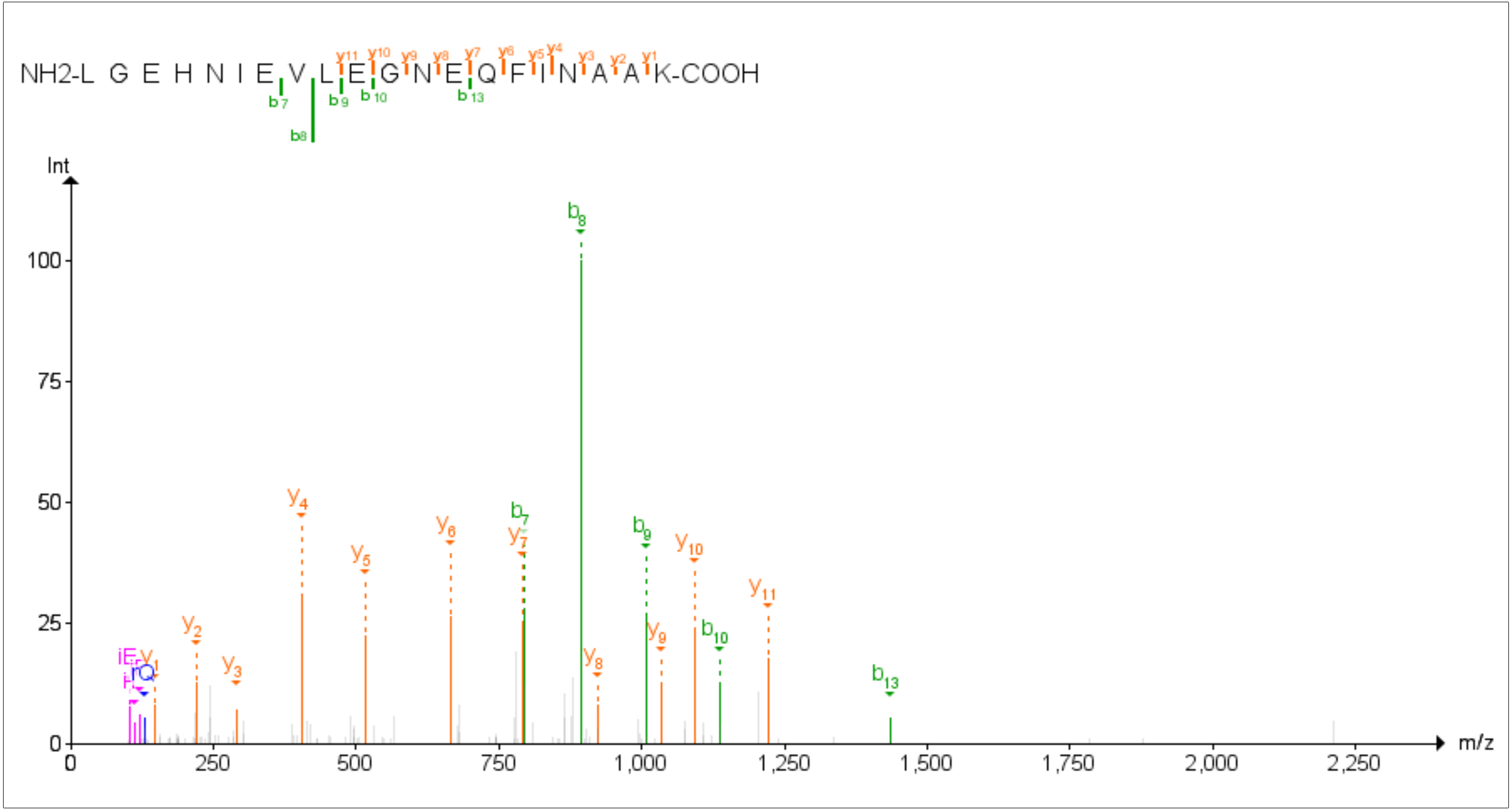
NH2-L G E H N I E V L E G N E Q F I N A A K-COOH
y11 y10 y9 y8 y7 y6 y5 y4 y3 y2 y1
b7 b8 b9 b10 b13
Int
100
75
50
25
0
b8
y4
b7
y7
y6
y5
b9
y10
y11
y2
y3
y1
iE
rQ
y9
b10
y8
b13
0
250
500
750
1,000
1,250
1,500
1,750
2,000
2,250
m/z

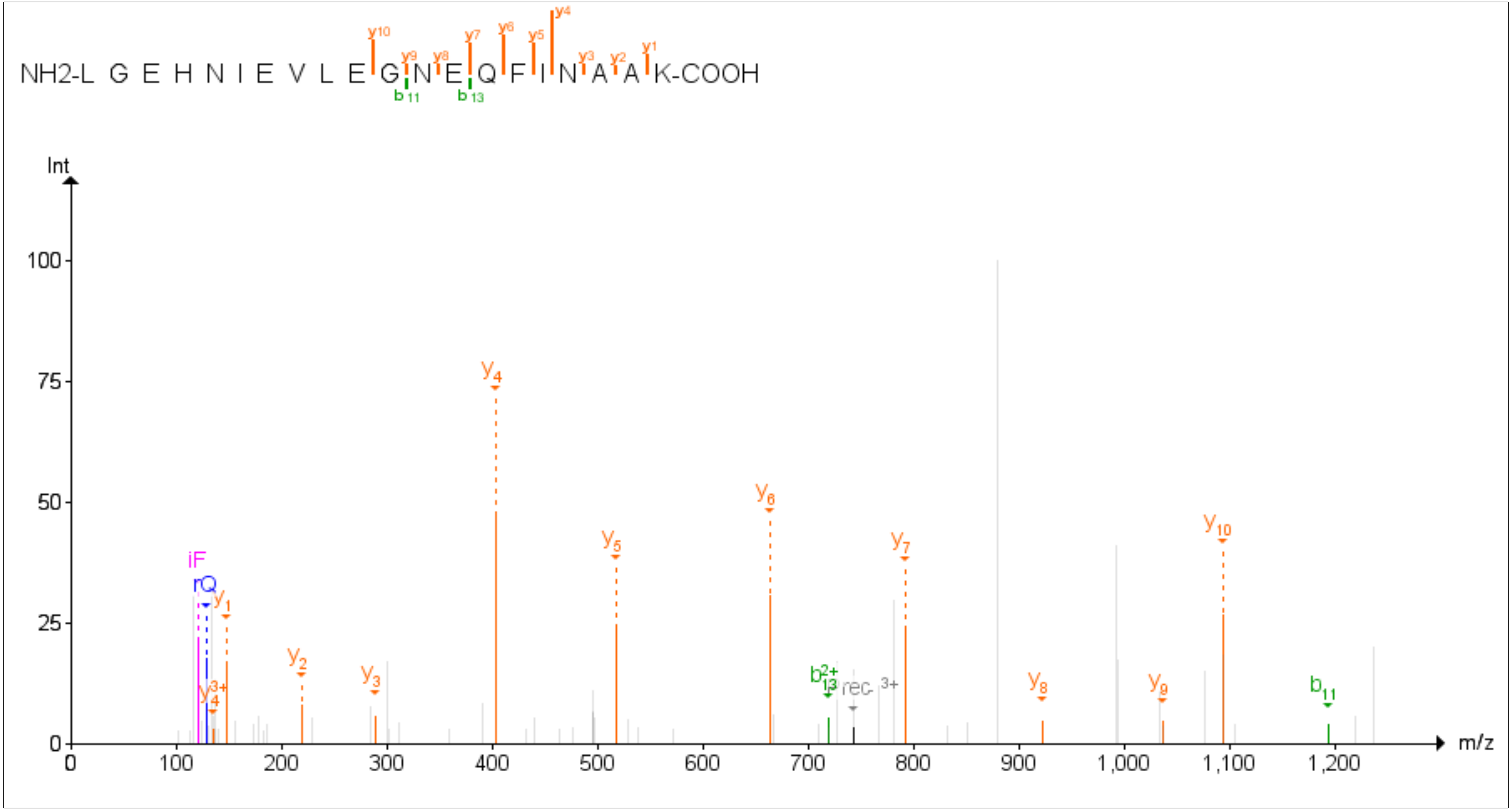

NH2-L G E H N I E V L E G N E Q F I N A A K-COOH
Int
m/z
iF
rQ

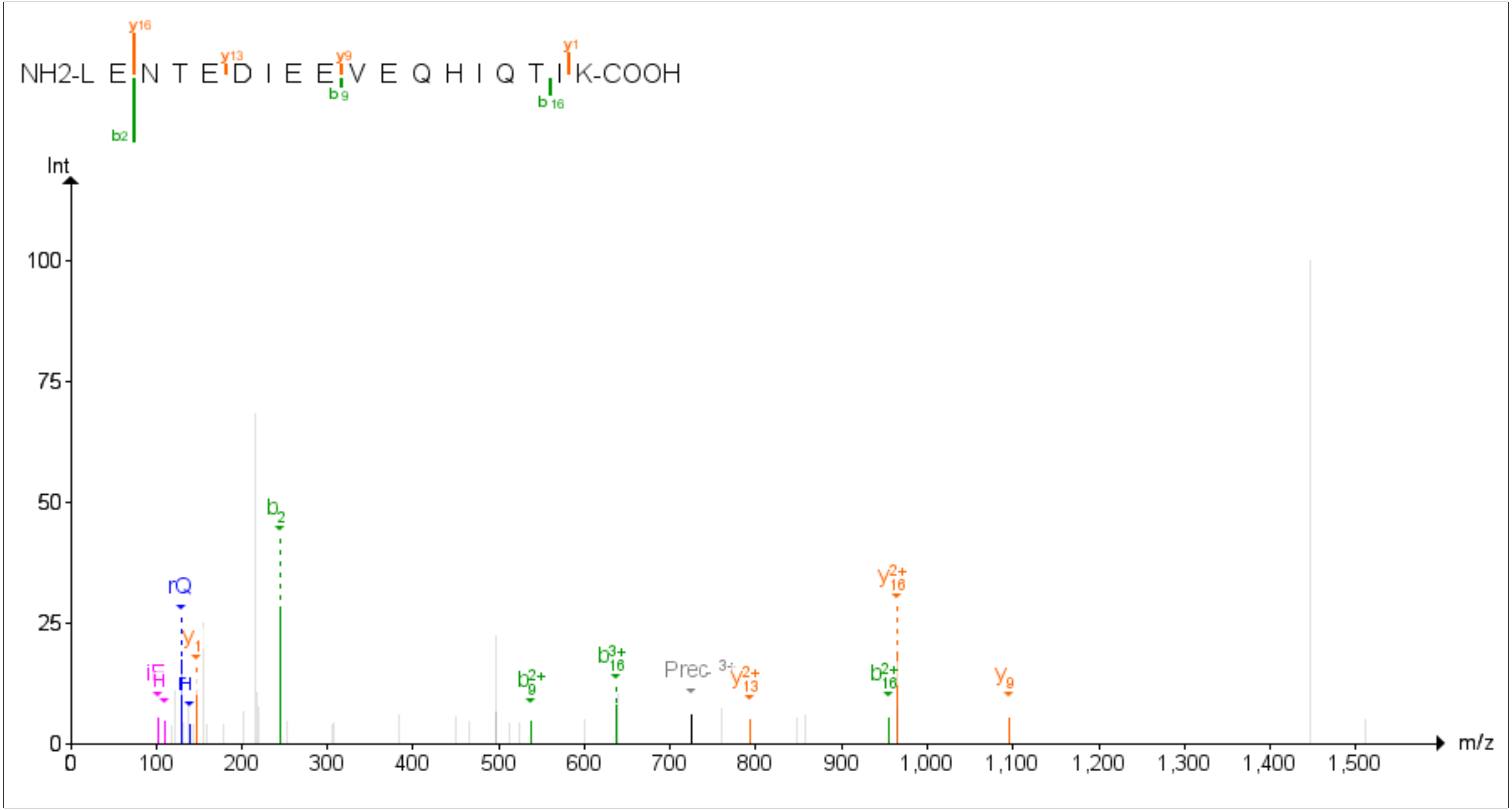

NH2-L E N T E D I E E V E Q H I Q T I K-COOH
y16
y13
y9
y1
b2
b9
b16
Int
100
75
50
25
0
b2
rQ
y1
iH
H
b9 2+
b16 3+
Prec 3+
y13 2+
y16 2+
b16 2+
y9
0
100
200
300
400
500
600
700
800
900
1,000
1,100
1,200
1,300
1,400
1,500
m/z

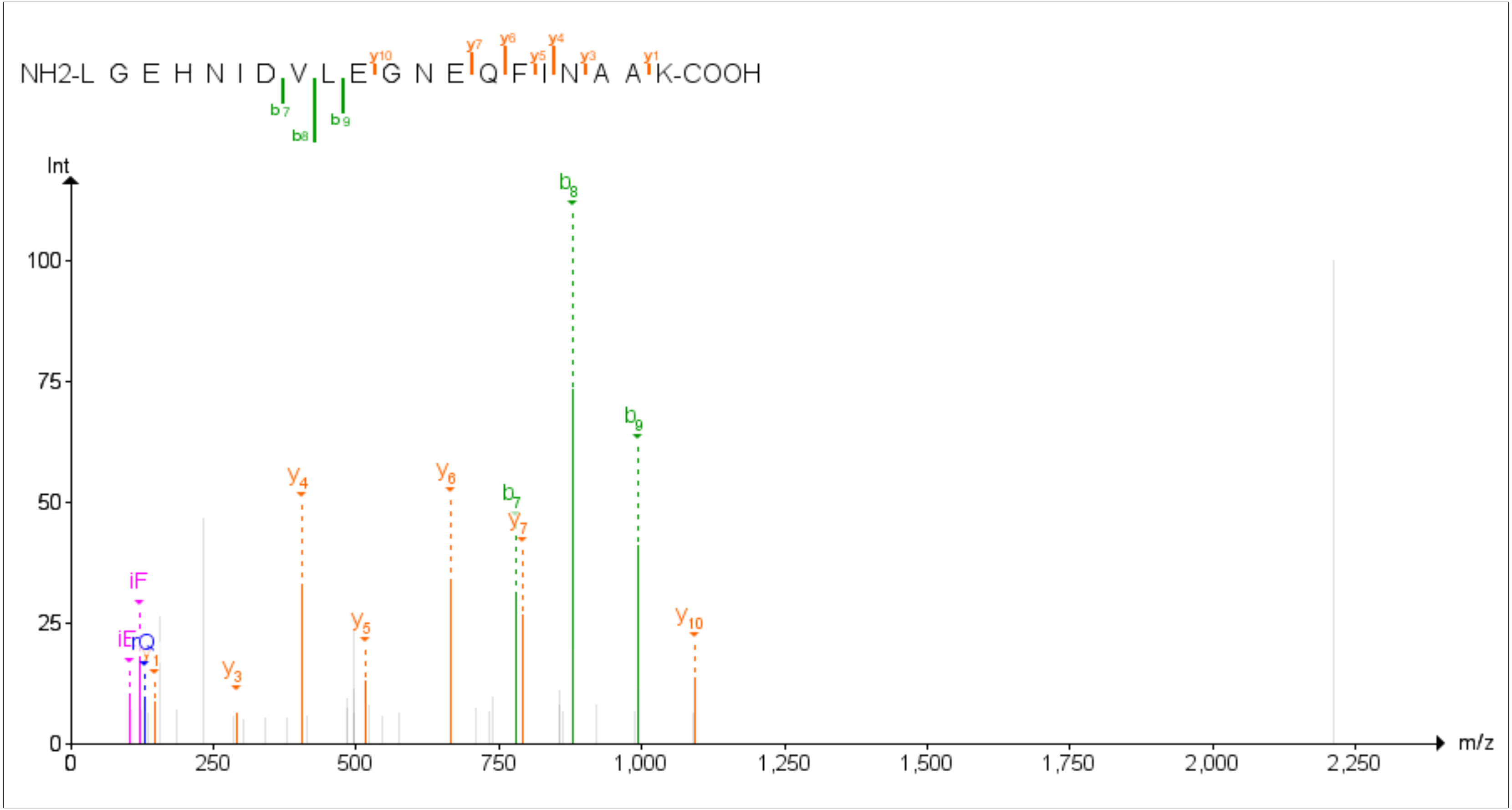

NH2-L G E H N I D V L E G N E Q F I N A A K-COOH
b7
b8
b9
y10
y7
y6
y5
y4
y3
y1
Int
m/z
100
75
50
25
0
250
500
750
1,000
1,250
1,500
1,750
2,000
2,250
b8
b9
b7
y4
y6
y7
y5
y10
y3
y1
iF
iE
rQ

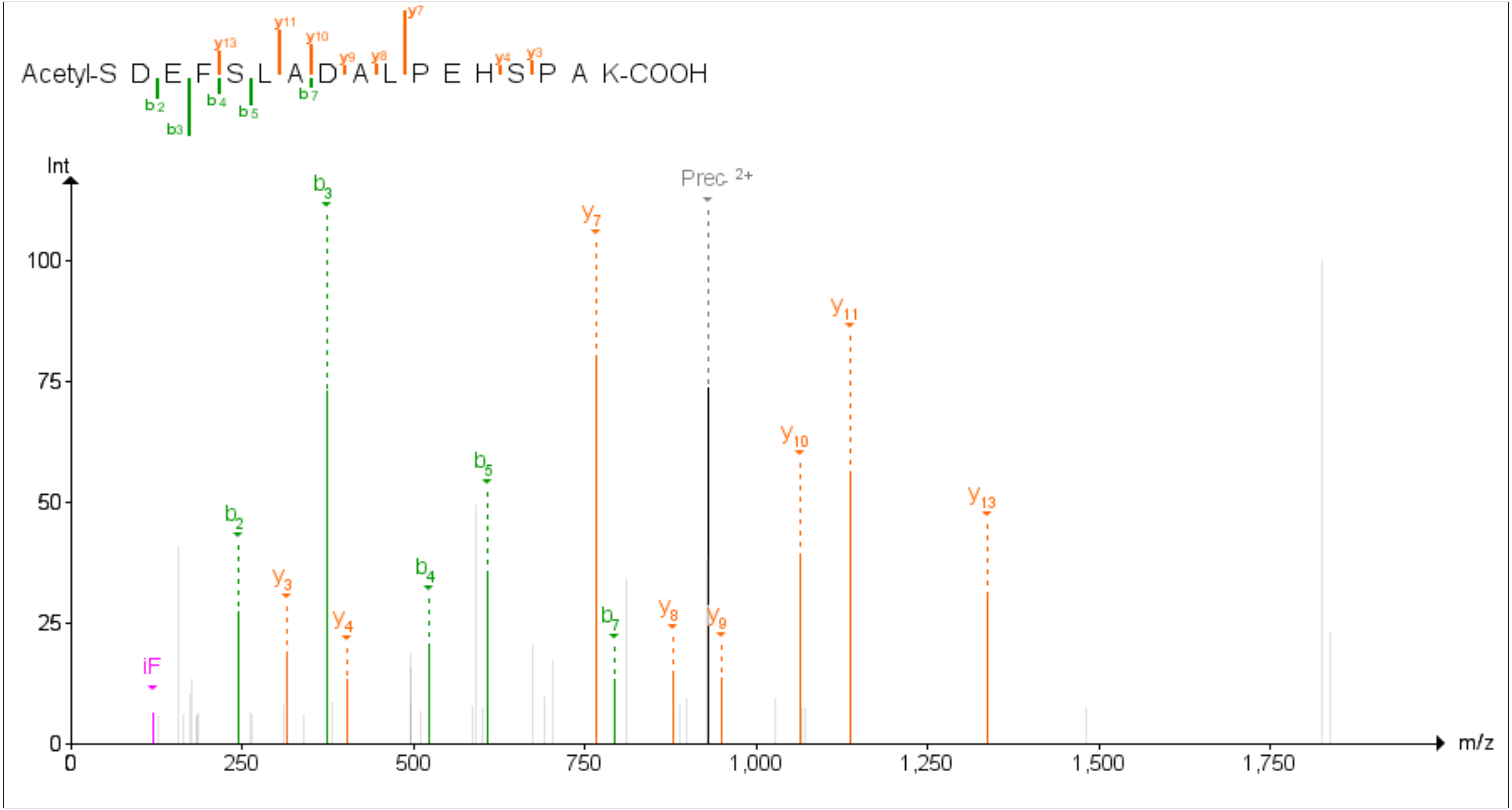

Acetyl-S D E F S L A D A L P E H S P A K-COOH
b2
b3
b4
b5
b7
y13
y11
y10
y9
y8
y7
y4
y3
Int
Prec. 2+
m/z
iF
0
25
50
75
100
250
500
750
1,000
1,250
1,500
1,750

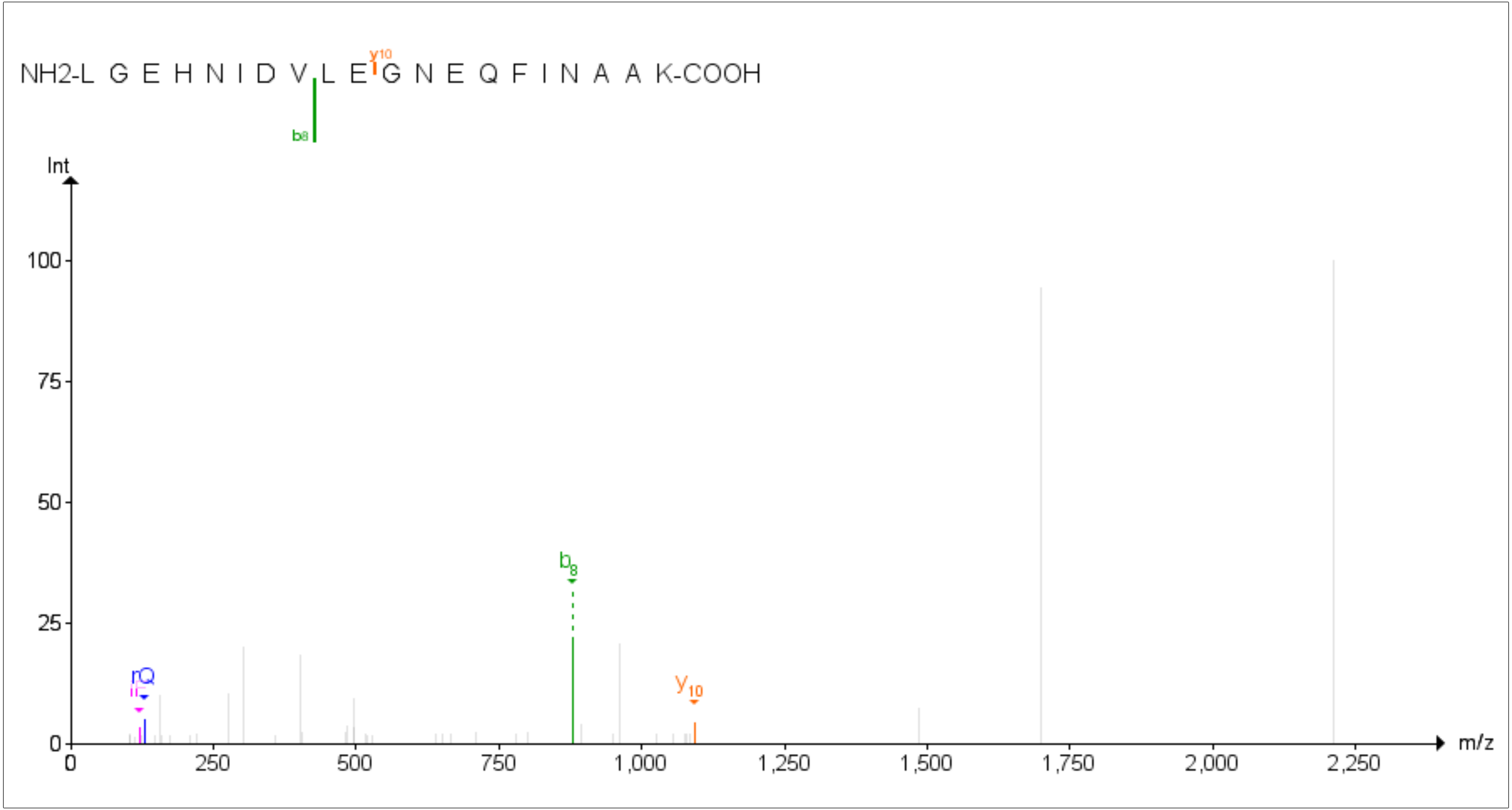

NH2-L G E H N I D V L E G N E Q F I N A A K-COOH
y10
b8
Int
100
75
50
25
0
b8
nQ
y10
0
250
500
750
1,000
1,250
1,500
1,750
2,000
2,250
m/z

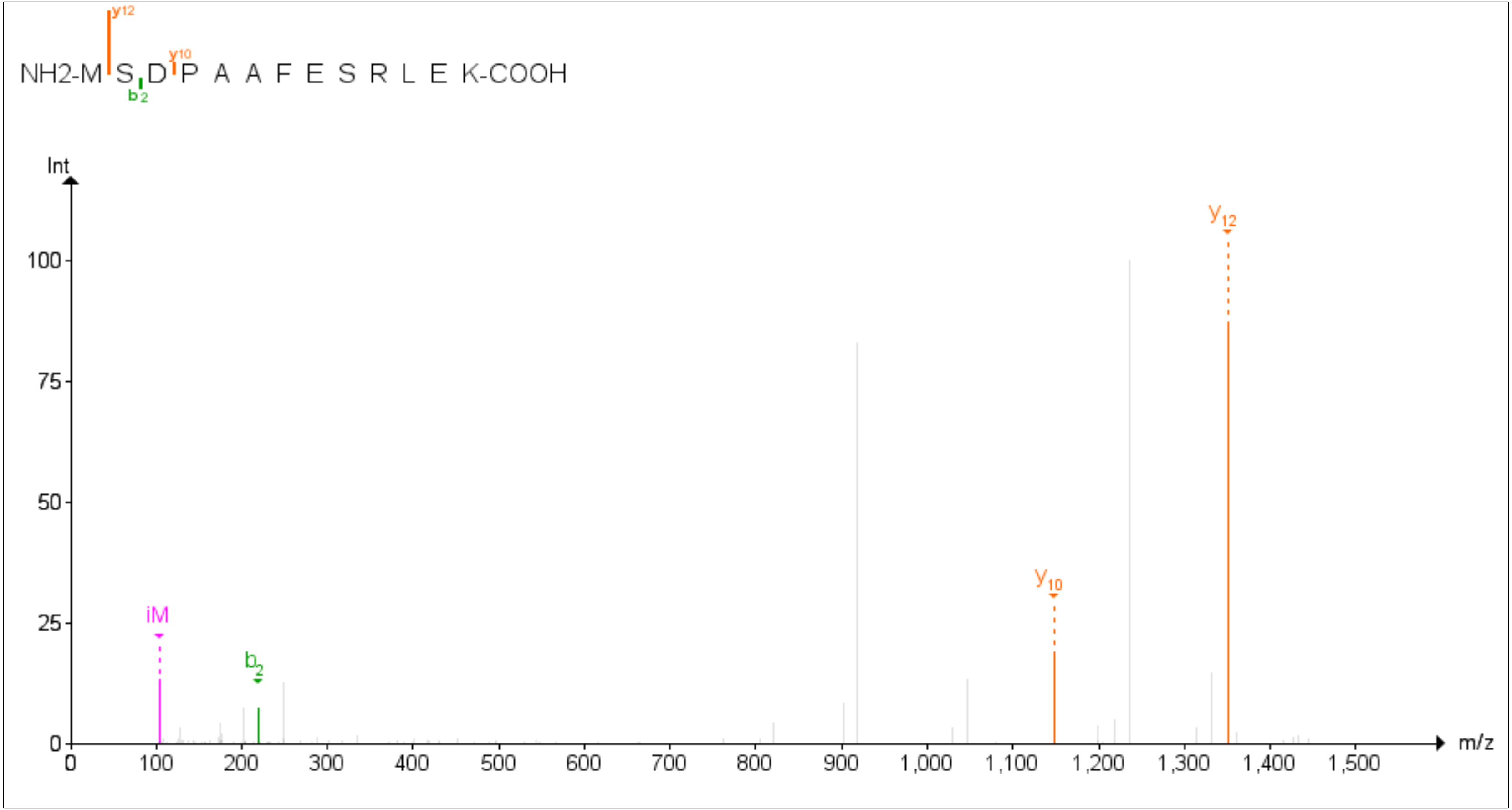

NH2-M S D P A A F E S R L E K-COOH
y12
y10
b2
Int
100
75
50
25
0
iM
b2
y10
y12
0
100
200
300
400
500
600
700
800
900
1,000
1,100
1,200
1,300
1,400
1,500
m/z

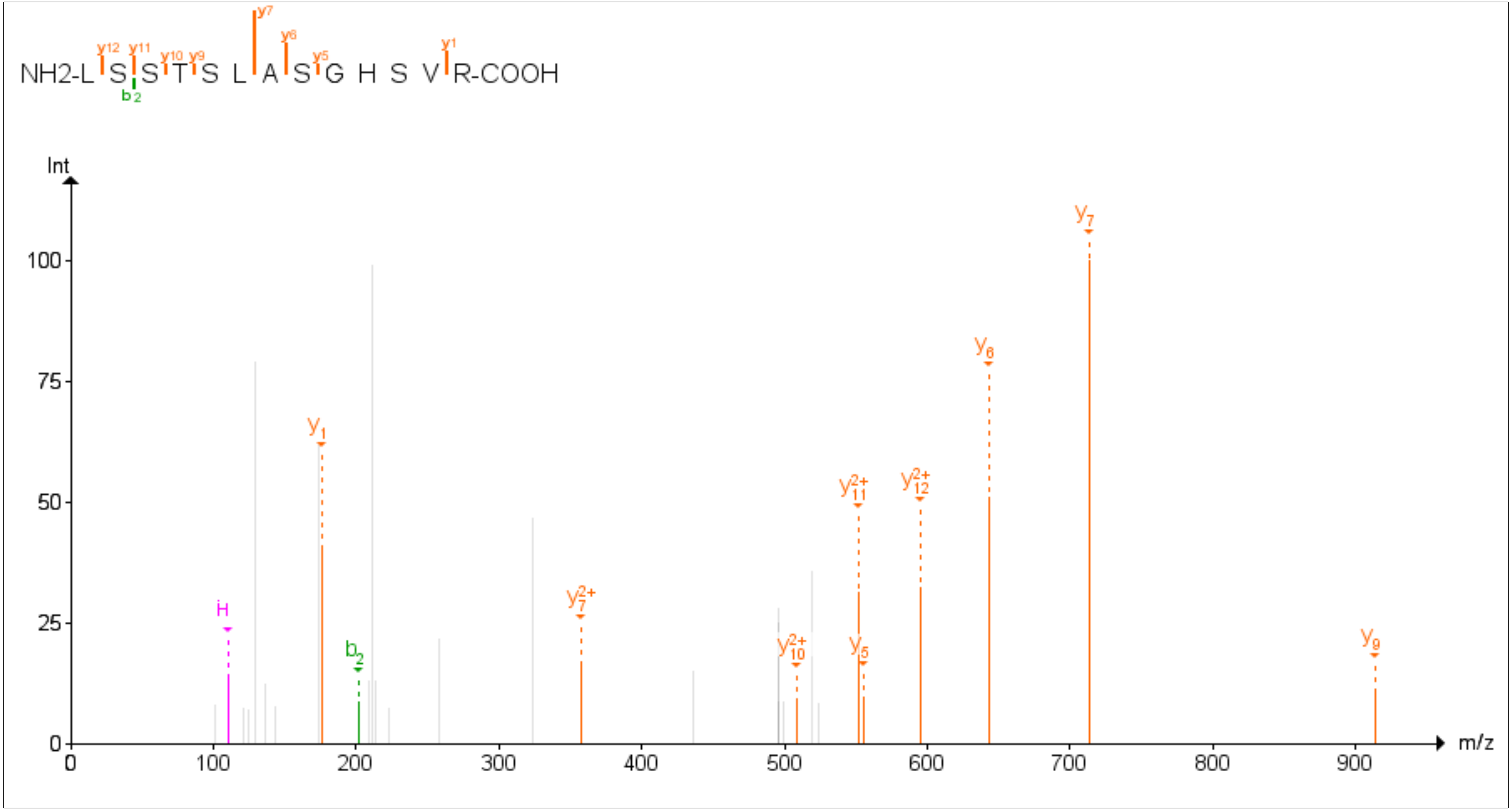

NH2-L S S T S L A S G H S V R-COOH
y12 y11 y10 y9 y7 y6 y5 y1
b2
Int
100
75
50
25
0
0 100 200 300 400 500 600 700 800 900
m/z
y1
H
b2
y7 2+
y10 2+
y11 2+
y5
y12 2+
y6
y7
y9

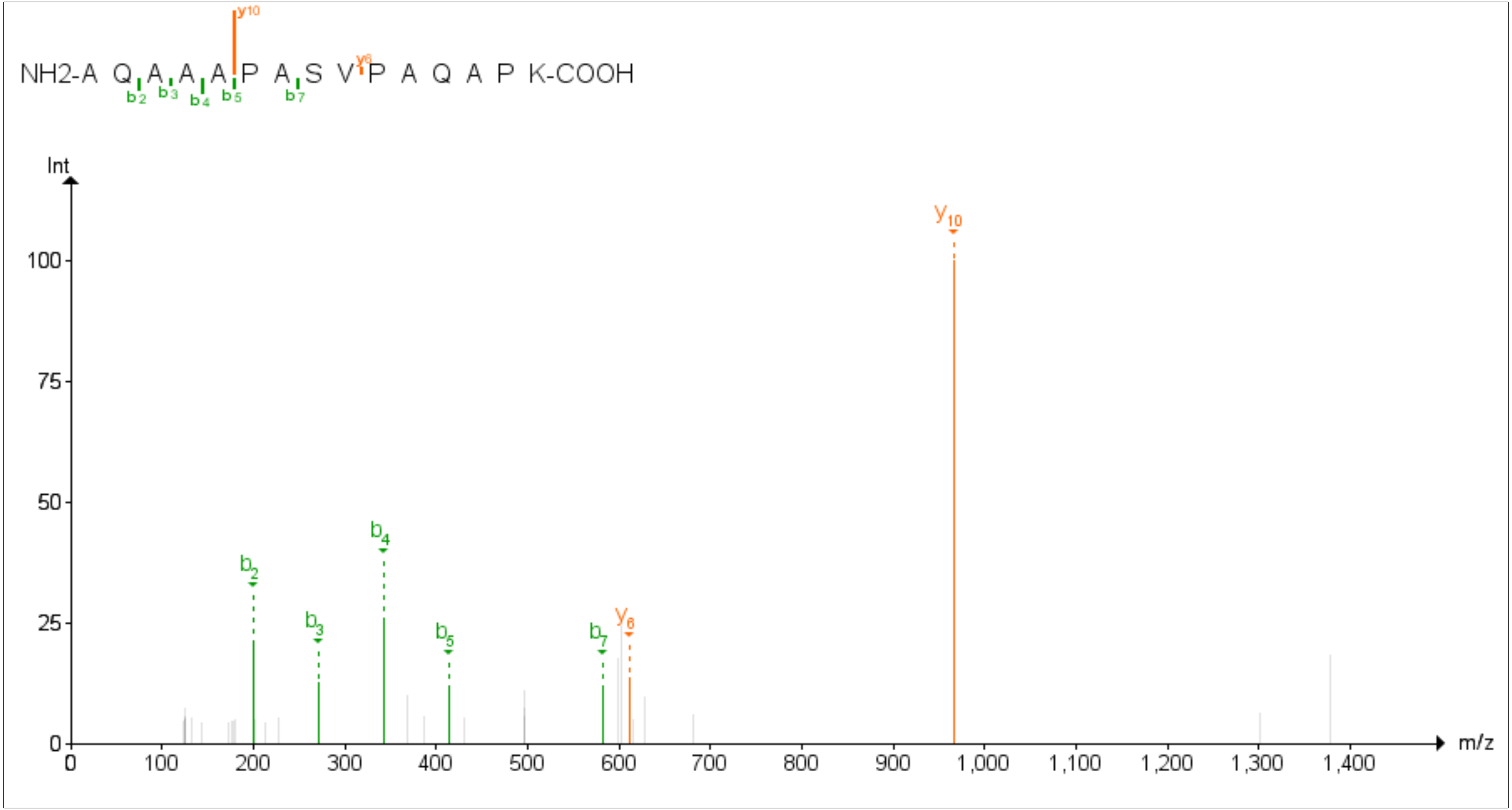

y10
y6
NH2-A Q A A A P A S V P A Q A P K-COOH
b2 b3 b4 b5 b7
Int
100
75
50
25
0
y10
b2
b3
b4
b5
b7
y6
0 100 200 300 400 500 600 700 800 900 1,000 1,100 1,200 1,300 1,400
m/z

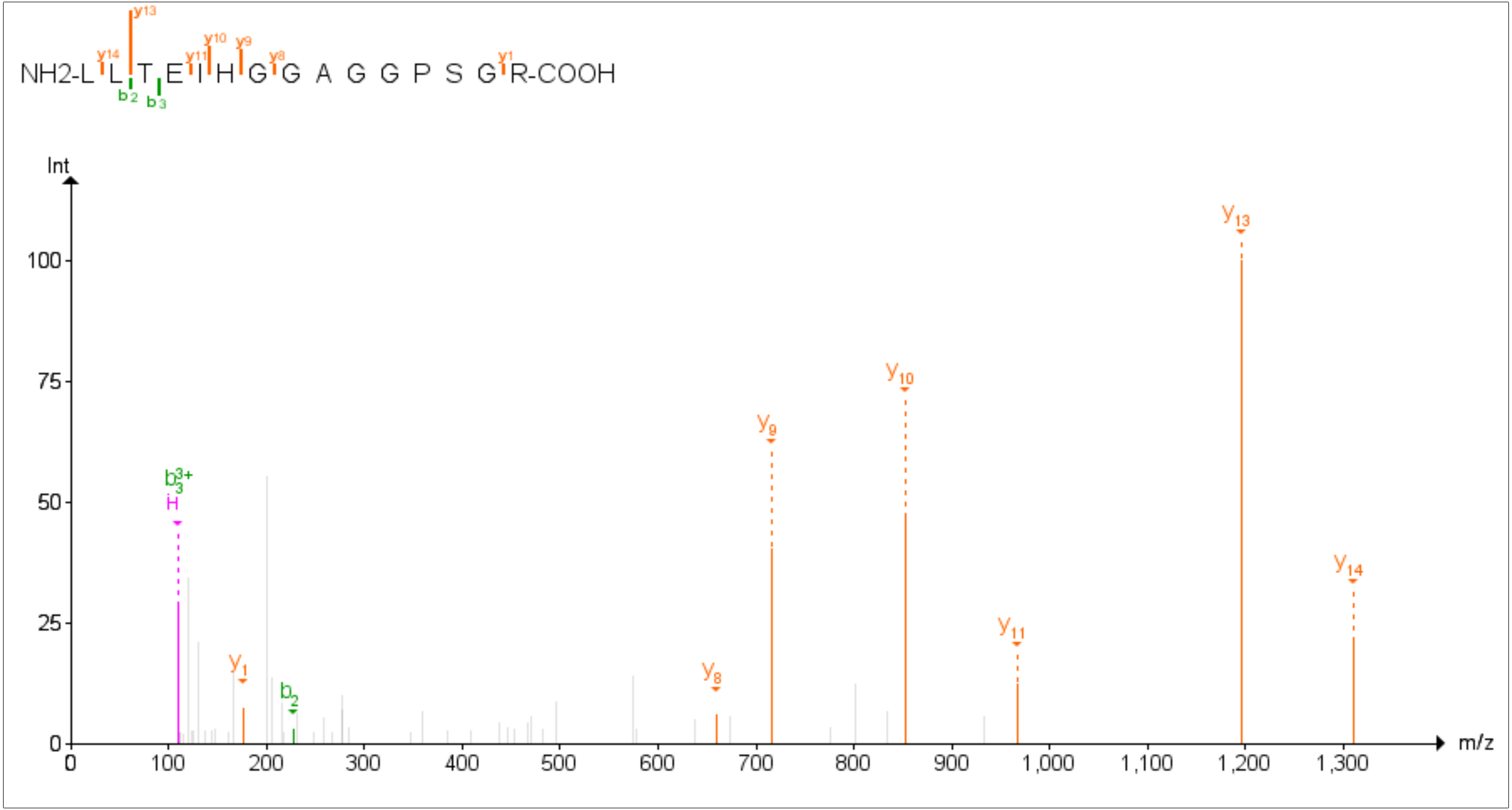

NH2-L L T E I H G G A G G P S G R-COOH
y14
y13
y11
y10
y9
y8
y1
b2
b3
Int
100
75
50
25
0
b_3^{3+}
H
y_1
b_2
y_8
y_9
y_{10}
y_{11}
y_{13}
y_{14}
0
100
200
300
400
500
600
700
800
900
1,000
1,100
1,200
1,300
m/z

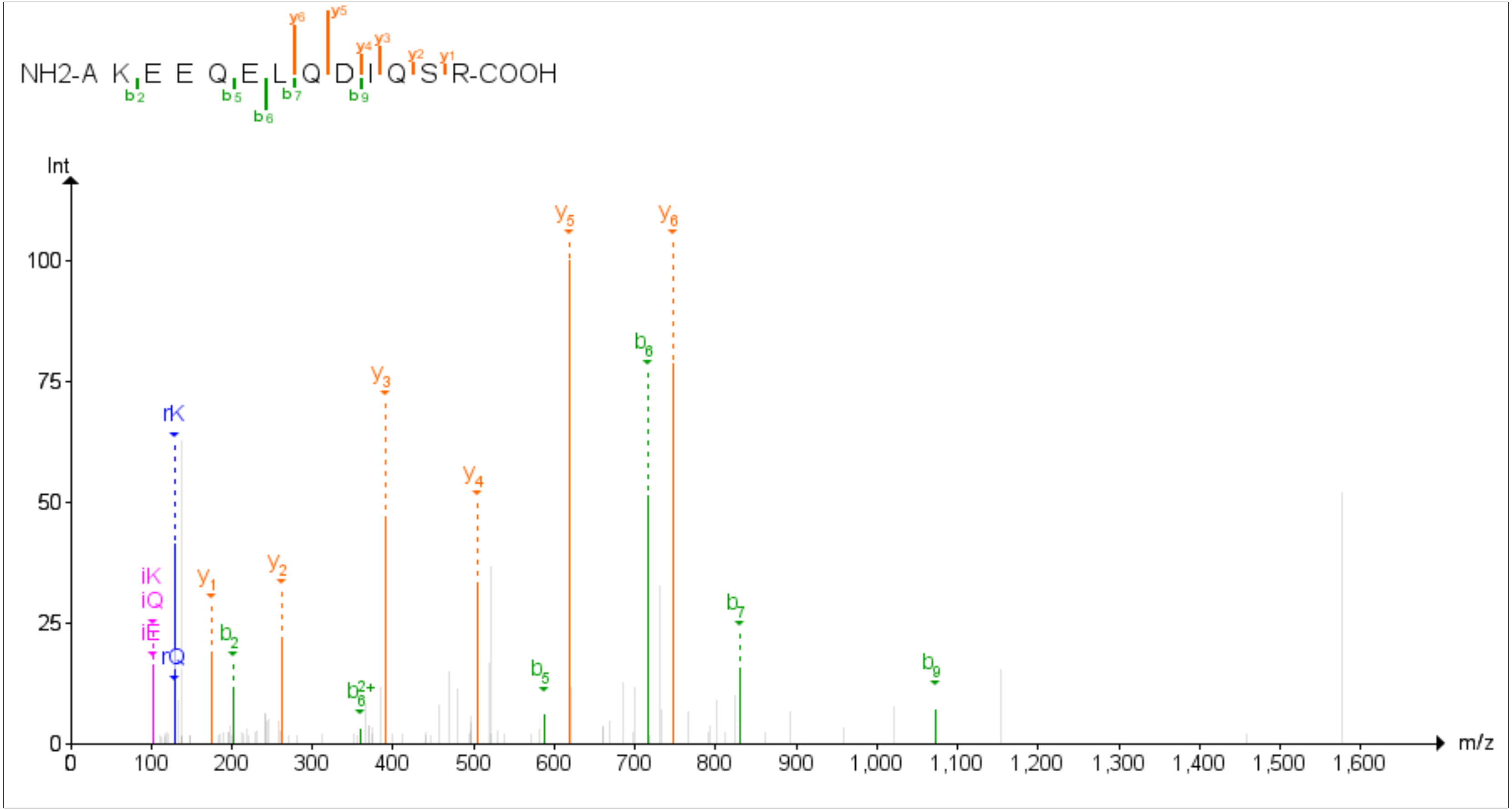

NH2-A K E E Q E L Q D I Q S R-COOH
y6 y5 y4 y3 y2 y1
b2 b5 b6 b7 b9
Int
m/z
y5
y6
b6
y3
rK
y4
iK
iQ
iE
y1
y2
b2
rQ
b6 2+
b5
b7
b9
0
25
50
75
100
0
100
200
300
400
500
600
700
800
900
1,000
1,100
1,200
1,300
1,400
1,500
1,600

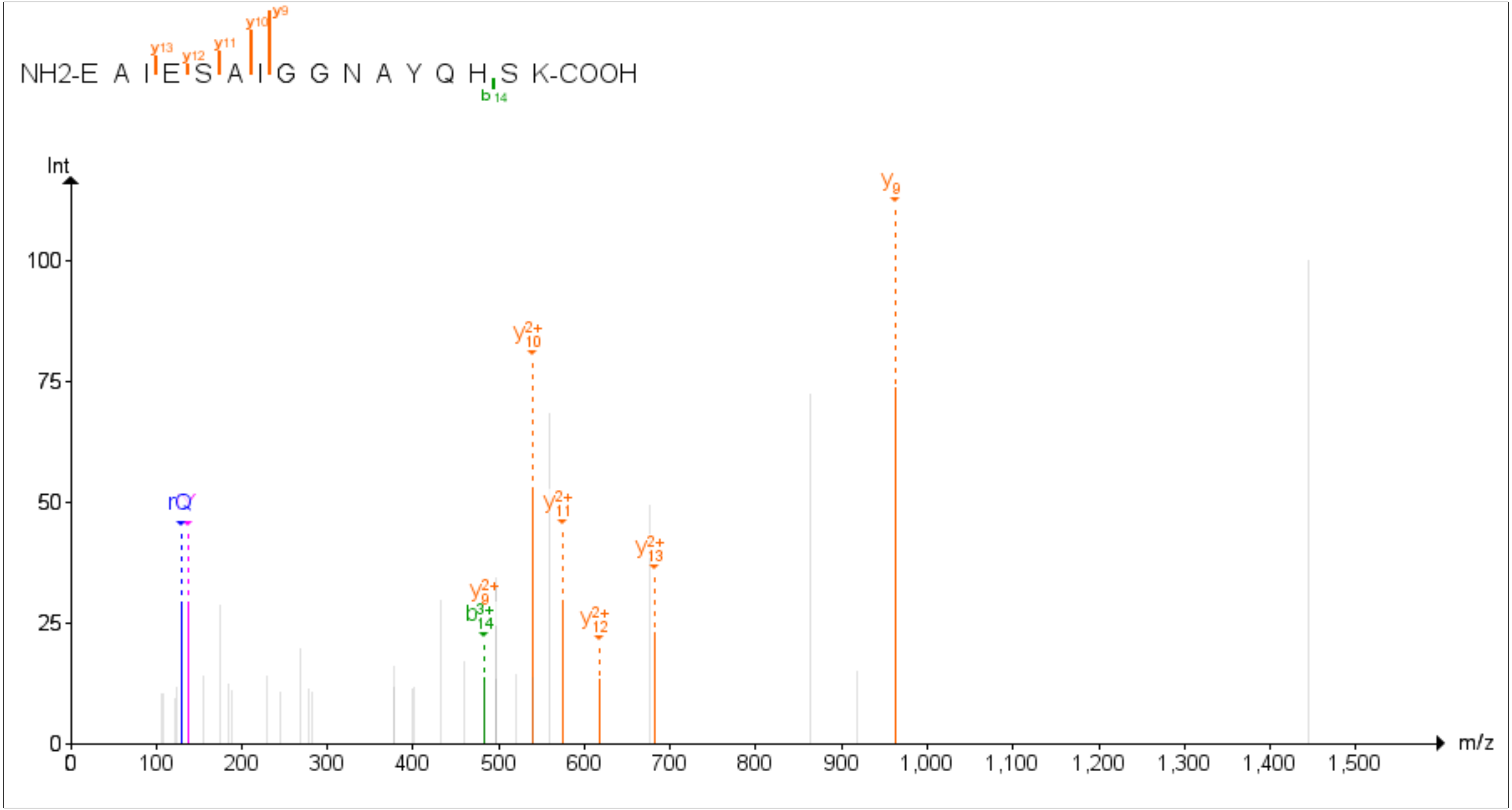

NH2-E A I E S A I G G N A Y Q H S K-COOH
y13
y12
y11
y10
y9
b14
Int
100
75
50
25
0
rQ
y9
y10 2+
y11 2+
y13 2+
y12 2+
y9 2+
b14 3+
0
100
200
300
400
500
600
700
800
900
1,000
1,100
1,200
1,300
1,400
1,500
m/z

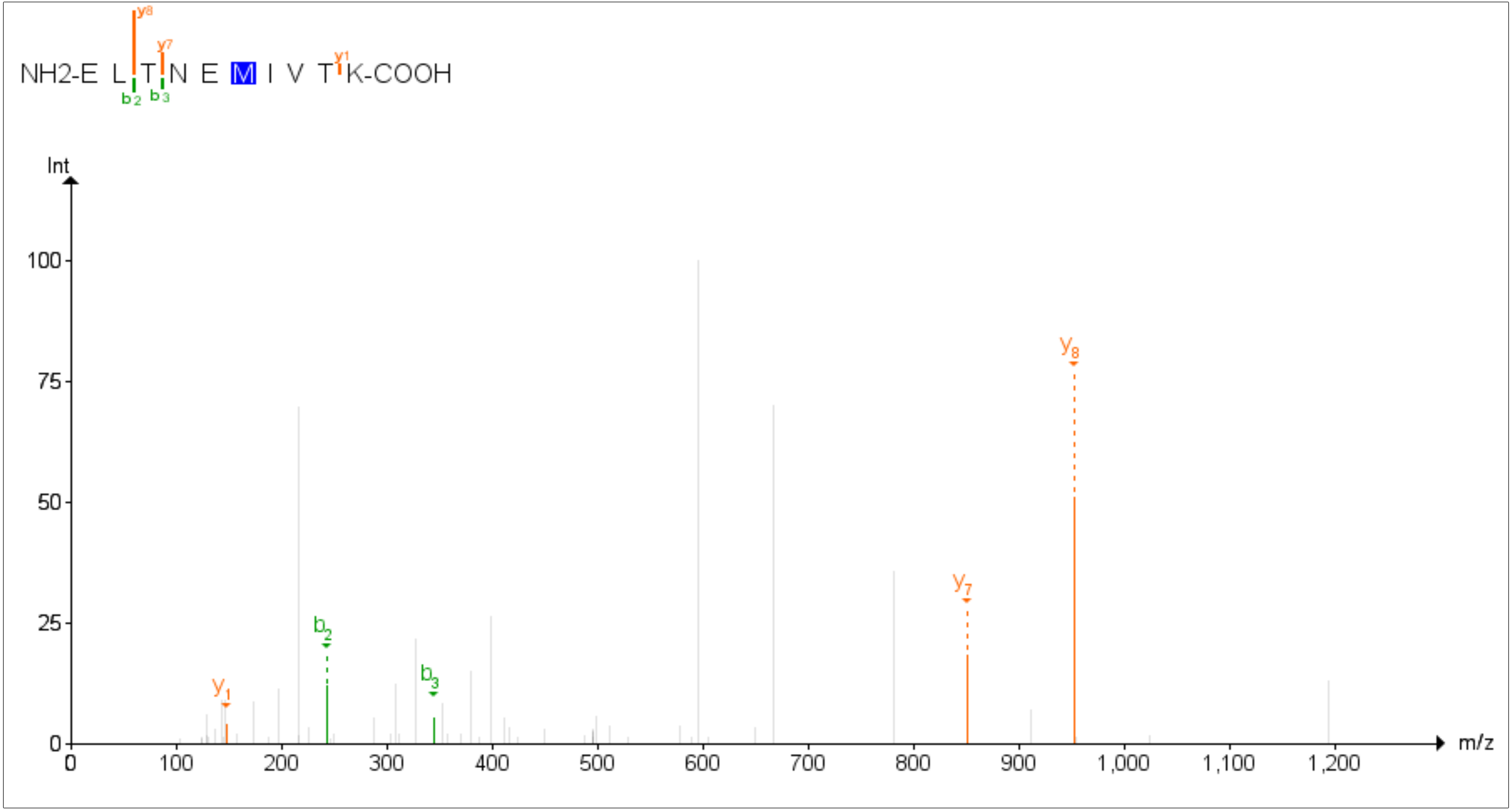

NH2-E L T N E M I V T K-COOH
y8
y7
y1
b2
b3
Int
100
75
50
25
0
y8
y7
b2
b3
y1
m/z
0
100
200
300
400
500
600
700
800
900
1,000
1,100
1,200

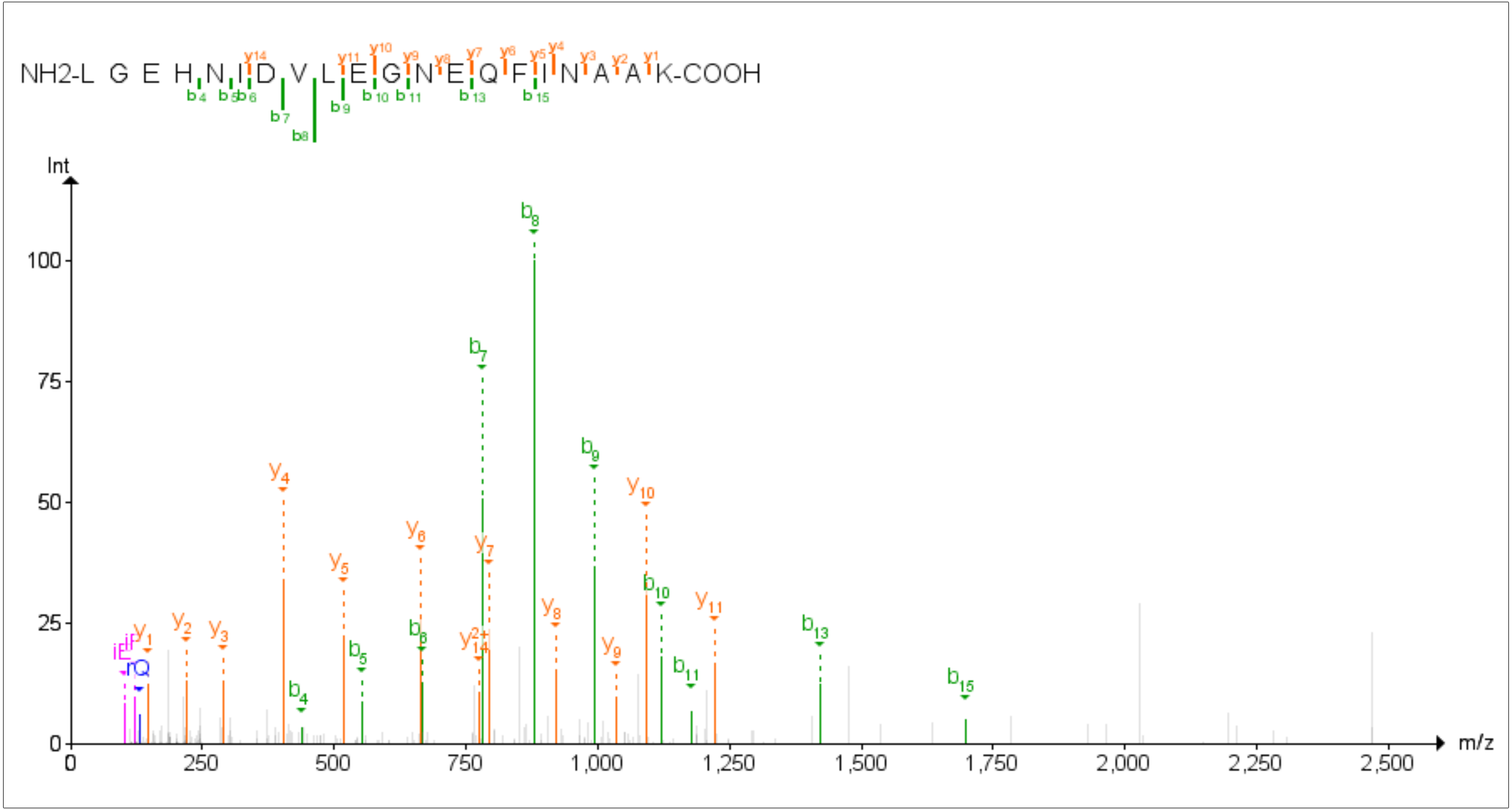

NH2-L G E H N I D V L E G N E Q F I N A A K-COOH
Int
m/z

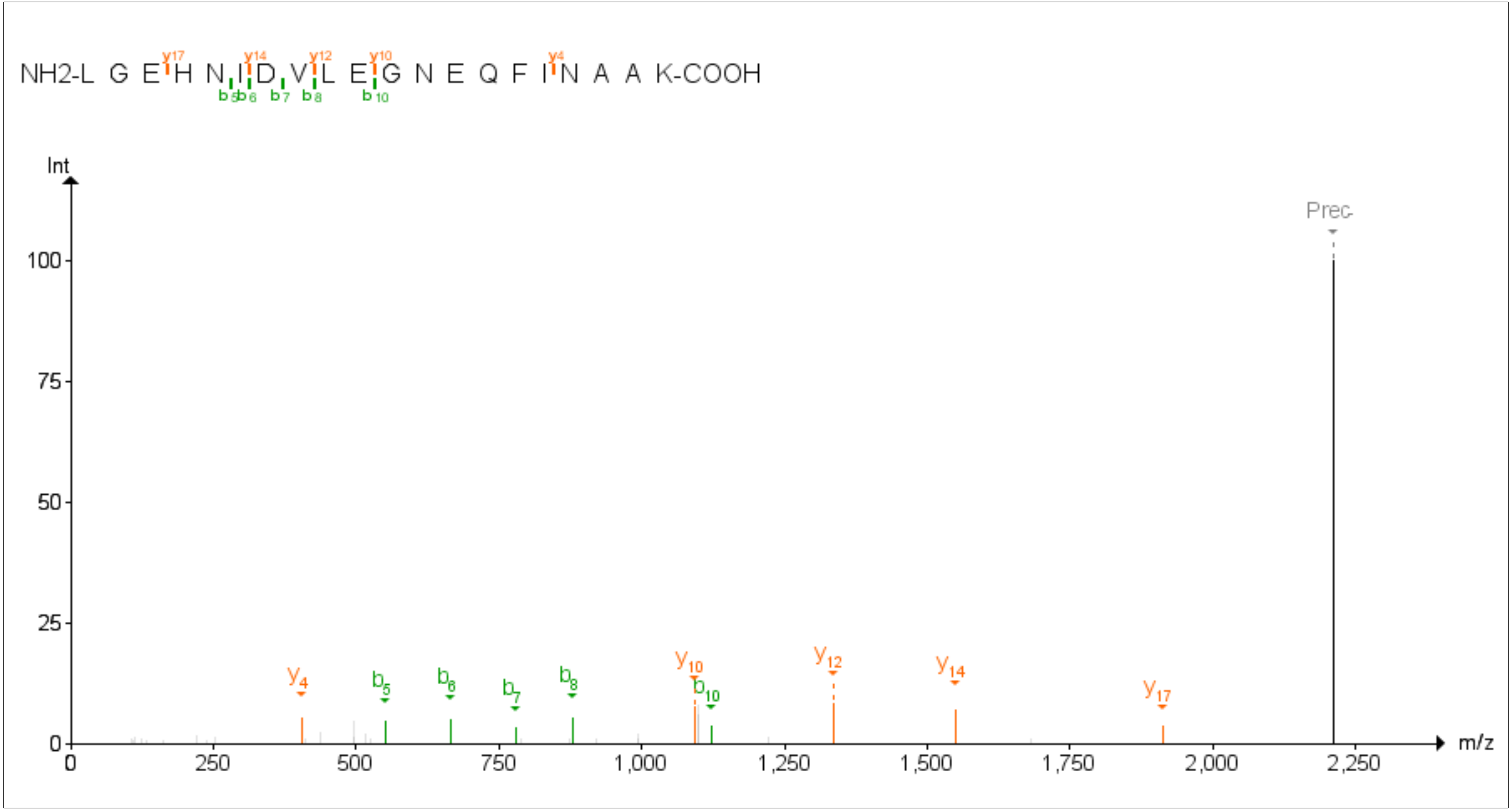

NH2-L G E H N I D V L E G N E Q F I N A A K-COOH
y17 y14 y12 y10 y4
b5 b6 b7 b8 b10
Int
100
75
50
25
0
Prec.
y4
b5
b6
b7
b8
y10
b10
y12
y14
y17
0
250
500
750
1,000
1,250
1,500
1,750
2,000
2,250
m/z

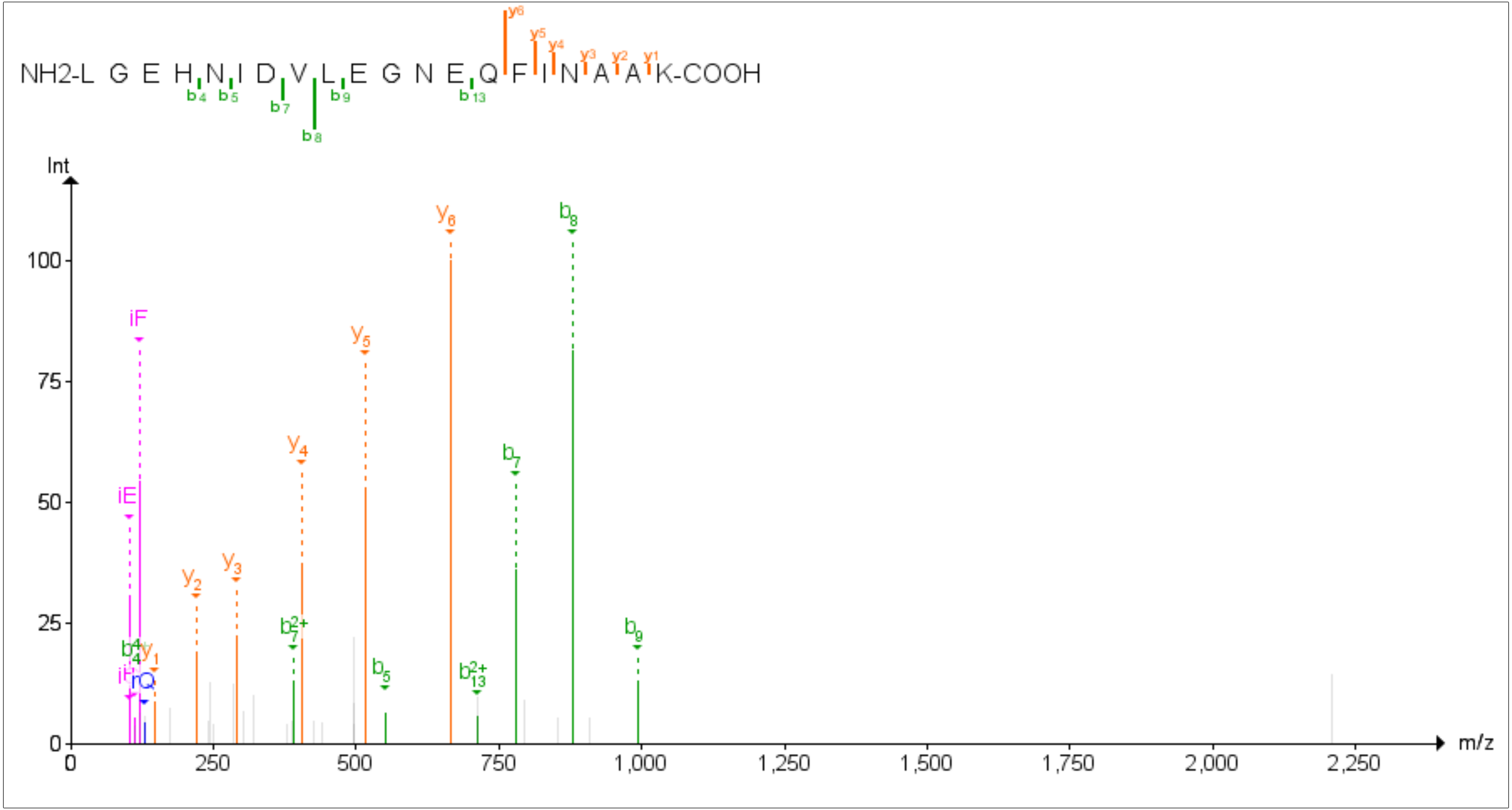

NH2-L G E H N I D V L E G N E Q F I N A A K-COOH
b4 b5 b7 b8 b9 b13
y6 y5 y4 y3 y2 y1
Int
m/z
0
25
50
75
100
0
250
500
750
1,000
1,250
1,500
1,750
2,000
2,250
iF
iE
iH
rQ
b_4^{2+}
y_1
y_2
y_3
y_4
y_5
y_6
b_7^{2+}
b_5
b_{13}^{2+}
b_7
b_8
b_9

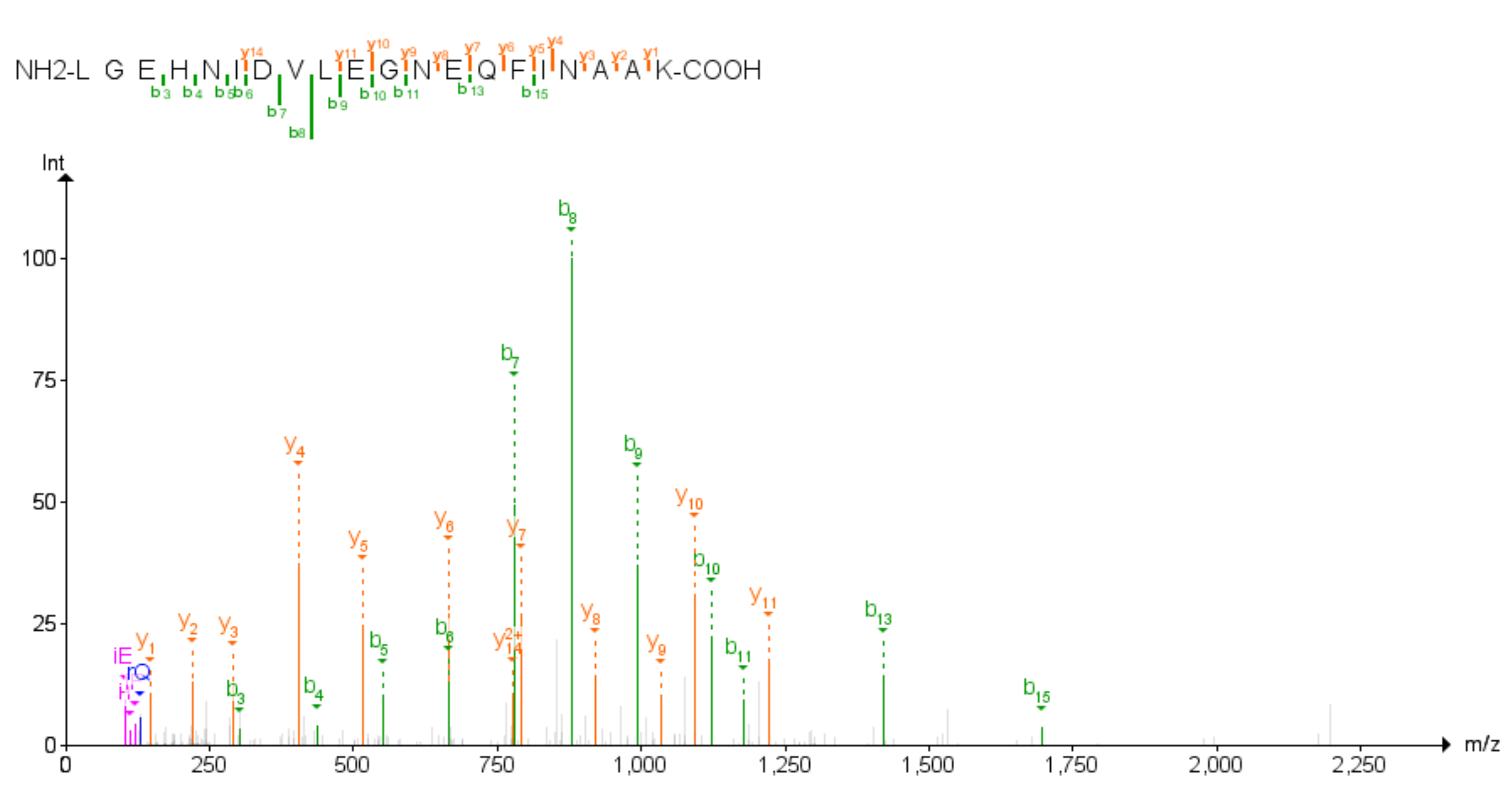

NH2-L G E H N I D V L E G N E Q F I N A A K-COOH
Int
m/z
0
25
50
75
100
250
500
750
1,000
1,250
1,500
1,750
2,000
2,250

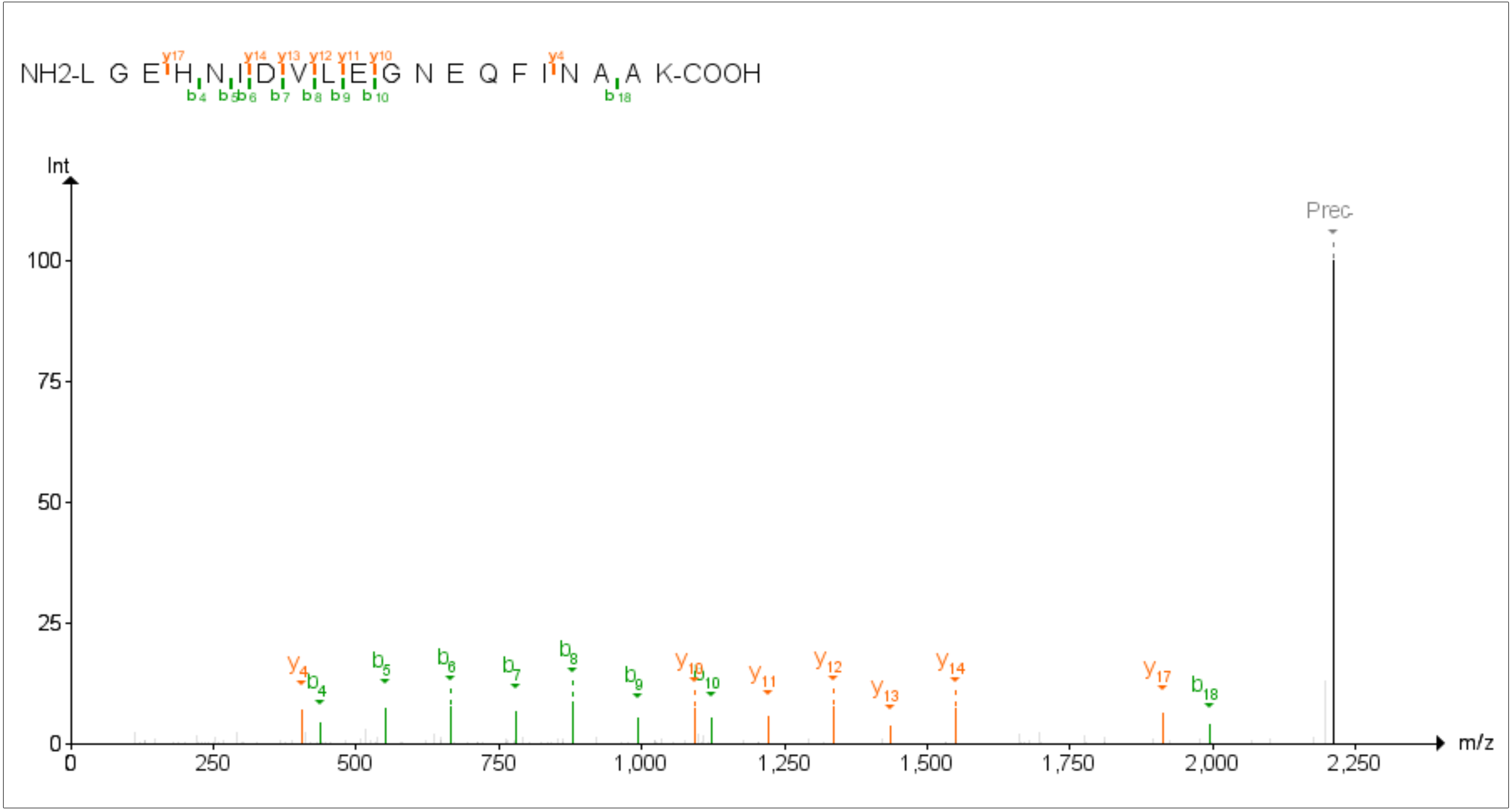
NH2-L G E H N I D V L E G N E Q F I N A A K-COOH
y17 y14 y13 y12 y11 y10 y4
b4 b5 b6 b7 b8 b9 b10 b18
Int
Prec.
100
75
50
25
0
0 250 500 750 1,000 1,250 1,500 1,750 2,000 2,250
m/z
y4 b4 b5 b6 b7 b8 b9 y10 b10 y11 y12 y13 y14 y17 b18

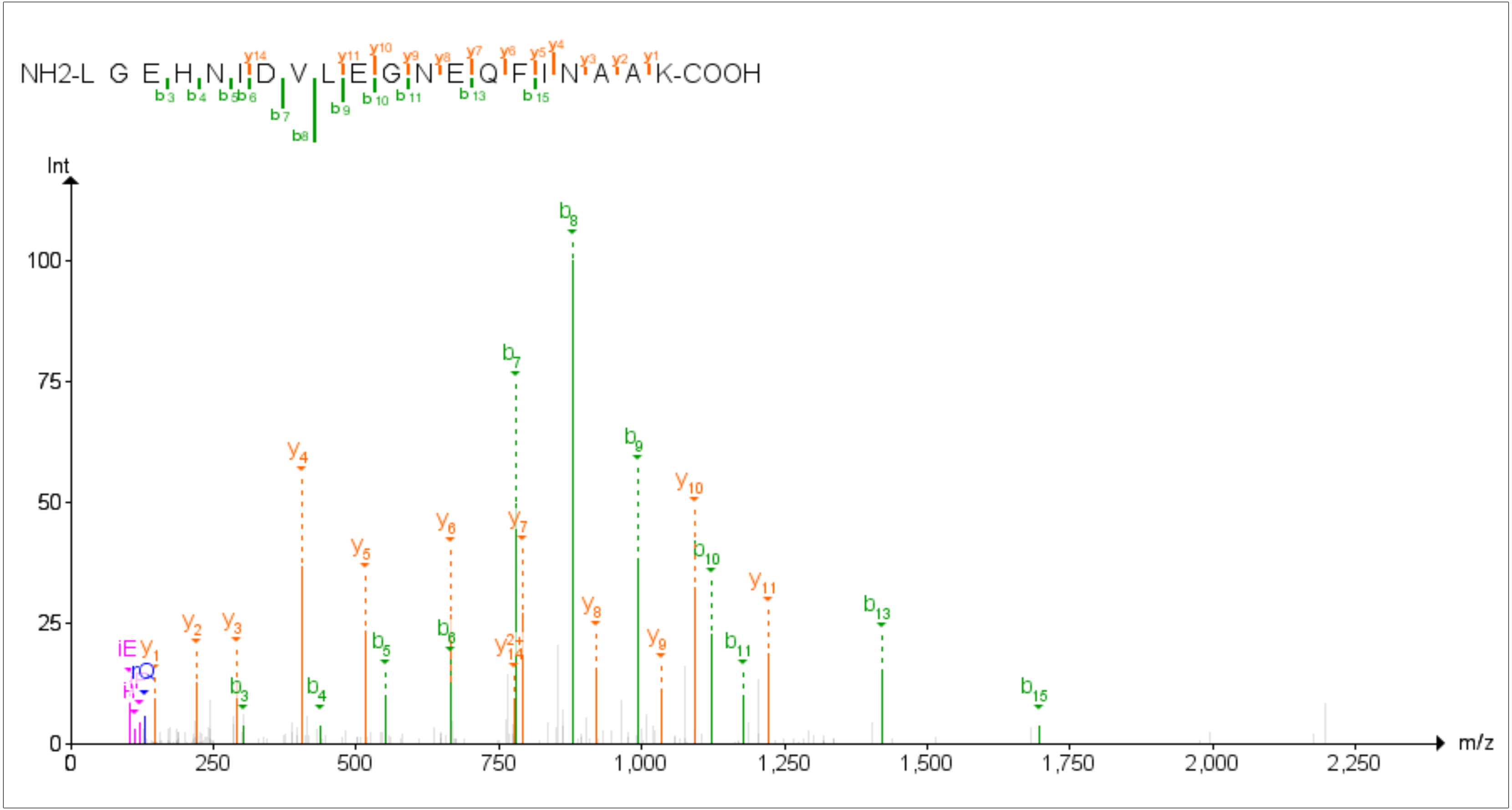
NH2-L G E H N I D V L E G N E Q F I N A A K-COOH
Int
m/z
0
25
50
75
100
250
500
750
1,000
1,250
1,500
1,750
2,000
2,250

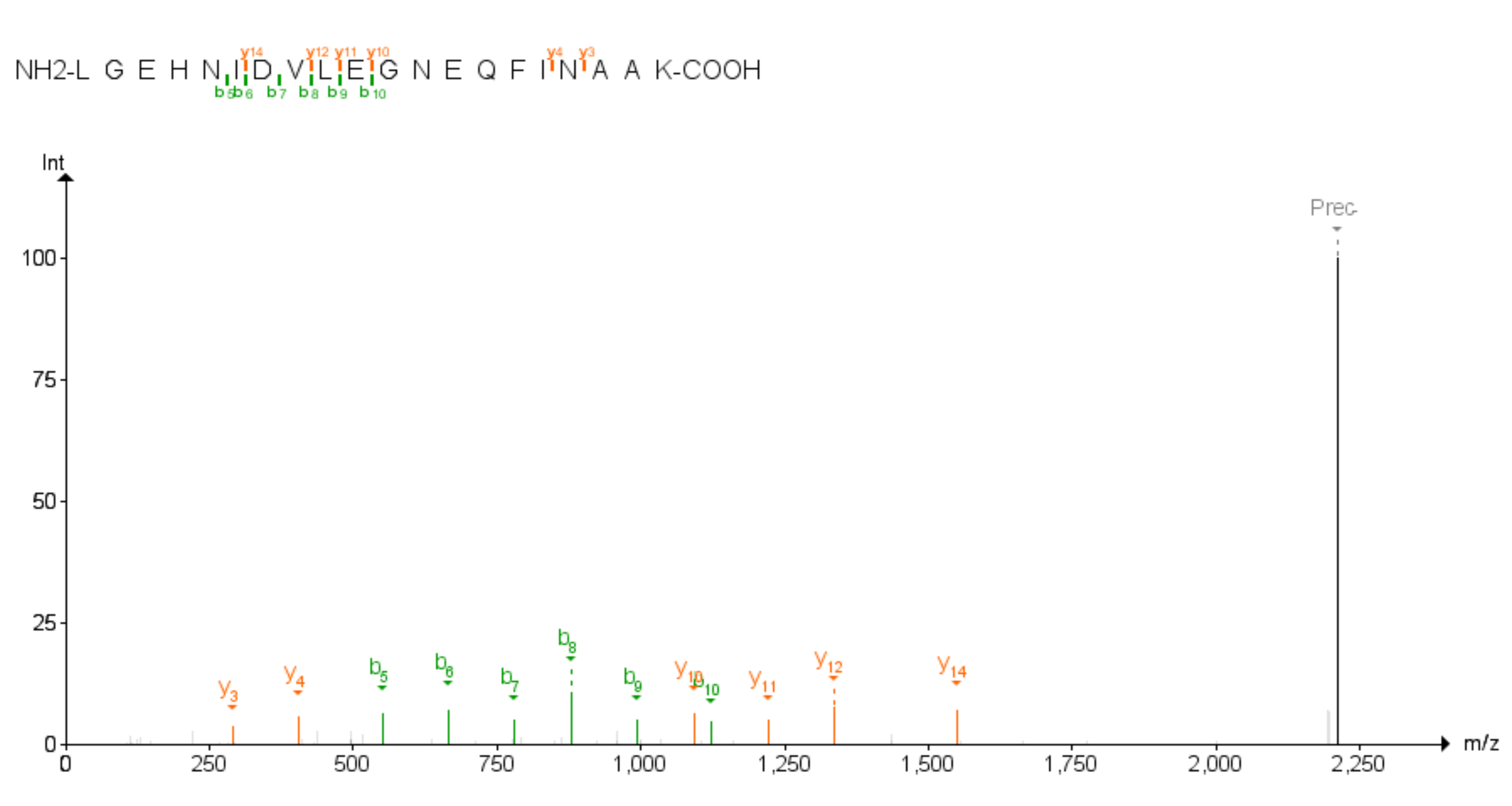

NH2-L G E H N I D V L E G N E Q F I N A A K-COOH
Int
100
75
50
25
0
m/z
0
250
500
750
1,000
1,250
1,500
1,750
2,000
2,250
Prec

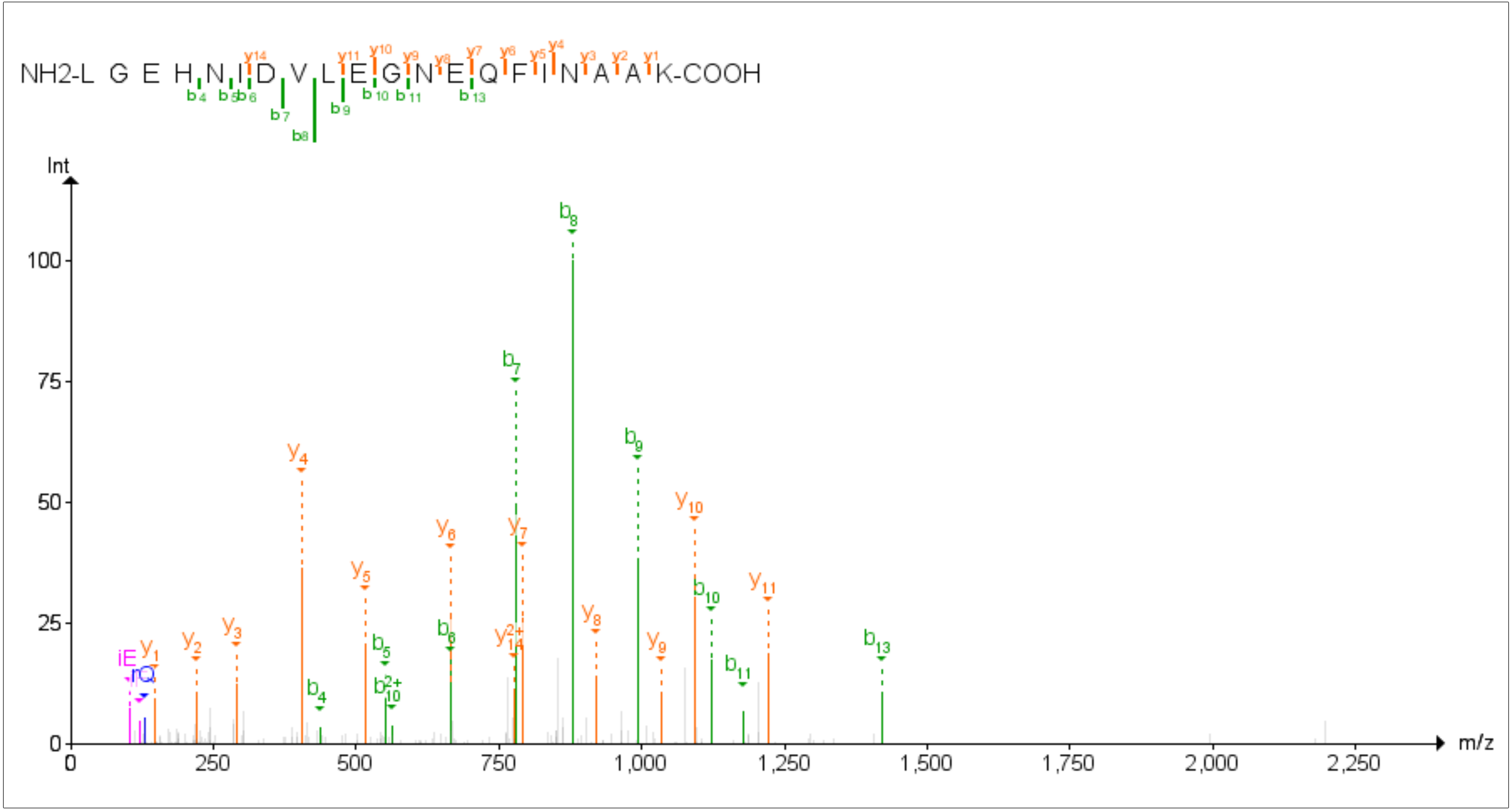
NH2-L G E H N I D V L E G N E Q F I N A A K-COOH
Int
m/z
100
75
50
25
0
250
500
750
1,000
1,250
1,500
1,750
2,000
2,250

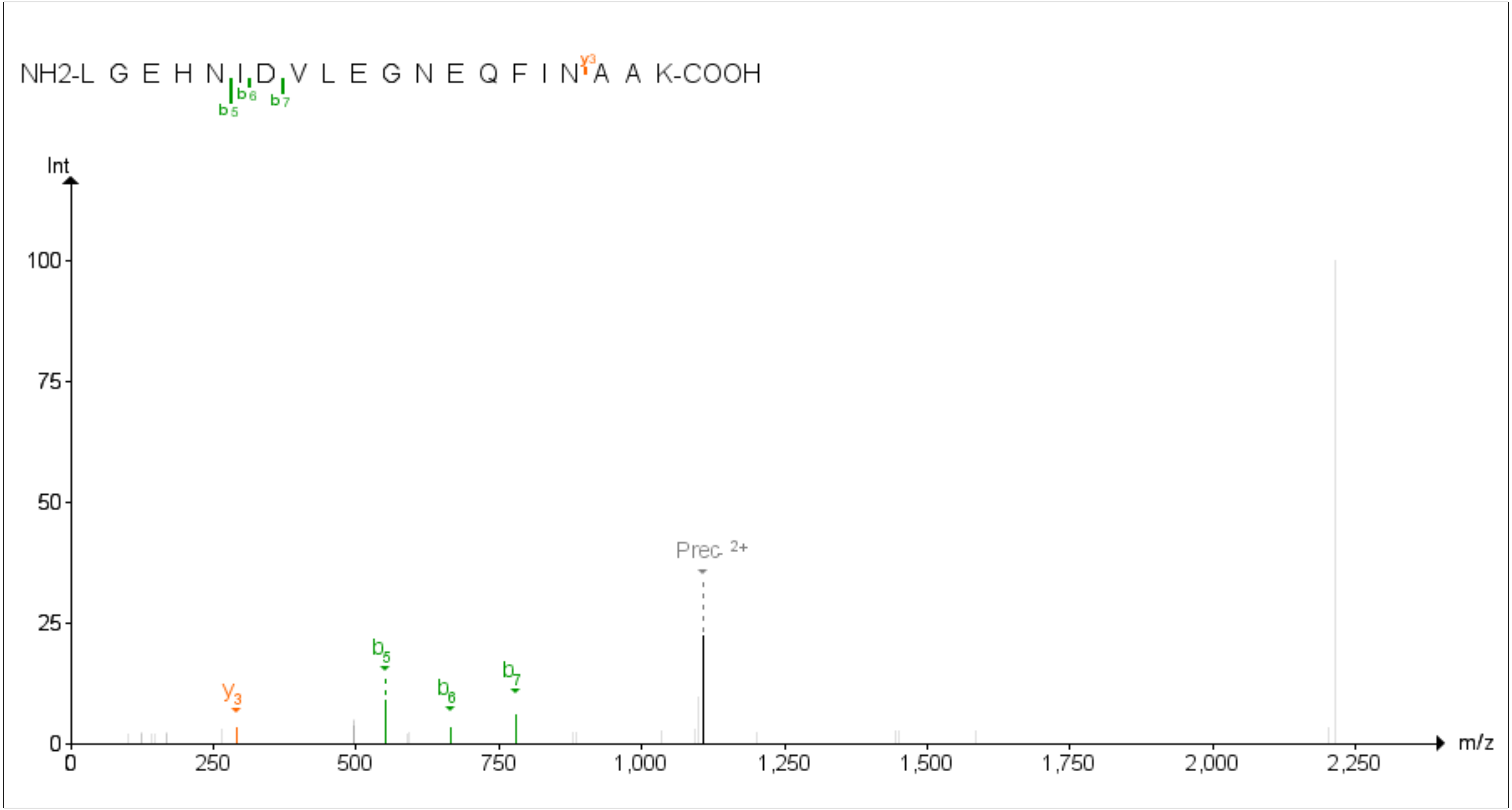

NH2-L G E H N I D V L E G N E Q F I N A A K-COOH
b5
b6
b7
y3
Int
100
75
50
25
0
Prec- 2+
m/z
0
250
500
750
1,000
1,250
1,500
1,750
2,000
2,250

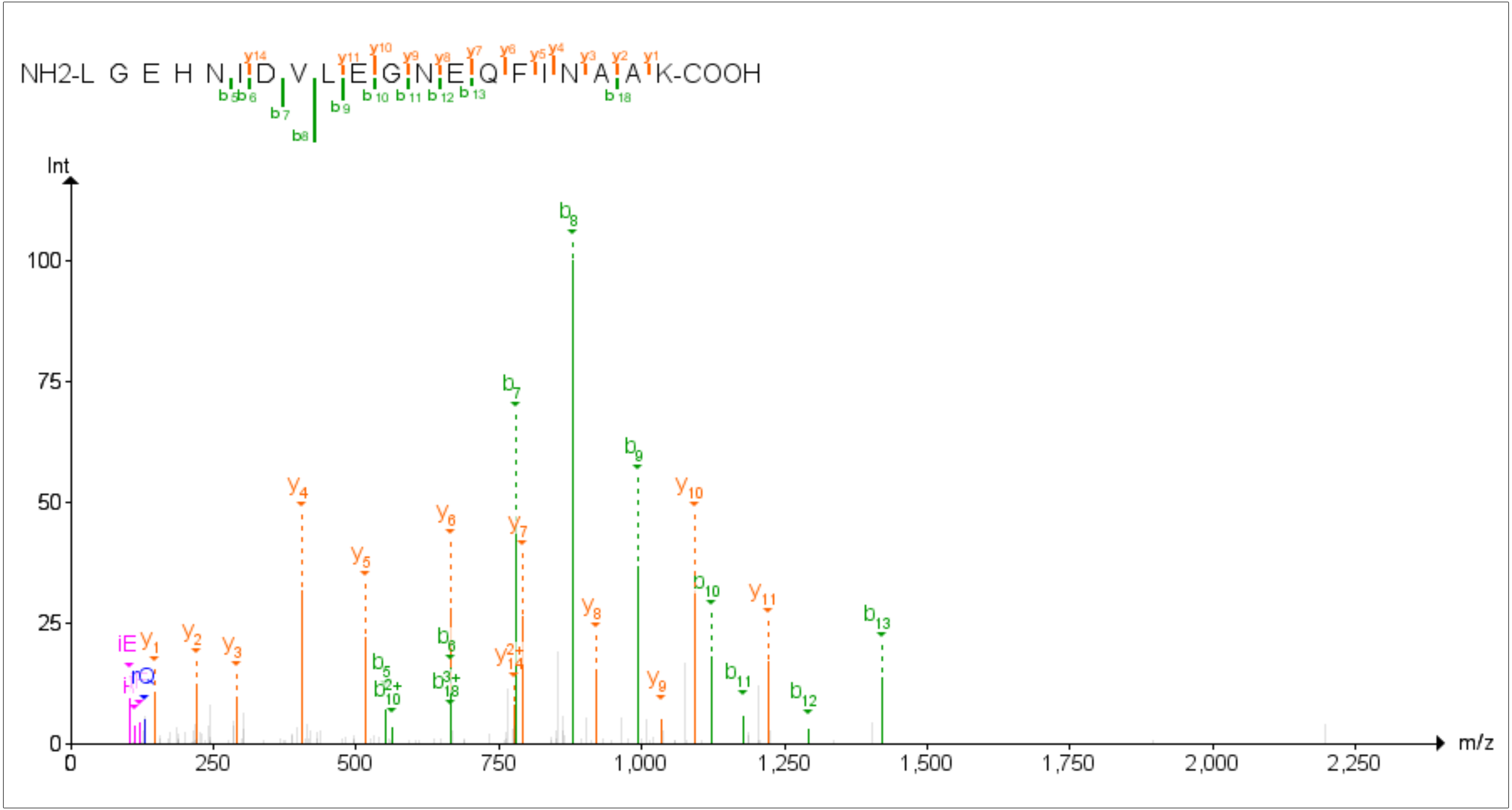
NH2-L G E H N I D V L E G N E Q F I N A A K-COOH
Int
m/z
0
25
50
75
100
250
500
750
1,000
1,250
1,500
1,750
2,000
2,250
b8
b7
b9
y4
y10
y6
y7
y5
b10
y11
b13
y8
iE
y1
y2
y3
b5
b6
y14 2+
b10 2+
b18 3+
y9
b11
b12
iQ

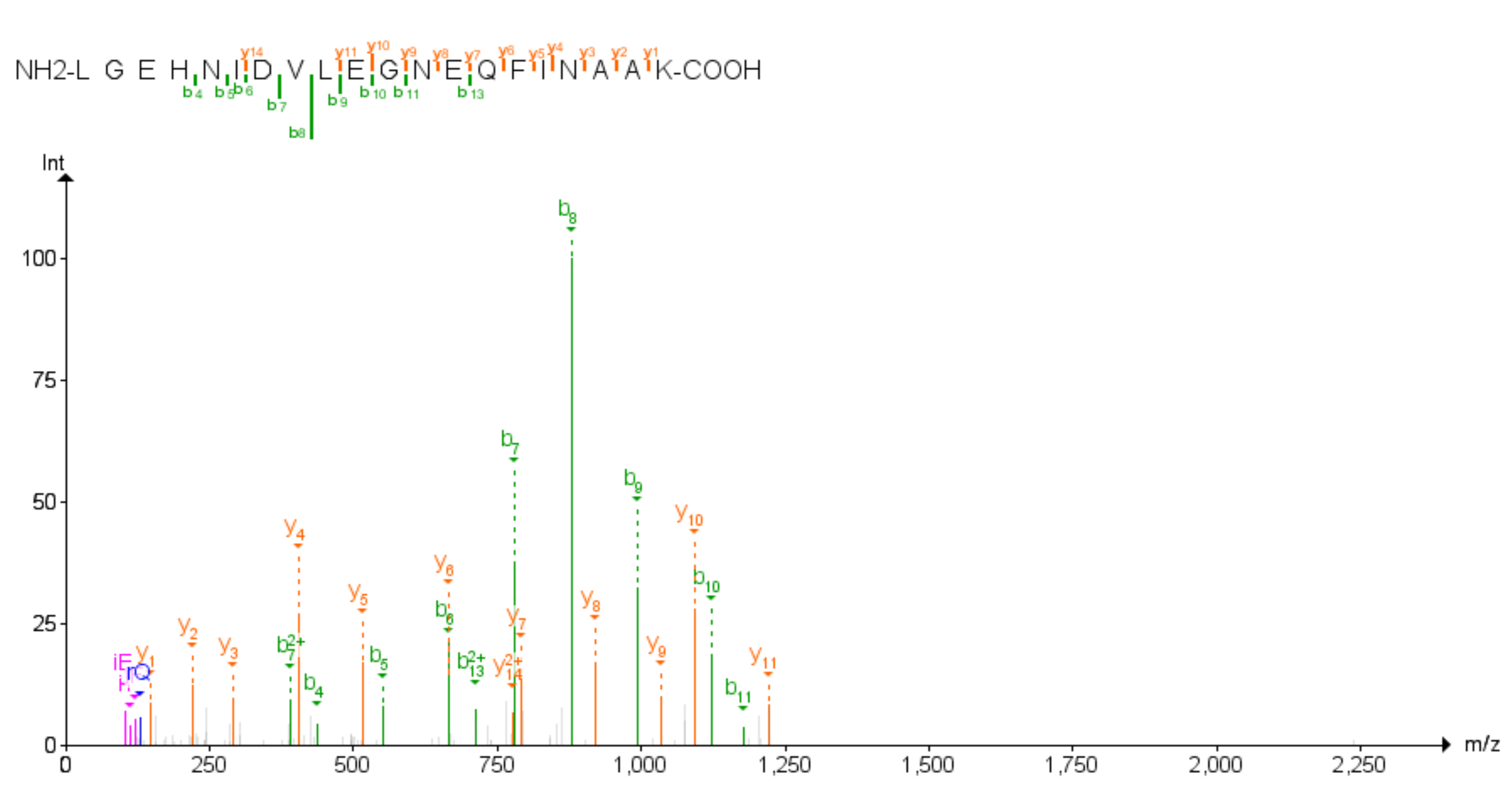

NH2-L G E H N I D V L E G N E Q F I N A A K-COOH
Int
m/z
0
25
50
75
100
250
500
750
1,000
1,250
1,500
1,750
2,000
2,250

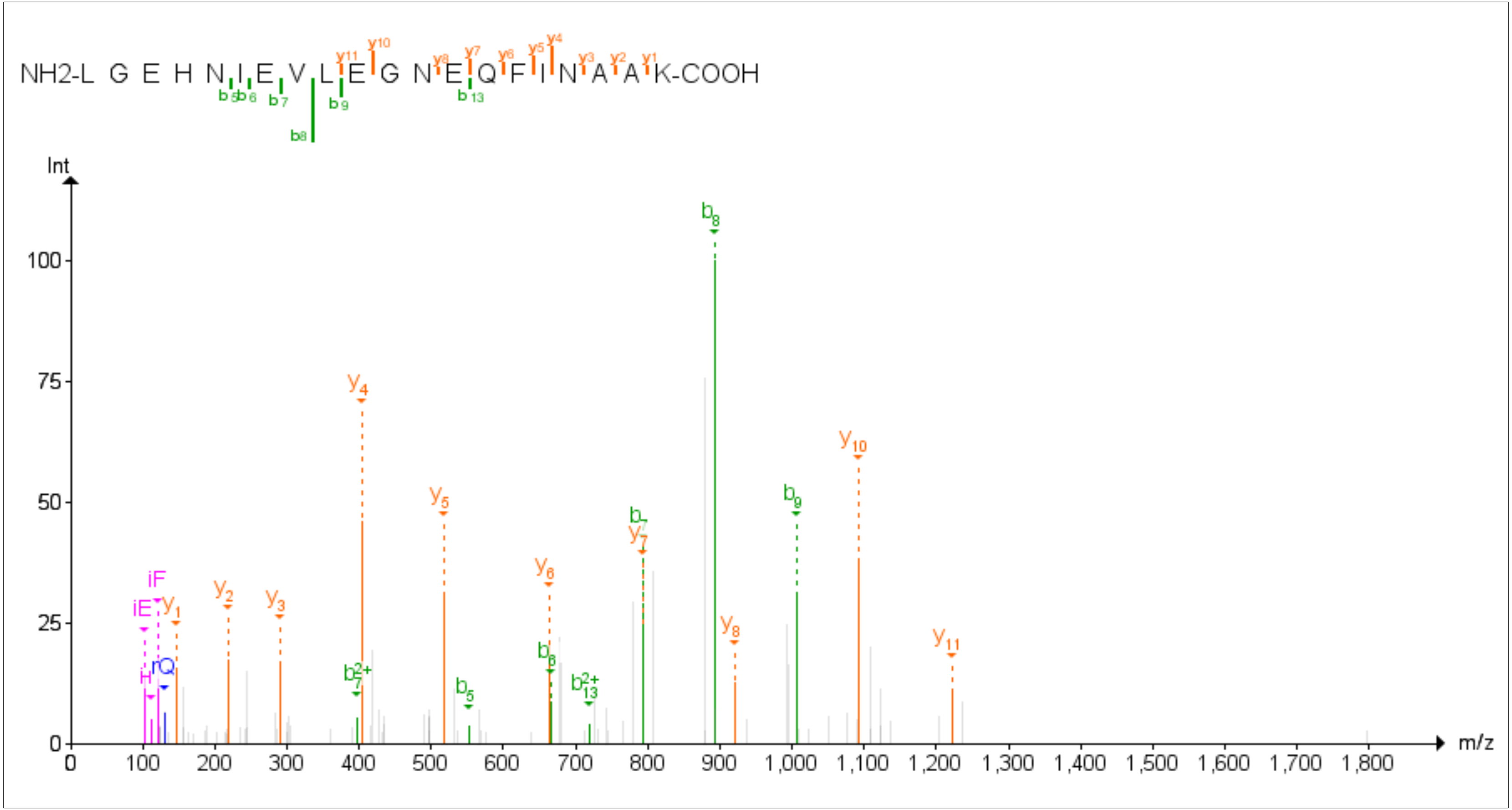

NH2-L G E H N I E V L E G N E Q F I N A A K-COOH
b5 b6 b7 b8 b9 b13
y11 y10 y8 y7 y6 y5 y4 y3 y2 y1
Int
100
75
50
25
0
m/z
0 100 200 300 400 500 600 700 800 900 1,000 1,100 1,200 1,300 1,400 1,500 1,600 1,700 1,800

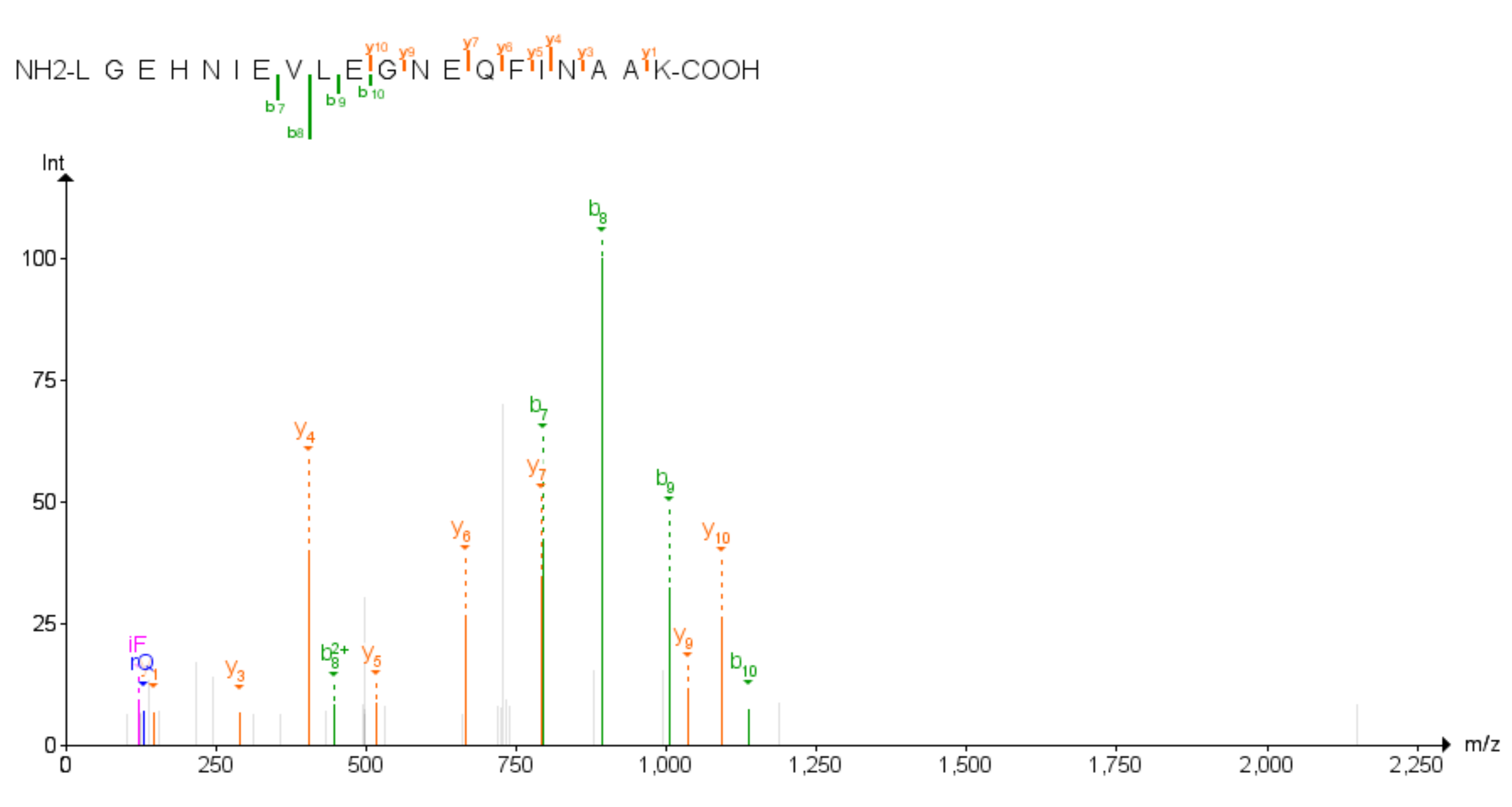

NH2-L G E H N I E V L E G N E Q F I N A A K-COOH
Int
m/z
b8
b7
y4
y7
b9
y10
y6
y9
b10
y5
b8 2+
y3
iF
rQ
0
25
50
75
100
250
500
750
1,000
1,250
1,500
1,750
2,000
2,250

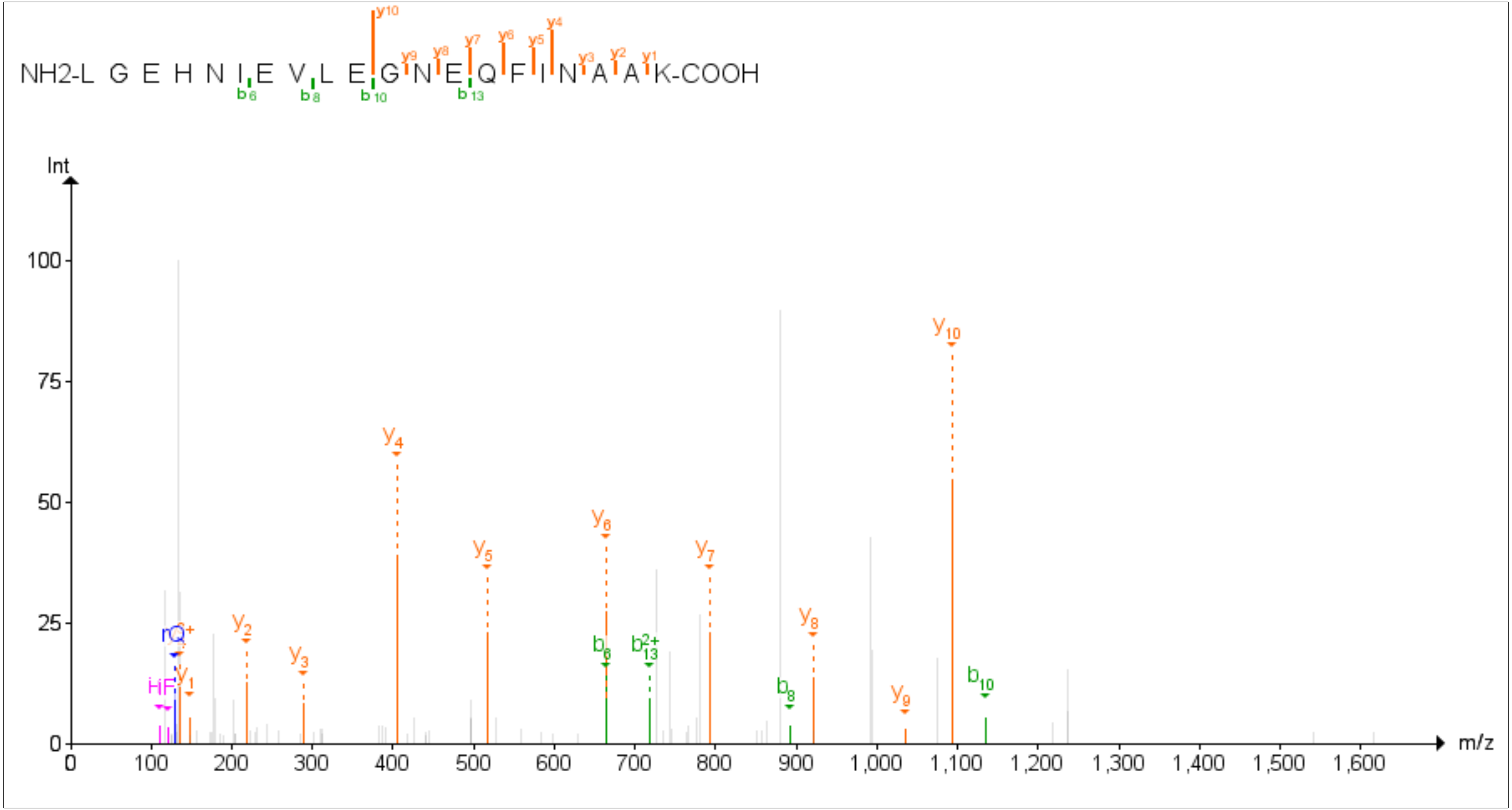

NH2-L G E H N I E V L E G N E Q F I N A A K-COOH
Int
m/z
y10
y4
y6
y5
y7
y8
y2
y3
y9
y1
b6
b8
b10
b13
HF

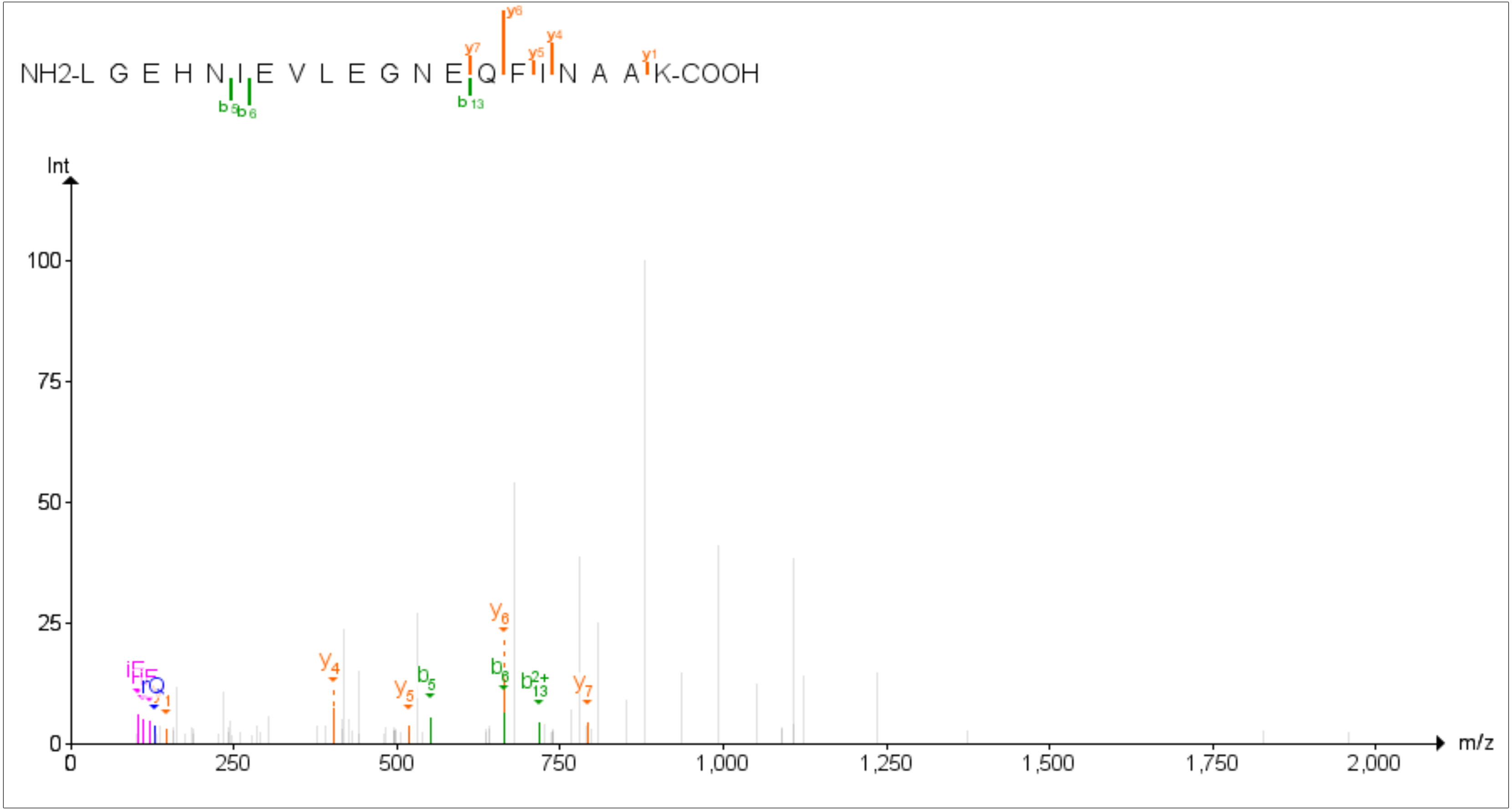

NH2-L G E H N I E V L E G N E Q F I N A A K-COOH
y8
y7
y5
y4
y1
b5
b6
b13
Int
100
75
50
25
0
y4
y5
b5
y6
b6
b2+13
y7
rQ
y1
0
250
500
750
1,000
1,250
1,500
1,750
2,000
m/z

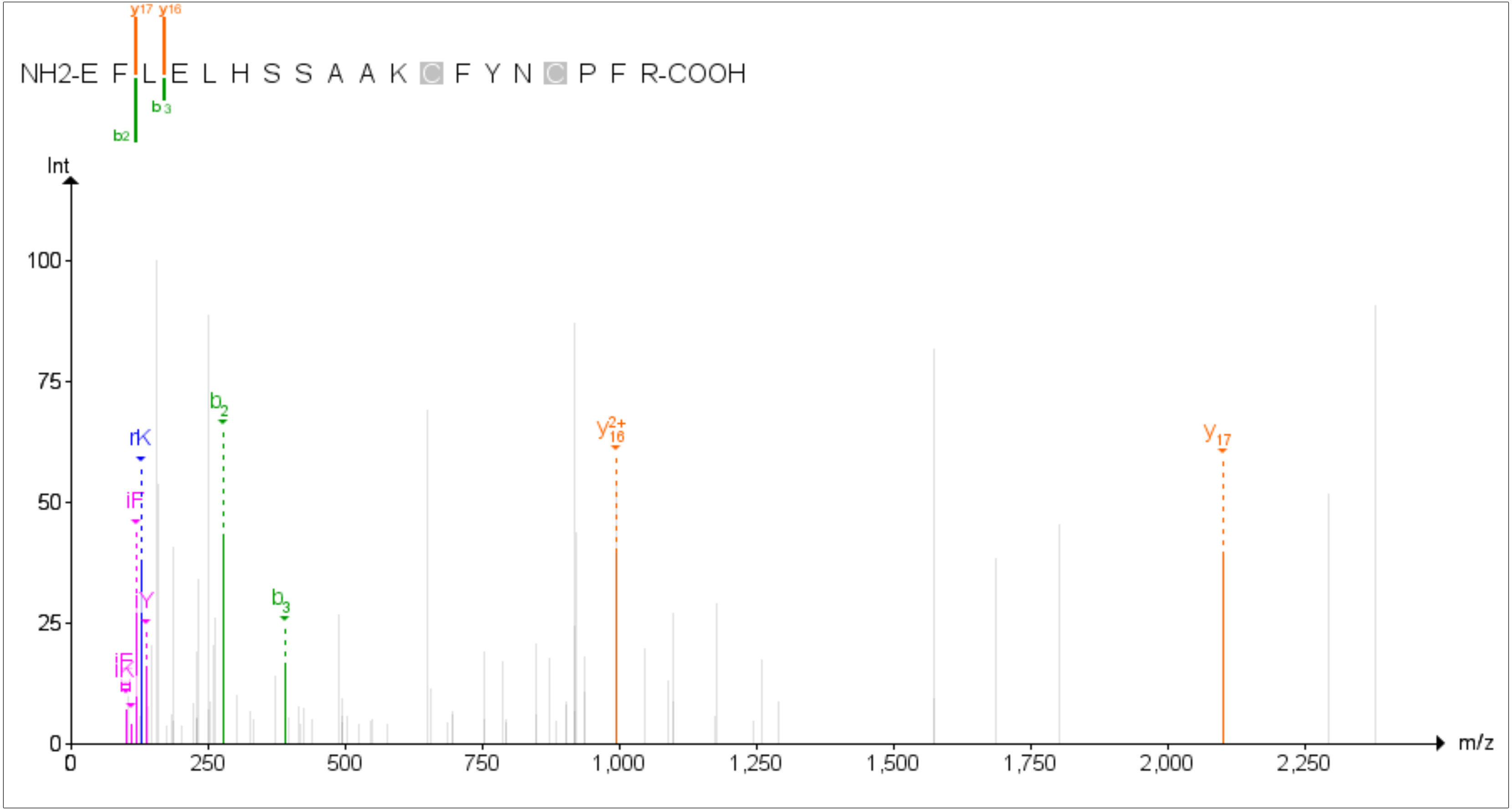

y17 y16
NH2-E F L E L H S S A A K C F Y N C P F R-COOH
b2 b3
Int
100
75
50
25
0
rK
iF
iY
iK
b2
b3
y16 2+
y17
0
250
500
750
1,000
1,250
1,500
1,750
2,000
2,250
m/z

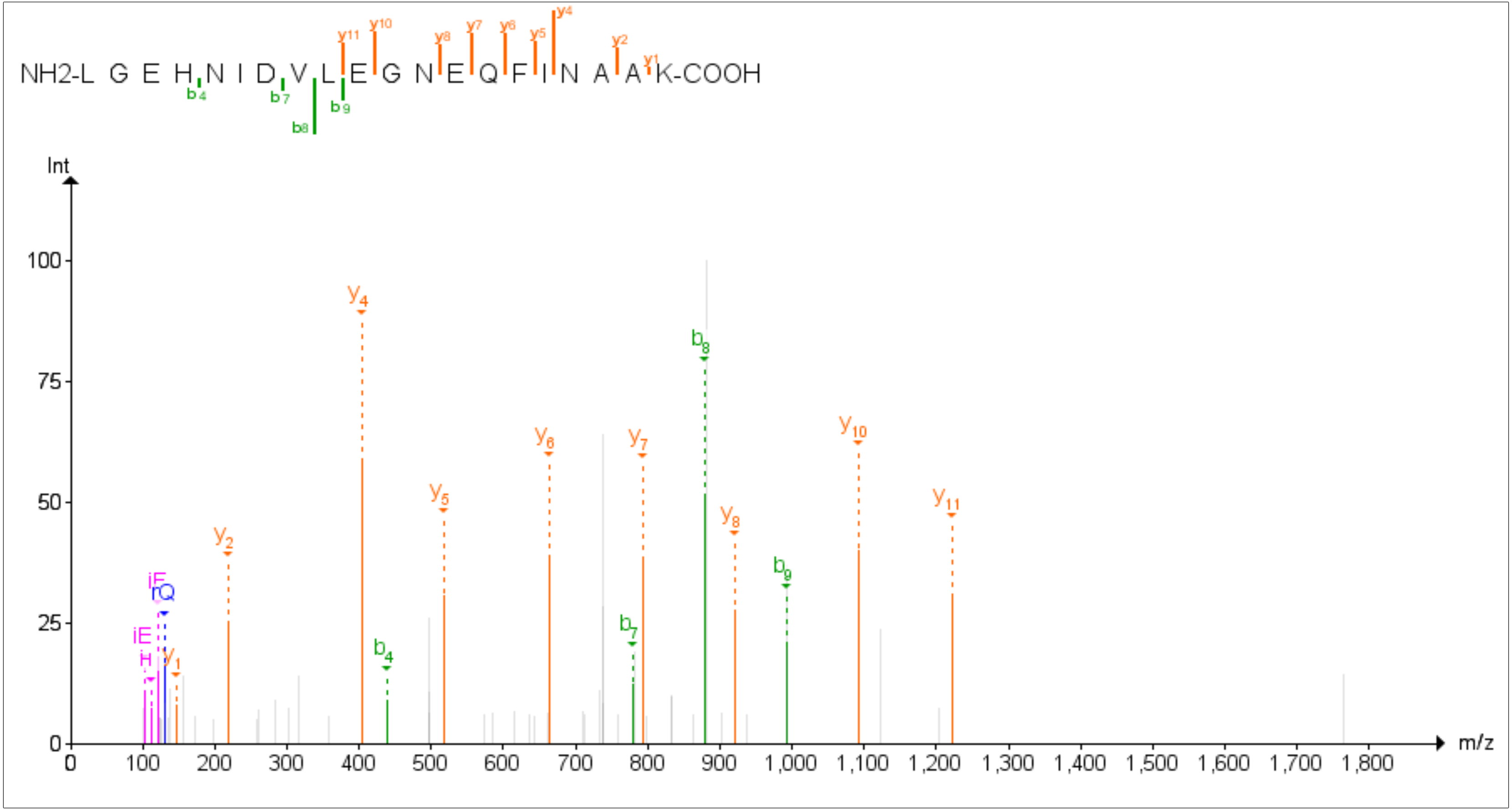

NH2-L G E H N I D V L E G N E Q F I N A A K-COOH
Int
m/z

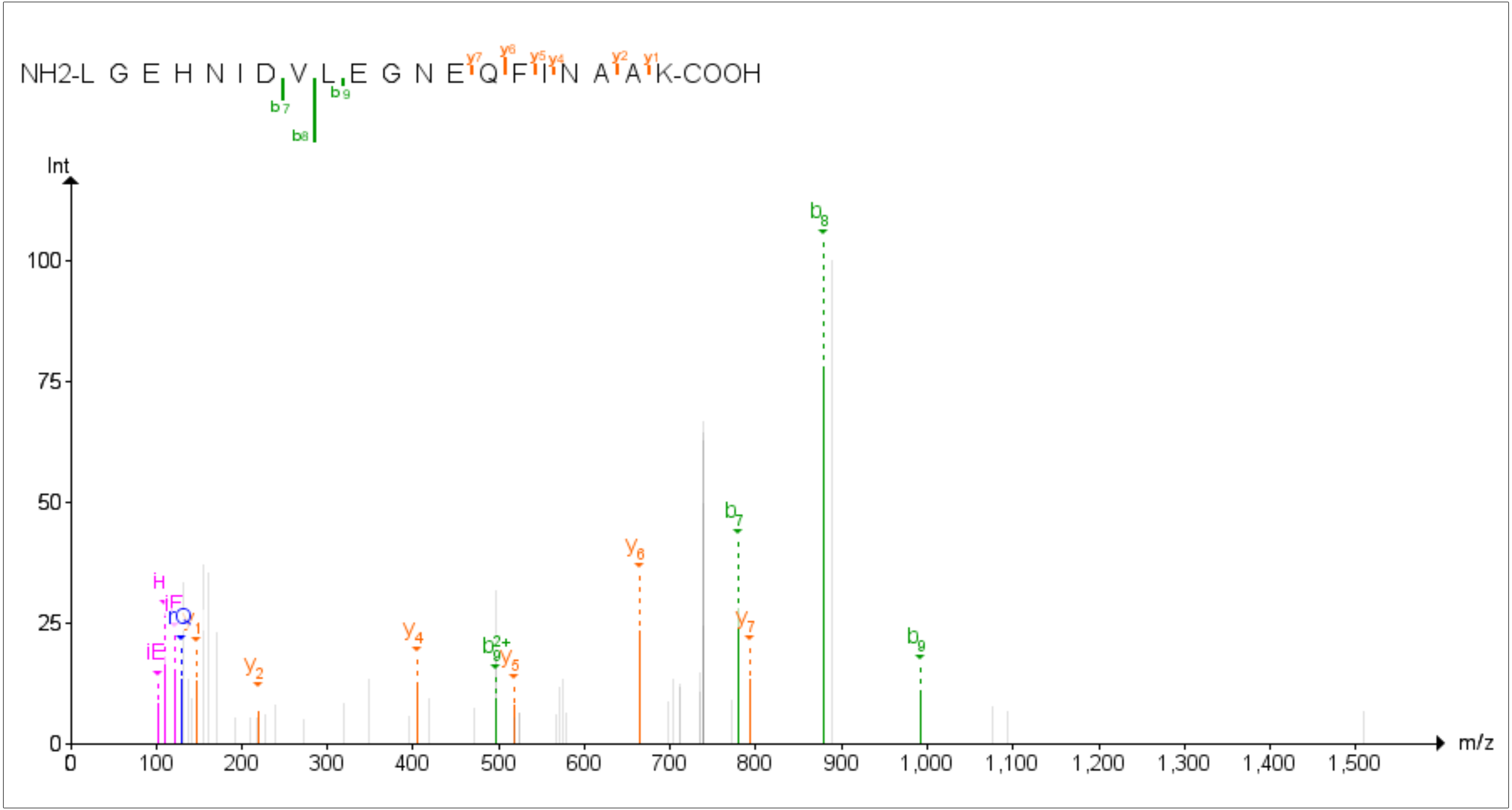

NH2-L G E H N I D V L E G N E Q F I N A A K-COOH
Int
m/z

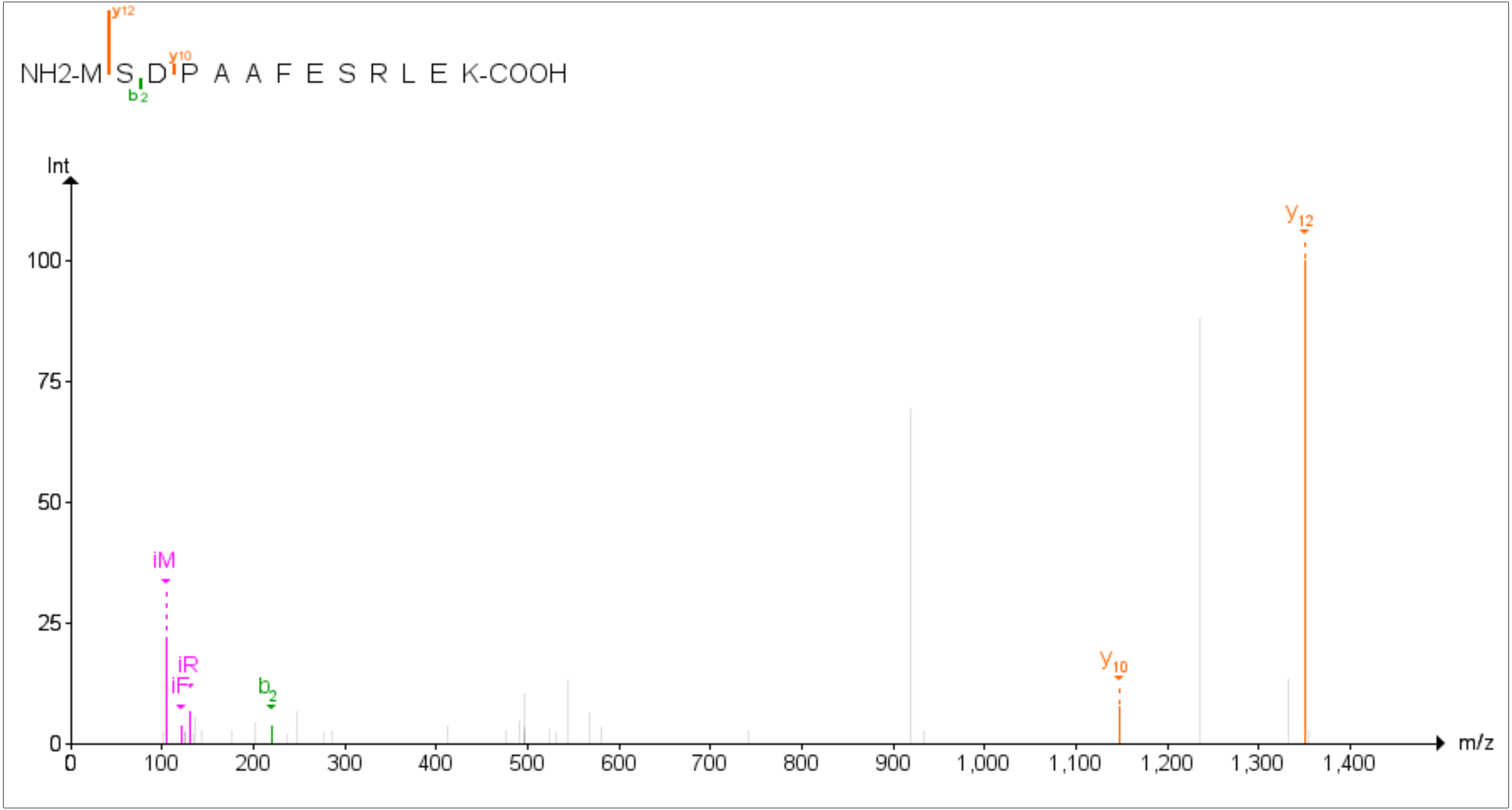
NH2-M S D P A A F E S R L E K-COOH
y12
y10
b2
Int
100
75
50
25
0
iM
iR
iF
b2
y10
y12
0
100
200
300
400
500
600
700
800
900
1,000
1,100
1,200
1,300
1,400
m/z

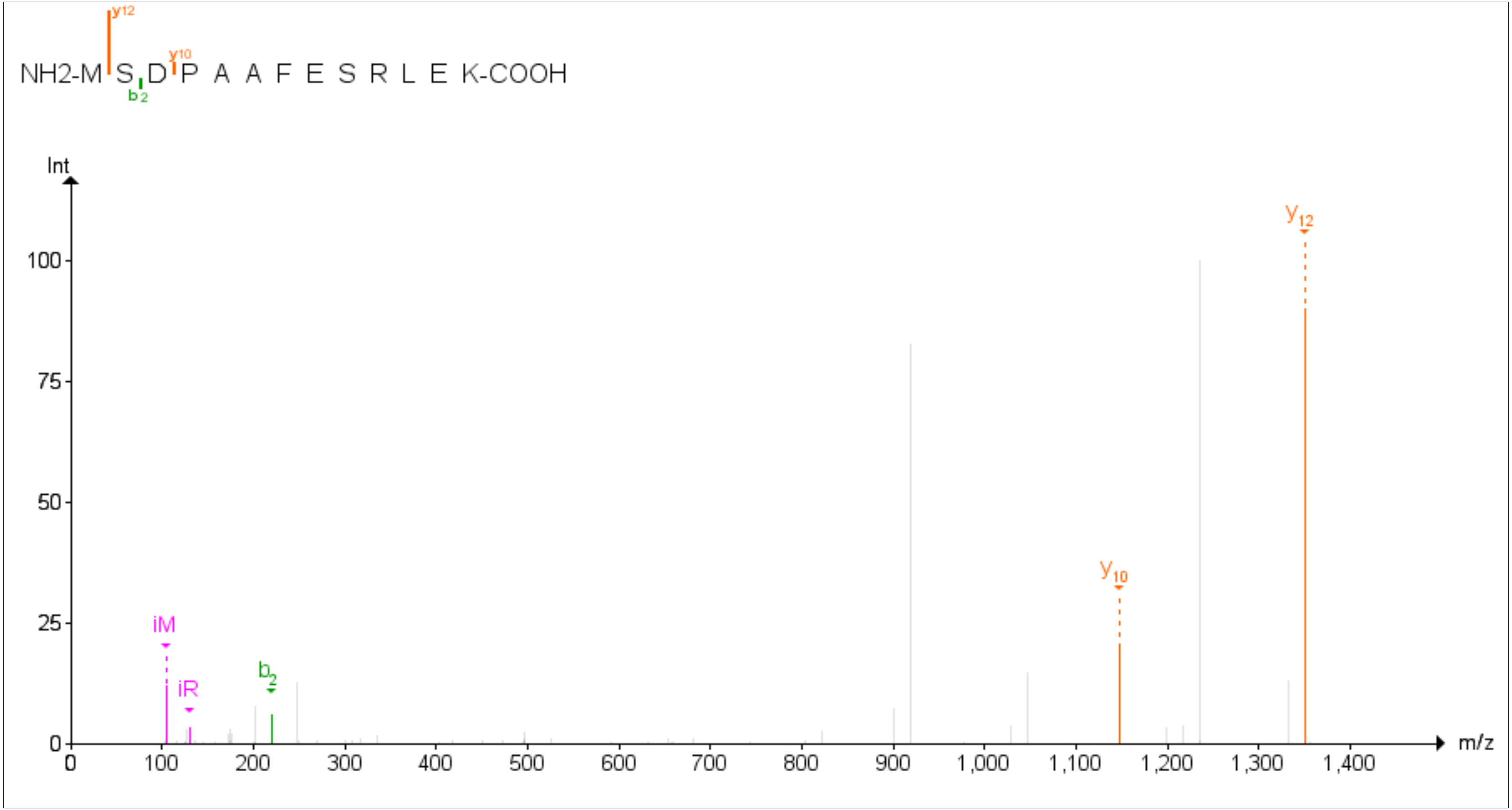

NH2-M S D P A A F E S R L E K-COOH
y12
y10
b2
Int
100
75
50
25
0
iM
iR
b2
y10
y12
0
100
200
300
400
500
600
700
800
900
1,000
1,100
1,200
1,300
1,400
m/z

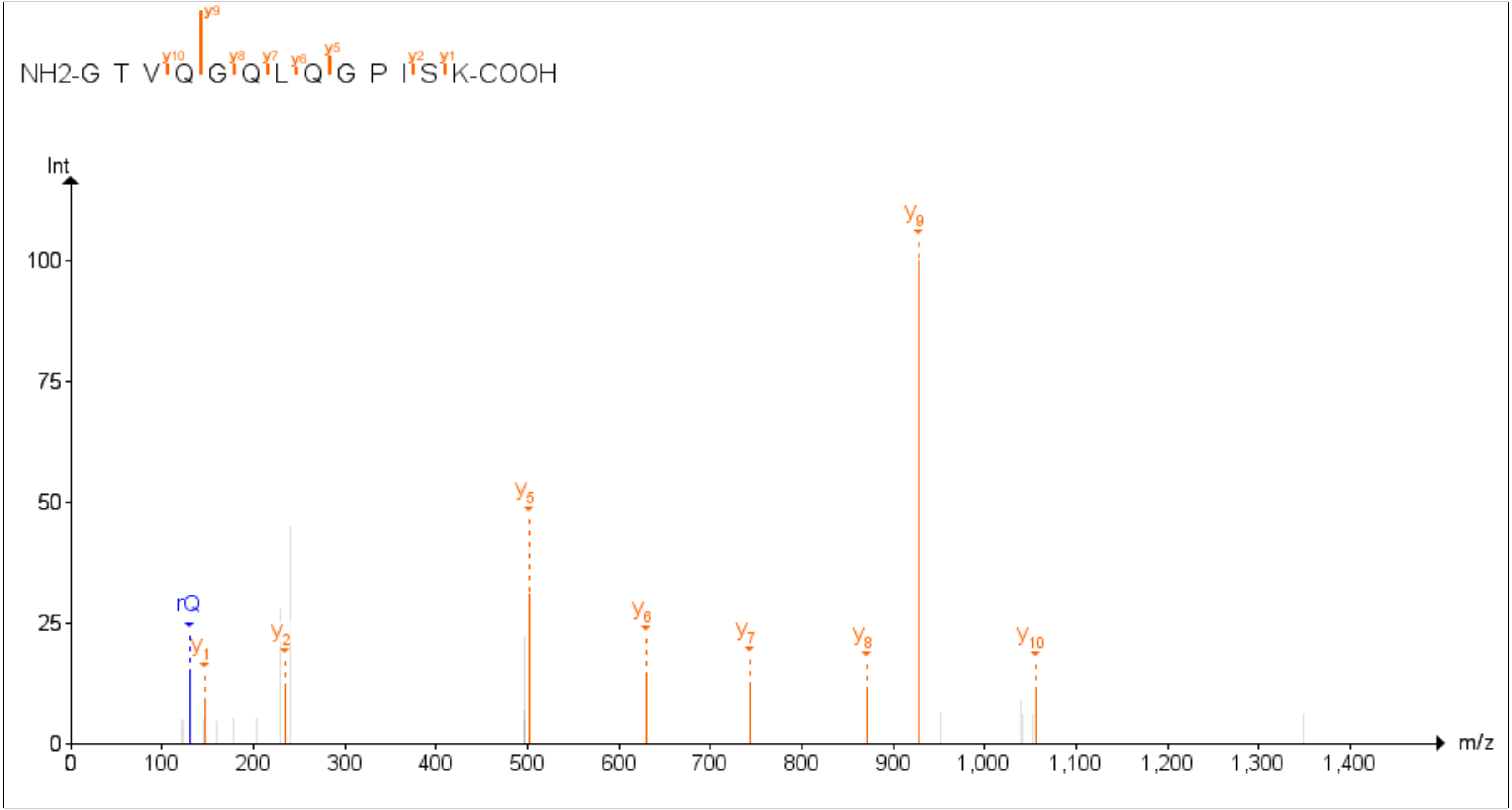

NH2-G T V Q G Q L Q G P I S K-COOH
y10 y9 y8 y7 y6 y5 y2 y1
Int
100
75
50
25
0
rQ
y1
y2
y5
y6
y7
y8
y9
y10
0 100 200 300 400 500 600 700 800 900 1,000 1,100 1,200 1,300 1,400
m/z

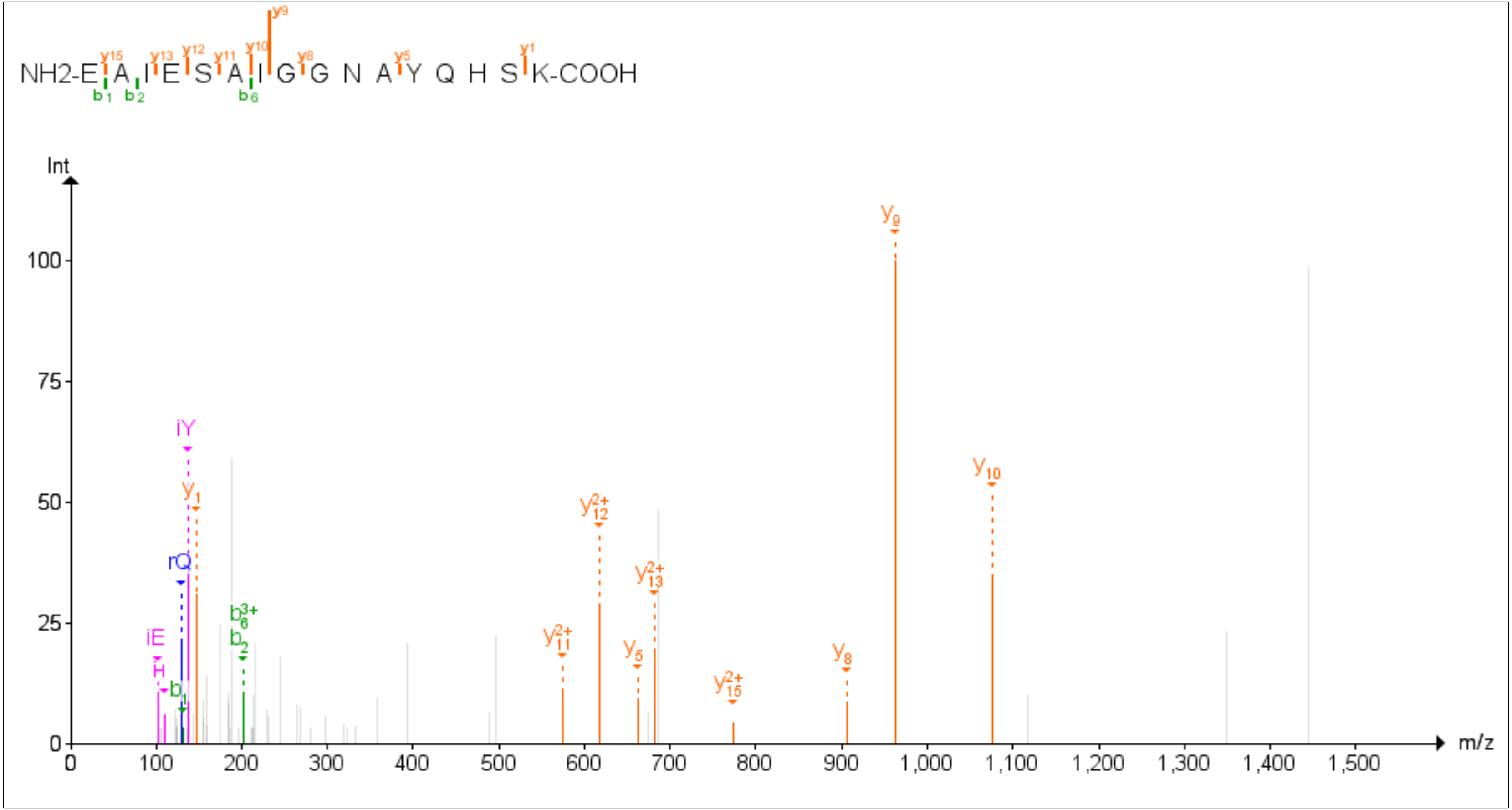

NH2-E A I E S A I G G N A Y Q H S K-COOH
y15 y13 y12 y11 y10 y9 y8 y5 y1
b1 b2 b6
Int
m/z
y9
y10
iY
y1
y_{12}^{2+}
y_{13}^{2+}
rQ
b_6^{3+}
b_2
iE
H
b_1
y_{11}^{2+}
y_5
y_{15}^{2+}
y_8

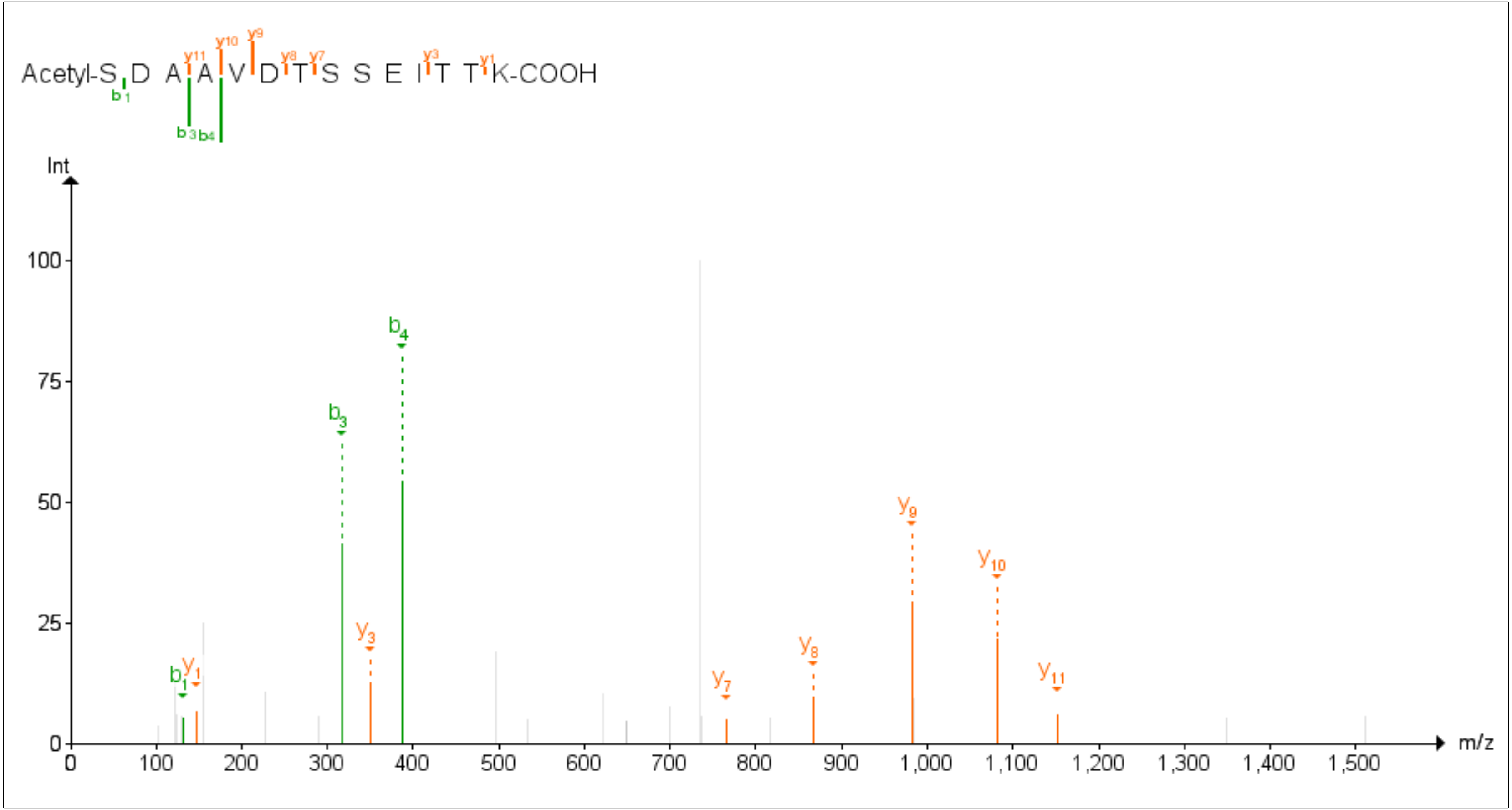

Acetyl-S D A A V D T S S E I T T K-COOH
b1
b3
b4
y11
y10
y9
y8
y7
y3
y1
Int
100
75
50
25
0
b4
b3
y9
y10
y3
b1
y1
y7
y8
y11
0
100
200
300
400
500
600
700
800
900
1,000
1,100
1,200
1,300
1,400
1,500
m/z

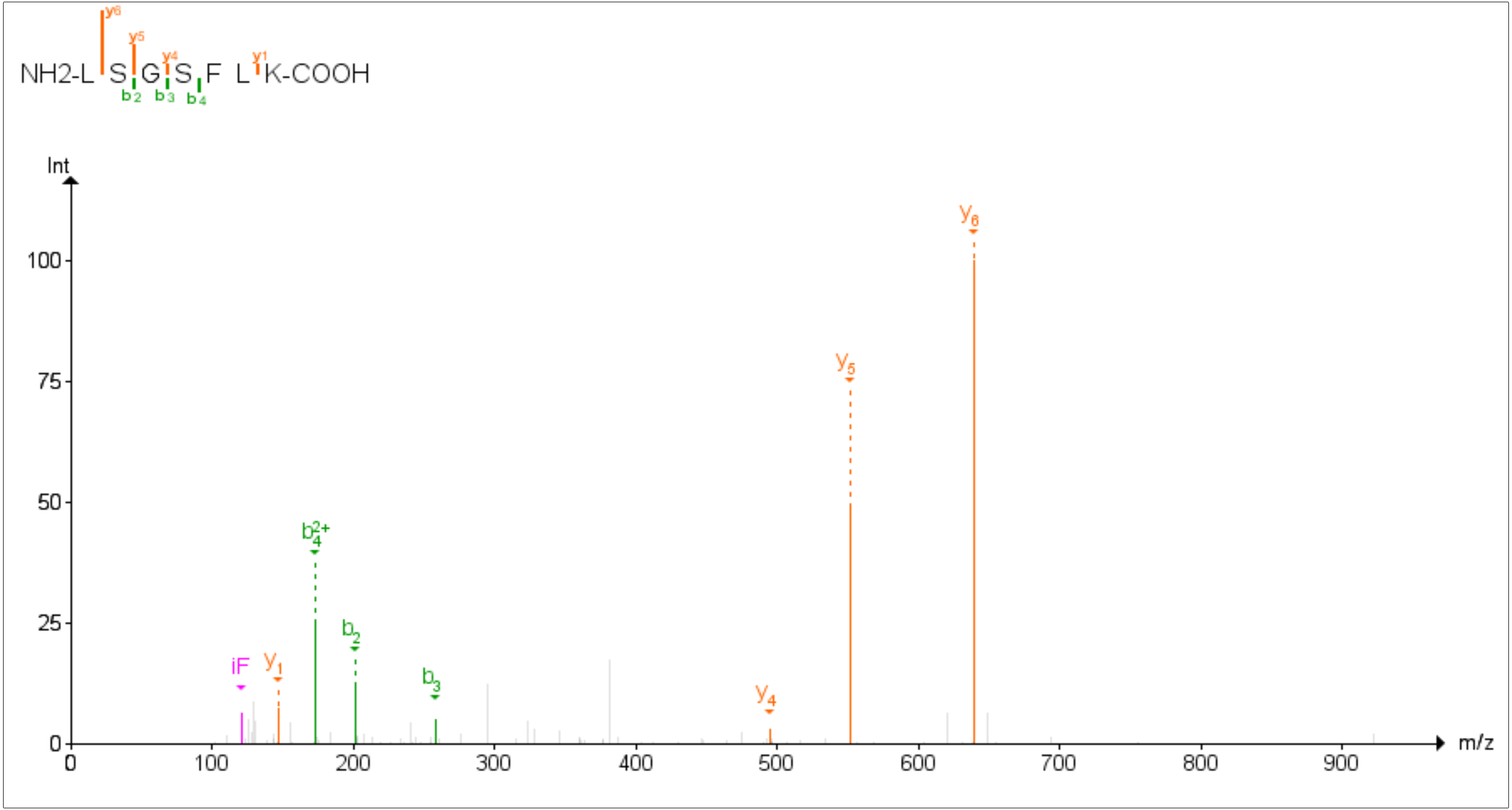
NH2-L S G S F L K-COOH
y6
y5
y4
y1
b2
b3
b4
Int
100
75
50
25
0
y6
y5
y4
y1
b4 2+
b2
b3
iF
0
100
200
300
400
500
600
700
800
900
m/z

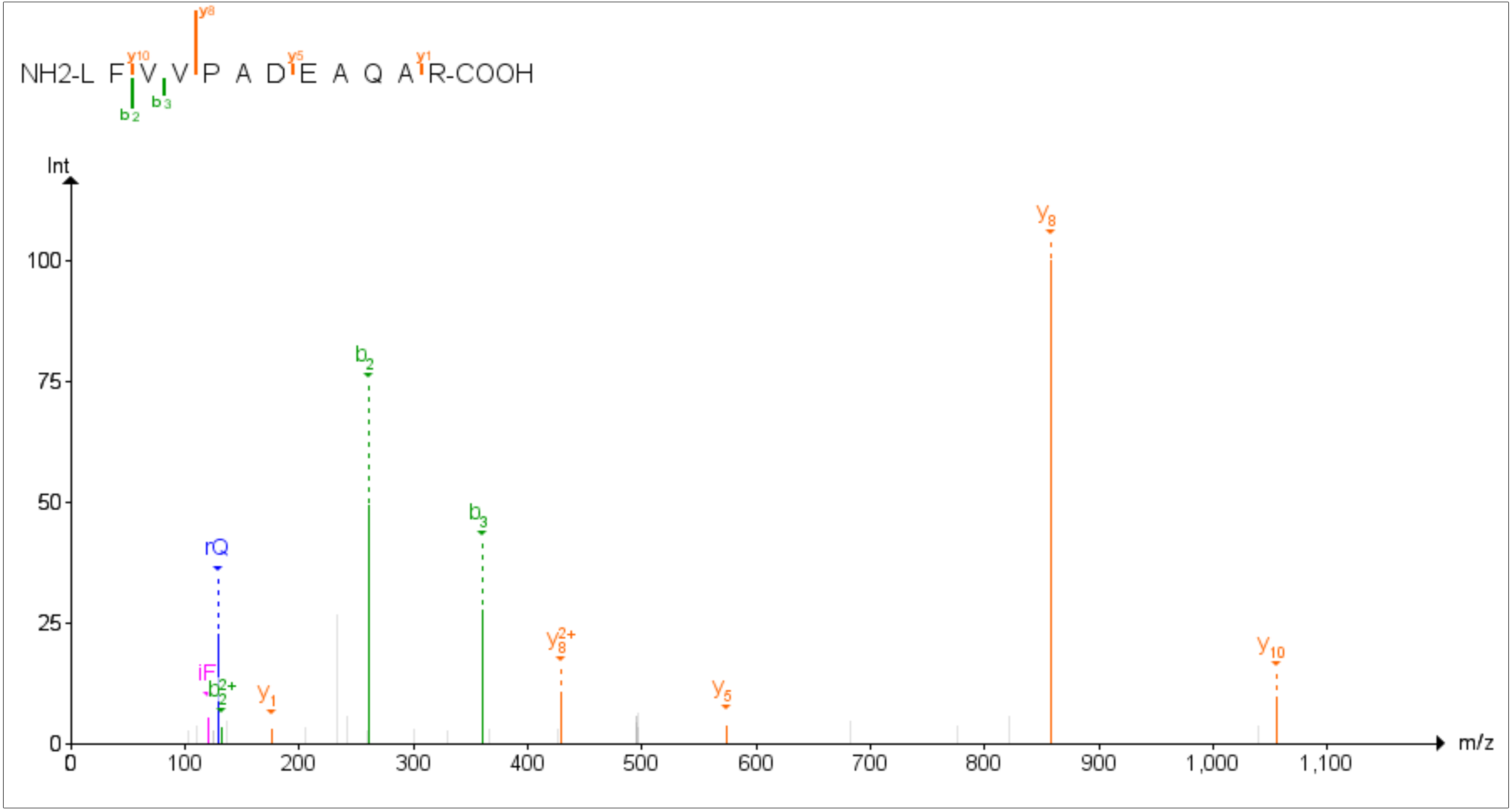

NH2-L F V V P A D E A Q A R-COOH
y8
y10
y5
y1
b 2
b 3
Int
100
75
50
25
0
y8
b2
b3
rQ
iF
b2 2+
y1
y8 2+
y5
y10
0
100
200
300
400
500
600
700
800
900
1,000
1,100
m/z

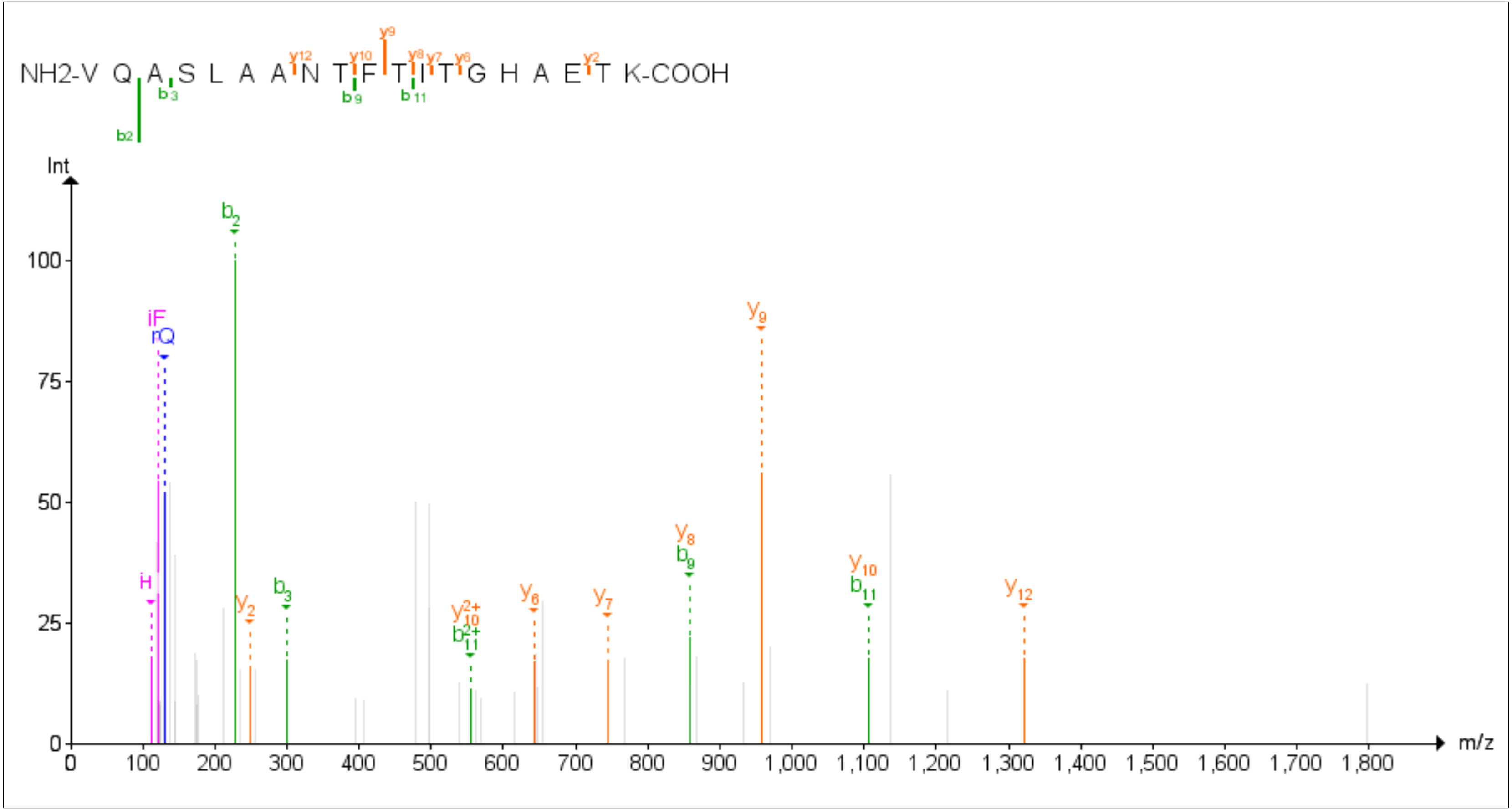
NH2-V Q A S L A A N T F T I T G H A E T K-COOH
b2
b3
b9
b11
y12
y10
y9
y8
y7
y6
y2
Int
m/z
iF
rQ
iH
b2
b3
y2
y9
y8
b9
y10
b11
y12
y6
y7
y10 2+
b11 2+
0
25
50
75
100
0
100
200
300
400
500
600
700
800
900
1,000
1,100
1,200
1,300
1,400
1,500
1,600
1,700
1,800

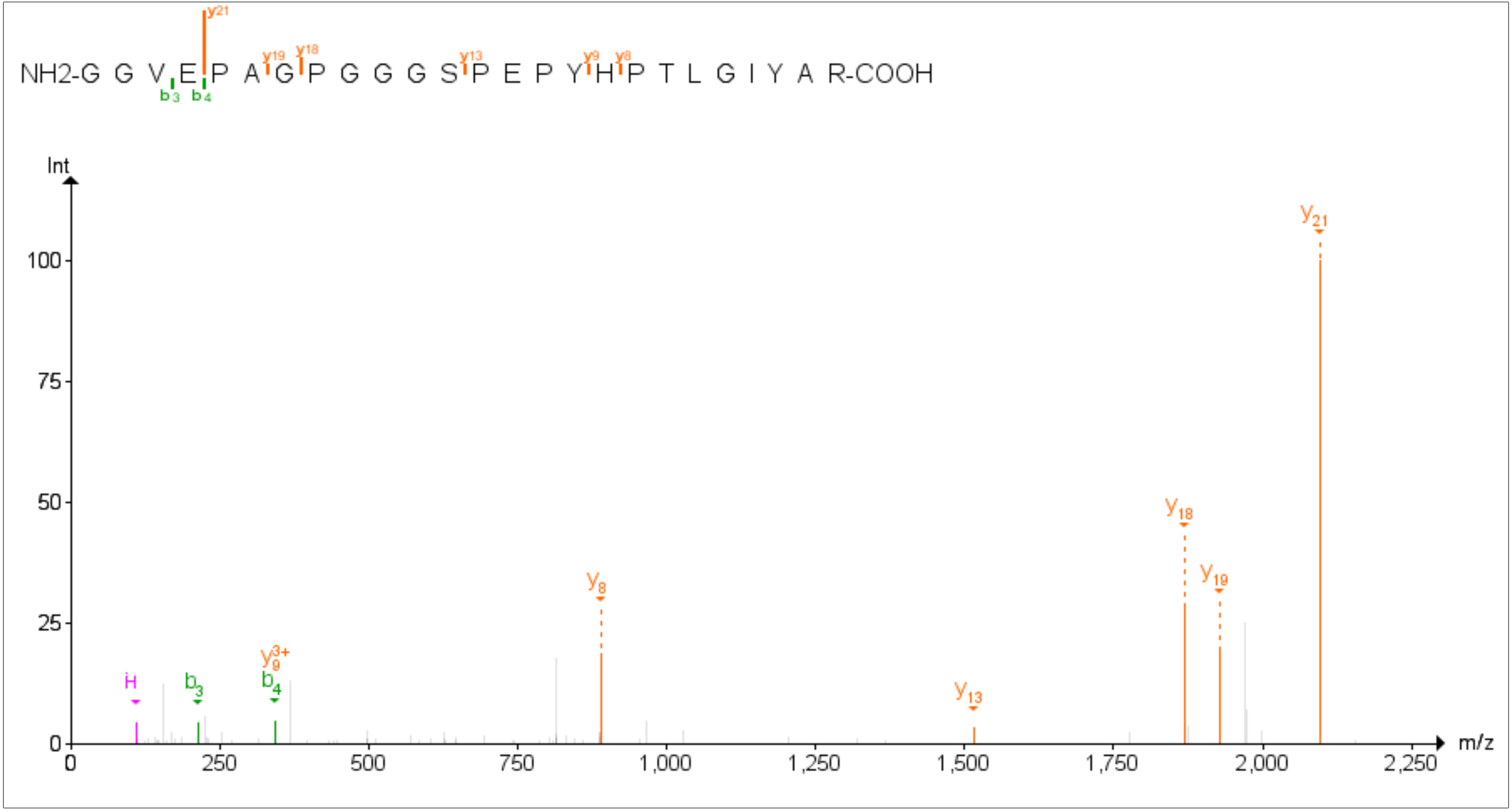
NH2-G G V E P A G P G G G S P E P Y H P T L G I Y A R-COOH
y21
y19
y18
y13
y9
y8
b3
b4
Int
100
75
50
25
0
y21
y18
y19
y8
y13
H
b3
b4
y9 3+
0
250
500
750
1,000
1,250
1,500
1,750
2,000
2,250
m/z

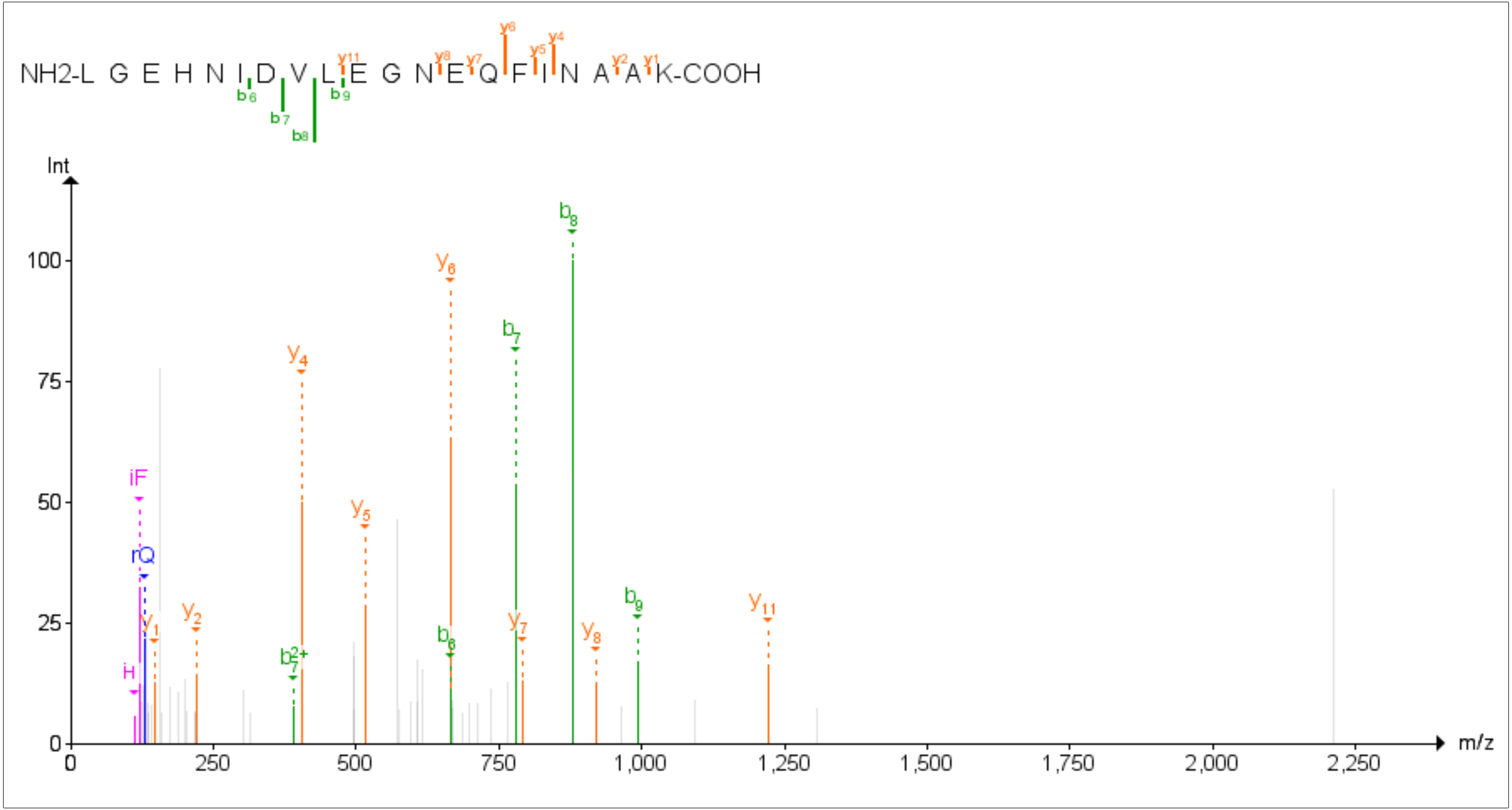

NH2-L G E H N I D V L E G N E Q F I N A A K-COOH
b6 b7 b8 b9
y11 y8 y7 y6 y5 y4 y2 y1
Int
100
75
50
25
0
iF
rQ
iH
y1
y2
b7 2+
y4
y5
b6
y6
b7
y7
b8
y8
b9
y11
0
250
500
750
1,000
1,250
1,500
1,750
2,000
2,250
m/z

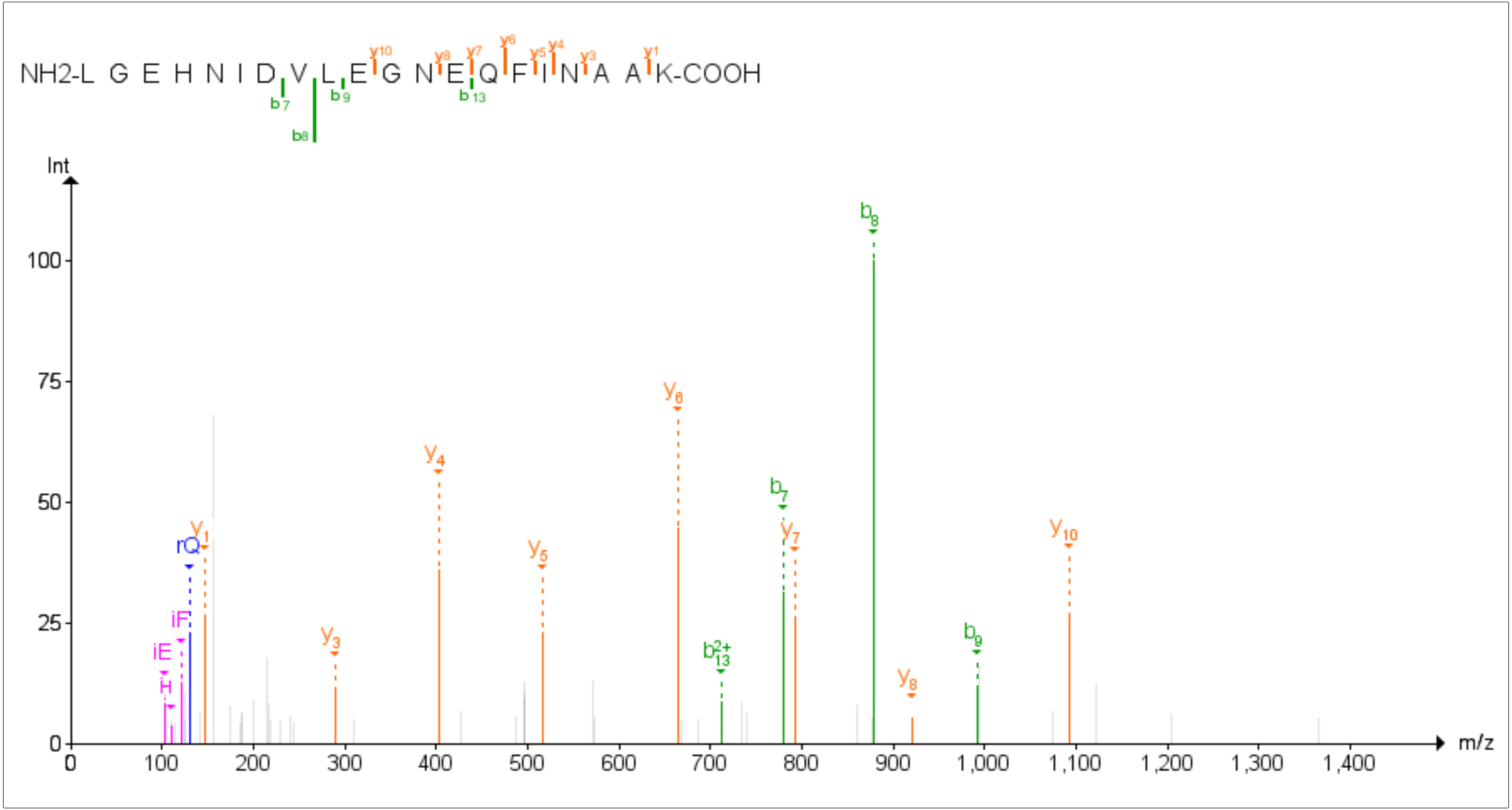

NH2-L G E H N I D V L E G N E Q F I N A A K-COOH
y10
y8
y7
y6
y5
y4
y3
y1
b7
b8
b9
b13
Int
m/z
0
25
50
75
100
0
100
200
300
400
500
600
700
800
900
1,000
1,100
1,200
1,300
1,400
b8
y6
y4
b7
y7
y10
y1
rQ
y5
iF
iE
H
y3
b2+13
b9
y8

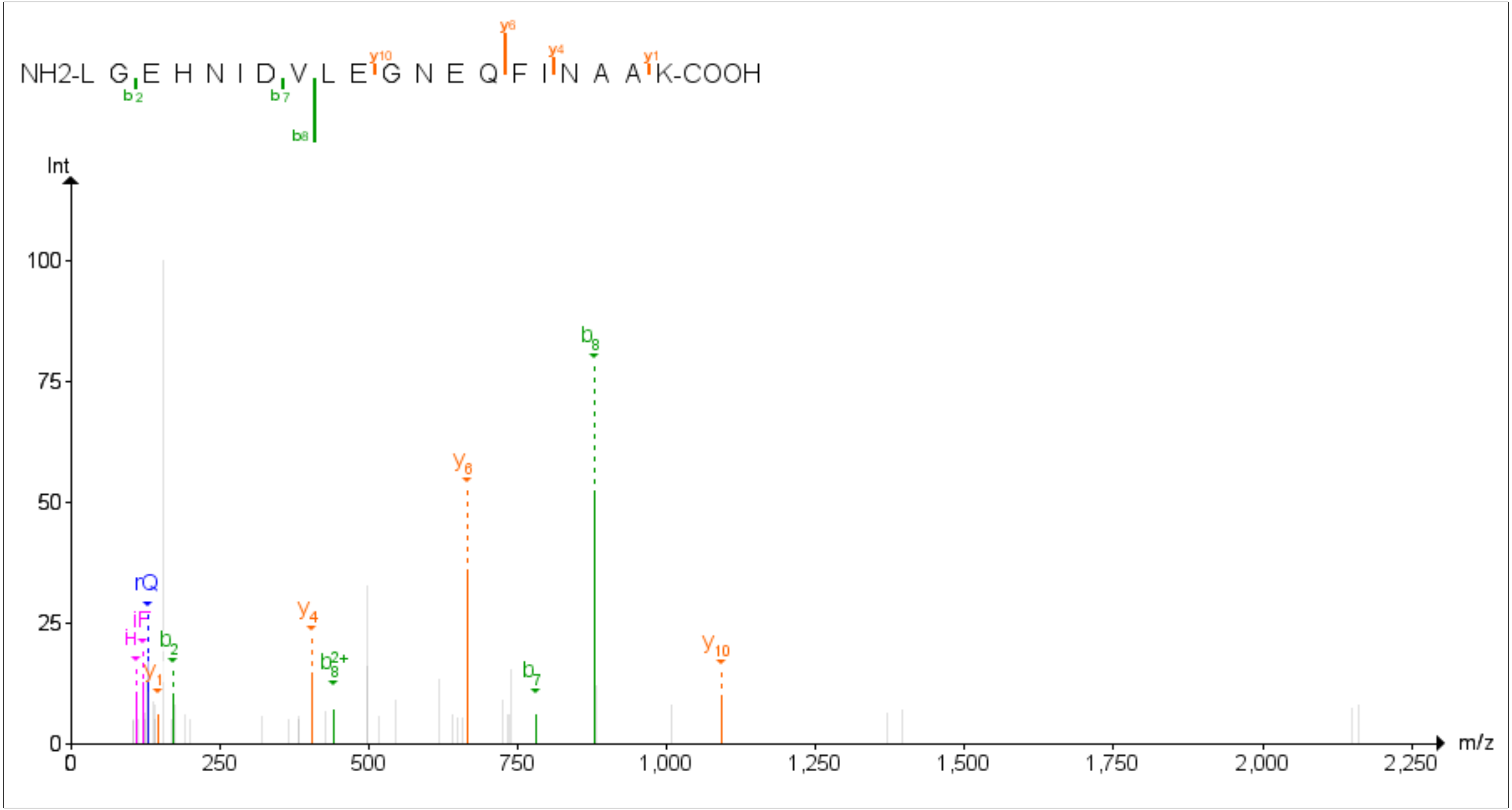
NH2-L G E H N I D V L E G N E Q F I N A A K-COOH
b2
b7
b8
y10
y6
y4
y1
Int
100
75
50
25
0
rQ
iF
iH
y1
b2
y4
b8 2+
y6
b7
b8
y10
0
250
500
750
1,000
1,250
1,500
1,750
2,000
2,250
m/z

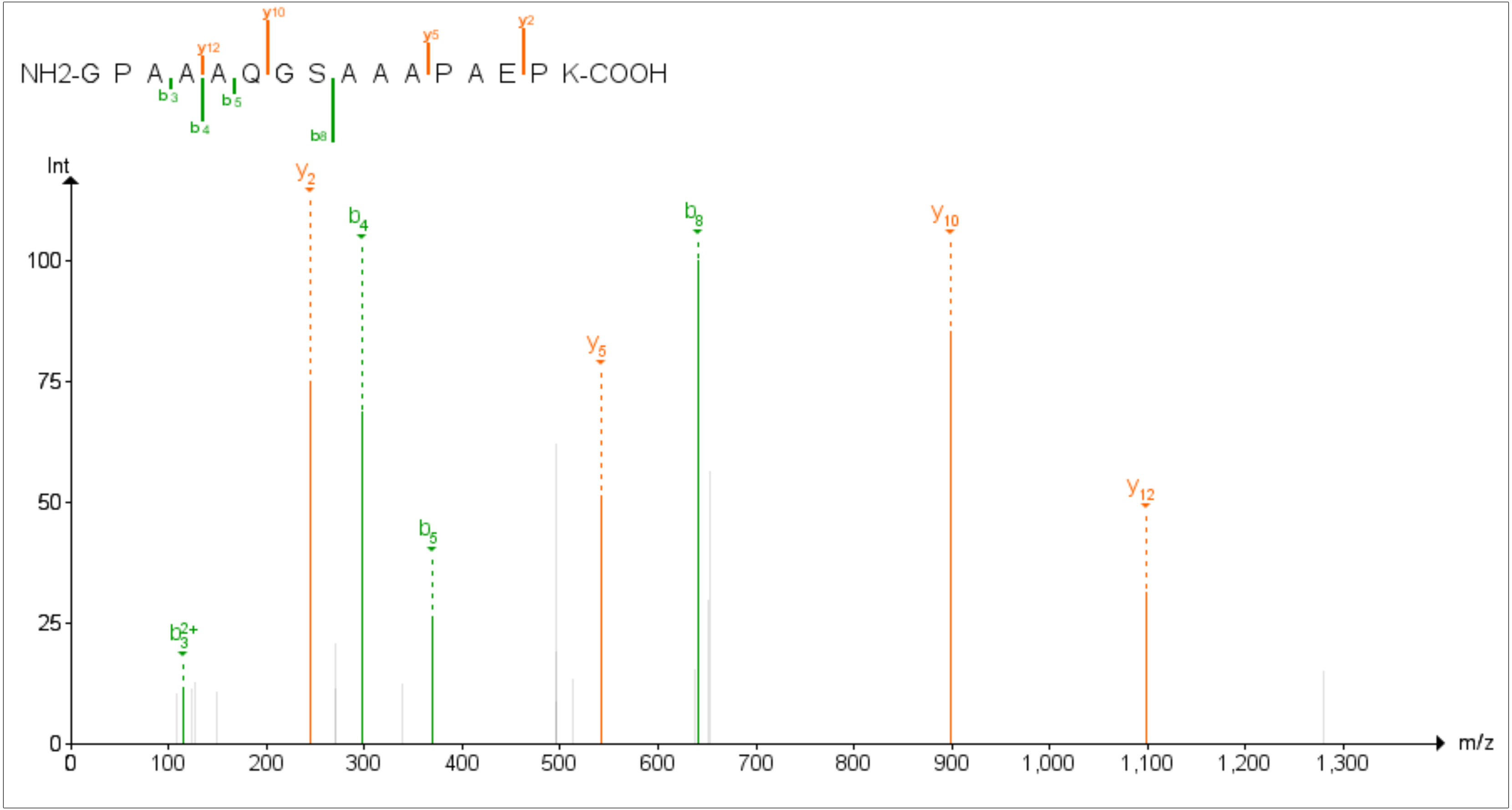

NH2-G P A A A Q G S A A A P A E P K-COOH
y10
y12
y5
y2
b3
b4
b5
b8
Int
m/z
y2
b4
b8
y10
y5
b5
y12
b3 2+
0
25
50
75
100
0
100
200
300
400
500
600
700
800
900
1,000
1,100
1,200
1,300

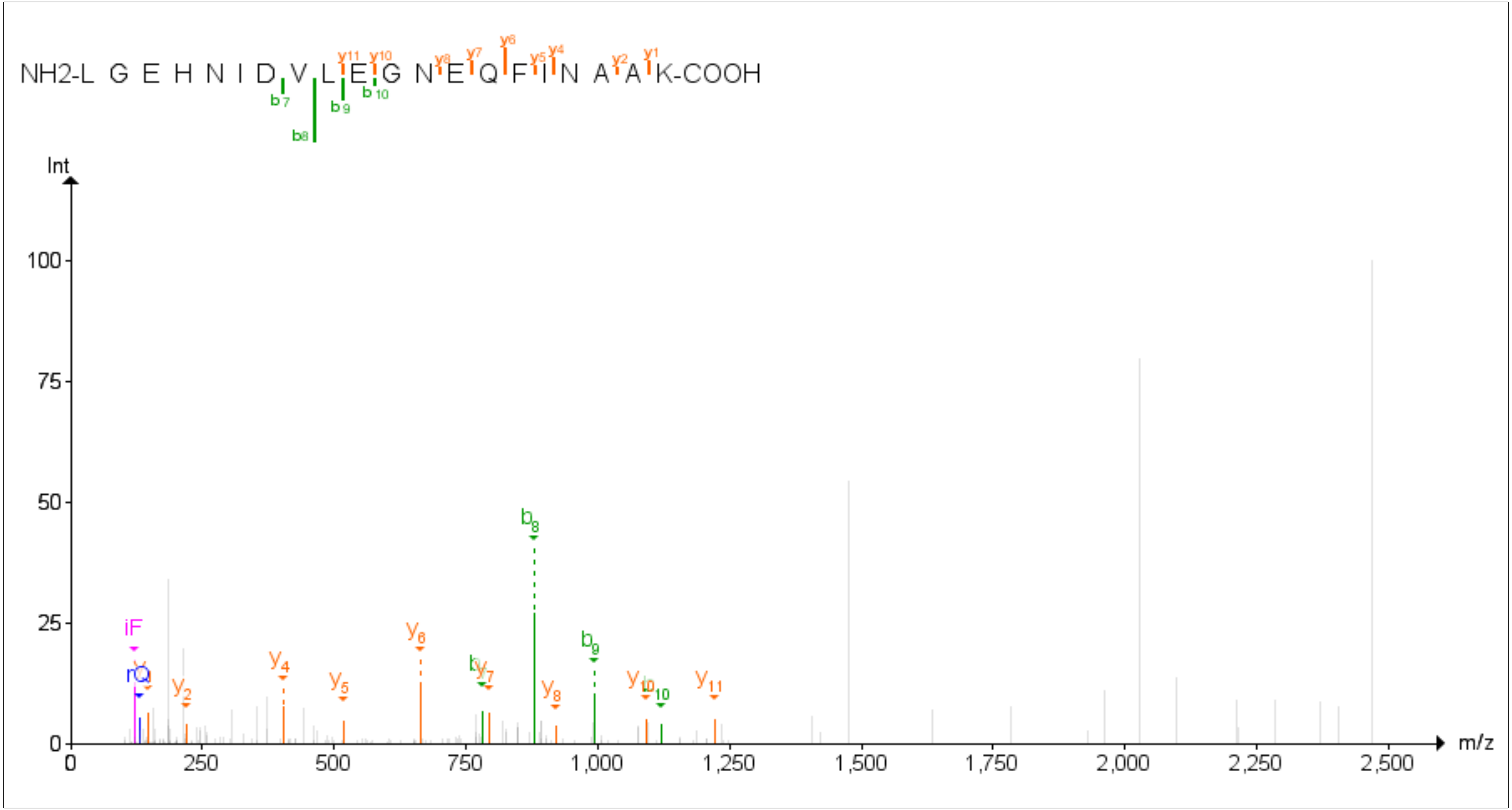

NH2-L G E H N I D V L E G N E Q F I N A A K-COOH
b7
b8
b9
b10
y11
y10
y8
y7
y6
y5
y4
y2
y1
Int
100
75
50
25
0
iF
rQ
y2
y4
y5
y6
y7
b8
y8
b9
y10
b10
y11
0
250
500
750
1,000
1,250
1,500
1,750
2,000
2,250
2,500
m/z

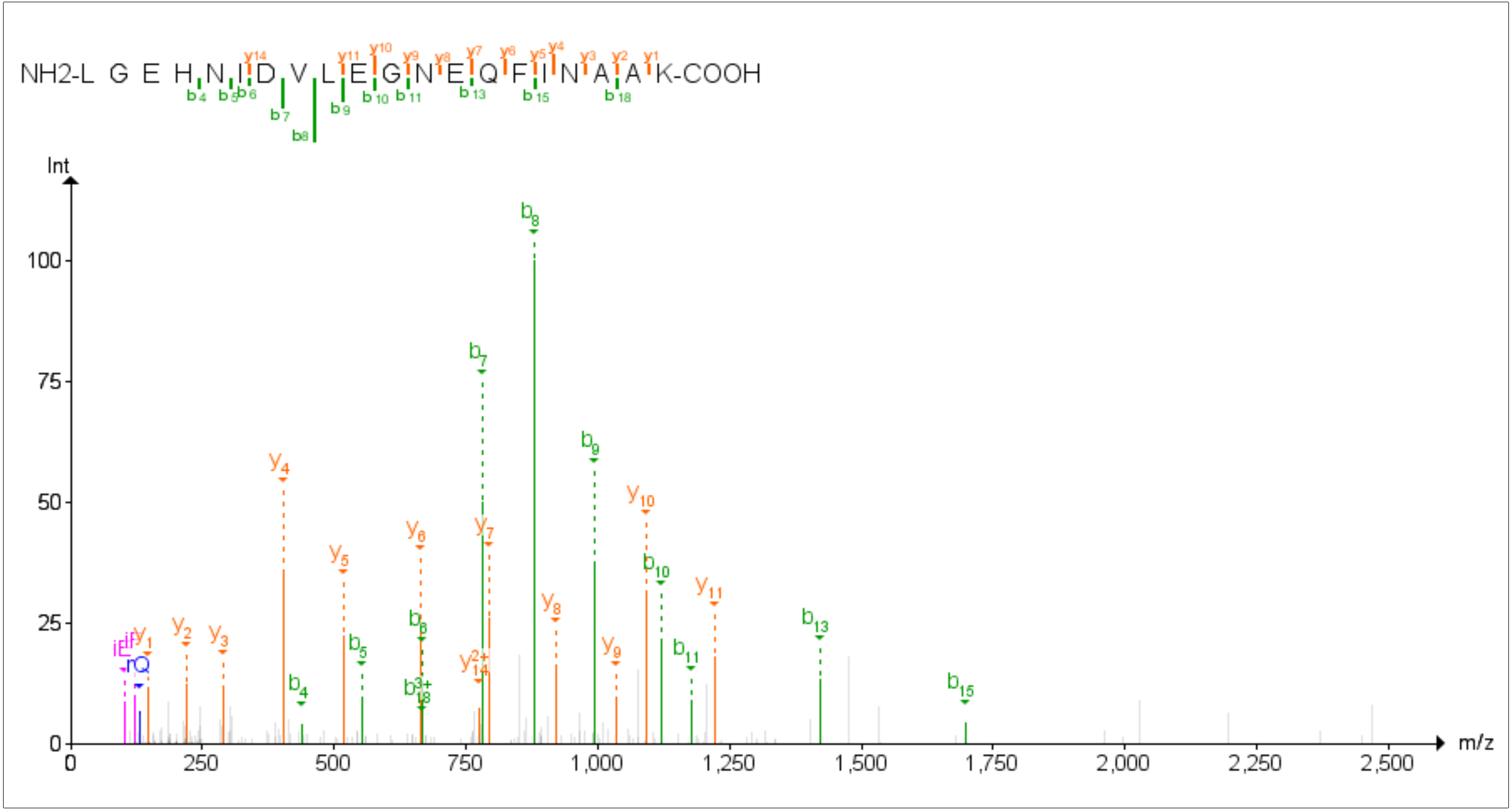
NH2-L G E H N I D V L E G N E Q F I N A A K-COOH
Int
m/z
0
25
50
75
100
250
500
750
1,000
1,250
1,500
1,750
2,000
2,250
2,500

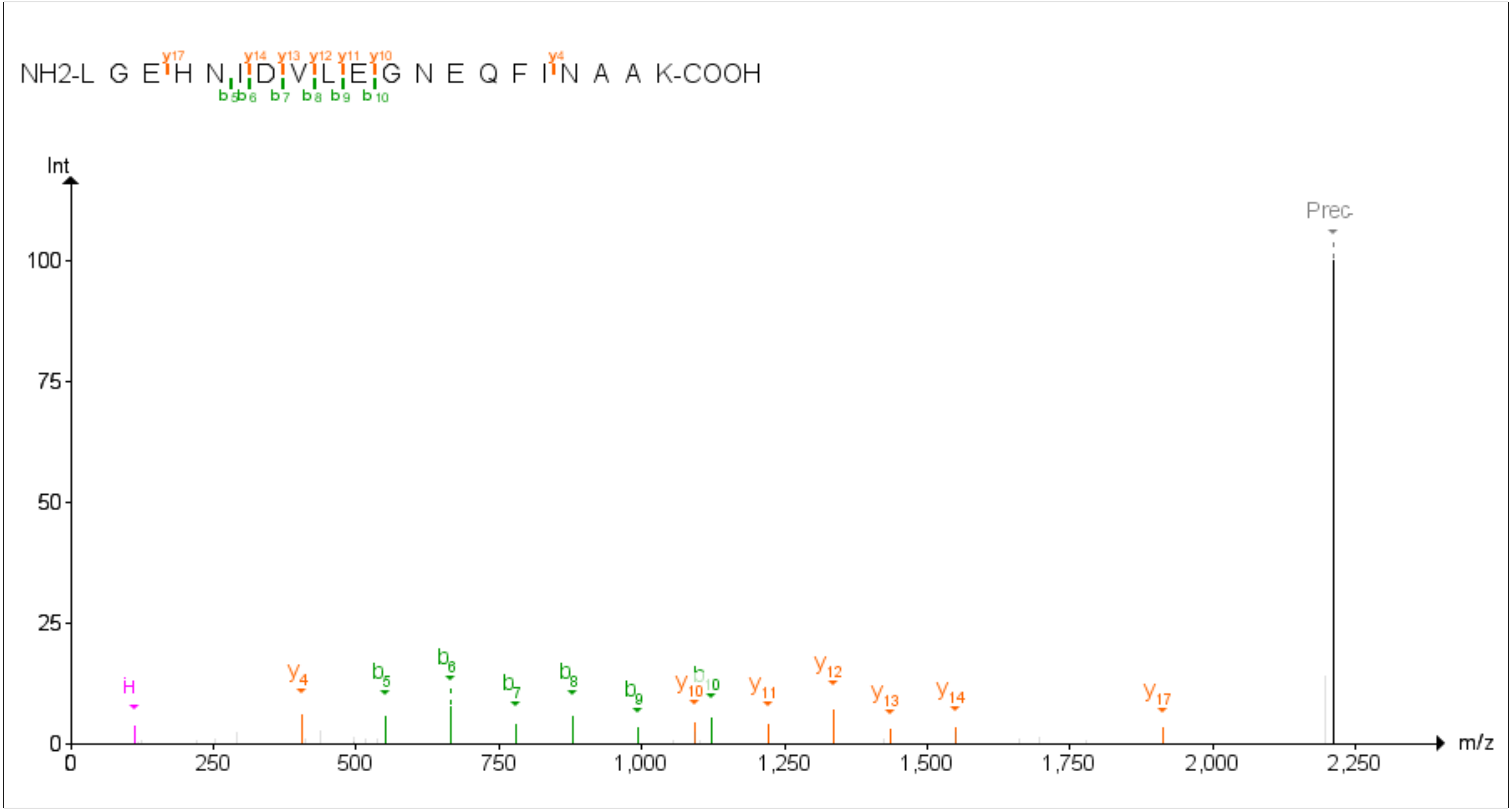
NH2-L G E H N I D V L E G N E Q F I N A A K-COOH
y17 y14 y13 y12 y11 y10 y4
b5 b6 b7 b8 b9 b10
Int
100
75
50
25
0
Prec
H
y4
b5
b6
b7
b8
b9
y10
b10
y11
y12
y13
y14
y17
0
250
500
750
1,000
1,250
1,500
1,750
2,000
2,250
m/z

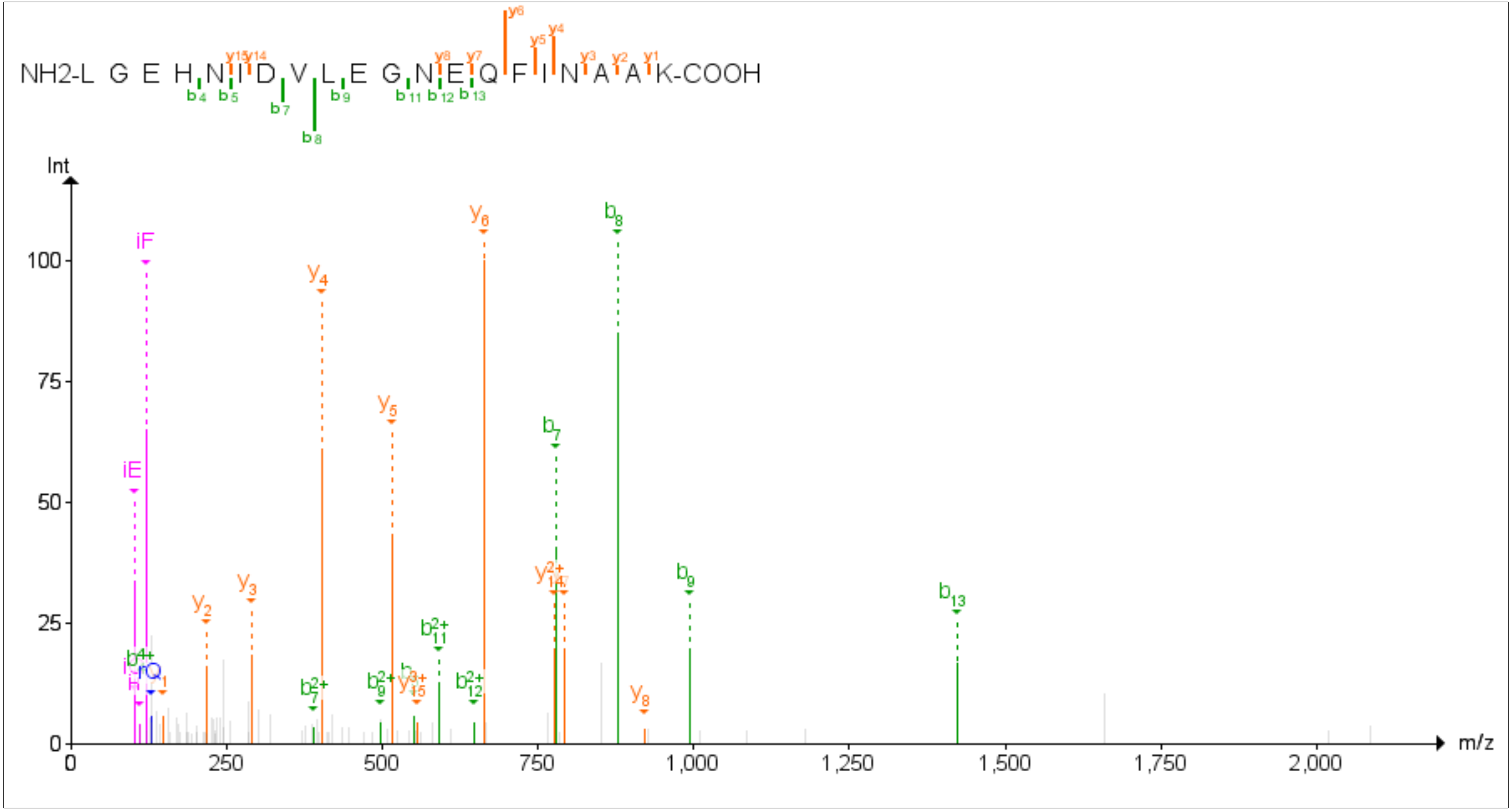
NH2-L G E H N I D V L E G N E Q F I N A A K-COOH
Int
100
75
50
25
0
m/z
0
250
500
750
1,000
1,250
1,500
1,750
2,000
iF
iE
y4
y5
y6
b8
b7
y3
y2
b9
b13
y8

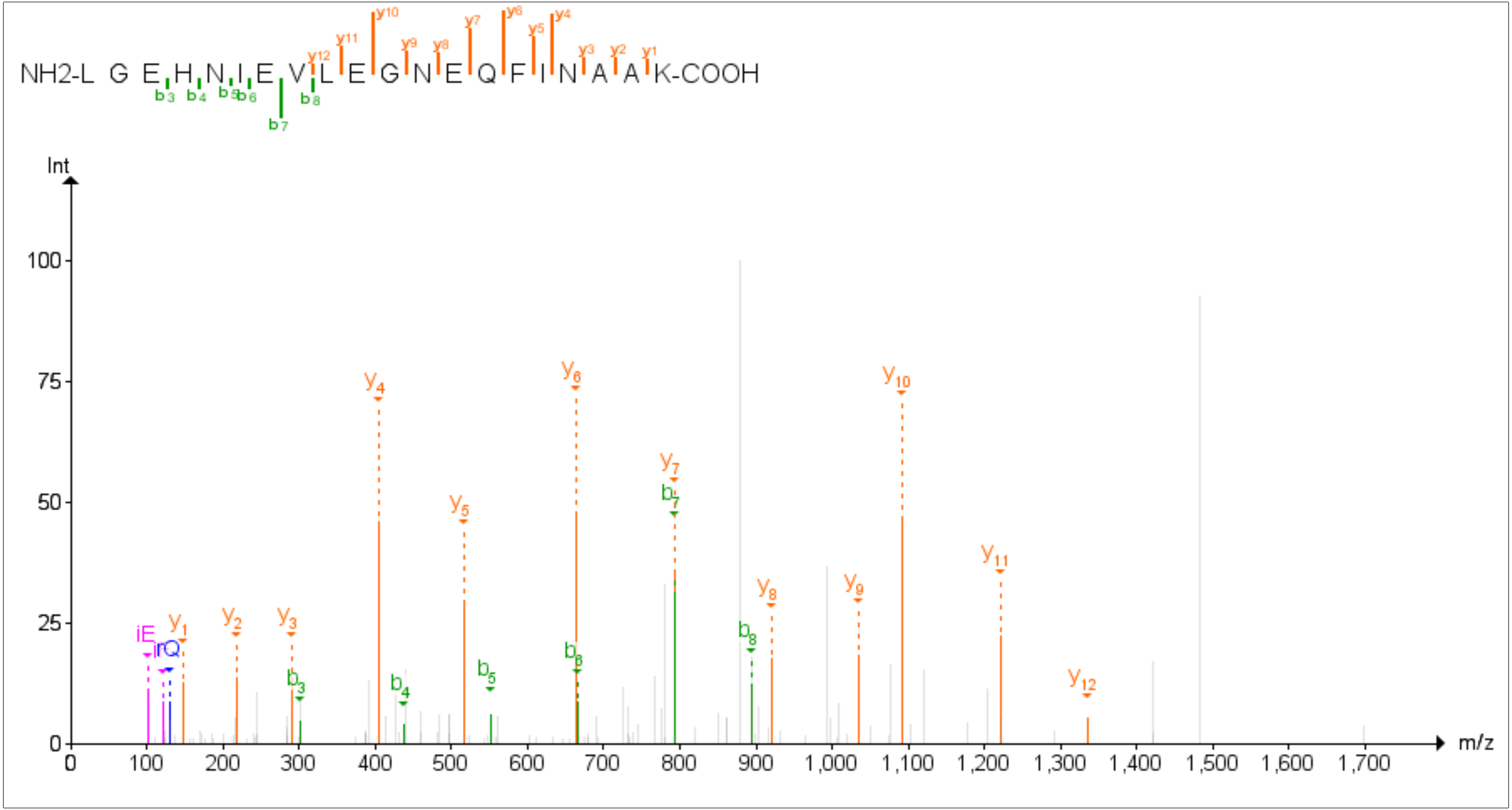

NH2-L G E H N I E V L E G N E Q F I N A A K-COOH
b3 b4 b5 b6 b7 b8
y12 y11 y10 y9 y8 y7 y6 y5 y4 y3 y2 y1
Int
100
75
50
25
0
m/z
0 100 200 300 400 500 600 700 800 900 1,000 1,100 1,200 1,300 1,400 1,500 1,600 1,700
iE
iQ

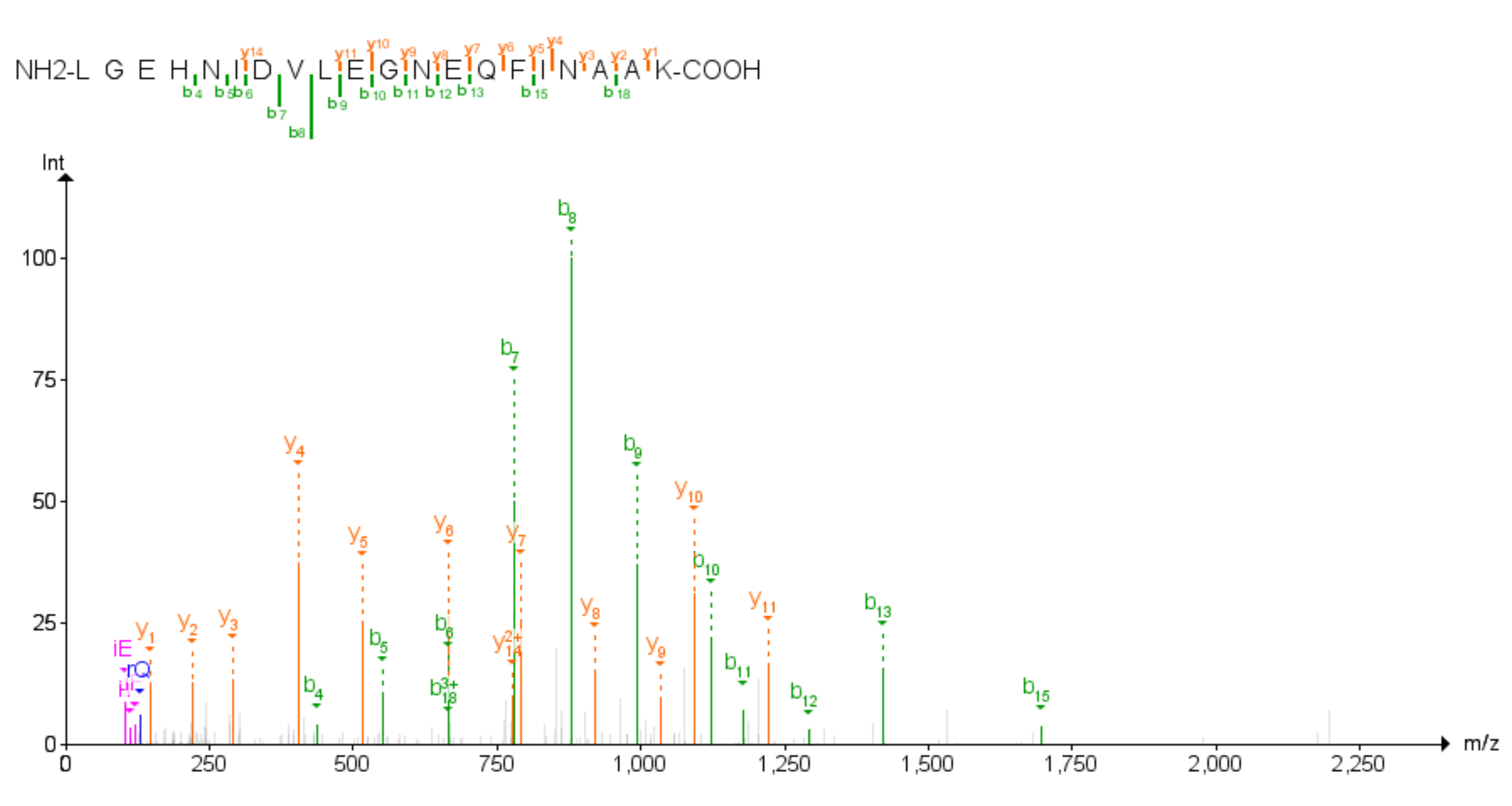

NH2-L G E H N I D V L E G N E Q F I N A A K-COOH
y14 y11 y10 y9 y8 y7 y6 y5 y4 y3 y2 y1
b4 b5 b6 b7 b8 b9 b10 b11 b12 b13 b15 b18
Int
m/z
0
25
50
75
100
250
500
750
1,000
1,250
1,500
1,750
2,000
2,250
b8
b7
y4
b9
y10
y5
y6
y7
b10
y8
y11
b13
y1
y2
y3
iE
b4
b5
b6
b3+ 18
y2+ 14
y9
b11
b12
b15

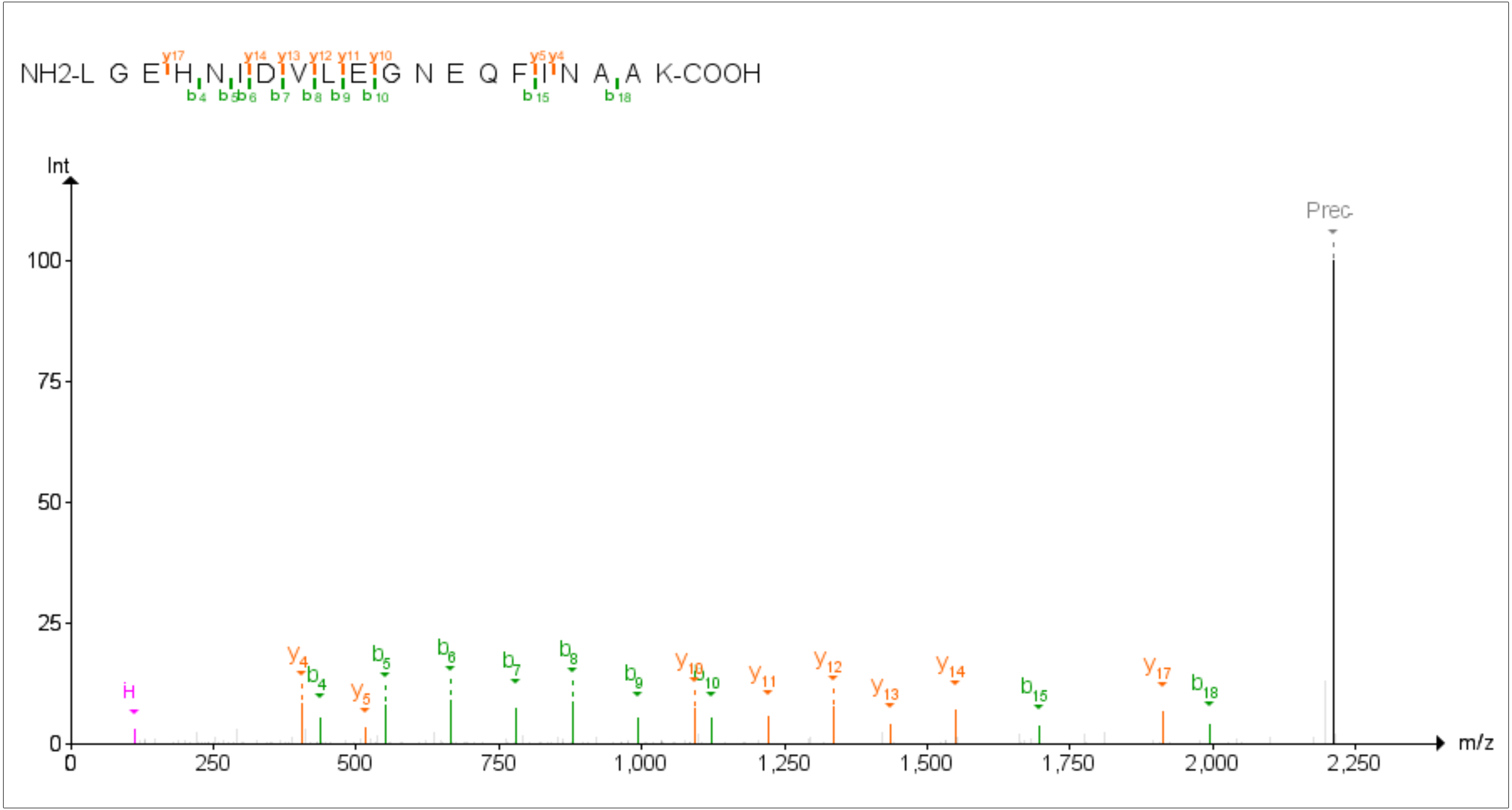

NH2-L G E H N I D V L E G N E Q F I N A A K-COOH
Int
Prec
m/z

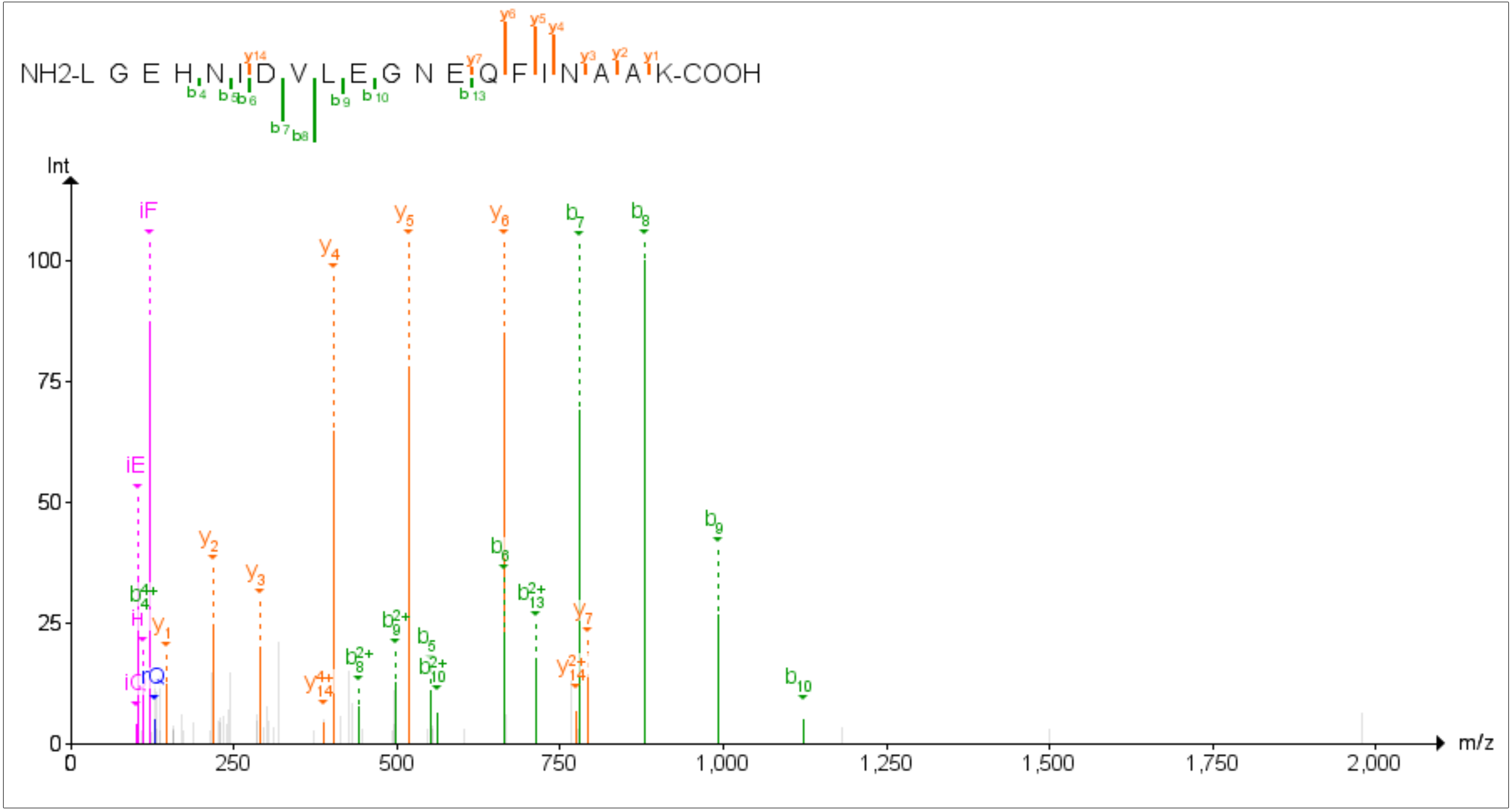

NH2-L G E H N I D V L E G N E Q F I N A A K-COOH
Int
m/z
0
25
50
75
100
250
500
750
1,000
1,250
1,500
1,750
2,000

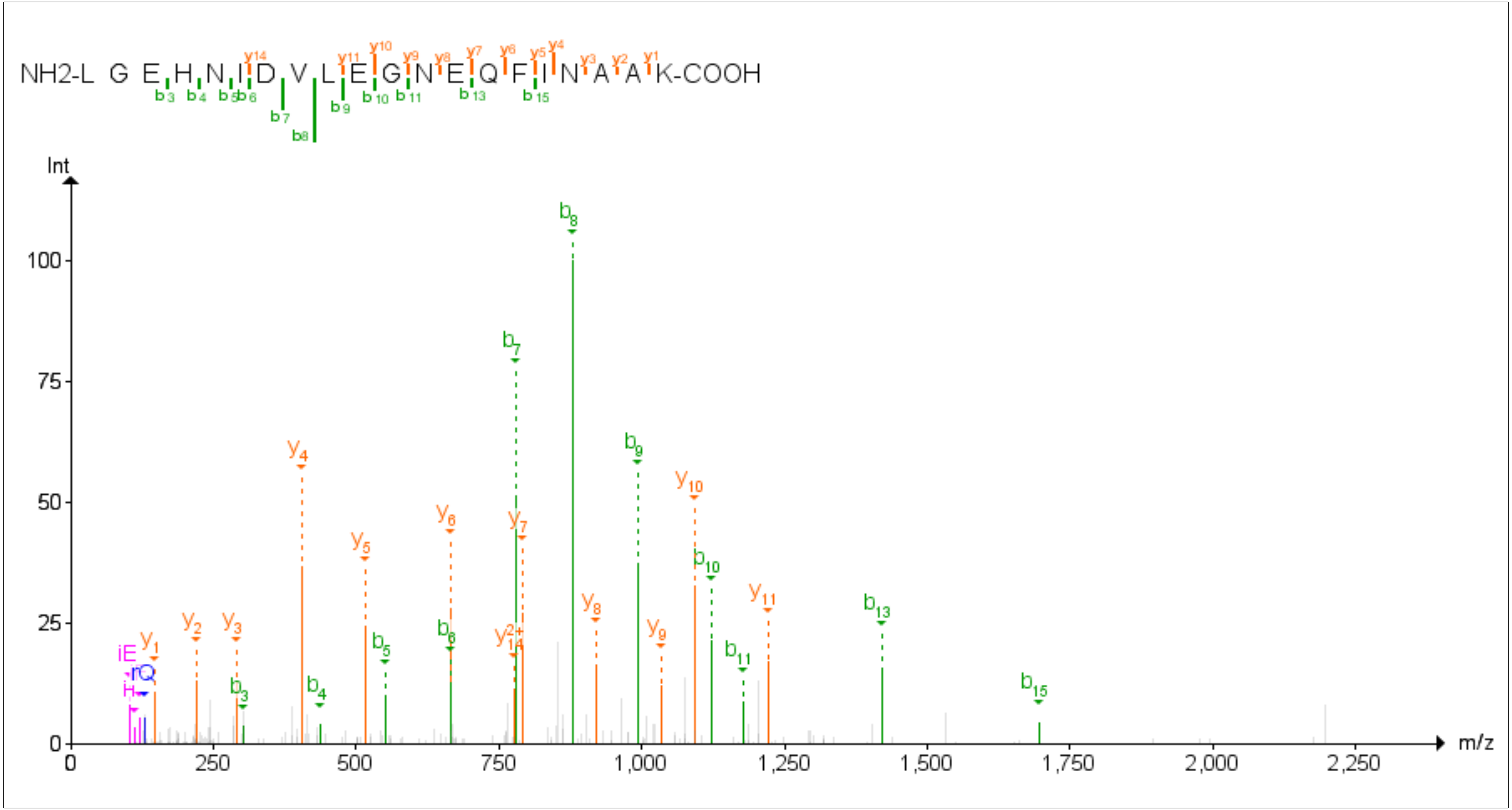

NH2-L G E H N I D V L E G N E Q F I N A A K-COOH
Int
m/z
0
25
50
75
100
250
500
750
1,000
1,250
1,500
1,750
2,000
2,250

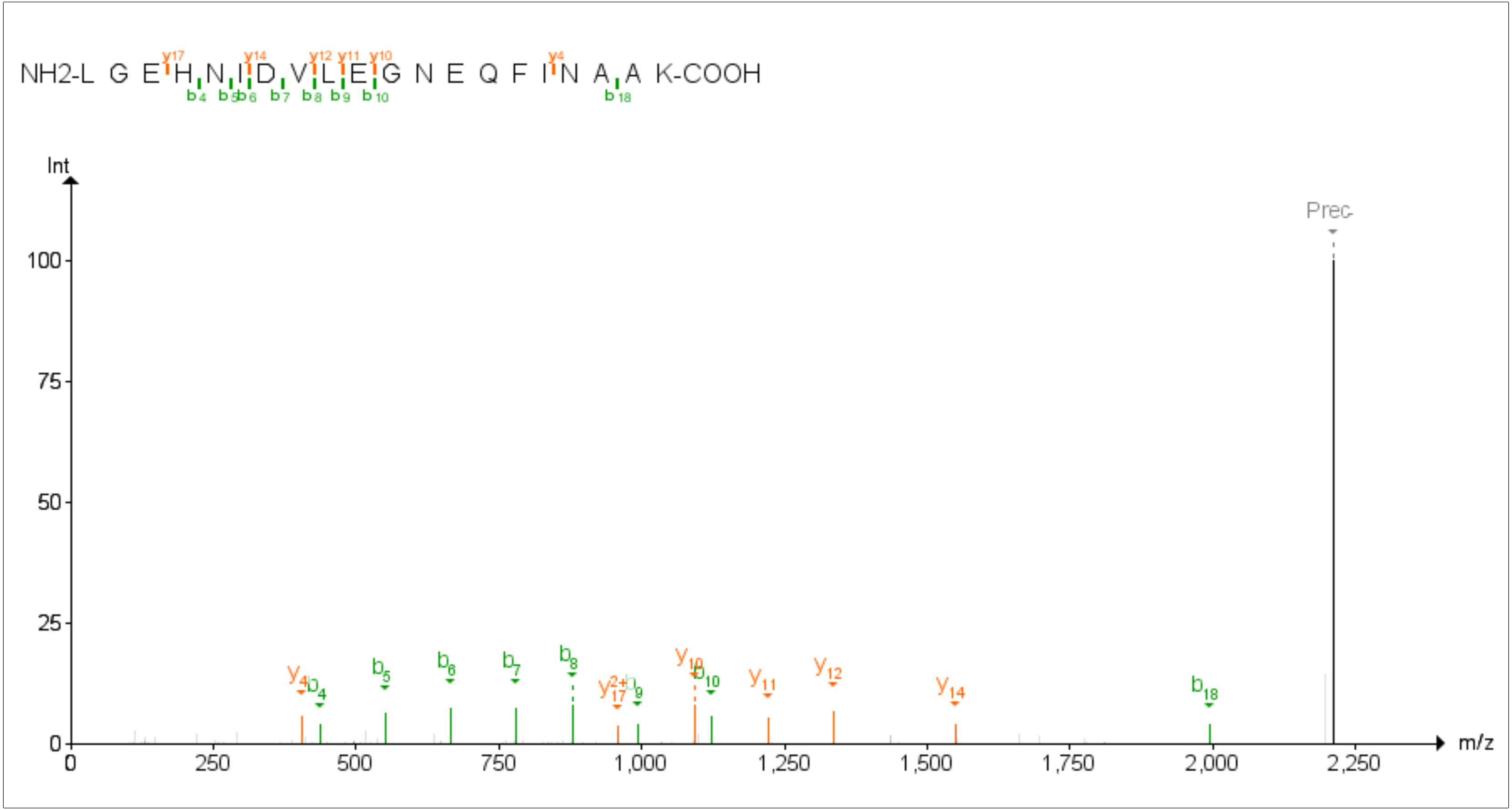
NH2-L G E H N I D V L E G N E Q F I N A A K-COOH
y17 y14 y12 y11 y10 y4
b4 b5 b6 b7 b8 b9 b10 b18
Int
Prec
100
75
50
25
0
0
250
500
750
1,000
1,250
1,500
1,750
2,000
2,250
m/z

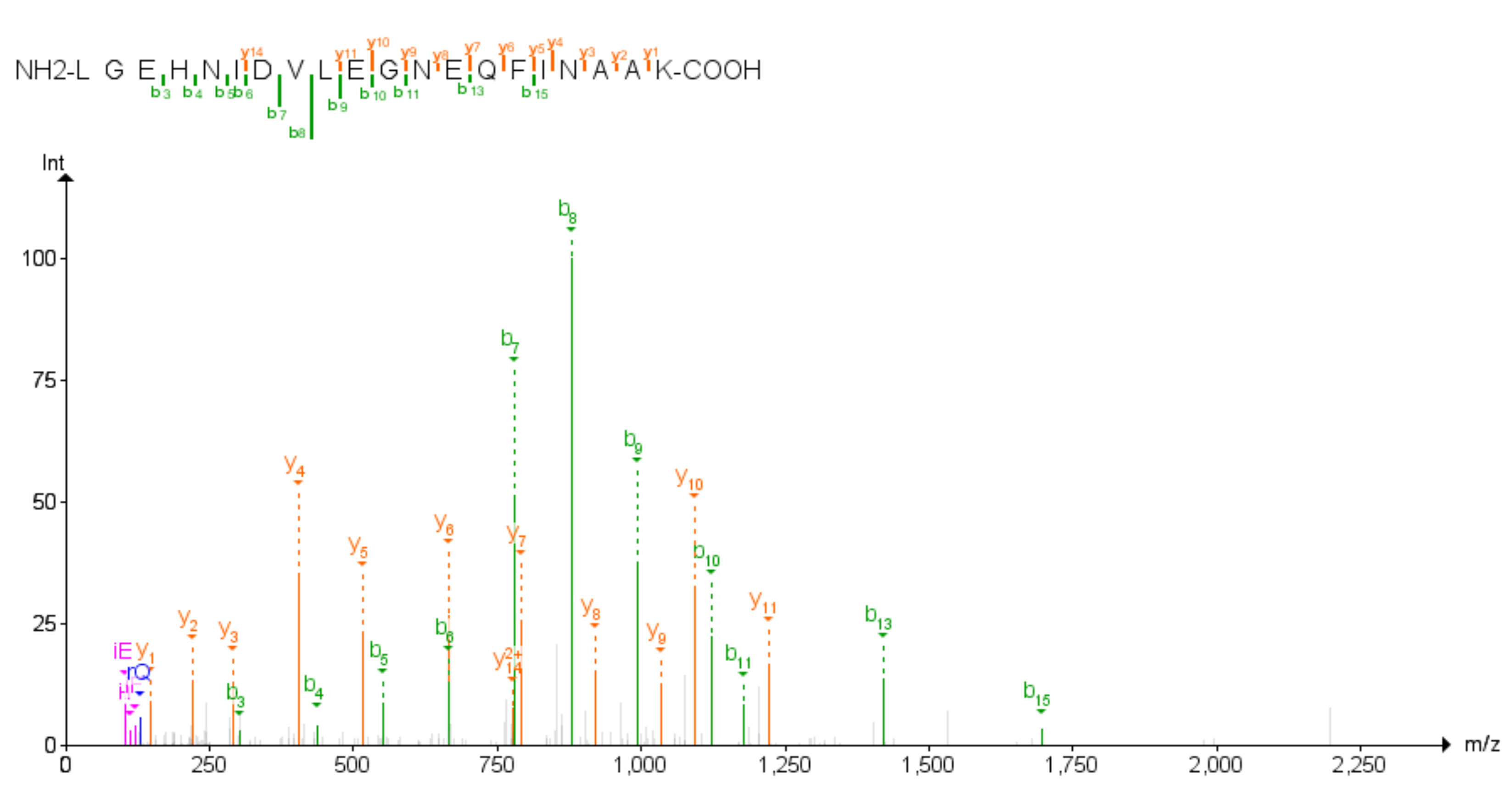

NH2-L G E H N I D V L E G N E Q F I N A A K-COOH
Int
m/z
0
25
50
75
100
250
500
750
1,000
1,250
1,500
1,750
2,000
2,250

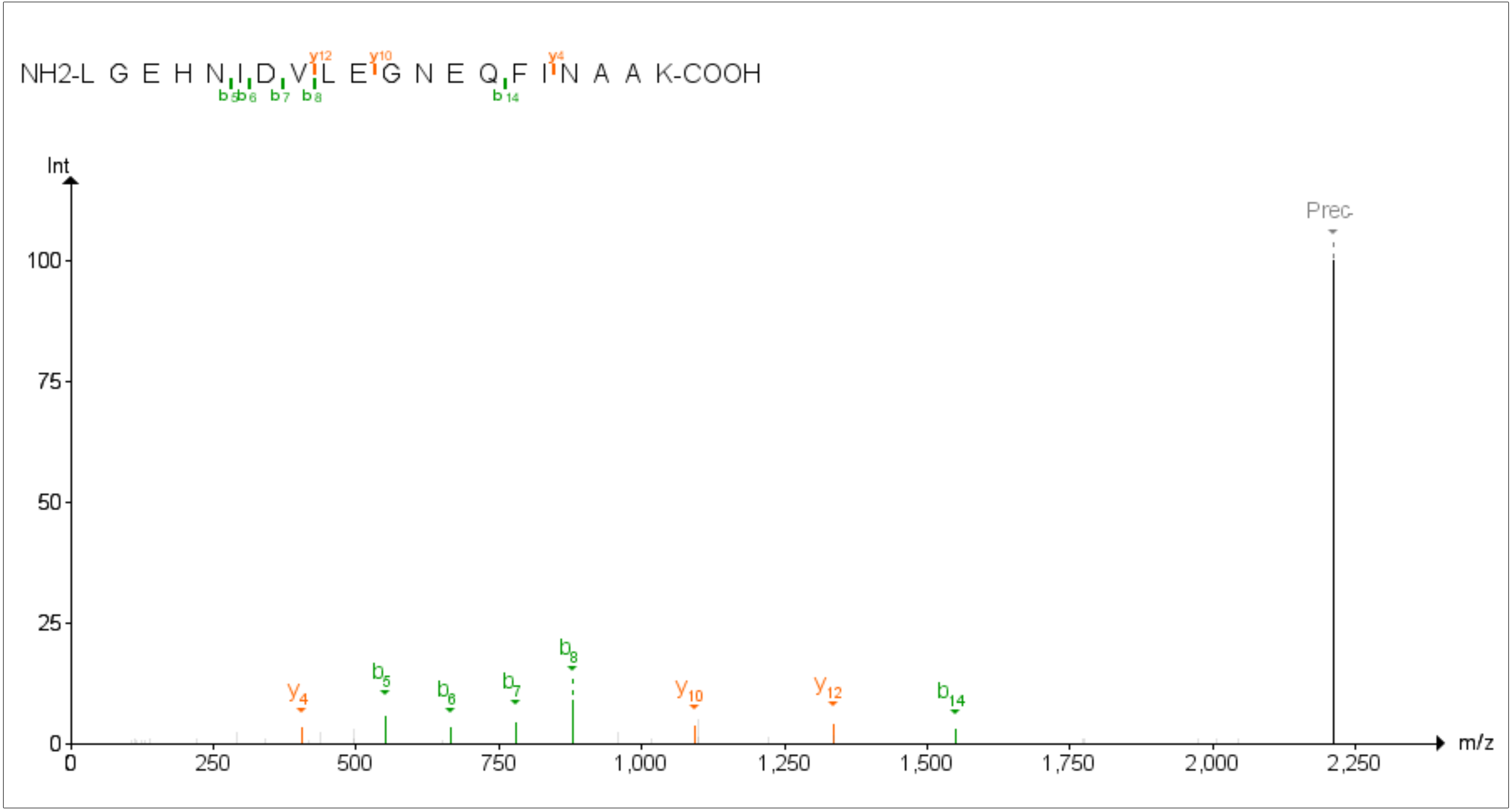

NH2-L G E H N I D V I L E G N E Q F I N A A K-COOH
y12
y10
y4
b5
b6
b7
b8
b14
Int
Prec
100
75
50
25
0
y4
b5
b6
b7
b8
y10
y12
b14
0
250
500
750
1,000
1,250
1,500
1,750
2,000
2,250
m/z

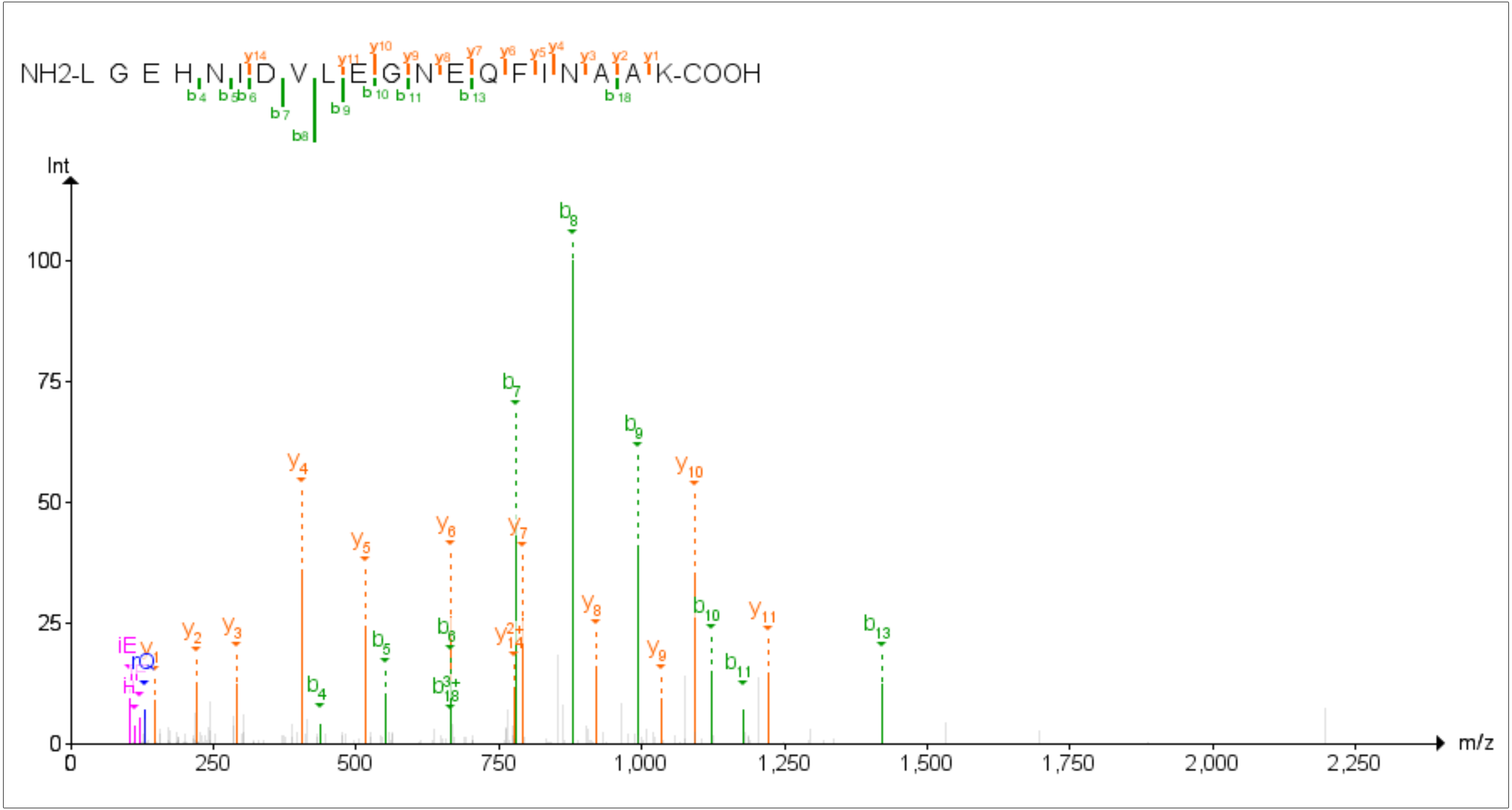
NH2-L G E H N I D V L E G N E Q F I N A A K-COOH
Int
m/z
0
25
50
75
100
250
500
750
1,000
1,250
1,500
1,750
2,000
2,250

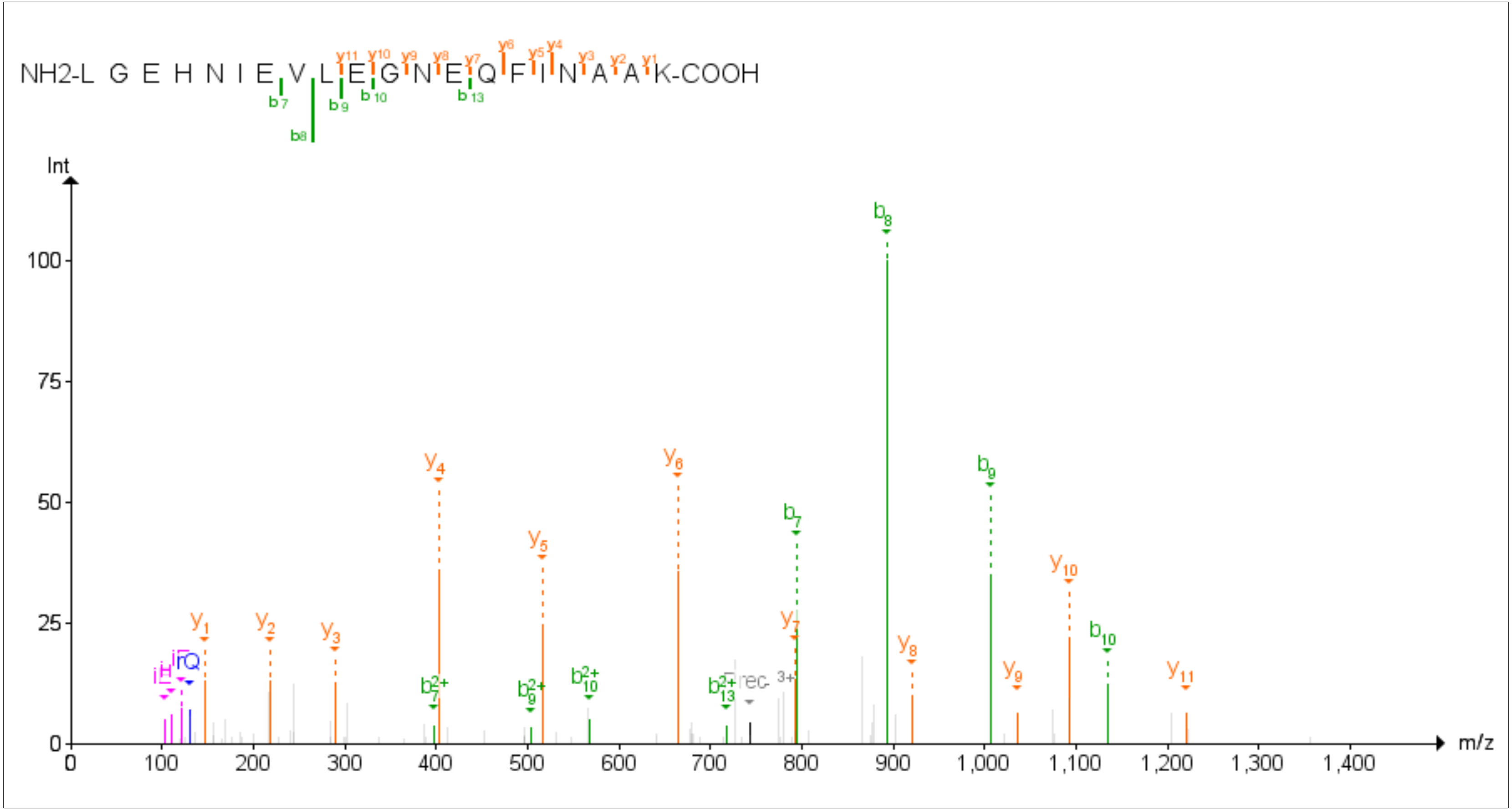

NH2-L G E H N I E V L E G N E Q F I N A A K-COOH
y11 y10 y9 y8 y7 y6 y5 y4 y3 y2 y1
b 7 b8 b 9 b 10 b 13
Int
100
75
50
25
0
m/z
0 100 200 300 400 500 600 700 800 900 1,000 1,100 1,200 1,300 1,400
b8
y4
y6
b9
b7
y5
y10
y1
y2
y3
y7
y8
b10
y9
y11
iQ
iH
b2+ 7
b2+ 9
b2+ 10
b2+ 13
Prec 3+

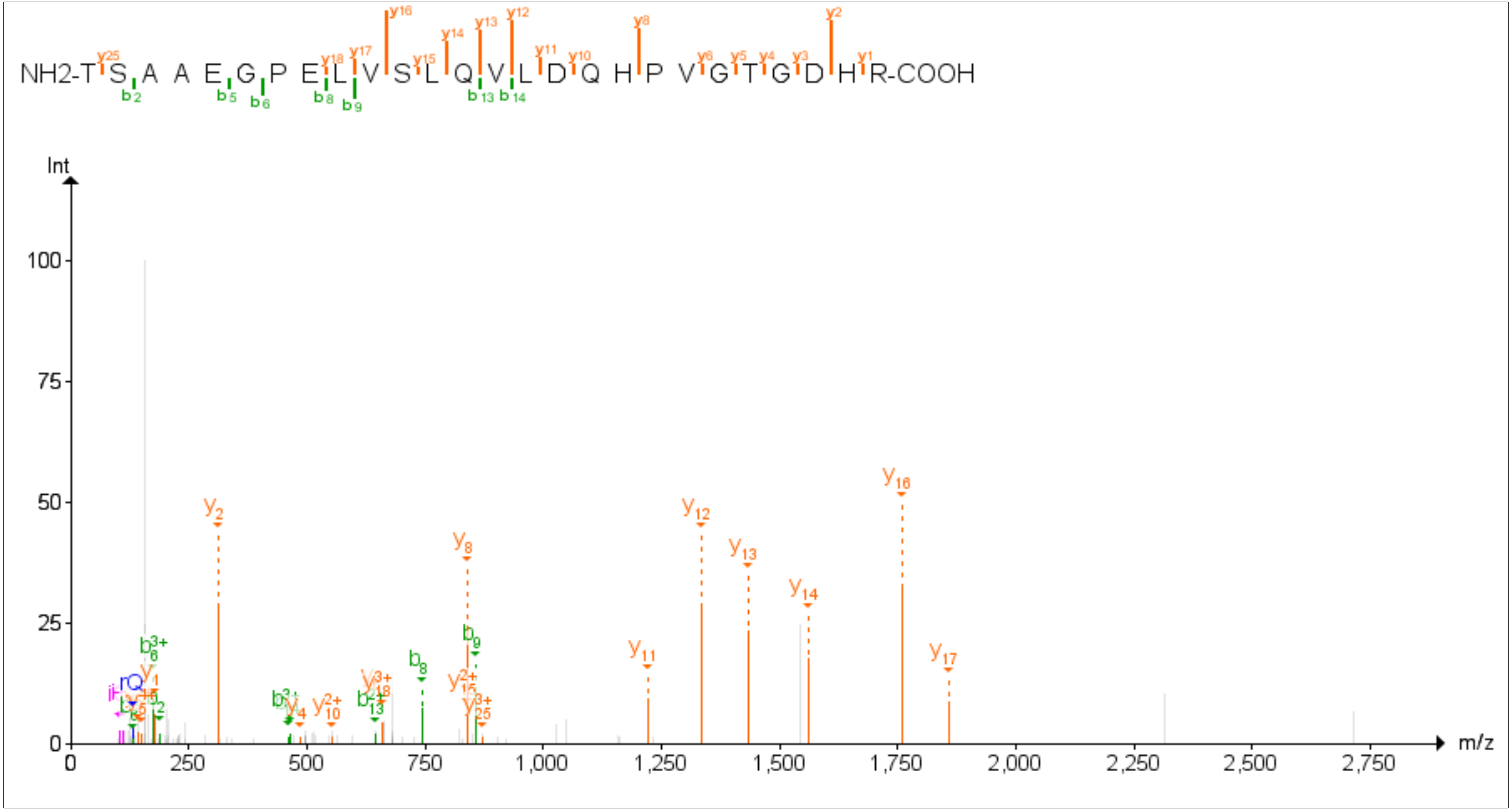
NH2-T S A A E G P E I L V S L Q V L D Q H P V G T G D H R-COOH
Int
100
75
50
25
0
m/z
0
250
500
750
1,000
1,250
1,500
1,750
2,000
2,250
2,500
2,750
y2
y8
b9
b8
y11
y12
y13
y14
y16
y17

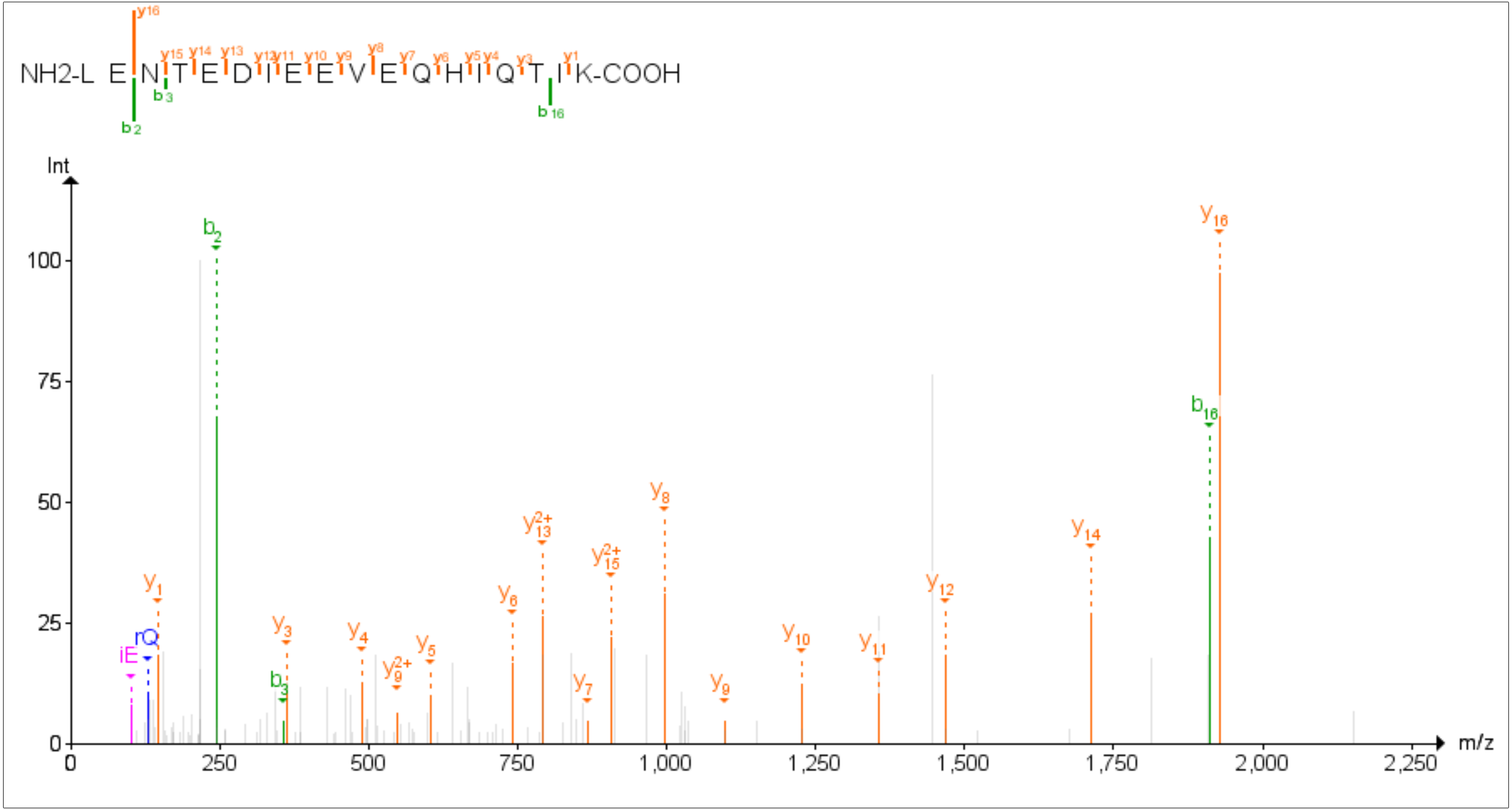

NH2-L E N T E D I E E V E Q H I Q T I K-COOH
Int
m/z
0
25
50
75
100
250
500
750
1,000
1,250
1,500
1,750
2,000
2,250
b2
b3
b16
y1
y3
y4
y5
y6
y7
y8
y9
y10
y11
y12
y14
y16
y9 2+
y13 2+
y15 2+
iE
rQ

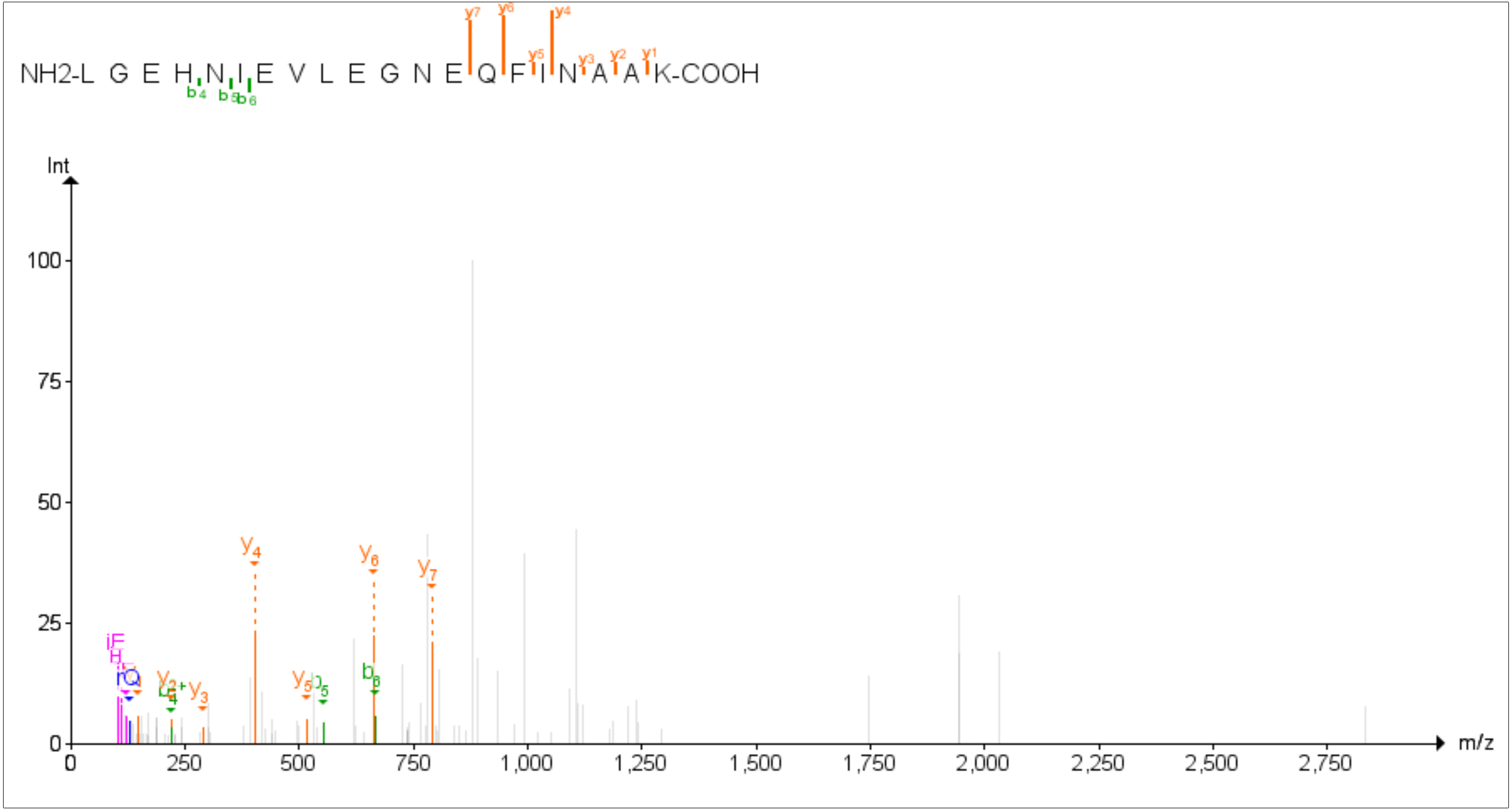

NH2-L G E H N I E V L E G N E Q F I N A A K-COOH
y7
y6
y4
y5
y3
y2
y1
b4
b5
b6
Int
100
75
50
25
0
y4
y6
y7
y5
b5
b6
y3
rQ
iF
m/z
0
250
500
750
1,000
1,250
1,500
1,750
2,000
2,250
2,500
2,750

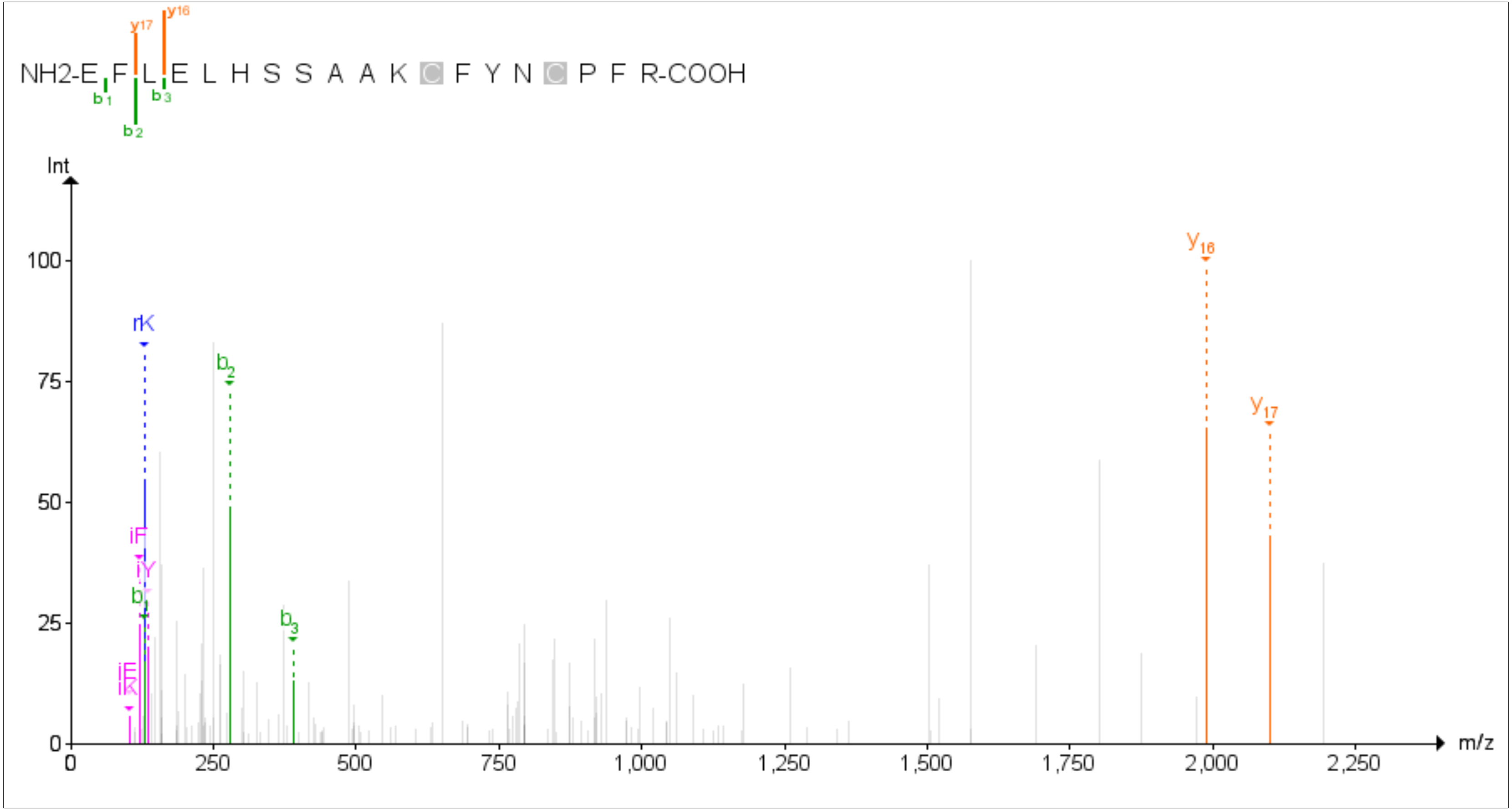

NH2-E F L E L H S S A A K C F Y N C P F R-COOH
y17
y16
b1
b2
b3
Int
100
75
50
25
0
rK
b2
iF
iY
b1
b3
iE
iK
y16
y17
0
250
500
750
1,000
1,250
1,500
1,750
2,000
2,250
m/z

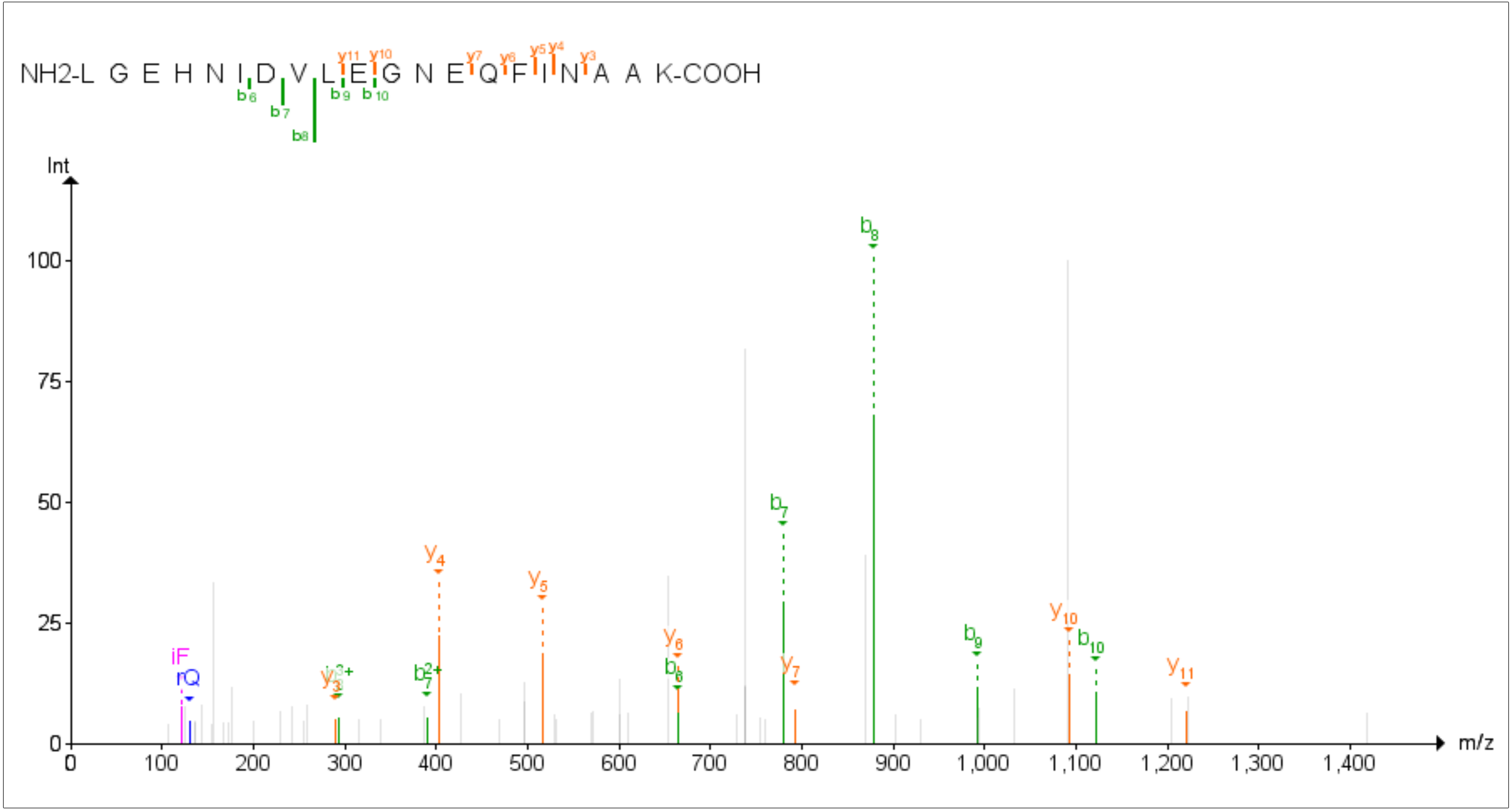

NH2-L G E H N I D V L E G N E Q F I N A A K-COOH
b6 b7 b8 b9 b10
y11 y10 y7 y6 y5 y4 y3
Int
m/z
b8
b7
y4
y5
y6
b6
y7
b9
y10
b10
y11
iF
rQ
y3
b7 2+

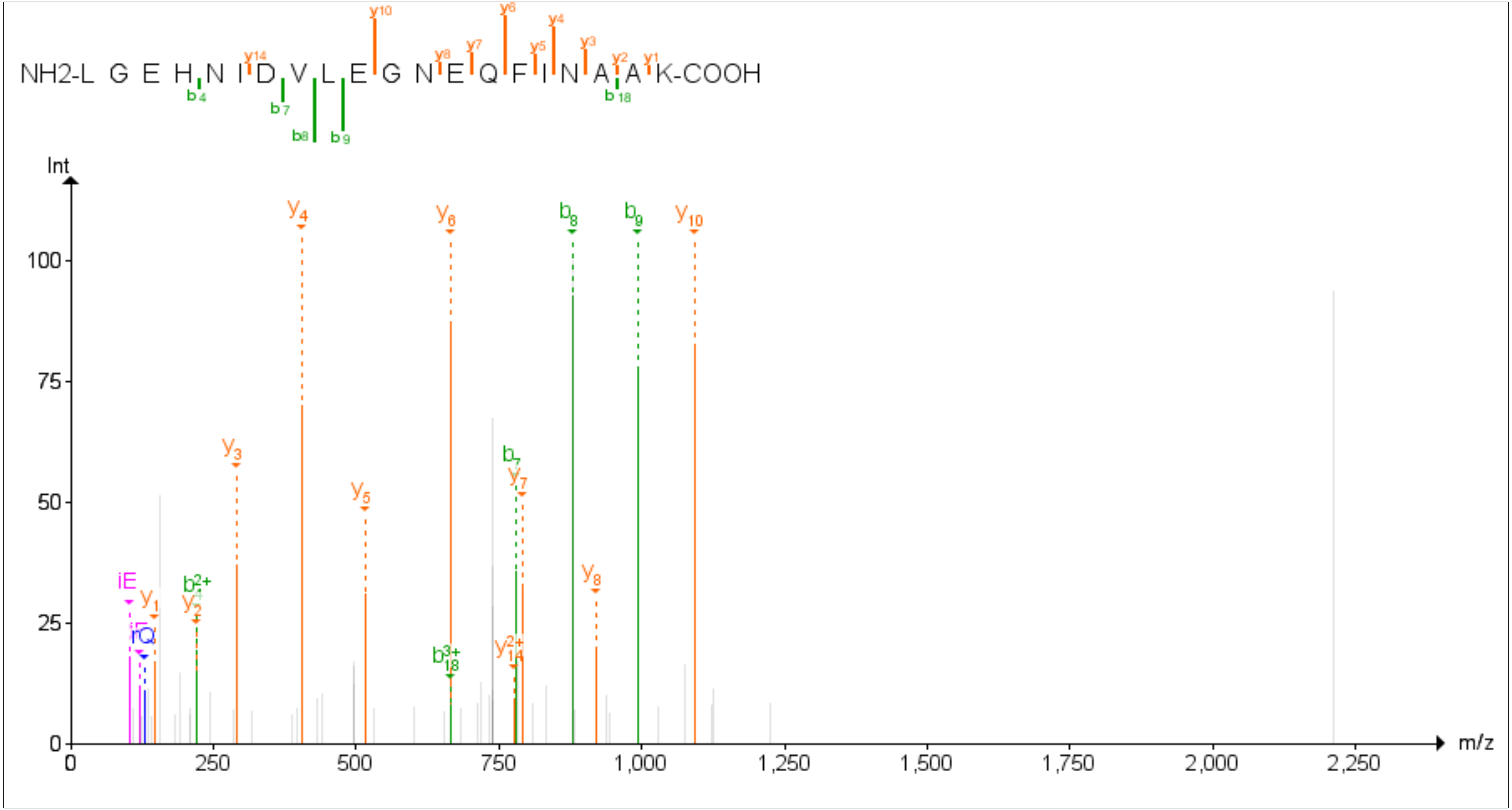

NH2-L G E H N I D V L E G N E Q F I N A A K-COOH
Int
m/z
y4
y6
b8
b9
y10
y3
y5
b7
y7
y8
iE
y1
y2
rQ

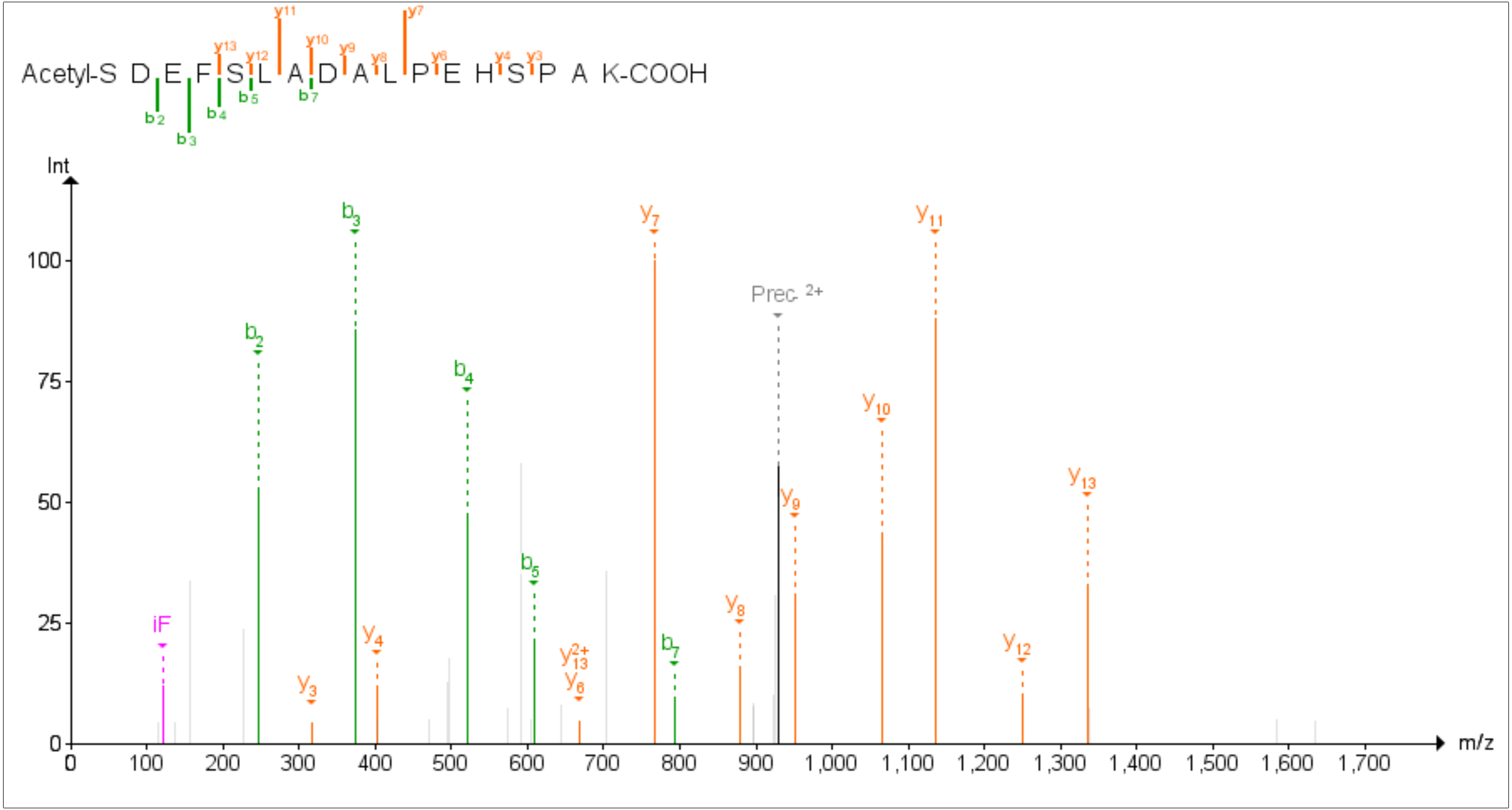
Acetyl-S D E F S L A D A L P E H S P A K-COOH
b2 b3 b4 b5 b7
y13 y12 y11 y10 y9 y8 y7 y6 y4 y3
Int
m/z
Prec 2+
iF
0
25
50
75
100
0
100
200
300
400
500
600
700
800
900
1,000
1,100
1,200
1,300
1,400
1,500
1,600
1,700

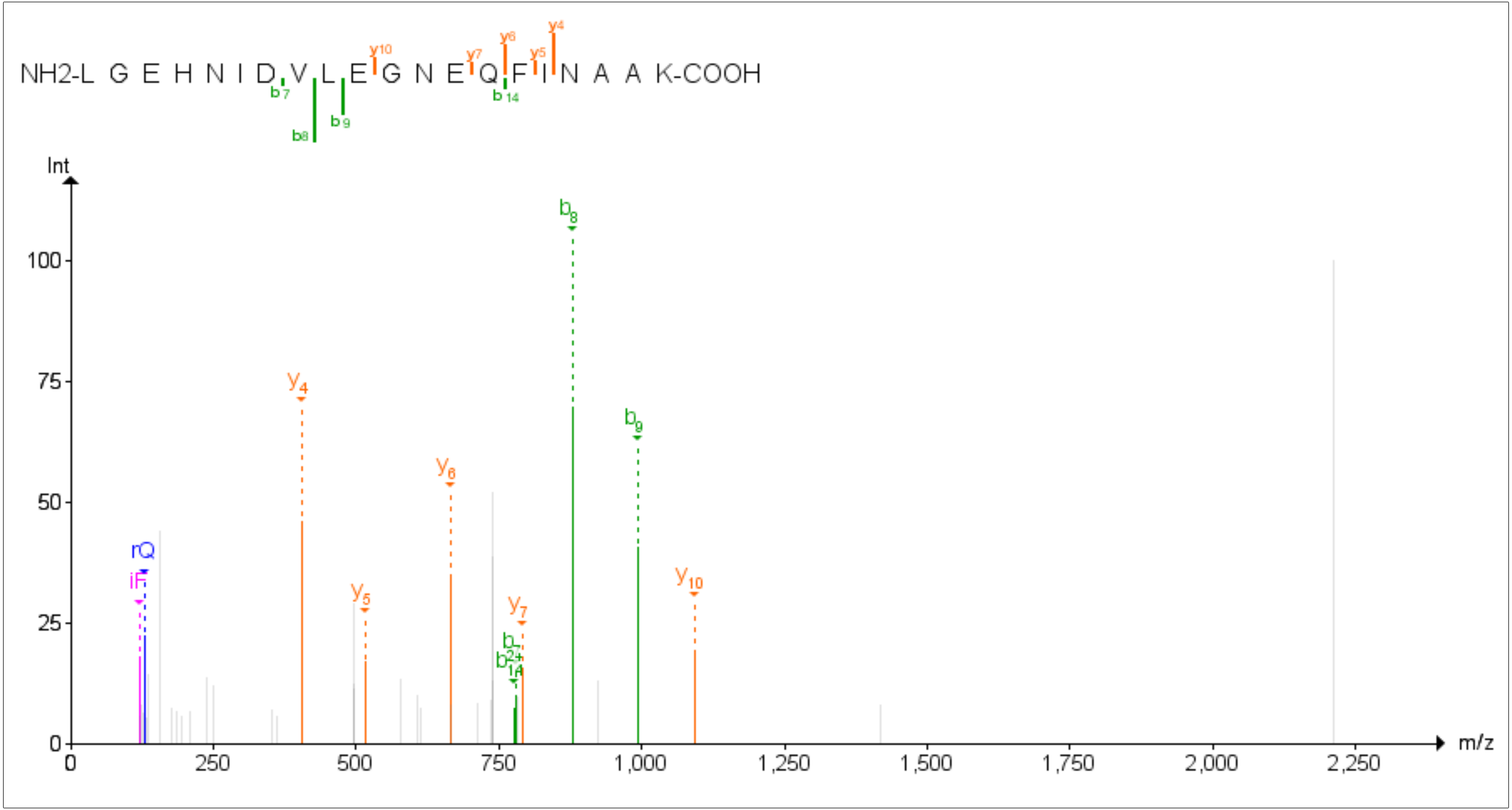

NH2-L G E H N I D V L E G N E Q F I N A A K-COOH
b7
b8
b9
b14
y10
y7
y6
y5
y4
Int
100
75
50
25
0
b8
y4
b9
y6
rQ
iF
y5
y7
b
b14 2+
y10
0
250
500
750
1,000
1,250
1,500
1,750
2,000
2,250
m/z

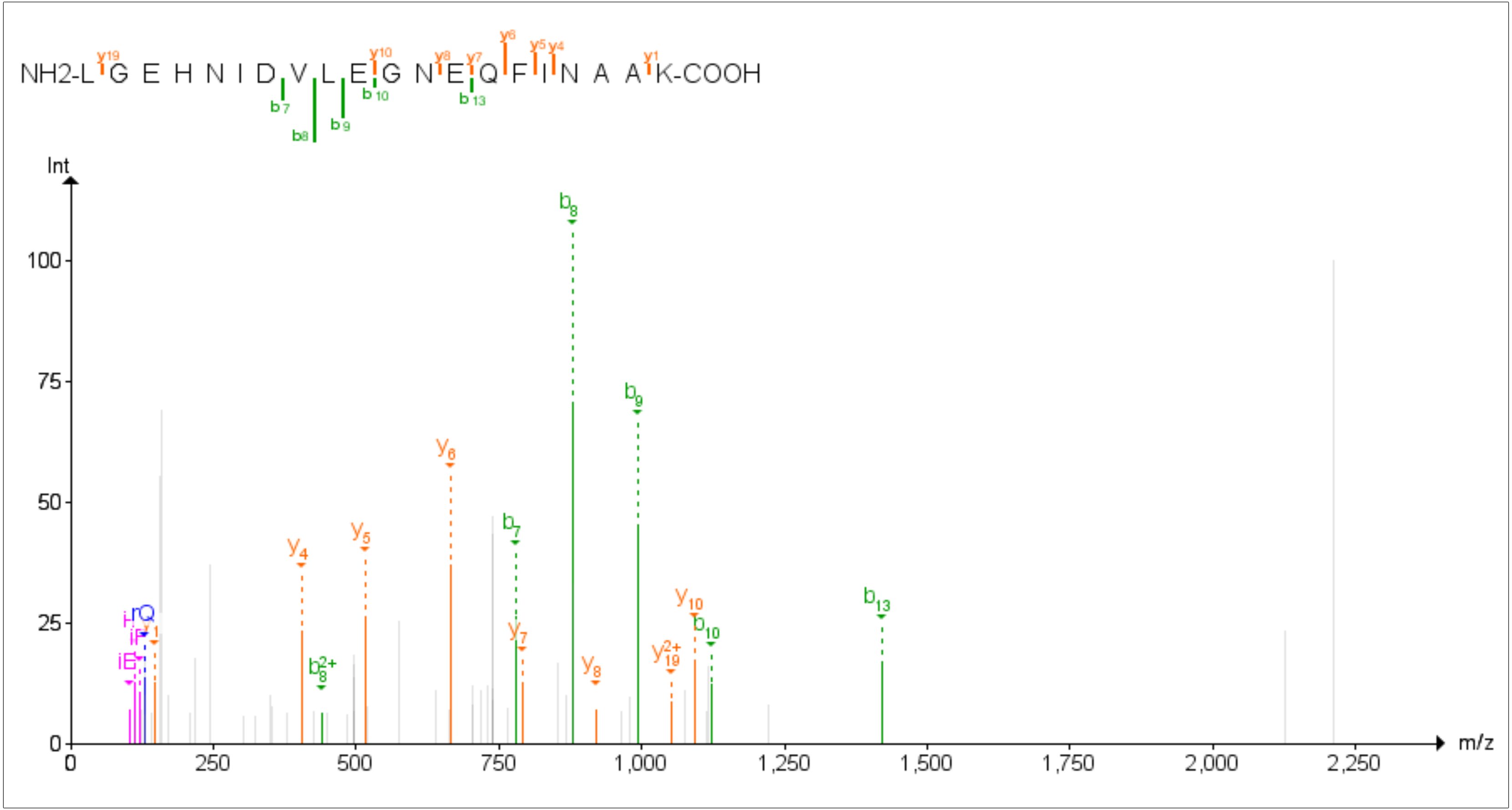

NH2-L G E H N I D V L E G N E Q F I N A A K-COOH
y19 y10 y8 y7 y6 y5 y4 y1
b7 b8 b9 b10 b13
Int
100
75
50
25
0
m/z
0
250
500
750
1,000
1,250
1,500
1,750
2,000
2,250
b8
b9
y6
b7
y5
y4
y7
y10
b10
b13
y8
y19 2+
b8 2+
y1
iH
iF
iE
nQ

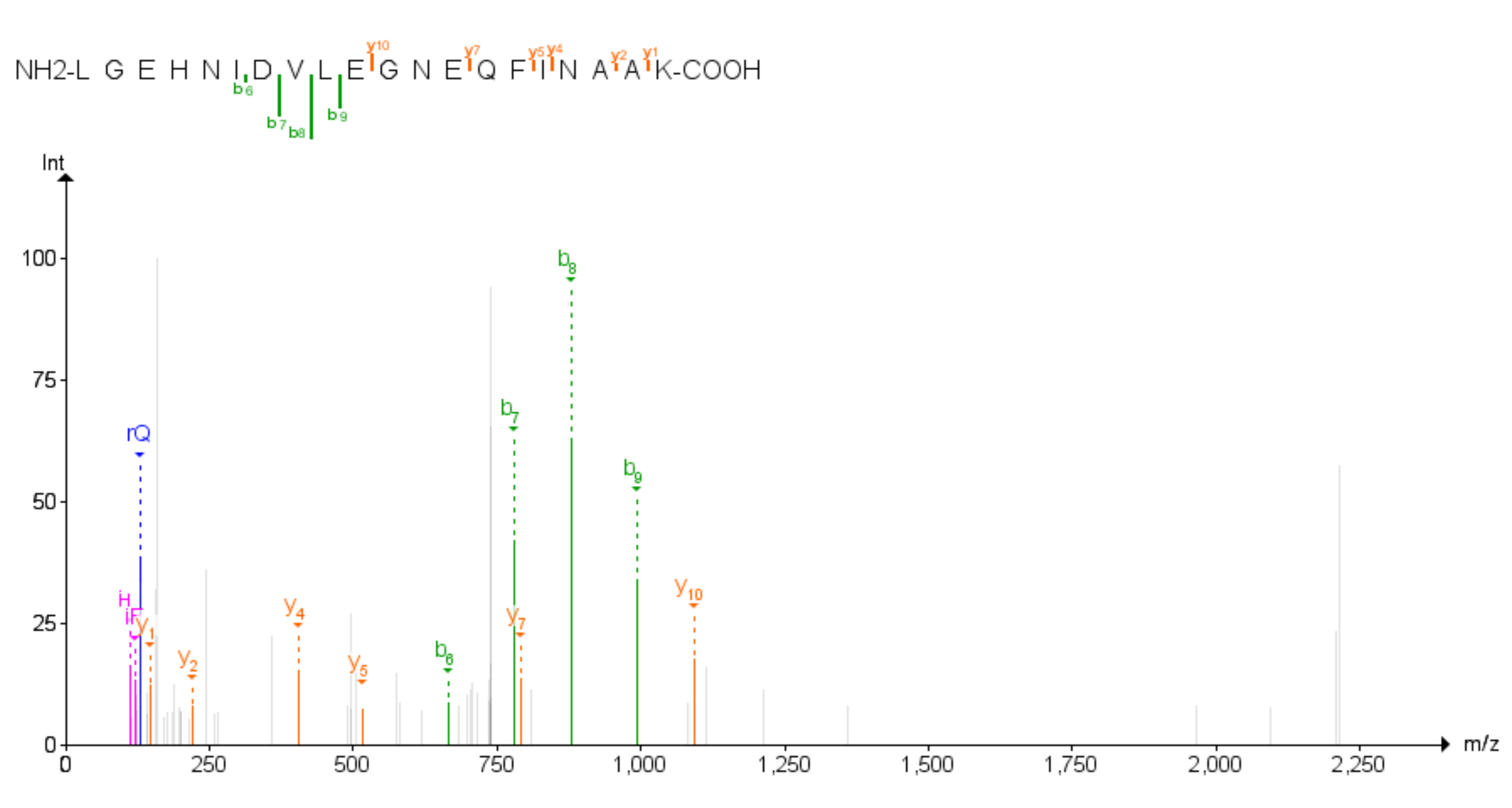
NH2-L G E H N I D V L E G N E Q F I N A A K-COOH
y10
y7
y5
y4
y2
y1
b6
b7
b8
b9
Int
100
75
50
25
0
rQ
b8
b7
b9
y10
y4
y7
y1
y2
y5
b6
m/z
0
250
500
750
1,000
1,250
1,500
1,750
2,000
2,250

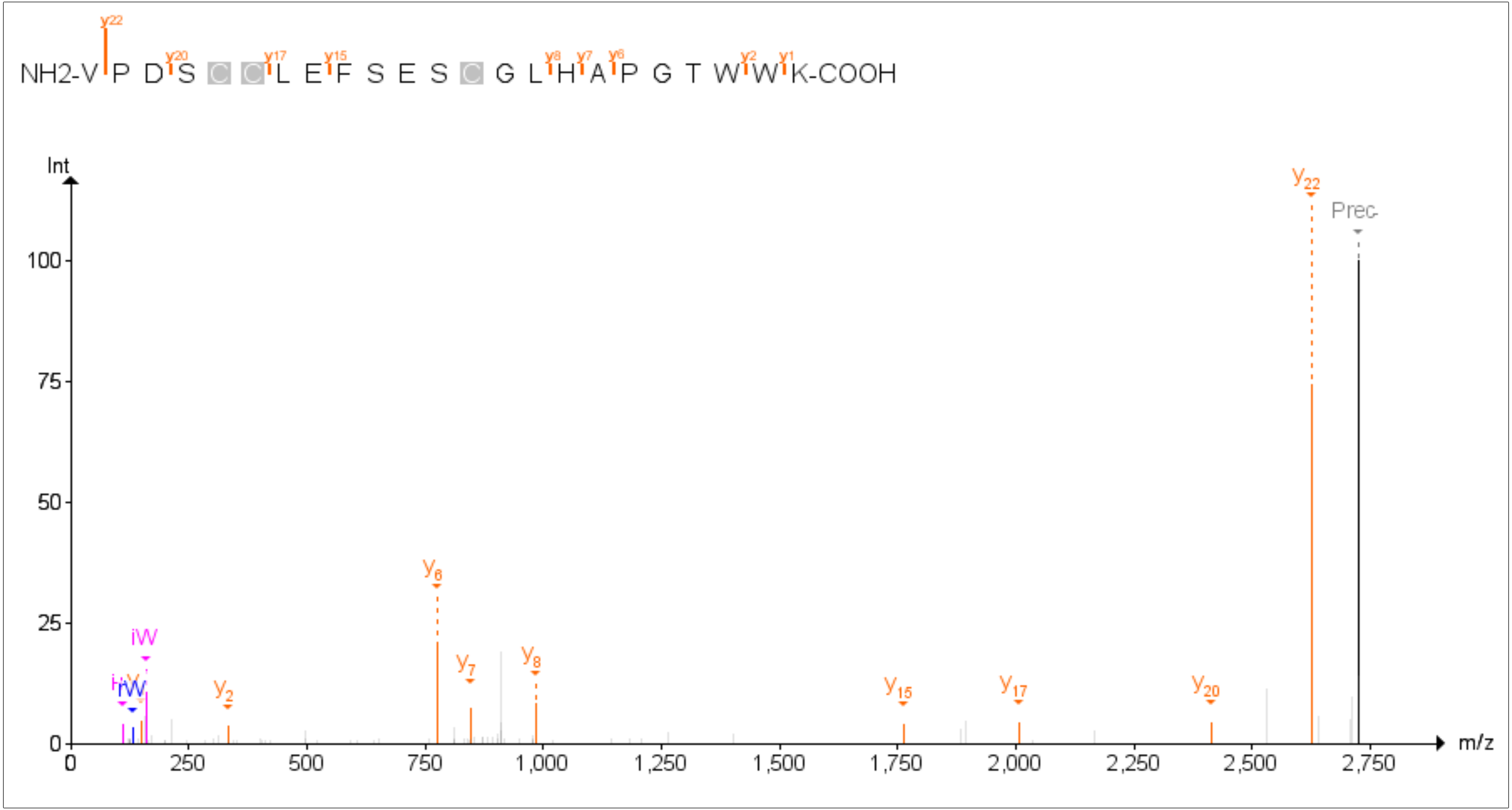

NH2-V P D S C C L E F S E S C G L H A P G T W W K-COOH
y22
y20
y17
y15
y8
y7
y6
y2
y1
Int
100
75
50
25
0
m/z
0
250
500
750
1,000
1,250
1,500
1,750
2,000
2,250
2,500
2,750
Prec
iW
iW

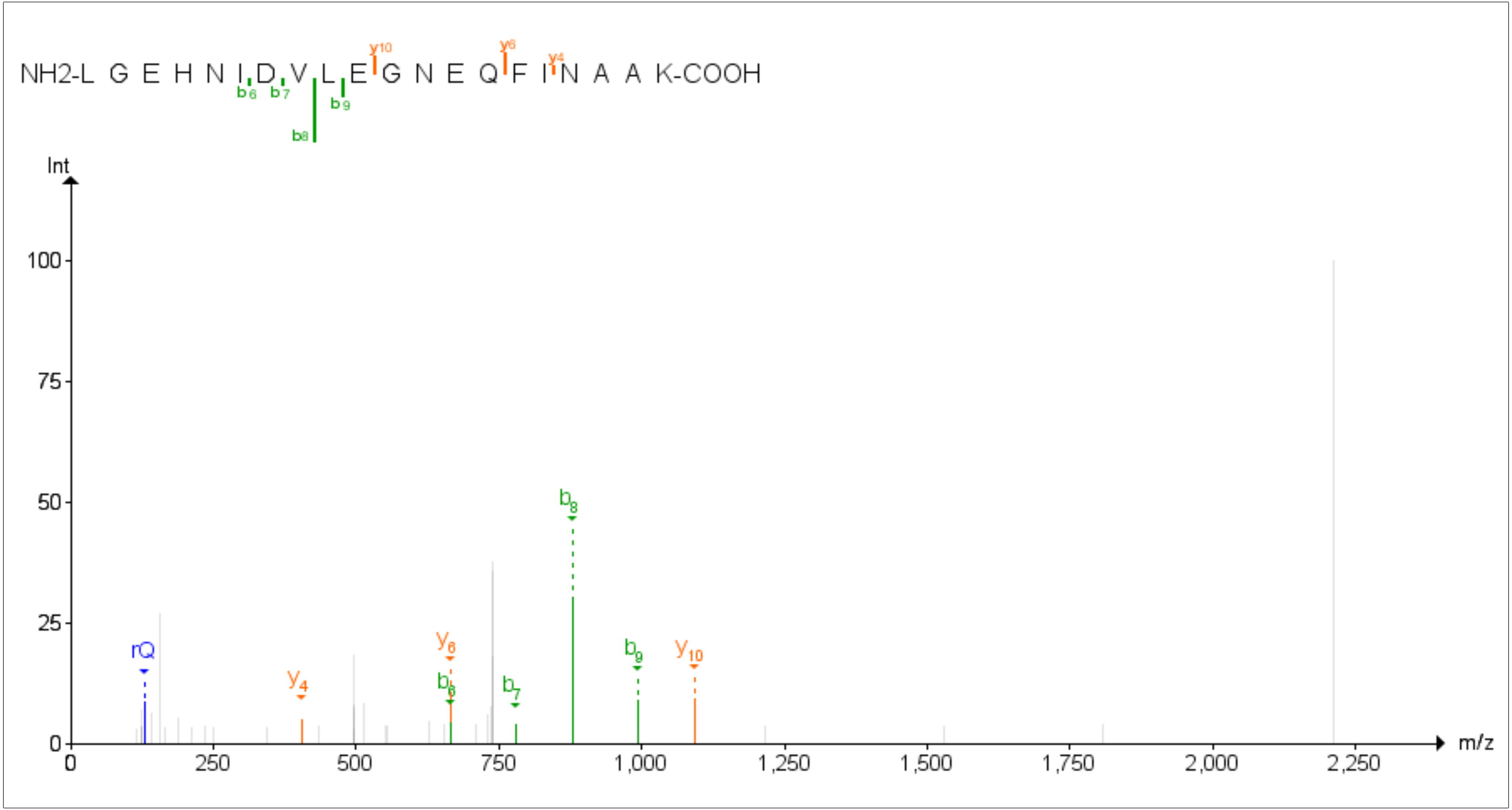

NH2-L G E H N I D V L E G N E Q F I N A A K-COOH
y10
y6
y4
b6
b7
b9
b8
Int
100
75
50
25
0
rQ
y4
y6
b6
b7
b8
b9
y10
0
250
500
750
1,000
1,250
1,500
1,750
2,000
2,250
m/z

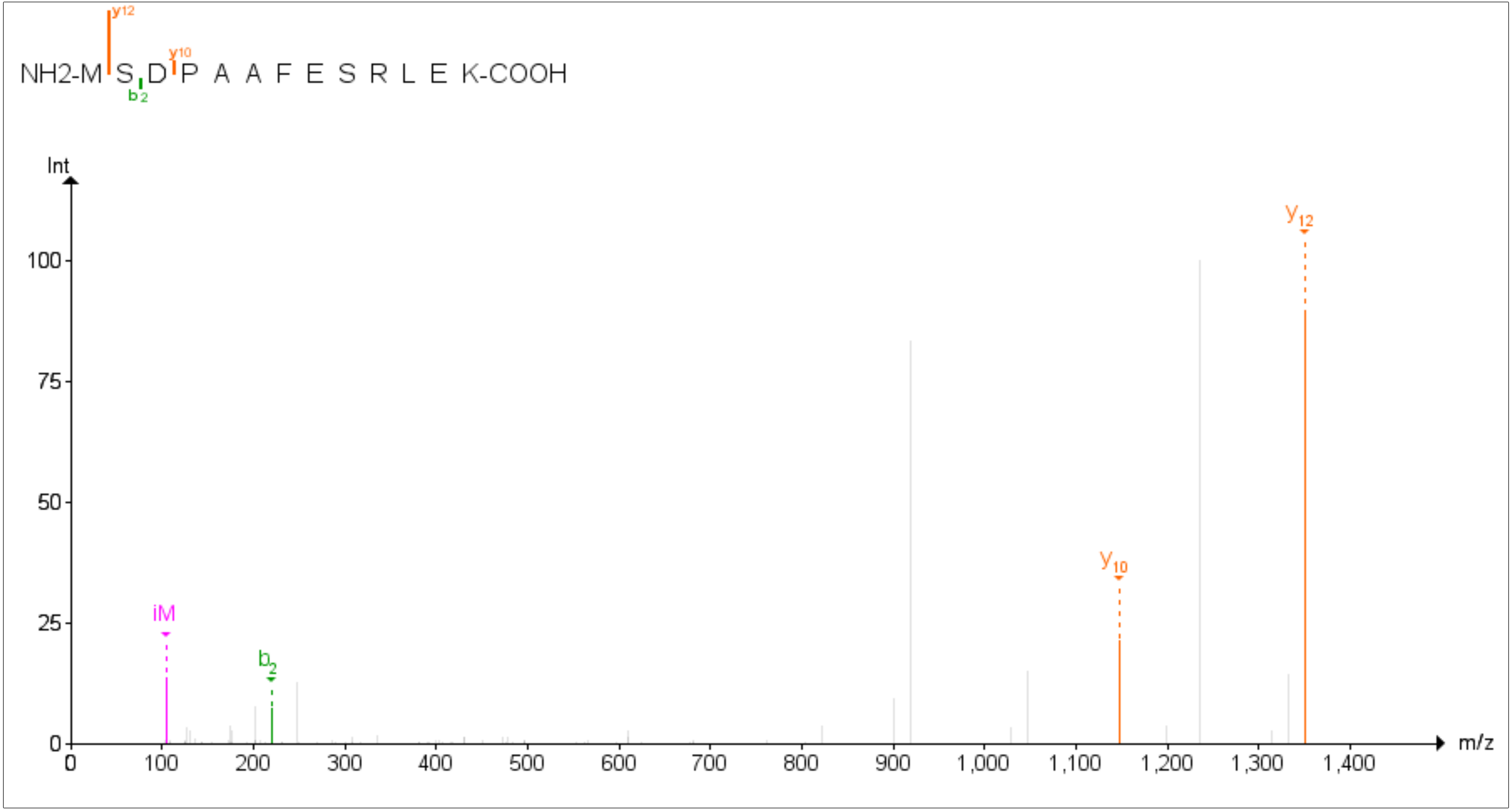

NH2-M S D P A A F E S R L E K-COOH
y12
y10
b2
Int
100
75
50
25
0
iM
b2
y10
y12
m/z
0
100
200
300
400
500
600
700
800
900
1,000
1,100
1,200
1,300
1,400

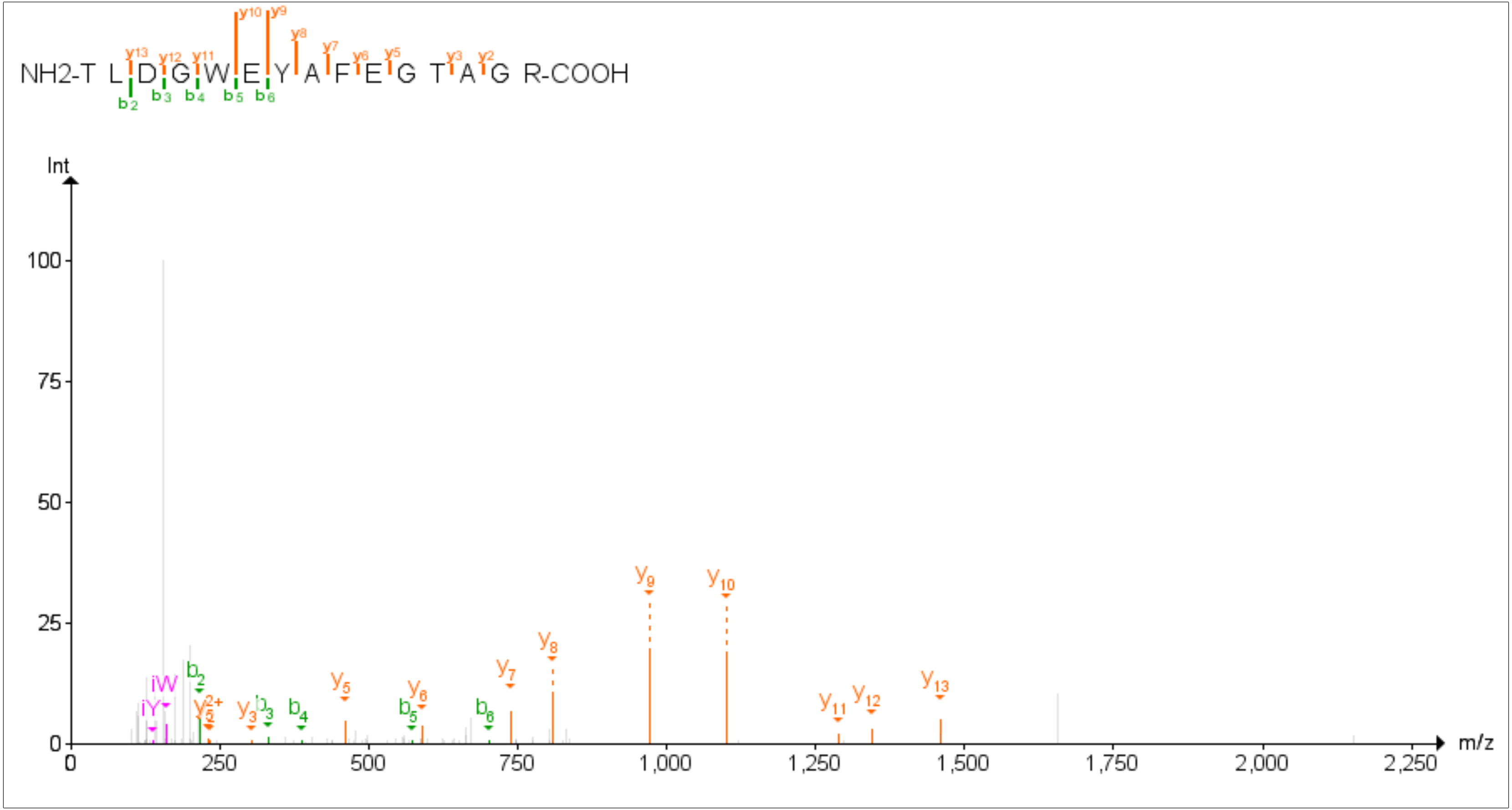

NH2-T L D G W E Y A F E G T A G R-COOH
Int
100
75
50
25
0
m/z
0
250
500
750
1,000
1,250
1,500
1,750
2,000
2,250

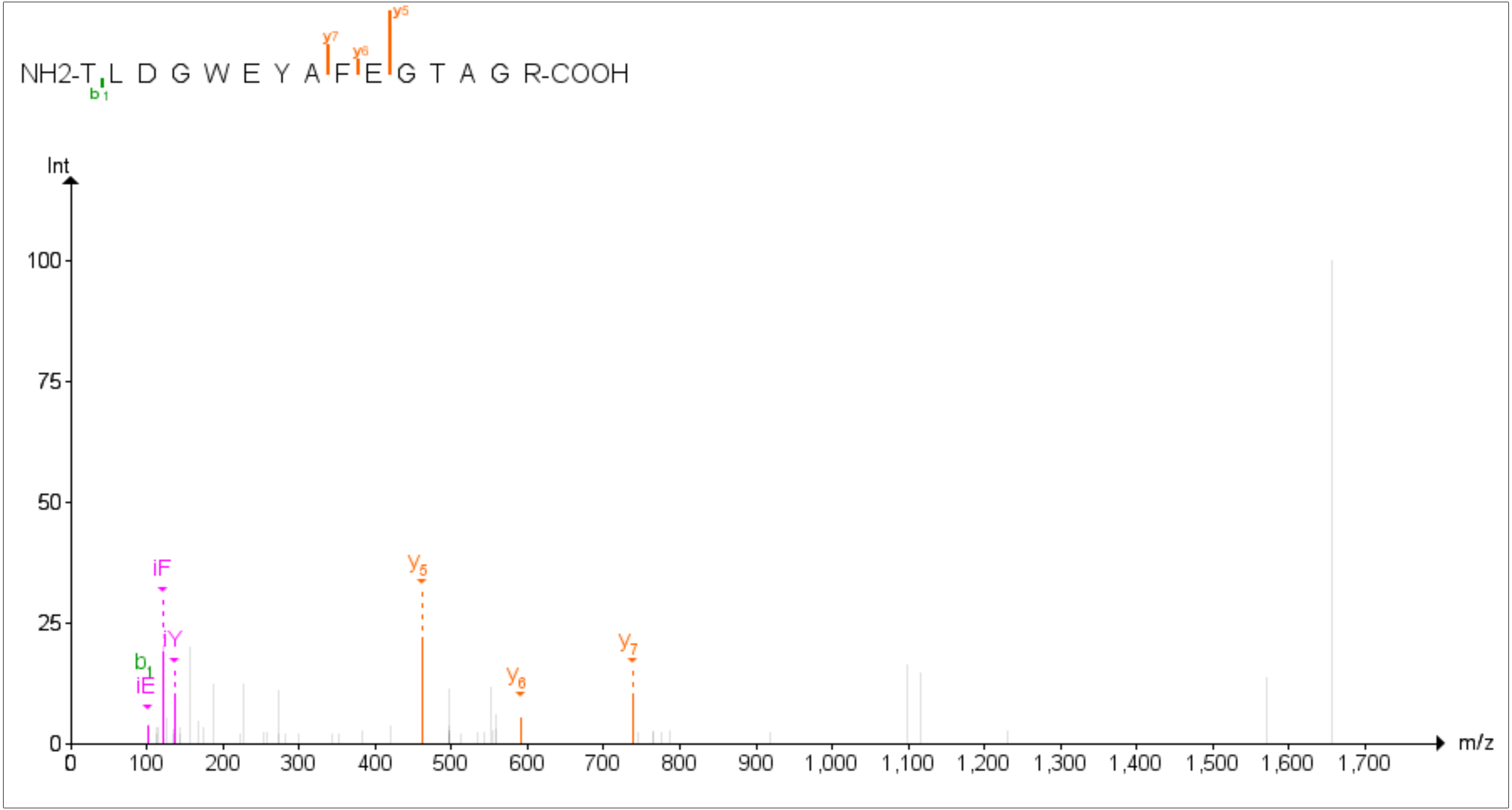

NH2-T L D G W E Y A F E G T A G R-COOH
b1
y7
y6
y5
Int
m/z
iF
iY
b1
iE
y5
y6
y7

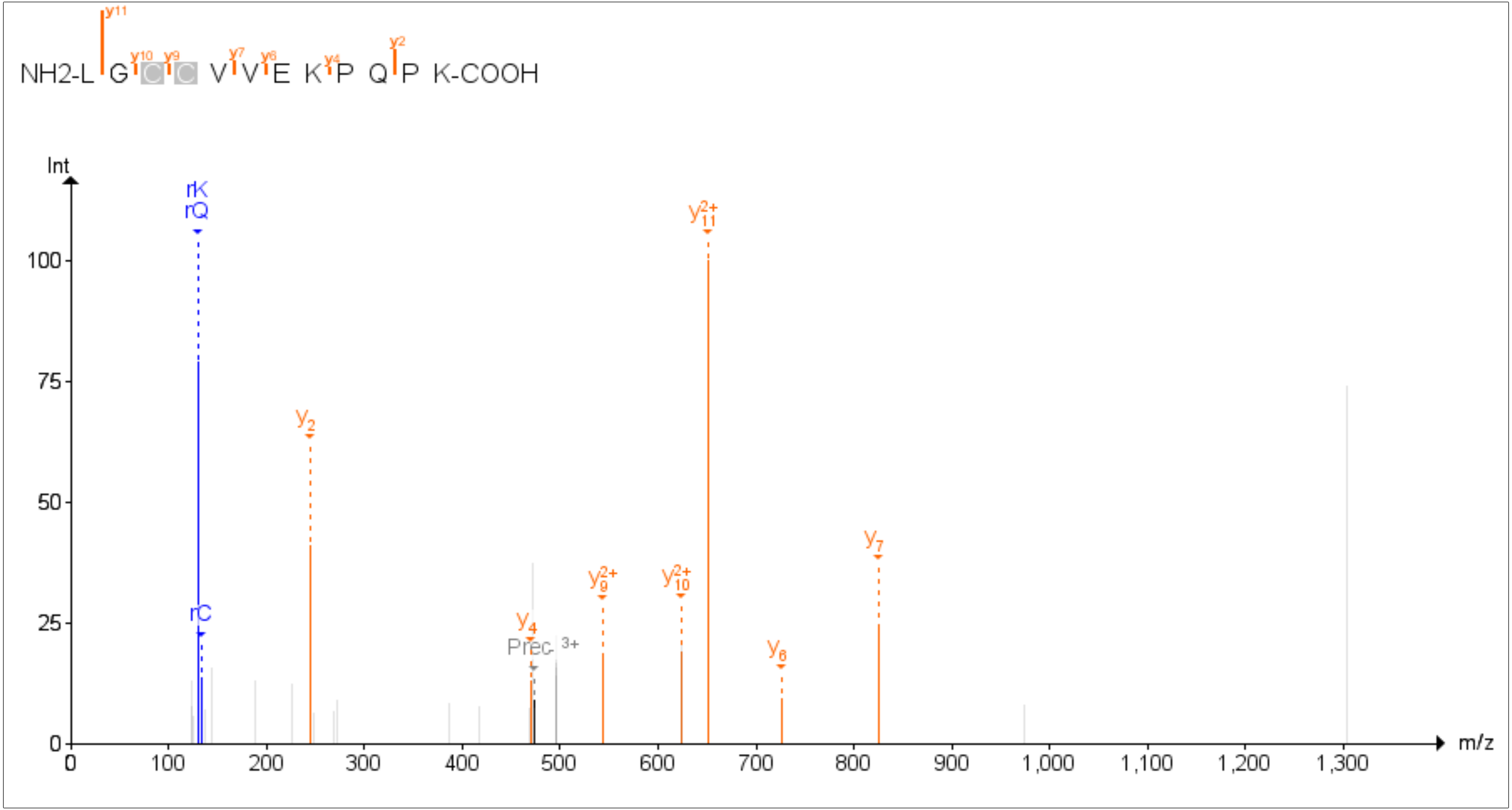

NH2-L G C C V V E K P Q P K-COOH
y11
y10
y9
y7
y6
y4
y2
Int
rK
rQ
rC
y2
y4
Prec- 3+
y9 2+
y10 2+
y11 2+
y6
y7
100
75
50
25
0
0
100
200
300
400
500
600
700
800
900
1,000
1,100
1,200
1,300
m/z

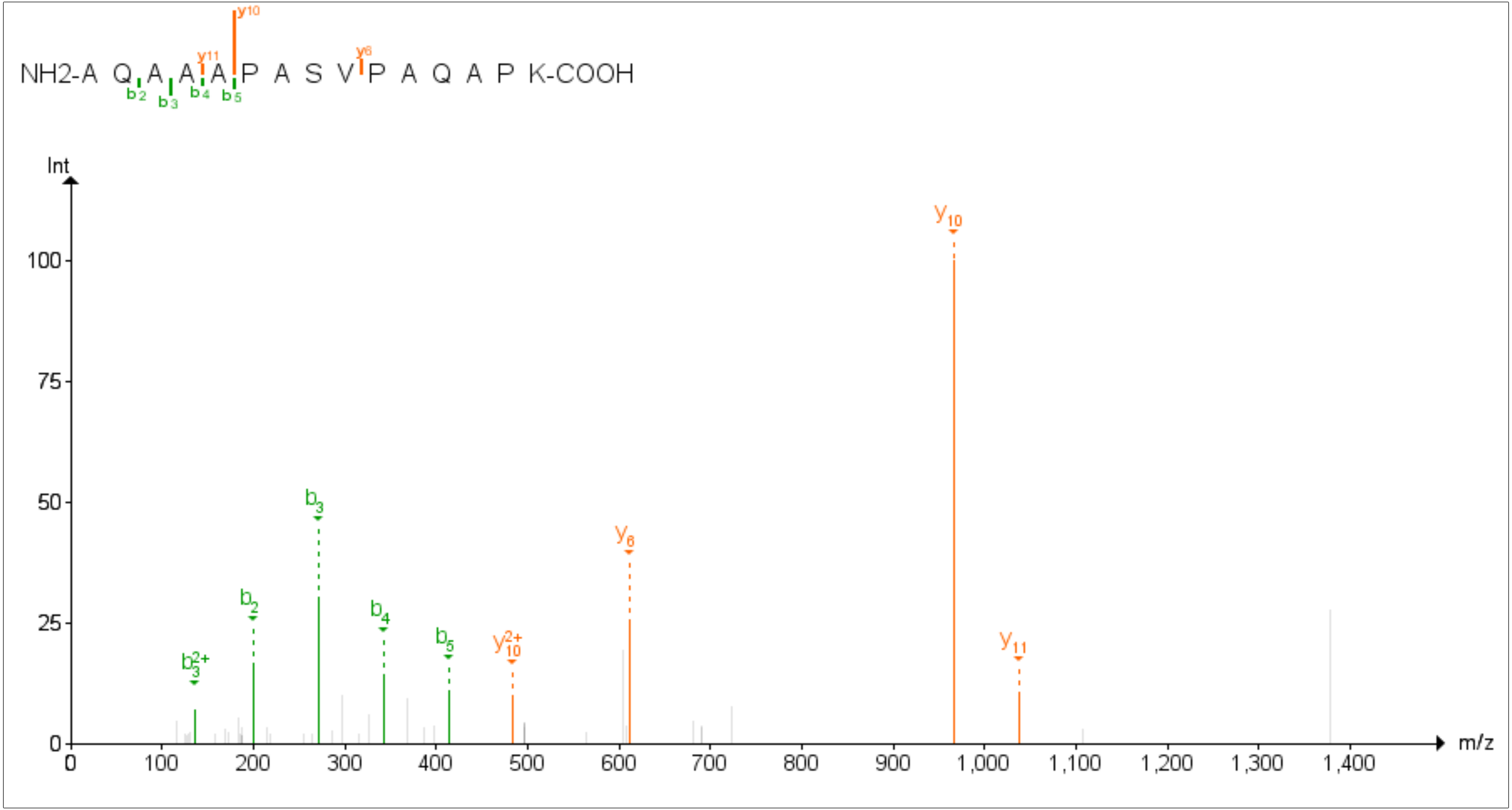

NH2-A Q A A A P A S V P A Q A P K-COOH
y10
y11
y6
b2
b3
b4
b5
Int
100
75
50
25
0
m/z
0
100
200
300
400
500
600
700
800
900
1,000
1,100
1,200
1,300
1,400
y_{10}
b_3
y_6
b_2
b_4
b_5
b_3^{2+}
y_{10}^{2+}
y_{11}

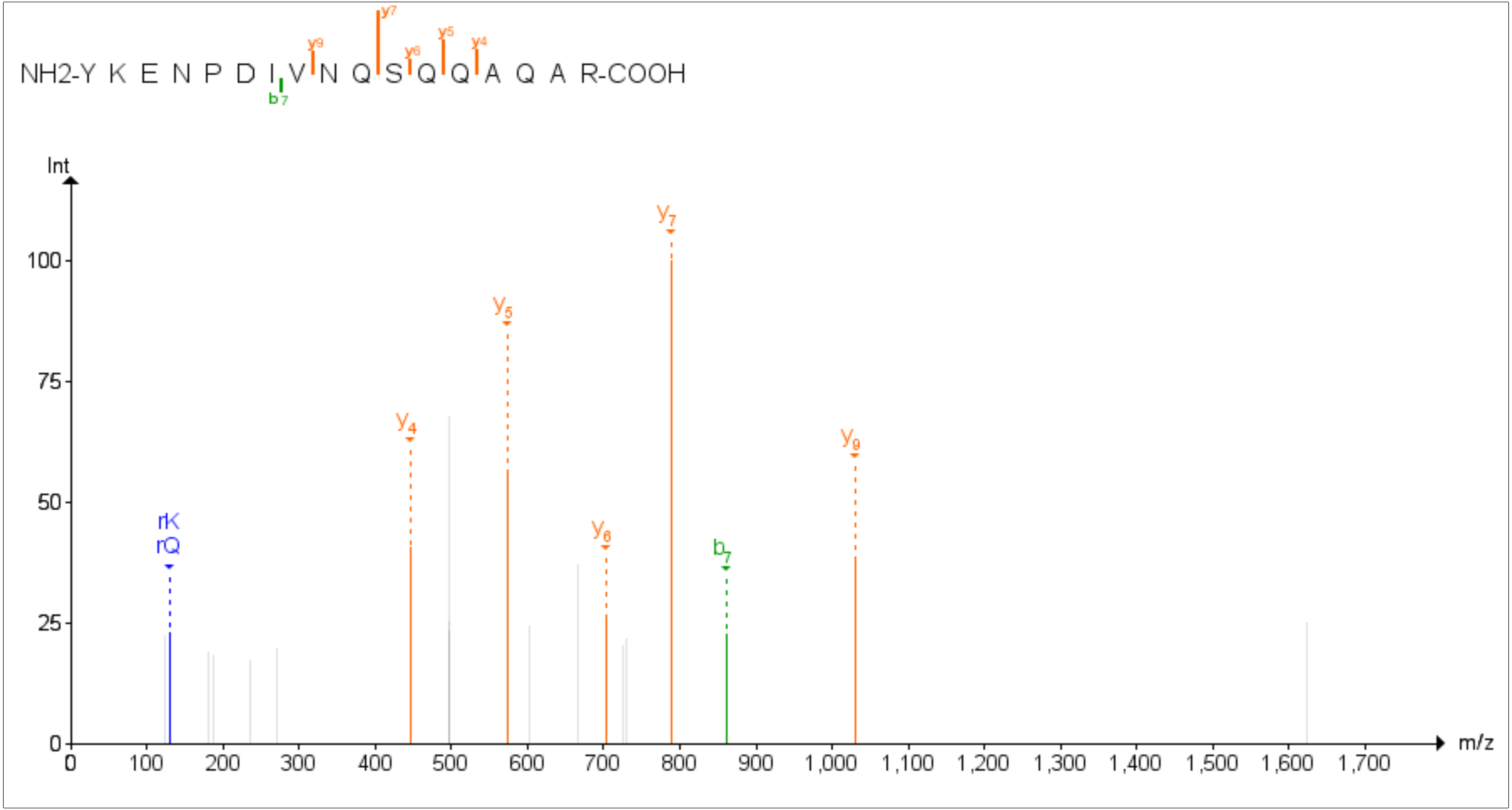

NH2-Y K E N P D I V N Q S Q Q A Q A R-COOH
y9
y7
y6
y5
y4
b7
Int
100
75
50
25
0
rK
rQ
y4
y5
y6
y7
b7
y9
0
100
200
300
400
500
600
700
800
900
1,000
1,100
1,200
1,300
1,400
1,500
1,600
1,700
m/z

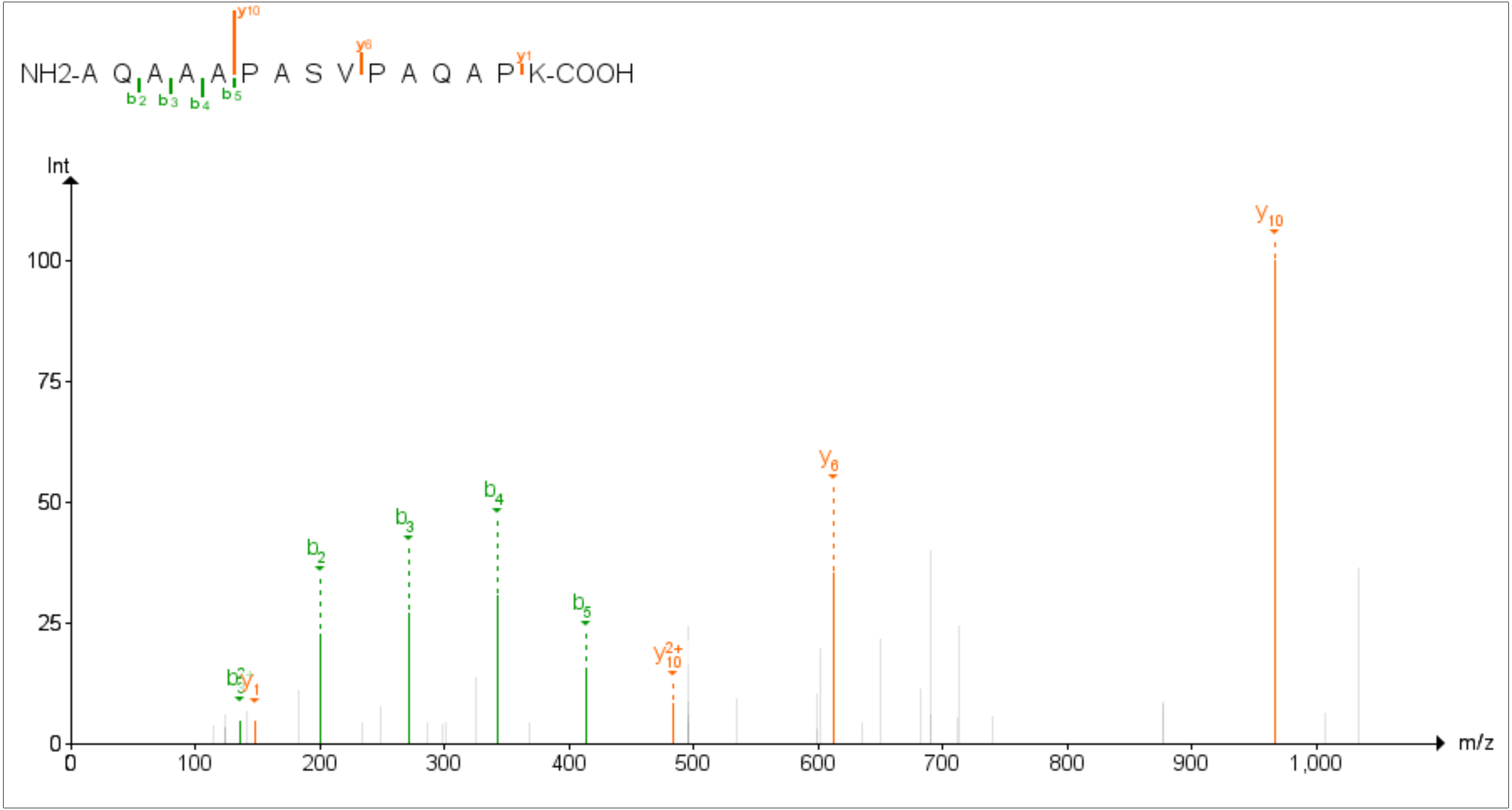

NH2-A Q A A A P A S V P A Q A P K-COOH
y10
y6
y1
b2
b3
b4
b5
Int
100
75
50
25
0
m/z
0
100
200
300
400
500
600
700
800
900
1,000
y_{10}
y_6
b_4
b_3
b_2
b_5
y_{10}^{2+}
b_3^{2+}
y_1

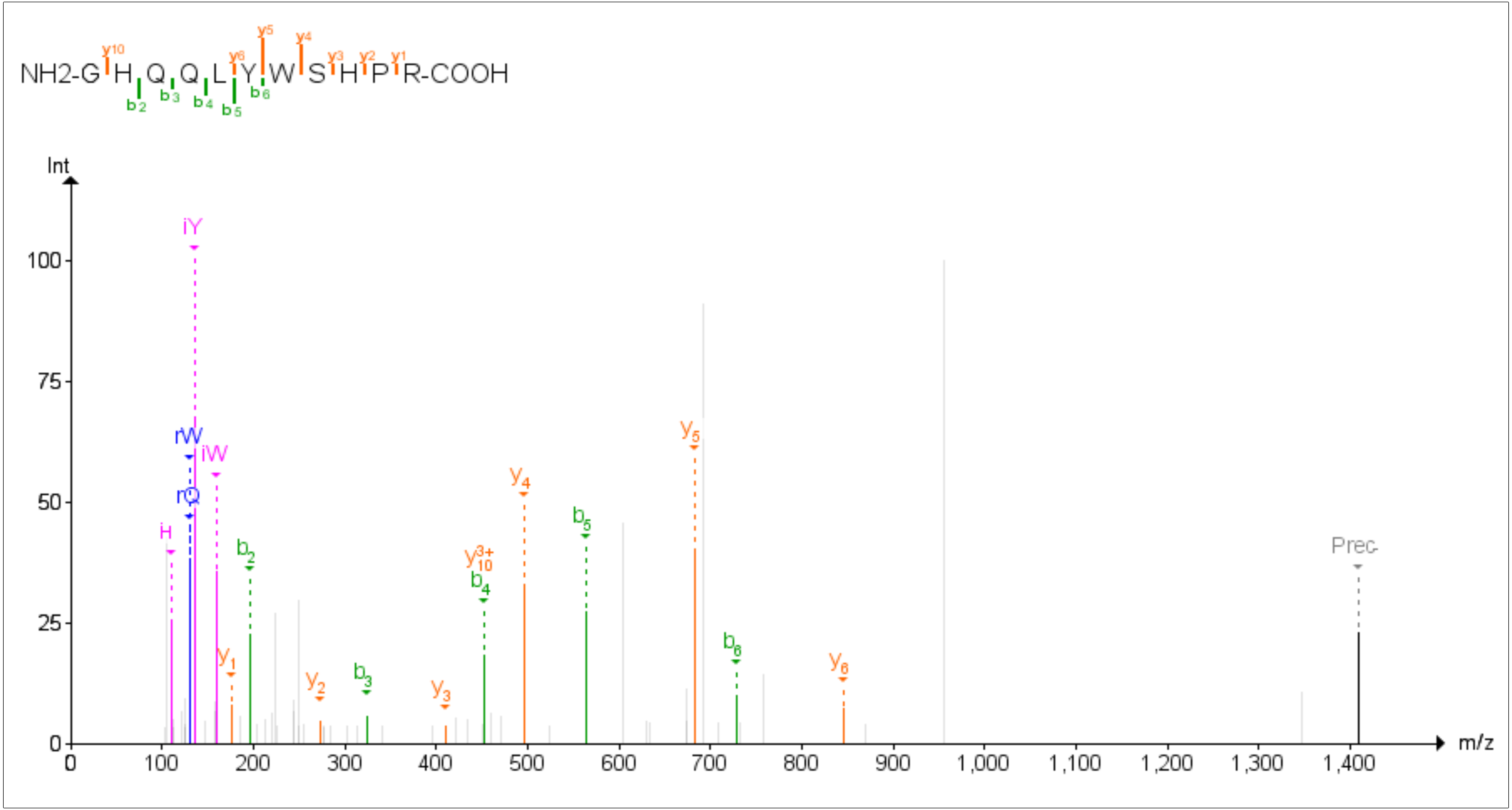

NH2-G H Q Q L Y W S H P R-COOH
y10 y6 y5 y4 y3 y2 y1
b2 b3 b4 b5 b6
Int
iY
rW
iW
rQ
iH
b2
y1
y2
b3
y3
y10 3+
b4
y4
b5
y5
b6
y6
Prec
m/z

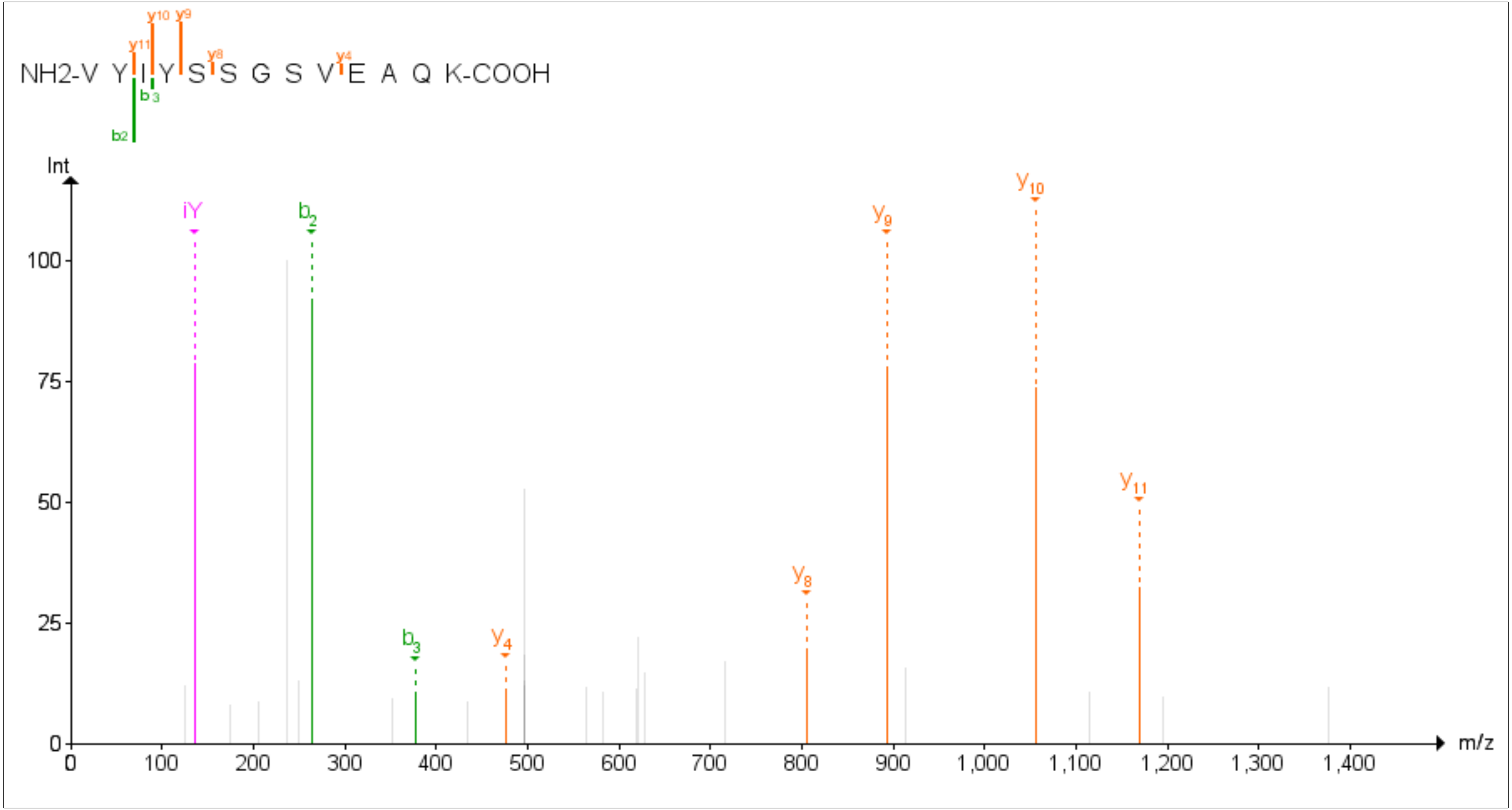

NH2-V Y I Y S S G S V E A Q K-COOH
y10 y9 y11 y8 y4
b3 b2
Int
iY
b2
y10
y9
y11
y8
b3
y4
100
75
50
25
0
0 100 200 300 400 500 600 700 800 900 1,000 1,100 1,200 1,300 1,400
m/z

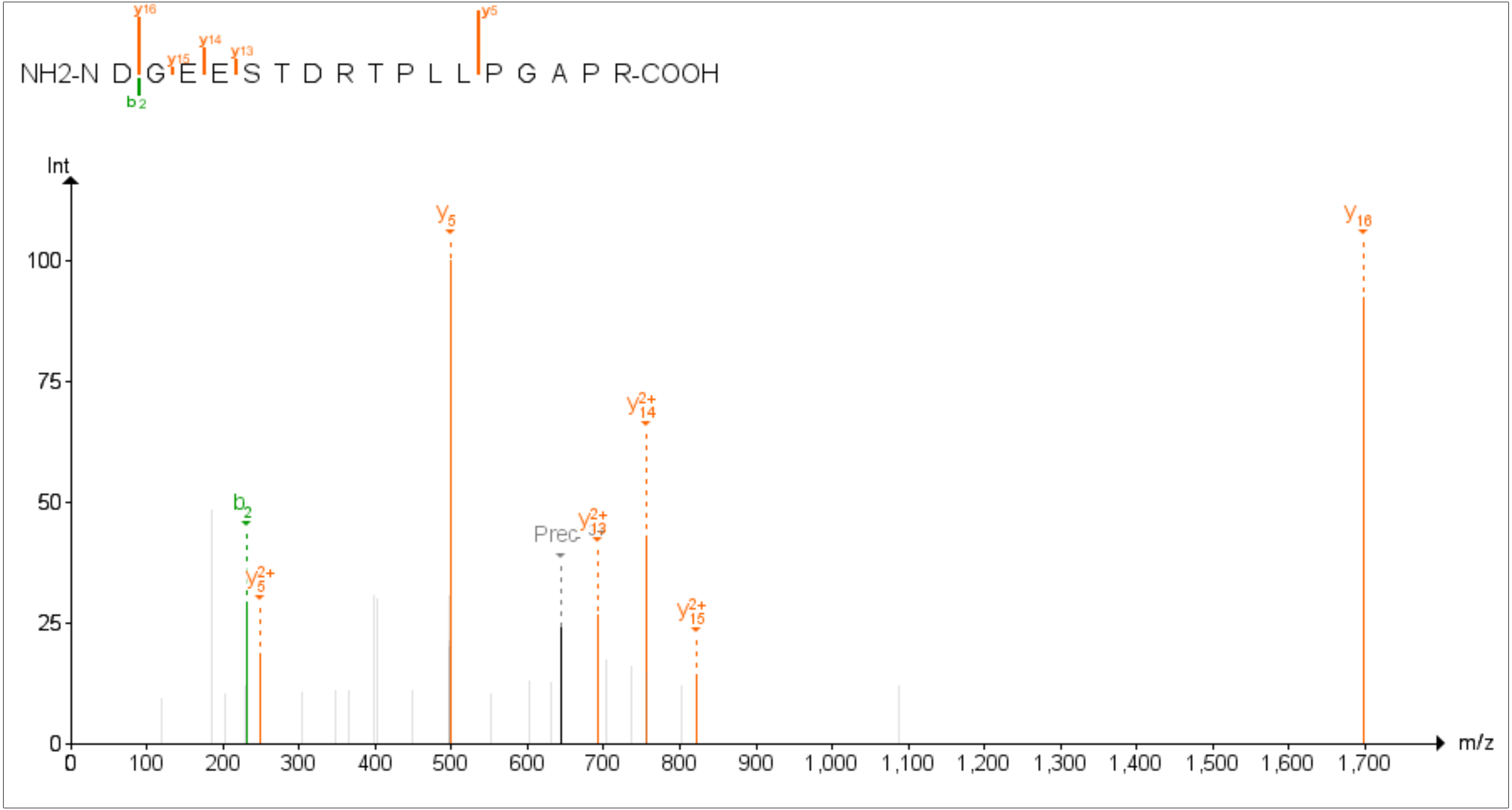

NH2-N D G E E S T D R T P L L P G A P R-COOH
y16
y15
y14
y13
y5
b2
Int
100
75
50
25
0
y5
y16
b2
y5 2+
Prec
y13 2+
y14 2+
y15 2+
0
100
200
300
400
500
600
700
800
900
1,000
1,100
1,200
1,300
1,400
1,500
1,600
1,700
m/z

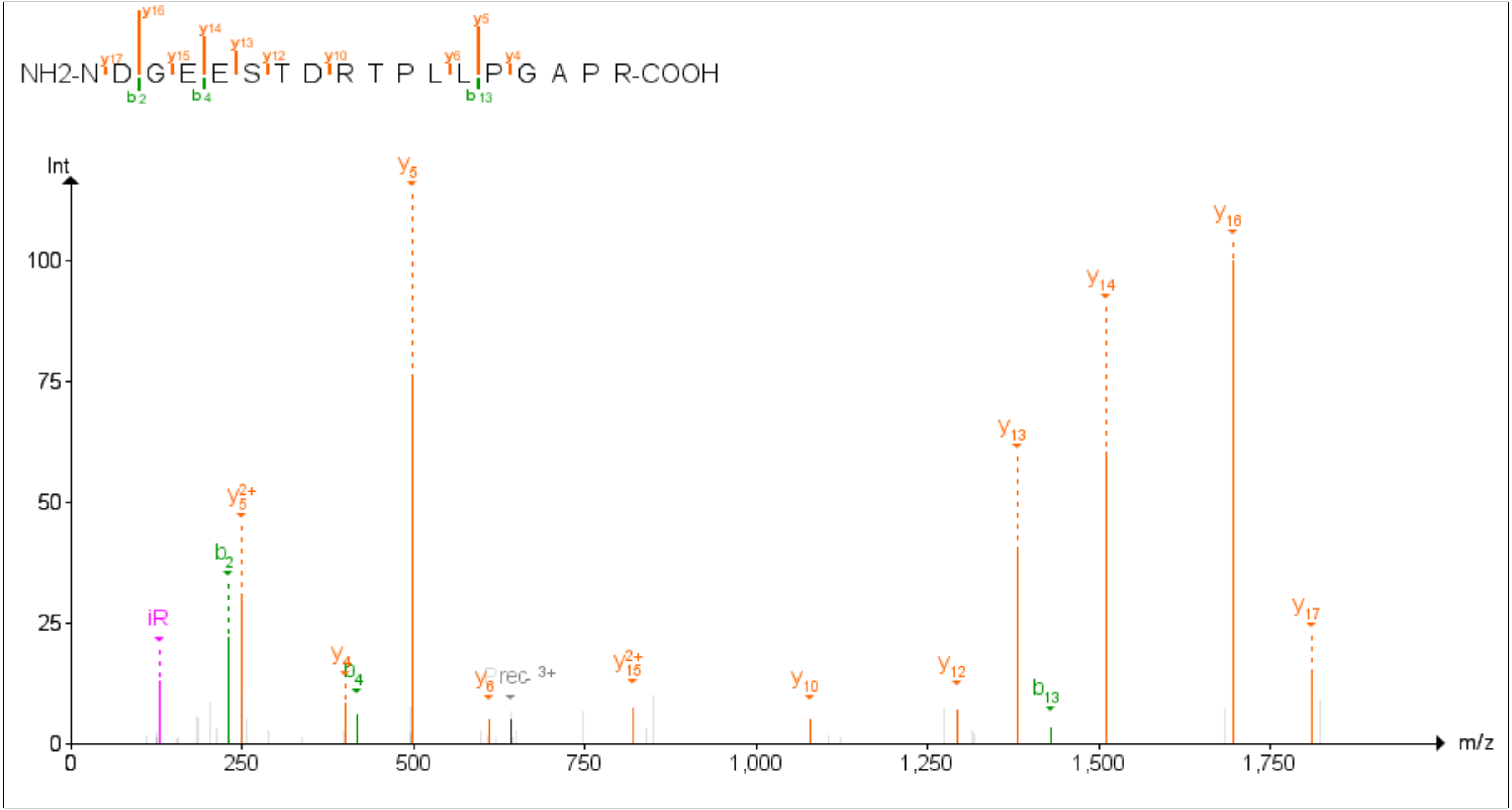

NH2-N D G E E S T D R T P L L P G A P R-COOH
y17 y16 y15 y14 y13 y12 y10 y6 y5 y4
b2 b4 b13
Int
m/z
y5
y16
y14
y13
y5 2+
b2
iR
y4
b4
y6
Prec 3+
y15 2+
y10
y12
b13
y17
0
25
50
75
100
0
250
500
750
1,000
1,250
1,500
1,750

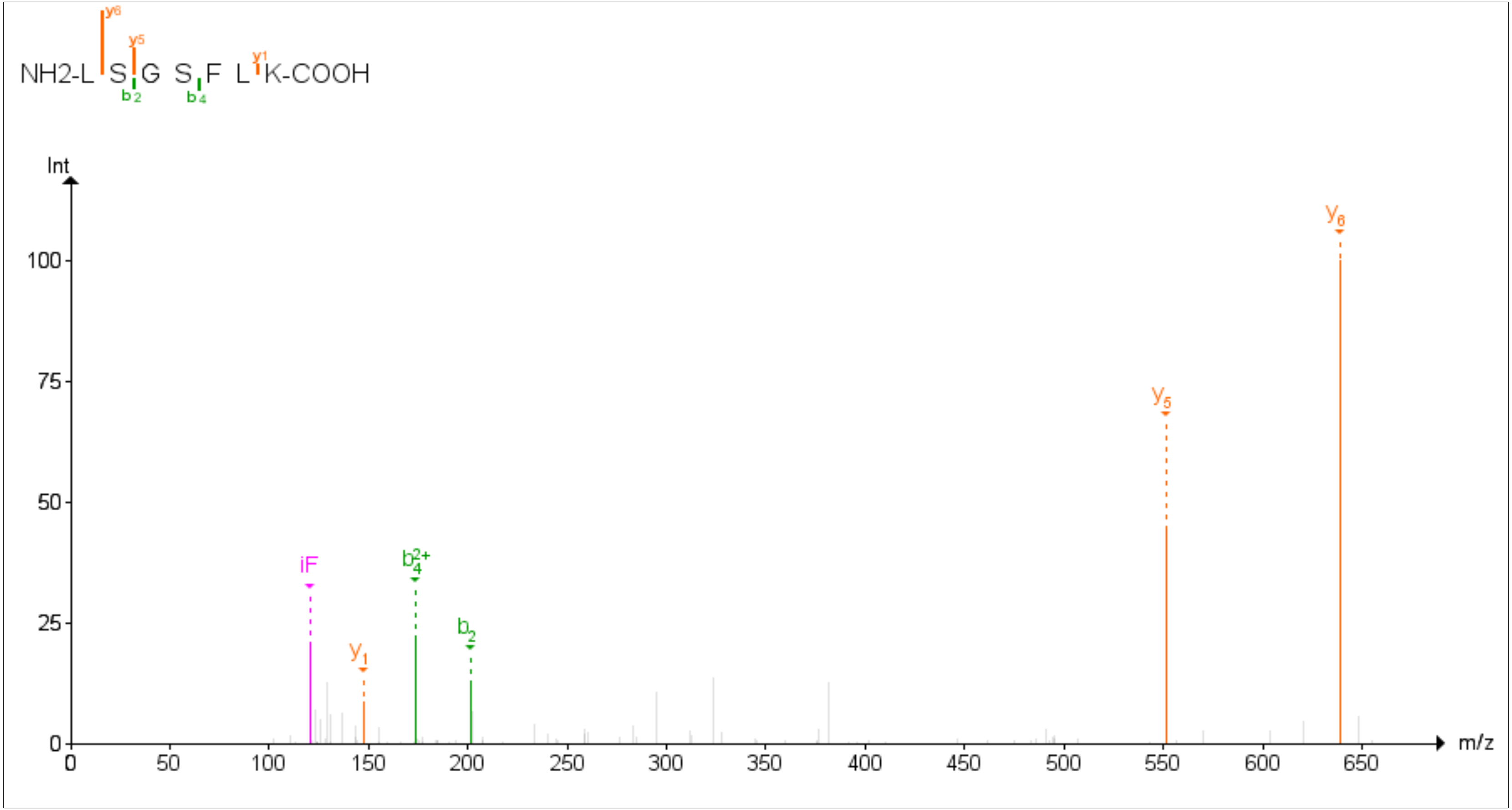

NH2-L S G S F L K-COOH
y6
y5
y1
b2
b4
Int
100
75
50
25
0
iF
y1
b_4^{2+}
b2
y5
y6
0 50 100 150 200 250 300 350 400 450 500 550 600 650
m/z

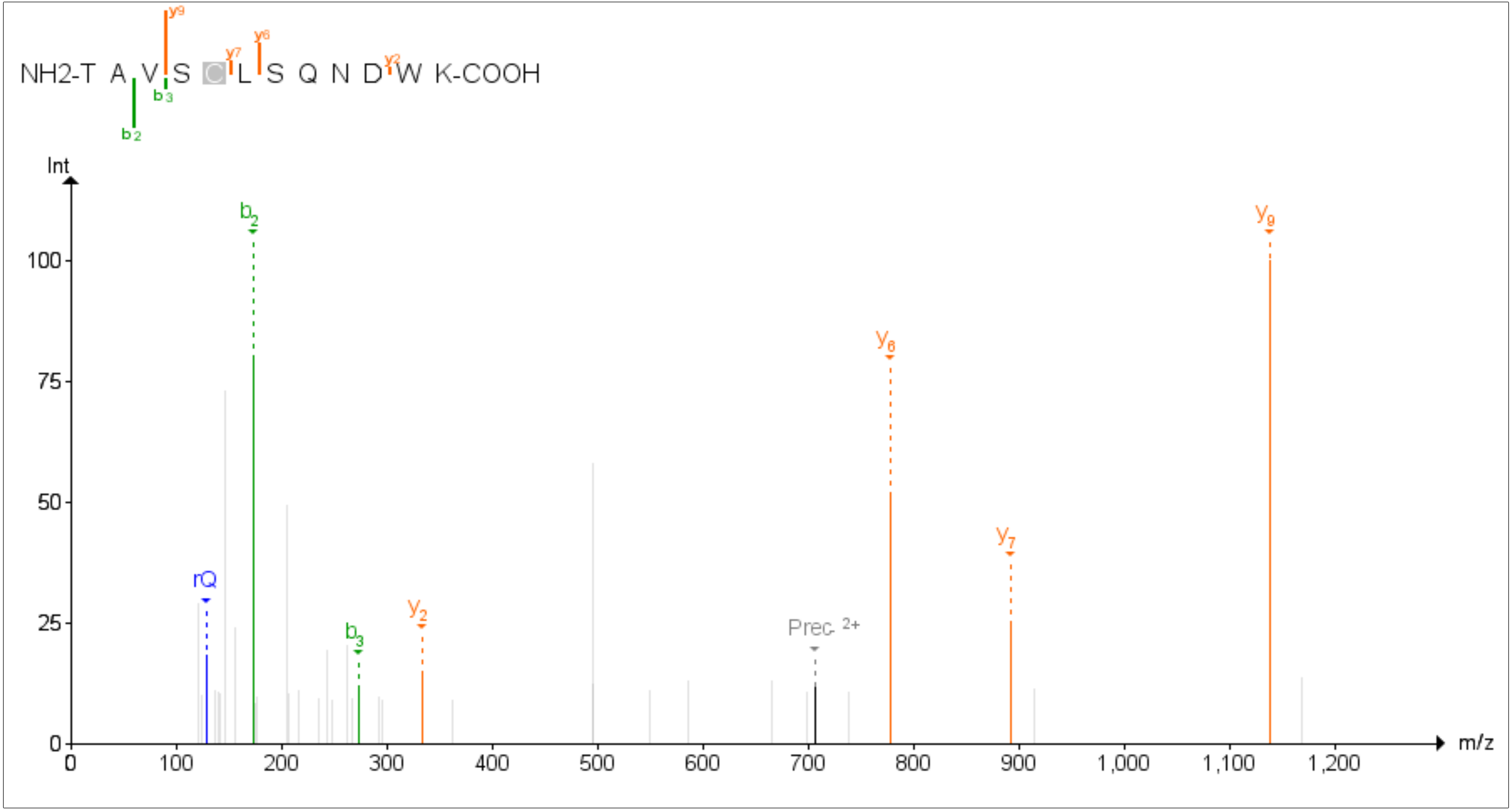

NH2-T A V S C L S Q N D W K-COOH
y9
y7
y6
y2
b2
b3
Int
100
75
50
25
0
rQ
b2
b3
y2
Prec 2+
y6
y7
y9
0
100
200
300
400
500
600
700
800
900
1,000
1,100
1,200
m/z

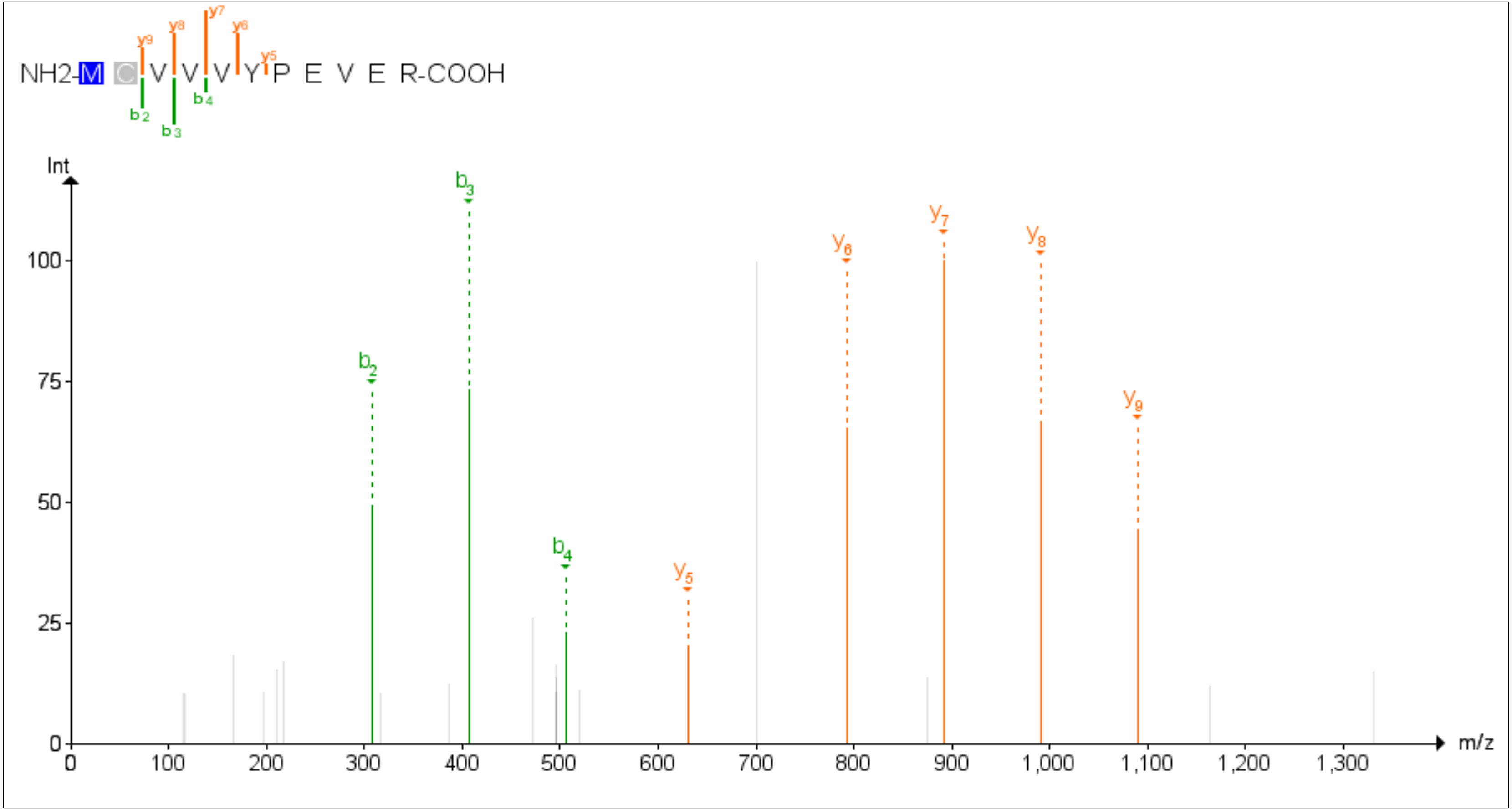
NH2-M C V V V Y P E V E R-COOH
y9 y8 y7 y6 y5
b2 b3 b4
Int
m/z
0
25
50
75
100
0
100
200
300
400
500
600
700
800
900
1,000
1,100
1,200
1,300
b_2
b_3
b_4
y_5
y_6
y_7
y_8
y_9

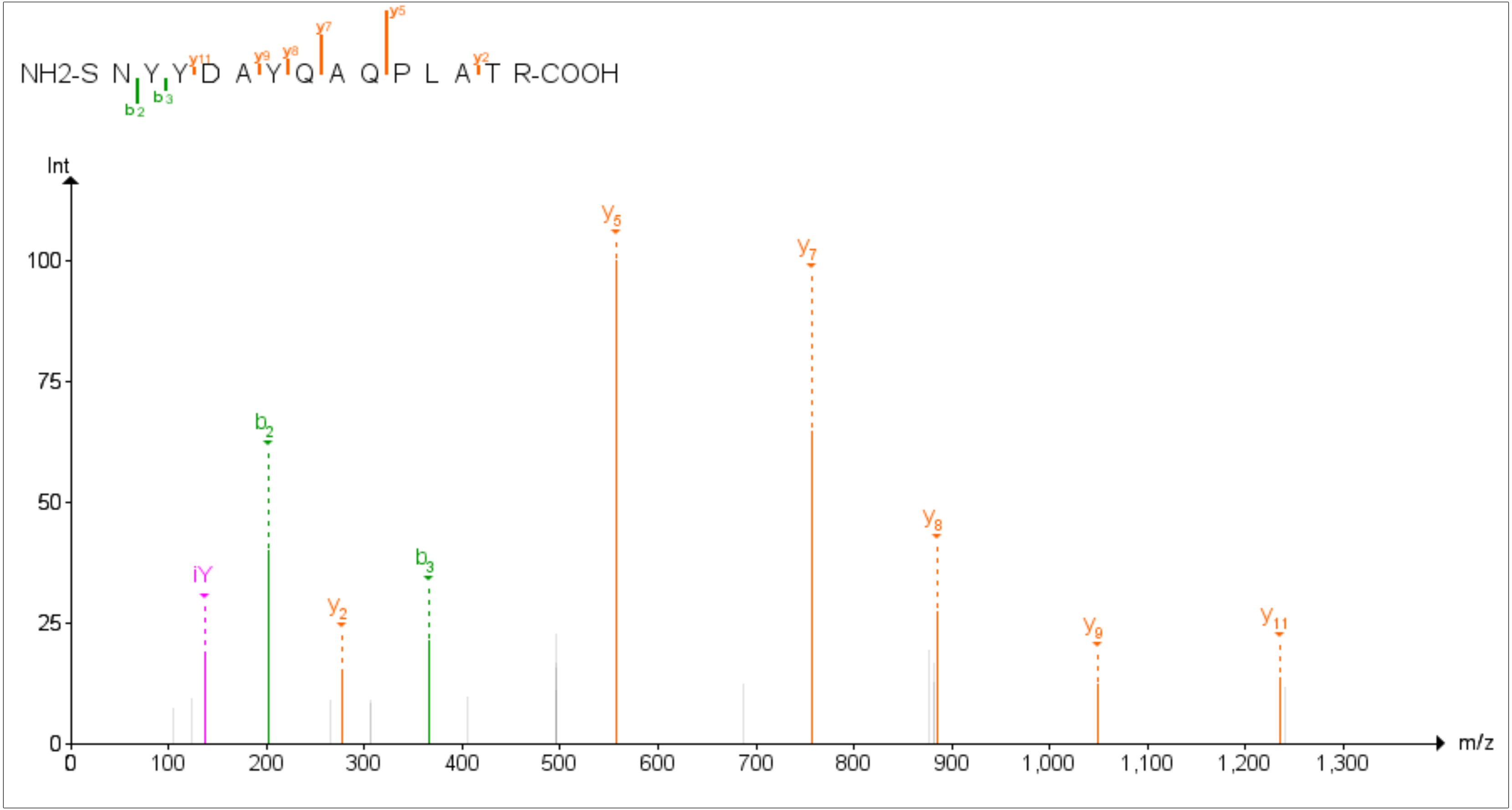

NH2-S N Y Y D A Y Q A Q P L A T R-COOH
b2
b3
y11
y9
y8
y7
y5
y2
Int
100
75
50
25
0
iY
b2
y2
b3
y5
y7
y8
y9
y11
0
100
200
300
400
500
600
700
800
900
1,000
1,100
1,200
1,300
m/z

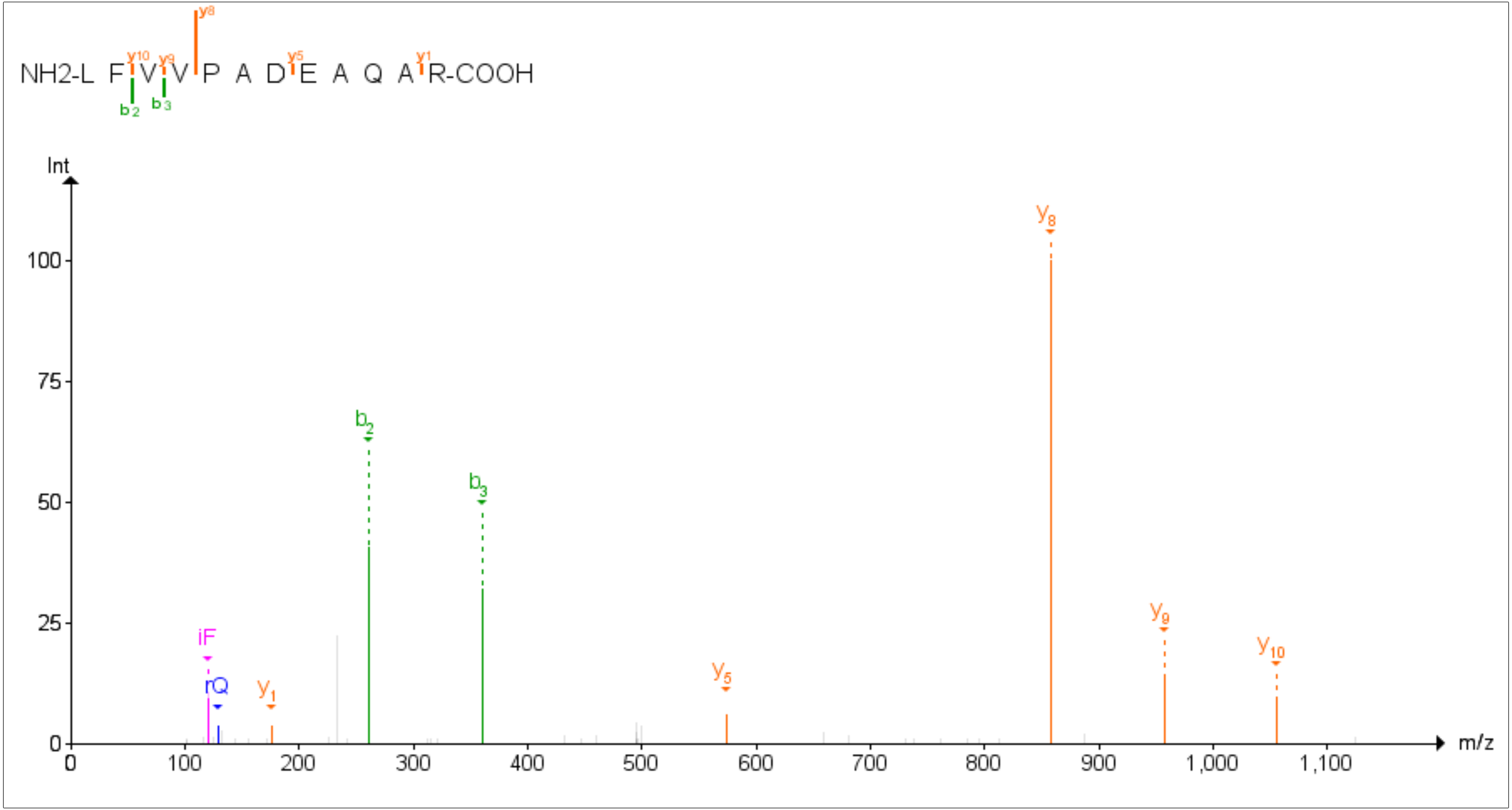

NH2-L F V V P A D E A Q A R-COOH
y10
y9
y8
y5
y1
b2
b3
Int
100
75
50
25
0
iF
rQ
y1
b2
b3
y5
y8
y9
y10
0
100
200
300
400
500
600
700
800
900
1,000
1,100
m/z

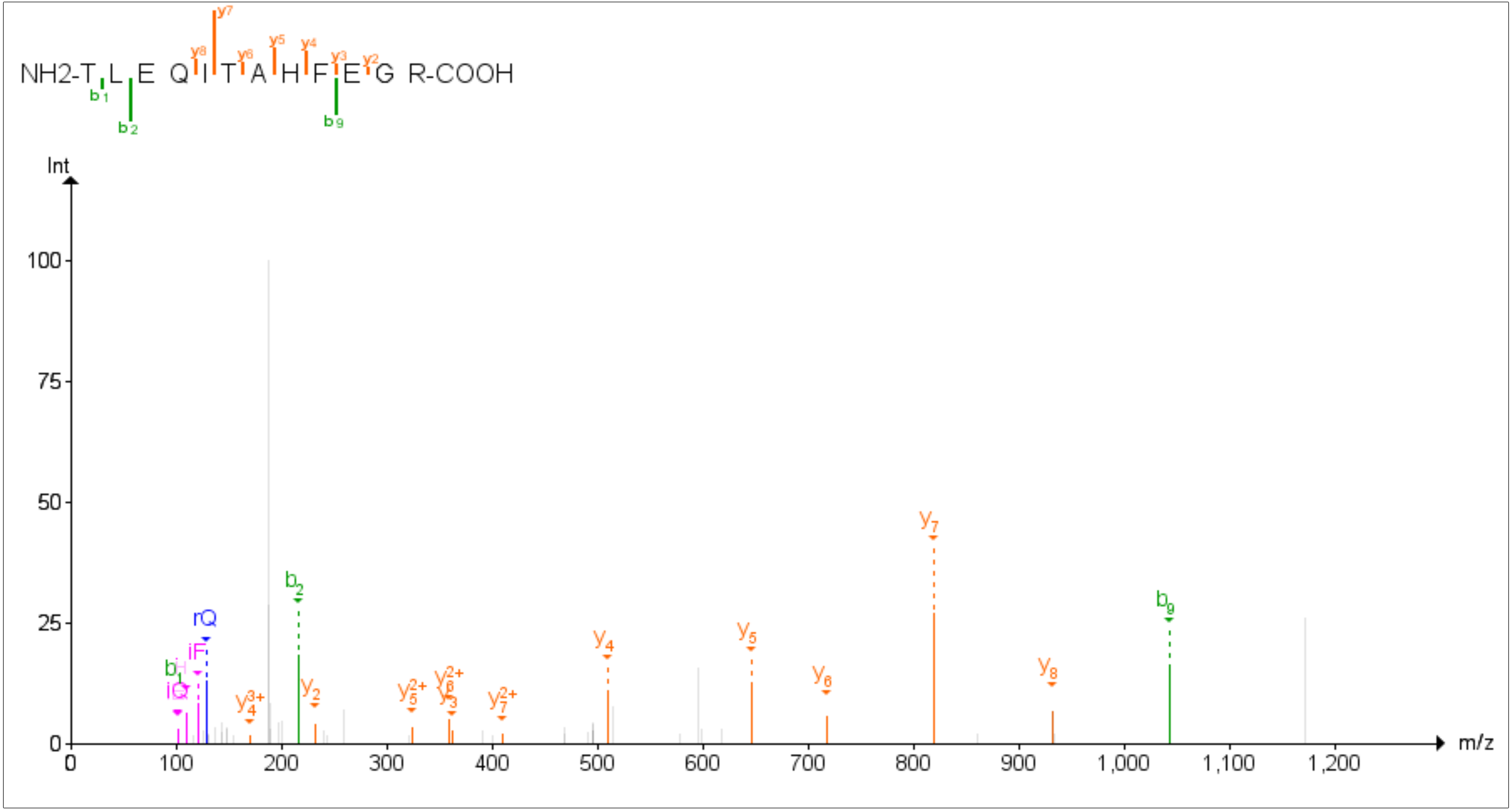

NH2-T L E Q I T A H F E G R-COOH
b1
b2
b9
y8
y7
y6
y5
y4
y3
y2
Int
100
75
50
25
0
m/z
0
100
200
300
400
500
600
700
800
900
1,000
1,100
1,200
rQ
iF
iQ
b2
y2
y4
y5
y6
y7
y8
b9

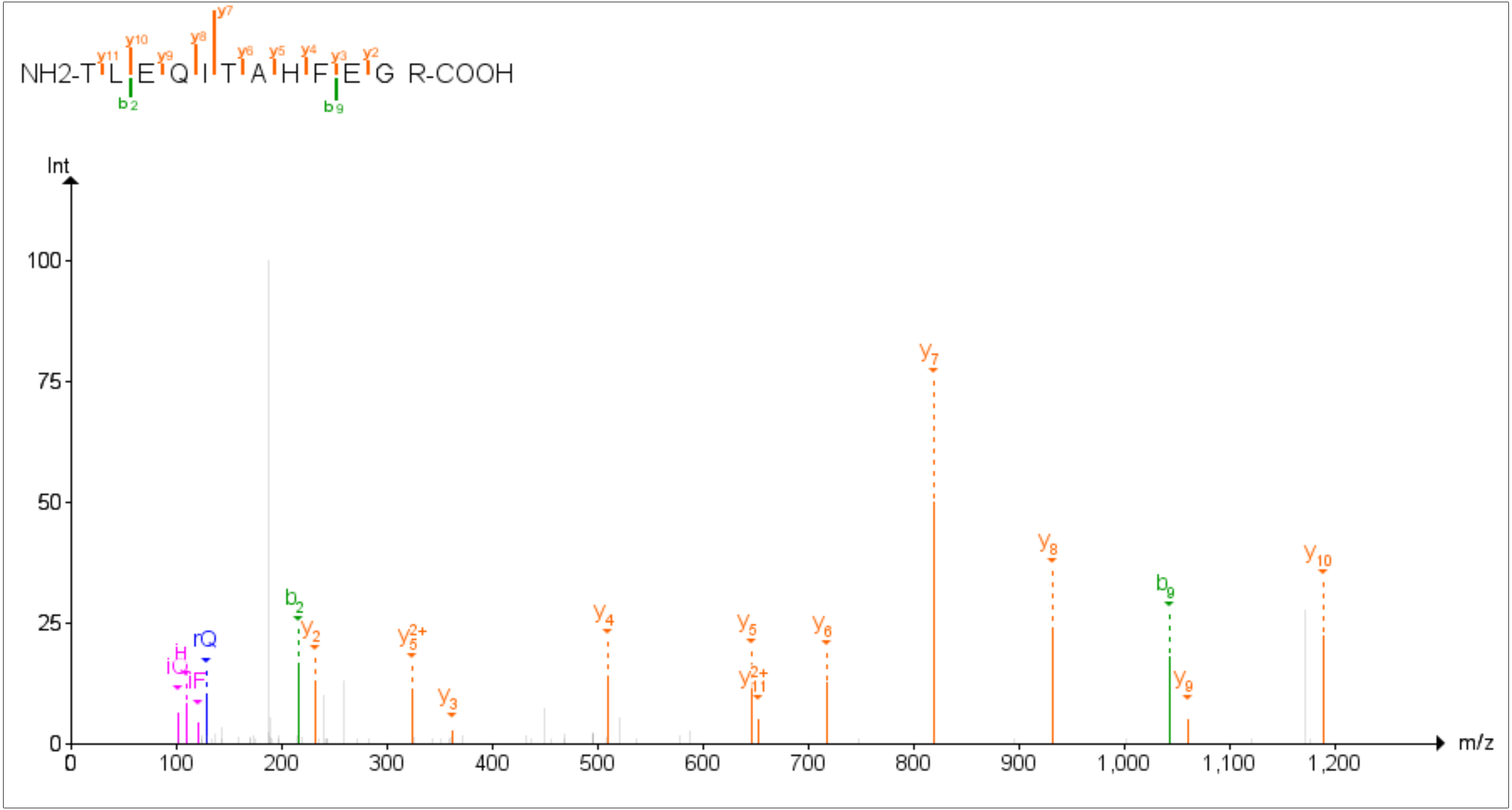

NH2-T L E Q I T A H F E G R-COOH
y11 y10 y9 y8 y7 y6 y5 y4 y3 y2
b2 b9
Int
m/z
y7
y8
y10
b9
y9
y4
y5
y6
y11 2+
y5 2+
y3
y2
b2
rQ
iH
iQ
iF
0
25
50
75
100
0
100
200
300
400
500
600
700
800
900
1,000
1,100
1,200

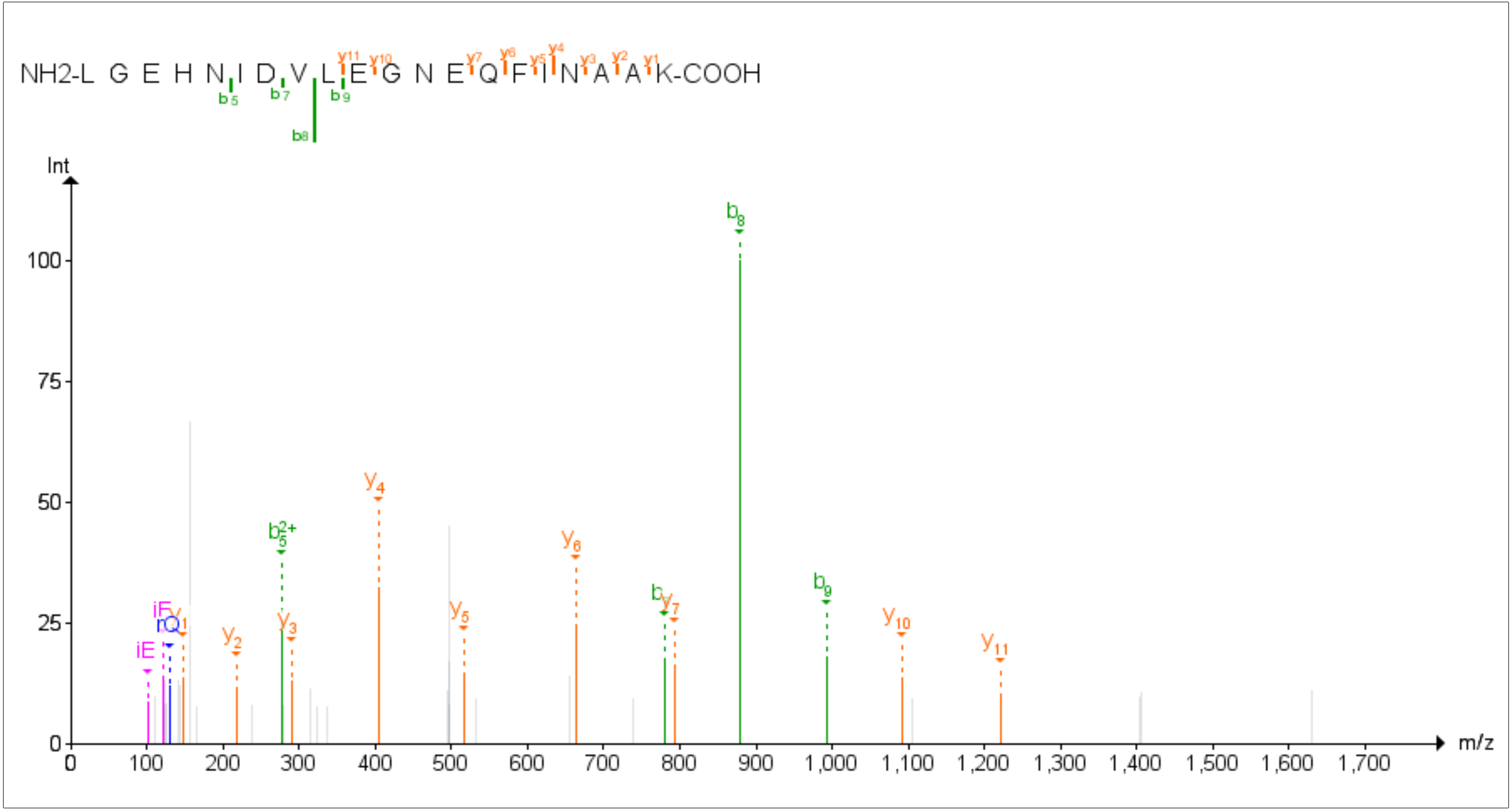

NH2-L G E H N I D V L I E G N E Q F I N A A K-COOH
b5 b7 b8 b9
y11 y10 y7 y6 y5 y4 y3 y2 y1
Int
m/z

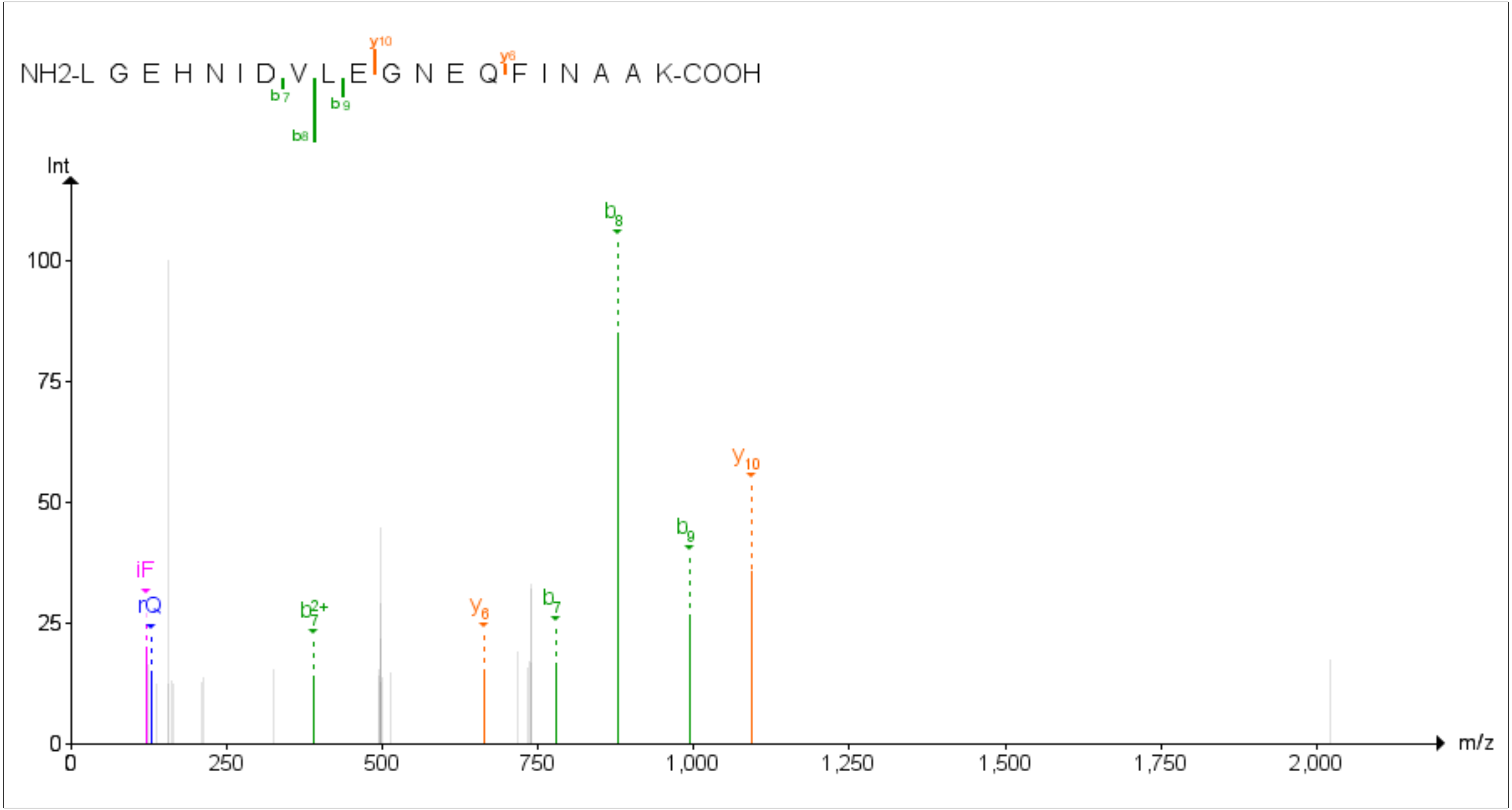

NH2-L G E H N I D V L E G N E Q F I N A A K-COOH
y10
y6
b7
b8
b9
Int
100
75
50
25
0
b8
y10
b9
iF
rQ
b7 2+
y6
b7
0
250
500
750
1,000
1,250
1,500
1,750
2,000
m/z

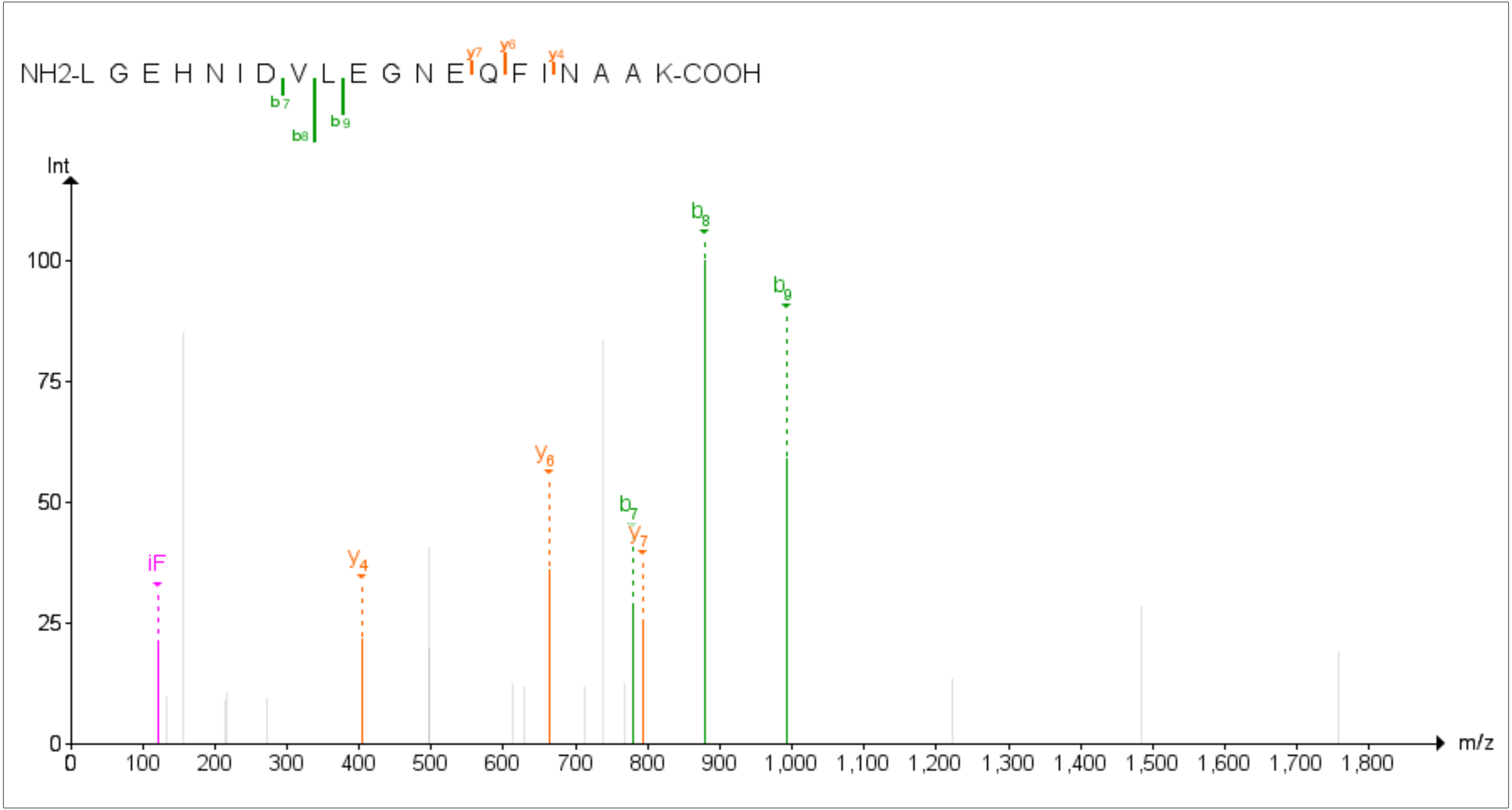
NH2-L G E H N I D V L E G N E Q F I N A A K-COOH
b7
b8
b9
y7
y6
y4
Int
m/z
0
25
50
75
100
100
200
300
400
500
600
700
800
900
1,000
1,100
1,200
1,300
1,400
1,500
1,600
1,700
1,800
iF
y4
y6
b7
y7
b8
b9

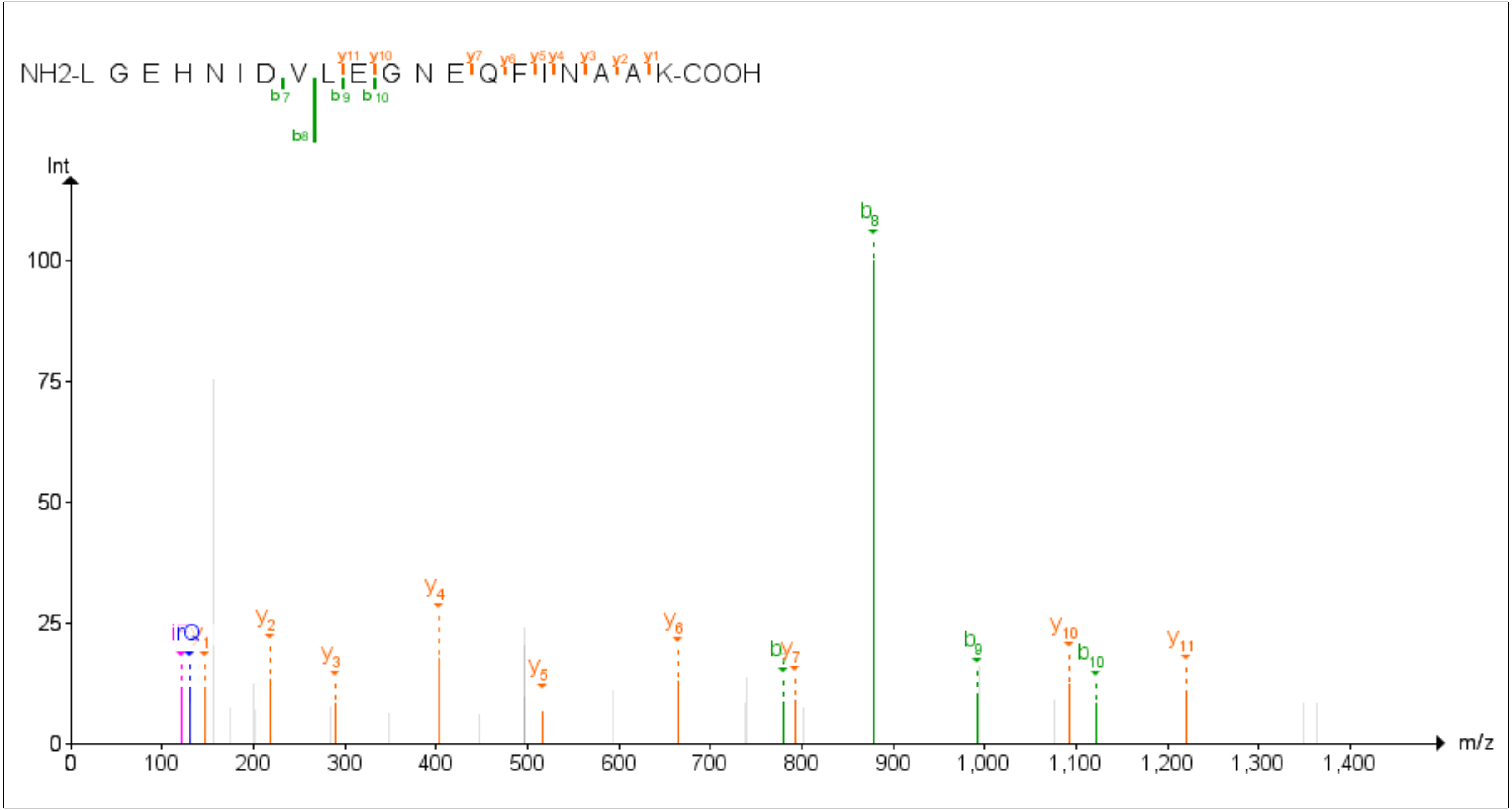

NH2-L G E H N I D V L E G N E Q F I N A A K-COOH
y11 y10 y7 y6 y5 y4 y3 y2 y1
b7 b8 b9 b10
Int
100
75
50
25
0
b8
y4
y2
iQ
y1
y3
y5
y6
b7
y7
b9
y10
b10
y11
0 100 200 300 400 500 600 700 800 900 1,000 1,100 1,200 1,300 1,400
m/z

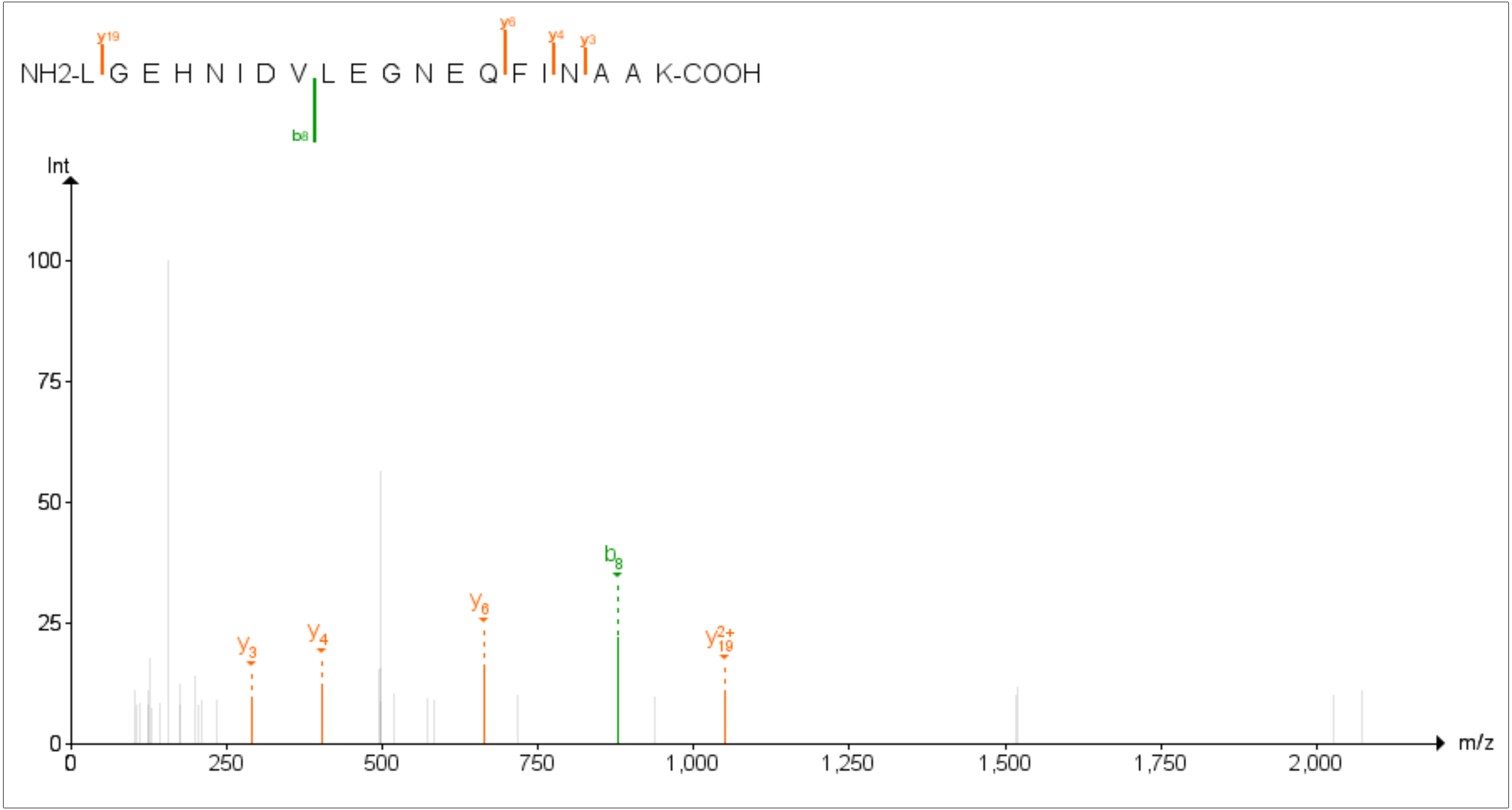

NH2-L G E H N I D V L E G N E Q F I N A A K-COOH
y19
y6
y4
y3
b8
Int
100
75
50
25
0
y3
y4
y6
b8
y19 2+
0
250
500
750
1,000
1,250
1,500
1,750
2,000
m/z

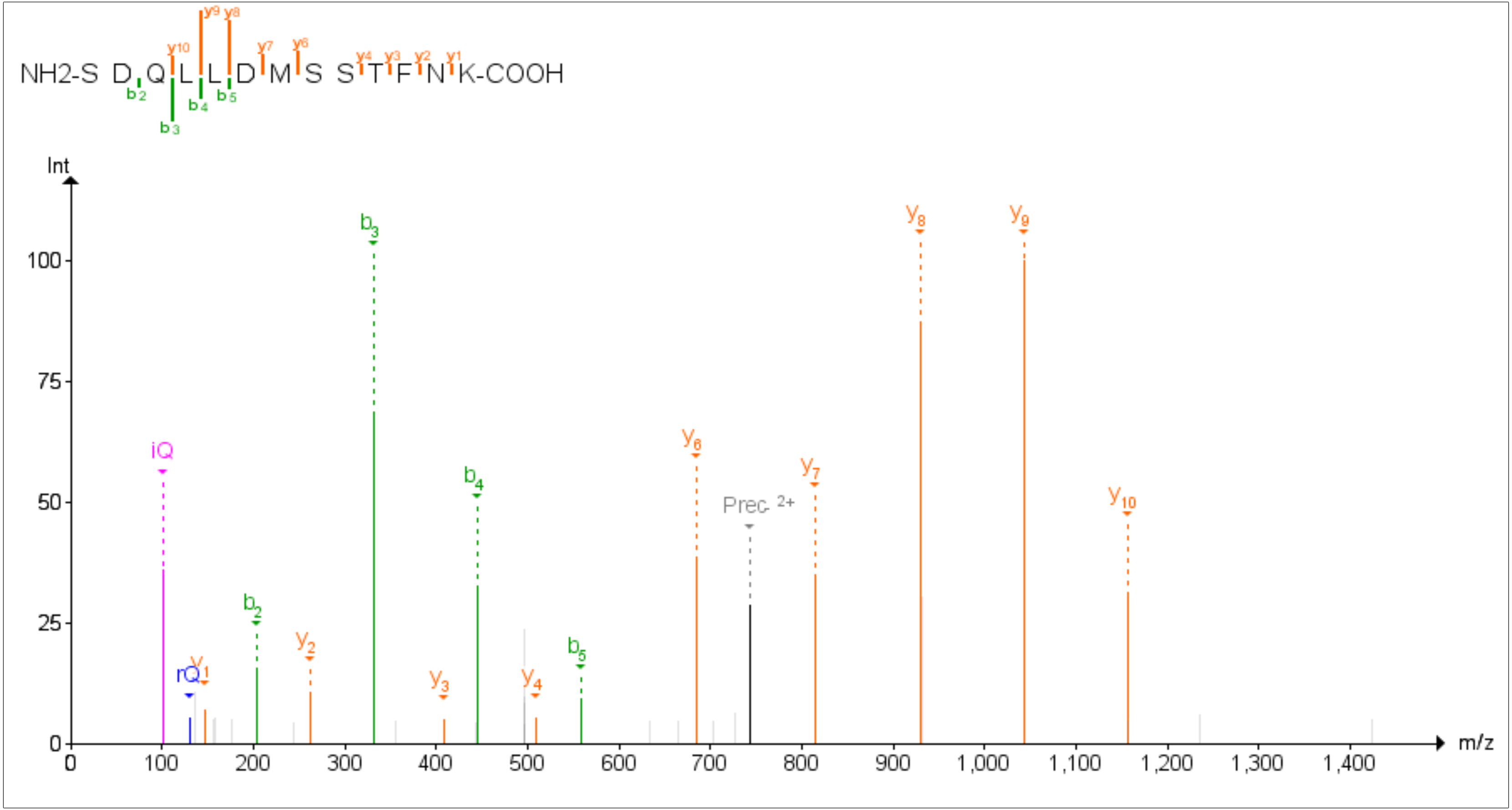

NH2-S D Q L L D M S S T F N K-COOH
y10 y9 y8 y7 y6 y4 y3 y2 y1
b2 b3 b4 b5
Int
100
75
50
25
0
m/z
0
100
200
300
400
500
600
700
800
900
1,000
1,100
1,200
1,300
1,400
iQ
rQ
y1
b2
y2
b3
y3
b4
y4
b5
y6
Prec 2+
y7
y8
y9
y10

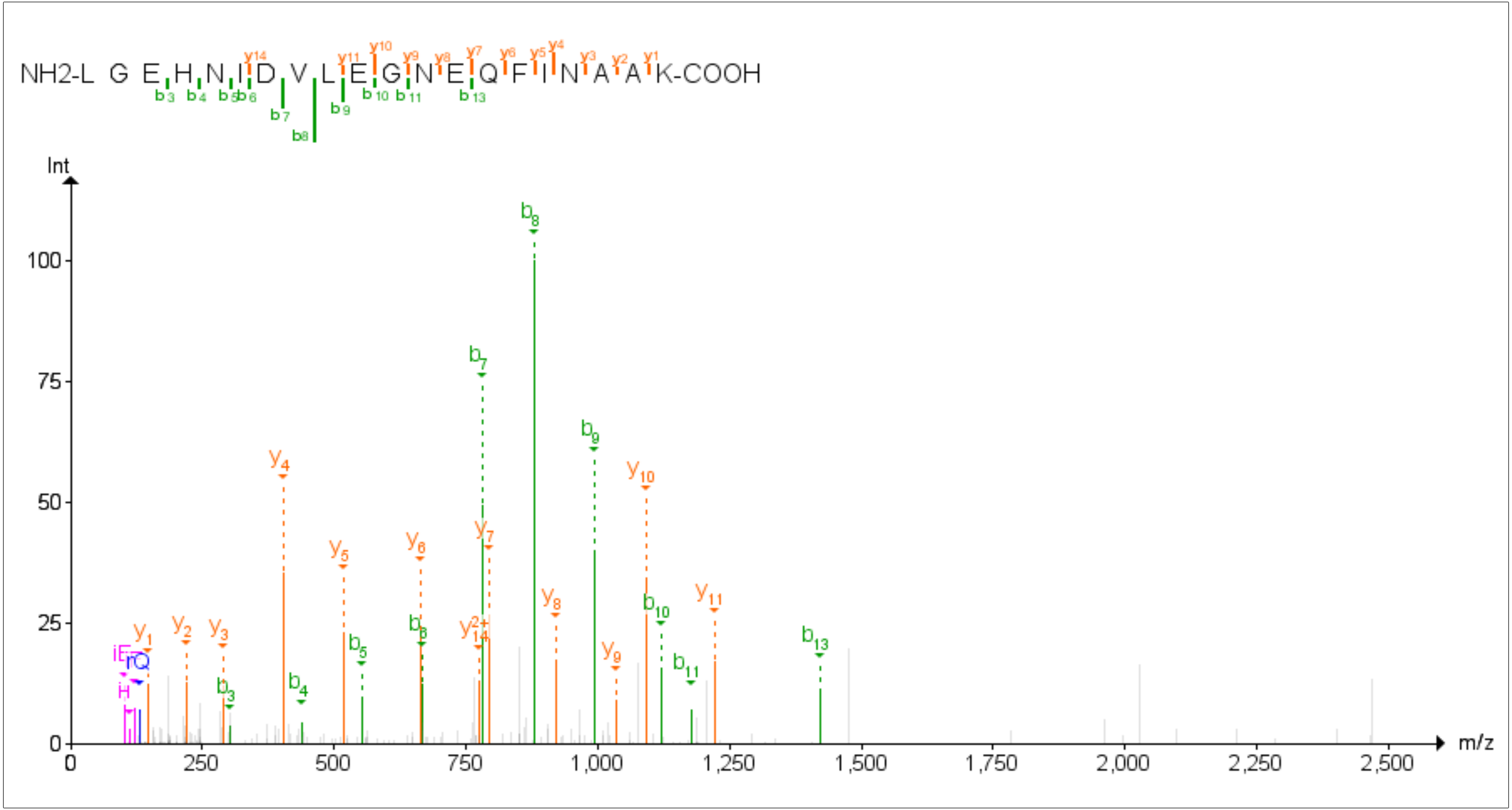

NH2-L G E H N I D V L E G N E Q F I N A A K-COOH
Int
m/z
b8
b7
b9
y4
y10
y5
y6
y7
b6
y8
b10
y11
y2
y3
y1
b5
y14 2+
b13
y9
b11
b4
b3
iE
rQ
iH

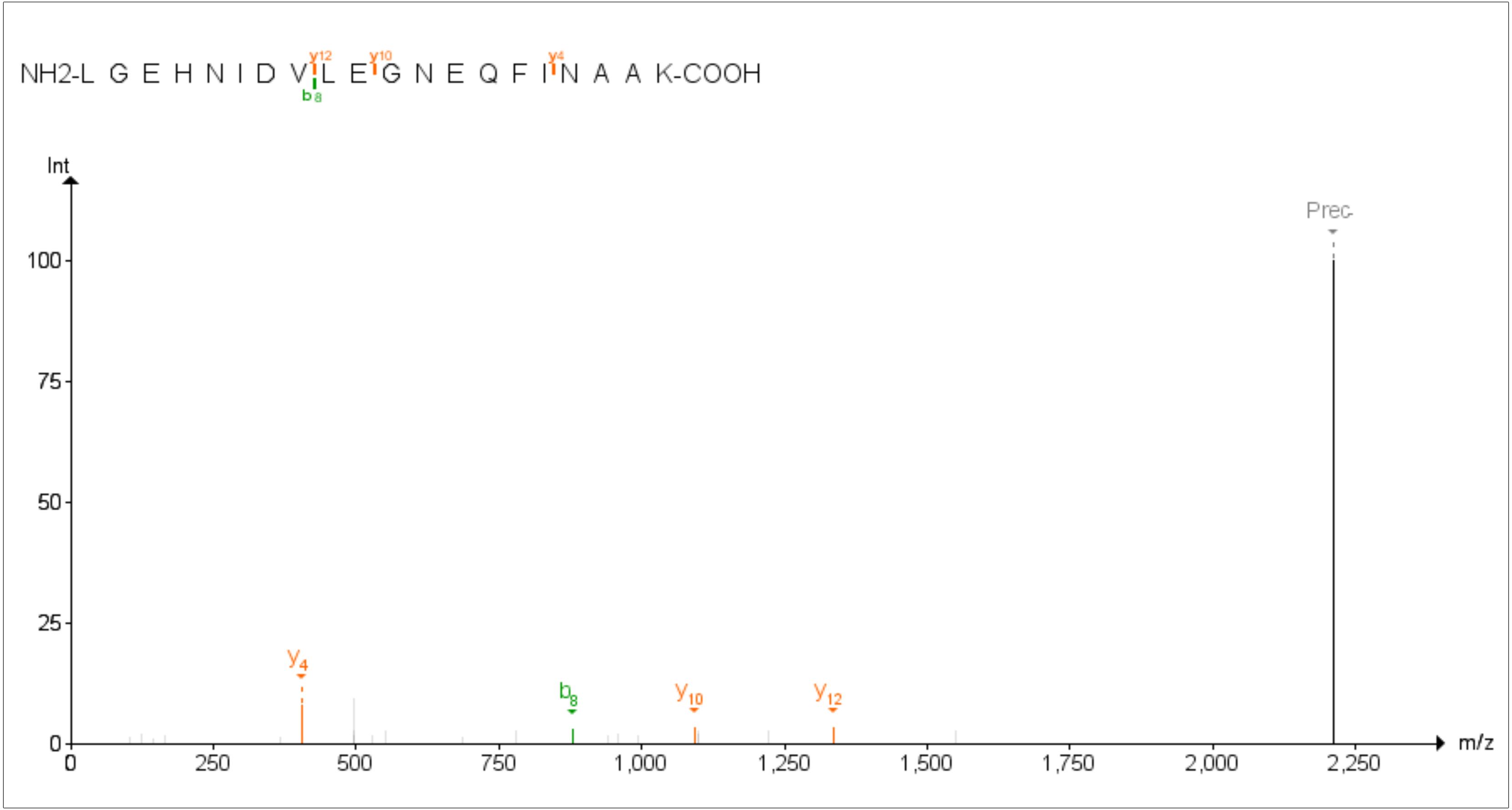

NH2-L G E H N I D V I L E G N E Q F I N A A K-COOH
y12
y10
y4
b8
Int
100
75
50
25
0
Prec.
y4
b8
y10
y12
0
250
500
750
1,000
1,250
1,500
1,750
2,000
2,250
m/z

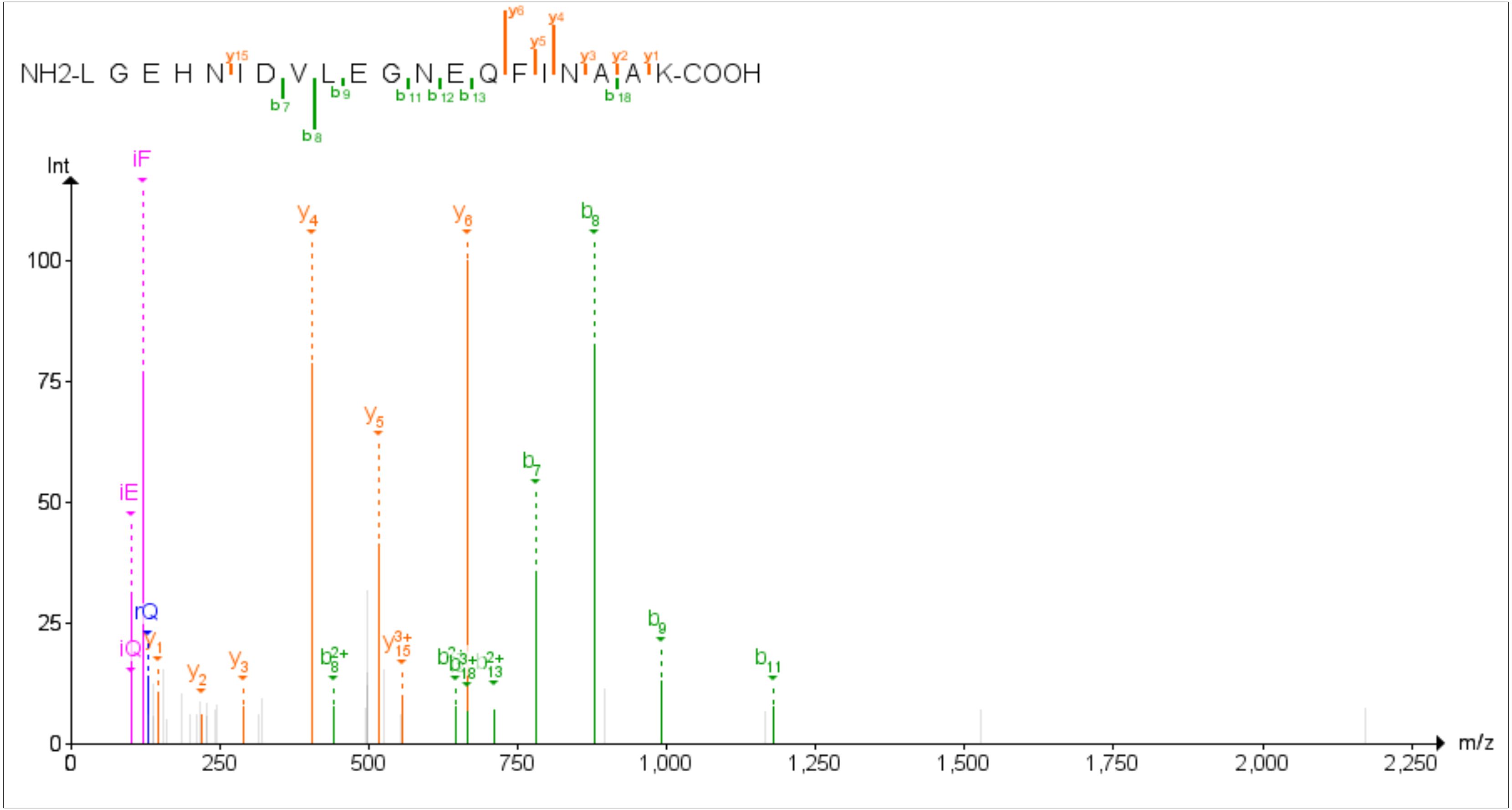

NH2-L G E H N I D V L E G N E Q F I N A A K-COOH
y15 y6 y5 y4 y3 y2 y1
b7 b8 b9 b11 b12 b13 b18
Int
m/z
iF iE iQ rQ
y1 y2 y3 y4 y5 y6
b7 b8 b9 b11
0 25 50 75 100
0 250 500 750 1,000 1,250 1,500 1,750 2,000 2,250

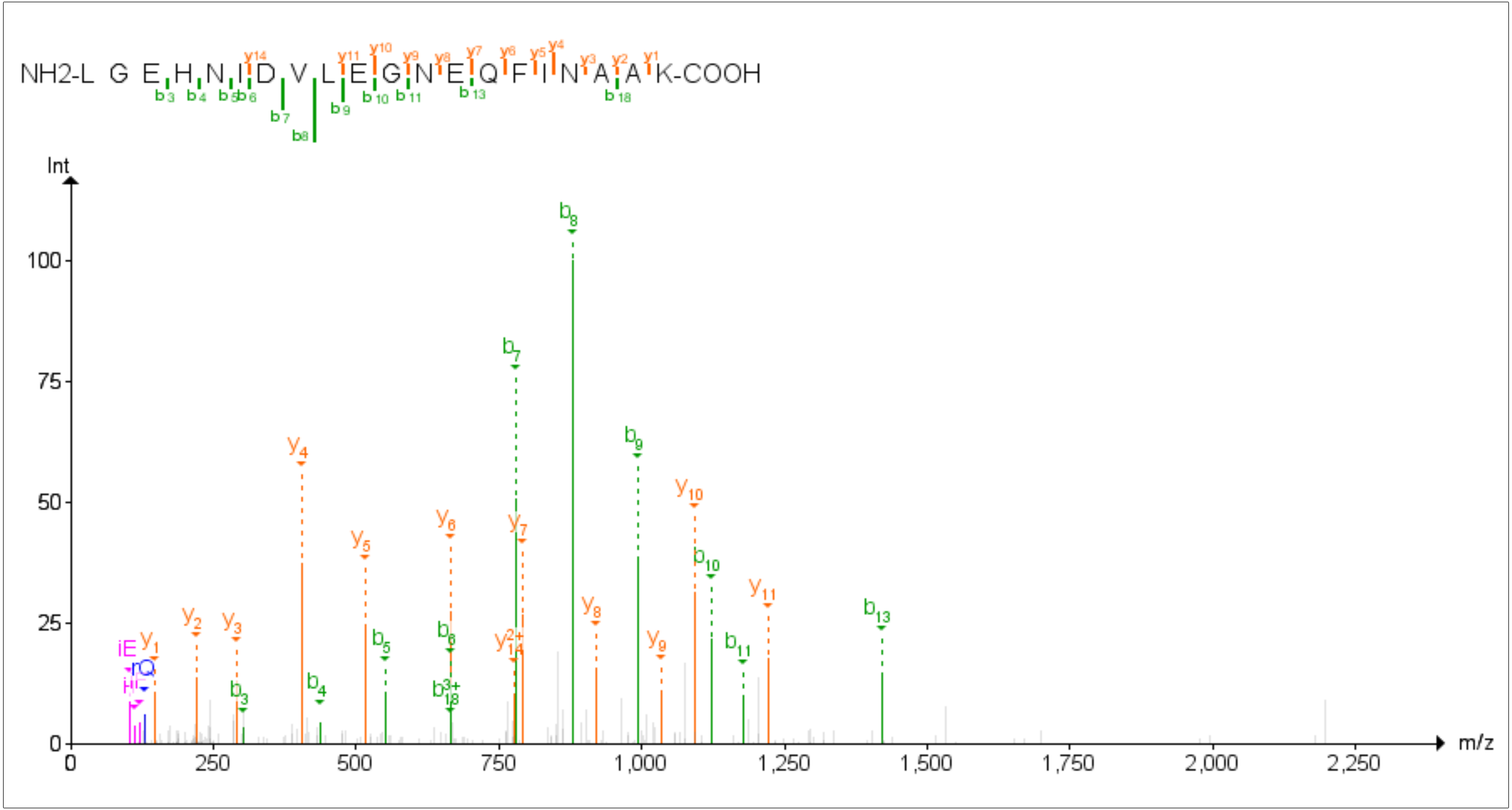

NH2-L G E H N I D V L E G N E Q F I N A A K-COOH
Int
m/z

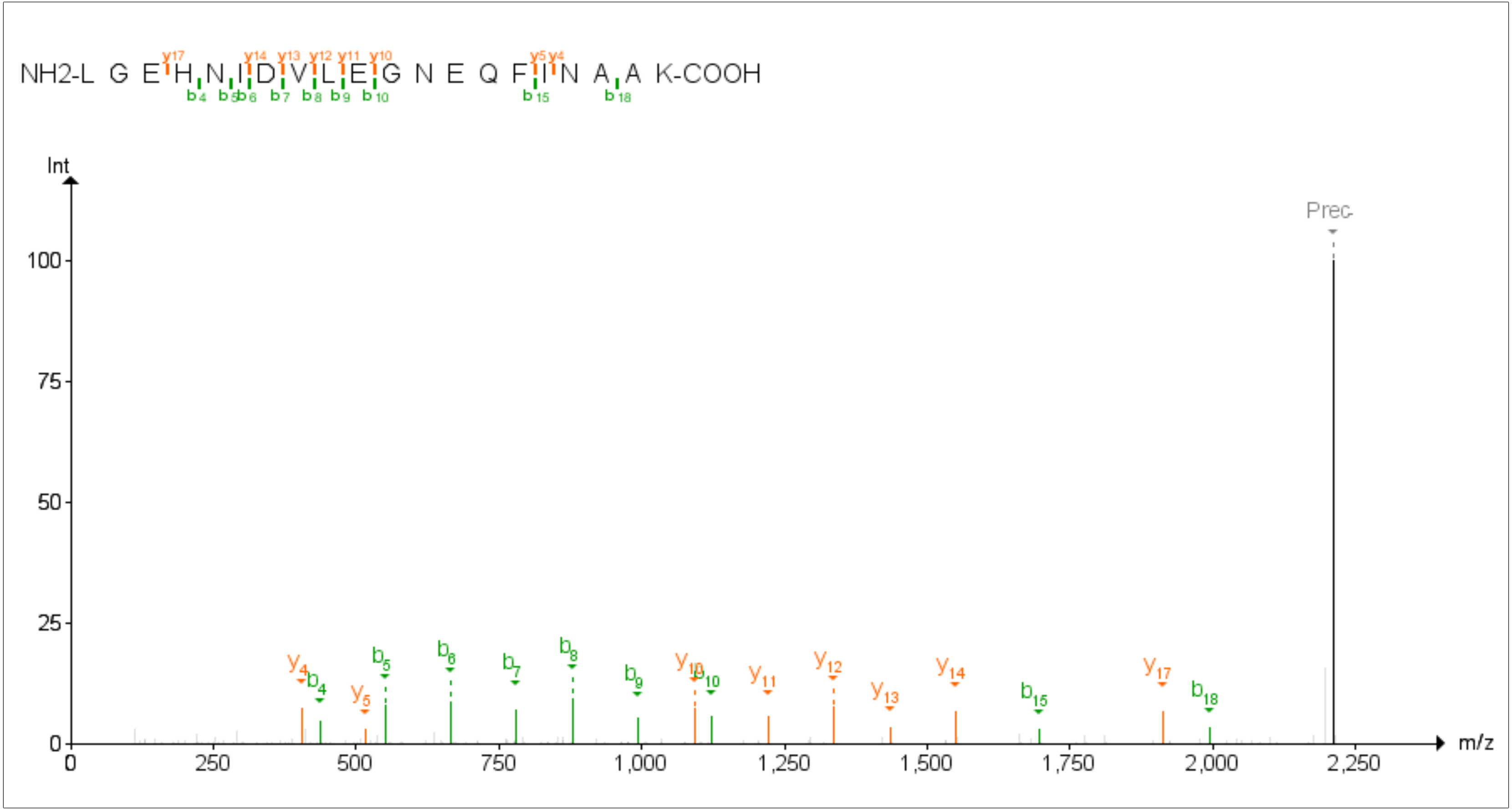

NH2-L G E H N I D V L E G N E Q F I N A A K-COOH
Int
Prec
m/z
0
25
50
75
100
250
500
750
1,000
1,250
1,500
1,750
2,000
2,250

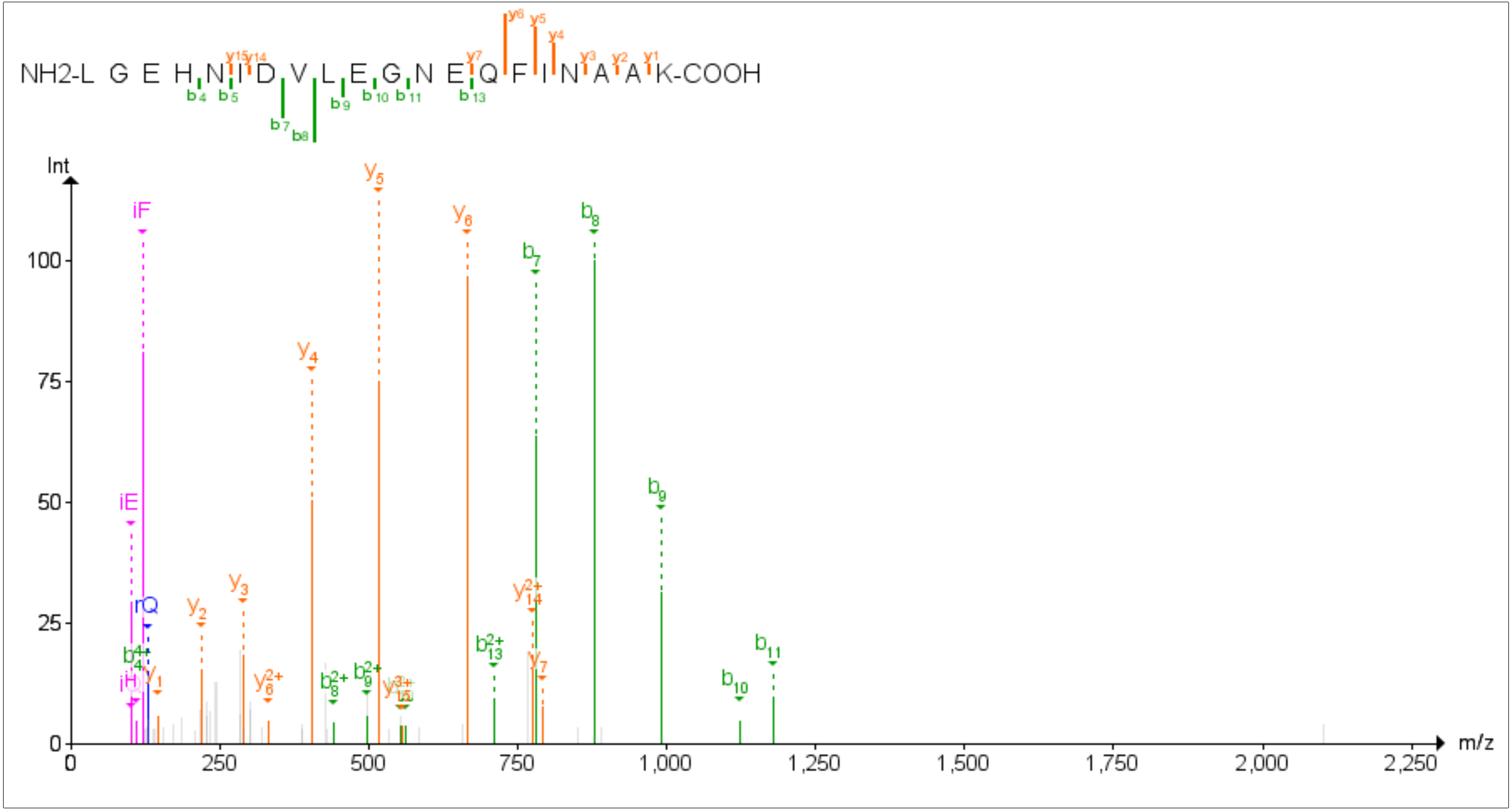

NH2-L G E H N I D V L E G N E Q F I N A A K-COOH
y15 y14 y7 y6 y5 y4 y3 y2 y1
b4 b5 b7 b8 b9 b10 b11 b13
Int
100
75
50
25
0
iF
iE
iH
rQ
y5
y6
y4
y3
y2
y1
b8
b7
b9
b10
b11
m/z
0
250
500
750
1,000
1,250
1,500
1,750
2,000
2,250

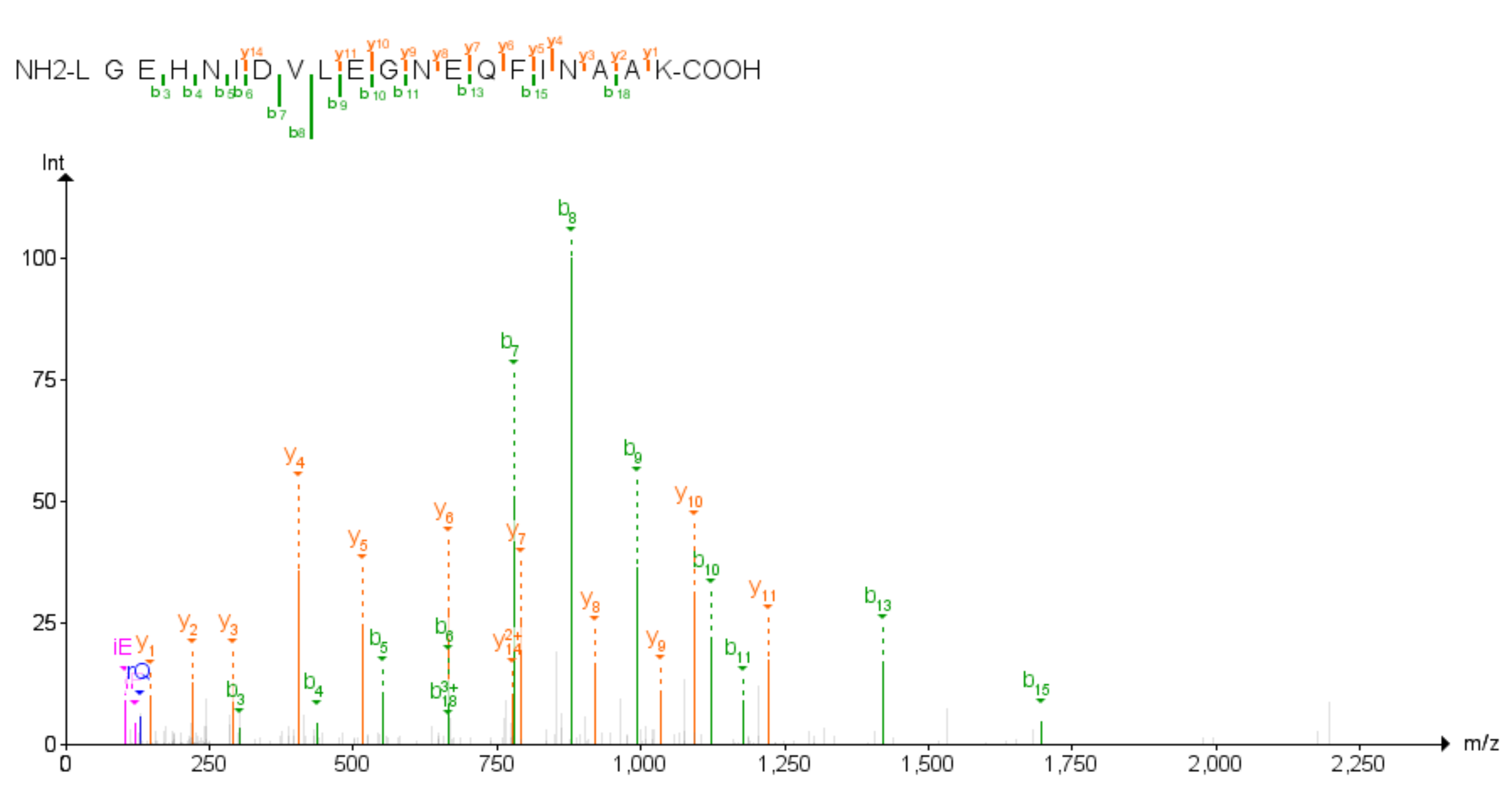

NH2-L G E H N I D V L E G N E Q F I N A A K-COOH
b3 b4 b5 b6 b7 b8 b9 b10 b11 b13 b15 b18
y14 y11 y10 y9 y8 y7 y6 y5 y4 y3 y2 y1
Int
m/z
0
25
50
75
100
250
500
750
1,000
1,250
1,500
1,750
2,000
2,250
b8
b7
b9
y4
y10
y6
y7
y5
b10
y11
b13
y8
y9
b11
b15
b5
b6
y14 2+
y2
y3
y1
iE
b3
b4
b18 3+

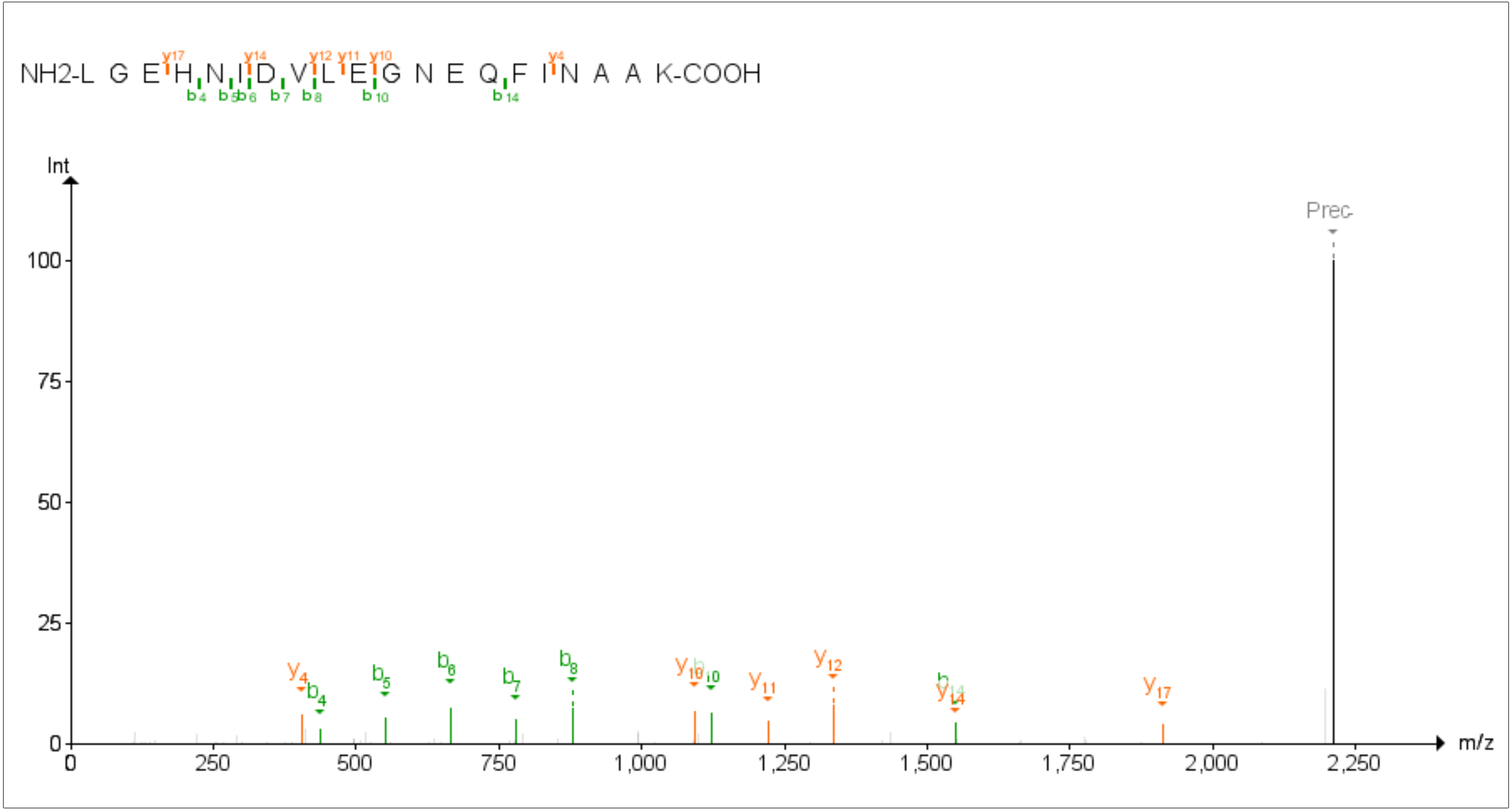

NH2-L G E H N I D V L E G N E Q F I N A A K-COOH
y17 y14 y12 y11 y10 y4
b4 b5 b6 b7 b8 b10 b14
Int
100
75
50
25
0
Prec
y4 b4 b5 b6 b7 b8 y10 b10 y11 y12 b14 y14 y17
0
250
500
750
1,000
1,250
1,500
1,750
2,000
2,250
m/z

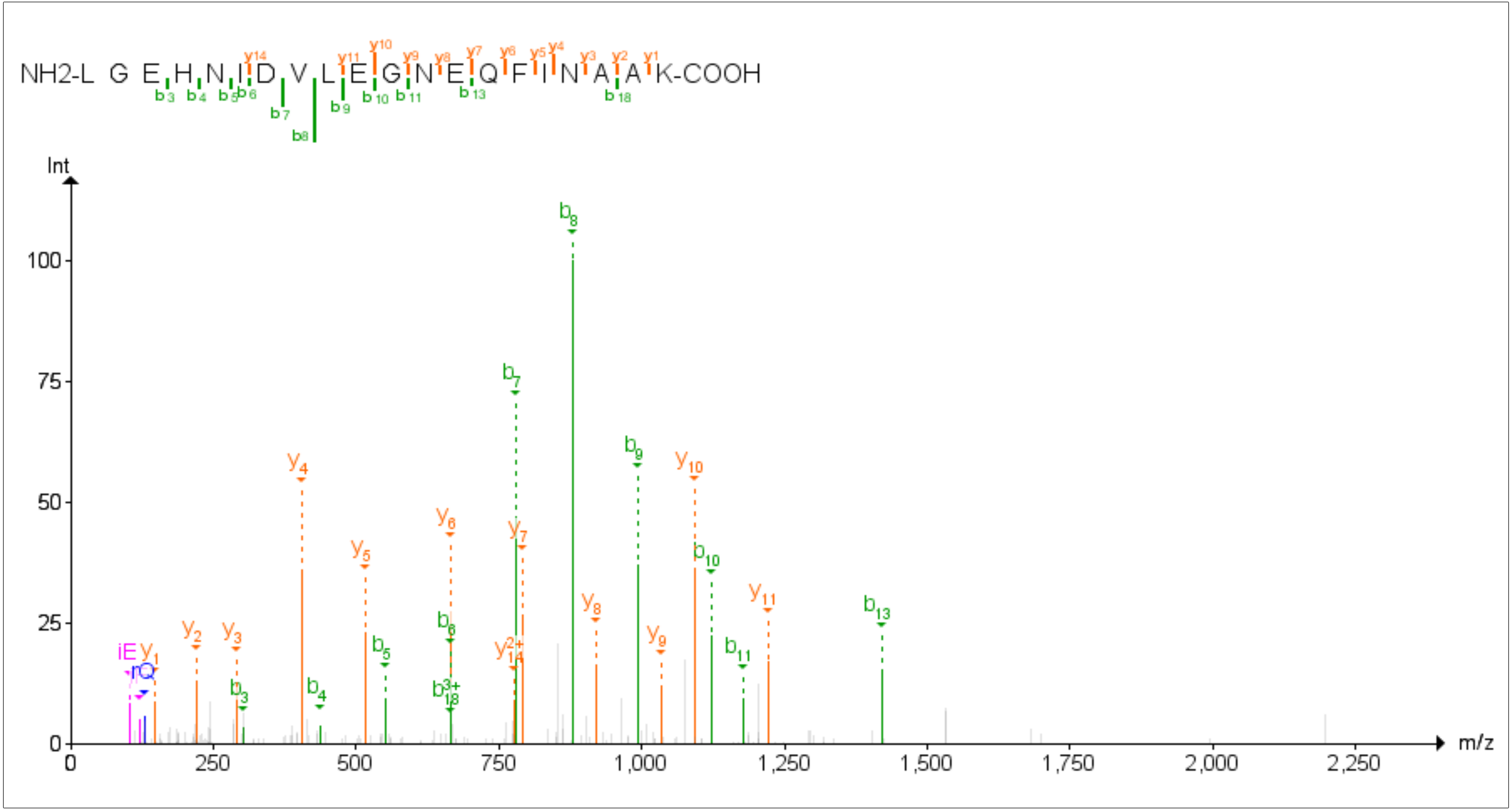

NH2-L G E H N I D V L E G N E Q F I N A A K-COOH
Int
m/z
b8
b7
b9
y10
y4
y6
y7
y5
b10
y11
b13
y8
b6
b5
y9
b11
y2
y3
iE
y1
rQ
b3
b4
0
25
50
75
100
250
500
750
1,000
1,250
1,500
1,750
2,000
2,250

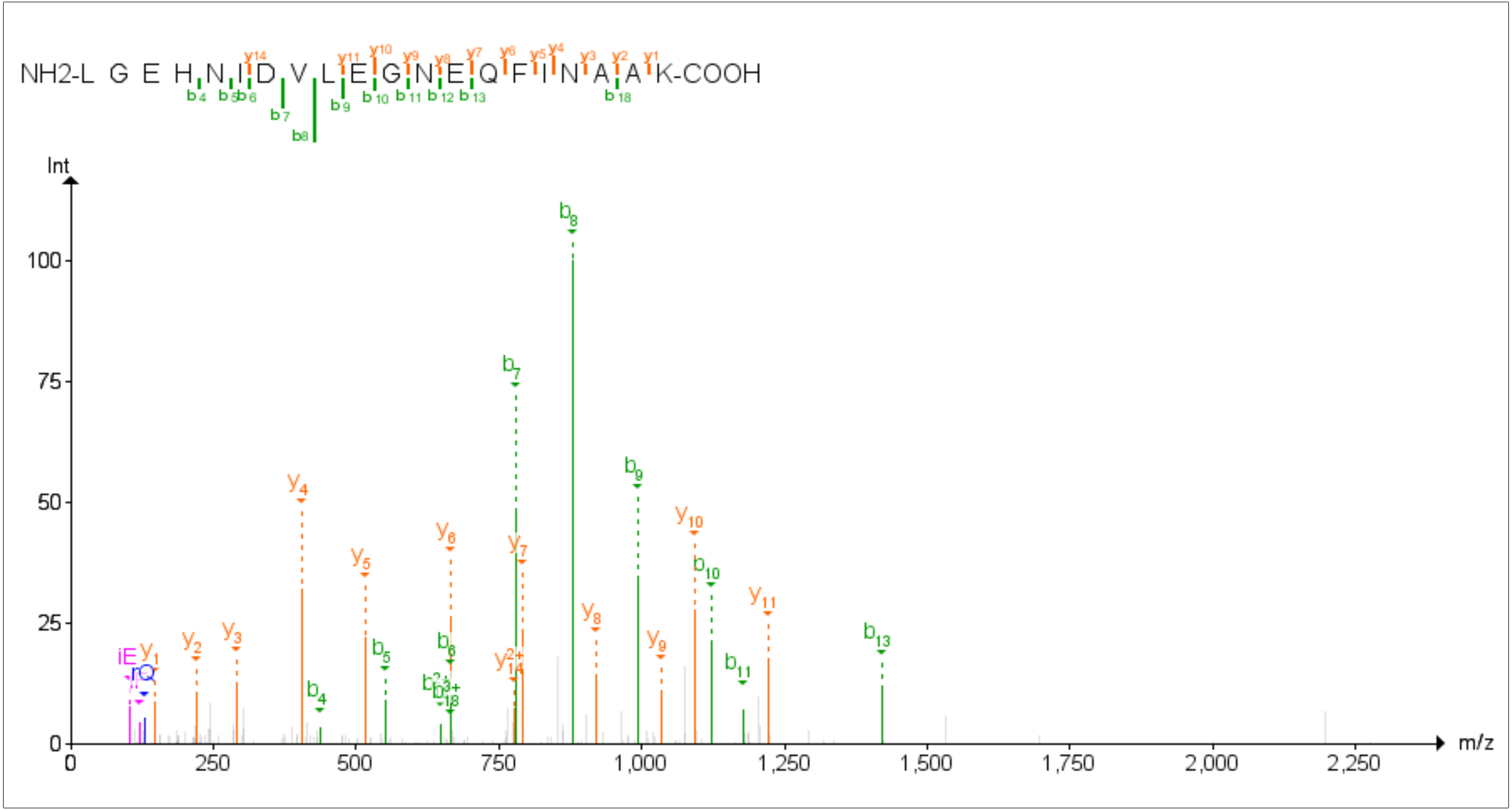
NH2-L G E H N I D V L E G N E Q F I N A A K-COOH
Int
m/z
0
25
50
75
100
250
500
750
1,000
1,250
1,500
1,750
2,000
2,250

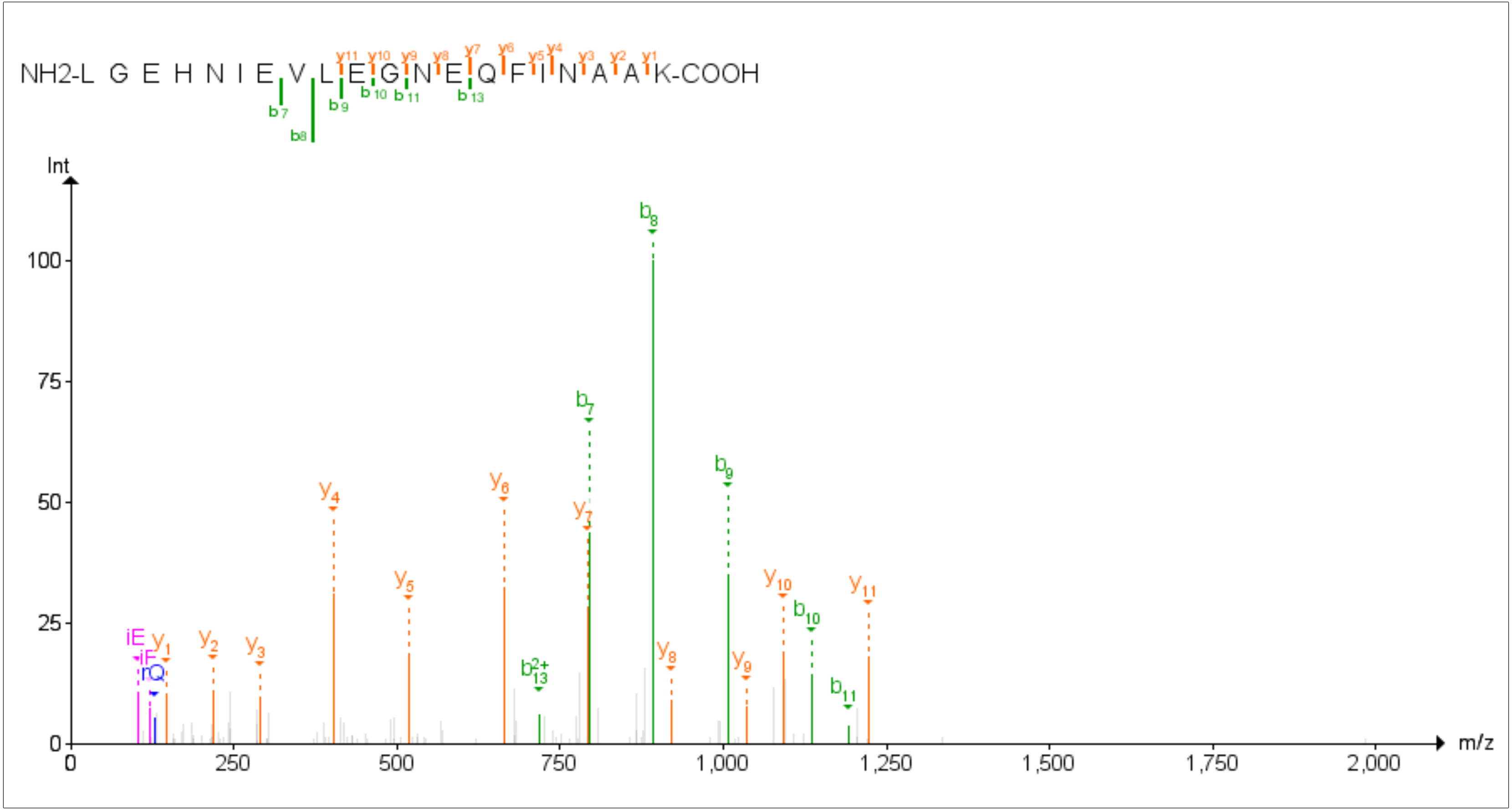

NH2-L G E H N I E V L E G N E Q F I N A A K-COOH
y11 y10 y9 y8 y7 y6 y5 y4 y3 y2 y1
b7 b8 b9 b10 b11 b13
Int
100
75
50
25
0
m/z
0
250
500
750
1,000
1,250
1,500
1,750
2,000
b8
b7
b9
y4
y6
y7
y5
y10
b10
y11
y8
y9
b11
b13 2+
iE
iF
rQ
y1
y2
y3

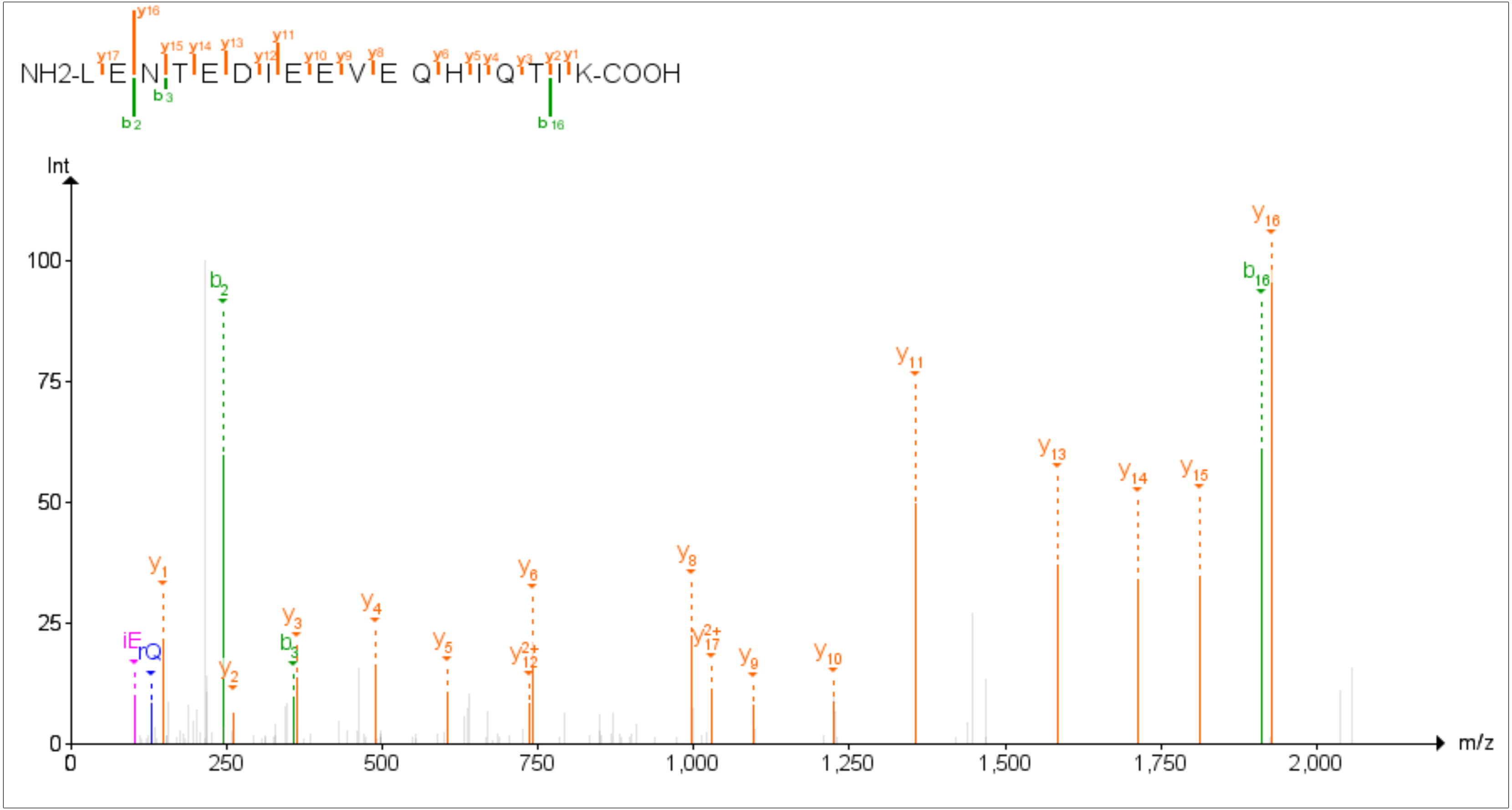
NH2-L E N T E D I E E V E Q H I Q T I K-COOH
y17 y16 y15 y14 y13 y12 y11 y10 y9 y8 y6 y5 y4 y3 y2 y1
b 2 b 3 b 16
Int
m/z
0
250
500
750
1,000
1,250
1,500
1,750
2,000
25
50
75
100
iE
rQ
y1
b2
y2
b3
y3
y4
y5
y12 2+
y6
y8
y17 2+
y9
y10
y11
y13
y14
y15
b16
y16

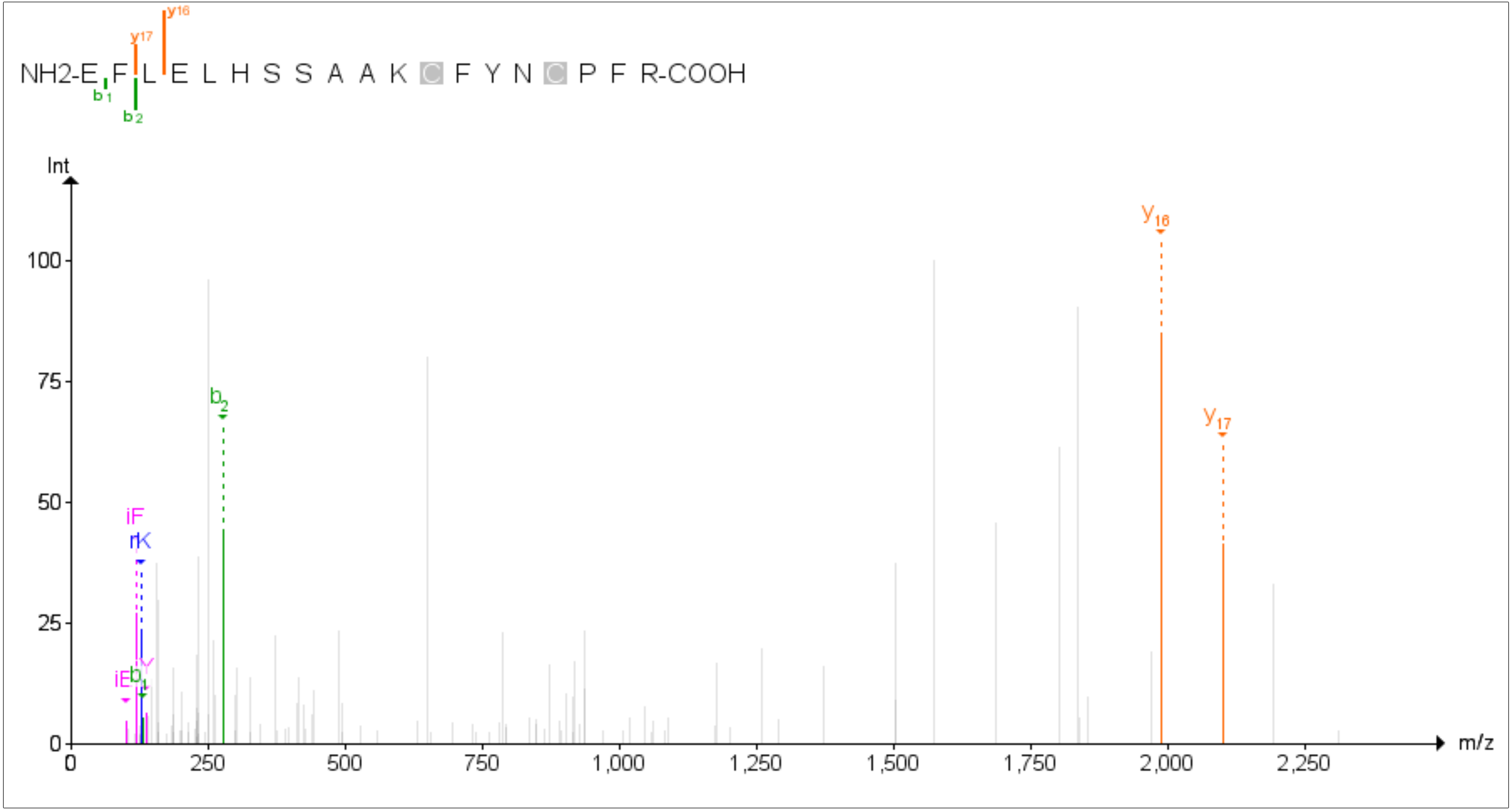

NH2-E F L E L H S S A A K C F Y N C P F R-COOH
y16
y17
b1
b2
Int
100
75
50
25
0
m/z
0
250
500
750
1,000
1,250
1,500
1,750
2,000
2,250
y16
y17
b2
iF
rK
iE
b1

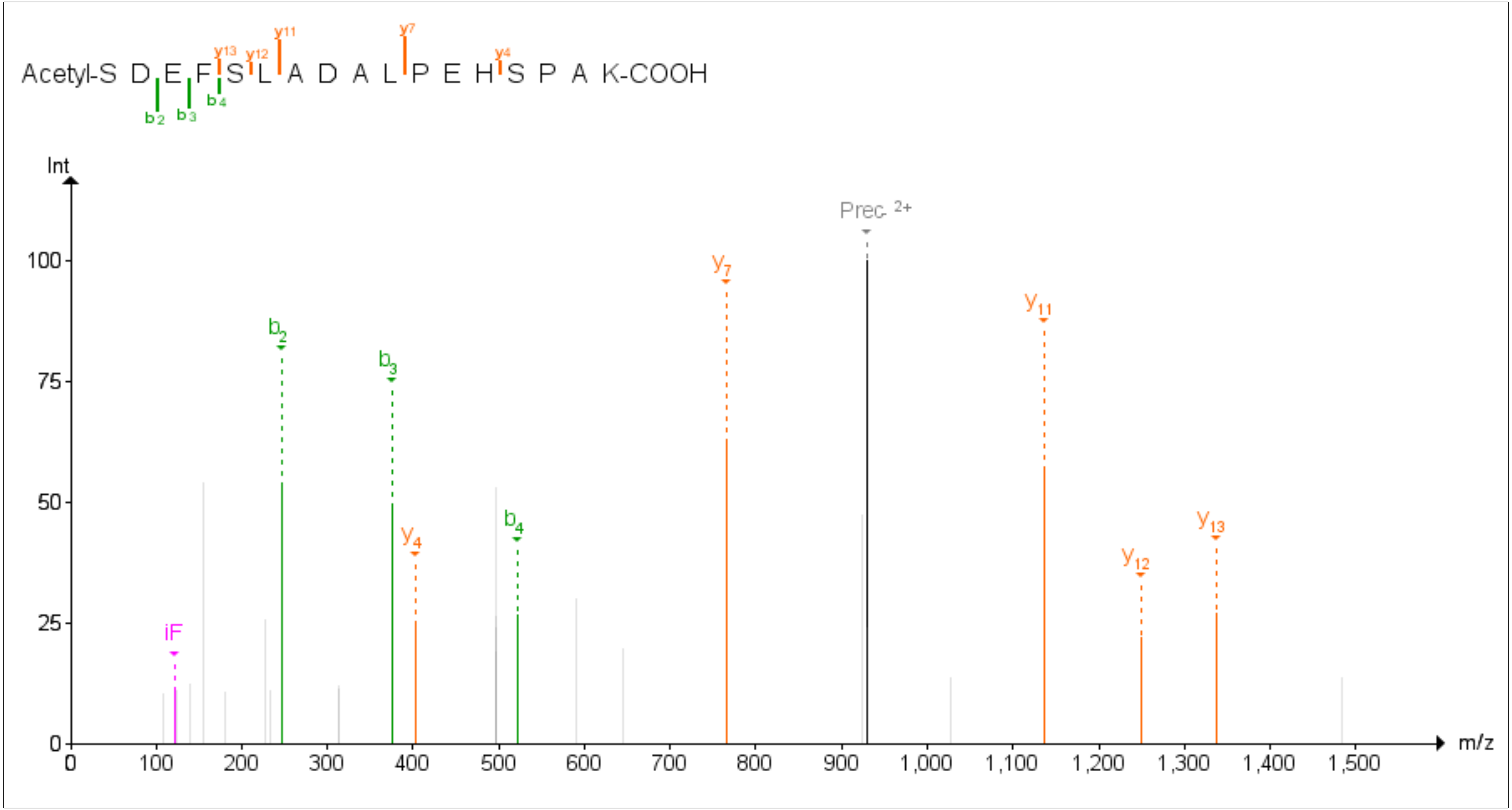

Acetyl-S D E F S L A D A L P E H S P A K-COOH
y13
y12
y11
y7
y4
b2
b3
b4
Int
Prec. 2+
y7
y11
b2
b3
y4
b4
y12
y13
iF
100
75
50
25
0
0
100
200
300
400
500
600
700
800
900
1,000
1,100
1,200
1,300
1,400
1,500
m/z

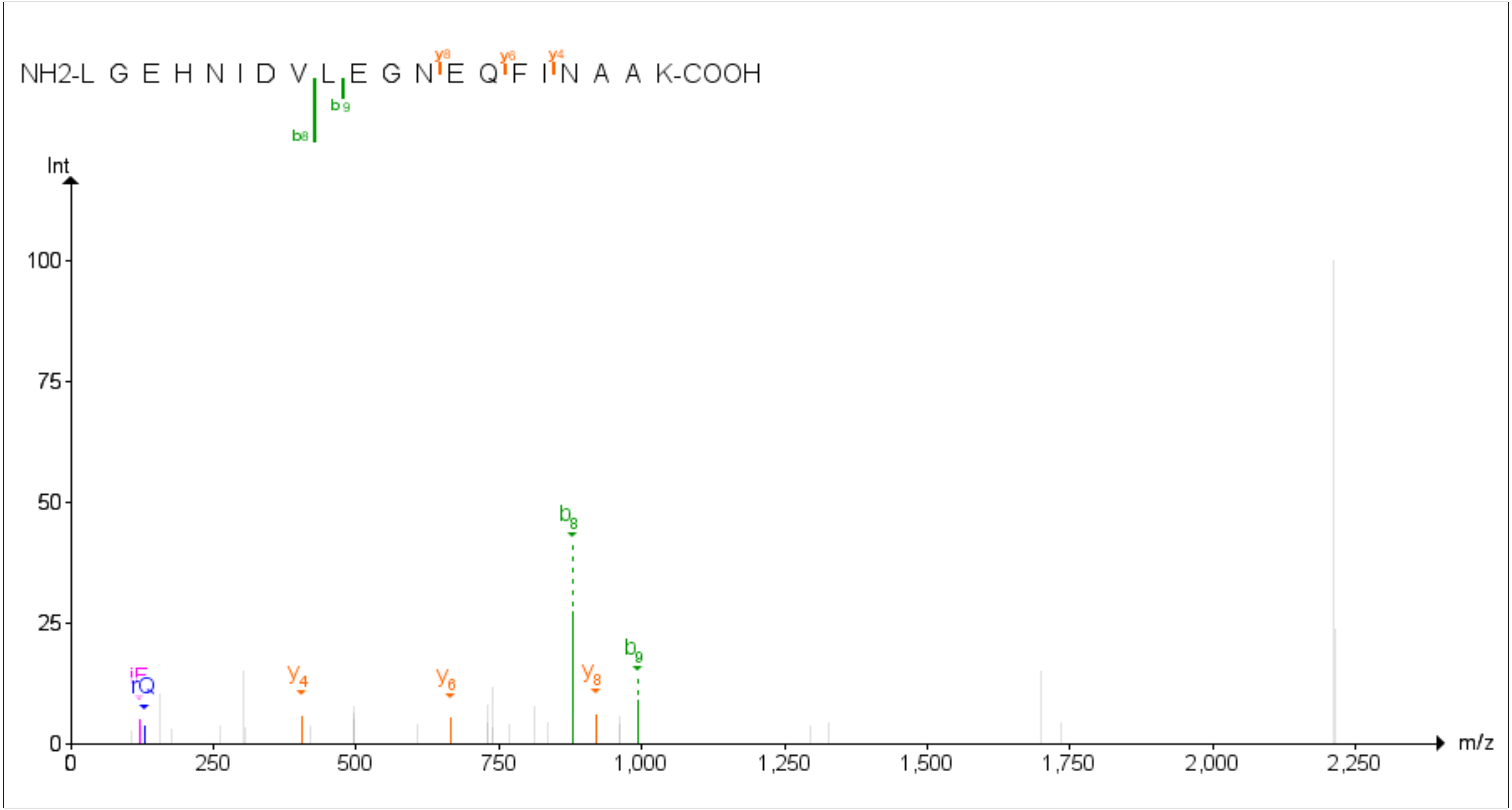

NH2-L G E H N I D V L E G N E Q F I N A A K-COOH
y8
y6
y4
b8
b9
Int
100
75
50
25
0
m/z
0
250
500
750
1,000
1,250
1,500
1,750
2,000
2,250
iF
rQ
y4
y6
b8
y8
b9

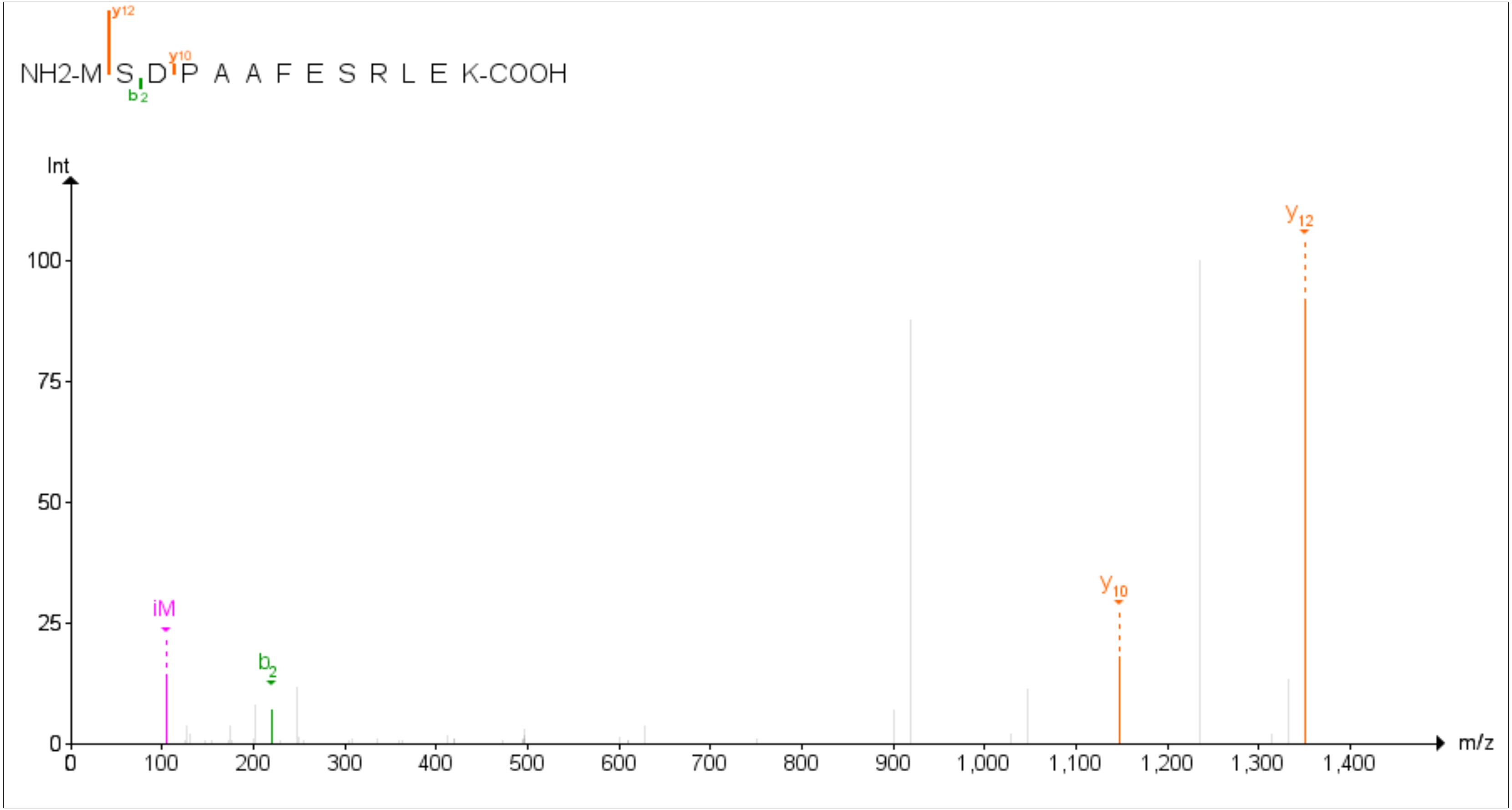

NH2-M S D P A A F E S R L E K-COOH
y12
y10
b2
Int
100
75
50
25
0
iM
b2
y10
y12
0
100
200
300
400
500
600
700
800
900
1,000
1,100
1,200
1,300
1,400
m/z

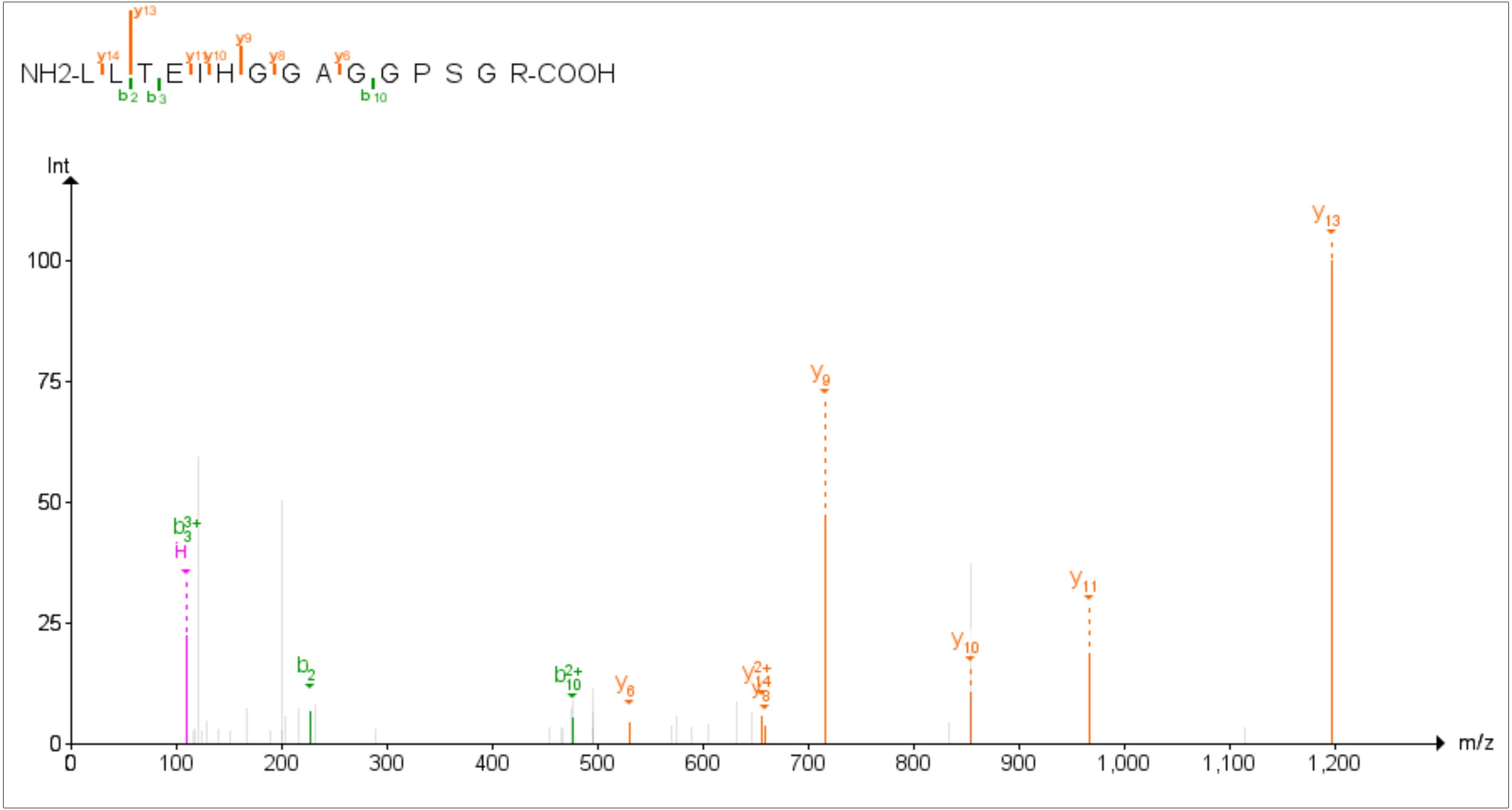

NH2-L L T E I H G G A G G P S G R-COOH
y13 y14 y11 y10 y9 y8 y6
b2 b3 b10
Int
100
75
50
25
0
b_3^{3+}
H
b_2
b_{10}^{2+}
y_6
y_{14}^{2+}
y_8
y_9
y_{10}
y_{11}
y_{13}
0 100 200 300 400 500 600 700 800 900 1,000 1,100 1,200
m/z

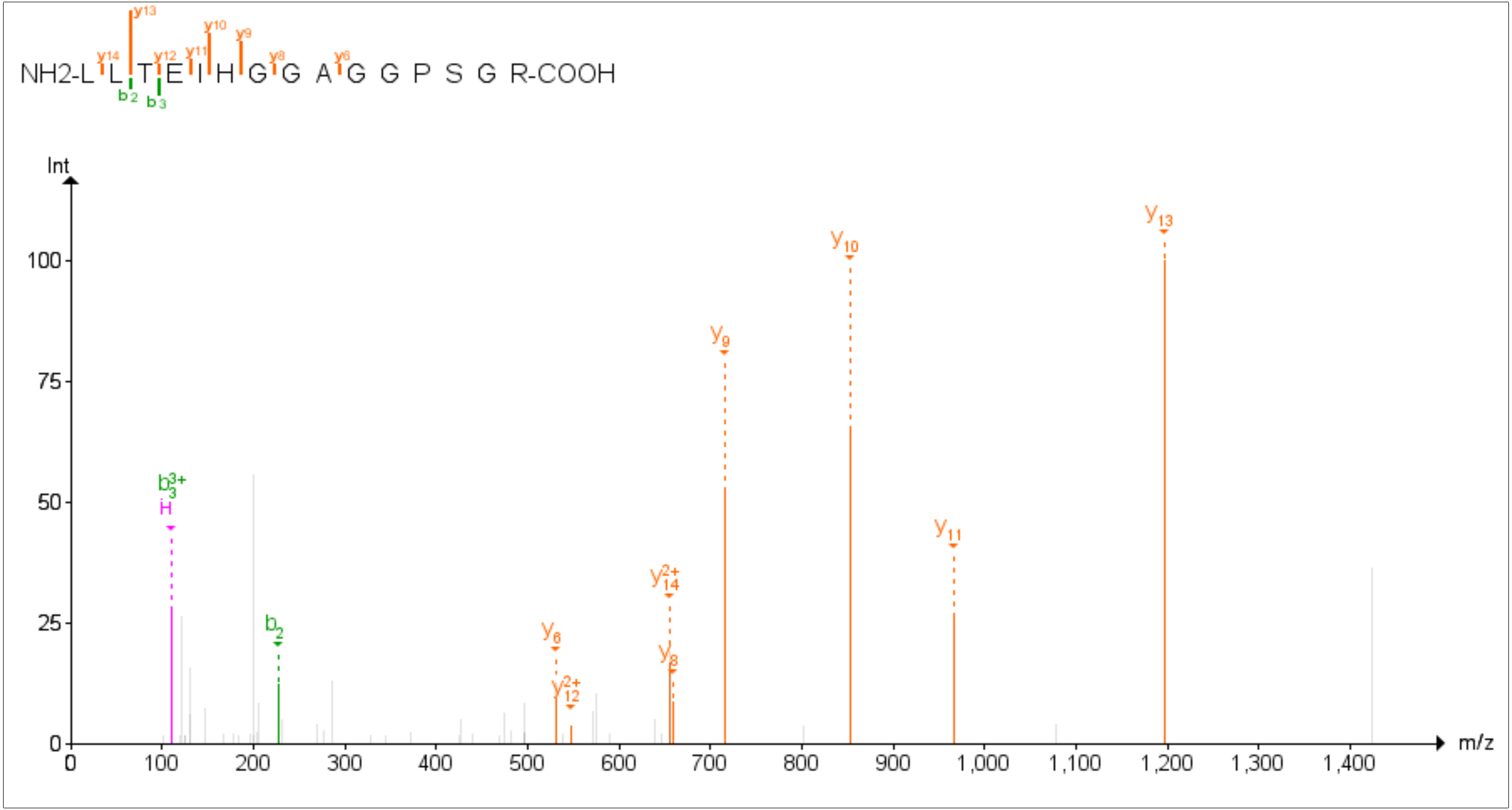

NH2-L L T E I H G G A G G P S G R-COOH
y14 y13 y12 y11 y10 y9 y8 y6
b2 b3
Int
m/z
y13
y10
y9
y11
y14 2+
y8
y6
y12 2+
b2
b3 3+
H
0
25
50
75
100
0
100
200
300
400
500
600
700
800
900
1,000
1,100
1,200
1,300
1,400

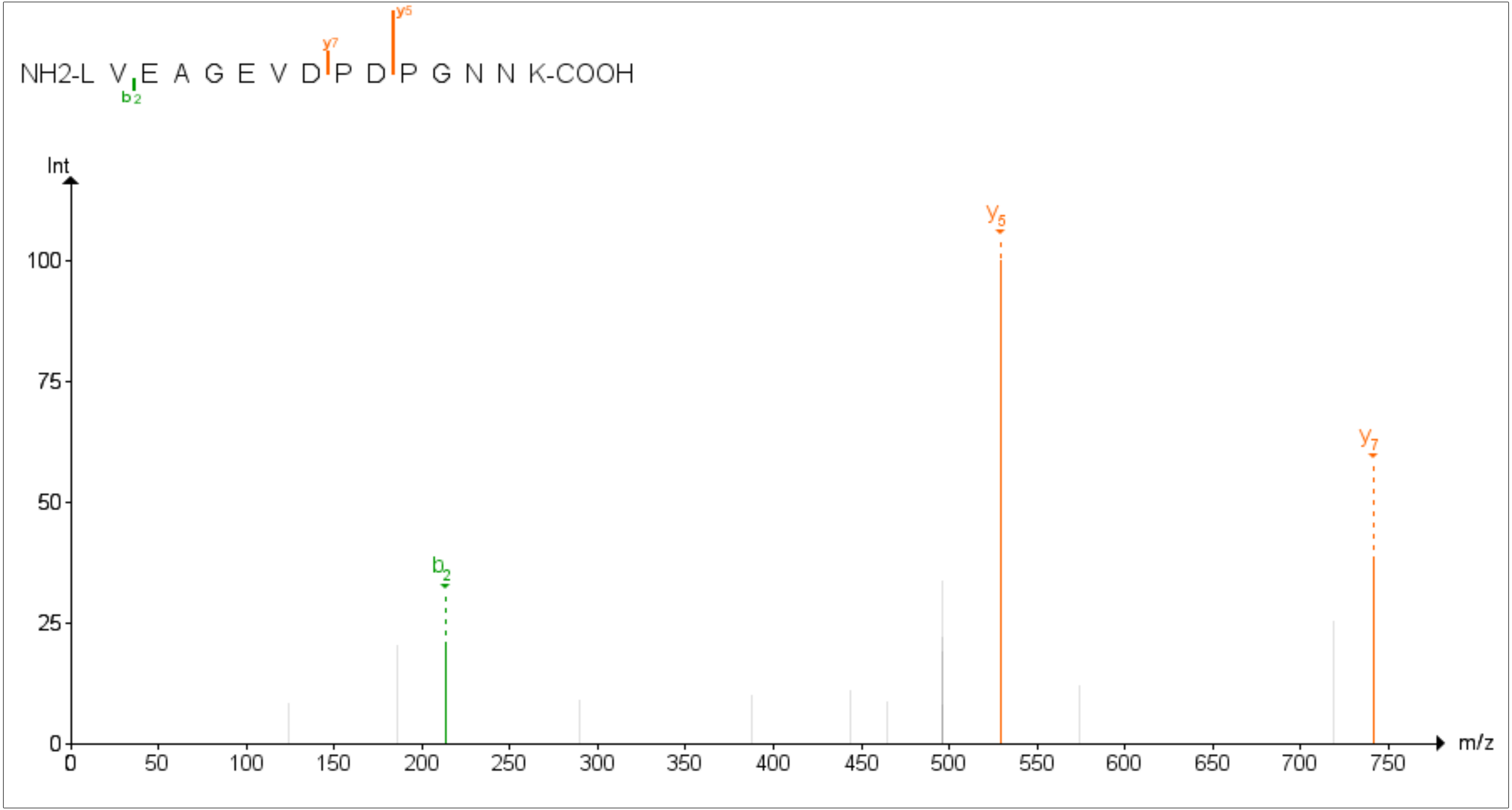

NH2-L V E A G E V D P D P G N N K-COOH
b2
y7
y5
Int
100
75
50
25
0
m/z
0
50
100
150
200
250
300
350
400
450
500
550
600
650
700
750

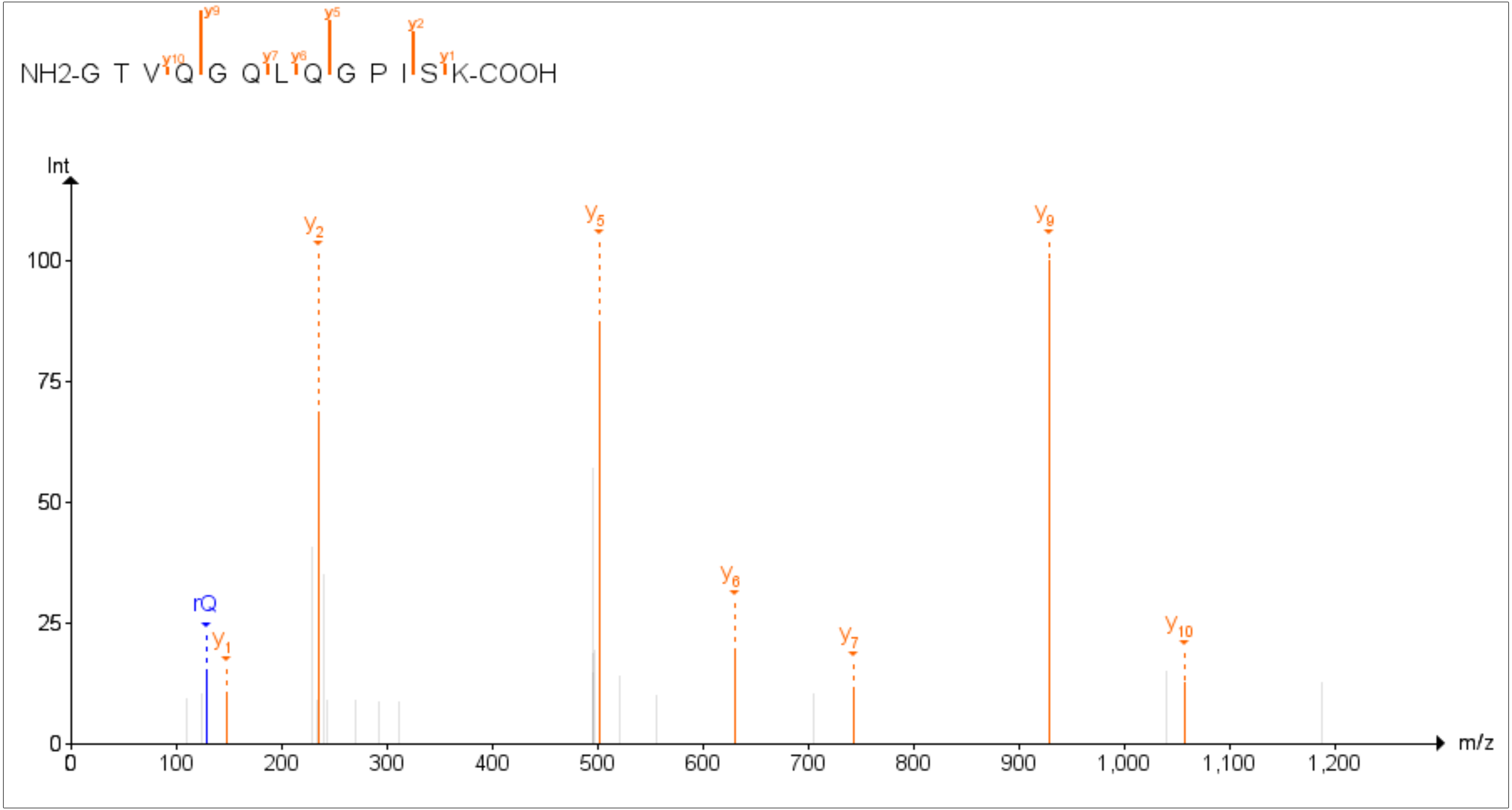

NH2-G T V Q G Q L Q G P I S K-COOH
y10 y9 y7 y6 y5 y2 y1
Int
m/z
0
25
50
75
100
0
100
200
300
400
500
600
700
800
900
1,000
1,100
1,200
rQ
y1
y2
y5
y6
y7
y9
y10

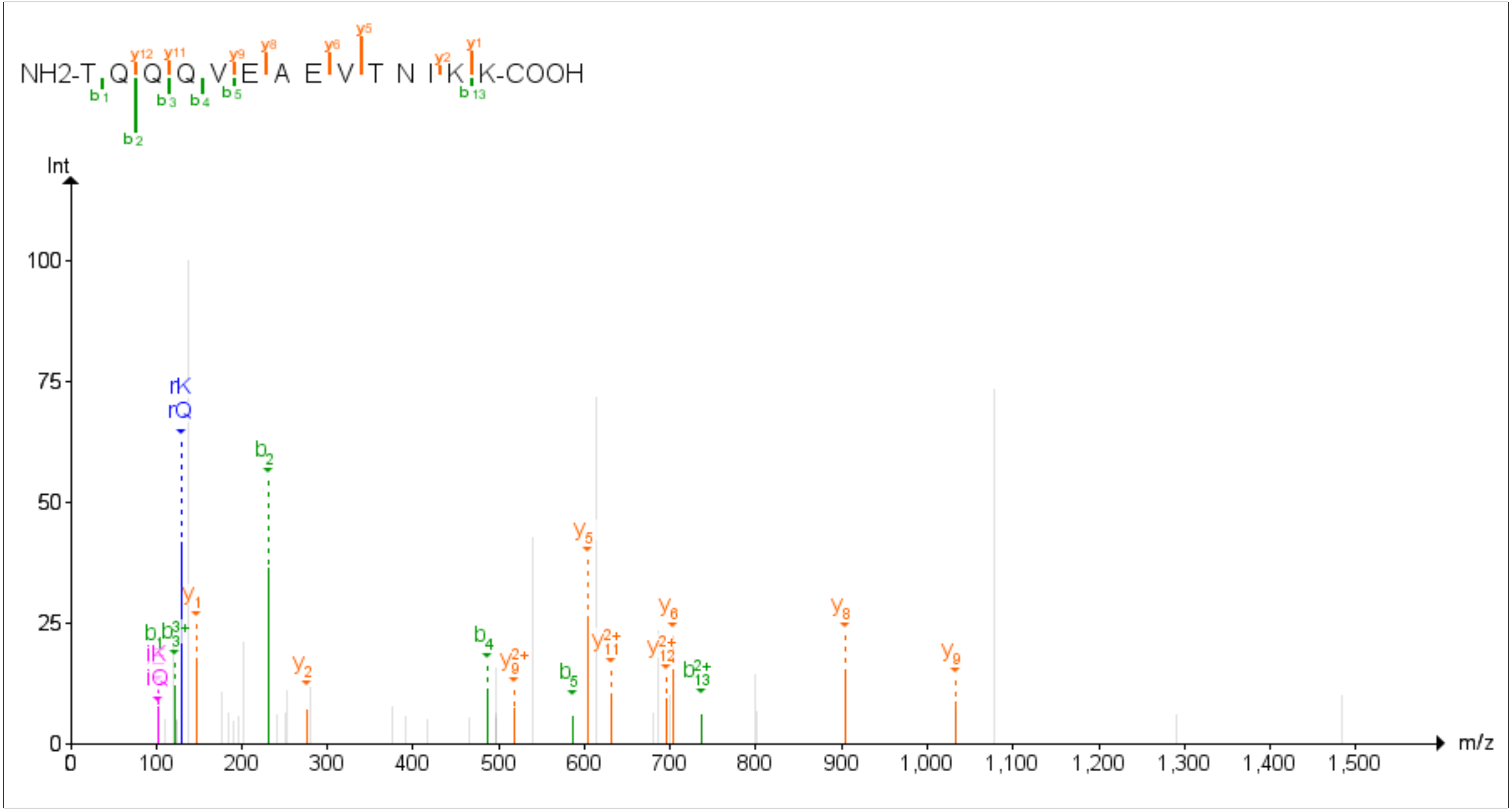

NH2-T Q Q Q V E A E V T N I K K-COOH
y12 y11 y9 y8 y6 y5 y2 y1
b1 b2 b3 b4 b5 b13
Int
100
75
50
25
0
rK
rQ
b2
y5
y1
b1 b3 3+
iK
iQ
y2
b4
y9 2+
b5
y11 2+
y6
y12 2+
b13 2+
y8
y9
0
100
200
300
400
500
600
700
800
900
1,000
1,100
1,200
1,300
1,400
1,500
m/z

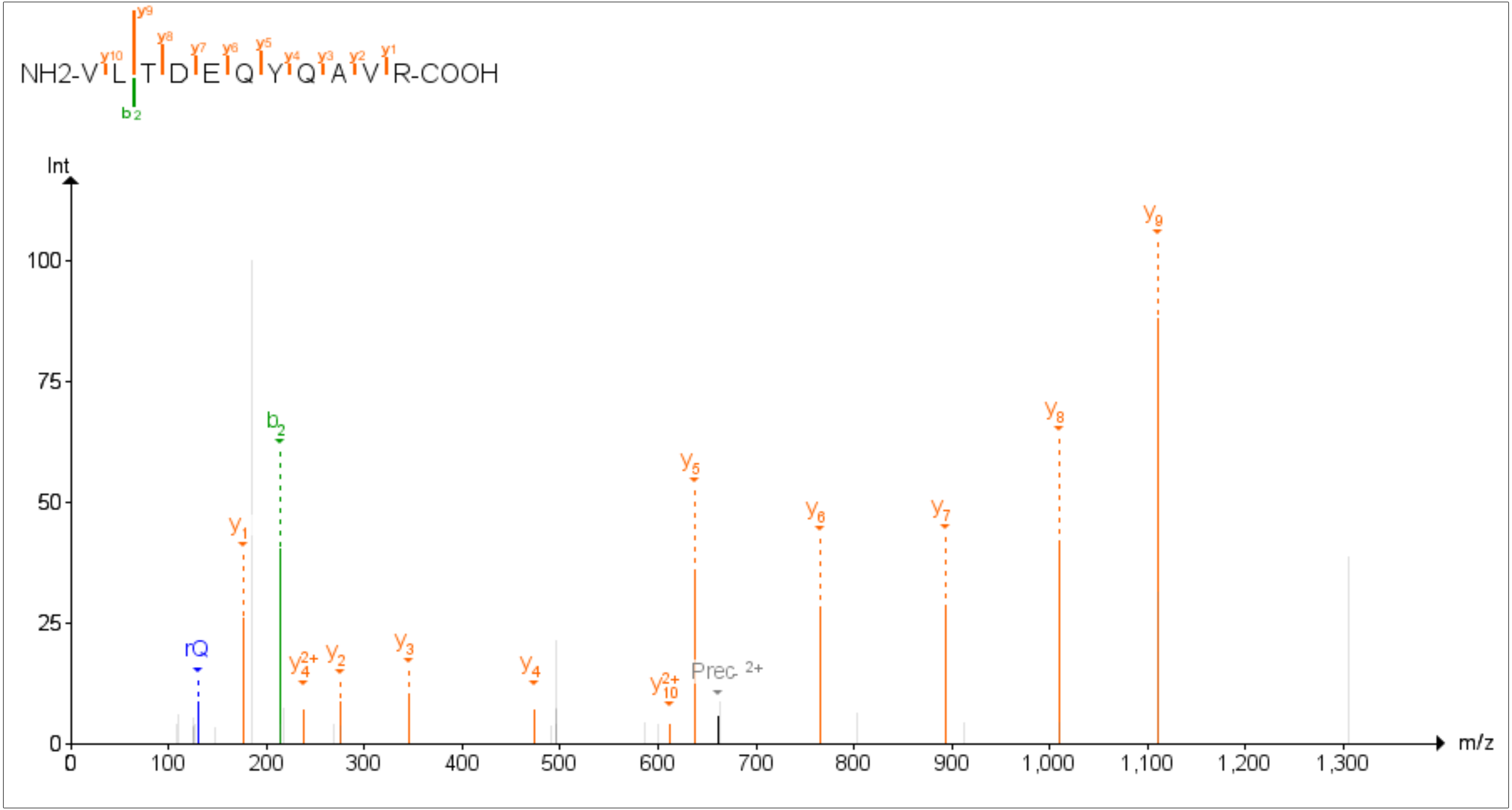

NH2-V L T D E Q Y Q A V R-COOH
y10 y9 y8 y7 y6 y5 y4 y3 y2 y1
b2
Int
m/z
0
25
50
75
100
100
200
300
400
500
600
700
800
900
1,000
1,100
1,200
1,300
rQ
y1
b2
y4 2+
y2
y3
y4
y10 2+
Prec. 2+
y5
y6
y7
y8
y9

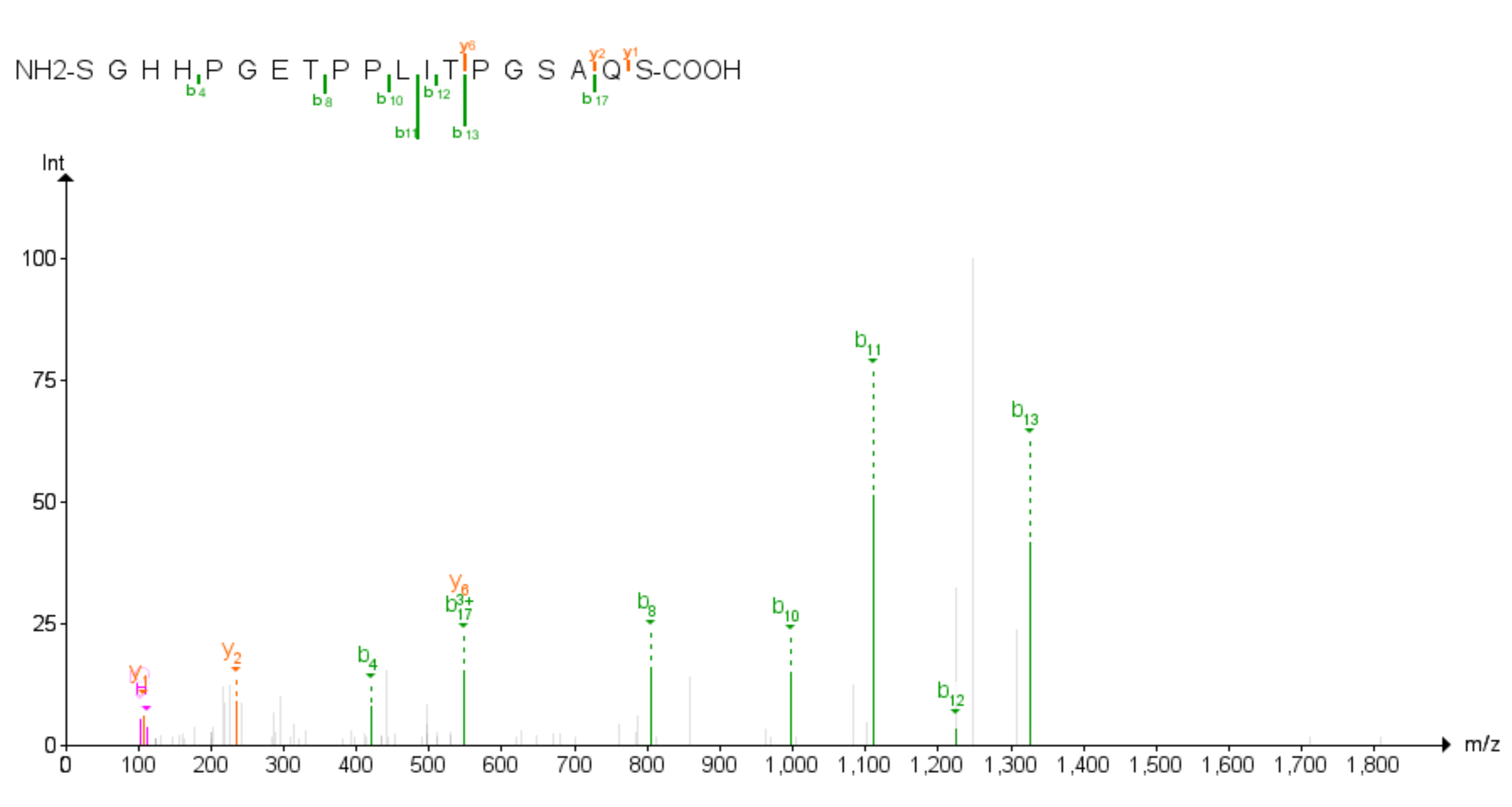

NH2-S G H H P G E T P P L I T P G S A Q S-COOH
b4
b8
b10
b11
b12
b13
b17
y6
y2
y1
Int
m/z
100
75
50
25
0
100
200
300
400
500
600
700
800
900
1,000
1,100
1,200
1,300
1,400
1,500
1,600
1,700
1,800
b_{11}
b_{13}
y_6
b_{17}^{3+}
b_8
b_{10}
y_2
y_1
b_4
b_{12}

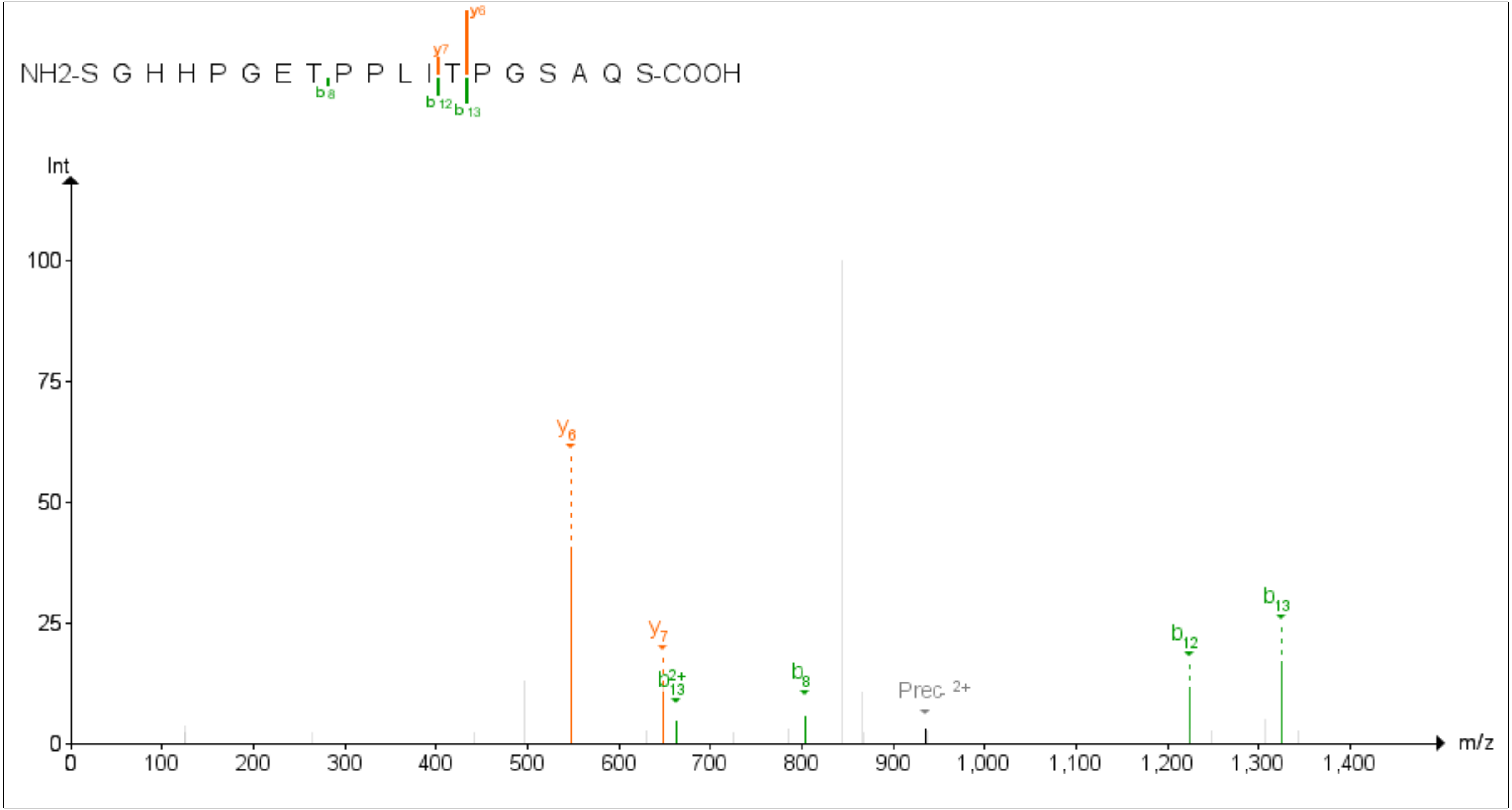

NH2-S G H H P G E T P P L I T P G S A Q S-COOH
y6
y7
b 8
b 12
b 13
Int
100
75
50
25
0
y6
y7
b2+ 13
b8
Prec. 2+
b12
b13
0
100
200
300
400
500
600
700
800
900
1,000
1,100
1,200
1,300
1,400
m/z

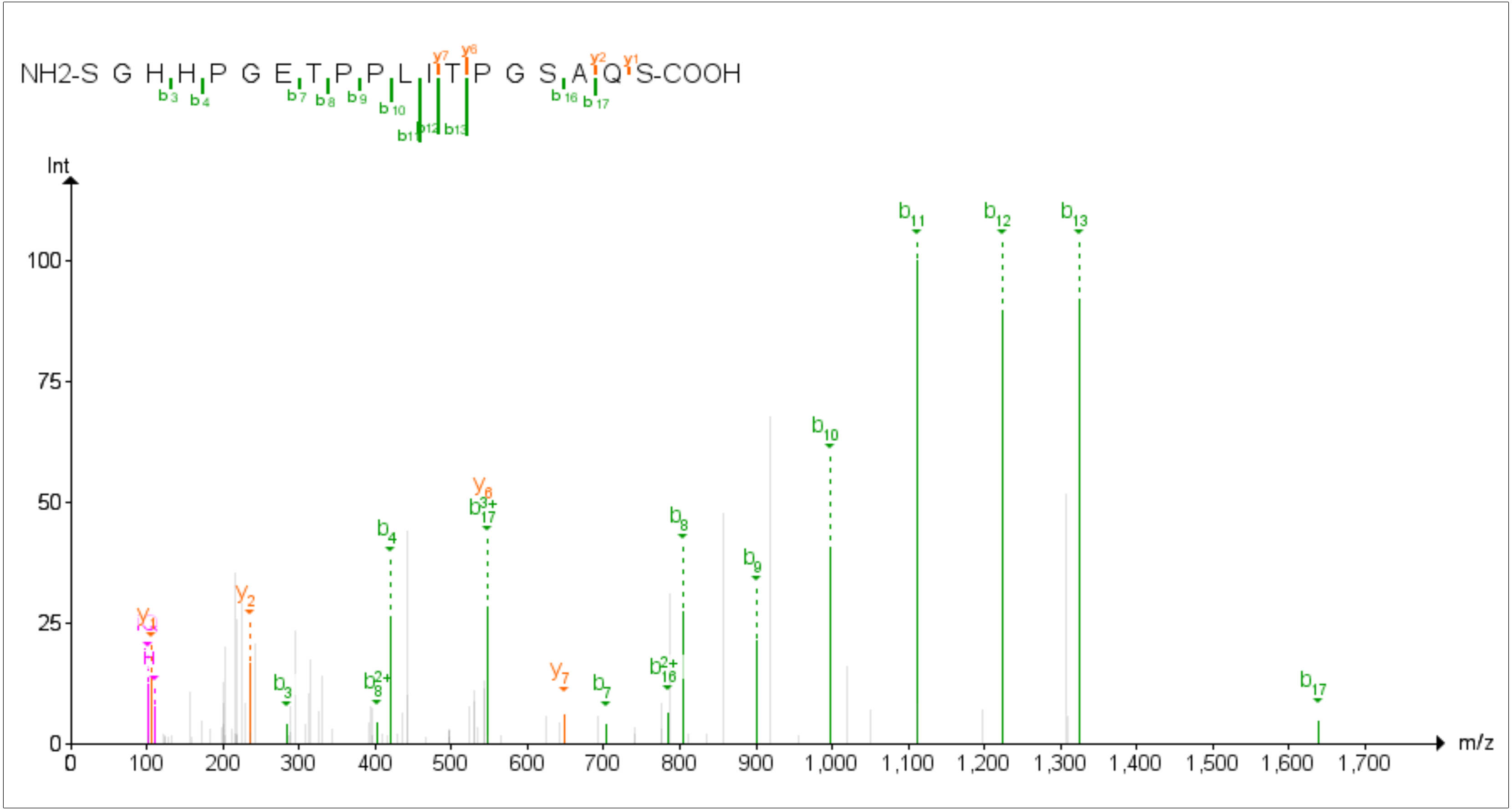

NH2-S G H H P G E T P P L I T P G S A Q S-COOH
Int
m/z
b3 b4 b7 b8 b9 b10 b11 b12 b13 b16 b17
y1 y2 y6 y7

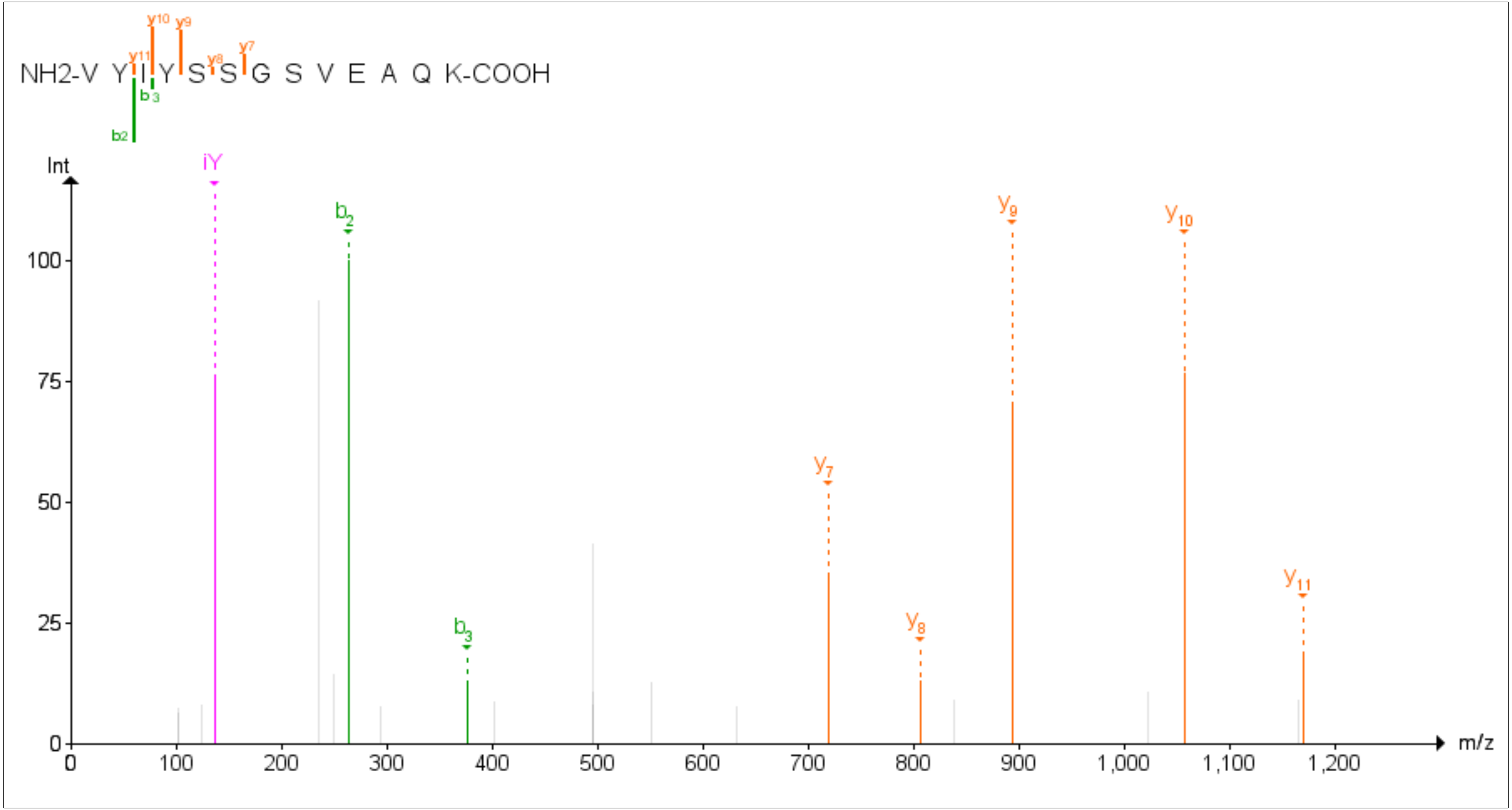

NH2-V Y I Y S S G S V E A Q K-COOH
y10
y9
y11
y8
y7
b2
b3
Int
iY
b2
y9
y10
y7
y11
b3
y8
100
75
50
25
0
0
100
200
300
400
500
600
700
800
900
1,000
1,100
1,200
m/z

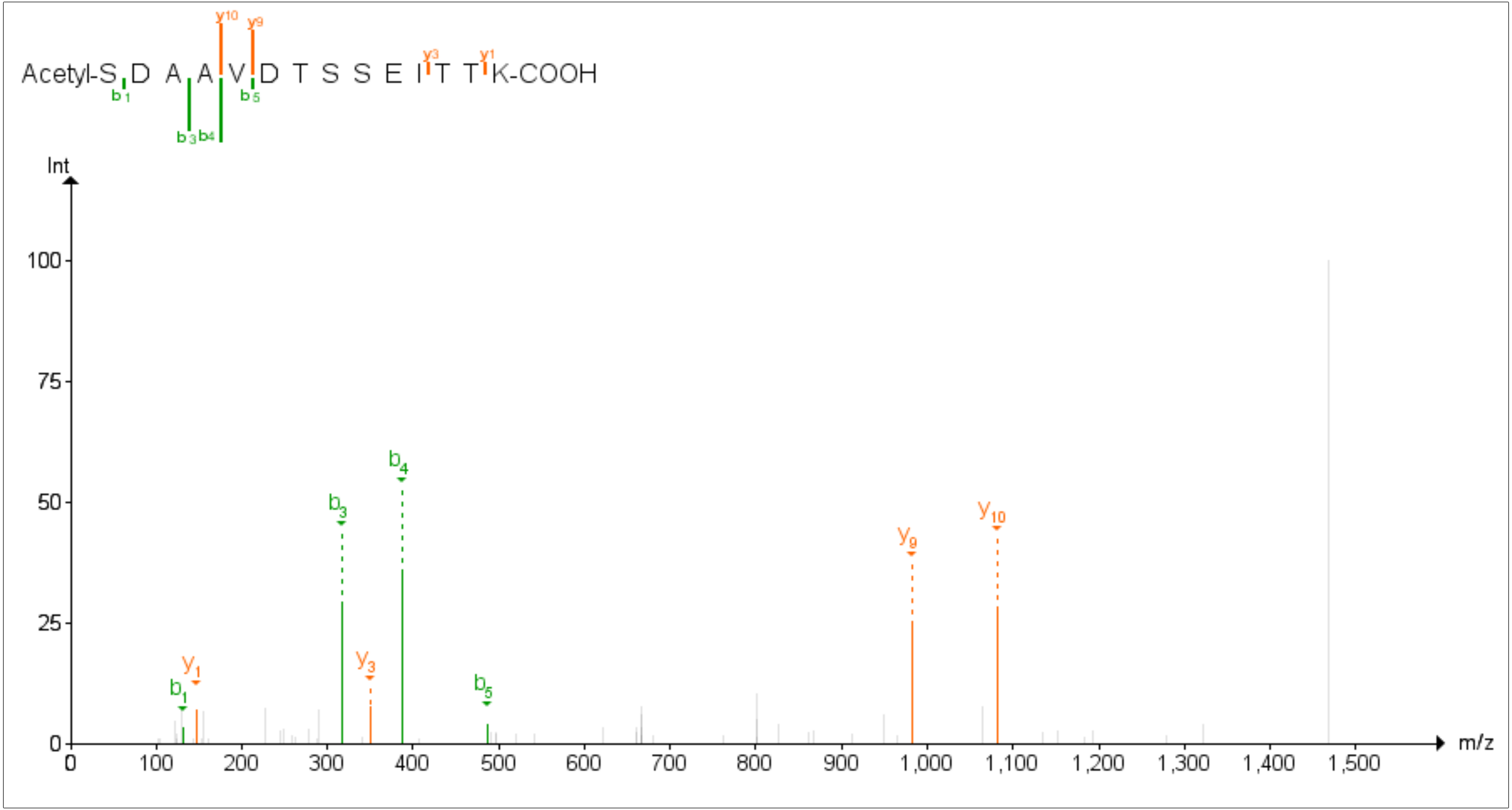

Acetyl-S D A A V D T S S E I T T K-COOH
b1
b3
b4
b5
y10
y9
y3
y1
Int
100
75
50
25
0
m/z
0
100
200
300
400
500
600
700
800
900
1,000
1,100
1,200
1,300
1,400
1,500

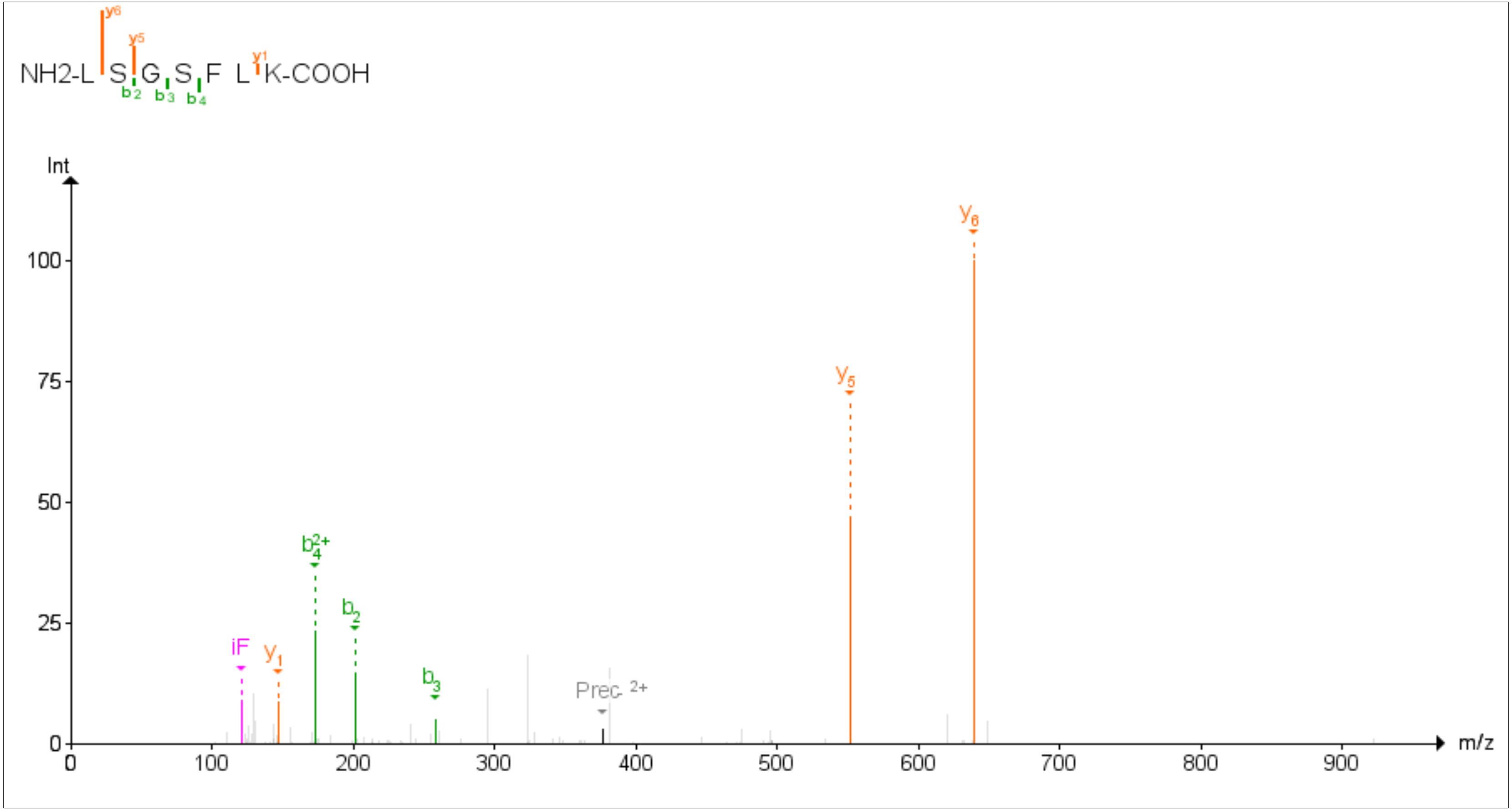

NH2-L S G S F L K-COOH
y6
y5
y1
b2
b3
b4
Int
100
75
50
25
0
y6
y5
b4 2+
b2
iF
y1
b3
Prec 2+
m/z
0
100
200
300
400
500
600
700
800
900

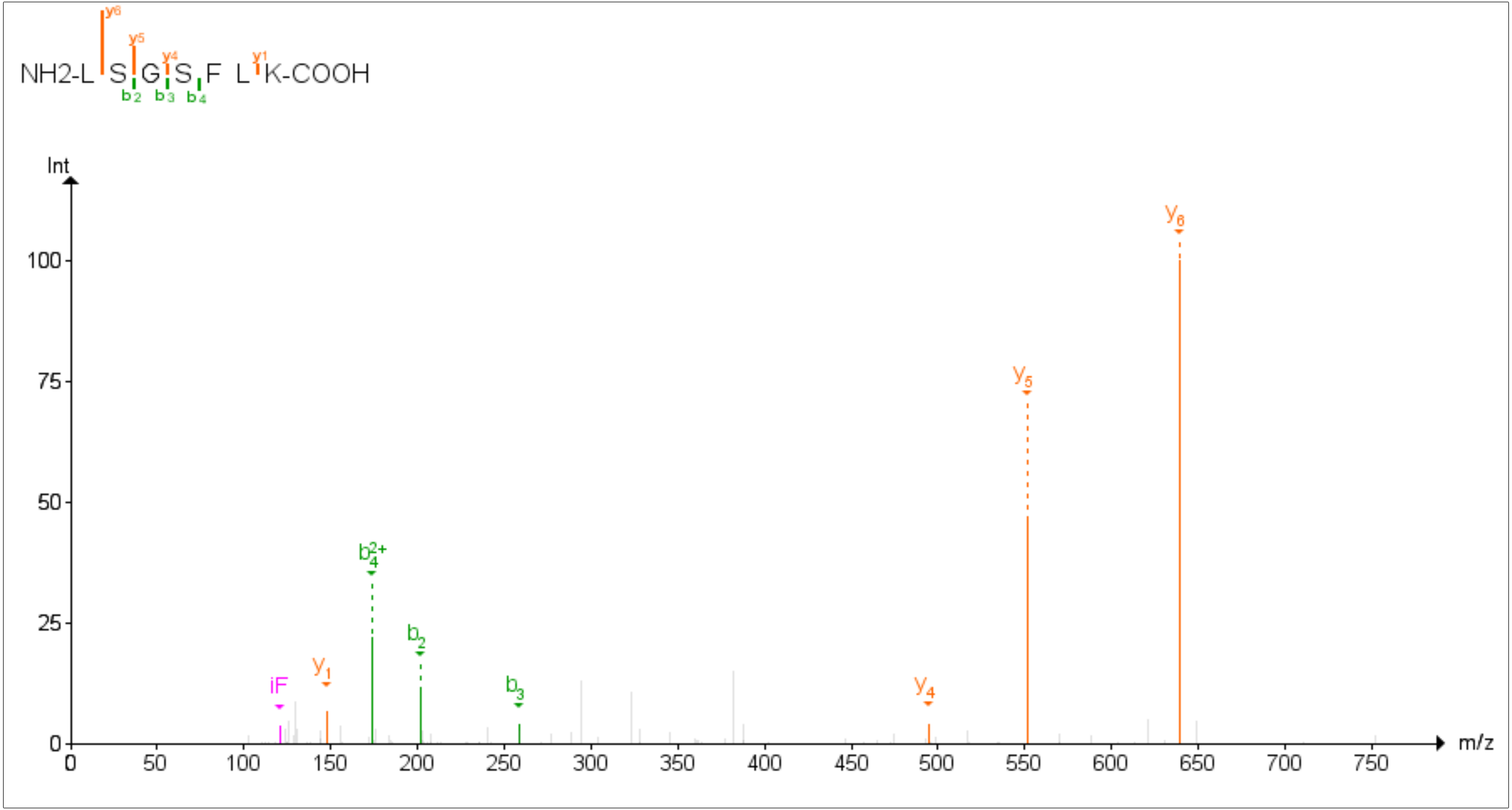

NH2-L S G S F L K-COOH
y6
y5
y4
y1
b2
b3
b4
Int
m/z
y6
y5
y4
y1
b4 2+
b2
b3
iF

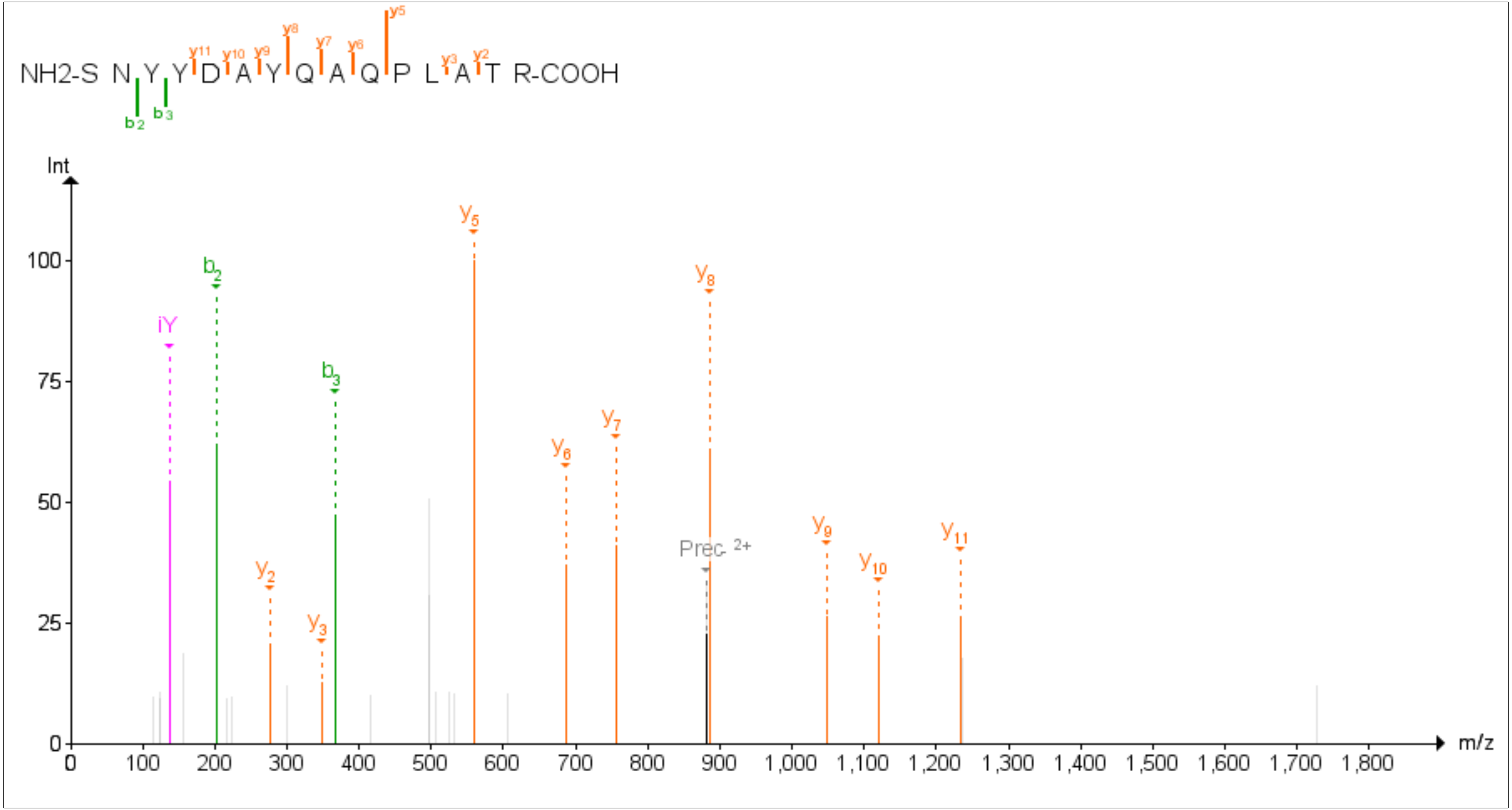

NH2-S N Y Y D A Y Q A Q P L A T R-COOH
y11 y10 y9 y8 y7 y6 y5 y3 y2
b2 b3
Int
m/z
iY
b2
b3
y2
y3
y5
y6
y7
y8
Prec. 2+
y9
y10
y11
0
25
50
75
100
0 100 200 300 400 500 600 700 800 900 1,000 1,100 1,200 1,300 1,400 1,500 1,600 1,700 1,800

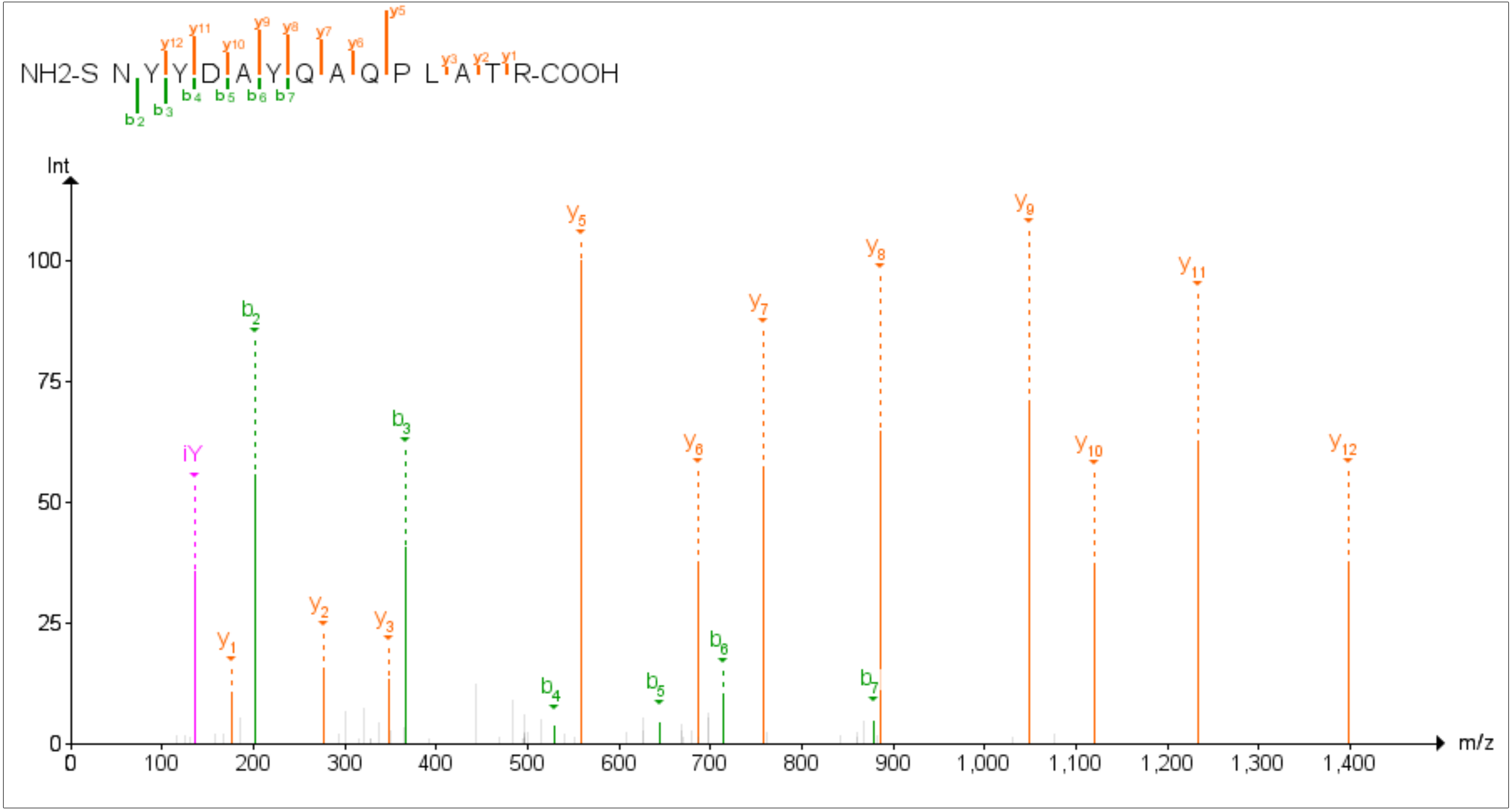
NH2-S N Y Y D A Y Q A Q P L A T R-COOH
y12 y11 y10 y9 y8 y7 y6 y5 y3 y2 y1
b2 b3 b4 b5 b6 b7
Int
100
75
50
25
0
iY
y1
b2
y2
y3
b3
b4
y5
b5
y6
b6
y7
b7
y8
y9
y10
y11
y12
0 100 200 300 400 500 600 700 800 900 1,000 1,100 1,200 1,300 1,400
m/z

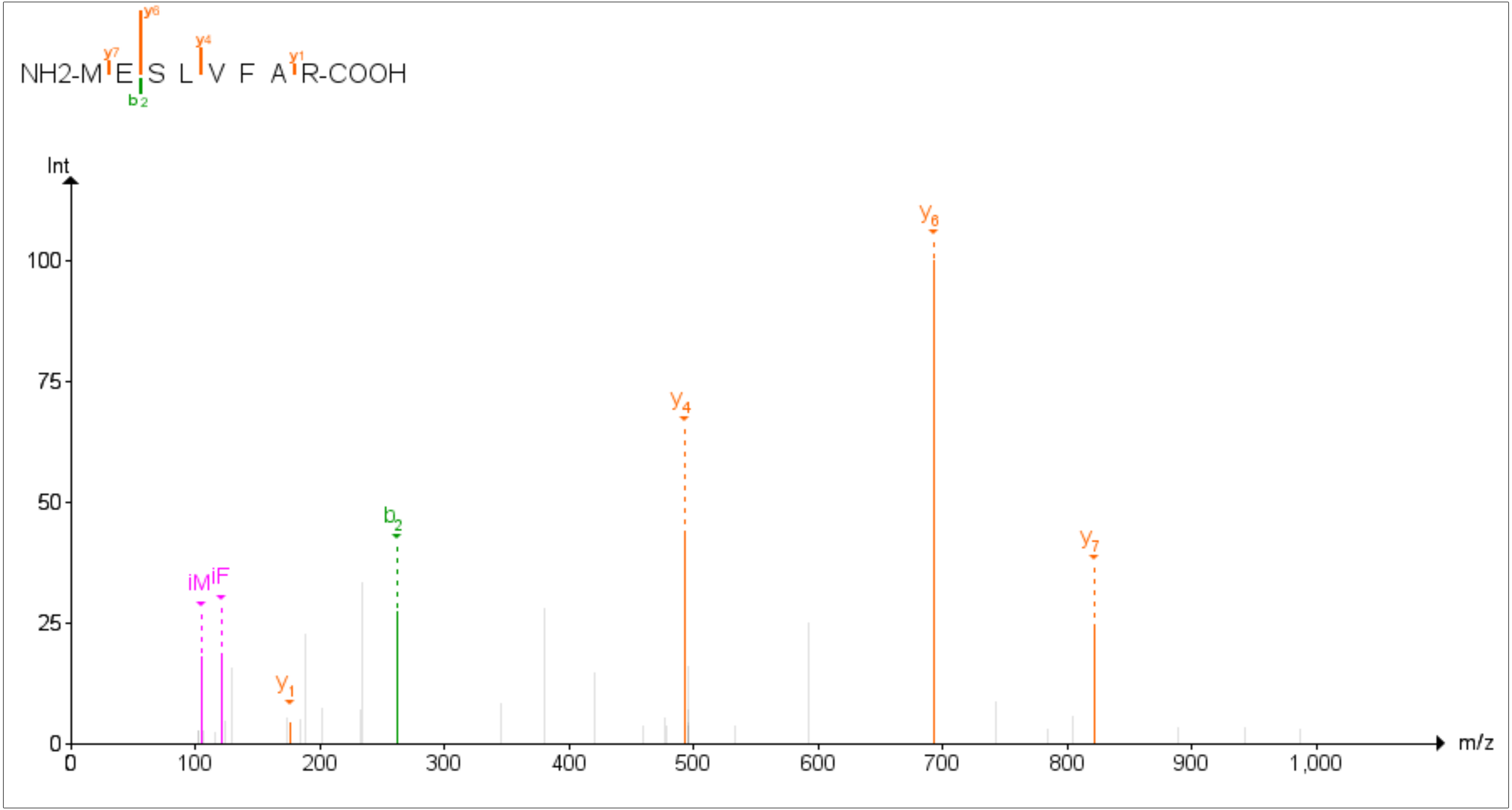

NH2-M E S L V F A R-COOH
y7
y6
y4
y1
b2
Int
100
75
50
25
0
y6
y4
b2
y7
iM
iF
y1
0
100
200
300
400
500
600
700
800
900
1,000
m/z

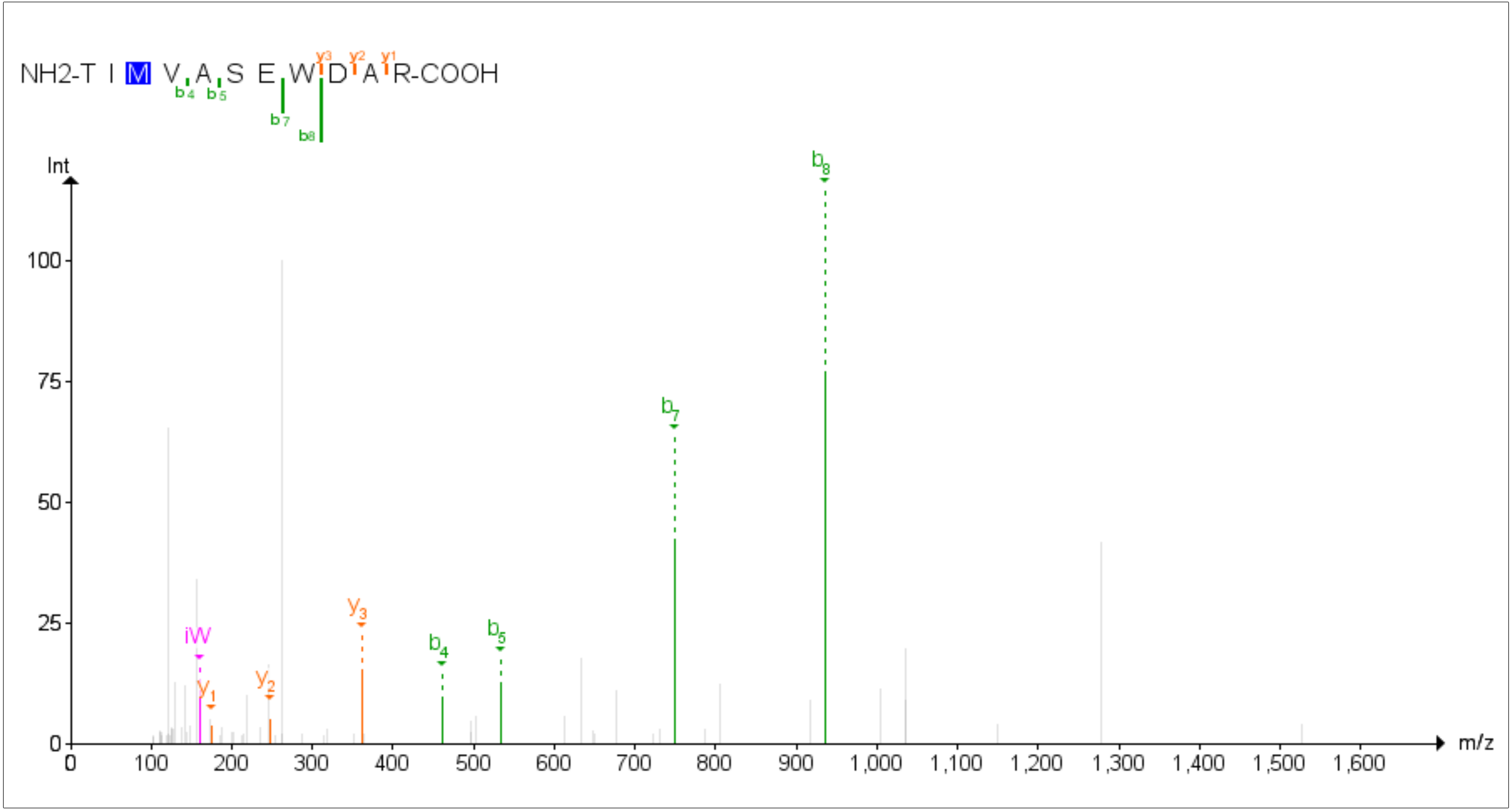
NH2-T I M V A S E W D A R-COOH
y3 y2 y1
b4 b5 b7 b8
Int
100
75
50
25
0
b8
b7
y3
iW
y1
y2
b4
b5
0 100 200 300 400 500 600 700 800 900 1,000 1,100 1,200 1,300 1,400 1,500 1,600
m/z

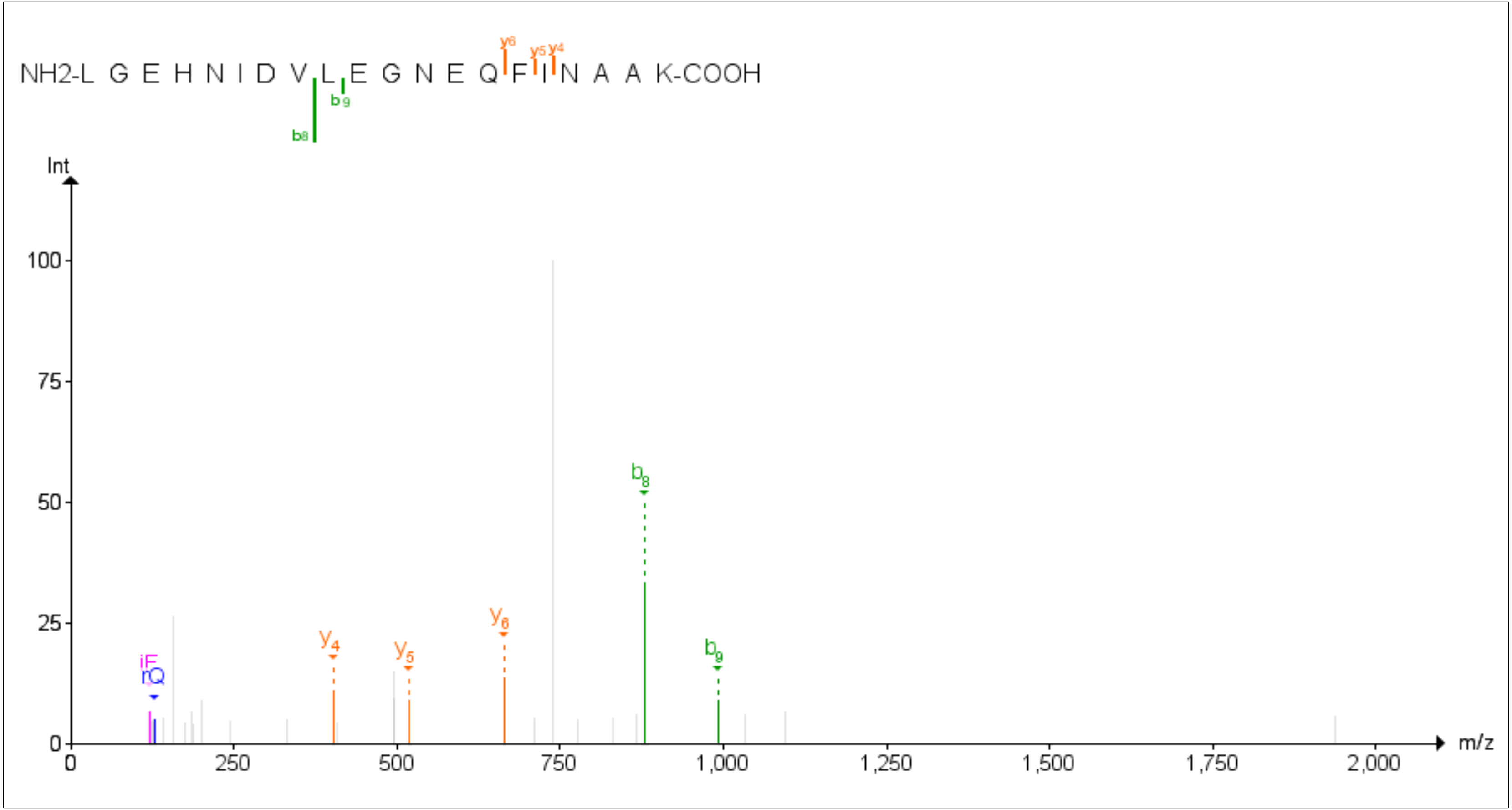

NH2-L G E H N I D V L E G N E Q F I N A A K-COOH
y6
y5
y4
b8
b9
Int
100
75
50
25
0
iF
rQ
y4
y5
y6
b8
b9
0
250
500
750
1,000
1,250
1,500
1,750
2,000
m/z

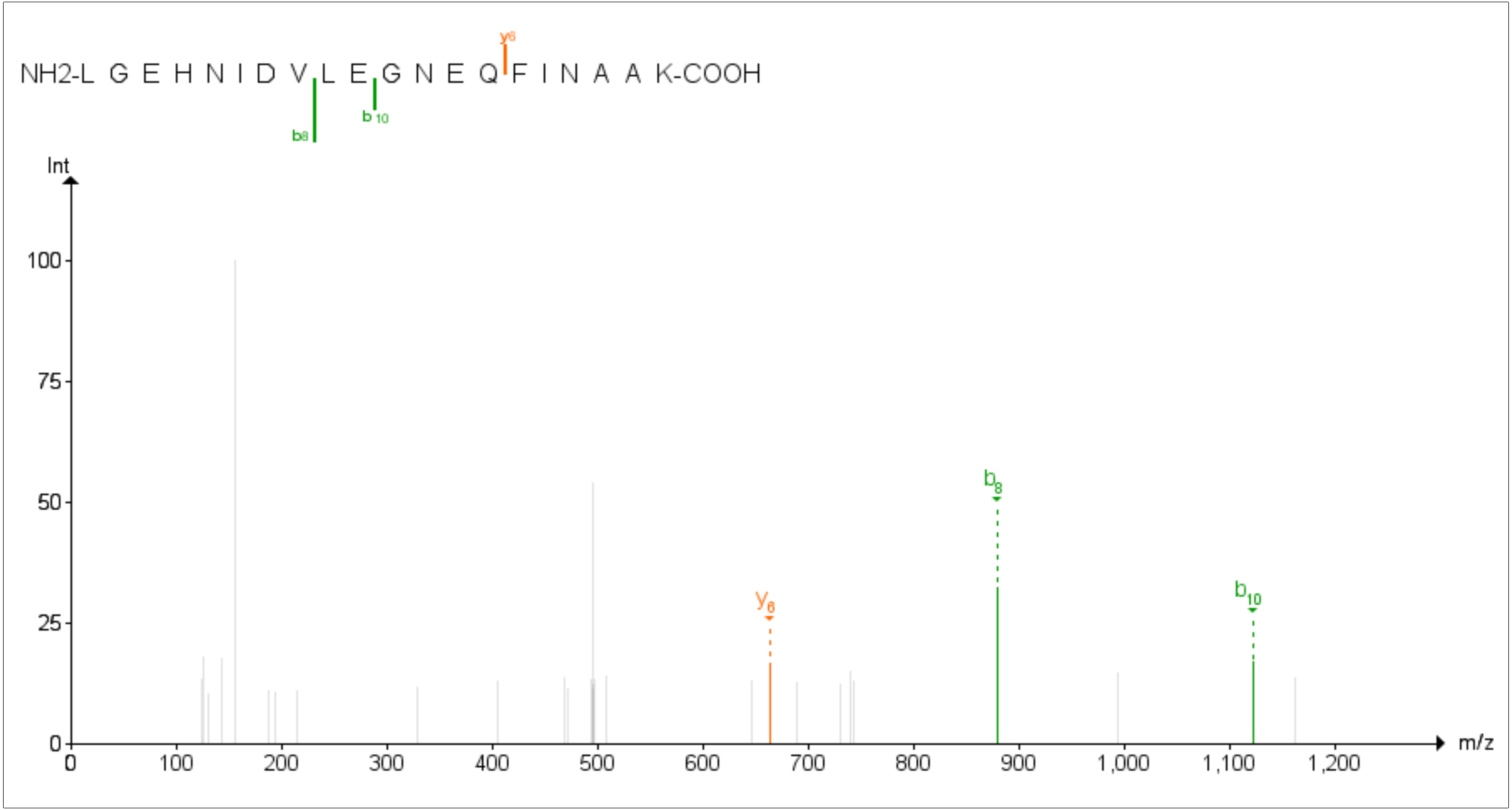
NH2-L G E H N I D V L E G N E Q F I N A A K-COOH
y6
b8
b 10
Int
100
75
50
25
0
b8
y6
b10
0
100
200
300
400
500
600
700
800
900
1,000
1,100
1,200
m/z

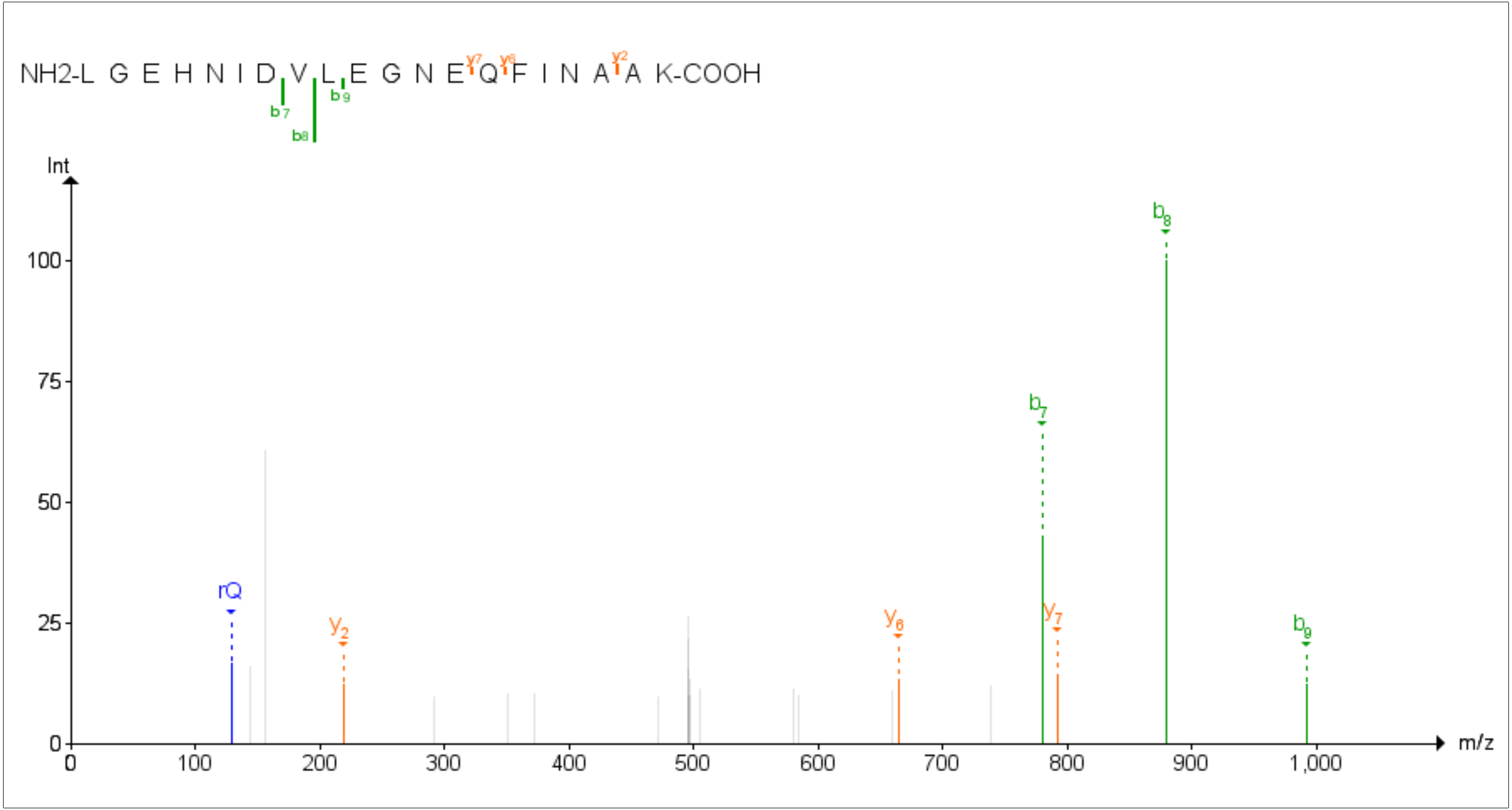
NH2-L G E H N I D V L E G N E Q F I N A A K-COOH
b7
b8
b9
y7
y6
y2
Int
100
75
50
25
0
rQ
y2
y6
y7
b7
b8
b9
0
100
200
300
400
500
600
700
800
900
1,000
m/z

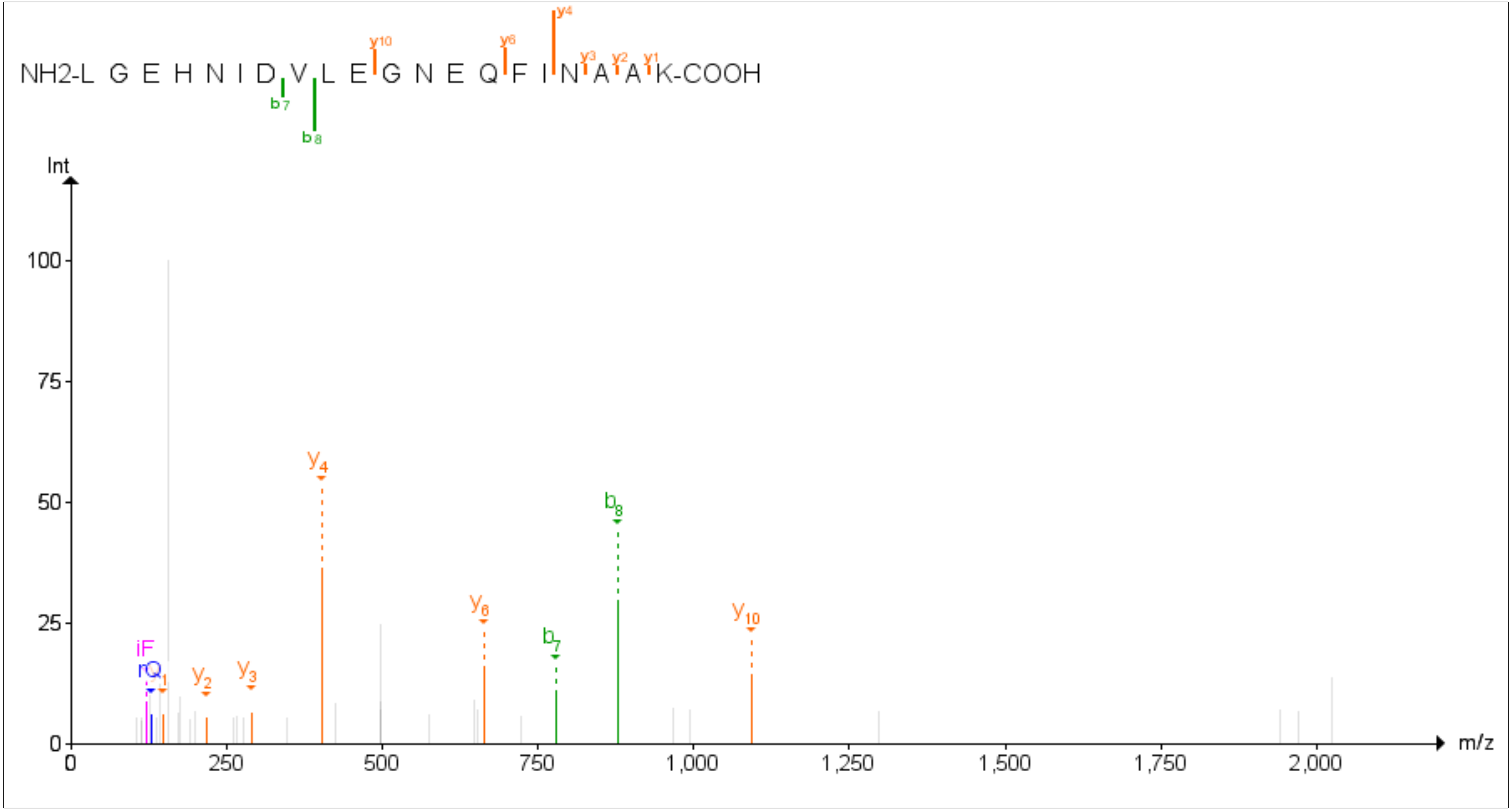

NH2-L G E H N I D V L E G N E Q F I N A A K-COOH
y10
y6
y4
y3
y2
y1
b7
b8
Int
100
75
50
25
0
y4
b8
y6
y10
b7
iF
rQ
y2
y3
0
250
500
750
1,000
1,250
1,500
1,750
2,000
m/z

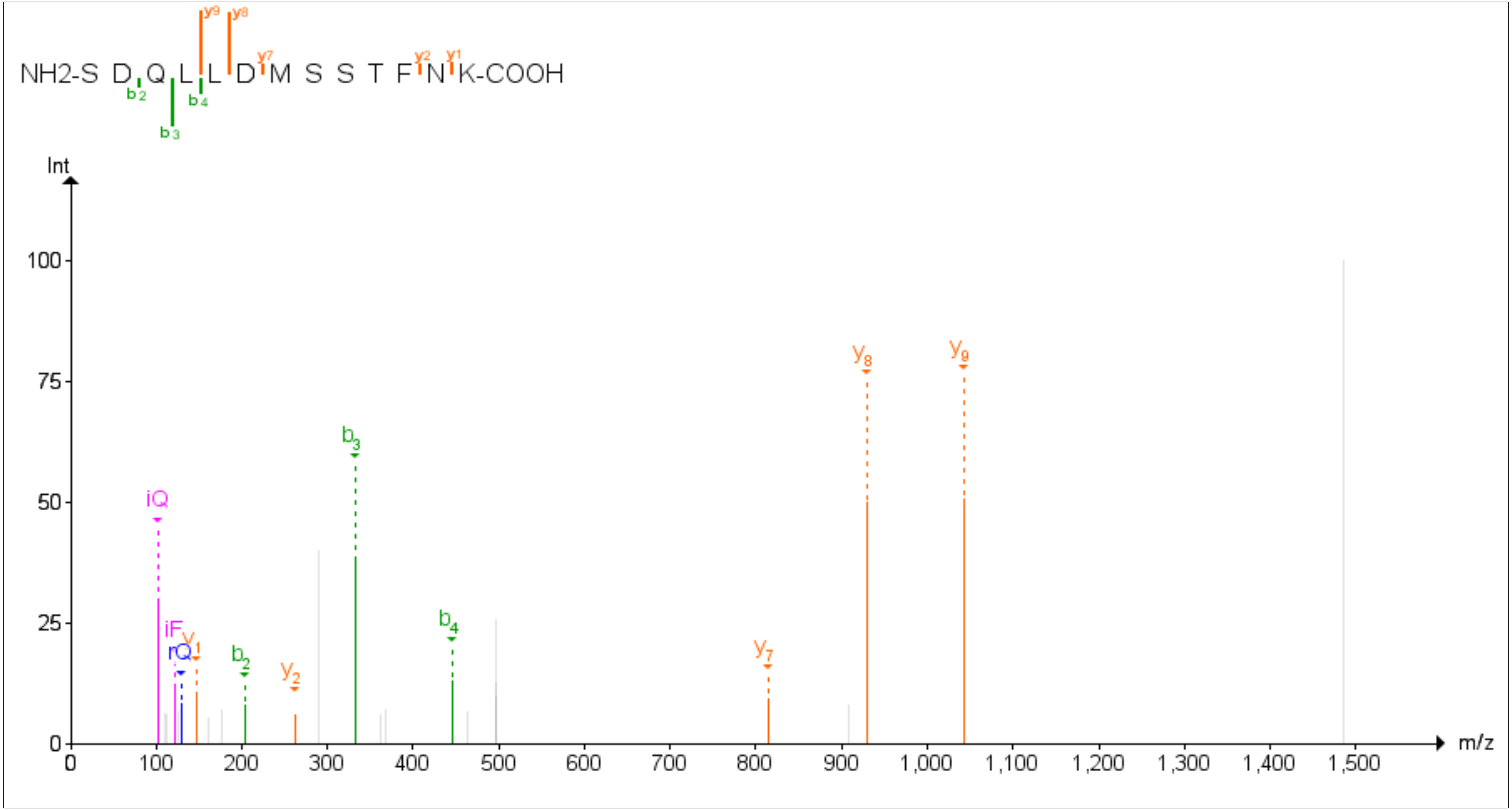

NH2-S D Q L L D M S S T F N K-COOH
b2
b3
b4
y9
y8
y7
y2
y1
Int
100
75
50
25
0
m/z
0
100
200
300
400
500
600
700
800
900
1,000
1,100
1,200
1,300
1,400
1,500
iQ
iF
y1
rQ
b2
y2
b3
b4
y7
y8
y9

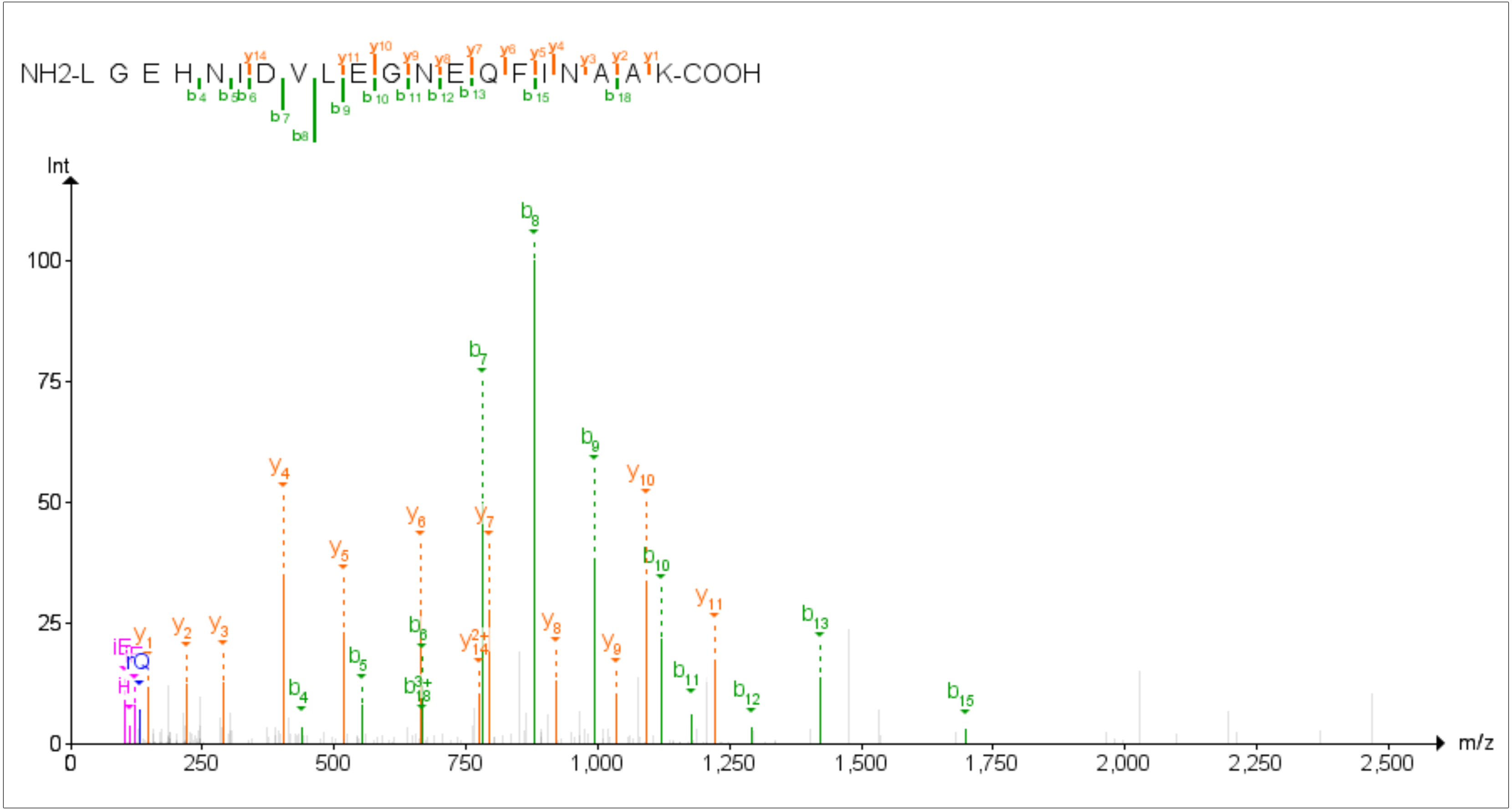
NH2-L G E H N I D V L E G N E Q F I N A A K-COOH
Int
m/z
0
25
50
75
100
250
500
750
1,000
1,250
1,500
1,750
2,000
2,250
2,500

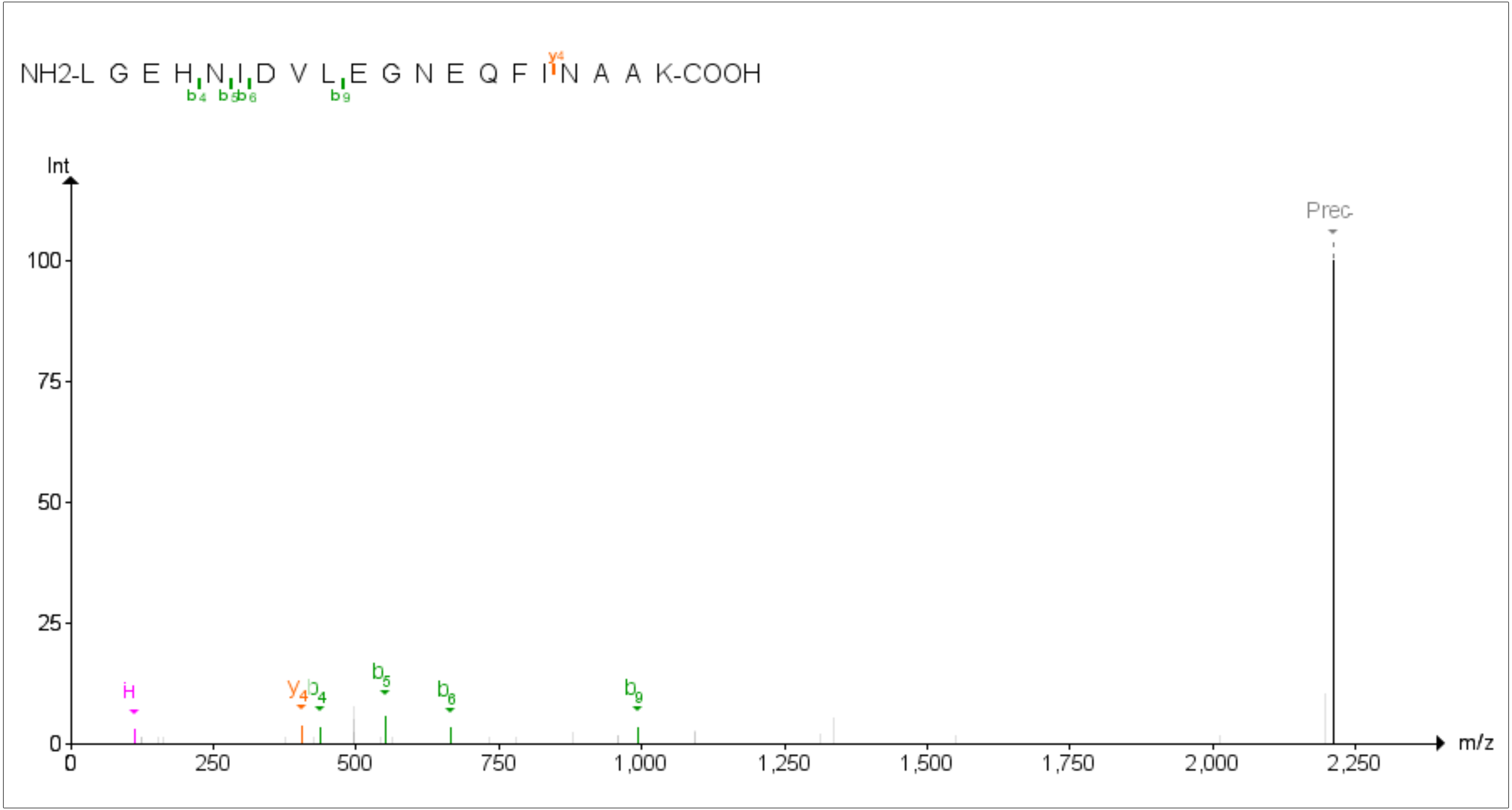
NH2-L G E H N I D V L E G N E Q F I N A A K-COOH
b4 b5 b6 b9 y4
Int
100
75
50
25
0
Prec
H
y4 b4
b5
b6
b9
0 250 500 750 1,000 1,250 1,500 1,750 2,000 2,250
m/z

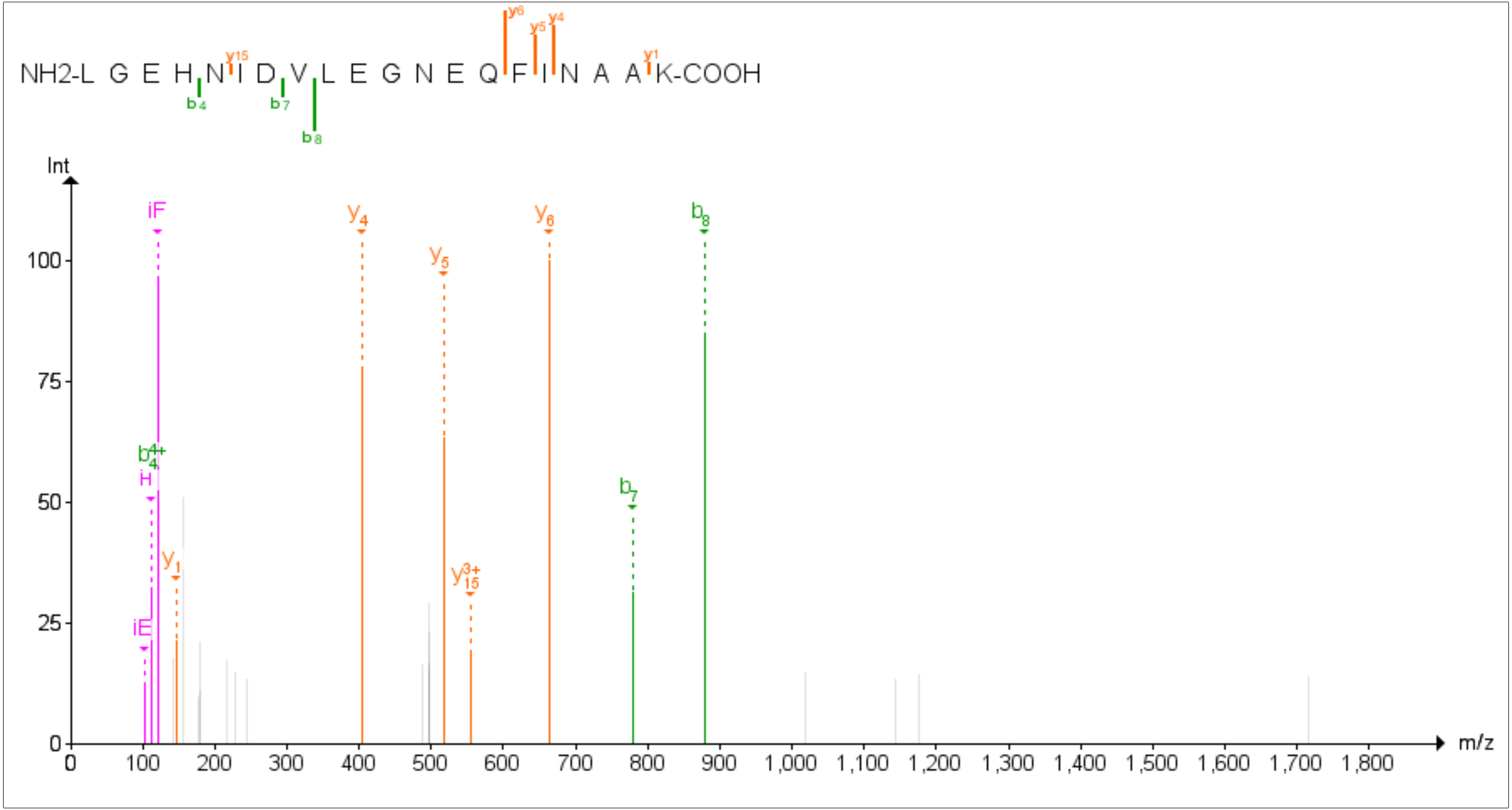
NH2-L G E H N I D V L E G N E Q F I N A A K-COOH
b4
b7
b8
y15
y6
y5
y4
y1
Int
iF
y4
y6
b8
y5
b_4^{4+}
iH
y1
y_{15}^{3+}
b7
iE
m/z
0
25
50
75
100
0
100
200
300
400
500
600
700
800
900
1,000
1,100
1,200
1,300
1,400
1,500
1,600
1,700
1,800

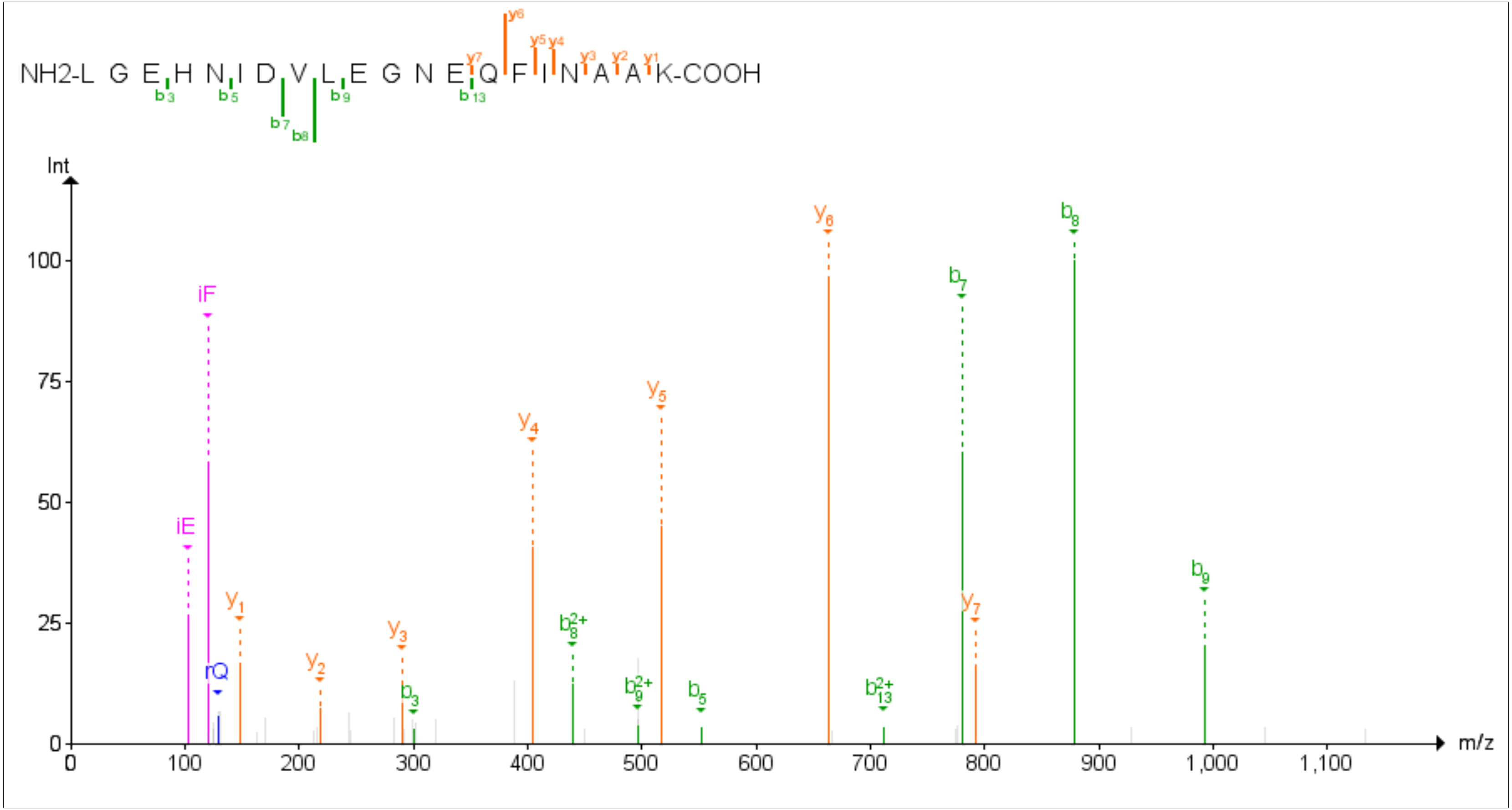

NH2-L G E H N I D V L E G N E Q F I N A A K-COOH
b3
b5
b7
b8
b9
b13
y7
y6
y5
y4
y3
y2
y1
Int
100
75
50
25
0
iF
iE
rQ
y1
y2
y3
b3
y4
b8 2+
b9 2+
y5
b5
y6
b13 2+
b7
y7
b8
b9
0
100
200
300
400
500
600
700
800
900
1,000
1,100
m/z

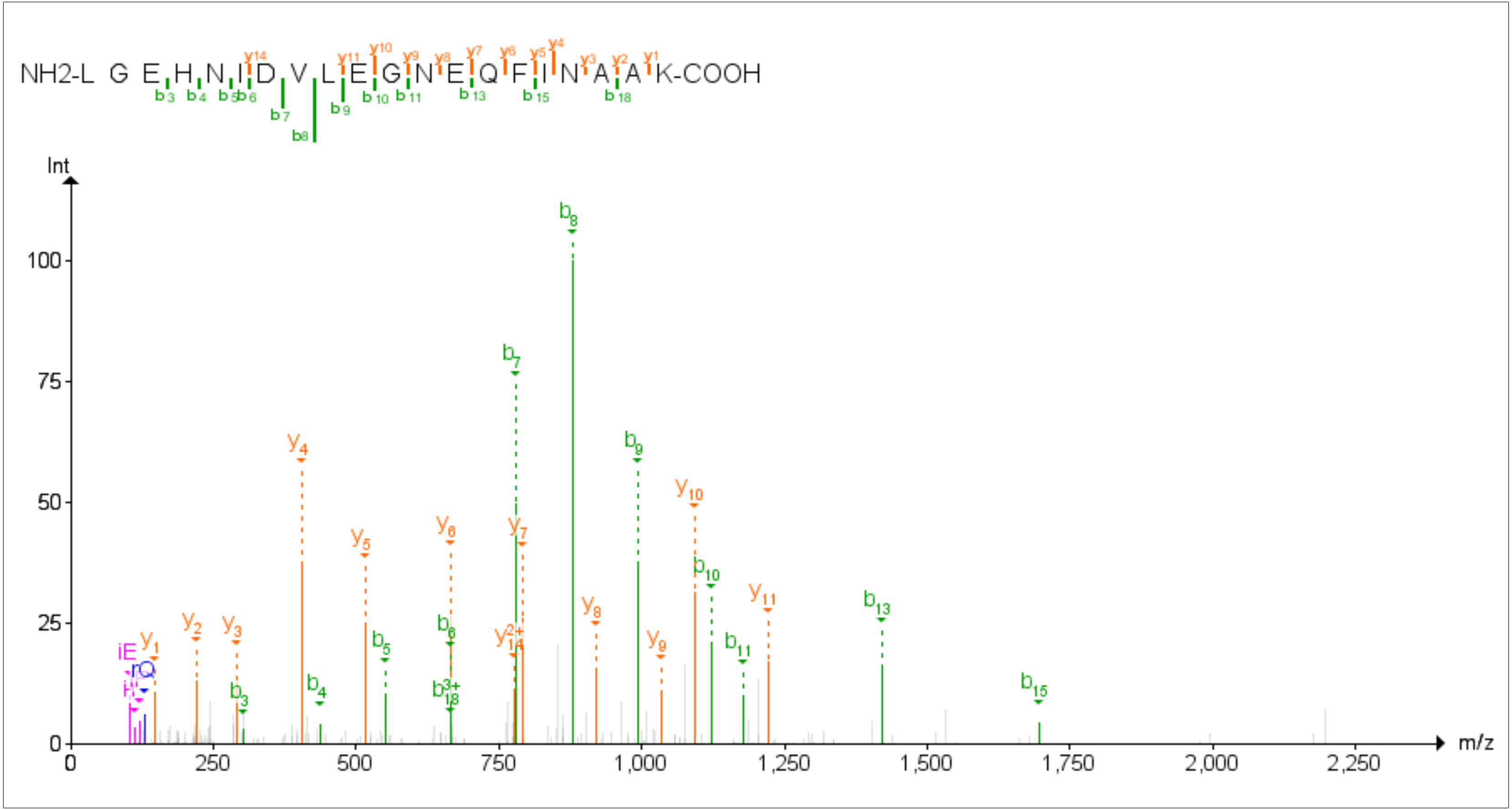

NH2-L G E H N I D V L E G N E Q F I N A A K-COOH
Int
m/z

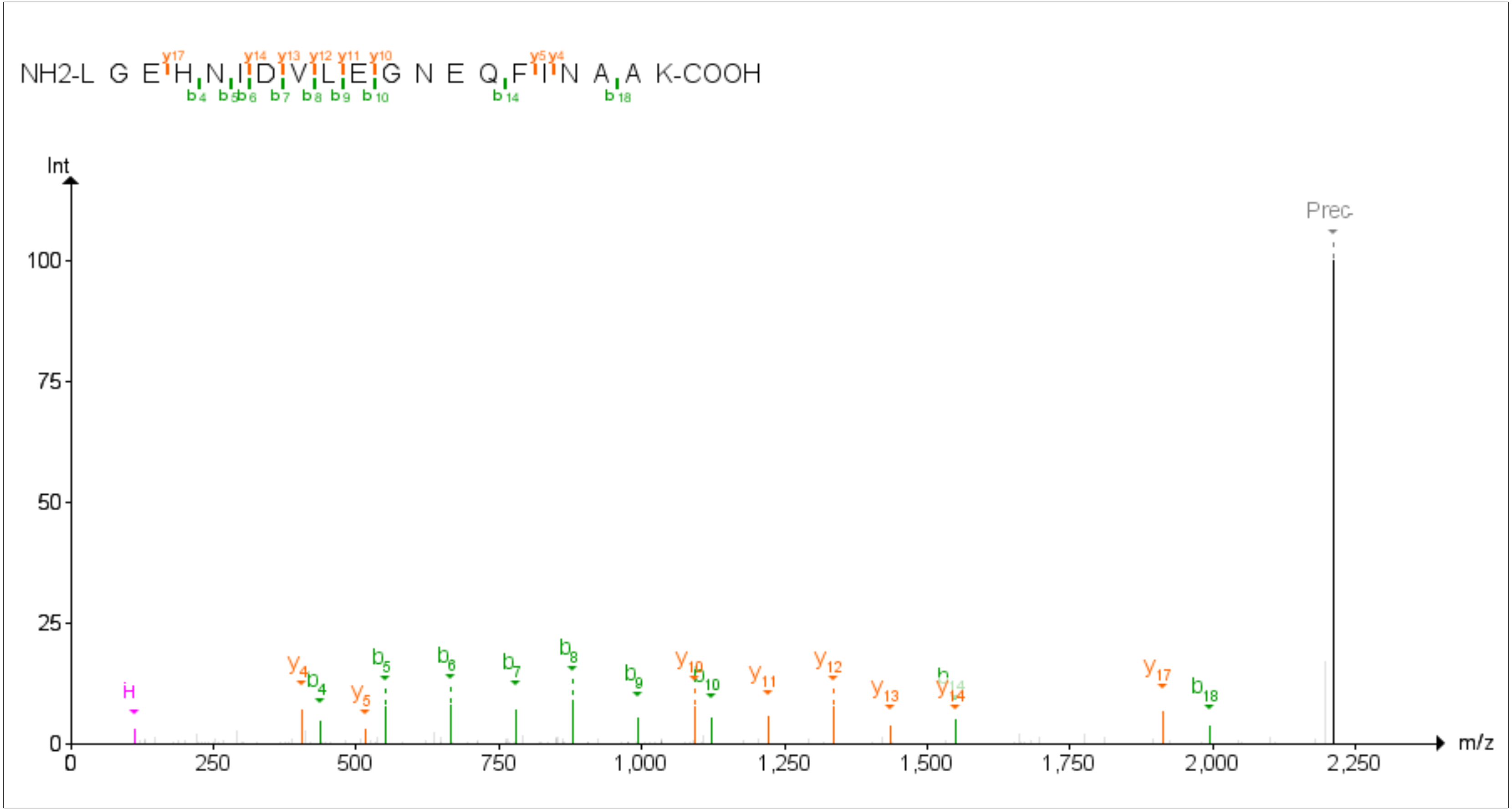

NH2-L G E H N I D V L E G N E Q F I N A A K-COOH
y17 y14 y13 y12 y11 y10 y5 y4
b4 b5 b6 b7 b8 b9 b10 b14 b18
Int
100
75
50
25
0
Prec
m/z
0
250
500
750
1,000
1,250
1,500
1,750
2,000
2,250

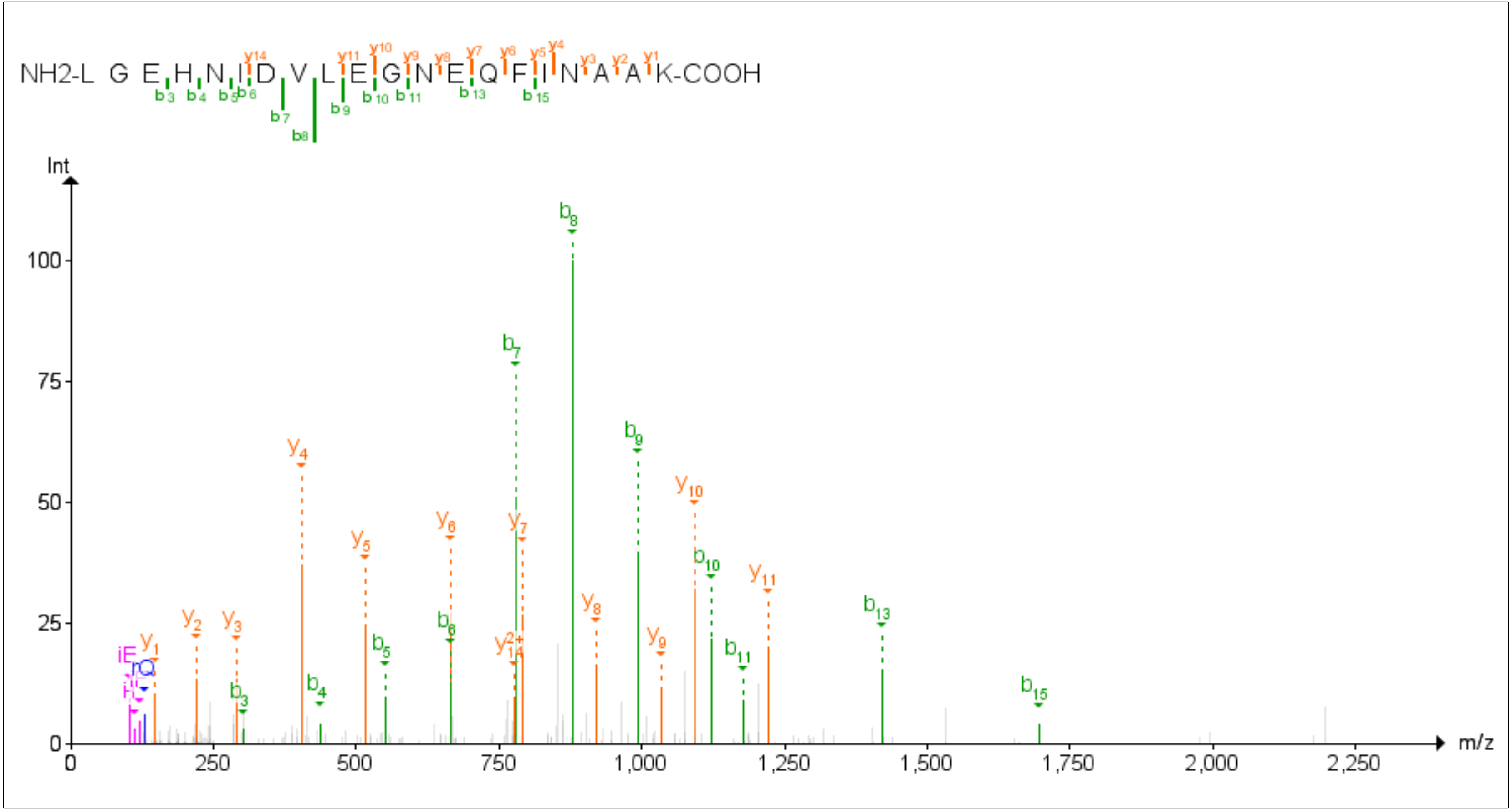

NH2-L G E H N I D V L E G N E Q F I N A A K-COOH
Int
m/z
0
25
50
75
100
250
500
750
1,000
1,250
1,500
1,750
2,000
2,250

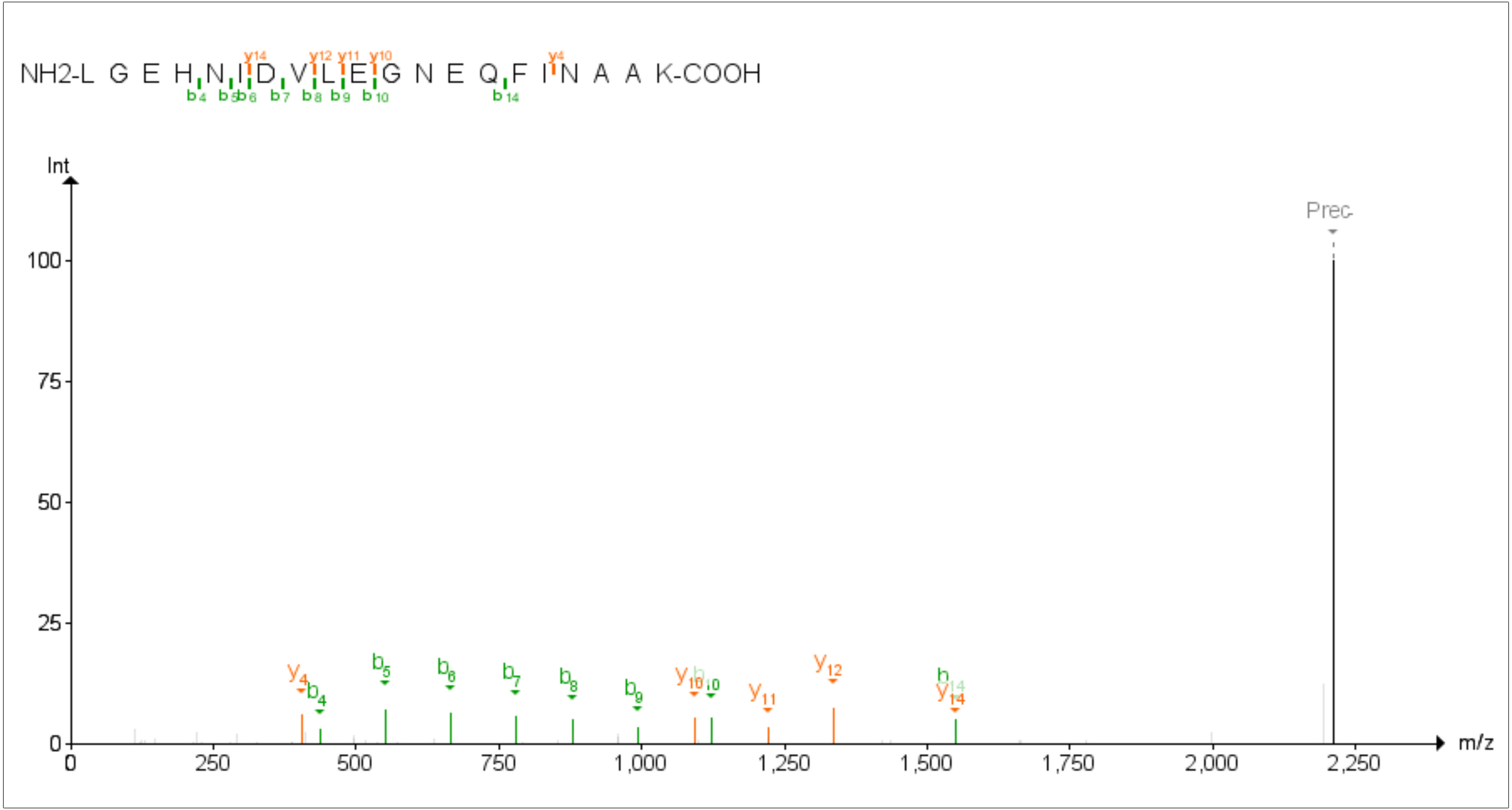

NH2-L G E H N I D V L E G N E Q F I N A A K-COOH
y14 y12 y11 y10 y4
b4 b5 b6 b7 b8 b9 b10 b14
Int
100
75
50
25
0
Prec.
y4 b4 b5 b6 b7 b8 b9 y10 b10 y11 y12 b14 y14
0
250
500
750
1,000
1,250
1,500
1,750
2,000
2,250
m/z

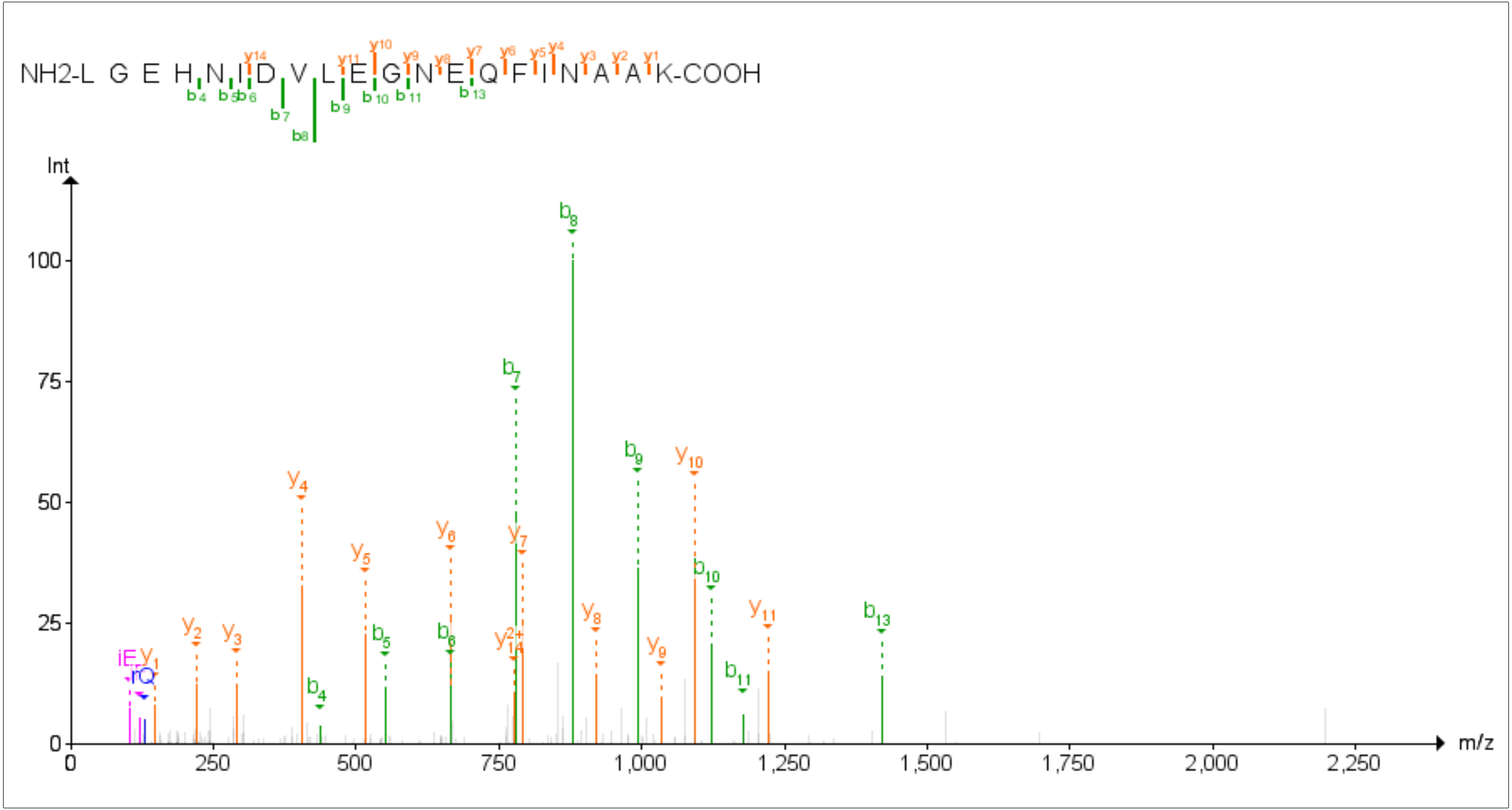
NH2-L G E H N I D V L E G N E Q F I N A A K-COOH
y14 y11 y10 y9 y8 y7 y6 y5 y4 y3 y2 y1
b4 b5 b6 b7 b8 b9 b10 b11 b13
Int
100
75
50
25
0
m/z
0
250
500
750
1,000
1,250
1,500
1,750
2,000
2,250
iE
rQ
y1
y2
y3
y4
y5
y6
y7
y8
y9
y10
y11
y14 2+
b4
b5
b6
b7
b8
b9
b10
b11
b13

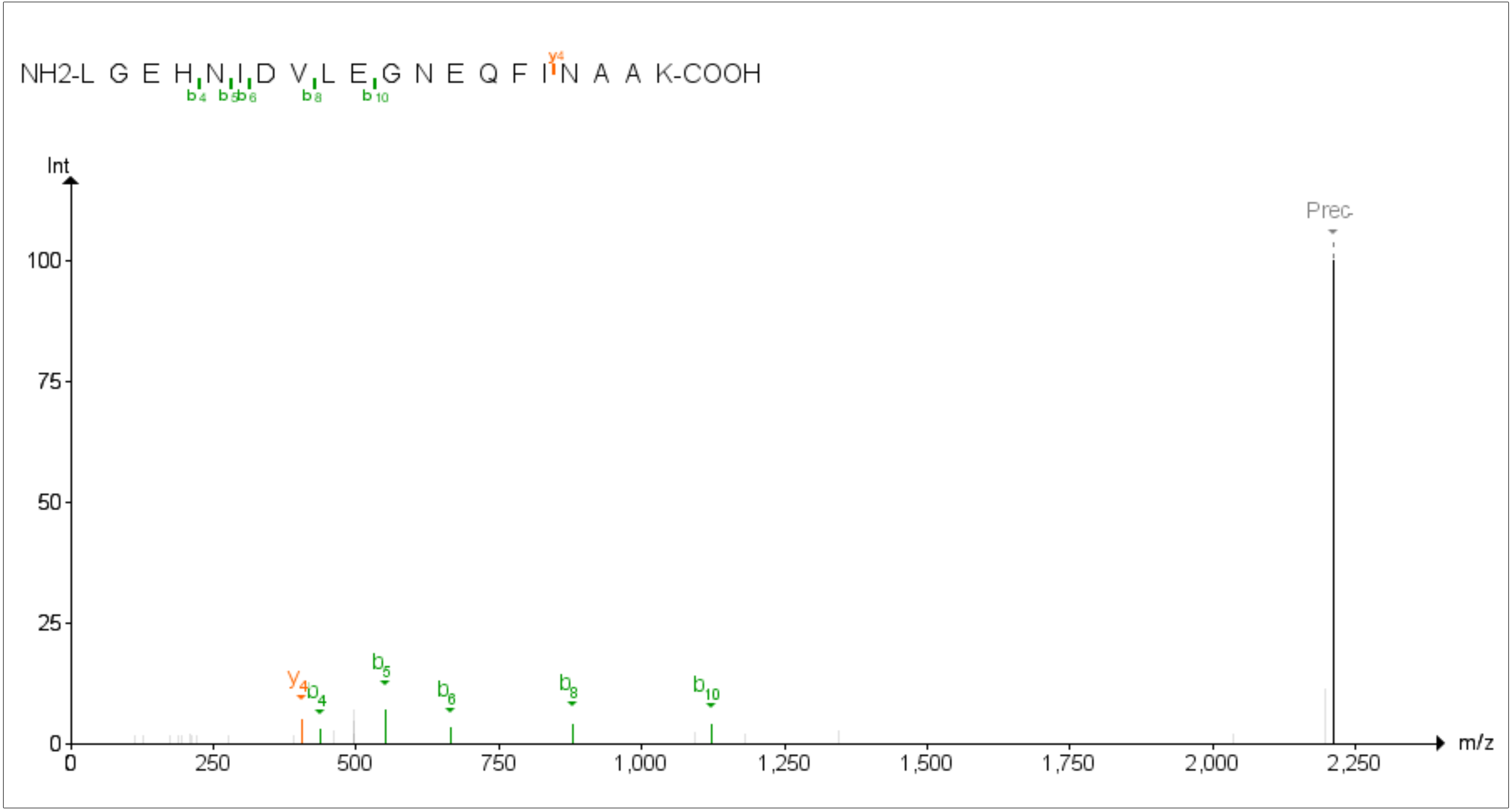
NH2-L G E H N I D V L E G N E Q F I N A A K-COOH
y4
b4 b5 b6 b8 b10
Int
100
75
50
25
0
Prec
y4
b4
b5
b6
b8
b10
0
250
500
750
1,000
1,250
1,500
1,750
2,000
2,250
m/z

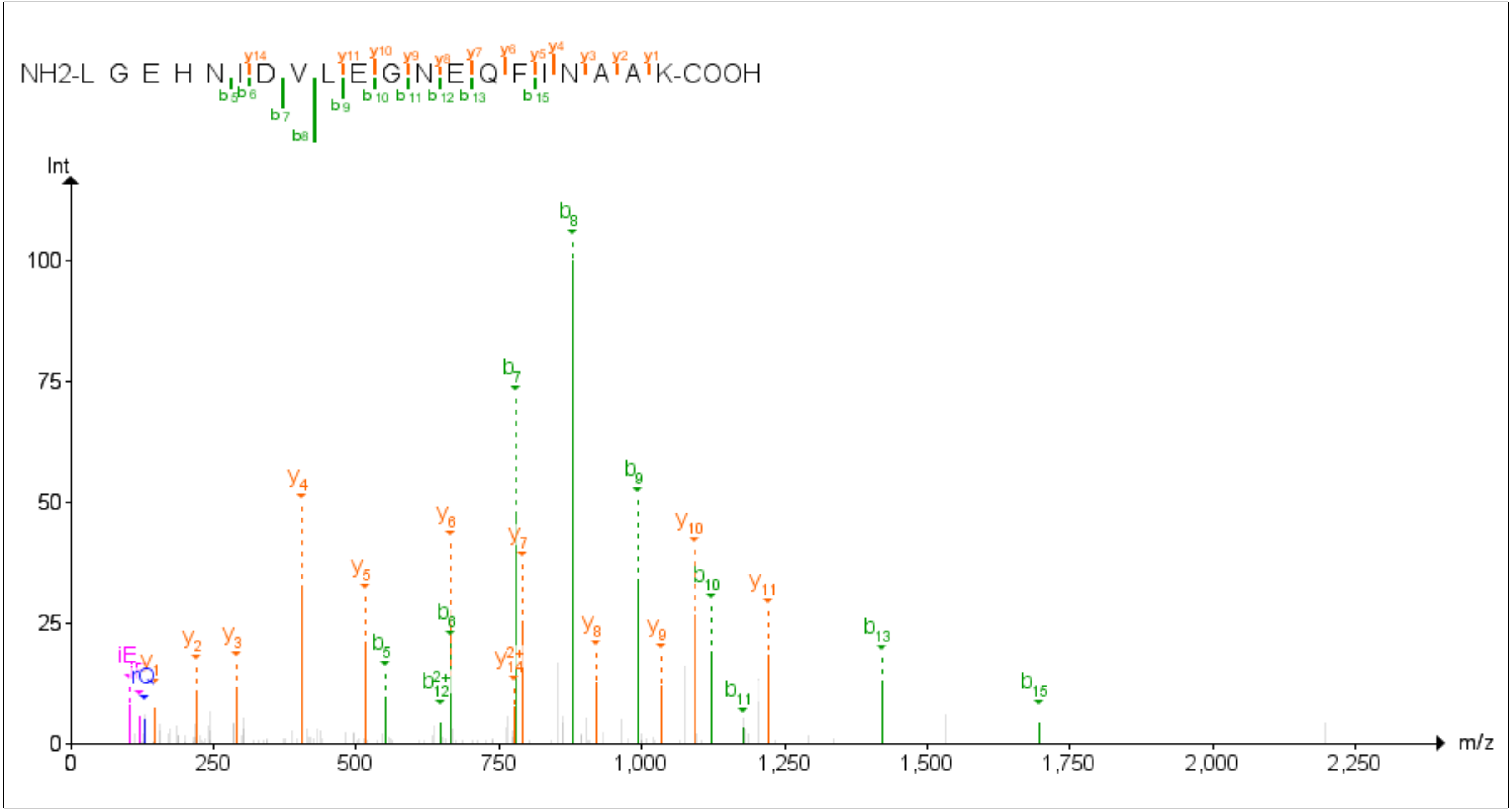

NH2-L G E H N I D V L E G N E Q F I N A A K-COOH
Int
m/z
0
25
50
75
100
250
500
750
1,000
1,250
1,500
1,750
2,000
2,250

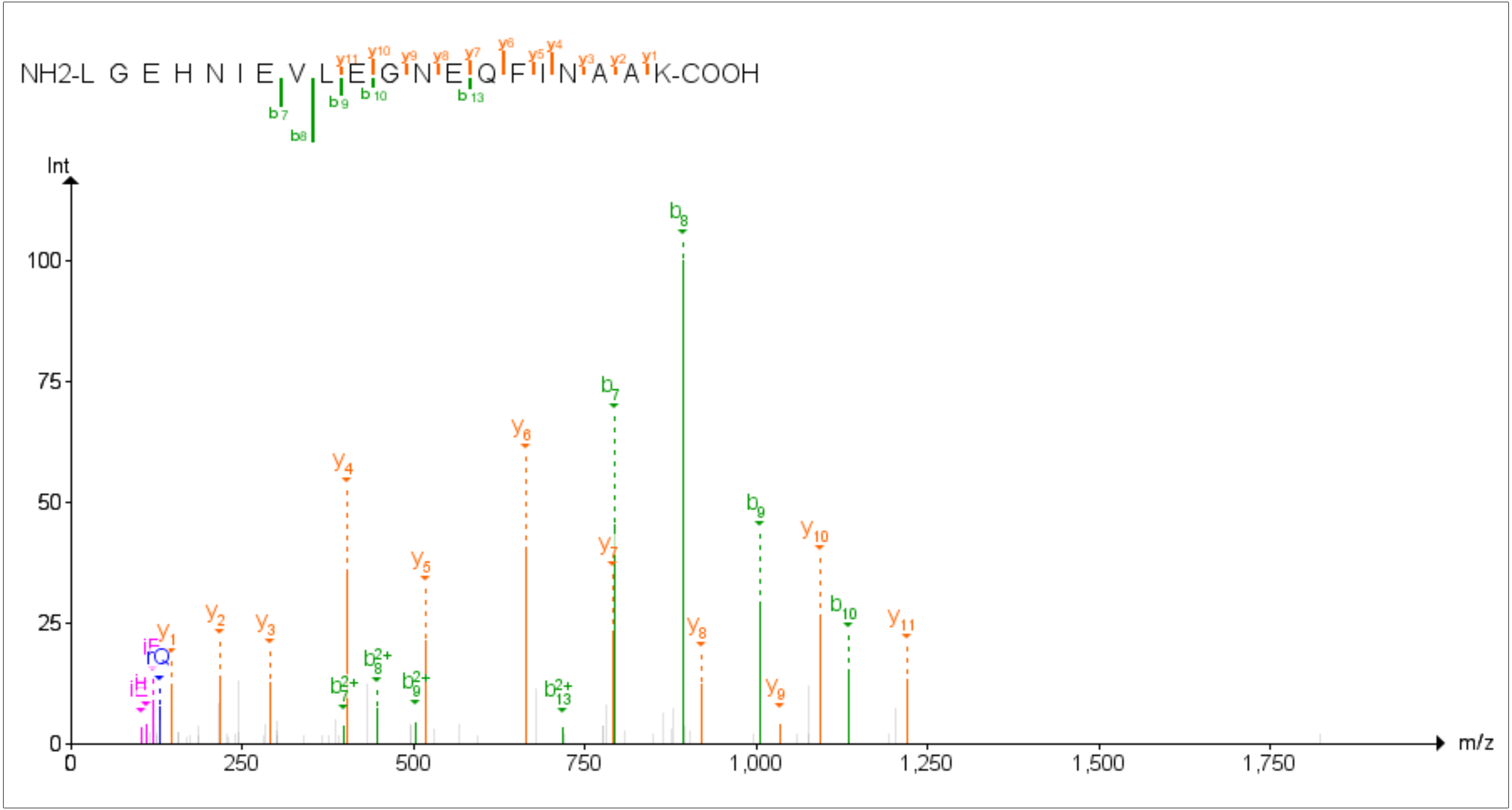

NH2-L G E H N I E V L E G N E Q F I N A A K-COOH
Int
m/z
0
25
50
75
100
250
500
750
1,000
1,250
1,500
1,750

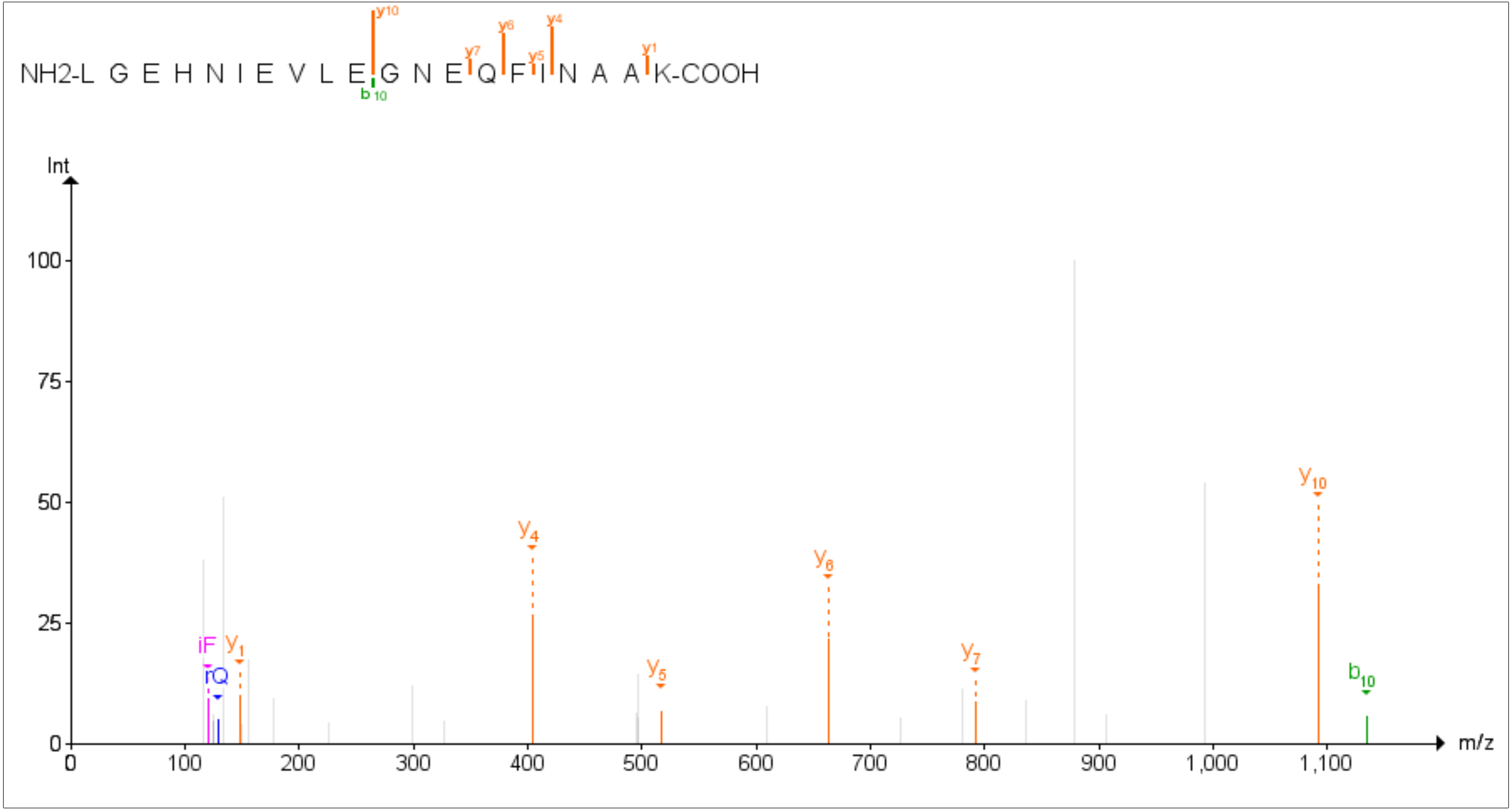
NH2-L G E H N I E V L E G N E Q F I N A A K-COOH
y10
y7
y6
y5
y4
y1
b 10
Int
100
75
50
25
0
m/z
0
100
200
300
400
500
600
700
800
900
1,000
1,100
iF
rQ
y1
y4
y5
y6
y7
y10
b10

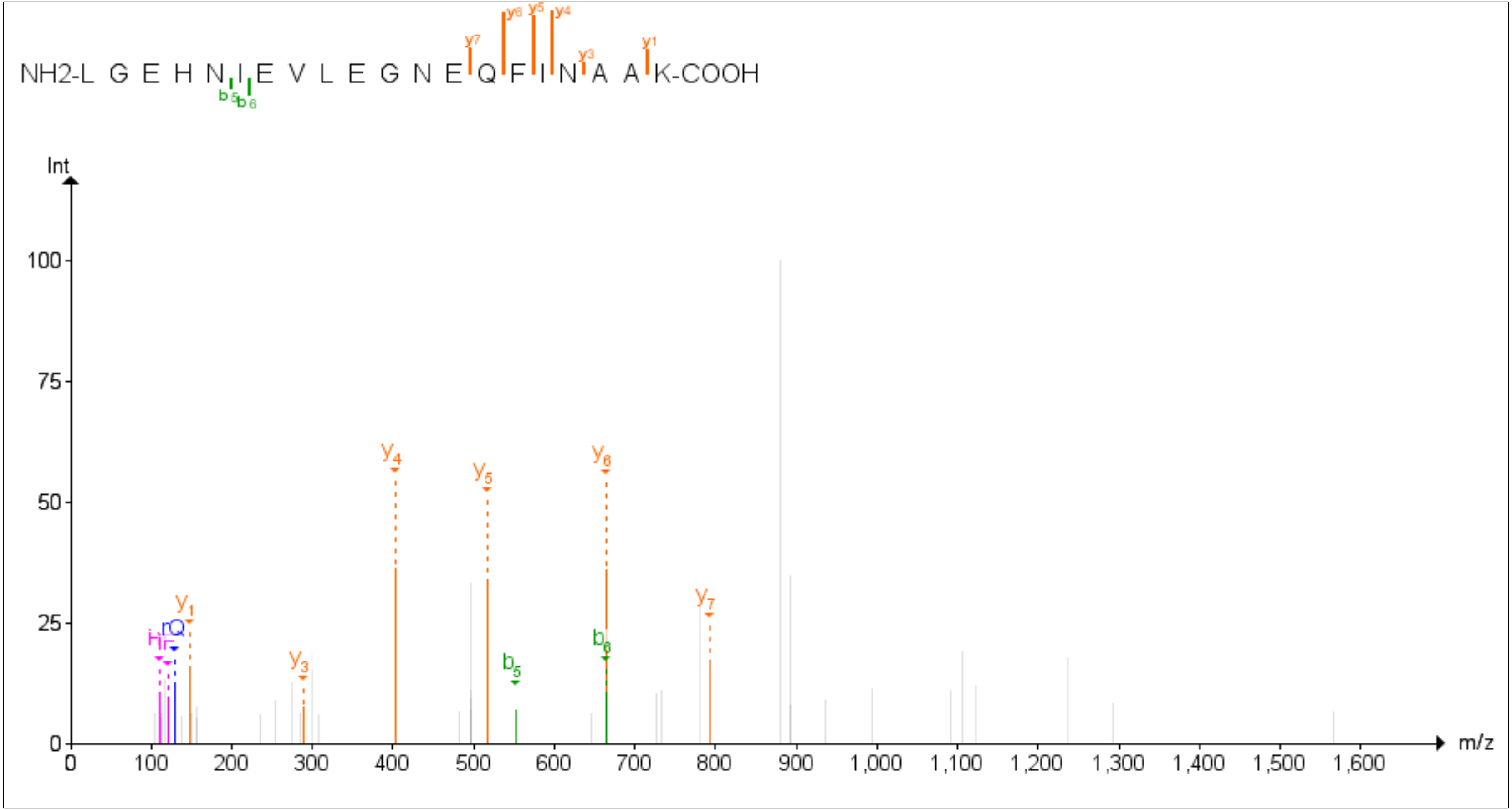
NH2-L G E H N I E V L E G N E Q F I N A A K-COOH
b5 b6
y7 y6 y5 y4 y3 y1
Int
100
75
50
25
0
m/z
0
100
200
300
400
500
600
700
800
900
1,000
1,100
1,200
1,300
1,400
1,500
1,600
y4
y5
y6
y7
y1
rQ
y3
b5
b6

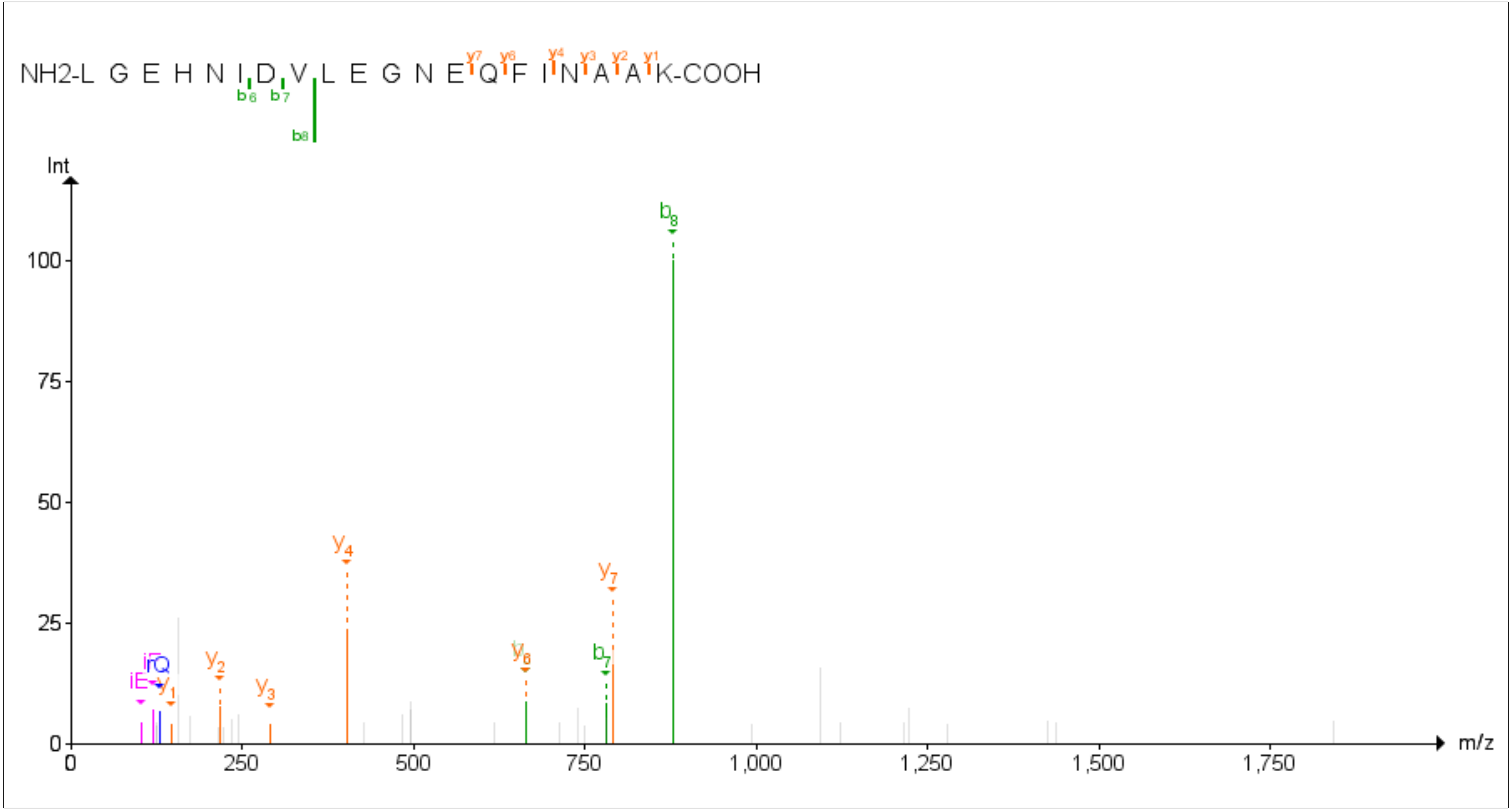

NH2-L G E H N I D V L E G N E Q F I N A A K-COOH
y7 y6 y4 y3 y2 y1
b6 b7 b8
Int
100
75
50
25
0
b8
y4
y7
y6
b7
y2
y3
y1
iE
rQ
0
250
500
750
1,000
1,250
1,500
1,750
m/z

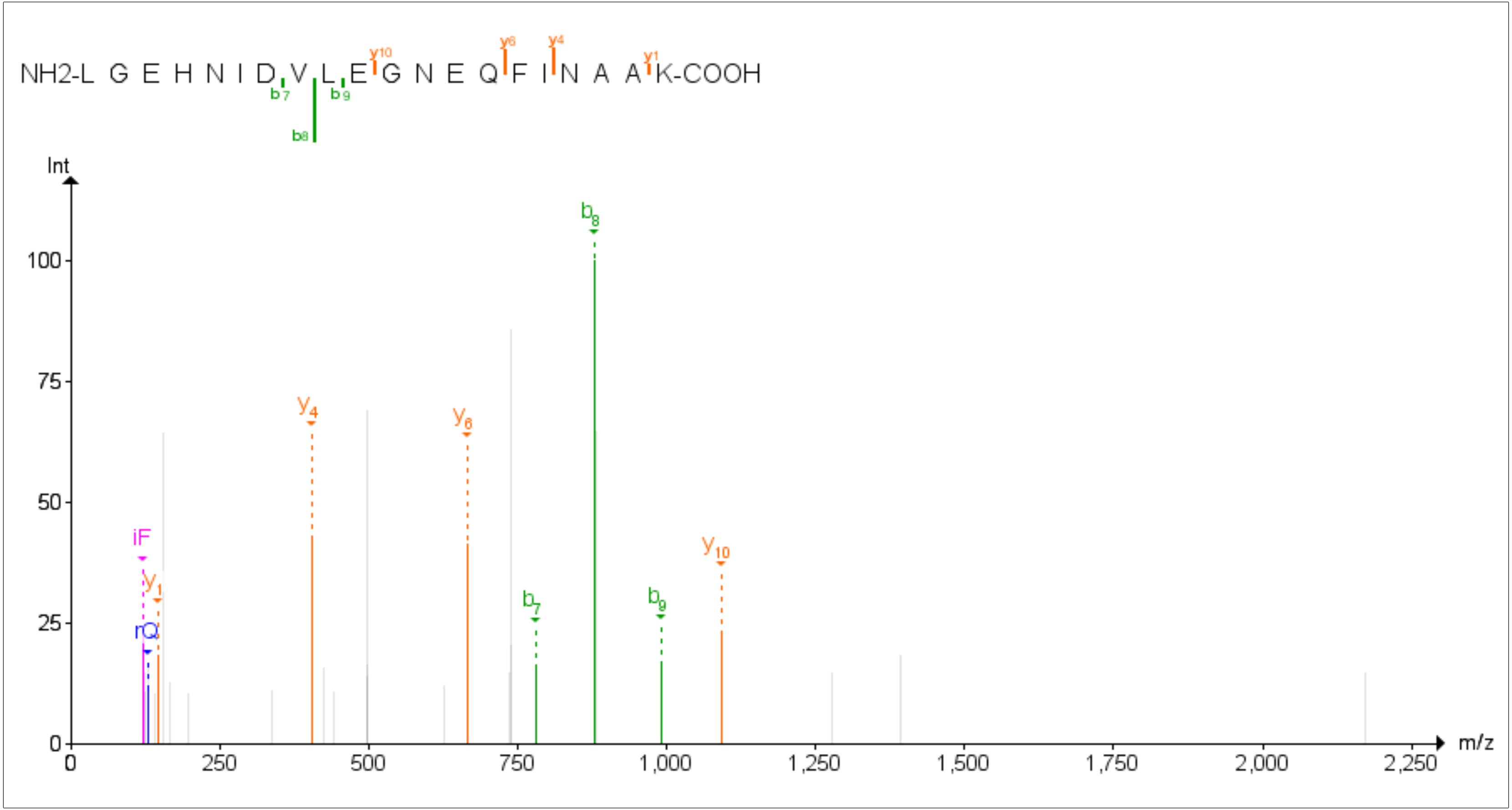

NH2-L G E H N I D V L E G N E Q F I N A A K-COOH
b7
b8
b9
y10
y6
y4
y1
Int
100
75
50
25
0
iF
y1
rQ
y4
y6
b7
b8
b9
y10
0
250
500
750
1,000
1,250
1,500
1,750
2,000
2,250
m/z

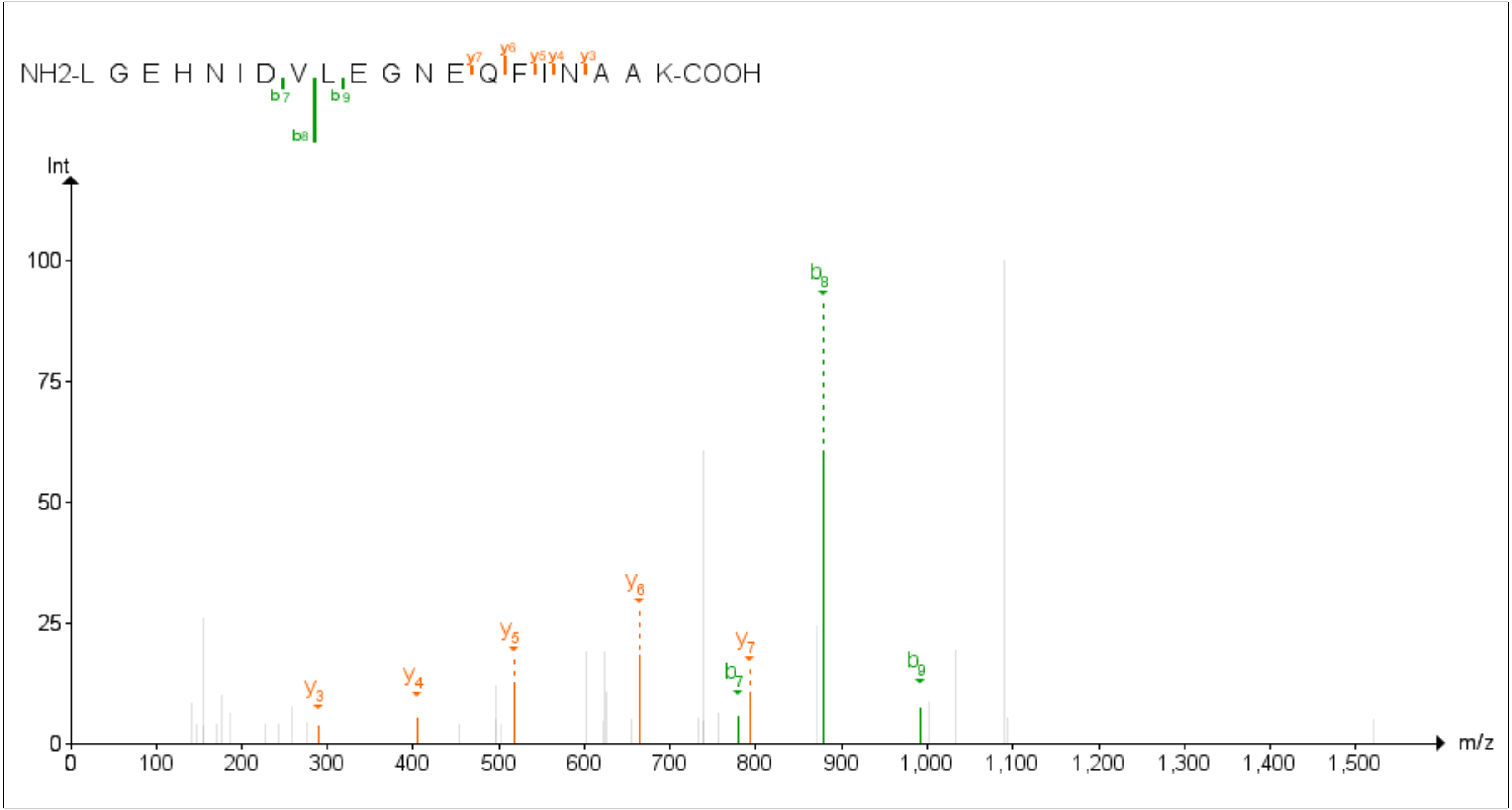

NH2-L G E H N I D V L E G N E Q F I N A A K-COOH
y7 y6 y5 y4 y3
b7 b9 b8
Int
100
75
50
25
0
b8
y6
y5
y7
b7
b9
y3
y4
0 100 200 300 400 500 600 700 800 900 1,000 1,100 1,200 1,300 1,400 1,500
m/z

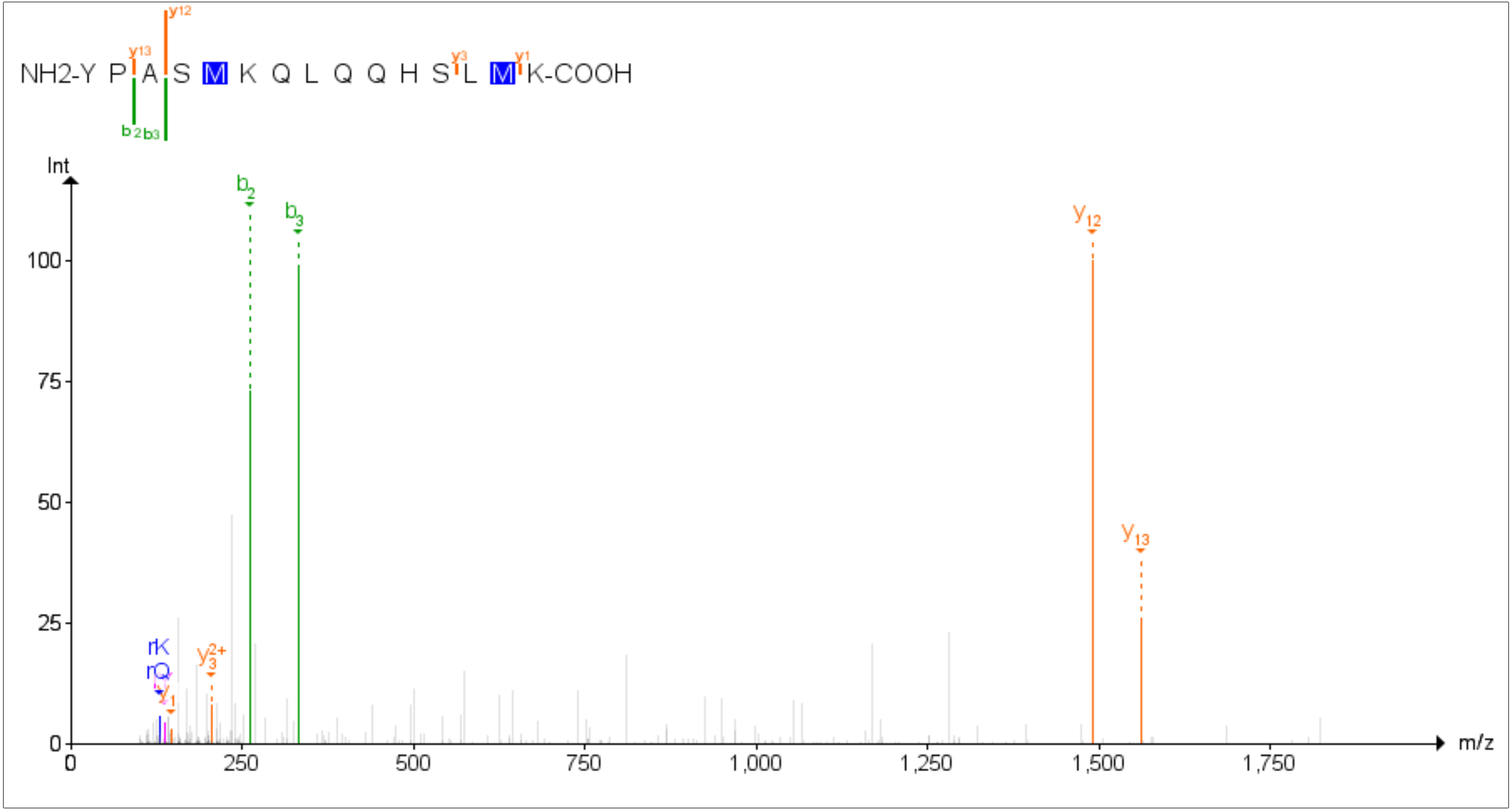

NH2-Y P A S M K Q L Q Q H S L M K-COOH
y12
y13
y3
y1
b2
b3
Int
m/z
100
75
50
25
0
250
500
750
1,000
1,250
1,500
1,750
b2
b3
y12
y13
rK
rQ
y1
y3 2+

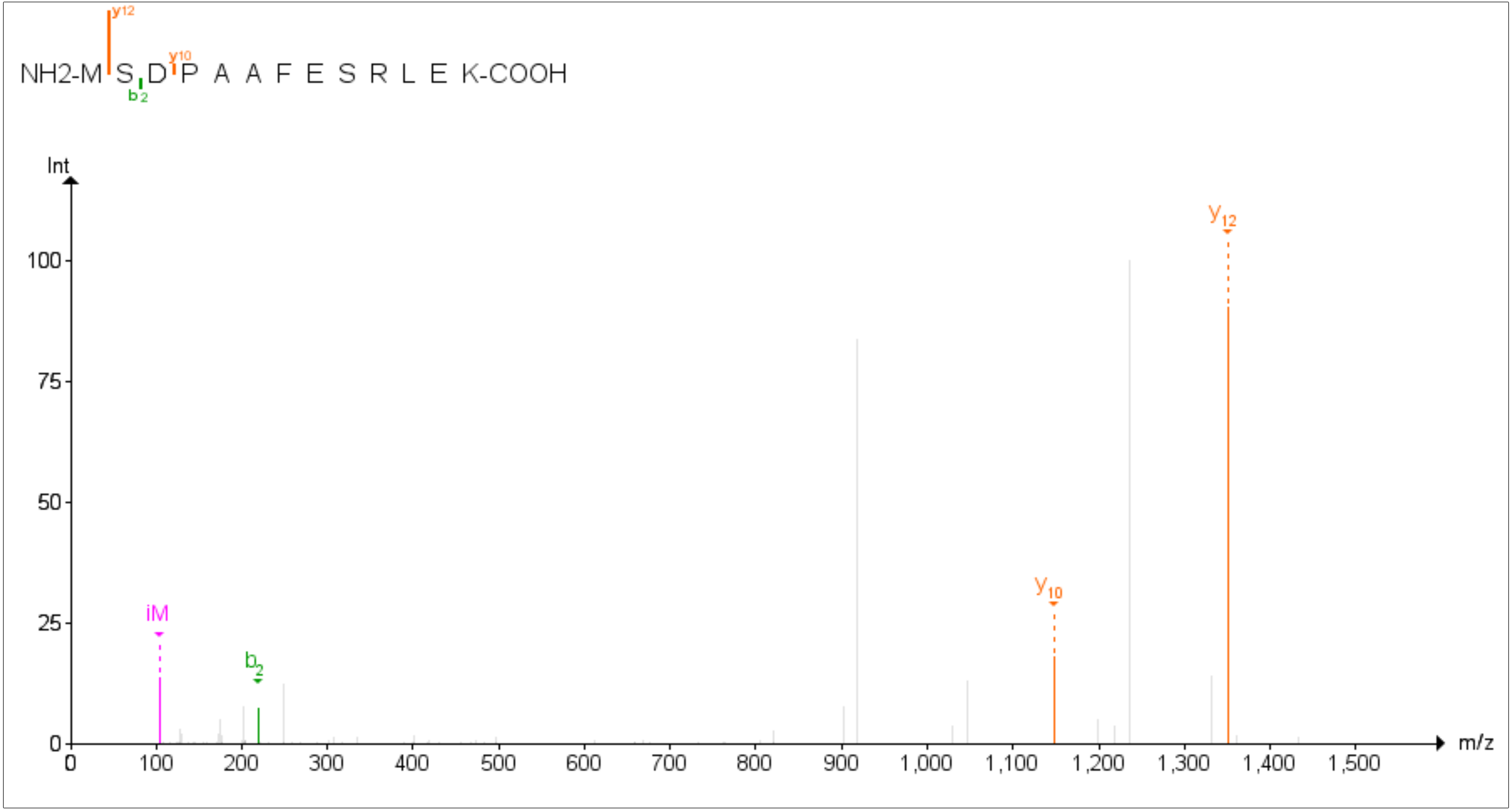

y12
y10
NH2-M S D P A A F E S R L E K-COOH
b2
Int
100
75
50
25
0
iM
b2
y10
y12
0
100
200
300
400
500
600
700
800
900
1,000
1,100
1,200
1,300
1,400
1,500
m/z

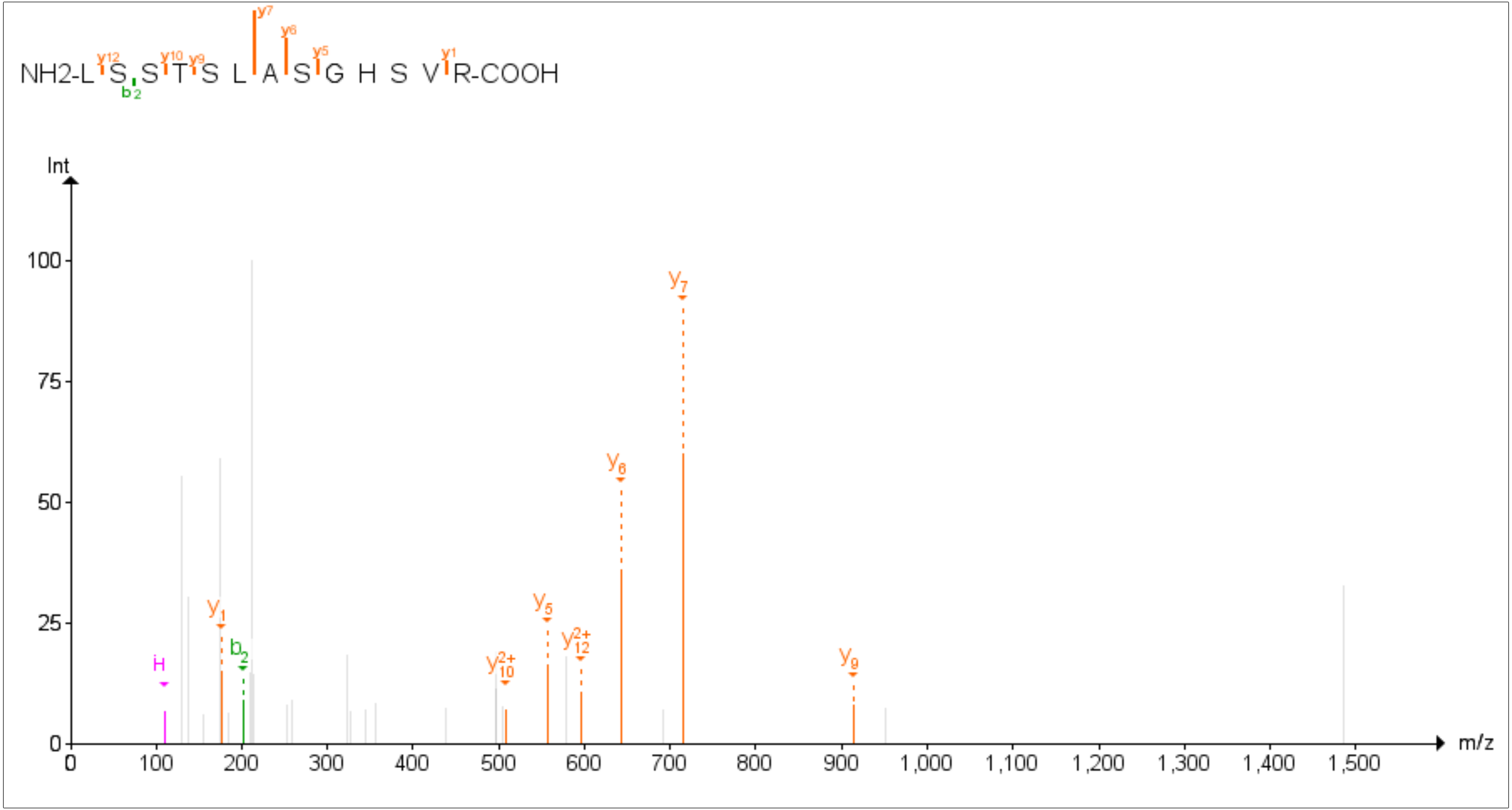

NH2-L S S T S L A S G H S V R-COOH
y12 y10 y9 y7 y6 y5 y1
b2
Int
100
75
50
25
0
m/z
0 100 200 300 400 500 600 700 800 900 1,000 1,100 1,200 1,300 1,400 1,500
H
y1
b2
y10 2+
y5
y12 2+
y6
y7
y9

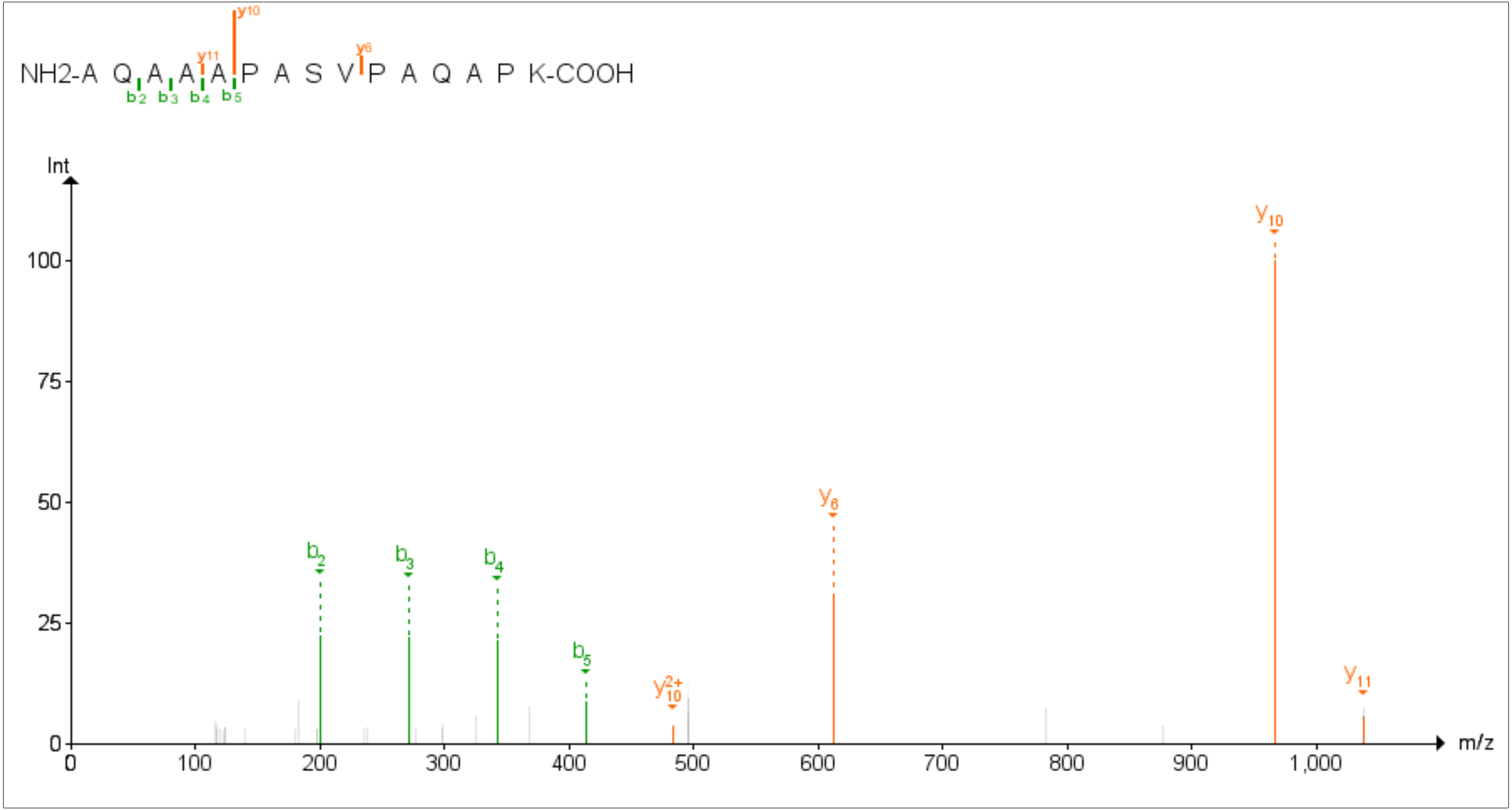

NH2-A Q A A A P A S V P A Q A P K-COOH
b2 b3 b4 b5
y11 y10 y6
Int
100
75
50
25
0
m/z
0
100
200
300
400
500
600
700
800
900
1,000
b2
b3
b4
b5
y_{10}^{2+}
y6
y10
y11

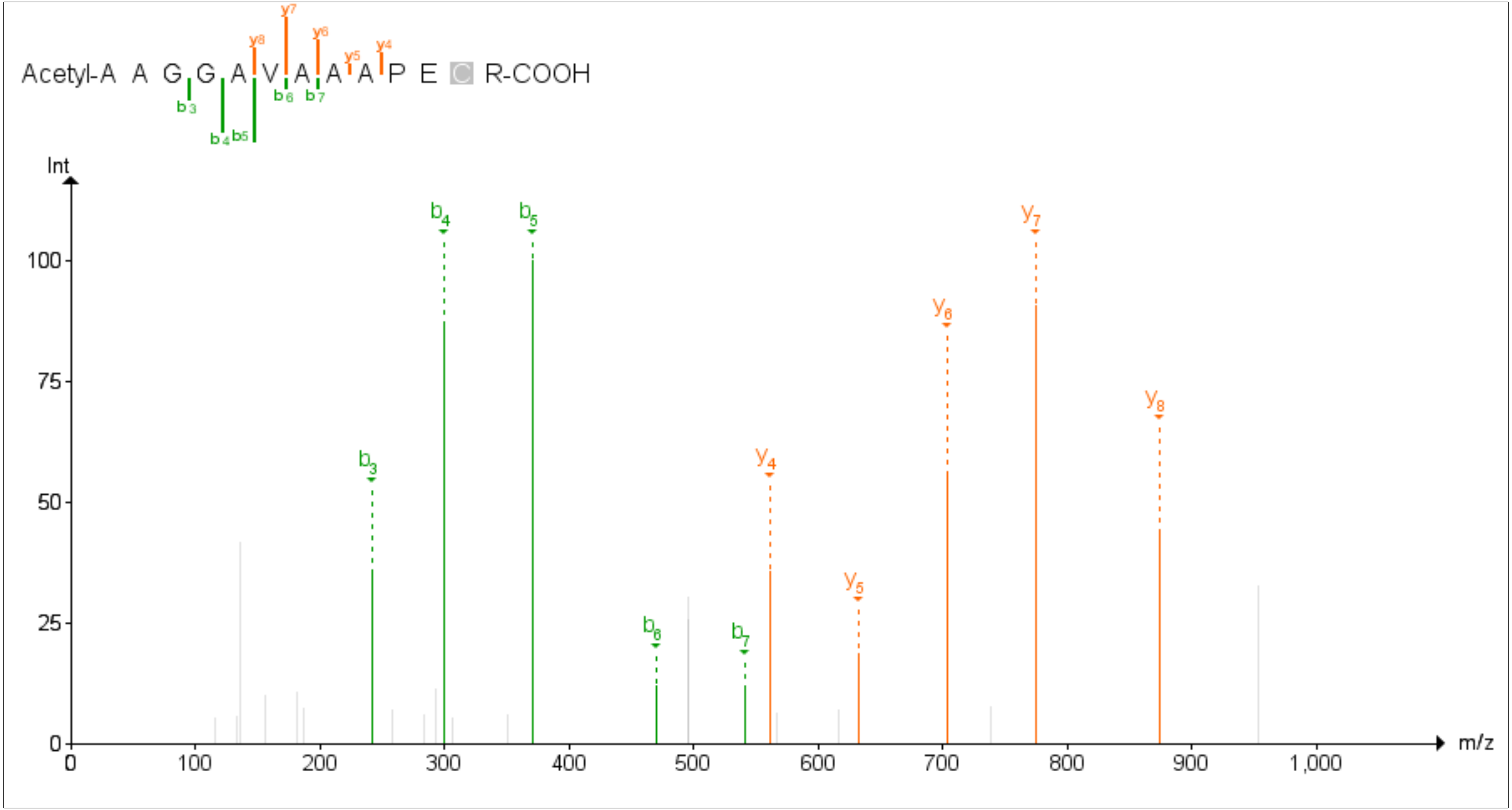

Acetyl-A A G G A V A A A A P E C R-COOH
b3
b4
b5
b6
b7
y8
y7
y6
y5
y4
Int
100
75
50
25
0
m/z
0
100
200
300
400
500
600
700
800
900
1,000

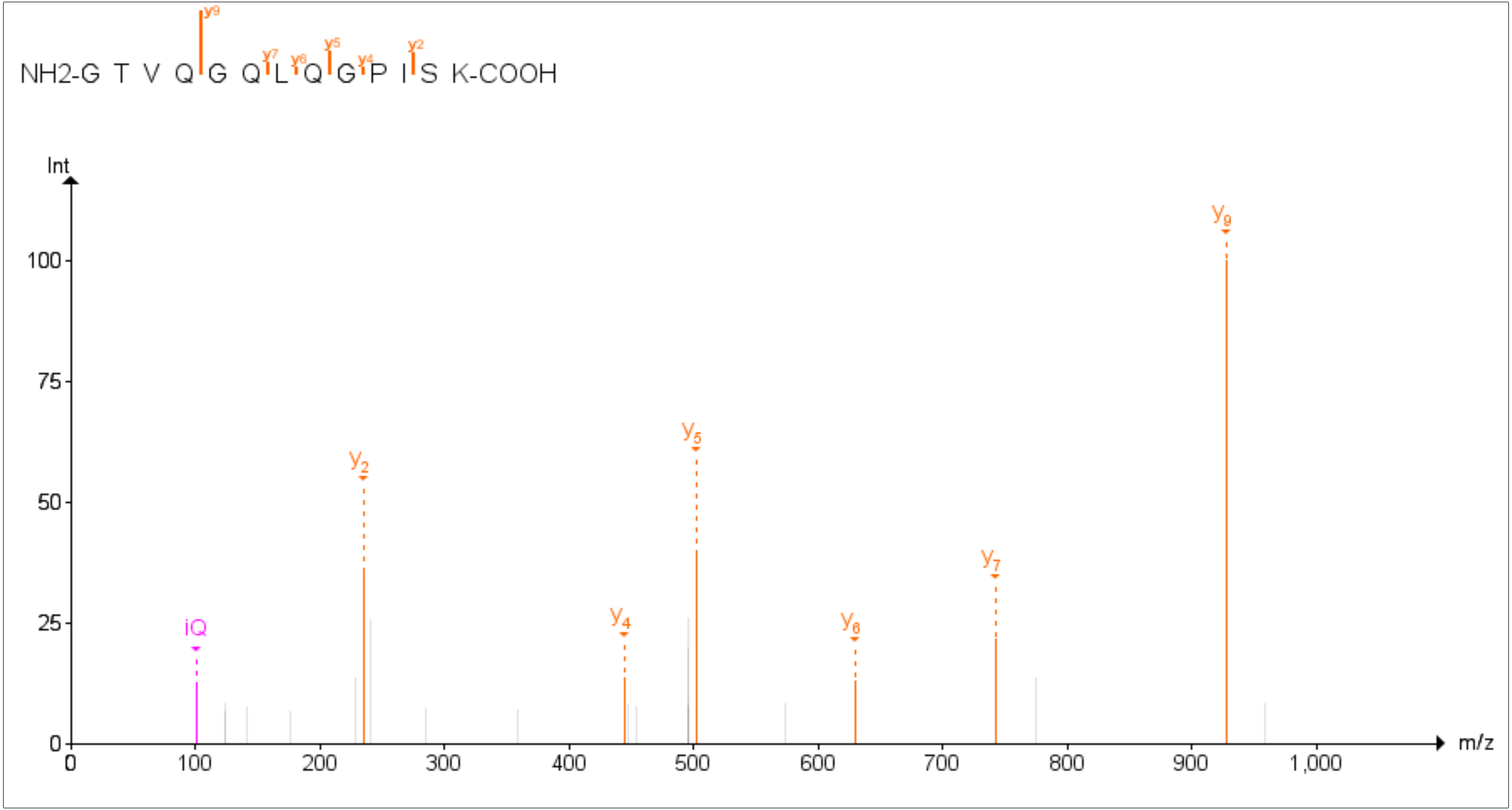
NH2-G T V Q G Q L Q G P I S K-COOH
y9
y7
y6
y5
y4
y2
Int
100
75
50
25
0
m/z
0
100
200
300
400
500
600
700
800
900
1,000
iQ
y2
y4
y5
y6
y7
y9

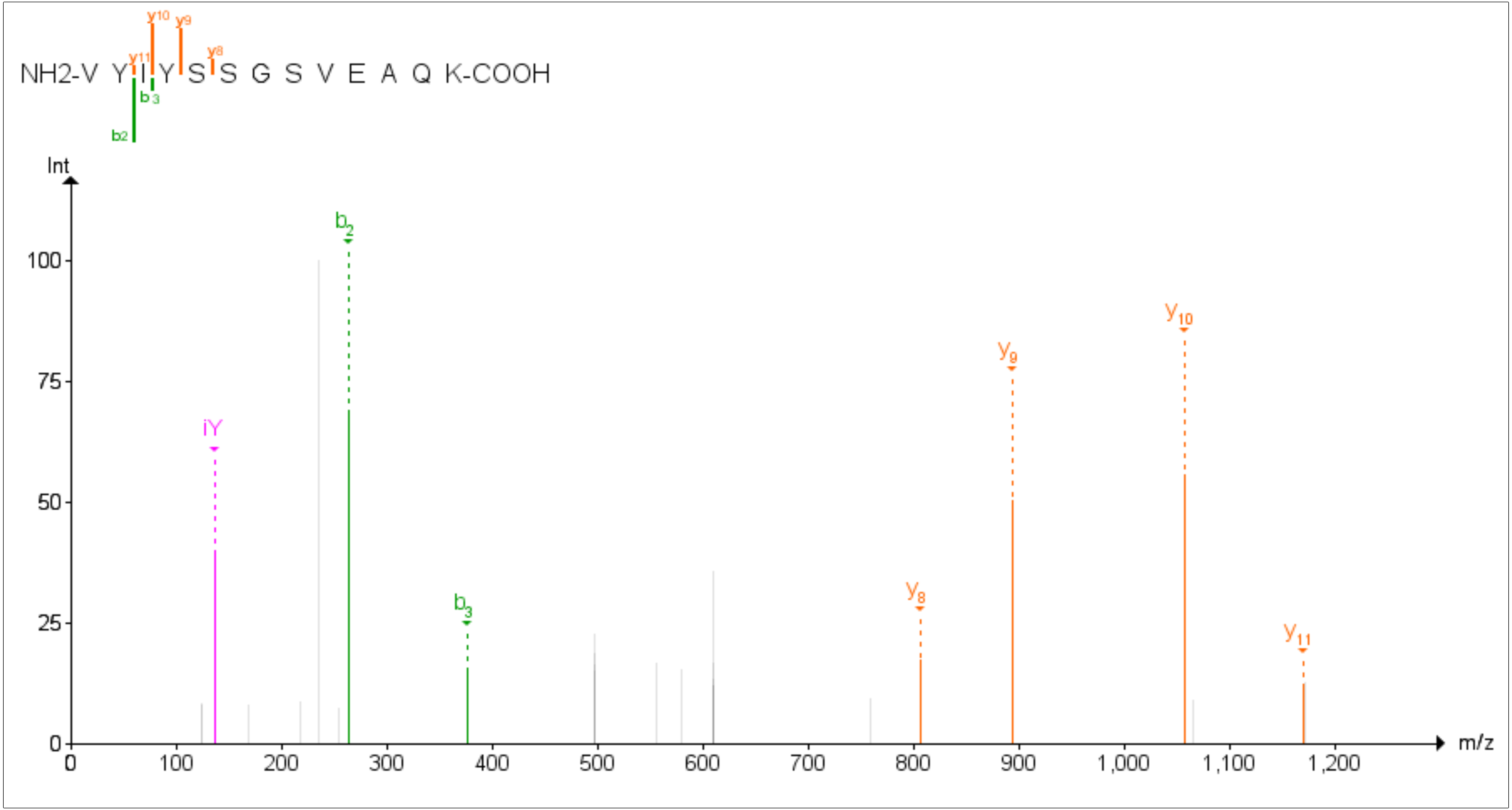

NH2-V Y I Y S S G S V E A Q K-COOH
y10
y9
y11
y8
b3
b2
Int
100
75
50
25
0
b2
iY
b3
y8
y9
y10
y11
m/z
0
100
200
300
400
500
600
700
800
900
1,000
1,100
1,200

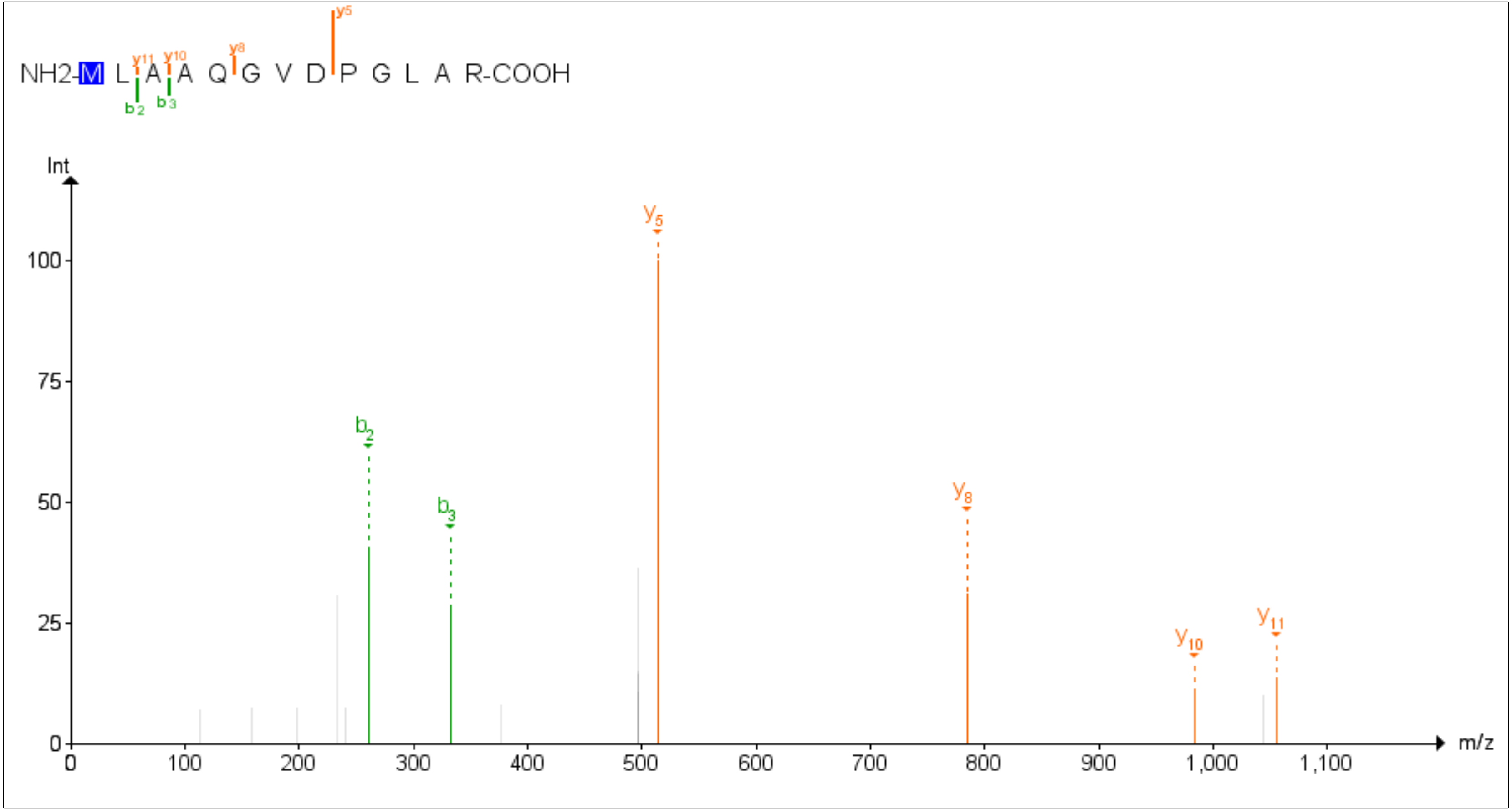

NH2-M L A A Q G V D P G L A R-COOH
y11 y10 y8 y5
b2 b3
Int
100
75
50
25
0
m/z
0 100 200 300 400 500 600 700 800 900 1,000 1,100
y5
b2
b3
y8
y10
y11

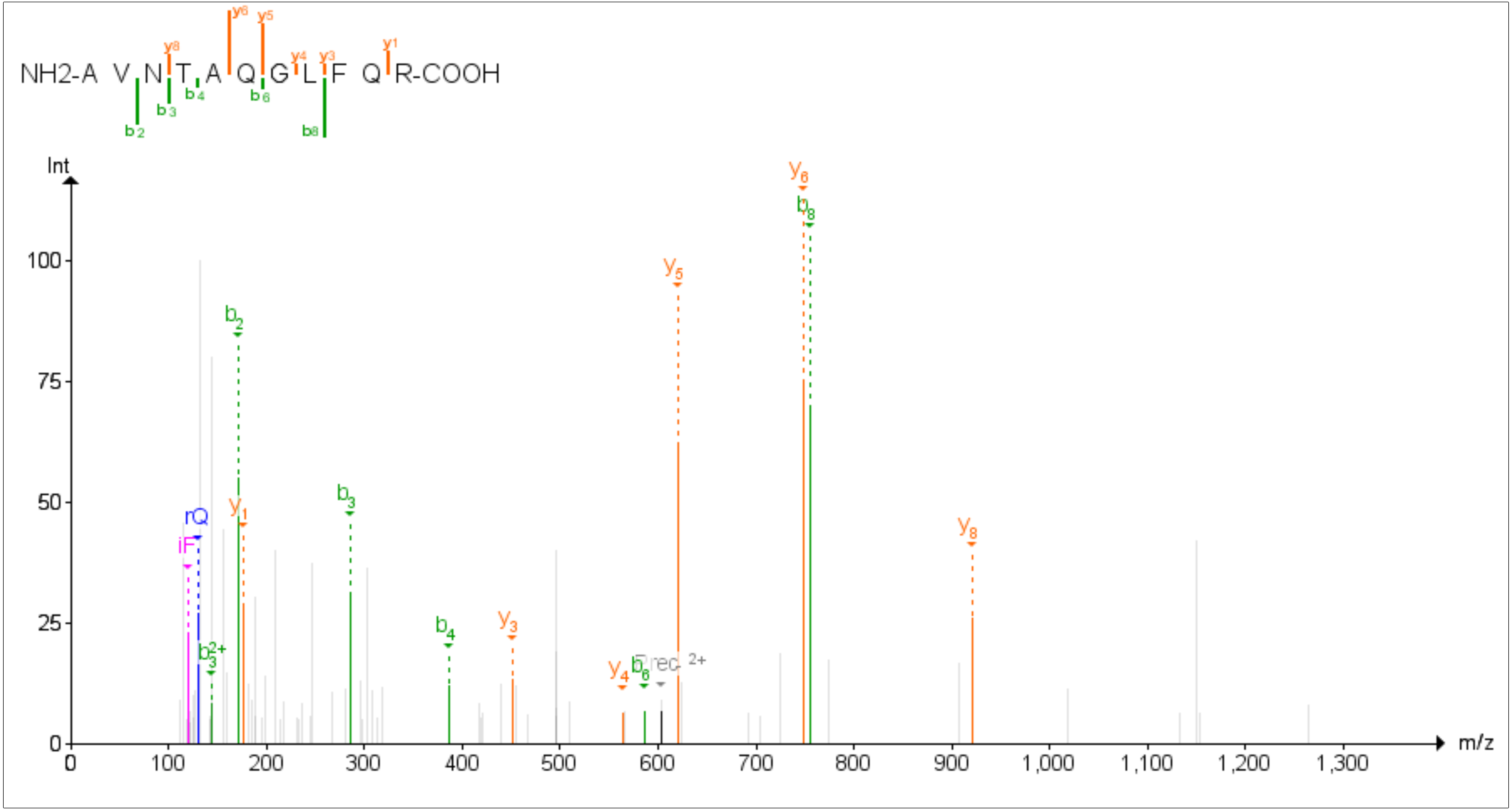

NH2-A V N T A Q G L F Q R-COOH
y8 y6 y5 y4 y3 y1
b2 b3 b4 b6 b8
Int
100
75
50
25
0
m/z
0 100 200 300 400 500 600 700 800 900 1,000 1,100 1,200 1,300
y6
b8
y5
b2
b3
rQ
y1
iF
y8
b4
y3
b3 2+
y4
b6
Prec 2+

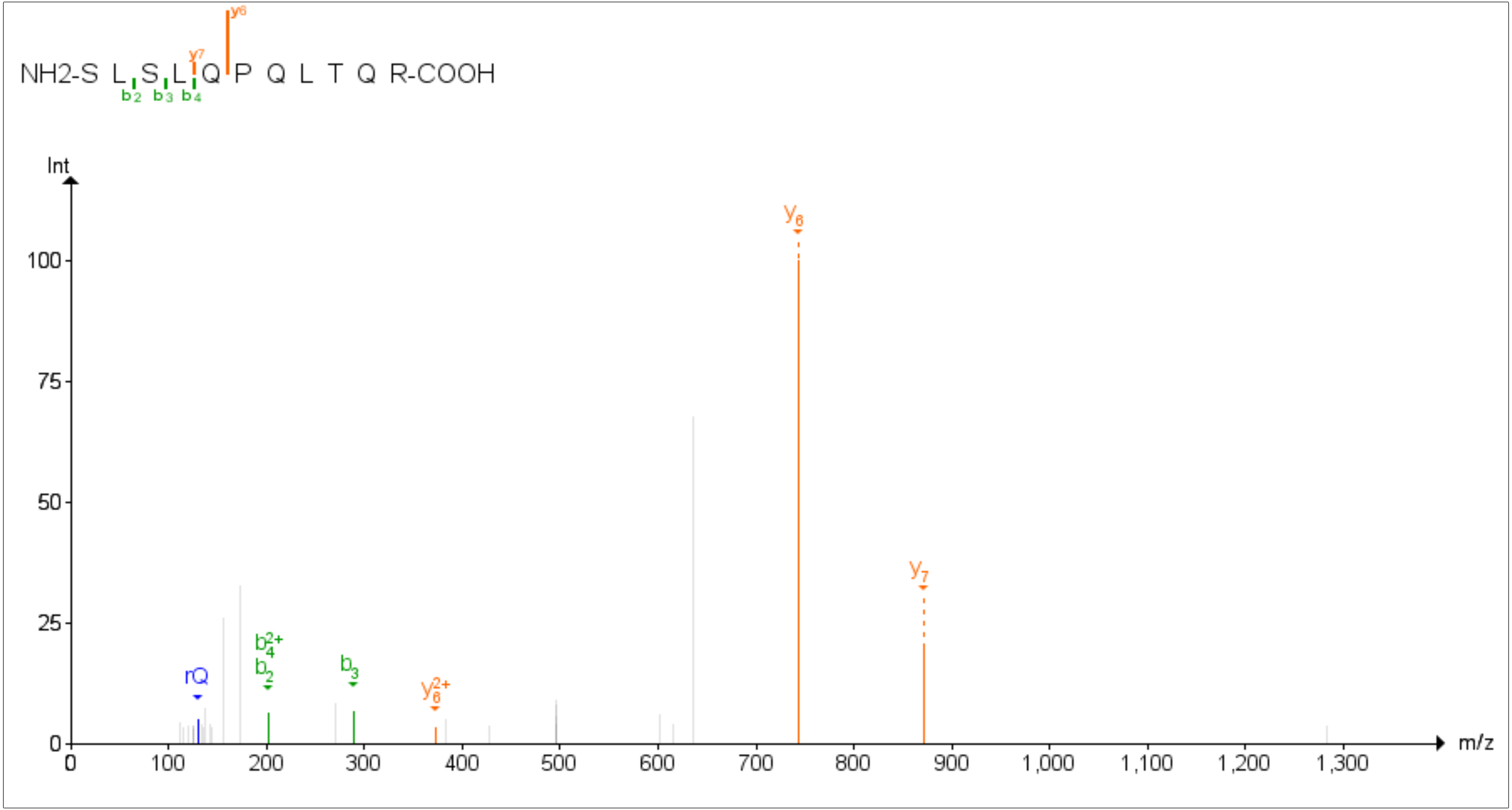
NH2-S L S L Q P Q L T Q R-COOH
b2 b3 b4
y7 y6
Int
100
75
50
25
0
y6
y7
rQ
b4 2+
b2
b3
y6 2+
0 100 200 300 400 500 600 700 800 900 1,000 1,100 1,200 1,300
m/z

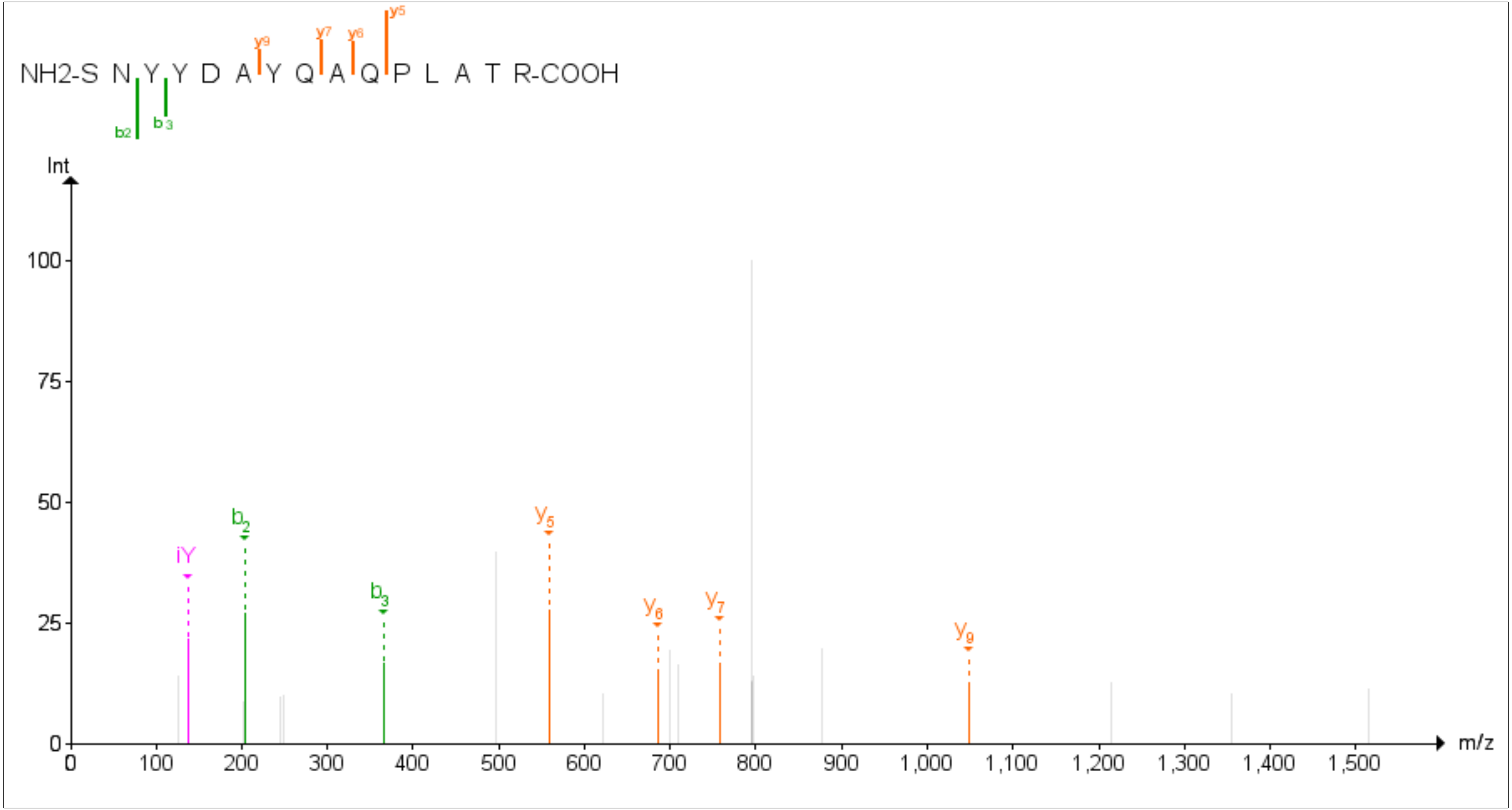
NH2-S N Y Y D A Y Q A Q P L A T R-COOH
y9
y7
y6
y5
b2
b3
Int
100
75
50
25
0
iY
b2
b3
y5
y6
y7
y9
0
100
200
300
400
500
600
700
800
900
1,000
1,100
1,200
1,300
1,400
1,500
m/z

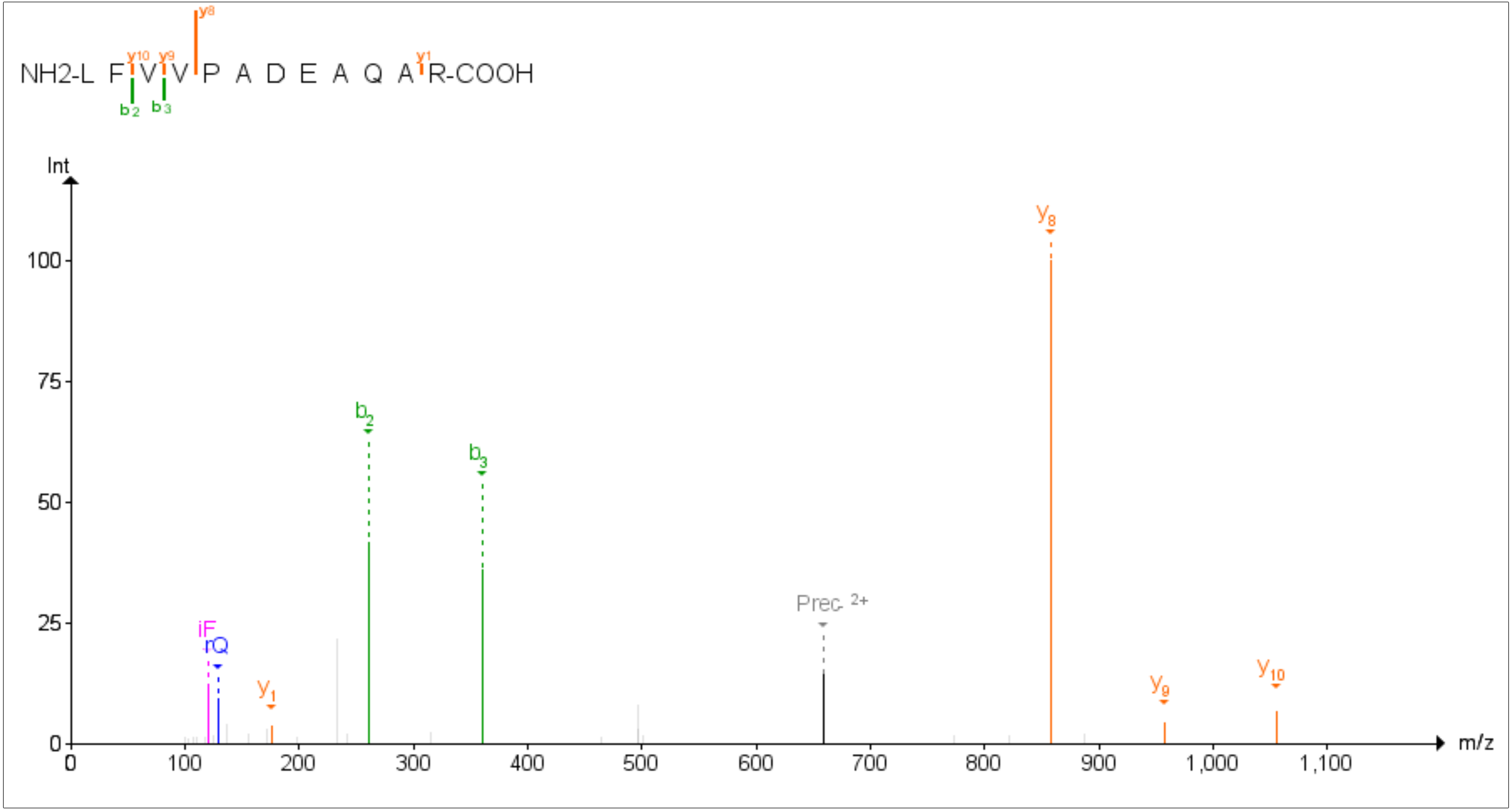

NH2-L F V V P A D E A Q A R-COOH
y8
y10
y9
y1
b2
b3
Int
100
75
50
25
0
y8
b2
b3
Prec 2+
iF
rQ
y1
y9
y10
0
100
200
300
400
500
600
700
800
900
1,000
1,100
m/z

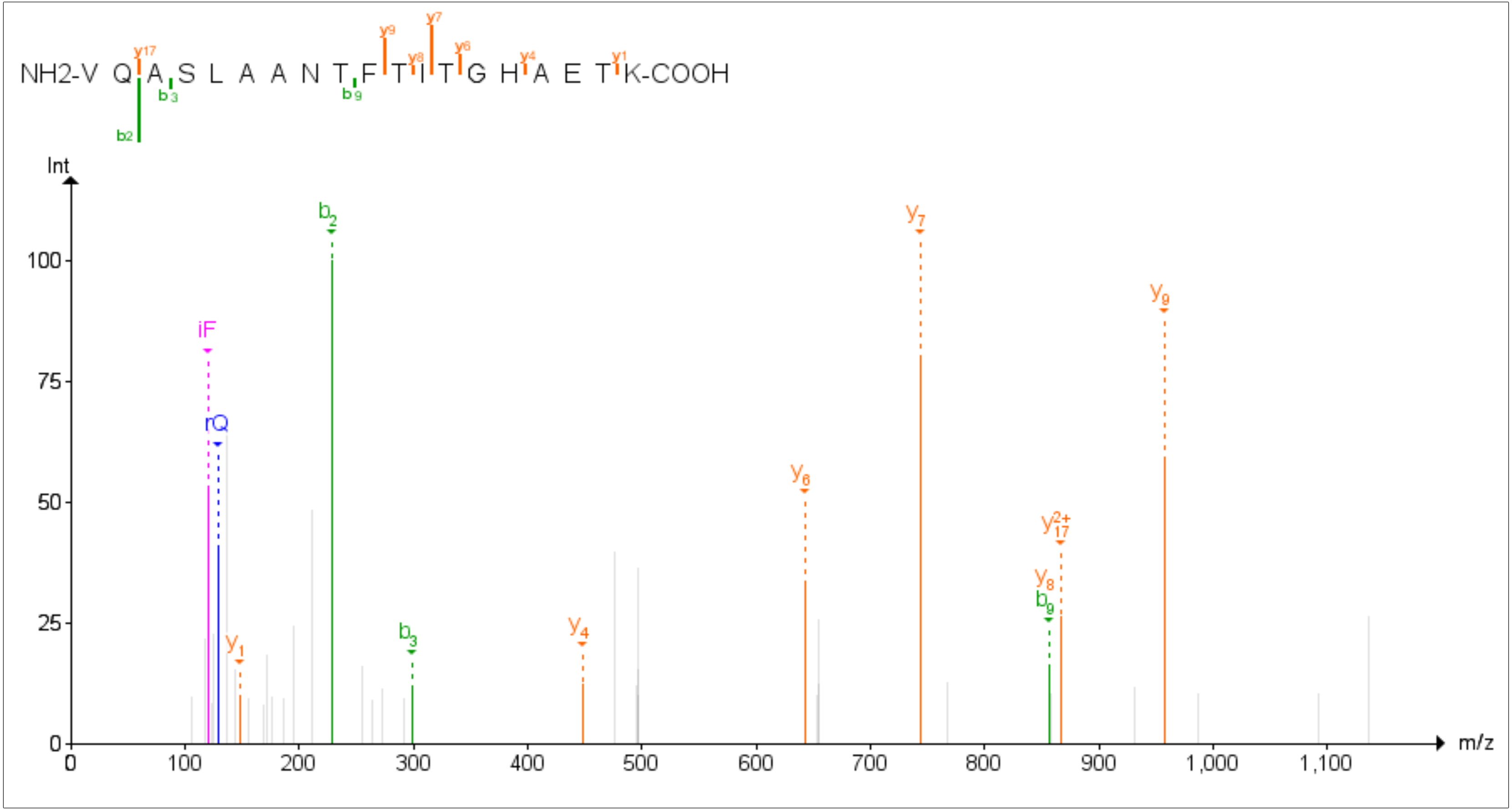

NH2-V Q A S L A A N T F T I T G H A E T K-COOH
y17
y9
y8
y7
y6
y4
y1
b2
b3
b9
Int
m/z
iF
rQ
y1
b2
b3
y4
y6
y7
y8
b9
y17 2+
y9
0
25
50
75
100
0
100
200
300
400
500
600
700
800
900
1,000
1,100

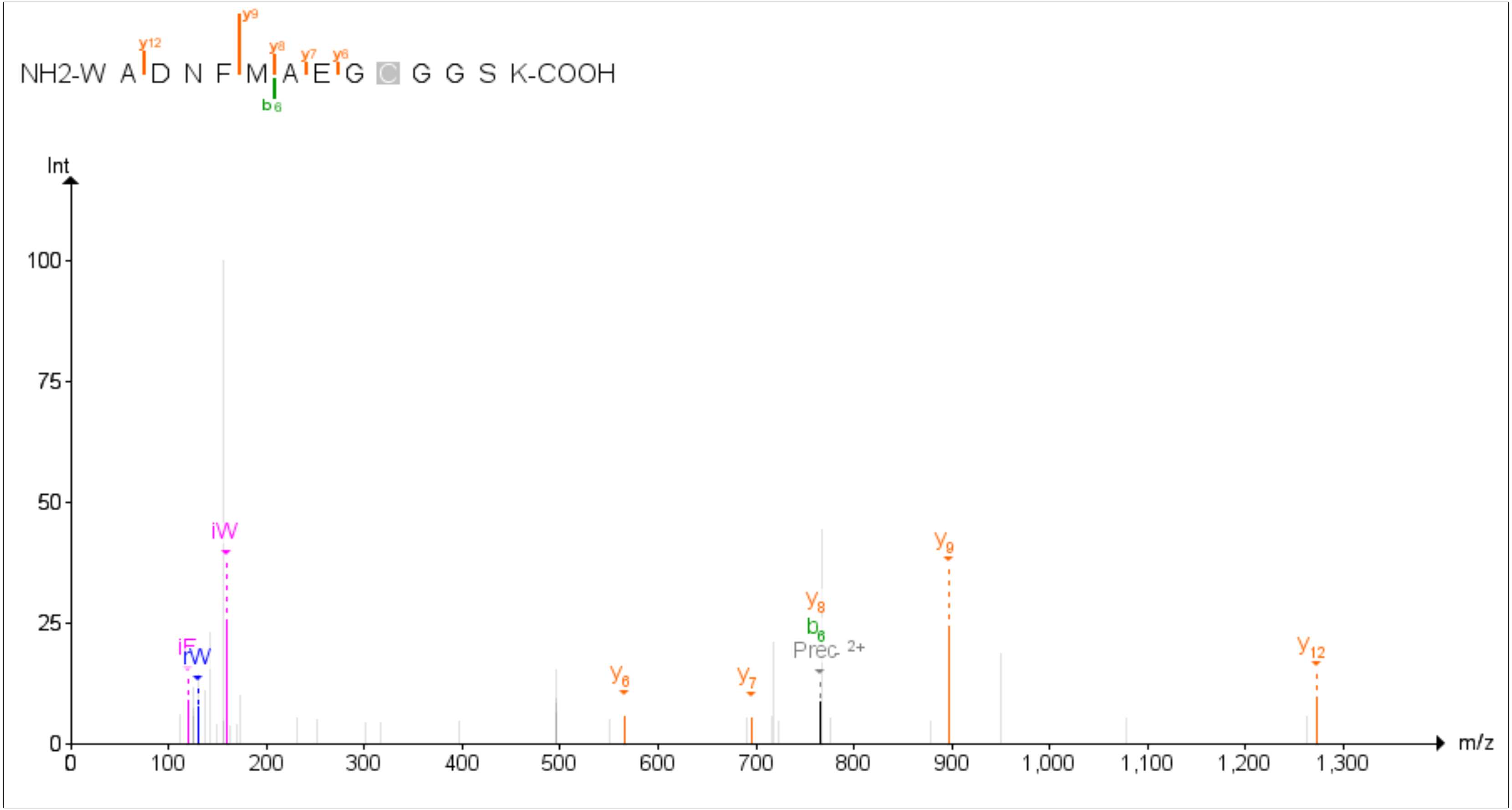

NH2-W A D N F M A E G C G G S K-COOH
y12
y9
y8
y7
y6
b6
Int
m/z
iW
iF
rW
y6
y7
y8
b6
Prec 2+
y9
y12

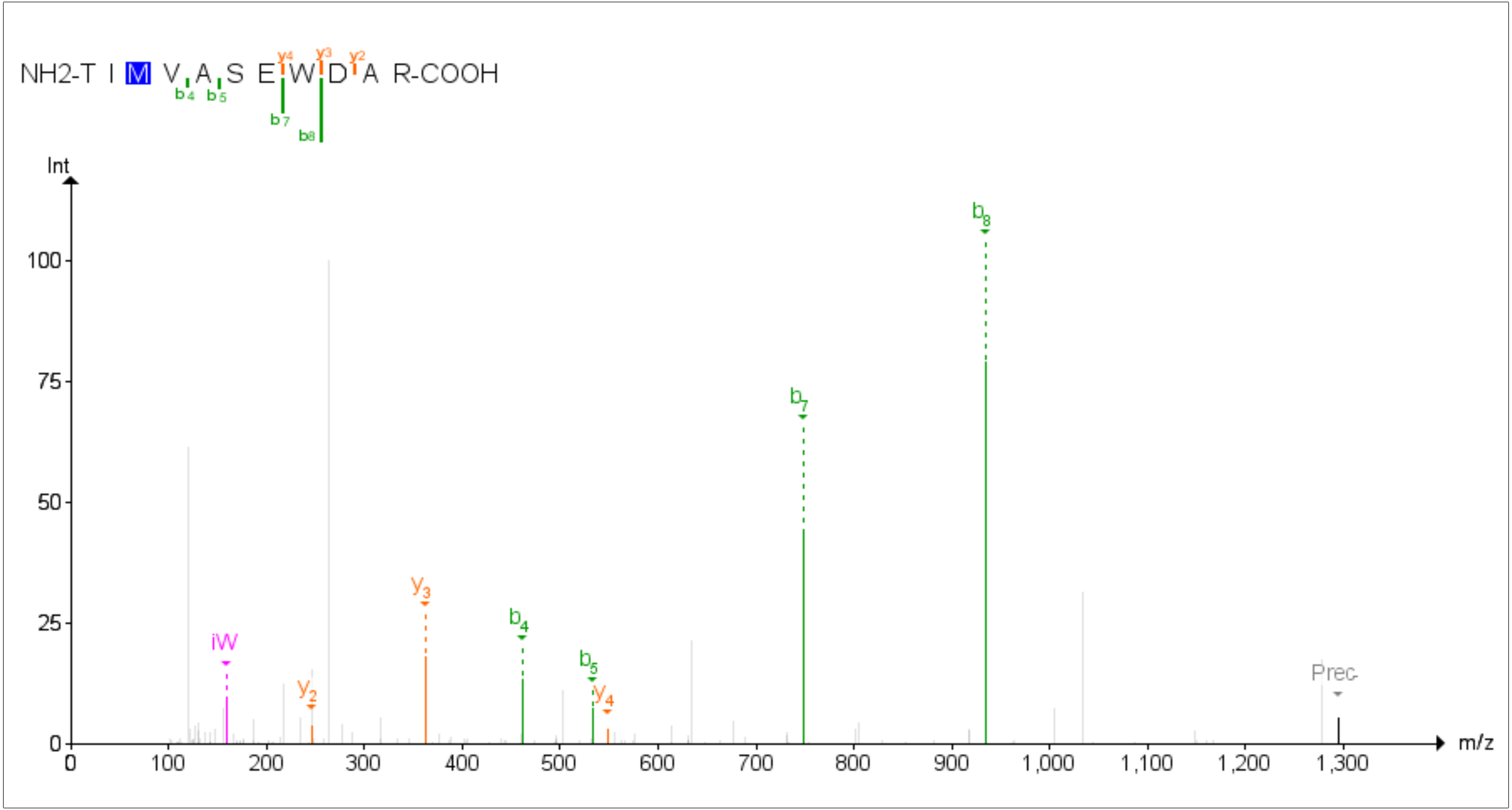

NH2-T I M V A S E W D A R-COOH
b4
b5
b7
b8
y4
y3
y2
Int
m/z
0
25
50
75
100
0
100
200
300
400
500
600
700
800
900
1,000
1,100
1,200
1,300
iW
y2
y3
b4
b5
y4
b7
b8
Prec

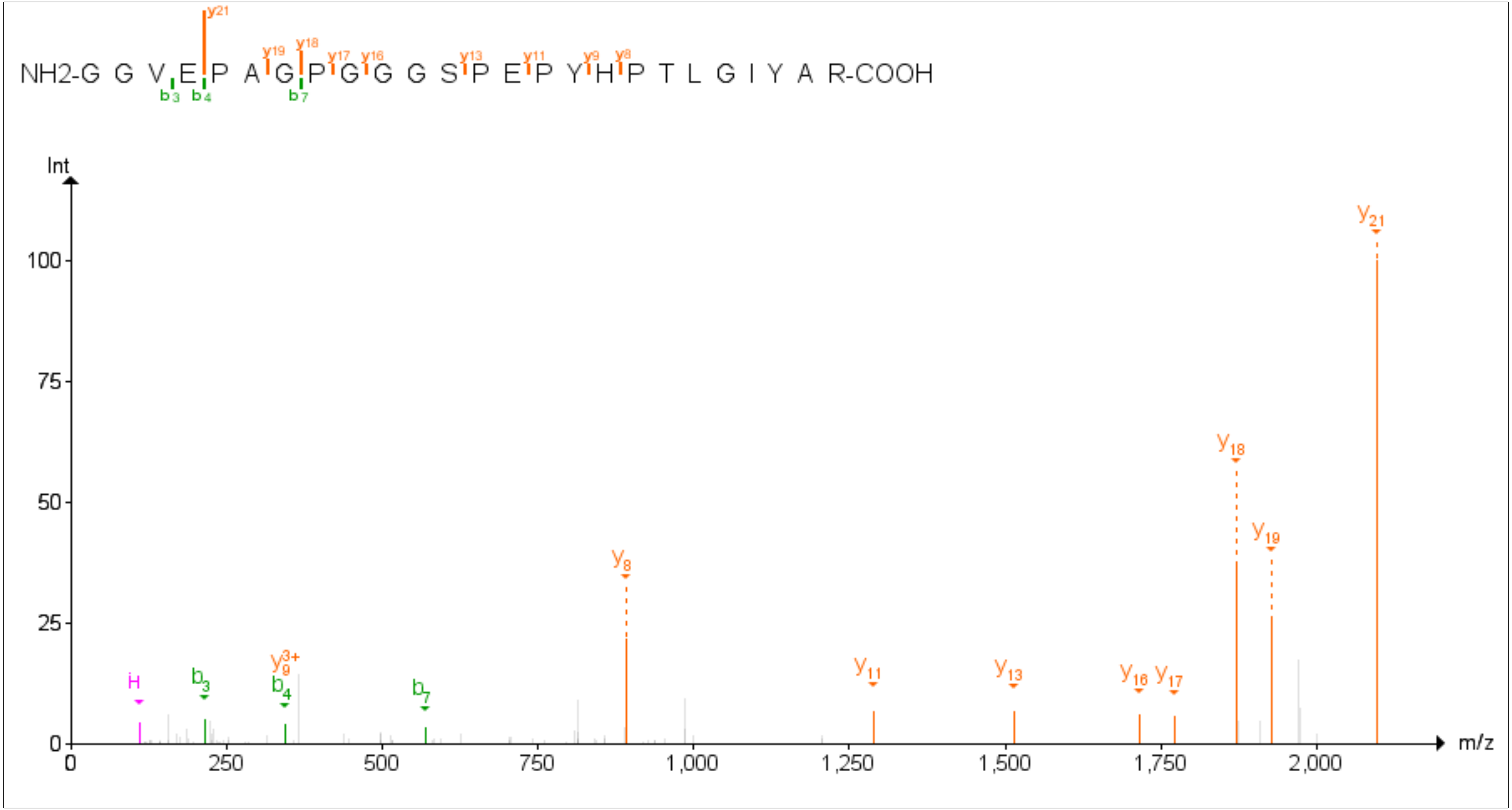

NH2-G G V E P A G P G G G S P E P Y H P T L G I Y A R-COOH
y21 y19 y18 y17 y16 y13 y11 y9 y8
b3 b4 b7
Int
100
75
50
25
0
0 250 500 750 1,000 1,250 1,500 1,750 2,000
m/z
H
b3
b4
y_9^{3+}
b7
y8
y11
y13
y16
y17
y18
y19
y21

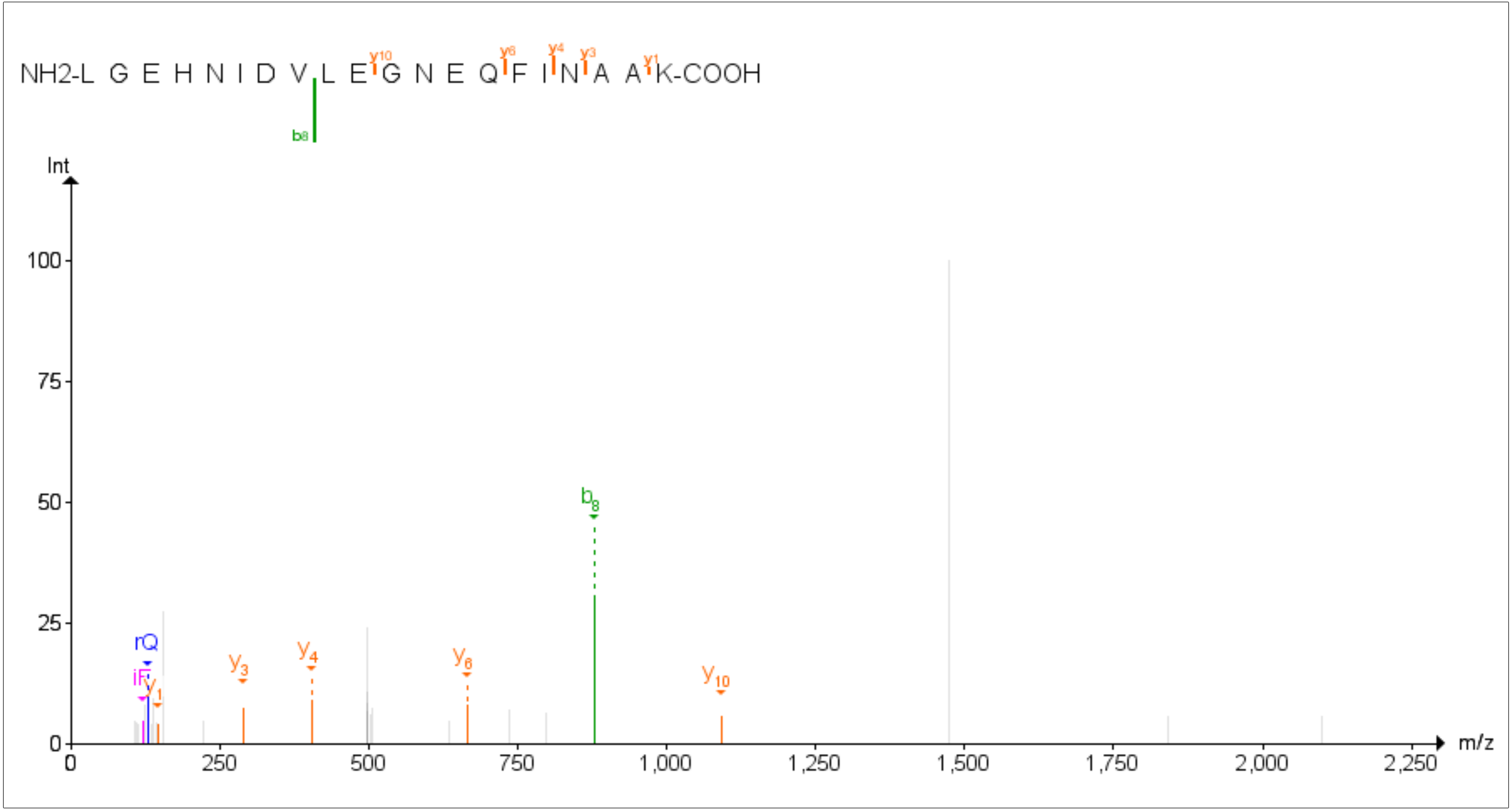
NH2-L G E H N I D V L E G N E Q F I N A A K-COOH
y10
y6
y4
y3
y1
b8
Int
100
75
50
25
0
rQ
iF
y1
y3
y4
y6
b8
y10
0
250
500
750
1,000
1,250
1,500
1,750
2,000
2,250
m/z

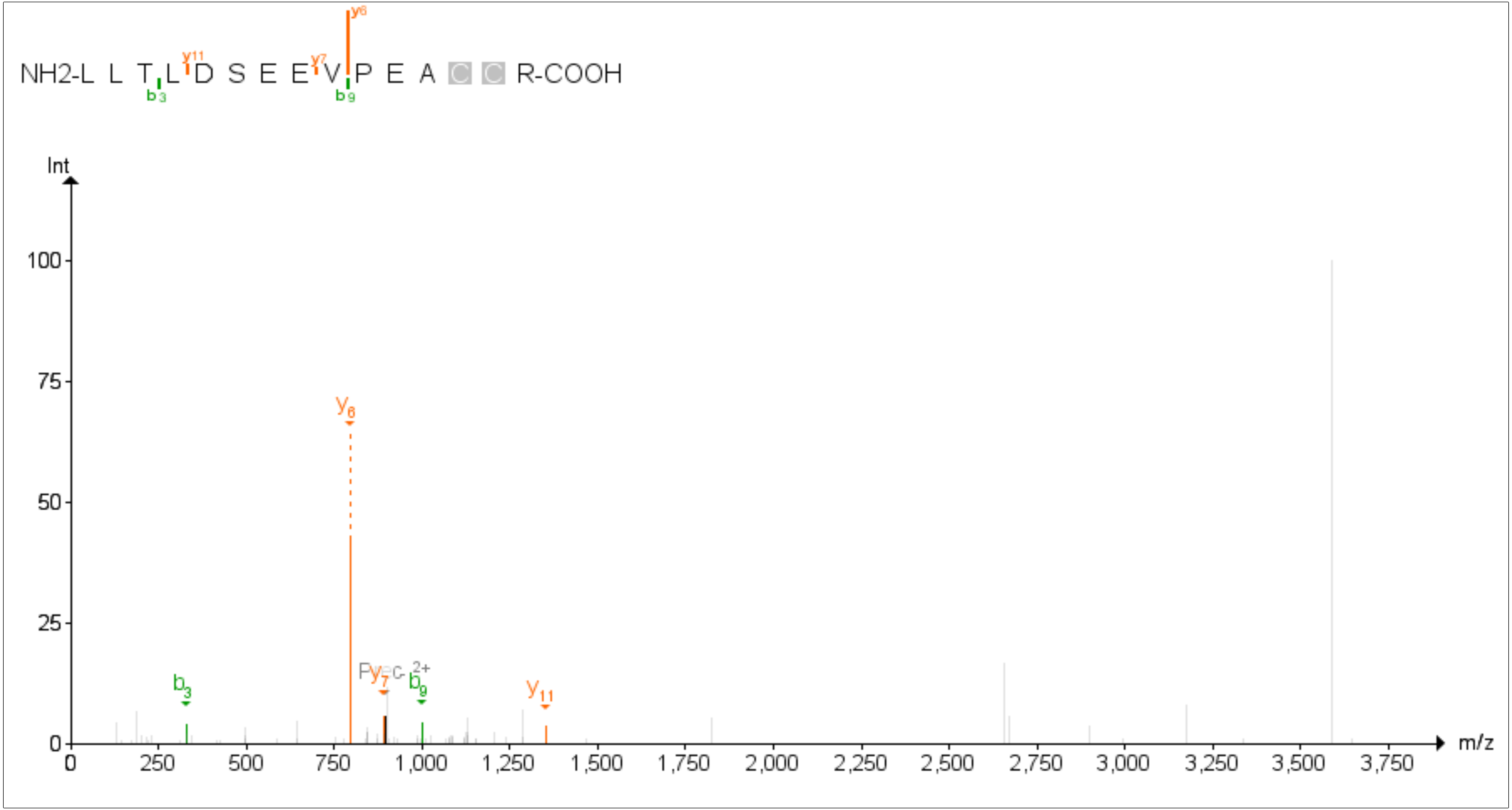

NH2-L L T L D S E E V P E A C C R-COOH
y11
y7
y6
b3
b9
Int
100
75
50
25
0
y6
b3
y7
b9
y11
0
250
500
750
1,000
1,250
1,500
1,750
2,000
2,250
2,500
2,750
3,000
3,250
3,500
3,750
m/z

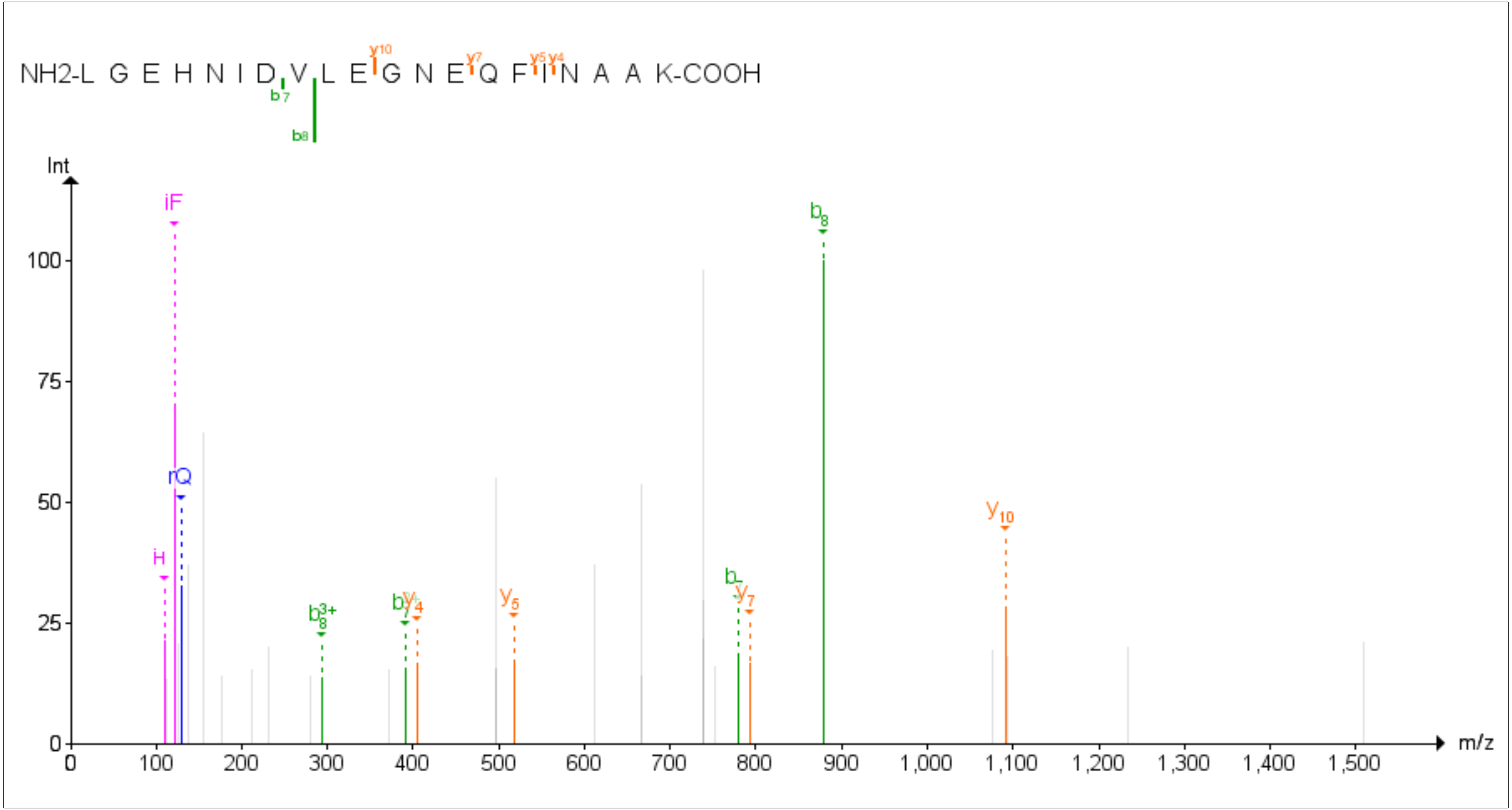

NH2-L G E H N I D V L E G N E Q F I N A A K-COOH
Int
m/z
iF
rQ
H
b8
y10
y5
y7
y4
0
25
50
75
100
0
100
200
300
400
500
600
700
800
900
1,000
1,100
1,200
1,300
1,400
1,500

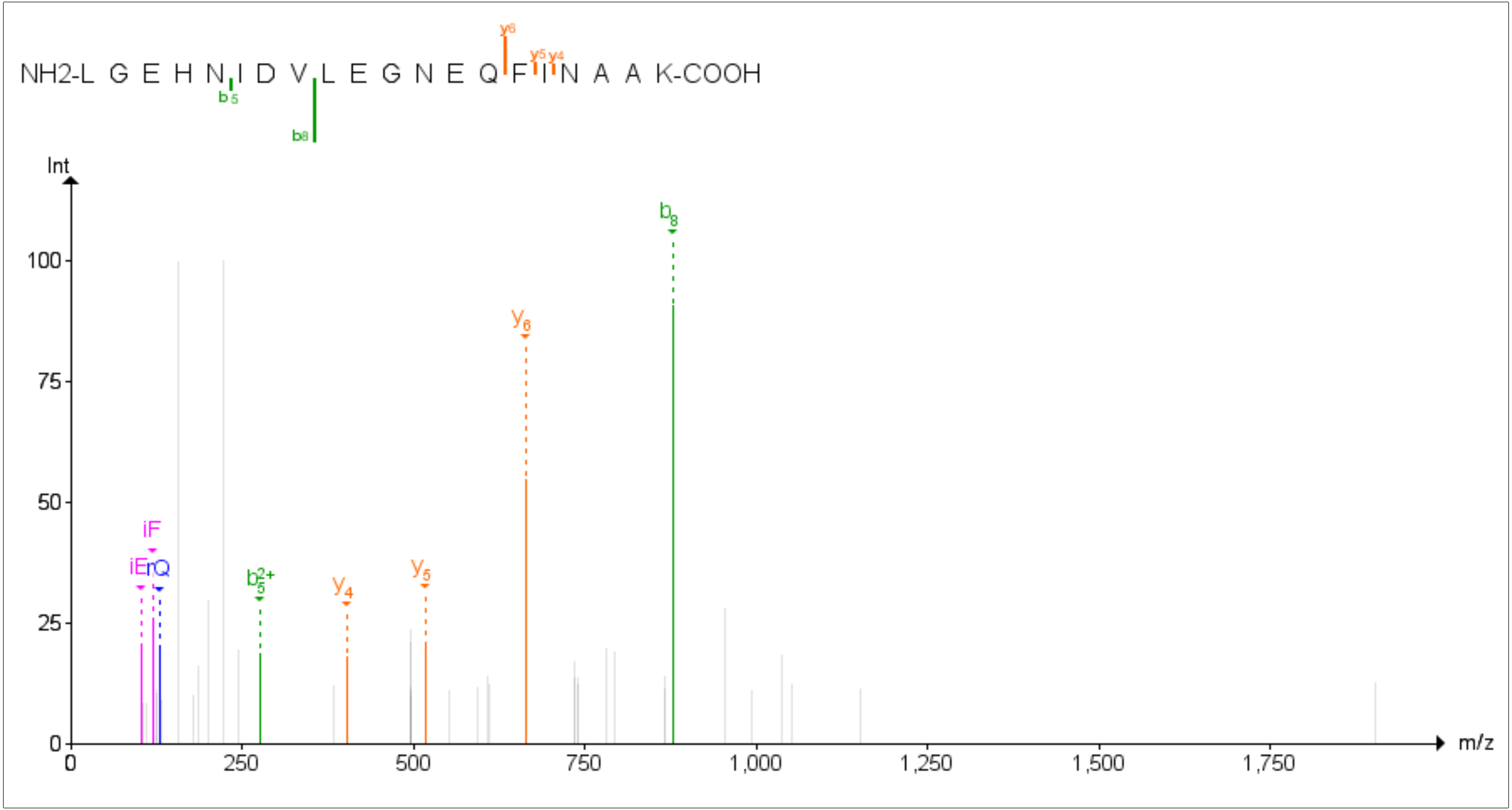

NH2-L G E H N I D V L E G N E Q F I N A A K-COOH
b5
b8
y6
y5
y4
Int
100
75
50
25
0
b8
y6
iF
iE
rQ
b_5^{2+}
y4
y5
0
250
500
750
1,000
1,250
1,500
1,750
m/z

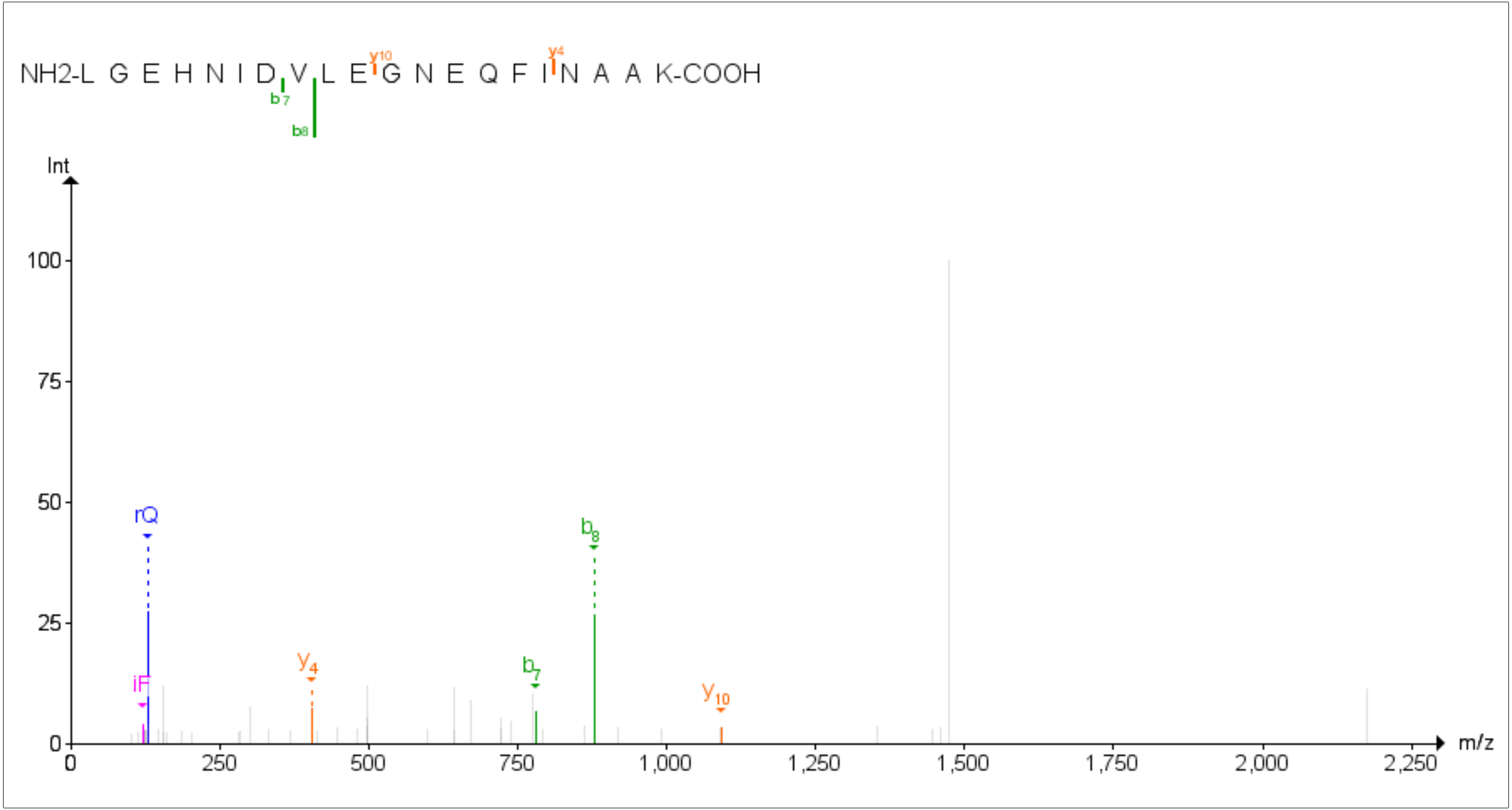

NH2-L G E H N I D V L E G N E Q F I N A A K-COOH
b7
b8
y10
y4
Int
100
75
50
25
0
rQ
iF
y4
b7
b8
y10
0
250
500
750
1,000
1,250
1,500
1,750
2,000
2,250
m/z

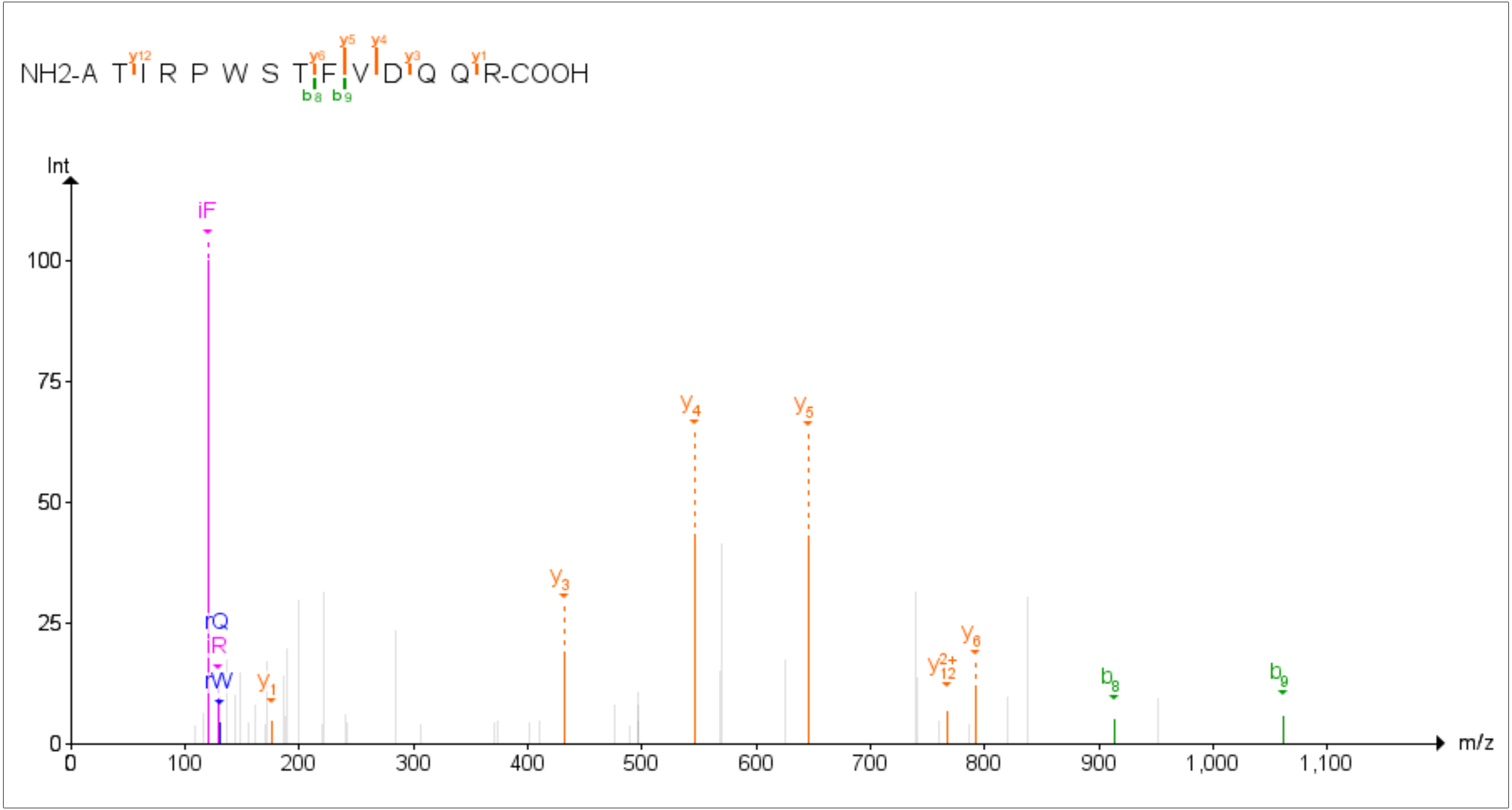
NH2-A T I R P W S T F V D Q Q R-COOH
y12 y6 y5 y4 y3 y1
b8 b9
Int
m/z
iF
rQ
iR
rW
y1
y3
y4
y5
y12 2+
y6
b8
b9
0
25
50
75
100
0
100
200
300
400
500
600
700
800
900
1,000
1,100

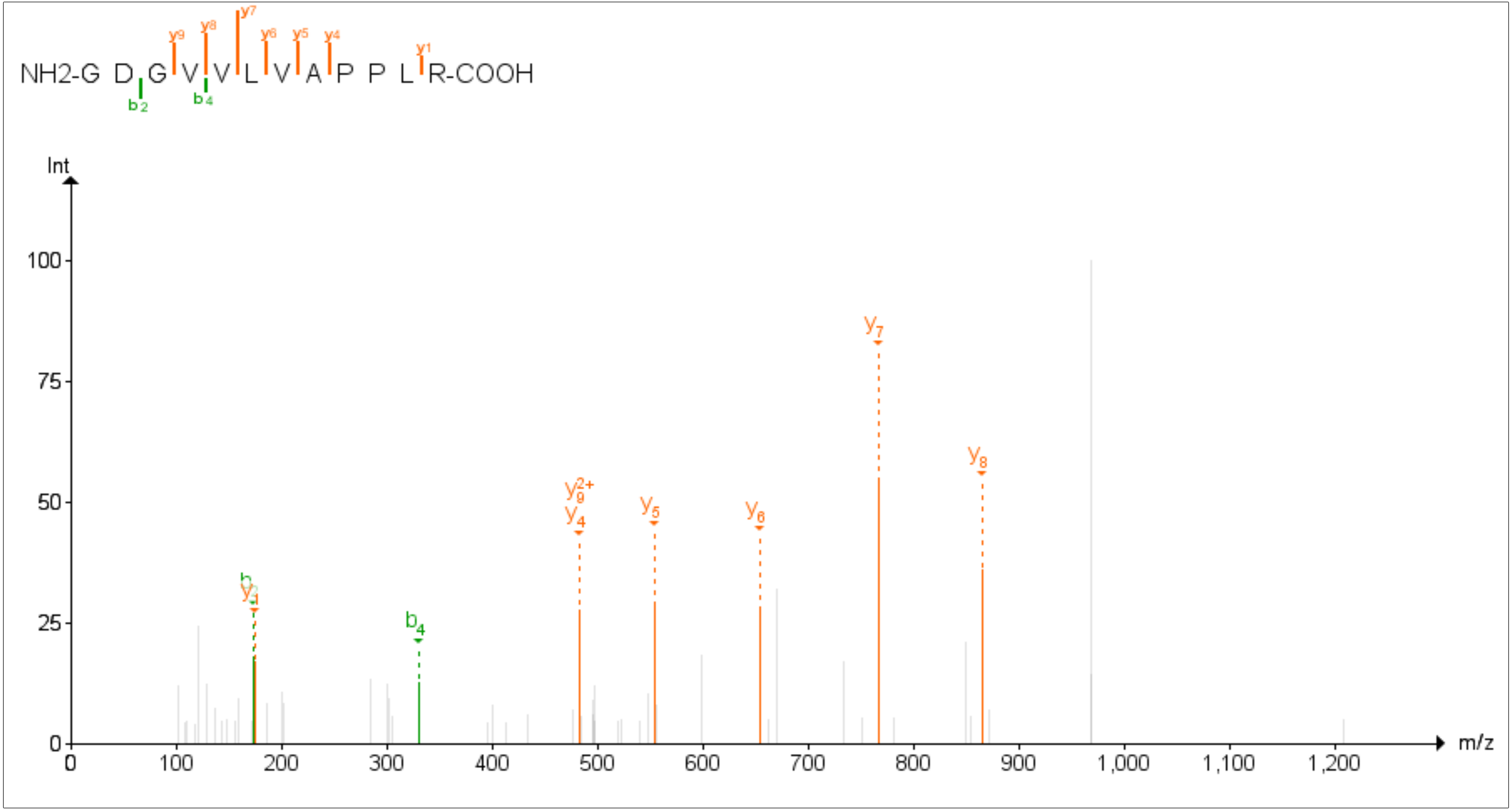

NH2-G D G V V L V A P P L R-COOH
y9 y8 y7 y6 y5 y4 y1
b2 b4
Int
m/z
0
25
50
75
100
0
100
200
300
400
500
600
700
800
900
1,000
1,100
1,200
y7
y8
y9 2+
y4
y5
y6
b4

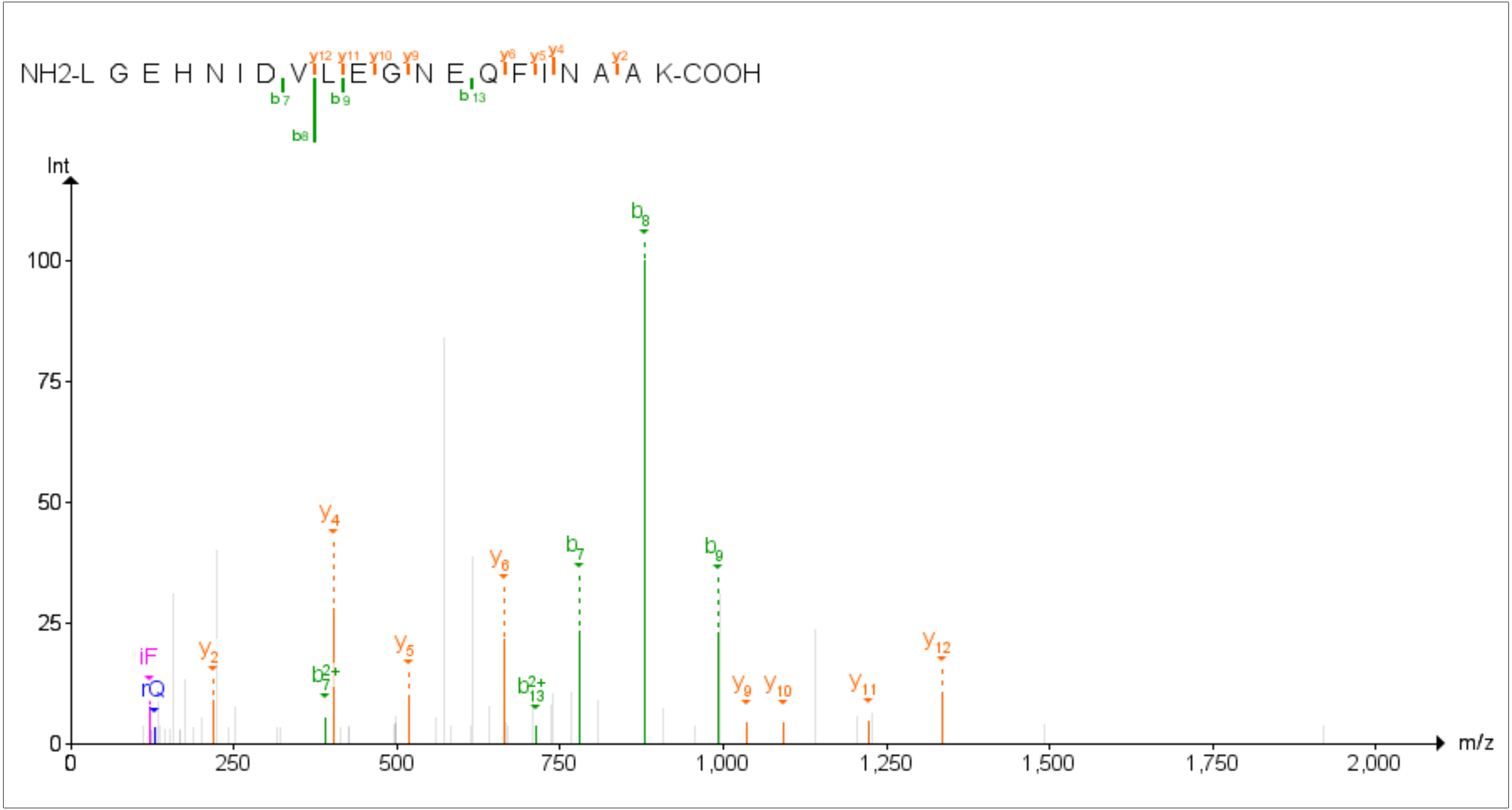
NH2-L G E H N I D V L E G N E Q F I N A A K-COOH
y12 y11 y10 y9 y6 y5 y4 y2
b7 b8 b9 b13
Int
m/z
b8
y4
y6
b7
b9
y2
y5
y9
y10
y11
y12
iF
rQ
b7 2+
b13 2+
0
25
50
75
100
250
500
750
1,000
1,250
1,500
1,750
2,000

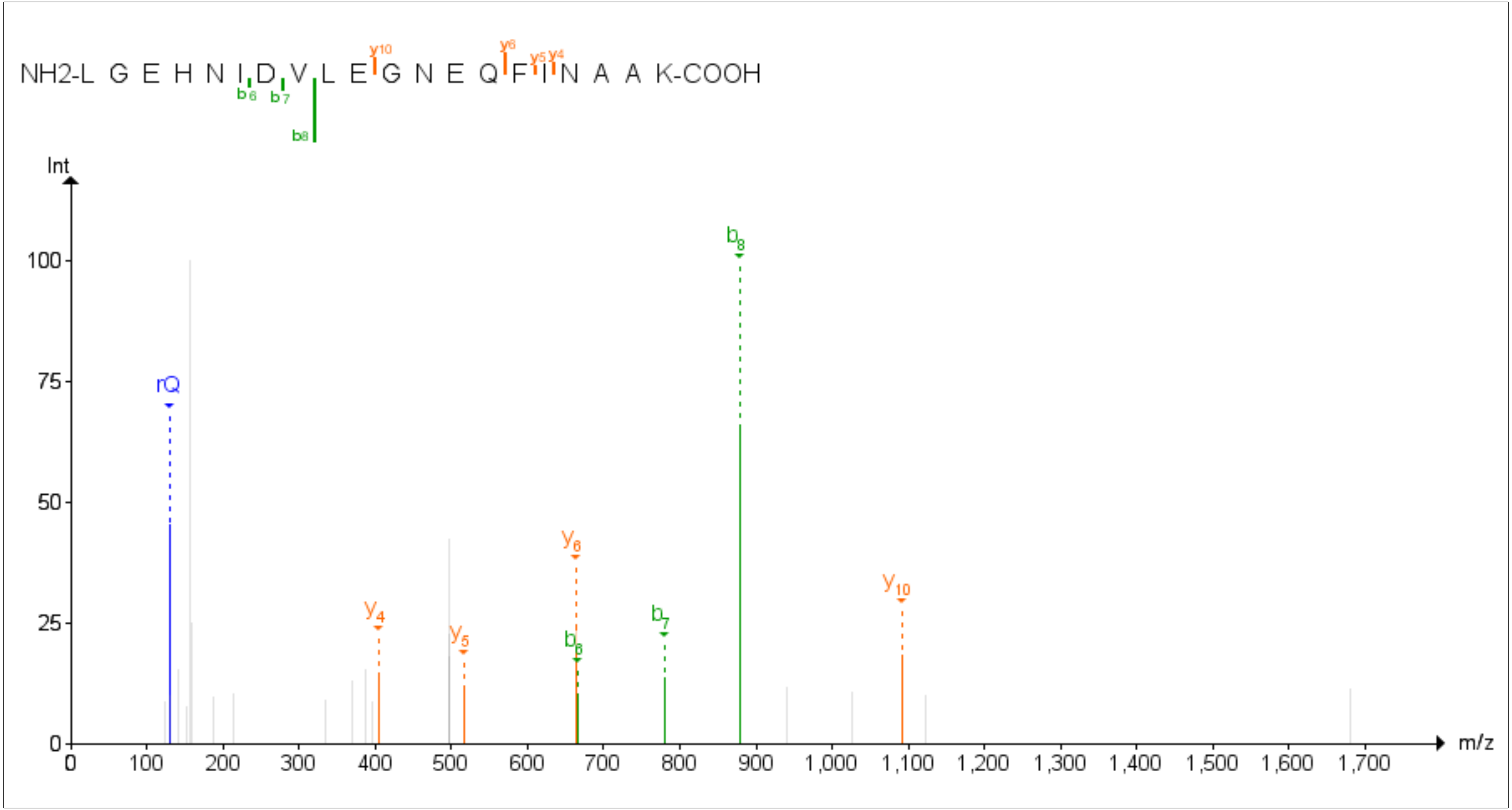

NH2-L G E H N I D V L E G N E Q F I N A A K-COOH
b6
b7
b8
y10
y6
y5
y4
Int
100
75
50
25
0
rQ
y4
y5
y6
b6
b7
b8
y10
0
100
200
300
400
500
600
700
800
900
1,000
1,100
1,200
1,300
1,400
1,500
1,600
1,700
m/z

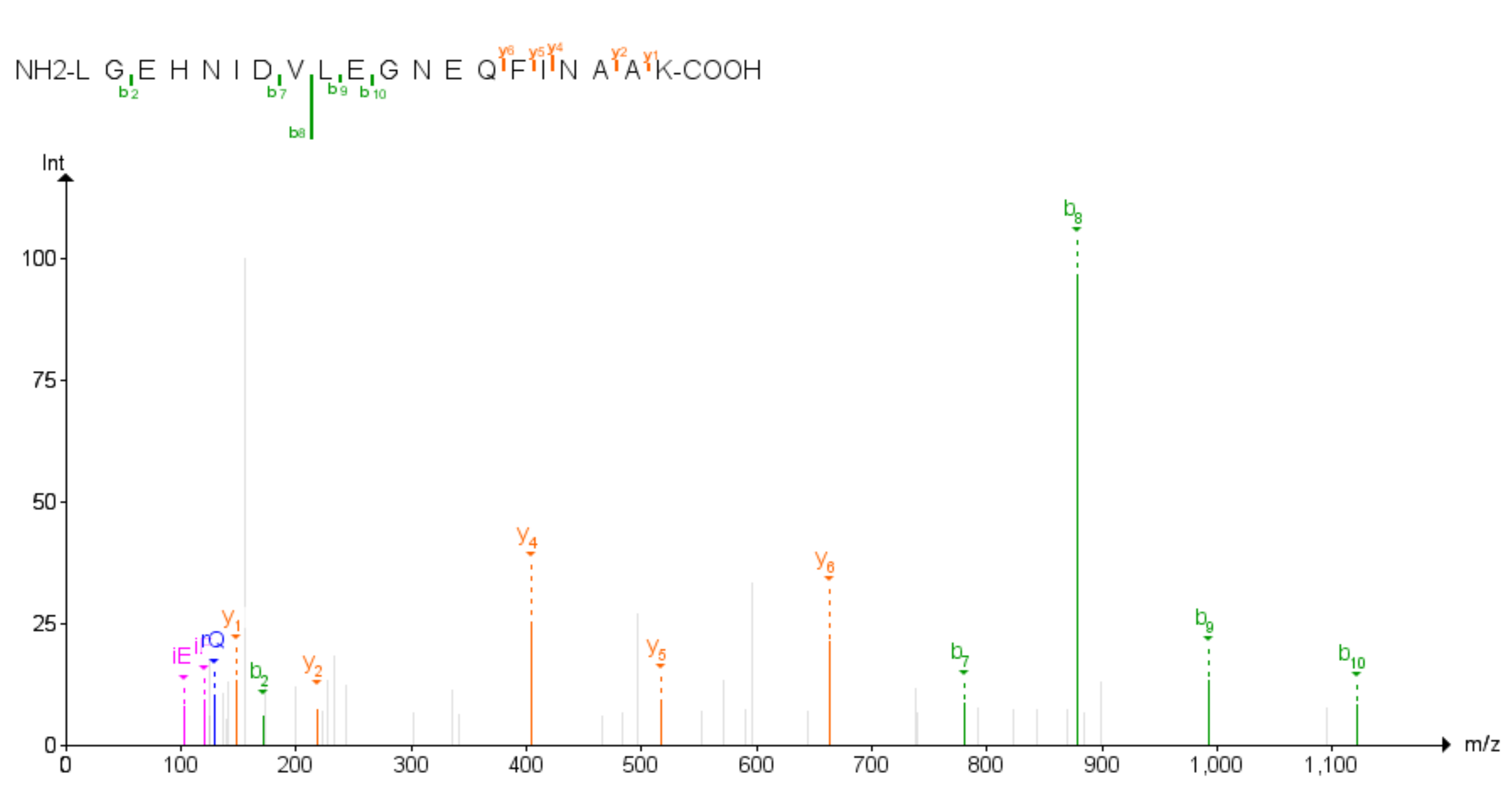

NH2-L G E H N I D V L E G N E Q F I N A A K-COOH
b2 b7 b8 b9 b10
y6 y5 y4 y2 y1
Int
100
75
50
25
0
m/z
0 100 200 300 400 500 600 700 800 900 1,000 1,100
iE
iI
rQ
y1
b2
y2
y4
y5
y6
b7
b8
b9
b10

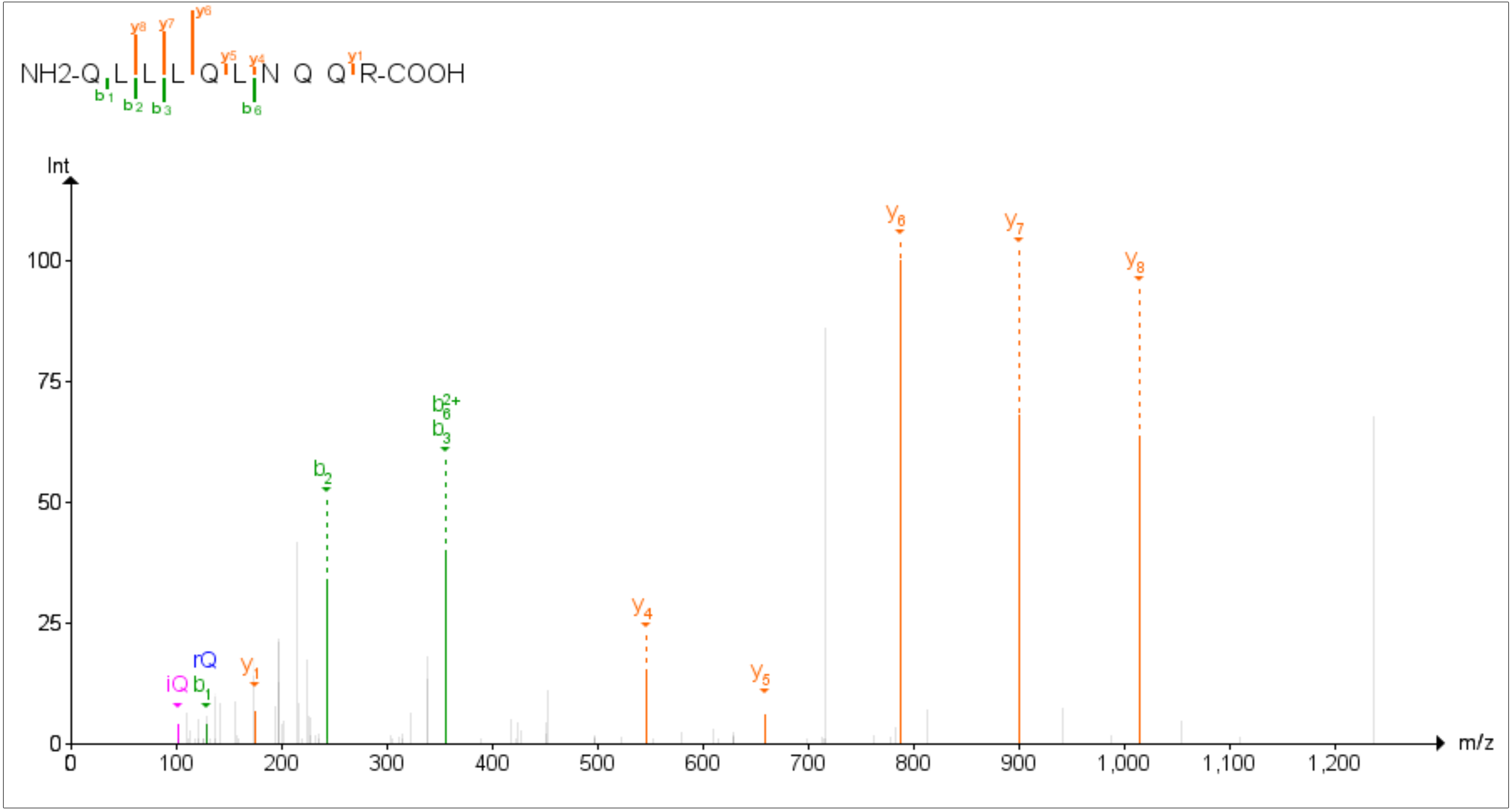

NH2-Q L L L Q L N Q Q R-COOH
y8 y7 y6 y5 y4 y1
b1 b2 b3 b6
Int
m/z
y6
y7
y8
b6 2+
b3
b2
y4
y5
rQ
iQ
b1
y1

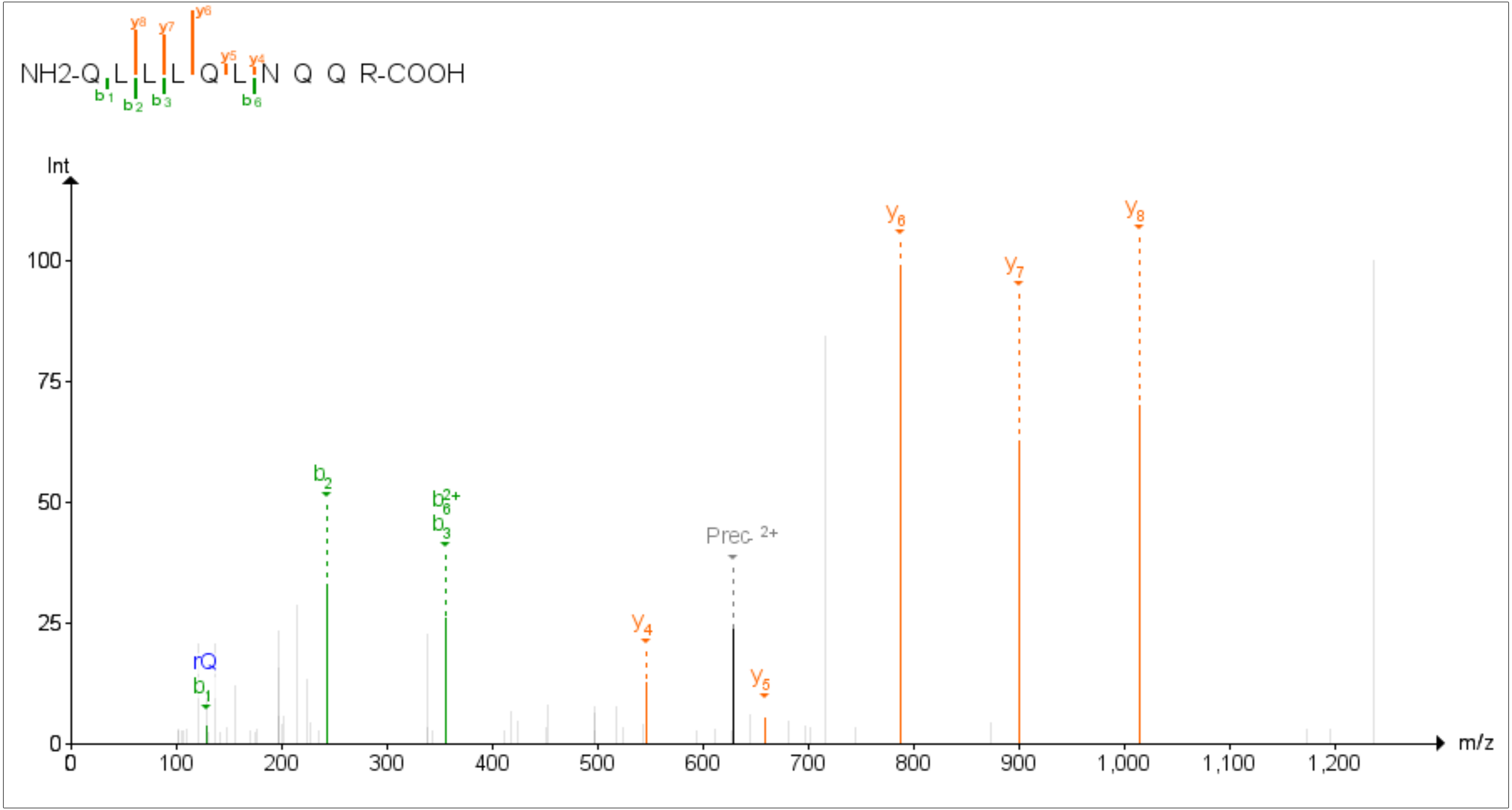

NH2-Q L L L Q L N Q Q R-COOH
y8 y7 y6 y5 y4
b1 b2 b3 b6
Int
m/z
y6
y8
y7
b2
b6 2+
b3
Prec 2+
y4
y5
rQ
b1

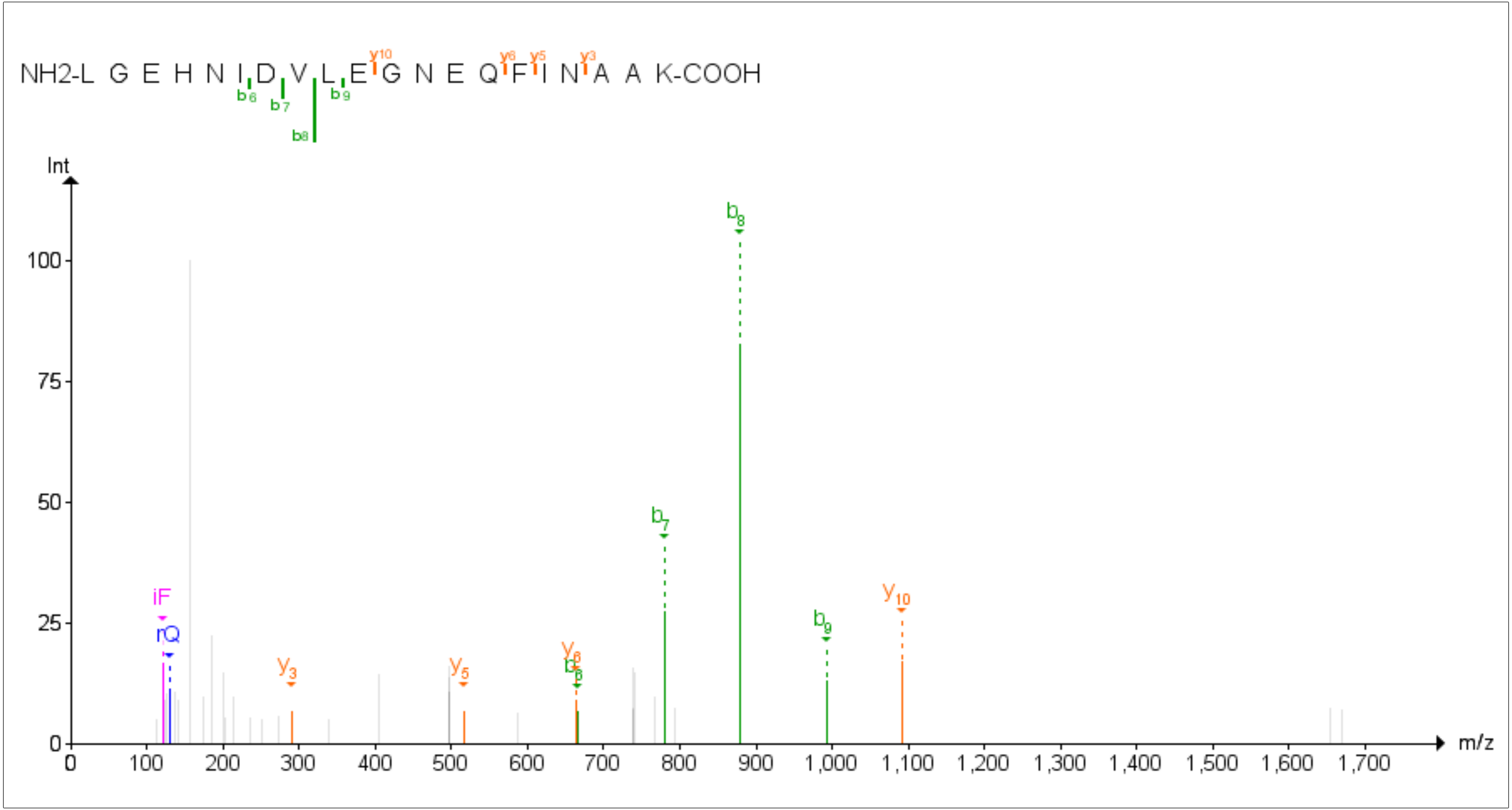
NH2-L G E H N I D V L E G N E Q F I N A A K-COOH
b6
b7
b8
b9
y10
y6
y5
y3
Int
100
75
50
25
0
m/z
0
100
200
300
400
500
600
700
800
900
1,000
1,100
1,200
1,300
1,400
1,500
1,600
1,700
iF
rQ
y3
y5
y6
b6
b7
b8
b9
y10

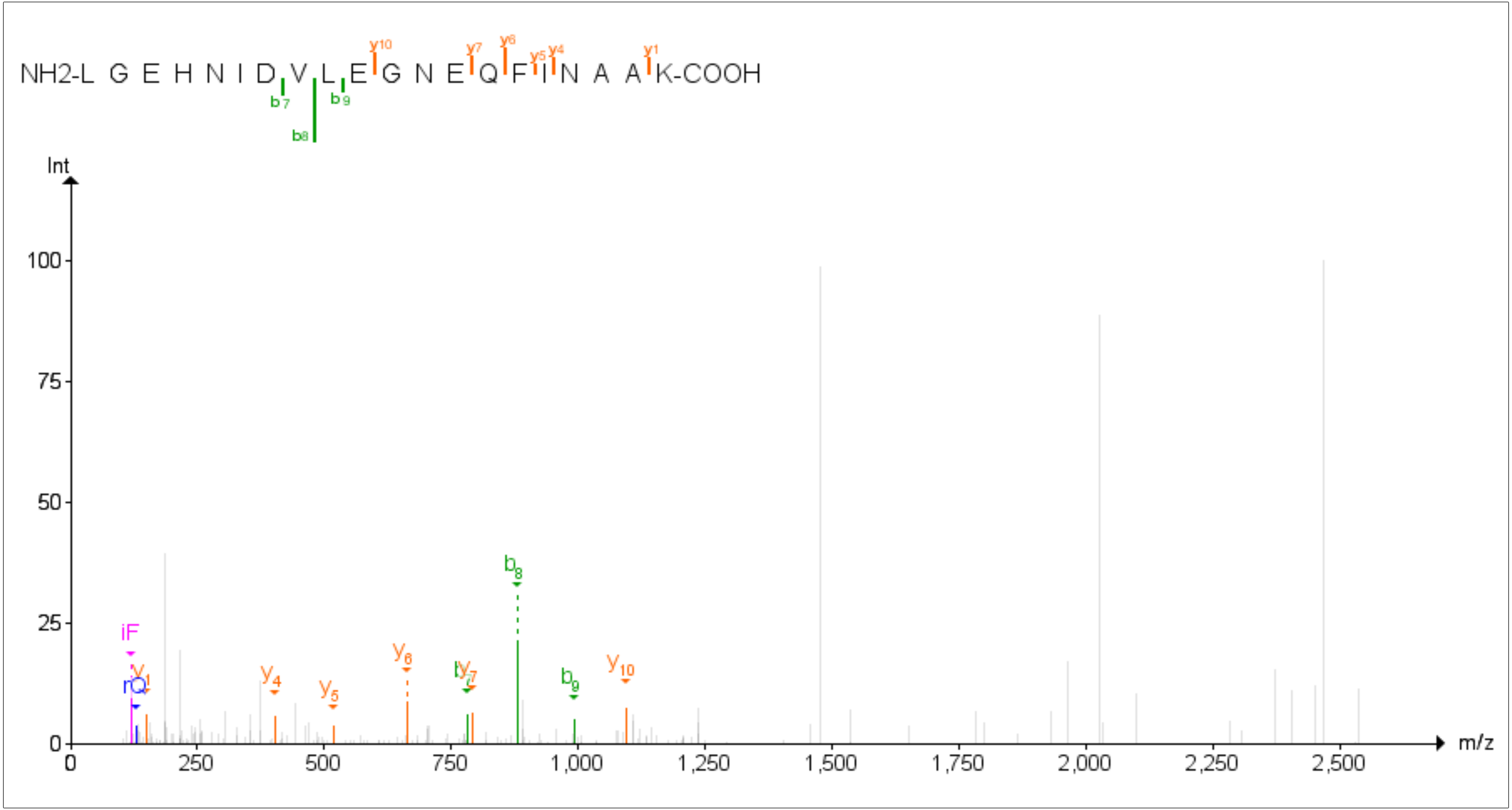

NH2-L G E H N I D V L E G N E Q F I N A A K-COOH
y10 y7 y6 y5 y4 y1
b7 b8 b9
Int
100
75
50
25
0
m/z
0 250 500 750 1,000 1,250 1,500 1,750 2,000 2,250 2,500
iF
nQ
y1
y4
y5
y6
b7 y7
b8
b9
y10

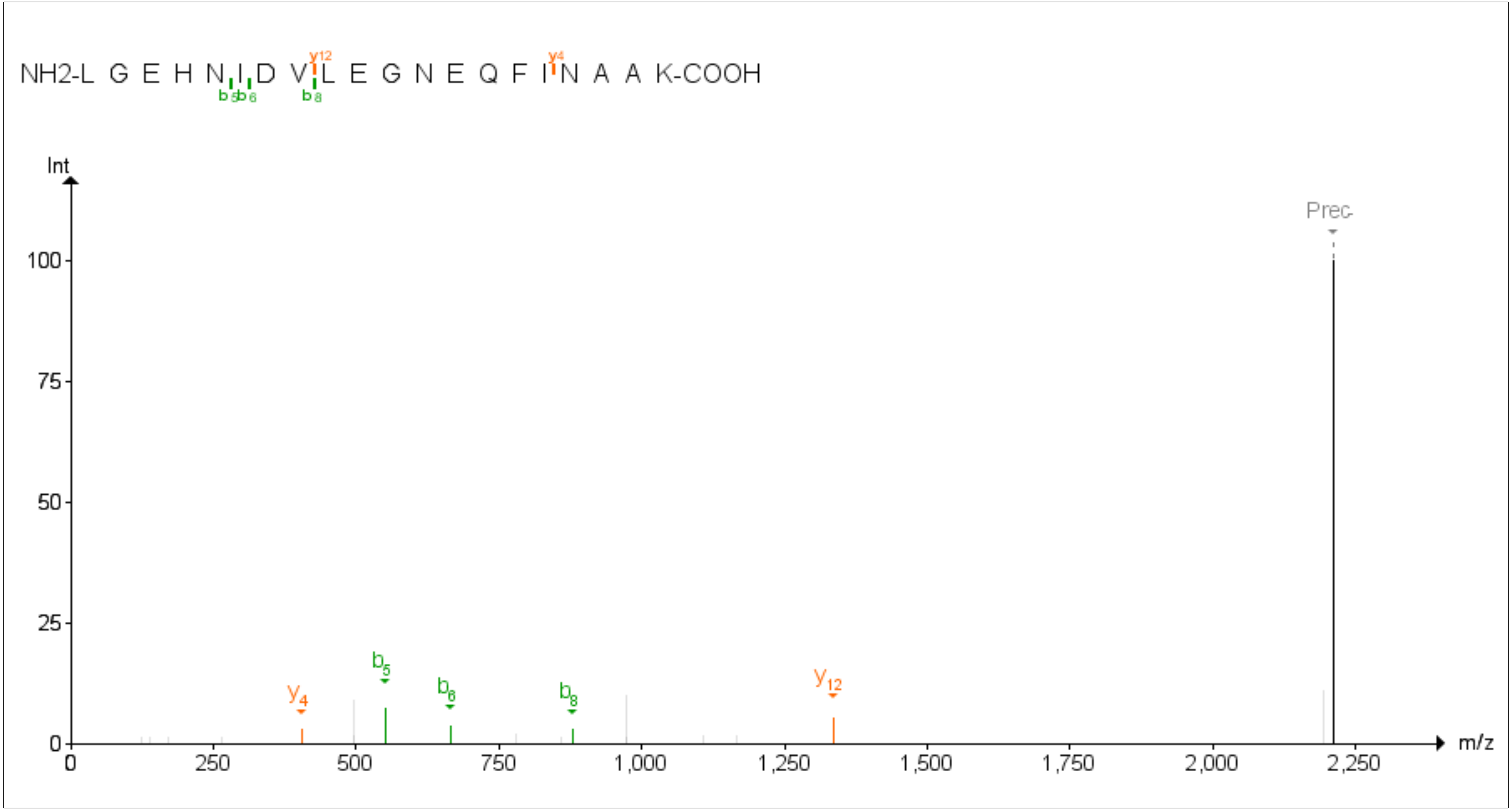
NH2-L G E H N I D V L E G N E Q F I N A A K-COOH
y12
y4
b5
b6
b8
Int
Prec
100
75
50
25
0
y4
b5
b6
b8
y12
0
250
500
750
1,000
1,250
1,500
1,750
2,000
2,250
m/z

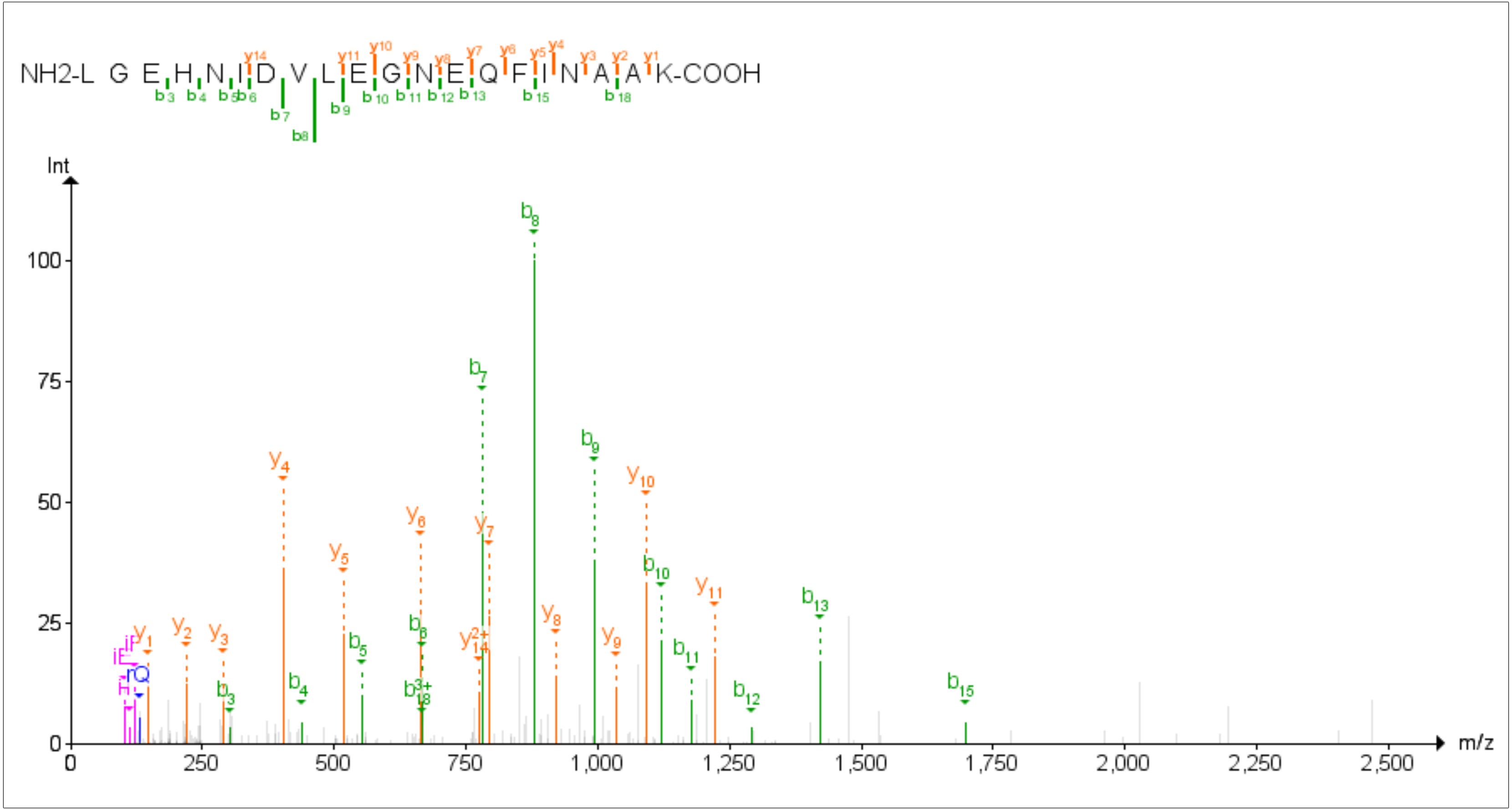

NH2-L G E H N I D V L E G N E Q F I N A A K-COOH
Int
m/z
0
25
50
75
100
250
500
750
1,000
1,250
1,500
1,750
2,000
2,250
2,500

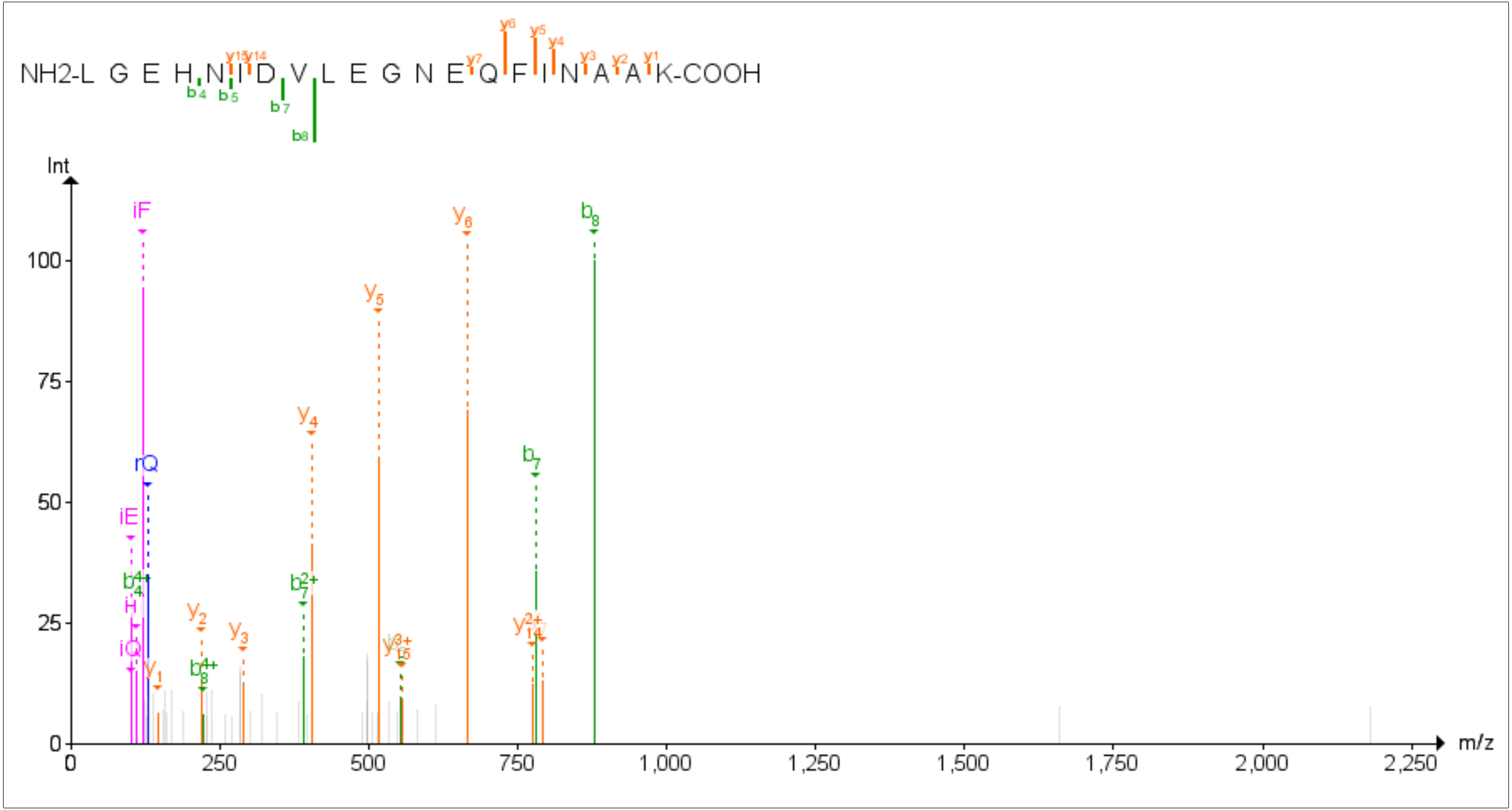

NH2-L G E H N I D V L E G N E Q F I N A A K-COOH
b4
b5
b7
b8
y15
y14
y7
y6
y5
y4
y3
y2
y1
Int
iF
y6
b8
y5
y4
rQ
b7
iE
y2
y3
y1
iQ
m/z
0
25
50
75
100
0
250
500
750
1,000
1,250
1,500
1,750
2,000
2,250

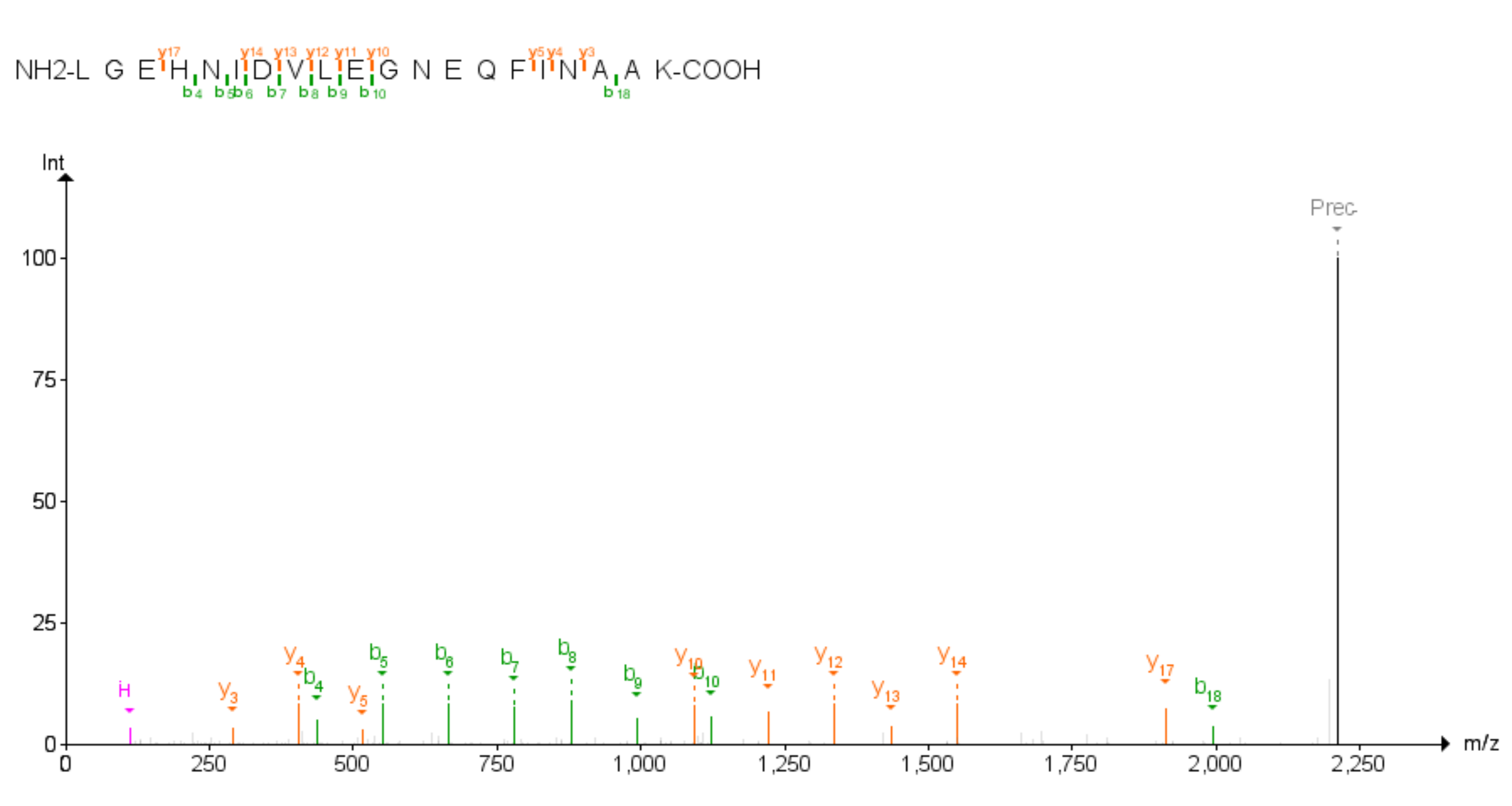

NH2-L G E H N I D V L E G N E Q F I N A A K-COOH
Int
Prec
m/z
0
25
50
75
100
250
500
750
1,000
1,250
1,500
1,750
2,000
2,250

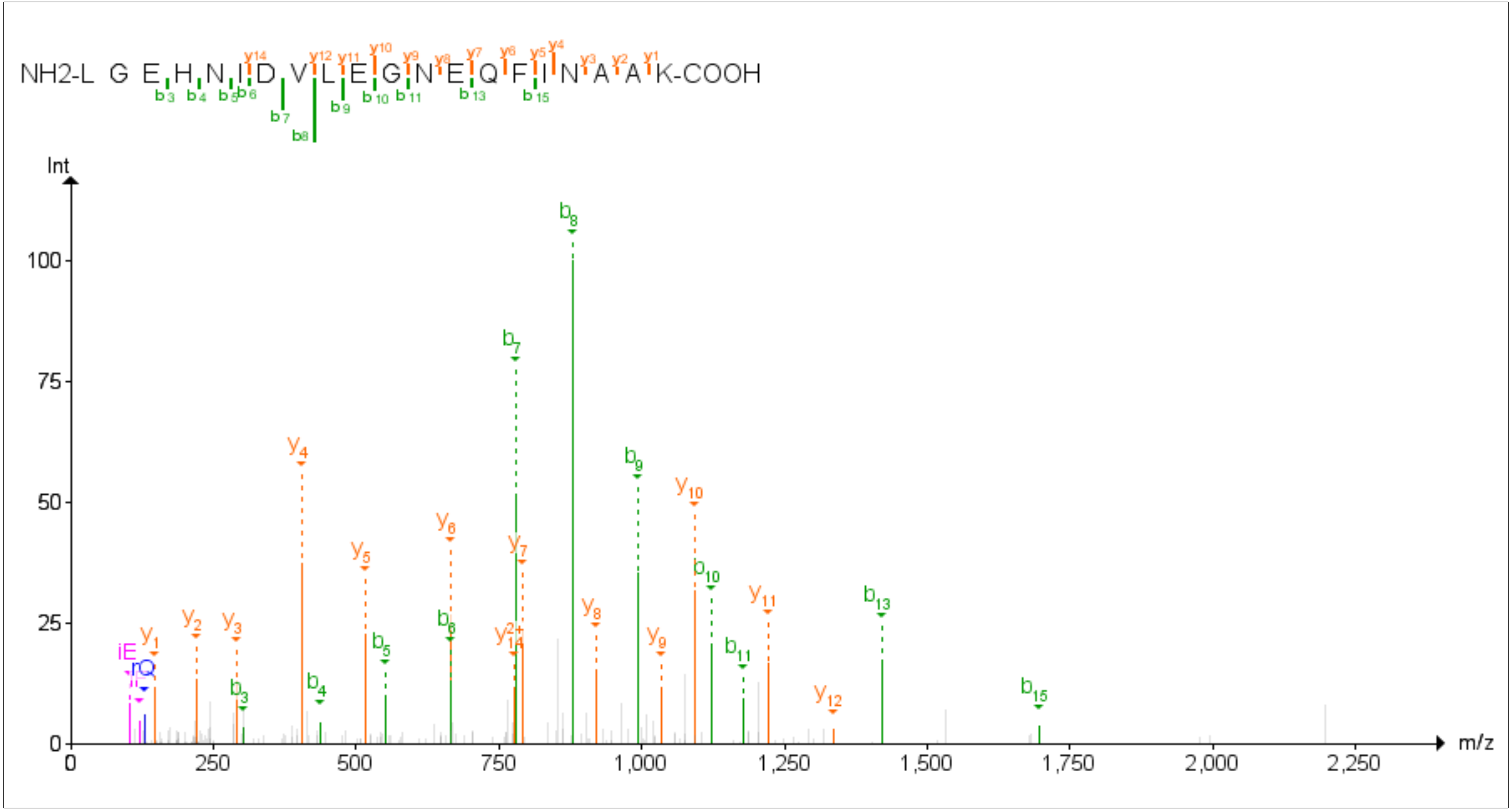

NH2-L G E H N I D V L E G N E Q F I N A A K-COOH
y14 y12 y11 y10 y9 y8 y7 y6 y5 y4 y3 y2 y1
b3 b4 b5 b6 b7 b8 b9 b10 b11 b13 b15
Int
m/z
0
25
50
75
100
250
500
750
1,000
1,250
1,500
1,750
2,000
2,250
iE
nQ

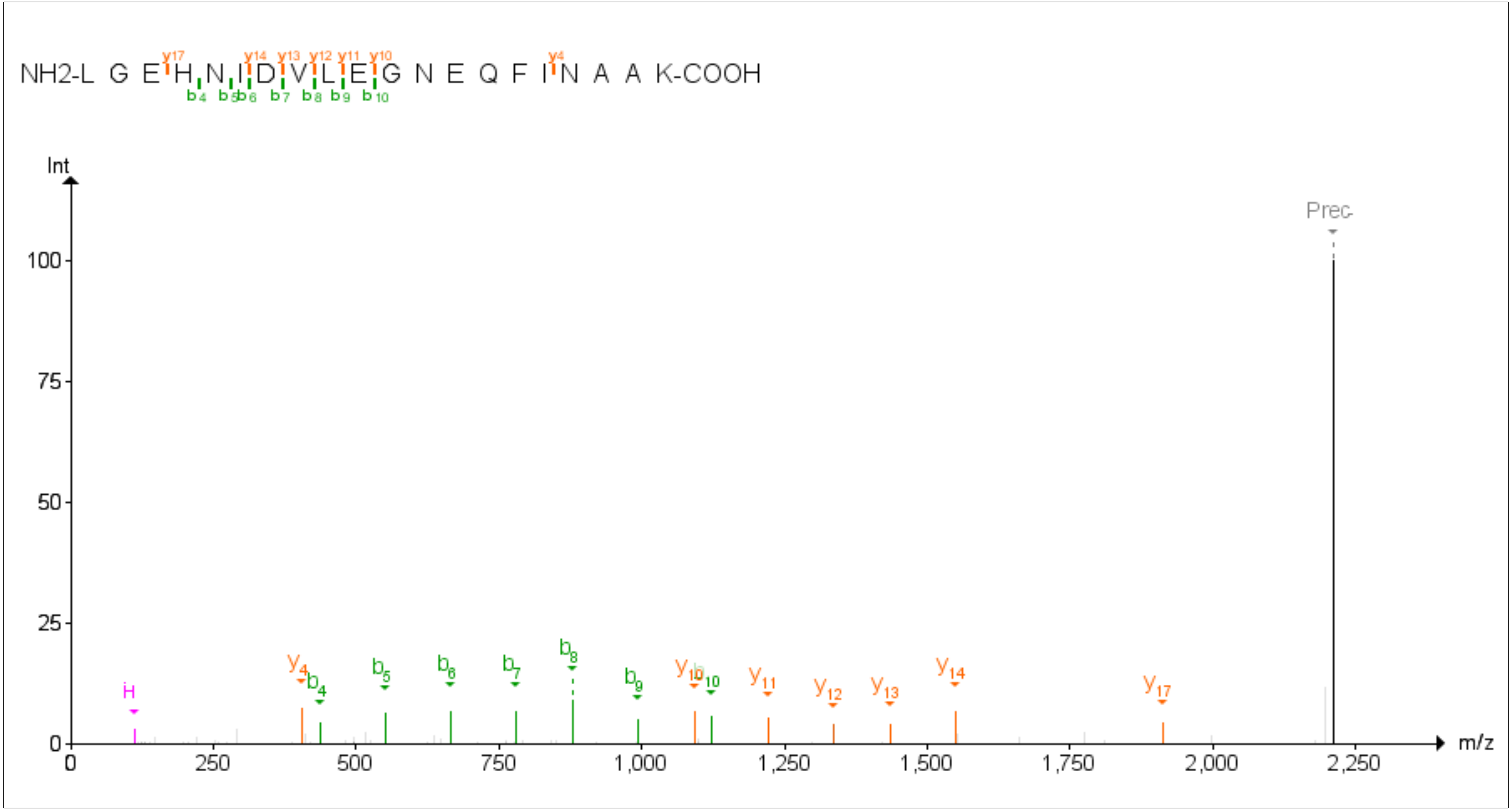

NH2-L G E H N I D V L E G N E Q F I N A A K-COOH
y17 y14 y13 y12 y11 y10 y4
b4 b5 b6 b7 b8 b9 b10
Int
100
75
50
25
0
Prec
H
y4 b4 b5 b6 b7 b8 b9 y10 b10 y11 y12 y13 y14 y17
0
250
500
750
1,000
1,250
1,500
1,750
2,000
2,250
m/z

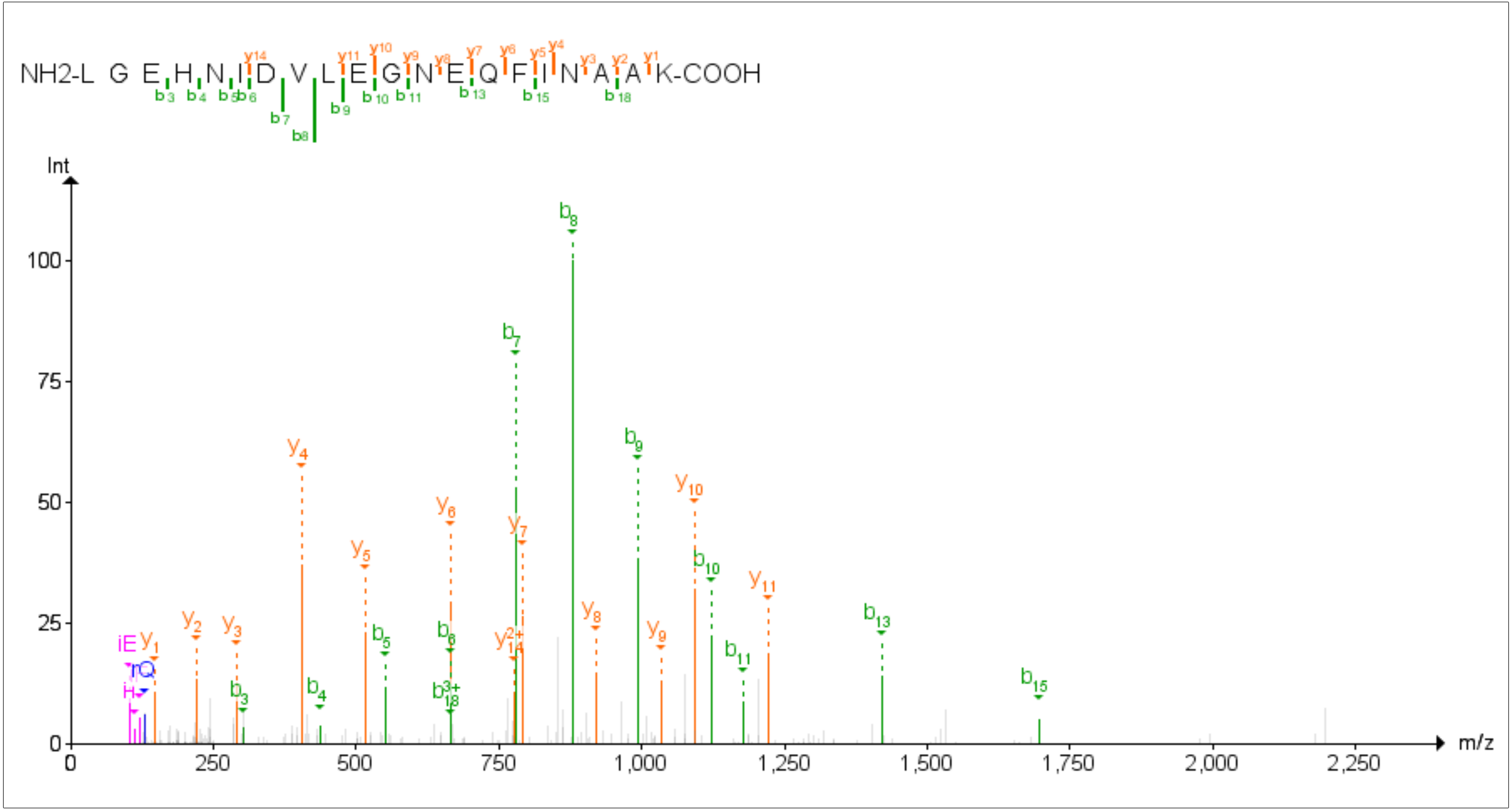
NH2-L G E H N I D V L E G N E Q F I N A A K-COOH
Int
m/z
0
25
50
75
100
250
500
750
1,000
1,250
1,500
1,750
2,000
2,250

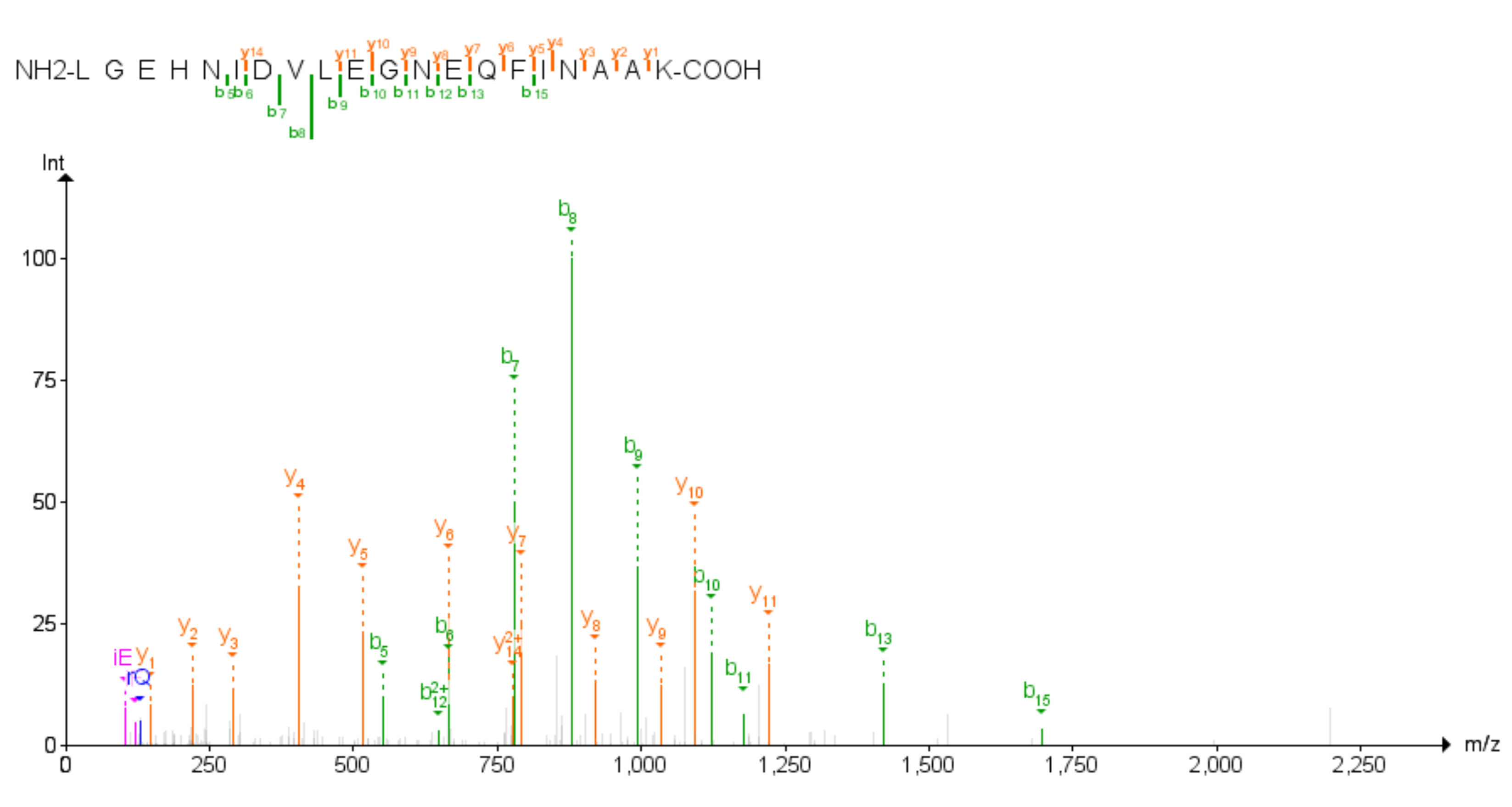
NH2-L G E H N I D V L E G N E Q F I N A A K-COOH
Int
m/z
100
75
50
25
0
0
250
500
750
1,000
1,250
1,500
1,750
2,000
2,250

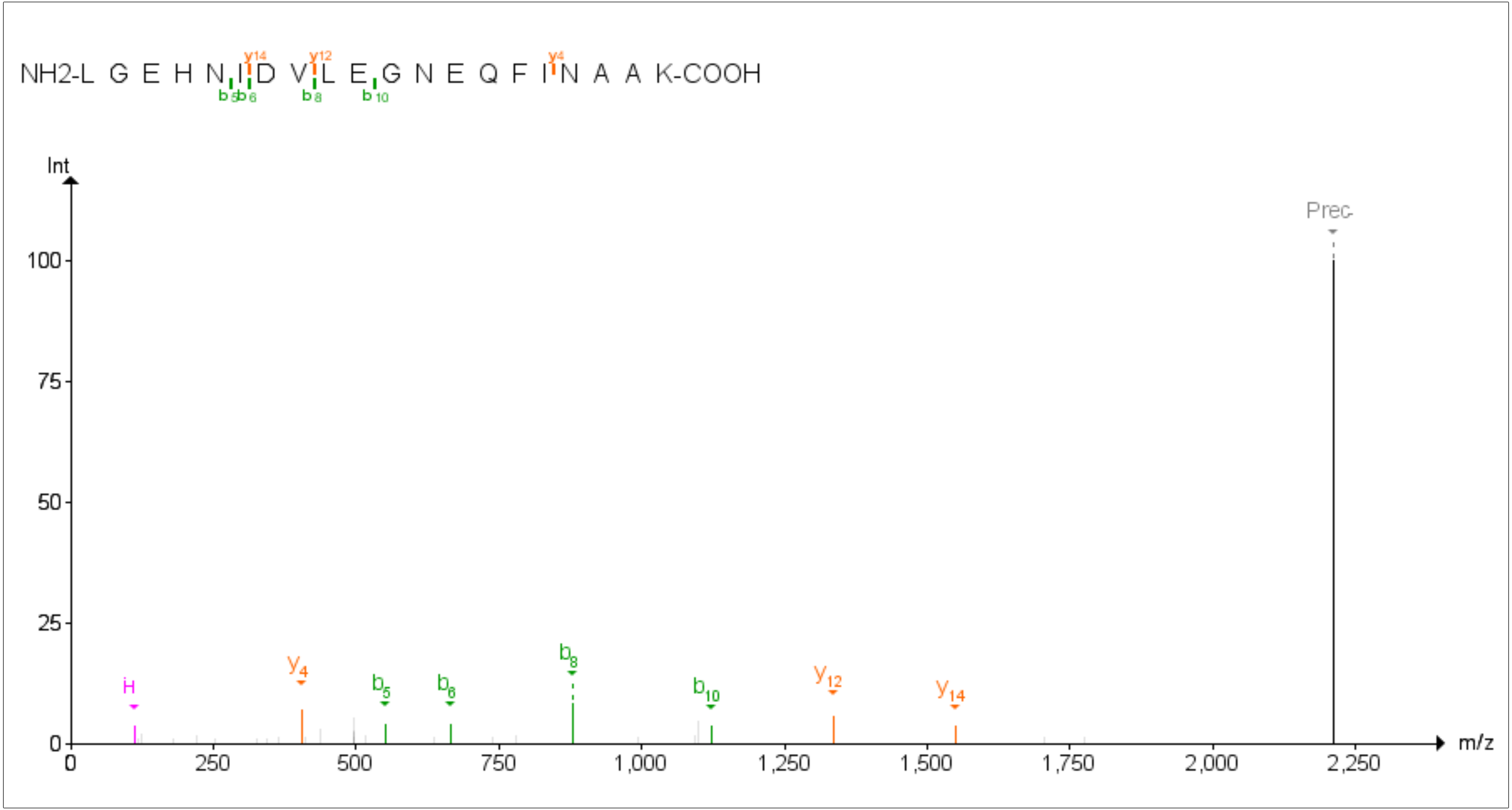

NH2-L G E H N I D V L E G N E Q F I N A A K-COOH
y14 y12 y4
b5 b6 b8 b10
Int
100
75
50
25
0
Prec
H
y4
b5
b6
b8
b10
y12
y14
0 250 500 750 1,000 1,250 1,500 1,750 2,000 2,250
m/z

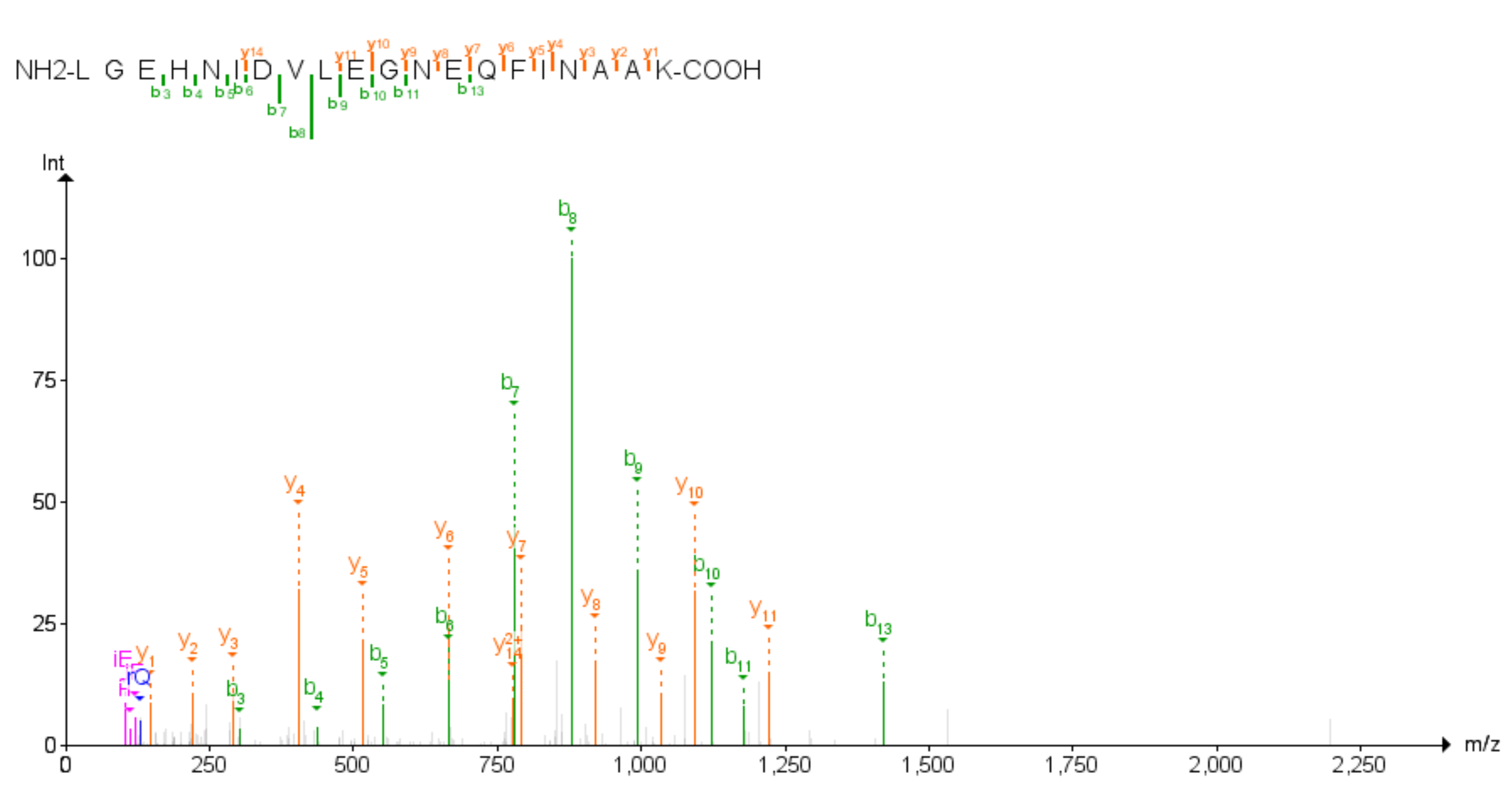

NH2-L G E H N I D V L E G N E Q F I N A A K-COOH
Int
m/z
0
25
50
75
100
250
500
750
1,000
1,250
1,500
1,750
2,000
2,250

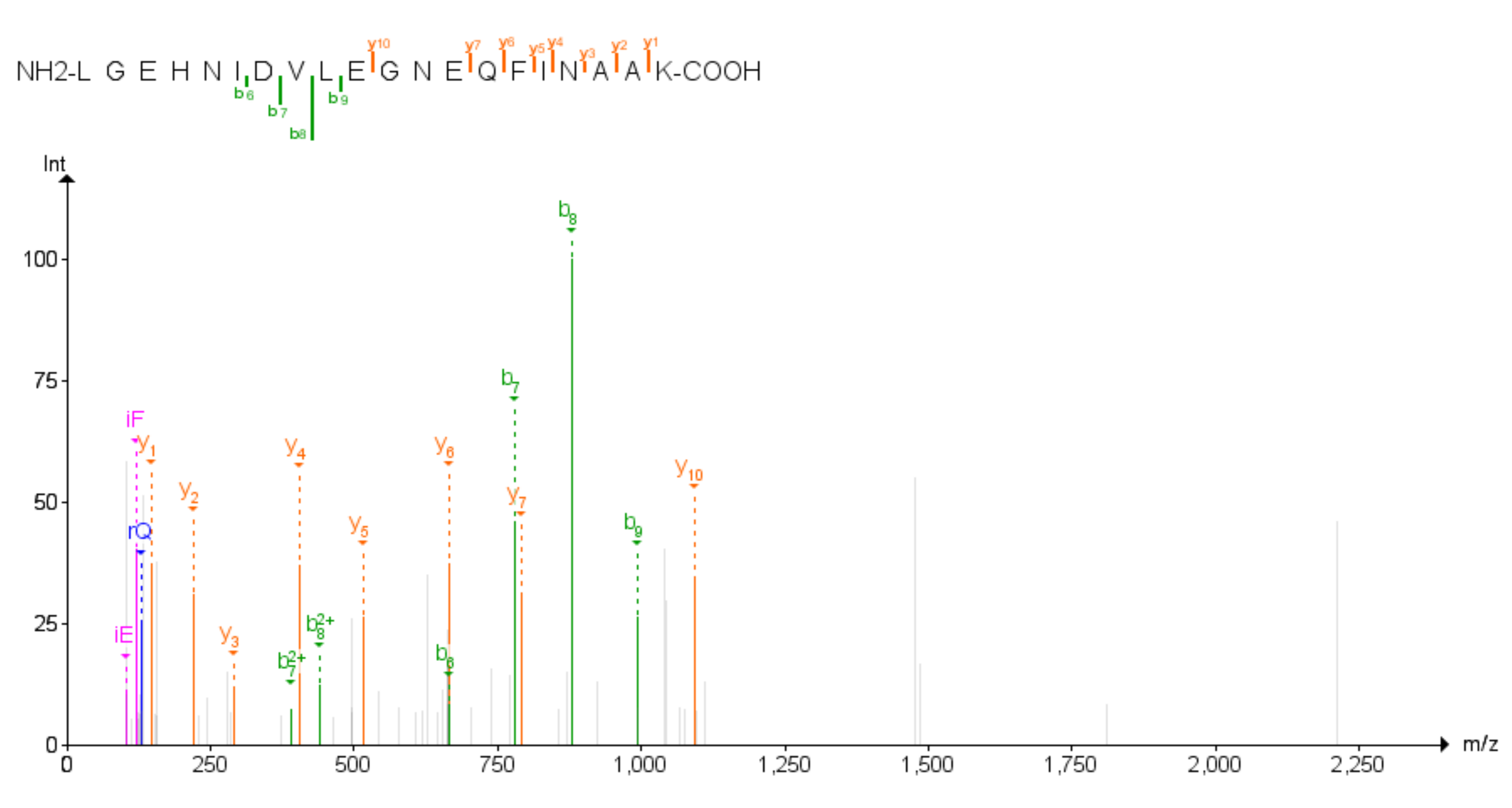

NH2-L G E H N I D V L E G N E Q F I N A A K-COOH
y10 y7 y6 y5 y4 y3 y2 y1
b6 b7 b8 b9
Int
100
75
50
25
0
m/z
0
250
500
750
1,000
1,250
1,500
1,750
2,000
2,250
b8
b7
iF
y1
y4
y6
y10
y2
y7
rQ
y5
b9
iE
y3
b8 2+
b7 2+
b6

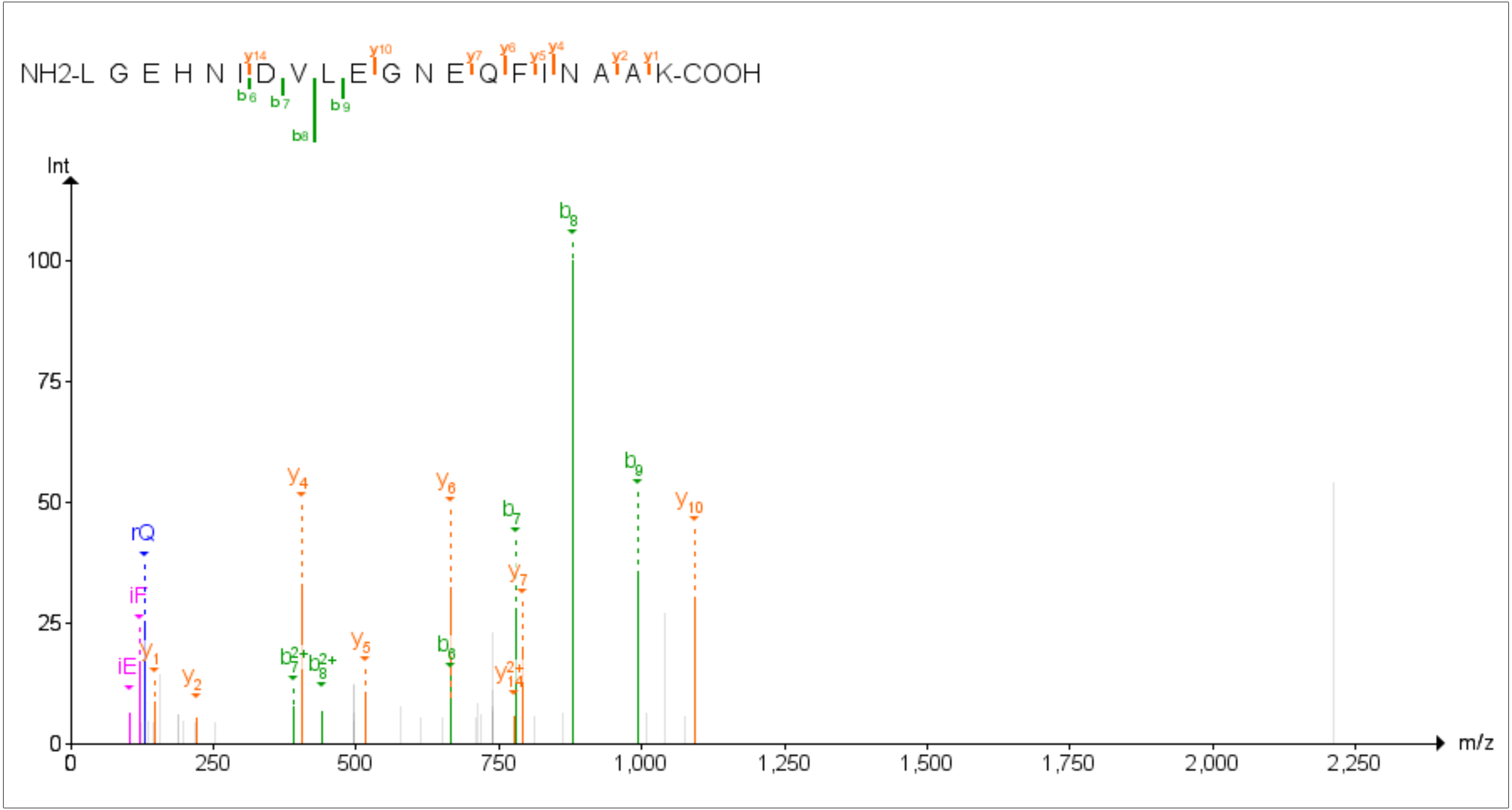

NH2-L G E H N I D V L E G N E Q F I N A A K-COOH
y14 y10 y7 y6 y5 y4 y2 y1
b6 b7 b8 b9
Int
100
75
50
25
0
b8
b9
y4
y6
b7
y10
y7
rQ
iF
y1
iE
y2
b7 2+
b8 2+
y5
b6
y14 2+
0
250
500
750
1,000
1,250
1,500
1,750
2,000
2,250
m/z

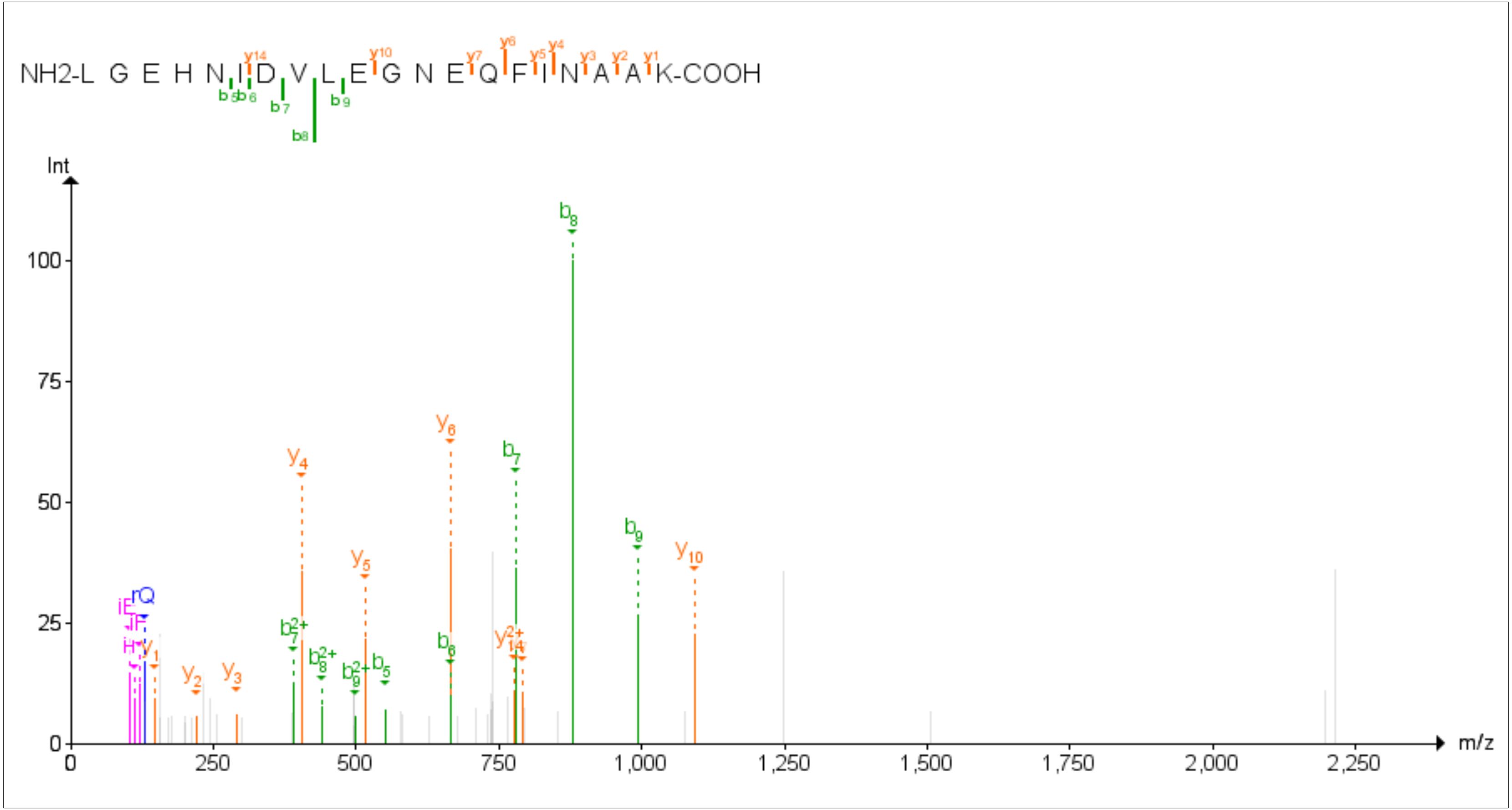

NH2-L G E H N I D V L E G N E Q F I N A A K-COOH
y14 y10 y7 y6 y5 y4 y3 y2 y1
b5 b6 b7 b8 b9
Int
m/z
0 25 50 75 100
0 250 500 750 1,000 1,250 1,500 1,750 2,000 2,250
b8 b7 b9 y10 y6 y4 y5 b7 2+ b8 2+ b9 2+ b5 b6 y14 2+ y7 y1 y2 y3 rQ iE iF iH

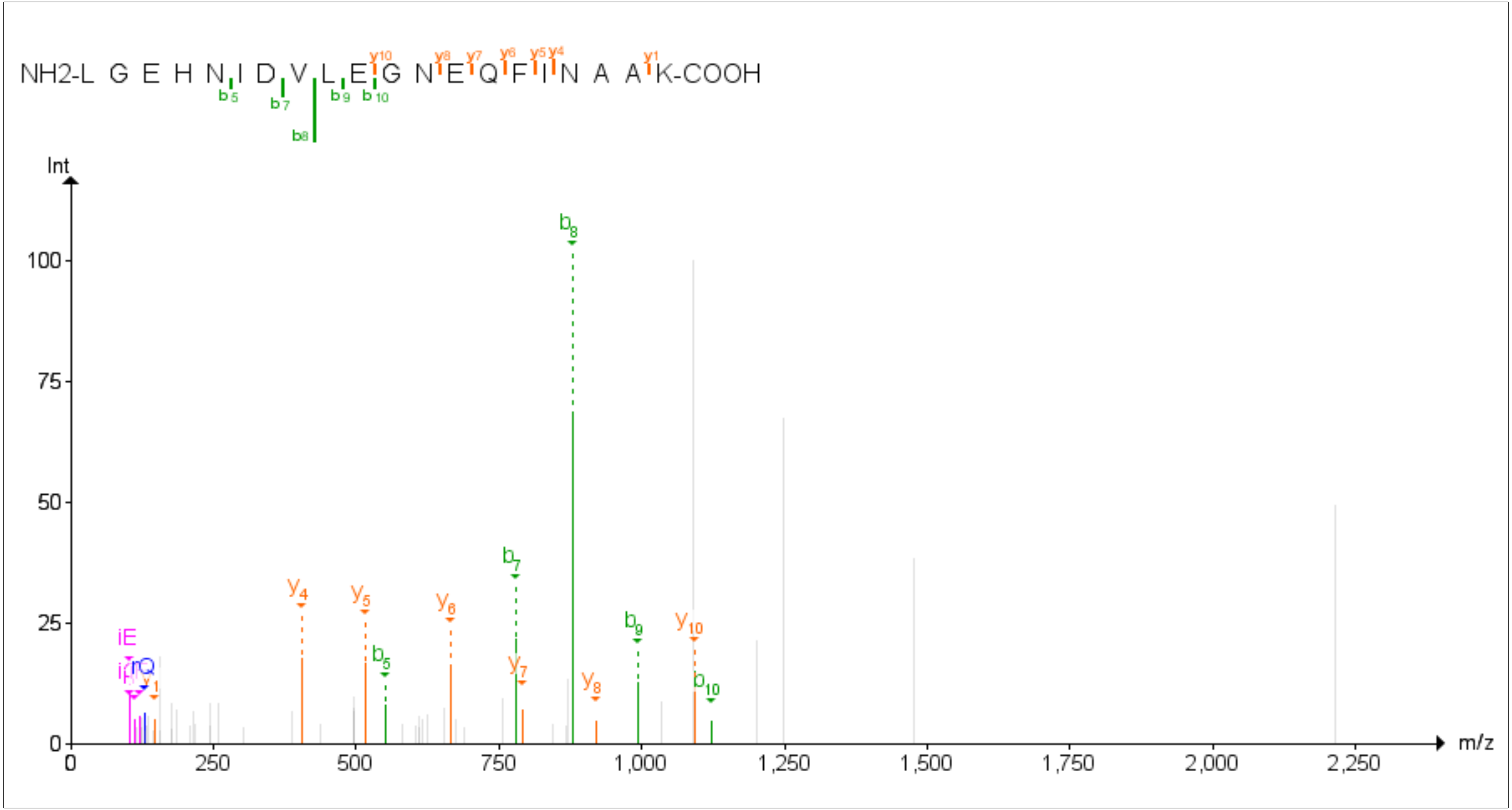

NH2-L G E H N I D V L E G N E Q F I N A A K-COOH
b5 b7 b8 b9 b10
y10 y8 y7 y6 y5 y4 y1
Int
100
75
50
25
0
m/z
0
250
500
750
1,000
1,250
1,500
1,750
2,000
2,250
b8
b7
b5
b9
b10
y4
y5
y6
y7
y8
y10
y1
iE
iQ

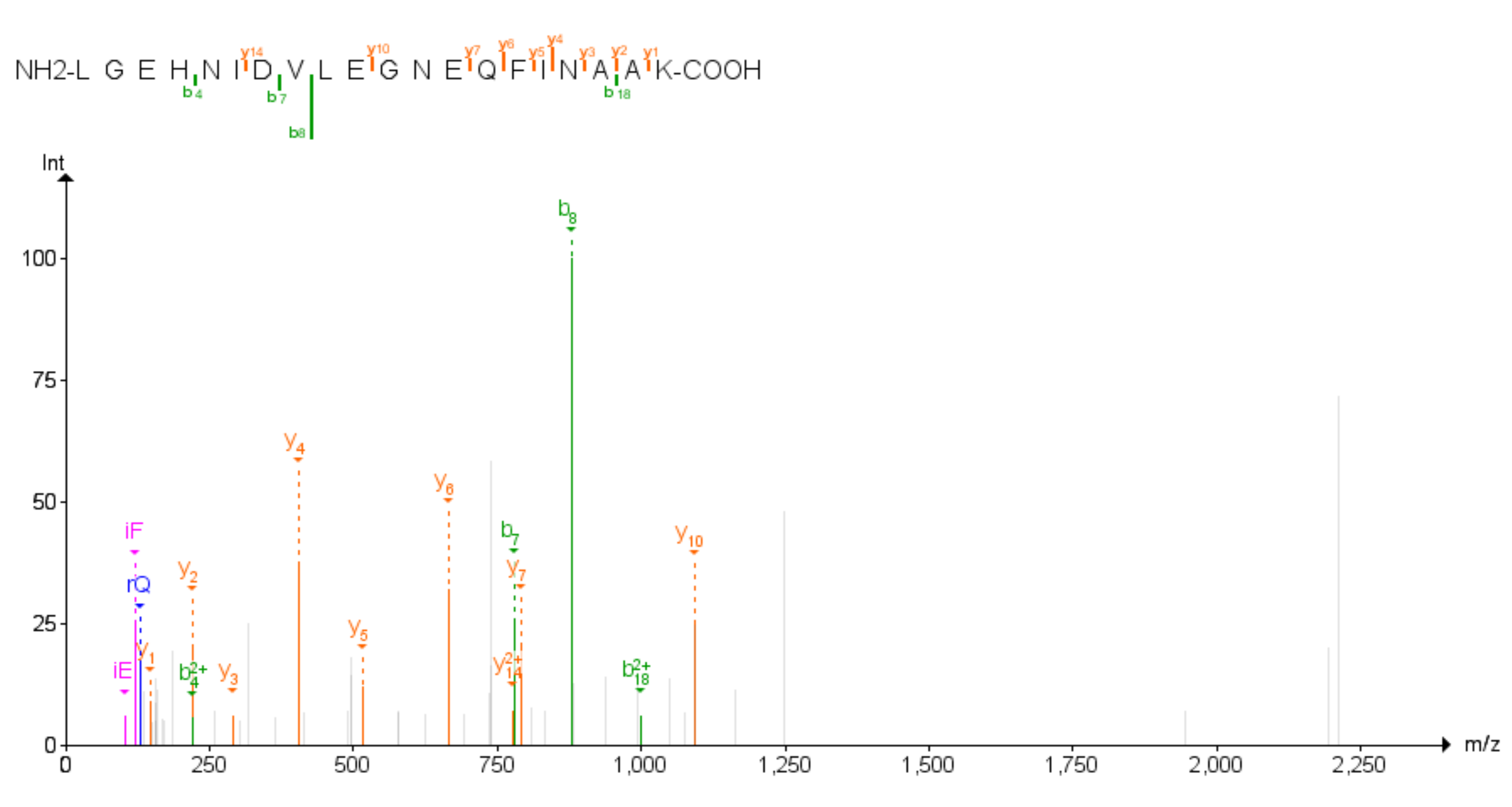

NH2-L G E H N I D V L E G N E Q F I N A A K-COOH
y14 y10 y7 y6 y5 y4 y3 y2 y1
b4 b7 b8 b18
Int
100
75
50
25
0
0 250 500 750 1,000 1,250 1,500 1,750 2,000 2,250
m/z
b8
y4
y6
b7
y7
y10
iF
rQ
y2
y5
y1
iE
b4 2+
y3
y14 2+
b18 2+

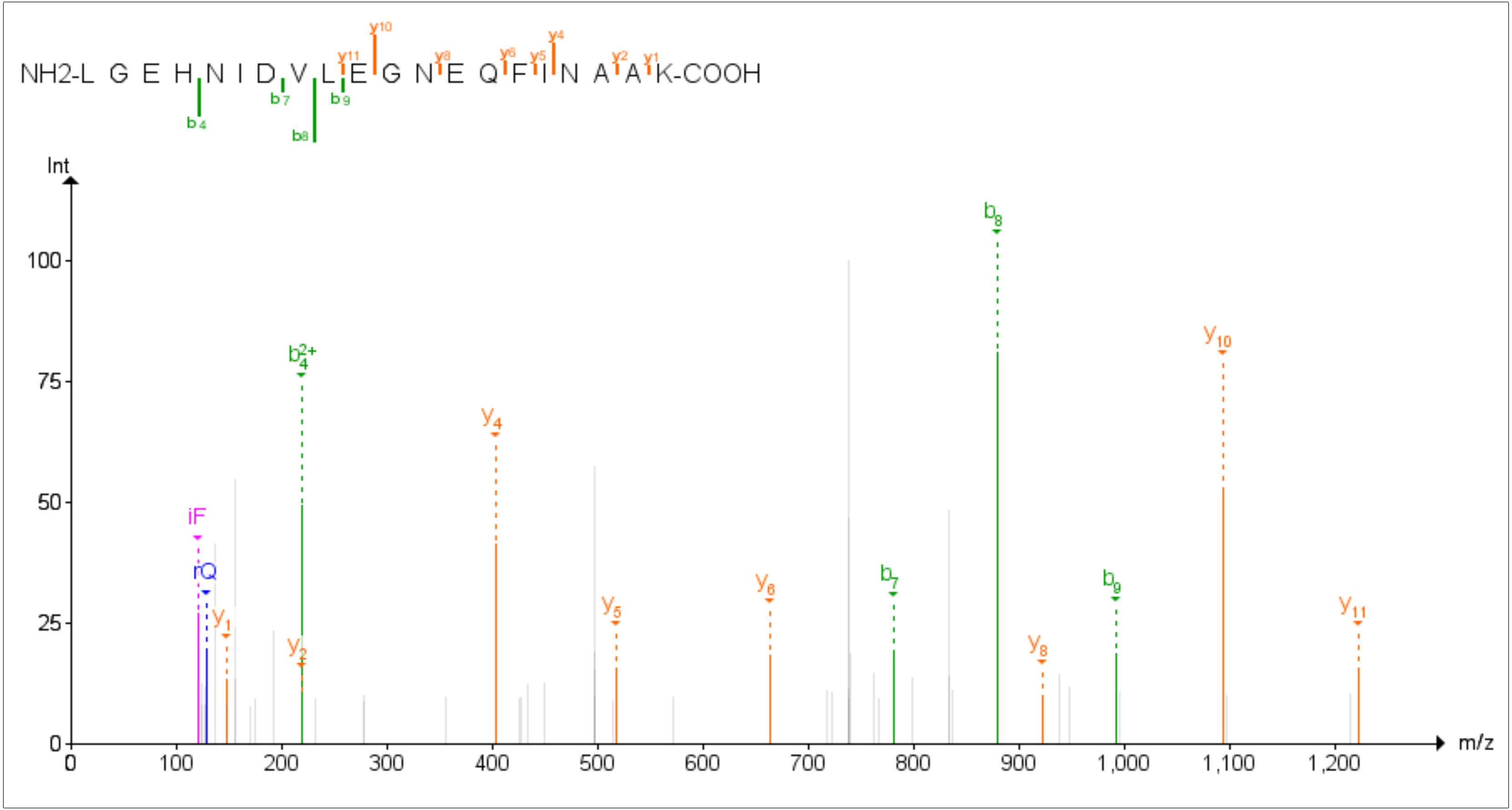

NH2-L G E H N I D V L E G N E Q F I N A A K-COOH
y10
y11
y8
y6
y5
y4
y2
y1
b4
b7
b8
b9
Int
100
75
50
25
0
m/z
0
100
200
300
400
500
600
700
800
900
1,000
1,100
1,200
iF
rQ
y1
y2
b4 2+
y4
y5
y6
b7
b8
y8
b9
y10
y11

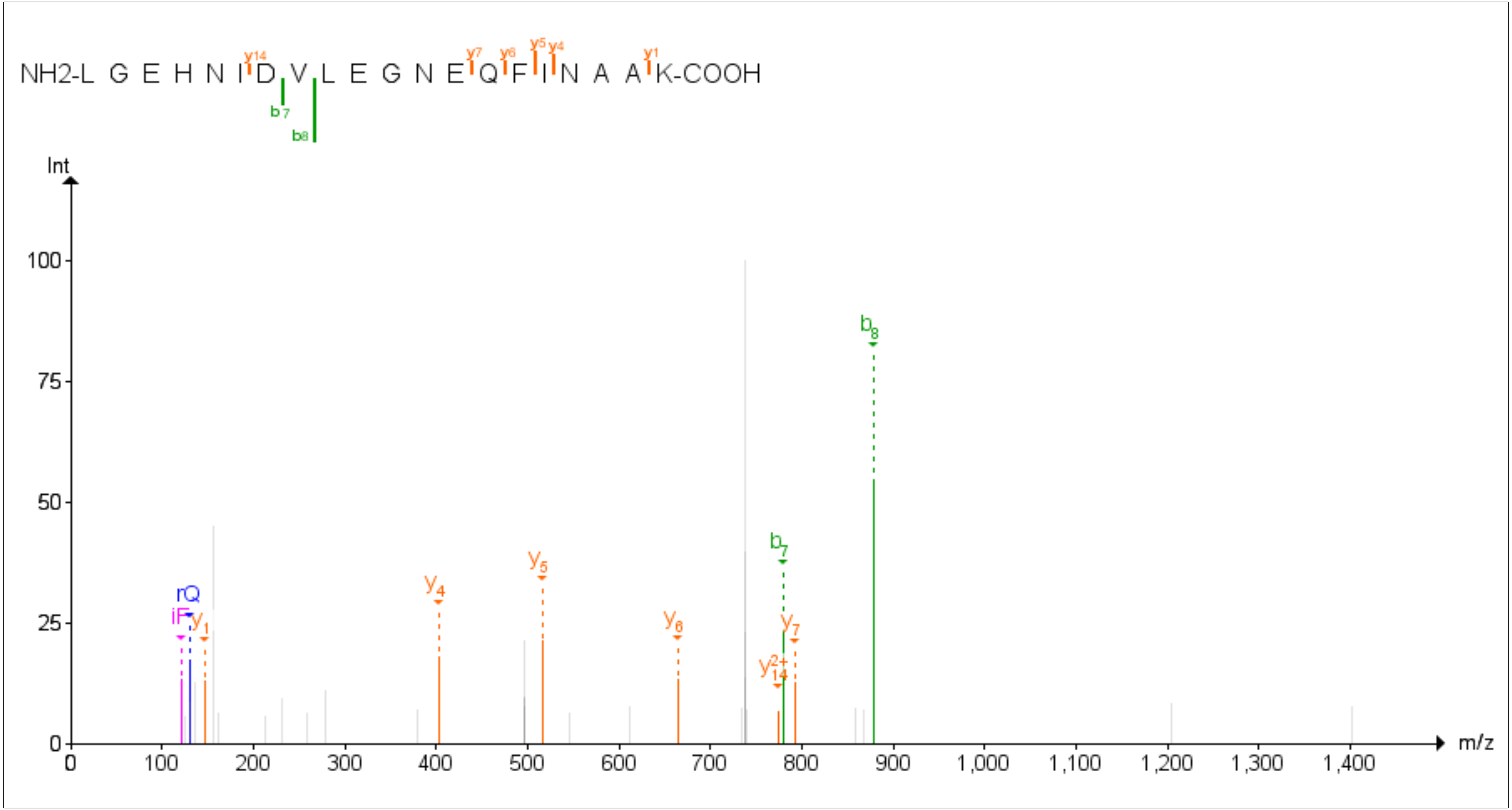
NH2-L G E H N I D V L E G N E Q F I N A A K-COOH
y14
y7
y6
y5
y4
y1
b7
b8
Int
100
75
50
25
0
rQ
iF
y1
y4
y5
y6
b7
y7
y14 2+
b8
0
100
200
300
400
500
600
700
800
900
1,000
1,100
1,200
1,300
1,400
m/z

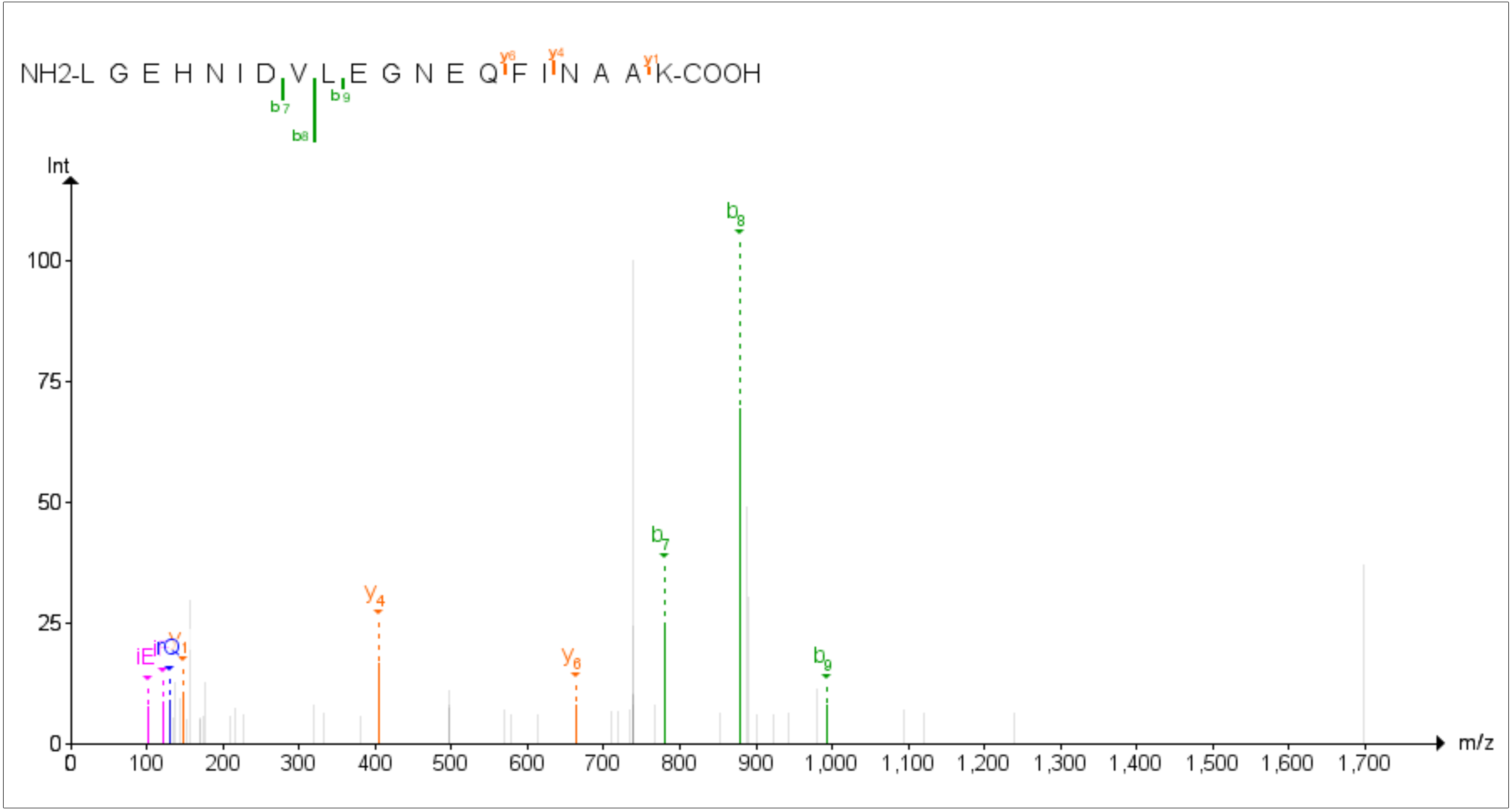

NH2-L G E H N I D V L E G N E Q F I N A A K-COOH
b7
b8
b9
y6
y4
y1
Int
m/z
iE
iQ
y1
y4
y6
b7
b8
b9
0
25
50
75
100
0
100
200
300
400
500
600
700
800
900
1,000
1,100
1,200
1,300
1,400
1,500
1,600
1,700

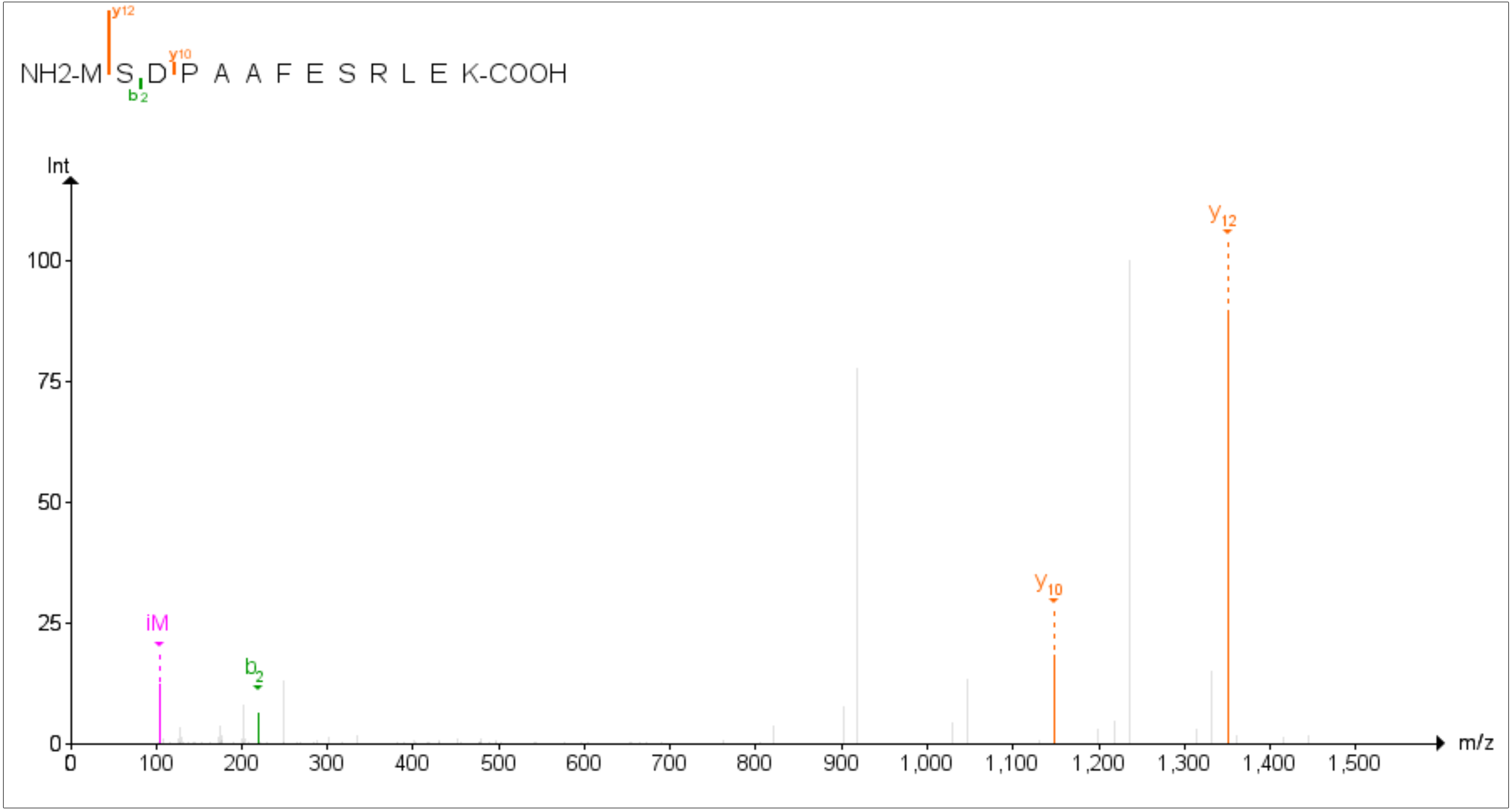
NH2-M S D P A A F E S R L E K-COOH
y12
y10
b2
Int
100
75
50
25
0
iM
b2
y10
y12
0
100
200
300
400
500
600
700
800
900
1,000
1,100
1,200
1,300
1,400
1,500
m/z

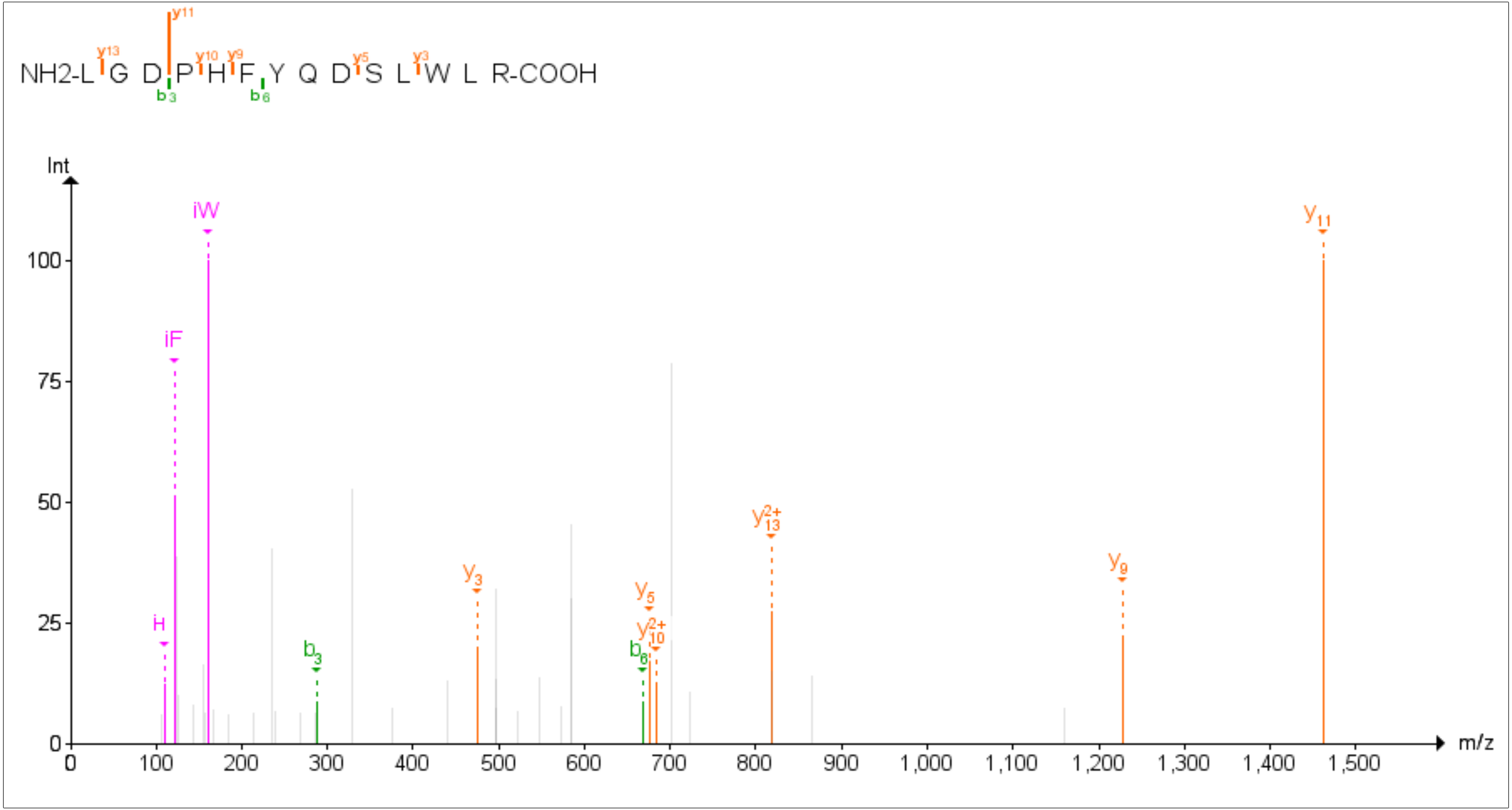

NH2-L G D P H F Y Q D S L W L R-COOH
y13
y11
y10
y9
y5
y3
b3
b6
Int
iW
iF
iH
b3
y3
y5
b6
y10 2+
y13 2+
y9
y11
0
25
50
75
100
0
100
200
300
400
500
600
700
800
900
1,000
1,100
1,200
1,300
1,400
1,500
m/z

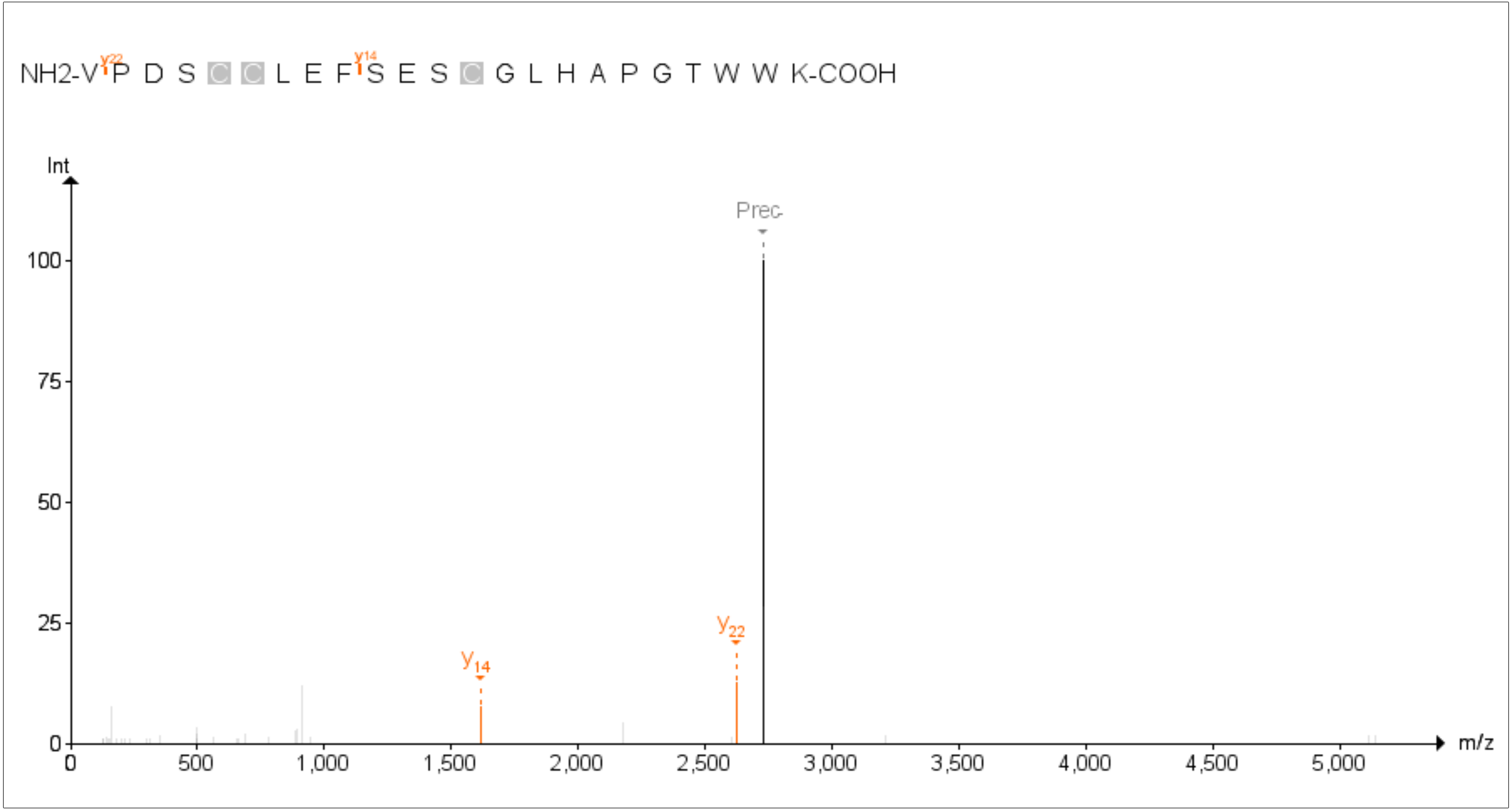

NH2-V P D S C C L E F S E S C G L H A P G T W W K-COOH
y22
y14
Int
100
75
50
25
0
Prec
y14
y22
0
500
1,000
1,500
2,000
2,500
3,000
3,500
4,000
4,500
5,000
m/z

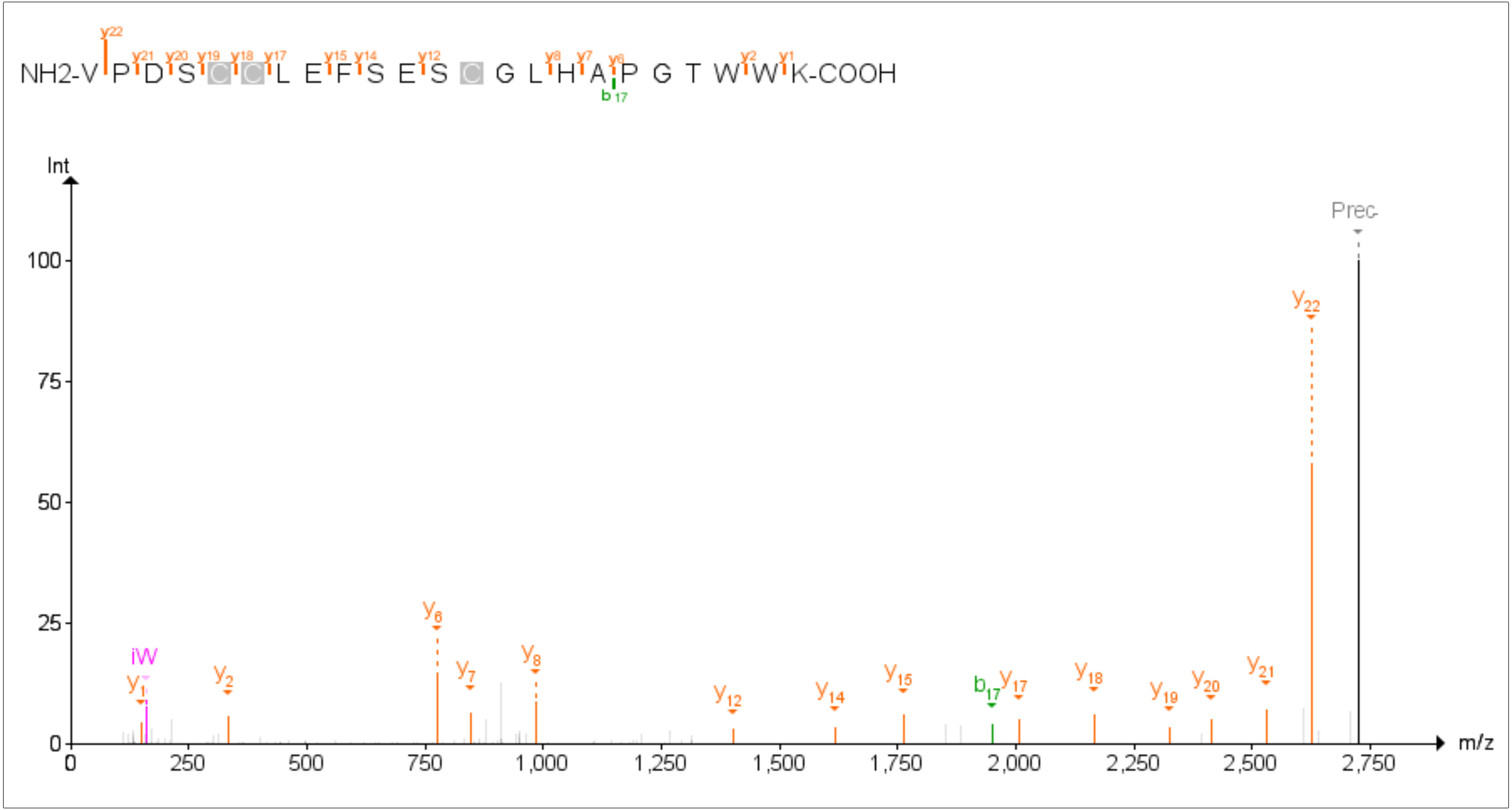

NH2-V P D S C C L E F S E S C G L H A P G T W W K-COOH
y22 y21 y20 y19 y18 y17 y15 y14 y12 y8 y7 y6 y2 y1
b17
Int
100
75
50
25
0
Prec.
iW
m/z
0
250
500
750
1,000
1,250
1,500
1,750
2,000
2,250
2,500
2,750

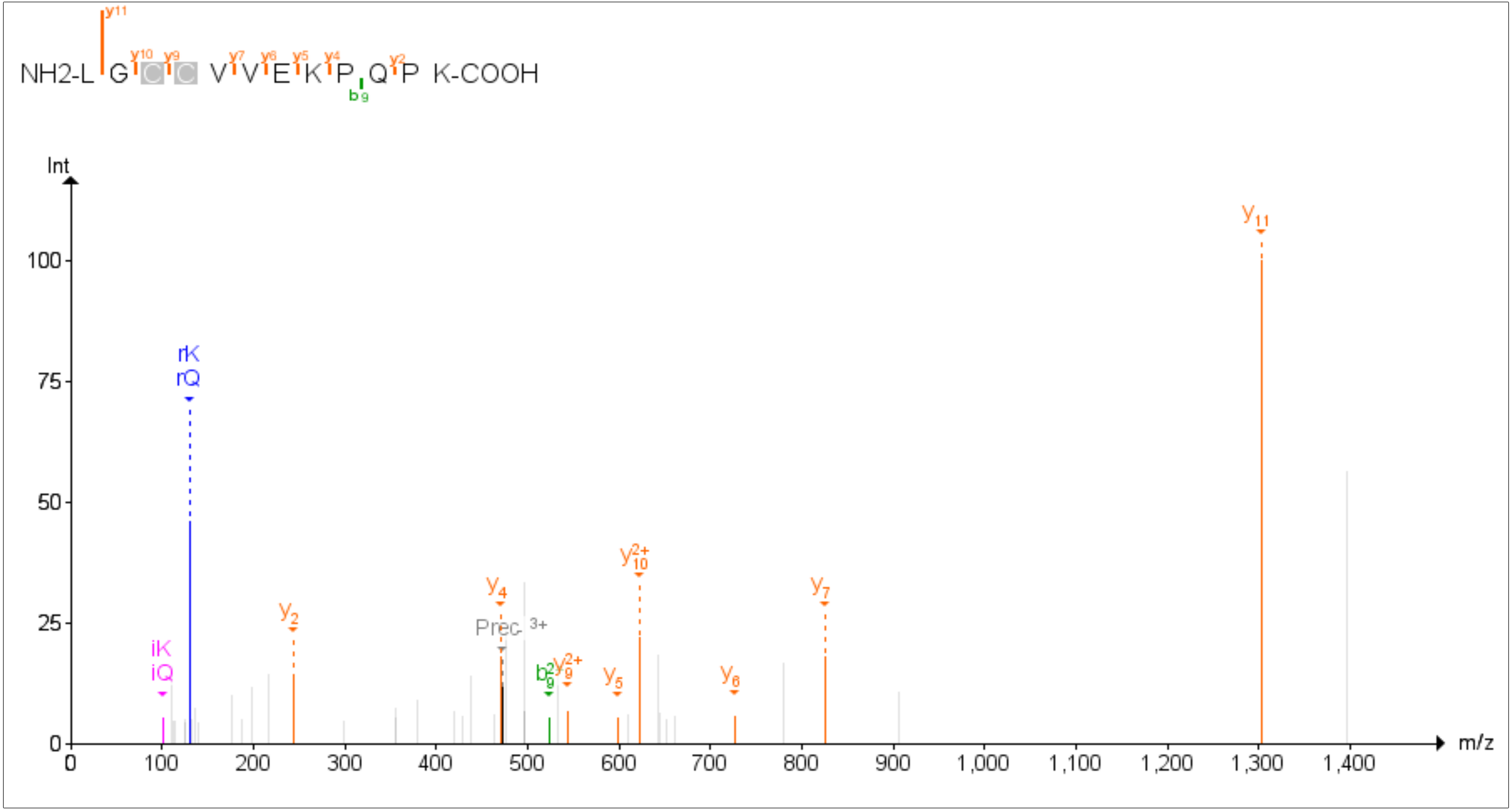

NH2-L G C C V V E K P Q P K-COOH
y11 y10 y9 y7 y6 y5 y4 y2
b9
Int
100
75
50
25
0
rK
rQ
iK
iQ
y2
y4
Prec 3+
b9 2+
y9 2+
y5
y10 2+
y6
y7
y11
0 100 200 300 400 500 600 700 800 900 1,000 1,100 1,200 1,300 1,400
m/z

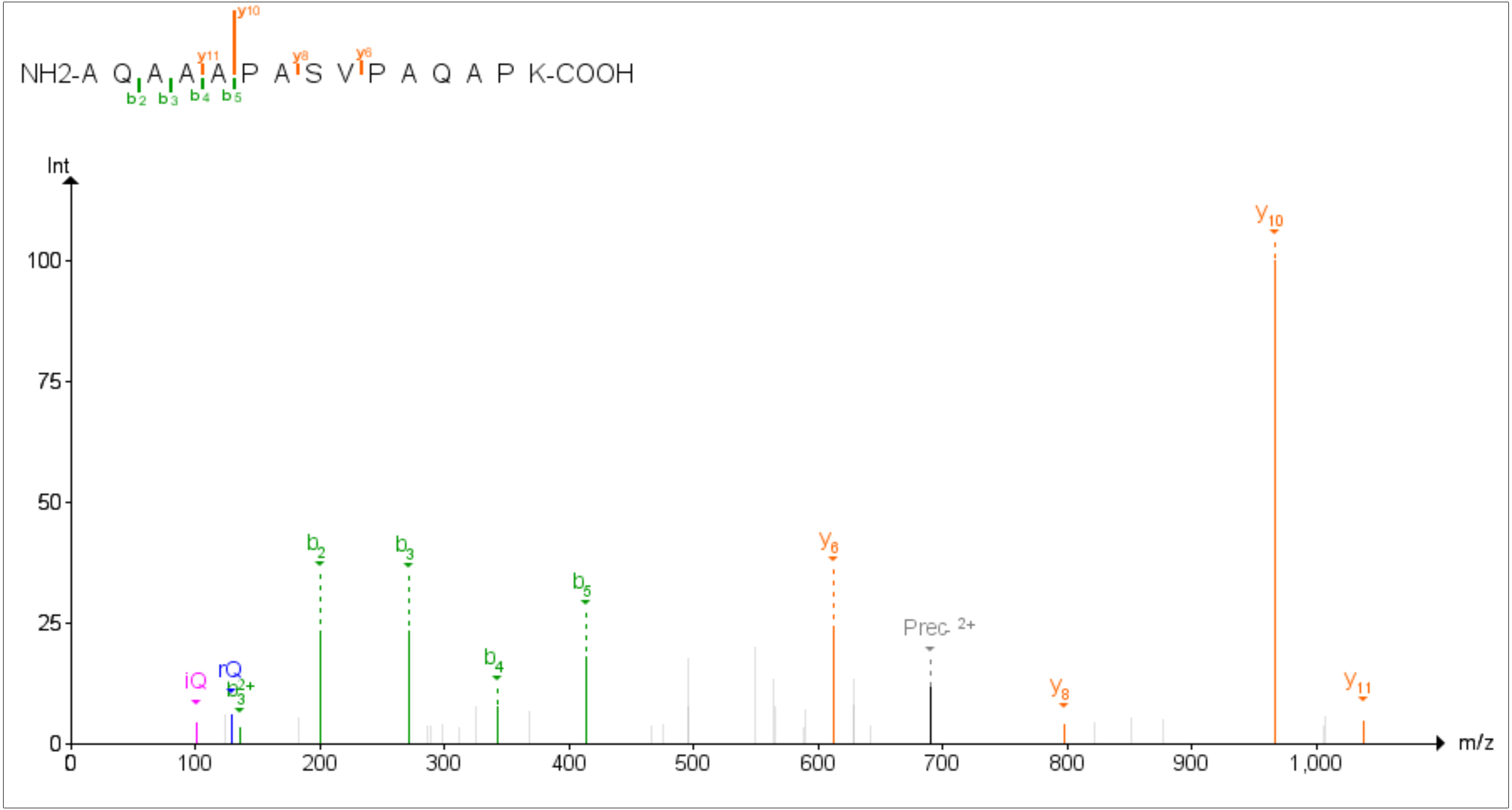

NH2-A Q A A A P A S V P A Q A P K-COOH
y10
y11
y8
y6
b2
b3
b4
b5
Int
100
75
50
25
0
m/z
0
100
200
300
400
500
600
700
800
900
1,000
iQ
rQ
b3 2+
b2
b3
b4
b5
y6
Prec 2+
y8
y10
y11

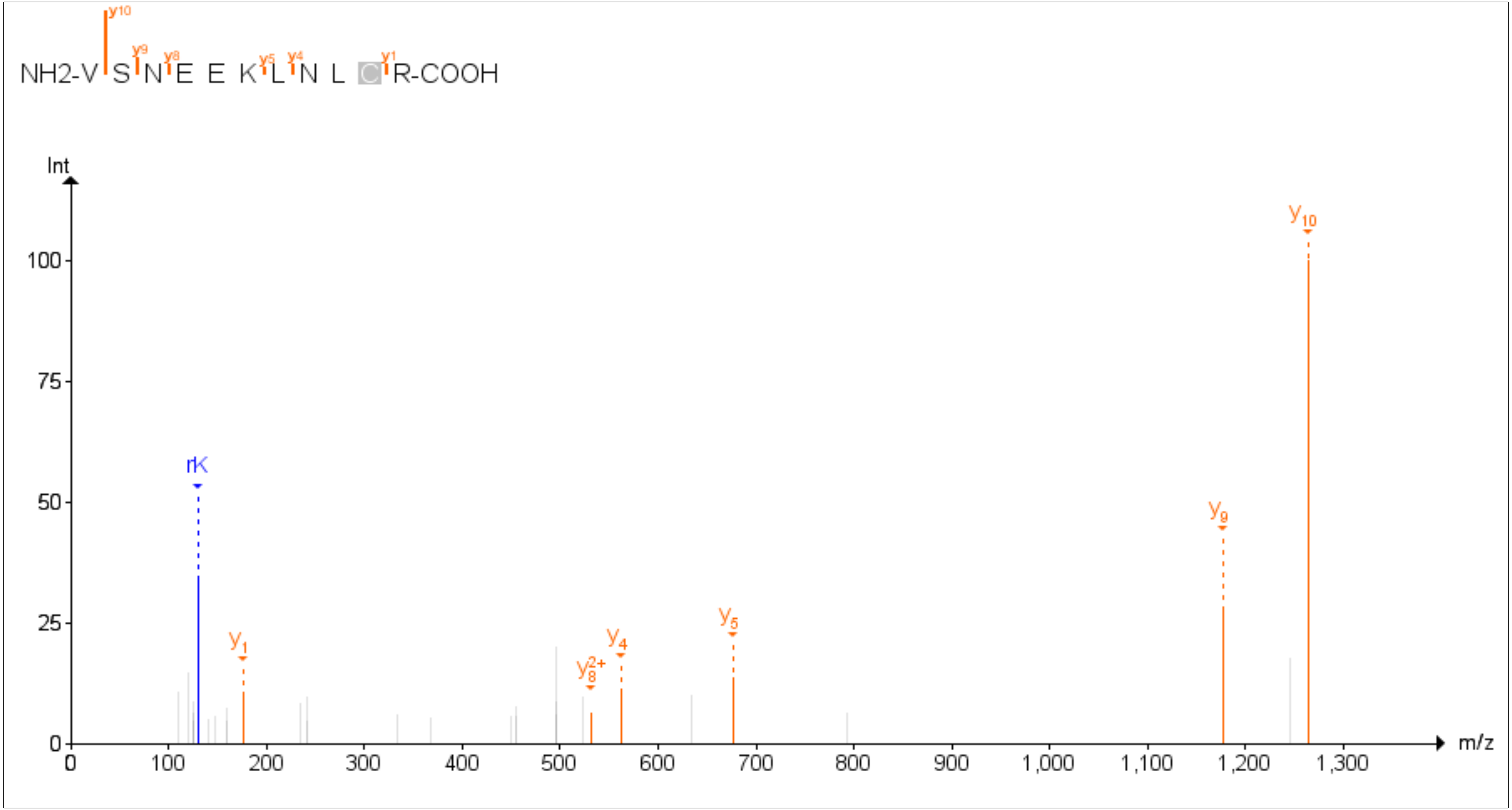

y10
y9
y8
y5
y4
y1
NH2-V S N E E K L N L C R-COOH
Int
100
75
50
25
0
rK
y1
y8 2+
y4
y5
y9
y10
0
100
200
300
400
500
600
700
800
900
1,000
1,100
1,200
1,300
m/z

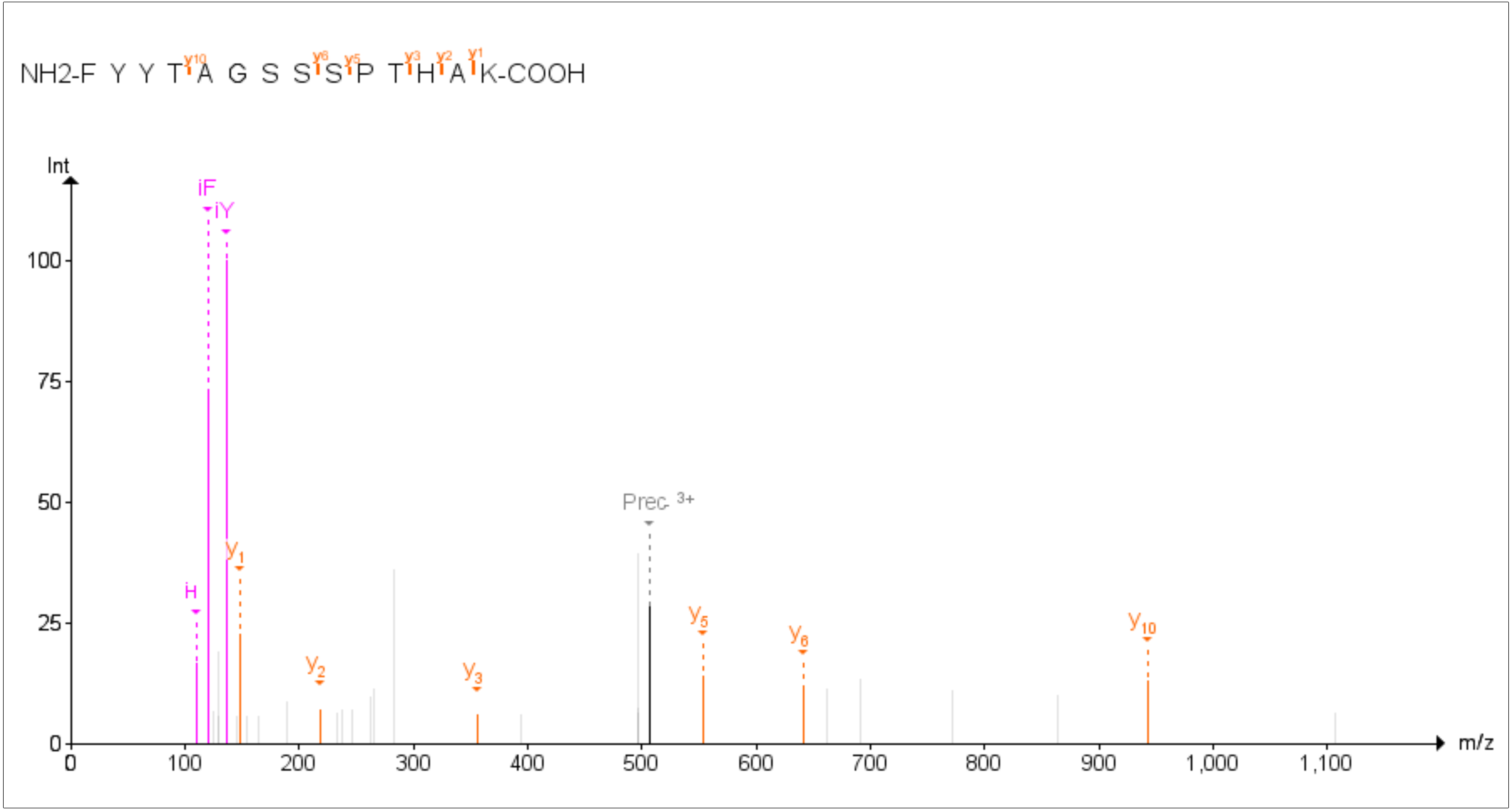

NH2-F Y Y T A G S S S P T H A K-COOH
y10
y6
y5
y3
y2
y1
Int
iF
iY
iH
y1
y2
y3
Prec 3+
y5
y6
y10
100
75
50
25
0
0
100
200
300
400
500
600
700
800
900
1,000
1,100
m/z

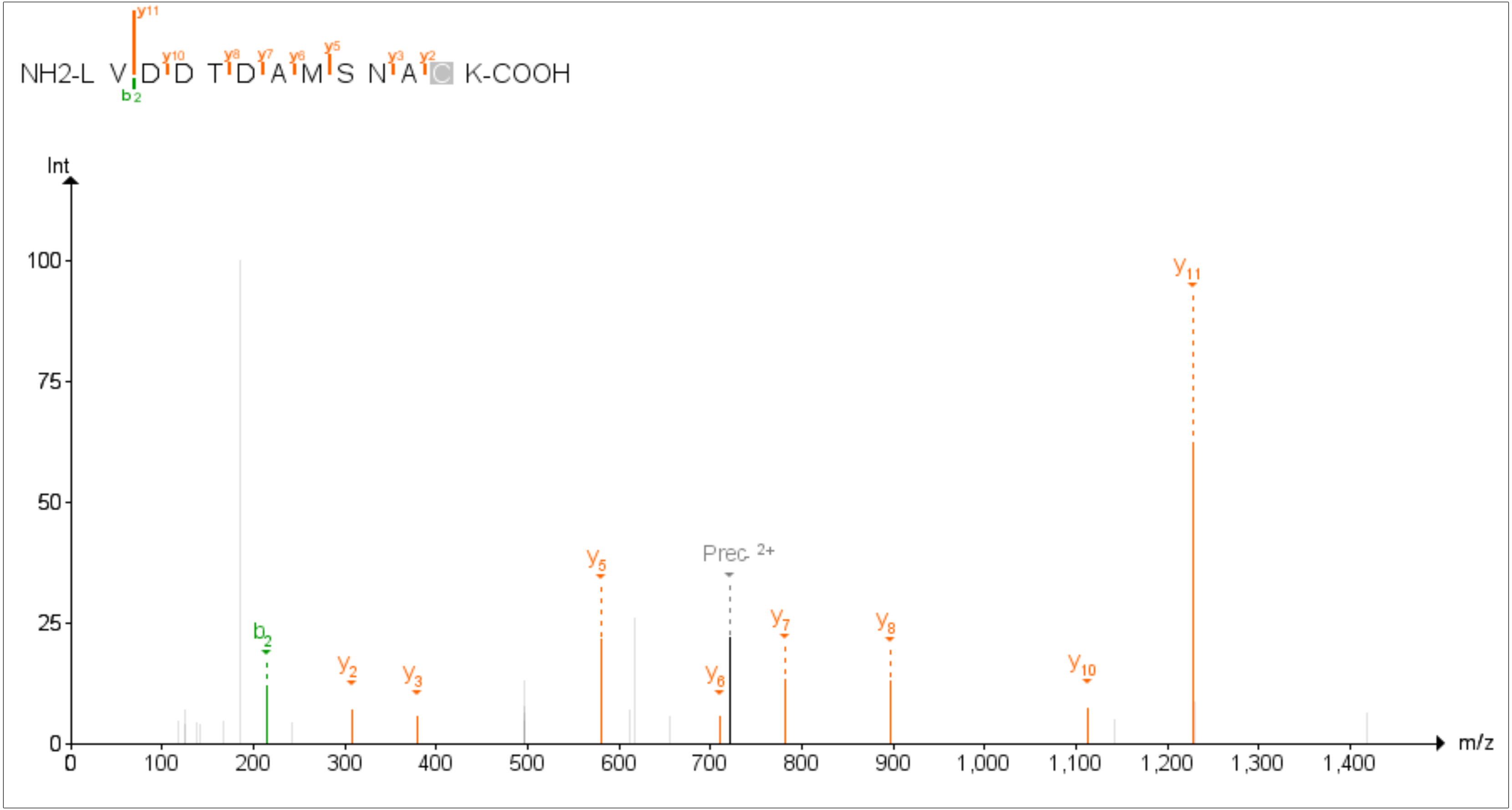

NH2-L V D D T D A M S N A C K-COOH
y11 y10 y8 y7 y6 y5 y3 y2
b2
Int
100
75
50
25
0
0 100 200 300 400 500 600 700 800 900 1,000 1,100 1,200 1,300 1,400
m/z
y11
y5
Prec 2+
y7
y8
b2
y2
y3
y6
y10

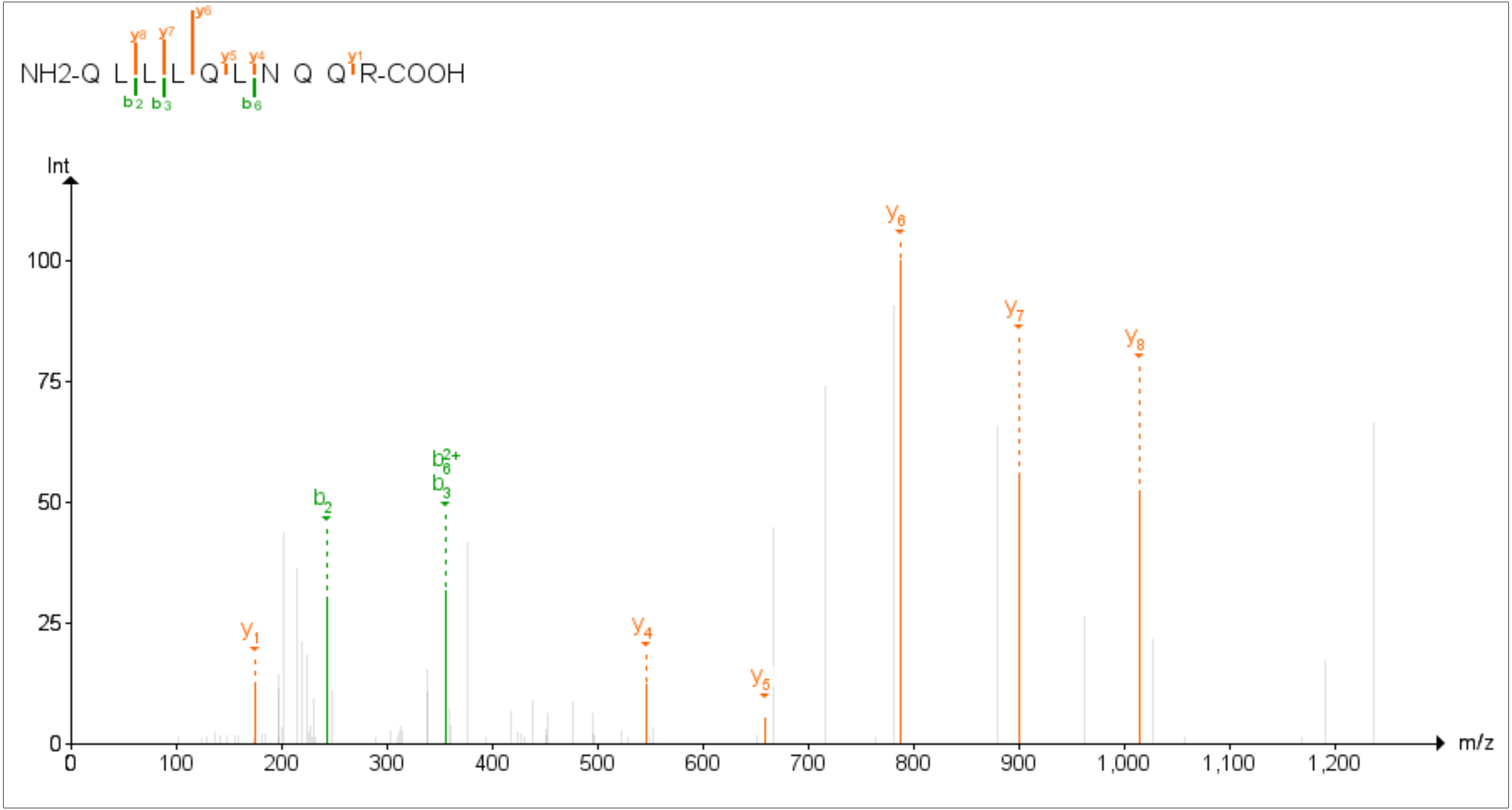

NH2-Q L L L Q L N Q Q R-COOH
y8 y7 y6 y5 y4 y1
b2 b3 b6
Int
100
75
50
25
0
y6
y7
y8
b6 2+
b3
b2
y1
y4
y5
0 100 200 300 400 500 600 700 800 900 1,000 1,100 1,200
m/z

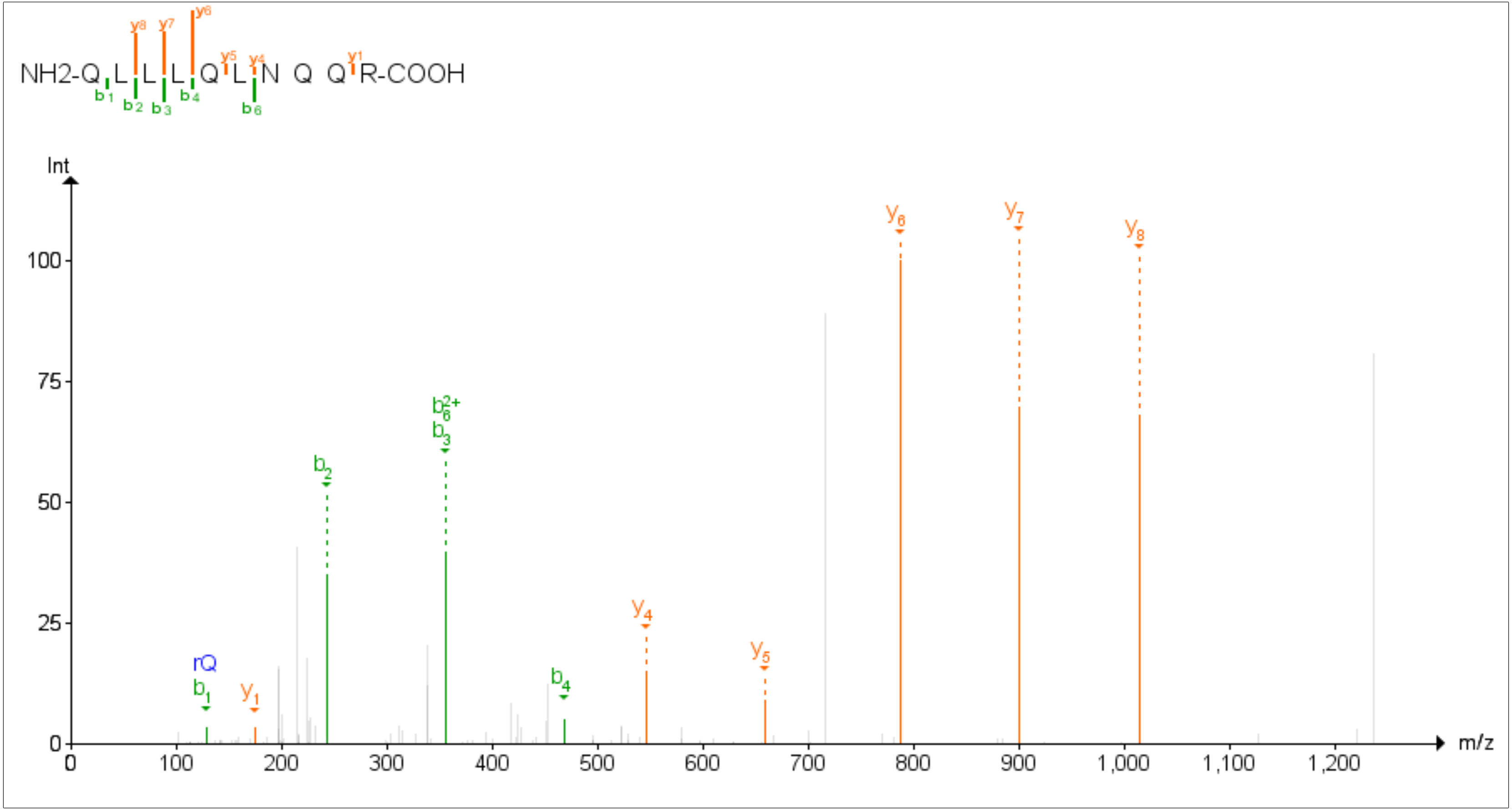

NH2-Q L L L Q L N Q Q R-COOH
y8 y7 y6 y5 y4 y1
b1 b2 b3 b4 b6
Int
100
75
50
25
0
rQ
b1
y1
b2
b6 2+
b3
b4
y4
y5
y6
y7
y8
0 100 200 300 400 500 600 700 800 900 1,000 1,100 1,200
m/z

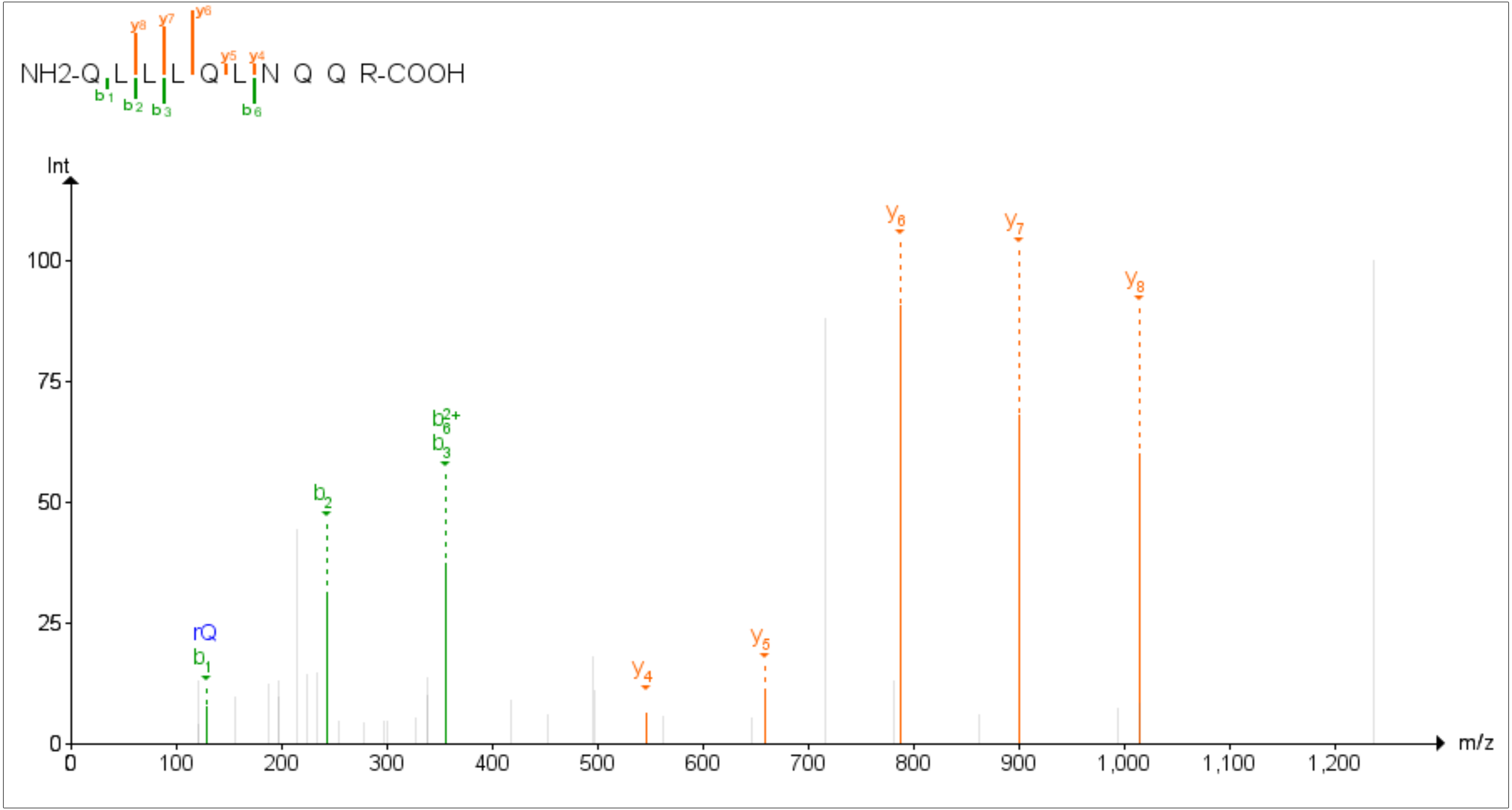
NH2-Q L L L Q L N Q Q R-COOH
y8 y7 y6 y5 y4
b1 b2 b3 b6
Int
m/z
rQ
b1
b2
b6 2+
b3
y4
y5
y6
y7
y8

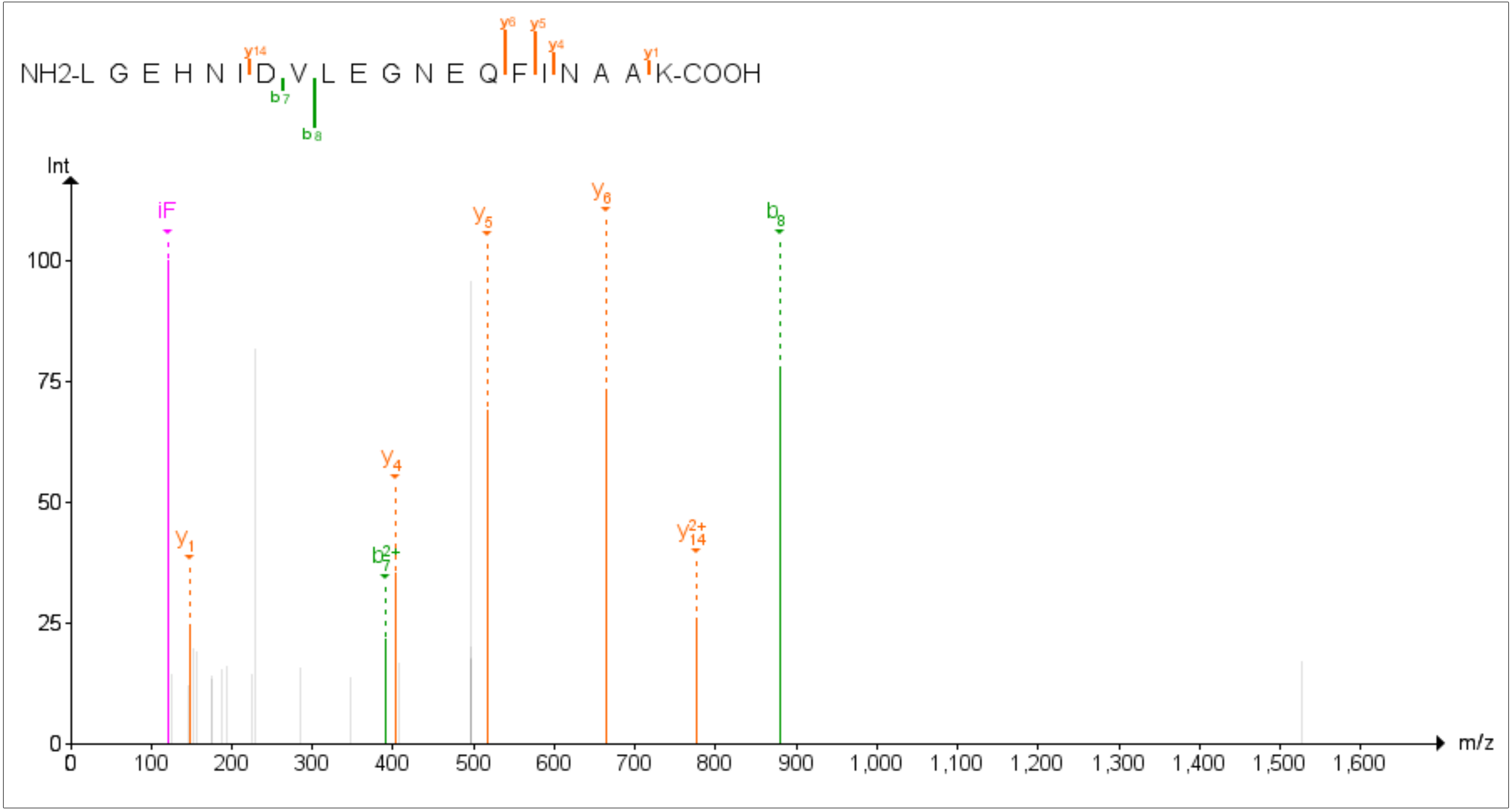

NH2-L G E H N I D V L E G N E Q F I N A A K-COOH
y14
y6
y5
y4
y1
b7
b8
Int
iF
y1
y4
b7 2+
y5
y6
y14 2+
b8
100
75
50
25
0
0
100
200
300
400
500
600
700
800
900
1,000
1,100
1,200
1,300
1,400
1,500
1,600
m/z

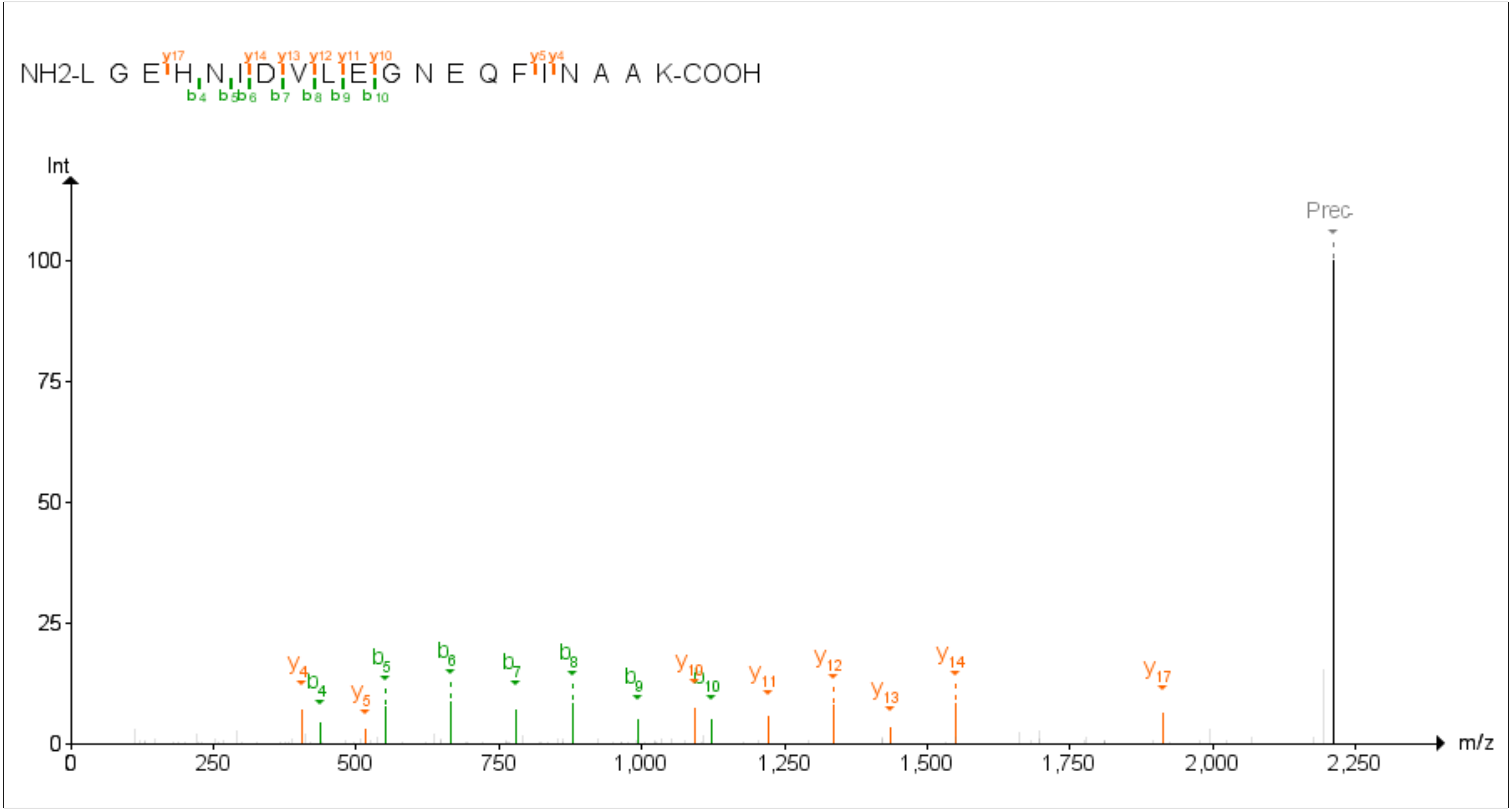

NH2-L G E H N I D V L E G N E Q F I N A A K-COOH
y17 y14 y13 y12 y11 y10 y5 y4
b4 b5 b6 b7 b8 b9 b10
Int
100
75
50
25
0
Prec
y4 b4 y5 b5 b6 b7 b8 b9 y10 b10 y11 y12 y13 y14 y17
0 250 500 750 1,000 1,250 1,500 1,750 2,000 2,250
m/z

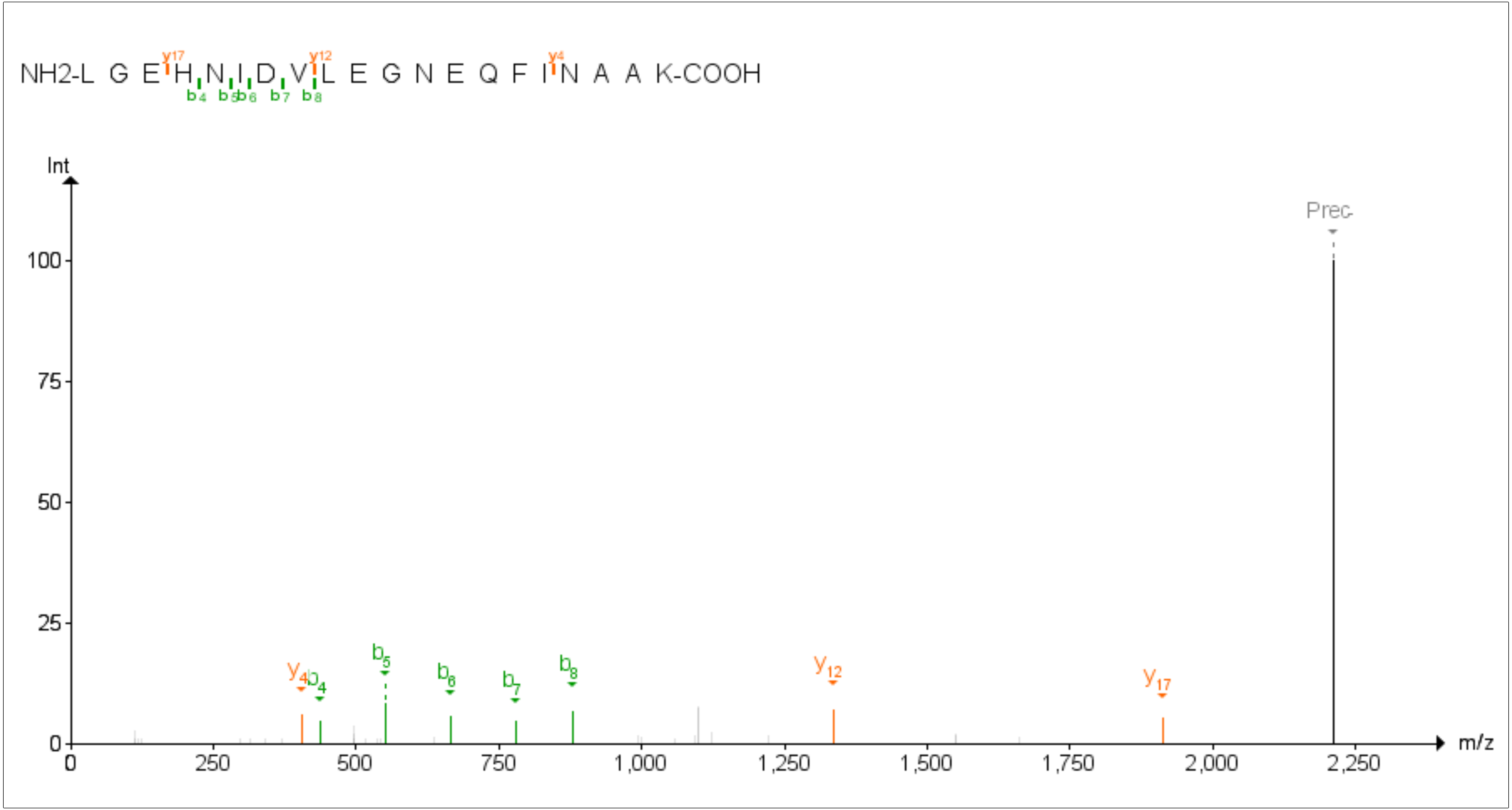

NH2-L G E H N I D V L E G N E Q F I N A A K-COOH
y17 y12 y4
b4 b5 b6 b7 b8
Int
100
75
50
25
0
Prec
y4 b4 b5 b6 b7 b8 y12 y17
0 250 500 750 1,000 1,250 1,500 1,750 2,000 2,250
m/z

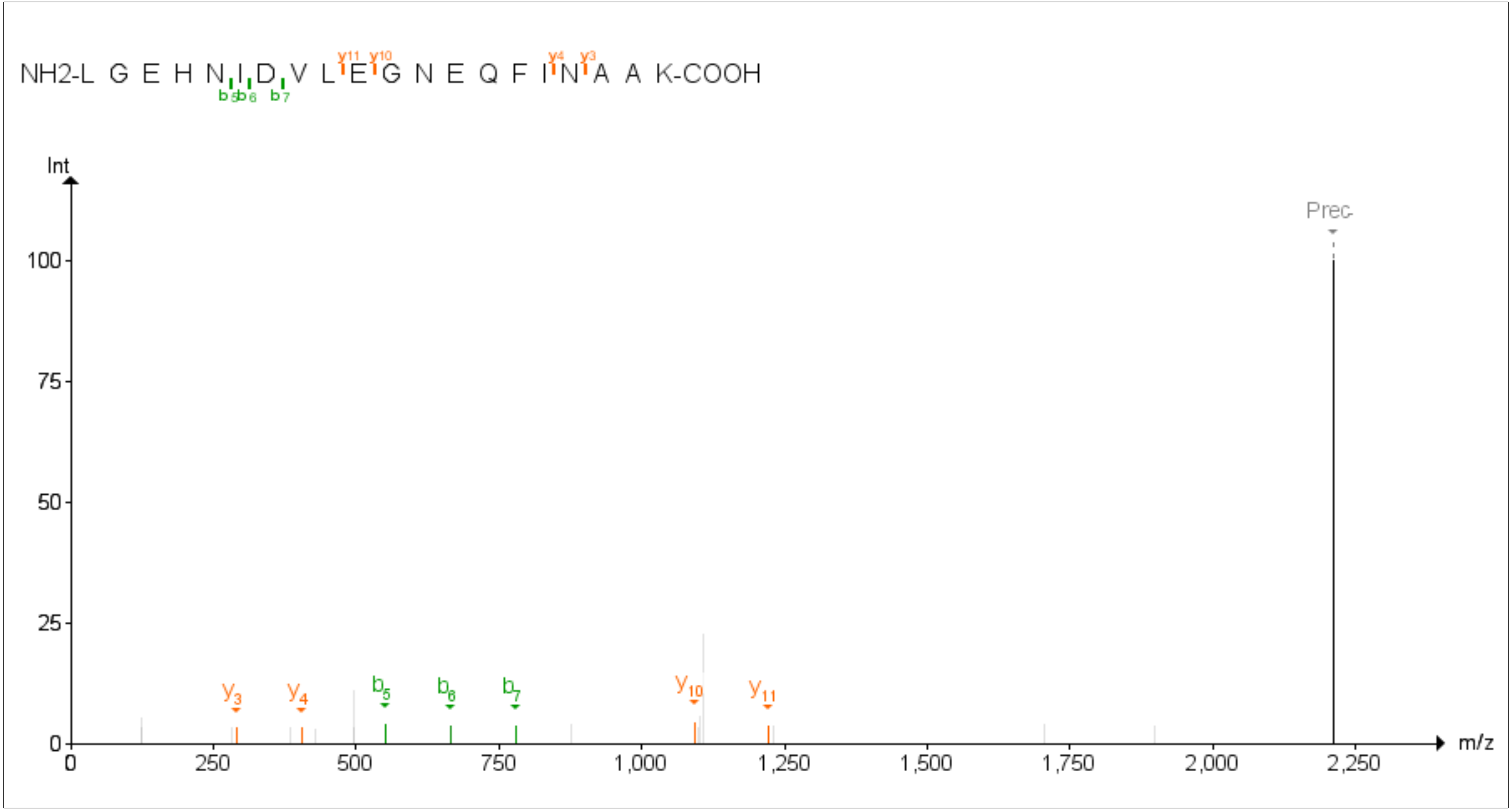

NH2-L G E H N I D V L E G N E Q F I N A A K-COOH
b5 b6 b7
y11 y10 y4 y3
Int
100
75
50
25
0
Prec
y3
y4
b5
b6
b7
y10
y11
0
250
500
750
1,000
1,250
1,500
1,750
2,000
2,250
m/z

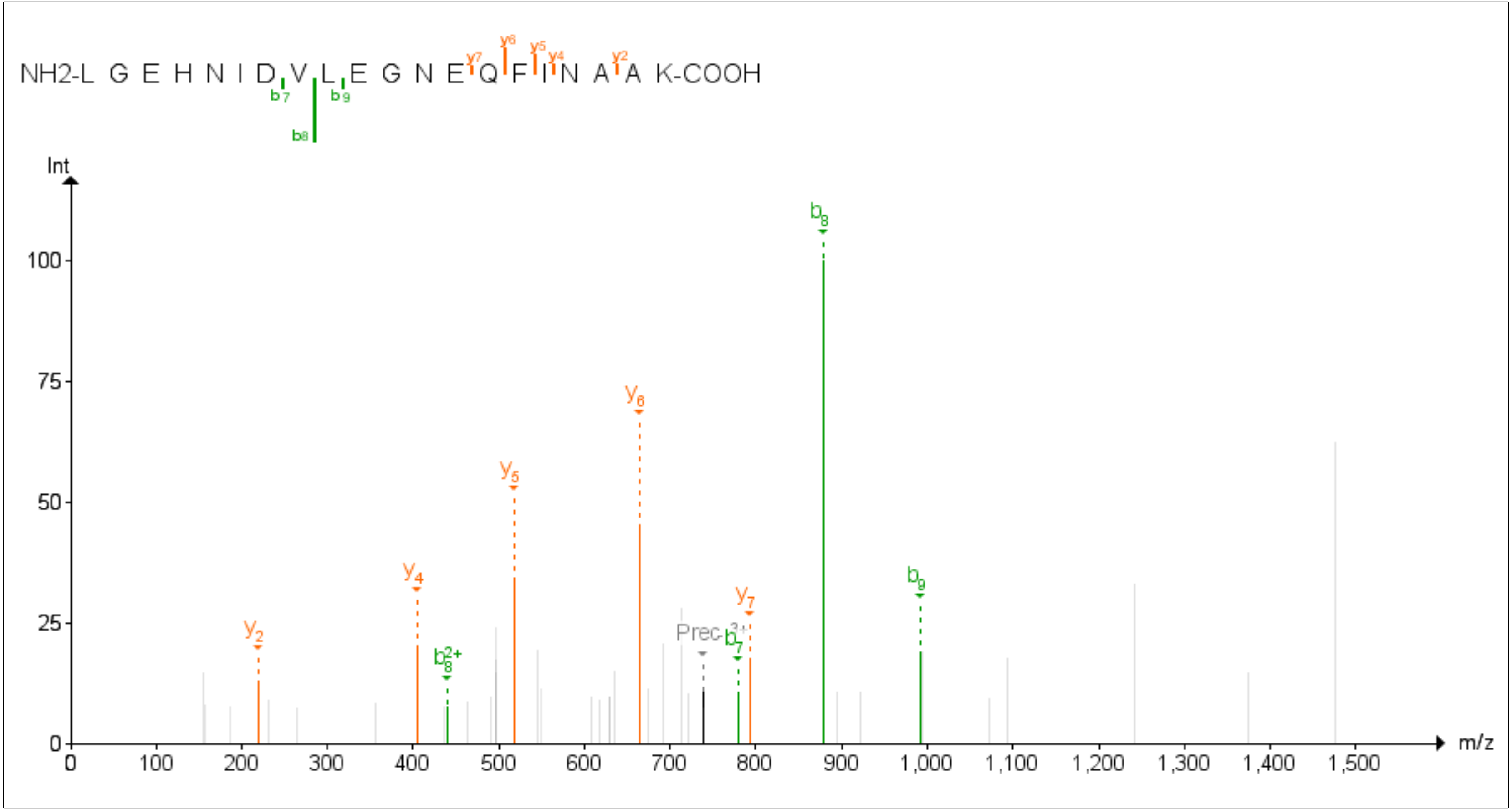

NH2-L G E H N I D V L E G N E Q F I N A A K-COOH
b7
b8
b9
y7
y6
y5
y4
y2
Int
m/z
Prec 3+

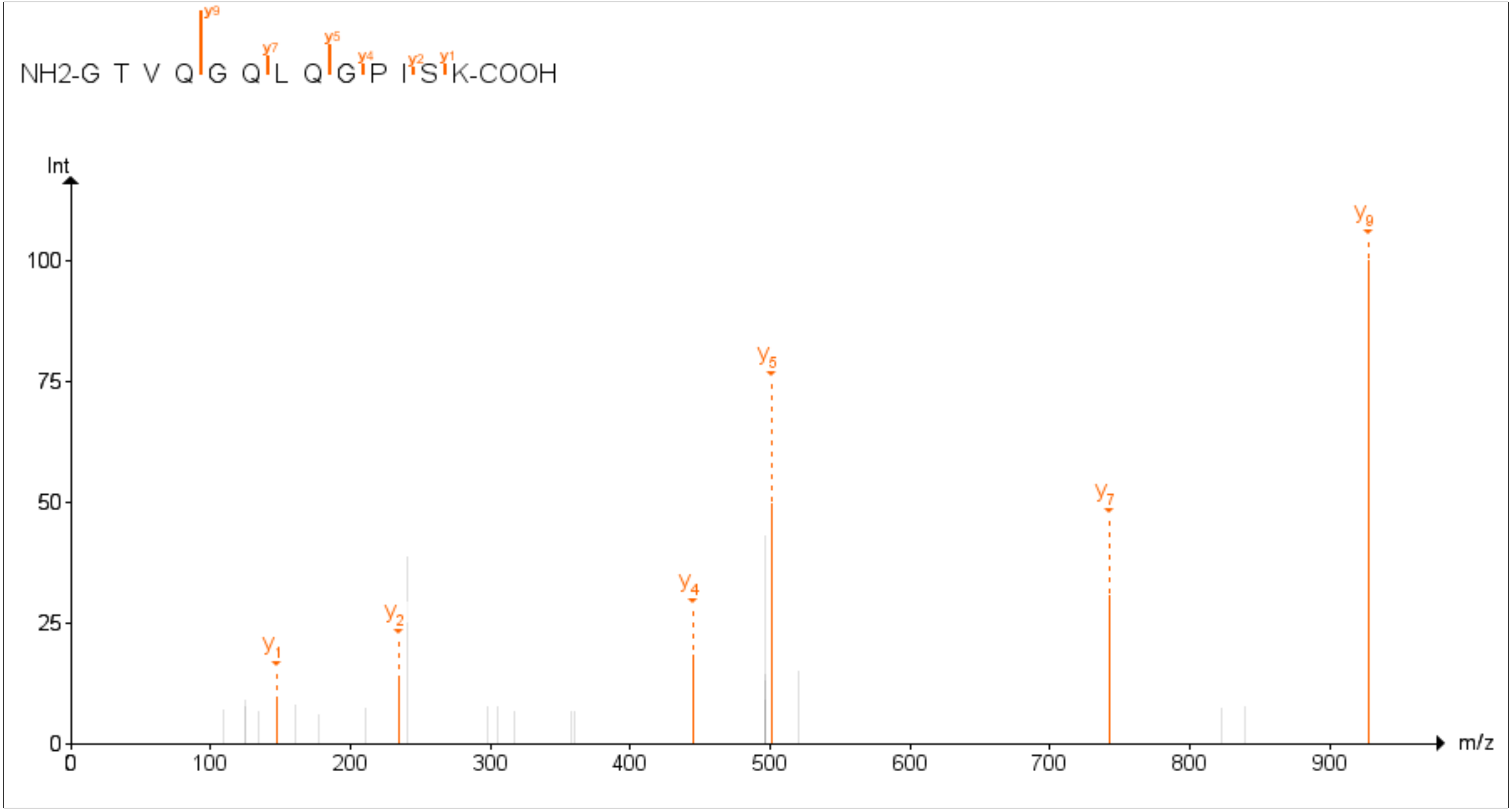

NH2-G T V Q G Q L Q G P I S K-COOH
y9
y7
y5
y4
y2
y1
Int
100
75
50
25
0
m/z
0
100
200
300
400
500
600
700
800
900

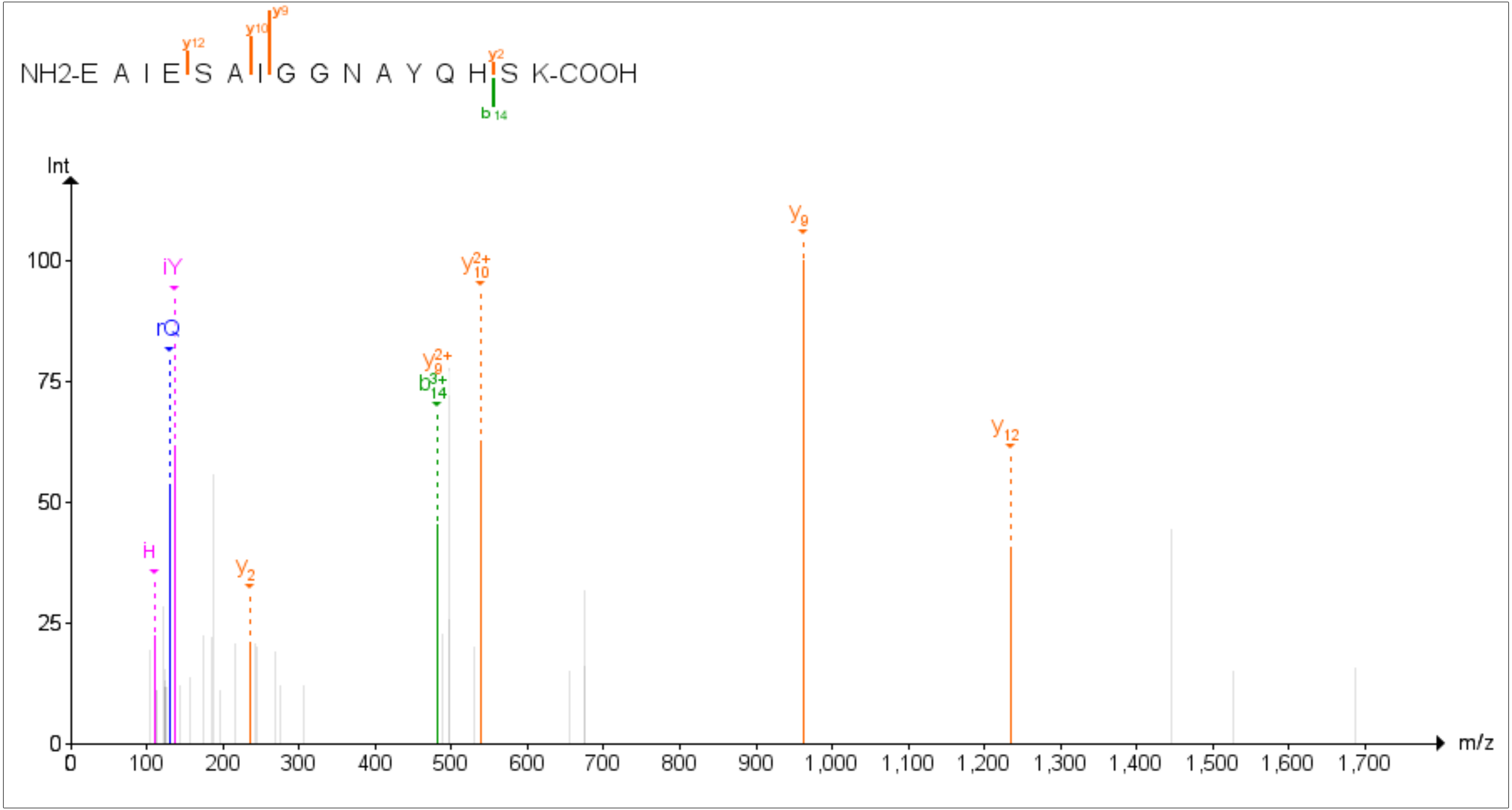

NH2-E A I E S A I G G N A Y Q H S K-COOH
y12
y10
y9
y2
b14
Int
100
75
50
25
0
iY
rQ
iH
y2
y9 2+
b14 3+
y10 2+
y9
y12
0
100
200
300
400
500
600
700
800
900
1,000
1,100
1,200
1,300
1,400
1,500
1,600
1,700
m/z

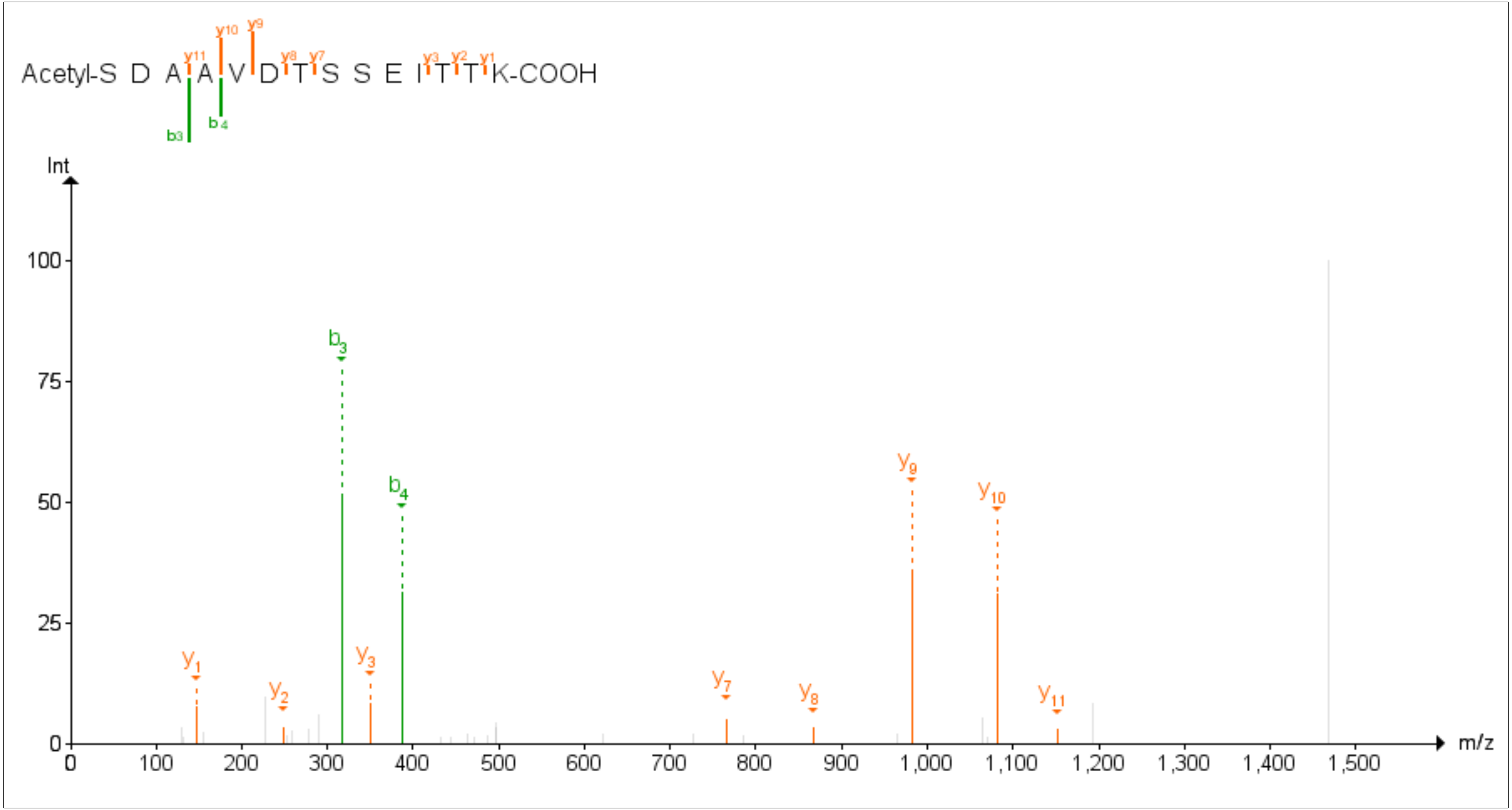

Acetyl-S D A A V D T S S E I T T K-COOH
y11
y10
y9
y8
y7
y3
y2
y1
b3
b4
Int
100
75
50
25
0
b3
b4
y1
y2
y3
y7
y8
y9
y10
y11
0
100
200
300
400
500
600
700
800
900
1,000
1,100
1,200
1,300
1,400
1,500
m/z

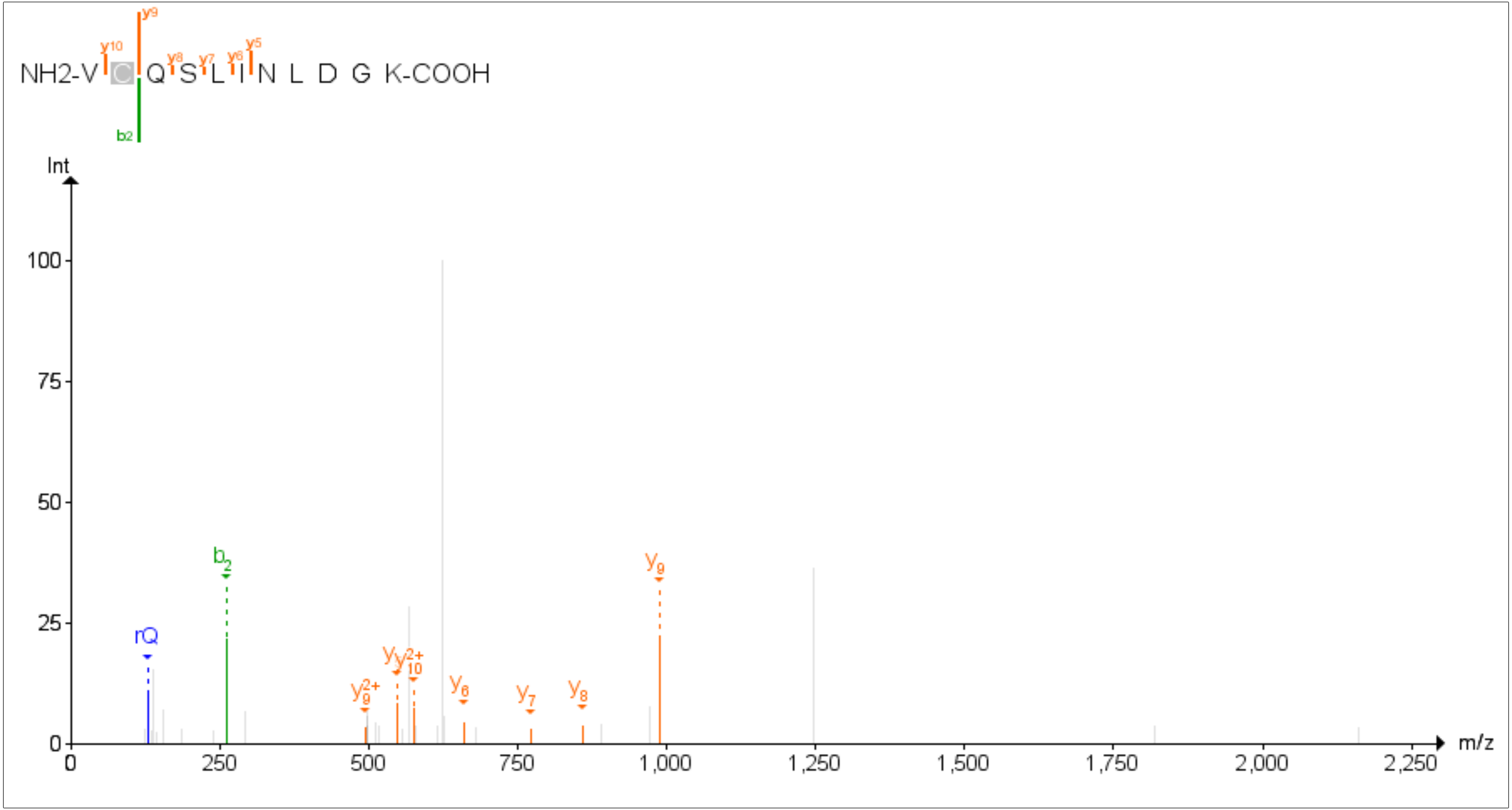

NH2-V C Q S L I N L D G K-COOH
y10
y9
y8
y7
y6
y5
b2
Int
100
75
50
25
0
rQ
b2
y9 2+
y y10 2+
y6
y7
y8
y9
0
250
500
750
1,000
1,250
1,500
1,750
2,000
2,250
m/z

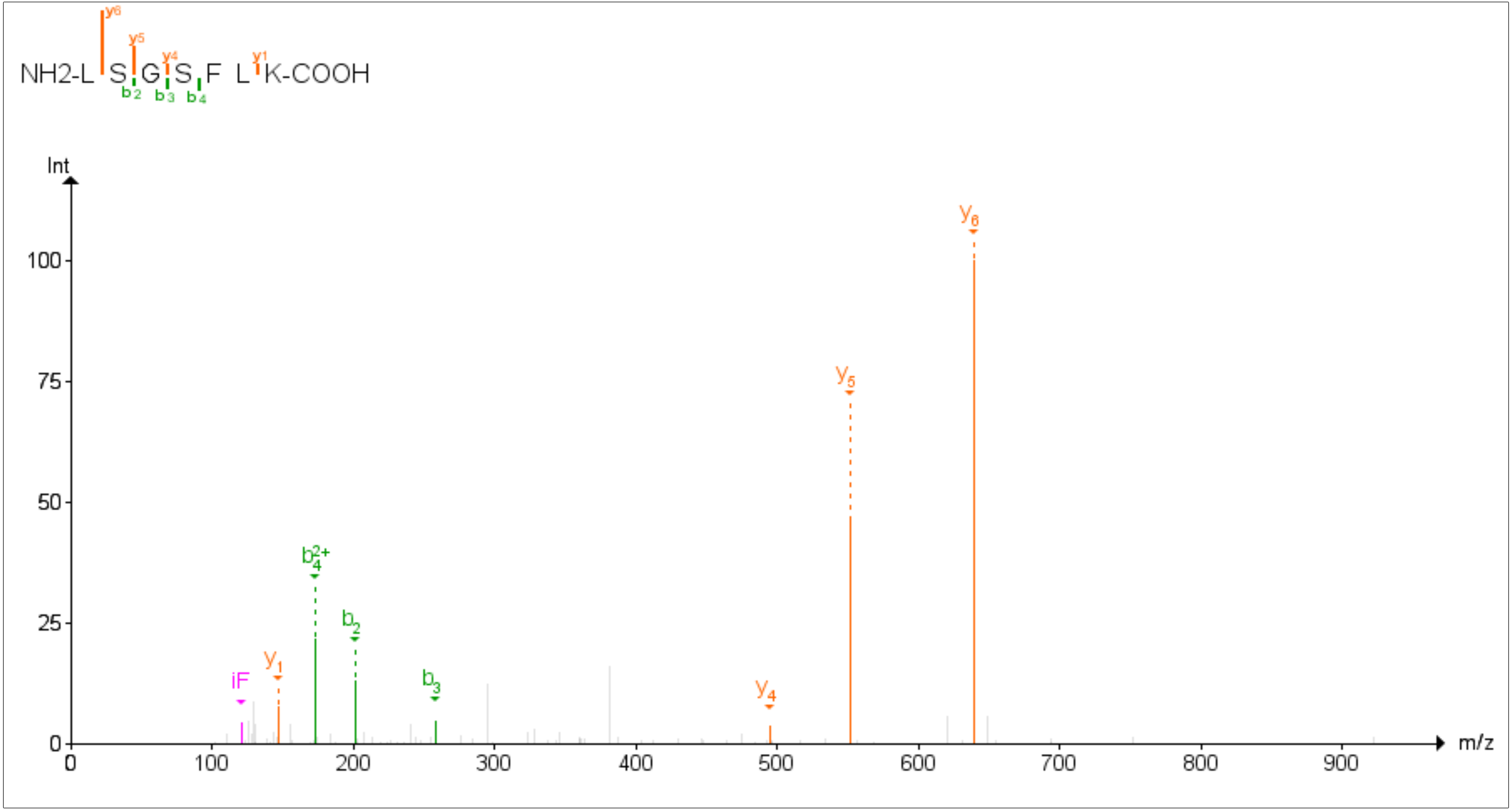

NH2-L S G S F L K-COOH
y6
y5
y4
y1
b2
b3
b4
Int
100
75
50
25
0
y6
y5
b4 2+
b2
y1
iF
b3
y4
0
100
200
300
400
500
600
700
800
900
m/z

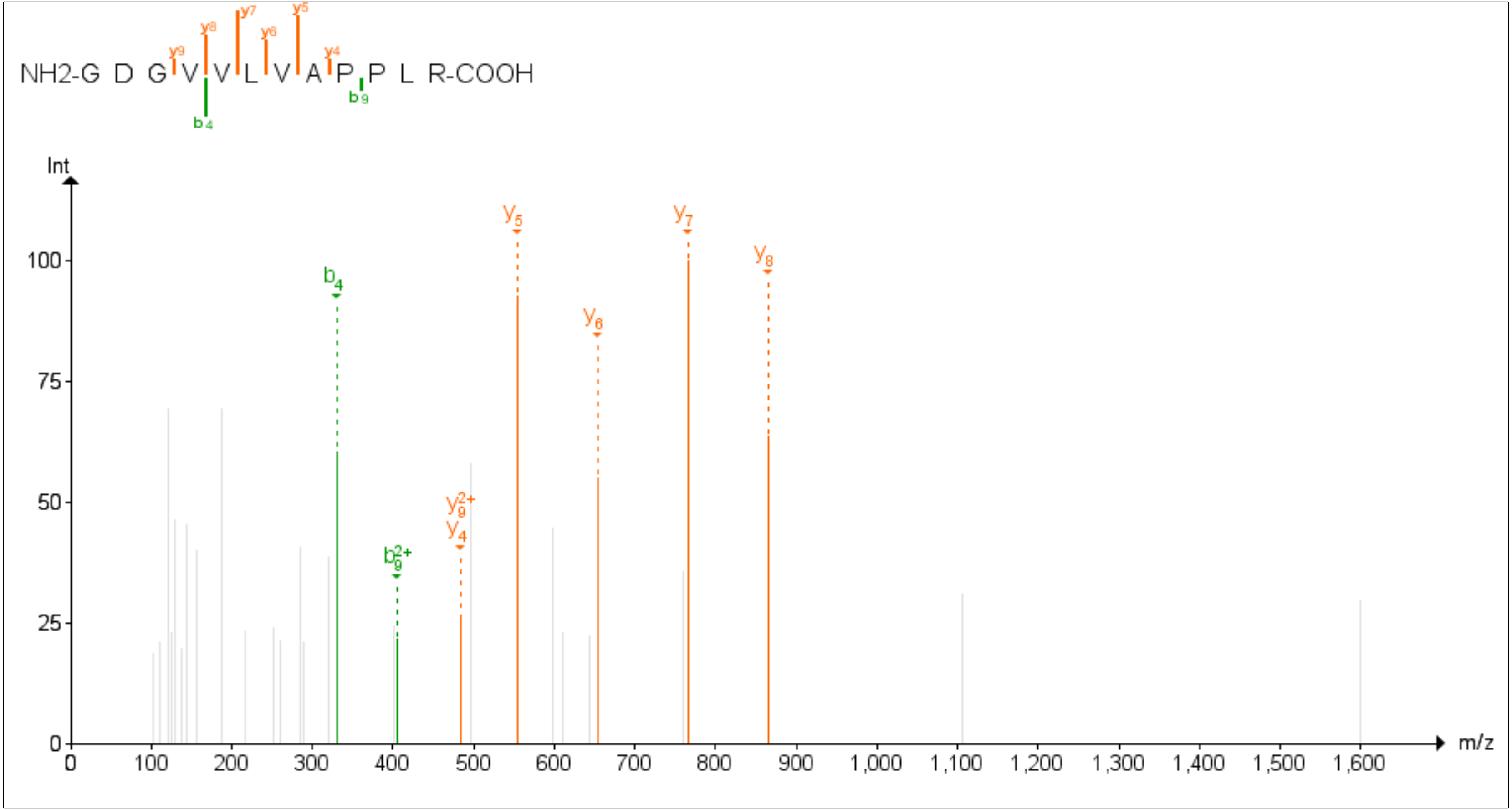

NH2-G D G V V L V A P P L R-COOH
y9 y8 y7 y6 y5 y4
b4 b9
Int
100
75
50
25
0
b4
b_9^{2+}
y_9^{2+}
y4
y5
y6
y7
y8
0 100 200 300 400 500 600 700 800 900 1,000 1,100 1,200 1,300 1,400 1,500 1,600
m/z

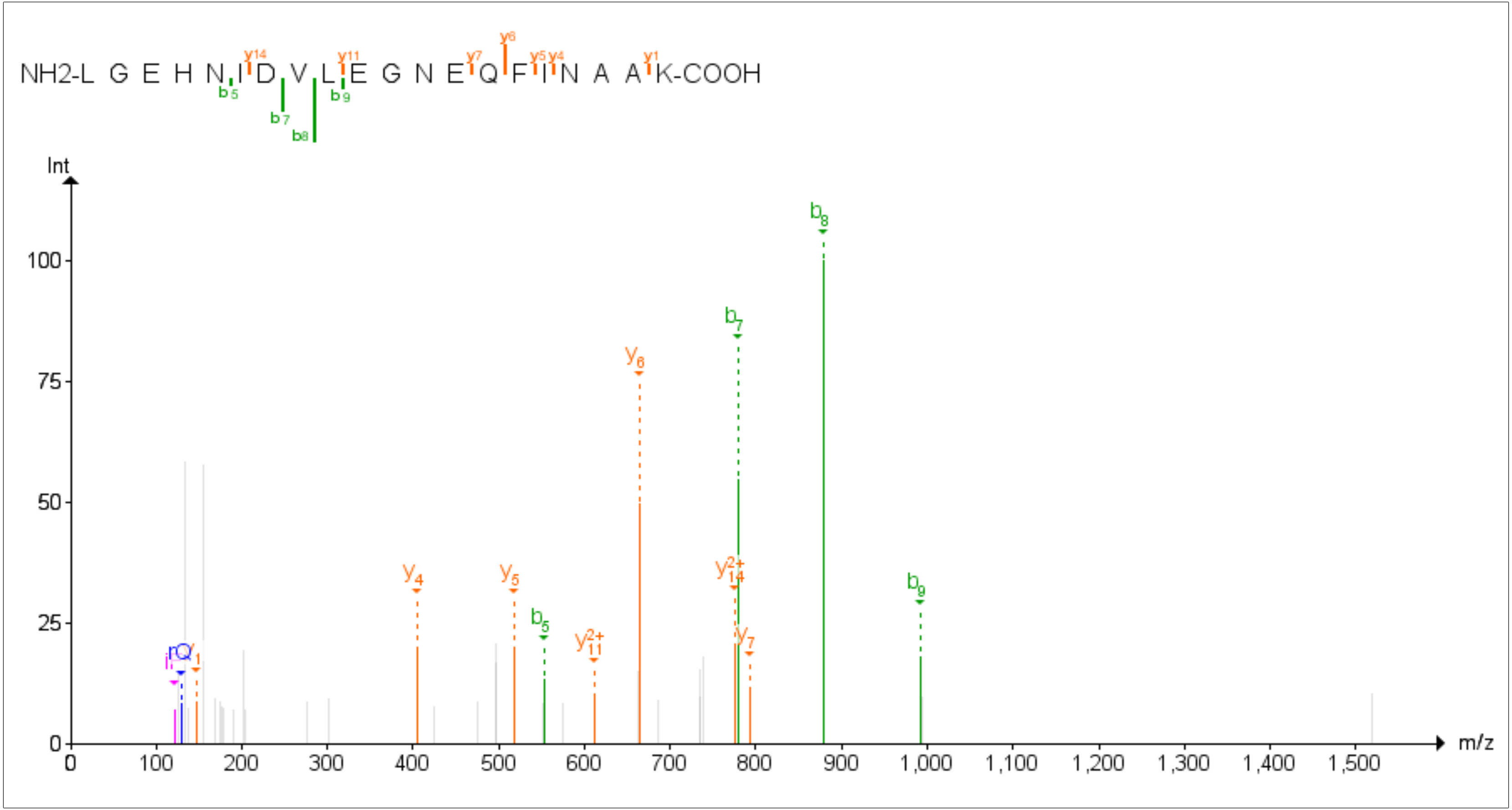

NH2-L G E H N I D V L E G N E Q F I N A A K-COOH
y14 y11 y7 y6 y5 y4 y1
b5 b7 b8 b9
Int
m/z
b8
b7
y6
y4
y5
b5
y2+ 11
y2+ 14
y7
b9
y1

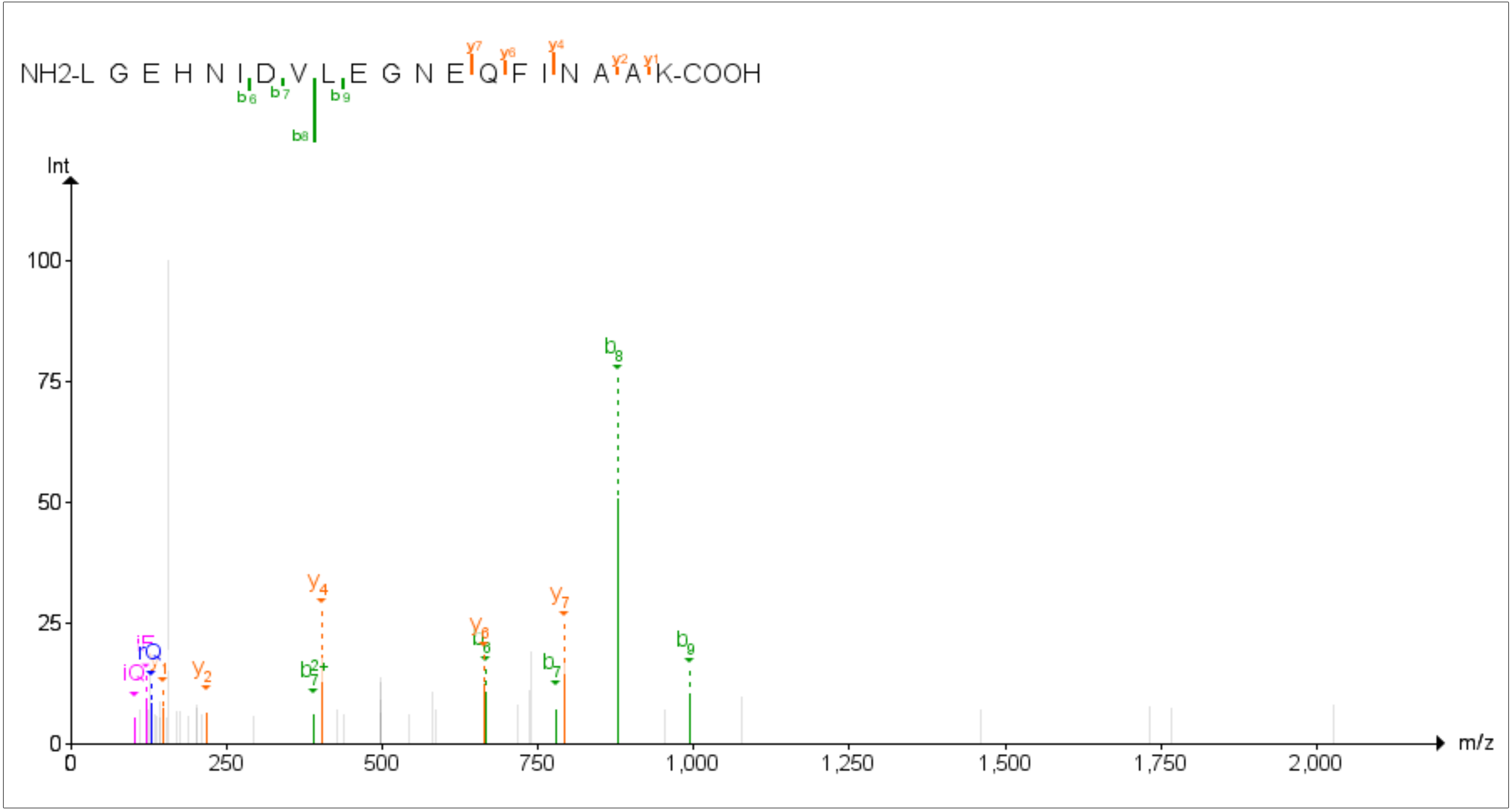

NH2-L G E H N I D V L E G N E Q F I N A A K-COOH
b6 b7 b8 b9
y7 y6 y4 y2 y1
Int
100
75
50
25
0
m/z
0
250
500
750
1,000
1,250
1,500
1,750
2,000
b8
y4
y7
y6
b6
b9
b7
y2
iQ
rQ
iF

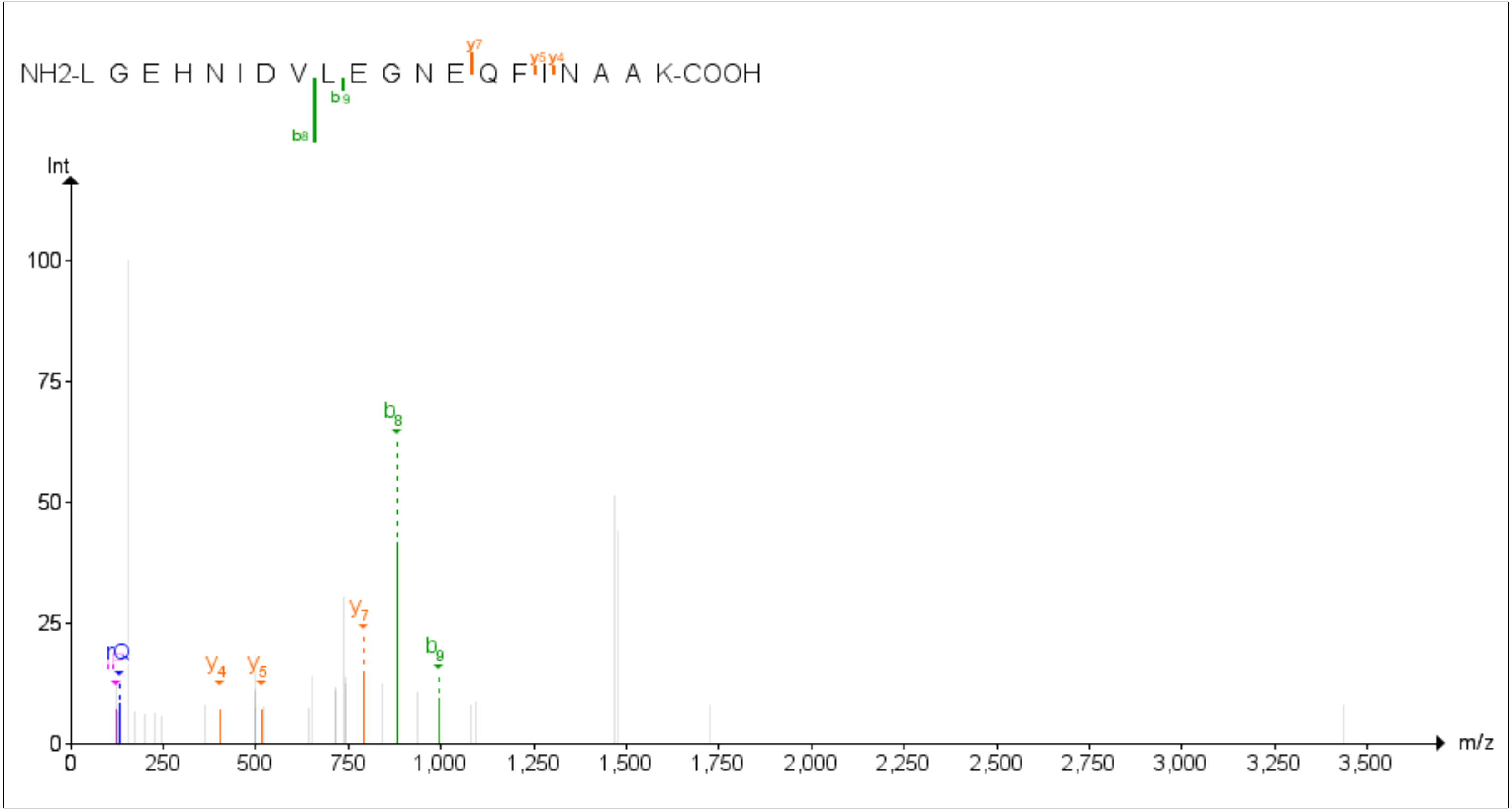
NH2-L G E H N I D V L E G N E Q F I N A A K-COOH
y7
y5
y4
b9
b8
Int
100
75
50
25
0
b8
y7
b9
nQ
y4
y5
0
250
500
750
1,000
1,250
1,500
1,750
2,000
2,250
2,500
2,750
3,000
3,250
3,500
m/z

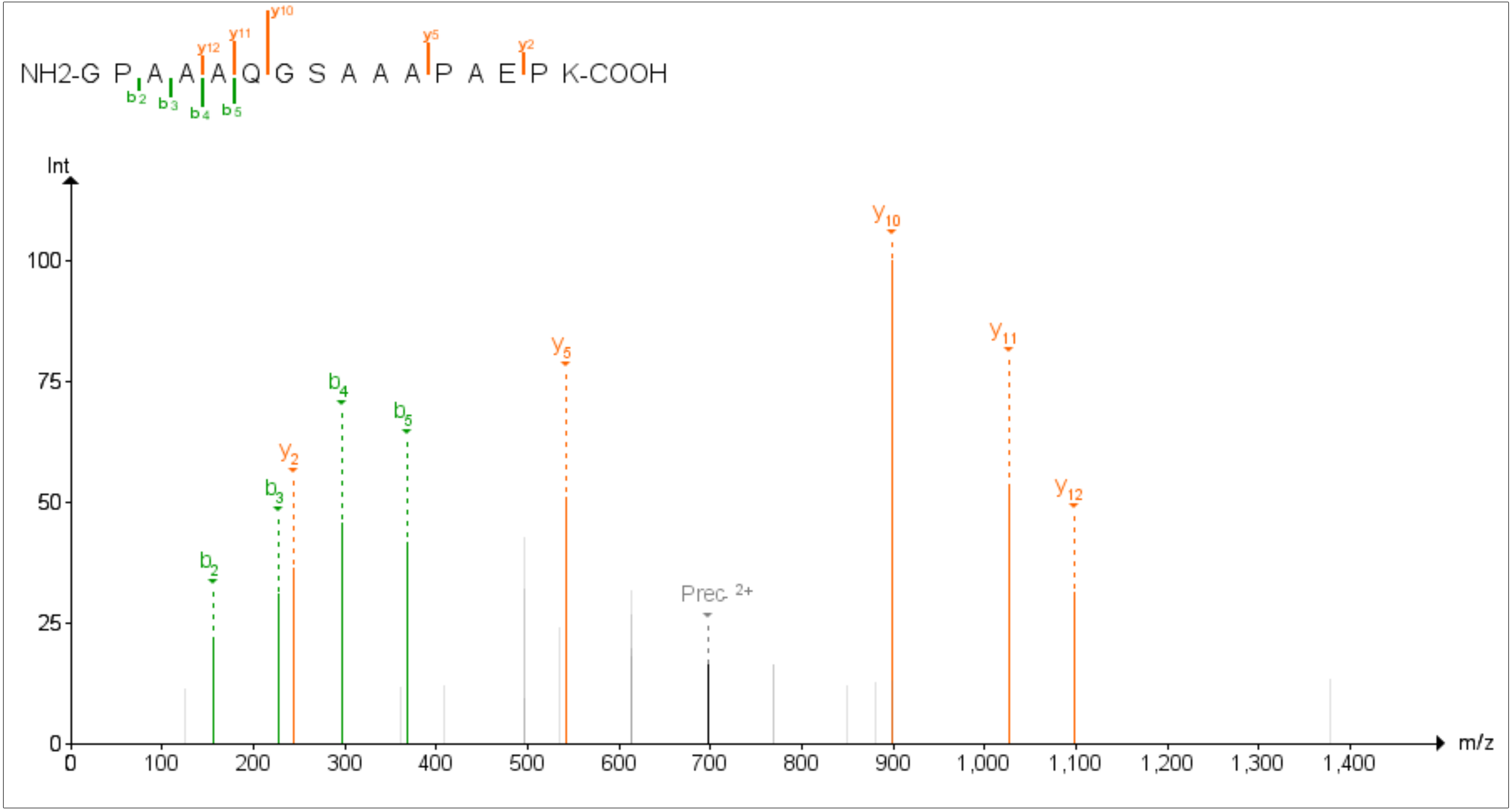

NH2-G P A A A Q G S A A A P A E P K-COOH
y12
y11
y10
y5
y2
b2
b3
b4
b5
Int
100
75
50
25
0
b2
b3
y2
b4
b5
y5
y10
y11
y12
Prec 2+
0
100
200
300
400
500
600
700
800
900
1,000
1,100
1,200
1,300
1,400
m/z

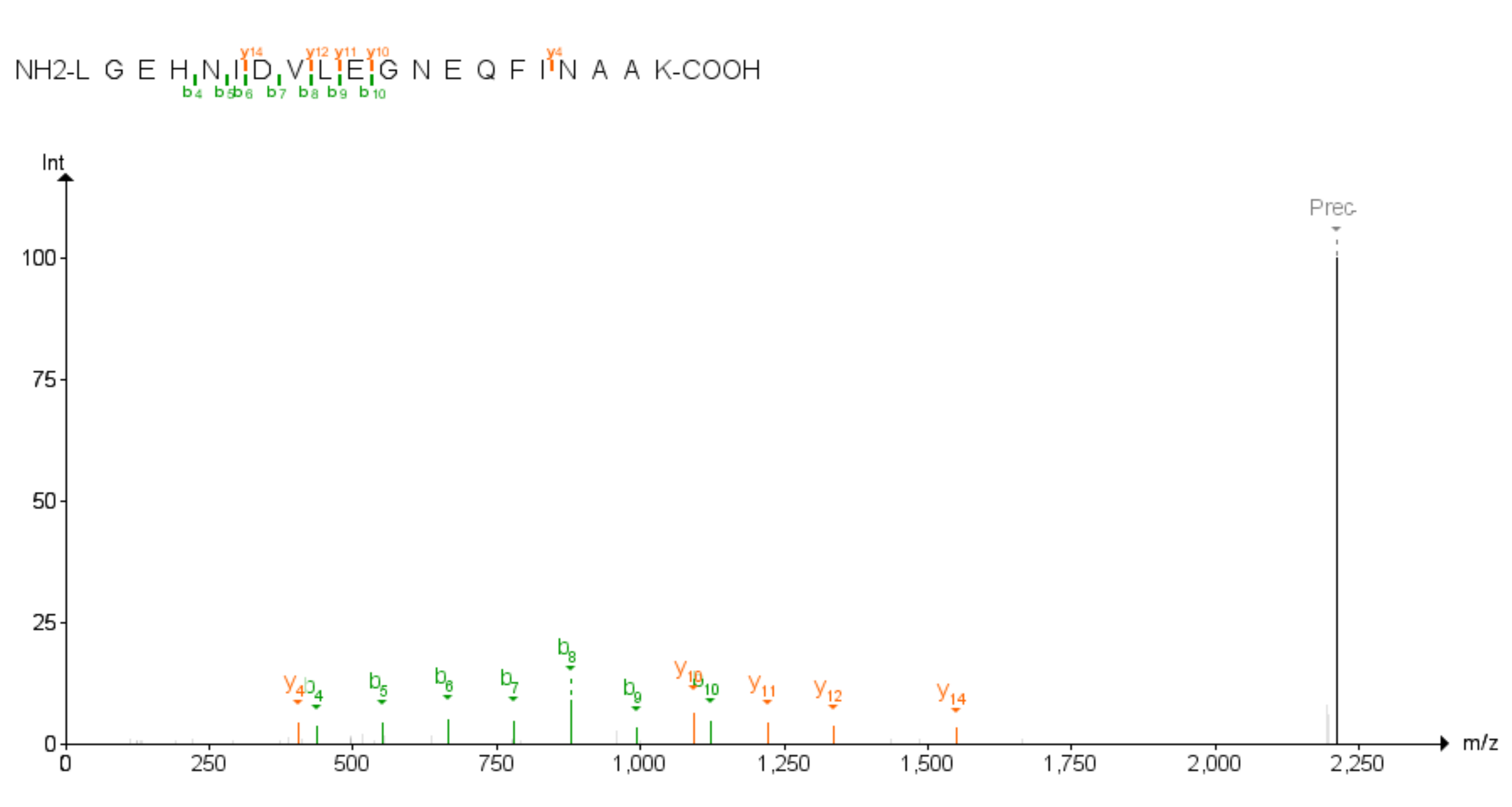
NH2-L G E H N I D V L E G N E Q F I N A A K-COOH
y14 y12 y11 y10 y4
b4 b5 b6 b7 b8 b9 b10
Int
100
75
50
25
0
Prec
y4 b4 b5 b6 b7 b8 b9 y10 b10 y11 y12 y14
0
250
500
750
1,000
1,250
1,500
1,750
2,000
2,250
m/z

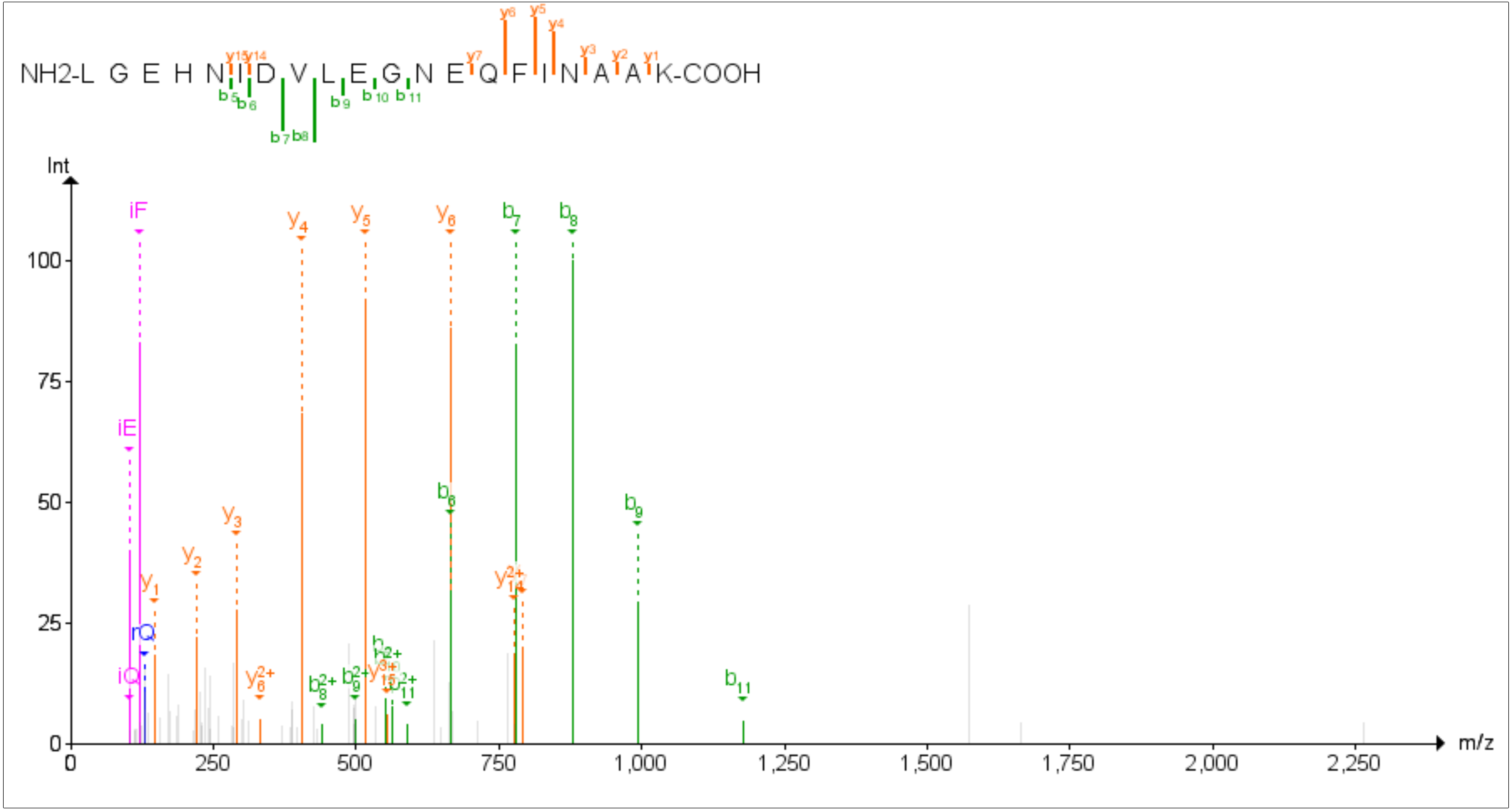
NH2-L G E H N I I D V L E G N E Q F I N A A K-COOH
Int
m/z
0
25
50
75
100
250
500
750
1,000
1,250
1,500
1,750
2,000
2,250
iF
iE
iQ
rQ
y1
y2
y3
y4
y5
y6
b6
b7
b8
b9
b11

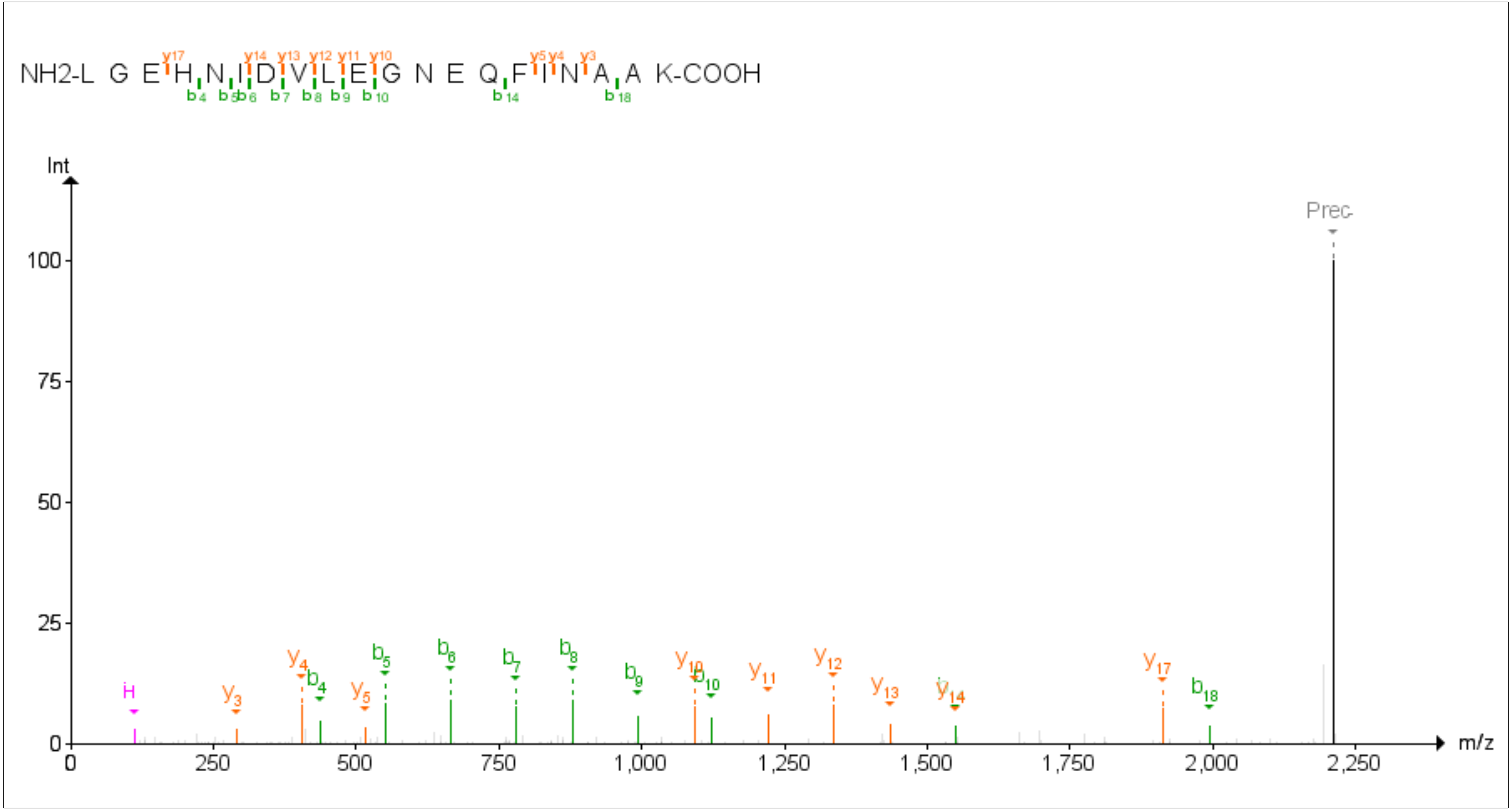
NH2-L G E H N I D V L E G N E Q F I N A A K-COOH
y17 y14 y13 y12 y11 y10 y5 y4 y3
b4 b5 b6 b7 b8 b9 b10 b14 b18
Int
100
75
50
25
0
Prec
H
y3 y4 b4 y5 b5 b6 b7 b8 b9 y10 b10 y11 y12 y13 y14 y17 b18
0
250
500
750
1,000
1,250
1,500
1,750
2,000
2,250
m/z

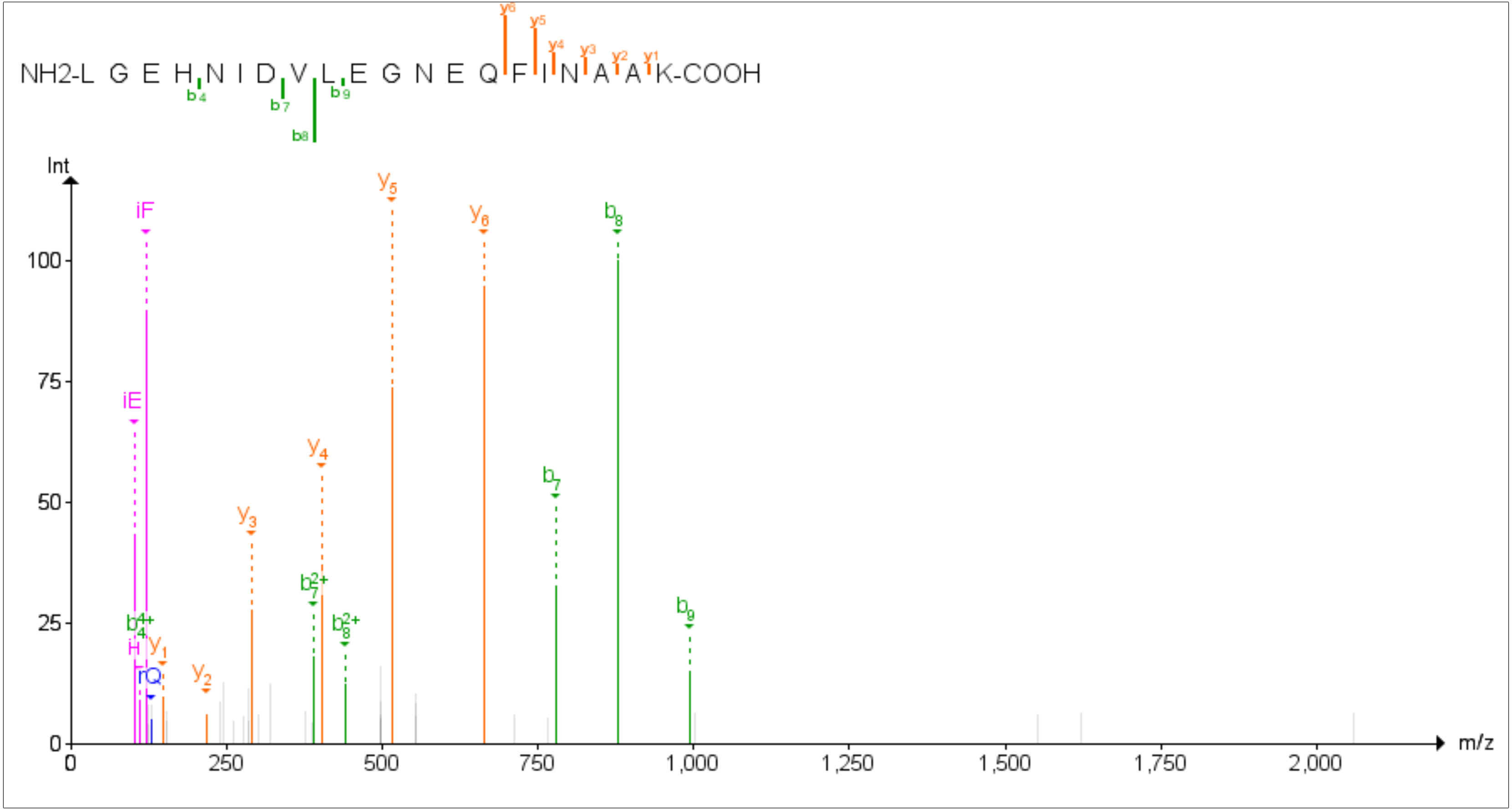
NH2-L G E H N I D V L E G N E Q F I N A A K-COOH
Int
m/z
iF
iE
iH
rQ
y1
y2
y3
y4
y5
y6
b4 2+
b7 2+
b8 2+
b7
b8
b9
0
25
50
75
100
250
500
750
1,000
1,250
1,500
1,750
2,000

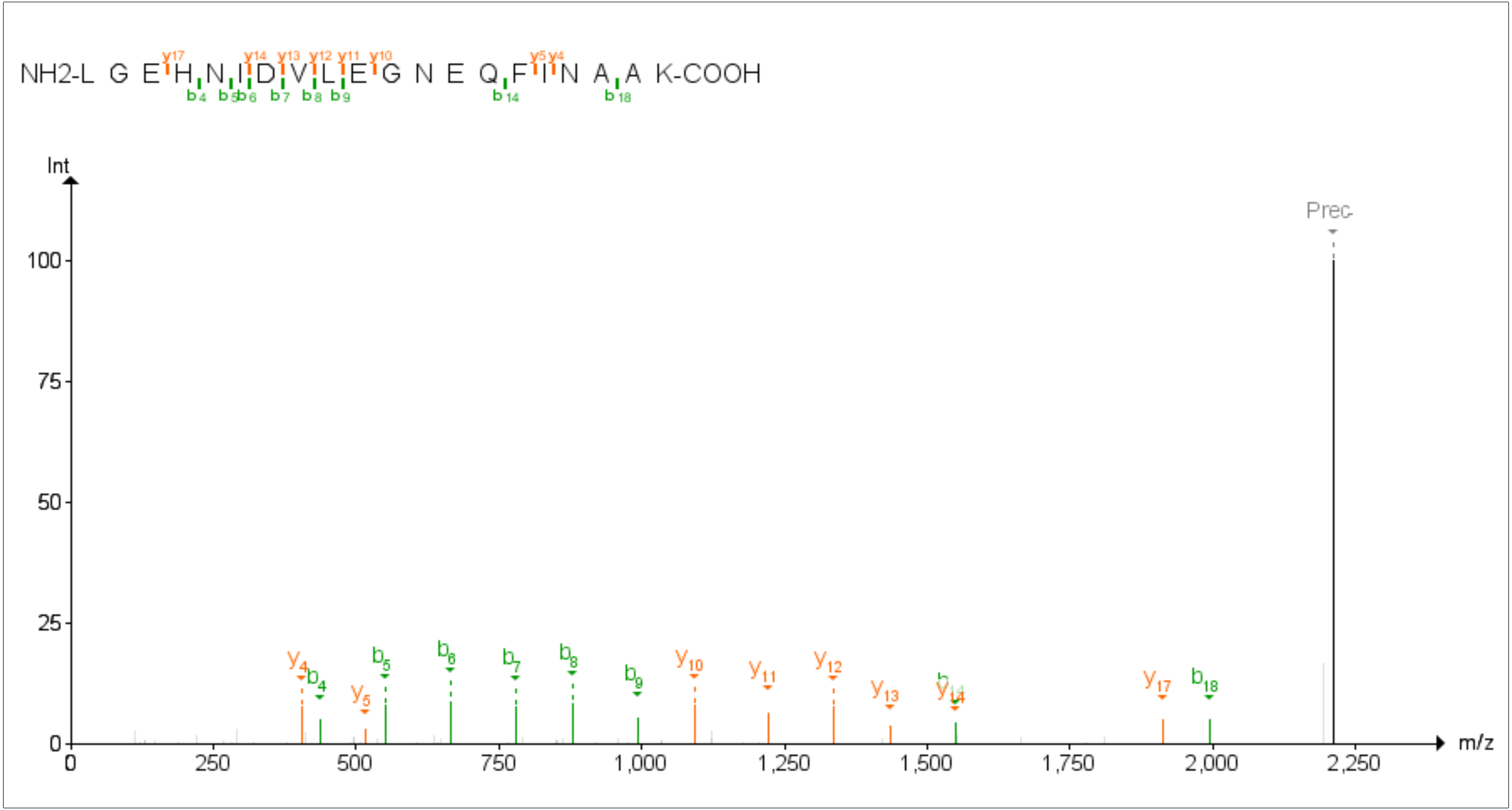

NH2-L G E H N I D V L E G N E Q F I N A A K-COOH
y17 y14 y13 y12 y11 y10 y5 y4
b4 b5 b6 b7 b8 b9 b14 b18
Int
Prec
100
75
50
25
0
y4 b4 y5 b5 b6 b7 b8 b9 y10 y11 y12 y13 b14 y14 y17 b18
0
250
500
750
1,000
1,250
1,500
1,750
2,000
2,250
m/z

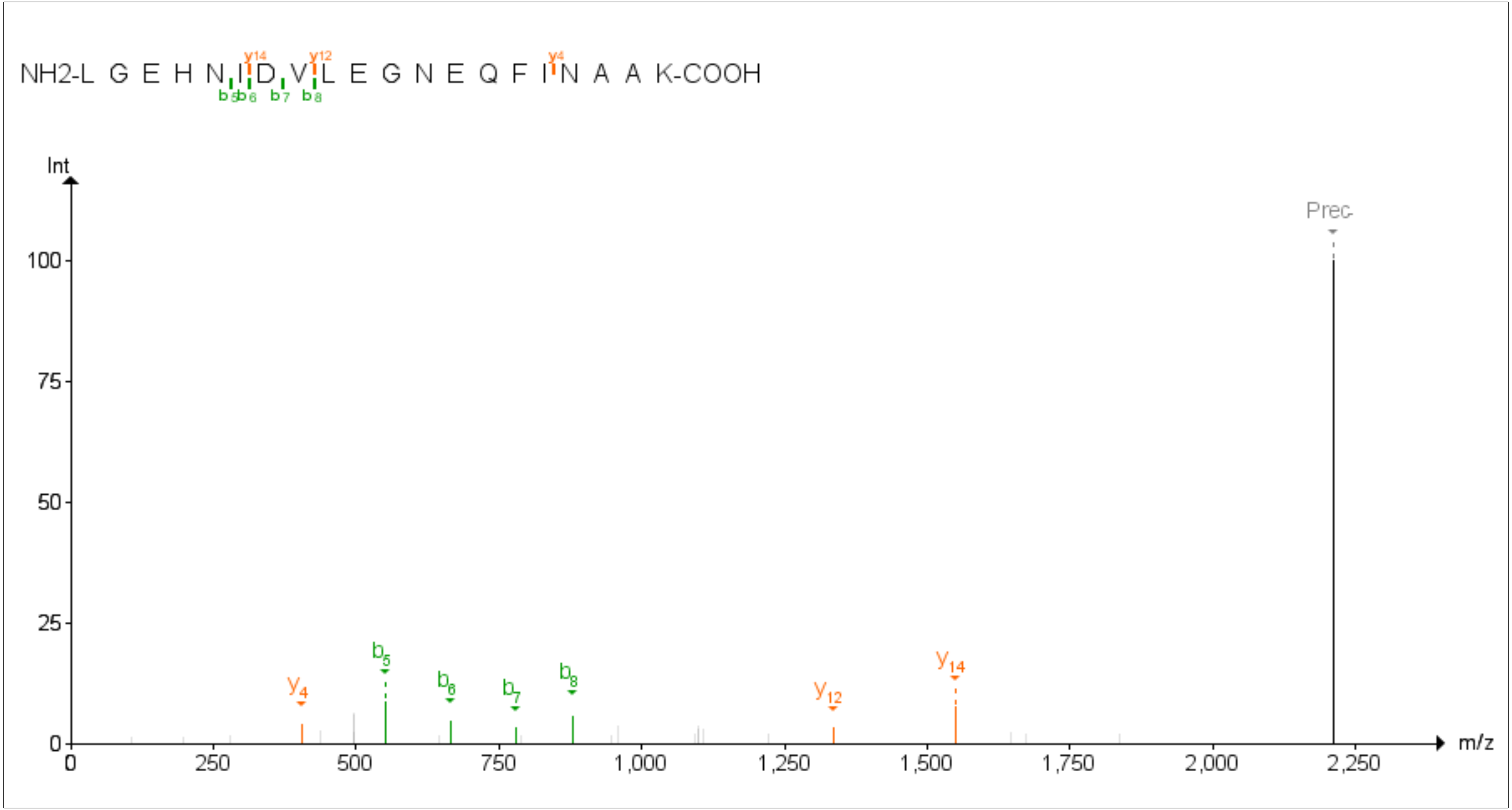

NH2-L G E H N I D V L E G N E Q F I N A A K-COOH
y14
y12
y4
b5 b6 b7 b8
Int
100
75
50
25
0
Prec
y4
b5
b6
b7
b8
y12
y14
0
250
500
750
1,000
1,250
1,500
1,750
2,000
2,250
m/z

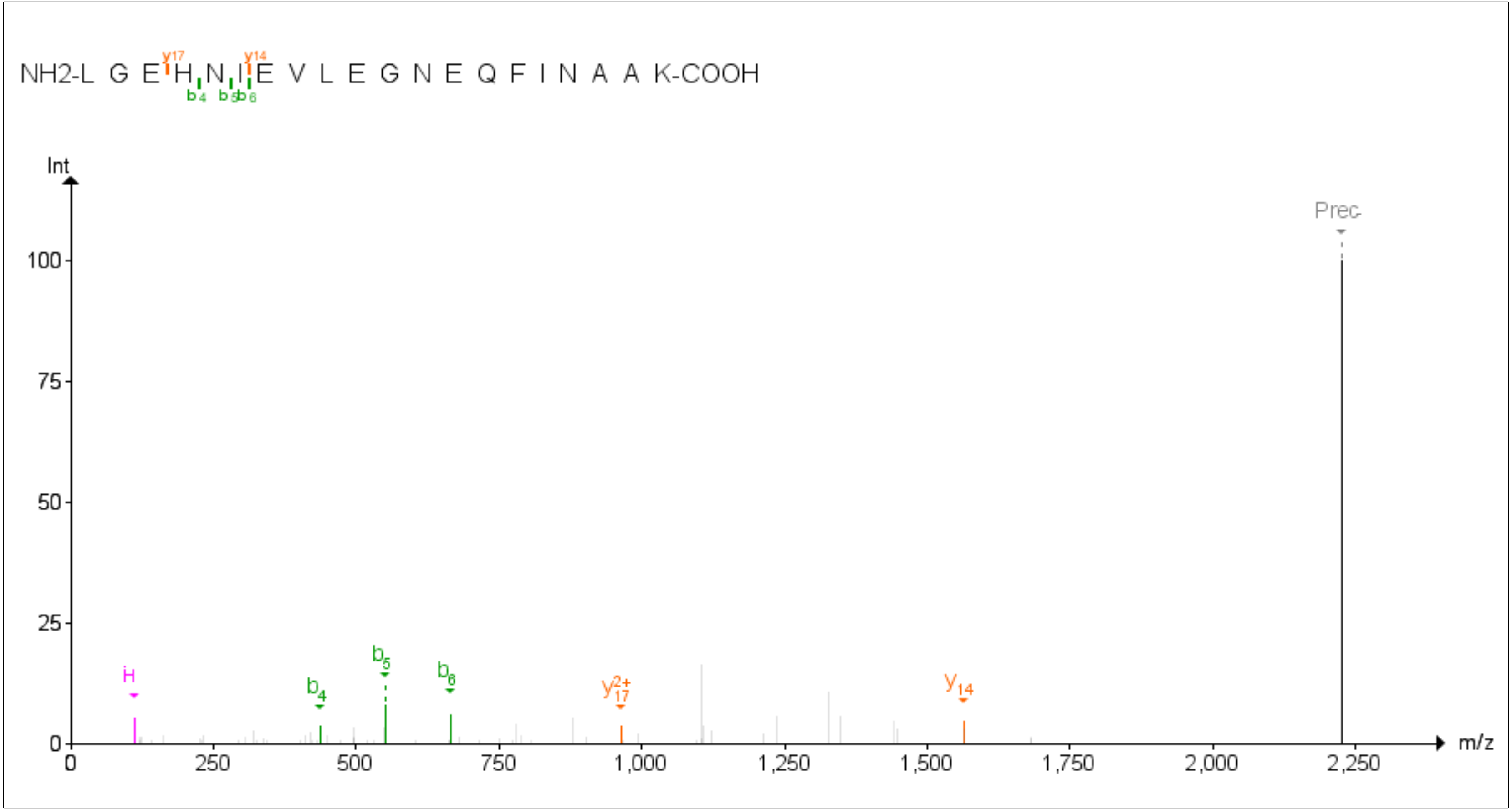

NH2-L G E H N I E V L E G N E Q F I N A A K-COOH
y17
y14
b4
b5
b6
Int
Prec.
100
75
50
25
0
H
b4
b5
b6
y17 2+
y14
0
250
500
750
1,000
1,250
1,500
1,750
2,000
2,250
m/z

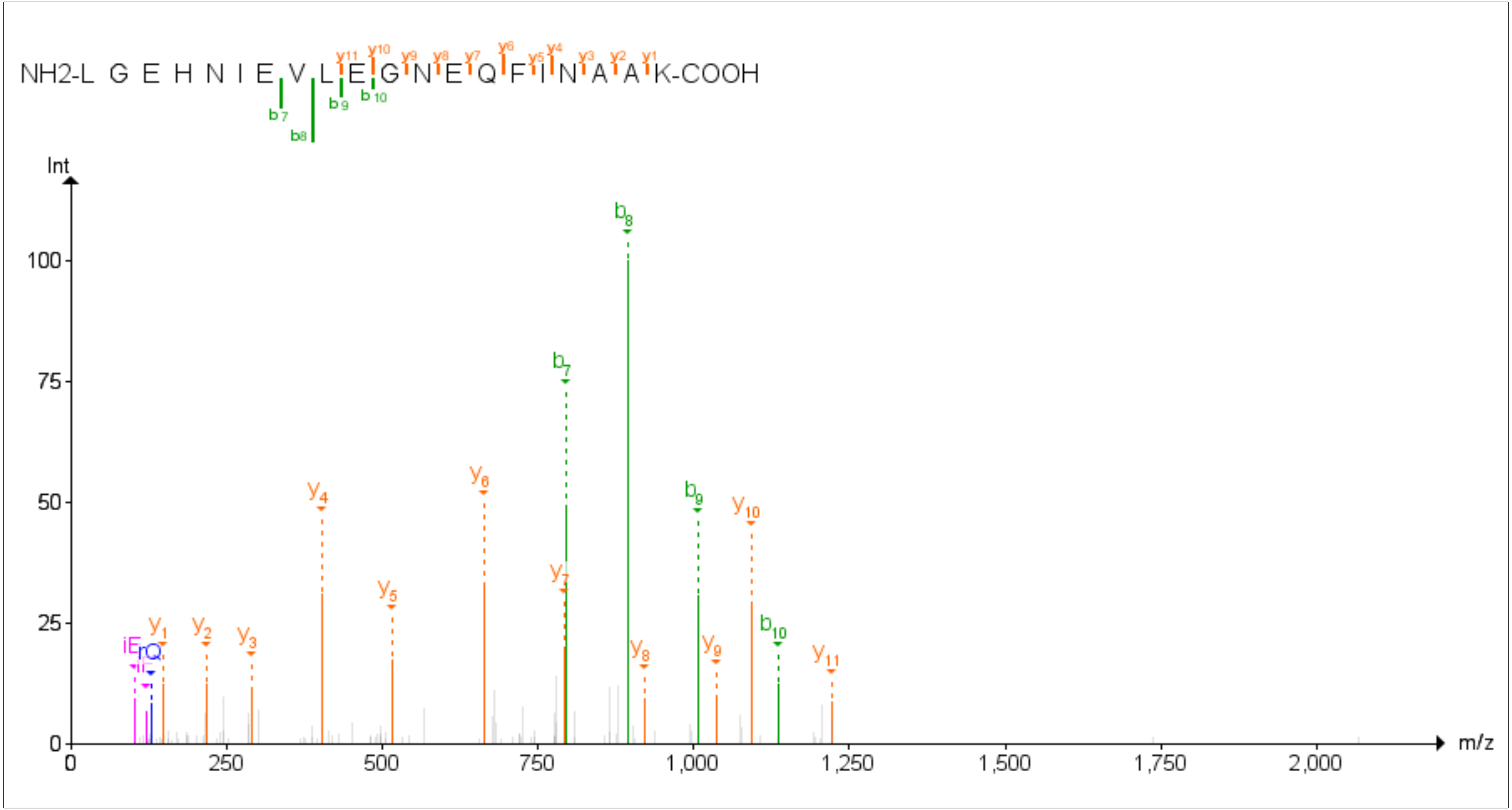
NH2-L G E H N I E V L E G N E Q F I N A A K-COOH
y11 y10 y9 y8 y7 y6 y5 y4 y3 y2 y1
b7 b8 b9 b10
Int
100
75
50
25
0
m/z
0
250
500
750
1,000
1,250
1,500
1,750
2,000
b8
b7
b9
b10
y1
y2
y3
y4
y5
y6
y7
y8
y9
y10
y11
iE
rQ
iF

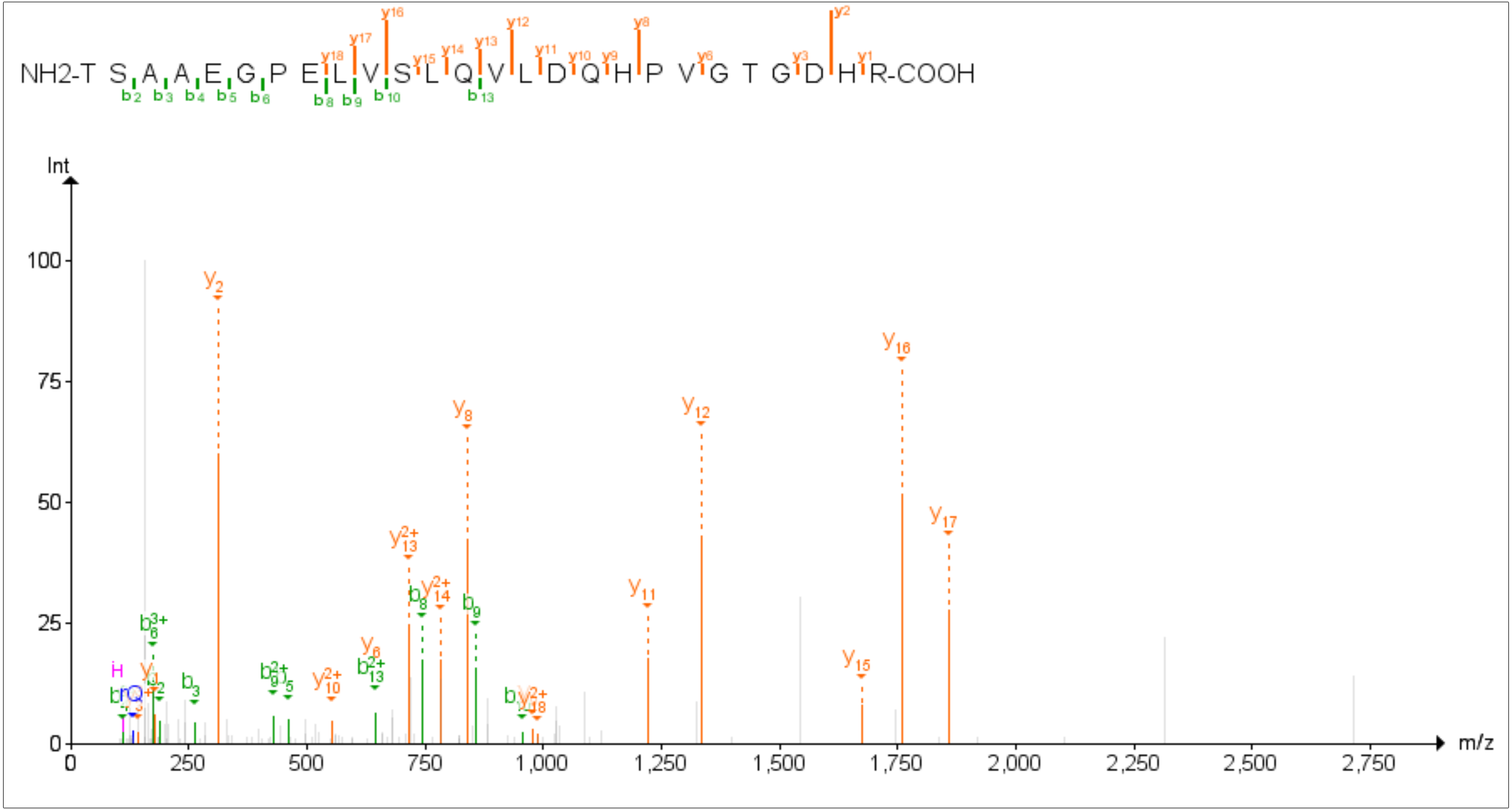
NH2-T S A A E G P E L V S L Q V L D Q H P V G T G D H R-COOH
Int
m/z
100
75
50
25
0
250
500
750
1,000
1,250
1,500
1,750
2,000
2,250
2,500
2,750

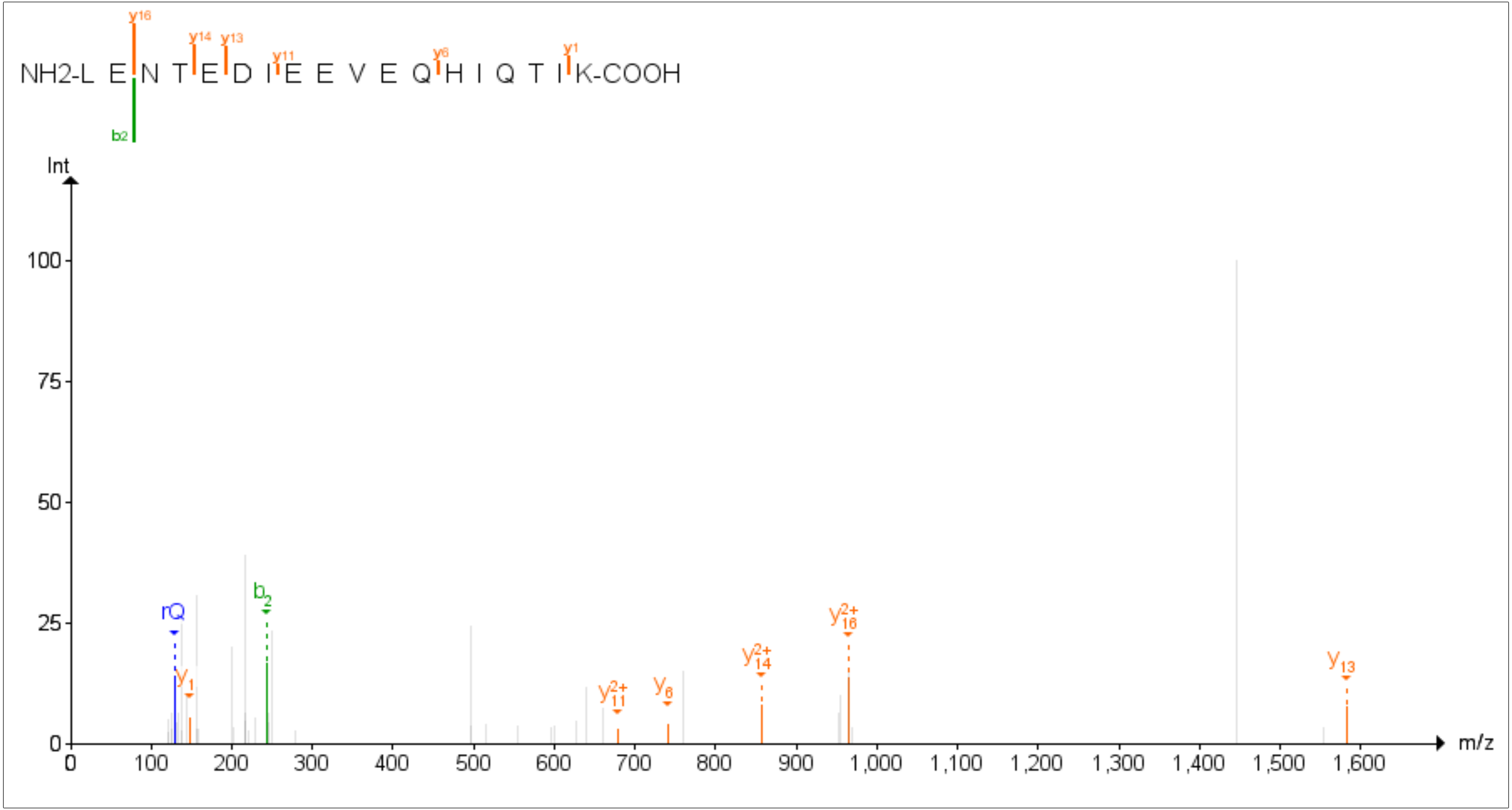

NH2-L E N T E D I E E V E Q H I Q T I K-COOH
y16
y14
y13
y11
y6
y1
b2
Int
100
75
50
25
0
rQ
b2
y1
y11 2+
y6
y14 2+
y16 2+
y13
0
100
200
300
400
500
600
700
800
900
1,000
1,100
1,200
1,300
1,400
1,500
1,600
m/z

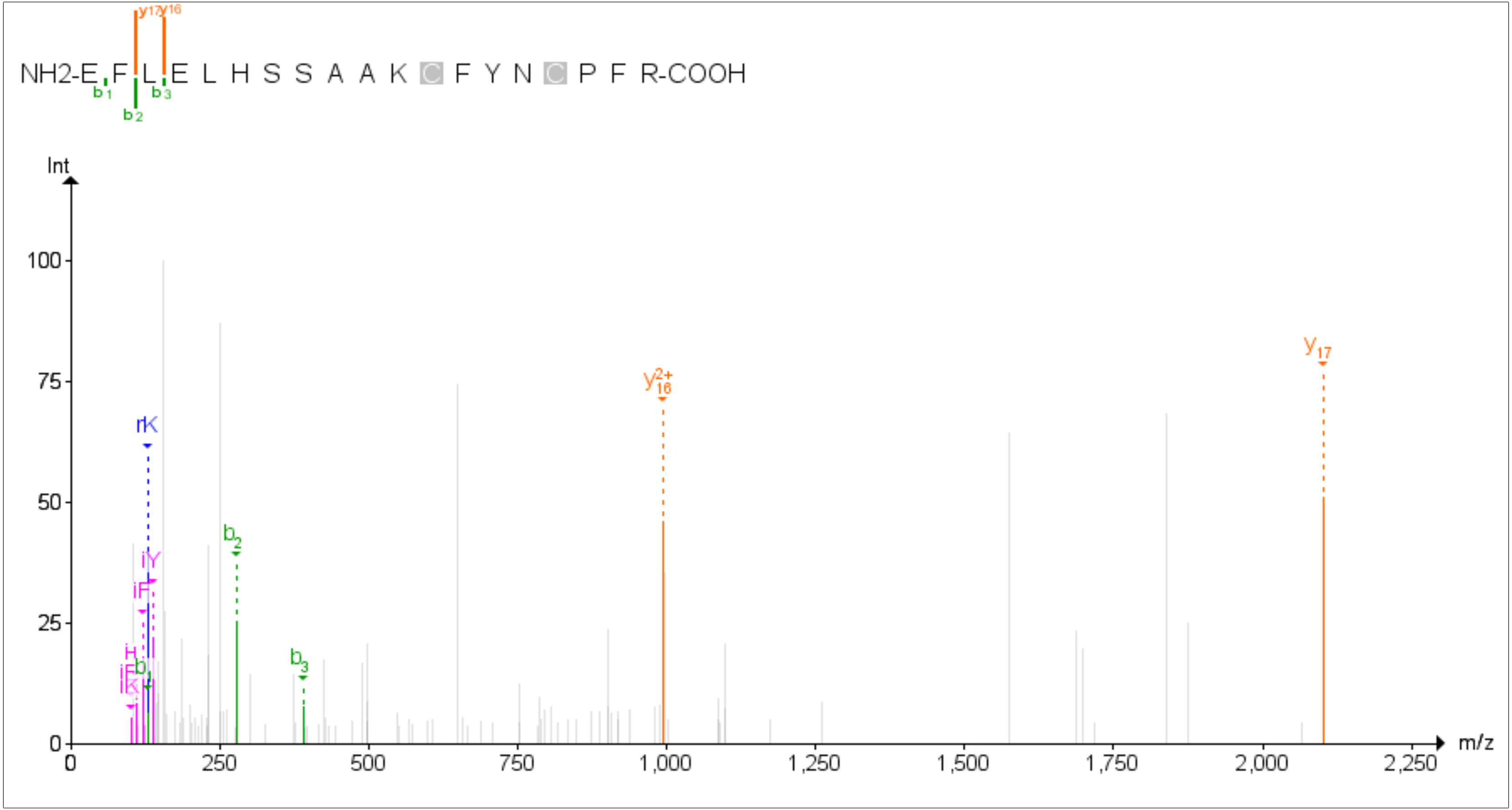

NH2-E F L E L H S S A A K C F Y N C P F R-COOH
y17 y16
b1 b2 b3
Int
100
75
50
25
0
rK
iY
iF
iH
iF
iK
b1
b2
b3
y16 2+
y17
0
250
500
750
1,000
1,250
1,500
1,750
2,000
2,250
m/z

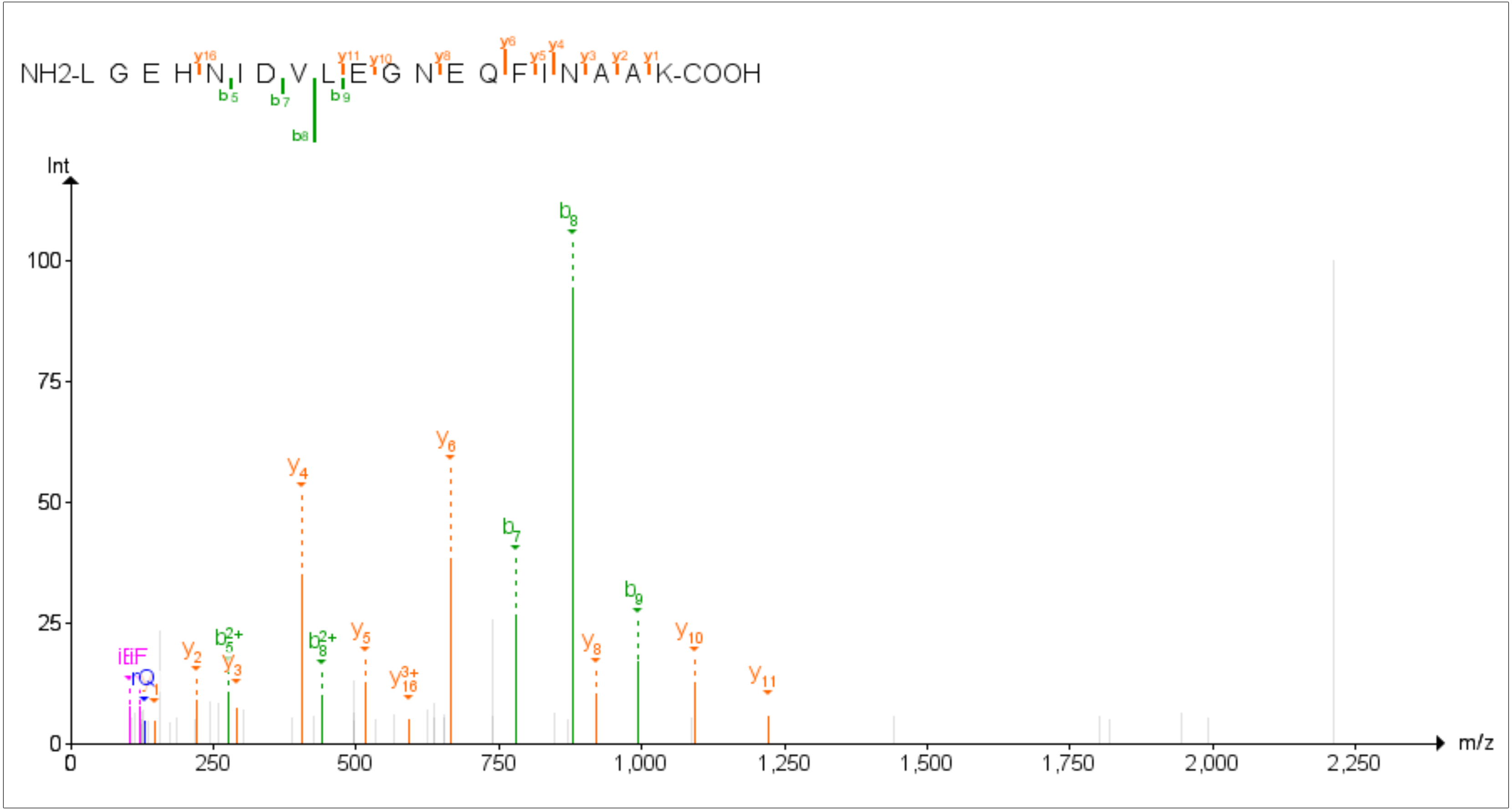
NH2-L G E H N I D V L E G N E Q F I N A A K-COOH
y16 y11 y10 y8 y6 y5 y4 y3 y2 y1
b5 b7 b8 b9
Int
m/z
0
25
50
75
100
0
250
500
750
1,000
1,250
1,500
1,750
2,000
2,250
b8
y6
y4
b7
b9
y10
y8
y11
y5
y16 3+
b8 2+
b5 2+
y2
y3
iEiF
rQ

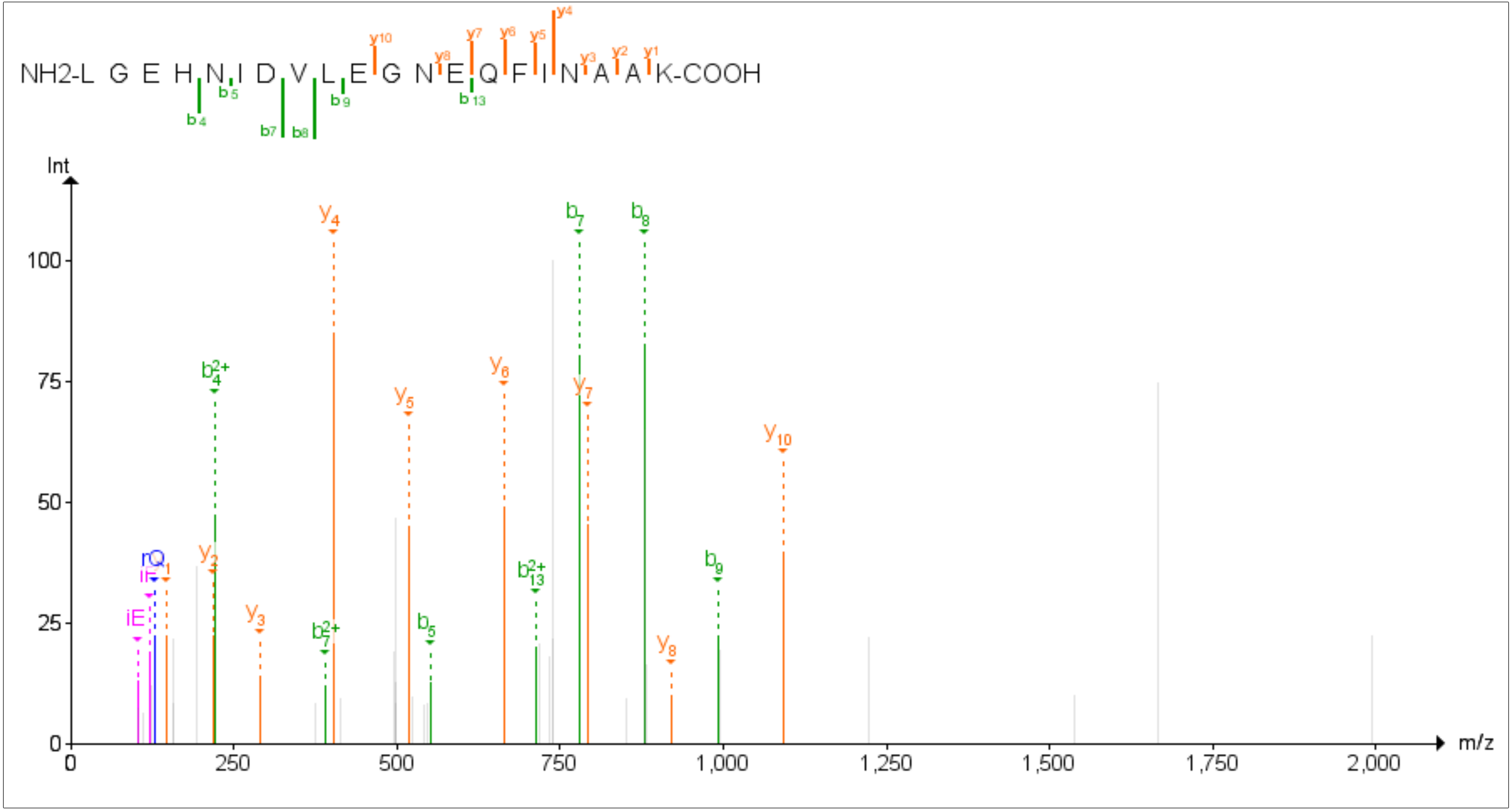

NH2-L G E H N I D V L E G N E Q F I N A A K-COOH
Int
m/z
0
25
50
75
100
250
500
750
1,000
1,250
1,500
1,750
2,000

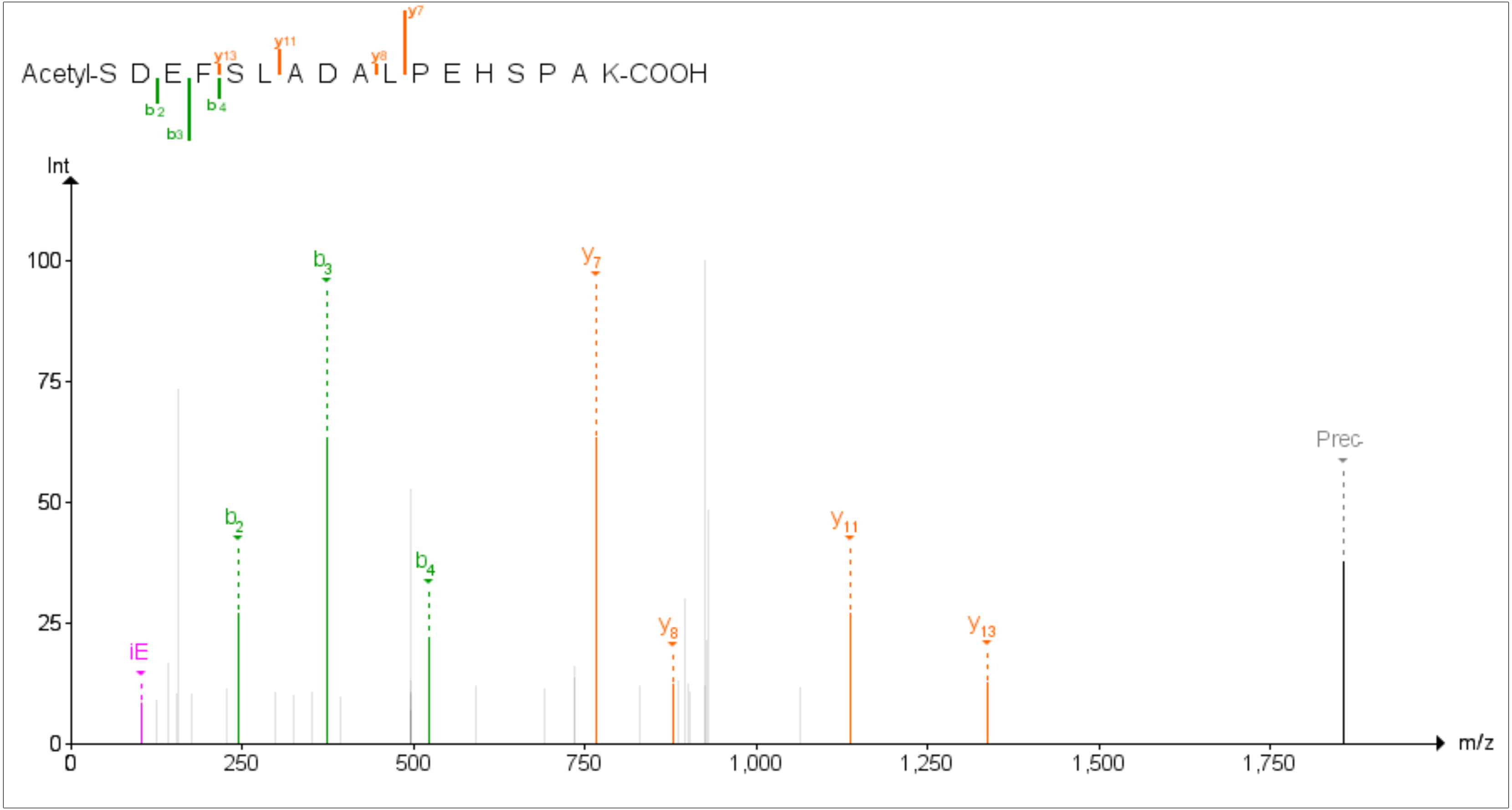

Acetyl-S D E F S L A D A L P E H S P A K-COOH
y7
y11
y13
y8
b2
b3
b4
Int
100
75
50
25
0
b3
y7
b2
b4
y11
y8
y13
iE
Prec
0
250
500
750
1,000
1,250
1,500
1,750
m/z

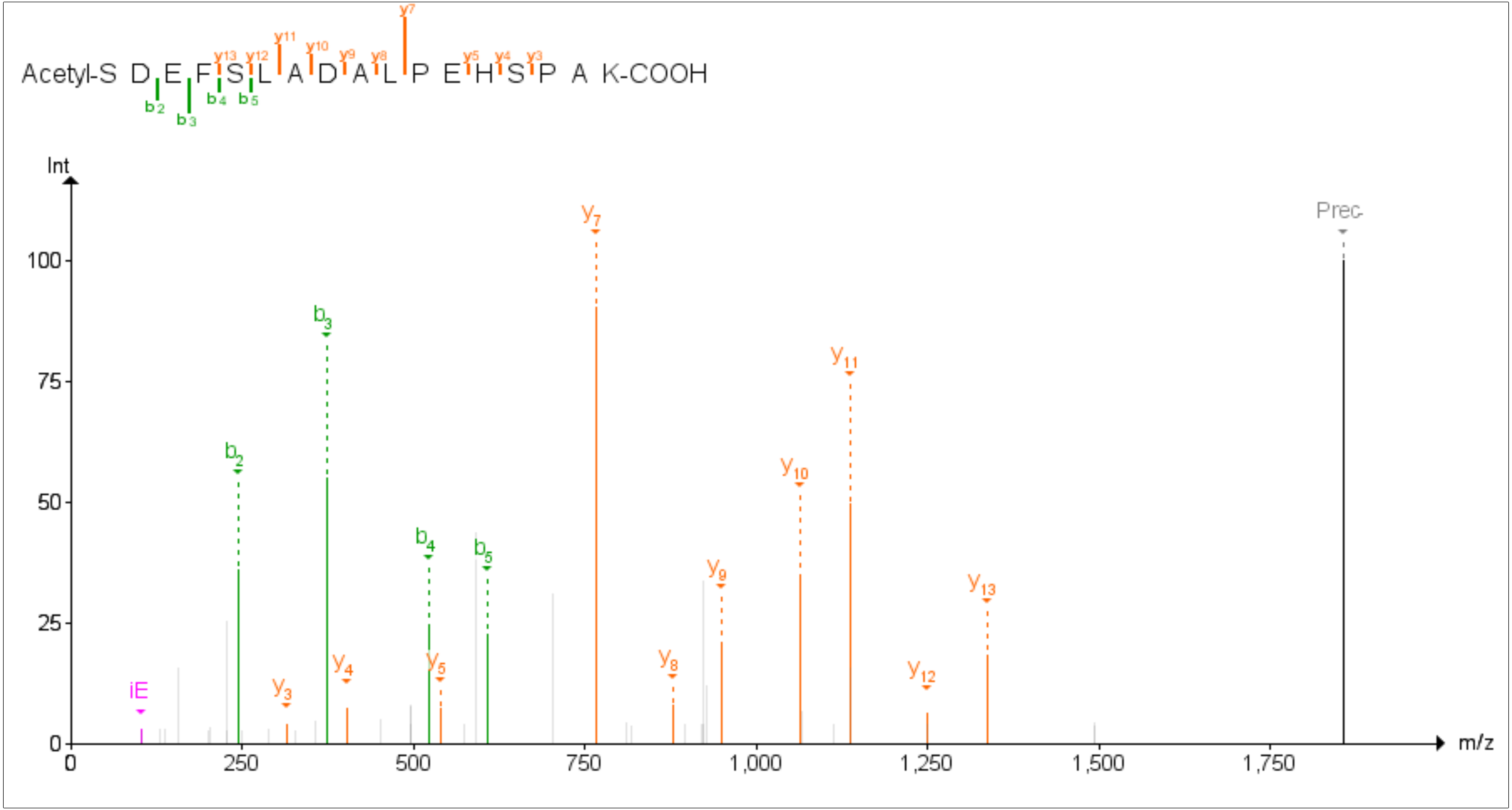

Acetyl-S D E F S L A D A L P E H S P A K-COOH
y13 y12 y11 y10 y9 y8 y7 y5 y4 y3
b2 b3 b4 b5
Int
m/z
Prec.
y7 y11 y10 y9 y13 y8 y12 y3 y4 y5
b2 b3 b4 b5
iE
0 25 50 75 100
0 250 500 750 1,000 1,250 1,500 1,750

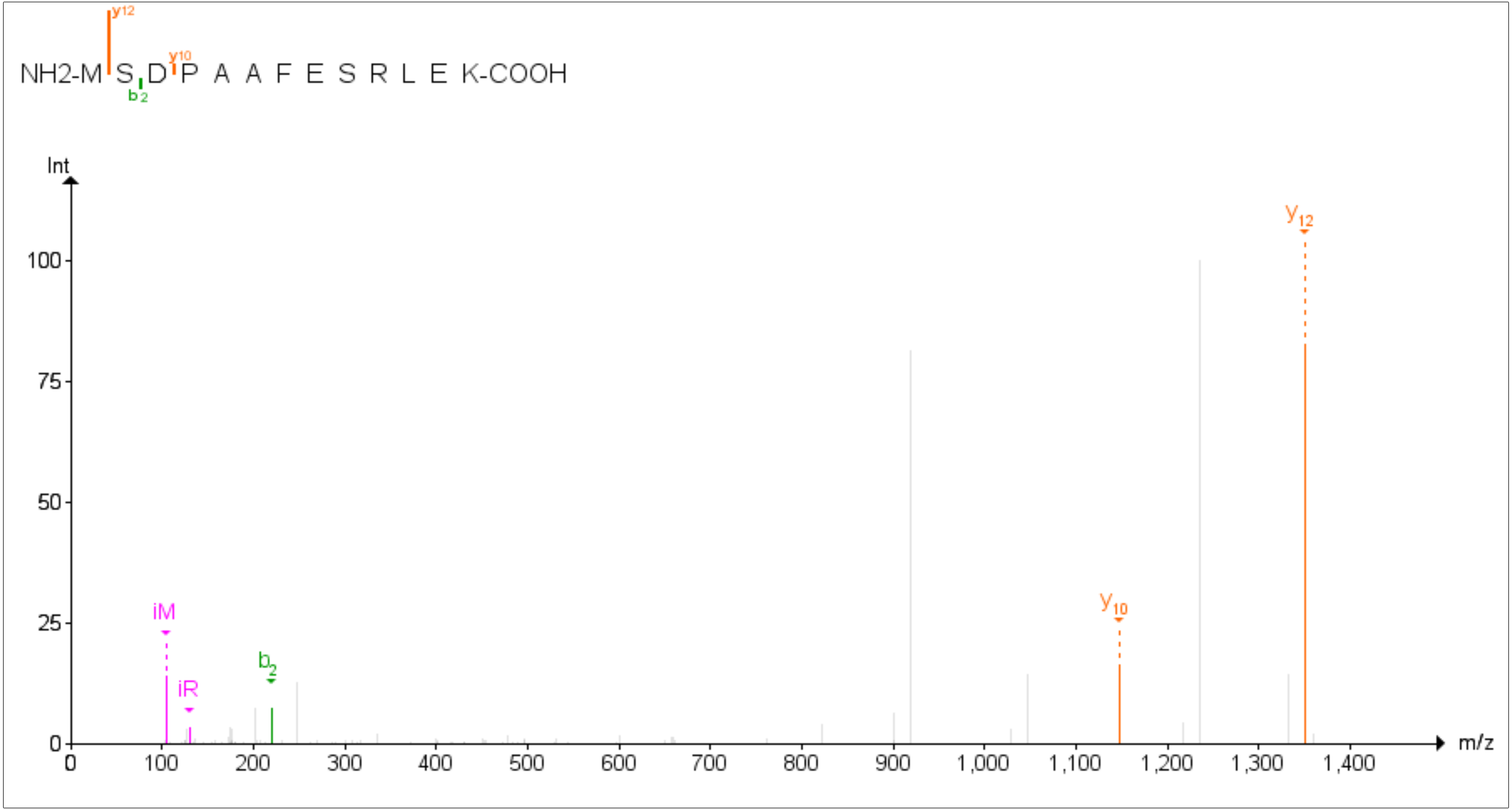

y12
y10
NH2-M S D P A A F E S R L E K-COOH
b 2
Int
100
75
50
25
0
y12
y10
iM
iR
b2
0
100
200
300
400
500
600
700
800
900
1,000
1,100
1,200
1,300
1,400
m/z

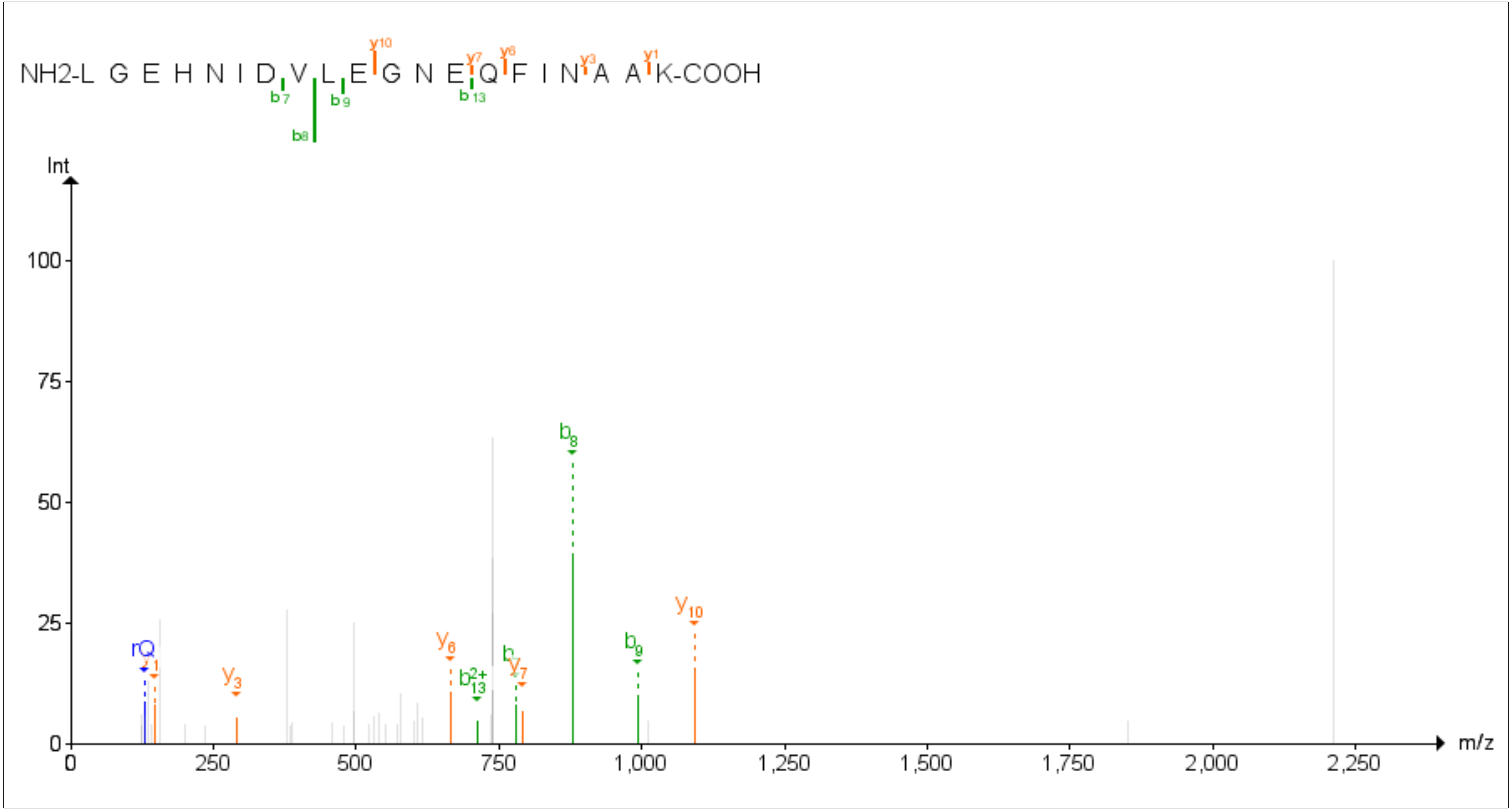
NH2-L G E H N I D V L E G N E Q F I N A A K-COOH
Int
m/z
100
75
50
25
0
250
500
750
1,000
1,250
1,500
1,750
2,000
2,250
b8
b9
b7
y10
y6
y7
y3
rQ

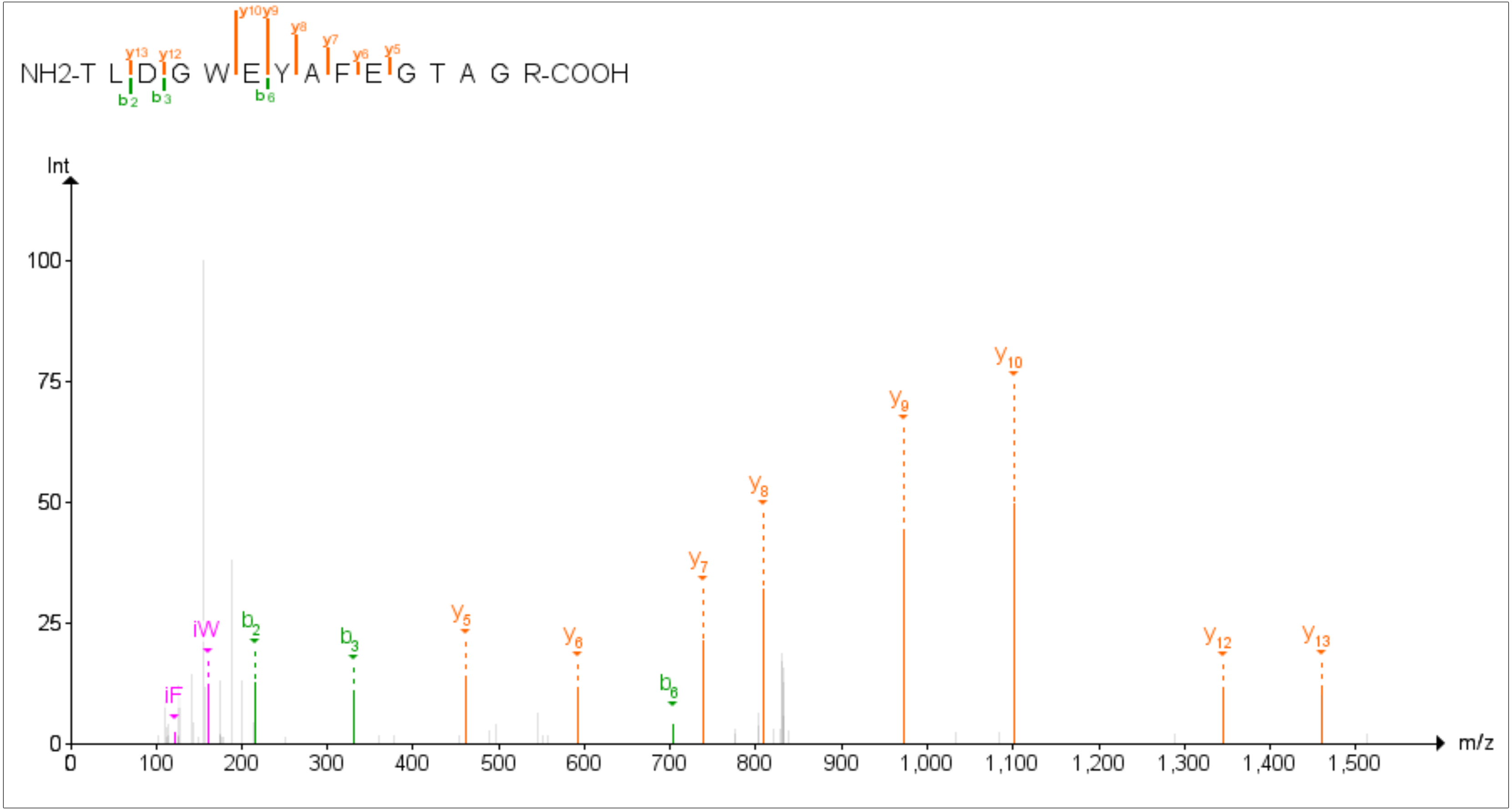

NH2-T L D G W E Y A F E G T A G R-COOH
y13 y12 y10 y9 y8 y7 y6 y5
b2 b3 b6
Int
100
75
50
25
0
iF
iW
b2
b3
y5
y6
b6
y7
y8
y9
y10
y12
y13
0 100 200 300 400 500 600 700 800 900 1,000 1,100 1,200 1,300 1,400 1,500
m/z

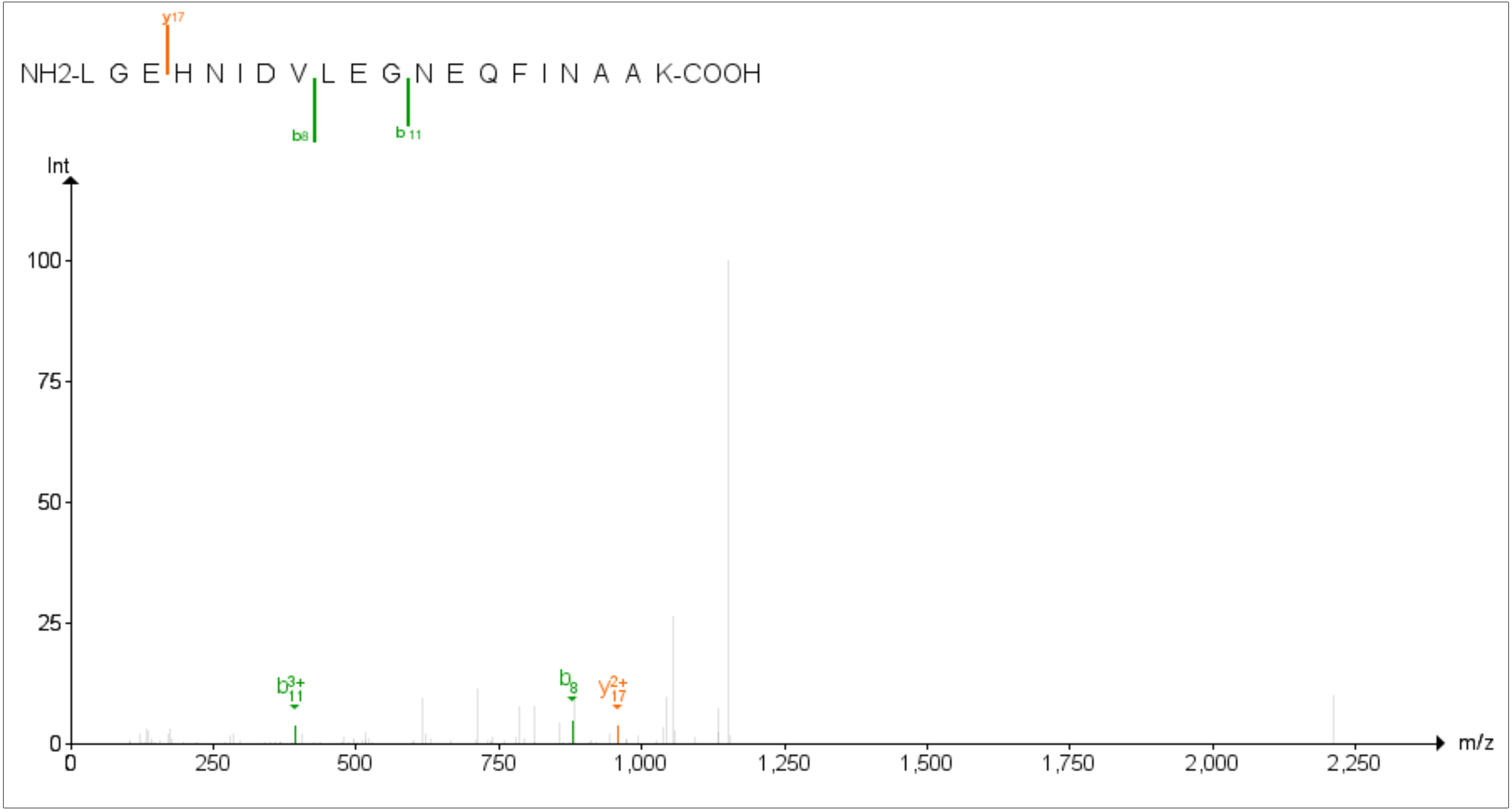

y17
NH2-L G E H N I D V L E G N E Q F I N A A K-COOH
b8
b 11
Int
100
75
50
25
0
b$_{11}^{3+}$
b$_8$
y$_{17}^{2+}$
0
250
500
750
1,000
1,250
1,500
1,750
2,000
2,250
m/z

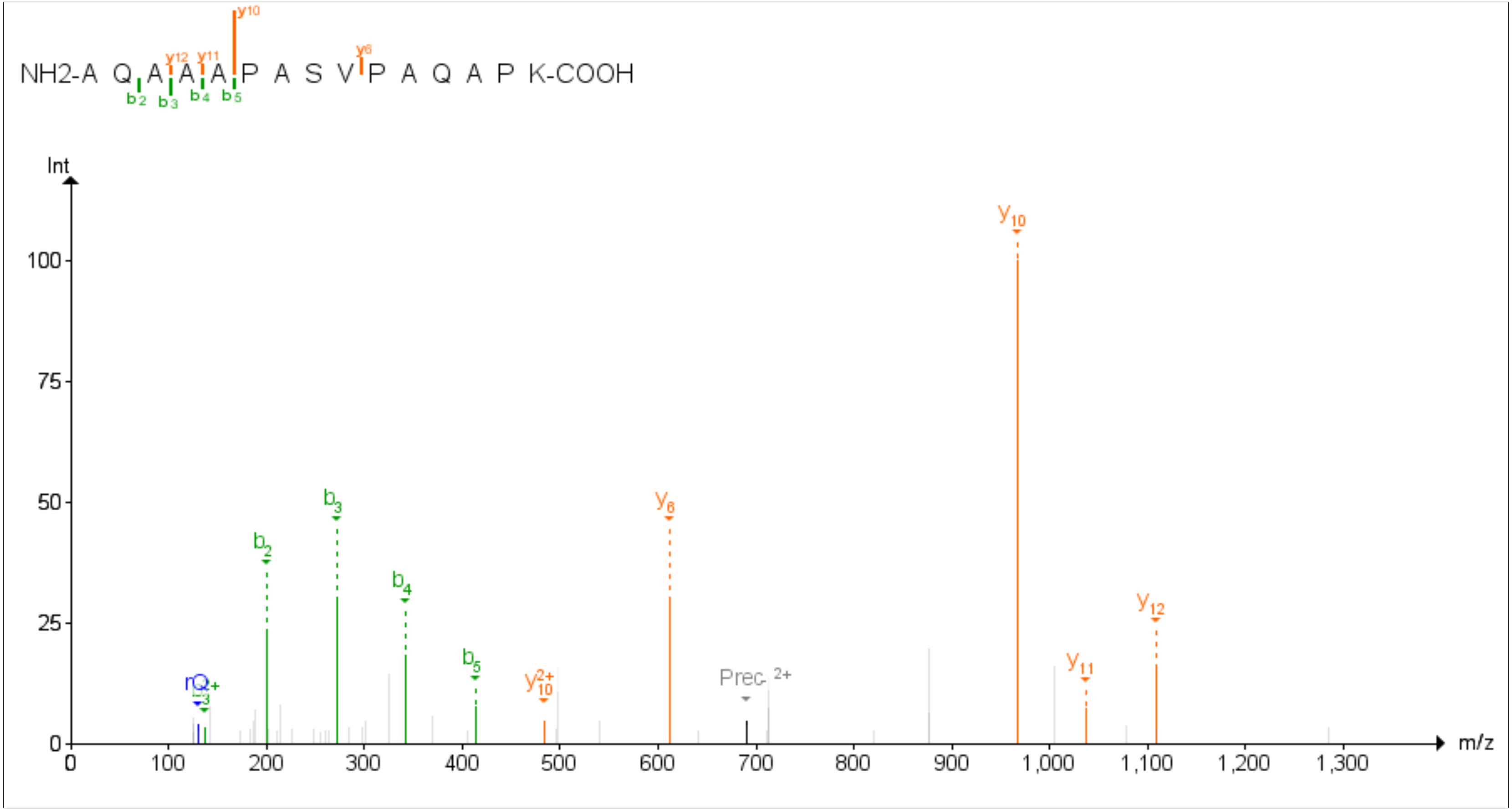

NH2-A Q A A A P A S V P A Q A P K-COOH
y10
y12
y11
y6
b2
b3
b4
b5
Int
100
75
50
25
0
y10
b3
y6
b2
b4
y12
b5
y11
y10 2+
Prec- 2+
m/z
0
100
200
300
400
500
600
700
800
900
1,000
1,100
1,200
1,300

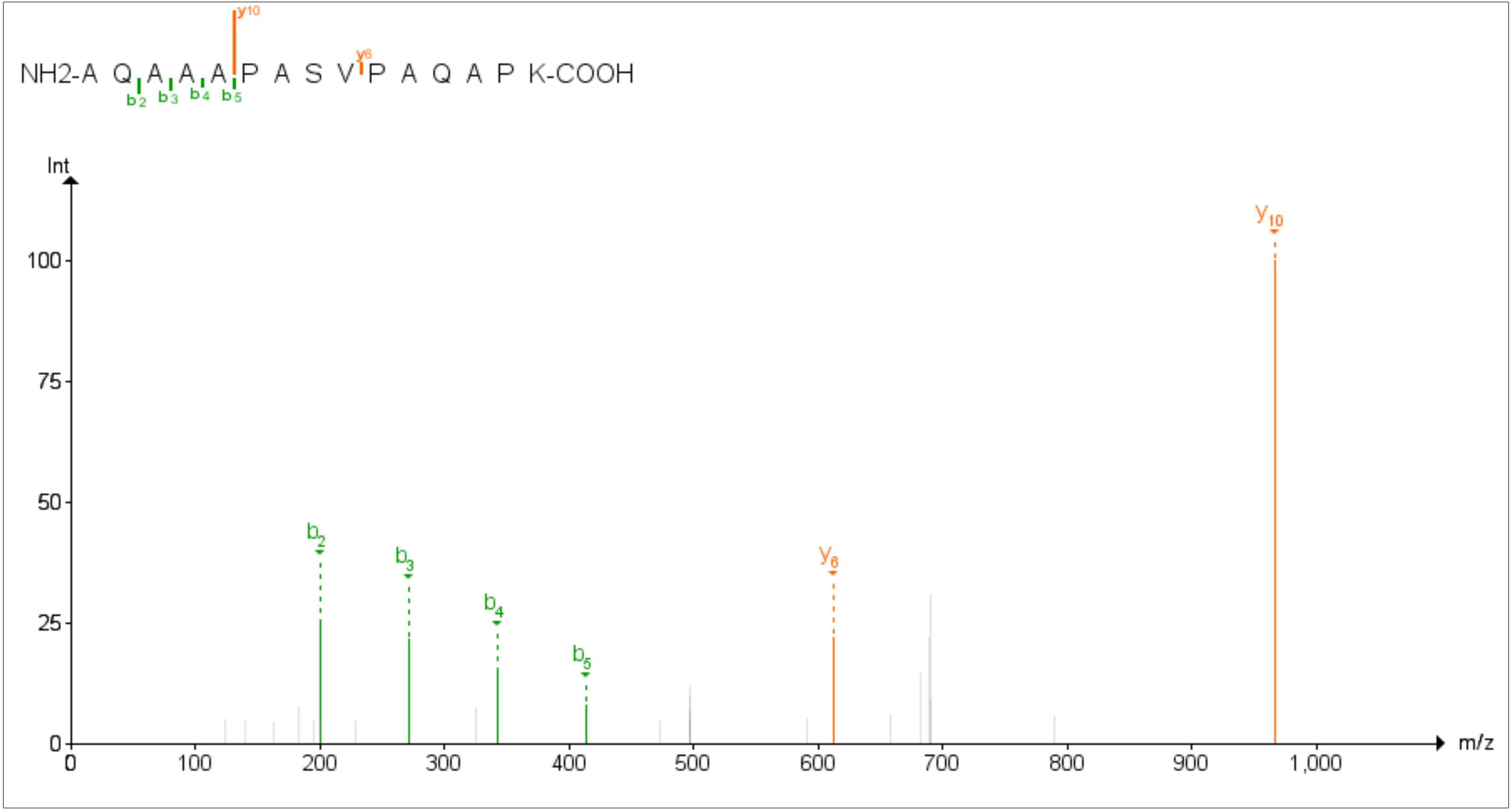
NH2-A Q A A A P A S V P A Q A P K-COOH
y10
y6
b2 b3 b4 b5
Int
100
75
50
25
0
m/z
0
100
200
300
400
500
600
700
800
900
1,000
b2
b3
b4
b5
y6
y10

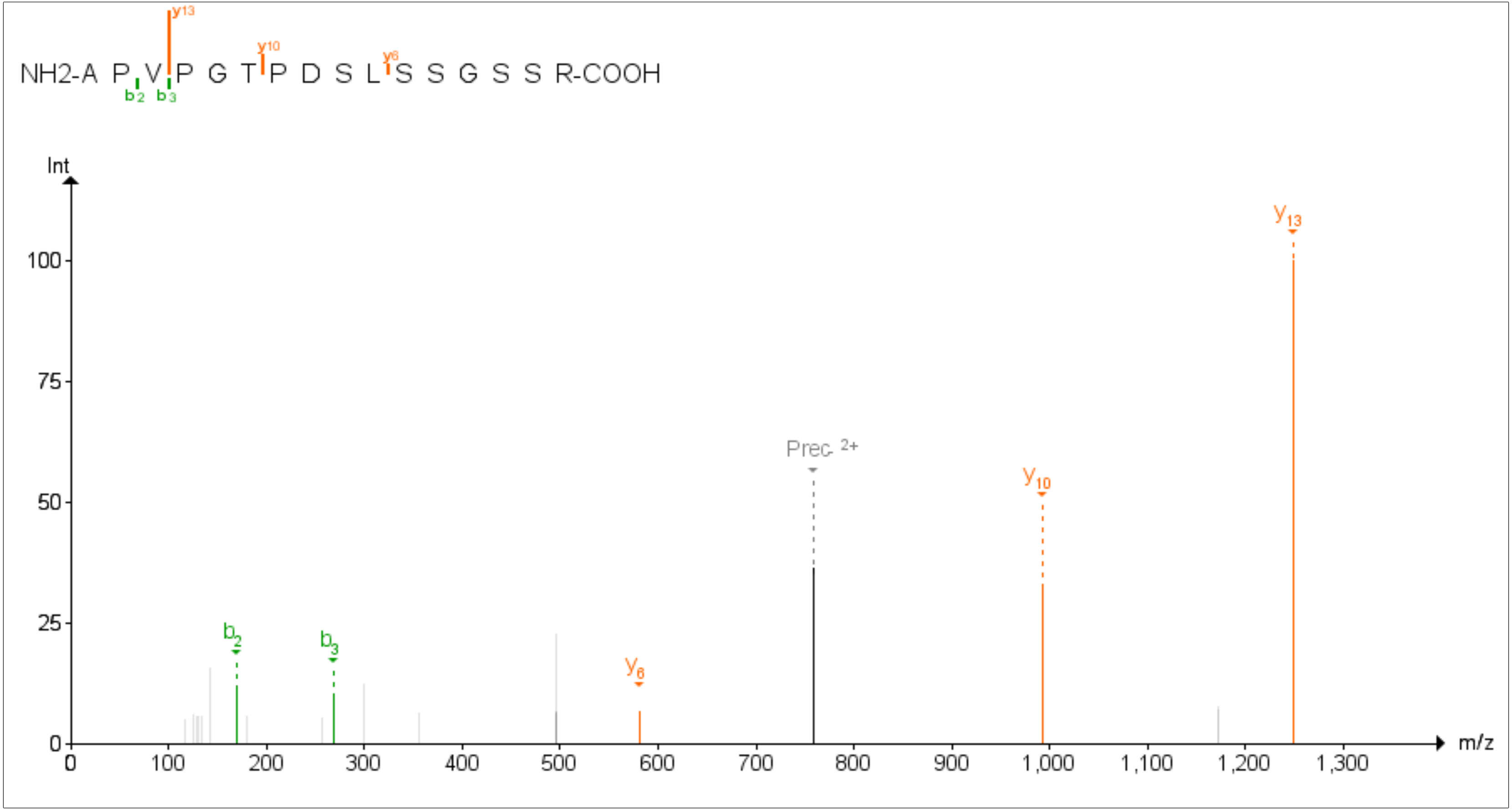

NH2-A P V P G T P D S L S S G S S R-COOH
y13
y10
y6
b2
b3
Int
100
75
50
25
0
y13
Prec 2+
y10
b2
b3
y6
0
100
200
300
400
500
600
700
800
900
1,000
1,100
1,200
1,300
m/z

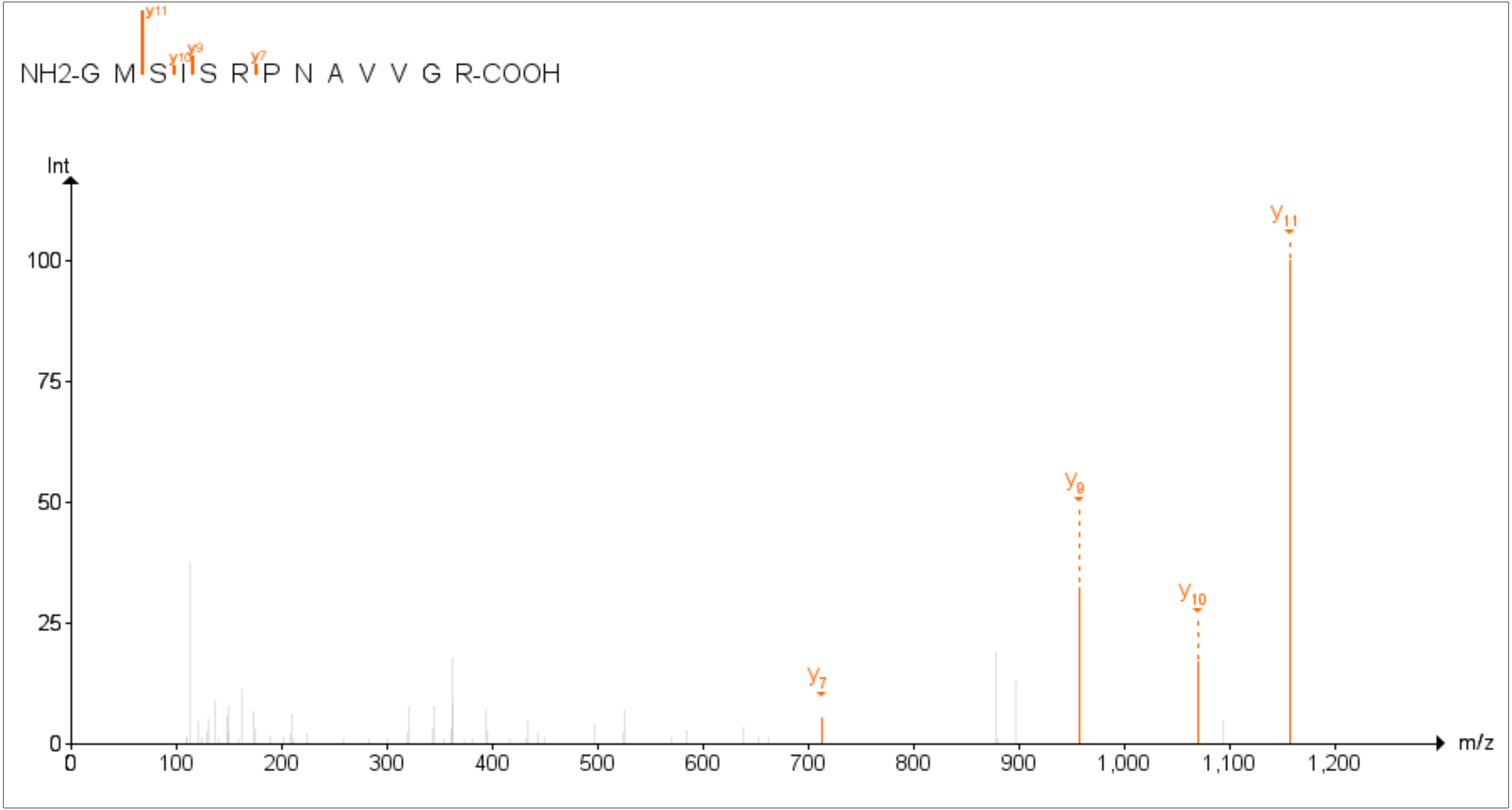

NH2-G M S I S R P N A V V G R-COOH
y11
y10
y9
y7
Int
100
75
50
25
0
m/z
0
100
200
300
400
500
600
700
800
900
1,000
1,100
1,200
y7
y9
y10
y11

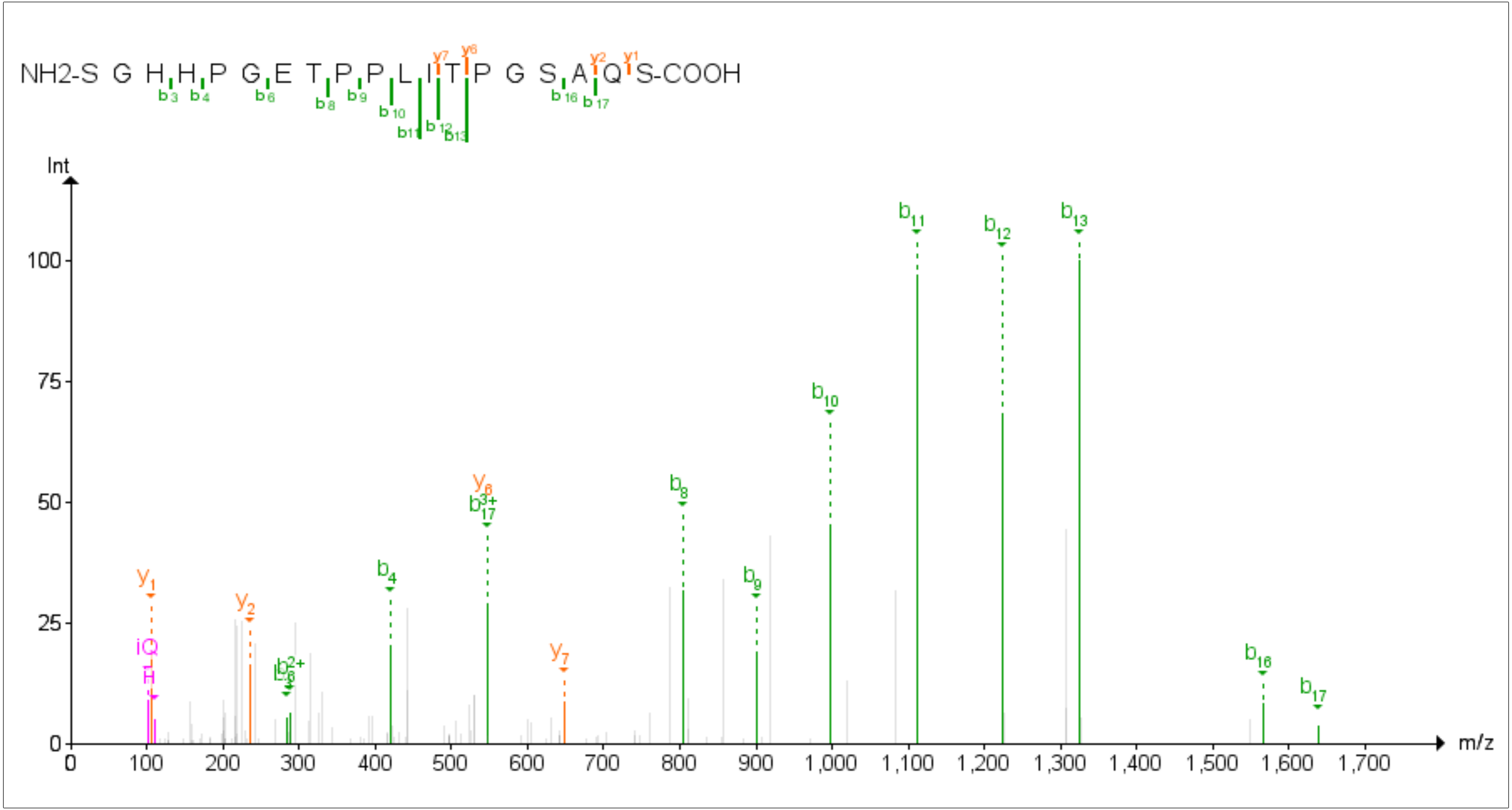
NH2-S G H H P G E T P P L I T P G S A Q S-COOH
b3 b4 b6 b8 b9 b10 b11 b12 b13 b16 b17
y7 y6 y2 y1
Int
m/z
0 25 50 75 100
0 100 200 300 400 500 600 700 800 900 1,000 1,100 1,200 1,300 1,400 1,500 1,600 1,700

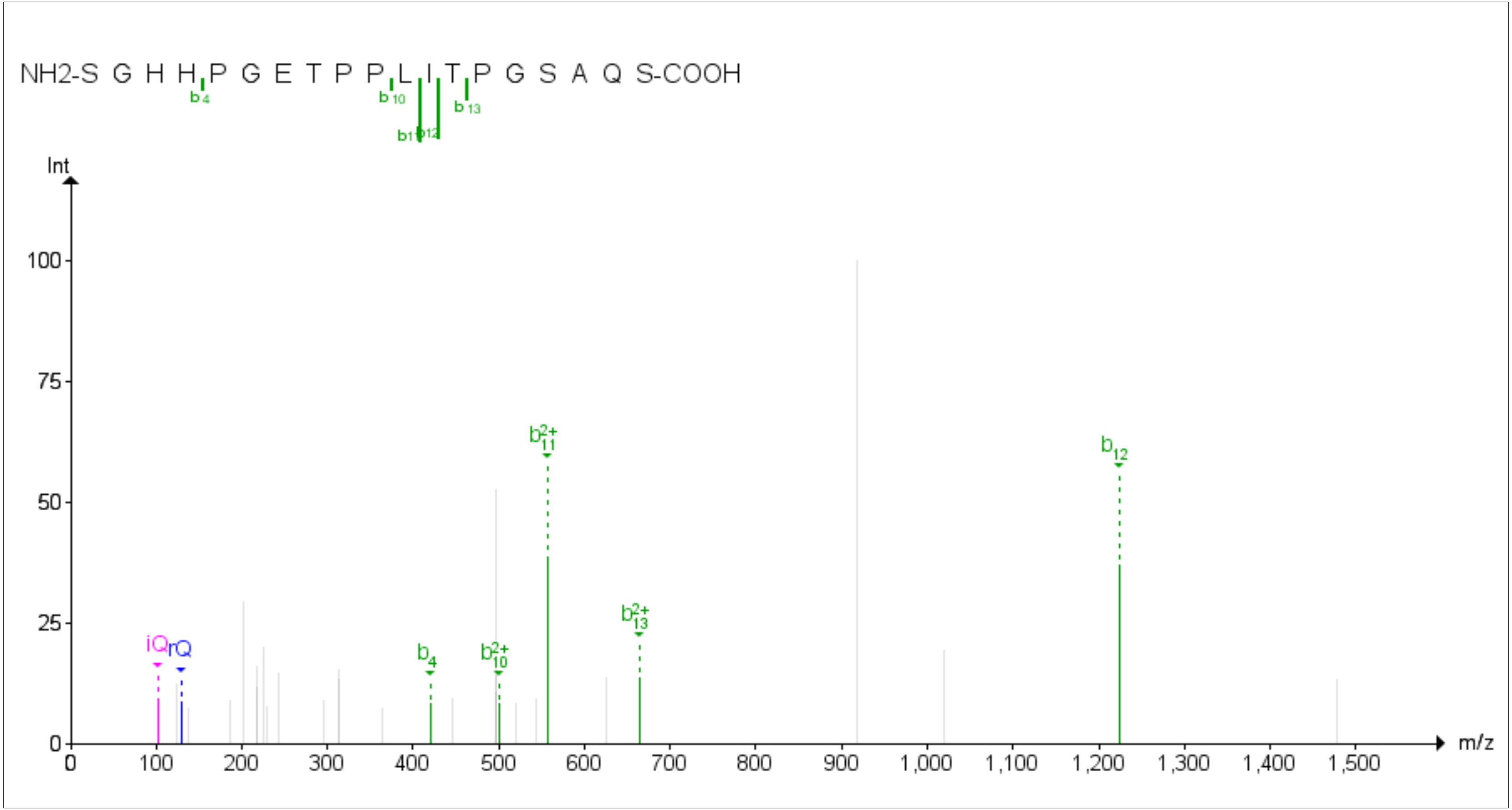

NH2-S G H H P G E T P P L I T P G S A Q S-COOH
b4
b10
b11
b12
b13
Int
100
75
50
25
0
iQ
rQ
b4
b2+10
b2+11
b2+13
b12
0
100
200
300
400
500
600
700
800
900
1,000
1,100
1,200
1,300
1,400
1,500
m/z

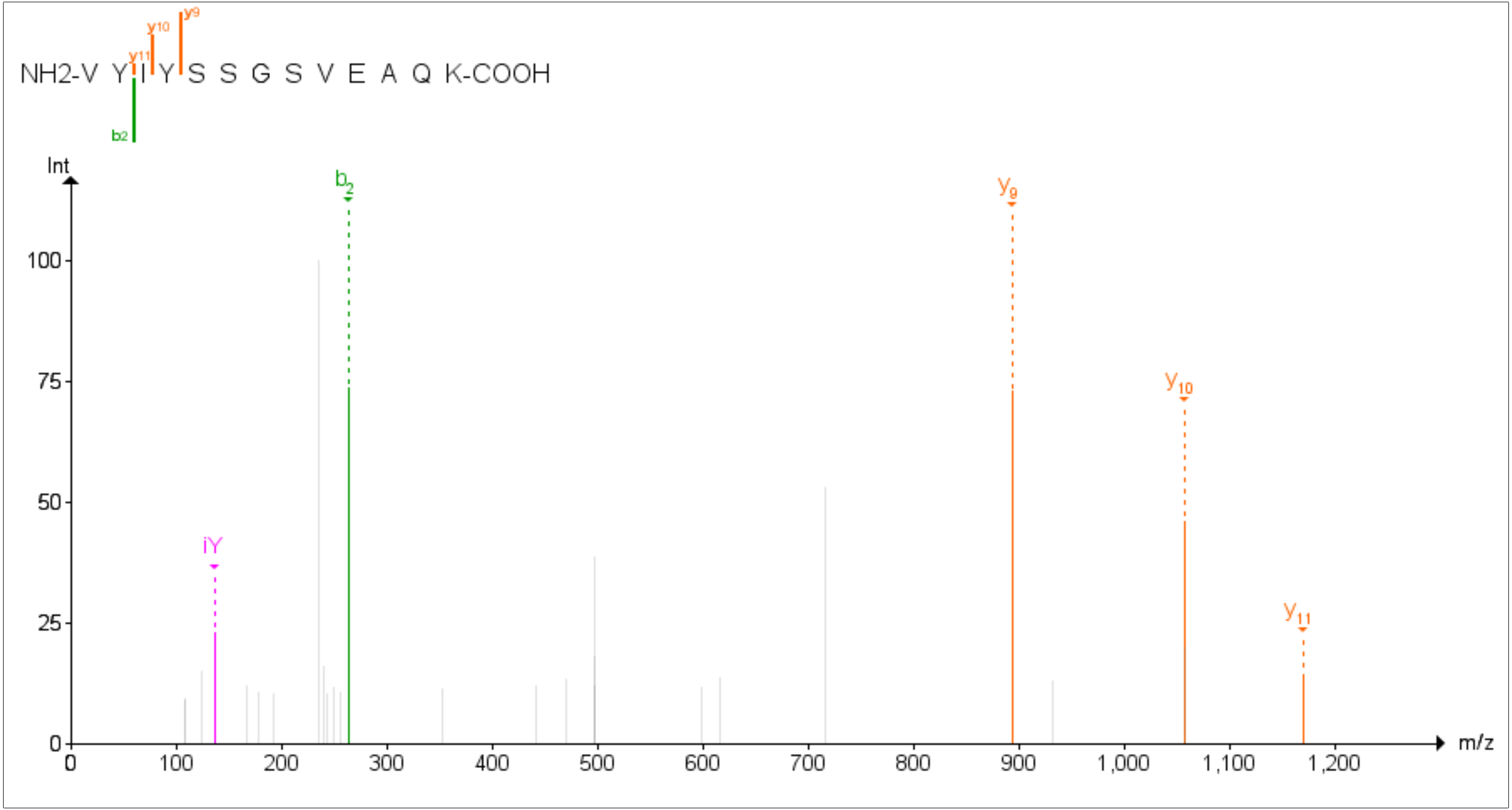

NH2-V Y I Y S S G S V E A Q K-COOH
y9
y10
y11
b2
Int
100
75
50
25
0
iY
b2
y9
y10
y11
0
100
200
300
400
500
600
700
800
900
1,000
1,100
1,200
m/z

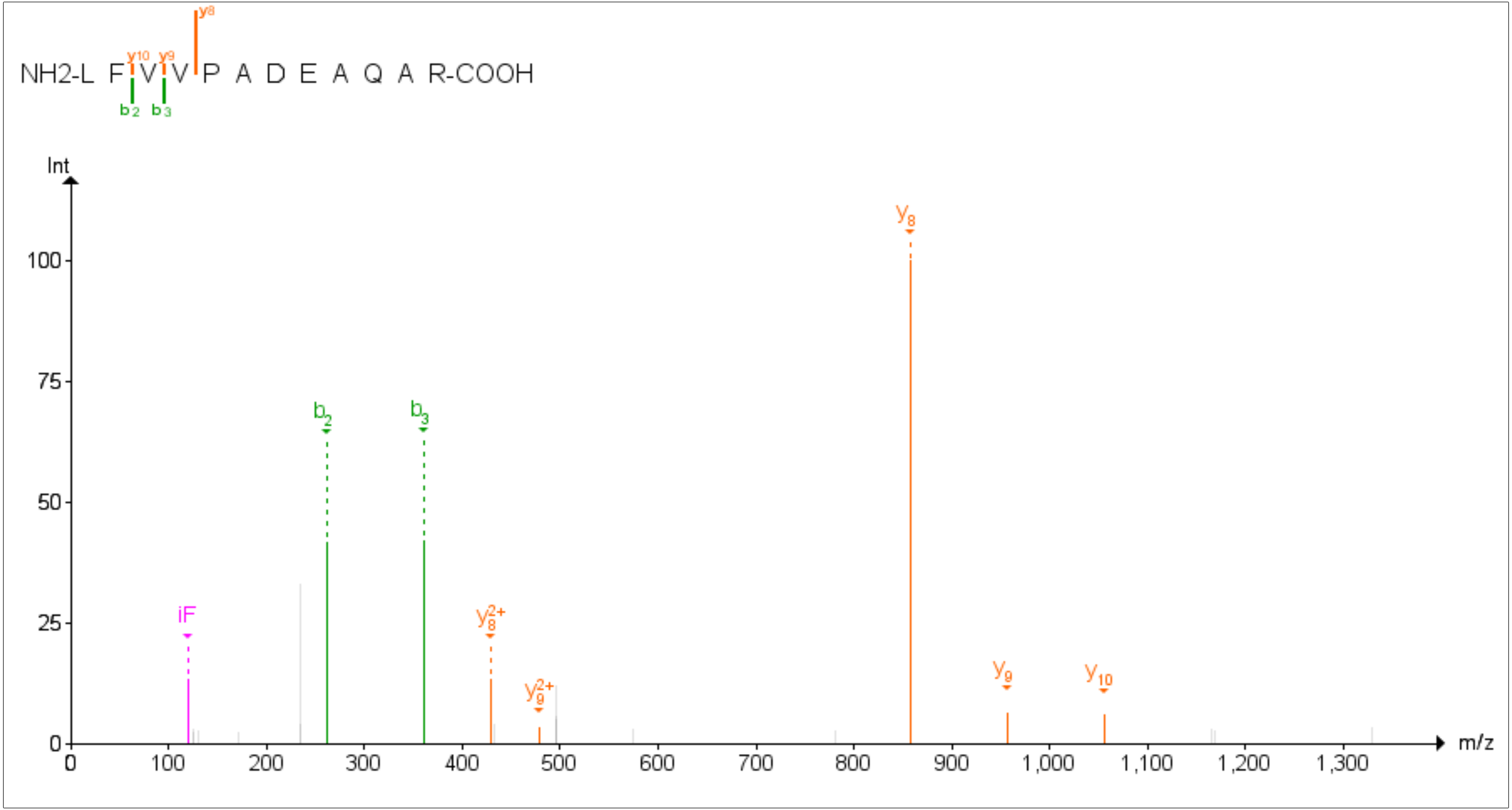

NH2-L F V V P A D E A Q A R-COOH
y10 y9 y8
b2 b3
Int
100
75
50
25
0
y8
b2
b3
iF
y8 2+
y9 2+
y9
y10
0 100 200 300 400 500 600 700 800 900 1,000 1,100 1,200 1,300
m/z

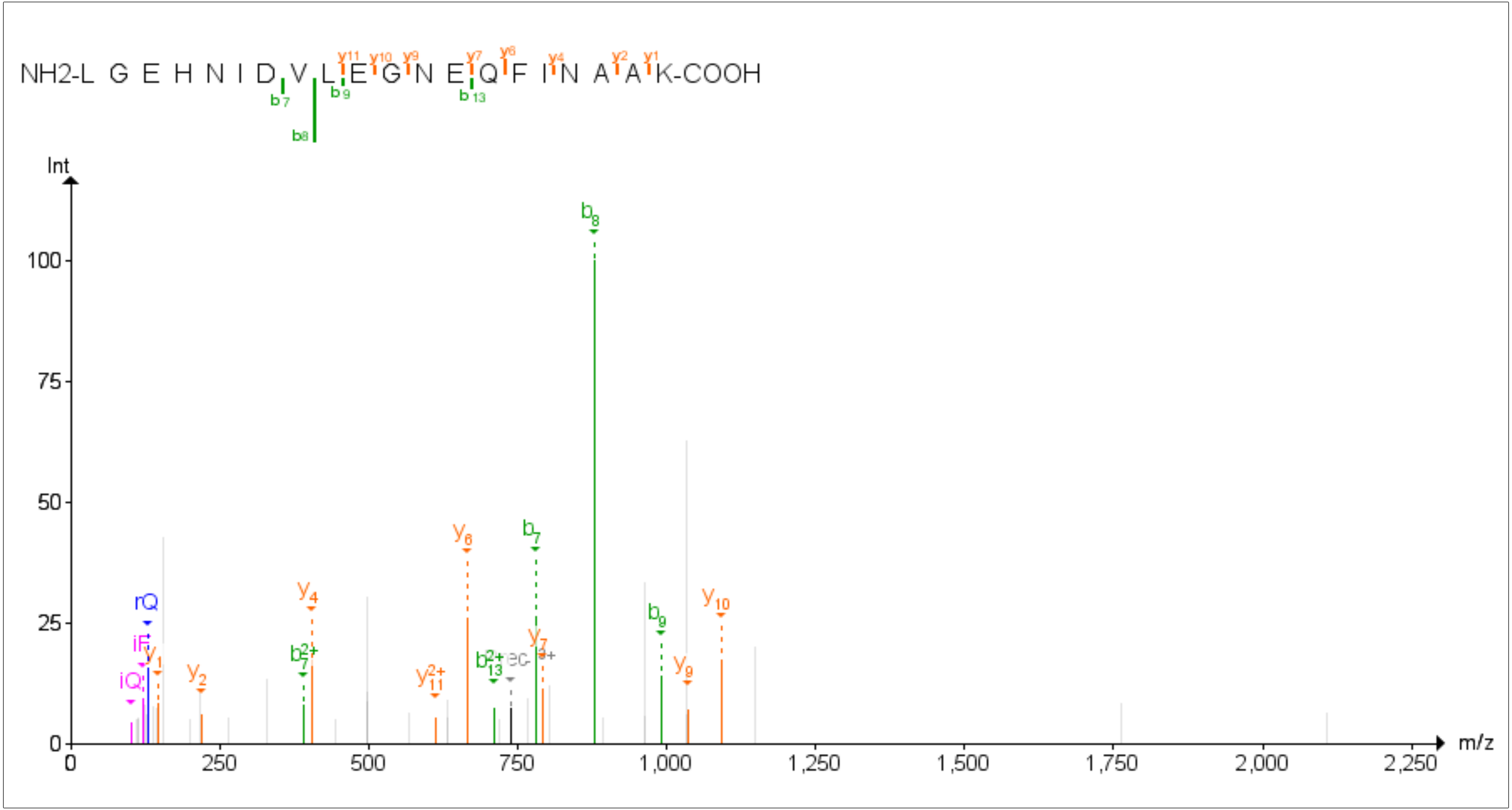

NH2-L G E H N I D V L E G N E Q F I N A A K-COOH
y11 y10 y9 y7 y6 y4 y2 y1
b7 b8 b9 b13
Int
100
75
50
25
0
m/z
0
250
500
750
1,000
1,250
1,500
1,750
2,000
2,250
b8
y6
b7
y4
rQ
iF
iQ
y1
y2
b7 2+
y11 2+
b13 2+
y7
b9
y9
y10

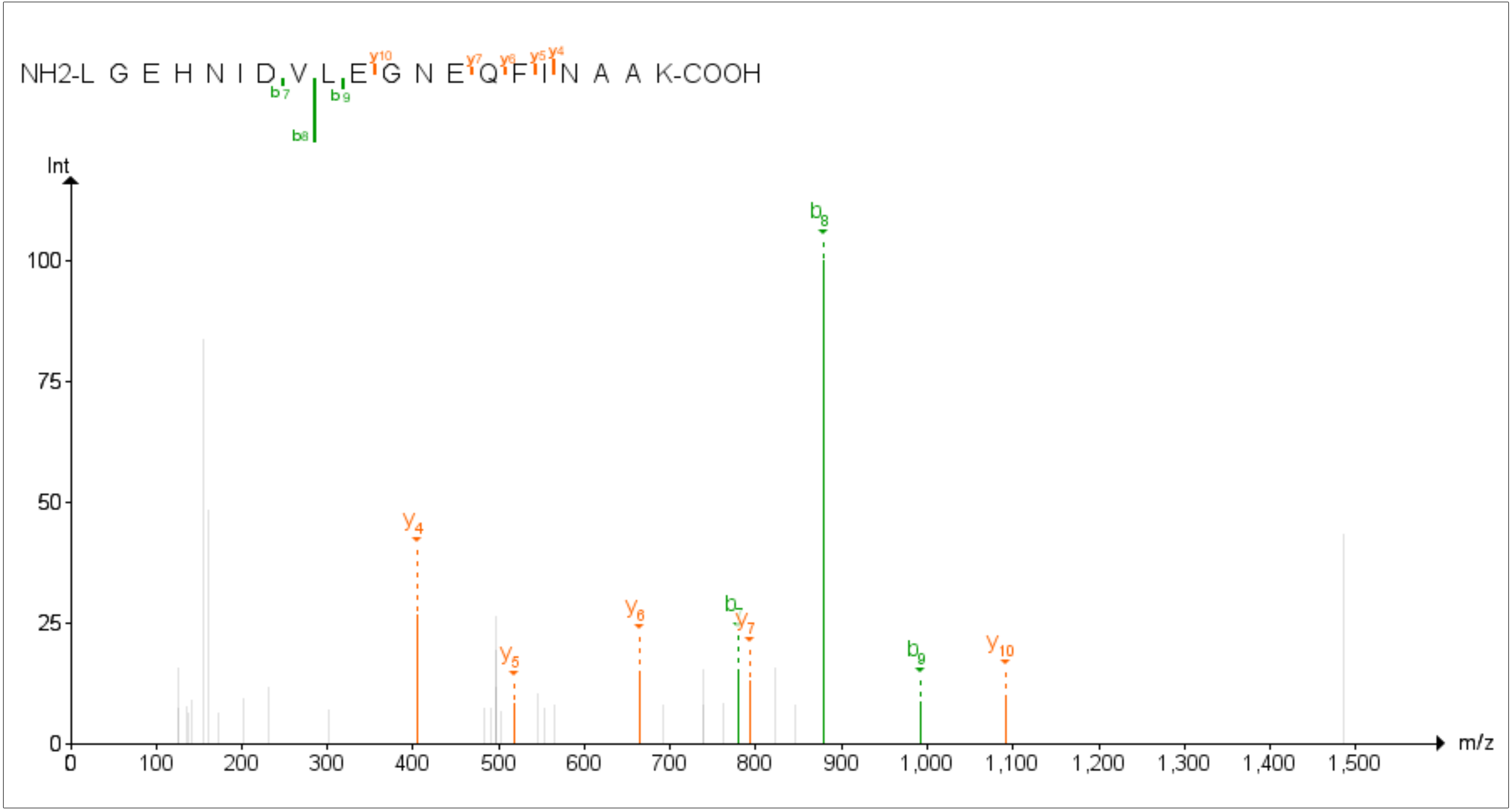

NH2-L G E H N I D V L E G N E Q F I N A A K-COOH
b7
b8
b9
y10
y7
y6
y5
y4
Int
100
75
50
25
0
m/z
0
100
200
300
400
500
600
700
800
900
1,000
1,100
1,200
1,300
1,400
1,500

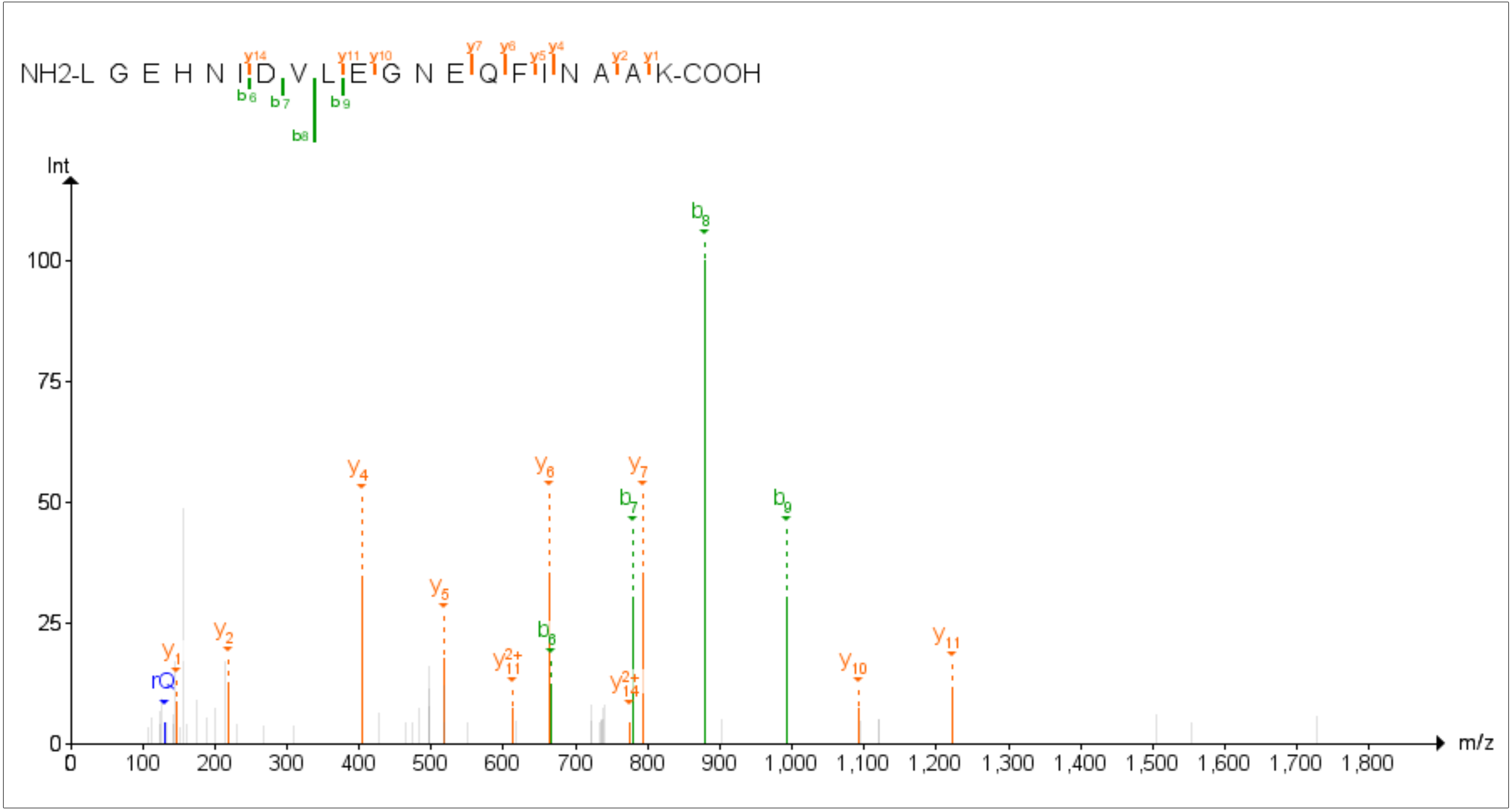

NH2-L G E H N I D V L E G N E Q F I N A A K-COOH
y14 y11 y10 y7 y6 y5 y4 y2 y1
b6 b7 b8 b9
Int
m/z
rQ
y1 y2 y4 y5 y6 y7 y10 y11
y11 2+
y14 2+
b6 b7 b8 b9
0 25 50 75 100
0 100 200 300 400 500 600 700 800 900 1,000 1,100 1,200 1,300 1,400 1,500 1,600 1,700 1,800

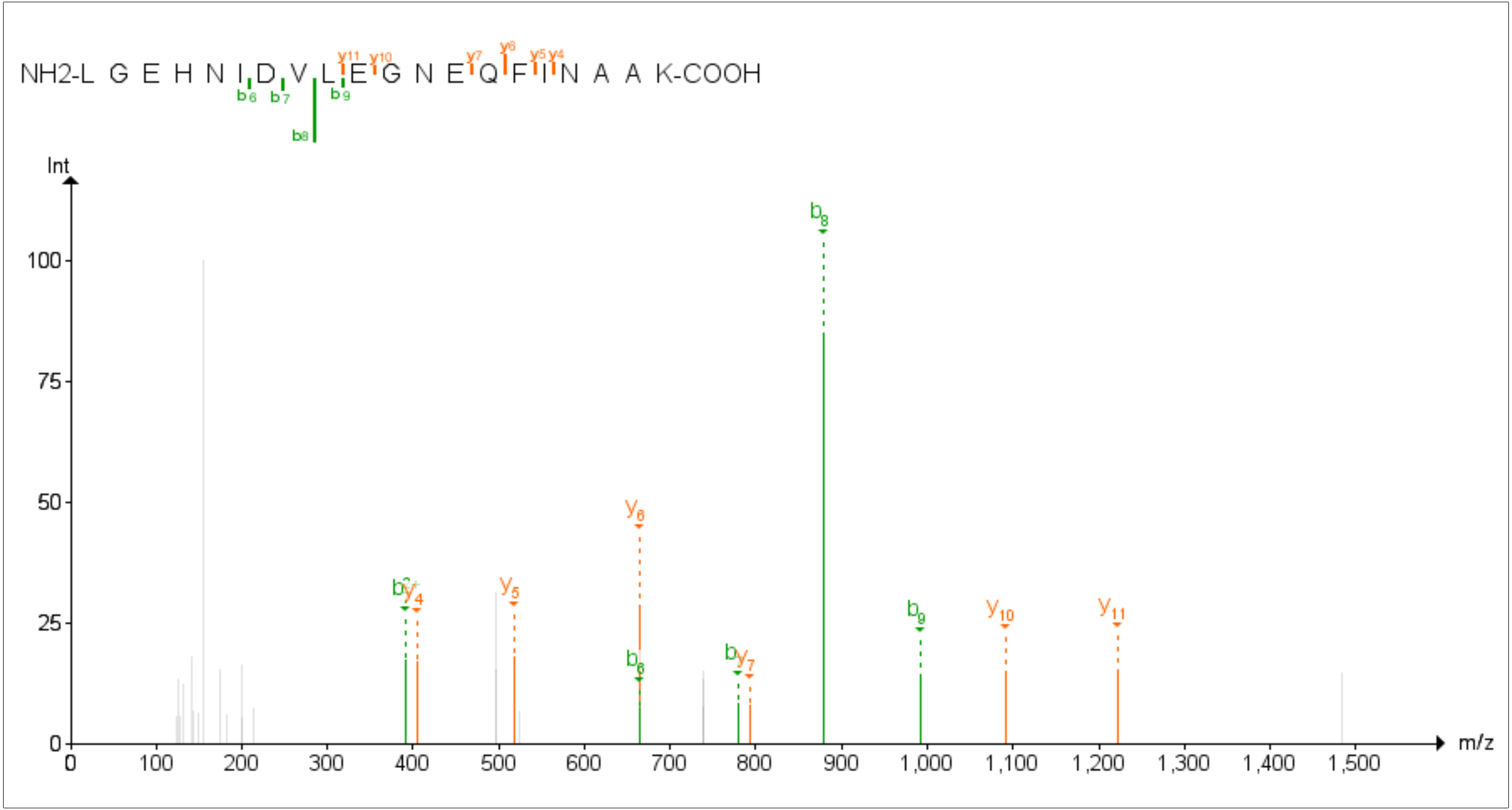
NH2-L G E H N I D V L E G N E Q F I N A A K-COOH
y11 y10 y7 y6 y5 y4
b6 b7 b8 b9
Int
100
75
50
25
0
m/z
0 100 200 300 400 500 600 700 800 900 1,000 1,100 1,200 1,300 1,400 1,500
b8
y6
y5
y4
b9
y10
y11
b6
y7

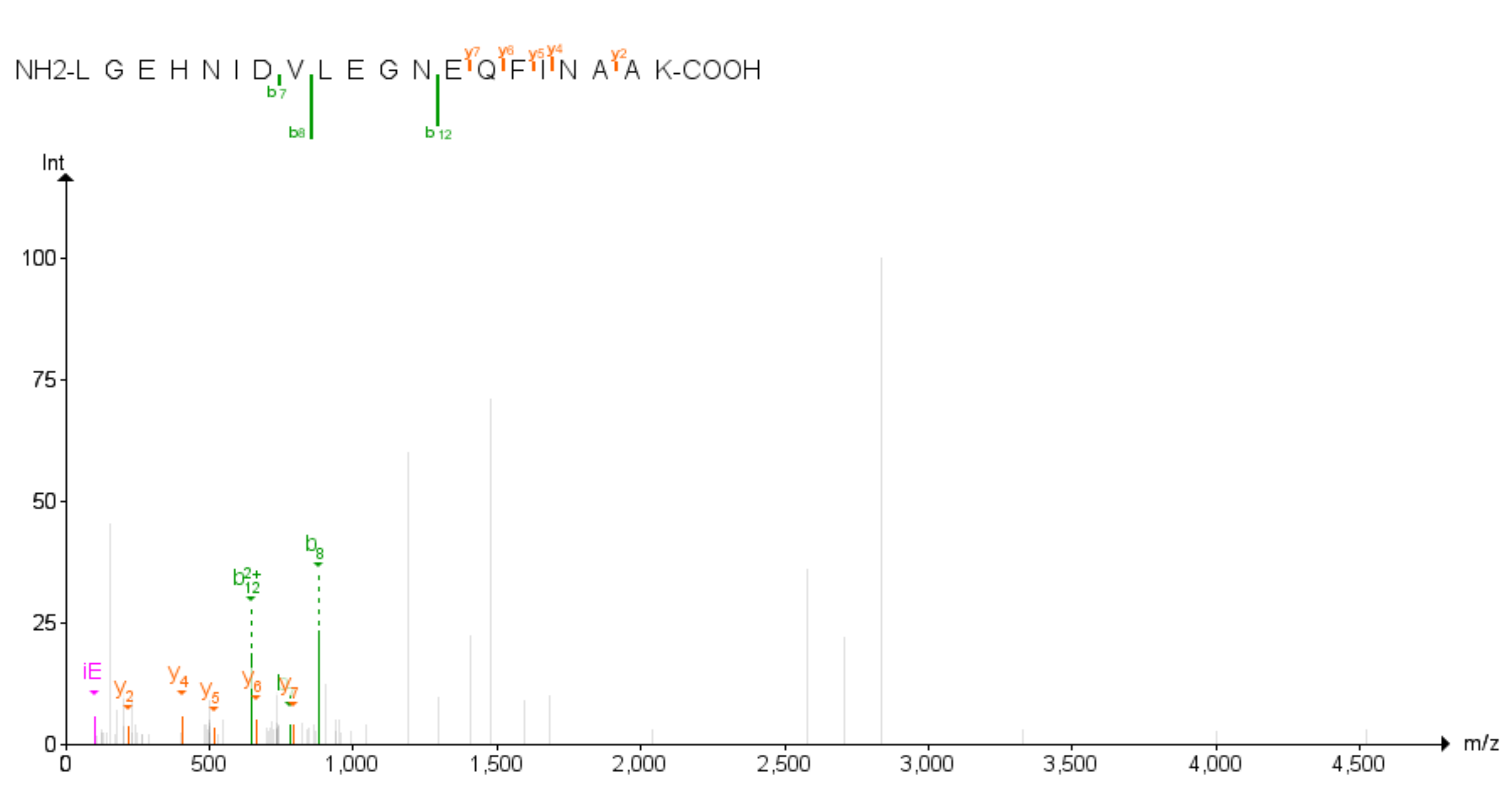

NH2-L G E H N I D V L E G N E Q F I N A A K-COOH
b7
b8
b12
y7
y6
y5
y4
y2
Int
100
75
50
25
0
iE
y2
y4
y5
y6
y7
b12 2+
b8
0
500
1,000
1,500
2,000
2,500
3,000
3,500
4,000
4,500
m/z

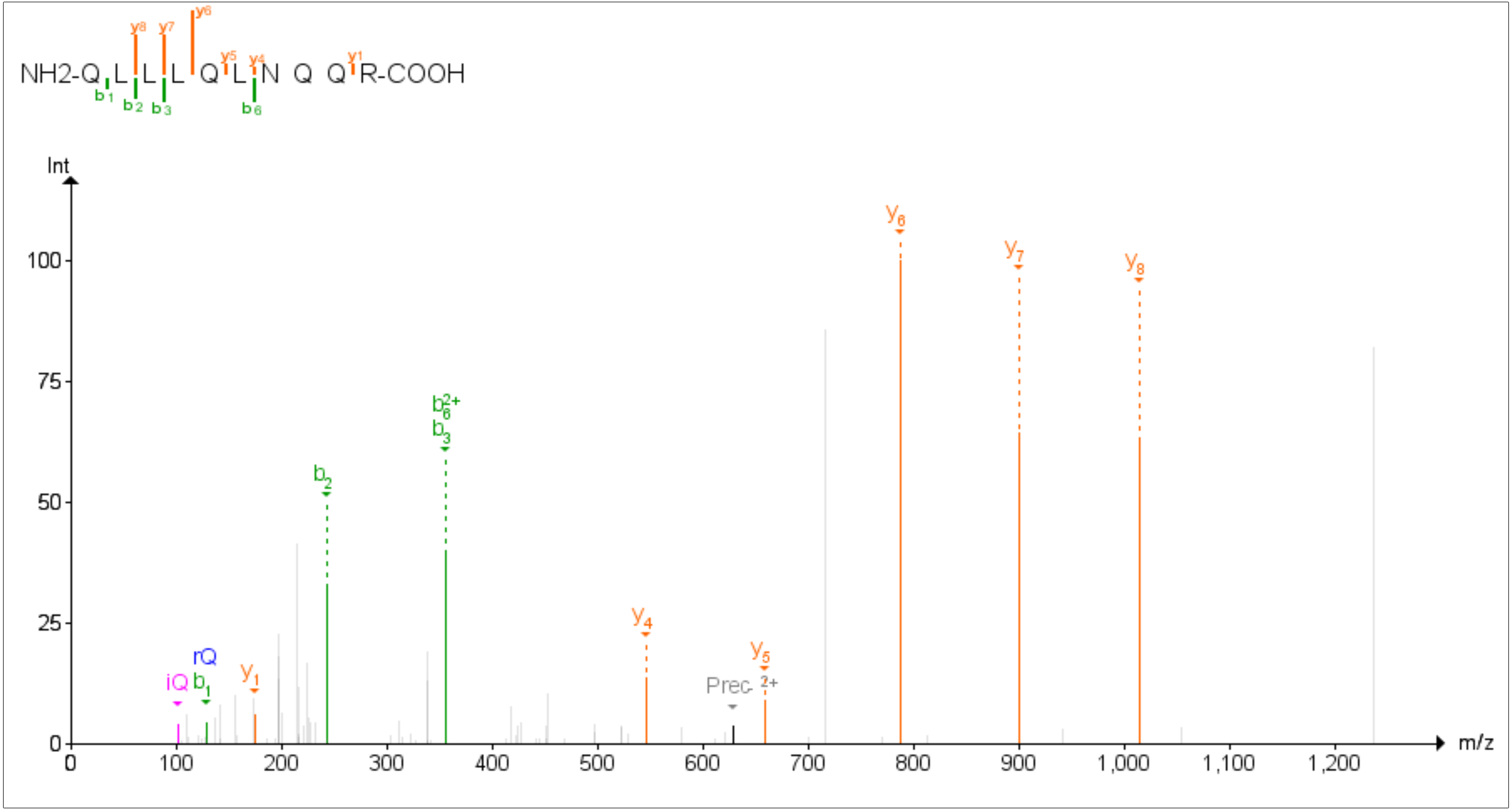

NH2-Q L L L Q L N Q Q R-COOH
y8 y7 y6 y5 y4 y1
b1 b2 b3 b6
Int
100
75
50
25
0
m/z
0
100
200
300
400
500
600
700
800
900
1,000
1,100
1,200
y6
y7
y8
b6 2+
b3
b2
y4
y5
Prec- 2+
rQ
iQ
b1
y1

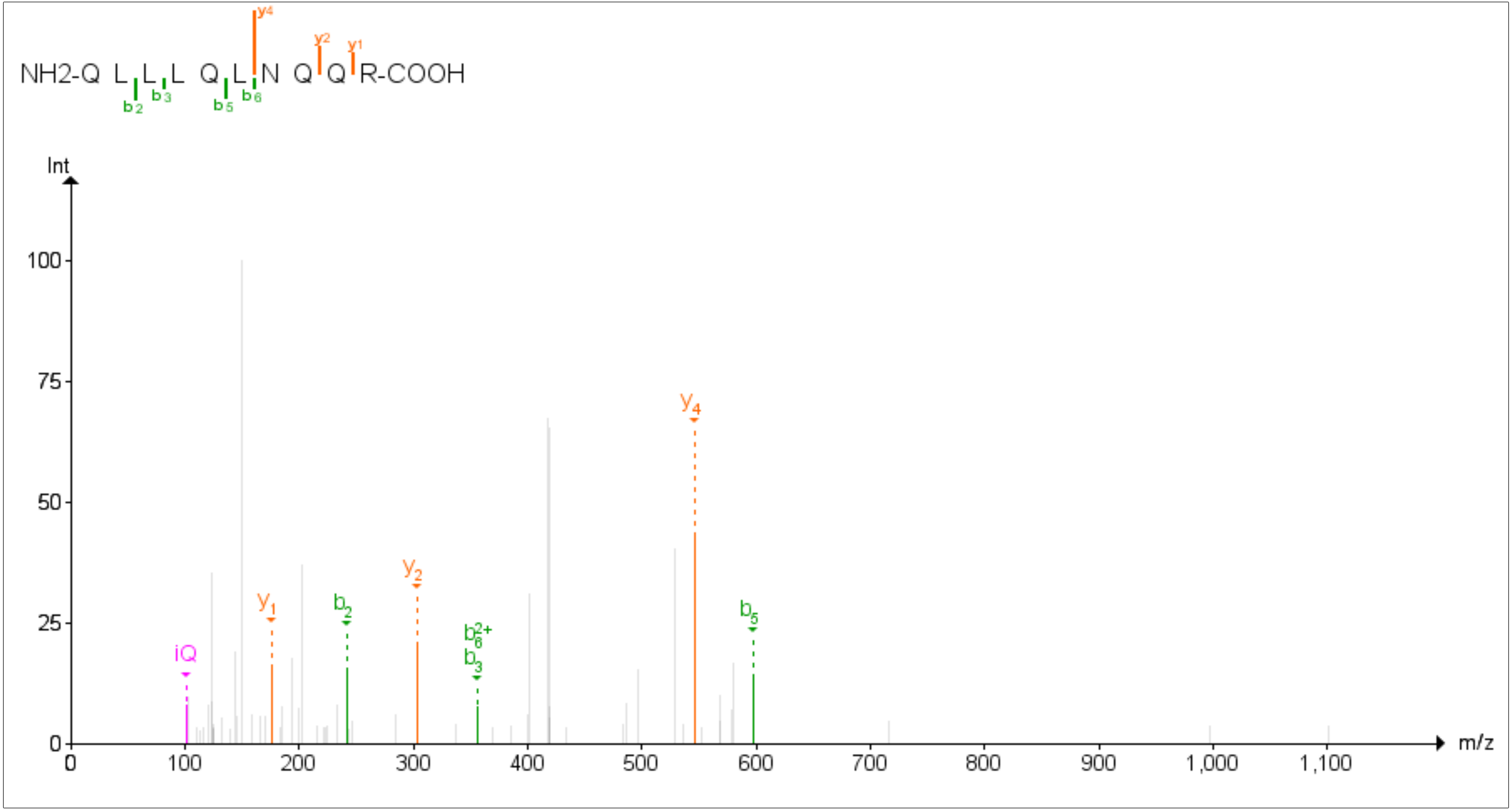

NH2-Q L L L Q L N Q Q R-COOH
y4
y2
y1
b2
b3
b5
b6
Int
100
75
50
25
0
iQ
y1
b2
y2
b6 2+
b3
y4
b5
0
100
200
300
400
500
600
700
800
900
1,000
1,100
m/z

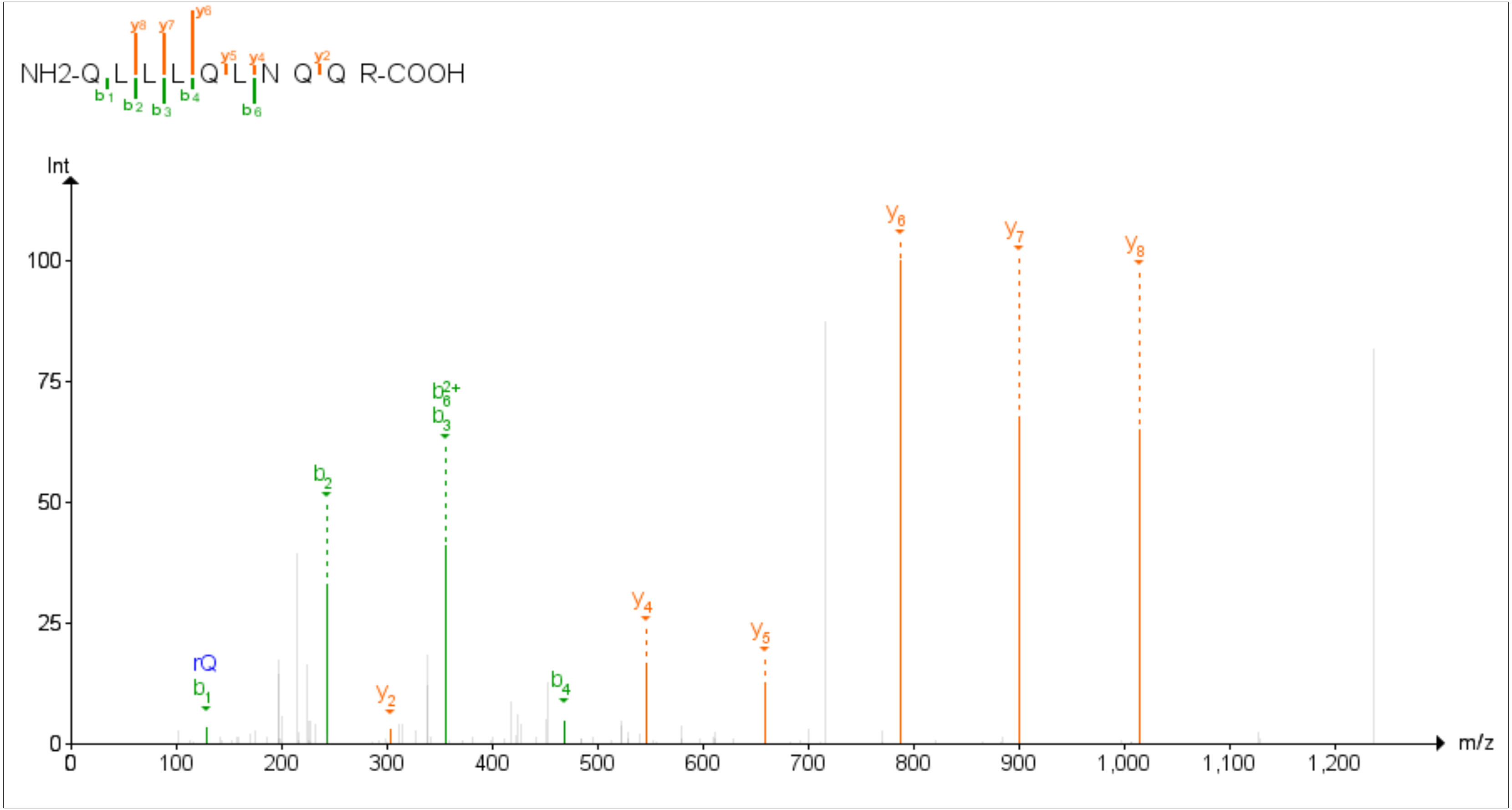

NH2-Q L L L Q L N Q Q R-COOH
y8 y7 y6 y5 y4 y2
b1 b2 b3 b4 b6
Int
100
75
50
25
0
b6 2+
b3
b2
rQ
b1
y2
b4
y4
y5
y6
y7
y8
0 100 200 300 400 500 600 700 800 900 1,000 1,100 1,200
m/z

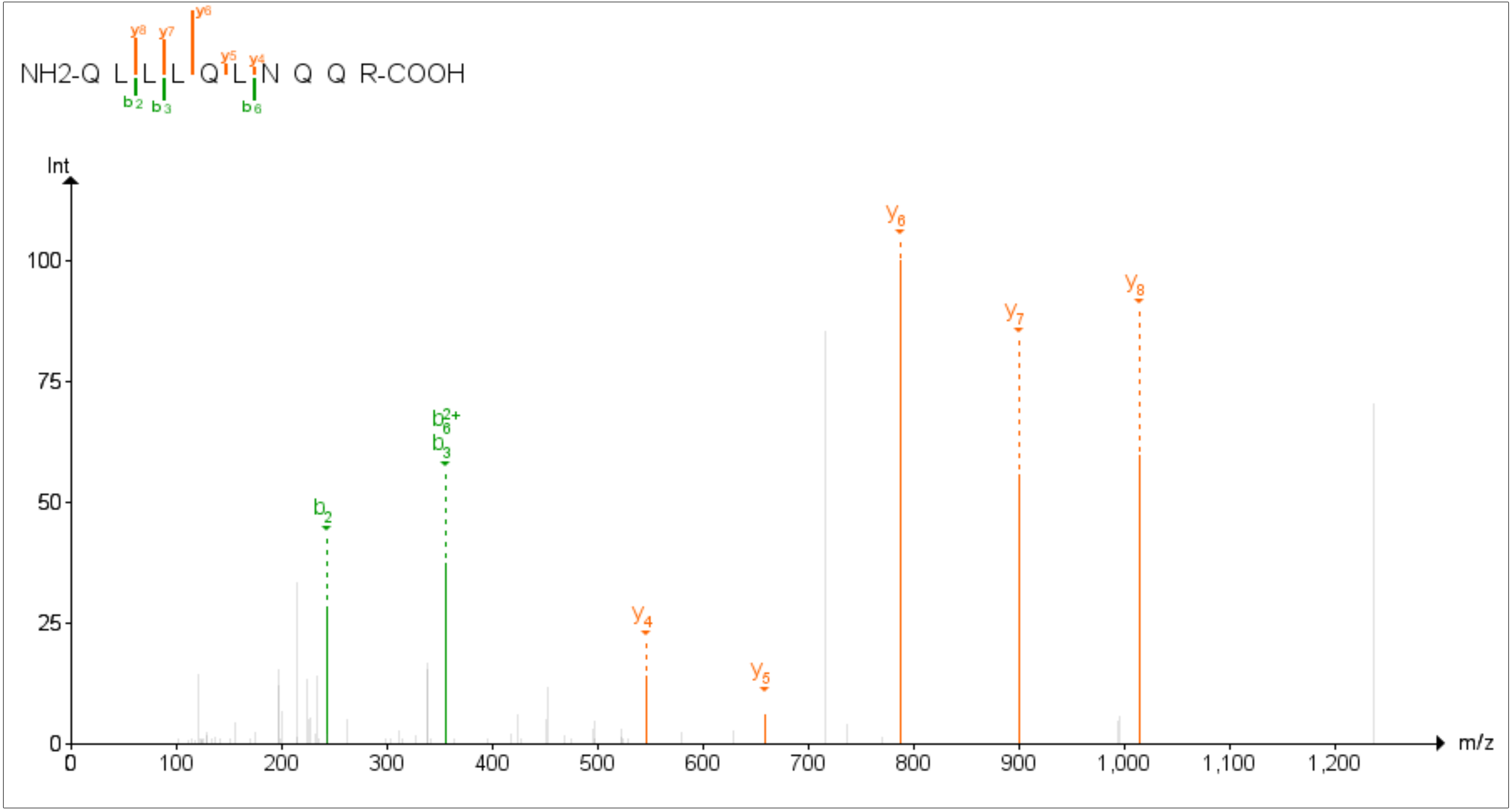

NH2-Q L L L Q L N Q Q R-COOH
y8 y7 y6 y5 y4
b2 b3 b6
Int
m/z
y6
y7
y8
b_6^{2+}
b_3
b_2
y_4
y_5

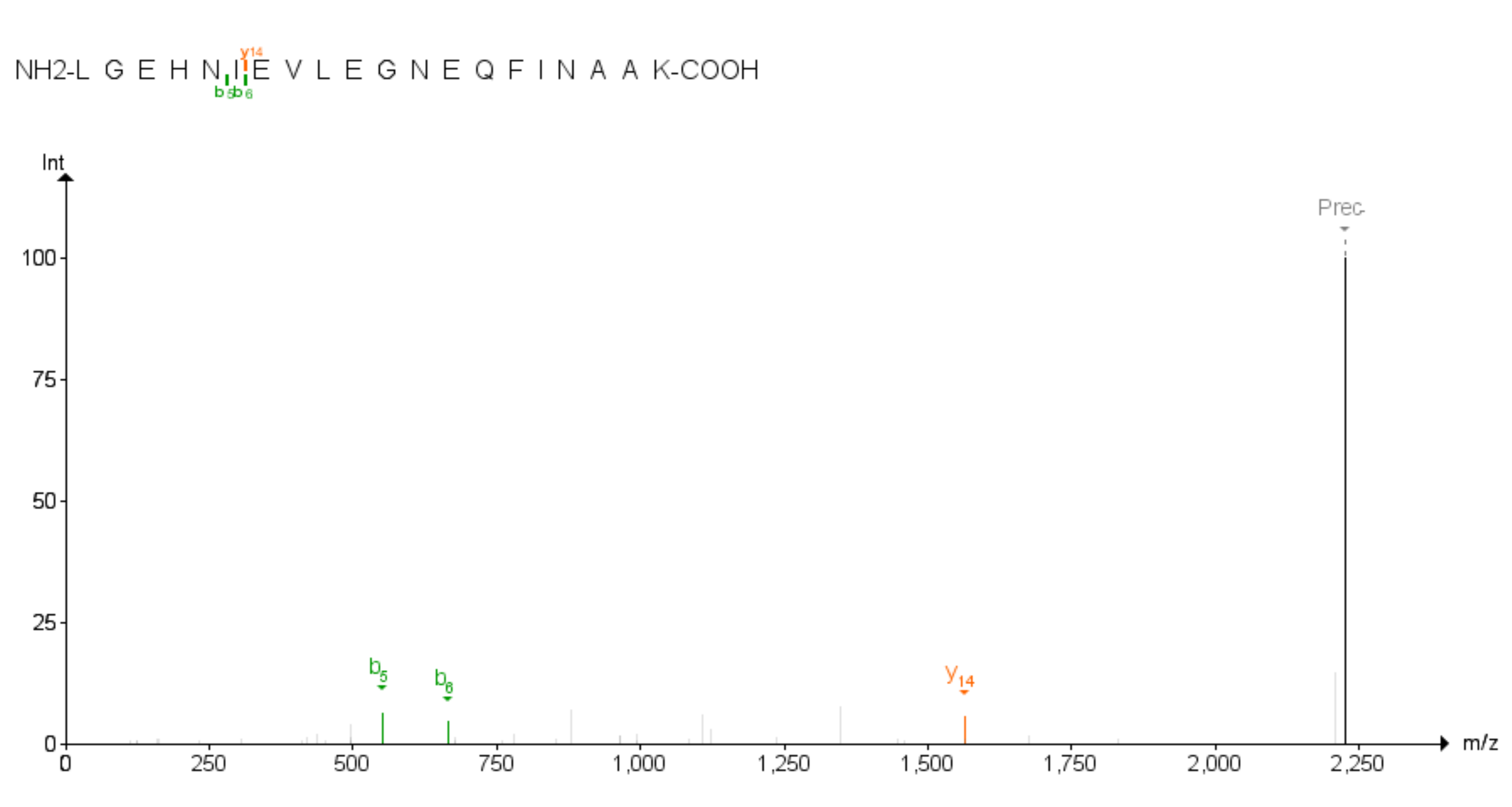

NH2-L G E H N I E V L E G N E Q F I N A A K-COOH
y14
b5 b6
Int
100
75
50
25
0
Prec
b5
b6
y14
0
250
500
750
1,000
1,250
1,500
1,750
2,000
2,250
m/z

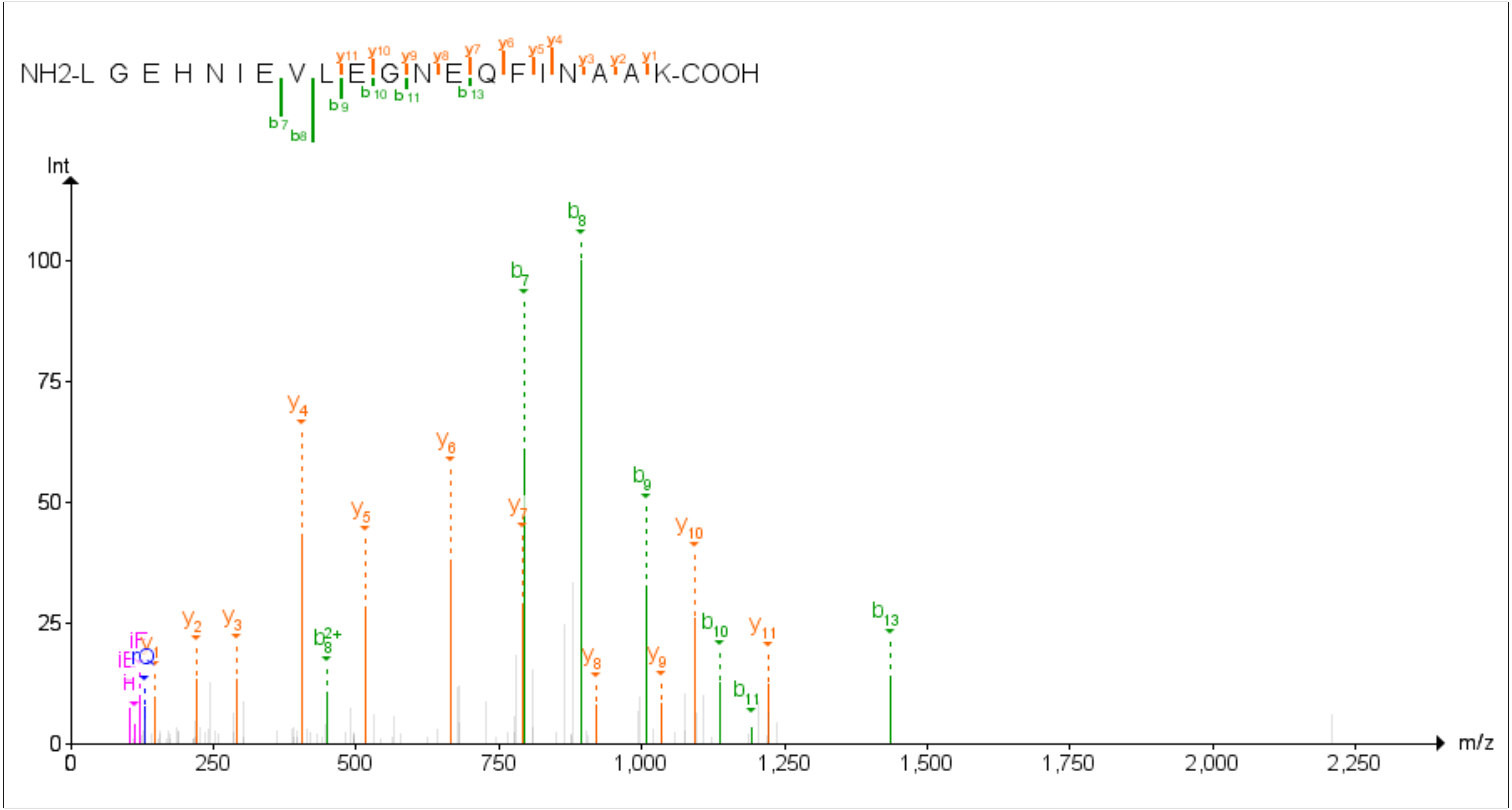

NH2-L G E H N I E V L E G N E Q F I N A A K-COOH
Int
m/z

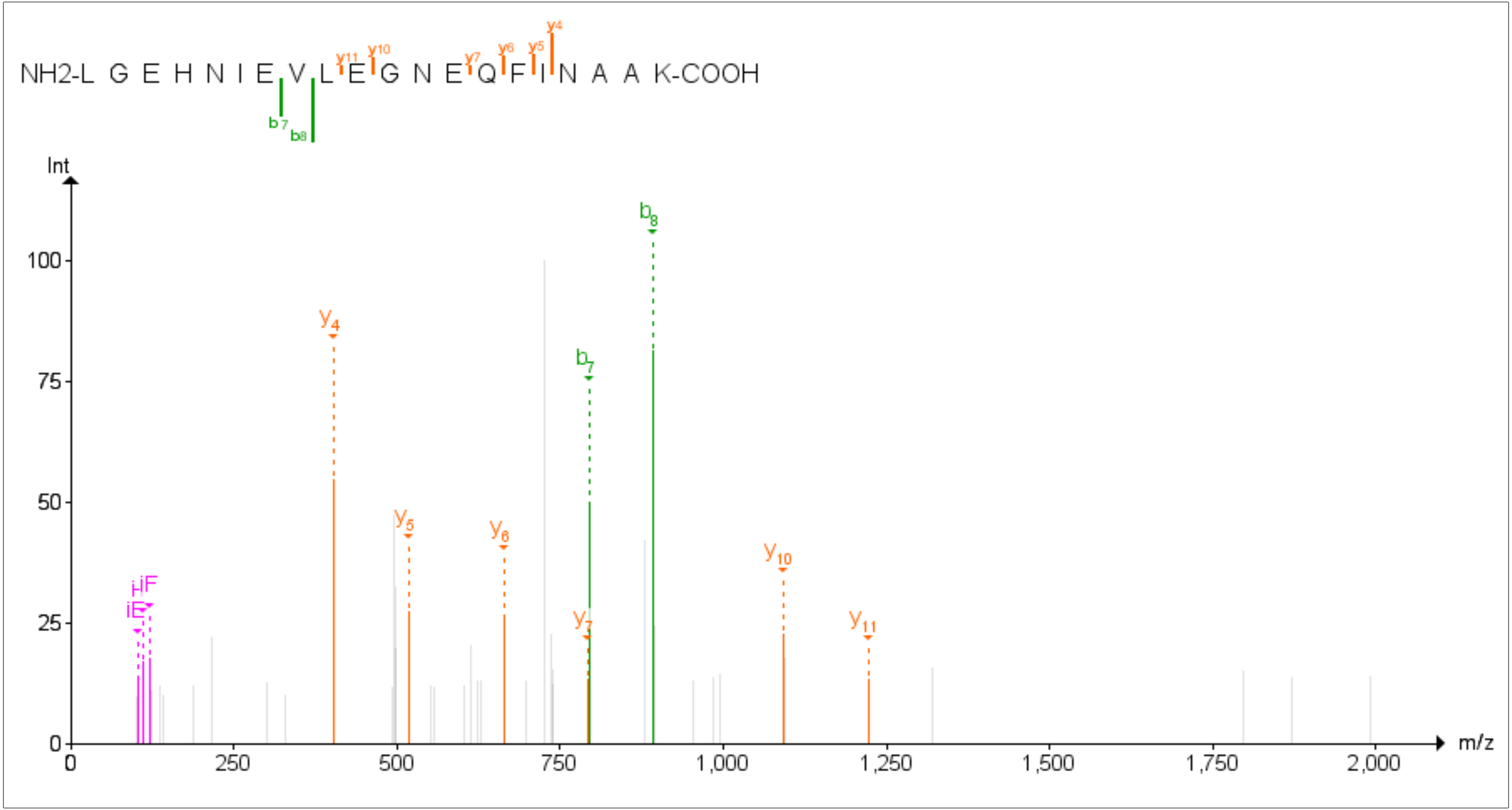

NH2-L G E H N I E V L E G N E Q F I N A A K-COOH
b7
b8
y11
y10
y7
y6
y5
y4
Int
m/z
iE
iF
y4
b8
b7
y5
y6
y7
y10
y11
0
25
50
75
100
0
250
500
750
1,000
1,250
1,500
1,750
2,000

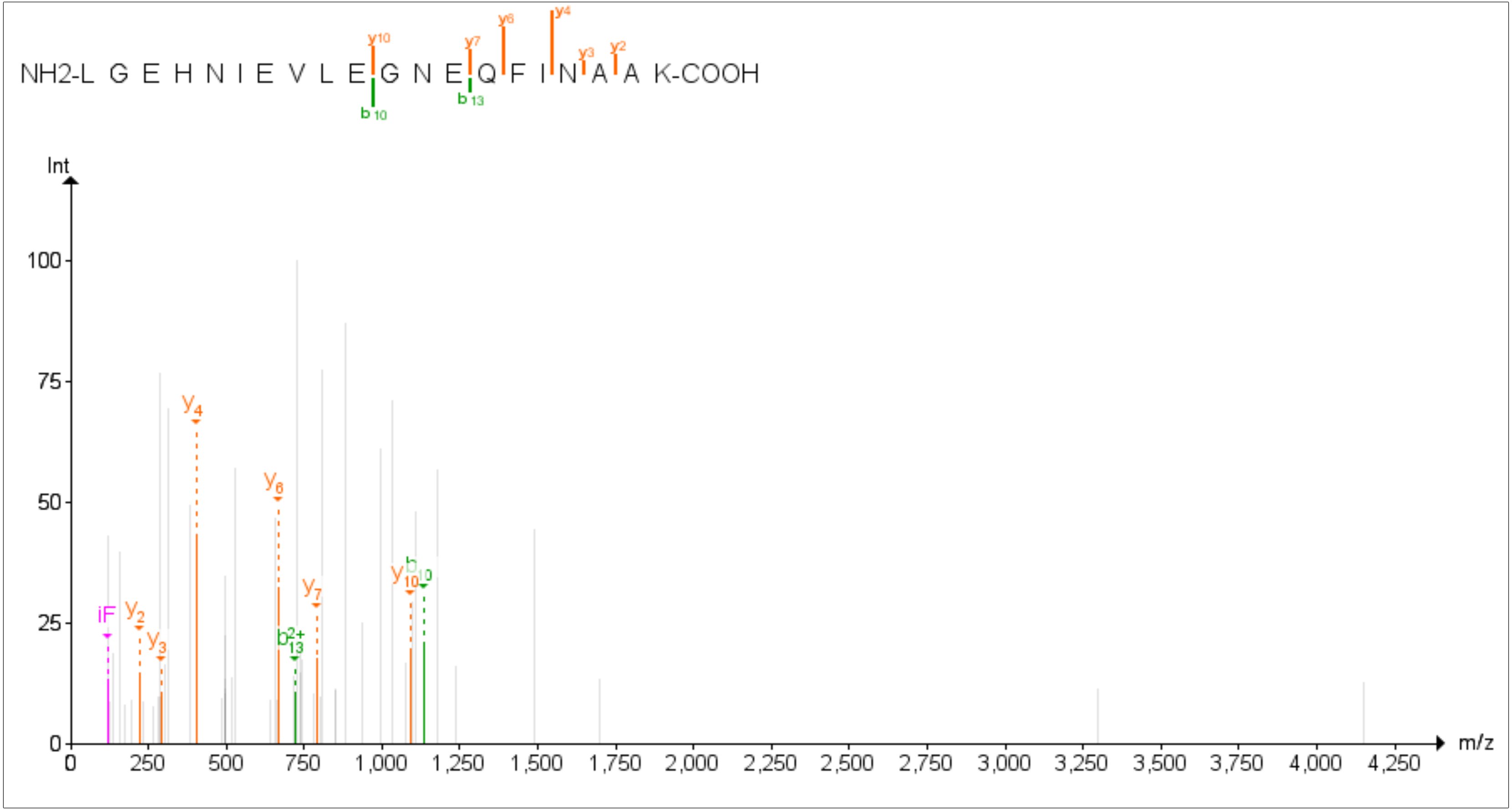
NH2-L G E H N I E V L E G N E Q F I N A A K-COOH
y10
y7
y6
y4
y3
y2
b 10
b 13
Int
100
75
50
25
0
y4
y6
y7
y10
b10
iF
y2
y3
b2+ 13
m/z
0
250
500
750
1,000
1,250
1,500
1,750
2,000
2,250
2,500
2,750
3,000
3,250
3,500
3,750
4,000
4,250

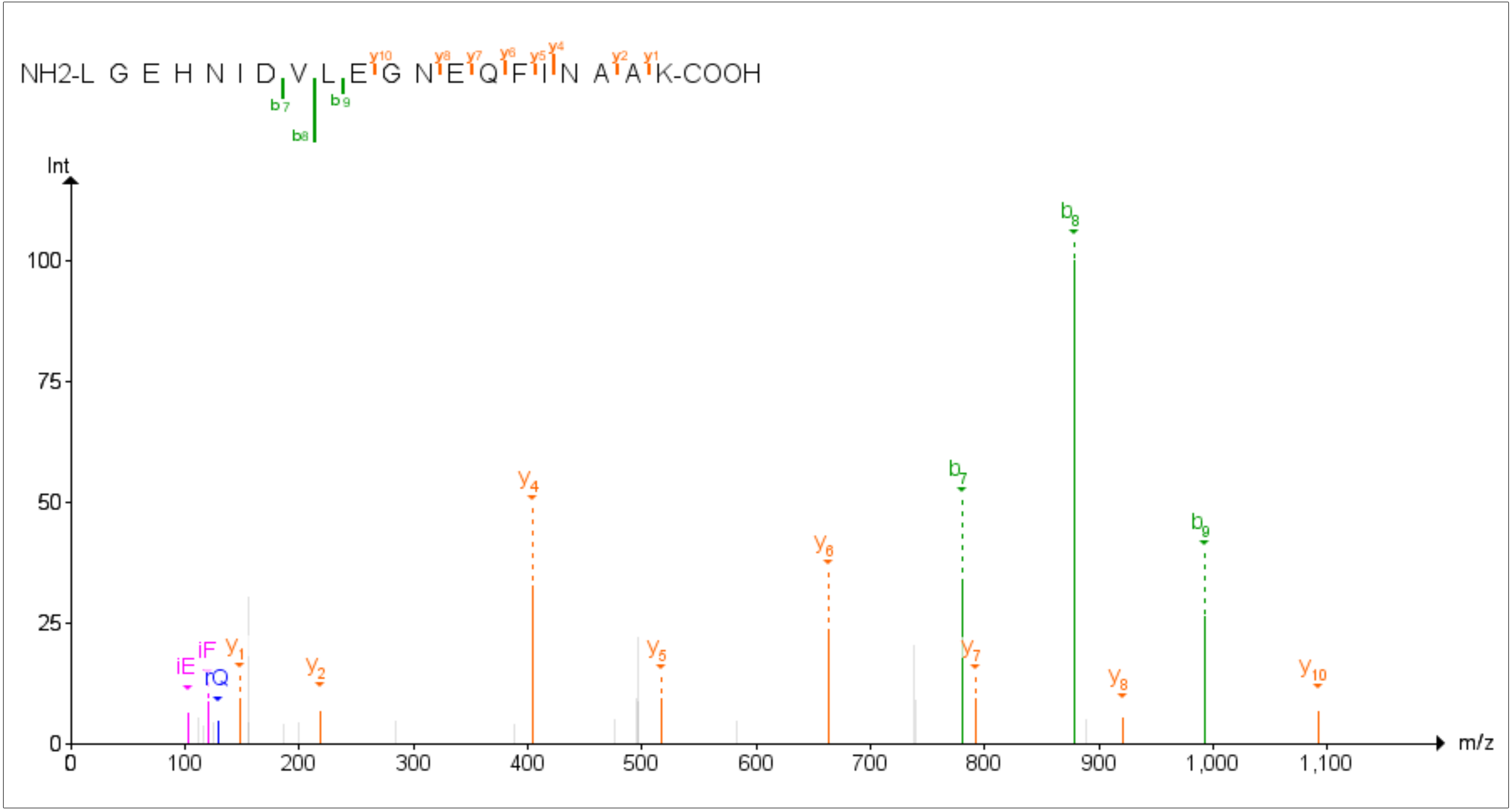
NH2-L G E H N I D V L E G N E Q F I N A A K-COOH
b7
b8
b9
y10
y8
y7
y6
y5
y4
y2
y1
Int
m/z
100
75
50
25
0
0
100
200
300
400
500
600
700
800
900
1,000
1,100
iE
iF
rQ

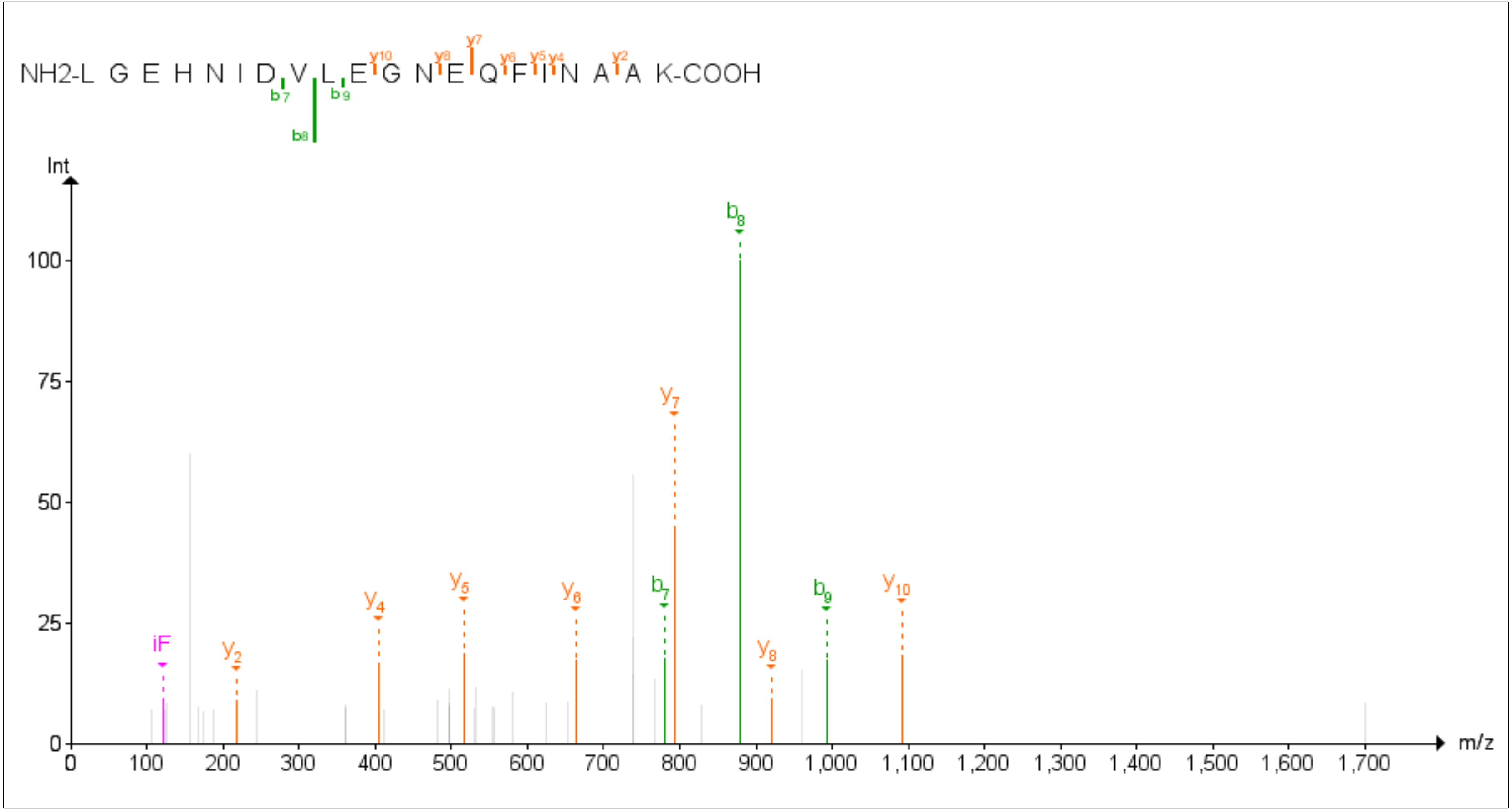

NH2-L G E H N I D V L E G N E Q F I N A A K-COOH
b7
b8
b9
y10
y8
y7
y6
y5
y4
y2
Int
100
75
50
25
0
iF
y2
y4
y5
y6
b7
y7
b8
y8
b9
y10
0
100
200
300
400
500
600
700
800
900
1,000
1,100
1,200
1,300
1,400
1,500
1,600
1,700
m/z

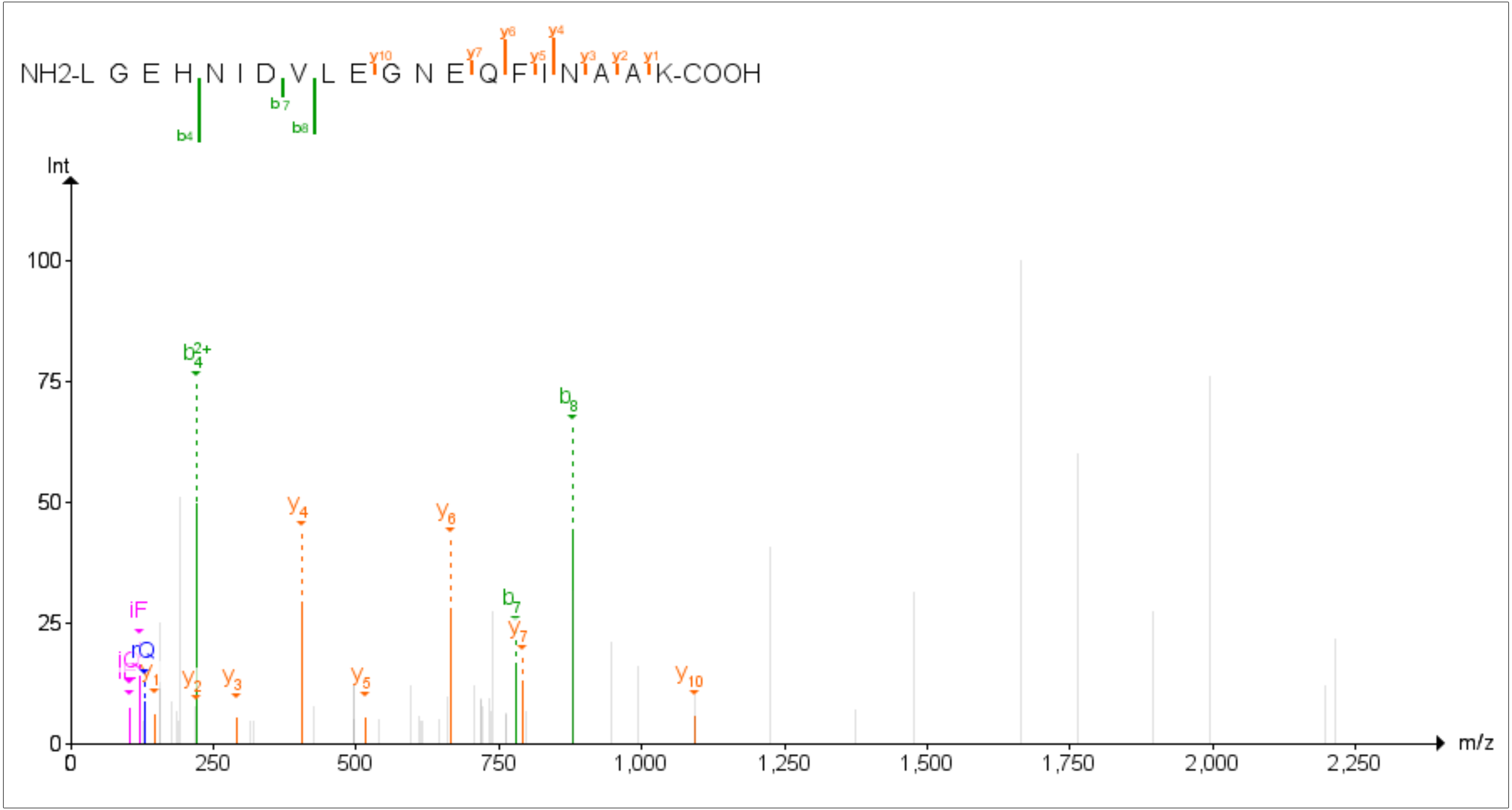

NH2-L G E H N I D V L E G N E Q F I N A A K-COOH
y10 y7 y6 y5 y4 y3 y2 y1
b4 b7 b8
Int
100
75
50
25
0
0 250 500 750 1,000 1,250 1,500 1,750 2,000 2,250
m/z
b4 2+
b8
y4
y6
b7
y7
iF
rQ
y1 y2 y3 y5 y10

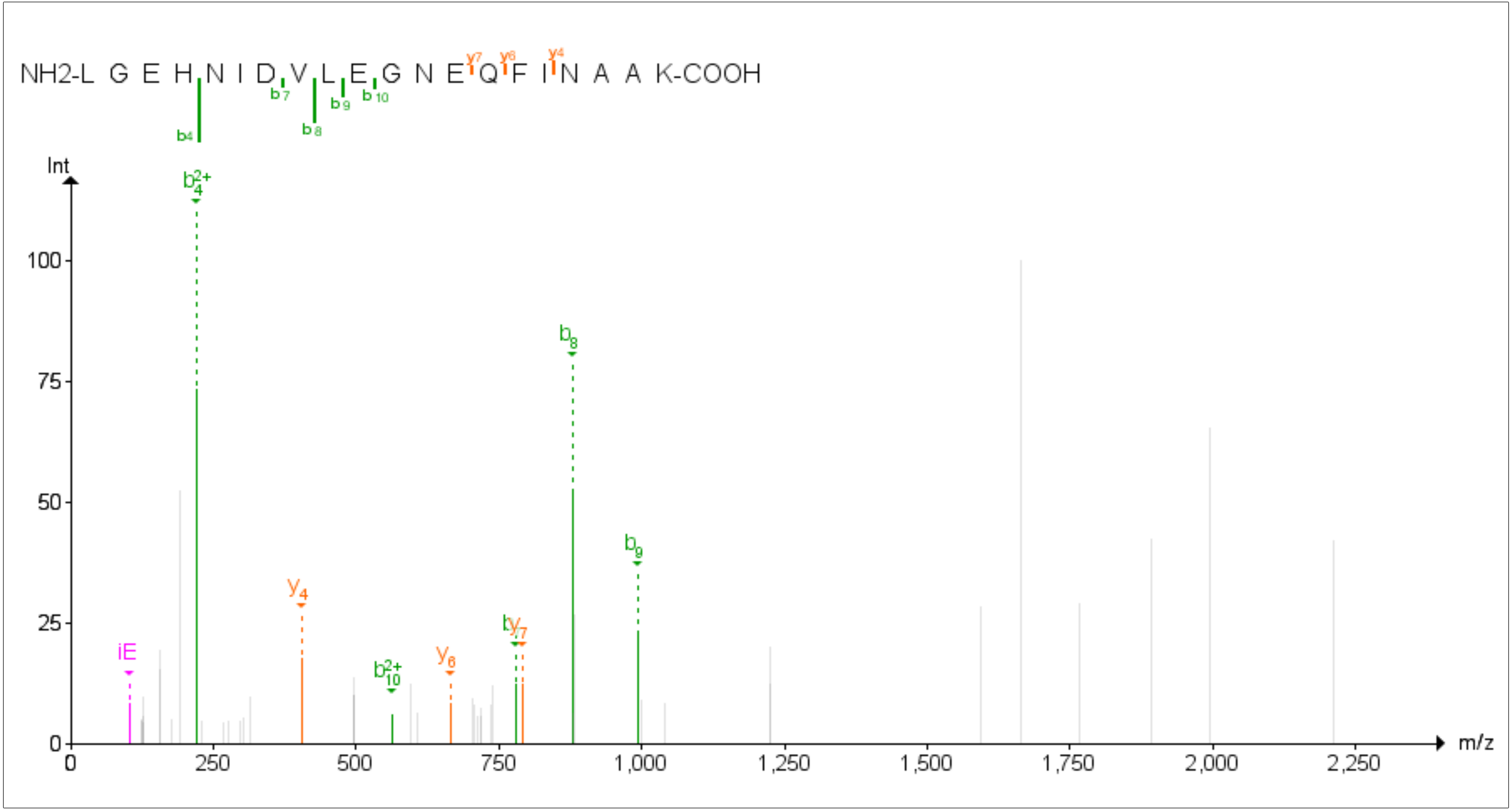

NH2-L G E H N I D V L E G N E Q F I N A A K-COOH
b4
b7
b8
b9
b10
y7
y6
y4
Int
b_4^{2+}
b_8
b_9
y_4
iE
b_{10}^{2+}
y_6
b
y_7
100
75
50
25
0
0
250
500
750
1,000
1,250
1,500
1,750
2,000
2,250
m/z

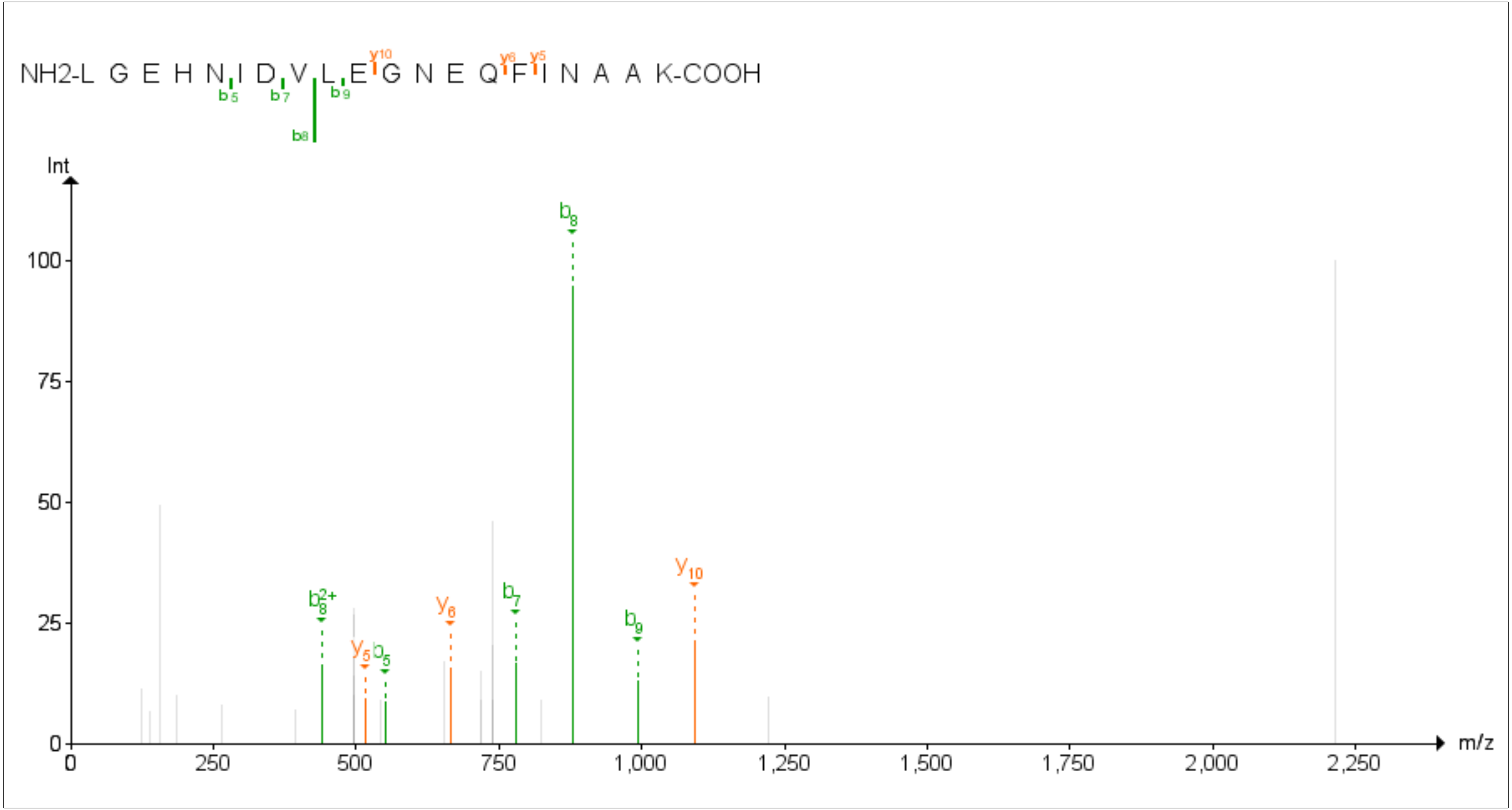
NH2-L G E H N I D V L E G N E Q F I N A A K-COOH
b5
b7
b8
b9
y10
y6
y5
Int
100
75
50
25
0
0
250
500
750
1,000
1,250
1,500
1,750
2,000
2,250
m/z
b8
y10
b8 2+
y6
b7
b9
y5
b5

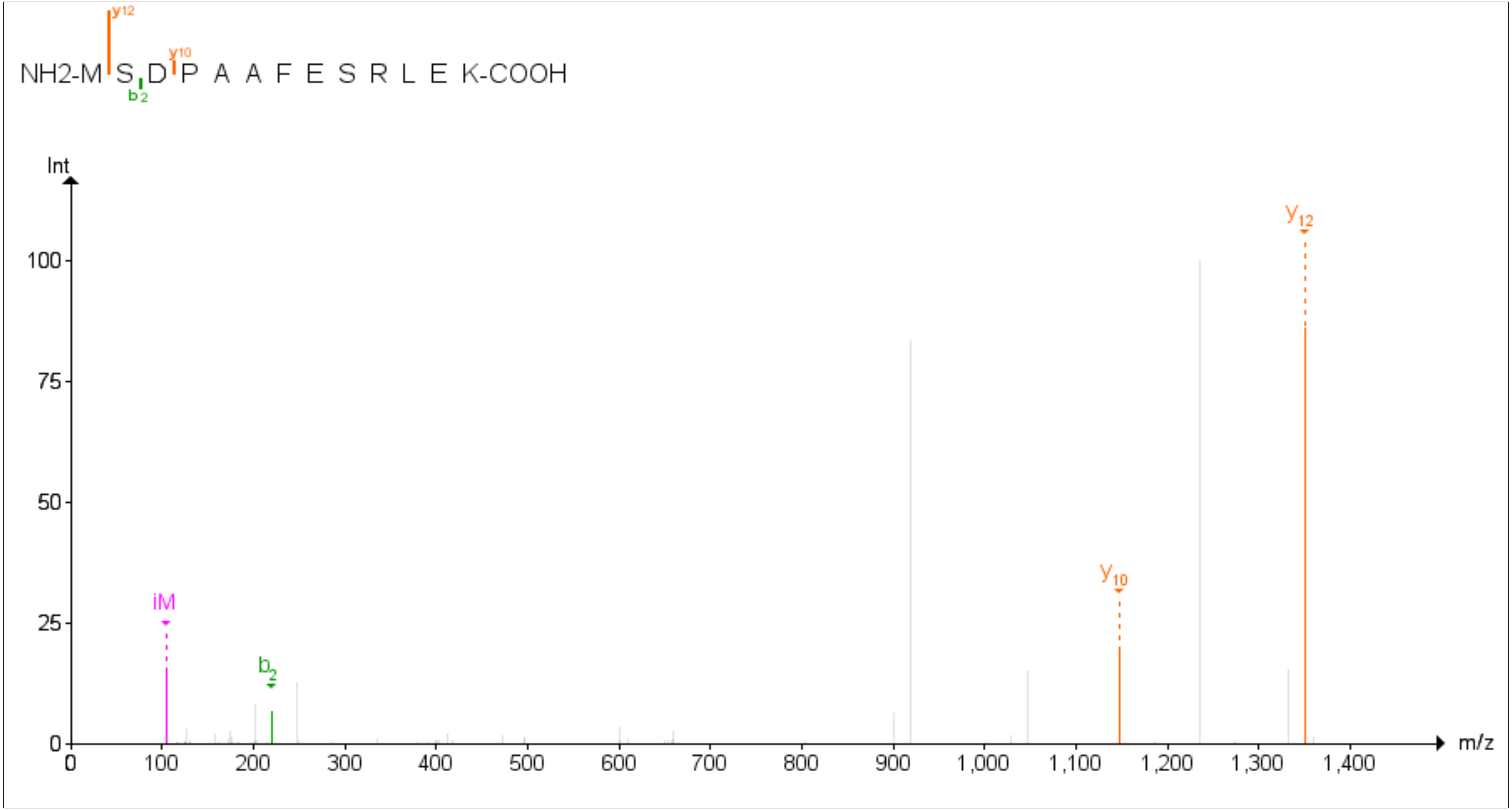

y12
y10
NH2-M S D P A A F E S R L E K-COOH
b2
Int
100
75
50
25
0
y12
y10
iM
b2
0
100
200
300
400
500
600
700
800
900
1,000
1,100
1,200
1,300
1,400
m/z

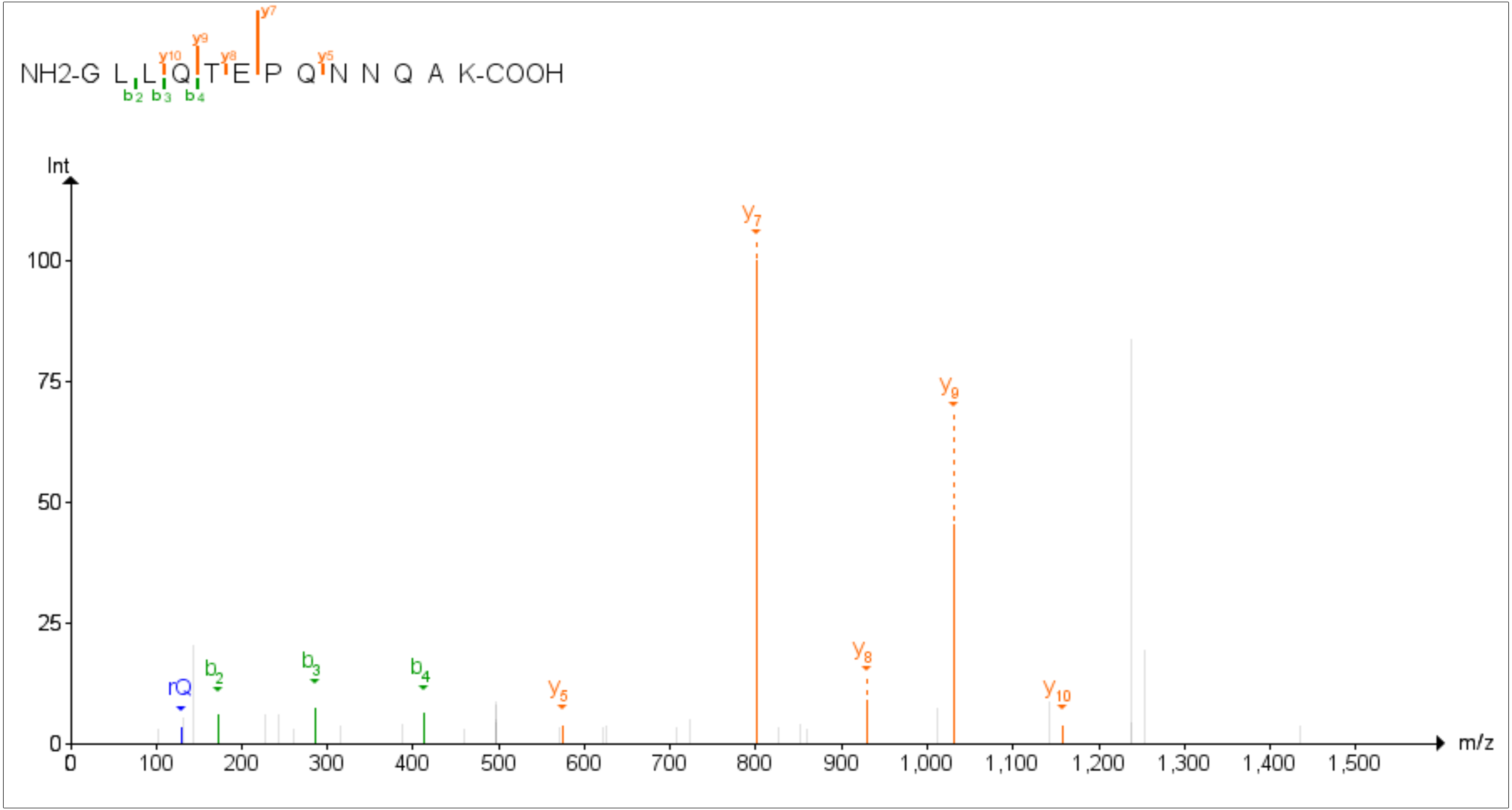
NH2-G L L Q T E P Q N N Q A K-COOH
b2 b3 b4
y10 y9 y8 y7 y5
Int
100
75
50
25
0
y7
y9
y8
y10
y5
rQ
b2
b3
b4
0 100 200 300 400 500 600 700 800 900 1,000 1,100 1,200 1,300 1,400 1,500
m/z

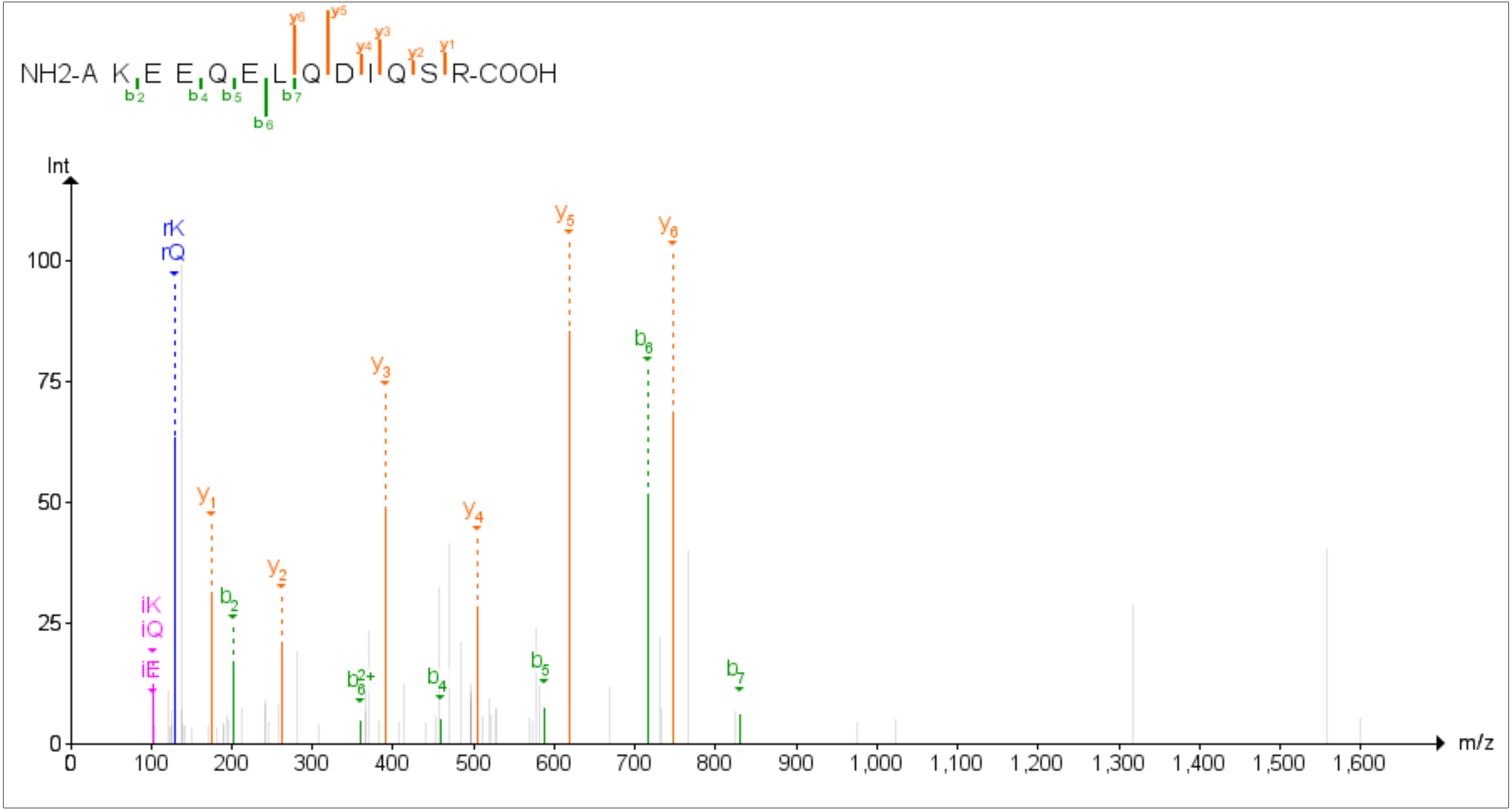

NH2-A K E E Q E L Q D I Q S R-COOH
y6 y5 y4 y3 y2 y1
b2 b4 b5 b6 b7
Int
m/z
rK
rQ
iK
iQ
iE
y1 y2 y3 y4 y5 y6
b2 b4 b5 b6 b7
b_6^{2+}
0
25
50
75
100
0 100 200 300 400 500 600 700 800 900 1,000 1,100 1,200 1,300 1,400 1,500 1,600

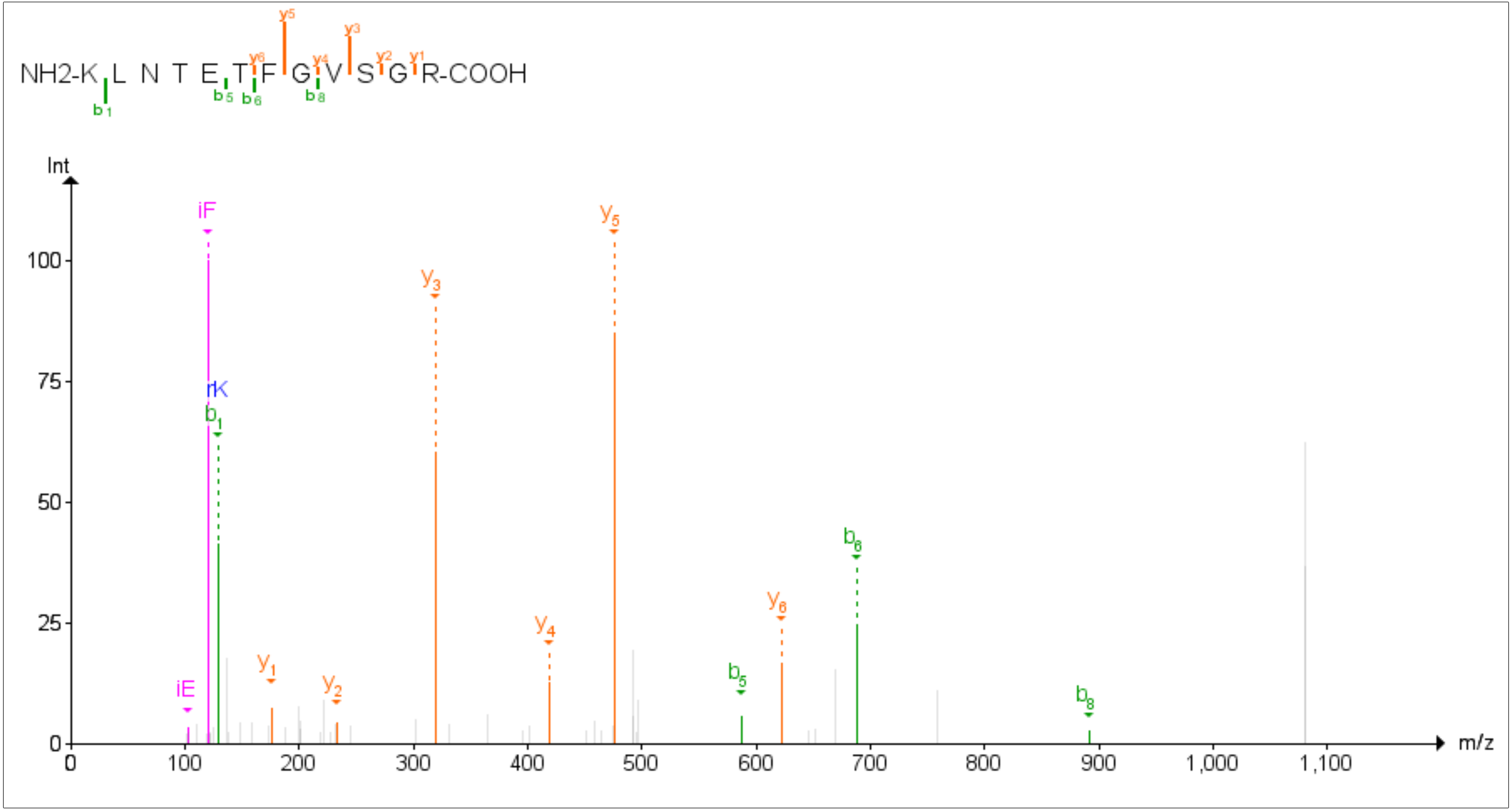

NH2-K L N T E T F G V S G R-COOH
y5 y3 y6 y4 y2 y1
b1 b5 b6 b8
Int
m/z
iF
y5
y3
rK
b1
b6
y6
y4
y1
y2
iE
b5
b8
0
25
50
75
100
0
100
200
300
400
500
600
700
800
900
1,000
1,100

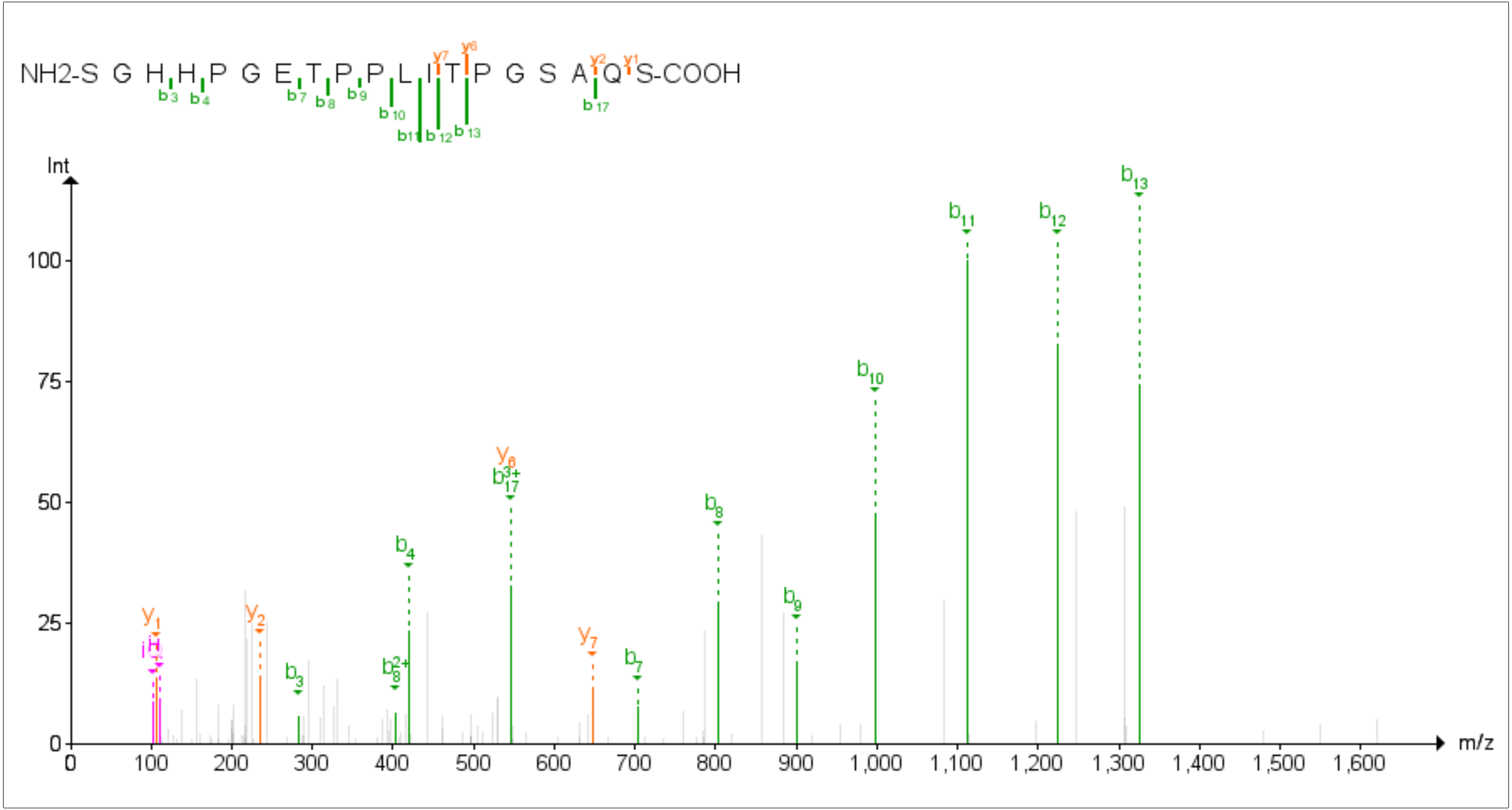
NH2-S G H H P G E T P P L I T P G S A Q S-COOH
Int
m/z

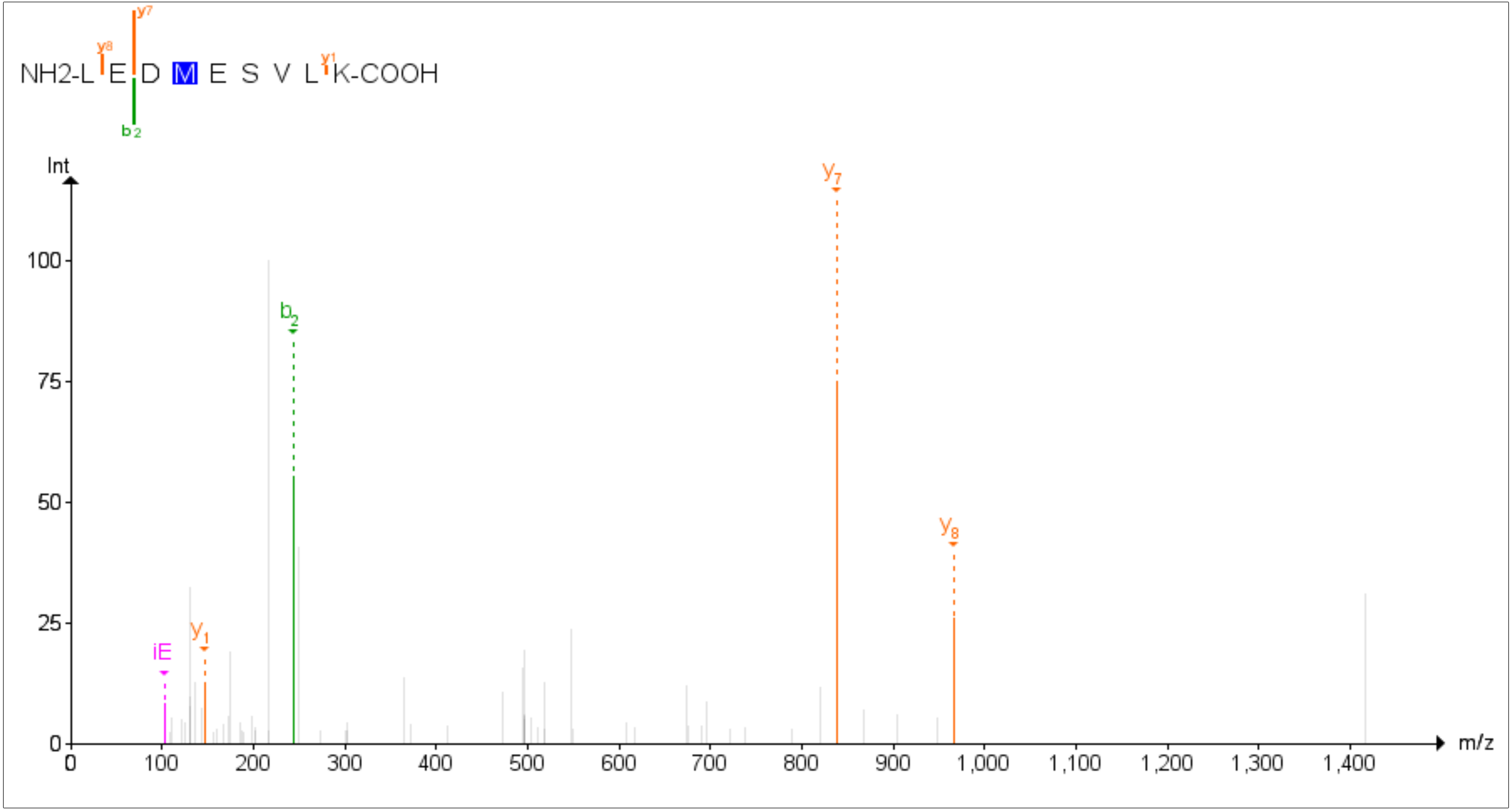

NH2-L E D M E S V L K-COOH
y8
y7
y1
b2
Int
m/z
y7
b2
y8
y1
iE
0
25
50
75
100
0
100
200
300
400
500
600
700
800
900
1,000
1,100
1,200
1,300
1,400

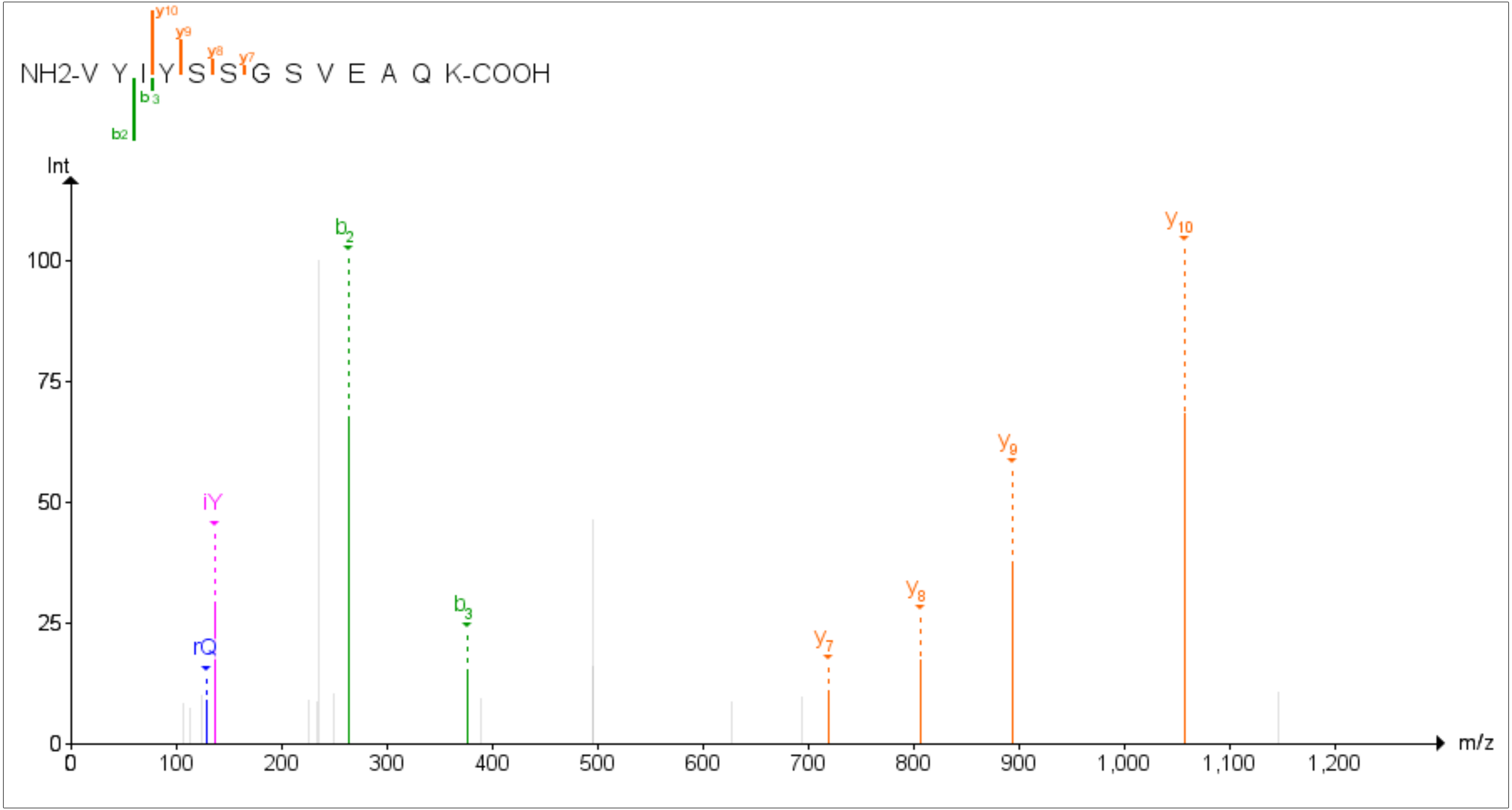

NH2-V Y I Y S S G S V E A Q K-COOH
y10
y9
y8
y7
b2
b3
Int
100
75
50
25
0
iY
rQ
b2
b3
y7
y8
y9
y10
0
100
200
300
400
500
600
700
800
900
1,000
1,100
1,200
m/z

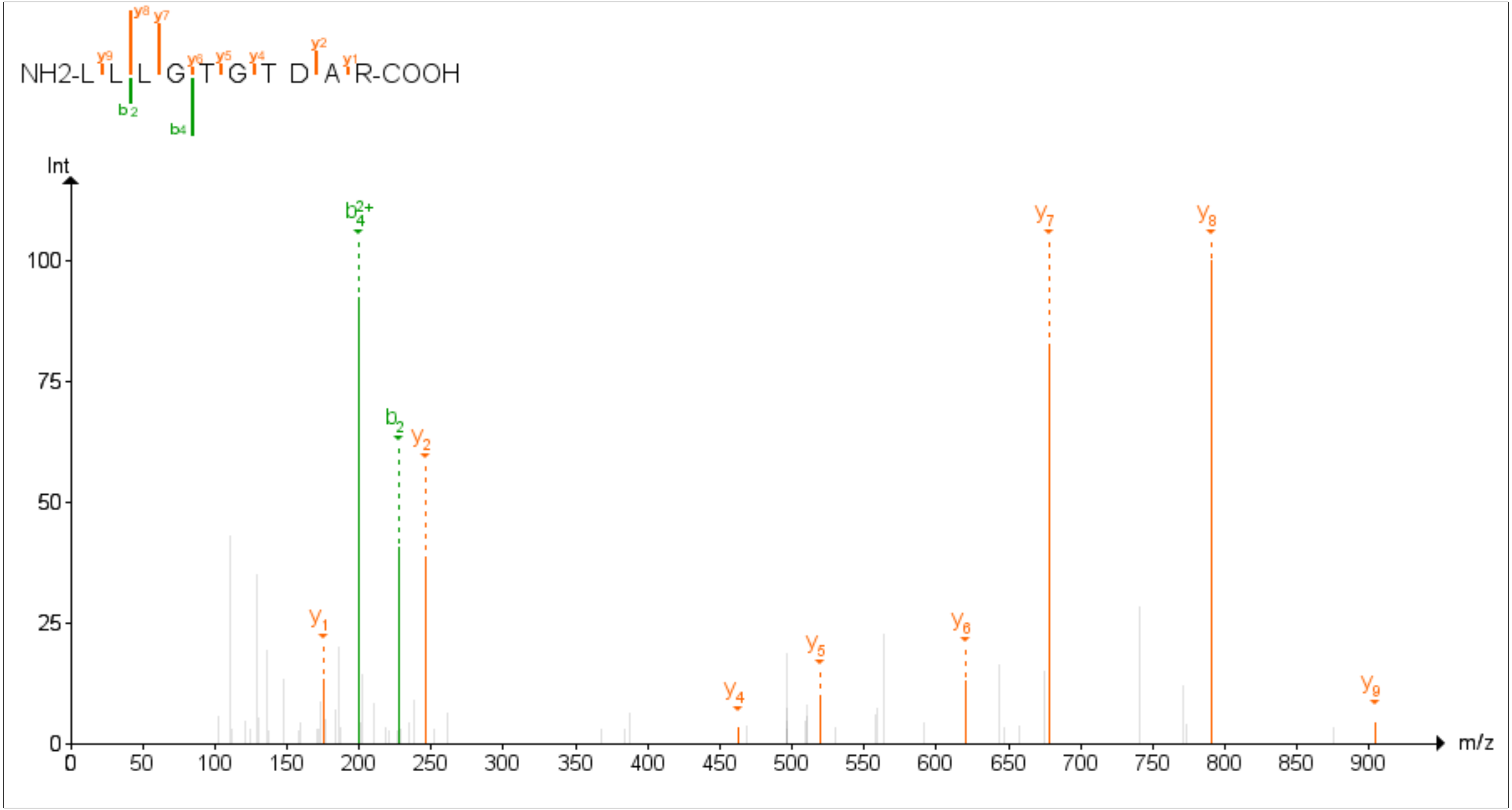

NH2-L L L G T G T D A R-COOH
y9 y8 y7 y6 y5 y4 y2 y1
b2 b4
Int
m/z
b_4^{2+}
b_2
y_1 y_2 y_4 y_5 y_6 y_7 y_8 y_9
0 25 50 75 100
0 50 100 150 200 250 300 350 400 450 500 550 600 650 700 750 800 850 900

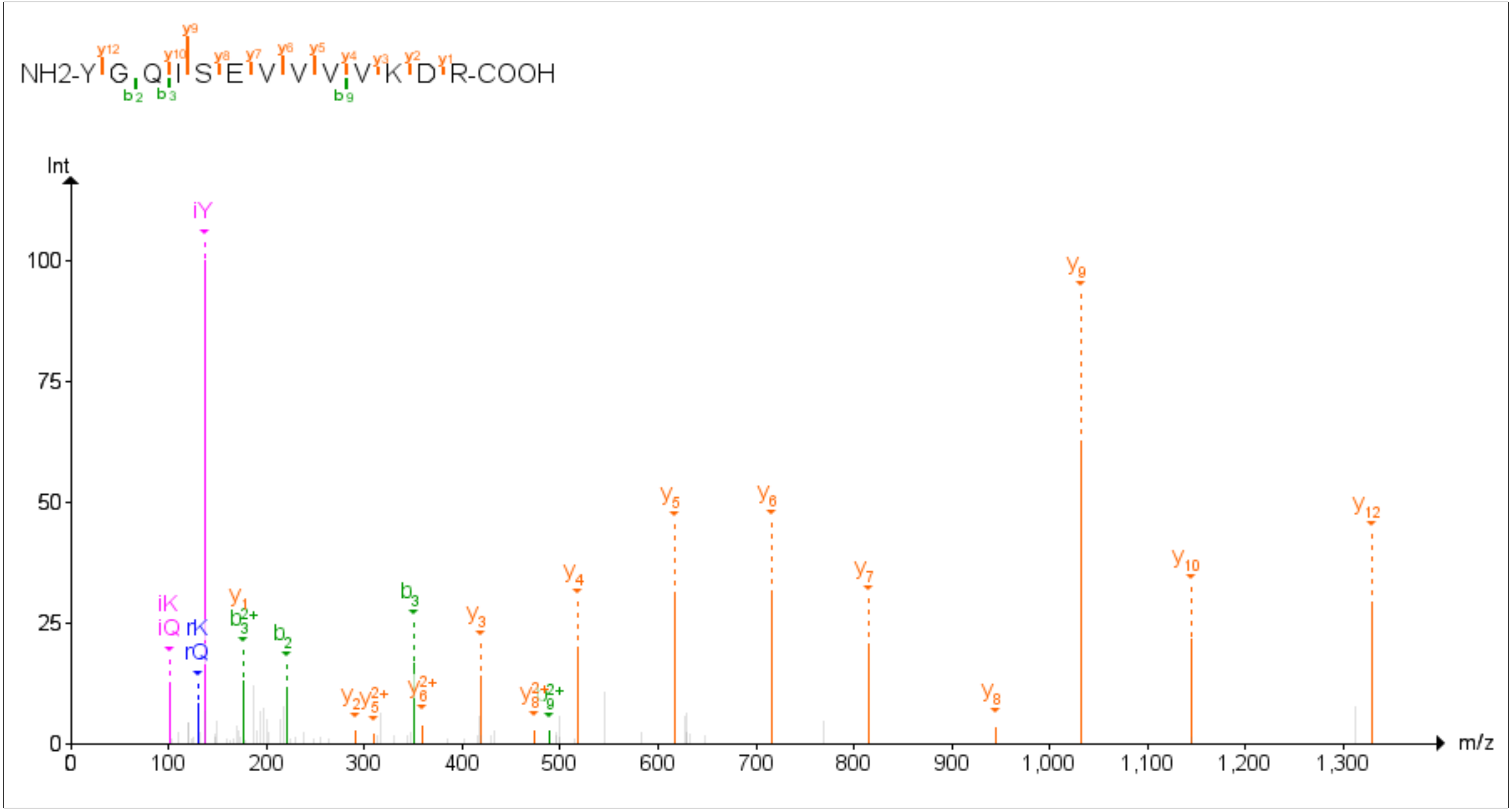
NH2-YGQIISEVVVVKDR-COOH
Int
m/z
iY
y9
y5
y6
y12
y10
y4
y7
y3
b3
y1
b3 2+
b2
iK
iQ
rK
rQ
y8
y2
y5 2+
y6 2+
y8 2+
y9 2+
b9

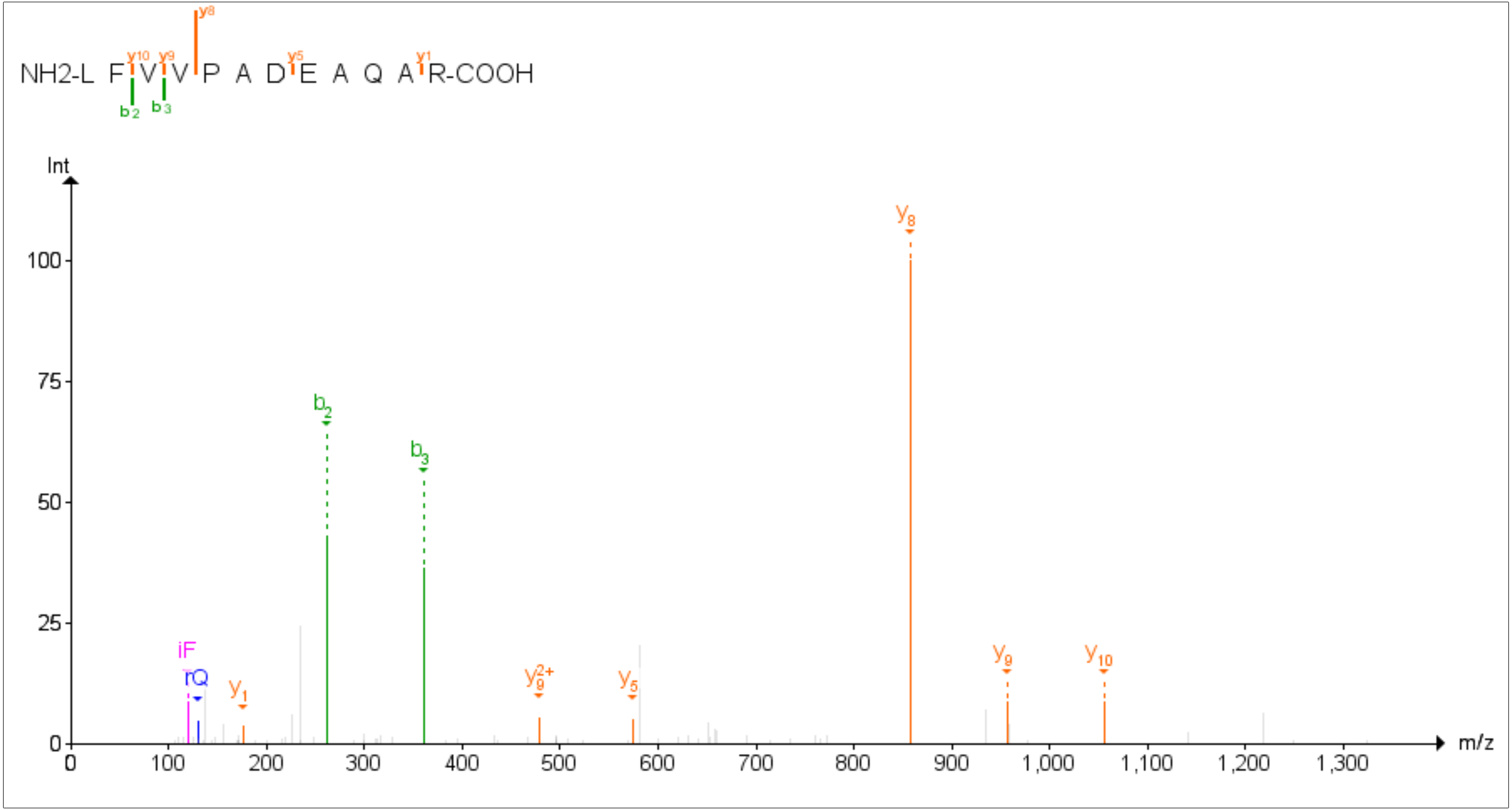

NH2-L F V V P A D E A Q A R-COOH
y10 y9 y8 y5 y1
b2 b3
Int
100
75
50
25
0
y8
b2
b3
iF
rQ
y1
y9 2+
y5
y9
y10
0 100 200 300 400 500 600 700 800 900 1,000 1,100 1,200 1,300
m/z

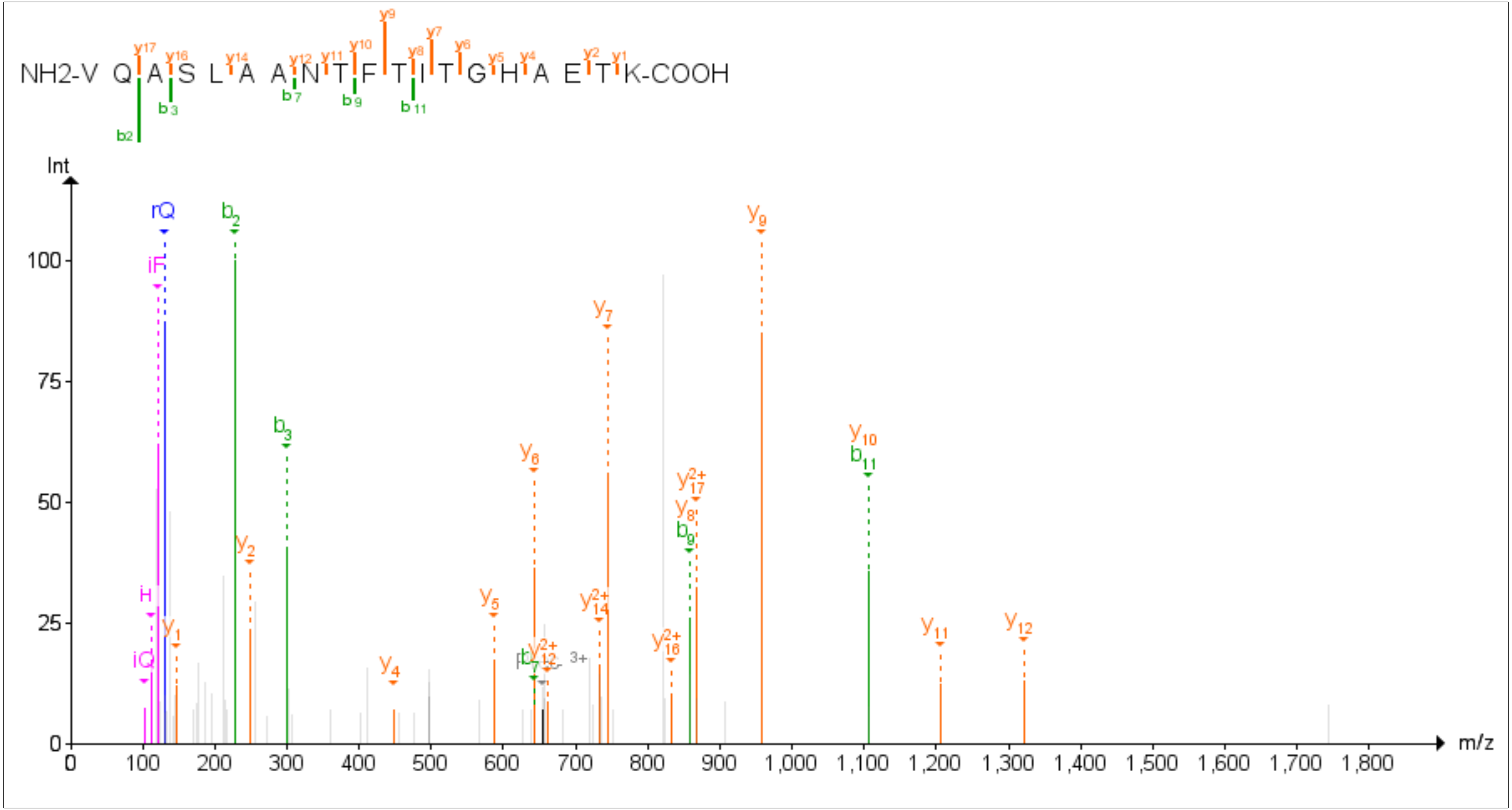
NH2-V Q A S L A A N T F T I T G H A E T K-COOH
Int
m/z
rQ
iF
b2
y9
y7
b3
y6
y10
b11
y2+17
y8
b9
y2
y5
y2+14
iH
y1
iQ
y2+16
y11
y12
y4
b7
y2+12
3+

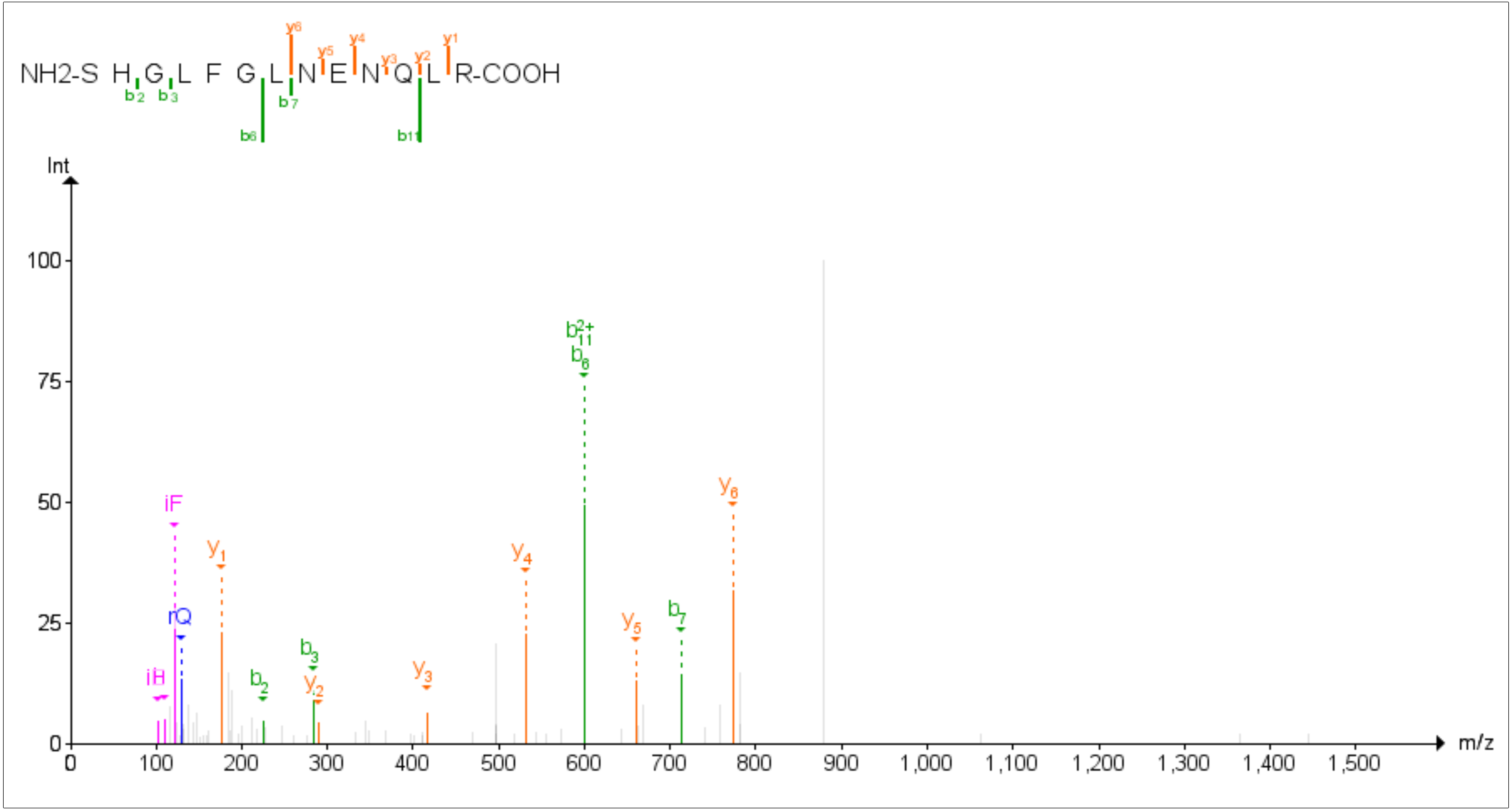

NH2-S H G L F G L N E N Q L R-COOH
y6 y5 y4 y3 y2 y1
b2 b3 b6 b7 b11
Int
m/z
iF
rQ
iH
y1
b2
b3
y2
y3
y4
b11 2+
b6
y5
b7
y6

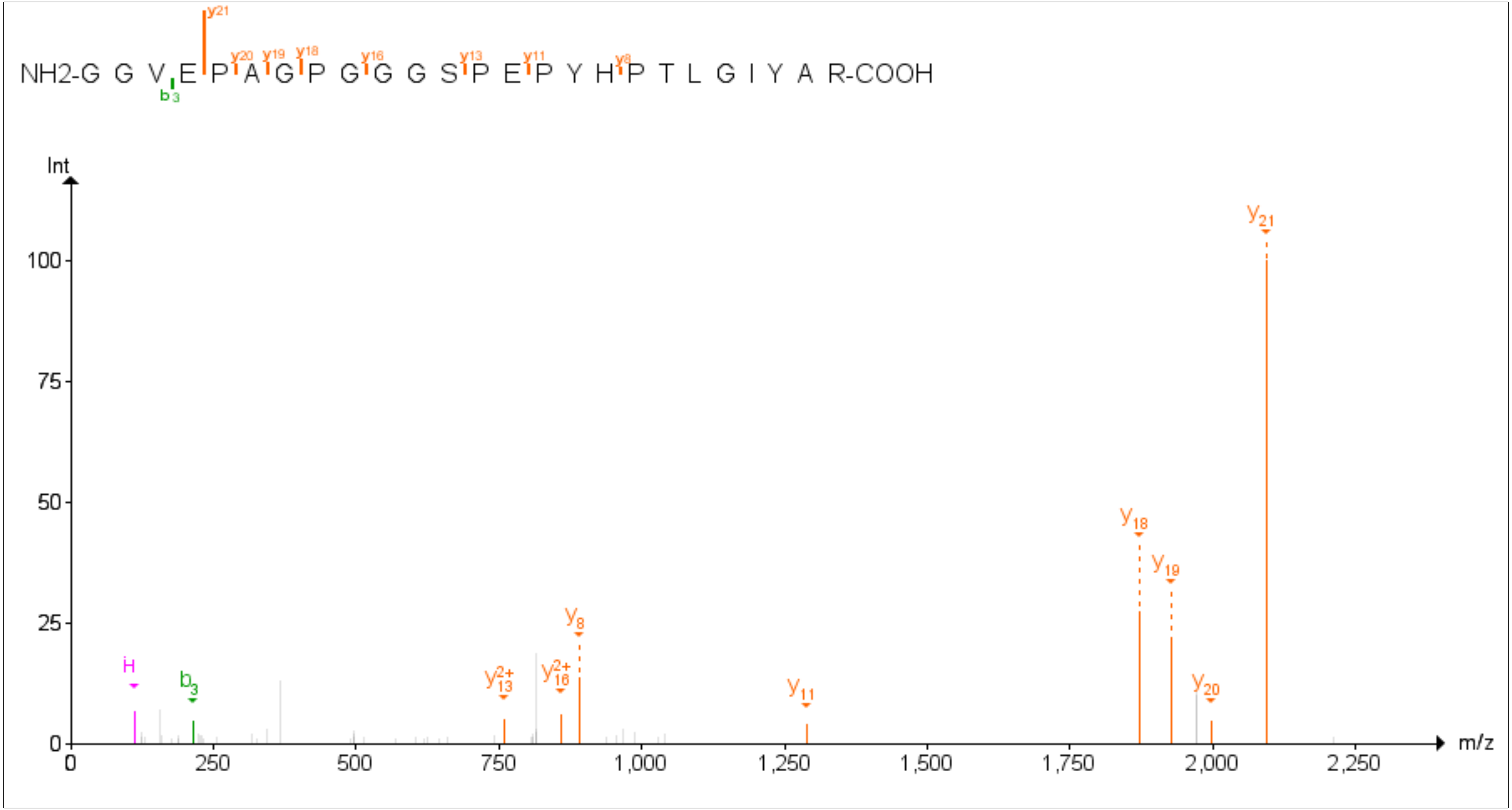

NH2-G G V E P A G P G G G S P E P Y H P T L G I Y A R-COOH
Int
m/z
y21
y20
y19
y18
y16
y13
y11
y8
b3
H
y2+13
y2+16
0
25
50
75
100
250
500
750
1,000
1,250
1,500
1,750
2,000
2,250

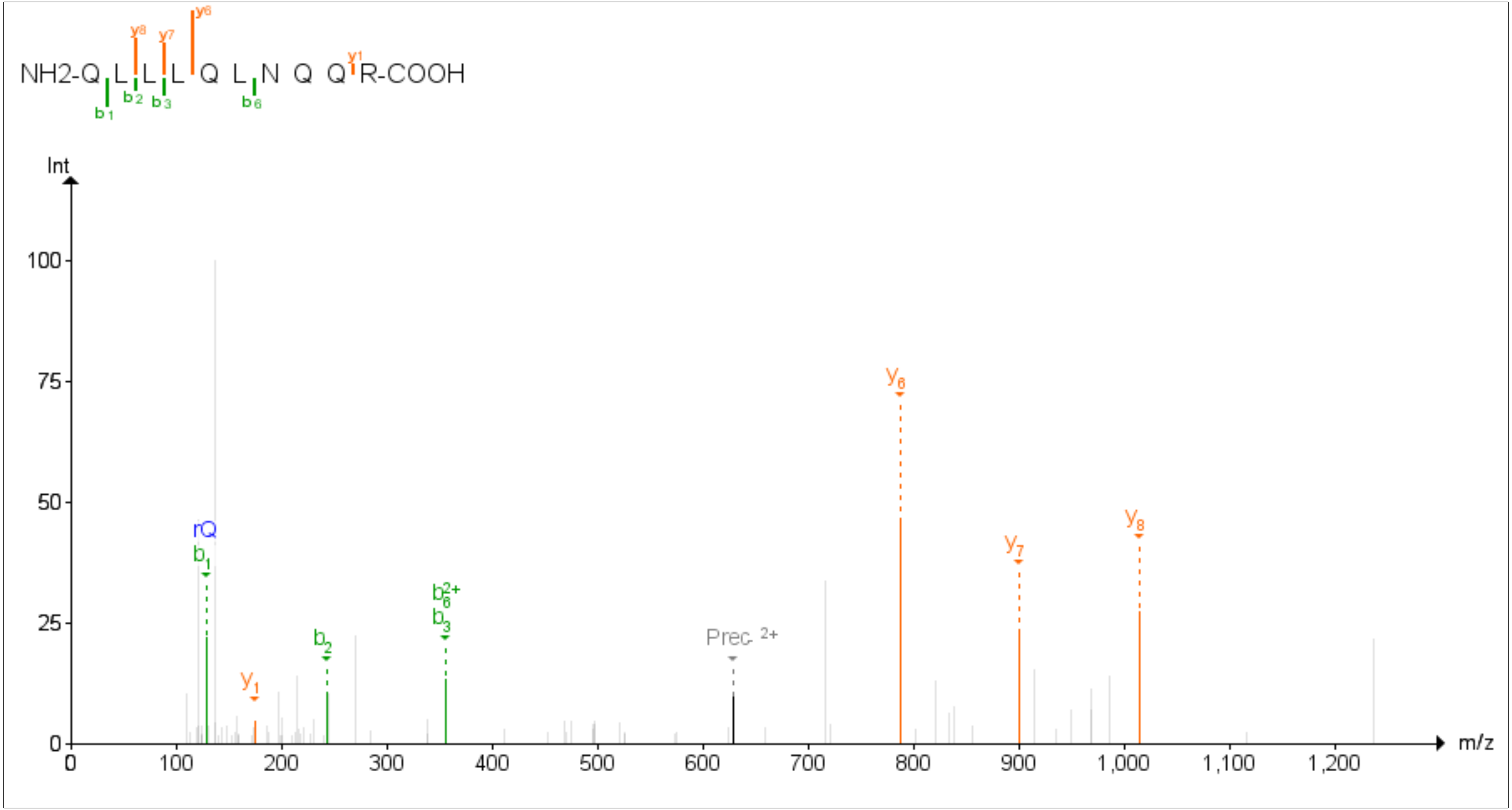

NH2-Q L L L Q L N Q Q R-COOH
y8
y7
y6
y1
b 1
b 2
b 3
b 6
Int
100
75
50
25
0
rQ
b1
y1
b2
b6 2+
b3
Prec 2+
y6
y7
y8
0
100
200
300
400
500
600
700
800
900
1,000
1,100
1,200
m/z

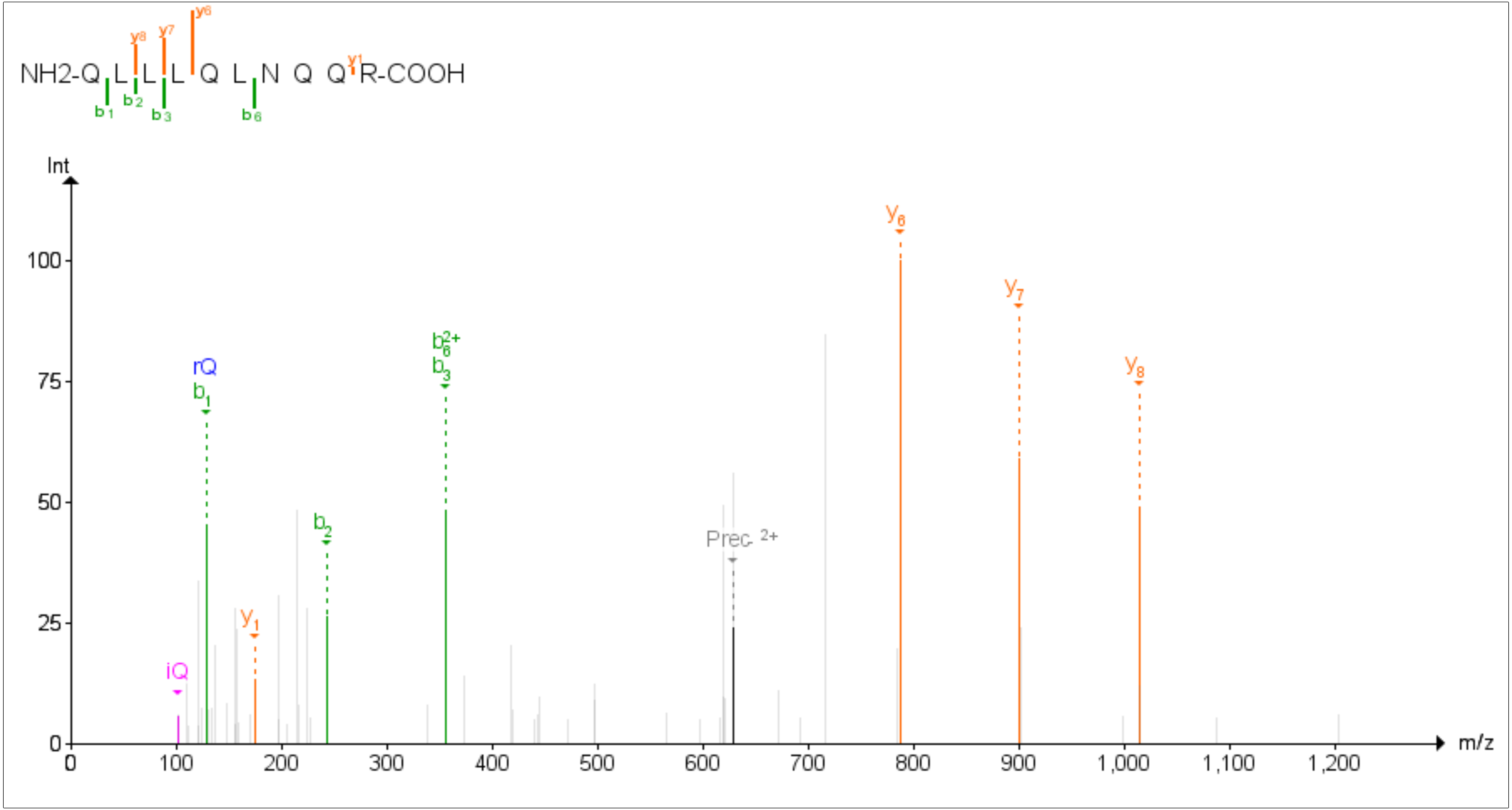

NH2-Q L L L Q L N Q Q R-COOH
y8
y7
y6
y1
b 1
b 2
b 3
b 6
Int
100
75
50
25
0
rQ
b1
iQ
y1
b2
b6 2+
b3
Prec 2+
y6
y7
y8
0
100
200
300
400
500
600
700
800
900
1,000
1,100
1,200
m/z

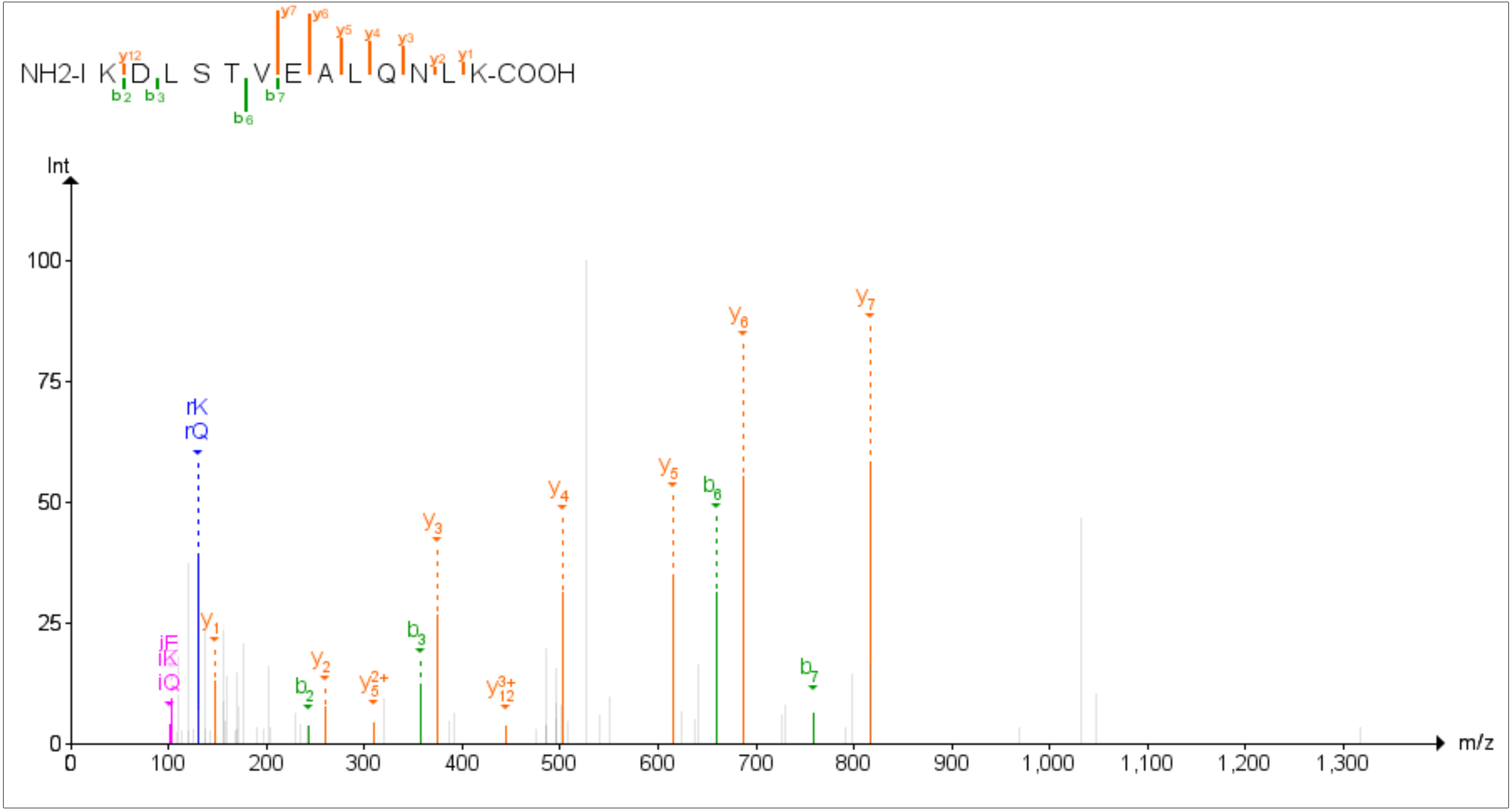

NH2-I K D L S T V E A L Q N L K-COOH
y12 y7 y6 y5 y4 y3 y2 y1
b2 b3 b6 b7
Int
m/z
rK
rQ
iF
iK
iQ
y1 y2 y3 y4 y5 y6 y7
y_5^{2+}
y_{12}^{3+}
b2 b3 b6 b7
0 25 50 75 100
0 100 200 300 400 500 600 700 800 900 1,000 1,100 1,200 1,300

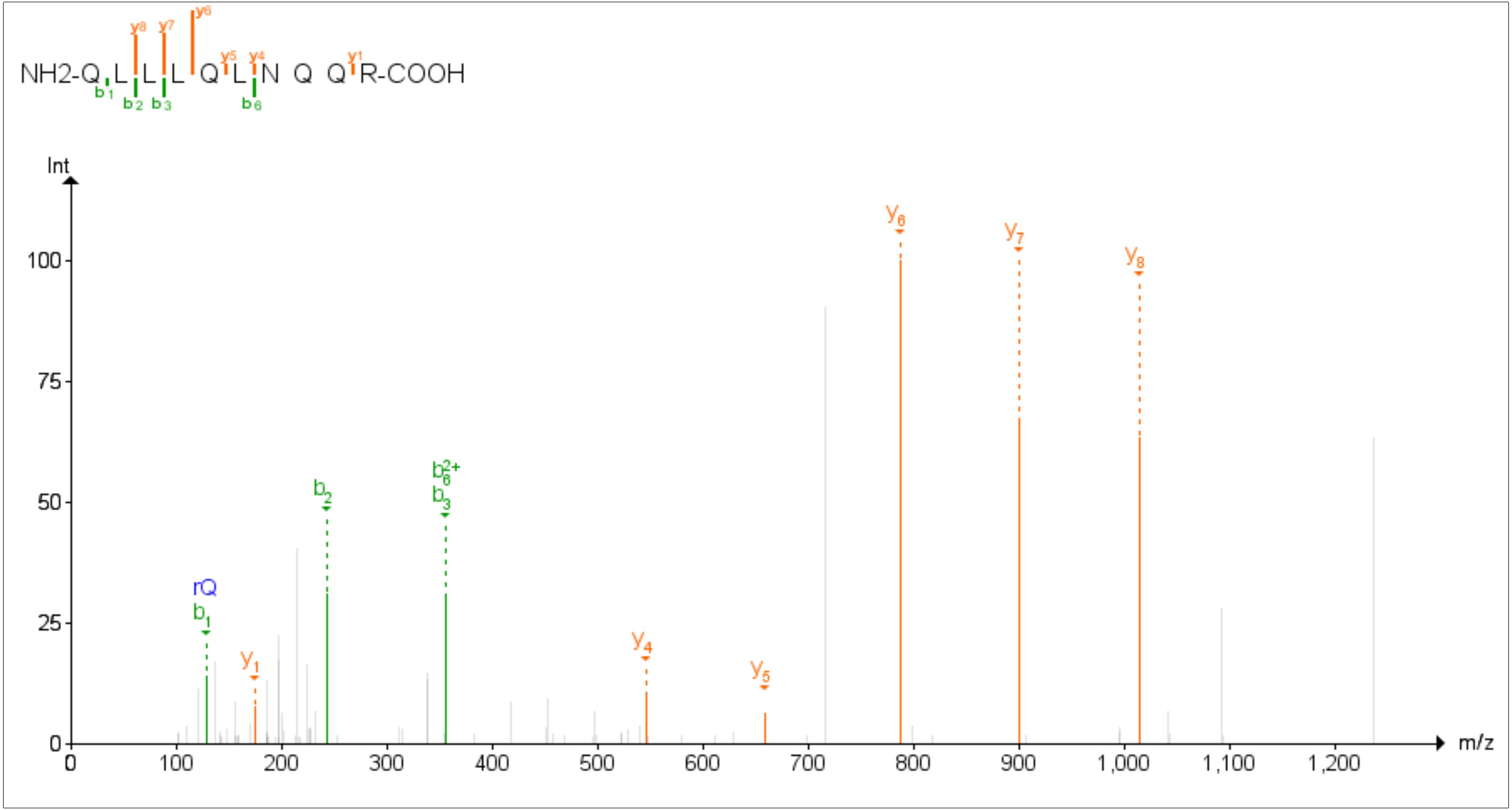
NH2-Q L L L Q L N Q Q R-COOH
y8 y7 y6 y5 y4 y1
b1 b2 b3 b6
Int
100
75
50
25
0
rQ
b1
y1
b2
b6 2+
b3
y4
y5
y6
y7
y8
0 100 200 300 400 500 600 700 800 900 1,000 1,100 1,200
m/z

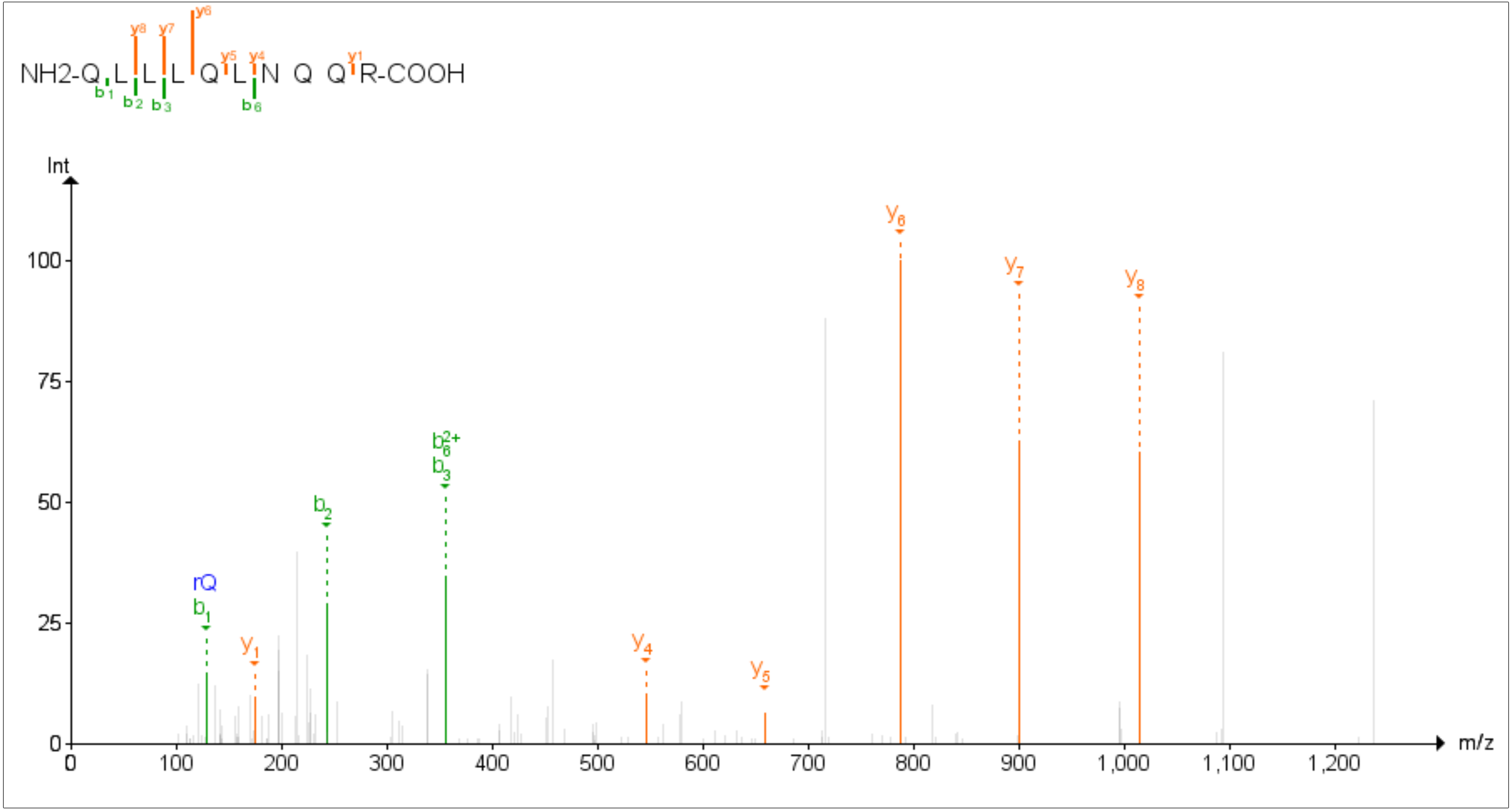
NH2-Q L L L Q L N Q Q R-COOH
y8 y7 y6 y5 y4 y1
b1 b2 b3 b6
Int
100
75
50
25
0
rQ
b1
y1
b2
b6 2+
b3
y4
y5
y6
y7
y8
0 100 200 300 400 500 600 700 800 900 1,000 1,100 1,200
m/z

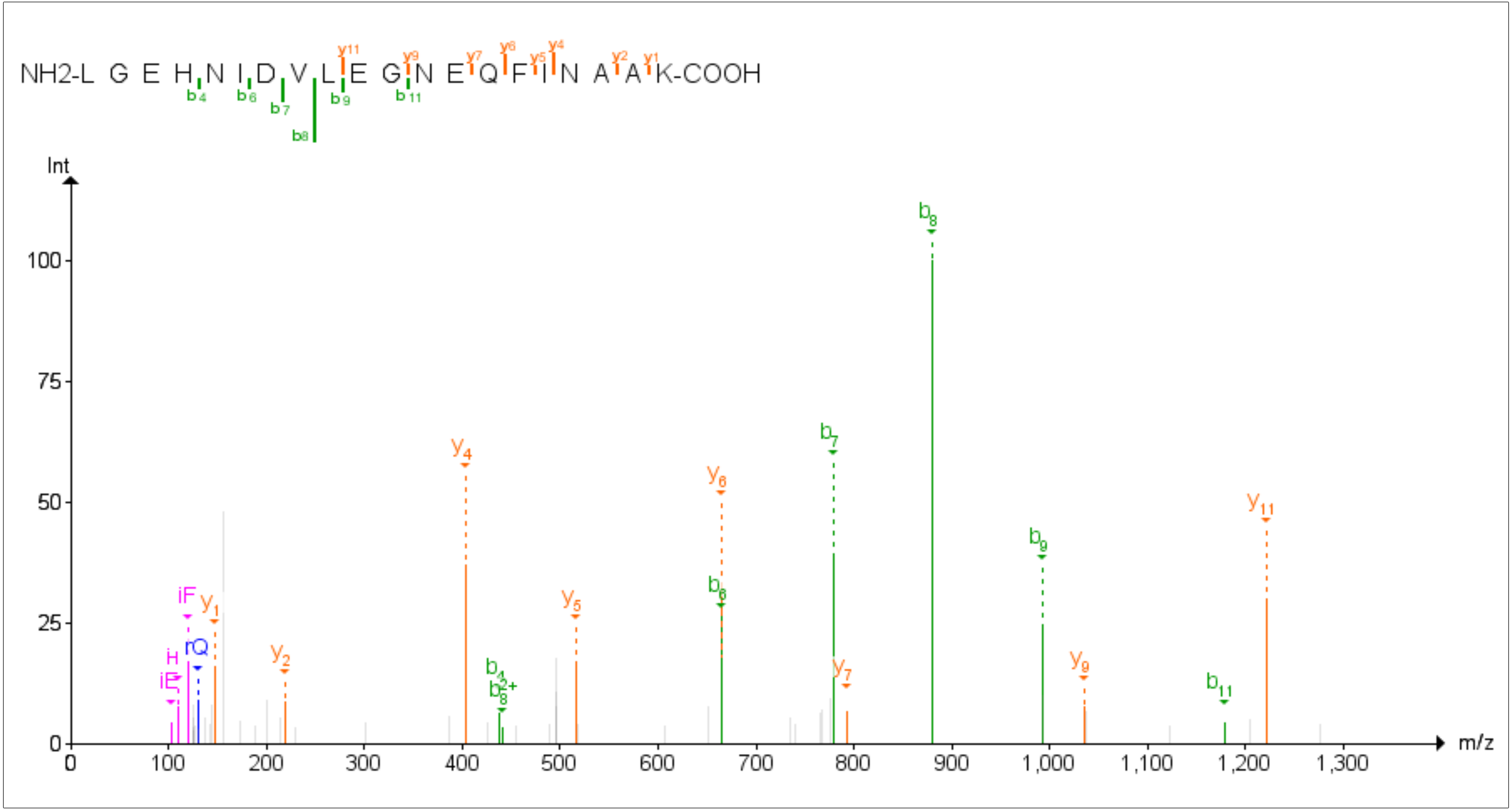

NH2-L G E H N I D V L E G N E Q F I N A A K-COOH
b4 b6 b7 b8 b9 b11
y11 y9 y7 y6 y5 y4 y2 y1
Int
100
75
50
25
0
m/z
0 100 200 300 400 500 600 700 800 900 1,000 1,100 1,200 1,300
b8
b7
b9
b6
b4
b11
y4
y6
y11
y5
y1
y2
y7
y9
iF
iH
iE
rQ

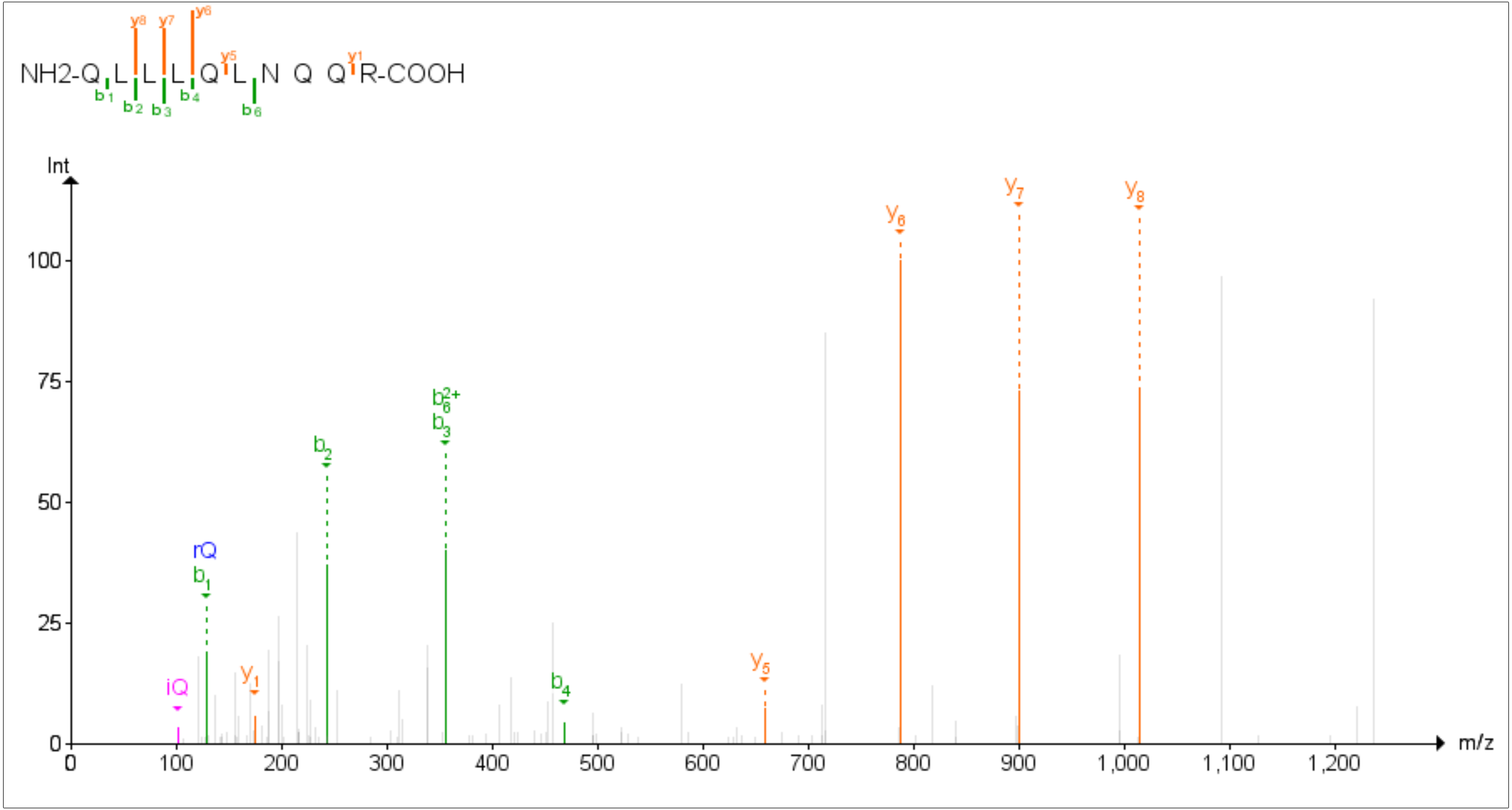

NH2-Q L L L Q L N Q Q R-COOH
y8 y7 y6 y5 y1
b1 b2 b3 b4 b6
Int
100
75
50
25
0
m/z
0 100 200 300 400 500 600 700 800 900 1,000 1,100 1,200
y6
y7
y8
b2
b3
b6 2+
rQ
b1
iQ
y1
b4
y5

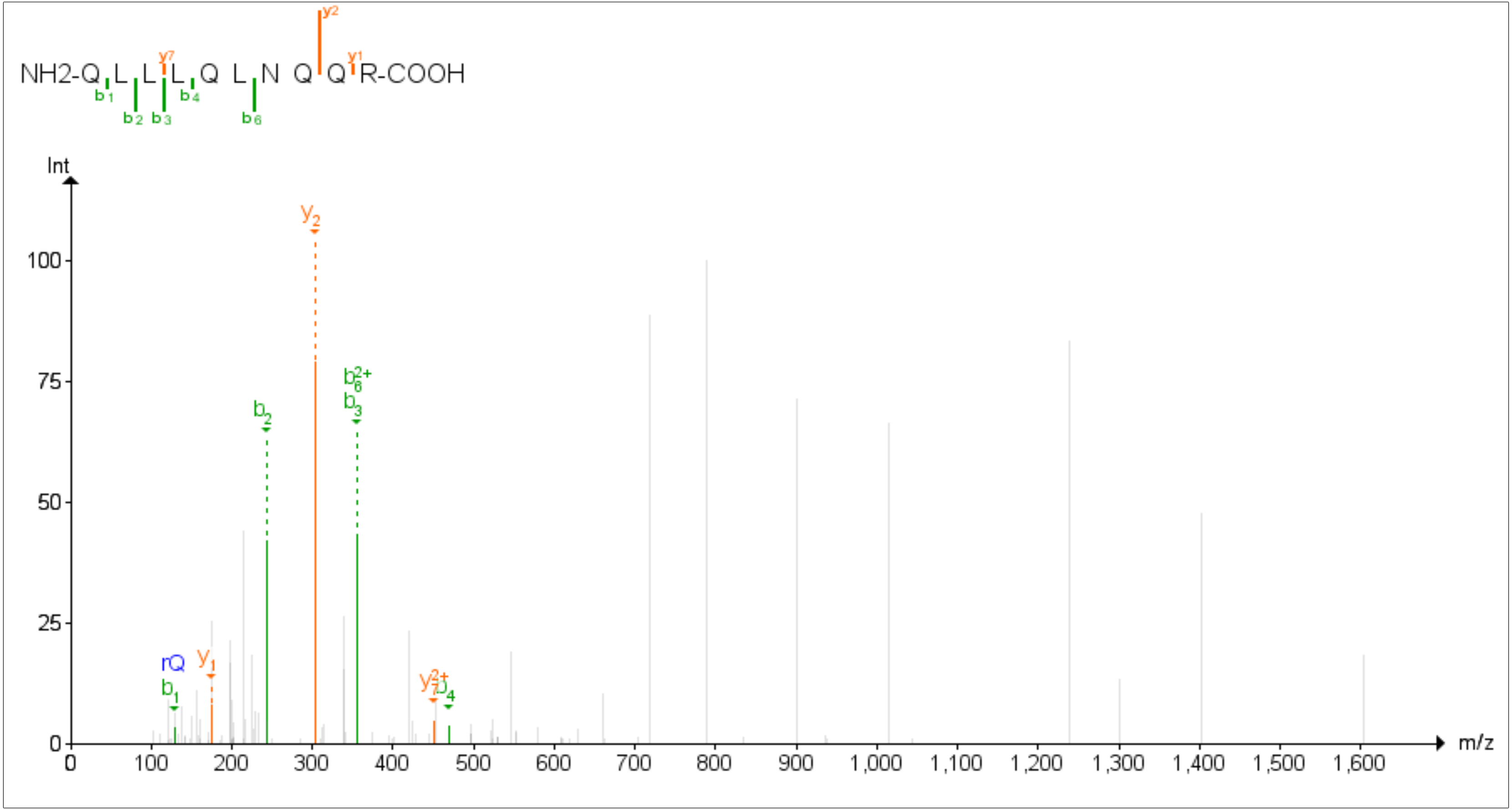

NH2-Q L L L Q L N Q Q R-COOH
b1
b2
b3
b4
b6
y7
y2
y1
Int
m/z
y2
b2
b6 2+
b3
rQ
b1
y1
y7 2+
b4
0
25
50
75
100
0
100
200
300
400
500
600
700
800
900
1,000
1,100
1,200
1,300
1,400
1,500
1,600

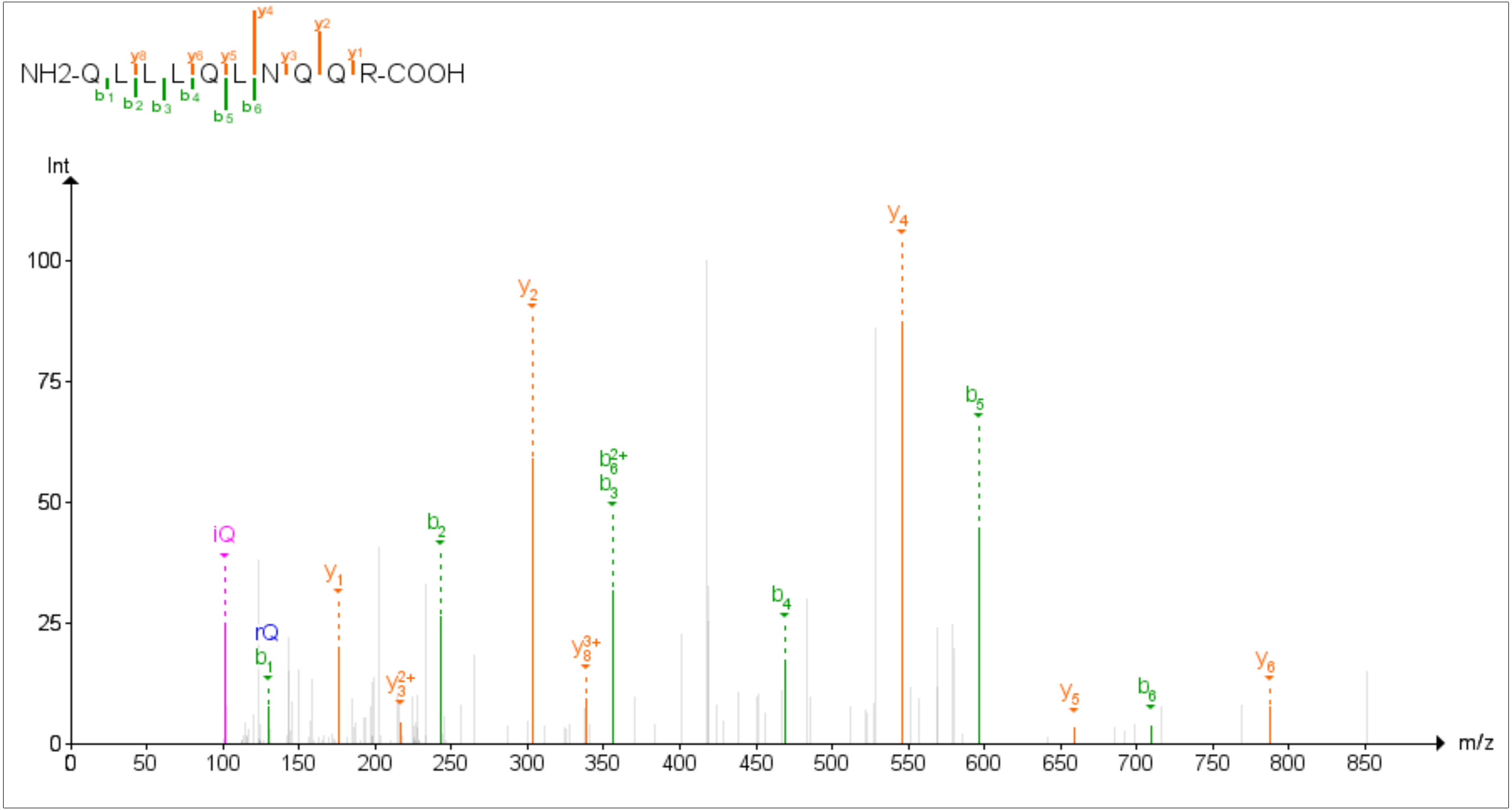

NH2-Q L L L Q L N Q Q R-COOH
y8 y6 y5 y4 y3 y2 y1
b1 b2 b3 b4 b5 b6
Int
100
75
50
25
0
m/z
0 50 100 150 200 250 300 350 400 450 500 550 600 650 700 750 800 850
iQ
rQ
b1
y1
y3 2+
b2
y2
y8 3+
b6 2+
b3
b4
y4
b5
y5
b6
y6

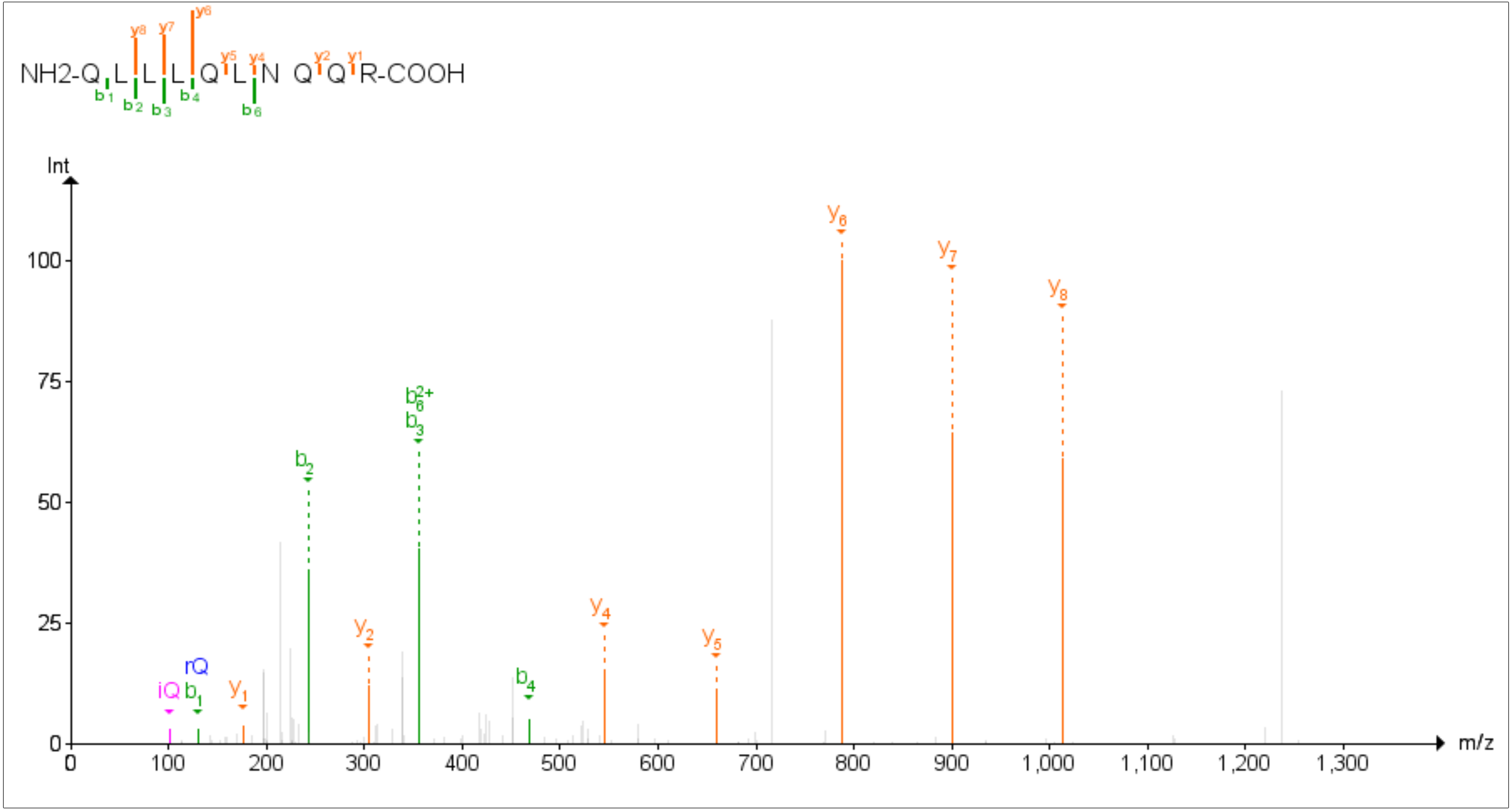

NH2-Q L L L Q L N Q Q R-COOH
y8 y7 y6 y5 y4 y2 y1
b1 b2 b3 b4 b6
Int
m/z
0
25
50
75
100
0
100
200
300
400
500
600
700
800
900
1,000
1,100
1,200
1,300
iQ
rQ
b1
y1
b2
y2
b6 2+
b3
b4
y4
y5
y6
y7
y8

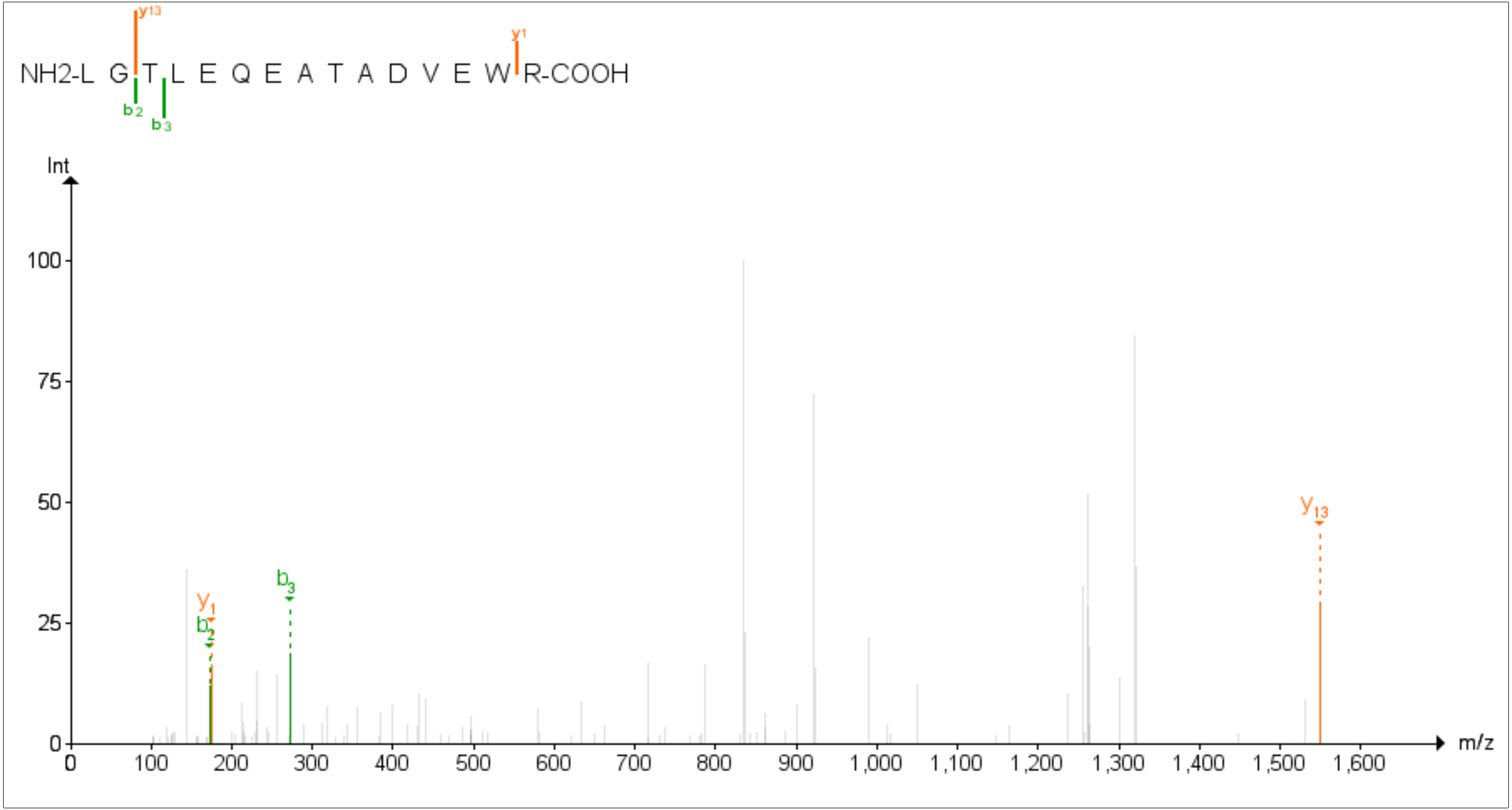

NH2-L G T L E Q E A T A D V E W R-COOH
y13
y1
b2
b3
Int
100
75
50
25
0
m/z
0
100
200
300
400
500
600
700
800
900
1,000
1,100
1,200
1,300
1,400
1,500
1,600
y1
b2
b3
y13

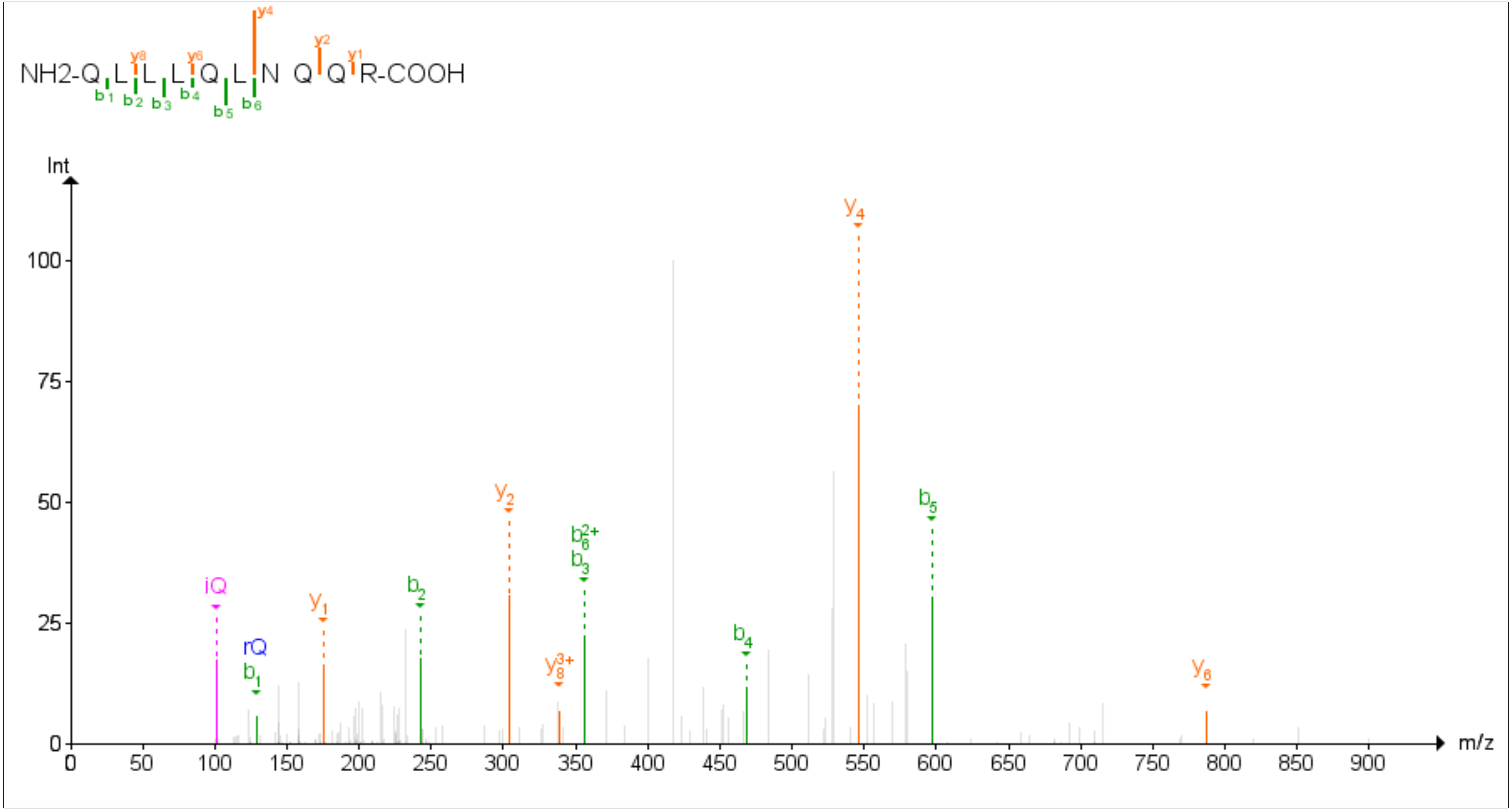
NH2-Q L L L Q L N Q Q R-COOH
y8 y6 y4 y2 y1
b1 b2 b3 b4 b5 b6
Int
m/z
iQ
rQ
b1
y1
b2
y2
y8 3+
b6 2+
b3
b4
y4
b5
y6

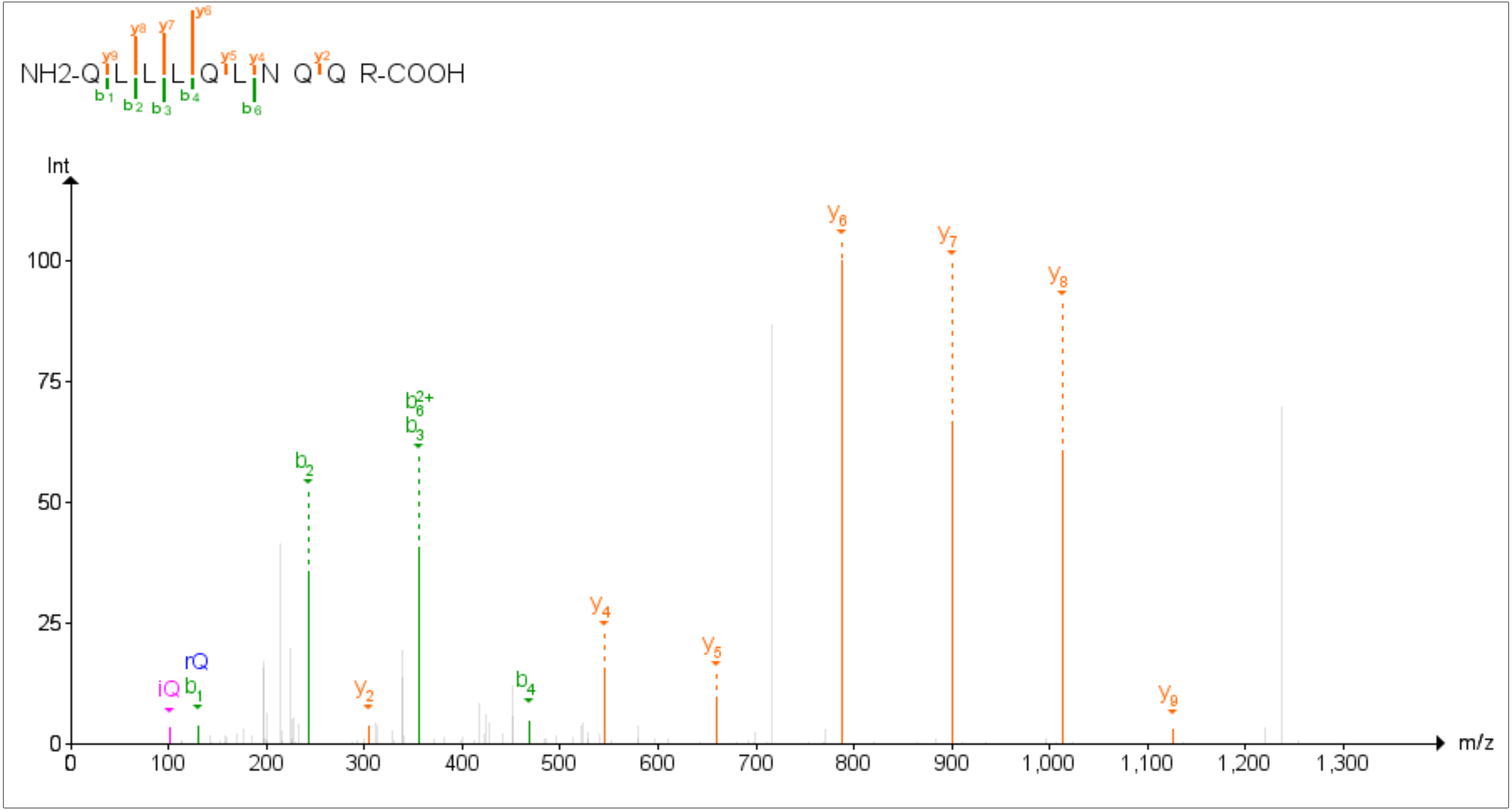
NH2-Q L L L L Q L N Q Q R-COOH
y9 y8 y7 y6 y5 y4 y2
b1 b2 b3 b4 b6
Int
m/z
y6
y7
y8
b6 2+
b3
b2
y4
y5
y9
y2
b4
rQ
iQ
b1
0
25
50
75
100
0
100
200
300
400
500
600
700
800
900
1,000
1,100
1,200
1,300

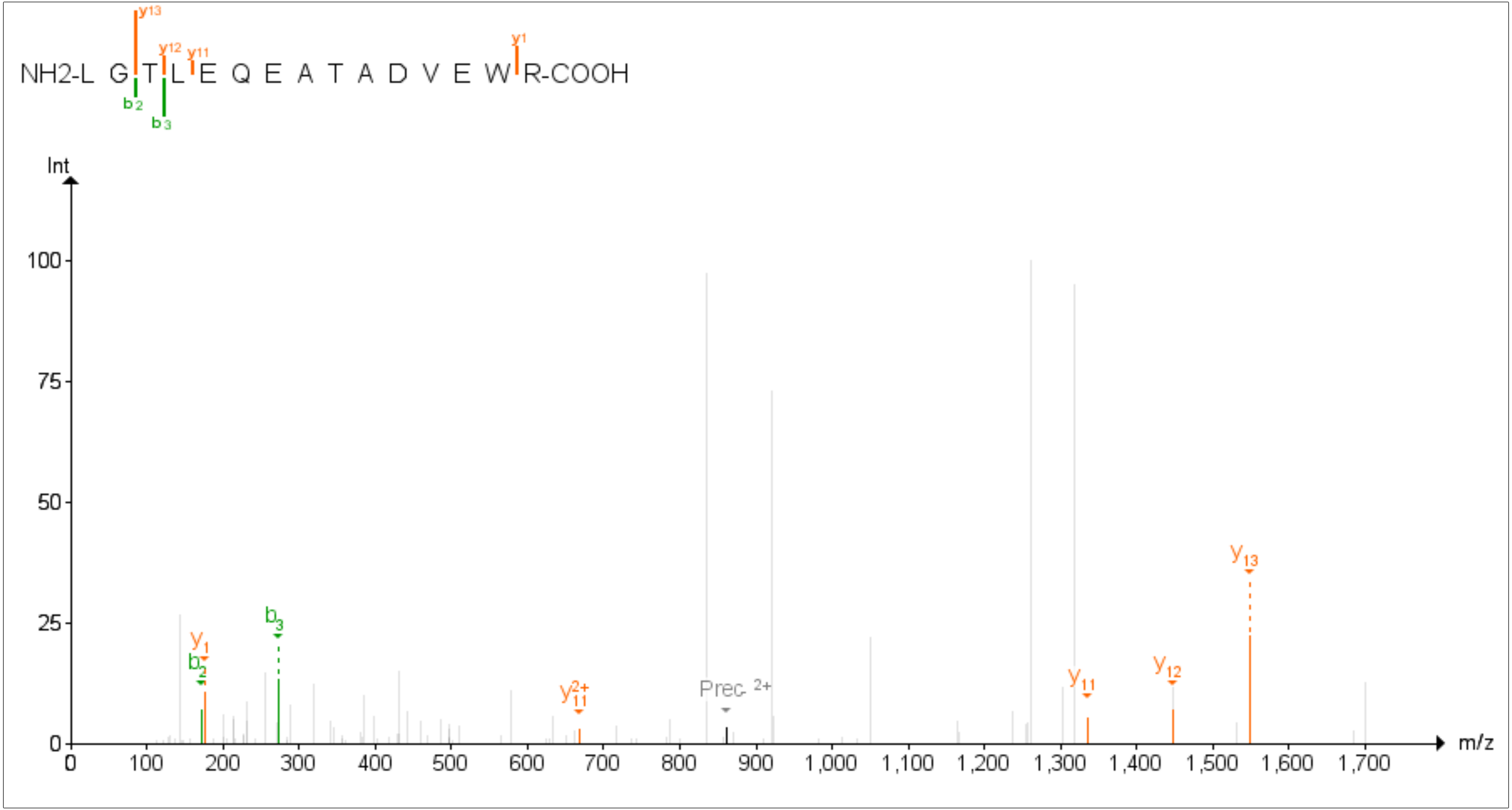

NH2-L G T L E Q E A T A D V E W R-COOH
y13
y12
y11
y1
b2
b3
Int
100
75
50
25
0
m/z
0
100
200
300
400
500
600
700
800
900
1,000
1,100
1,200
1,300
1,400
1,500
1,600
1,700
y1
b2
b3
y11 2+
Prec 2+
y11
y12
y13

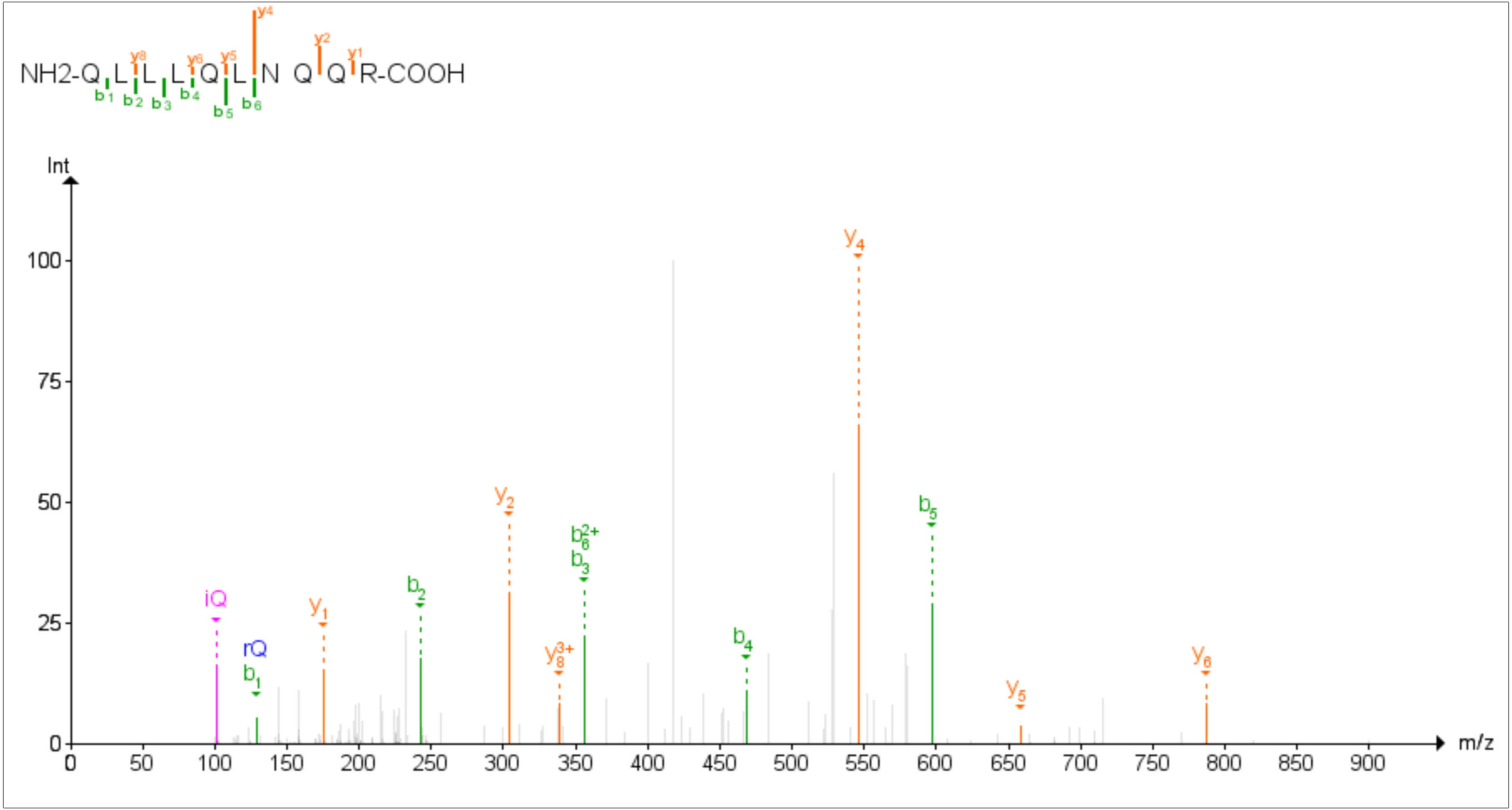

NH2-Q L L L Q L N Q Q R-COOH
y8 y6 y5 y4 y2 y1
b1 b2 b3 b4 b5 b6
Int
m/z
iQ
rQ
b1
y1
b2
y2
b6 2+
b3
y8 3+
b4
y4
b5
y5
y6

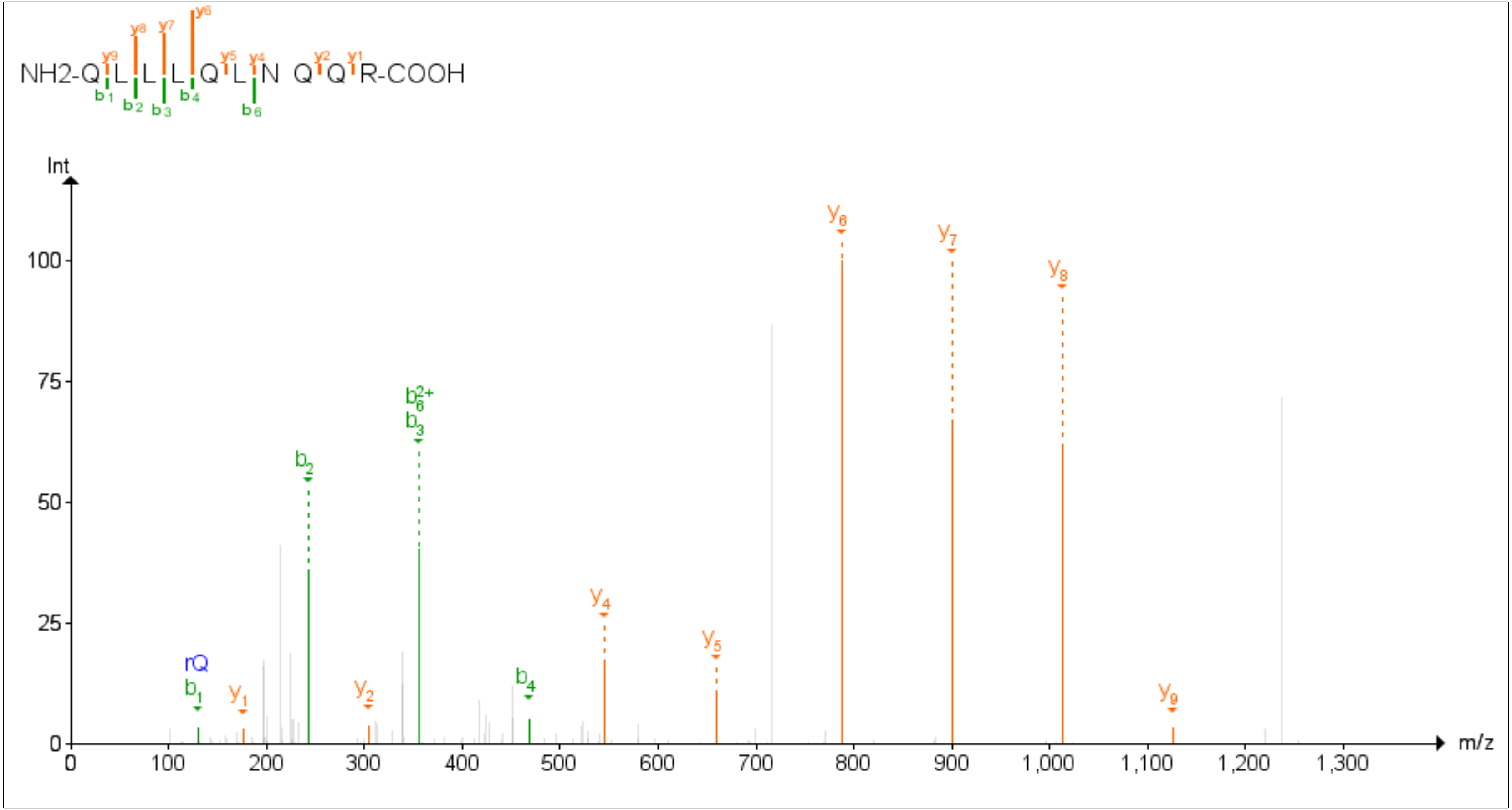
NH2-Q L L L L Q L N Q Q R-COOH
y9 y8 y7 y6 y5 y4 y2 y1
b1 b2 b3 b4 b6
Int
100
75
50
25
0
m/z
0 100 200 300 400 500 600 700 800 900 1,000 1,100 1,200 1,300
rQ
b1
y1
b2
y2
b6 2+
b3
b4
y4
y5
y6
y7
y8
y9

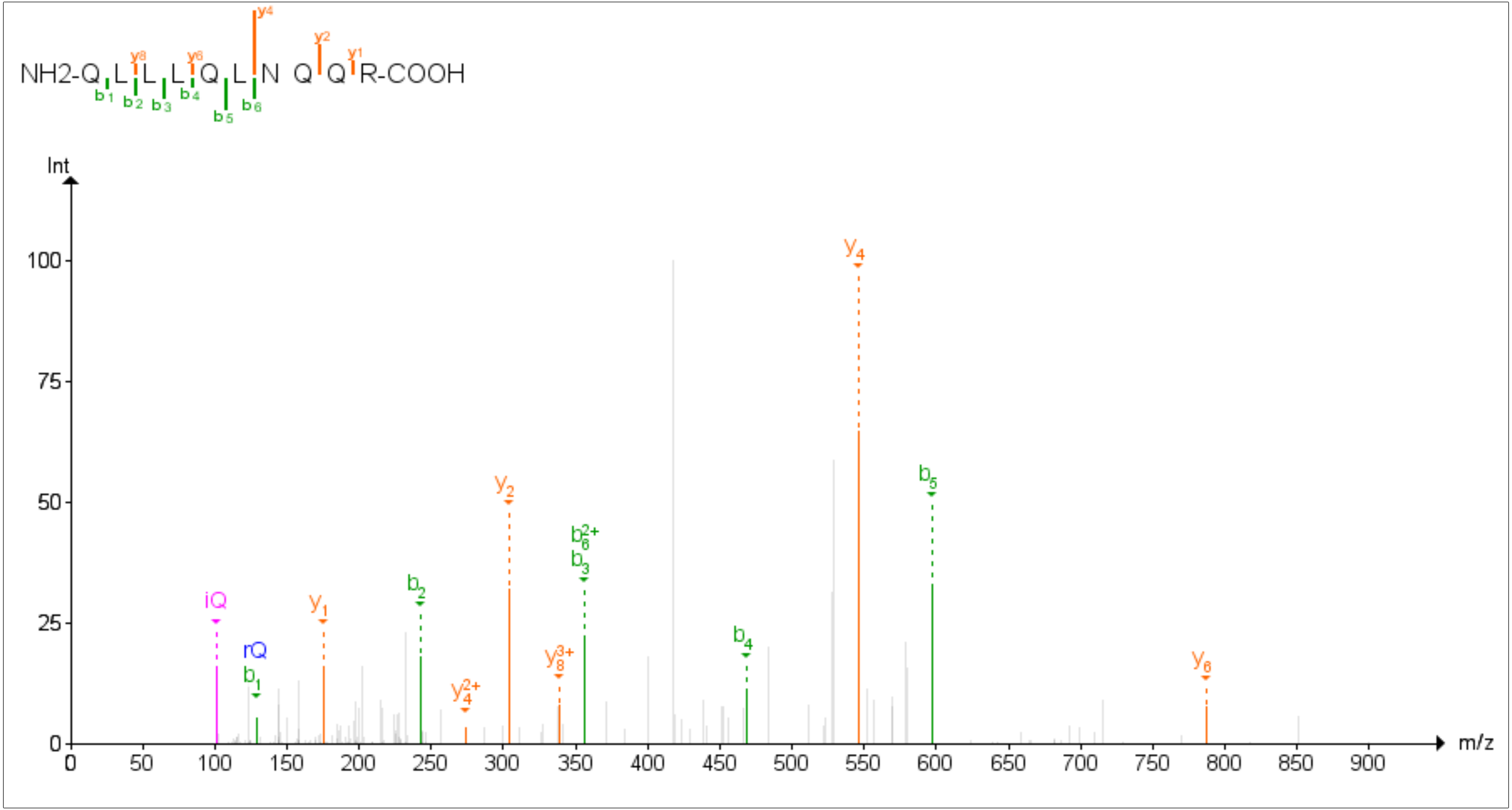

NH2-Q L L L Q L N Q Q R-COOH
y8 y6 y4 y2 y1
b1 b2 b3 b4 b5 b6
Int
100
75
50
25
0
iQ
rQ
b1
y1
b2
y4 2+
y2
y8 3+
b6 2+
b3
b4
y4
b5
y6
0 50 100 150 200 250 300 350 400 450 500 550 600 650 700 750 800 850 900
m/z

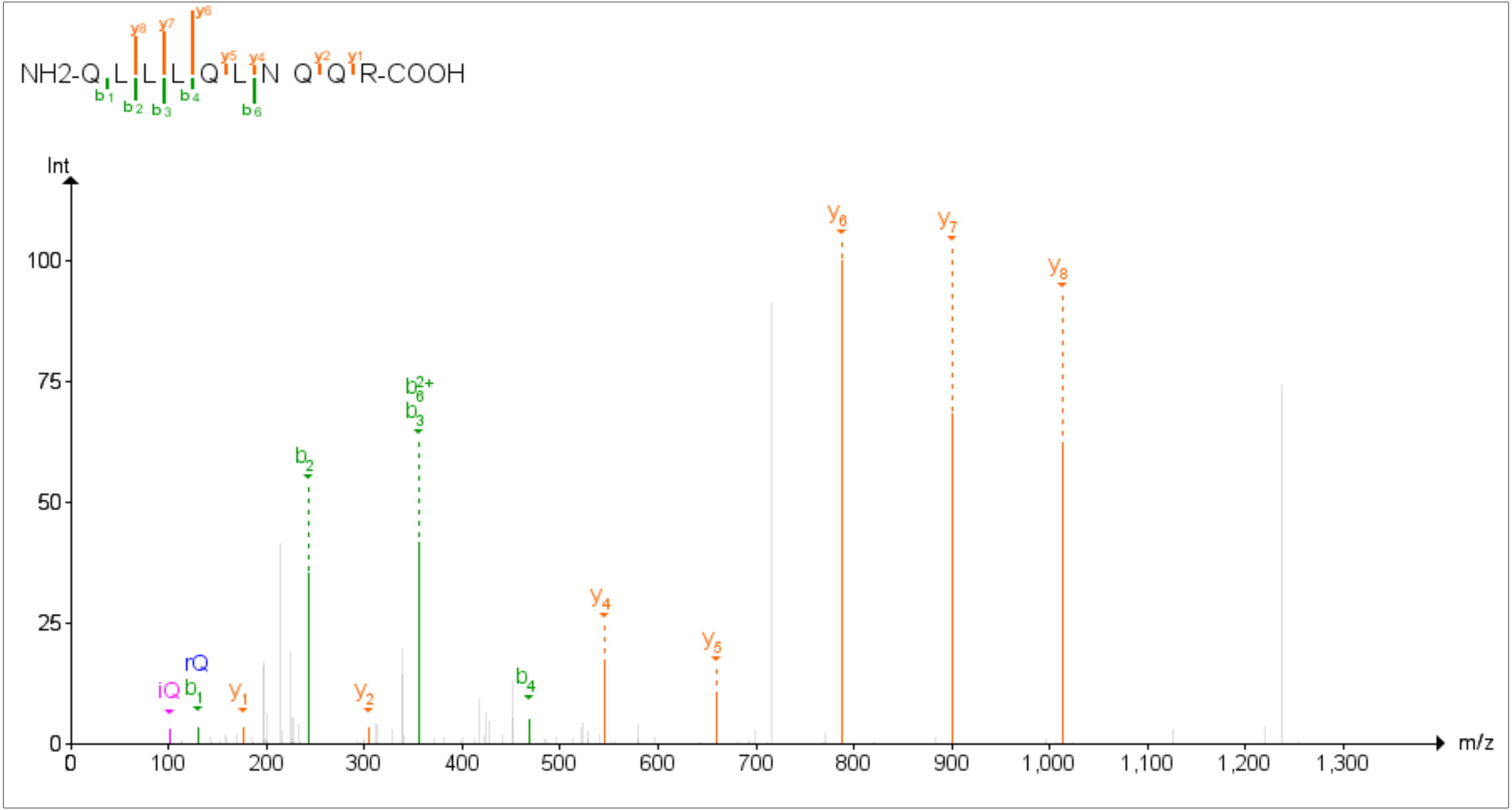

NH2-Q L L L Q L N Q Q R-COOH
y8 y7 y6 y5 y4 y2 y1
b1 b2 b3 b4 b6
Int
m/z
y6
y7
y8
b6 2+
b3
b2
y4
y5
b4
rQ
iQ
b1
y1
y2
0
25
50
75
100
0
100
200
300
400
500
600
700
800
900
1,000
1,100
1,200
1,300

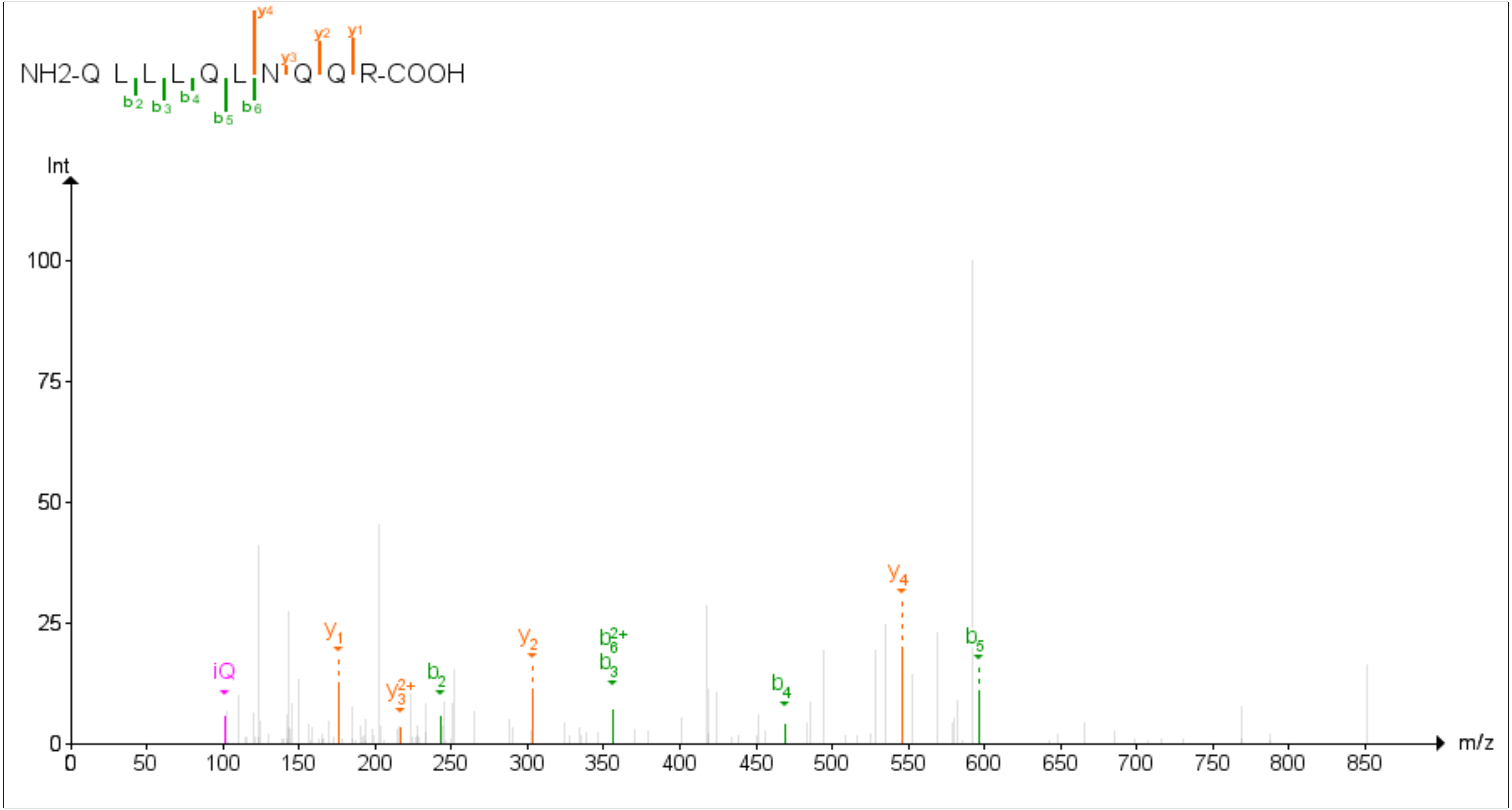

NH2-Q L L L Q L N Q Q R-COOH
y4
y3
y2
y1
b2
b3
b4
b5
b6
Int
100
75
50
25
0
iQ
y1
y3 2+
b2
y2
b6 2+
b3
b4
y4
b5
0
50
100
150
200
250
300
350
400
450
500
550
600
650
700
750
800
850
m/z

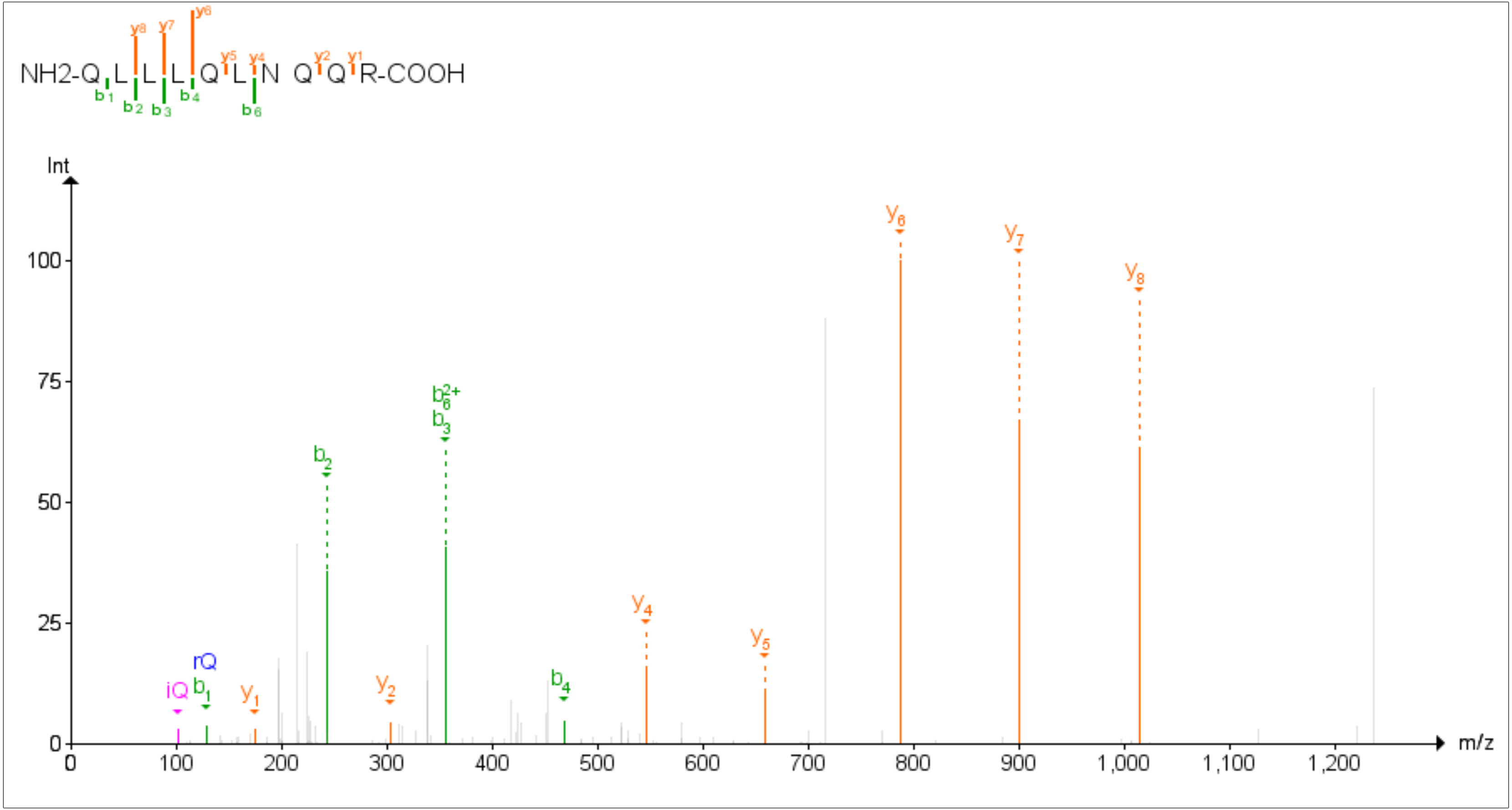

NH2-Q L L L Q L N Q Q R-COOH
y8 y7 y6 y5 y4 y2 y1
b1 b2 b3 b4 b6
Int
100
75
50
25
0
y_6
y_7
y_8
b_6^{2+}
b_3
b_2
y_4
y_5
b_4
y_2
y_1
rQ
iQ
b_1
0
100
200
300
400
500
600
700
800
900
1,000
1,100
1,200
m/z

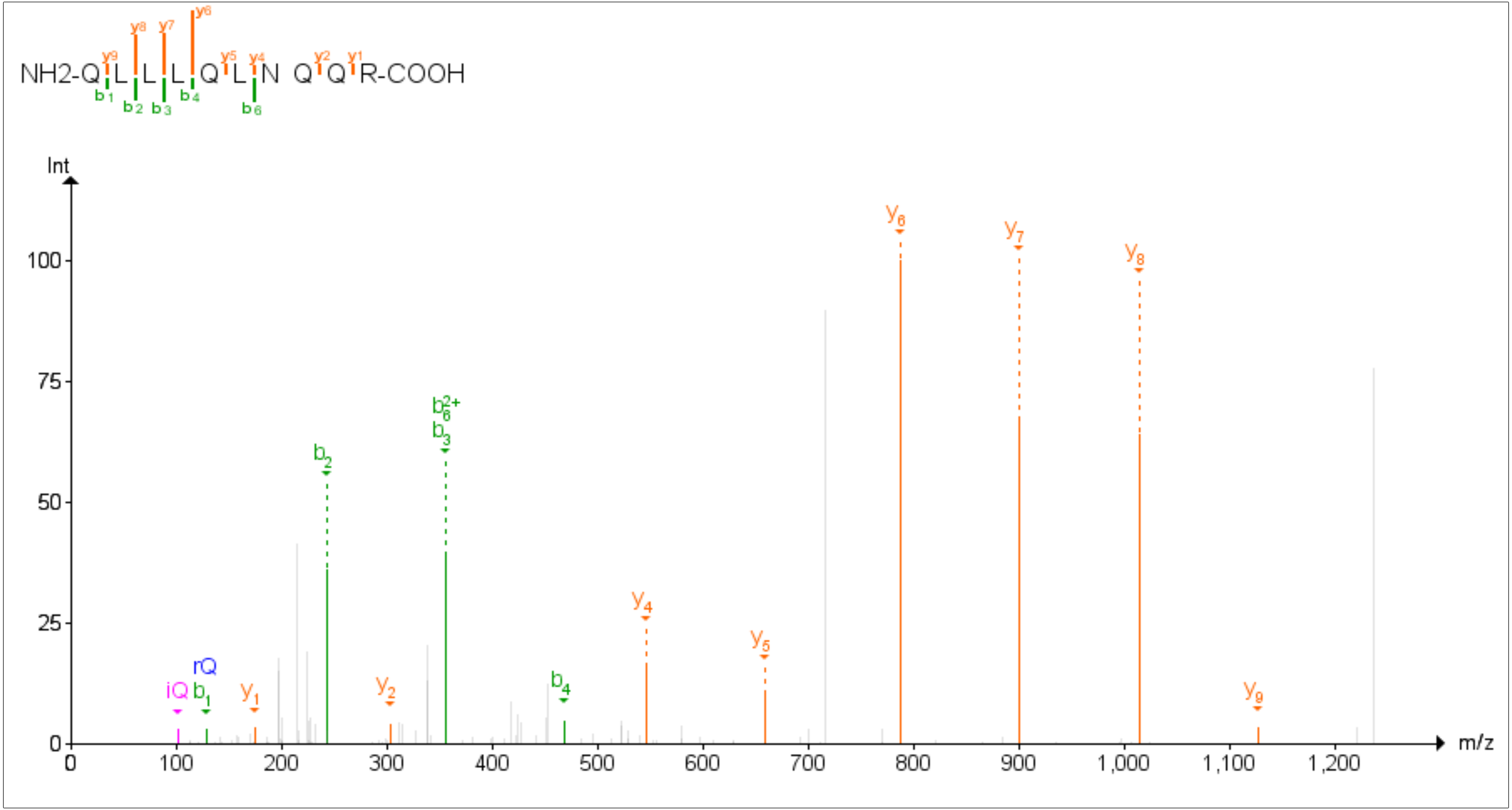

NH2-Q L L L Q L N Q Q R-COOH
y9 y8 y7 y6 y5 y4 y2 y1
b1 b2 b3 b4 b6
Int
m/z
y6
y7
y8
b6 2+
b3
b2
y4
y5
b4
y9
y2
y1
b1
rQ
iQ
0
25
50
75
100
0
100
200
300
400
500
600
700
800
900
1,000
1,100
1,200

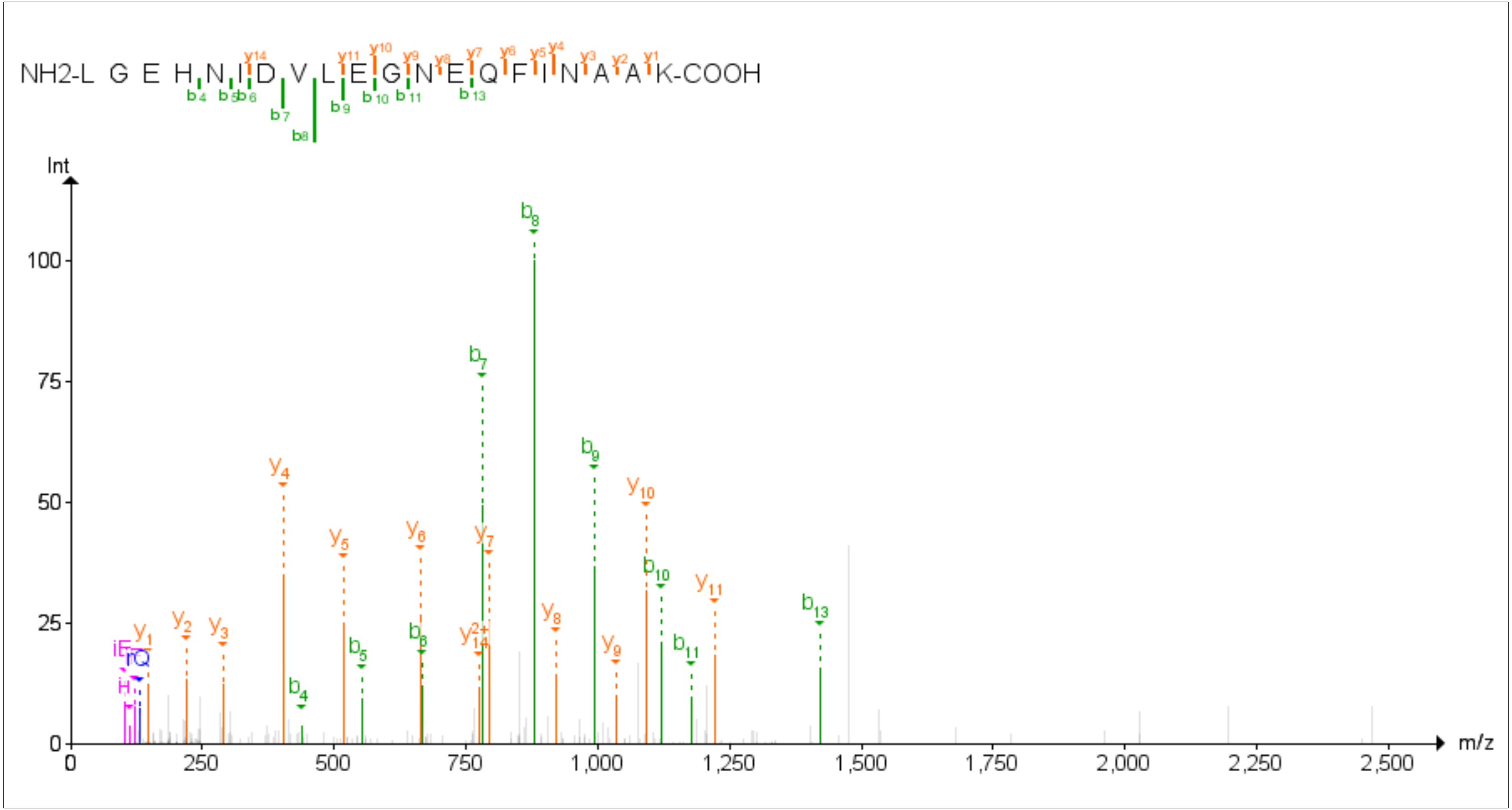
NH2-L G E H N I D V L E G N E Q F I N A A K-COOH
Int
m/z
0
25
50
75
100
0
250
500
750
1,000
1,250
1,500
1,750
2,000
2,250
2,500

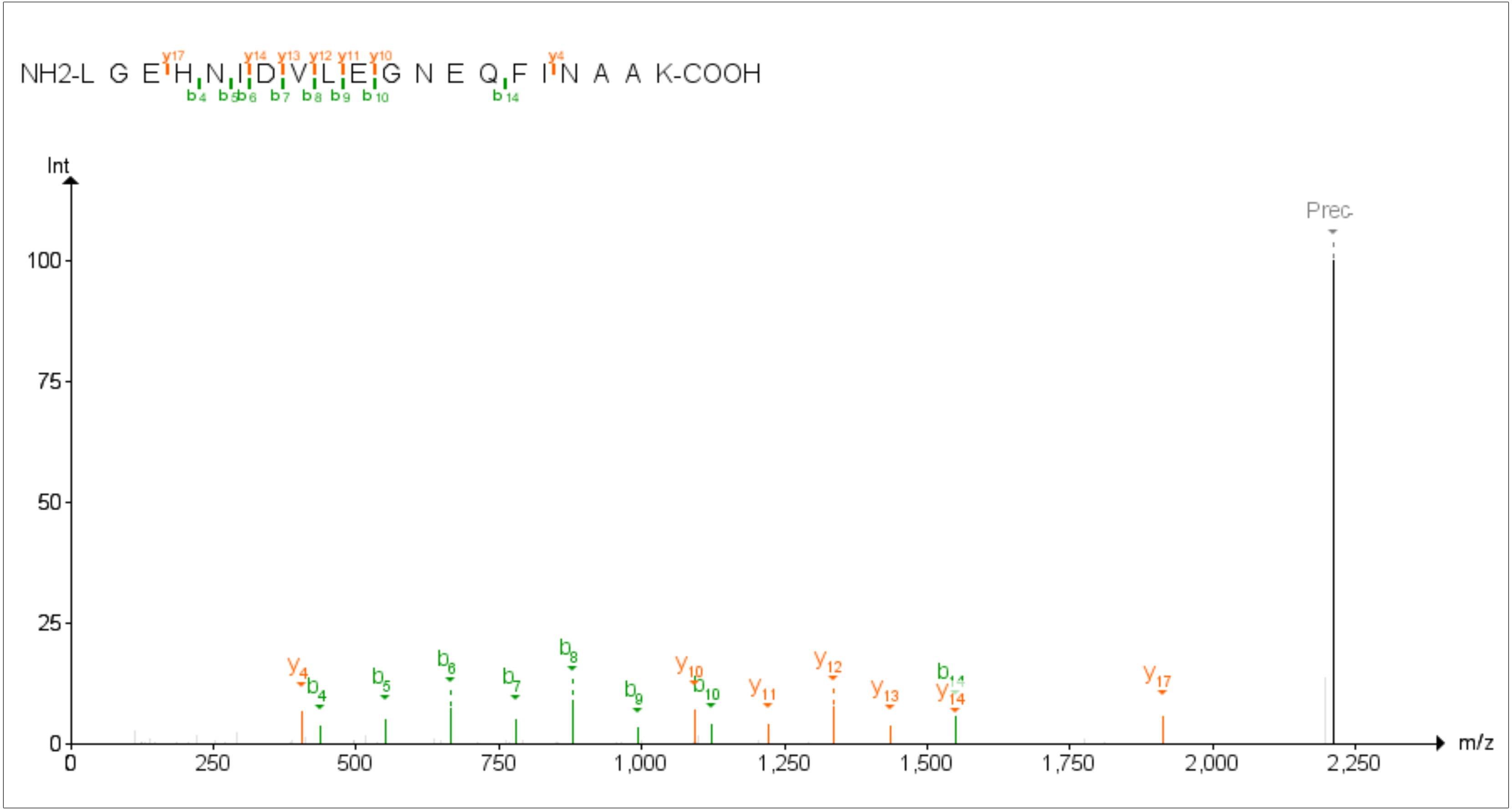

NH2-L G E H N I D V L E G N E Q F I N A A K-COOH
y17 y14 y13 y12 y11 y10 y4
b4 b5 b6 b7 b8 b9 b10 b14
Int
100
75
50
25
0
Prec
y4 b4 b5 b6 b7 b8 b9 y10 b10 y11 y12 y13 b14 y14 y17
0
250
500
750
1,000
1,250
1,500
1,750
2,000
2,250
m/z

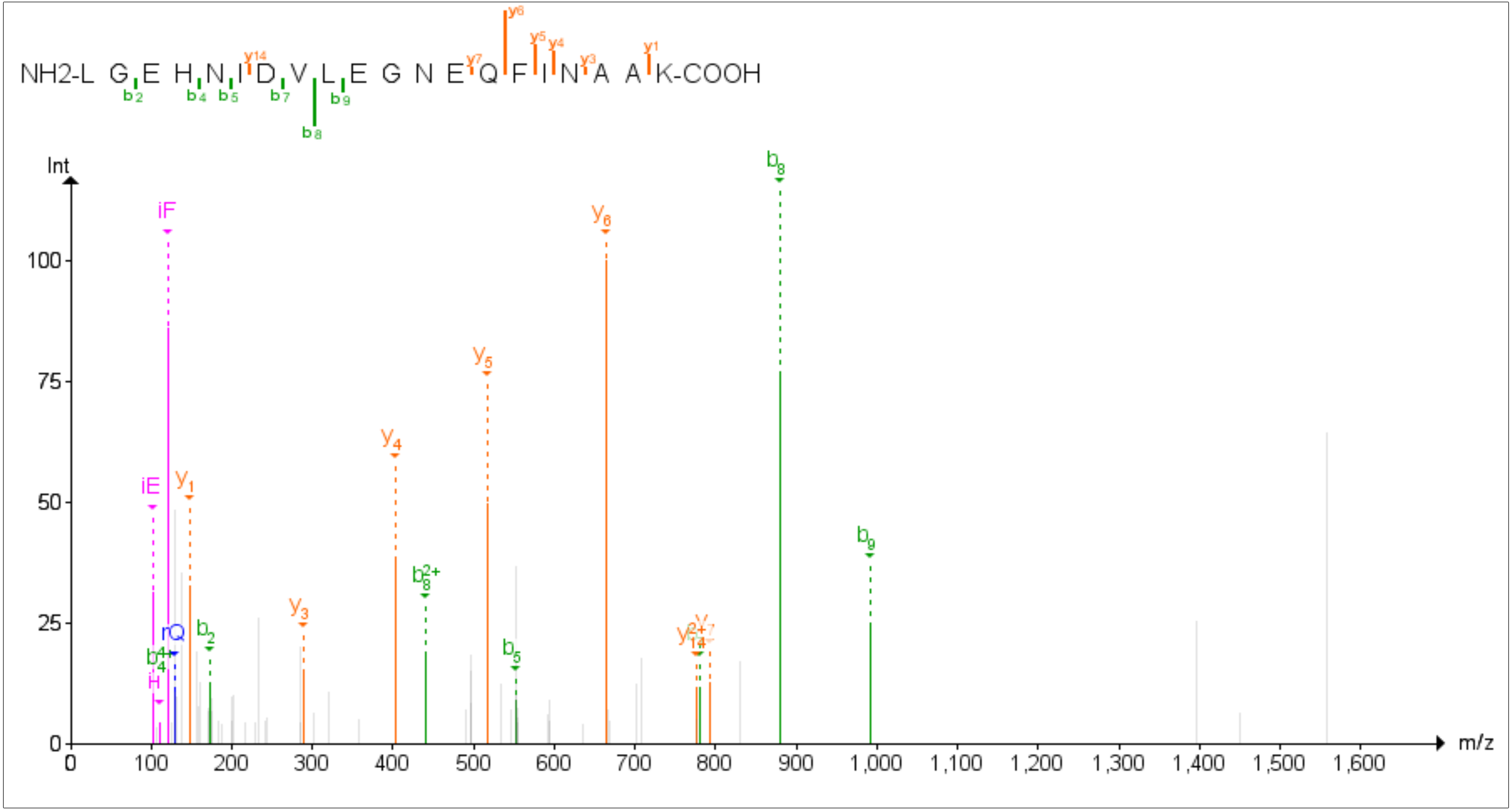
NH2-L G E H N I D V L E G N E Q F I N A A K-COOH
Int
m/z
iF
iE
iH
rQ
y1
y3
y4
y5
y6
b2
b5
b8
b9
0
25
50
75
100
0
100
200
300
400
500
600
700
800
900
1,000
1,100
1,200
1,300
1,400
1,500
1,600

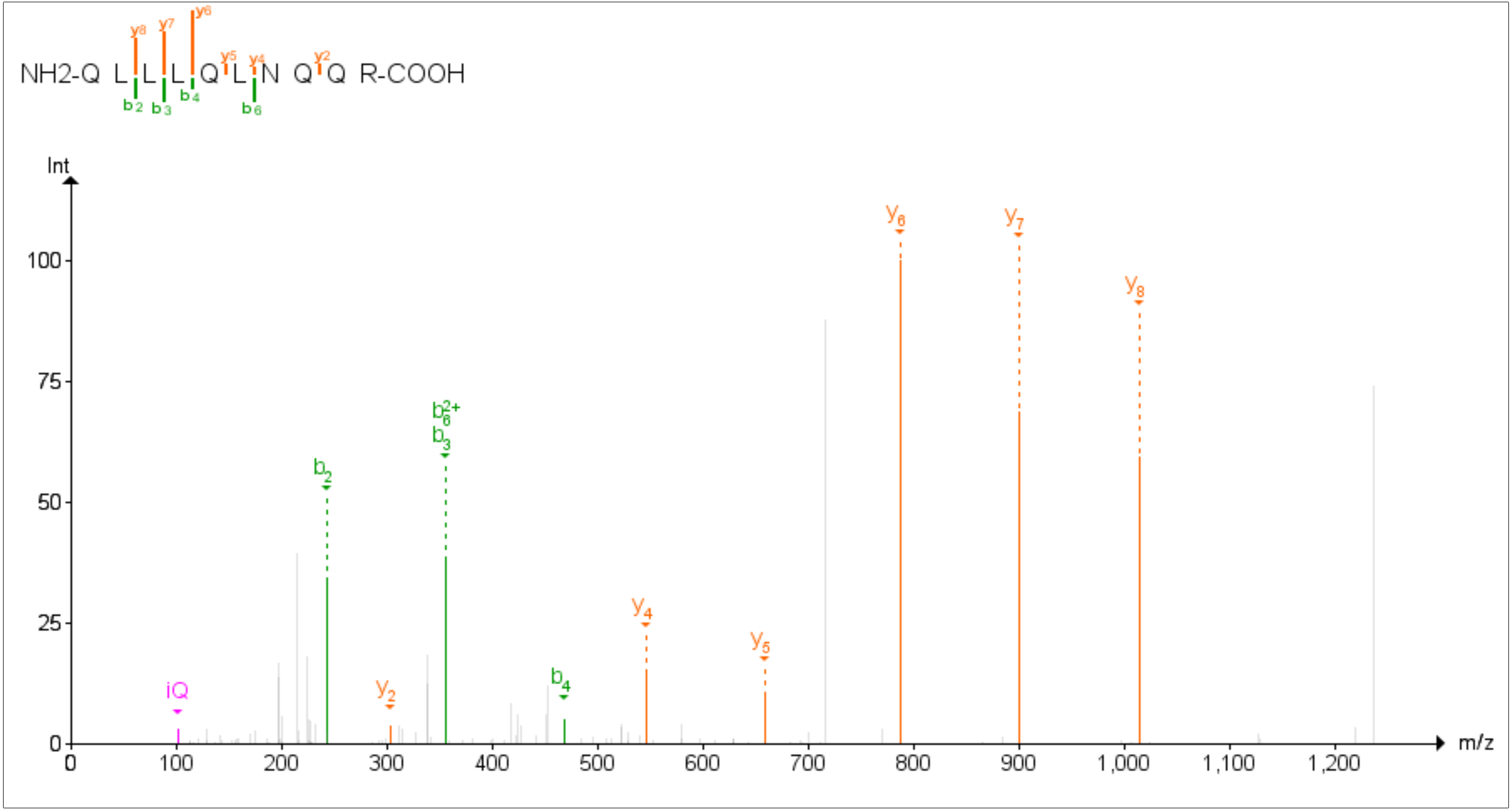

NH2-Q L L L Q L N Q Q R-COOH
y8 y7 y6 y5 y4 y2
b2 b3 b4 b6
Int
m/z
y6
y7
y8
b_6^{2+}
b_3
b_2
b_4
y_4
y_5
y_2
iQ
0
25
50
75
100
0
100
200
300
400
500
600
700
800
900
1,000
1,100
1,200

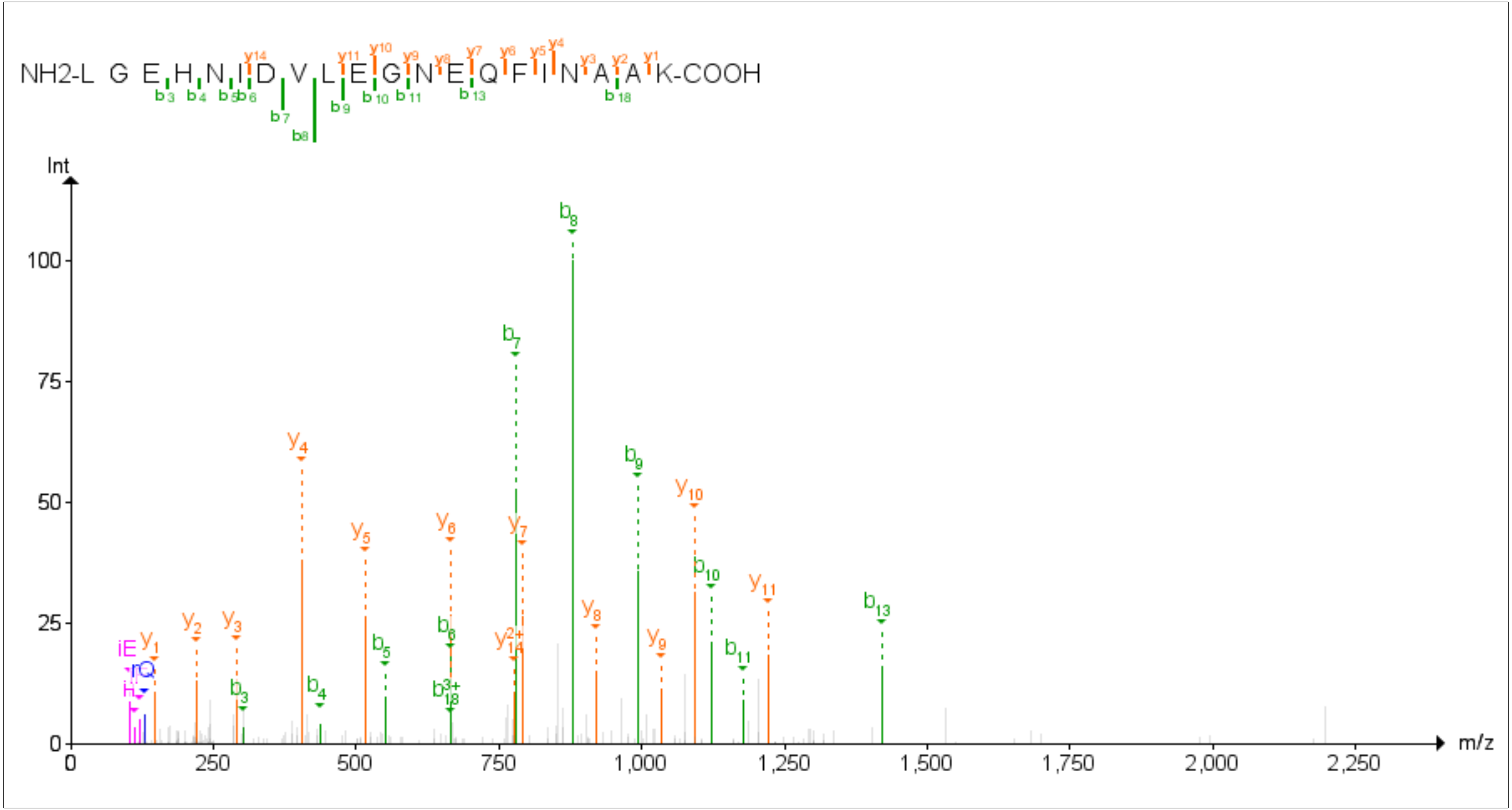

NH2-L G E H N I D V L E G N E Q F I N A A K-COOH
Int
m/z
0
25
50
75
100
250
500
750
1,000
1,250
1,500
1,750
2,000
2,250

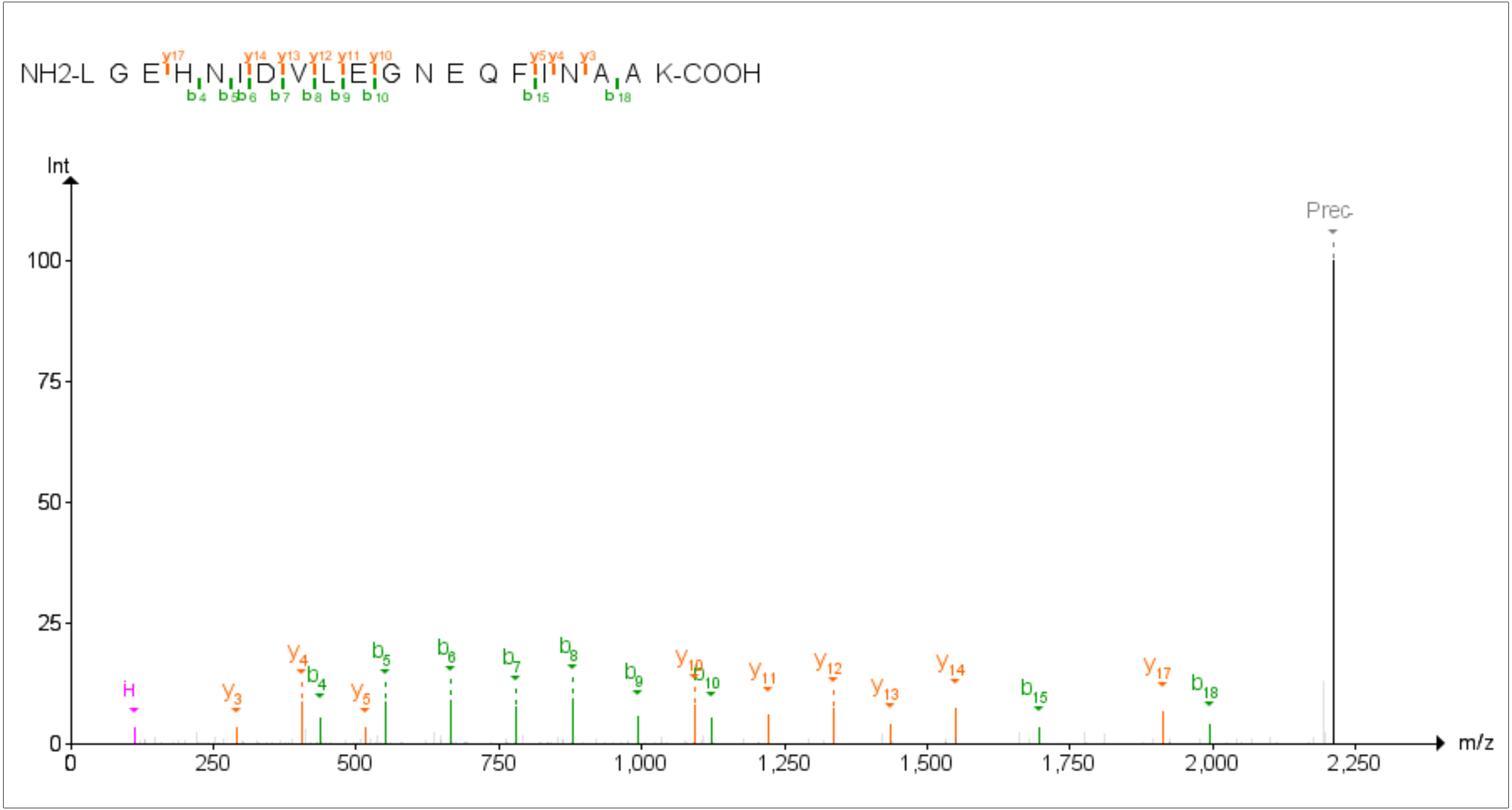

NH2-L G E H N I D V L E G N E Q F I N A A K-COOH
y17 y14 y13 y12 y11 y10 y5 y4 y3
b4 b5 b6 b7 b8 b9 b10 b15 b18
Int
Prec
100
75
50
25
0
H
y3 y4 b4 y5 b5 b6 b7 b8 b9 y10 b10 y11 y12 y13 y14 b15 y17 b18
0
250
500
750
1,000
1,250
1,500
1,750
2,000
2,250
m/z

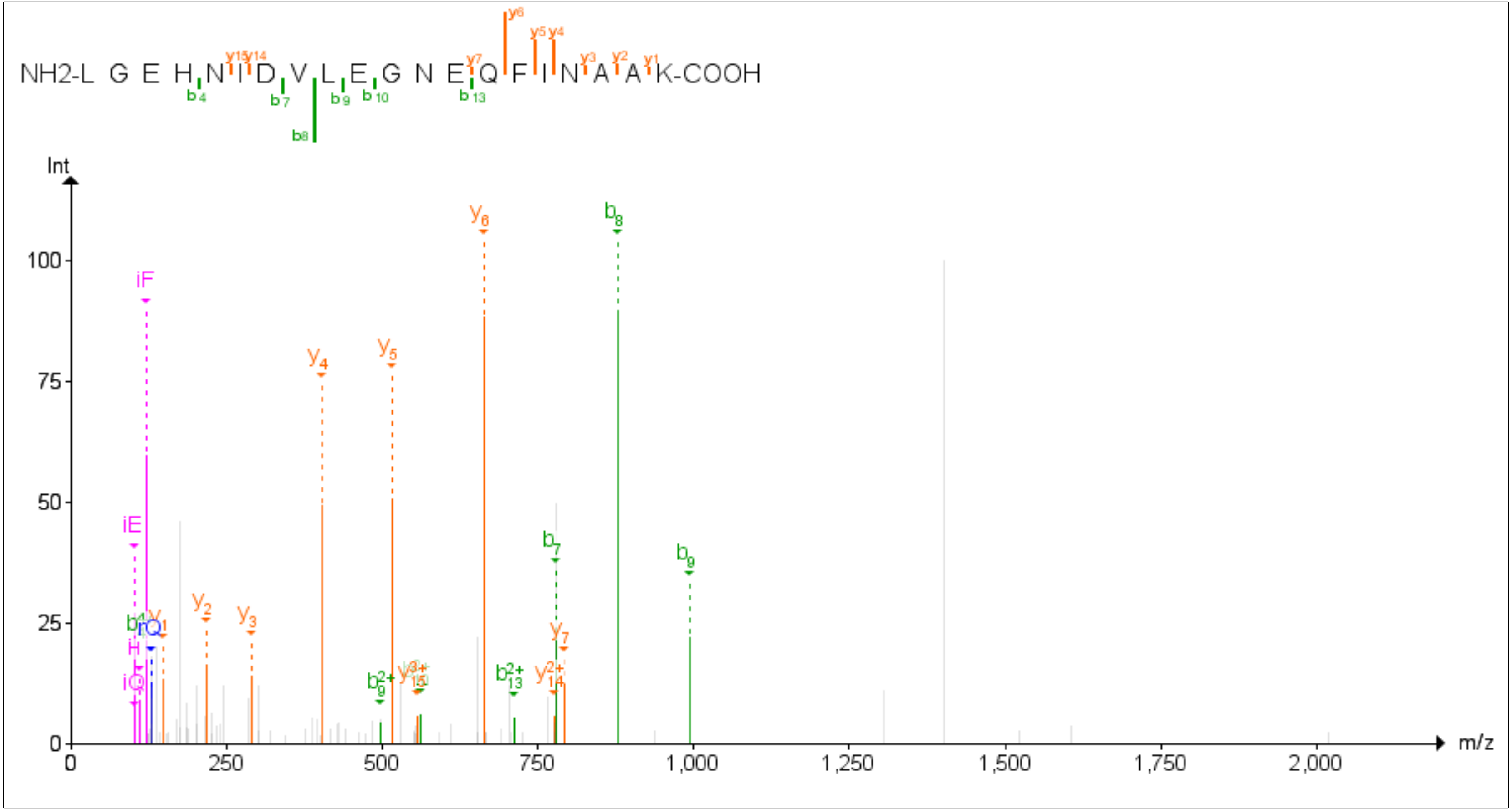

NH2-L G E H N I D V L E G N E Q F I N A A K-COOH
Int
100
75
50
25
0
m/z
0
250
500
750
1,000
1,250
1,500
1,750
2,000

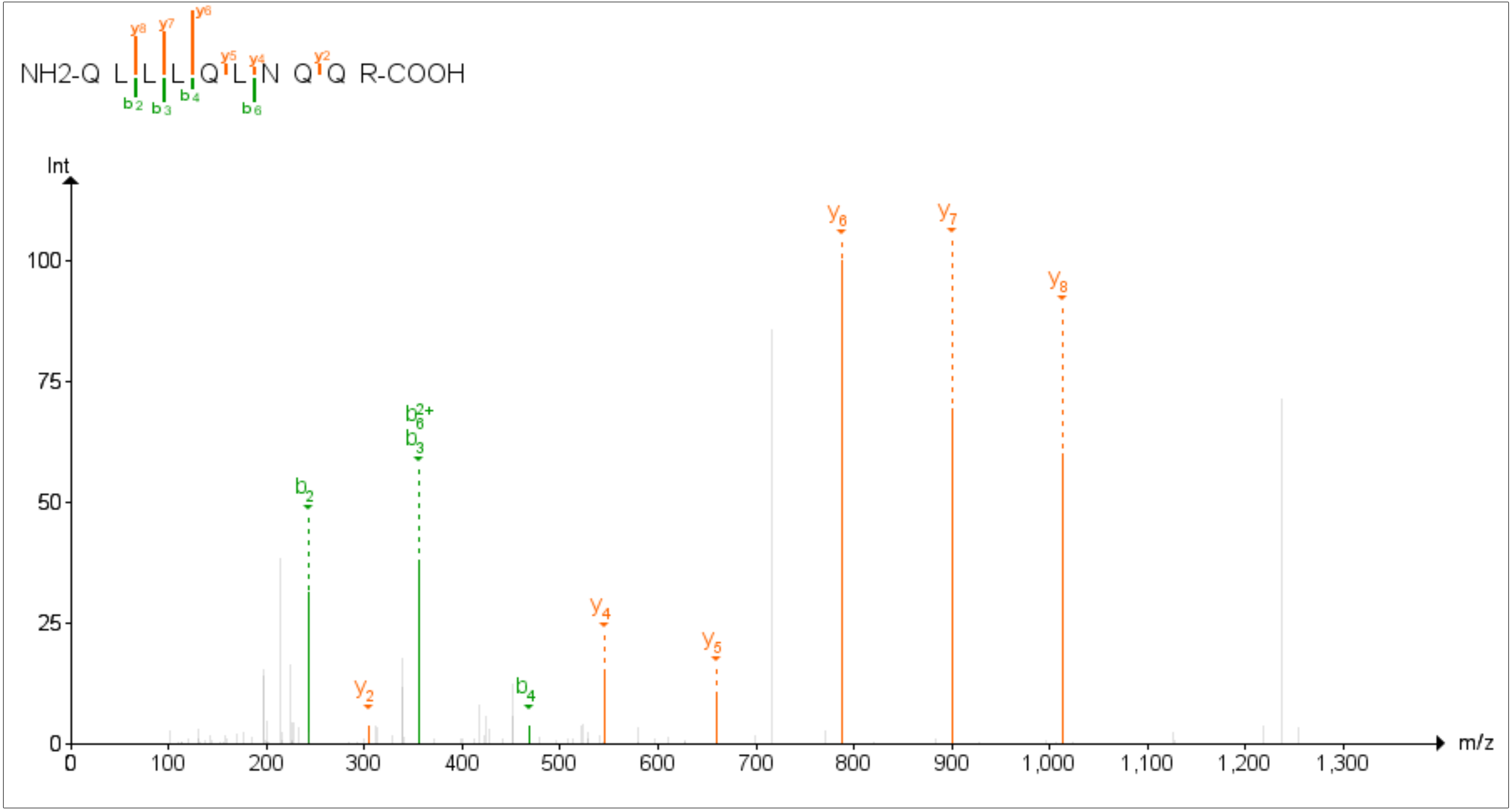

NH2-Q L L L Q L N Q Q R-COOH
y8 y7 y6 y5 y4 y2
b2 b3 b4 b6
Int
100
75
50
25
0
y6 y7 y8
b6 2+
b3
b2
y2
b4
y4
y5
0 100 200 300 400 500 600 700 800 900 1,000 1,100 1,200 1,300
m/z

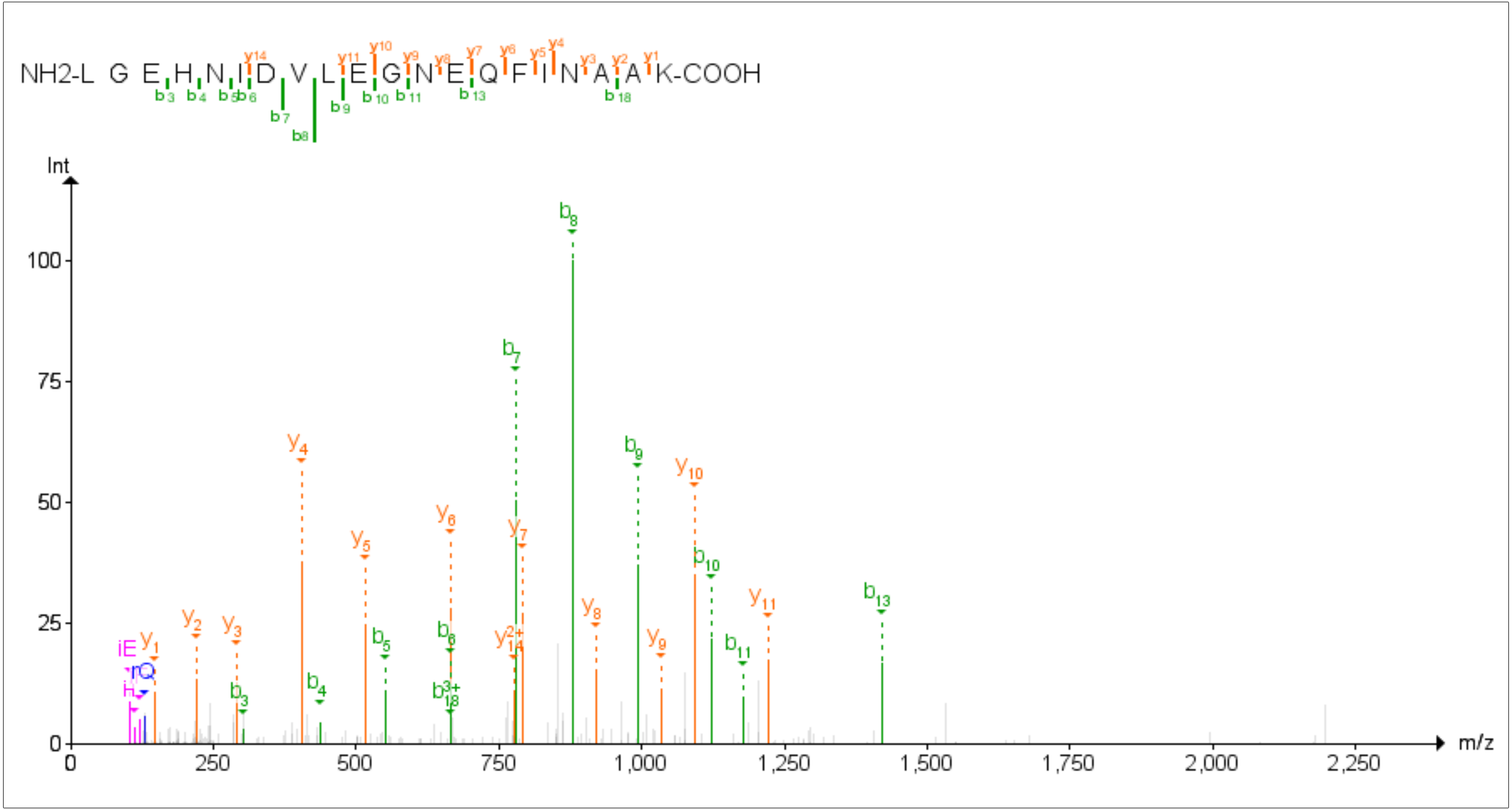

NH2-L G E H N I D V L E G N E Q F I N A A K-COOH
Int
m/z

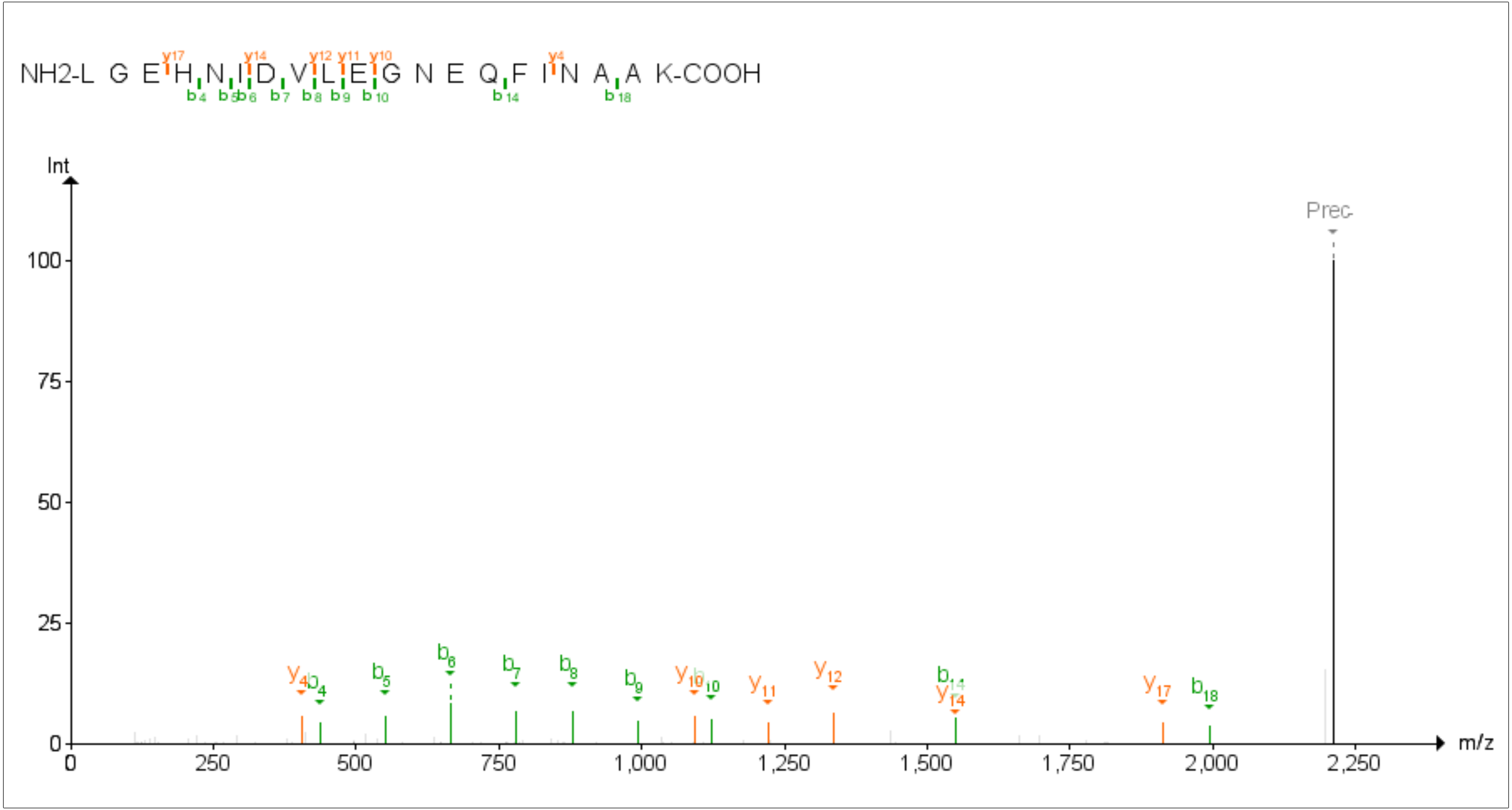

NH2-L G E H N I D V L E G N E Q F I N A A K-COOH
y17 y14 y12 y11 y10 y4
b4 b5 b6 b7 b8 b9 b10 b14 b18
Int
100
75
50
25
0
Prec
y4 b4 b5 b6 b7 b8 b9 y10 b10 y11 y12 b14 y14 y17 b18
0
250
500
750
1,000
1,250
1,500
1,750
2,000
2,250
m/z

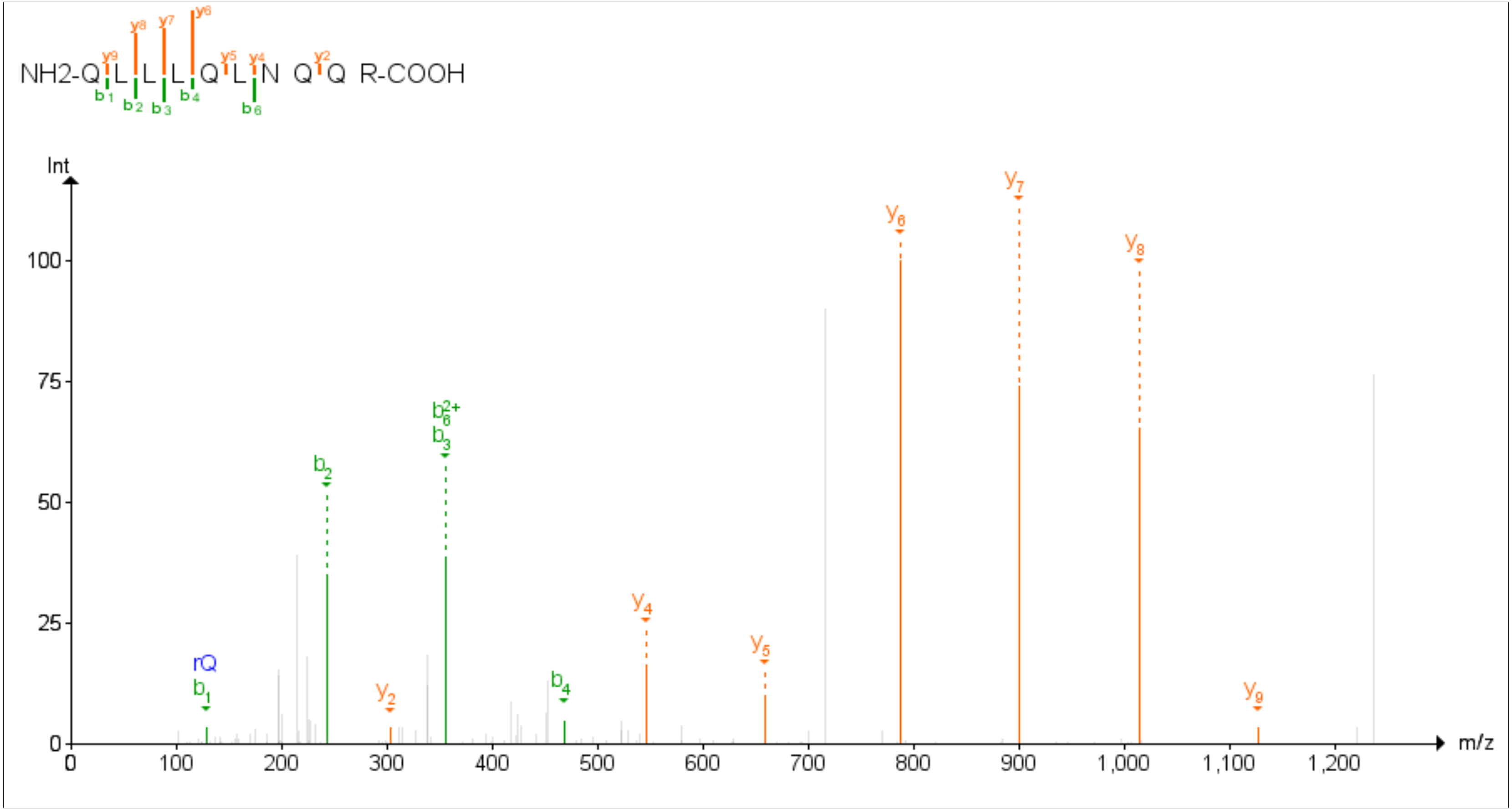
NH2-Q L L L L Q L N Q Q R-COOH
y9 y8 y7 y6 y5 y4 y2
b1 b2 b3 b4 b6
Int
100
75
50
25
0
m/z
0
100
200
300
400
500
600
700
800
900
1,000
1,100
1,200
rQ
b1
b2
b6 2+
b3
y2
b4
y4
y5
y6
y7
y8
y9

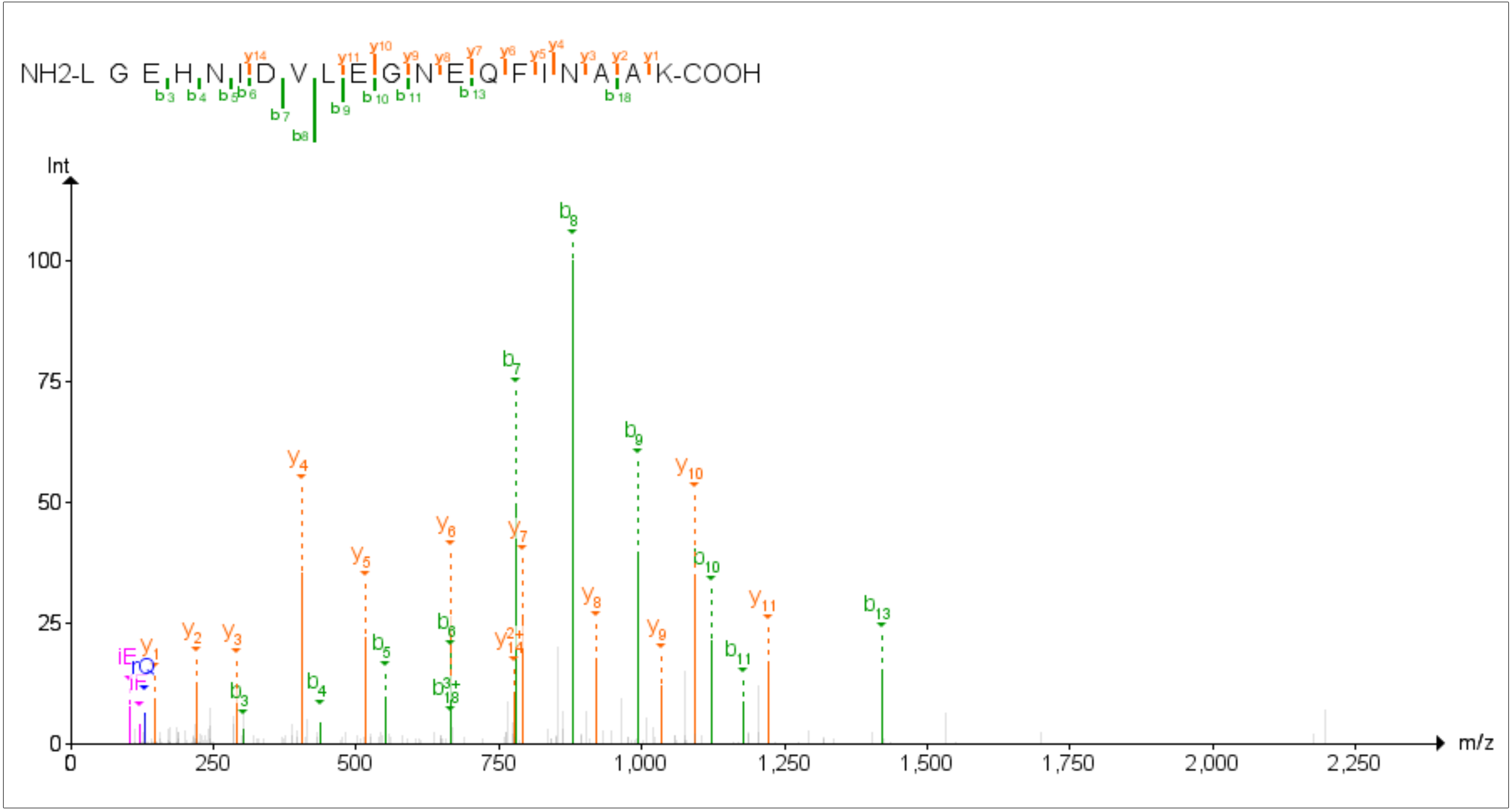

NH2-L G E H N I D V L E G N E Q F I N A A K-COOH
Int
m/z
b8
b7
b9
y10
y4
y6
y7
y5
b10
y8
y11
b13
b6
b5
y9
b11
b4
b3
y1
y2
y3
iE
nQ

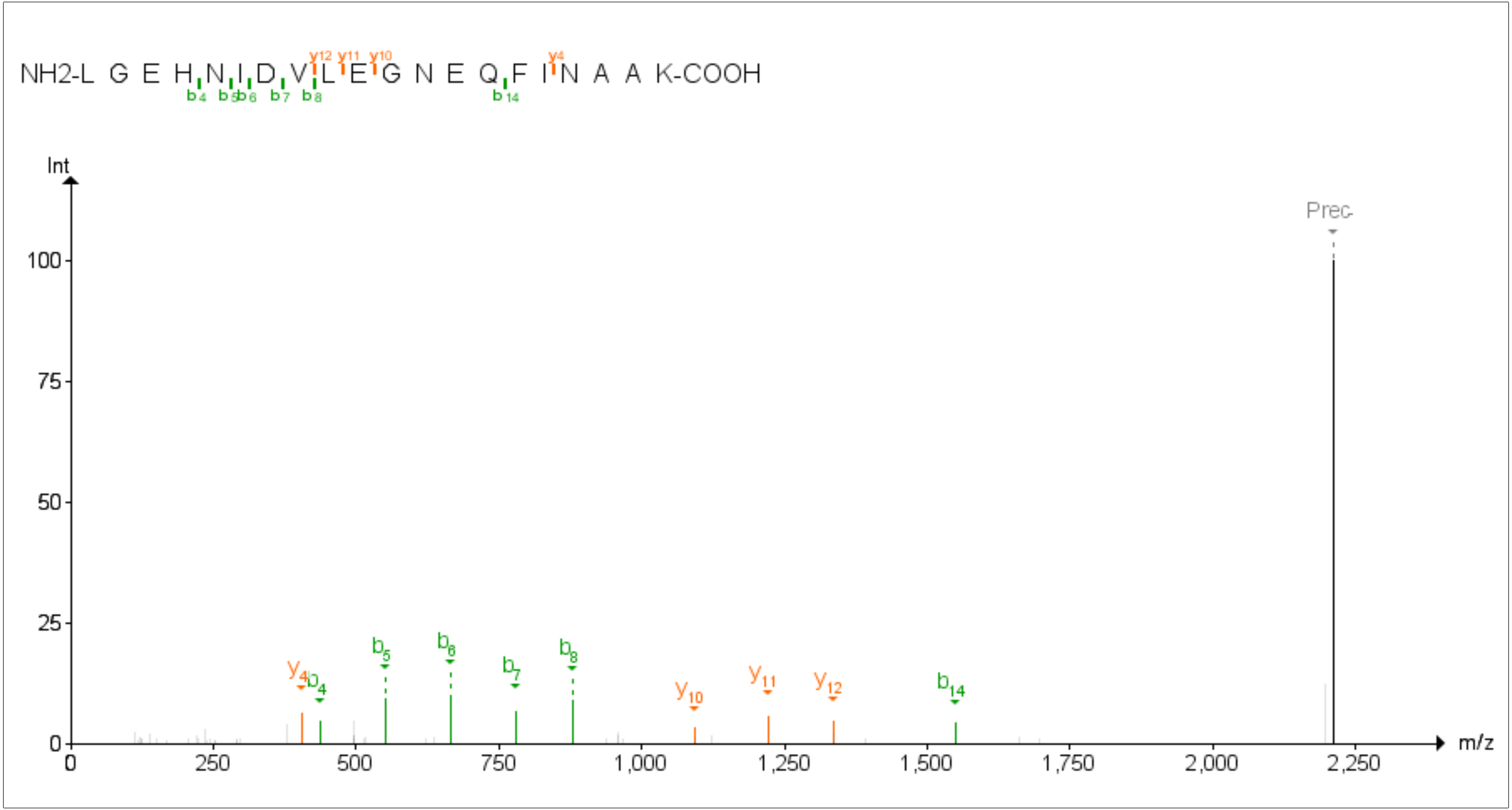

NH2-L G E H N I D V L E G N E Q F I N A A K-COOH
y12 y11 y10 y4
b4 b5 b6 b7 b8 b14
Int
100
75
50
25
0
Prec.
y4
b4
b5
b6
b7
b8
y10
y11
y12
b14
0
250
500
750
1,000
1,250
1,500
1,750
2,000
2,250
m/z

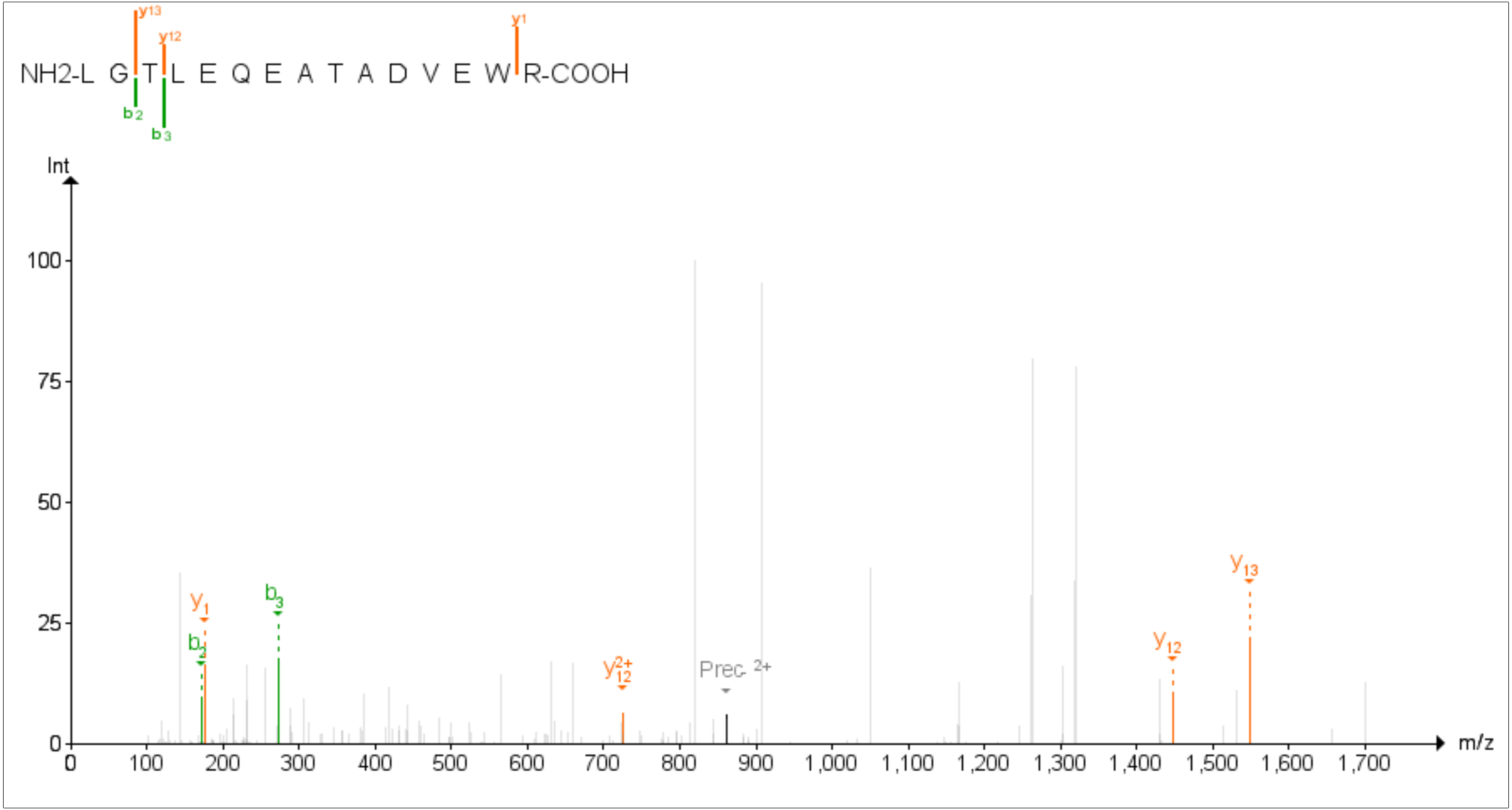

NH2-L G T L E Q E A T A D V E W R-COOH
y13
y12
y1
b2
b3
Int
100
75
50
25
0
y1
b2
b3
y12 2+
Prec 2+
y12
y13
0
100
200
300
400
500
600
700
800
900
1,000
1,100
1,200
1,300
1,400
1,500
1,600
1,700
m/z

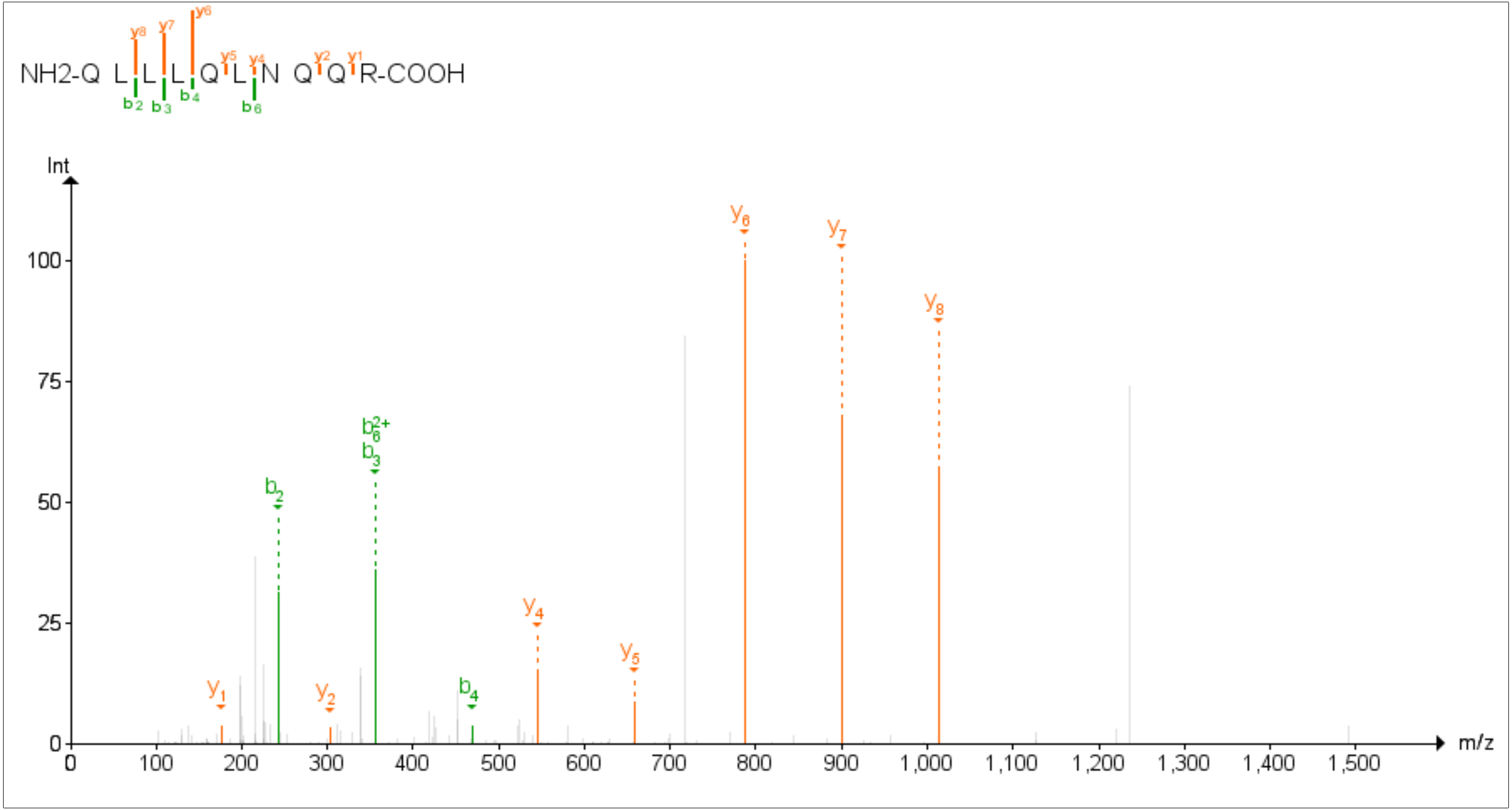

NH2-Q L L L Q L N Q Q R-COOH
y8 y7 y6 y5 y4 y2 y1
b2 b3 b4 b6
Int
100
75
50
25
0
m/z
0 100 200 300 400 500 600 700 800 900 1,000 1,100 1,200 1,300 1,400 1,500
y6
y7
y8
b6 2+
b3
b2
y4
y5
y1
y2
b4

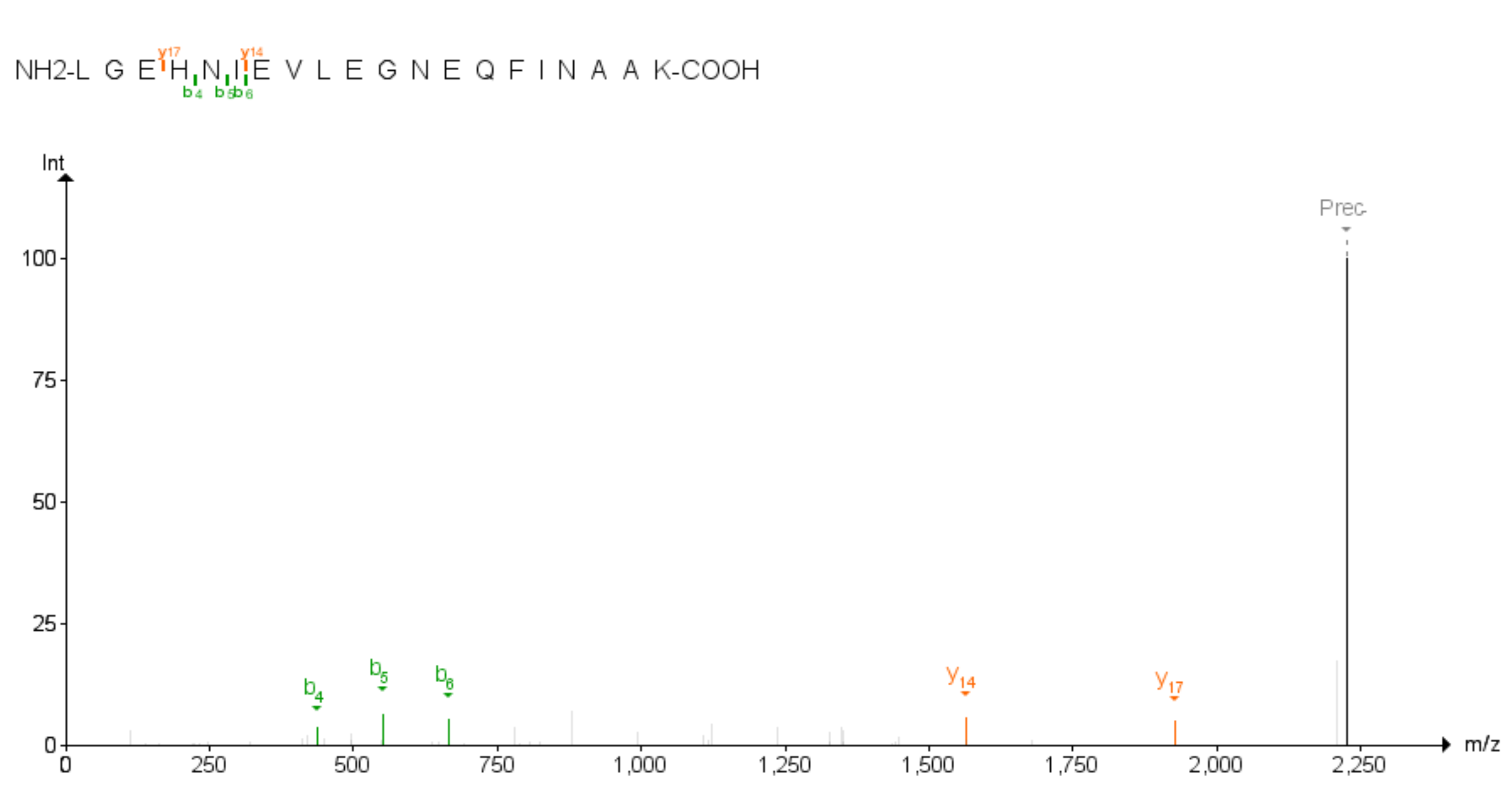

NH2-L G E H N I E V L E G N E Q F I N A A K-COOH
y17
y14
b4
b5
b6
Int
100
75
50
25
0
Prec.
b4
b5
b6
y14
y17
0
250
500
750
1,000
1,250
1,500
1,750
2,000
2,250
m/z

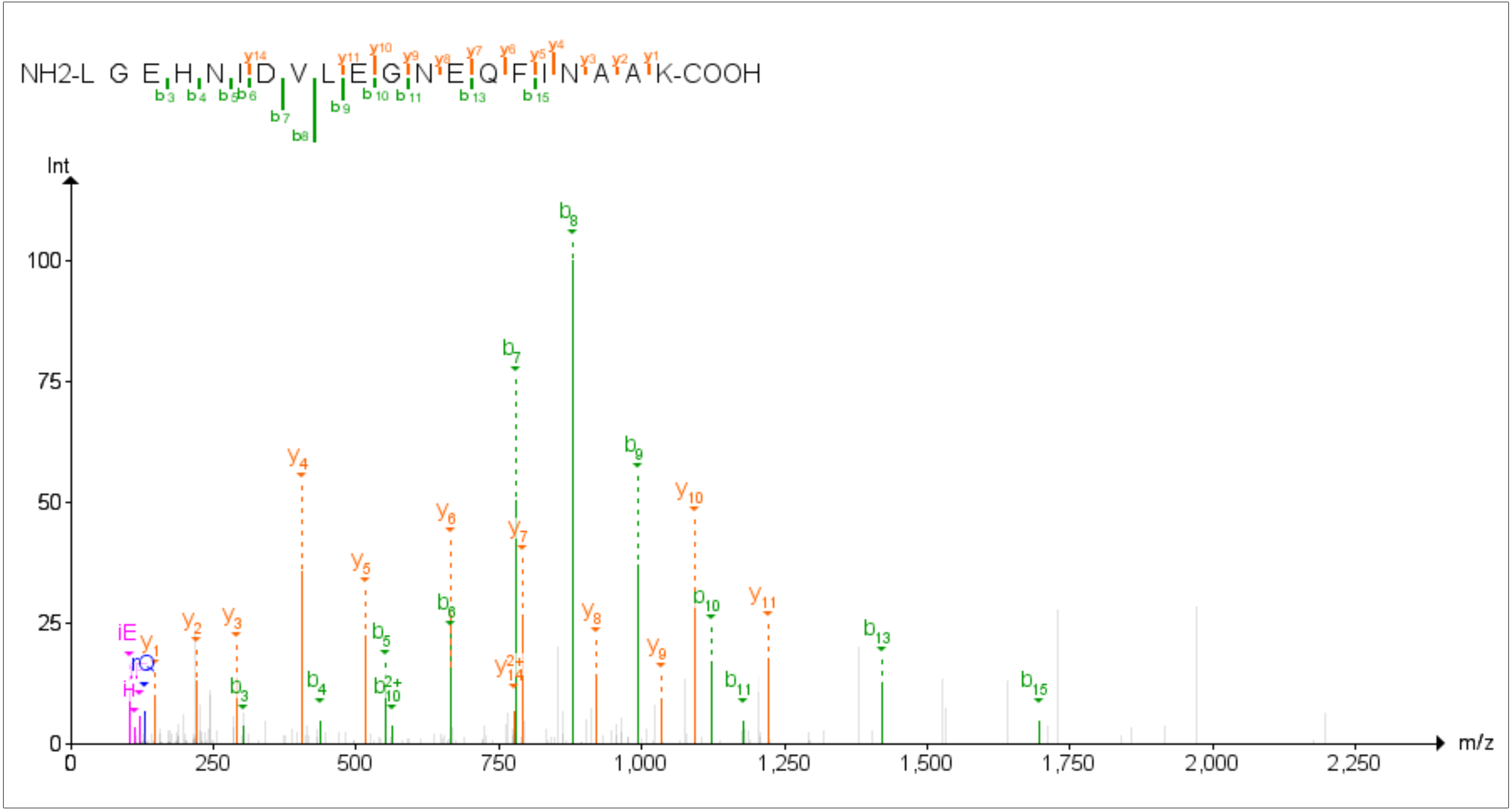

NH2-L G E H N I D V L E G N E Q F I N A A K-COOH
Int
100
75
50
25
0
m/z
0
250
500
750
1,000
1,250
1,500
1,750
2,000
2,250

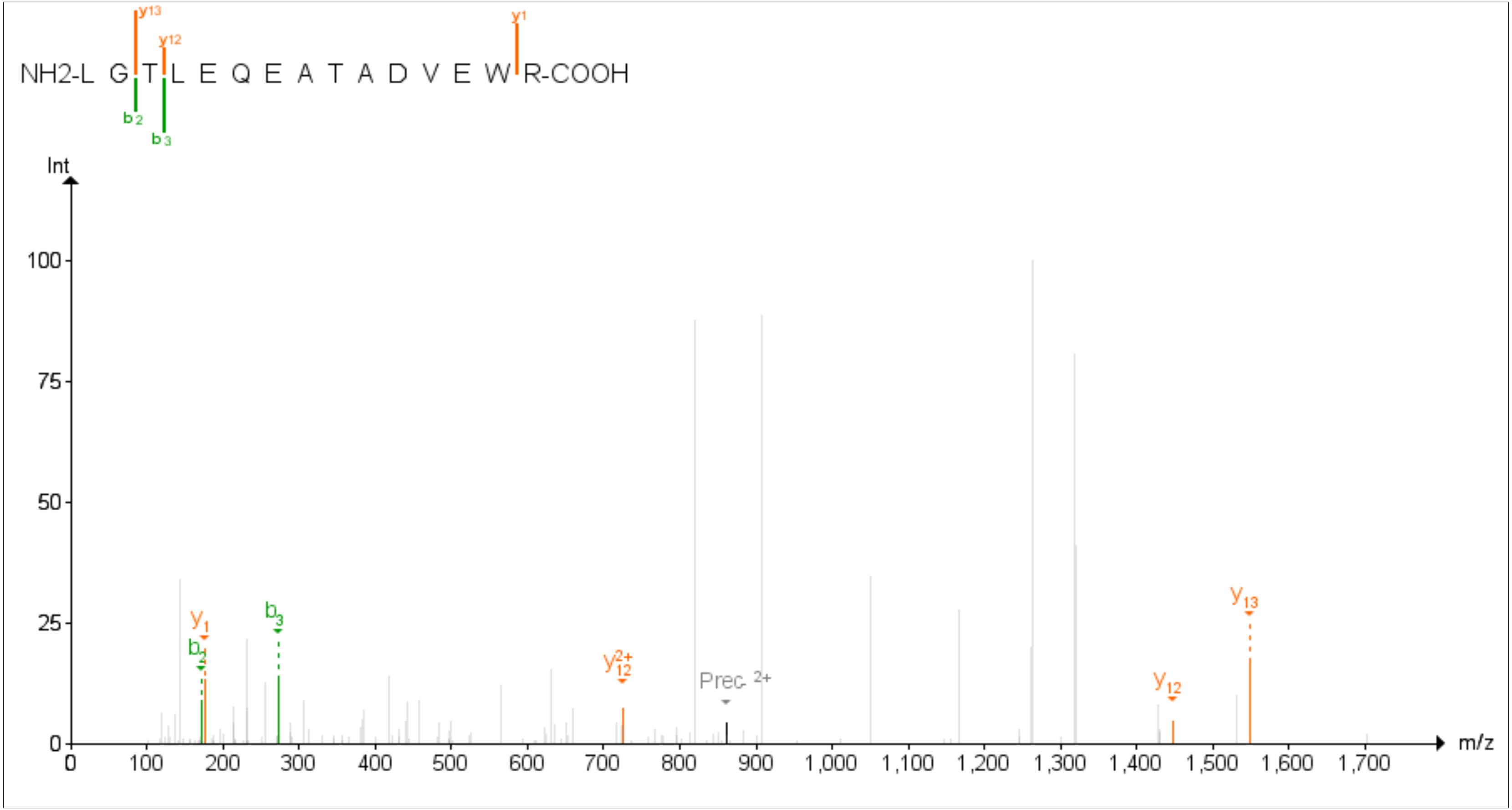
NH2-L G T L E Q E A T A D V E W R-COOH
y13
y12
y1
b2
b3
Int
100
75
50
25
0
m/z
0
100
200
300
400
500
600
700
800
900
1,000
1,100
1,200
1,300
1,400
1,500
1,600
1,700
y1
b2
b3
y12 2+
Prec. 2+
y12
y13

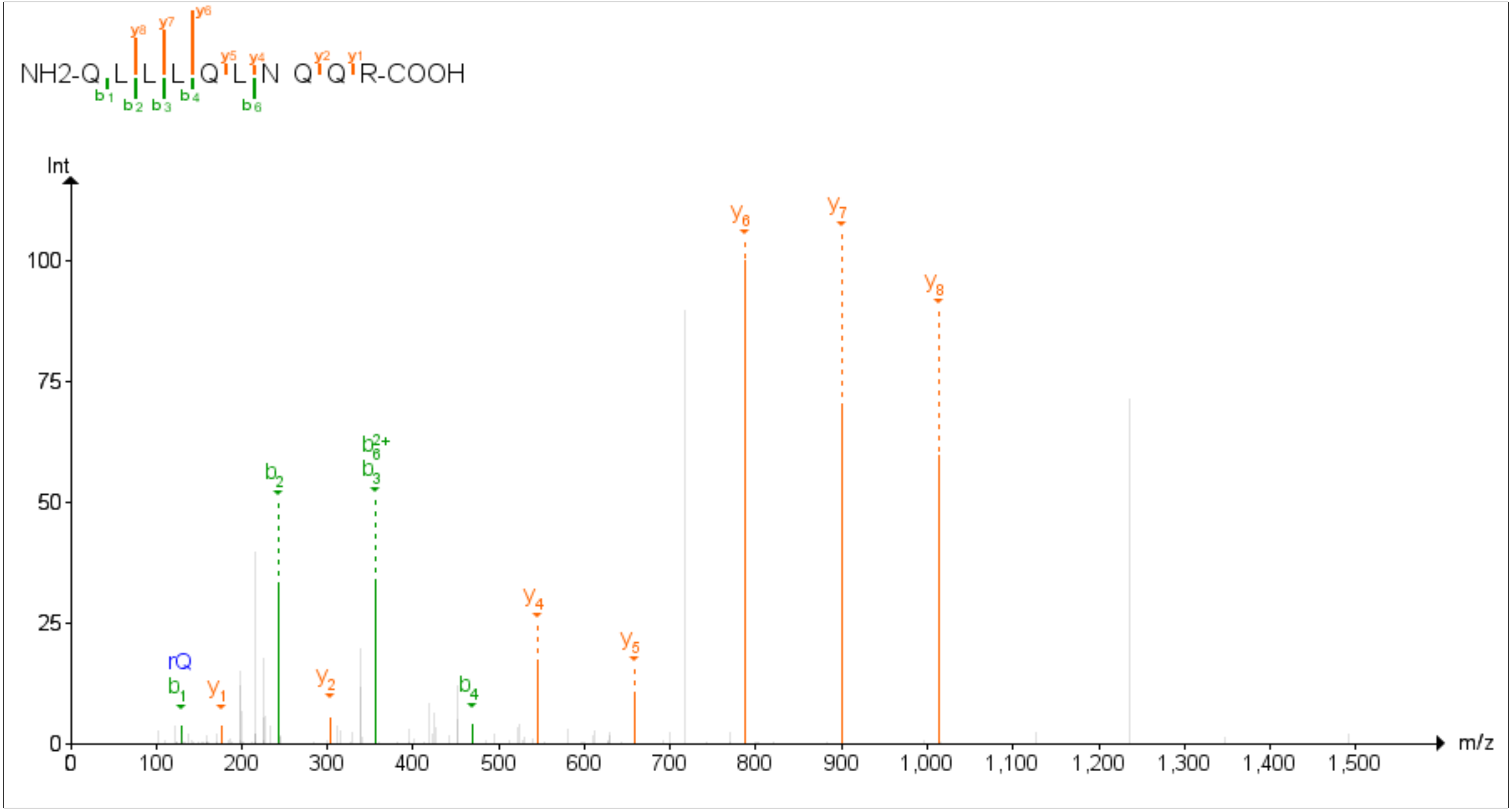

NH2-Q L L L Q L N Q Q R-COOH
y8 y7 y6 y5 y4 y2 y1
b1 b2 b3 b4 b6
Int
100
75
50
25
0
m/z
0 100 200 300 400 500 600 700 800 900 1,000 1,100 1,200 1,300 1,400 1,500
rQ
b1
y1
b2
y2
b6 2+
b3
b4
y4
y5
y6
y7
y8

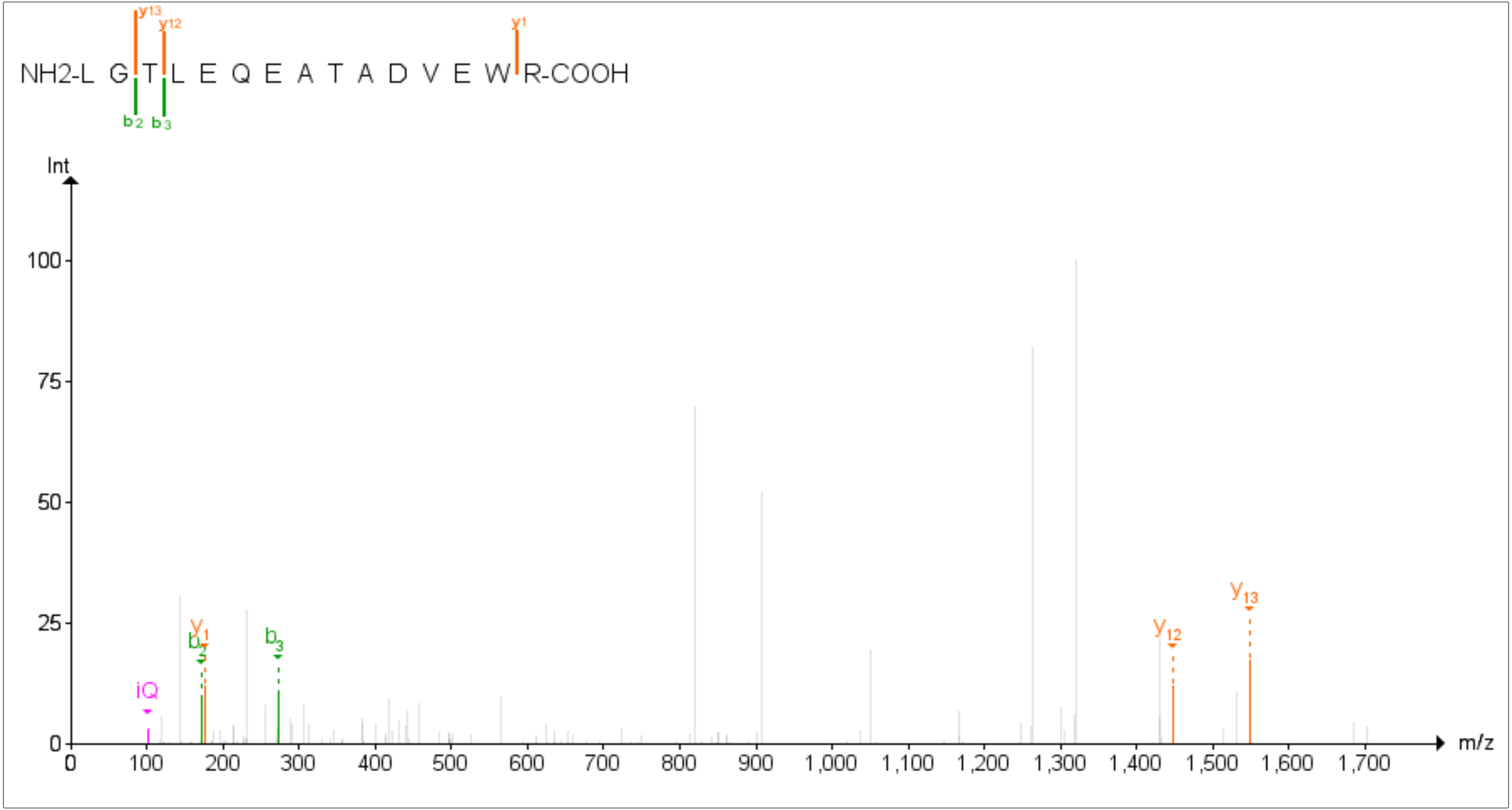

y13
y12
y1
NH2-L G T L E Q E A T A D V E W R-COOH
b2
b3
Int
100
75
50
25
0
iQ
y1
b2
b3
y12
y13
0
100
200
300
400
500
600
700
800
900
1,000
1,100
1,200
1,300
1,400
1,500
1,600
1,700
m/z

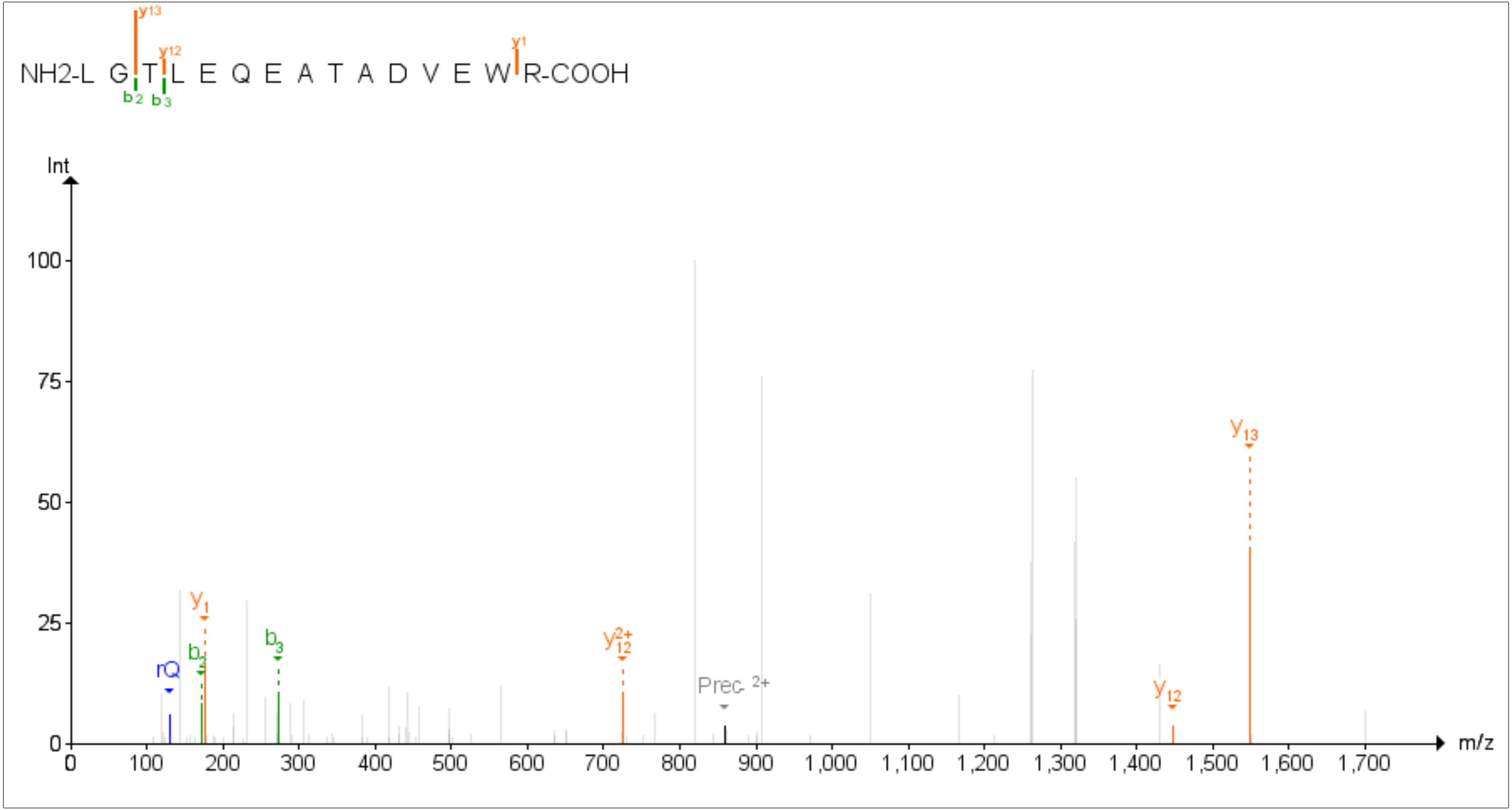

NH2-L G T L E Q E A T A D V E W R-COOH
y13
y12
y1
b2
b3
Int
m/z
y1
rQ
b2
b3
y_{12}^{2+}
Prec 2+
y_{12}
y_{13}

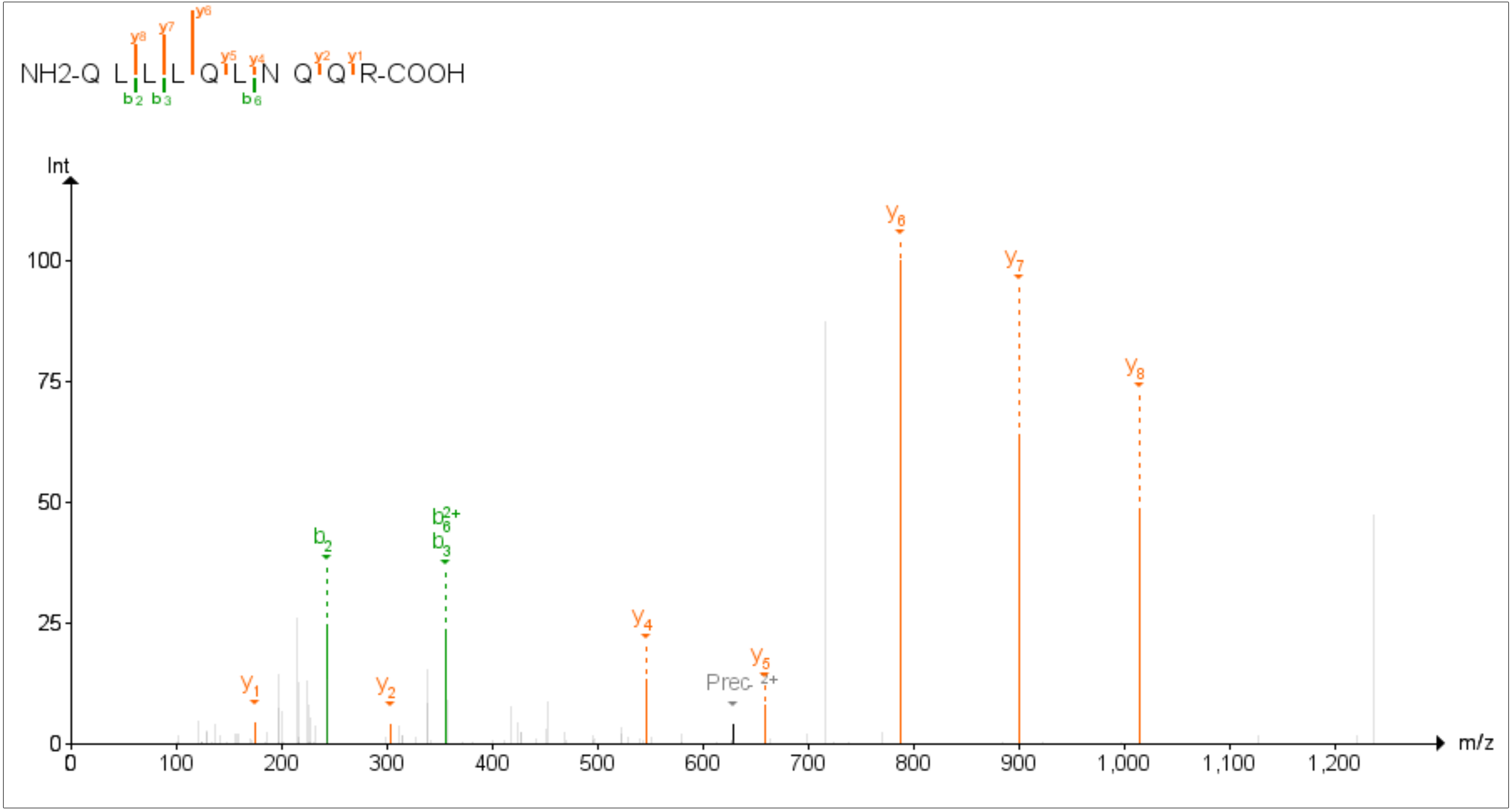

NH2-Q L L L Q L N Q Q R-COOH
y8 y7 y6 y5 y4 y2 y1
b2 b3 b6
Int
100
75
50
25
0
y6
y7
y8
b2
b6 2+
b3
y4
y5
Prec 2+
y1
y2
0 100 200 300 400 500 600 700 800 900 1,000 1,100 1,200
m/z

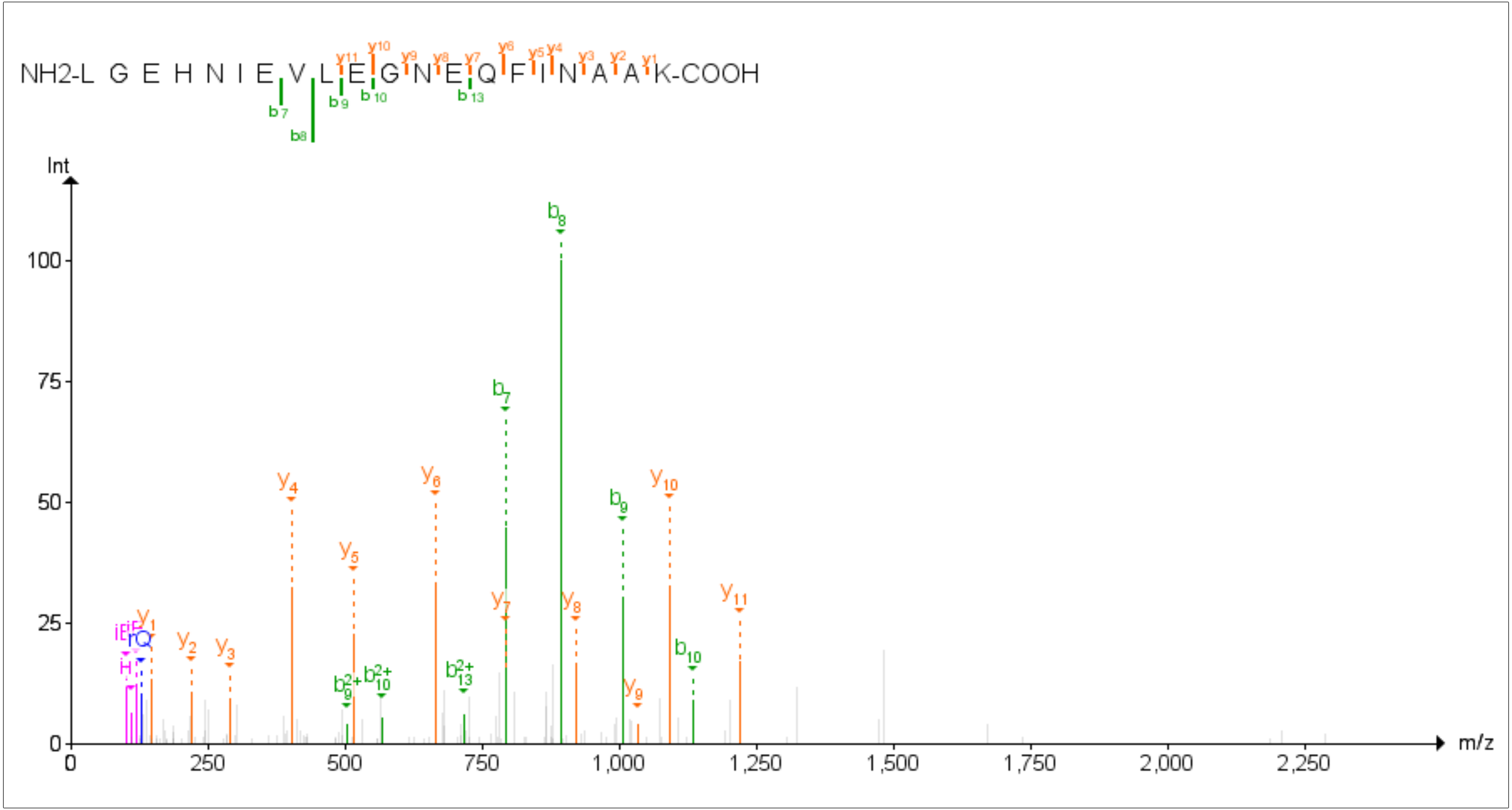

NH2-L G E H N I E V L E G N E Q F I N A A K-COOH
Int
m/z
b8
b7
y4
y6
y10
b9
y5
y11
y7
y8
y1
y2
y3
b10
y9
rQ
H
0
25
50
75
100
250
500
750
1,000
1,250
1,500
1,750
2,000
2,250

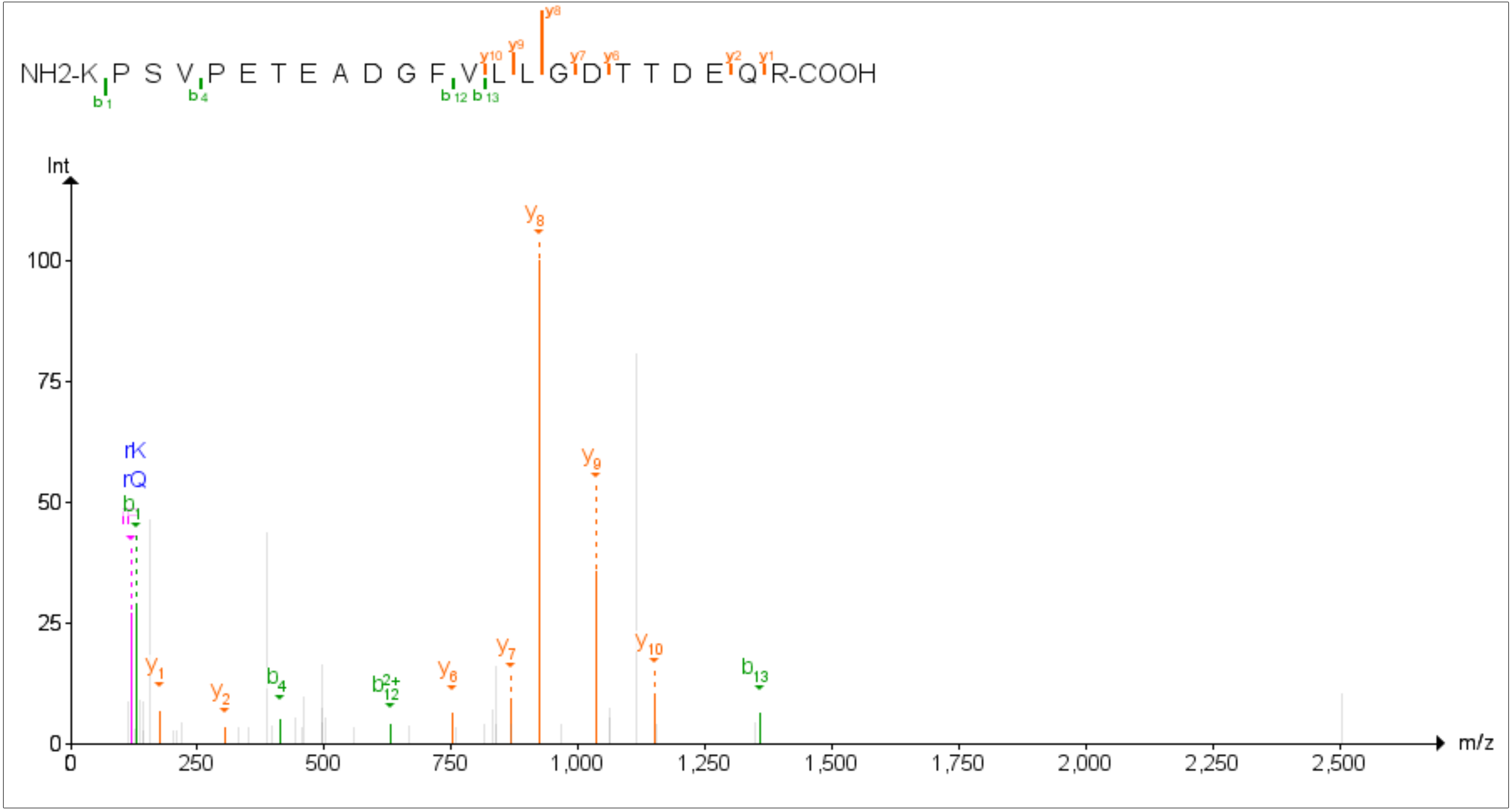

NH2-K P S V P E T E A D G F V L L G D T T D E Q R-COOH
b1
b4
b12
b13
y10
y9
y8
y7
y6
y2
y1
Int
m/z
rK
rQ
b1
y1
y2
b4
b12 2+
y6
y7
y8
y9
y10
b13
0
25
50
75
100
0
250
500
750
1,000
1,250
1,500
1,750
2,000
2,250
2,500

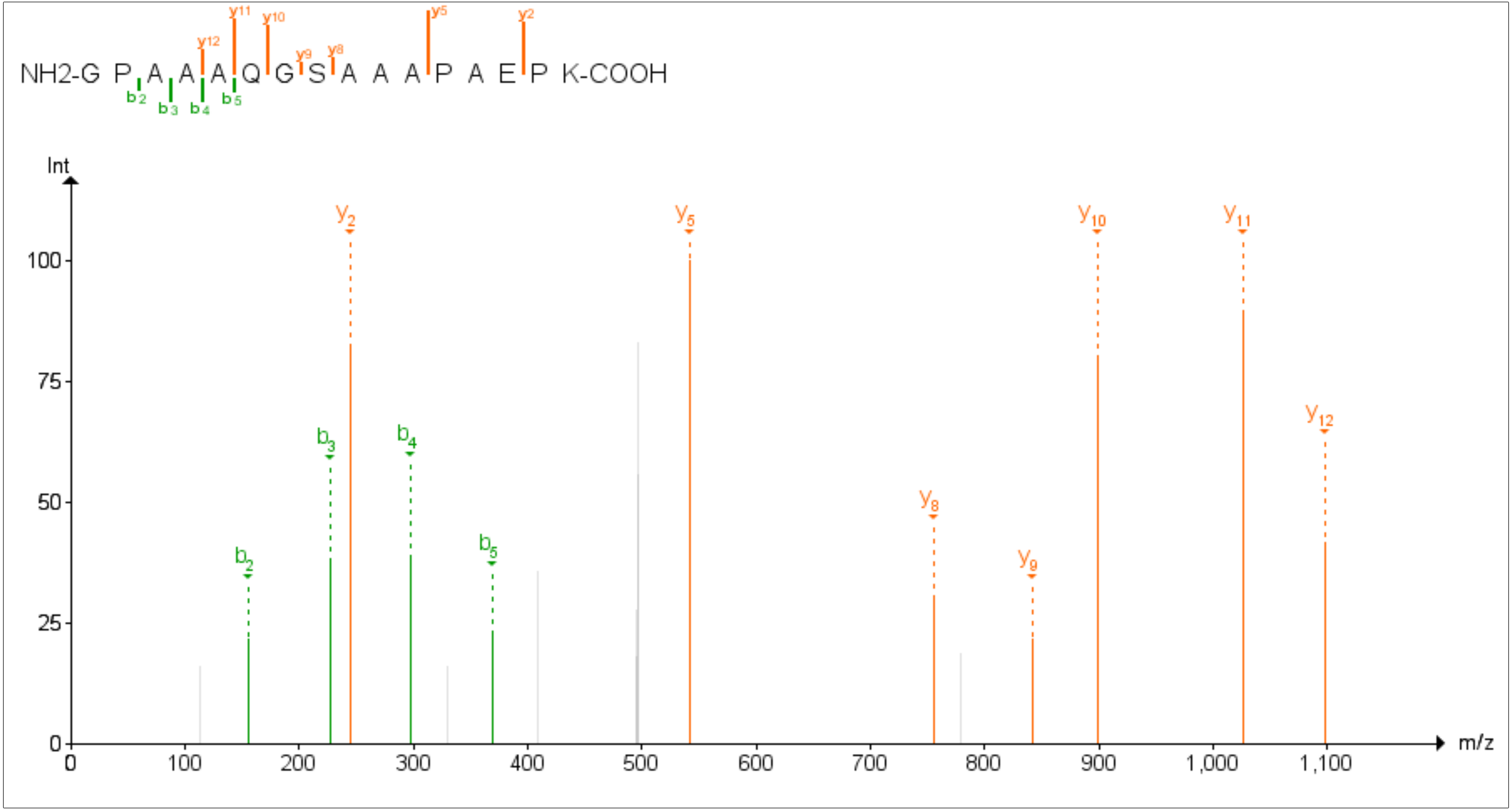
NH2-G P A A A Q G S A A A P A E P K-COOH
y12 y11 y10 y9 y8 y5 y2
b2 b3 b4 b5
Int
100
75
50
25
0
y2 y5 y10 y11 y12 b3 b4 b2 b5 y8 y9
0 100 200 300 400 500 600 700 800 900 1,000 1,100
m/z

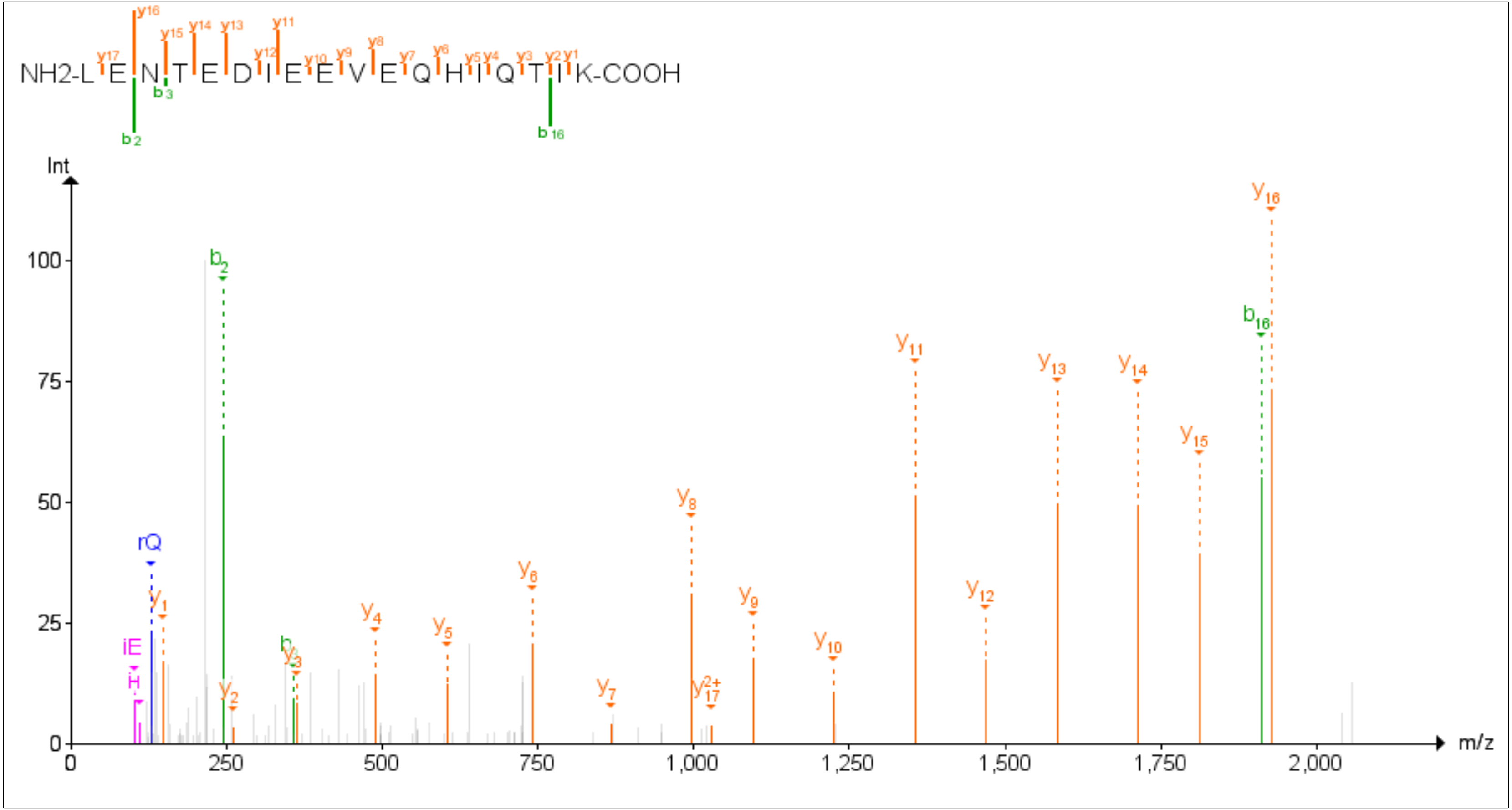

NH2-L E N T E D I E E V E Q H I Q T I K-COOH
y17 y16 y15 y14 y13 y12 y11 y10 y9 y8 y7 y6 y5 y4 y3 y2 y1
b2 b3 b16
Int
100
75
50
25
0
0 250 500 750 1,000 1,250 1,500 1,750 2,000
m/z
b2
b16
y16
y11
y13
y14
y15
y8
y12
y9
y10
y6
y4
y5
y7
y2+17
y2
y3
b3
y1
rQ
iE
iH

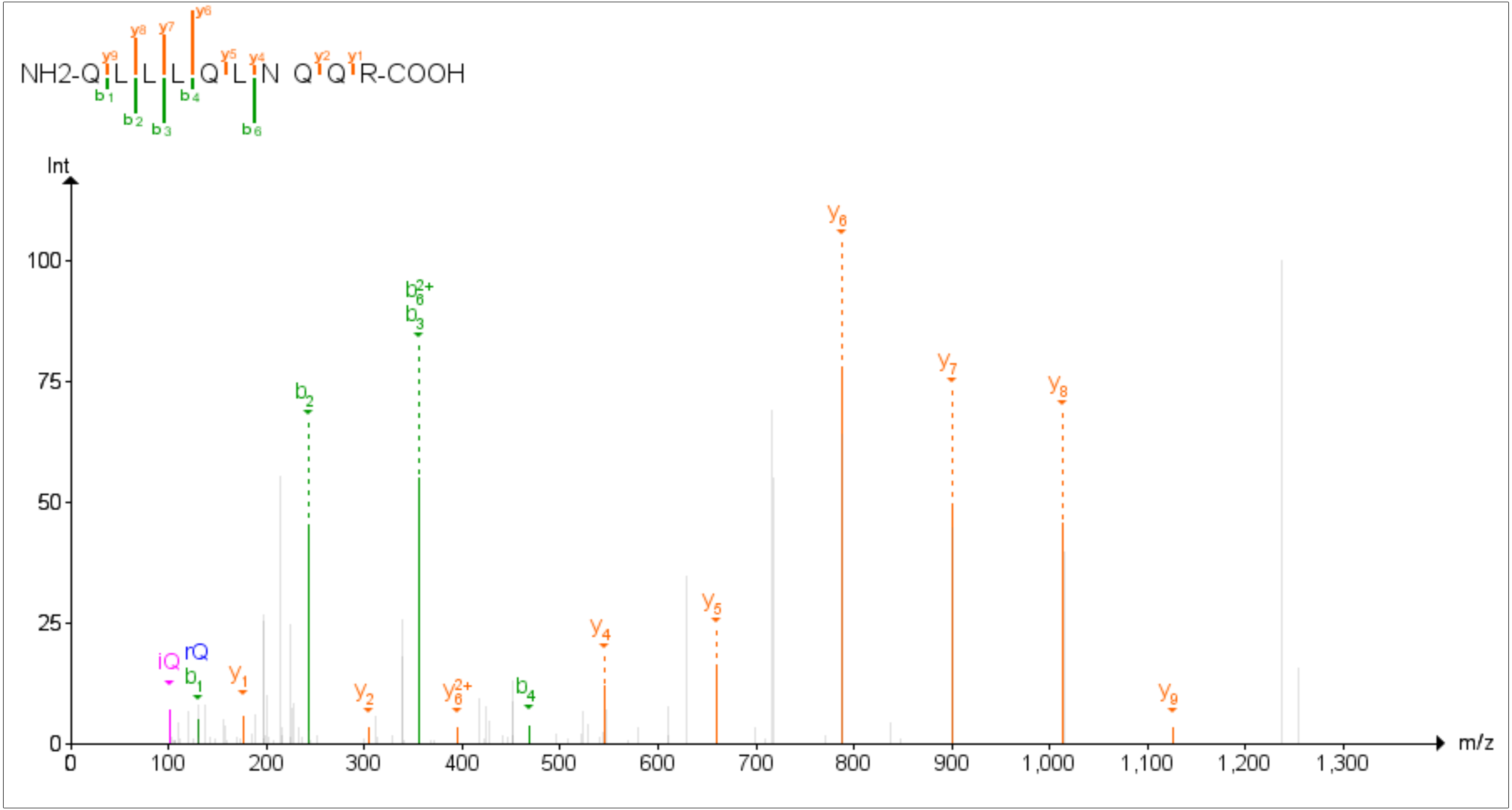

NH2-Q L L L Q L N Q Q R-COOH
y9 y8 y7 y6 y5 y4 y2 y1
b1 b2 b3 b4 b6
Int
m/z
100
75
50
25
0
0 100 200 300 400 500 600 700 800 900 1,000 1,100 1,200 1,300

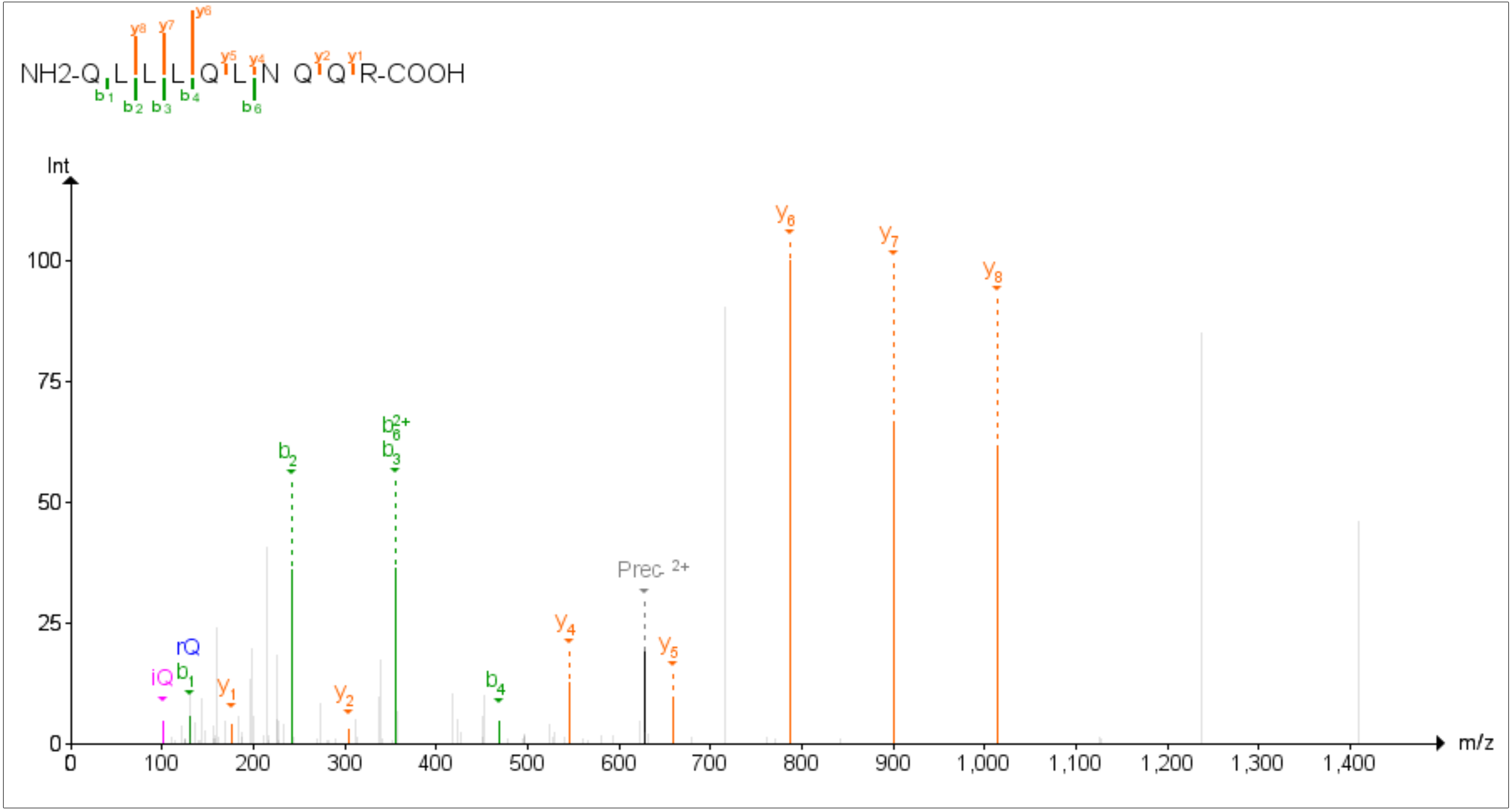

NH2-Q L L L Q L N Q Q R-COOH
y8 y7 y6 y5 y4 y2 y1
b1 b2 b3 b4 b6
Int
m/z
Prec. 2+
iQ
rQ

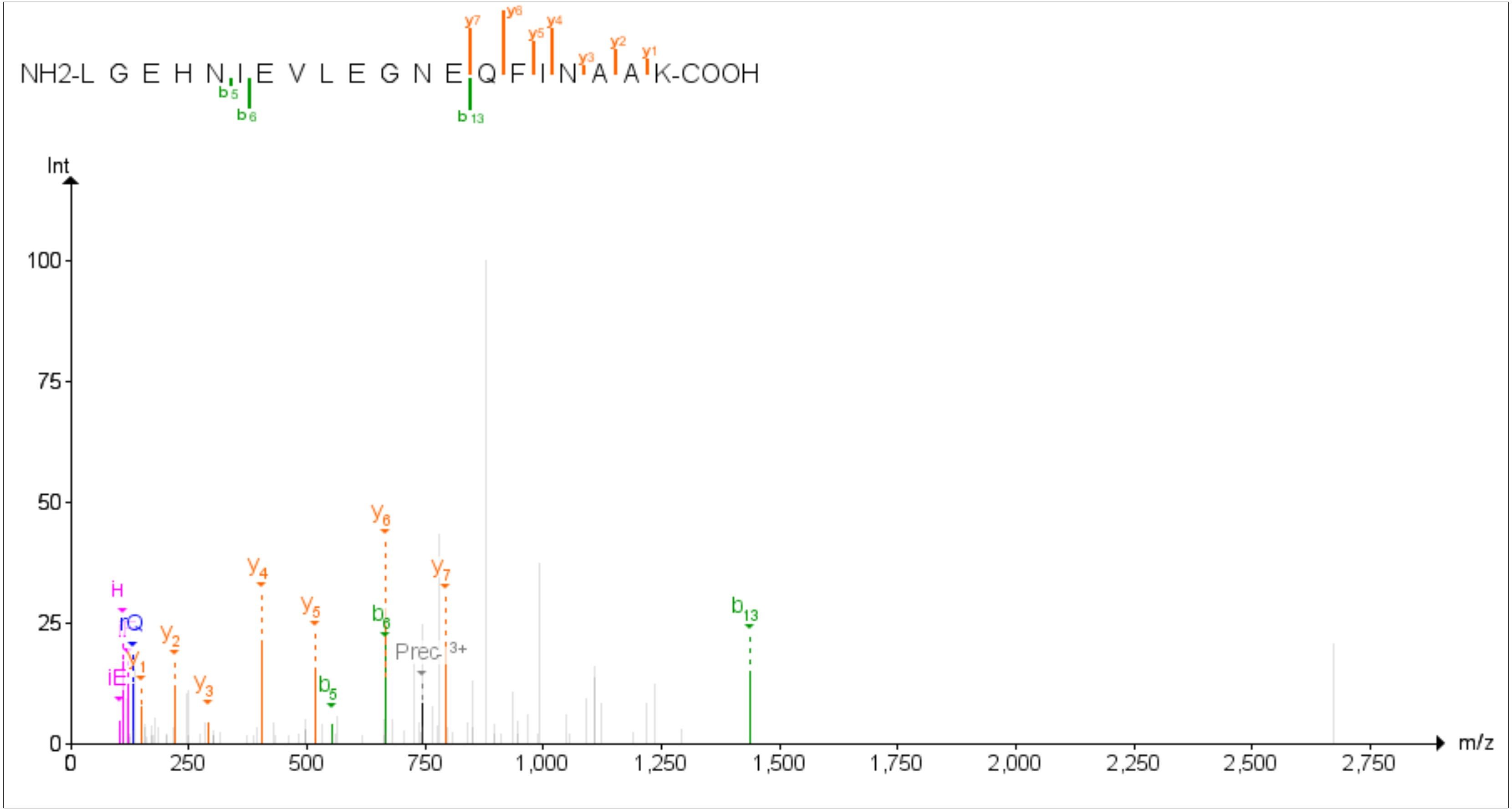

NH2-L G E H N I E V L E G N E Q F I N A A K-COOH
y7 y6 y5 y4 y3 y2 y1
b5 b6 b13
Int
100
75
50
25
0
m/z
0 250 500 750 1,000 1,250 1,500 1,750 2,000 2,250 2,500 2,750
y6 y4 y7 y5 b6 b13 y2 y1 y3 b5
iH rQ iE
Prec 3+

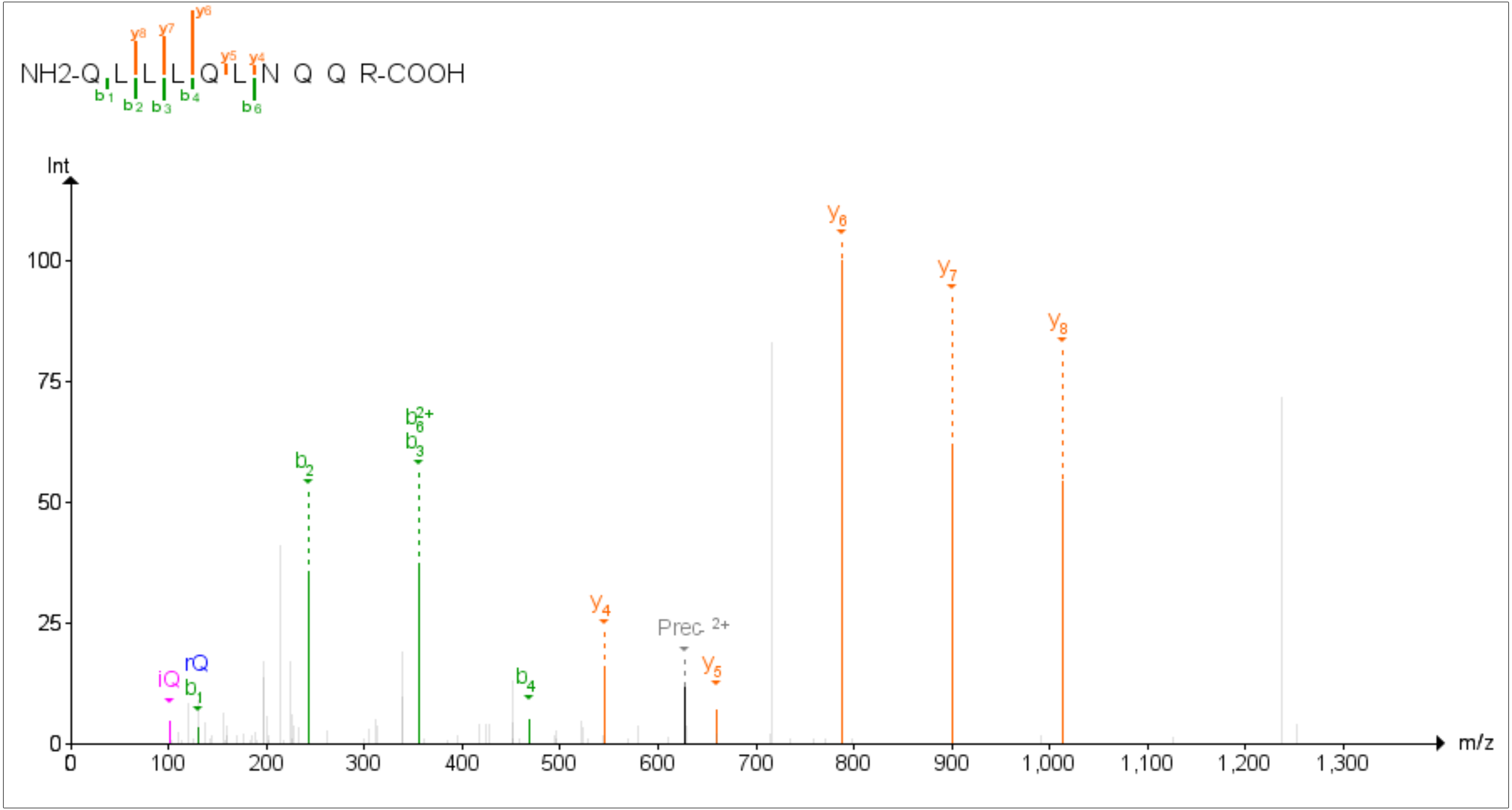
NH2-Q L L L Q L N Q Q R-COOH
y8 y7 y6 y5 y4
b1 b2 b3 b4 b6
Int
m/z
0
25
50
75
100
0
100
200
300
400
500
600
700
800
900
1,000
1,100
1,200
1,300
iQ
rQ
b1
b2
b6 2+
b3
b4
y4
Prec- 2+
y5
y6
y7
y8

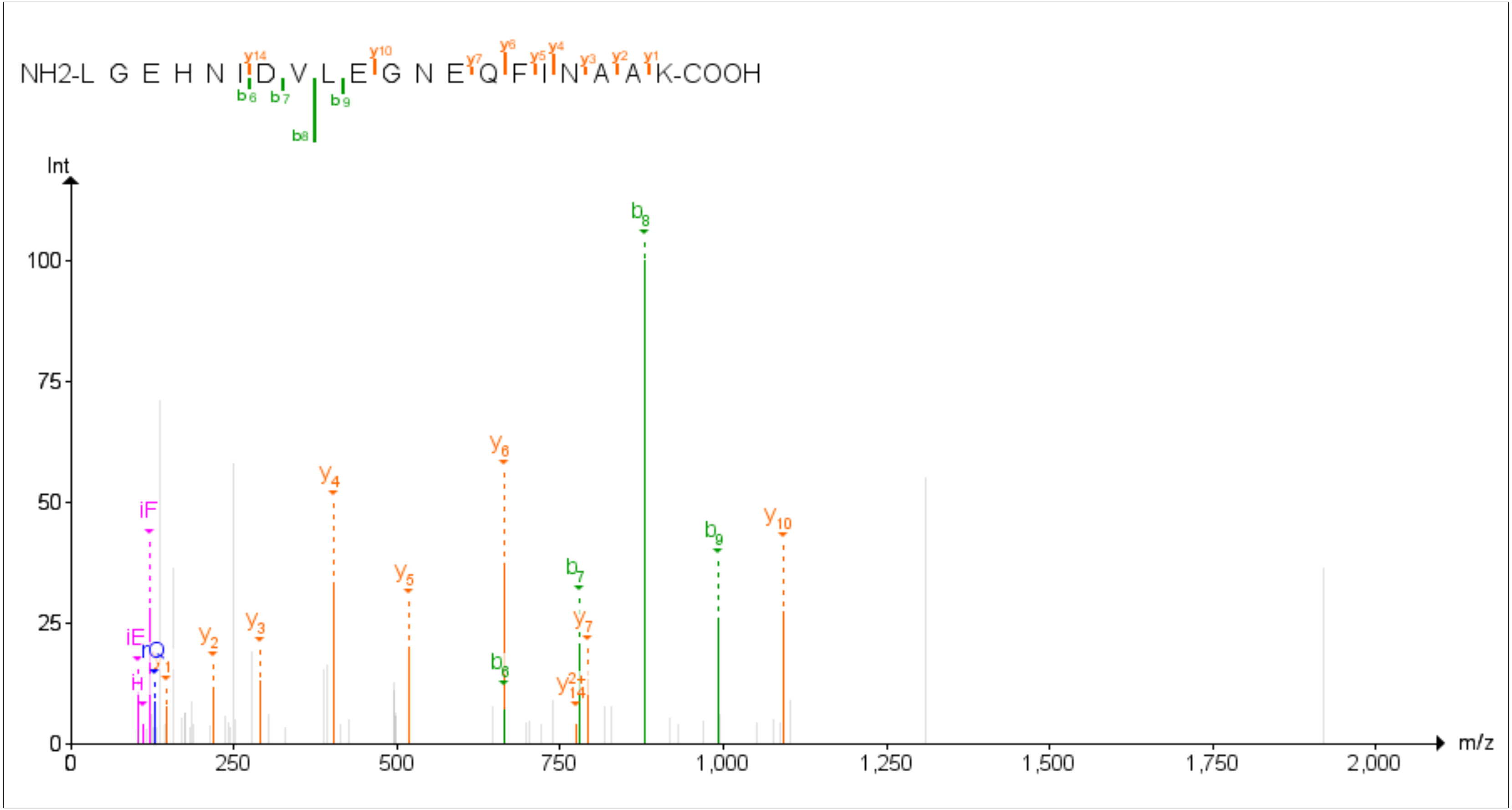

NH2-L G E H N I D V L E G N E Q F I N A A K-COOH
y14 y10 y7 y6 y5 y4 y3 y2 y1
b6 b7 b8 b9
Int
m/z
0
25
50
75
100
250
500
750
1,000
1,250
1,500
1,750
2,000
b8
y6
y4
iF
y10
b9
b7
y5
y7
y3
y2
iE
rQ
b6
y1
iH
y2+ 14

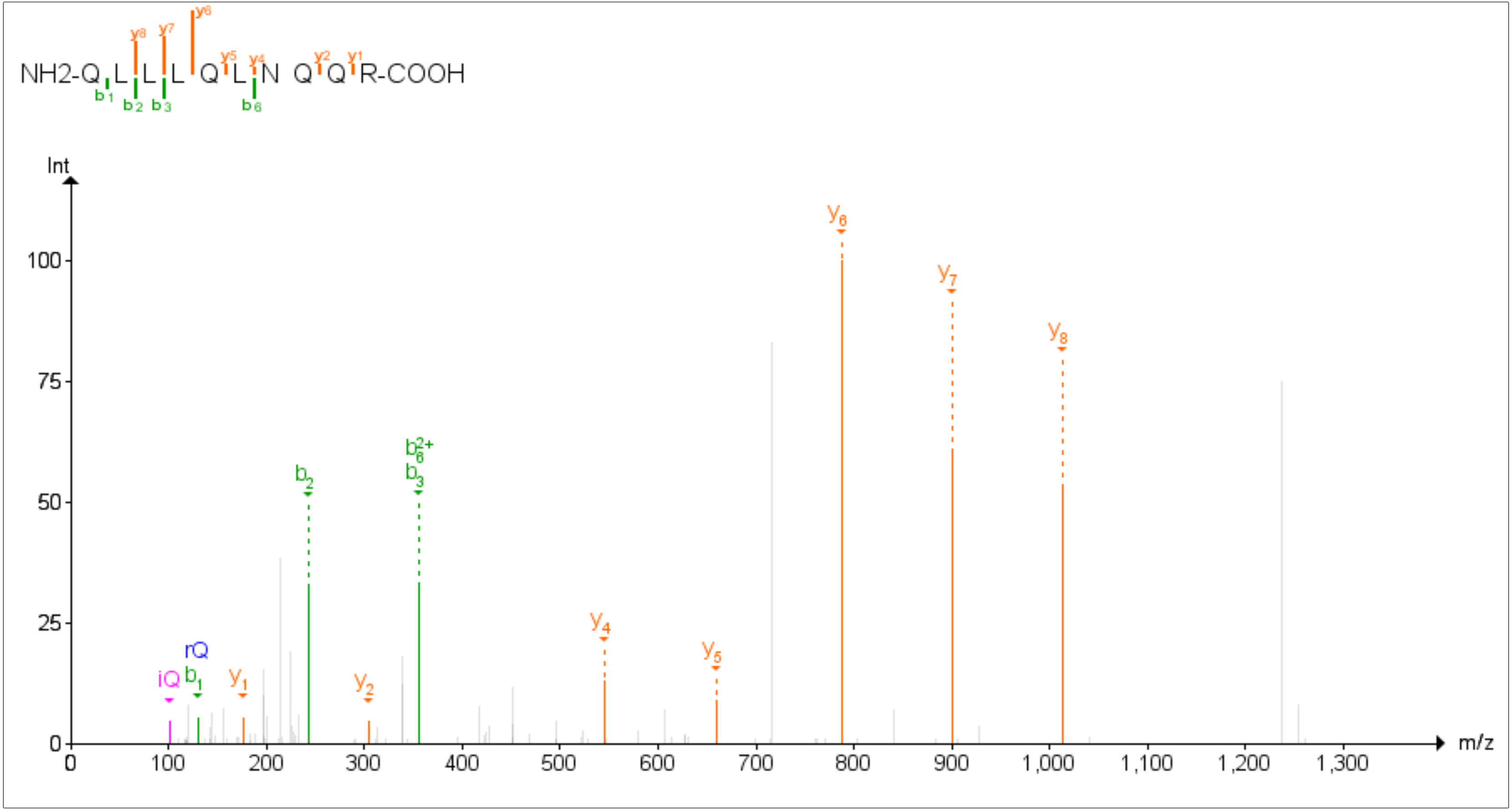

NH2-Q L L L Q L N Q Q R-COOH
y8 y7 y6 y5 y4 y2 y1
b1 b2 b3 b6
Int
m/z
y6
y7
y8
b2
b6 2+
b3
y4
y5
rQ
iQ
b1
y1
y2
0
25
50
75
100
0
100
200
300
400
500
600
700
800
900
1,000
1,100
1,200
1,300

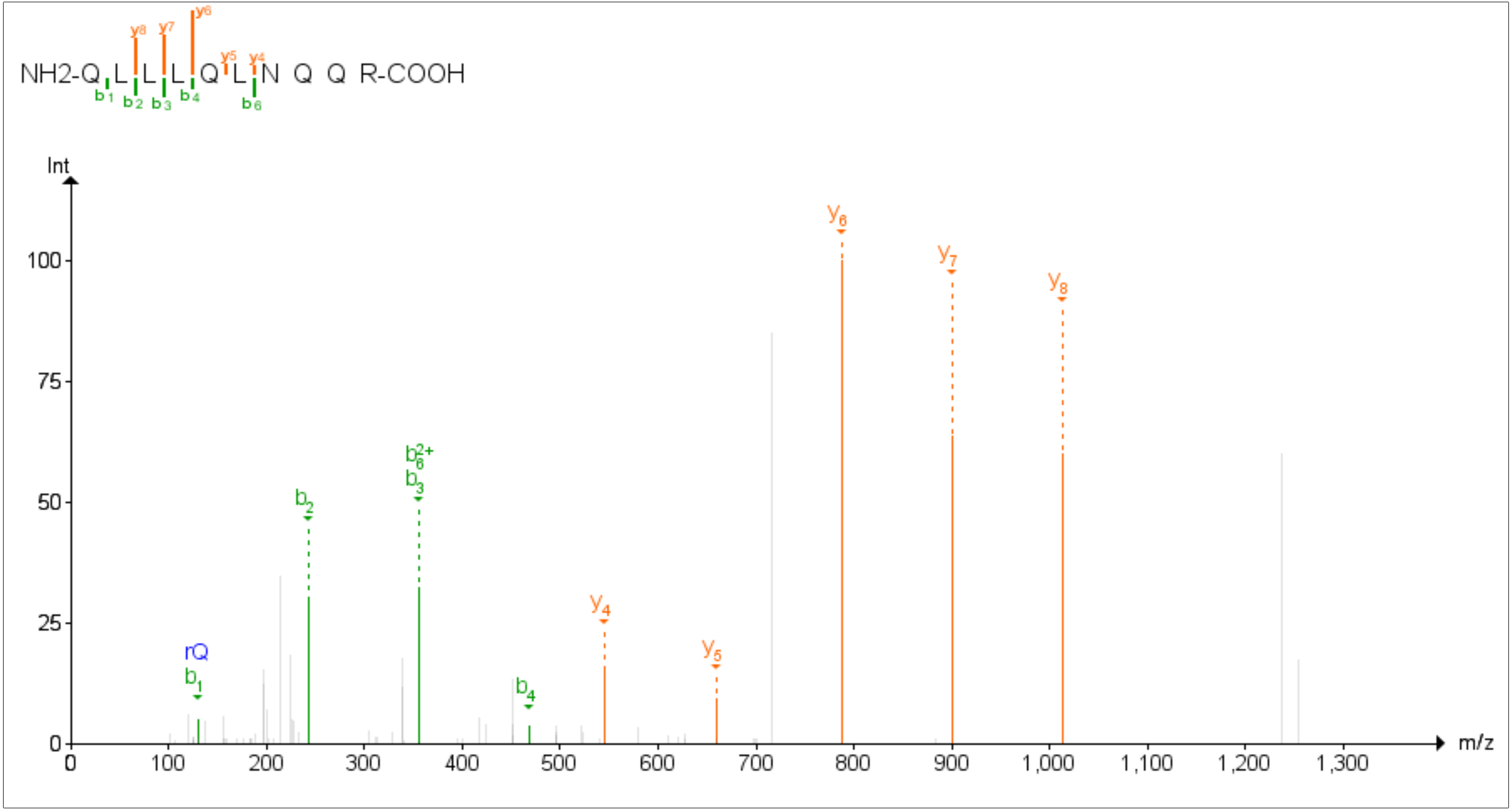

NH2-Q L L L Q L N Q Q R-COOH
y8 y7 y6 y5 y4
b1 b2 b3 b4 b6
Int
100
75
50
25
0
y6
y7
y8
b6 2+
b3
b2
y4
y5
rQ
b1
b4
0 100 200 300 400 500 600 700 800 900 1,000 1,100 1,200 1,300
m/z

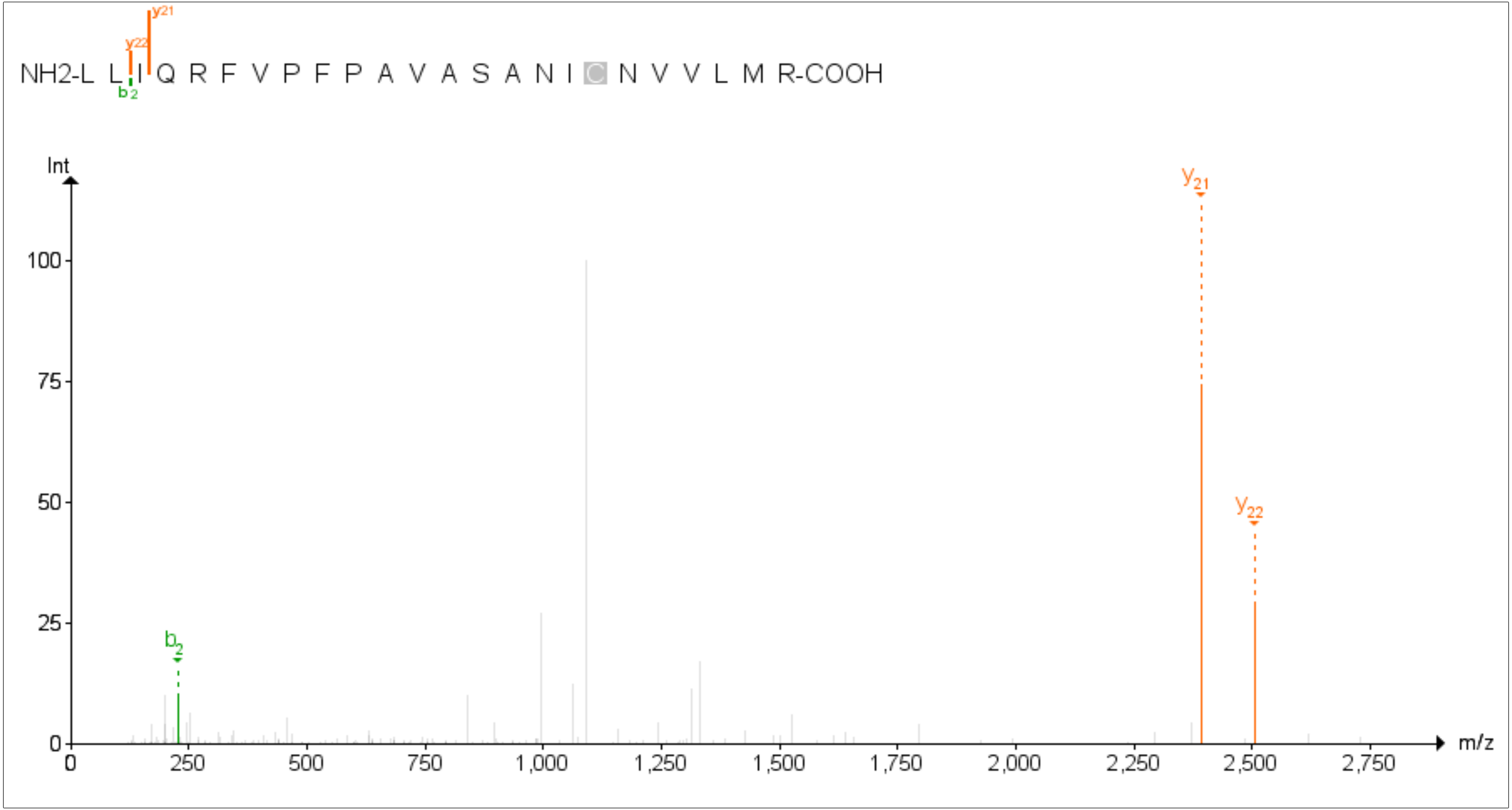

NH2-L L I Q R F V P F P A V A S A N I C N V V L M R-COOH
y21
y22
b2
Int
100
75
50
25
0
0
250
500
750
1,000
1,250
1,500
1,750
2,000
2,250
2,500
2,750
m/z

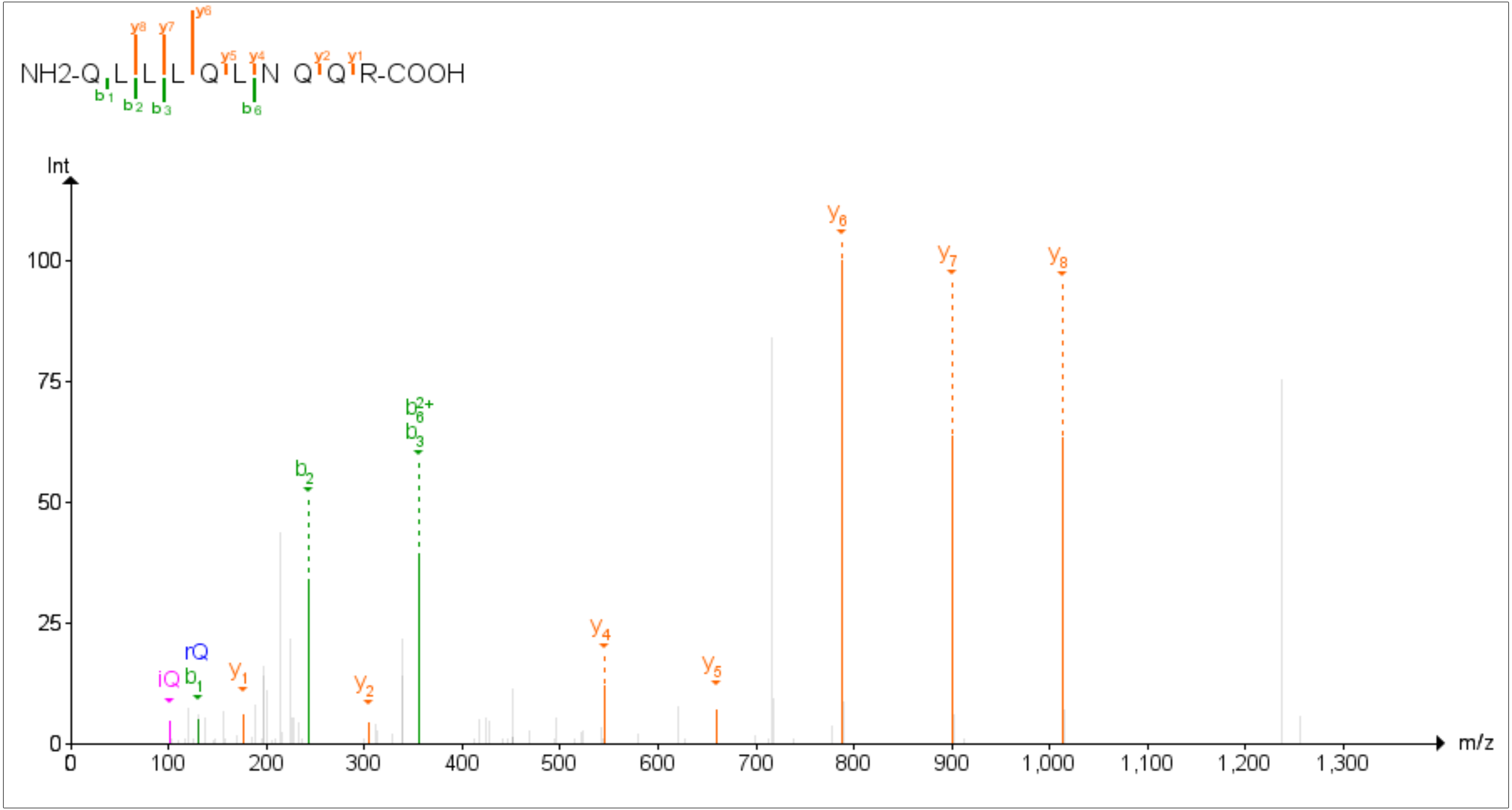

NH2-Q L L L Q L N Q Q R-COOH
y8 y7 y6 y5 y4 y2 y1
b1 b2 b3 b6
Int
100
75
50
25
0
y6
y7
y8
b6 2+
b3
b2
y4
y5
rQ
iQ
b1
y1
y2
0 100 200 300 400 500 600 700 800 900 1,000 1,100 1,200 1,300
m/z

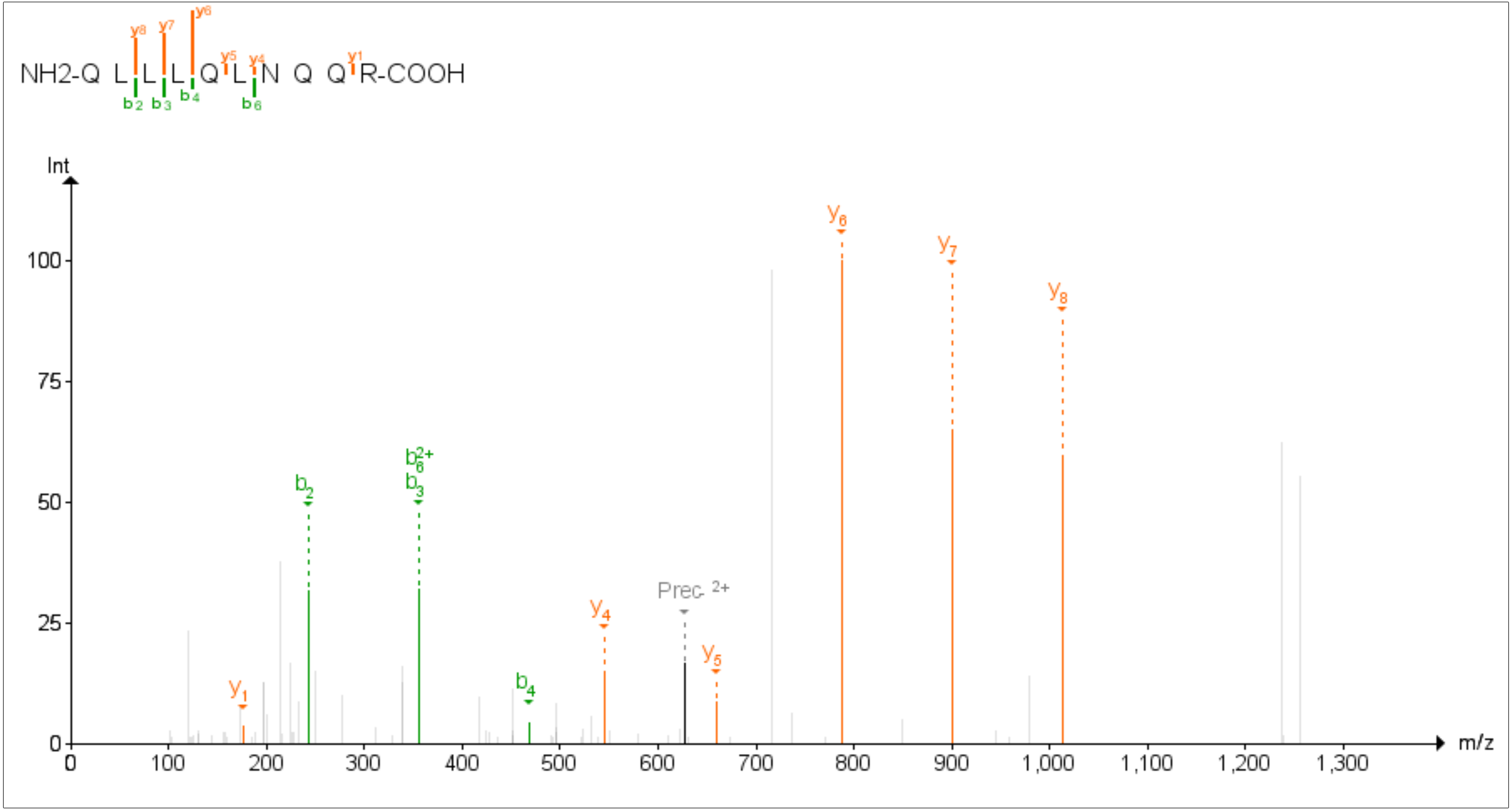

NH2-Q L L L Q L N Q Q R-COOH
y8 y7 y6 y5 y4 y1
b2 b3 b4 b6
Int
100
75
50
25
0
y1
b2
b6 2+
b3
b4
y4
Prec 2+
y5
y6
y7
y8
0
100
200
300
400
500
600
700
800
900
1,000
1,100
1,200
1,300
m/z

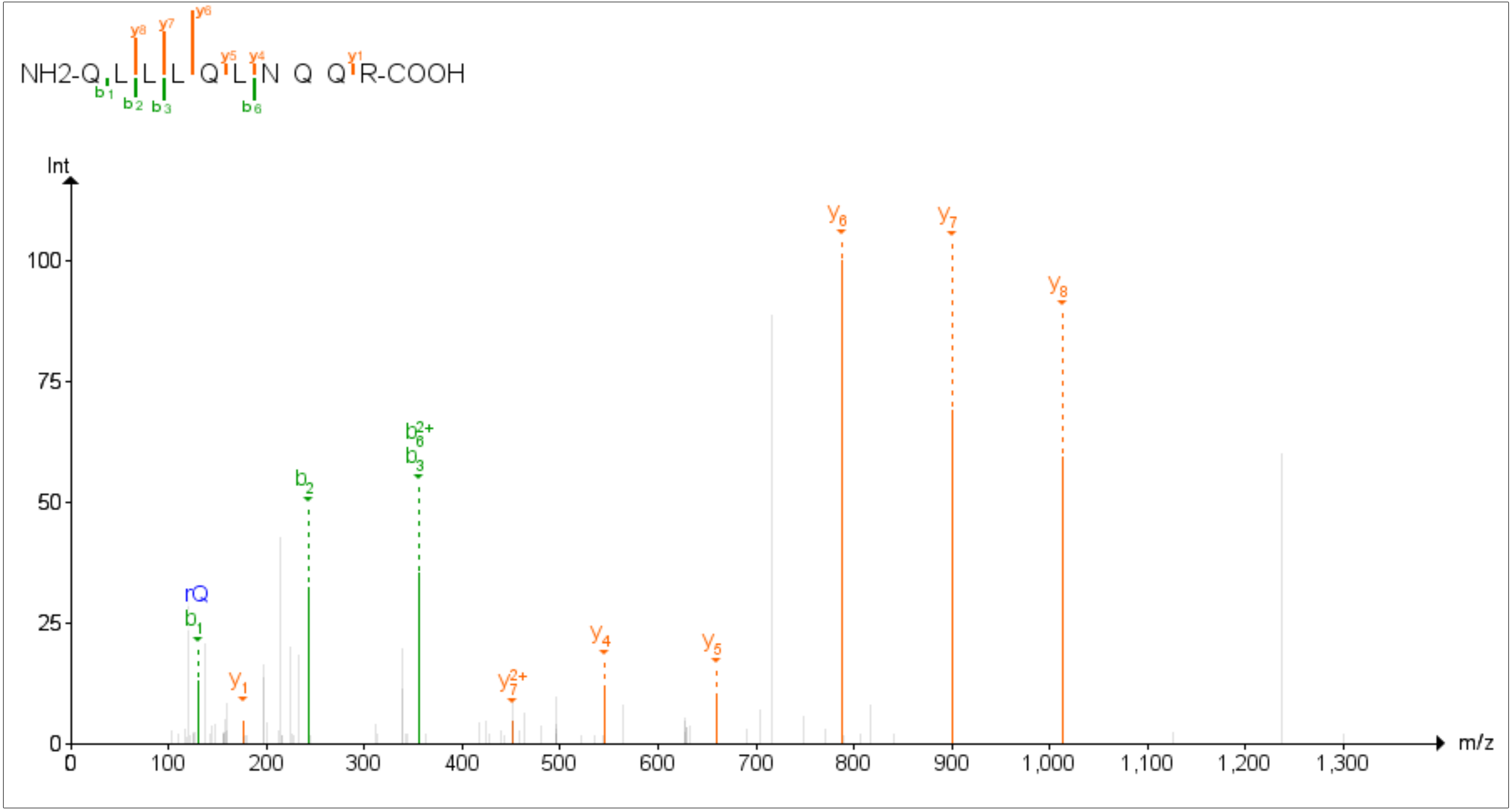

NH2-Q L L L Q L N Q Q R-COOH
y8 y7 y6 y5 y4 y1
b1 b2 b3 b6
Int
m/z
rQ
b1
y1
b2
b6 2+
b3
y7 2+
y4
y5
y6
y7
y8
0
25
50
75
100
100
200
300
400
500
600
700
800
900
1,000
1,100
1,200
1,300

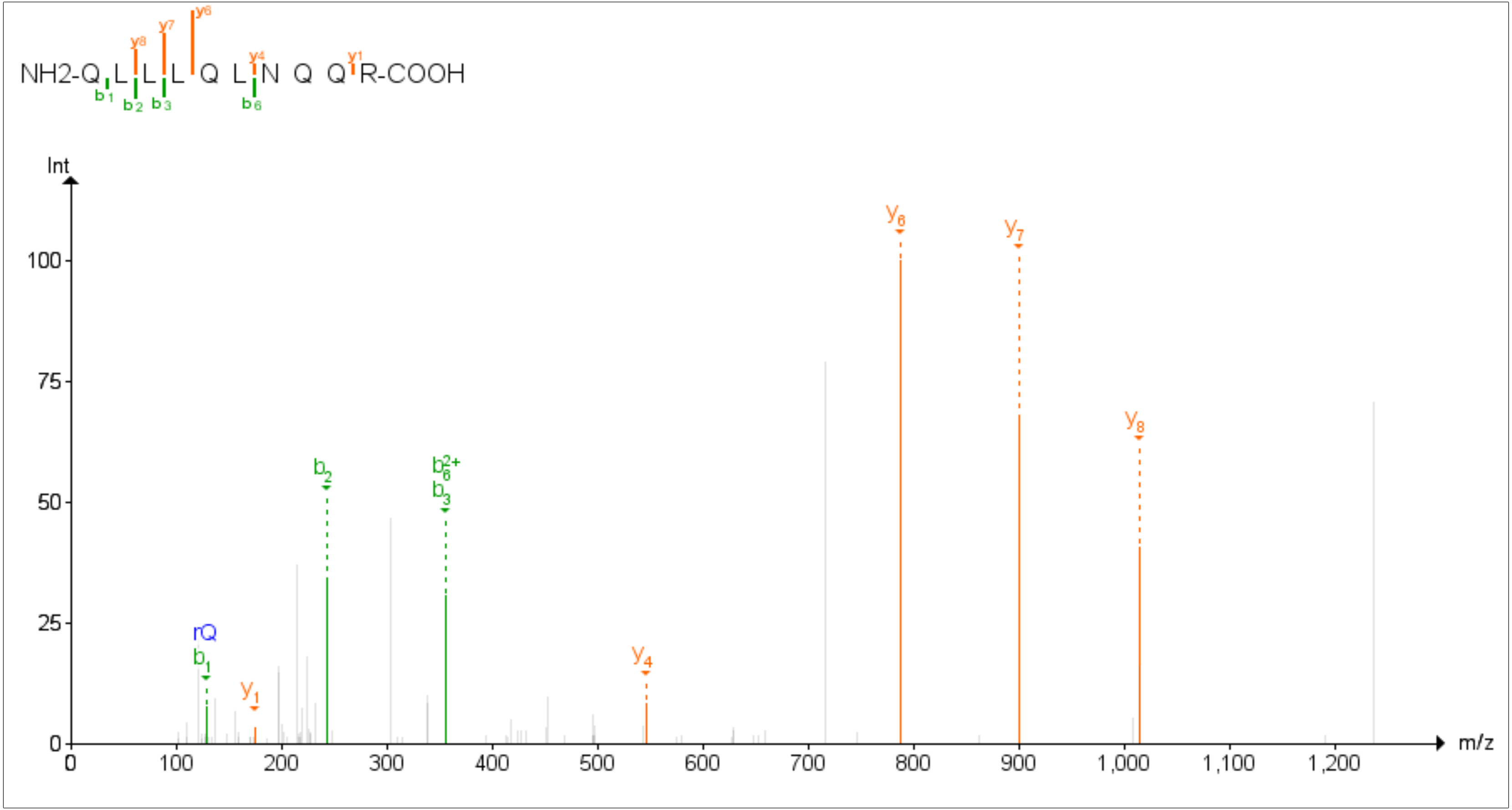

NH2-Q L L L Q L N Q Q R-COOH
b1
b2
b3
b6
y8
y7
y6
y4
y1
Int
m/z
rQ
b1
y1
b2
b6 2+
b3
y4
y6
y7
y8
0
25
50
75
100
0
100
200
300
400
500
600
700
800
900
1,000
1,100
1,200

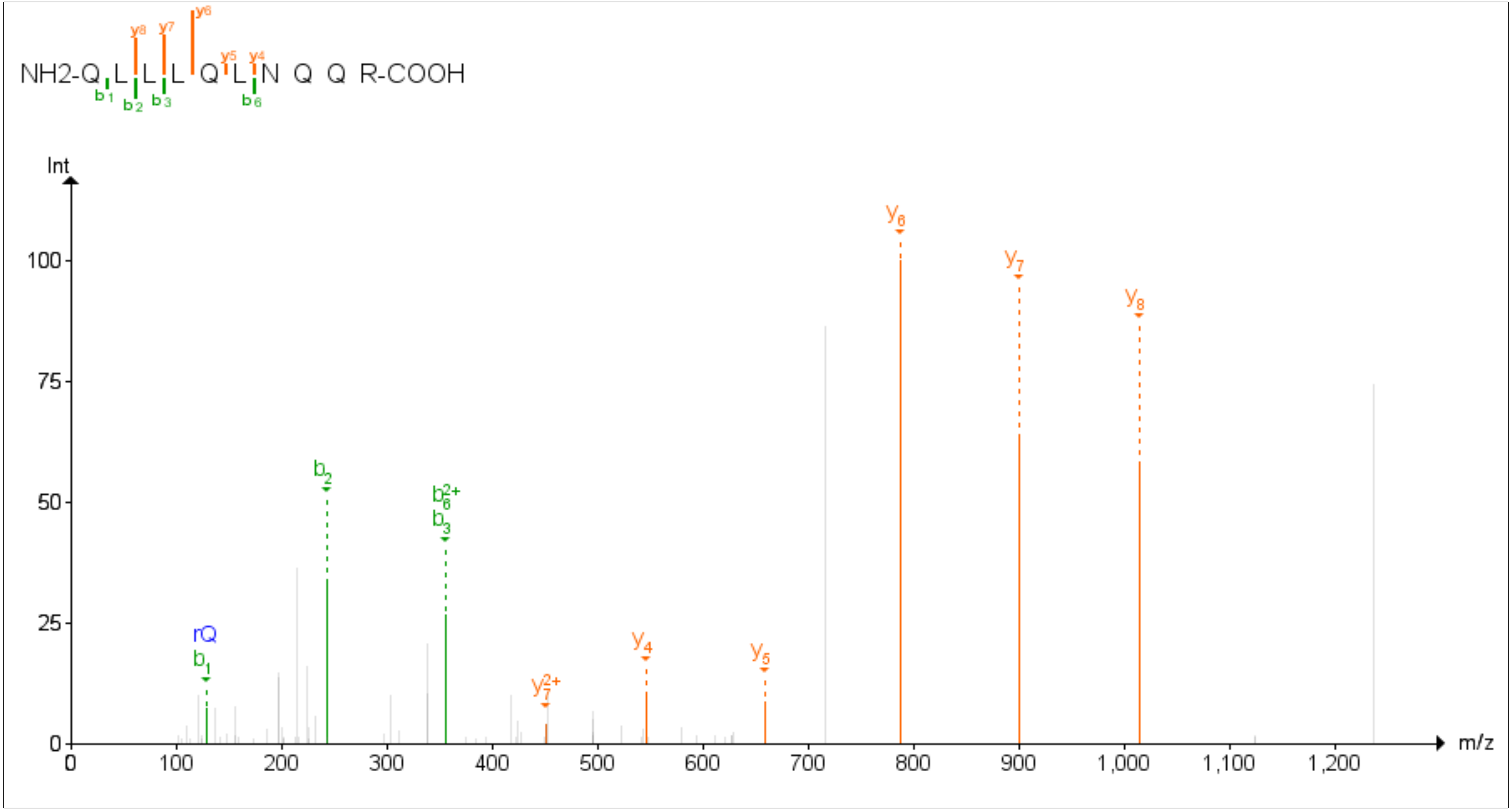
NH2-Q L L L Q L N Q Q R-COOH
y8 y7 y6 y5 y4
b1 b2 b3 b6
Int
100
75
50
25
0
rQ
b1
b2
b6 2+
b3
y7 2+
y4
y5
y6
y7
y8
0 100 200 300 400 500 600 700 800 900 1,000 1,100 1,200
m/z

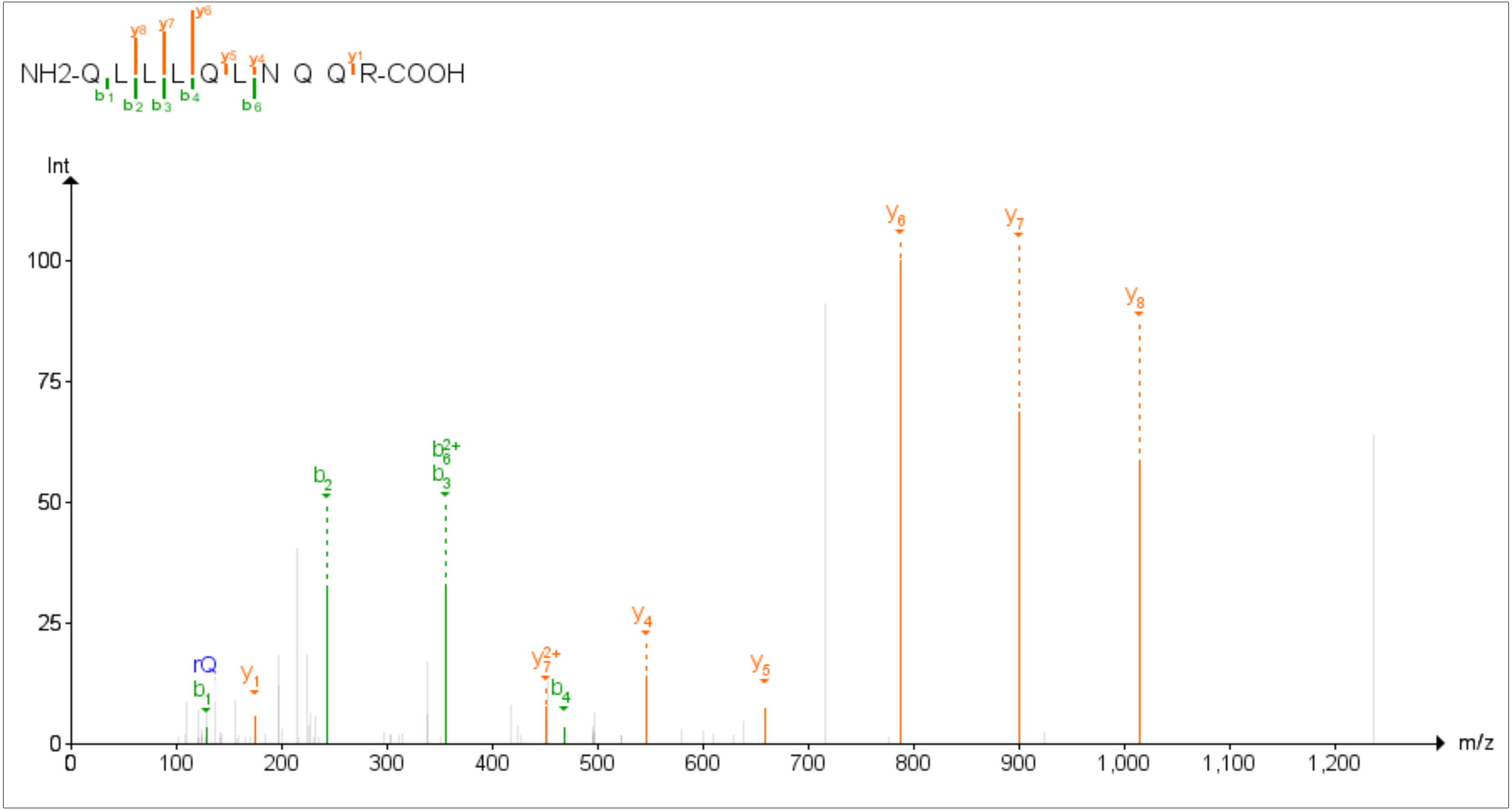
NH2-Q L L L Q L N Q Q R-COOH
y8 y7 y6 y5 y4 y1
b1 b2 b3 b4 b6
Int
100
75
50
25
0
m/z
0 100 200 300 400 500 600 700 800 900 1,000 1,100 1,200
rQ
b1
y1
b2
b6 2+
b3
y7 2+
b4
y4
y5
y6
y7
y8

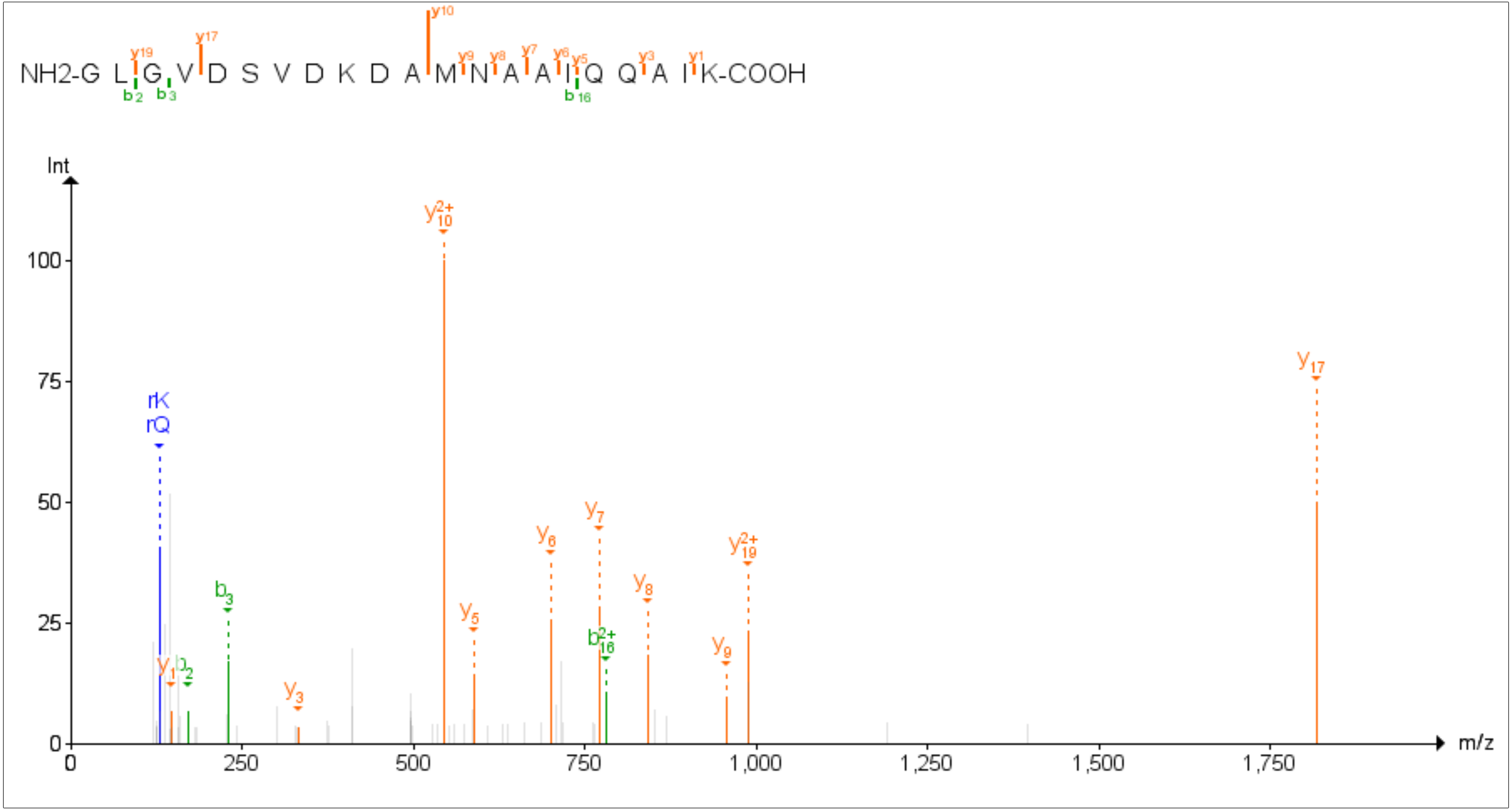

NH2-G L G V D S V D K D A M N A A I Q Q A I K-COOH
y19
y17
y10
y9
y8
y7
y6
y5
y3
y1
b2
b3
b16
Int
m/z
0
25
50
75
100
250
500
750
1,000
1,250
1,500
1,750
rK
rQ
y10 2+
y17
y7
y6
y19 2+
y8
y5
b16 2+
b3
y9
y1
b2
y3

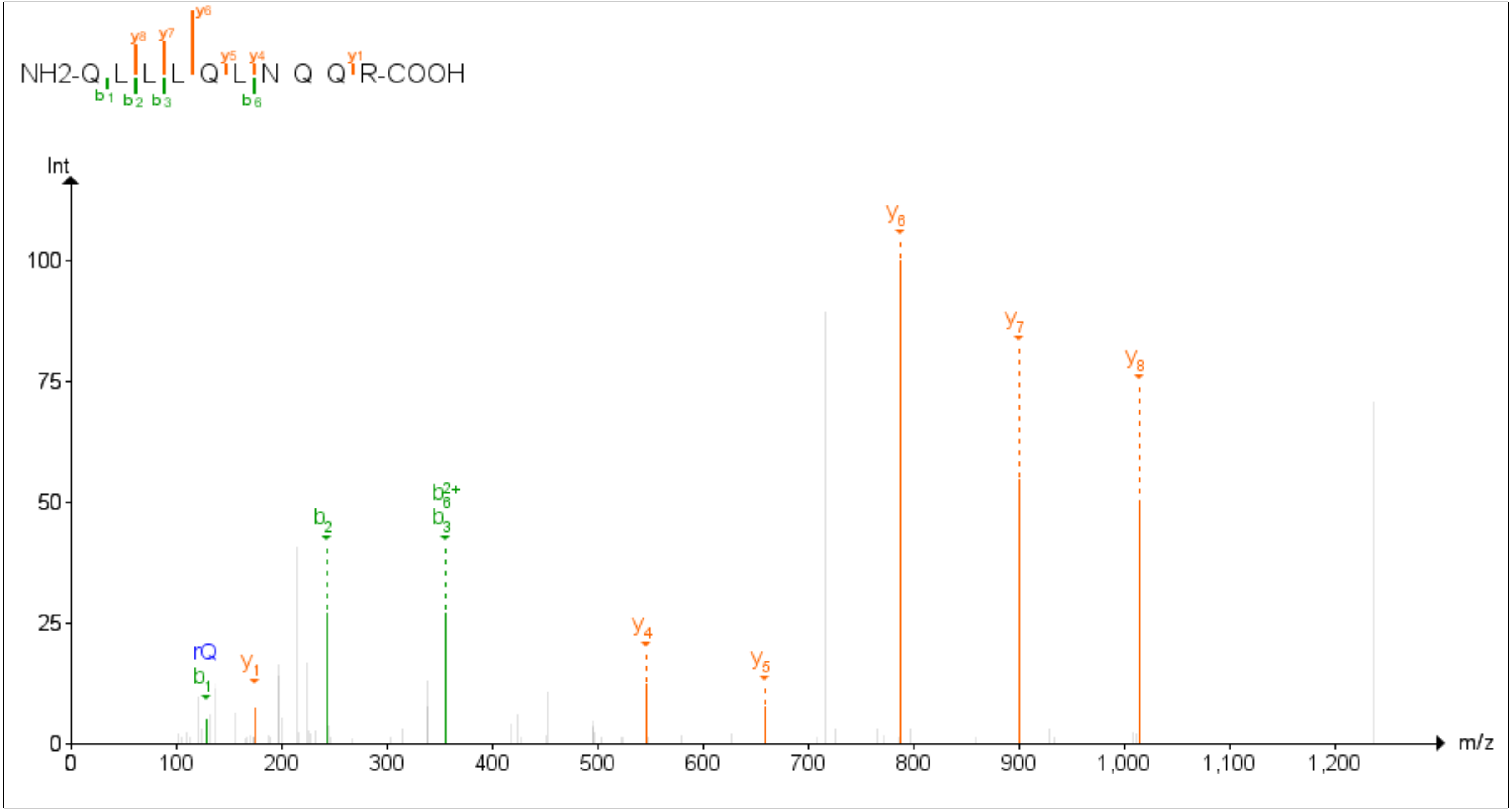

NH2-Q L L L Q L N Q Q R-COOH
b1 b2 b3 b6
y8 y7 y6 y5 y4 y1
Int
m/z
rQ
b1
y1
b2
b6 2+
b3
y4
y5
y6
y7
y8

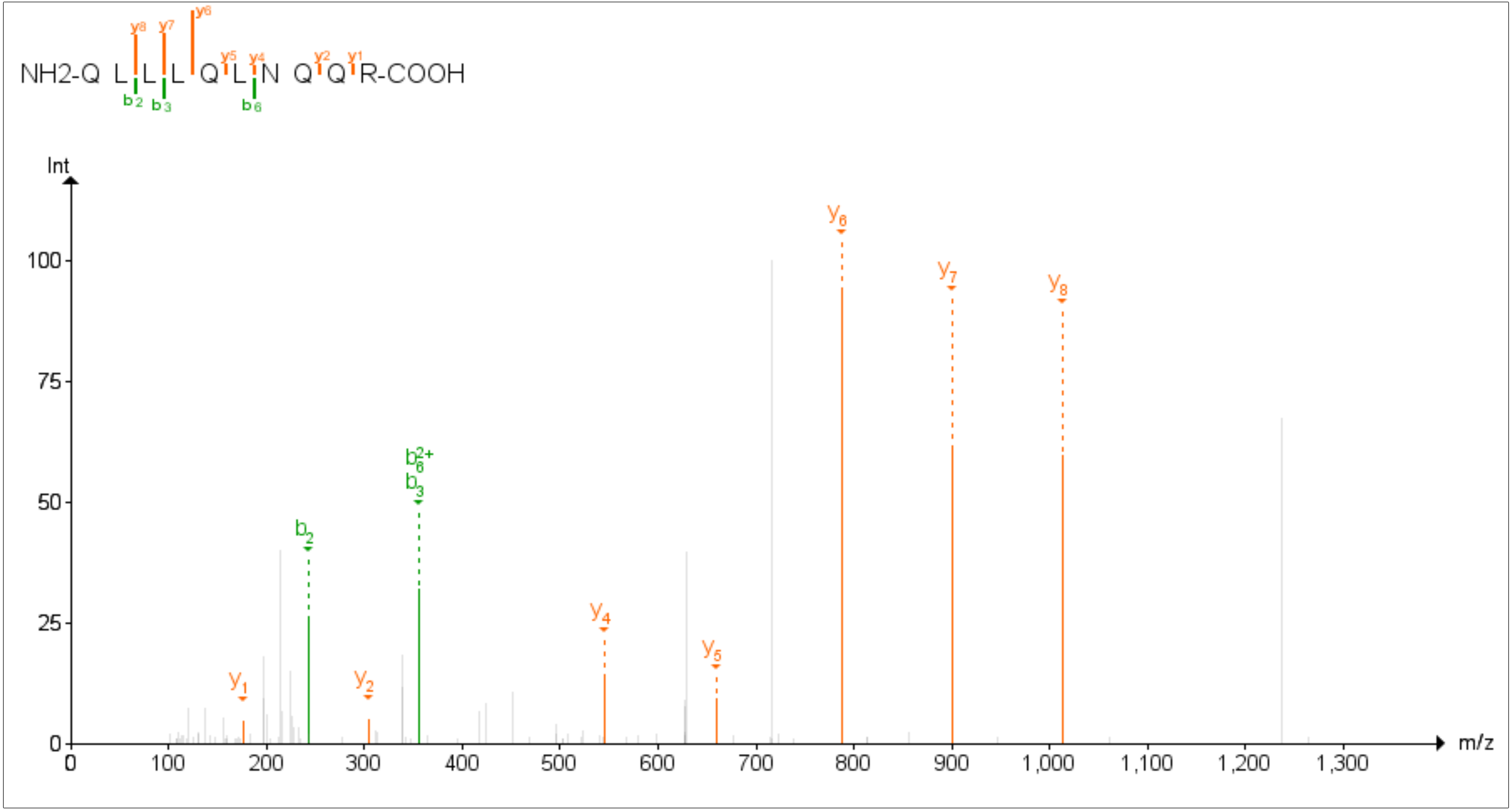

NH2-Q L L L Q L N Q Q R-COOH
y8 y7 y6 y5 y4 y2 y1
b2 b3 b6
Int
m/z
y6
y7
y8
b6 2+
b3
b2
y4
y5
y1
y2
0
25
50
75
100
0
100
200
300
400
500
600
700
800
900
1,000
1,100
1,200
1,300

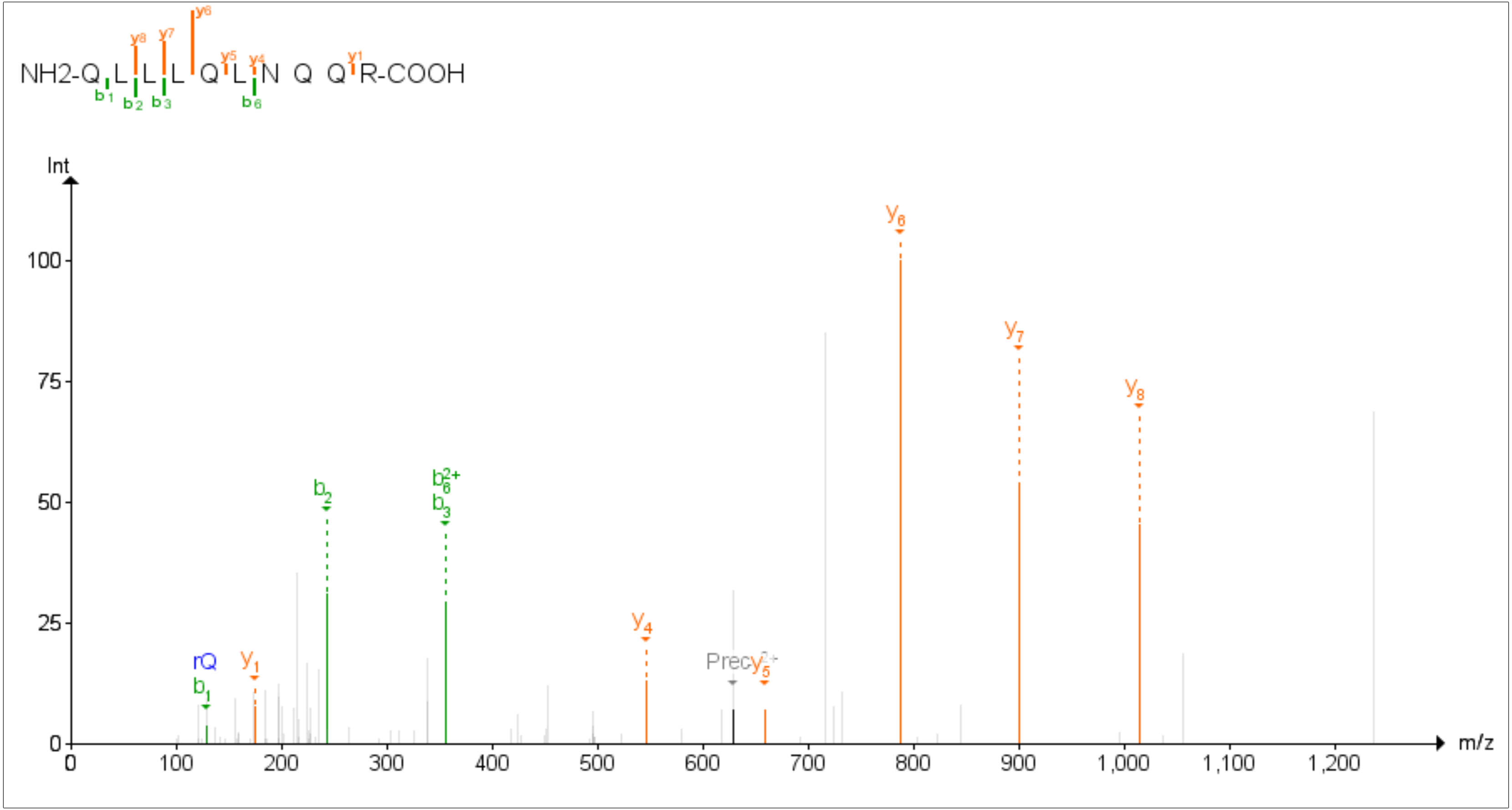

NH2-Q L L L Q L N Q Q R-COOH
y8 y7 y6 y5 y4 y1
b1 b2 b3 b6
Int
m/z
y6
y7
y8
b2
b6 2+
b3
y4
rQ
b1
y1
Prec 2+
y5
0
25
50
75
100
0
100
200
300
400
500
600
700
800
900
1,000
1,100
1,200

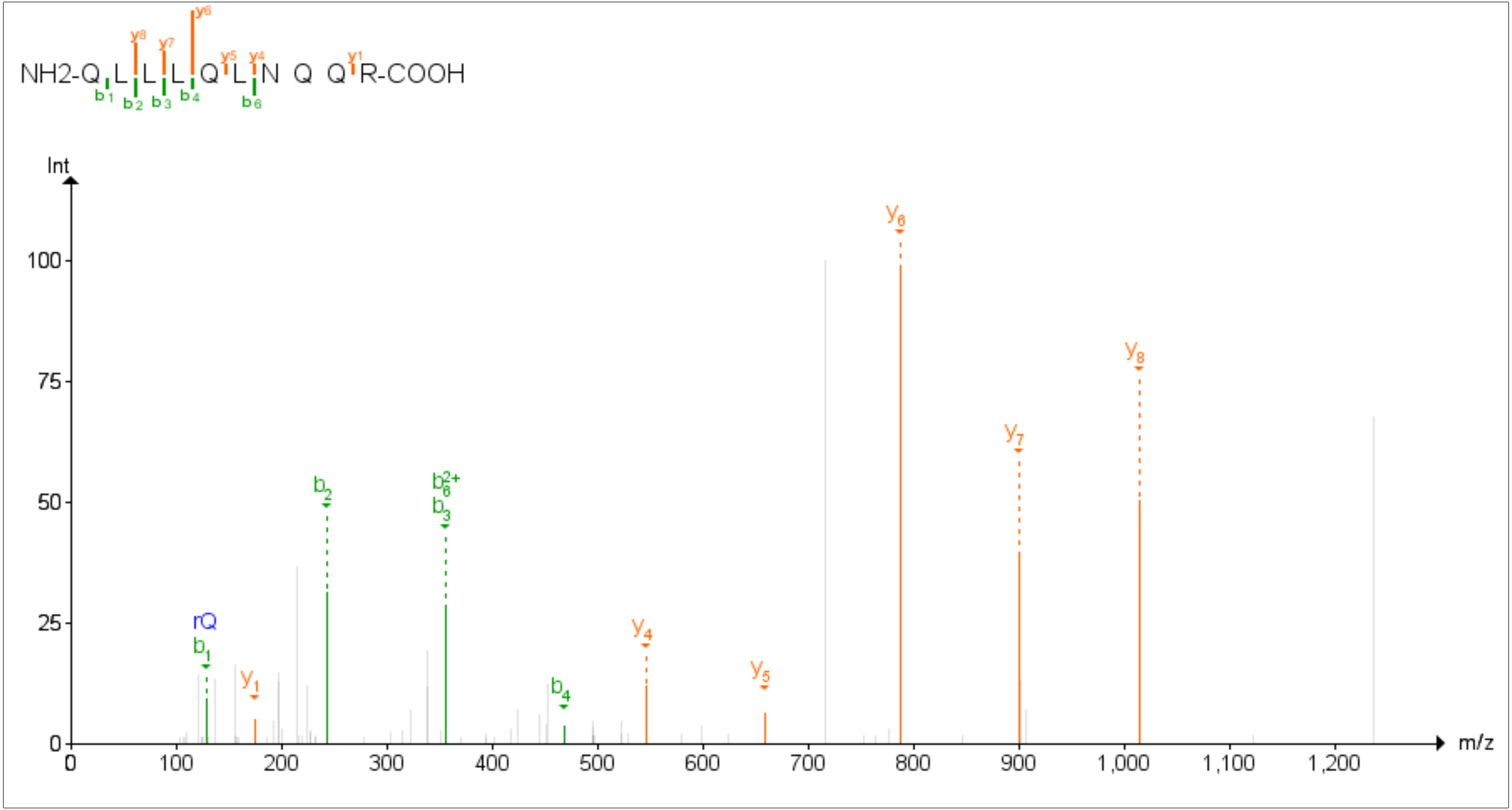

NH2-Q L L L Q L N Q Q R-COOH
y8 y7 y6 y5 y4 y1
b1 b2 b3 b4 b6
Int
100
75
50
25
0
rQ
b1
y1
b2
b6 2+
b3
b4
y4
y5
y6
y7
y8
0 100 200 300 400 500 600 700 800 900 1,000 1,100 1,200
m/z

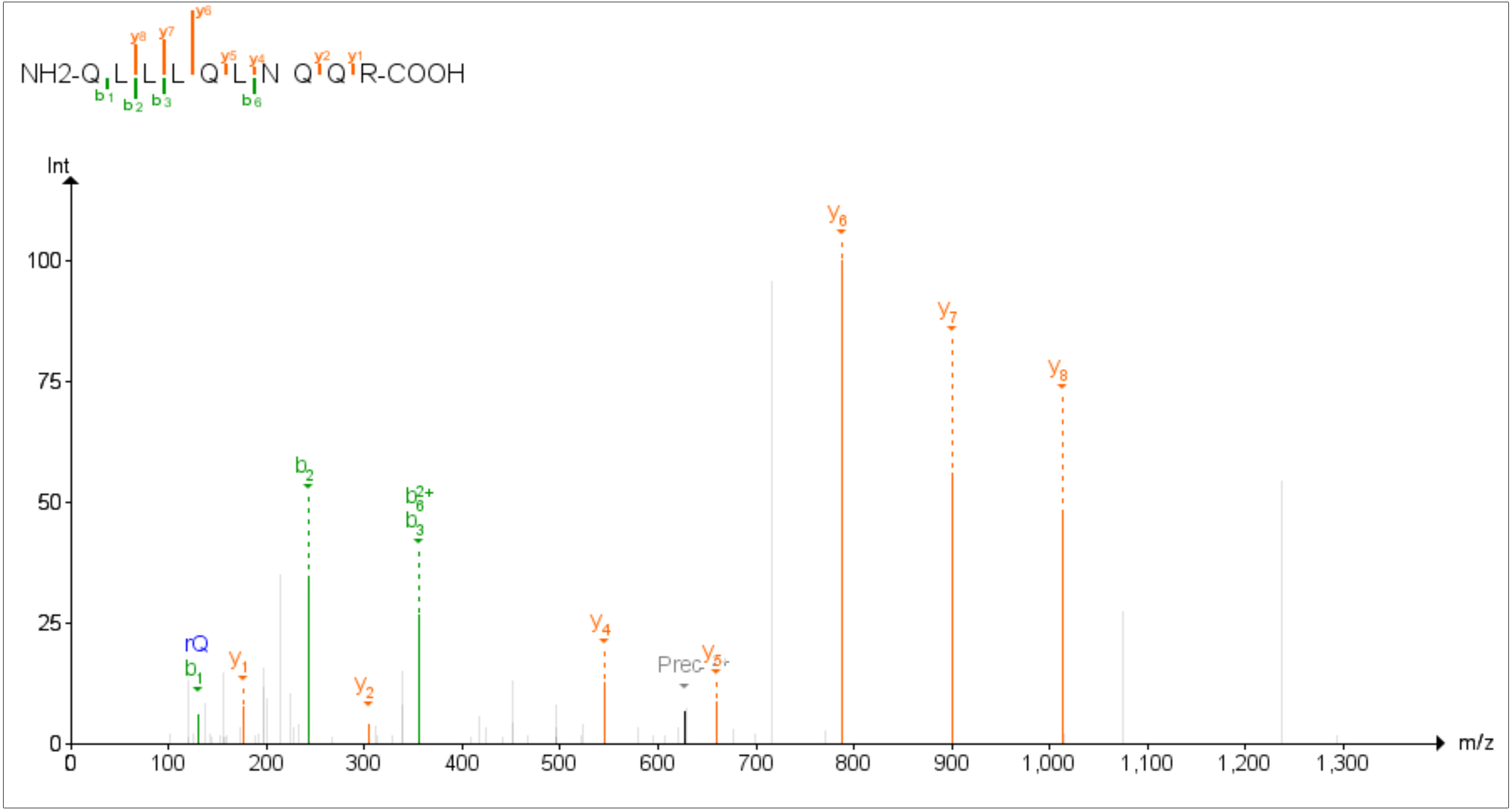

NH2-Q L L L Q L N Q Q R-COOH
y8 y7 y6 y5 y4 y2 y1
b1 b2 b3 b6
Int
100
75
50
25
0
m/z
0
100
200
300
400
500
600
700
800
900
1,000
1,100
1,200
1,300
y6
y7
y8
b2
b6 2+
b3
y4
Prec
y5
rQ
b1
y1
y2

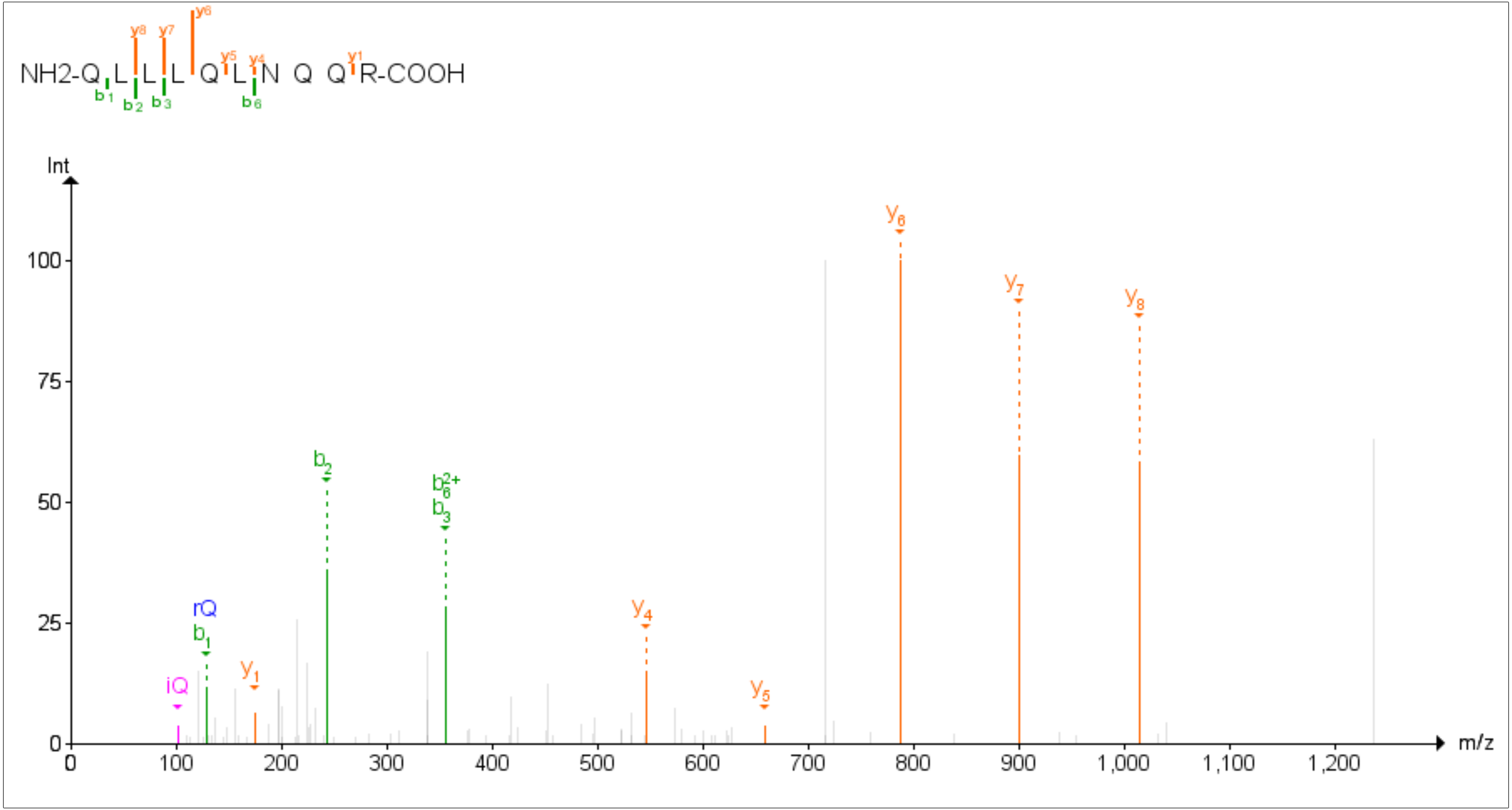
NH2-Q L L L Q L N Q Q R-COOH
b1 b2 b3 b6
y8 y7 y6 y5 y4 y1
Int
m/z
iQ
rQ
b1
y1
b2
b6 2+
b3
y4
y5
y6
y7
y8

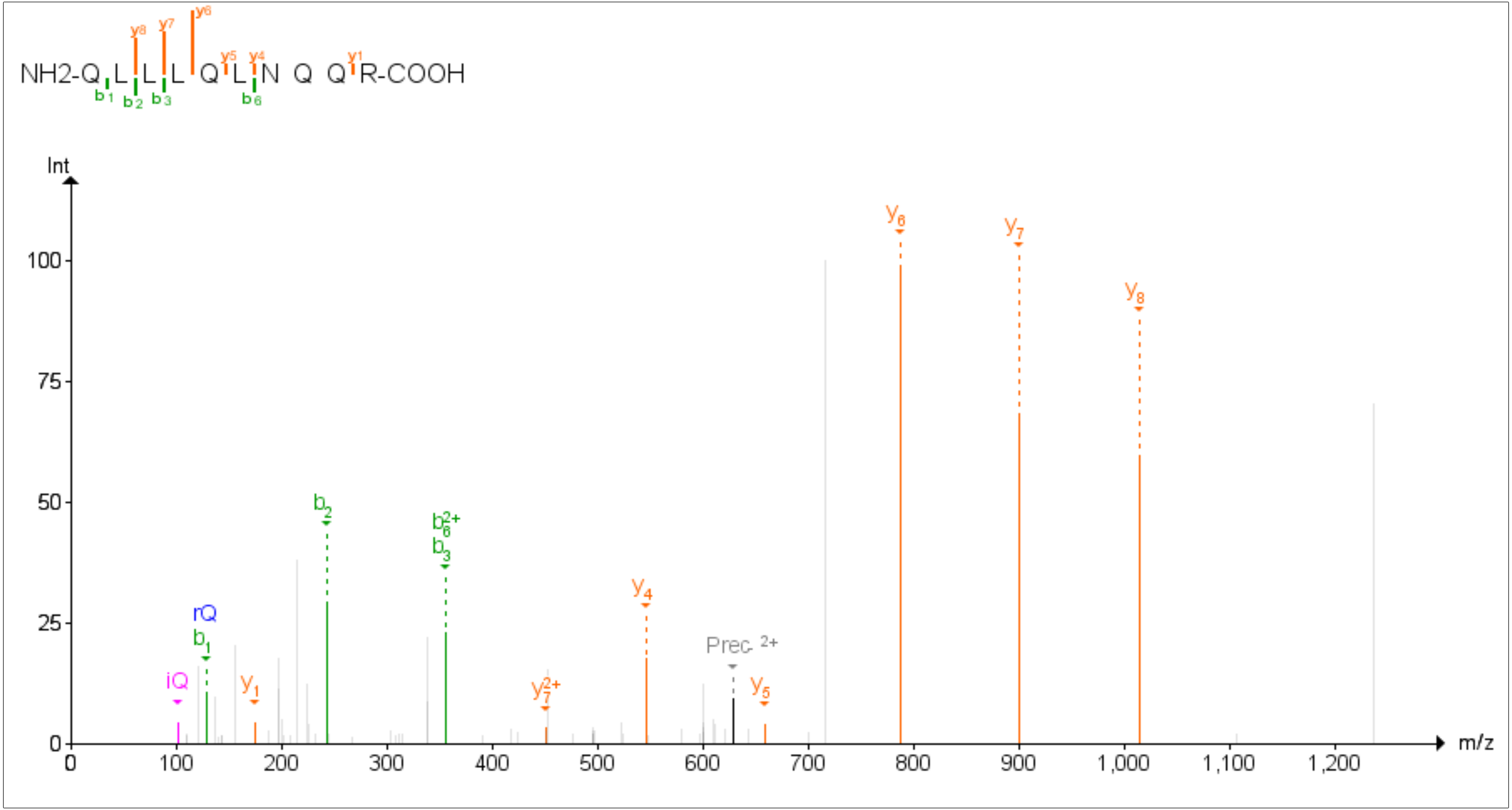

NH2-Q L L L Q L N Q Q R-COOH
y8 y7 y6 y5 y4 y1
b 1 b 2 b 3 b 6
Int
100
75
50
25
0
y6
y7
y8
b2
b6 2+
b3
y4
rQ
b1
iQ
y1
y7 2+
Prec 2+
y5
0 100 200 300 400 500 600 700 800 900 1,000 1,100 1,200
m/z

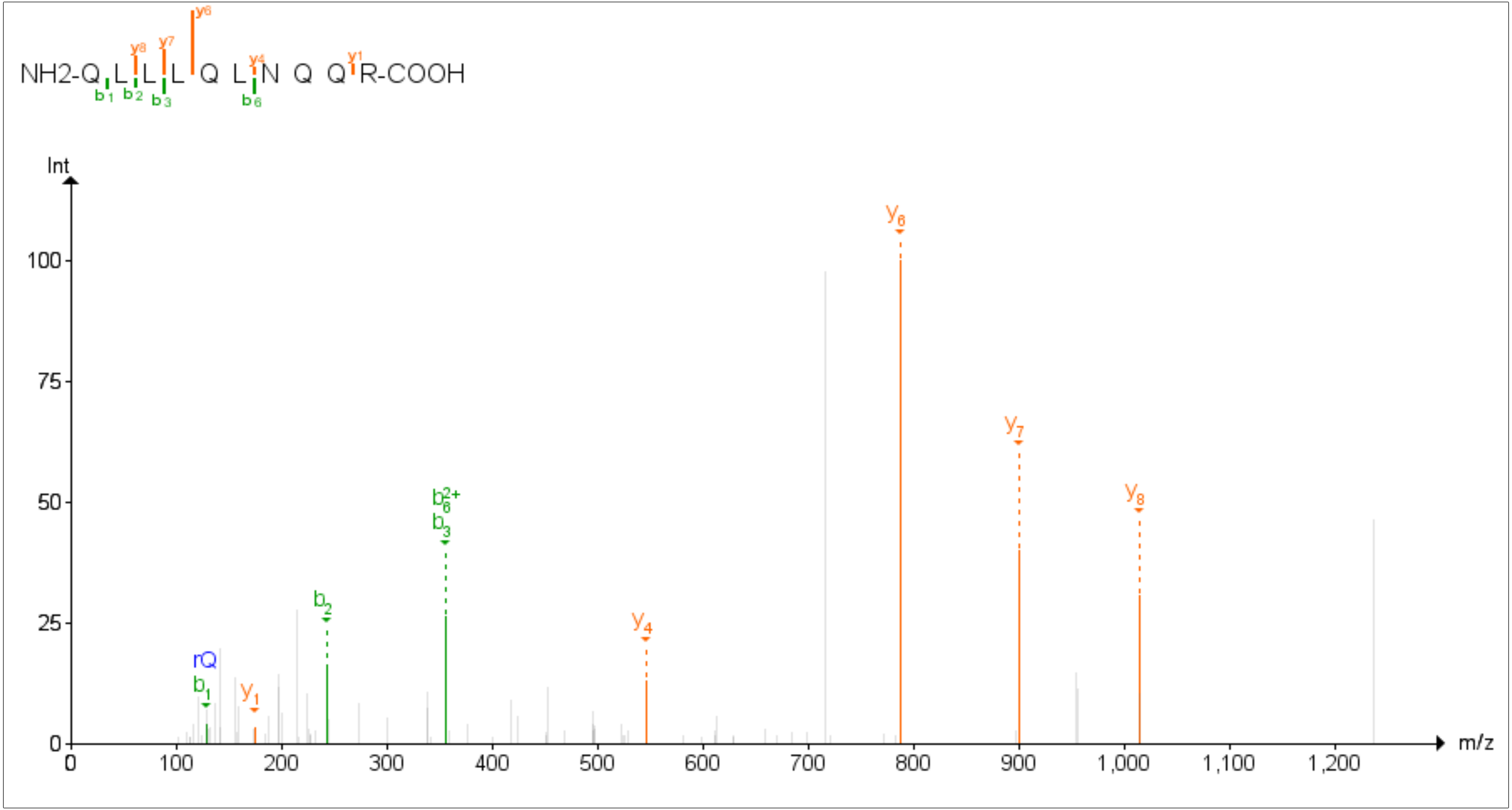
NH2-Q L L L Q L N Q Q R-COOH
y8 y7 y6 y4 y1
b1 b2 b3 b6
Int
m/z
rQ
b1
y1
b2
b$_6^{2+}$
b3
y4
y6
y7
y8
0
25
50
75
100
0
100
200
300
400
500
600
700
800
900
1,000
1,100
1,200

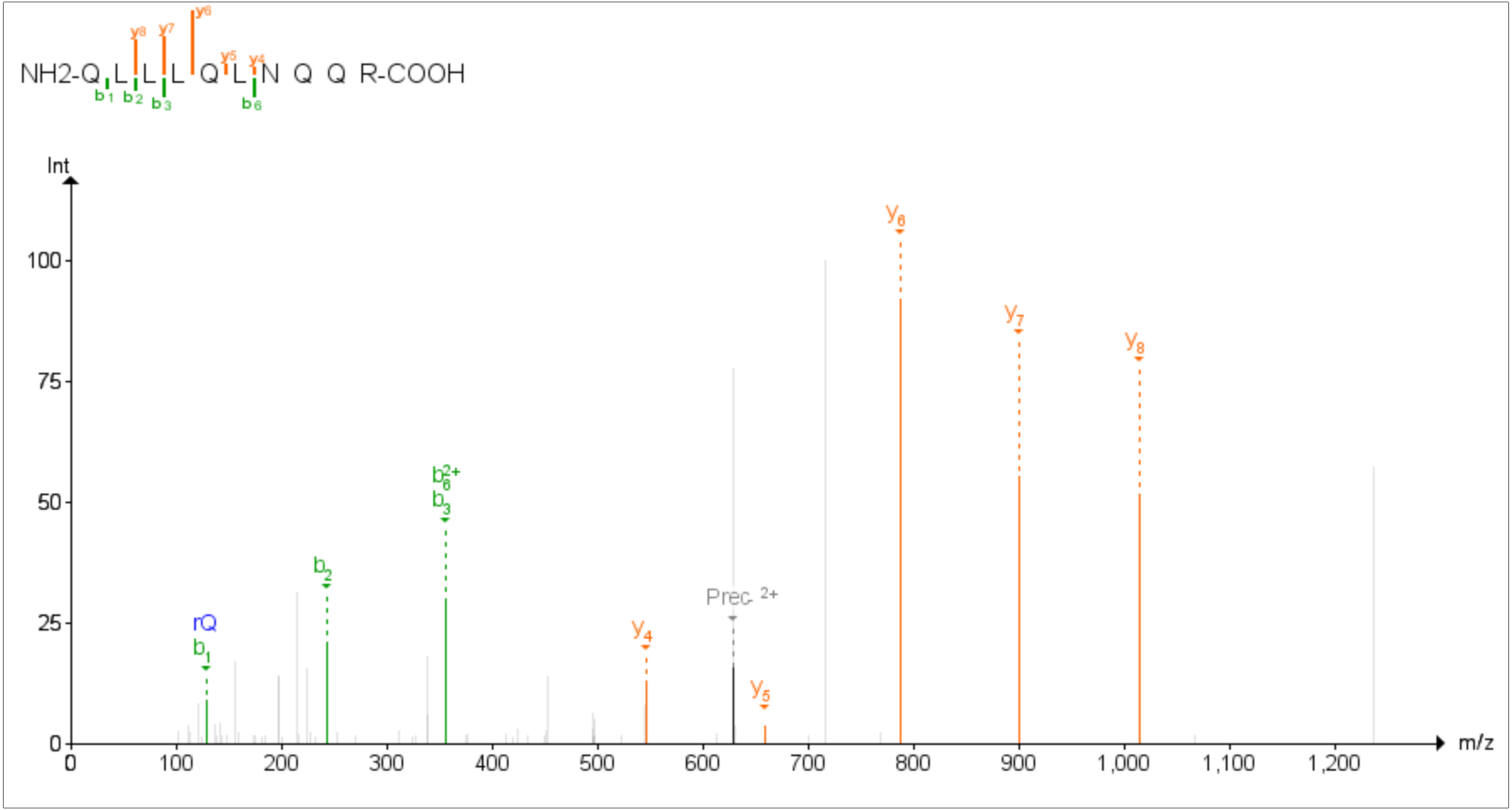
NH2-Q L L L Q L N Q Q R-COOH
y8 y7 y6 y5 y4
b1 b2 b3 b6
Int
100
75
50
25
0
rQ
b1
b2
b6 2+
b3
y4
Prec 2+
y5
y6
y7
y8
0 100 200 300 400 500 600 700 800 900 1,000 1,100 1,200
m/z

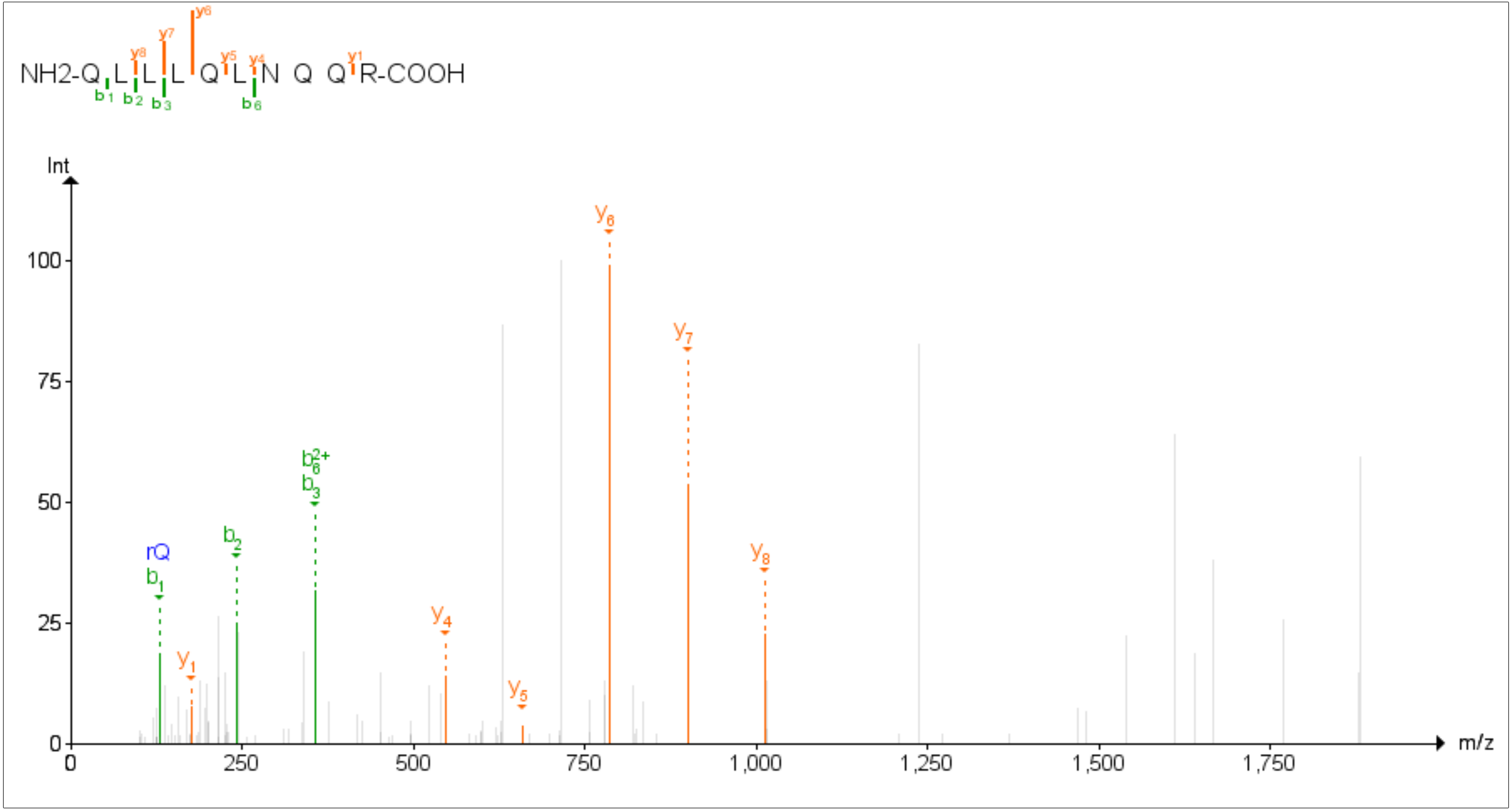

NH2-Q L L L Q L N Q Q R-COOH
y8 y7 y6 y5 y4 y1
b1 b2 b3 b6
Int
100
75
50
25
0
rQ
b1
y1
b2
b3
b6 2+
y4
y5
y6
y7
y8
0
250
500
750
1,000
1,250
1,500
1,750
m/z

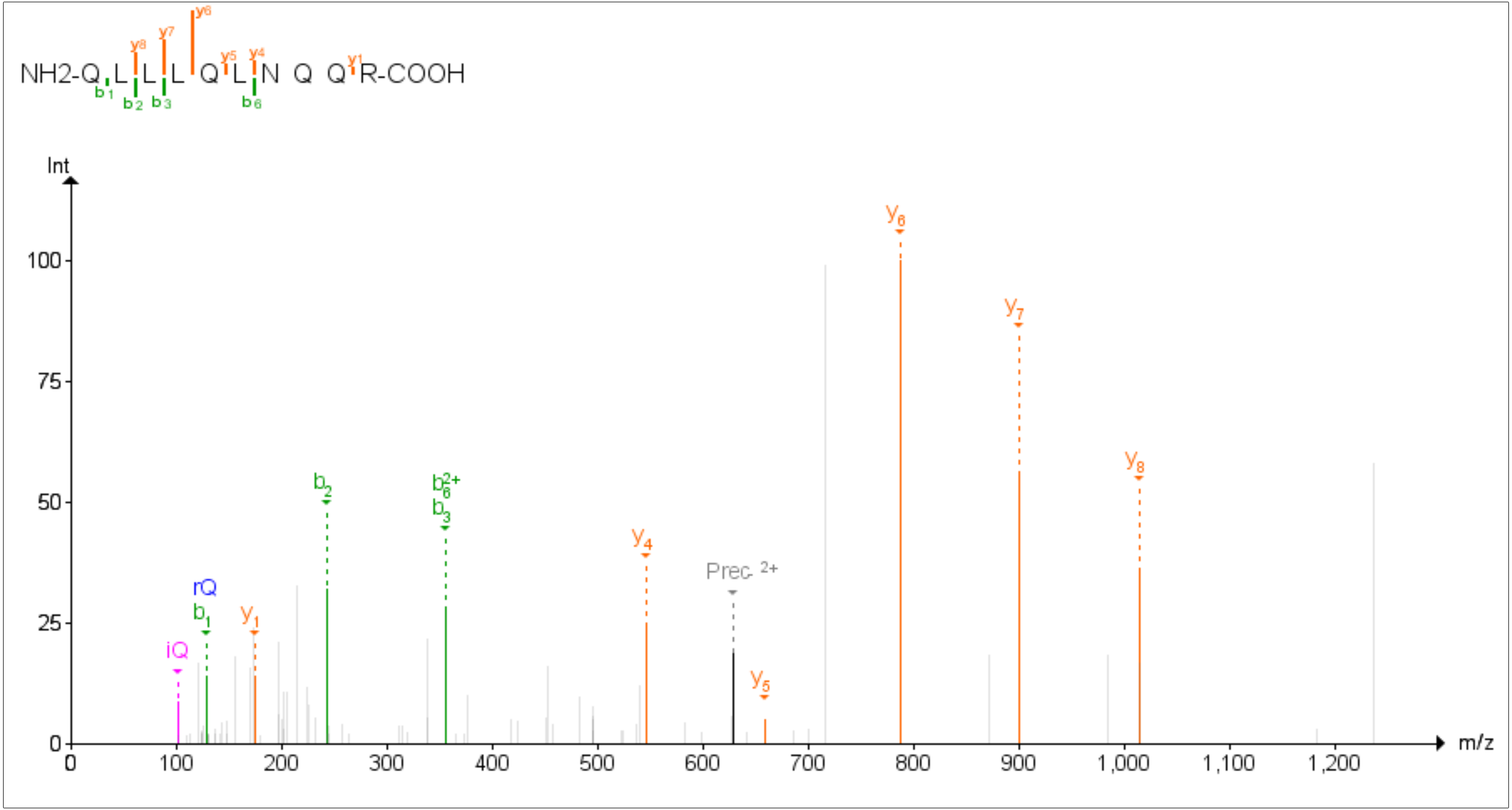

NH2-Q L L L Q L N Q Q R-COOH
y8 y7 y6 y5 y4 y1
b1 b2 b3 b6
Int
100
75
50
25
0
y6
y7
y8
b2
b6 2+
b3
y4
Prec 2+
rQ
b1
y1
iQ
y5
0 100 200 300 400 500 600 700 800 900 1,000 1,100 1,200
m/z

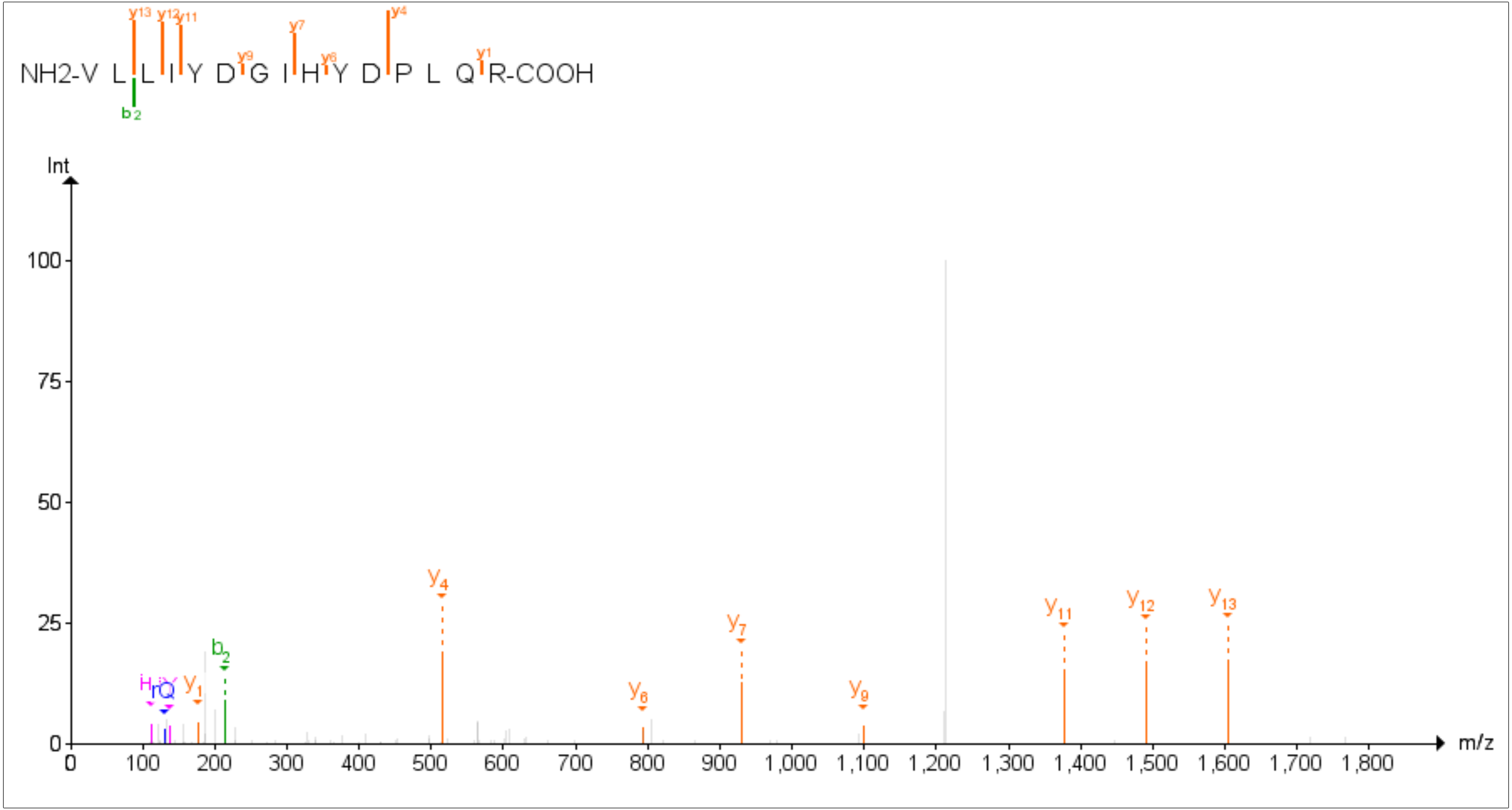

NH2-V L L I Y D G I H Y D P L Q R-COOH
y13 y12 y11 y9 y7 y6 y4 y1
b2
Int
100
75
50
25
0
m/z
0 100 200 300 400 500 600 700 800 900 1,000 1,100 1,200 1,300 1,400 1,500 1,600 1,700 1,800
y4 y6 y7 y9 y11 y12 y13 y1 b2 rQ

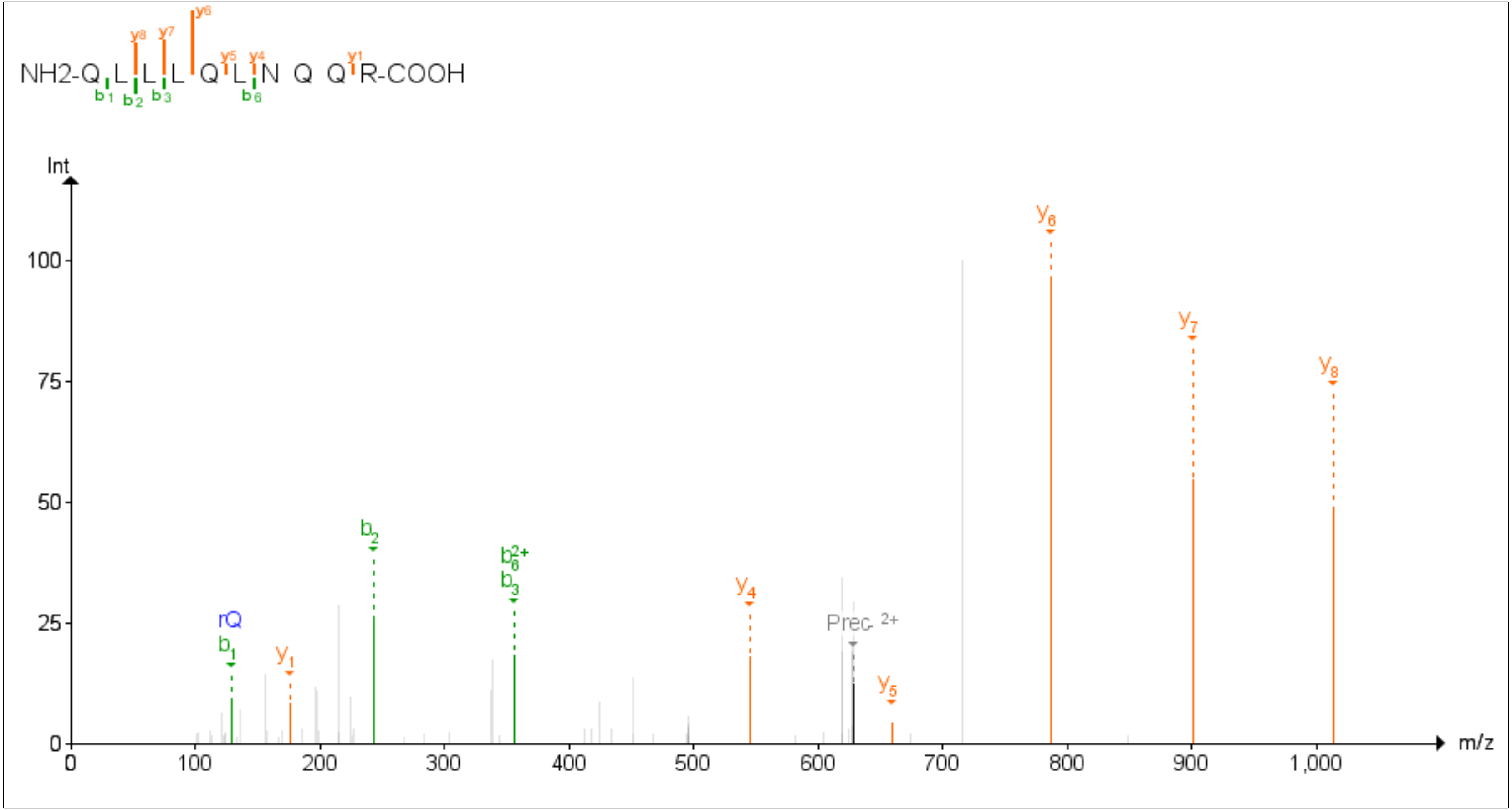
NH2-Q L L L Q L N Q Q R-COOH
y8 y7 y6 y5 y4 y1
b1 b2 b3 b6
Int
100
75
50
25
0
rQ
b1
y1
b2
b6 2+
b3
y4
Prec 2+
y5
y6
y7
y8
0
100
200
300
400
500
600
700
800
900
1,000
m/z

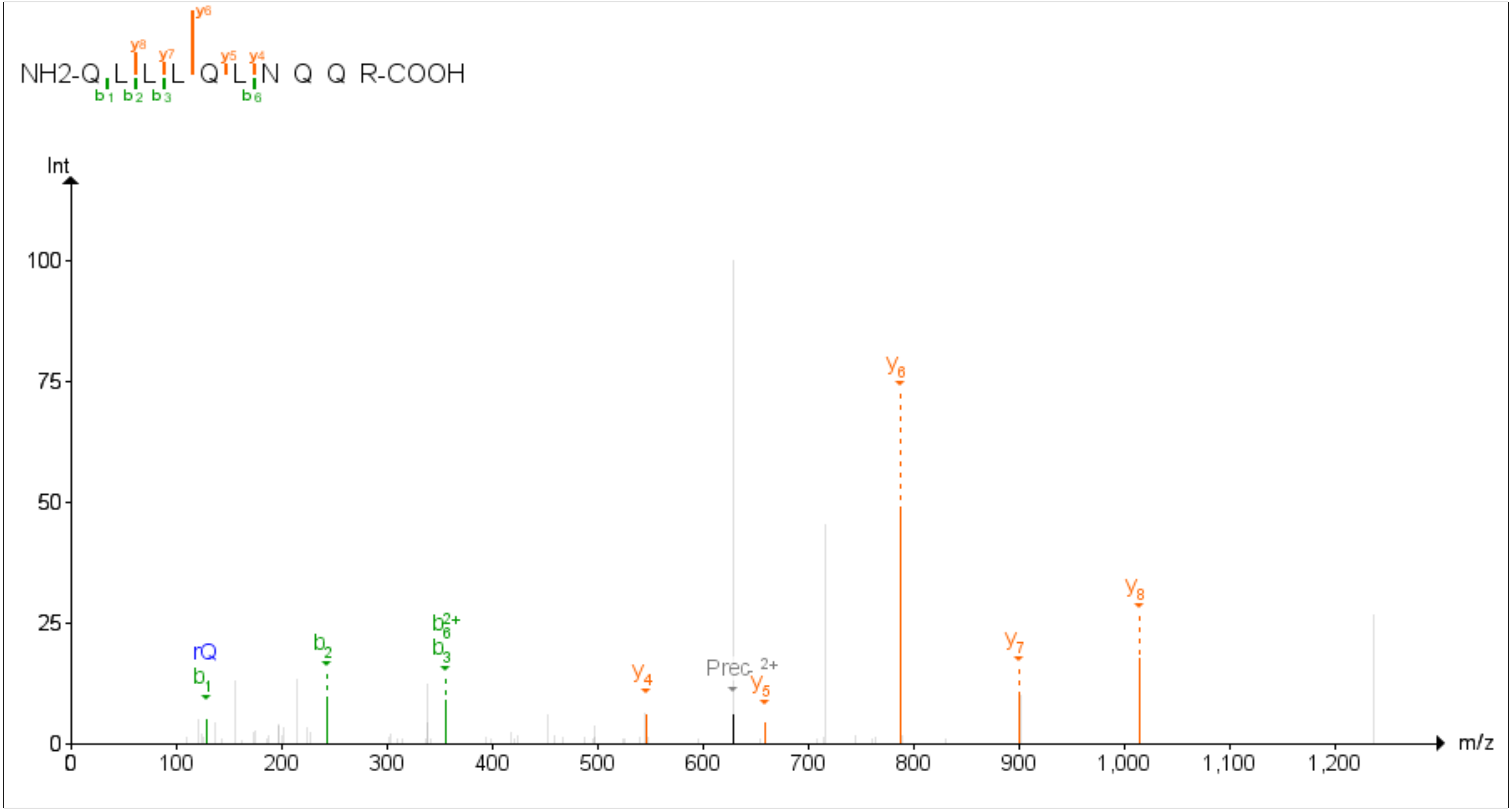

NH2-Q L L L Q L N Q Q R-COOH
y8 y7 y6 y5 y4
b1 b2 b3 b6
Int
100
75
50
25
0
rQ
b1
b2
b6 2+
b3
y4
Prec 2+
y5
y6
y7
y8
0 100 200 300 400 500 600 700 800 900 1,000 1,100 1,200
m/z

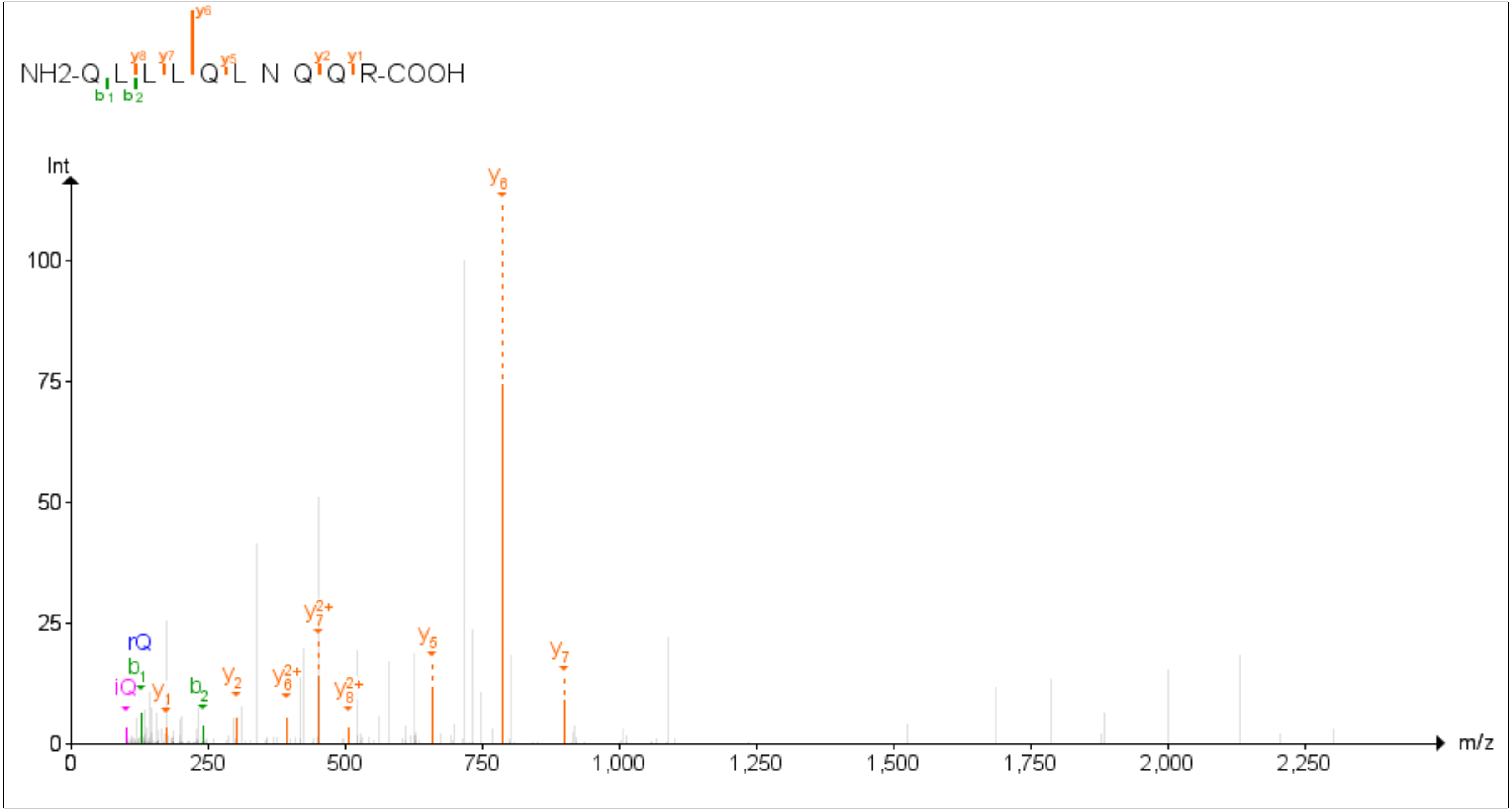

NH2-Q L L L Q L N Q Q R-COOH
b1 b2
y8 y7 y6 y5 y2 y1
Int
100
75
50
25
0
rQ
b1
iQ
y1
b2
y2
y6 2+
y7 2+
y8 2+
y5
y6
y7
0
250
500
750
1,000
1,250
1,500
1,750
2,000
2,250
m/z

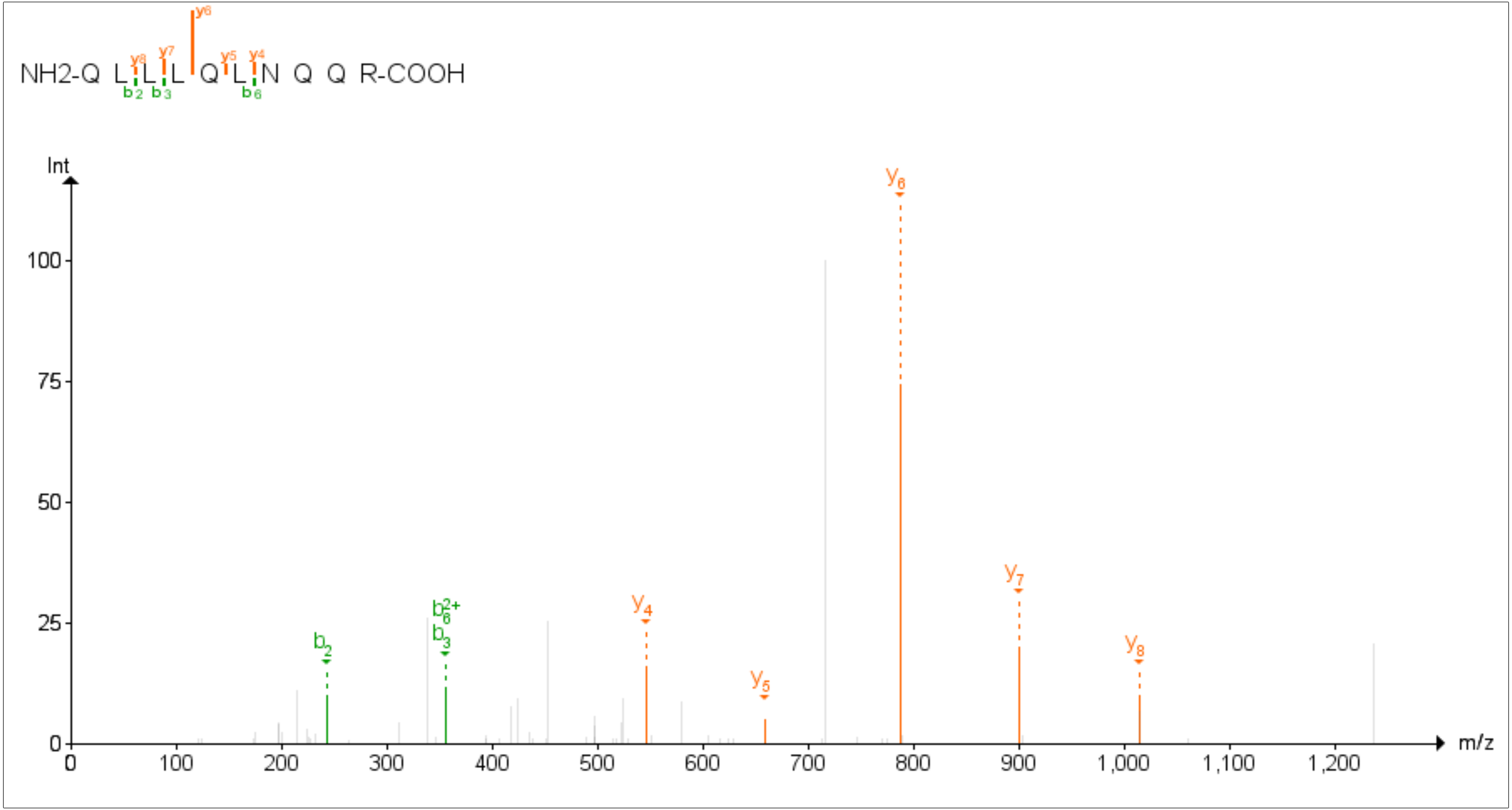

NH2-Q L L L Q L N Q Q R-COOH
y8 y7 y6 y5 y4
b2 b3 b6
Int
100
75
50
25
0
b2
b6 2+
b3
y4
y5
y6
y7
y8
0 100 200 300 400 500 600 700 800 900 1,000 1,100 1,200
m/z

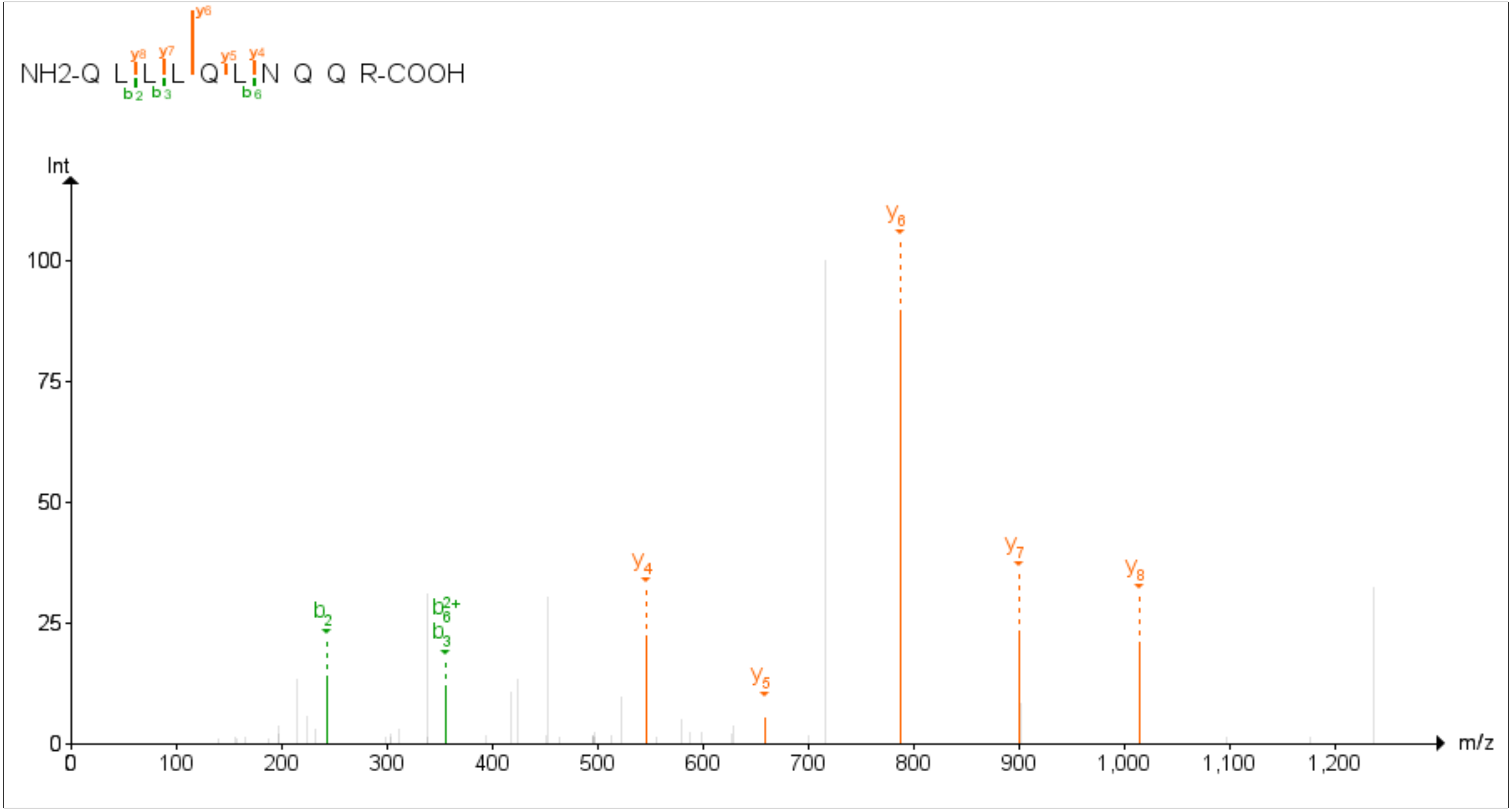

NH2-Q L L L Q L N Q Q R-COOH
y8 y7 y6 y5 y4
b2 b3 b6
Int
100
75
50
25
0
b2
b6 2+
b3
y4
y5
y6
y7
y8
0 100 200 300 400 500 600 700 800 900 1,000 1,100 1,200
m/z

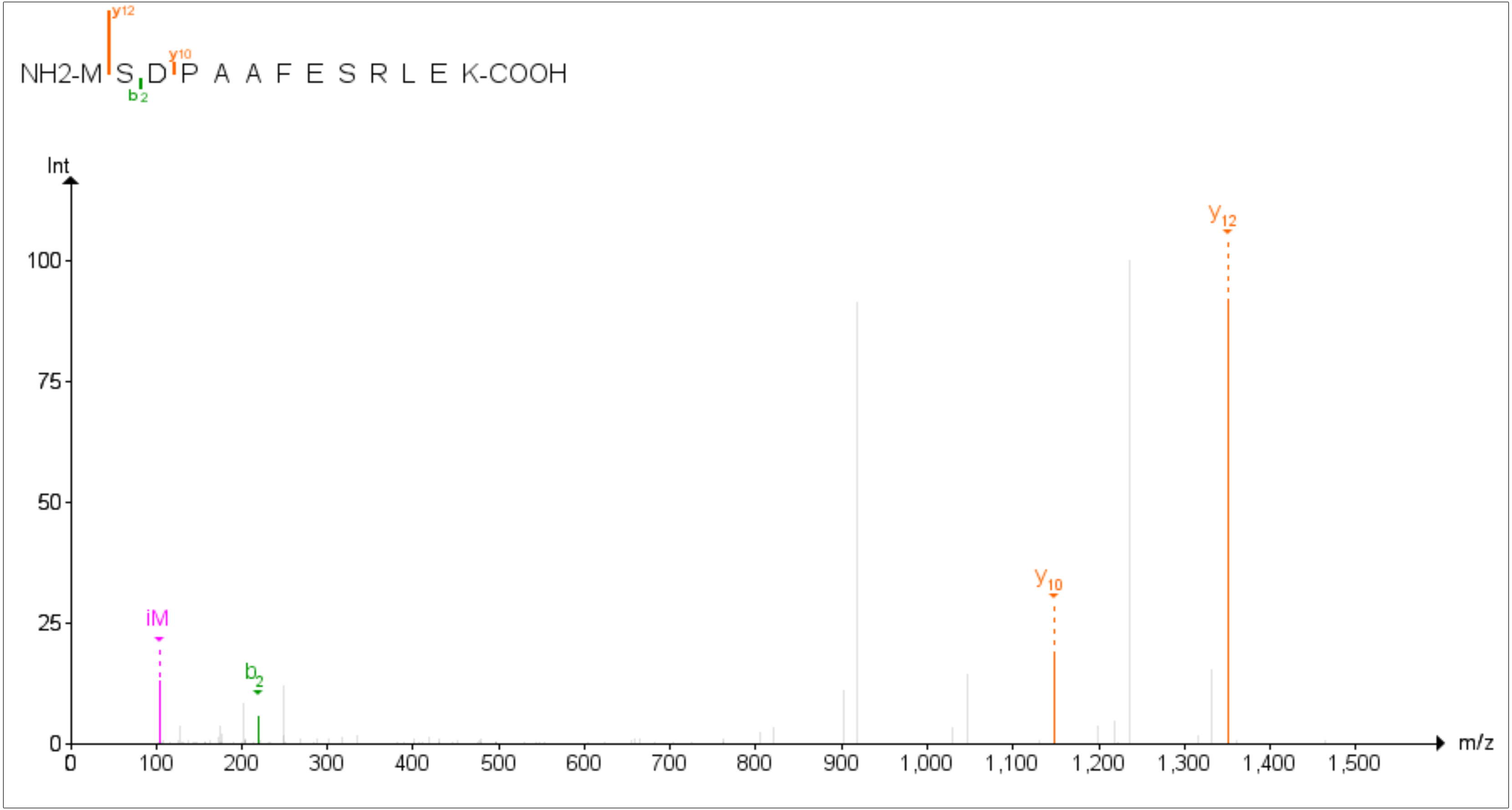

NH2-M S D P A A F E S R L E K-COOH
y12
y10
b2
Int
100
75
50
25
0
iM
b2
y10
y12
0
100
200
300
400
500
600
700
800
900
1,000
1,100
1,200
1,300
1,400
1,500
m/z

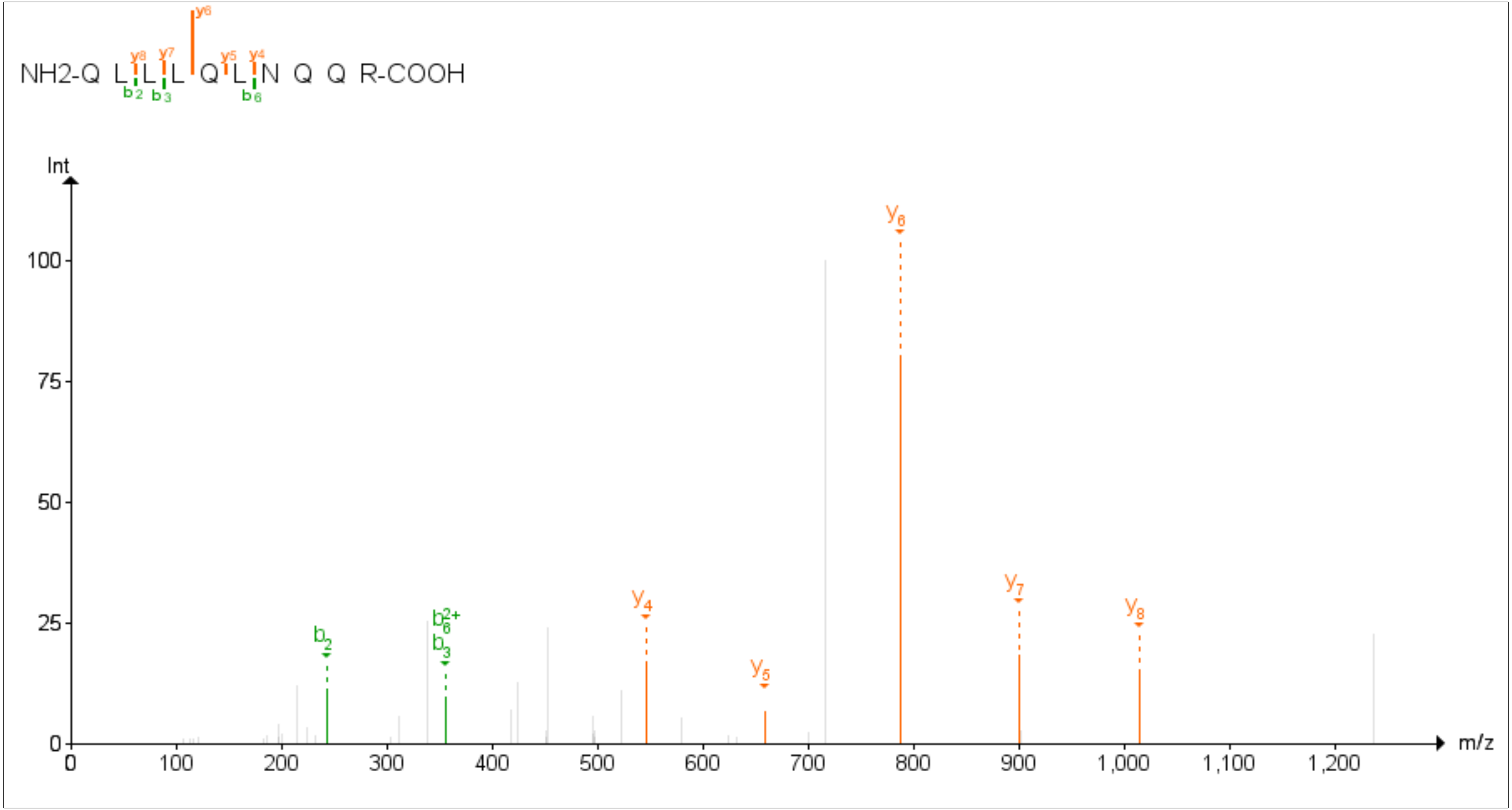

NH2-Q L L L Q L N Q Q R-COOH
y8 y7 y6 y5 y4
b2 b3 b6
Int
100
75
50
25
0
m/z
0 100 200 300 400 500 600 700 800 900 1,000 1,100 1,200
b_2
b_6^{2+}
b_3
y_4
y_5
y_6
y_7
y_8

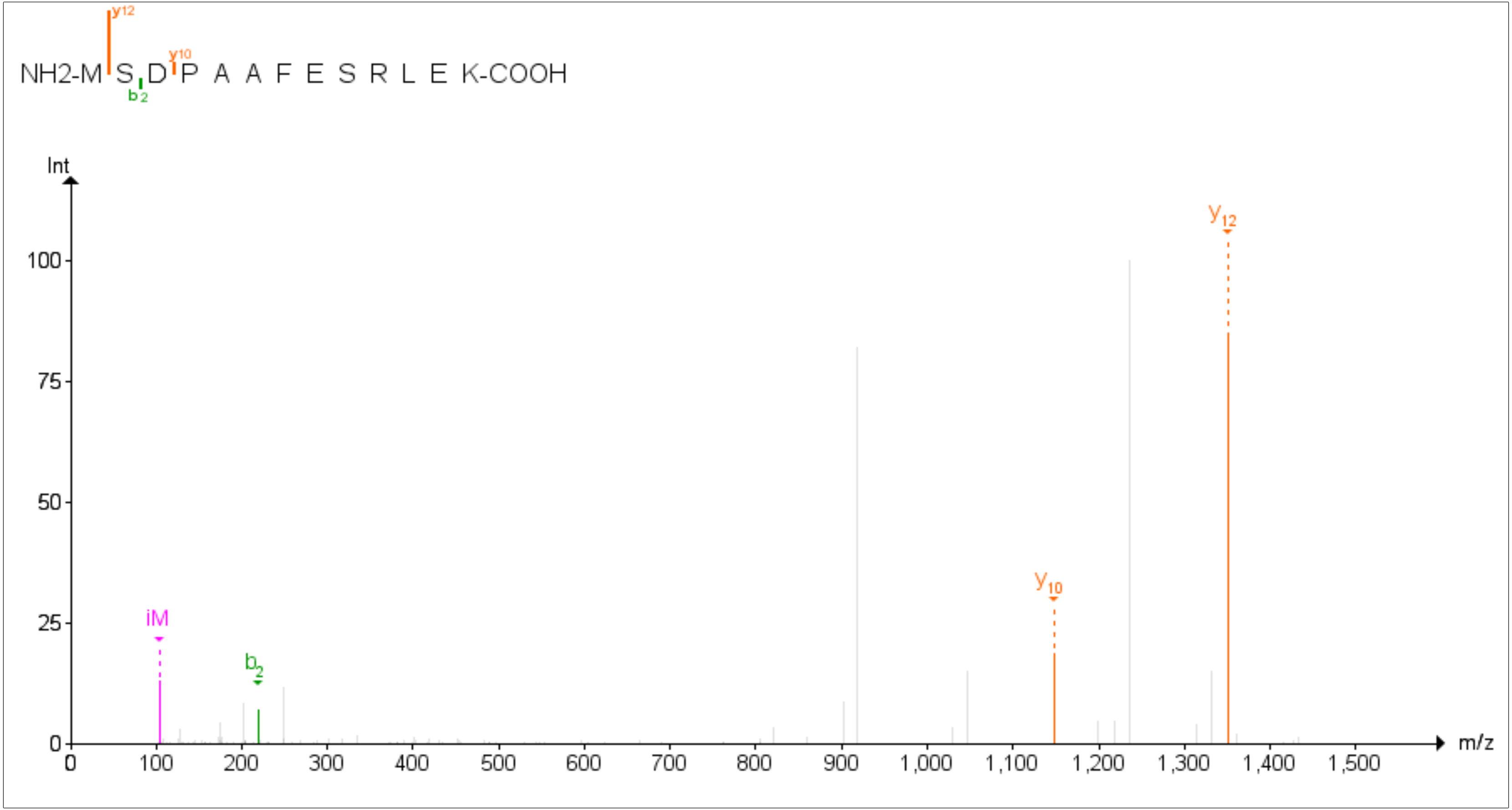

NH2-M S D P A A F E S R L E K-COOH
y12
y10
b2
Int
100
75
50
25
0
iM
b2
y10
y12
0
100
200
300
400
500
600
700
800
900
1,000
1,100
1,200
1,300
1,400
1,500
m/z

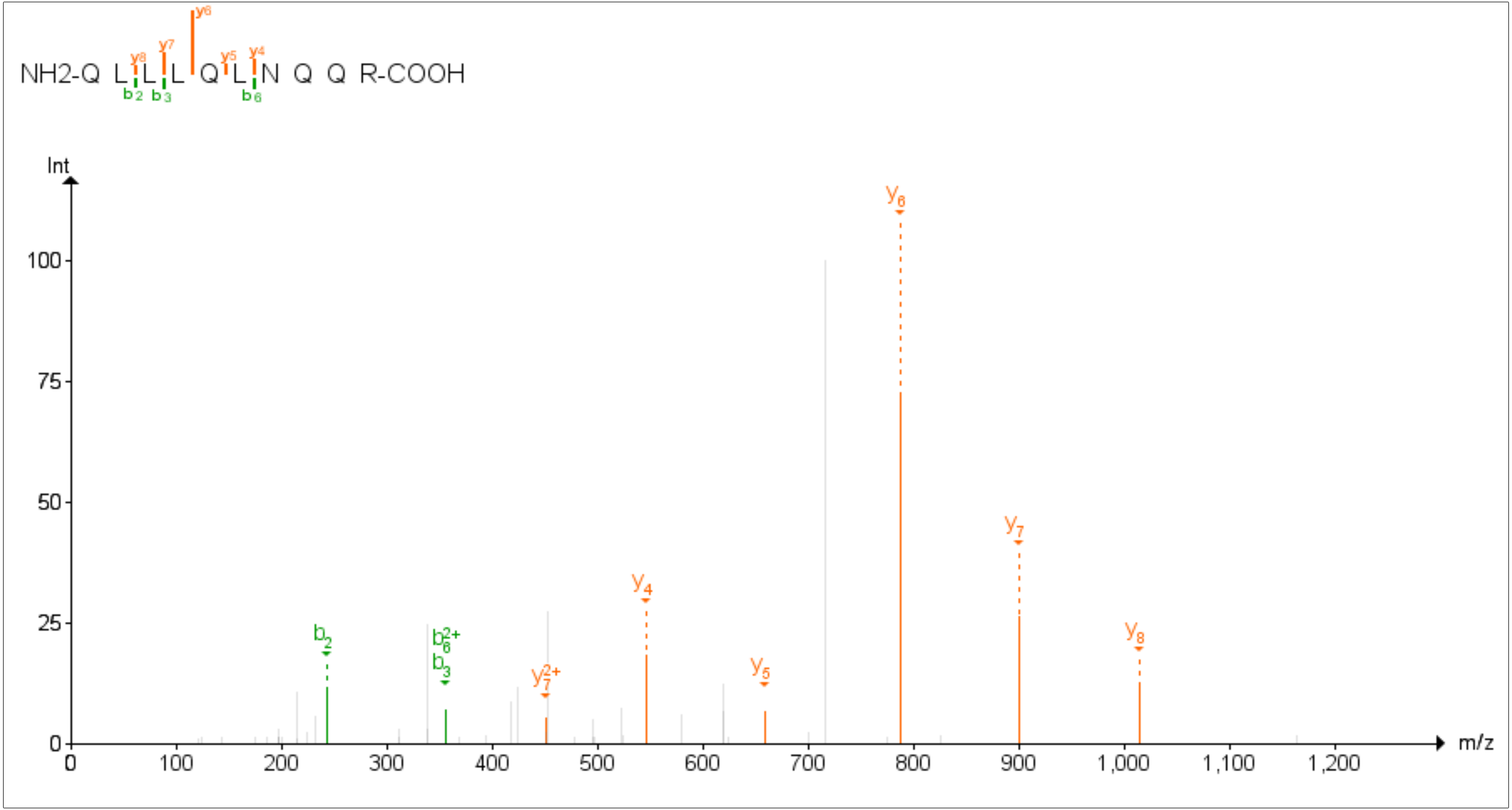

NH2-Q L L L Q L N Q Q R-COOH
y8 y7 y6 y5 y4
b2 b3 b6
Int
100
75
50
25
0
m/z
0
100
200
300
400
500
600
700
800
900
1,000
1,100
1,200
y6
y7
y4
y8
b2
b6 2+
b3
y7 2+
y5

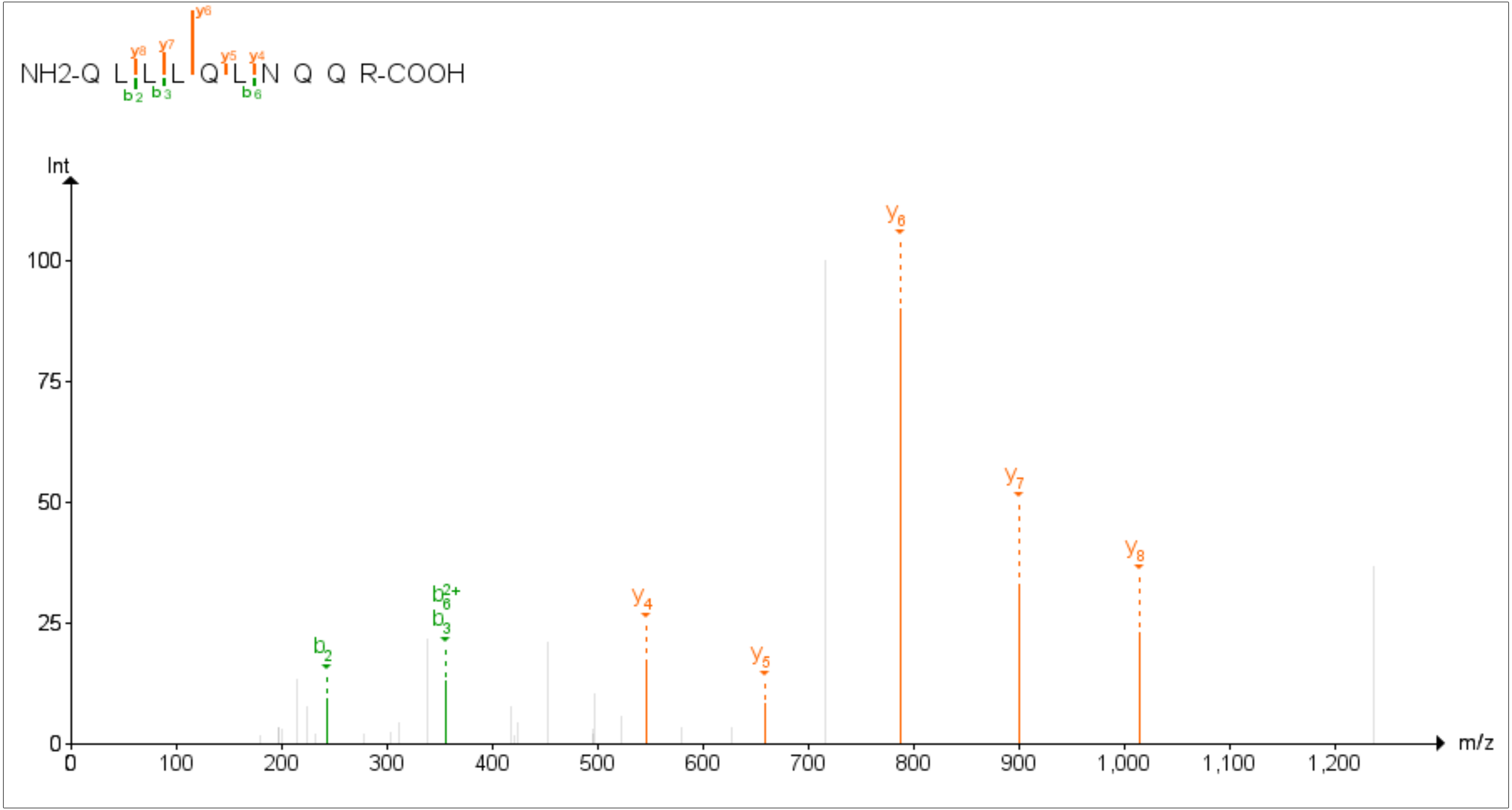

NH2-Q L L L Q L N Q Q R-COOH
y6
y8
y7
y5
y4
b2
b3
b6
Int
100
75
50
25
0
m/z
0
100
200
300
400
500
600
700
800
900
1,000
1,100
1,200
b2
b6 2+
b3
y4
y5
y6
y7
y8

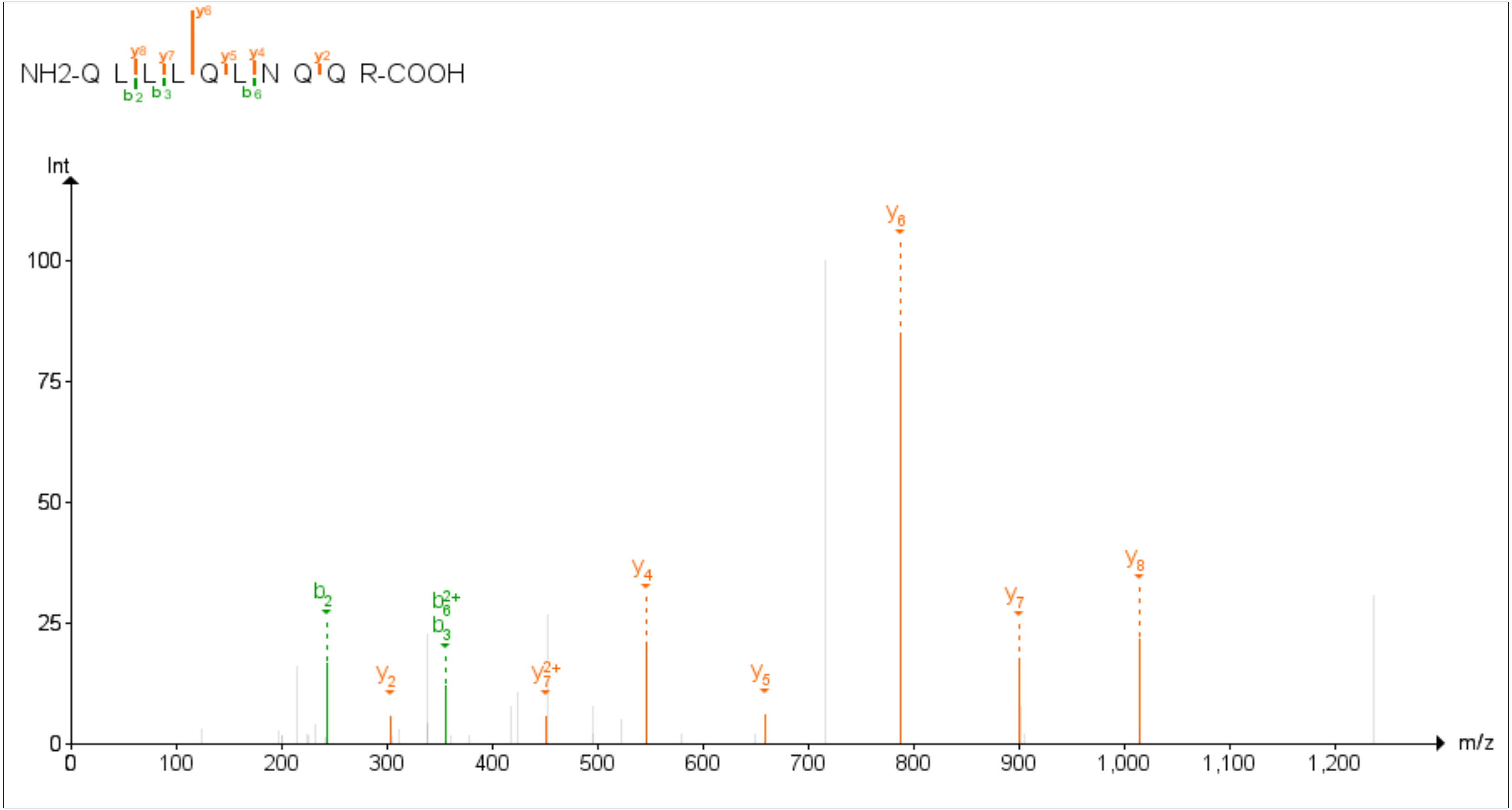

NH2-Q L L L Q L N Q Q R-COOH
y8 y7 y6 y5 y4 y2
b2 b3 b6
Int
100
75
50
25
0
b2
y2
b6 2+
b3
y7 2+
y4
y5
y6
y7
y8
0 100 200 300 400 500 600 700 800 900 1,000 1,100 1,200
m/z

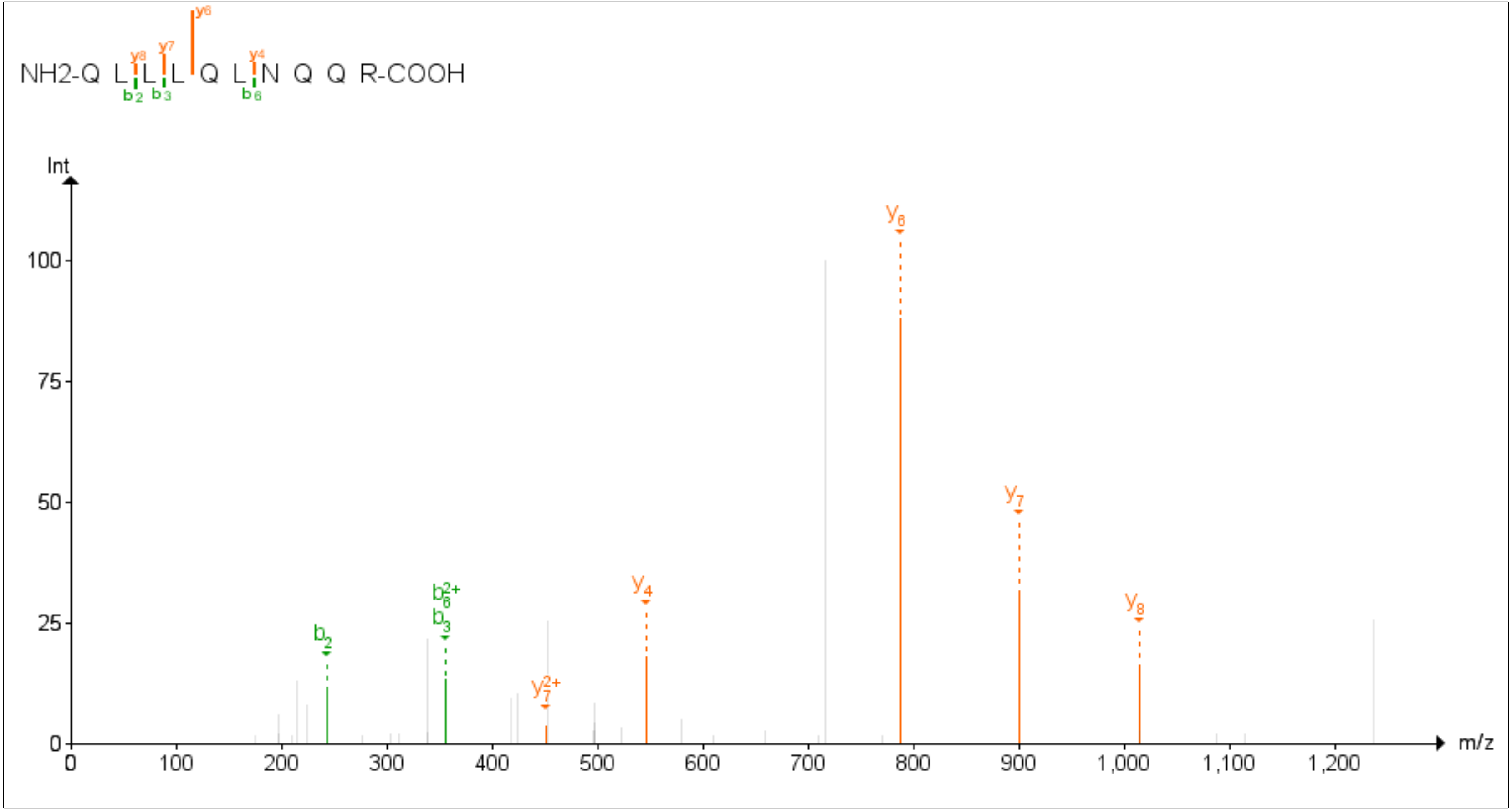

NH2-Q L L L Q L N Q Q R-COOH
y8 y7 y6 y4
b2 b3 b6
Int
100
75
50
25
0
0 100 200 300 400 500 600 700 800 900 1,000 1,100 1,200
m/z
y6
y7
y8
y4
b2
b3
b6 2+
y7 2+

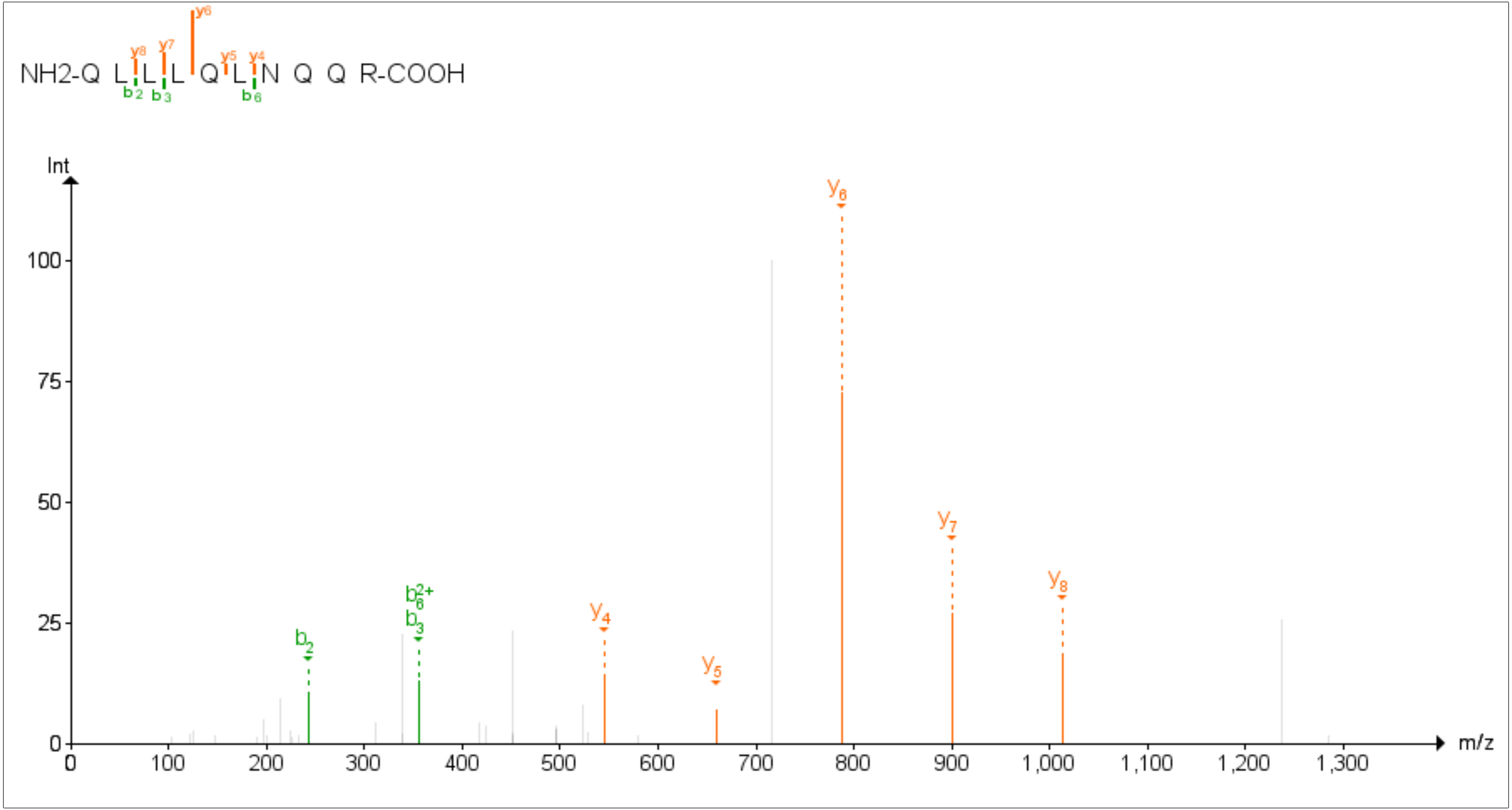

NH2-Q L L L Q L N Q Q R-COOH
y8 y7 y6 y5 y4
b2 b3 b6
Int
100
75
50
25
0
0 100 200 300 400 500 600 700 800 900 1,000 1,100 1,200 1,300
m/z
y6
y7
y8
y4
y5
b2
b3
b6 2+

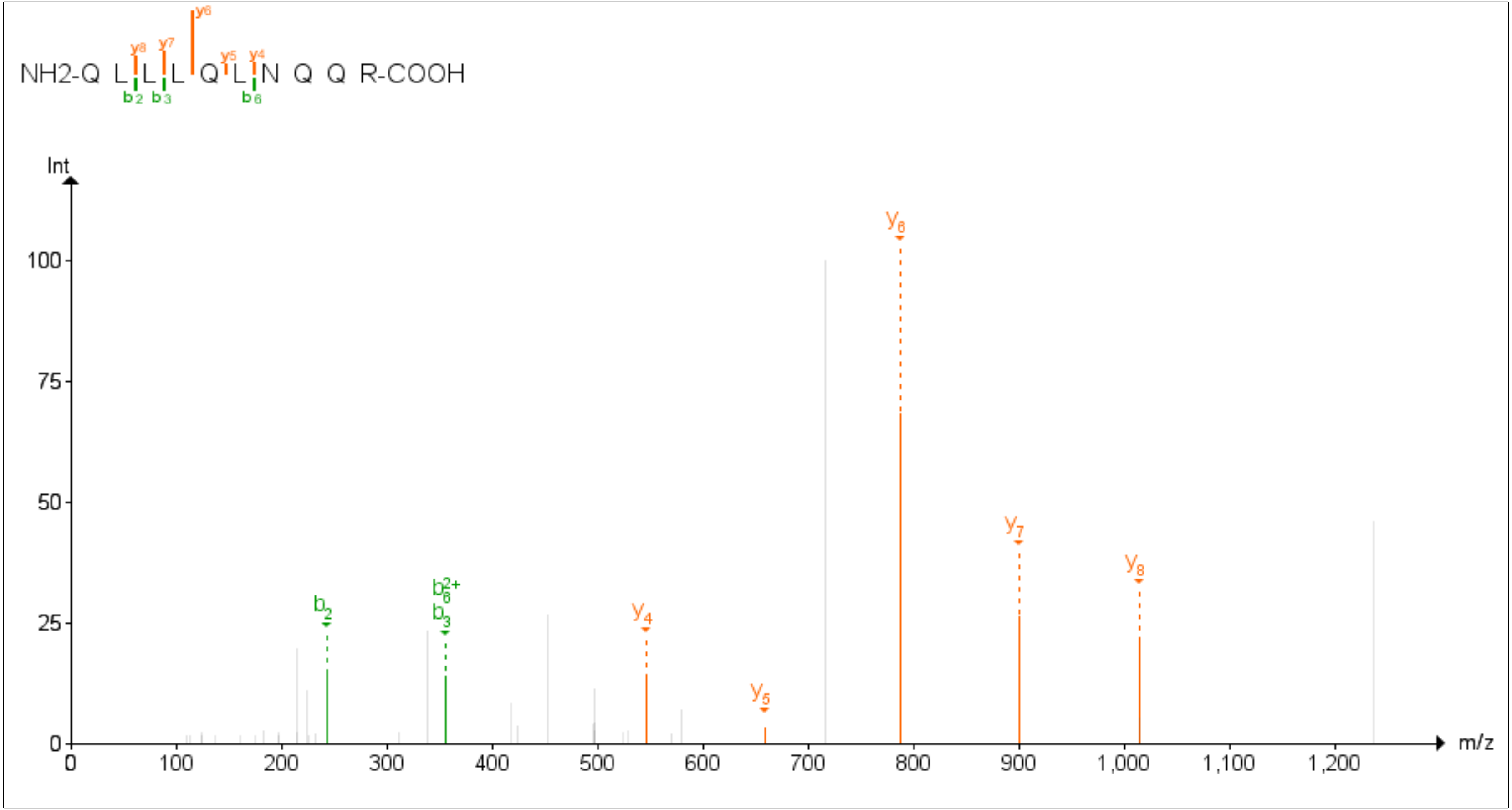

NH2-Q L L L Q L N Q Q R-COOH
y8 y7 y6 y5 y4
b2 b3 b6
Int
100
75
50
25
0
b2
b6 2+
b3
y4
y5
y6
y7
y8
0 100 200 300 400 500 600 700 800 900 1,000 1,100 1,200
m/z

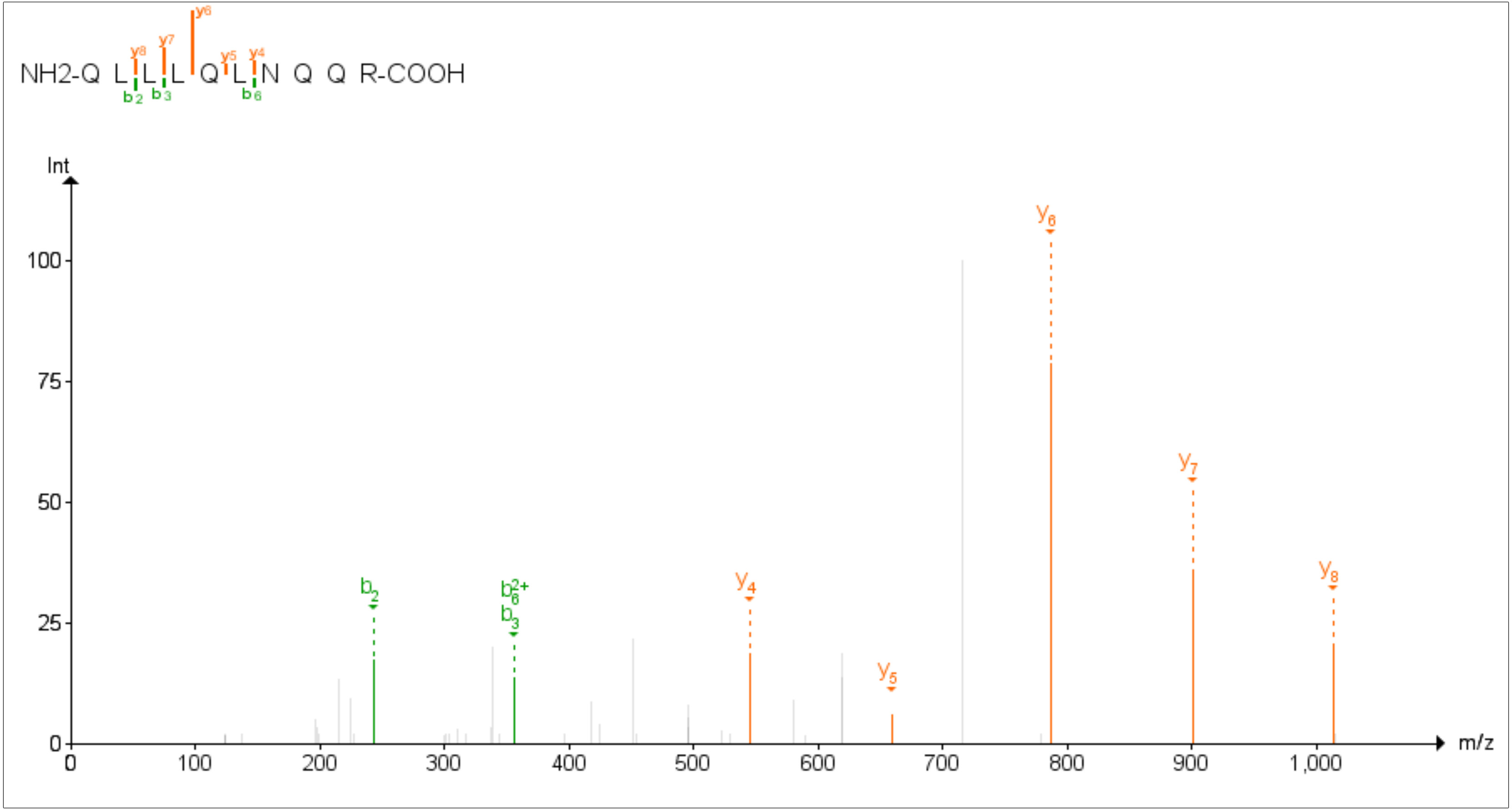

NH2-Q L L L Q L N Q Q R-COOH
y8
y7
y6
y5
y4
b2
b3
b6
Int
100
75
50
25
0
b2
b6 2+
b3
y4
y5
y6
y7
y8
0
100
200
300
400
500
600
700
800
900
1,000
m/z

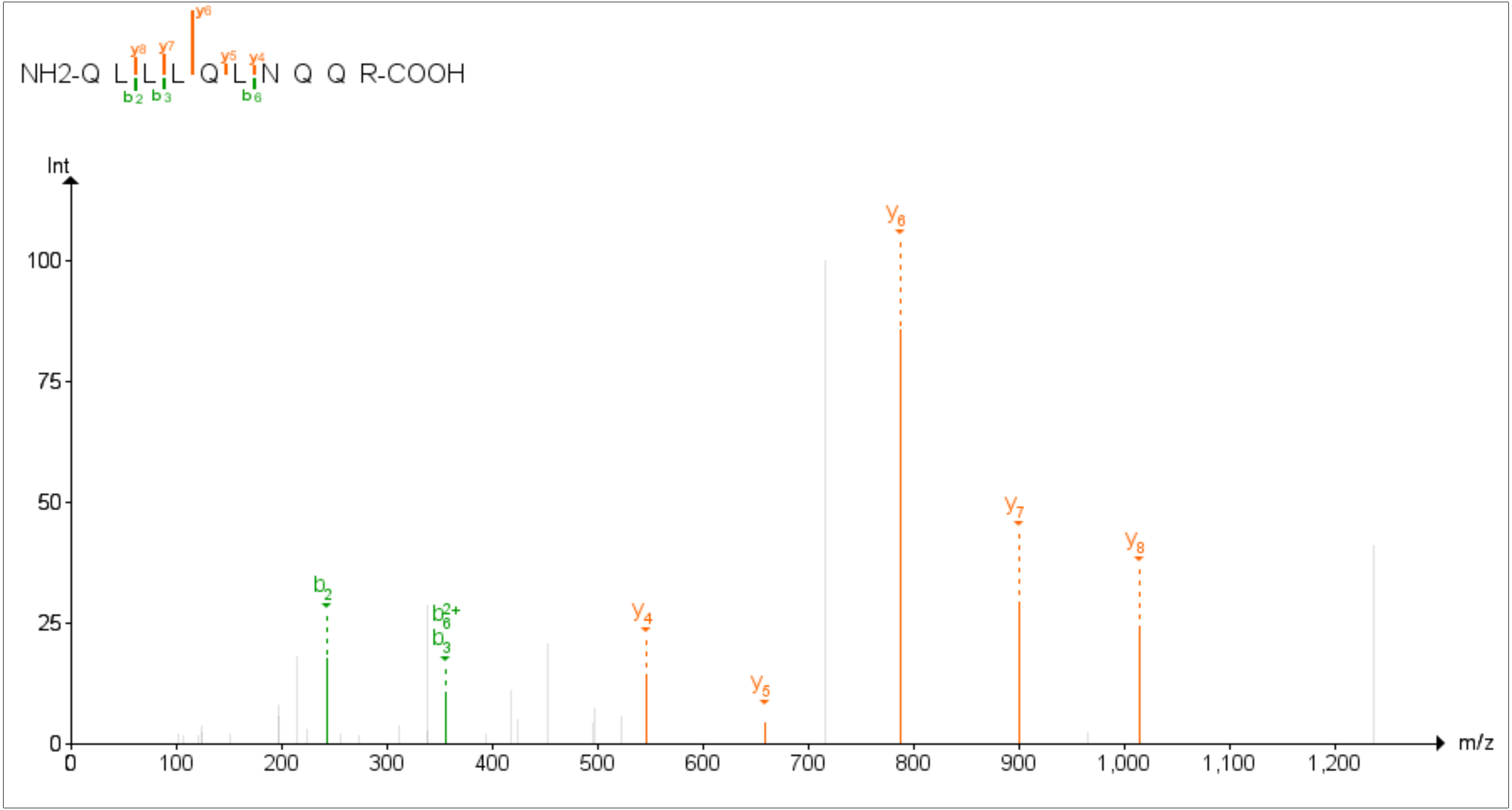
NH2-Q L L L Q L N Q Q R-COOH
y8 y7 y6 y5 y4
b2 b3 b6
Int
100
75
50
25
0
y6
y7
y8
b2
b6 2+
b3
y4
y5
0 100 200 300 400 500 600 700 800 900 1,000 1,100 1,200
m/z

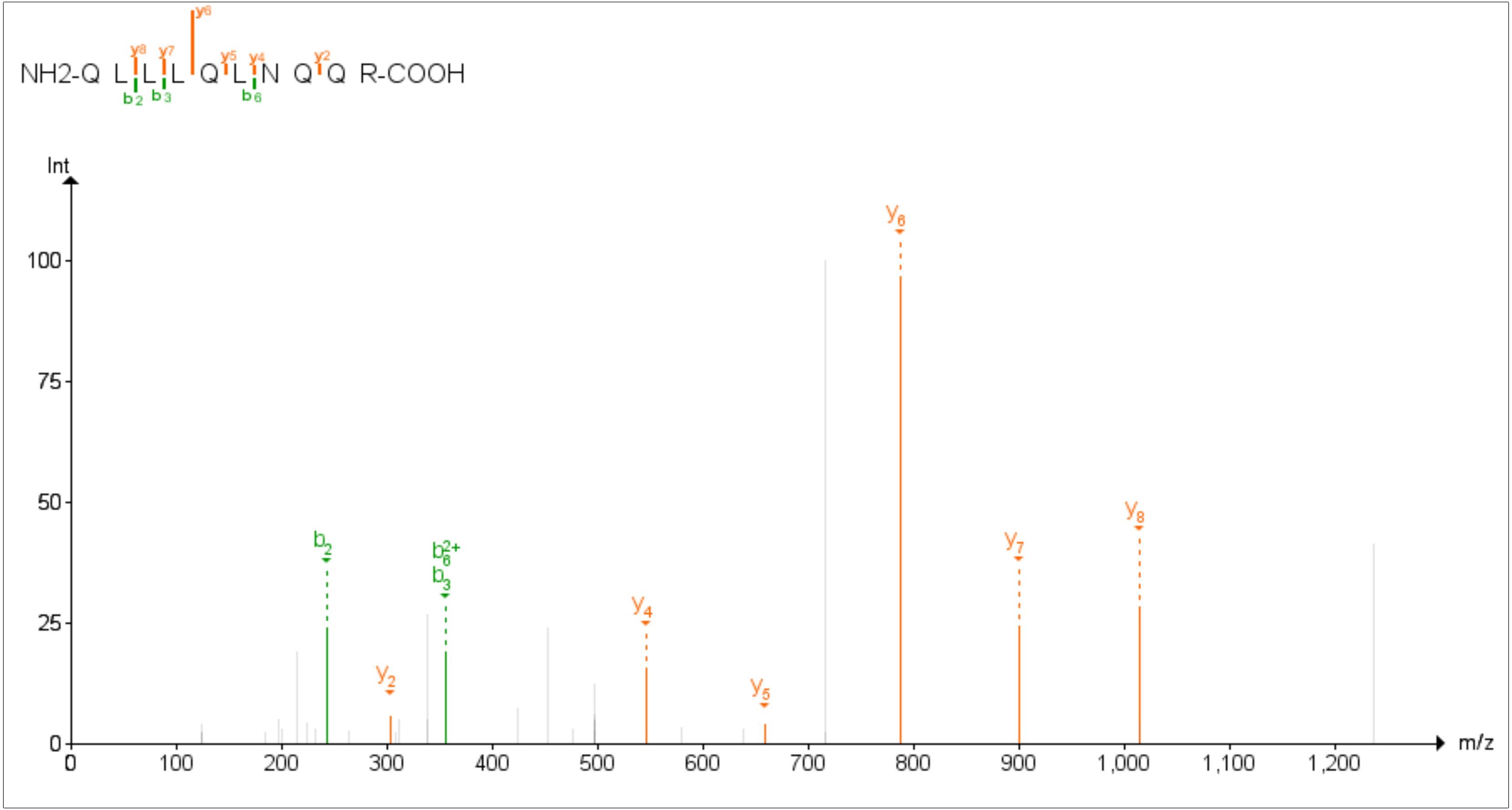

NH2-Q L L L Q L N Q Q R-COOH
y8 y7 y6 y5 y4 y2
b2 b3 b6
Int
100
75
50
25
0
m/z
0 100 200 300 400 500 600 700 800 900 1,000 1,100 1,200
y6
y8
y7
b2
b6 2+
b3
y4
y2
y5

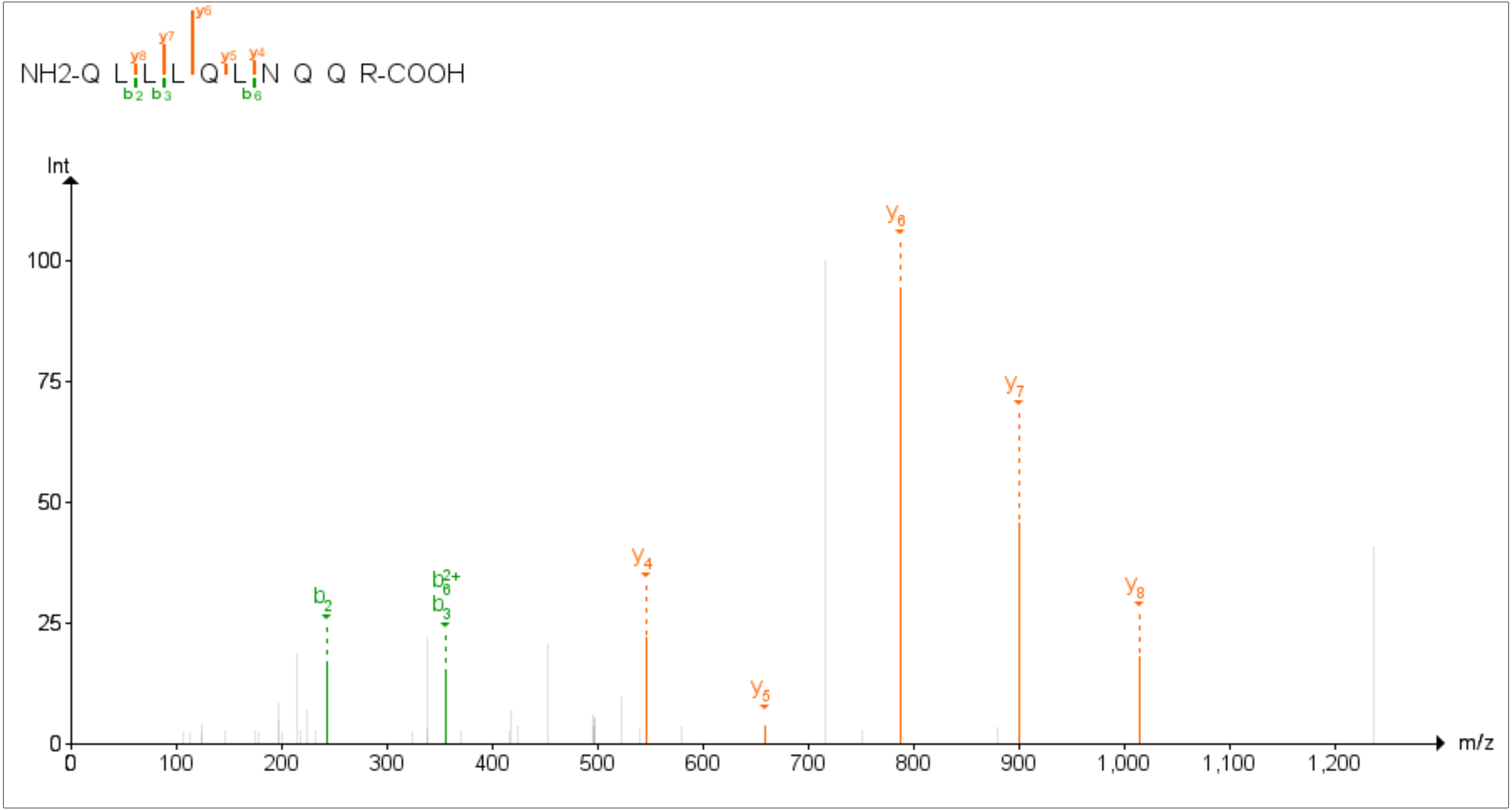
NH2-Q L L L Q L N Q Q R-COOH
y8 y7 y6 y5 y4
b2 b3 b6
Int
100
75
50
25
0
m/z
0
100
200
300
400
500
600
700
800
900
1,000
1,100
1,200
b2
b6 2+
b3
y4
y5
y6
y7
y8

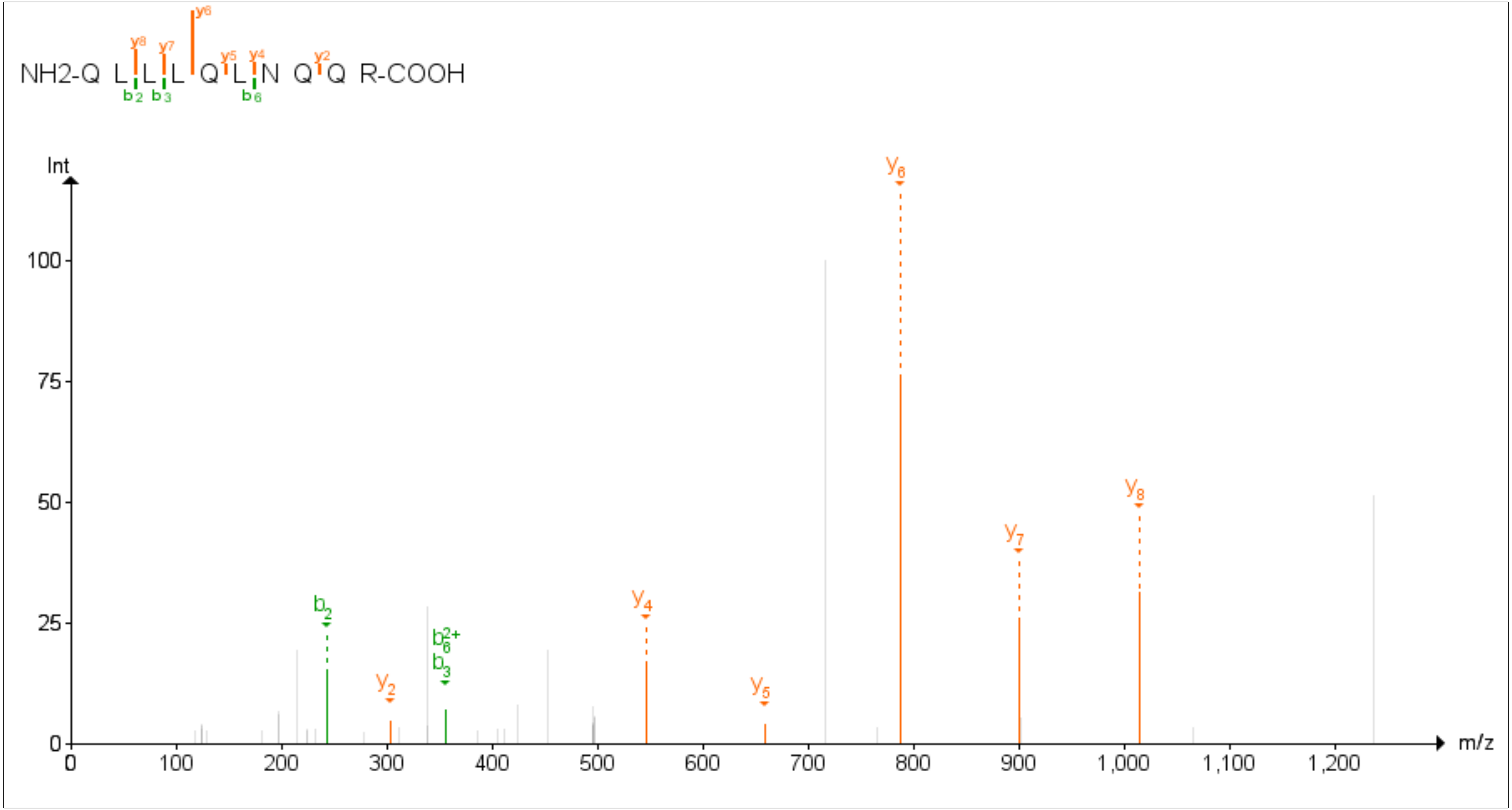

NH2-Q L L L Q L N Q Q R-COOH
y8 y7 y6 y5 y4 y2
b2 b3 b6
Int
100
75
50
25
0
y6
y8
y7
y4
b2
b6 2+
b3
y2
y5
0 100 200 300 400 500 600 700 800 900 1,000 1,100 1,200
m/z

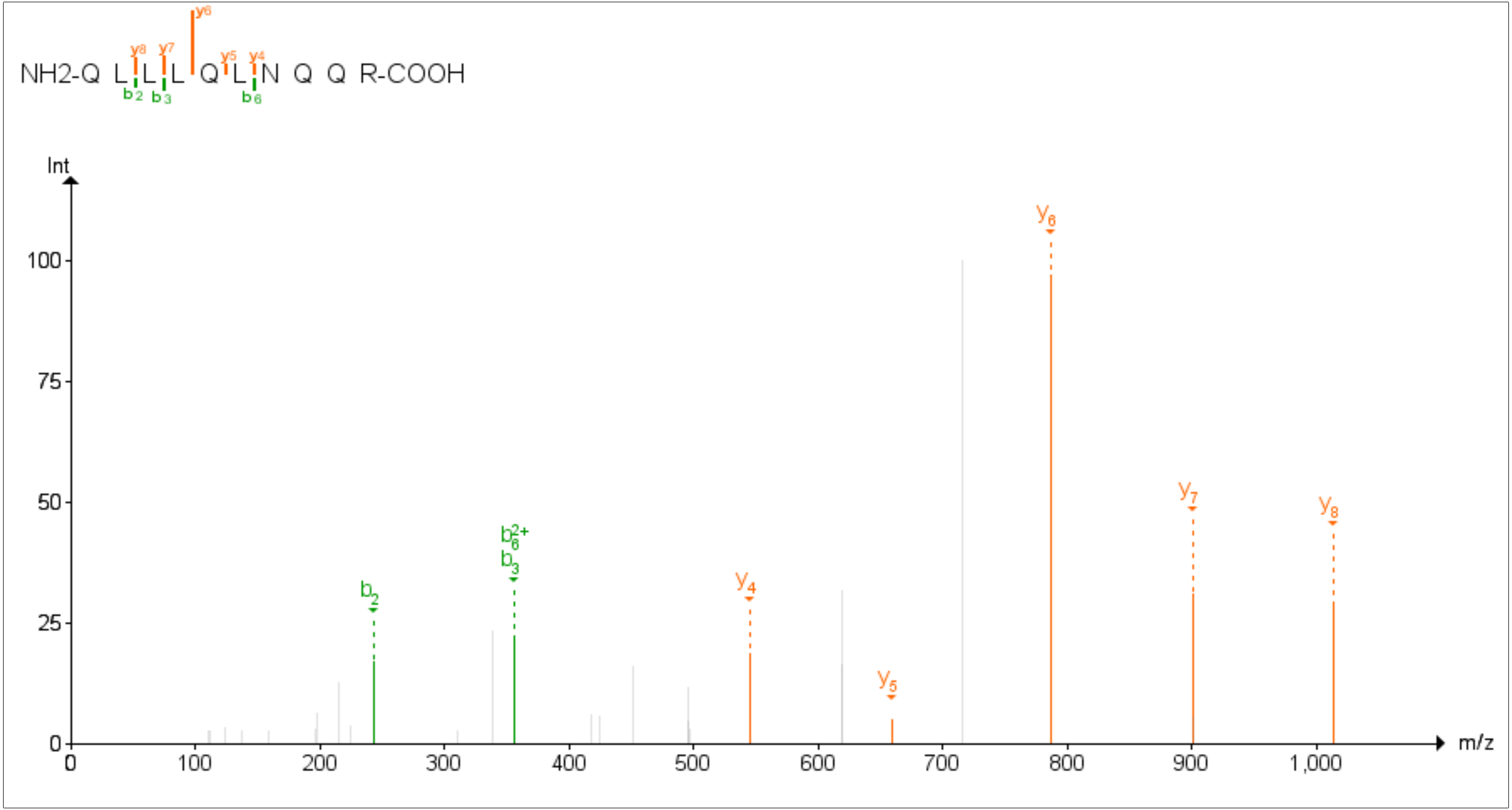

NH2-Q L L L Q L N Q Q R-COOH
y8 y7 y6 y5 y4
b2 b3 b6
Int
100
75
50
25
0
m/z
0 100 200 300 400 500 600 700 800 900 1,000
b2
b6 2+
b3
y4
y5
y6
y7
y8

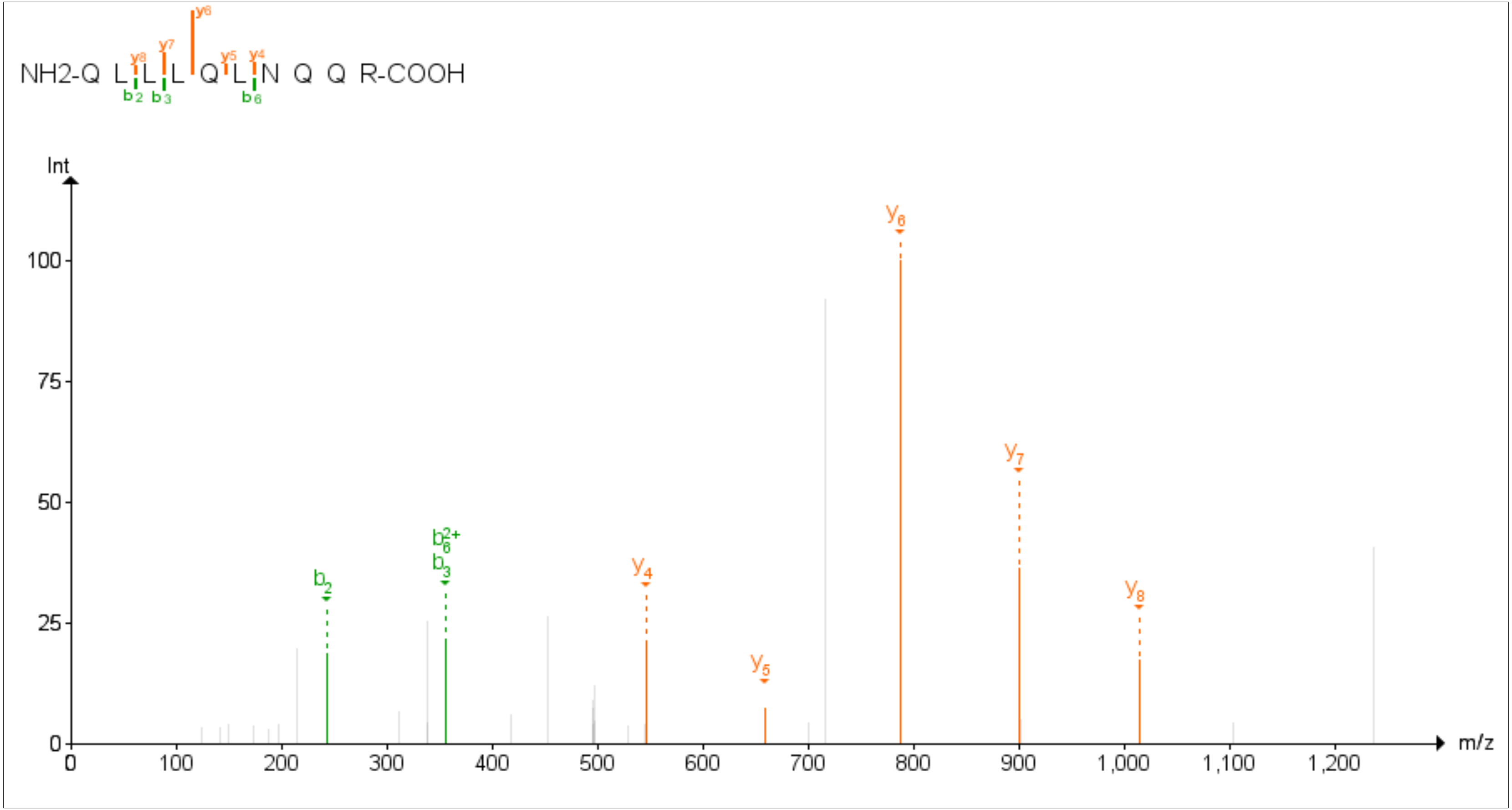

NH2-Q L L L Q L N Q Q R-COOH
y8 y7 y6 y5 y4
b2 b3 b6
Int
100
75
50
25
0
m/z
0 100 200 300 400 500 600 700 800 900 1,000 1,100 1,200
b2
b6 2+
b3
y4
y5
y6
y7
y8

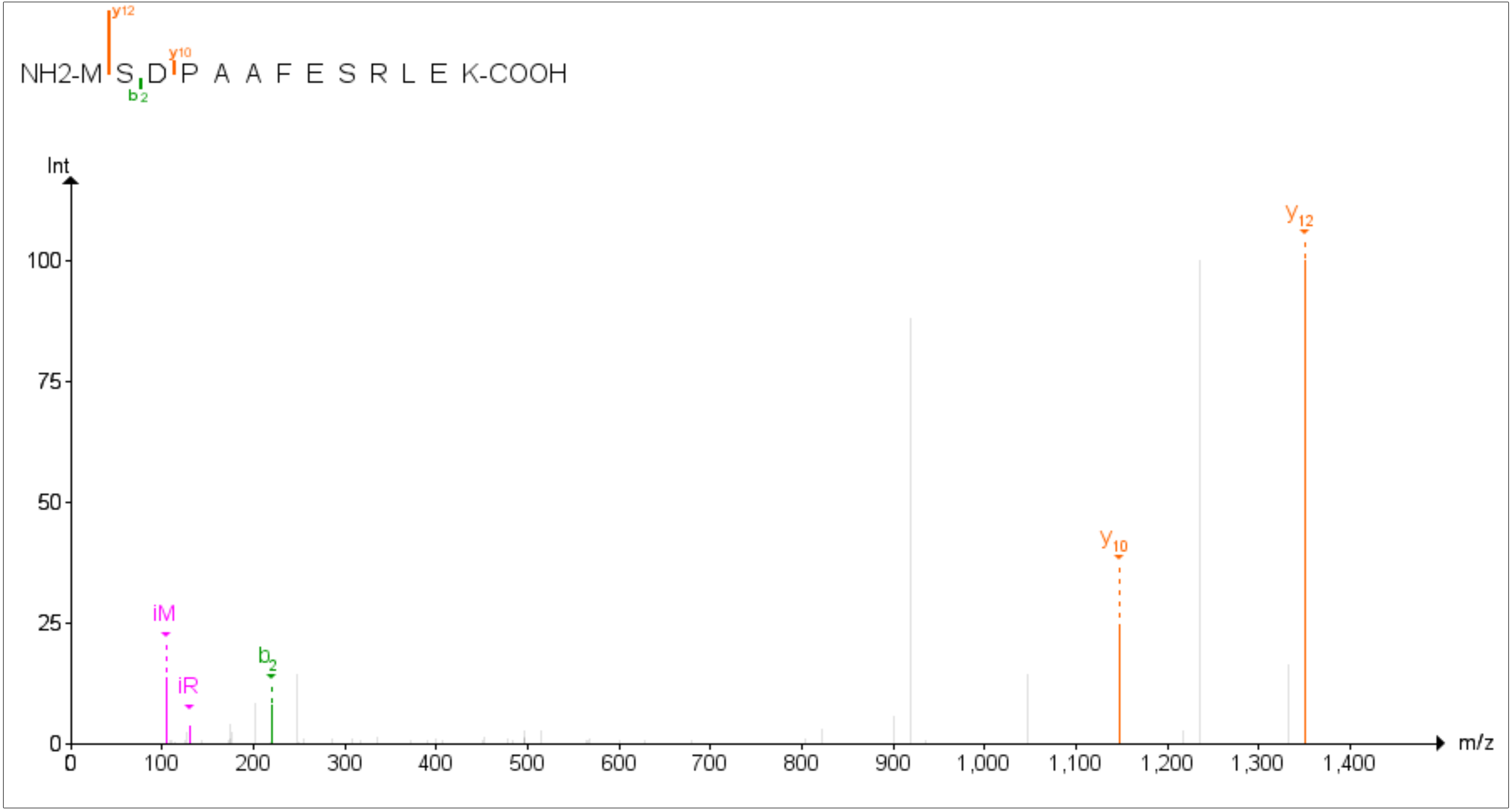

NH2-M S D P A A F E S R L E K-COOH
y12
y10
b2
Int
m/z
iM
iR
b2
y10
y12
0
25
50
75
100
0
100
200
300
400
500
600
700
800
900
1,000
1,100
1,200
1,300
1,400

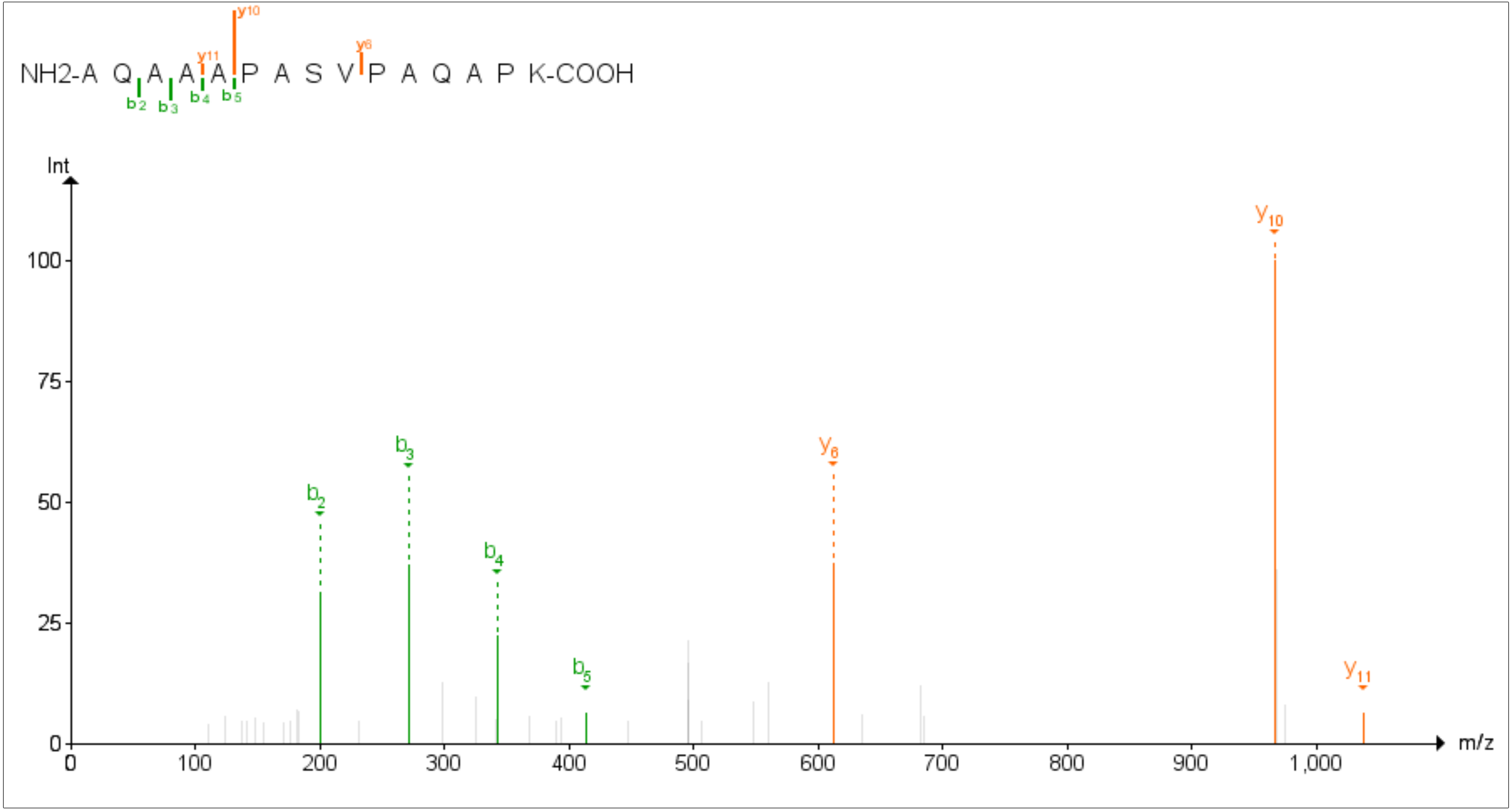
NH2-A Q A A A P A S V P A Q A P K-COOH
y10
y11
y6
b2
b3
b4
b5
Int
100
75
50
25
0
b2
b3
b4
b5
y6
y10
y11
0
100
200
300
400
500
600
700
800
900
1,000
m/z

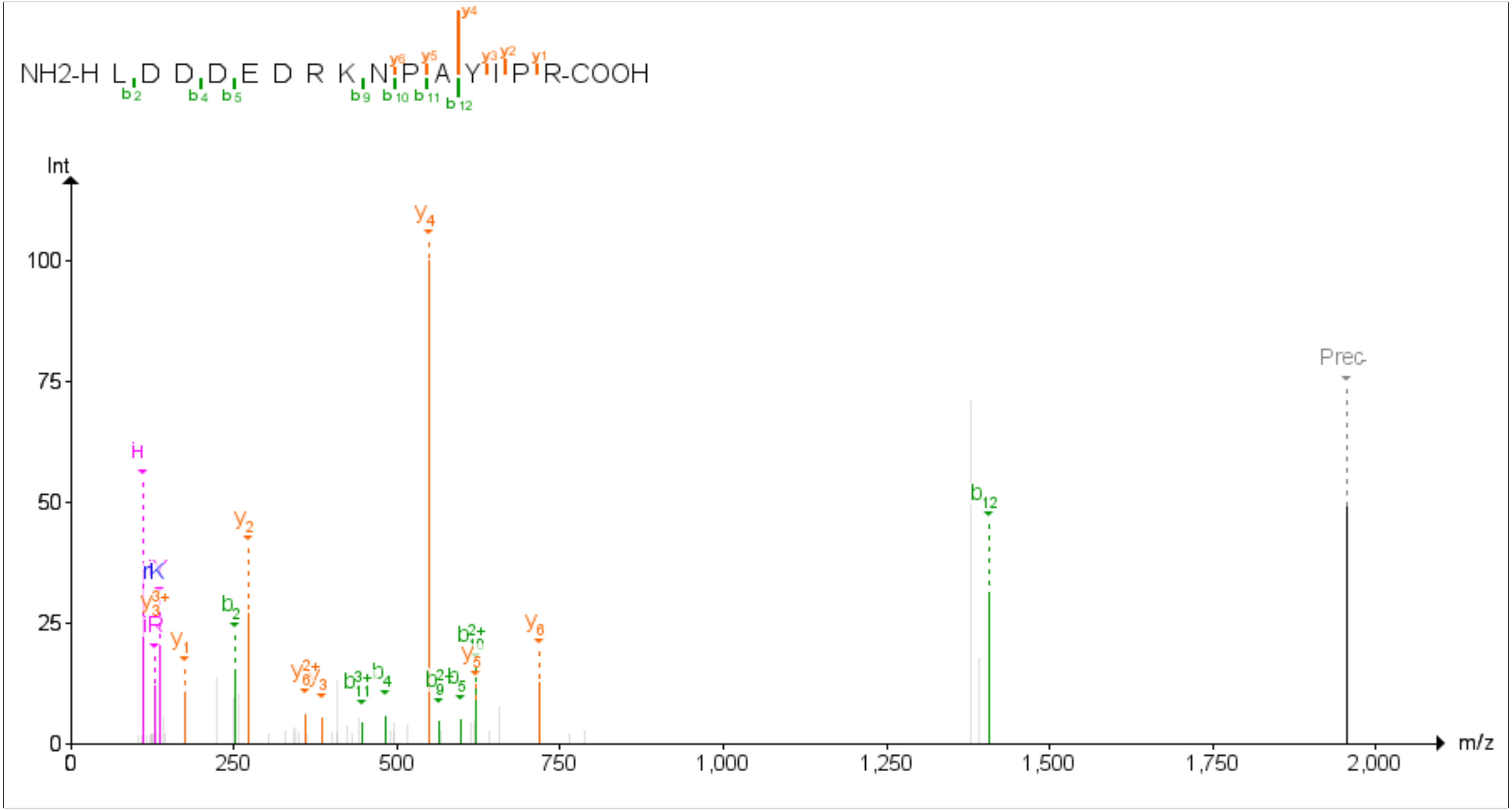

NH2-H L D D D E D R K N P A Y I P R-COOH
Int
100
75
50
25
0
m/z
0
250
500
750
1,000
1,250
1,500
1,750
2,000
y4
Prec
b12
y2
b2
y1
y6

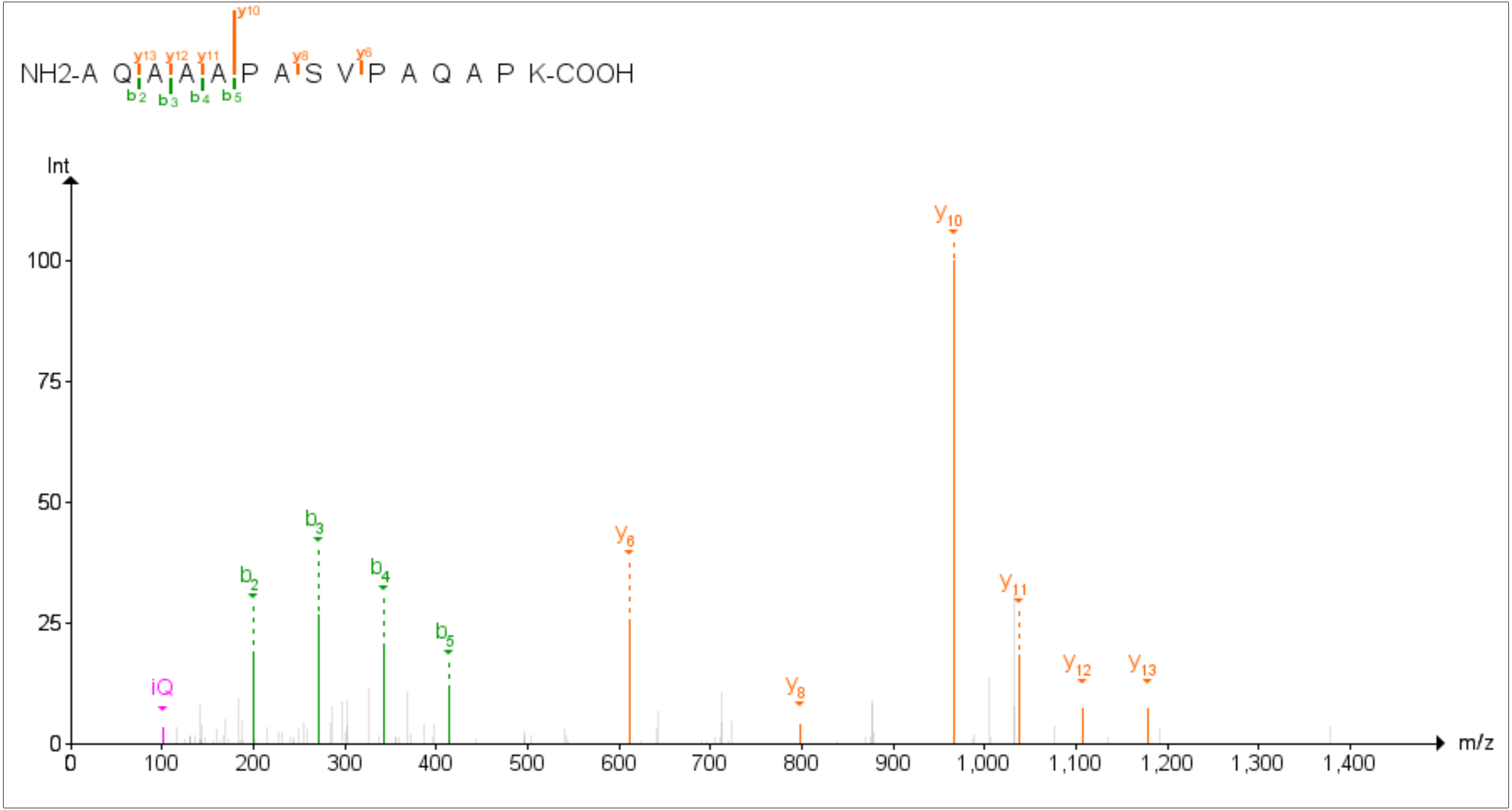

NH2-A Q A A A P A S V P A Q A P K-COOH
y10
y13 y12 y11 y8 y6
b2 b3 b4 b5
Int
100
75
50
25
0
iQ
b2
b3
b4
b5
y6
y8
y10
y11
y12
y13
0 100 200 300 400 500 600 700 800 900 1,000 1,100 1,200 1,300 1,400
m/z

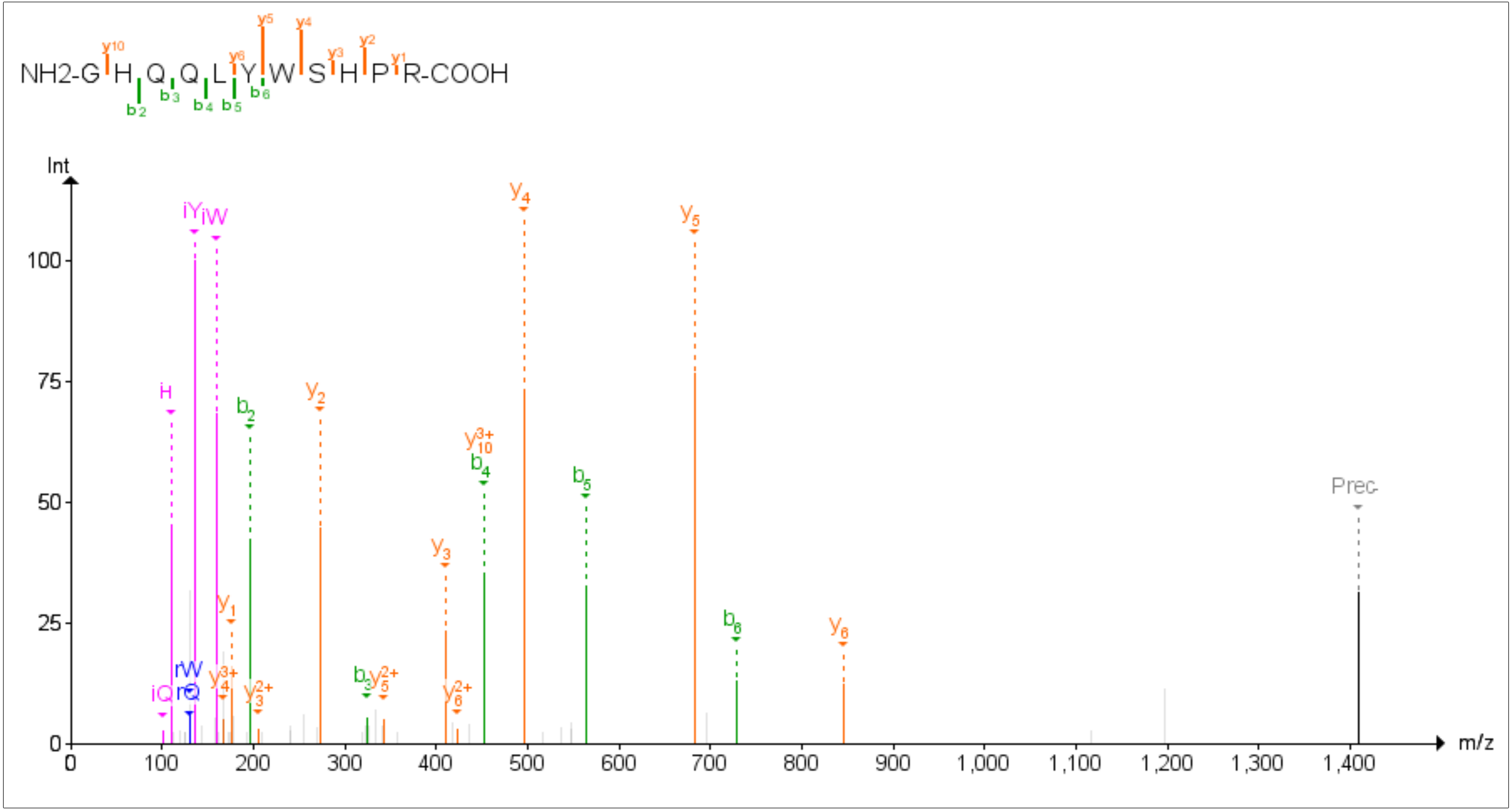

NH2-G H Q Q L Y W S H P R-COOH
y10 y6 y5 y4 y3 y2 y1
b2 b3 b4 b5 b6
Int
m/z
0
25
50
75
100
0 100 200 300 400 500 600 700 800 900 1,000 1,100 1,200 1,300 1,400
iY iW
iH
iQ
rW
rQ
y4
y5
y2
b2
y10 3+
b4
b5
Prec.
y3
y1
y4 3+
y3 2+
b3
y5 2+
y6 2+
b6
y6

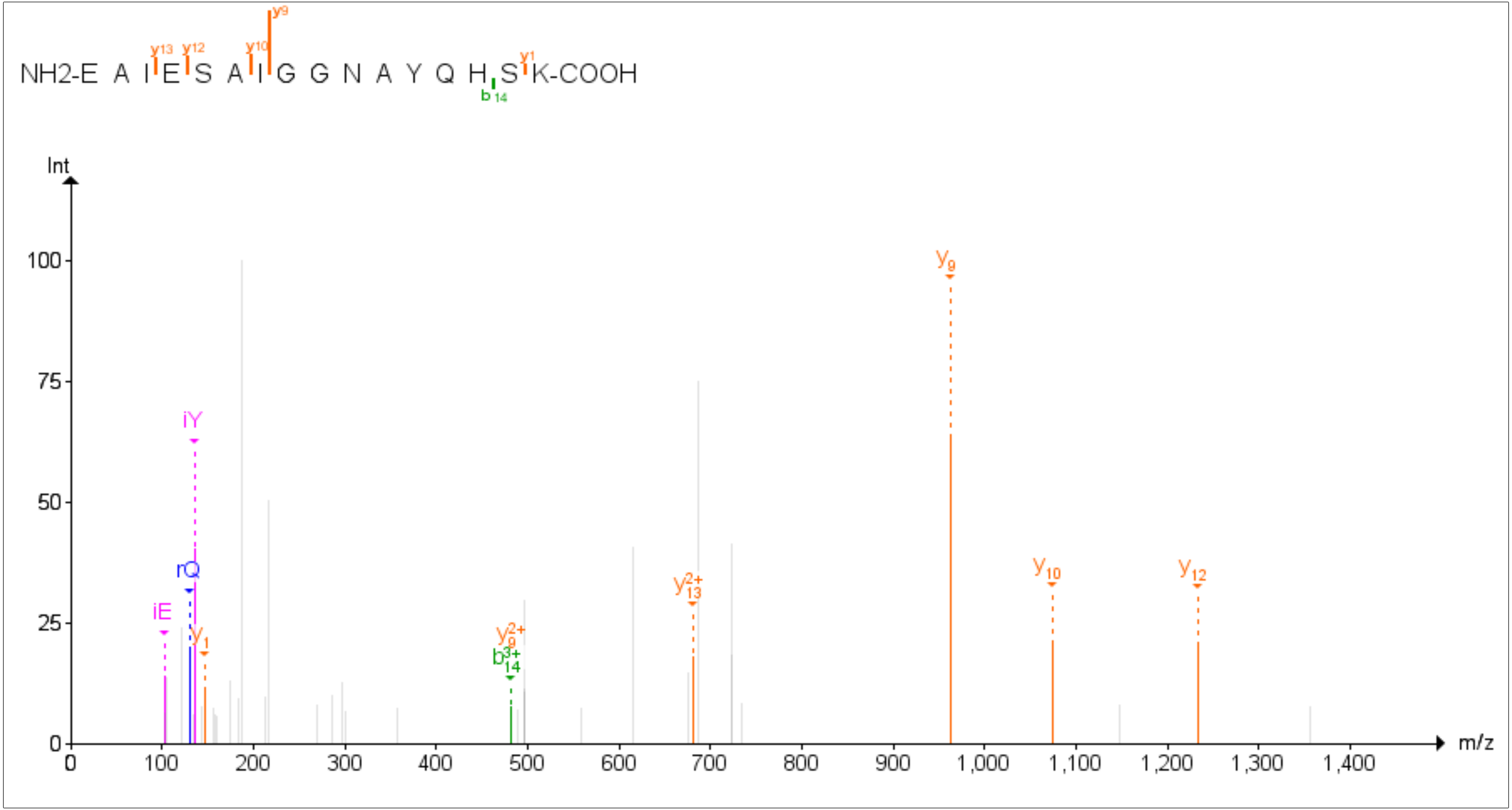
NH2-E A I E S A I G G N A Y Q H S K-COOH
y13
y12
y10
y9
y1
b14
Int
100
75
50
25
0
iY
rQ
iE
y1
y9 2+
b14 3+
y13 2+
y9
y10
y12
0
100
200
300
400
500
600
700
800
900
1,000
1,100
1,200
1,300
1,400
m/z

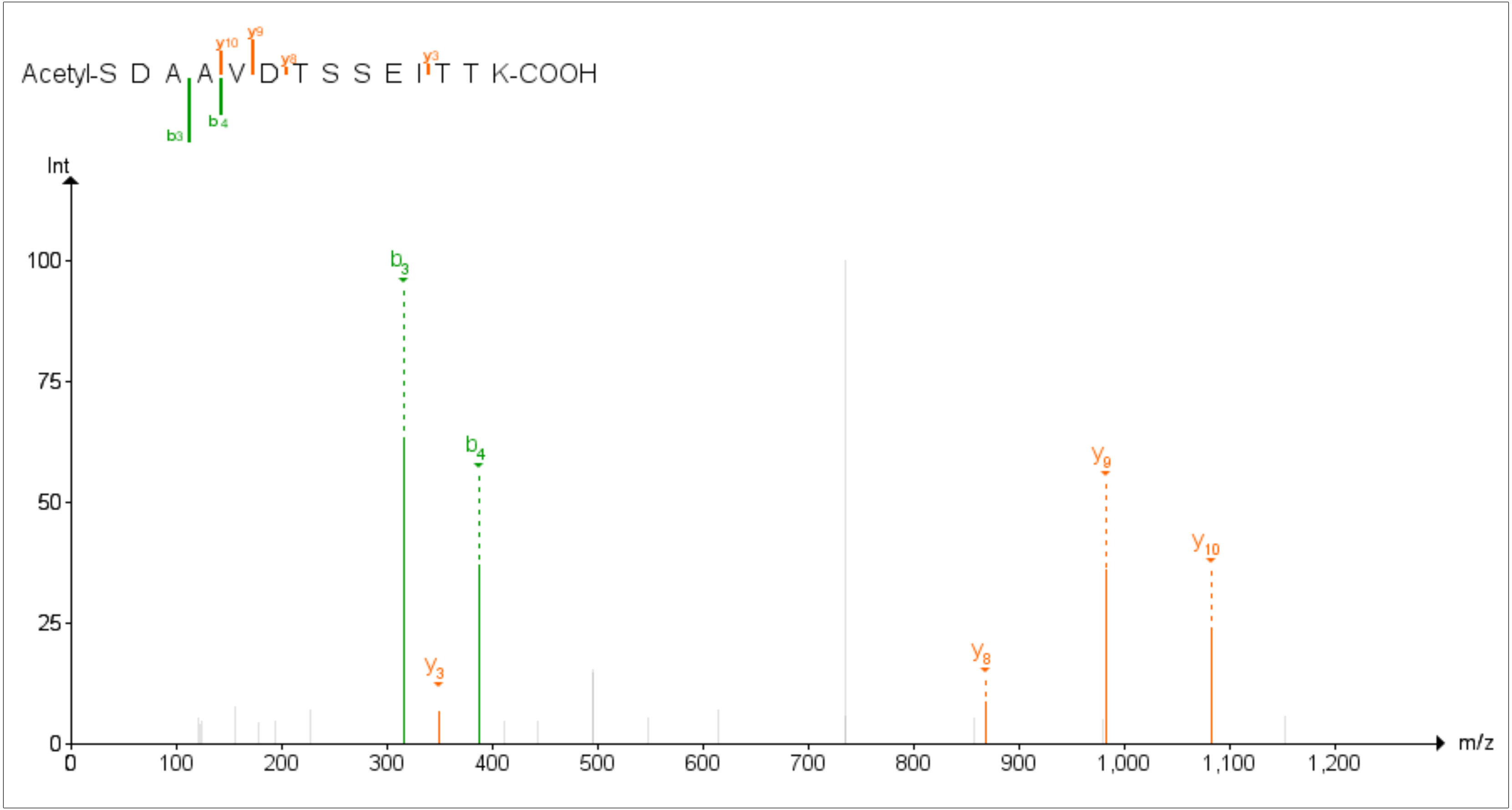
Acetyl-S D A A V D T S S E I T T K-COOH
y10
y9
y8
y3
b3
b4
Int
100
75
50
25
0
b3
b4
y3
y8
y9
y10
m/z
0
100
200
300
400
500
600
700
800
900
1,000
1,100
1,200

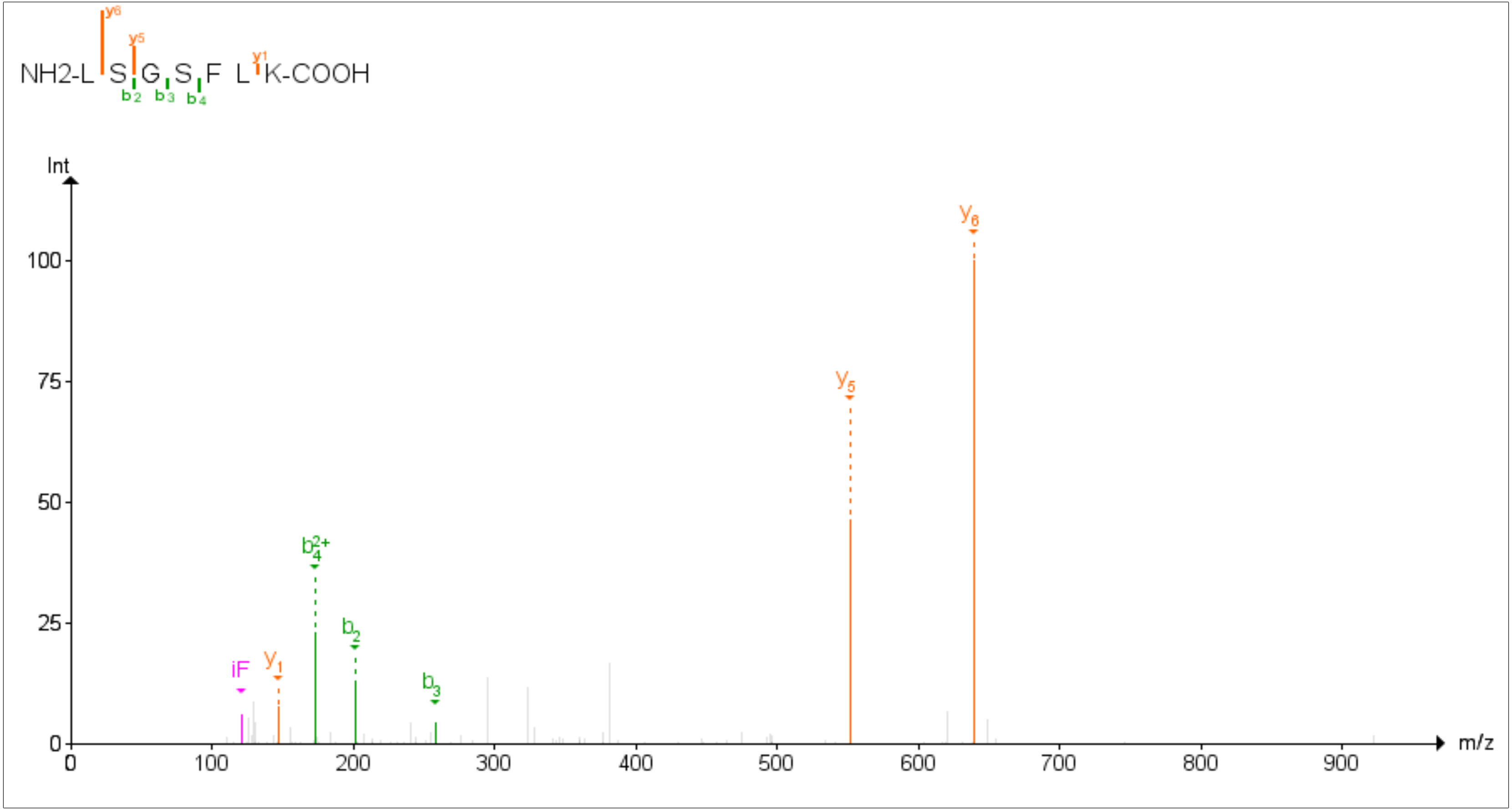

NH2-L S G S F L K-COOH
y6
y5
y1
b2
b3
b4
Int
100
75
50
25
0
y6
y5
b4 2+
b2
b3
iF
y1
0
100
200
300
400
500
600
700
800
900
m/z

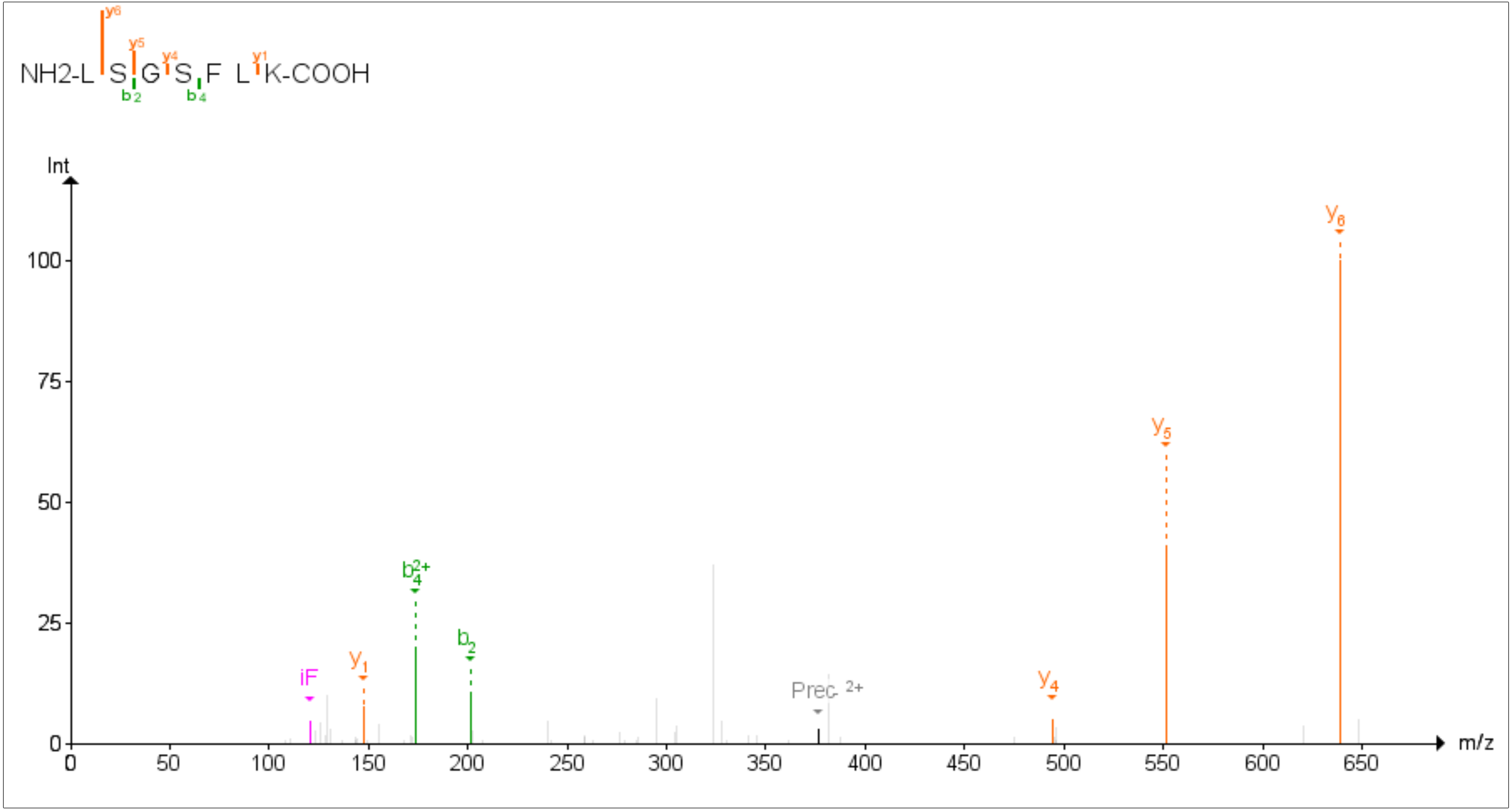
NH2-L S G S F L K-COOH
y6
y5
y4
y1
b2
b4
Int
100
75
50
25
0
y6
y5
b4 2+
b2
y1
iF
Prec 2+
y4
0
50
100
150
200
250
300
350
400
450
500
550
600
650
m/z

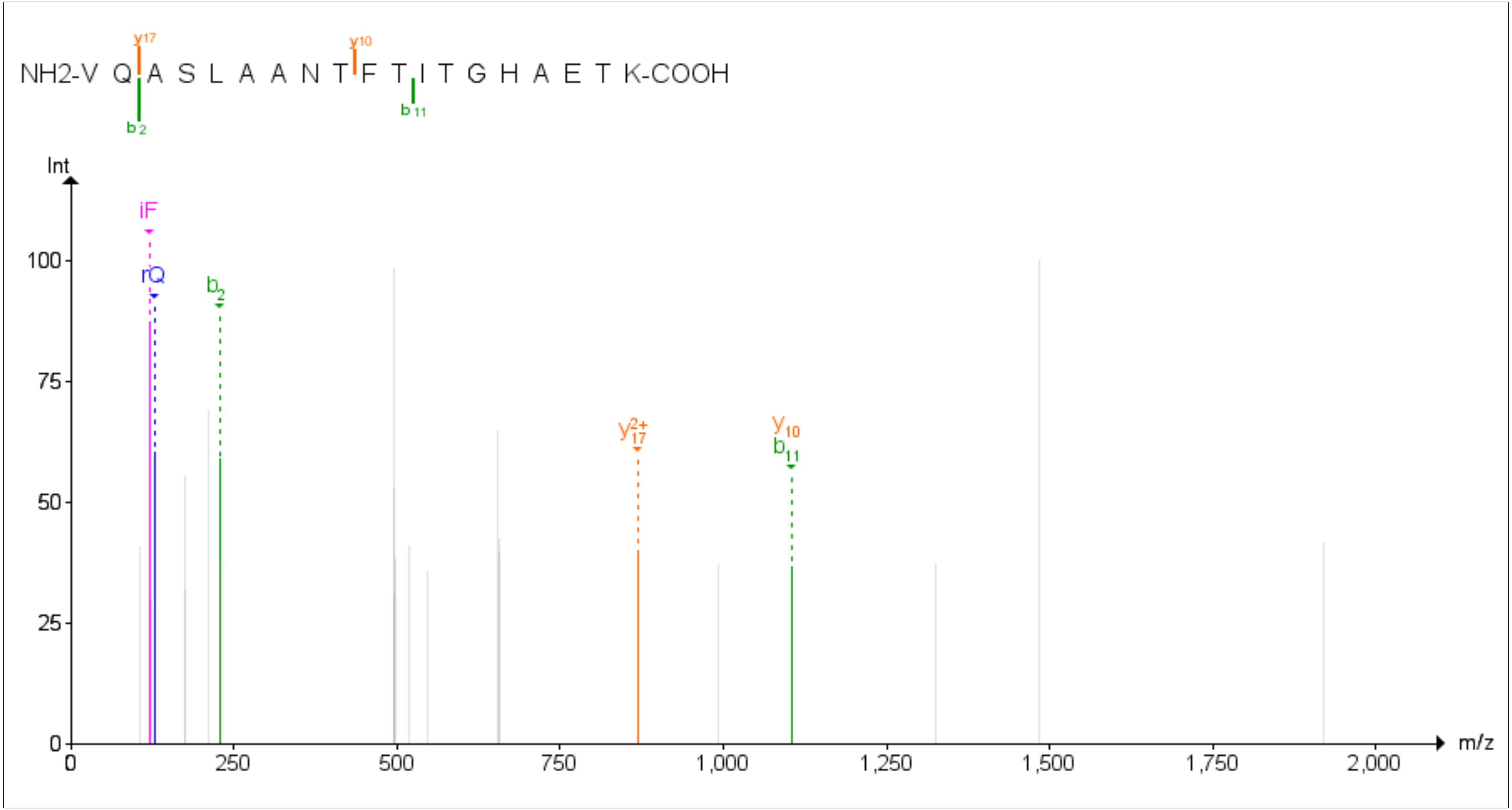

NH2-V Q A S L A A N T F T I T G H A E T K-COOH
y17
y10
b2
b11
Int
iF
rQ
b2
y17 2+
y10
b11
100
75
50
25
0
0
250
500
750
1,000
1,250
1,500
1,750
2,000
m/z

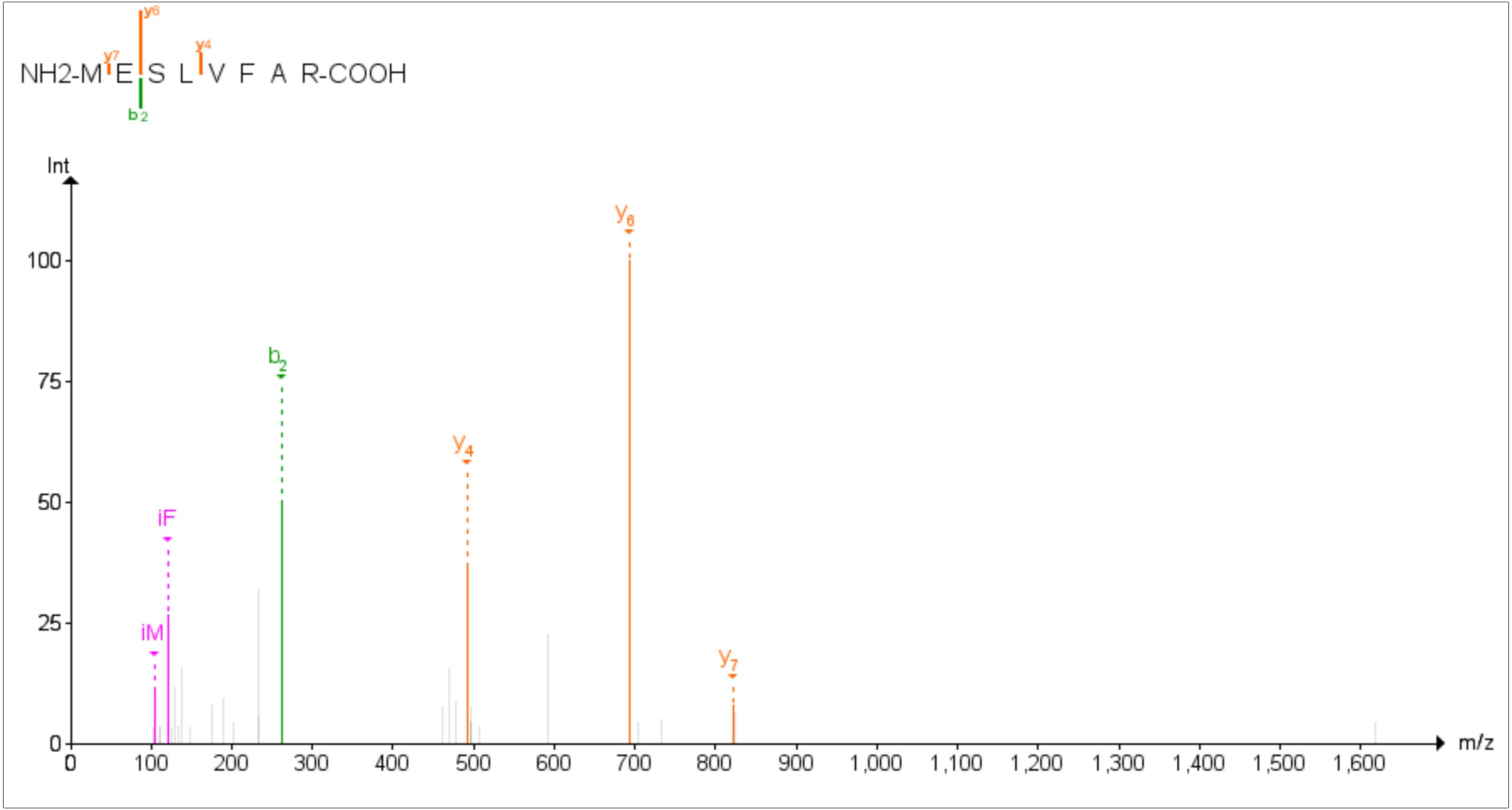

NH2-M E S L V F A R-COOH
y7
y6
y4
b 2
Int
100
75
50
25
0
y6
b2
y4
iF
iM
y7
0
100
200
300
400
500
600
700
800
900
1,000
1,100
1,200
1,300
1,400
1,500
1,600
m/z

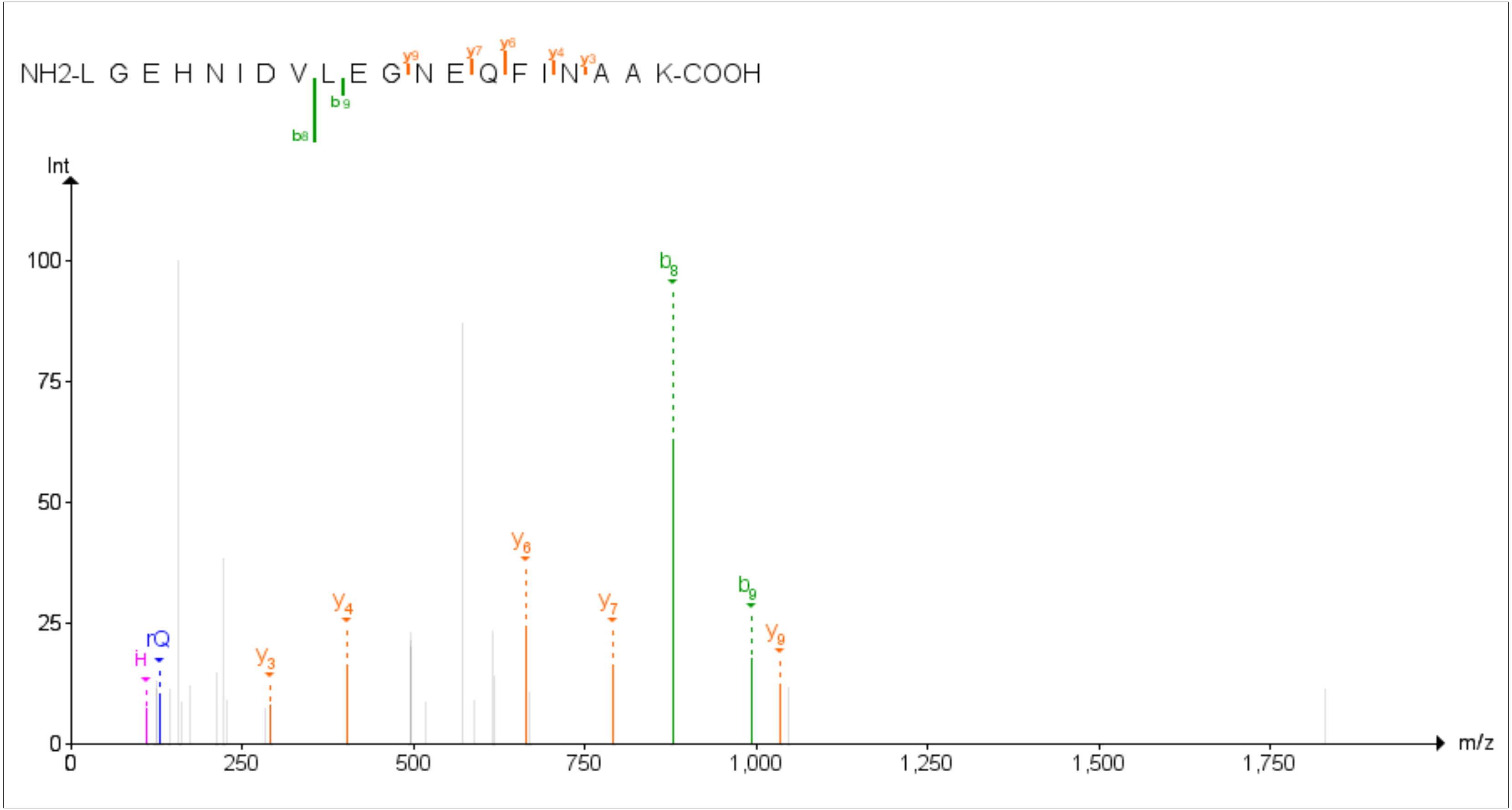

NH2-L G E H N I D V L E G N E Q F I N A A K-COOH
y9 y7 y6 y4 y3
b8 b9
Int
100
75
50
25
0
H
rQ
y3
y4
y6
y7
b8
b9
y9
0
250
500
750
1,000
1,250
1,500
1,750
m/z

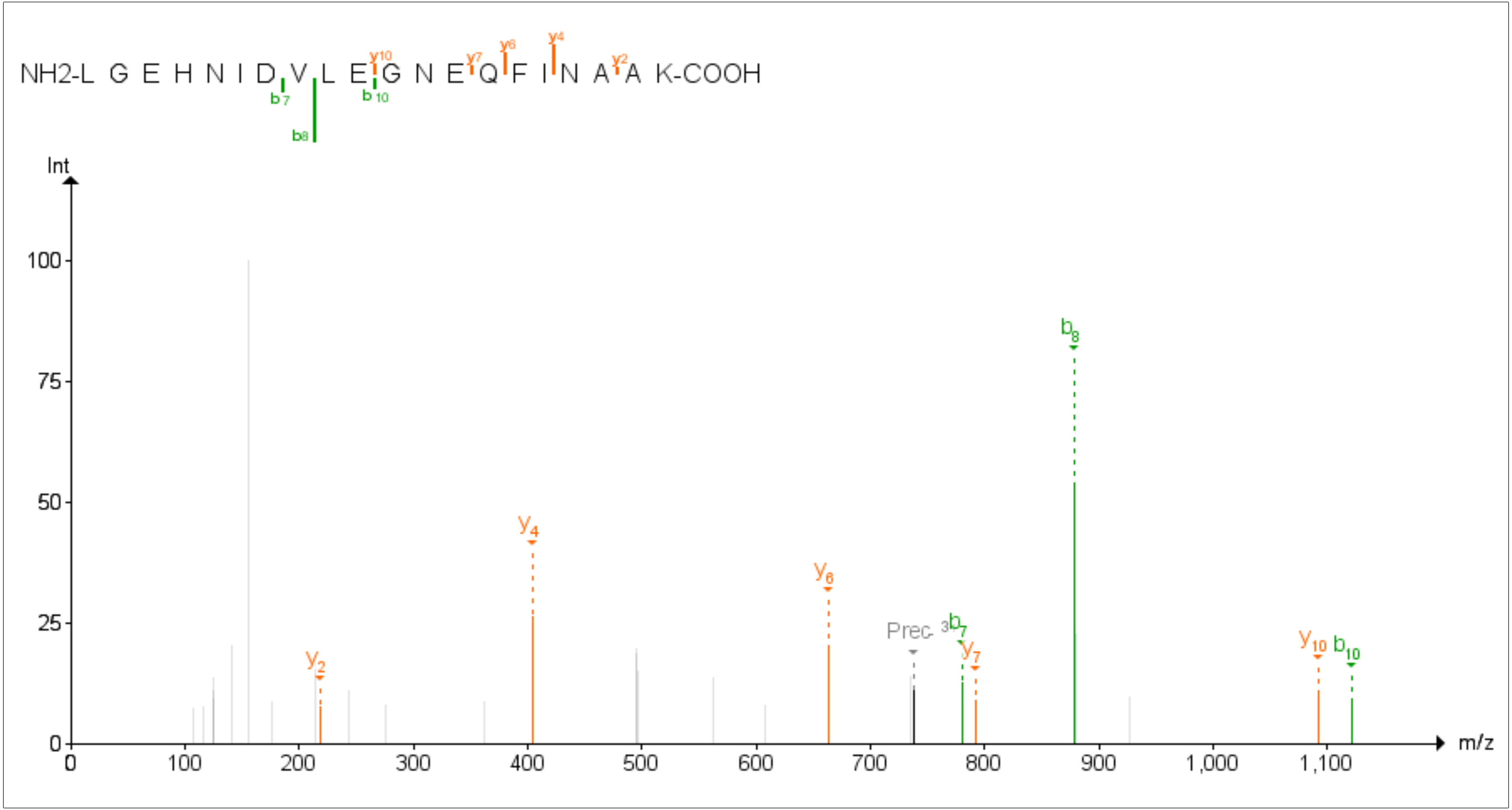

NH2-L G E H N I D V L E G N E Q F I N A A K-COOH
y10
y7
y6
y4
y2
b7
b10
b8
Int
100
75
50
25
0
y2
y4
y6
Prec
b7
y7
b8
y10
b10
0
100
200
300
400
500
600
700
800
900
1,000
1,100
m/z

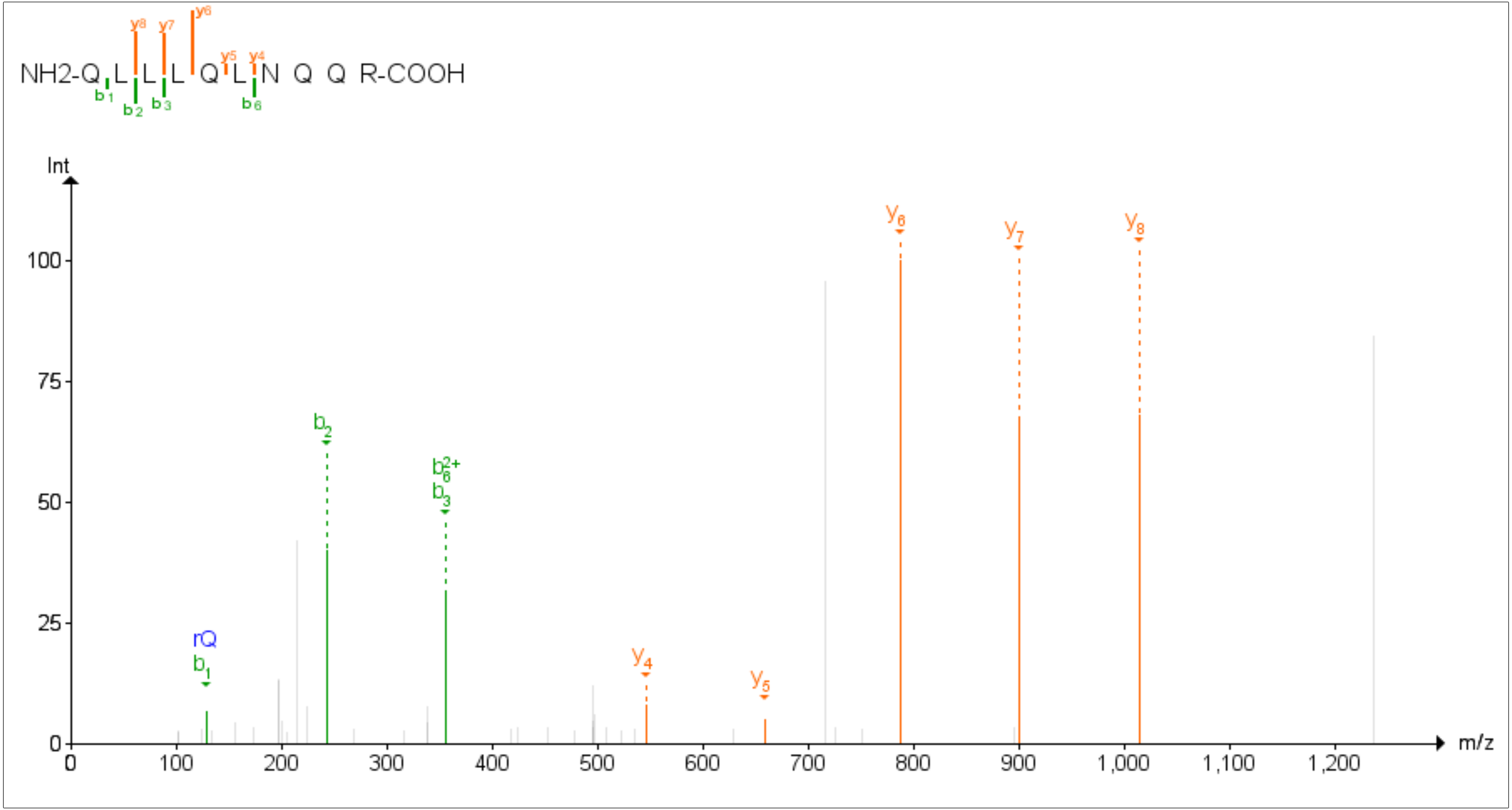
NH2-Q L L L Q L N Q Q R-COOH
y8 y7 y6 y5 y4
b1 b2 b3 b6
Int
100
75
50
25
0
rQ
b1
b2
b6 2+
b3
y4
y5
y6
y7
y8
m/z
0 100 200 300 400 500 600 700 800 900 1,000 1,100 1,200

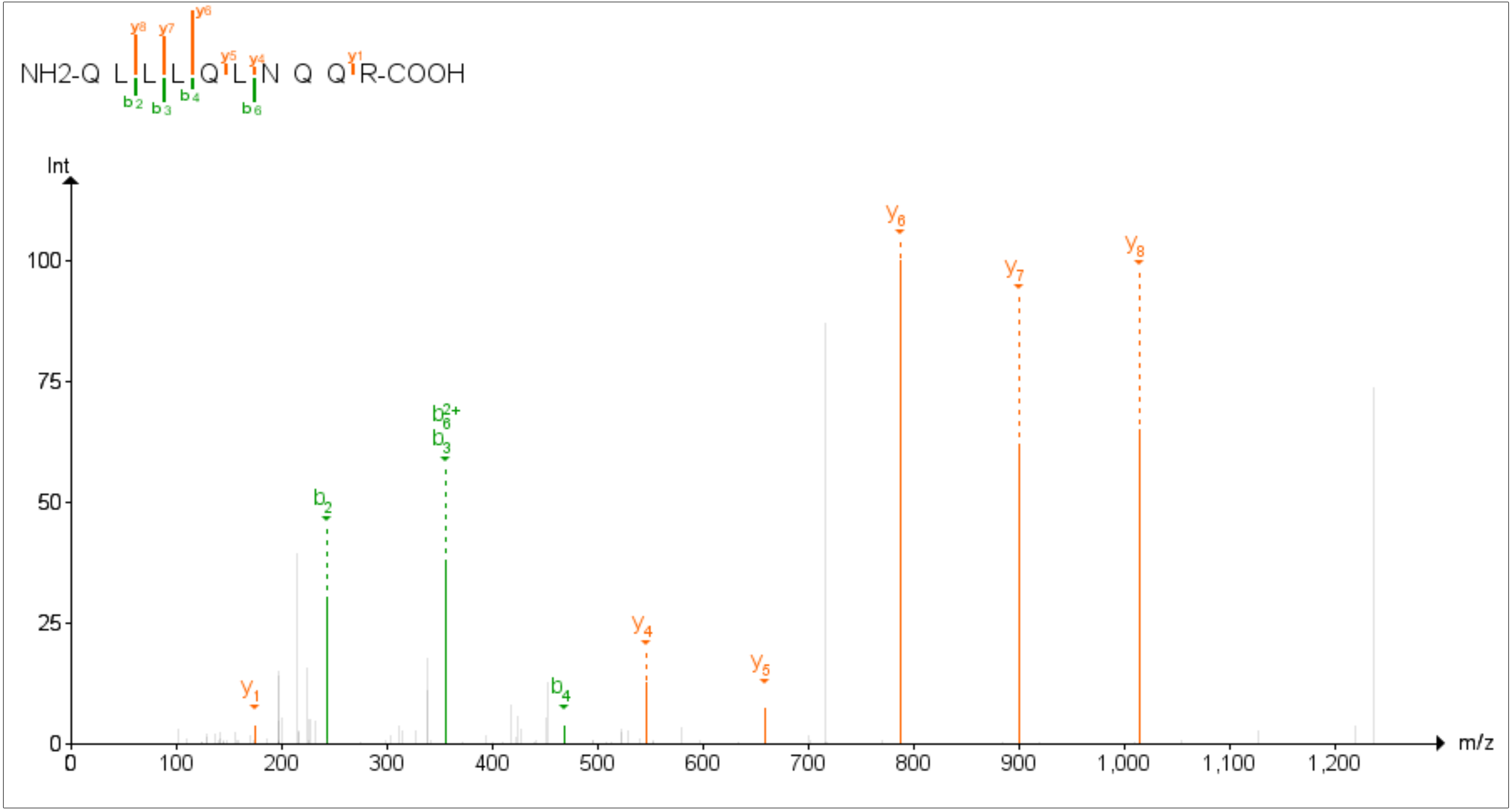

NH2-Q L L L Q L N Q Q R-COOH
y8 y7 y6 y5 y4 y1
b2 b3 b4 b6
Int
m/z
y6
y7
y8
b2
b6 2+
b3
y1
b4
y4
y5

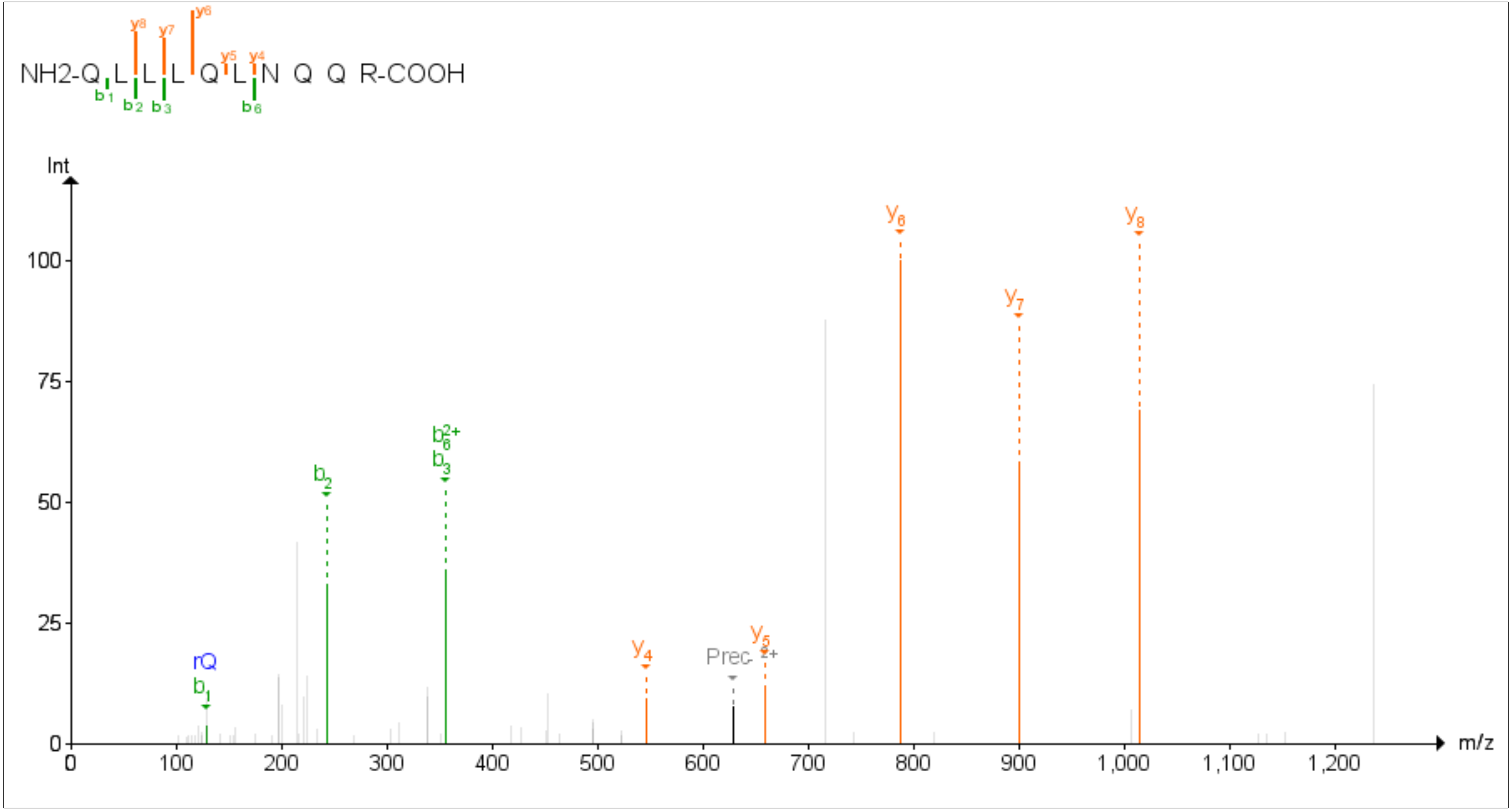

NH2-Q L L L Q L N Q Q R-COOH
y8 y7 y6 y5 y4
b1 b2 b3 b6
Int
m/z
y6
y8
y7
b6 2+
b3
b2
rQ
b1
y4
Prec 2+
y5

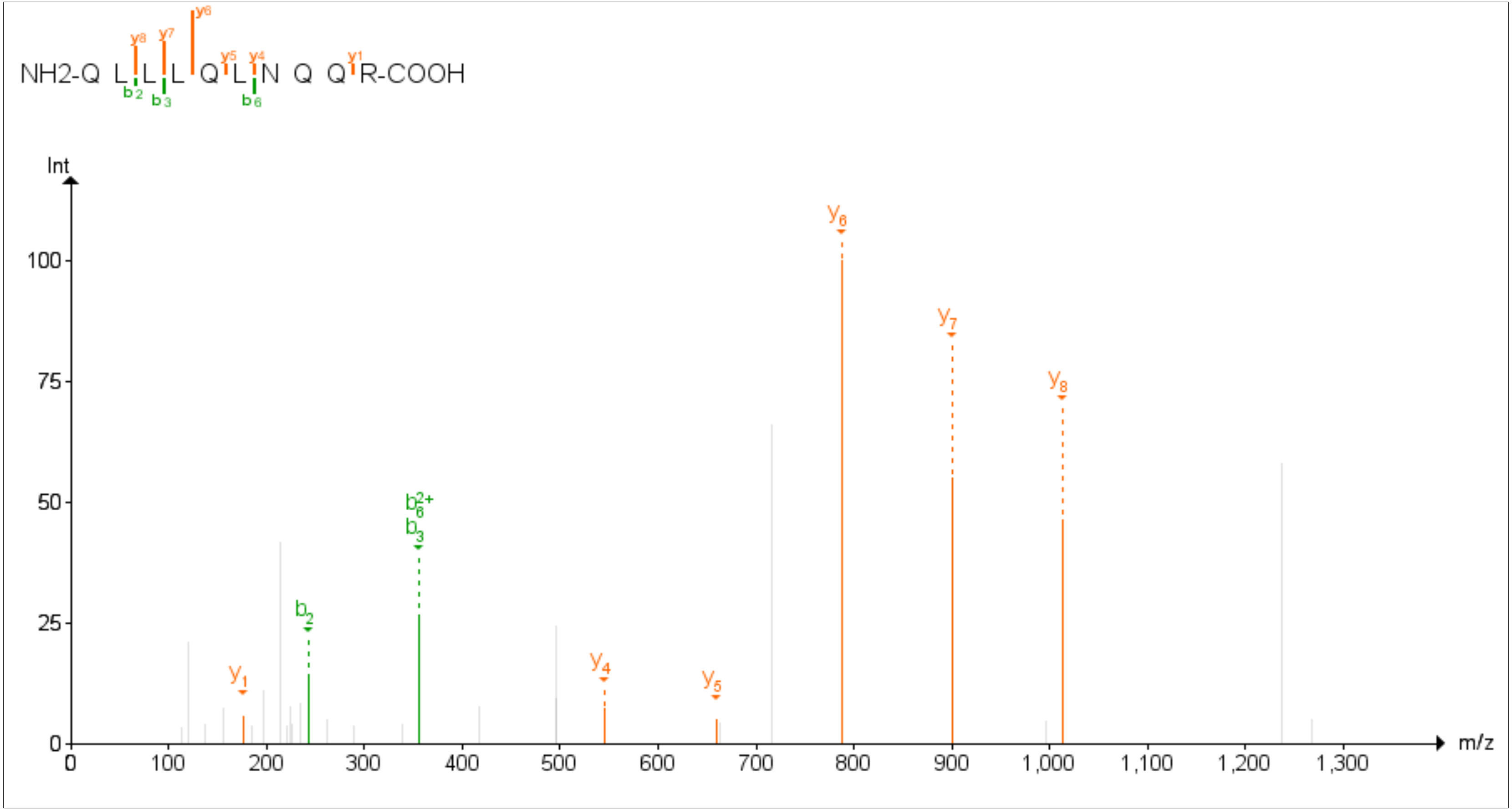
NH2-Q L L L Q L N Q Q R-COOH
y8 y7 y6 y5 y4 y1
b2 b3 b6
Int
100
75
50
25
0
y6
y7
y8
b6 2+
b3
b2
y1
y4
y5
0 100 200 300 400 500 600 700 800 900 1,000 1,100 1,200 1,300
m/z

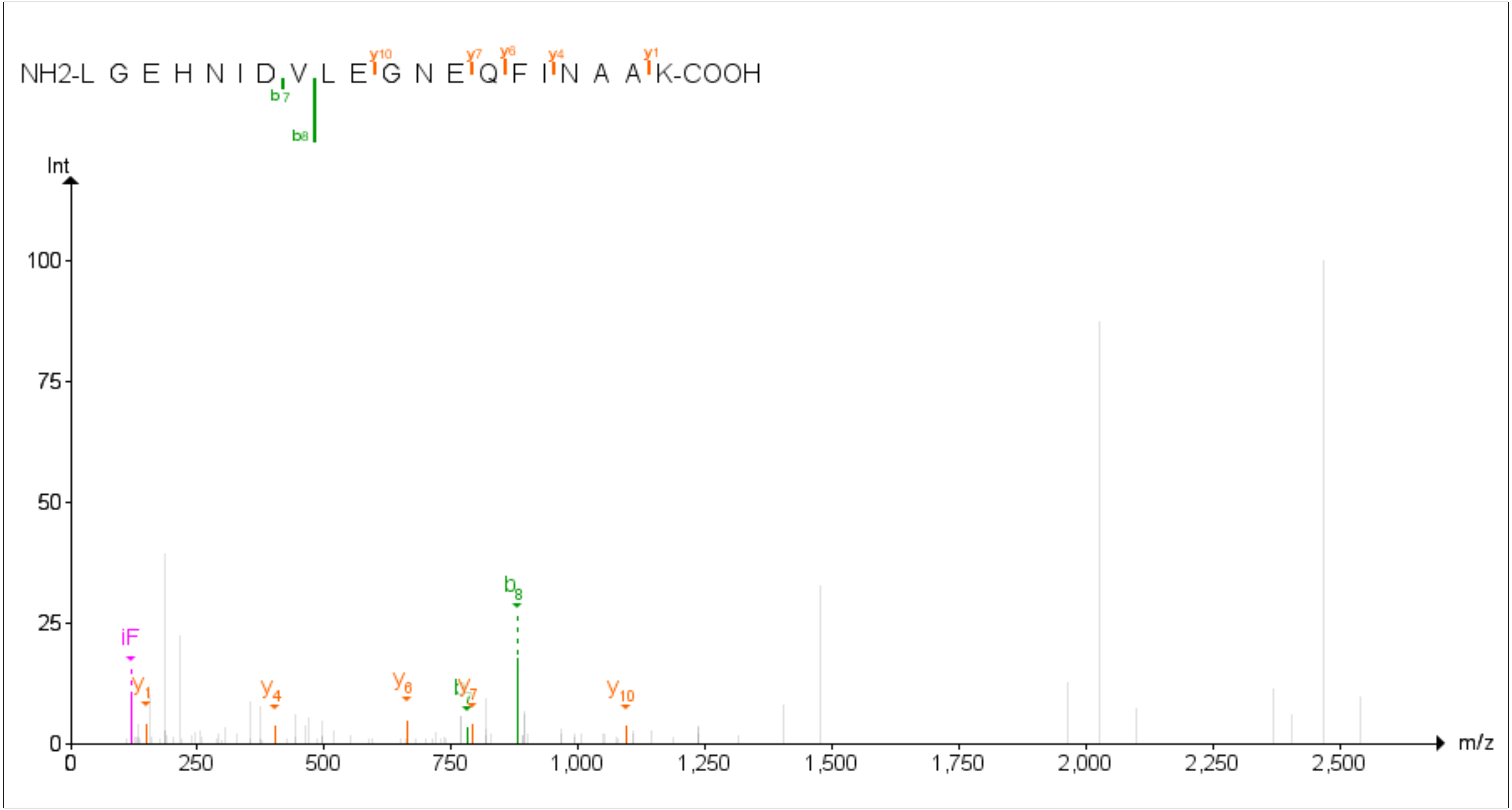

NH2-L G E H N I D V L E G N E Q F I N A A K-COOH
y10
y7
y6
y4
y1
b7
b8
Int
100
75
50
25
0
iF
y1
y4
y6
y7
b8
y10
0
250
500
750
1,000
1,250
1,500
1,750
2,000
2,250
2,500
m/z

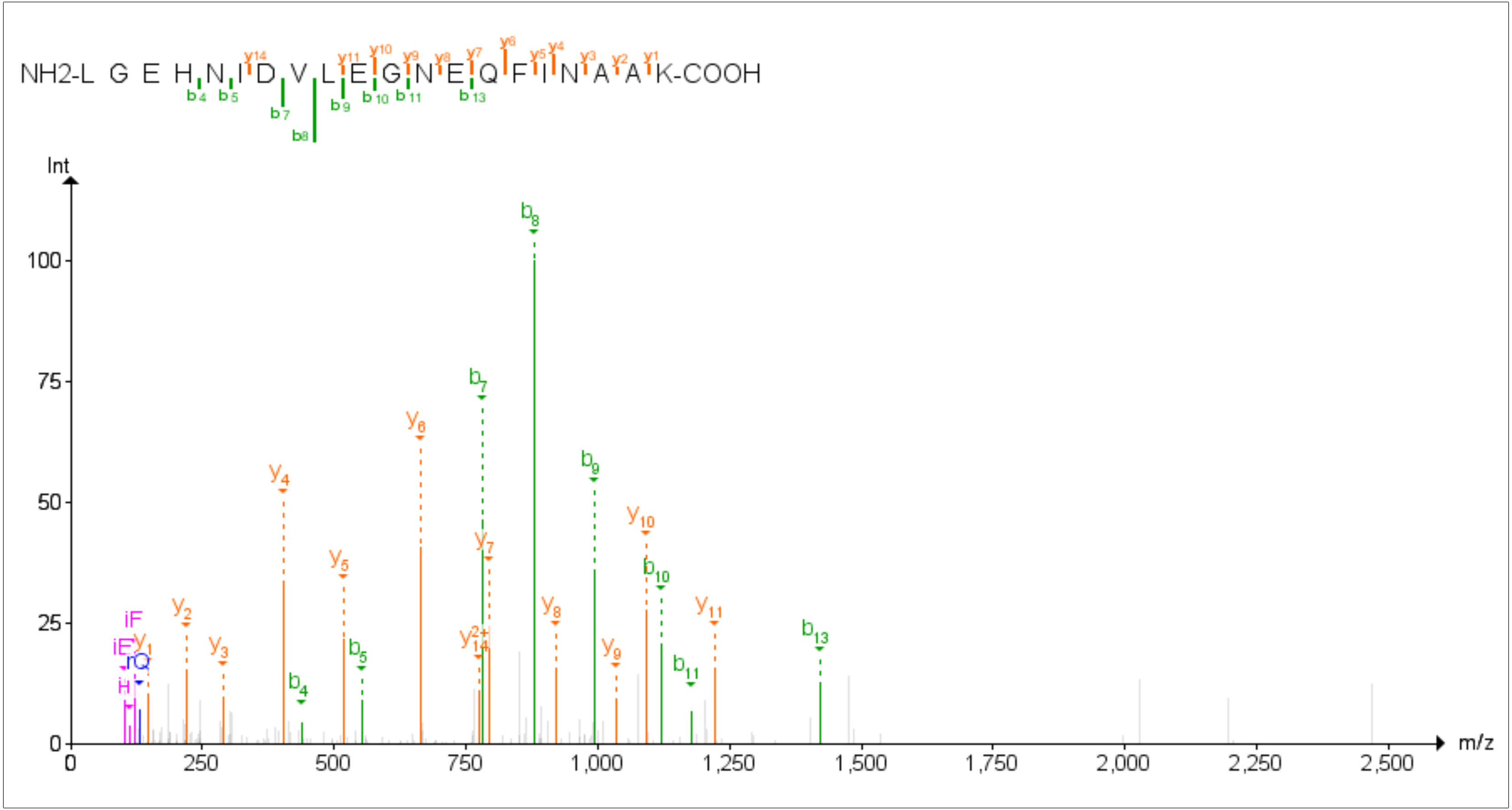

NH2-L G E H N I D V L E G N E Q F I N A A K-COOH
Int
m/z
0
25
50
75
100
250
500
750
1,000
1,250
1,500
1,750
2,000
2,250
2,500

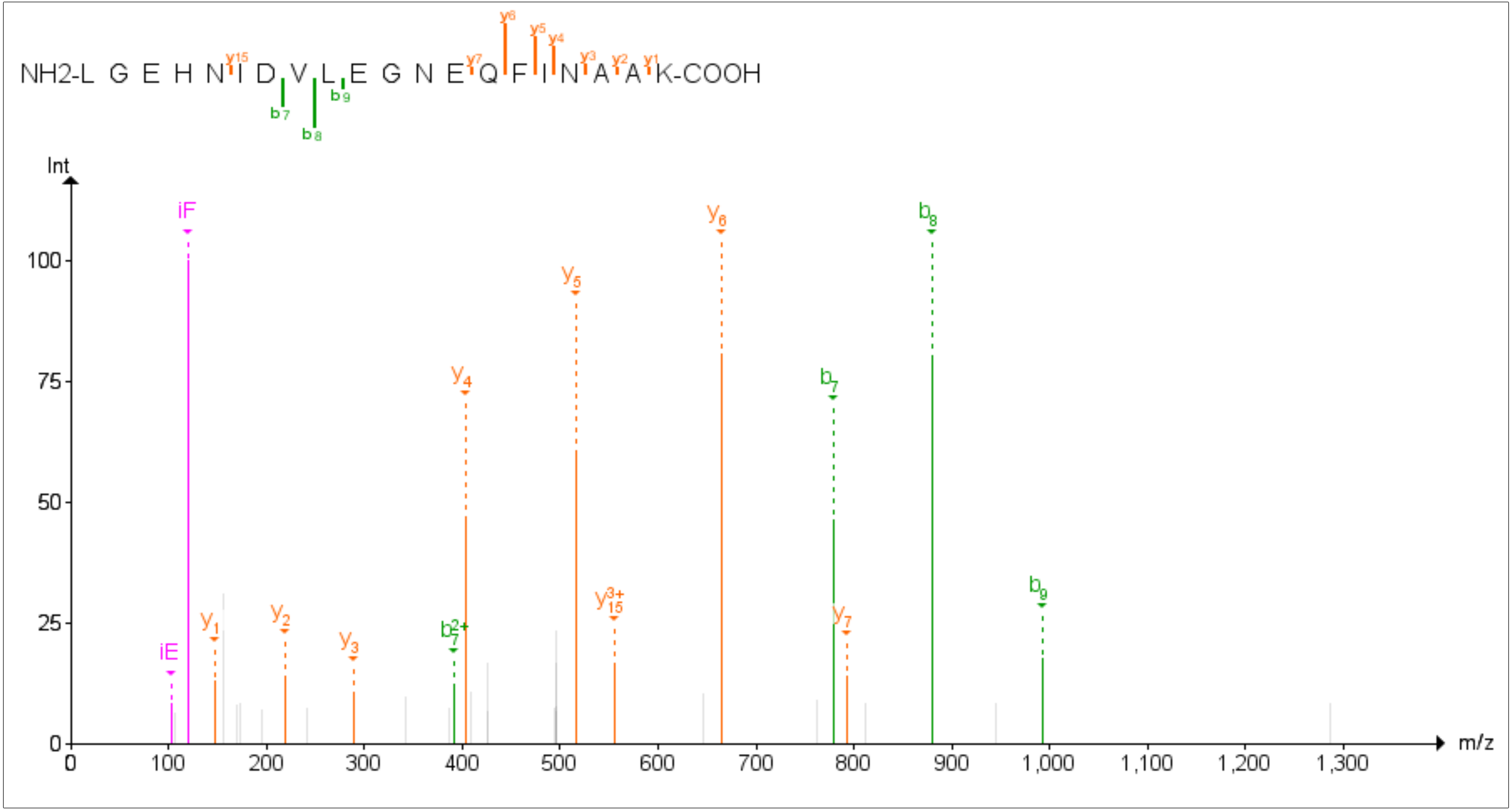
NH2-L G E H N I D V L E G N E Q F I N A A K-COOH
y15 y7 y6 y5 y4 y3 y2 y1
b7 b8 b9
Int
100
75
50
25
0
iF
iE
y1
y2
y3
b7 2+
y4
y5
y15 3+
y6
b7
y7
b8
b9
0 100 200 300 400 500 600 700 800 900 1,000 1,100 1,200 1,300
m/z

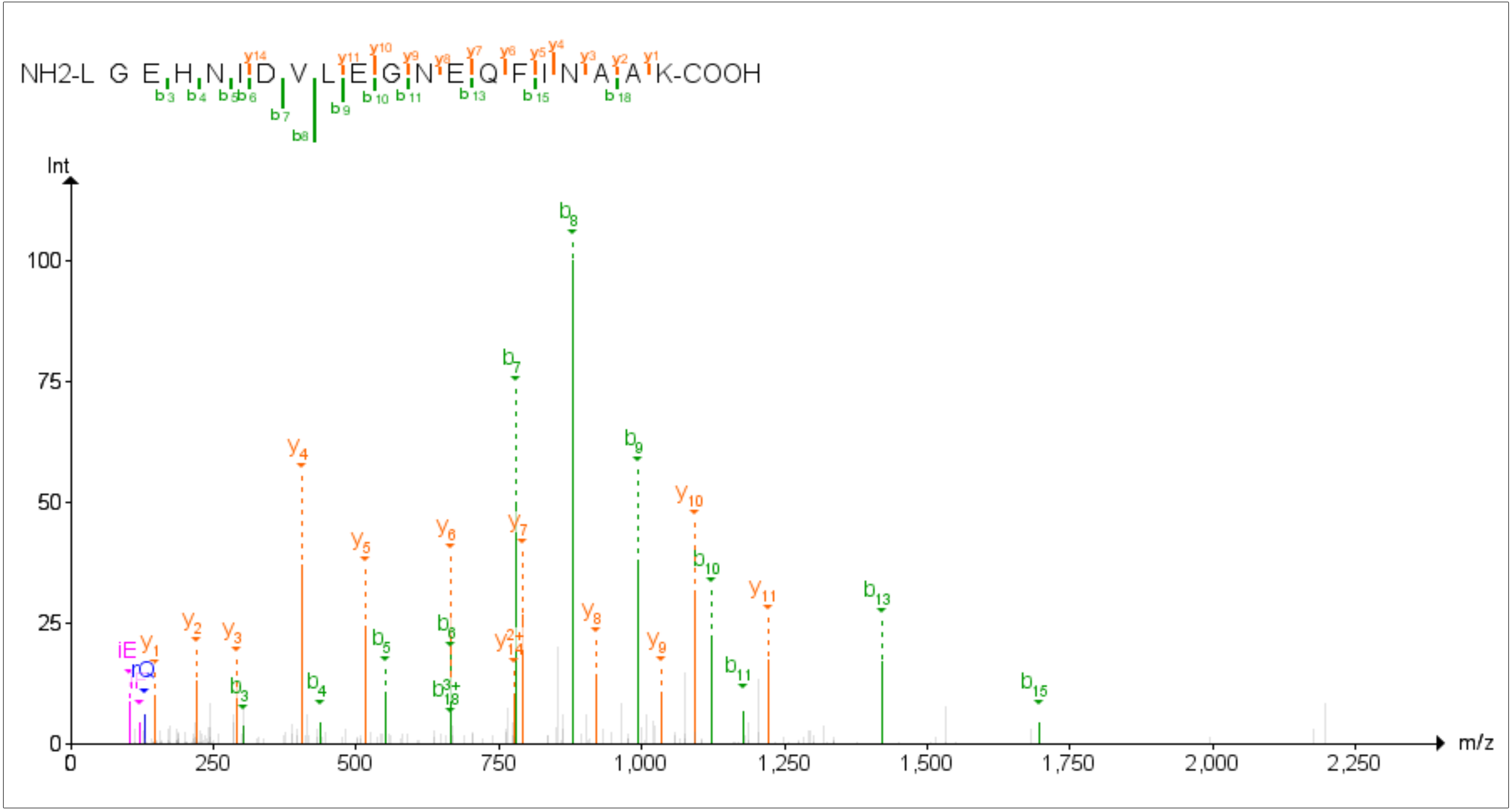

NH2-L G E H N I D V L E G N E Q F I N A A K-COOH
Int
m/z
0
25
50
75
100
250
500
750
1,000
1,250
1,500
1,750
2,000
2,250

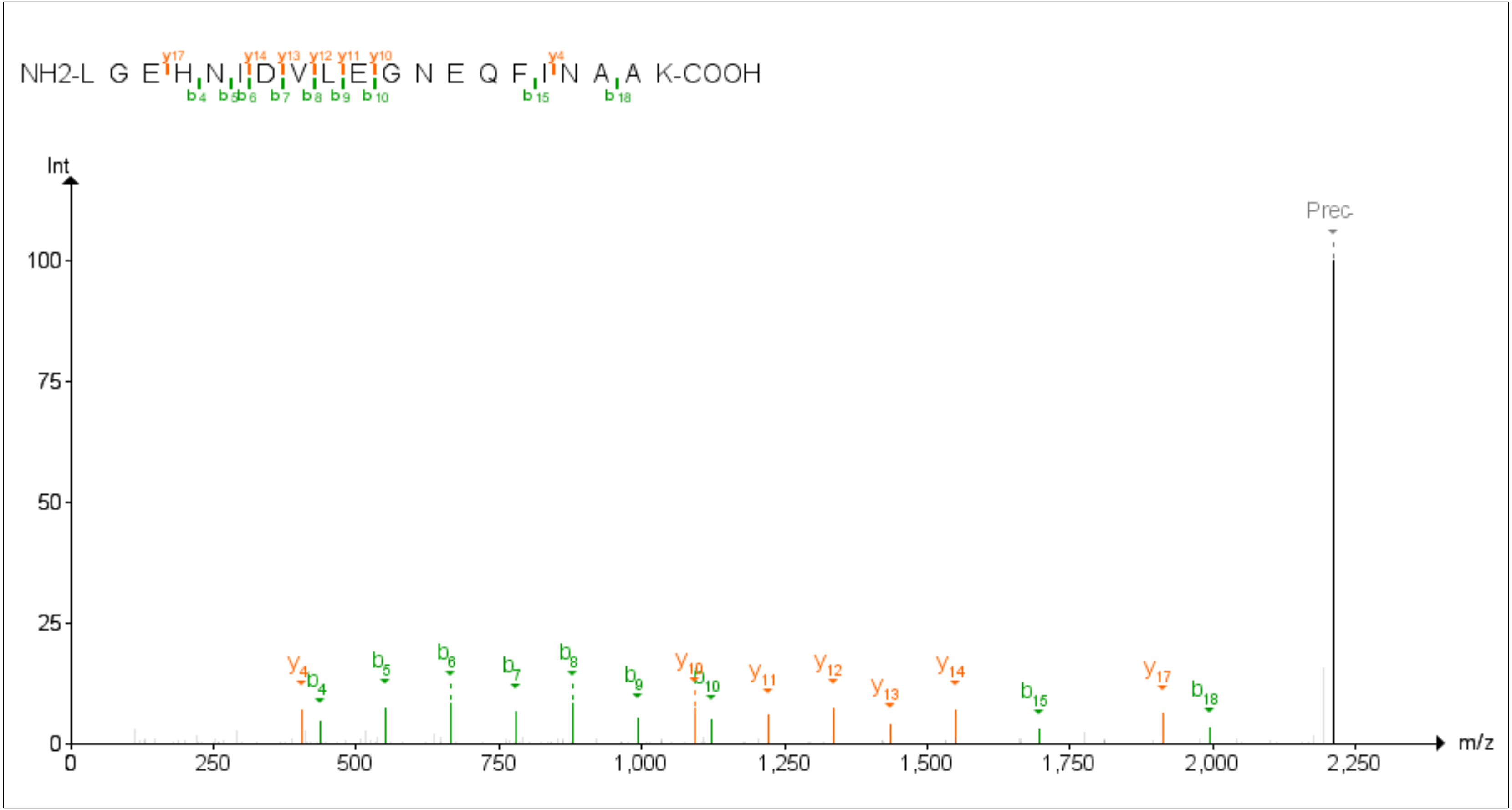

NH2-L G E H N I D V L E G N E Q F I N A A K-COOH
y17 y14 y13 y12 y11 y10 y4
b4 b5 b6 b7 b8 b9 b10 b15 b18
Int
100
75
50
25
0
Prec
m/z
0
250
500
750
1,000
1,250
1,500
1,750
2,000
2,250

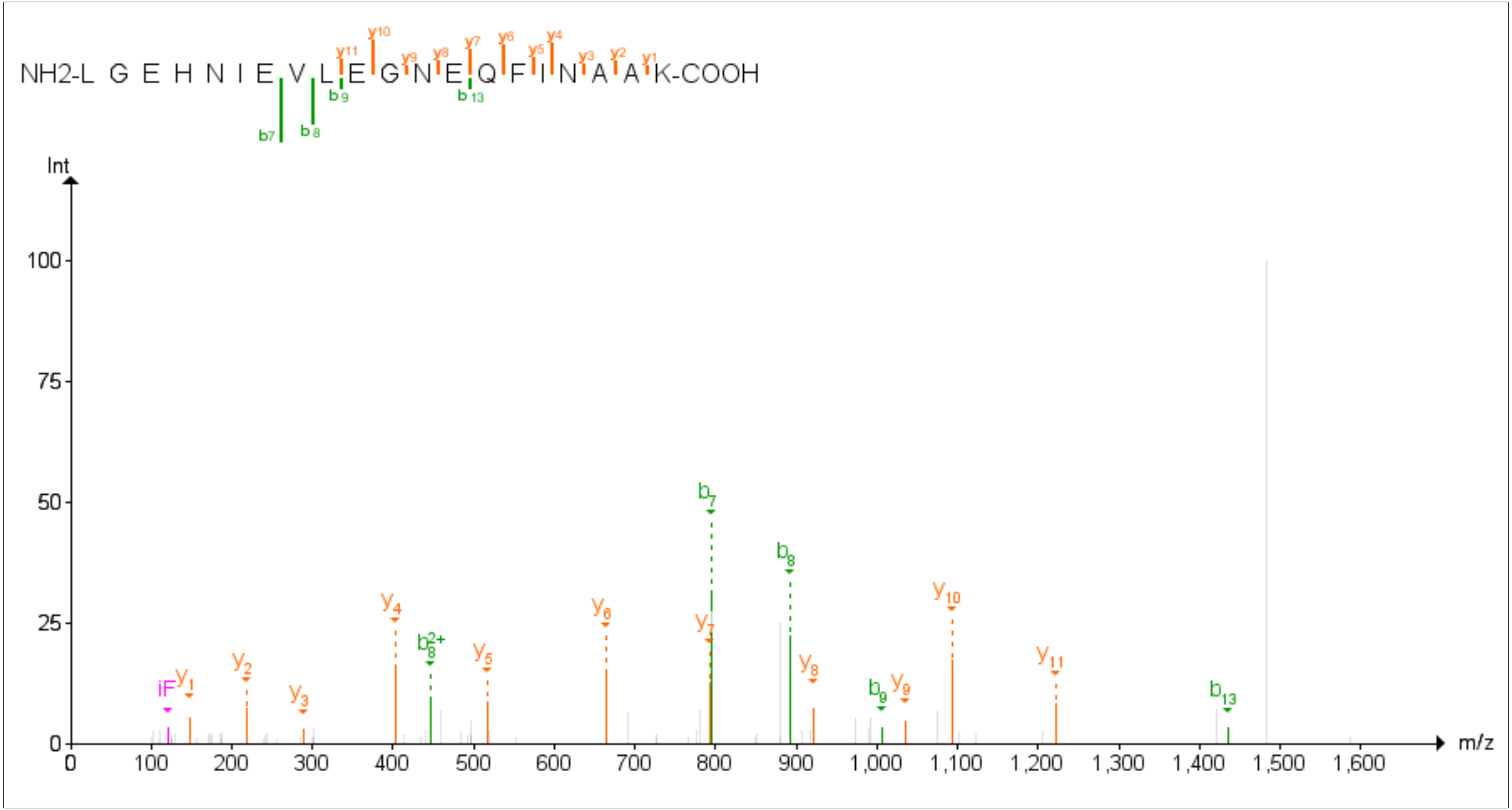

NH2-L G E H N I E V L E G N E Q F I N A A K-COOH
y10 y11 y9 y8 y7 y6 y5 y4 y3 y2 y1
b7 b8 b9 b13
Int
100
75
50
25
0
0 100 200 300 400 500 600 700 800 900 1,000 1,100 1,200 1,300 1,400 1,500 1,600
m/z
iF
y1 y2 y3 y4 y5 y6 y7 y8 y9 y10 y11
b8 2+
b7 b8 b9 b13

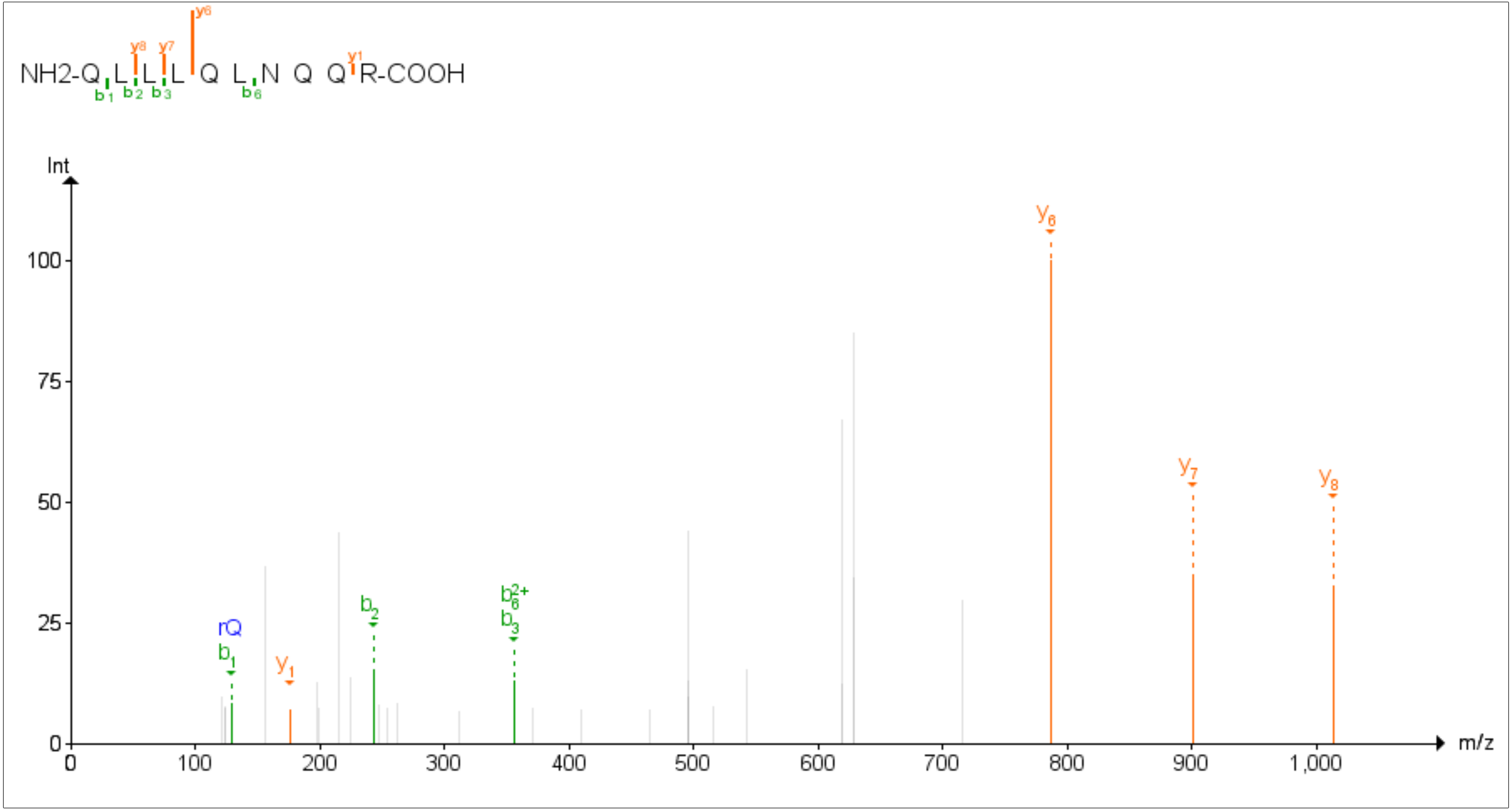

NH2-Q L L L Q L N Q Q R-COOH
y8 y7 y6 y1
b1 b2 b3 b6
Int
100
75
50
25
0
rQ
b1
y1
b2
b6 2+
b3
y6
y7
y8
0 100 200 300 400 500 600 700 800 900 1,000
m/z

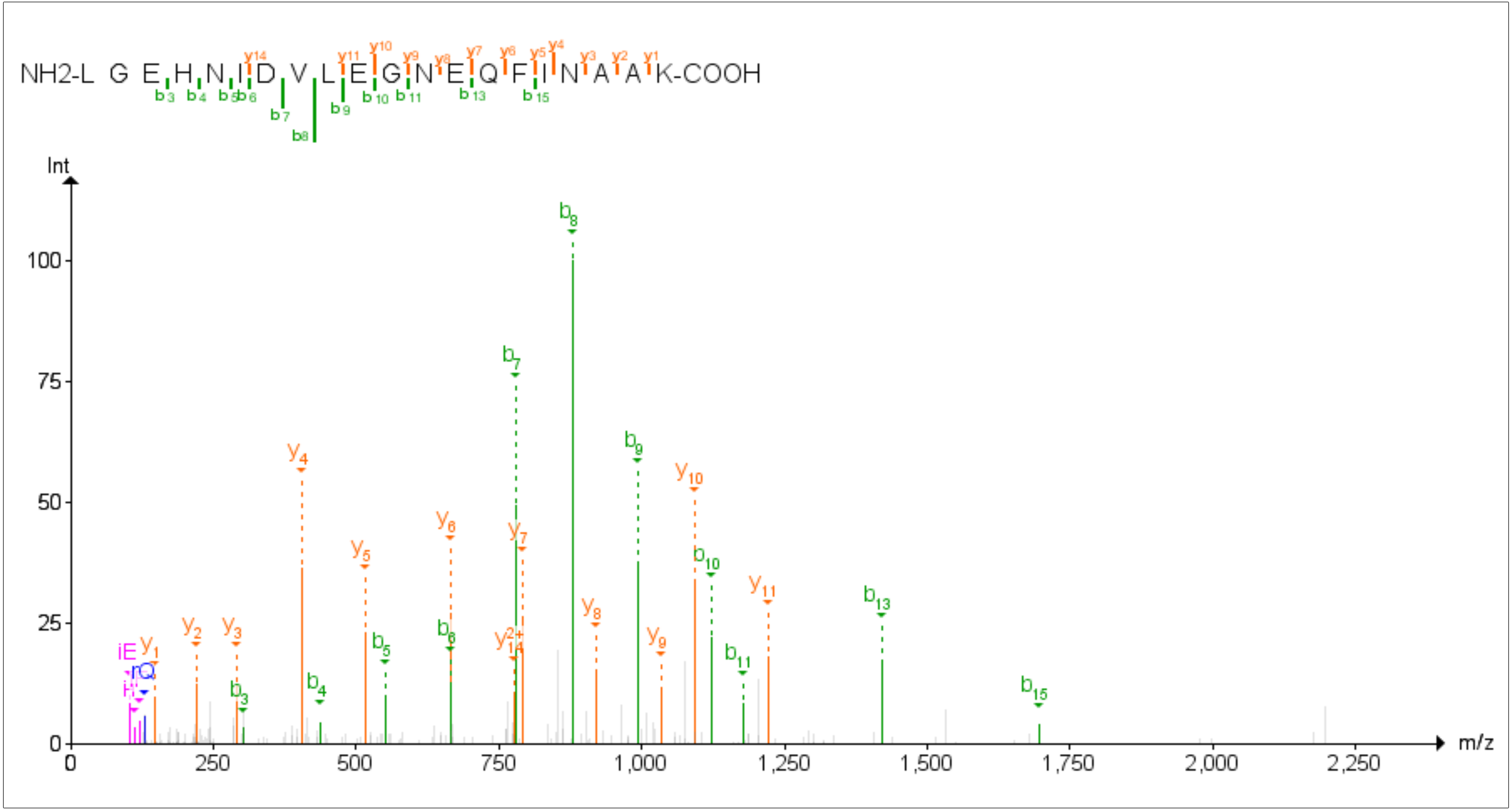
NH2-L G E H N I D V L E G N E Q F I N A A K-COOH
Int
m/z
0
25
50
75
100
250
500
750
1,000
1,250
1,500
1,750
2,000
2,250

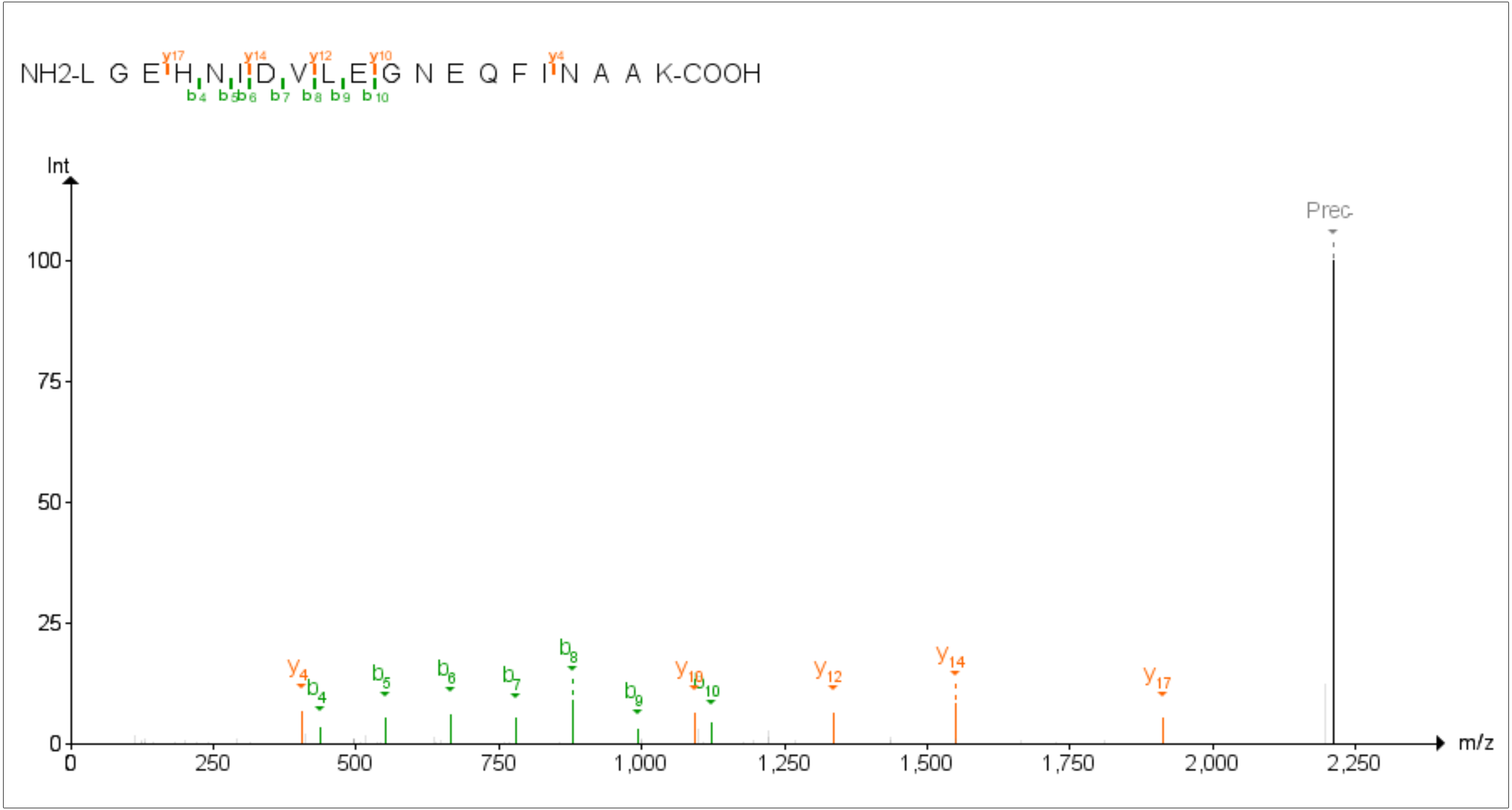
NH2-L G E H N I D V L E G N E Q F I N A A K-COOH
y17 y14 y12 y10 y4
b4 b5 b6 b7 b8 b9 b10
Int
Prec
100
75
50
25
0
0 250 500 750 1,000 1,250 1,500 1,750 2,000 2,250
m/z

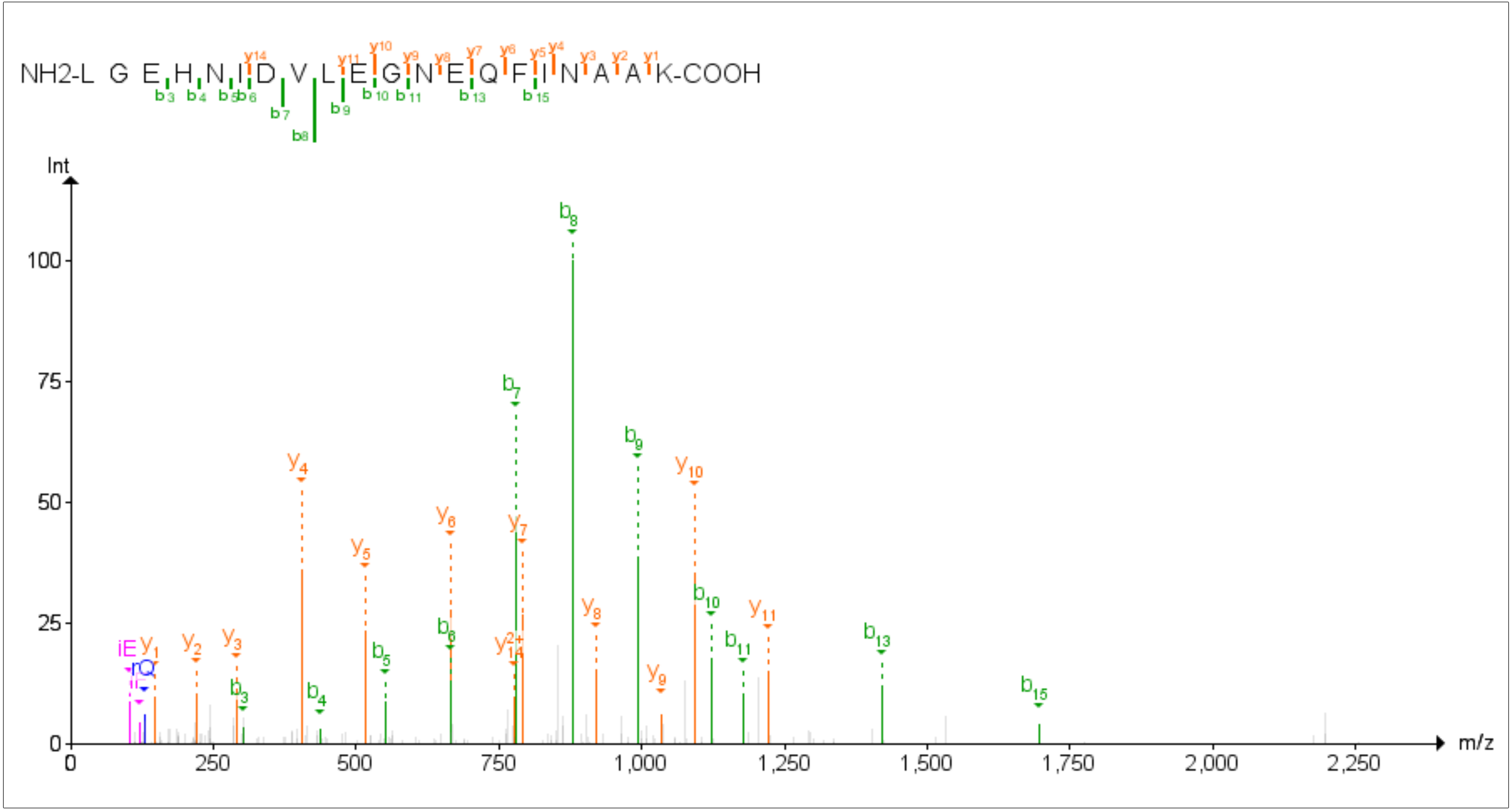

NH2-L G E H N I D V L E G N E Q F I N A A K-COOH
Int
m/z
0
25
50
75
100
250
500
750
1,000
1,250
1,500
1,750
2,000
2,250

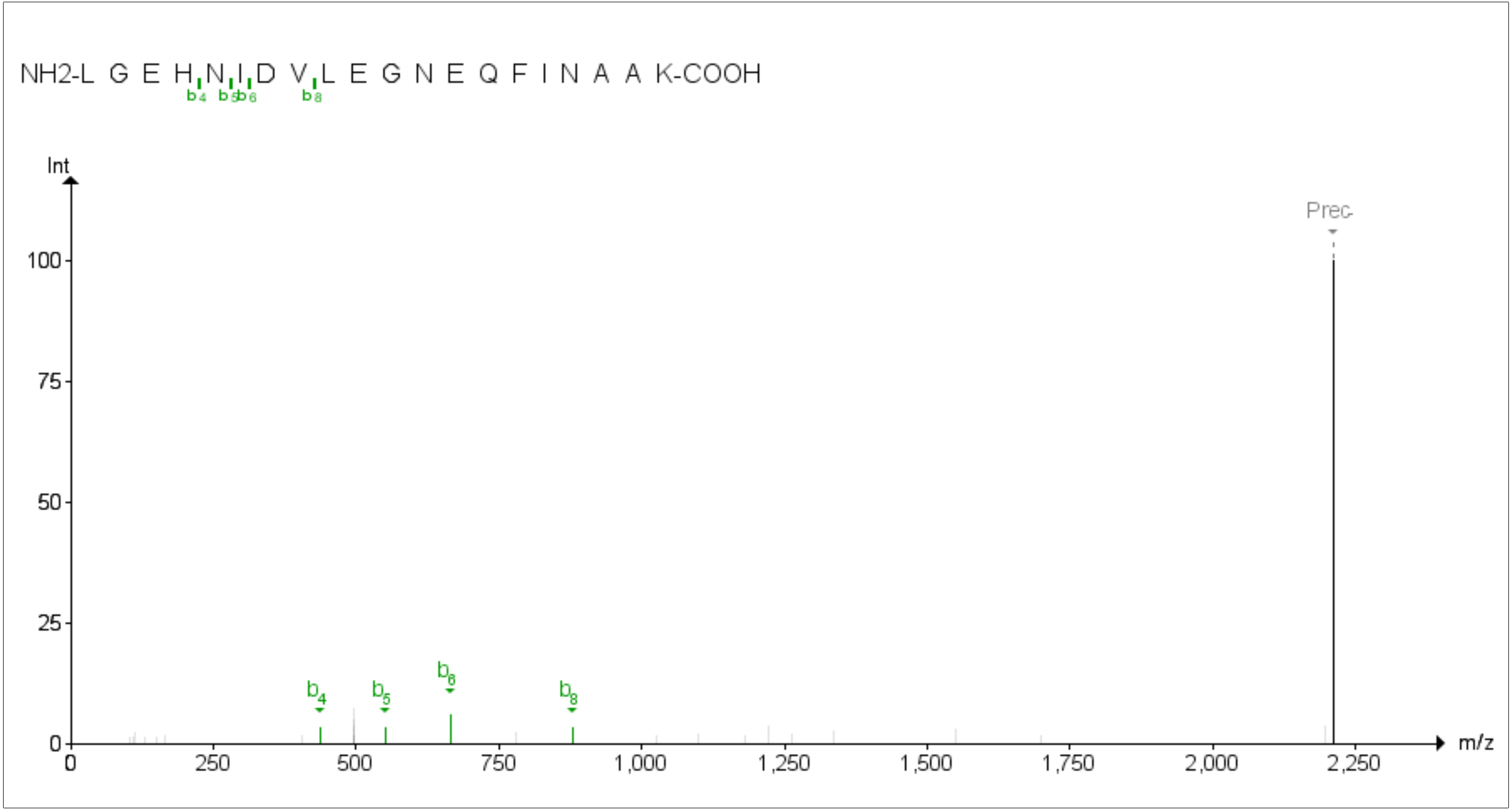

NH2-L G E H N I D V L E G N E Q F I N A A K-COOH
b4 b5 b6 b8
Int
100
75
50
25
0
Prec
b4
b5
b6
b8
0
250
500
750
1,000
1,250
1,500
1,750
2,000
2,250
m/z

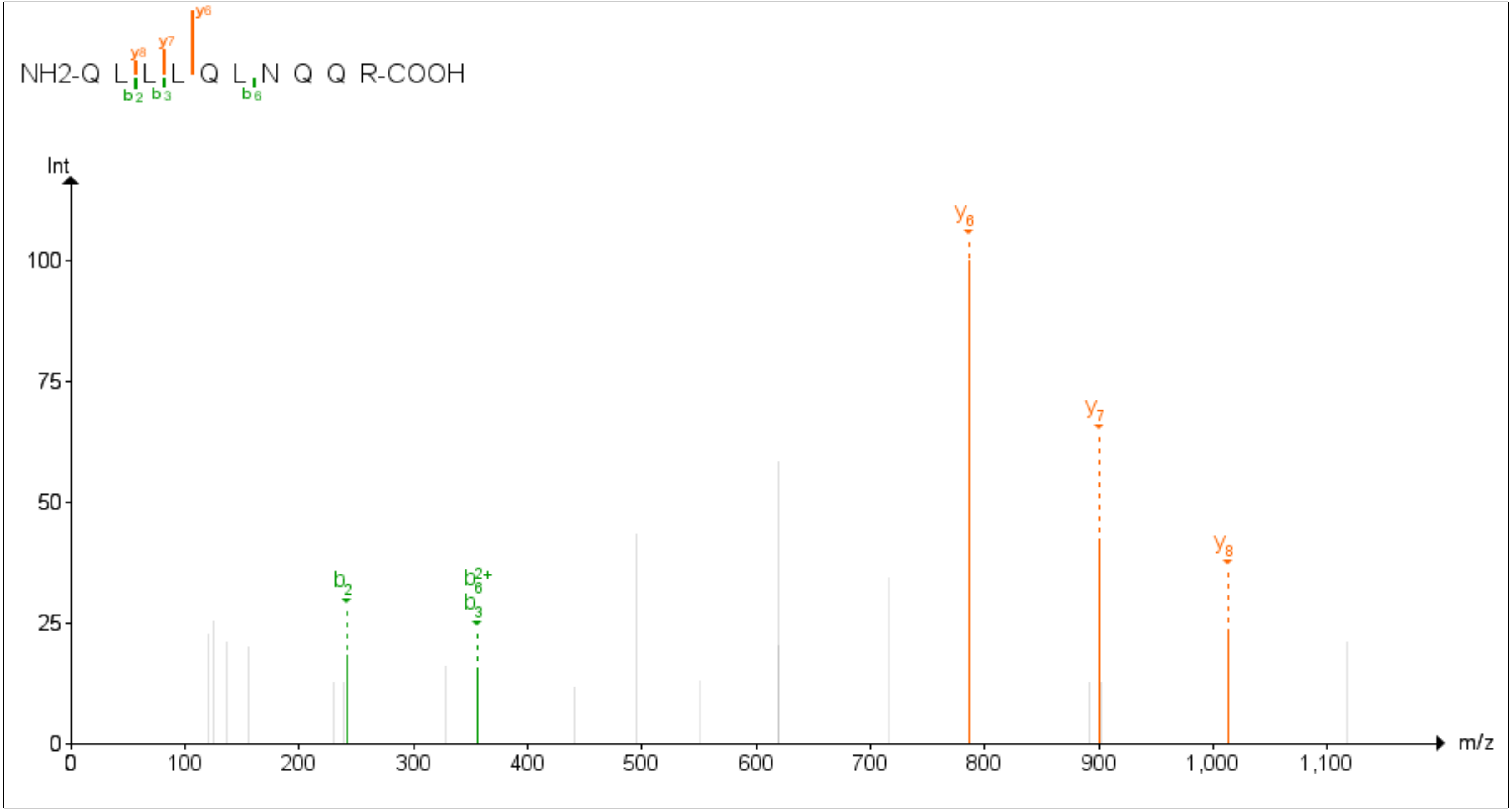
NH2-Q L L L Q L N Q Q R-COOH
y8
y7
y6
b2
b3
b6
Int
100
75
50
25
0
y6
y7
y8
b2
b6 2+
b3
0
100
200
300
400
500
600
700
800
900
1,000
1,100
m/z

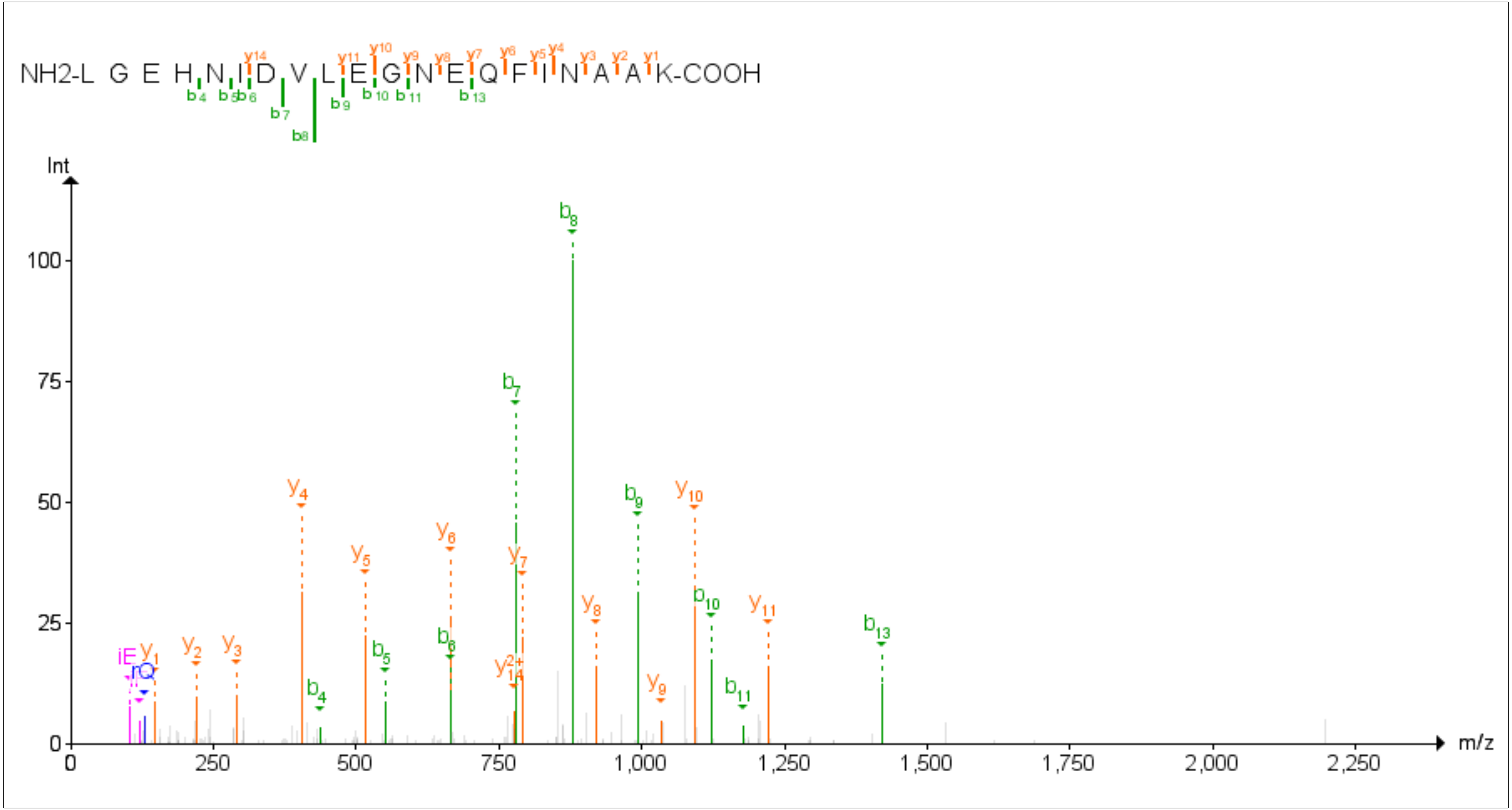

NH2-L G E H N I D V L E G N E Q F I N A A K-COOH
b4 b5 b6 b7 b8 b9 b10 b11 b13
y14 y11 y10 y9 y8 y7 y6 y5 y4 y3 y2 y1
Int
100
75
50
25
0
m/z
0
250
500
750
1,000
1,250
1,500
1,750
2,000
2,250
iE
rQ

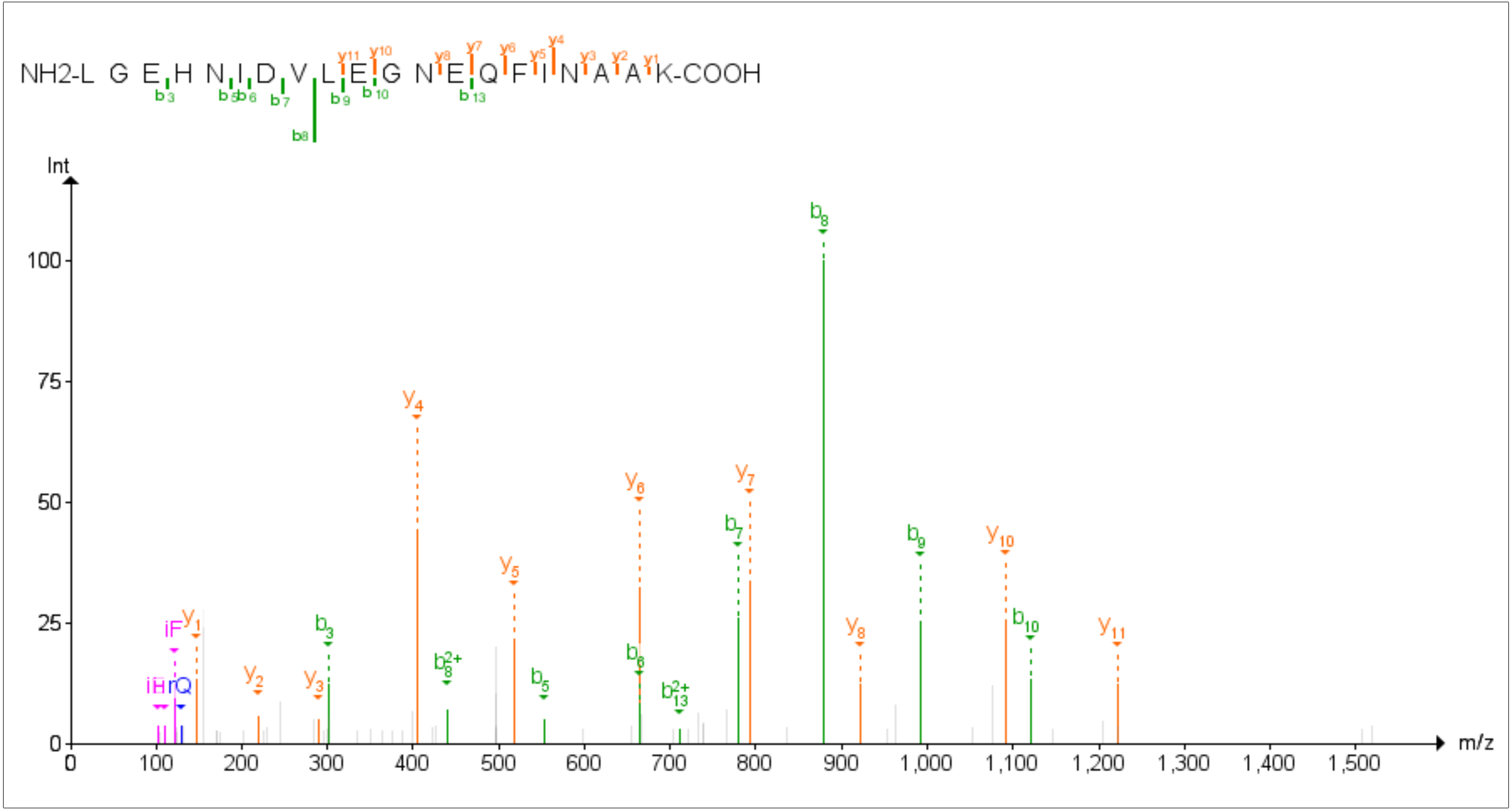
NH2-L G E H N I D V L I E G N E Q F I N A A K-COOH
b3 b5 b6 b7 b8 b9 b10 b13
y11 y10 y8 y7 y6 y5 y4 y3 y2 y1
Int
100
75
50
25
0
m/z
0 100 200 300 400 500 600 700 800 900 1,000 1,100 1,200 1,300 1,400 1,500
iF
iH
rQ
y1 y2 y3 y4 y5 y6 y7 y8 y10 y11
b3 b5 b6 b7 b8 b9 b10
b8 2+
b13 2+

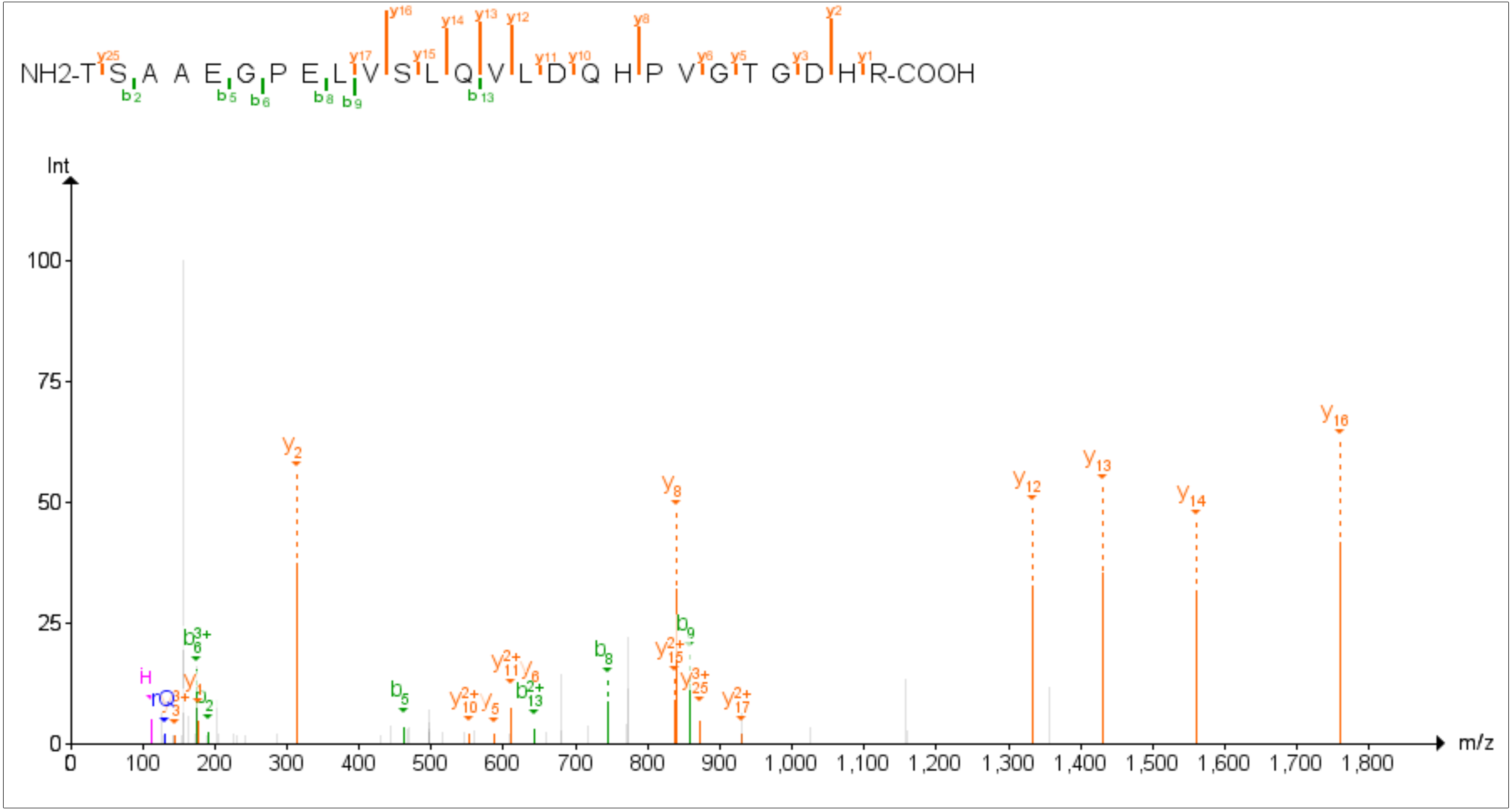

NH2-T S A A E G P E L V S L Q V L D Q H P V G T G D H R-COOH
Int
m/z
y2
y8
y12
y13
y14
y16
b5
b8
b9

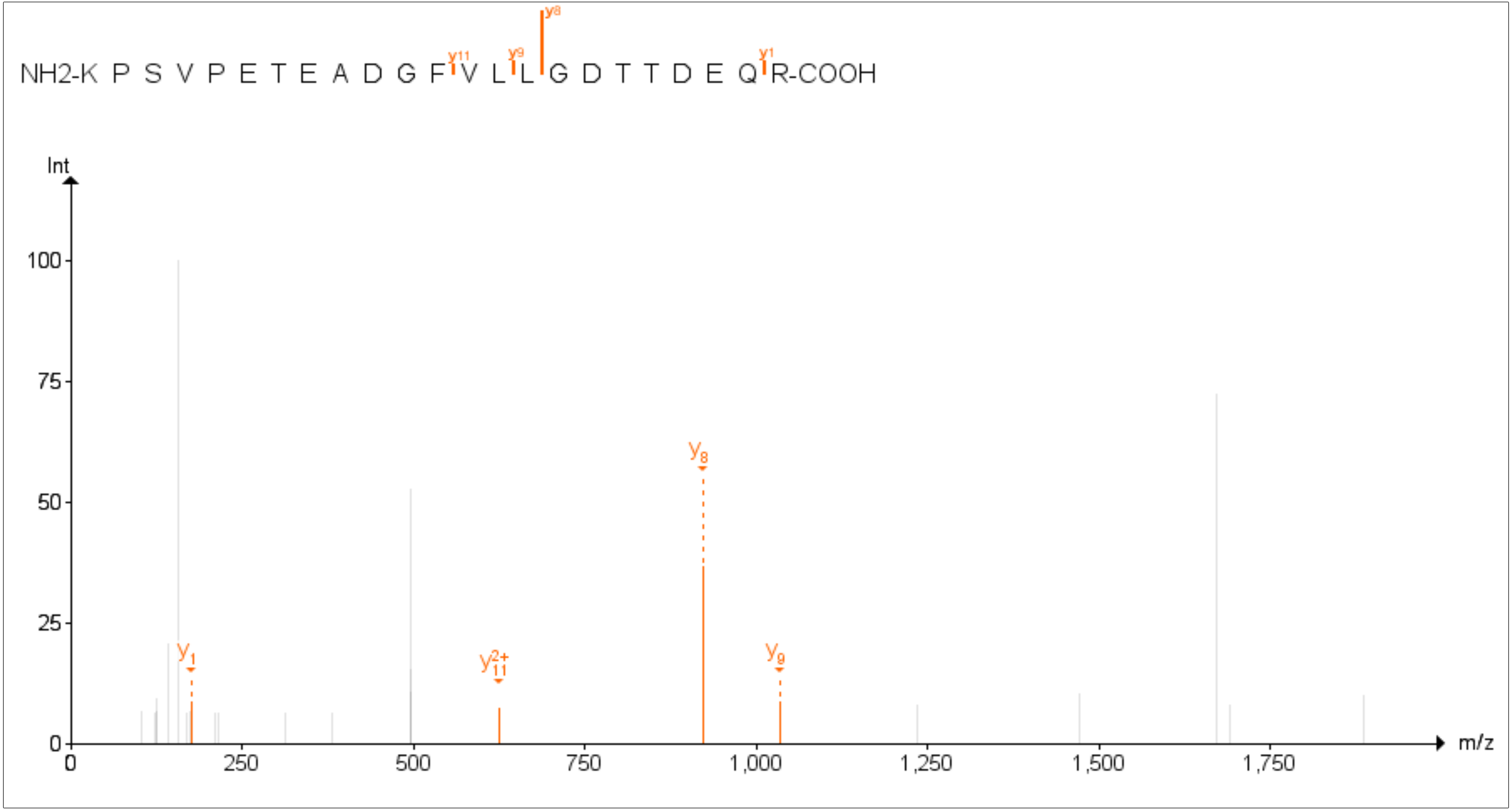

NH2-K P S V P E T E A D G F V L L G D T T D E Q R-COOH
y11
y9
y8
y1
Int
100
75
50
25
0
y8
y1
y11 2+
y9
0
250
500
750
1,000
1,250
1,500
1,750
m/z

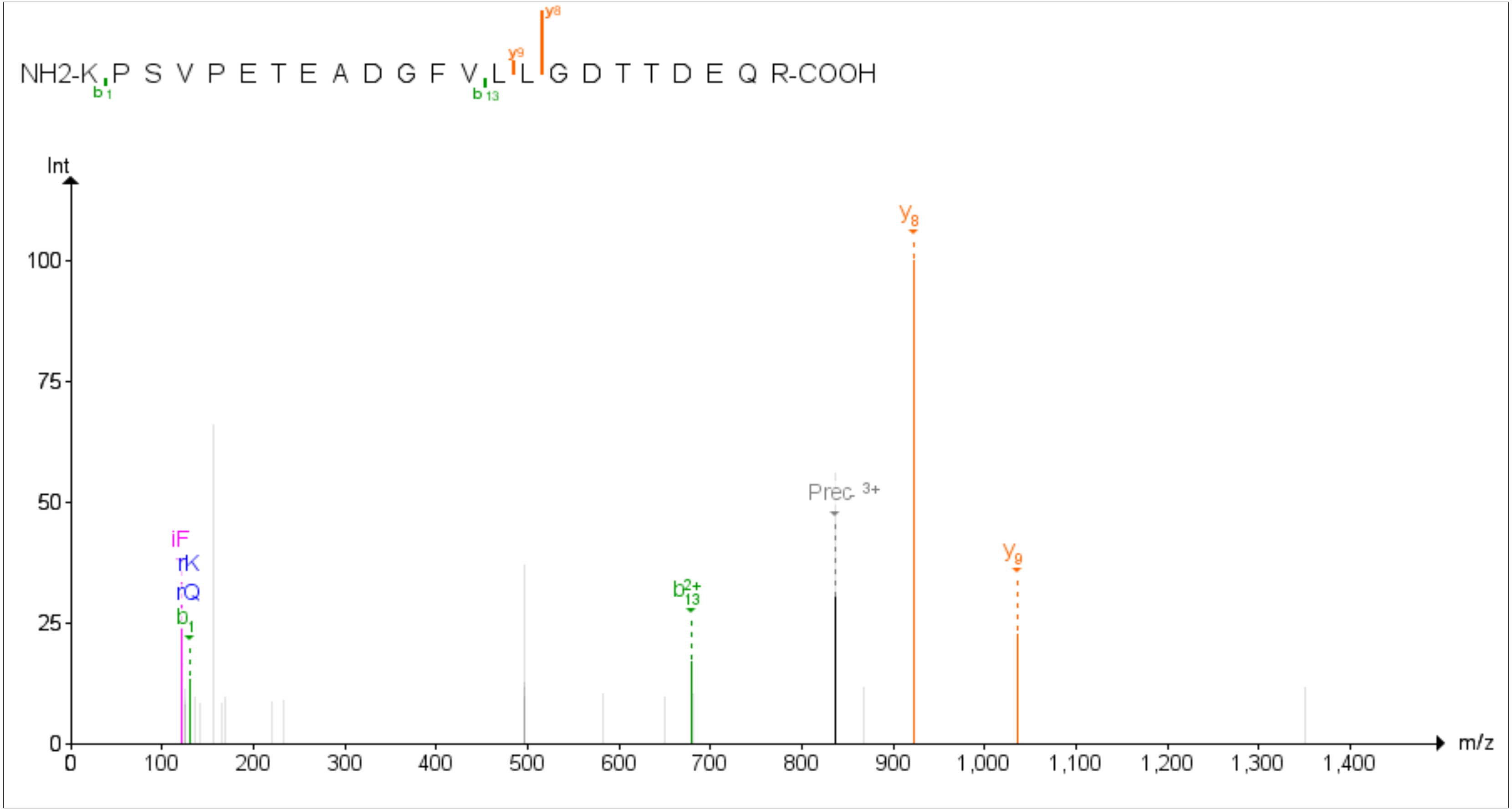

NH2-K P S V P E T E A D G F V L L G D T T D E Q R-COOH
b1
b13
y9
y8
Int
100
75
50
25
0
iF
rK
rQ
b1
b_{13}^{2+}
Prec. 3+
y8
y9
0
100
200
300
400
500
600
700
800
900
1,000
1,100
1,200
1,300
1,400
m/z

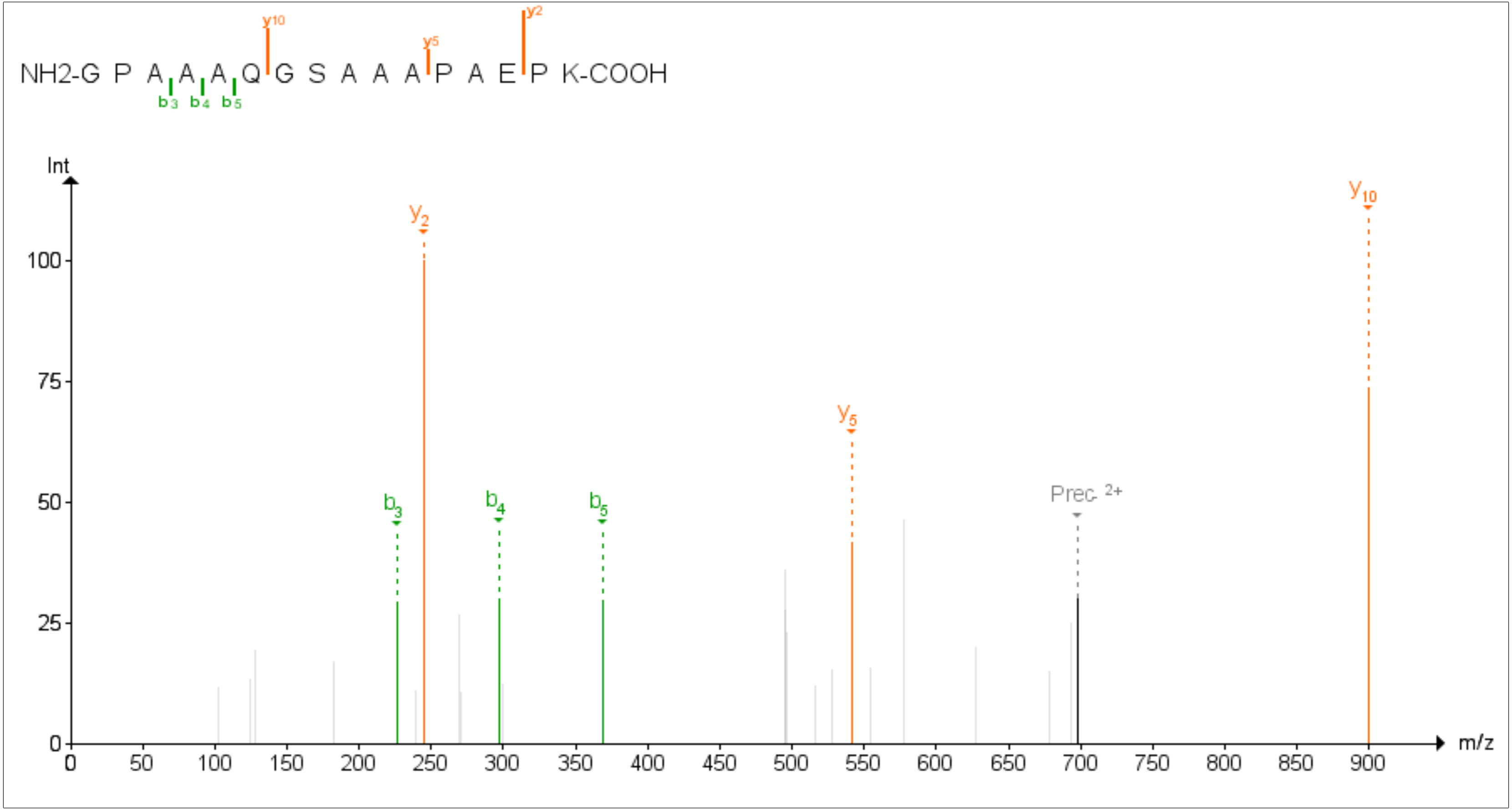

NH2-G P A A A Q G S A A A P A E P K-COOH
y10
y5
y2
b3
b4
b5
Int
100
75
50
25
0
y2
y10
y5
b3
b4
b5
Prec 2+
m/z
0 50 100 150 200 250 300 350 400 450 500 550 600 650 700 750 800 850 900

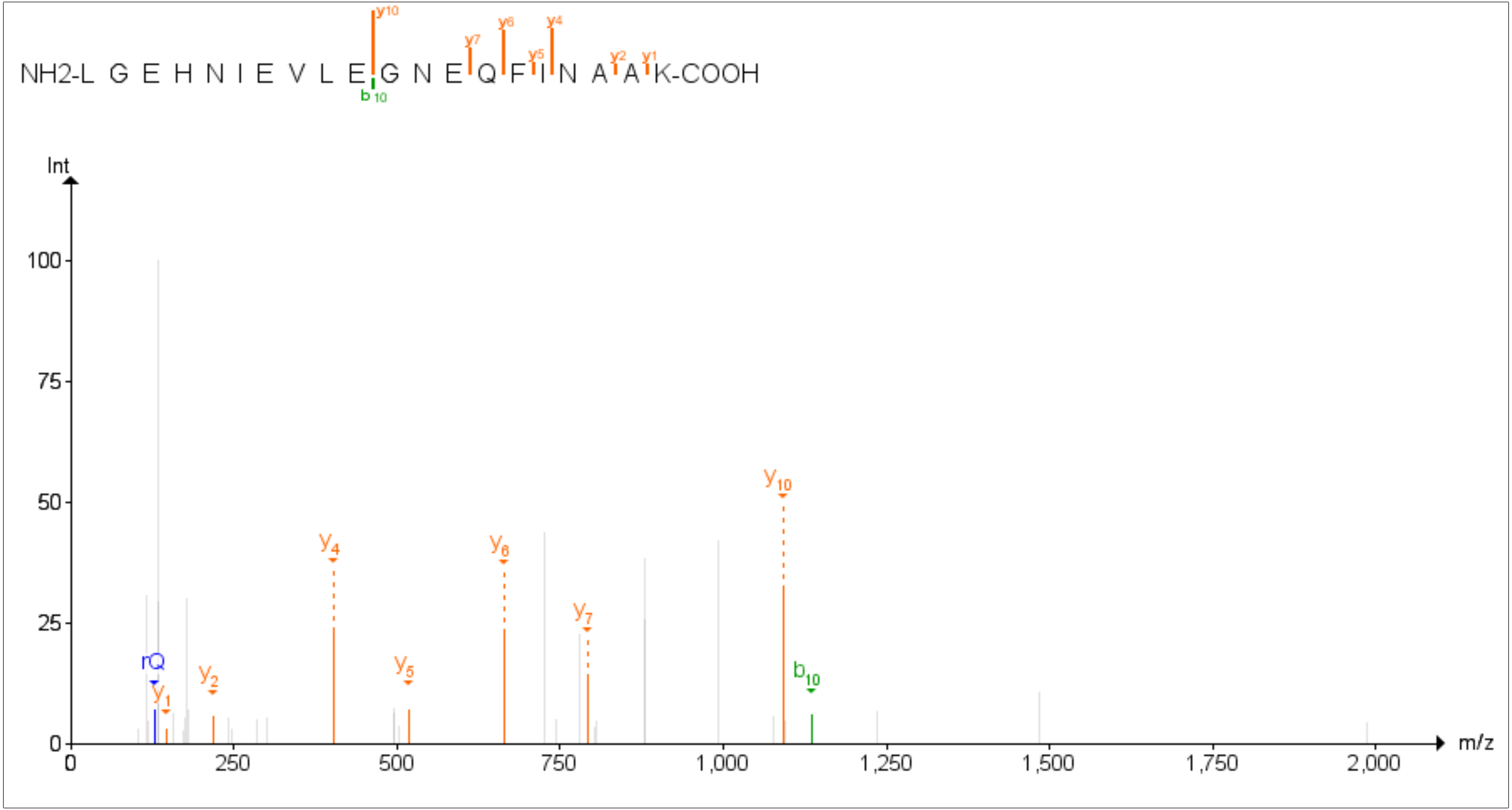

NH2-L G E H N I E V L E G N E Q F I N A A K-COOH
y10
y7
y6
y5
y4
y2
y1
b10
Int
100
75
50
25
0
rQ
y1
y2
y4
y5
y6
y7
y10
b10
0
250
500
750
1,000
1,250
1,500
1,750
2,000
m/z

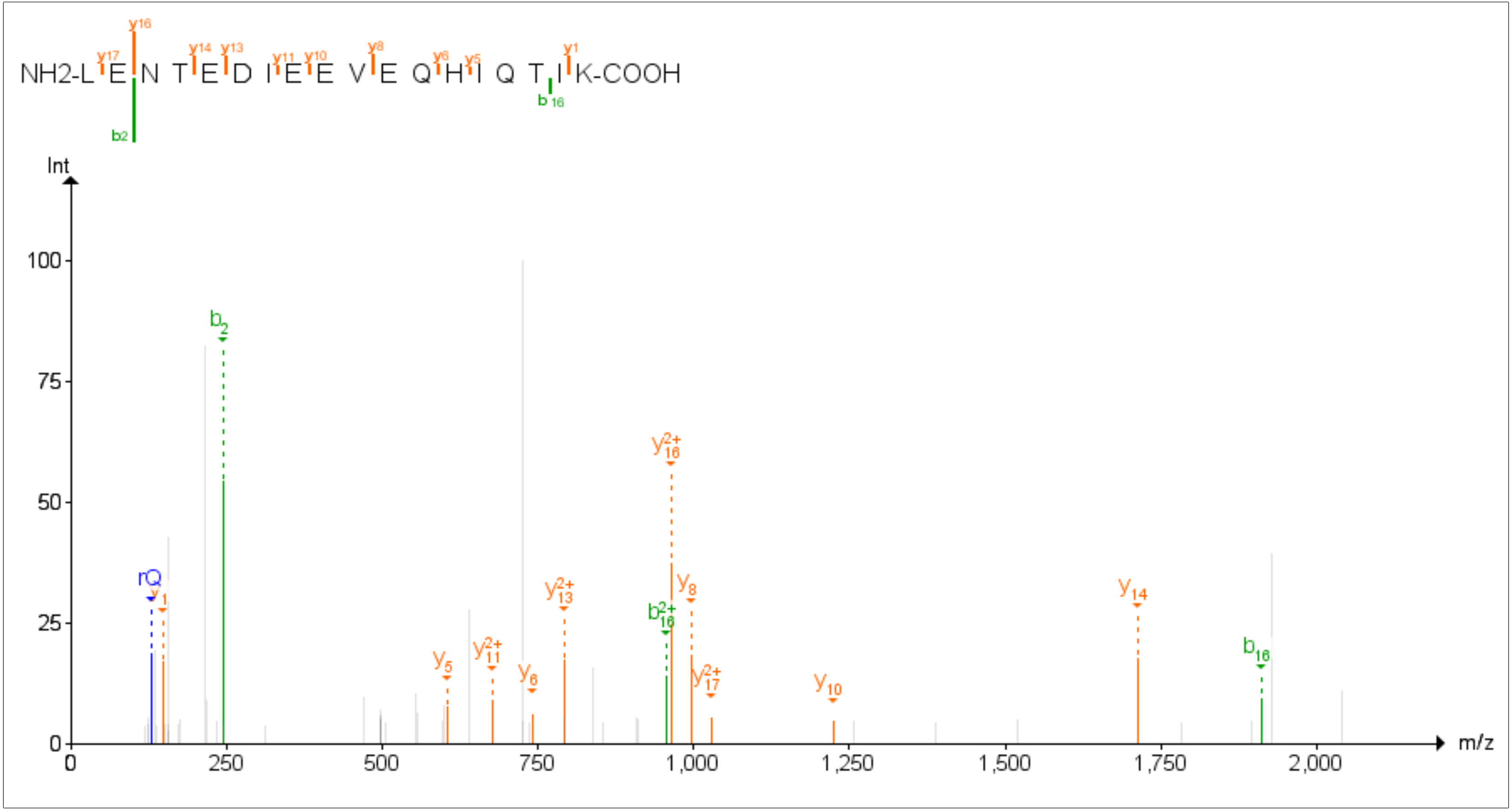

NH2-L E N T E D I E E V E Q H I Q T I K-COOH
y17
y16
y14
y13
y11
y10
y8
y6
y5
y1
b2
b 16
Int
m/z
rQ
y1
b2
y5
y11 2+
y6
y13 2+
b16 2+
y16 2+
y8
y17 2+
y10
y14
b16
0
25
50
75
100
250
500
750
1,000
1,250
1,500
1,750
2,000

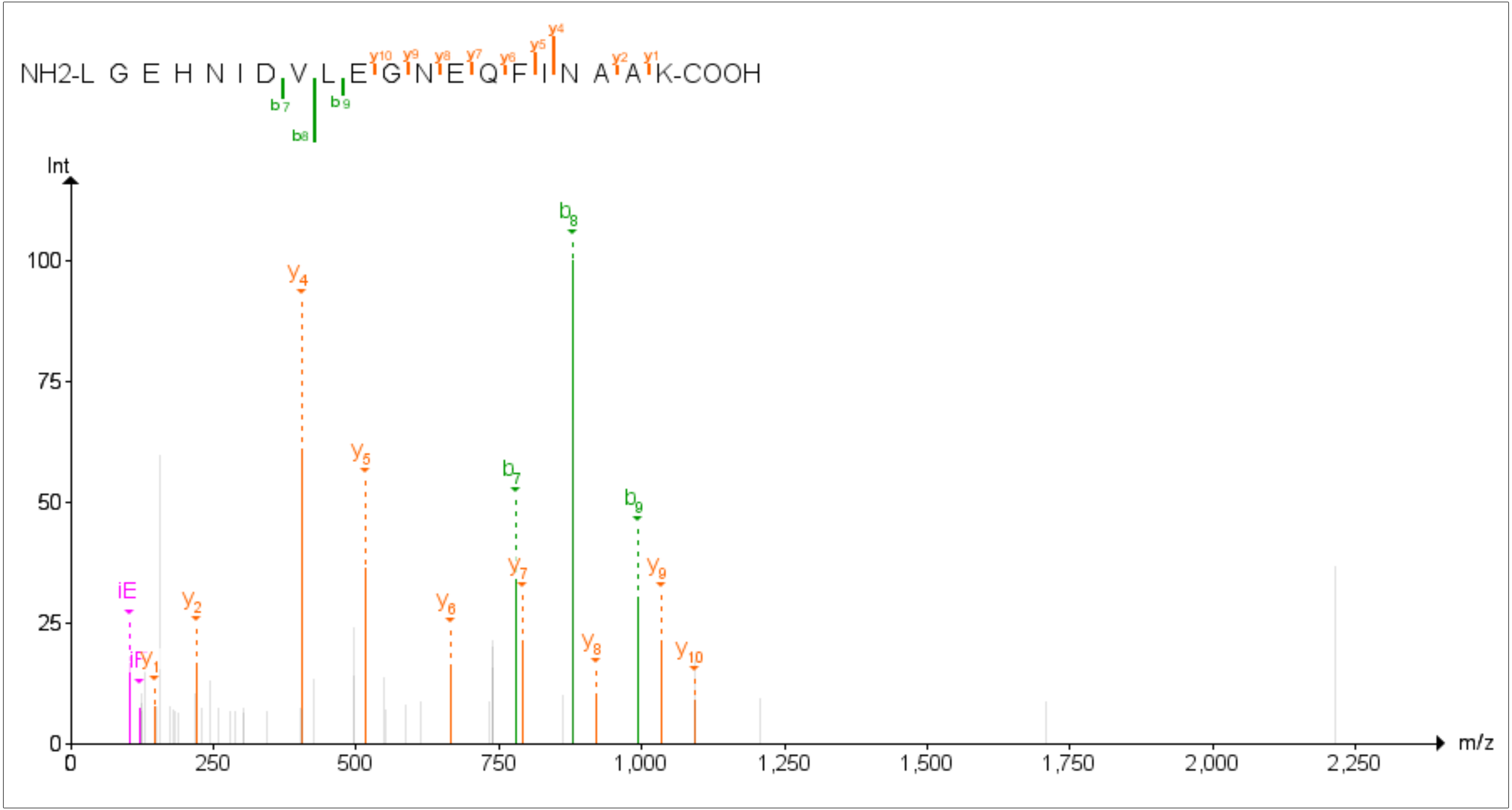

NH2-L G E H N I D V L E G N E Q F I N A A K-COOH
b7
b8
b9
y10
y9
y8
y7
y6
y5
y4
y2
y1
Int
m/z
iE
iF

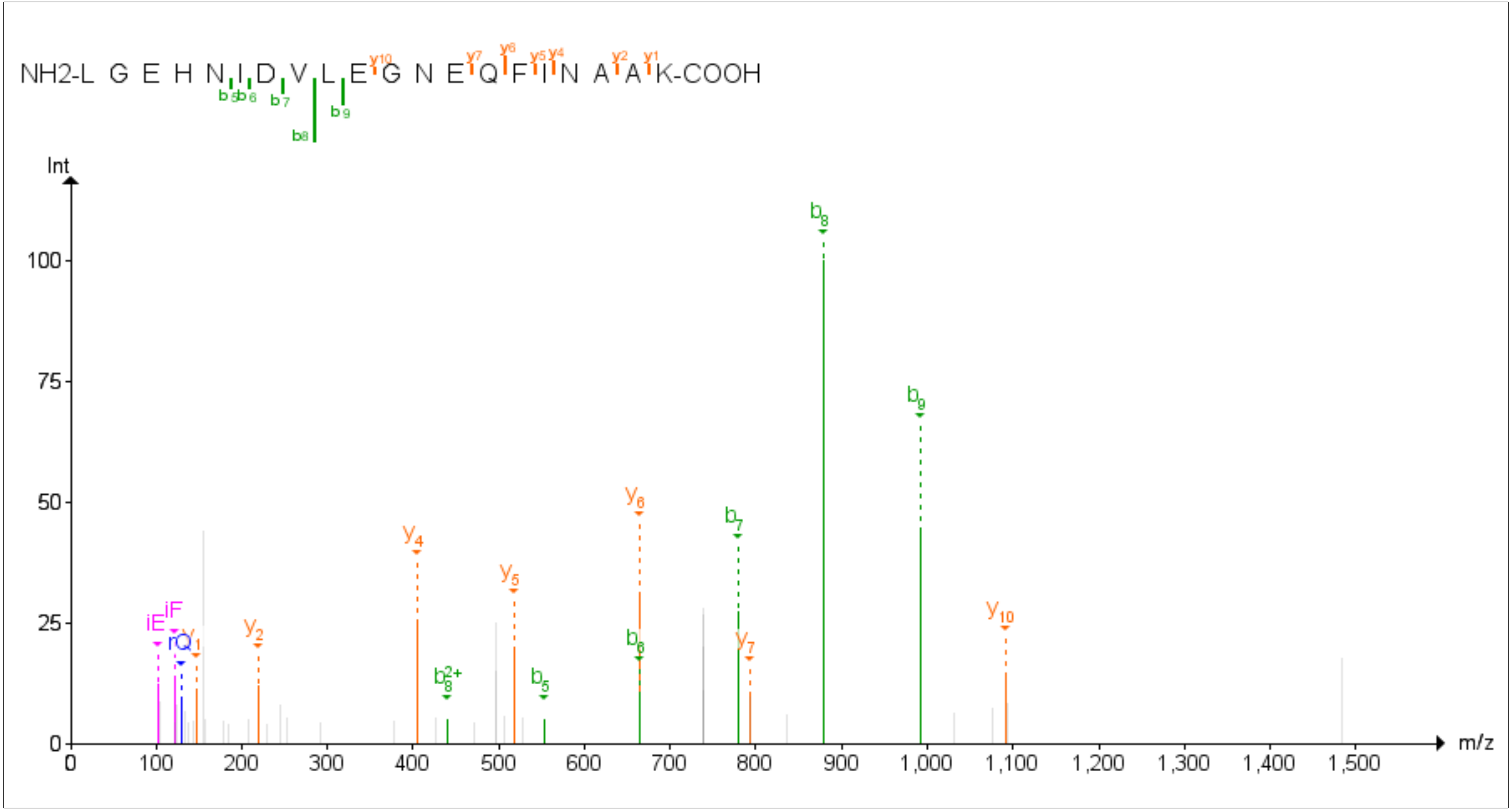

NH2-L G E H N I D V L E G N E Q F I N A A K-COOH
b5 b6 b7 b8 b9
y10 y7 y6 y5 y4 y2 y1
Int
m/z
100
75
50
25
0
0 100 200 300 400 500 600 700 800 900 1,000 1,100 1,200 1,300 1,400 1,500
iE iF rQ y1 y2 y4 b8 2+ y5 b5 y6 b6 b7 y7 b8 b9 y10

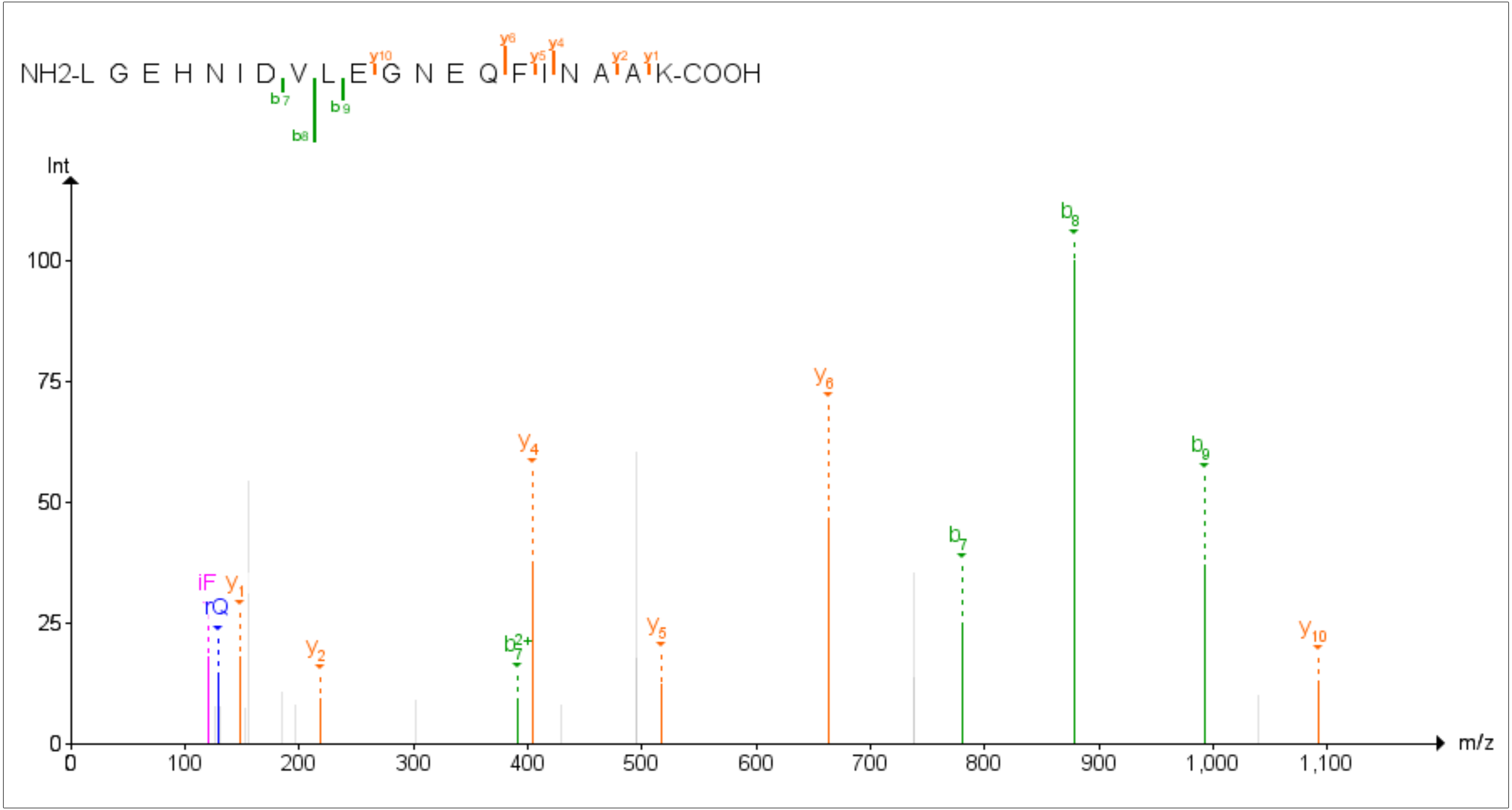

NH2-L G E H N I D V L E G N E Q F I N A A K-COOH
y10
y6
y5
y4
y2
y1
b7
b8
b9
Int
100
75
50
25
0
iF
y1
rQ
y2
b7 2+
y4
y5
y6
b7
b8
b9
y10
0
100
200
300
400
500
600
700
800
900
1,000
1,100
m/z

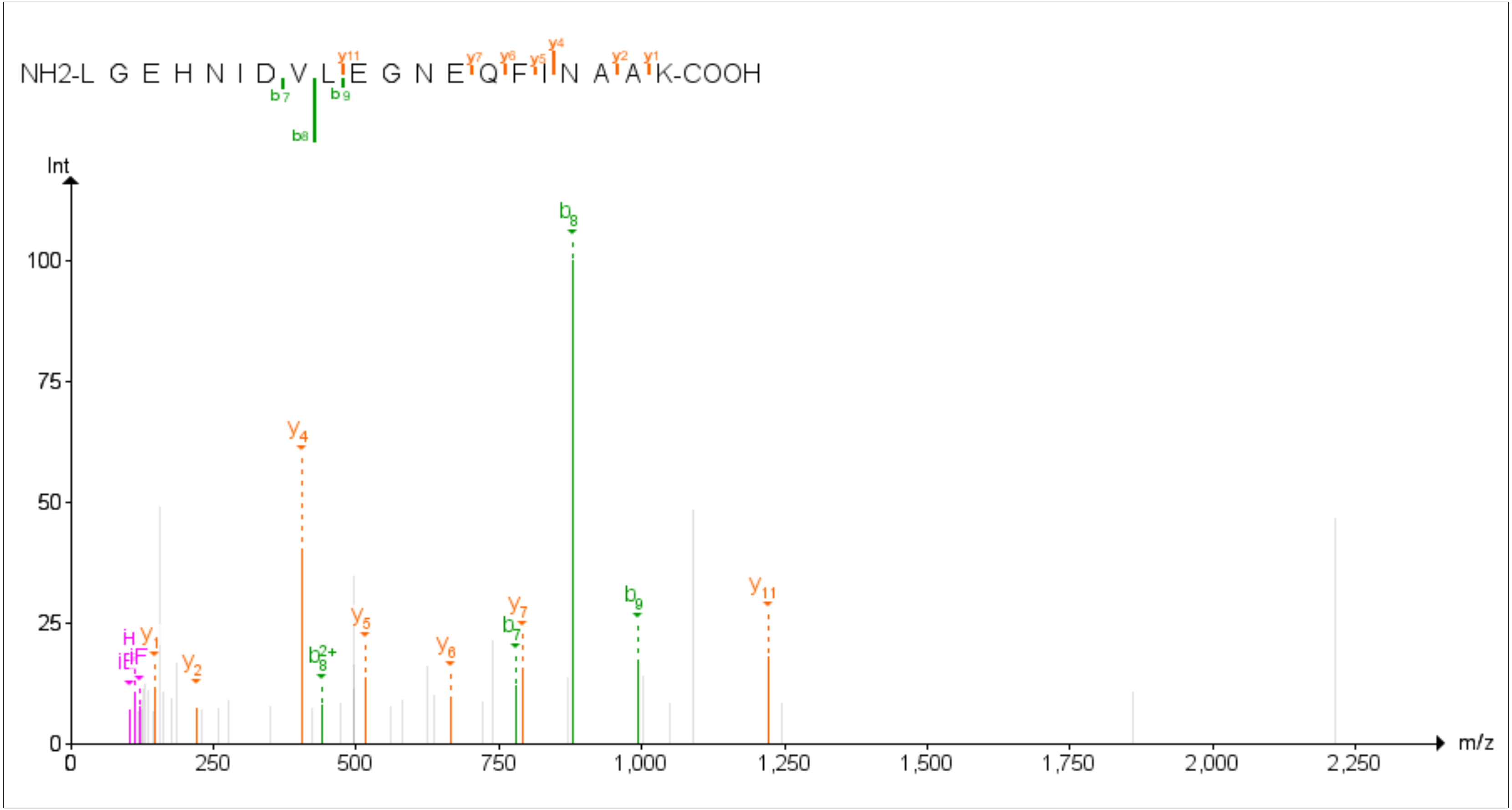

NH2-L G E H N I D V L E G N E Q F I N A A K-COOH
b7
b8
b9
y11
y7
y6
y5
y4
y2
y1
Int
100
75
50
25
0
b8
y4
y5
y6
y7
b7
b9
y11
y1
y2
iH
iE
iF
0
250
500
750
1,000
1,250
1,500
1,750
2,000
2,250
m/z

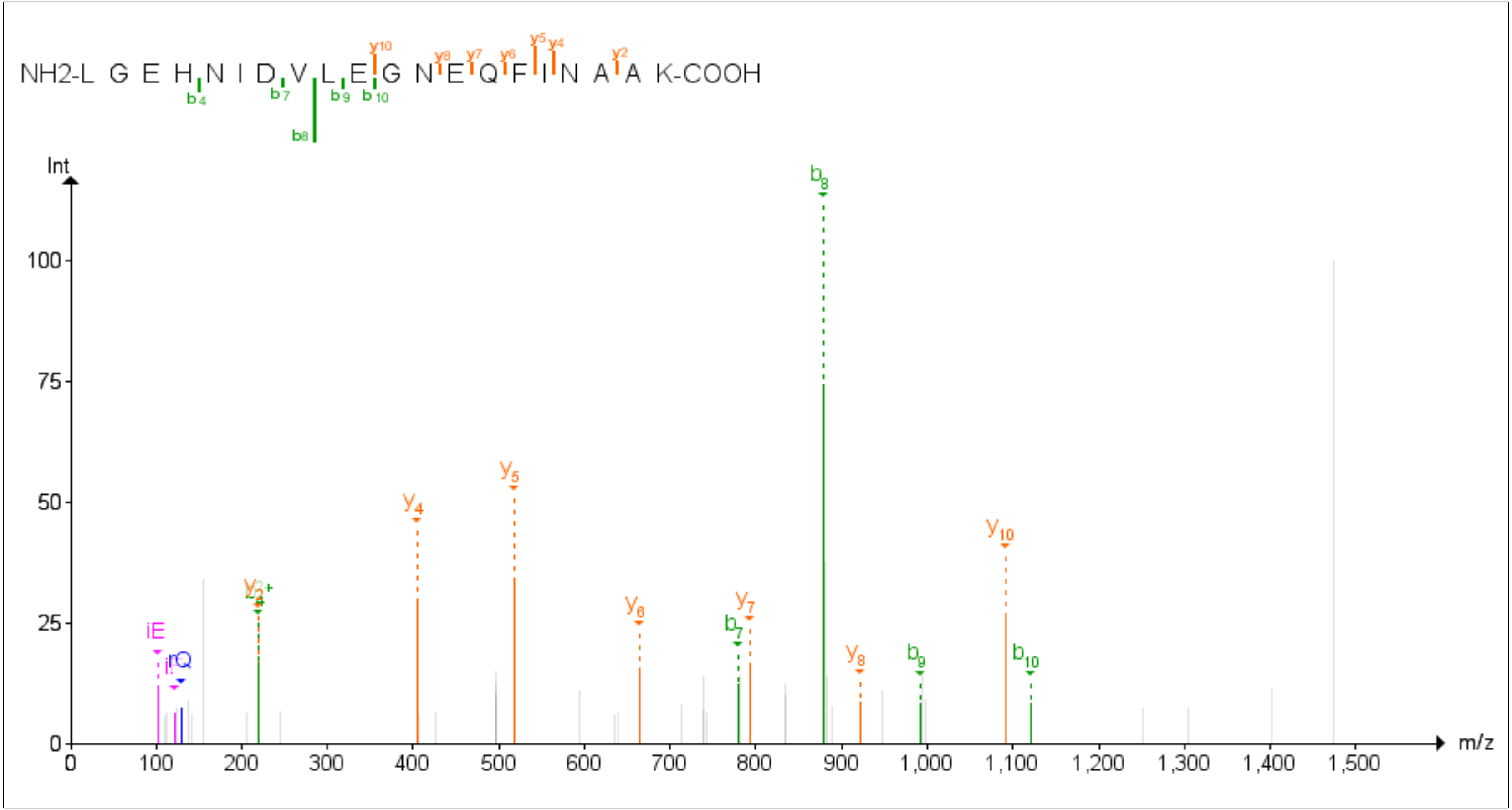

NH2-L G E H N I D V L E G N E Q F I N A A K-COOH
b4
b7
b8
b9
b10
y10
y8
y7
y6
y5
y4
y2
Int
100
75
50
25
0
b8
y5
y4
y10
y6
y7
b7
y8
b9
b10
iE
iQ
m/z
0
100
200
300
400
500
600
700
800
900
1,000
1,100
1,200
1,300
1,400
1,500

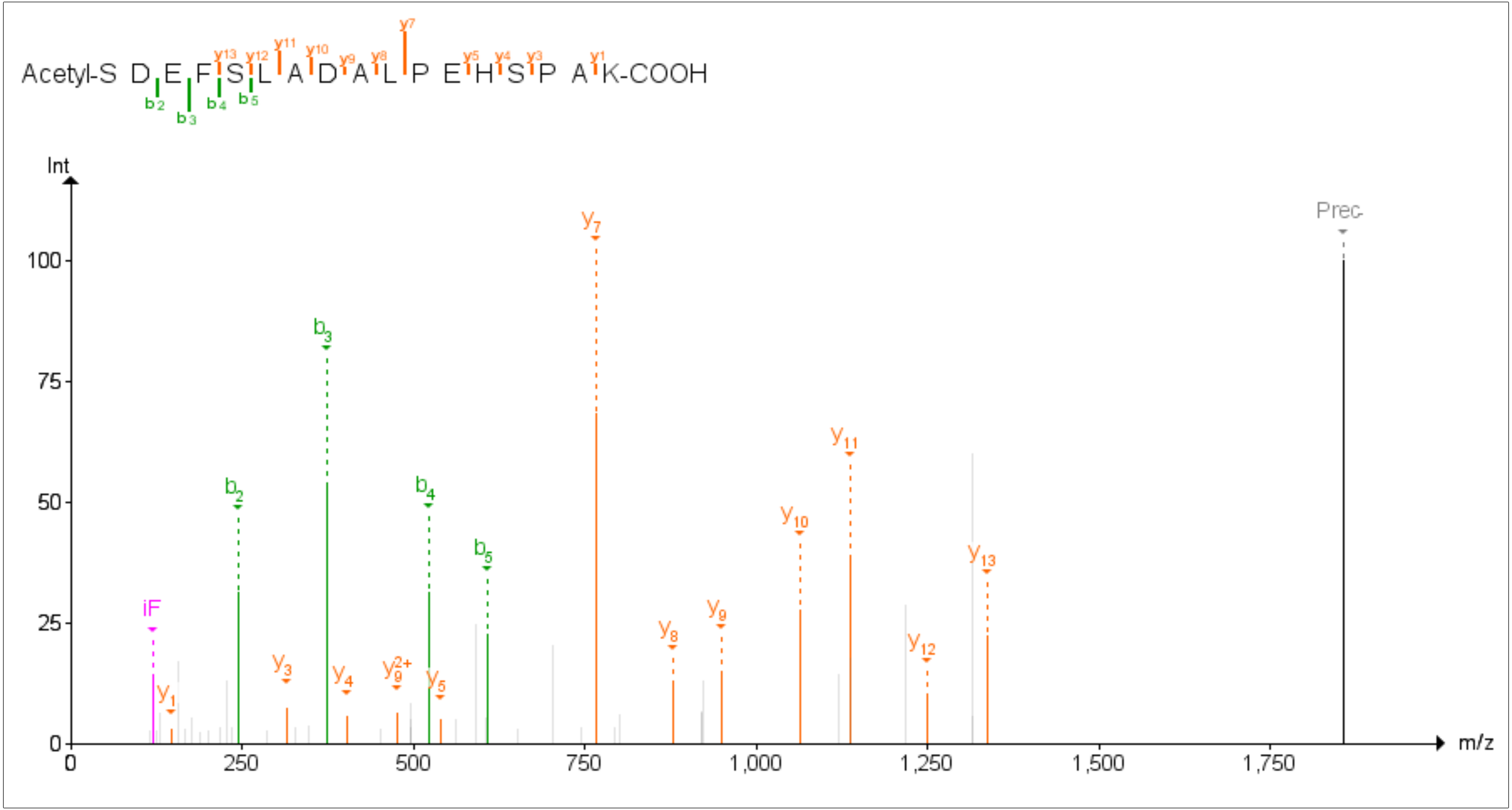

Acetyl-S D E F S L A D A L P E H S P A K-COOH
b2
b3
b4
b5
y13
y12
y11
y10
y9
y8
y7
y5
y4
y3
y1
Int
m/z
Prec.
iF
0
25
50
75
100
250
500
750
1,000
1,250
1,500
1,750

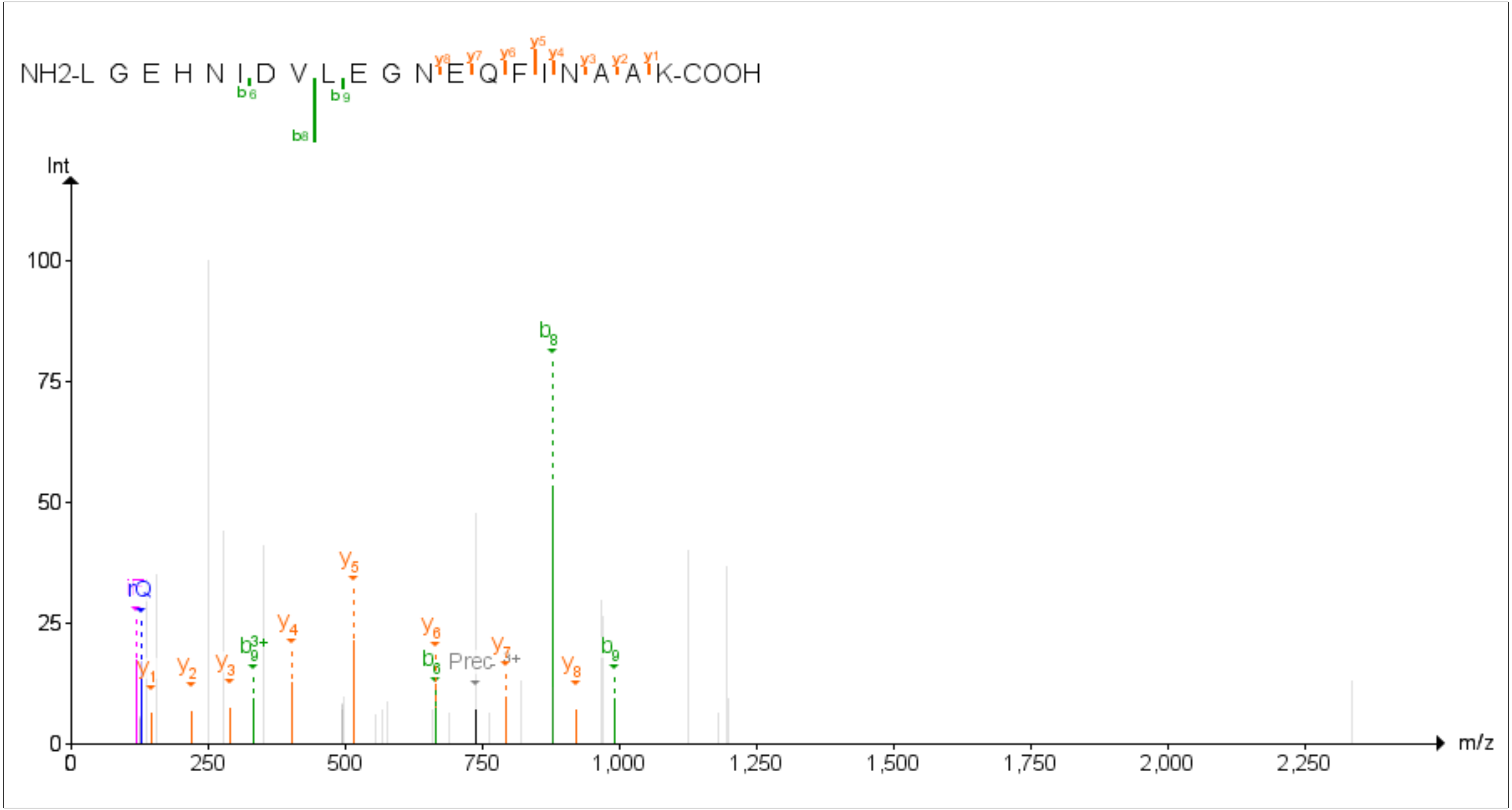

NH2-L G E H N I D V L E G N E Q F I N A A K-COOH
b6
b9
b8
y8 y7 y6 y5 y4 y3 y2 y1
Int
100
75
50
25
0
iQ
y1
y2
y3
b9 3+
y4
y5
y6
b6
Prec 3+
y7
b8
y8
b9
0
250
500
750
1,000
1,250
1,500
1,750
2,000
2,250
m/z

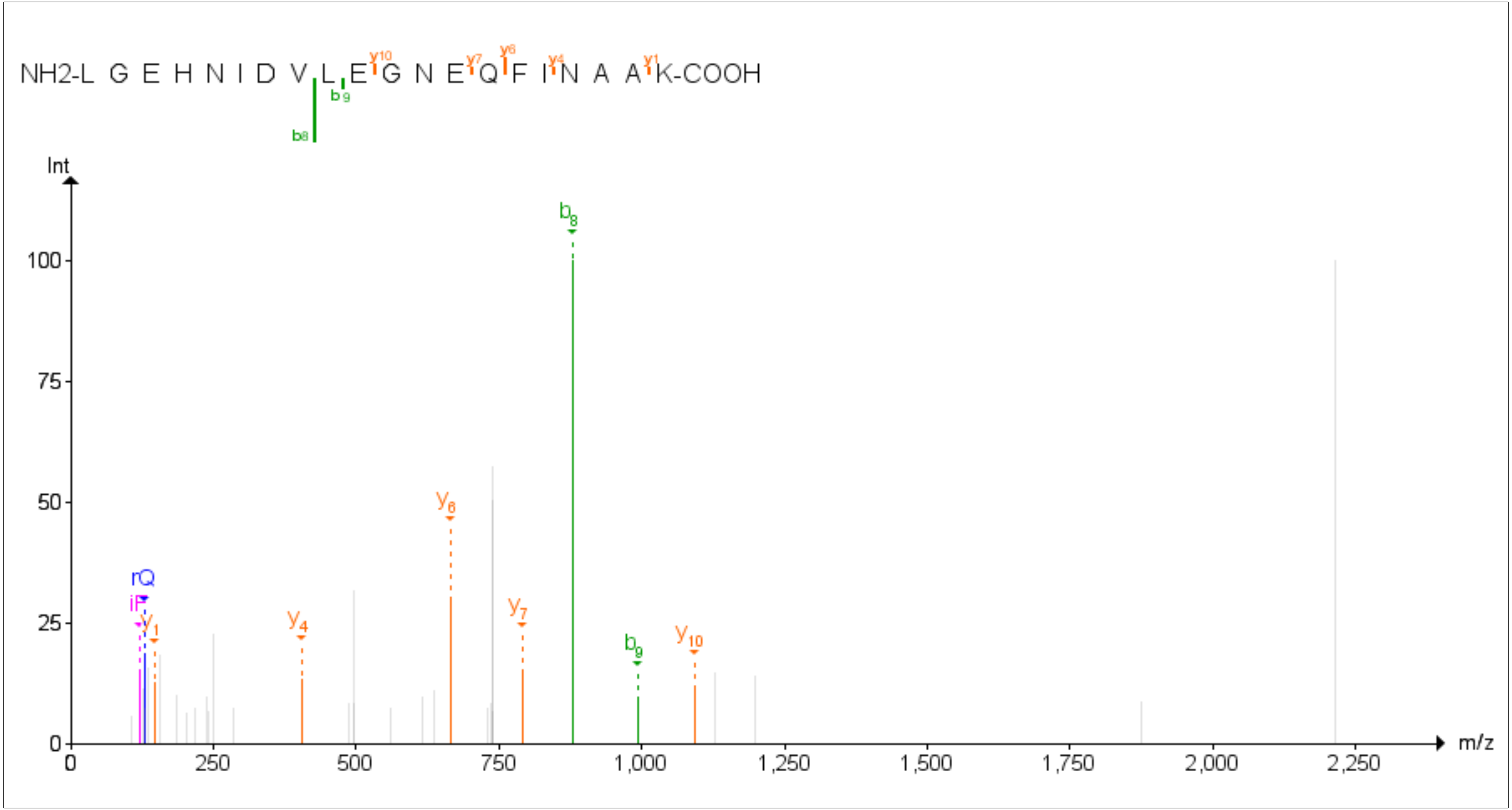
NH2-L G E H N I D V L E G N E Q F I N A A K-COOH
y10 y7 y6 y4 y1
b8 b9
Int
m/z
b8
y6
rQ
iF
y1
y4
y7
b9
y10
0
25
50
75
100
0
250
500
750
1,000
1,250
1,500
1,750
2,000
2,250

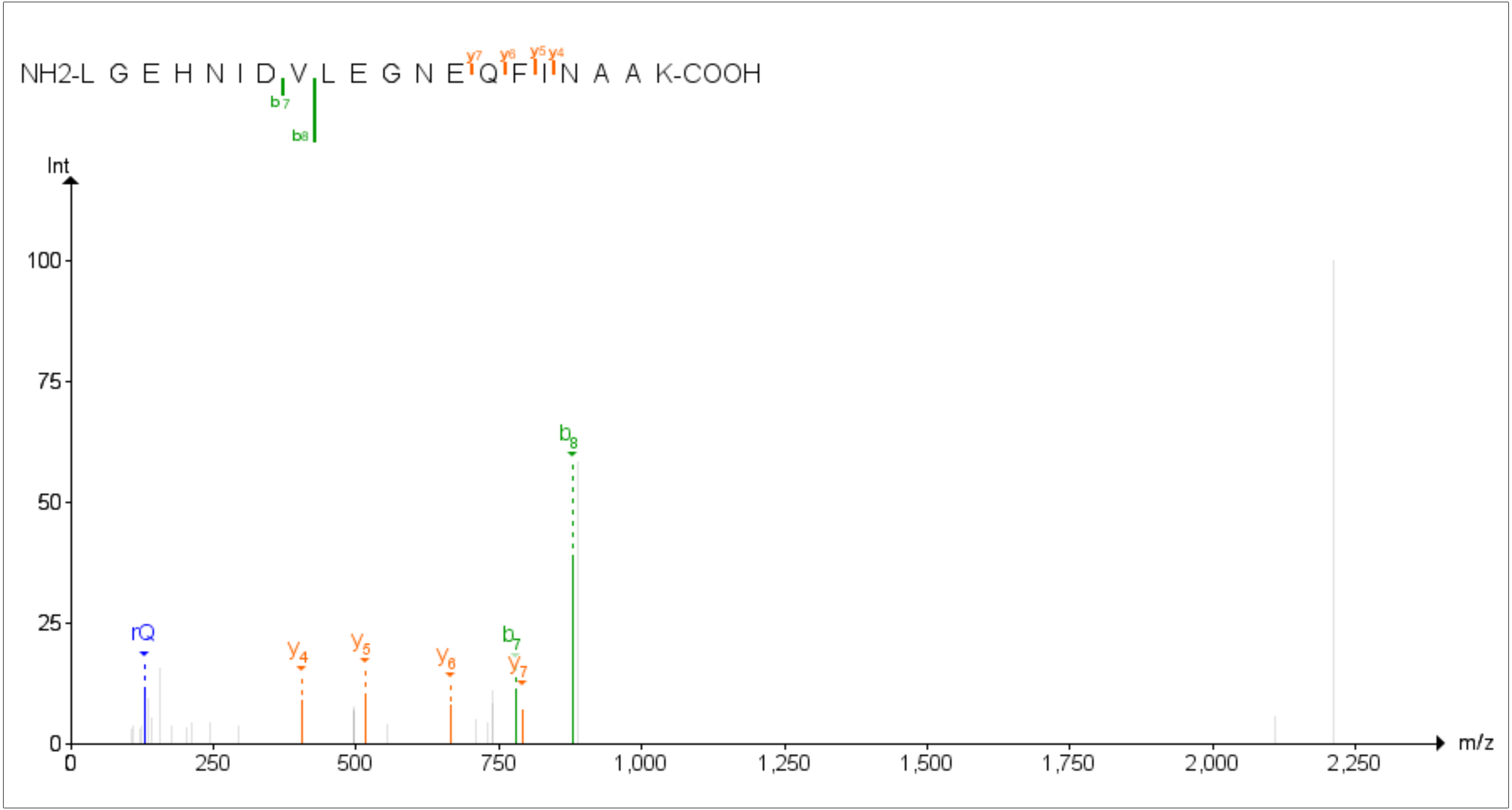
NH2-L G E H N I D V L E G N E Q F I N A A K-COOH
y7 y6 y5 y4
b7
b8
Int
100
75
50
25
0
rQ
y4
y5
y6
b7
y7
b8
0
250
500
750
1,000
1,250
1,500
1,750
2,000
2,250
m/z

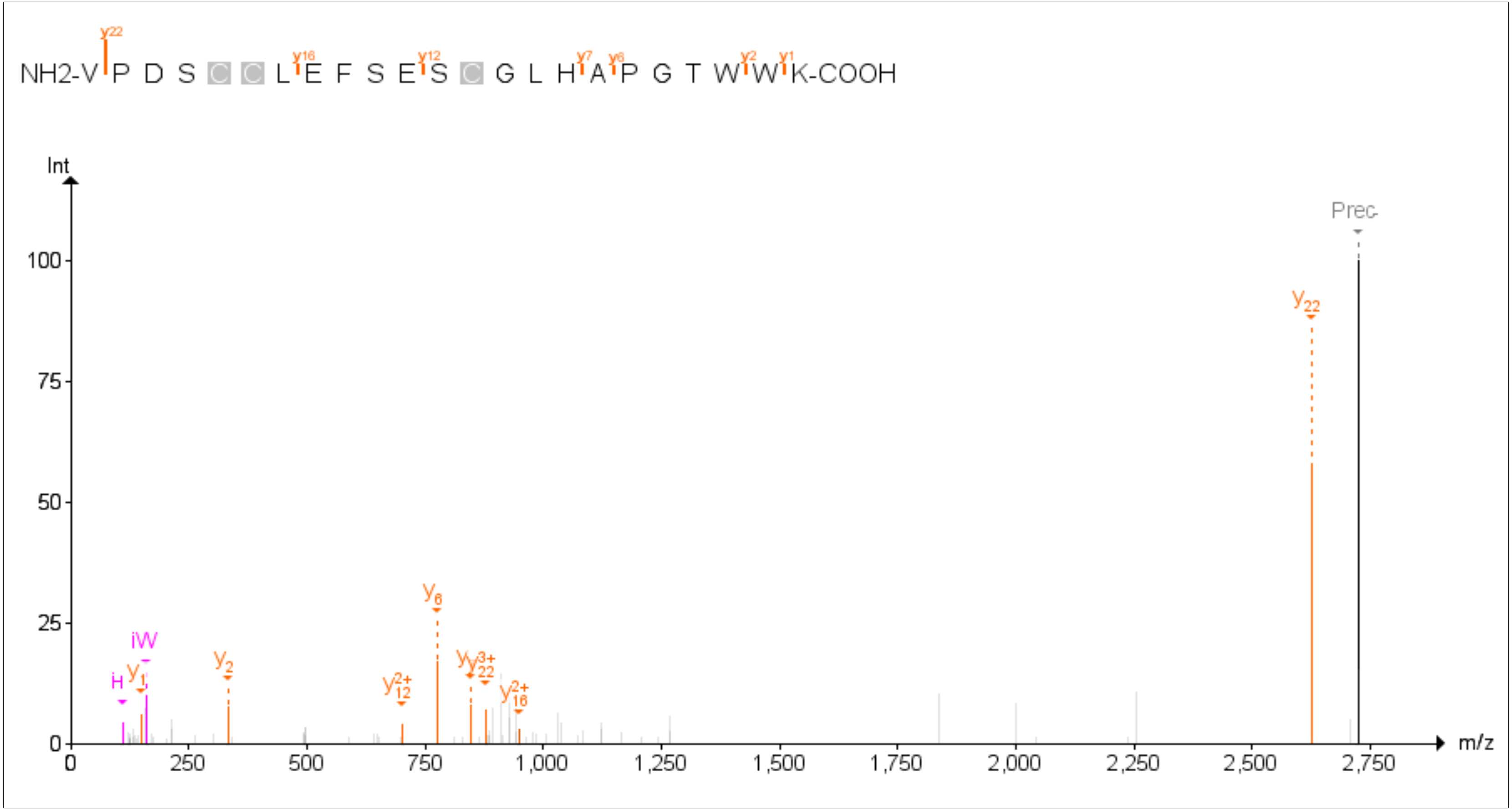
NH2-V P D S C C L E F S E S C G L H A P G T W W K-COOH
y22
y16
y12
y7
y6
y2
y1
Int
100
75
50
25
0
Prec.
H
iW
m/z
0
250
500
750
1,000
1,250
1,500
1,750
2,000
2,250
2,500
2,750

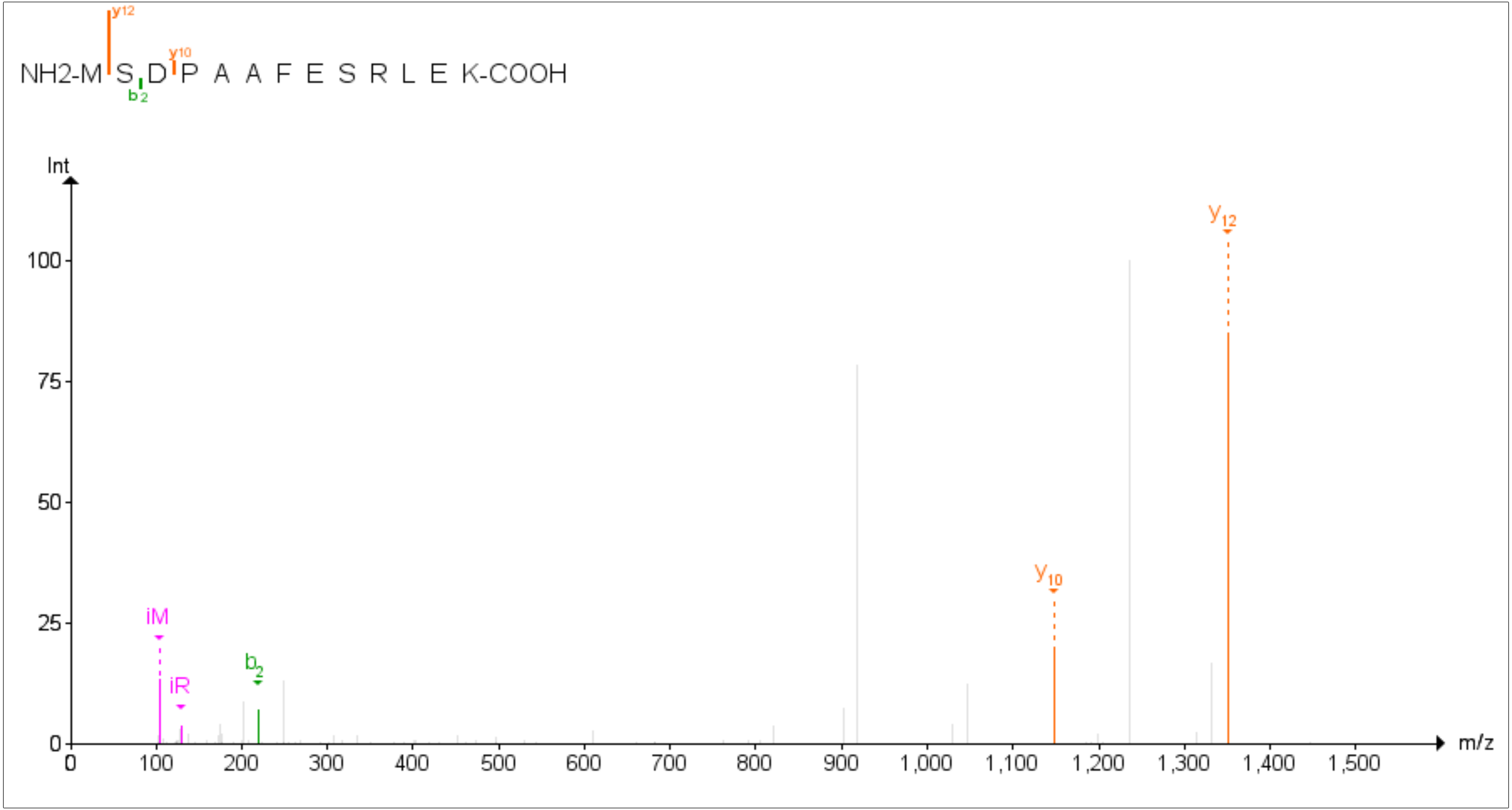

NH2-M S D P A A F E S R L E K-COOH
y12
y10
b2
Int
100
75
50
25
0
iM
iR
b2
y10
y12
0
100
200
300
400
500
600
700
800
900
1,000
1,100
1,200
1,300
1,400
1,500
m/z

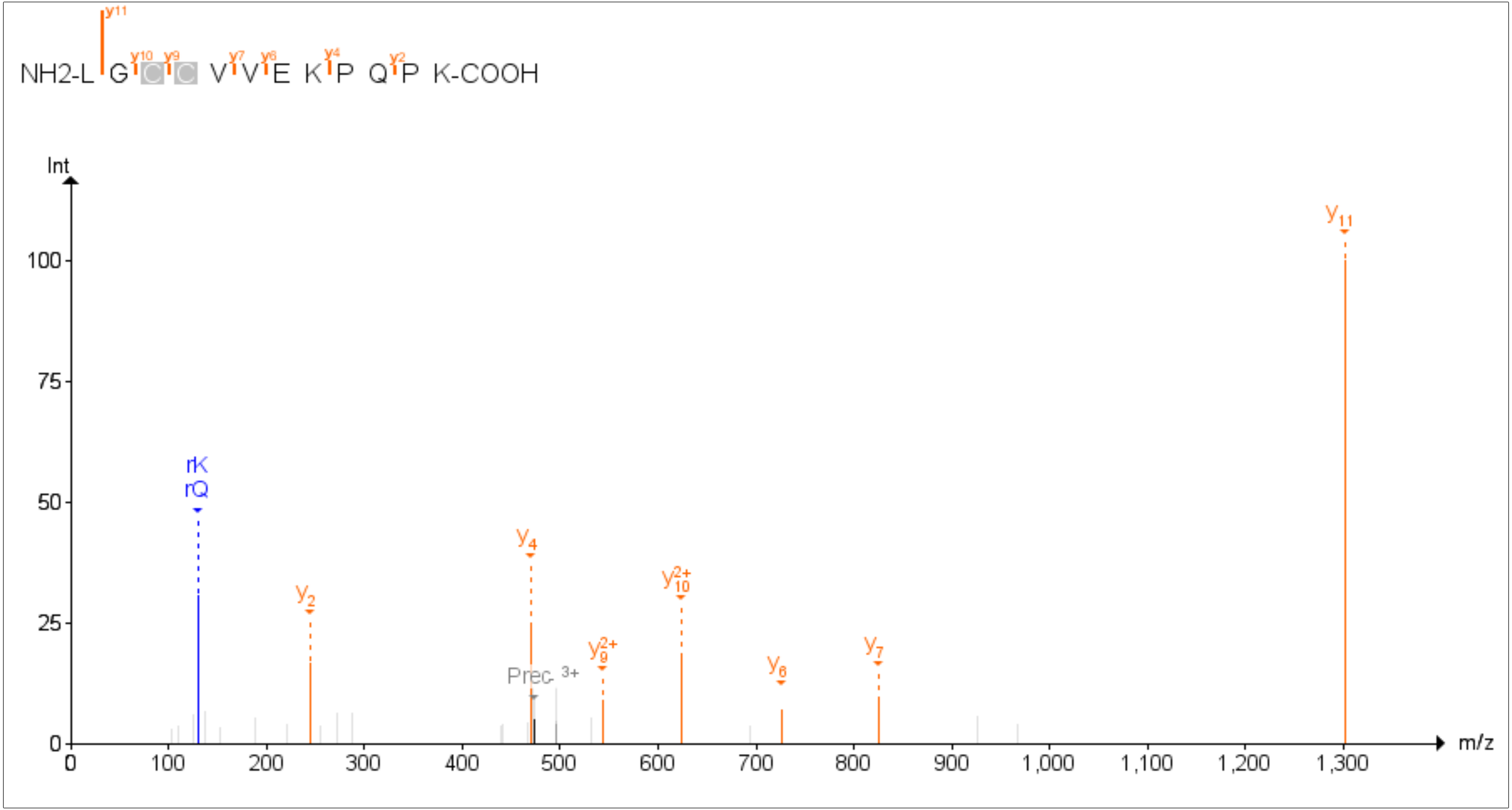

NH2-L G C C V V E K P Q P K-COOH
y11 y10 y9 y7 y6 y4 y2
Int
m/z
rK
rQ
y2
y4
Prec 3+
y9 2+
y10 2+
y6
y7
y11
0
25
50
75
100
0
100
200
300
400
500
600
700
800
900
1,000
1,100
1,200
1,300

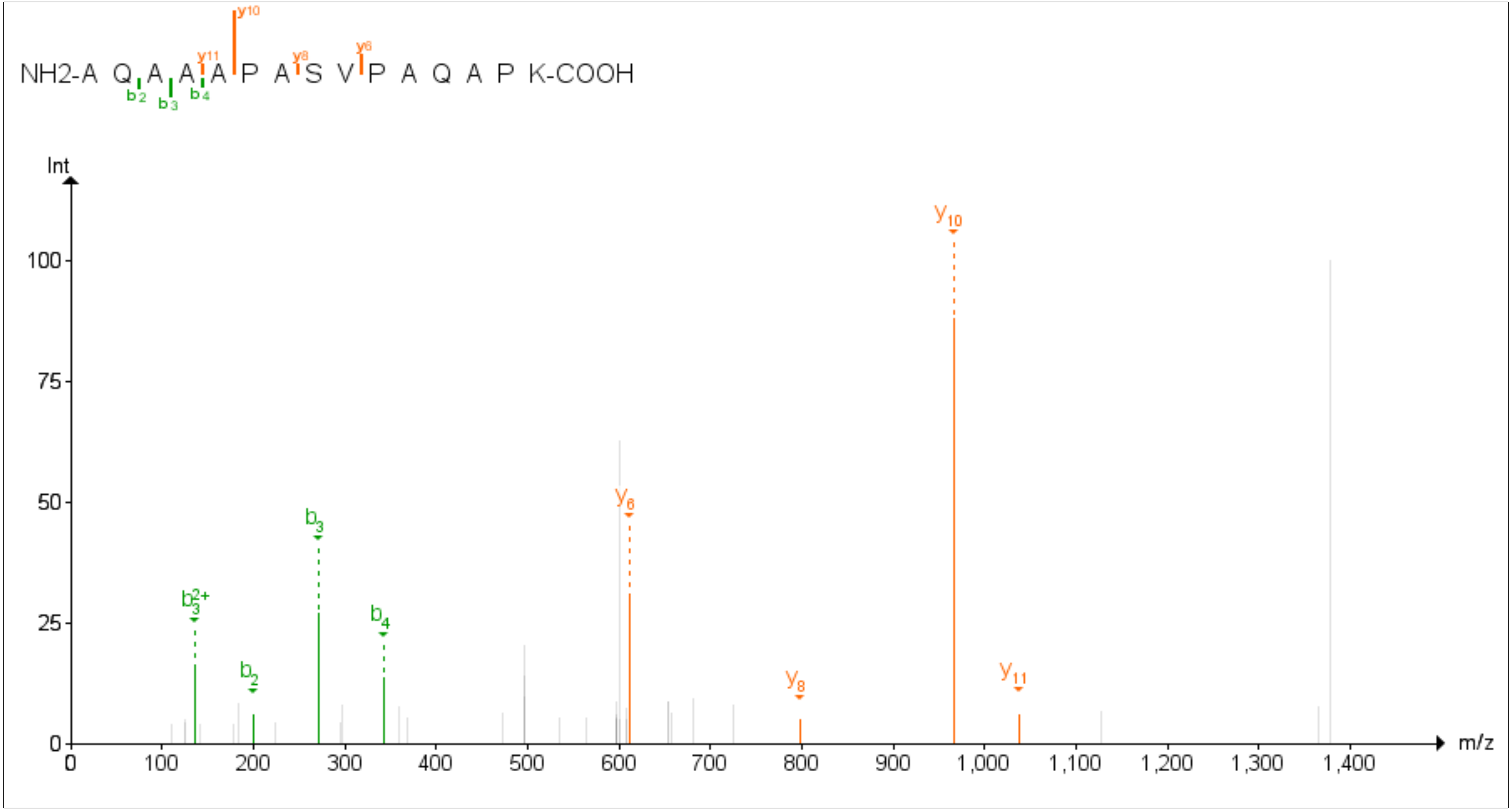

NH2-A Q A A A P A S V P A Q A P K-COOH
y10
y11
y8
y6
b2
b3
b4
Int
100
75
50
25
0
y10
y6
b3
b3 2+
b4
b2
y8
y11
0
100
200
300
400
500
600
700
800
900
1,000
1,100
1,200
1,300
1,400
m/z

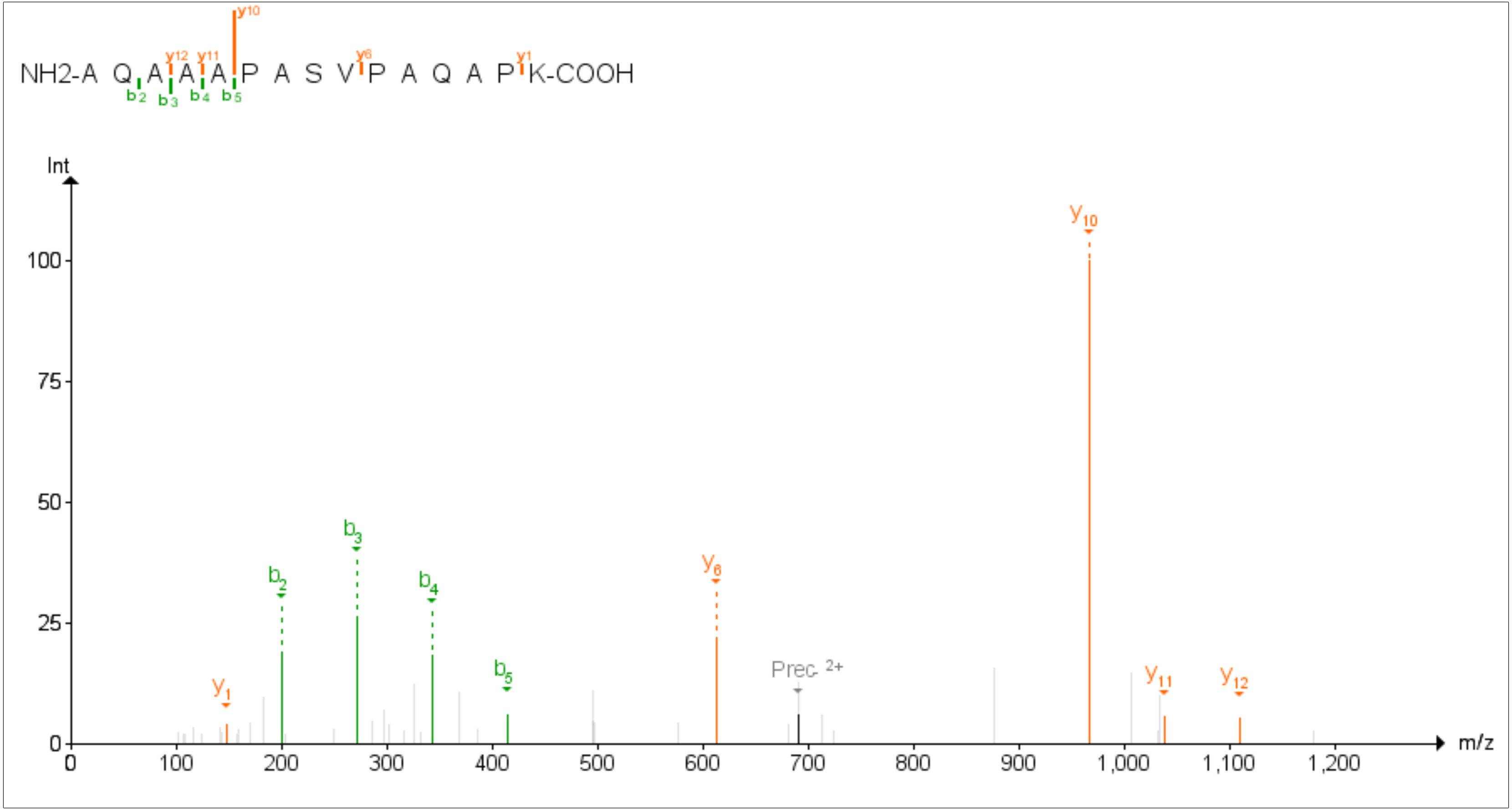

NH2-A Q A A A P A S V P A Q A P K-COOH
y10
y12
y11
y6
y1
b2
b3
b4
b5
Int
100
75
50
25
0
y10
b3
b2
b4
y6
b5
y1
Prec 2+
y11
y12
0
100
200
300
400
500
600
700
800
900
1,000
1,100
1,200
m/z

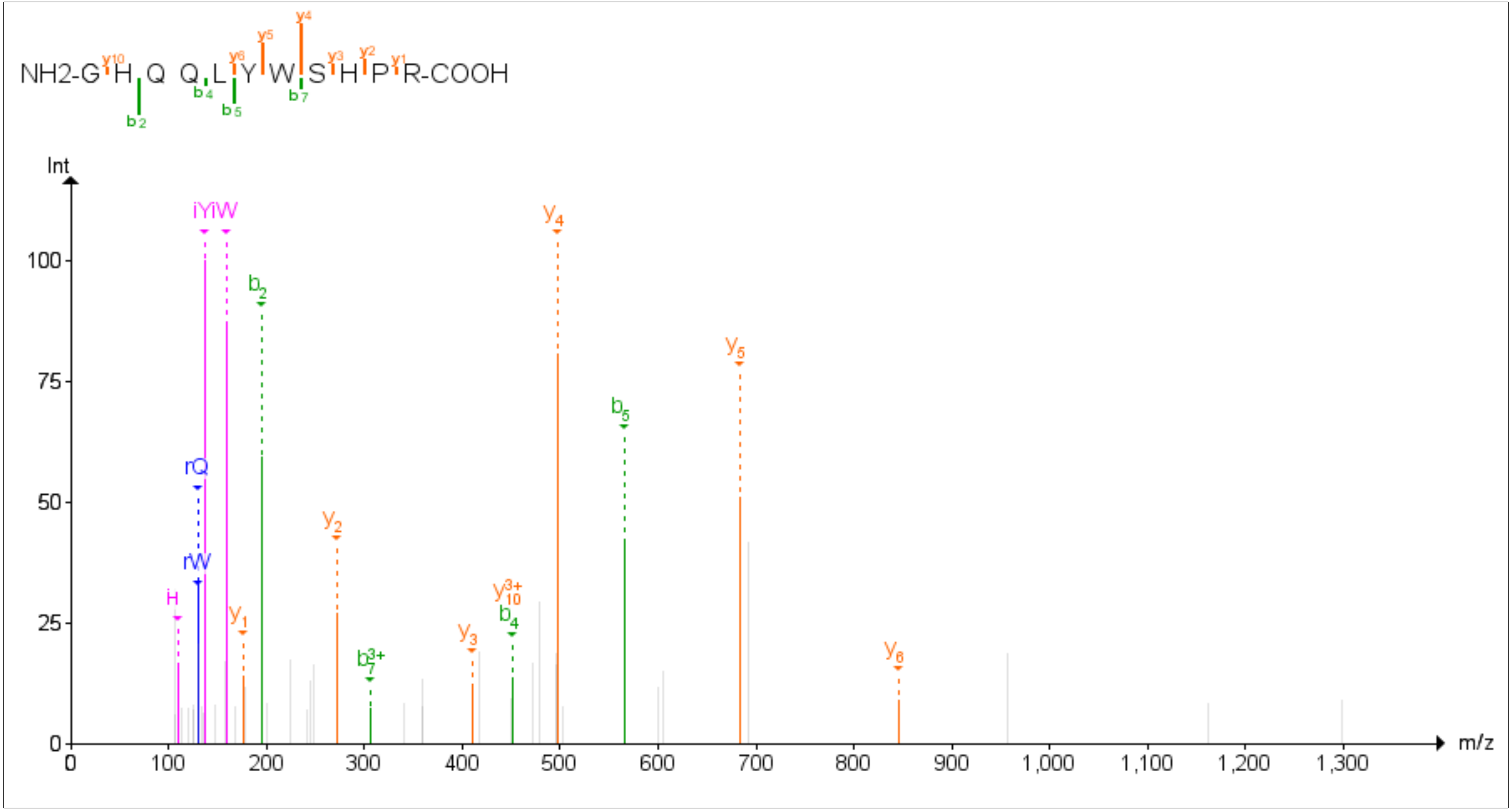
NH2-G H Q Q L Y W S H P R-COOH
Int
m/z
iYiW
rQ
rW
iH

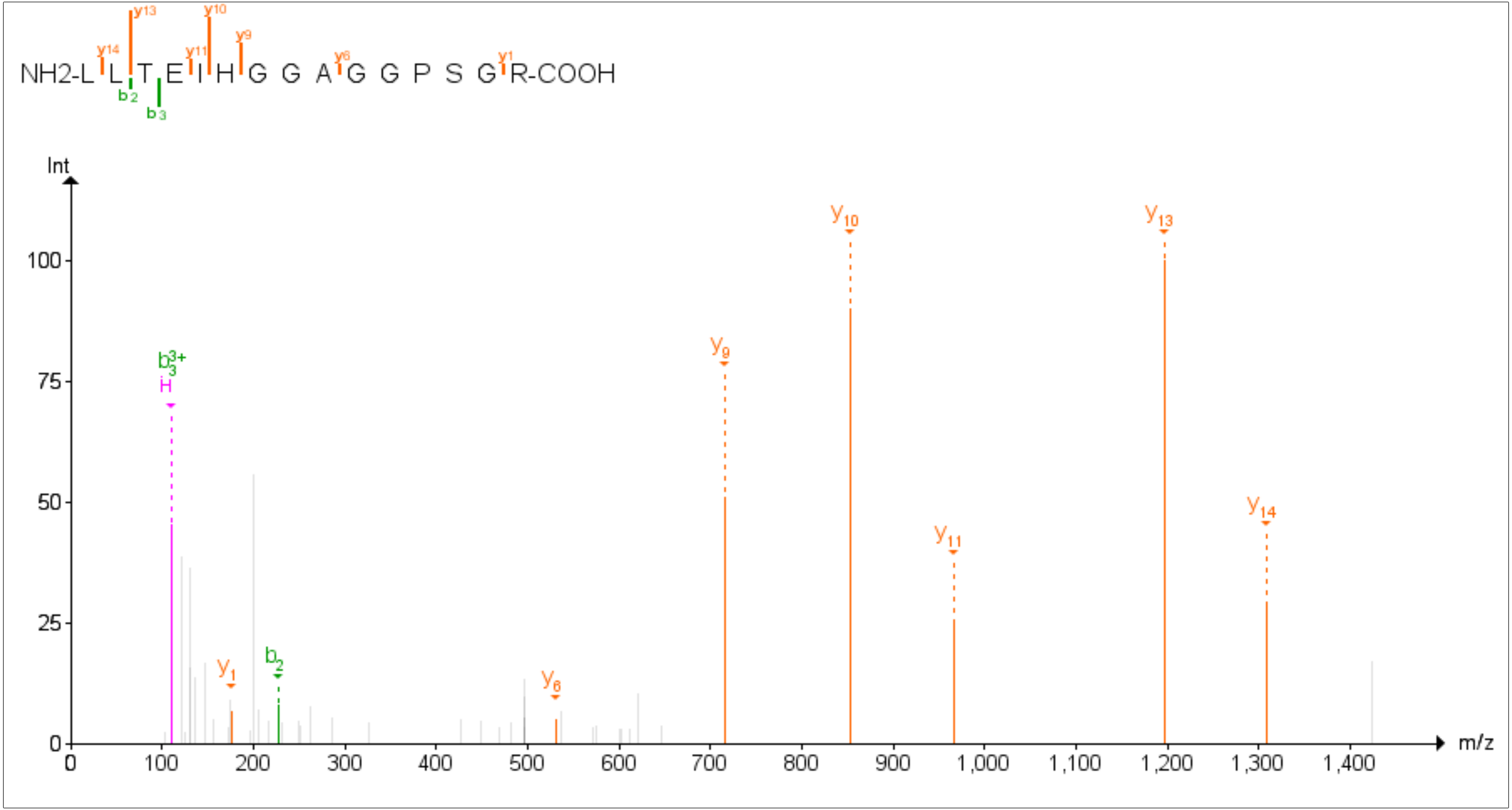

NH2-L L T E I H G G A G G P S G R-COOH
y14 y13 y11 y10 y9 y6 y1
b2 b3
Int
100
75
50
25
0
m/z
0 100 200 300 400 500 600 700 800 900 1,000 1,100 1,200 1,300 1,400
b3 3+
H
y1
b2
y6
y9
y10
y11
y13
y14

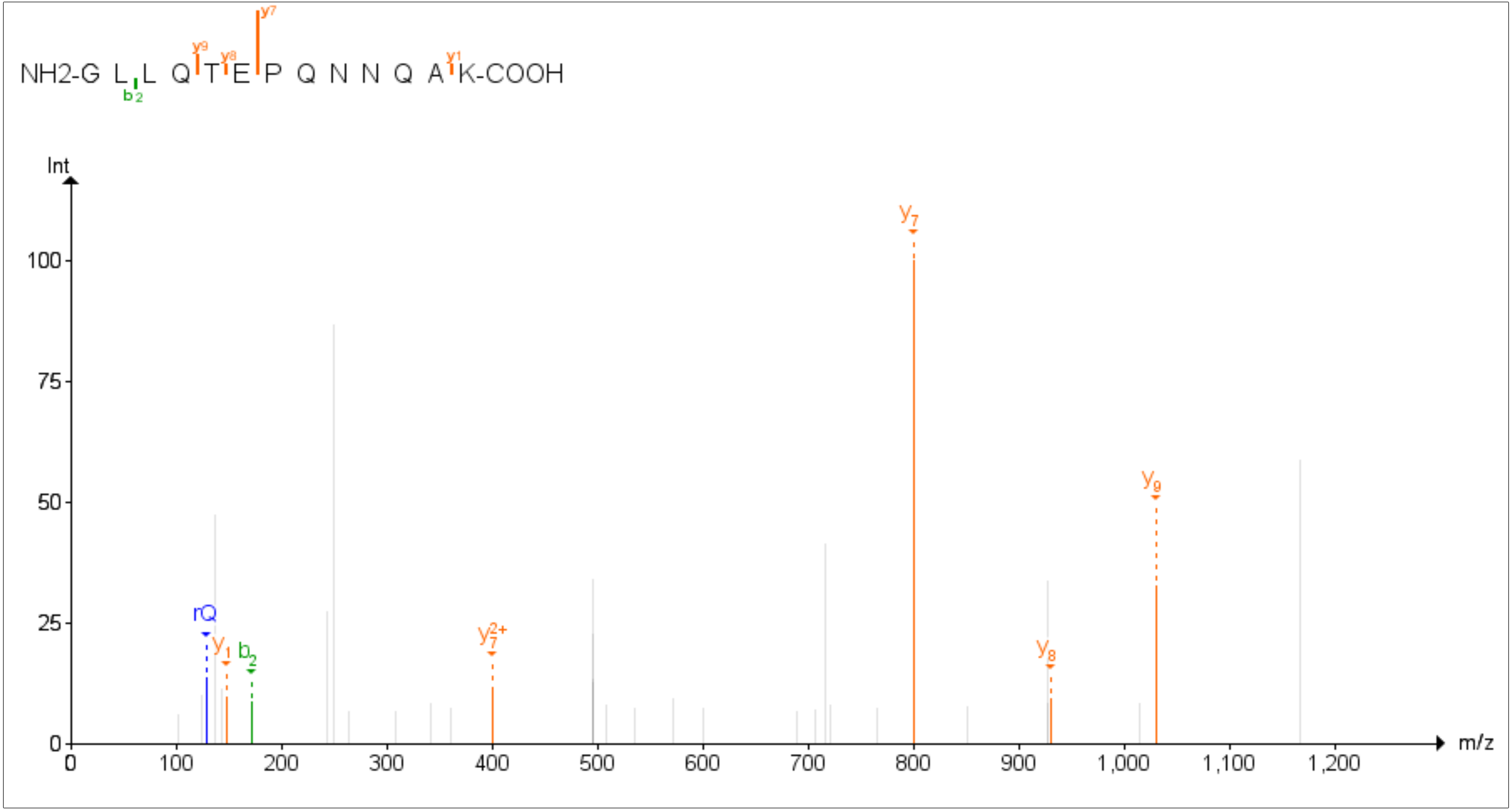
NH2-G L L Q T E P Q N N Q A K-COOH
y7
y9
y8
y1
b2
Int
100
75
50
25
0
m/z
0
100
200
300
400
500
600
700
800
900
1,000
1,100
1,200
rQ
y1
b2
y7 2+
y7
y8
y9

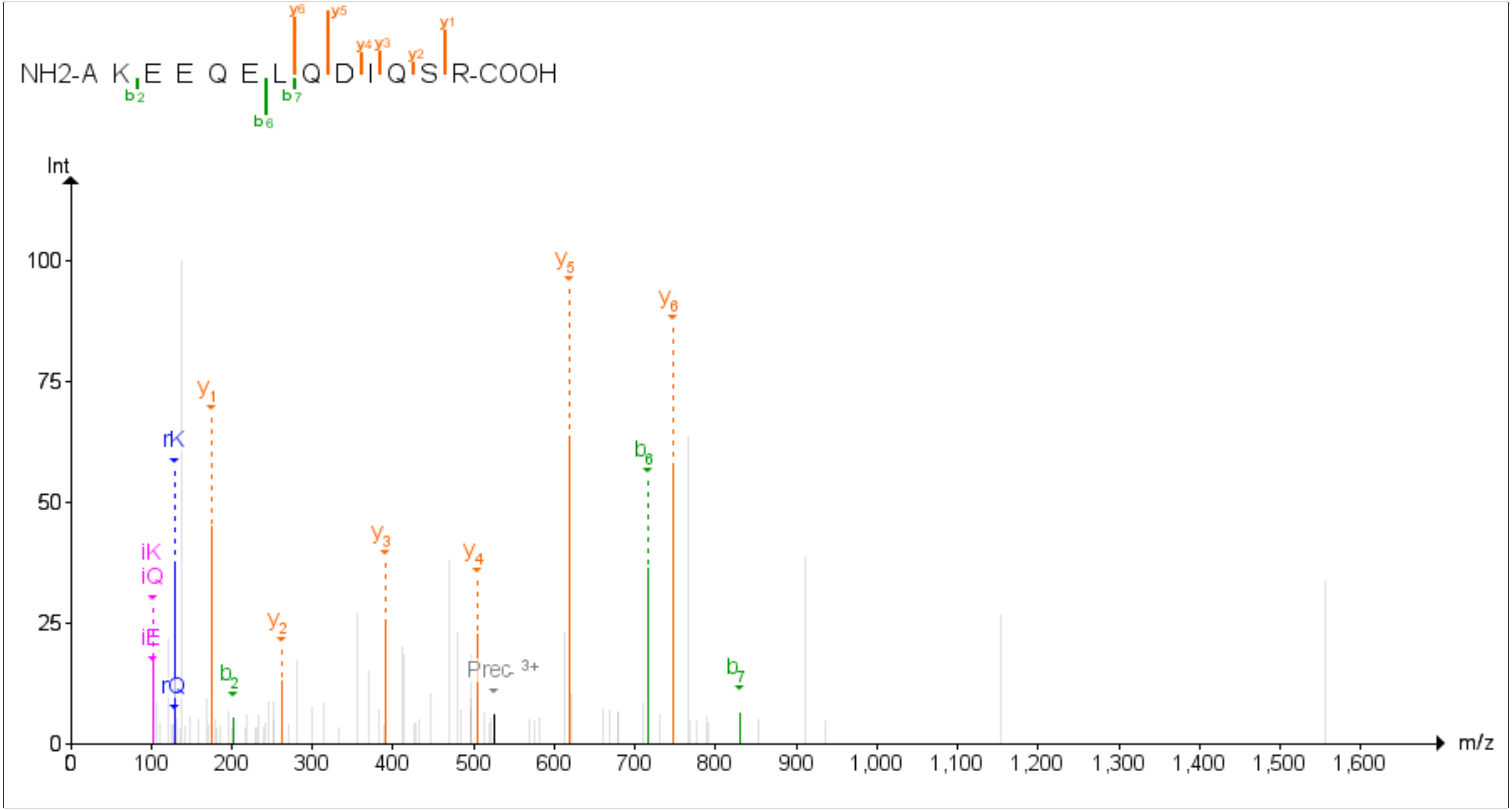

NH2-A K E E Q E L Q D I Q S R-COOH
y6 y5 y4 y3 y2 y1
b2 b6 b7
Int
100
75
50
25
0
y5
y6
y1
rK
b6
iK
iQ
y3
y4
y2
iE
b2
rQ
Prec 3+
b7
0 100 200 300 400 500 600 700 800 900 1,000 1,100 1,200 1,300 1,400 1,500 1,600
m/z

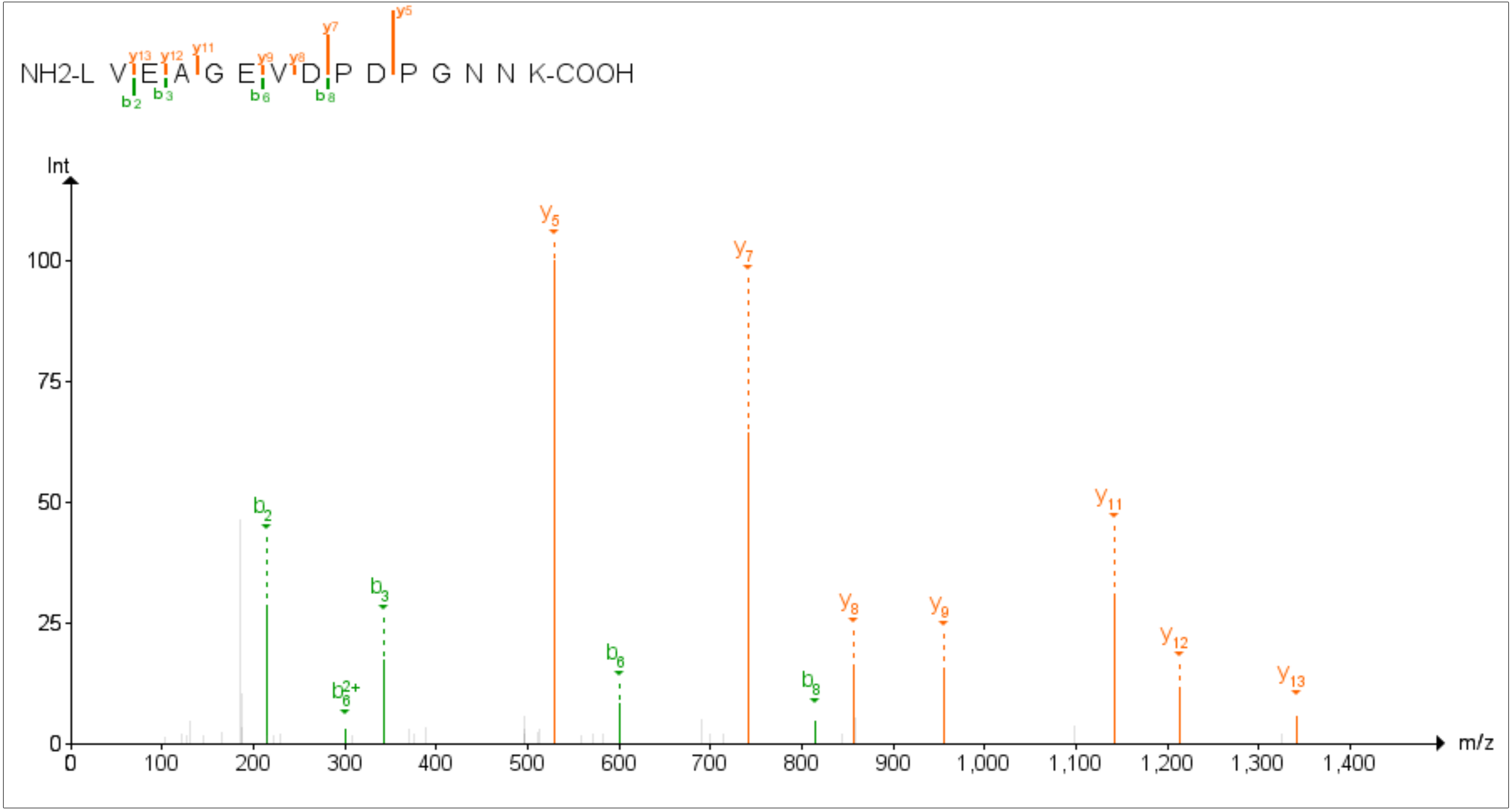
NH2-L V E A G E V D P D P G N N K-COOH
y13 y12 y11 y9 y8 y7 y5
b2 b3 b6 b8
Int
m/z
y5
y7
y11
y8
y9
y12
y13
b2
b3
b6
b8
b6 2+
0
25
50
75
100
0
100
200
300
400
500
600
700
800
900
1,000
1,100
1,200
1,300
1,400

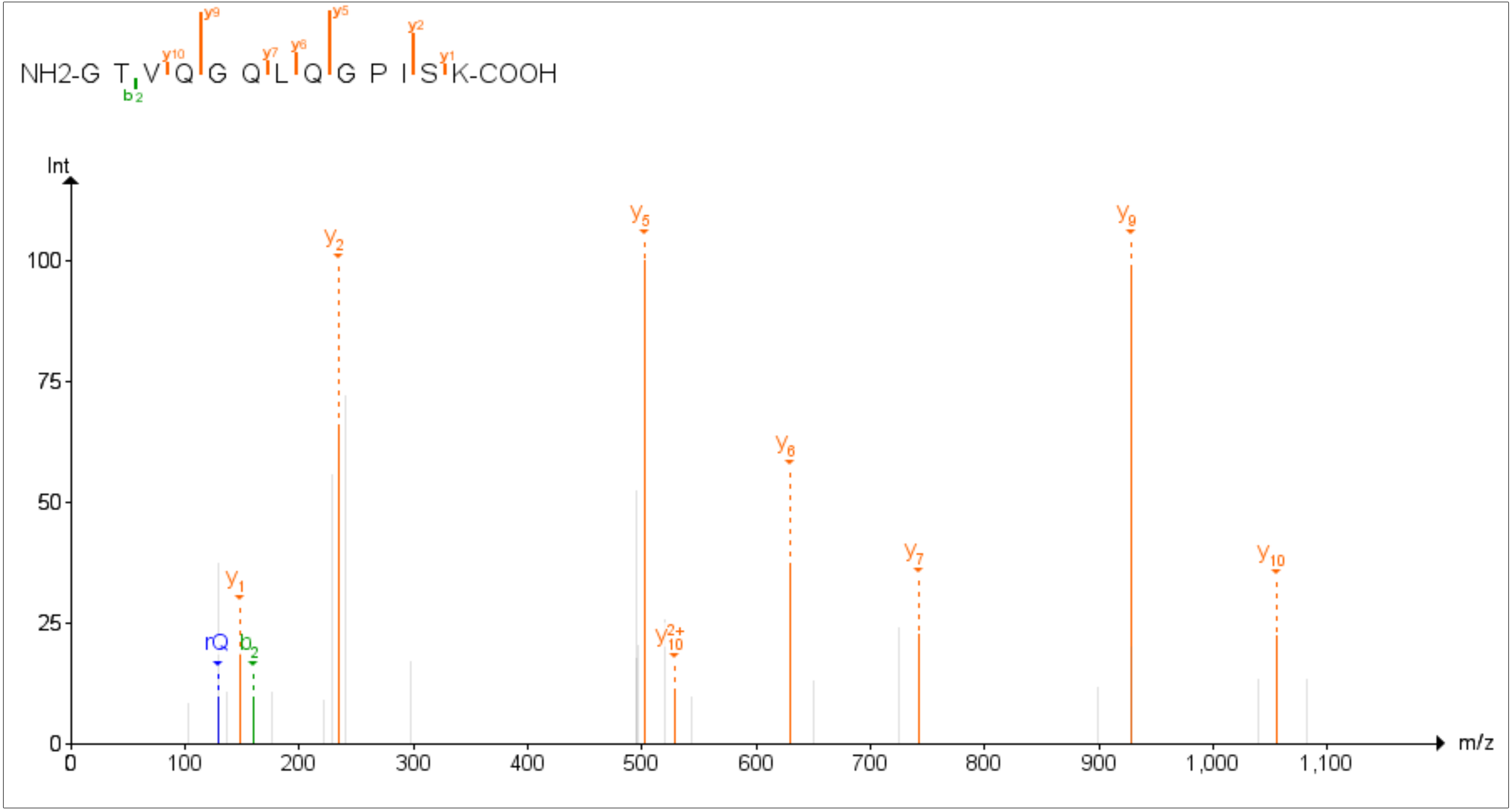
NH2-G T V Q G Q L Q G P I S K-COOH
y9
y5
y2
y10
y7
y6
y1
b2
Int
100
75
50
25
0
y2
y5
y9
y6
y7
y10
y1
rQ
b2
y10 2+
m/z
0
100
200
300
400
500
600
700
800
900
1,000
1,100

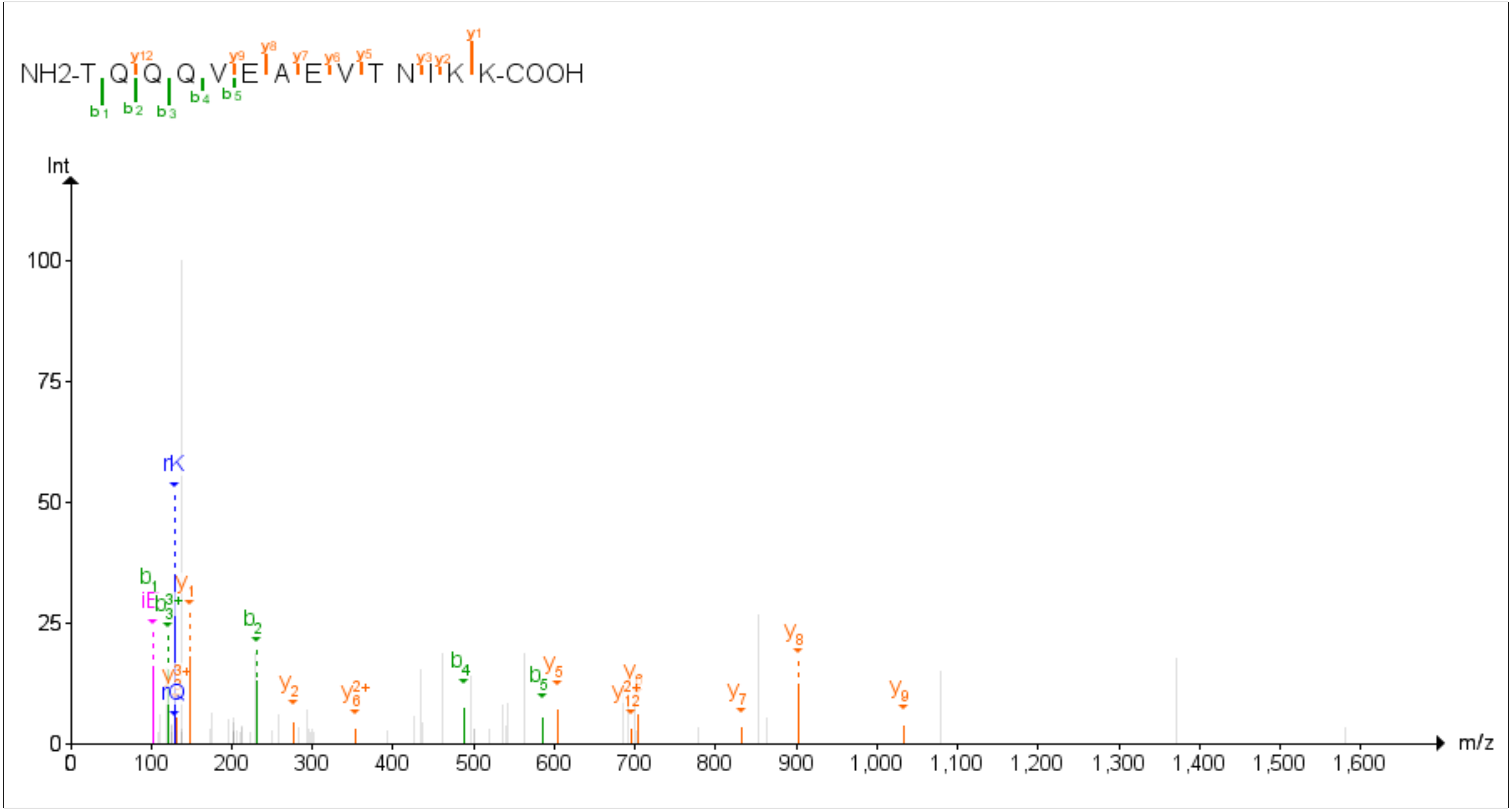
NH2-T Q Q Q V E A E V T N I K K-COOH
y12 y9 y8 y7 y6 y5 y3 y2 y1
b1 b2 b3 b4 b5
Int
100
75
50
25
0
rK
b1
y1
iE
b3 3+
b2
y3+
rQ
y2
y6 2+
b4
b5
y5
y6
y12 2+
y7
y8
y9
0 100 200 300 400 500 600 700 800 900 1,000 1,100 1,200 1,300 1,400 1,500 1,600
m/z

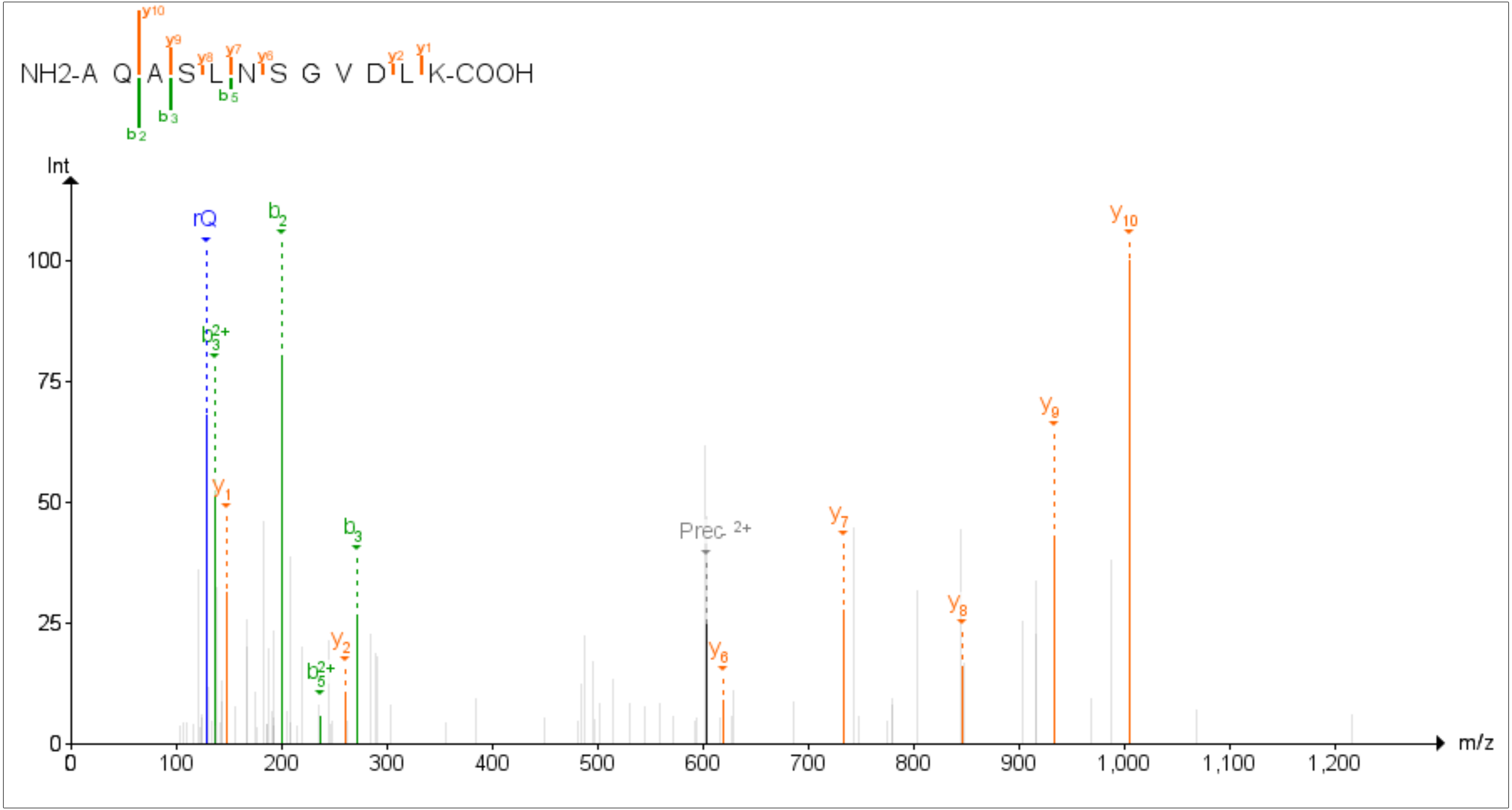

NH2-A Q A S L N S G V D L K-COOH
y10 y9 y8 y7 y6 y2 y1
b2 b3 b5
Int
rQ
b2
y10
b3 2+
y1
y9
b3
Prec. 2+
y7
y8
y2
b5 2+
y6
0
25
50
75
100
0 100 200 300 400 500 600 700 800 900 1,000 1,100 1,200
m/z

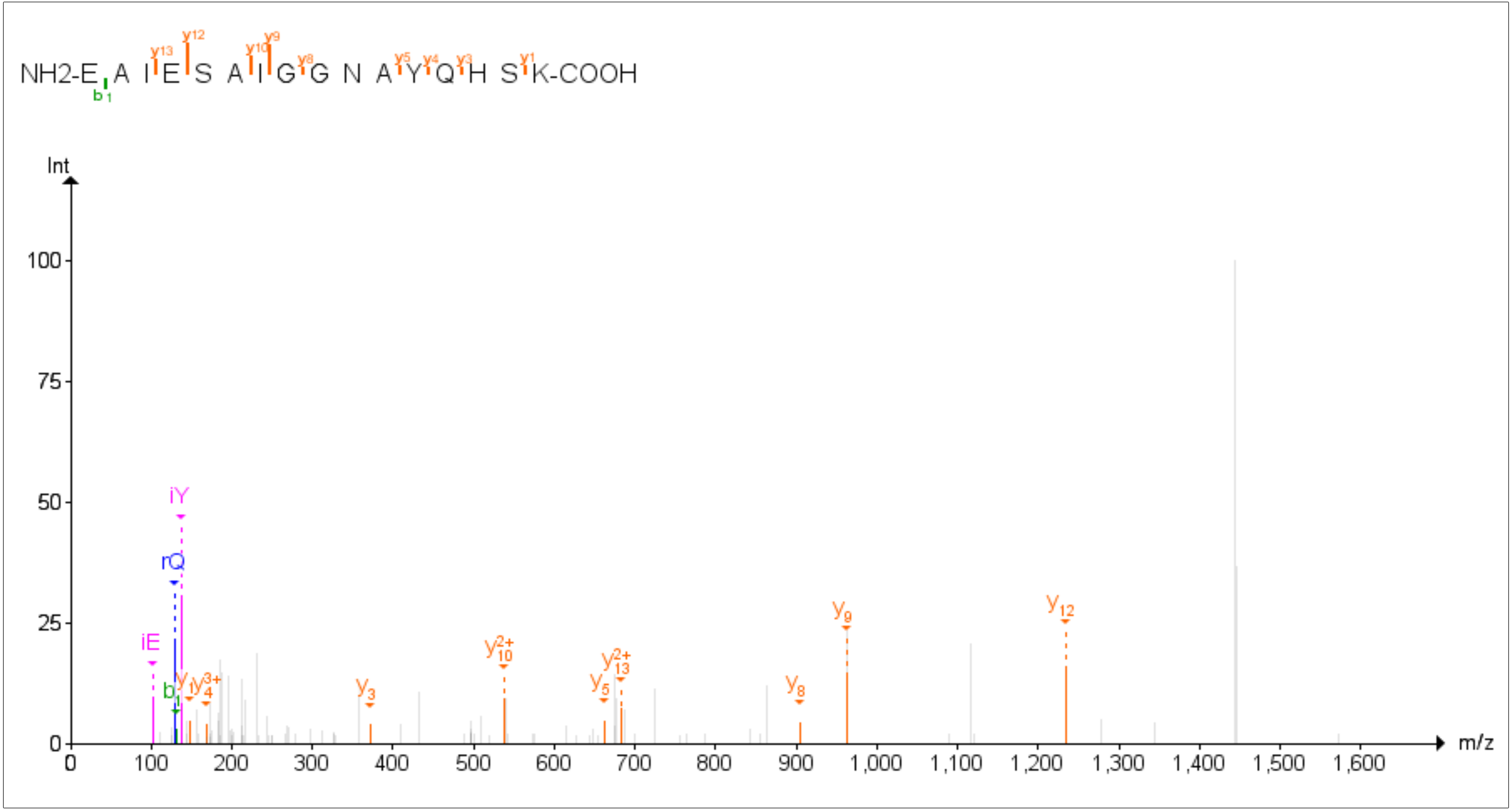
NH2-E A I E S A I G G N A Y Q H S K-COOH
b1
y13
y12
y10
y9
y8
y5
y4
y3
y1
Int
m/z
iY
rQ
iE
b1
y1
y4 3+
y3
y10 2+
y5
y13 2+
y8
y9
y12

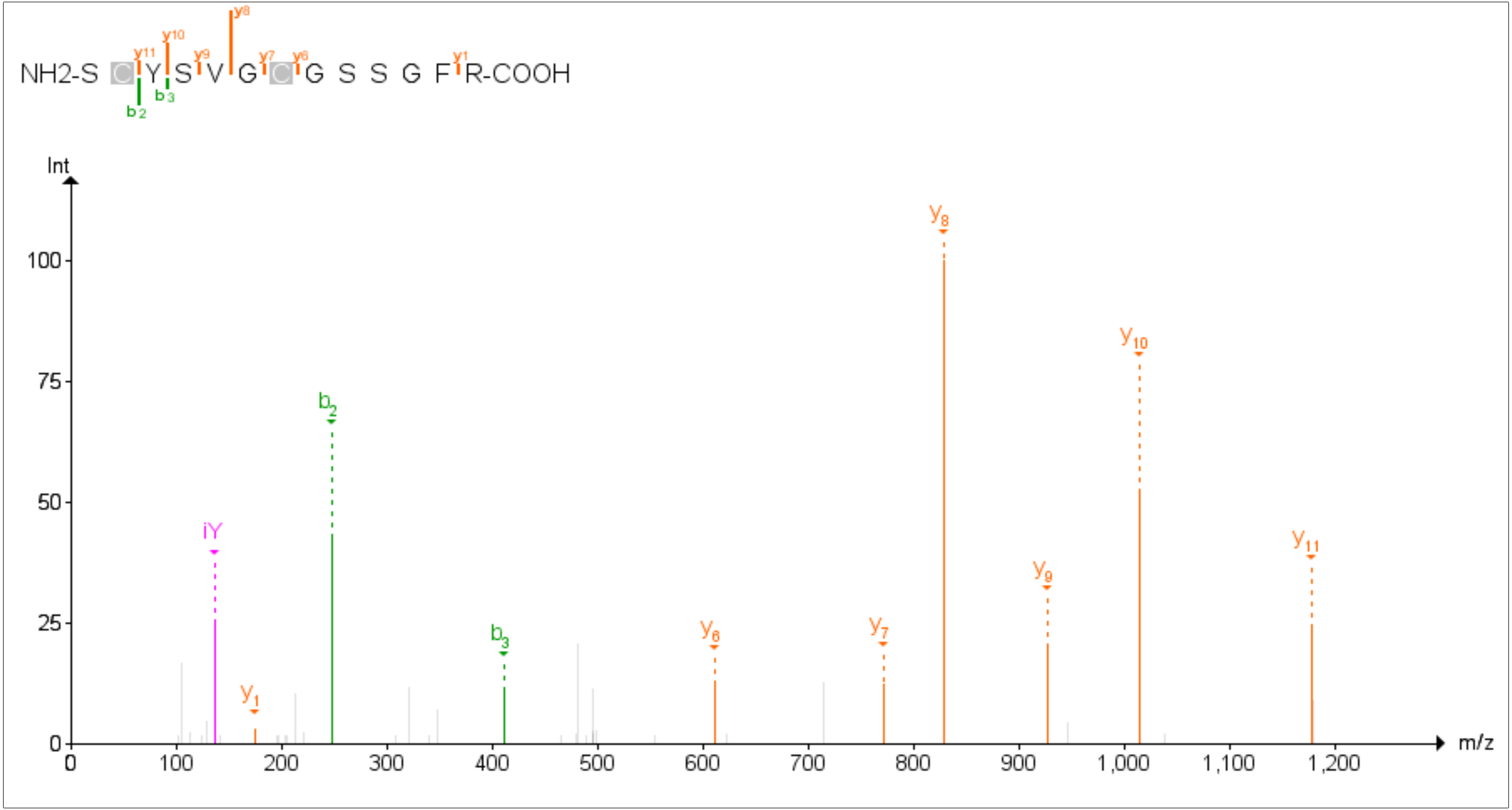

NH2-S C Y S V G C G S S G F R-COOH
y8
y10
y11
y9
y7
y6
y1
b2
b3
Int
100
75
50
25
0
iY
b2
b3
y1
y6
y7
y8
y9
y10
y11
0
100
200
300
400
500
600
700
800
900
1,000
1,100
1,200
m/z

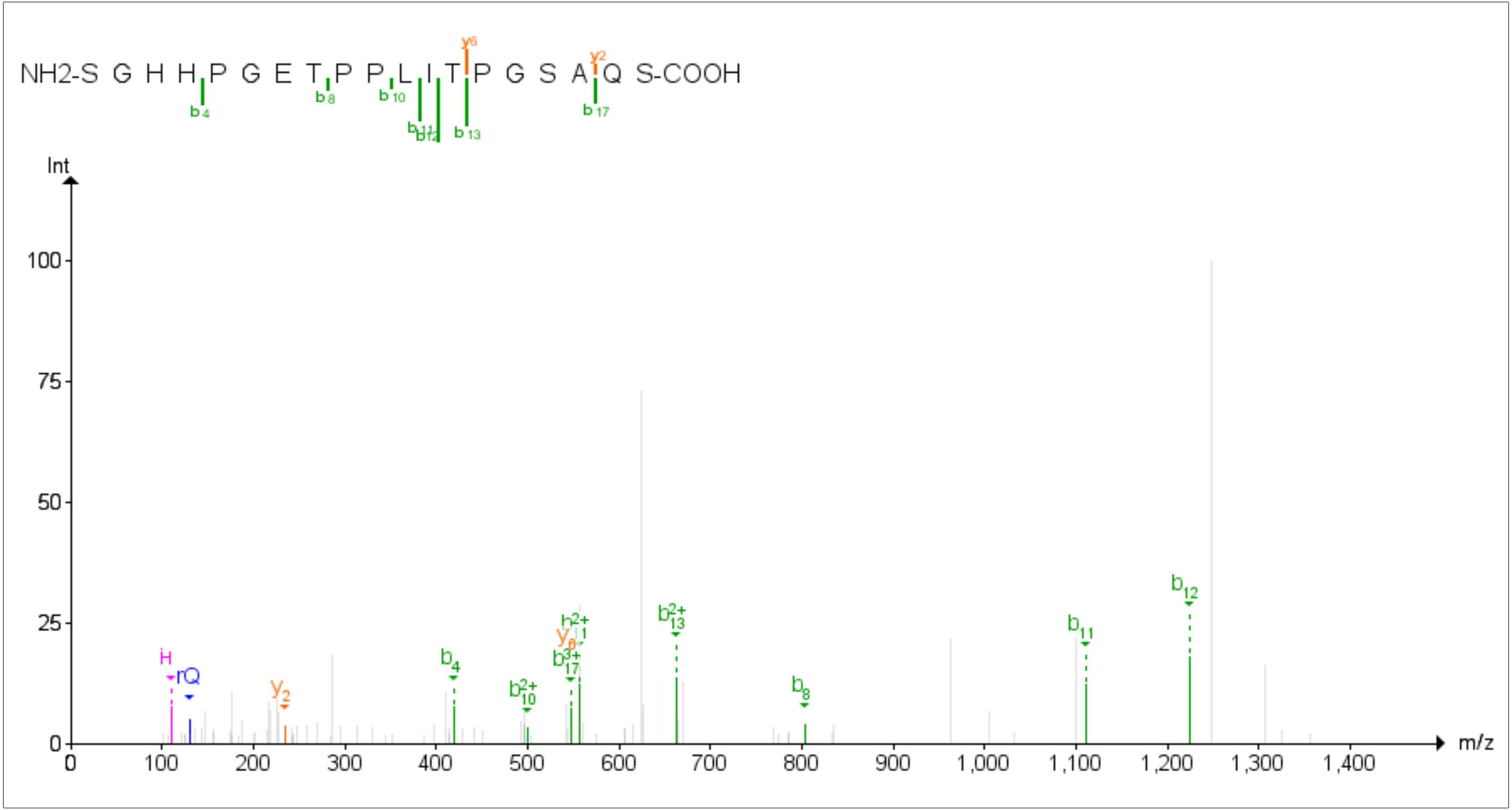

NH2-S G H H P G E T P P L I T P G S A Q S-COOH
b4
b8
b10
b11
b12
b13
b17
y6
y2
Int
m/z
iH
rQ
y2
b4
b2+ 10
b3+ 17
y6
b2+ 11
b2+ 13
b8
b11
b12

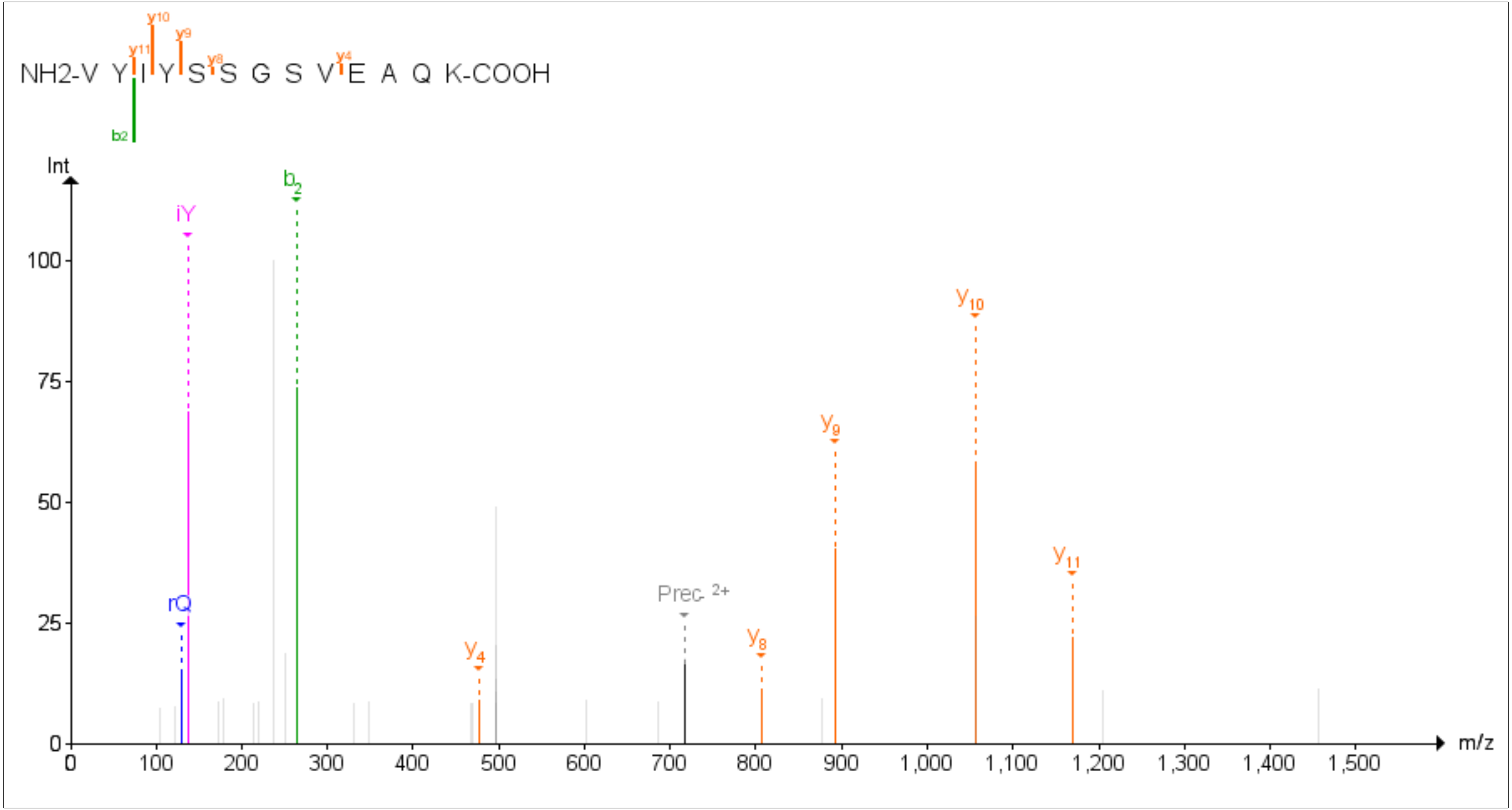

NH2-V Y I Y S S G S V E A Q K-COOH
y10
y9
y11
y8
y4
b2
Int
iY
b₂
rQ
Prec 2+
y₄
y₈
y₉
y₁₀
y₁₁
100
75
50
25
0
0
100
200
300
400
500
600
700
800
900
1,000
1,100
1,200
1,300
1,400
1,500
m/z

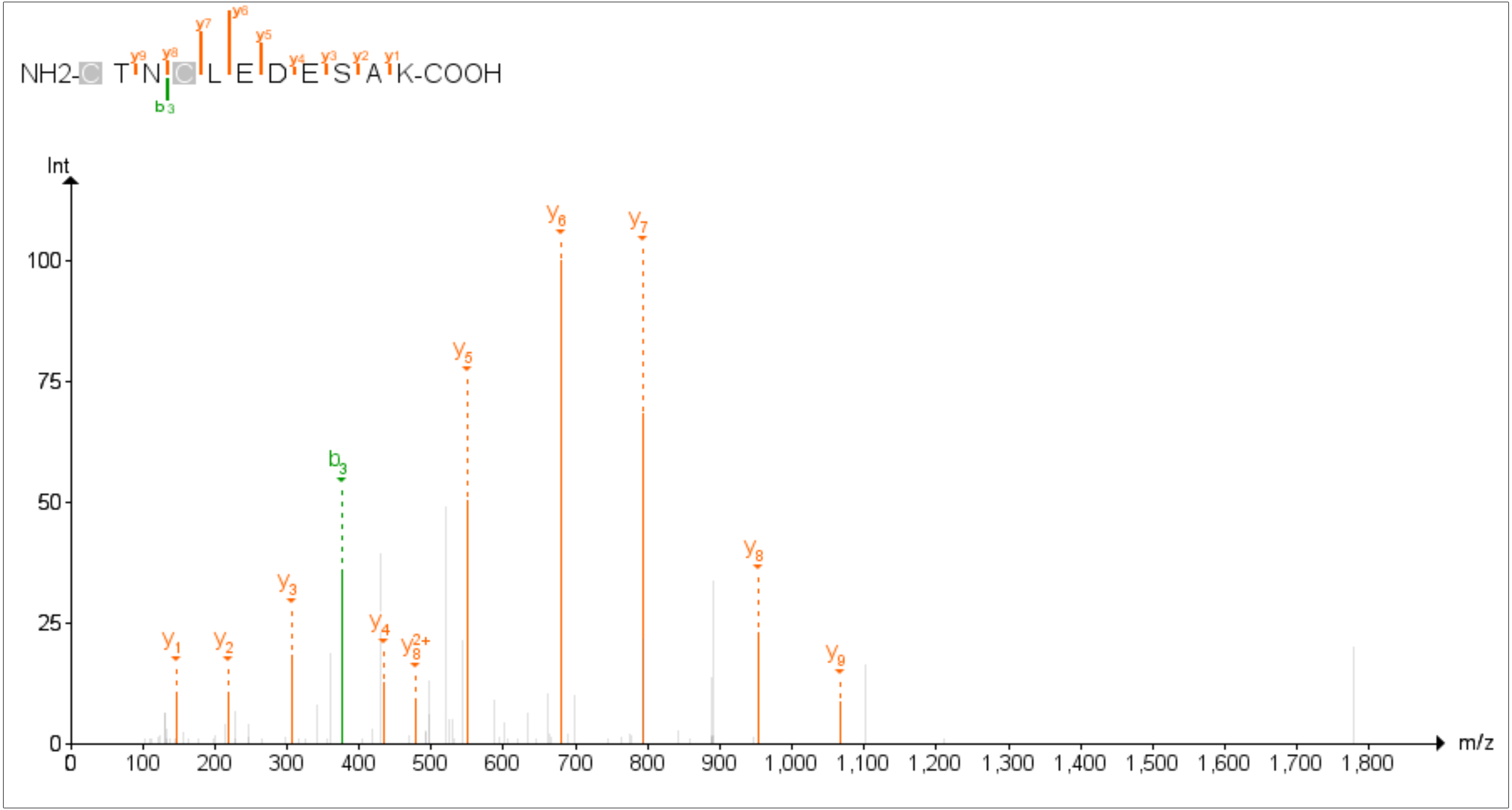

NH2-C T N C L E D E S A K-COOH
y9 y8 y7 y6 y5 y4 y3 y2 y1
b3
Int
100
75
50
25
0
y6
y7
y5
b3
y8
y3
y4
y1
y2
y8 2+
y9
m/z
0 100 200 300 400 500 600 700 800 900 1,000 1,100 1,200 1,300 1,400 1,500 1,600 1,700 1,800

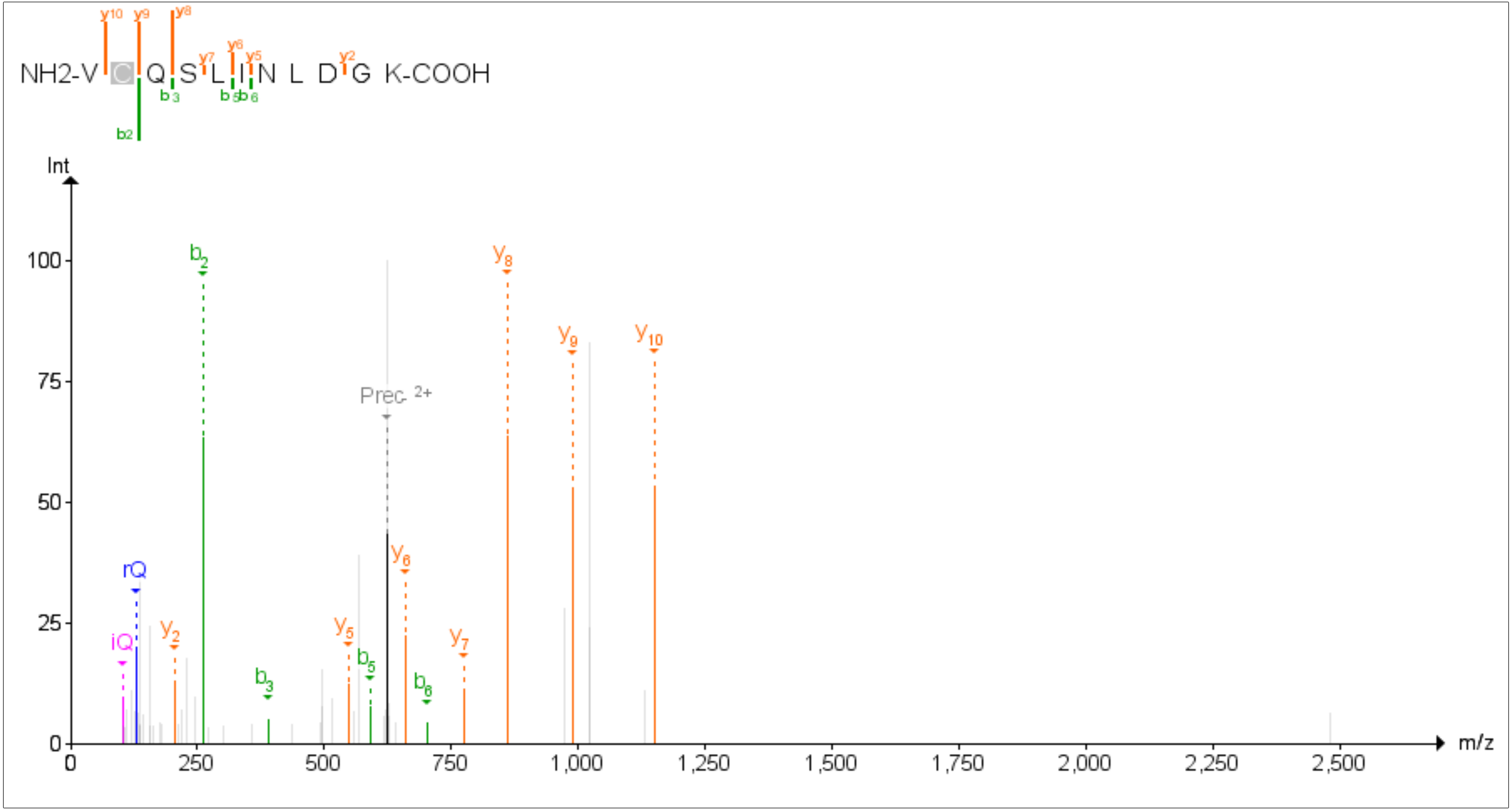

y10 y9 y8 y7 y6 y5 y2
NH2-V C Q S L I N L D G K-COOH
b2 b3 b5 b6
Int
100
75
50
25
0
b2
y8
y9
y10
Prec 2+
y6
rQ
iQ
y2
y5
b5
y7
b3
b6
0 250 500 750 1,000 1,250 1,500 1,750 2,000 2,250 2,500
m/z

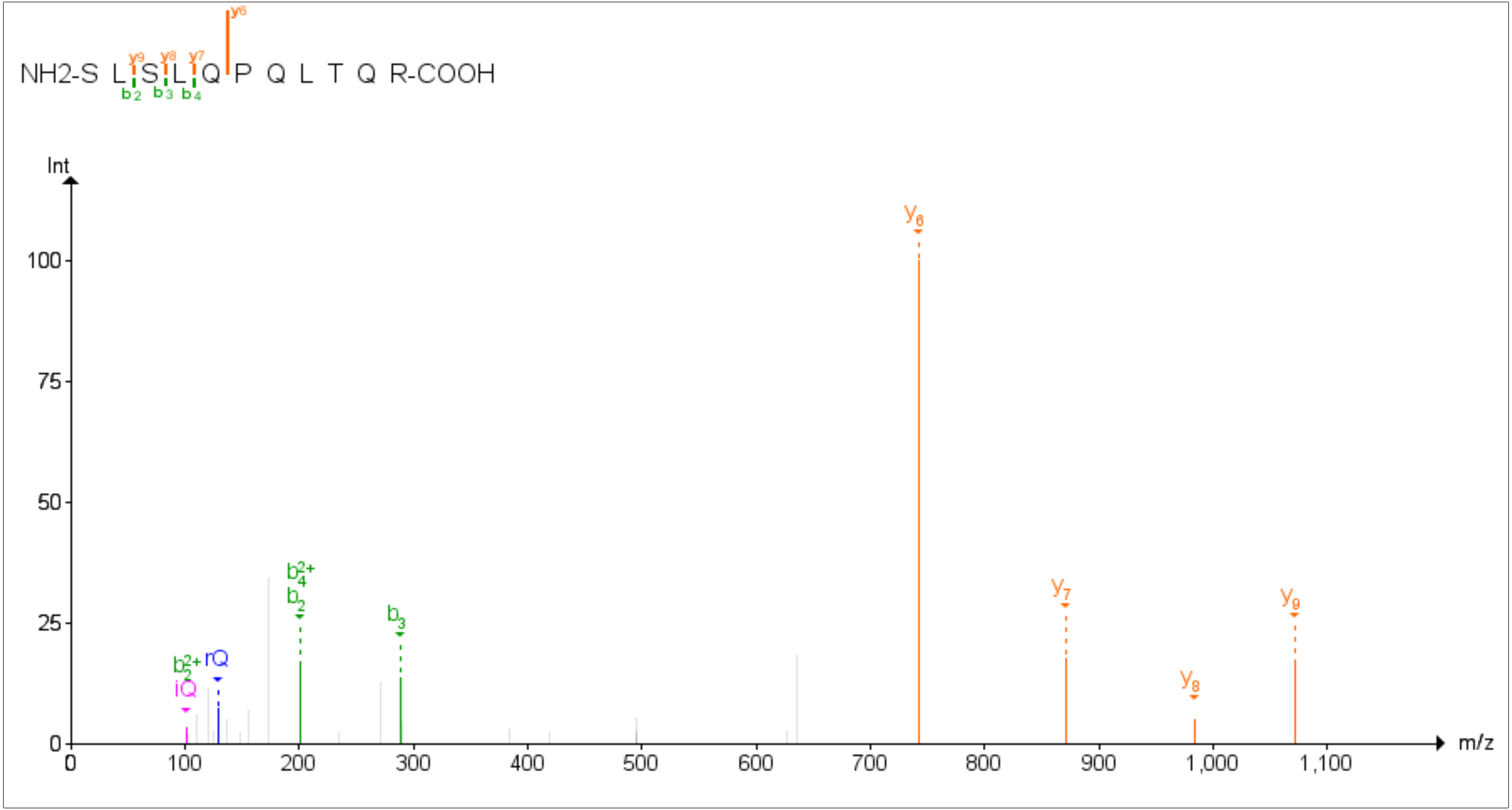

NH2-S L S L Q P Q L T Q R-COOH
y9 y8 y7 y6
b2 b3 b4
Int
100
75
50
25
0
m/z
0
100
200
300
400
500
600
700
800
900
1,000
1,100
y6
y7
y8
y9
b4 2+
b2
b3
b2 2+
rQ
iQ

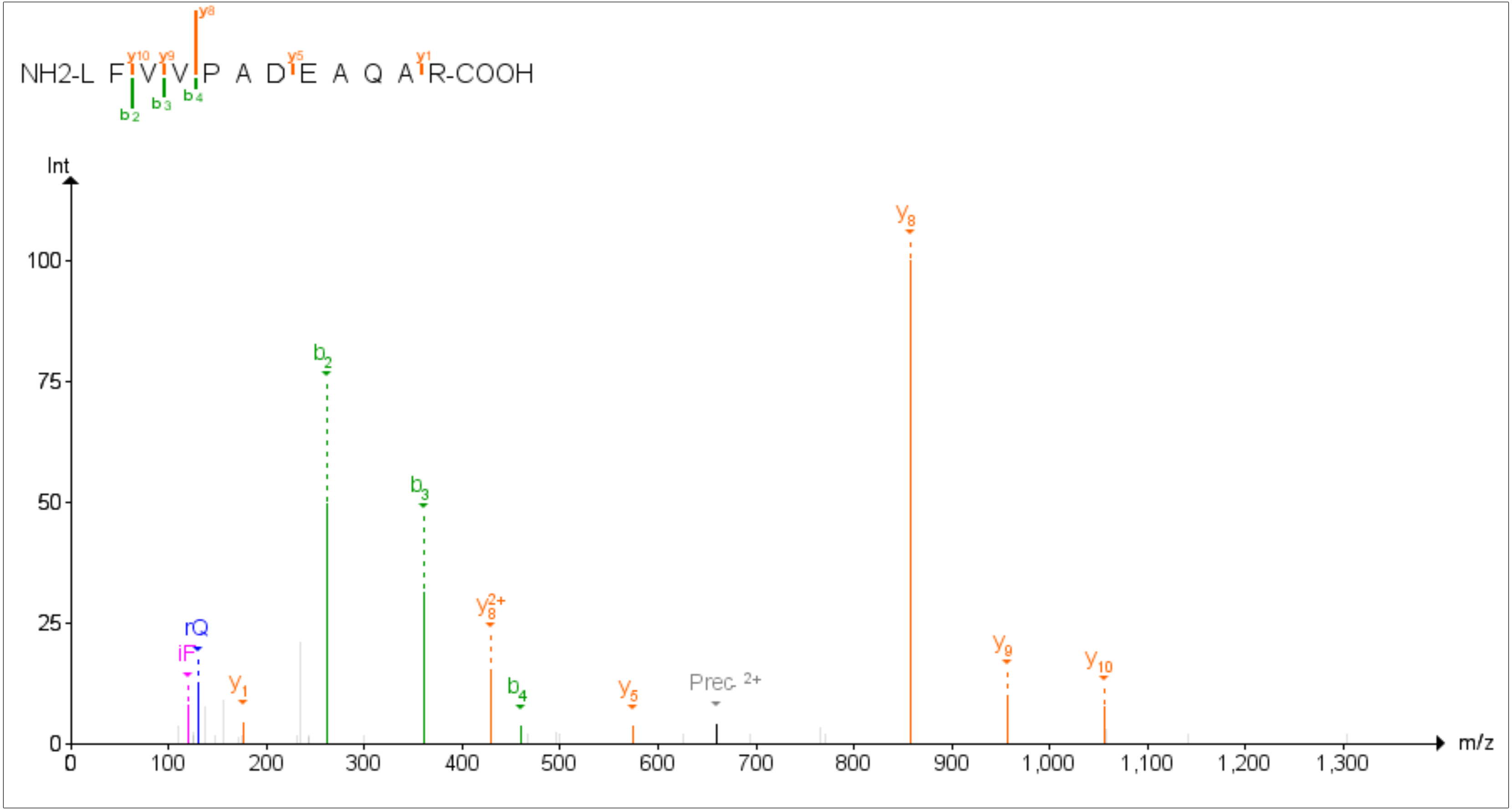

NH2-L F V V P A D E A Q A R-COOH
y10 y9 y8 y5 y1
b2 b3 b4
Int
100
75
50
25
0
y8
b2
b3
y8 2+
rQ
iF
y1
b4
y5
Prec 2+
y9
y10
0 100 200 300 400 500 600 700 800 900 1,000 1,100 1,200 1,300
m/z

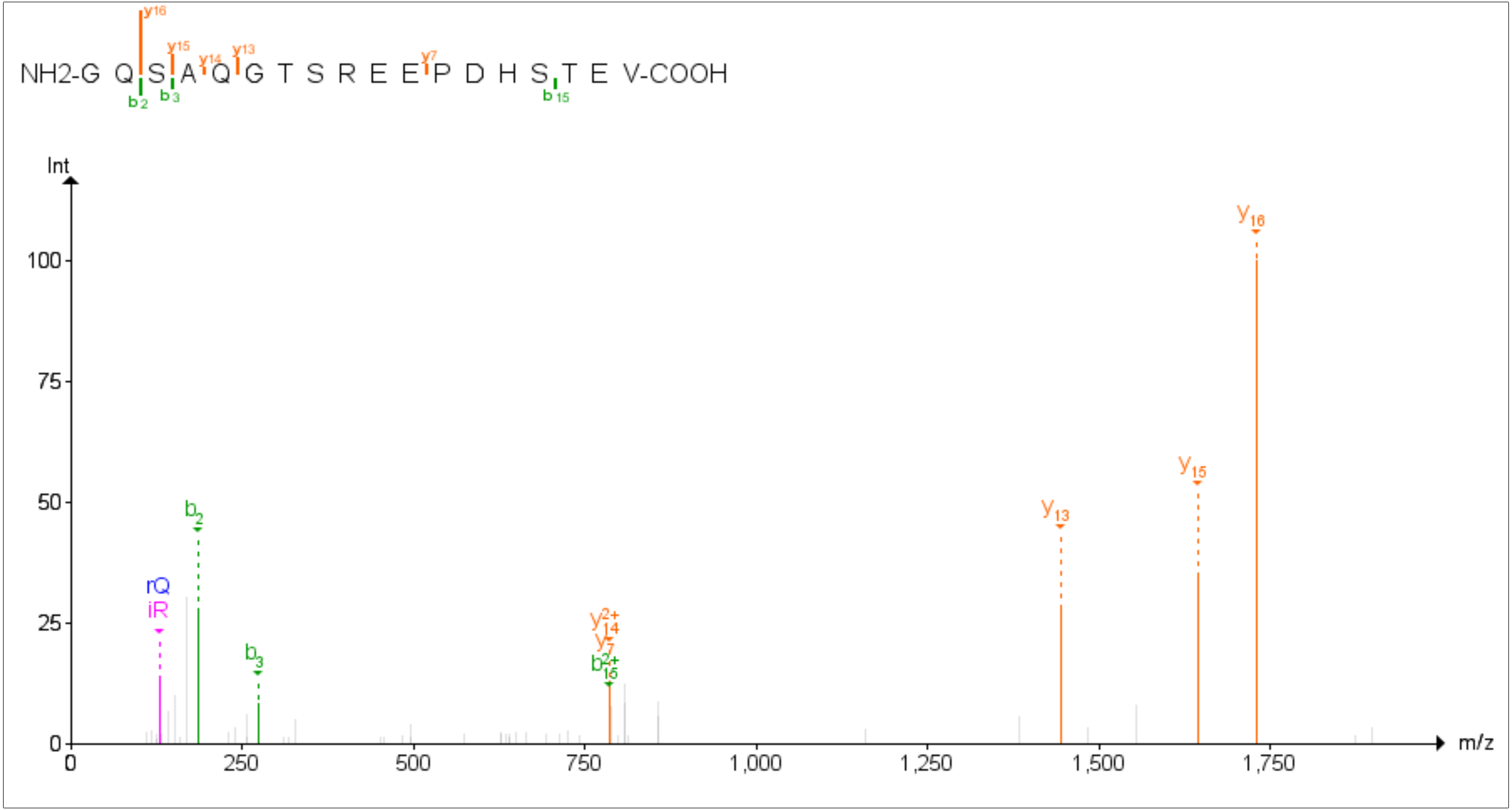

NH2-G Q S A Q G T S R E E P D H S T E V-COOH
y16
y15
y14
y13
y7
b2
b3
b15
Int
m/z
100
75
50
25
0
250
500
750
1,000
1,250
1,500
1,750
rQ
iR
b2
b3
y2+14
y7
b2+15
y13
y15
y16

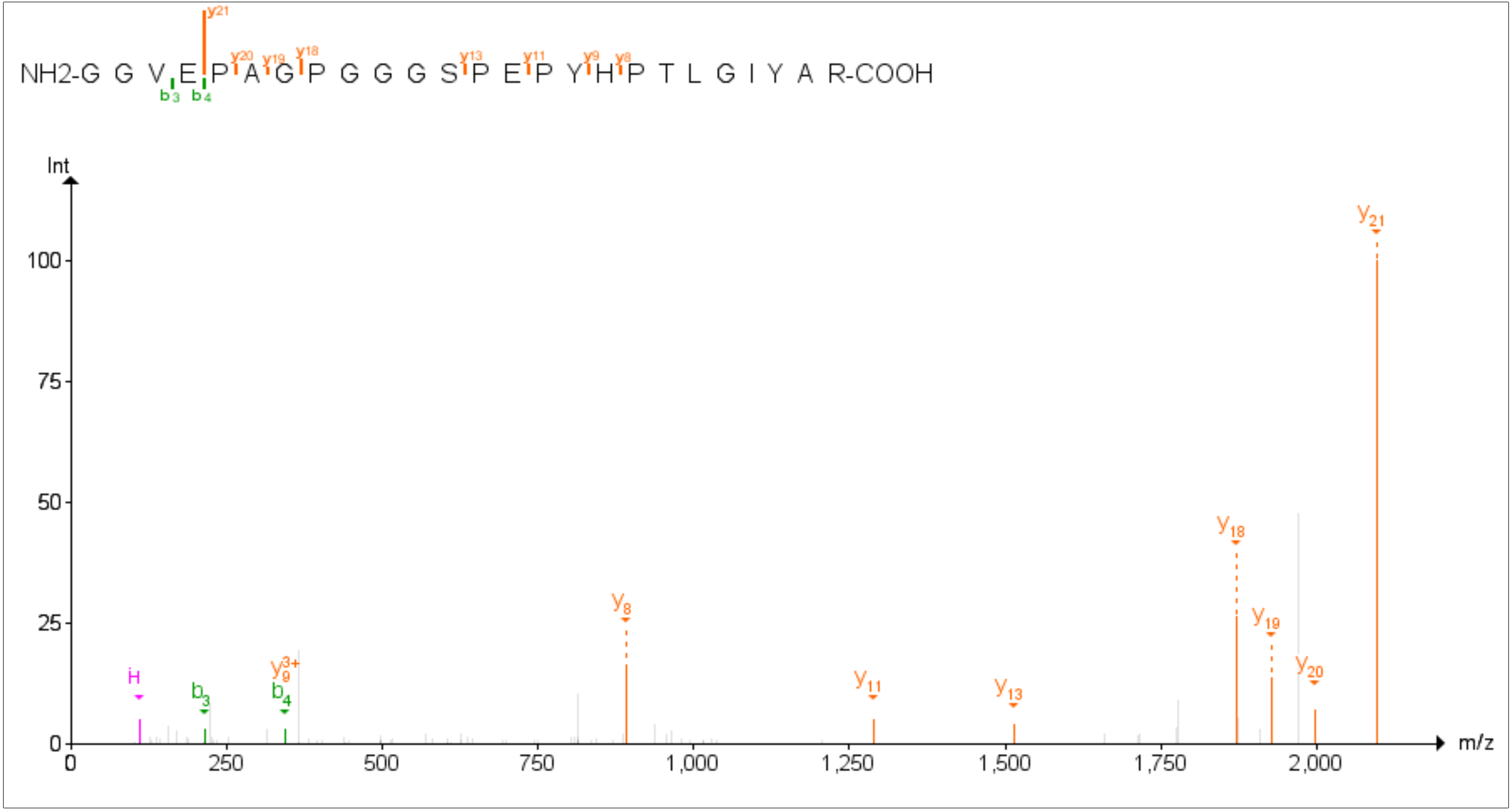

NH2-G G V E P A G P G G G S P E P Y H P T L G I Y A R-COOH
y21
y20
y19
y18
y13
y11
y9
y8
b3
b4
Int
100
75
50
25
0
m/z
0
250
500
750
1,000
1,250
1,500
1,750
2,000
H
b3
b4
y9 3+
y8
y11
y13
y18
y19
y20
y21

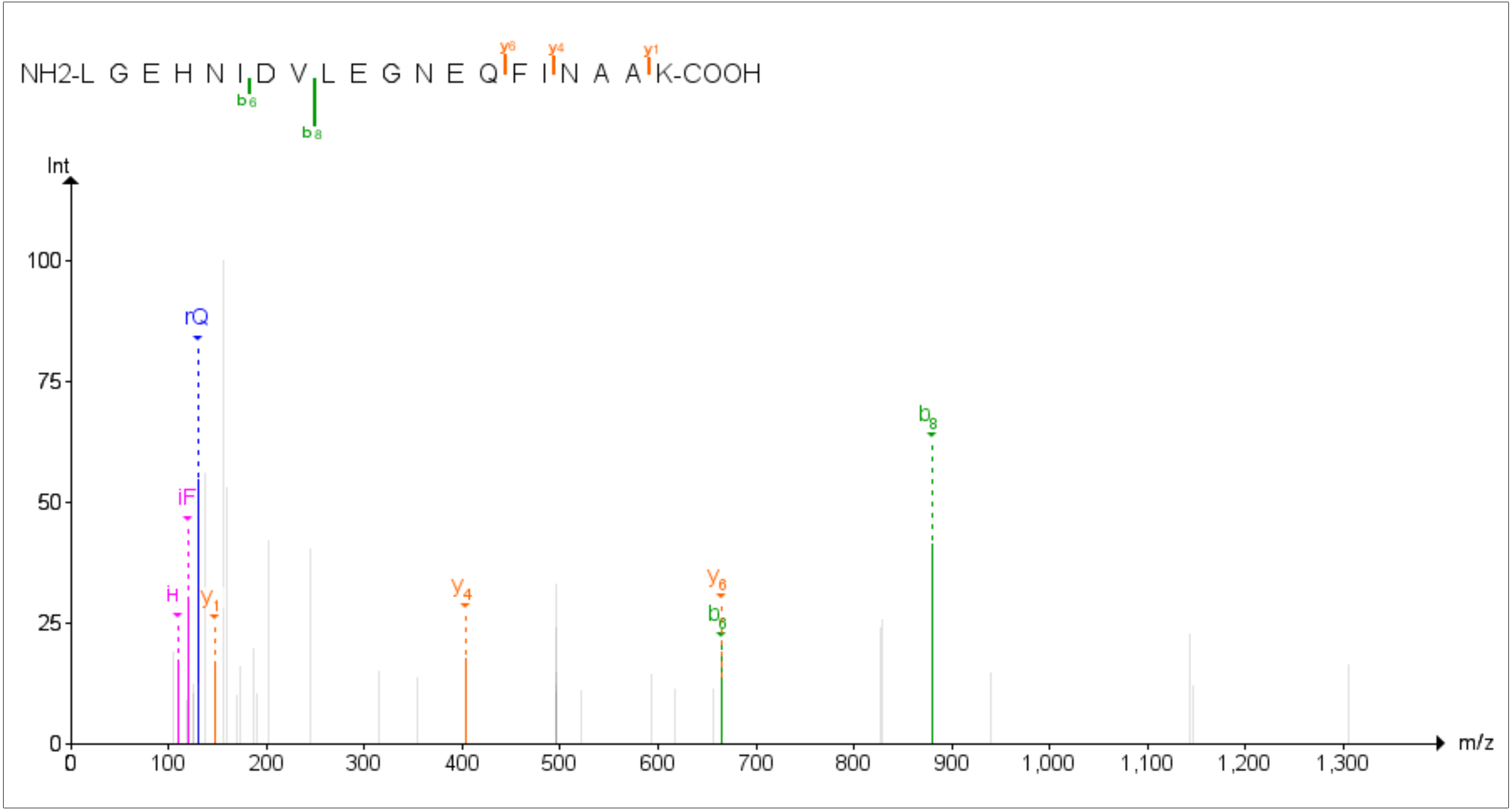

NH2-L G E H N I D V L E G N E Q F I N A A K-COOH
b6
b8
y6
y4
y1
Int
100
75
50
25
0
rQ
iF
iH
y1
y4
y6
b6
b8
0
100
200
300
400
500
600
700
800
900
1,000
1,100
1,200
1,300
m/z

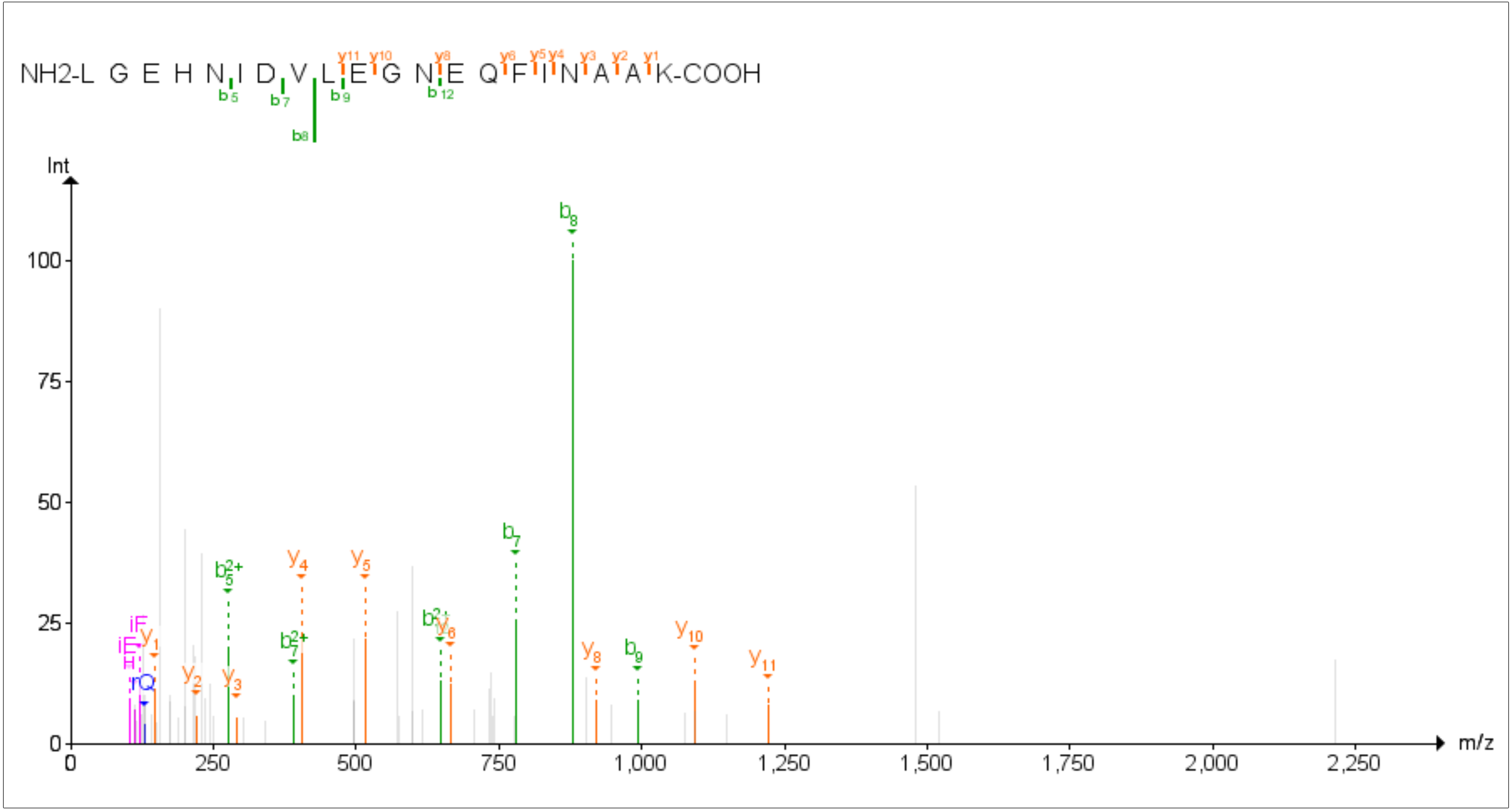

NH2-L G E H N I D V L E G N E Q F I N A A K-COOH
y11 y10 y8 y6 y5 y4 y3 y2 y1
b5 b7 b8 b9 b12
Int
100
75
50
25
0
b8
b7
b5 2+
b7 2+
y4
y5
y1
y2
y3
y6
y8
b9
y10
y11
iF
rQ
m/z
0
250
500
750
1,000
1,250
1,500
1,750
2,000
2,250

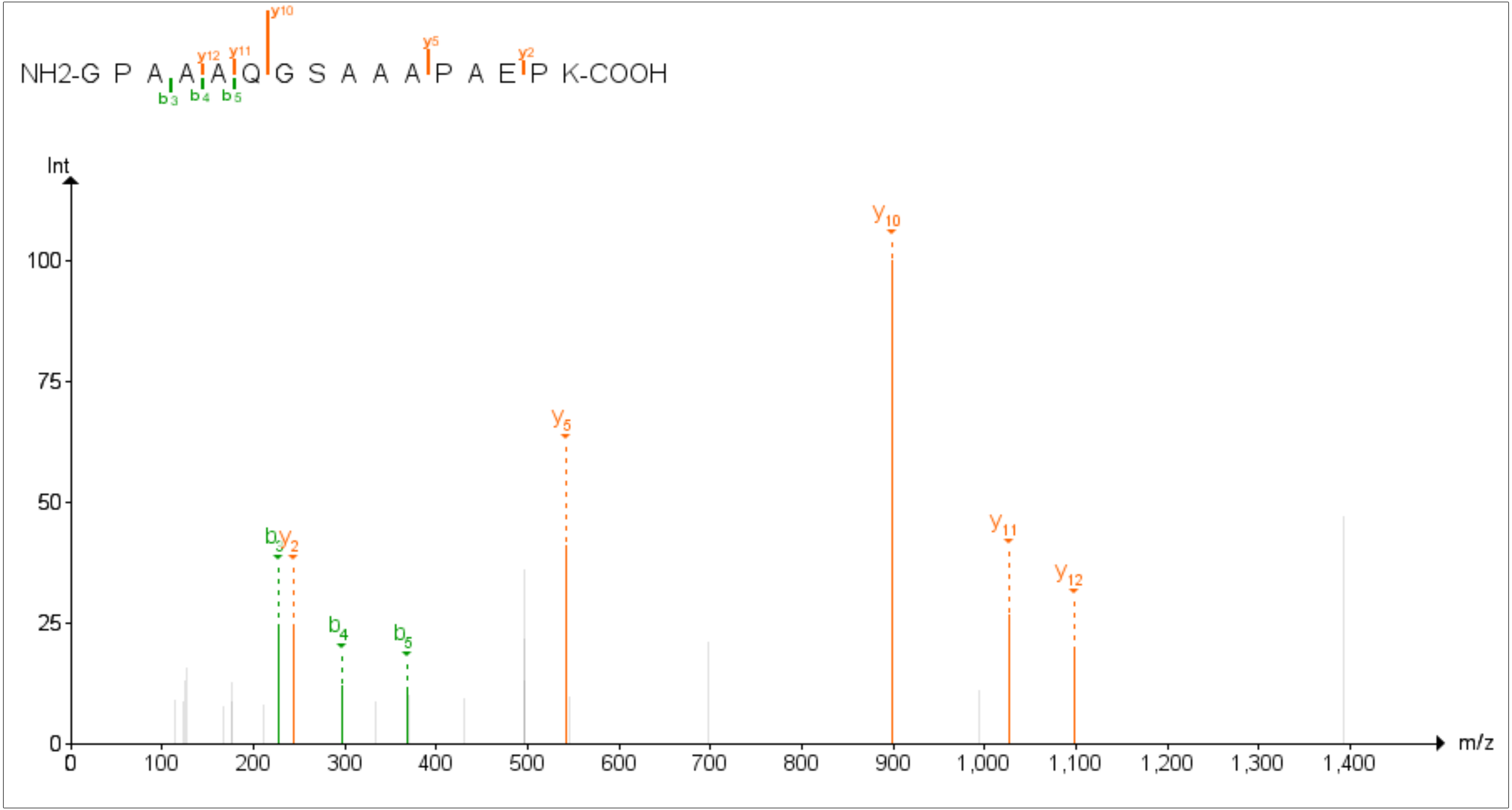

NH2-G P A A A Q G S A A A P A E P K-COOH
y10
y12
y11
y5
y2
b3
b4
b5
Int
100
75
50
25
0
y10
y5
b3
y2
b4
b5
y11
y12
m/z
0
100
200
300
400
500
600
700
800
900
1,000
1,100
1,200
1,300
1,400

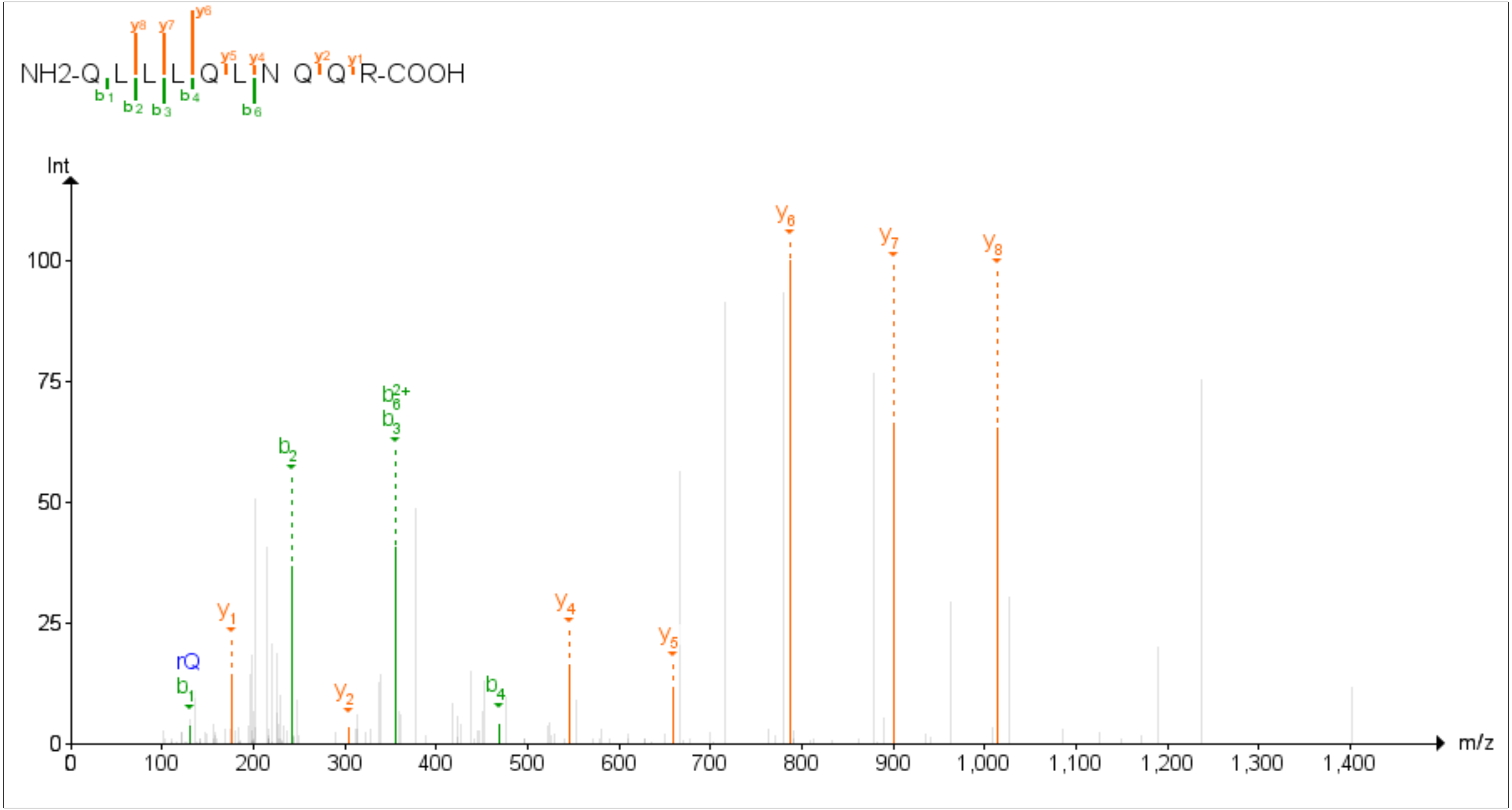
NH2-Q L L L Q L N Q Q R-COOH
y8 y7 y6 y5 y4 y2 y1
b1 b2 b3 b4 b6
Int
m/z
y6
y7
y8
b6 2+
b3
b2
rQ
b1
y1
y2
b4
y4
y5

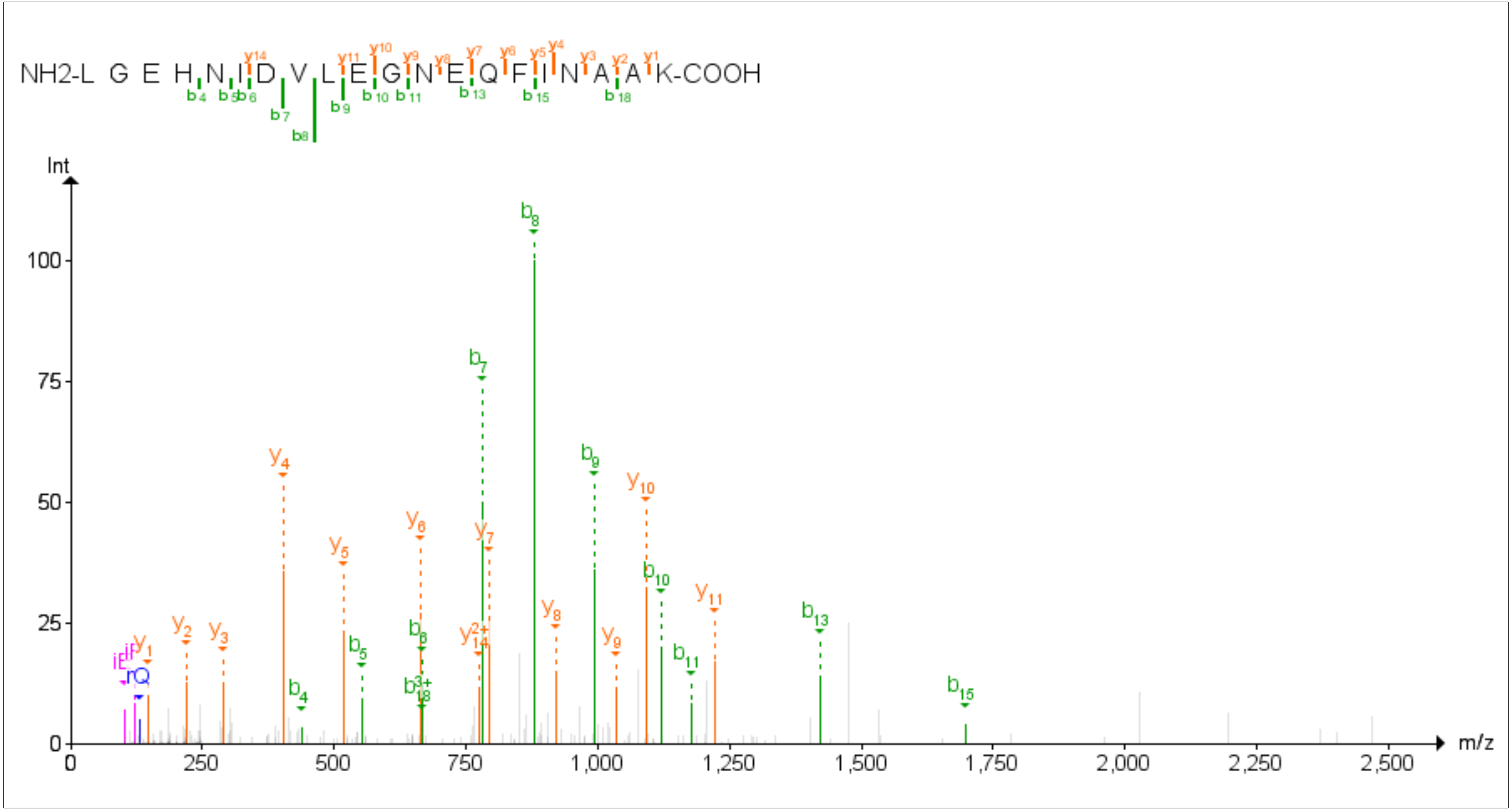

NH2-L G E H N I D V L E G N E Q F I N A A K-COOH
Int
m/z

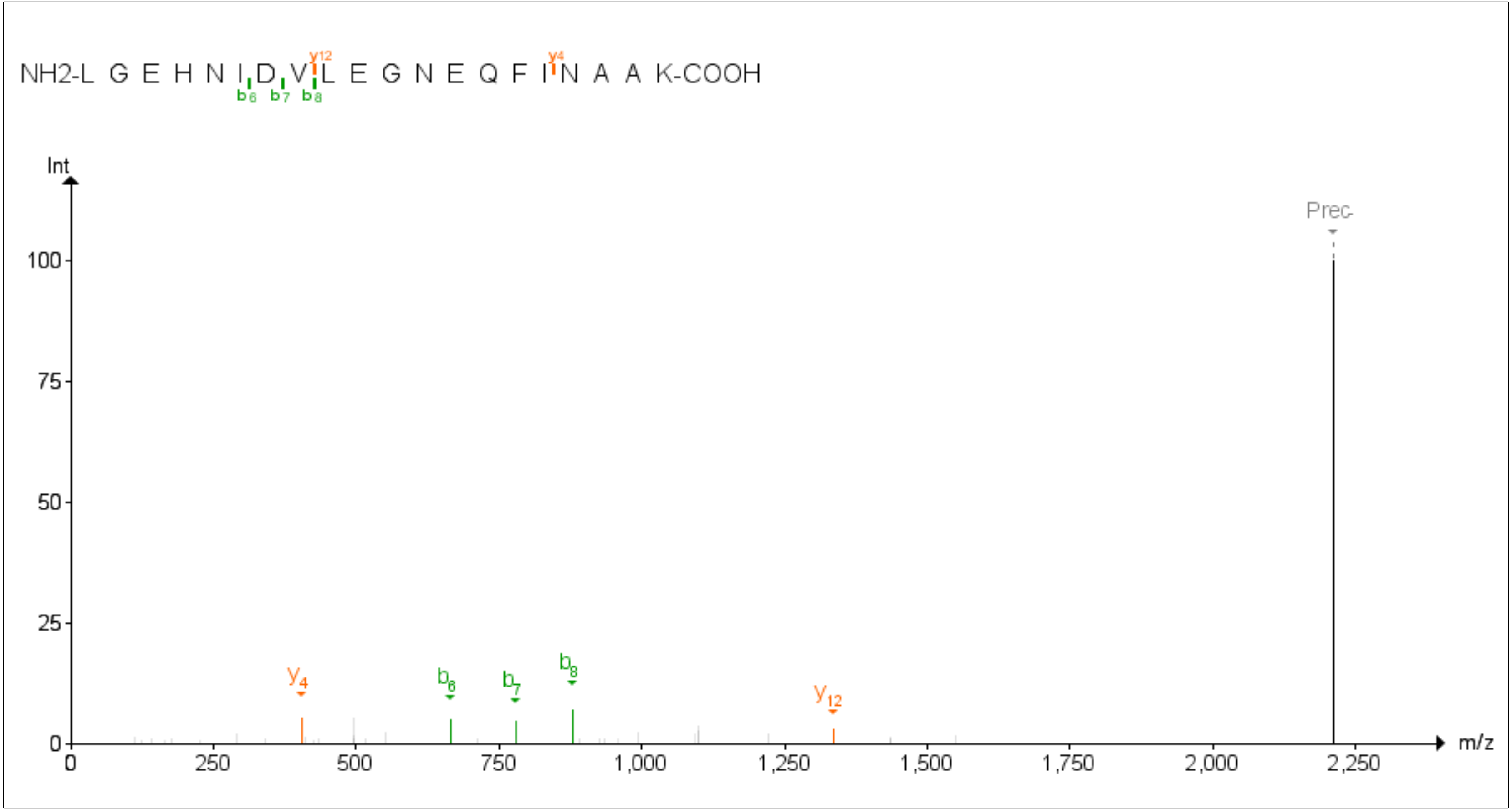
NH2-L G E H N I D V L E G N E Q F I N A A K-COOH
y12
y4
b6
b7
b8
Int
Prec
100
75
50
25
0
y4
b6
b7
b8
y12
0
250
500
750
1,000
1,250
1,500
1,750
2,000
2,250
m/z

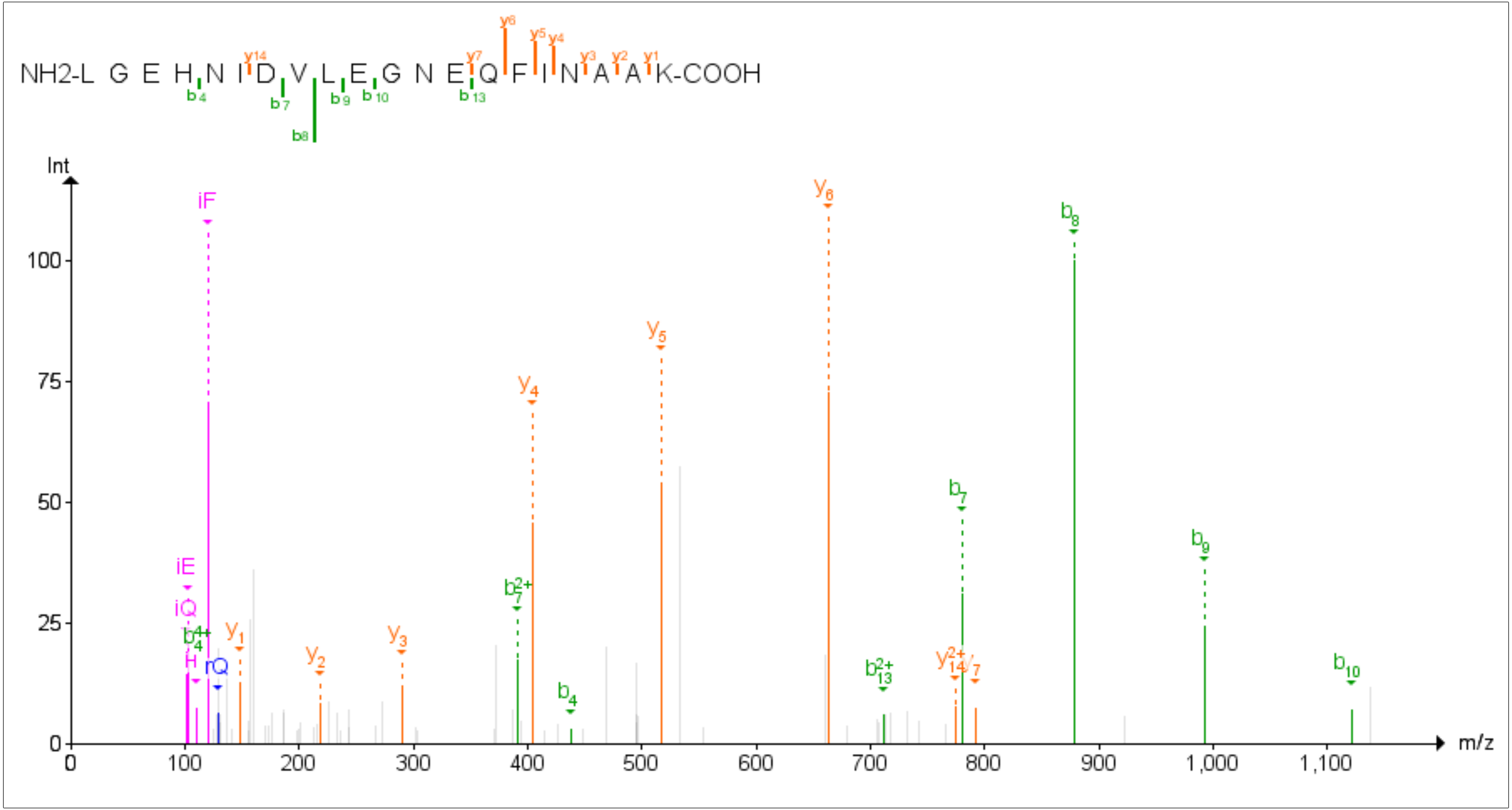

NH2-L G E H N I D V L E G N E Q F I N A A K-COOH
Int
m/z
iF
iE
iQ
H
rQ
y6
y5
y4
y3
y2
y1
b8
b7
b9
b10
b4

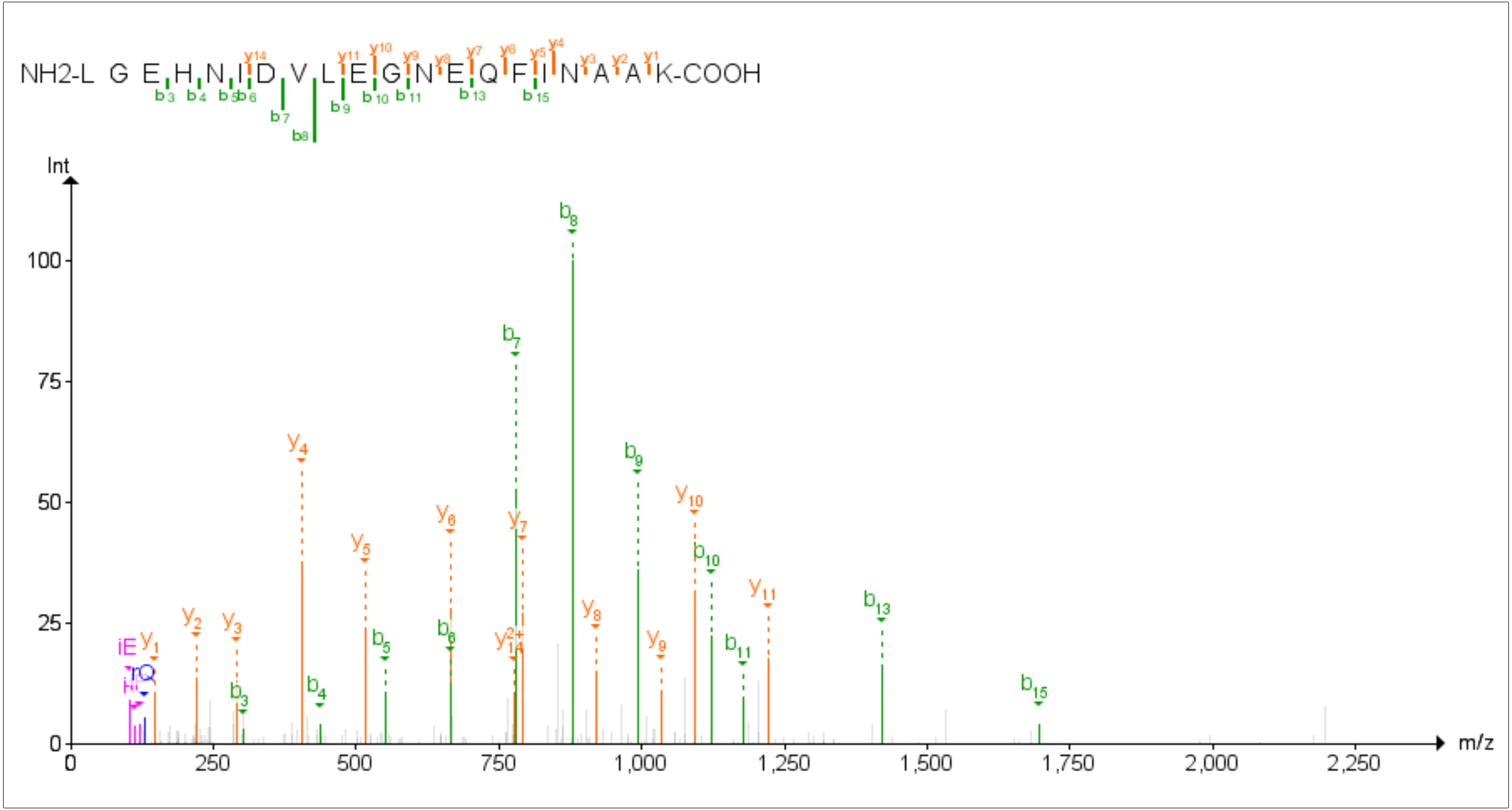
NH2-L G E H N I D V L E G N E Q F I N A A K-COOH
Int
m/z
0
25
50
75
100
250
500
750
1,000
1,250
1,500
1,750
2,000
2,250

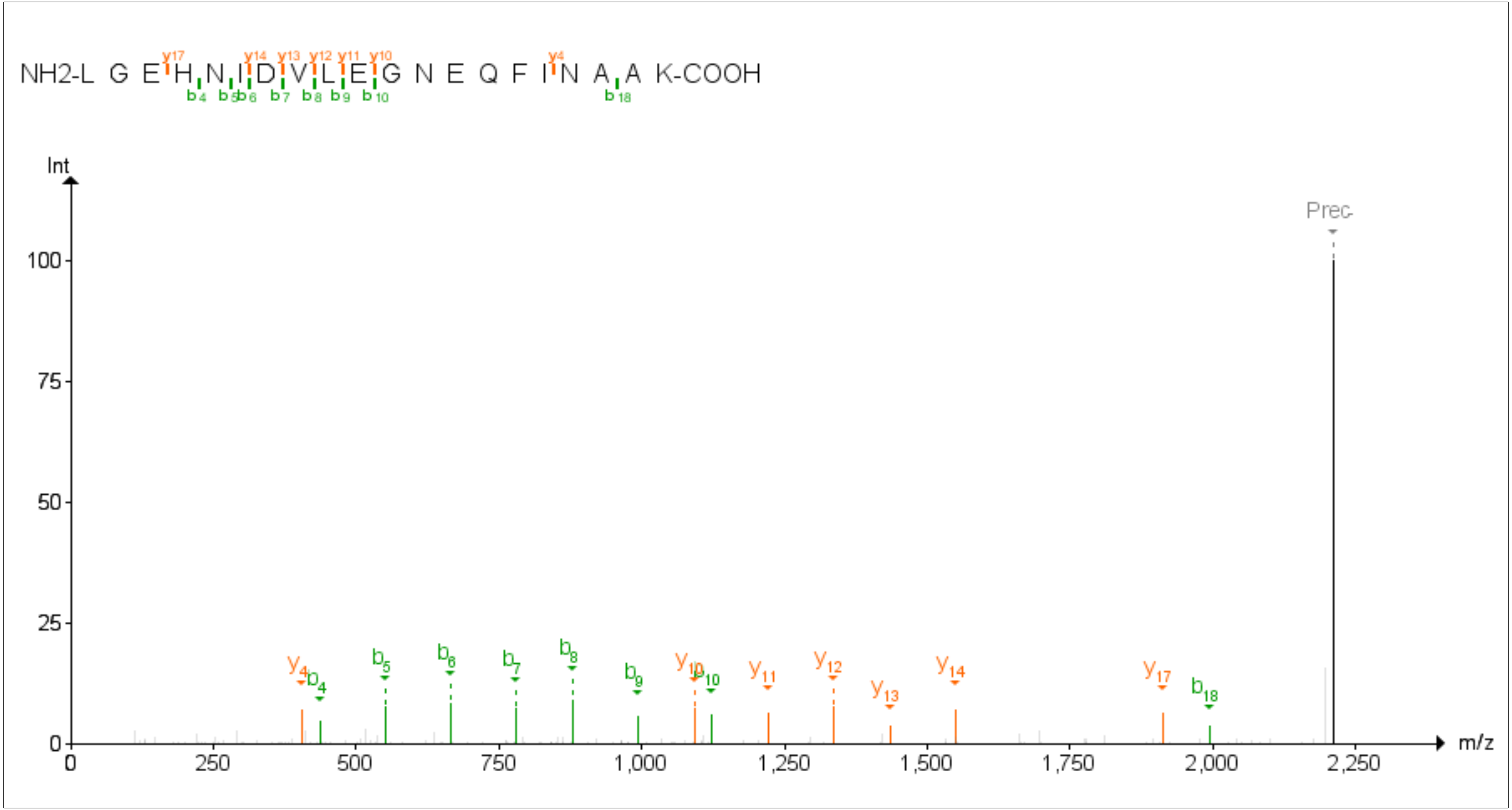

NH2-L G E H N I D V L E G N E Q F I N A A K-COOH
Int
100
75
50
25
0
Prec
m/z
0
250
500
750
1,000
1,250
1,500
1,750
2,000
2,250

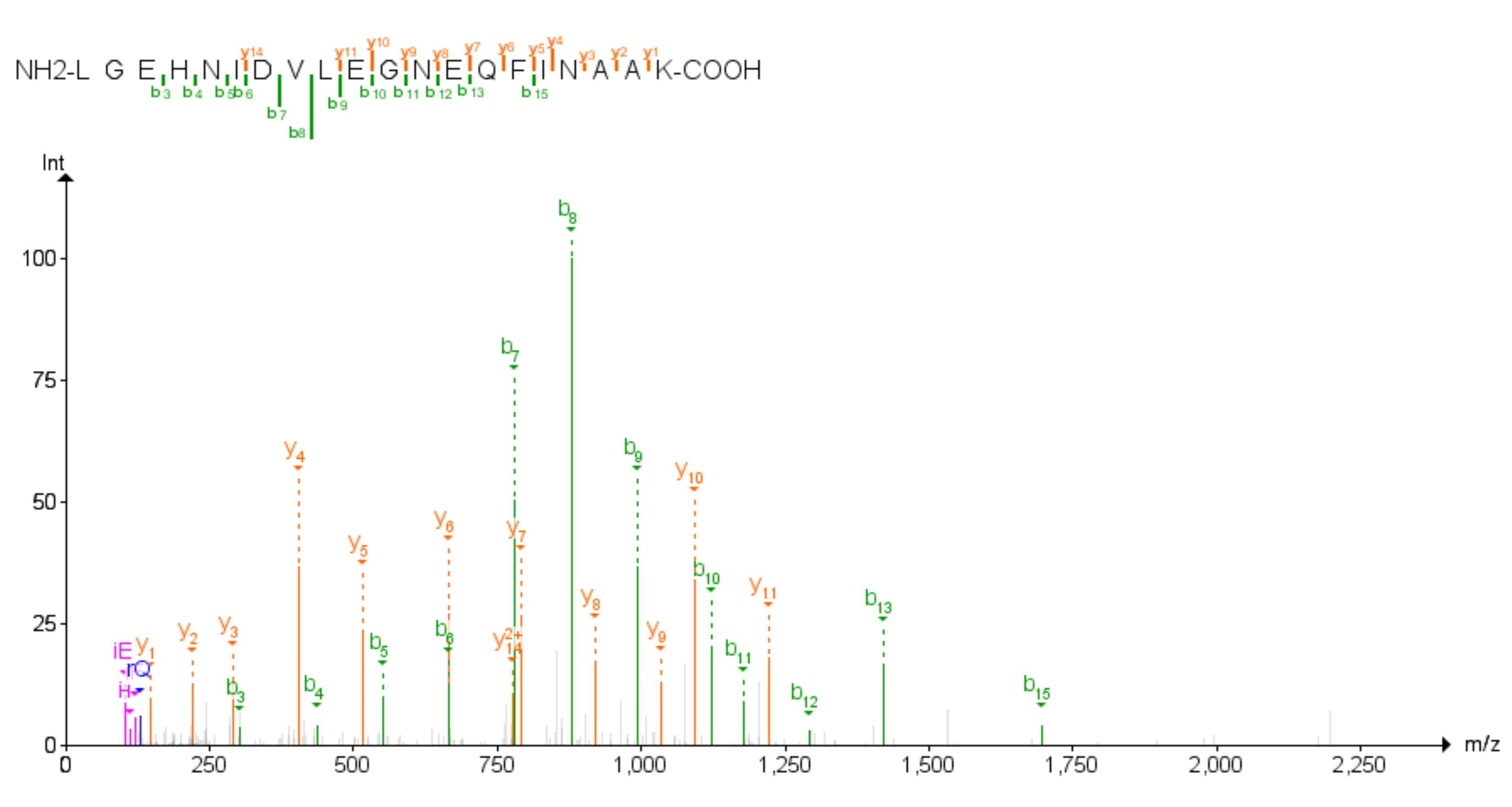

NH2-L G E H N I D V L E G N E Q F I N A A K-COOH
Int
m/z
0
25
50
75
100
250
500
750
1,000
1,250
1,500
1,750
2,000
2,250

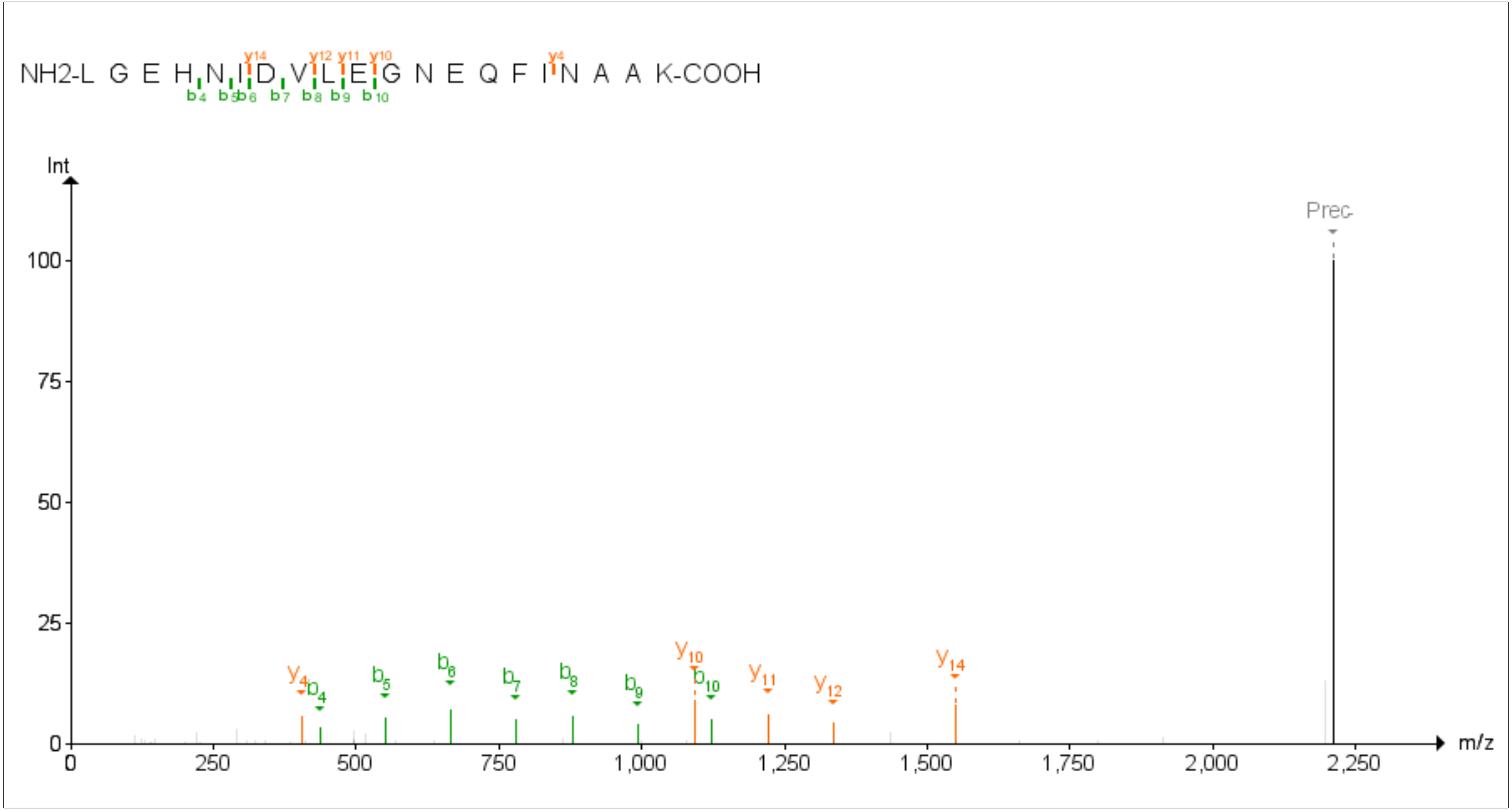

NH2-L G E H N I D V L E G N E Q F I N A A K-COOH
y14 y12 y11 y10 y4
b4 b5 b6 b7 b8 b9 b10
Int
100
75
50
25
0
Prec
y4 b4 b5 b6 b7 b8 b9 y10 b10 y11 y12 y14
0 250 500 750 1,000 1,250 1,500 1,750 2,000 2,250
m/z

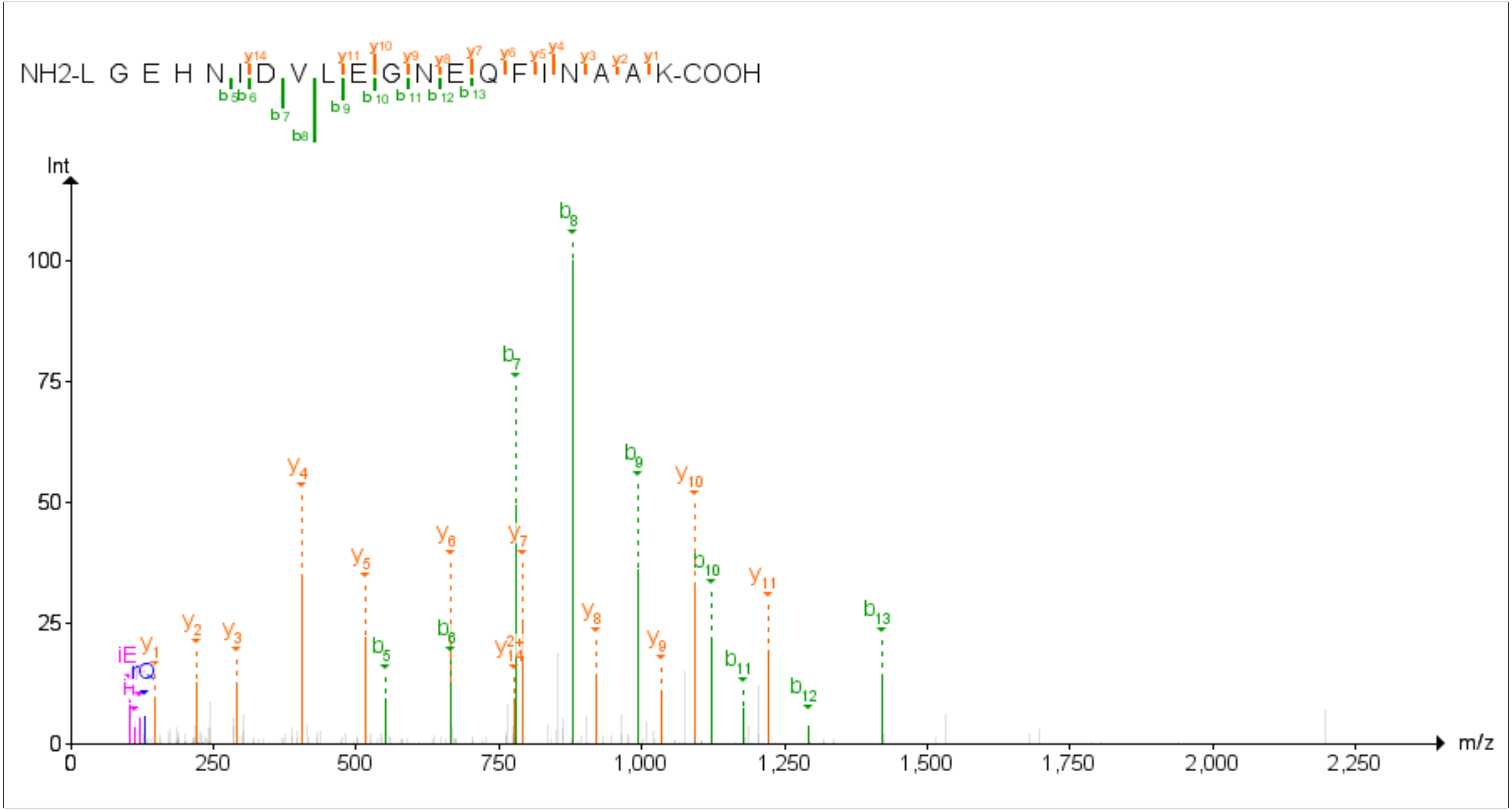
NH2-L G E H N I D V L E G N E Q F I N A A K-COOH
Int
m/z
b8
b7
b9
y10
y4
y6
y7
y5
b10
y11
b13
y8
b6
b5
y9
b11
b12
y2
y3
y1
iE
rQ
iH
0
25
50
75
100
250
500
750
1,000
1,250
1,500
1,750
2,000
2,250

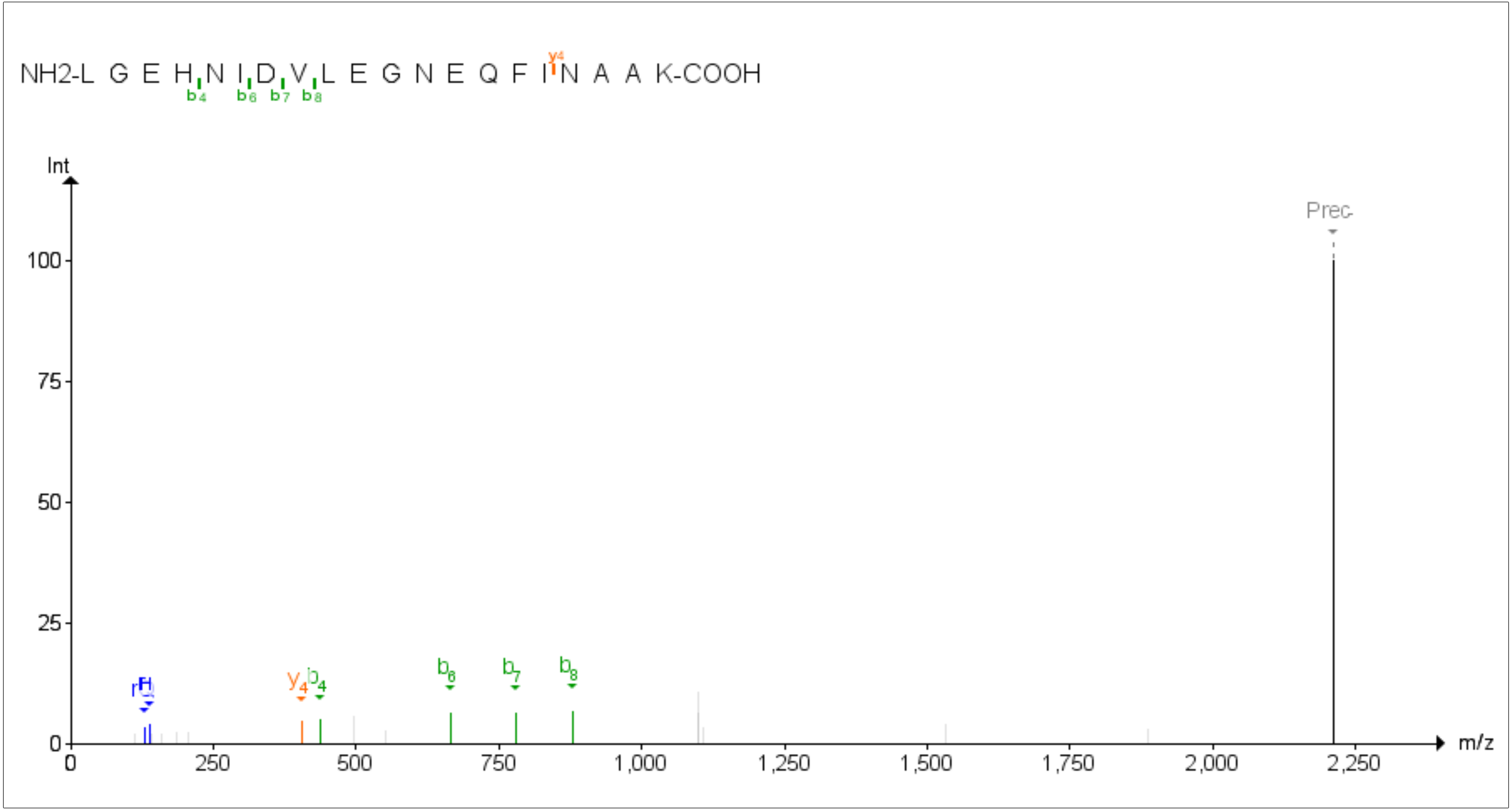

NH2-L G E H N I D V L E G N E Q F I N A A K-COOH
b4
b6
b7
b8
y4
Int
100
75
50
25
0
Prec
y4
b4
b6
b7
b8
0
250
500
750
1,000
1,250
1,500
1,750
2,000
2,250
m/z

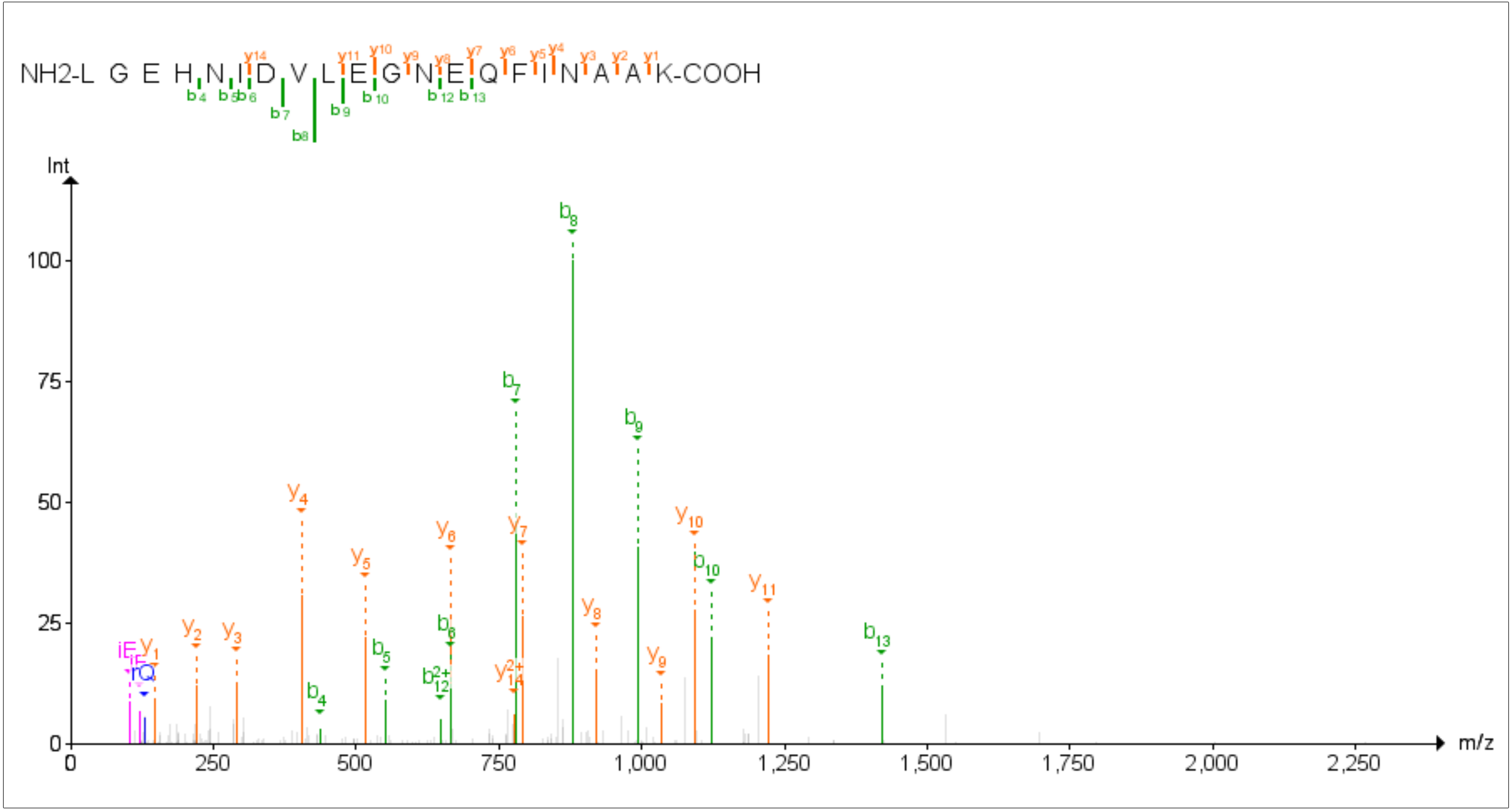

NH2-L G E H N I D V L E G N E Q F I N A A K-COOH
Int
m/z
b8
b7
b9
y4
y10
y7
y6
b10
y5
y11
y8
b6
b5
b13
y9
y2
y3
y1
rQ
b4
0
25
50
75
100
250
500
750
1,000
1,250
1,500
1,750
2,000
2,250

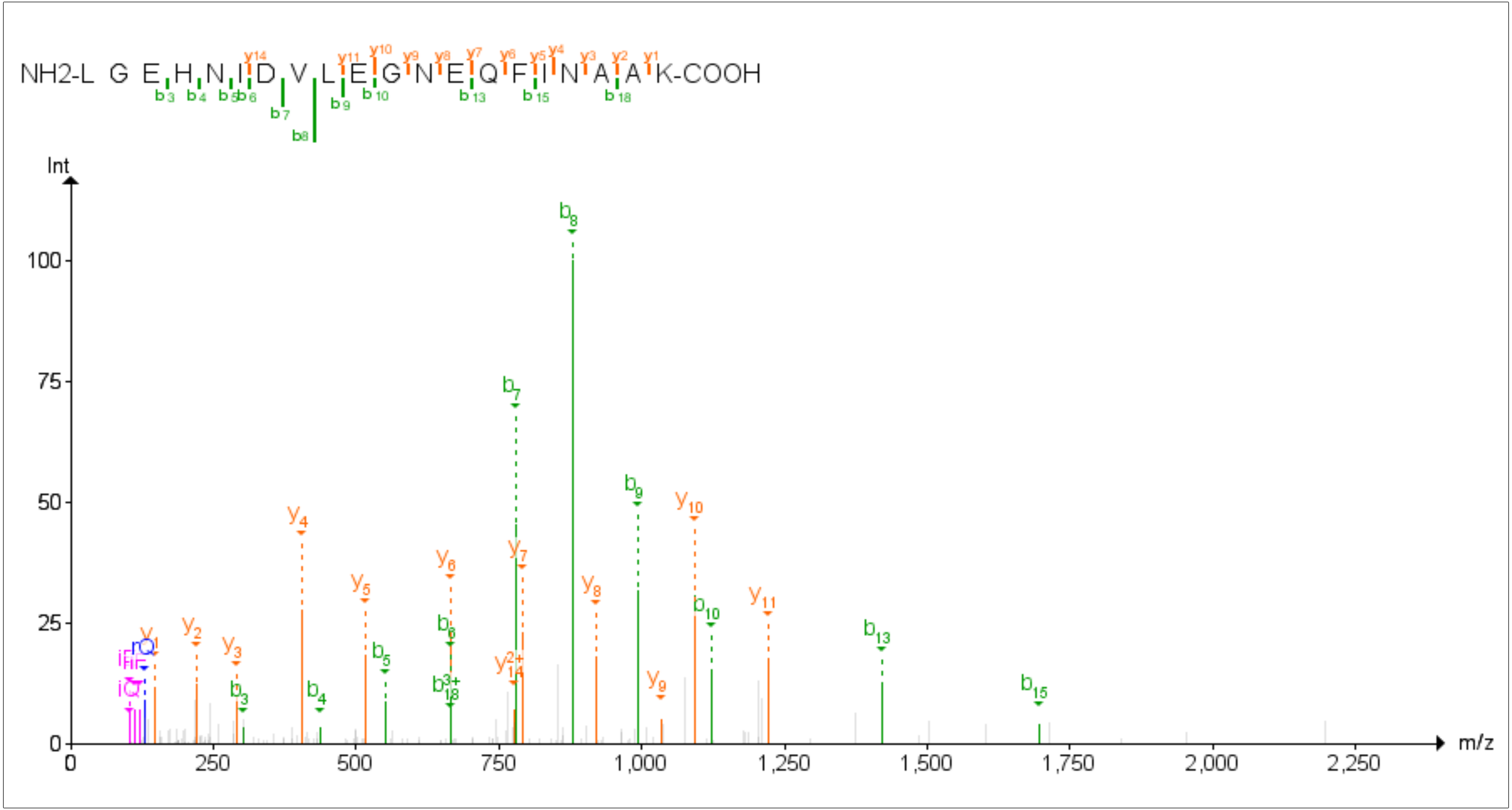

NH2-L G E H N I D V L E G N E Q F I N A A K-COOH
Int
m/z
b8
b7
b9
y10
y4
y6
y7
y5
y8
b10
y11
b6
b13
y2
y3
b5
y14 2+
b18 3+
y9
b3
b4
b15

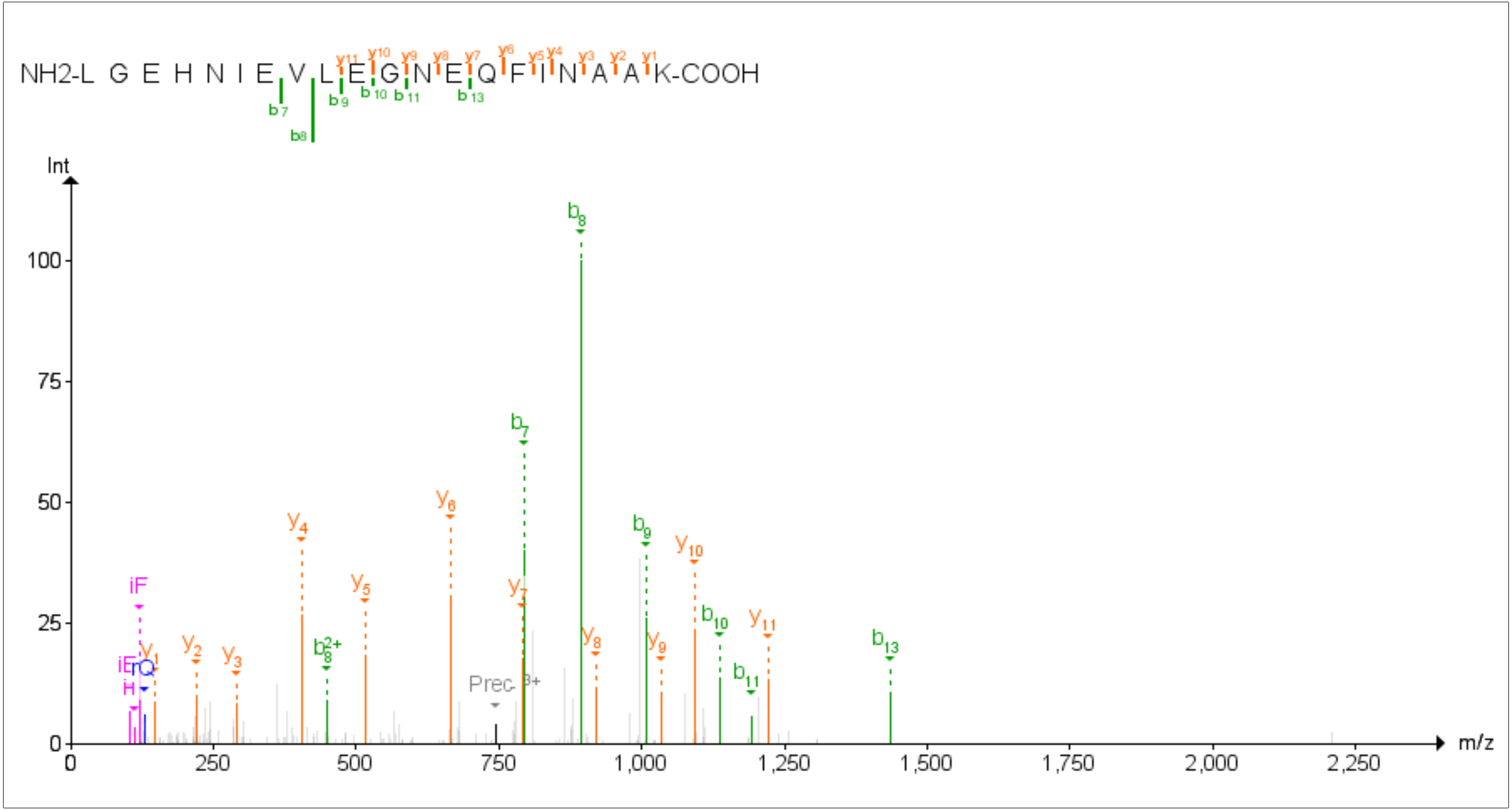

NH2-L G E H N I E V L E G N E Q F I N A A K-COOH
Int
m/z
0
25
50
75
100
250
500
750
1,000
1,250
1,500
1,750
2,000
2,250
Prec 3+

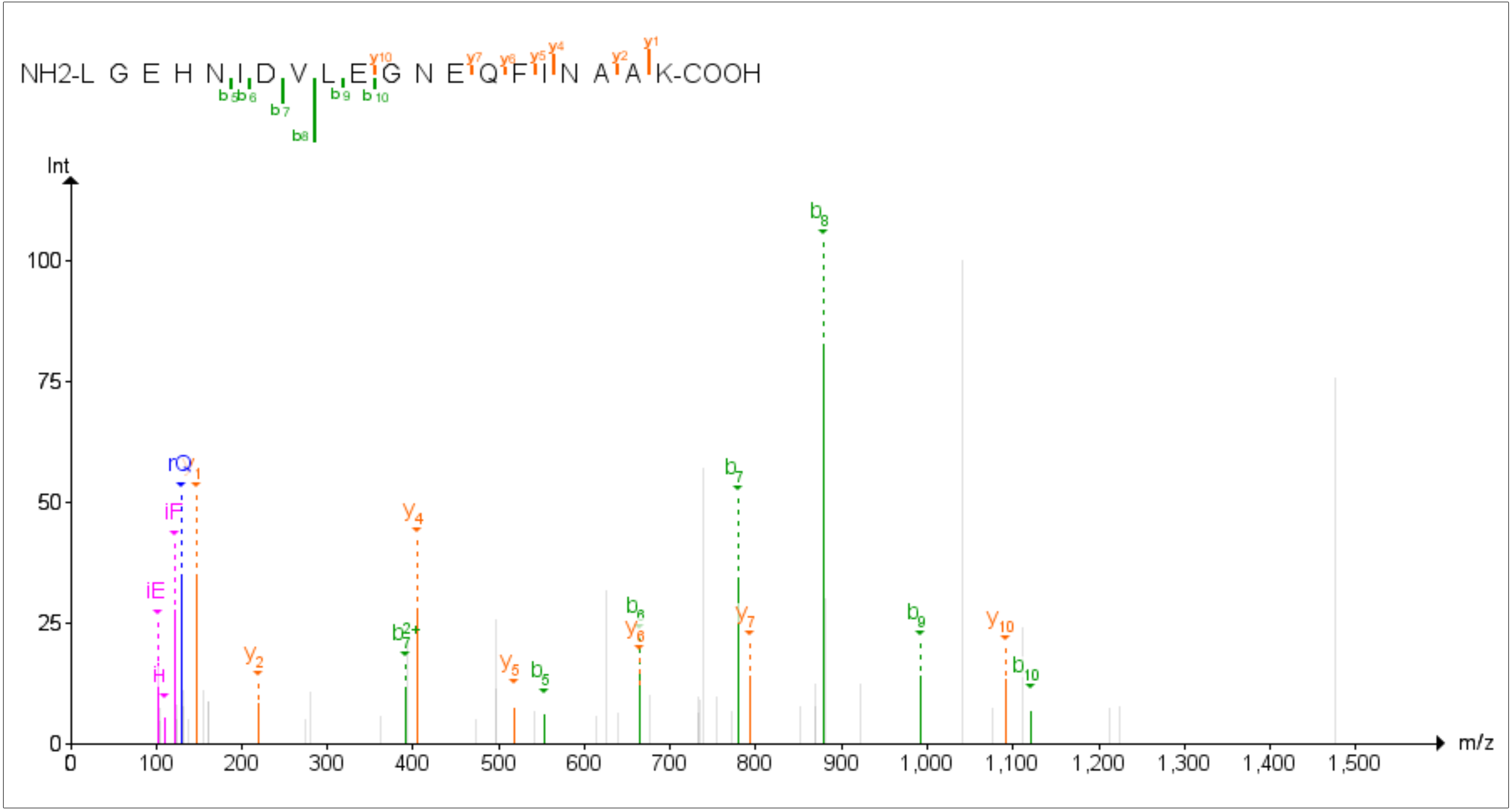
NH2-L G E H N I D V L E G N E Q F I N A A K-COOH
Int
m/z
b8
b7
b9
b10
b5
y10
y7
y4
y5
y2
y1
rQ
iF
iE
iH
y4
b7 2+
y2
y5
b5
b6
y6
y7
b9
y10
b10

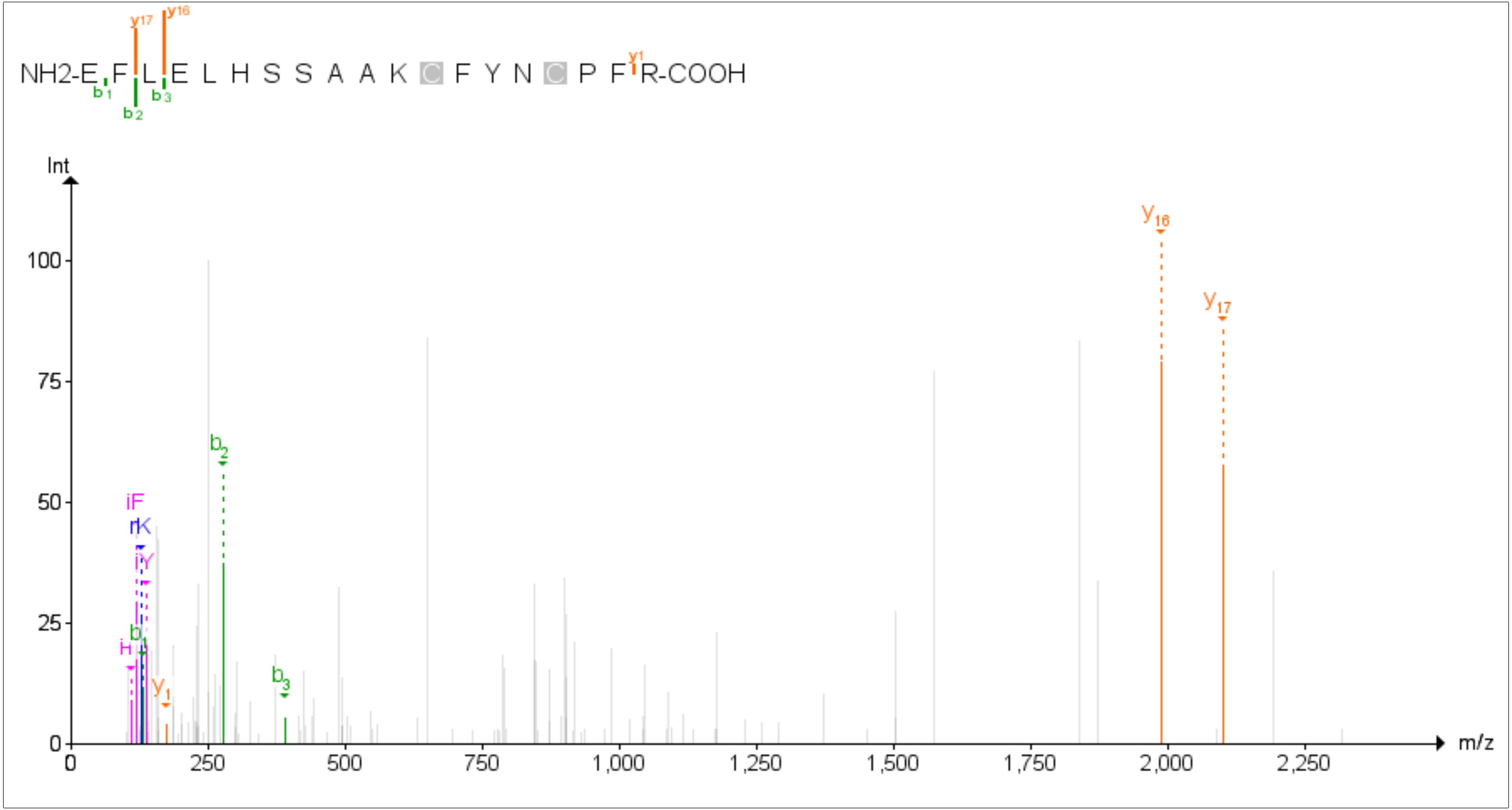
NH2-E F L E L H S S A A K C F Y N C P F R-COOH
y17
y16
y1
b1
b2
b3
Int
100
75
50
25
0
y16
y17
b2
iF
rK
iY
b1
iH
y1
b3
0
250
500
750
1,000
1,250
1,500
1,750
2,000
2,250
m/z

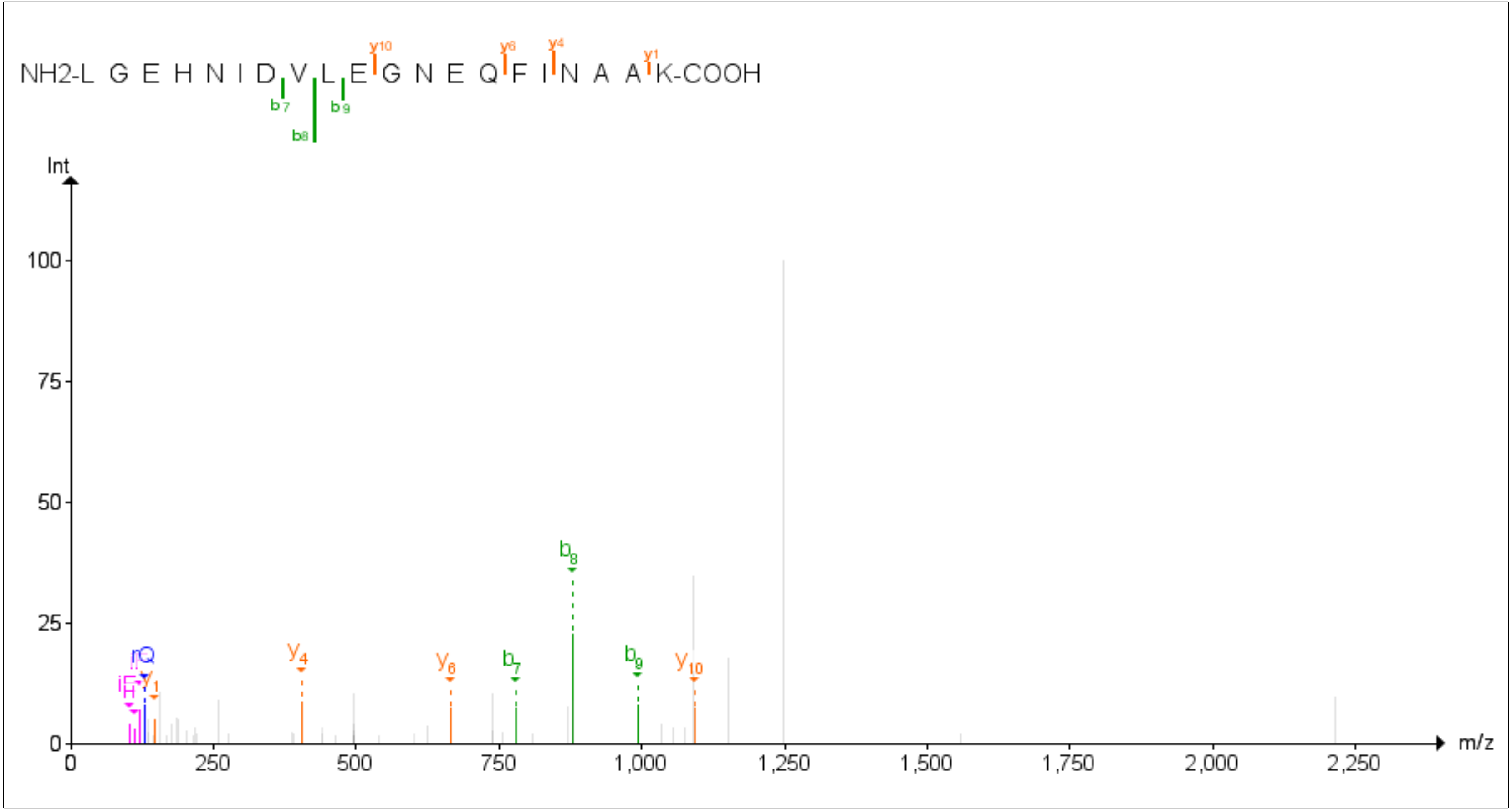

NH2-L G E H N I D V L E G N E Q F I N A A K-COOH
y10
y6
y4
y1
b7
b8
b9
Int
100
75
50
25
0
b8
y4
y6
b7
b9
y10
nQ
iF
iH
y1
m/z
0
250
500
750
1,000
1,250
1,500
1,750
2,000
2,250

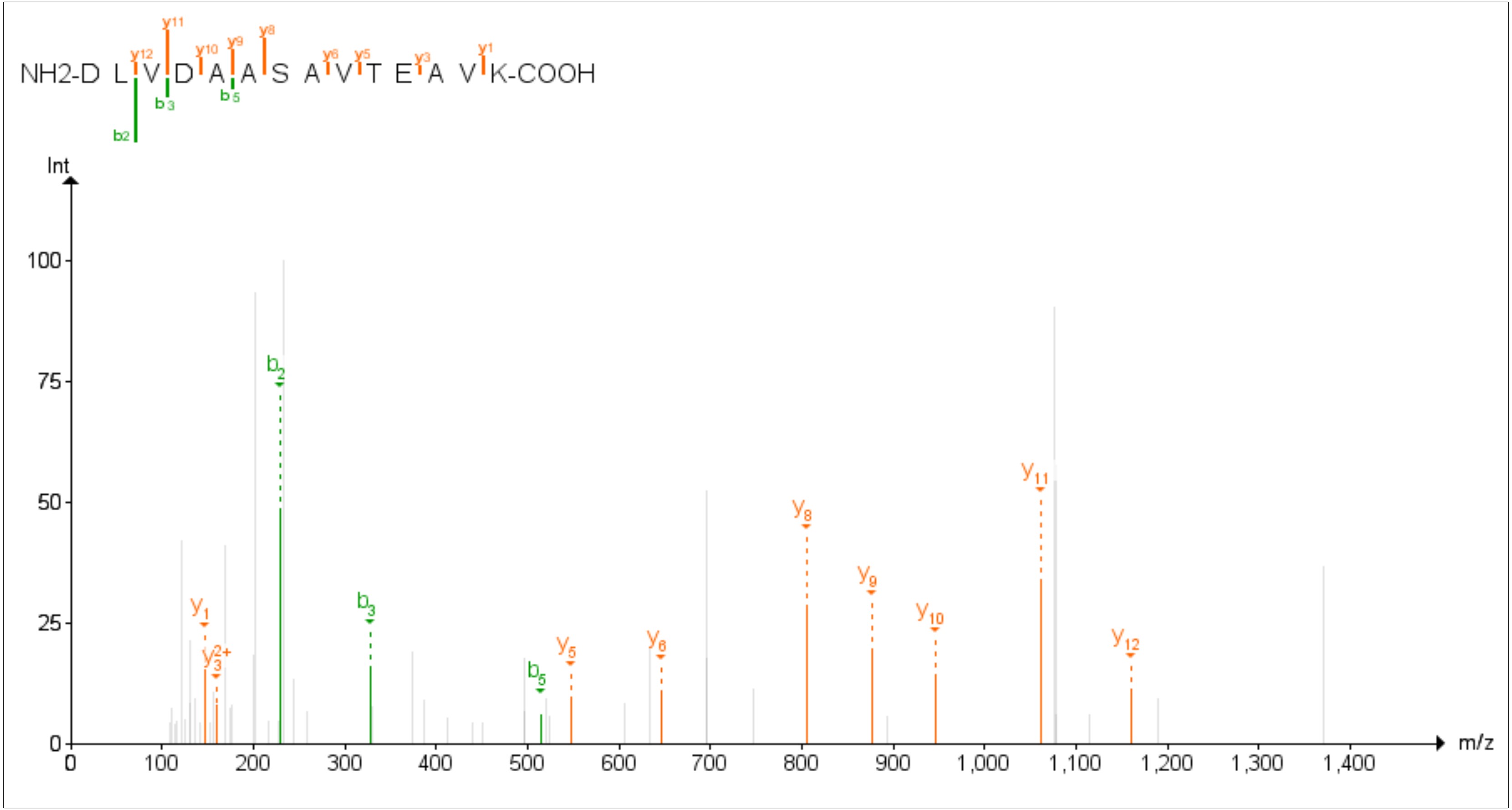

NH2-D L V D A A S A V T E A V K-COOH
y12 y11 y10 y9 y8 y6 y5 y3 y1
b2 b3 b5
Int
100
75
50
25
0
m/z
0 100 200 300 400 500 600 700 800 900 1,000 1,100 1,200 1,300 1,400
b2
b3
b5
y1
y3 2+
y5
y6
y8
y9
y10
y11
y12

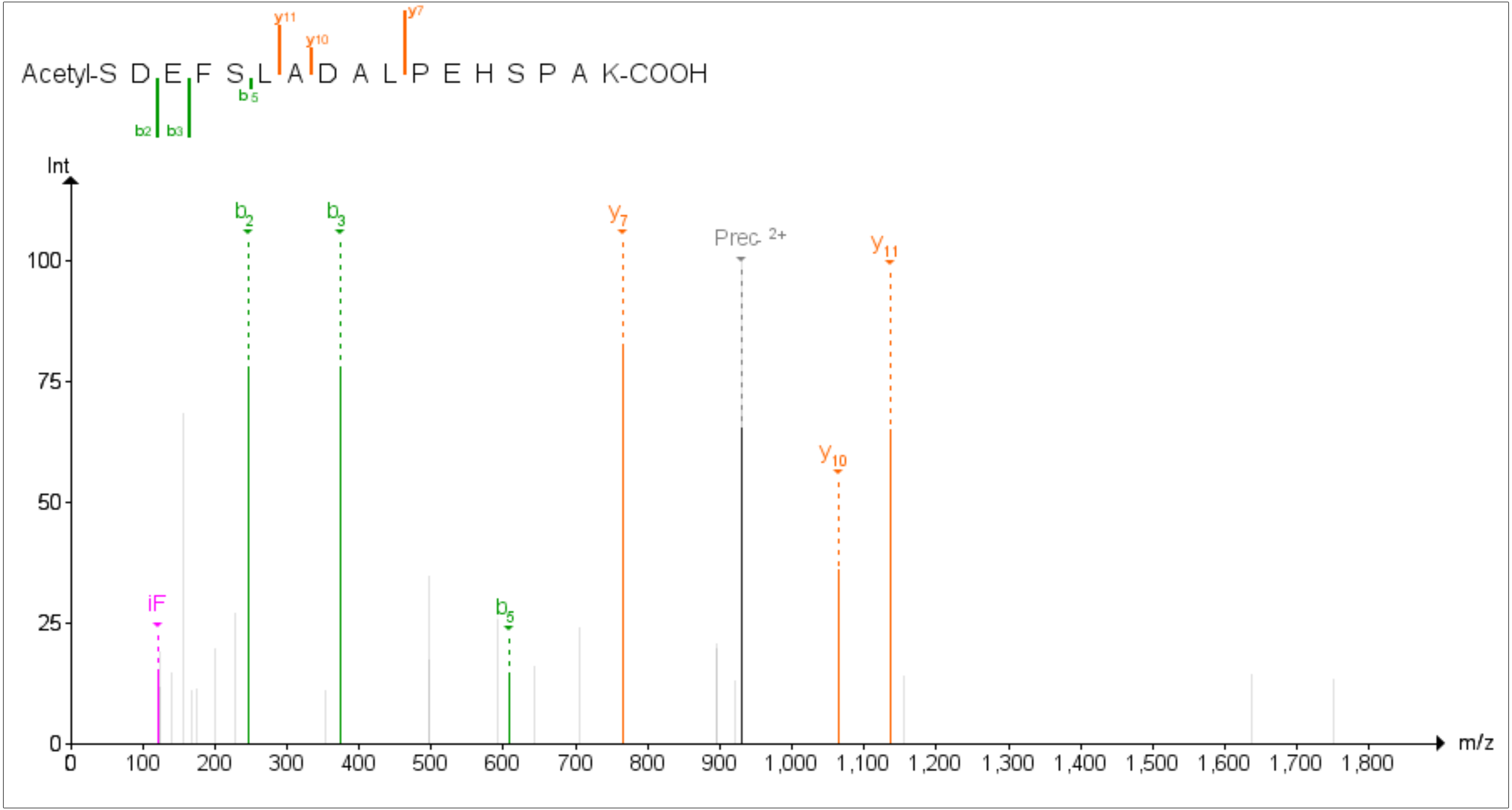

Acetyl-S D E F S L A D A L P E H S P A K-COOH
y11
y10
y7
b2
b3
b5
Int
m/z
iF
Prec 2+
0
25
50
75
100
0
100
200
300
400
500
600
700
800
900
1,000
1,100
1,200
1,300
1,400
1,500
1,600
1,700
1,800

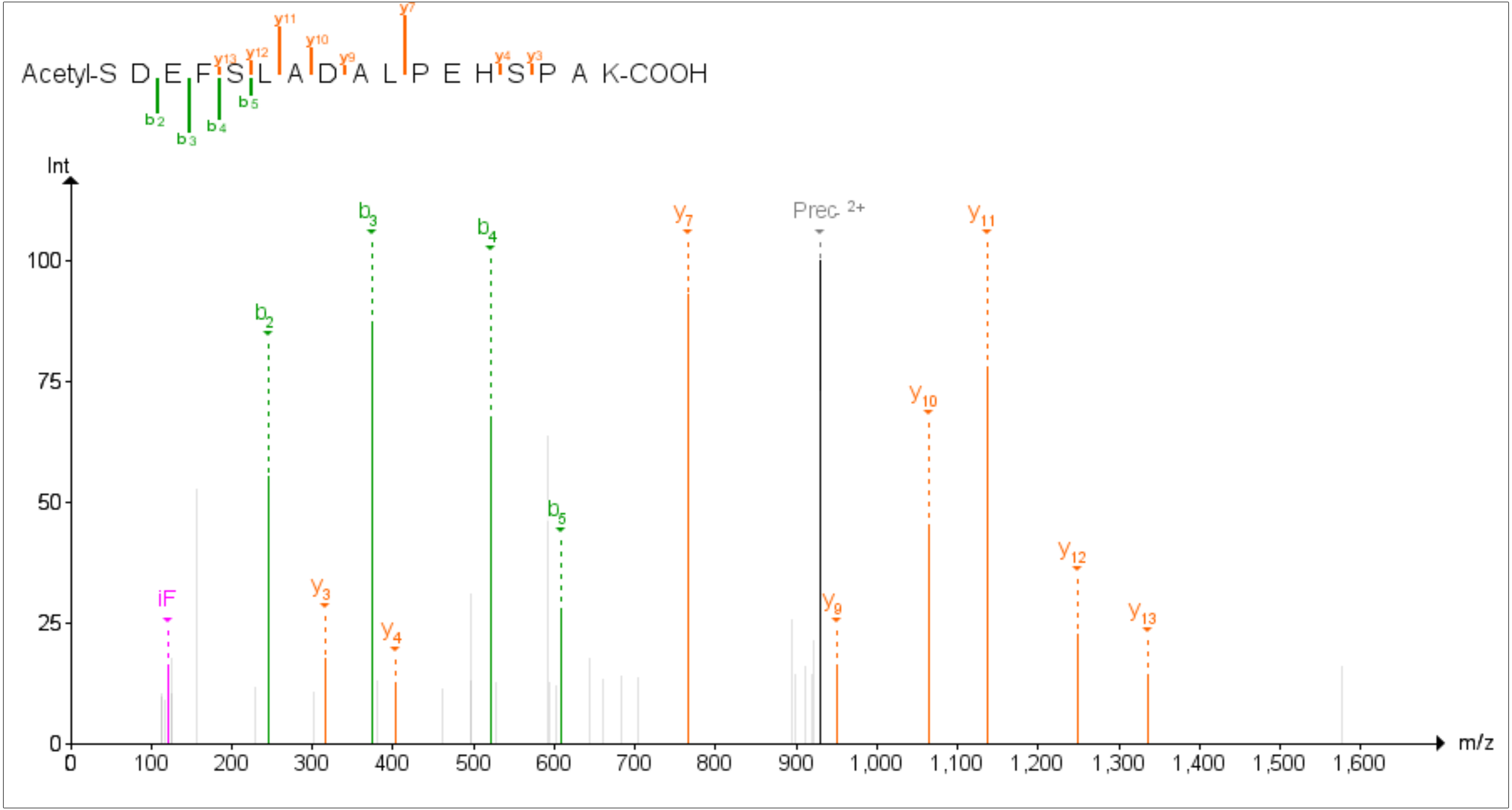

Acetyl-S D E F S L A D A L P E H S P A K-COOH
b2
b3
b4
b5
y13
y12
y11
y10
y9
y7
y4
y3
Int
m/z
iF
Prec. 2+
0
25
50
75
100
0
100
200
300
400
500
600
700
800
900
1,000
1,100
1,200
1,300
1,400
1,500
1,600

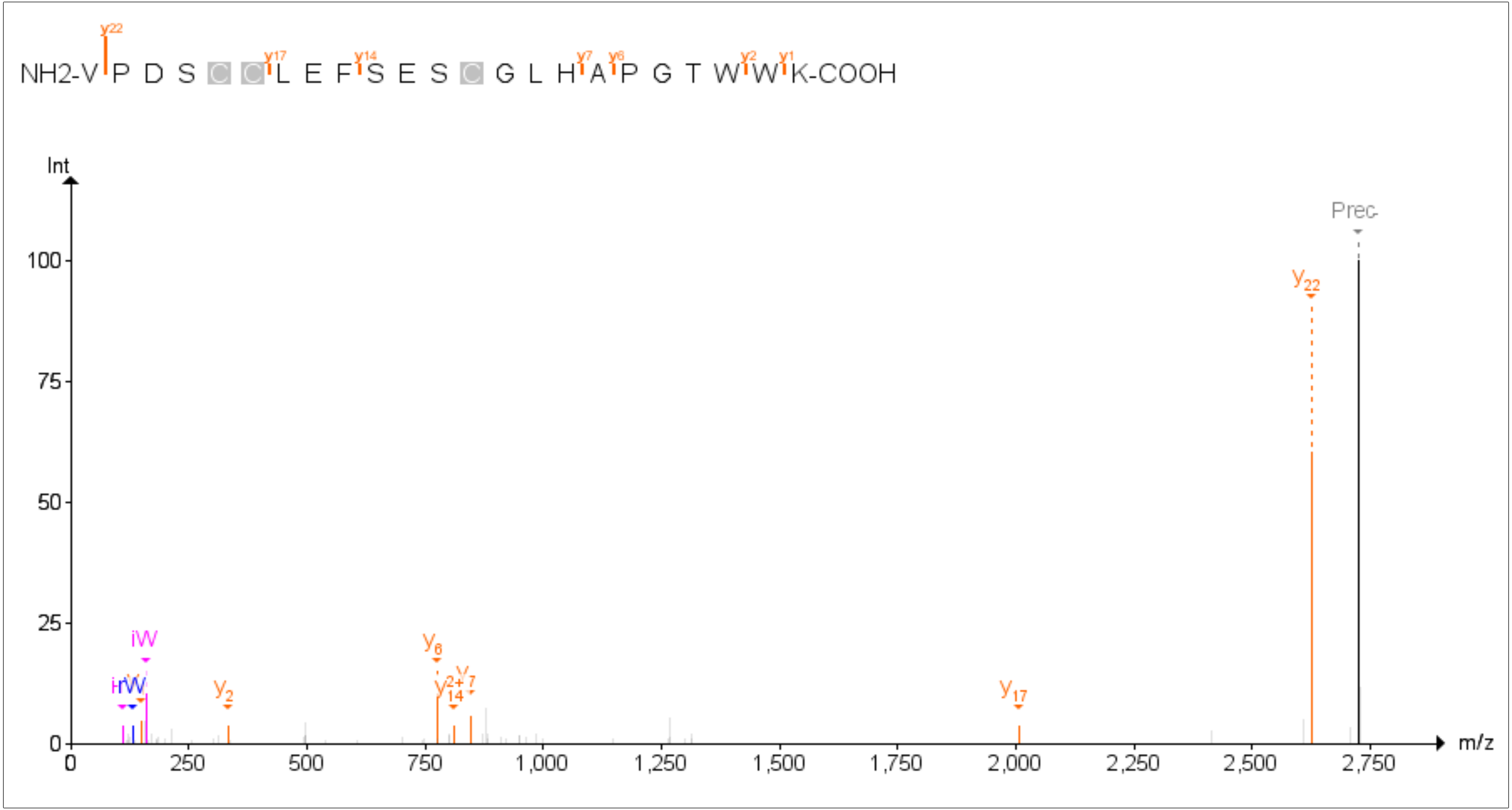

NH2-V P D S C C L E F S E S C G L H A P G T W W K-COOH
y22
y17
y14
y7
y6
y2
y1
Int
100
75
50
25
0
Prec.
y22
iW
y6
y7
y14 2+
y2
y17
0
250
500
750
1,000
1,250
1,500
1,750
2,000
2,250
2,500
2,750
m/z

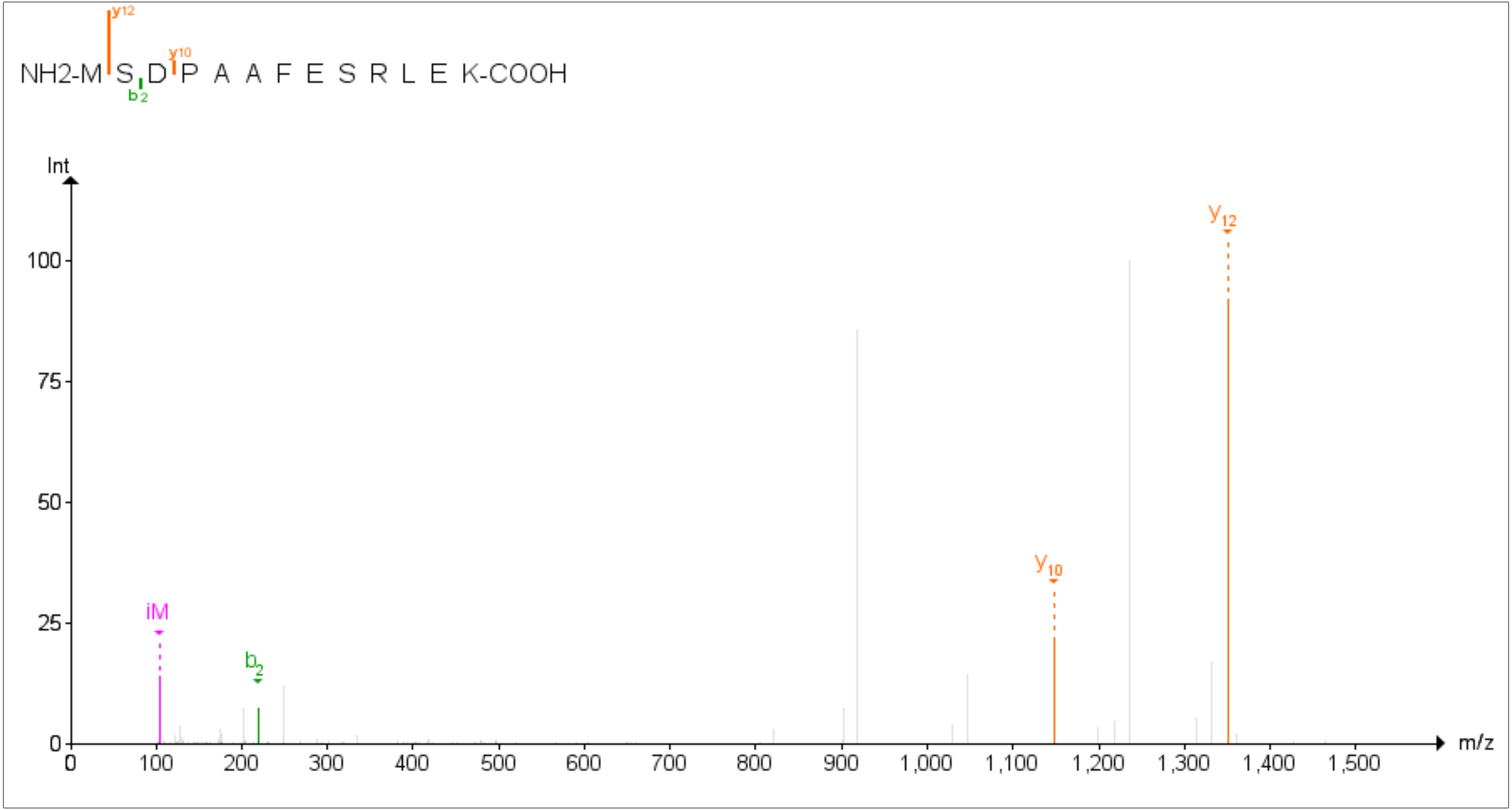

NH2-M S D P A A F E S R L E K-COOH
y12
y10
b2
Int
100
75
50
25
0
iM
b2
y10
y12
0
100
200
300
400
500
600
700
800
900
1,000
1,100
1,200
1,300
1,400
1,500
m/z

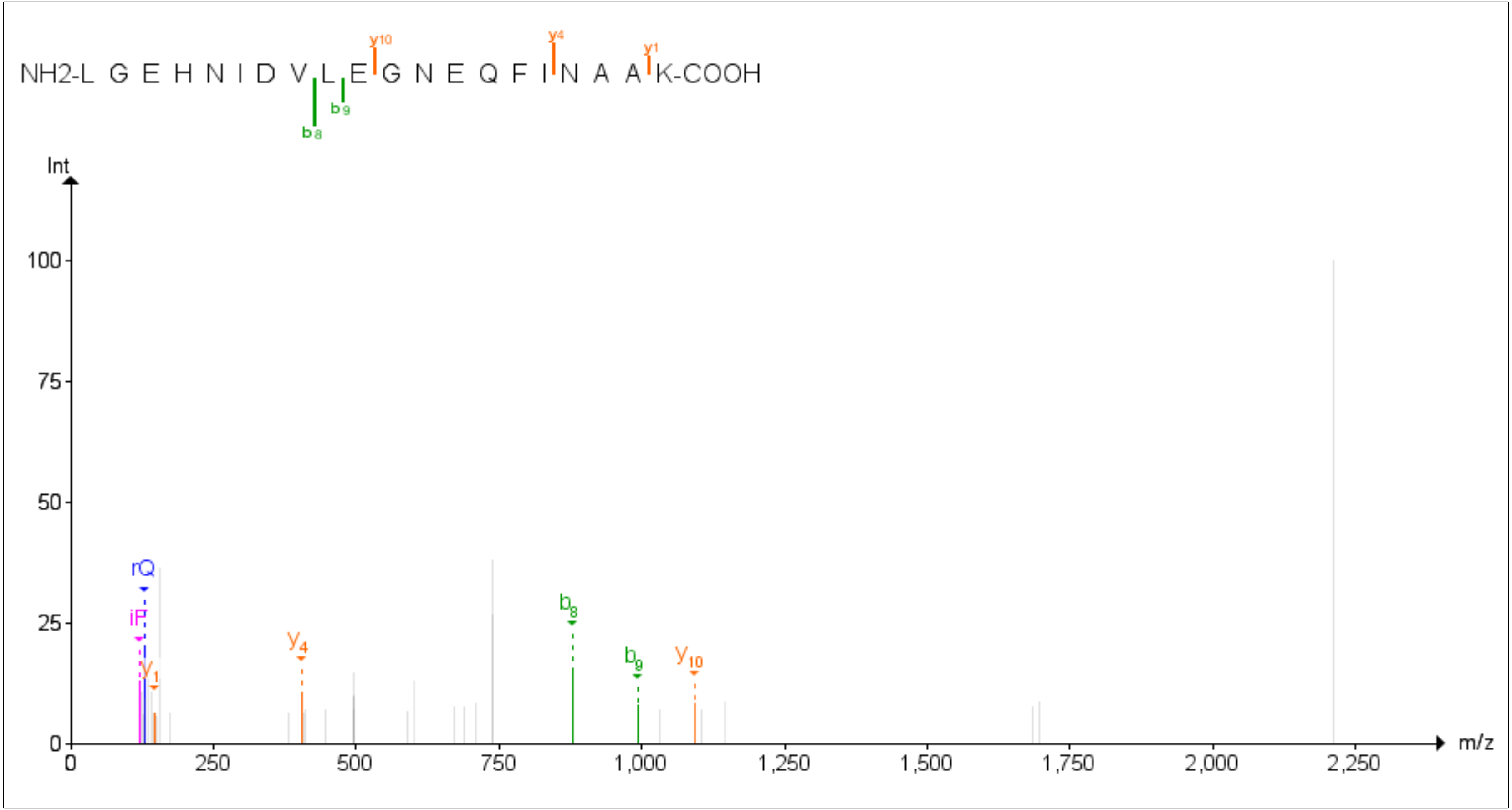

NH2-L G E H N I D V L E G N E Q F I N A A K-COOH
y10
y4
y1
b8
b9
Int
100
75
50
25
0
rQ
iF
y1
y4
b8
b9
y10
0
250
500
750
1,000
1,250
1,500
1,750
2,000
2,250
m/z

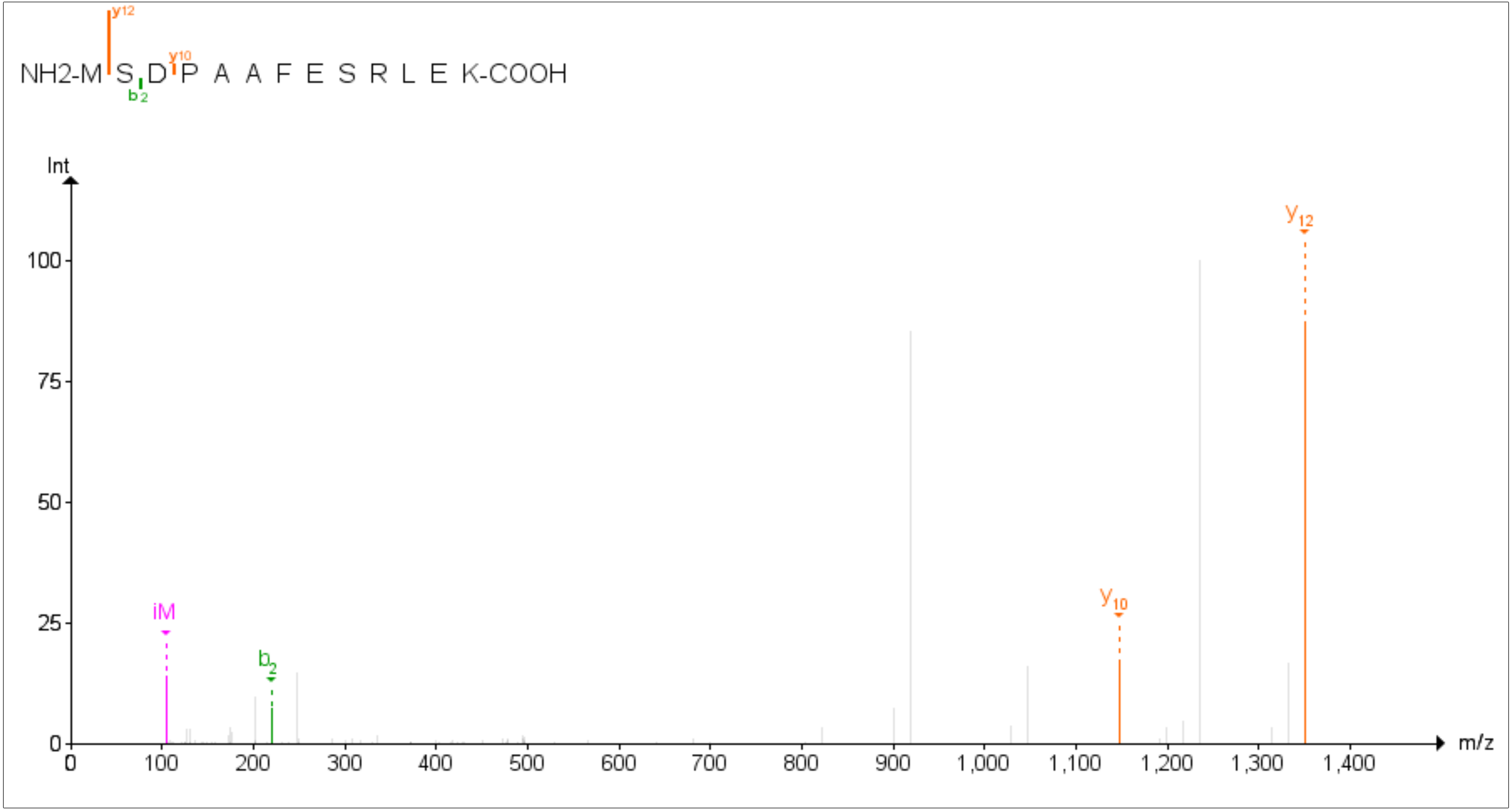

NH2-M S D P A A F E S R L E K-COOH
y12
y10
b2
Int
100
75
50
25
0
iM
b2
y10
y12
0
100
200
300
400
500
600
700
800
900
1,000
1,100
1,200
1,300
1,400
m/z

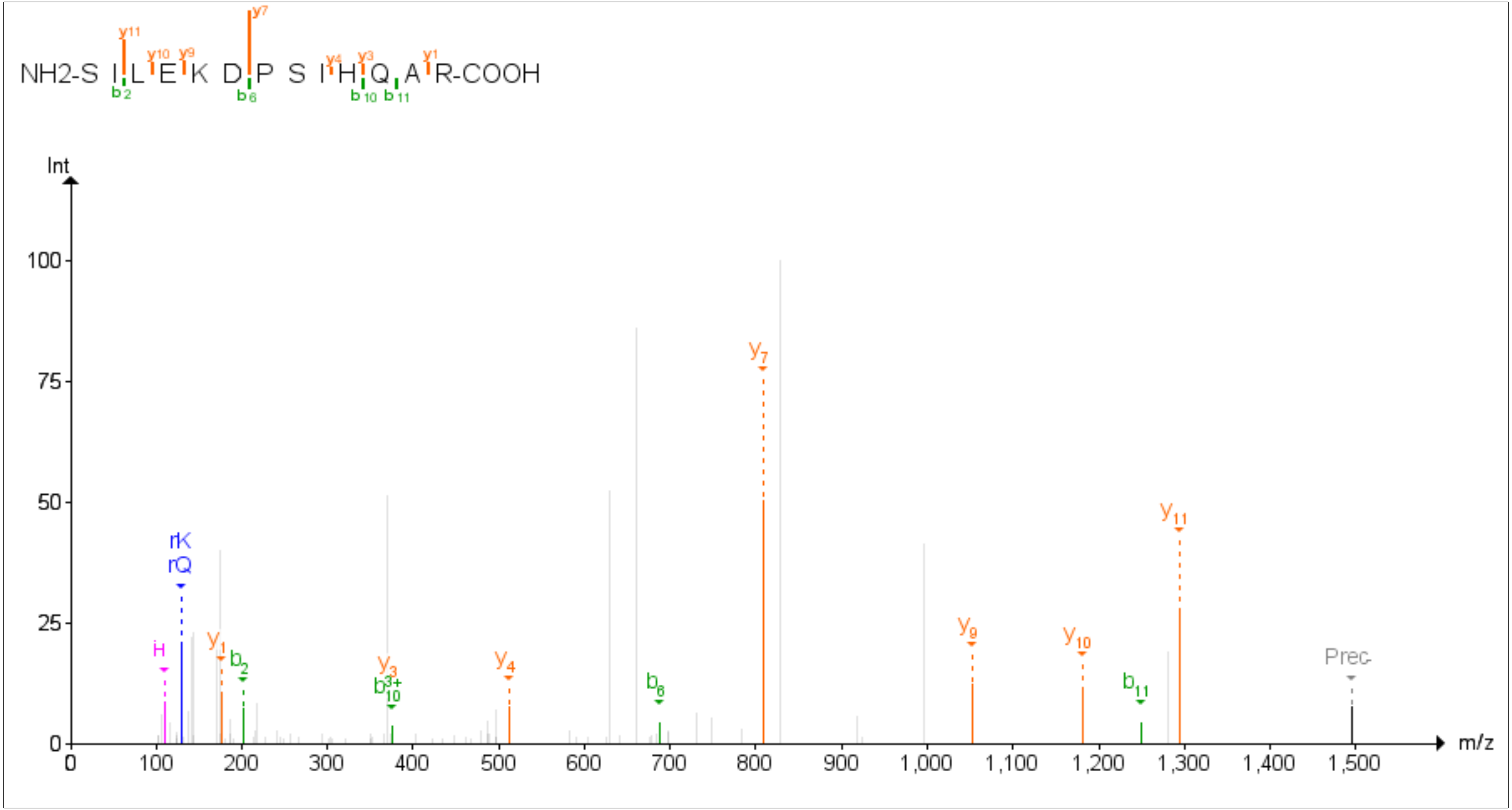

NH2-S I L E K D P S I H Q A R-COOH
y11 y10 y9 y7 y4 y3 y1
b2 b6 b10 b11
Int
100
75
50
25
0
rK
rQ
H
y1
b2
y3
b3+10
y4
b6
y7
y9
y10
b11
y11
Prec.
0 100 200 300 400 500 600 700 800 900 1,000 1,100 1,200 1,300 1,400 1,500
m/z

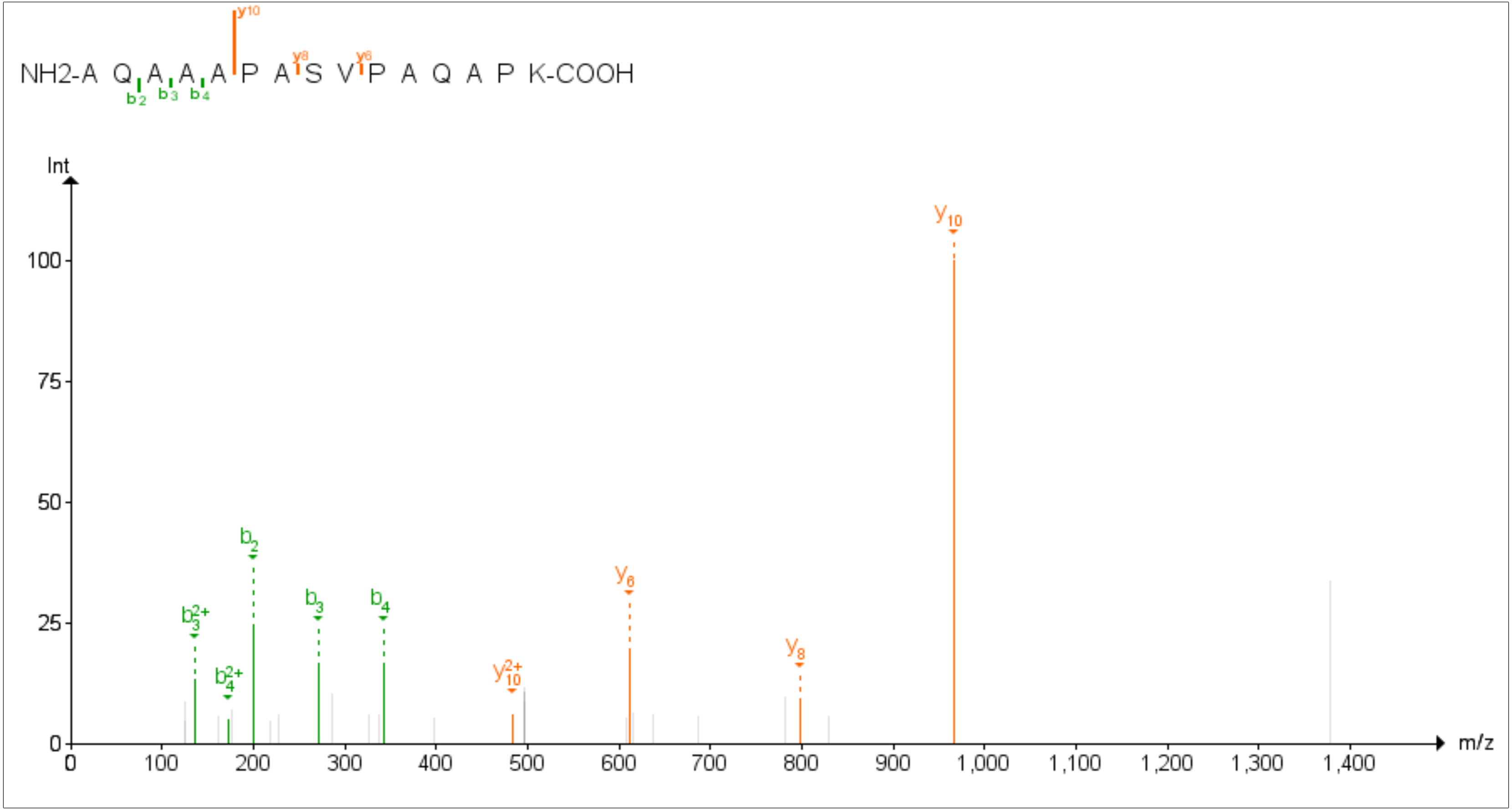
NH2-A Q A A A P A S V P A Q A P K-COOH
y10
y8
y6
b2
b3
b4
Int
100
75
50
25
0
y10
b2
b3
b4
y6
y8
b3 2+
b4 2+
y10 2+
0
100
200
300
400
500
600
700
800
900
1,000
1,100
1,200
1,300
1,400
m/z

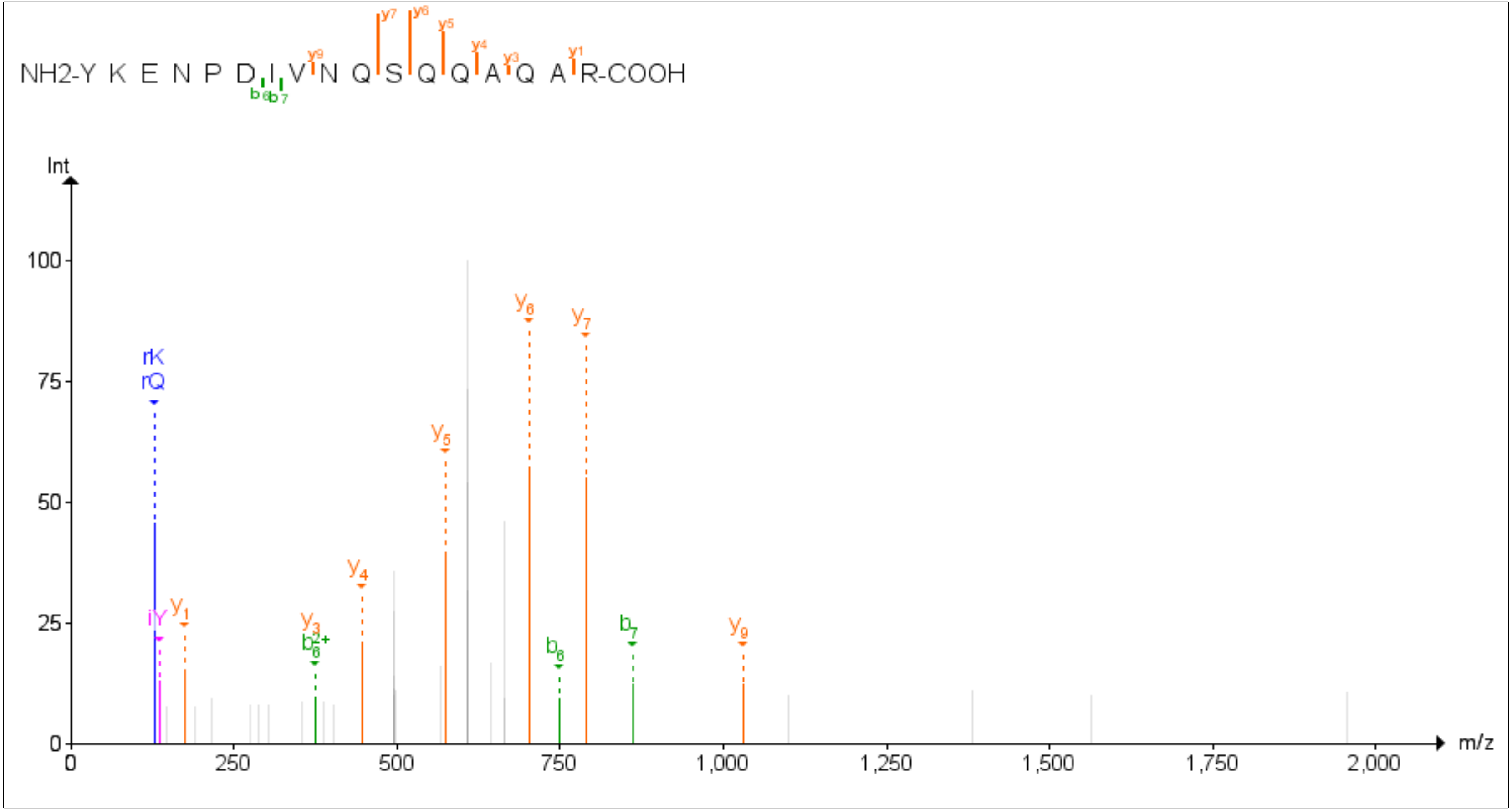

NH2-Y K E N P D I V N Q S Q Q A Q A R-COOH
y9
y7
y6
y5
y4
y3
y1
b6
b7
Int
100
75
50
25
0
rK
rQ
iY
y1
y3
b6 2+
y4
y5
y6
y7
b6
b7
y9
0
250
500
750
1,000
1,250
1,500
1,750
2,000
m/z

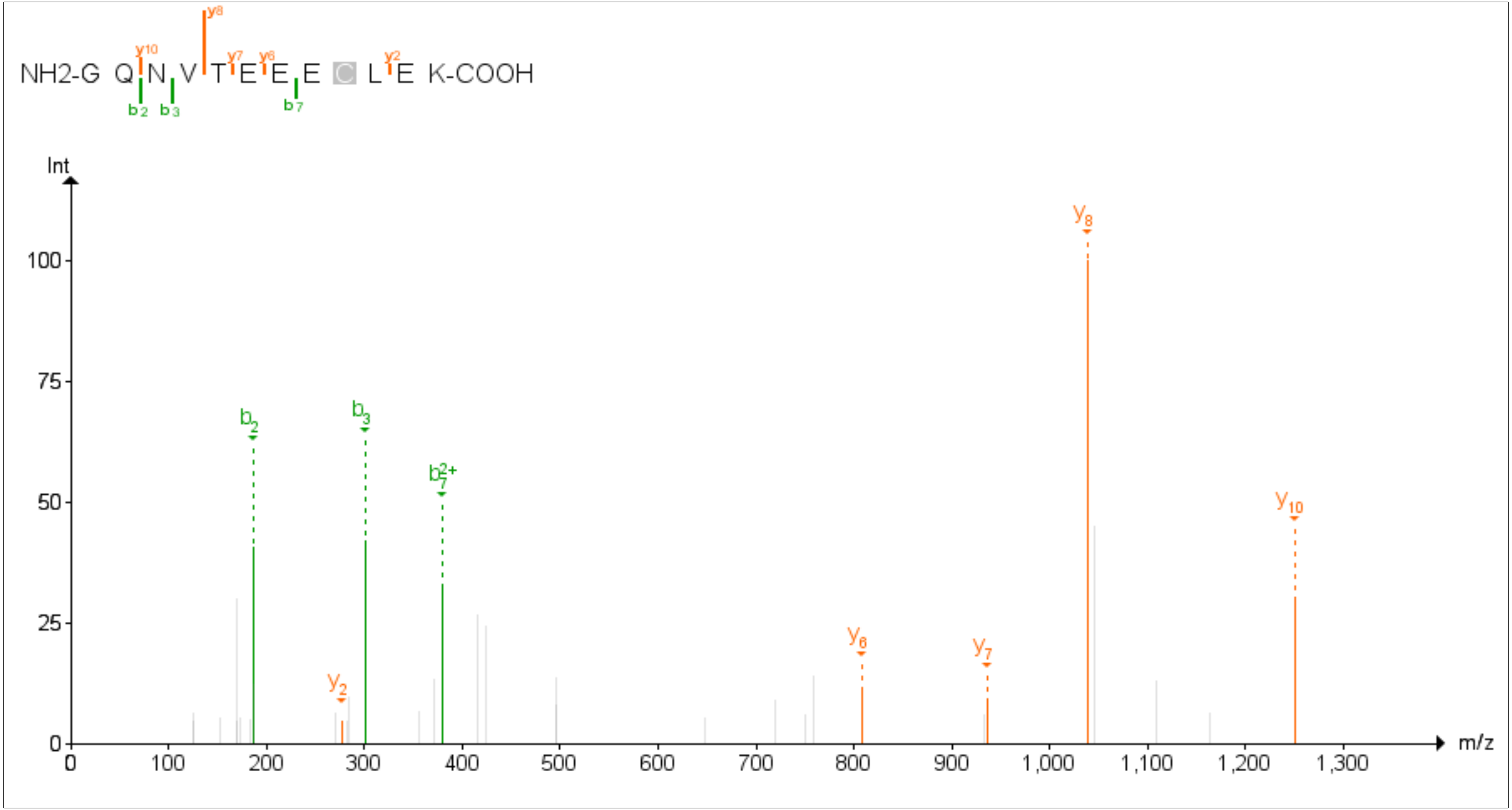

NH2-G Q N V T E E E C L E K-COOH
y8
y10
y7
y6
y2
b2
b3
b7
Int
100
75
50
25
0
b2
b3
b7 2+
y2
y6
y7
y8
y10
0
100
200
300
400
500
600
700
800
900
1,000
1,100
1,200
1,300
m/z

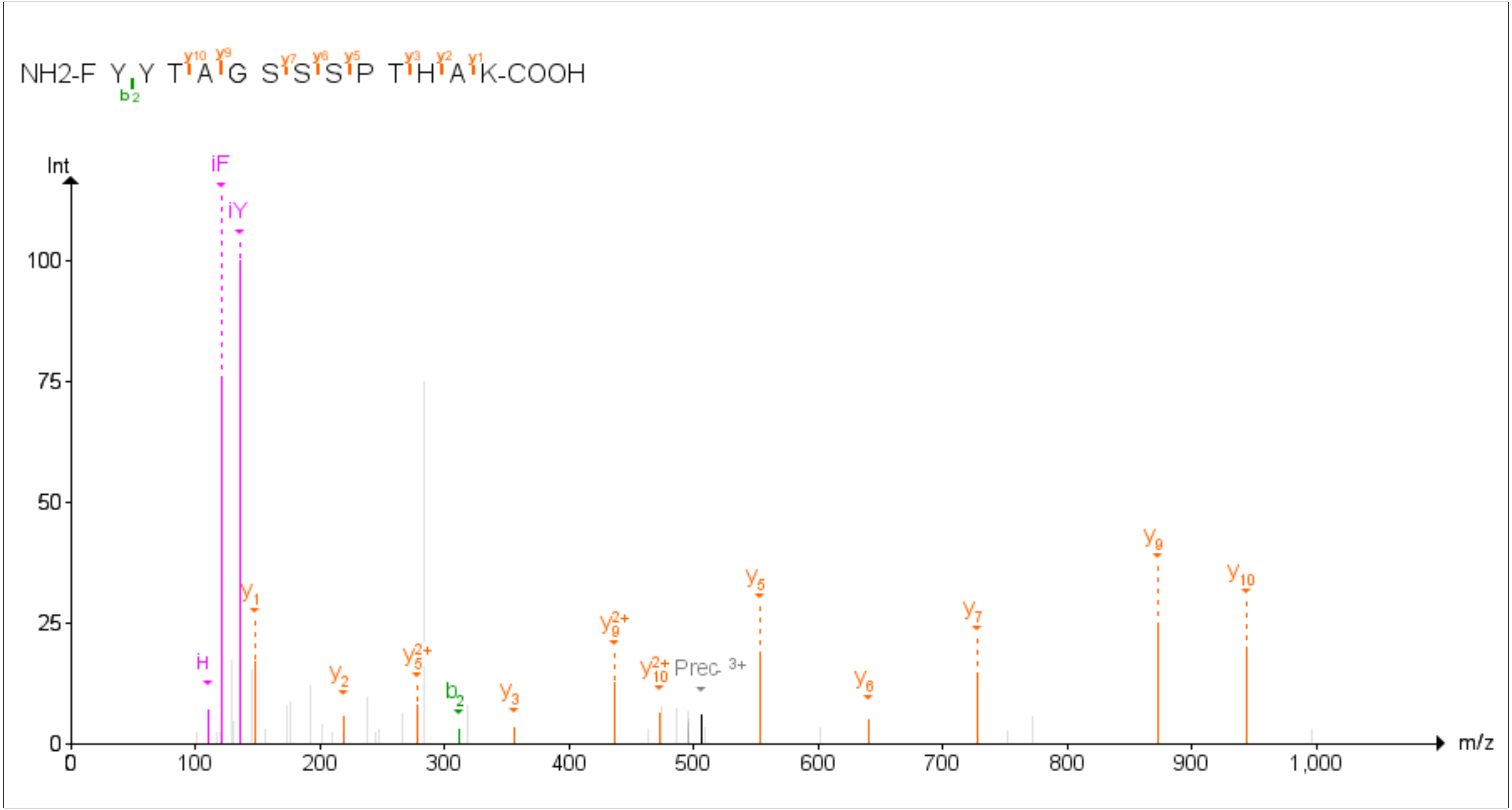

NH2-F Y Y T A G S S S P T H A K-COOH
b2
y10 y9 y7 y6 y5 y3 y2 y1
Int
100
75
50
25
0
iF
iY
iH
y1
y2
y5 2+
b2
y3
y9 2+
y10 2+
Prec 3+
y5
y6
y7
y9
y10
0
100
200
300
400
500
600
700
800
900
1,000
m/z

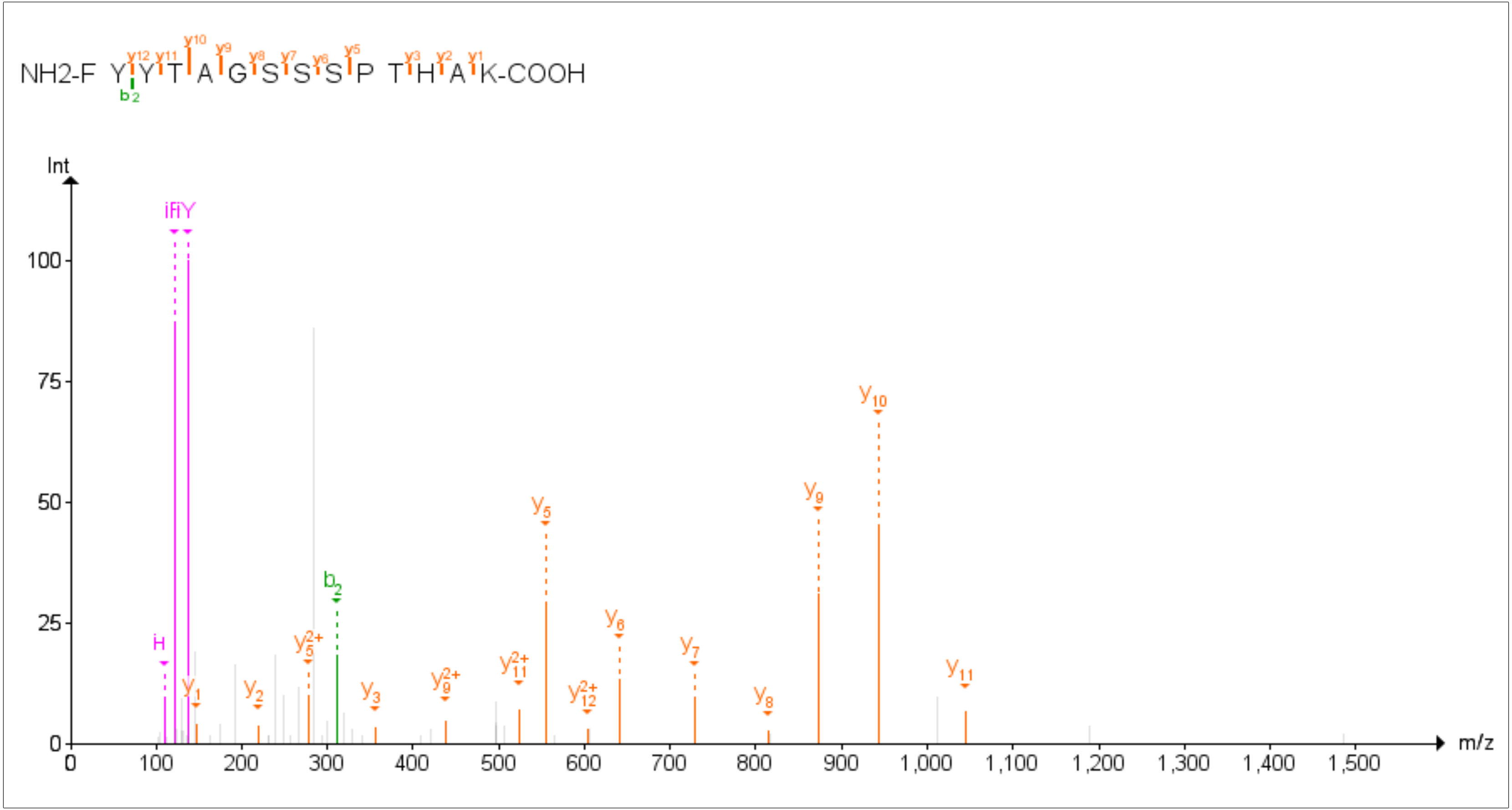

NH2-F Y Y T A G S S S P T H A K-COOH
y12 y11 y10 y9 y8 y7 y6 y5 y3 y2 y1
b2
Int
m/z
iFiY
iH
y1
y2
y5 2+
b2
y3
y9 2+
y11 2+
y5
y12 2+
y6
y7
y8
y9
y10
y11
0
25
50
75
100
0
100
200
300
400
500
600
700
800
900
1,000
1,100
1,200
1,300
1,400
1,500

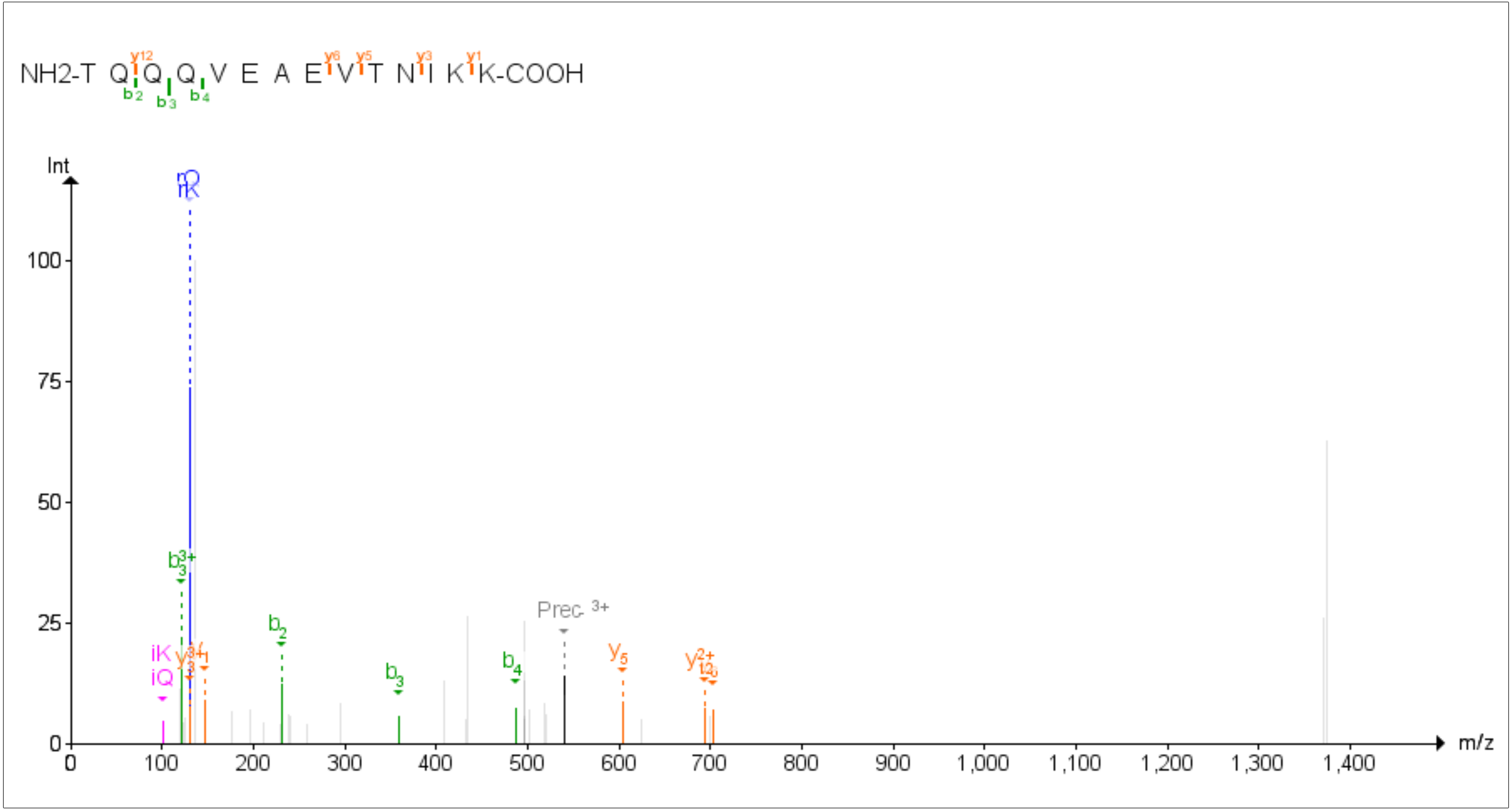

NH2-T Q Q Q V E A E V T N I K K-COOH
y12 y6 y5 y3 y1
b2 b3 b4
Int
iQ
iK
b3 3+
b2
b3
b4
Prec 3+
y5
iK
iQ
m/z
0
25
50
75
100
0
100
200
300
400
500
600
700
800
900
1,000
1,100
1,200
1,300
1,400

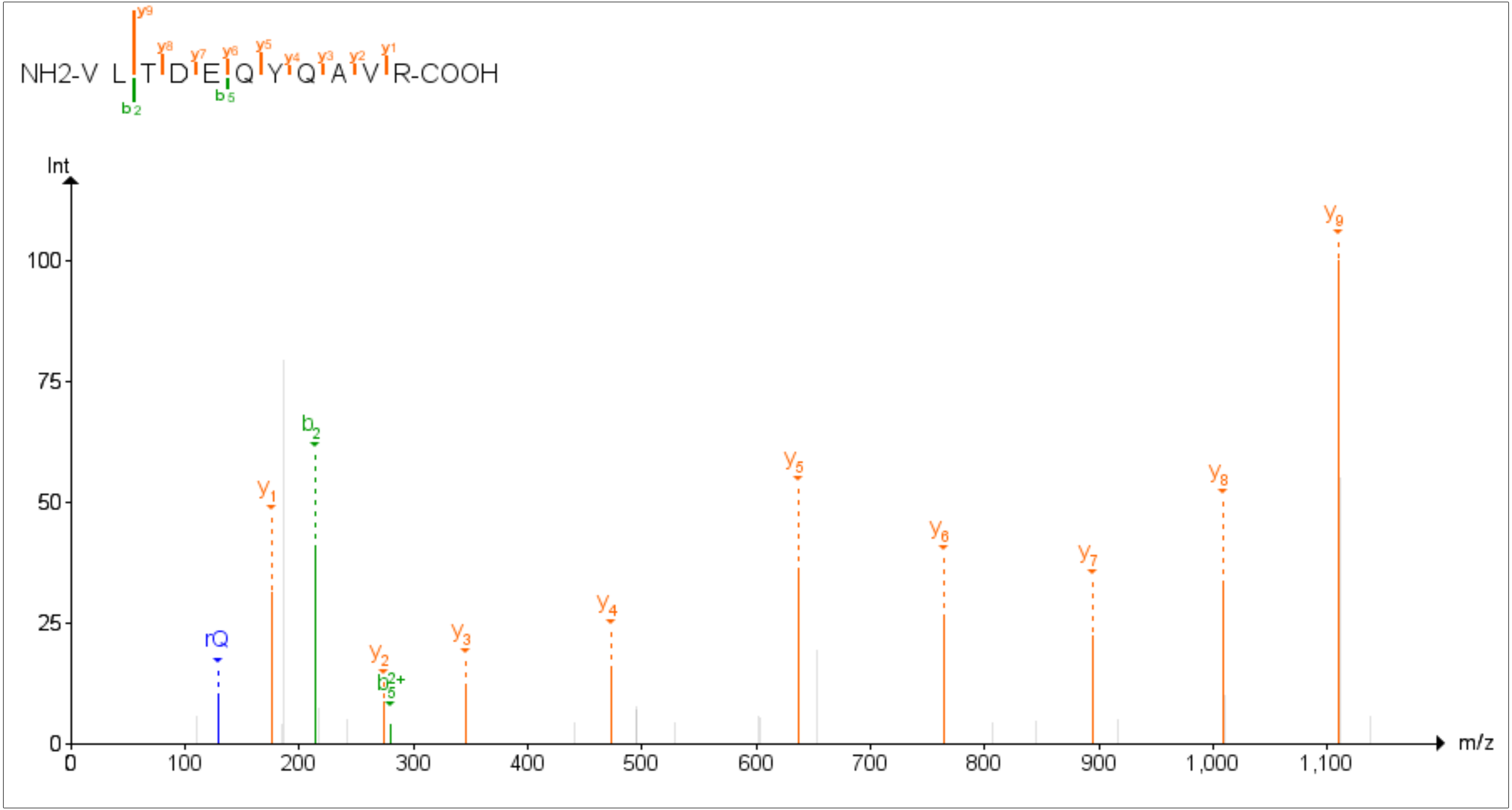
NH2-V L T D E Q Y Q A V R-COOH
y9 y8 y7 y6 y5 y4 y3 y2 y1
b2 b5
Int
m/z
rQ
y1
b2
y2
b5 2+
y3
y4
y5
y6
y7
y8
y9
0 25 50 75 100
0 100 200 300 400 500 600 700 800 900 1,000 1,100

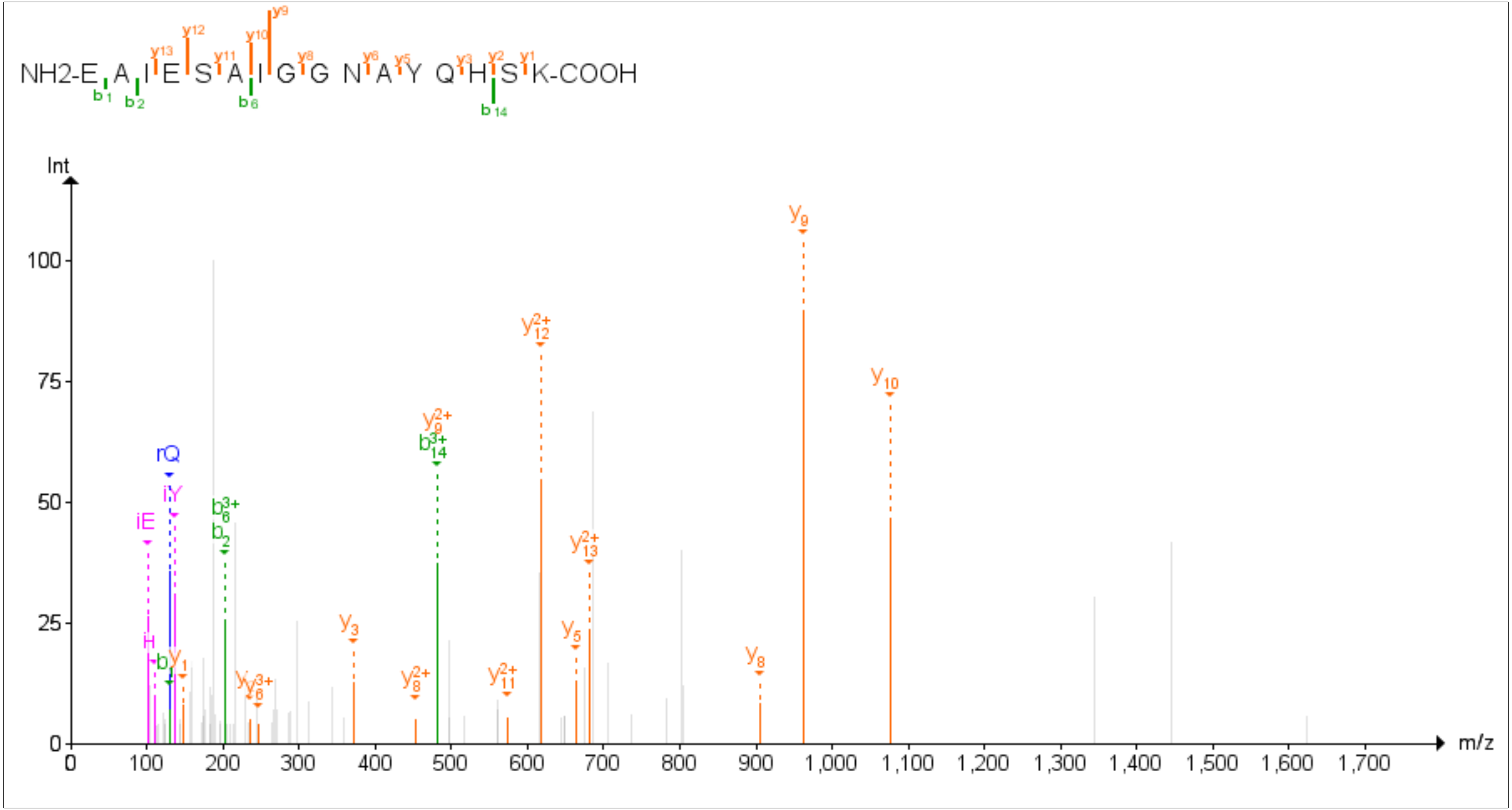
NH2-E A I E S A I G G N A Y Q H S K-COOH
Int
m/z
y9
y10
y12
y13
y11
y8
y6
y5
y3
y2
y1
b1
b2
b6
b14
rQ
iY
iE
iH
y1
0
25
50
75
100
0
100
200
300
400
500
600
700
800
900
1,000
1,100
1,200
1,300
1,400
1,500
1,600
1,700

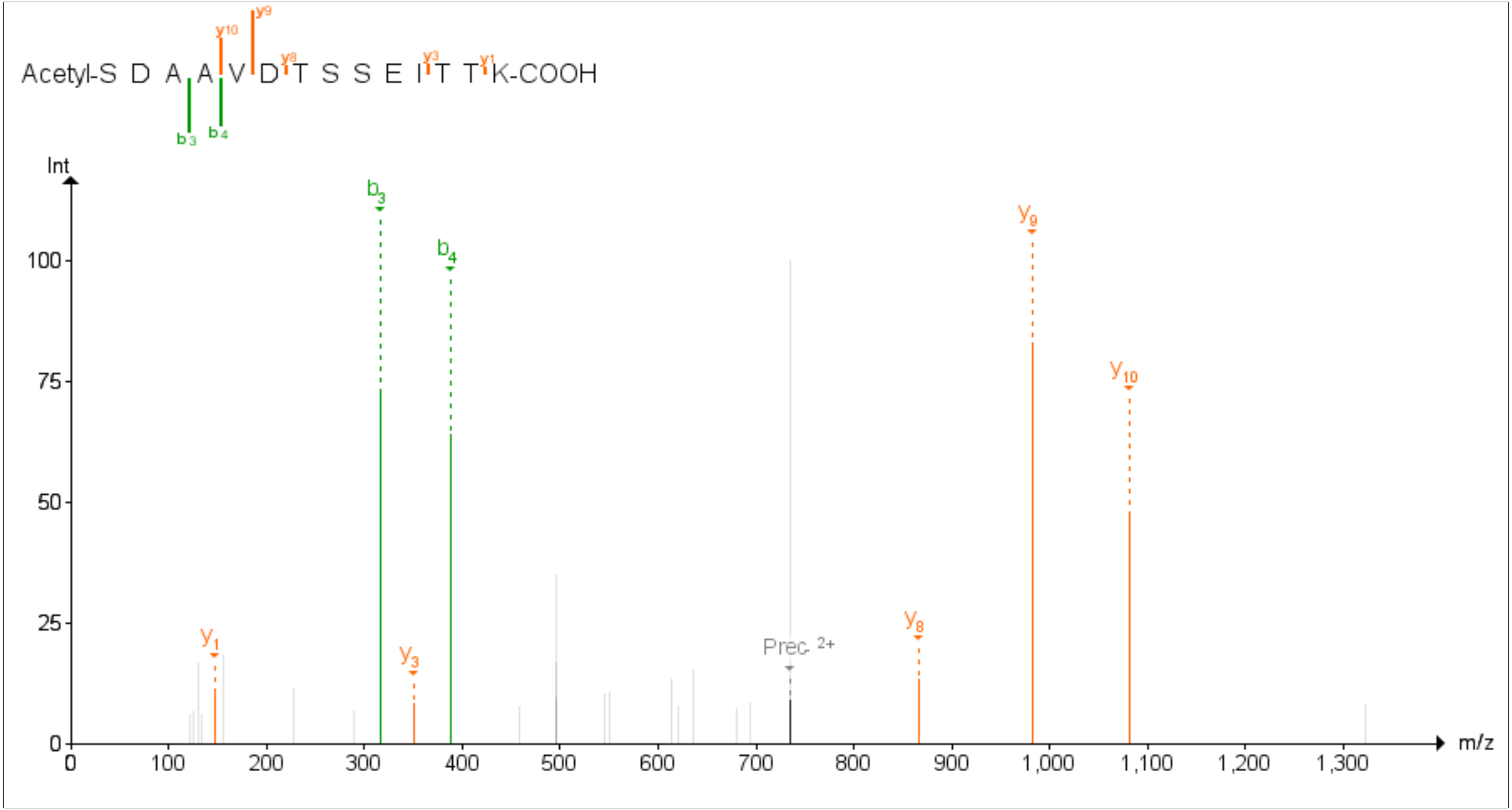

Acetyl-S D A A V D T S S E I T T K-COOH
y10
y9
y8
y3
y1
b3
b4
Int
100
75
50
25
0
b3
b4
y9
y10
y1
y3
Prec- 2+
y8
0
100
200
300
400
500
600
700
800
900
1,000
1,100
1,200
1,300
m/z

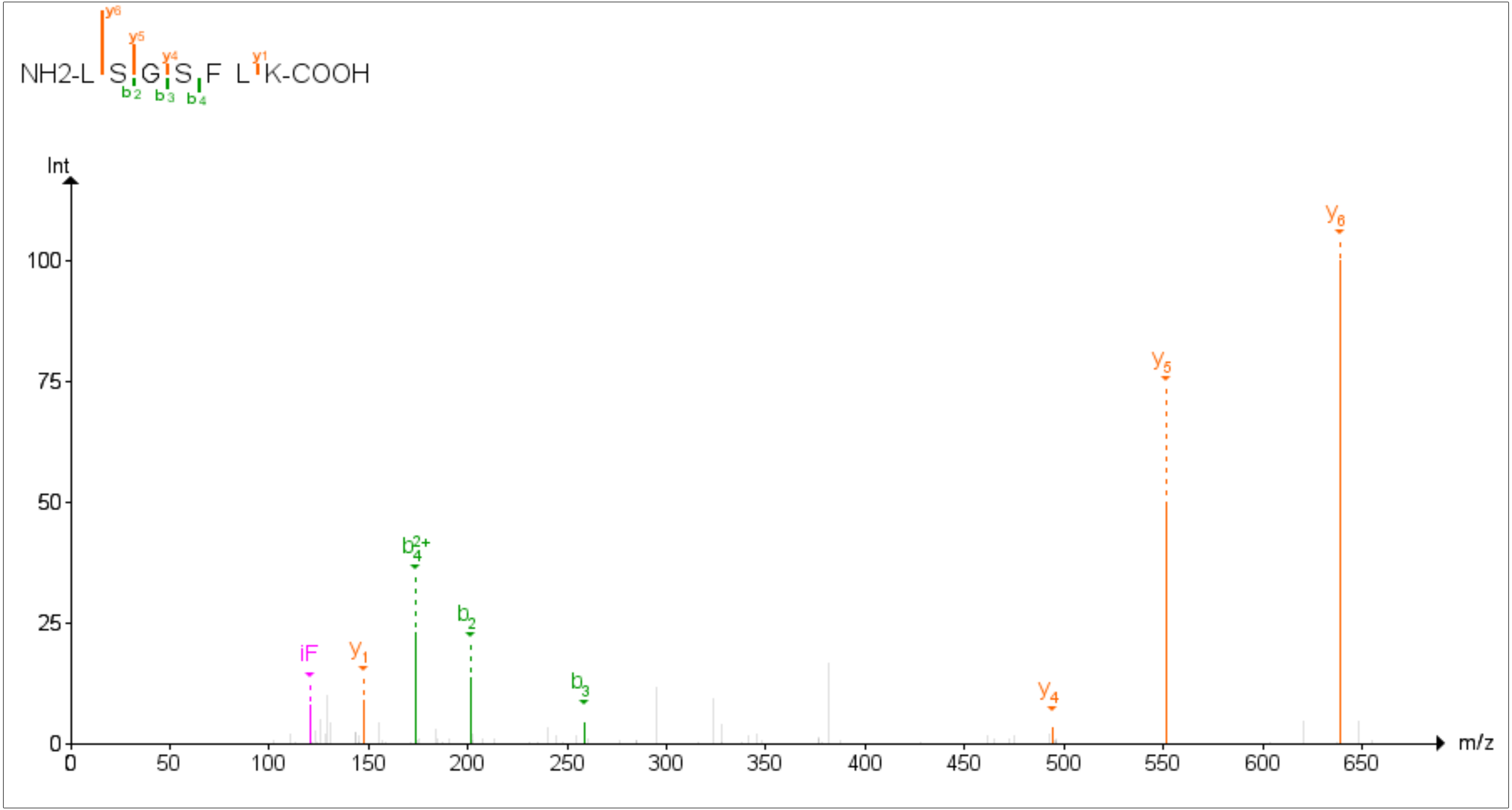
NH2-L S G S F L K-COOH
y6
y5
y4
y1
b2
b3
b4
Int
100
75
50
25
0
iF
y1
b4 2+
b2
b3
y4
y5
y6
0
50
100
150
200
250
300
350
400
450
500
550
600
650
m/z

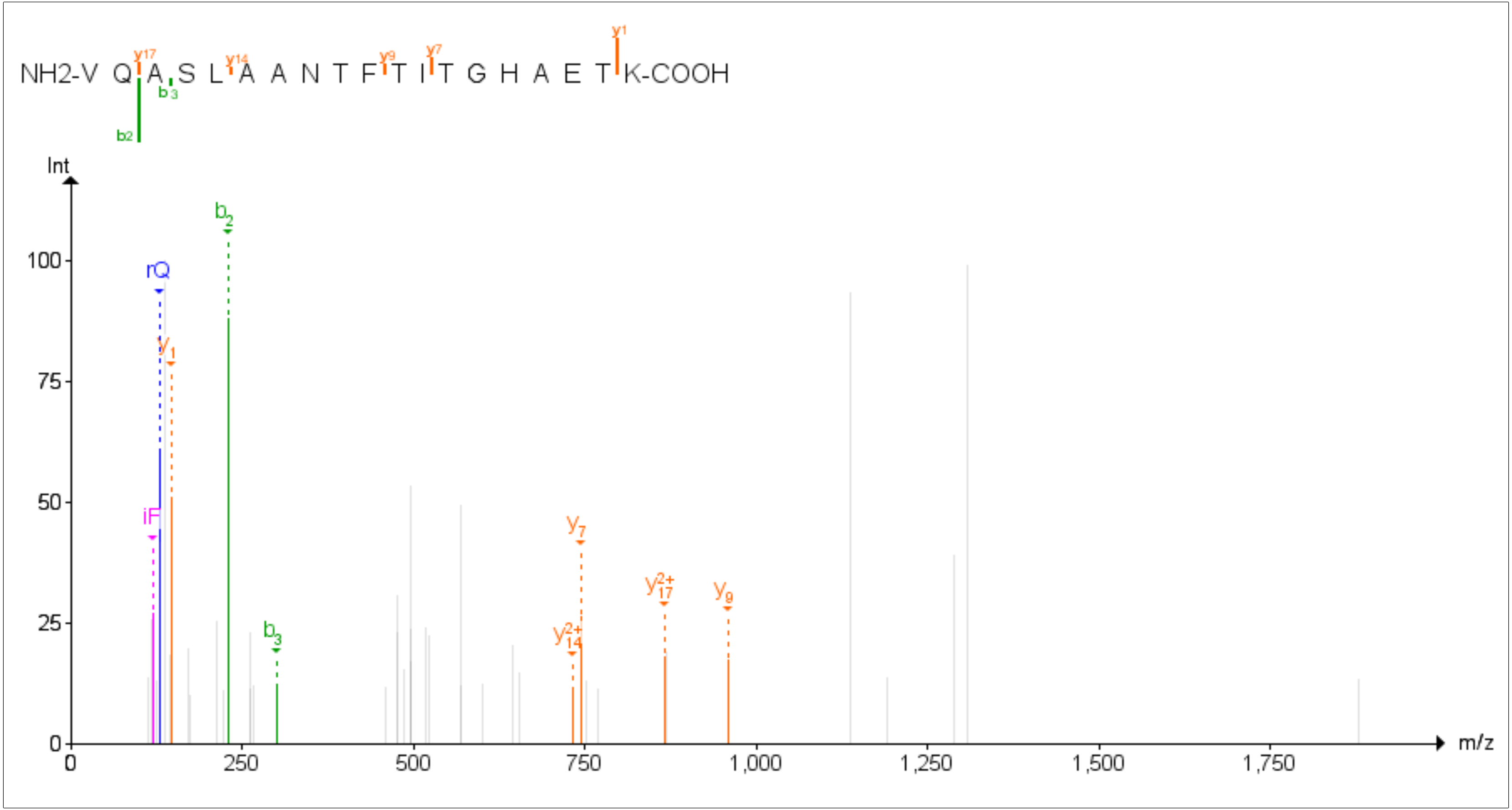
NH2-V Q A S L A A N T F T I T G H A E T K-COOH
y17
y14
y9
y7
y1
b2
b3
Int
m/z
b2
rQ
y1
iF
b3
y7
y2+ 14
y2+ 17
y9
0
25
50
75
100
0
250
500
750
1,000
1,250
1,500
1,750

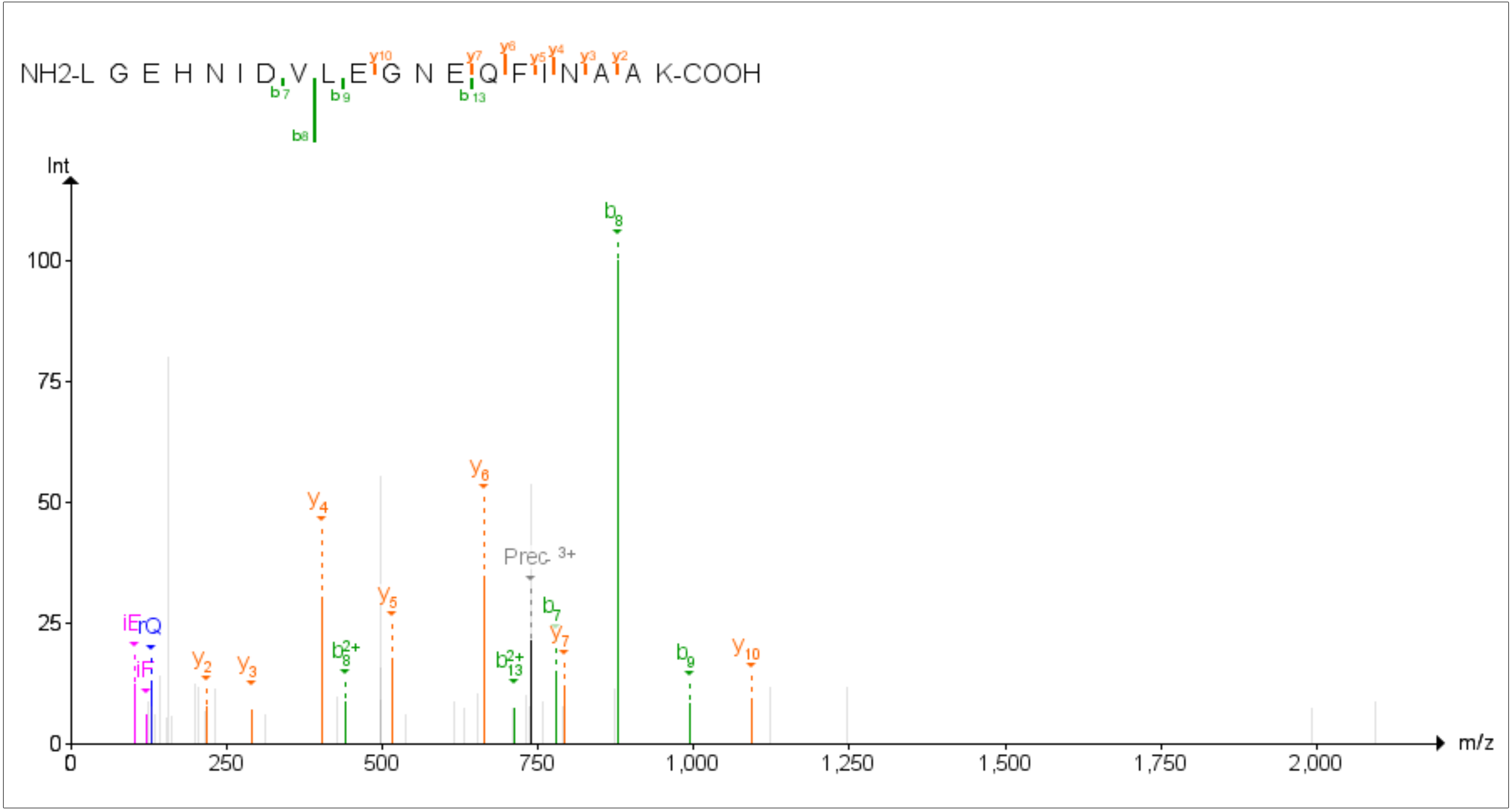

NH2-L G E H N I D V L E G N E Q F I N A A K-COOH
b7
b8
b9
b13
y10
y7
y6
y5
y4
y3
y2
Int
m/z
Prec 3+

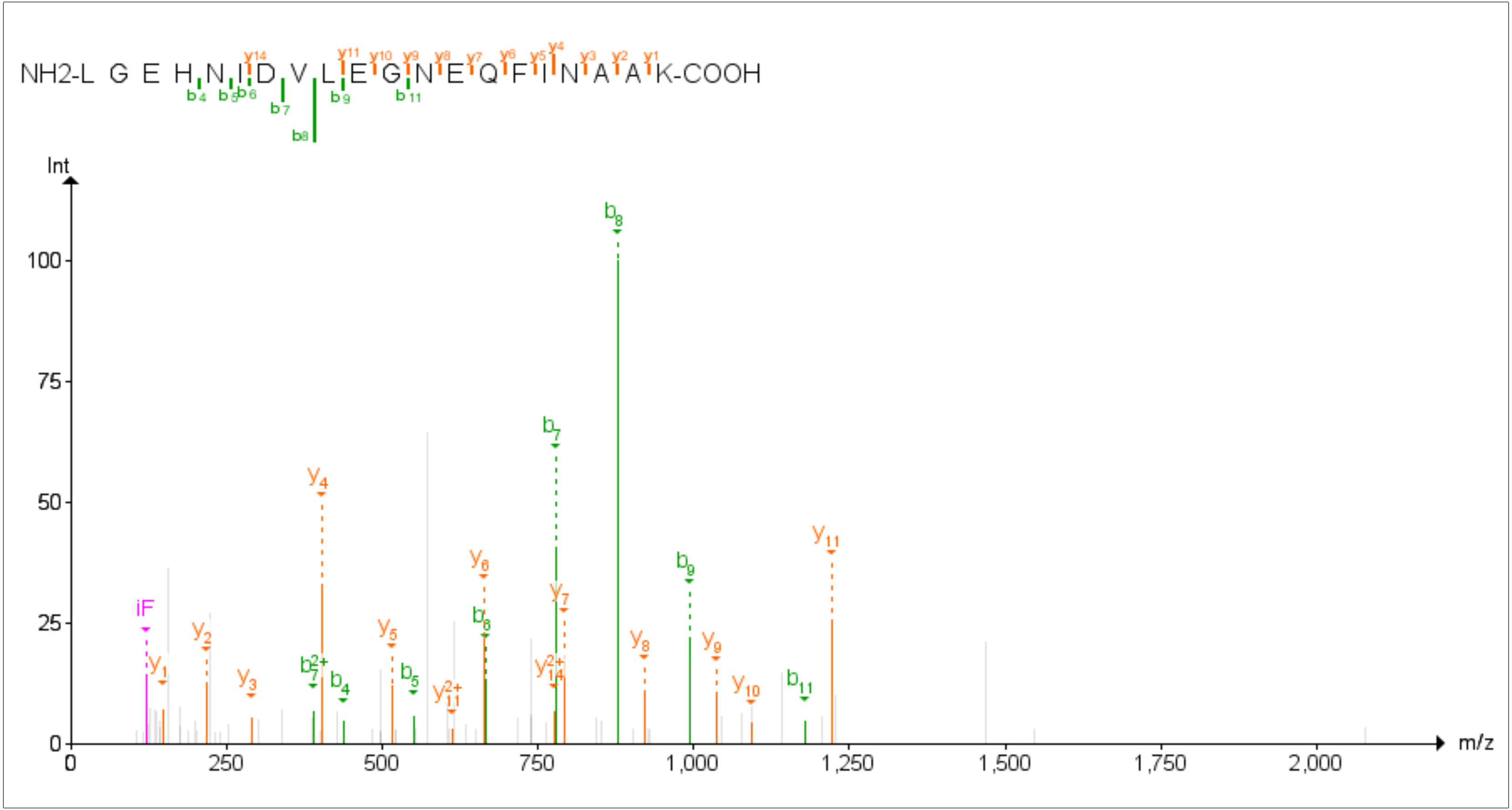

NH2-L G E H N I D V L E G N E Q F I N A A K-COOH
y14 y11 y10 y9 y8 y7 y6 y5 y4 y3 y2 y1
b4 b5 b6 b7 b8 b9 b11
Int
100
75
50
25
0
m/z
0
250
500
750
1,000
1,250
1,500
1,750
2,000
b8
b7
y4
y11
b9
y6
y7
b6
iF
y2
y1
y3
b7 2+
b4
y5
b5
y11 2+
y14 2+
y8
y9
y10
b11

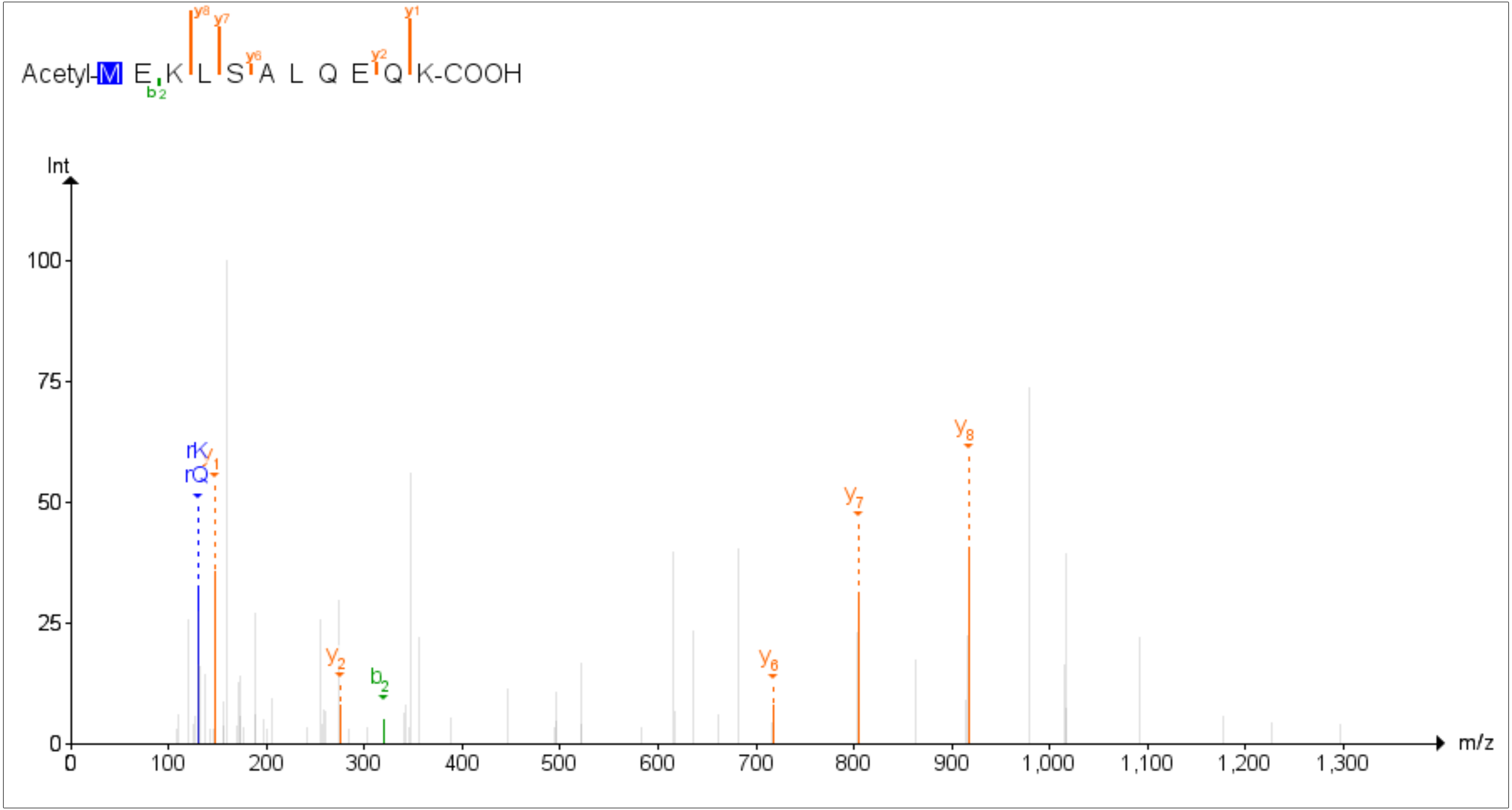

Acetyl-M E K L S A L Q E Q K-COOH
y8 y7 y6 y2 y1
b2
Int
100
75
50
25
0
rK
rQ
y1
y2
b2
y6
y7
y8
0 100 200 300 400 500 600 700 800 900 1,000 1,100 1,200 1,300
m/z

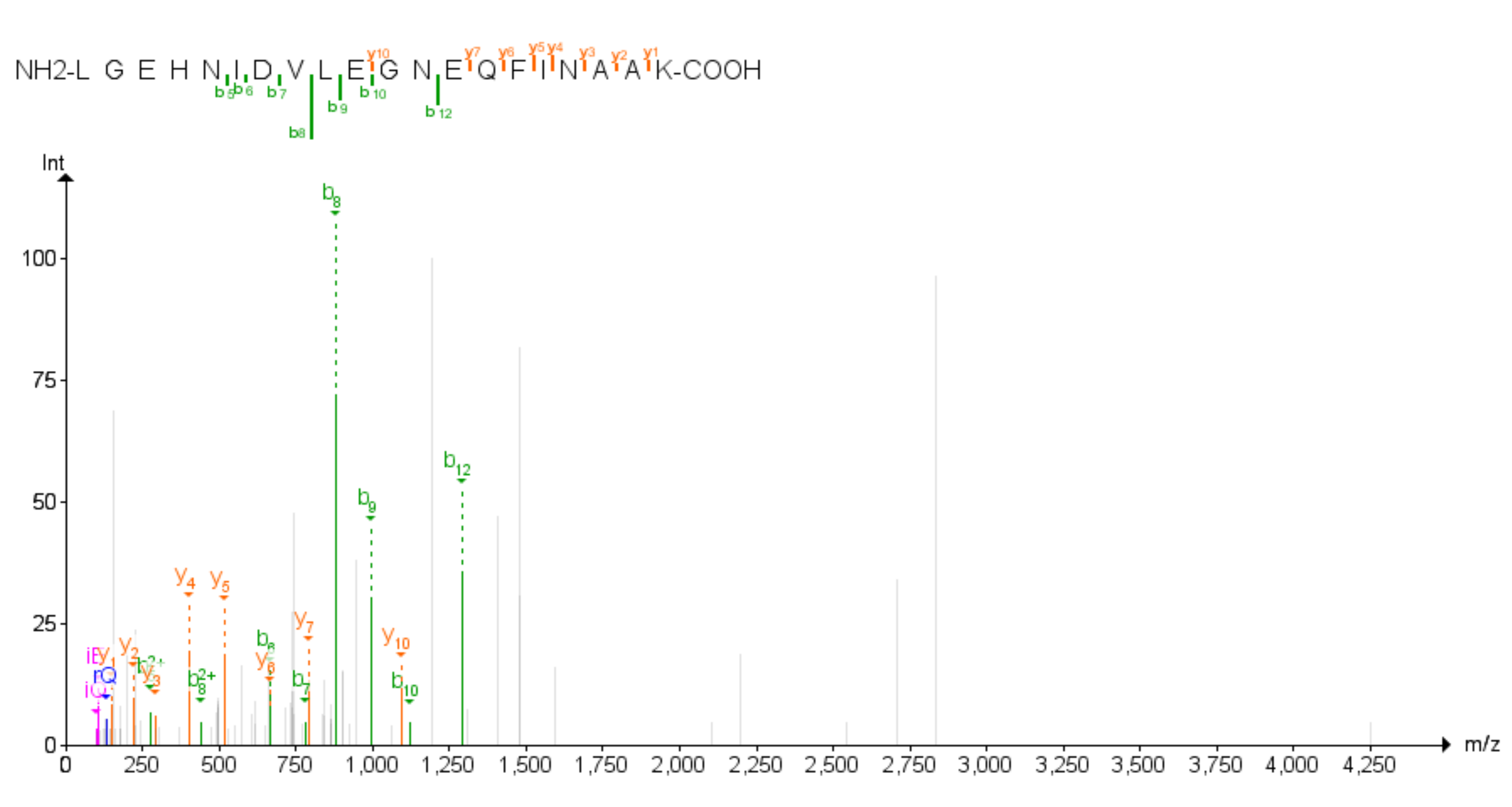
NH2-L G E H N I D V L E G N E Q F I N A A K-COOH
b5 b6 b7 b8 b9 b10 b12
y10 y7 y6 y5 y4 y3 y2 y1
Int
m/z
0
25
50
75
100
0 250 500 750 1,000 1,250 1,500 1,750 2,000 2,250 2,500 2,750 3,000 3,250 3,500 3,750 4,000 4,250
b8
b12
b9
y4
y5
y7
y10
b10
b7
y1
y2
y3
rQ

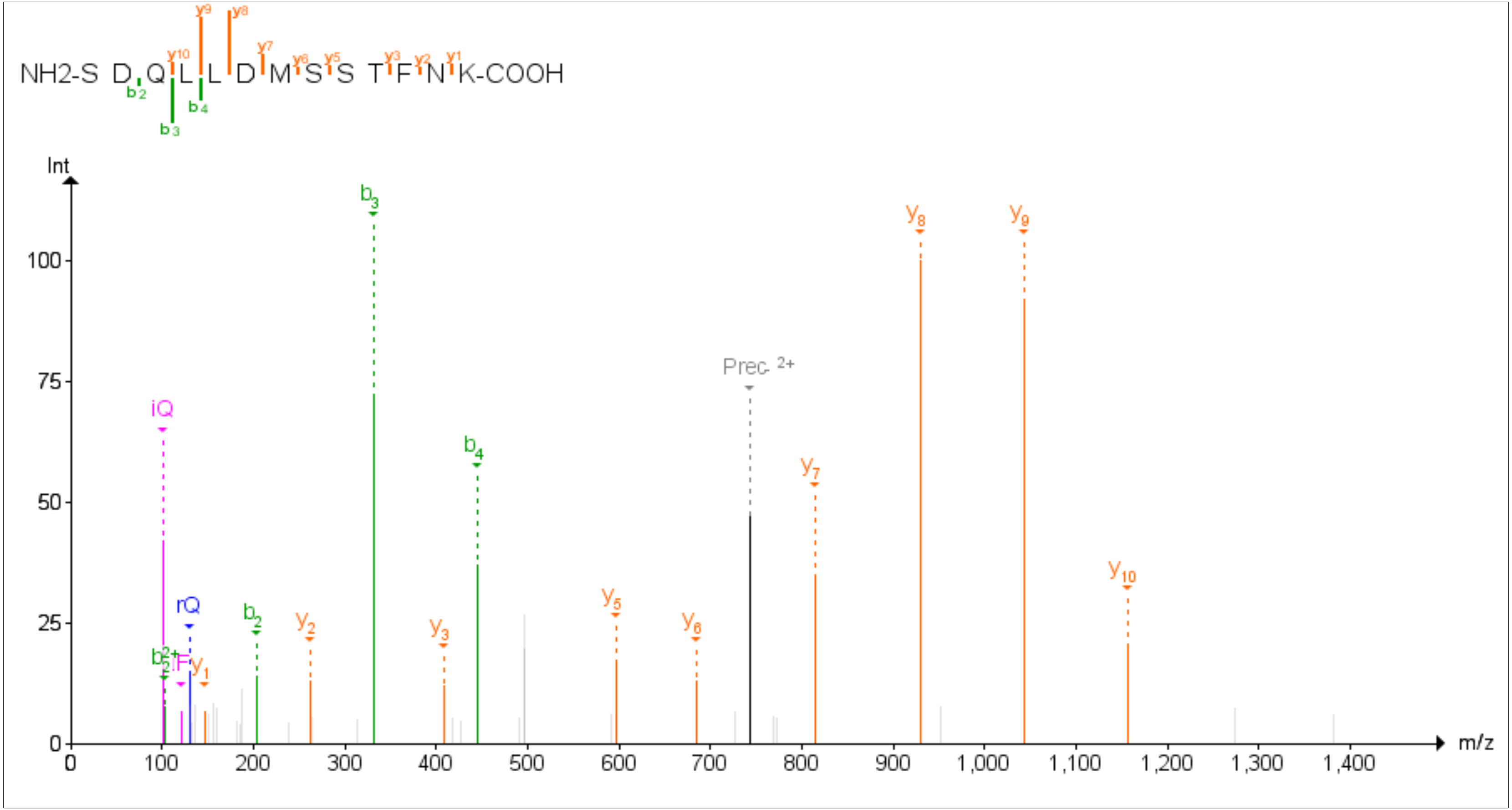

NH2-S D Q L L D M S S T F N K-COOH
Int
m/z
Prec. 2+
b3
b4
b2
iQ
rQ
y1
y2
y3
y5
y6
y7
y8
y9
y10

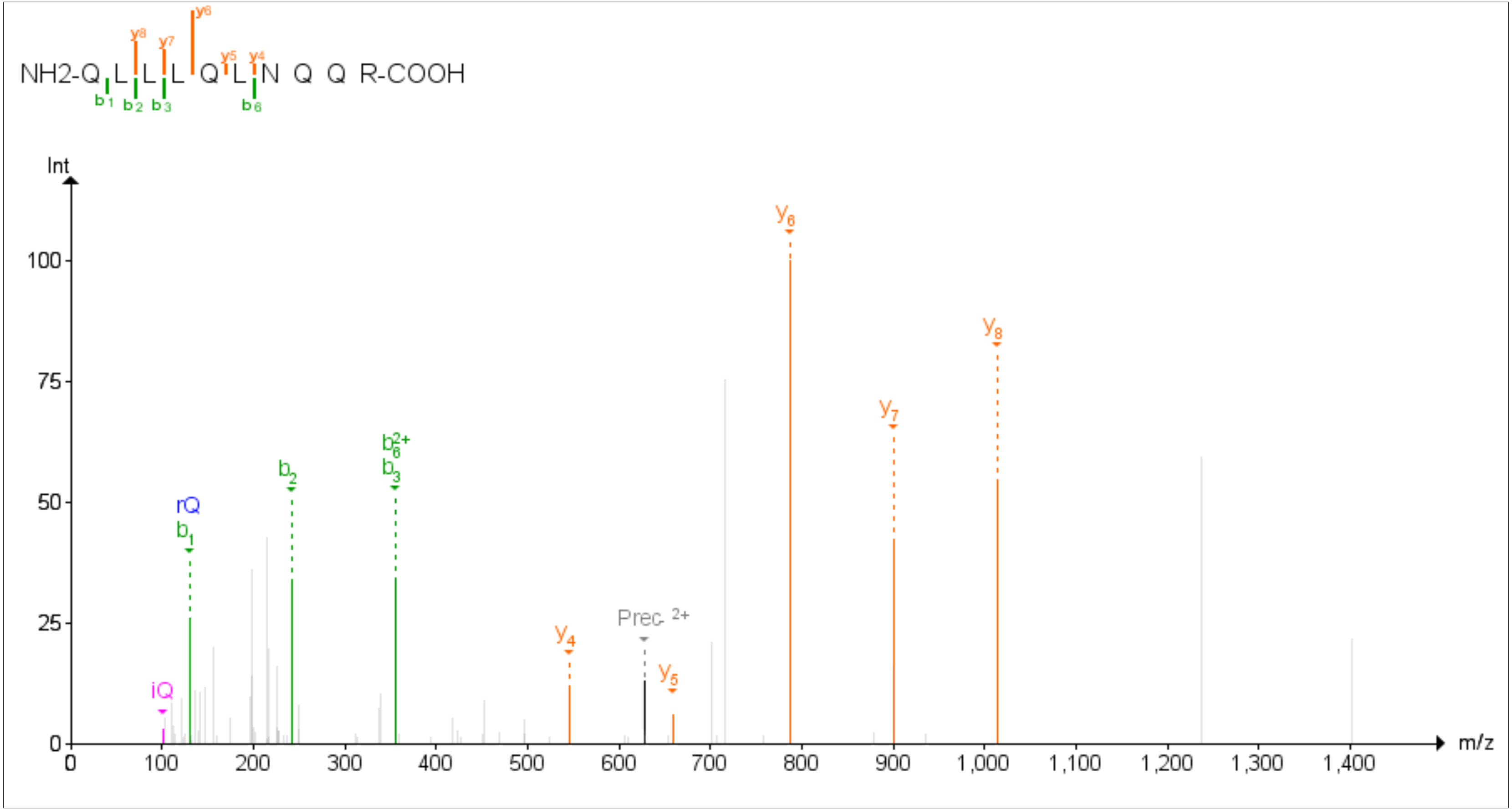

NH2-Q L L L Q L N Q Q R-COOH
b1
b2
b3
b6
y8
y7
y6
y5
y4
Int
100
75
50
25
0
rQ
b1
iQ
b2
b6 2+
b3
y4
Prec 2+
y5
y6
y7
y8
0
100
200
300
400
500
600
700
800
900
1,000
1,100
1,200
1,300
1,400
m/z

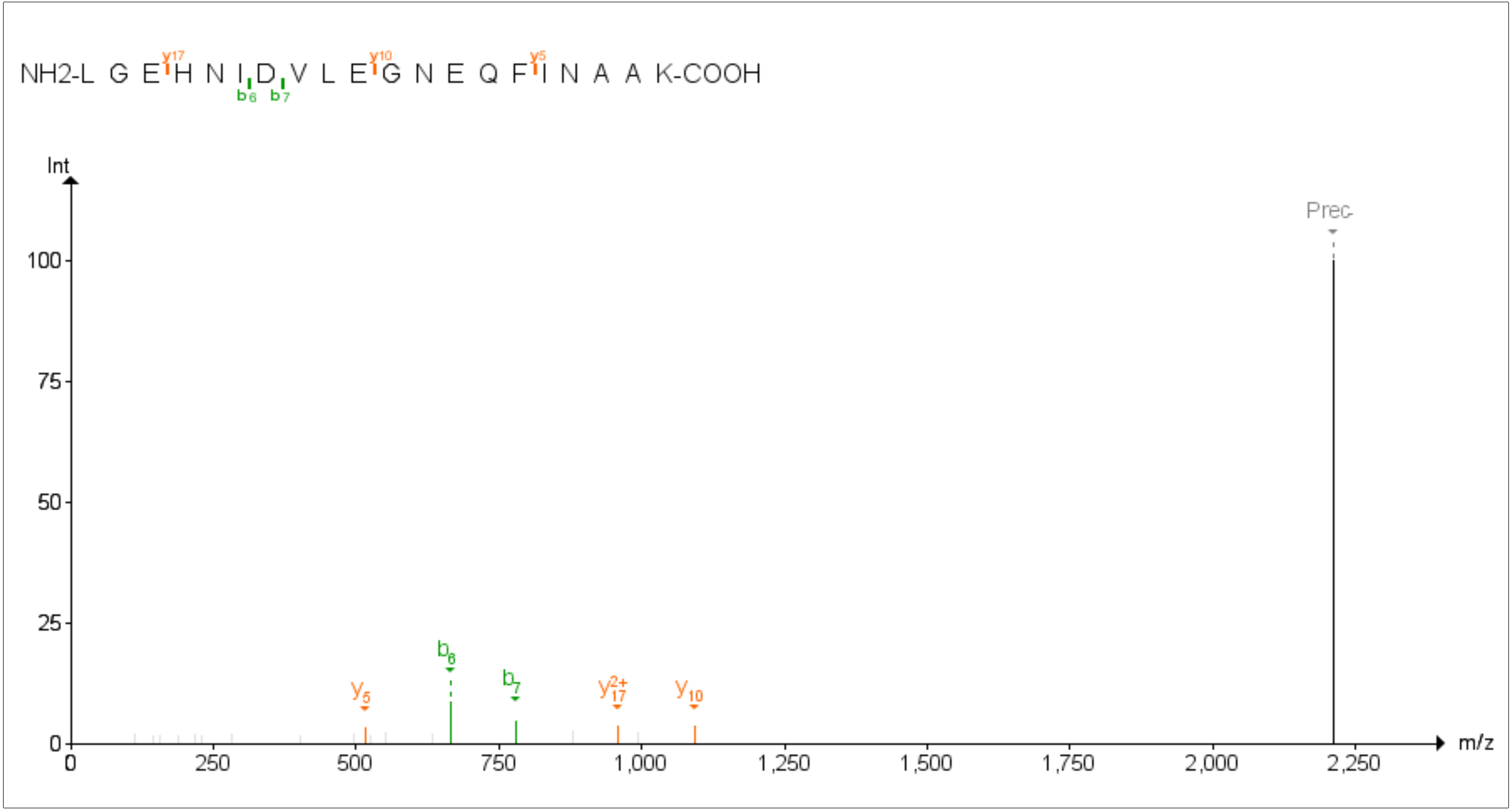

NH2-L G E H N I D V L E G N E Q F I N A A K-COOH
y17
y10
y5
b6
b7
Int
Prec
m/z
100
75
50
25
0
250
500
750
1,000
1,250
1,500
1,750
2,000
2,250

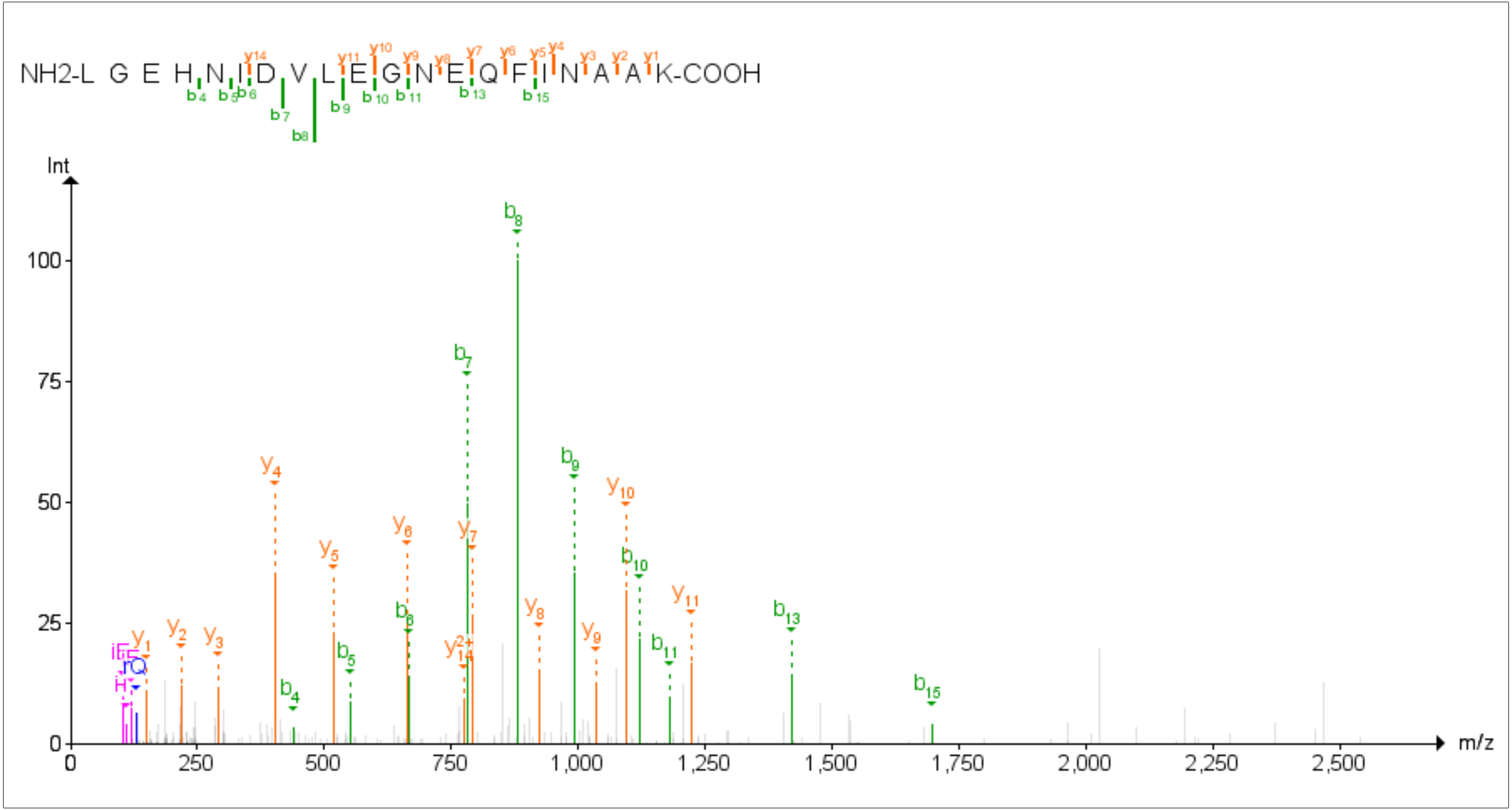

NH2-L G E H N I D V L E G N E Q F I N A A K-COOH
y14 y11 y10 y9 y8 y7 y6 y5 y4 y3 y2 y1
b4 b5 b6 b7 b8 b9 b10 b11 b13 b15
Int
m/z
0
25
50
75
100
250
500
750
1,000
1,250
1,500
1,750
2,000
2,250
2,500

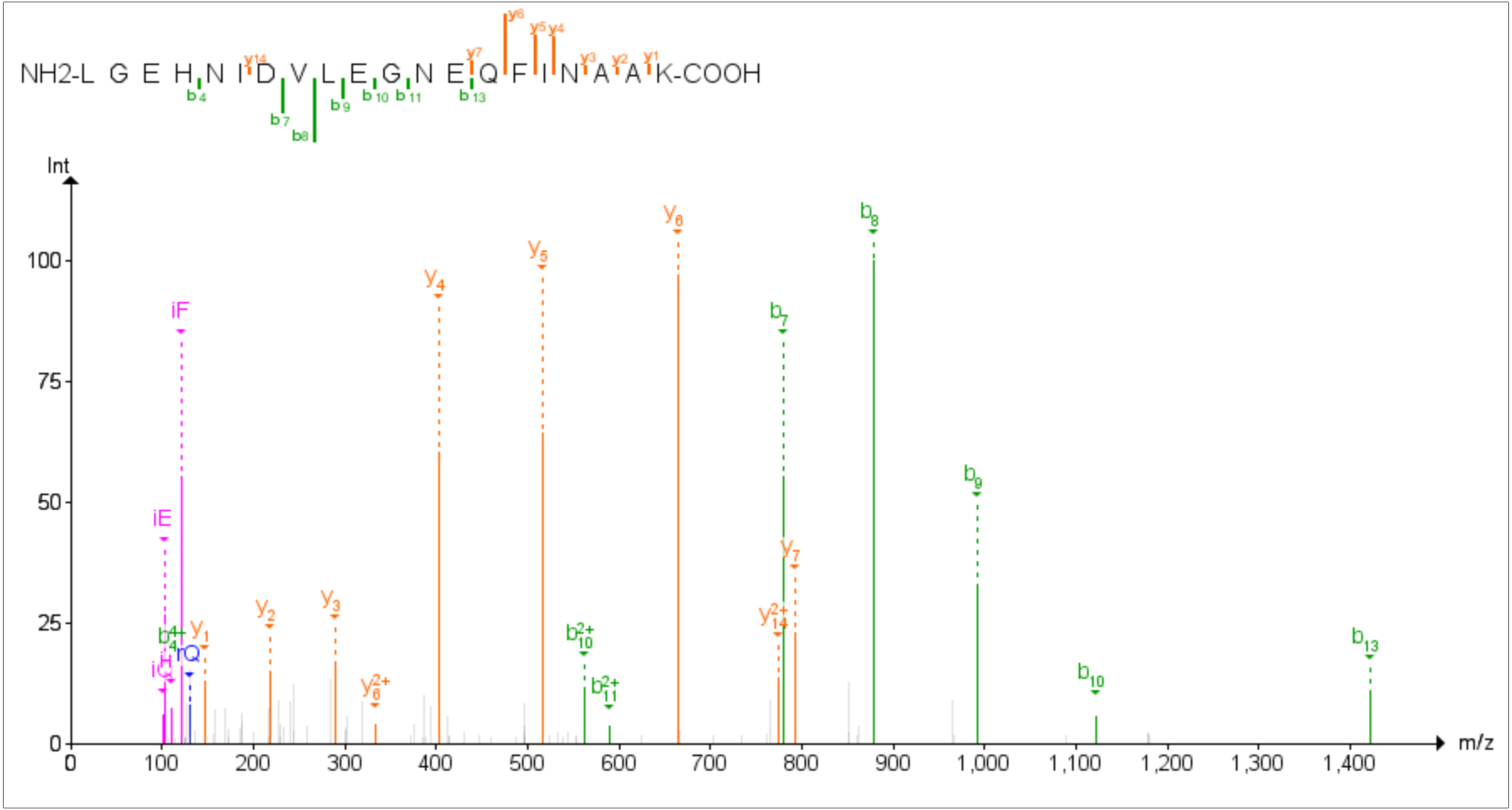

NH2-L G E H N I D V L E G N E Q F I N A A K-COOH
y14 y7 y6 y5 y4 y3 y2 y1
b4 b7 b8 b9 b10 b11 b13
Int
m/z

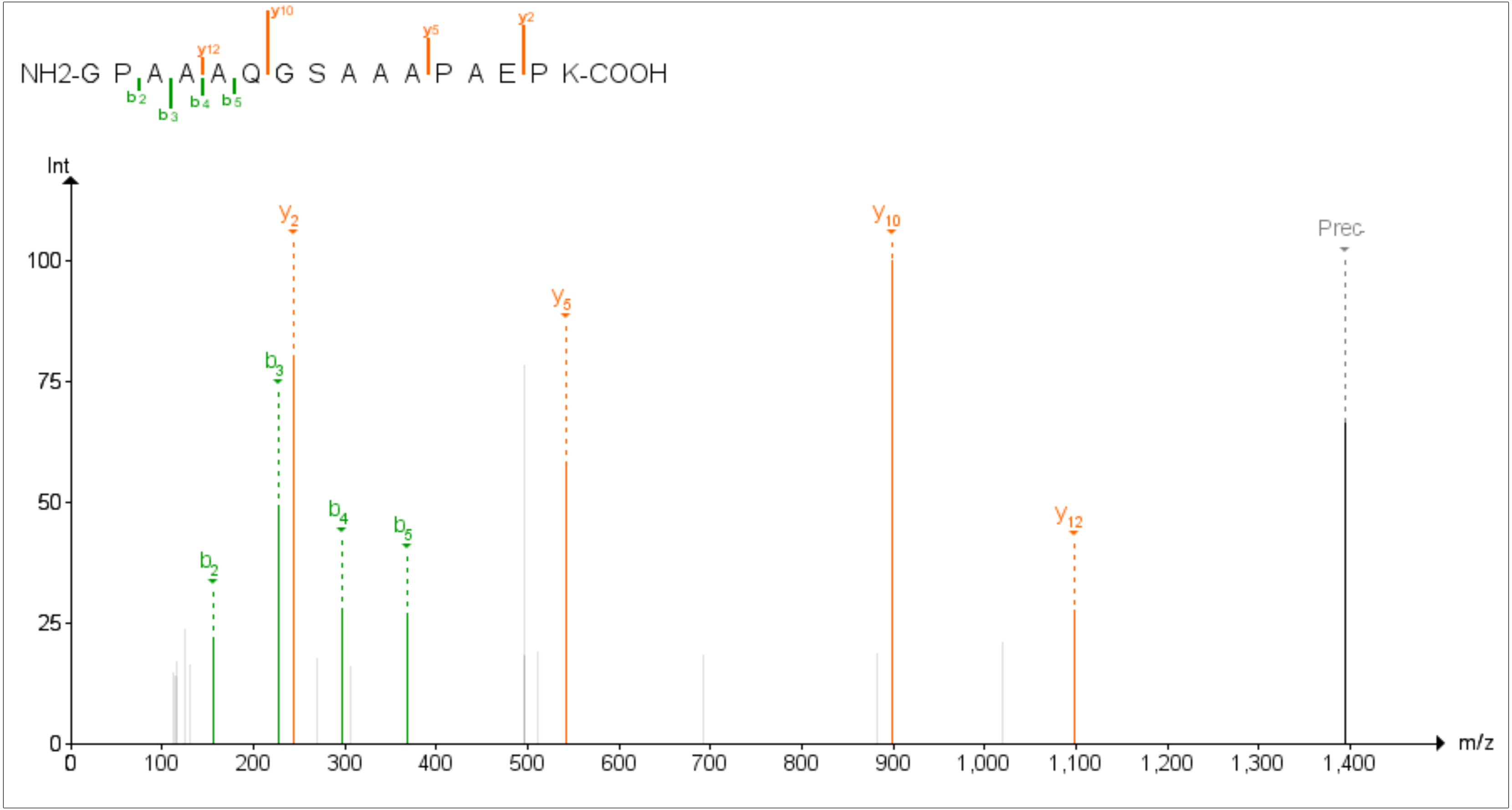

NH2-G P A A A Q G S A A A P A E P K-COOH
y10
y12
y5
y2
b2
b3
b4
b5
Int
m/z
Prec
y2
y10
y5
b3
b4
b5
b2
y12
0
25
50
75
100
0
100
200
300
400
500
600
700
800
900
1,000
1,100
1,200
1,300
1,400

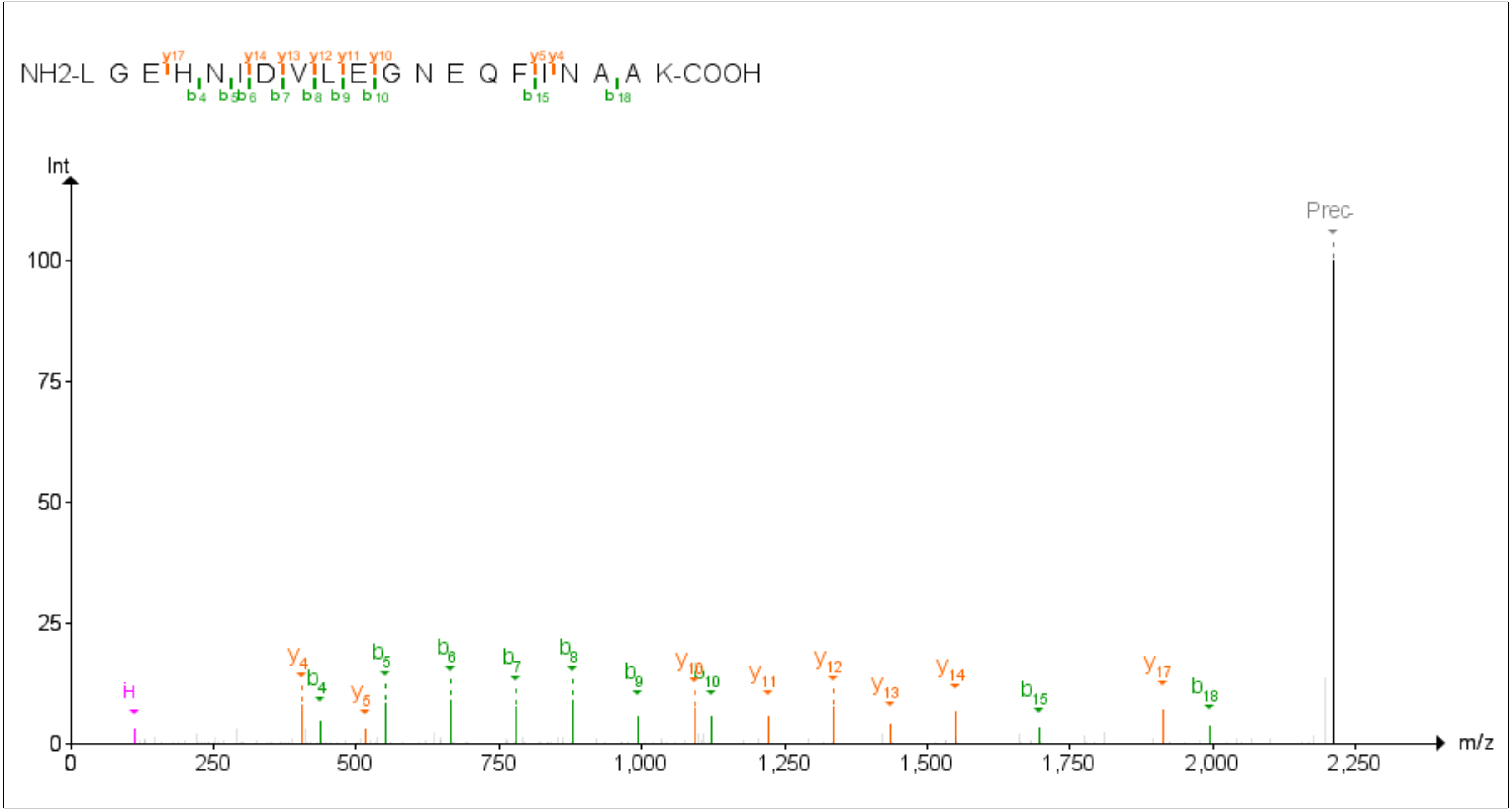

NH2-L G E H N I D V L E G N E Q F I N A A K-COOH
Int
Prec
m/z
0
25
50
75
100
250
500
750
1,000
1,250
1,500
1,750
2,000
2,250

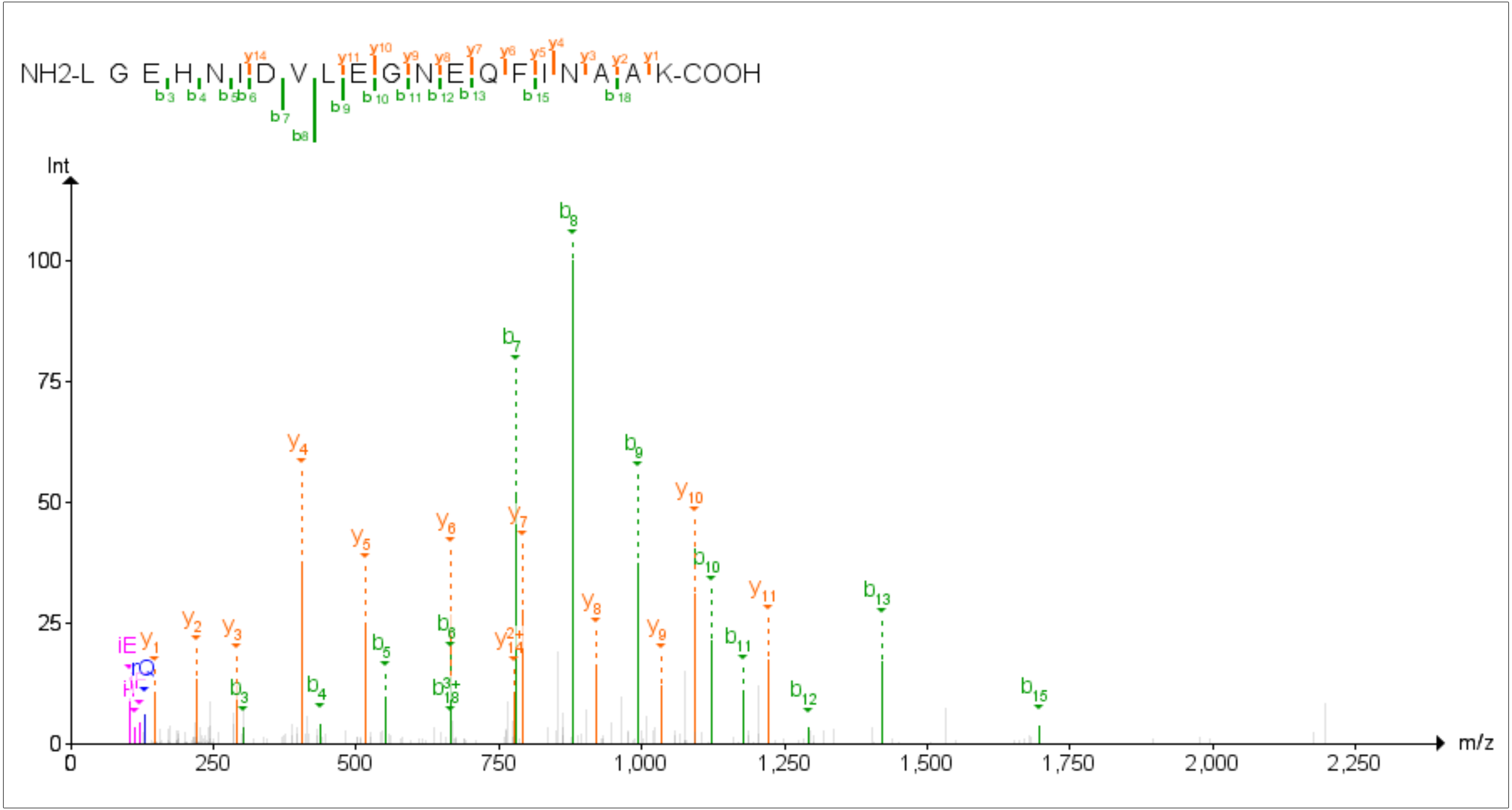

NH2-L G E H N I D V L E G N E Q F I N A A K-COOH
Int
m/z
0
25
50
75
100
250
500
750
1,000
1,250
1,500
1,750
2,000
2,250

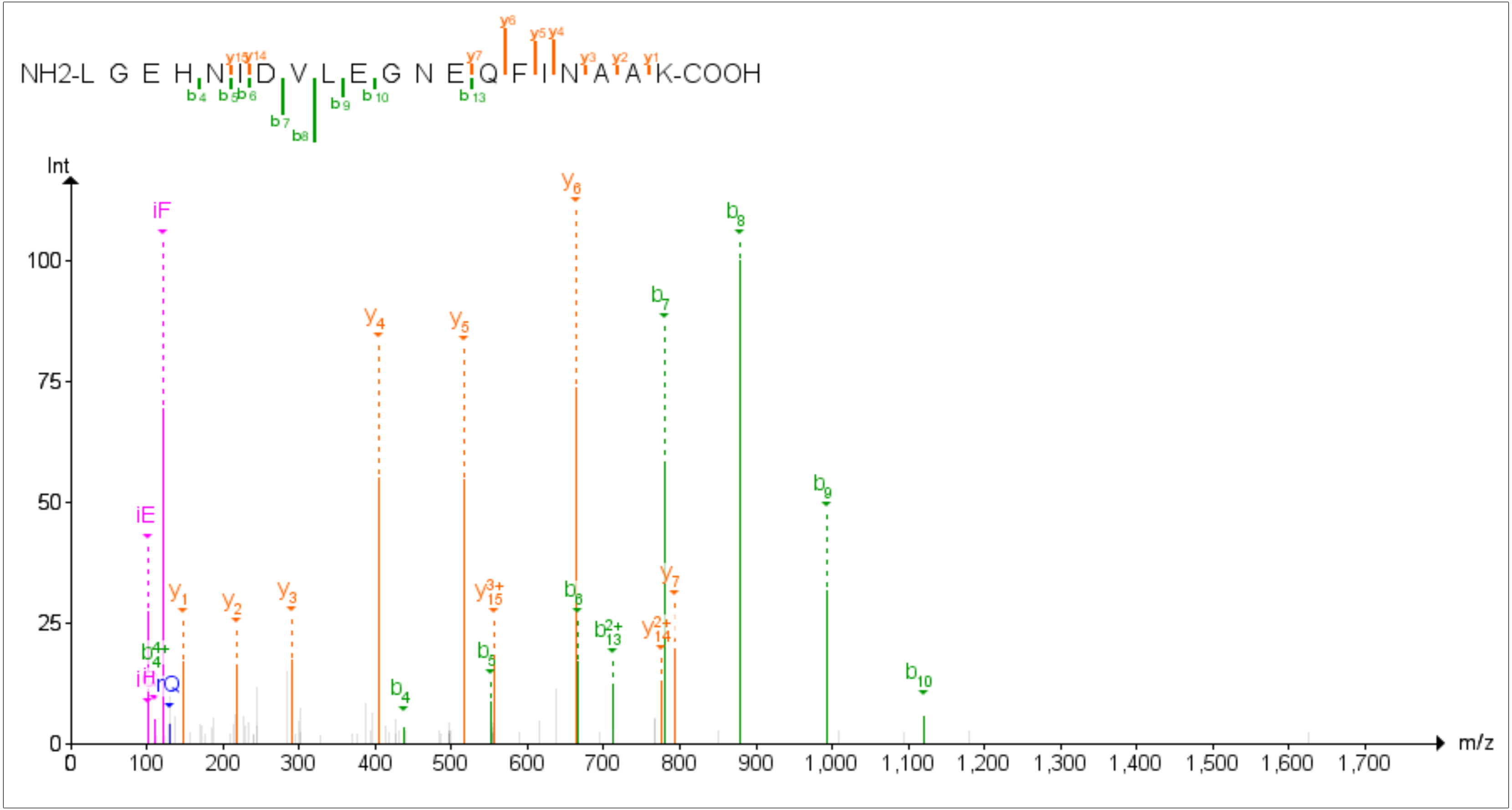

NH2-L G E H N I D V L E G N E Q F I N A A K-COOH
Int
m/z
iF
iE
iH
rQ
y6
b8
b7
y4
y5
b9
y1
y2
y3
b10
b4
b5
b6

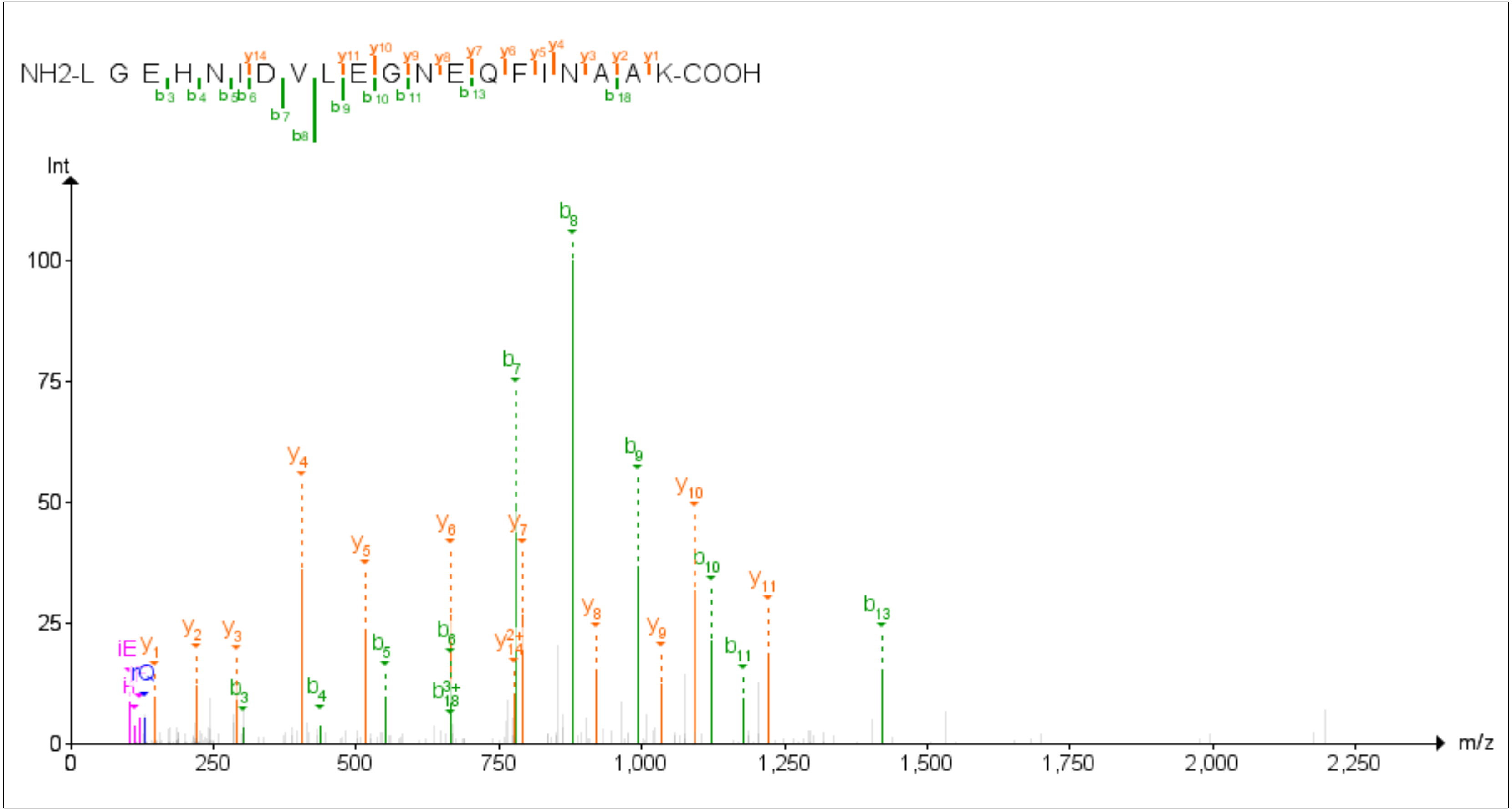

NH2-L G E H N I D V L E G N E Q F I N A A K-COOH
y14 y11 y10 y9 y8 y7 y6 y5 y4 y3 y2 y1
b3 b4 b5 b6 b7 b8 b9 b10 b11 b13 b18
Int
m/z
0
25
50
75
100
250
500
750
1,000
1,250
1,500
1,750
2,000
2,250

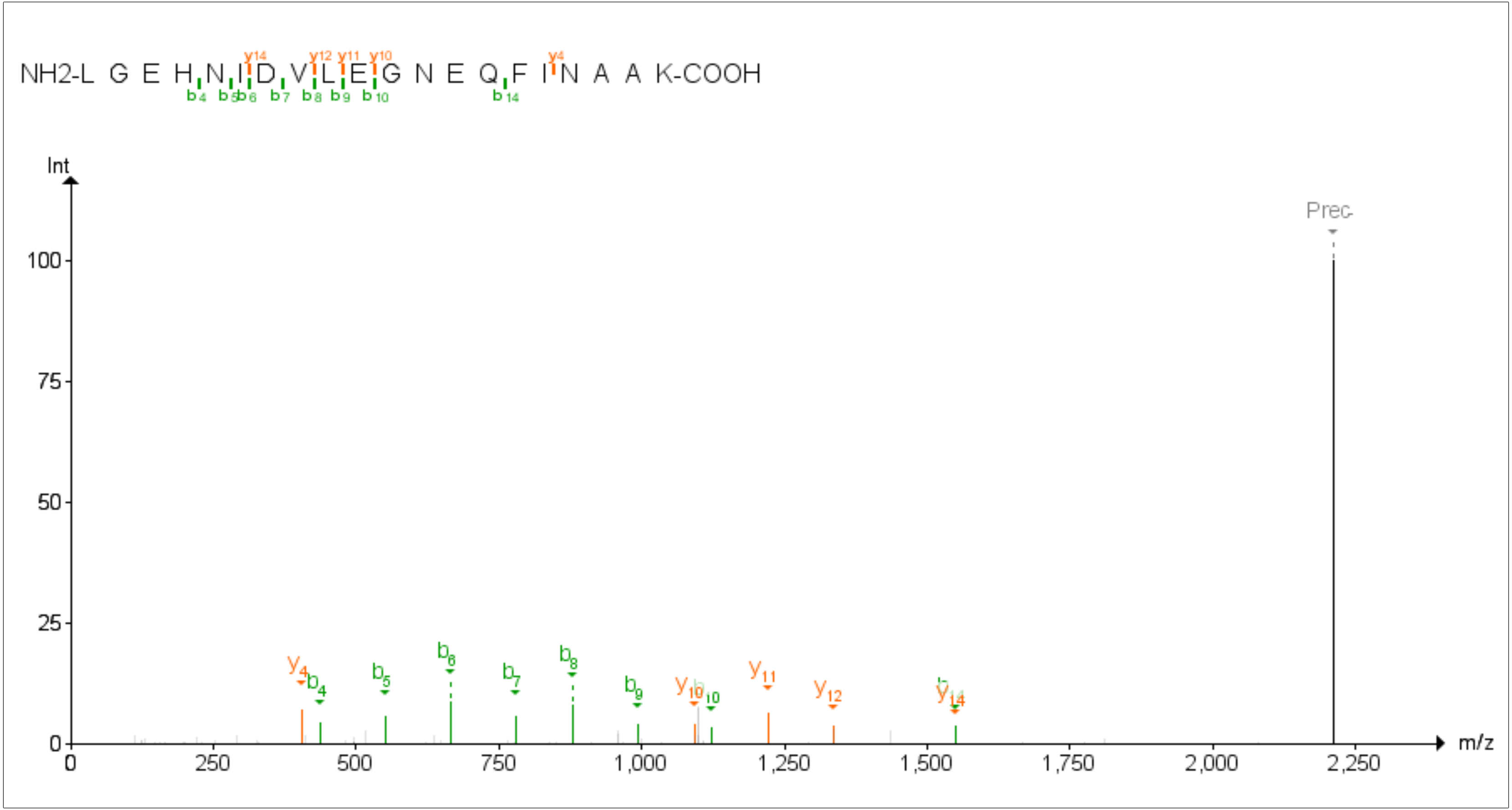
NH2-L G E H N I D V L E G N E Q F I N A A K-COOH
y14 y12 y11 y10 y4
b4 b5 b6 b7 b8 b9 b10 b14
Int
100
75
50
25
0
Prec
y4
b4
b5
b6
b7
b8
b9
y10
b10
y11
y12
y14
0
250
500
750
1,000
1,250
1,500
1,750
2,000
2,250
m/z

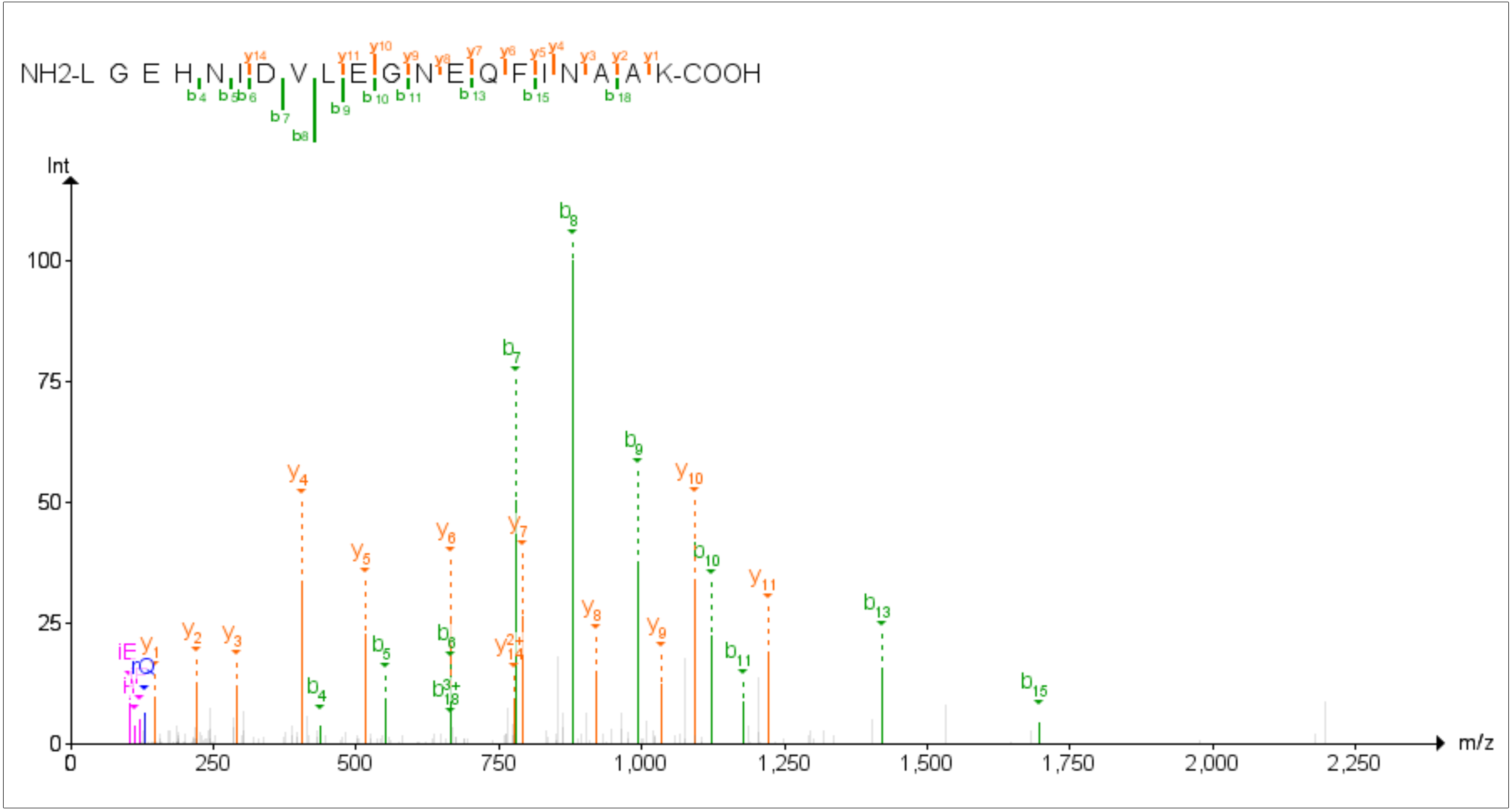

NH2-L G E H N I D V L E G N E Q F I N A A K-COOH
Int
m/z
b8
b7
b9
y10
y4
b10
y11
b13
y5
y6
y7
y8
y9
b11
b15
b4
b5
b6
y1
y2
y3
iE
rQ

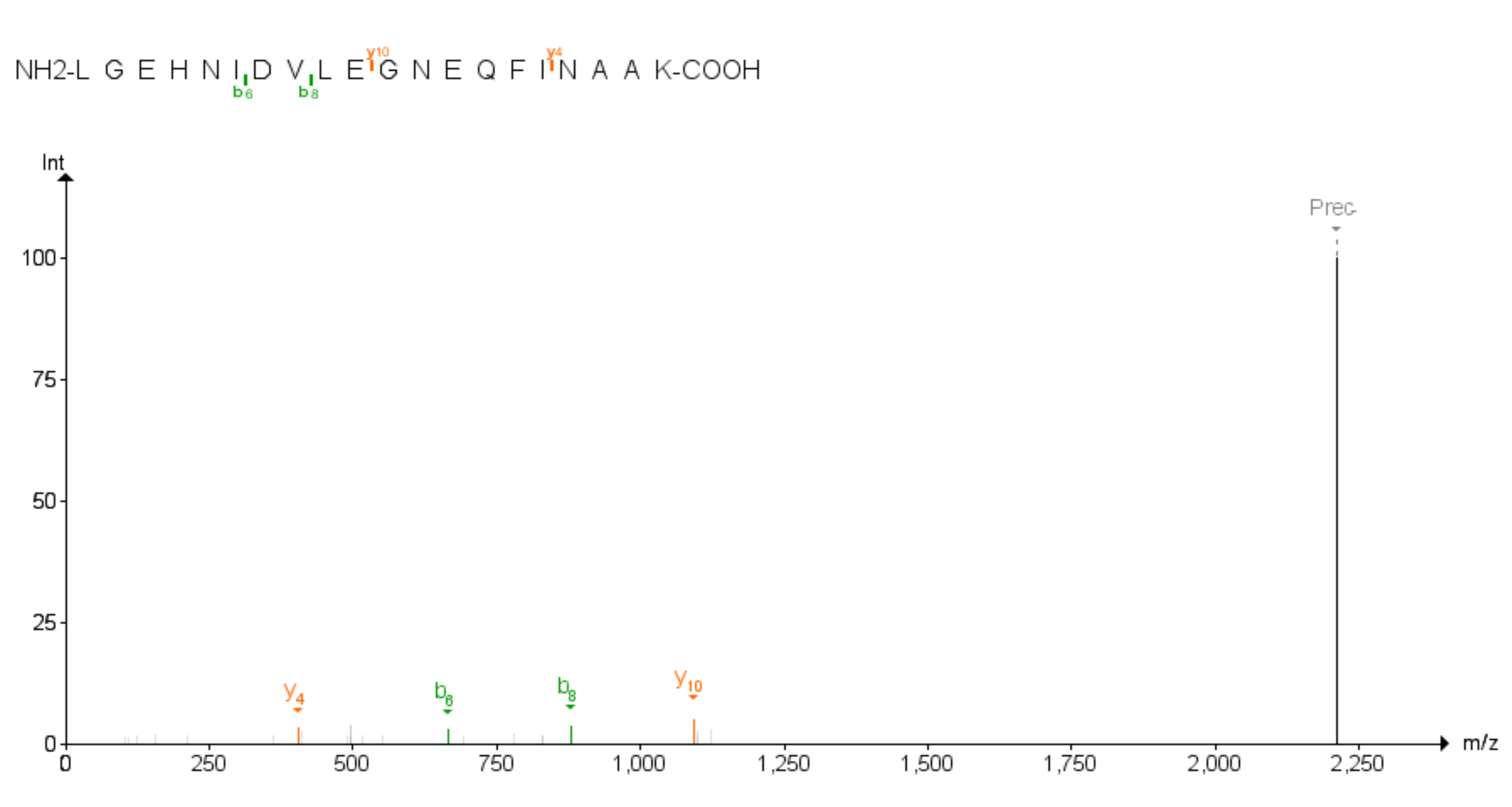

NH2-L G E H N I D V L E G N E Q F I N A A K-COOH
b6
b8
y10
y4
Int
100
75
50
25
0
Prec
y4
b6
b8
y10
0
250
500
750
1,000
1,250
1,500
1,750
2,000
2,250
m/z

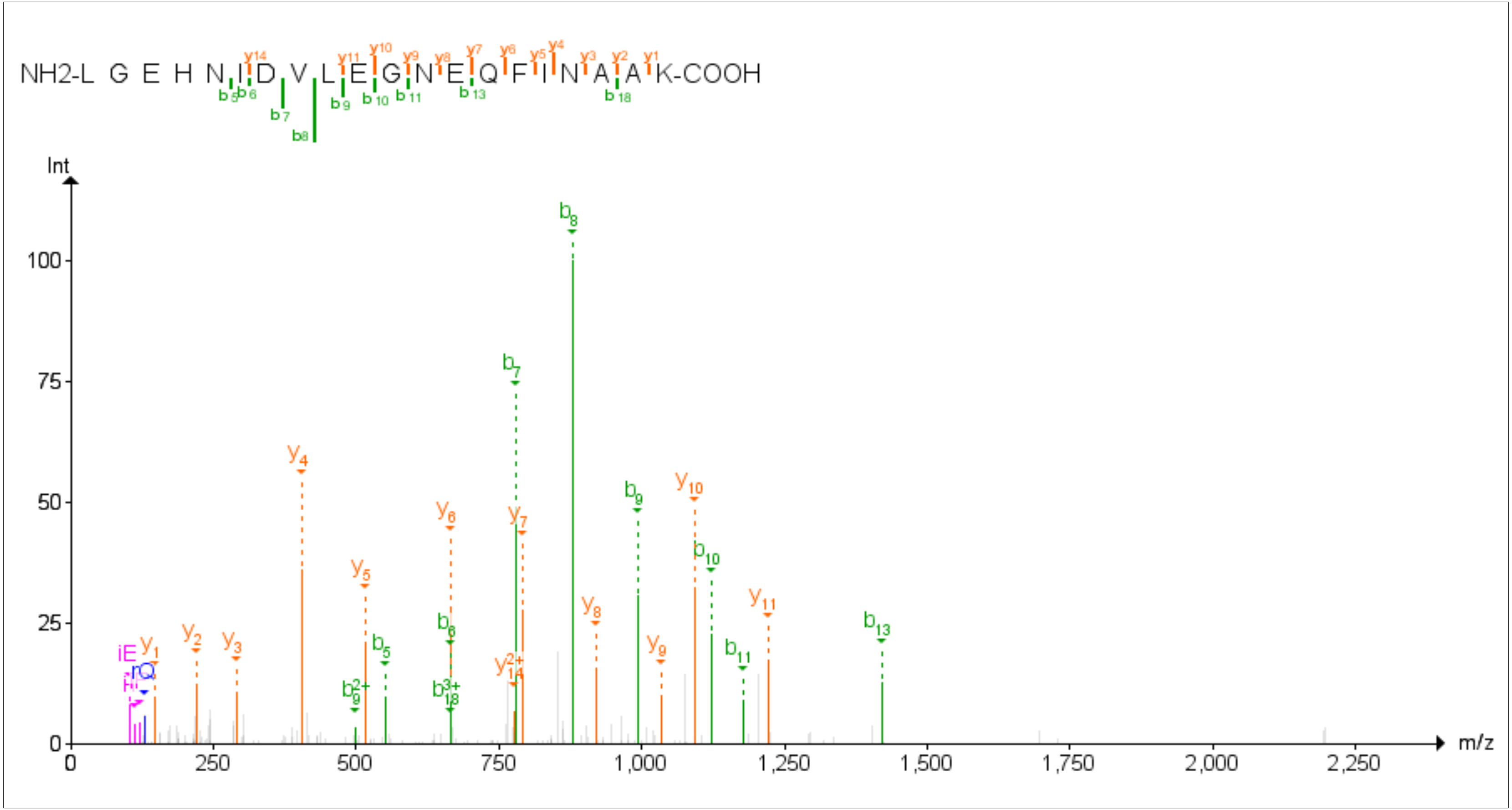
NH2-L G E H N I D V L E G N E Q F I N A A K-COOH
Int
m/z
b8
b7
y4
b9
y10
y6
y7
b10
y5
y11
b13
y8
b6
y9
b11
y1
y2
y3
iE
b5
b9 2+
b18 3+
y14 2+
0
25
50
75
100
0
250
500
750
1,000
1,250
1,500
1,750
2,000
2,250

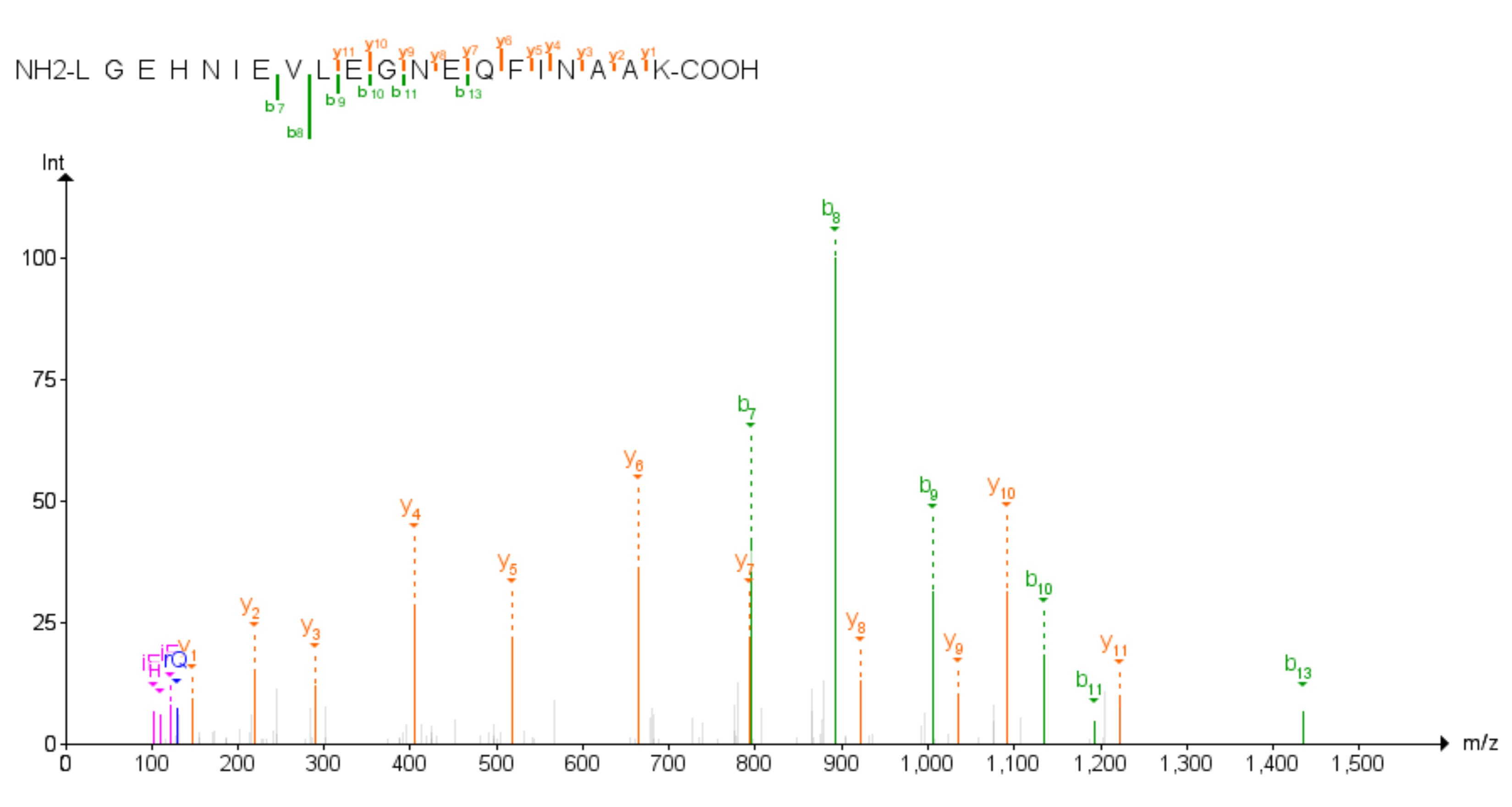

NH2-L G E H N I E V L E G N E Q F I N A A K-COOH
Int
m/z

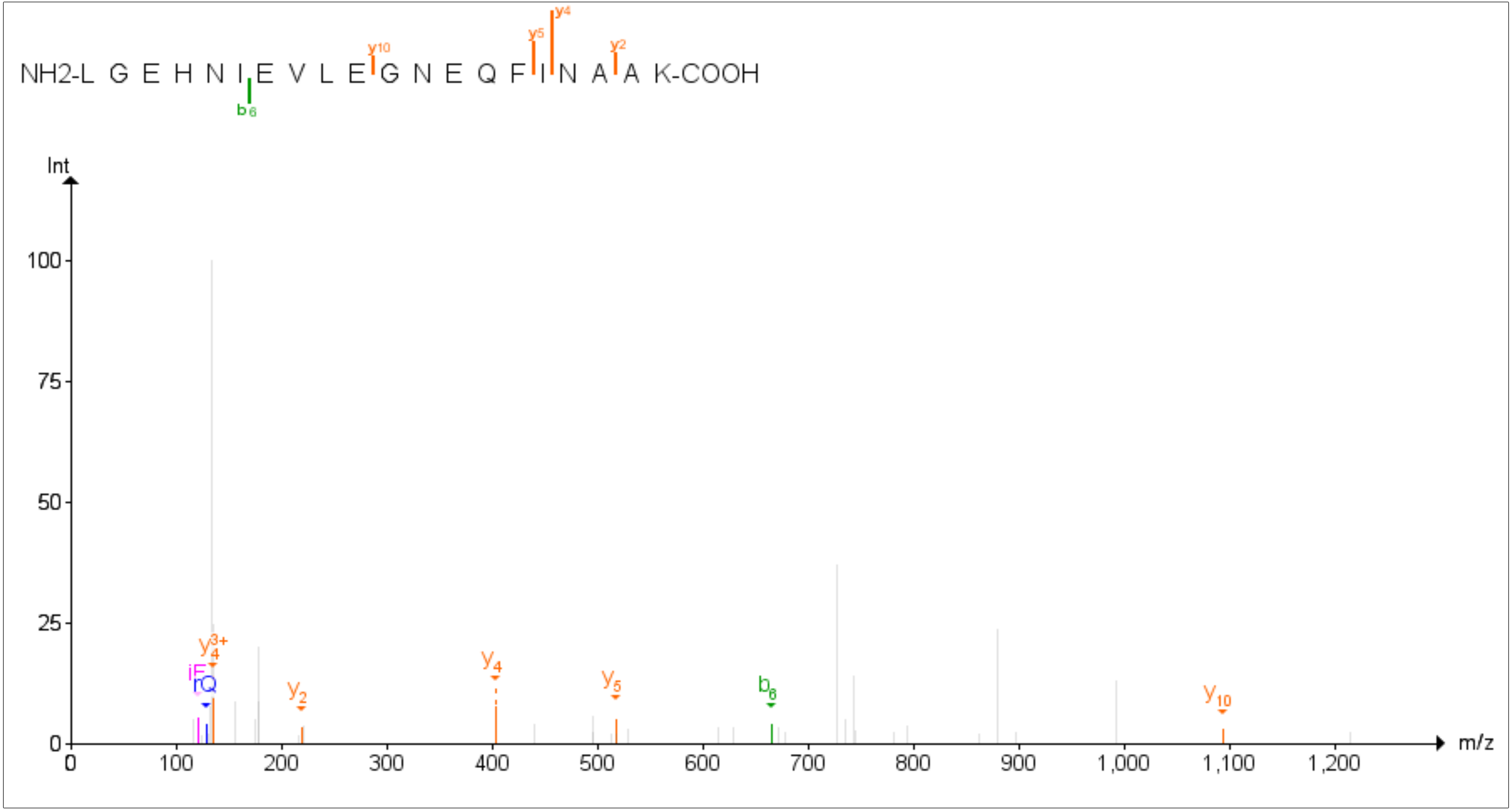

NH2-L G E H N I E V L E G N E Q F I N A A K-COOH
y10
y5
y4
y2
b6
Int
100
75
50
25
0
m/z
0
100
200
300
400
500
600
700
800
900
1,000
1,100
1,200
y4 3+
iF
rQ
y2
y4
y5
b6
y10

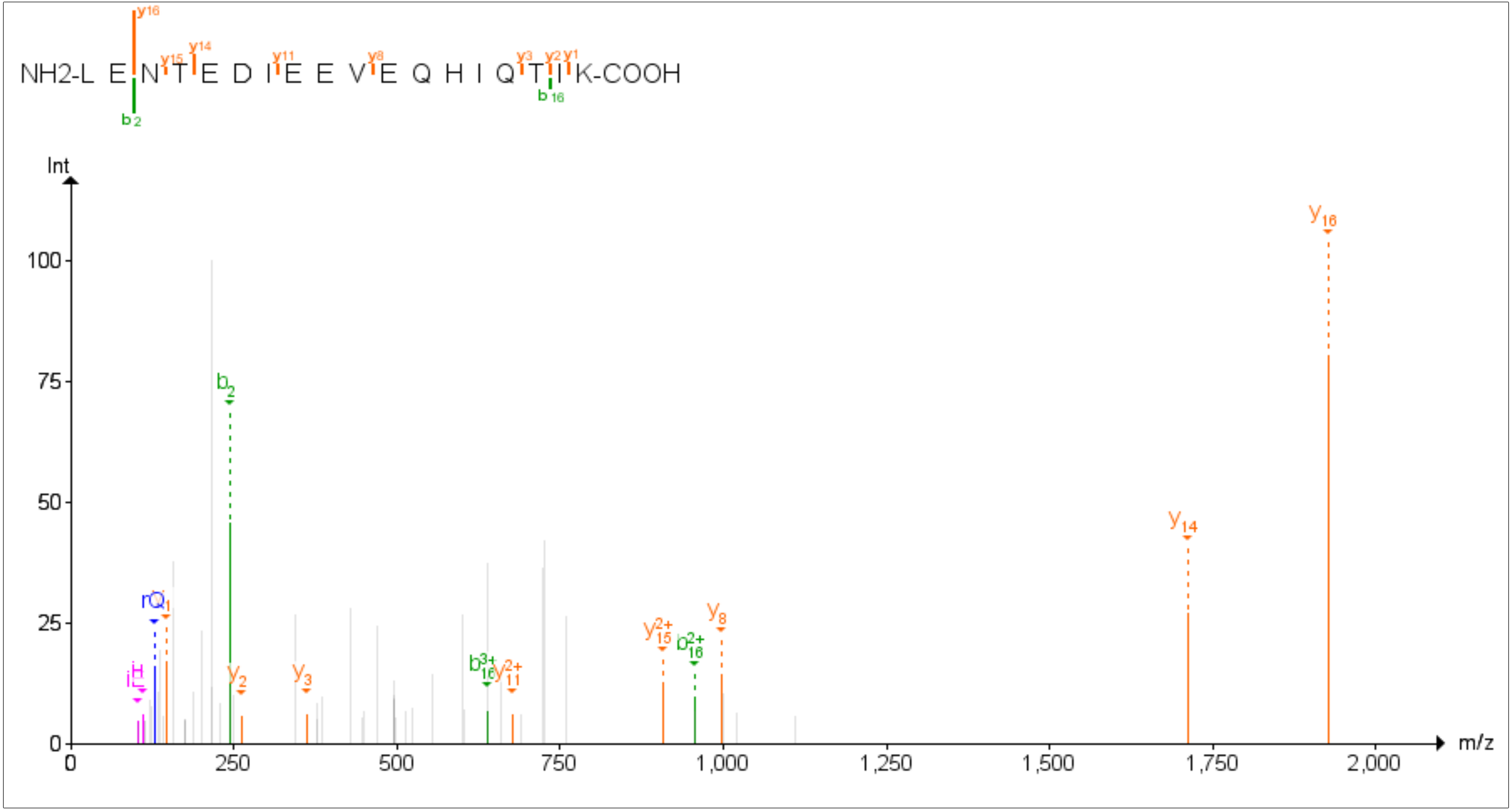

NH2-L E N T E D I E E V E Q H I Q T I K-COOH
y16
y15
y14
y11
y8
y3
y2
y1
b2
b16
Int
m/z
0
25
50
75
100
250
500
750
1,000
1,250
1,500
1,750
2,000

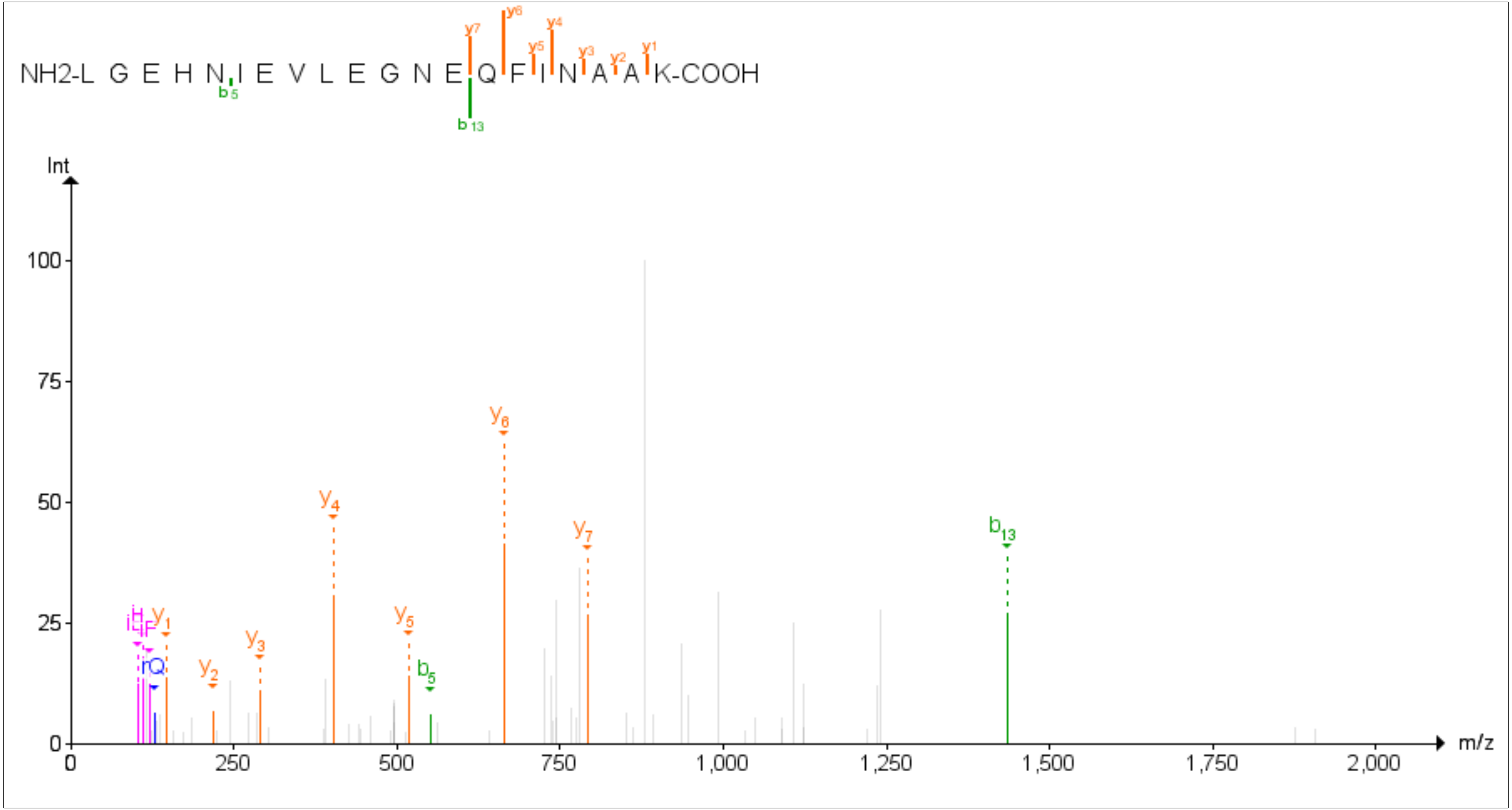

NH2-L G E H N I E V L E G N E Q F I N A A K-COOH
y7 y6 y5 y4 y3 y2 y1
b5 b13
Int
100
75
50
25
0
m/z
0 250 500 750 1,000 1,250 1,500 1,750 2,000
y6 y4 y7 b13 y1 y5 y3 y2 b5 rQ

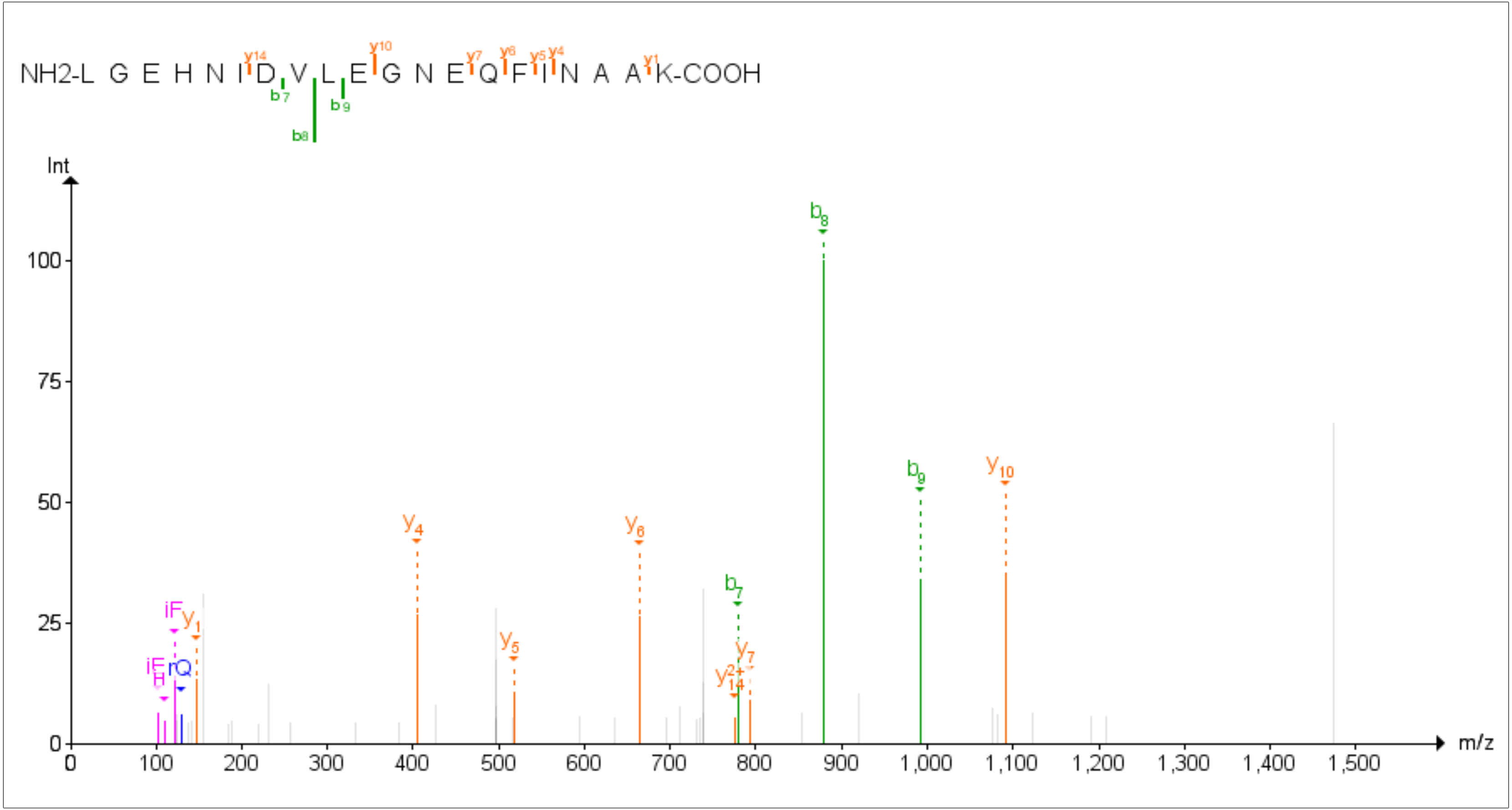
NH2-L G E H N I D V L E G N E Q F I N A A K-COOH
y14 y10 y7 y6 y5 y4 y1
b7 b8 b9
Int
m/z
iF
y1
iF
H
rQ
y4
y5
y6
b7
y7
y14 2+
b8
b9
y10

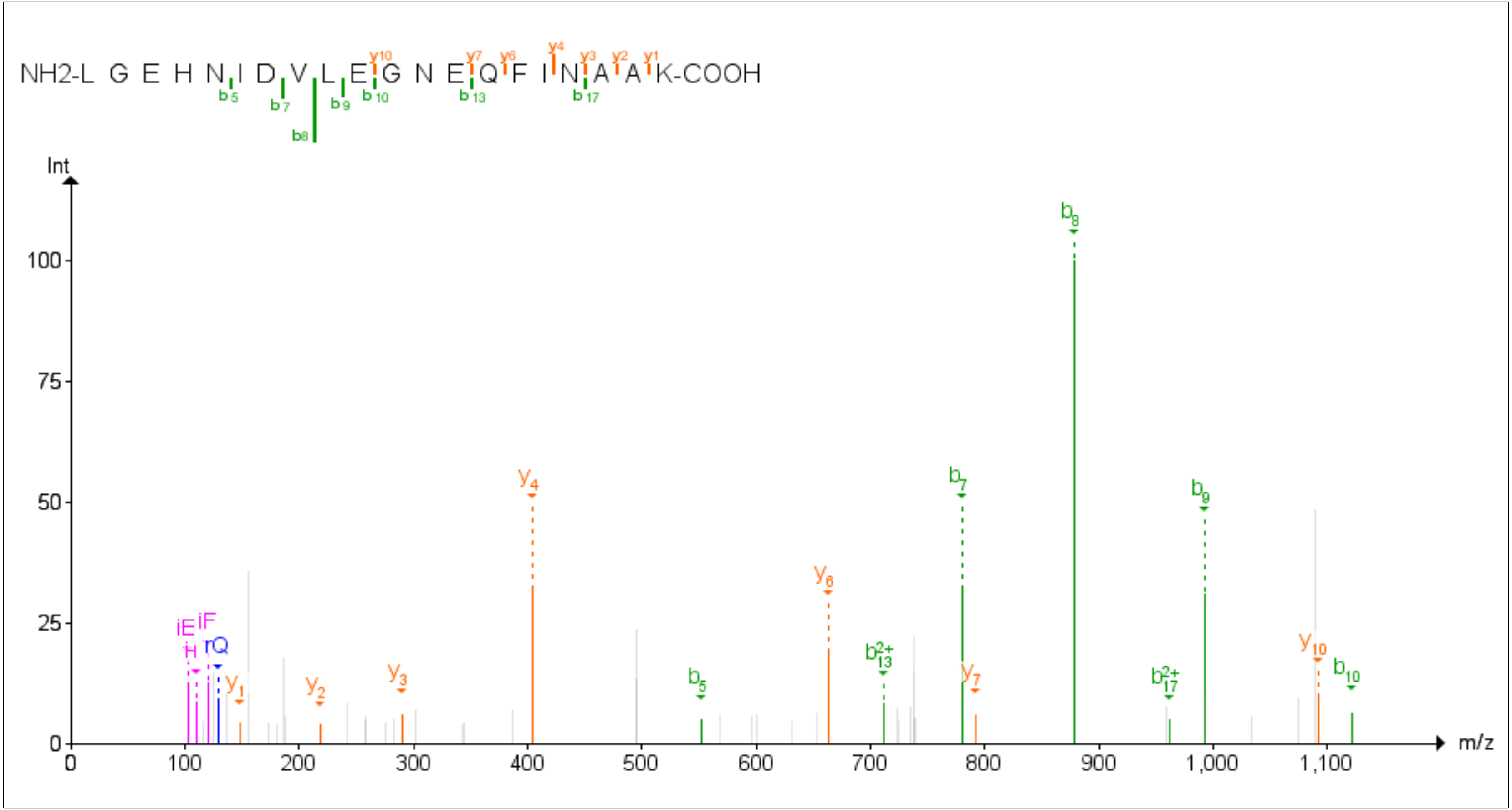

NH2-L G E H N I D V L E G N E Q F I N A A K-COOH
y10 y7 y6 y4 y3 y2 y1
b5 b7 b8 b9 b10 b13 b17
Int
m/z
iE iF H rQ
y_1 y_2 y_3 y_4 y_6 y_7 y_{10}
b_5 b_{13}^{2+} b_7 b_8 b_{17}^{2+} b_9 b_{10}

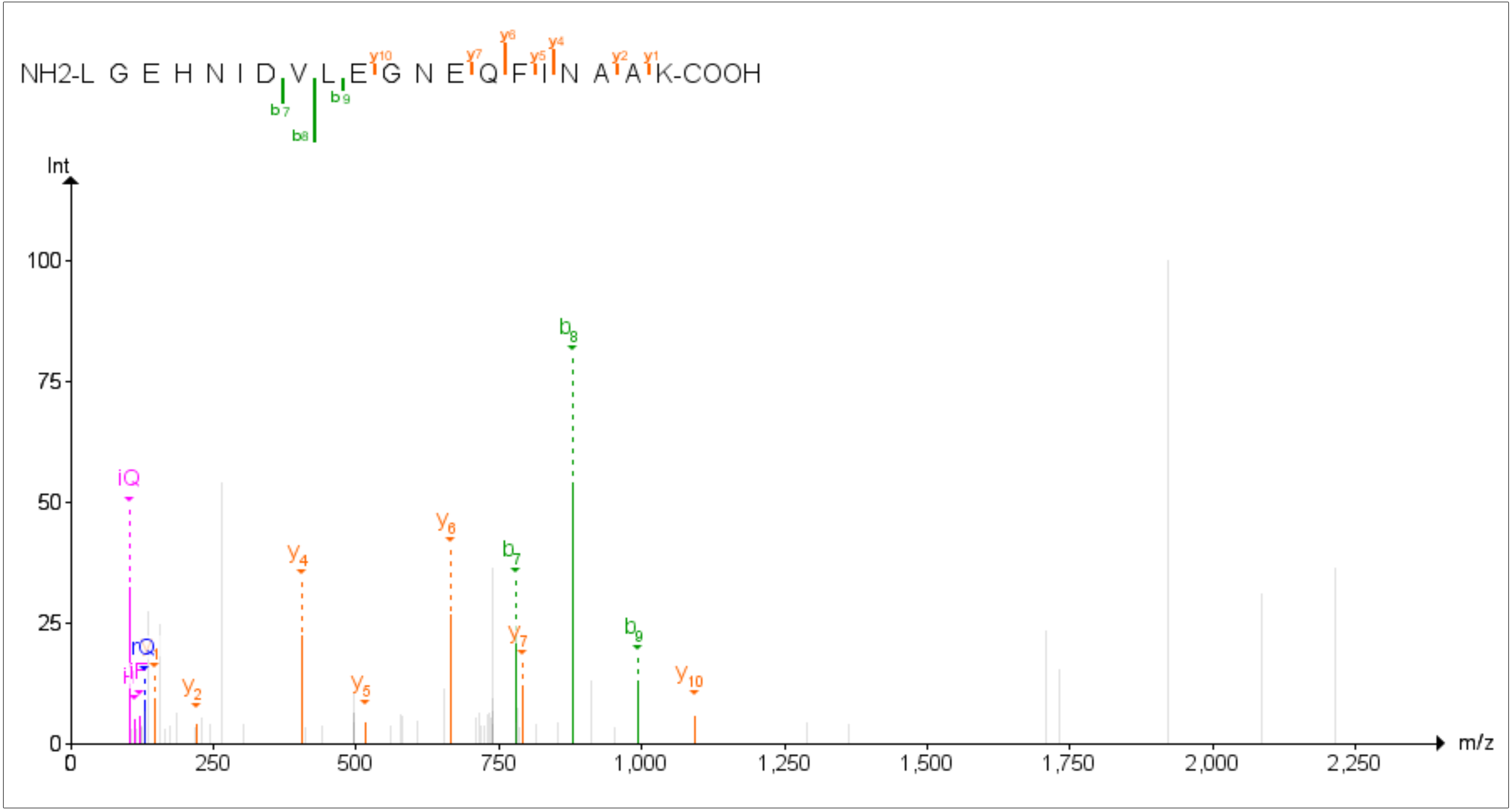
NH2-L G E H N I D V L E G N E Q F I N A A K-COOH
b7 b8 b9
y10 y7 y6 y5 y4 y2 y1
Int
100
75
50
25
0
iQ
y4
y6
b7
b8
y7
b9
y10
y2
y5
iF
nQ
0
250
500
750
1,000
1,250
1,500
1,750
2,000
2,250
m/z

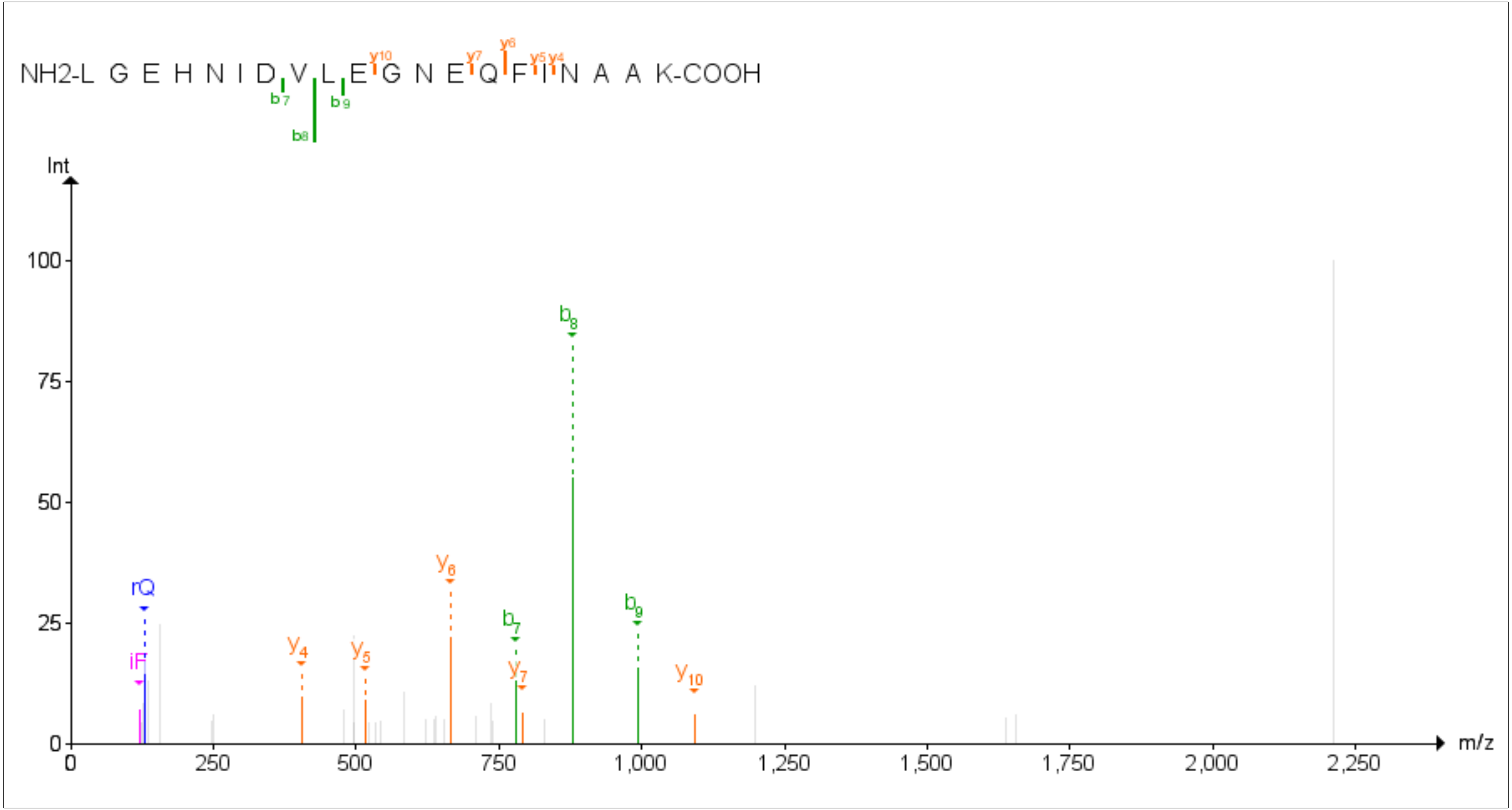

NH2-L G E H N I D V L E G N E Q F I N A A K-COOH
b7
b8
b9
y10
y7
y6
y5
y4
Int
100
75
50
25
0
rQ
iF
y4
y5
y6
b7
y7
b8
b9
y10
0
250
500
750
1,000
1,250
1,500
1,750
2,000
2,250
m/z

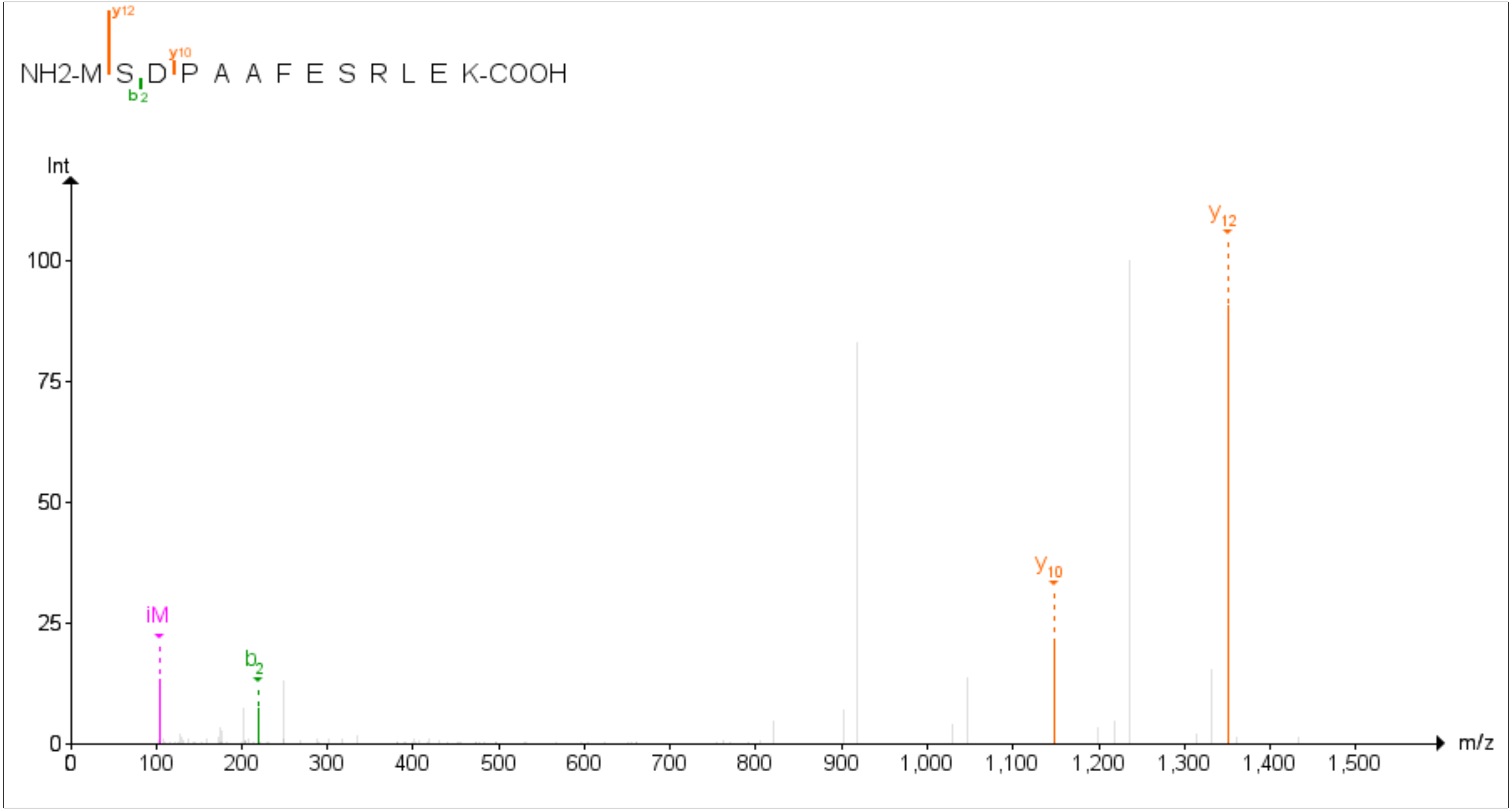

NH2-M S D P A A F E S R L E K-COOH
y12
y10
b2
Int
100
75
50
25
0
iM
b2
y10
y12
0
100
200
300
400
500
600
700
800
900
1,000
1,100
1,200
1,300
1,400
1,500
m/z

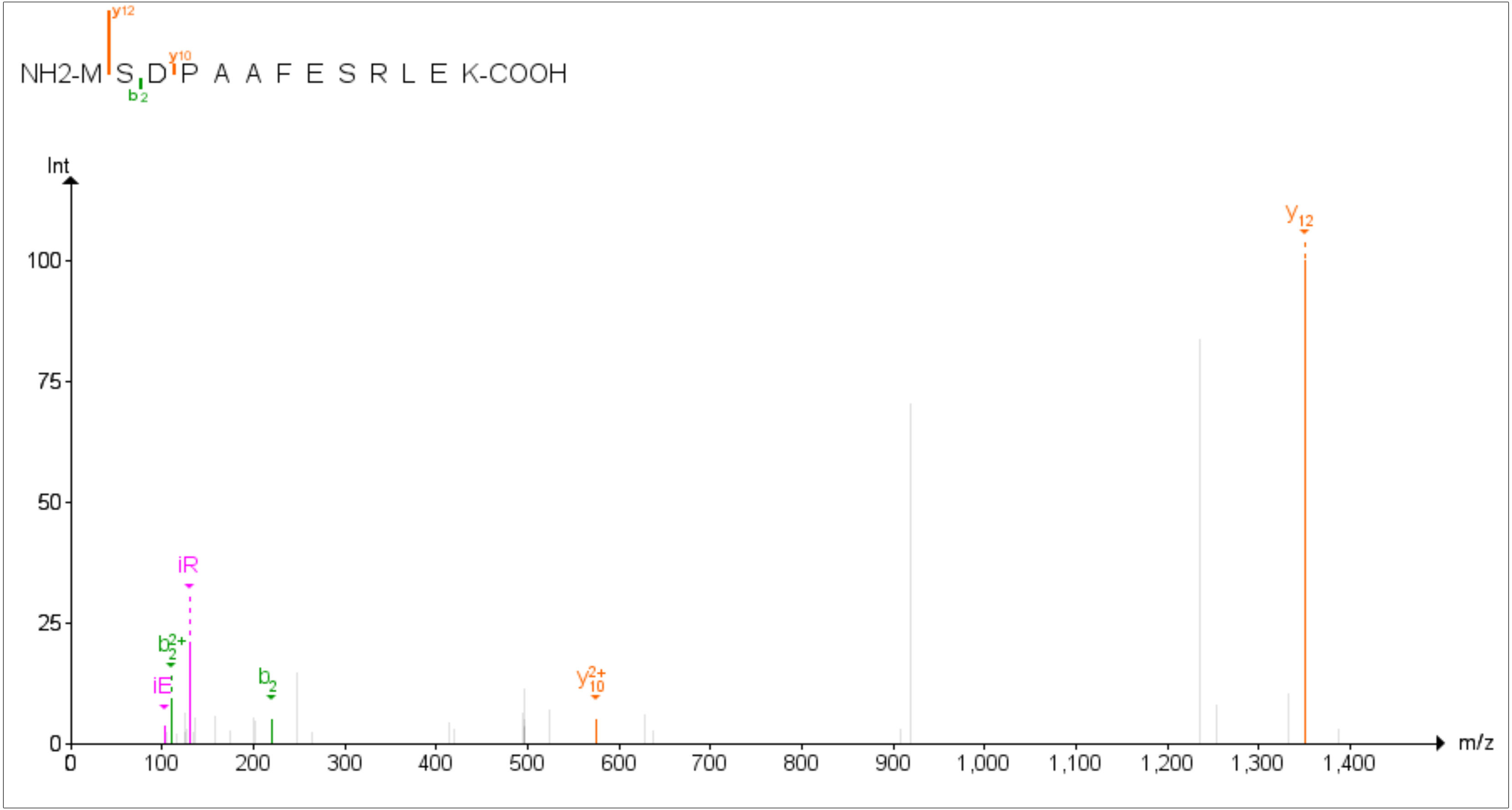

y12
y10
NH2-M S D P A A F E S R L E K-COOH
b 2
Int
100
75
50
25
0
y12
iR
b2 2+
iE
b2
y10 2+
0
100
200
300
400
500
600
700
800
900
1,000
1,100
1,200
1,300
1,400
m/z

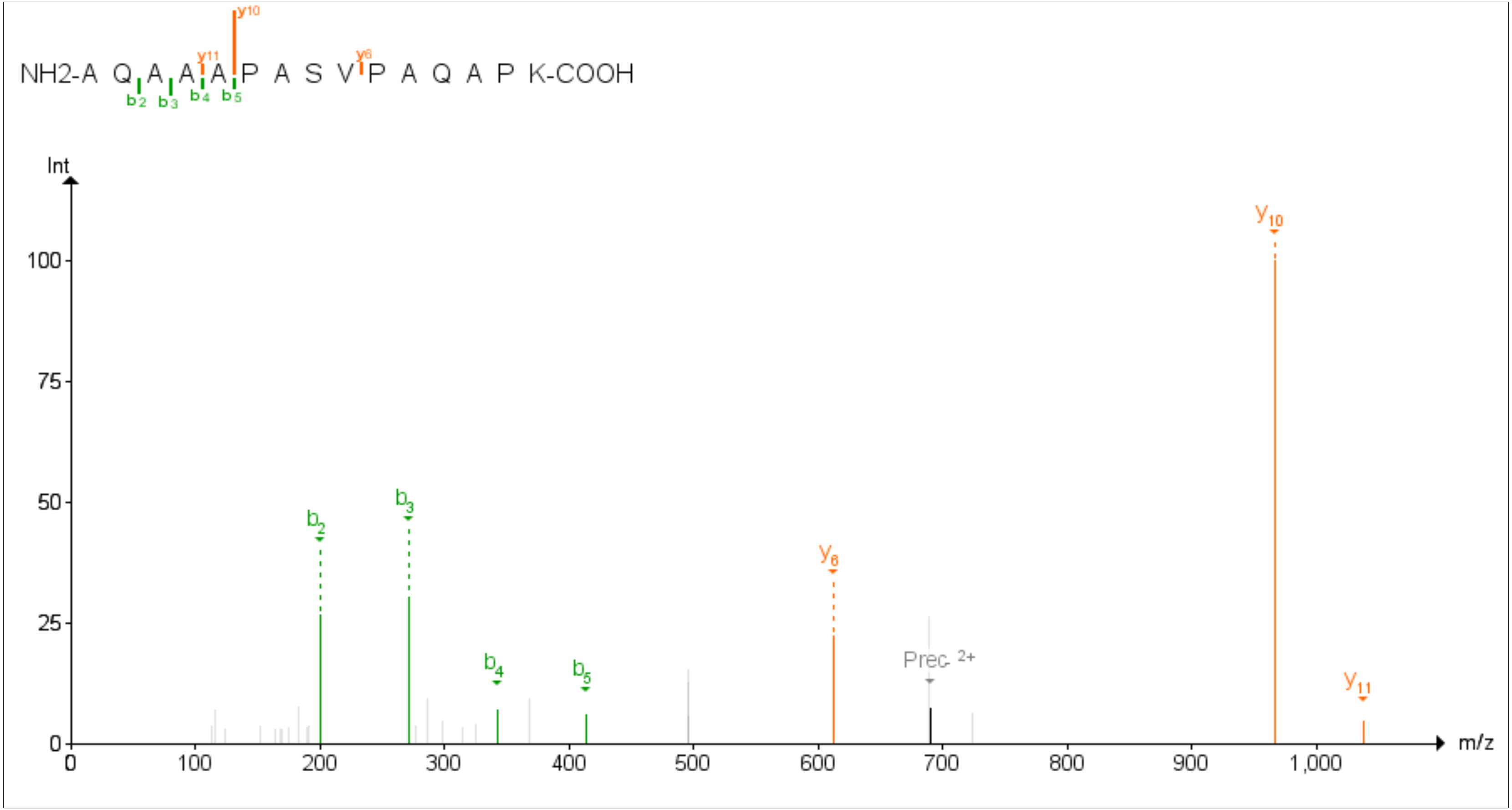

NH2-A Q A A A P A S V P A Q A P K-COOH
y10
y11
y6
b2
b3
b4
b5
Int
100
75
50
25
0
y10
b2
b3
y6
b4
b5
Prec 2+
y11
0
100
200
300
400
500
600
700
800
900
1,000
m/z

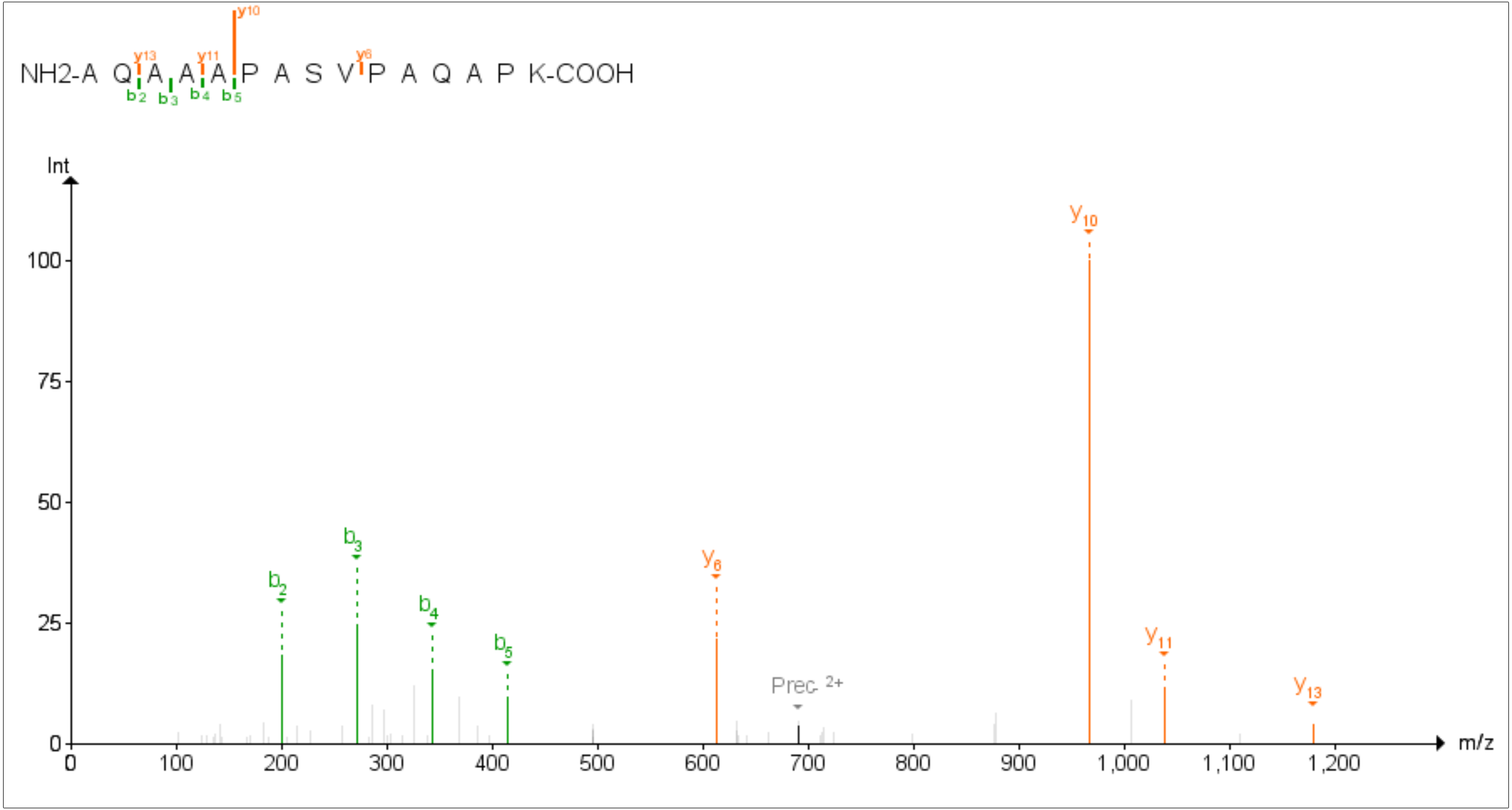

NH2-A Q A A A P A S V P A Q A P K-COOH
y13 y11 y10 y6
b2 b3 b4 b5
Int
100
75
50
25
0
y10
b3
b2
b4
b5
y6
Prec 2+
y11
y13
0 100 200 300 400 500 600 700 800 900 1,000 1,100 1,200
m/z

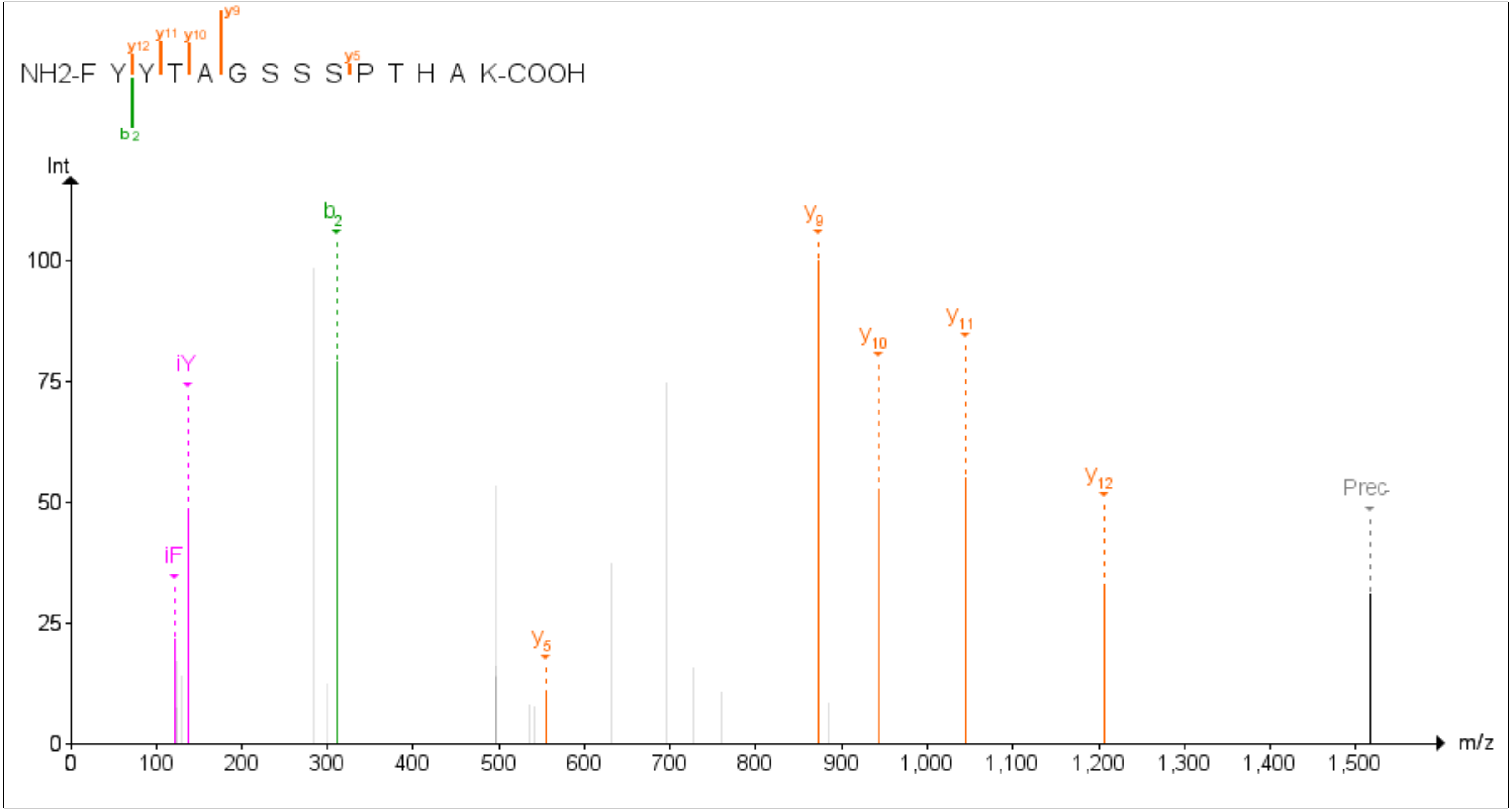

NH2-F Y Y T A G S S S P T H A K-COOH
y12
y11
y10
y9
y5
b2
Int
b2
y9
y10
y11
y12
iY
iF
y5
Prec
100
75
50
25
0
0
100
200
300
400
500
600
700
800
900
1,000
1,100
1,200
1,300
1,400
1,500
m/z

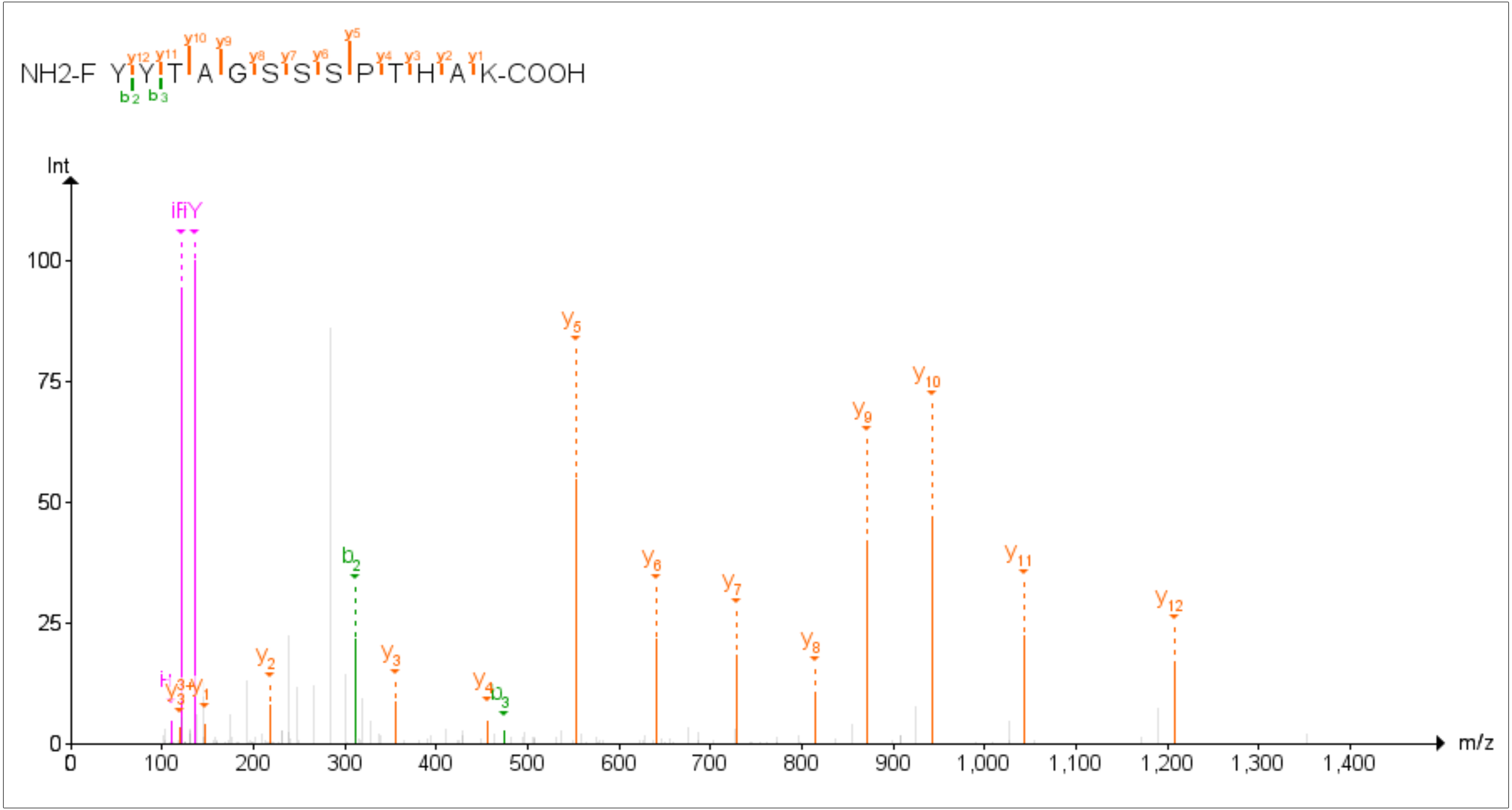

NH2-F Y Y T A G S S S P T H A K-COOH
Int
m/z

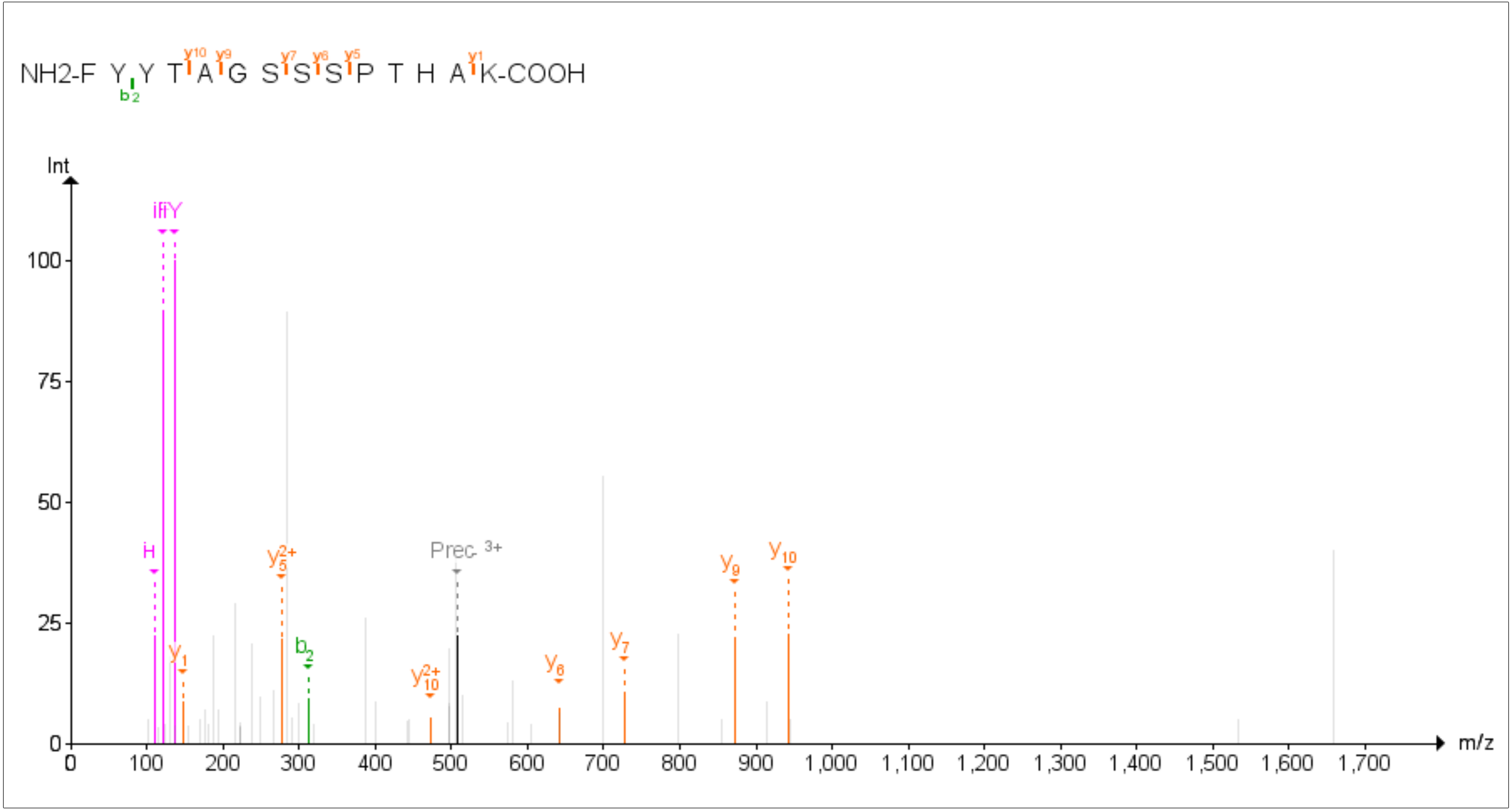
NH2-F Y Y T A G S S S P T H A K-COOH
b2
y10 y9 y7 y6 y5 y1
Int
m/z
ifY
iH
y1
y5 2+
b2
Prec 3+
y10 2+
y6
y7
y9
y10
0
25
50
75
100
0
100
200
300
400
500
600
700
800
900
1,000
1,100
1,200
1,300
1,400
1,500
1,600
1,700

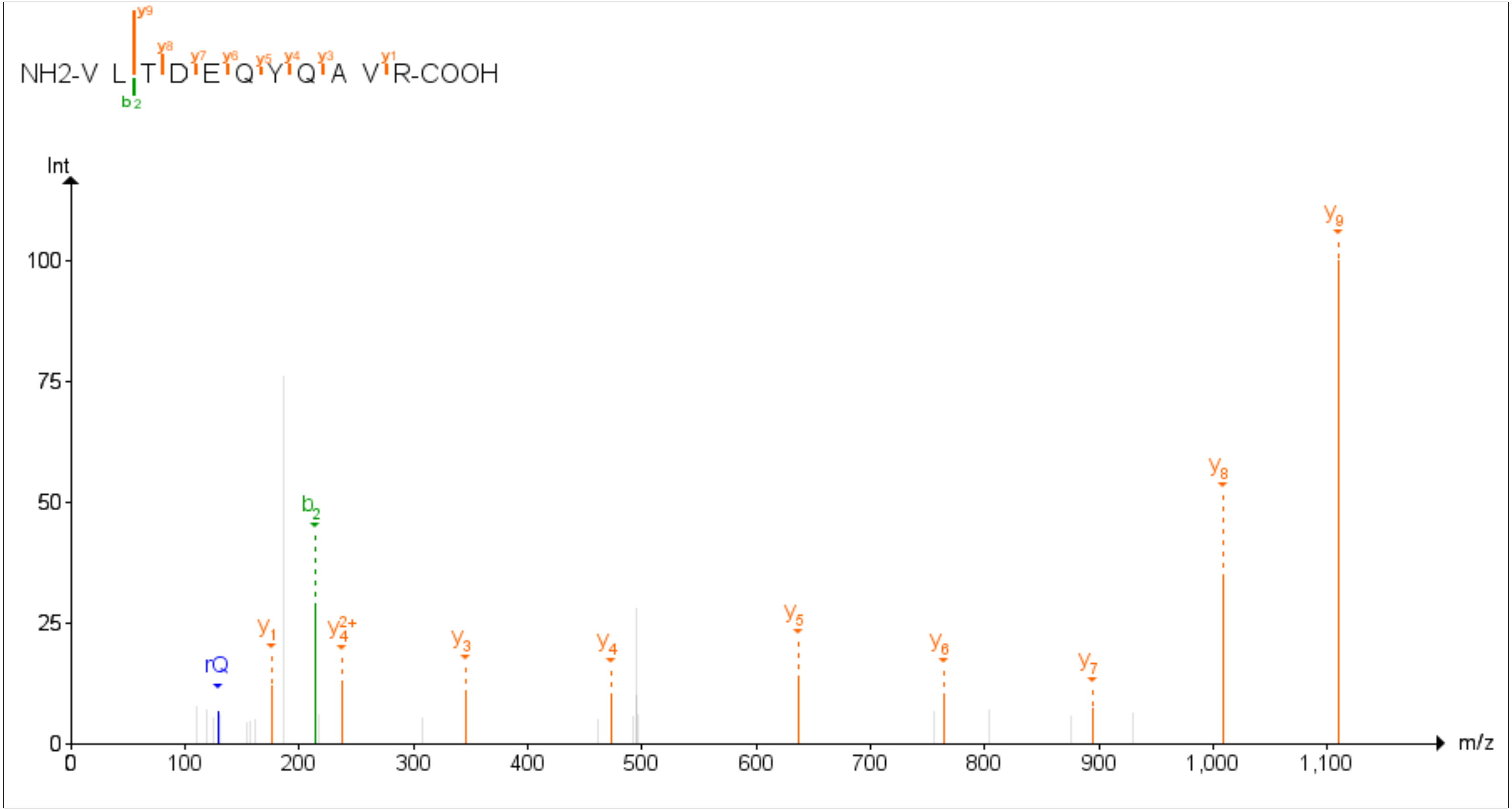
NH2-V L T D E Q Y Q A V R-COOH
y9
y8
y7
y6
y5
y4
y3
y1
b2
Int
100
75
50
25
0
m/z
0
100
200
300
400
500
600
700
800
900
1,000
1,100
rQ
y1
b2
y4 2+
y3
y4
y5
y6
y7
y8
y9

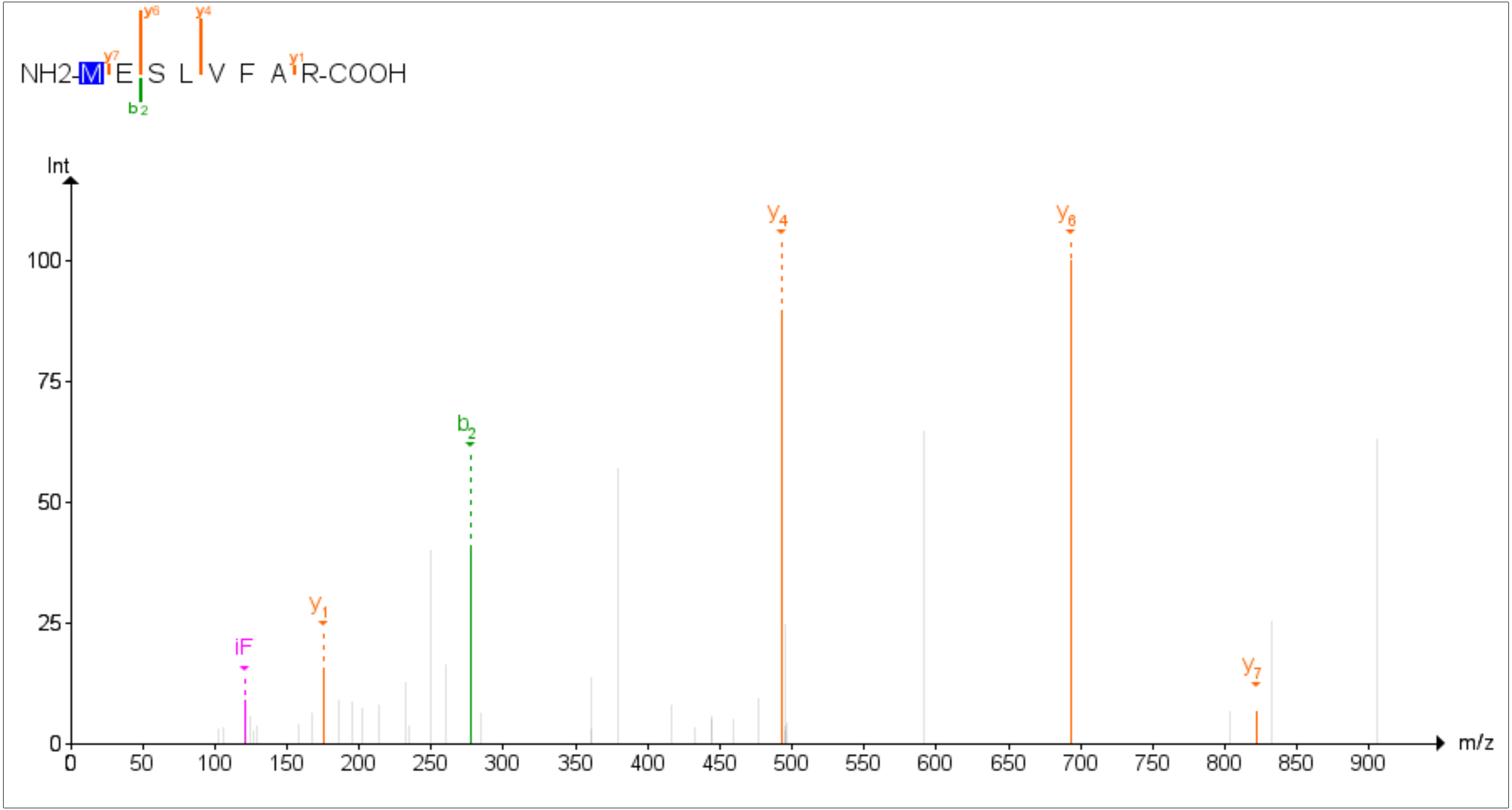

NH2-M E S L V F A R-COOH
y7
y6
y4
y1
b2
Int
100
75
50
25
0
y4
y6
b2
y1
iF
y7
0
50
100
150
200
250
300
350
400
450
500
550
600
650
700
750
800
850
900
m/z

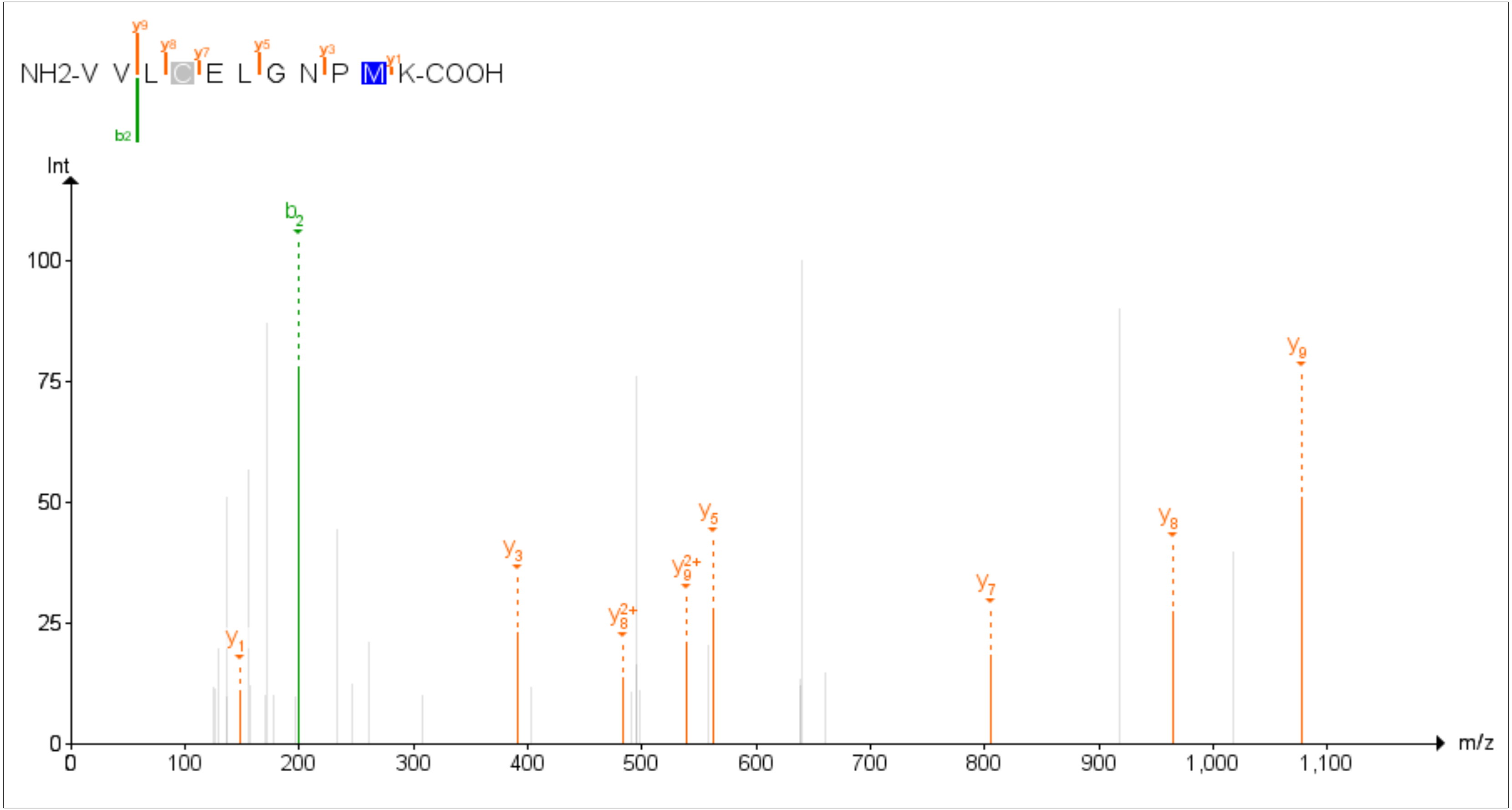
NH2-V V L C E L G N P M K-COOH
y9
y8
y7
y5
y3
y1
b2
Int
100
75
50
25
0
b2
y1
y3
y8 2+
y9 2+
y5
y7
y8
y9
0
100
200
300
400
500
600
700
800
900
1,000
1,100
m/z

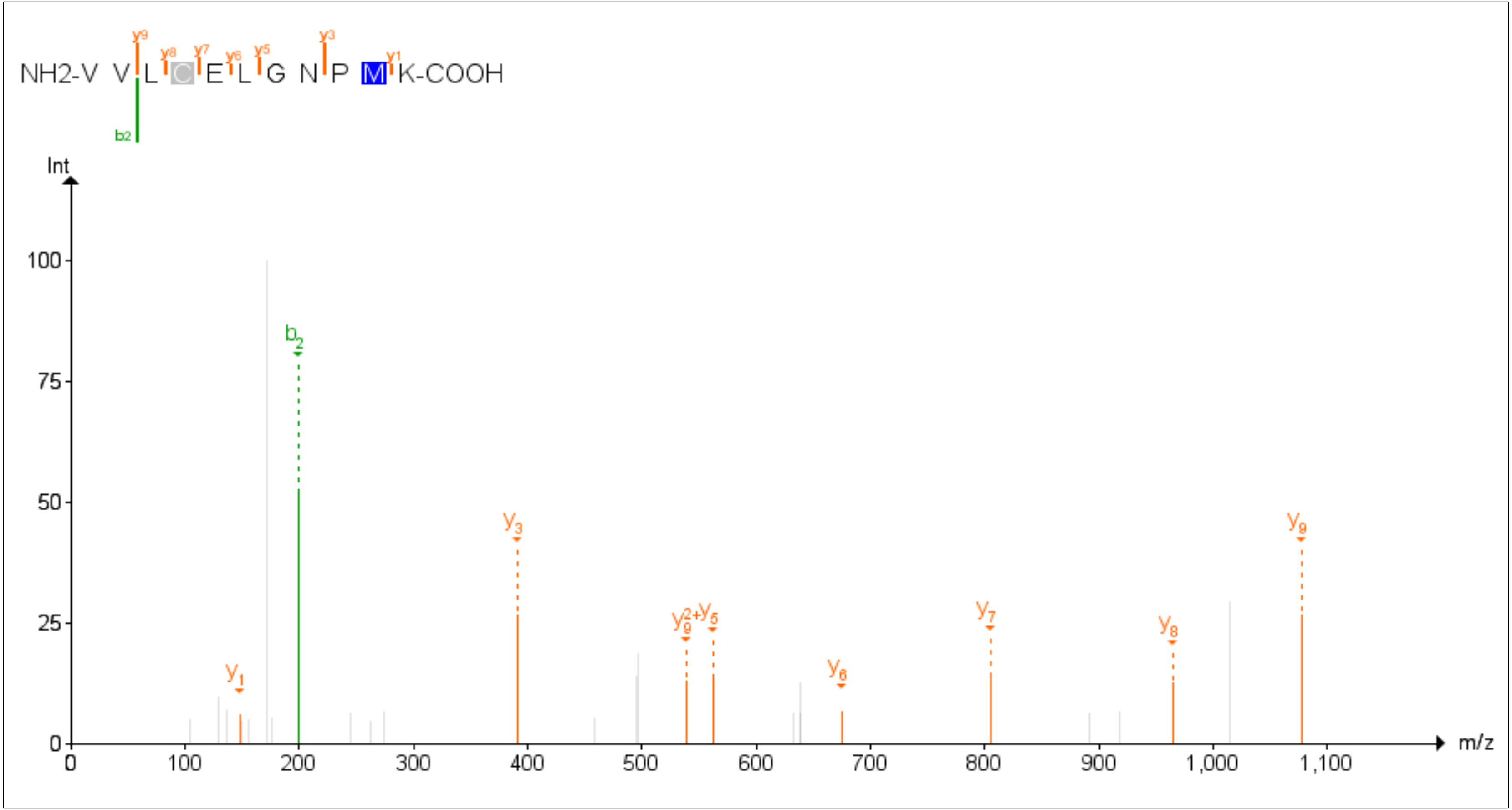
NH2-V V L C E L G N P M K-COOH
y9
y8
y7
y6
y5
y3
y1
b2
Int
m/z
100
75
50
25
0
0
100
200
300
400
500
600
700
800
900
1,000
1,100
b2
y1
y3
y9 2+ y5
y6
y7
y8
y9

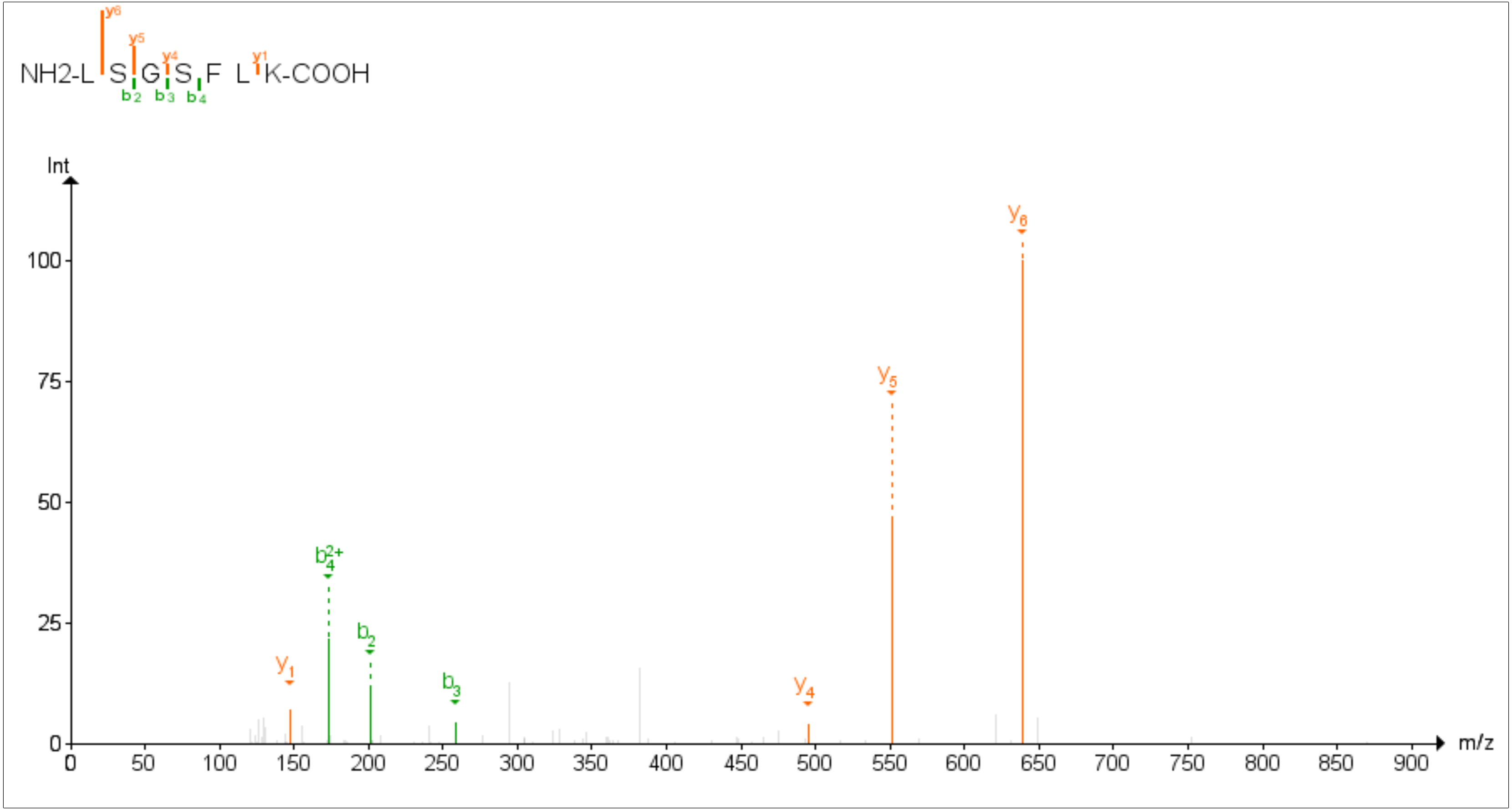

NH2-L S G S F L K-COOH
y6
y5
y4
y1
b2
b3
b4
Int
100
75
50
25
0
y6
y5
b_4^{2+}
b2
y1
b3
y4
0
50
100
150
200
250
300
350
400
450
500
550
600
650
700
750
800
850
900
m/z

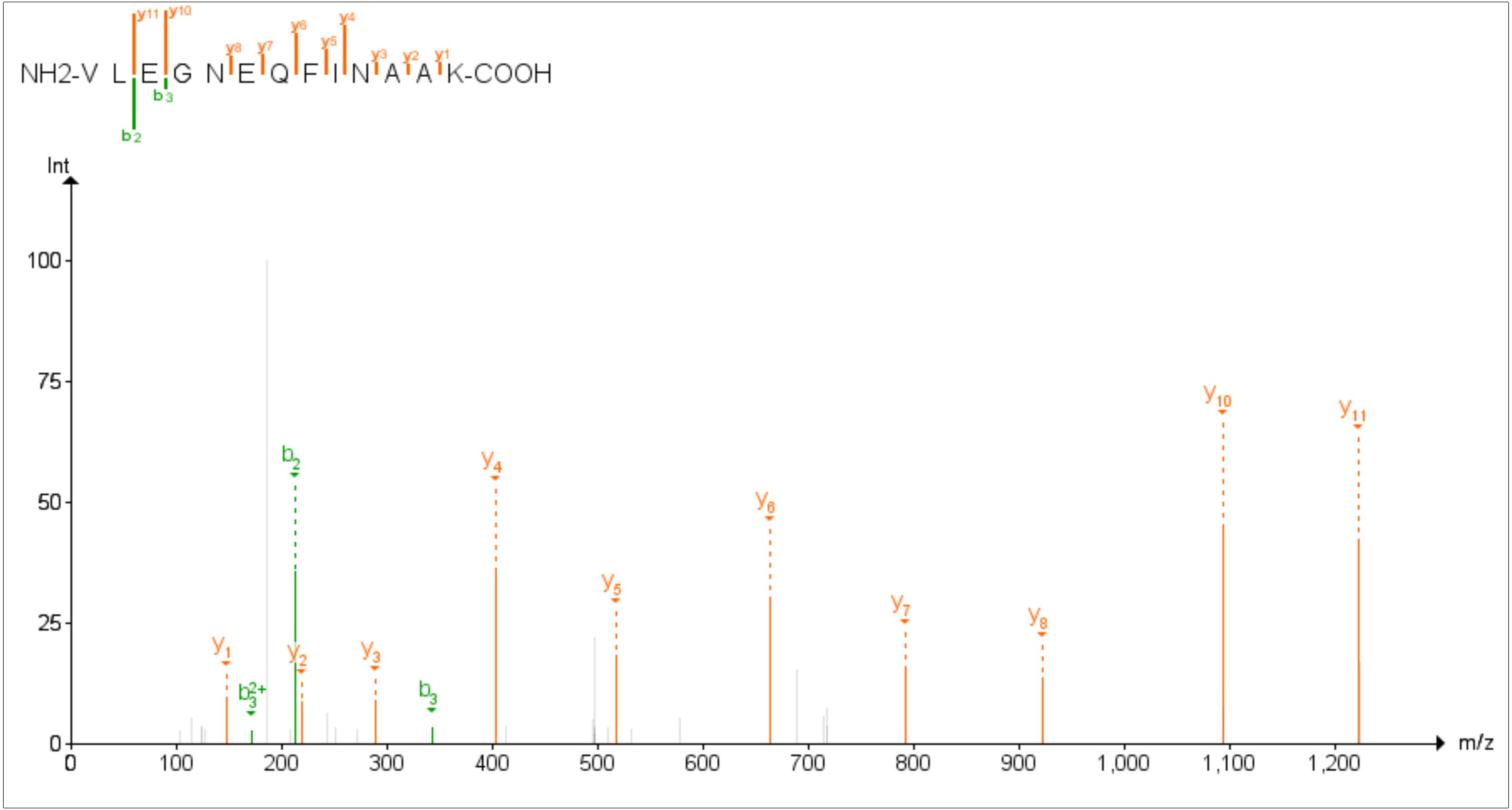
NH2-V L E G N E Q F I N A A K-COOH
y11 y10 y8 y7 y6 y5 y4 y3 y2 y1
b2 b3
Int
100
75
50
25
0
m/z
0 100 200 300 400 500 600 700 800 900 1,000 1,100 1,200
y1 b3 2+ b2 y2 y3 b3 y4 y5 y6 y7 y8 y10 y11

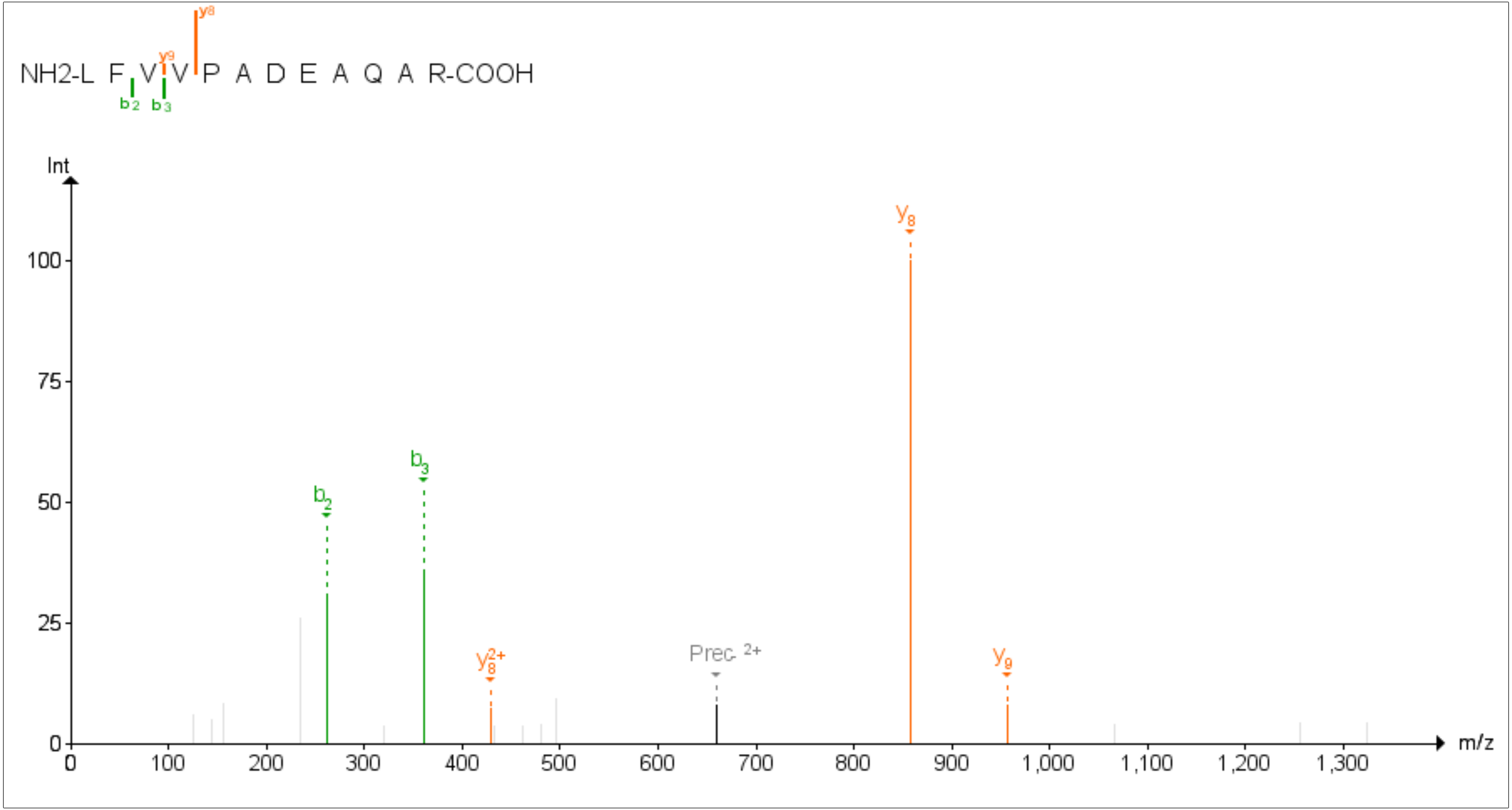

NH2-L F V V P A D E A Q A R-COOH
y8
y9
b2
b3
Int
m/z
y8
b3
b2
y8 2+
Prec 2+
y9

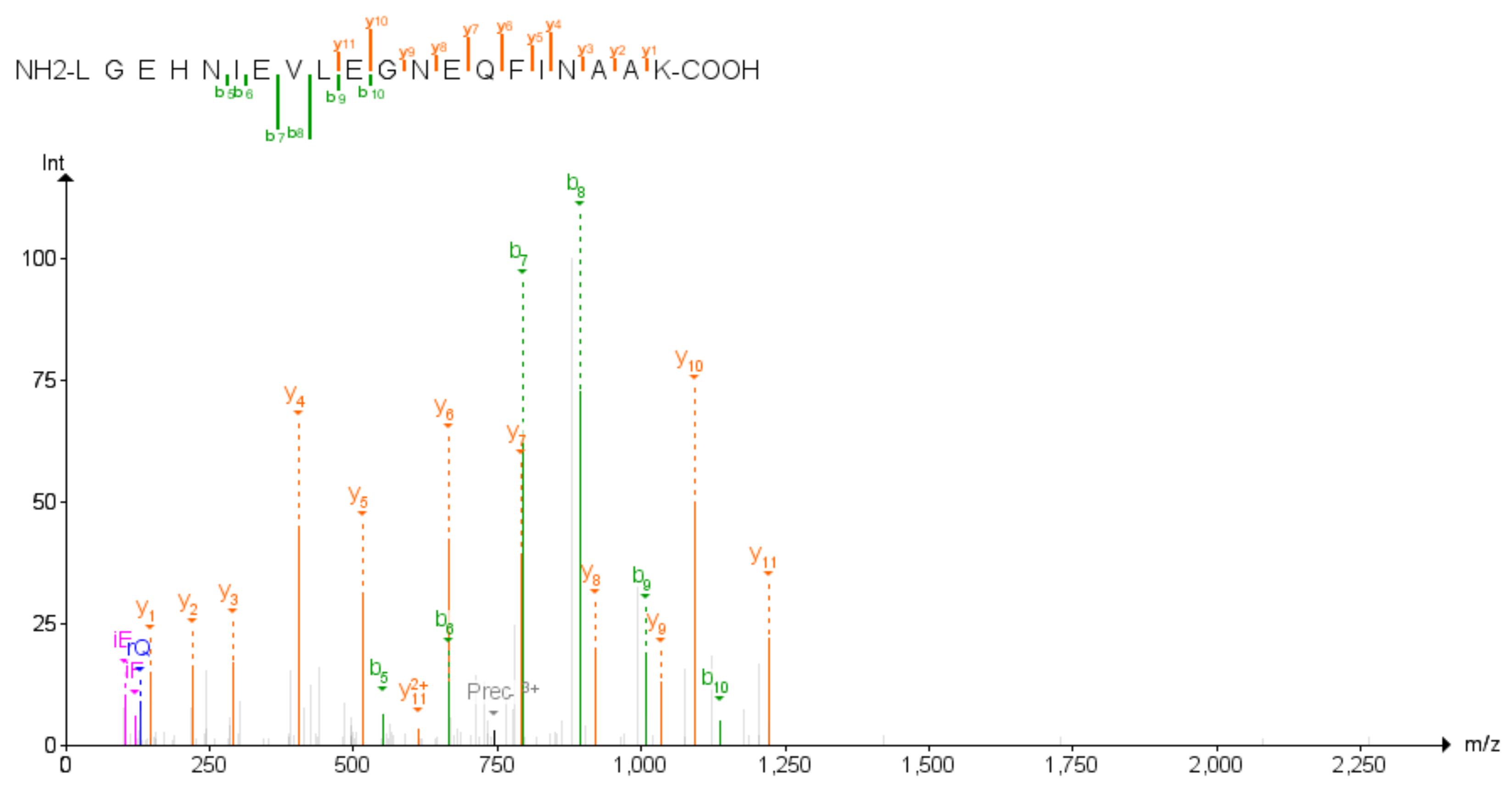

NH2-L G E H N I E V L E G N E Q F I N A A K-COOH
Int
m/z
Prec 3+

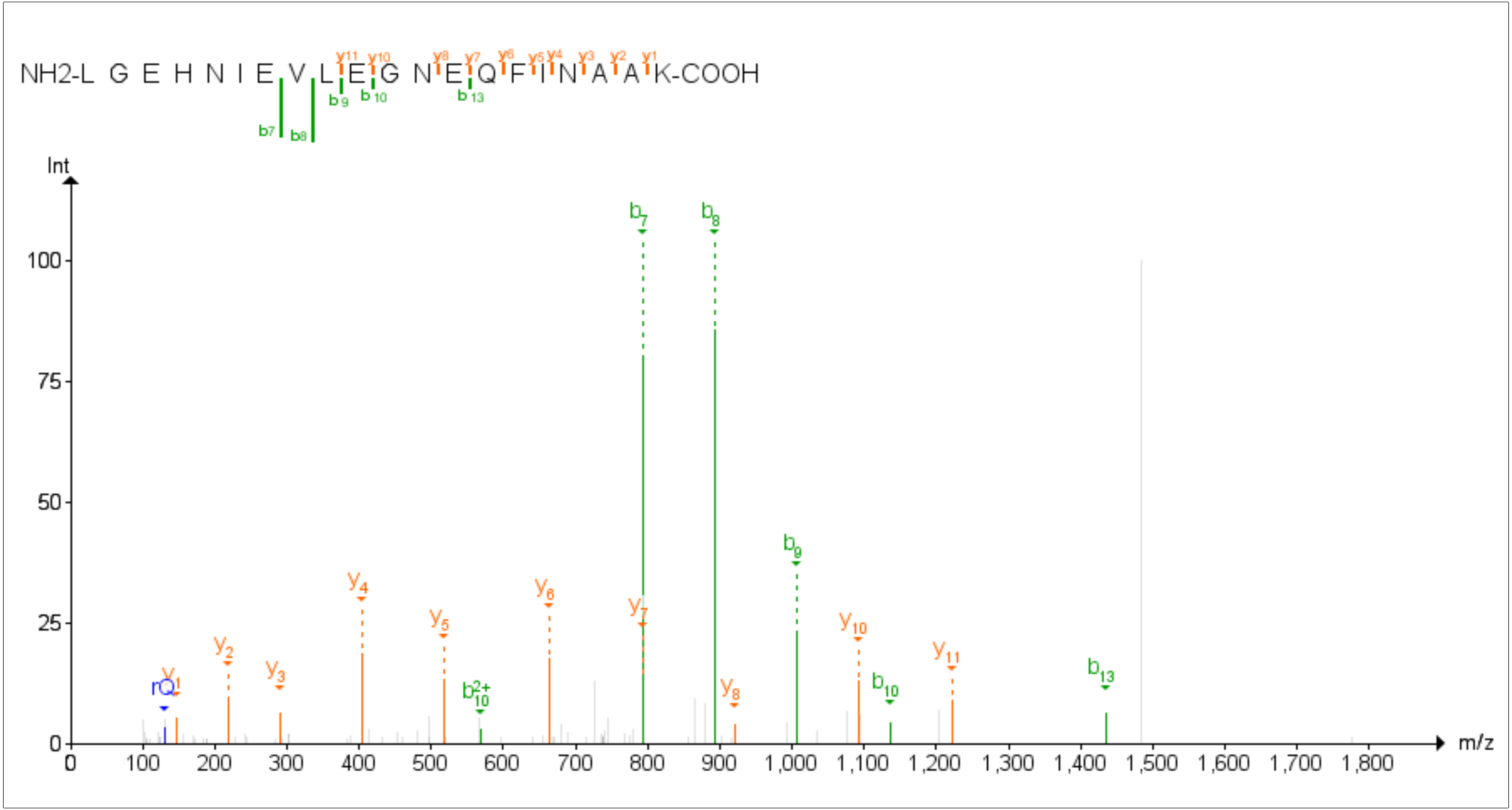

NH2-L G E H N I E V L E G N E Q F I N A A K-COOH
Int
m/z

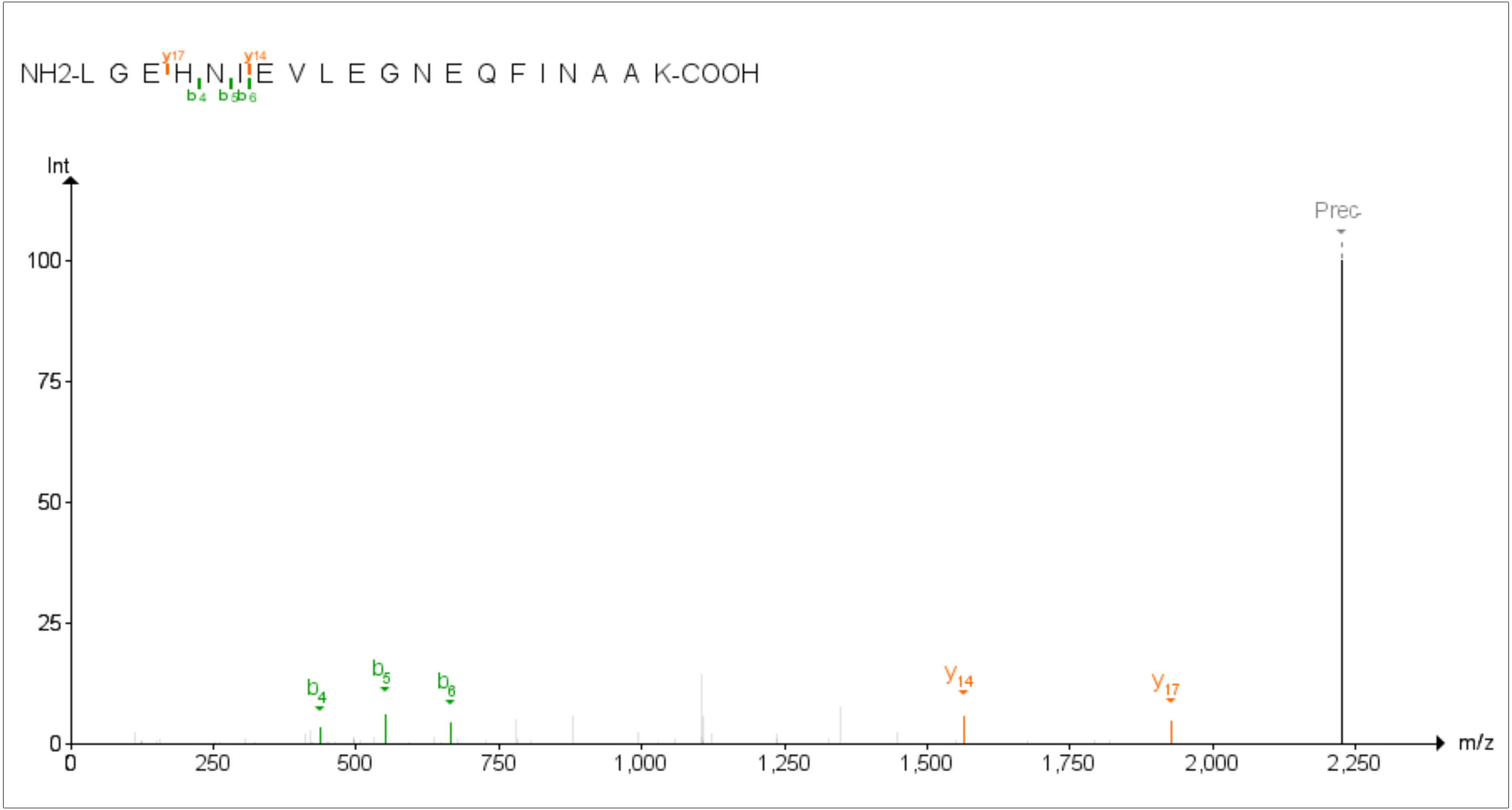

NH2-L G E H N I E V L E G N E Q F I N A A K-COOH
y17
y14
b4
b5
b6
Int
m/z
Prec.
100
75
50
25
0
250
500
750
1,000
1,250
1,500
1,750
2,000
2,250

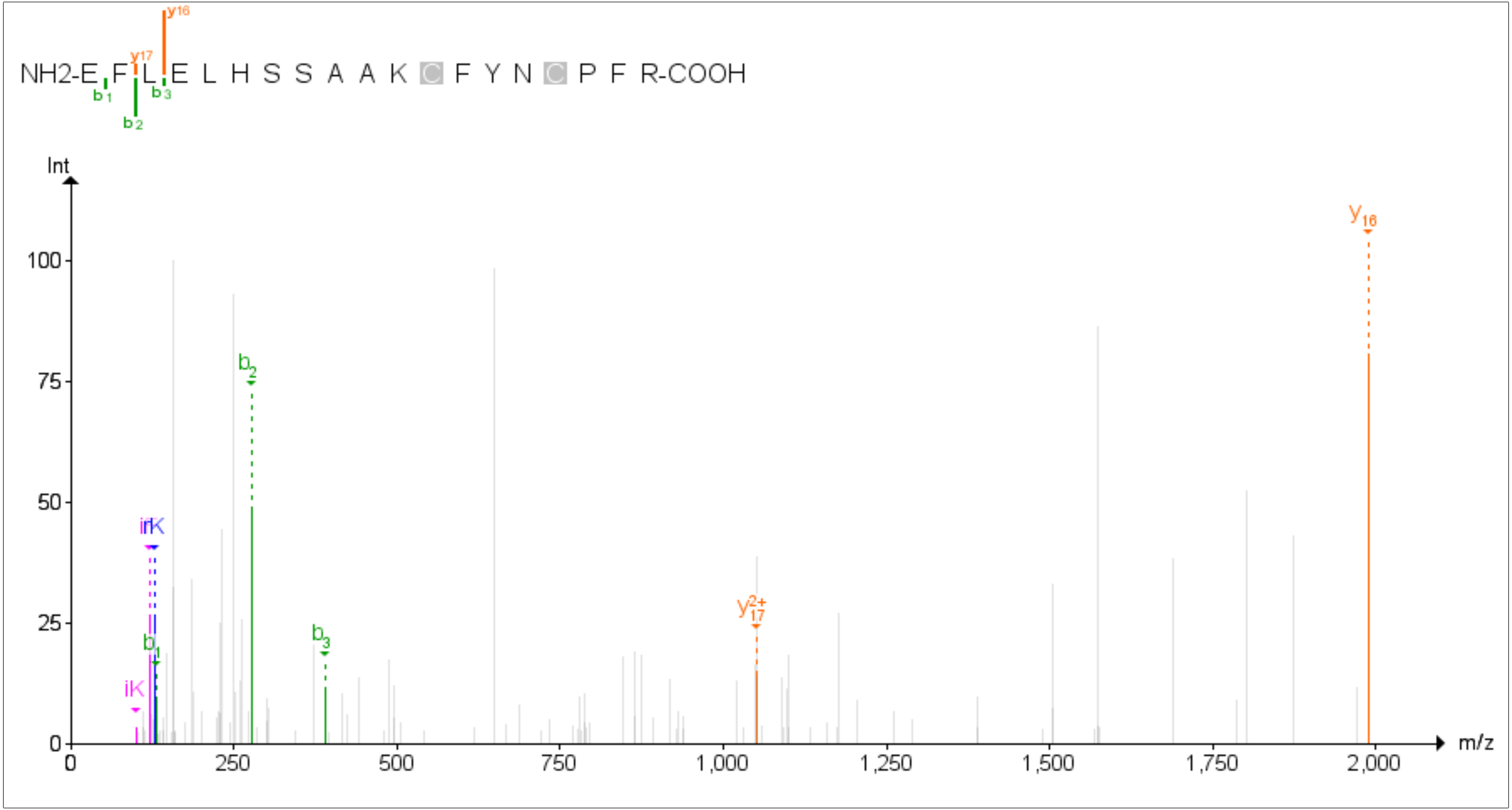
NH2-E F L E L H S S A A K C F Y N C P F R-COOH
y16
y17
b1
b2
b3
Int
100
75
50
25
0
0
250
500
750
1,000
1,250
1,500
1,750
2,000
m/z
y16
b2
iK
b1
iK
b3
y17 2+

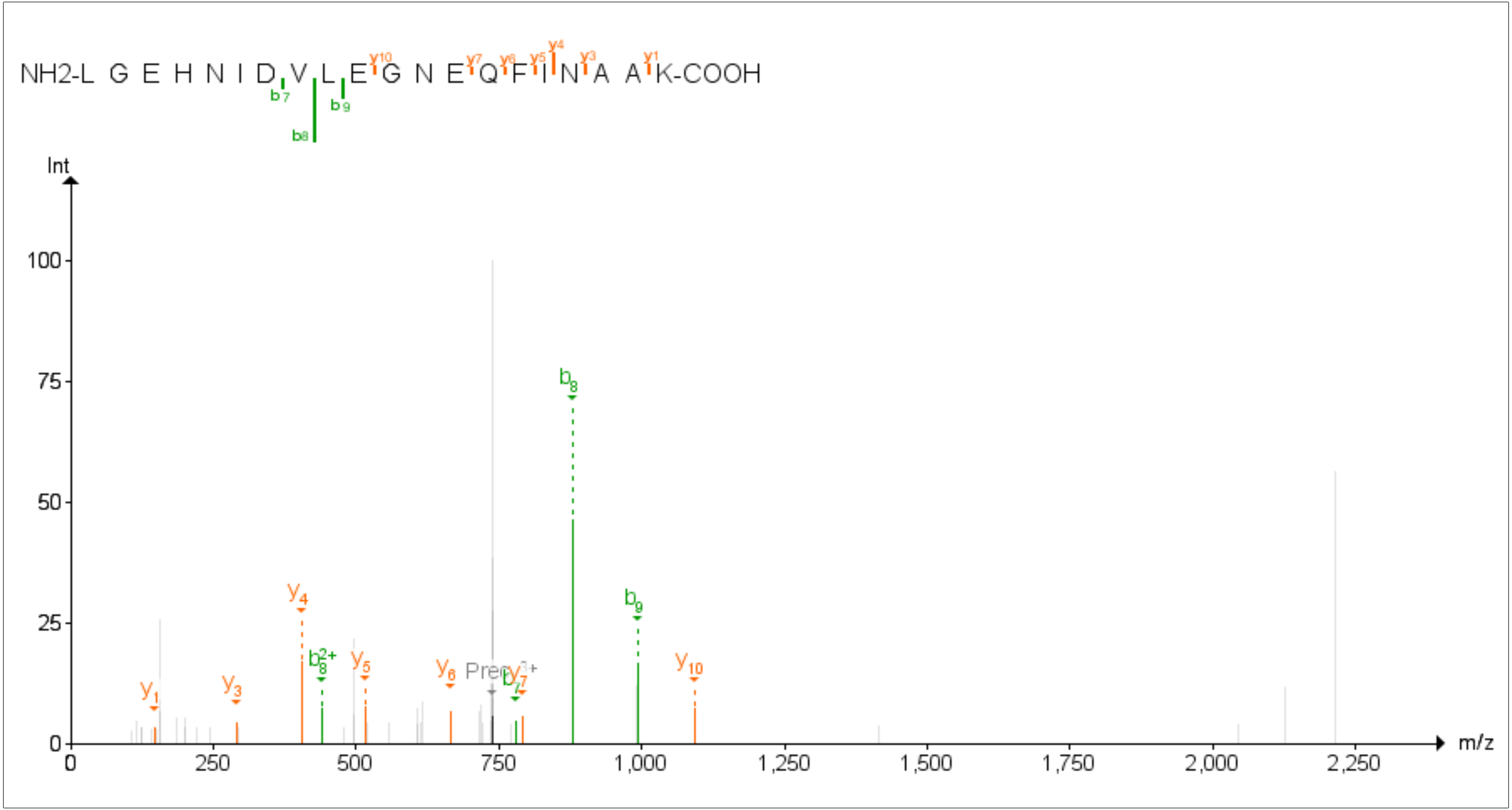
NH2-L G E H N I D V L E G N E Q F I N A A K-COOH
b7
b8
b9
y10
y7
y6
y5
y4
y3
y1
Int
100
75
50
25
0
b8
b9
y4
y1
y3
y5
y6
y7
y10
Prec
0
250
500
750
1,000
1,250
1,500
1,750
2,000
2,250
m/z

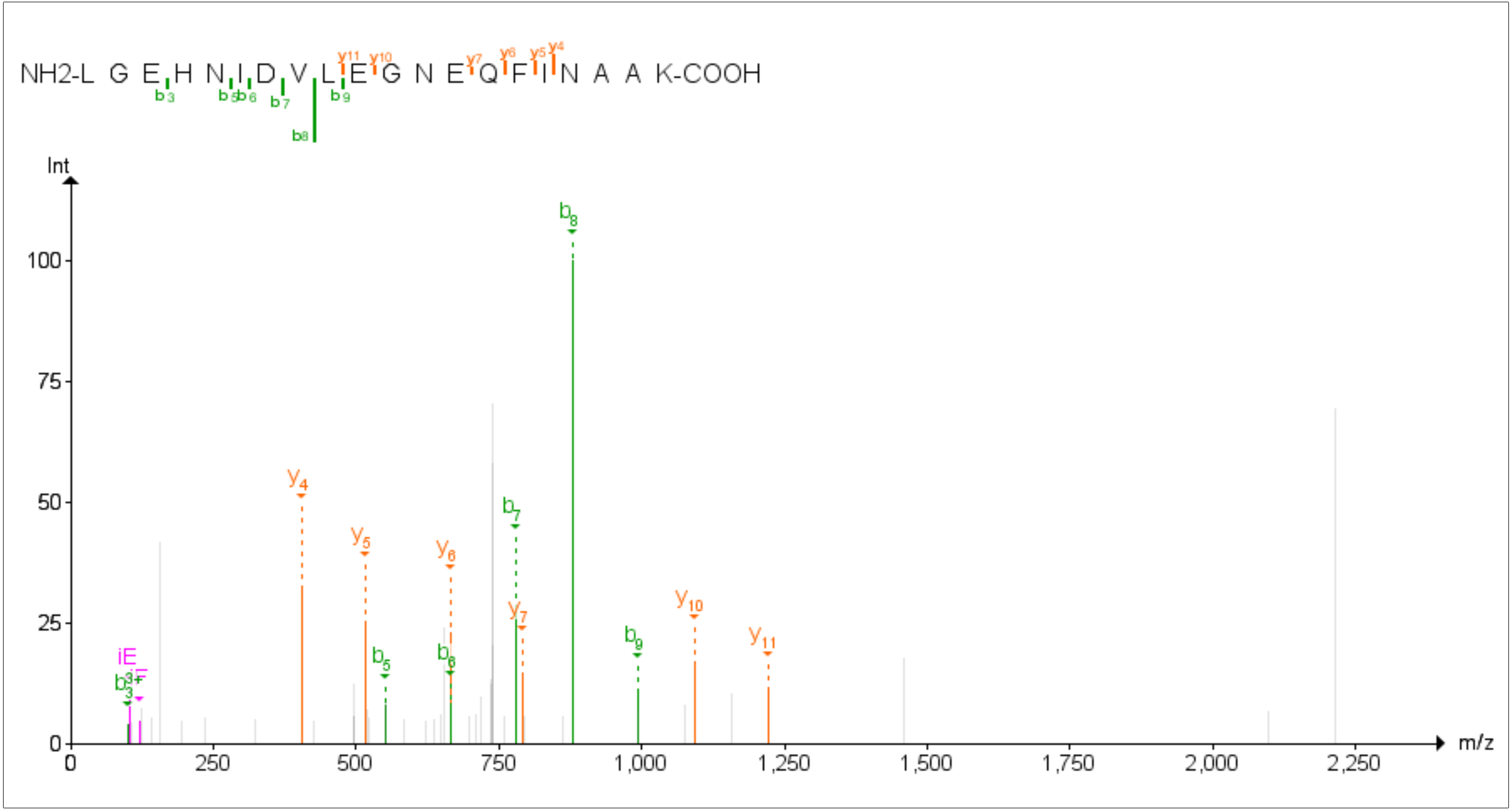

NH2-L G E H N I D V L E G N E Q F I N A A K-COOH
y11 y10 y7 y6 y5 y4
b3 b5 b6 b7 b8 b9
Int
100
75
50
25
0
m/z
0
250
500
750
1,000
1,250
1,500
1,750
2,000
2,250
b8
y4
b7
y5
y6
y7
b5
b6
b9
y10
y11
iE
iF
b3

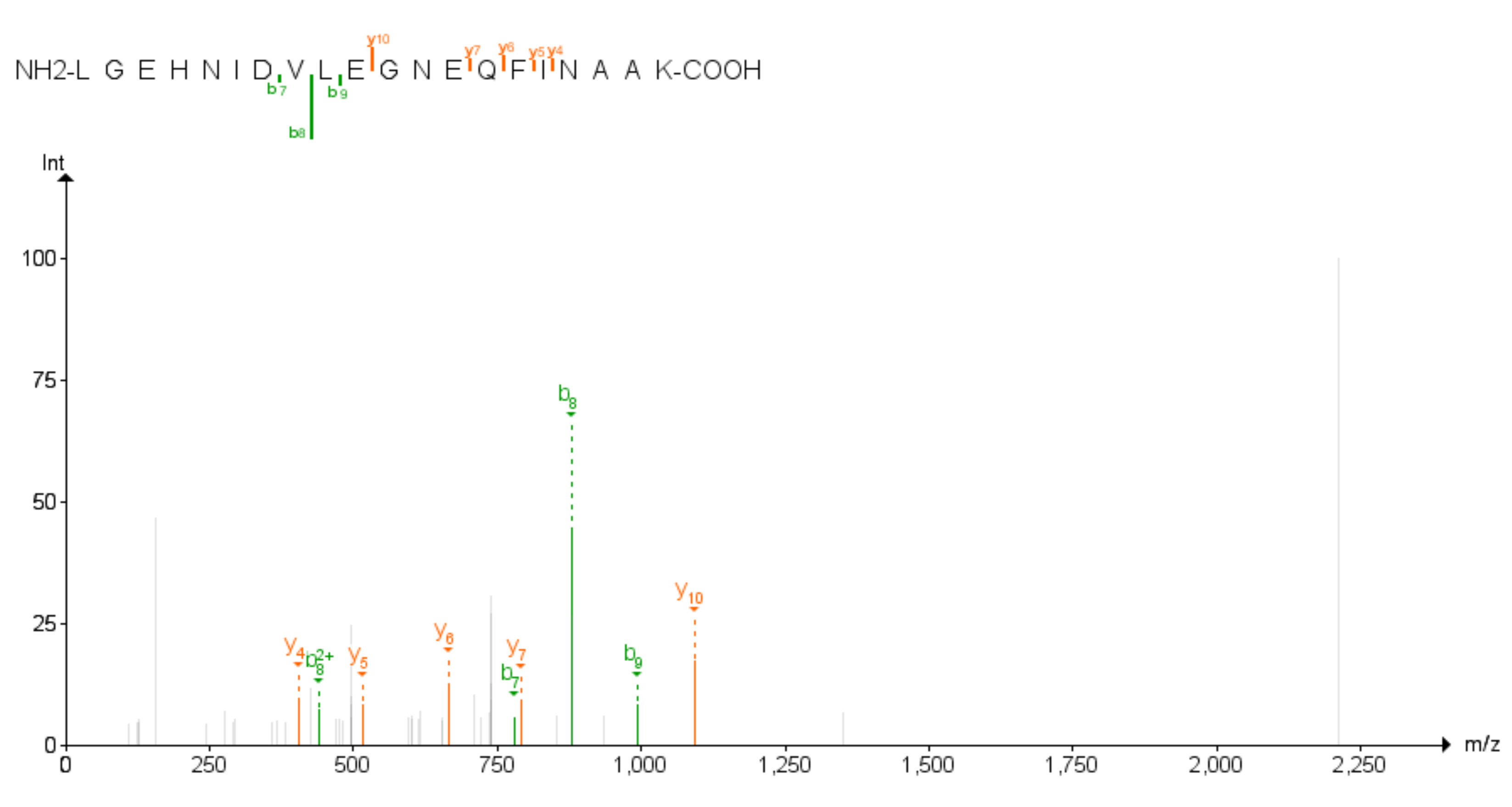
NH2-L G E H N I D V L E G N E Q F I N A A K-COOH
y10
y7
y6
y5
y4
b7
b9
b8
Int
100
75
50
25
0
b8
y10
y4
b8 2+
y5
y6
y7
b7
b9
0
250
500
750
1,000
1,250
1,500
1,750
2,000
2,250
m/z

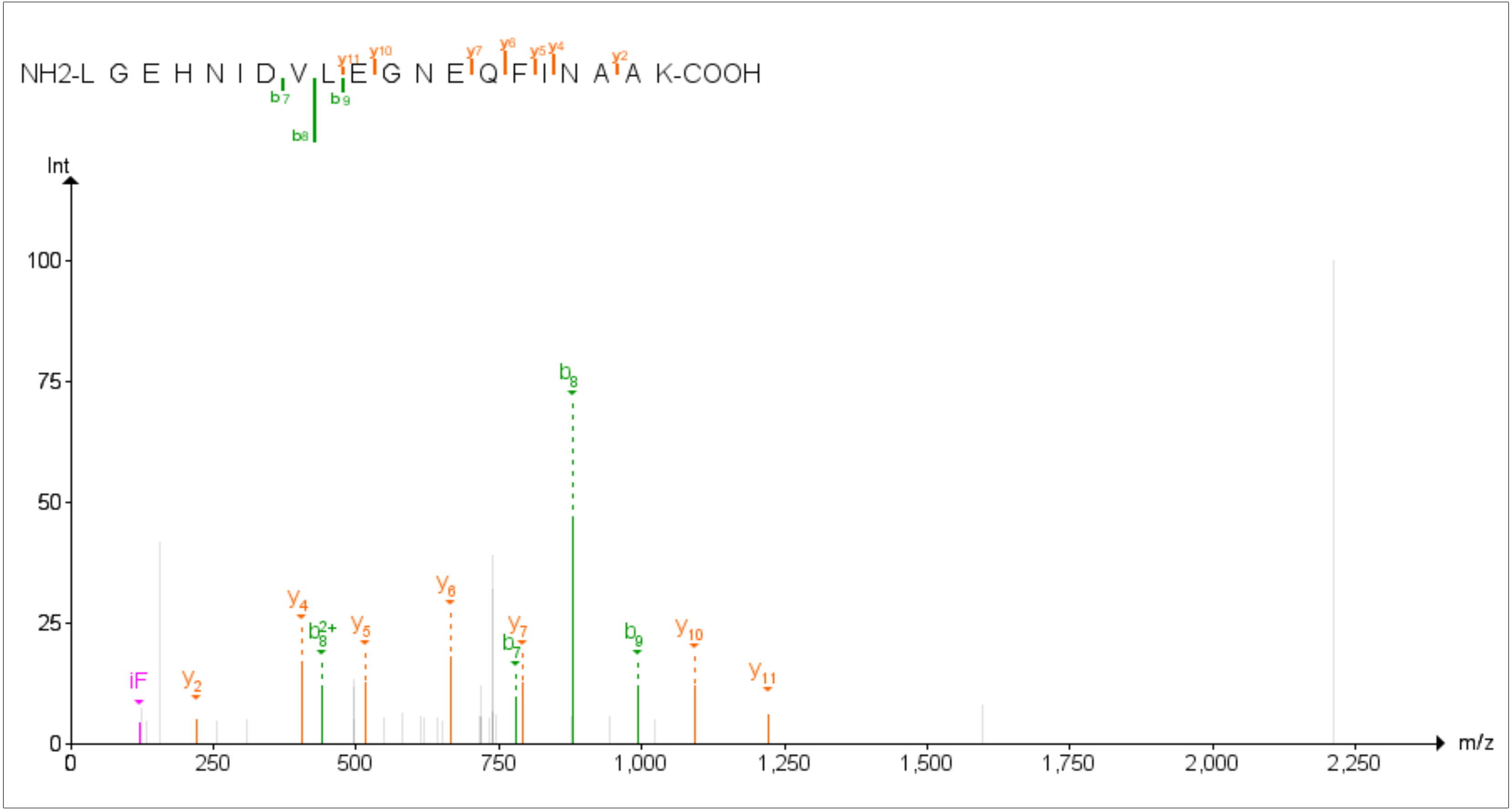

NH2-L G E H N I D V L E G N E Q F I N A A K-COOH
b7
b8
b9
y11
y10
y7
y6
y5
y4
y2
Int
100
75
50
25
0
m/z
0
250
500
750
1,000
1,250
1,500
1,750
2,000
2,250
iF
y2
y4
b8 2+
y5
y6
b7
y7
b8
b9
y10
y11

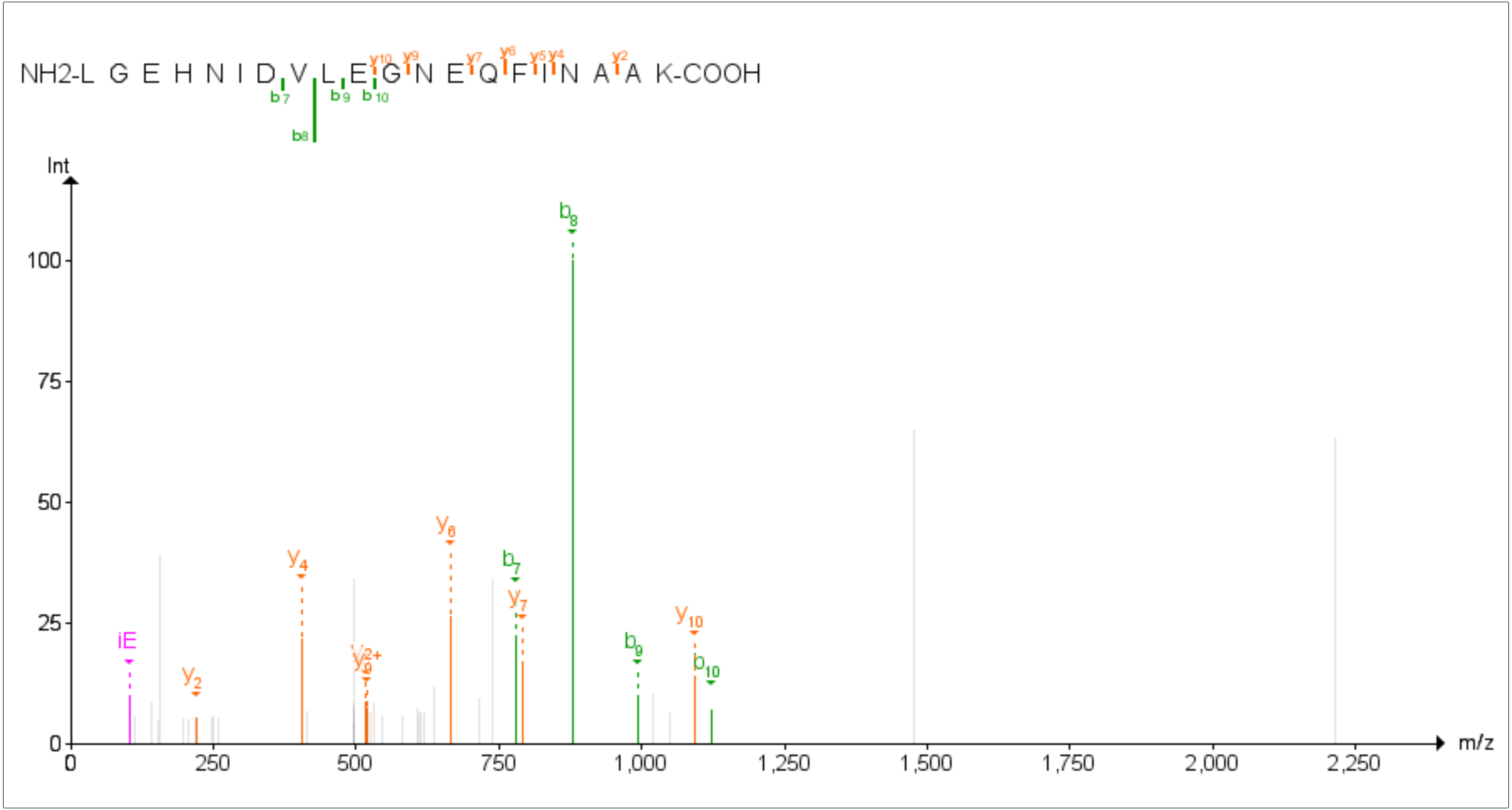

NH2-L G E H N I D V L E G N E Q F I N A A K-COOH
y10 y9 y7 y6 y5 y4 y2
b7 b9 b10
b8
Int
100
75
50
25
0
b8
y6
y4
b7
y7
iE
y2
y9 2+
b9
y10
b10
0
250
500
750
1,000
1,250
1,500
1,750
2,000
2,250
m/z

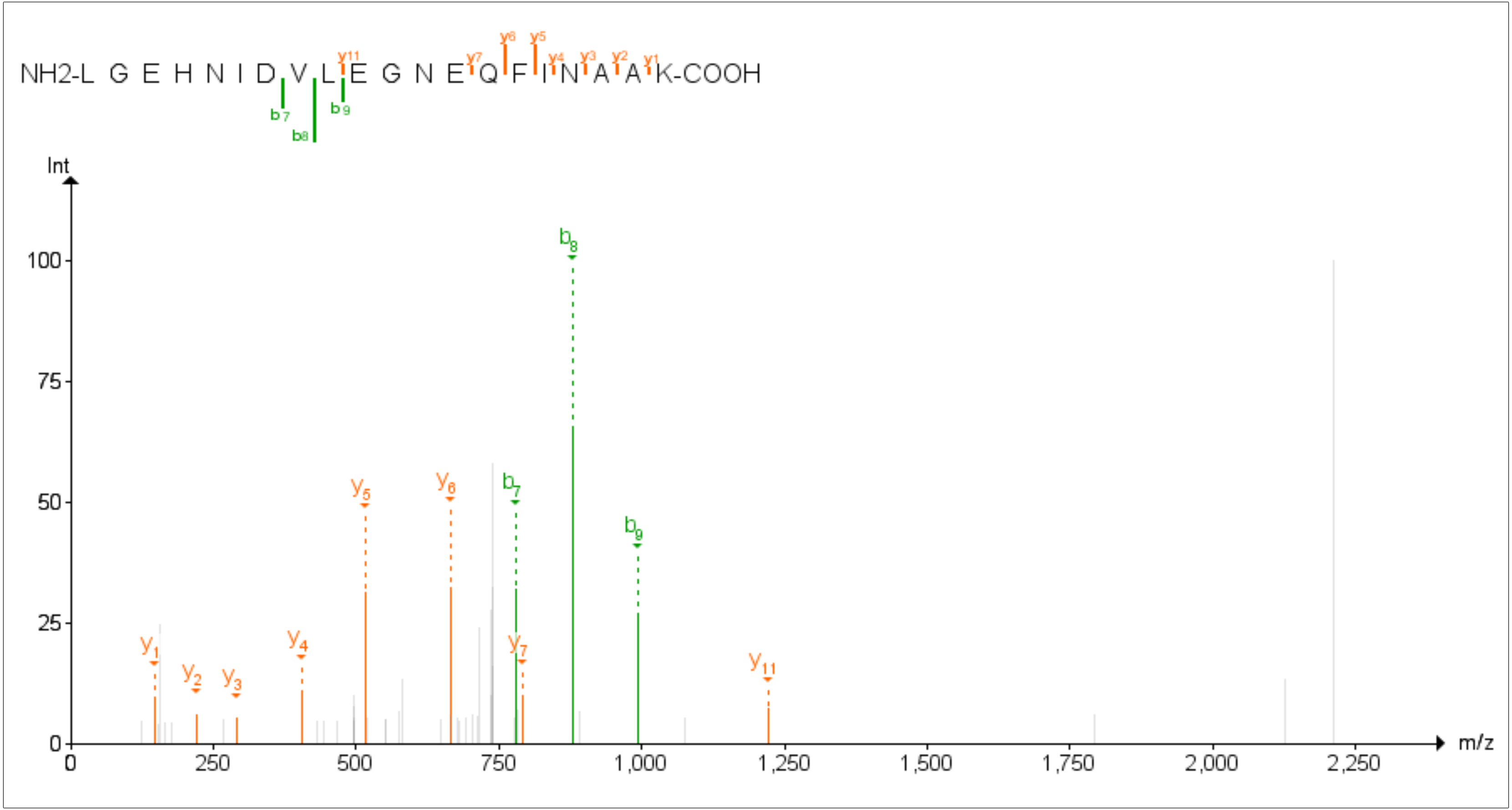

NH2-L G E H N I D V L E G N E Q F I N A A K-COOH
y11 y7 y6 y5 y4 y3 y2 y1
b7 b8 b9
Int
100
75
50
25
0
b8
y5
y6
b7
b9
y1
y2
y3
y4
y7
y11
0
250
500
750
1,000
1,250
1,500
1,750
2,000
2,250
m/z

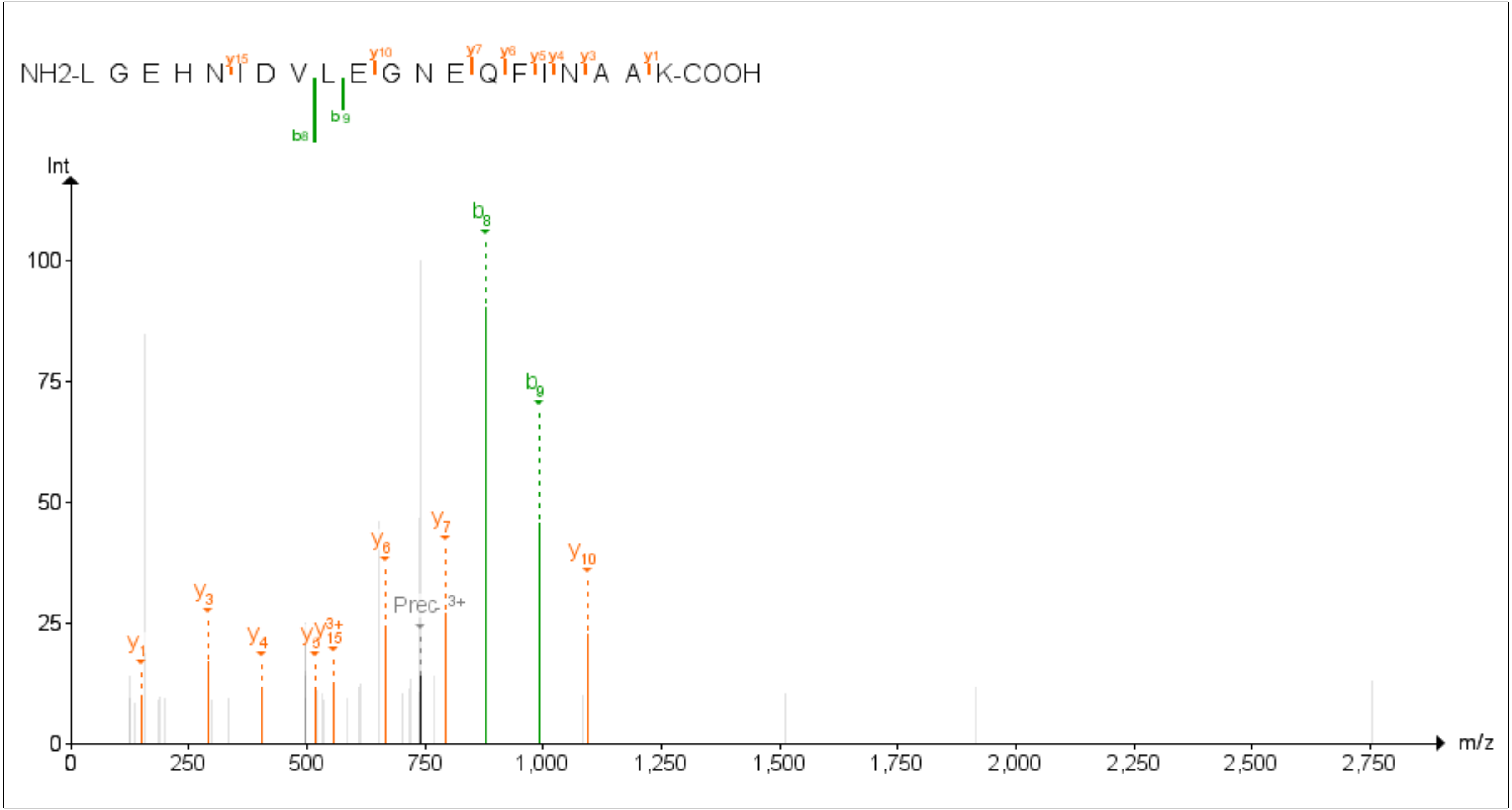

NH2-L G E H N I D V L E G N E Q F I N A A K-COOH
y15
y10
y7
y6
y5
y4
y3
y1
b8
b9
Int
100
75
50
25
0
m/z
0
250
500
750
1,000
1,250
1,500
1,750
2,000
2,250
2,500
2,750
Prec 3+

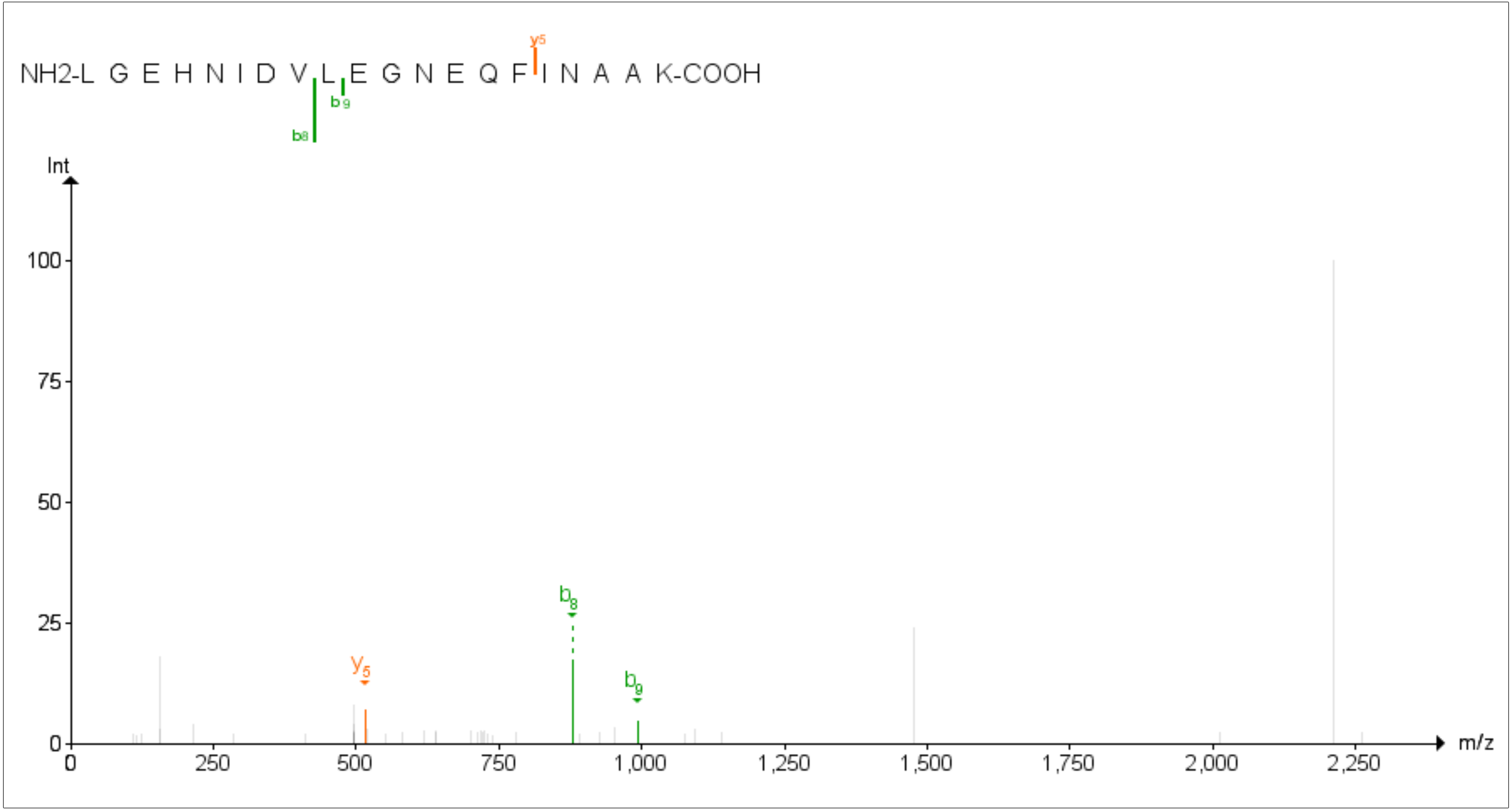

NH2-L G E H N I D V L E G N E Q F I N A A K-COOH
y5
b9
b8
Int
100
75
50
25
0
y5
b8
b9
0
250
500
750
1,000
1,250
1,500
1,750
2,000
2,250
m/z

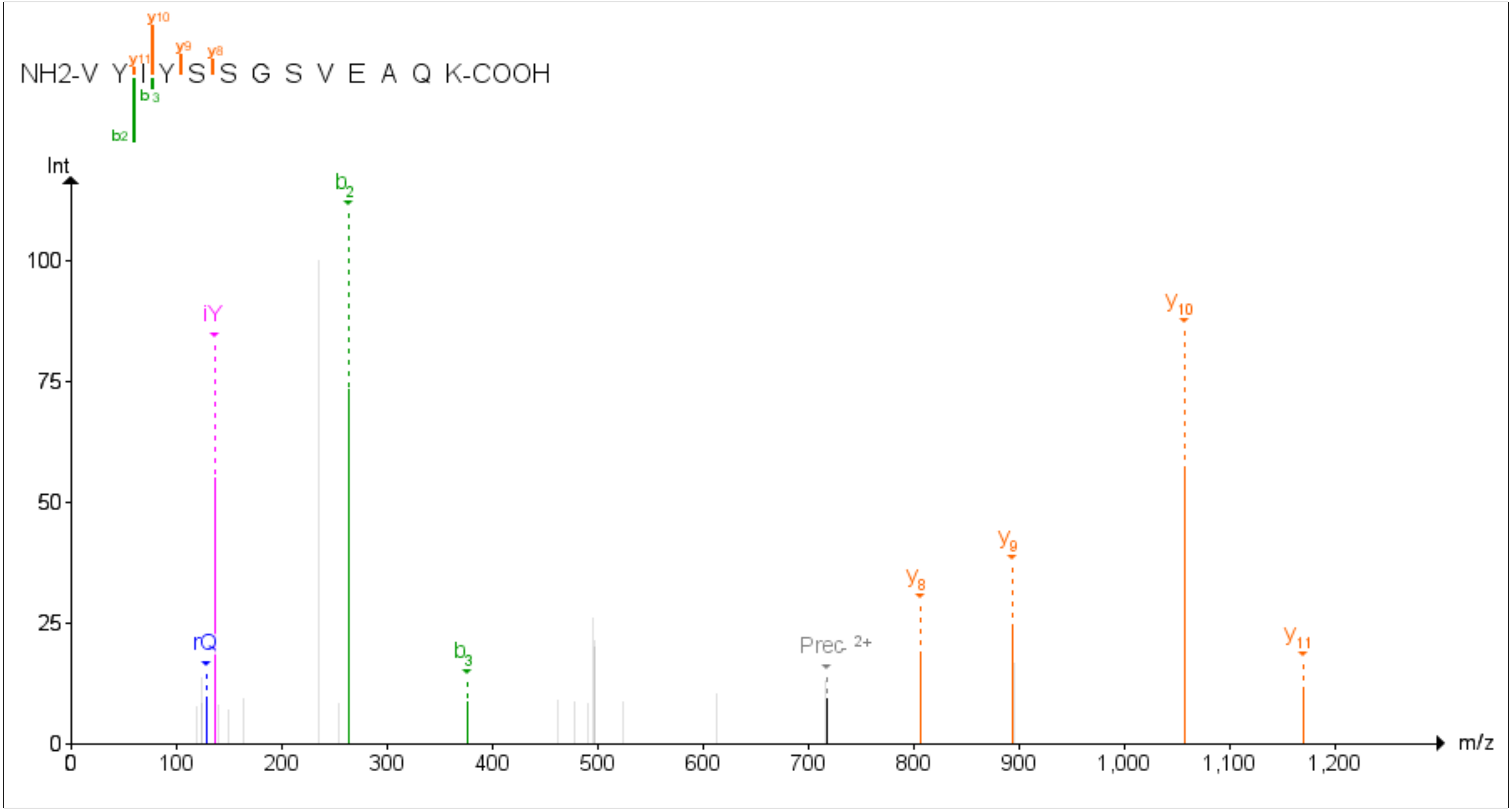

NH2-V Y I Y S S G S V E A Q K-COOH
y10
y11
y9
y8
b3
b2
Int
100
75
50
25
0
b2
iY
y10
y9
y8
rQ
b3
Prec 2+
y11
0
100
200
300
400
500
600
700
800
900
1,000
1,100
1,200
m/z

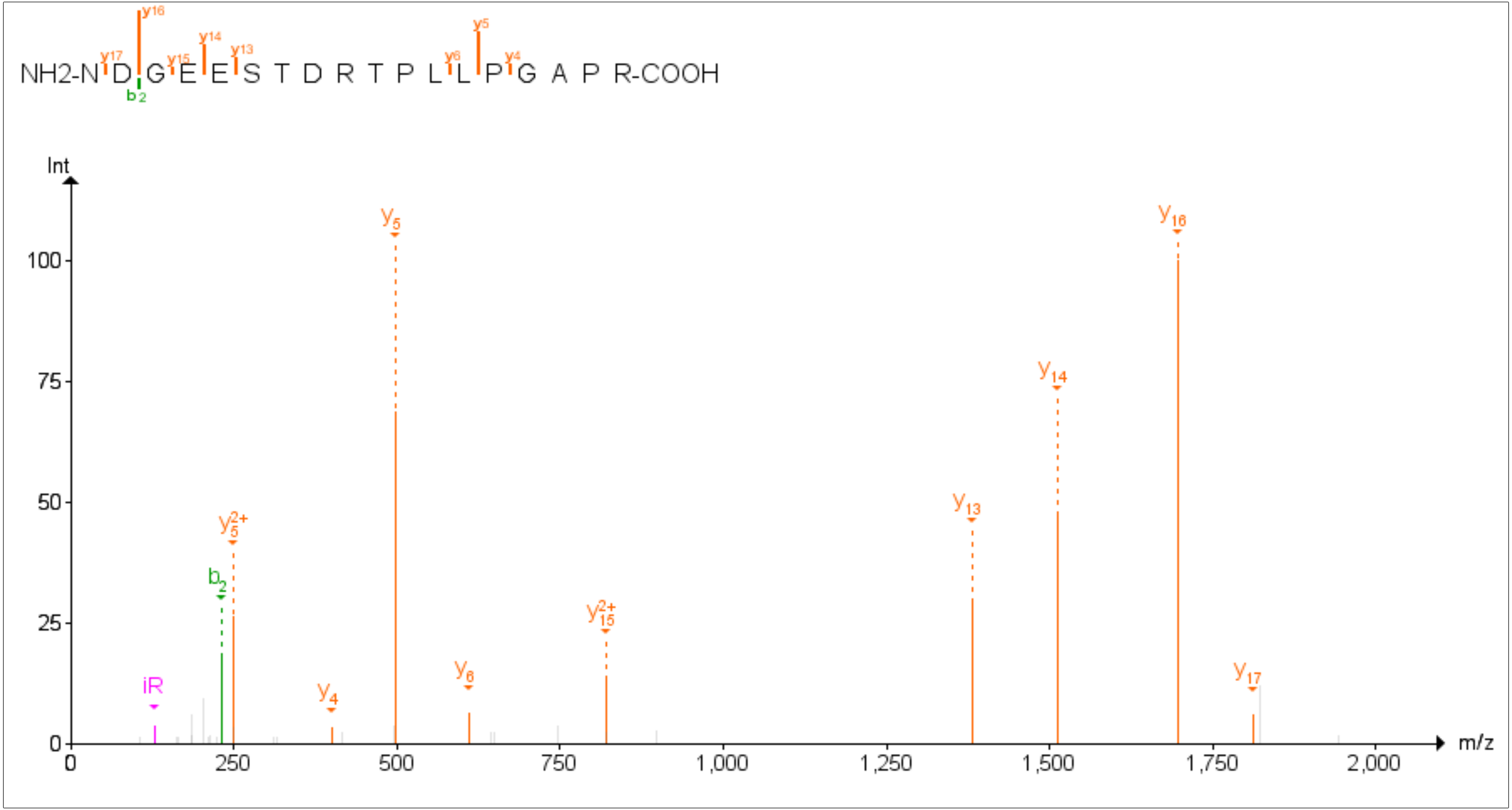
NH2-N D G E E S T D R T P L L P G A P R-COOH
y17
y16
y15
y14
y13
y6
y5
y4
b2
Int
100
75
50
25
0
iR
y5 2+
y4
y5
y6
y15 2+
y13
y14
y16
y17
0
250
500
750
1,000
1,250
1,500
1,750
2,000
m/z

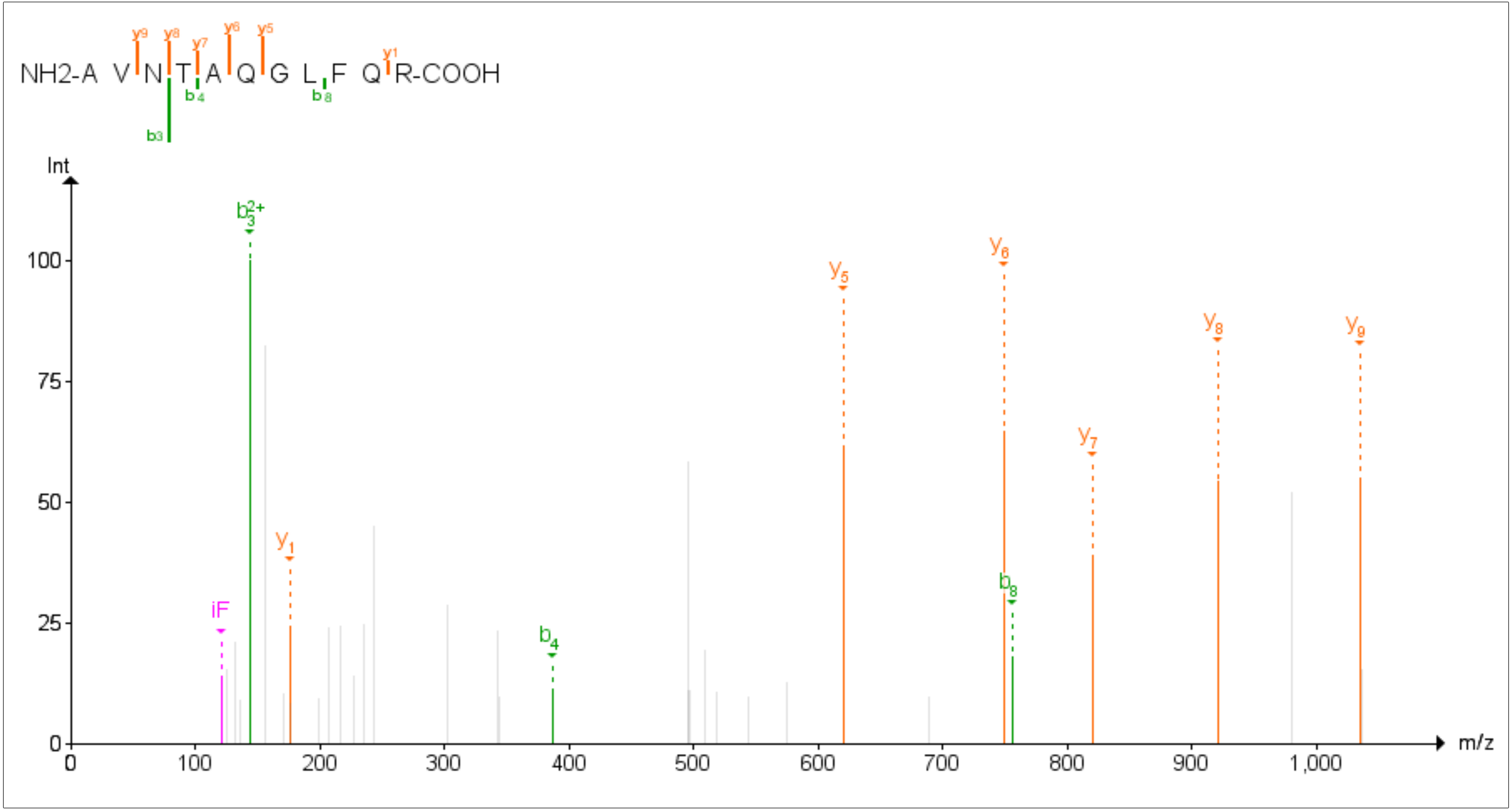

NH2-A V N T A Q G L F Q R-COOH
y9 y8 y7 y6 y5 y1
b3 b4 b8
Int
100
75
50
25
0
m/z
0 100 200 300 400 500 600 700 800 900 1,000
b3 2+
y1
iF
b4
y5
y6
b8
y7
y8
y9

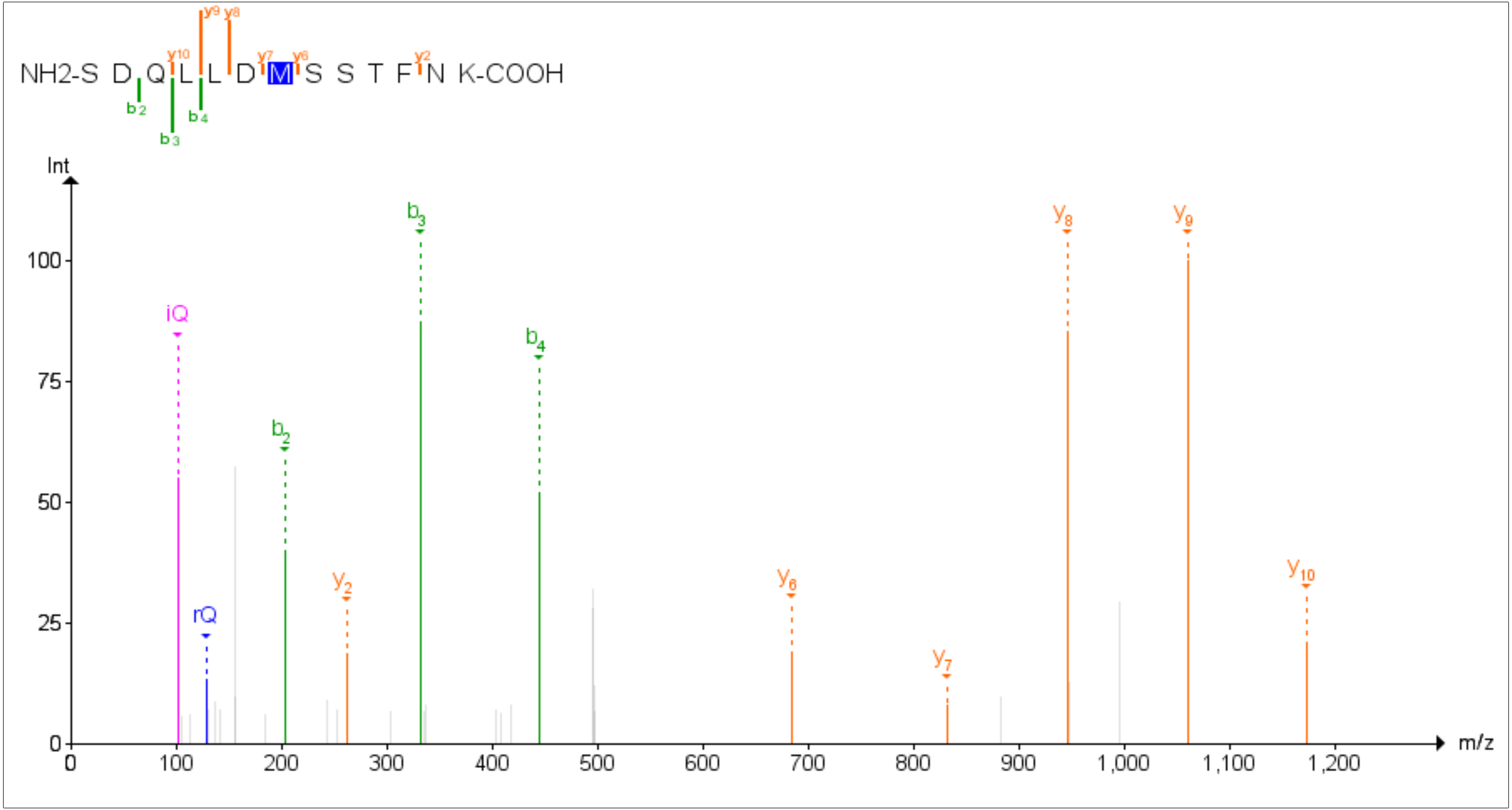

NH2-S D Q L L D M S S T F N K-COOH
y9 y8 y10 y7 y6 y2
b2 b3 b4
Int
100
75
50
25
0
iQ
rQ
b2
b3
b4
y2
y6
y7
y8
y9
y10
0
100
200
300
400
500
600
700
800
900
1,000
1,100
1,200
m/z

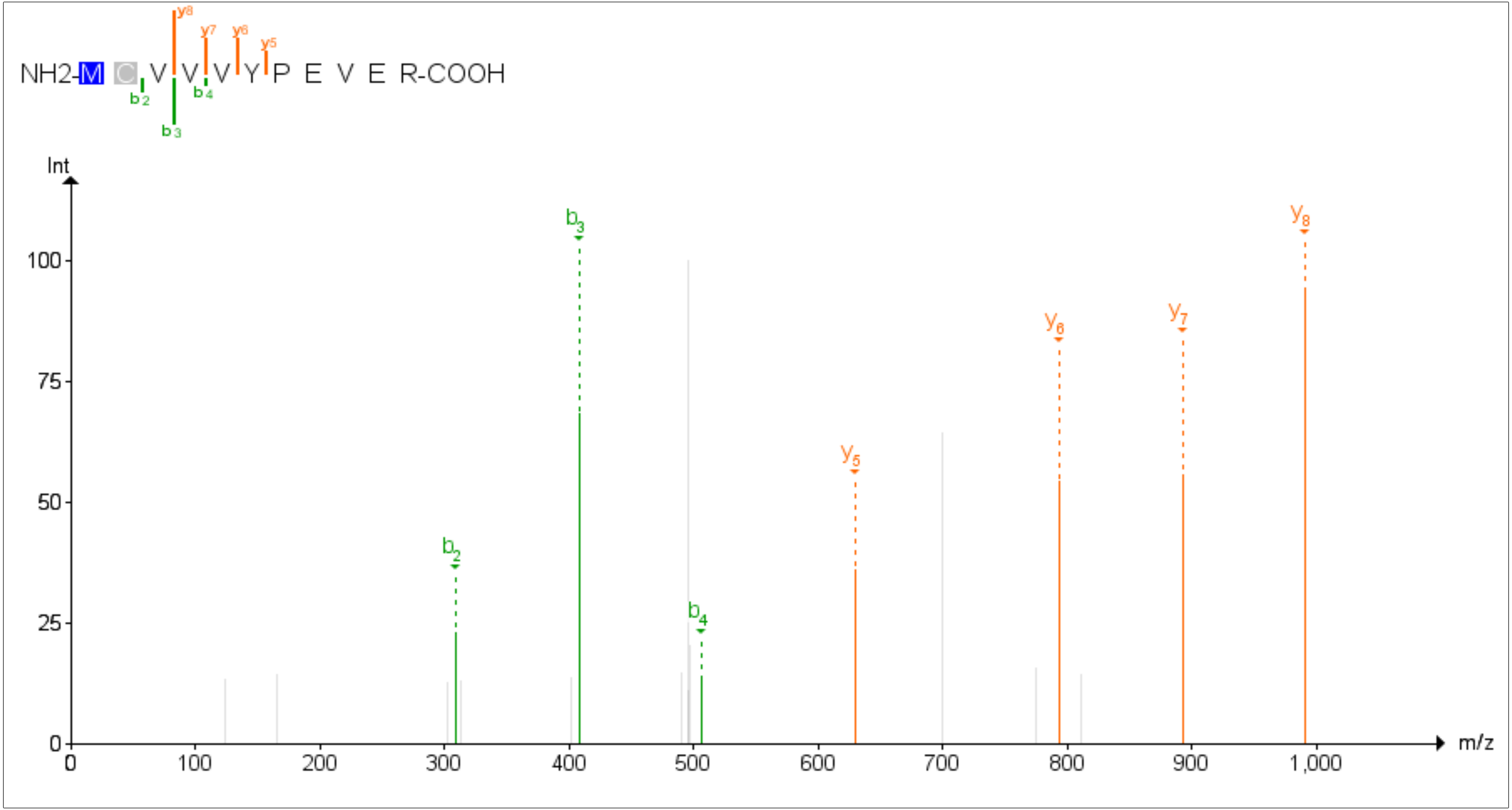

NH2-M C V V V Y P E V E R-COOH
y8
y7
y6
y5
b2
b3
b4
Int
100
75
50
25
0
b3
y8
y6
y7
y5
b2
b4
0
100
200
300
400
500
600
700
800
900
1,000
m/z

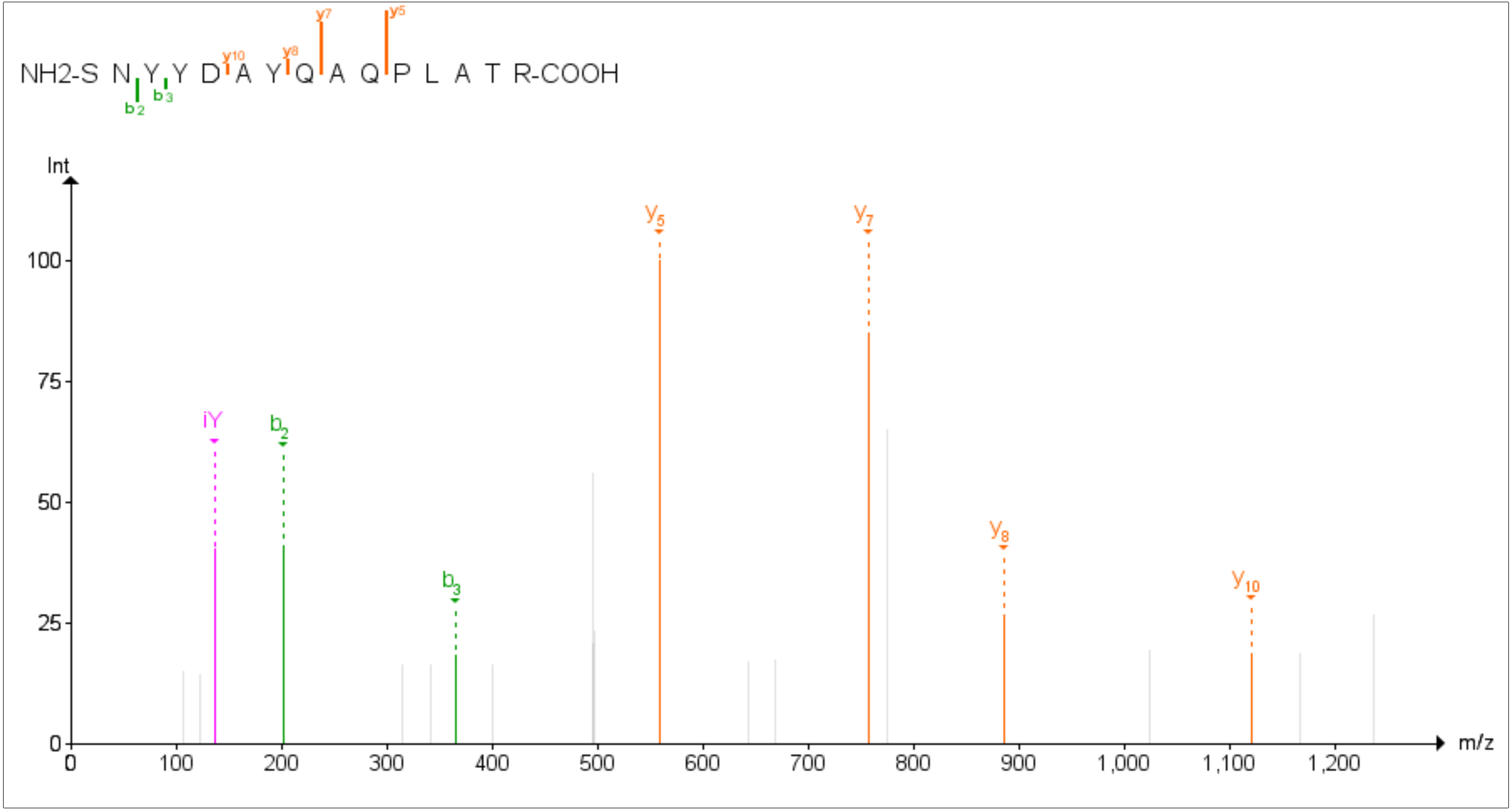

NH2-S N Y Y D A Y Q A Q P L A T R-COOH
y10
y8
y7
y5
b2
b3
Int
100
75
50
25
0
iY
b2
b3
y5
y7
y8
y10
0
100
200
300
400
500
600
700
800
900
1,000
1,100
1,200
m/z

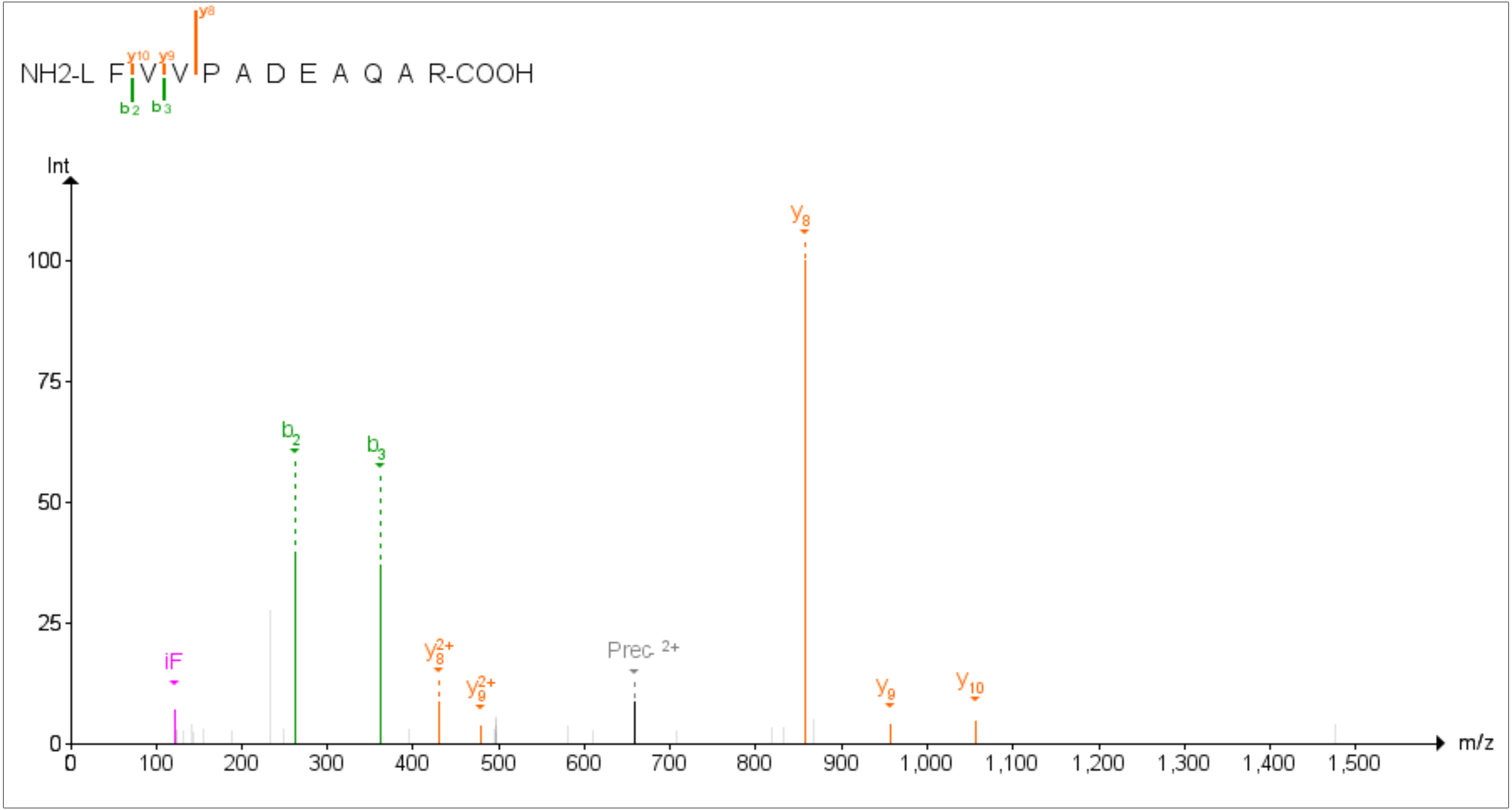

NH2-L F V V P A D E A Q A R-COOH
y10 y9 y8
b2 b3
Int
100
75
50
25
0
iF
b2
b3
y8 2+
y9 2+
Prec. 2+
y8
y9
y10
0 100 200 300 400 500 600 700 800 900 1,000 1,100 1,200 1,300 1,400 1,500
m/z

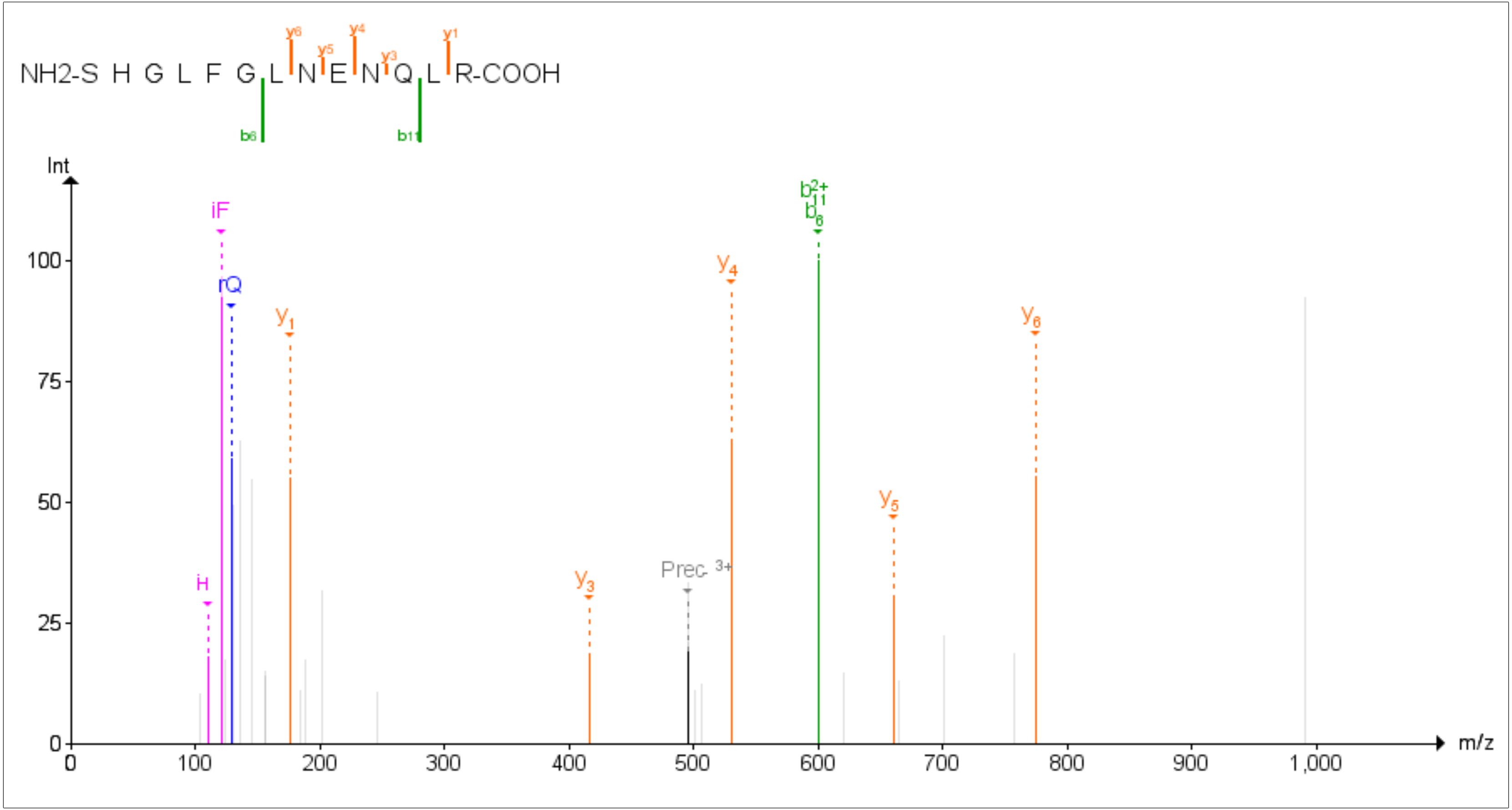
NH2-S H G L F G L N E N Q L R-COOH
y6
y5
y4
y3
y1
b6
b11
Int
iF
rQ
iH
y1
y3
Prec- 3+
y4
b2+ 11
b6
y5
y6
0
25
50
75
100
0
100
200
300
400
500
600
700
800
900
1,000
m/z

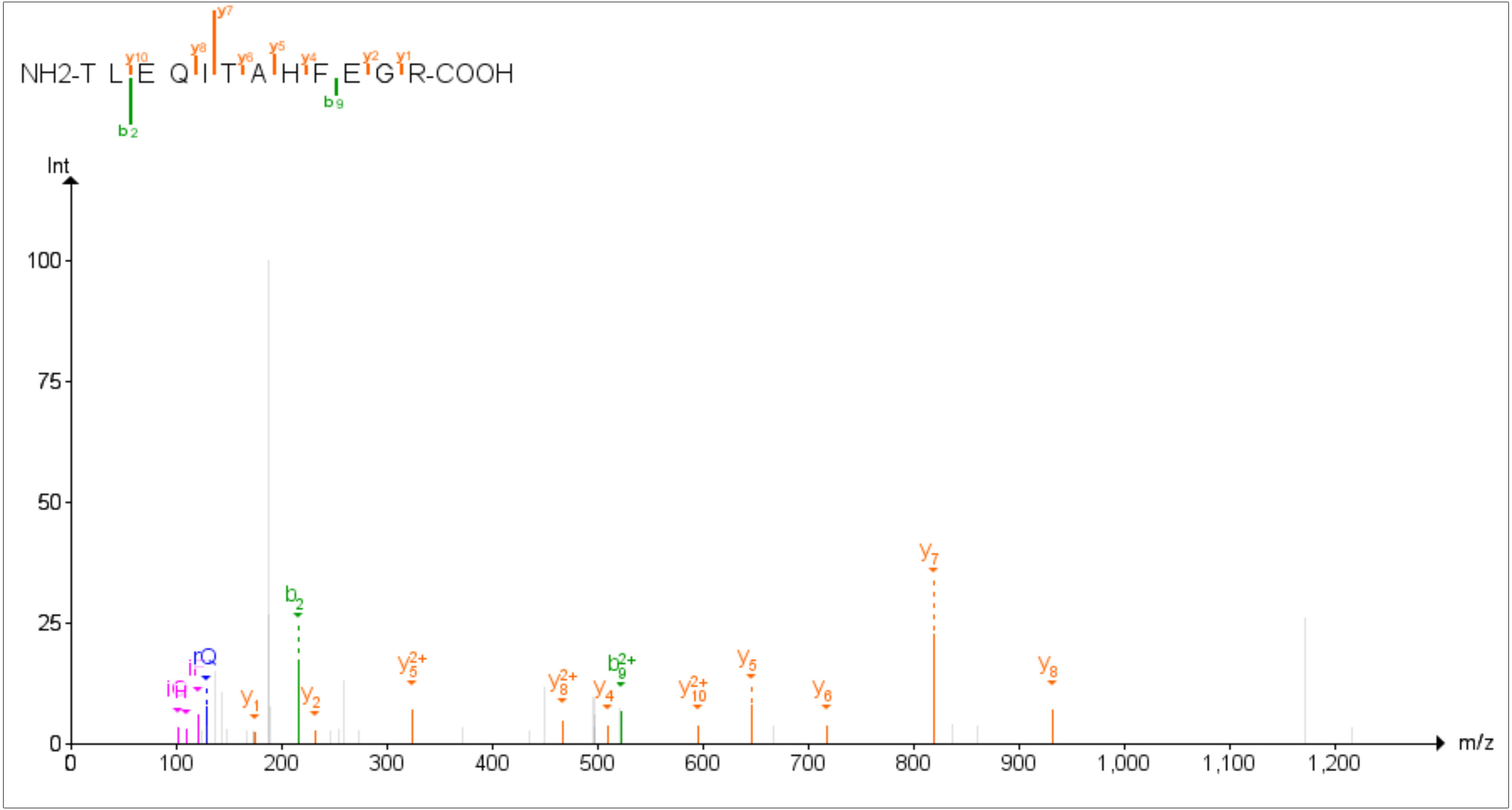
NH2-T L E Q I T A H F E G R-COOH
y10 y8 y7 y6 y5 y4 y2 y1
b2 b9
Int
100
75
50
25
0
m/z
0
100
200
300
400
500
600
700
800
900
1,000
1,100
1,200
y7
b2
rQ
y1
y2
y5 2+
y8 2+
b9 2+
y4
y10 2+
y5
y6
y8

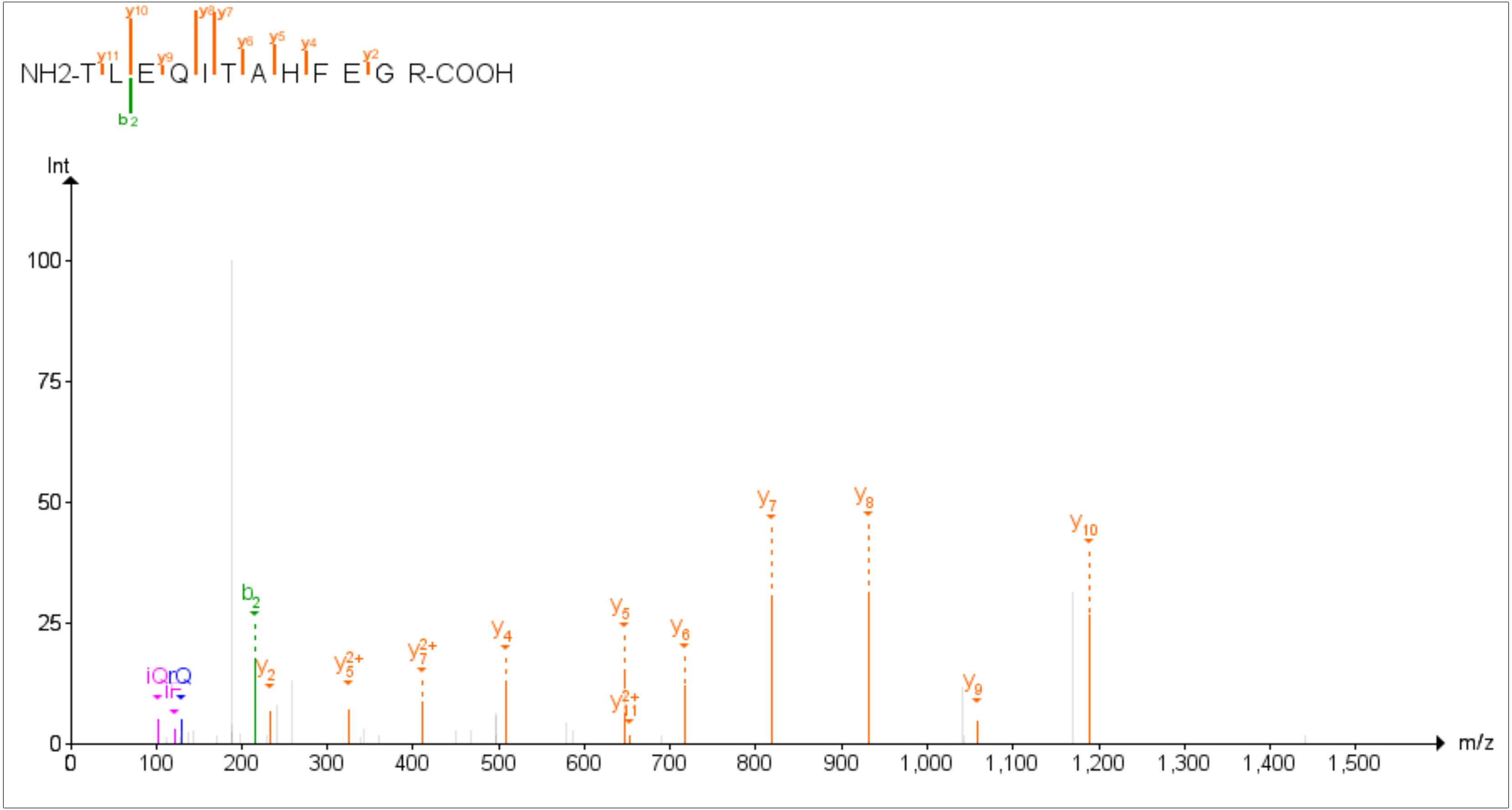
NH2-T L E Q I T A H F E G R-COOH
y11 y10 y9 y8 y7 y6 y5 y4 y2
b2
Int
100
75
50
25
0
m/z
0 100 200 300 400 500 600 700 800 900 1,000 1,100 1,200 1,300 1,400 1,500
iQ rQ IF
b2 y2 y5 2+ y7 2+ y4 y5 y11 2+ y6 y7 y8 y9 y10

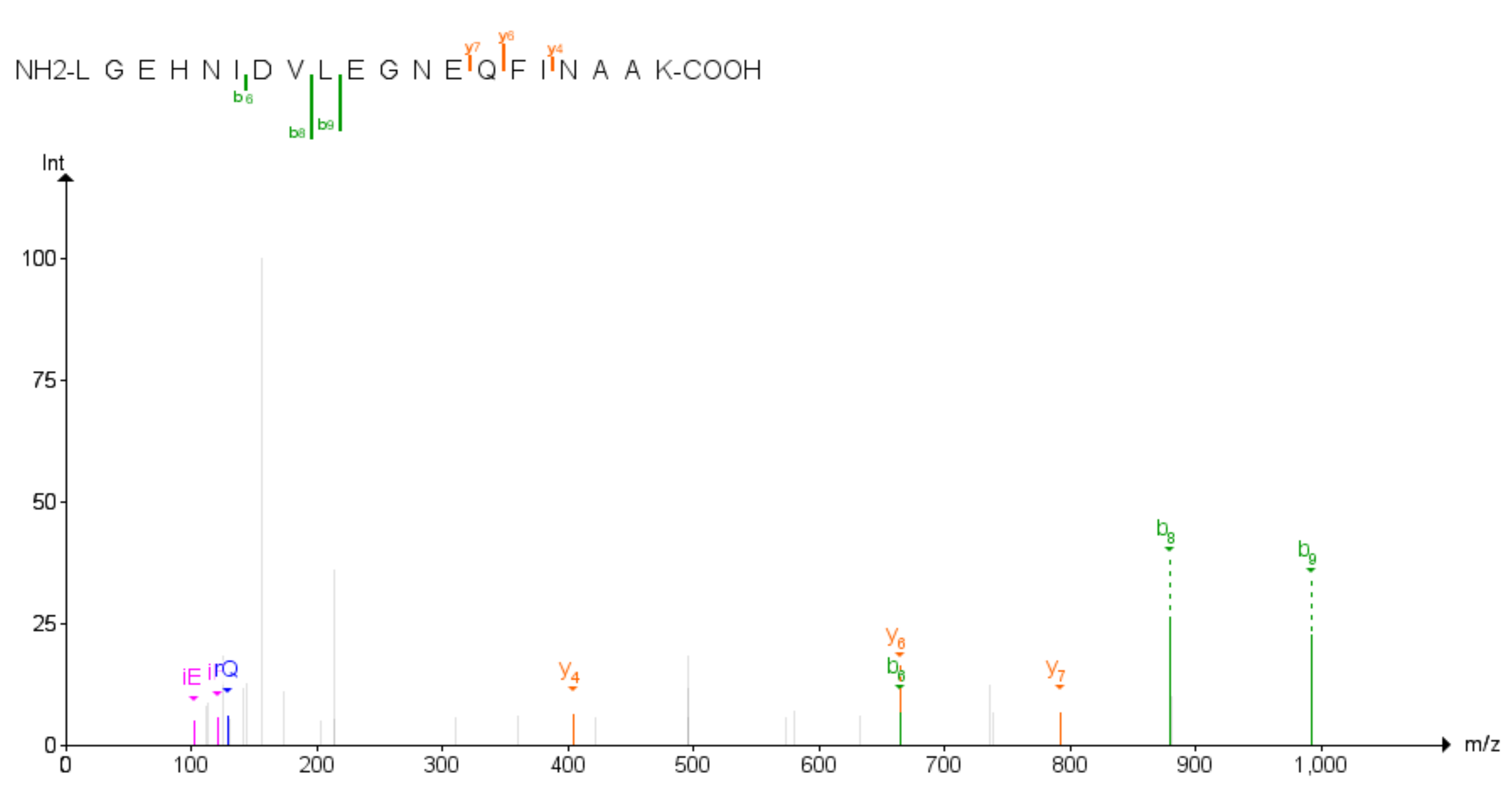

NH2-L G E H N I D V L E G N E Q F I N A A K-COOH
b6
b8
b9
y7
y6
y4
Int
100
75
50
25
0
iE
iQ
y4
y6
b6
y7
b8
b9
0
100
200
300
400
500
600
700
800
900
1,000
m/z

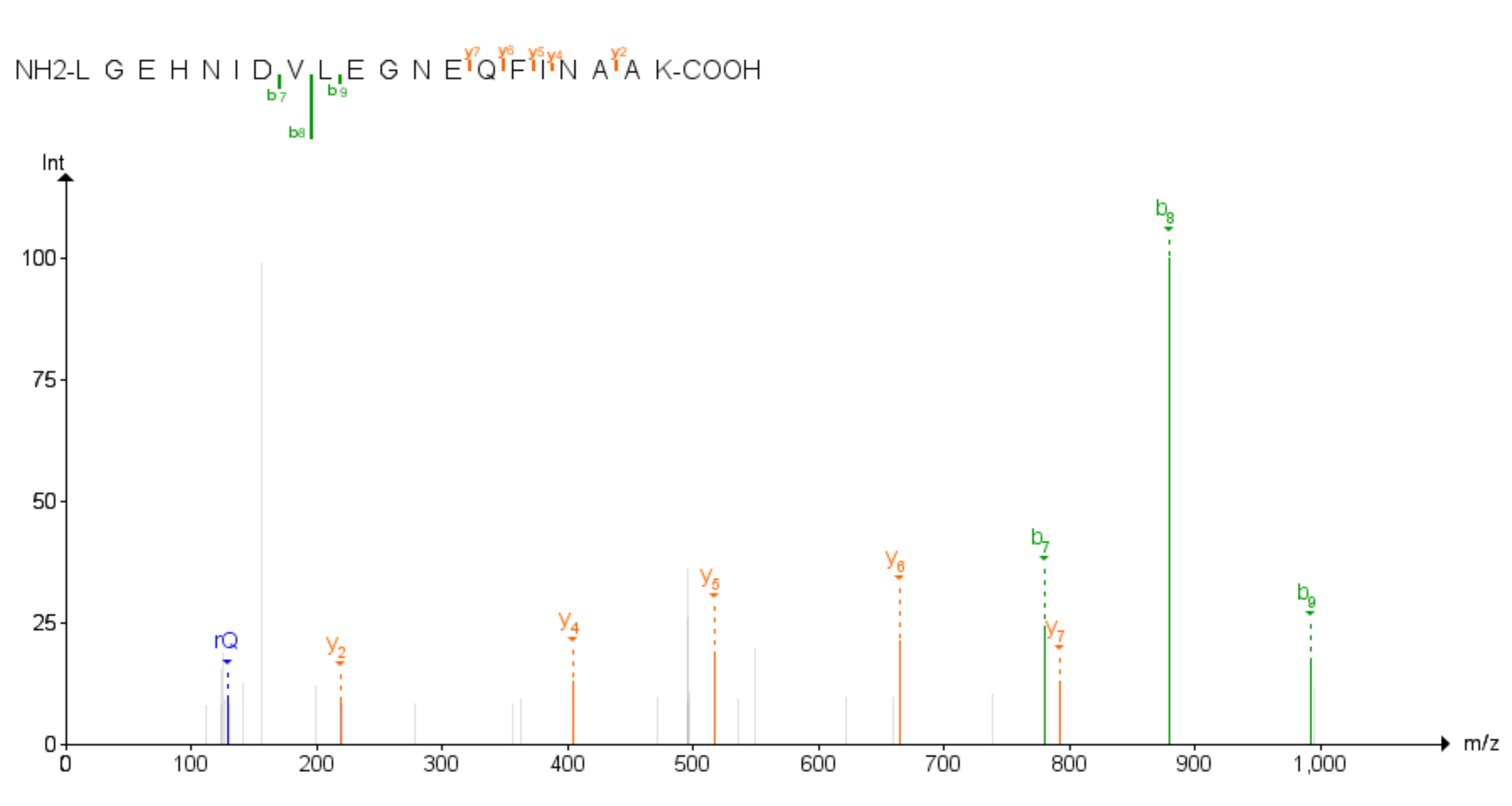
NH2-L G E H N I D V L E G N E Q F I N A A K-COOH
b7
b8
b9
y7
y6
y5
y4
y2
Int
100
75
50
25
0
rQ
y2
y4
y5
y6
y7
b7
b8
b9
0
100
200
300
400
500
600
700
800
900
1,000
m/z

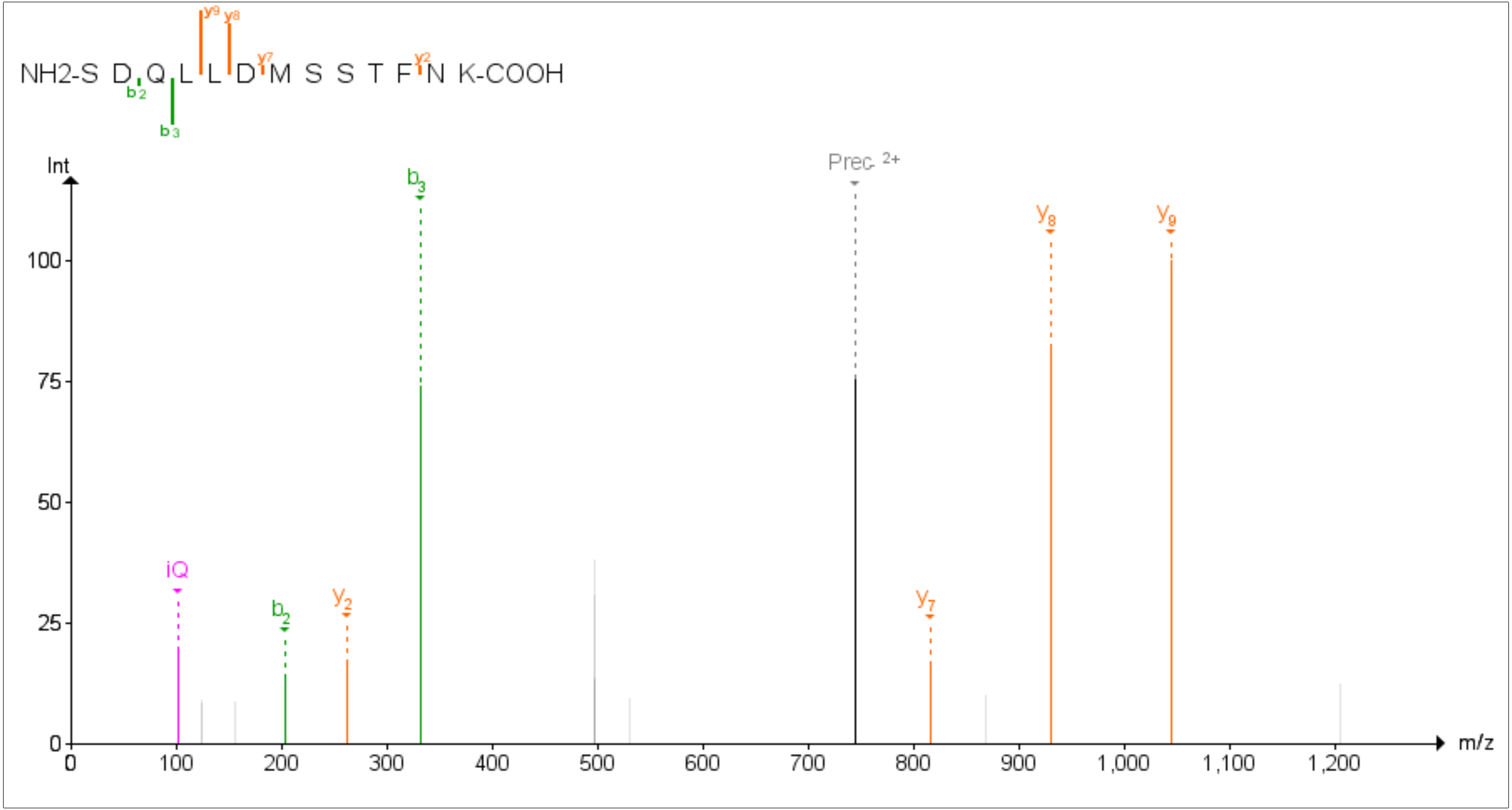

NH2-S D Q L L D M S S T F N K-COOH
y9 y8 y7 y2
b2 b3
Int
Prec 2+
b3
y8
y9
iQ
b2
y2
y7
0
25
50
75
100
0 100 200 300 400 500 600 700 800 900 1,000 1,100 1,200
m/z

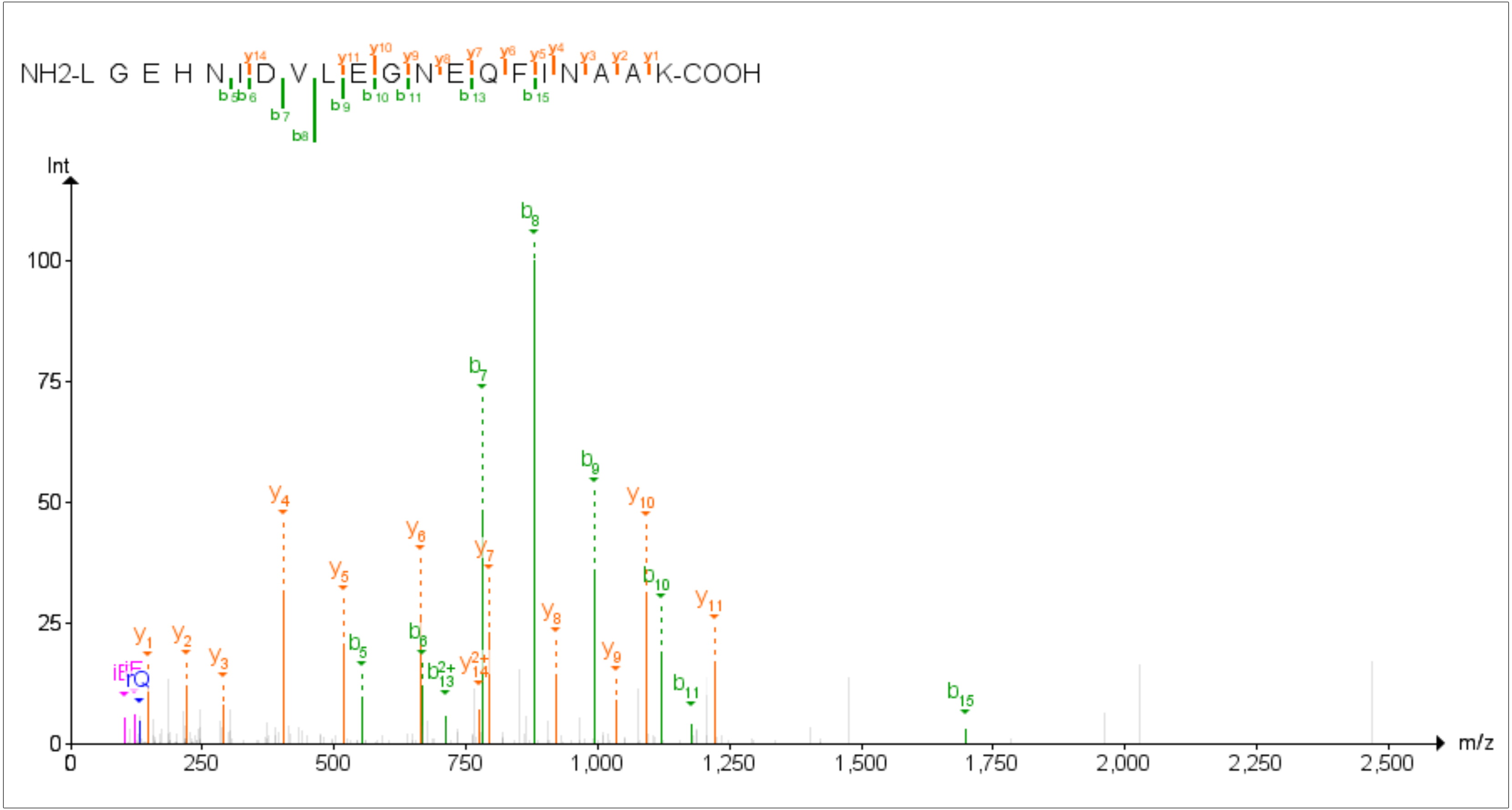
NH2-L G E H N I D V L E G N E Q F I N A A K-COOH
Int
m/z
b8
b7
b9
y10
y4
y6
y7
y5
b10
y11
y8
b6
b5
y1
y2
y3
y9
b11
b15
rQ
100
75
50
25
0
250
500
750
1,000
1,250
1,500
1,750
2,000
2,250
2,500

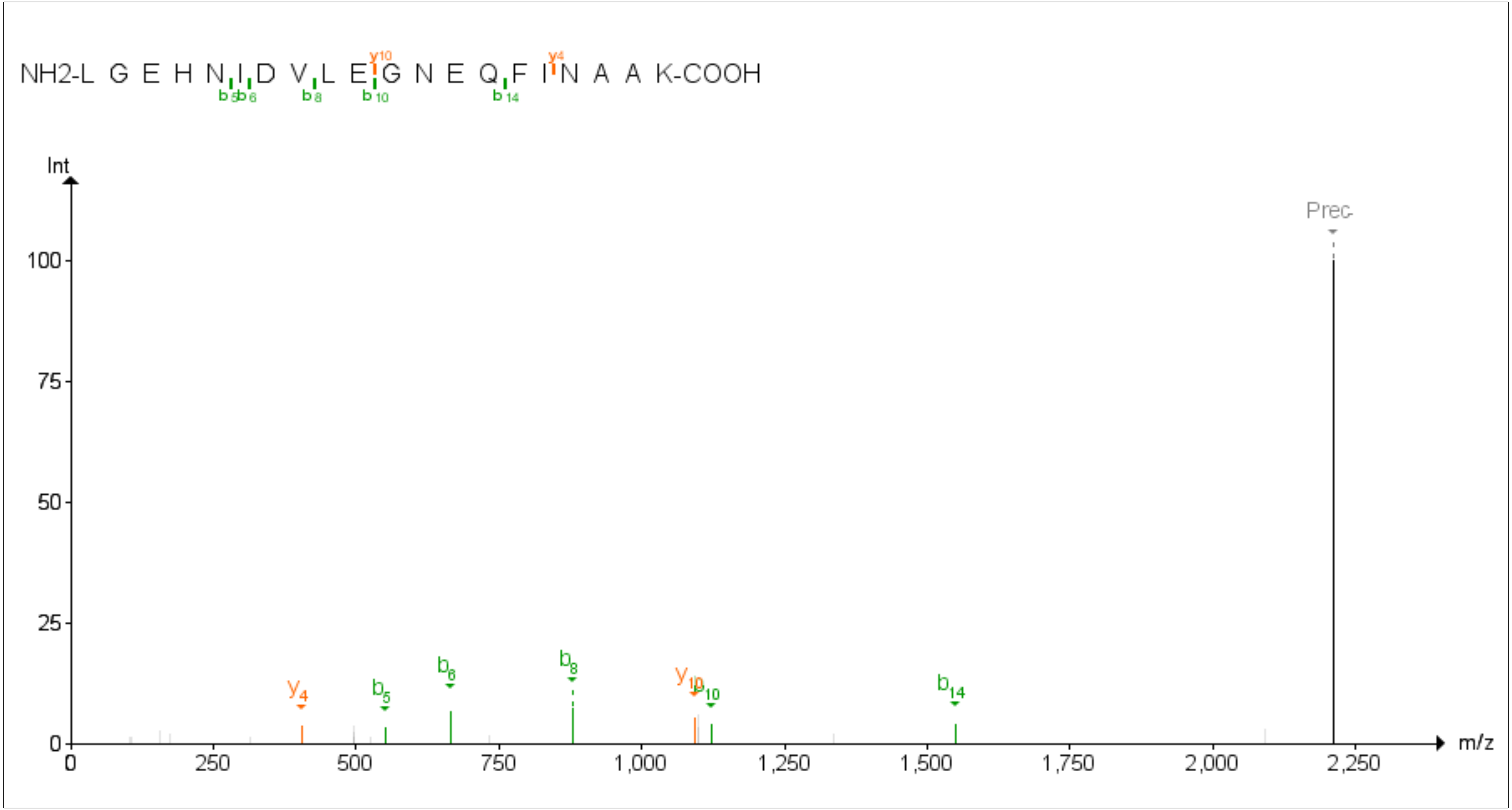

NH2-L G E H N I D V L E G N E Q F I N A A K-COOH
y10
y4
b5
b6
b8
b10
b14
Int
100
75
50
25
0
Prec
y4
b5
b6
b8
y10
b10
b14
0
250
500
750
1,000
1,250
1,500
1,750
2,000
2,250
m/z

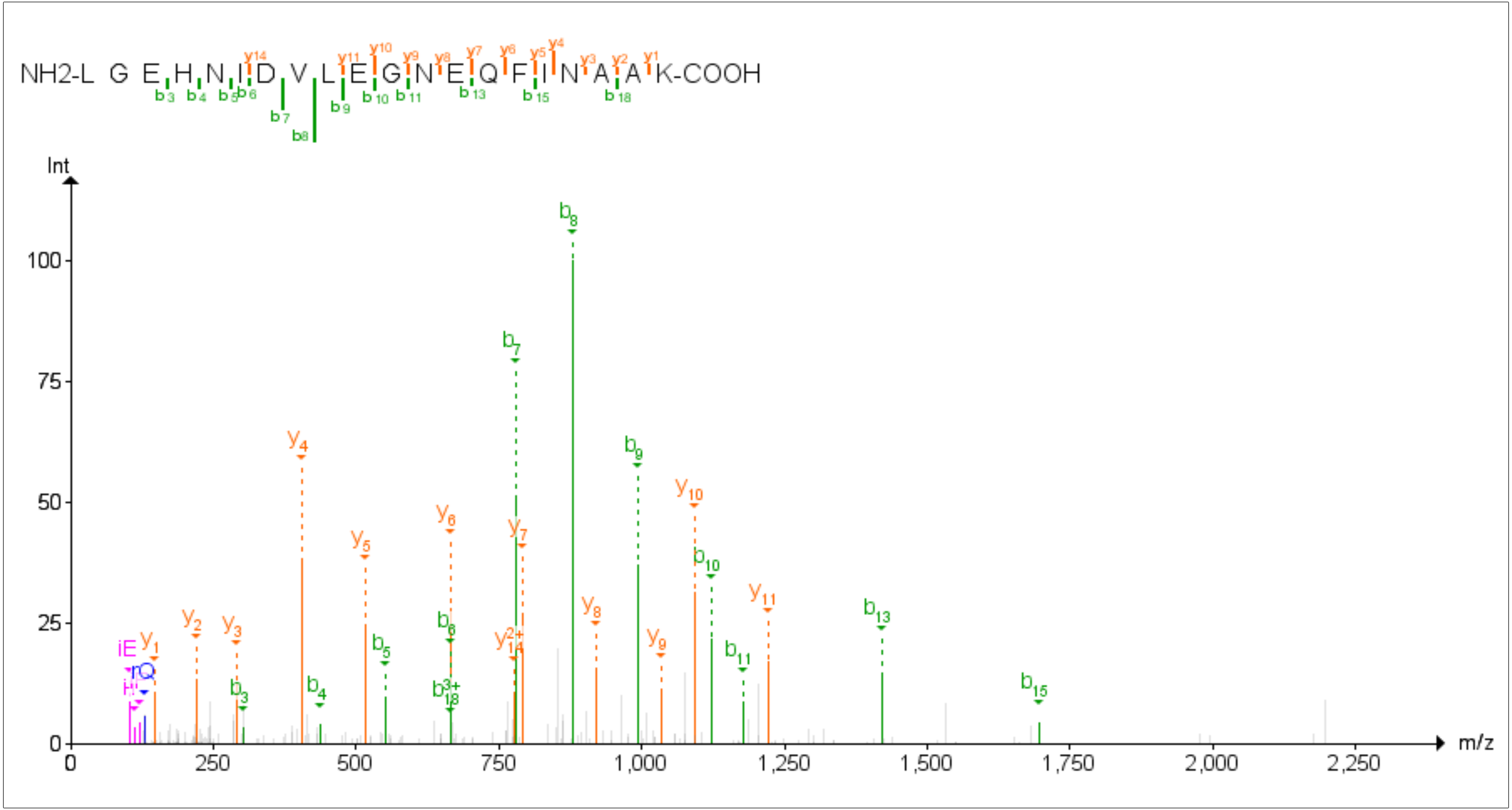
NH2-L G E H N I D V L E G N E Q F I N A A K-COOH
Int
m/z
0
25
50
75
100
250
500
750
1,000
1,250
1,500
1,750
2,000
2,250

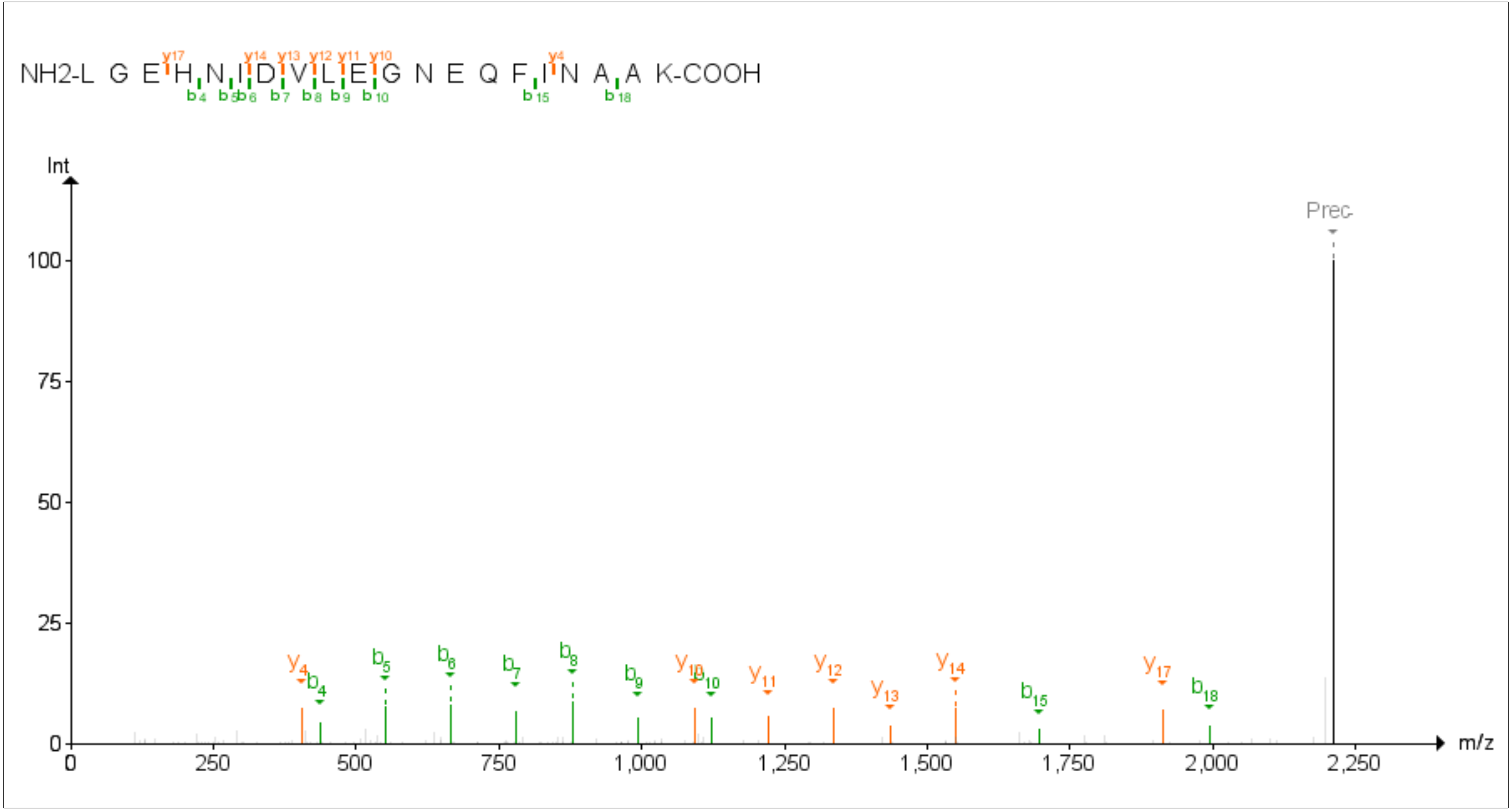
NH2-L G E H N I D V L E G N E Q F I N A A K-COOH
Int
100
75
50
25
0
Prec
m/z
0
250
500
750
1,000
1,250
1,500
1,750
2,000
2,250

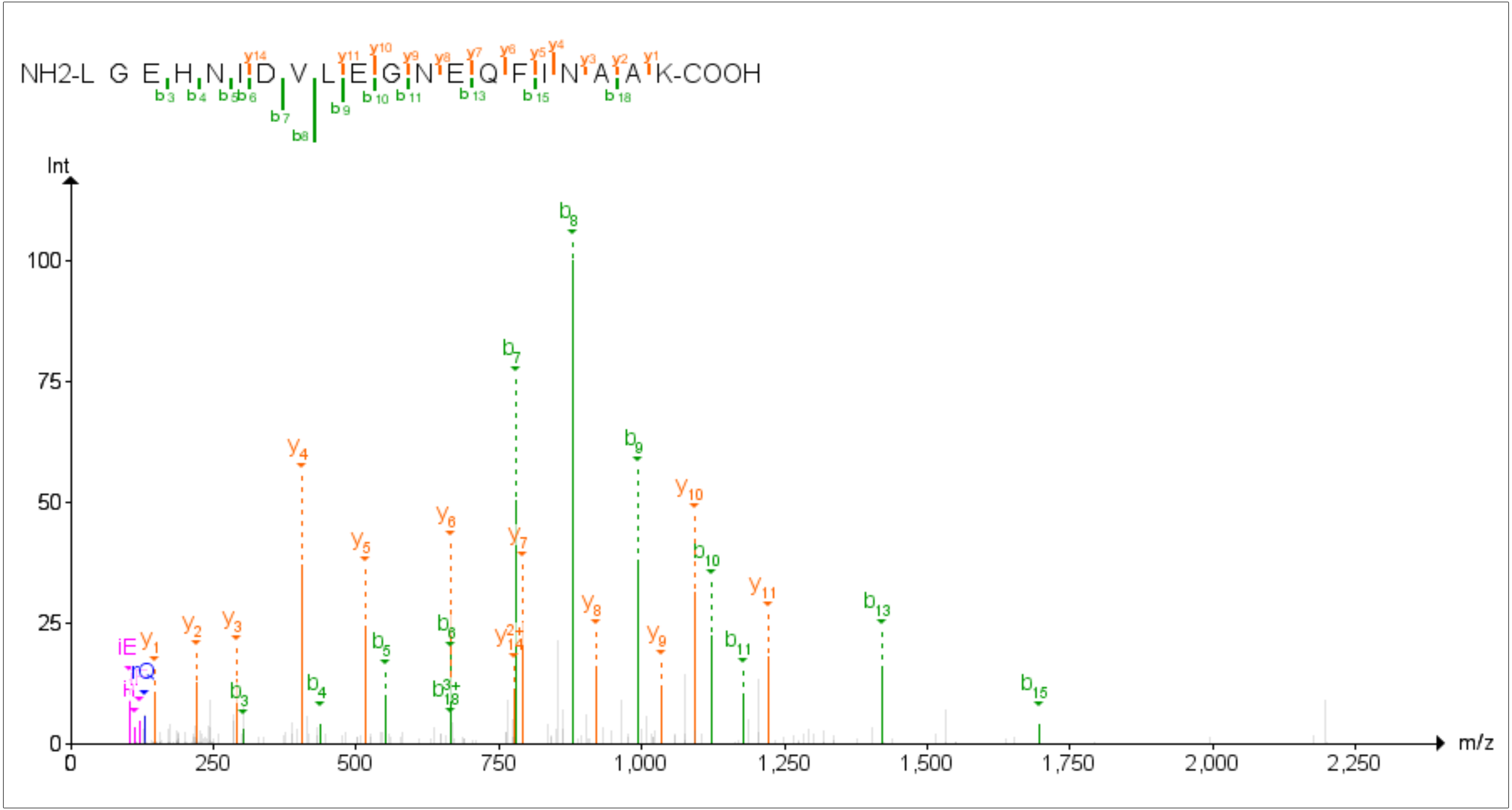
NH2-L G E H N I D V L E G N E Q F I N A A K-COOH
Int
m/z
0
25
50
75
100
250
500
750
1,000
1,250
1,500
1,750
2,000
2,250

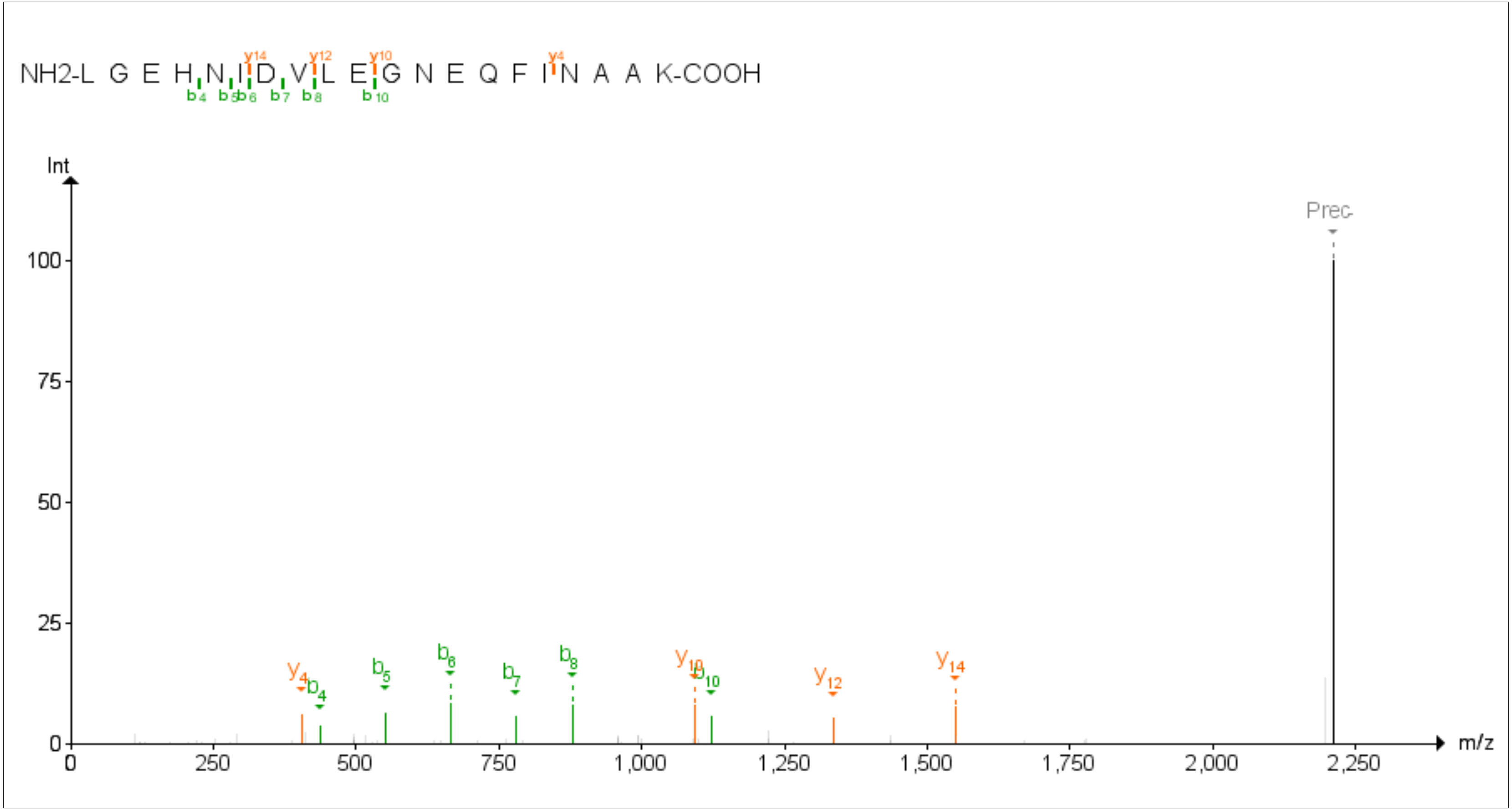

NH2-L G E H N I D V L E G N E Q F I N A A K-COOH
y14 y12 y10 y4
b4 b5 b6 b7 b8 b10
Int
100
75
50
25
0
Prec
y4 b4 b5 b6 b7 b8 y10 b10 y12 y14
0 250 500 750 1,000 1,250 1,500 1,750 2,000 2,250
m/z

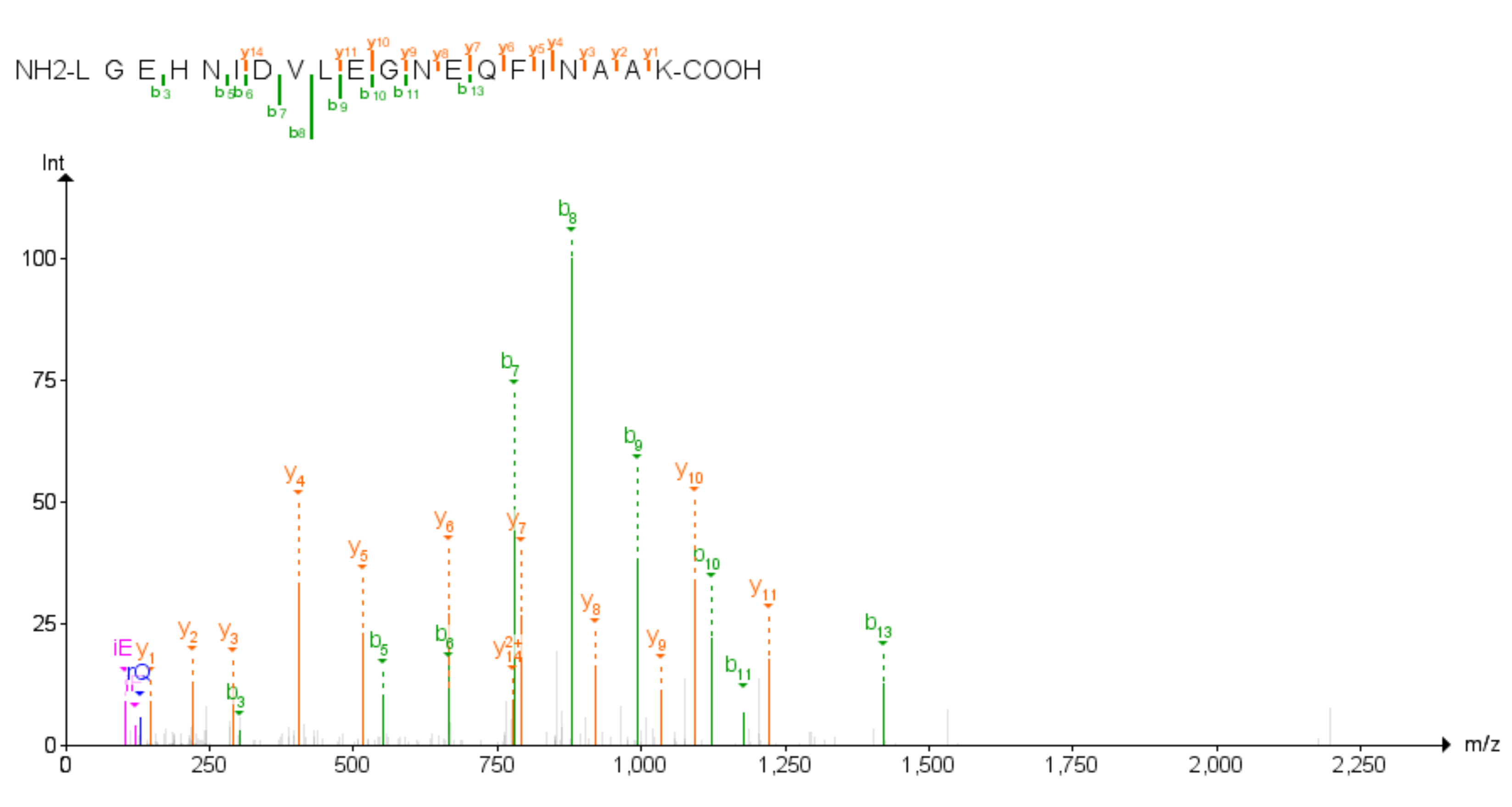

NH2-L G E H N I D V L E G N E Q F I N A A K-COOH
Int
m/z
b8
b7
b9
y10
y4
y6
y7
b10
y5
y11
y8
b13
y9
b5
b6
y2
y3
b11
iE
y1
nQ
b3

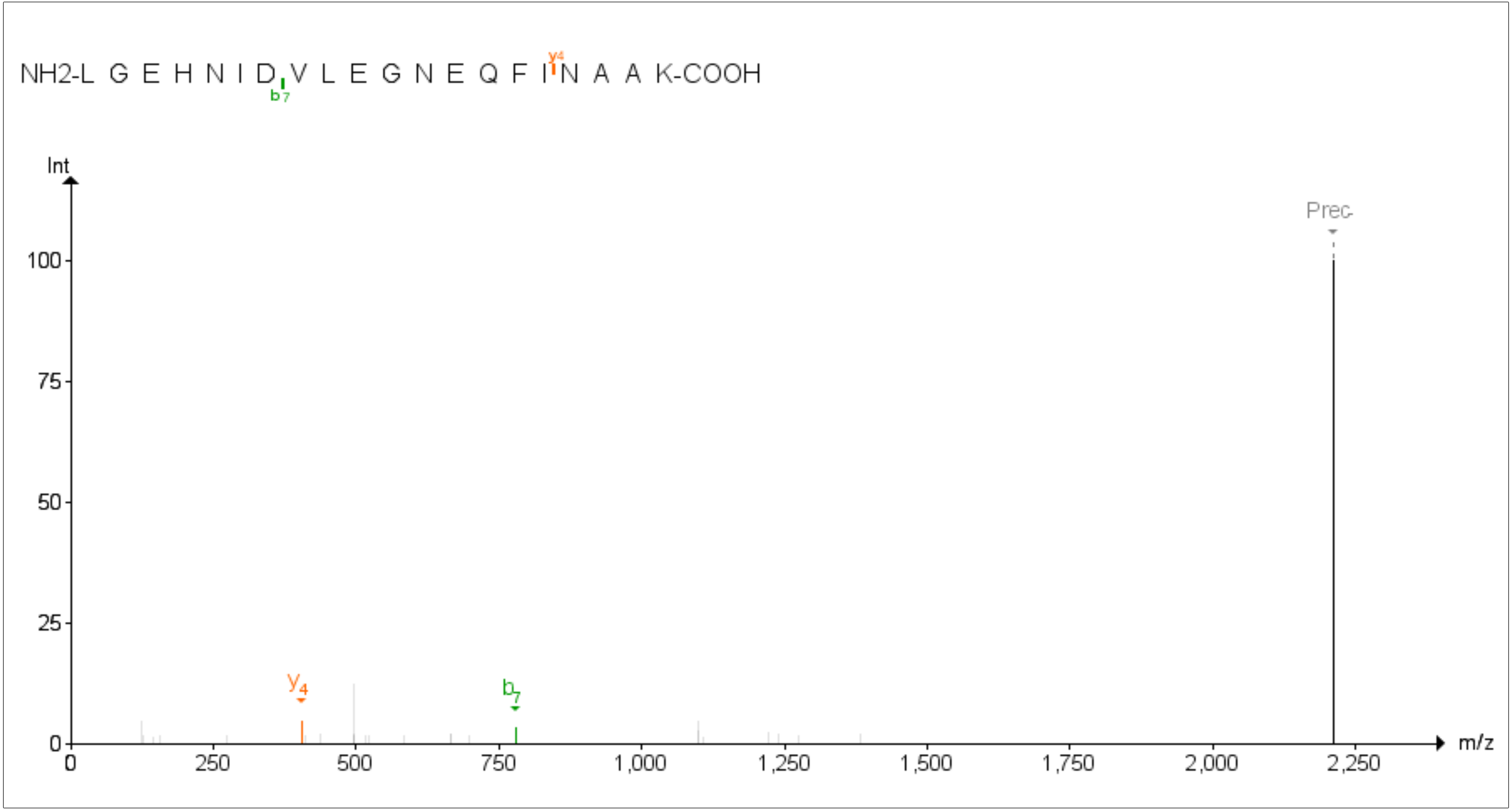

NH2-L G E H N I D V L E G N E Q F I N A A K-COOH
b7
y4
Int
100
75
50
25
0
Prec.
y4
b7
0
250
500
750
1,000
1,250
1,500
1,750
2,000
2,250
m/z

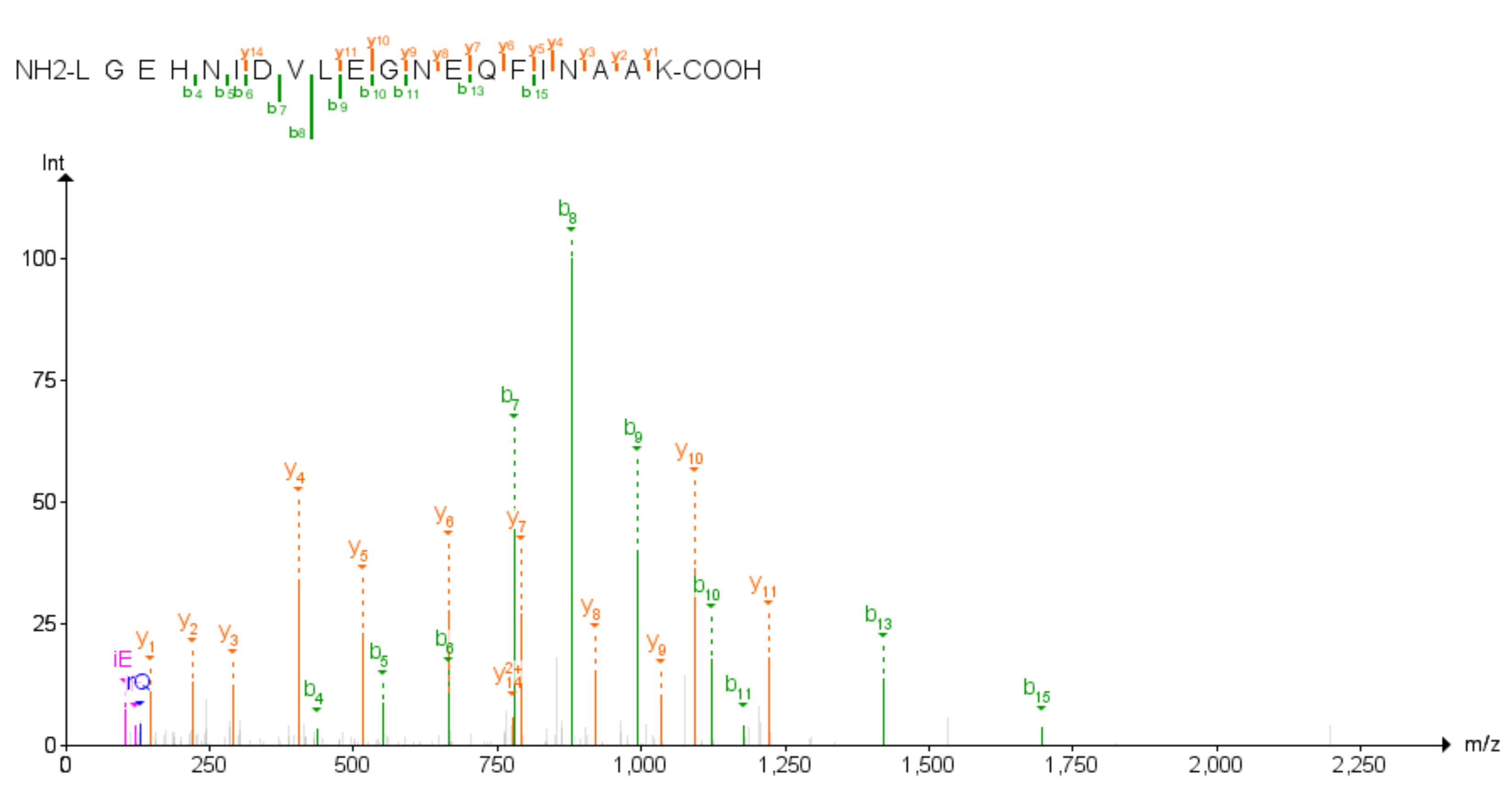
NH2-L G E H N I D V L E G N E Q F I N A A K-COOH
Int
m/z
0
25
50
75
100
0
250
500
750
1,000
1,250
1,500
1,750
2,000
2,250

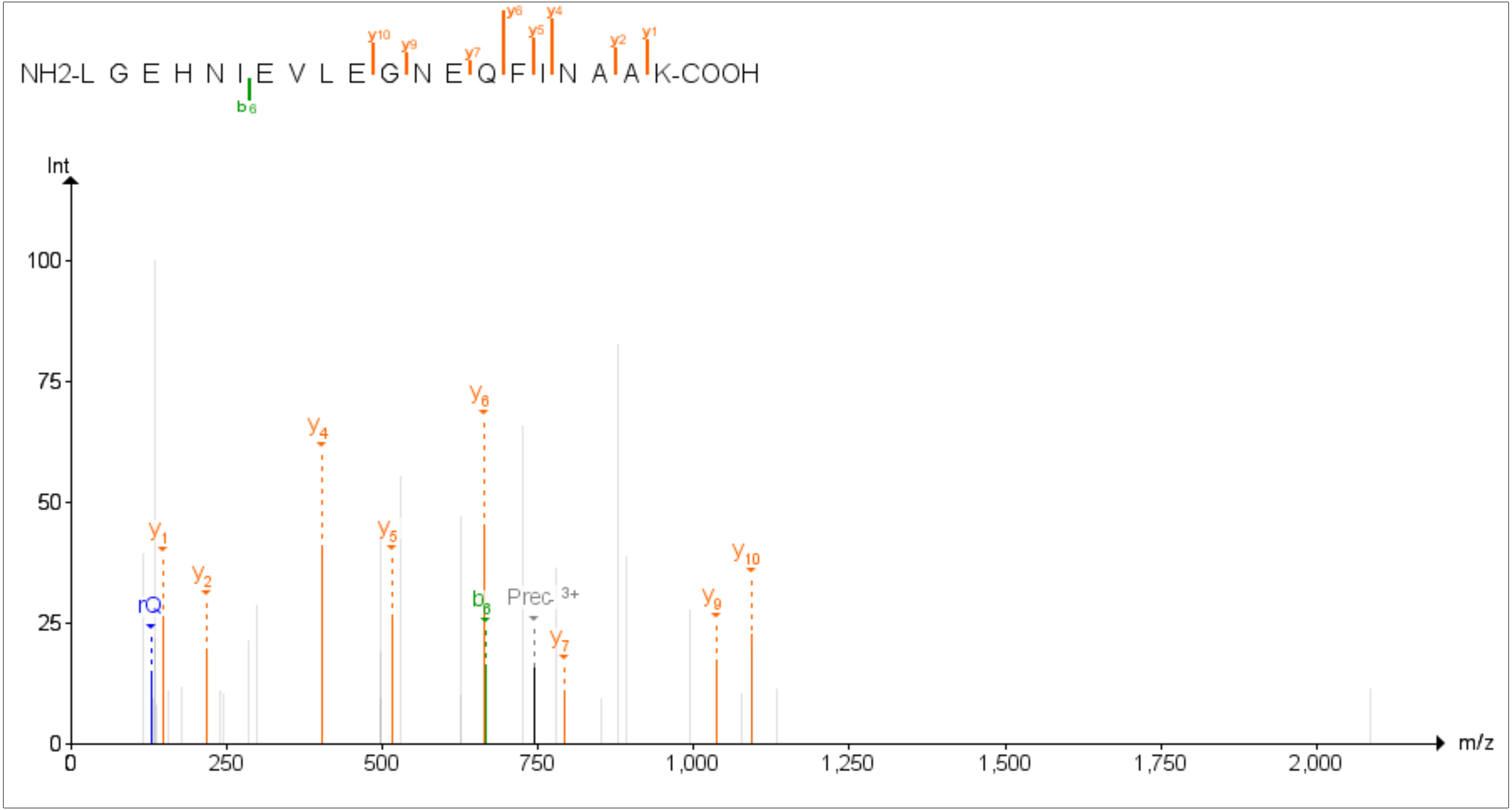
NH2-L G E H N I E V L E G N E Q F I N A A K-COOH
y10 y9 y7 y6 y5 y4 y2 y1
b6
Int
100
75
50
25
0
0 250 500 750 1,000 1,250 1,500 1,750 2,000
m/z
rQ
y1
y2
y4
y5
y6
b6
Prec 3+
y7
y9
y10

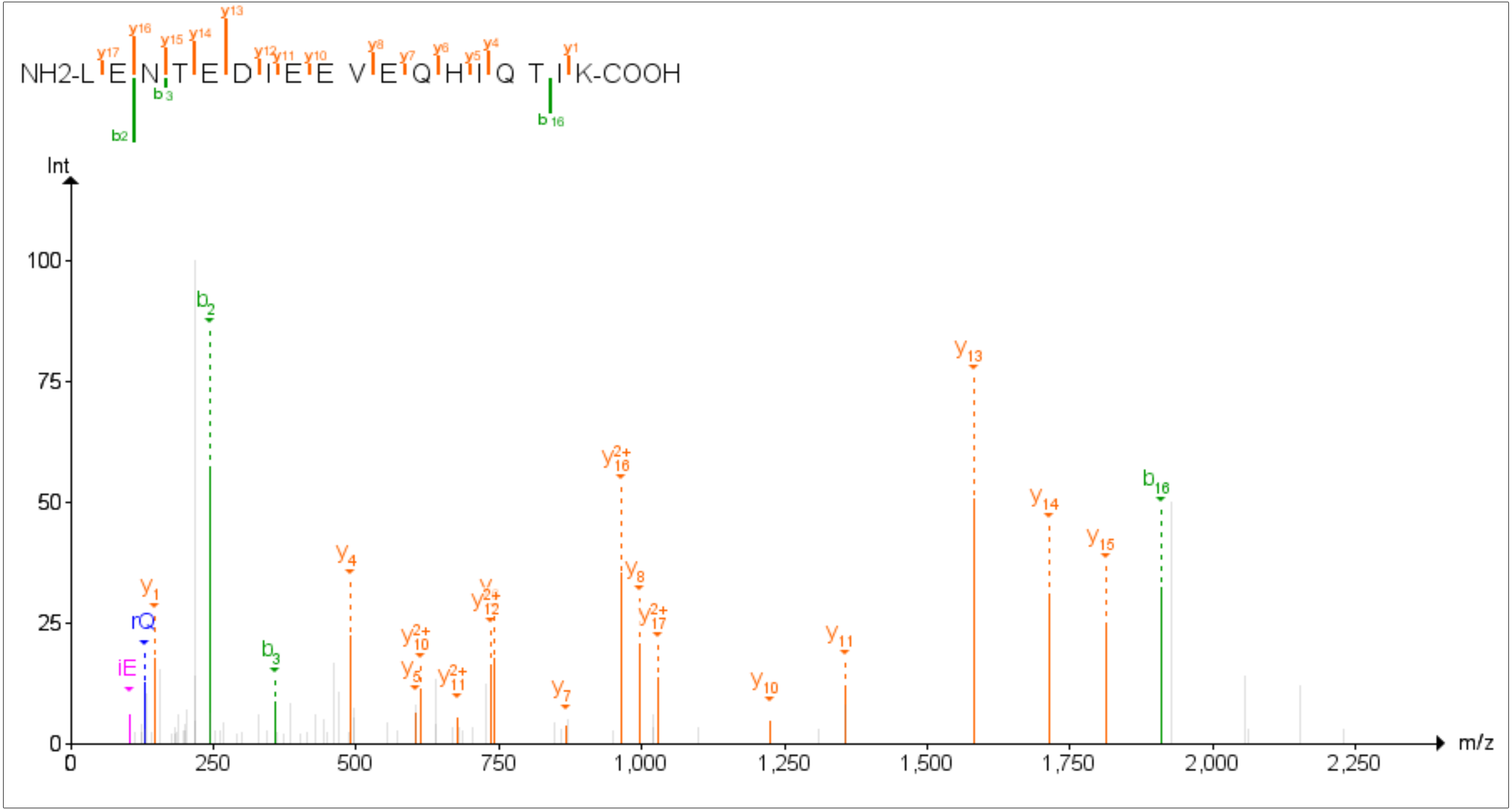
NH2-L E N T E D I E E V E Q H I Q T I K-COOH
y17 y16 y15 y14 y13 y12 y11 y10 y8 y7 y6 y5 y4 y1
b2 b3 b16
Int
100
75
50
25
0
m/z
0
250
500
750
1,000
1,250
1,500
1,750
2,000
2,250

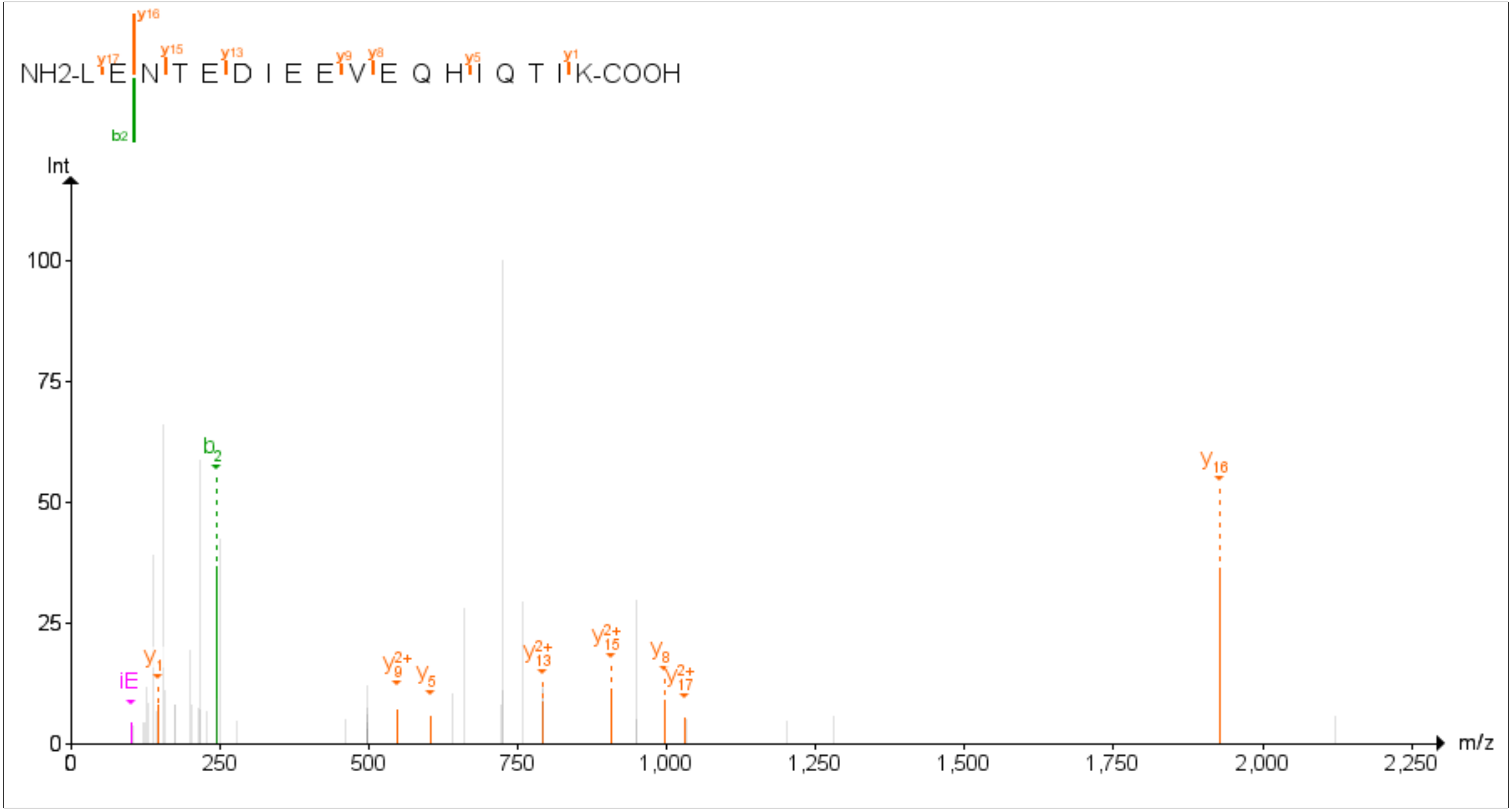

NH2-L E N T E D I E E V E Q H I Q T I K-COOH
y17
y16
y15
y13
y9
y8
y5
y1
b2
Int
100
75
50
25
0
iE
y1
b2
y9 2+
y5
y13 2+
y15 2+
y8
y17 2+
y16
m/z
0
250
500
750
1,000
1,250
1,500
1,750
2,000
2,250

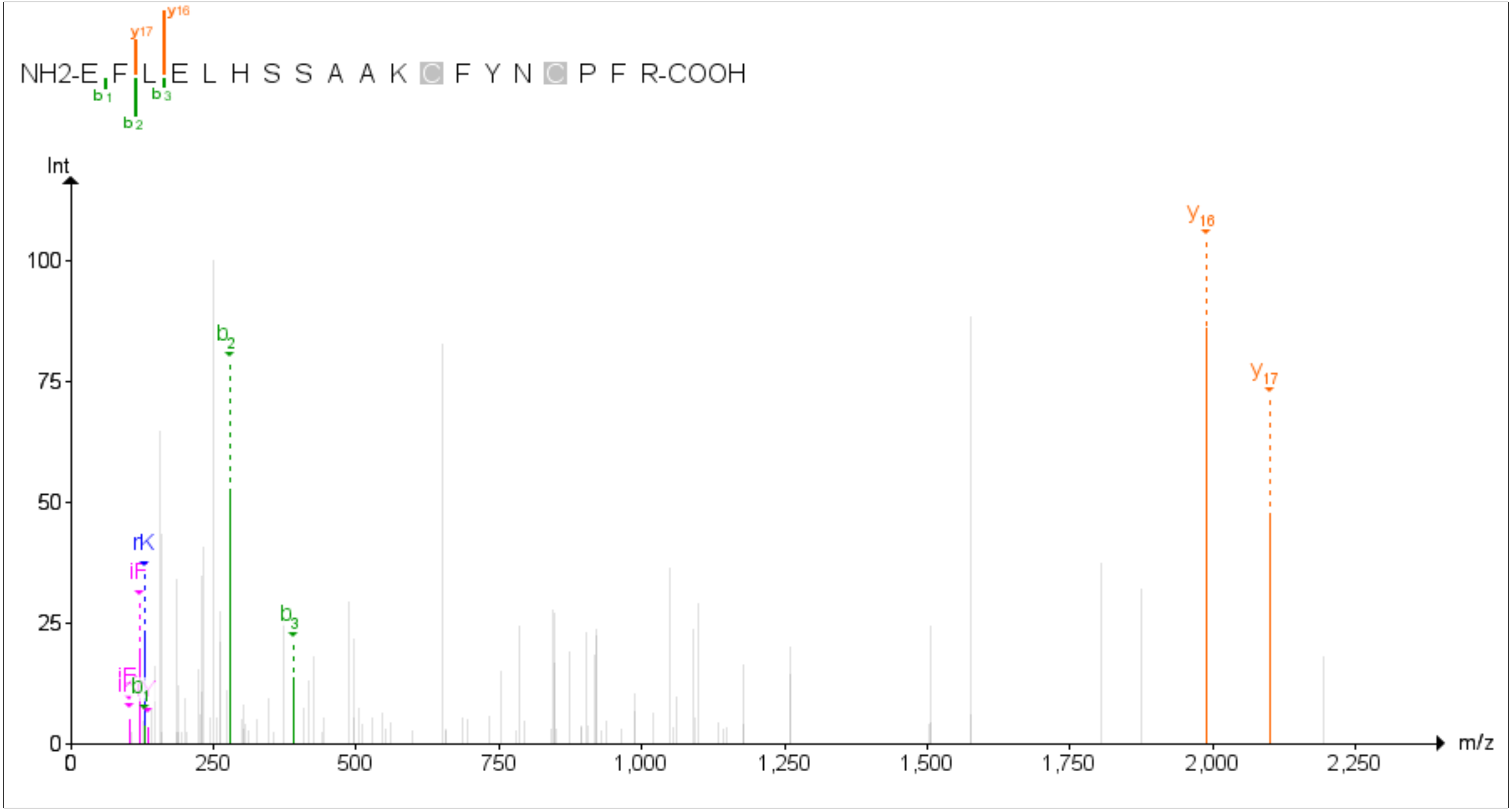

NH2-E F L E L H S S A A K C F Y N C P F R-COOH
y17
y16
b1
b2
b3
Int
100
75
50
25
0
m/z
0
250
500
750
1,000
1,250
1,500
1,750
2,000
2,250
y16
y17
b2
b3
rK
iF
iF
b1

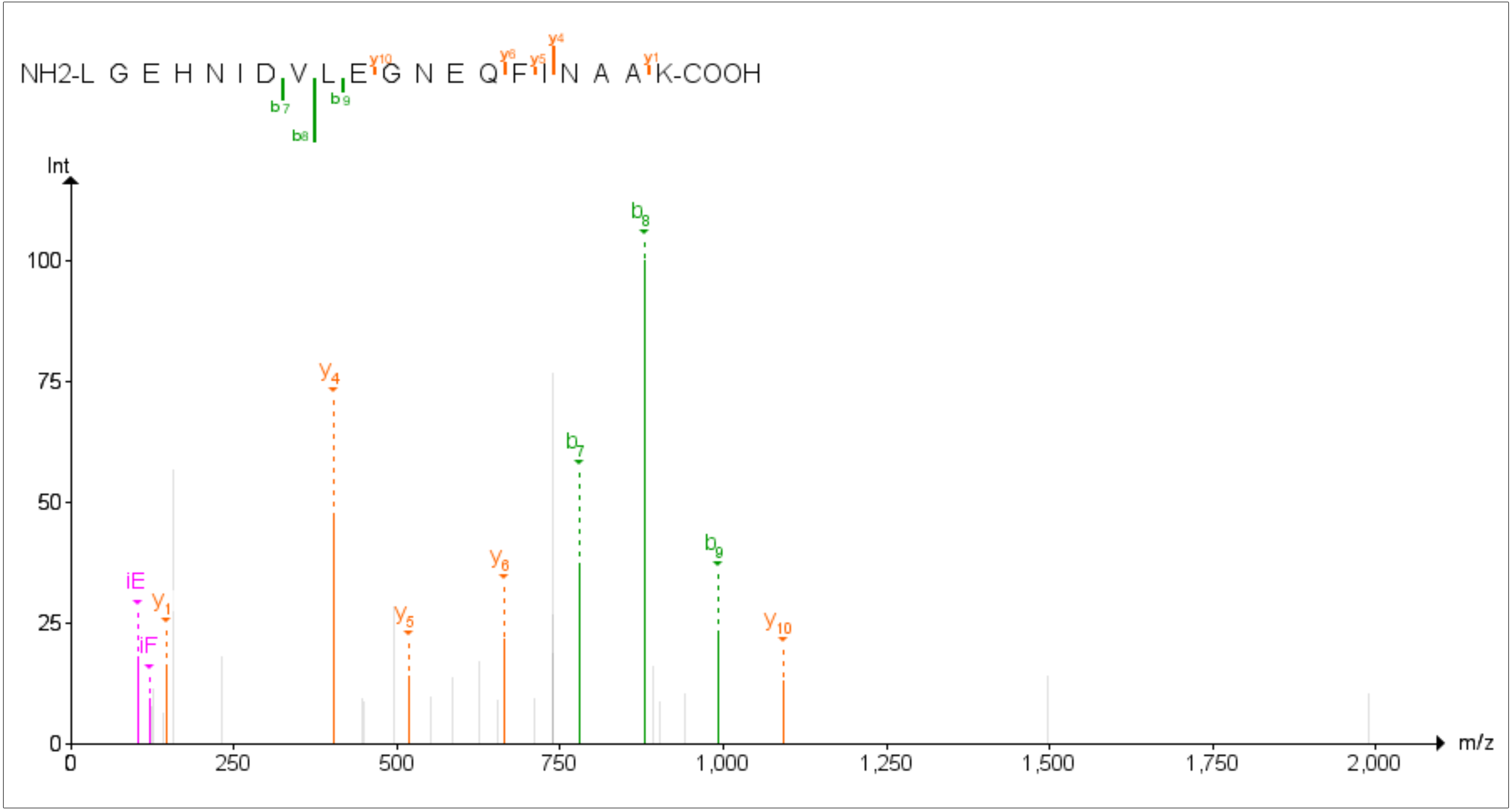

NH2-L G E H N I D V L E G N E Q F I N A A K-COOH
b7
b8
b9
y10
y6
y5
y4
y1
Int
100
75
50
25
0
b8
y4
b7
b9
y6
y5
y10
iE
y1
iF
0
250
500
750
1,000
1,250
1,500
1,750
2,000
m/z

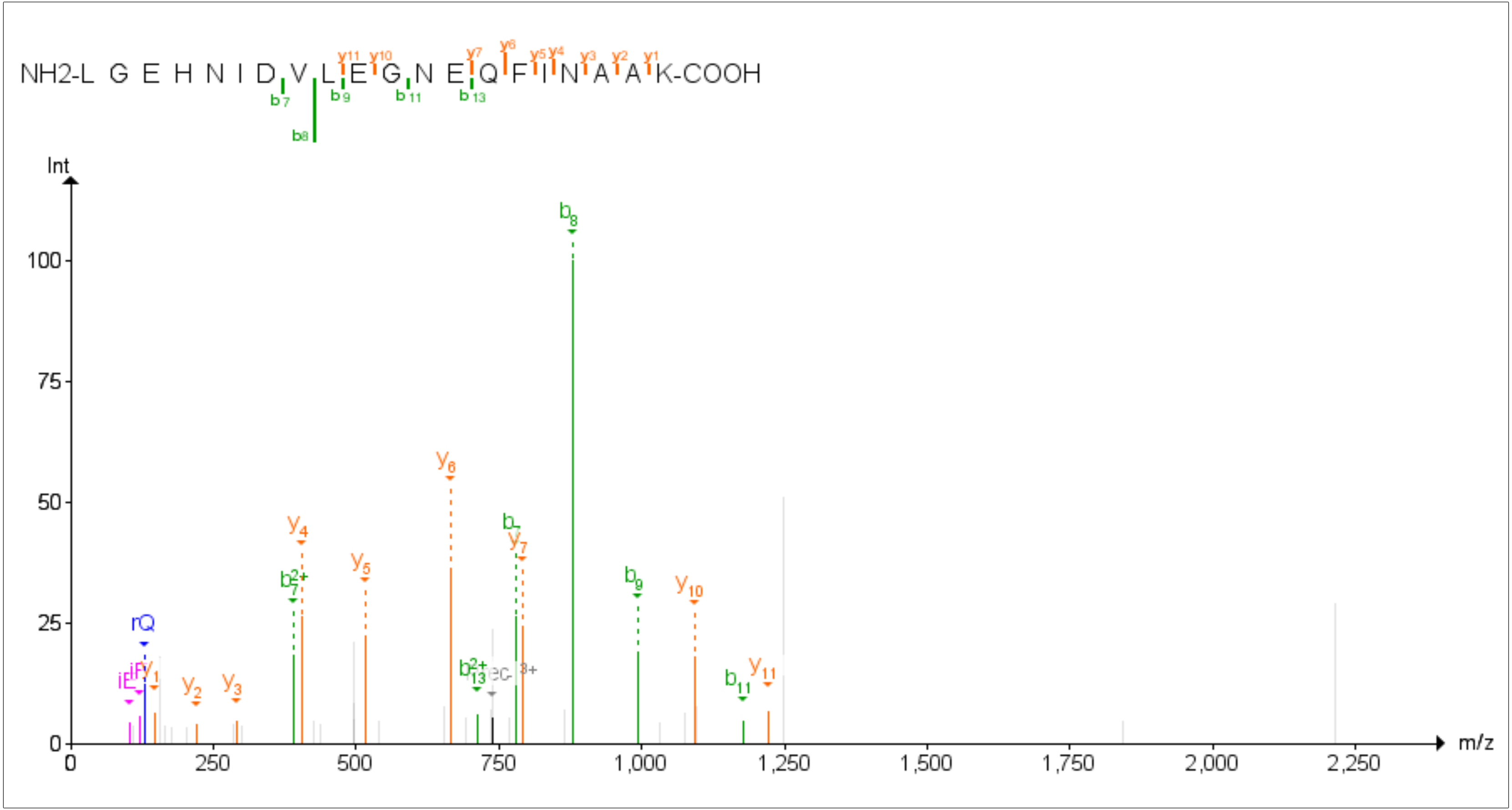

NH2-L G E H N I D V L E G N E Q F I N A A K-COOH
Int
m/z
0
25
50
75
100
250
500
750
1,000
1,250
1,500
1,750
2,000
2,250

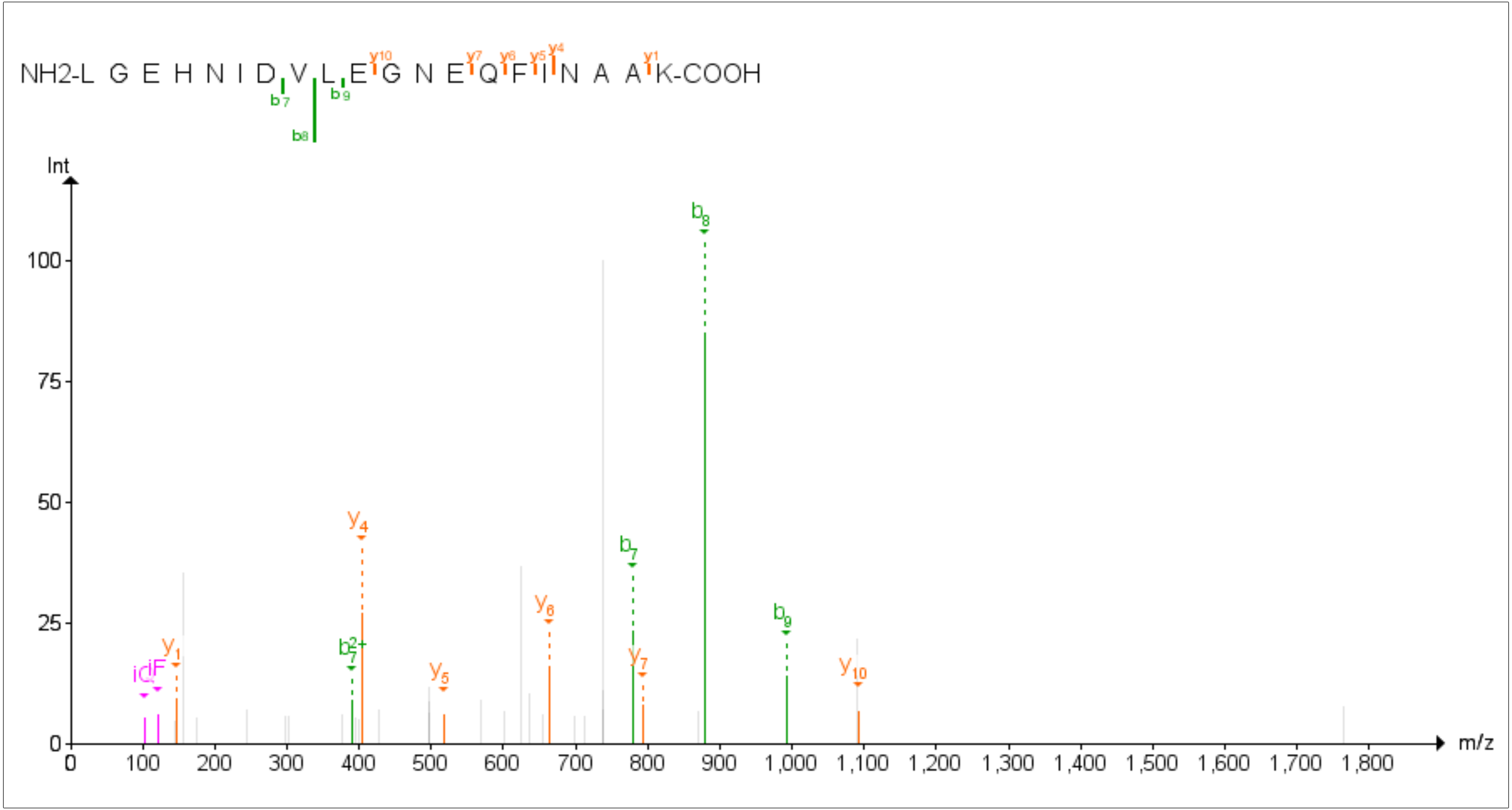
NH2-L G E H N I D V L E G N E Q F I N A A K-COOH
Int
m/z
b8
b7
b9
y4
y6
y7
y1
y5
y10
iQ
iF
100
75
50
25
0
0 100 200 300 400 500 600 700 800 900 1,000 1,100 1,200 1,300 1,400 1,500 1,600 1,700 1,800

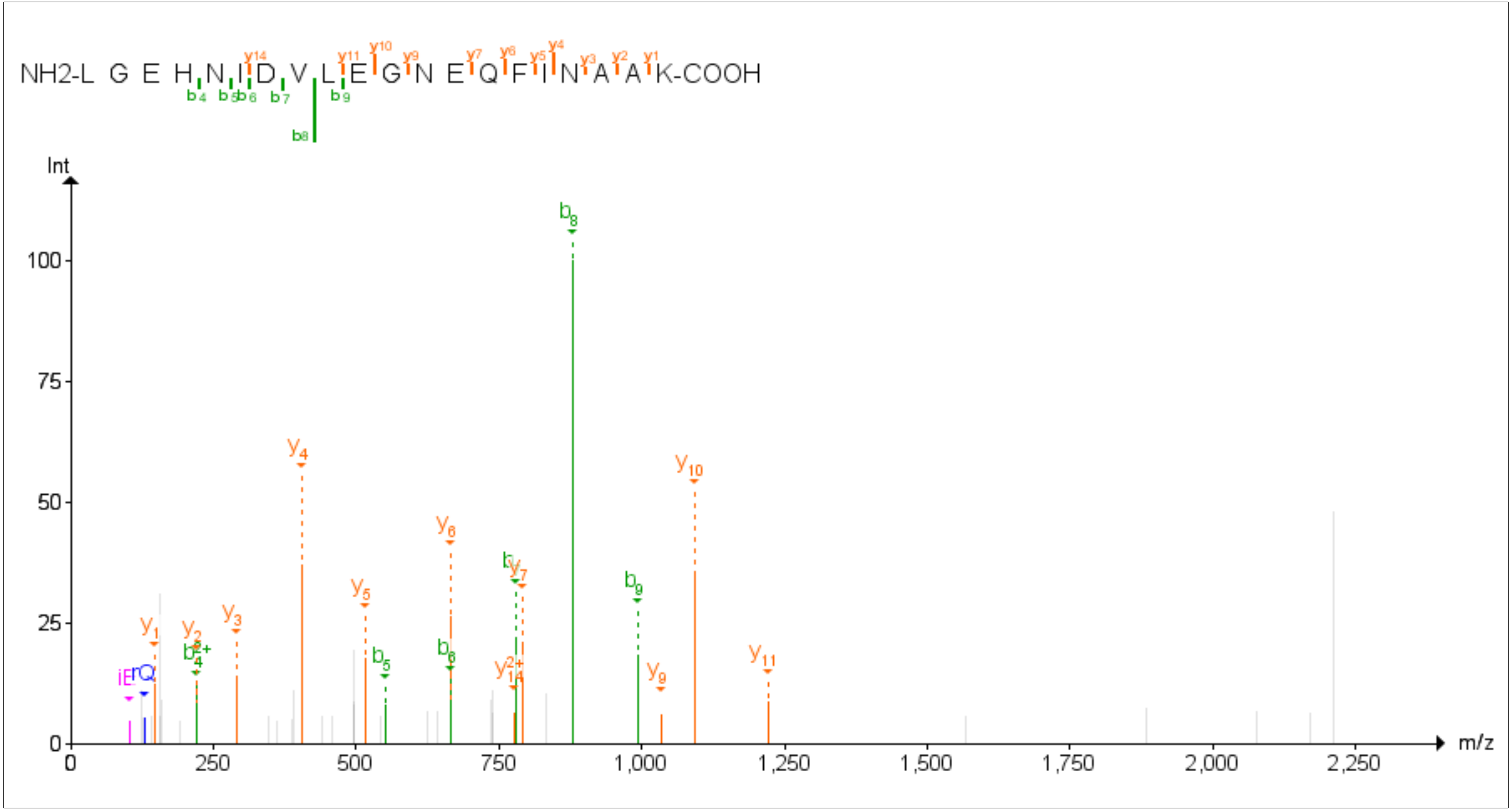

NH2-L G E H N I D V L E G N E Q F I N A A K-COOH
Int
m/z
0
25
50
75
100
250
500
750
1,000
1,250
1,500
1,750
2,000
2,250

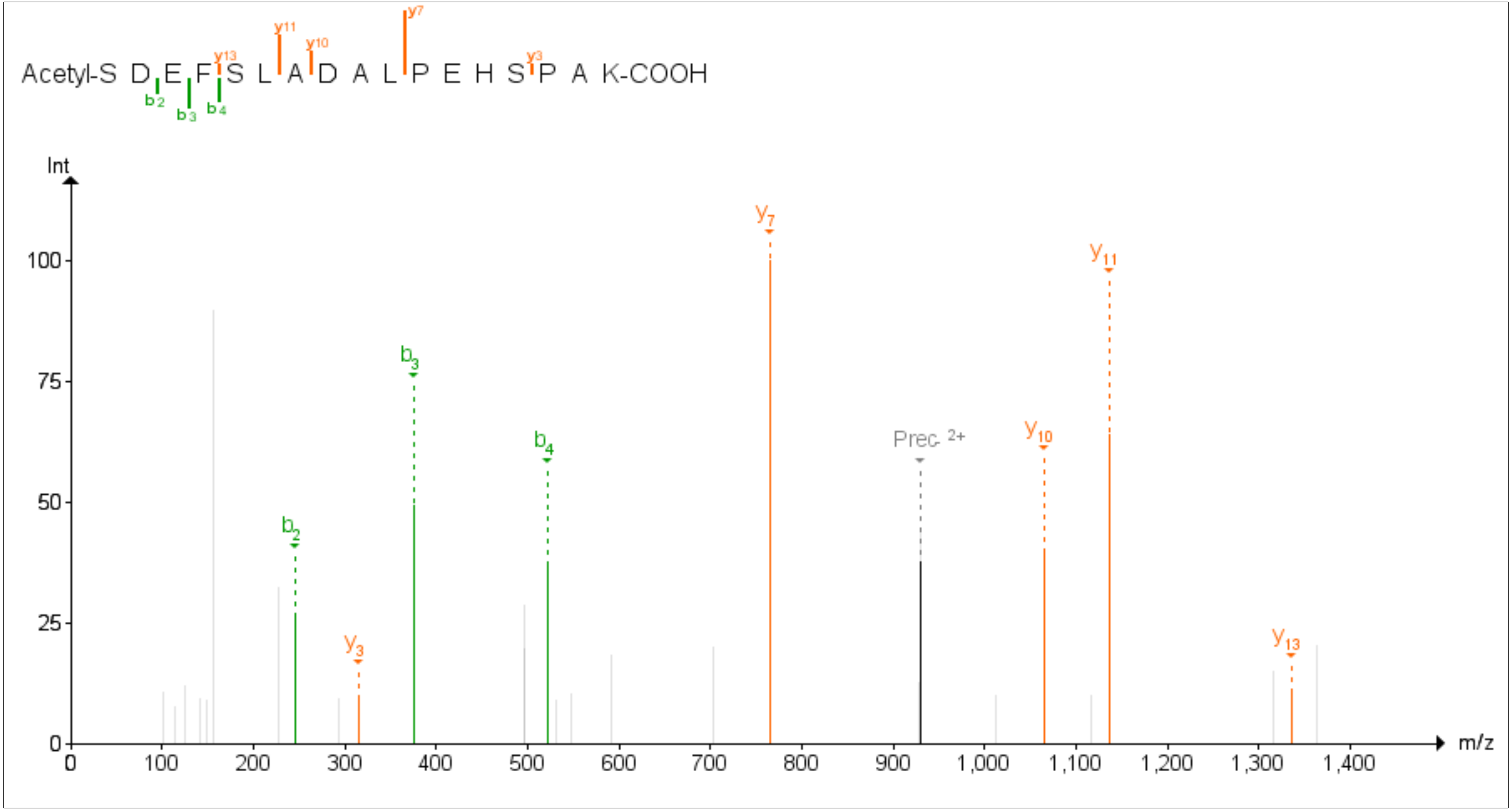

Acetyl-S D E F S L A D A L P E H S P A K-COOH
y13 y11 y10 y7 y3
b2 b3 b4
Int
100
75
50
25
0
0 100 200 300 400 500 600 700 800 900 1,000 1,100 1,200 1,300 1,400
m/z
b2 y3 b3 b4 y7 Prec 2+ y10 y11 y13

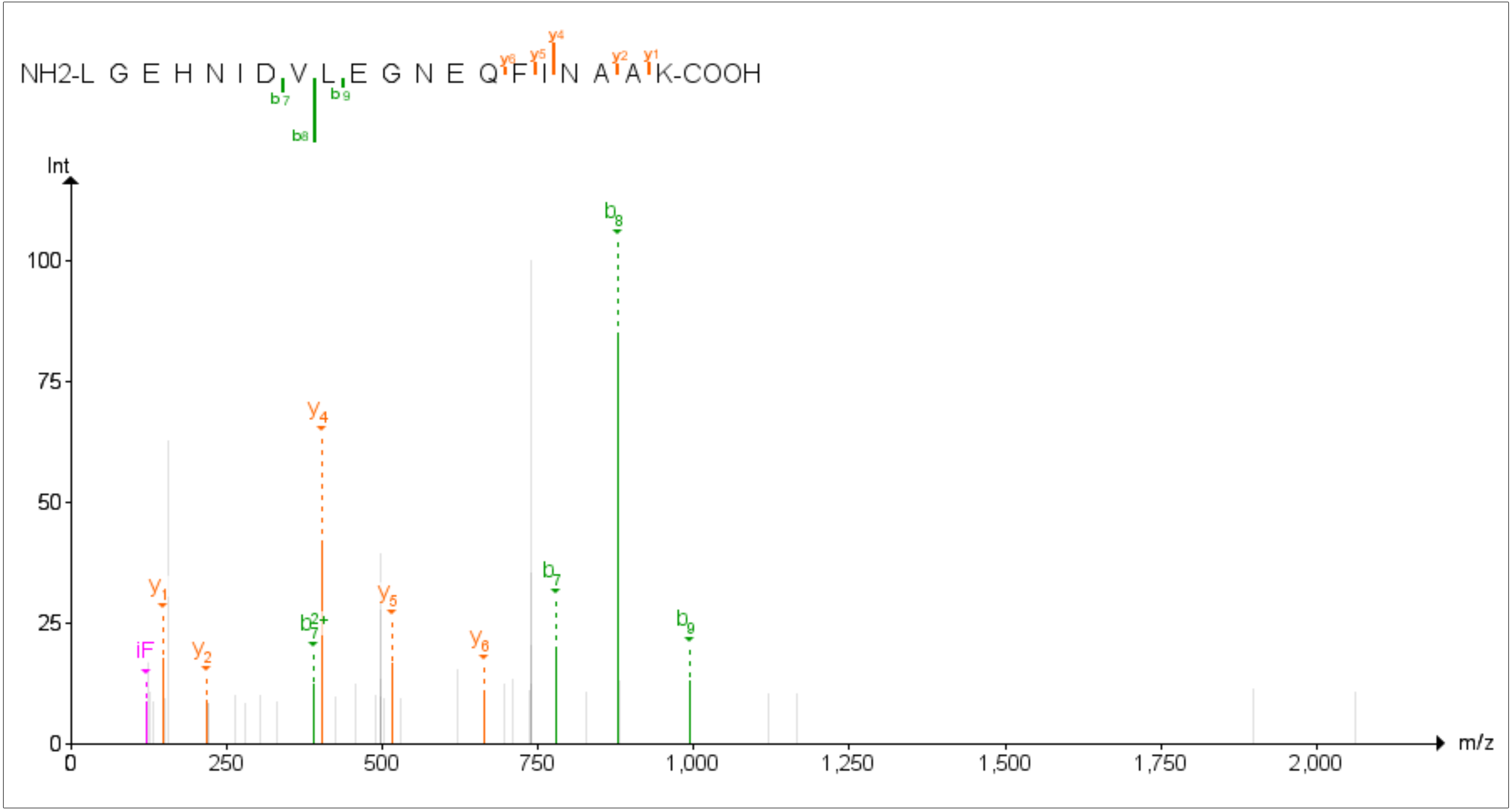

NH2-L G E H N I D V L E G N E Q F I N A A K-COOH
b7
b8
b9
y6
y5
y4
y2
y1
Int
100
75
50
25
0
b8
y4
y1
iF
y2
b7 2+
y5
y6
b7
b9
0
250
500
750
1,000
1,250
1,500
1,750
2,000
m/z

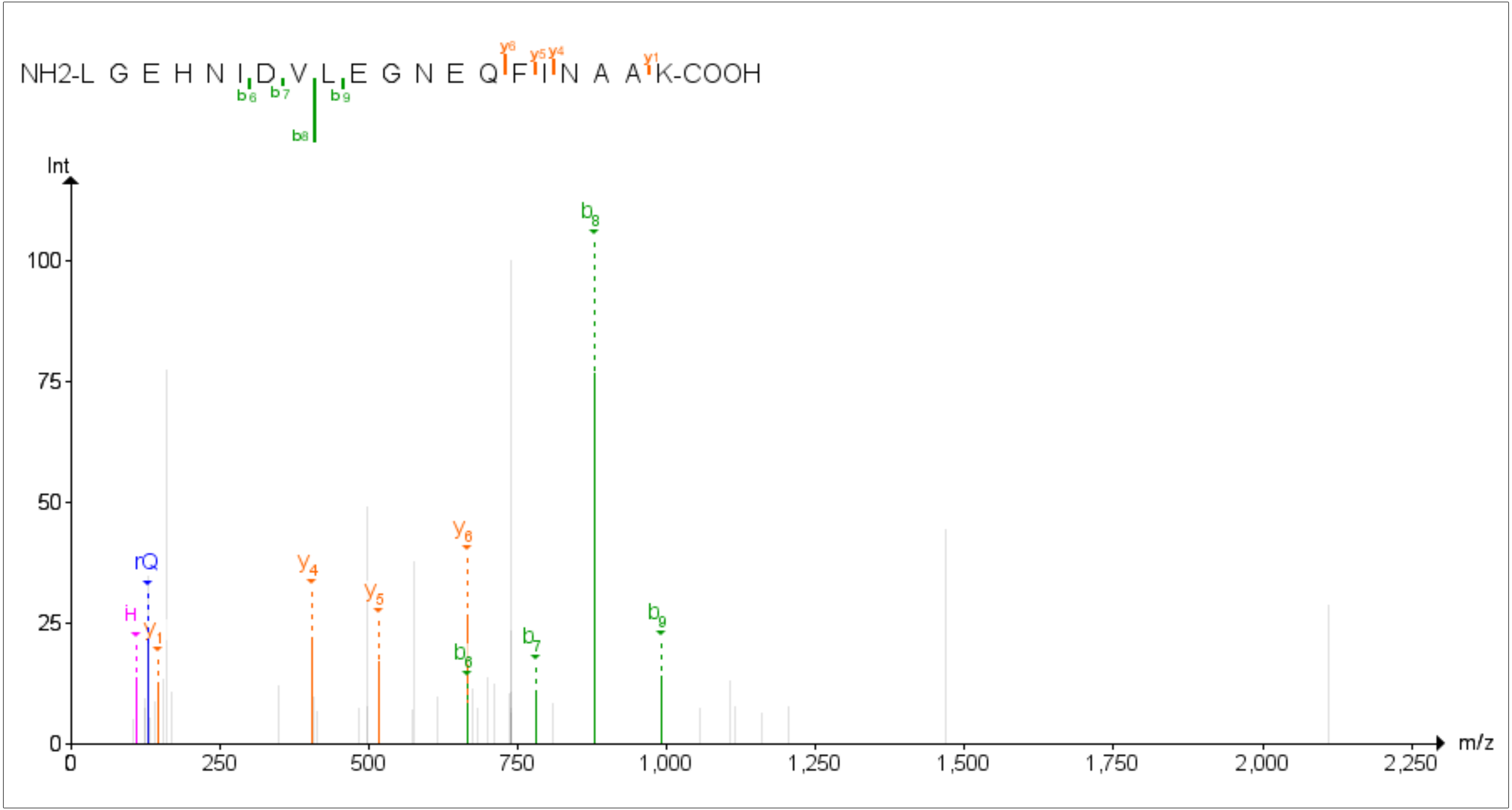

NH2-L G E H N I D V L E G N E Q F I N A A K-COOH
y6 y5 y4 y1
b6 b7 b9 b8
Int
100
75
50
25
0
m/z
0
250
500
750
1,000
1,250
1,500
1,750
2,000
2,250
b8
y6
rQ
y4
y5
H
y1
b6
b7
b9

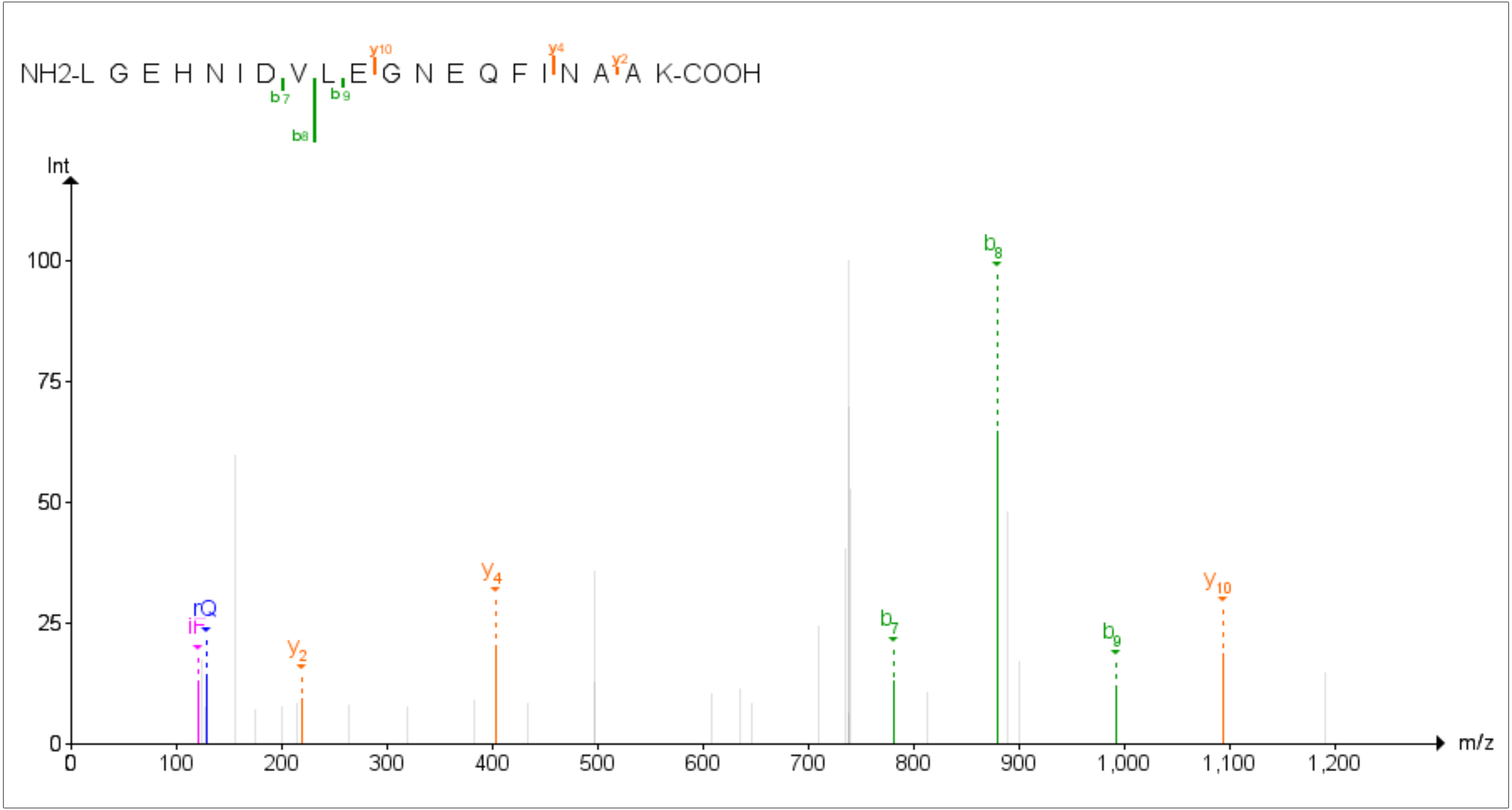
NH2-L G E H N I D V L E G N E Q F I N A A K-COOH
b7
b8
b9
y10
y4
y2
Int
100
75
50
25
0
m/z
0
100
200
300
400
500
600
700
800
900
1,000
1,100
1,200
rQ
iF
y2
y4
b7
b8
b9
y10

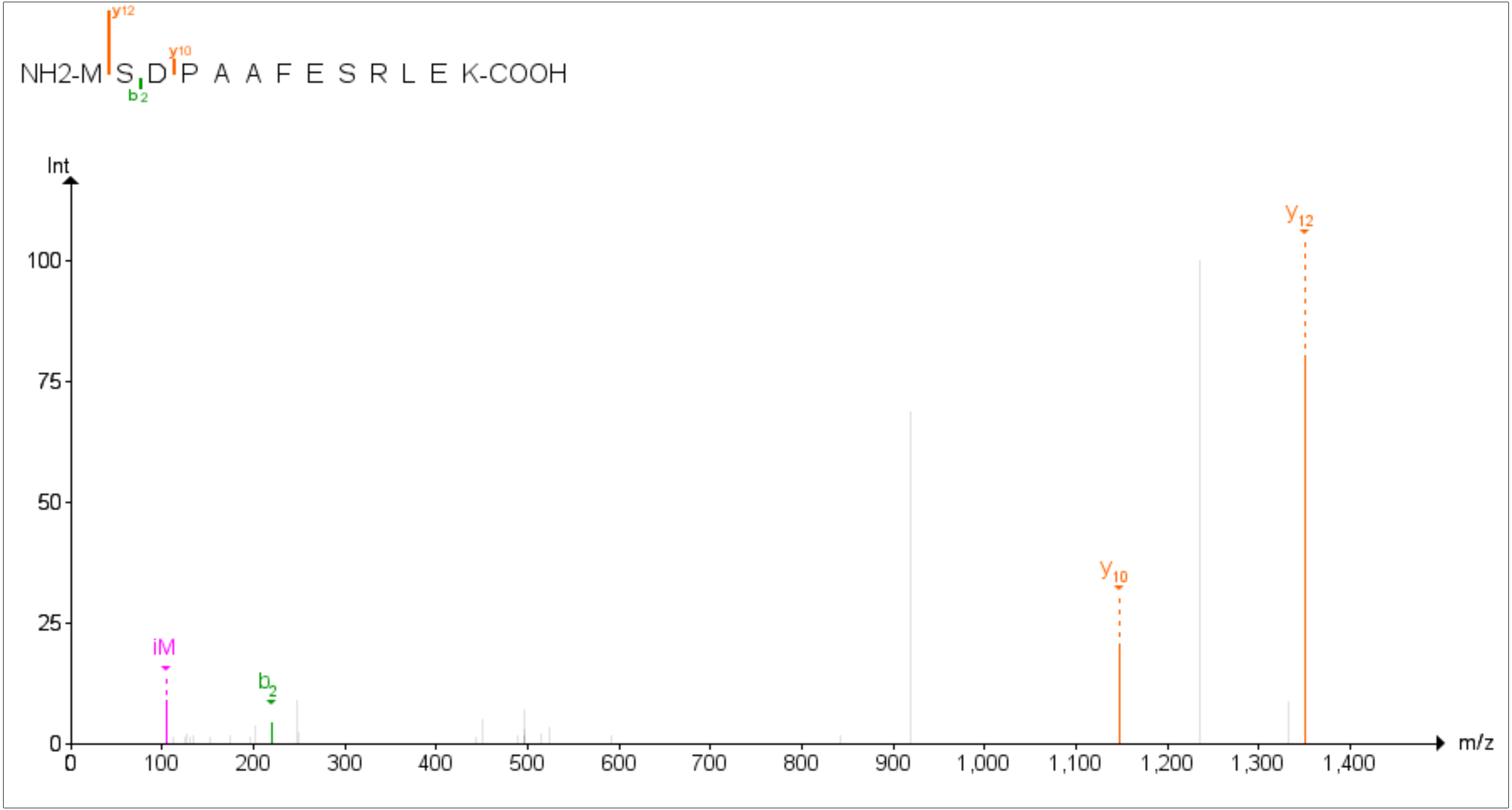
NH2-M S D P A A F E S R L E K-COOH
y12
y10
b2
Int
m/z
100
75
50
25
0
100
200
300
400
500
600
700
800
900
1,000
1,100
1,200
1,300
1,400
iM
b2
y10
y12

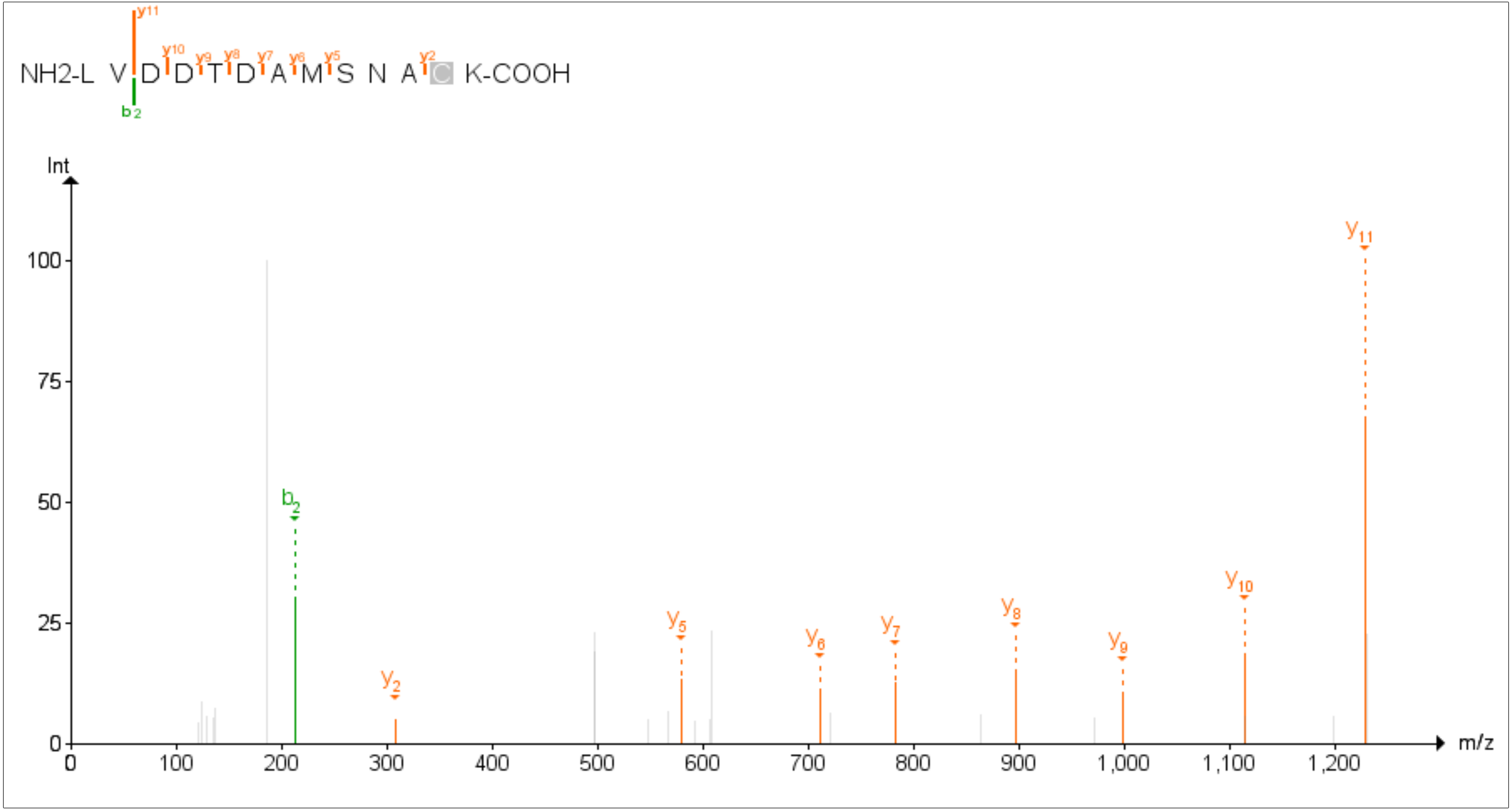

NH2-L V D D T D A M S N A C K-COOH
y11
y10
y9
y8
y7
y6
y5
y2
b2
Int
100
75
50
25
0
m/z
0
100
200
300
400
500
600
700
800
900
1,000
1,100
1,200

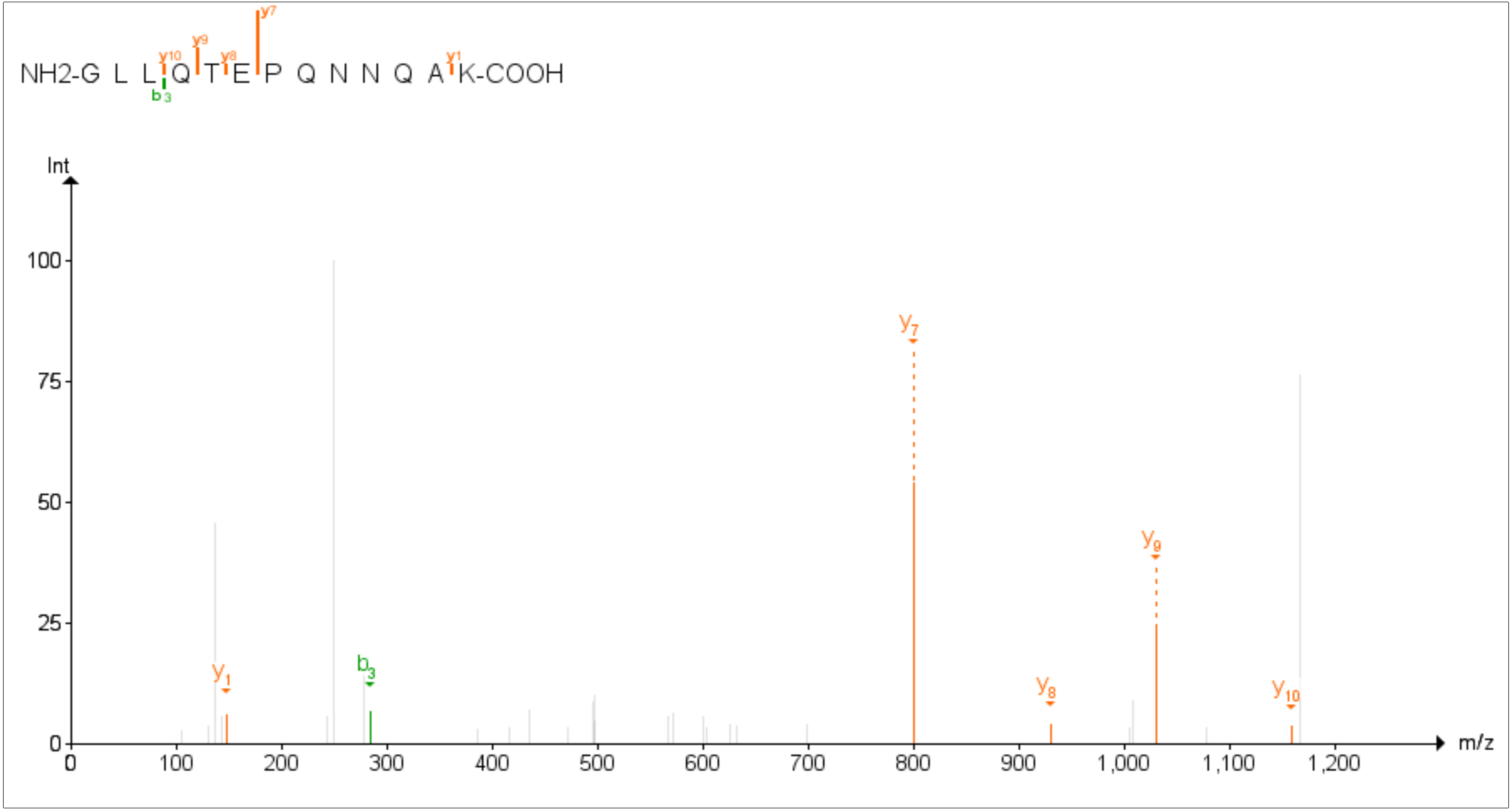

NH2-G L L Q T E P Q N N Q A K-COOH
y10
y9
y8
y7
y1
b3
Int
100
75
50
25
0
y1
b3
y7
y8
y9
y10
0
100
200
300
400
500
600
700
800
900
1,000
1,100
1,200
m/z

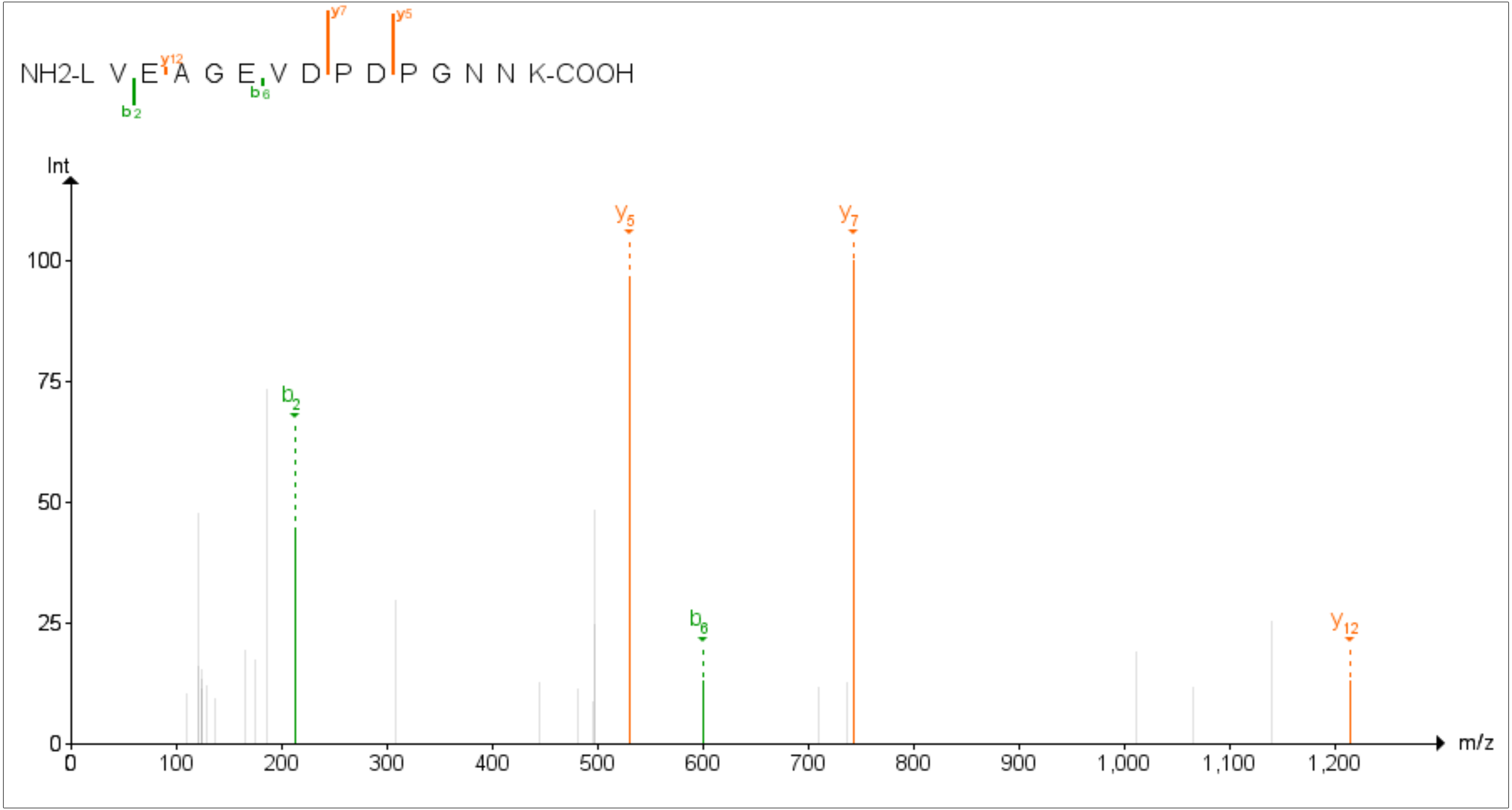
NH2-L V E A G E V D P D P G N N K-COOH
y7
y5
y12
b2
b6
Int
100
75
50
25
0
y5
y7
b2
b6
y12
0
100
200
300
400
500
600
700
800
900
1,000
1,100
1,200
m/z

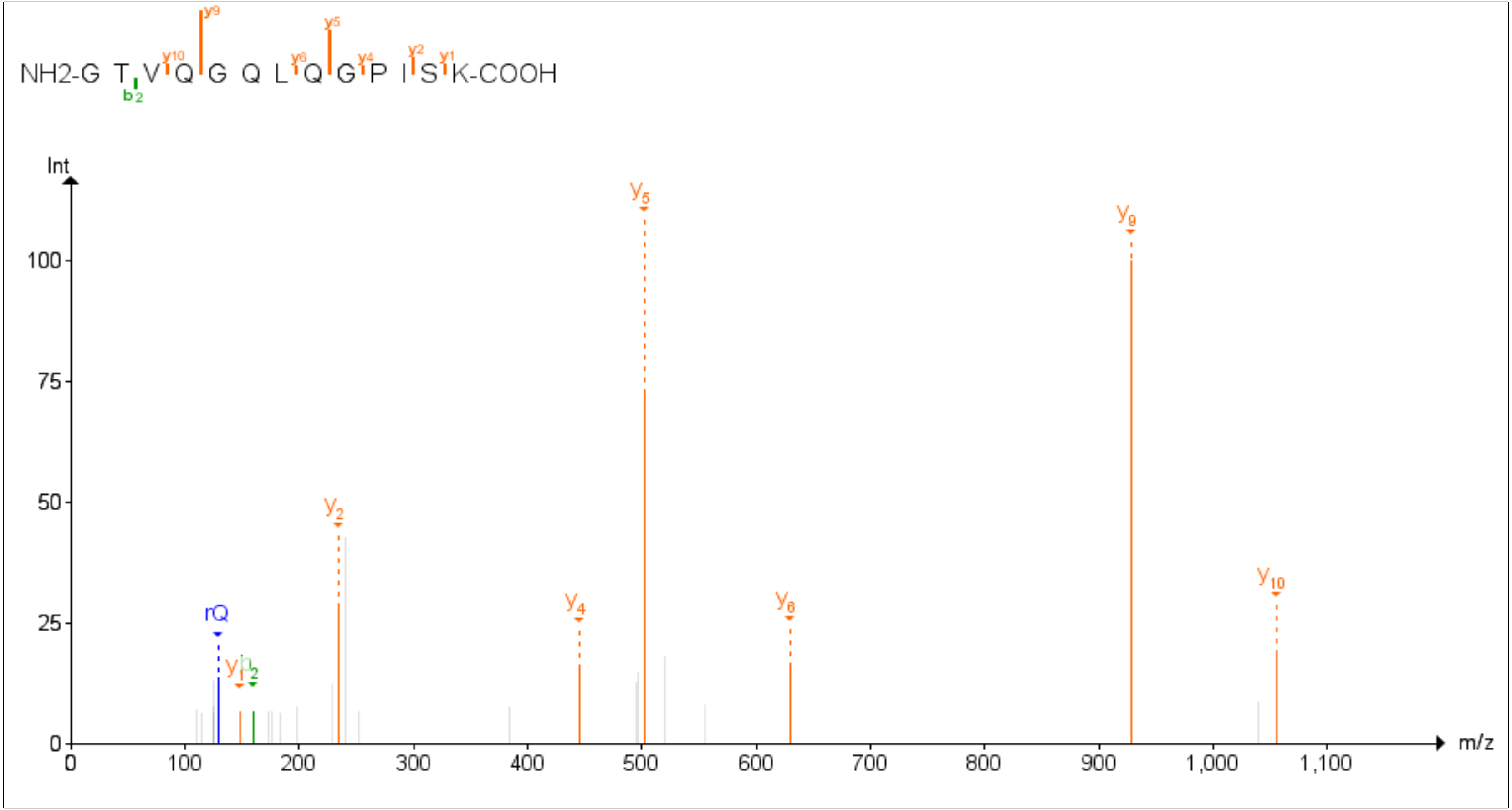

NH2-G T V Q G Q L Q G P I S K-COOH
y9
y10
y5
y6
y4
y2
y1
b2
Int
100
75
50
25
0
rQ
y1
b2
y2
y4
y5
y6
y9
y10
0
100
200
300
400
500
600
700
800
900
1,000
1,100
m/z

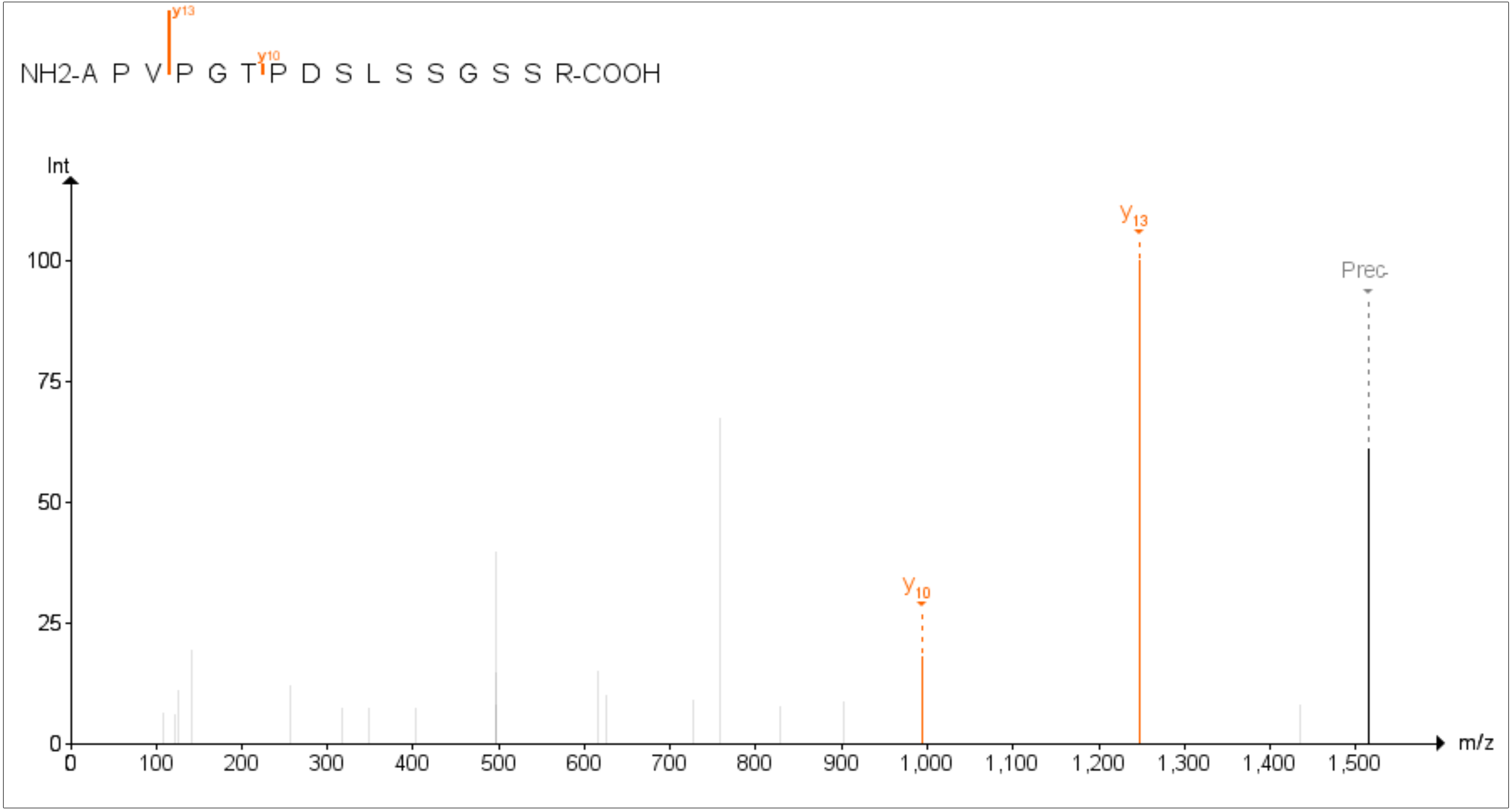

NH2-A P V P G T P D S L S S G S S R-COOH
y13
y10
Int
100
75
50
25
0
m/z
0
100
200
300
400
500
600
700
800
900
1,000
1,100
1,200
1,300
1,400
1,500
Prec

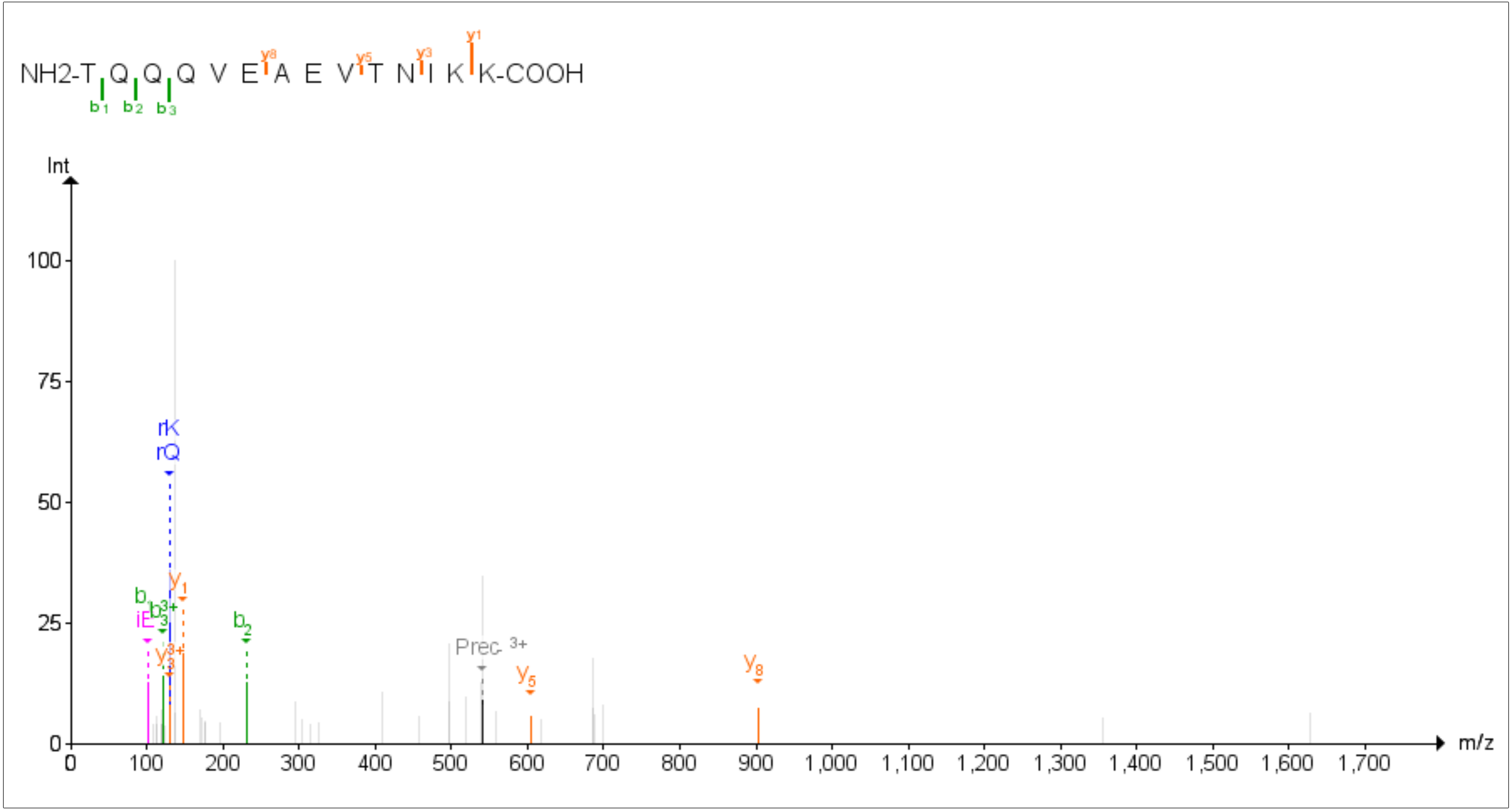

NH2-T Q Q Q V E A E V T N I K K-COOH
b1 b2 b3
y8 y5 y3 y1
Int
100
75
50
25
0
rK
rQ
y1
b1
b3 3+
iE
y3 3+
b2
Prec 3+
y5
y8
0
100
200
300
400
500
600
700
800
900
1,000
1,100
1,200
1,300
1,400
1,500
1,600
1,700
m/z

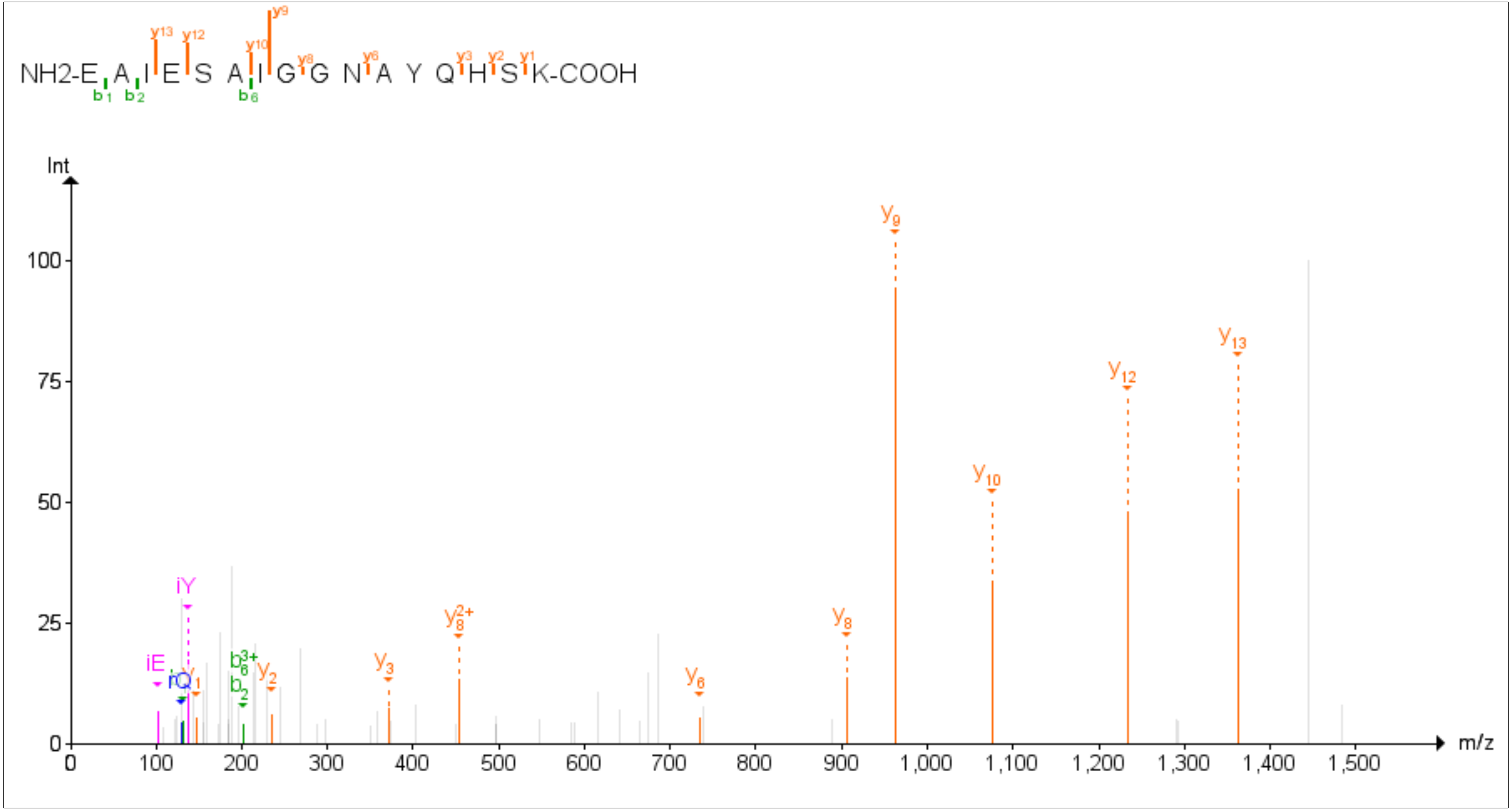

NH2-E A I E S A I G G N A Y Q H S K-COOH
y13 y12 y10 y9 y8 y6 y3 y2 y1
b1 b2 b6
Int
100
75
50
25
0
y9
y13
y12
y10
iY
y8
y8 2+
iE
rQ
y1
b6 3+
b2
y2
y3
y6
0 100 200 300 400 500 600 700 800 900 1,000 1,100 1,200 1,300 1,400 1,500
m/z

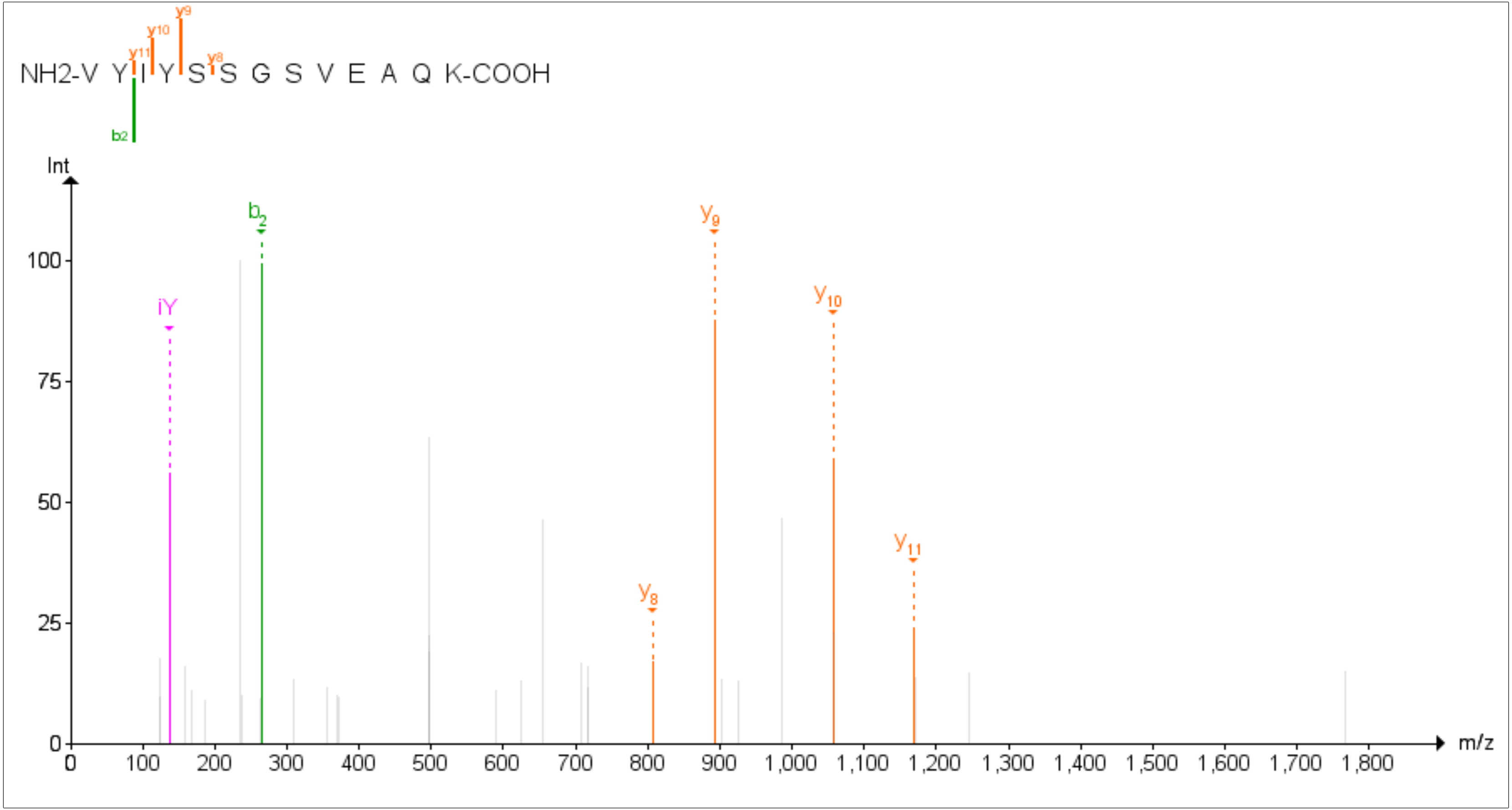

NH2-V Y I Y S S G S V E A Q K-COOH
y11
y10
y9
y8
b2
Int
iY
b_2
y_9
y_{10}
y_8
y_{11}
100
75
50
25
0
0 100 200 300 400 500 600 700 800 900 1,000 1,100 1,200 1,300 1,400 1,500 1,600 1,700 1,800
m/z

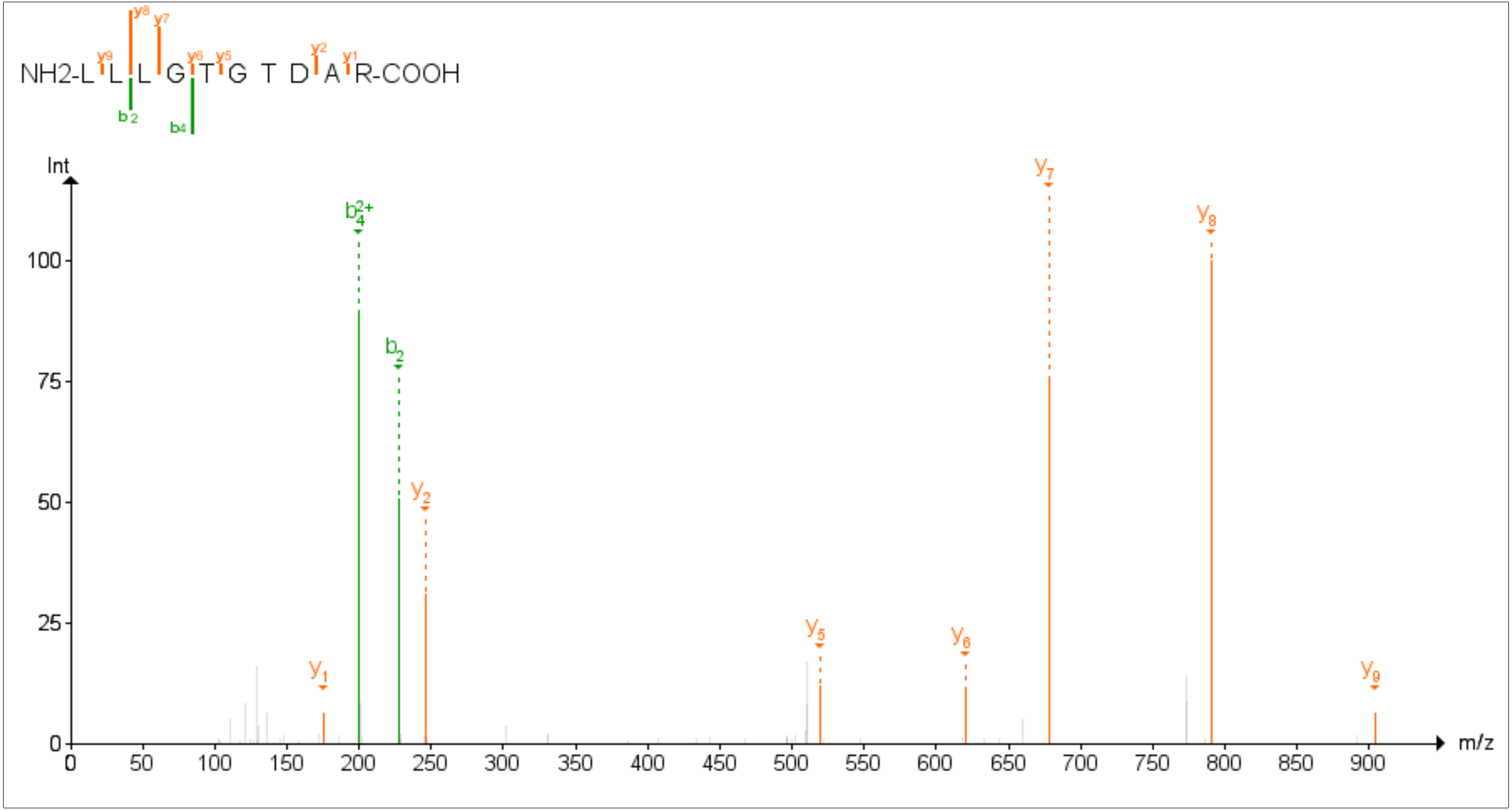

NH2-L L L G T G T D A R-COOH
y9 y8 y7 y6 y5 y2 y1
b2 b4
Int
m/z
0
25
50
75
100
0 50 100 150 200 250 300 350 400 450 500 550 600 650 700 750 800 850 900
b_4^{2+}
b_2
y_1
y_2
y_5
y_6
y_7
y_8
y_9

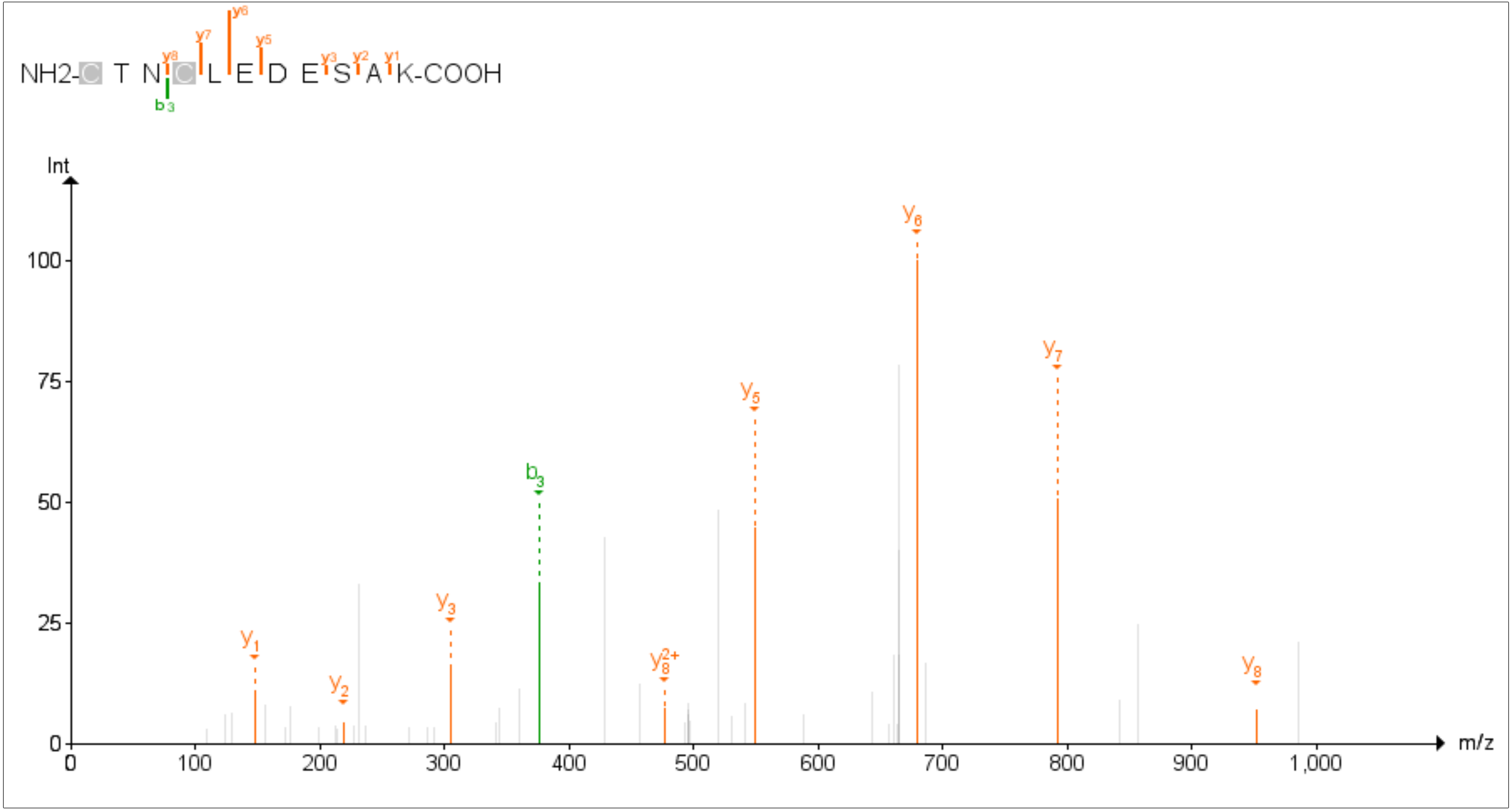

NH2-C T N C L E D E S A K-COOH
y8
y7
y6
y5
y3
y2
y1
b3
Int
100
75
50
25
0
m/z
0
100
200
300
400
500
600
700
800
900
1,000
y6
y7
y5
b3
y3
y1
y2
y8 2+
y8

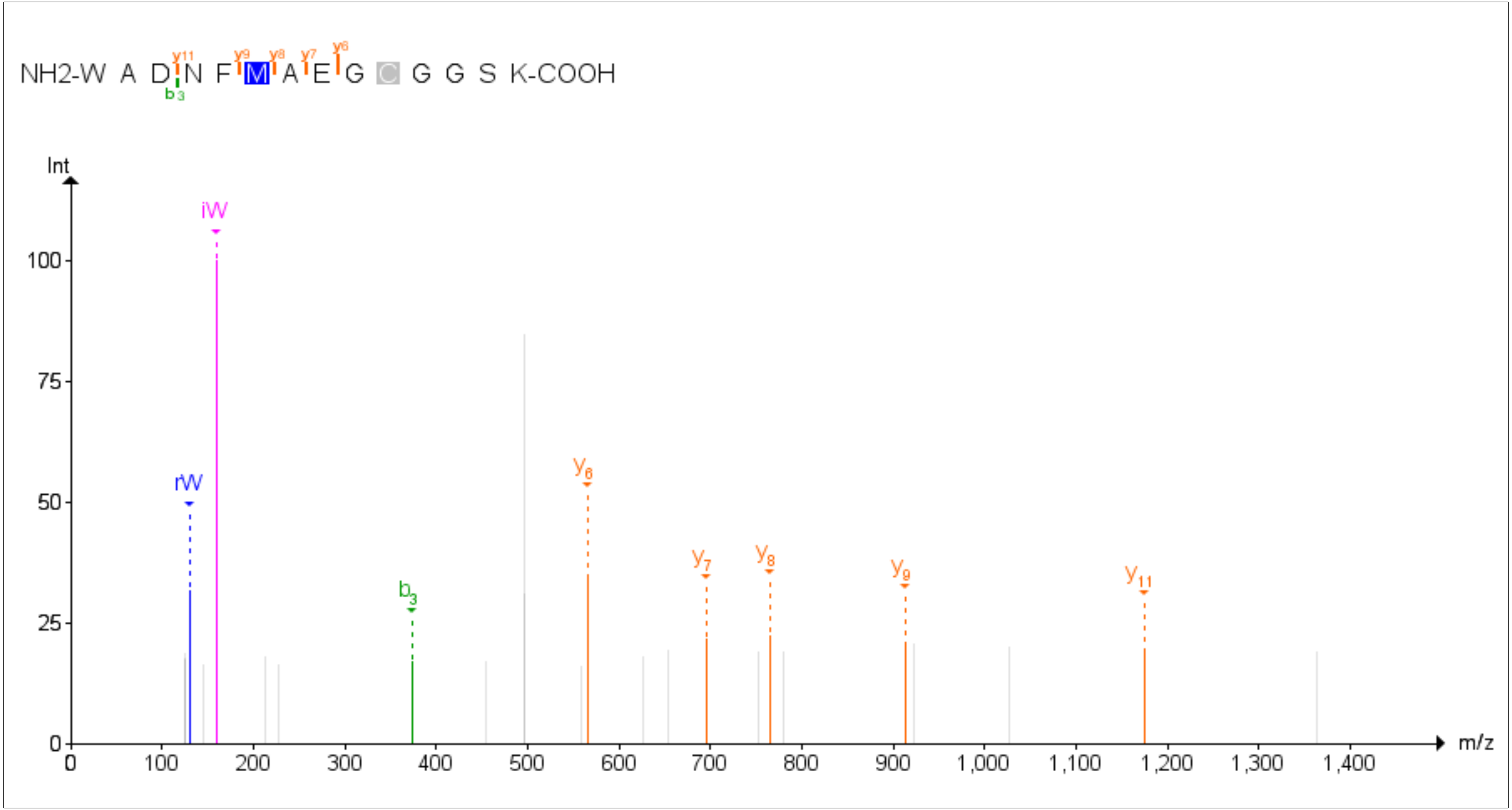

NH2-W A D N F M A E G C G G S K-COOH
y11 y9 y8 y7 y6
b3
Int
100
75
50
25
0
iW
rW
b3
y6
y7
y8
y9
y11
0 100 200 300 400 500 600 700 800 900 1,000 1,100 1,200 1,300 1,400
m/z

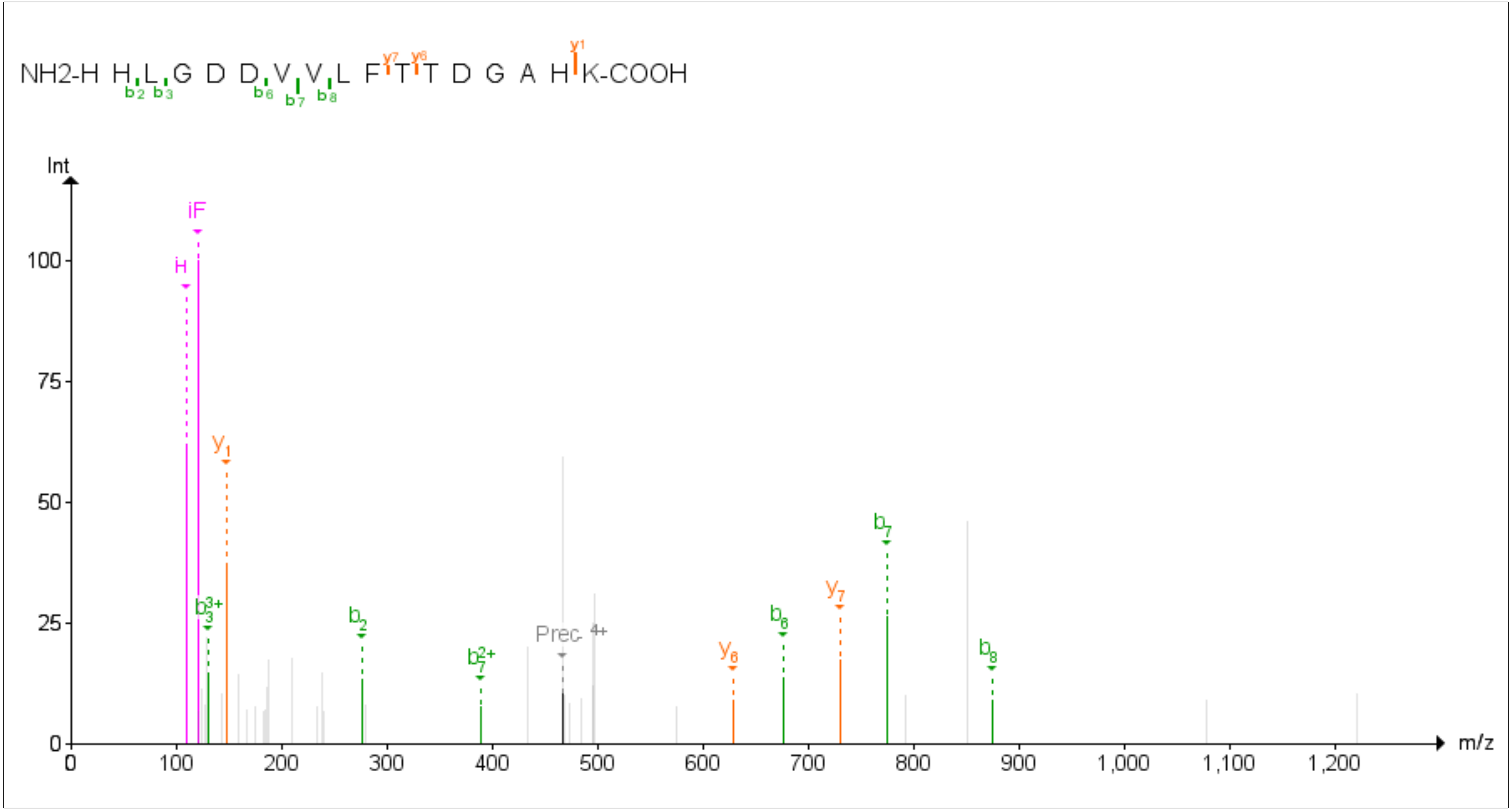

NH2-H H L G D D V V L F T T D G A H K-COOH
b2 b3 b6 b7 b8
y7 y6 y1
Int
iF
H
y1
b3 3+
b2
b7 2+
Prec 4+
y6
b6
y7
b7
b8
0
25
50
75
100
0
100
200
300
400
500
600
700
800
900
1,000
1,100
1,200
m/z

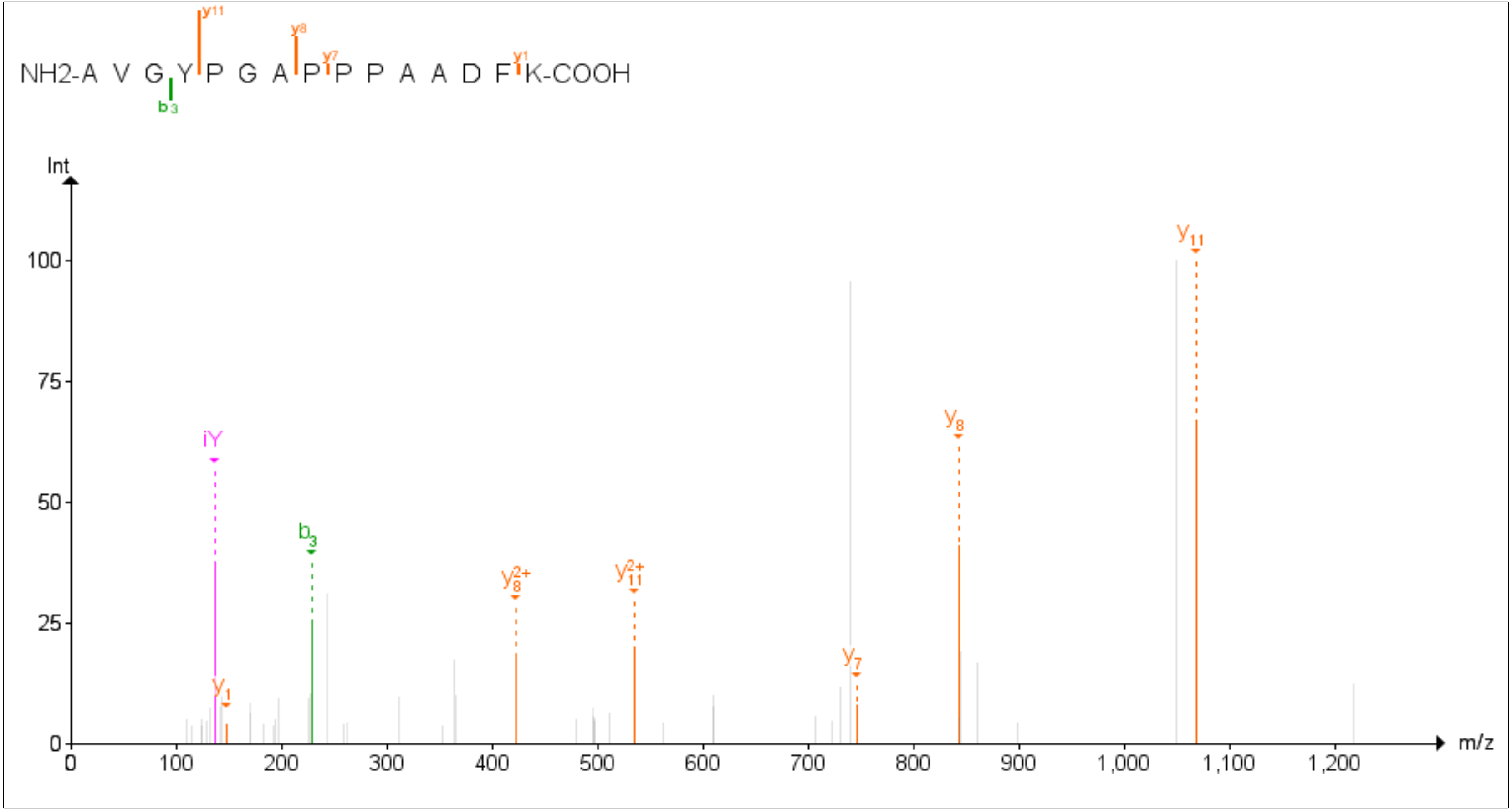

NH2-A V G Y P G A P P P A A D F K-COOH
y11
y8
y7
y1
b3
Int
100
75
50
25
0
iY
b3
y1
y8 2+
y11 2+
y7
y8
y11
0
100
200
300
400
500
600
700
800
900
1,000
1,100
1,200
m/z

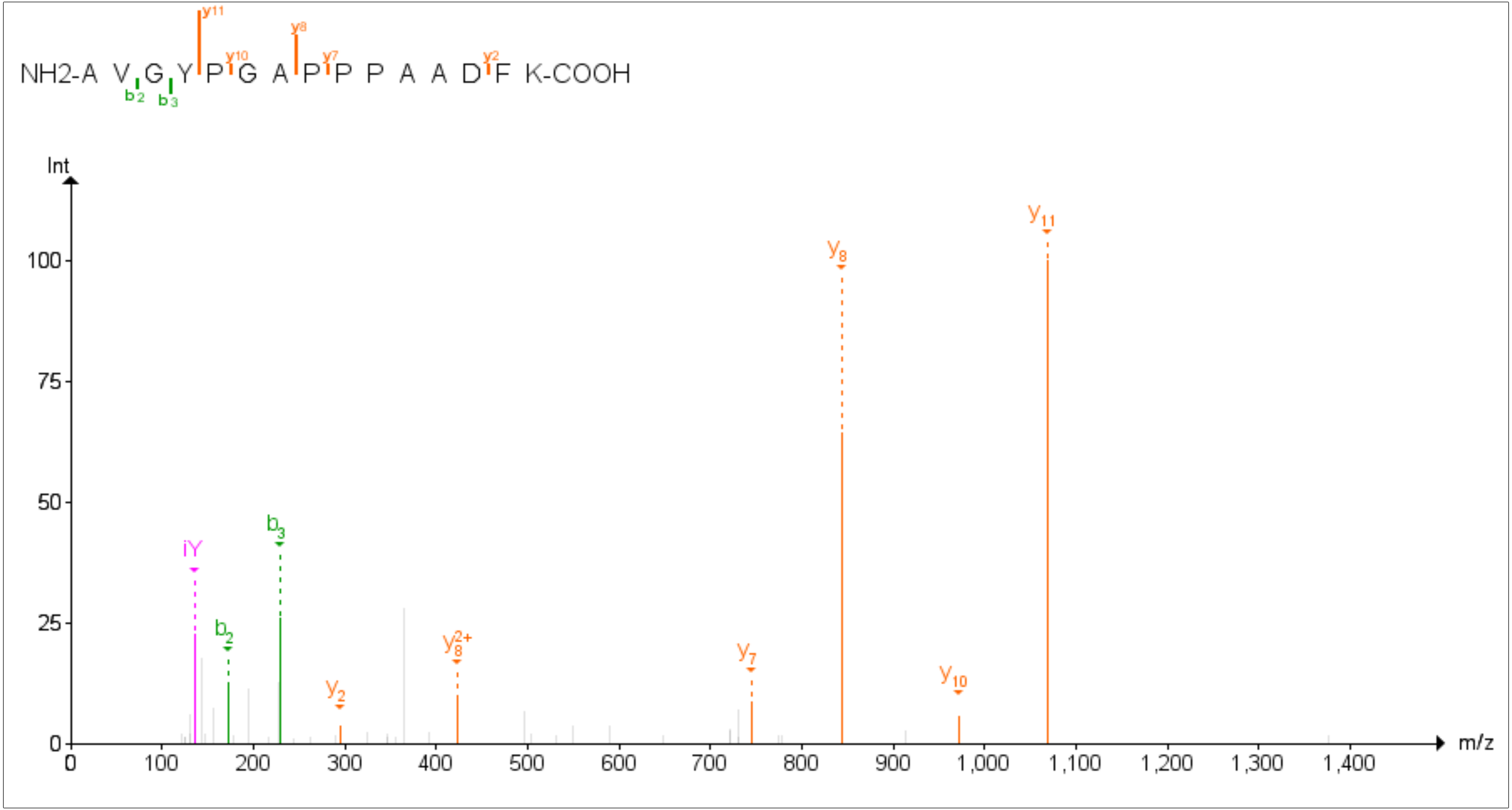
NH2-A V G Y P G A P P P A A D F K-COOH
y11
y10
y8
y7
y2
b2
b3
Int
100
75
50
25
0
iY
b2
b3
y2
y8 2+
y7
y8
y10
y11
0
100
200
300
400
500
600
700
800
900
1,000
1,100
1,200
1,300
1,400
m/z

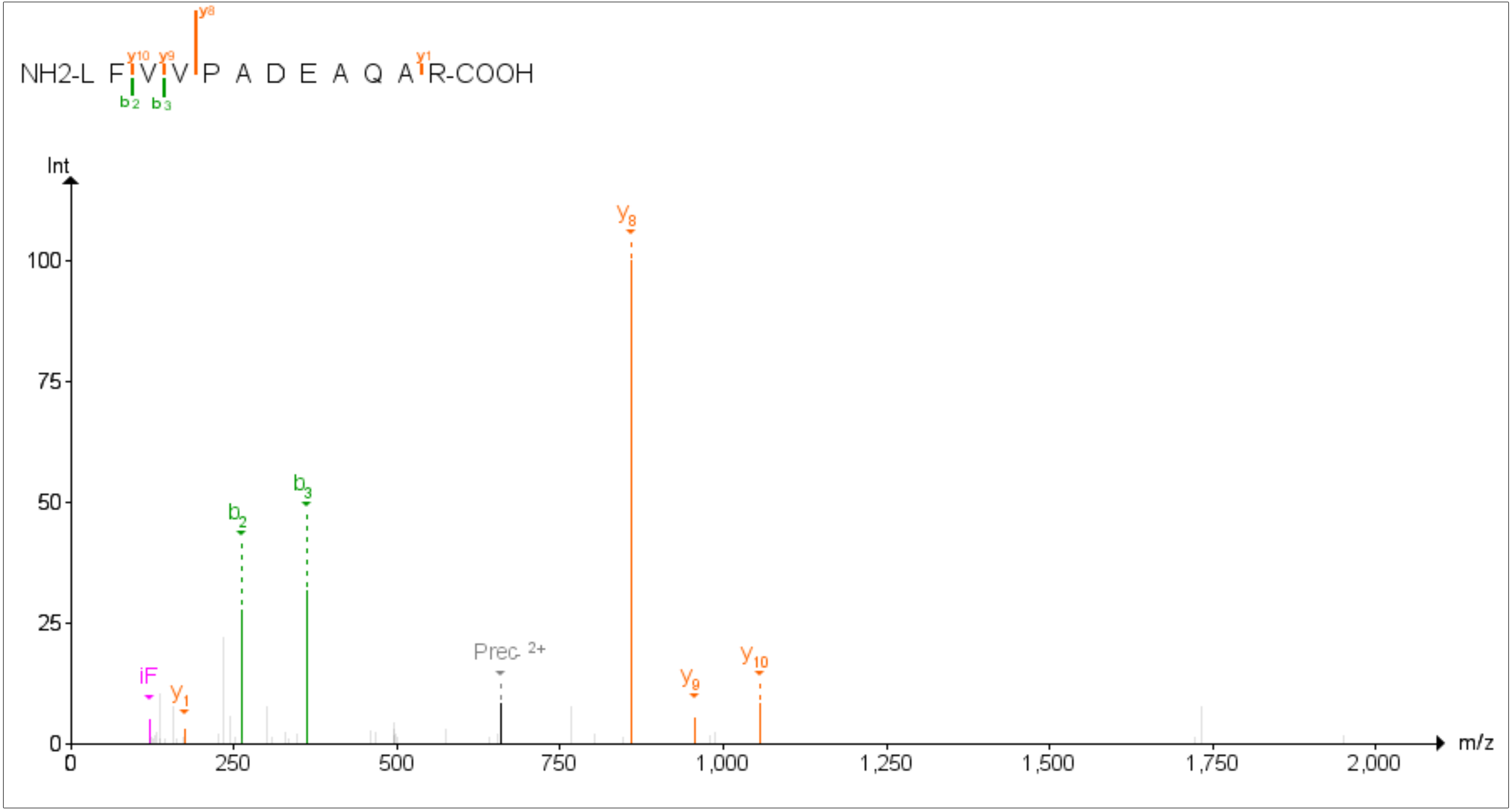

NH2-L F V V P A D E A Q A R-COOH
y8
y10
y9
y1
b2
b3
Int
100
75
50
25
0
y8
b3
b2
Prec 2+
iF
y1
y9
y10
0
250
500
750
1,000
1,250
1,500
1,750
2,000
m/z

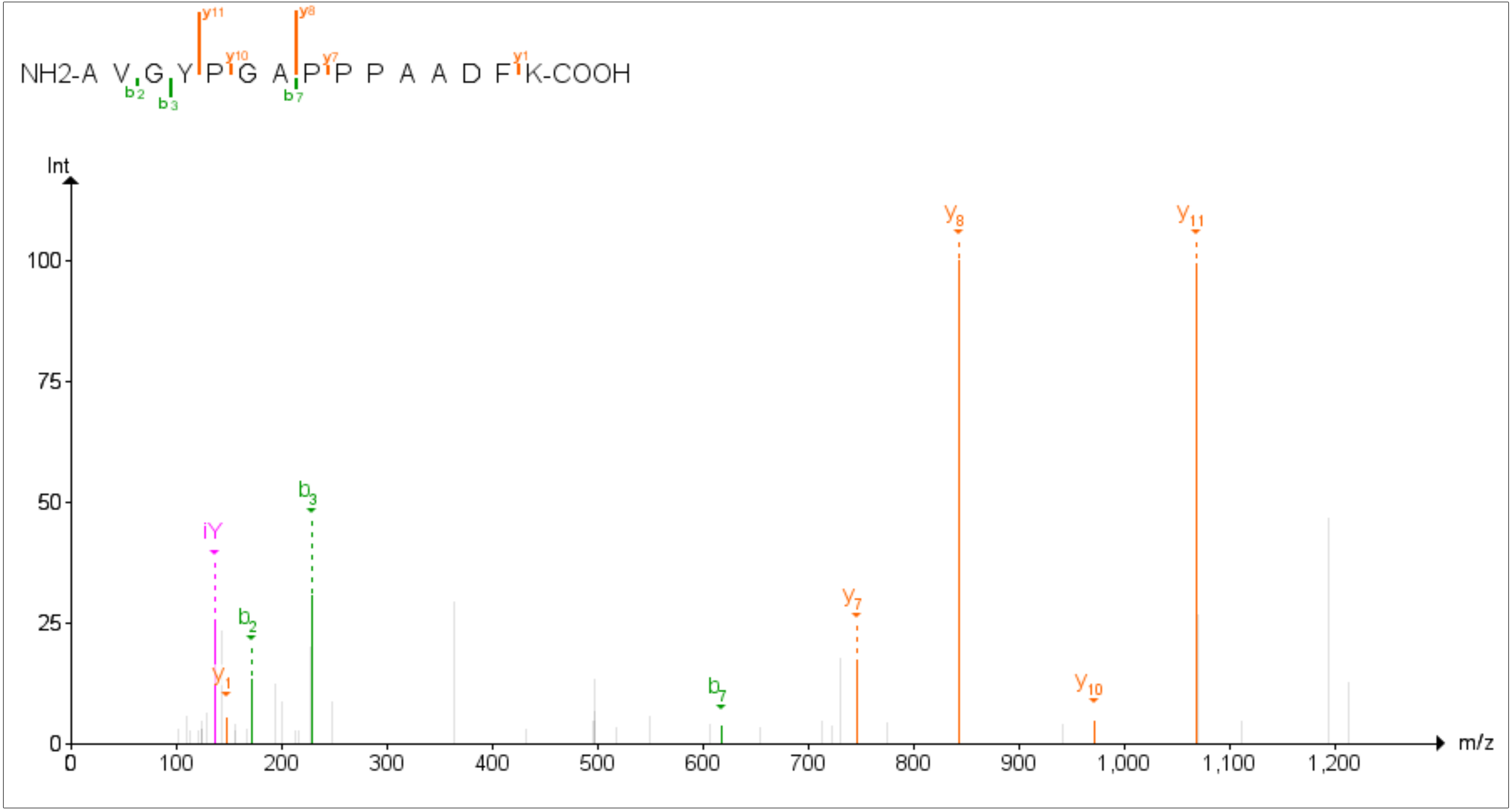
NH2-A V G Y P G A P P P A A D F K-COOH
b2
b3
b7
y11
y10
y8
y7
y1
Int
100
75
50
25
0
iY
y1
b2
b3
b7
y7
y8
y10
y11
0
100
200
300
400
500
600
700
800
900
1,000
1,100
1,200
m/z

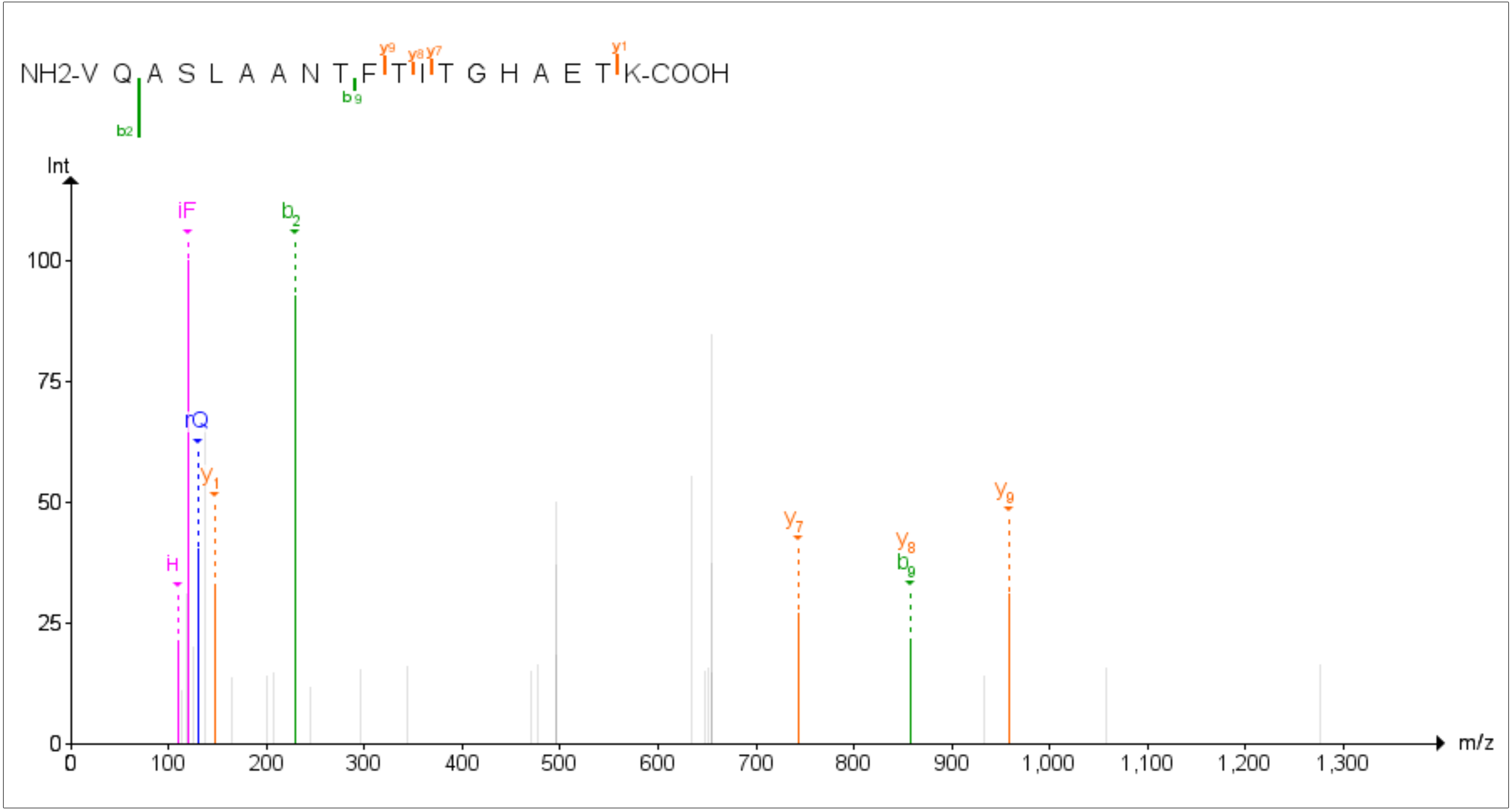

NH2-V Q A S L A A N T F T I T G H A E T K-COOH
b2
b9
y9
y8
y7
y1
Int
m/z
iF
b2
rQ
y1
iH
y7
y8
b9
y9
0
25
50
75
100
0
100
200
300
400
500
600
700
800
900
1,000
1,100
1,200
1,300

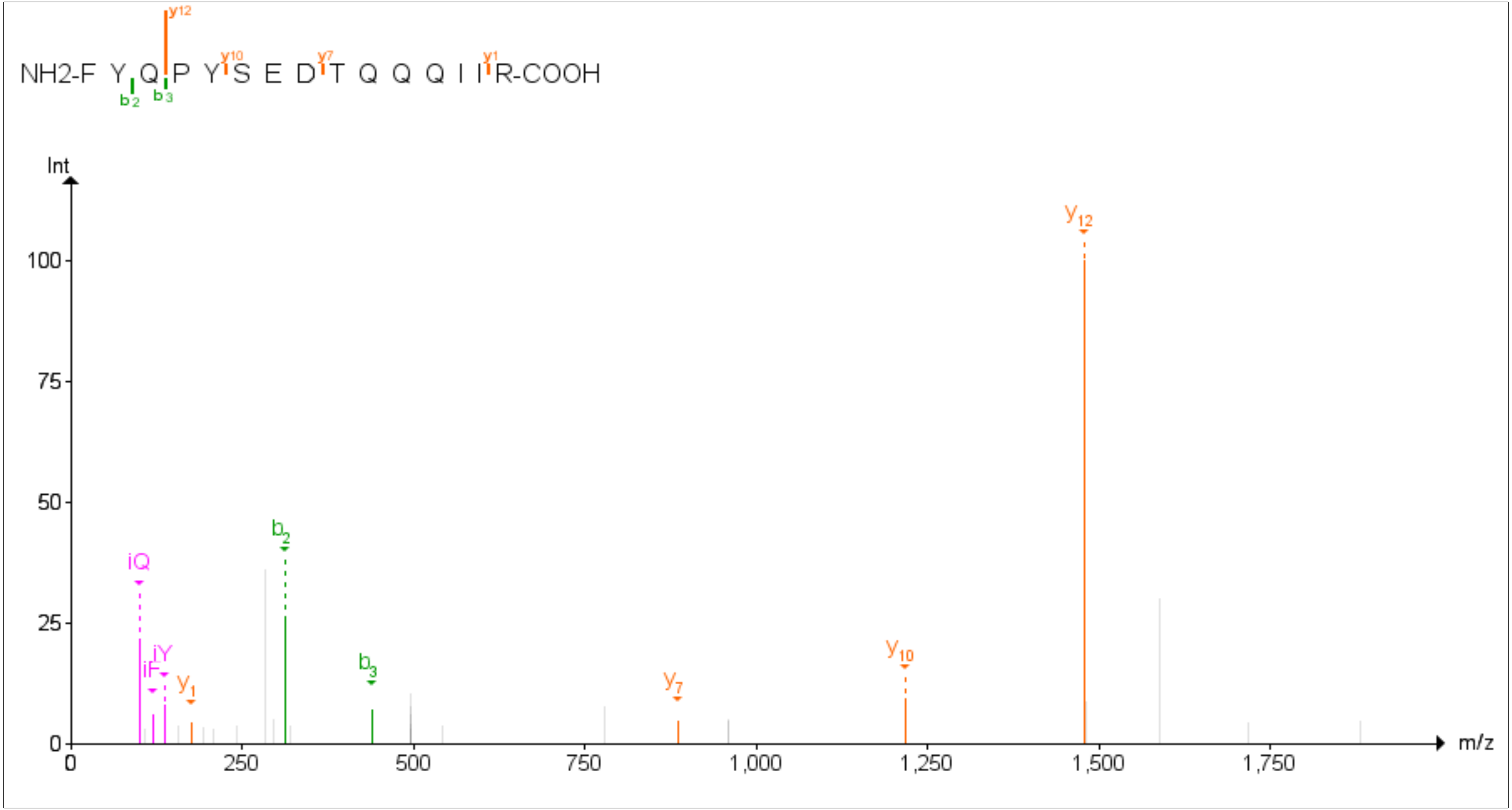

NH2-F Y Q P Y S E D T Q Q Q I I R-COOH
y12
y10
y7
y1
b2
b3
Int
100
75
50
25
0
m/z
0
250
500
750
1,000
1,250
1,500
1,750
iQ
iY
iF
y1
b2
b3
y7
y10
y12

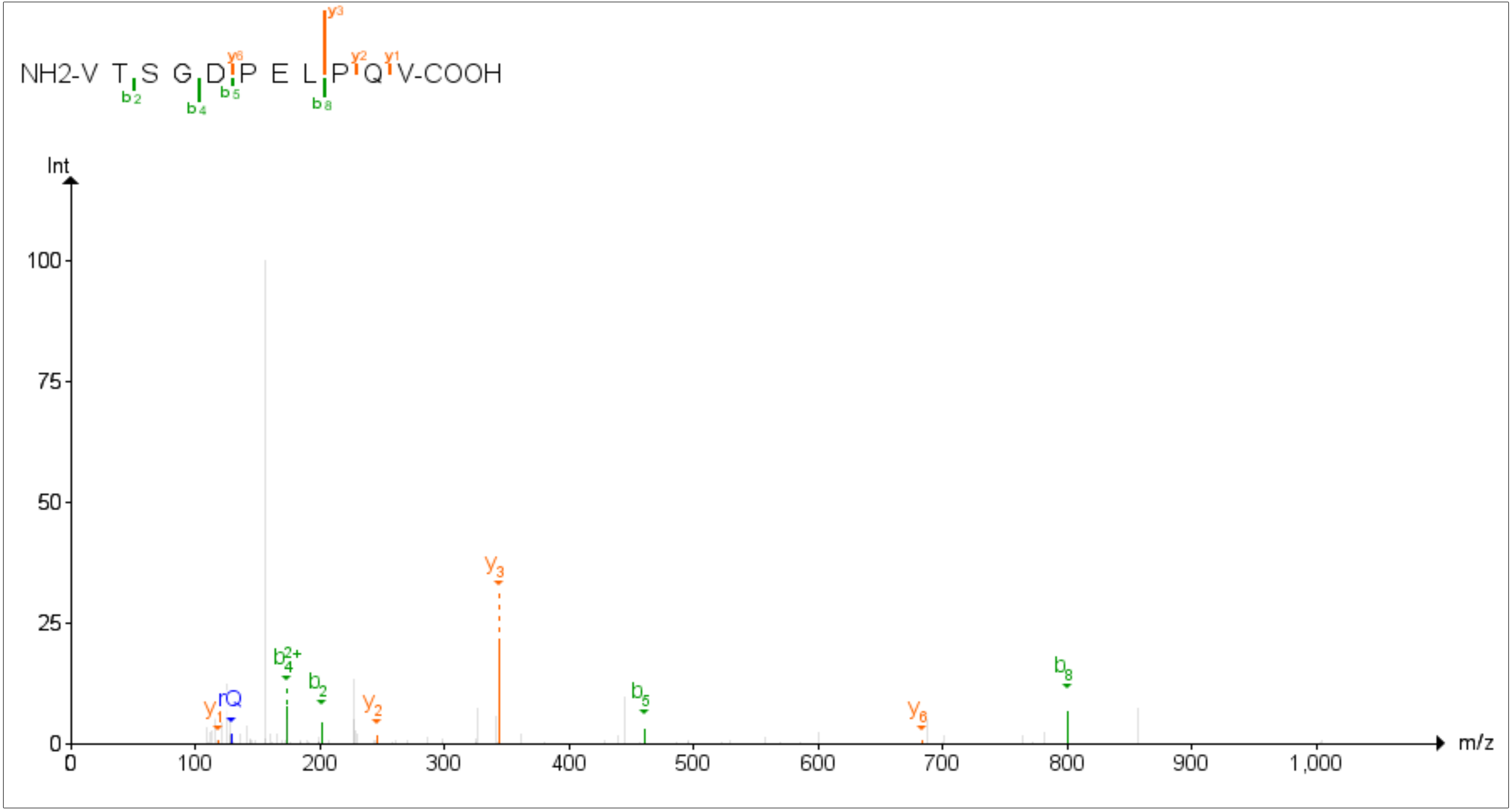
NH2-V T S G D P E L P Q V-COOH
b2
b4
b5
b8
y6
y3
y2
y1
Int
100
75
50
25
0
y1
rQ
b4 2+
b2
y2
y3
b5
y6
b8
0
100
200
300
400
500
600
700
800
900
1,000
m/z

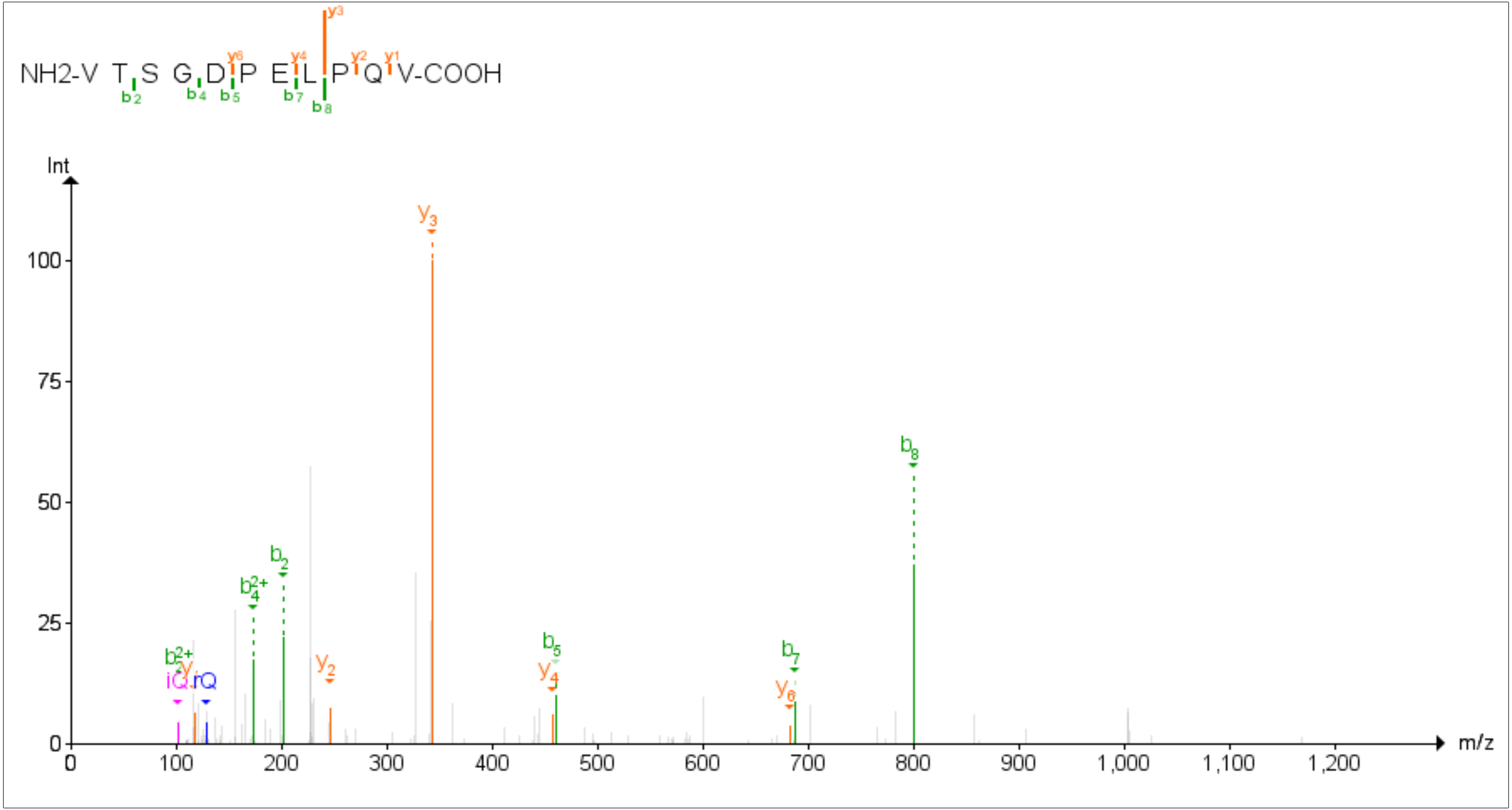

NH2-V T S G D P E L P Q V-COOH
y6 y4 y3 y2 y1
b2 b4 b5 b7 b8
Int
m/z
y3
b8
b2
b4 2+
b2 2+
y1
iQ
rQ
y2
b5
y4
b7
y6
0
25
50
75
100
0
100
200
300
400
500
600
700
800
900
1,000
1,100
1,200

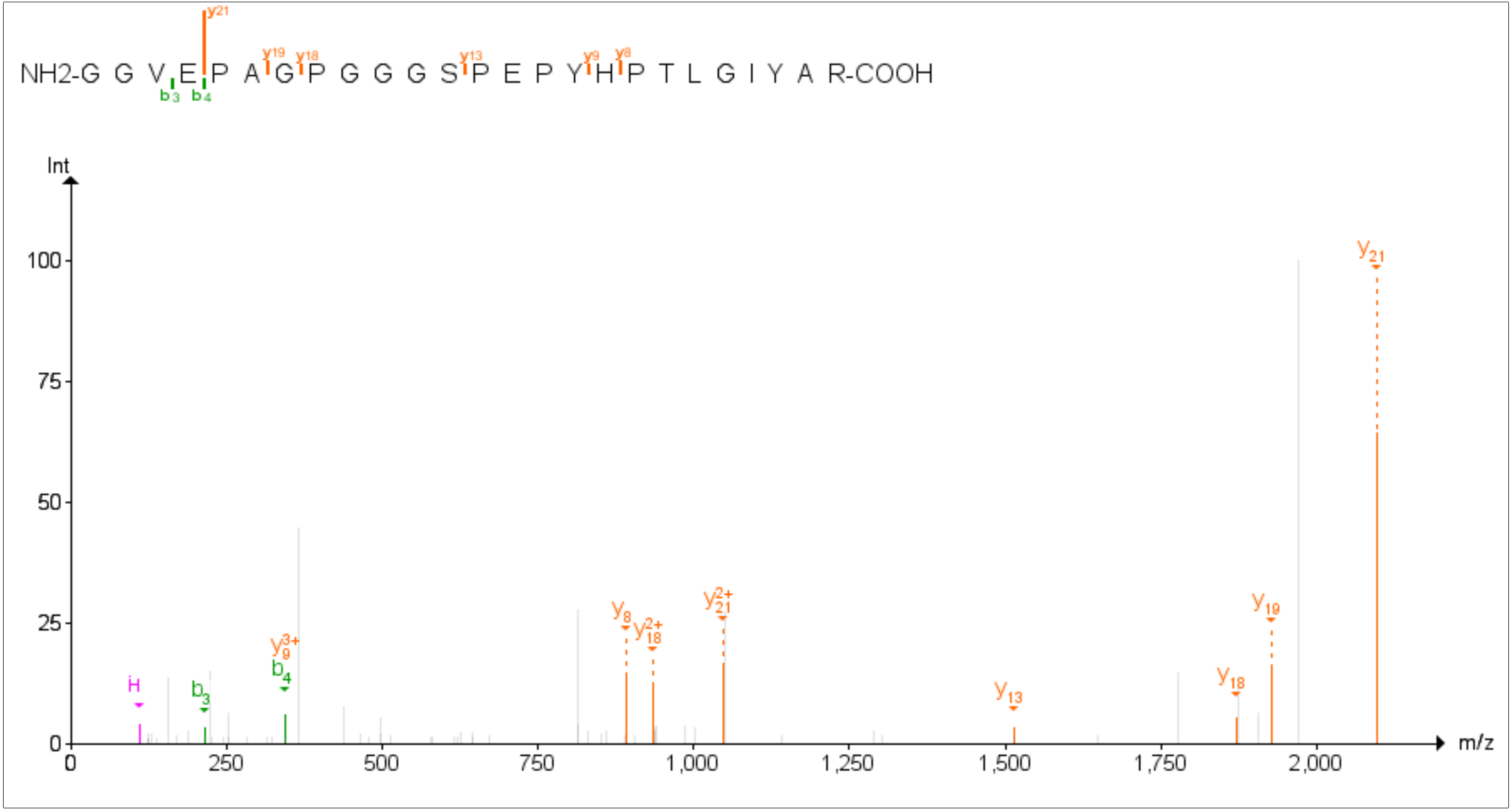
NH2-G G V E P A G P G G G S P E P Y H P T L G I Y A R-COOH
Int
100
75
50
25
0
m/z
0
250
500
750
1,000
1,250
1,500
1,750
2,000

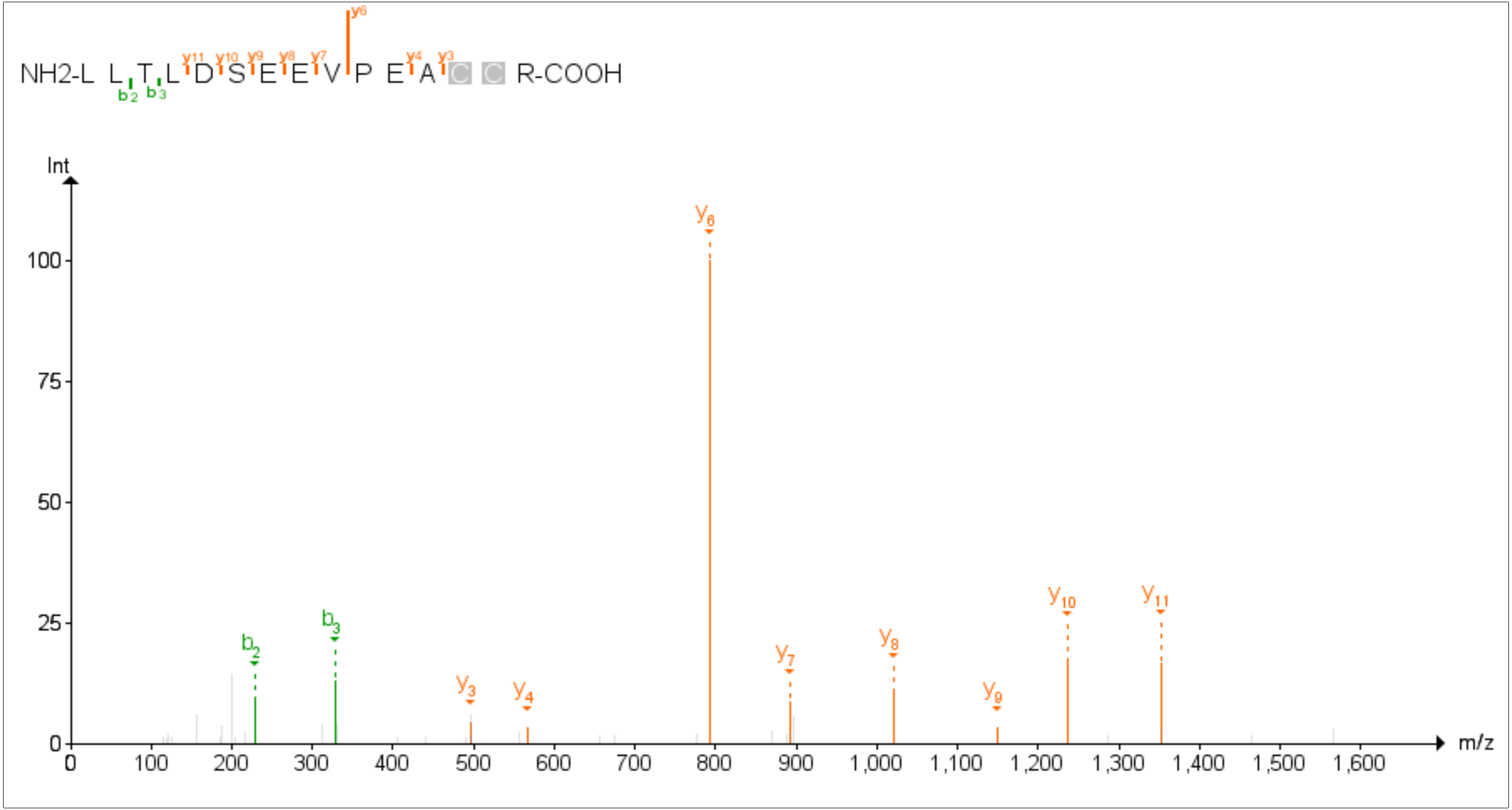
NH2-L L T L D S E E V P E A C C R-COOH
b2 b3
y11 y10 y9 y8 y7 y6 y4 y3
Int
100
75
50
25
0
0 100 200 300 400 500 600 700 800 900 1,000 1,100 1,200 1,300 1,400 1,500 1,600
m/z
y6
b2
b3
y3
y4
y7
y8
y9
y10
y11

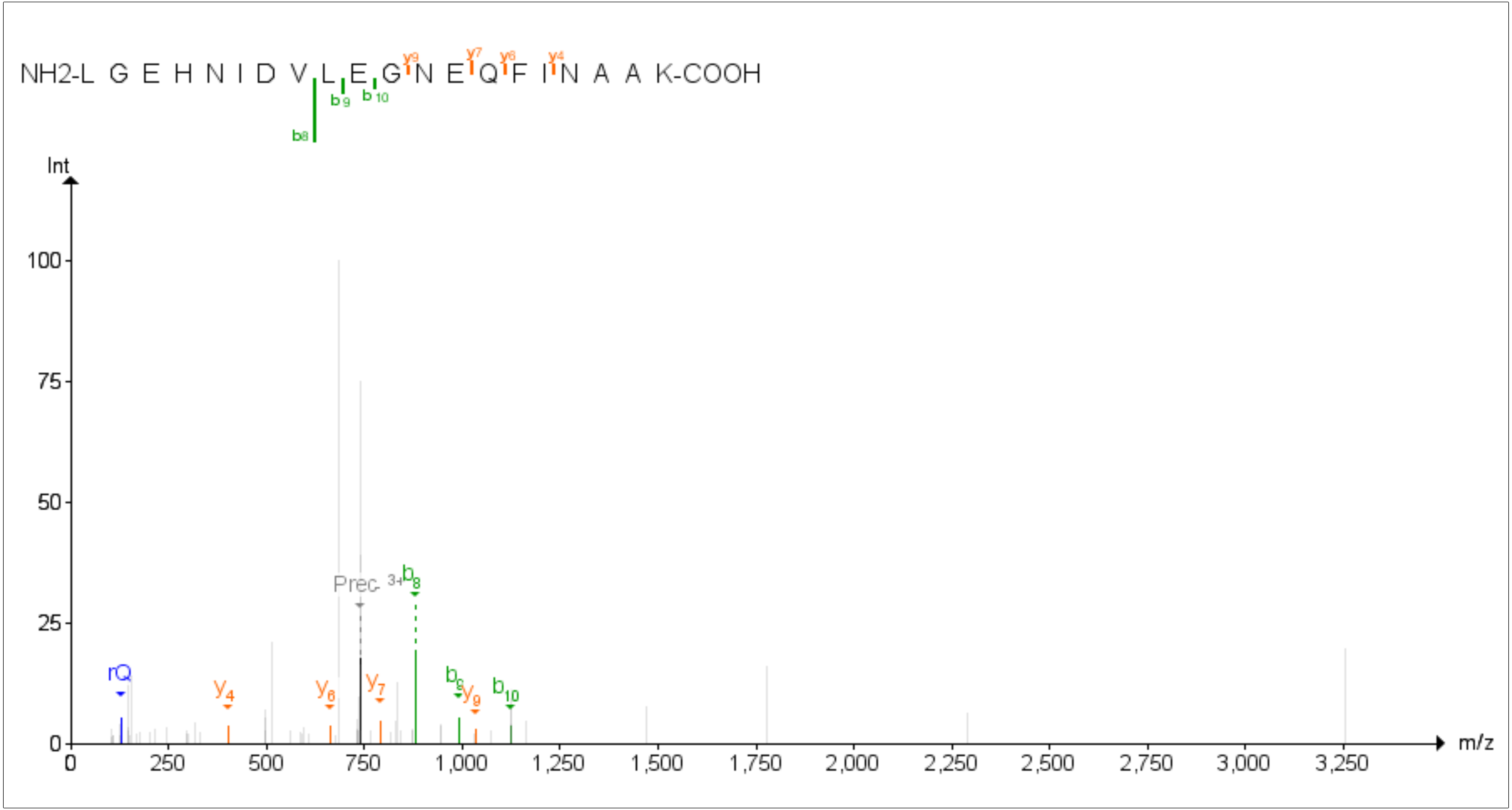
NH2-L G E H N I D V L E G N E Q F I N A A K-COOH
b8
b9
b10
y9
y7
y6
y4
Int
100
75
50
25
0
rQ
y4
y6
y7
Prec- 3+
b8
b9
y9
b10
0
250
500
750
1,000
1,250
1,500
1,750
2,000
2,250
2,500
2,750
3,000
3,250
m/z

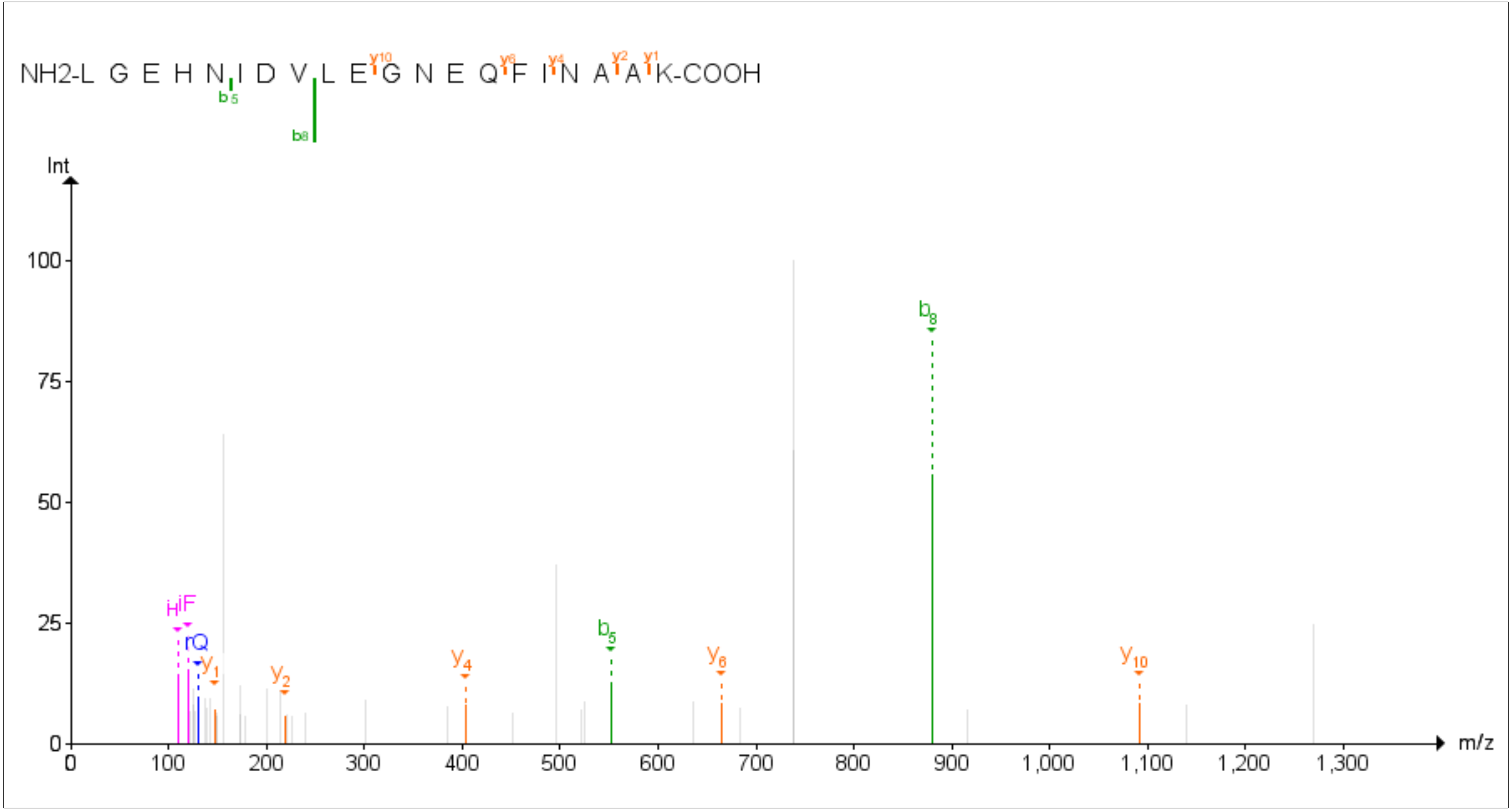

NH2-L G E H N I D V L E G N E Q F I N A A K-COOH
b5
b8
y10
y6
y4
y2
y1
Int
100
75
50
25
0
HiF
rQ
y1
y2
y4
b5
y6
b8
y10
0
100
200
300
400
500
600
700
800
900
1,000
1,100
1,200
1,300
m/z

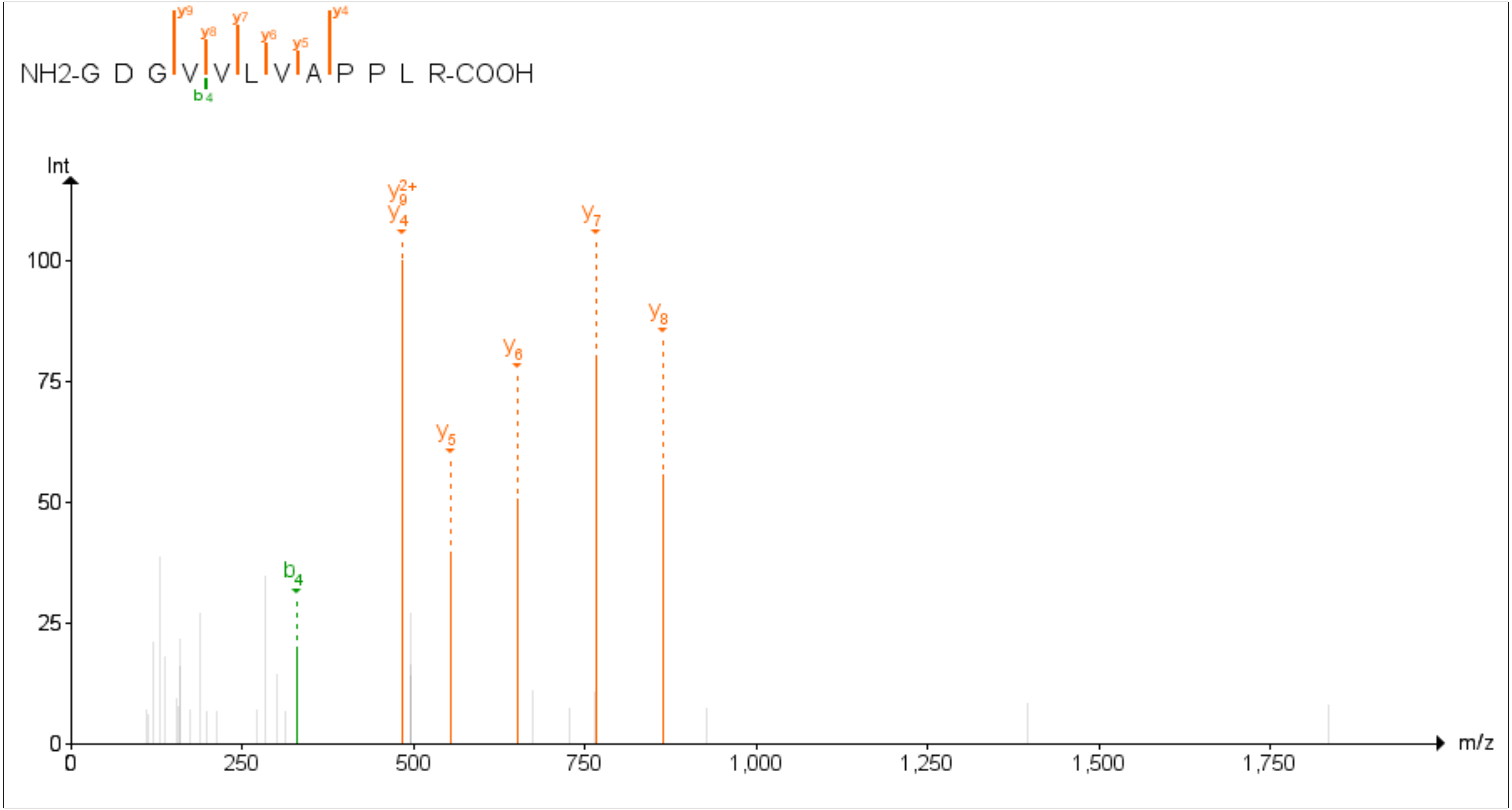
NH2-G D G V V L V A P P L R-COOH
y9
y8
y7
y6
y5
y4
b4
Int
100
75
50
25
0
y_9^{2+}
y_4
y_7
y_8
y_6
y_5
b_4
0
250
500
750
1,000
1,250
1,500
1,750
m/z

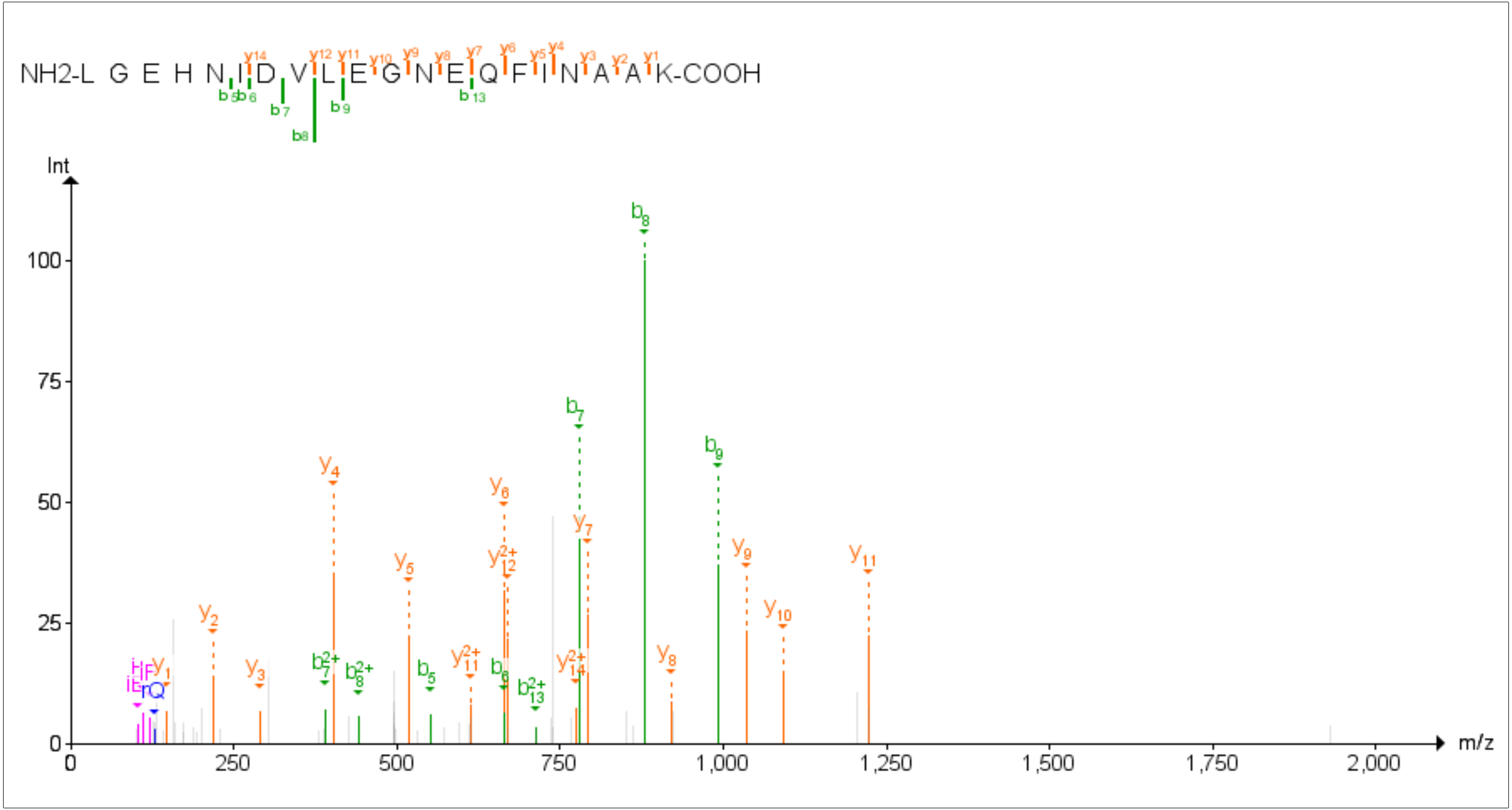
NH2-L G E H N I D V L E G N E Q F I N A A K-COOH
y14 y12 y11 y10 y9 y8 y7 y6 y5 y4 y3 y2 y1
b5 b6 b7 b8 b9 b13
Int
100
75
50
25
0
m/z
0
250
500
750
1,000
1,250
1,500
1,750
2,000

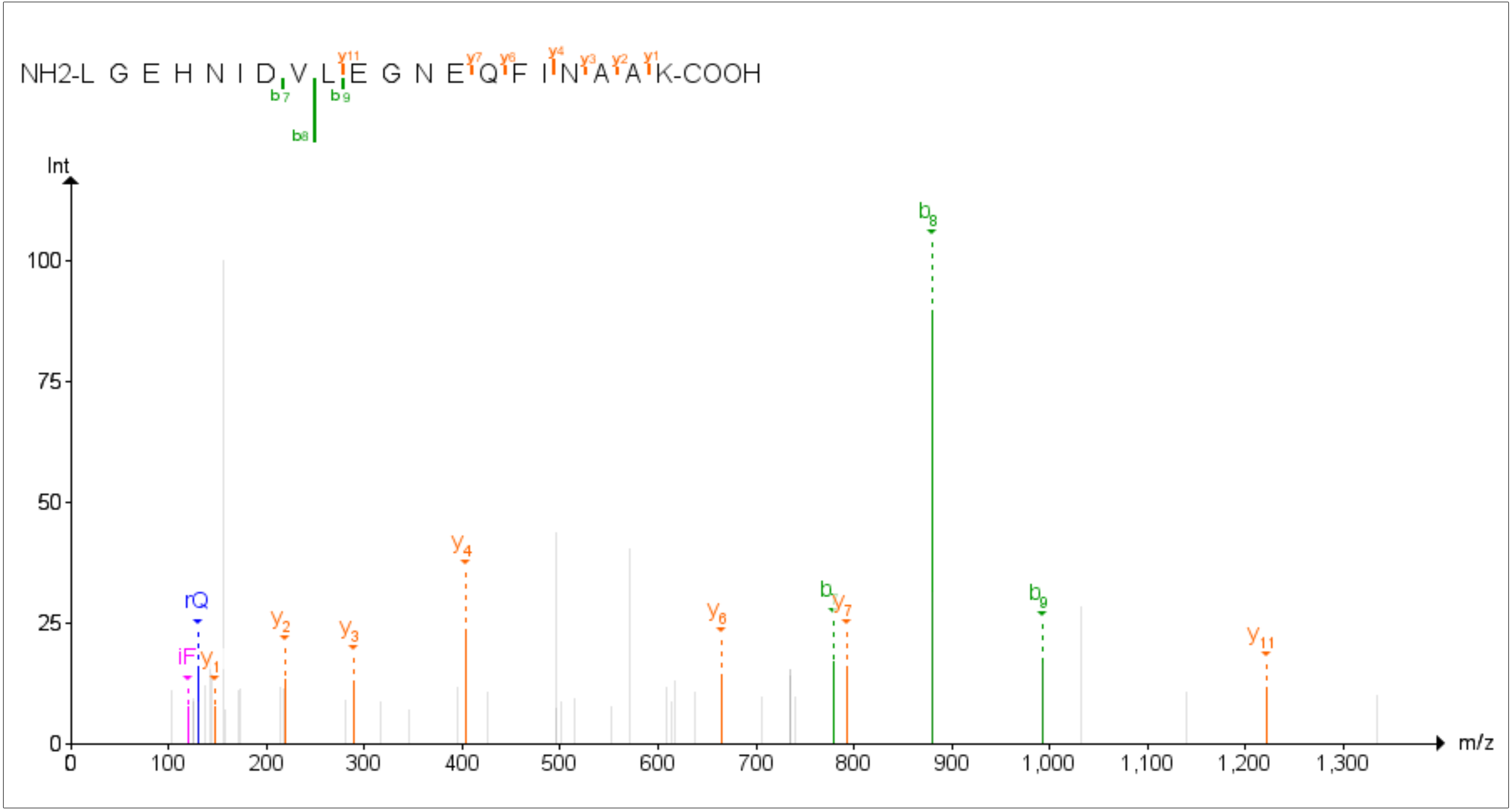
NH2-L G E H N I D V L E G N E Q F I N A A K-COOH
b7
b8
b9
y11
y7
y6
y4
y3
y2
y1
Int
100
75
50
25
0
rQ
iF
m/z
0
100
200
300
400
500
600
700
800
900
1,000
1,100
1,200
1,300

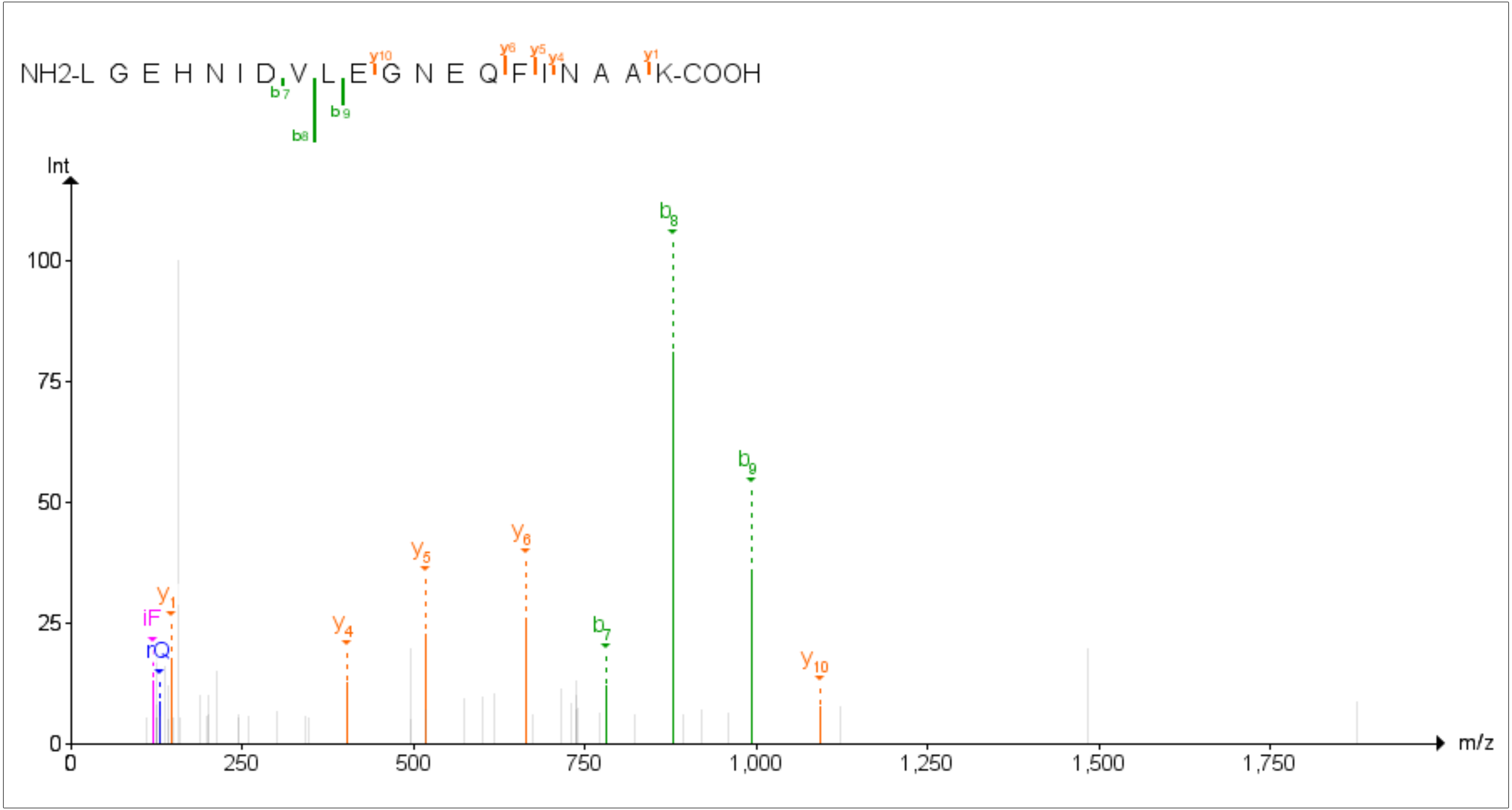

NH2-L G E H N I D V L E G N E Q F I N A A K-COOH
y10
y6
y5
y4
y1
b7
b8
b9
Int
100
75
50
25
0
b8
b9
y6
y5
y4
y1
iF
rQ
b7
y10
0
250
500
750
1,000
1,250
1,500
1,750
m/z

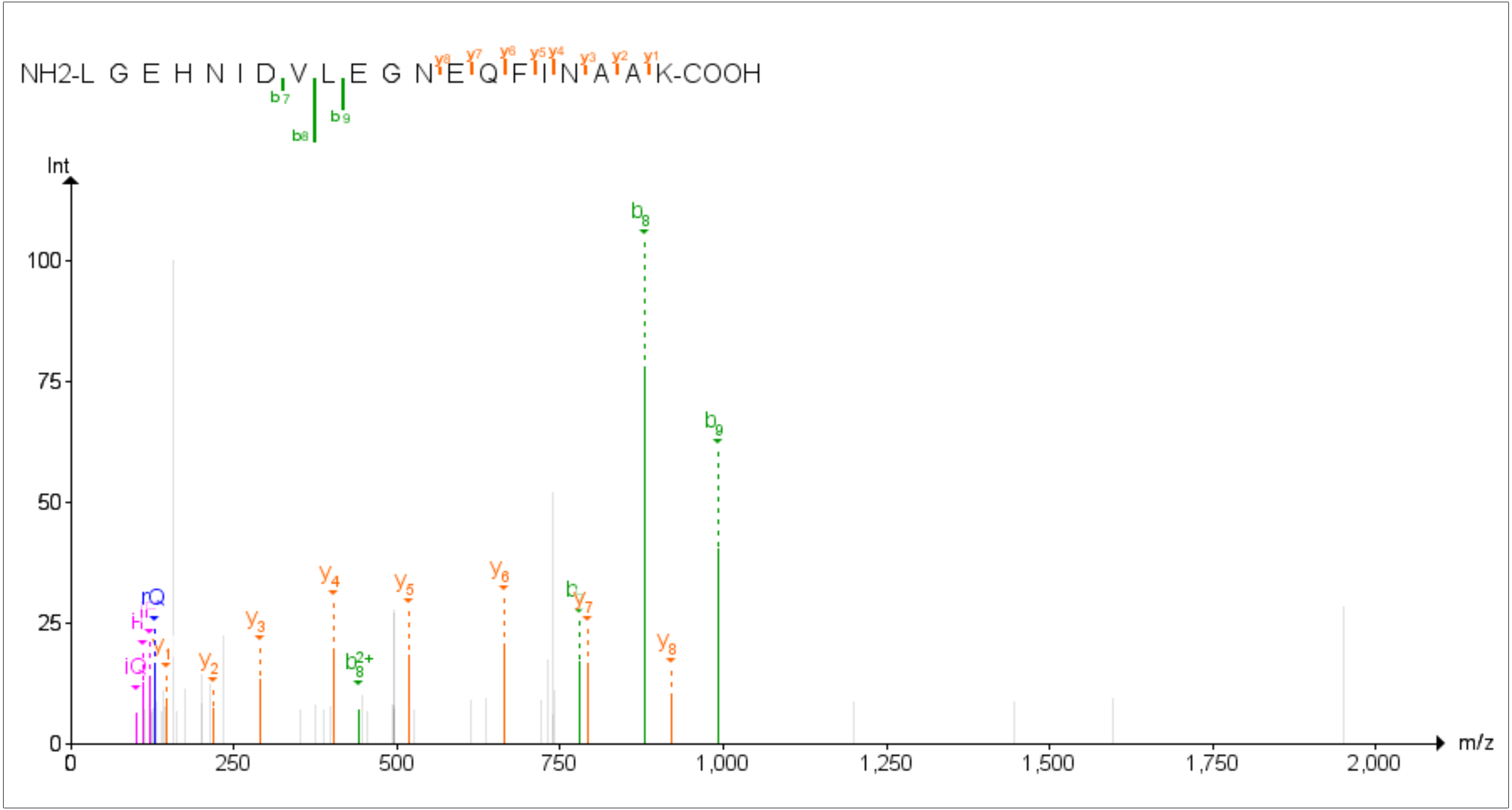
NH2-L G E H N I D V L E G N E Q F I N A A K-COOH
b7
b8
b9
y8 y7 y6 y5 y4 y3 y2 y1
Int
100
75
50
25
0
0
250
500
750
1,000
1,250
1,500
1,750
2,000
m/z
b8
b9
y1
y2
y3
y4
y5
y6
y7
y8
rQ
iQ

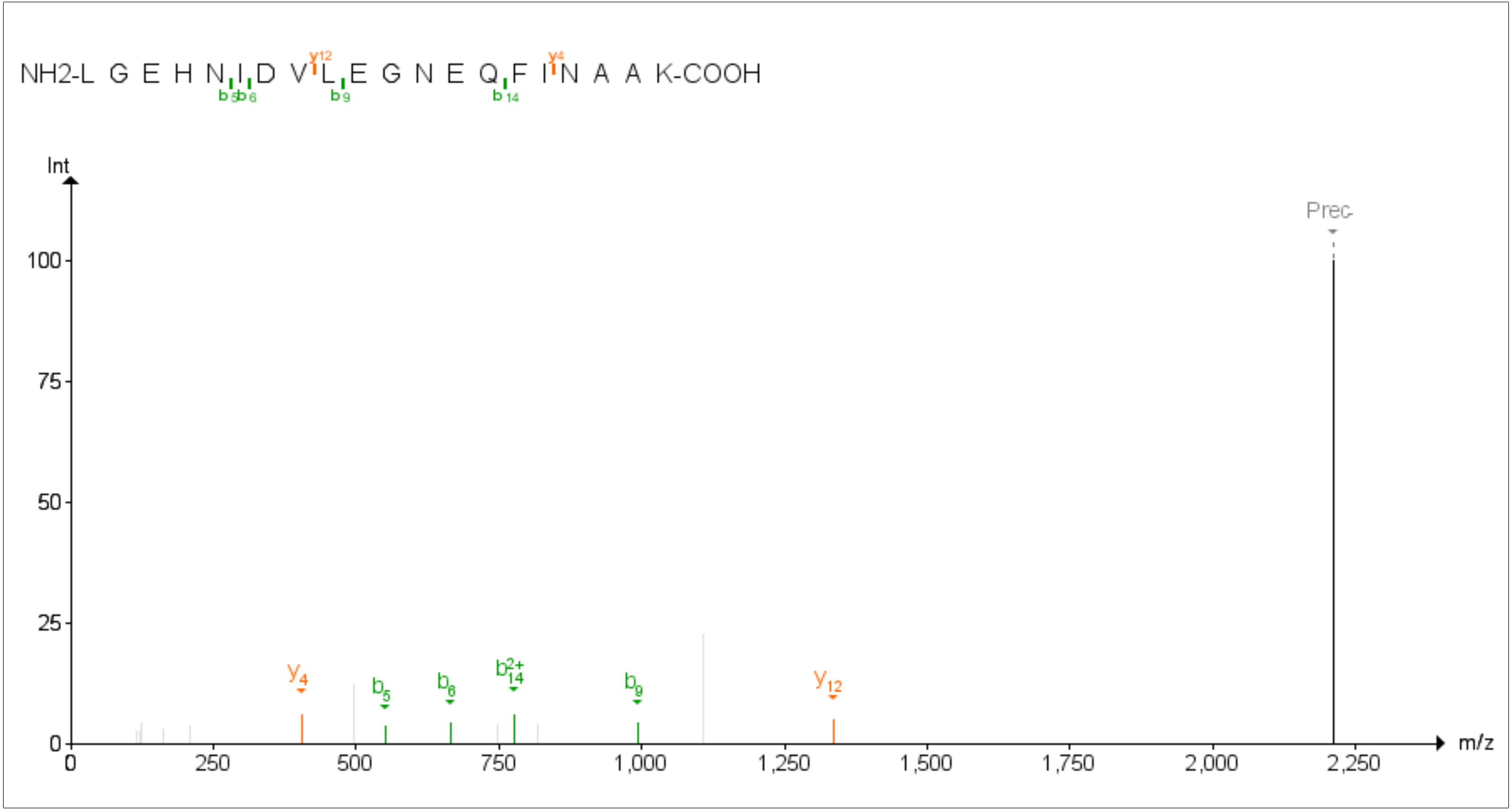
NH2-L G E H N I D V L E G N E Q F I N A A K-COOH
y12
y4
b5
b6
b9
b14
Int
Prec
100
75
50
25
0
y4
b5
b6
b14 2+
b9
y12
0
250
500
750
1,000
1,250
1,500
1,750
2,000
2,250
m/z

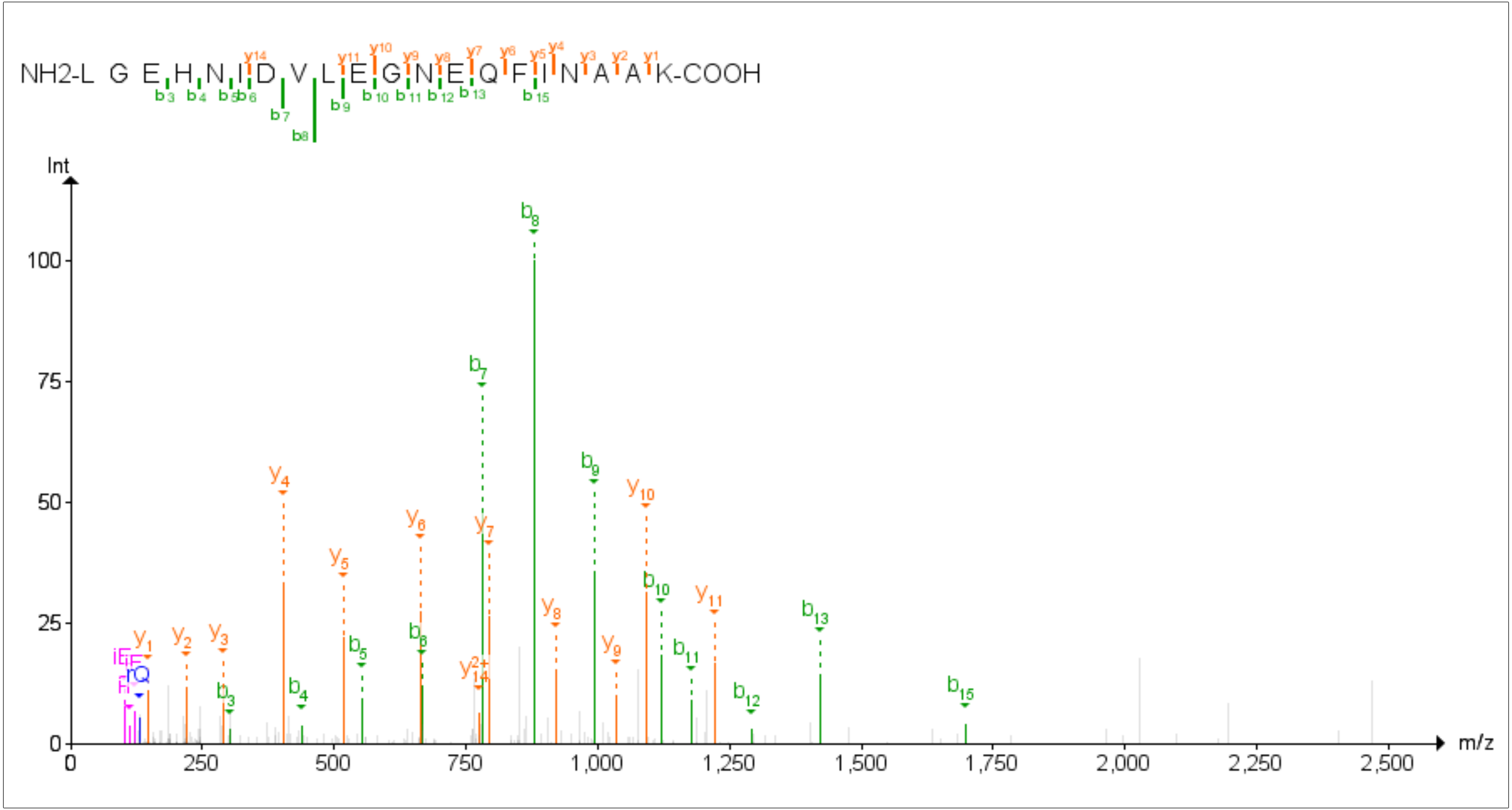

NH2-L G E H N I D V L E G N E Q F I N A A K-COOH
y14 y11 y10 y9 y8 y7 y6 y5 y4 y3 y2 y1
b3 b4 b5 b6 b7 b8 b9 b10 b11 b12 b13 b15
Int
100
75
50
25
0
m/z
0 250 500 750 1,000 1,250 1,500 1,750 2,000 2,250 2,500

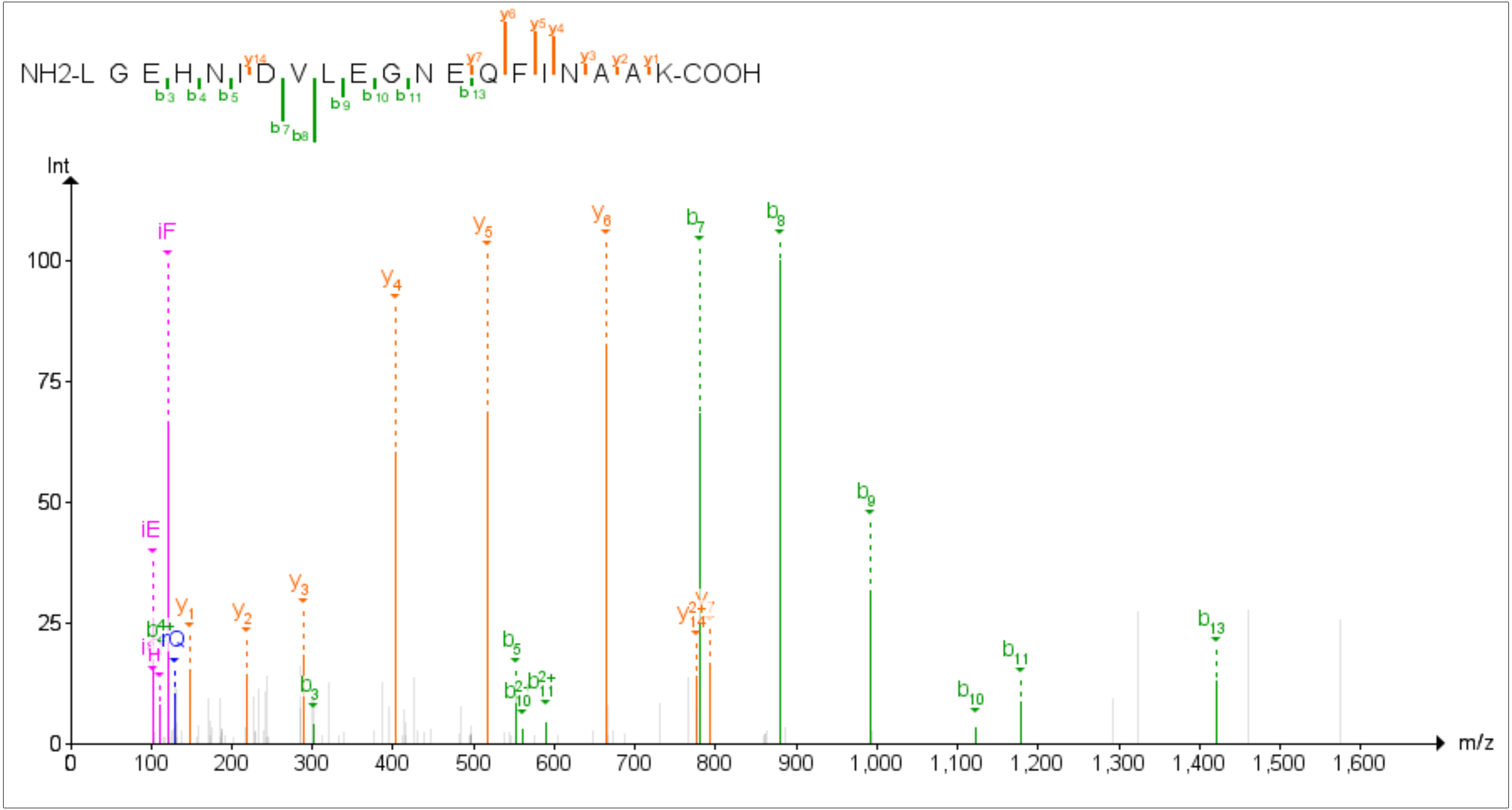

NH2-L G E H N I D V L E G N E Q F I N A A K-COOH
Int
m/z
iF
iE
iH
iQ
y1
y2
y3
y4
y5
y6
y7
y14 2+
b3
b4 2+
b5
b7
b8
b9
b10
b10 2+
b11
b11 2+
b13
0
25
50
75
100
0
100
200
300
400
500
600
700
800
900
1,000
1,100
1,200
1,300
1,400
1,500
1,600

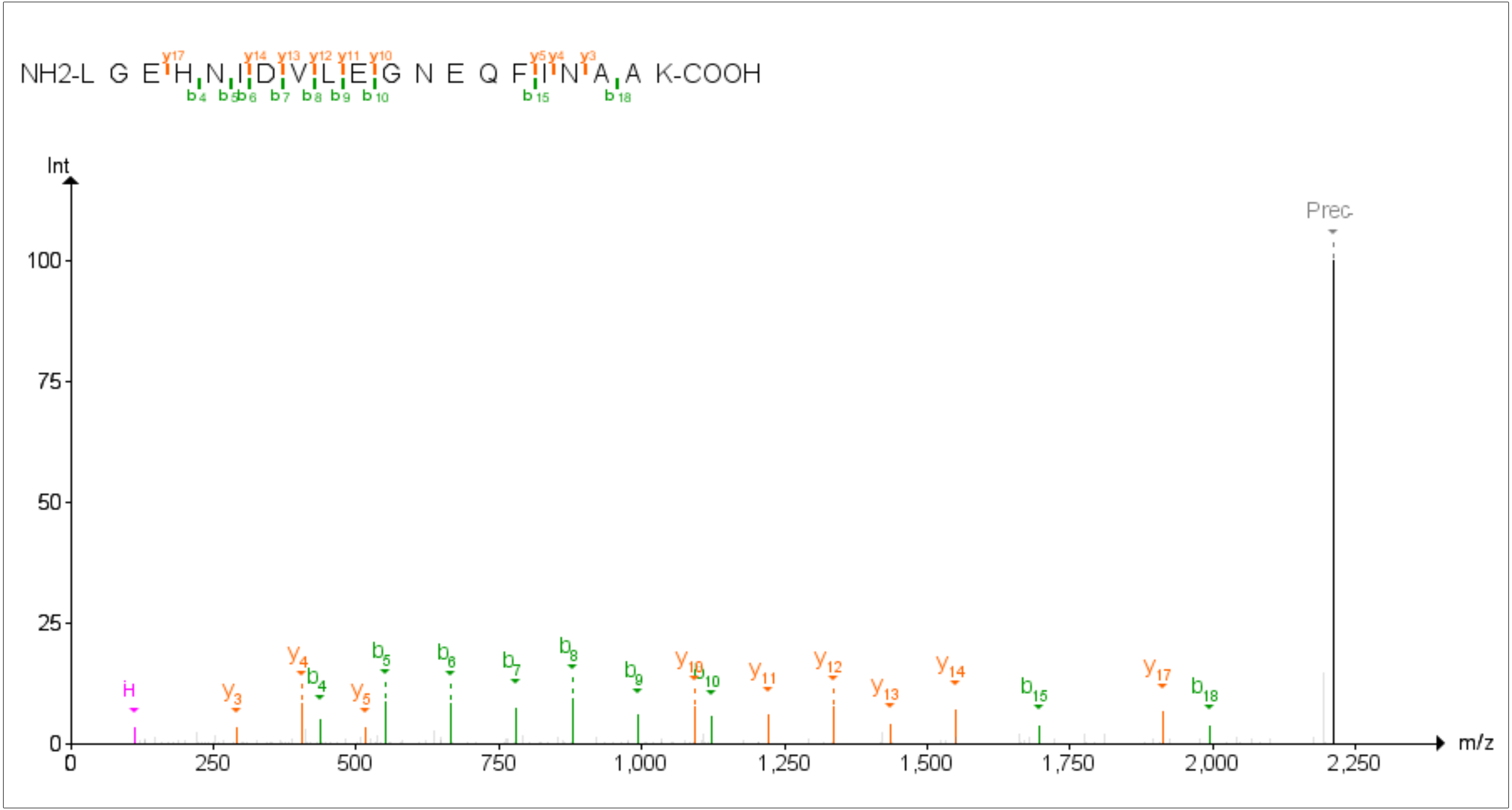

NH2-L G E H N I D V L E G N E Q F I N A A K-COOH
y17 y14 y13 y12 y11 y10 y5 y4 y3
b4 b5 b6 b7 b8 b9 b10 b15 b18
Int
100
75
50
25
0
Prec
H
y3 y4 b4 y5 b5 b6 b7 b8 b9 y10 b10 y11 y12 y13 y14 b15 y17 b18
0 250 500 750 1,000 1,250 1,500 1,750 2,000 2,250
m/z

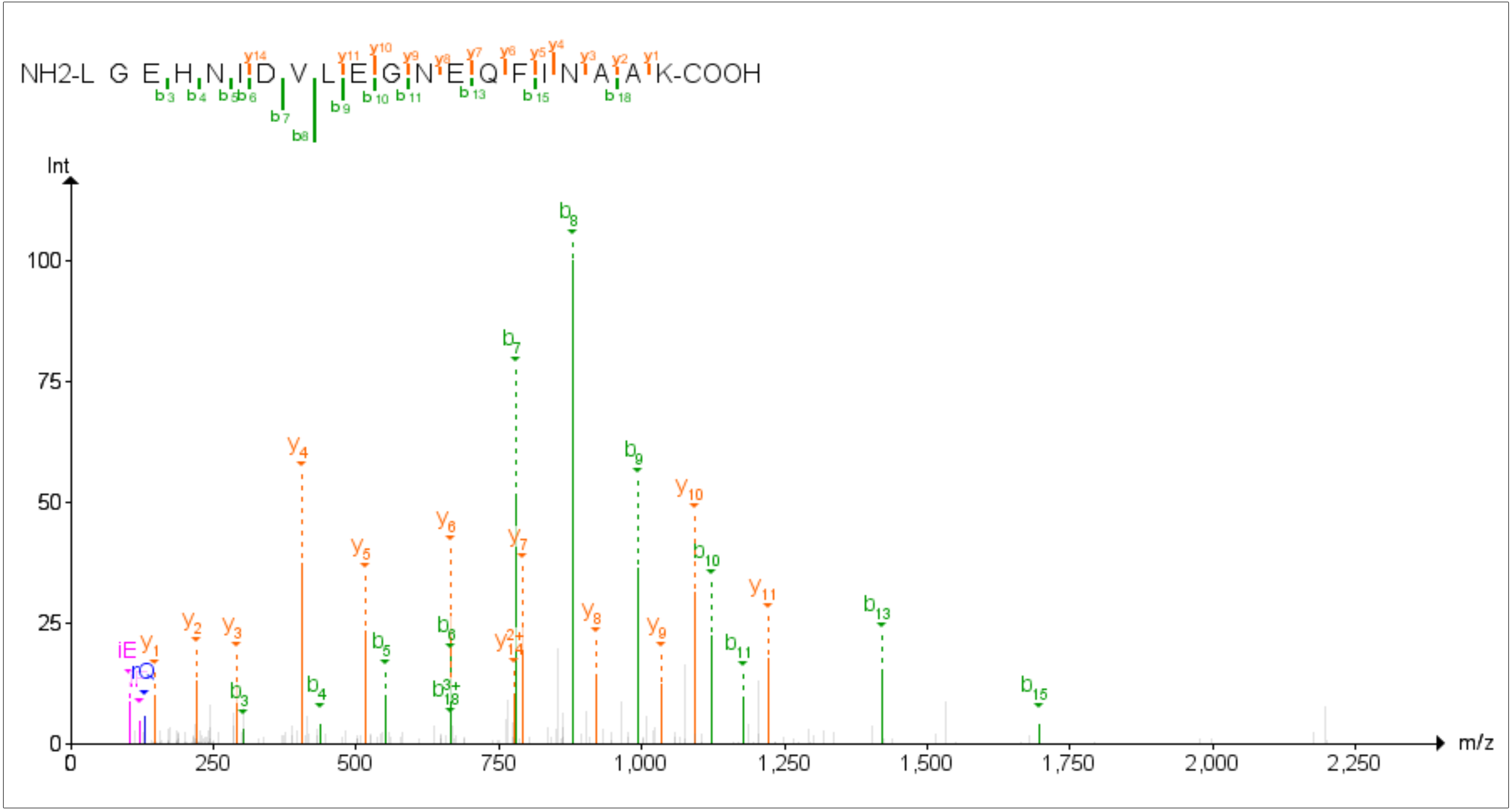

NH2-L G E H N I D V L E G N E Q F I N A A K-COOH
Int
m/z
b8
b7
b9
y4
y10
b10
y6
y7
y5
y11
b13
y8
y9
b11
b5
b6
y14 2+
y1
iE
nQ
y2
y3
b3
b4
b18 3+
b15
0
25
50
75
100
250
500
750
1,000
1,250
1,500
1,750
2,000
2,250

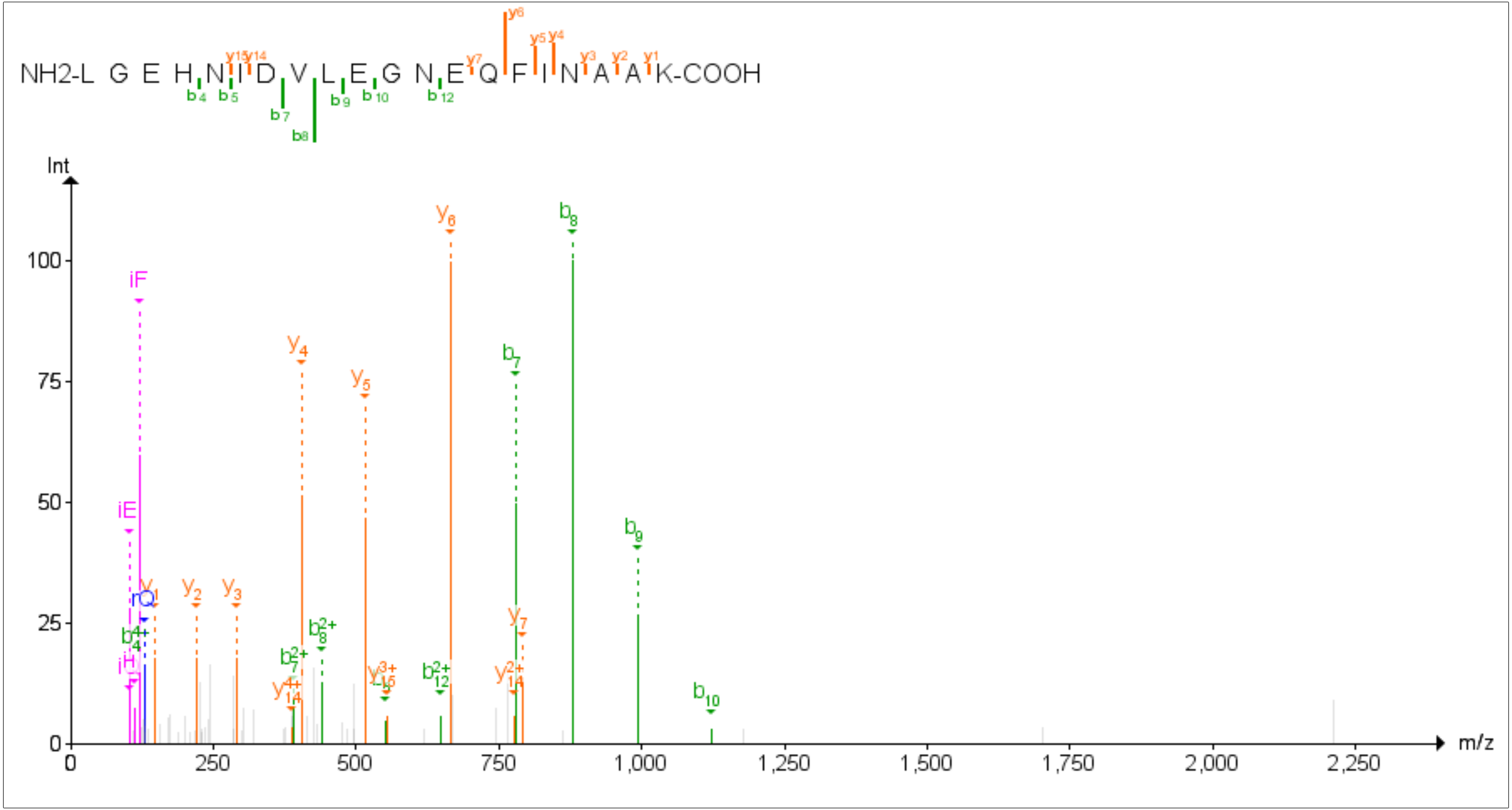

NH2-L G E H N I D V L E G N E Q F I N A A K-COOH
Int
m/z
0
25
50
75
100
250
500
750
1,000
1,250
1,500
1,750
2,000
2,250

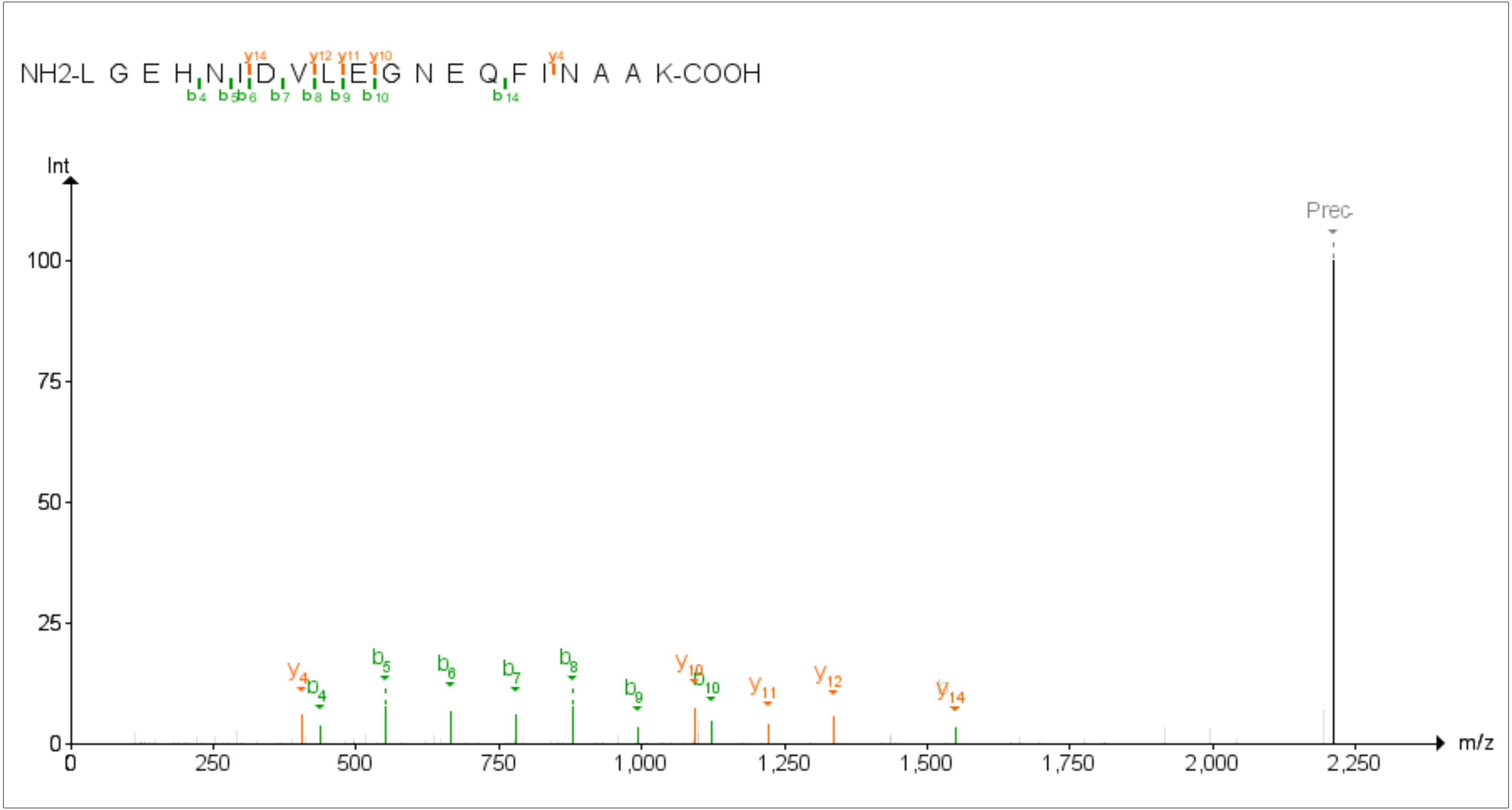

NH2-L G E H N I D V L E G N E Q F I N A A K-COOH
y14 y12 y11 y10 y4
b4 b5 b6 b7 b8 b9 b10 b14
Int
Prec
100
75
50
25
0
0 250 500 750 1,000 1,250 1,500 1,750 2,000 2,250
m/z
y4 b4 b5 b6 b7 b8 b9 y10 b10 y11 y12 y14

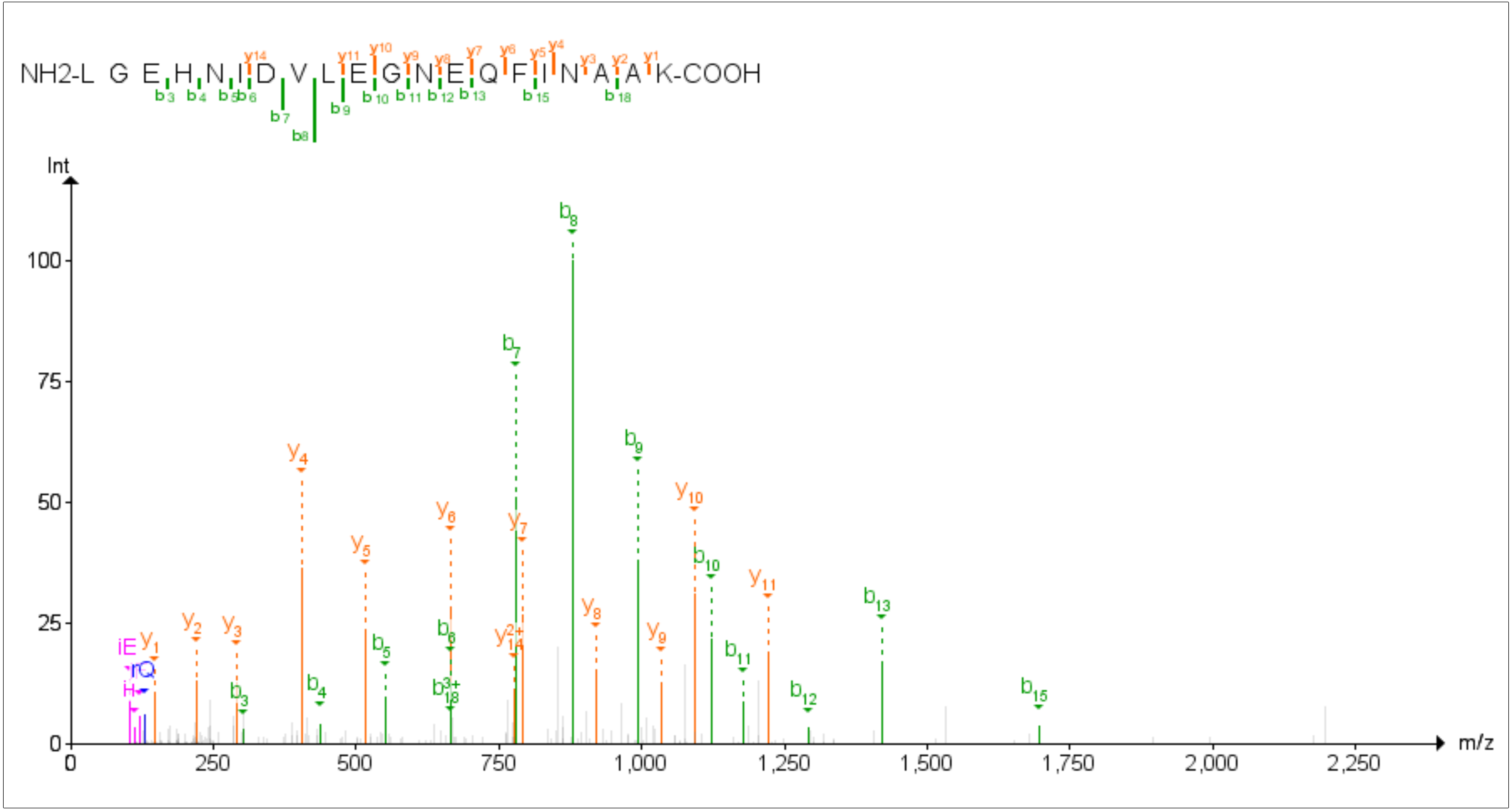
NH2-L G E H N I D V L E G N E Q F I N A A K-COOH
Int
m/z
0
25
50
75
100
250
500
750
1,000
1,250
1,500
1,750
2,000
2,250

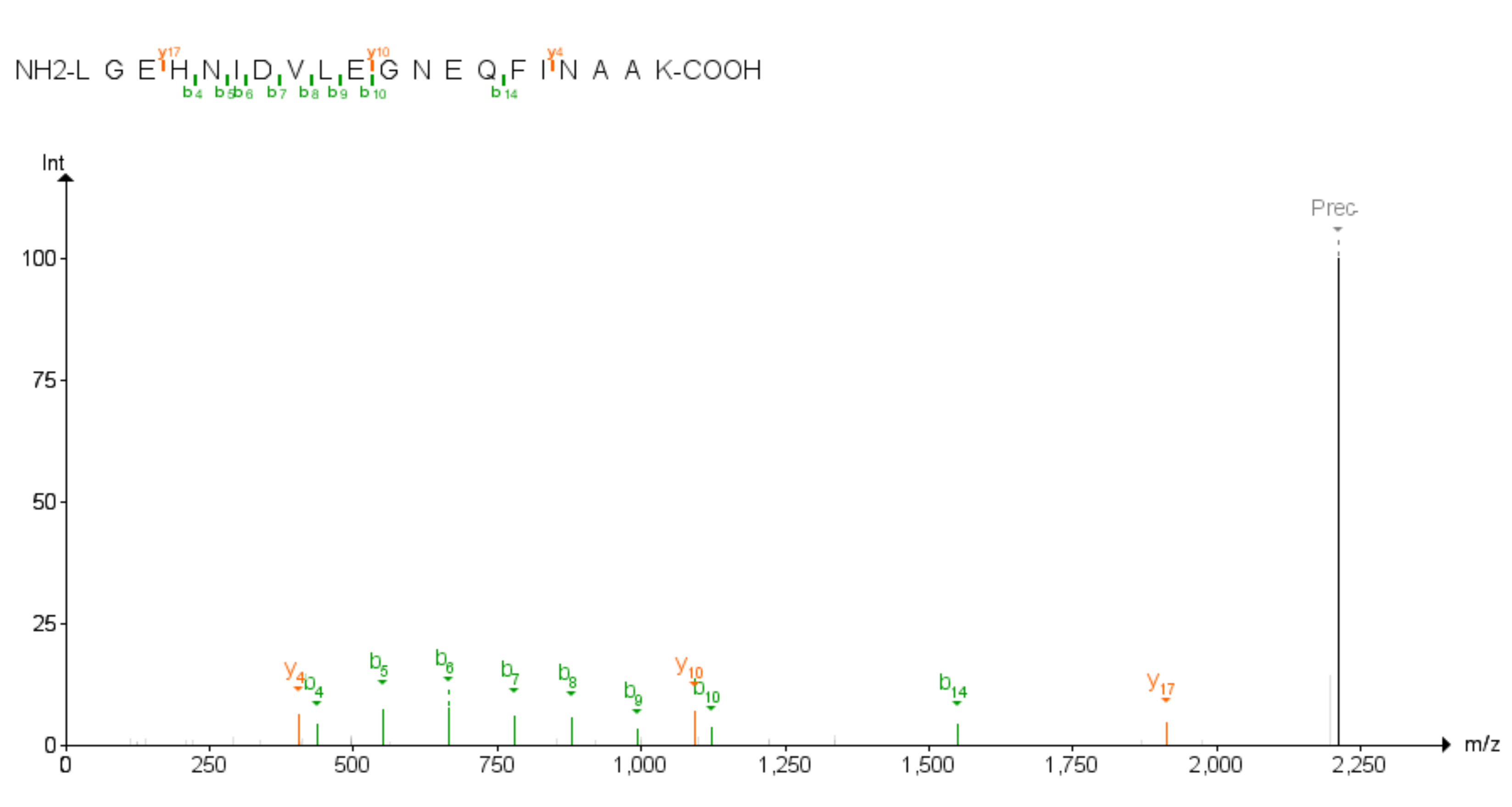

NH2-L G E H N I D V L E G N E Q F I N A A K-COOH
y17 y10 y4
b4 b5 b6 b7 b8 b9 b10 b14
Int
100
75
50
25
0
Prec
y4 b4 b5 b6 b7 b8 b9 y10 b10 b14 y17
0
250
500
750
1,000
1,250
1,500
1,750
2,000
2,250
m/z

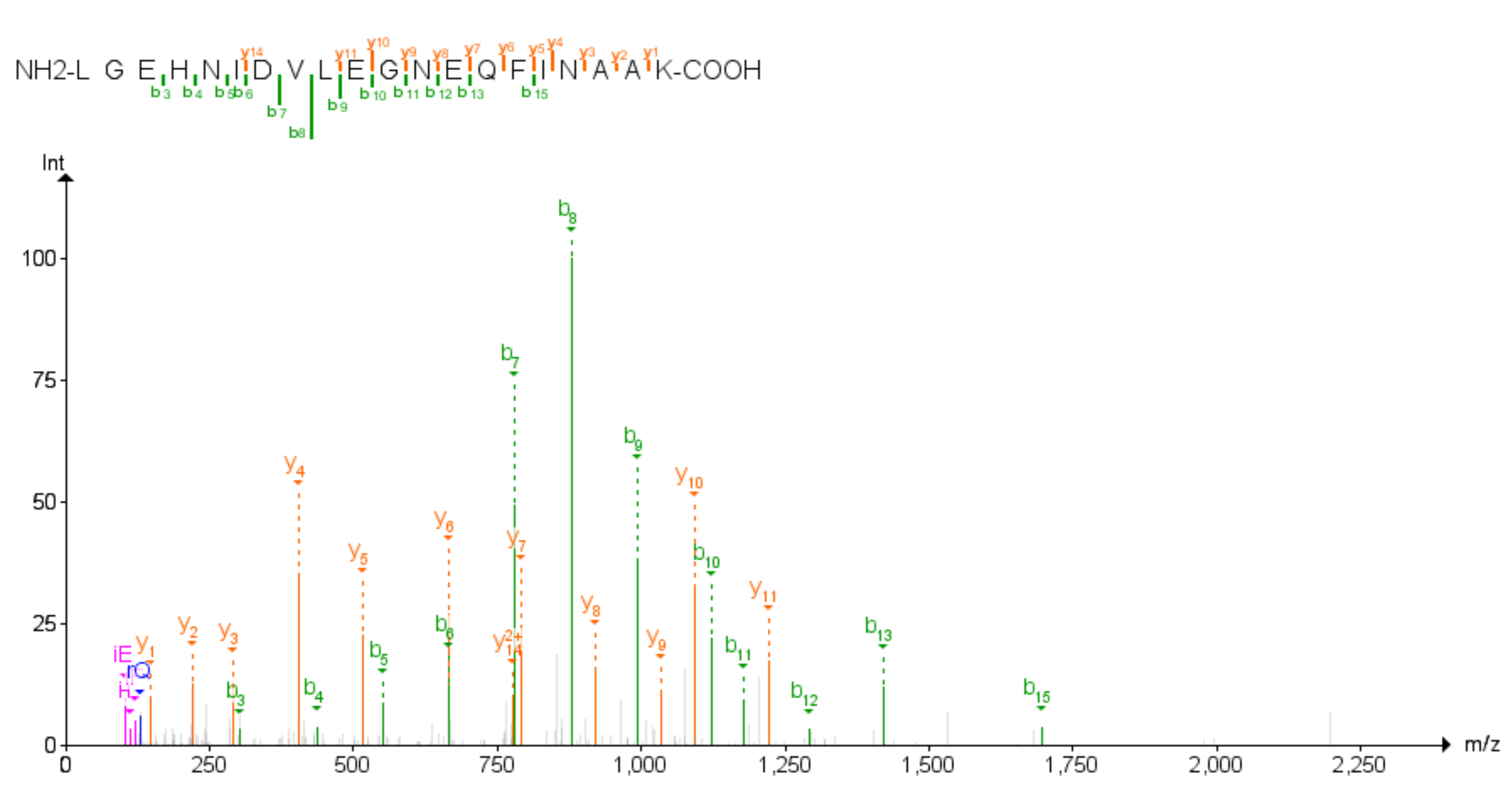

NH2-L G E H N I D V L E G N E Q F I N A A K-COOH
Int
m/z
0
25
50
75
100
250
500
750
1,000
1,250
1,500
1,750
2,000
2,250

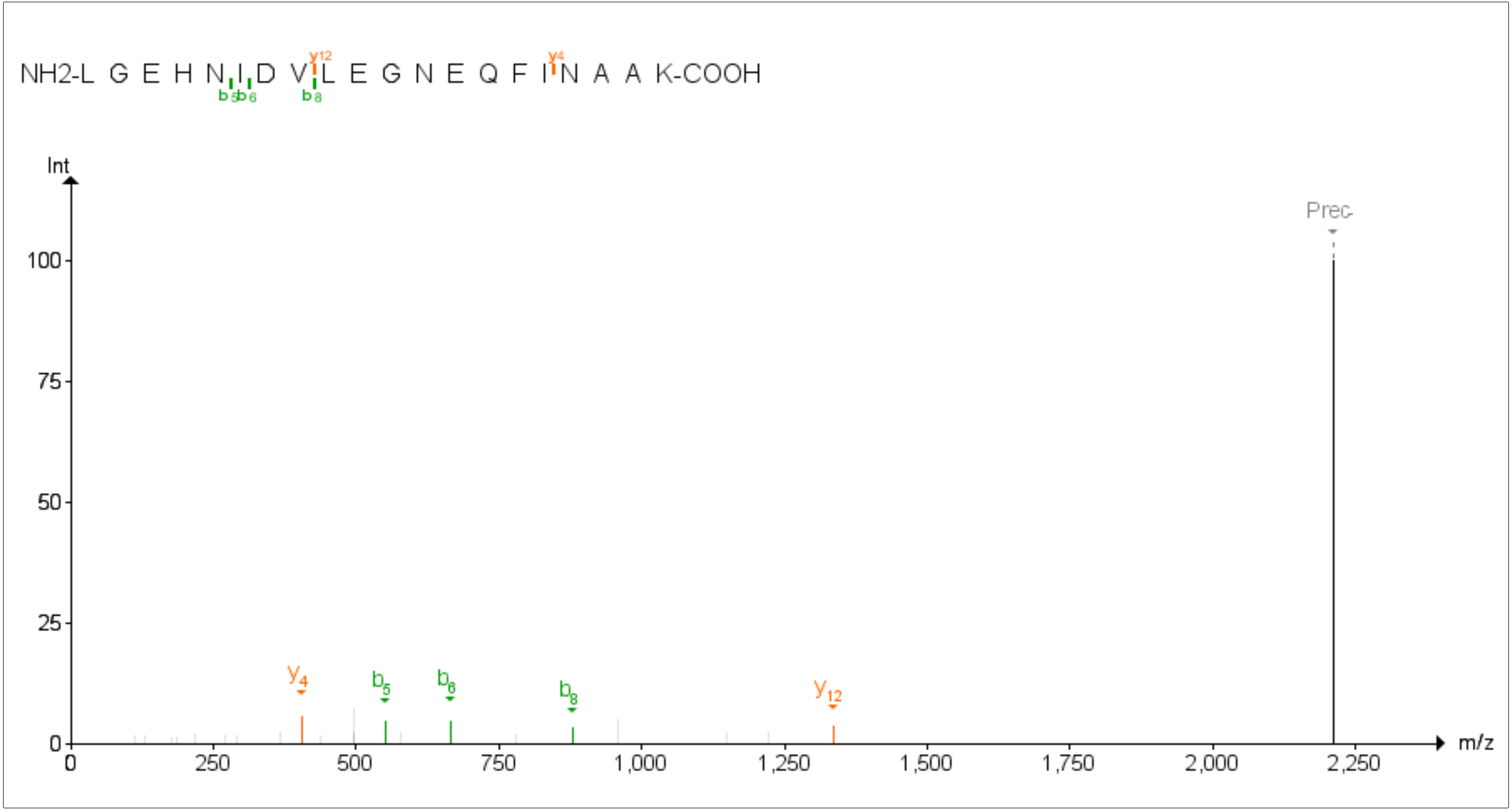
NH2-L G E H N I D V L E G N E Q F I N A A K-COOH
y12
y4
b5
b6
b8
Int
100
75
50
25
0
Prec
y4
b5
b6
b8
y12
0
250
500
750
1,000
1,250
1,500
1,750
2,000
2,250
m/z

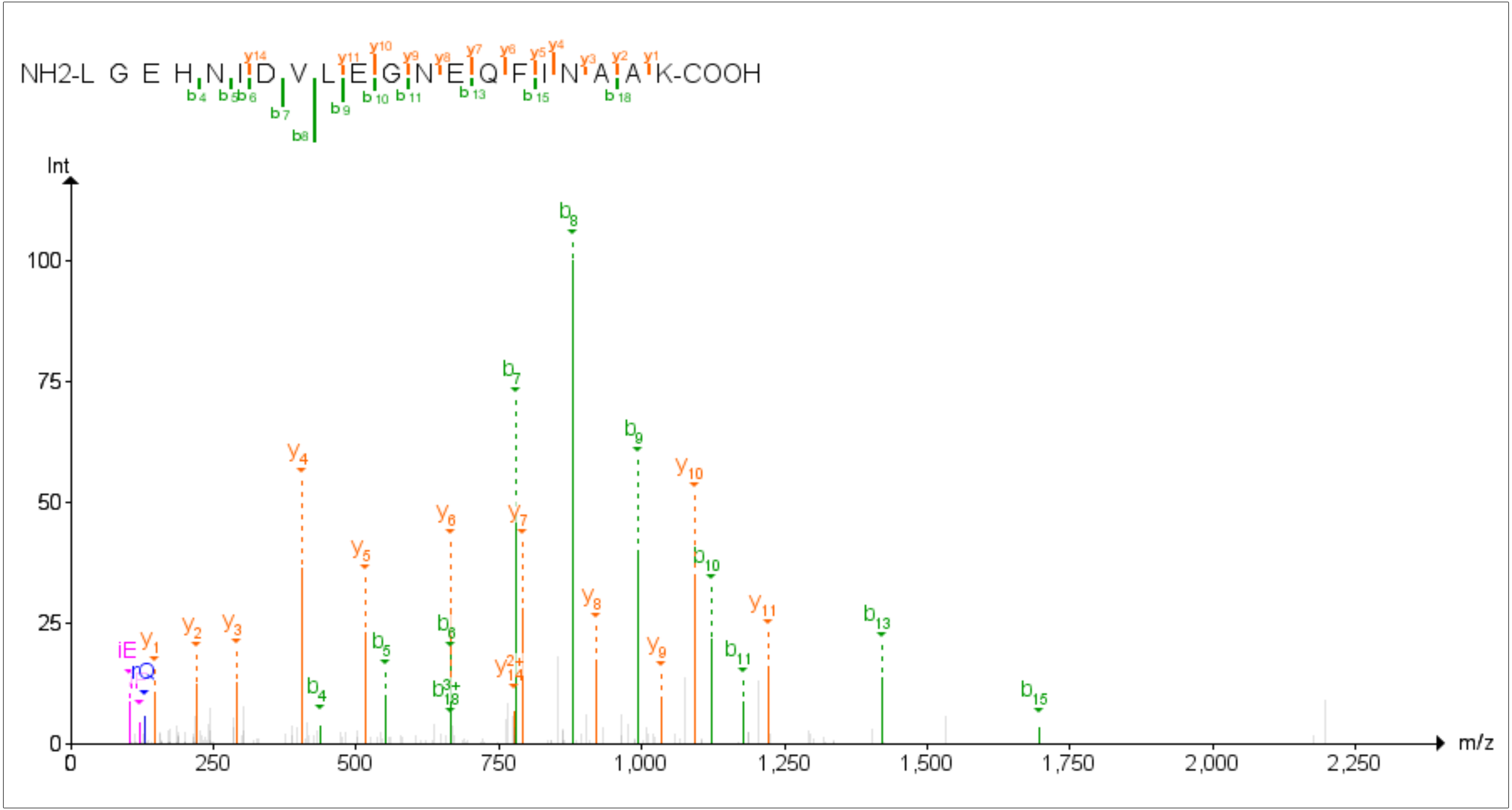
NH2-L G E H N I D V L E G N E Q F I N A A K-COOH
Int
m/z
0
25
50
75
100
250
500
750
1,000
1,250
1,500
1,750
2,000
2,250

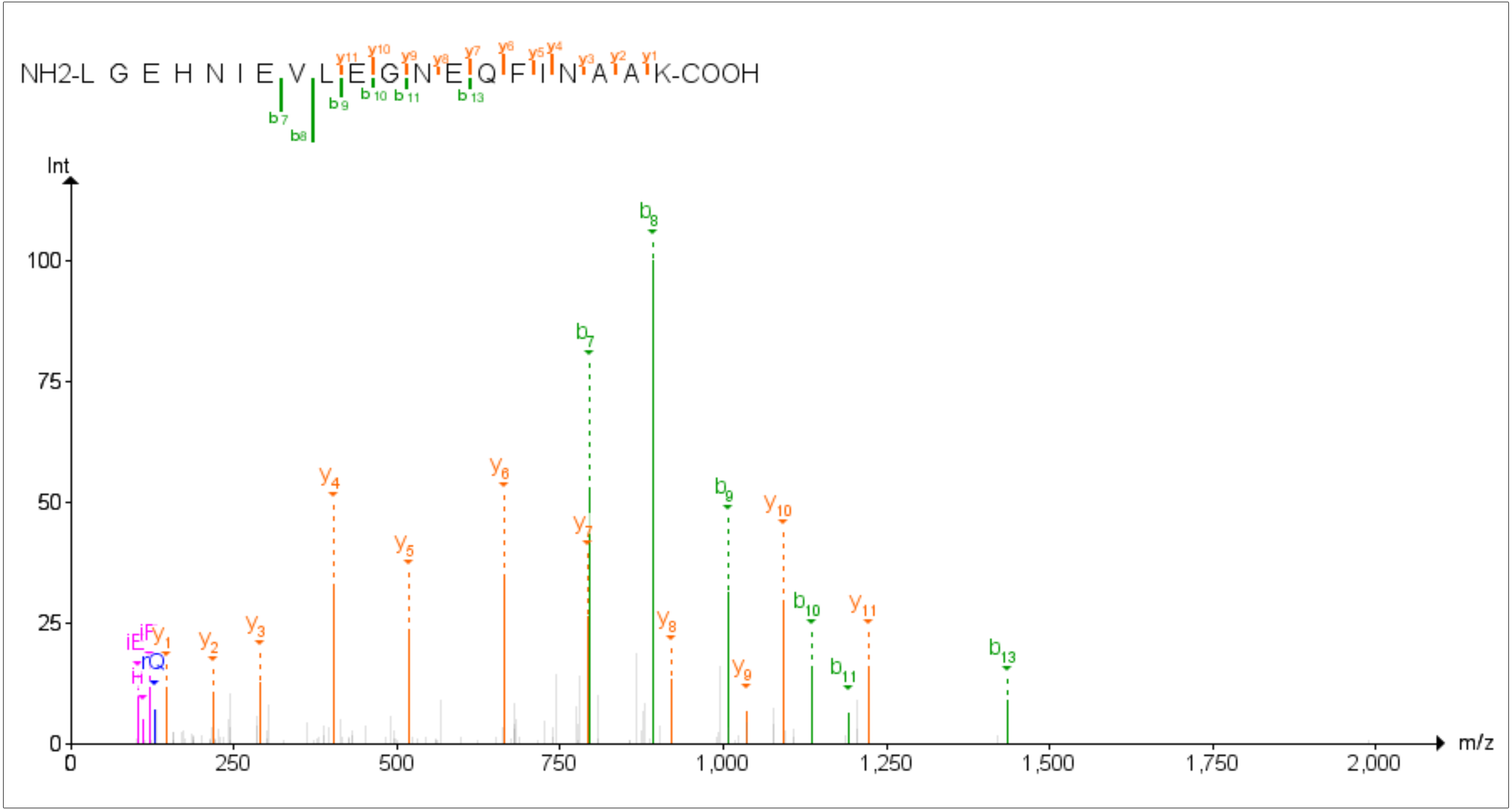
NH2-L G E H N I E V L E G N E Q F I N A A K-COOH
Int
m/z
0
25
50
75
100
250
500
750
1,000
1,250
1,500
1,750
2,000

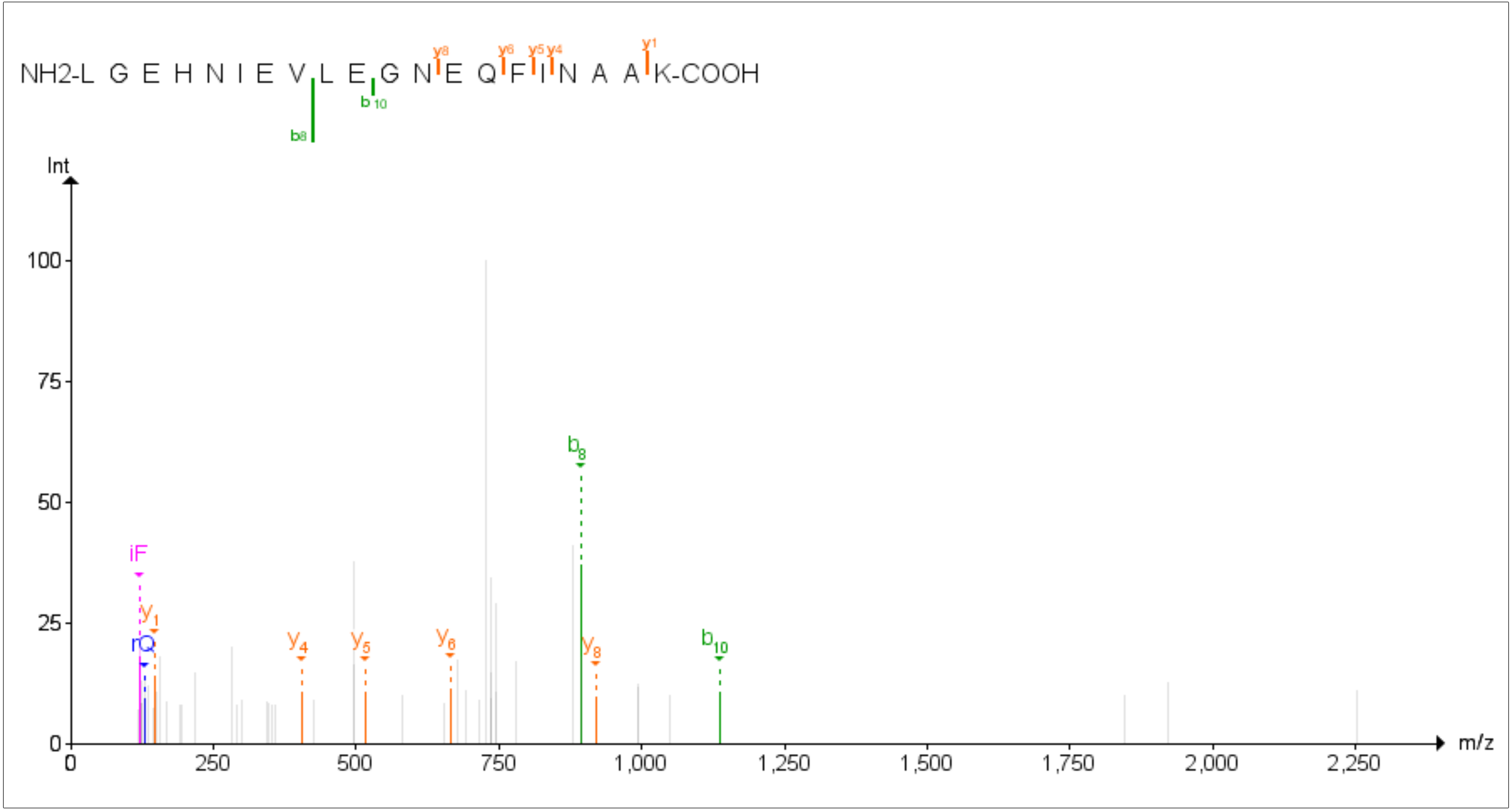
NH2-L G E H N I E V L E G N E Q F I N A A K-COOH
b8
b10
y8
y6
y5
y4
y1
Int
m/z
0
25
50
75
100
0
250
500
750
1,000
1,250
1,500
1,750
2,000
2,250
iF
rQ
y1
y4
y5
y6
y8
b8
b10

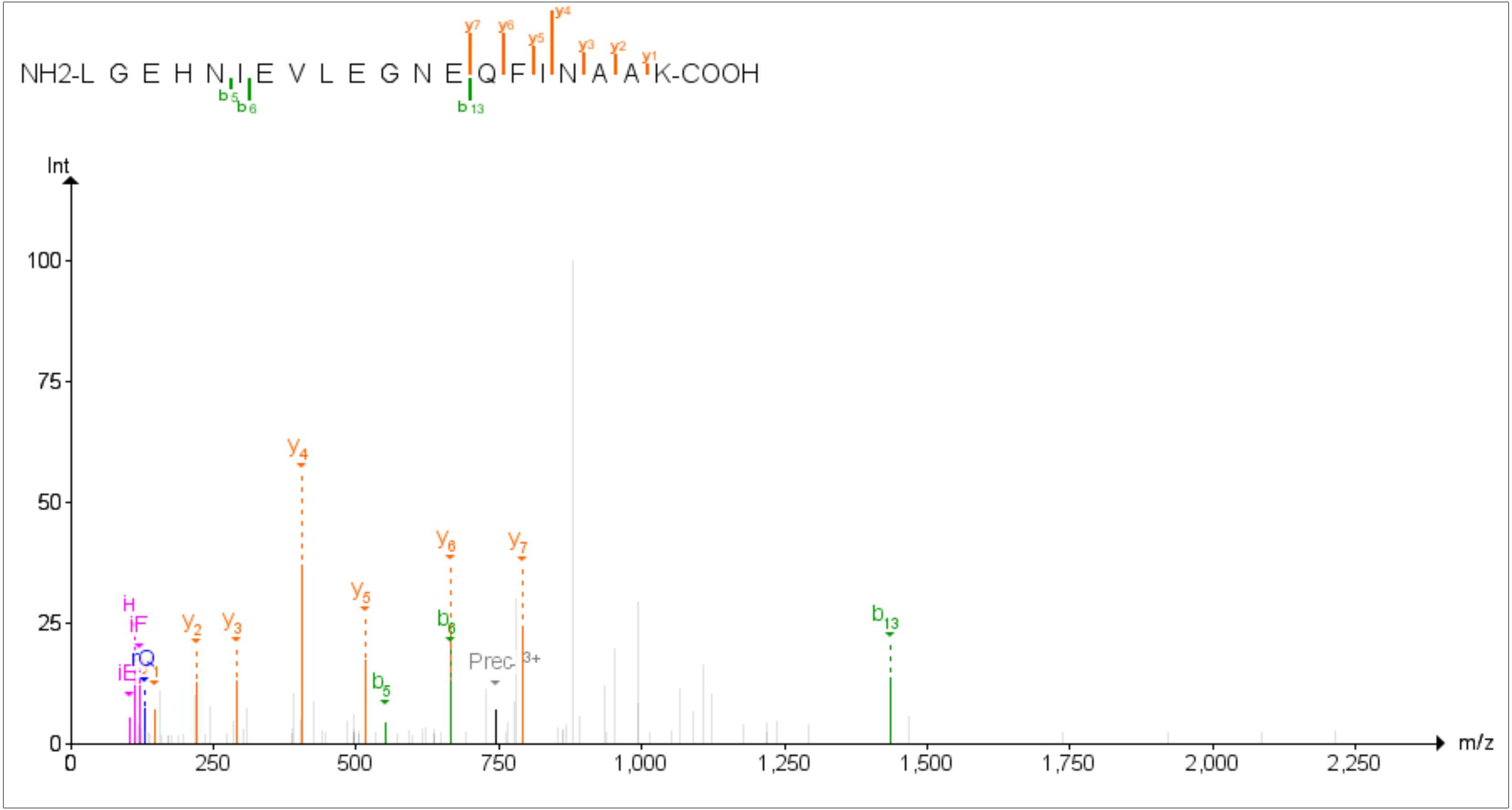
NH2-L G E H N I E V L E G N E Q F I N A A K-COOH
y7 y6 y5 y4 y3 y2 y1
b5 b6 b13
Int
100
75
50
25
0
m/z
0
250
500
750
1,000
1,250
1,500
1,750
2,000
2,250
y4
y6
y7
y5
y2
y3
b6
b5
b13
iH
iF
rQ
iE
Prec 3+

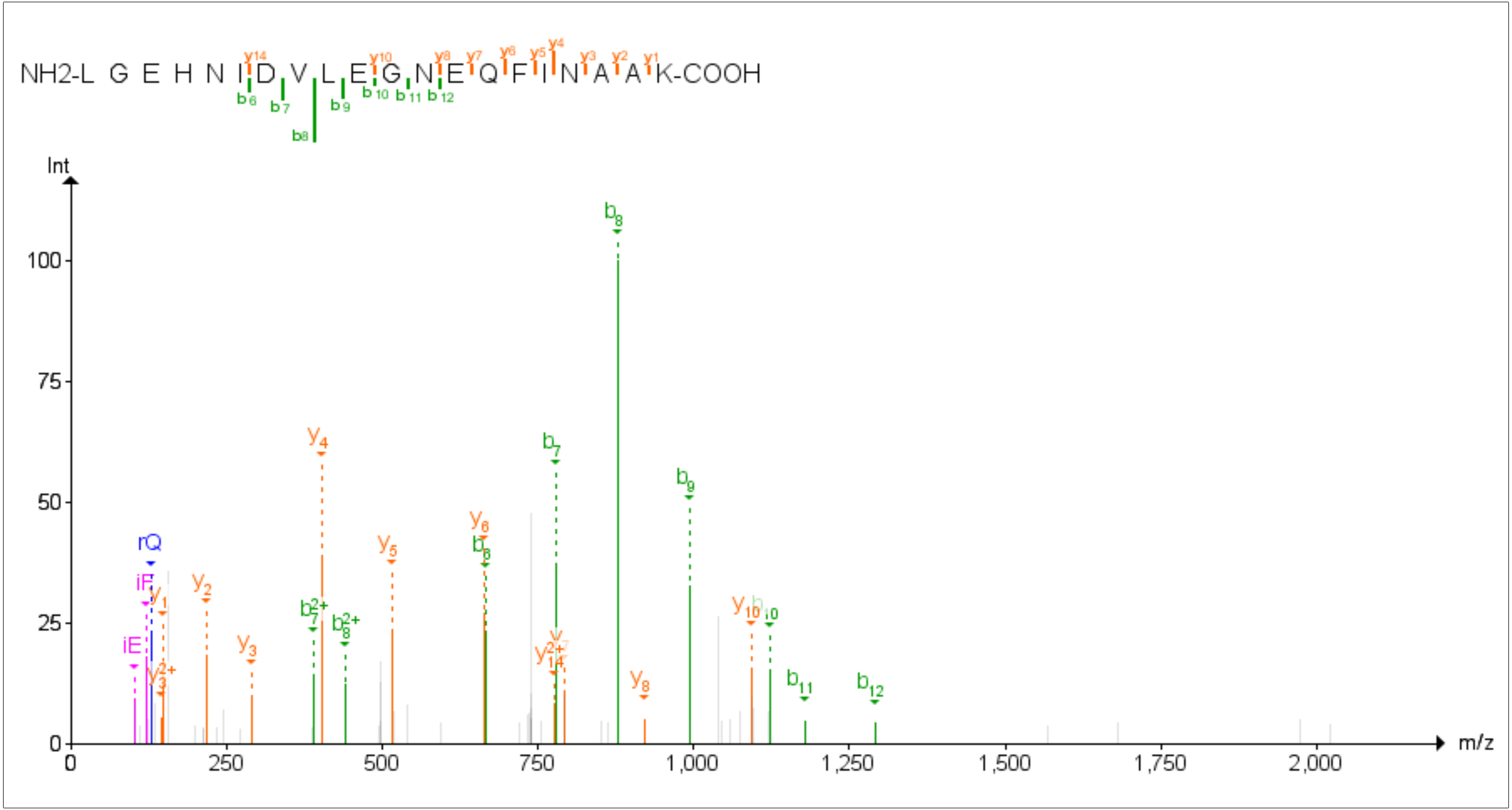
NH2-L G E H N I D V L E G N E Q F I N A A K-COOH
y14 y10 y8 y7 y6 y5 y4 y3 y2 y1
b6 b7 b8 b9 b10 b11 b12
Int
m/z
0
25
50
75
100
250
500
750
1,000
1,250
1,500
1,750
2,000
b8
y4
b7
b9
y6
b6
y5
rQ
iF
y1
y2
iE
y3
y3 2+
b7 2+
b8 2+
y14 2+
y8
y10
b10
b11
b12

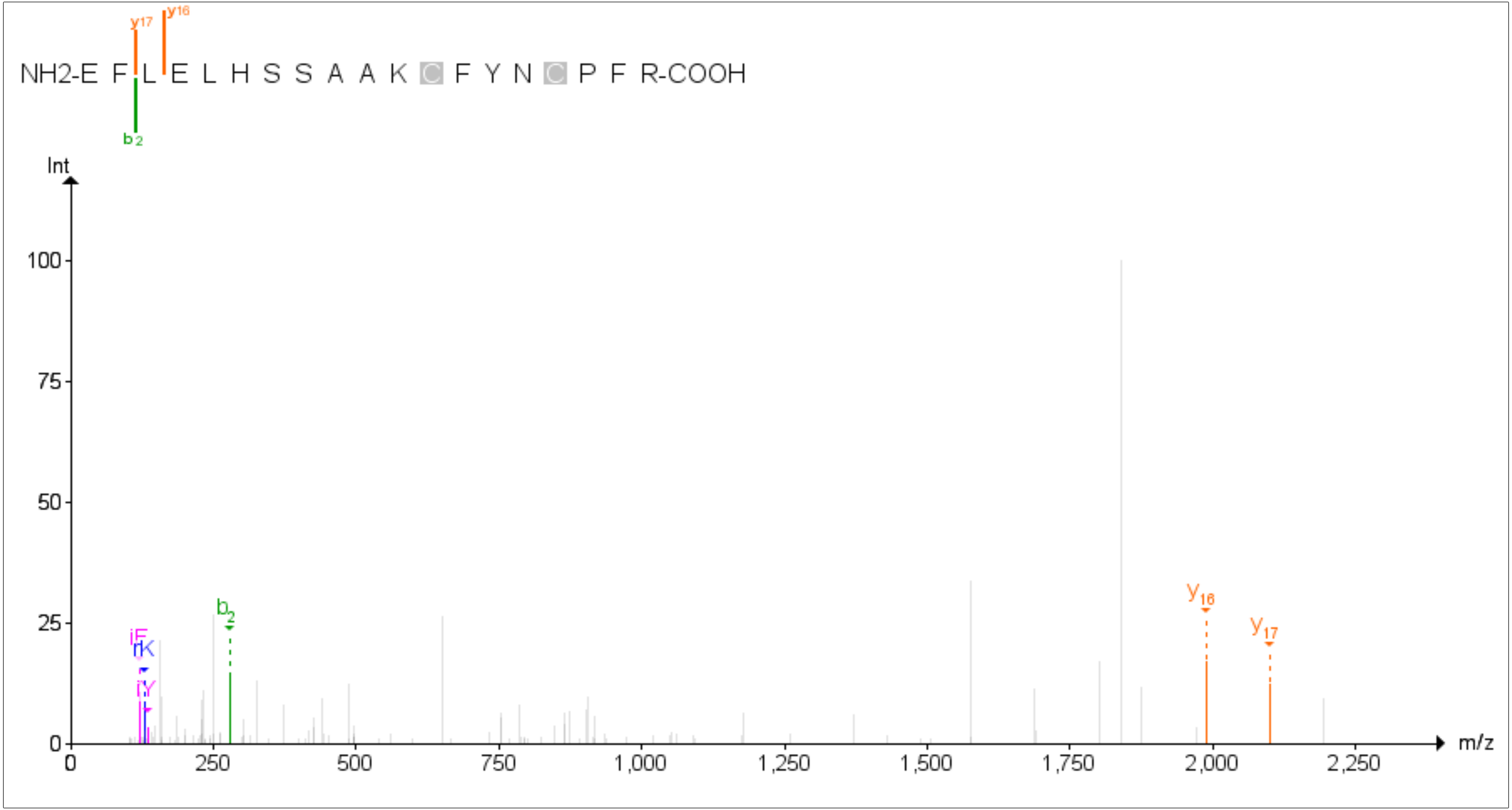

NH2-E F L E L H S S A A K C F Y N C P F R-COOH
y17
y16
b2
Int
100
75
50
25
0
m/z
0
250
500
750
1,000
1,250
1,500
1,750
2,000
2,250
iF
rK
iY
y16
y17

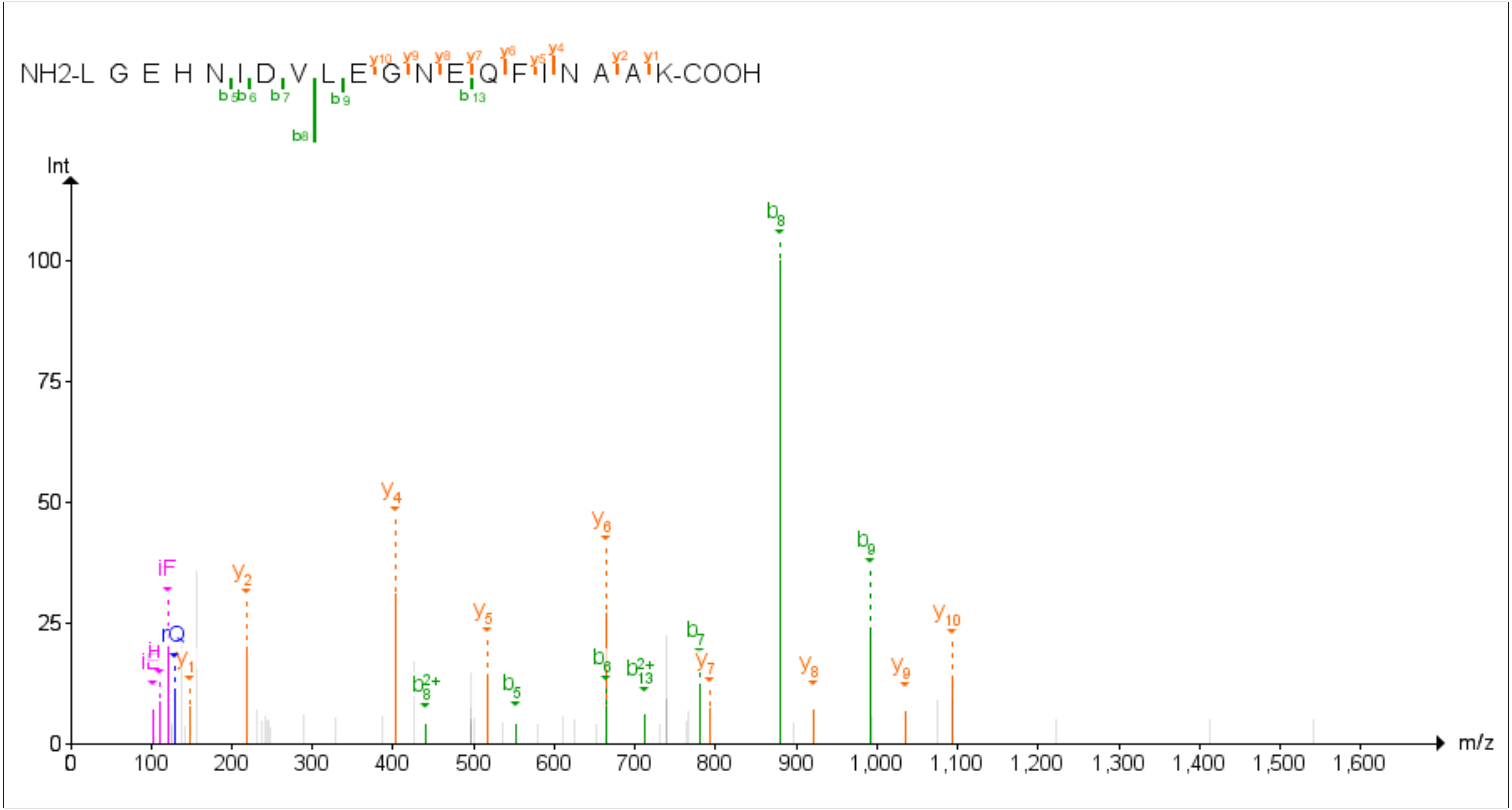

NH2-L G E H N I D V L E G N E Q F I N A A K-COOH
Int
m/z

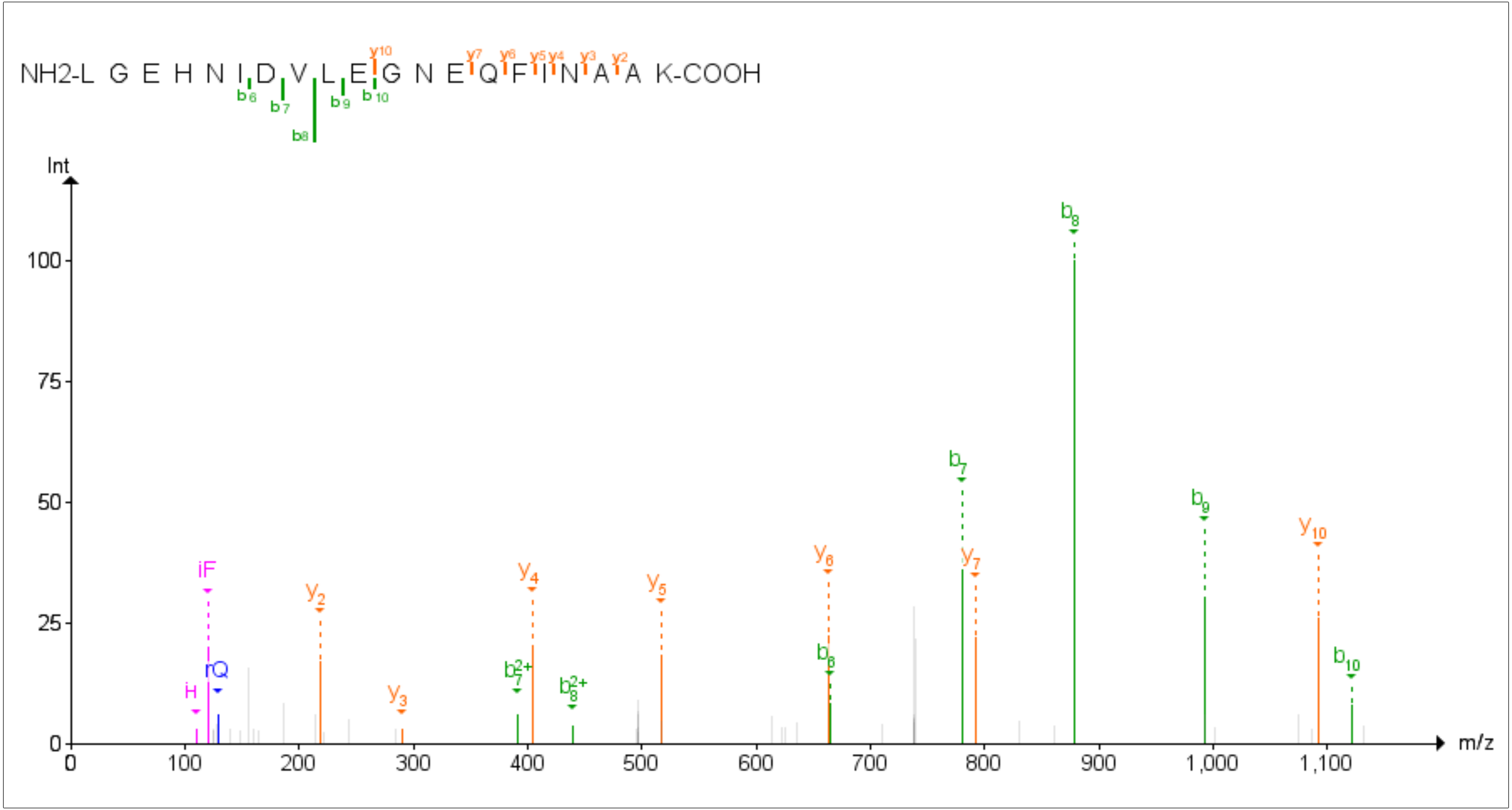

NH2-L G E H N I D V L E G N E Q F I N A A K-COOH
b6 b7 b8 b9 b10
y10 y7 y6 y5 y4 y3 y2
Int
100
75
50
25
0
m/z
0
100
200
300
400
500
600
700
800
900
1,000
1,100
b8
b7
b9
y10
b10
y6
y7
b6
iF
y2
y4
y5
y3
rQ
H
b7 2+
b8 2+

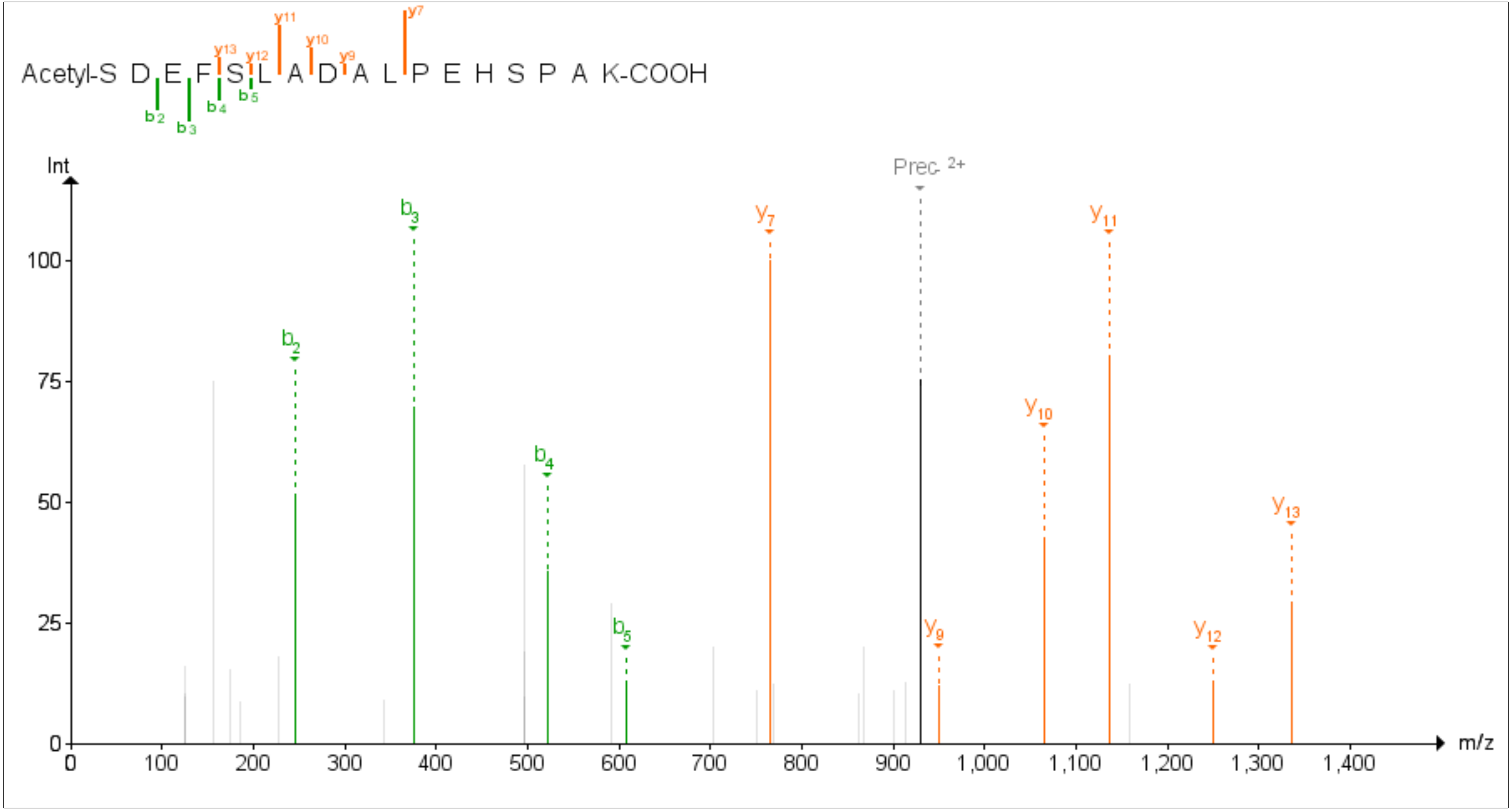

Acetyl-S D E F S L A D A L P E H S P A K-COOH
y13 y12 y11 y10 y9 y7
b2 b3 b4 b5
Int
100
75
50
25
0
Prec 2+
b2
b3
b4
b5
y7
y9
y10
y11
y12
y13
0 100 200 300 400 500 600 700 800 900 1,000 1,100 1,200 1,300 1,400
m/z

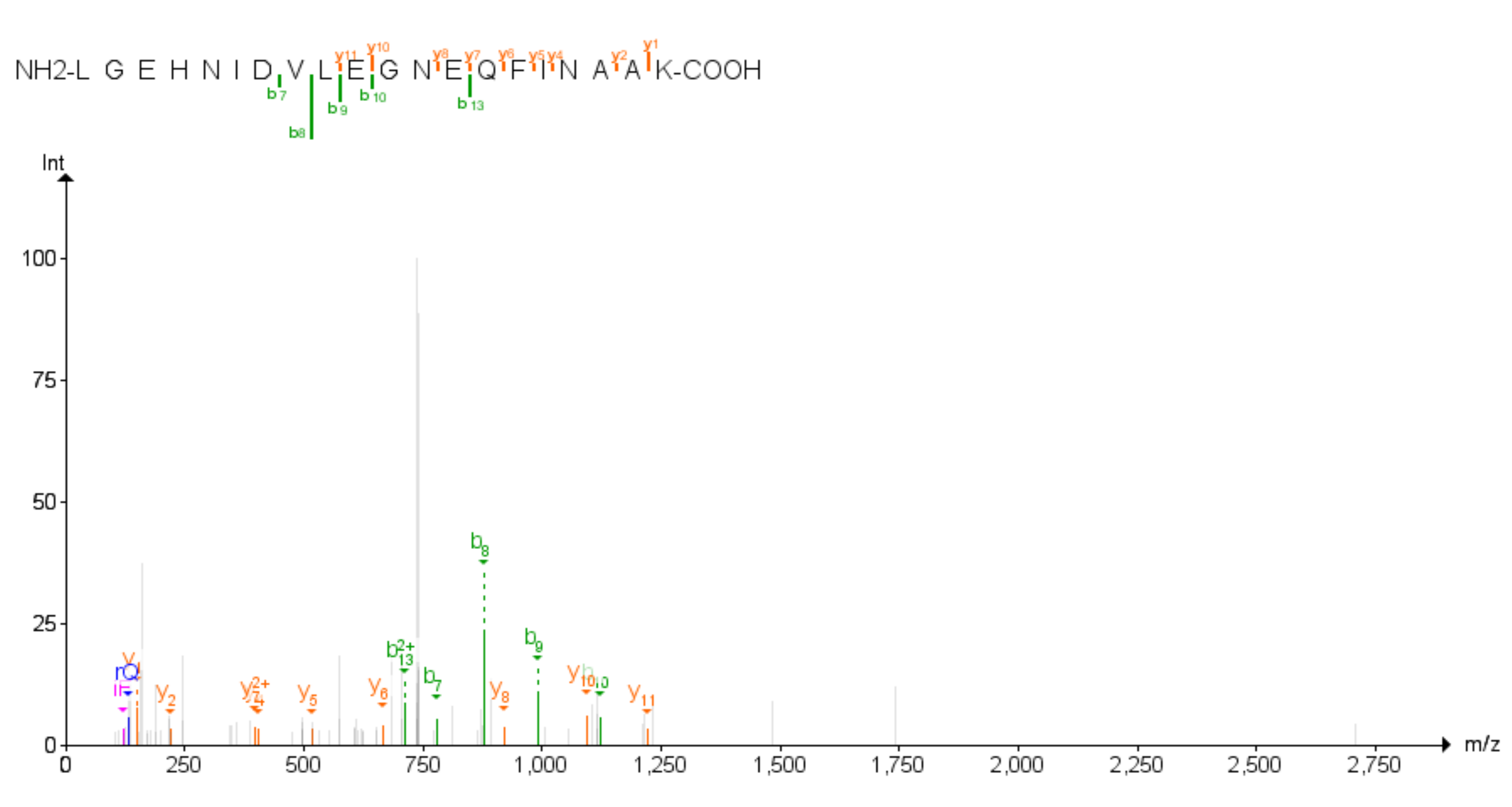

NH2-L G E H N I D V L E G N E Q F I N A A K-COOH
Int
100
75
50
25
0
m/z
250
500
750
1,000
1,250
1,500
1,750
2,000
2,250
2,500
2,750

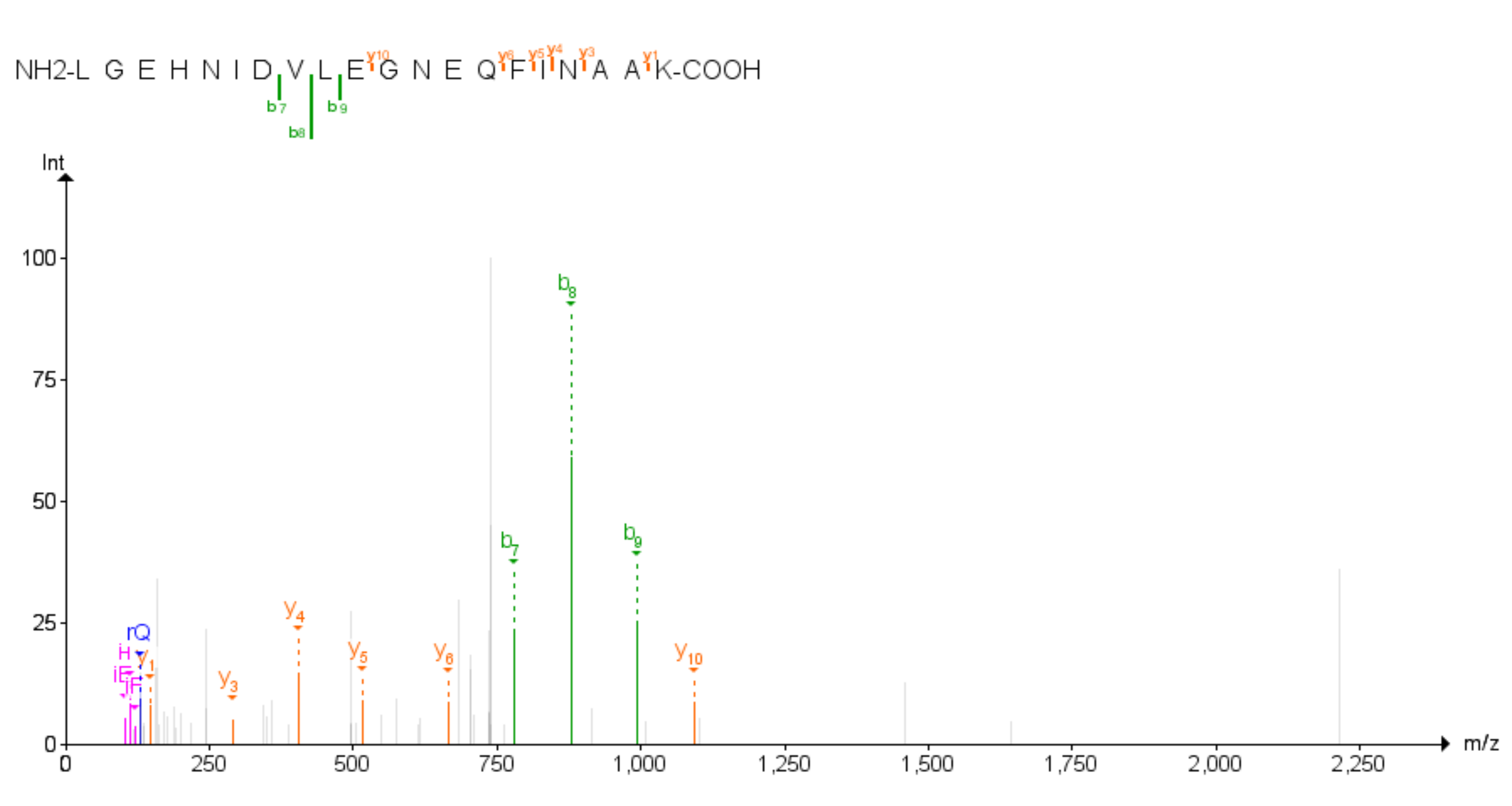

NH2-L G E H N I D V L E G N E Q F I N A A K-COOH
b7
b8
b9
y10
y6
y5
y4
y3
y1
Int
100
75
50
25
0
rQ
H
iE
iF
m/z
0
250
500
750
1,000
1,250
1,500
1,750
2,000
2,250

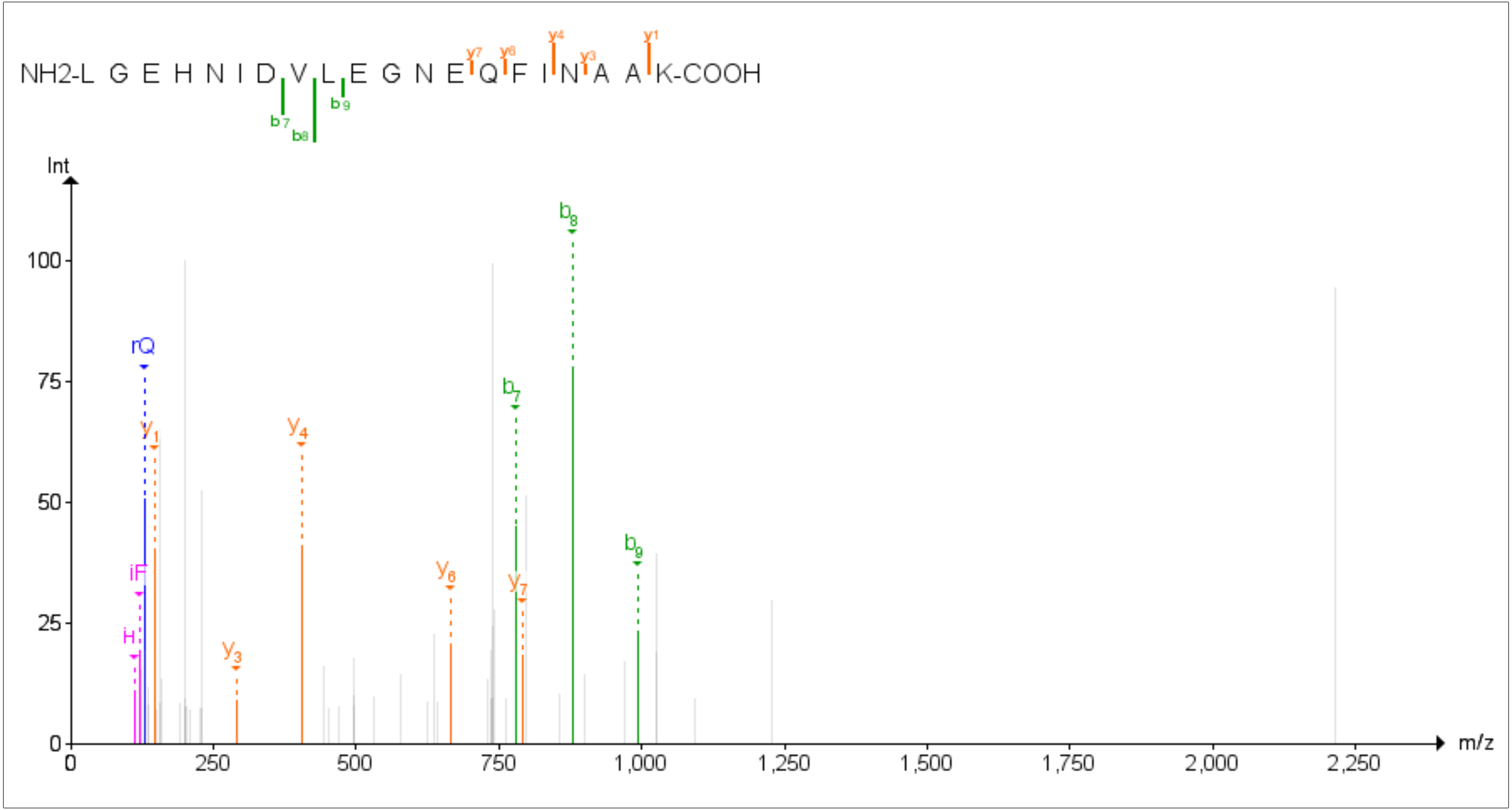

NH2-L G E H N I D V L E G N E Q F I N A A K-COOH
y7 y6 y4 y3 y1
b7 b8 b9
Int
m/z
0
25
50
75
100
250
500
750
1,000
1,250
1,500
1,750
2,000
2,250
rQ
y1
iF
iH
y3
y4
y6
y7
b7
b8
b9

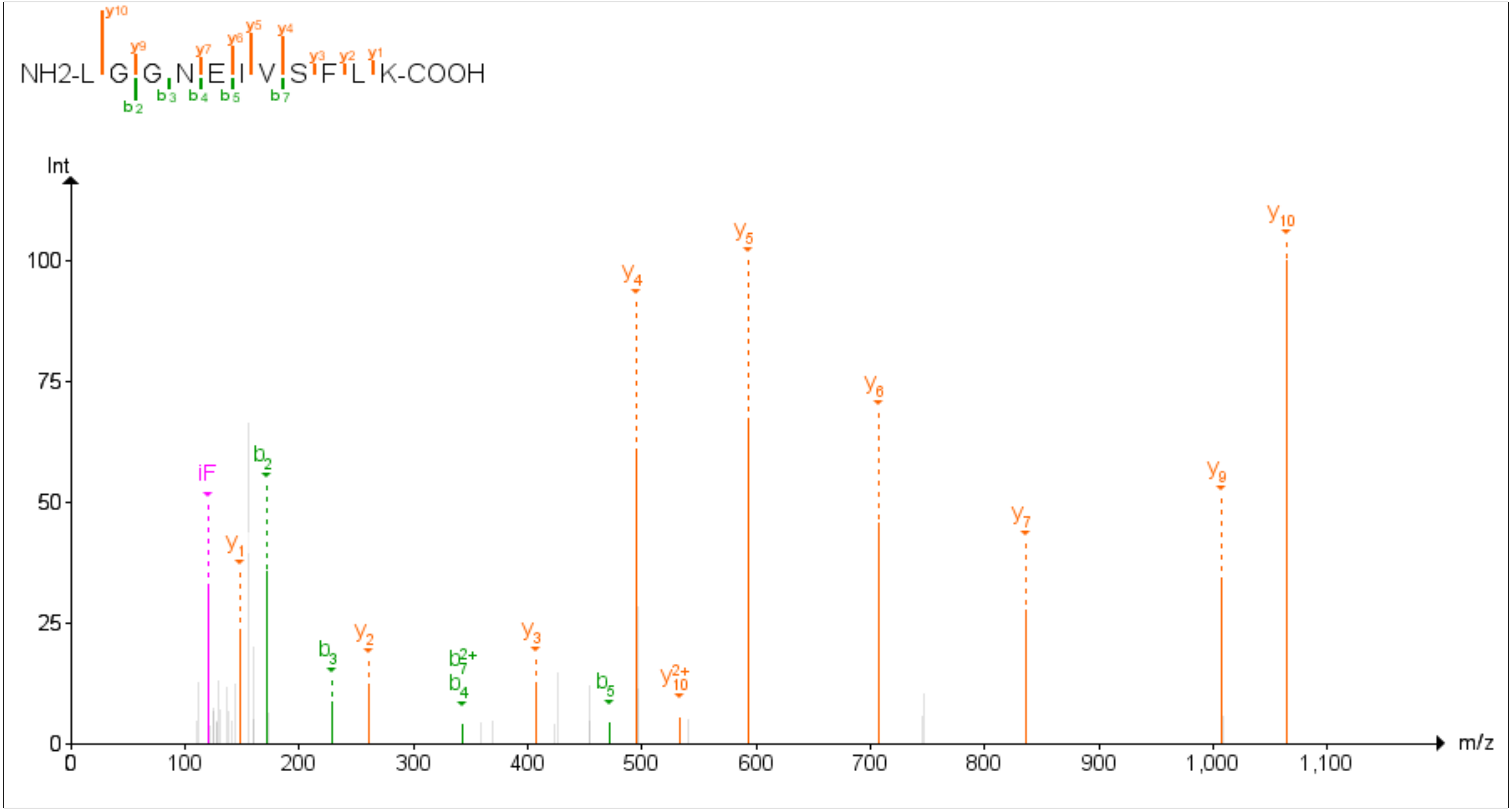

NH2-L G G N E I V S F L K-COOH
y10 y9 y7 y6 y5 y4 y3 y2 y1
b2 b3 b4 b5 b7
Int
m/z
iF
y1
b2
b3
y2
b7 2+
b4
y3
b5
y10 2+
y4
y5
y6
y7
y9
y10
0
25
50
75
100
0
100
200
300
400
500
600
700
800
900
1,000
1,100

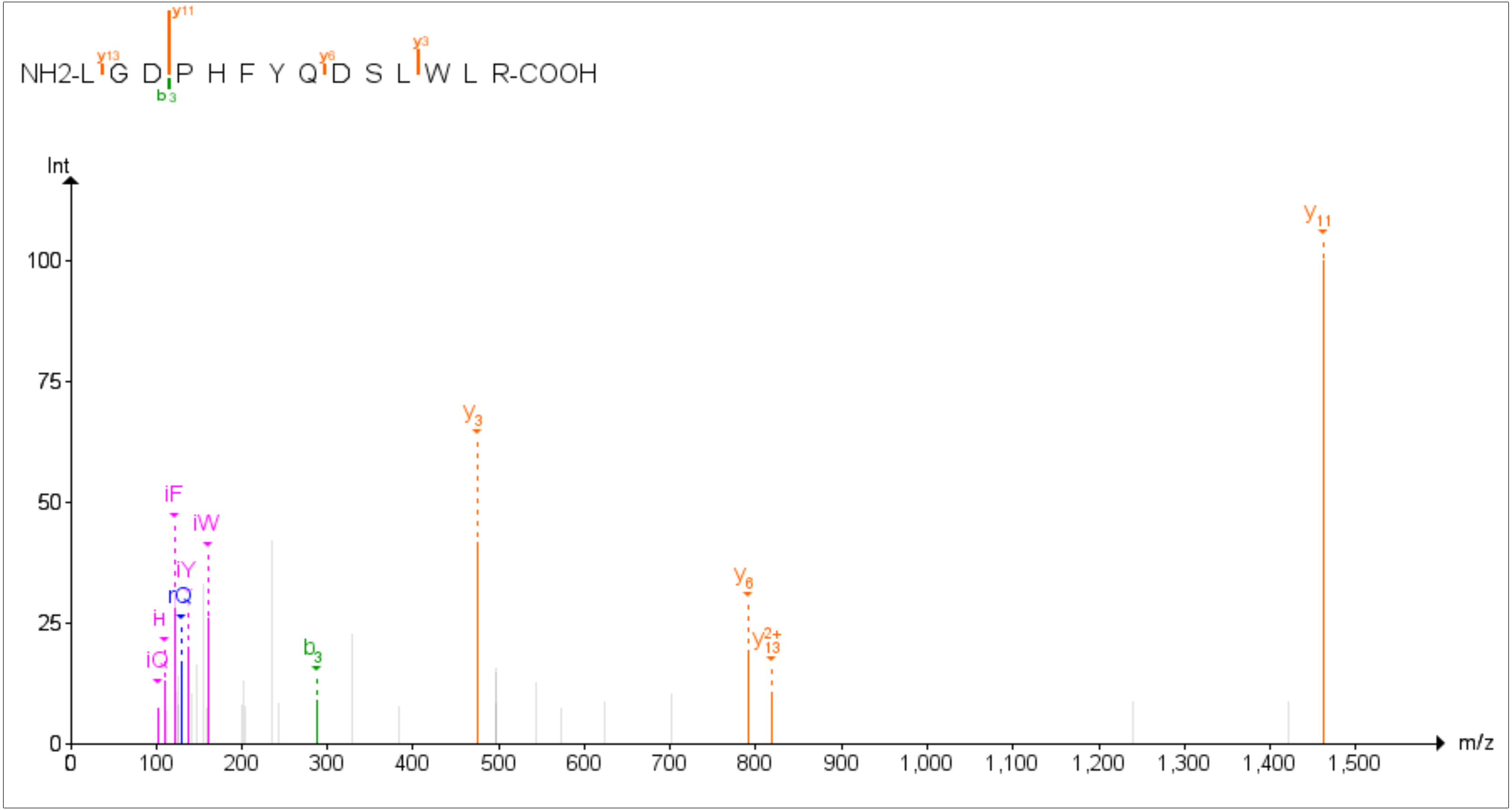

NH2-L G D P H F Y Q D S L W L R-COOH
y13
y11
b3
y6
y3
Int
100
75
50
25
0
y11
y3
iF
iW
iY
rQ
H
iQ
b3
y6
y13 2+
0
100
200
300
400
500
600
700
800
900
1,000
1,100
1,200
1,300
1,400
1,500
m/z

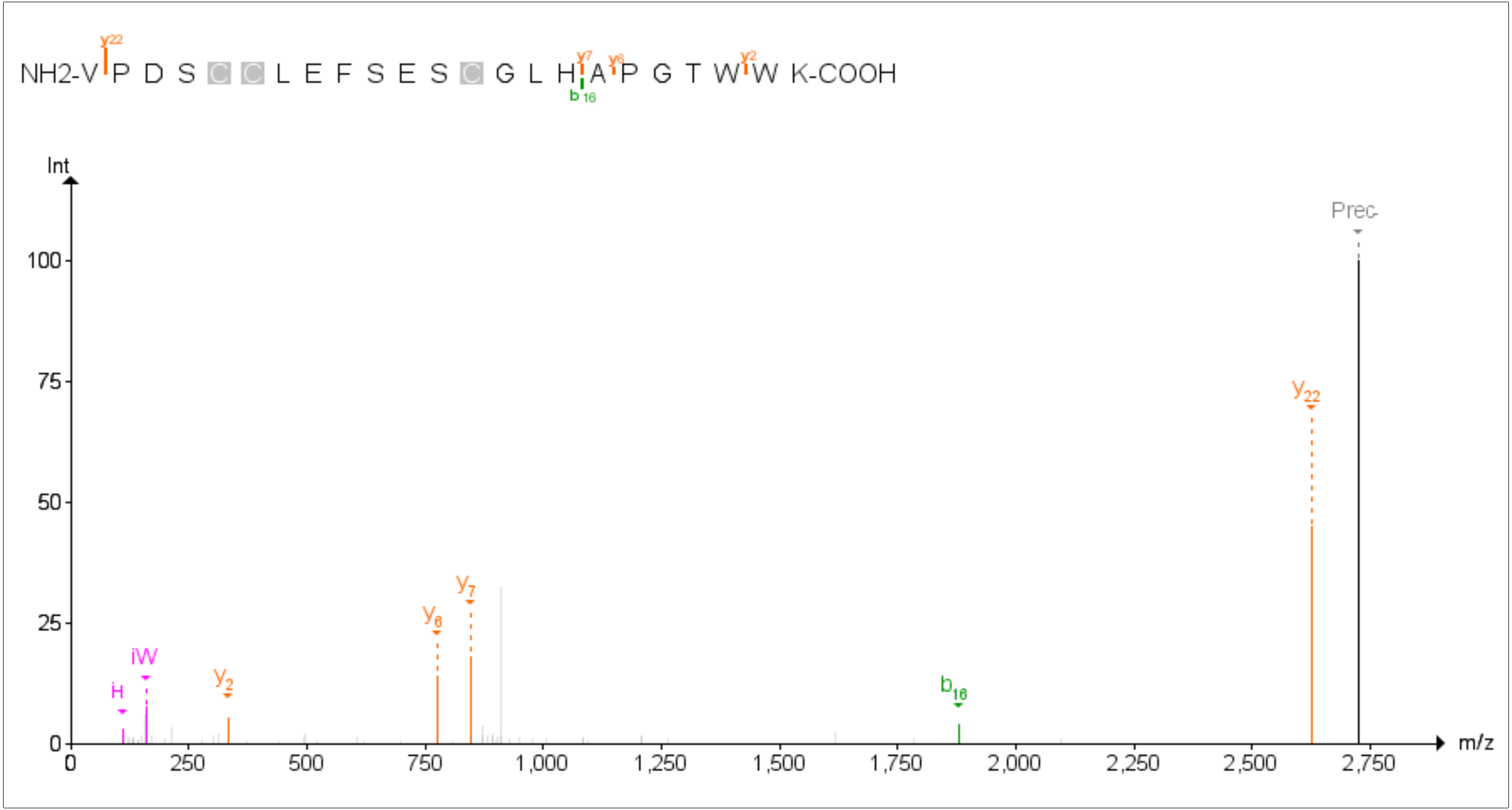
NH2-V P D S C C L E F S E S C G L H A P G T W W K-COOH
y22
y7
y6
y2
b16
Int
Prec.
100
75
50
25
0
y22
y7
y6
iW
H
y2
b16
0
250
500
750
1,000
1,250
1,500
1,750
2,000
2,250
2,500
2,750
m/z

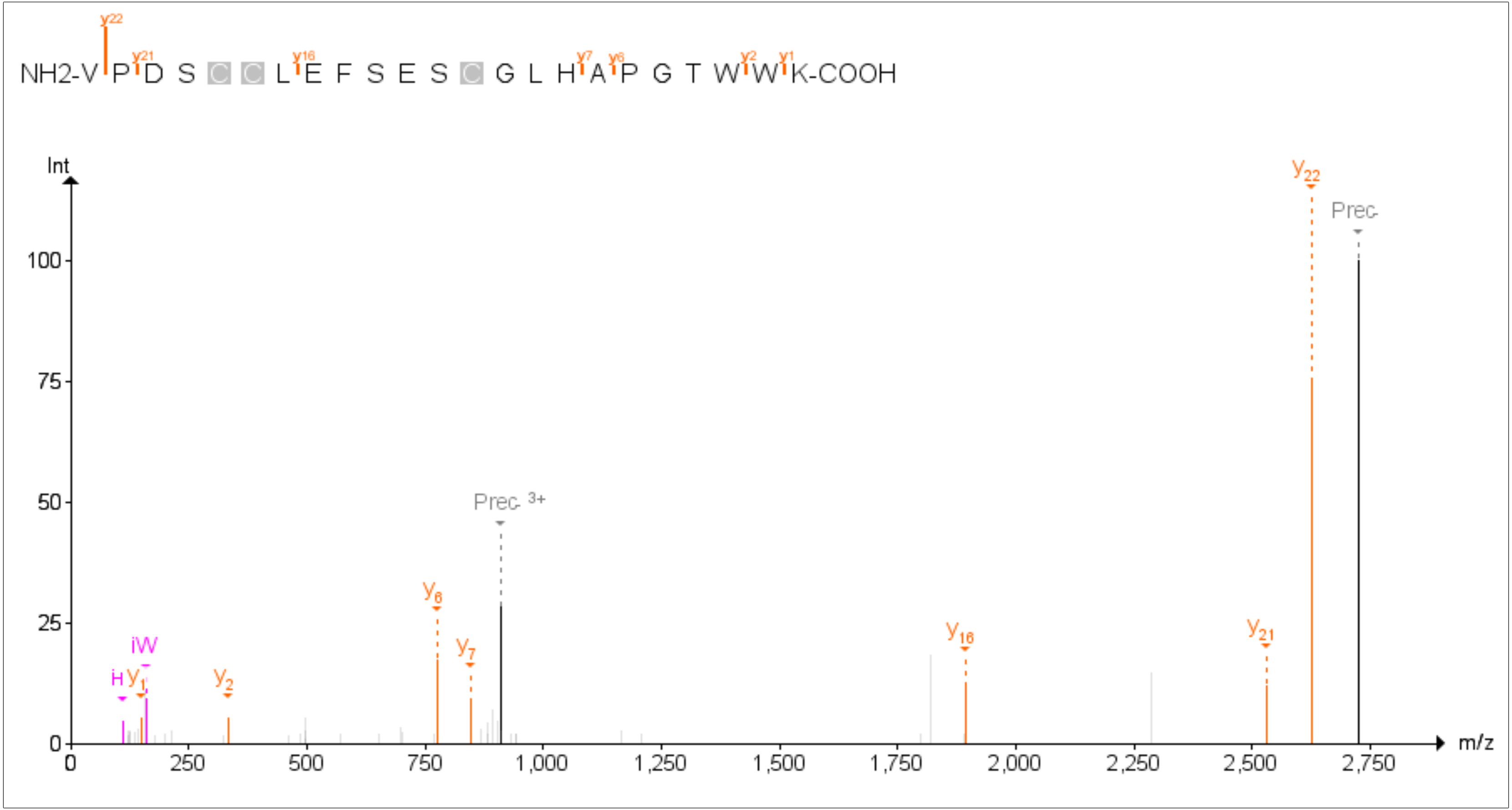

NH2-V P D S C C L E F S E S C G L H A P G T W W K-COOH
y22 y21 y16 y7 y6 y2 y1
Int
m/z
Prec.
Prec. 3+
y22
y21
y16
y7
y6
y2
y1
iW
iH
0
25
50
75
100
0
250
500
750
1,000
1,250
1,500
1,750
2,000
2,250
2,500
2,750

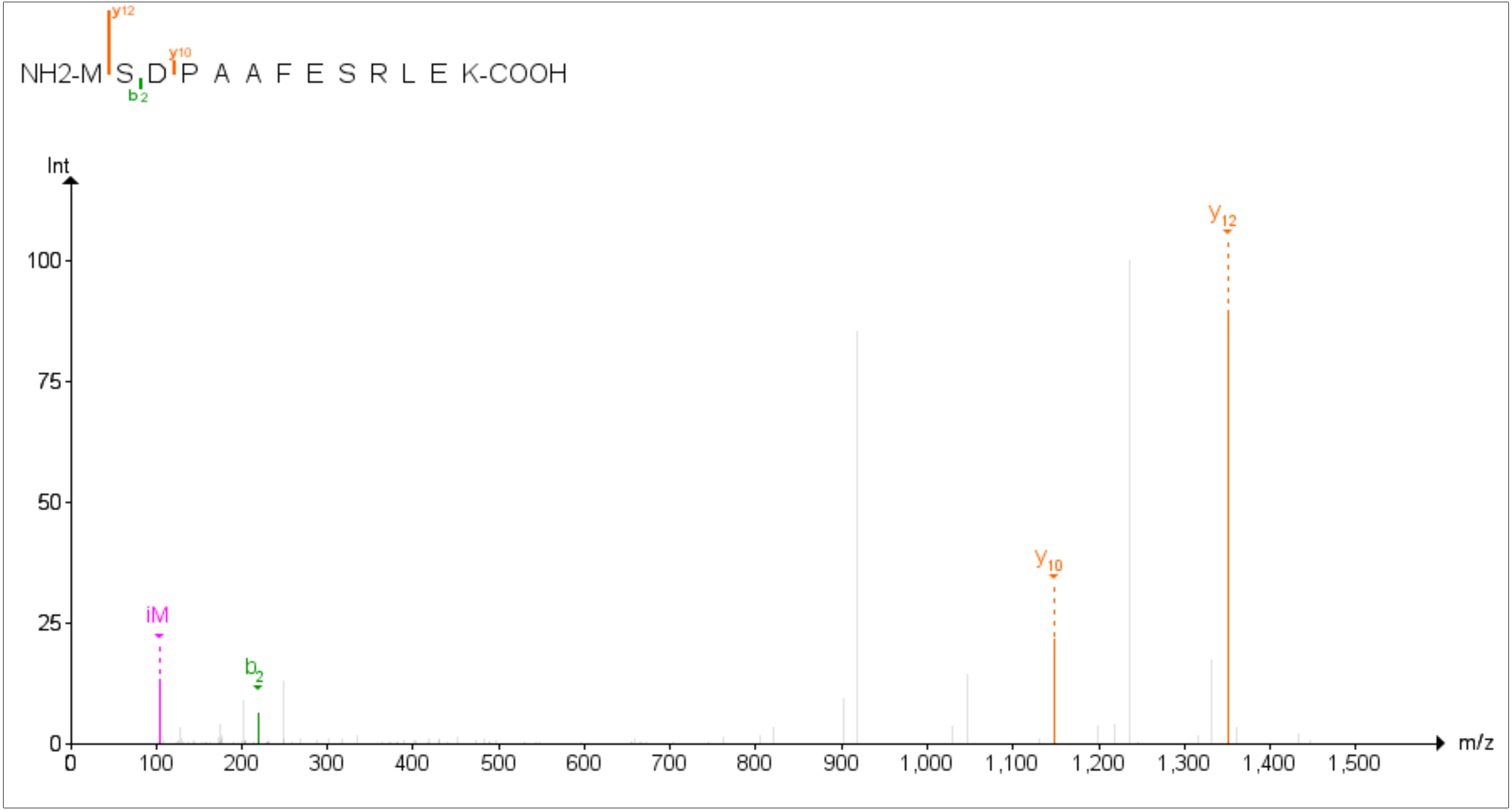
NH2-M S D P A A F E S R L E K-COOH
y12
y10
b2
Int
100
75
50
25
0
iM
b2
y10
y12
m/z
0
100
200
300
400
500
600
700
800
900
1,000
1,100
1,200
1,300
1,400
1,500

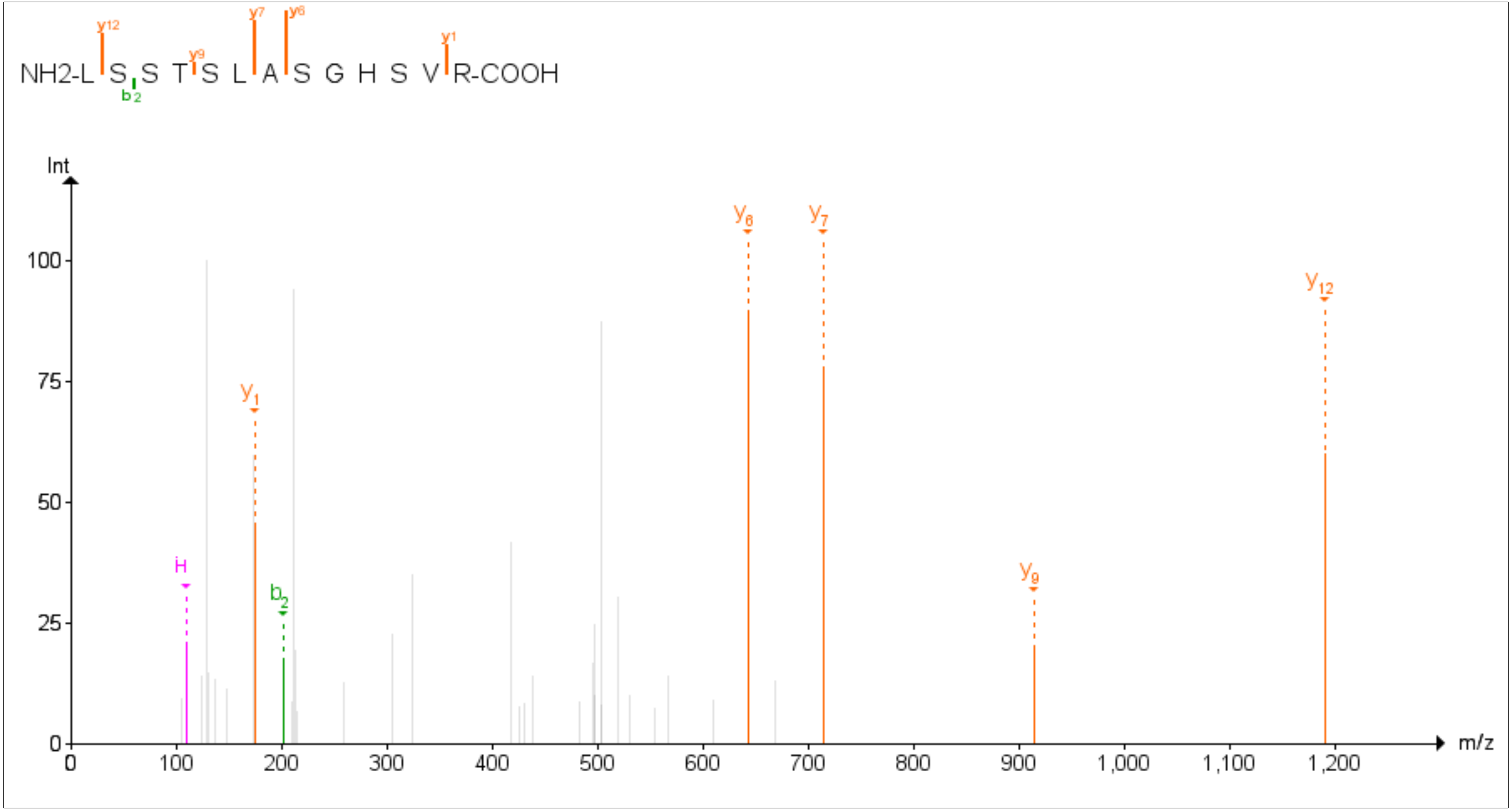

NH2-L S S T S L A S G H S V R-COOH
y12
y9
y7
y6
y1
b2
Int
100
75
50
25
0
y6
y7
y12
y1
H
b2
y9
0
100
200
300
400
500
600
700
800
900
1,000
1,100
1,200
m/z

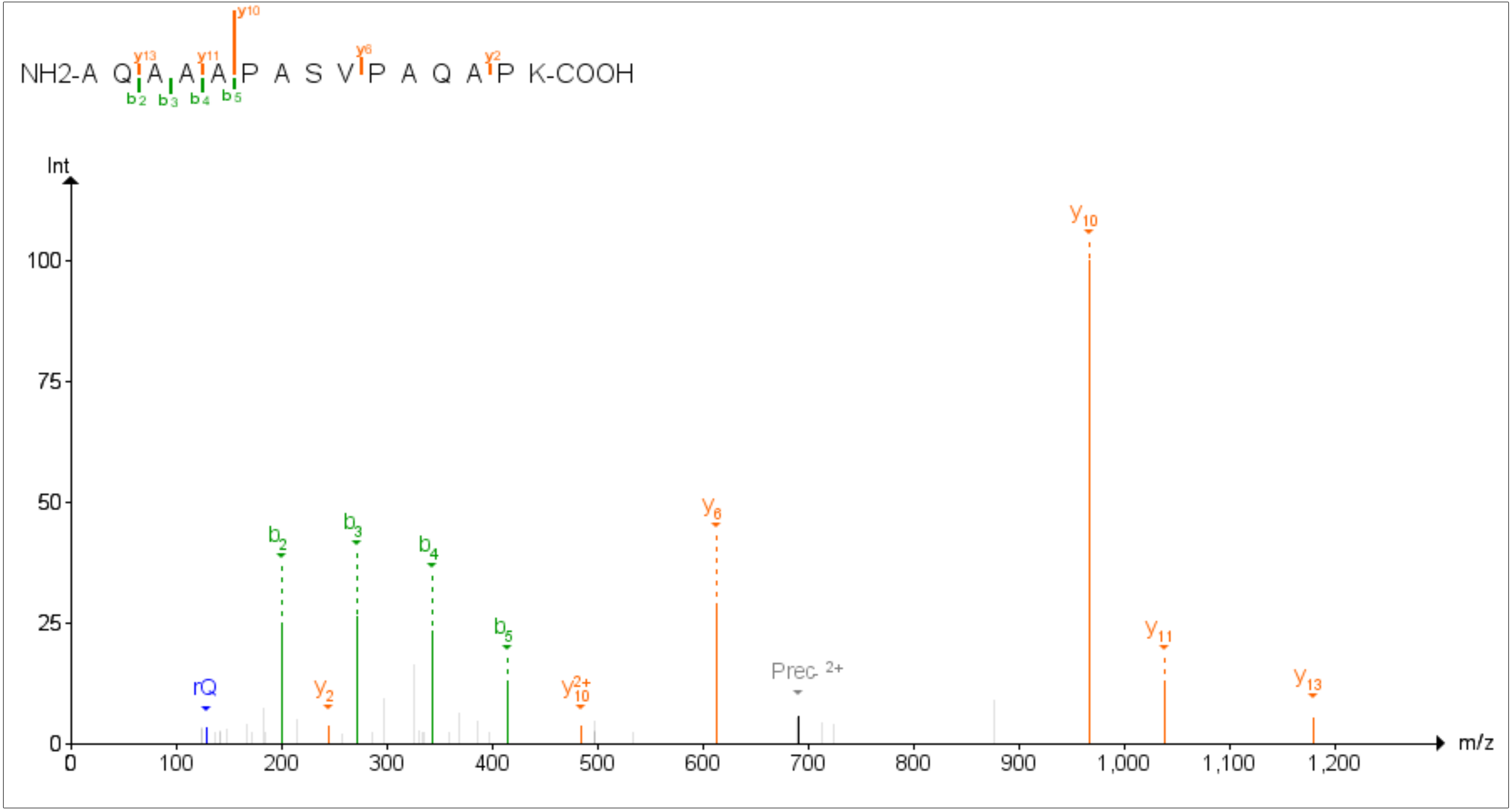

NH2-A Q A A A P A S V P A Q A P K-COOH
y13
y11
y10
y6
y2
b2
b3
b4
b5
Int
100
75
50
25
0
rQ
b2
y2
b3
b4
b5
y10 2+
y6
Prec 2+
y10
y11
y13
0
100
200
300
400
500
600
700
800
900
1,000
1,100
1,200
m/z

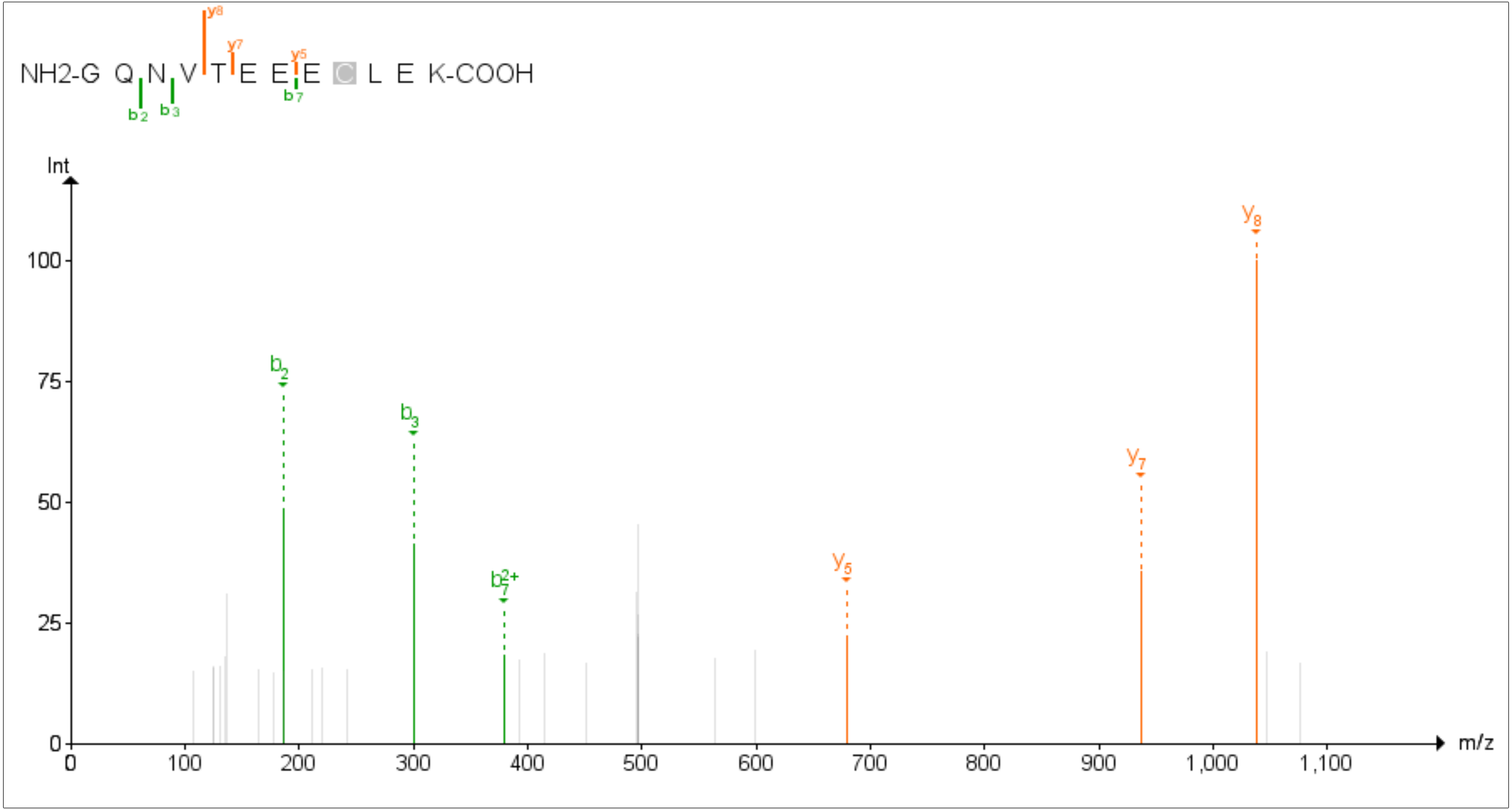
NH2-G Q N V T E E E C L E K-COOH
y8
y7
y5
b2
b3
b7
Int
100
75
50
25
0
b2
b3
b7 2+
y5
y7
y8
0
100
200
300
400
500
600
700
800
900
1,000
1,100
m/z

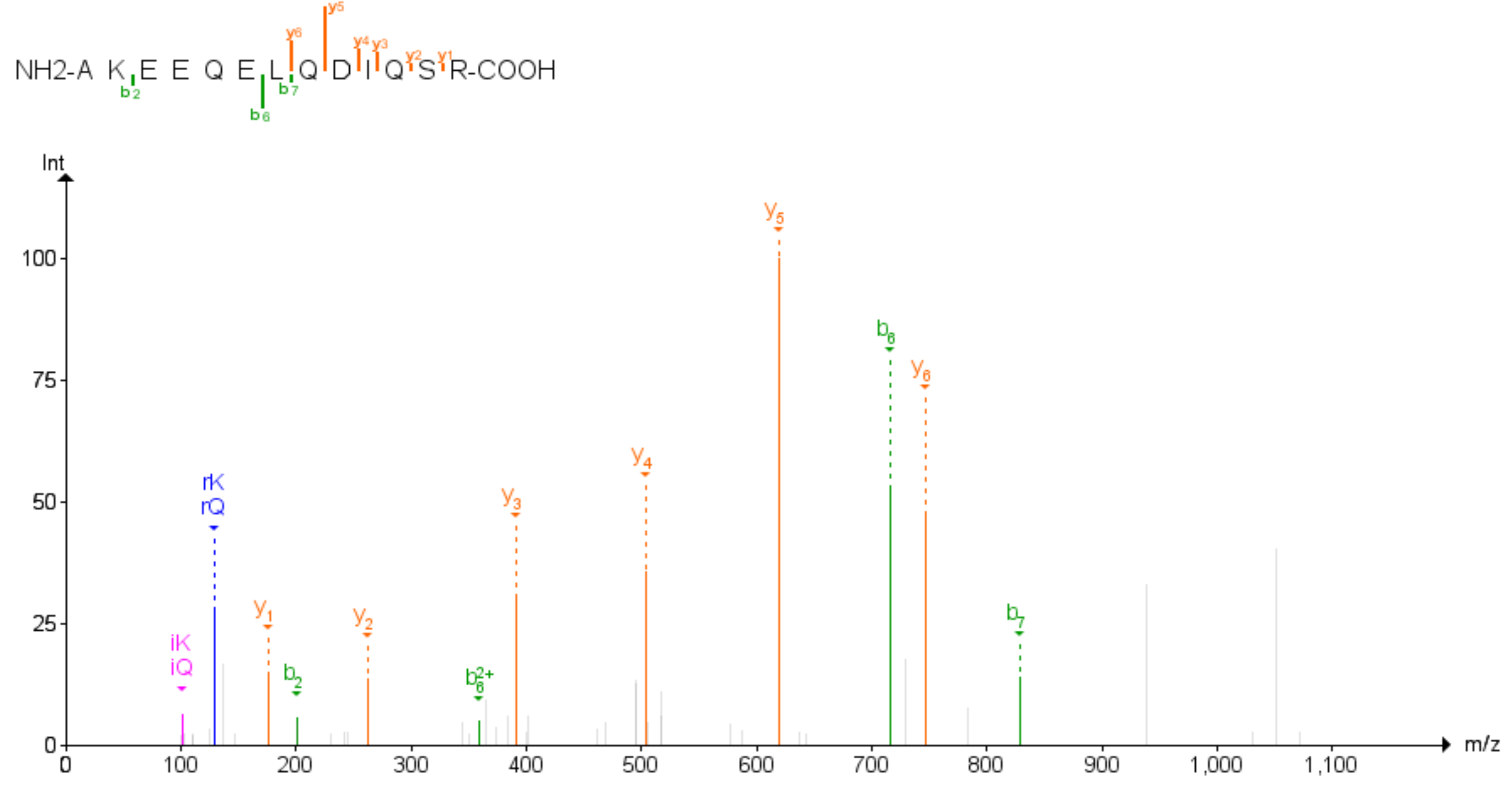

NH2-A K E E Q E L Q D I Q S R-COOH
y5
y6
y4
y3
y2
y1
b2
b7
b6
Int
100
75
50
25
0
y5
b6
y6
y4
y3
rK
rQ
y1
y2
iK
iQ
b2
b6 2+
b7
0
100
200
300
400
500
600
700
800
900
1,000
1,100
m/z

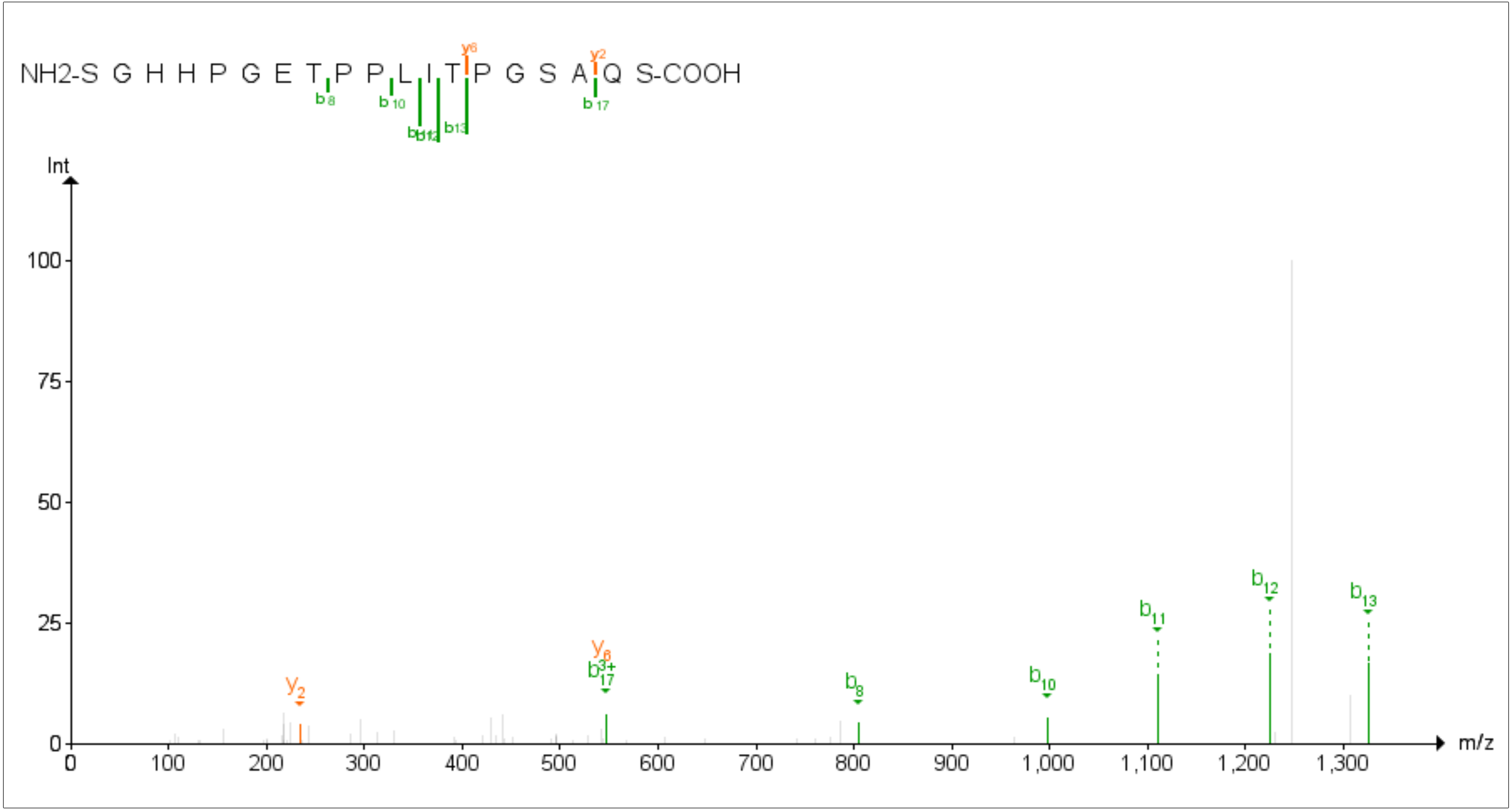

NH2-S G H H P G E T P P L I T P G S A Q S-COOH
y6
y2
b8
b10
b11
b12
b13
b17
Int
100
75
50
25
0
m/z
0
100
200
300
400
500
600
700
800
900
1,000
1,100
1,200
1,300
y_2
y_6
b^{3+}_{17}
b_8
b_{10}
b_{11}
b_{12}
b_{13}

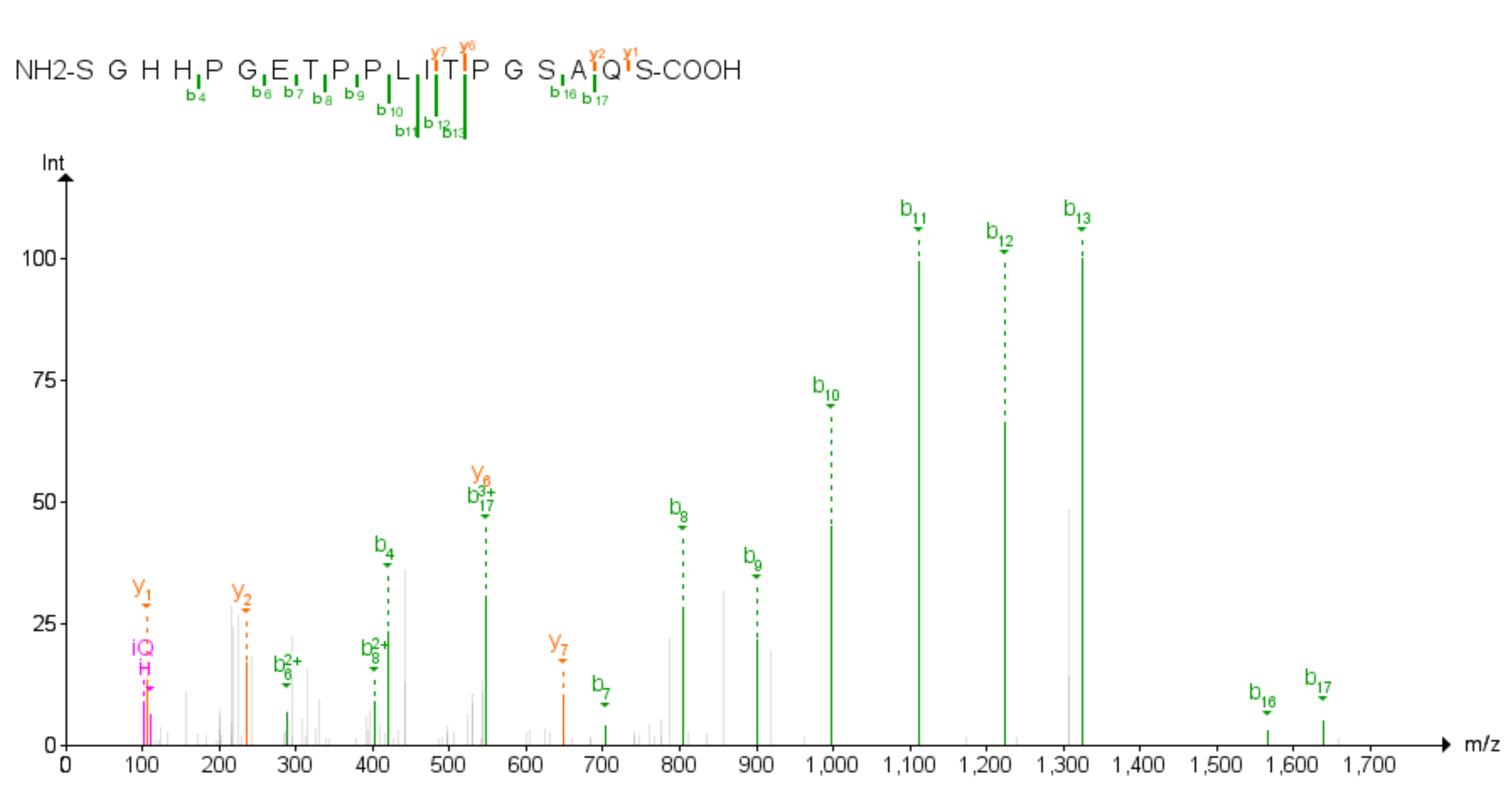

NH2-S G H H P G E T P P L I T P G S A Q S-COOH
Int
m/z

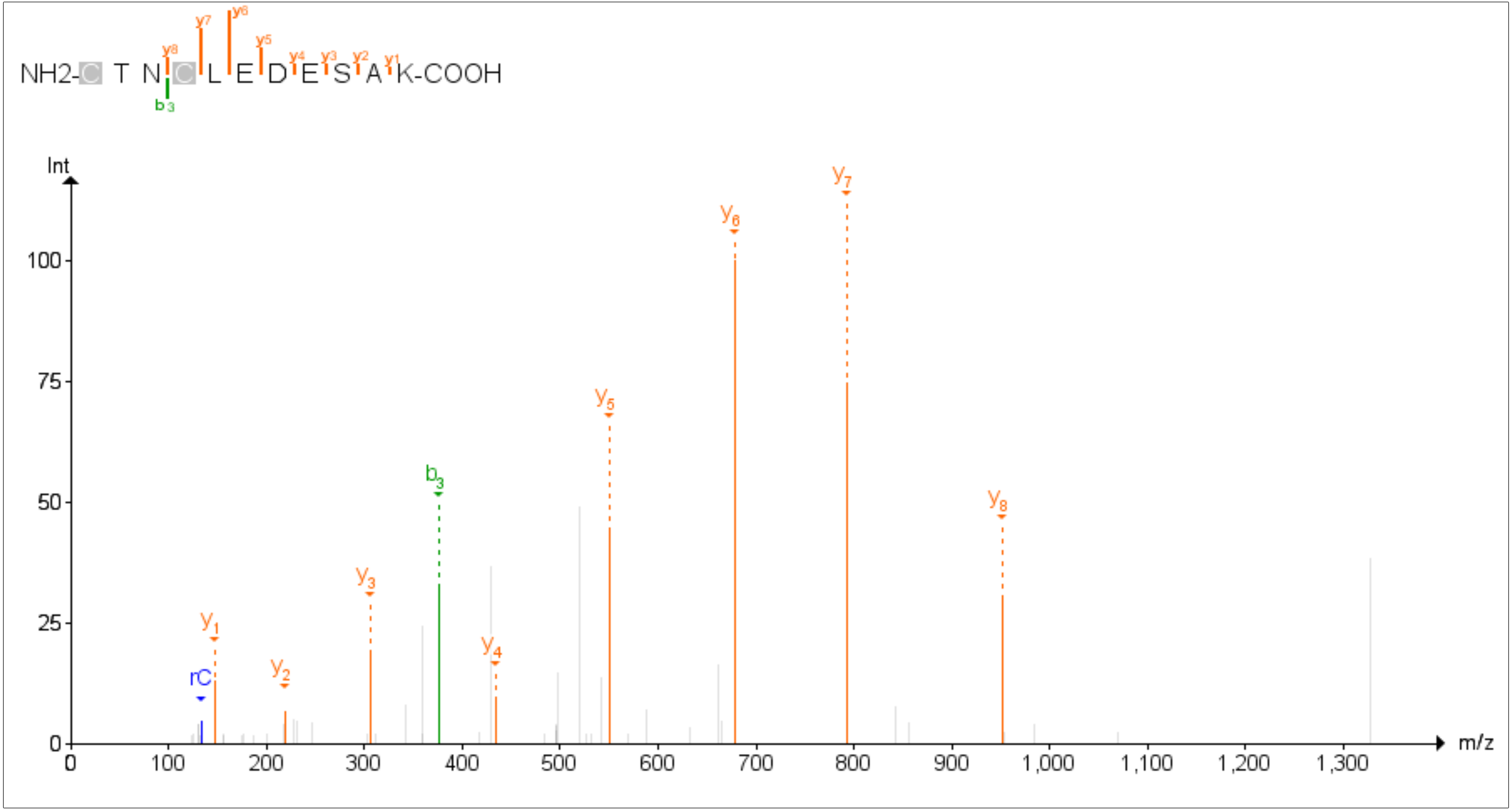

NH2-C T N C L E D E S A K-COOH
y8
y7
y6
y5
y4
y3
y2
y1
b3
Int
m/z
0
25
50
75
100
0
100
200
300
400
500
600
700
800
900
1,000
1,100
1,200
1,300
rC
y1
y2
y3
b3
y4
y5
y6
y7
y8

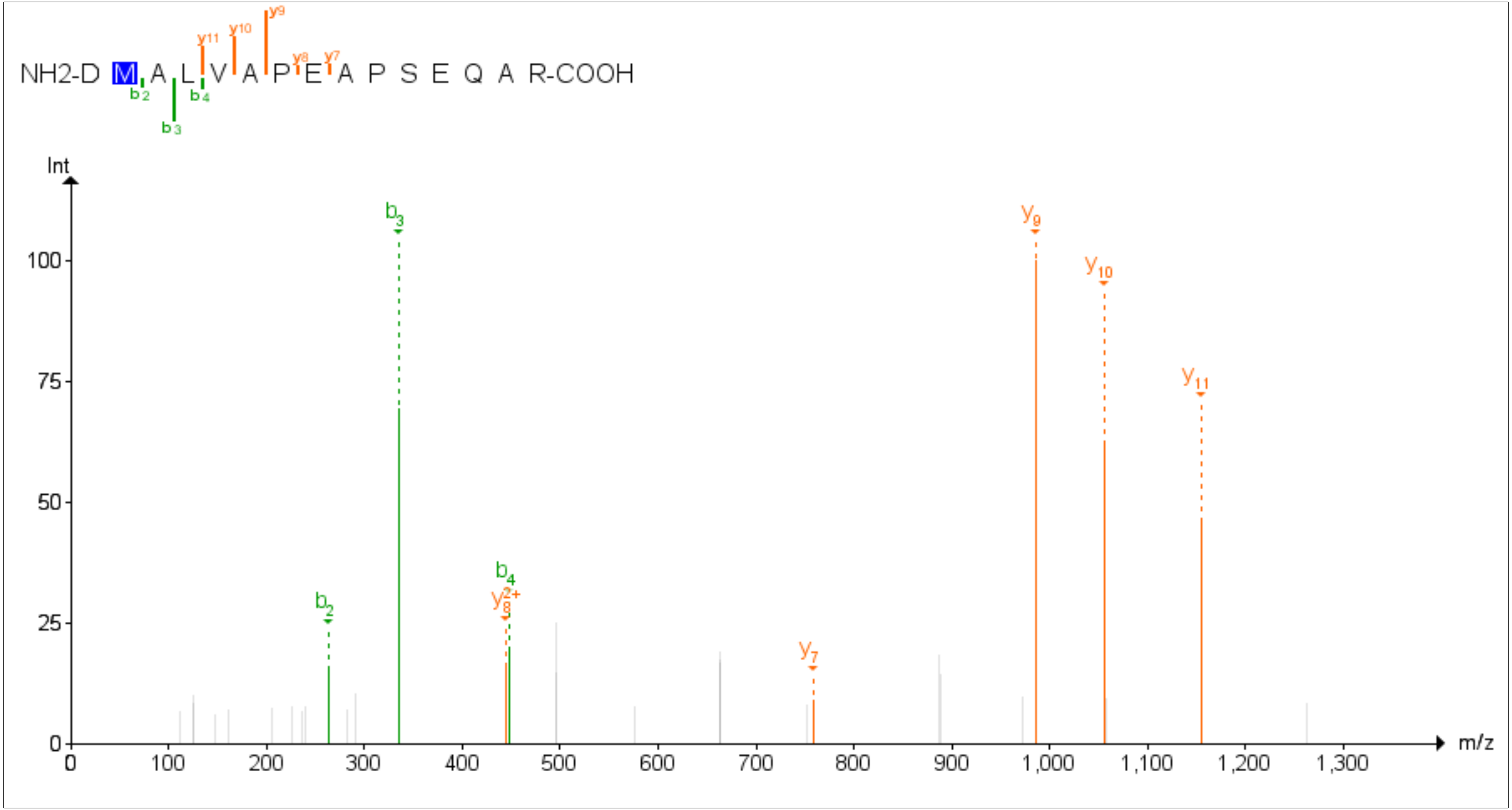
NH2-D M A L V A P E A P S E Q A R-COOH
b2 b3 b4 y11 y10 y9 y8 y7
Int
100
75
50
25
0
m/z
0 100 200 300 400 500 600 700 800 900 1,000 1,100 1,200 1,300
b3
b2
b4
y8 2+
y7
y9
y10
y11

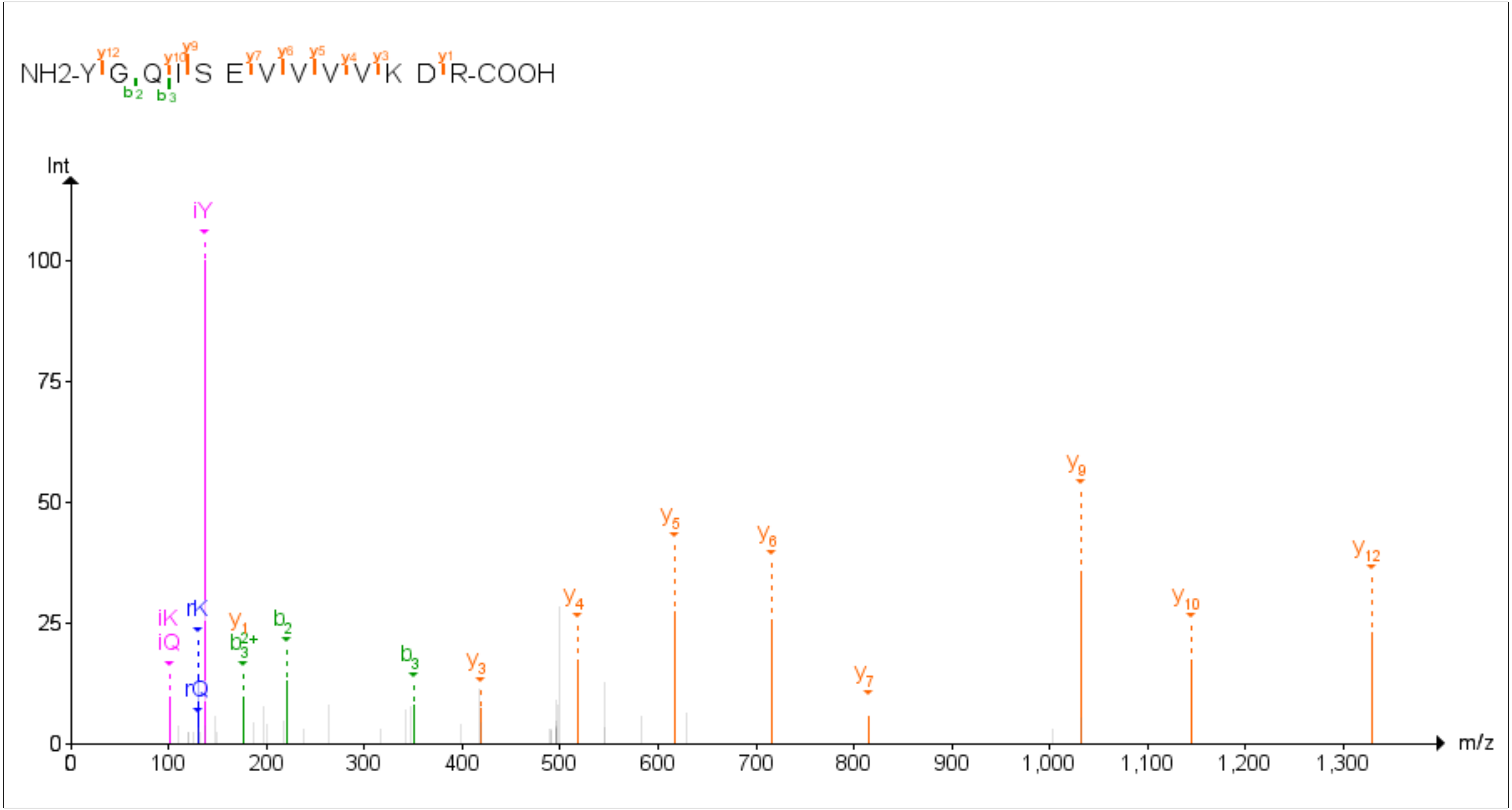

NH2-Y G Q I I S E V V V V K D R-COOH
y12 y10 y9 y7 y6 y5 y4 y3 y1
b2 b3
Int
100
75
50
25
0
iY
iK
iQ
rK
rQ
y1
b3 2+
b2
b3
y3
y4
y5
y6
y7
y9
y10
y12
0
100
200
300
400
500
600
700
800
900
1,000
1,100
1,200
1,300
m/z

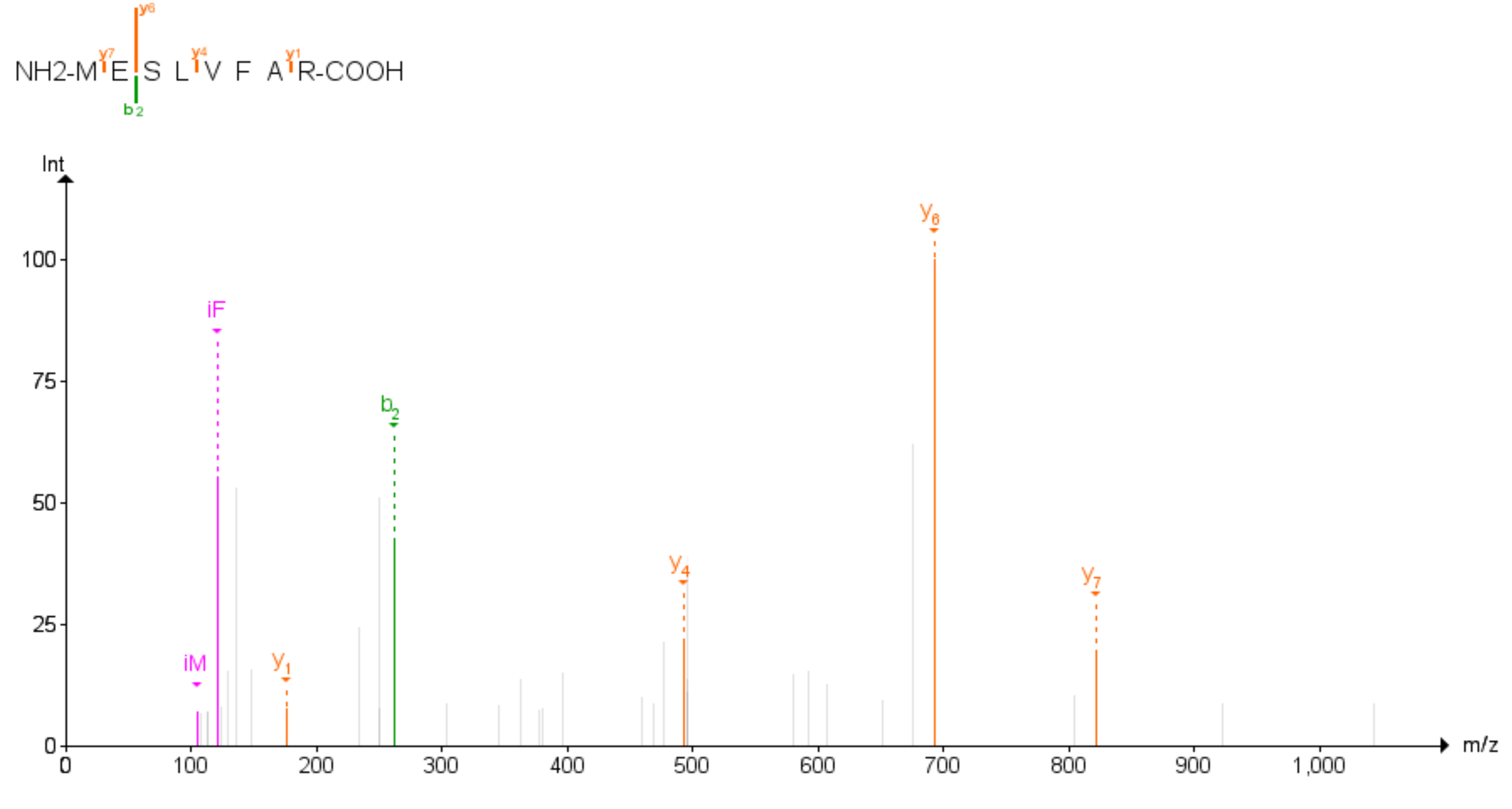
NH2-M E S L V F A R-COOH
y8
y7
y4
y1
b2
Int
100
75
50
25
0
iF
b2
y8
y4
y7
iM
y1
0
100
200
300
400
500
600
700
800
900
1,000
m/z

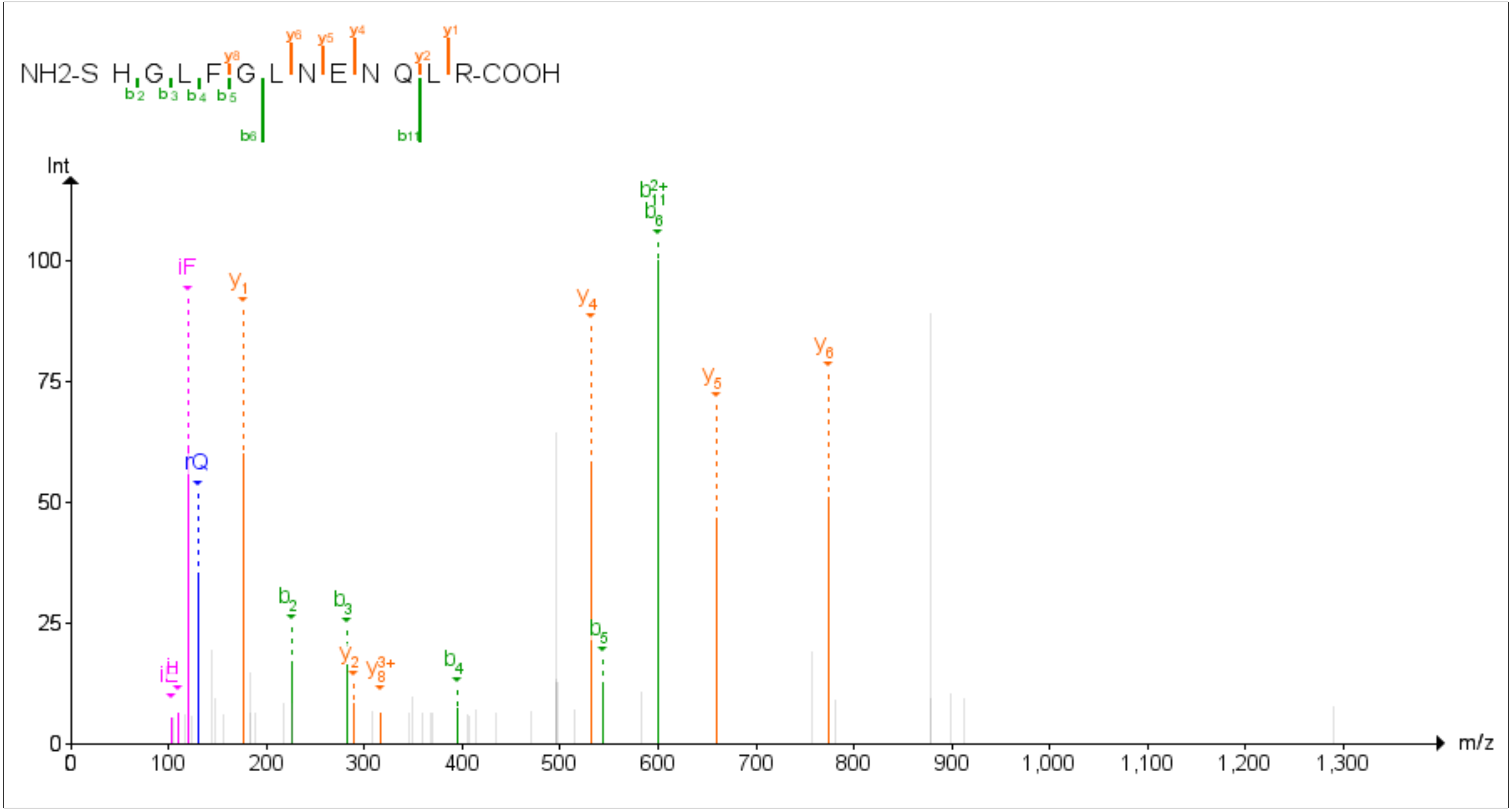

NH2-S H G L F G L N E N Q L R-COOH
b2 b3 b4 b5 b6 b11
y8 y6 y5 y4 y2 y1
Int
m/z
iF
y1
rQ
iL
iH
b2
b3
y2
y8 3+
b4
b5
b11 2+
b6
y4
y5
y6
0
25
50
75
100
0
100
200
300
400
500
600
700
800
900
1,000
1,100
1,200
1,300

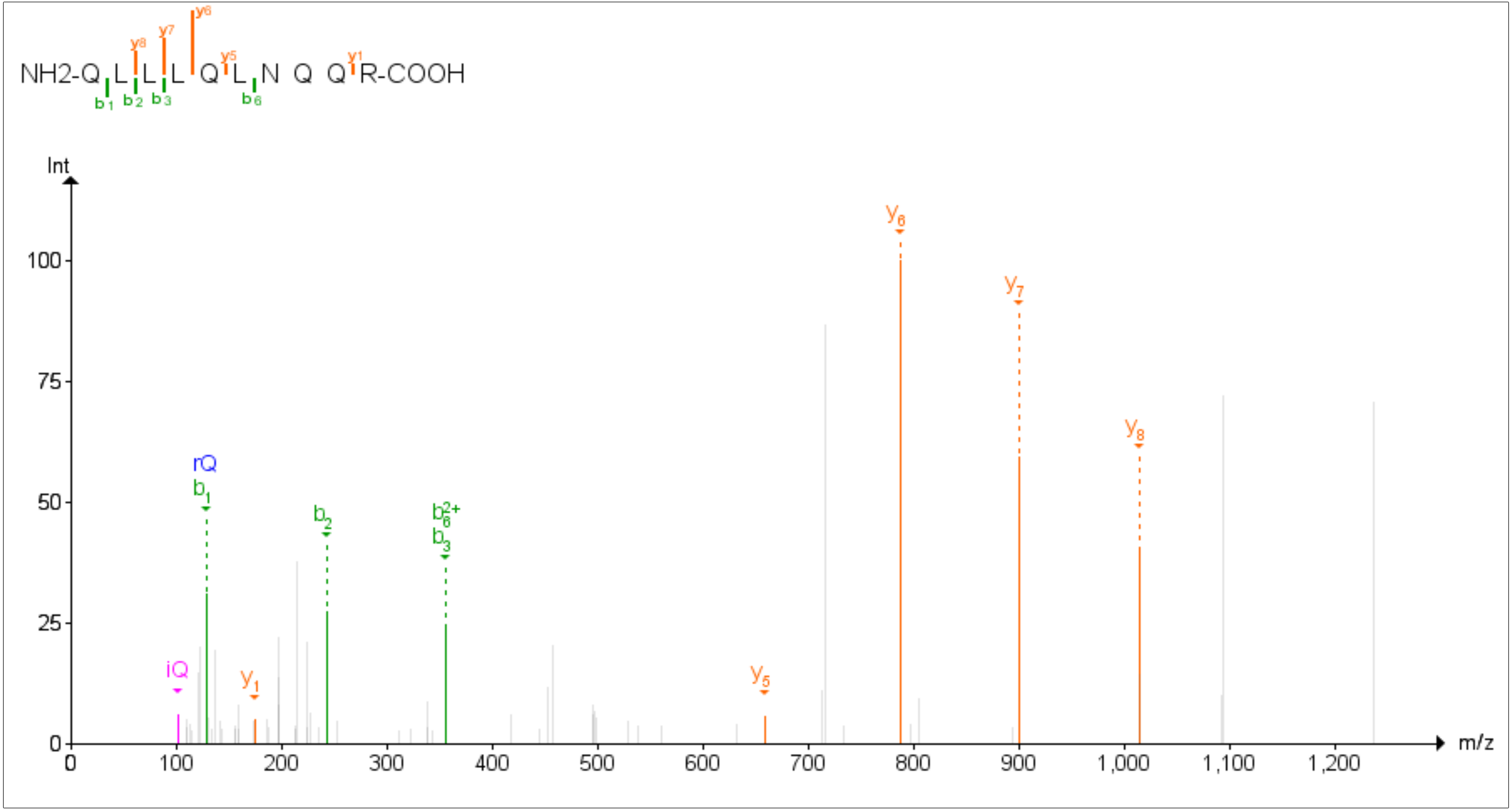

NH2-Q L L L Q L N Q Q R-COOH
y8 y7 y6 y5 y1
b1 b2 b3 b6
Int
m/z
rQ
b1
b2
b6 2+
b3
iQ
y1
y5
y6
y7
y8

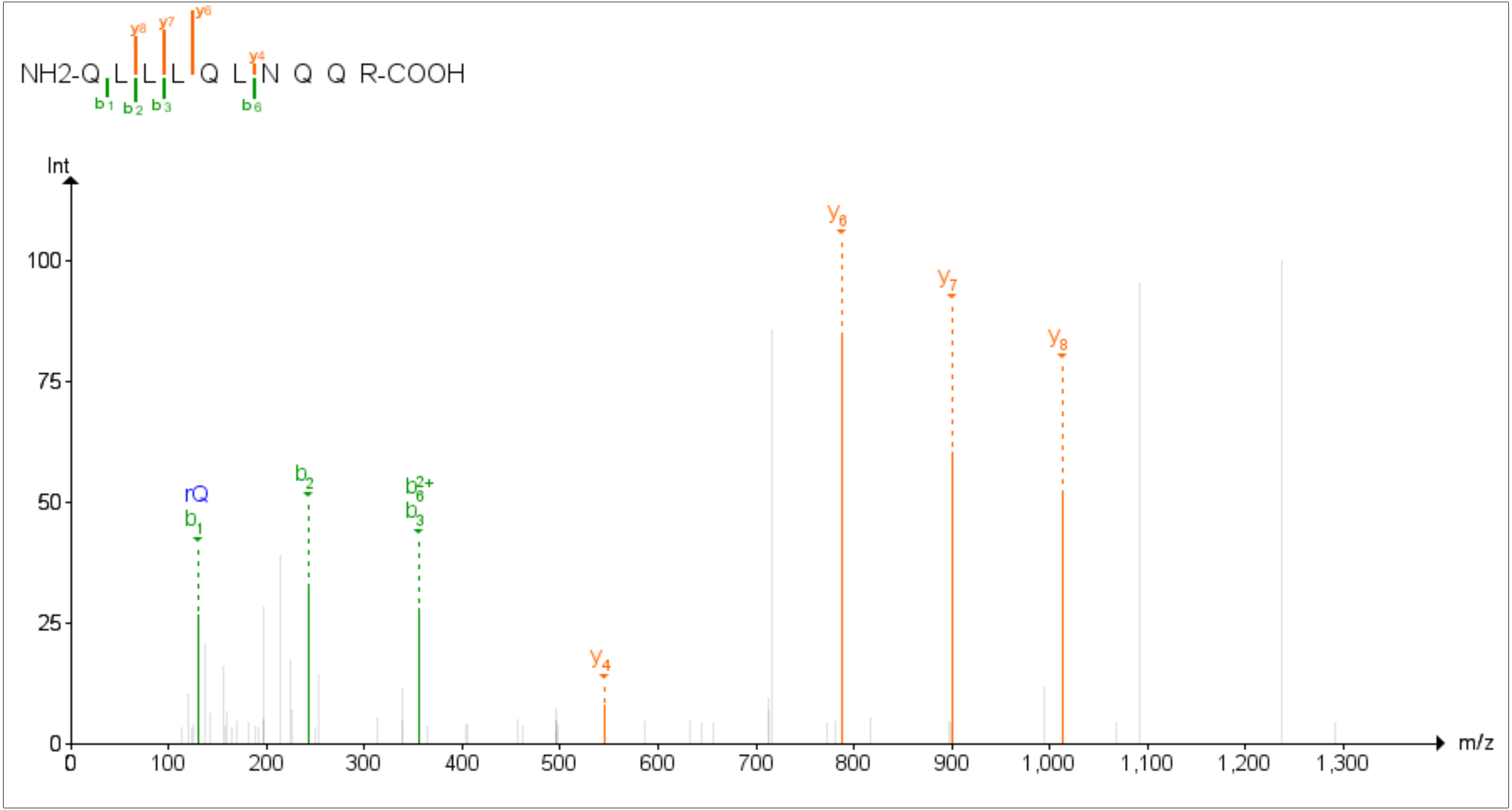

NH2-Q L L L Q L N Q Q R-COOH
y8
y7
y6
y4
b1
b2
b3
b6
Int
100
75
50
25
0
rQ
b1
b2
b6 2+
b3
y4
y6
y7
y8
0
100
200
300
400
500
600
700
800
900
1,000
1,100
1,200
1,300
m/z

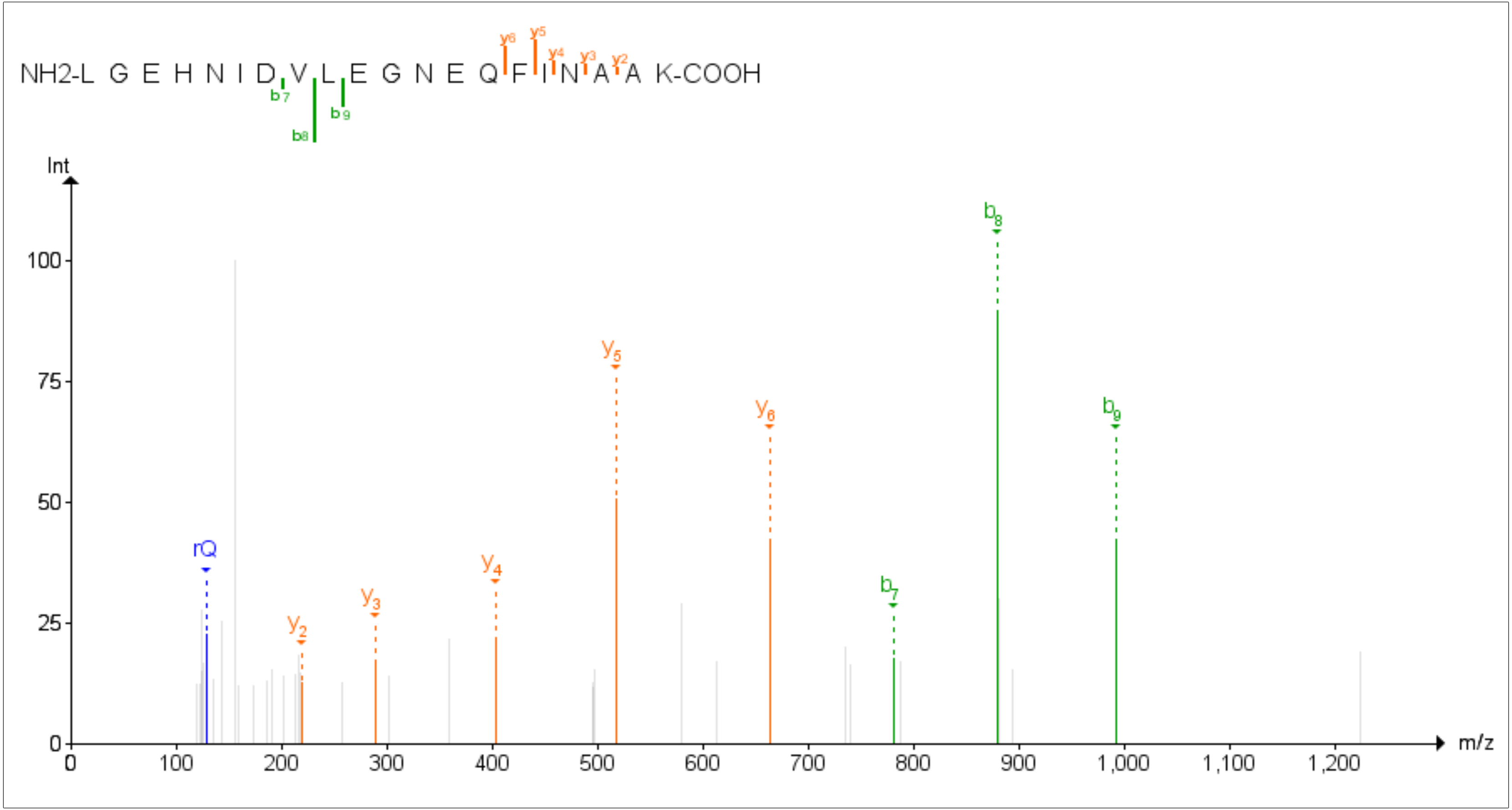
NH2-L G E H N I D V L E G N E Q F I N A A K-COOH
b7
b8
b9
y6
y5
y4
y3
y2
Int
100
75
50
25
0
rQ
y2
y3
y4
y5
y6
b7
b8
b9
0
100
200
300
400
500
600
700
800
900
1,000
1,100
1,200
m/z

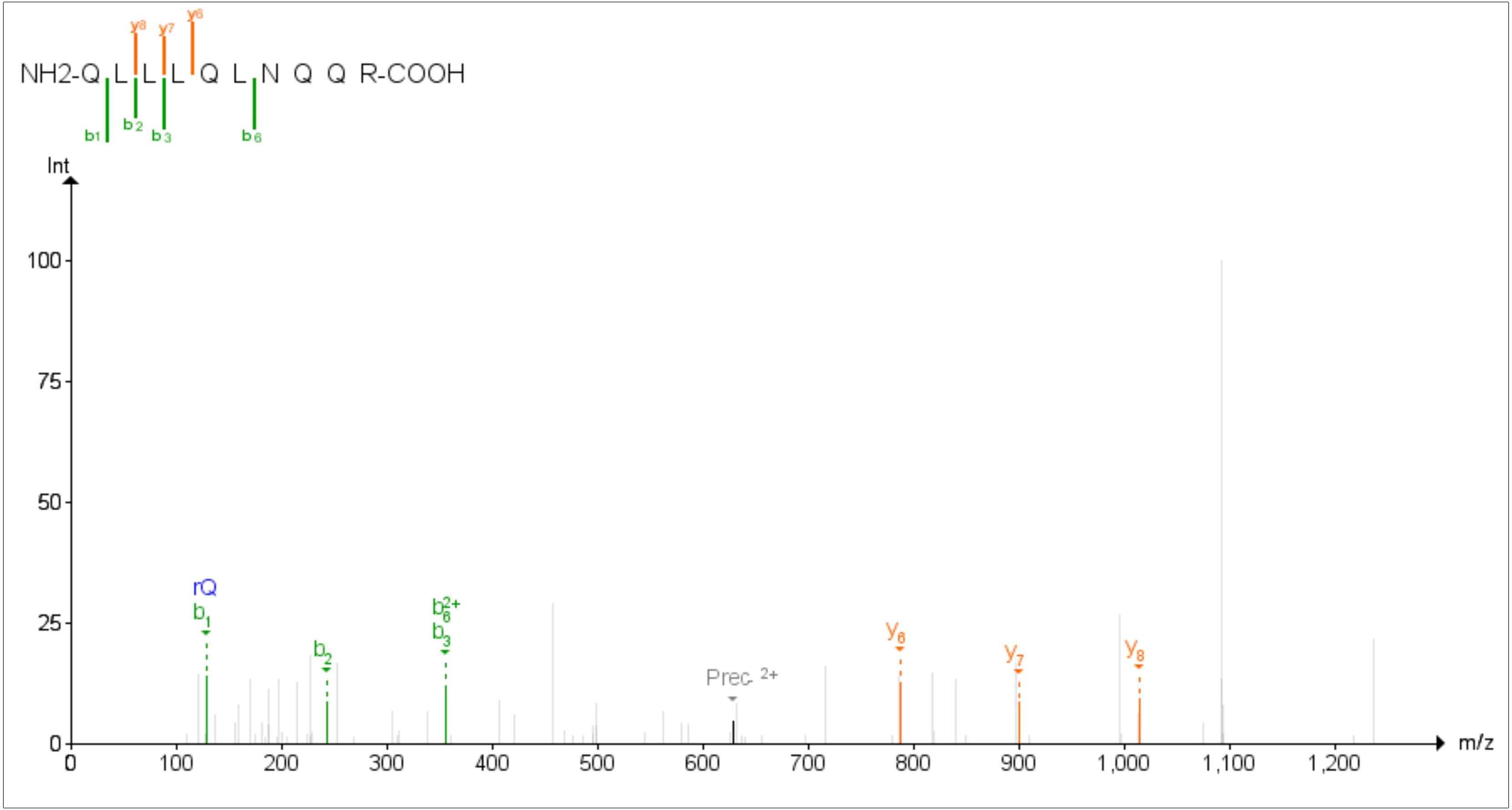

NH2-Q L L L Q L N Q Q R-COOH
y8
y7
y6
b1
b2
b3
b6
Int
100
75
50
25
0
rQ
b1
b2
b6 2+
b3
Prec 2+
y6
y7
y8
0
100
200
300
400
500
600
700
800
900
1,000
1,100
1,200
m/z

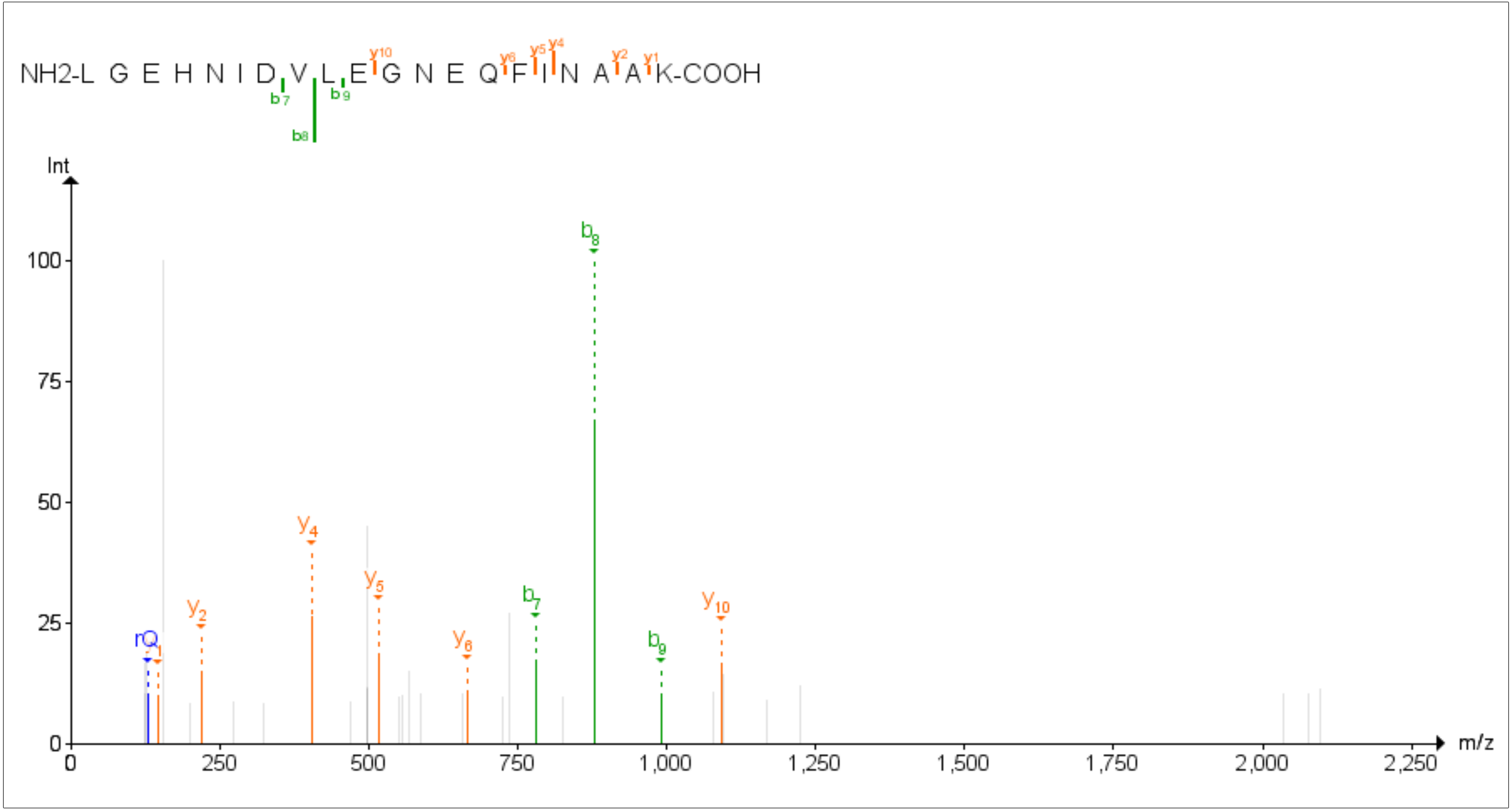

NH2-L G E H N I D V L E G N E Q F I N A A K-COOH
b7
b8
b9
y10
y6
y5
y4
y2
y1
Int
100
75
50
25
0
b8
y4
y5
b7
y10
y2
rQ
y1
y6
b9
0
250
500
750
1,000
1,250
1,500
1,750
2,000
2,250
m/z

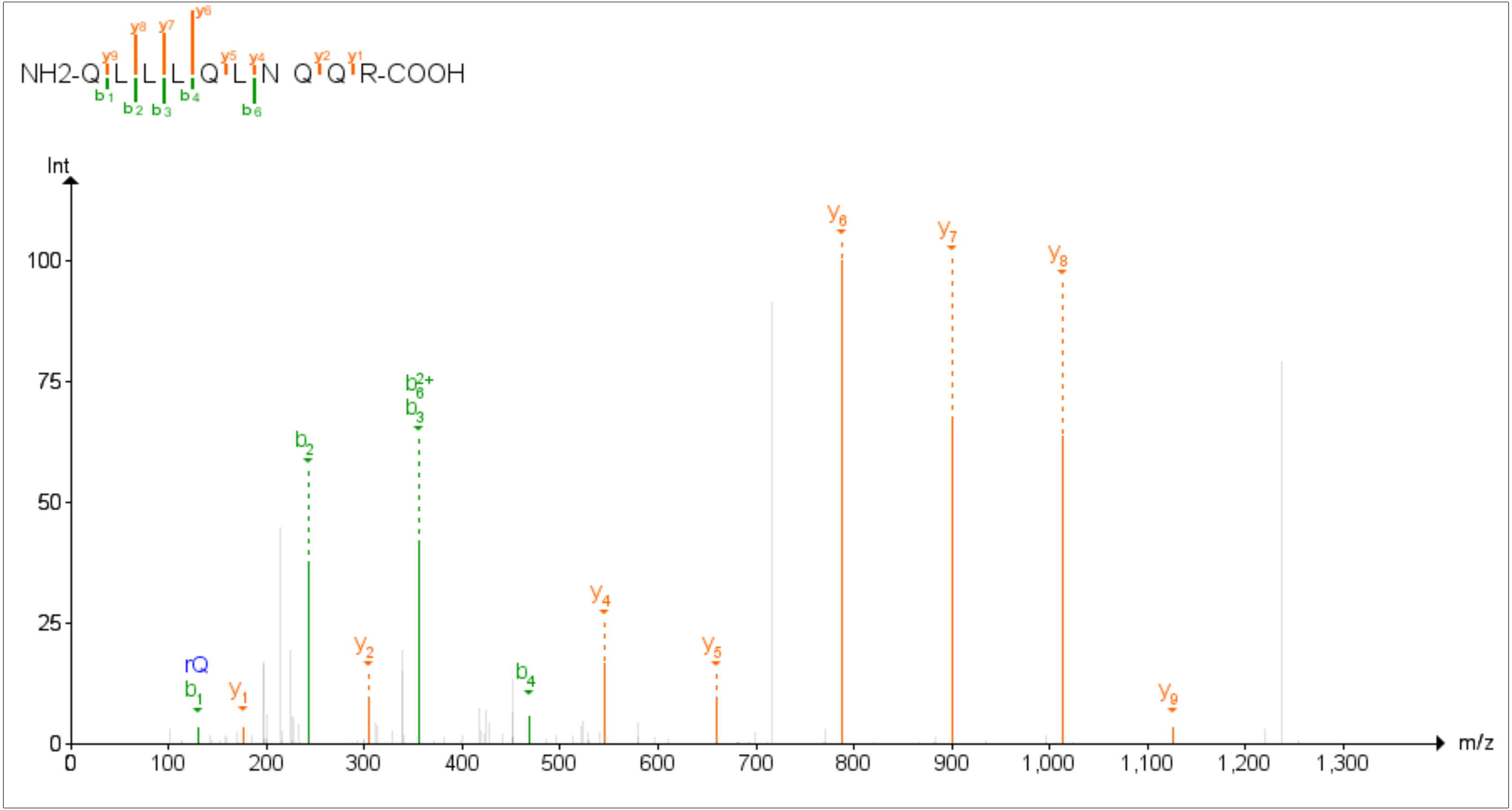

NH2-Q L L L Q L N Q Q R-COOH
y9 y8 y7 y6 y5 y4 y2 y1
b1 b2 b3 b4 b6
Int
100
75
50
25
0
m/z
0 100 200 300 400 500 600 700 800 900 1,000 1,100 1,200 1,300
rQ
b1
y1
b2
b6 2+
b3
y2
b4
y4
y5
y6
y7
y8
y9

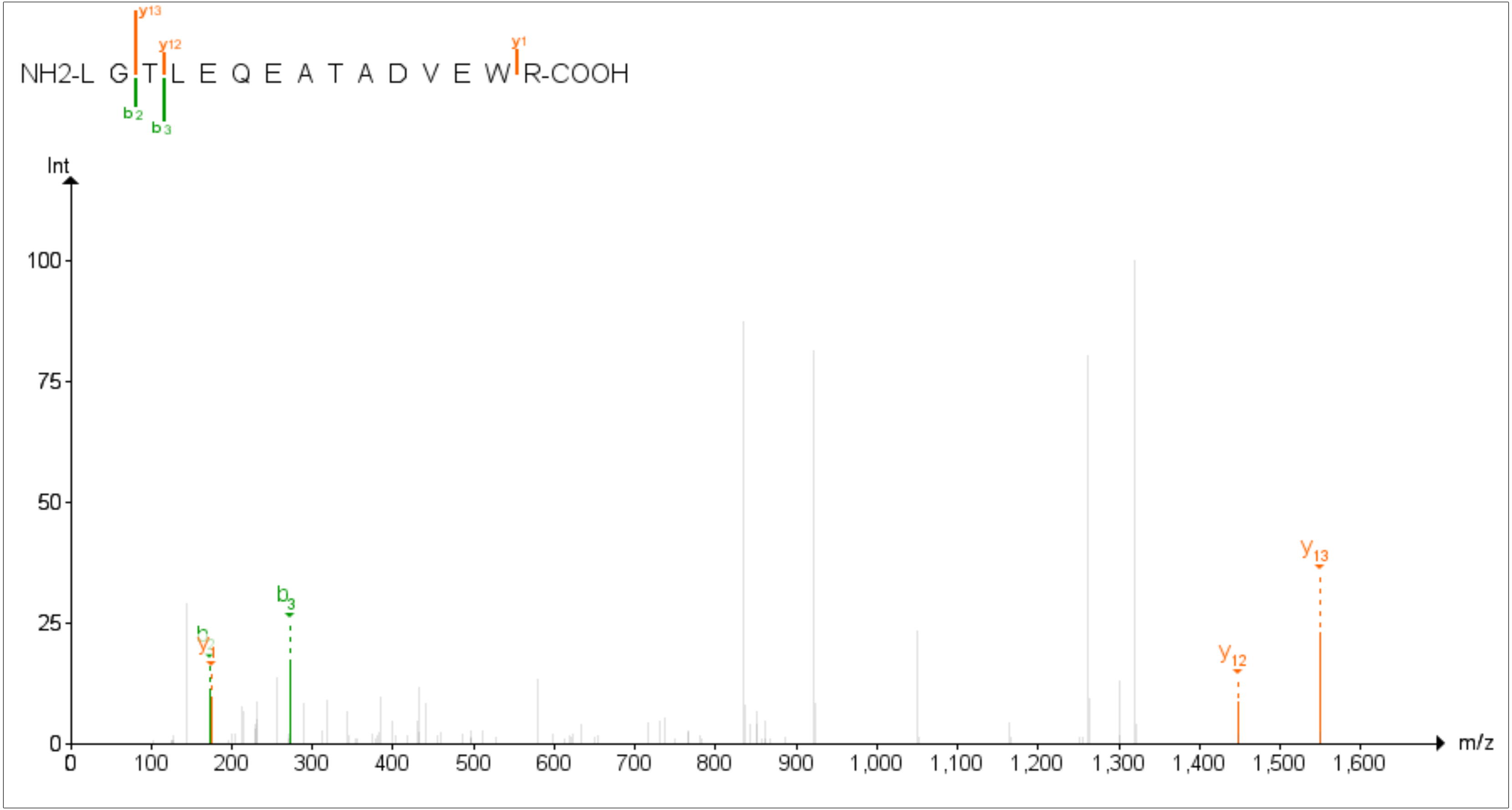

NH2-L G T L E Q E A T A D V E W R-COOH
y13
y12
y1
b2
b3
Int
100
75
50
25
0
m/z
0
100
200
300
400
500
600
700
800
900
1,000
1,100
1,200
1,300
1,400
1,500
1,600
b3
y13
y12

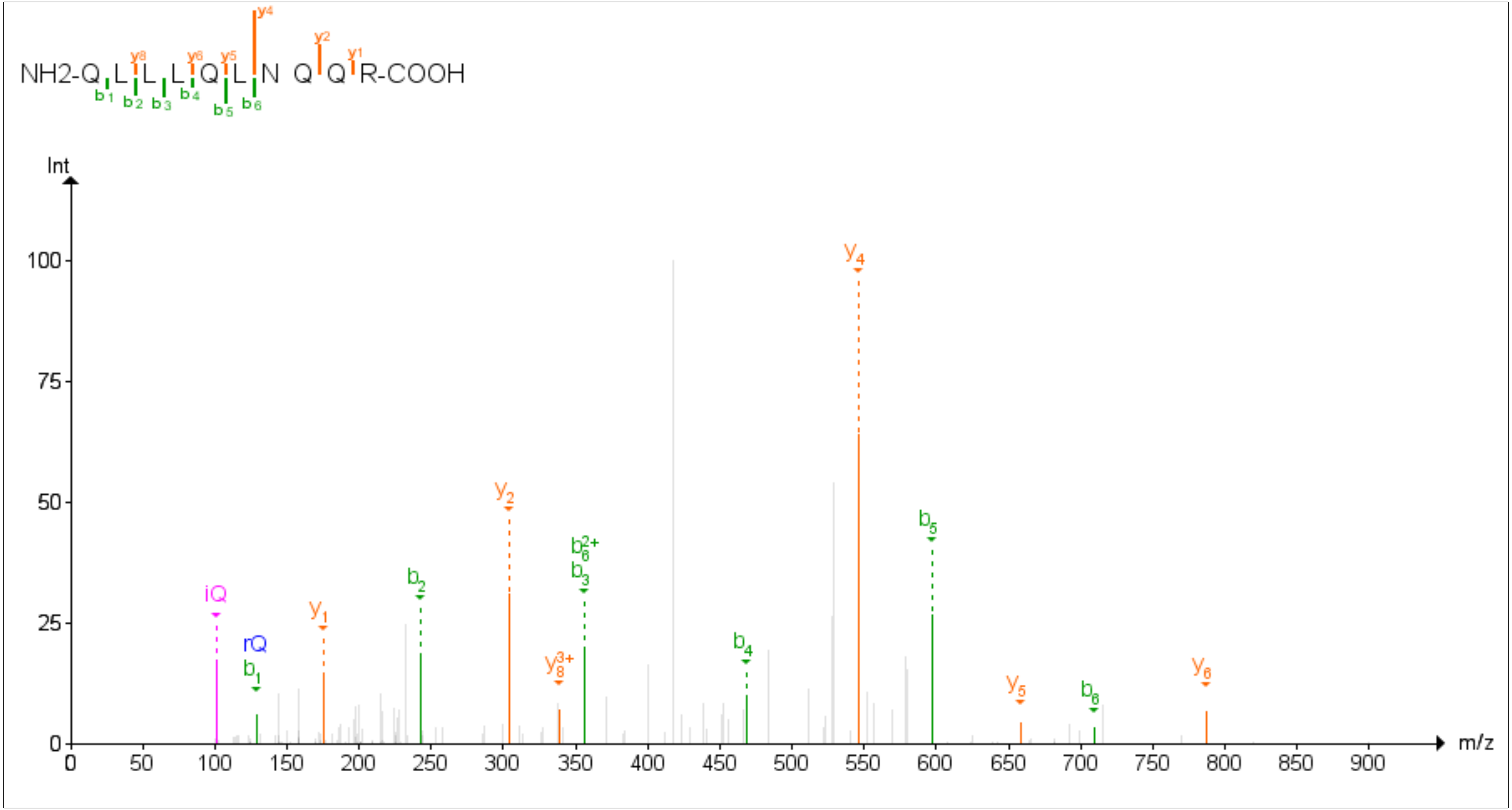

NH2-Q L L L L Q L N Q Q R-COOH
y8 y6 y5 y4 y2 y1
b1 b2 b3 b4 b5 b6
Int
100
75
50
25
0
iQ
rQ
b1
y1
b2
y2
y8 3+
b6 2+
b3
b4
y4
b5
y5
b6
y6
0 50 100 150 200 250 300 350 400 450 500 550 600 650 700 750 800 850 900
m/z

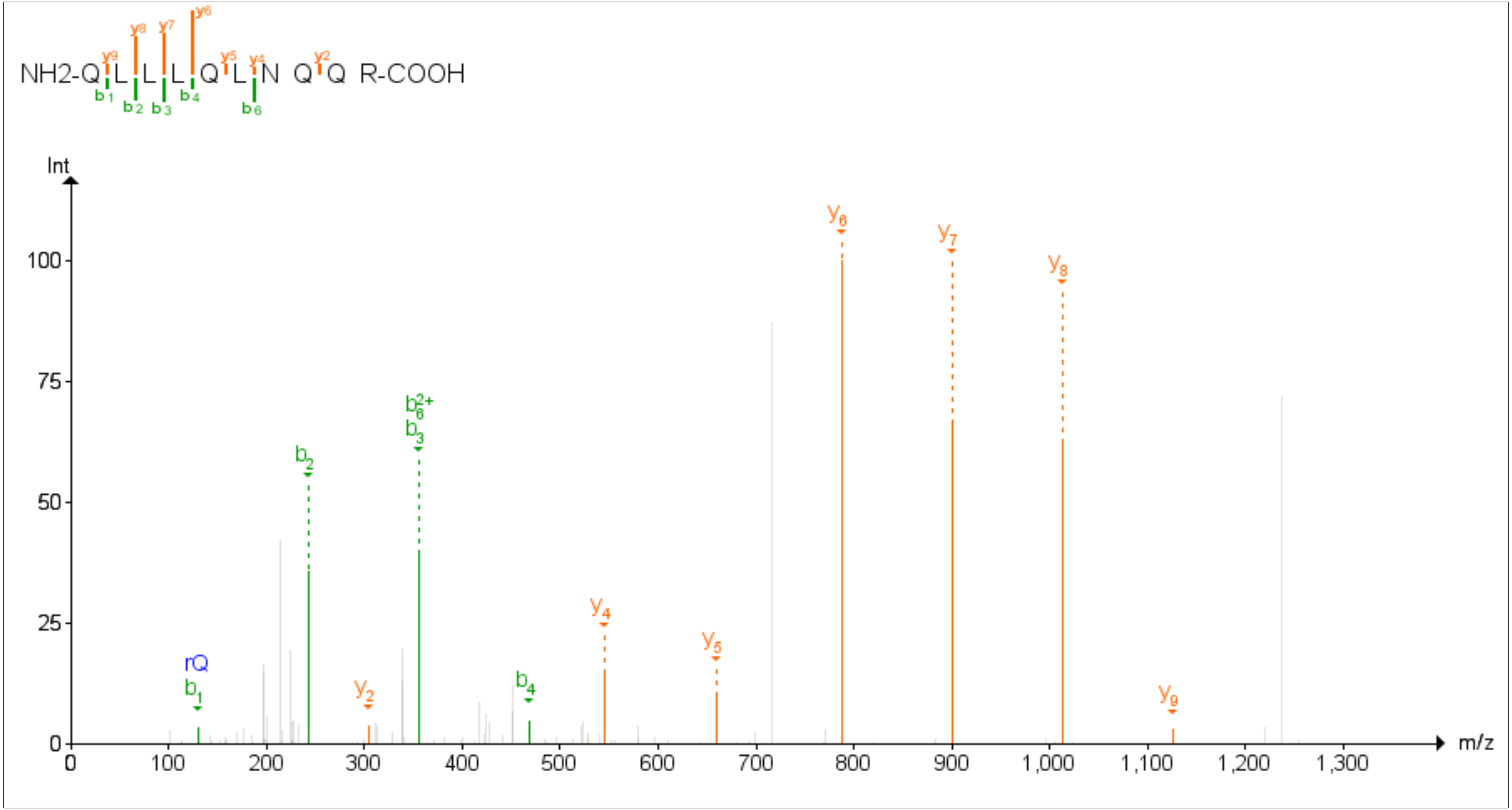

NH2-Q L L L Q L N Q Q R-COOH
y9 y8 y7 y6 y5 y4 y2
b1 b2 b3 b4 b6
Int
100
75
50
25
0
rQ
b1
b2
y2
b6 2+
b3
b4
y4
y5
y6
y7
y8
y9
0
100
200
300
400
500
600
700
800
900
1,000
1,100
1,200
1,300
m/z

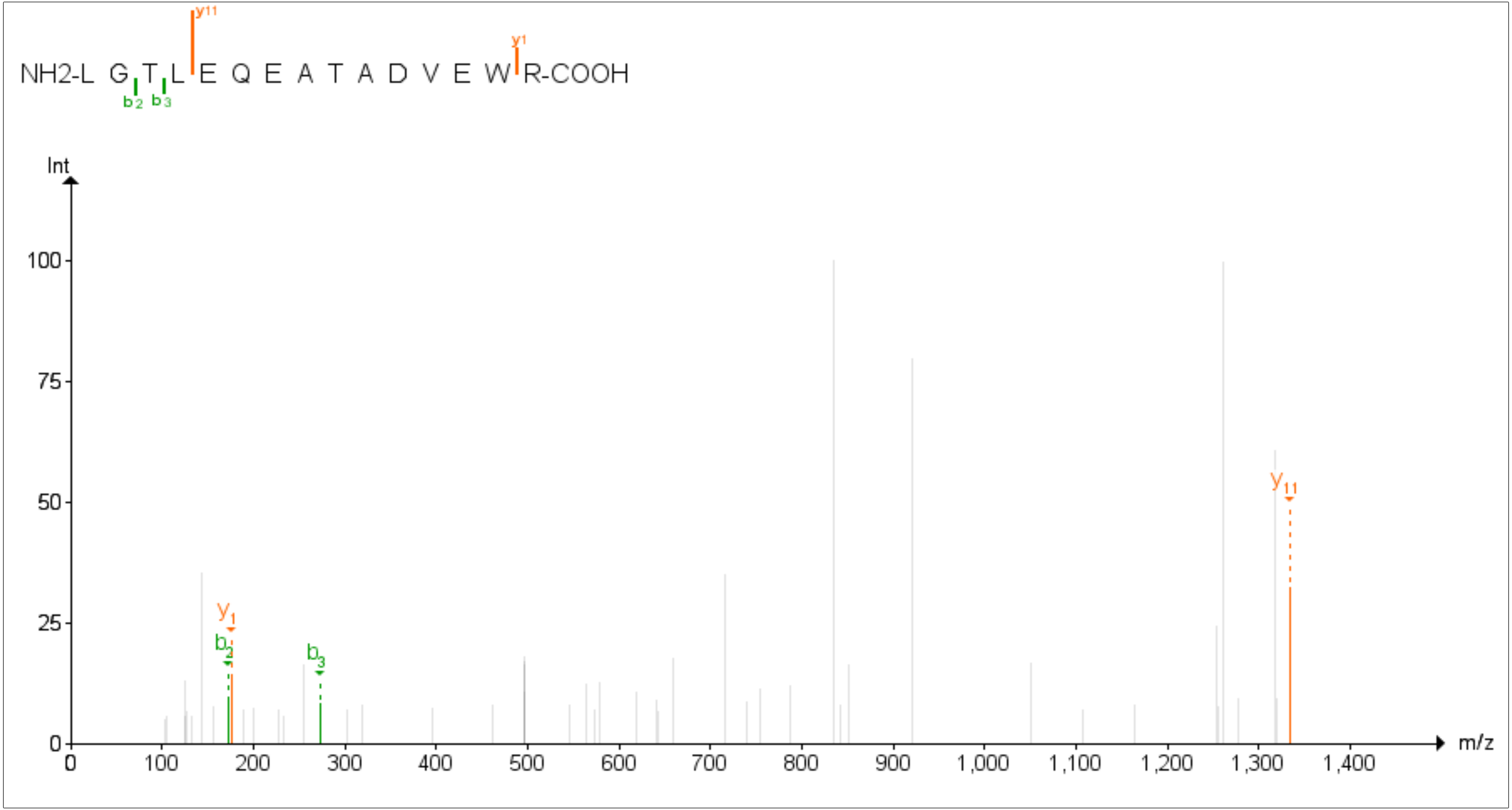

NH2-L G T L E Q E A T A D V E W R-COOH
y11
y1
b2
b3
Int
100
75
50
25
0
m/z
0
100
200
300
400
500
600
700
800
900
1,000
1,100
1,200
1,300
1,400

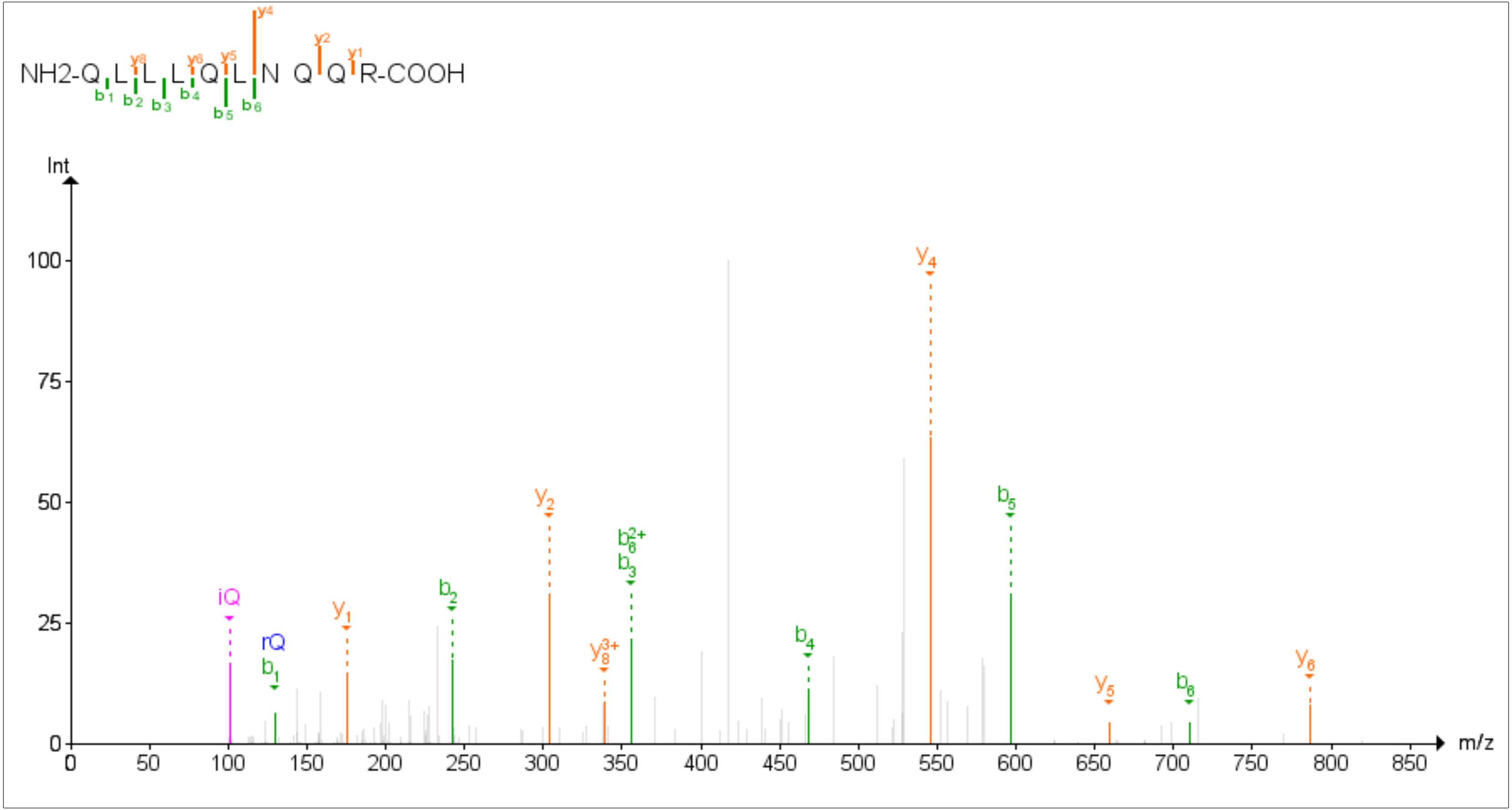

NH2-Q L L L Q L N Q Q R-COOH
b1 b2 b3 b4 b5 b6
y8 y6 y5 y4 y2 y1
Int
m/z
iQ
rQ
b1
y1
b2
y2
y8 3+
b6 2+
b3
b4
y4
b5
y5
b6
y6

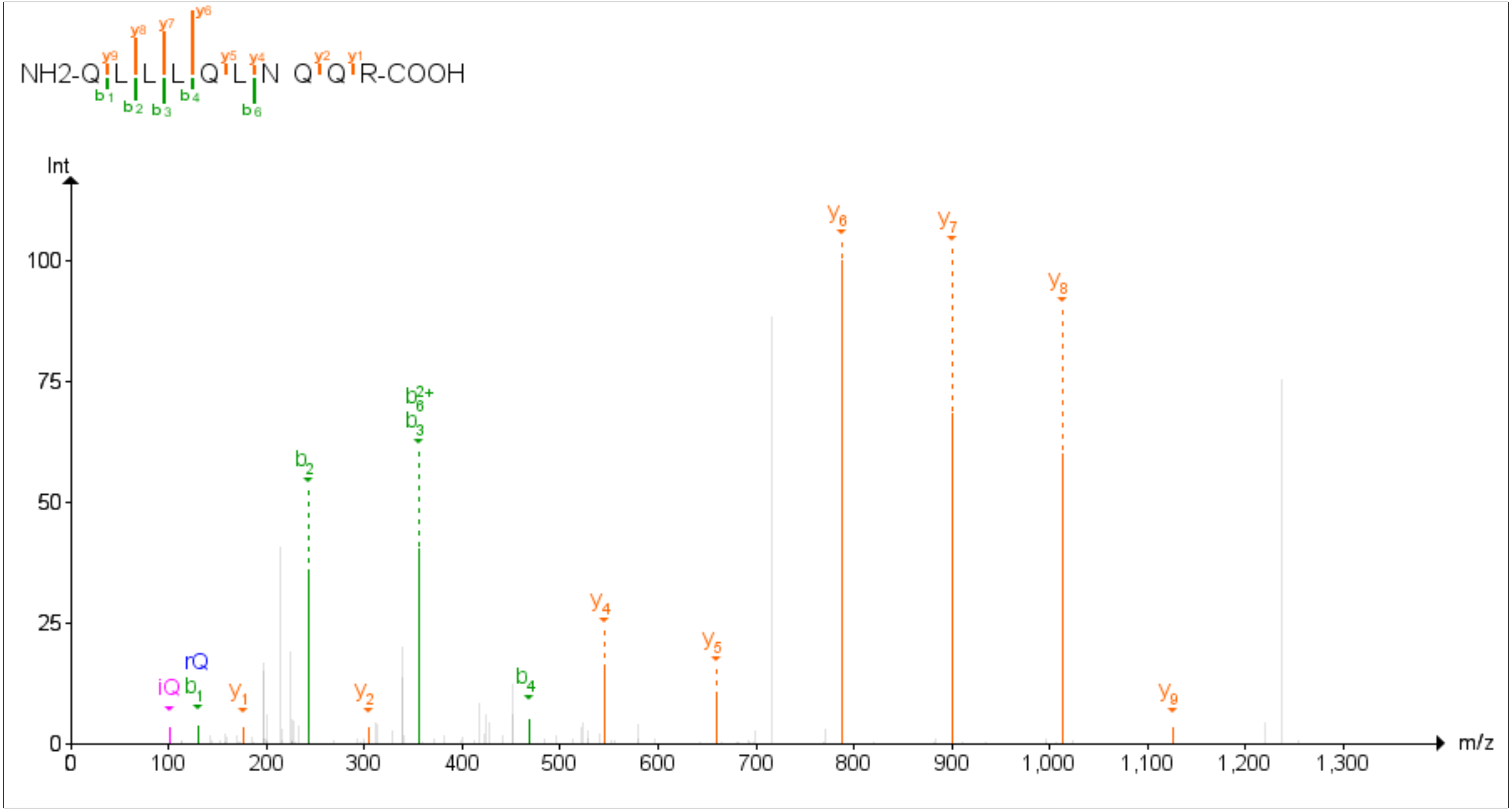
NH2-Q L L L Q L N Q Q R-COOH
y9 y8 y7 y6 y5 y4 y2 y1
b1 b2 b3 b4 b6
Int
100
75
50
25
0
y6
y7
y8
b_6^{2+}
b_3
b_2
y_4
y_5
rQ
iQ
b_1
y_1
y_2
b_4
y_9
0
100
200
300
400
500
600
700
800
900
1,000
1,100
1,200
1,300
m/z

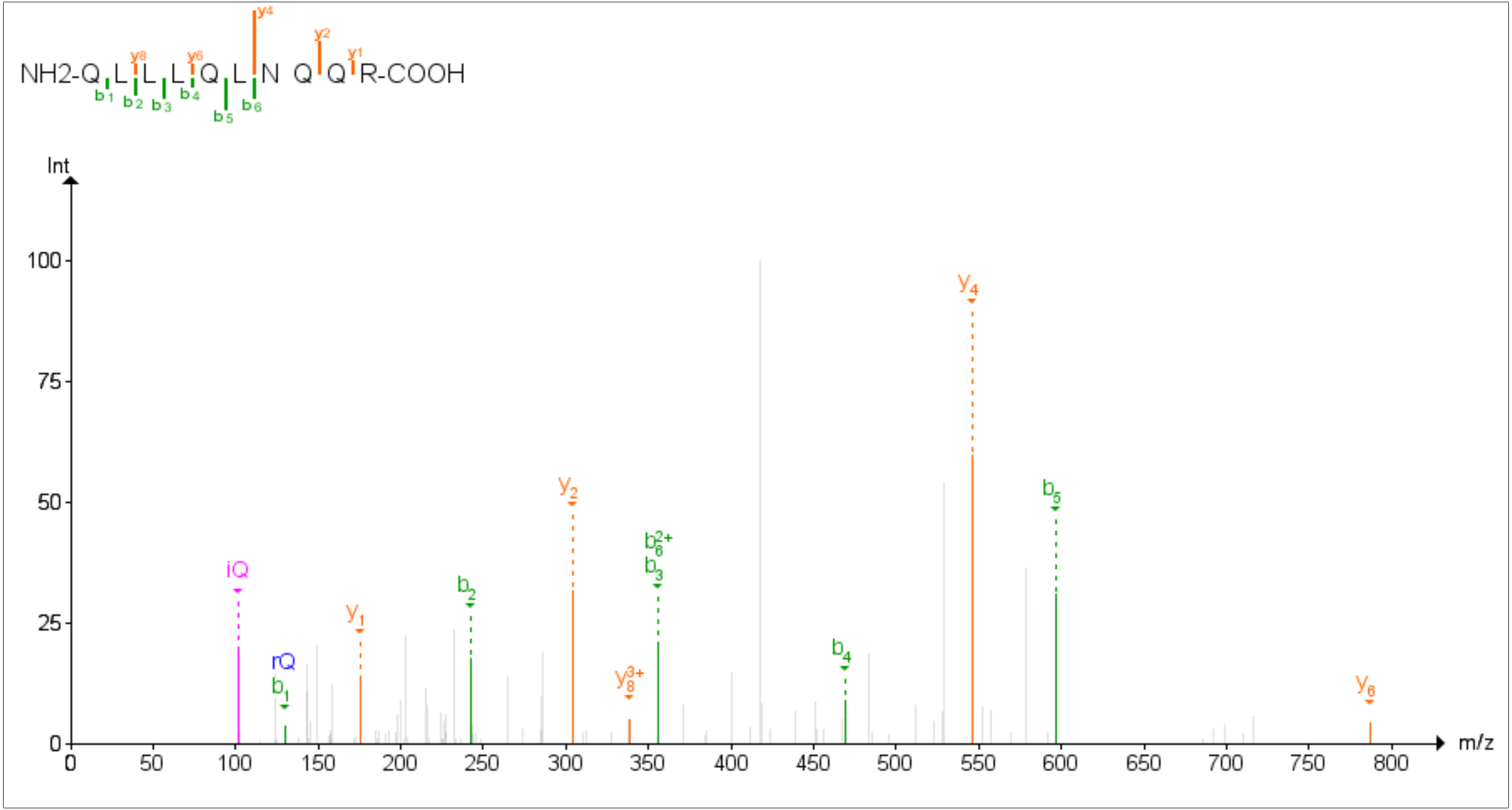

NH2-Q L L L L Q L N Q Q R-COOH
b1 b2 b3 b4 b5 b6
y8 y6 y4 y2 y1
Int
m/z
iQ
rQ
b1
y1
b2
y2
y8 3+
b6 2+
b3
b4
y4
b5
y6
0
25
50
75
100
0
50
100
150
200
250
300
350
400
450
500
550
600
650
700
750
800

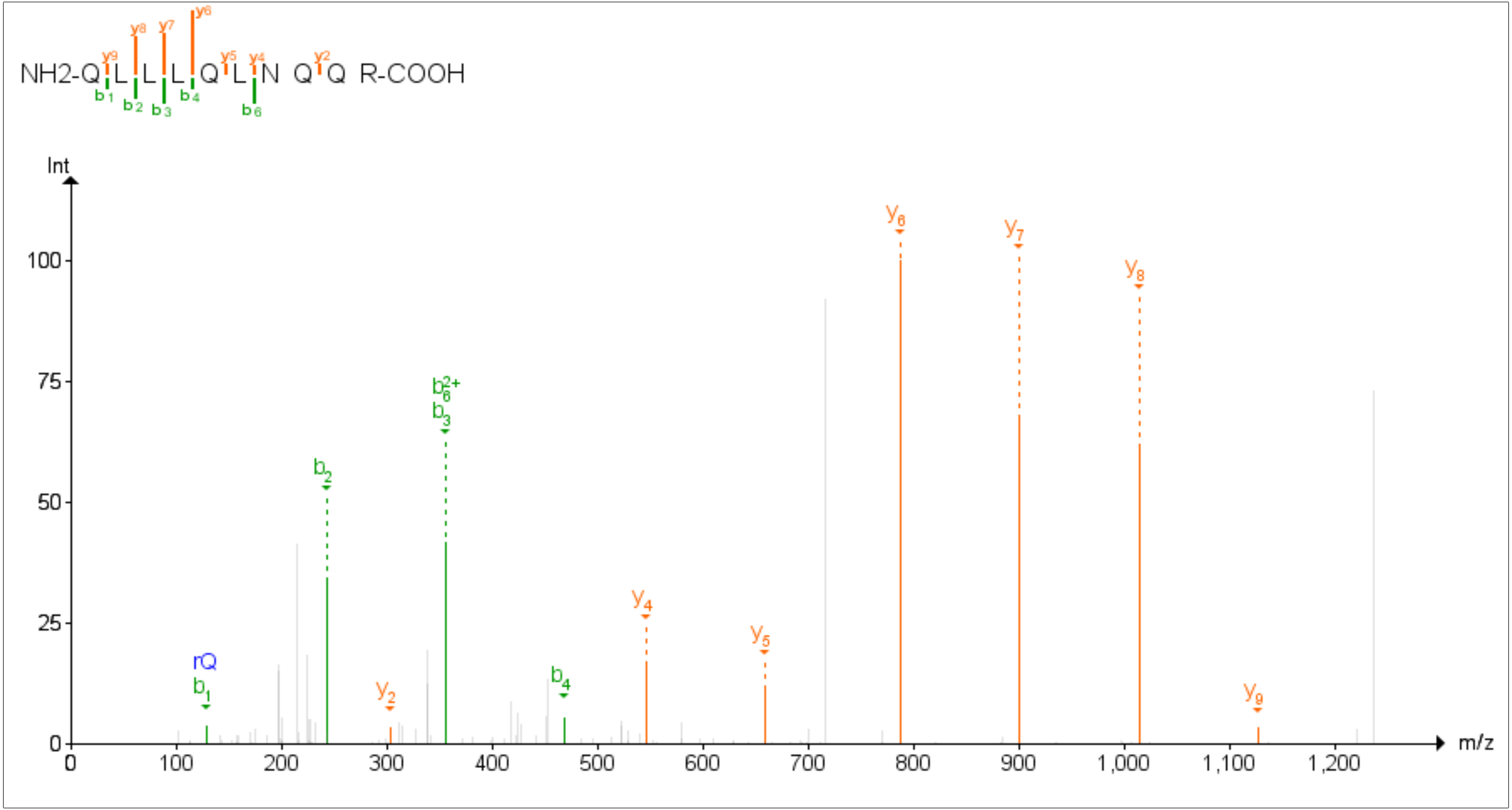
NH2-Q L L L Q L N Q Q R-COOH
y9 y8 y7 y6 y5 y4 y2
b1 b2 b3 b4 b6
Int
100
75
50
25
0
y6
y7
y8
b6 2+
b3
b2
y4
y5
rQ
b1
y2
b4
y9
0 100 200 300 400 500 600 700 800 900 1,000 1,100 1,200
m/z

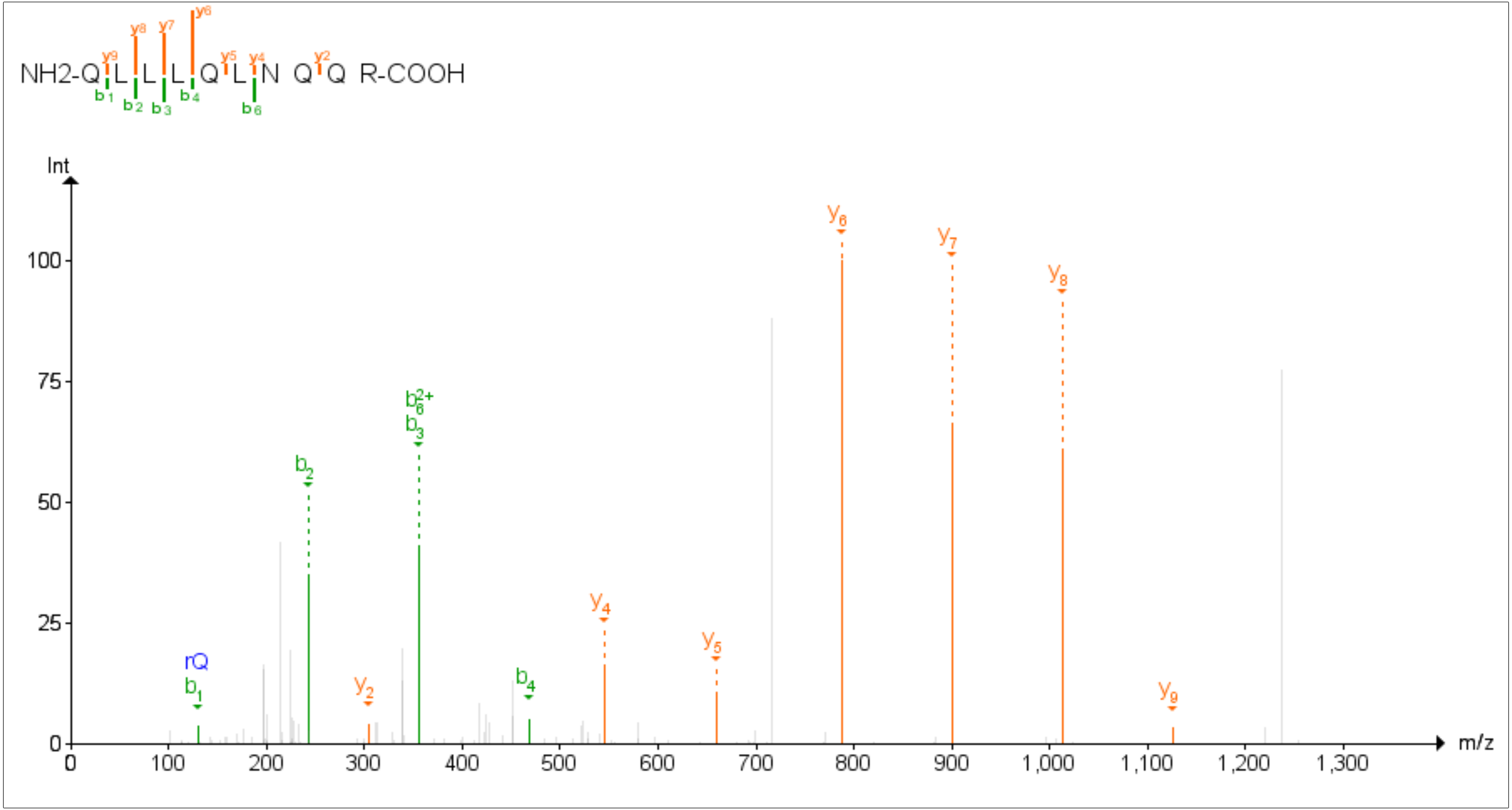

NH2-Q L L L Q L N Q Q R-COOH
y9 y8 y7 y6 y5 y4 y2
b1 b2 b3 b4 b6
Int
m/z
rQ
b1
b2
b6 2+
b3
y2
b4
y4
y5
y6
y7
y8
y9
0
25
50
75
100
0
100
200
300
400
500
600
700
800
900
1,000
1,100
1,200
1,300

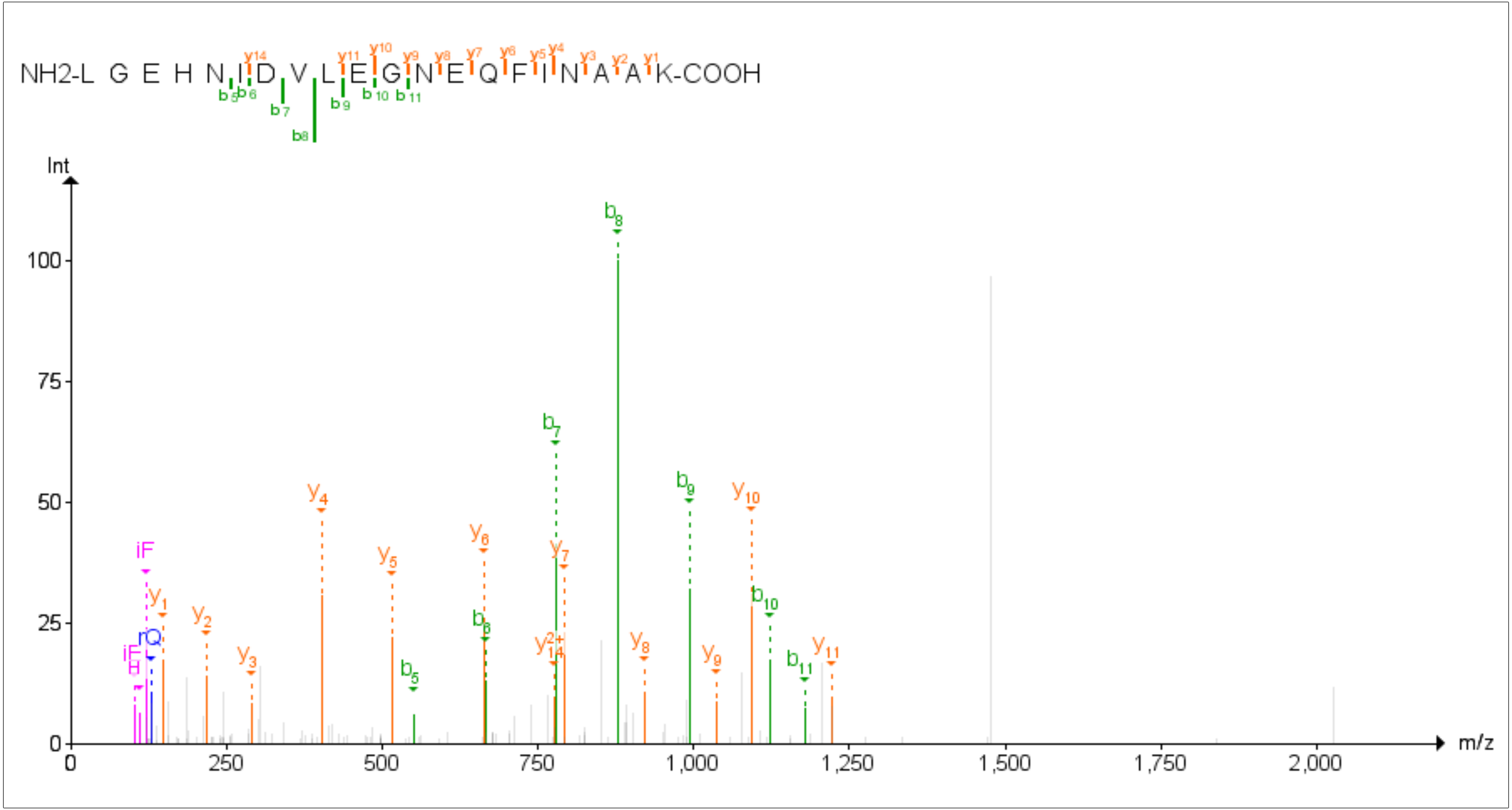
NH2-L G E H N I D V L E G N E Q F I N A A K-COOH
Int
m/z
0
25
50
75
100
250
500
750
1,000
1,250
1,500
1,750
2,000

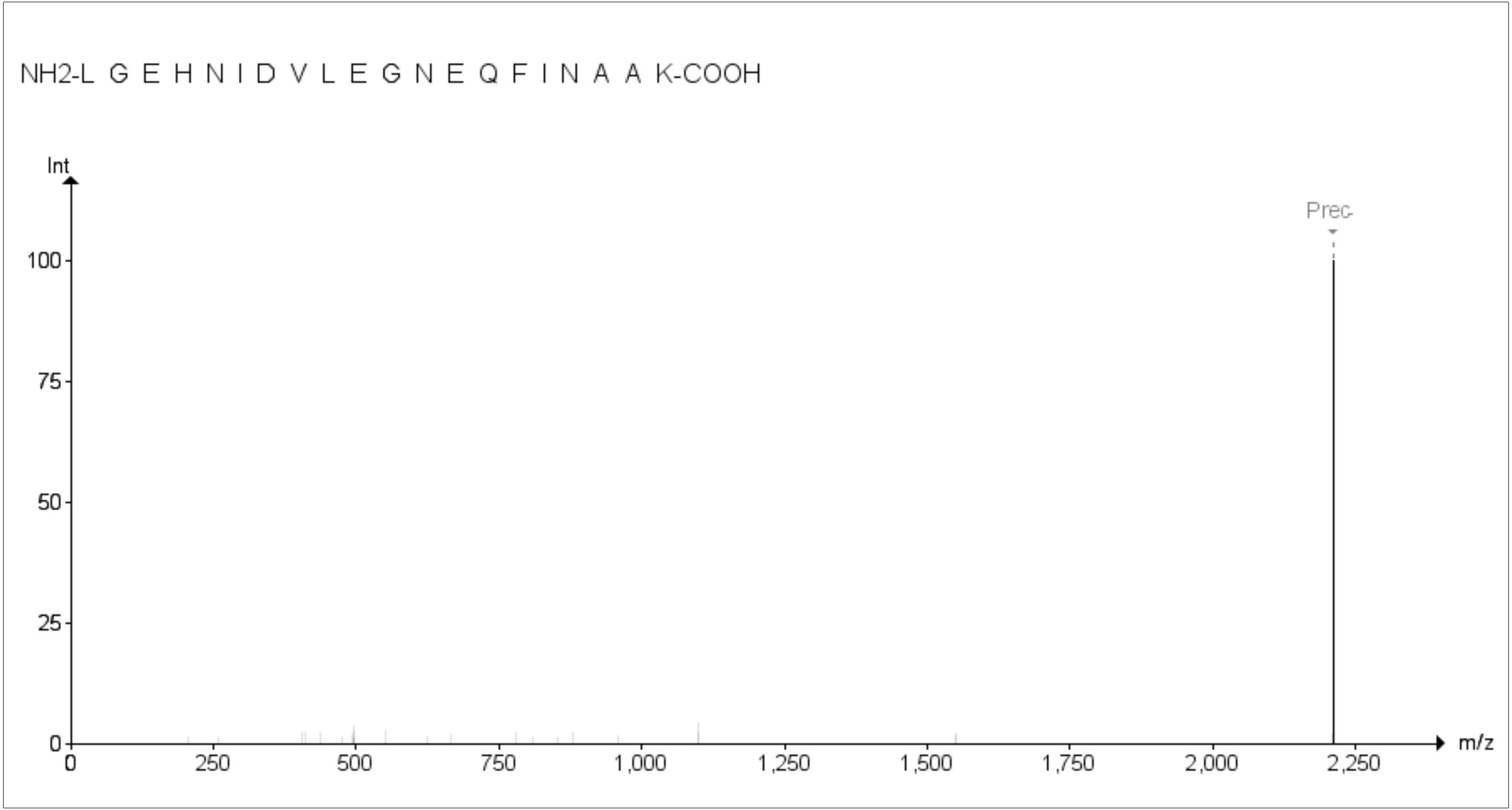
NH2-L G E H N I D V L E G N E Q F I N A A K-COOH
Int
Prec
100
75
50
25
0
0
250
500
750
1,000
1,250
1,500
1,750
2,000
2,250
m/z

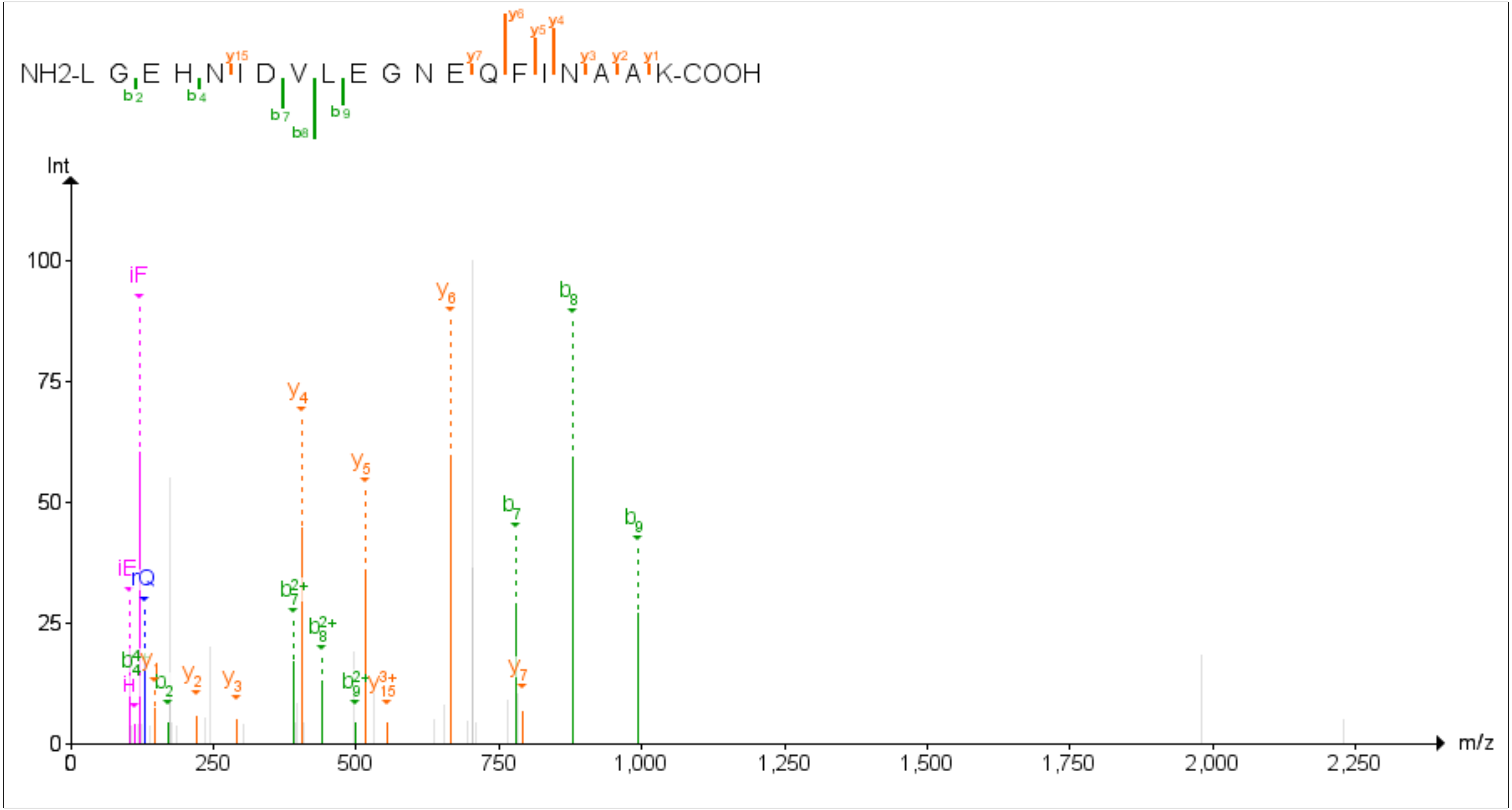

NH2-L G E H N I D V L E G N E Q F I N A A K-COOH
Int
m/z
0
25
50
75
100
250
500
750
1,000
1,250
1,500
1,750
2,000
2,250

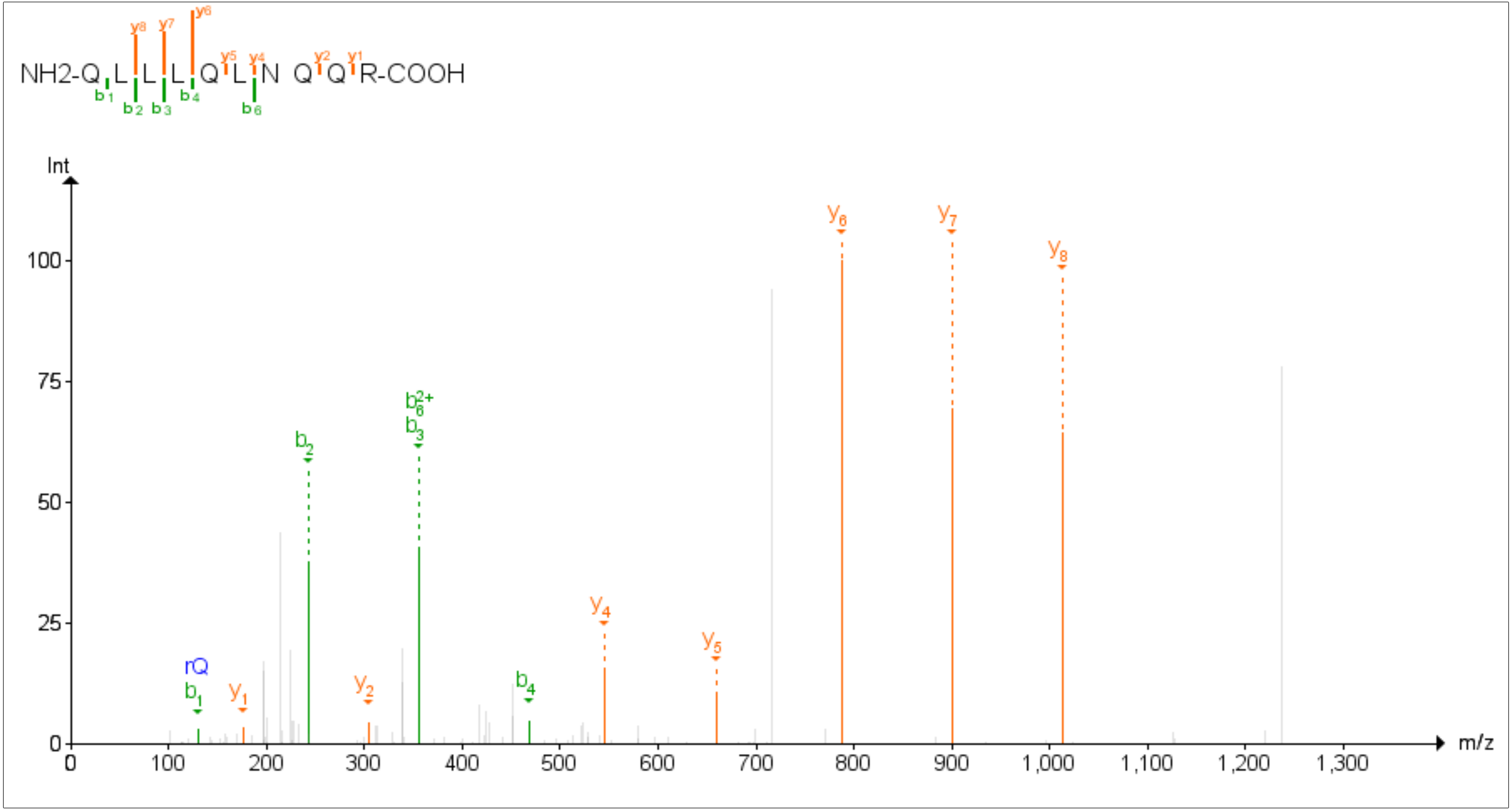

NH2-Q L L L Q L N Q Q R-COOH
y8 y7 y6 y5 y4 y2 y1
b1 b2 b3 b4 b6
Int
100
75
50
25
0
y6
y7
y8
b6 2+
b3
b2
y4
y5
rQ
b1
y1
y2
b4
0
100
200
300
400
500
600
700
800
900
1,000
1,100
1,200
1,300
m/z

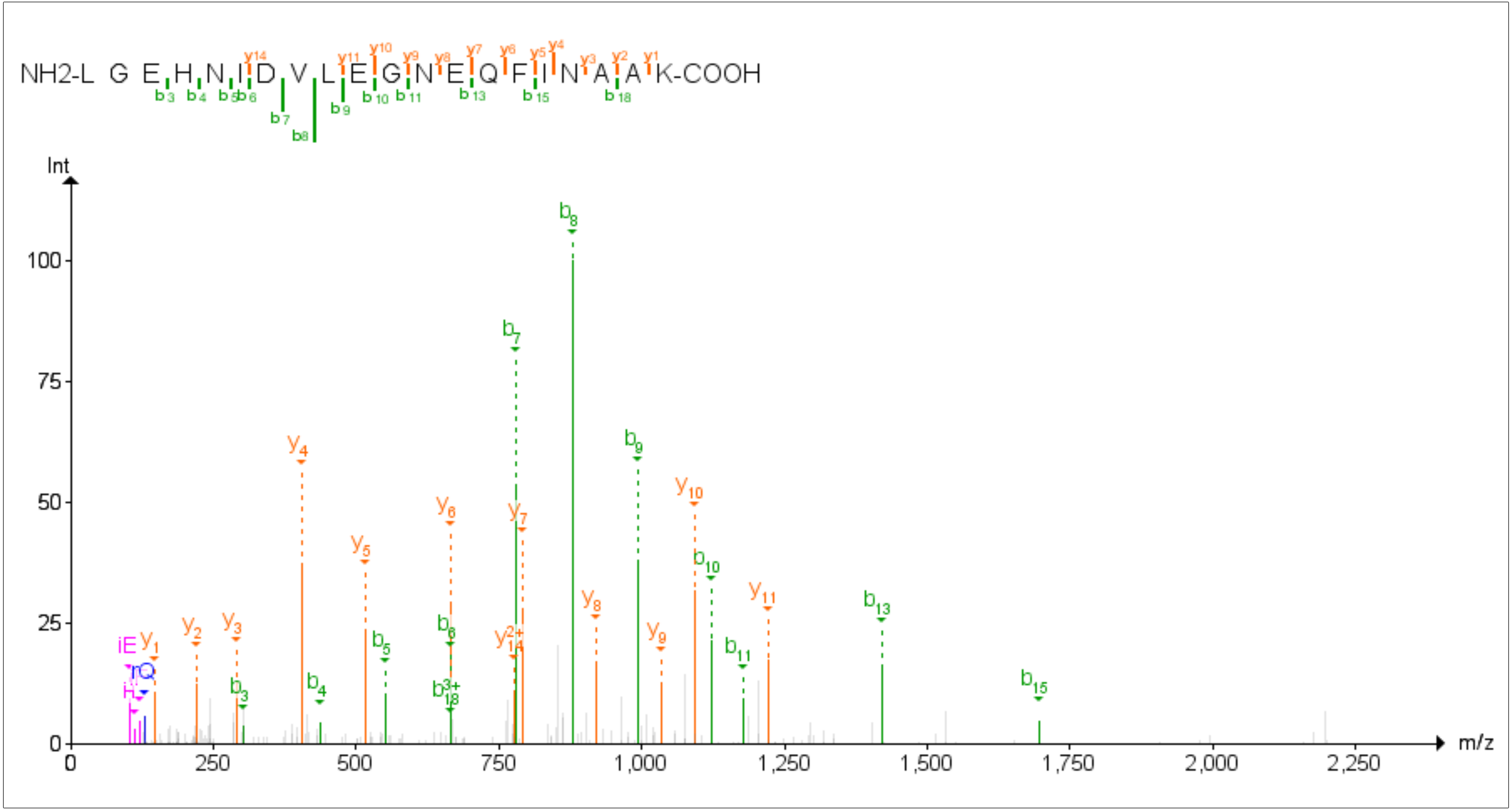

NH2-L G E H N I D V L E G N E Q F I N A A K-COOH
Int
m/z
0
25
50
75
100
250
500
750
1,000
1,250
1,500
1,750
2,000
2,250

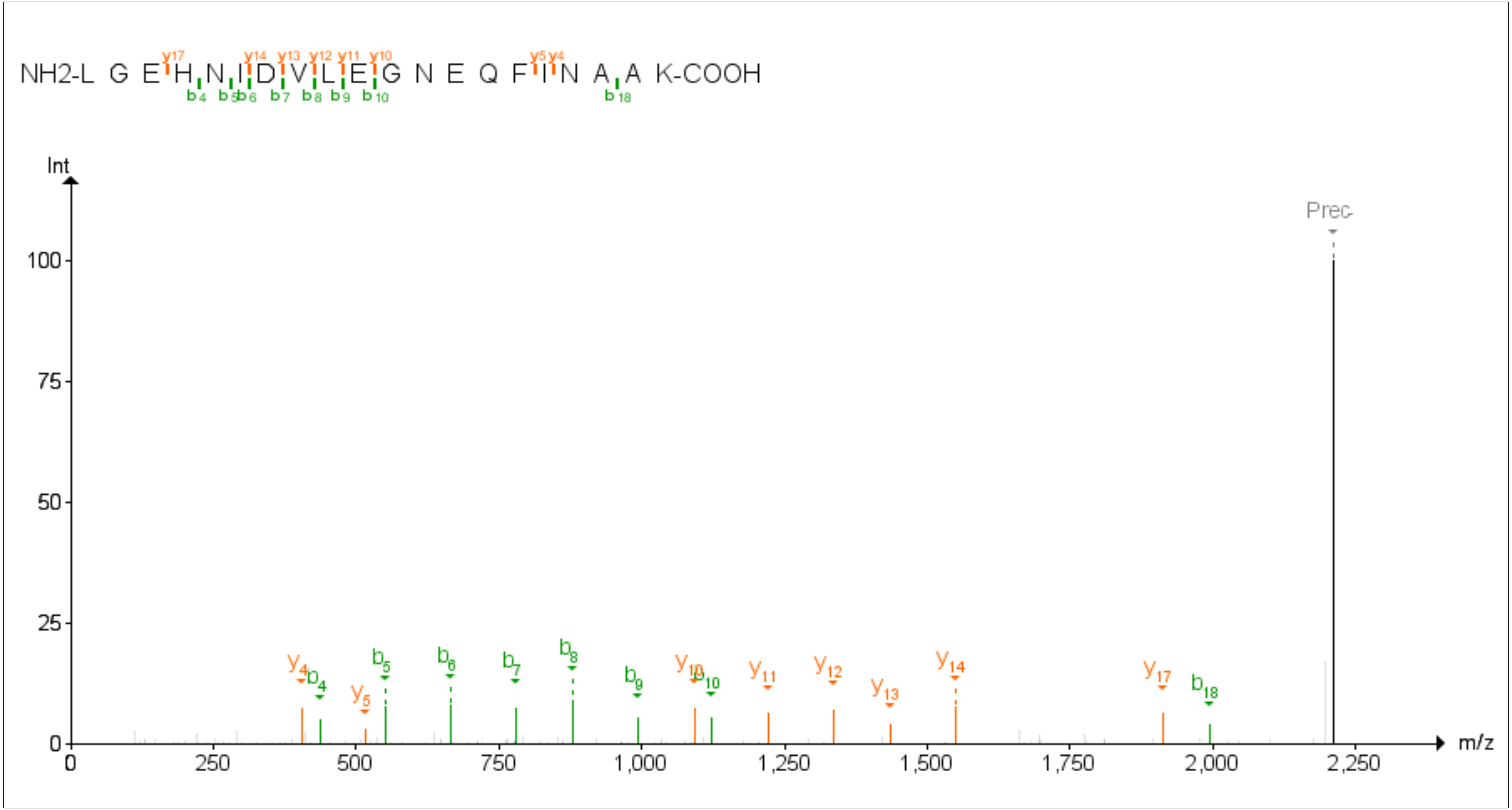
NH2-L G E H N I D V L E G N E Q F I N A A K-COOH
y17 y14 y13 y12 y11 y10 y5 y4
b4 b5 b6 b7 b8 b9 b10 b18
Int
100
75
50
25
0
Prec
m/z
0
250
500
750
1,000
1,250
1,500
1,750
2,000
2,250

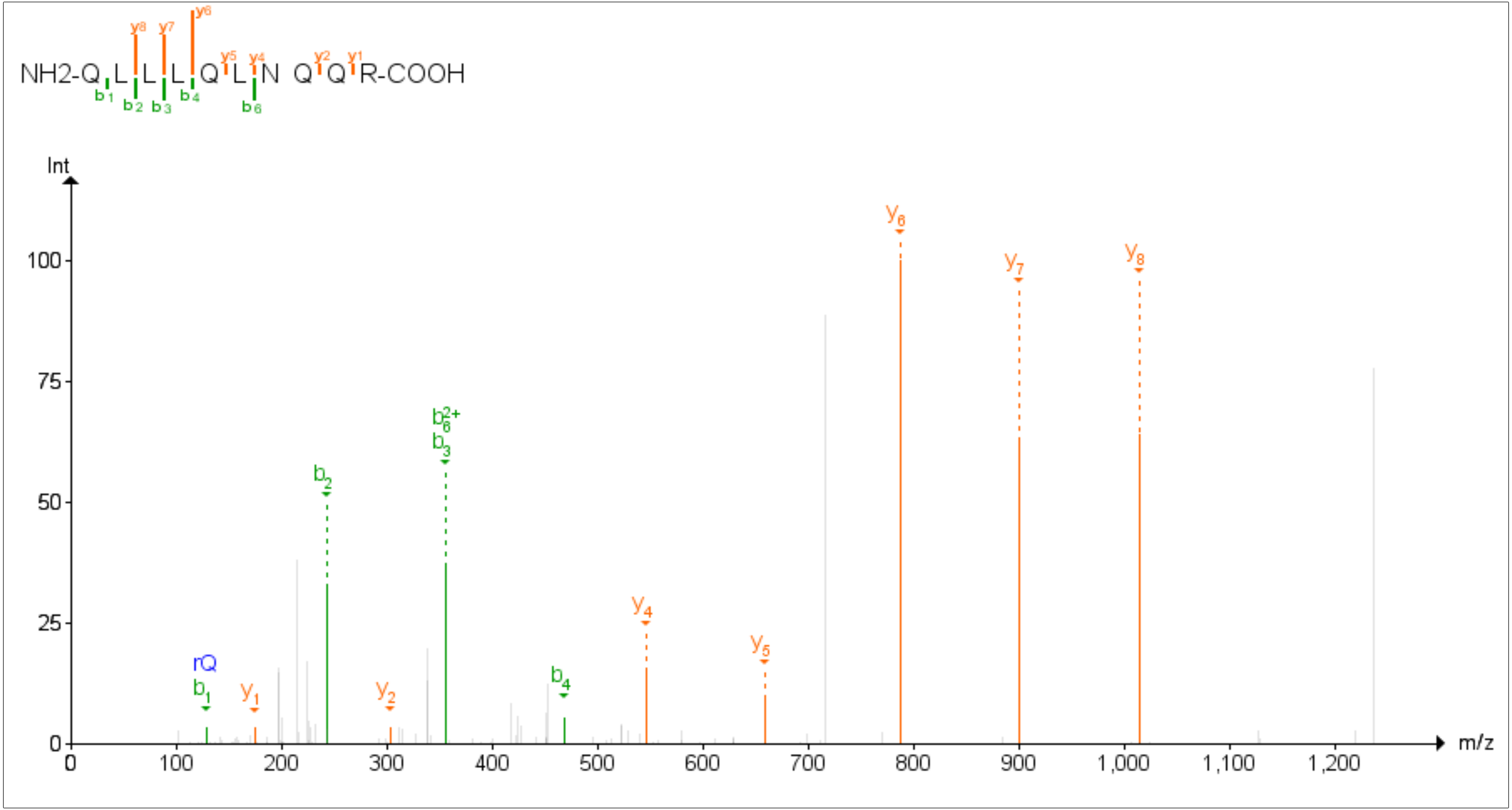
NH2-Q L L L Q L N Q Q R-COOH
y8 y7 y6 y5 y4 y2 y1
b1 b2 b3 b4 b6
Int
100
75
50
25
0
rQ
b1
y1
b2
y2
b6 2+
b3
b4
y4
y5
y6
y7
y8
0
100
200
300
400
500
600
700
800
900
1,000
1,100
1,200
m/z

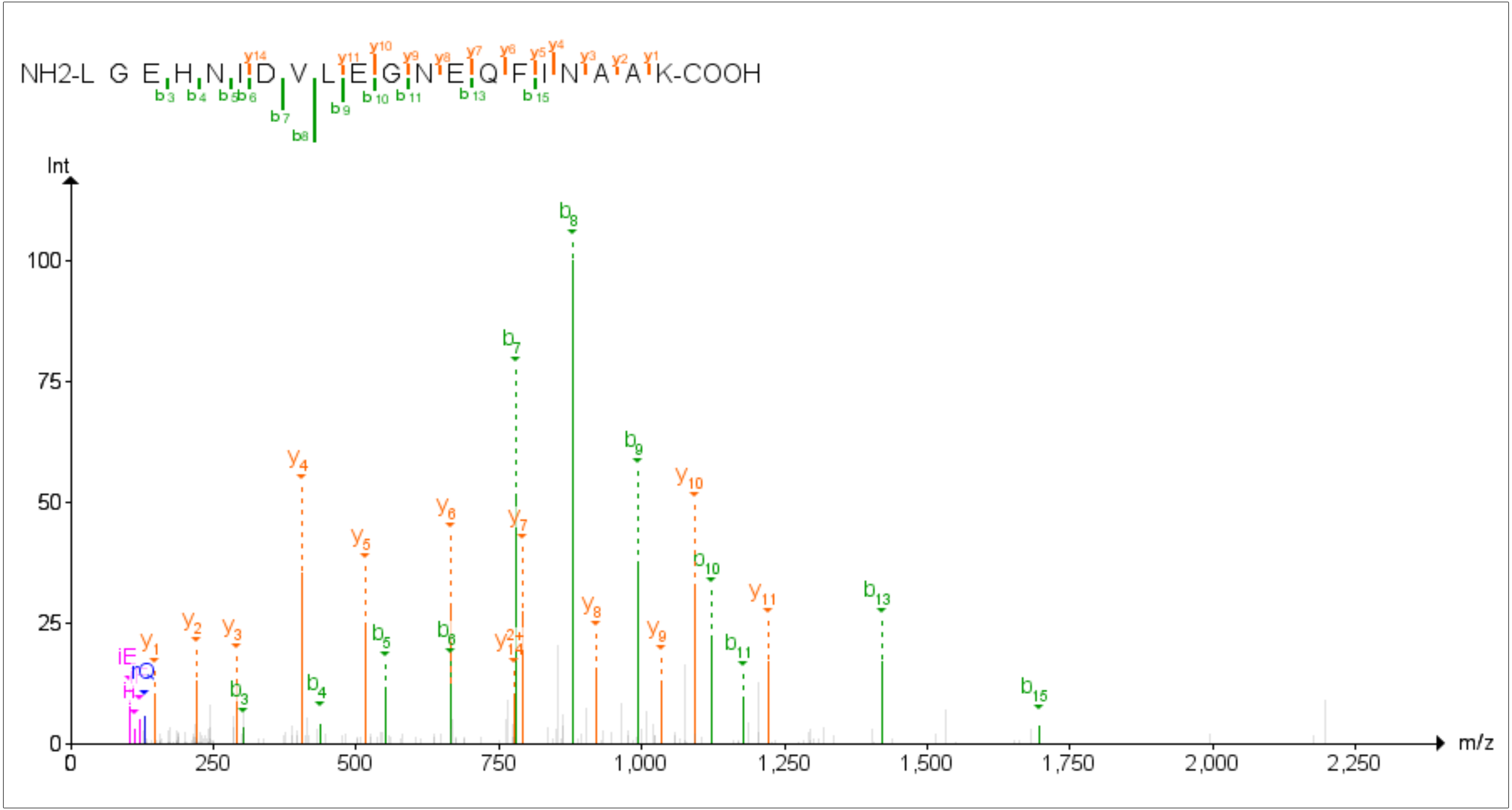
NH2-L G E H N I D V L E G N E Q F I N A A K-COOH
Int
m/z
0
25
50
75
100
250
500
750
1,000
1,250
1,500
1,750
2,000
2,250
iE
rQ
iH
y1 y2 y3 y4 y5 y6 y7 y8 y9 y10 y11
y14 2+
b3 b4 b5 b6 b7 b8 b9 b10 b11 b13 b15

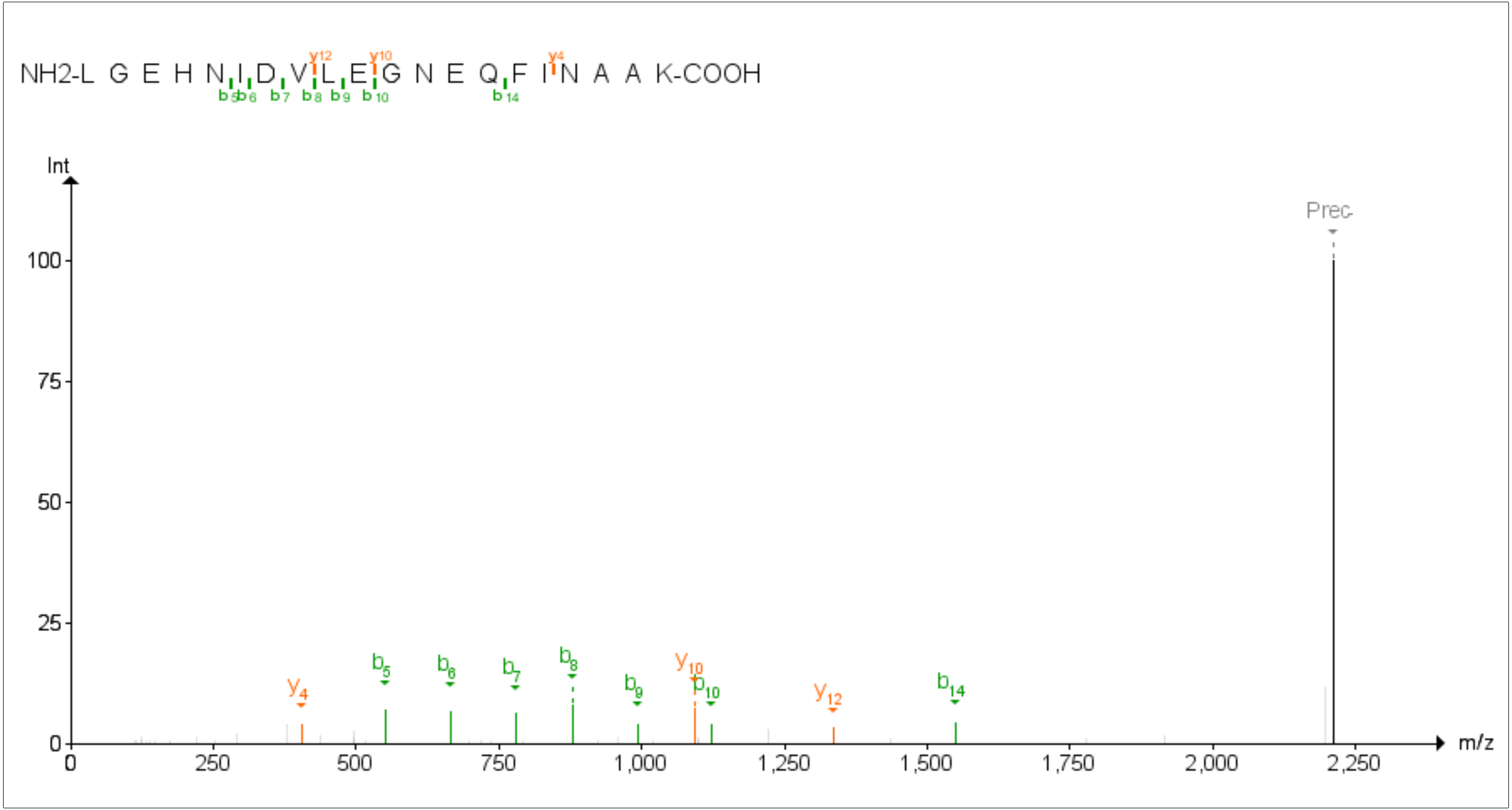

NH2-L G E H N I D V L E G N E Q F I N A A K-COOH
y12 y10 y4
b5 b6 b7 b8 b9 b10 b14
Int
Prec
100
75
50
25
0
y4 b5 b6 b7 b8 b9 y10 b10 y12 b14
0 250 500 750 1,000 1,250 1,500 1,750 2,000 2,250
m/z

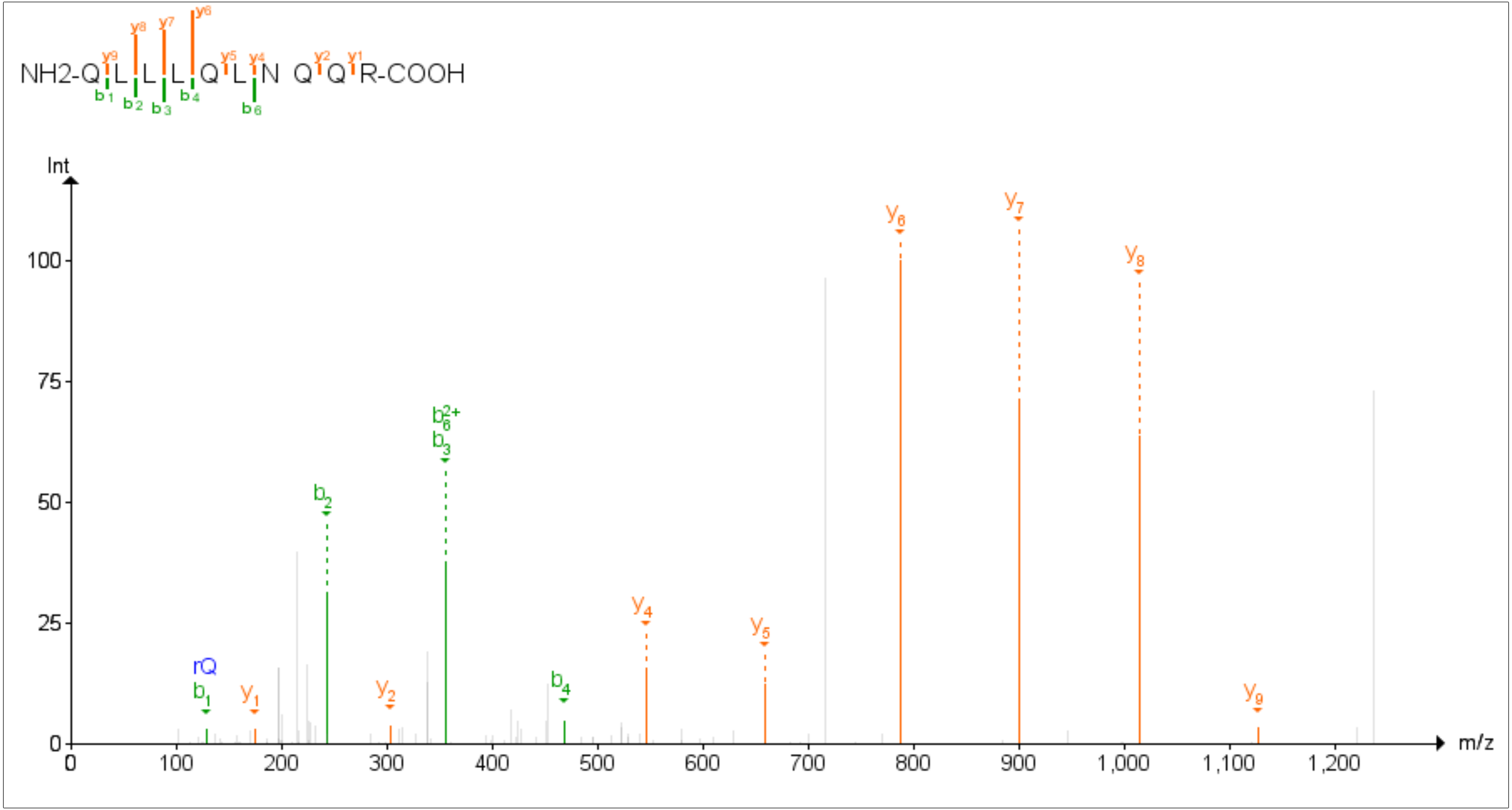

NH2-Q L L L Q L N Q Q R-COOH
y9 y8 y7 y6 y5 y4 y2 y1
b1 b2 b3 b4 b6
Int
100
75
50
25
0
rQ
b1
y1
b2
y2
b6 2+
b3
b4
y4
y5
y6
y7
y8
y9
0 100 200 300 400 500 600 700 800 900 1,000 1,100 1,200
m/z

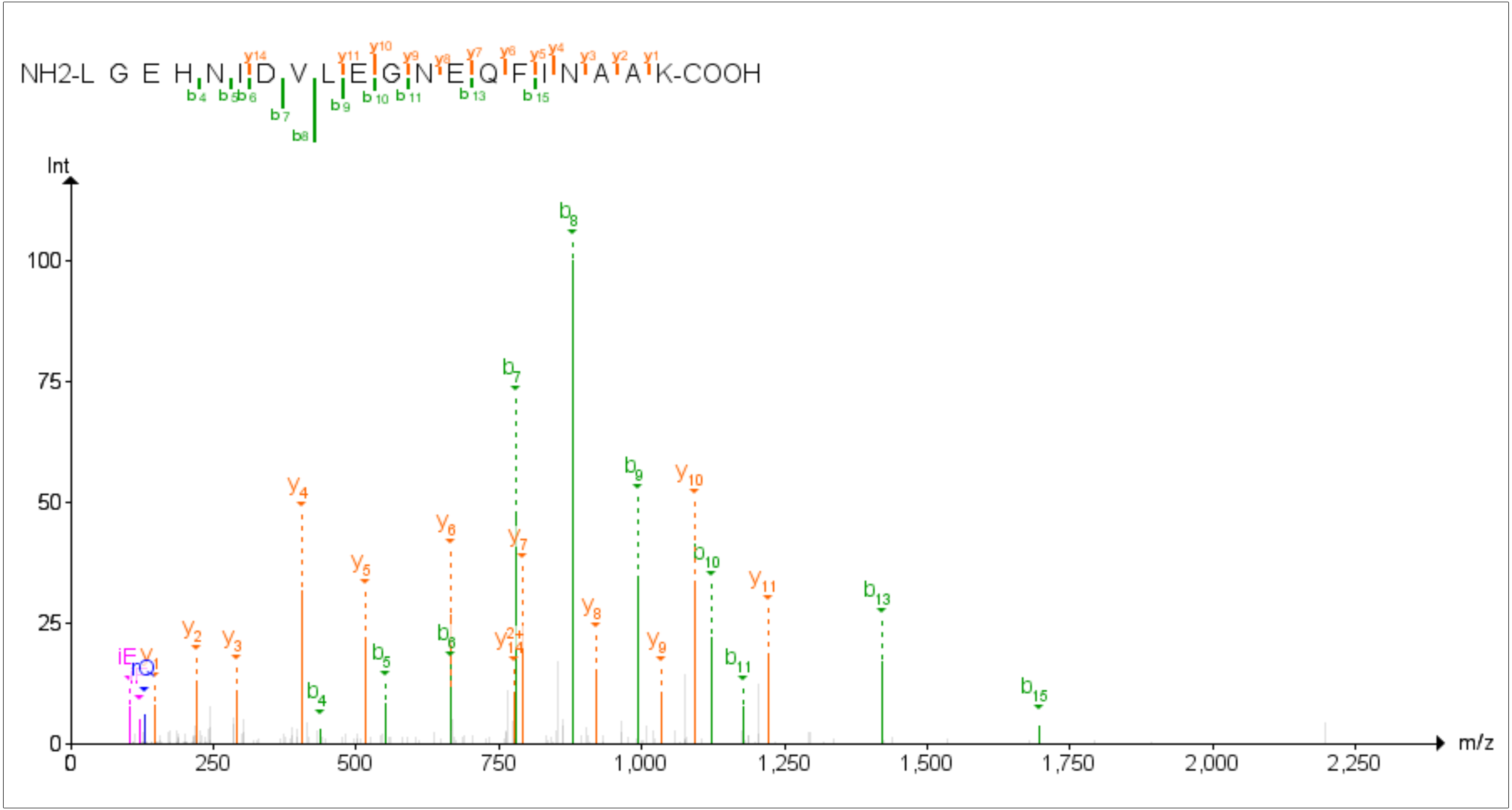
NH2-L G E H N I D V L E G N E Q F I N A A K-COOH
Int
m/z
b8
b7
b9
y10
y4
y6
y7
y5
b10
y11
b13
y8
y2
y3
b6
b5
y14 2+
y9
b11
b4
b15
iE
y1
nQ

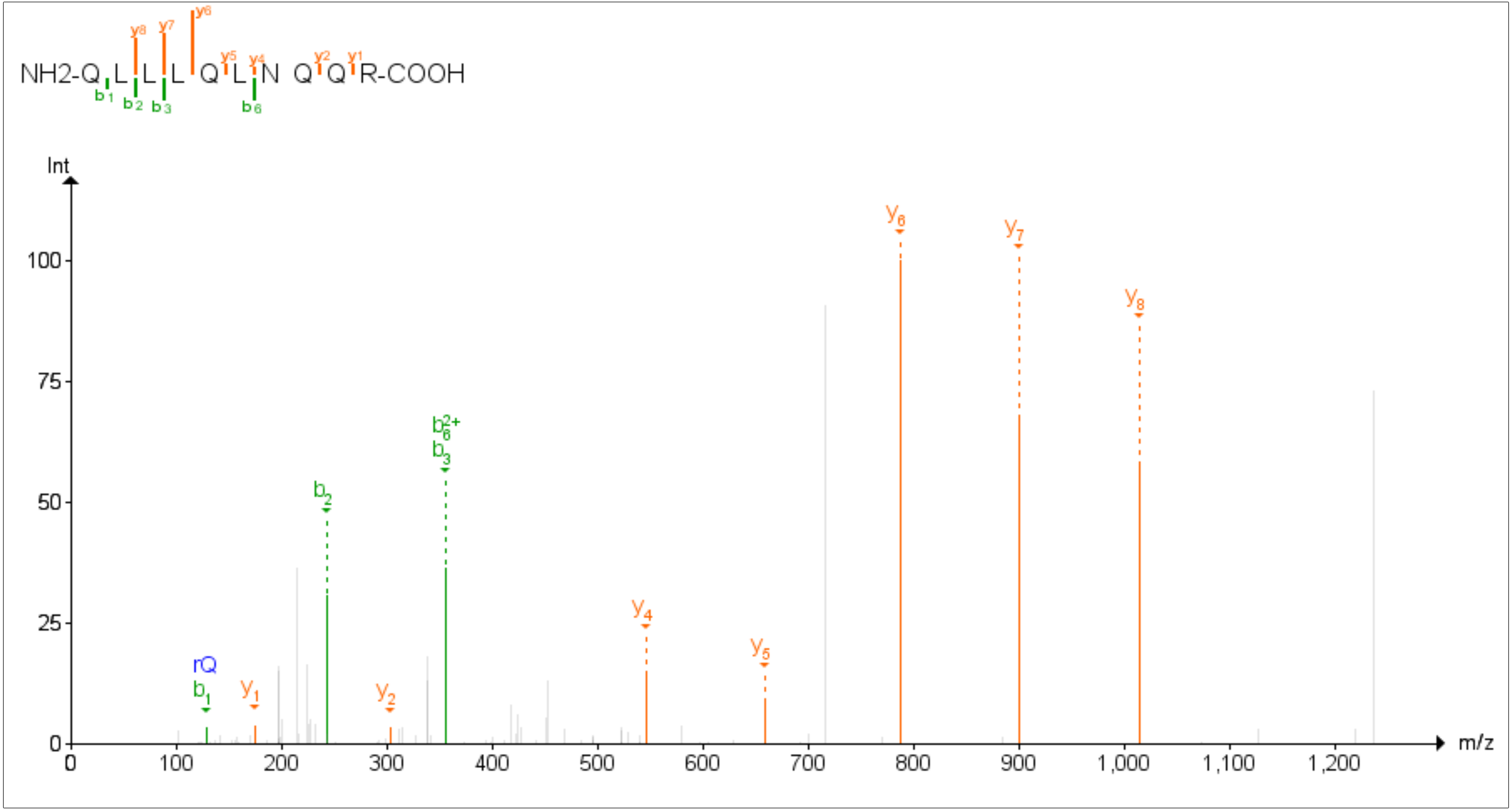

NH2-Q L L L Q L N Q Q R-COOH
y8 y7 y6 y5 y4 y2 y1
b1 b2 b3 b6
Int
100
75
50
25
0
rQ
b1
y1
b2
y2
b6 2+
b3
y4
y5
y6
y7
y8
0 100 200 300 400 500 600 700 800 900 1,000 1,100 1,200
m/z

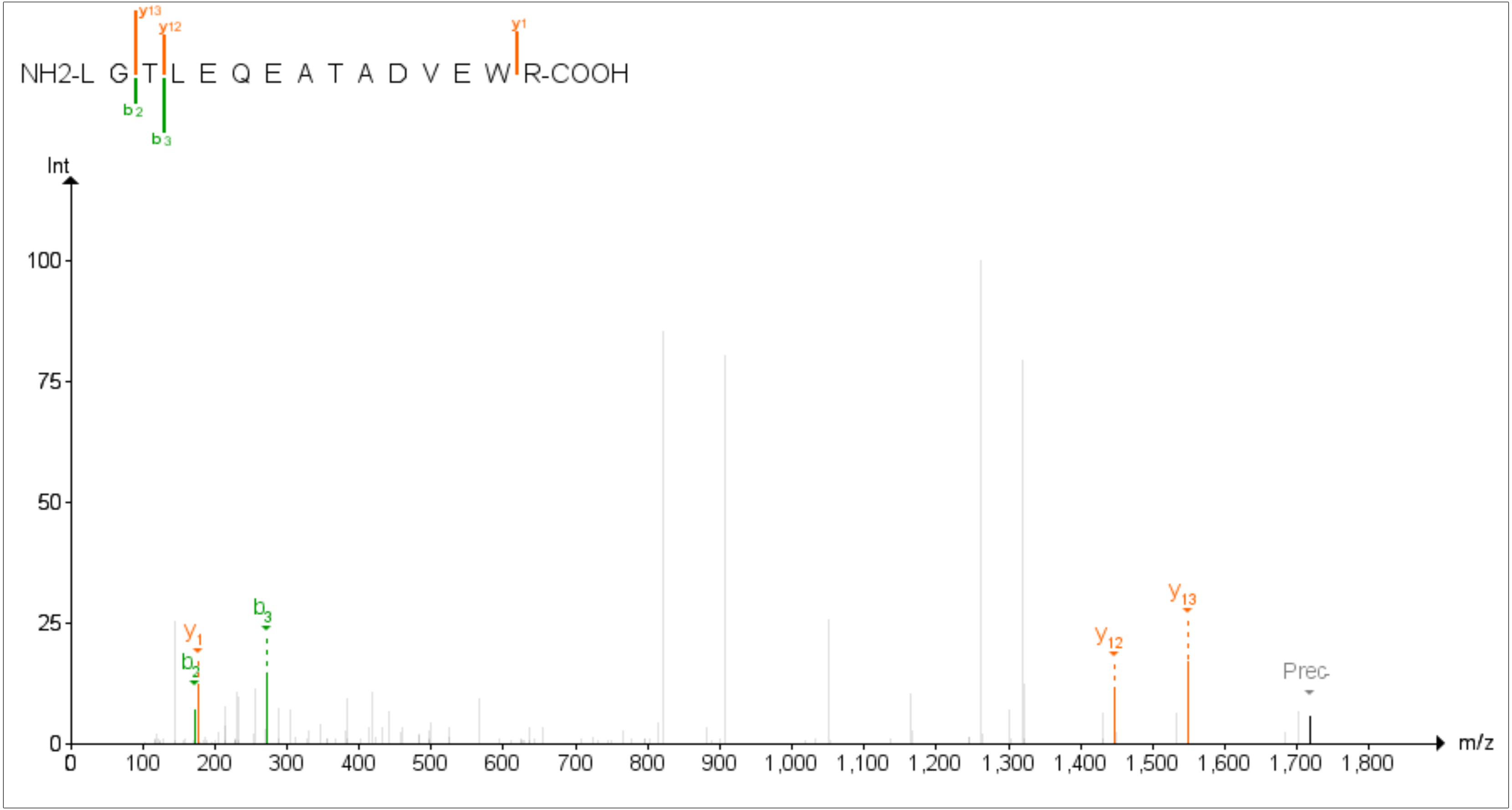

NH2-L G T L E Q E A T A D V E W R-COOH
y13
y12
y1
b2
b3
Int
100
75
50
25
0
m/z
0
100
200
300
400
500
600
700
800
900
1,000
1,100
1,200
1,300
1,400
1,500
1,600
1,700
1,800
Prec

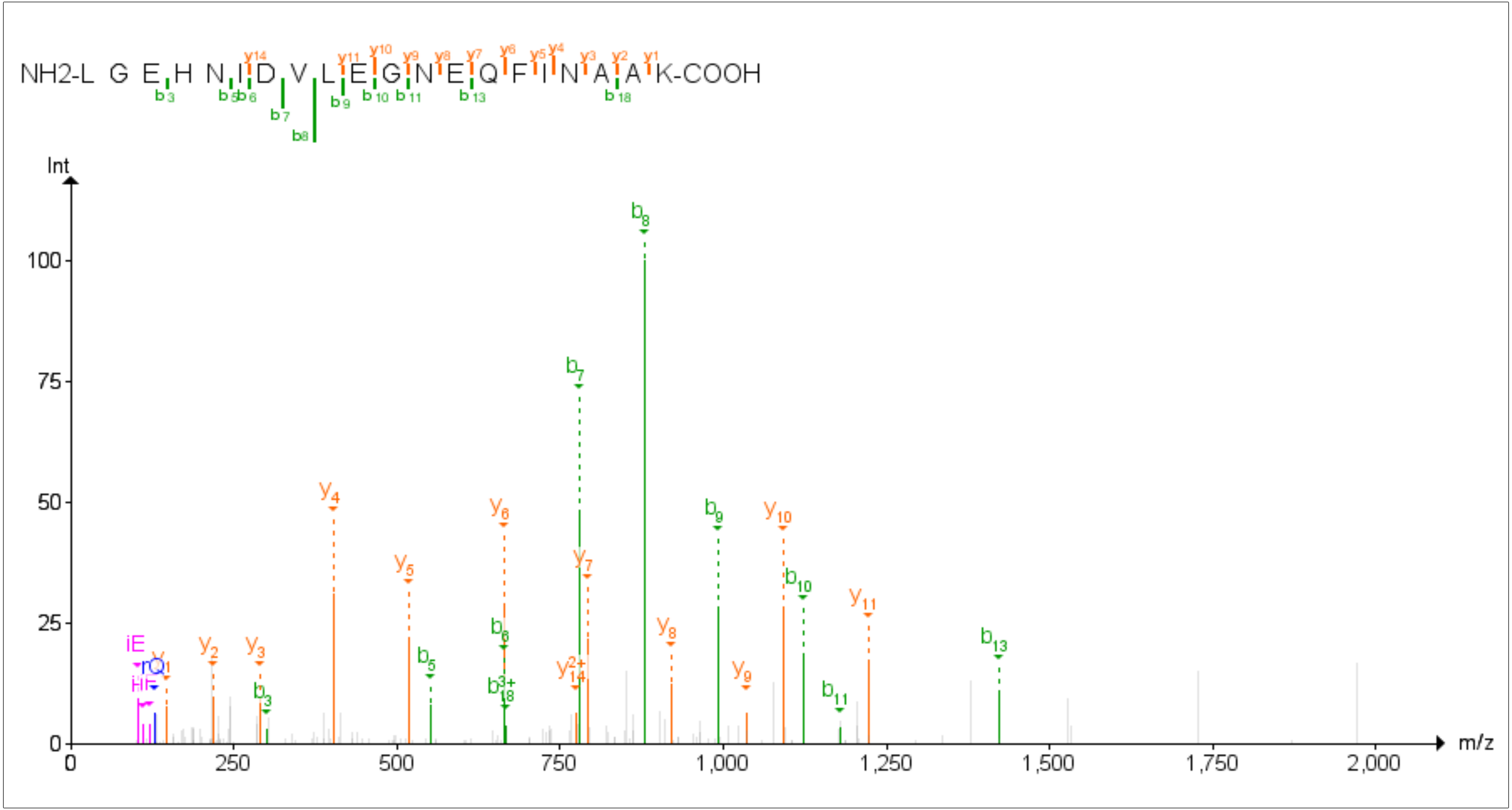
NH2-L G E H N I D V L E G N E Q F I N A A K-COOH
Int
m/z
0
25
50
75
100
250
500
750
1,000
1,250
1,500
1,750
2,000

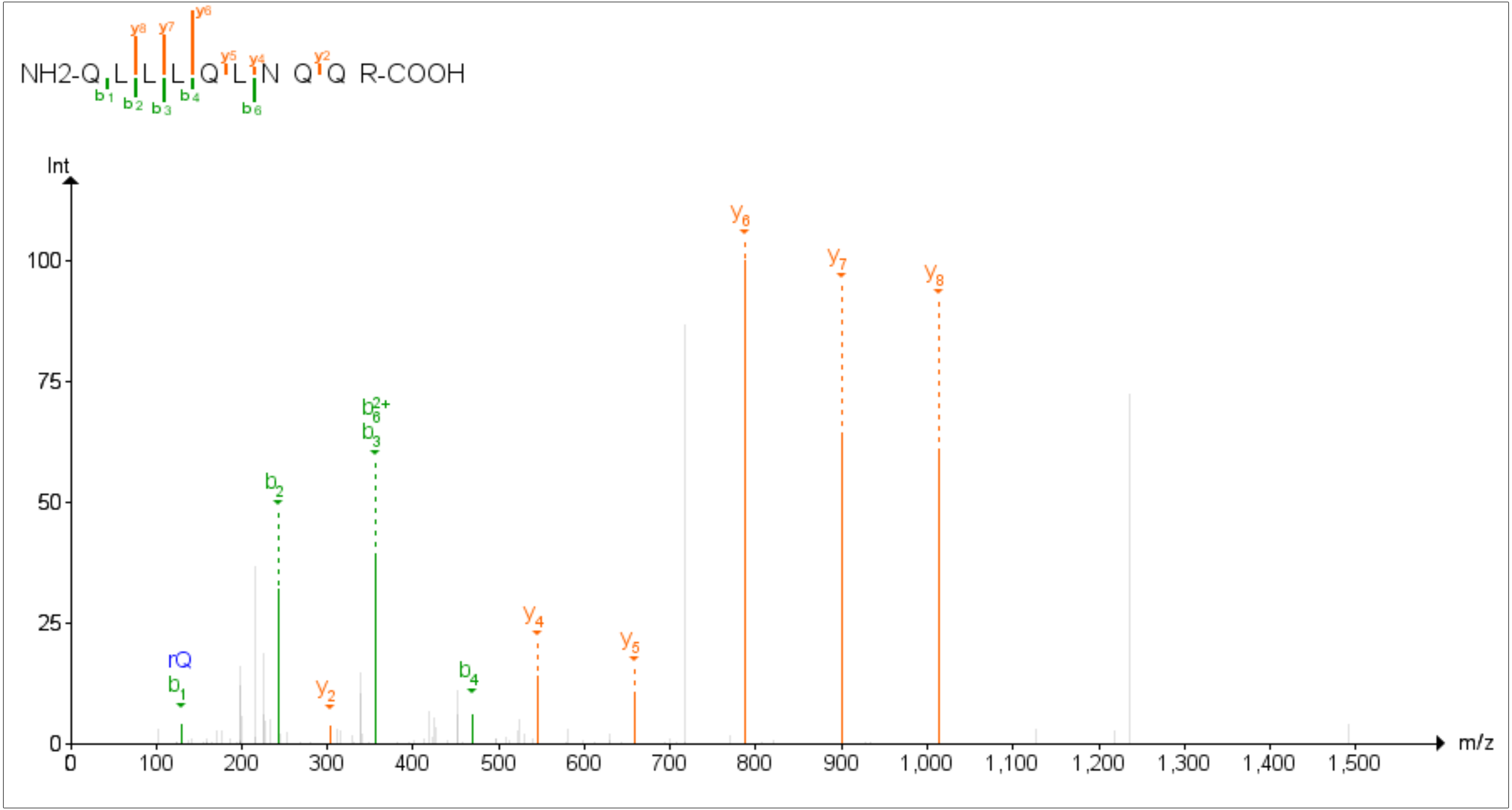
NH2-Q L L L Q L N Q Q R-COOH
y8 y7 y6 y5 y4 y2
b1 b2 b3 b4 b6
Int
100
75
50
25
0
m/z
0 100 200 300 400 500 600 700 800 900 1,000 1,100 1,200 1,300 1,400 1,500
rQ
b1
b2
b6 2+
b3
y2
b4
y4
y5
y6
y7
y8

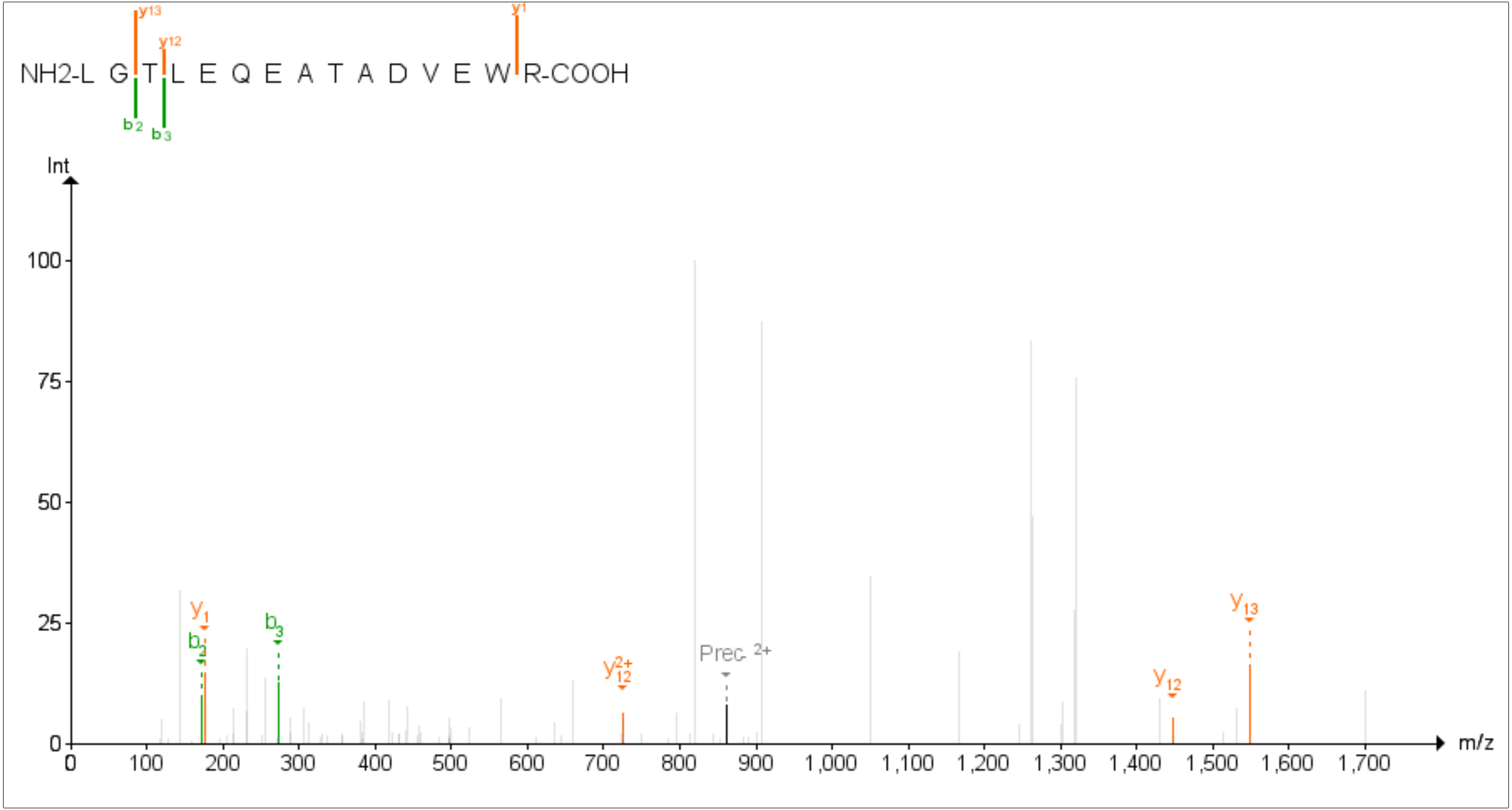

NH2-L G T L E Q E A T A D V E W R-COOH
y13
y12
y1
b2
b3
Int
100
75
50
25
0
y1
b2
b3
y12 2+
Prec. 2+
y12
y13
0
100
200
300
400
500
600
700
800
900
1,000
1,100
1,200
1,300
1,400
1,500
1,600
1,700
m/z

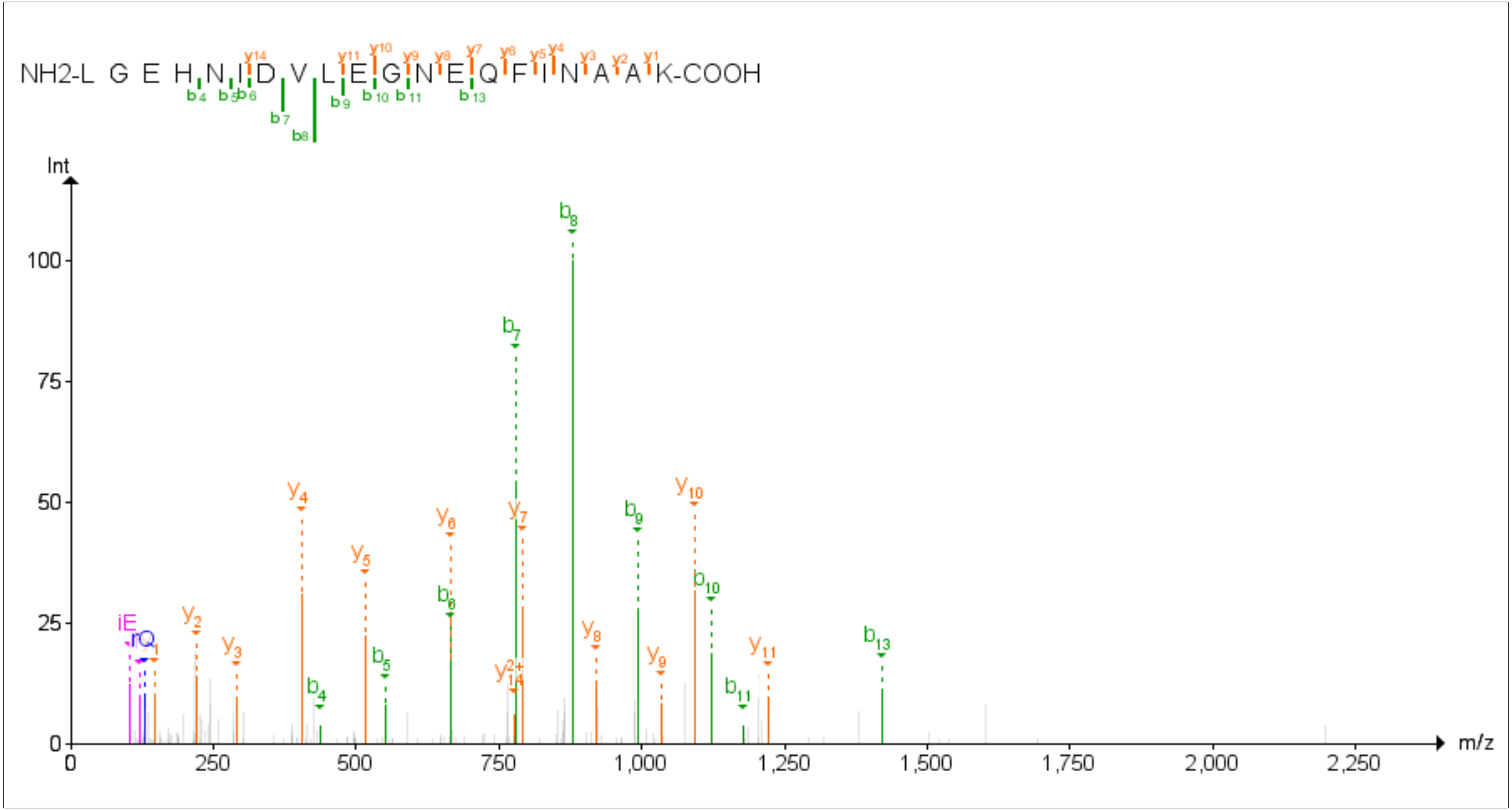

NH2-L G E H N I D V L E G N E Q F I N A A K-COOH
Int
m/z
b8
b7
y4
y10
y6
y7
b9
y5
b10
b6
iE
rQ
y2
y3
y8
b5
y9
y11
b13
b4
b11

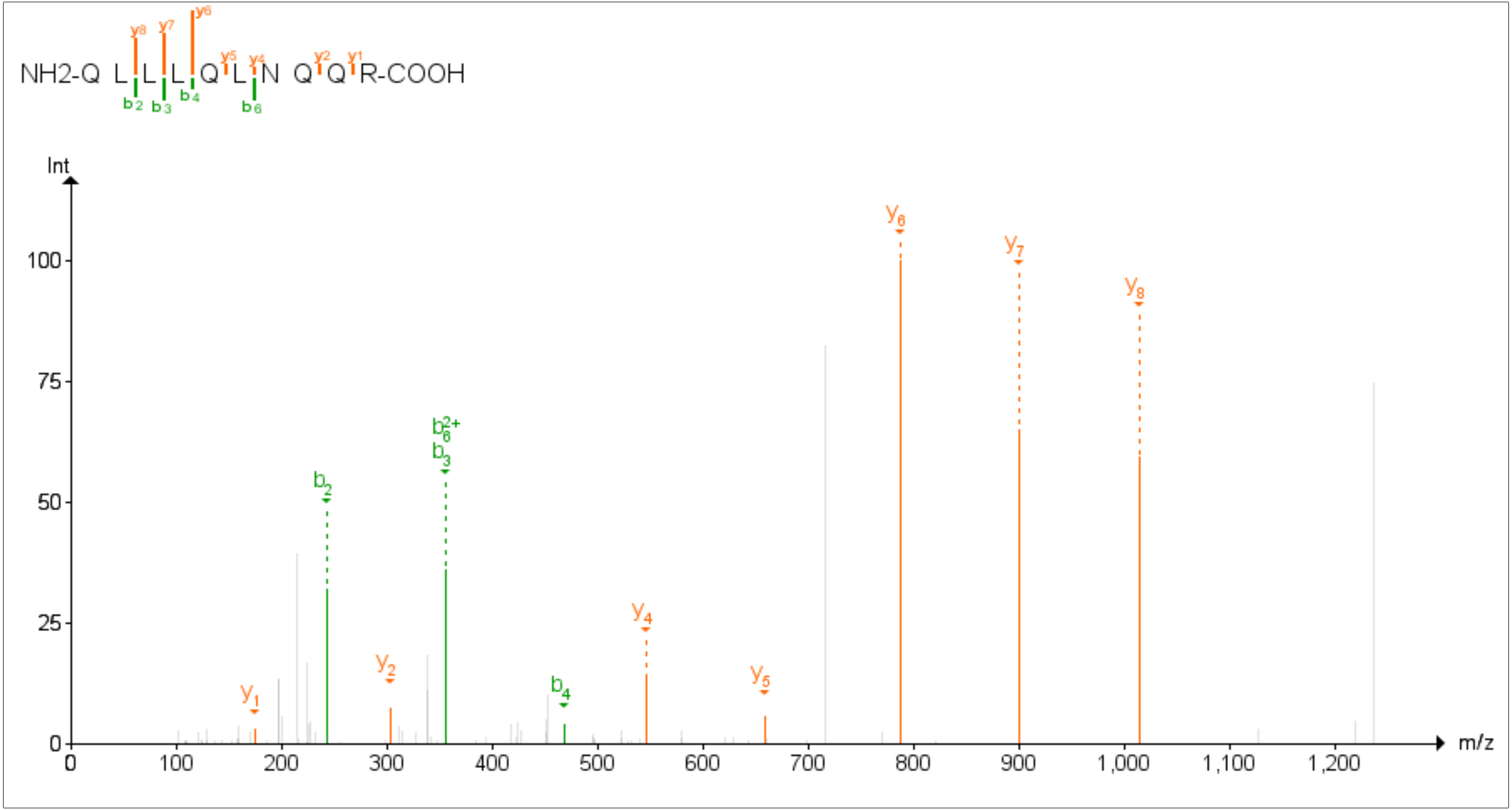

NH2-Q L L L Q L N Q Q R-COOH
y8 y7 y6 y5 y4 y2 y1
b2 b3 b4 b6
Int
100
75
50
25
0
y6
y7
y8
b2
b6 2+
b3
y1
y2
b4
y4
y5
0 100 200 300 400 500 600 700 800 900 1,000 1,100 1,200
m/z

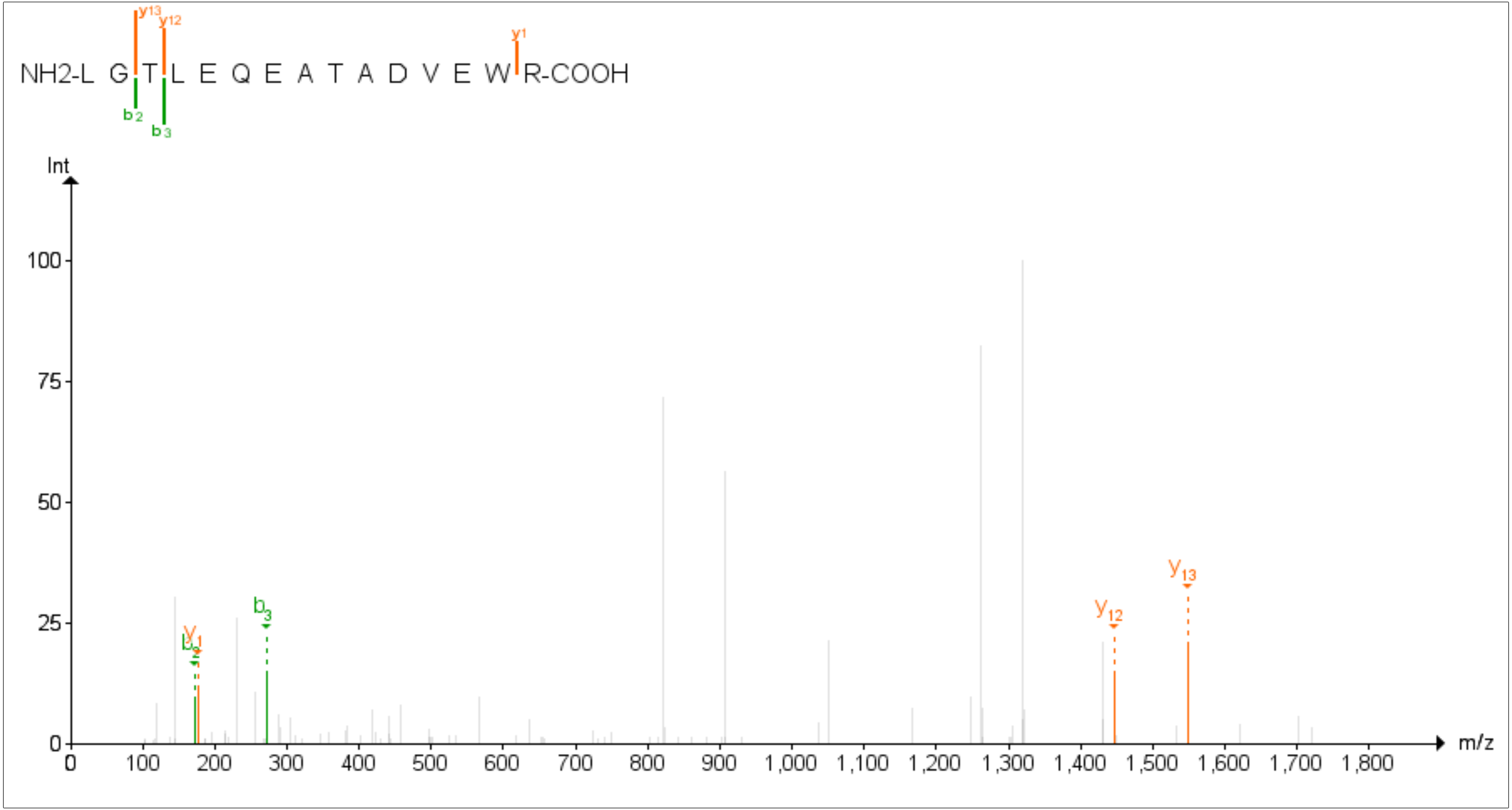

NH2-L G T L E Q E A T A D V E W R-COOH
y13
y12
y1
b2
b3
Int
m/z

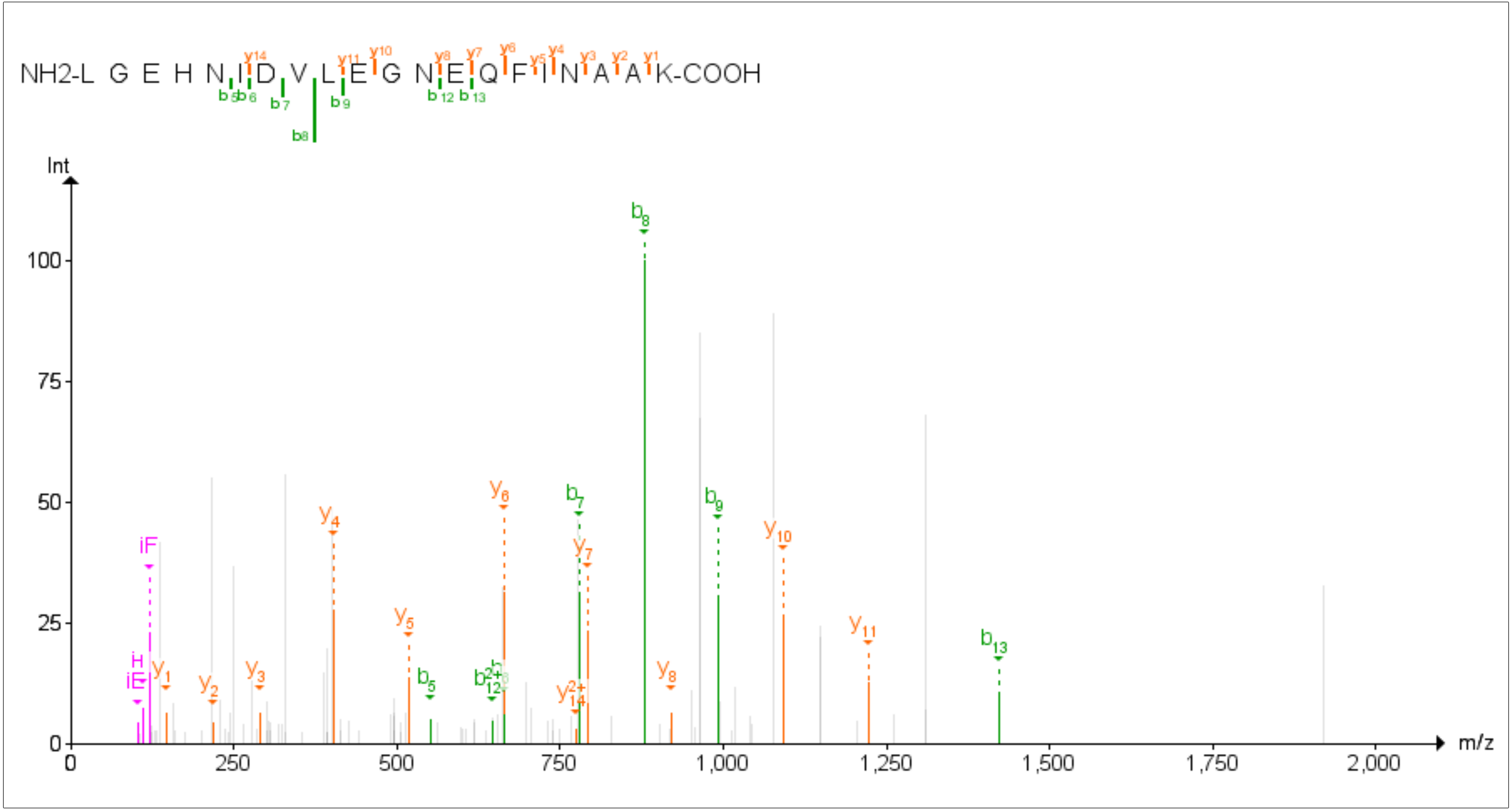

NH2-L G E H N I D V L E G N E Q F I N A A K-COOH
y14 y11 y10 y8 y7 y6 y5 y4 y3 y2 y1
b5 b6 b7 b8 b9 b12 b13
Int
100
75
50
25
0
0
250
500
750
1,000
1,250
1,500
1,750
2,000
m/z
b8
y6
b7
b9
y4
y10
y7
iF
y5
y11
b13
iH
iE
y1
y2
y3
b5
y8

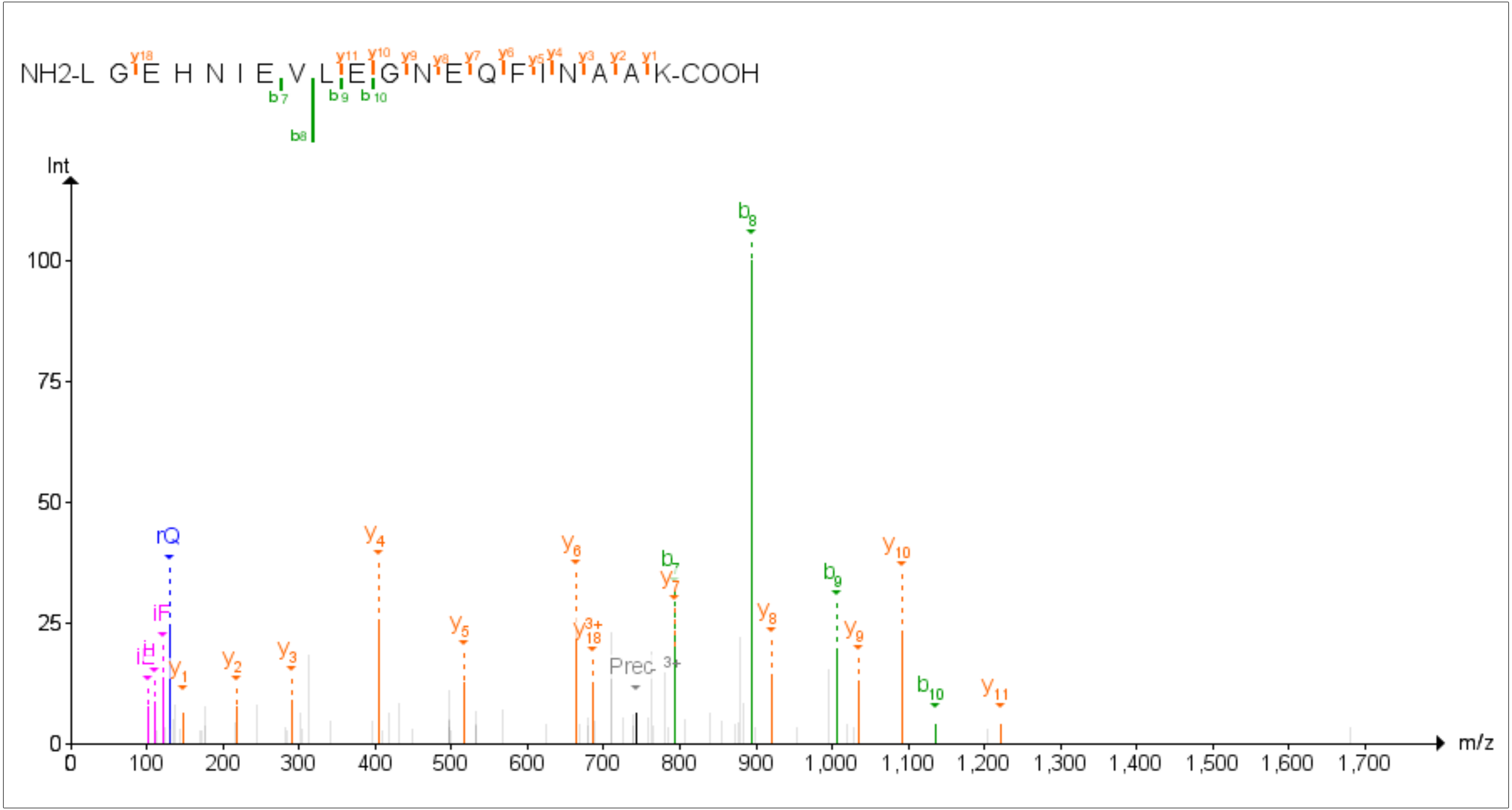
NH2-L G E H N I E V L E G N E Q F I N A A K-COOH
y18 y11 y10 y9 y8 y7 y6 y5 y4 y3 y2 y1
b7 b9 b10 b8
Int
m/z
rQ
iF
iL
iH
Prec 3+
b8
b7
b9
b10
y1 y2 y3 y4 y5 y6 y7 y8 y9 y10 y11
y18 3+
0 25 50 75 100
0 100 200 300 400 500 600 700 800 900 1,000 1,100 1,200 1,300 1,400 1,500 1,600 1,700

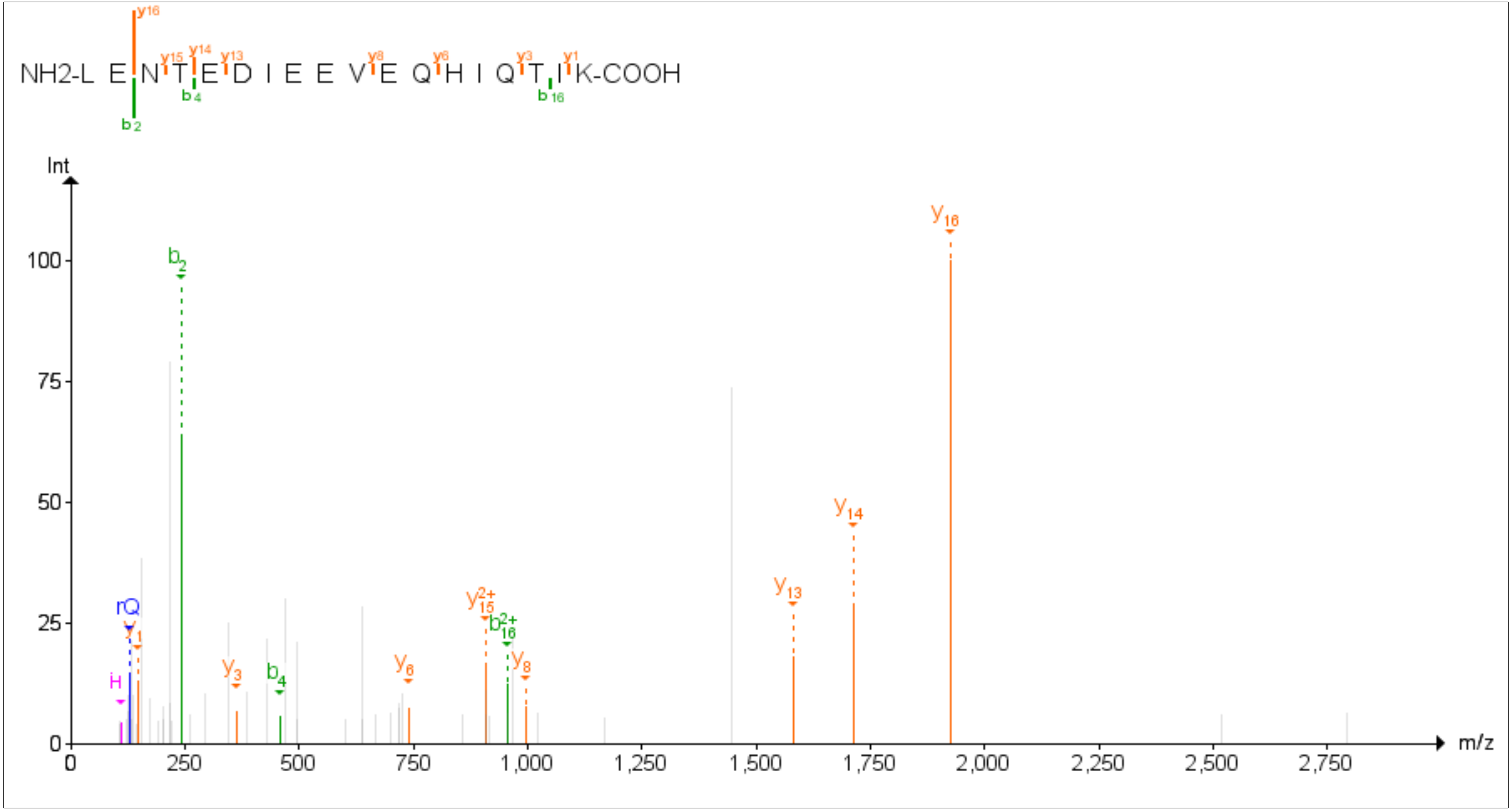

NH2-L E N T E D I E E V E Q H I Q T I K-COOH
y16
y15
y14
y13
y8
y6
y3
y1
b2
b4
b16
Int
100
75
50
25
0
m/z
0
250
500
750
1,000
1,250
1,500
1,750
2,000
2,250
2,500
2,750

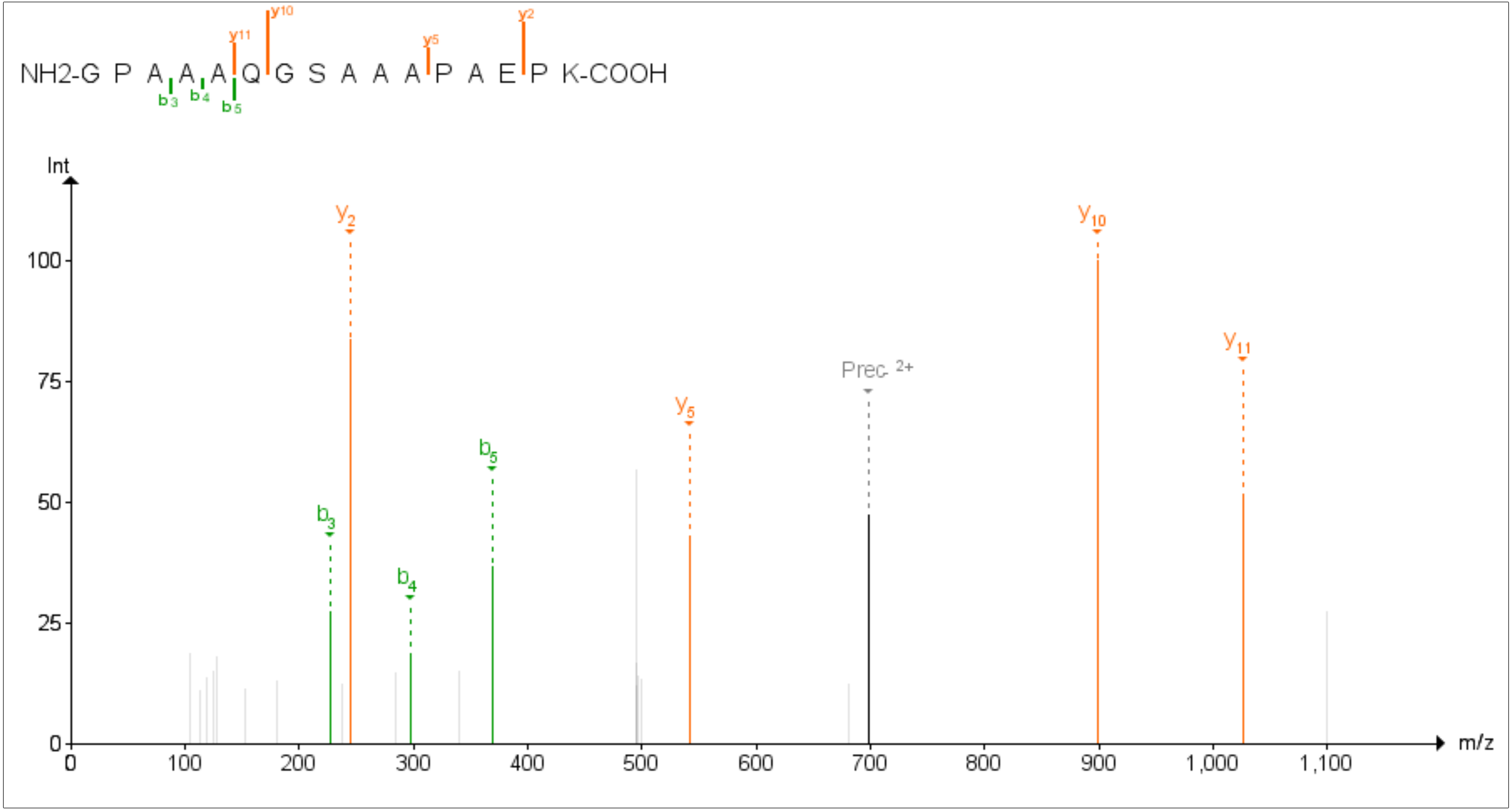

NH2-G P A A A Q G S A A A P A E P K-COOH
y11
y10
y5
y2
b3
b4
b5
Int
100
75
50
25
0
y2
y10
y11
Prec. 2+
y5
b5
b3
b4
m/z
0
100
200
300
400
500
600
700
800
900
1,000
1,100

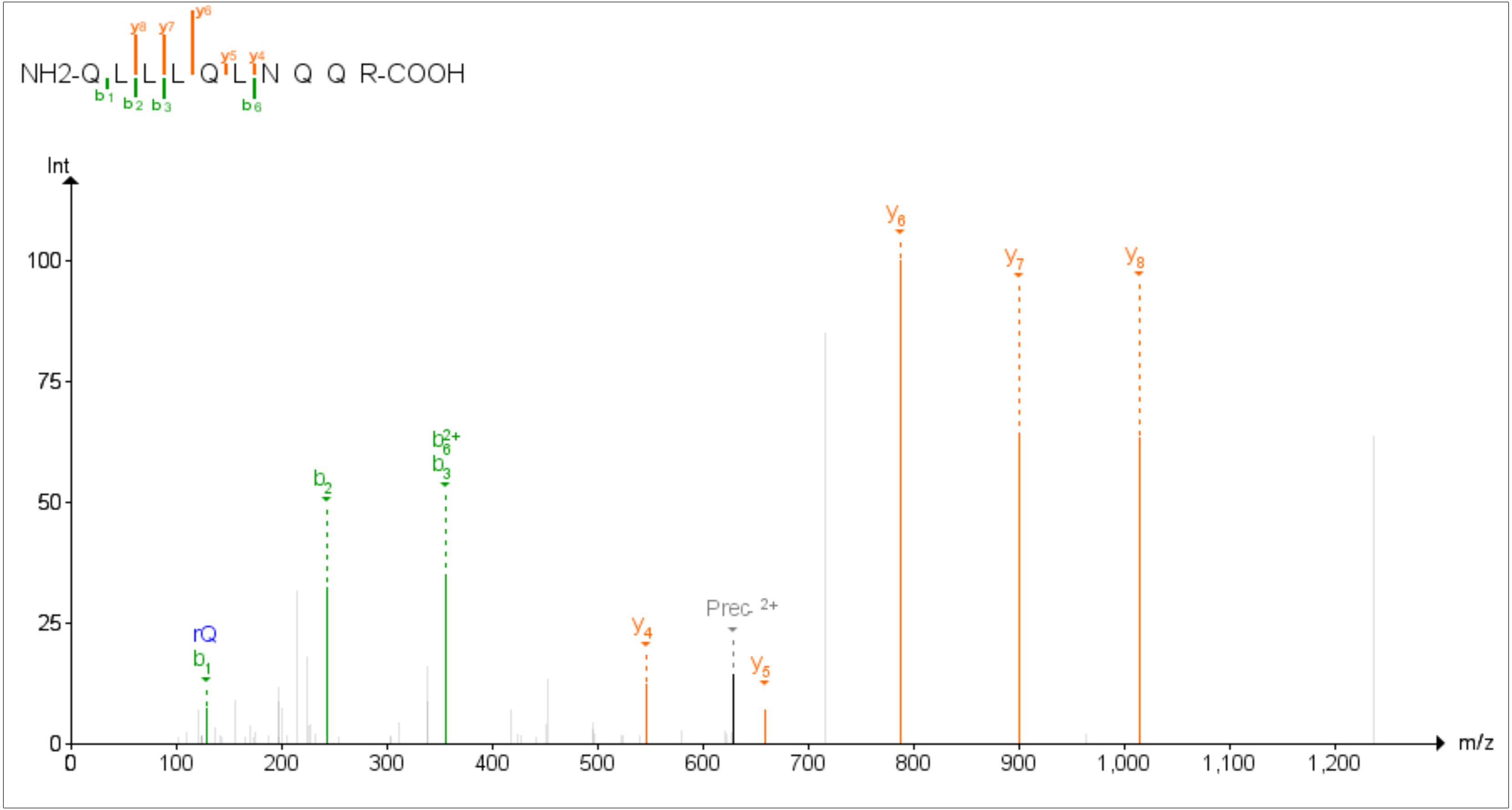

NH2-Q L L L Q L N Q Q R-COOH
y8 y7 y6 y5 y4
b1 b2 b3 b6
Int
100
75
50
25
0
y6
y7
y8
b6 2+
b3
b2
rQ
b1
y4
Prec. 2+
y5
0 100 200 300 400 500 600 700 800 900 1,000 1,100 1,200
m/z

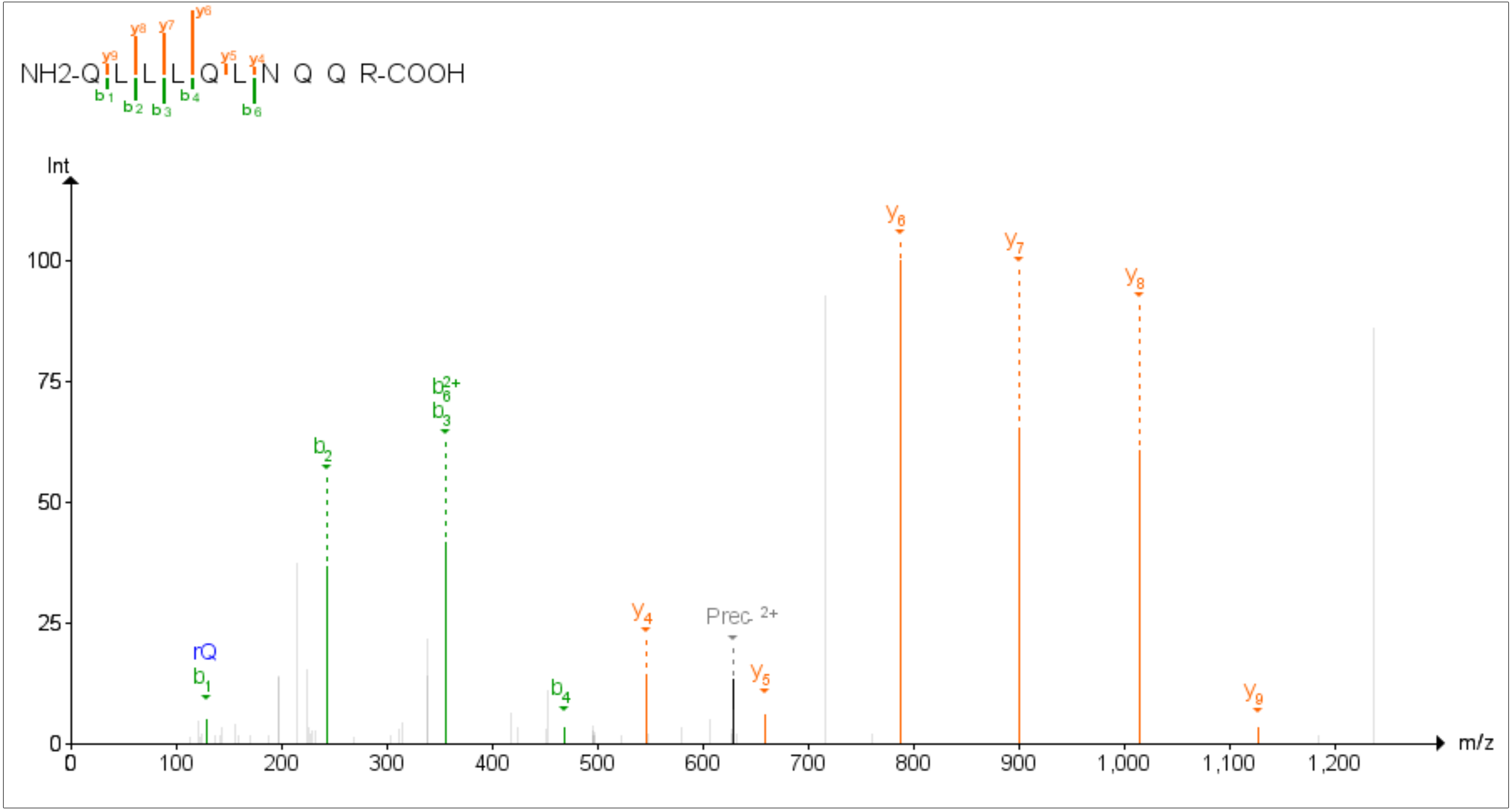

NH2-Q L L L Q L N Q Q R-COOH
y9 y8 y7 y6 y5 y4
b1 b2 b3 b4 b6
Int
100
75
50
25
0
rQ
b1
b2
b6 2+
b3
b4
y4
Prec 2+
y5
y6
y7
y8
y9
0 100 200 300 400 500 600 700 800 900 1,000 1,100 1,200
m/z

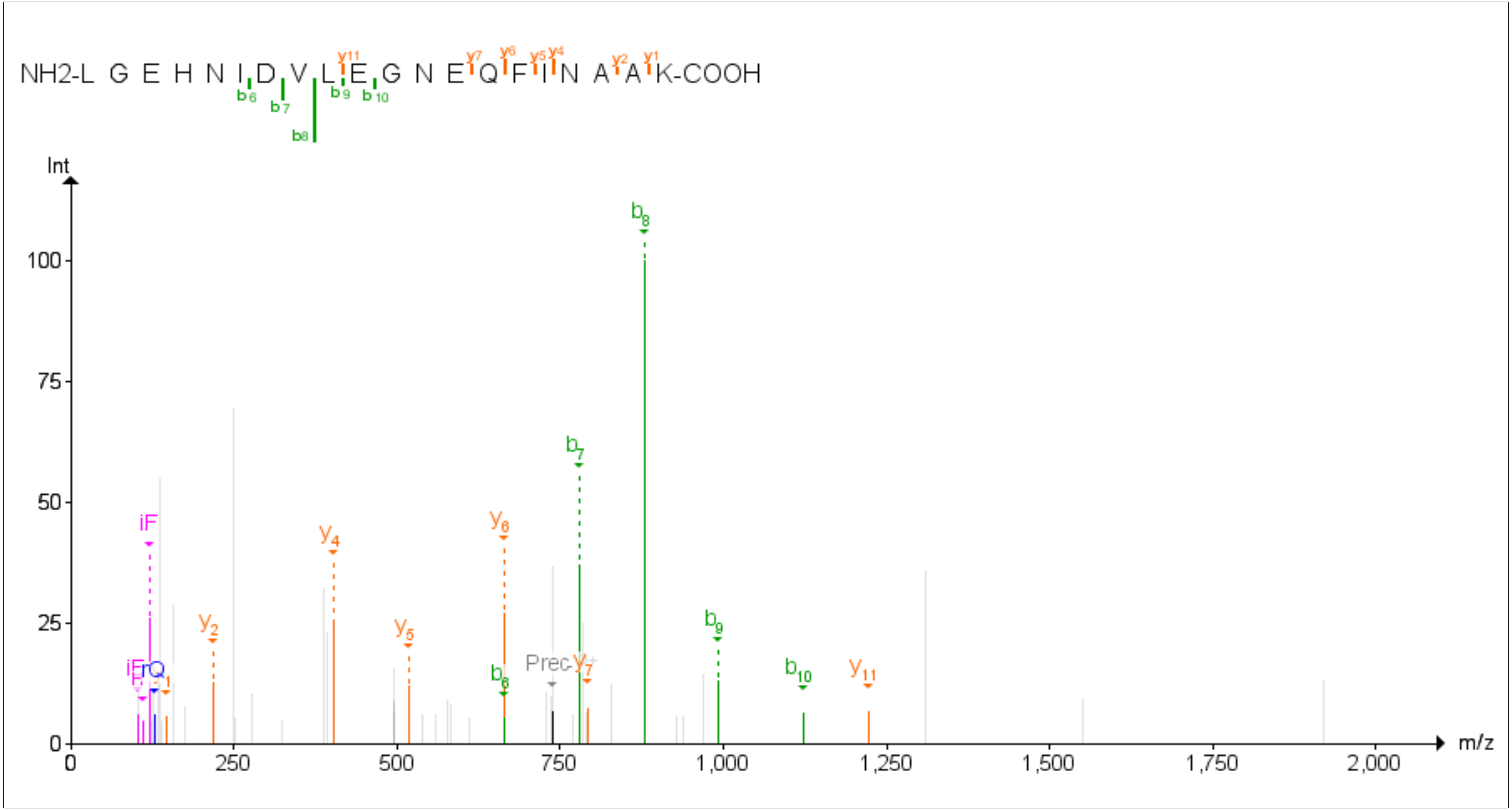

NH2-L G E H N I D V L E G N E Q F I N A A K-COOH
y11 y7 y6 y5 y4 y2 y1
b6 b7 b8 b9 b10
Int
100
75
50
25
0
m/z
0
250
500
750
1,000
1,250
1,500
1,750
2,000
b8
b7
iF
y4
y6
y2
y5
b9
iF
iQ
b6
Prec
y7
b10
y11

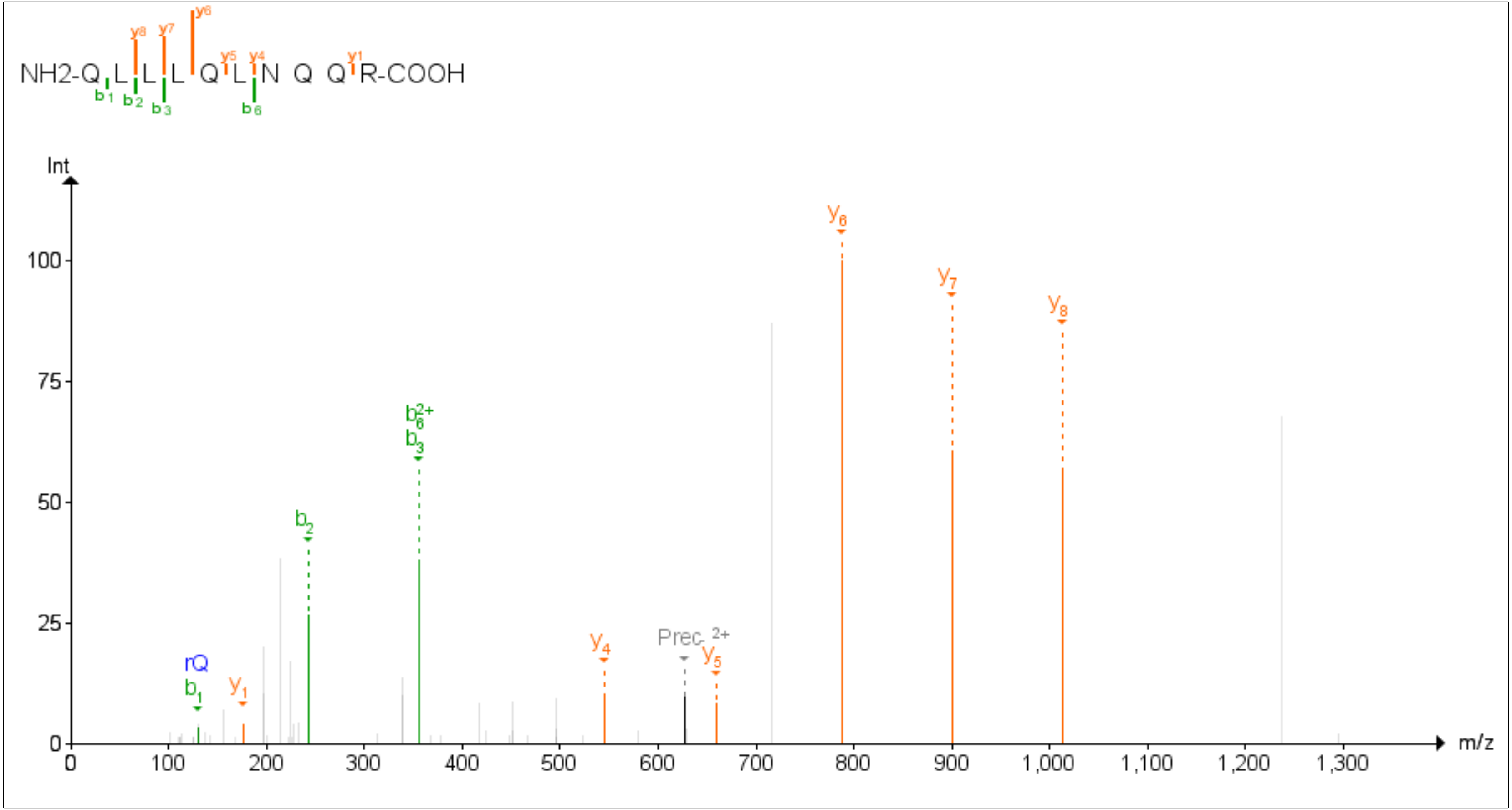

NH2-Q L L L Q L N Q Q R-COOH
y8 y7 y6 y5 y4 y1
b1 b2 b3 b6
Int
100
75
50
25
0
y6
y7
y8
b6 2+
b3
b2
rQ
b1
y1
y4
Prec 2+
y5
0 100 200 300 400 500 600 700 800 900 1,000 1,100 1,200 1,300
m/z

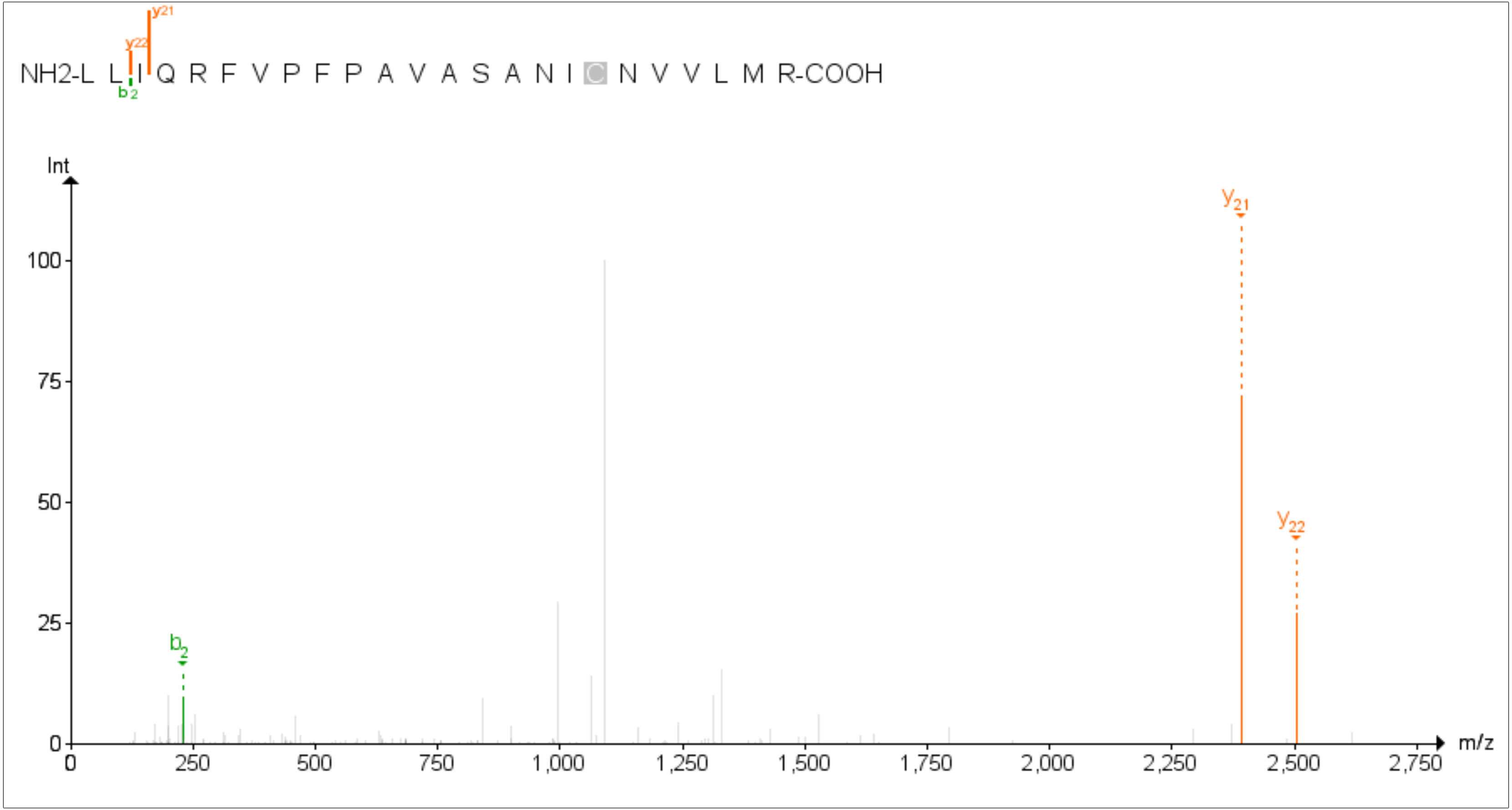

NH2-L L I Q R F V P F P A V A S A N I C N V V L M R-COOH
y21
y22
b2
Int
100
75
50
25
0
y21
y22
b2
0
250
500
750
1,000
1,250
1,500
1,750
2,000
2,250
2,500
2,750
m/z

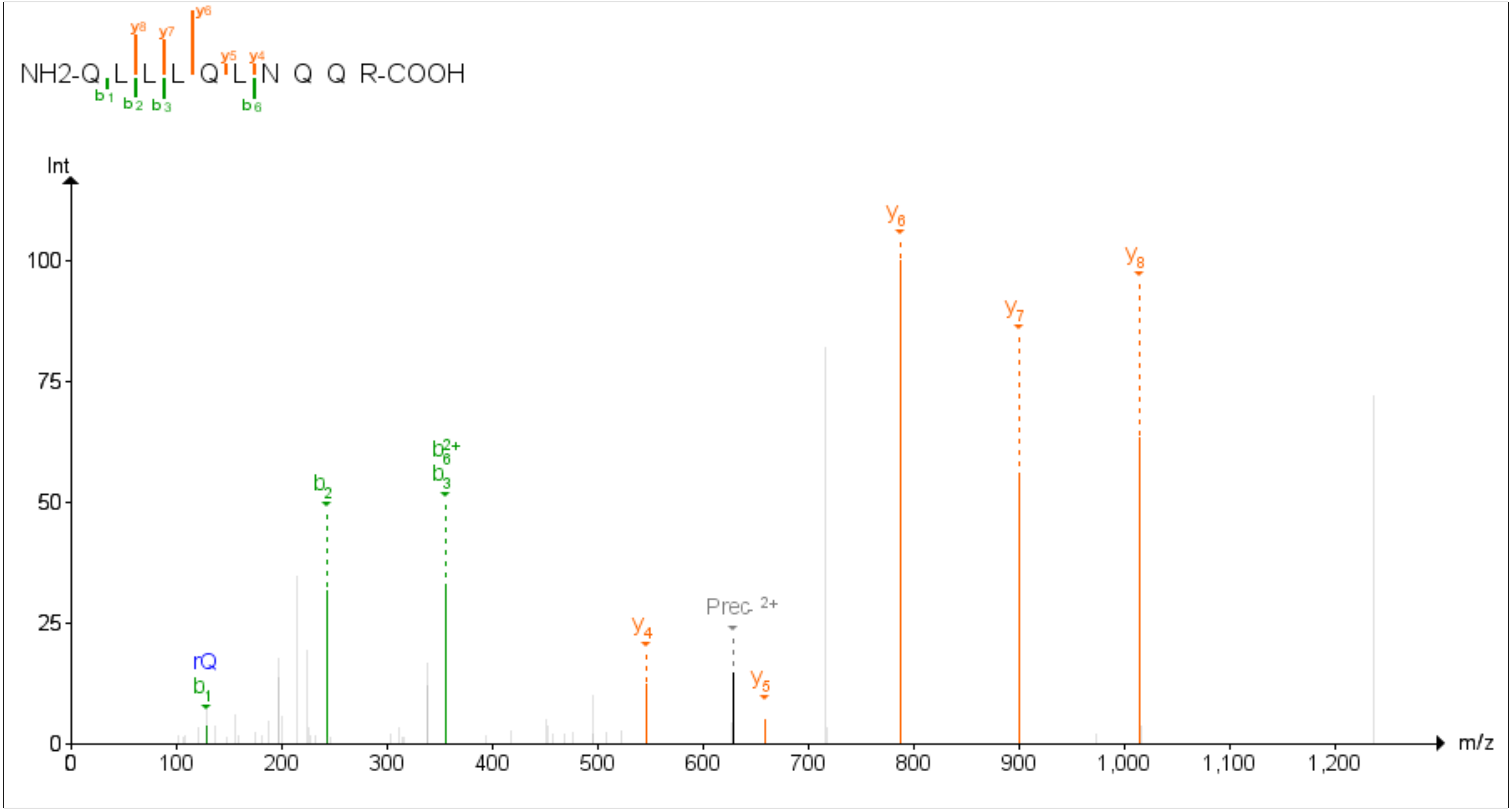

NH2-Q L L L Q L N Q Q R-COOH
y8 y7 y6 y5 y4
b1 b2 b3 b6
Int
100
75
50
25
0
rQ
b1
b2
b6 2+
b3
y4
Prec 2+
y5
y6
y7
y8
0 100 200 300 400 500 600 700 800 900 1,000 1,100 1,200
m/z

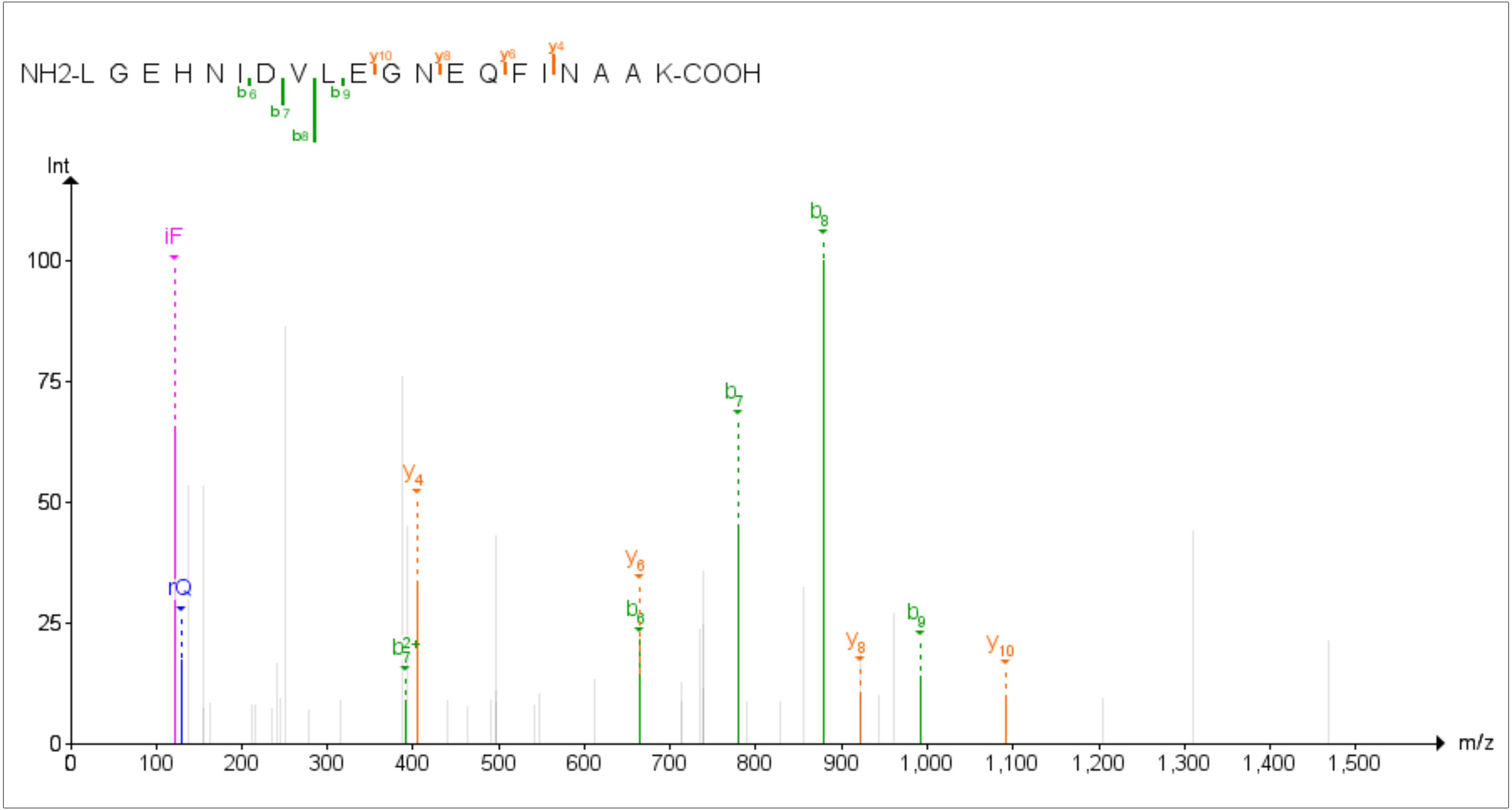

NH2-L G E H N I D V L E G N E Q F I N A A K-COOH
b6 b7 b8 b9
y10 y8 y6 y4
Int
m/z
iF
rQ
b7 2+
y4
y6
b6
b7
b8
y8
b9
y10
0
25
50
75
100
0
100
200
300
400
500
600
700
800
900
1,000
1,100
1,200
1,300
1,400
1,500

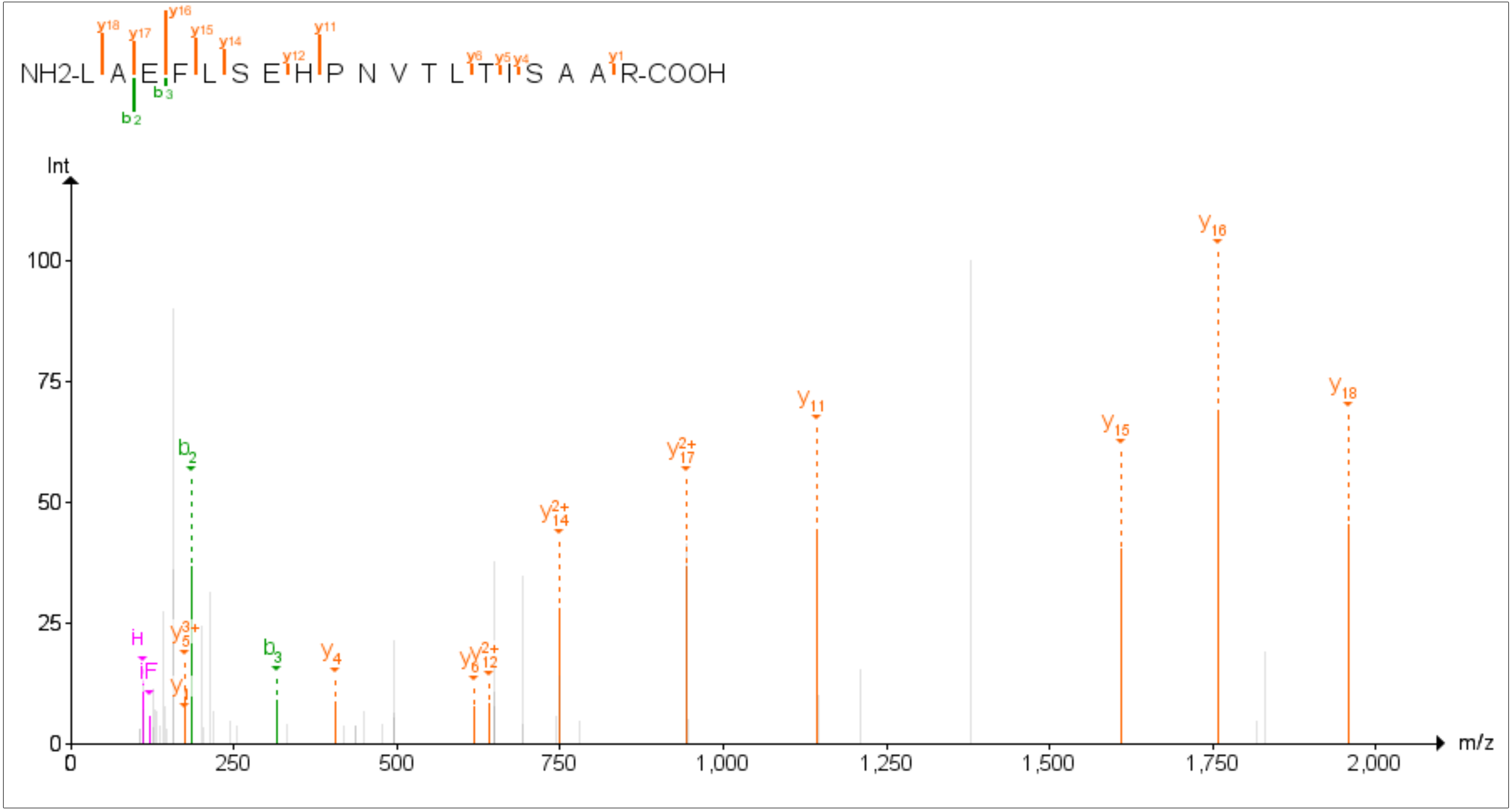

NH2-L A E F L S E H P N V T L T I S A A R-COOH
y18 y17 y16 y15 y14 y12 y11 y6 y5 y4 y1
b2 b3
Int
m/z
0
25
50
75
100
250
500
750
1,000
1,250
1,500
1,750
2,000
iH
iF
y1
y5 3+
b2
b3
y4
y6
y12 2+
y14 2+
y17 2+
y11
y15
y16
y18

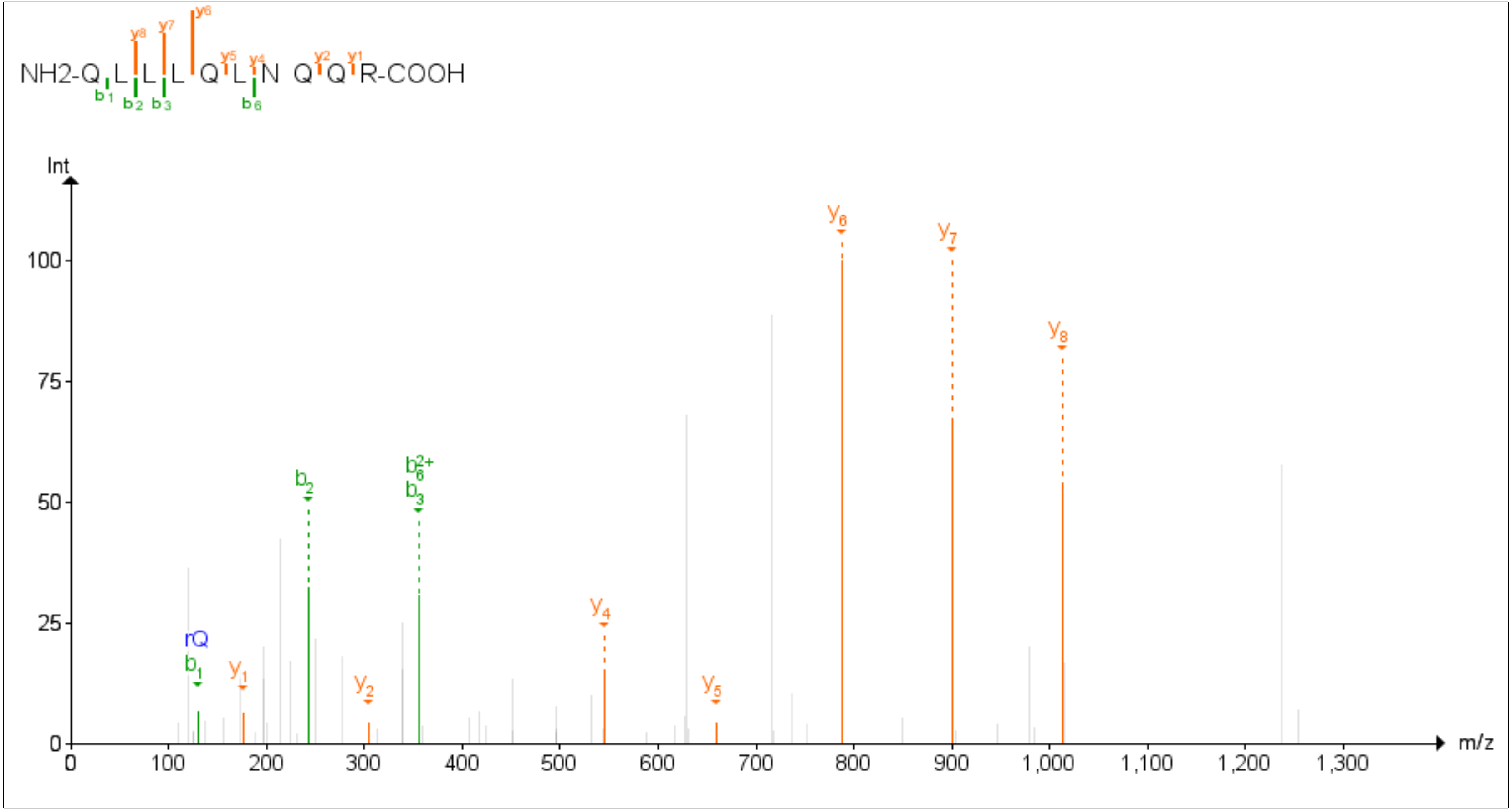
NH2-Q L L L Q L N Q Q R-COOH
y8 y7 y6 y5 y4 y2 y1
b1 b2 b3 b6
Int
m/z
rQ
b1
y1
b2
y2
b6 2+
b3
y4
y5
y6
y7
y8

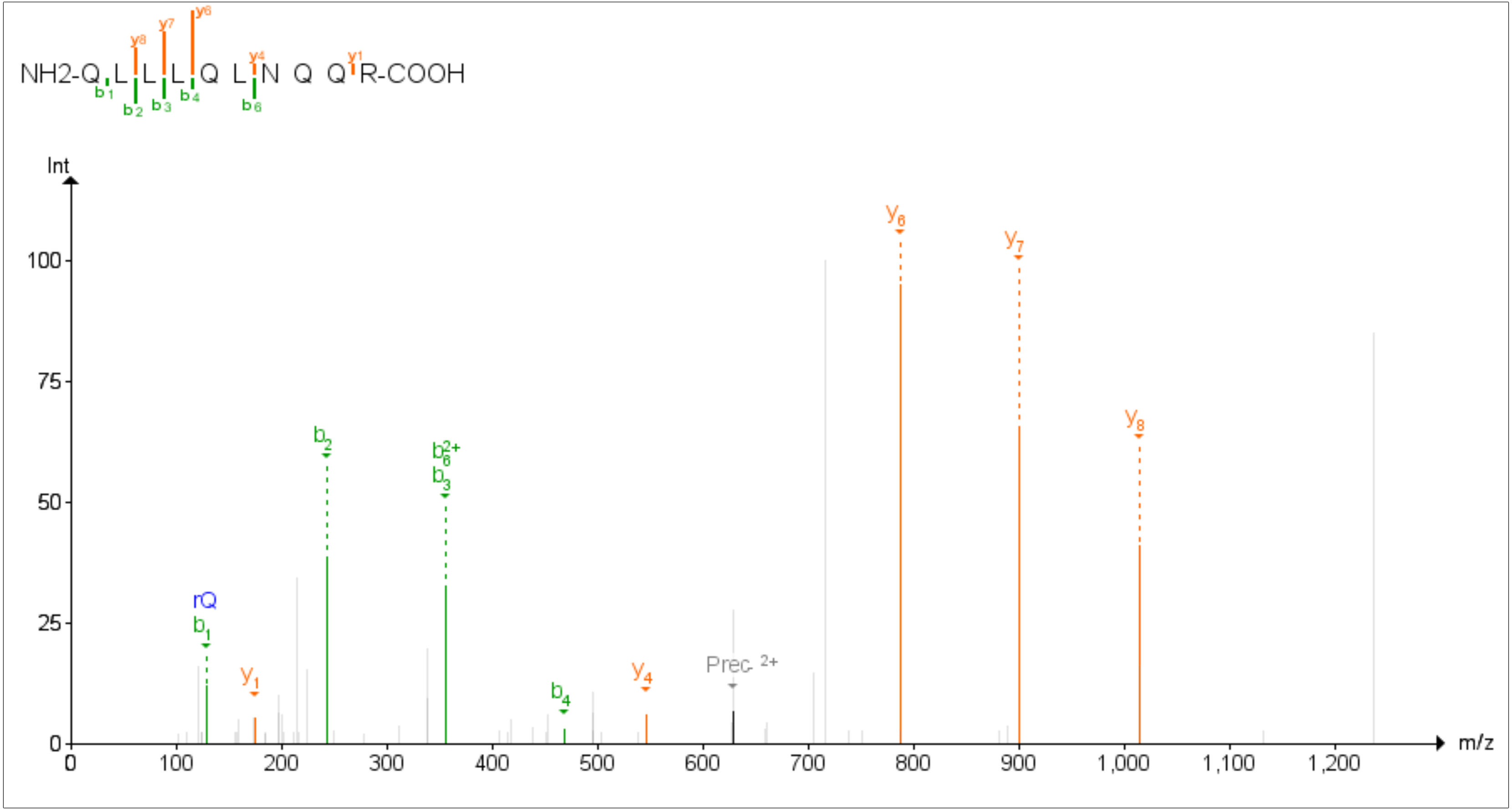

NH2-Q L L L Q L N Q Q R-COOH
y8
y7
y6
y4
y1
b1
b2
b3
b4
b6
Int
100
75
50
25
0
y6
y7
y8
b2
b6 2+
b3
rQ
b1
y1
b4
y4
Prec 2+
0
100
200
300
400
500
600
700
800
900
1,000
1,100
1,200
m/z

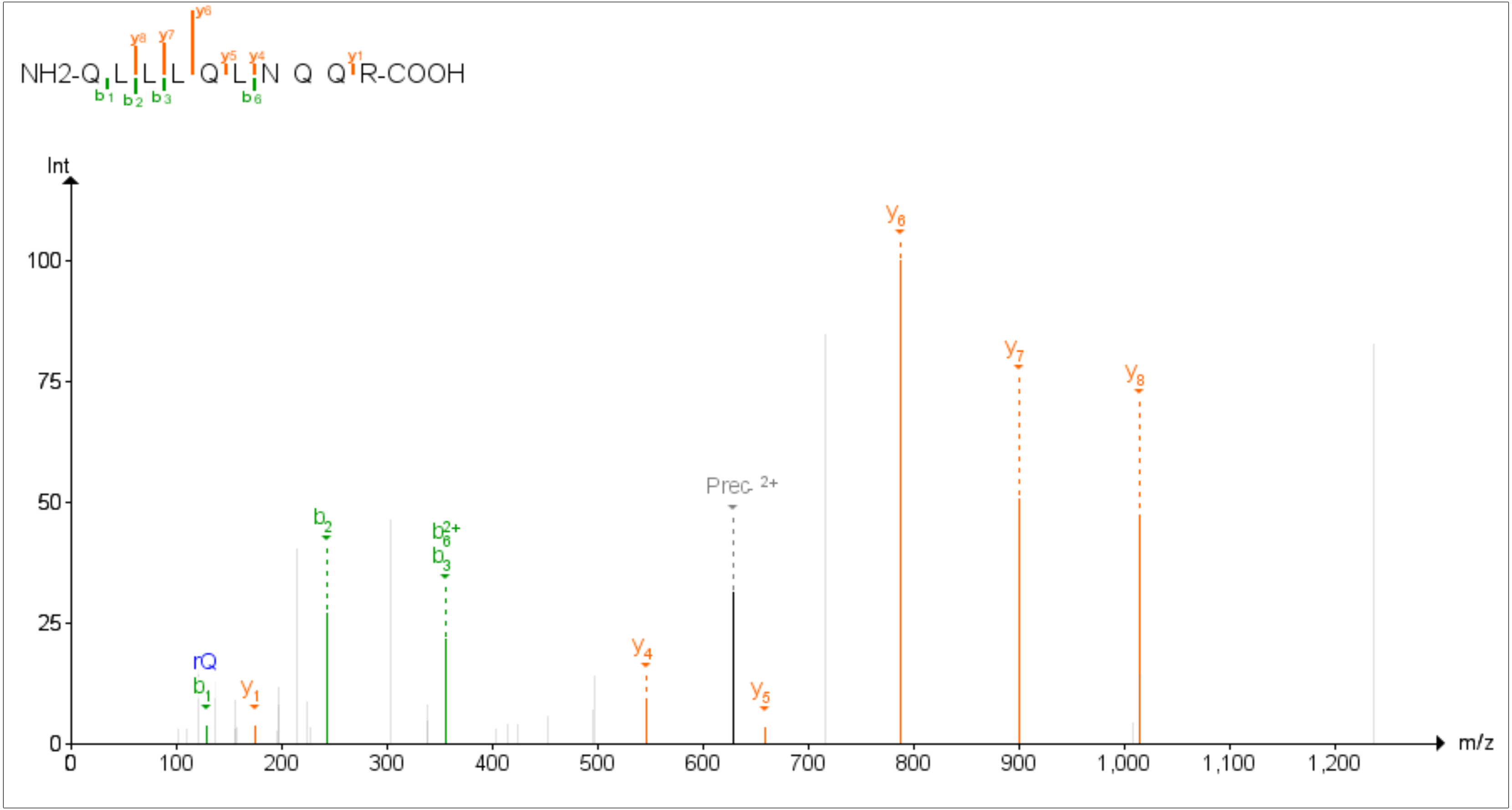

NH2-Q L L L Q L N Q Q R-COOH
y8 y7 y6 y5 y4 y1
b1 b2 b3 b6
Int
100
75
50
25
0
rQ
b1
y1
b2
b6 2+
b3
y4
Prec 2+
y5
y6
y7
y8
0 100 200 300 400 500 600 700 800 900 1,000 1,100 1,200
m/z

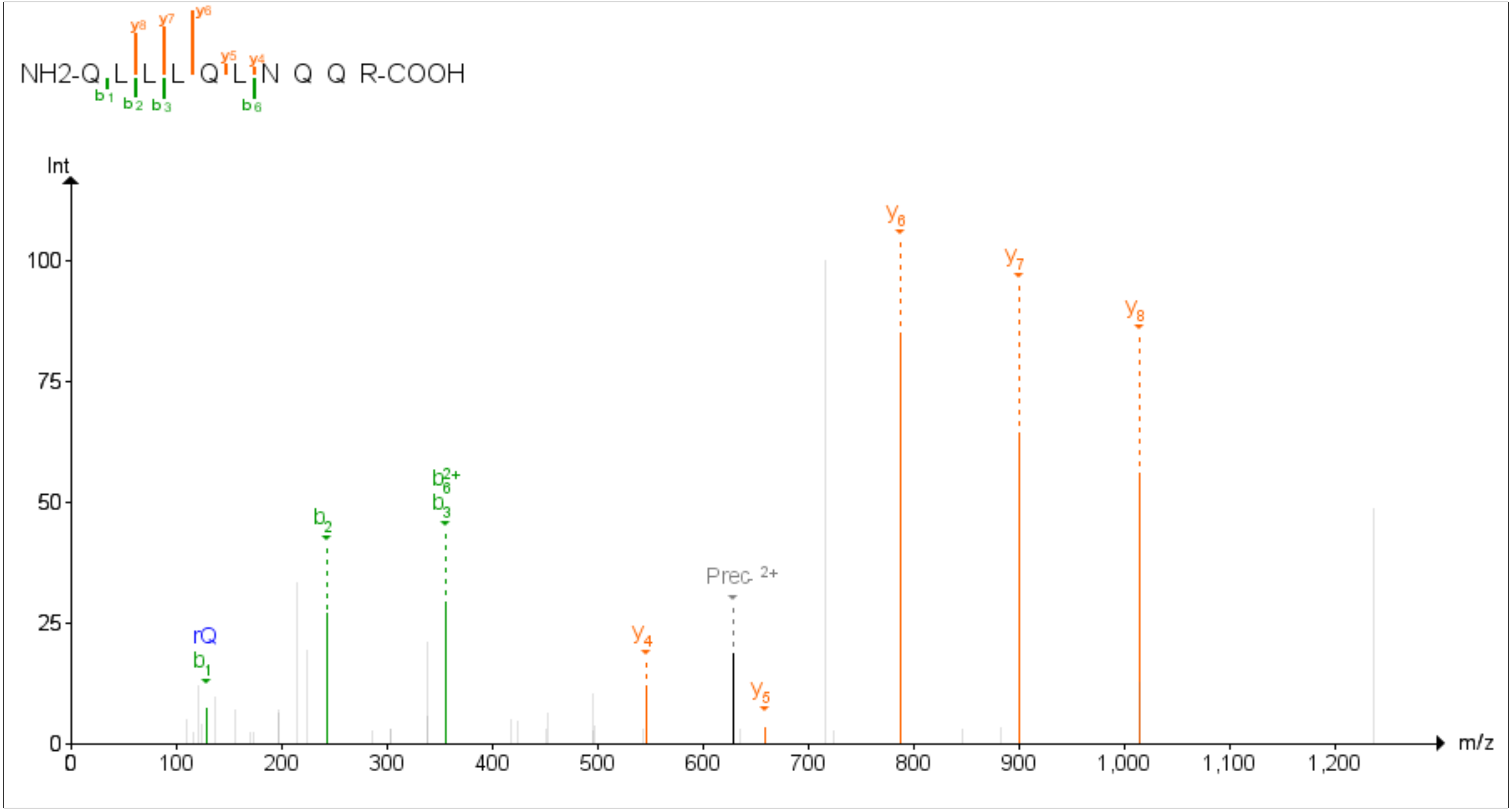

NH2-Q L L L Q L N Q Q R-COOH
y8 y7 y6 y5 y4
b1 b2 b3 b6
Int
m/z
y6
y7
y8
b6 2+
b3
b2
Prec 2+
y4
y5
rQ
b1
0
25
50
75
100
0
100
200
300
400
500
600
700
800
900
1,000
1,100
1,200

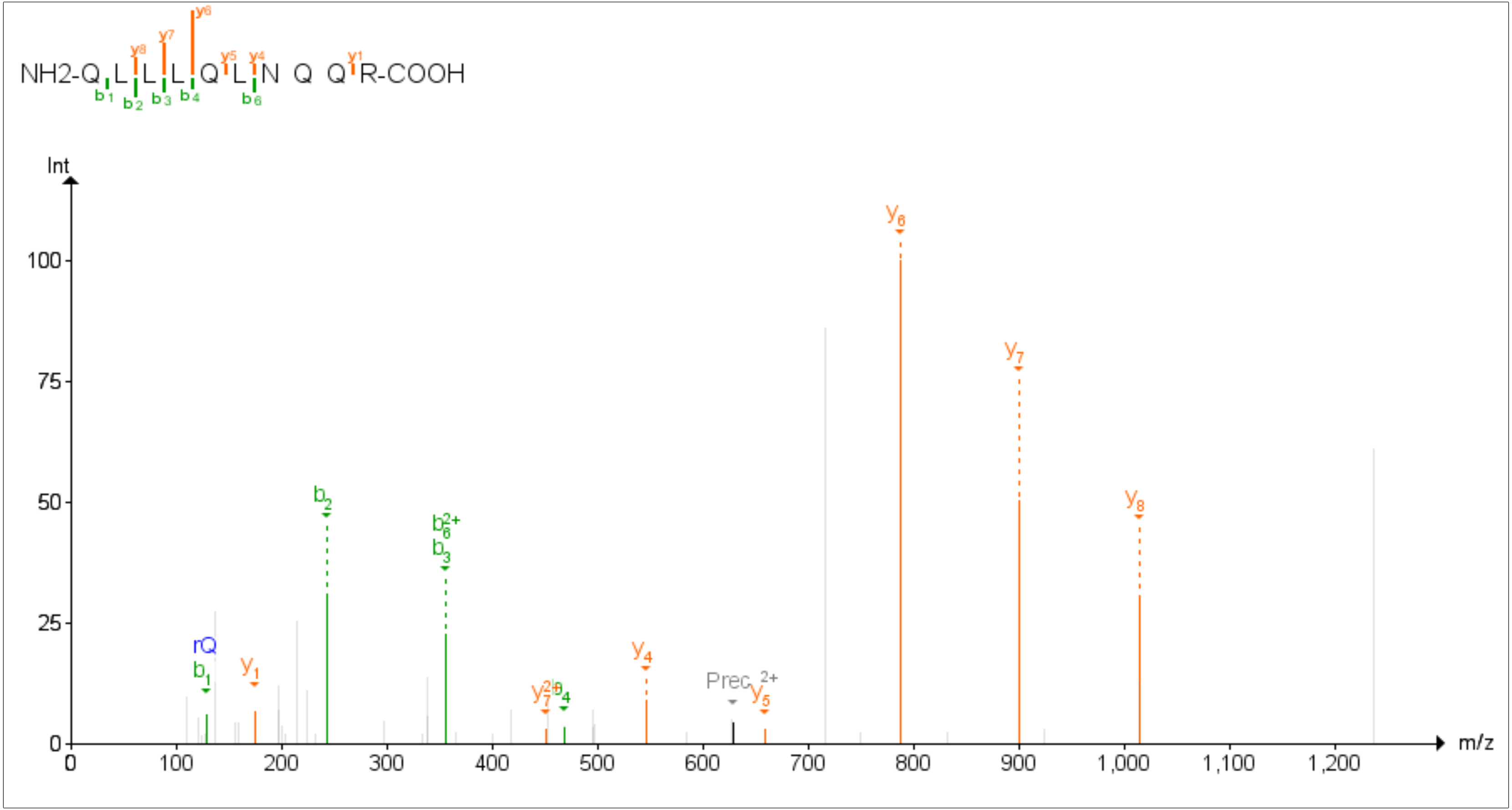

NH2-Q L L L Q L N Q Q R-COOH
y8 y7 y6 y5 y4 y1
b1 b2 b3 b4 b6
Int
m/z
y6
y7
y8
b2
b6 2+
b3
rQ
b1
y1
y7 2+
b4
y4
Prec 2+
y5

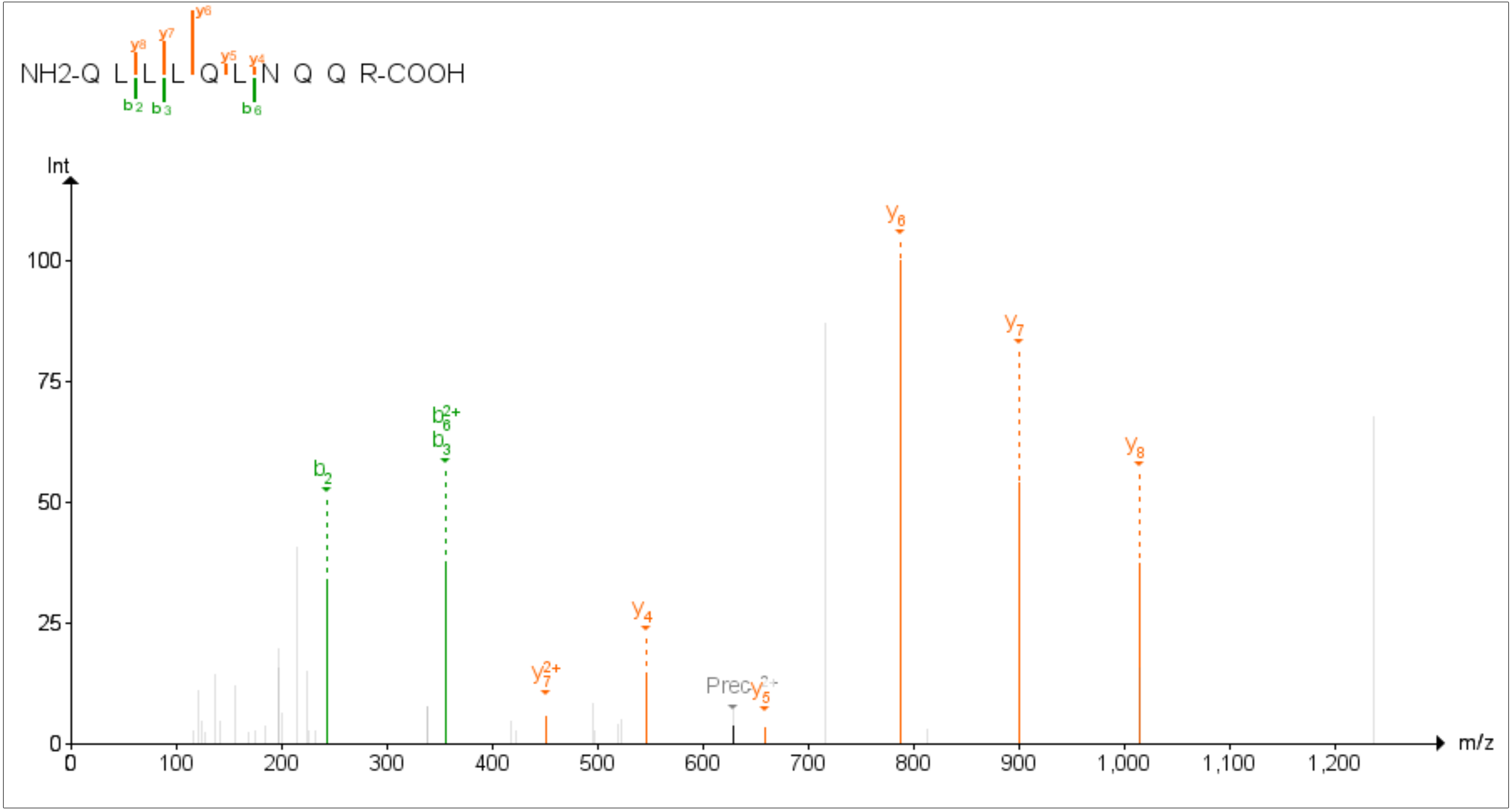

NH2-Q L L L Q L N Q Q R-COOH
y8
y7
y6
y5
y4
b2
b3
b6
Int
100
75
50
25
0
b2
b2+6
b3
y2+7
y4
Prec2+
y5
y6
y7
y8
0
100
200
300
400
500
600
700
800
900
1,000
1,100
1,200
m/z

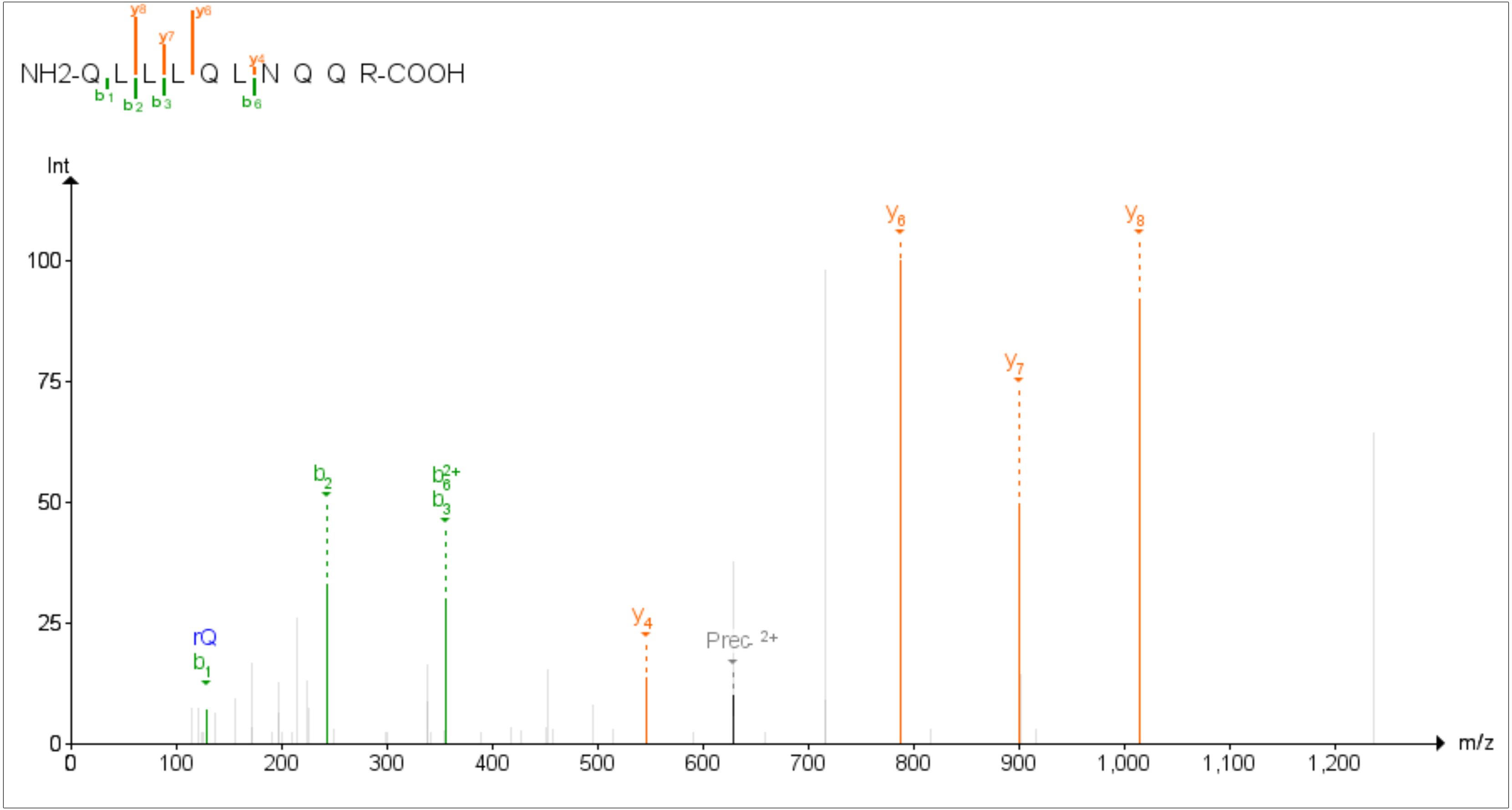

NH2-Q L L L Q L N Q Q R-COOH
y8
y7
y6
y4
b1
b2
b3
b6
Int
100
75
50
25
0
y6
y8
y7
b2
b6 2+
b3
rQ
b1
y4
Prec 2+
m/z
0
100
200
300
400
500
600
700
800
900
1,000
1,100
1,200

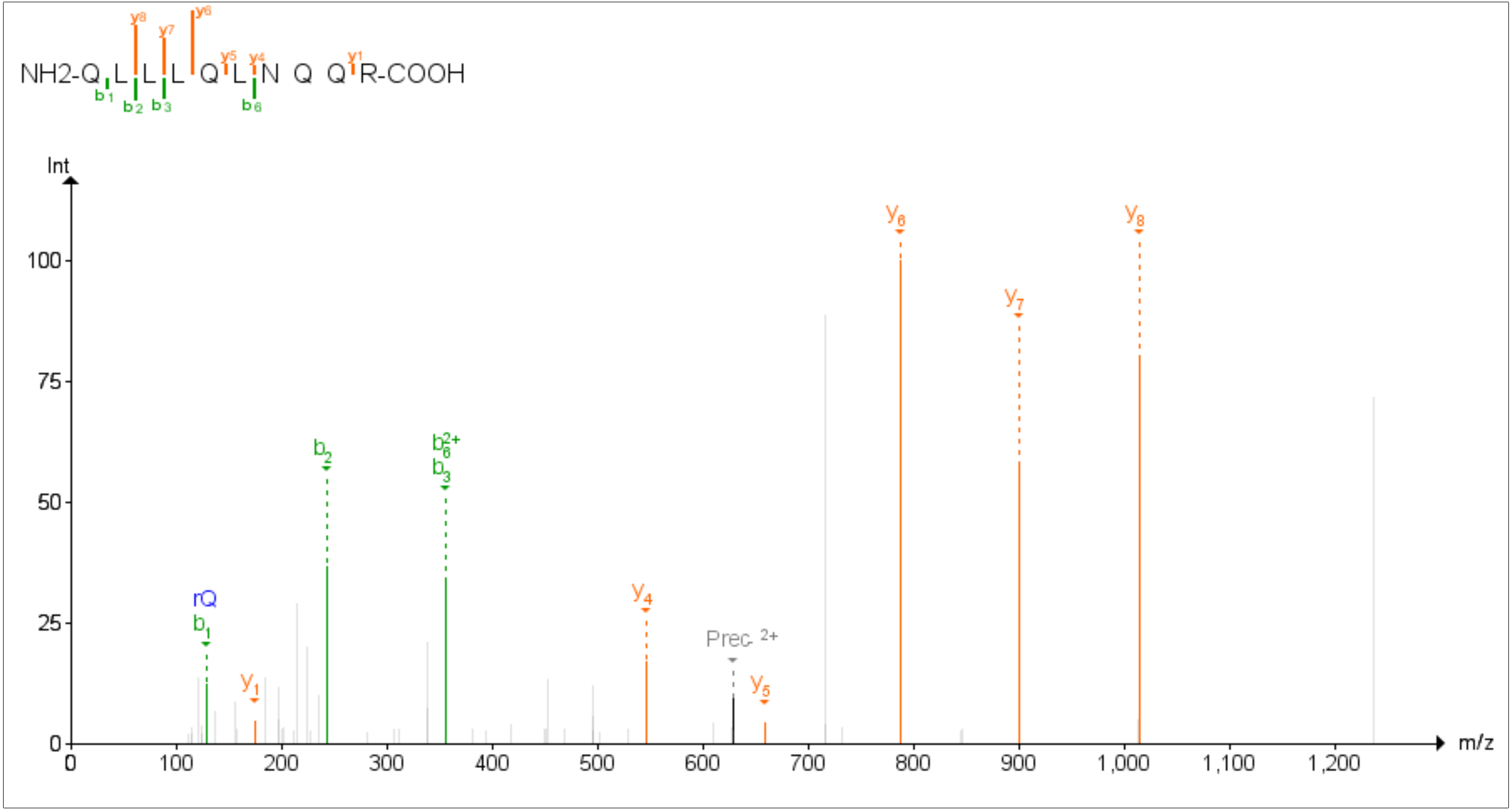

NH2-Q L L L Q L N Q Q R-COOH
y8 y7 y6 y5 y4 y1
b1 b2 b3 b6
Int
m/z
y6
y8
y7
b2
b6 2+
b3
rQ
b1
y1
y4
Prec 2+
y5
0
25
50
75
100
0
100
200
300
400
500
600
700
800
900
1,000
1,100
1,200

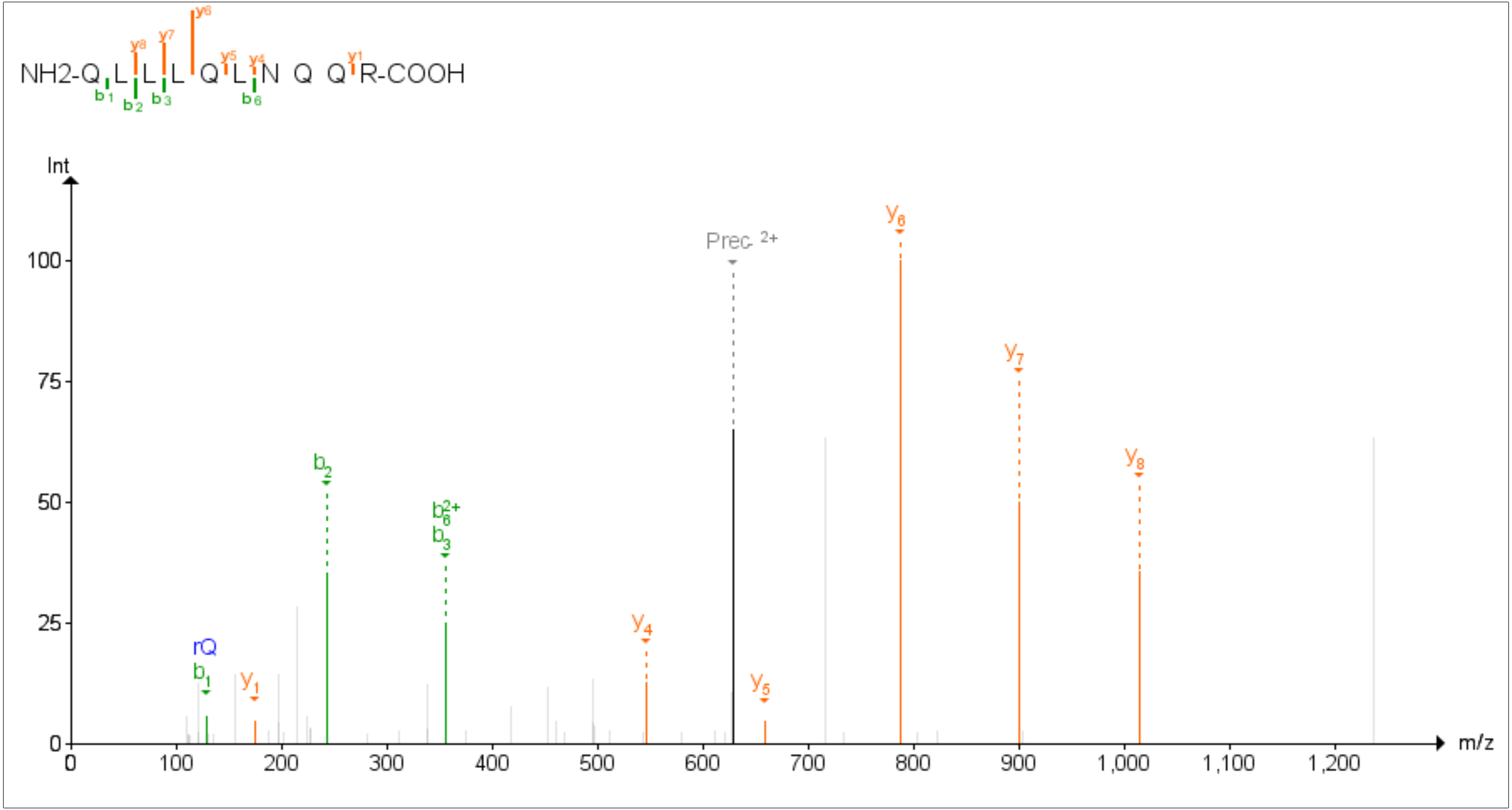

NH2-Q L L L Q L N Q Q R-COOH
y8 y7 y6 y5 y4 y1
b1 b2 b3 b6
Int
Prec 2+
y6
y7
y8
b2
b6 2+
b3
rQ
b1
y1
y4
y5
0
25
50
75
100
0 100 200 300 400 500 600 700 800 900 1,000 1,100 1,200
m/z

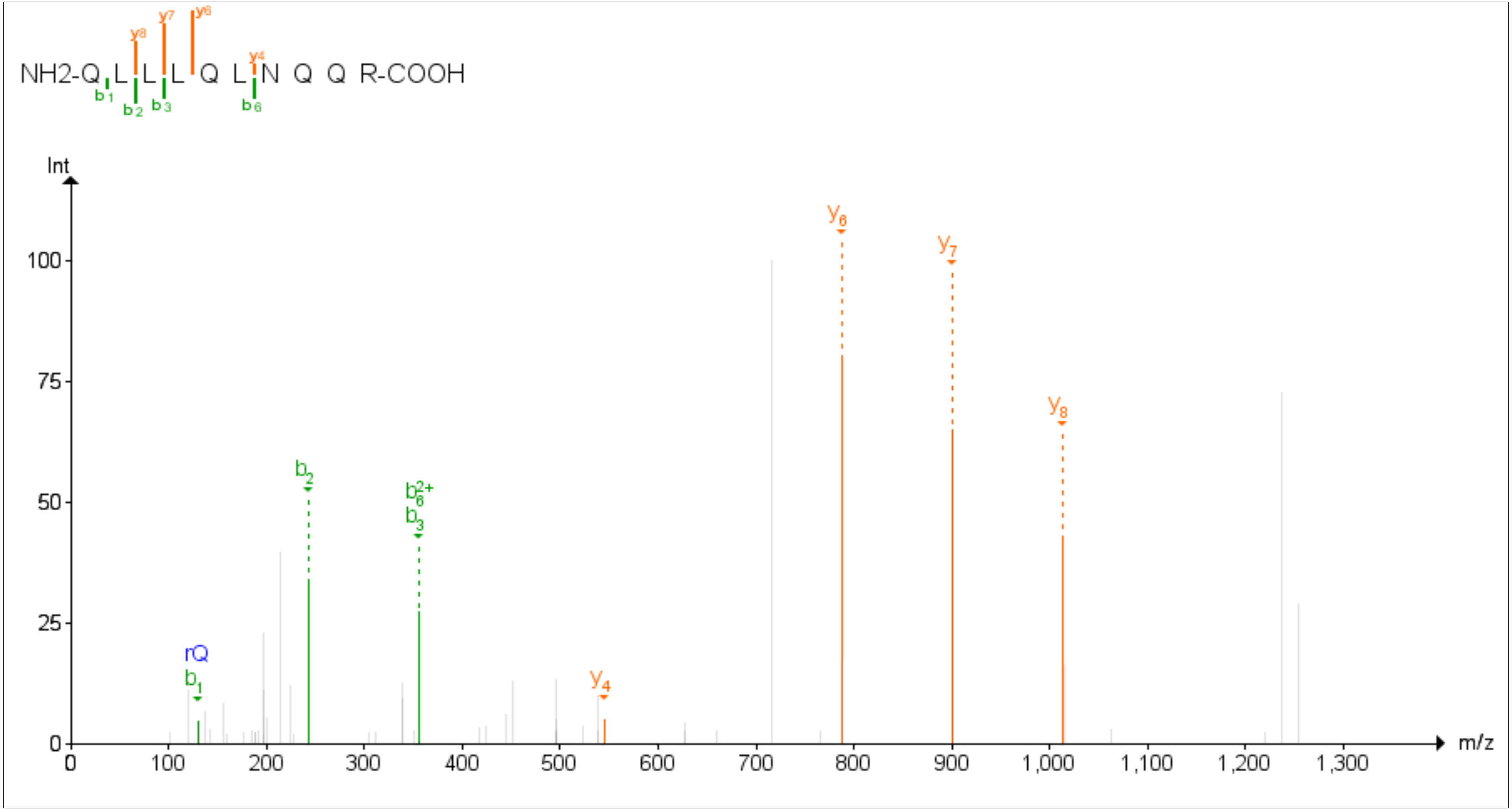

NH2-Q L L L Q L N Q Q R-COOH
y8
y7
y6
y4
b1
b2
b3
b6
Int
100
75
50
25
0
y6
y7
y8
b2
b6 2+
b3
rQ
b1
y4
0
100
200
300
400
500
600
700
800
900
1,000
1,100
1,200
1,300
m/z

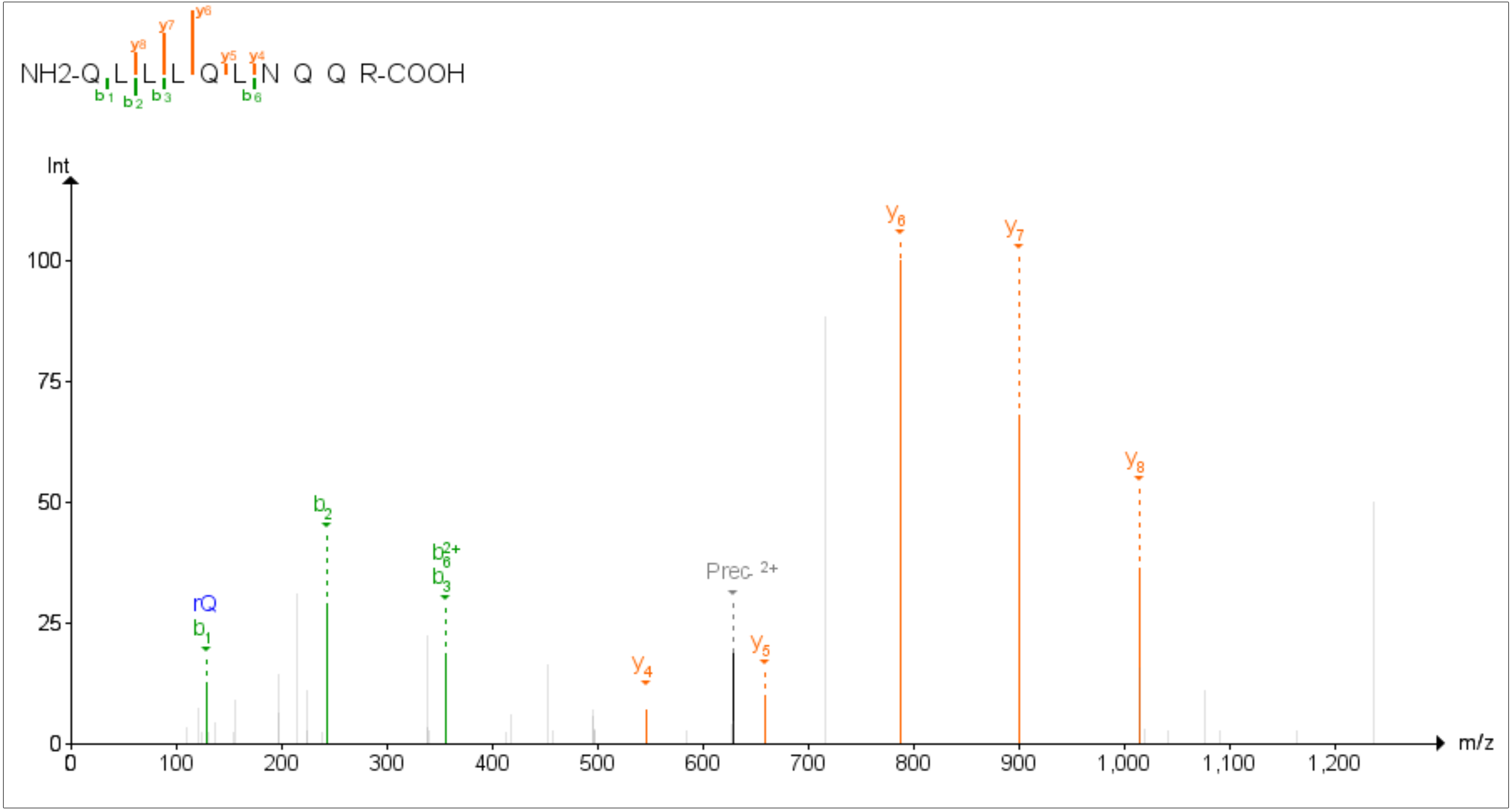
NH2-Q L L L Q L N Q Q R-COOH
y8 y7 y6 y5 y4
b1 b2 b3 b6
Int
100
75
50
25
0
y6
y7
y8
b2
b_6^{2+}
b3
rQ
b1
Prec 2+
y4
y5
m/z
0 100 200 300 400 500 600 700 800 900 1,000 1,100 1,200

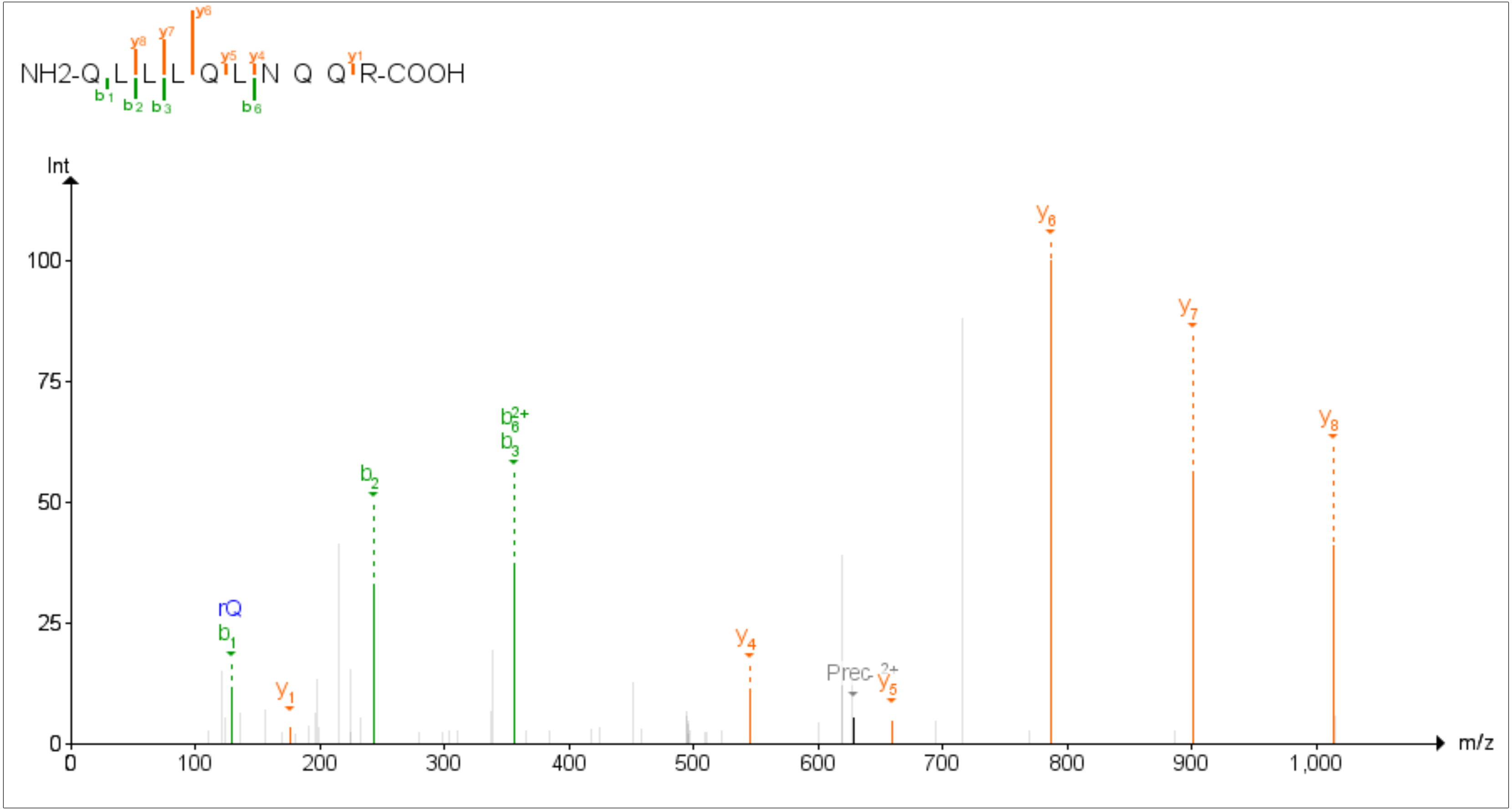

NH2-Q L L L Q L N Q Q R-COOH
b1 b2 b3 b6
y8 y7 y6 y5 y4 y1
Int
m/z
rQ
b1
y1
b2
b6 2+
b3
y4
Prec 2+
y5
y6
y7
y8

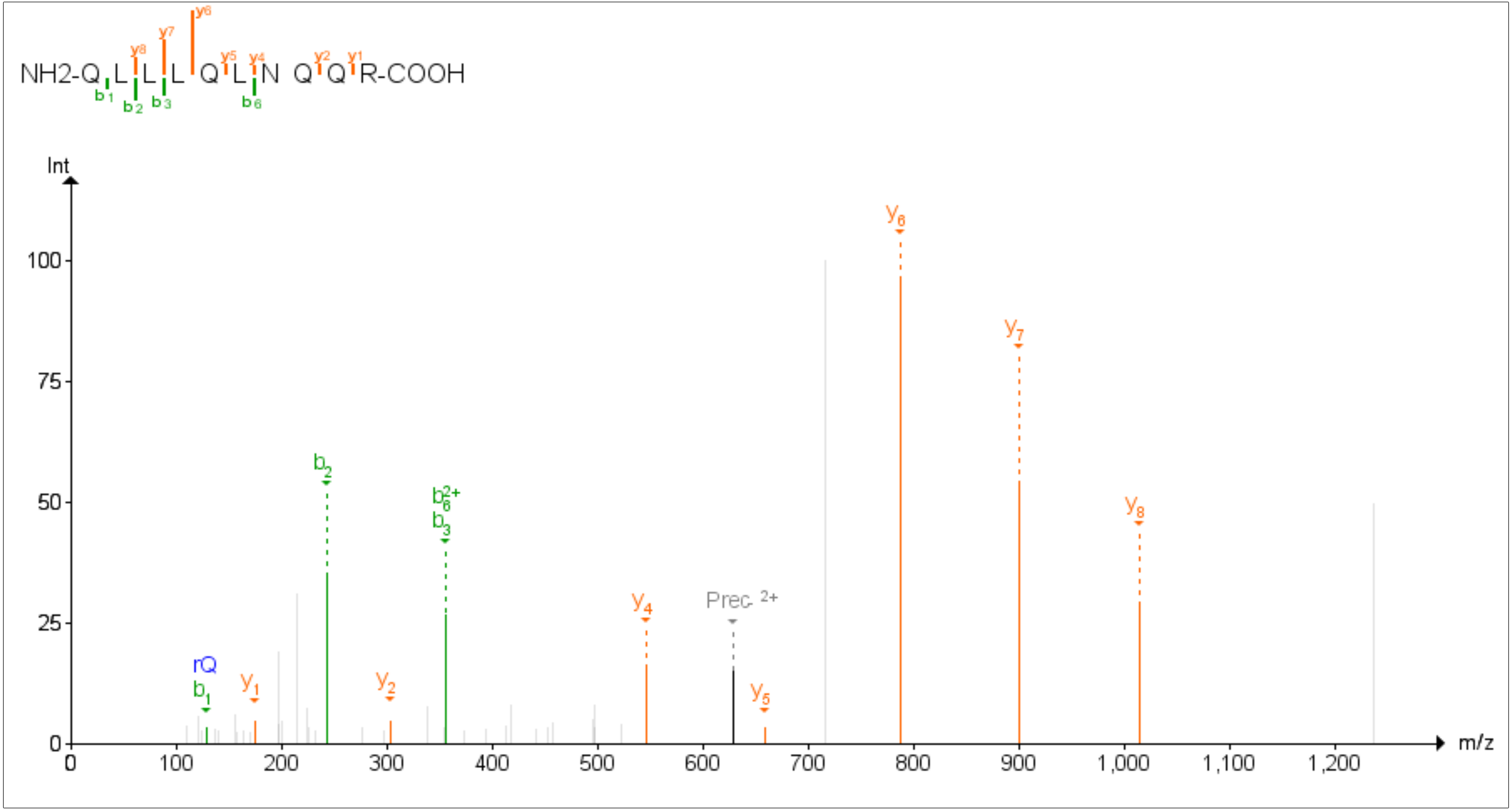

NH2-Q L L L Q L N Q Q R-COOH
y8 y7 y6 y5 y4 y2 y1
b1 b2 b3 b6
Int
100
75
50
25
0
rQ
b1
y1
b2
y2
b6 2+
b3
y4
Prec 2+
y5
y6
y7
y8
0
100
200
300
400
500
600
700
800
900
1,000
1,100
1,200
m/z

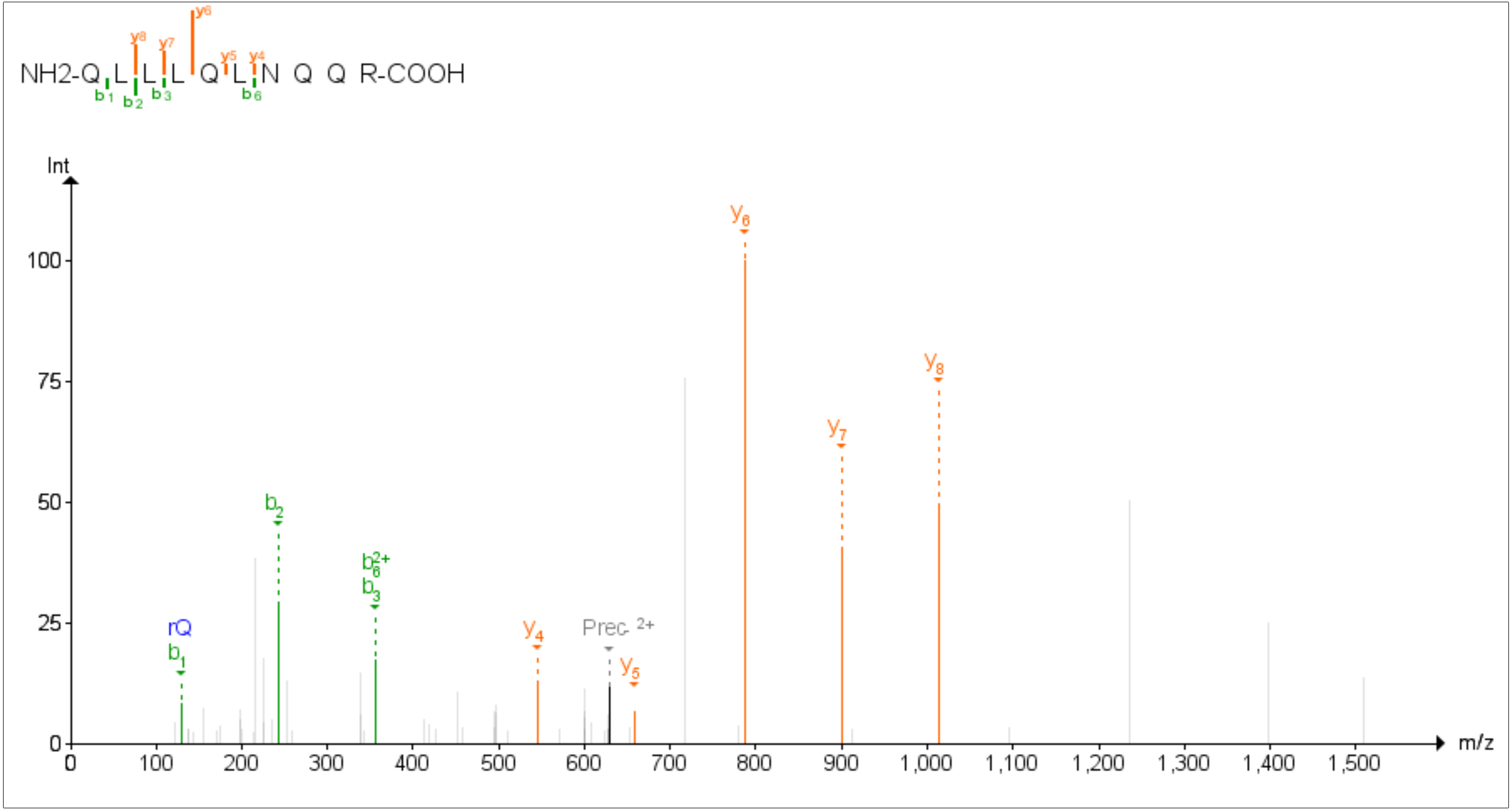
NH2-Q L L L Q L N Q Q R-COOH
y8 y7 y6 y5 y4
b1 b2 b3 b6
Int
100
75
50
25
0
y6
y8
y7
b2
b6 2+
b3
rQ
b1
y4
Prec 2+
y5
0 100 200 300 400 500 600 700 800 900 1,000 1,100 1,200 1,300 1,400 1,500
m/z

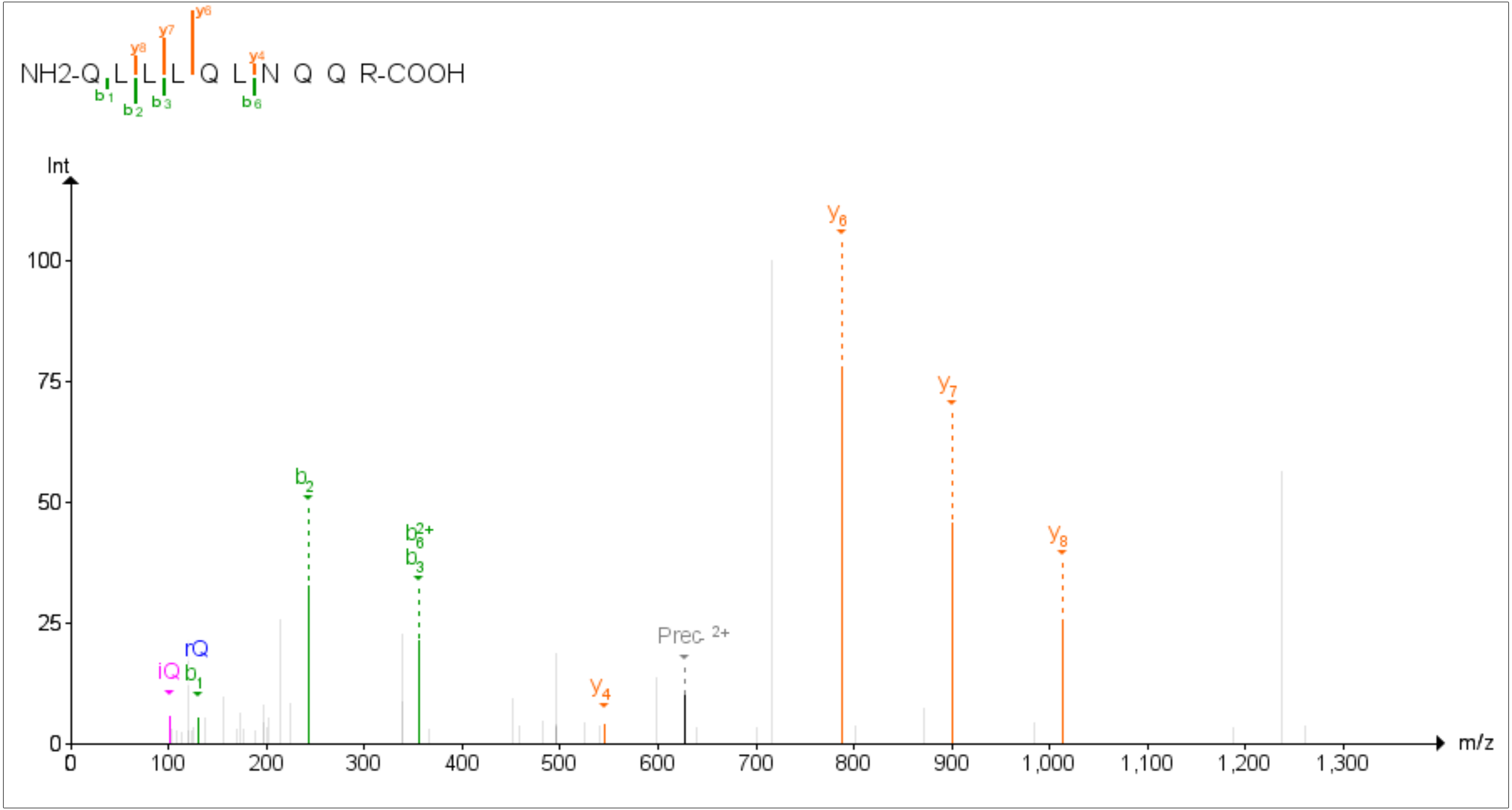

NH2-Q L L L Q L N Q Q R-COOH
y8
y7
y6
y4
b1
b2
b3
b6
Int
100
75
50
25
0
y6
y7
y8
b2
b6 2+
b3
iQ
rQ
b1
y4
Prec 2+
m/z
0
100
200
300
400
500
600
700
800
900
1,000
1,100
1,200
1,300

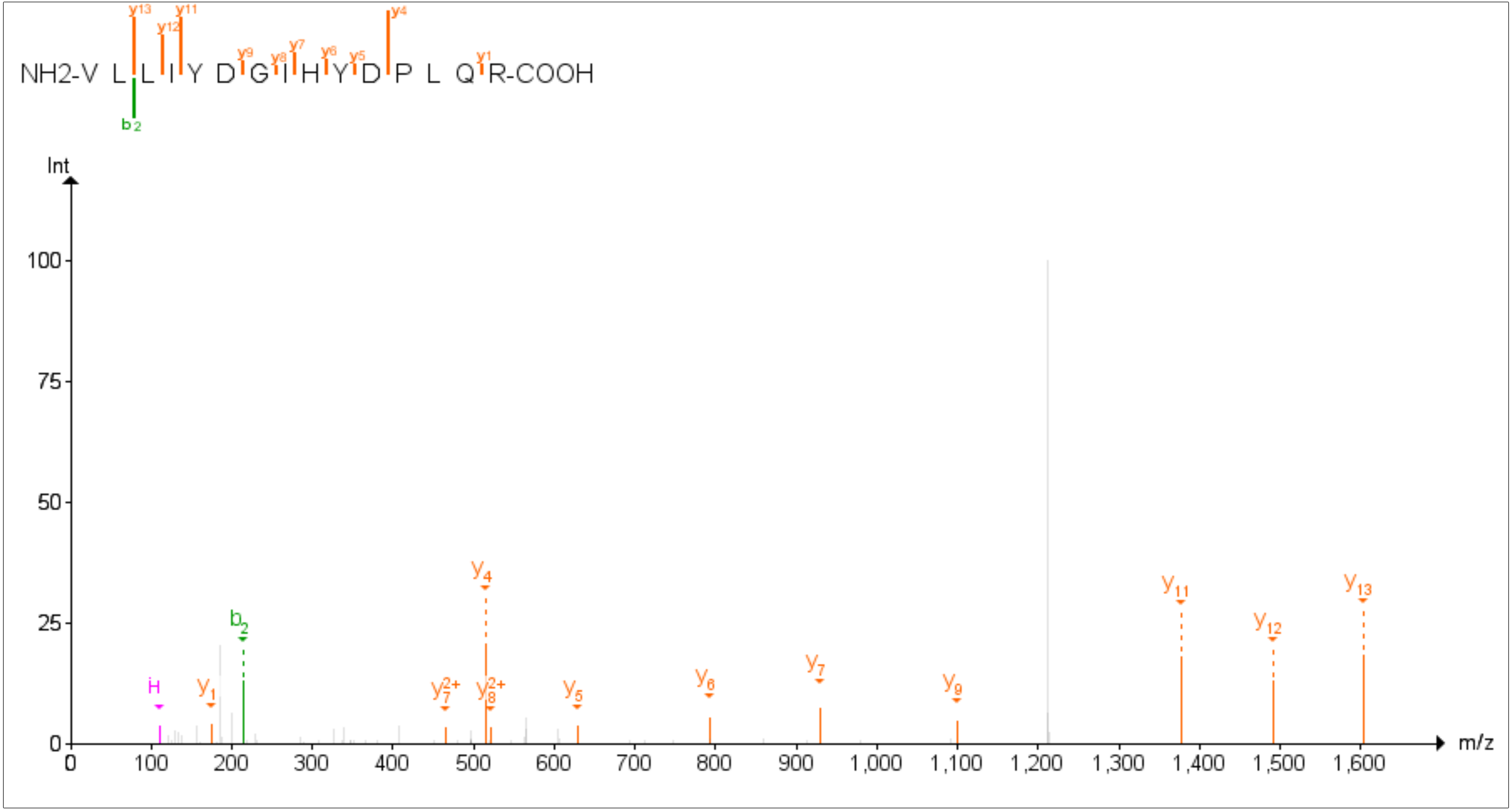

NH2-V L L I Y D G I H Y D P L Q R-COOH
y13
y12
y11
y9
y8
y7
y6
y5
y4
y1
b2
Int
100
75
50
25
0
H
y1
b2
y7 2+
y4
y8 2+
y5
y6
y7
y9
y11
y12
y13
m/z
0
100
200
300
400
500
600
700
800
900
1,000
1,100
1,200
1,300
1,400
1,500
1,600

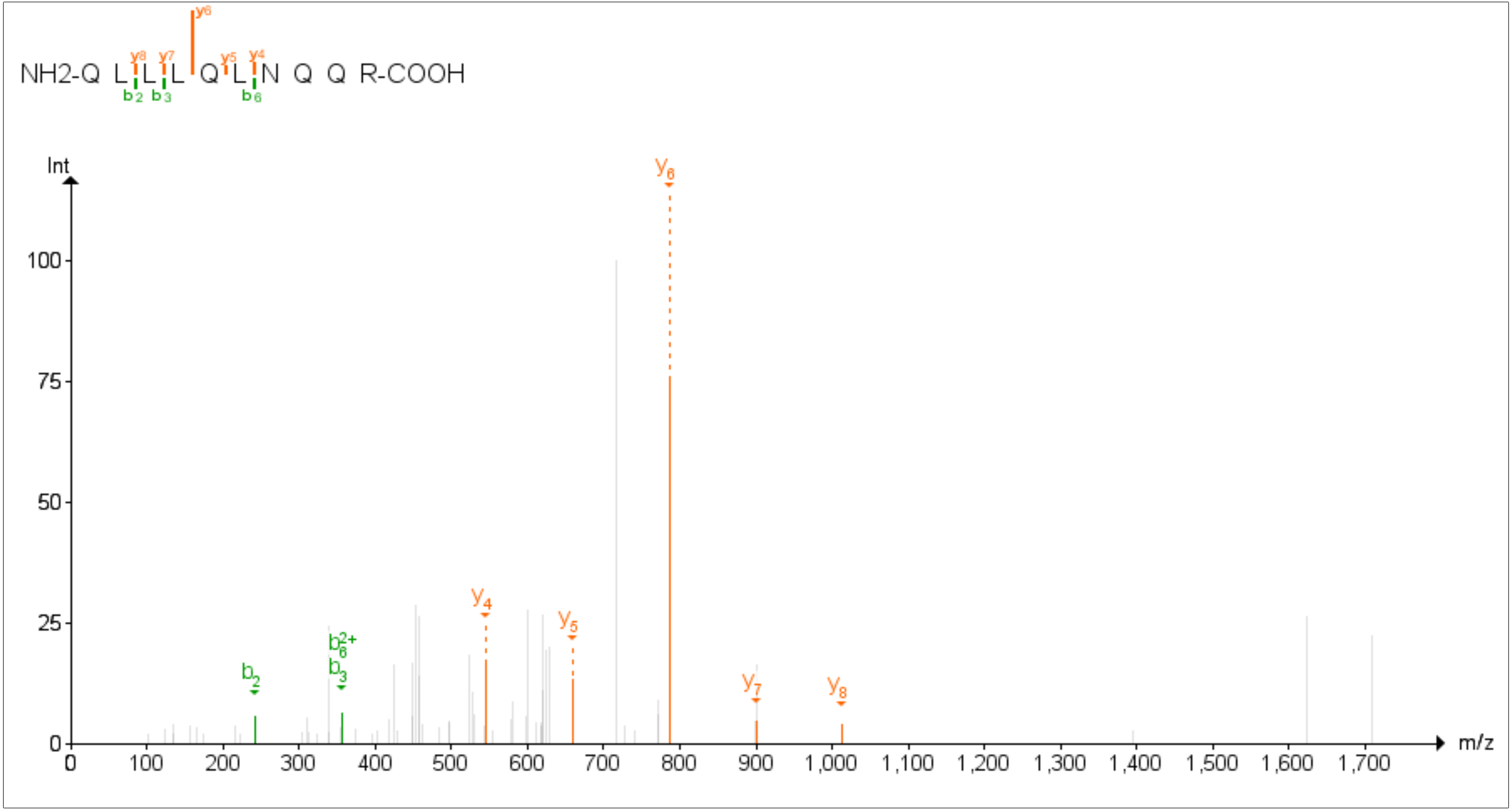

NH2-Q L L L Q L N Q Q R-COOH
y8 y7 y6 y5 y4
b2 b3 b6
Int
100
75
50
25
0
y6
y4
y5
y7
y8
b2
b6 2+
b3
0 100 200 300 400 500 600 700 800 900 1,000 1,100 1,200 1,300 1,400 1,500 1,600 1,700
m/z

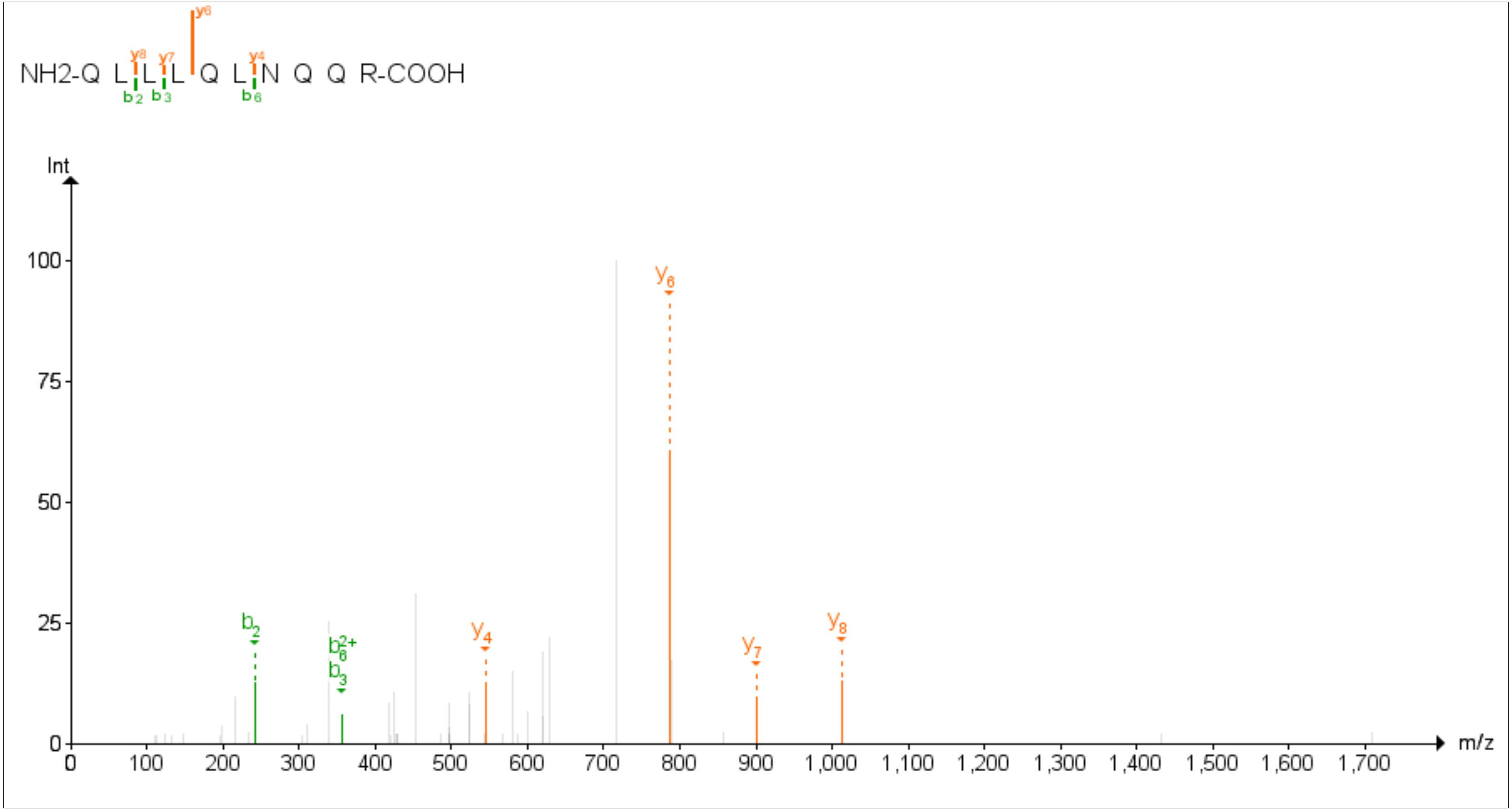

NH2-Q L L L Q L N Q Q R-COOH
y8 y7 y6 y4
b2 b3 b6
Int
100
75
50
25
0
b2
b6 2+
b3
y4
y6
y7
y8
0 100 200 300 400 500 600 700 800 900 1,000 1,100 1,200 1,300 1,400 1,500 1,600 1,700
m/z

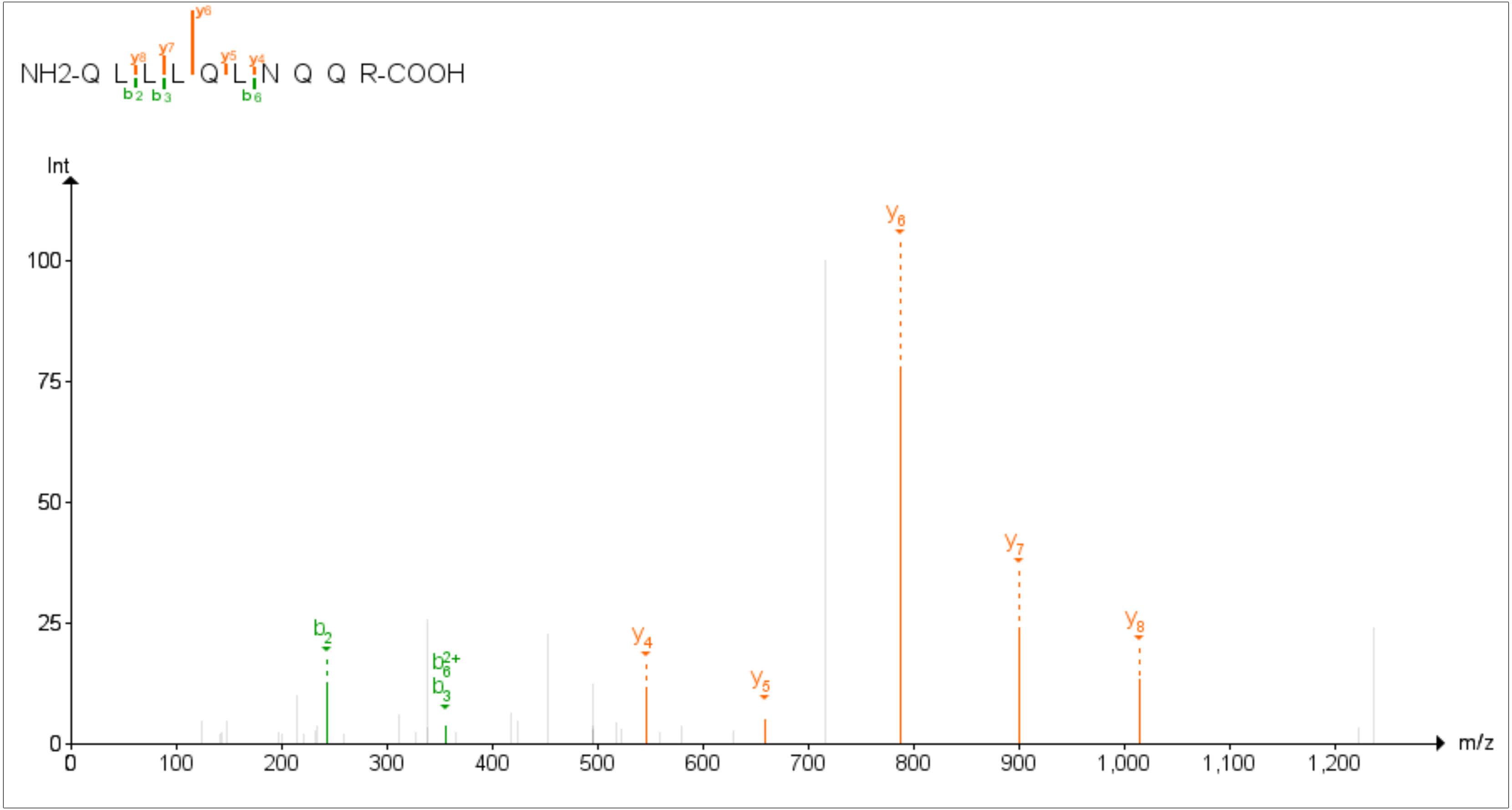
NH2-Q L L L Q L N Q Q R-COOH
y8 y7 y6 y5 y4
b2 b3 b6
Int
m/z
0
25
50
75
100
0
100
200
300
400
500
600
700
800
900
1,000
1,100
1,200
b2
b6 2+
b3
y4
y5
y6
y7
y8

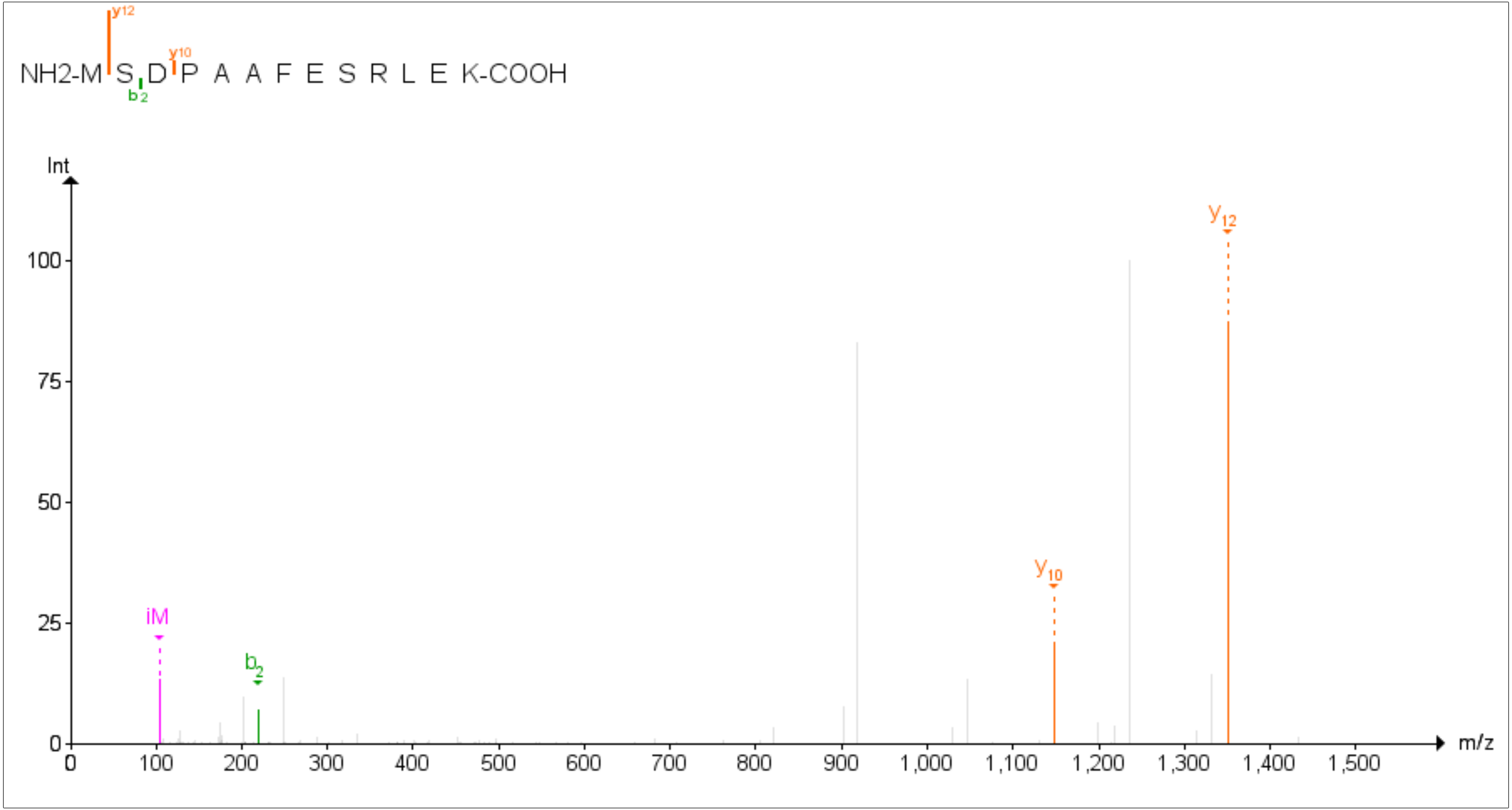

NH2-M S D P A A F E S R L E K-COOH
y12
y10
b2
Int
100
75
50
25
0
iM
b2
y10
y12
0
100
200
300
400
500
600
700
800
900
1,000
1,100
1,200
1,300
1,400
1,500
m/z

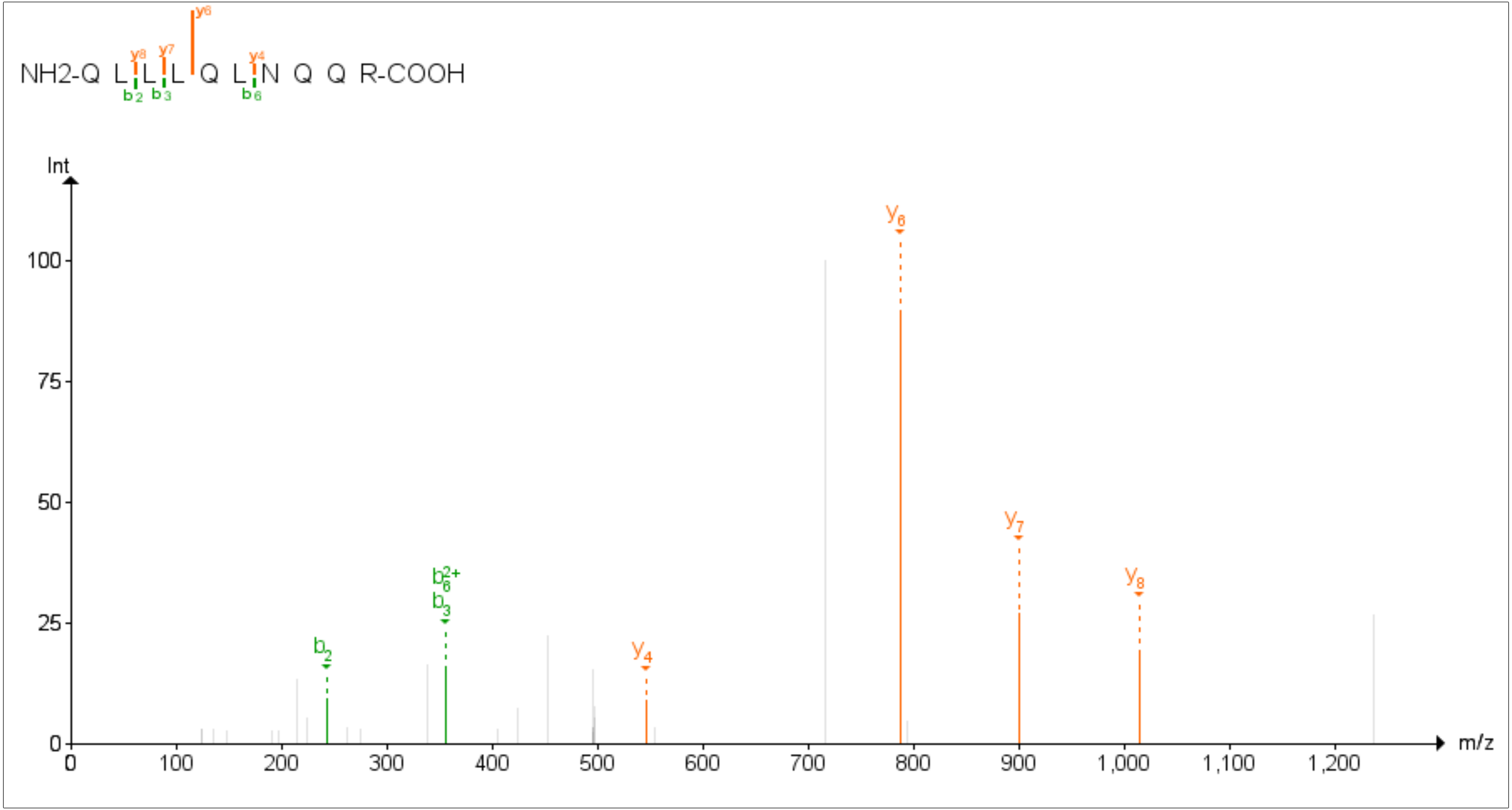

NH2-Q L L L Q L N Q Q R-COOH
y8 y7 y6 y4
b2 b3 b6
Int
100
75
50
25
0
y6
y7
y8
y4
b2
b3
b6 2+
m/z
0 100 200 300 400 500 600 700 800 900 1,000 1,100 1,200

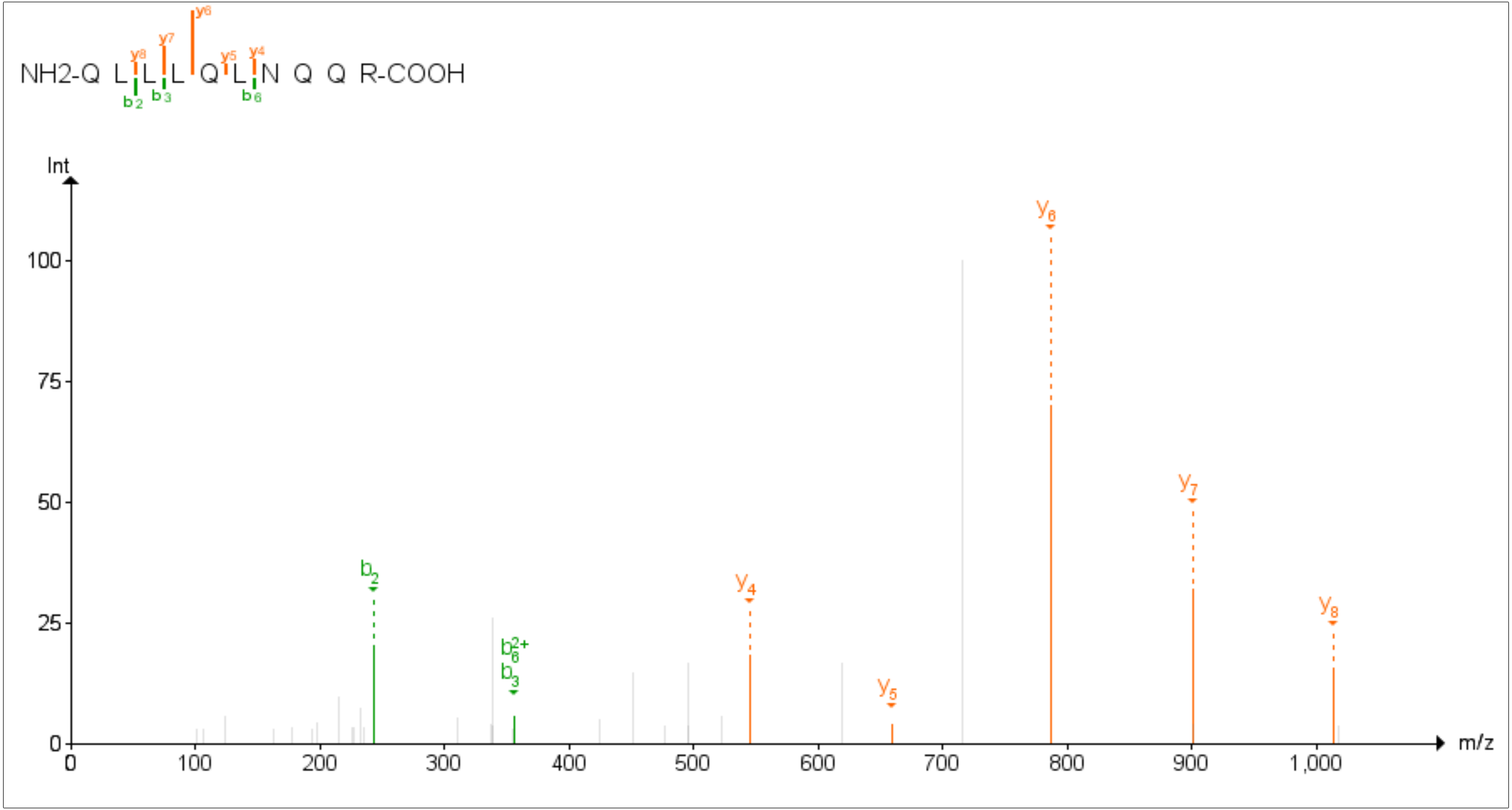

NH2-Q L L L Q L N Q Q R-COOH
y8
y7
y6
y5
y4
b2
b3
b6
Int
100
75
50
25
0
m/z
0
100
200
300
400
500
600
700
800
900
1,000
b2
b6 2+
b3
y4
y5
y6
y7
y8

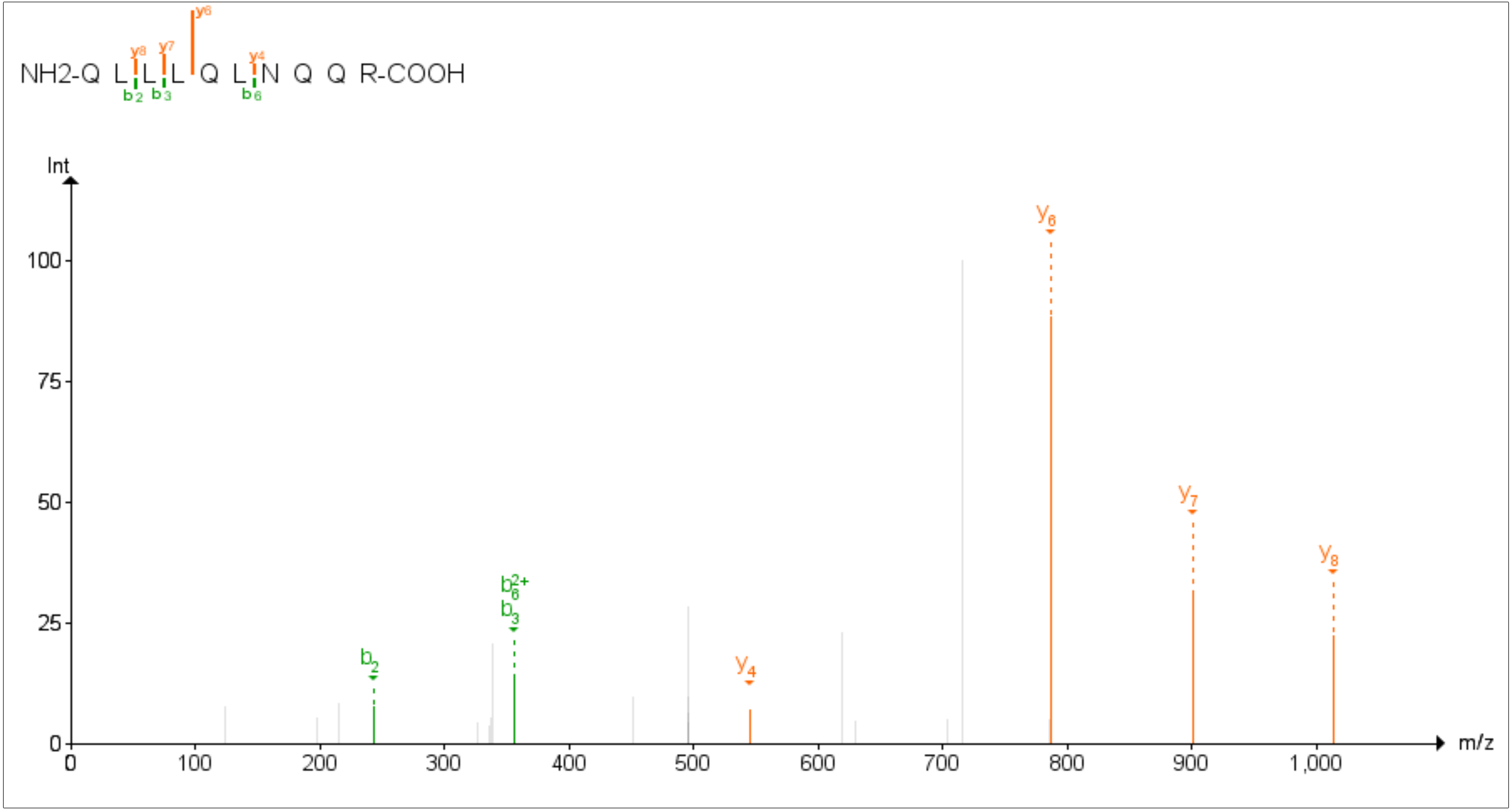

NH2-Q L L L Q L N Q Q R-COOH
y8
y7
y6
y4
b2
b3
b6
Int
100
75
50
25
0
y6
y7
y8
y4
b2
b6 2+
b3
0
100
200
300
400
500
600
700
800
900
1,000
m/z

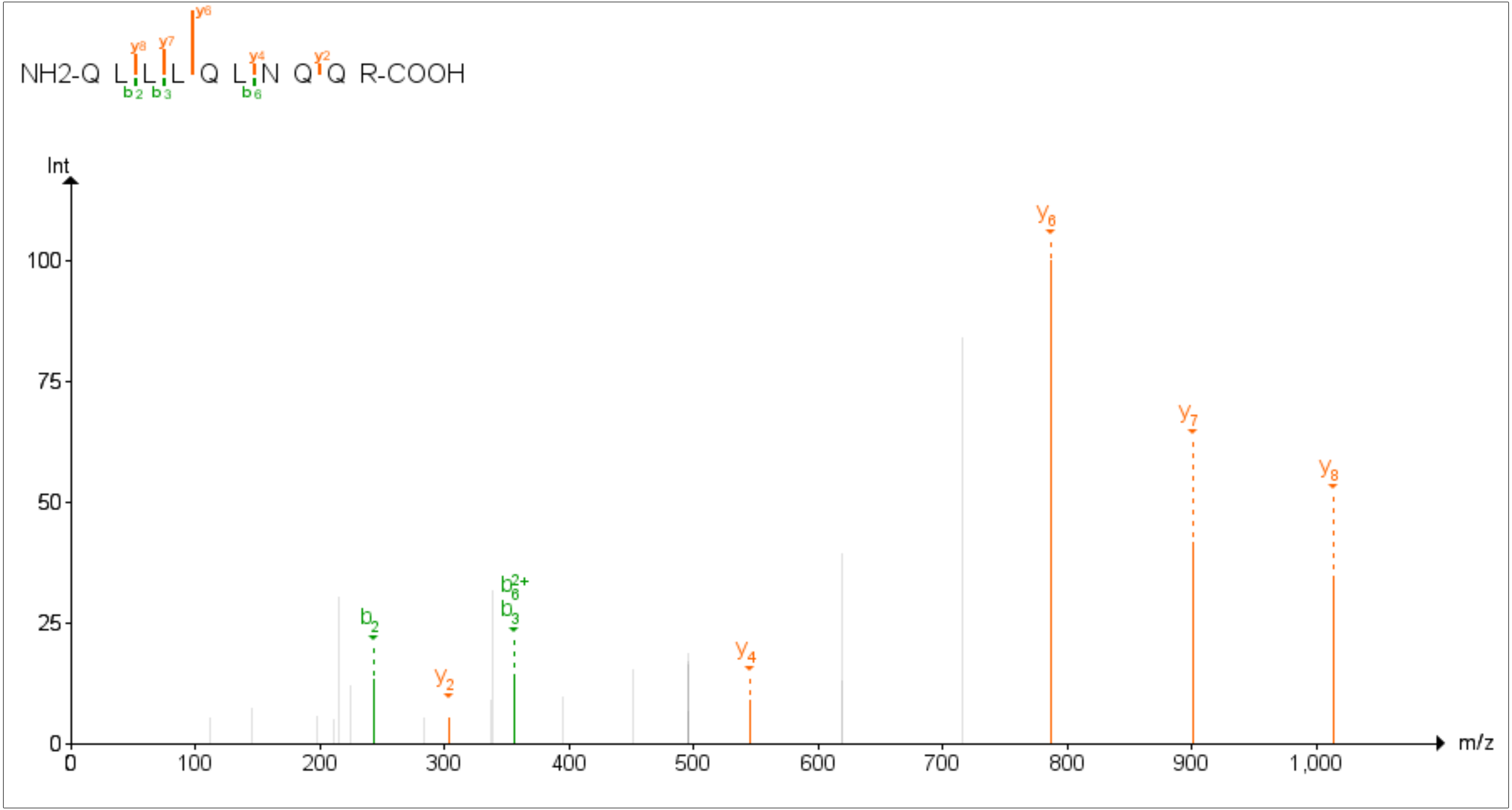
NH2-Q L L L Q L N Q Q R-COOH
y8 y7 y6 y4 y2
b2 b3 b6
Int
100
75
50
25
0
m/z
0 100 200 300 400 500 600 700 800 900 1,000
y6
y7
y8
y4
y2
b2
b6 2+
b3

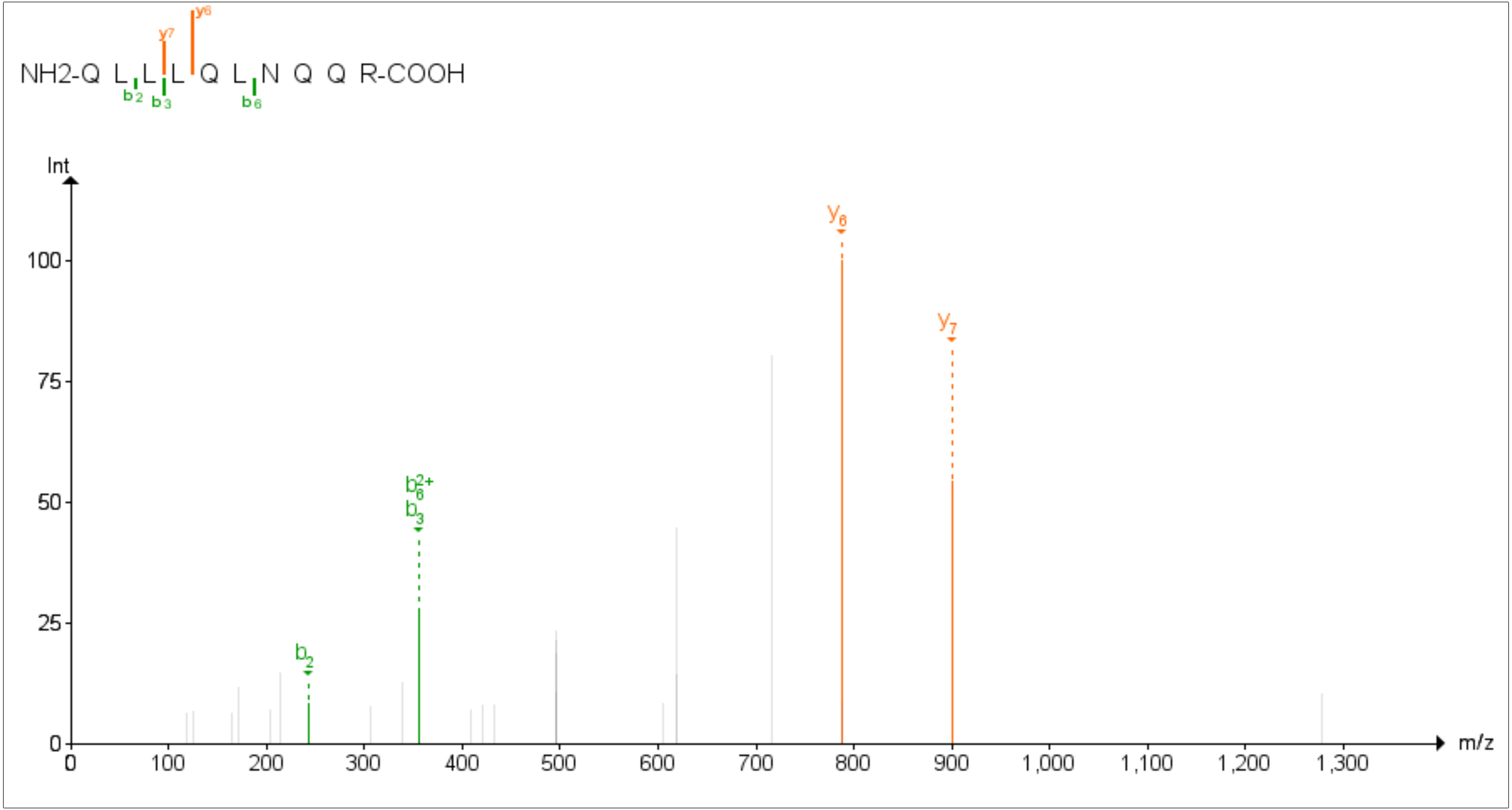

NH2-Q L L L Q L N Q Q R-COOH
y6
y7
b2
b3
b6
Int
100
75
50
25
0
y6
y7
b6 2+
b3
b2
0
100
200
300
400
500
600
700
800
900
1,000
1,100
1,200
1,300
m/z

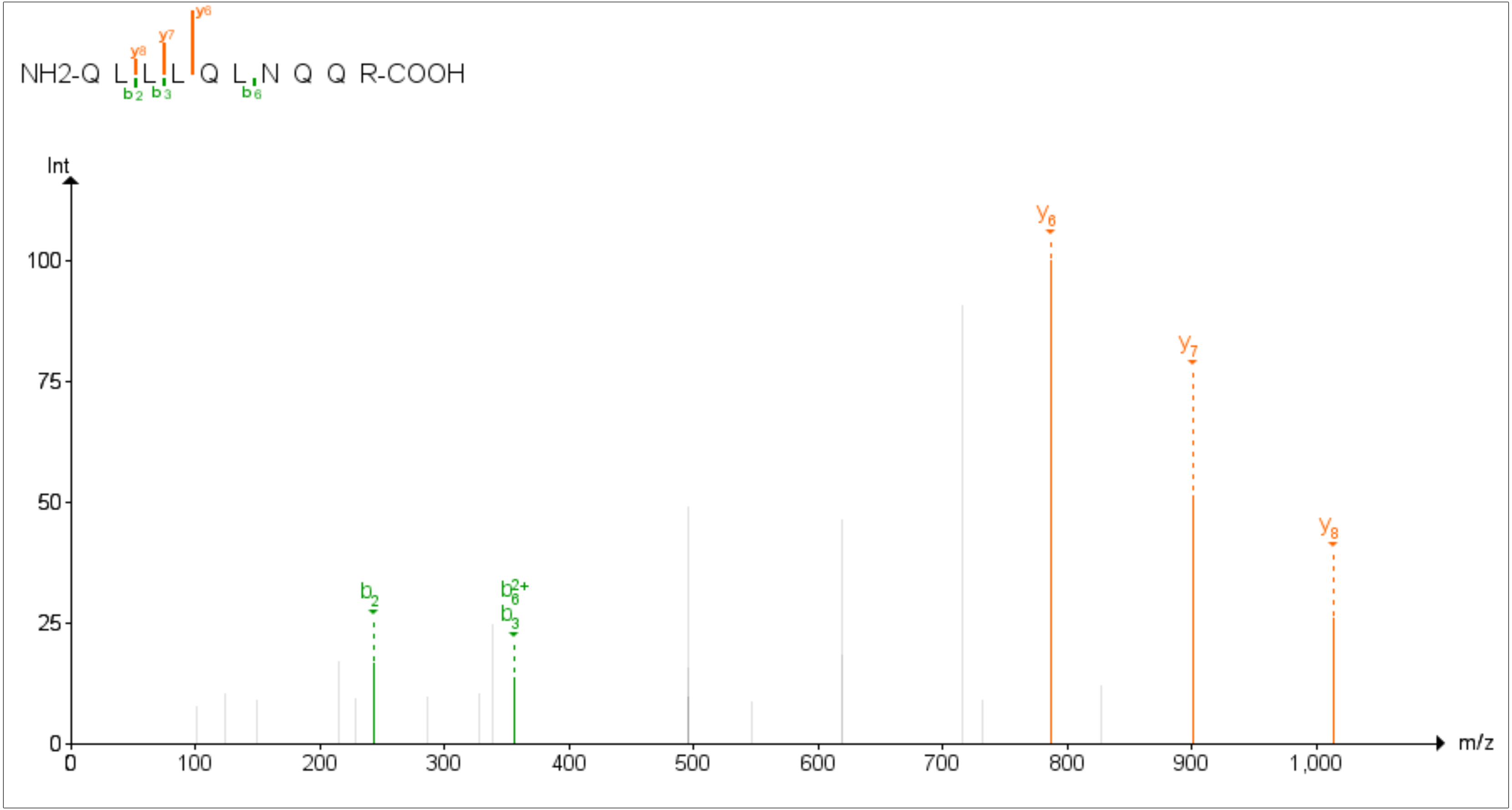

NH2-Q L L L Q L N Q Q R-COOH
y8
y7
y6
b2
b3
b6
Int
100
75
50
25
0
y6
y7
y8
b2
b6 2+
b3
0
100
200
300
400
500
600
700
800
900
1,000
m/z

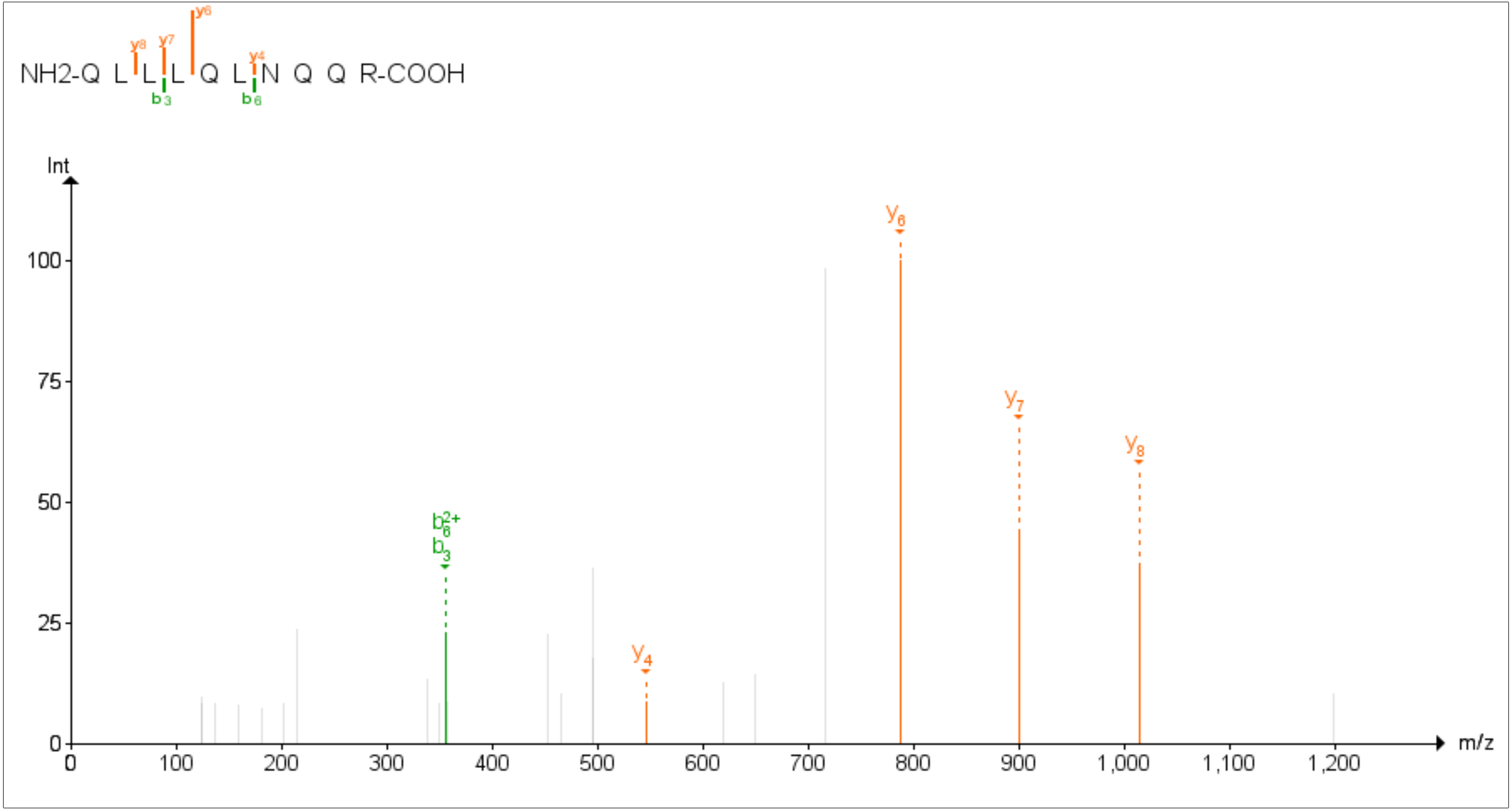

NH2-Q L L L Q L N Q Q R-COOH
y8 y7 y6 y4
b3 b6
Int
100
75
50
25
0
y6
y7
y8
y4
b6 2+
b3
0 100 200 300 400 500 600 700 800 900 1,000 1,100 1,200
m/z

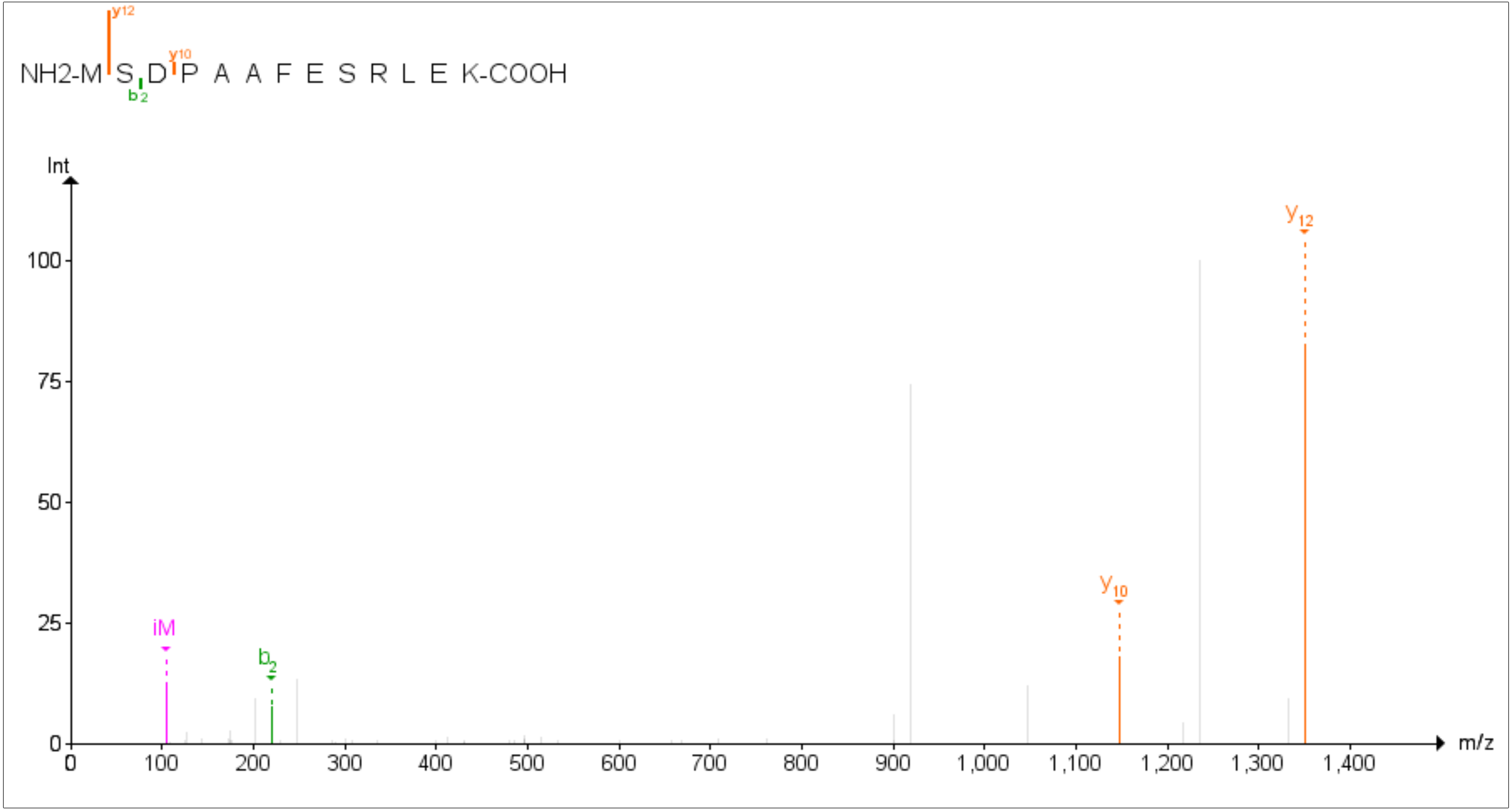

NH2-M S D P A A F E S R L E K-COOH
y12
y10
b2
Int
100
75
50
25
0
iM
b2
y10
y12
0
100
200
300
400
500
600
700
800
900
1,000
1,100
1,200
1,300
1,400
m/z

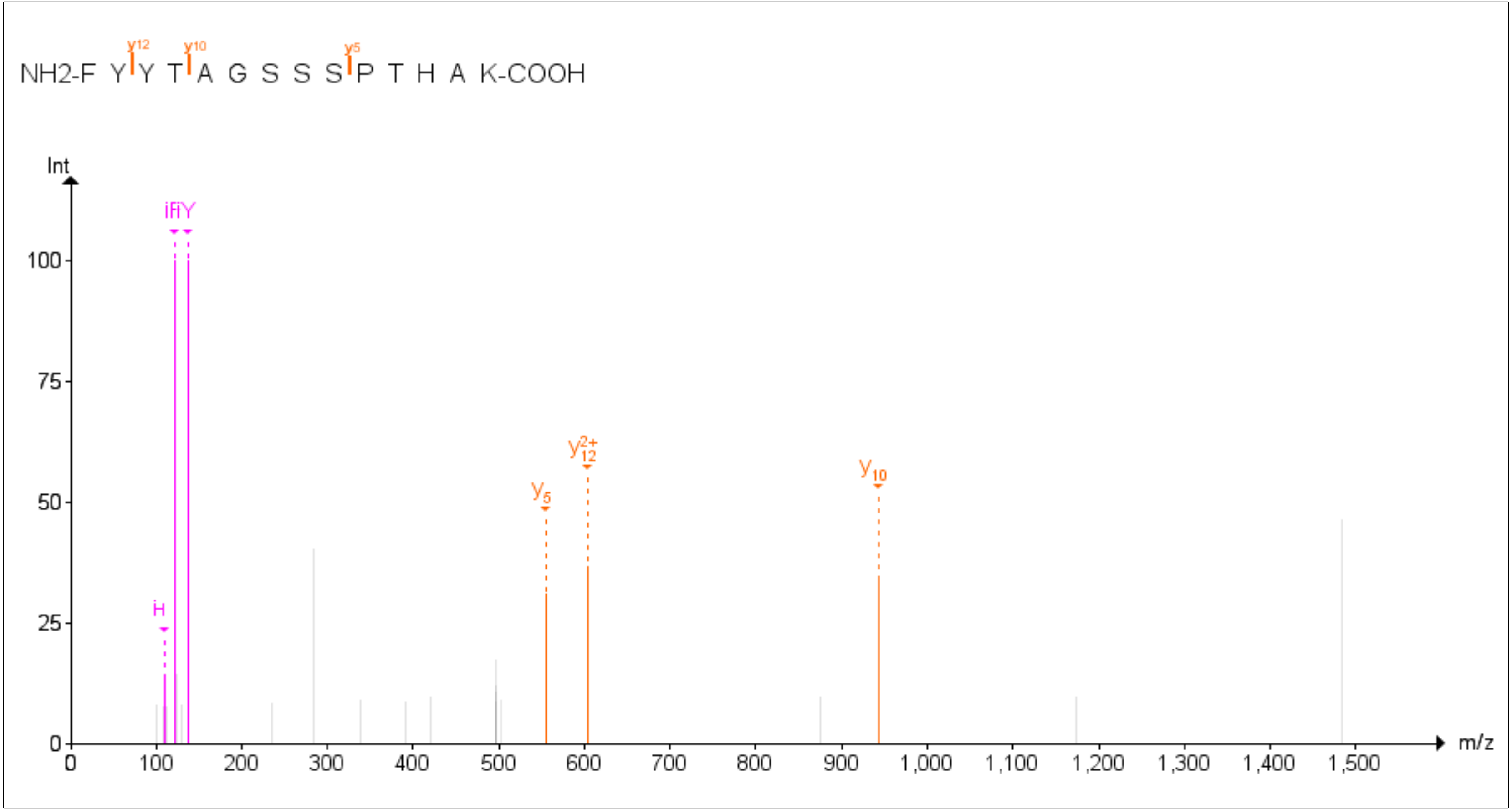

NH2-F Y Y T A G S S S P T H A K-COOH
y12
y10
y5
Int
100
75
50
25
0
iFiY
iH
y5
y12 2+
y10
m/z
0
100
200
300
400
500
600
700
800
900
1,000
1,100
1,200
1,300
1,400
1,500

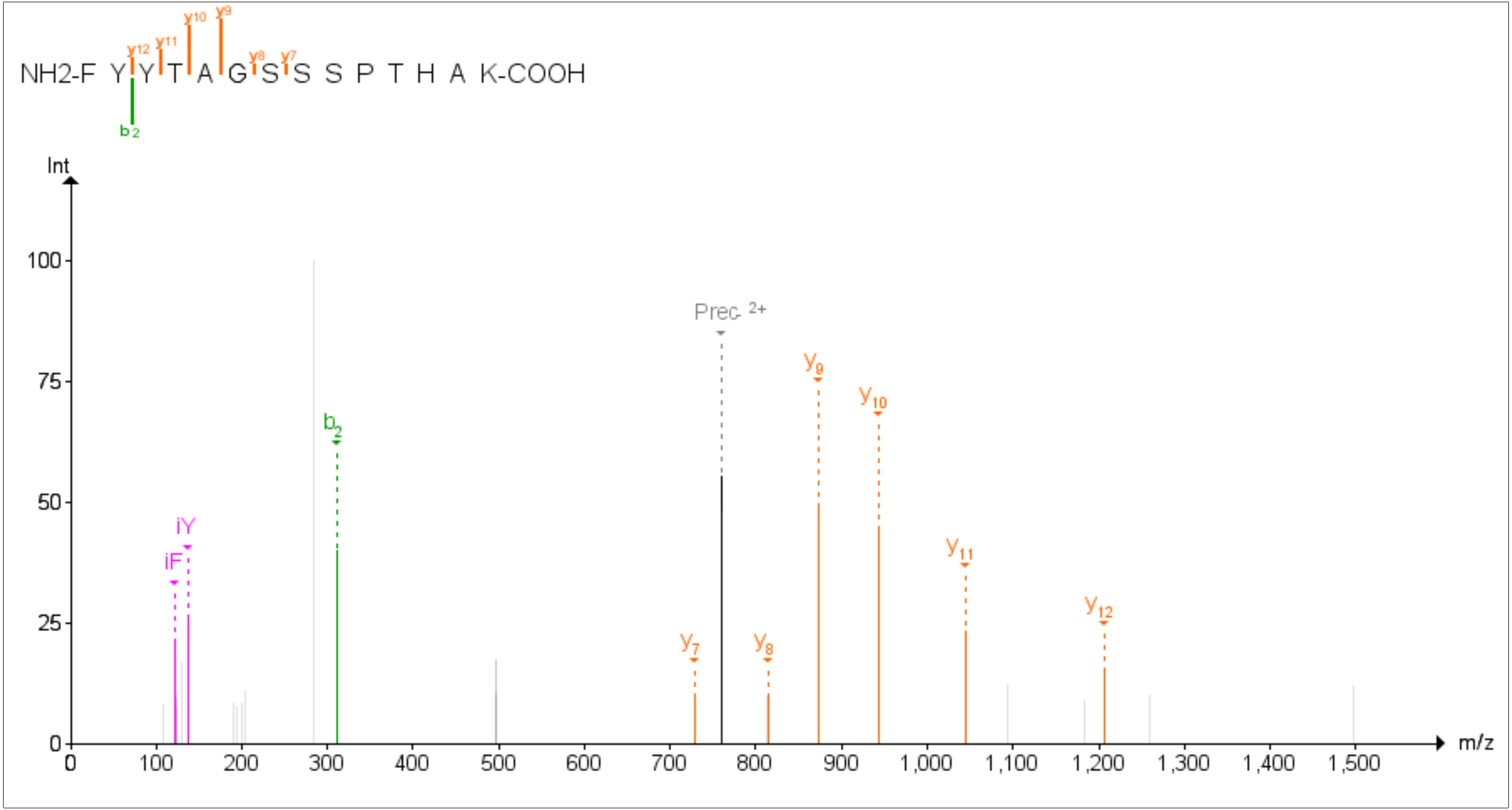
NH2-F Y Y T A G S S S P T H A K-COOH
y12 y11 y10 y9 y8 y7
b2
Int
100
75
50
25
0
iF
iY
b2
Prec 2+
y7
y8
y9
y10
y11
y12
0 100 200 300 400 500 600 700 800 900 1,000 1,100 1,200 1,300 1,400 1,500
m/z

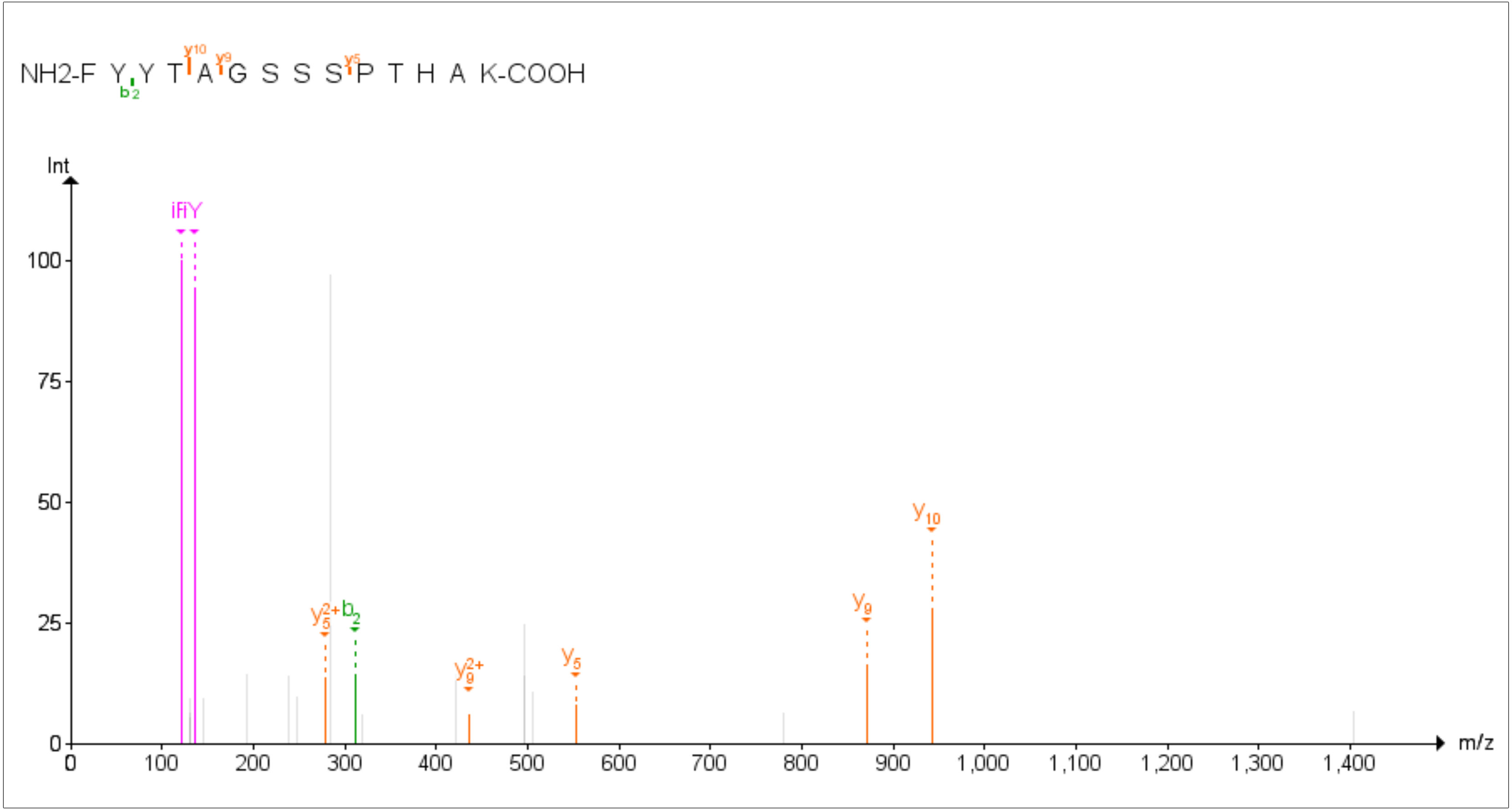
NH2-F Y Y T A G S S S P T H A K-COOH
b2
y10
y9
y5
Int
iF
iY
y5 2+
b2
y9 2+
y5
y9
y10
100
75
50
25
0
m/z
0
100
200
300
400
500
600
700
800
900
1,000
1,100
1,200
1,300
1,400

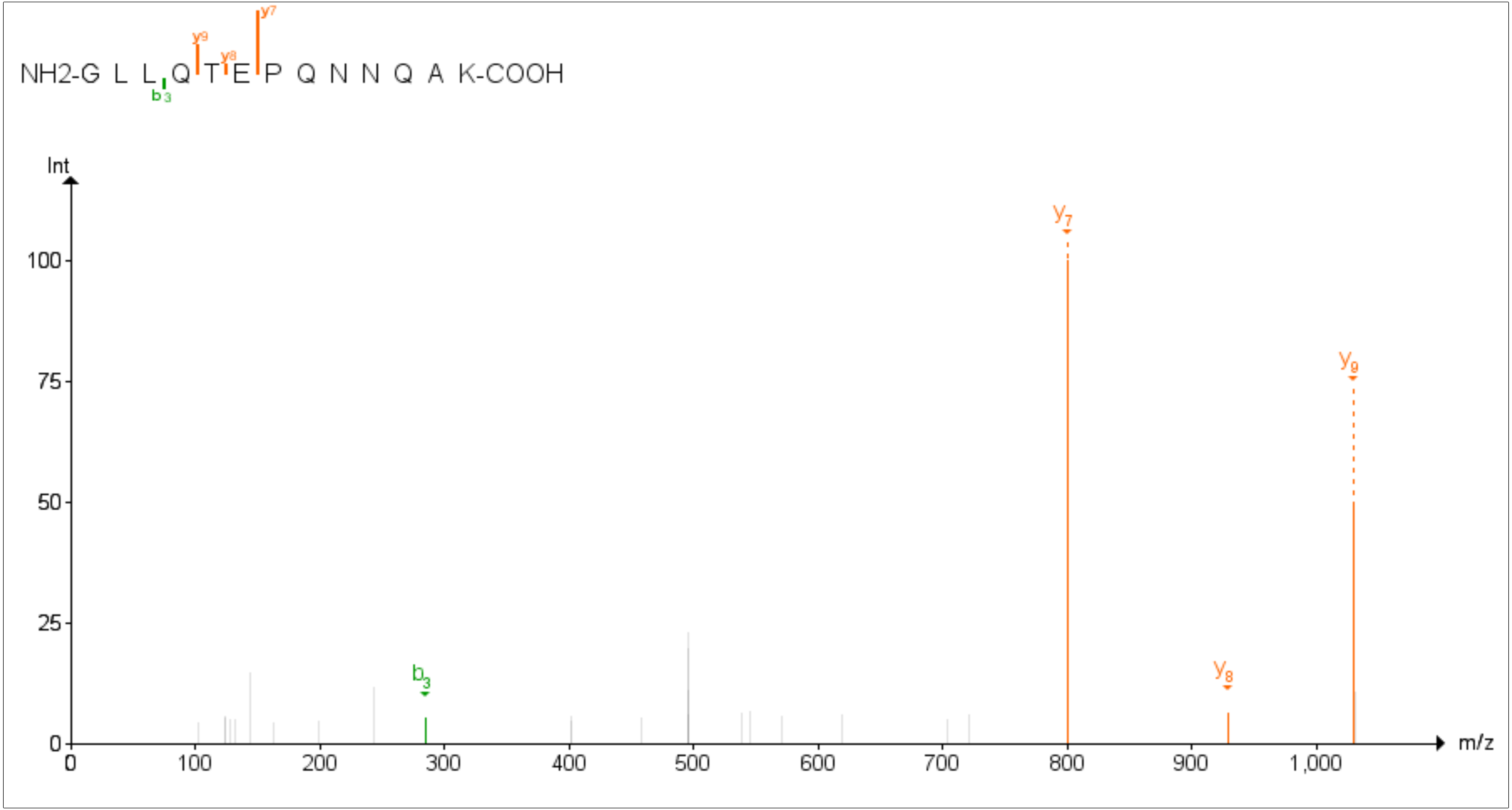

NH2-G L L Q T E P Q N N Q A K-COOH
y9
y8
y7
b3
Int
100
75
50
25
0
y7
y9
y8
b3
0
100
200
300
400
500
600
700
800
900
1,000
m/z

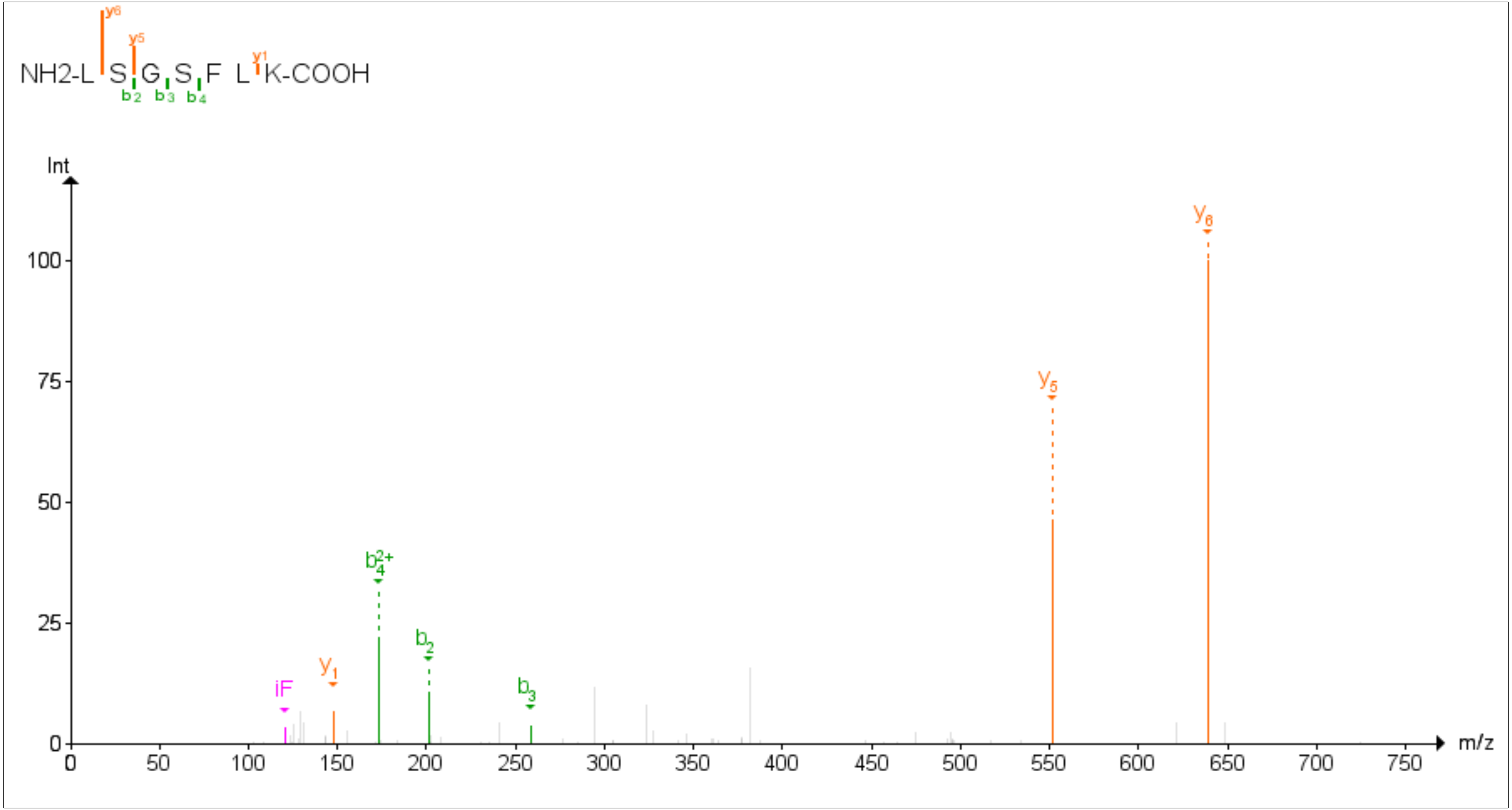

NH2-L S G S F L K-COOH
y6
y5
y1
b2
b3
b4
Int
100
75
50
25
0
m/z
0
50
100
150
200
250
300
350
400
450
500
550
600
650
700
750
iF
y1
b4 2+
b2
b3
y5
y6

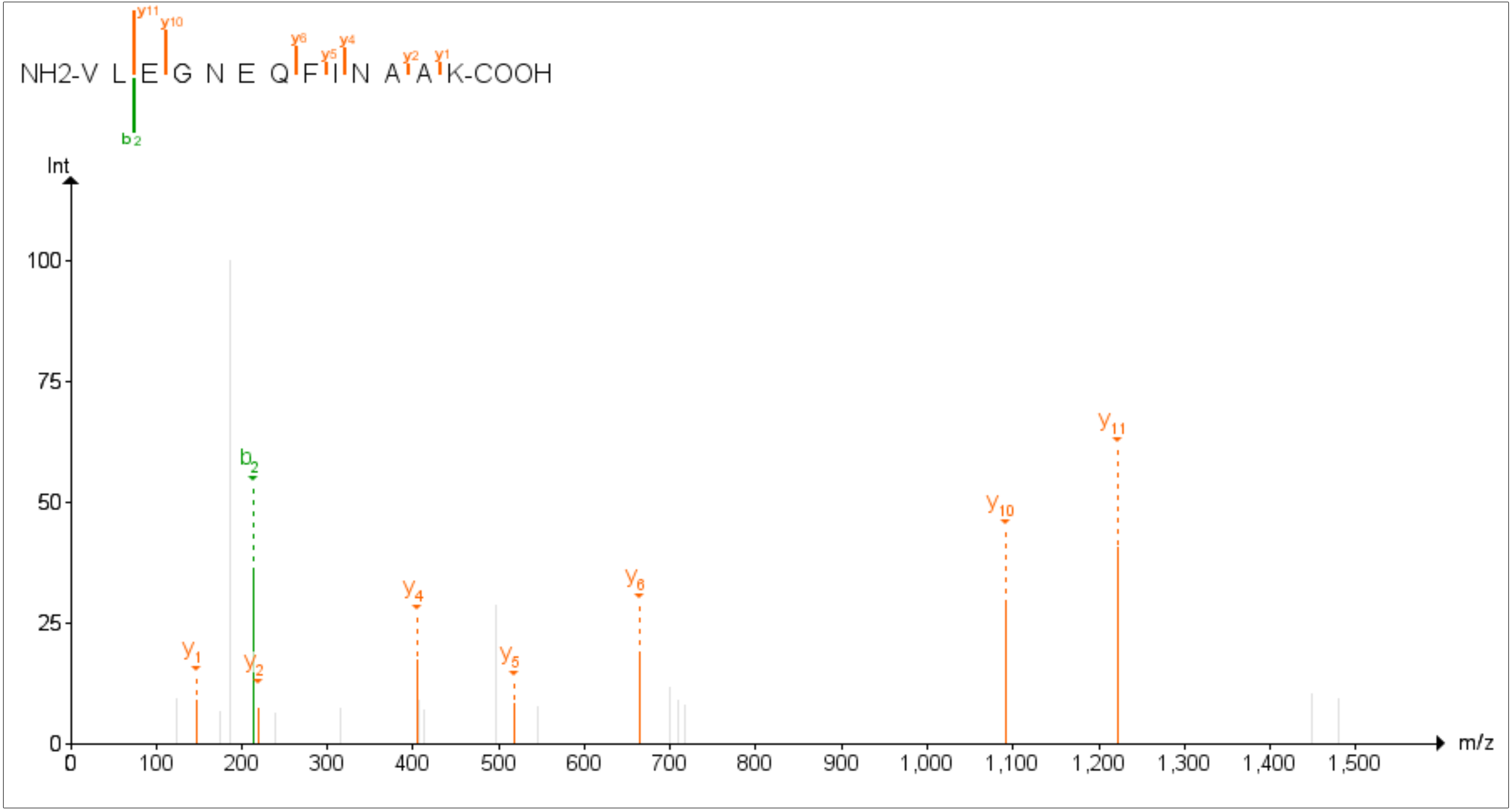

NH2-V L E G N E Q F I N A A K-COOH
y11
y10
y6
y5
y4
y2
y1
b2
Int
100
75
50
25
0
m/z
0
100
200
300
400
500
600
700
800
900
1,000
1,100
1,200
1,300
1,400
1,500
y1
b2
y2
y4
y5
y6
y10
y11

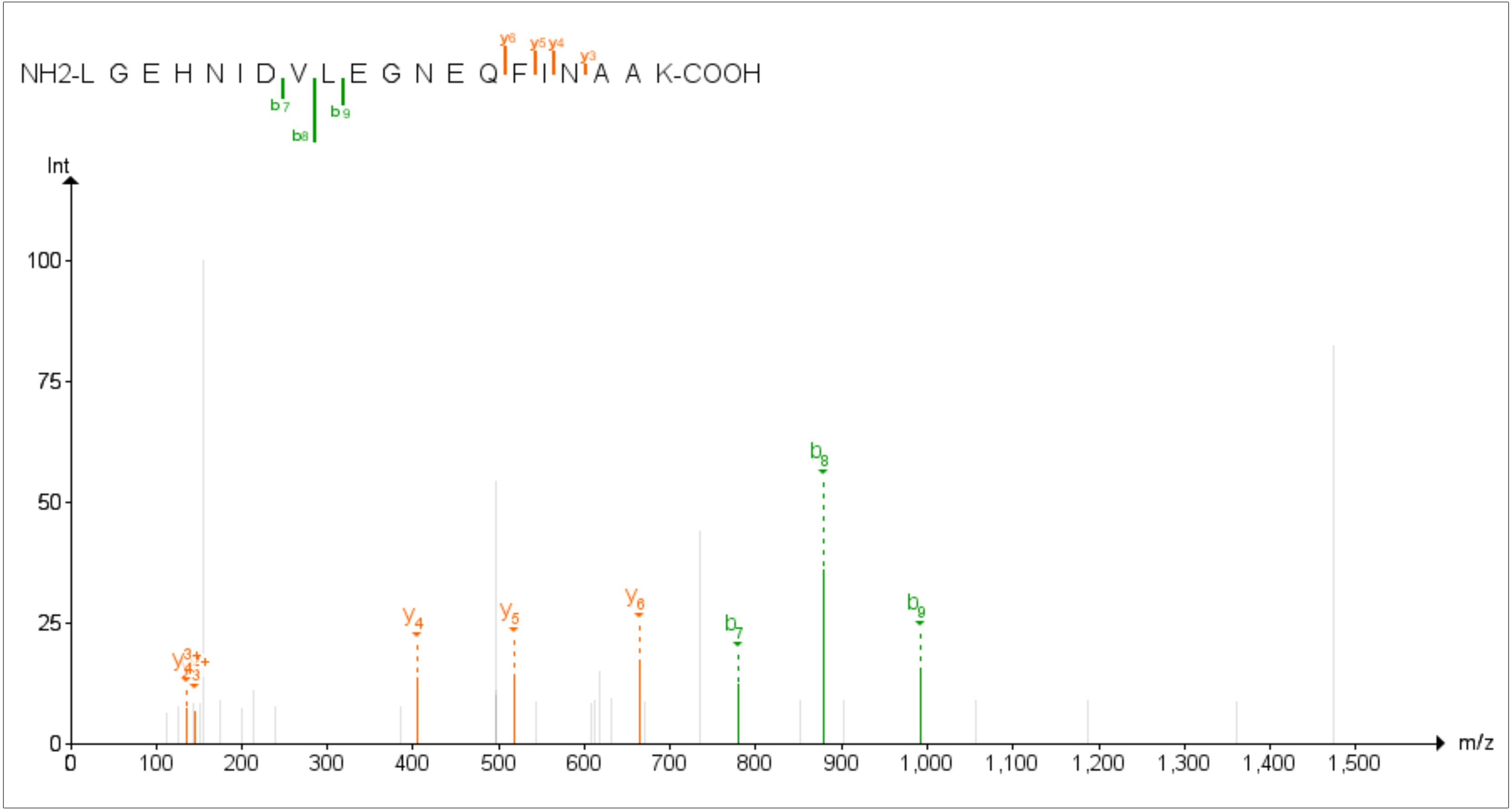
NH2-L G E H N I D V L E G N E Q F I N A A K-COOH
y6
y5
y4
y3
b7
b8
b9
Int
100
75
50
25
0
m/z
0
100
200
300
400
500
600
700
800
900
1,000
1,100
1,200
1,300
1,400
1,500
y4
y5
y6
b7
b8
b9

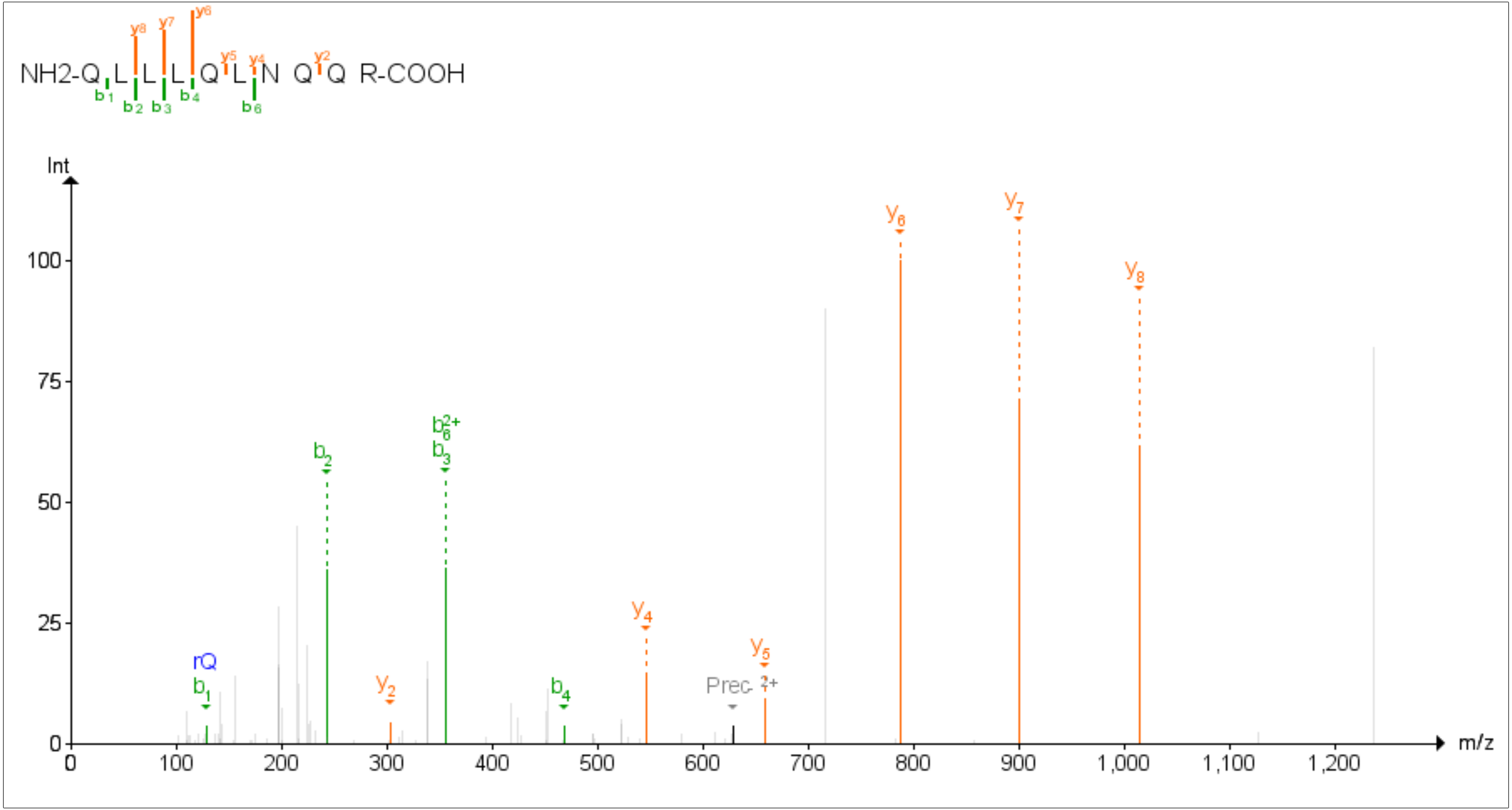

NH2-Q L L L Q L N Q Q R-COOH
y8 y7 y6 y5 y4 y2
b1 b2 b3 b4 b6
Int
m/z
y6
y7
y8
b2
b6 2+
b3
y4
y5
y2
rQ
b1
b4
Prec. 2+
0
25
50
75
100
0
100
200
300
400
500
600
700
800
900
1,000
1,100
1,200

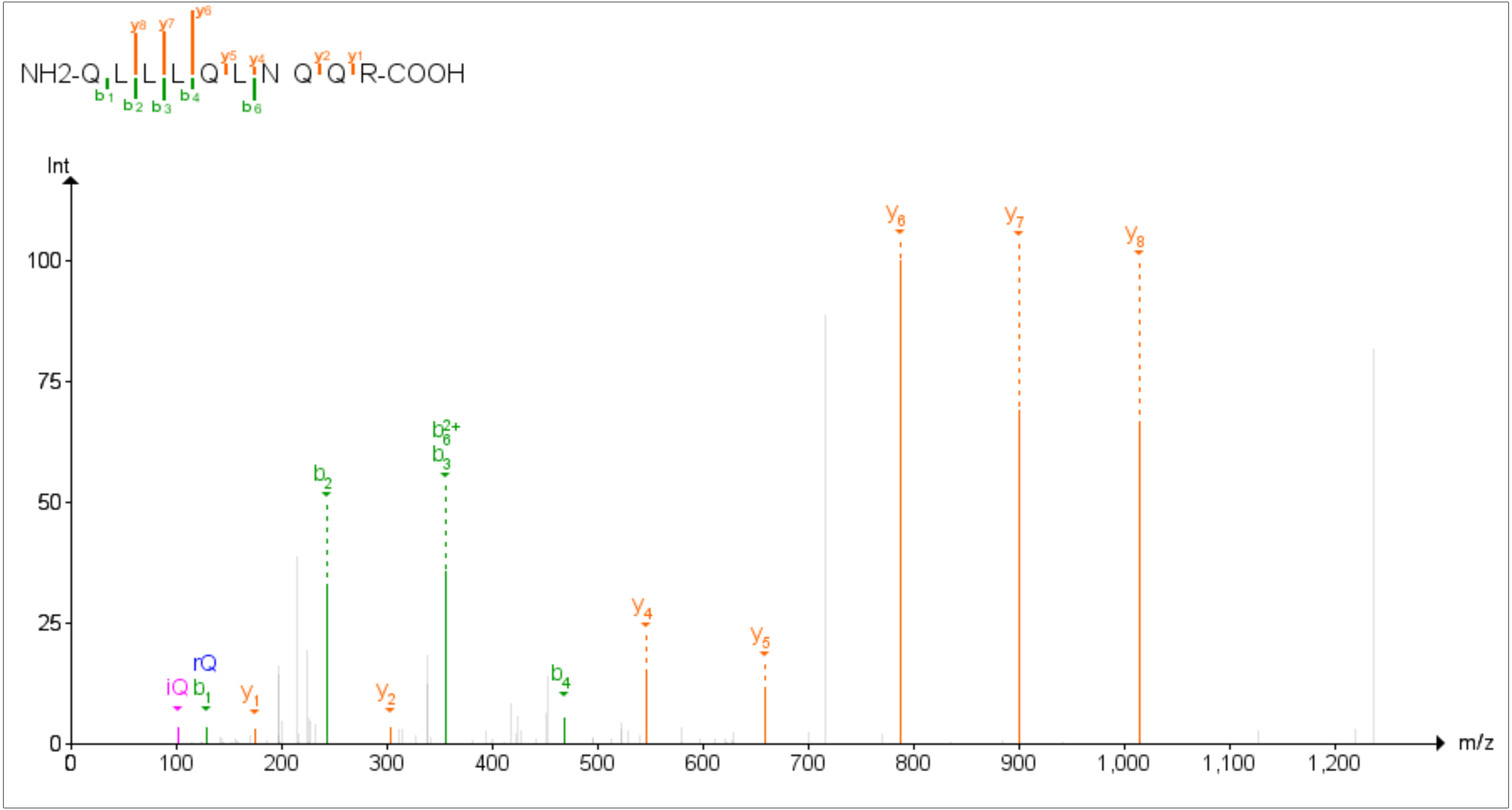

NH2-Q L L L Q L N Q Q R-COOH
y8 y7 y6 y5 y4 y2 y1
b1 b2 b3 b4 b6
Int
100
75
50
25
0
m/z
0 100 200 300 400 500 600 700 800 900 1,000 1,100 1,200
y6
y7
y8
b6 2+
b3
b2
y4
y5
b4
rQ
iQ
b1
y1
y2

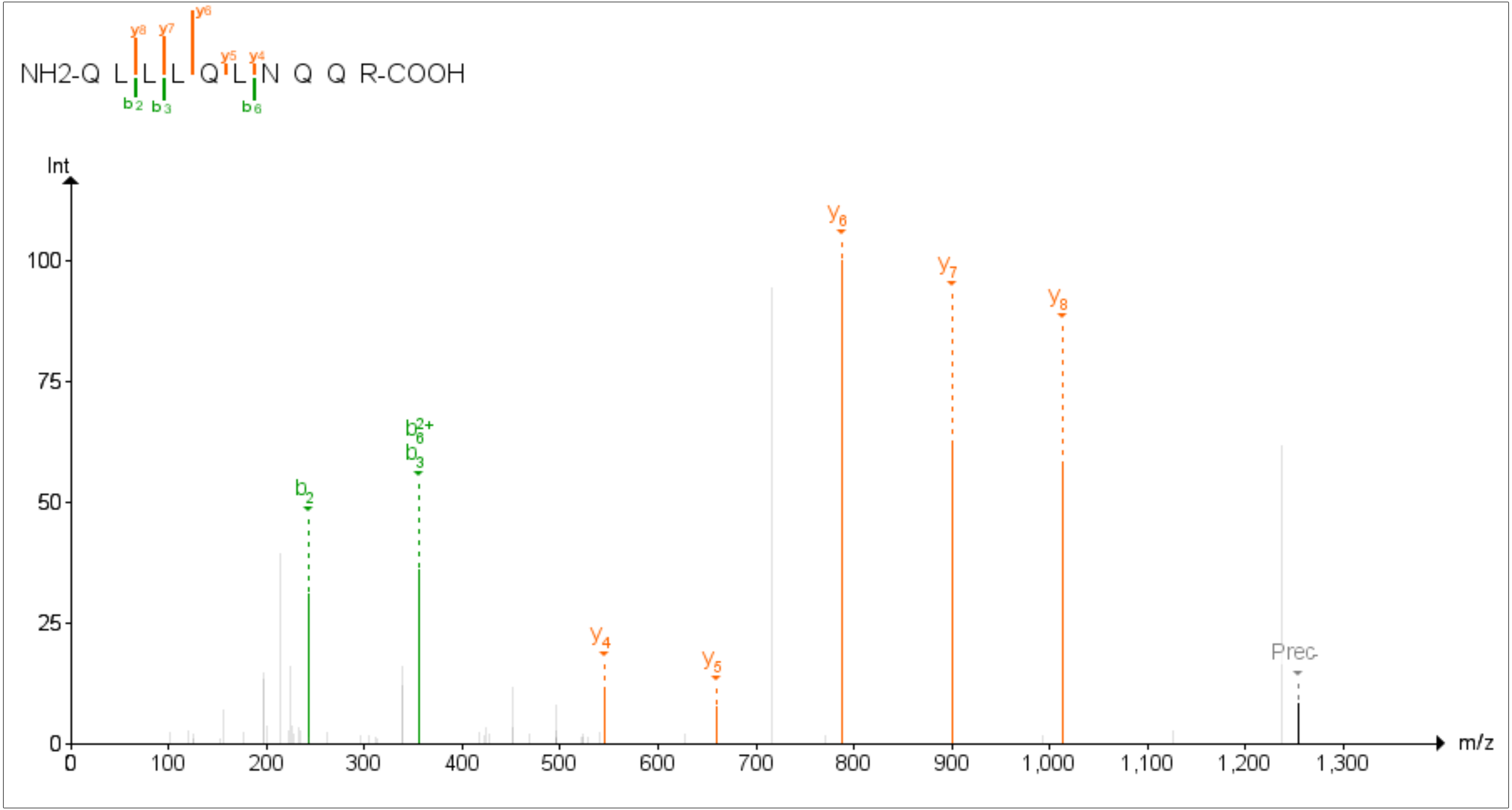

NH2-Q L L L Q L N Q Q R-COOH
y8 y7 y6 y5 y4
b2 b3 b6
Int
m/z
y6
y7
y8
b6 2+
b3
b2
y4
y5
Prec
0
25
50
75
100
0
100
200
300
400
500
600
700
800
900
1,000
1,100
1,200
1,300

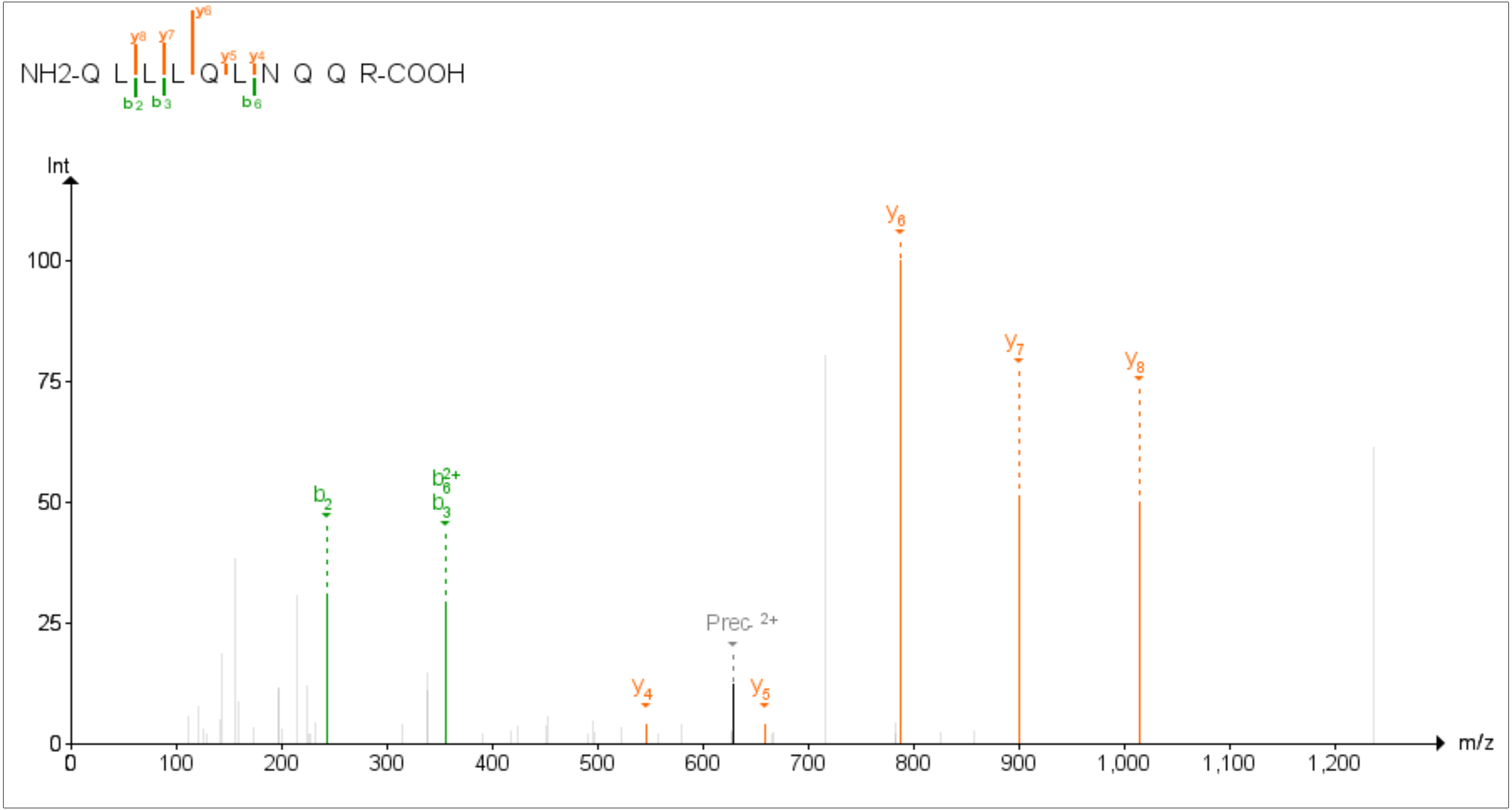

NH2-Q L L L Q L N Q Q R-COOH
y8 y7 y6 y5 y4
b2 b3 b6
Int
100
75
50
25
0
y6
y7
y8
b2
b6 2+
b3
Prec 2+
y4
y5
0 100 200 300 400 500 600 700 800 900 1,000 1,100 1,200
m/z

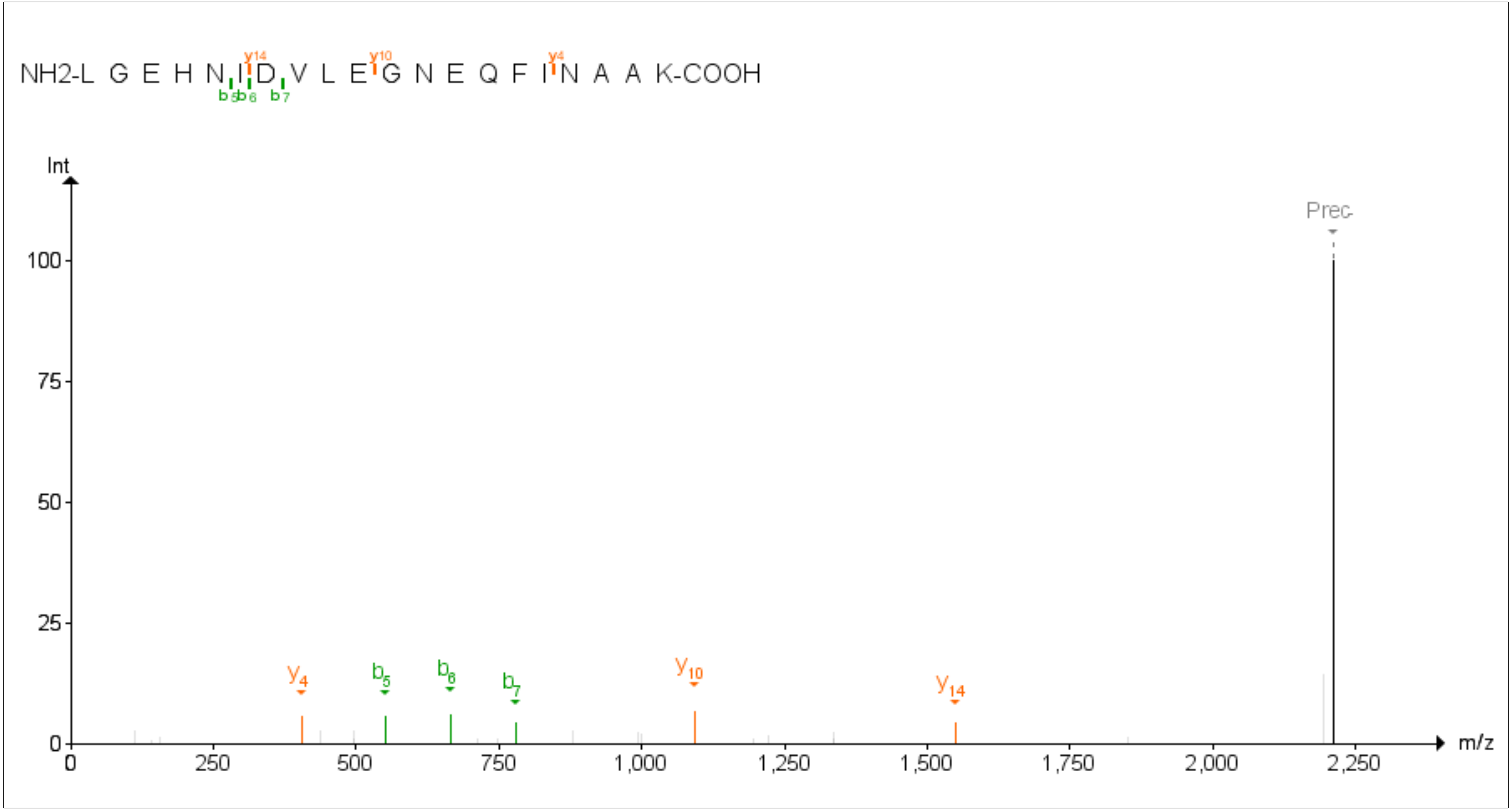

NH2-L G E H N I D V L E G N E Q F I N A A K-COOH
y14
y10
y4
b5
b6
b7
Int
100
75
50
25
0
Prec
y4
b5
b6
b7
y10
y14
0
250
500
750
1,000
1,250
1,500
1,750
2,000
2,250
m/z

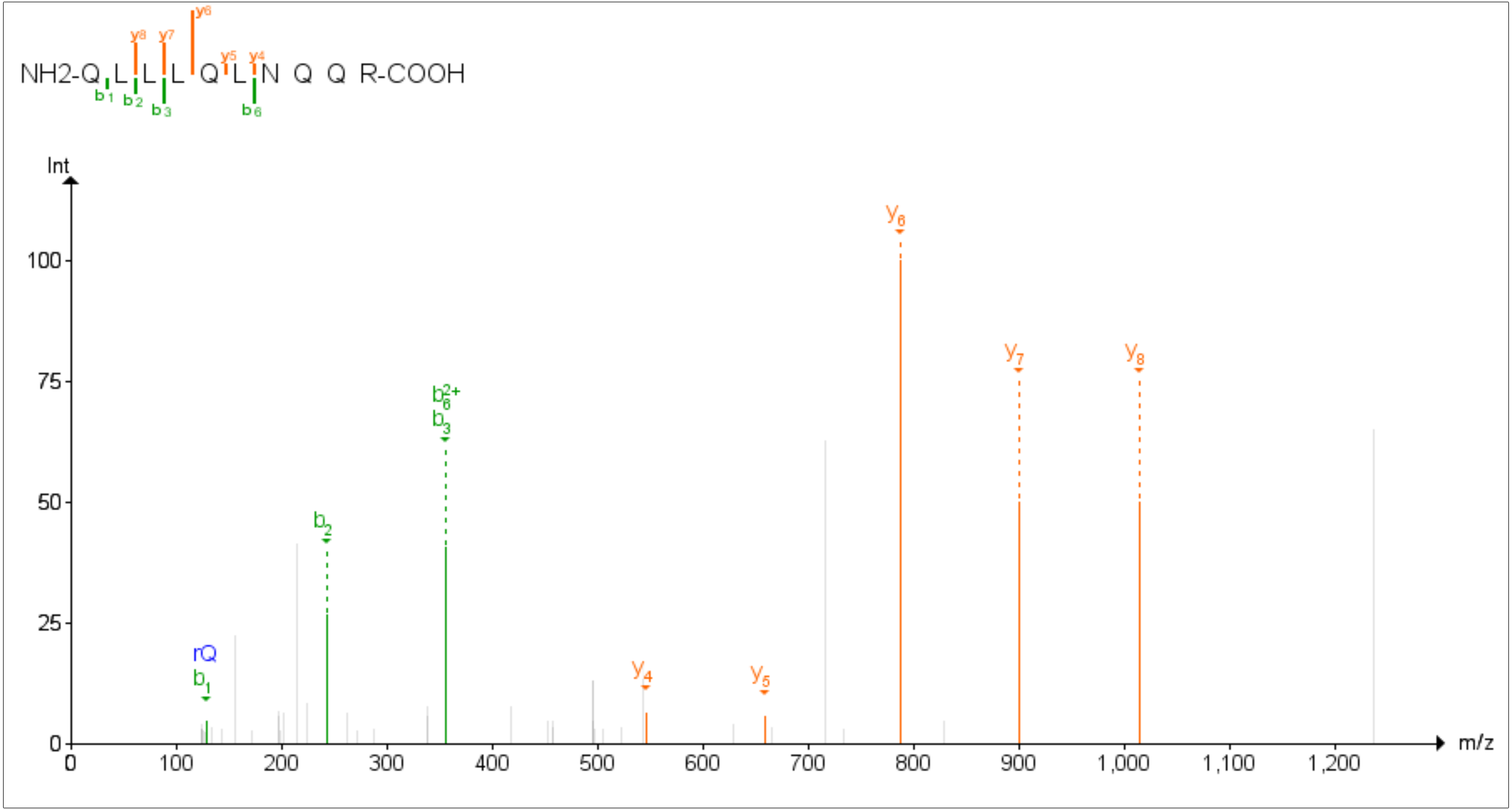

NH2-Q L L L Q L N Q Q R-COOH
b1 b2 b3 b6
y8 y7 y6 y5 y4
Int
m/z
rQ
b1
b2
b3
b6 2+
y4
y5
y6
y7
y8
0
25
50
75
100
0
100
200
300
400
500
600
700
800
900
1,000
1,100
1,200

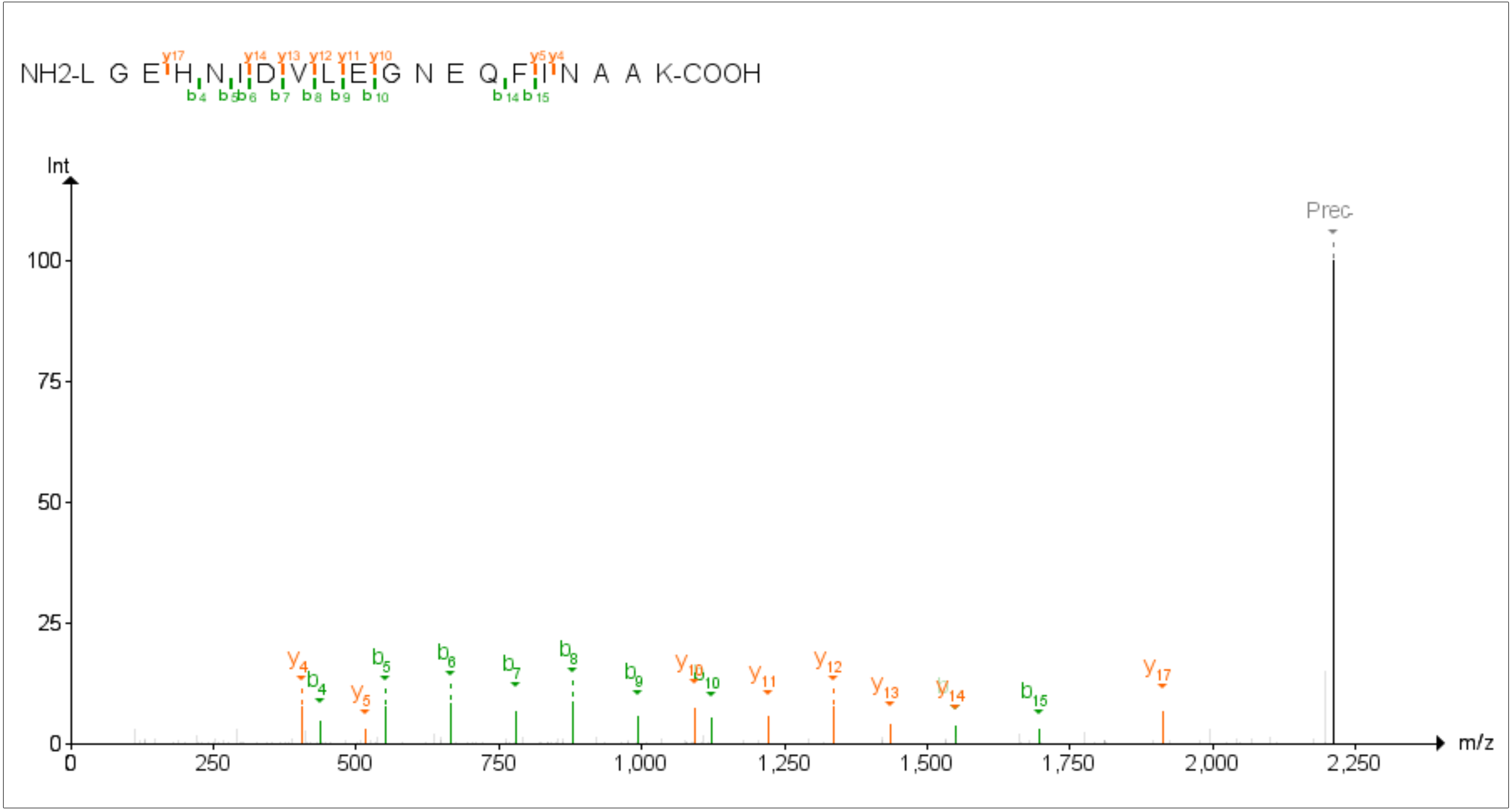

NH2-L G E H N I D V L E G N E Q F I N A A K-COOH
y17 y14 y13 y12 y11 y10 y5 y4
b4 b5 b6 b7 b8 b9 b10 b14 b15
Int
100
75
50
25
0
Prec
y4 b4 y5 b5 b6 b7 b8 b9 y10 b10 y11 y12 y13 y14 b15 y17
0
250
500
750
1,000
1,250
1,500
1,750
2,000
2,250
m/z

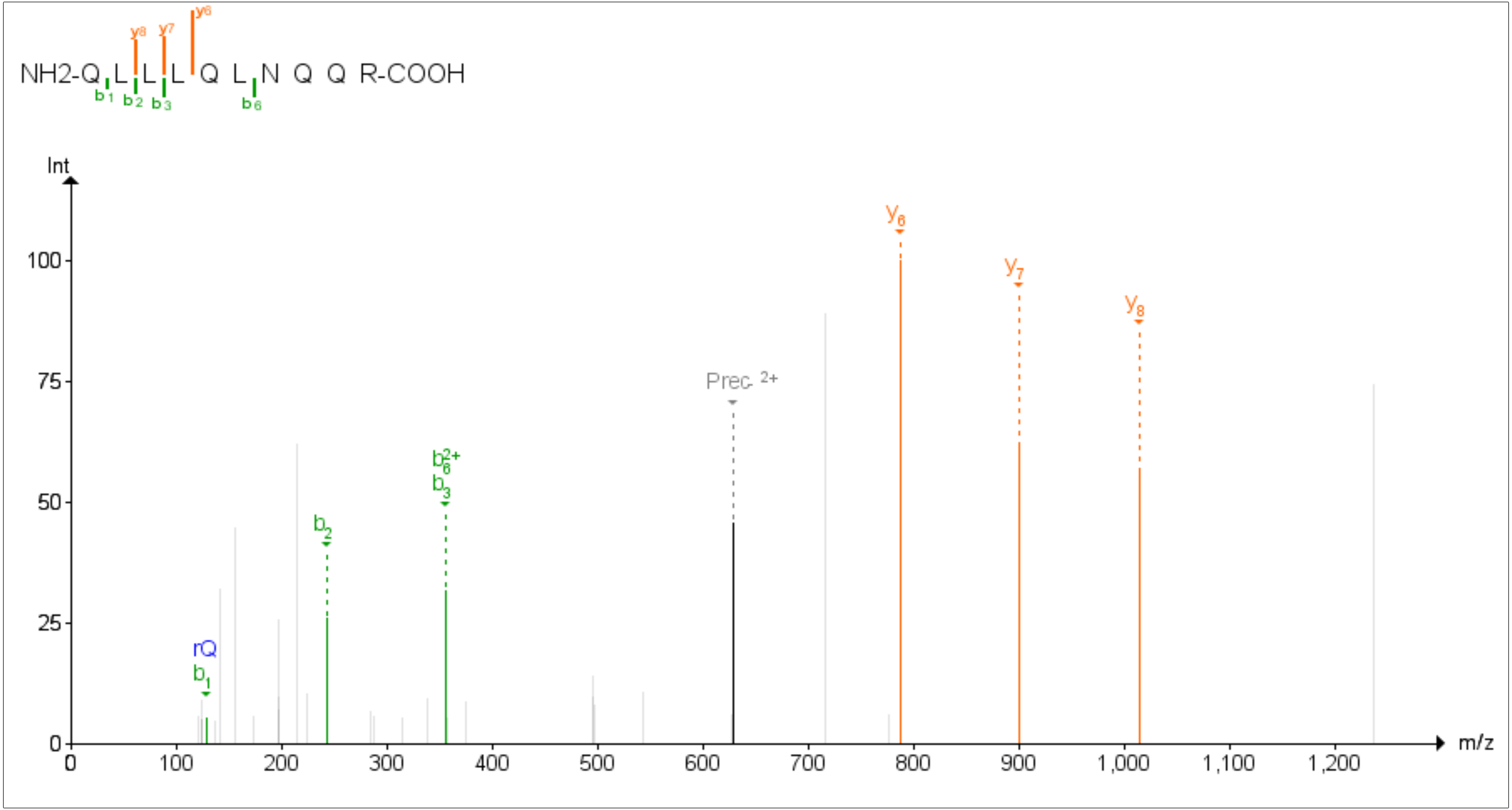

NH2-Q L L L Q L N Q Q R-COOH
y8
y7
y6
b1
b2
b3
b6
Int
100
75
50
25
0
rQ
b1
b2
b6 2+
b3
Prec 2+
y6
y7
y8
0
100
200
300
400
500
600
700
800
900
1,000
1,100
1,200
m/z

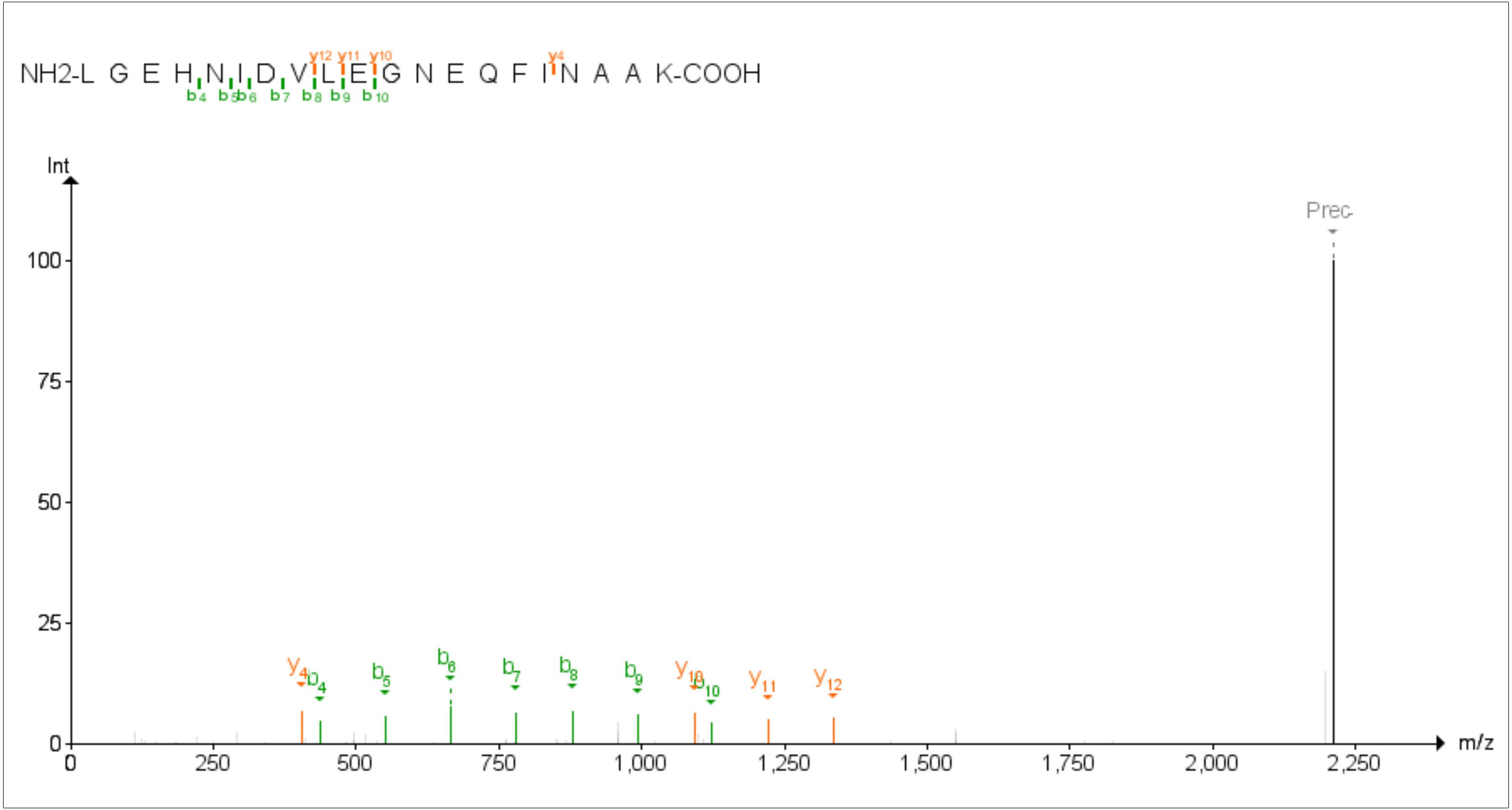
NH2-L G E H N I D V L E G N E Q F I N A A K-COOH
y12 y11 y10
y4
b4 b5 b6 b7 b8 b9 b10
Int
Prec
100
75
50
25
0
y4 b4 b5 b6 b7 b8 b9 y10 b10 y11 y12
0
250
500
750
1,000
1,250
1,500
1,750
2,000
2,250
m/z

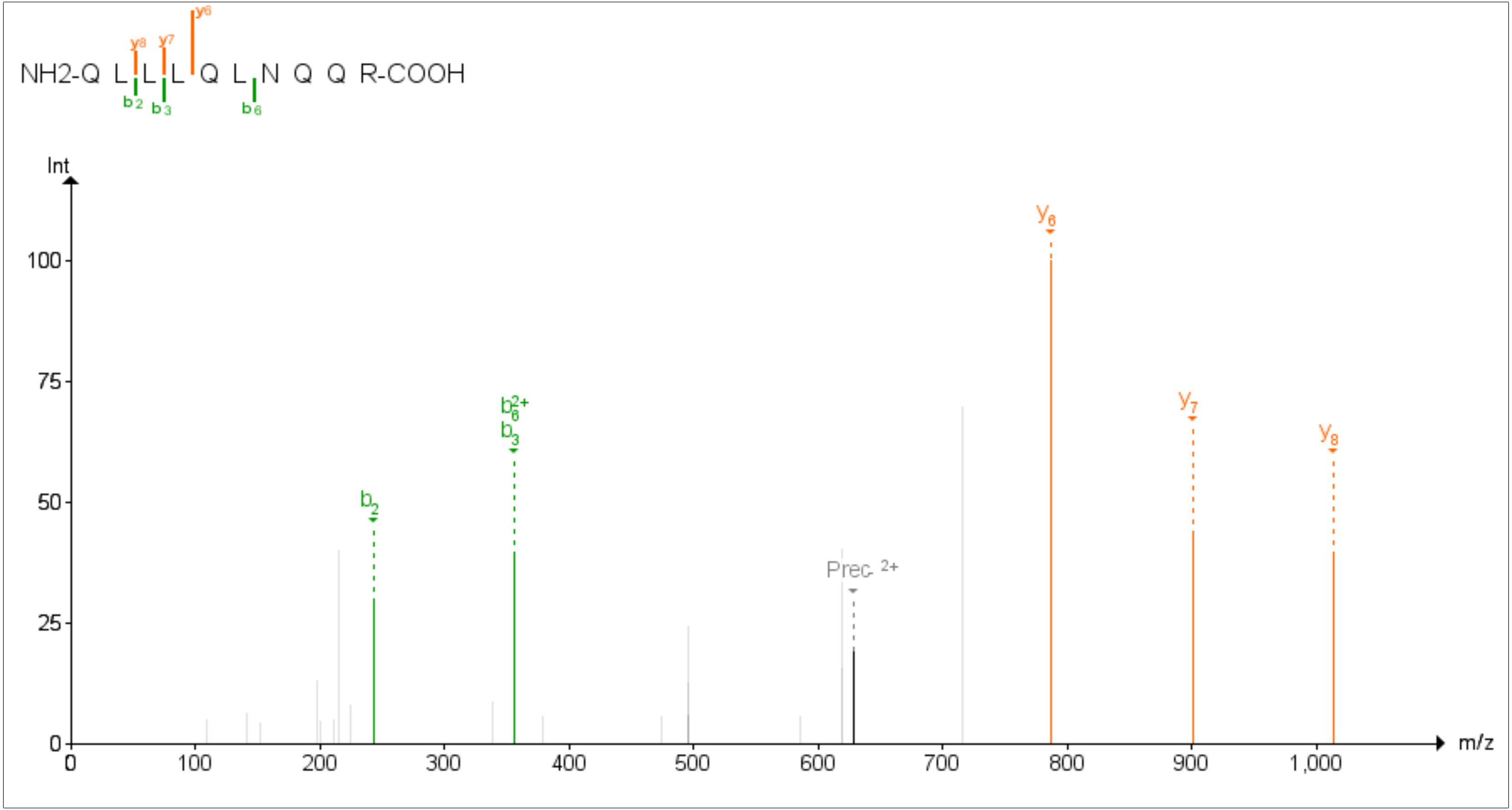
NH2-Q L L L Q L N Q Q R-COOH
y8
y7
y6
b2
b3
b6
Int
100
75
50
25
0
m/z
0
100
200
300
400
500
600
700
800
900
1,000
b2
b6 2+
b3
Prec 2+
y6
y7
y8

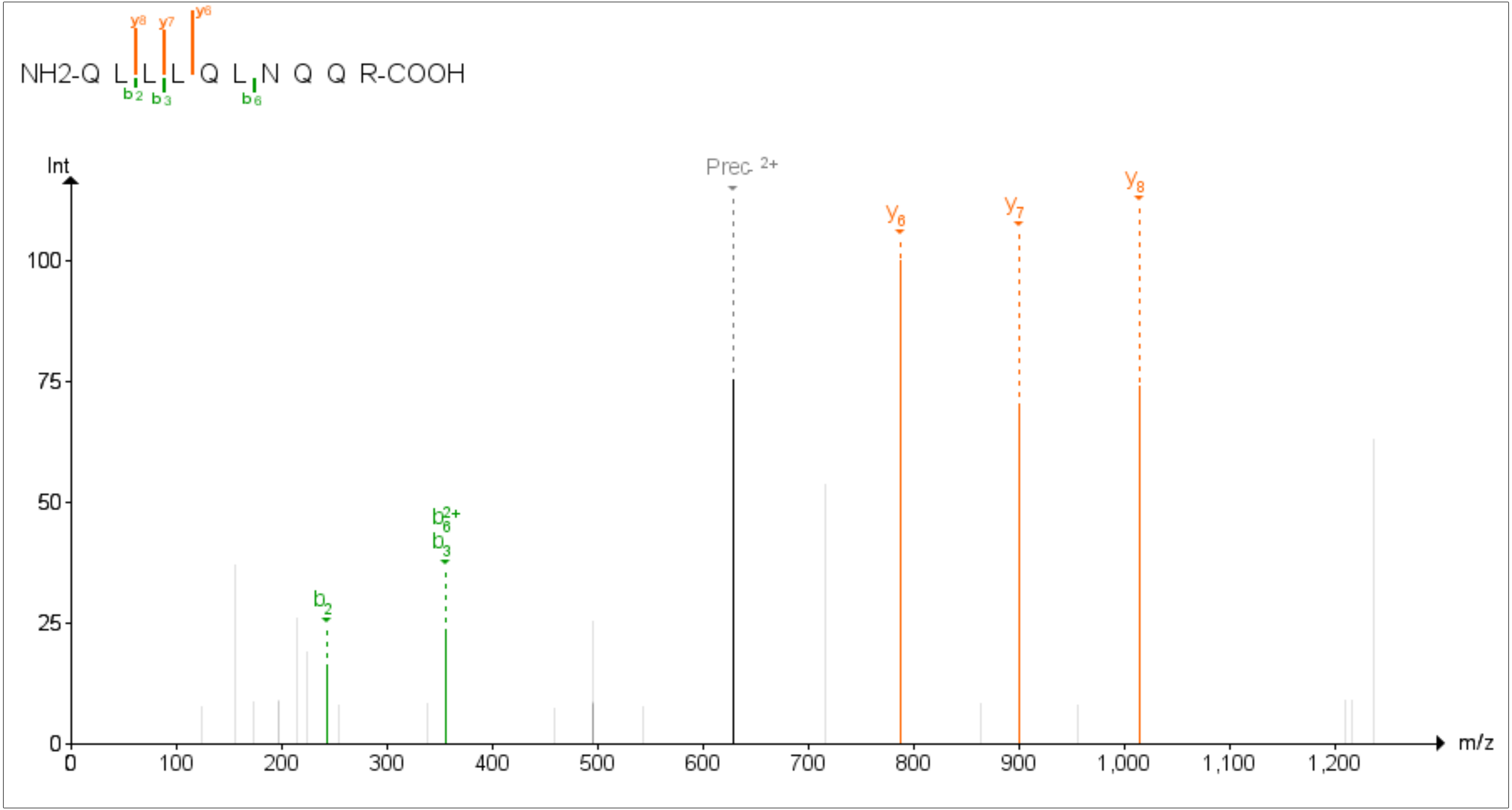

y8
y7
y6
NH2-Q L L L Q L N Q Q R-COOH
b2
b3
b6
Int
Prec 2+
y6
y7
y8
b6 2+
b3
b2
100
75
50
25
0
0
100
200
300
400
500
600
700
800
900
1,000
1,100
1,200
m/z

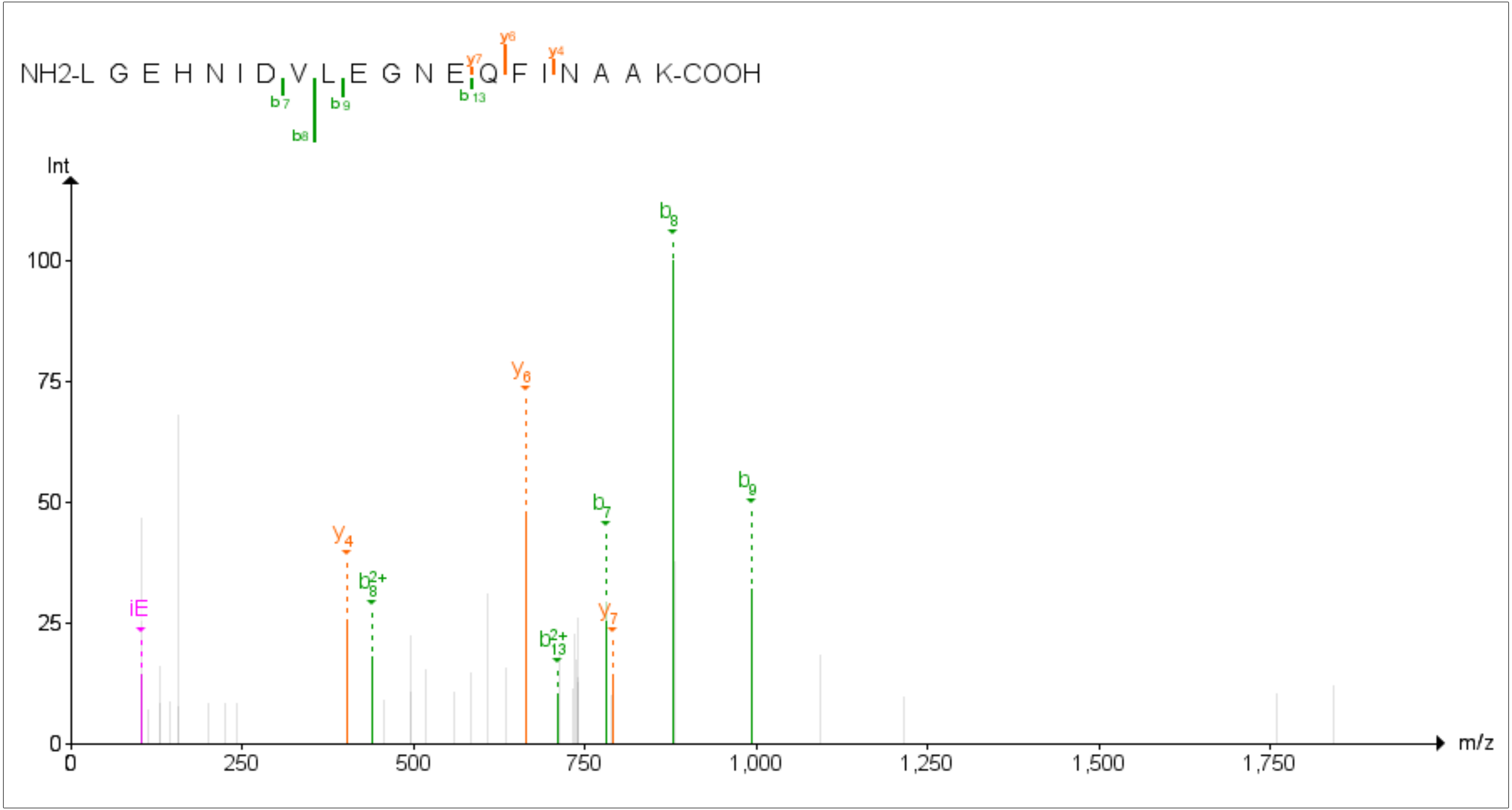

NH2-L G E H N I D V L E G N E Q F I N A A K-COOH
b7 b8 b9 b13 y7 y6 y4
Int
100
75
50
25
0
250
500
750
1,000
1,250
1,500
1,750
m/z
iE
y4
b8 2+
y6
b13 2+
b7
y7
b8
b9

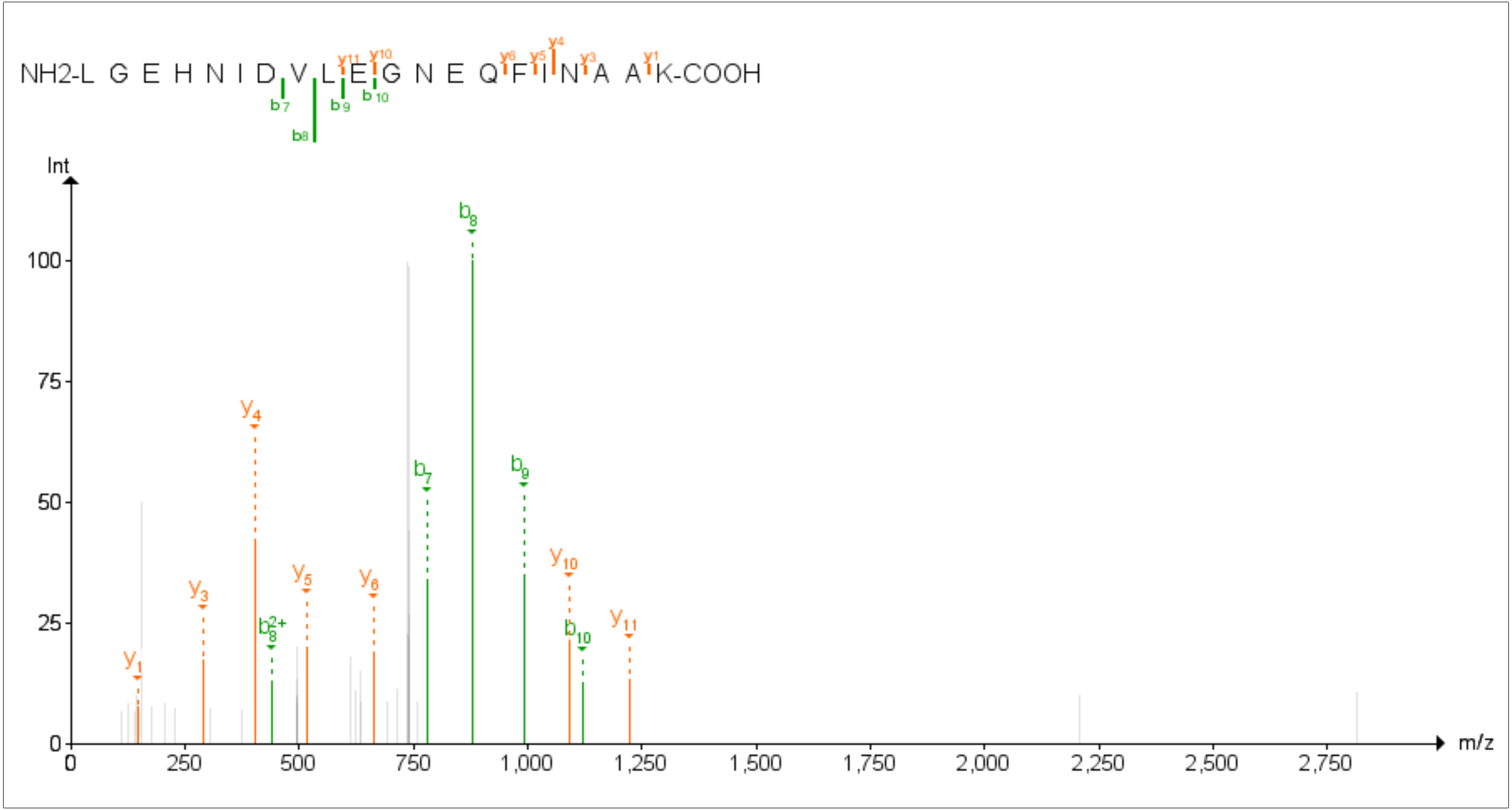

NH2-L G E H N I D V L E G N E Q F I N A A K-COOH
y11 y10 y6 y5 y4 y3 y1
b7 b8 b9 b10
Int
100
75
50
25
0
b8
y4
b7
b9
y10
y3
y5
y6
b8 2+
b10
y11
y1
0
250
500
750
1,000
1,250
1,500
1,750
2,000
2,250
2,500
2,750
m/z

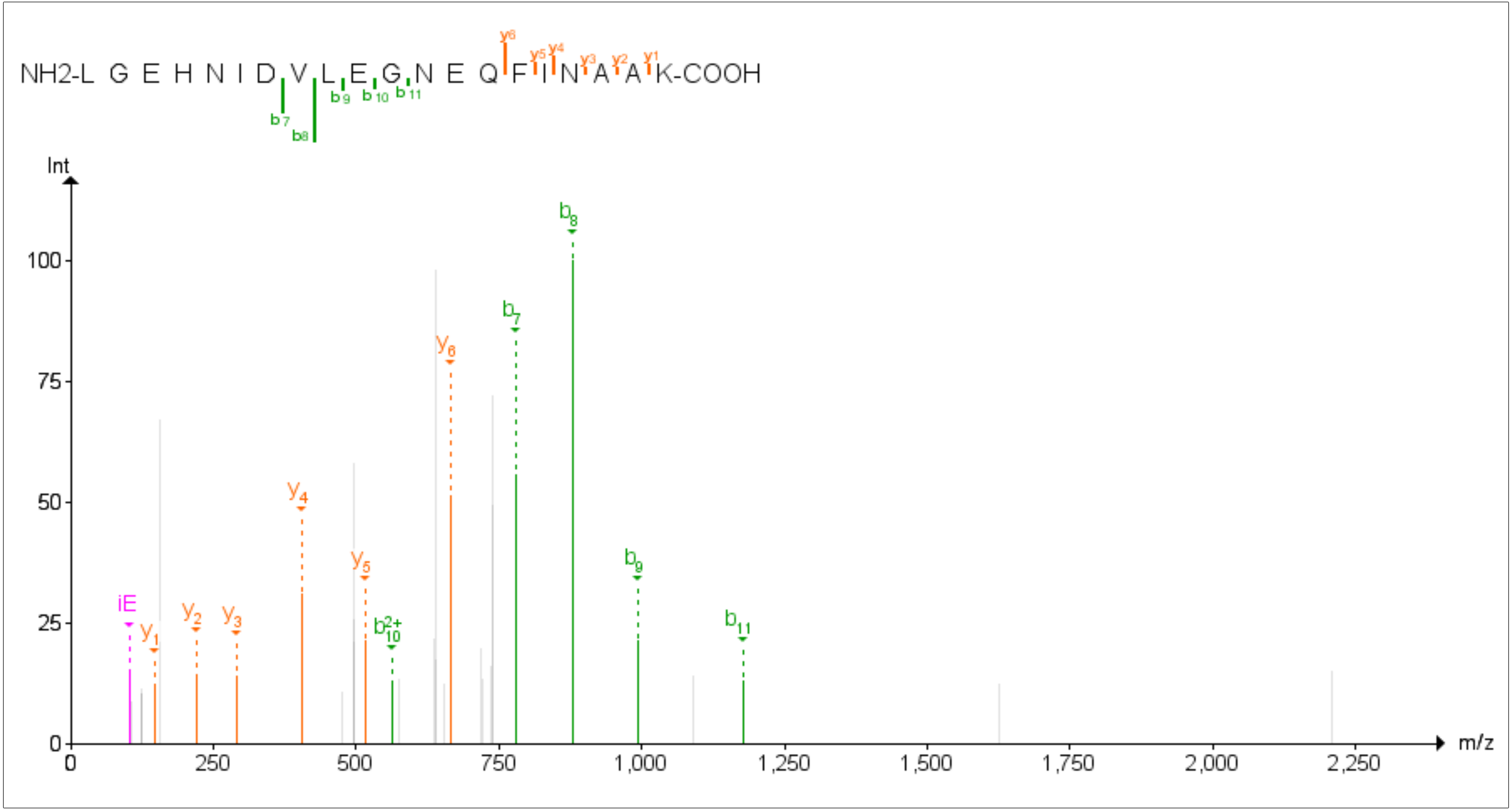
NH2-L G E H N I D V L E G N E Q F I N A A K-COOH
y6 y5 y4 y3 y2 y1
b7 b8 b9 b10 b11
Int
100
75
50
25
0
m/z
0
250
500
750
1,000
1,250
1,500
1,750
2,000
2,250
iE
y1
y2
y3
y4
y5
y6
b7
b8
b9
b10 2+
b11

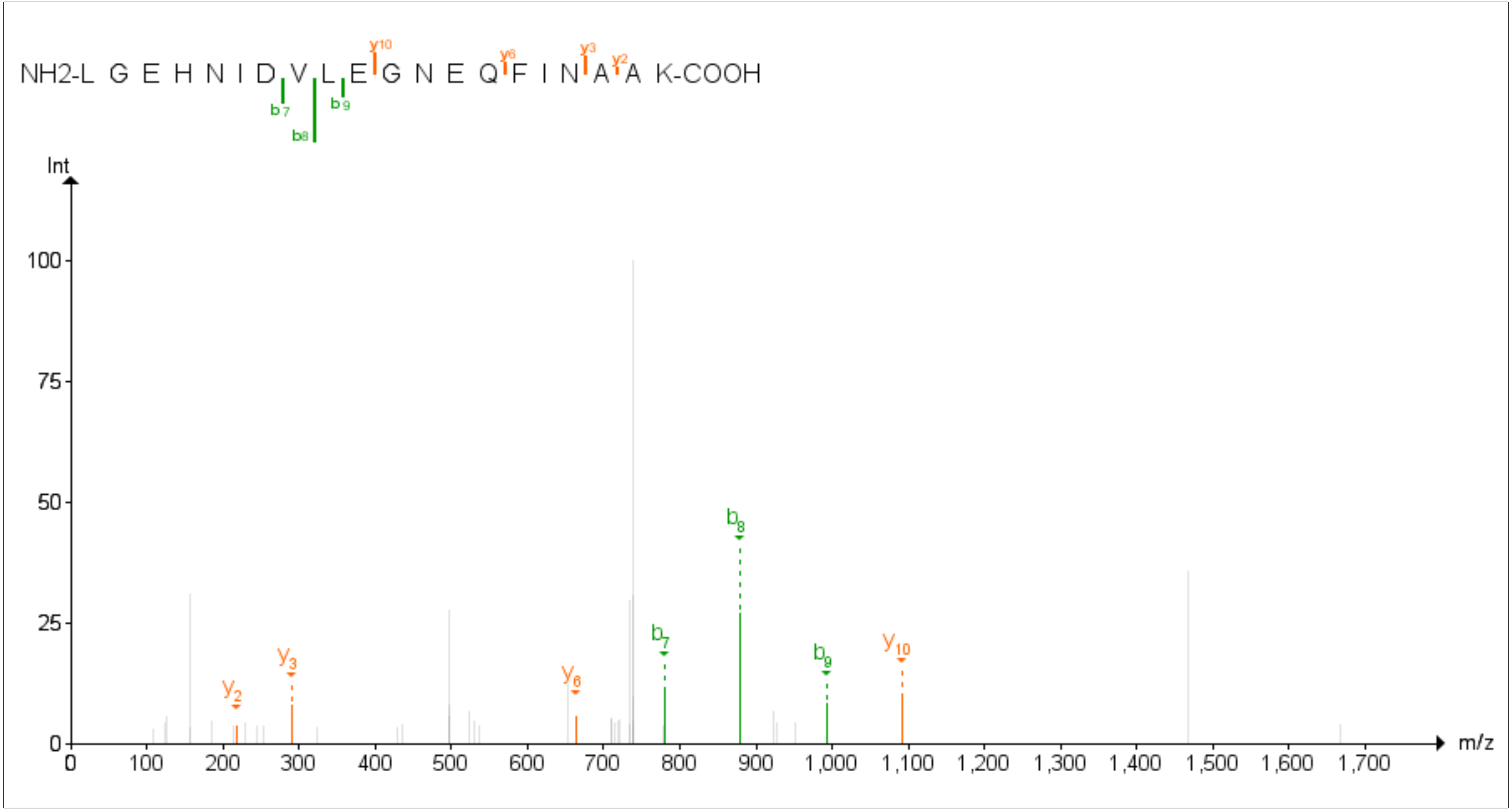
NH2-L G E H N I D V L E G N E Q F I N A A K-COOH
y10
y6
y3
y2
b7
b8
b9
Int
100
75
50
25
0
m/z
0
100
200
300
400
500
600
700
800
900
1,000
1,100
1,200
1,300
1,400
1,500
1,600
1,700
y2
y3
y6
b7
b8
b9
y10

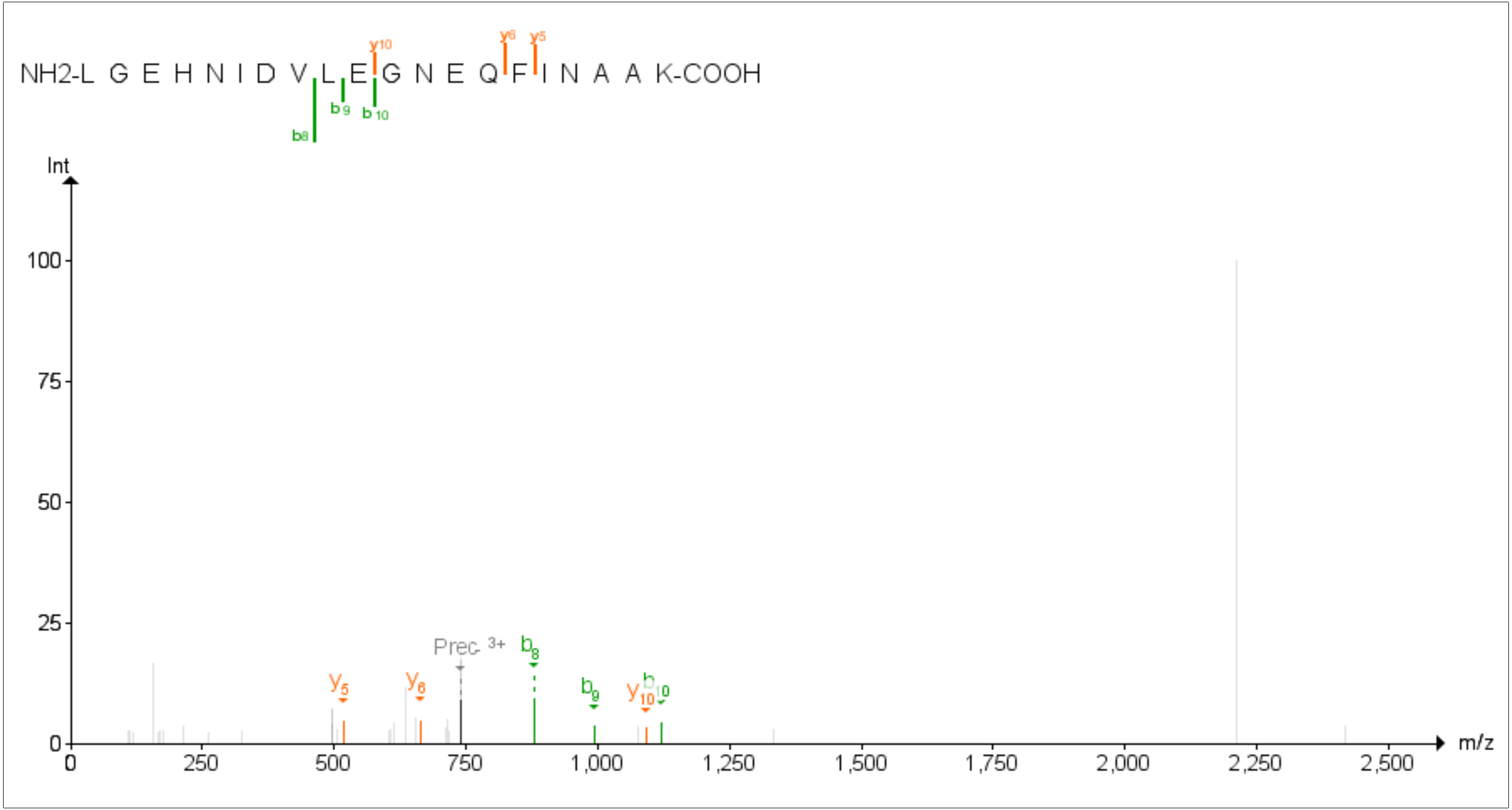

NH2-L G E H N I D V L E G N E Q F I N A A K-COOH
y10
y6
y5
b8
b9
b10
Int
100
75
50
25
0
Prec- 3+
y5
y6
b8
b9
y10
b10
m/z
0
250
500
750
1,000
1,250
1,500
1,750
2,000
2,250
2,500

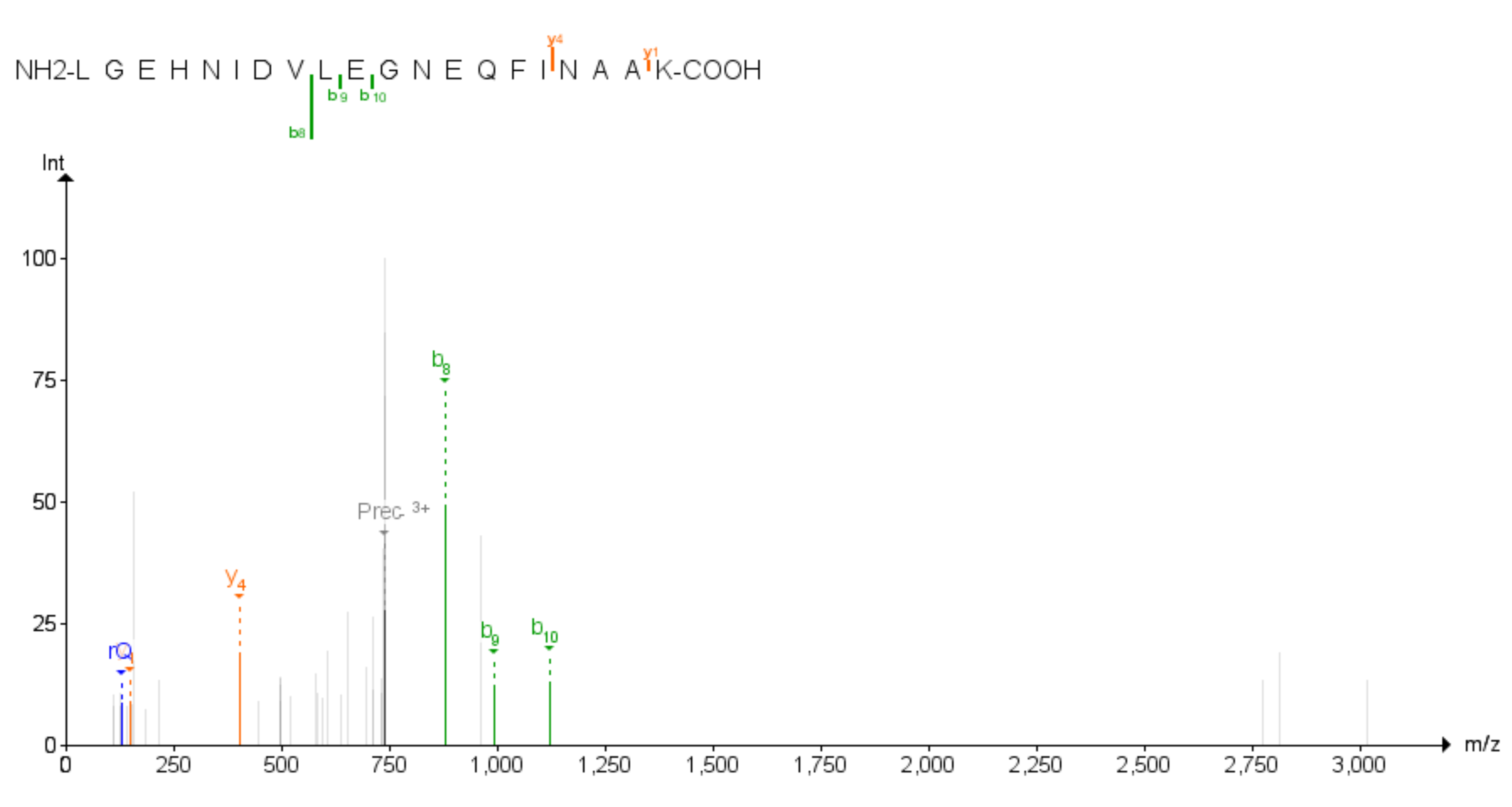

NH2-L G E H N I D V L E G N E Q F I N A A K-COOH
y4
y1
b8
b9
b10
Int
100
75
50
25
0
Prec 3+
b8
b9
b10
y4
rQ
0
250
500
750
1,000
1,250
1,500
1,750
2,000
2,250
2,500
2,750
3,000
m/z

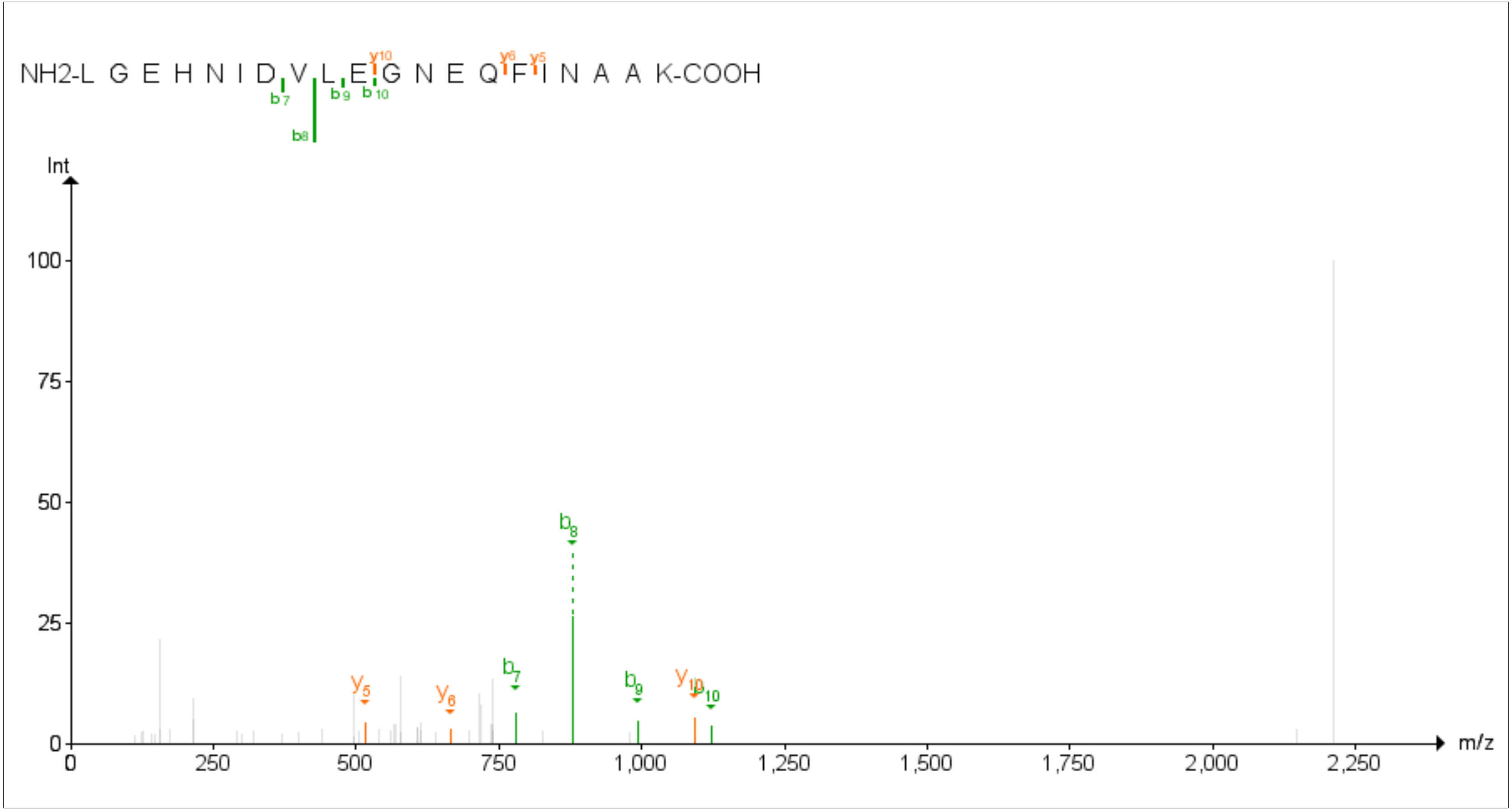

NH2-L G E H N I D V L E G N E Q F I N A A K-COOH
y10 y6 y5
b7 b9 b10 b8
Int
100
75
50
25
0
b8
y5 y6 b7 b9 y10 b10
0 250 500 750 1,000 1,250 1,500 1,750 2,000 2,250
m/z

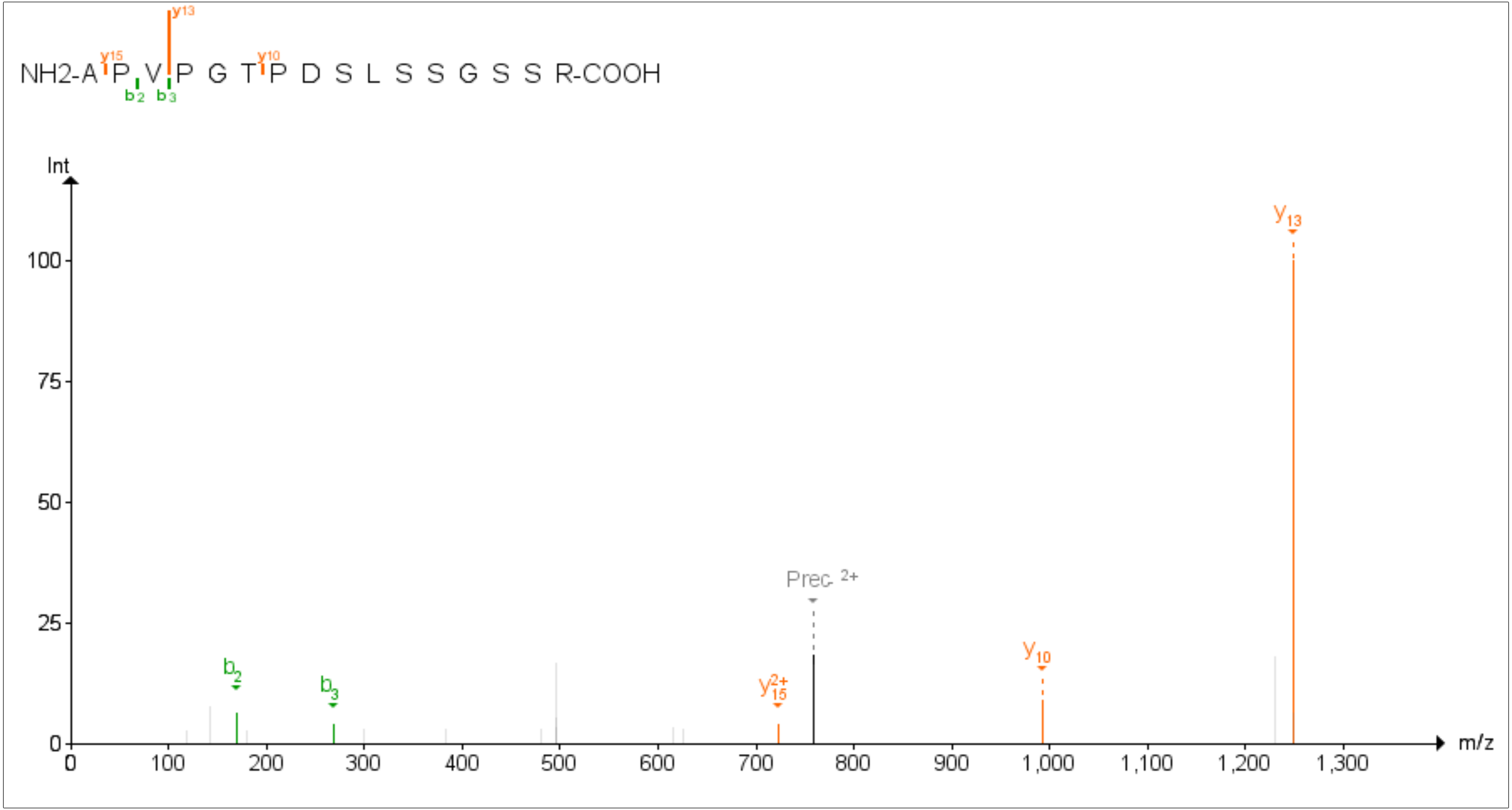

NH2-A P V P G T P D S L S S G S S R-COOH
y13
y15
y10
b2
b3
Int
100
75
50
25
0
y13
Prec 2+
y10
y15 2+
b2
b3
0
100
200
300
400
500
600
700
800
900
1,000
1,100
1,200
1,300
m/z

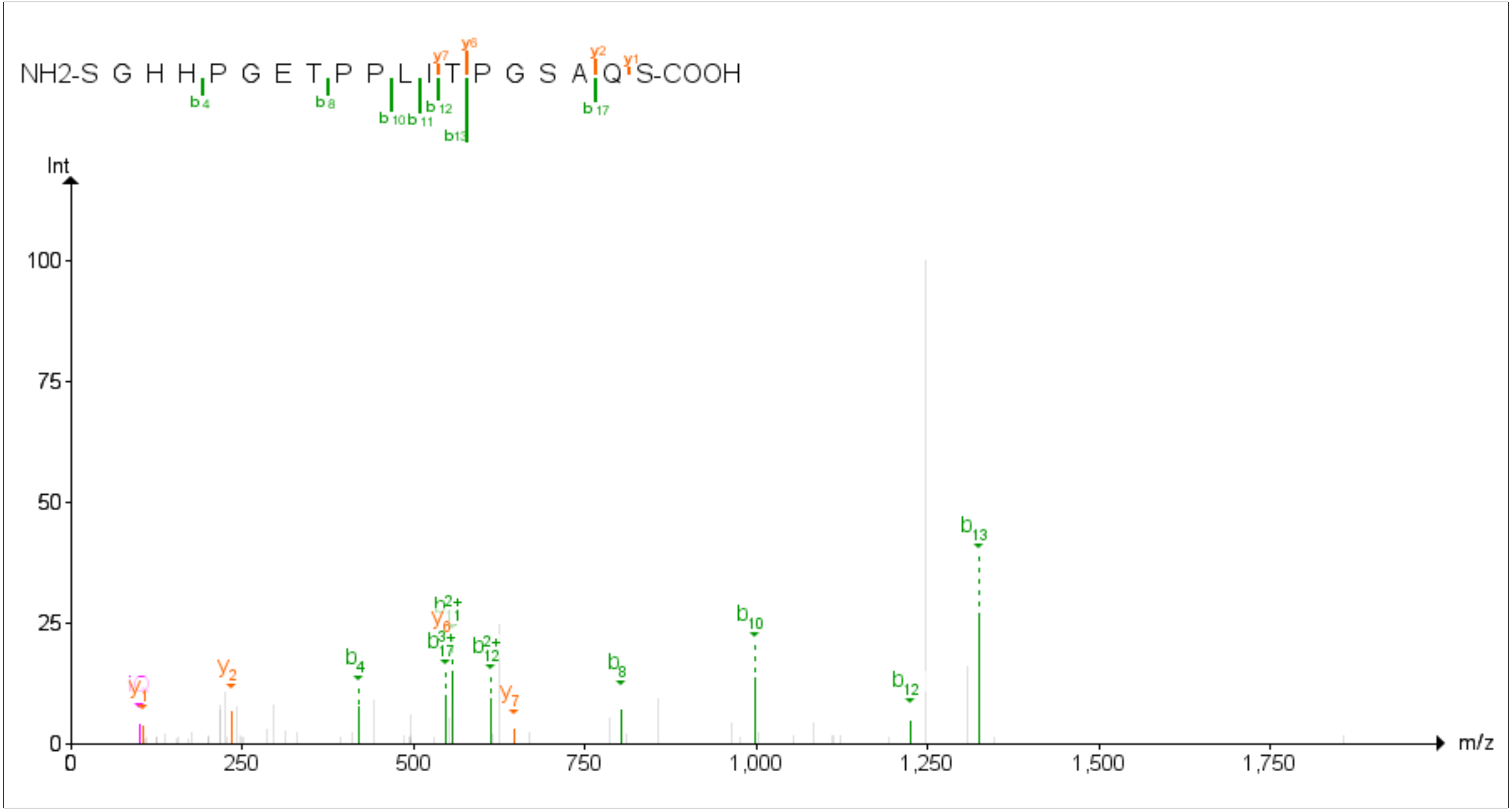

NH2-S G H H P G E T P P L I T P G S A Q S-COOH
b4
b8
b10
b11
b12
b13
b17
y7
y6
y2
y1
Int
100
75
50
25
0
m/z
0
250
500
750
1,000
1,250
1,500
1,750

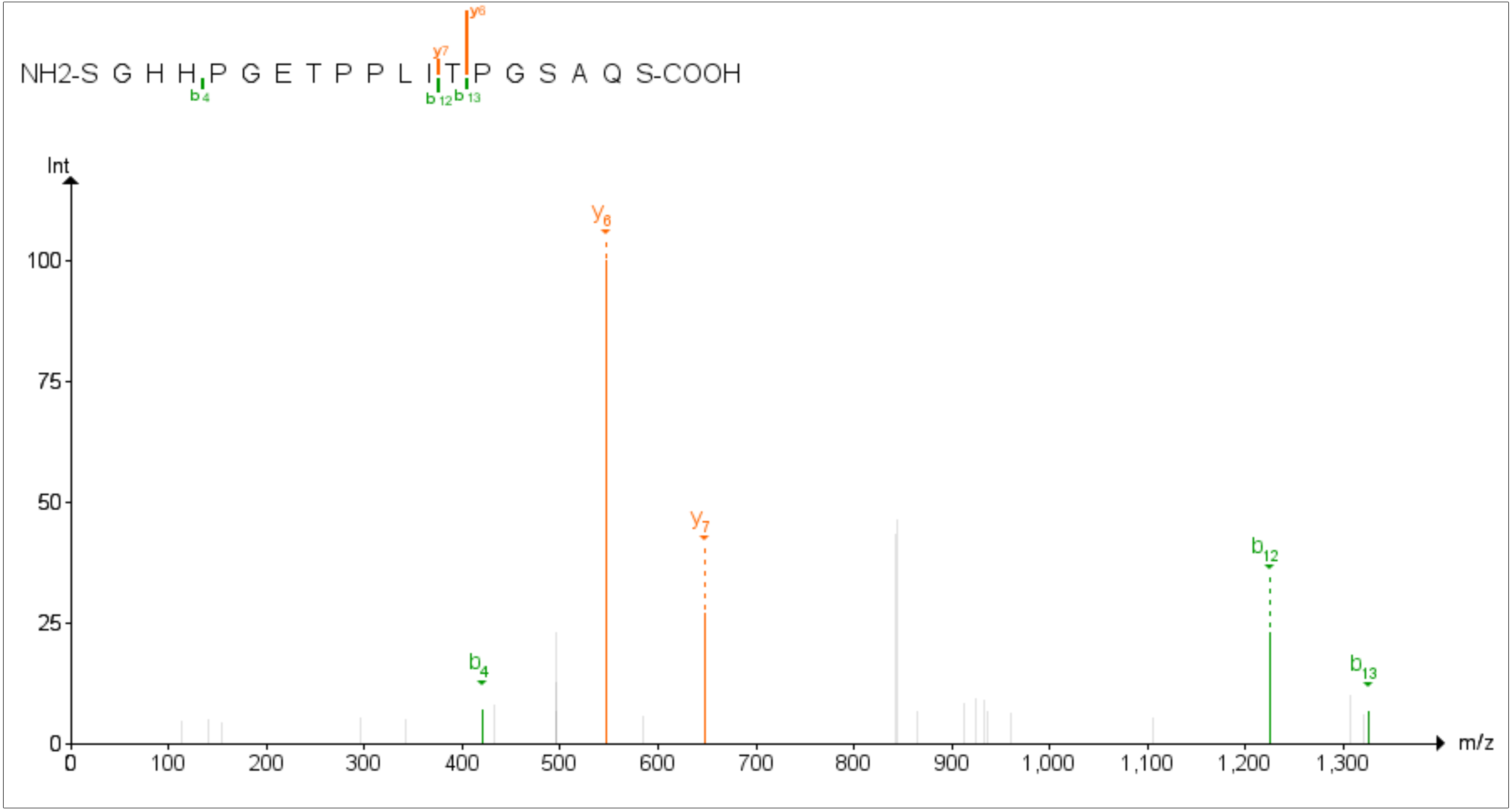

NH2-S G H H P G E T P P L I T P G S A Q S-COOH
b4
b12
b13
y7
y6
Int
100
75
50
25
0
y6
y7
b4
b12
b13
0
100
200
300
400
500
600
700
800
900
1,000
1,100
1,200
1,300
m/z

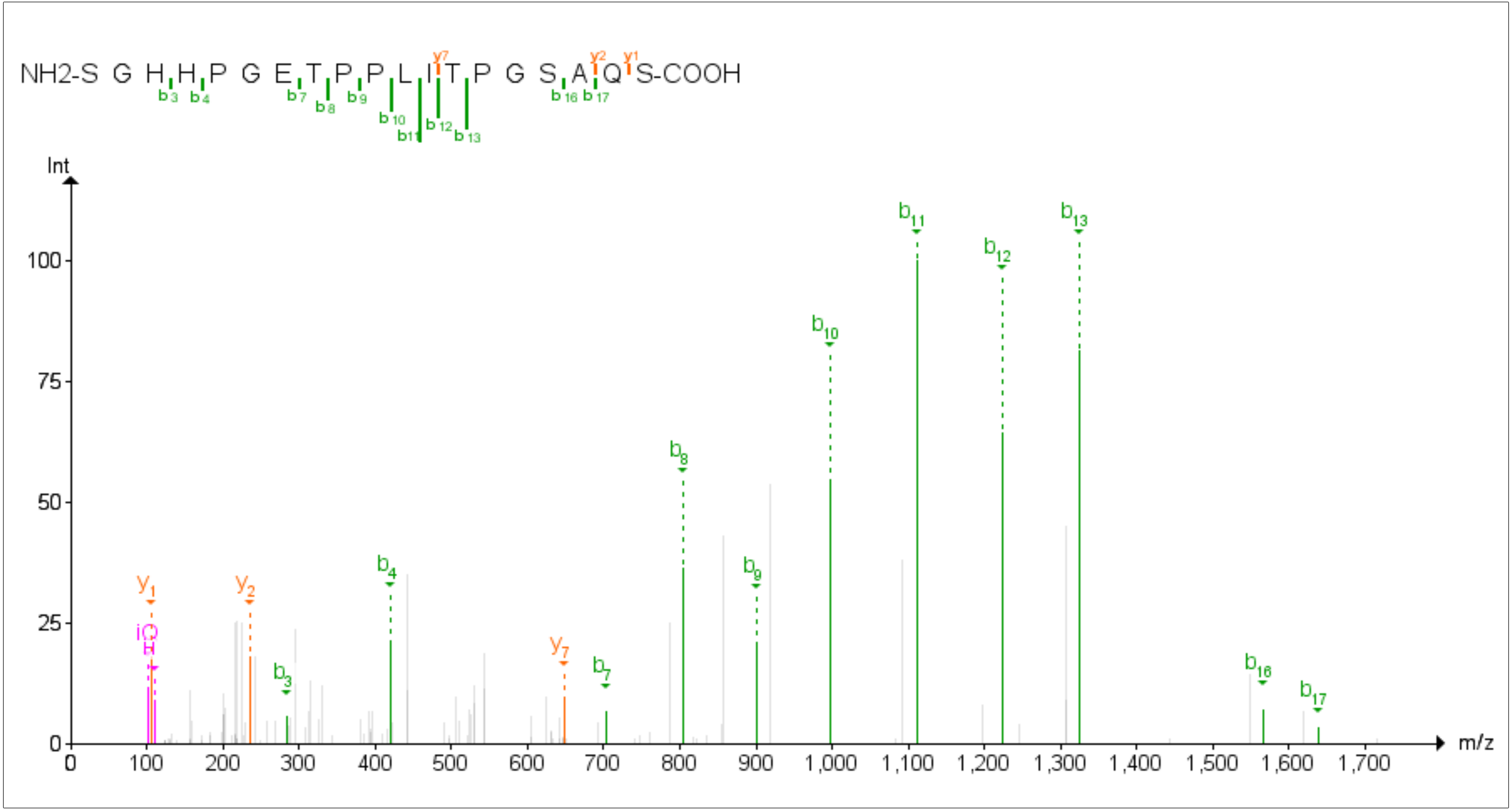
NH2-S G H H P G E T P P L I T P G S A Q S-COOH
Int
m/z
y1
y2
y7
iQ
H
b3
b4
b7
b8
b9
b10
b11
b12
b13
b16
b17

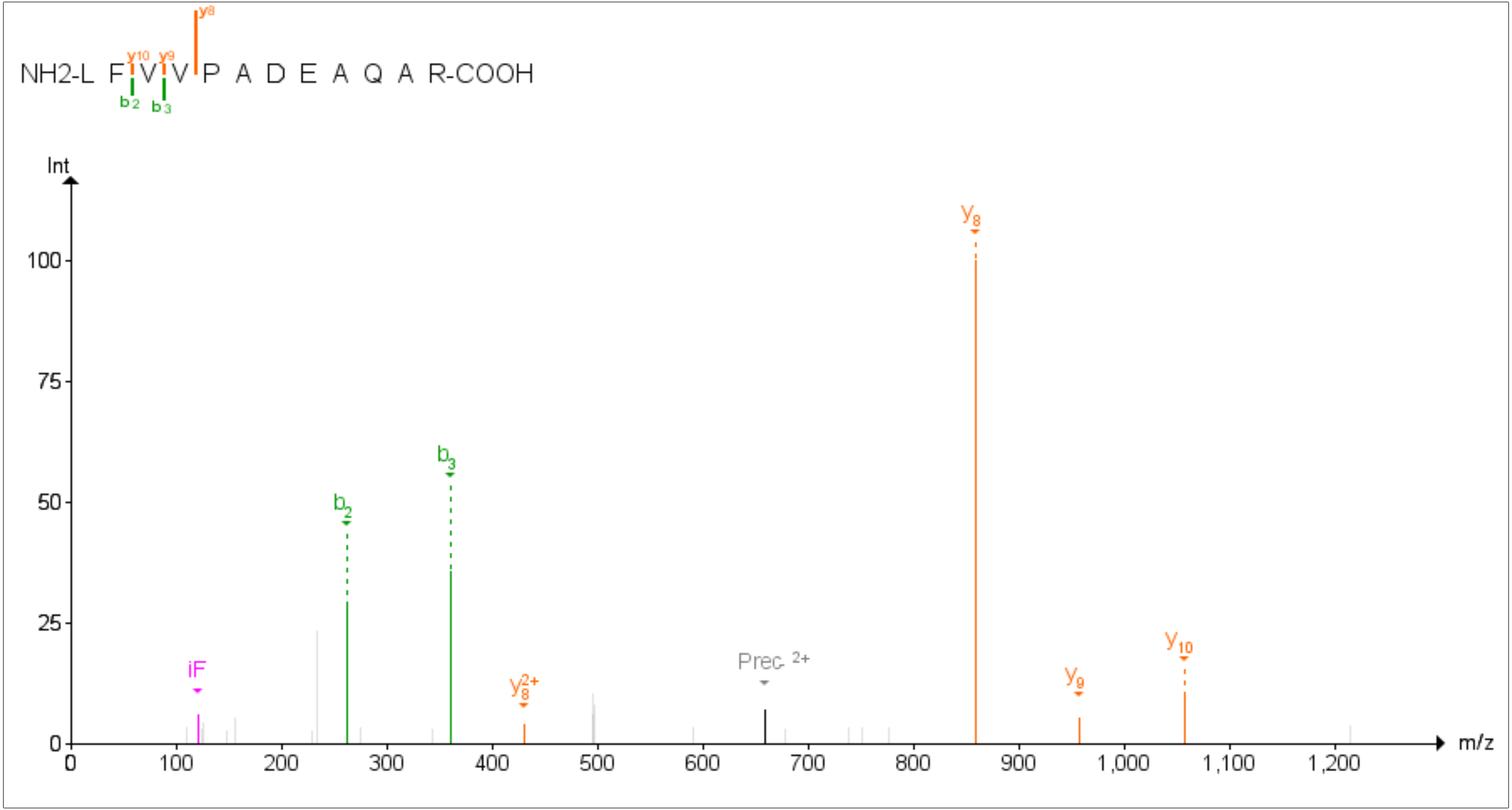

NH2-L F V V P A D E A Q A R-COOH
y10
y9
y8
b2
b3
Int
100
75
50
25
0
y8
b3
b2
iF
y8 2+
Prec 2+
y9
y10
0
100
200
300
400
500
600
700
800
900
1,000
1,100
1,200
m/z

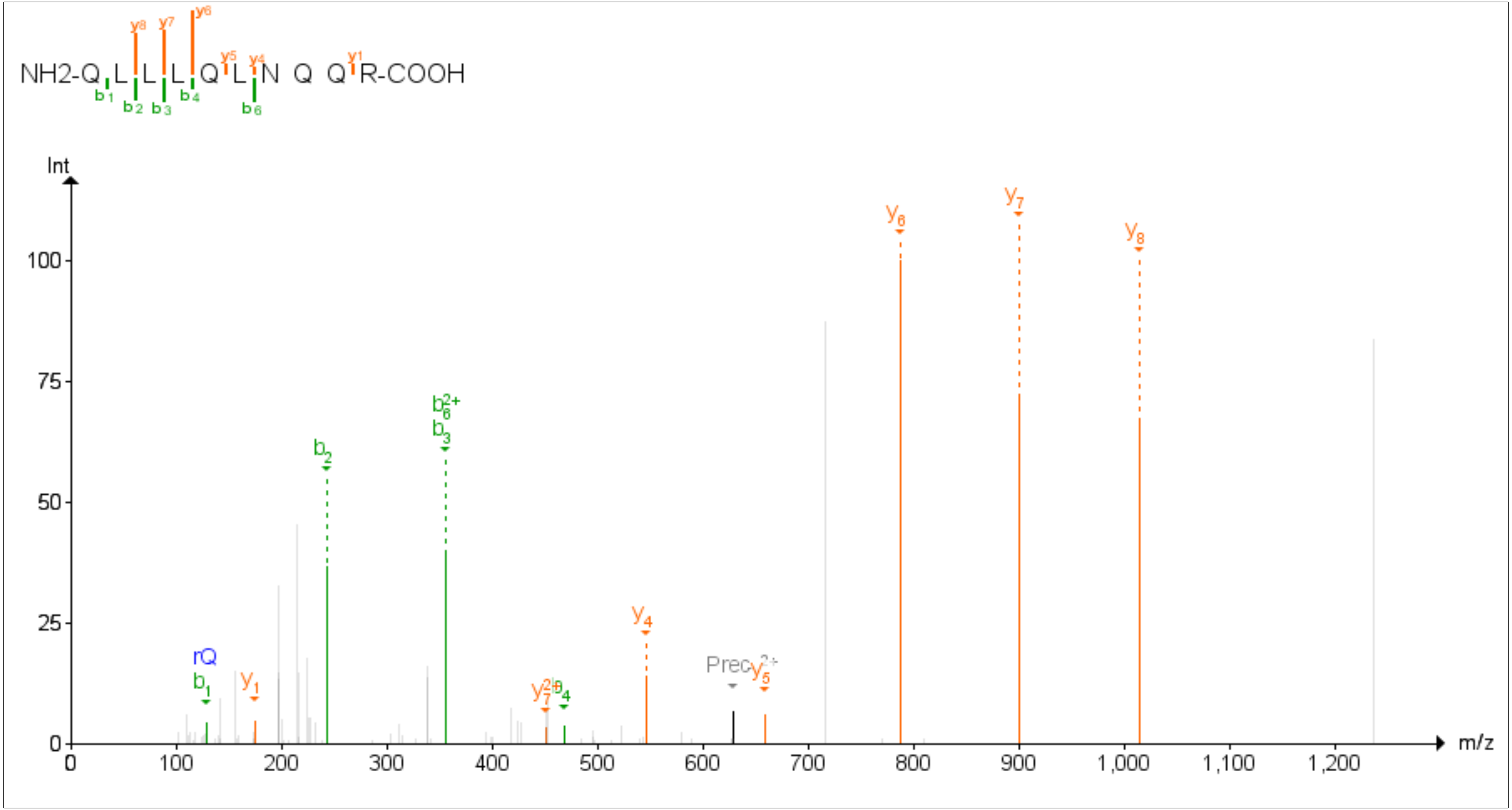

NH2-Q L L L Q L N Q Q R-COOH
y8 y7 y6 y5 y4 y1
b1 b2 b3 b4 b6
Int
100
75
50
25
0
m/z
0
100
200
300
400
500
600
700
800
900
1,000
1,100
1,200
y6
y7
y8
b6 2+
b3
b2
y4
rQ
b1
y1
y7 2+
b4
Prec 2+
y5

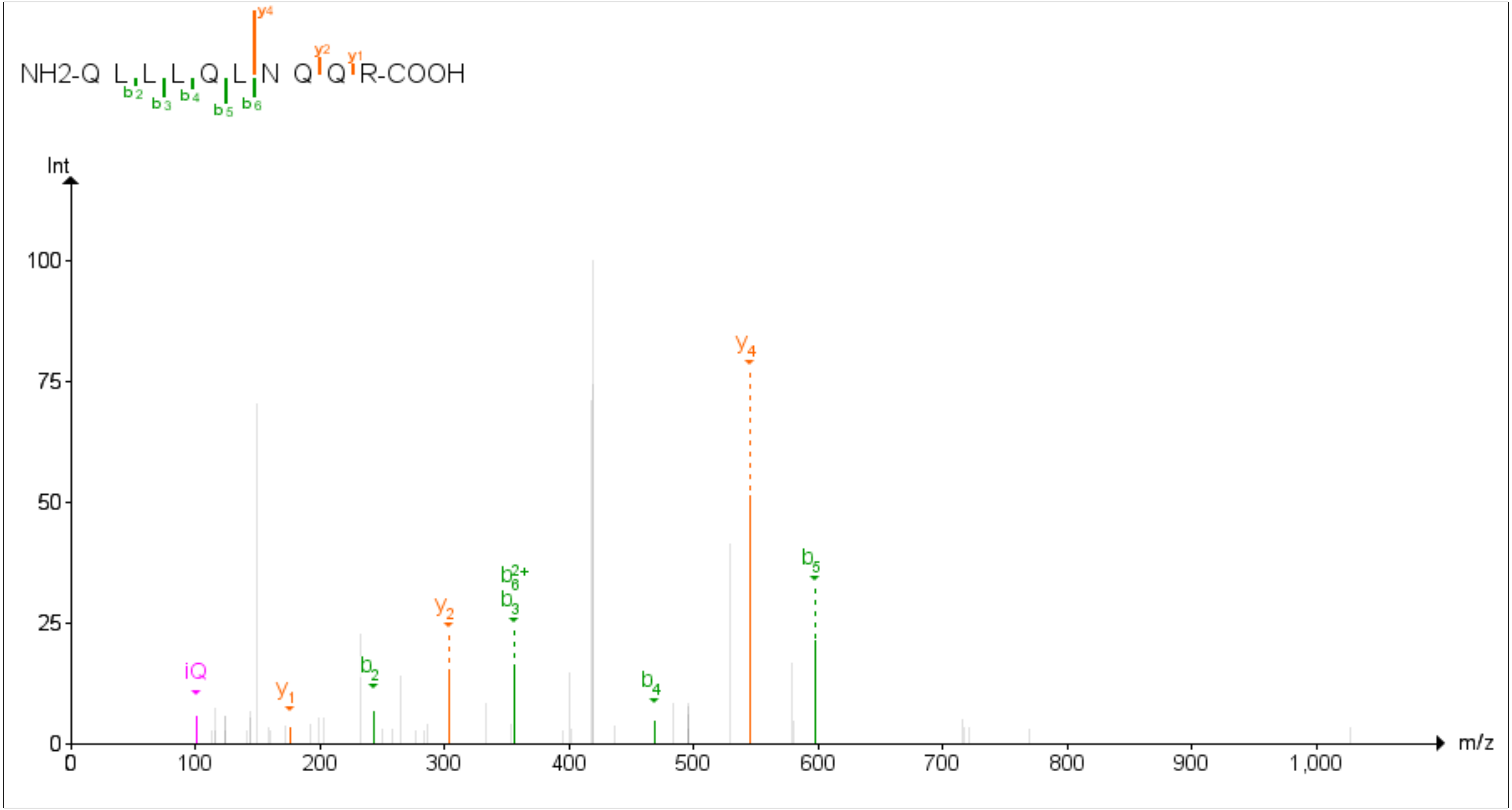

NH2-Q L L L Q L N Q Q R-COOH
y4
y2
y1
b2
b3
b4
b5
b6
Int
100
75
50
25
0
iQ
y_1
b_2
y_2
b_6^{2+}
b_3
b_4
y_4
b_5
0
100
200
300
400
500
600
700
800
900
1,000
m/z

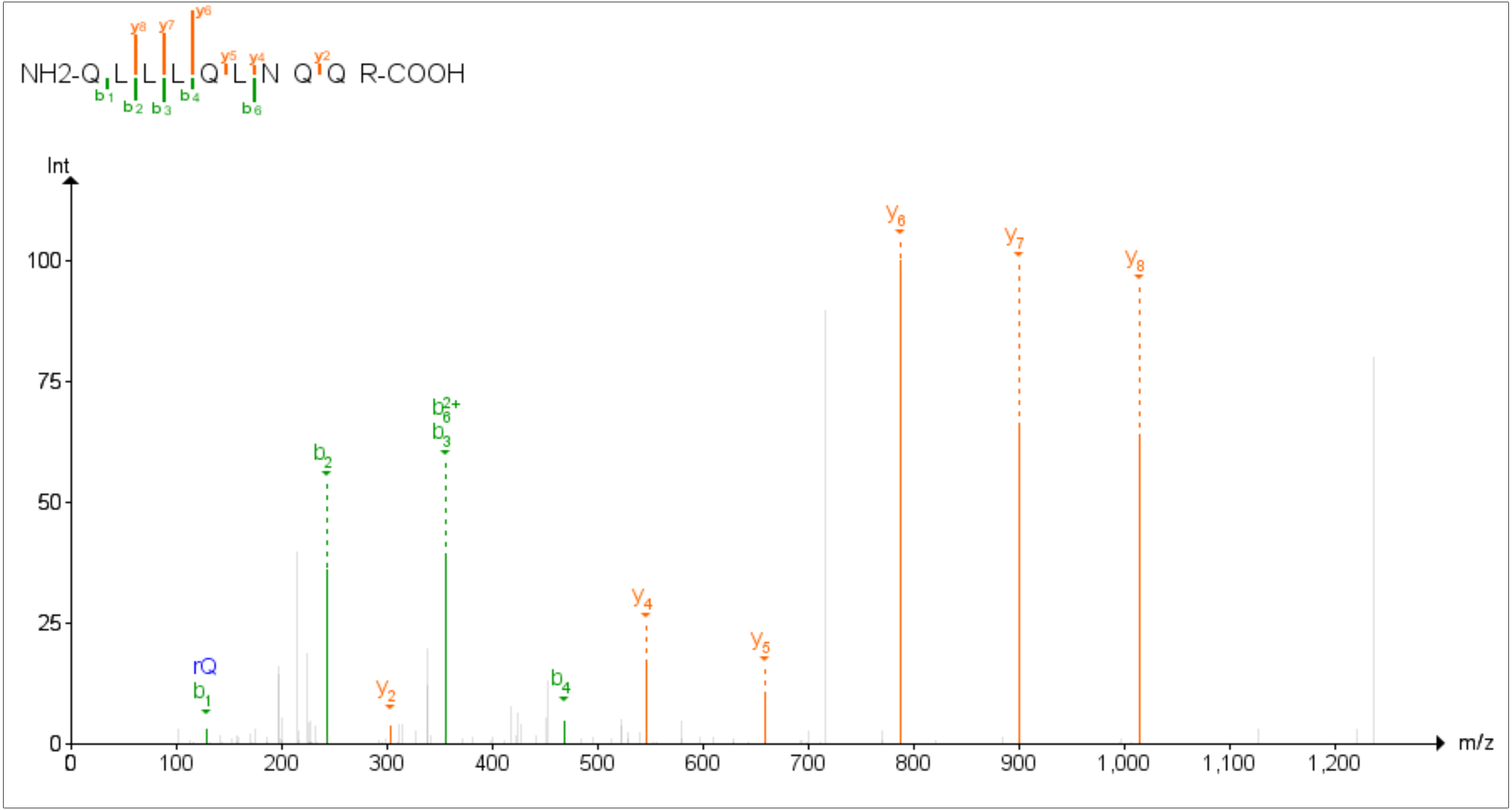
NH2-Q L L L Q L N Q Q R-COOH
y8 y7 y6 y5 y4 y2
b1 b2 b3 b4 b6
Int
100
75
50
25
0
rQ
b1
b2
y2
b3
b6 2+
b4
y4
y5
y6
y7
y8
0 100 200 300 400 500 600 700 800 900 1,000 1,100 1,200
m/z

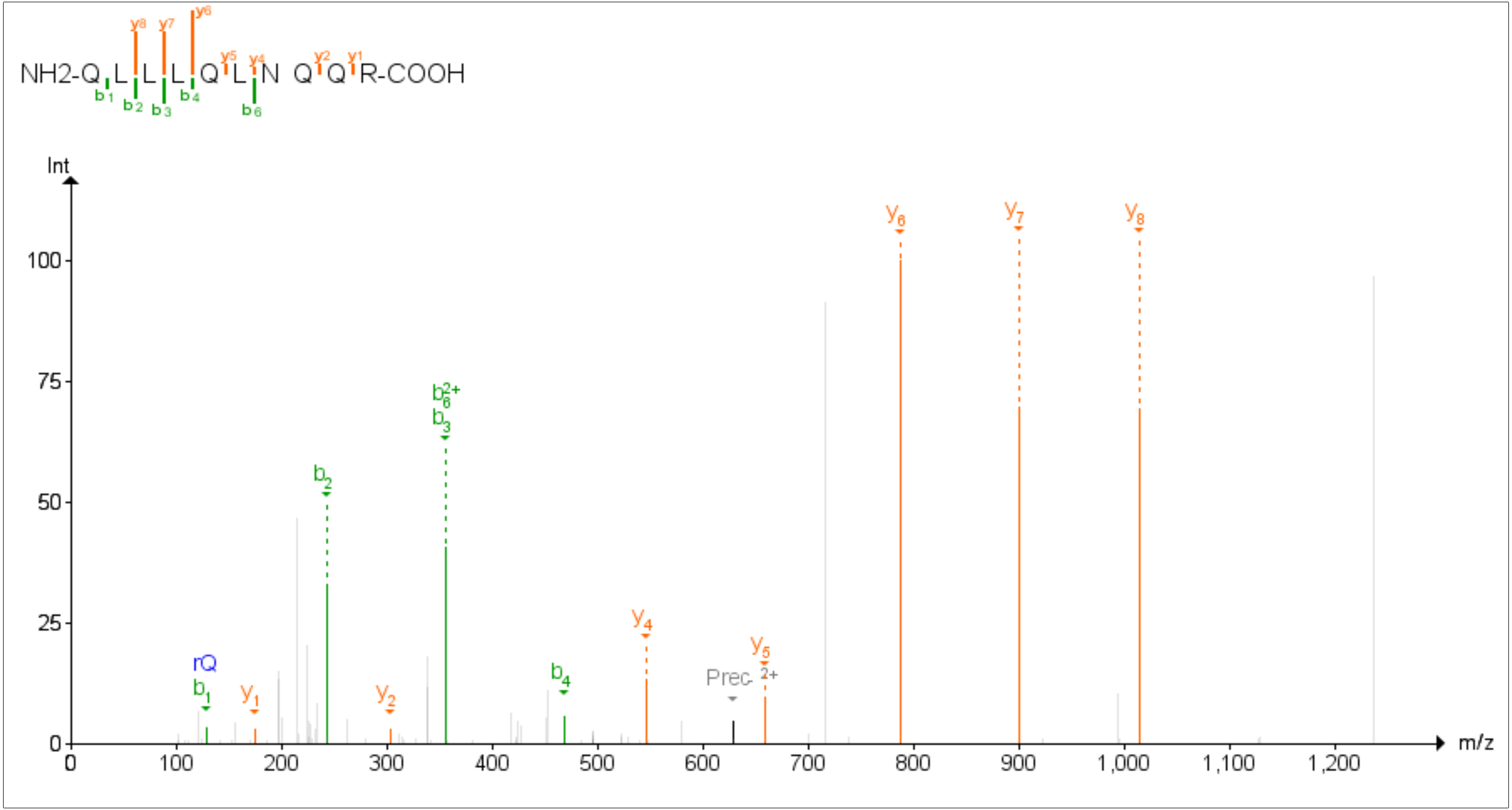

NH2-Q L L L Q L N Q Q R-COOH
y8 y7 y6 y5 y4 y2 y1
b1 b2 b3 b4 b6
Int
m/z
y6
y7
y8
b6 2+
b3
b2
rQ
b1
y1
y2
b4
y4
y5
Prec 2+
0
25
50
75
100
0
100
200
300
400
500
600
700
800
900
1,000
1,100
1,200

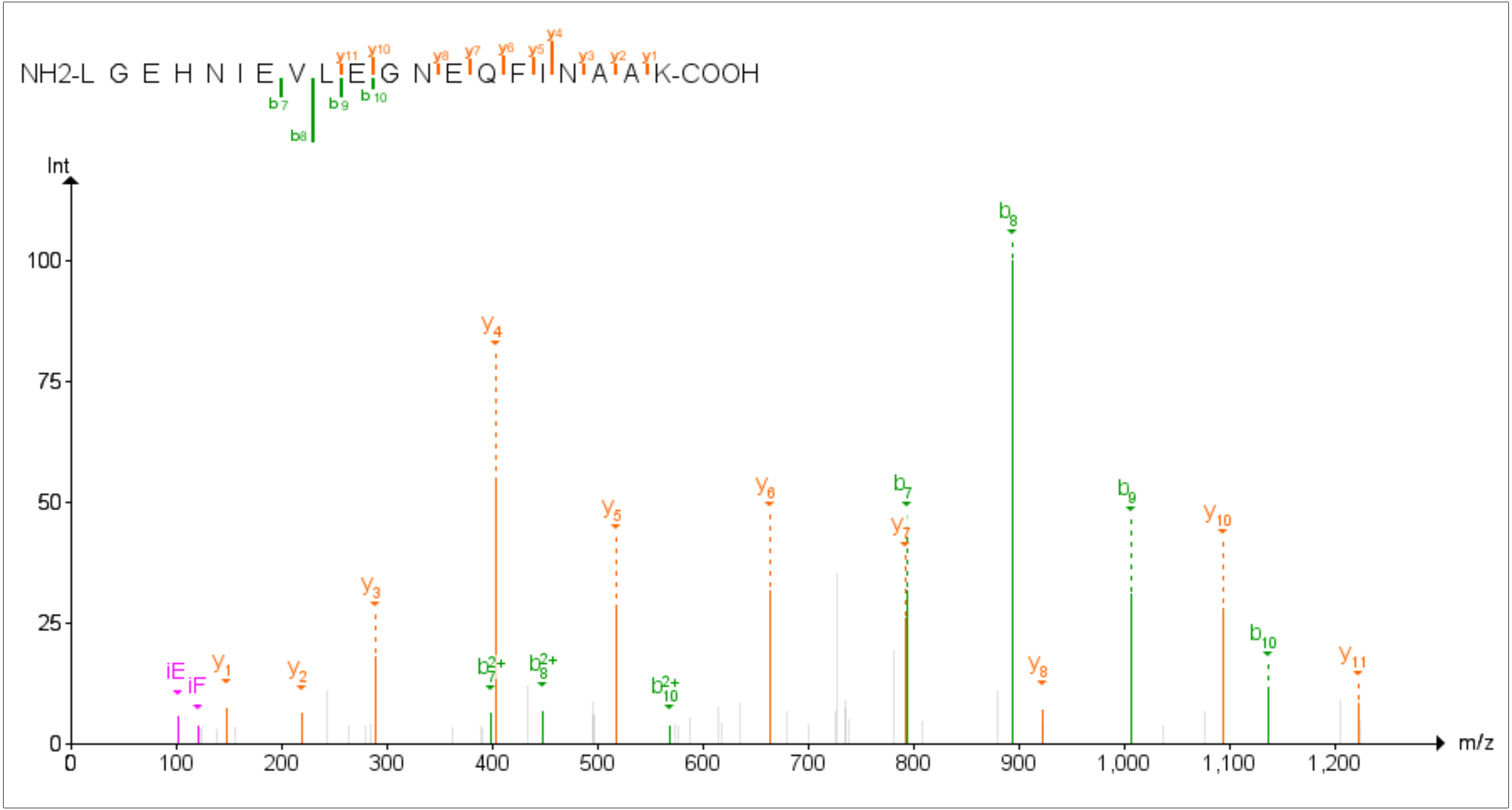

NH2-L G E H N I E V L E G N E Q F I N A A K-COOH
Int
100
75
50
25
0
m/z
0
100
200
300
400
500
600
700
800
900
1,000
1,100
1,200

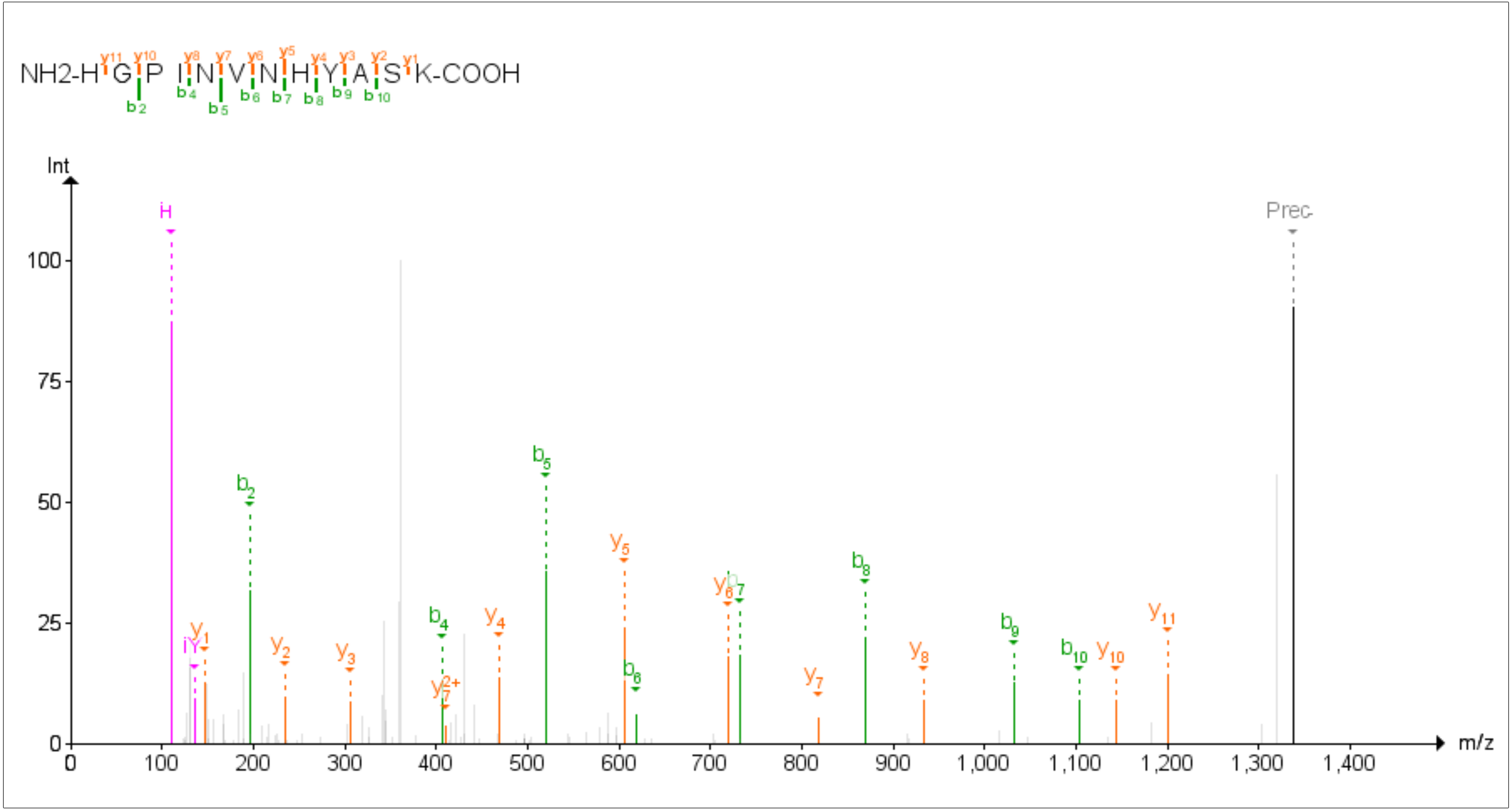

NH2-H G P I N V N H Y A S K-COOH
y11 y10 y8 y7 y6 y5 y4 y3 y2 y1
b2 b4 b5 b6 b7 b8 b9 b10
Int
100
75
50
25
0
iH
iY
Prec
b2 b4 b5 b6 b7 b8 b9 b10
y1 y2 y3 y4 y5 y6 y7 y8 y10 y11
y7 2+
0
100
200
300
400
500
600
700
800
900
1,000
1,100
1,200
1,300
1,400
m/z

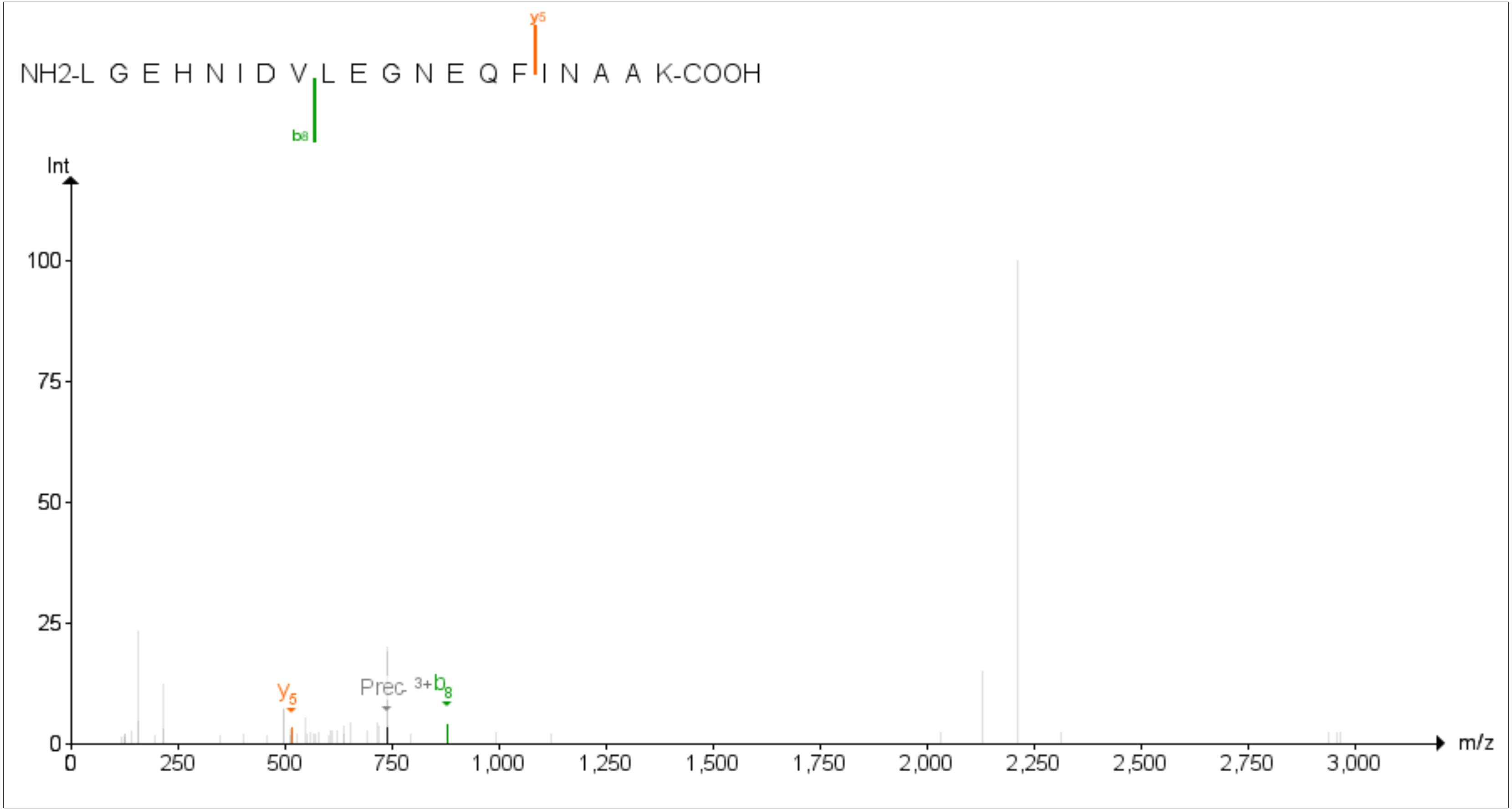

NH2-L G E H N I D V L E G N E Q F I N A A K-COOH
y5
b8
Int
100
75
50
25
0
y5
Prec- 3+
b8
0
250
500
750
1,000
1,250
1,500
1,750
2,000
2,250
2,500
2,750
3,000
m/z

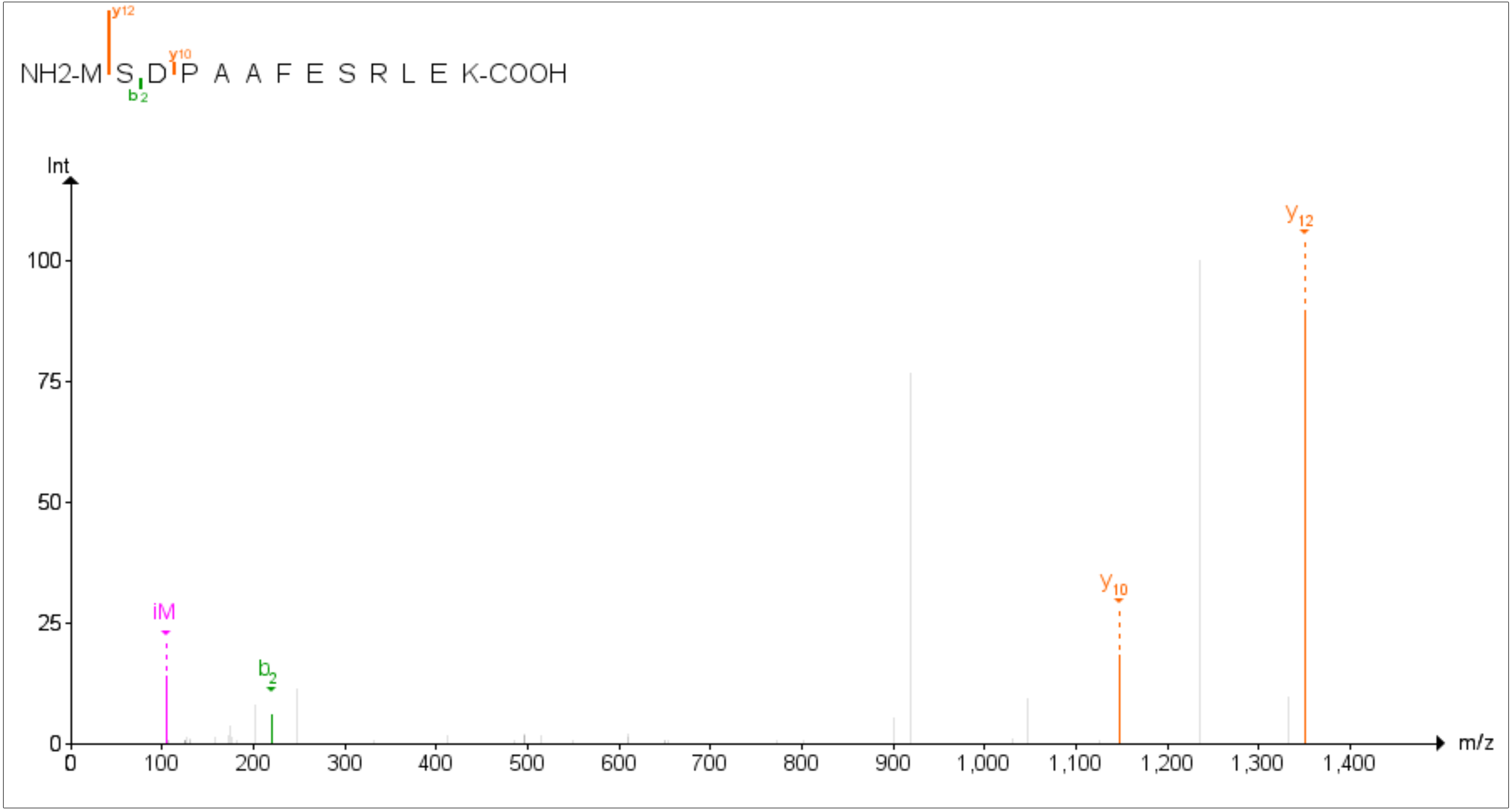

y12
y10
NH2-M S D P A A F E S R L E K-COOH
b2
Int
100
75
50
25
0
y12
y10
iM
b2
0
100
200
300
400
500
600
700
800
900
1,000
1,100
1,200
1,300
1,400
m/z

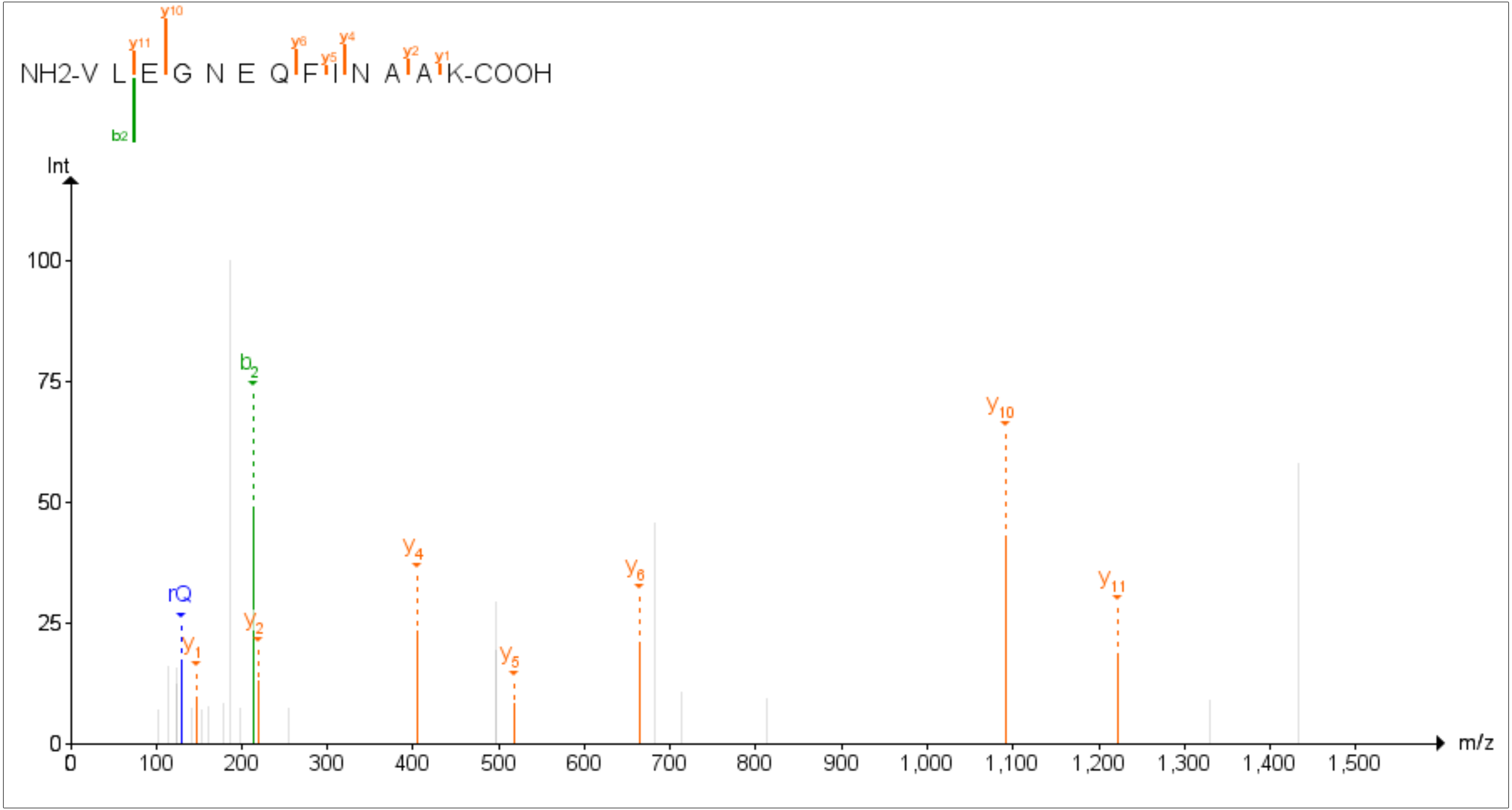

NH2-V L E G N E Q F I N A A K-COOH
y10
y11
y6
y5
y4
y2
y1
b2
Int
100
75
50
25
0
b2
y10
y4
y6
y11
rQ
y1
y2
y5
0
100
200
300
400
500
600
700
800
900
1,000
1,100
1,200
1,300
1,400
1,500
m/z

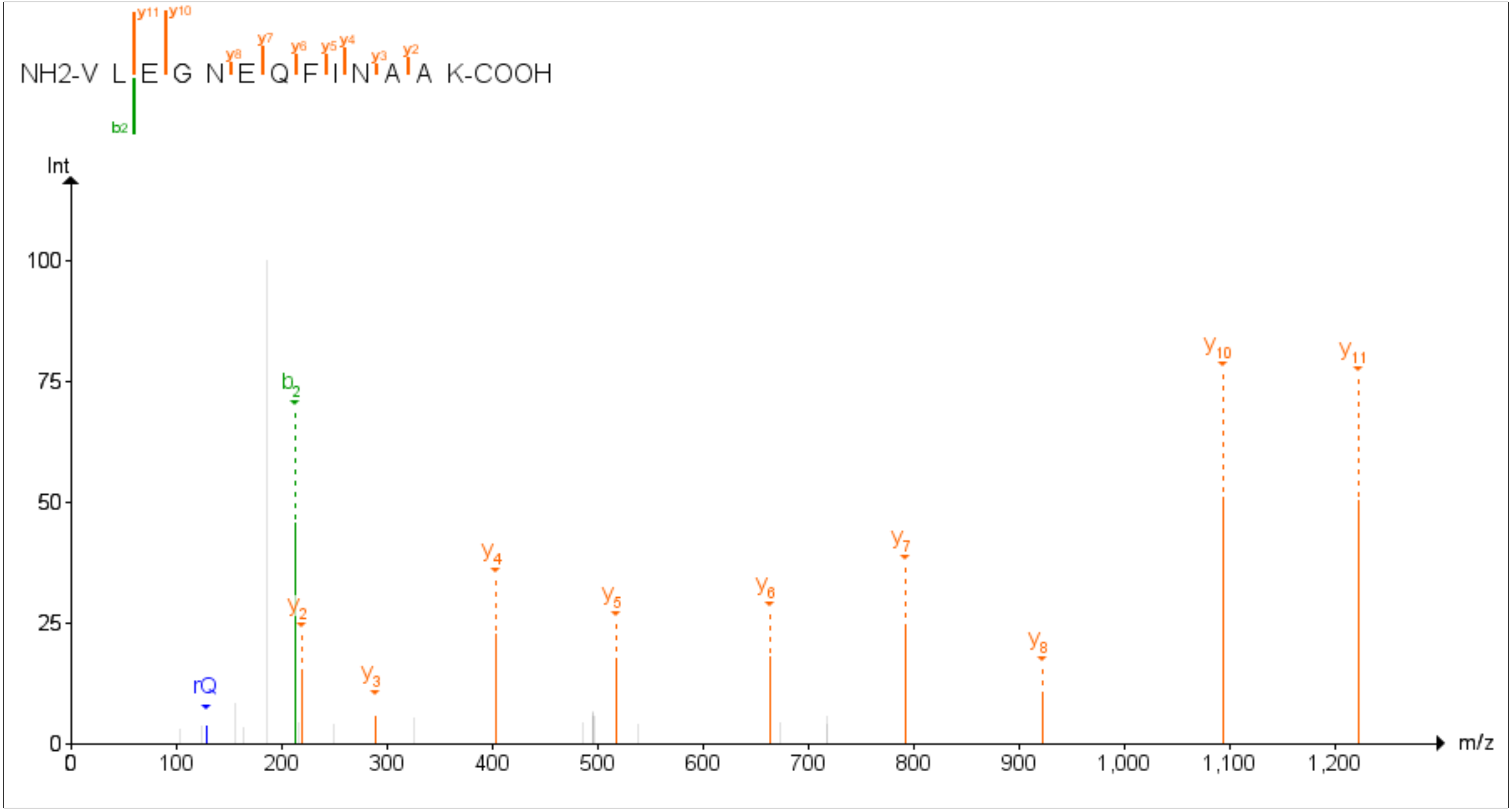

NH2-V L E G N E Q F I N A A K-COOH
y11 y10 y8 y7 y6 y5 y4 y3 y2
b2
Int
100
75
50
25
0
b2
y2
y3
y4
y5
y6
y7
y8
y10
y11
rQ
0 100 200 300 400 500 600 700 800 900 1,000 1,100 1,200
m/z

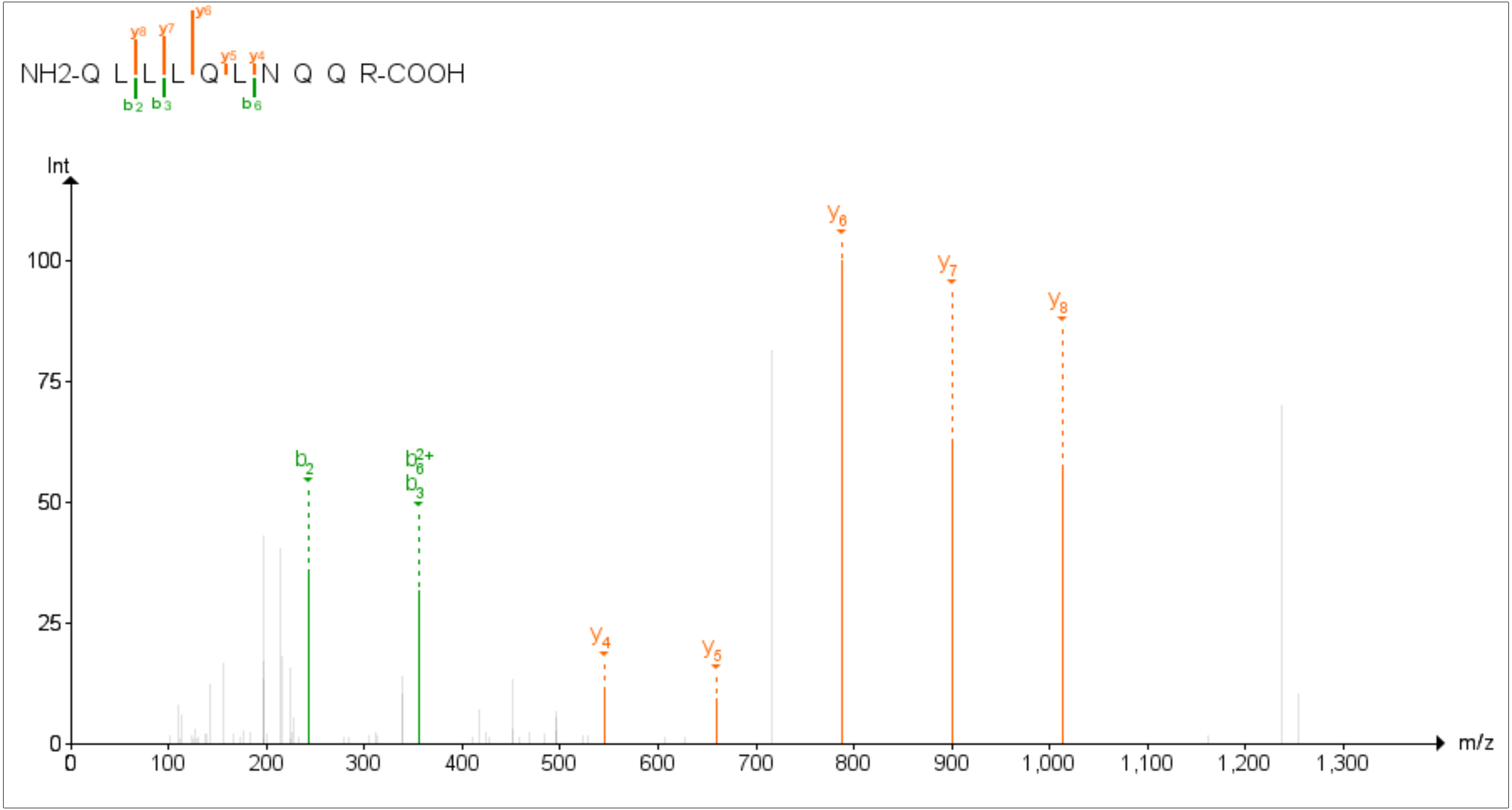

NH2-Q L L L Q L N Q Q R-COOH
y8 y7 y6 y5 y4
b2 b3 b6
Int
100
75
50
25
0
b2
b6 2+
b3
y4
y5
y6
y7
y8
0 100 200 300 400 500 600 700 800 900 1,000 1,100 1,200 1,300
m/z

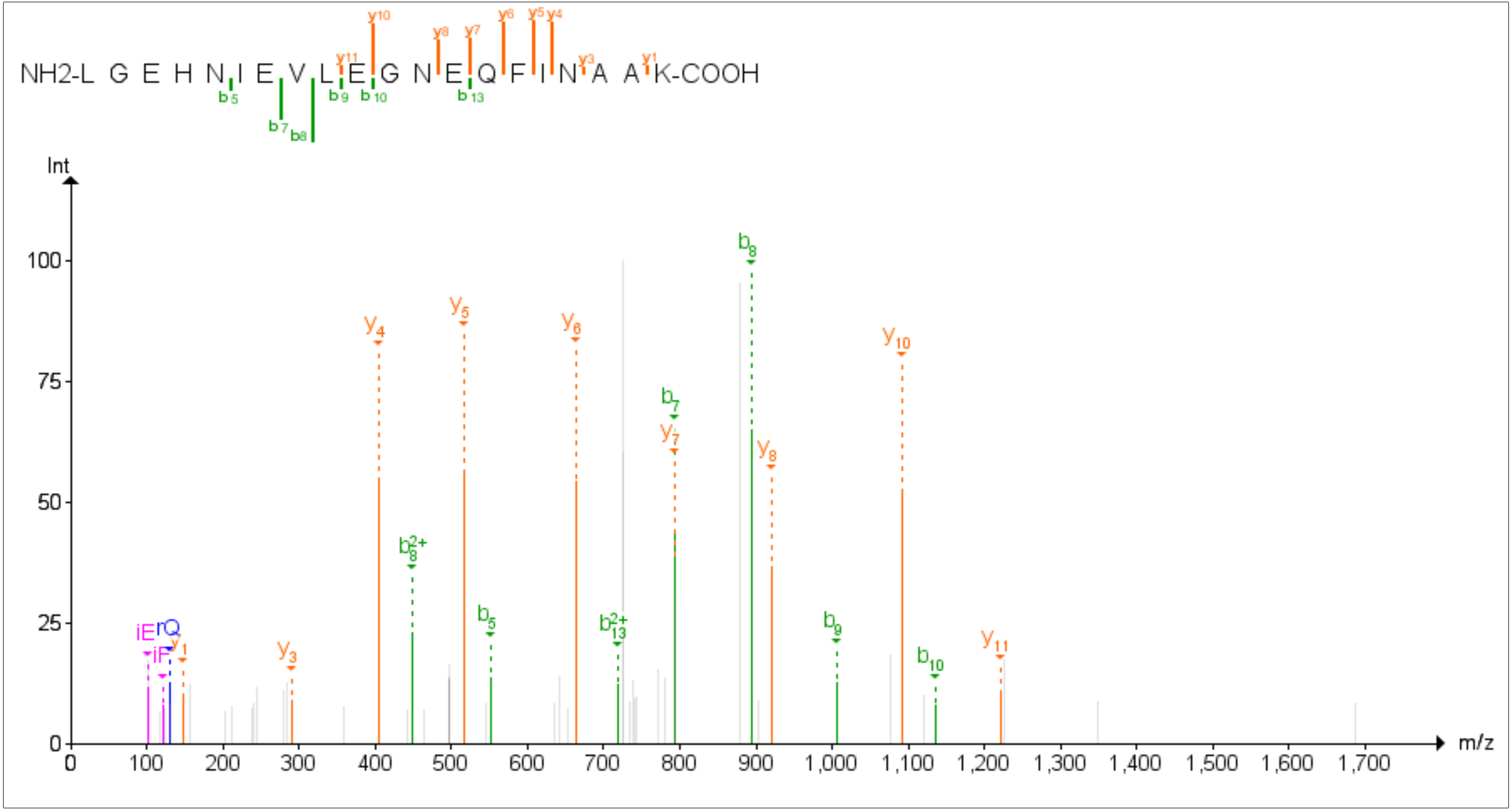
NH2-L G E H N I E V L E G N E Q F I N A A K-COOH
Int
m/z
b8
y4
y5
y6
y10
b7
y7
y8
b8 2+
b5
b13 2+
b9
b10
y11
y3
y1
iE
rQ
iF

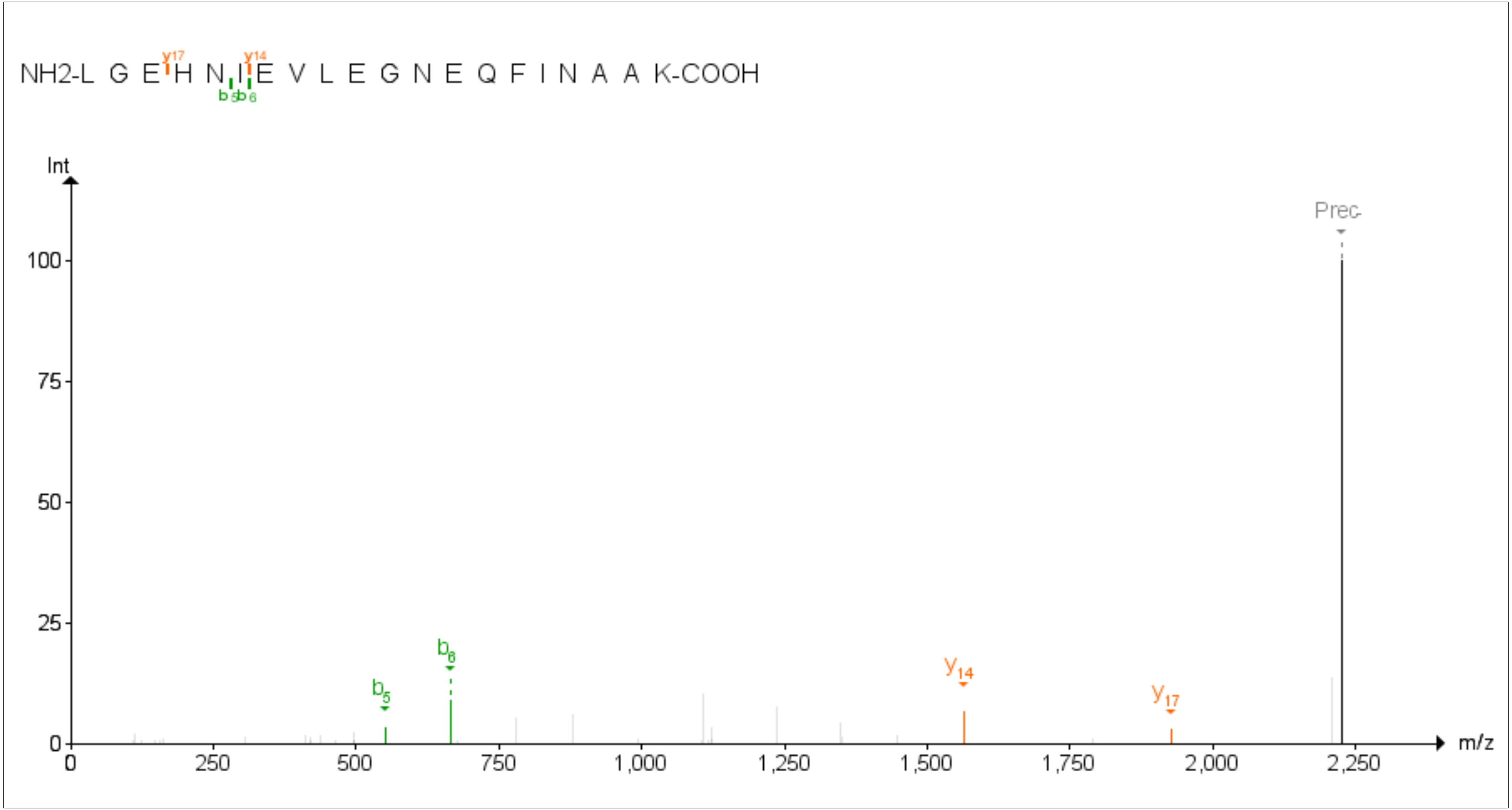
NH2-L G E H N I E V L E G N E Q F I N A A K-COOH
y17
y14
b5
b6
Int
100
75
50
25
0
Prec
b5
b6
y14
y17
0
250
500
750
1,000
1,250
1,500
1,750
2,000
2,250
m/z

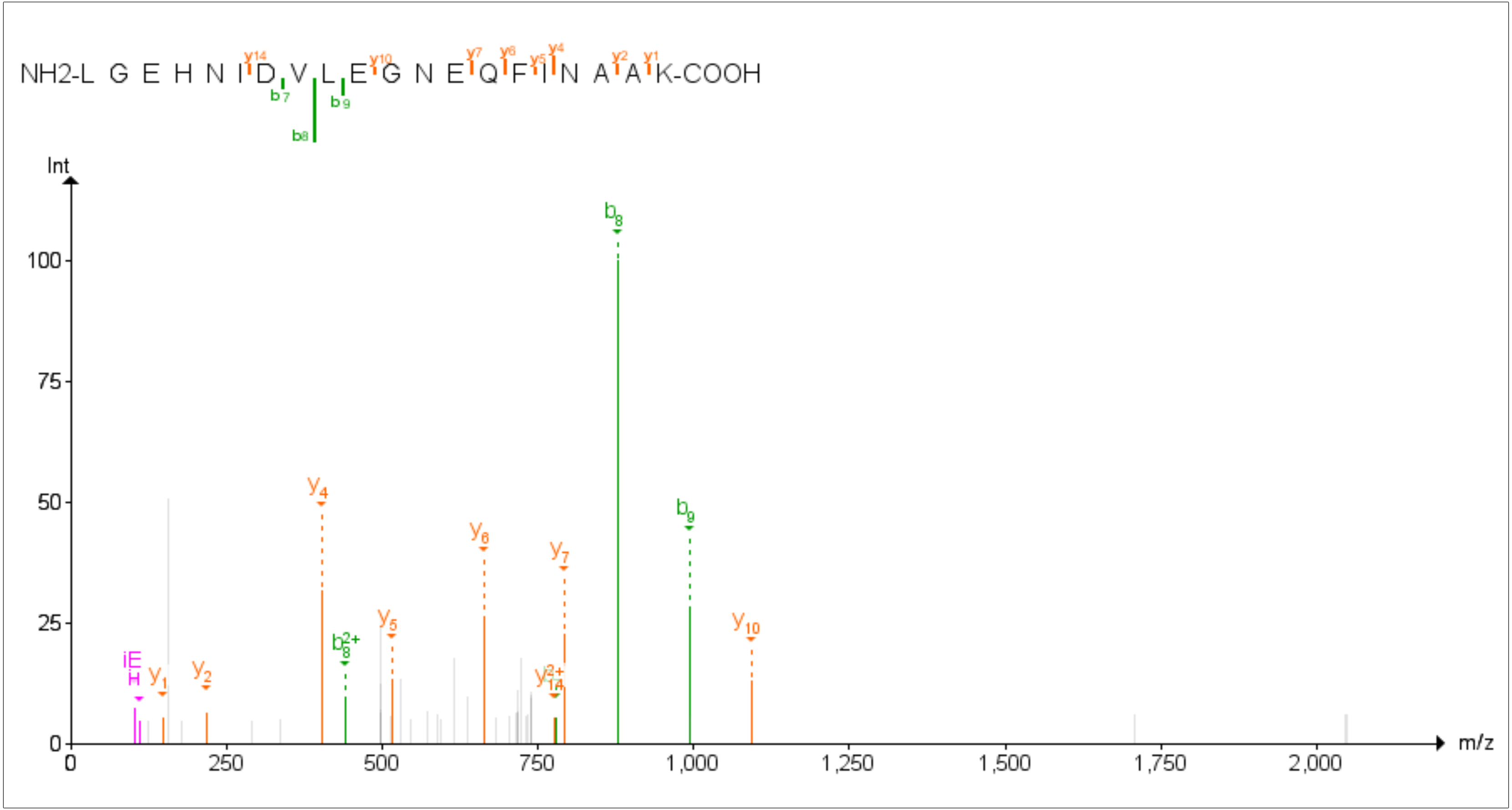
NH2-L G E H N I D V L E G N E Q F I N A A K-COOH
y14
y10
y7
y6
y5
y4
y2
y1
b7
b9
b8
Int
100
75
50
25
0
b8
b9
y4
y6
y7
y5
y10
b8 2+
y14 2+
iE
H
y1
y2
0
250
500
750
1,000
1,250
1,500
1,750
2,000
m/z

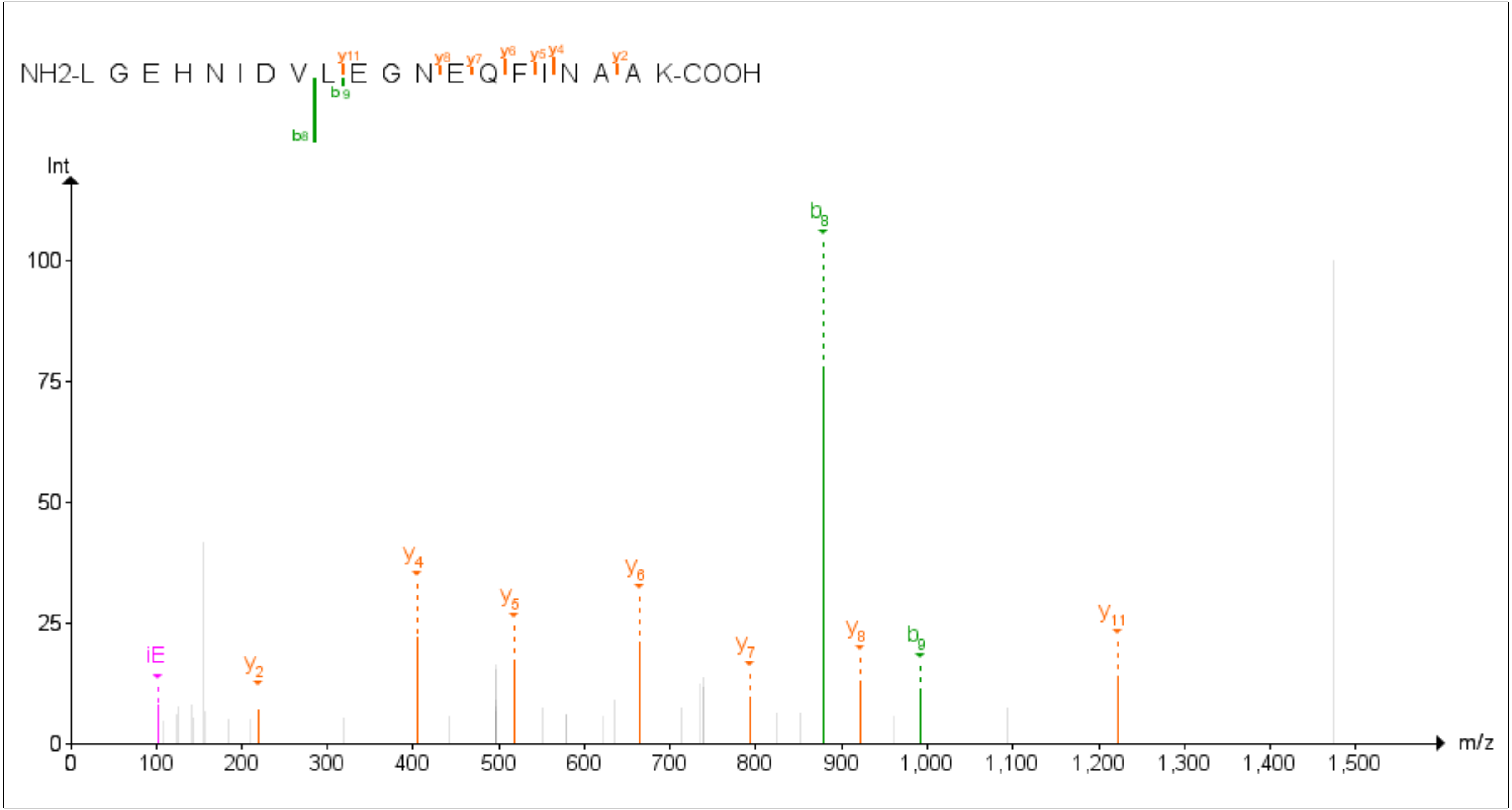

NH2-L G E H N I D V L I E G N E Q F I N A A K-COOH
y11
y8
y7
y6
y5
y4
y2
b9
b8
Int
100
75
50
25
0
b8
y4
y6
y5
y11
y8
b9
y7
iE
y2
0
100
200
300
400
500
600
700
800
900
1,000
1,100
1,200
1,300
1,400
1,500
m/z

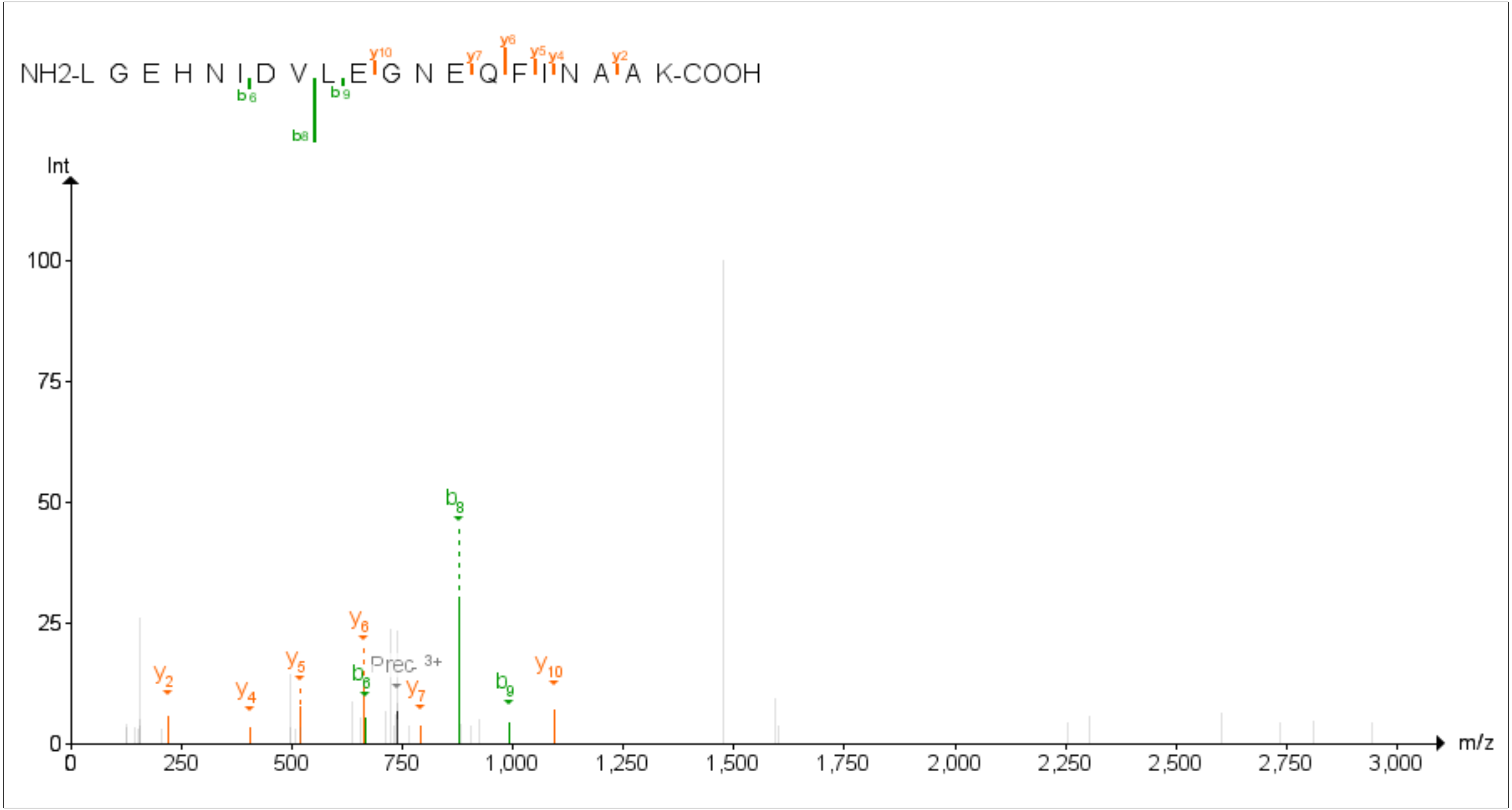
NH2-L G E H N I D V L E G N E Q F I N A A K-COOH
b6
b8
b9
y10
y7
y6
y5
y4
y2
Int
100
75
50
25
0
y2
y4
y5
y6
b6
Prec- 3+
y7
b8
b9
y10
0
250
500
750
1,000
1,250
1,500
1,750
2,000
2,250
2,500
2,750
3,000
m/z

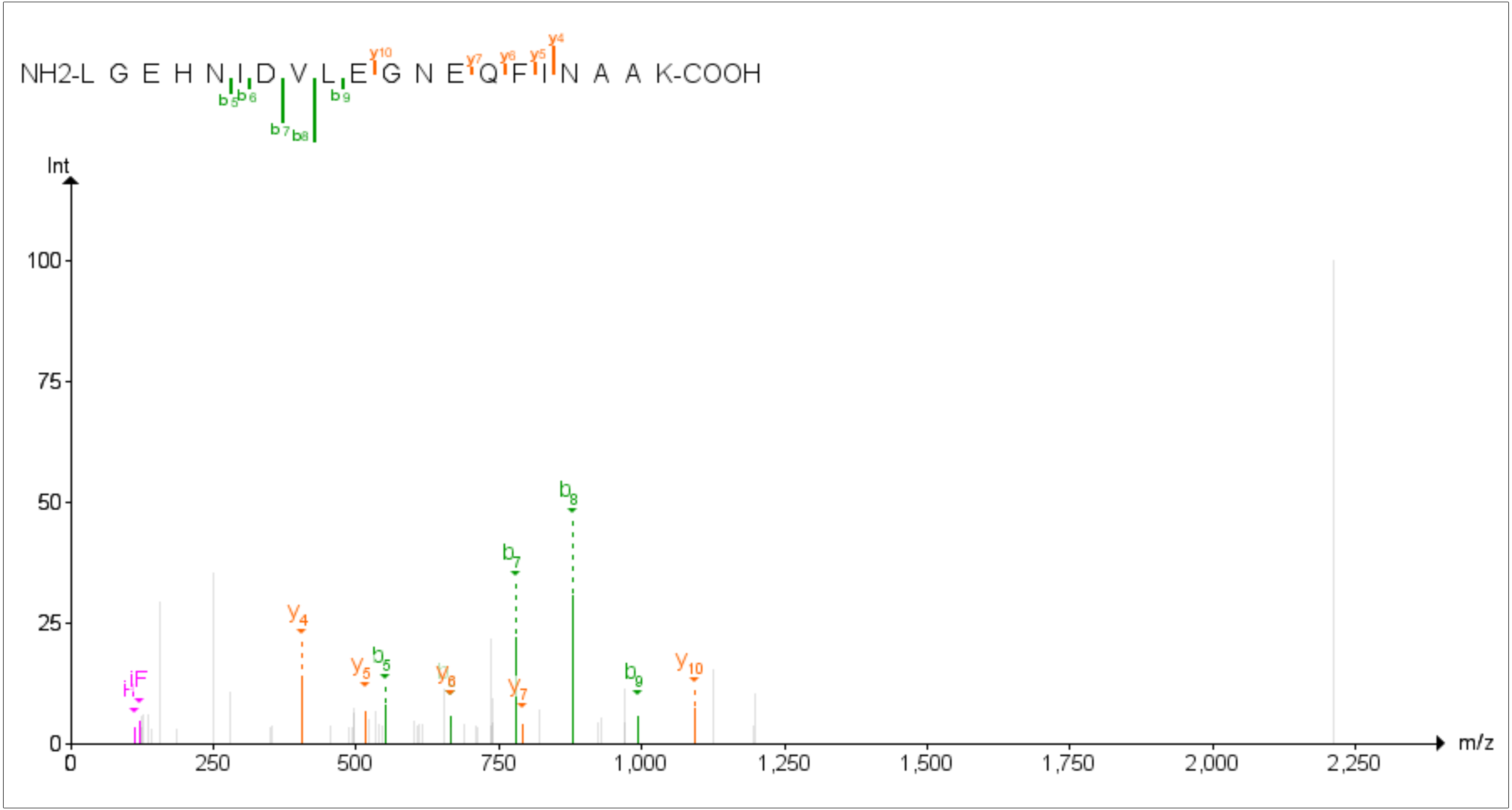
NH2-L G E H N I D V L E G N E Q F I N A A K-COOH
b5 b6 b7 b8 b9
y10 y7 y6 y5 y4
Int
100
75
50
25
0
m/z
0
250
500
750
1,000
1,250
1,500
1,750
2,000
2,250
iF
b8
b7
y4
y5
b5
y6
y7
b9
y10

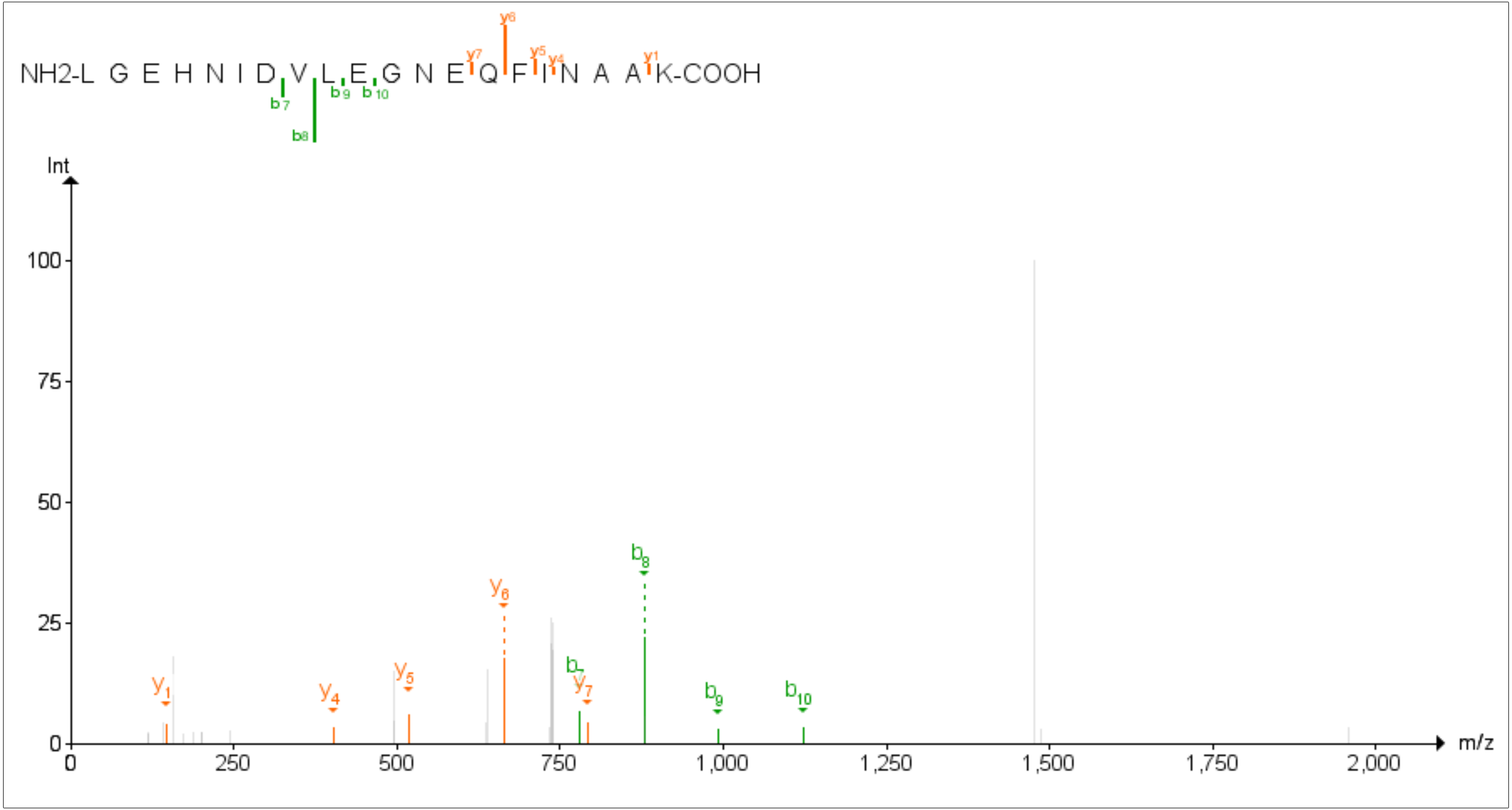
NH2-L G E H N I D V L E G N E Q F I N A A K-COOH
b7
b8
b9
b10
y7
y6
y5
y4
y1
Int
100
75
50
25
0
m/z
250
500
750
1,000
1,250
1,500
1,750
2,000
y1
y4
y5
y6
y7
b7
b8
b9
b10

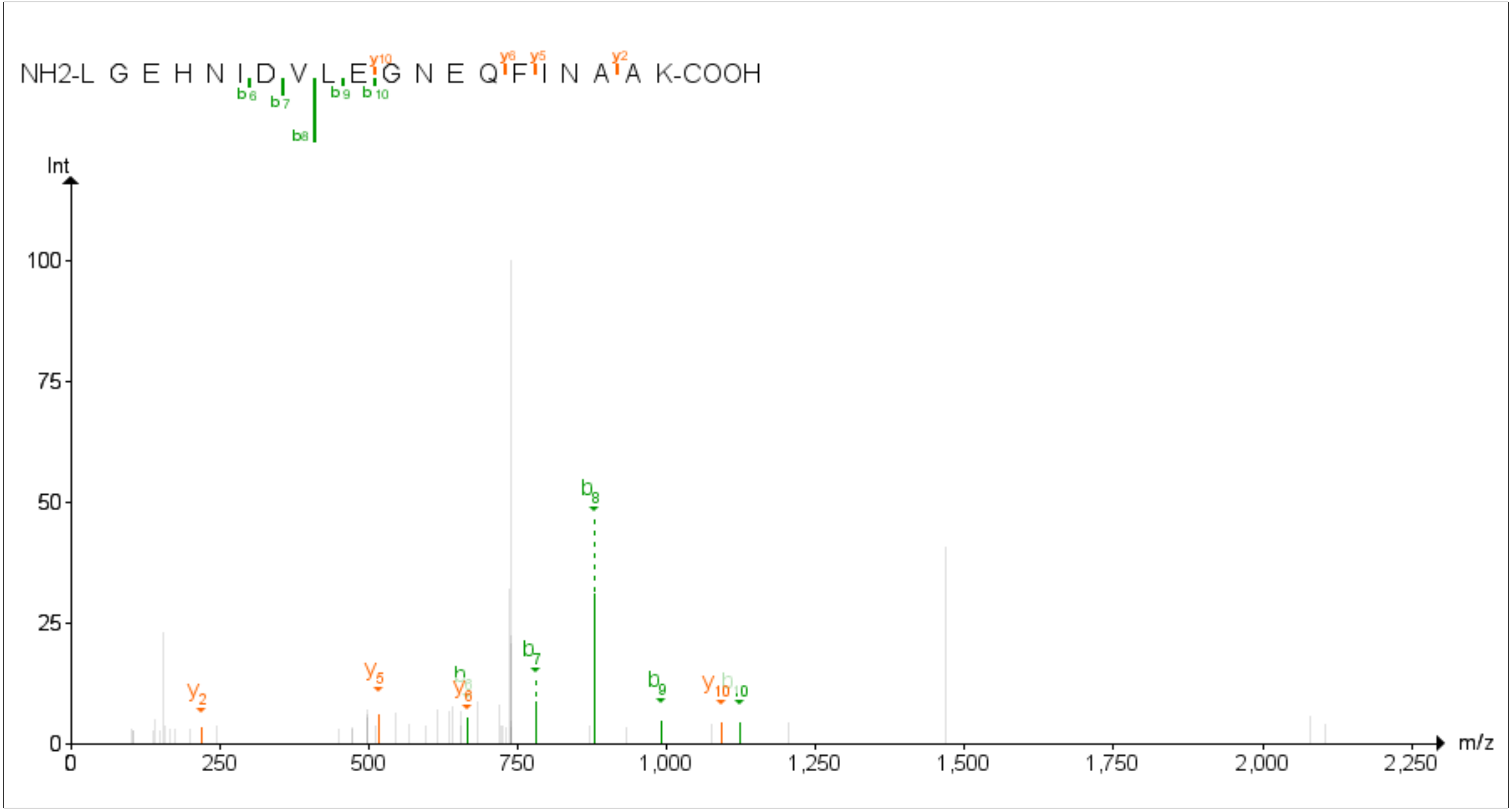

NH2-L G E H N I D V L E G N E Q F I N A A K-COOH
y10 y6 y5 y2
b6 b7 b8 b9 b10
Int
100
75
50
25
0
y2
y5
b8
y6
b7
b8
b9
y10
b10
0
250
500
750
1,000
1,250
1,500
1,750
2,000
2,250
m/z

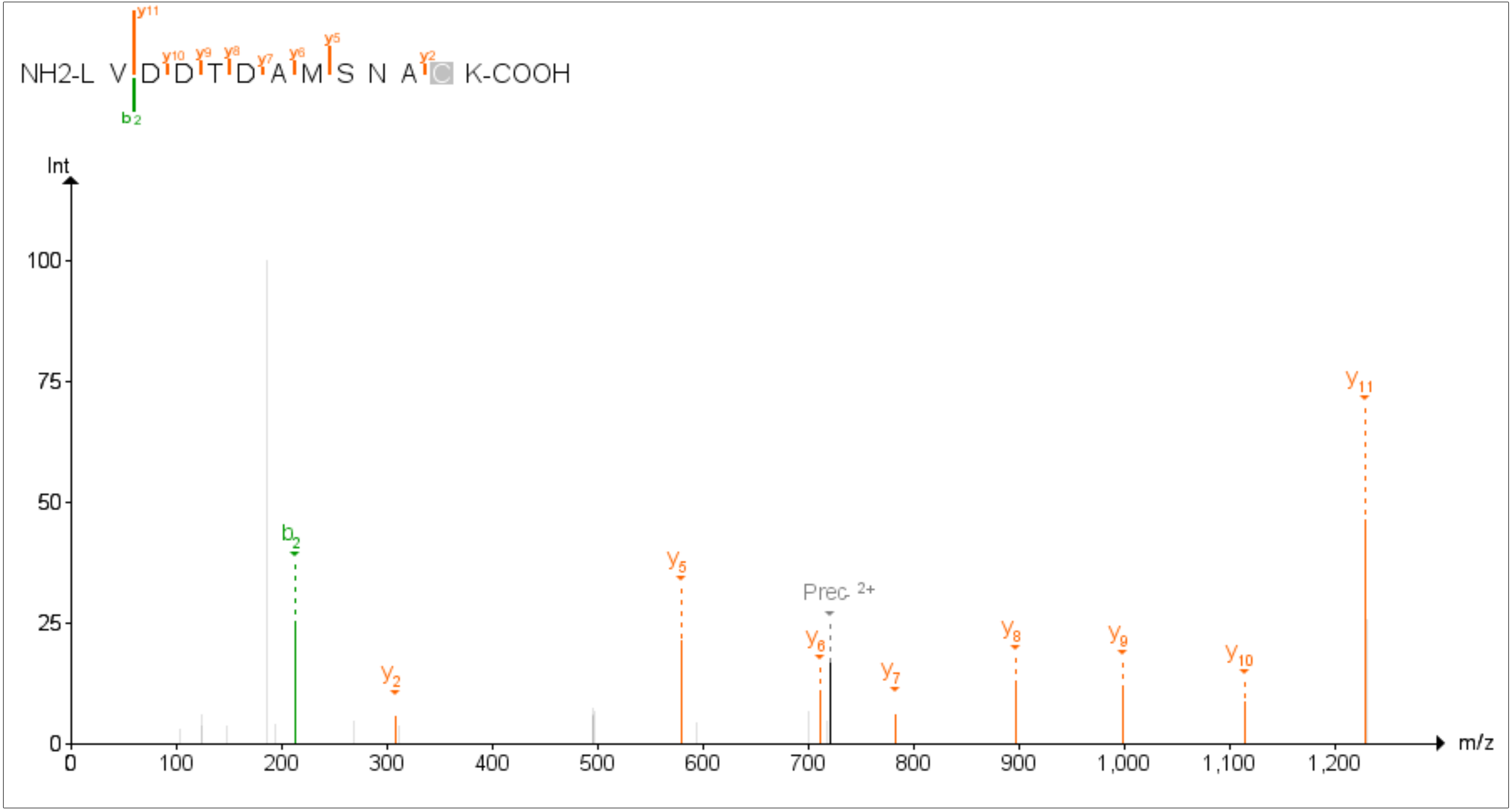
NH2-L V D D T D A M S N A C K-COOH
y11 y10 y9 y8 y7 y6 y5 y2
b2
Int
100
75
50
25
0
b2
y2
y5
Prec 2+
y6
y7
y8
y9
y10
y11
0 100 200 300 400 500 600 700 800 900 1,000 1,100 1,200
m/z

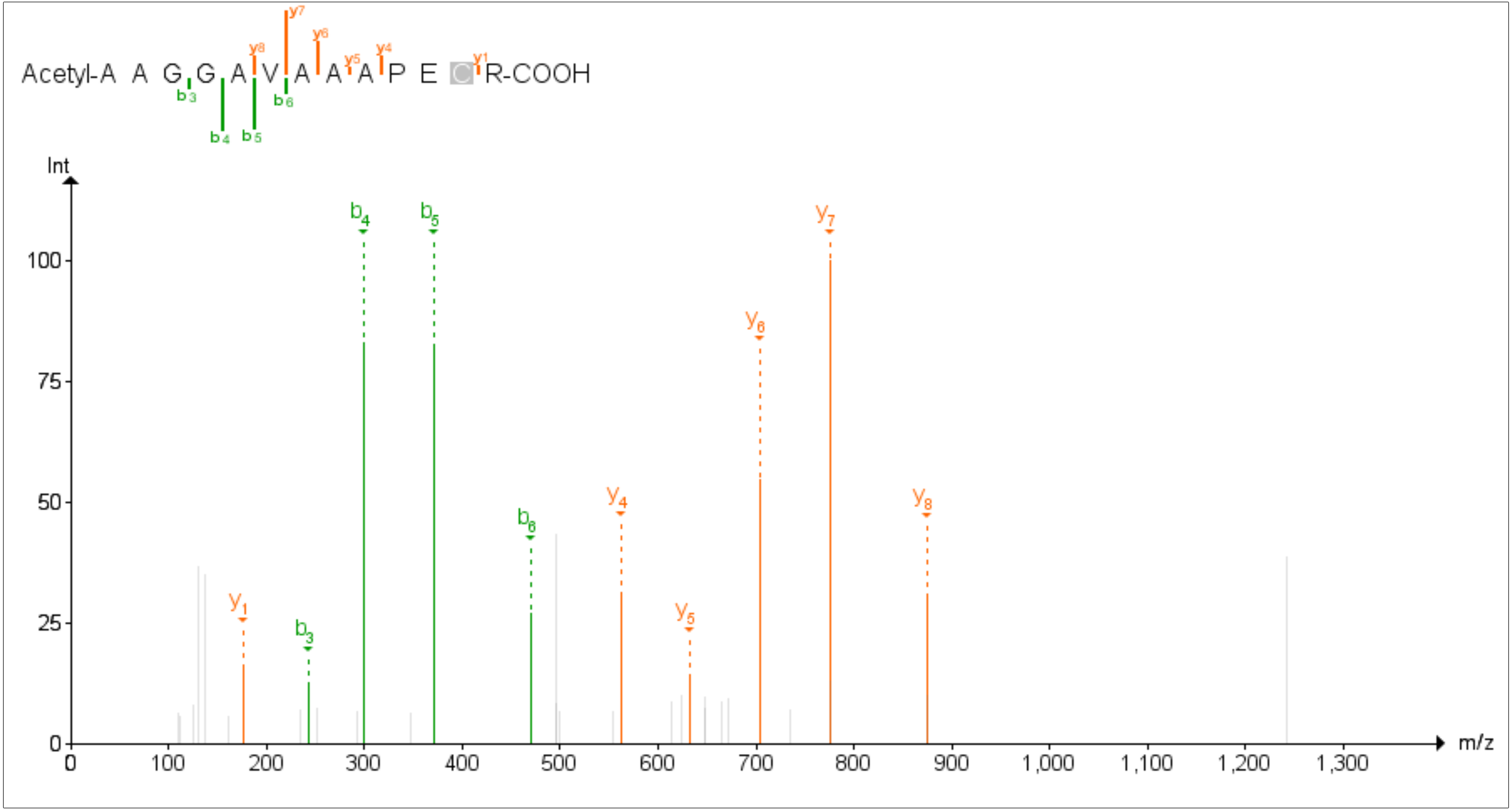

Acetyl-A A G G A V A A A P E C R-COOH
b3
b4
b5
b6
y8
y7
y6
y5
y4
y1
Int
100
75
50
25
0
b4
b5
y7
y6
b6
y4
y8
y1
b3
y5
0
100
200
300
400
500
600
700
800
900
1,000
1,100
1,200
1,300
m/z

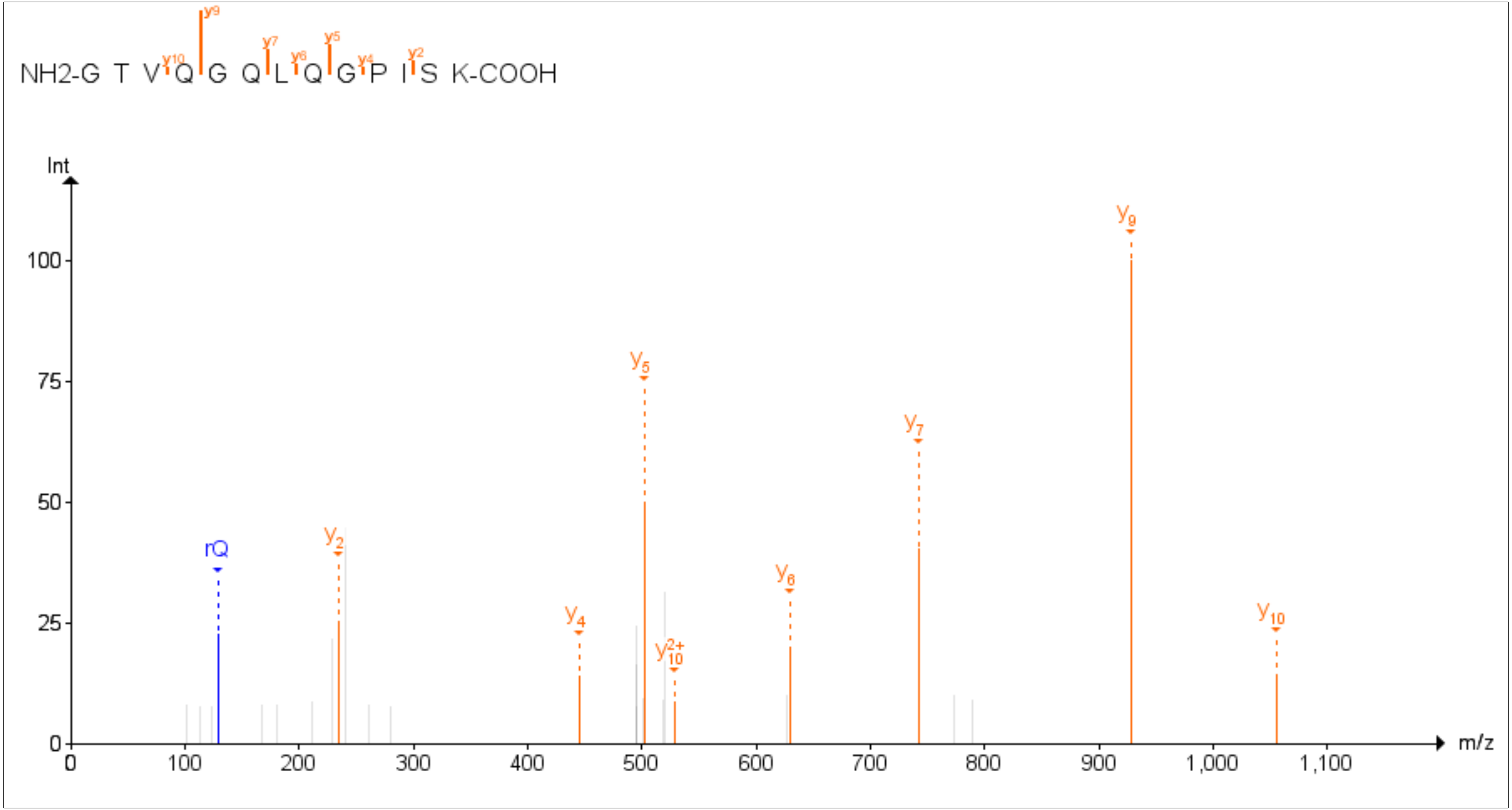

NH2-G T V Q G Q L Q G P I S K-COOH
y10
y9
y7
y6
y5
y4
y2
Int
100
75
50
25
0
rQ
y2
y4
y5
y10 2+
y6
y7
y9
y10
0
100
200
300
400
500
600
700
800
900
1,000
1,100
m/z

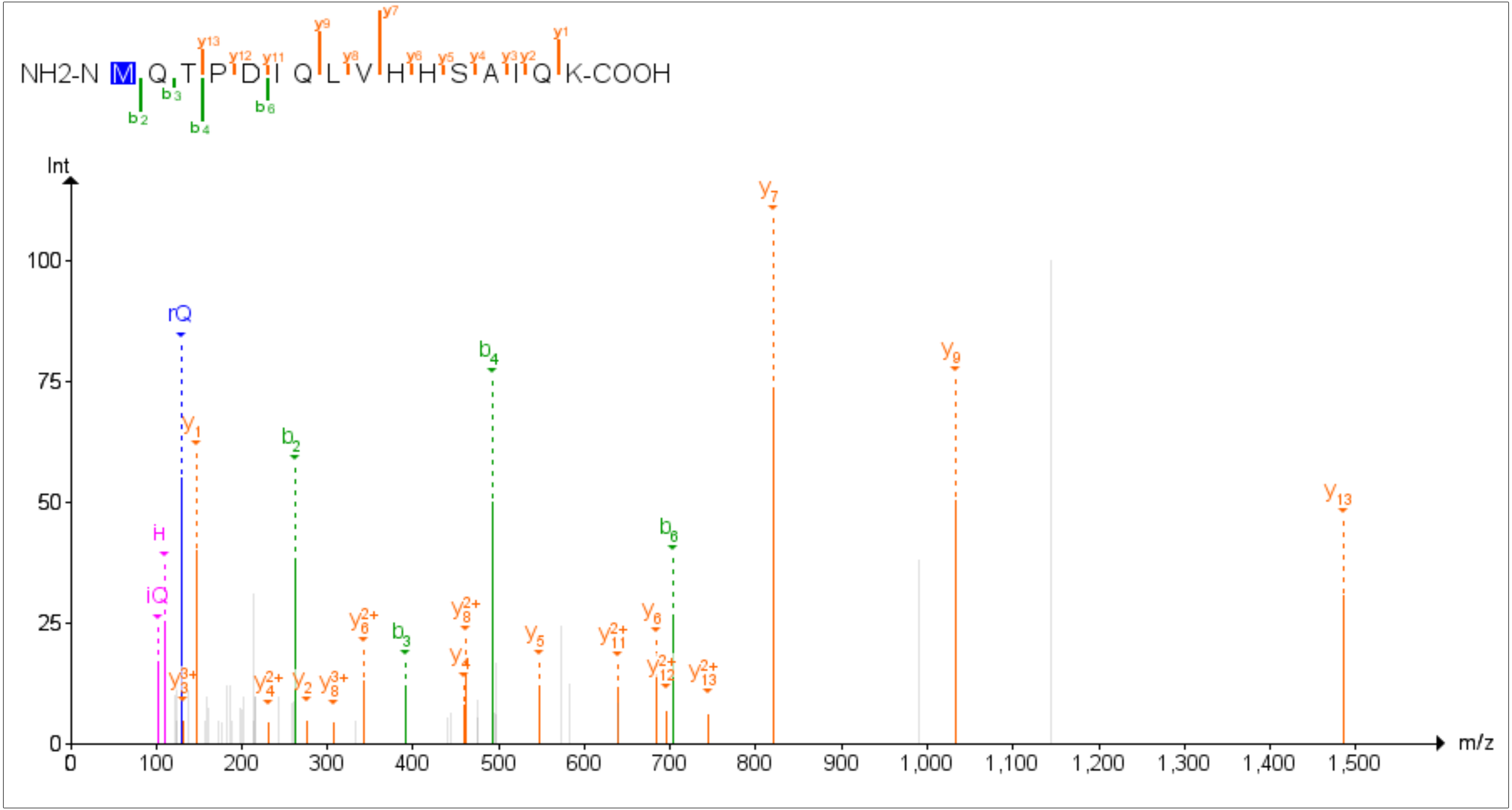

NH2-N M Q T P D I Q L V H H S A I Q K-COOH
y13 y12 y11 y9 y8 y7 y6 y5 y4 y3 y2 y1
b2 b3 b4 b6
Int
100
75
50
25
0
rQ
iQ
H
y1
b2
b4
y7
y9
y13
b6
y6
y5
y4
b3
y6 2+
y8 2+
y11 2+
y12 2+
y13 2+
y3 3+
y4 2+
y2
y8 3+
0 100 200 300 400 500 600 700 800 900 1,000 1,100 1,200 1,300 1,400 1,500
m/z

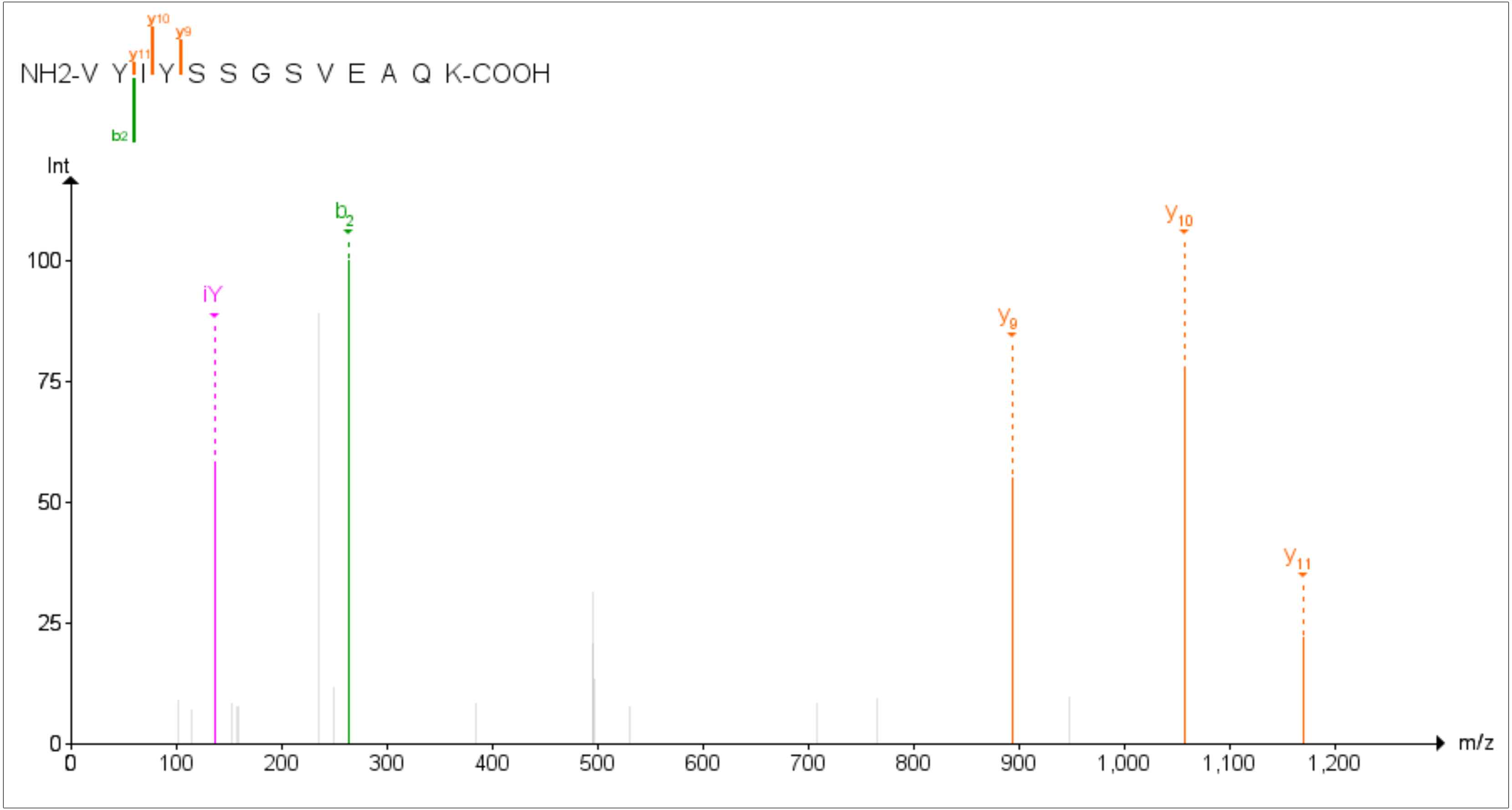

NH2-V Y I Y S S G S V E A Q K-COOH
y10
y9
y11
b2
Int
100
75
50
25
0
iY
y9
y10
y11
0
100
200
300
400
500
600
700
800
900
1,000
1,100
1,200
m/z

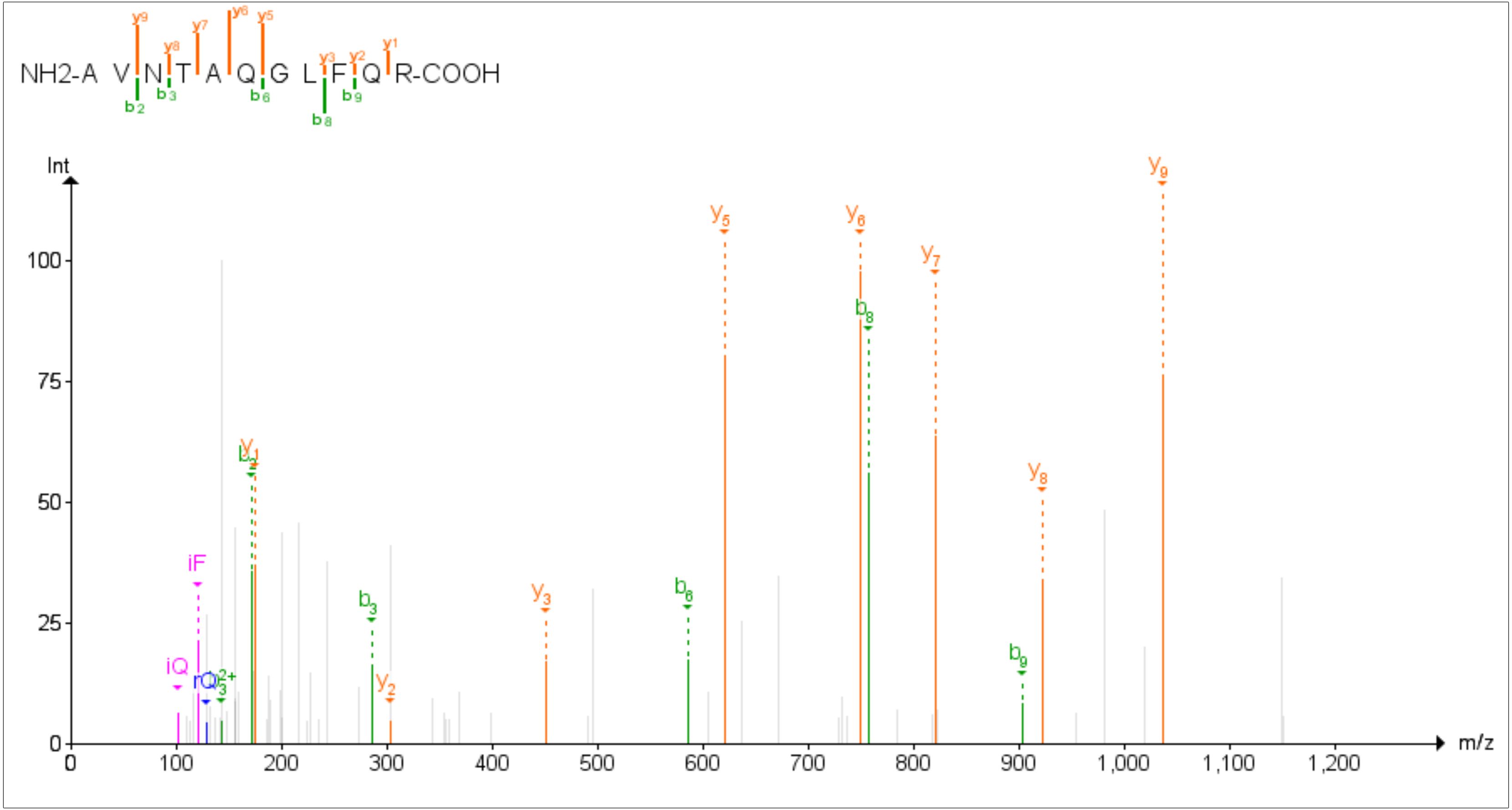

NH2-A V N T A Q G L F Q R-COOH
y9
y8
y7
y6
y5
y3
y2
y1
b2
b3
b6
b8
b9
Int
100
75
50
25
0
m/z
0
100
200
300
400
500
600
700
800
900
1,000
1,100
1,200
y5
y6
y7
y8
y9
b8
b6
b3
b9
y3
y2
y1
b2
iF
iQ
rQ

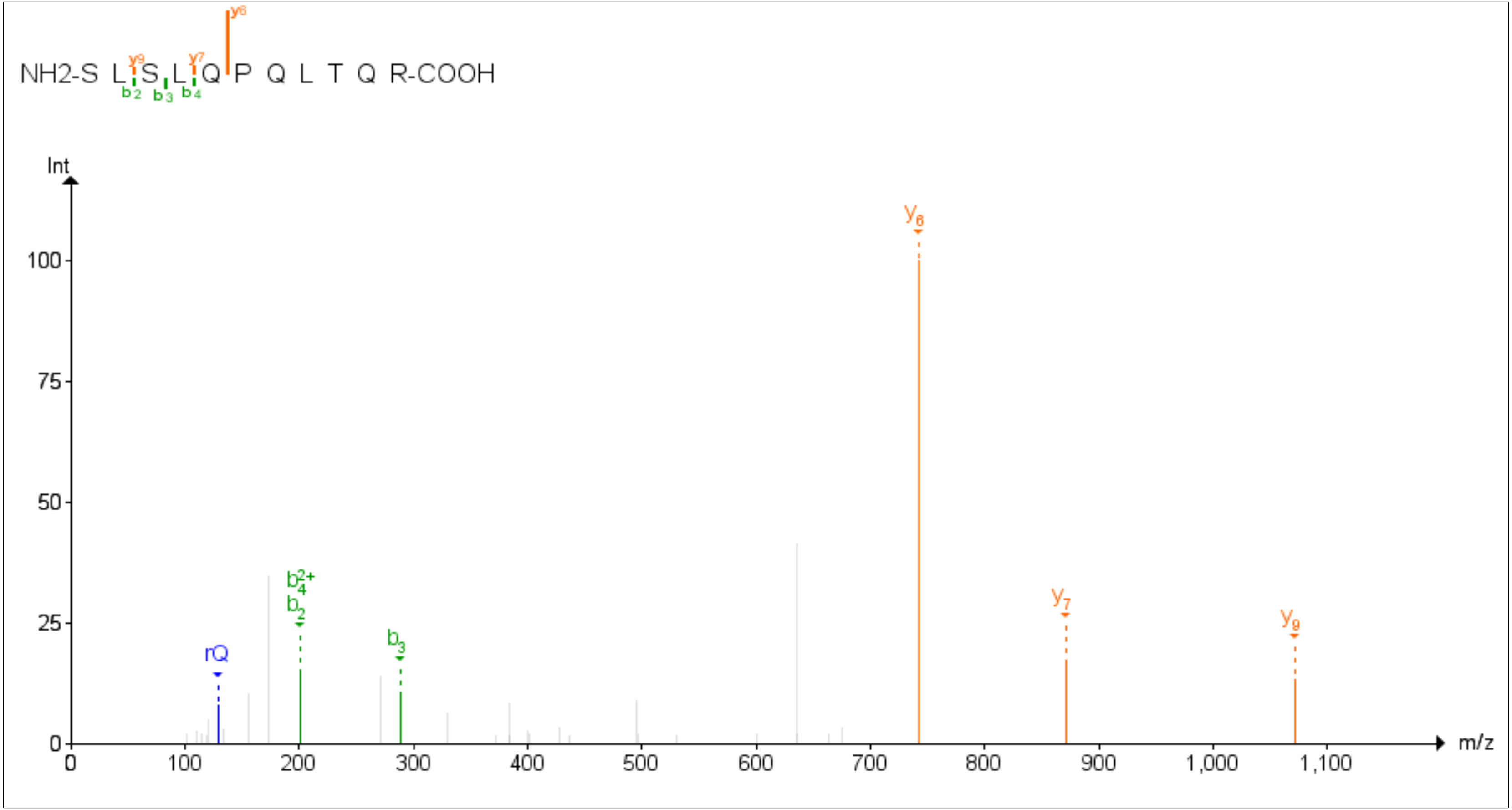

NH2-S L S L Q P Q L T Q R-COOH
y6
y9
y7
b2
b3
b4
Int
100
75
50
25
0
y6
y7
y9
rQ
b4 2+
b2
b3
0
100
200
300
400
500
600
700
800
900
1,000
1,100
m/z

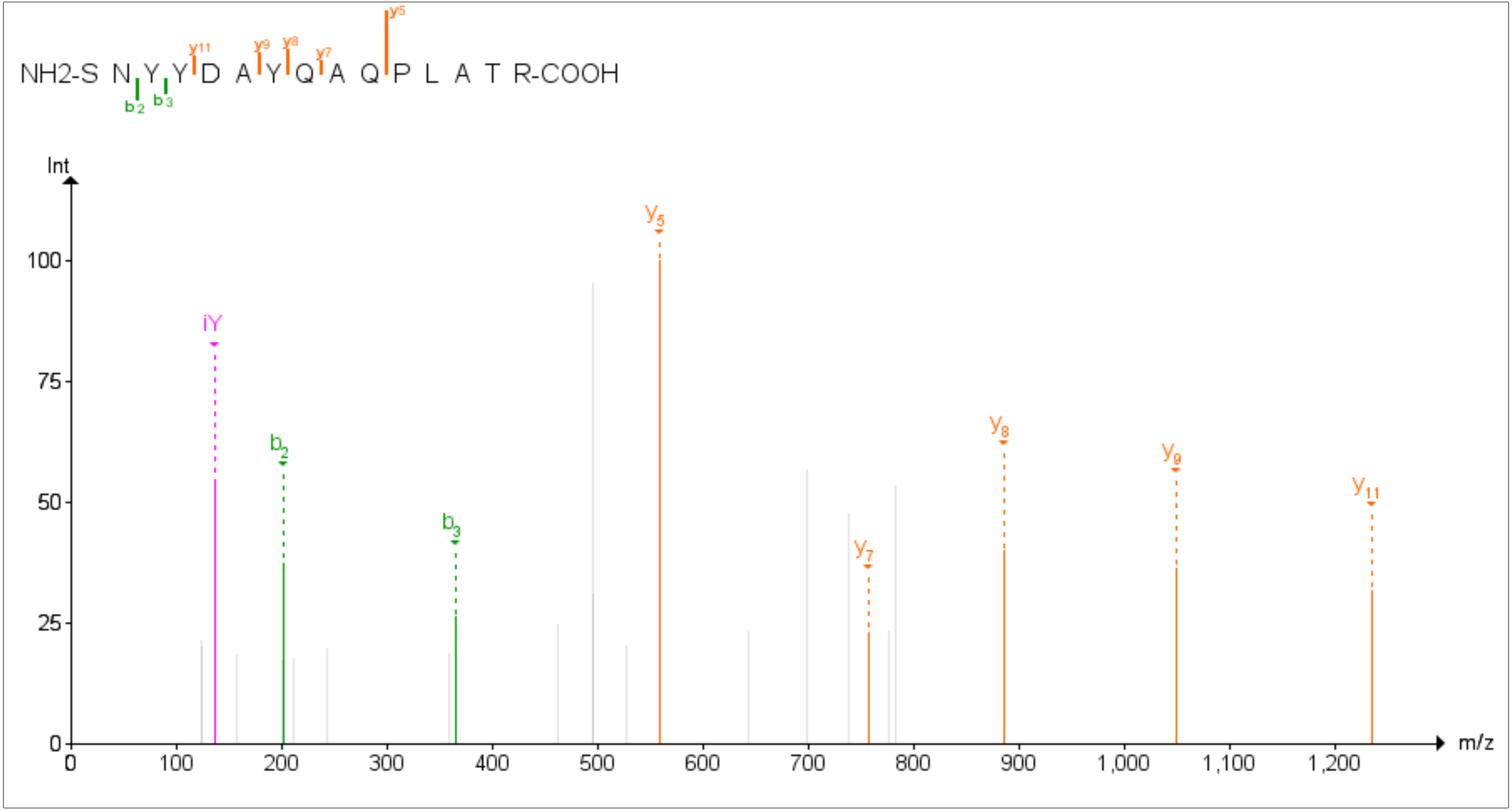

NH2-S N Y Y D A Y Q A Q P L A T R-COOH
b2
b3
y11
y9
y8
y7
y5
Int
100
75
50
25
0
iY
b2
b3
y5
y7
y8
y9
y11
0
100
200
300
400
500
600
700
800
900
1,000
1,100
1,200
m/z

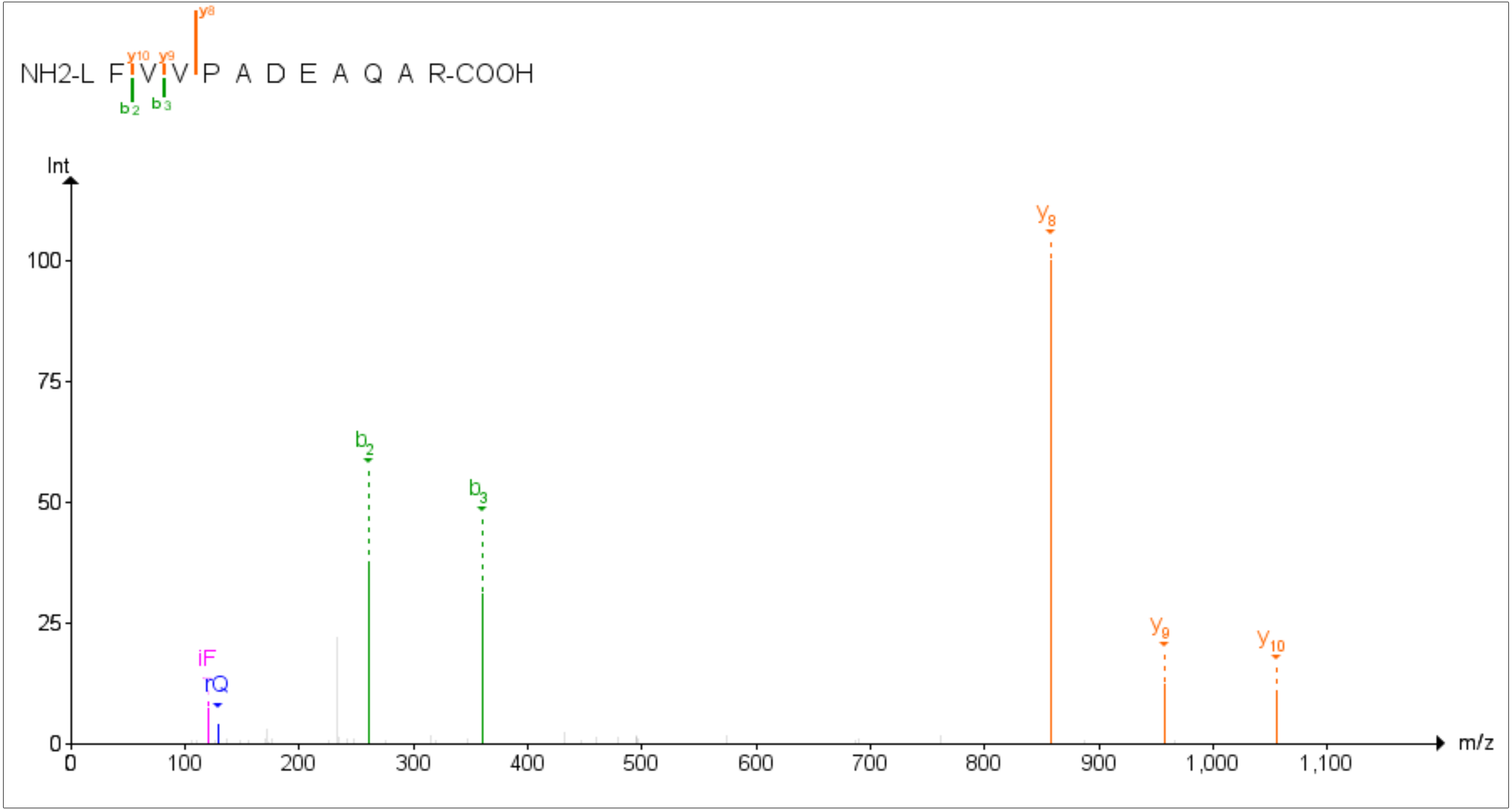

NH2-L F V V P A D E A Q A R-COOH
y10 y9 y8
b2 b3
Int
100
75
50
25
0
y8
b2
b3
y9
y10
iF
rQ
0 100 200 300 400 500 600 700 800 900 1,000 1,100
m/z

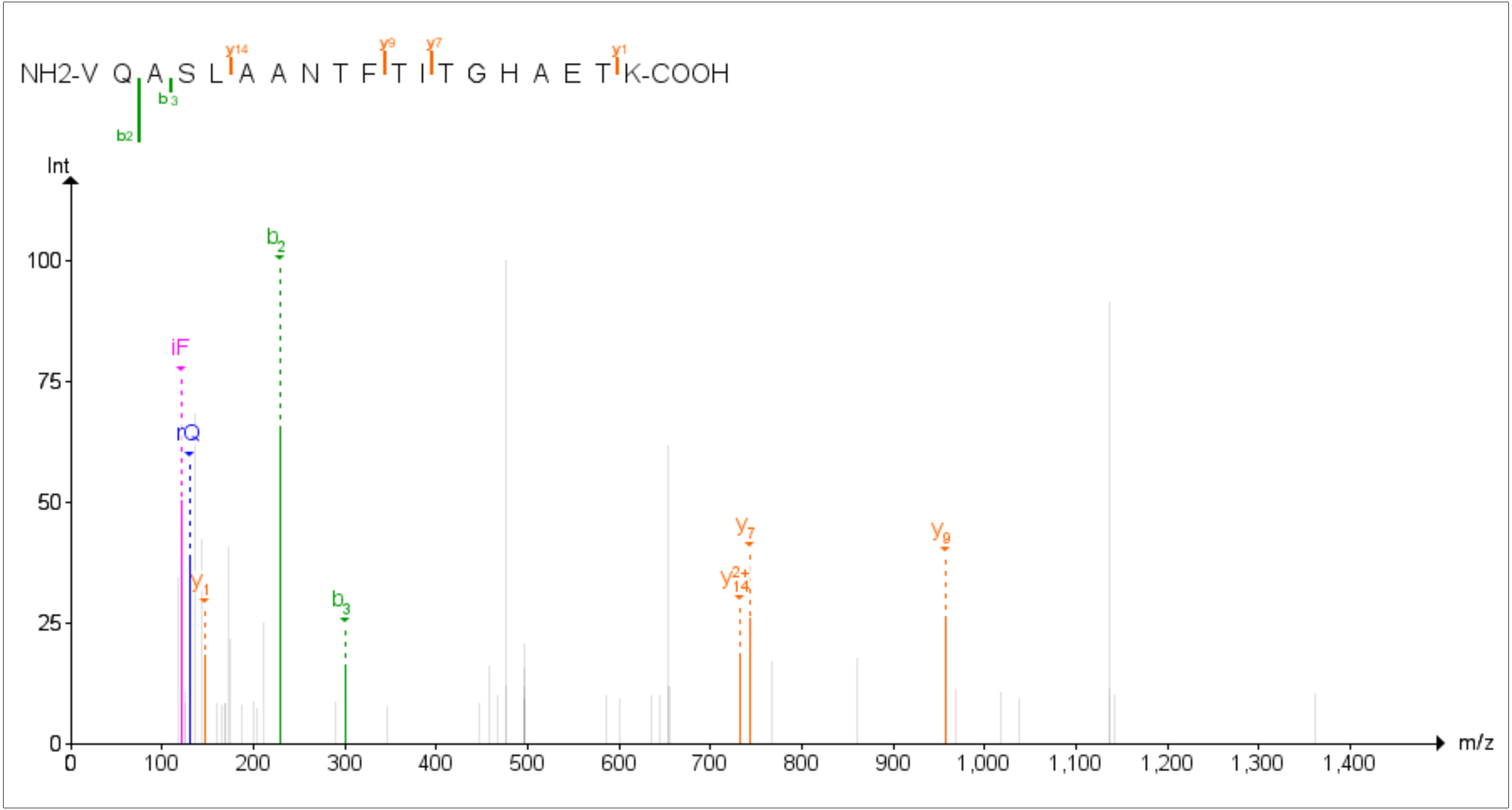

NH2-V Q A S L A A N T F T I T G H A E T K-COOH
y14
y9
y7
y1
b2
b3
Int
b2
iF
rQ
y1
b3
y7
y9
y14 2+
100
75
50
25
0
0
100
200
300
400
500
600
700
800
900
1,000
1,100
1,200
1,300
1,400
m/z

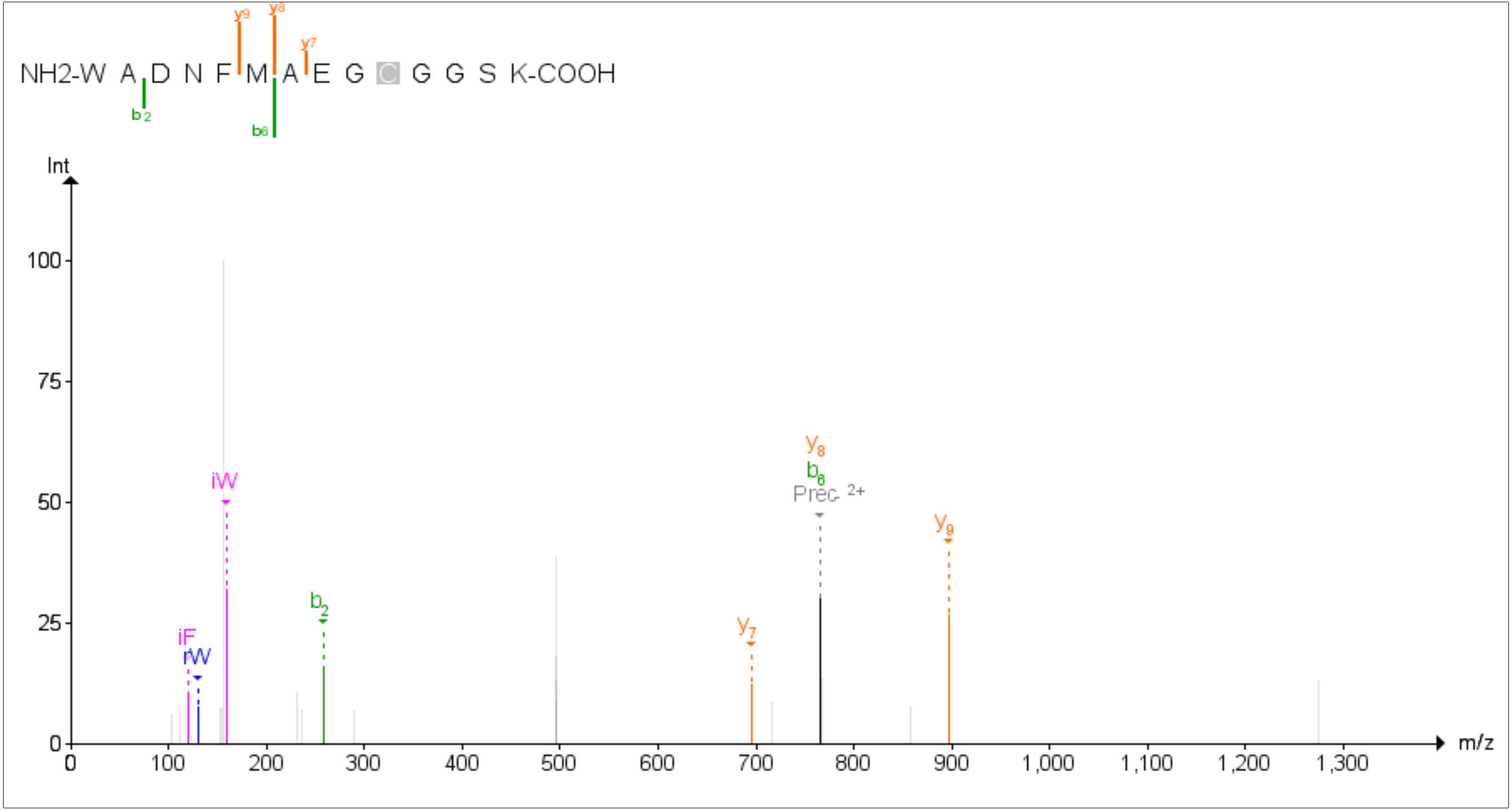

NH2-W A D N F M A E G C G G S K-COOH
y9
y8
y7
b2
b6
Int
100
75
50
25
0
iW
iF
rW
b2
y7
y8
b6
Prec 2+
y9
0
100
200
300
400
500
600
700
800
900
1,000
1,100
1,200
1,300
m/z

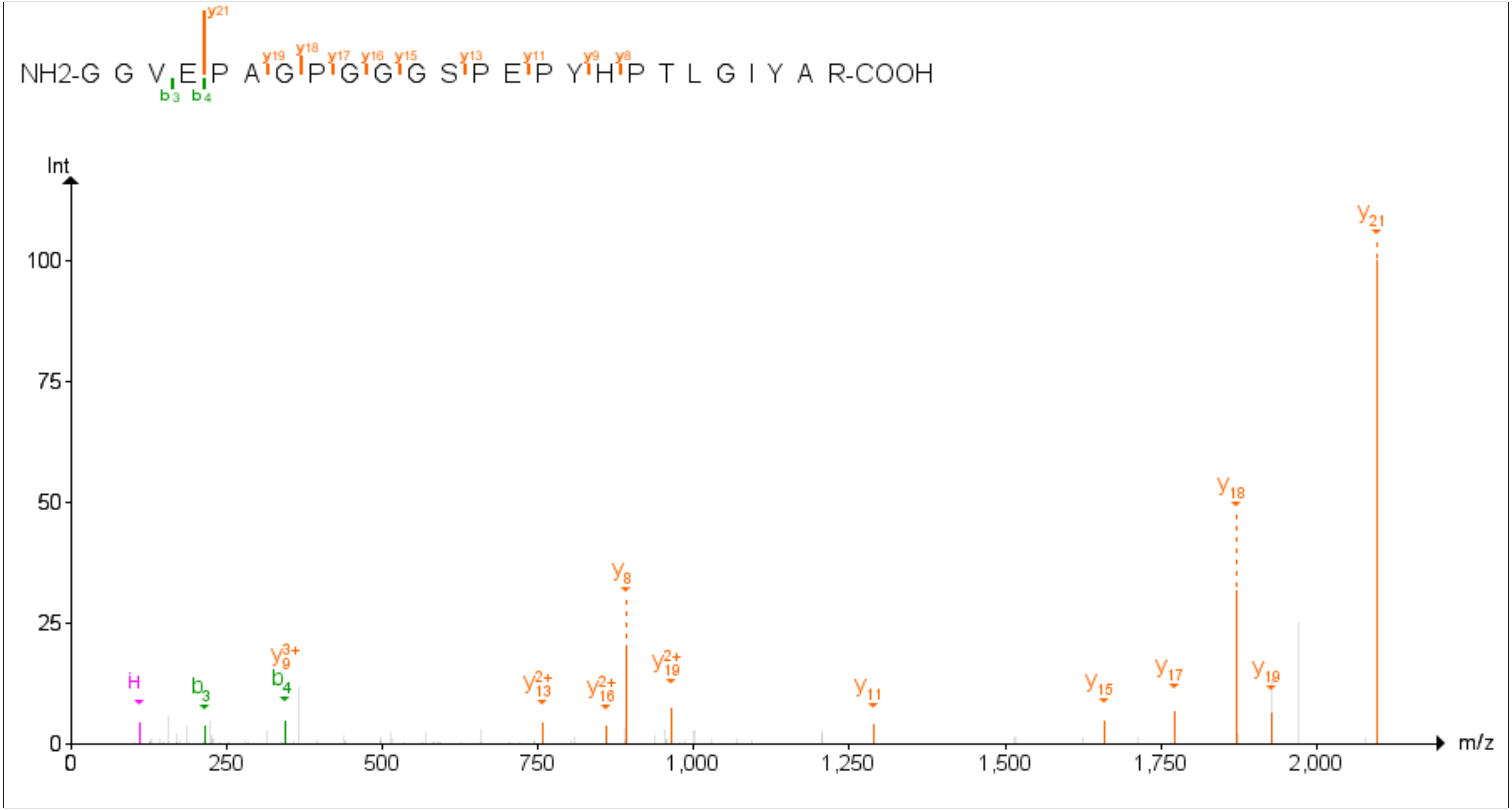

NH2-G G V E P A G P G G G G S P E P Y H P T L G I Y A R-COOH
b3
b4
y21
y19
y18
y17
y16
y15
y13
y11
y9
y8
Int
100
75
50
25
0
m/z
0
250
500
750
1,000
1,250
1,500
1,750
2,000
H
b_3
b_4
y_9^{3+}
y_{13}^{2+}
y_{16}^{2+}
y_8
y_{19}^{2+}
y_{11}
y_{15}
y_{17}
y_{18}
y_{19}
y_{21}

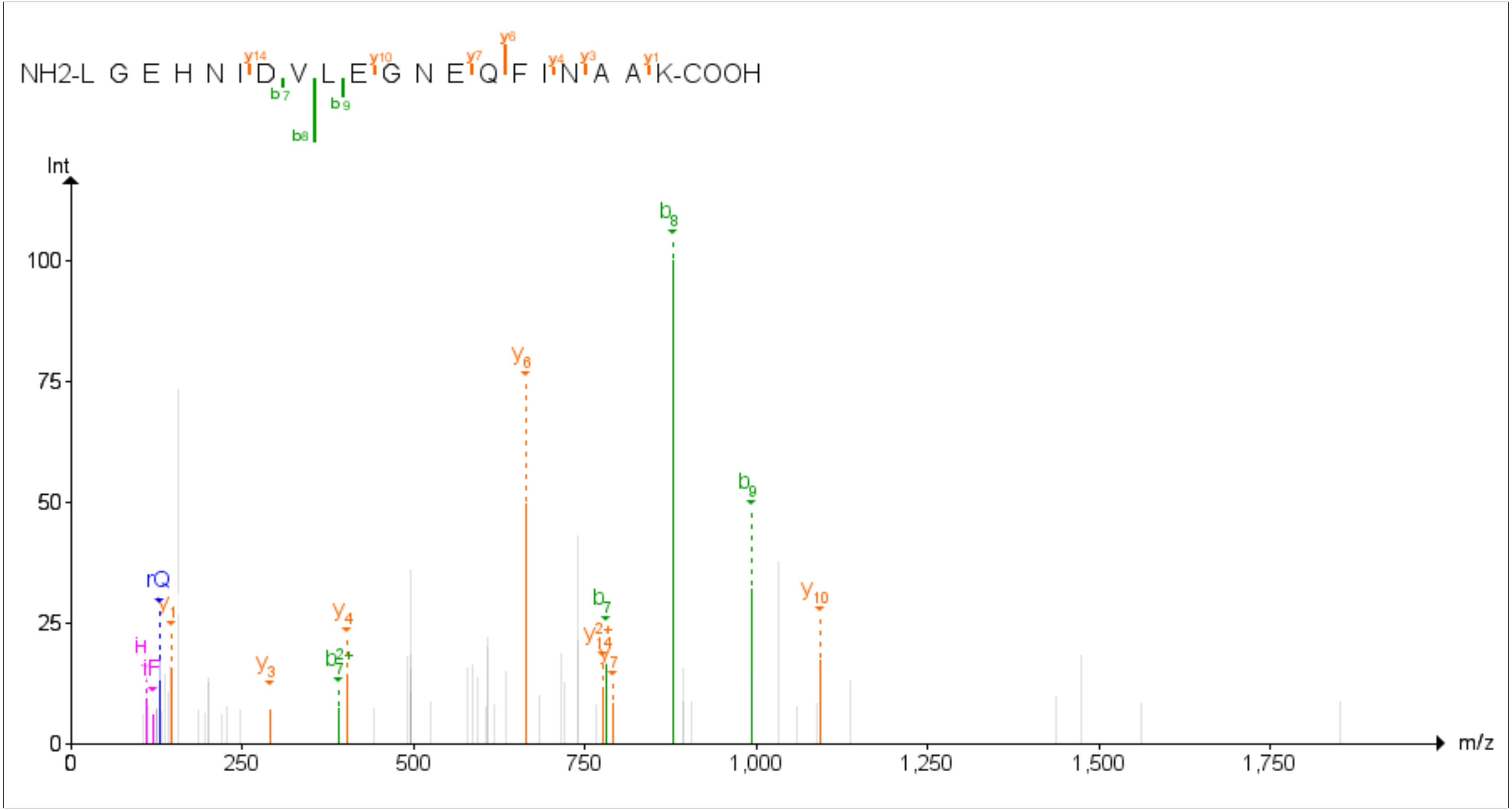

NH2-L G E H N I D V L E G N E Q F I N A A K-COOH
y14
y10
y7
y6
y4
y3
y1
b7
b9
b8
Int
100
75
50
25
0
b8
y6
b9
y10
b7
y14 2+
y7
y4
y3
b7 2+
rQ
y1
iH
iF
0
250
500
750
1,000
1,250
1,500
1,750
m/z

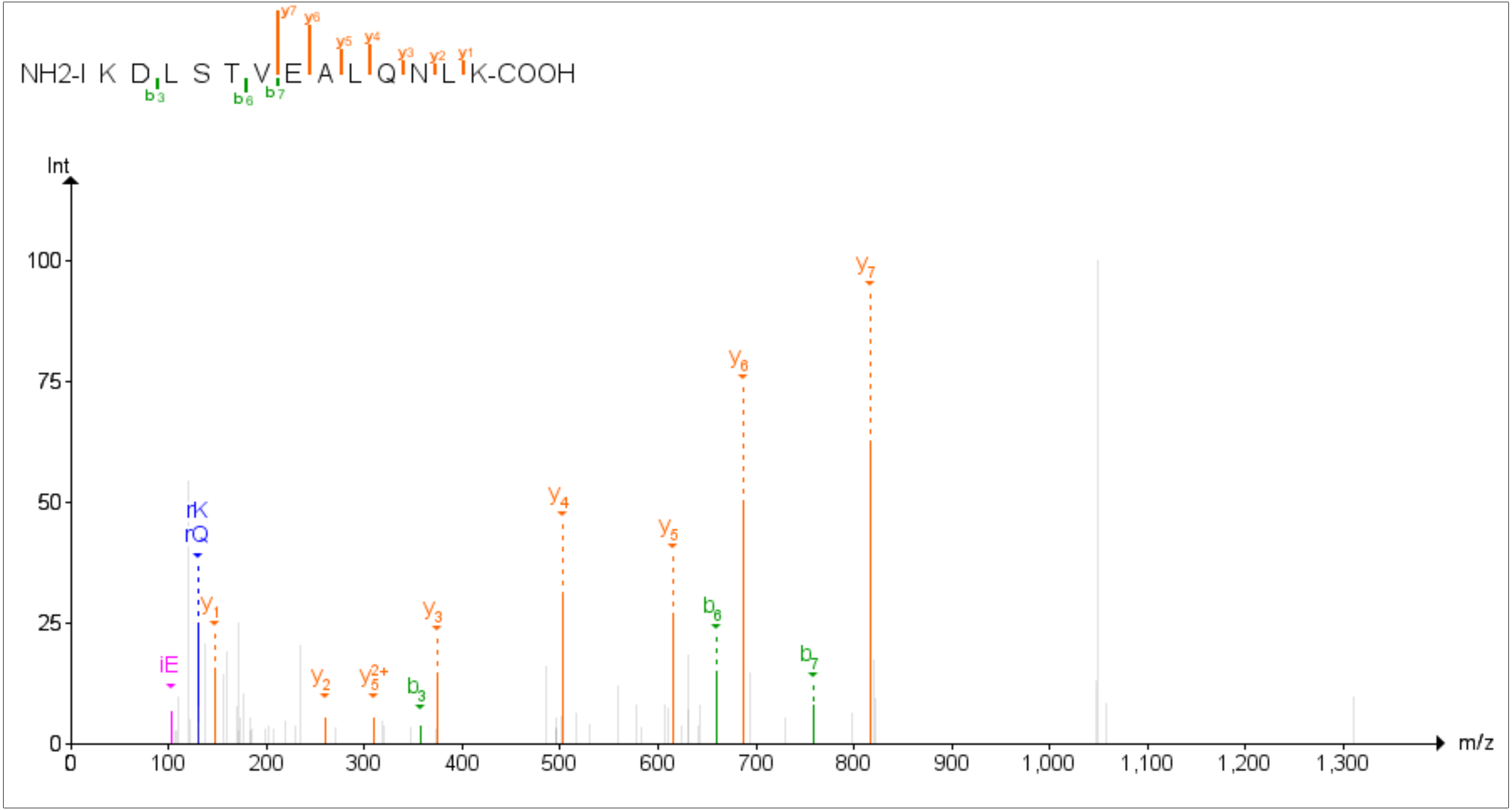

NH2-I K D L S T V E A L Q N L K-COOH
b3 b6 b7
y7 y6 y5 y4 y3 y2 y1
Int
100
75
50
25
0
iE
rK
rQ
y1
y2
y5 2+
b3
y3
y4
y5
b6
y6
b7
y7
0 100 200 300 400 500 600 700 800 900 1,000 1,100 1,200 1,300
m/z

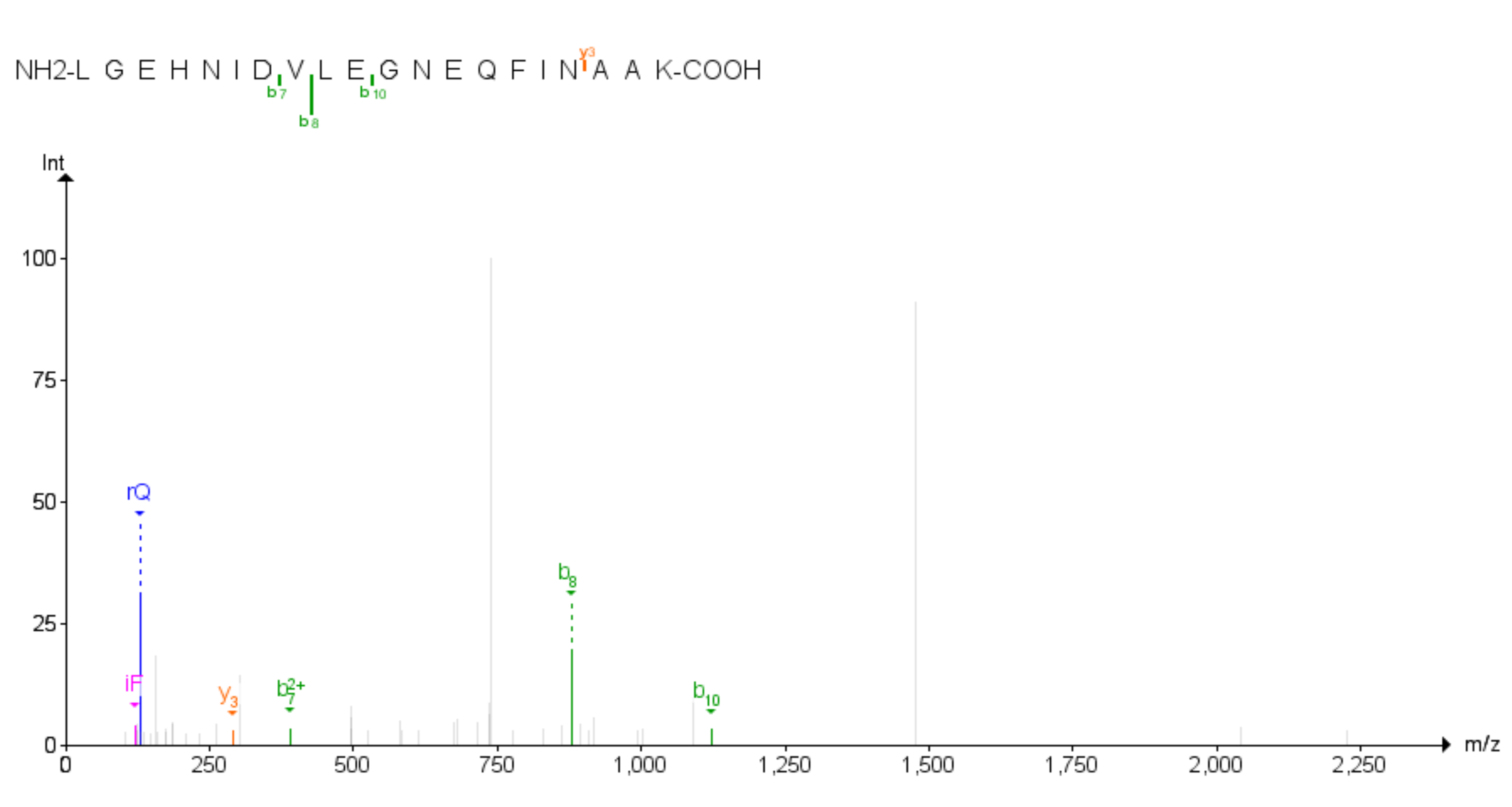

NH2-L G E H N I D V L E G N E Q F I N A A K-COOH
b7
b8
b10
y3
Int
100
75
50
25
0
rQ
iF
y3
b7 2+
b8
b10
0
250
500
750
1,000
1,250
1,500
1,750
2,000
2,250
m/z

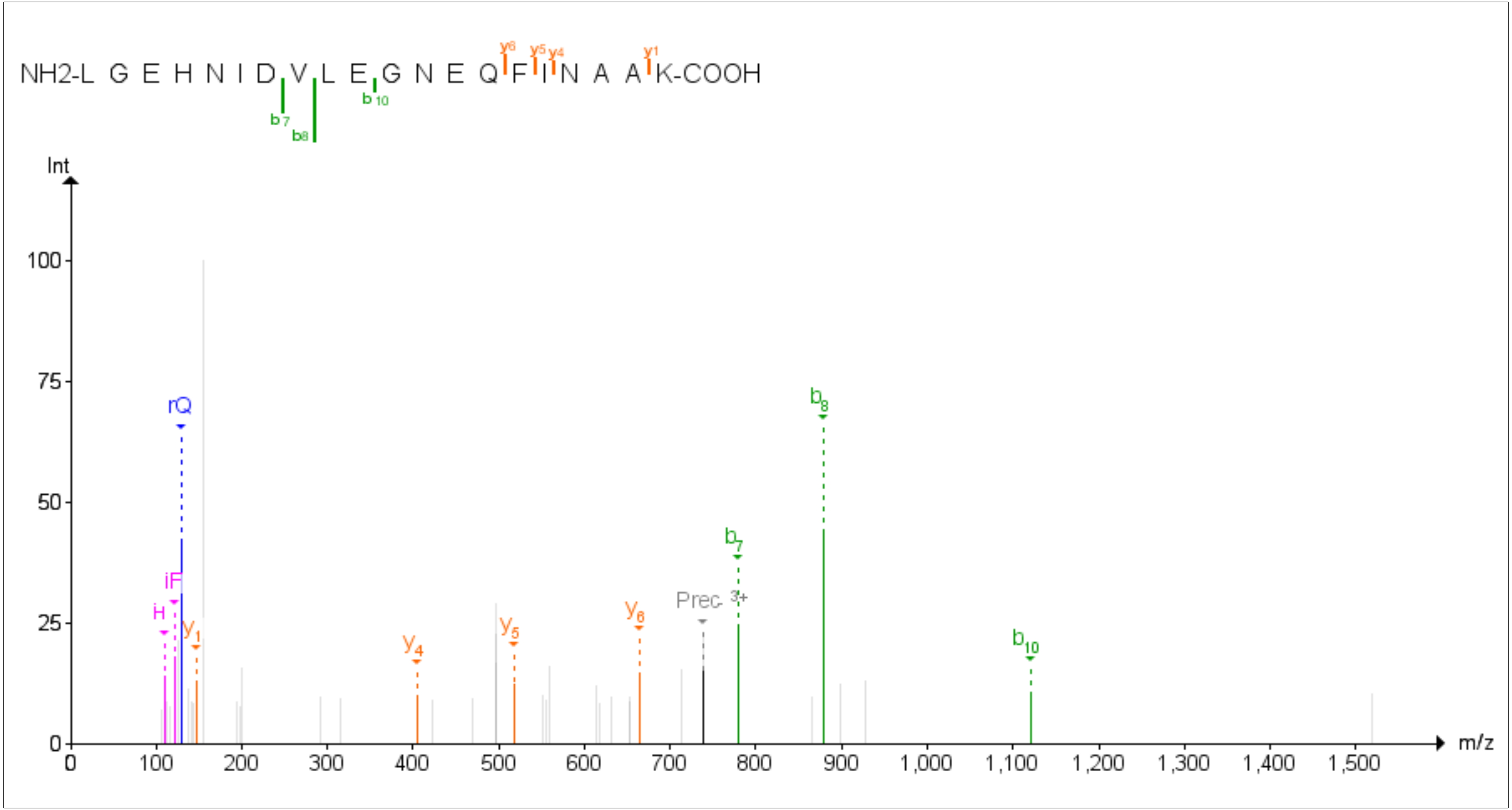
NH2-L G E H N I D V L E G N E Q F I N A A K-COOH
y6 y5 y4 y1
b7 b8 b10
Int
100
75
50
25
0
rQ
iF
iH
y1
y4
y5
y6
Prec 3+
b7
b8
b10
0 100 200 300 400 500 600 700 800 900 1,000 1,100 1,200 1,300 1,400 1,500
m/z

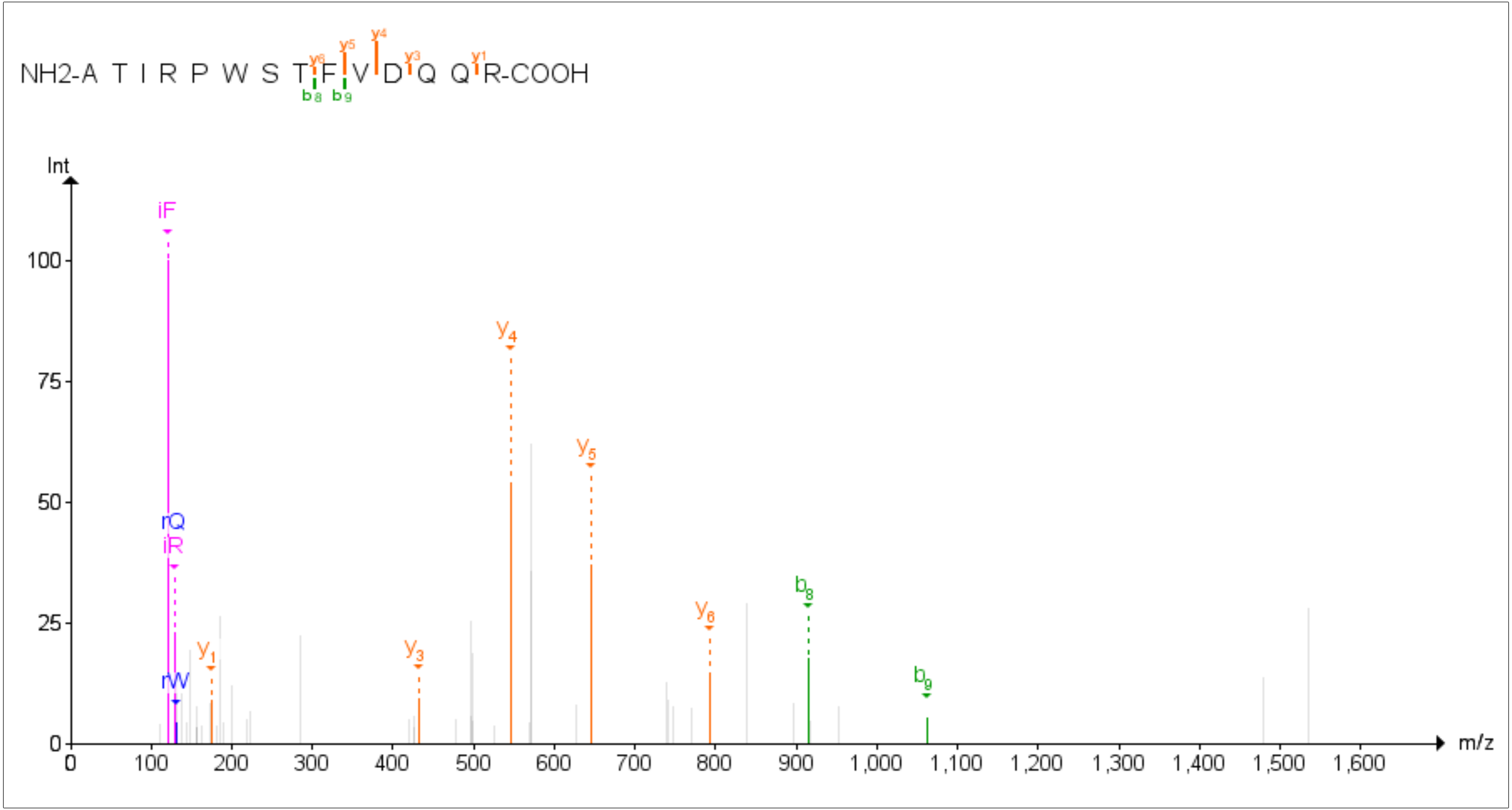

NH2-A T I R P W S T F V D Q Q R-COOH
y6
y5
y4
y3
y1
b8
b9
Int
100
75
50
25
0
iF
rQ
iR
rW
y1
y3
y4
y5
y6
b8
b9
0
100
200
300
400
500
600
700
800
900
1,000
1,100
1,200
1,300
1,400
1,500
1,600
m/z

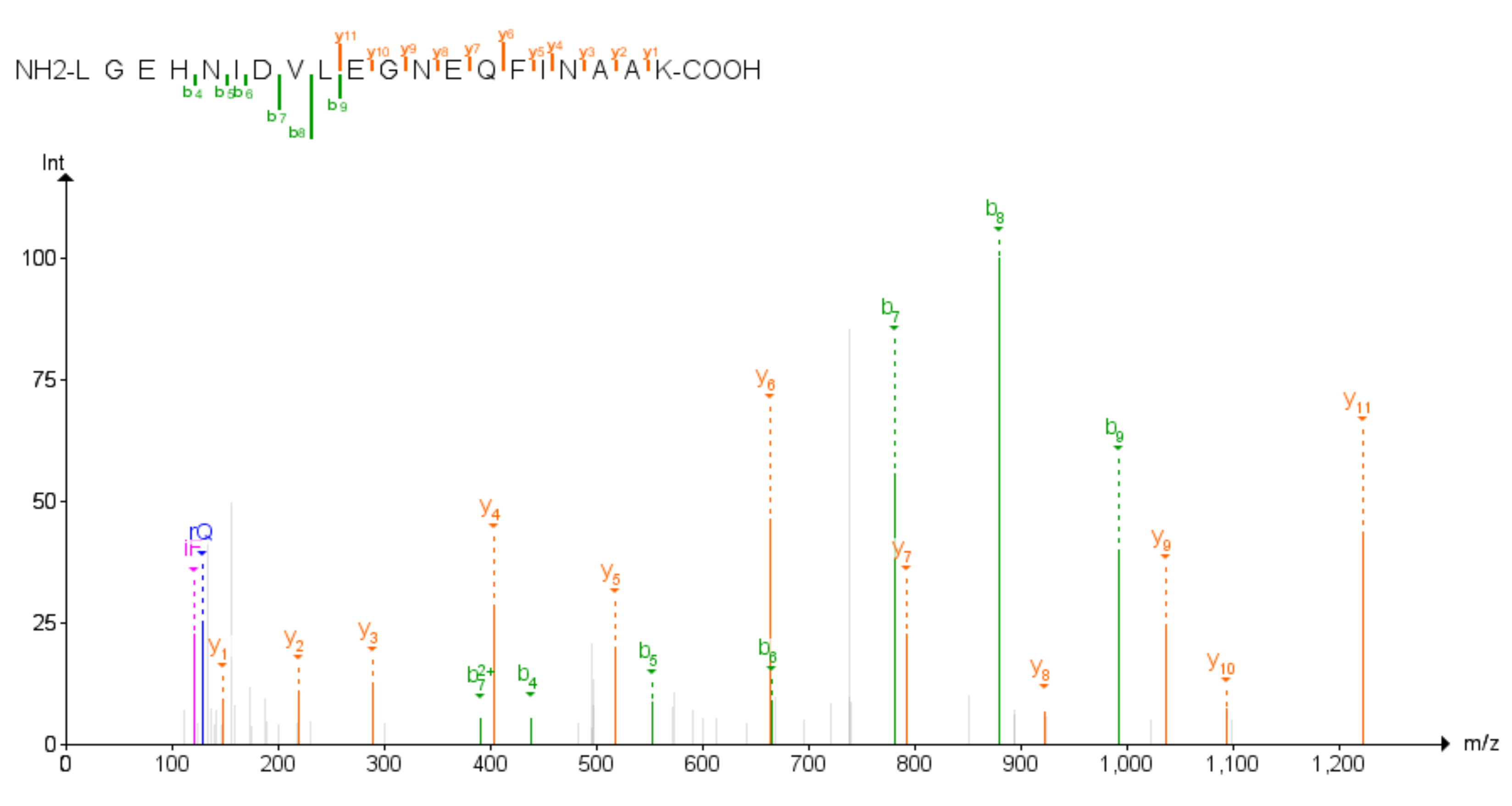

NH2-L G E H N I D V L E G N E Q F I N A A K-COOH
Int
m/z
0
25
50
75
100
100
200
300
400
500
600
700
800
900
1,000
1,100
1,200

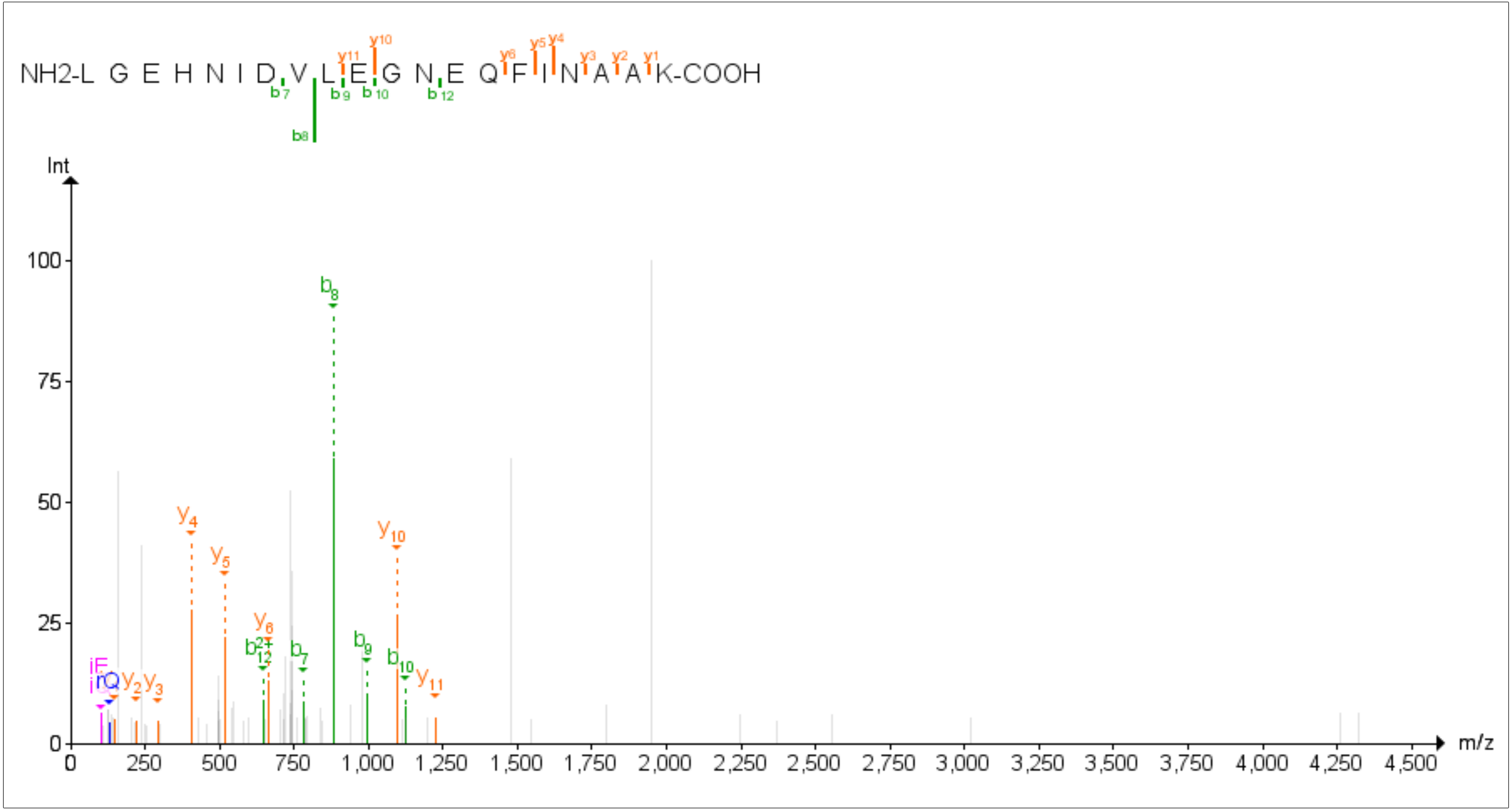

NH2-L G E H N I D V L E G N E Q F I N A A K-COOH
y10
y11
y6
y5
y4
y3
y2
y1
b7
b9
b10
b12
b8
Int
100
75
50
25
0
y4
y5
y6
b8
y10
b9
b10
y11
b7
y2
y3
m/z
0
250
500
750
1,000
1,250
1,500
1,750
2,000
2,250
2,500
2,750
3,000
3,250
3,500
3,750
4,000
4,250
4,500

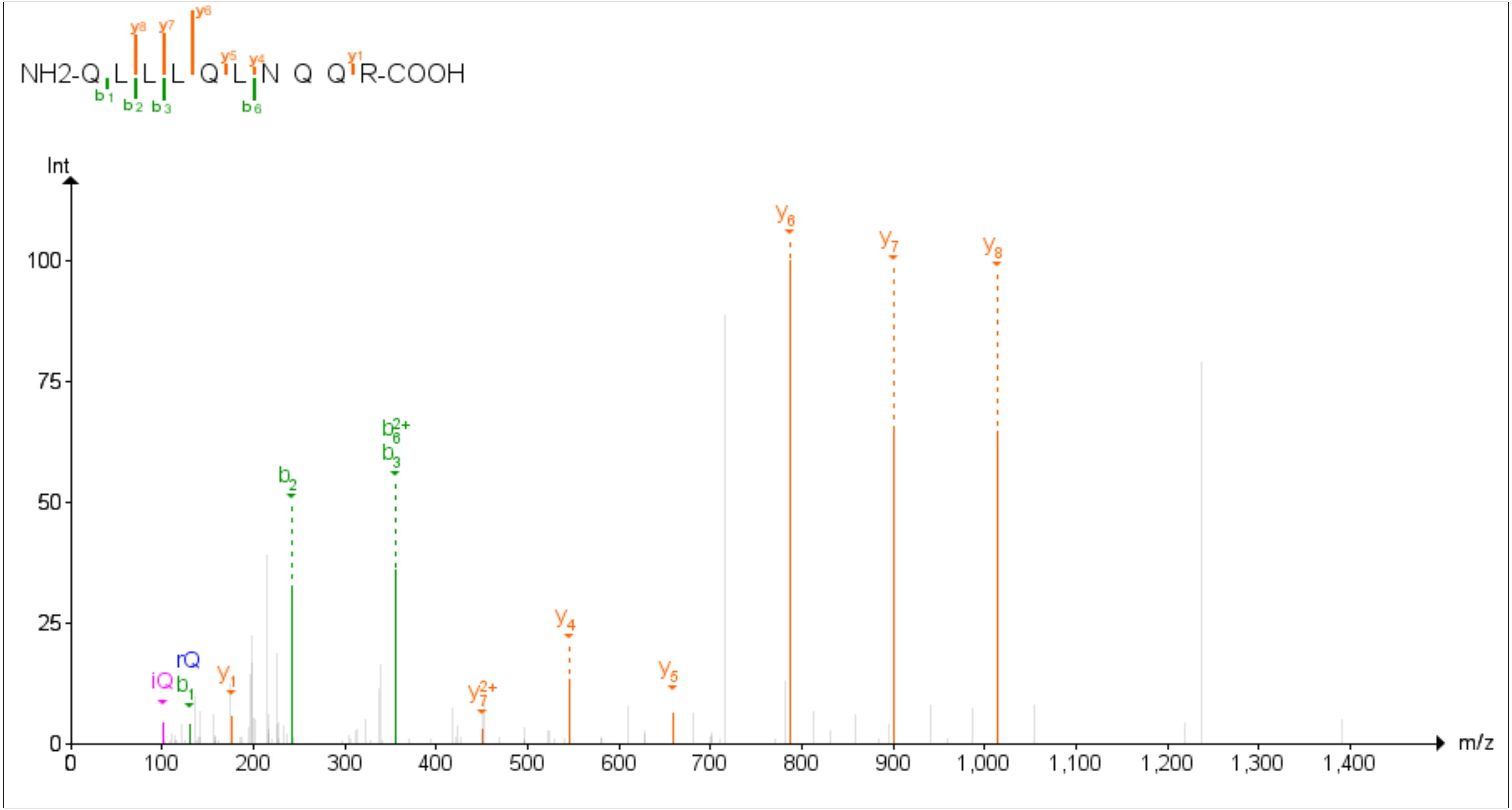

NH2-Q L L L Q L N Q Q R-COOH
y8 y7 y6 y5 y4 y1
b1 b2 b3 b6
Int
100
75
50
25
0
y6
y7
y8
b6 2+
b3
b2
y4
y5
y7 2+
y1
iQ
rQ
b1
0 100 200 300 400 500 600 700 800 900 1,000 1,100 1,200 1,300 1,400
m/z

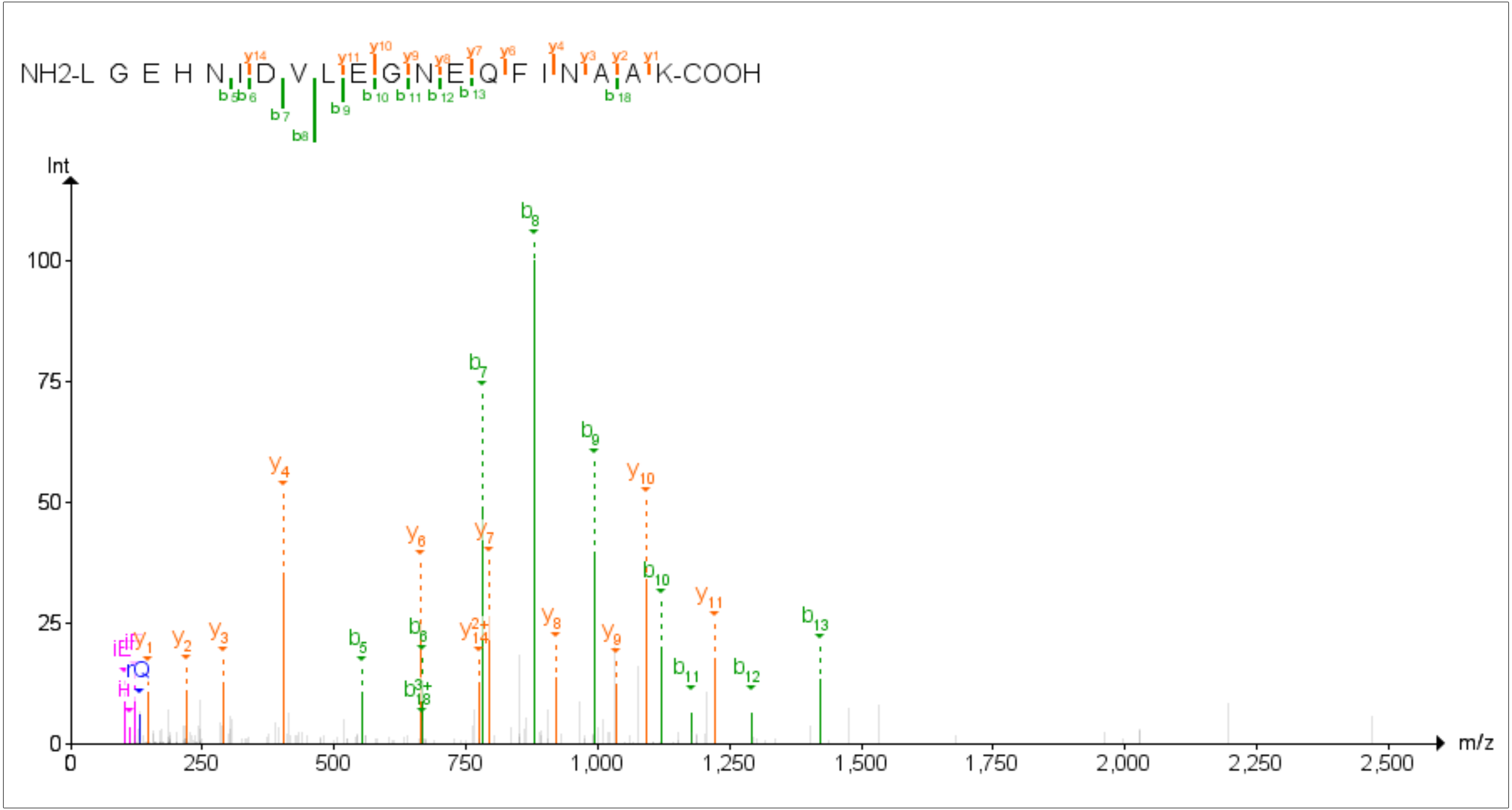

NH2-L G E H N I D V L E G N E Q F I N A A K-COOH
Int
m/z

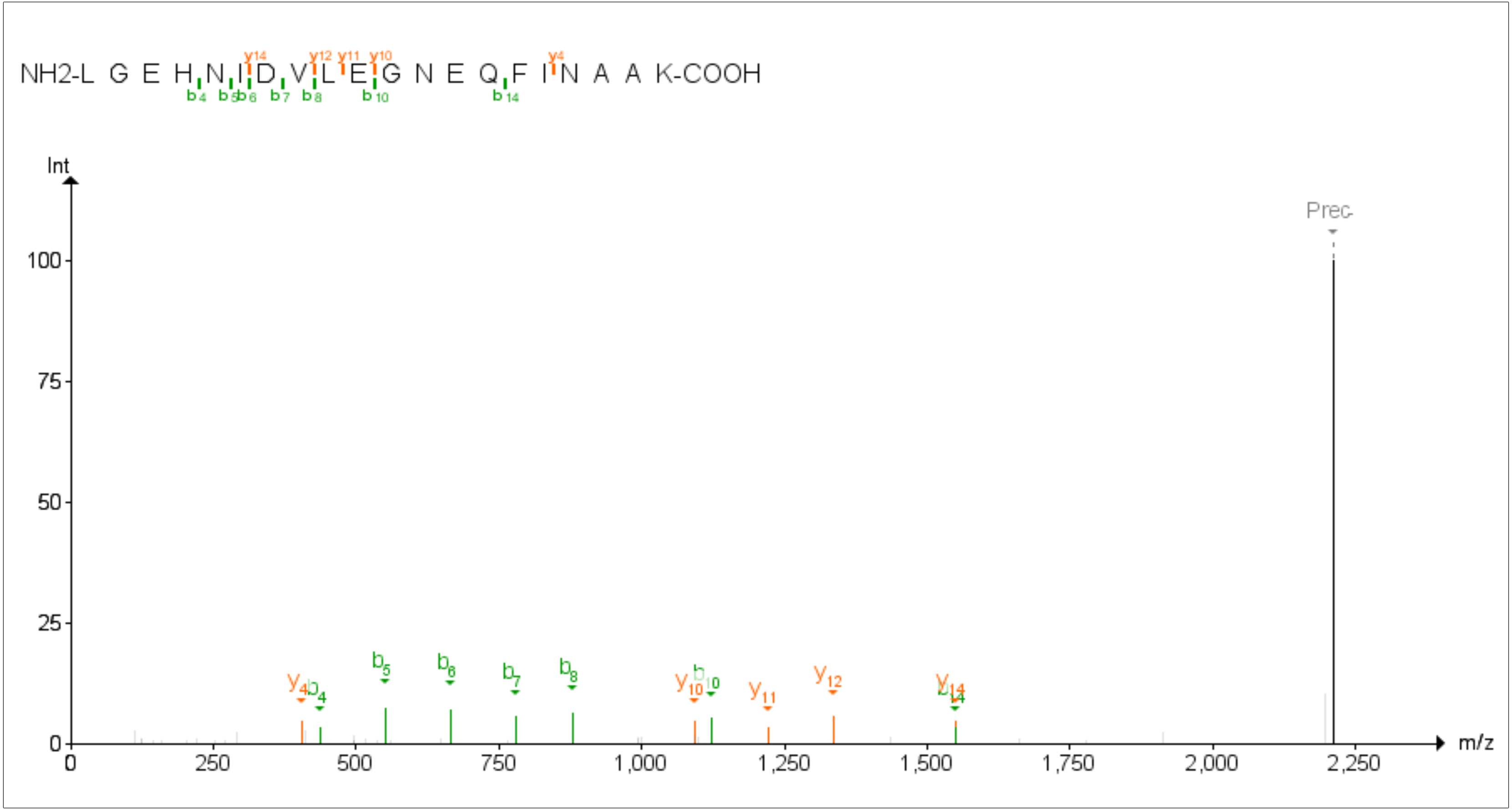

NH2-L G E H N I D V L E G N E Q F I N A A K-COOH
y14 y12 y11 y10 y4
b4 b5 b6 b7 b8 b10 b14
Int
Prec
100
75
50
25
0
y4 b4 b5 b6 b7 b8 y10 b10 y11 y12 y14 b14
0
250
500
750
1,000
1,250
1,500
1,750
2,000
2,250
m/z

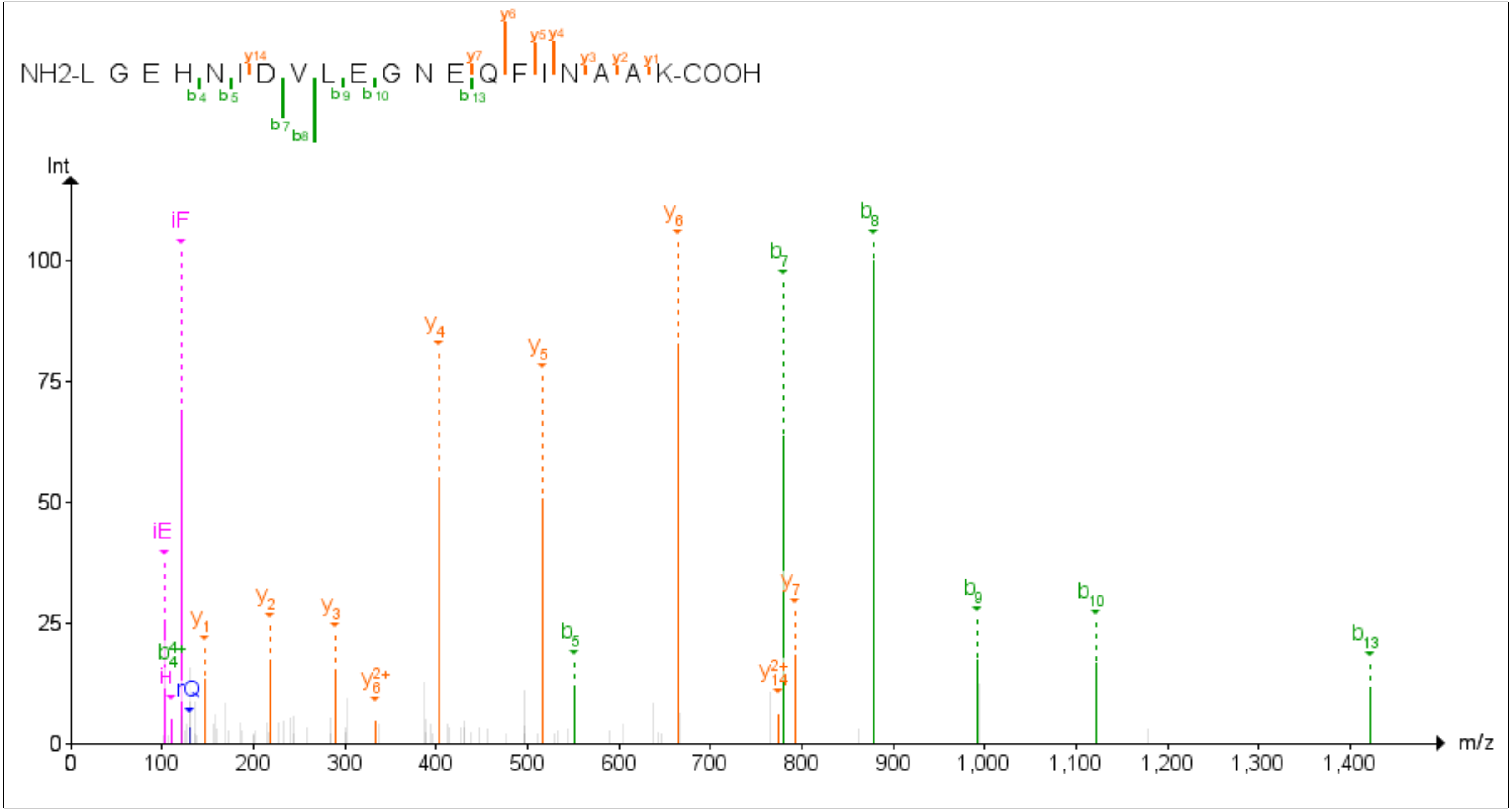

NH2-L G E H N I D V L E G N E Q F I N A A K-COOH
y14 y7 y6 y5 y4 y3 y2 y1
b4 b5 b7 b8 b9 b10 b13
Int
m/z
iF iE H rQ
y1 y2 y3 y4 y5 y6 y7
b4 b5 b7 b8 b9 b10 b13
0
25
50
75
100
0
100
200
300
400
500
600
700
800
900
1,000
1,100
1,200
1,300
1,400

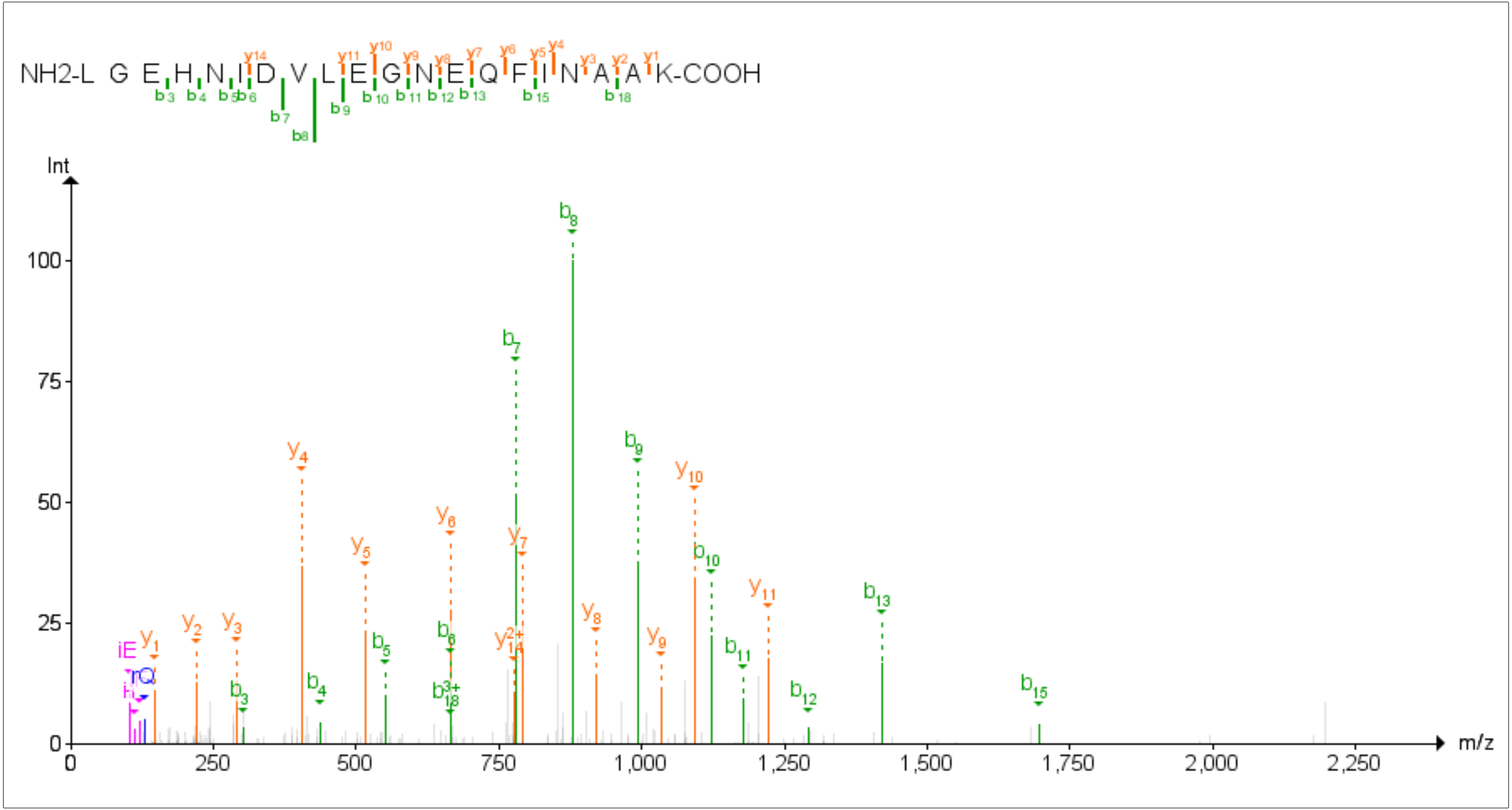

NH2-L G E H N I D V L E G N E Q F I N A A K-COOH
Int
m/z

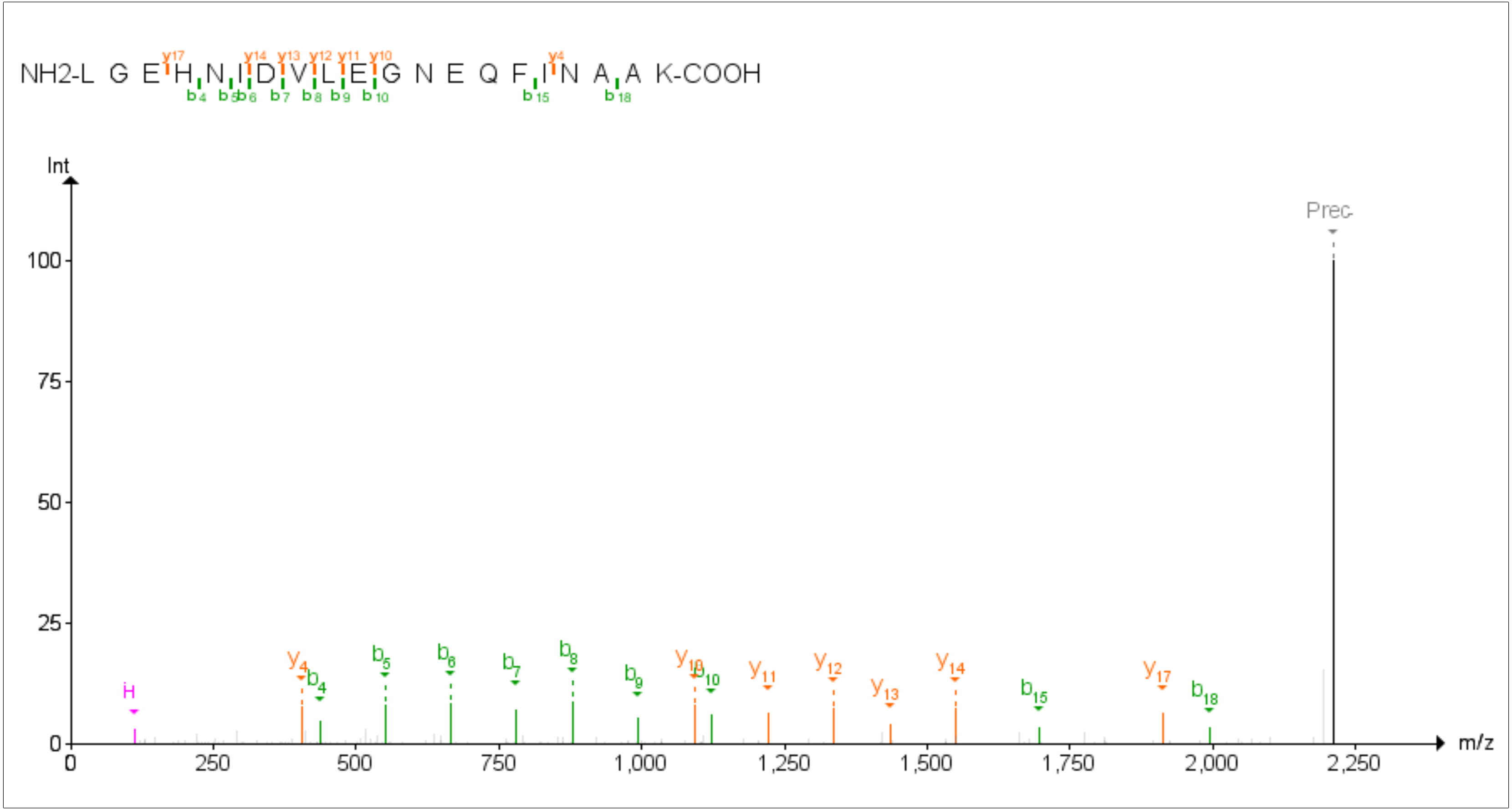

NH2-L G E H N I D V L E G N E Q F I N A A K-COOH
y17 y14 y13 y12 y11 y10 y4
b4 b5 b6 b7 b8 b9 b10 b15 b18
Int
Prec.
100
75
50
25
0
H
y4 b4 b5 b6 b7 b8 b9 y10 b10 y11 y12 y13 y14 b15 y17 b18
0 250 500 750 1,000 1,250 1,500 1,750 2,000 2,250
m/z

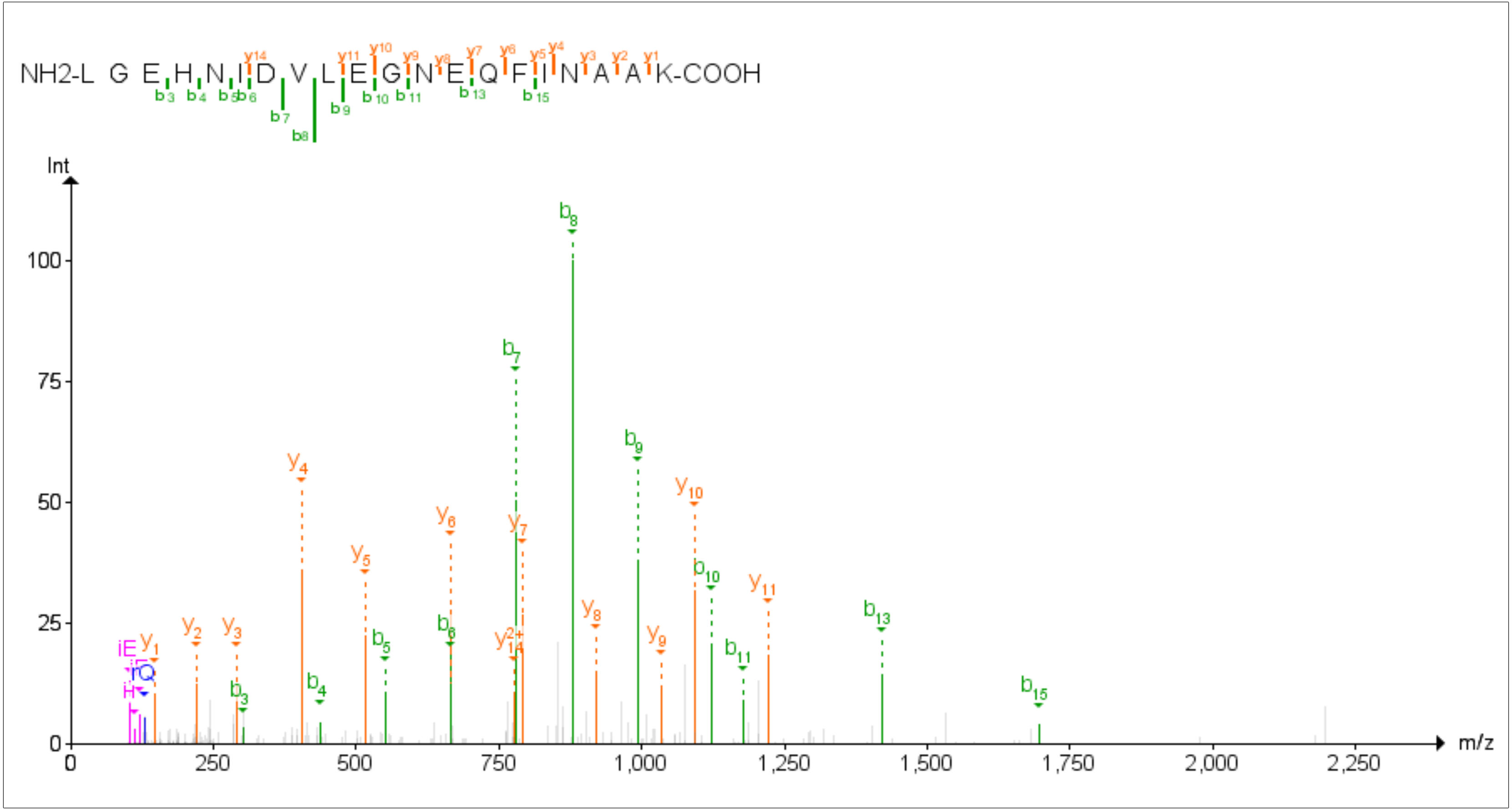
NH2-L G E H N I D V L E G N E Q F I N A A K-COOH
Int
m/z
100
75
50
25
0
250
500
750
1,000
1,250
1,500
1,750
2,000
2,250

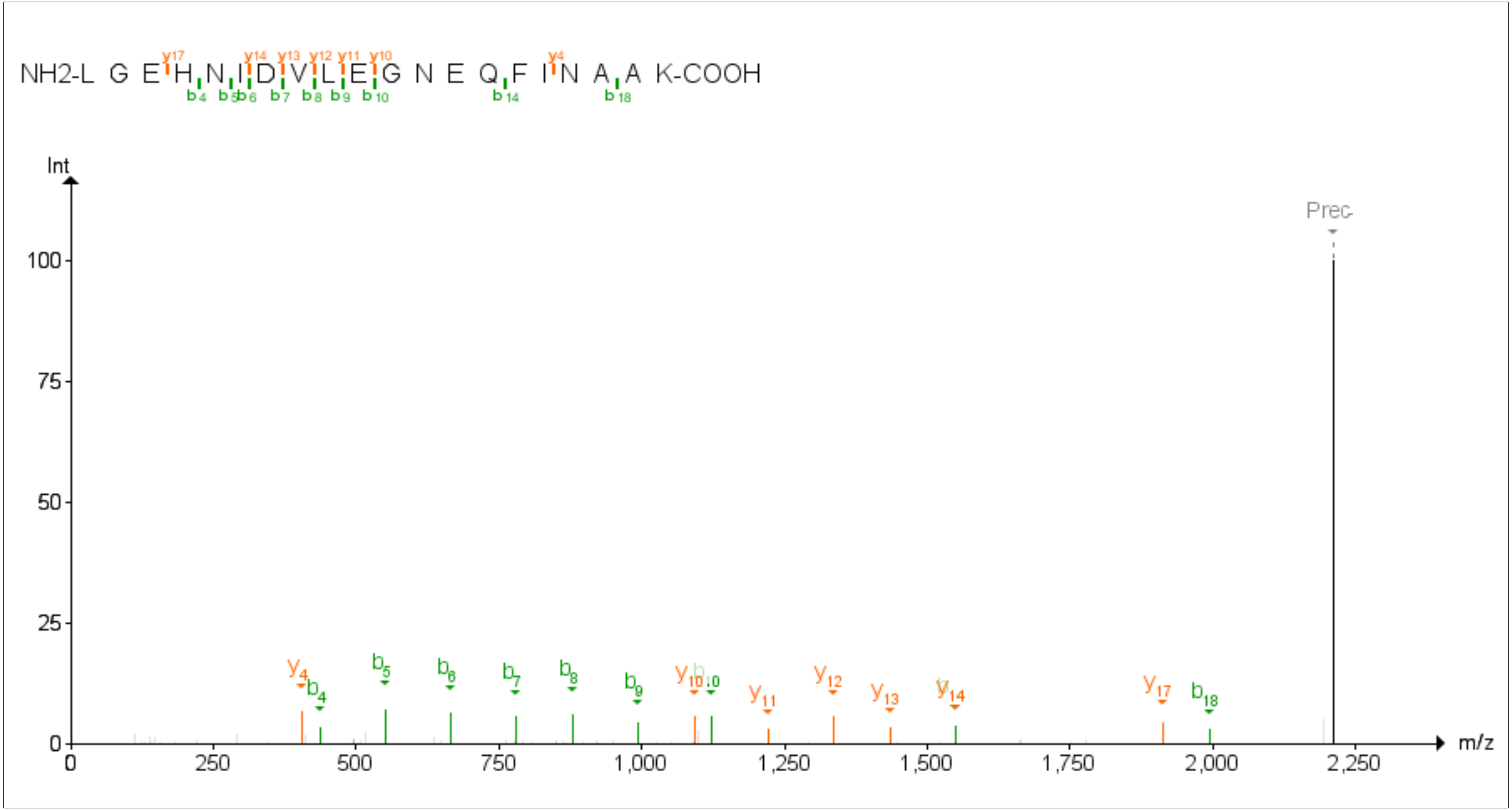
NH2-L G E H N I D V L E G N E Q F I N A A K-COOH
y17 y14 y13 y12 y11 y10 y4
b4 b5 b6 b7 b8 b9 b10 b14 b18
Int
Prec
m/z
0
25
50
75
100
0
250
500
750
1,000
1,250
1,500
1,750
2,000
2,250
y4
b4
b5
b6
b7
b8
b9
y10
y11
y12
y13
y14
y17
b18

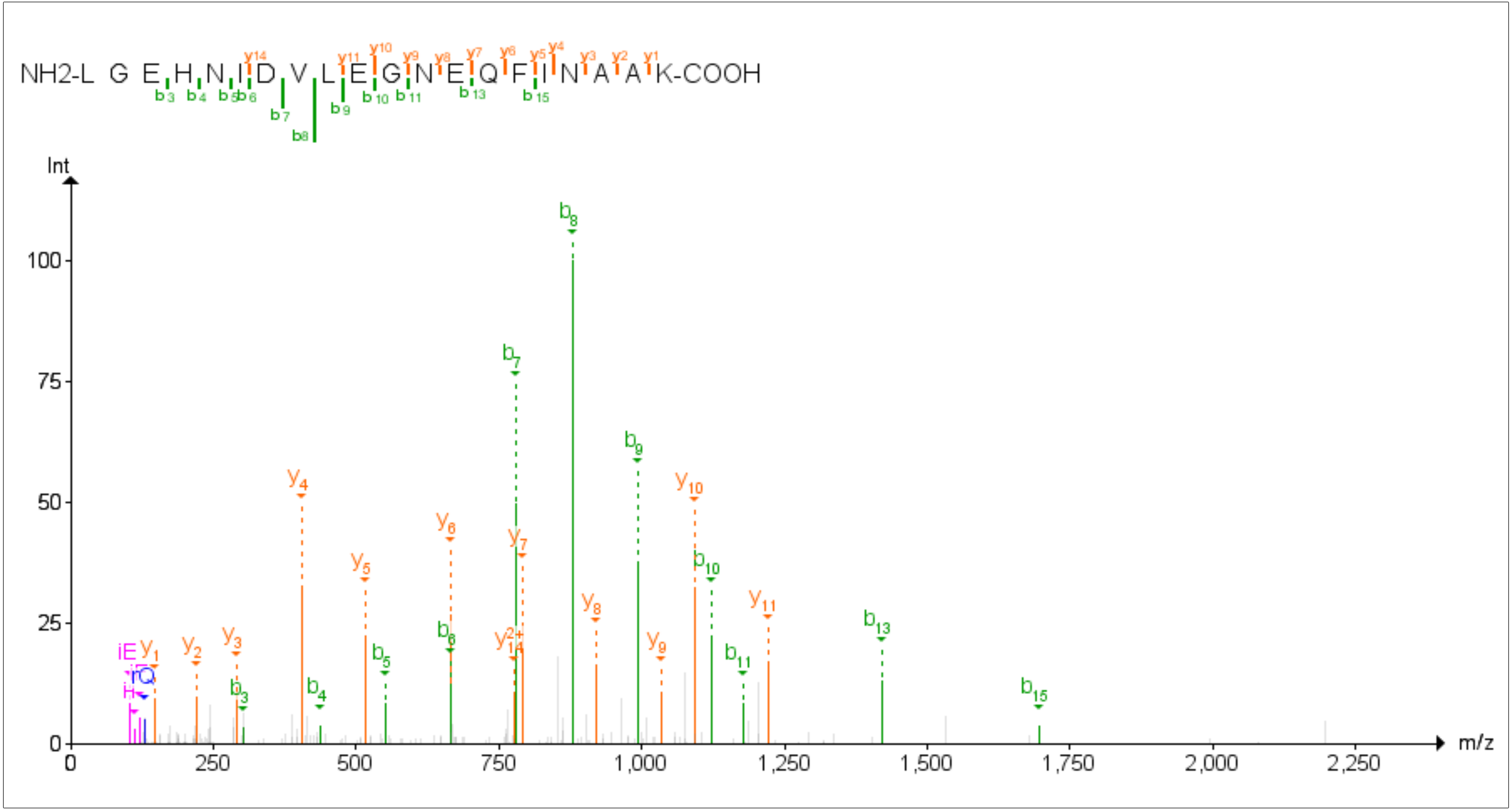
NH2-L G E H N I D V L E G N E Q F I N A A K-COOH
Int
m/z
0
25
50
75
100
250
500
750
1,000
1,250
1,500
1,750
2,000
2,250
b8
b7
b9
y4
y10
y6
y7
b10
y5
y8
y11
b13
b6
b5
y9
b11
y1
y2
y3
b3
b4
b15
iE
rQ
H

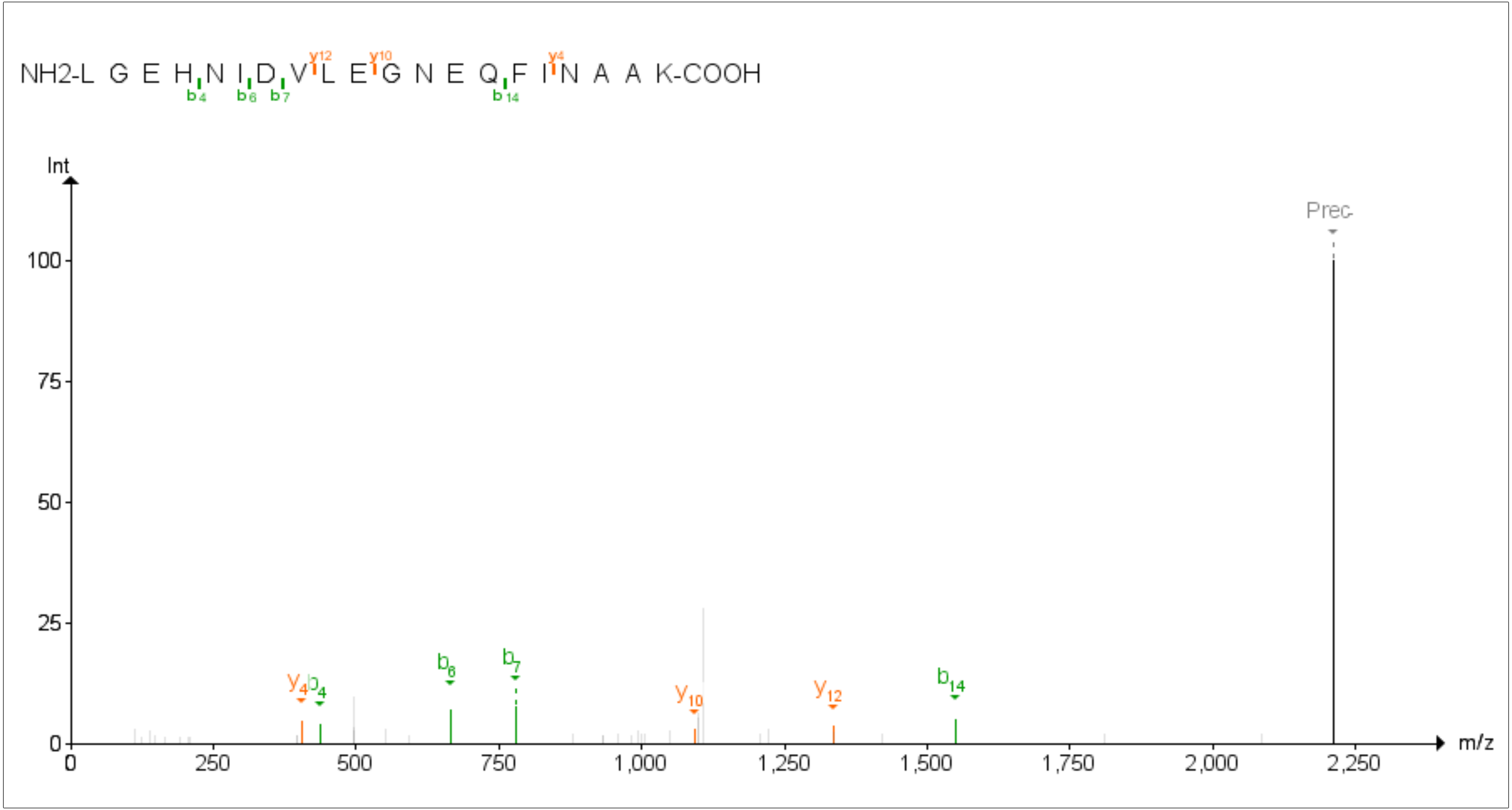

NH2-L G E H N I D V L E G N E Q F I N A A K-COOH
y12
y10
y4
b4
b6
b7
b14
Int
100
75
50
25
0
Prec
y4
b4
b6
b7
y10
y12
b14
0
250
500
750
1,000
1,250
1,500
1,750
2,000
2,250
m/z

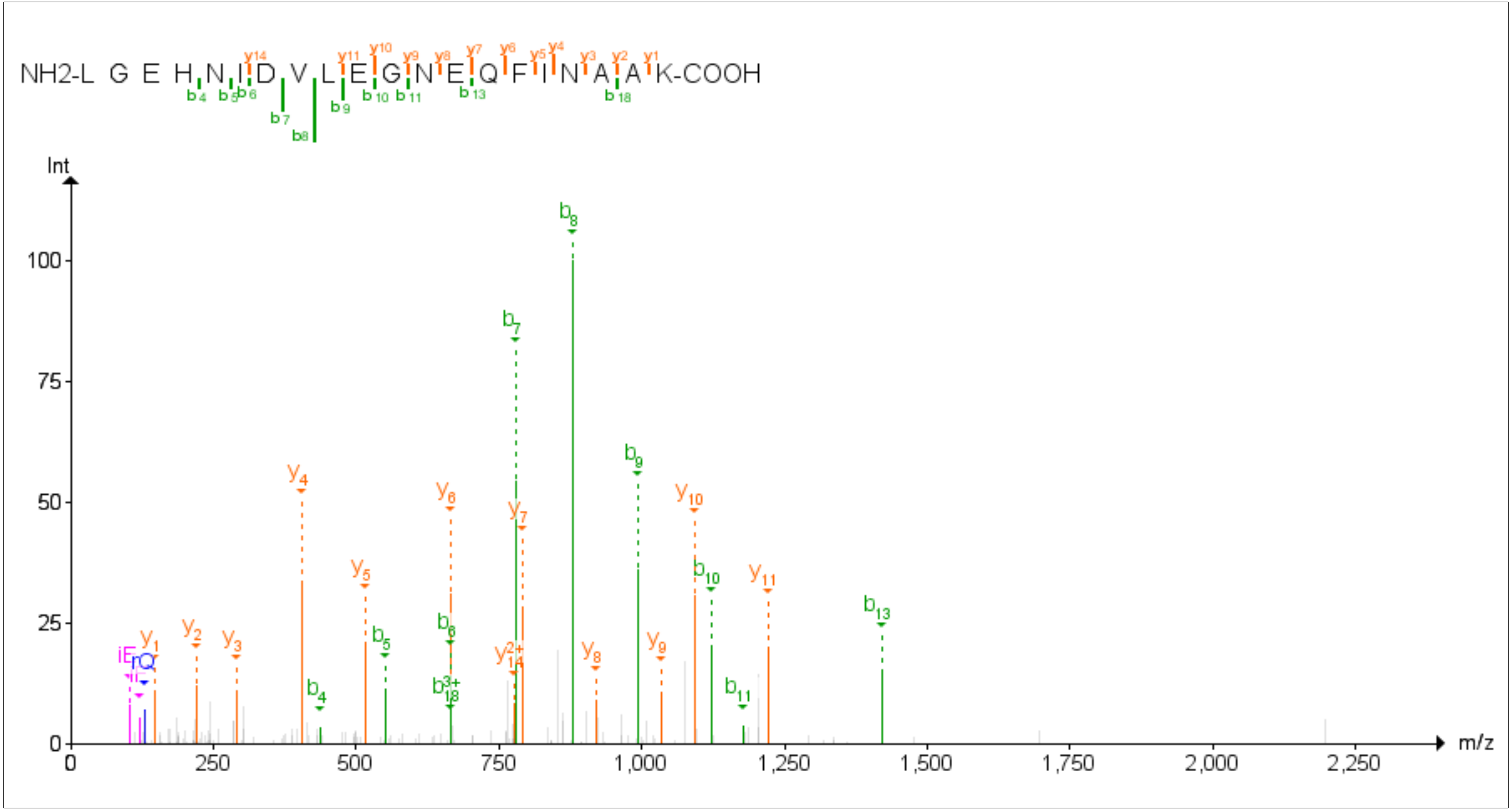
NH2-L G E H N I D V L E G N E Q F I N A A K-COOH
Int
m/z
b8
b7
b9
y4
y6
y7
y10
b10
y11
y5
b13
b5
b6
y1
y2
y3
iE
nQ
b4
b3+ 18
y2+ 14
y8
y9
b11
0
25
50
75
100
0
250
500
750
1,000
1,250
1,500
1,750
2,000
2,250

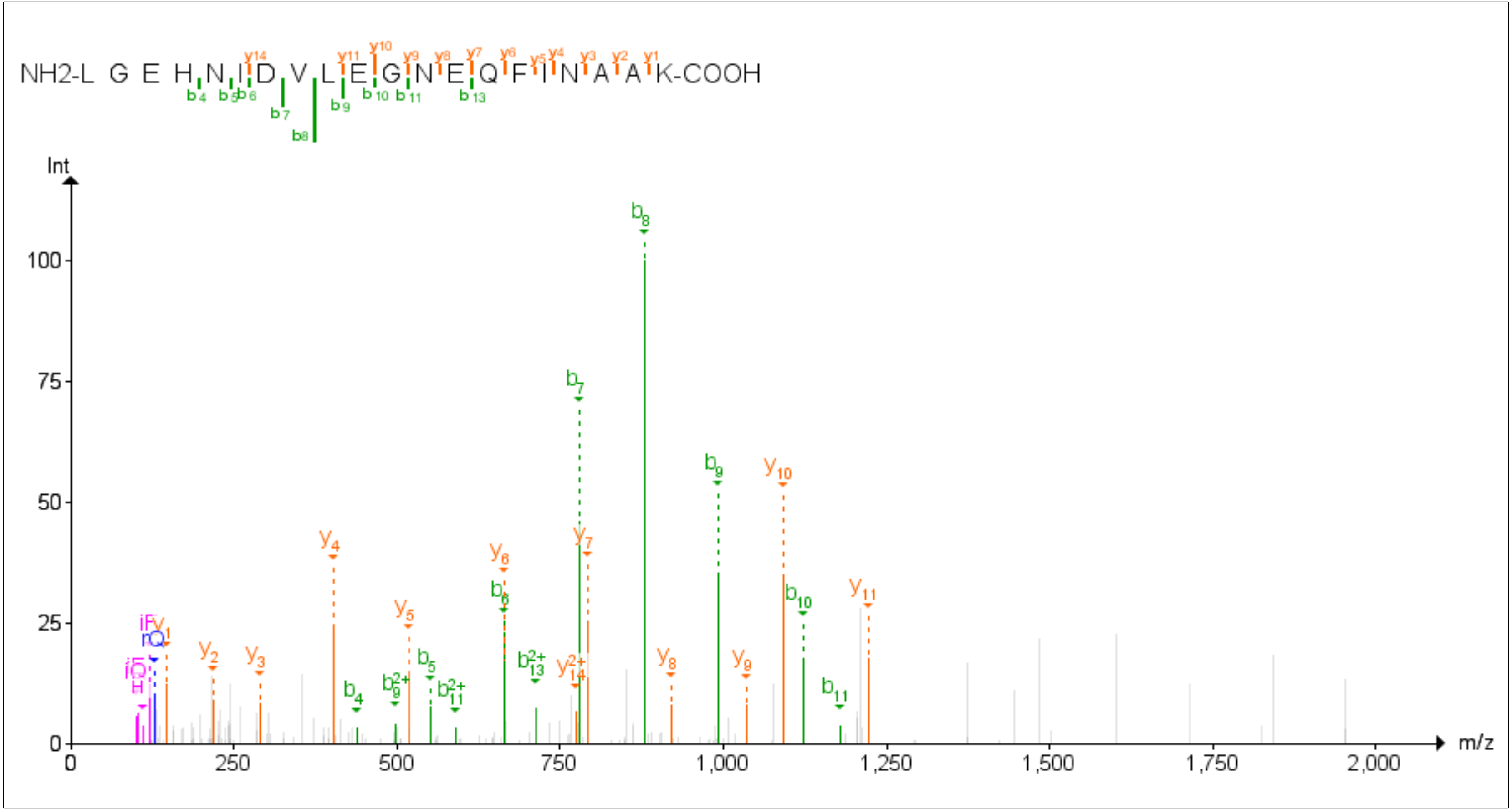

NH2-L G E H N I D V L E G N E Q F I N A A K-COOH
Int
m/z

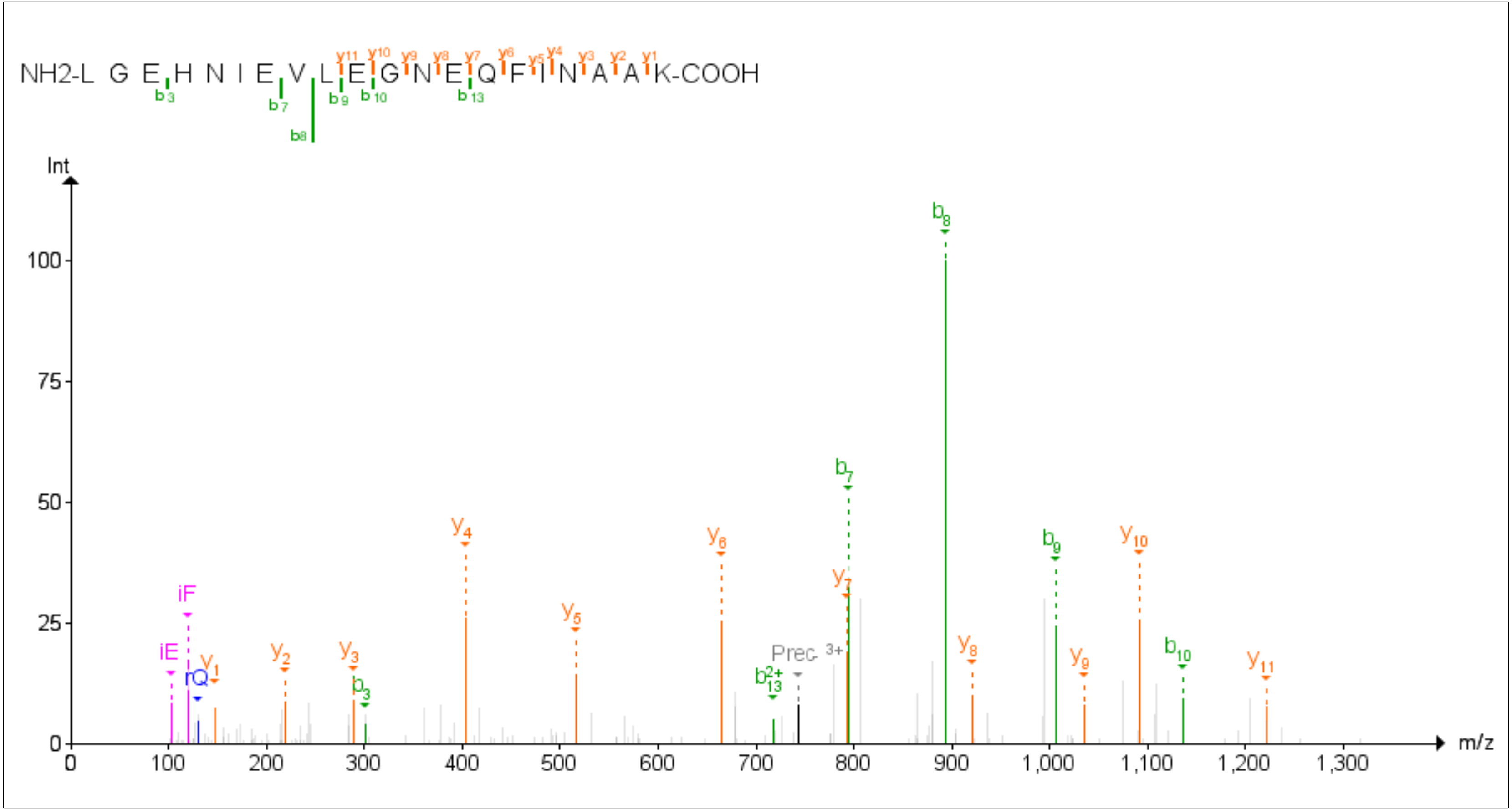

NH2-L G E H N I E V L E G N E Q F I N A A K-COOH
b3 b7 b8 b9 b10 b13
y11 y10 y9 y8 y7 y6 y5 y4 y3 y2 y1
Int
100
75
50
25
0
m/z
0 100 200 300 400 500 600 700 800 900 1,000 1,100 1,200 1,300
iF
iE
rQ
Prec- 3+

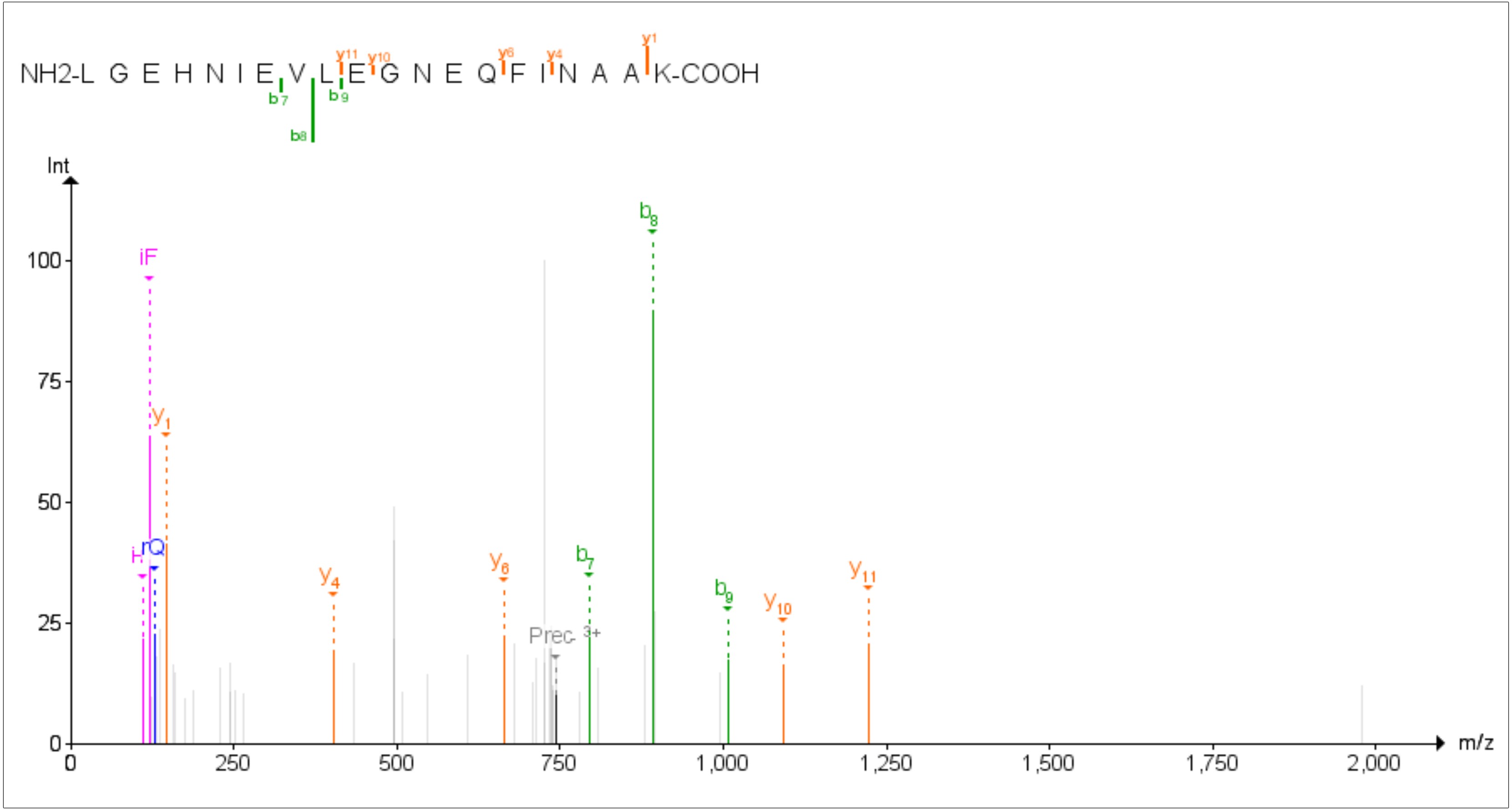

NH2-L G E H N I E V L E G N E Q F I N A A K-COOH
b7 b8 b9 y11 y10 y6 y4 y1
Int
m/z
iF
y1
iH
rQ
y4
y6
b7
b8
b9
y10
y11
Prec 3+
0
25
50
75
100
250
500
750
1,000
1,250
1,500
1,750
2,000

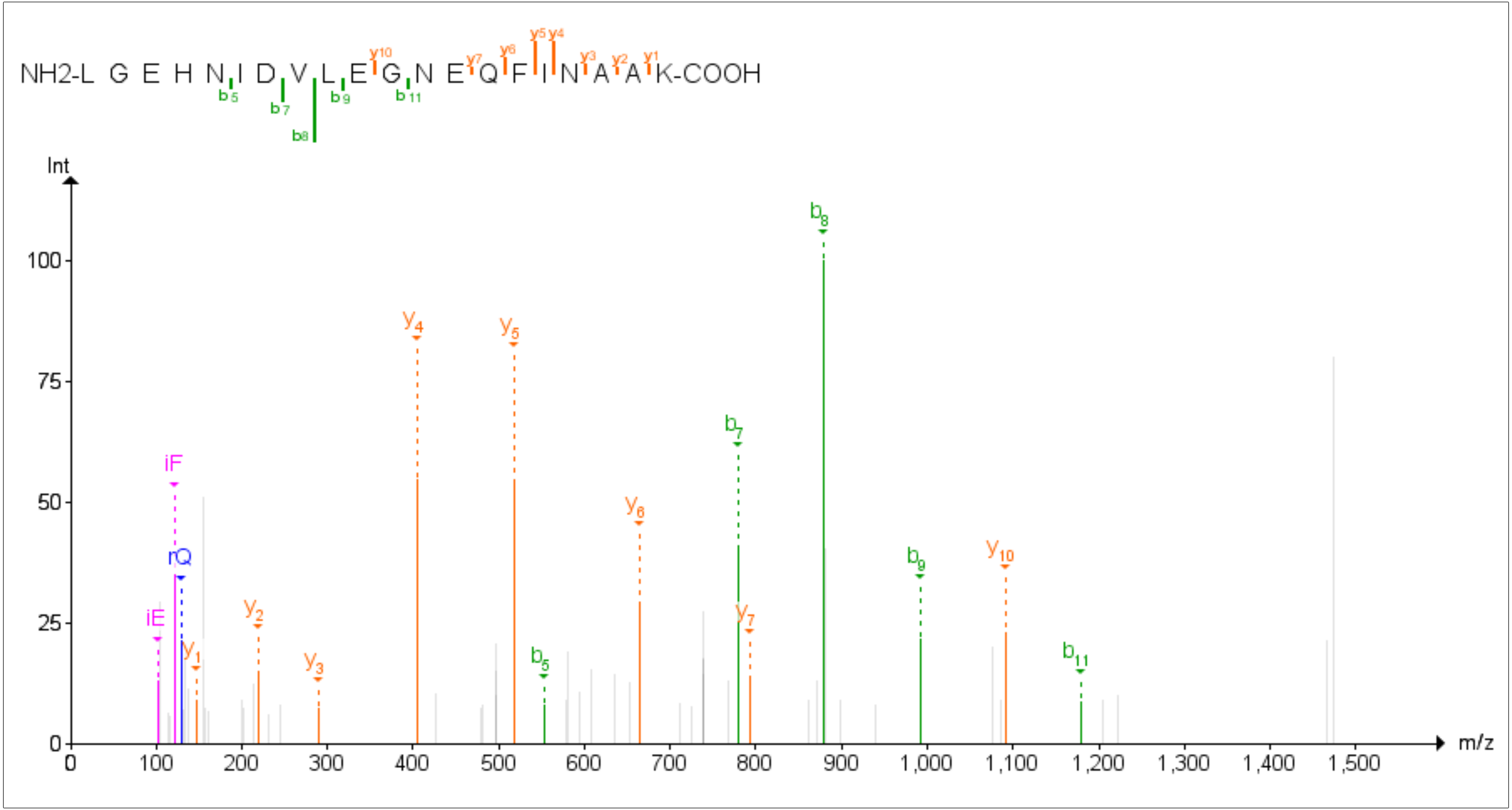

NH2-L G E H N I D V L E G N E Q F I N A A K-COOH
b5
b7
b8
b9
b11
y10
y7
y6
y5
y4
y3
y2
y1
Int
m/z
iF
rQ
iE

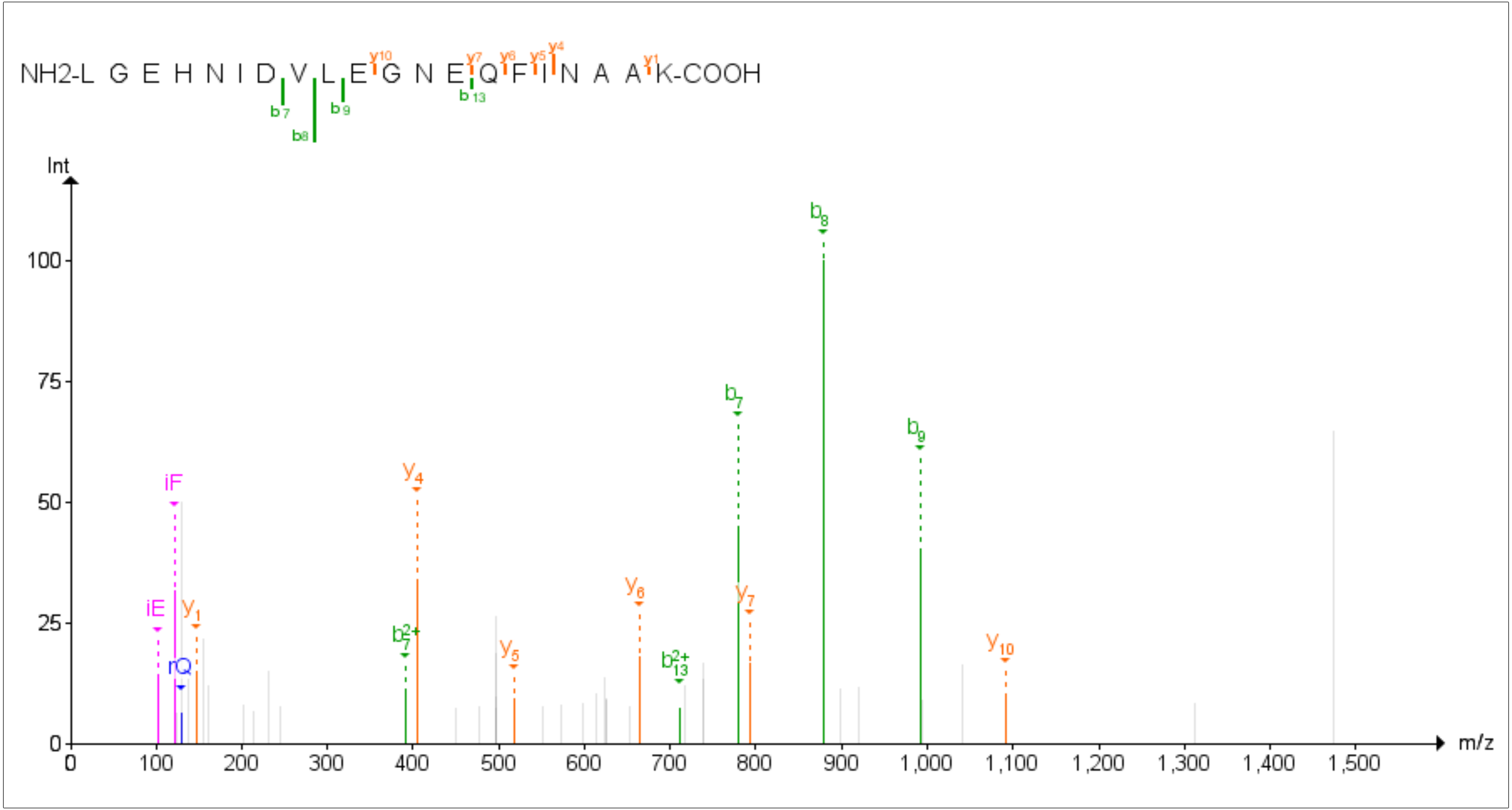

NH2-L G E H N I D V L E G N E Q F I N A A K-COOH
Int
m/z
b8
b7
b9
y4
iF
iE
y1
rQ
y6
y7
y5
y10

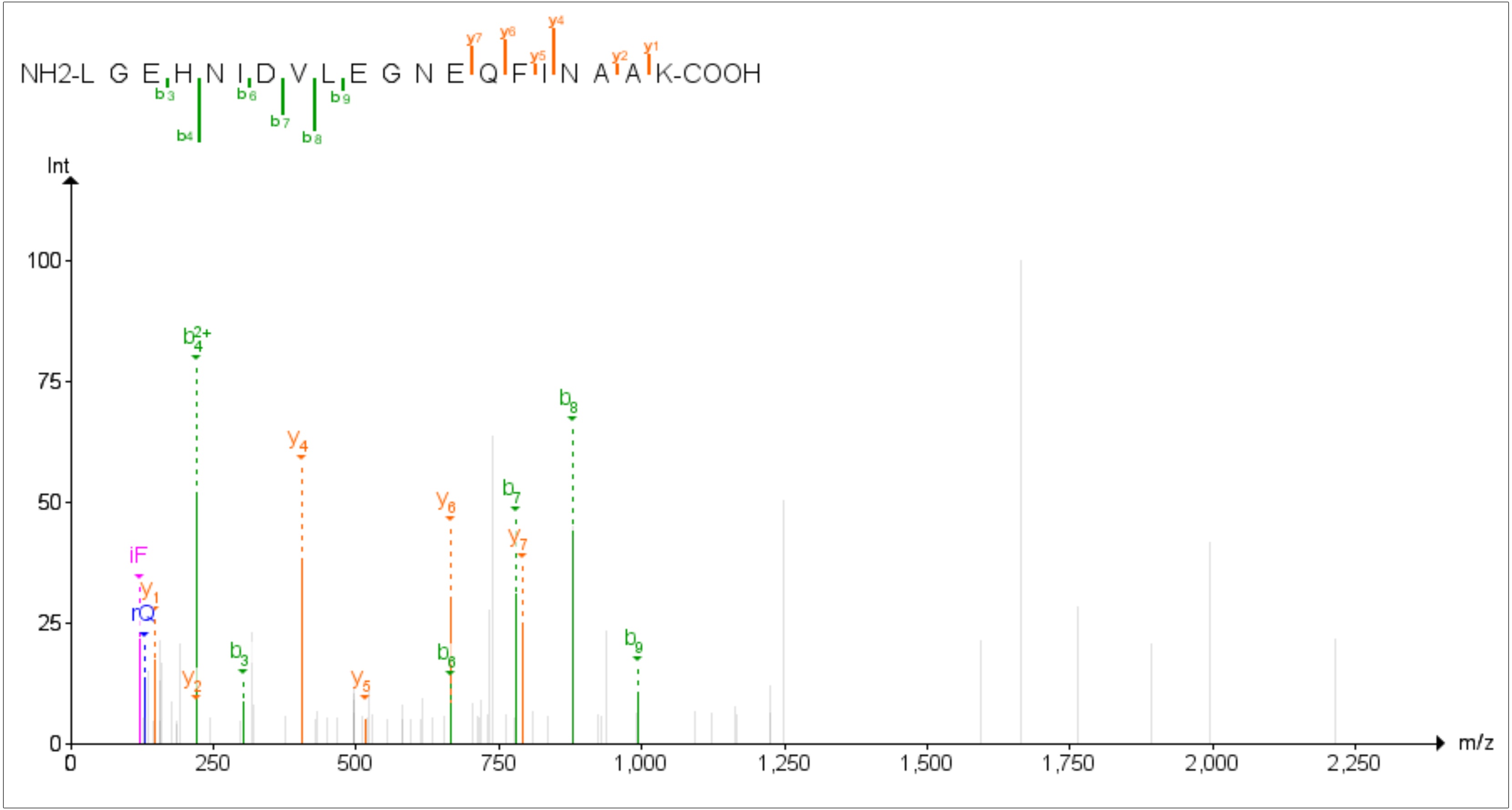
NH2-L G E H N I D V L E G N E Q F I N A A K-COOH
Int
m/z

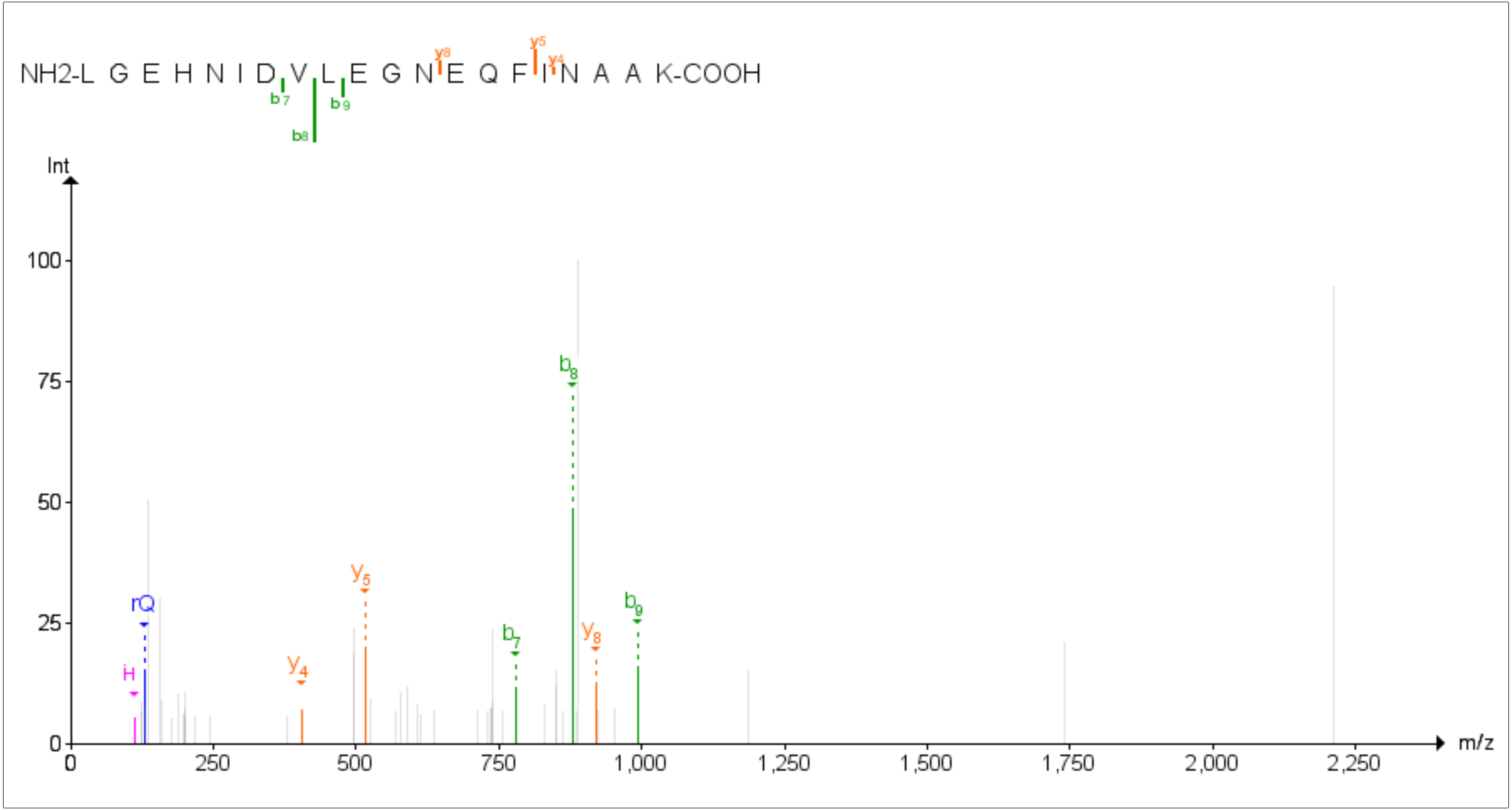

NH2-L G E H N I D V L E G N E Q F I N A A K-COOH
b7
b8
b9
y8
y5
y4
Int
100
75
50
25
0
rQ
H
y4
y5
b7
b8
y8
b9
0
250
500
750
1,000
1,250
1,500
1,750
2,000
2,250
m/z

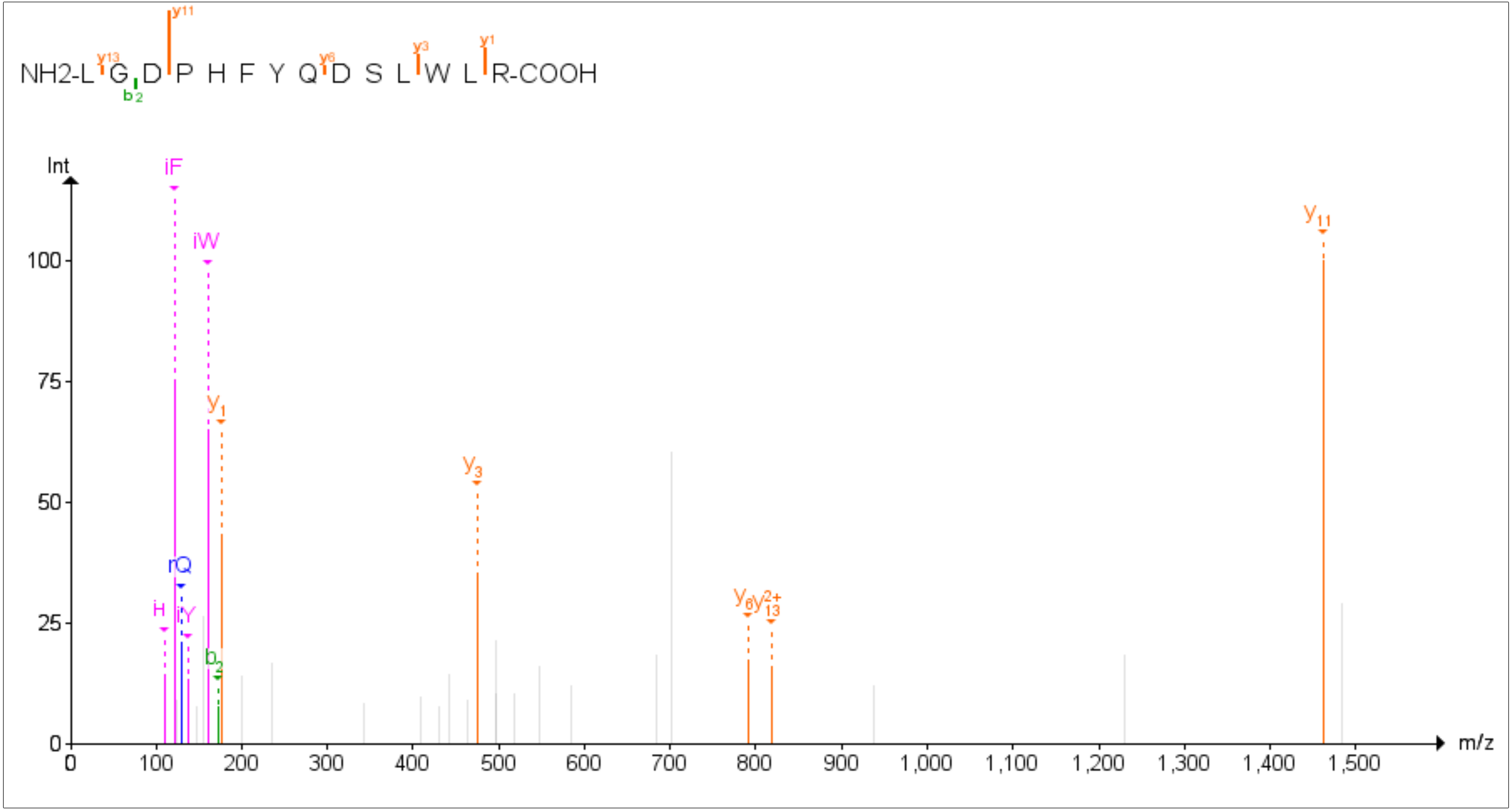

NH2-L G D P H F Y Q D S L W L R-COOH
y13
y11
y6
y3
y1
b2
Int
iF
iW
y11
y1
y3
rQ
iH
iY
b2
y6
y13 2+
m/z
0
25
50
75
100
0
100
200
300
400
500
600
700
800
900
1,000
1,100
1,200
1,300
1,400
1,500

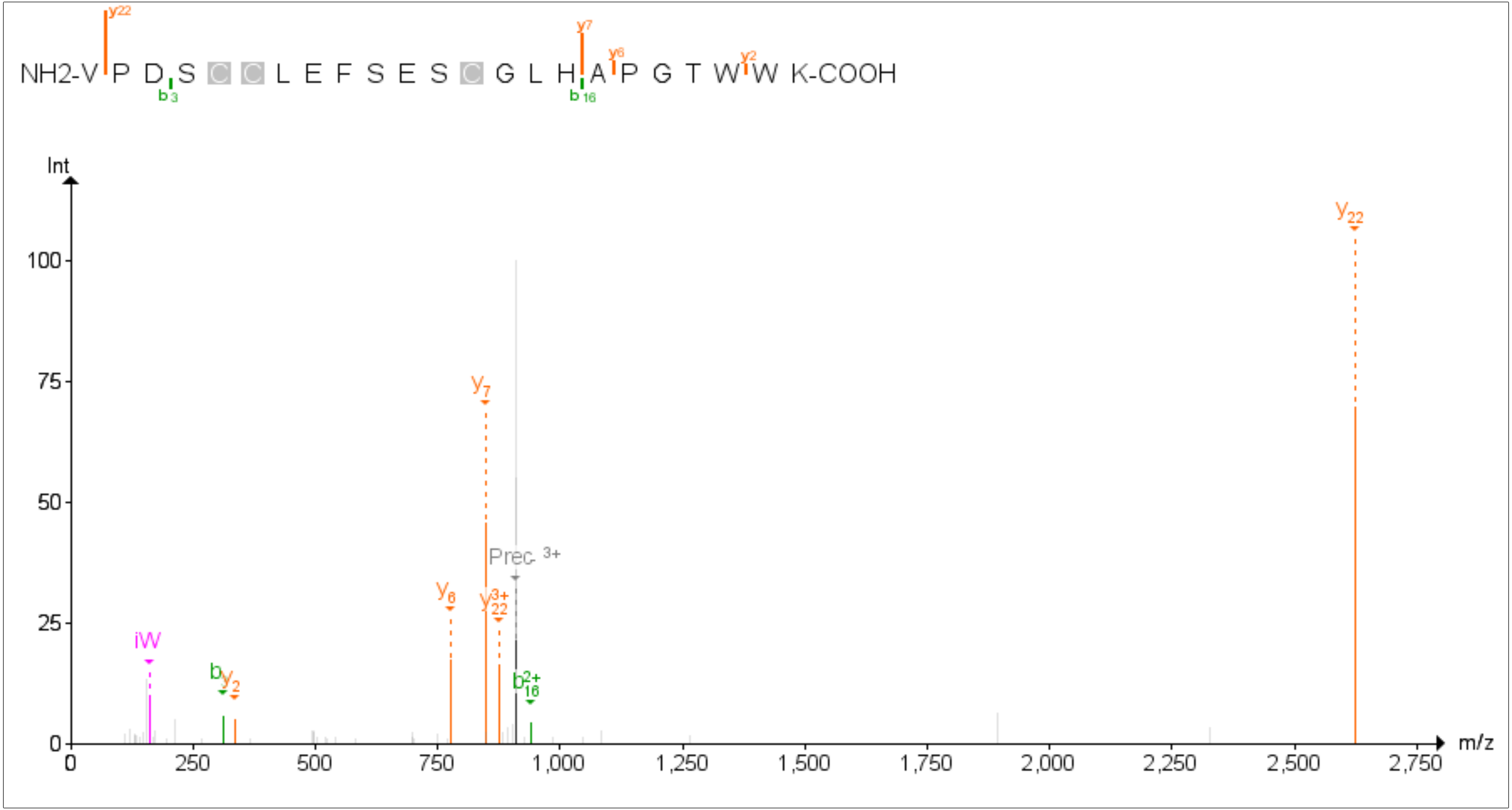

NH2-V P D S C C L E F S E S C G L H A P G T W W K-COOH
y22
y7
y6
y2
b3
b16
Int
100
75
50
25
0
y22
y7
Prec 3+
y6
y22 3+
iW
b
y2
b16 2+
0
250
500
750
1,000
1,250
1,500
1,750
2,000
2,250
2,500
2,750
m/z

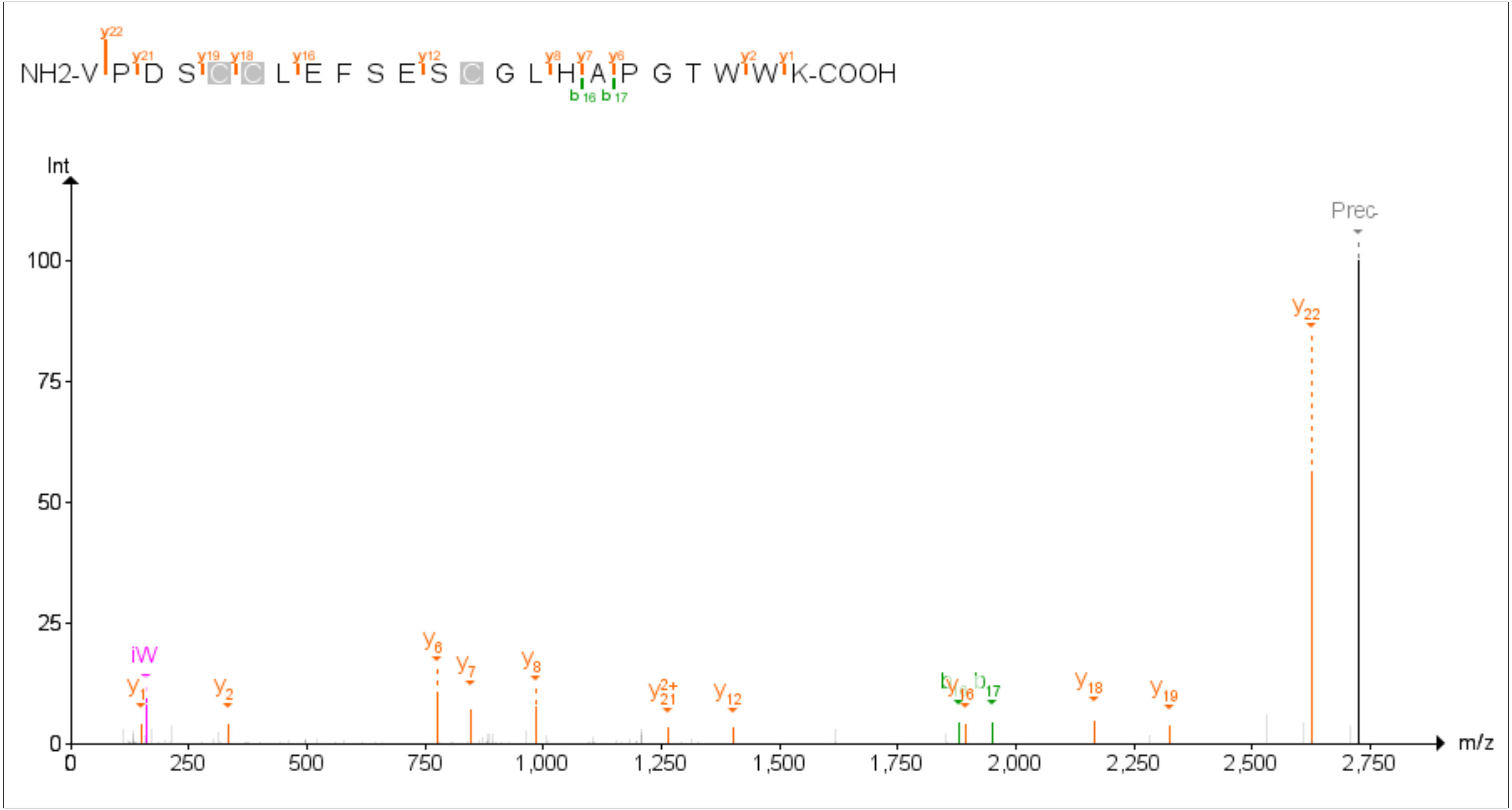

NH2-V P D S C C L E F S E S C G L H A P G T W W K-COOH
y22 y21 y19 y18 y16 y12 y8 y7 y6 y2 y1
b16 b17
Int
m/z
Prec.
y22
iW
y1
y2
y6
y7
y8
y21 2+
y12
b16 y16 b17
y18
y19
0
25
50
75
100
0
250
500
750
1,000
1,250
1,500
1,750
2,000
2,250
2,500
2,750

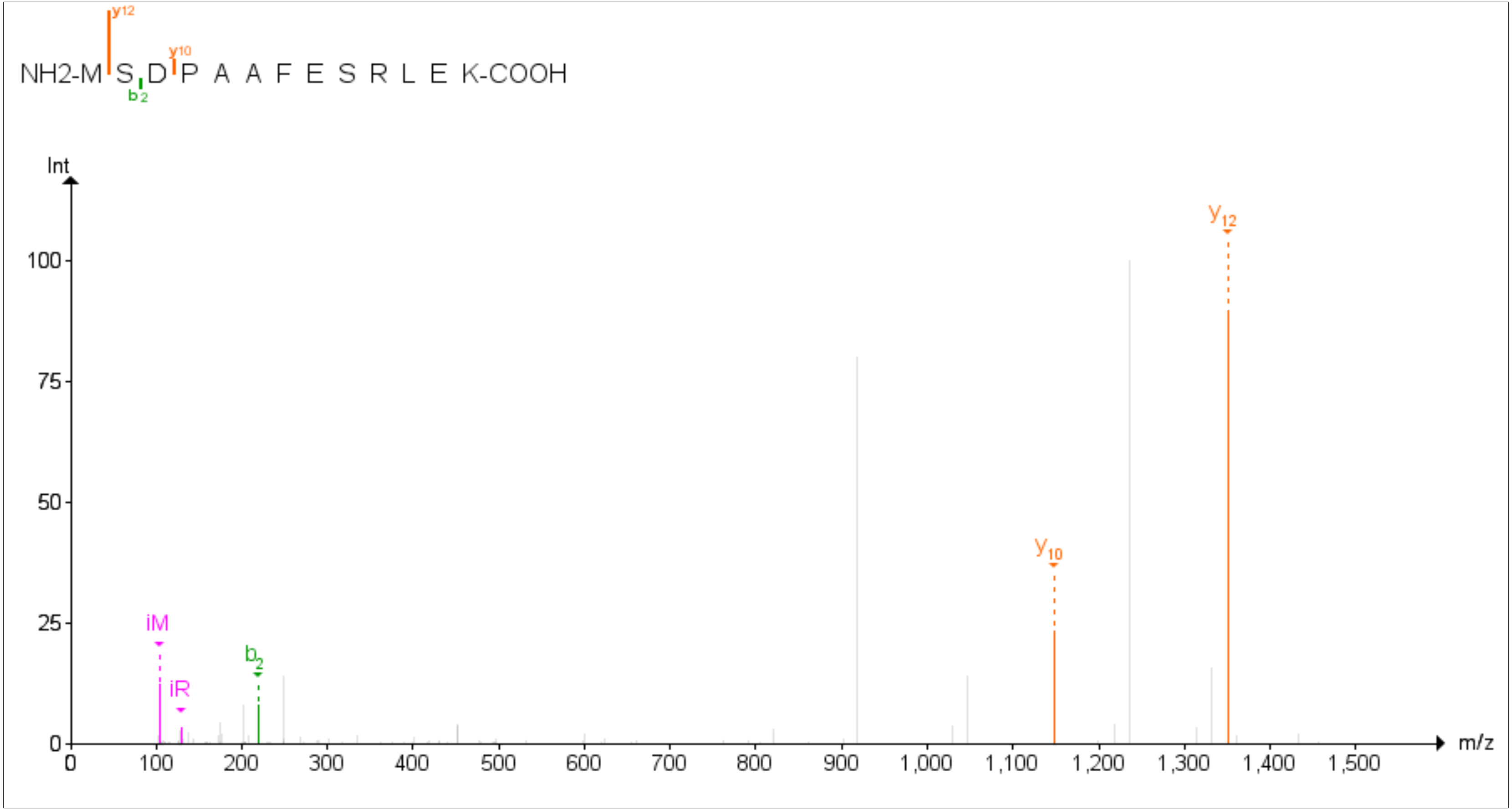

NH2-M S D P A A F E S R L E K-COOH
y12
y10
b2
Int
100
75
50
25
0
iM
iR
m/z
0
100
200
300
400
500
600
700
800
900
1,000
1,100
1,200
1,300
1,400
1,500

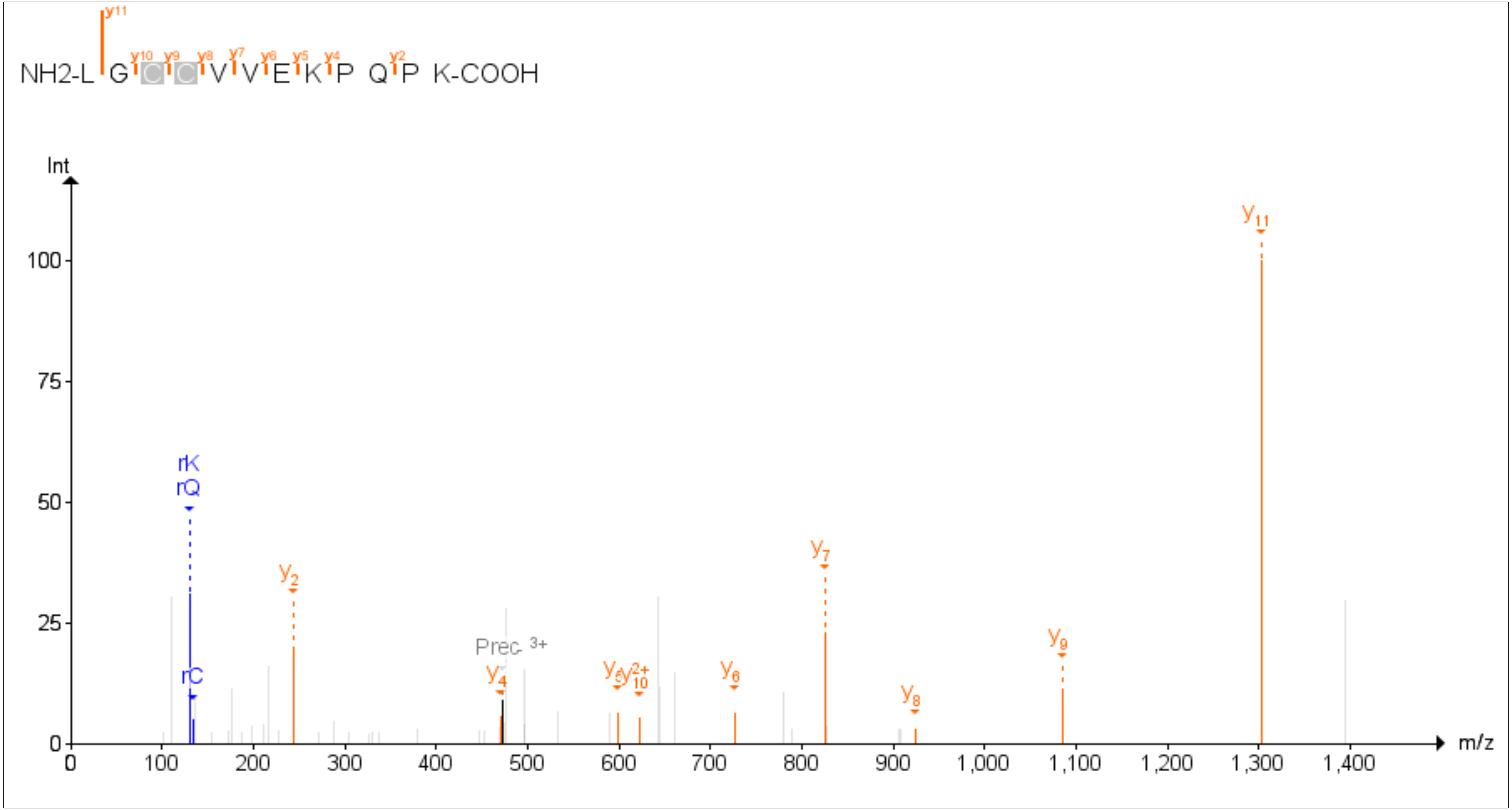

NH2-L G C C V V E K P Q P K-COOH
y11 y10 y9 y8 y7 y6 y5 y4 y2
Int
100
75
50
25
0
rK
rQ
rC
y2
Prec 3+
y4
y5
y2+ 10
y6
y7
y8
y9
y11
0 100 200 300 400 500 600 700 800 900 1,000 1,100 1,200 1,300 1,400
m/z

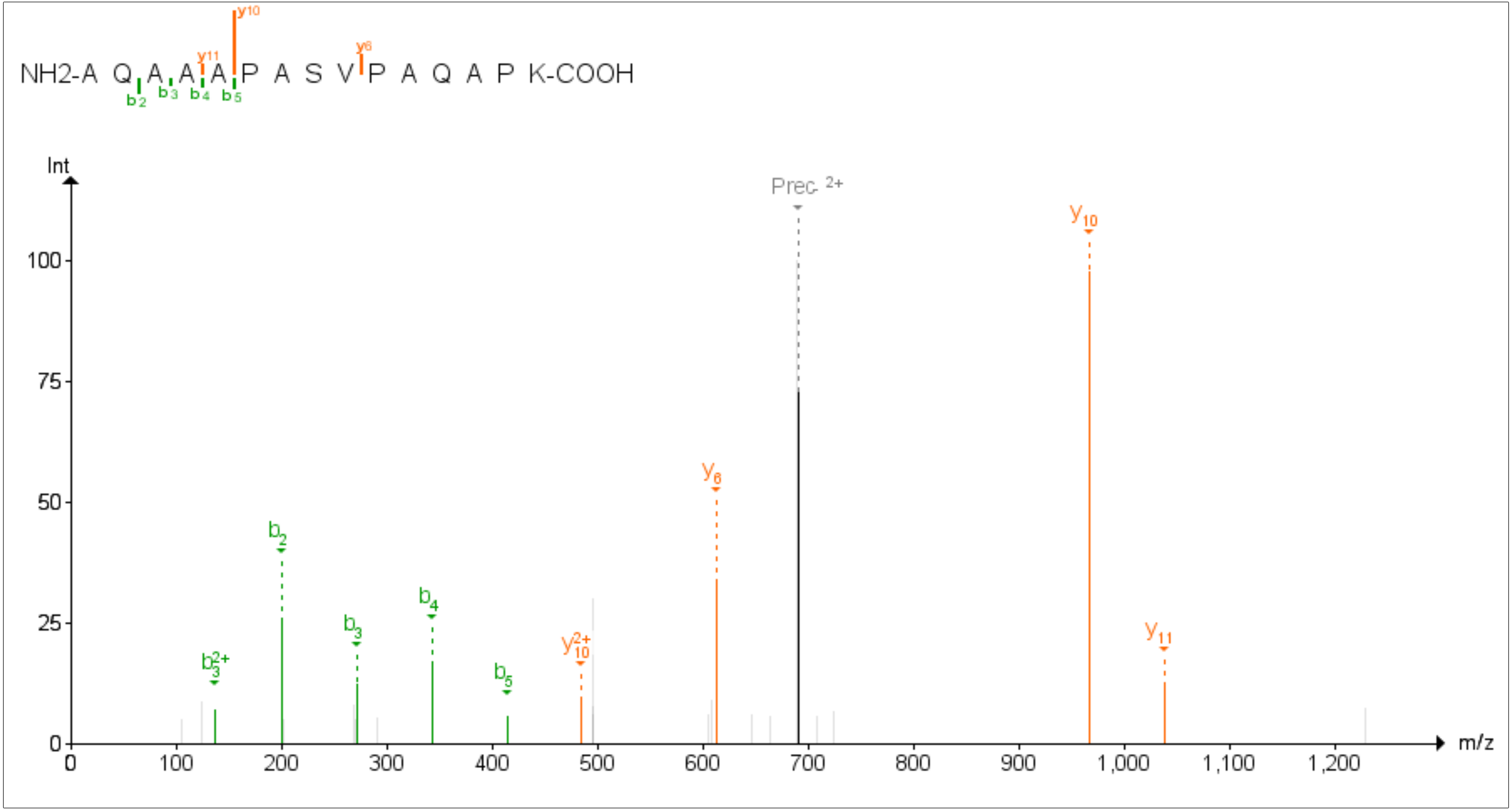

NH2-A Q A A A P A S V P A Q A P K-COOH
y10
y11
y6
b2
b3
b4
b5
Int
Prec 2+
y10
y6
b2
b4
b3
y11
b3 2+
b5
y10 2+
100
75
50
25
0
0
100
200
300
400
500
600
700
800
900
1,000
1,100
1,200
m/z

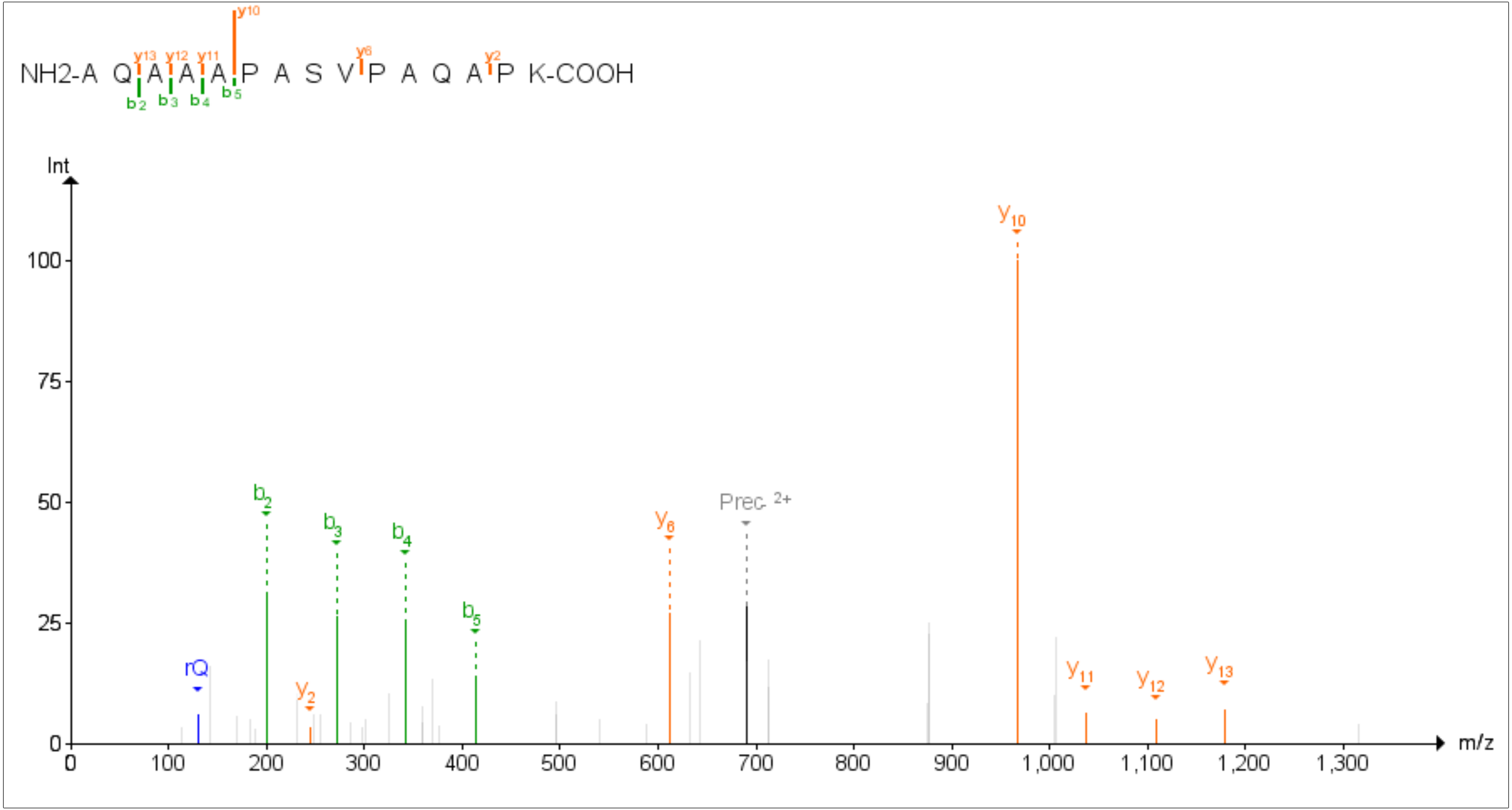

NH2-A Q A A A P A S V P A Q A P K-COOH
y10
y13 y12 y11
y6
y2
b2 b3 b4 b5
Int
100
75
50
25
0
y10
b2
b3
b4
b5
y6
Prec 2+
rQ
y2
y11
y12
y13
0 100 200 300 400 500 600 700 800 900 1,000 1,100 1,200 1,300
m/z

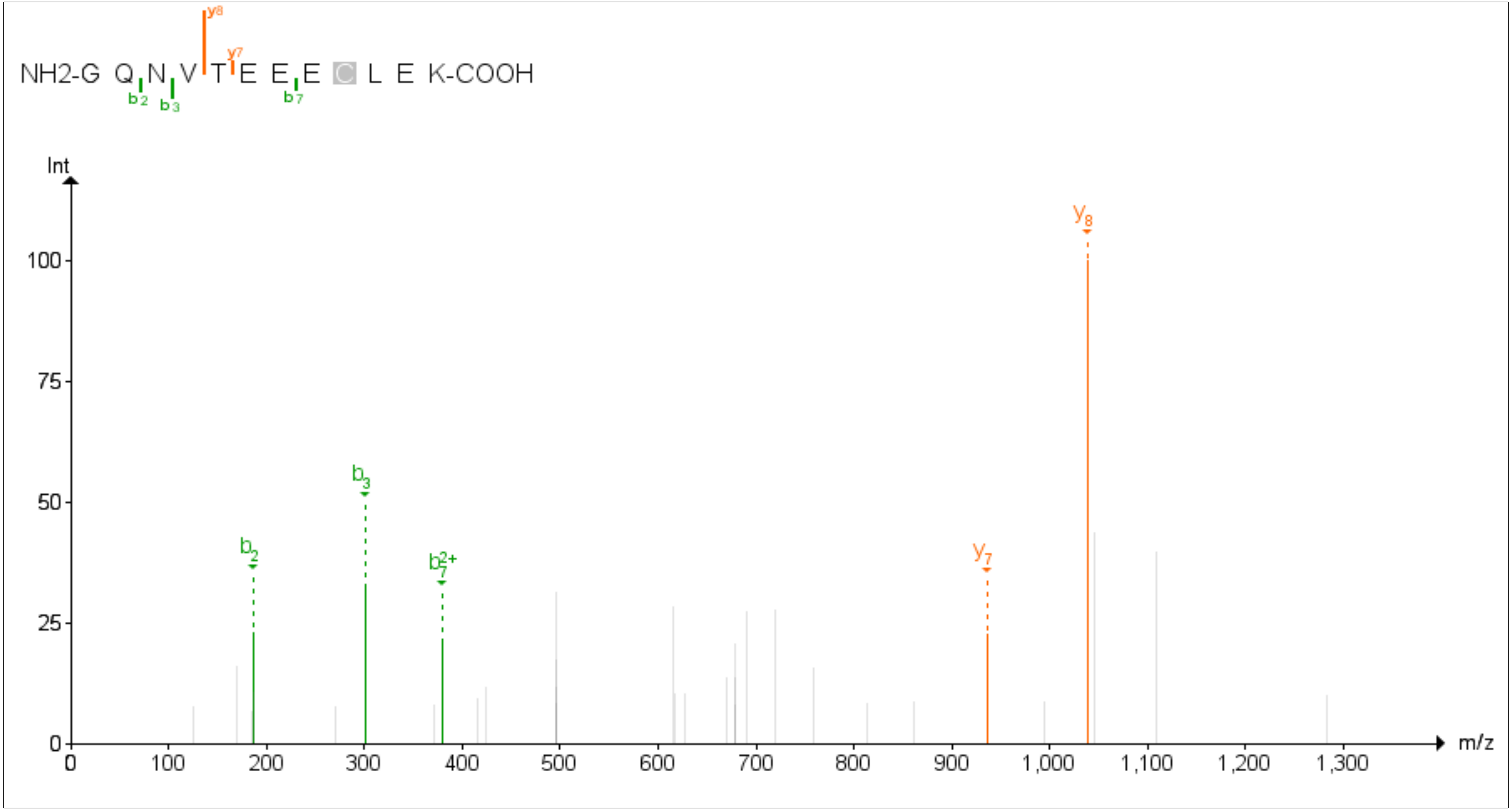

NH2-G Q N V T E E E C L E K-COOH
y8
y7
b2
b3
b7
Int
100
75
50
25
0
m/z
0
100
200
300
400
500
600
700
800
900
1,000
1,100
1,200
1,300

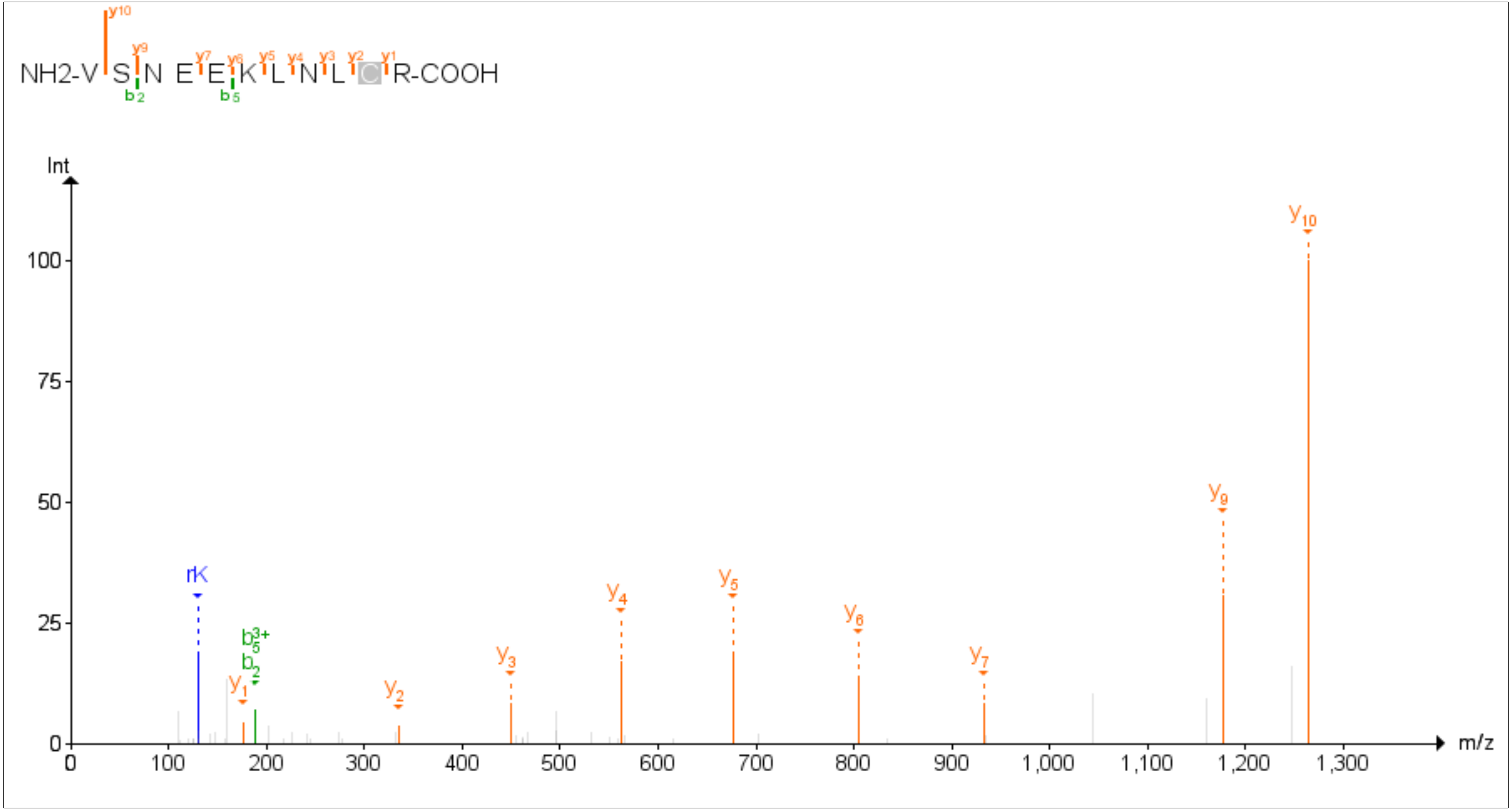

NH2-V S N E E K L N L C R-COOH
y10
y9
y7
y6
y5
y4
y3
y2
y1
b2
b5
Int
100
75
50
25
0
rK
b5 3+
b2
y1
y2
y3
y4
y5
y6
y7
y9
y10
0
100
200
300
400
500
600
700
800
900
1,000
1,100
1,200
1,300
m/z

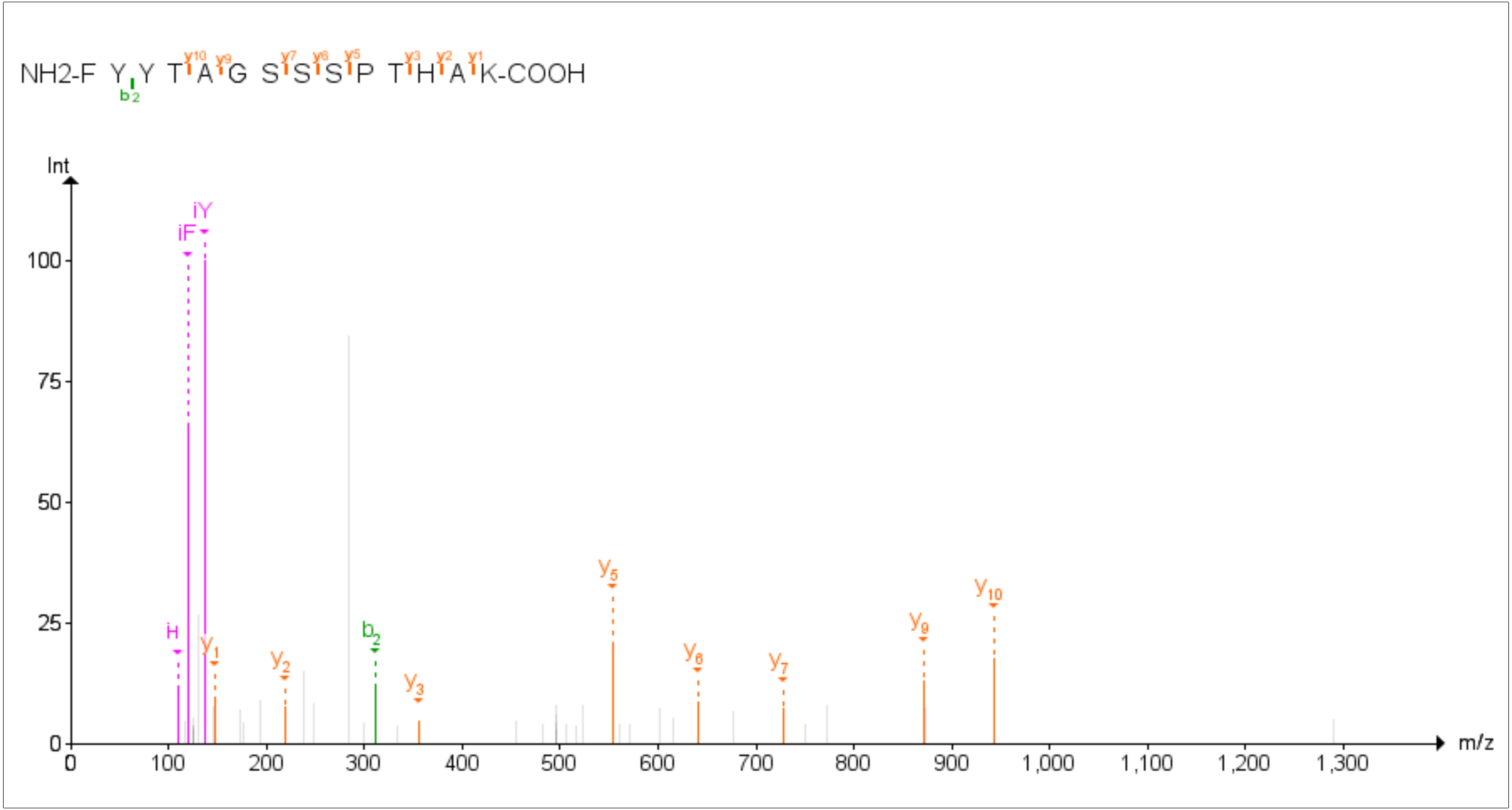

NH2-F Y Y T A G S S S P T H A K-COOH
b2
y10 y9 y7 y6 y5 y3 y2 y1
Int
m/z
iY
iF
iH
y1
y2
b2
y3
y5
y6
y7
y9
y10
0
25
50
75
100
0
100
200
300
400
500
600
700
800
900
1,000
1,100
1,200
1,300

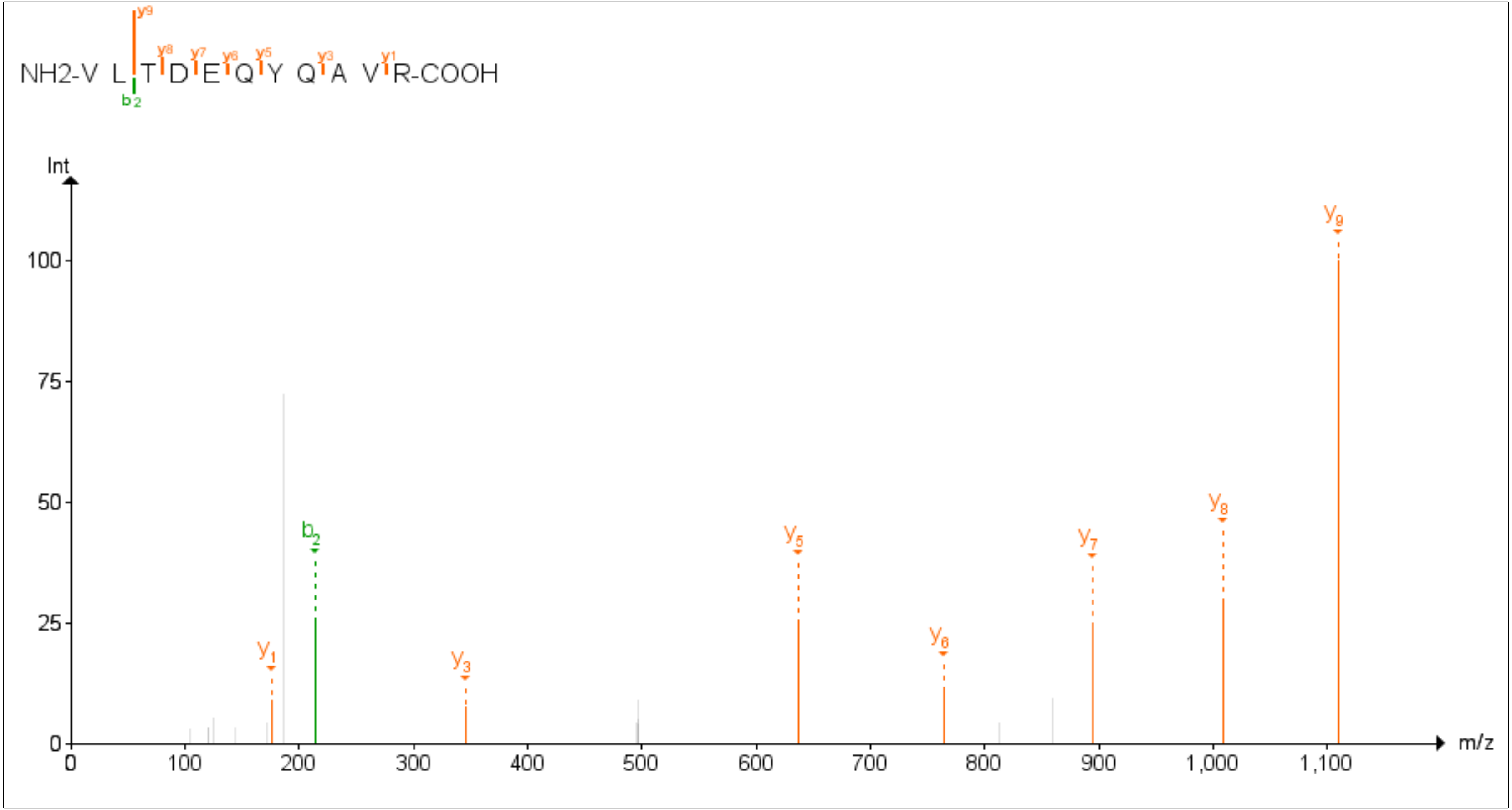
NH2-V L T D E Q Y Q A V R-COOH
y9
y8
y7
y6
y5
y3
y1
b2
Int
100
75
50
25
0
m/z
0
100
200
300
400
500
600
700
800
900
1,000
1,100
y9
y8
b2
y5
y7
y6
y1
y3

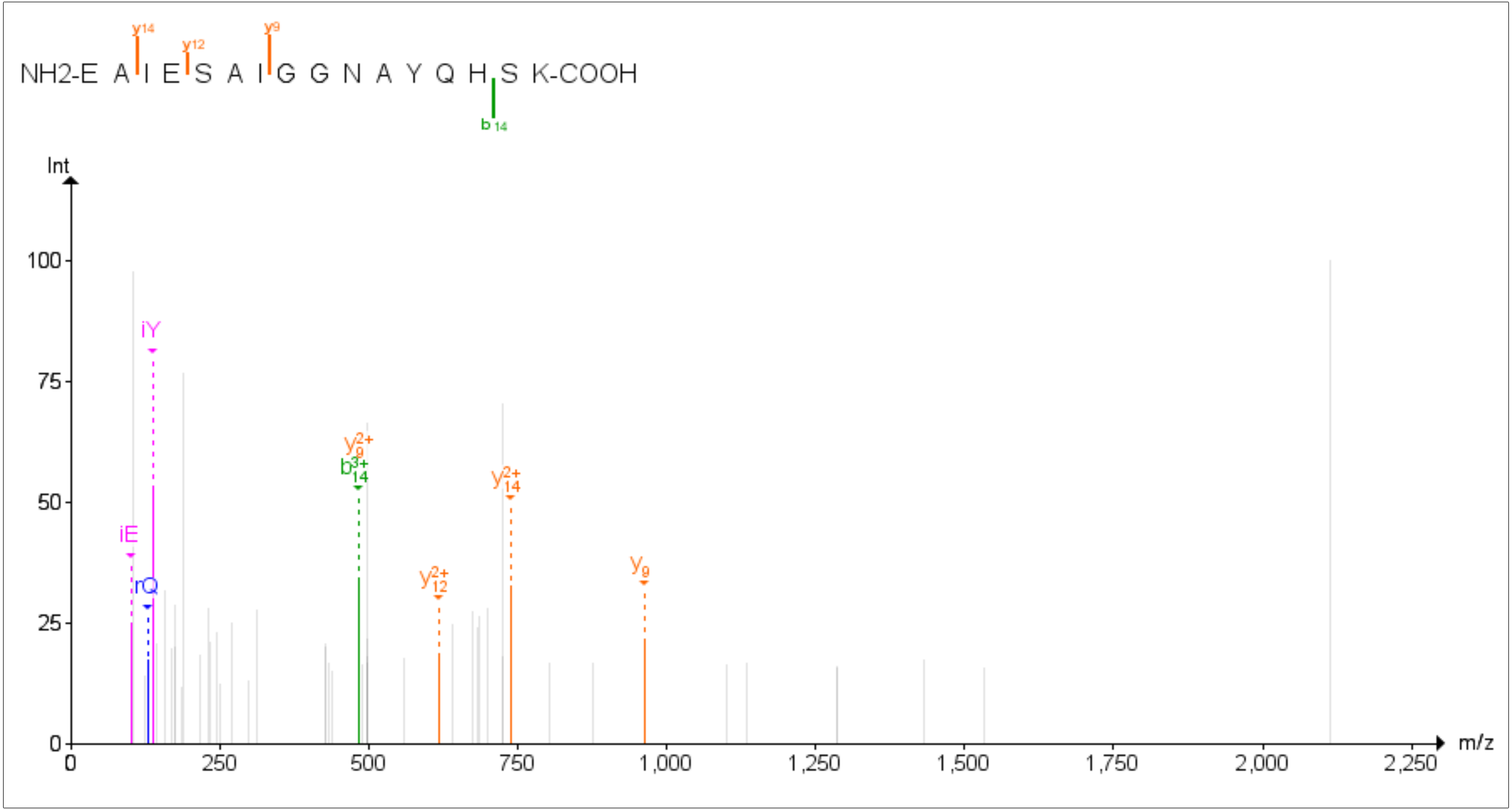
NH2-E A I E S A I G G N A Y Q H S K-COOH
y14
y12
y9
b 14
Int
100
75
50
25
0
iY
iE
rQ
y9 2+
b14 3+
y14 2+
y12 2+
y9
0
250
500
750
1,000
1,250
1,500
1,750
2,000
2,250
m/z

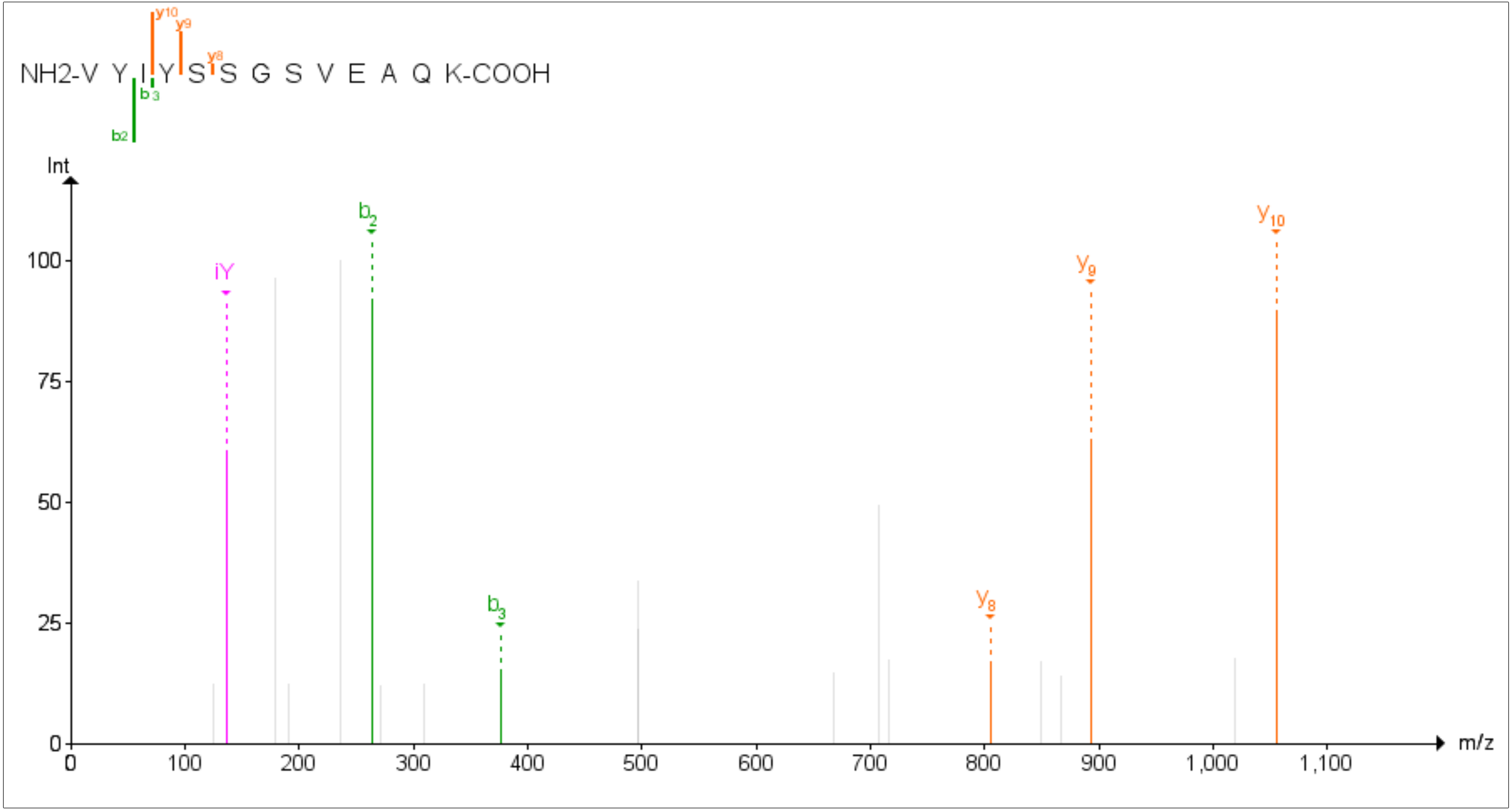
NH2-V Y I Y S S G S V E A Q K-COOH
y10
y9
y8
b3
b2
Int
iY
b2
b3
y8
y9
y10
100
75
50
25
0
0
100
200
300
400
500
600
700
800
900
1,000
1,100
m/z

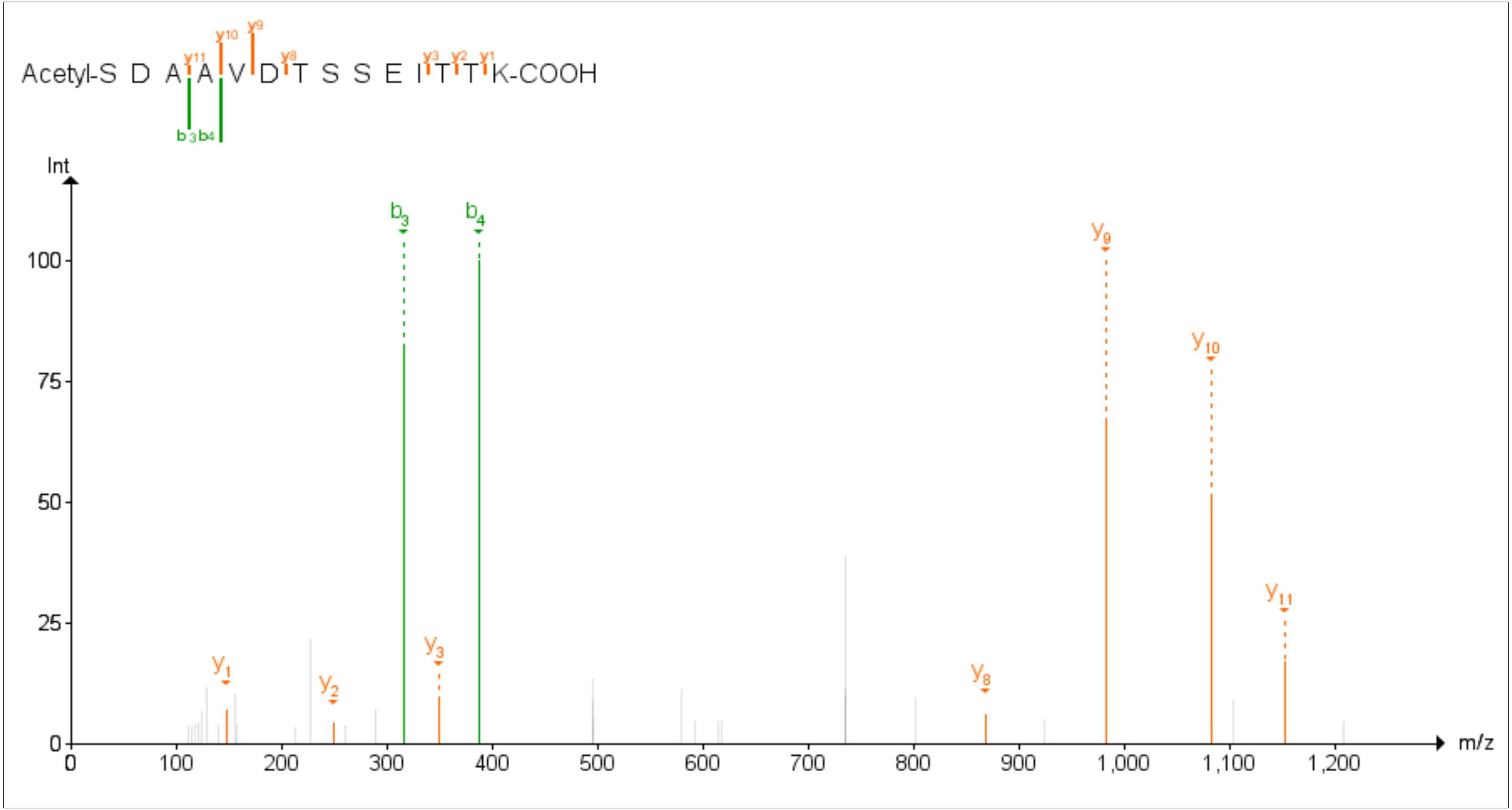
Acetyl-S D A A V D T S S E I T T K-COOH
y11 y10 y9 y8 y3 y2 y1
b3 b4
Int
100
75
50
25
0
0 100 200 300 400 500 600 700 800 900 1,000 1,100 1,200
m/z
b3 b4 y1 y2 y3 y8 y9 y10 y11

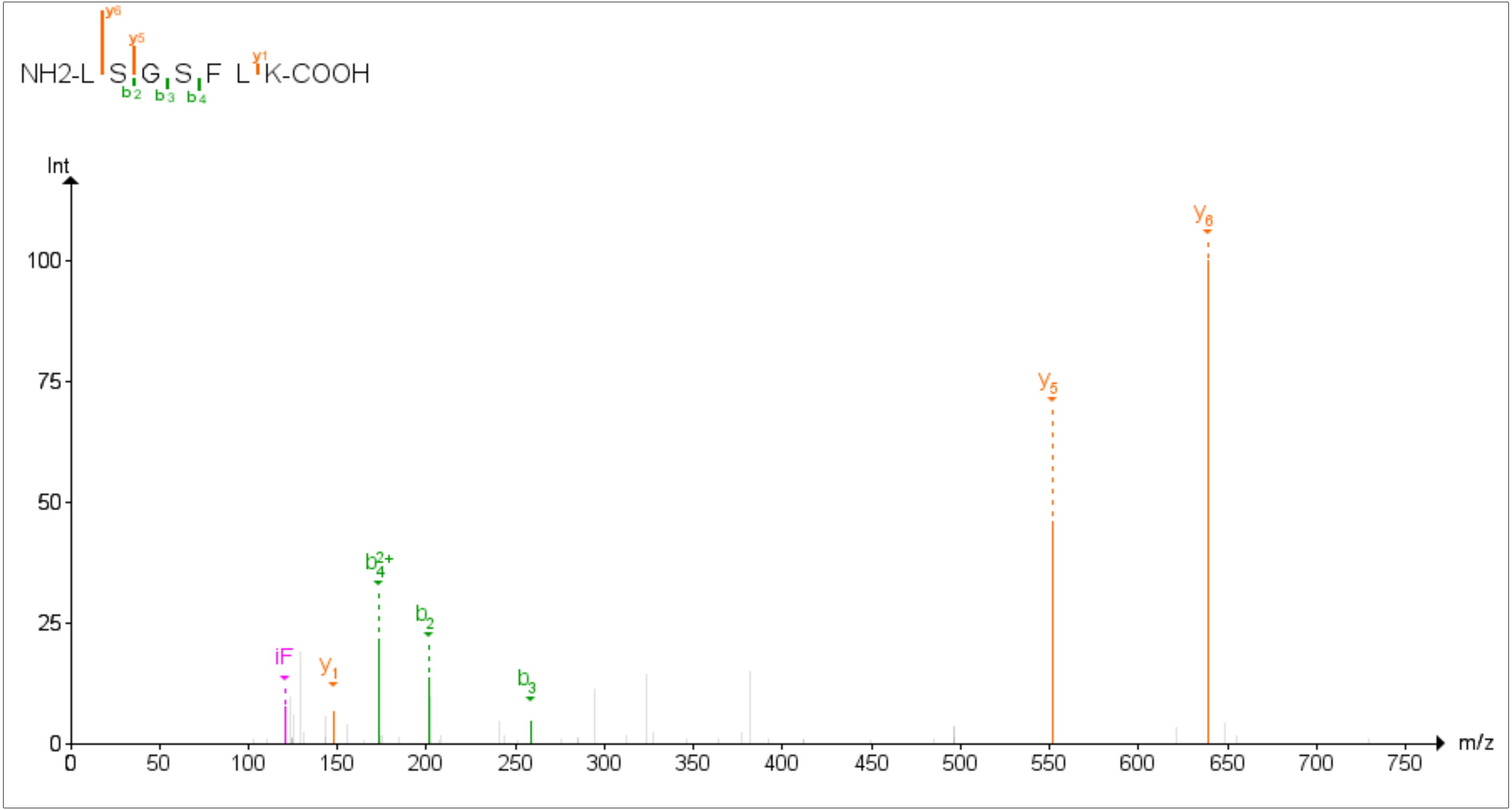
NH2-L S G S F L K-COOH
y6
y5
y1
b2
b3
b4
Int
100
75
50
25
0
y6
y5
b4 2+
b2
b3
iF
y1
0
50
100
150
200
250
300
350
400
450
500
550
600
650
700
750
m/z

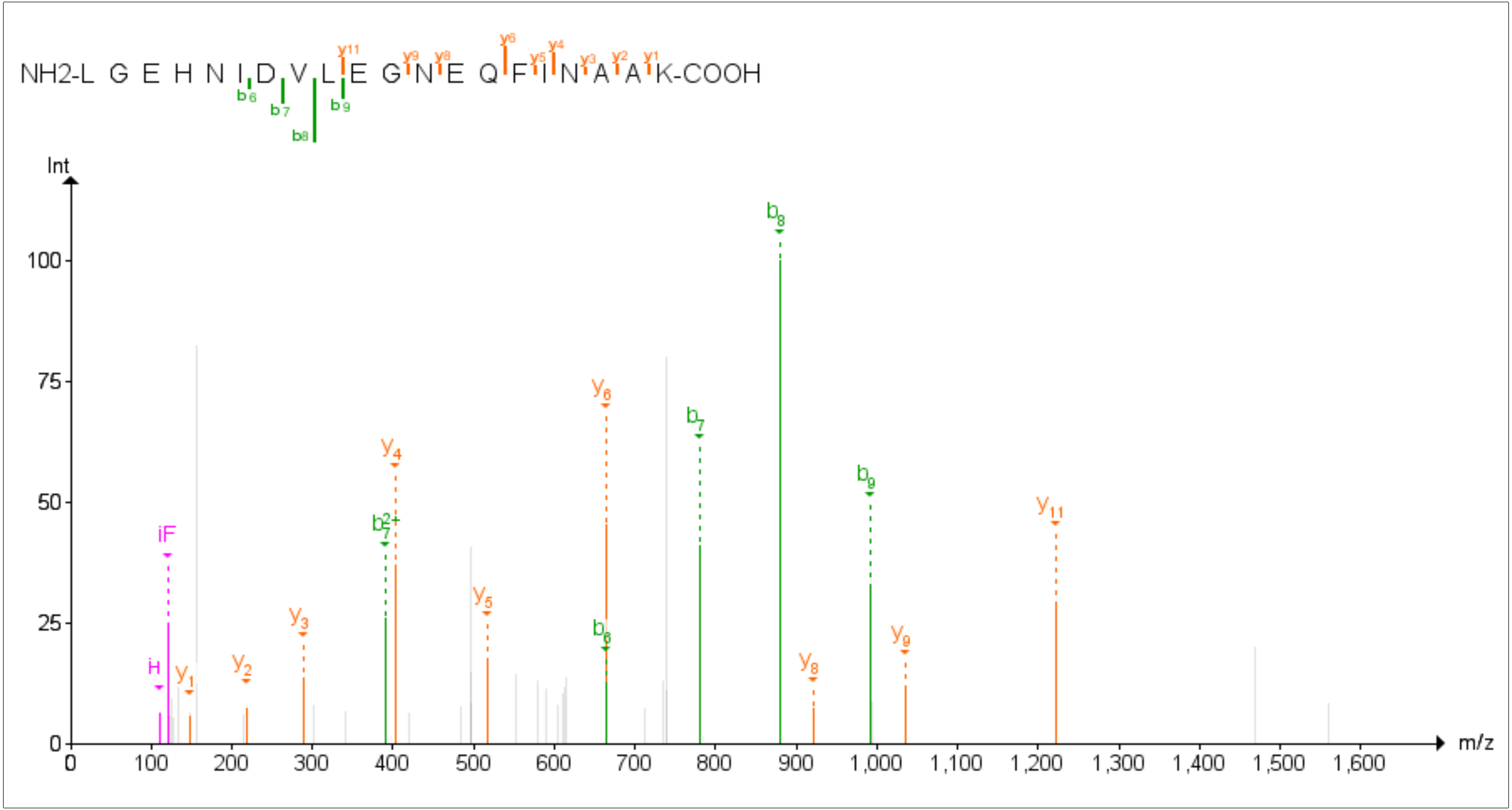

NH2-L G E H N I D V L E G N E Q F I N A A K-COOH
b6
b7
b8
b9
y11
y9
y8
y6
y5
y4
y3
y2
y1
Int
m/z
0
25
50
75
100
0
100
200
300
400
500
600
700
800
900
1,000
1,100
1,200
1,300
1,400
1,500
1,600
iH
iF
y_1
y_2
y_3
b_7^{2+}
y_4
y_5
b_6
y_6
b_7
b_8
y_8
b_9
y_9
y_{11}

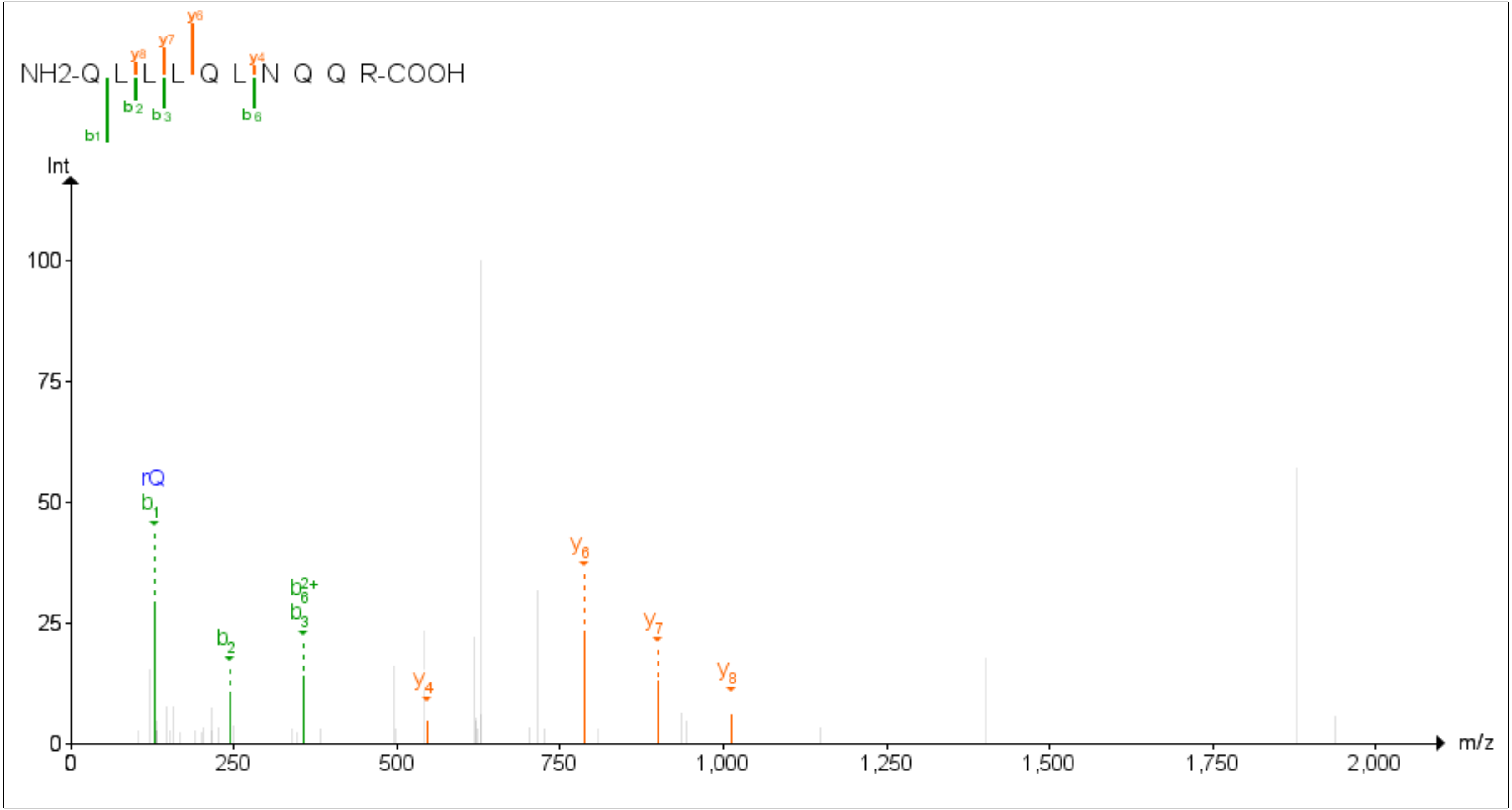

NH2-Q L L L Q L N Q Q R-COOH
y8
y7
y6
y4
b1
b2
b3
b6
Int
100
75
50
25
0
rQ
b1
b2
b6 2+
b3
y4
y6
y7
y8
0
250
500
750
1,000
1,250
1,500
1,750
2,000
m/z

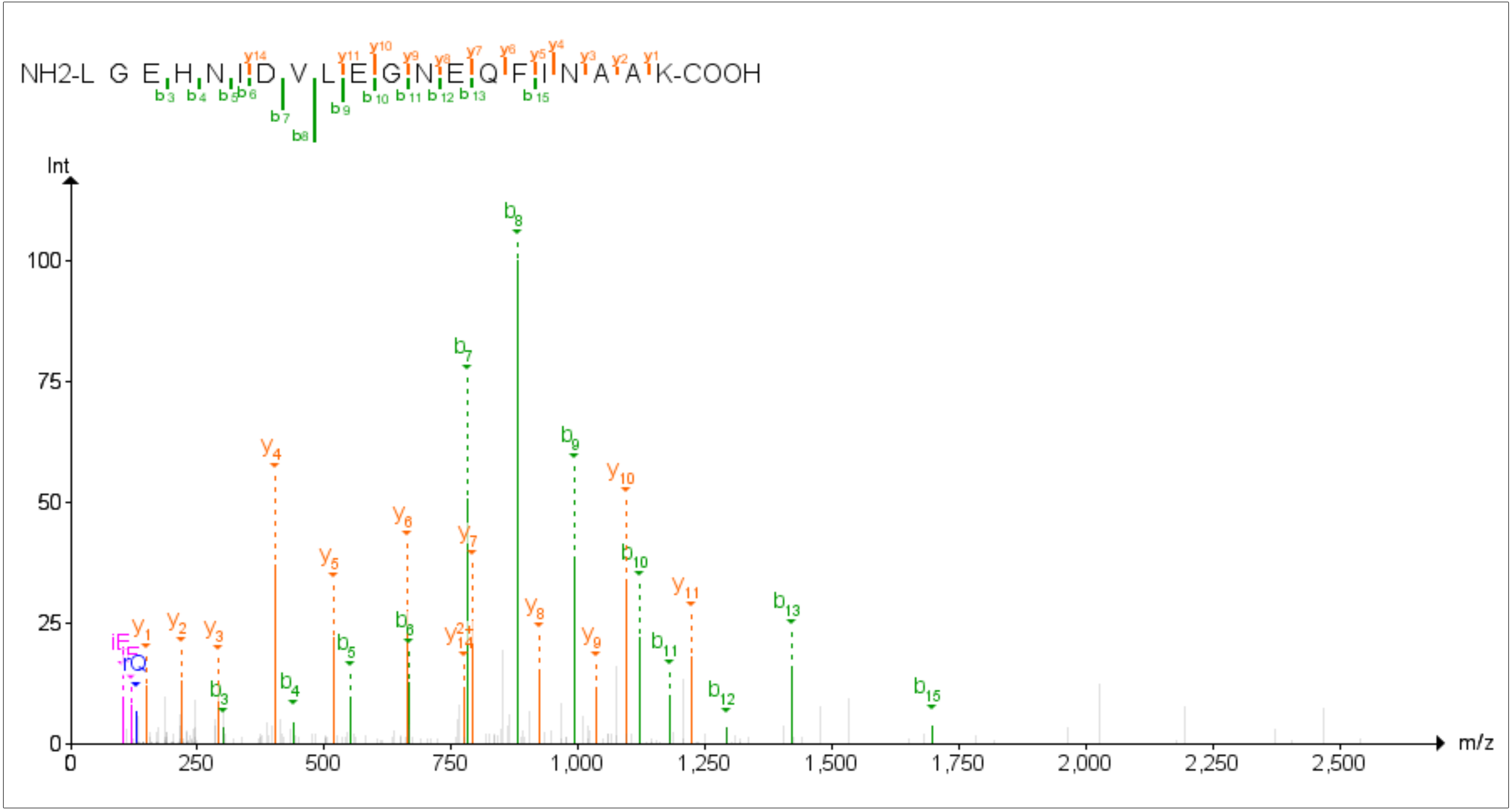

NH2-L G E H N I D V L E G N E Q F I N A A K-COOH
Int
m/z
0
25
50
75
100
250
500
750
1,000
1,250
1,500
1,750
2,000
2,250
2,500

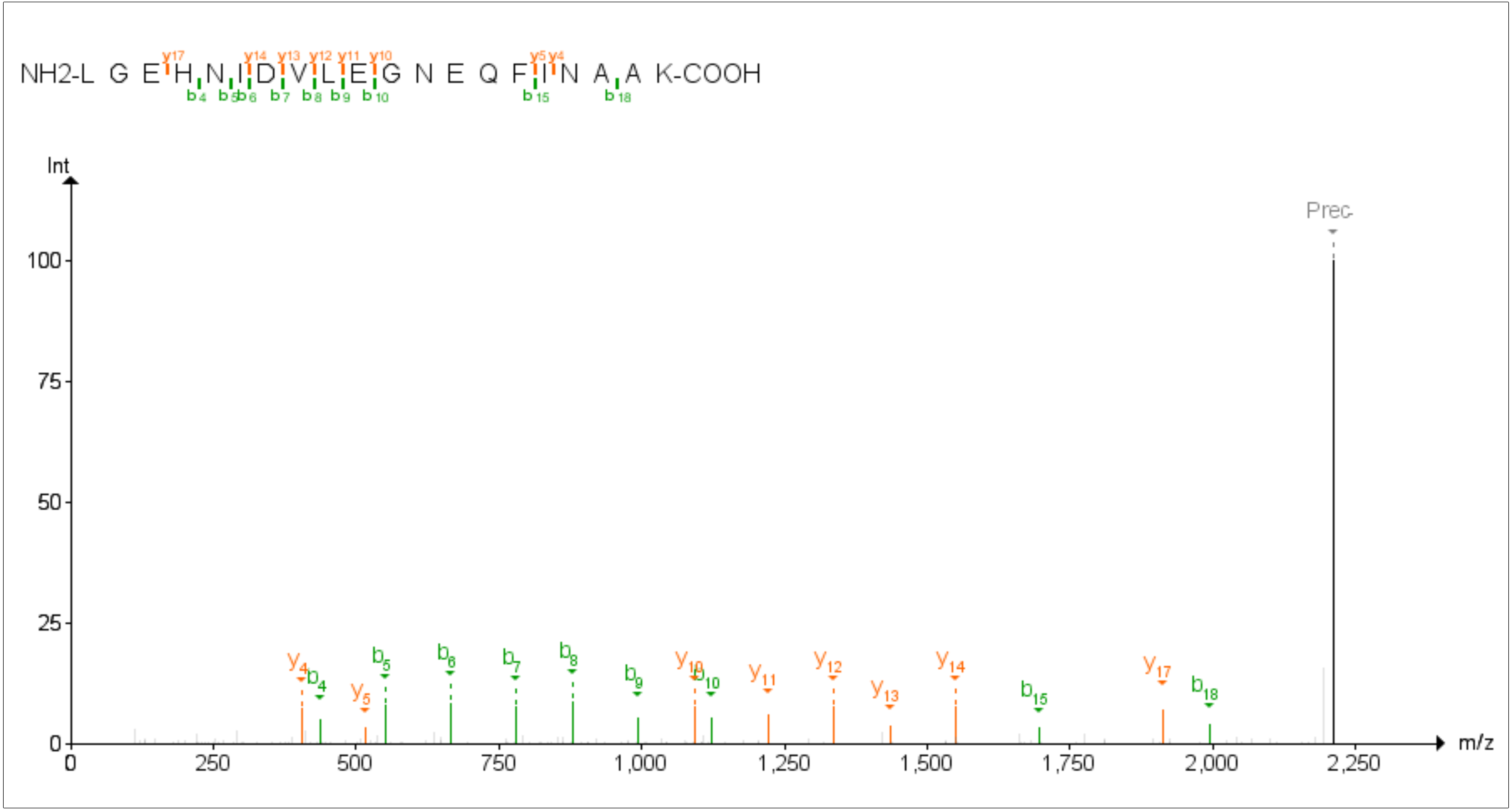

NH2-L G E H N I D V L E G N E Q F I N A A K-COOH
Int
Prec.
100
75
50
25
0
m/z
0
250
500
750
1,000
1,250
1,500
1,750
2,000
2,250

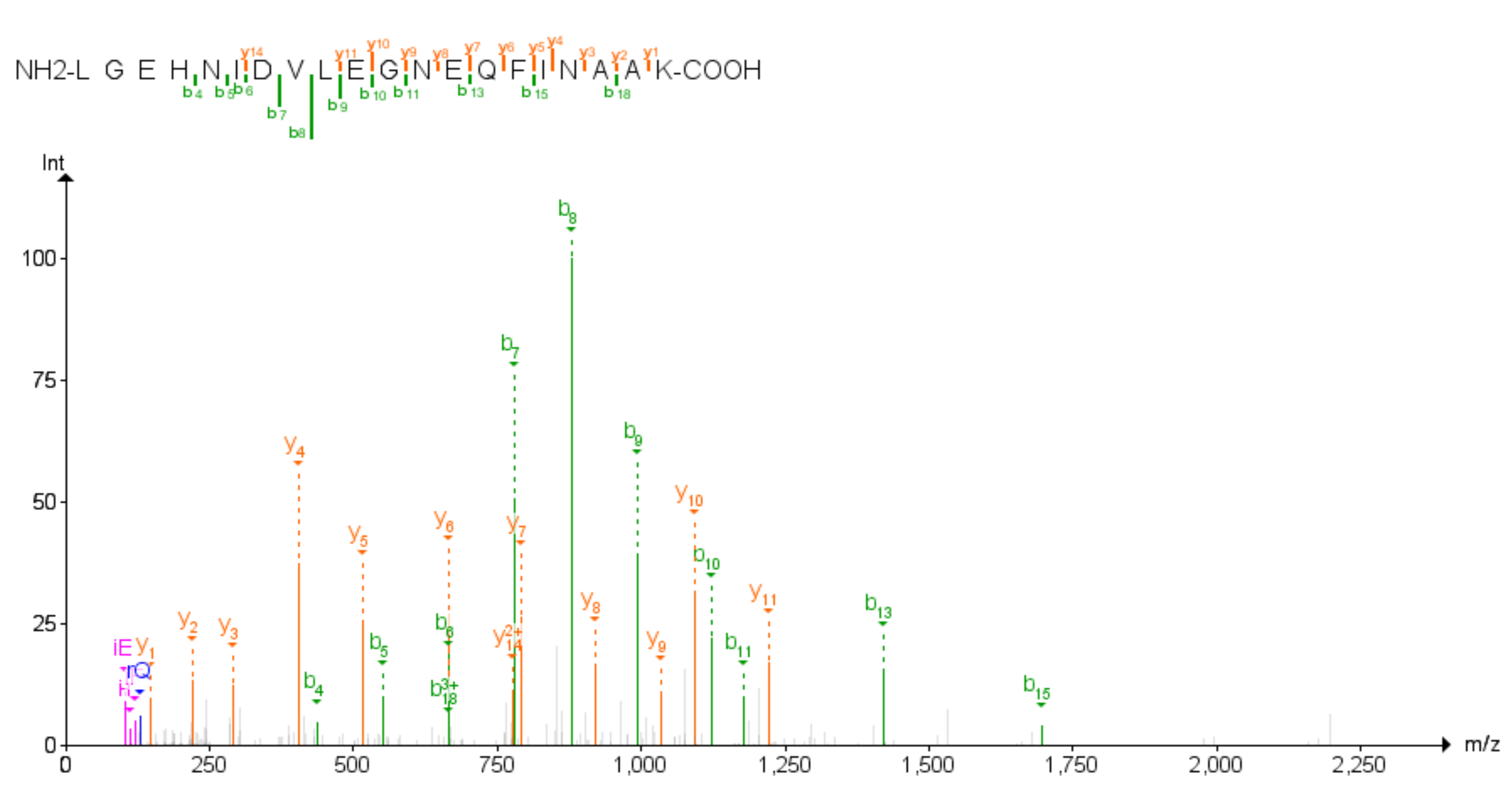

NH2-L G E H N I D V L E G N E Q F I N A A K-COOH
Int
m/z
0
250
500
750
1,000
1,250
1,500
1,750
2,000
2,250
25
50
75
100

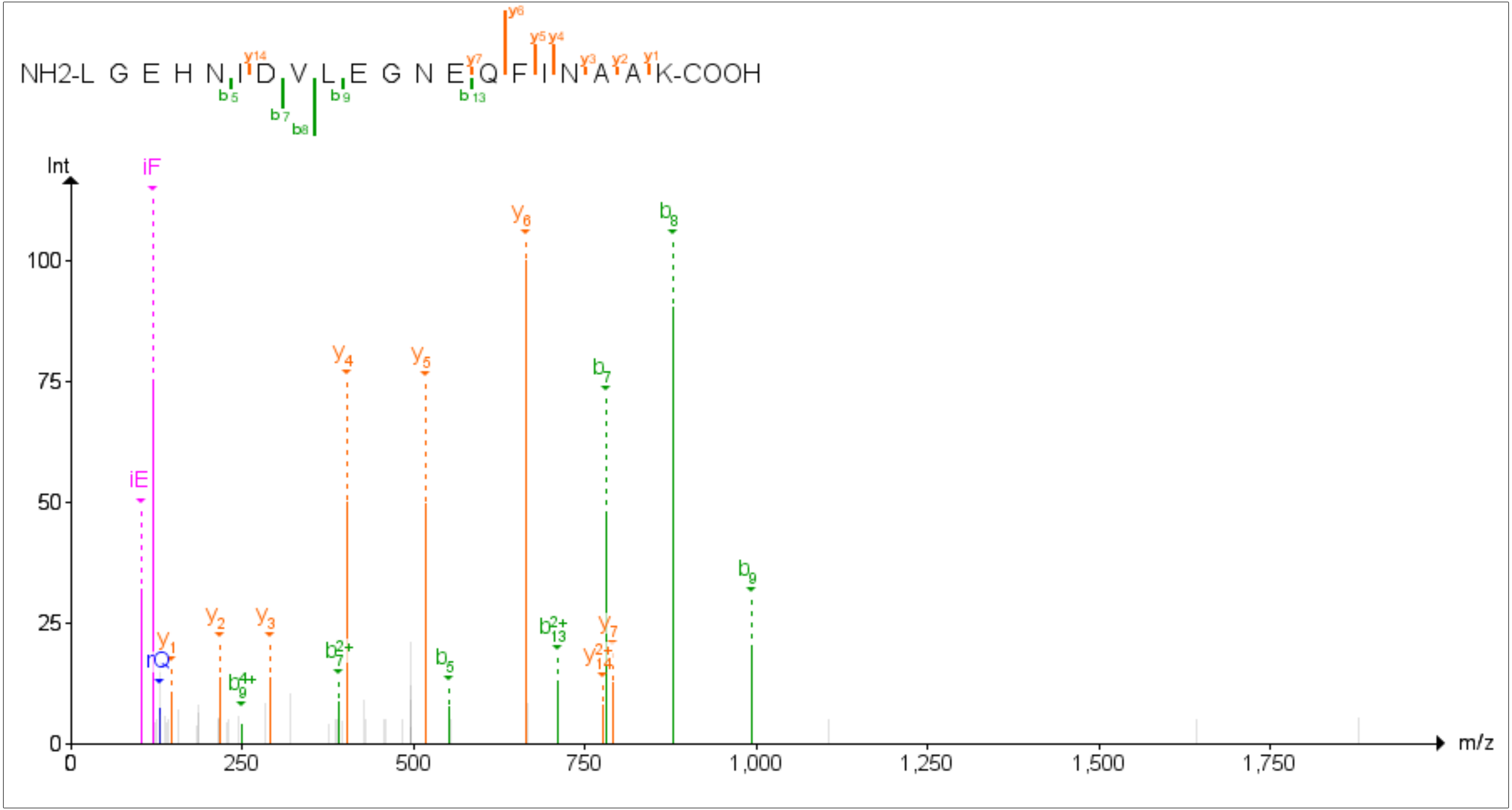

NH2-L G E H N I D V L E G N E Q F I N A A K-COOH
y14 y7 y6 y5 y4 y3 y2 y1
b5 b7 b8 b9 b13
Int
iF
iE
rQ
y1 y2 y3 y4 y5 y6 y7
b8 b7 b9 b5
b9 4+
b7 2+
b13 2+
y14 2+
100
75
50
25
0
250
500
750
1,000
1,250
1,500
1,750
m/z

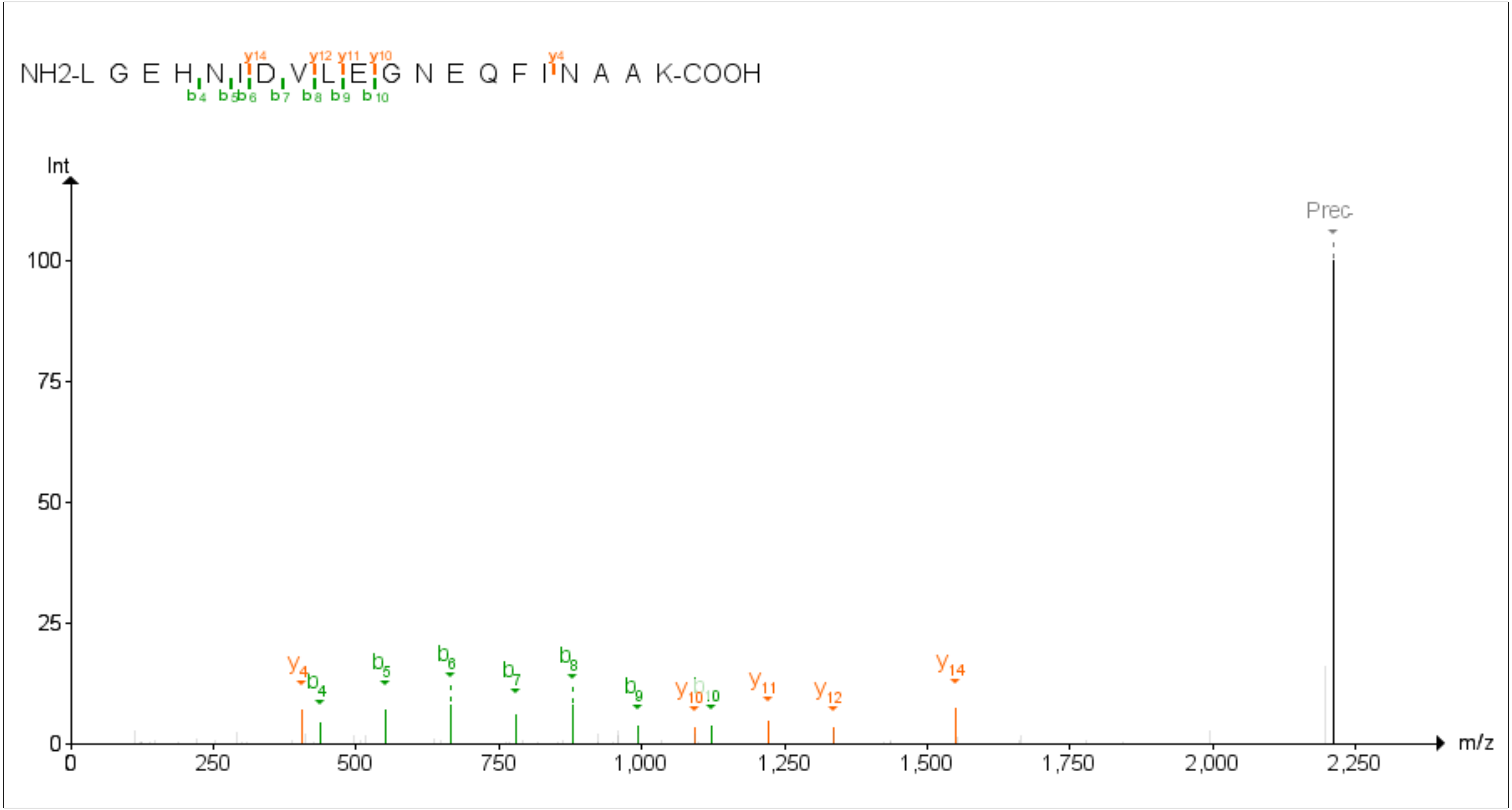
NH2-L G E H N I D V L E G N E Q F I N A A K-COOH
y14 y12 y11 y10 y4
b4 b5 b6 b7 b8 b9 b10
Int
100
75
50
25
0
Prec
y4 b4 b5 b6 b7 b8 b9 y10 b10 y11 y12 y14
0 250 500 750 1,000 1,250 1,500 1,750 2,000 2,250
m/z

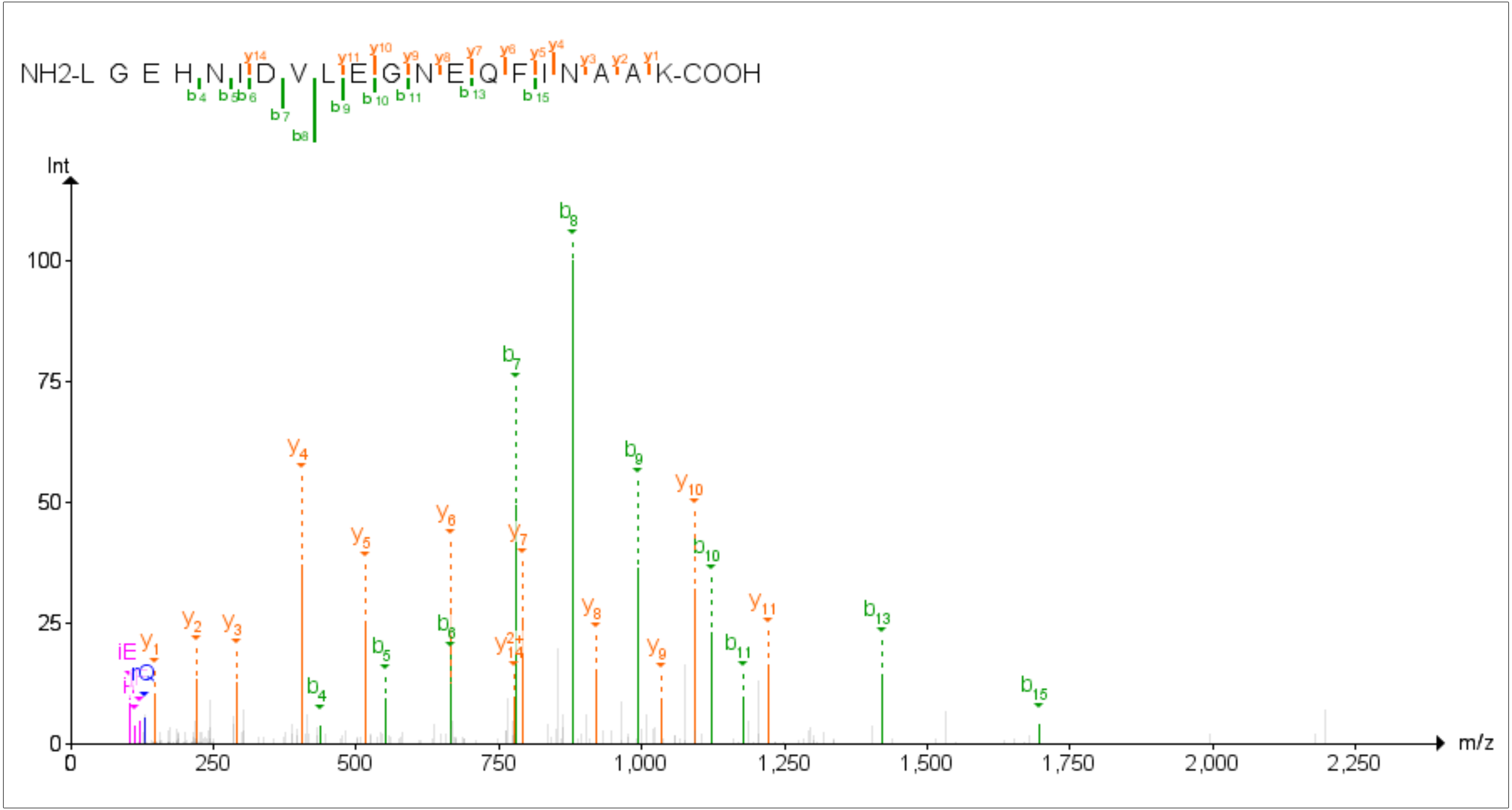

NH2-L G E H N I D V L E G N E Q F I N A A K-COOH
Int
m/z
0
25
50
75
100
250
500
750
1,000
1,250
1,500
1,750
2,000
2,250

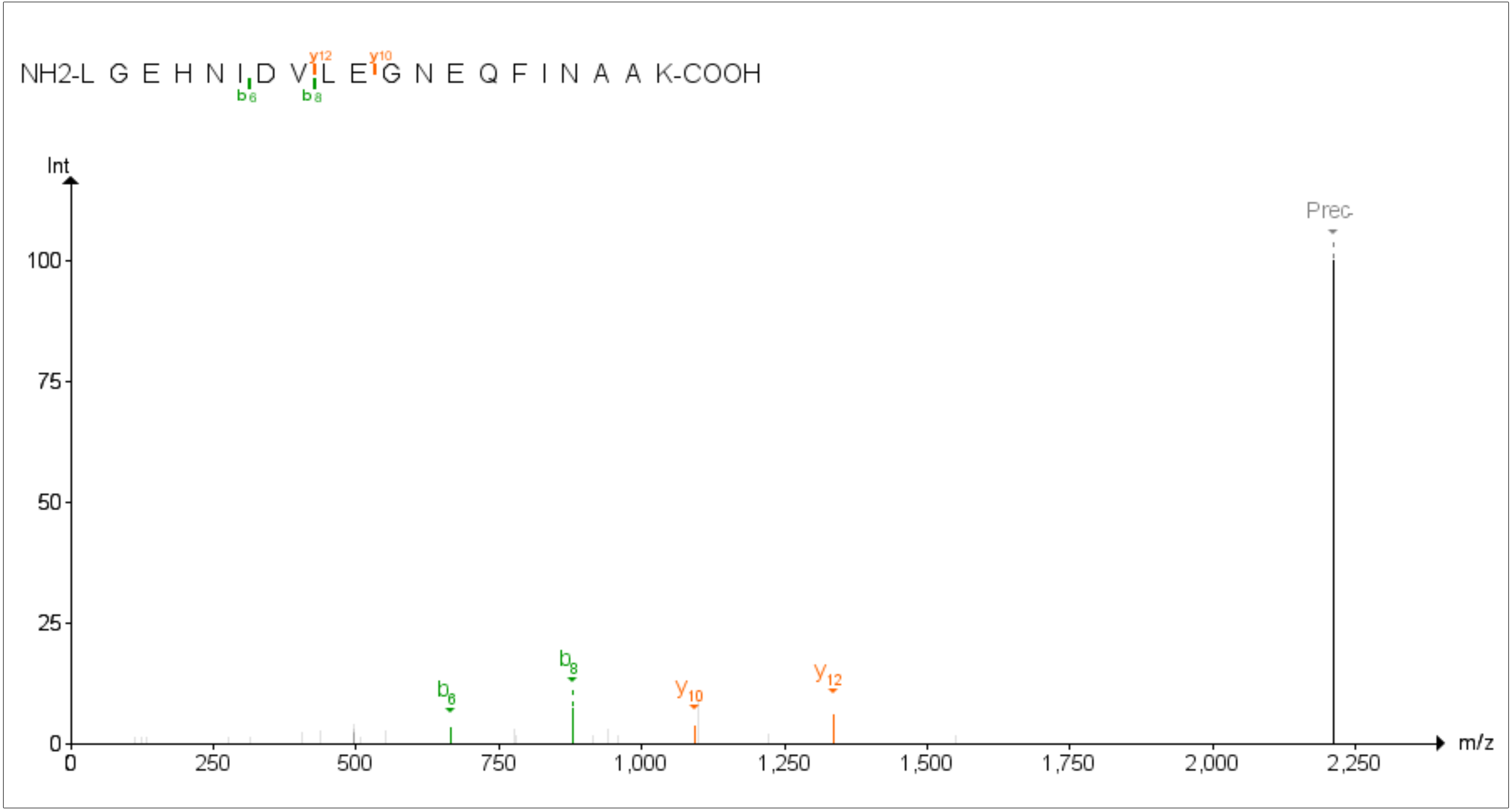

NH2-L G E H N I D V I L E G N E Q F I N A A K-COOH
y12
y10
b6
b8
Int
100
75
50
25
0
Prec
b6
b8
y10
y12
0
250
500
750
1,000
1,250
1,500
1,750
2,000
2,250
m/z

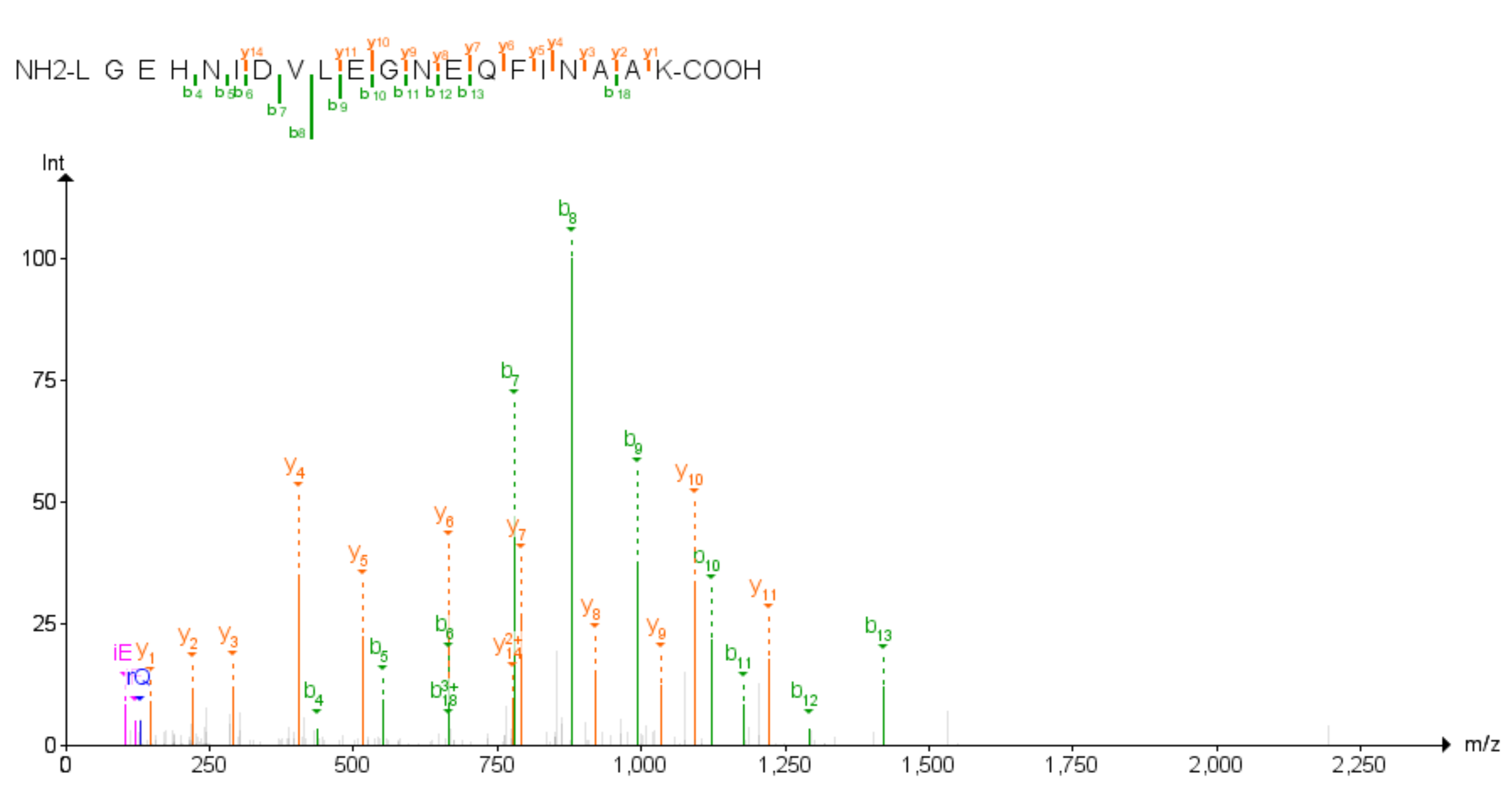

NH2-L G E H N I D V L E G N E Q F I N A A K-COOH
y14 y11 y10 y9 y8 y7 y6 y5 y4 y3 y2 y1
b4 b5 b6 b7 b8 b9 b10 b11 b12 b13 b18
Int
m/z
0
25
50
75
100
250
500
750
1,000
1,250
1,500
1,750
2,000
2,250
b8
b7
b9
y10
y4
y6
y7
y5
b10
y11
b13
y8
y9
b6
b5
y14 2+
iE
y1
y2
y3
iQ
b4
b3+ 18
b11
b12

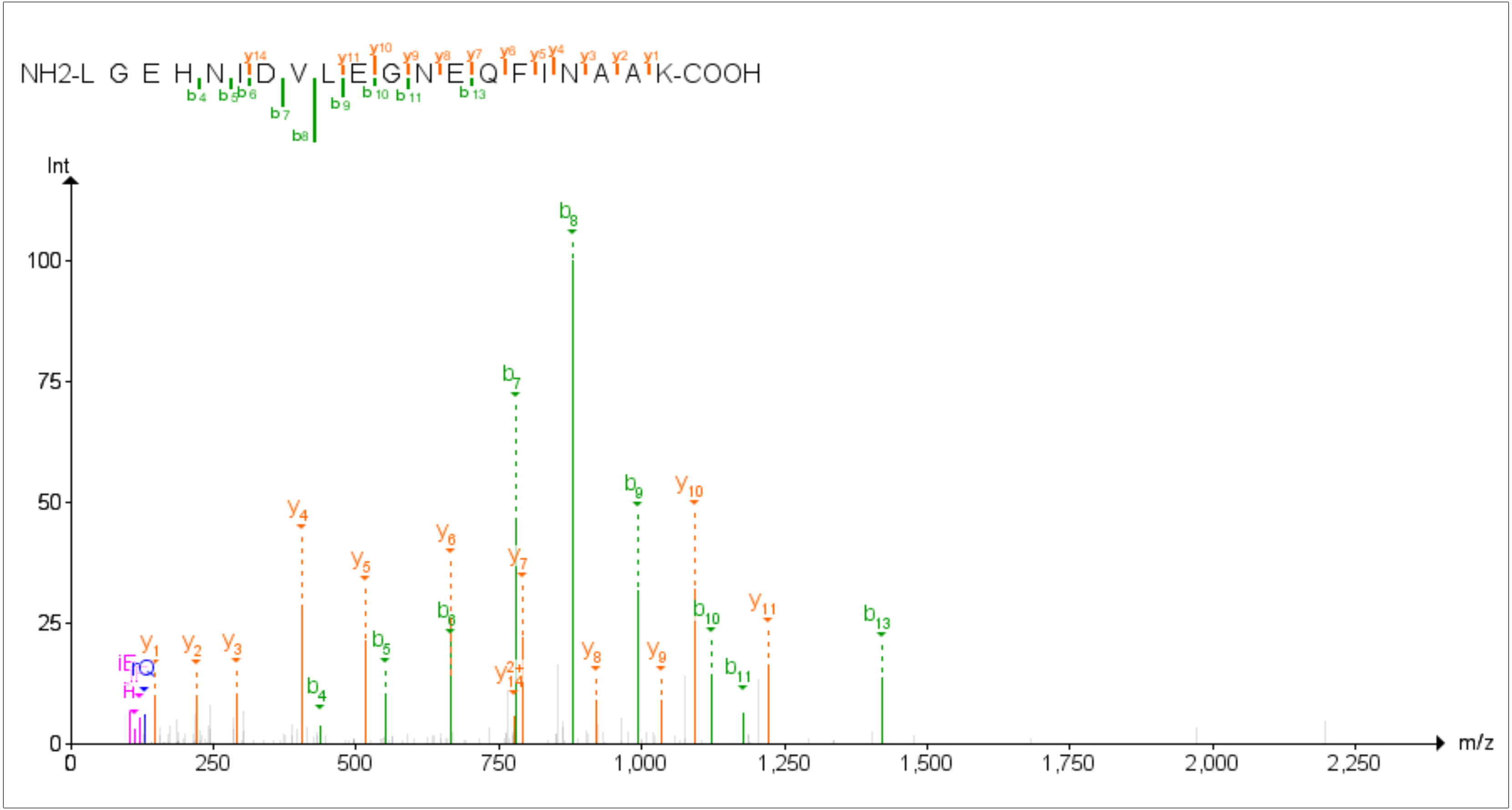
NH2-L G E H N I D V L E G N E Q F I N A A K-COOH
Int
m/z
b8
b7
b9
y10
y4
y6
y5
y7
b6
b5
b4
y1
y2
y3
b10
y11
b13
b11
y8
y9
y14 2+
0
25
50
75
100
0
250
500
750
1,000
1,250
1,500
1,750
2,000
2,250

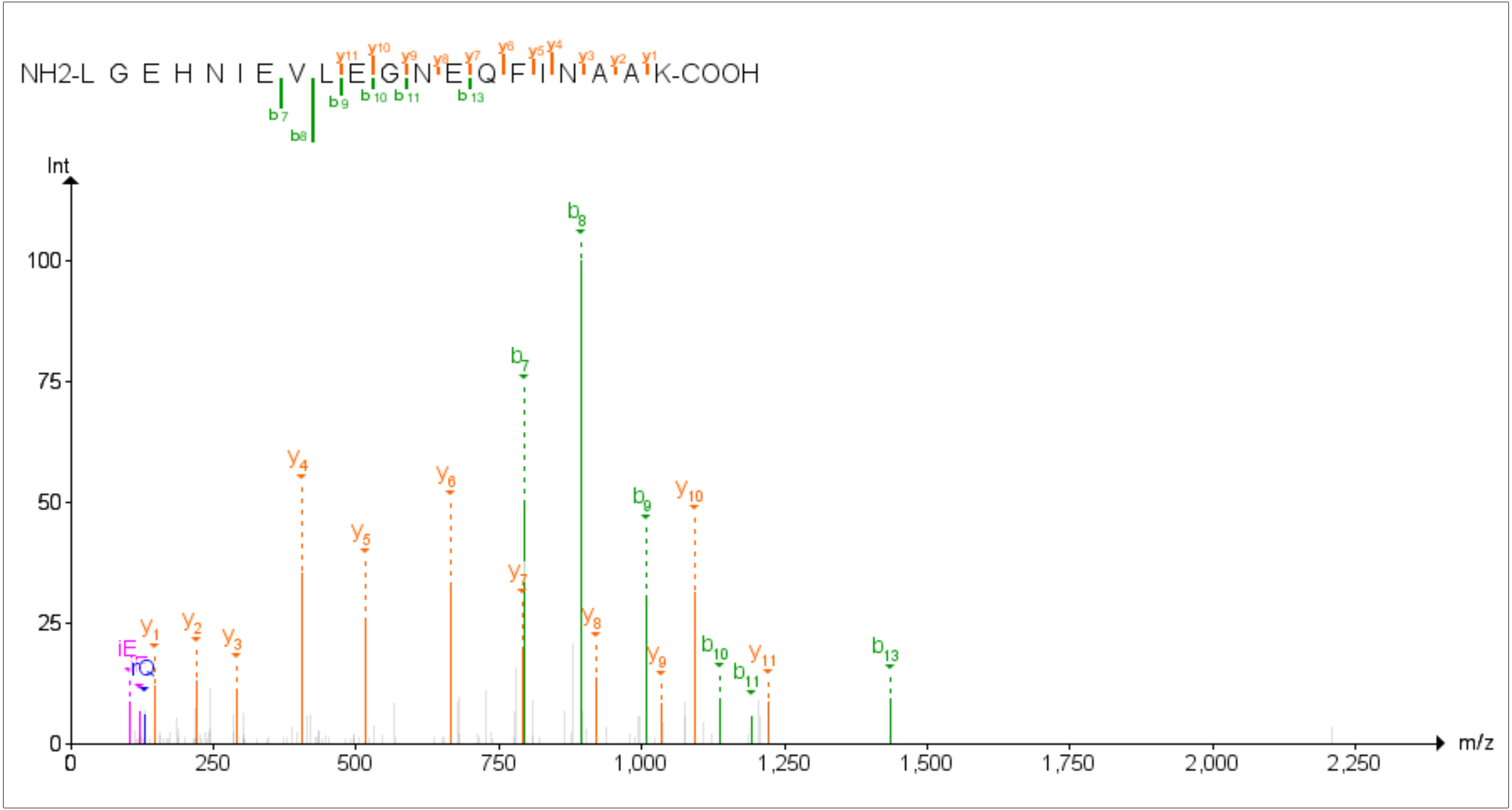

NH2-L G E H N I E V L E G N E Q F I N A A K-COOH
y11 y10 y9 y8 y7 y6 y5 y4 y3 y2 y1
b7 b8 b9 b10 b11 b13
Int
m/z
0
25
50
75
100
0
250
500
750
1,000
1,250
1,500
1,750
2,000
2,250
iE
rQ

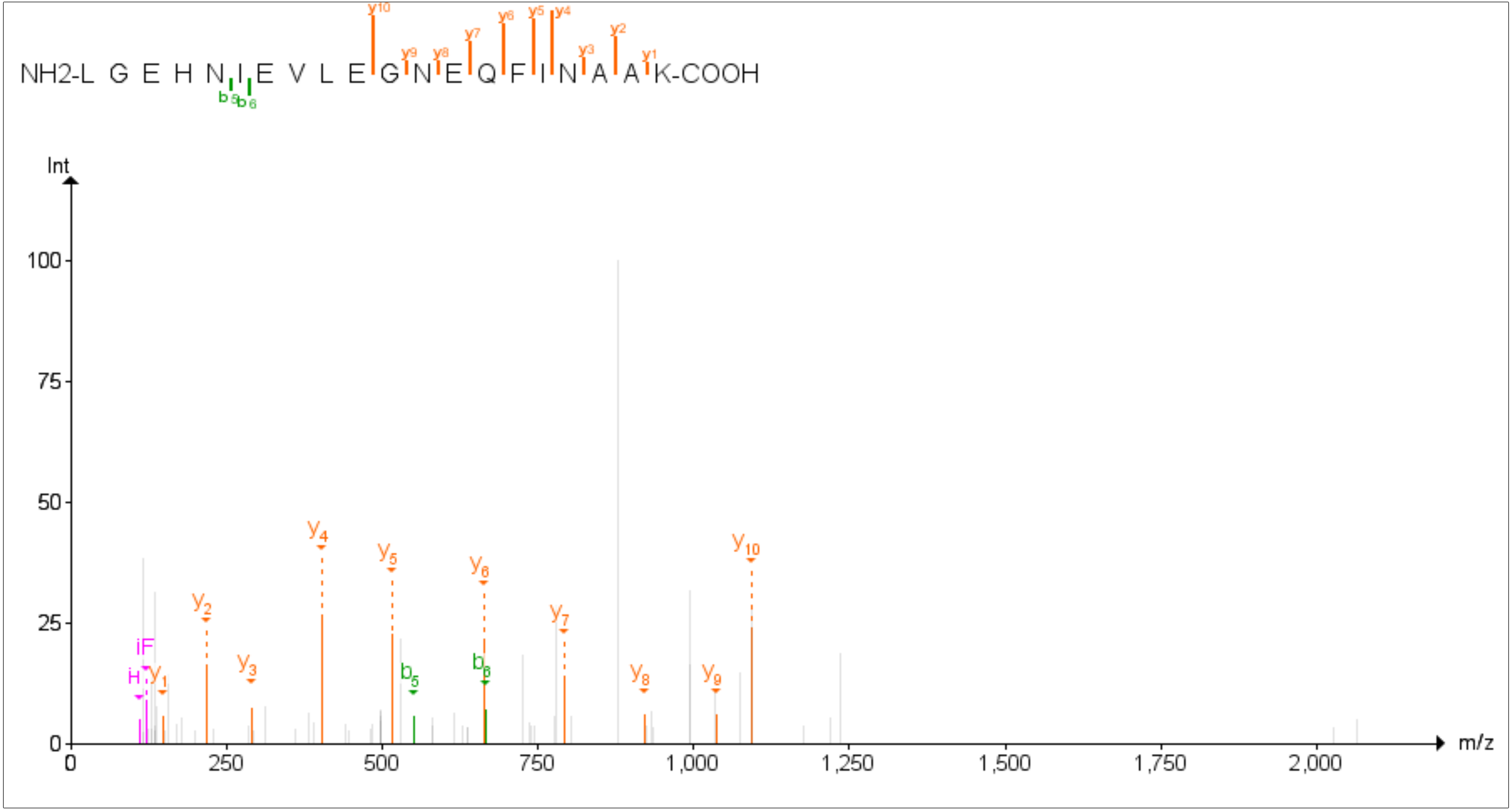

NH2-L G E H N I E V L E G N E Q F I N A A K-COOH
y10 y9 y8 y7 y6 y5 y4 y3 y2 y1
b5 b6
Int
100
75
50
25
0
y1 y2 y3 y4 y5 y6 y7 y8 y9 y10
b5 b6
iF
iH
0
250
500
750
1,000
1,250
1,500
1,750
2,000
m/z

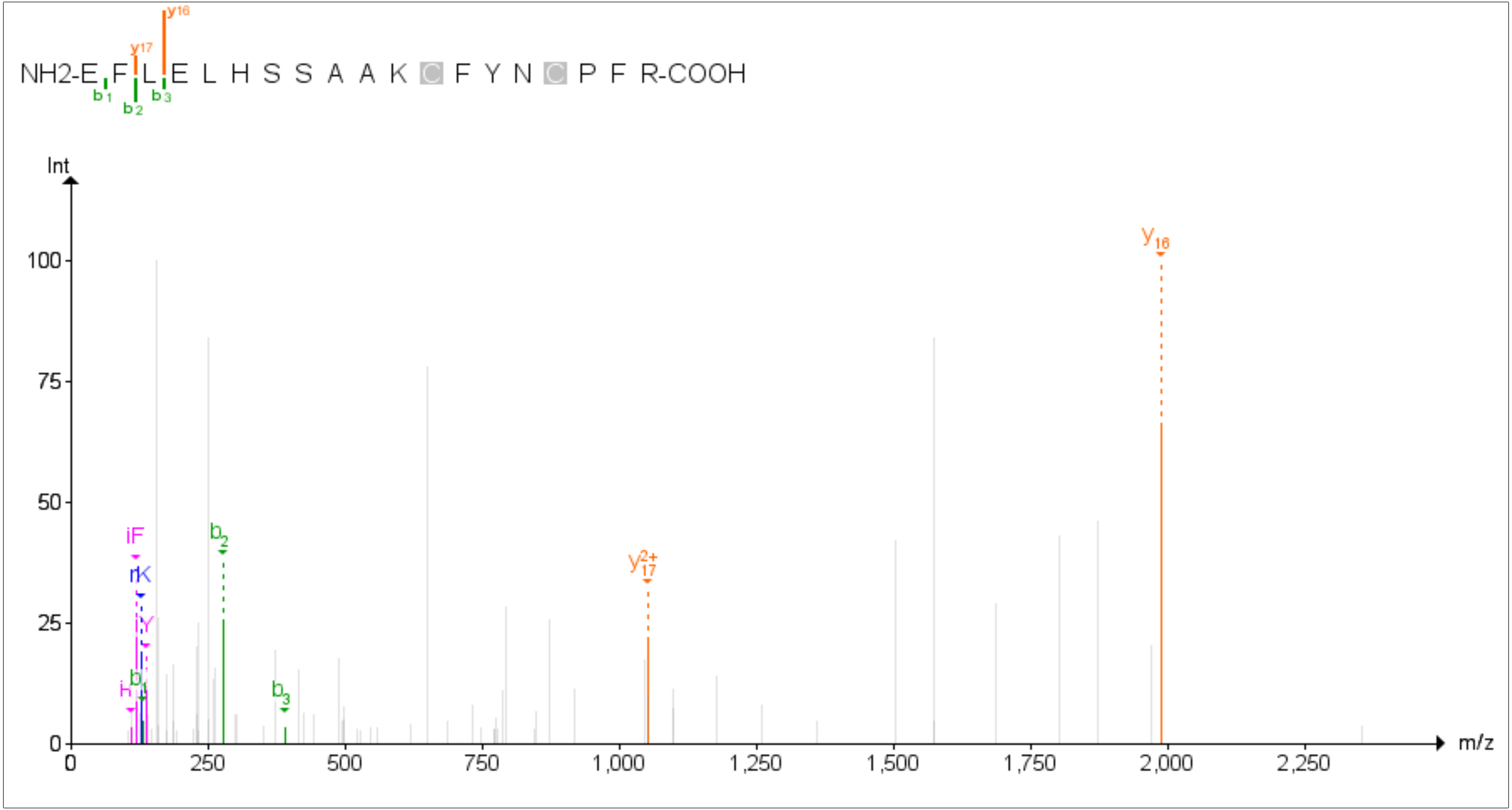

NH2-E F L E L H S S A A K C F Y N C P F R-COOH
y16
y17
b1
b2
b3
Int
100
75
50
25
0
iF
rK
iY
iH
b1
b2
b3
y17 2+
y16
0
250
500
750
1,000
1,250
1,500
1,750
2,000
2,250
m/z

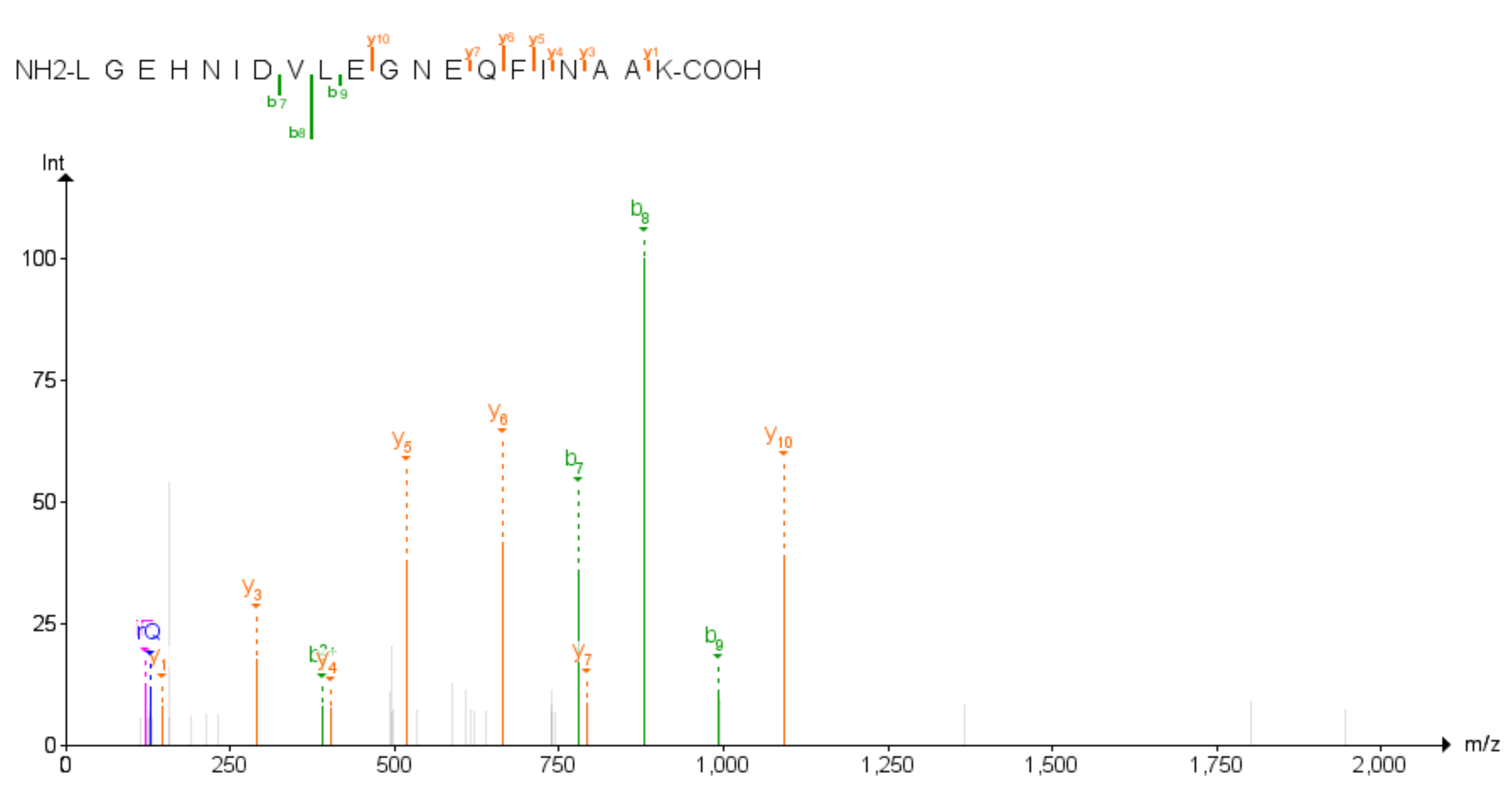

NH2-L G E H N I D V L E G N E Q F I N A A K-COOH
y10
y7
y6
y5
y4
y3
y1
b7
b8
b9
Int
100
75
50
25
0
m/z
0
250
500
750
1,000
1,250
1,500
1,750
2,000
b8
y6
y5
b7
y10
y3
rQ
y1
y4
y7
b9

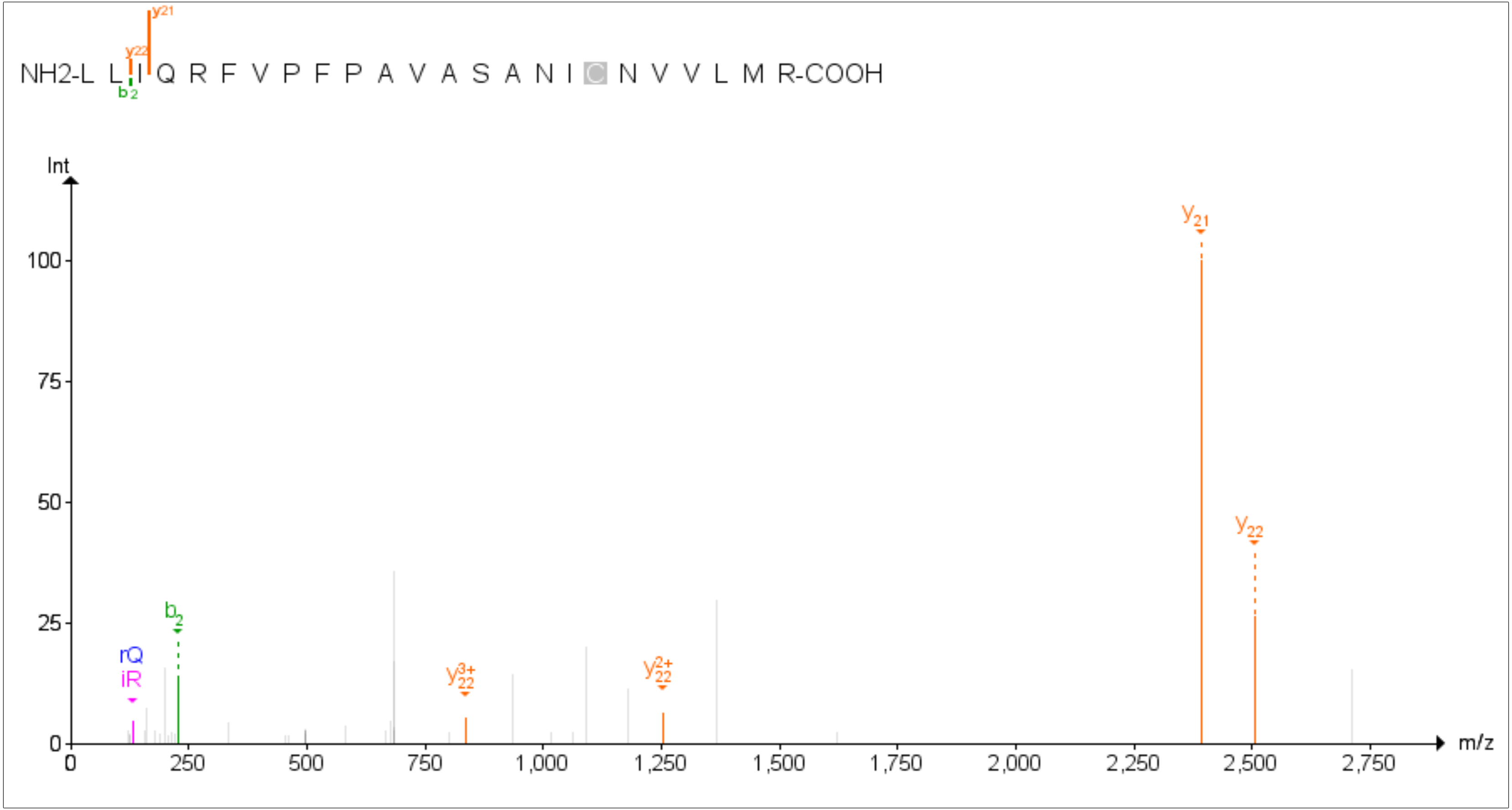

y21
y22
NH2-L L I Q R F V P F P A V A S A N I C N V V L M R-COOH
b2
Int
100
75
50
25
0
rQ
iR
b2
y22 3+
y22 2+
y21
y22
0
250
500
750
1,000
1,250
1,500
1,750
2,000
2,250
2,500
2,750
m/z

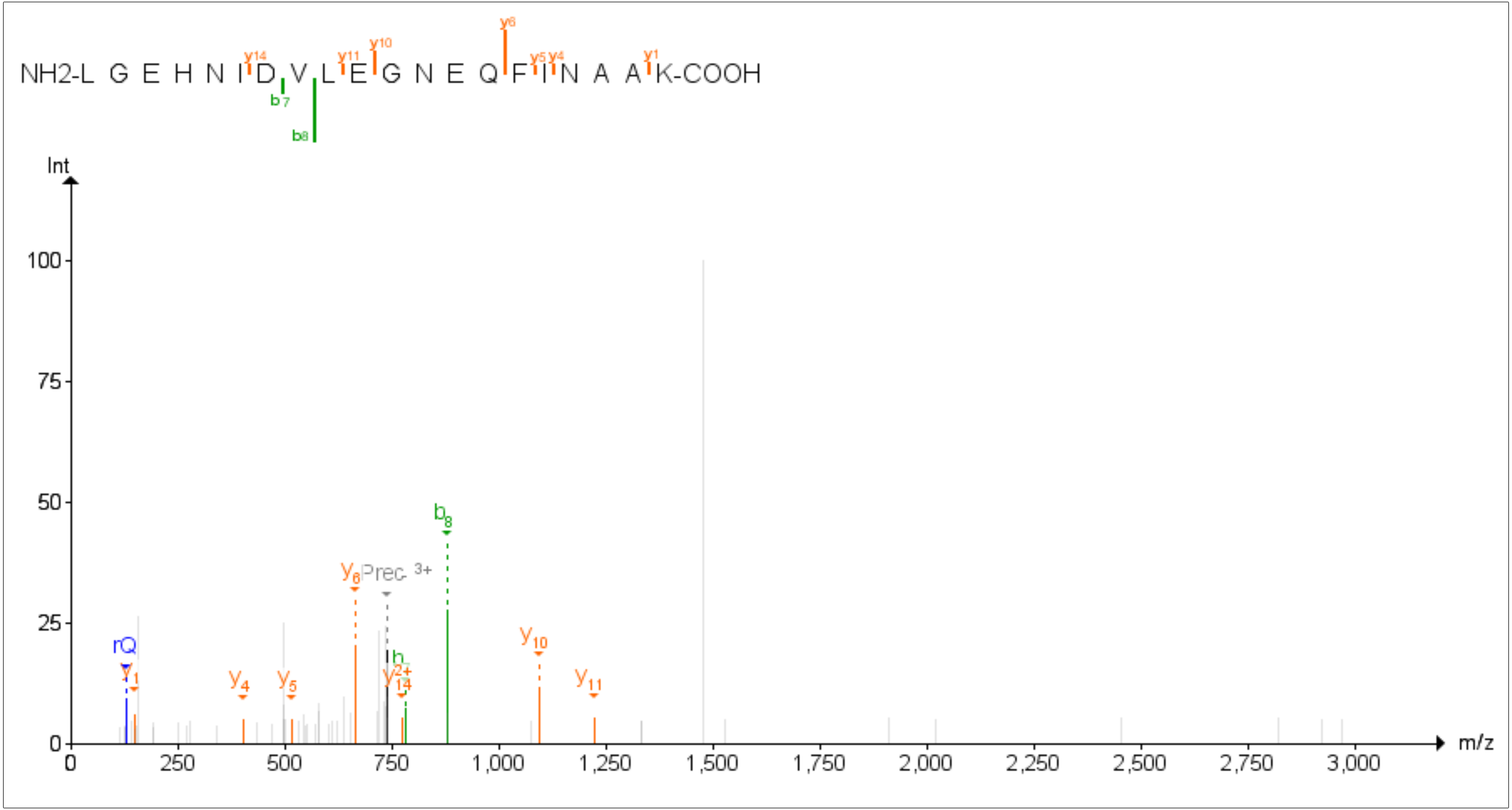
NH2-L G E H N I D V L E G N E Q F I N A A K-COOH
y14
y11
y10
y6
y5
y4
y1
b7
b8
Int
100
75
50
25
0
rQ
y1
y4
y5
y6
Prec 3+
b8
b7
y14 2+
y10
y11
0
250
500
750
1,000
1,250
1,500
1,750
2,000
2,250
2,500
2,750
3,000
m/z

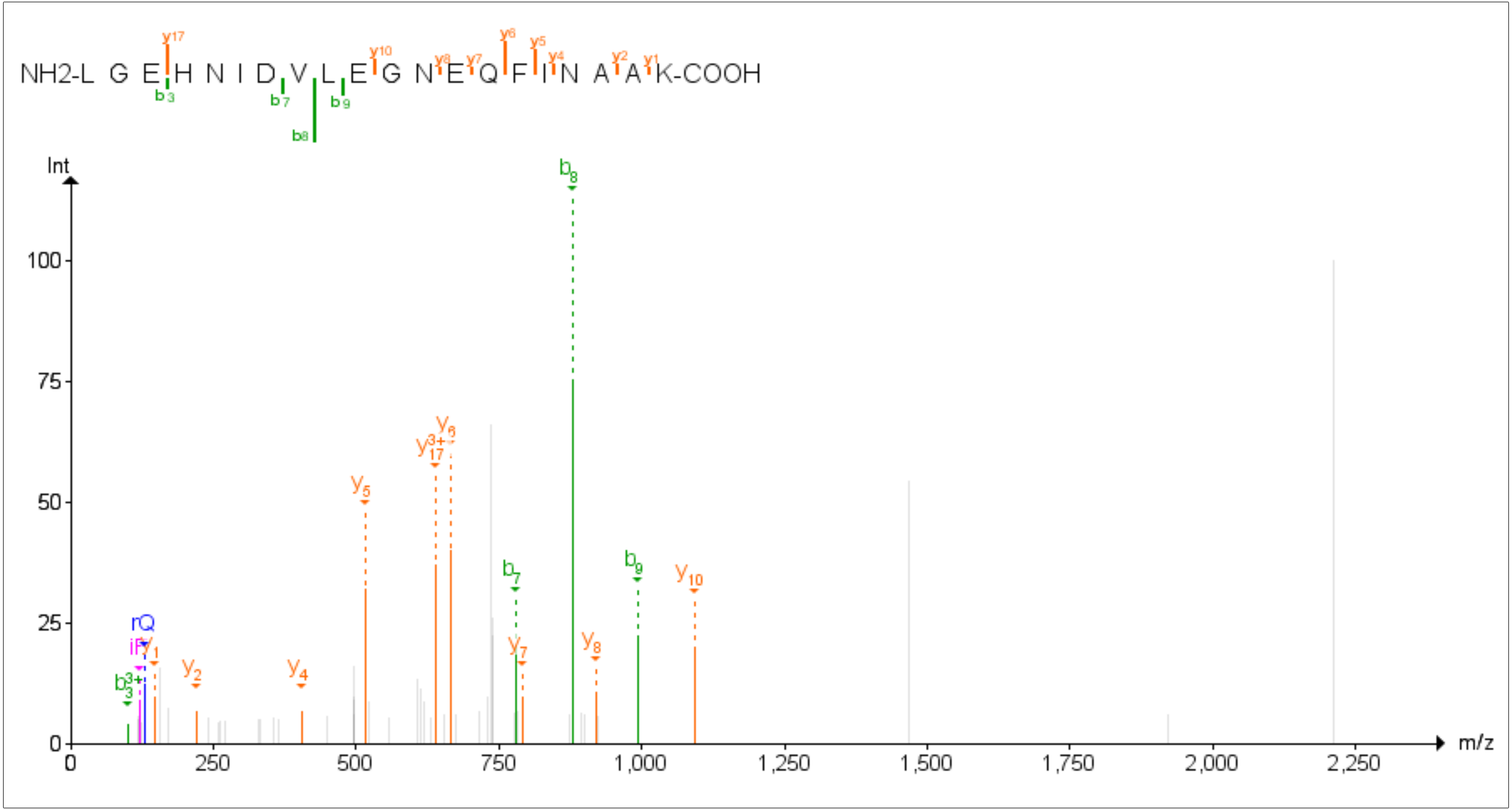

NH2-L G E H N I D V L E G N E Q F I N A A K-COOH
Int
m/z

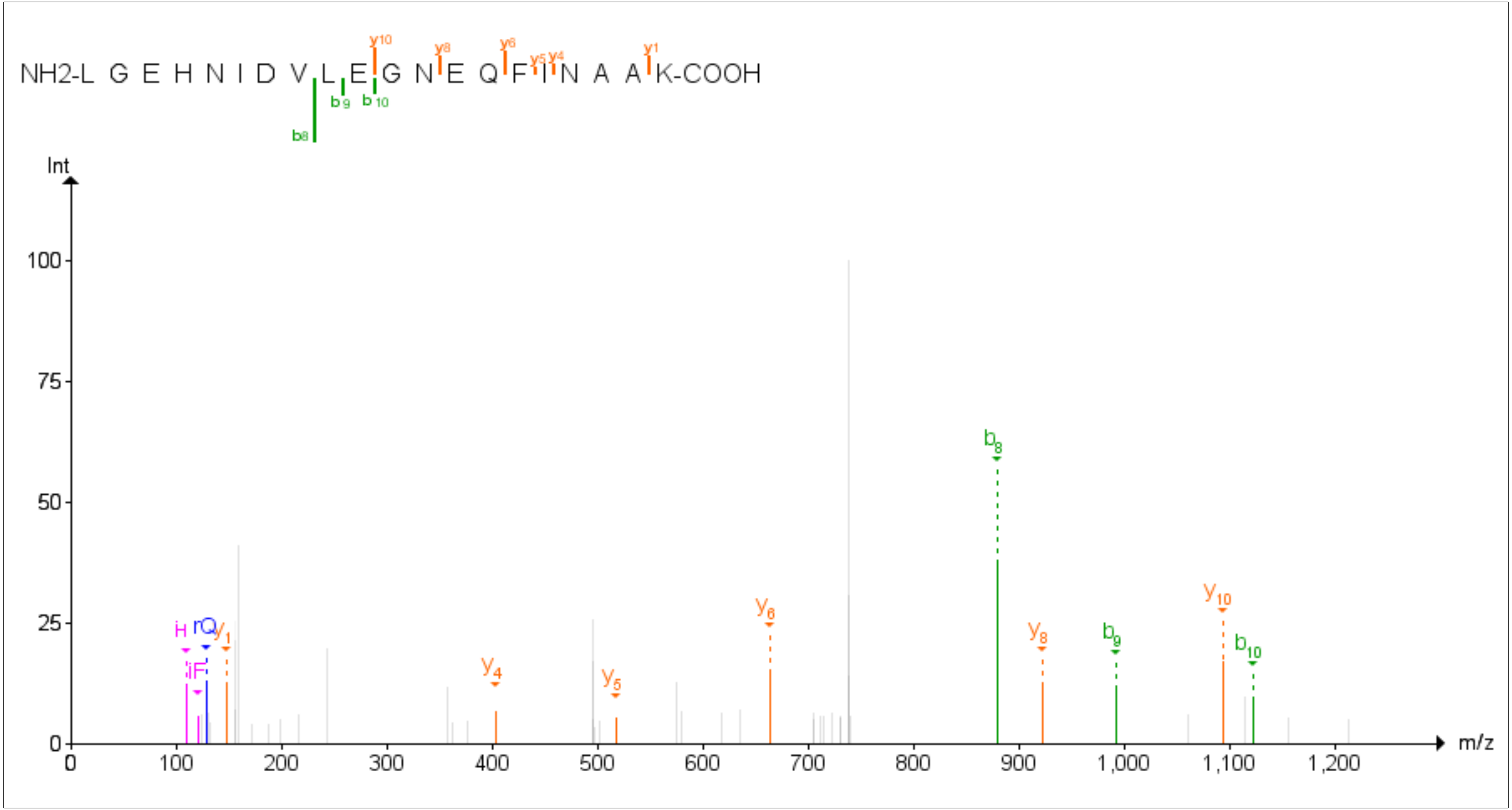

NH2-L G E H N I D V L E G N E Q F I N A A K-COOH
y10 y8 y6 y5 y4 y1
b8 b9 b10
Int
100
75
50
25
0
m/z
0 100 200 300 400 500 600 700 800 900 1,000 1,100 1,200
iH rQ y1 iF y4 y5 y6 b8 y8 b9 y10 b10

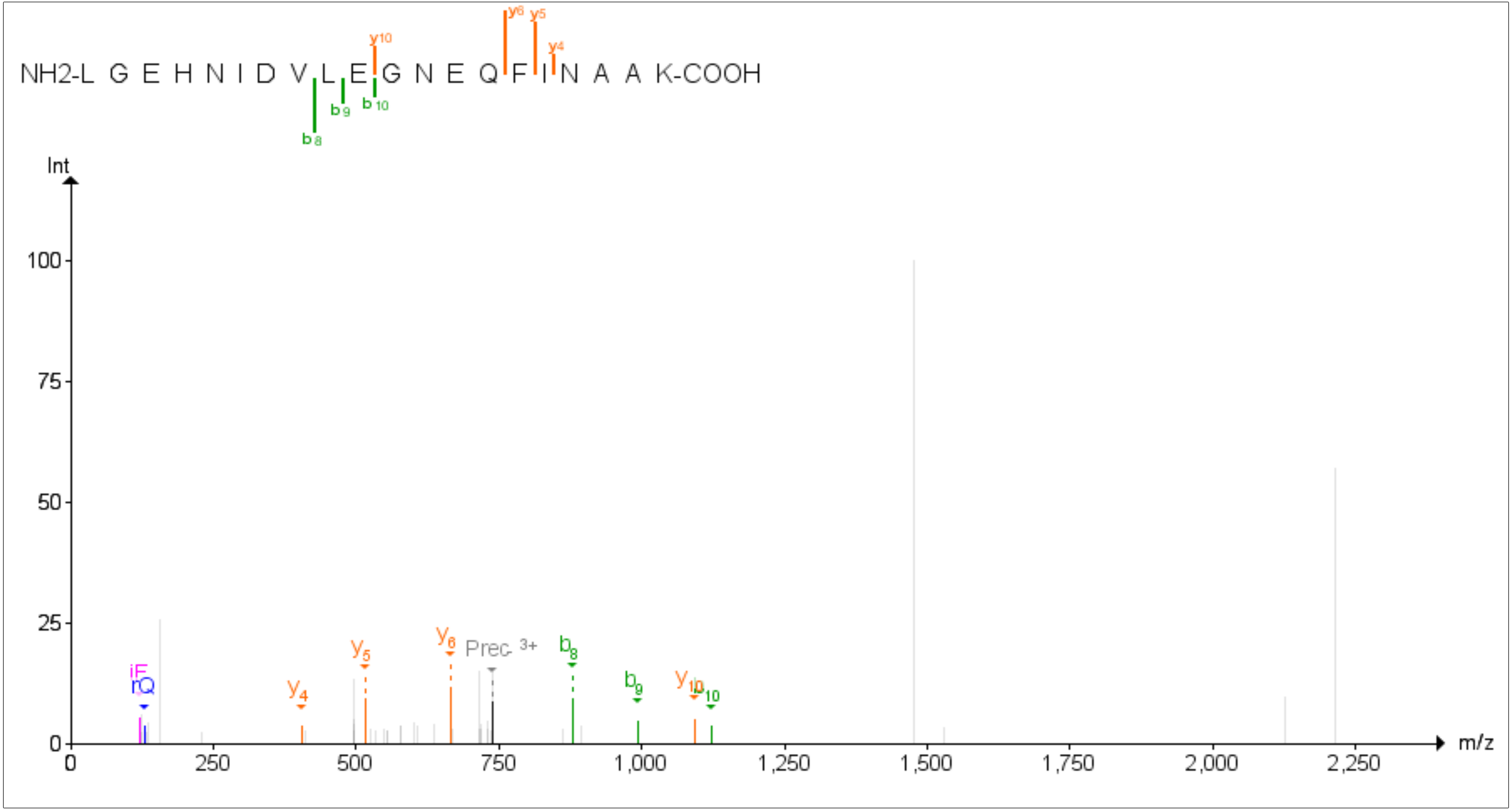

NH2-L G E H N I D V L E G N E Q F I N A A K-COOH
y10
y6
y5
y4
b8
b9
b10
Int
m/z
Prec 3+

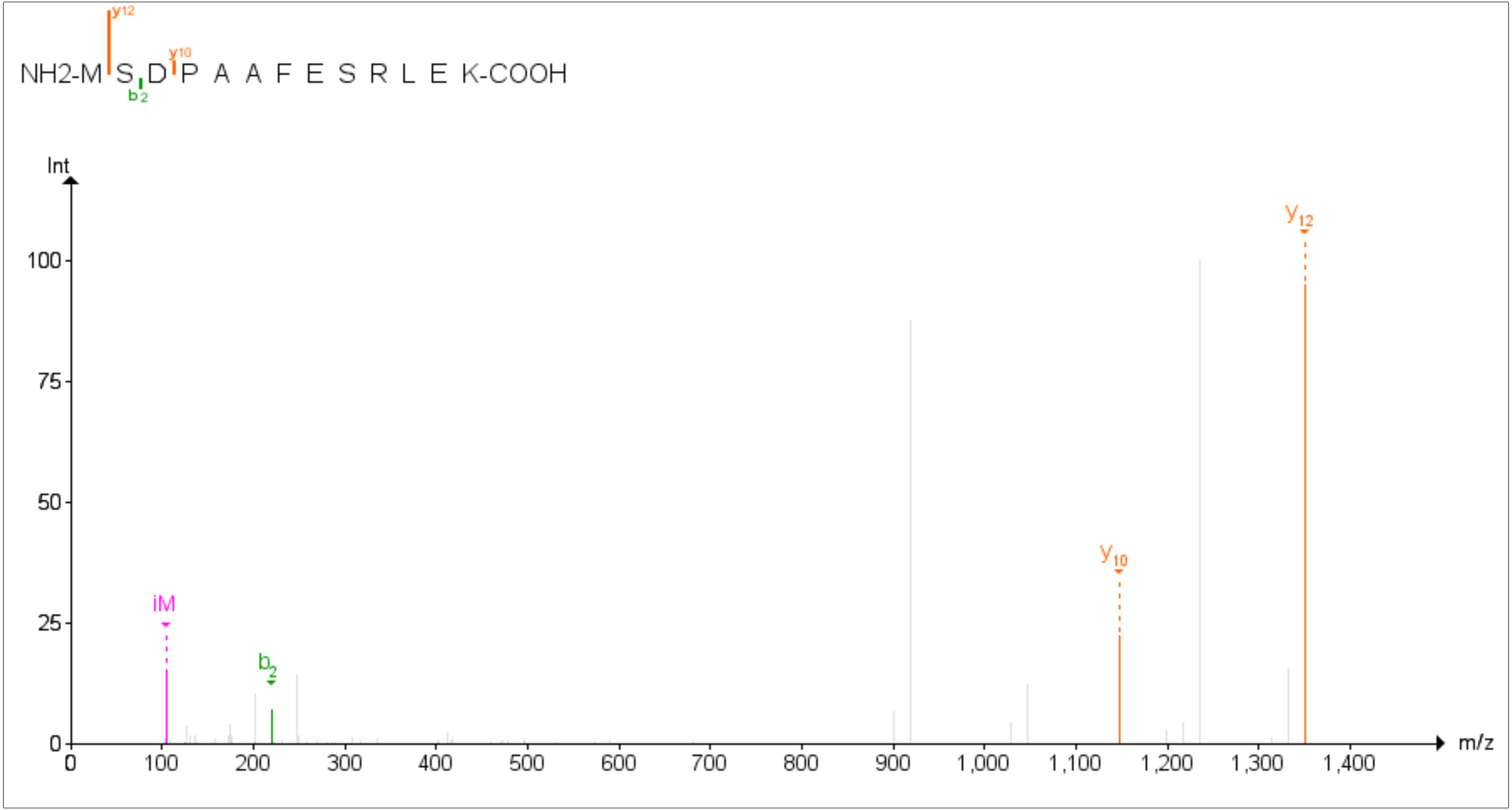

NH2-M S D P A A F E S R L E K-COOH
y12
y10
b2
Int
100
75
50
25
0
iM
b2
y10
y12
0
100
200
300
400
500
600
700
800
900
1,000
1,100
1,200
1,300
1,400
m/z

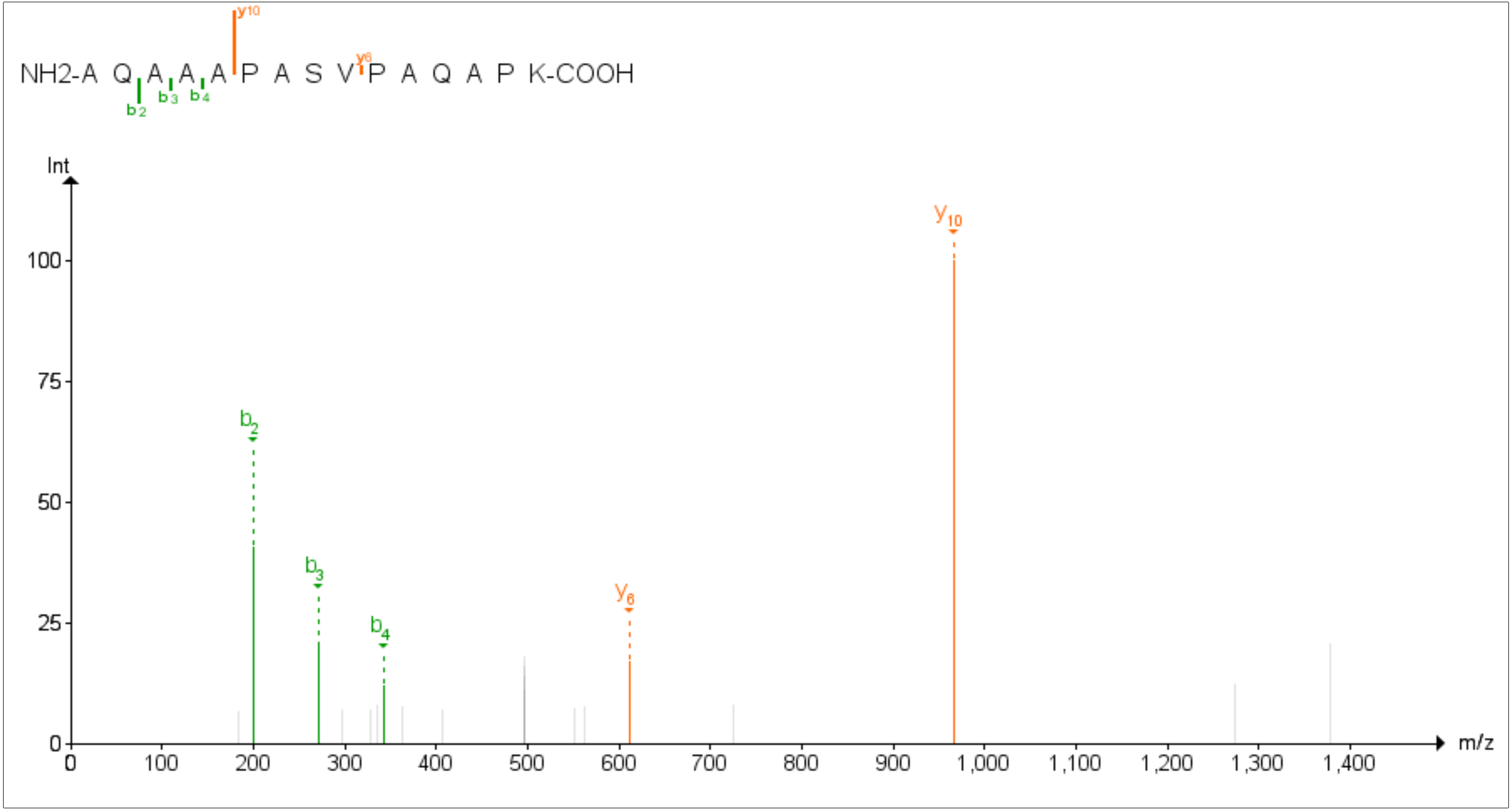

NH2-A Q A A A P A S V P A Q A P K-COOH
y10
y6
b2
b3
b4
Int
100
75
50
25
0
m/z
0
100
200
300
400
500
600
700
800
900
1,000
1,100
1,200
1,300
1,400

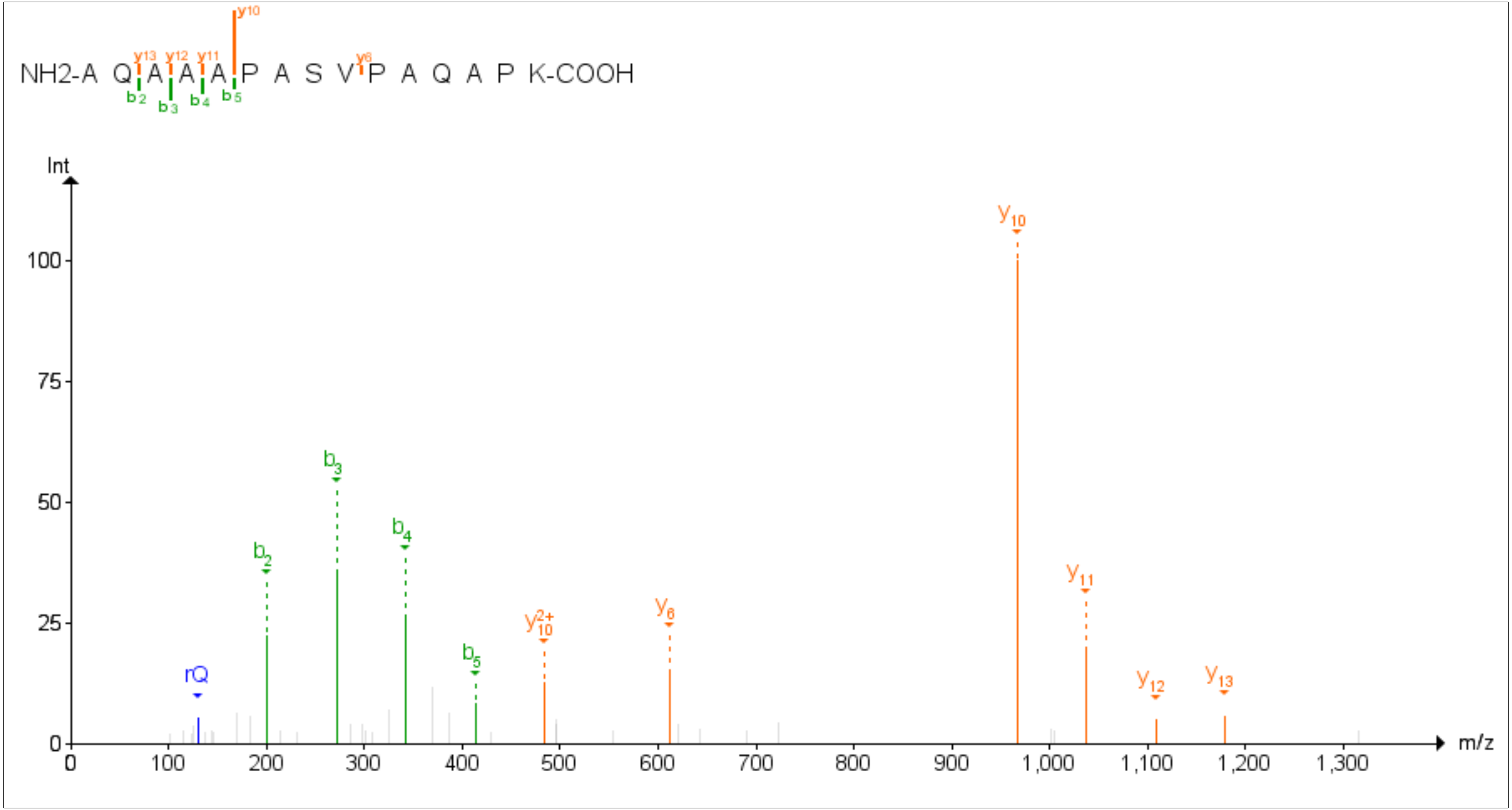
NH2-A Q A A A P A S V P A Q A P K-COOH
y10
y13
y12
y11
y6
b2
b3
b4
b5
Int
100
75
50
25
0
y10
b3
b4
b2
y11
y6
y10 2+
b5
rQ
y12
y13
0
100
200
300
400
500
600
700
800
900
1,000
1,100
1,200
1,300
m/z

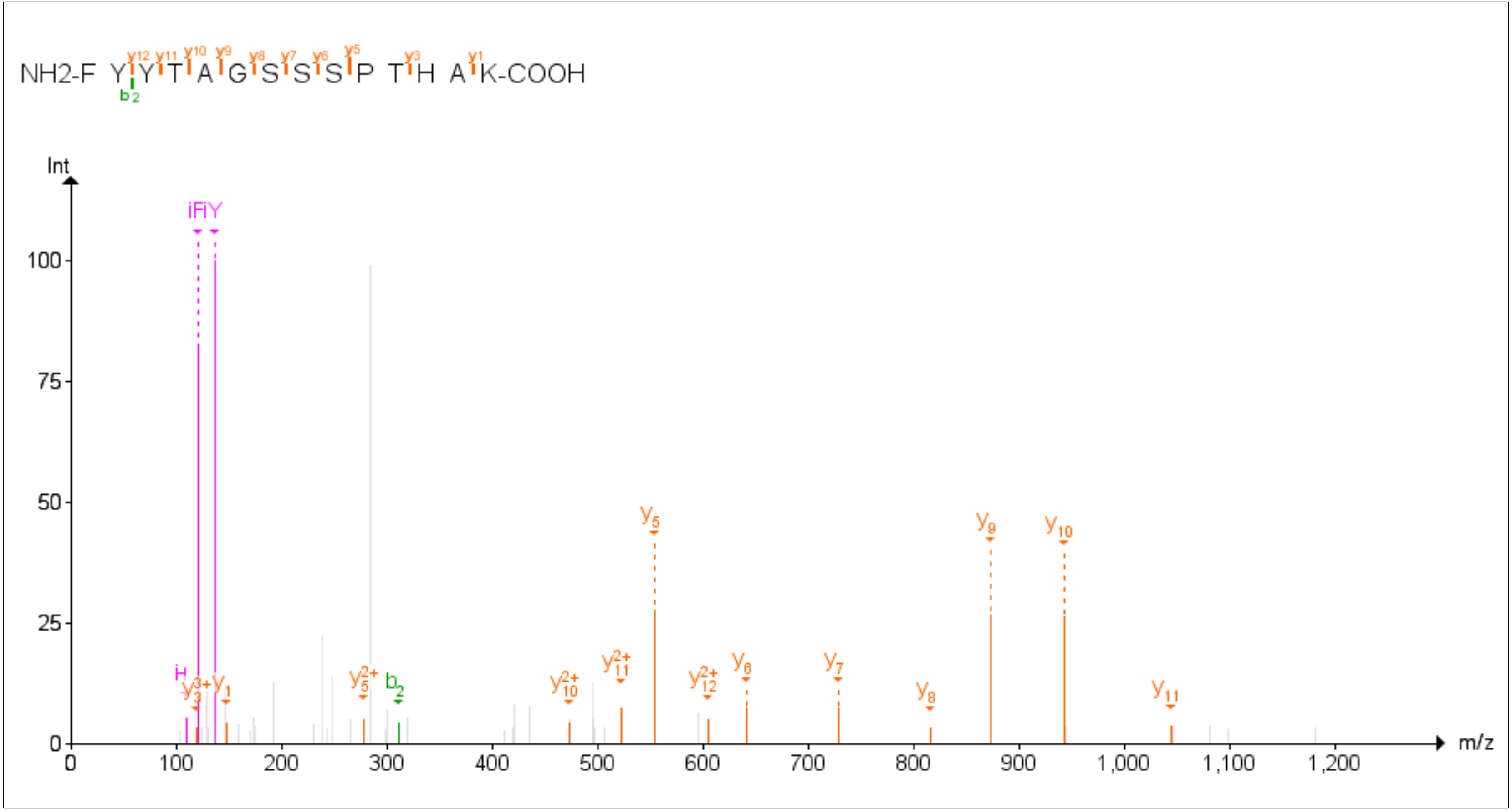

NH2-F Y Y T A G S S S P T H A K-COOH
y12 y11 y10 y9 y8 y7 y6 y5 y3 y1
b2
Int
iF iY
100
75
50
25
0
iH
y3 3+
y1
y5 2+
b2
y10 2+
y11 2+
y5
y12 2+
y6
y7
y8
y9
y10
y11
0 100 200 300 400 500 600 700 800 900 1,000 1,100 1,200
m/z

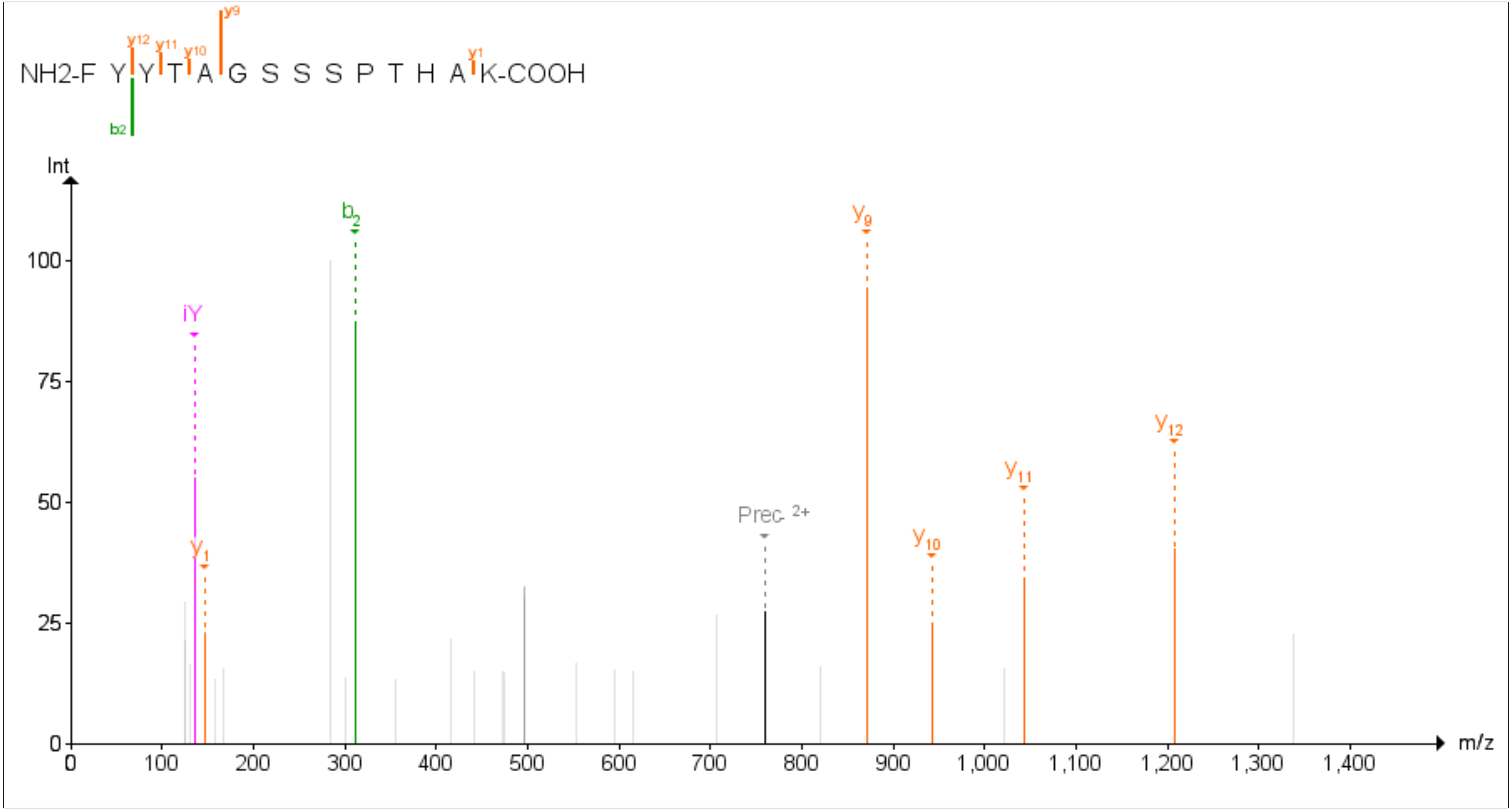

NH2-F Y Y T A G S S S P T H A K-COOH
y12 y11 y10 y9 y1
b2
Int
m/z
iY
b2
y1
Prec 2+
y9
y10
y11
y12

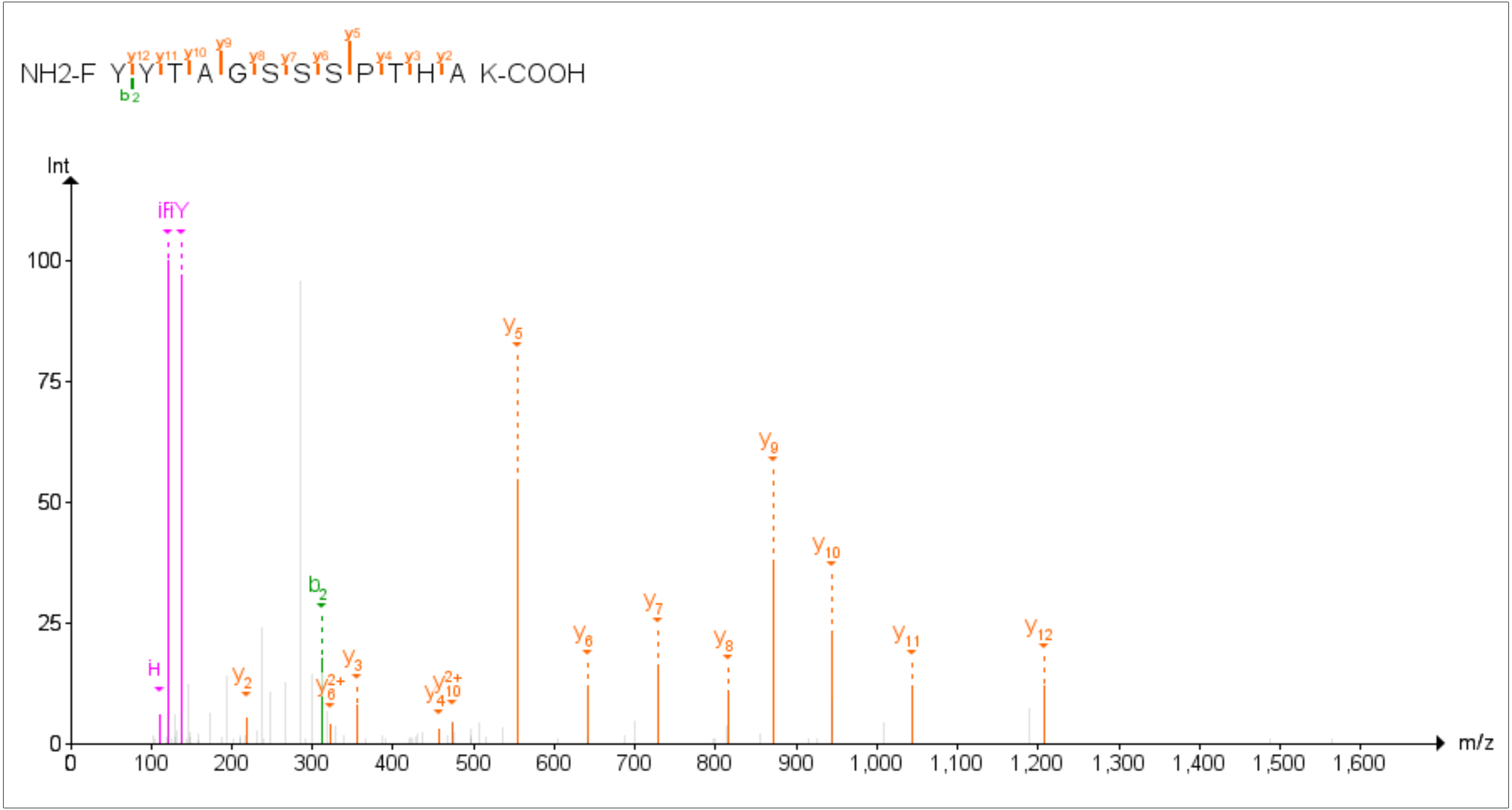
NH2-F Y Y T A G S S S P T H A K-COOH
y12 y11 y10 y9 y8 y7 y6 y5 y4 y3 y2
b2
Int
iFiY
H
y2
b2
y3
y6 2+
y4
y10 2+
y5
y6
y7
y8
y9
y10
y11
y12
0
25
50
75
100
0 100 200 300 400 500 600 700 800 900 1,000 1,100 1,200 1,300 1,400 1,500 1,600
m/z

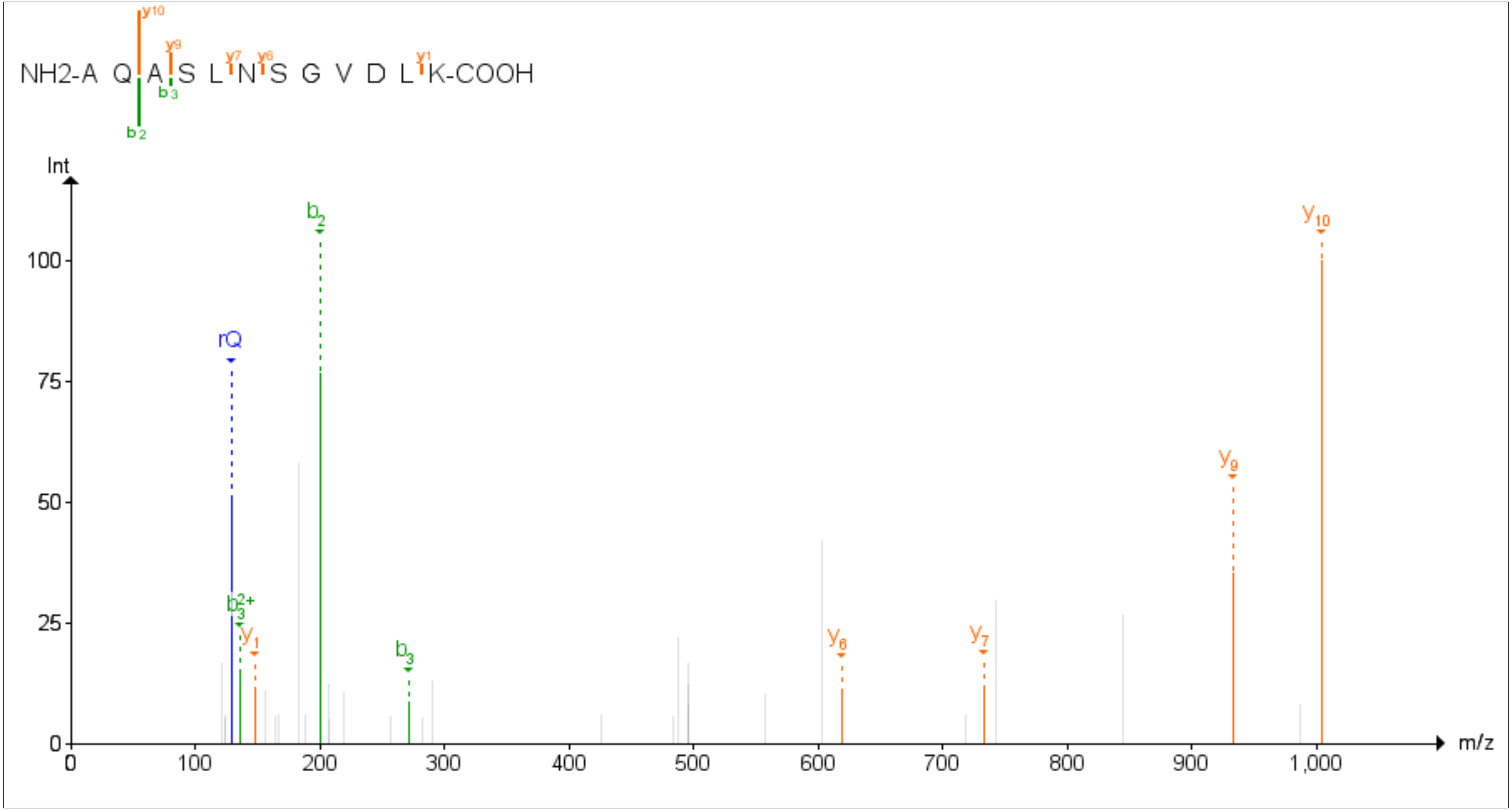

NH2-A Q A S L N S G V D L K-COOH
y10
y9
y7
y6
y1
b2
b3
Int
b2
y10
rQ
y9
b3 2+
y1
b3
y6
y7
0
25
50
75
100
0
100
200
300
400
500
600
700
800
900
1,000
m/z

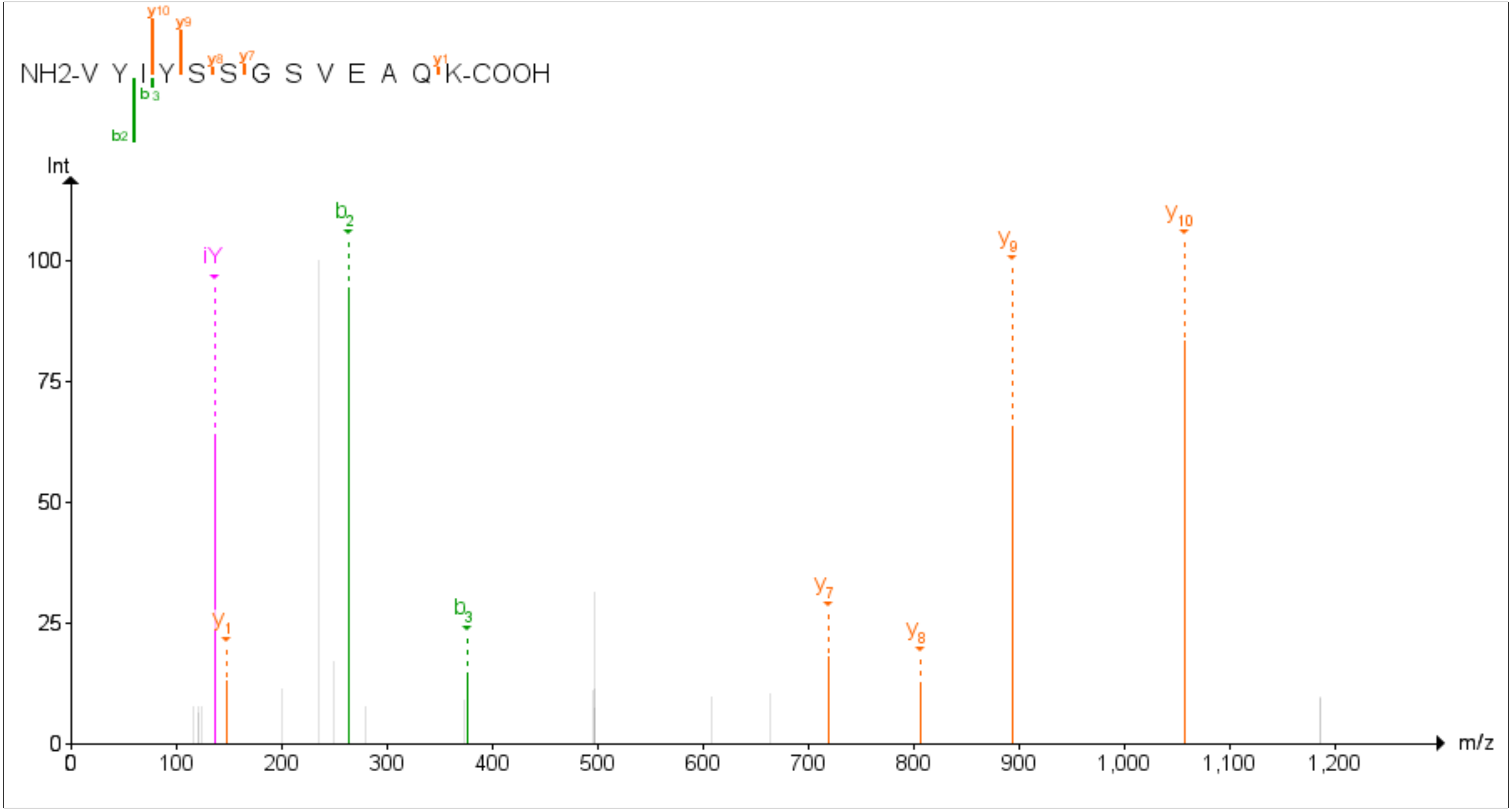
NH2-V Y I Y S S G S V E A Q K-COOH
y10
y9
y8
y7
y1
b2
b3
Int
100
75
50
25
0
iY
b2
b3
y1
y7
y8
y9
y10
0
100
200
300
400
500
600
700
800
900
1,000
1,100
1,200
m/z

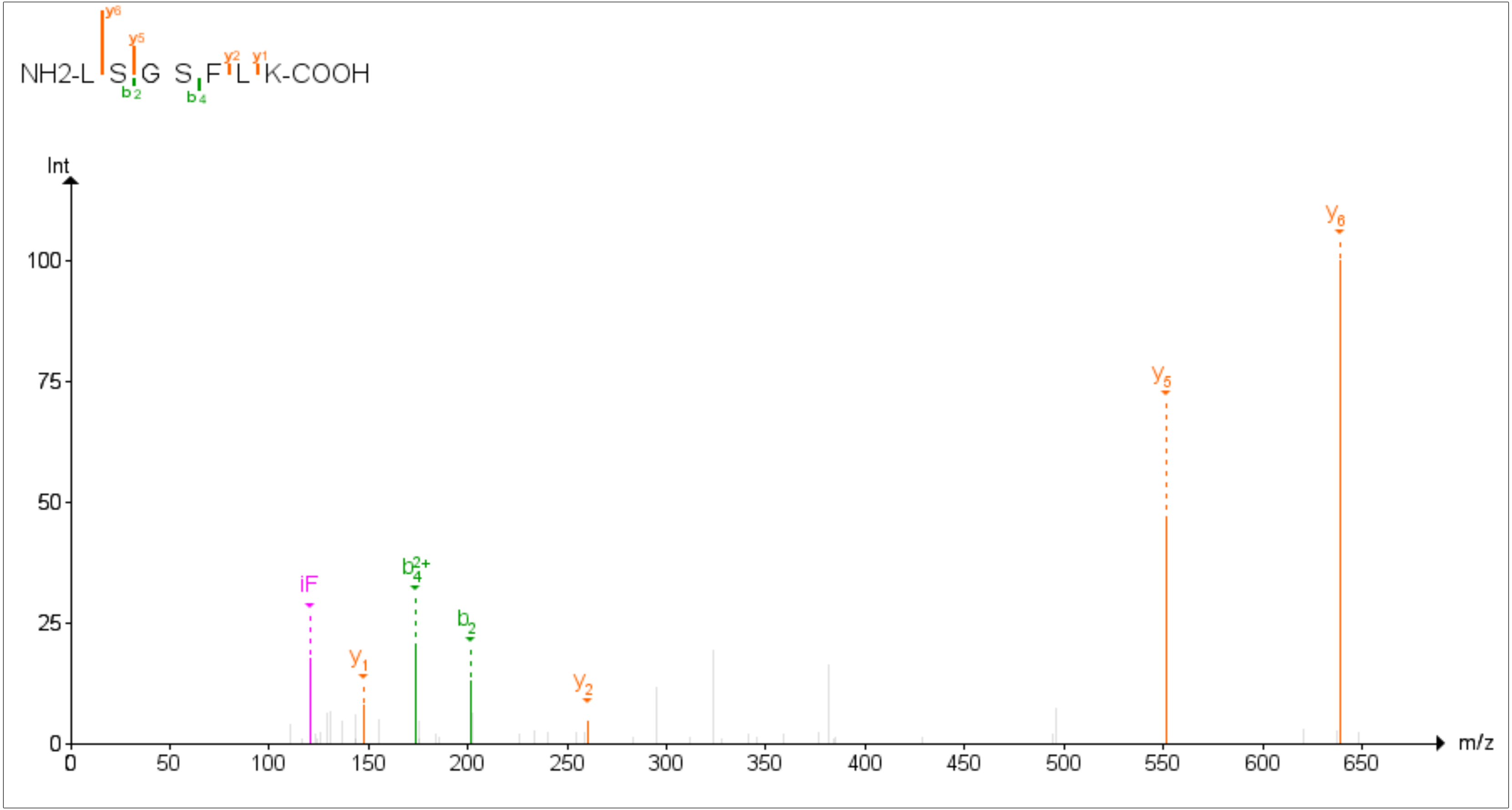
NH2-L S G S F L K-COOH
y6
y5
y2
y1
b2
b4
Int
m/z
iF
y1
b4 2+
b2
y2
y5
y6
0
25
50
75
100
0
50
100
150
200
250
300
350
400
450
500
550
600
650

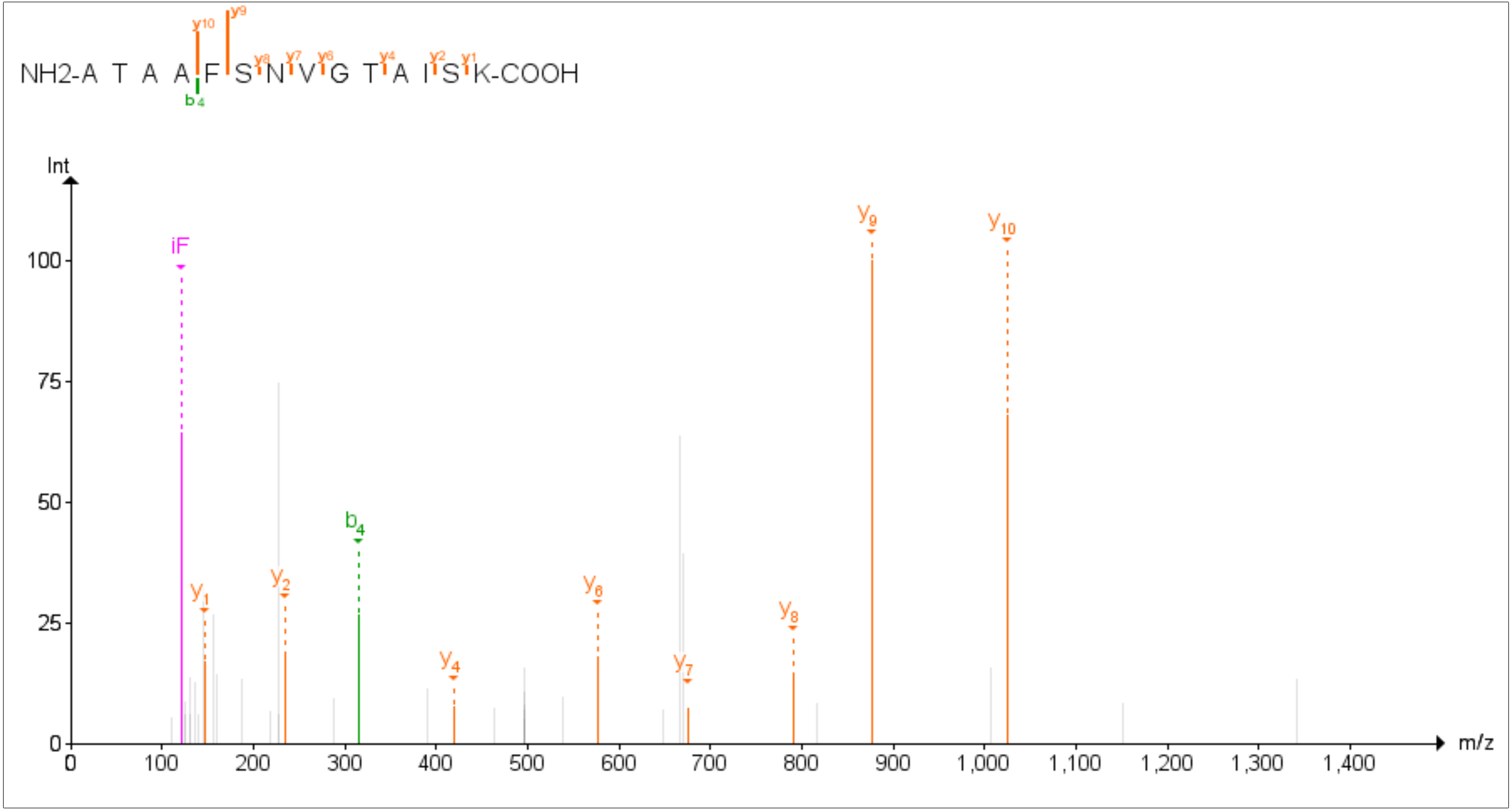

NH2-A T A A F S N V G T A I S K-COOH
y10
y9
y8
y7
y6
y4
y2
y1
b4
Int
100
75
50
25
0
iF
y1
y2
b4
y4
y6
y7
y8
y9
y10
0
100
200
300
400
500
600
700
800
900
1,000
1,100
1,200
1,300
1,400
m/z

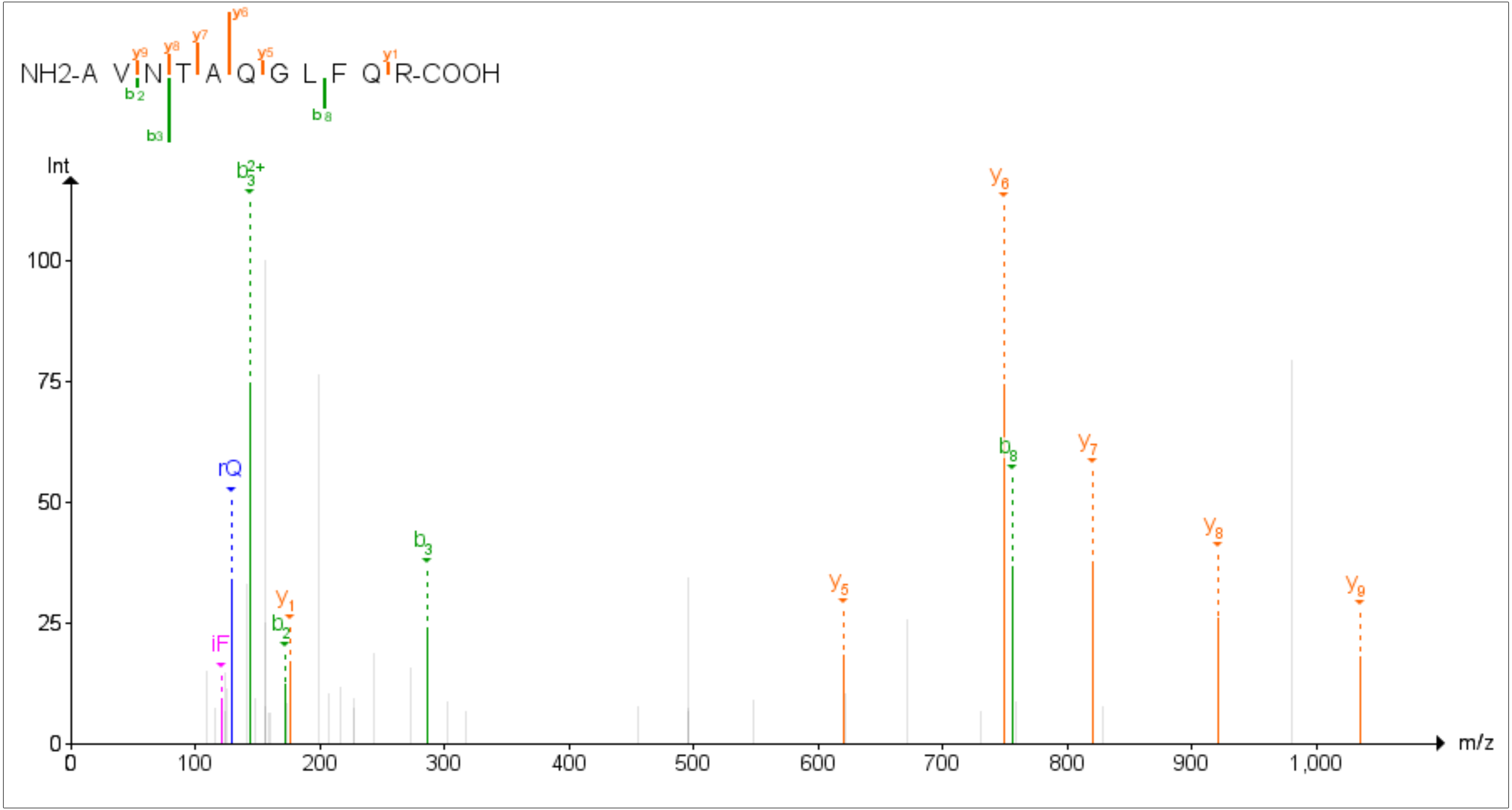

NH2-A V N T A Q G L F Q R-COOH
y9 y8 y7 y6 y5 y1
b2 b3 b8
Int
100
75
50
25
0
rQ
iF
b3 2+
y1
b2
b3
y5
y6
b8
y7
y8
y9
0 100 200 300 400 500 600 700 800 900 1,000
m/z

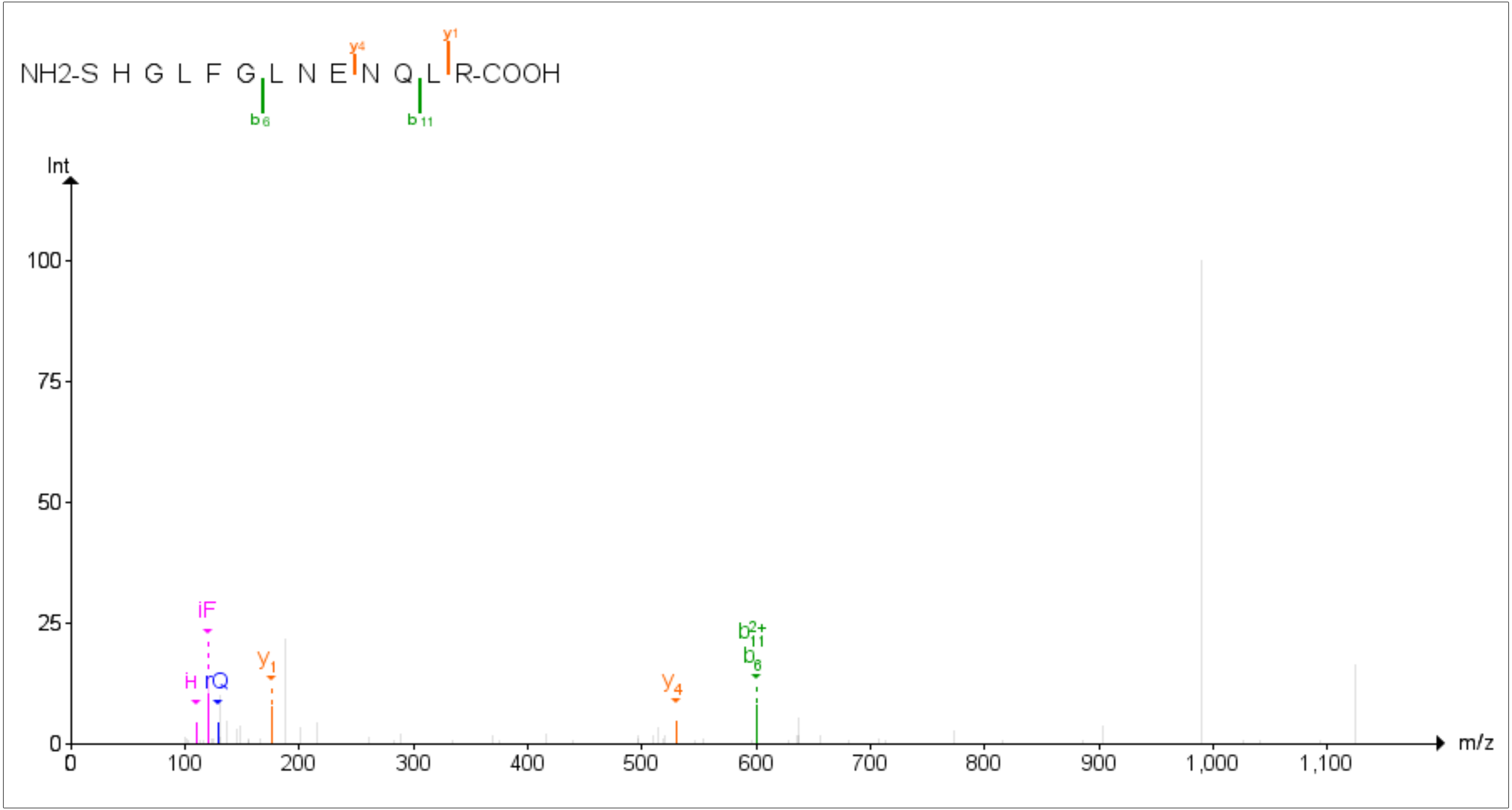

NH2-S H G L F G L N E N Q L R-COOH
y4
y1
b6
b11
Int
100
75
50
25
0
iF
H
rQ
y1
y4
b11 2+
b6
0
100
200
300
400
500
600
700
800
900
1,000
1,100
m/z

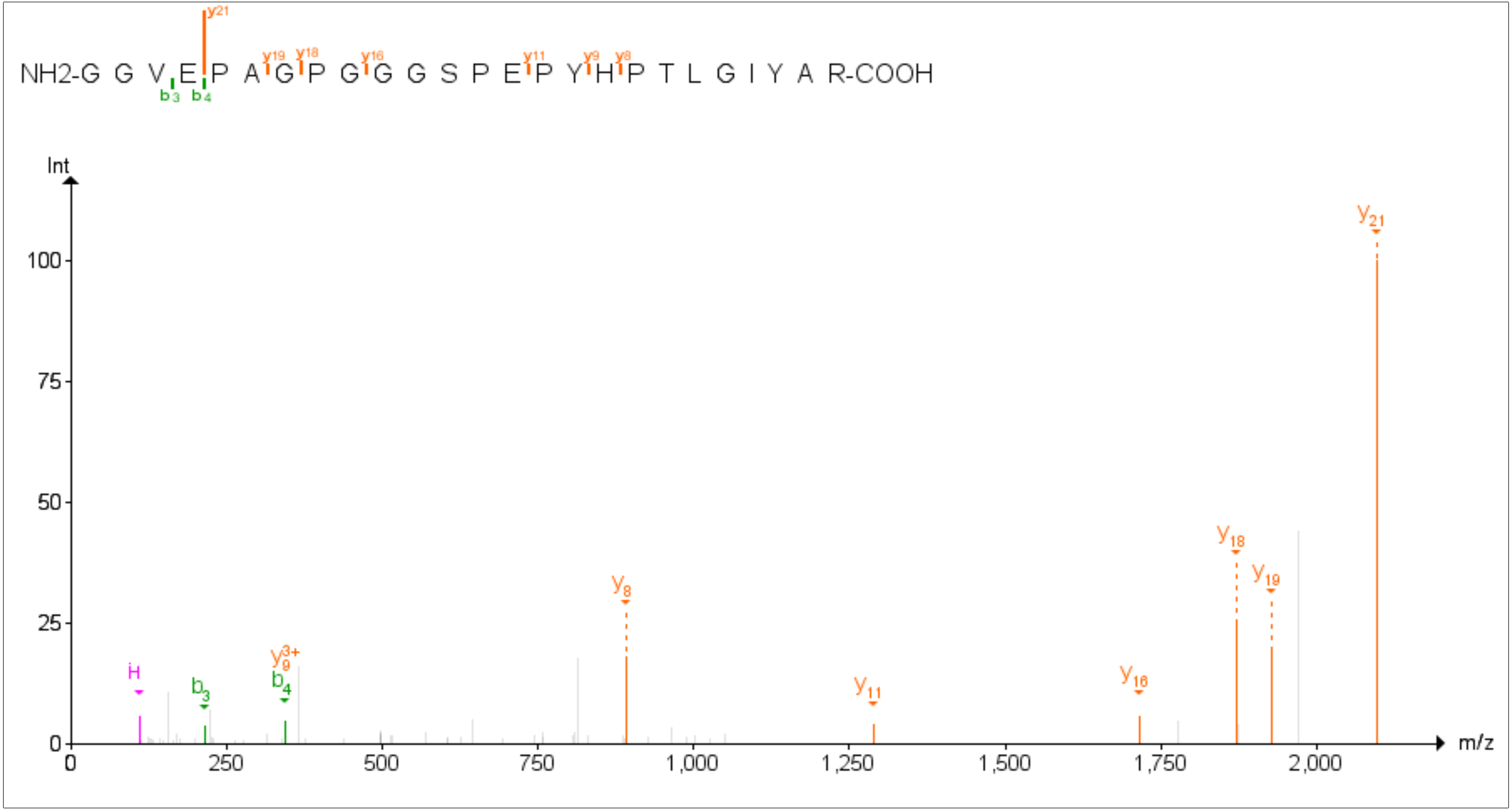

NH2-G G V E P A G P G G G S P E P Y H P T L G I Y A R-COOH
y21
y19
y18
y16
y11
y9
y8
b3
b4
Int
100
75
50
25
0
m/z
0
250
500
750
1,000
1,250
1,500
1,750
2,000
H
b3
b4
y9 3+
y8
y11
y16
y18
y19
y21

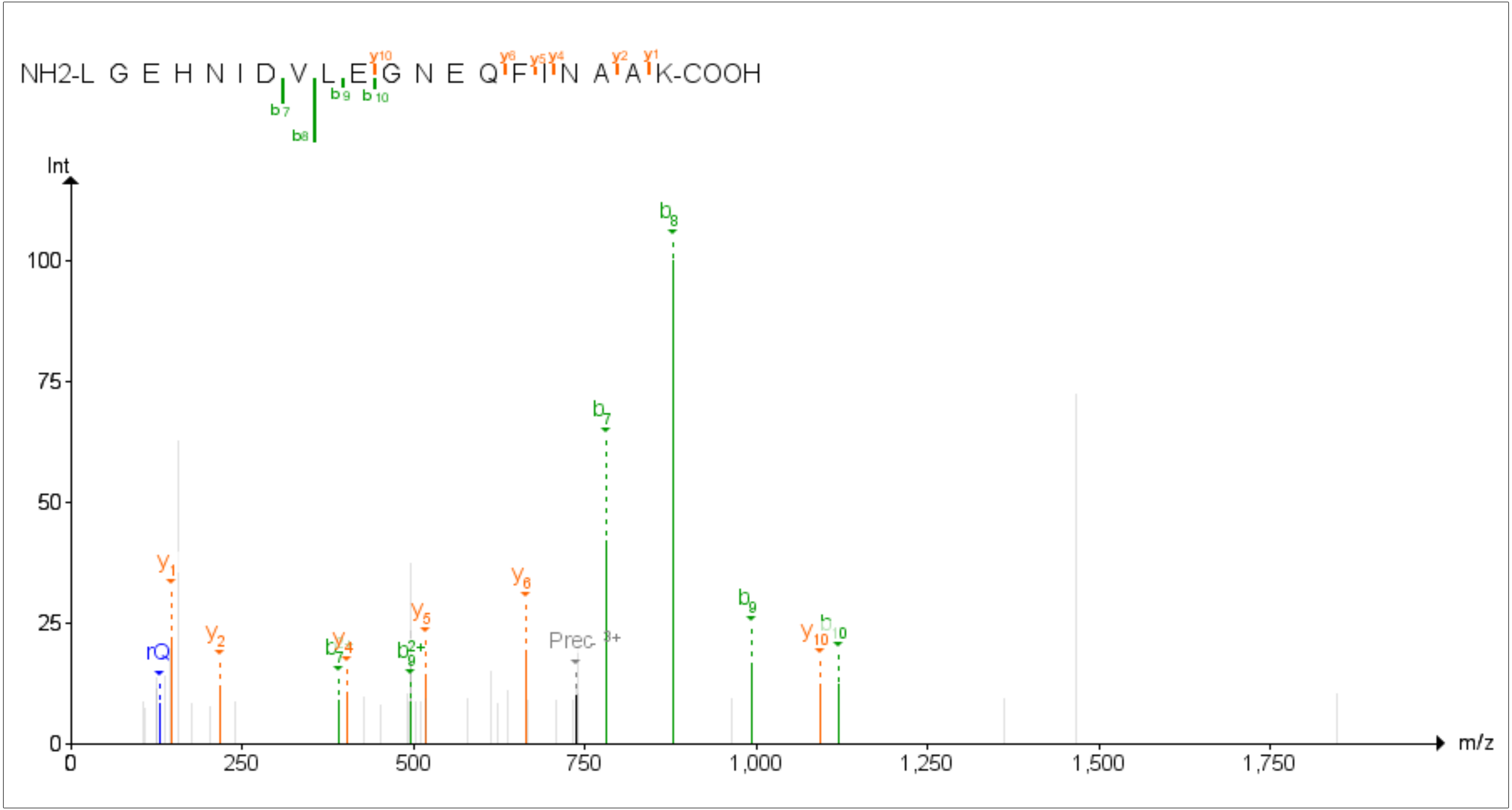
NH2-L G E H N I D V L E G N E Q F I N A A K-COOH
y10 y6 y5 y4 y2 y1
b7 b8 b9 b10
Int
100
75
50
25
0
m/z
0
250
500
750
1,000
1,250
1,500
1,750
b8
b7
y1
y2
rQ
y4
b7
b9 2+
y5
y6
Prec 3+
b9
y10
b10

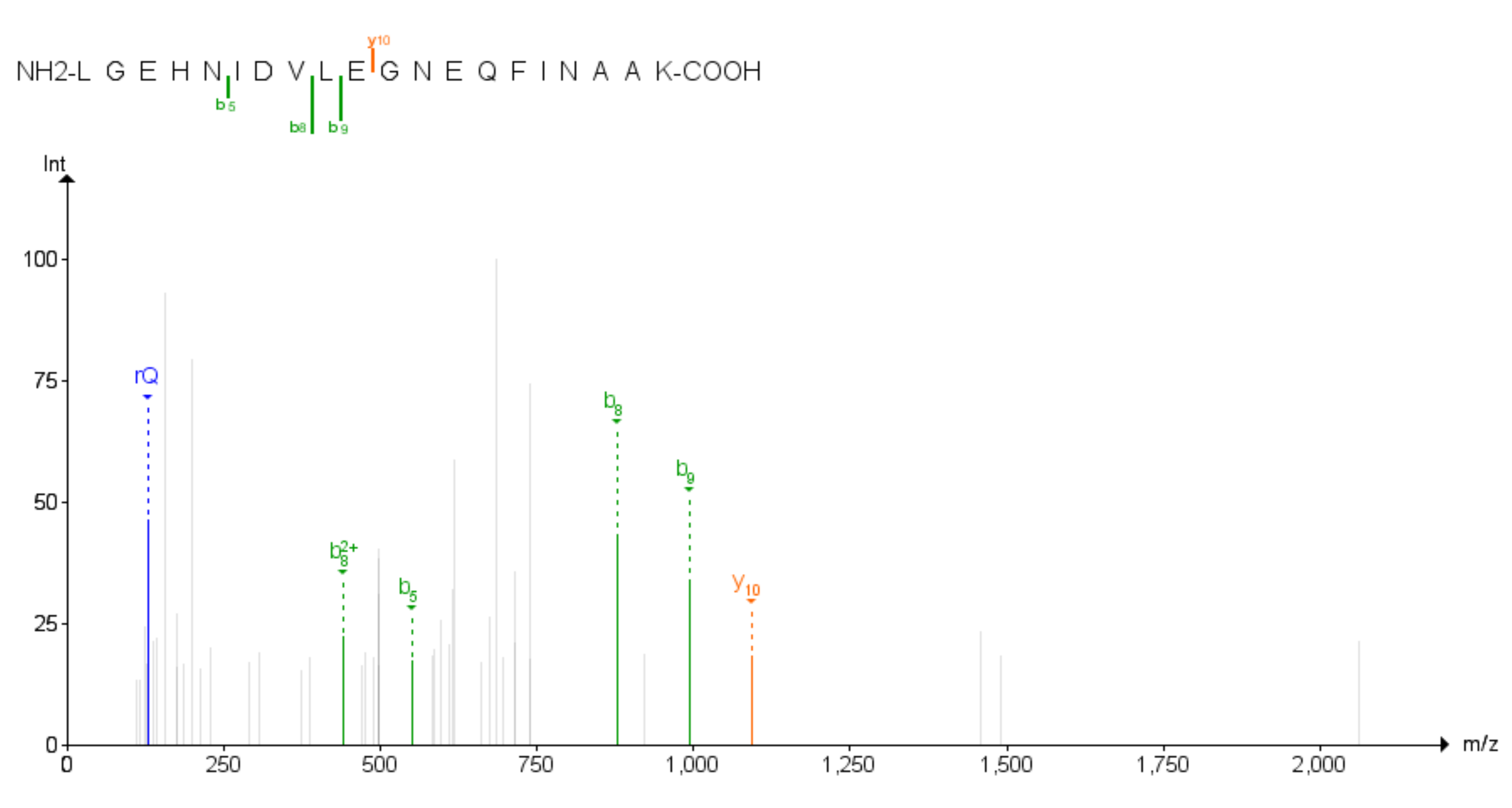
NH2-L G E H N I D V L E G N E Q F I N A A K-COOH
y10
b5
b8
b9
Int
100
75
50
25
0
rQ
b8
b9
b8 2+
b5
y10
0
250
500
750
1,000
1,250
1,500
1,750
2,000
m/z

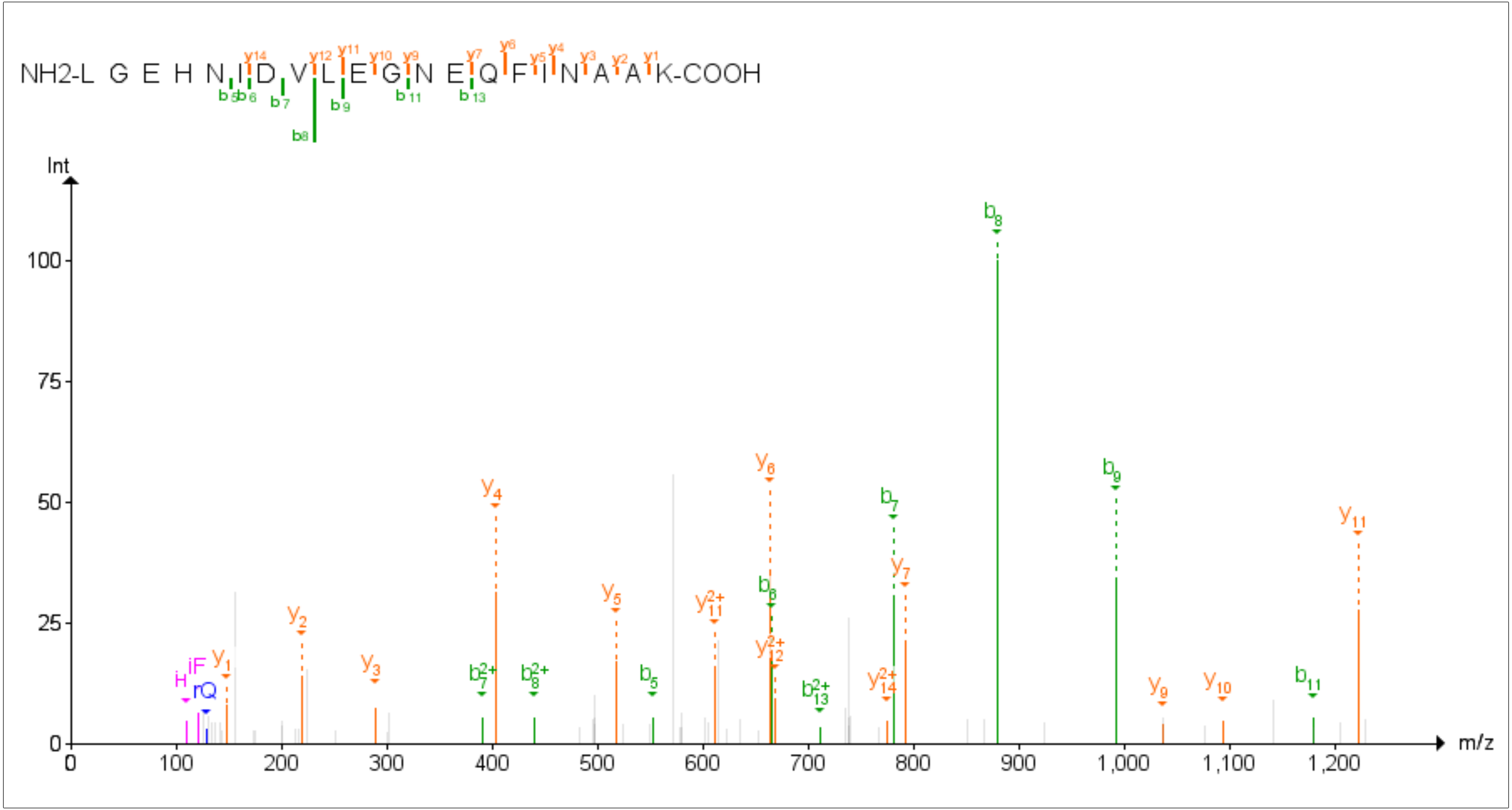

NH2-L G E H N I D V L E G I N E Q F I N A A K-COOH
Int
m/z
b8
b9
b7
y4
y6
y11
y7
y5
y2
y1
y3
b5
b6
y9
y10
b11

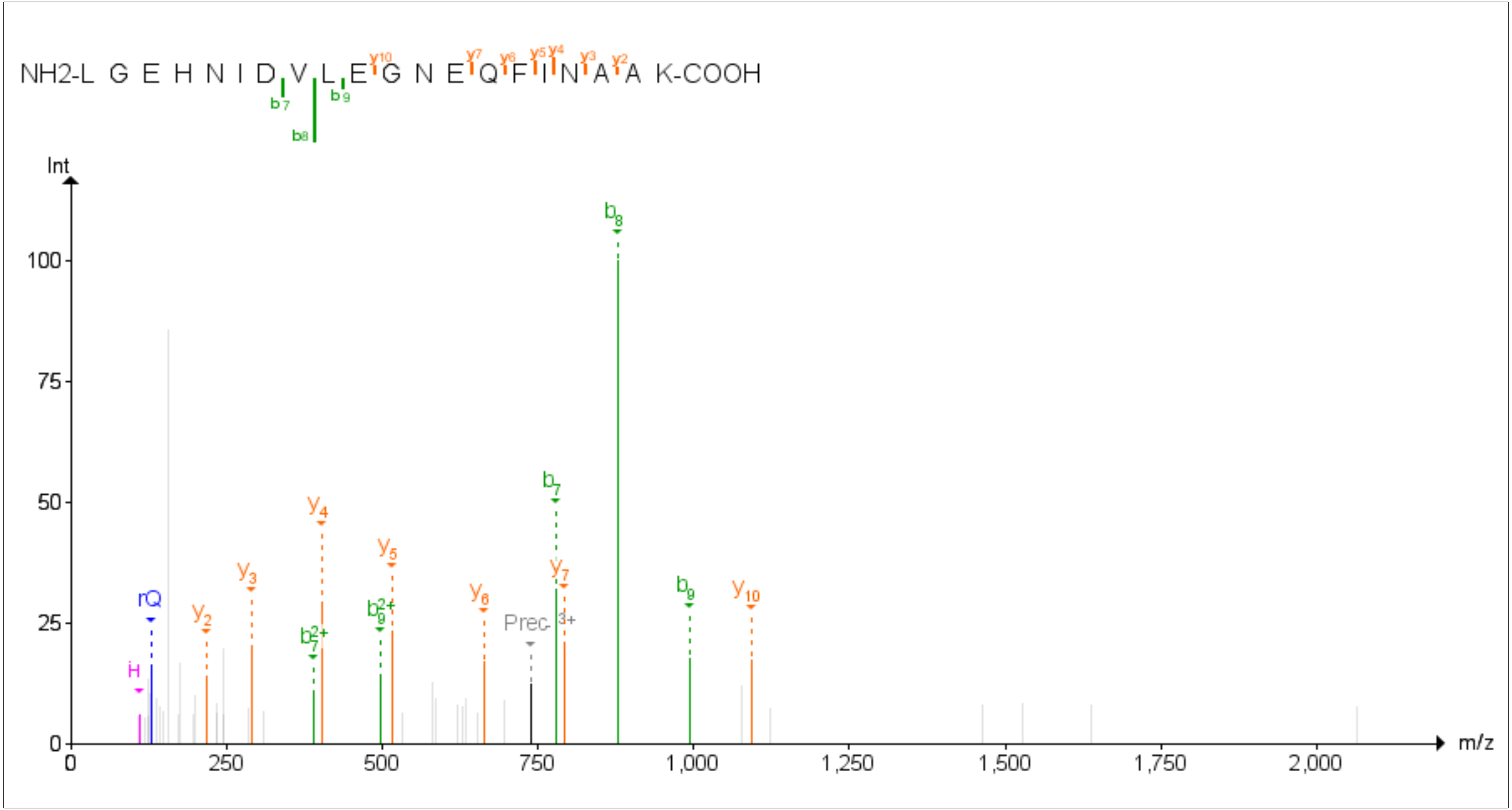
NH2-L G E H N I D V L E G N E Q F I N A A K-COOH
b7
b8
b9
y10
y7
y6
y5
y4
y3
y2
Int
100
75
50
25
0
b8
b7
y4
y5
y3
rQ
y2
b7 2+
b9 2+
y6
y7
Prec- 3+
b9
y10
H
m/z
0
250
500
750
1,000
1,250
1,500
1,750
2,000

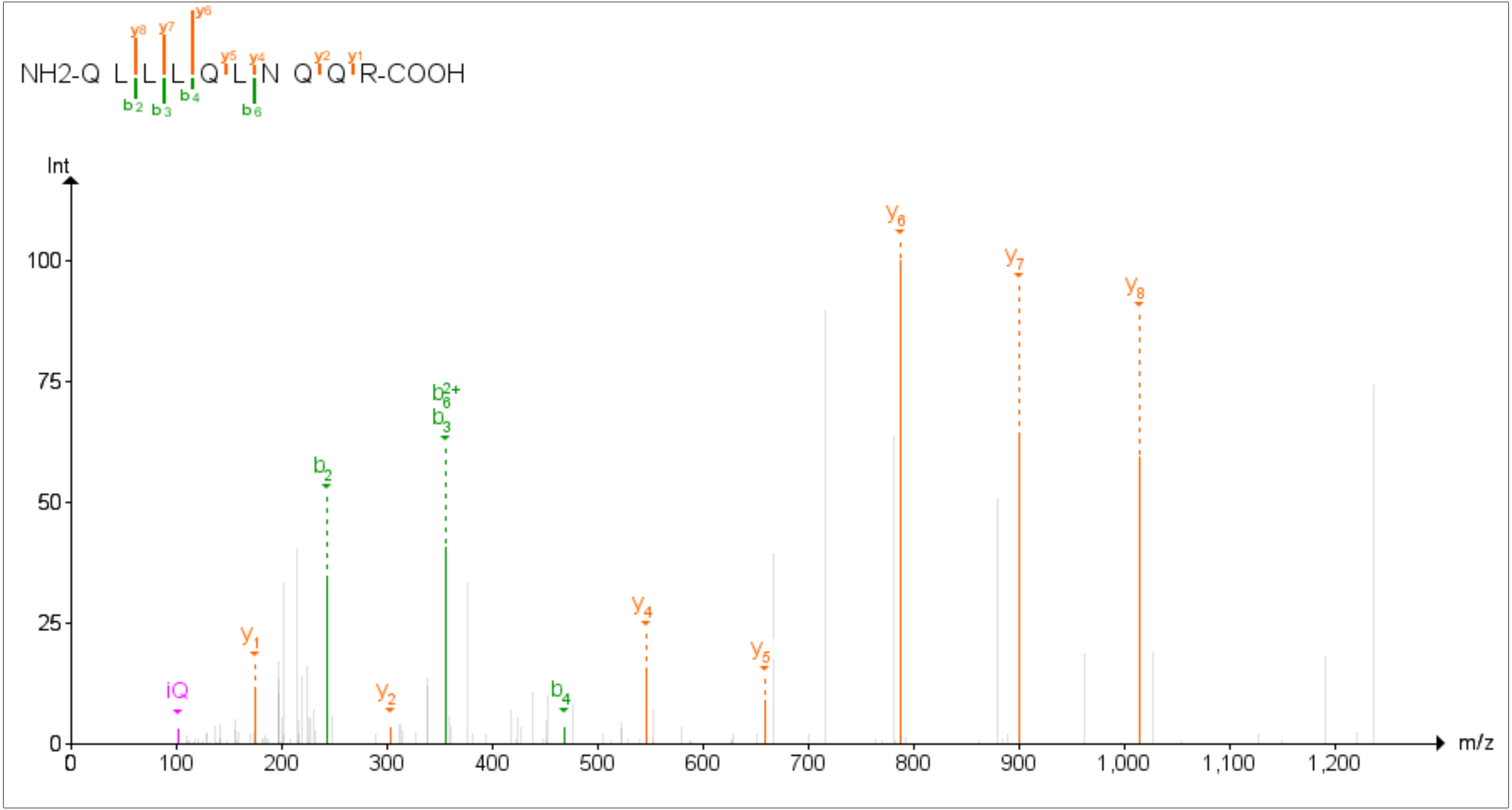
NH2-Q L L L Q L N Q Q R-COOH
Int
m/z
0
25
50
75
100
0
100
200
300
400
500
600
700
800
900
1,000
1,100
1,200
iQ
y1
b2
y2
b3
b6 2+
b4
y4
y5
y6
y7
y8

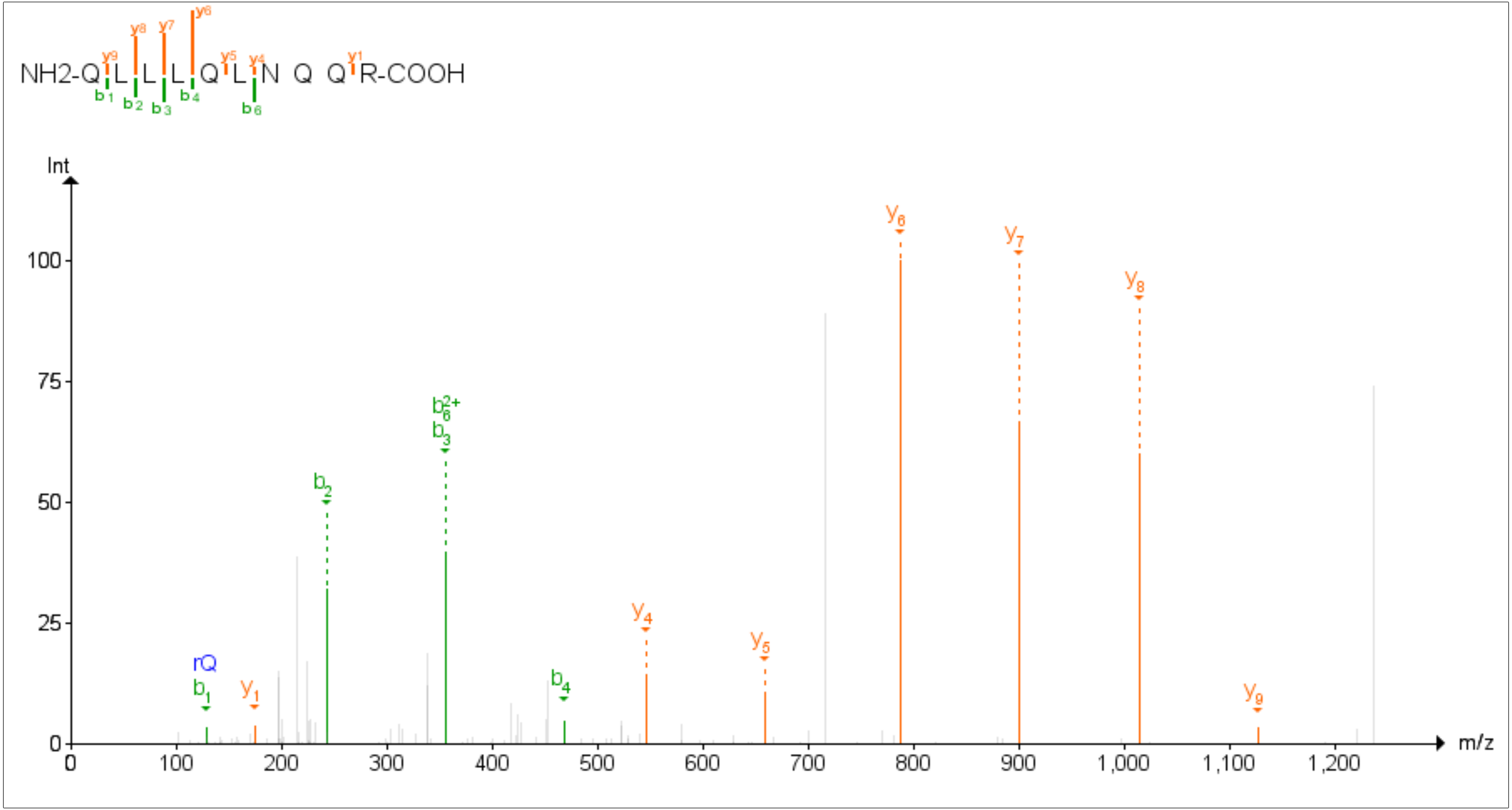

NH2-Q L L L Q L N Q Q R-COOH
Int
m/z
rQ
b1
y1
b2
b6 2+
b3
b4
y4
y5
y6
y7
y8
y9

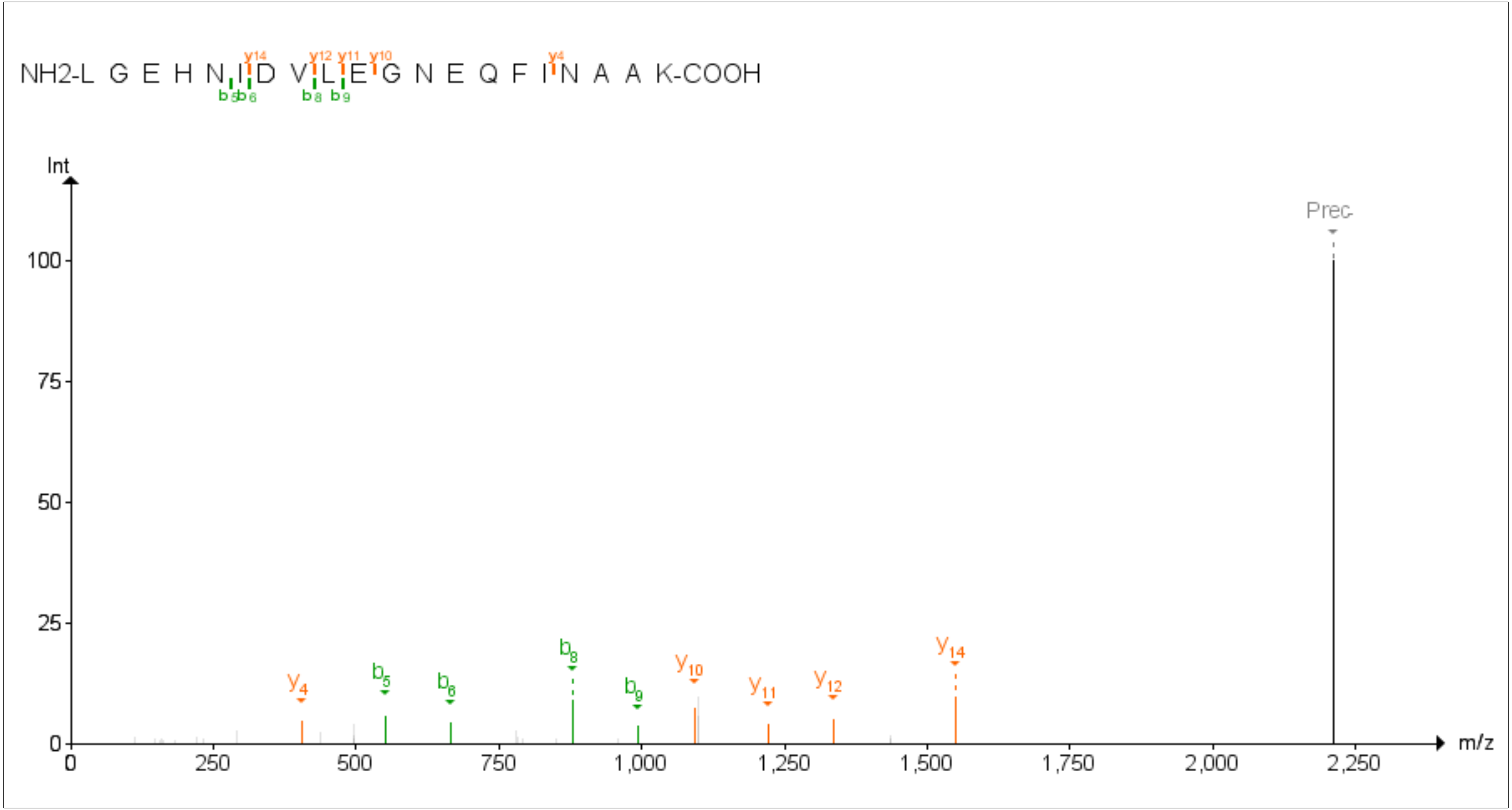
NH2-L G E H N I D V L E G N E Q F I N A A K-COOH
y14 y12 y11 y10 y4
b5 b6 b8 b9
Int
100
75
50
25
0
Prec
y4 b5 b6 b8 b9 y10 y11 y12 y14
0 250 500 750 1,000 1,250 1,500 1,750 2,000 2,250
m/z

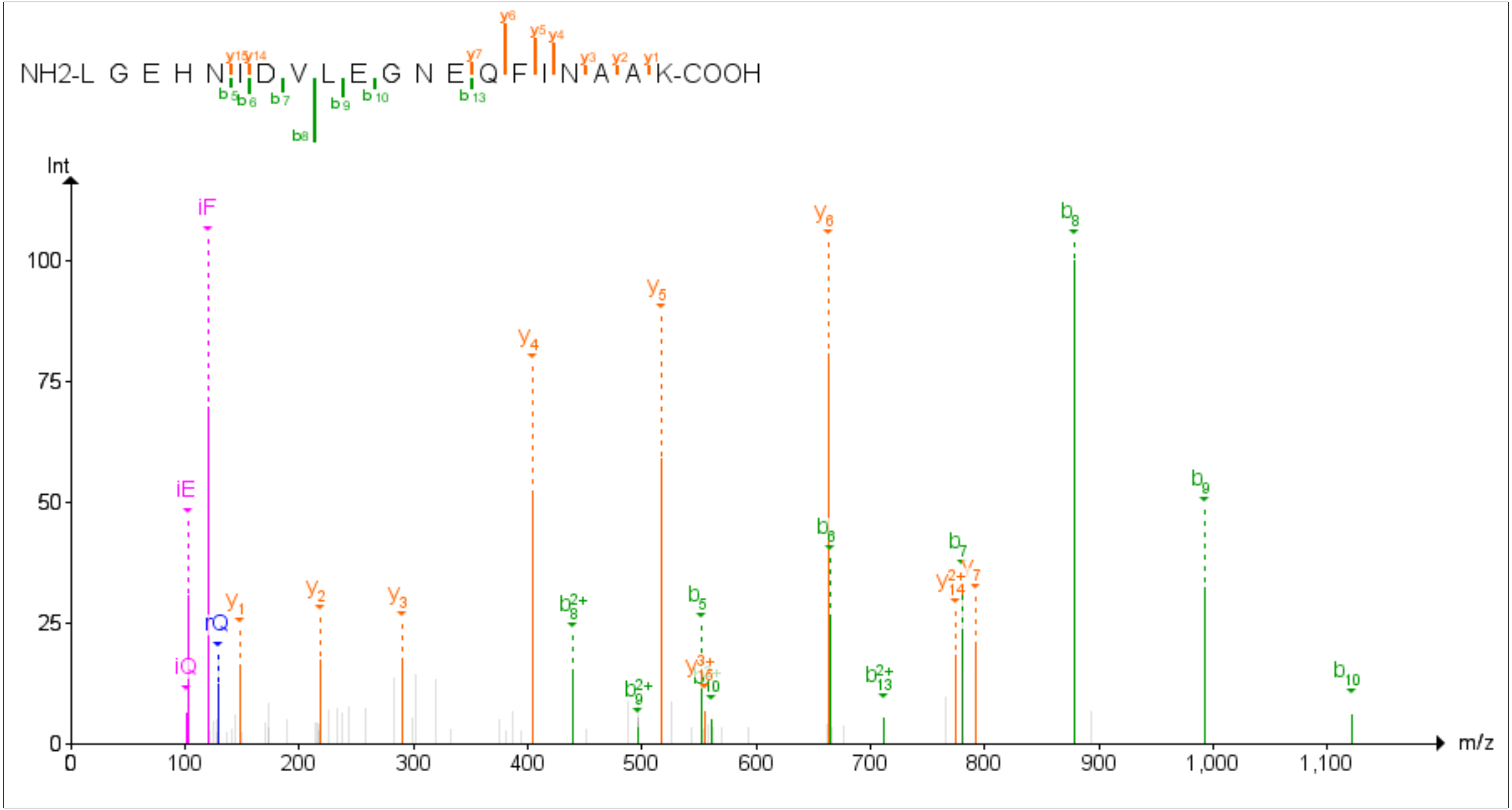

NH2-L G E H N I D V L E G N E Q F I N A A K-COOH
Int
m/z
iF
iE
iQ
rQ
y1
y2
y3
y4
y5
y6
y7
b8
b9
b10
b5
b6
b7

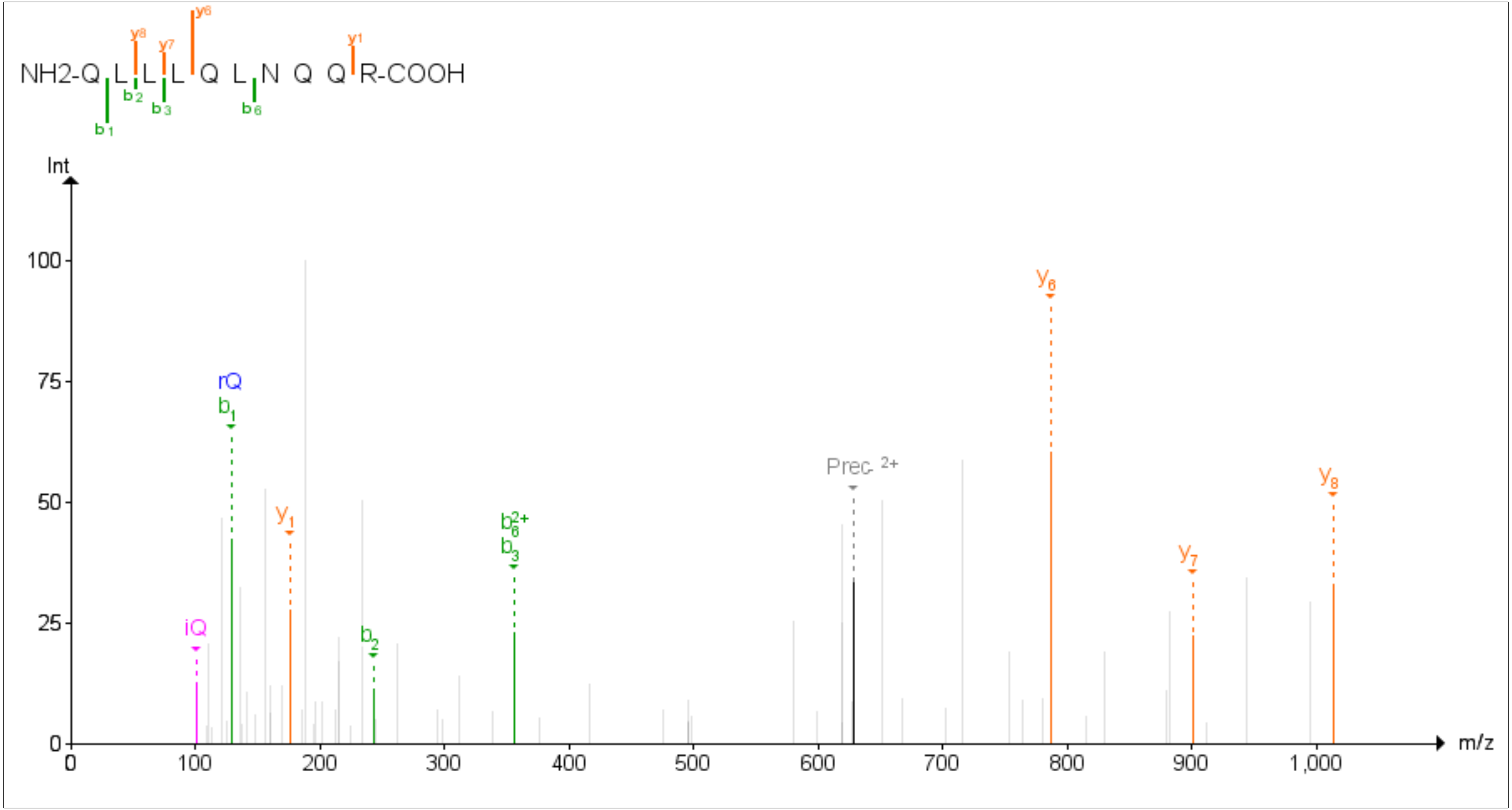

NH2-Q L L L Q L N Q Q R-COOH
y8
y7
y6
y1
b1
b2
b3
b6
Int
100
75
50
25
0
rQ
b1
y1
iQ
b2
b6 2+
b3
Prec 2+
y6
y7
y8
0
100
200
300
400
500
600
700
800
900
1,000
m/z

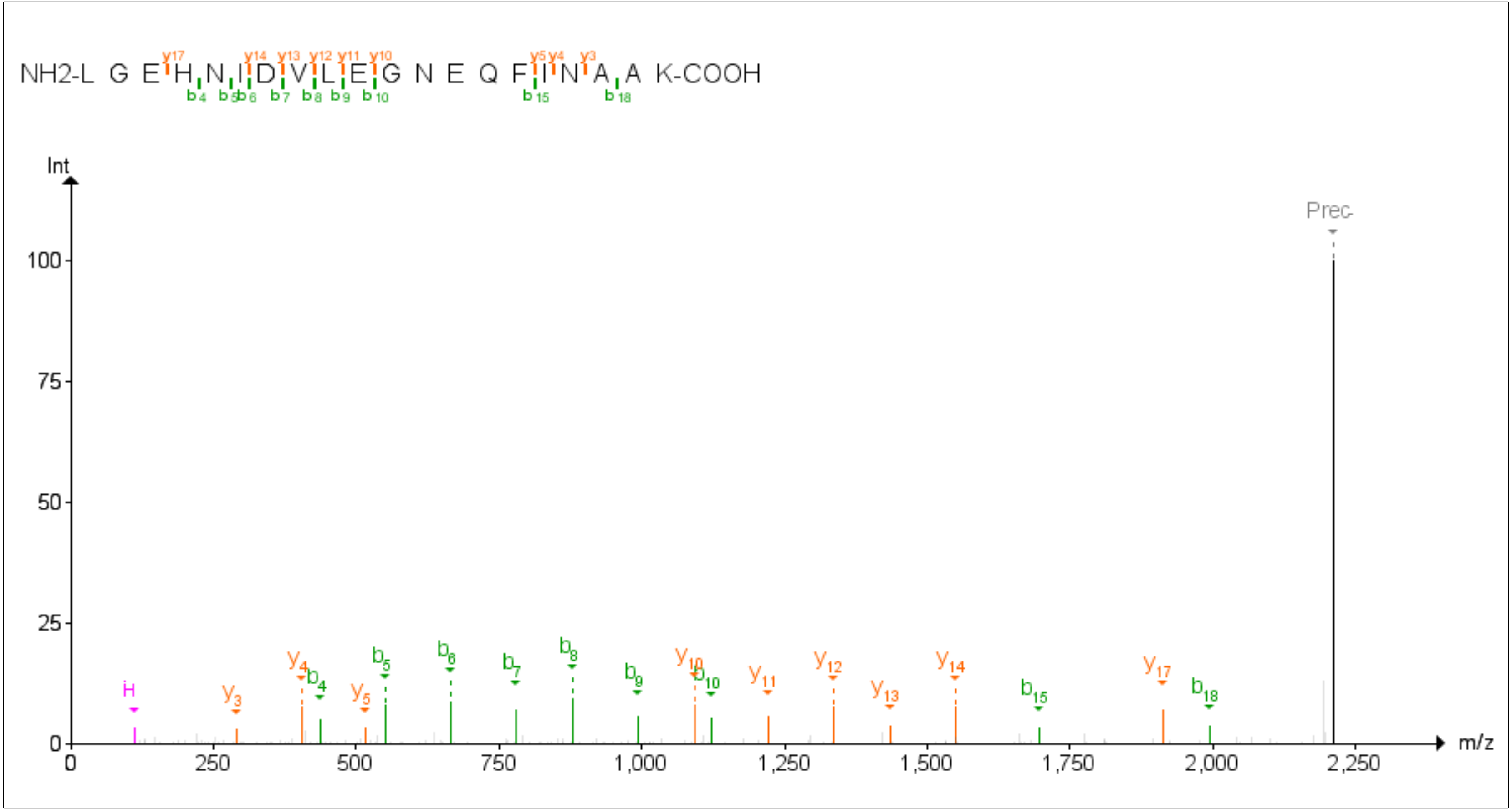

NH2-L G E H N I D V L E G N E Q F I N A A K-COOH
y17 y14 y13 y12 y11 y10 y5 y4 y3
b4 b5 b6 b7 b8 b9 b10 b15 b18
Int
100
75
50
25
0
Prec
H
y3 y4 b4 y5 b5 b6 b7 b8 b9 y10 b10 y11 y12 y13 y14 b15 y17 b18
0
250
500
750
1,000
1,250
1,500
1,750
2,000
2,250
m/z

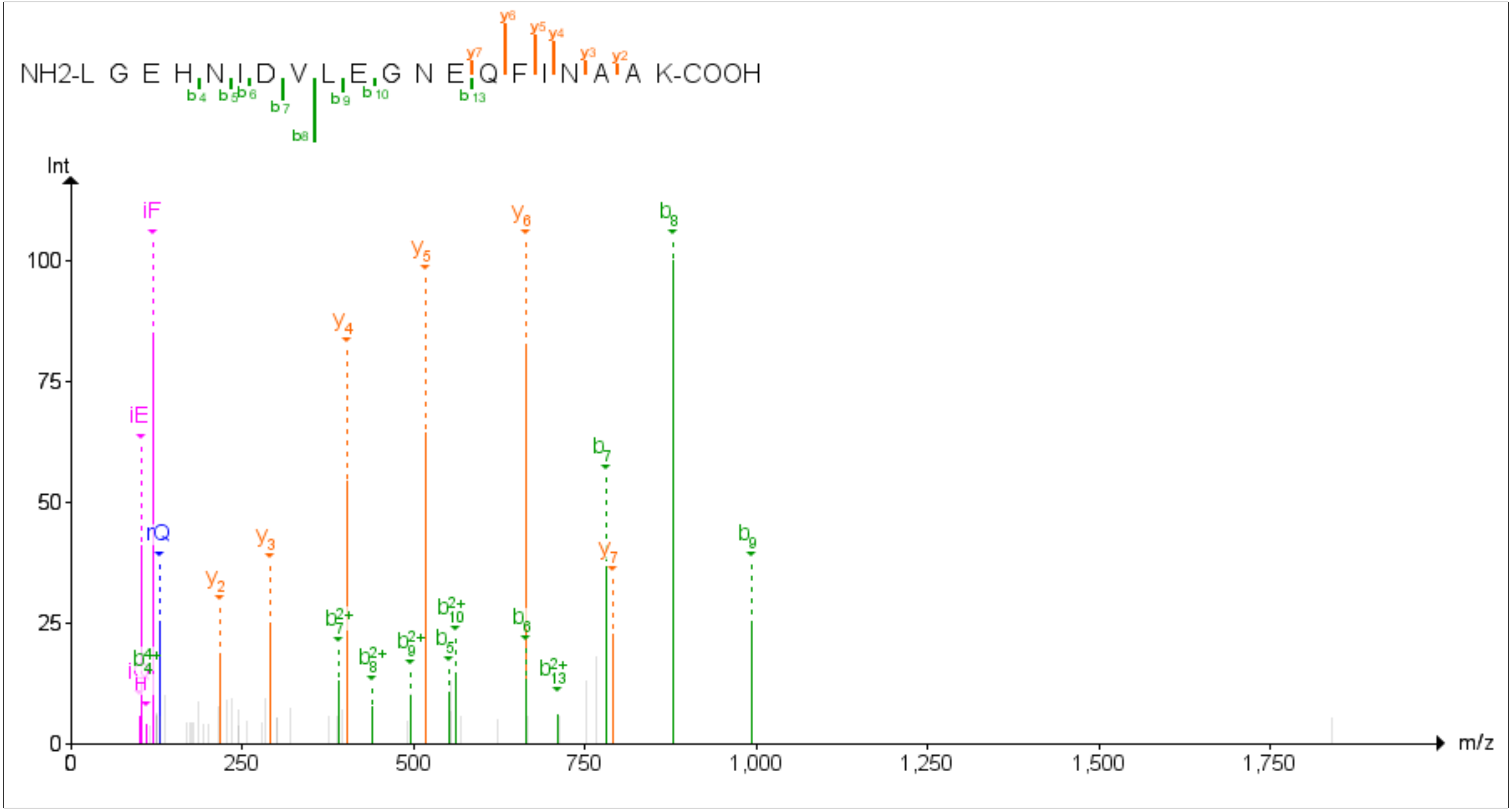

NH2-L G E H N I D V L E G N E Q F I N A A K-COOH
Int
m/z
iF
iE
rQ
y6
y5
y4
y3
y2
y7
b8
b7
b9
b6
b5

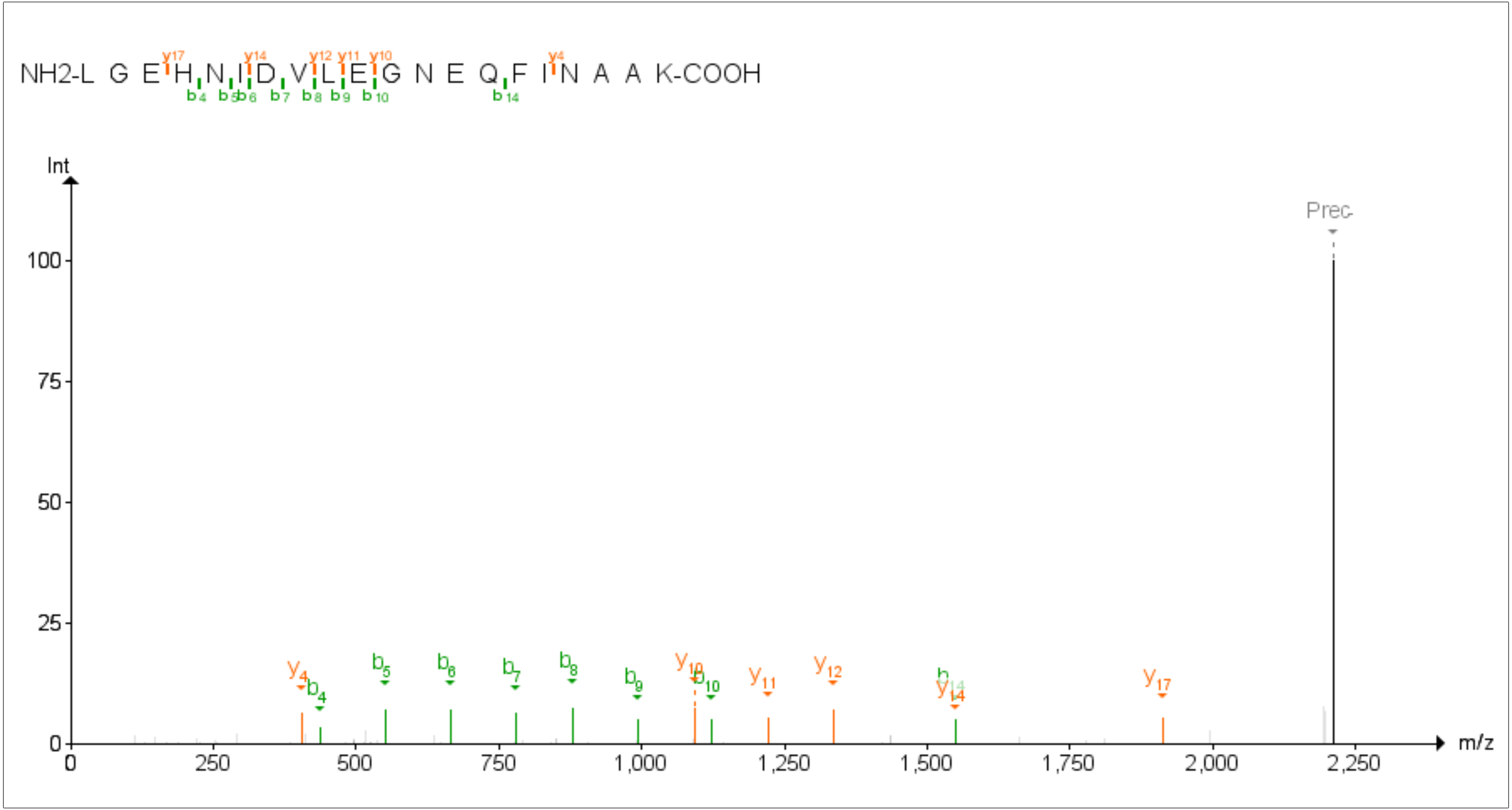

NH2-L G E H N I D V L E G N E Q F I N A A K-COOH
y17 y14 y12 y11 y10 y4
b4 b5 b6 b7 b8 b9 b10 b14
Int
Prec
100
75
50
25
0
y4 b4 b5 b6 b7 b8 b9 y10 b10 y11 y12 b14 y14 y17
0 250 500 750 1,000 1,250 1,500 1,750 2,000 2,250
m/z

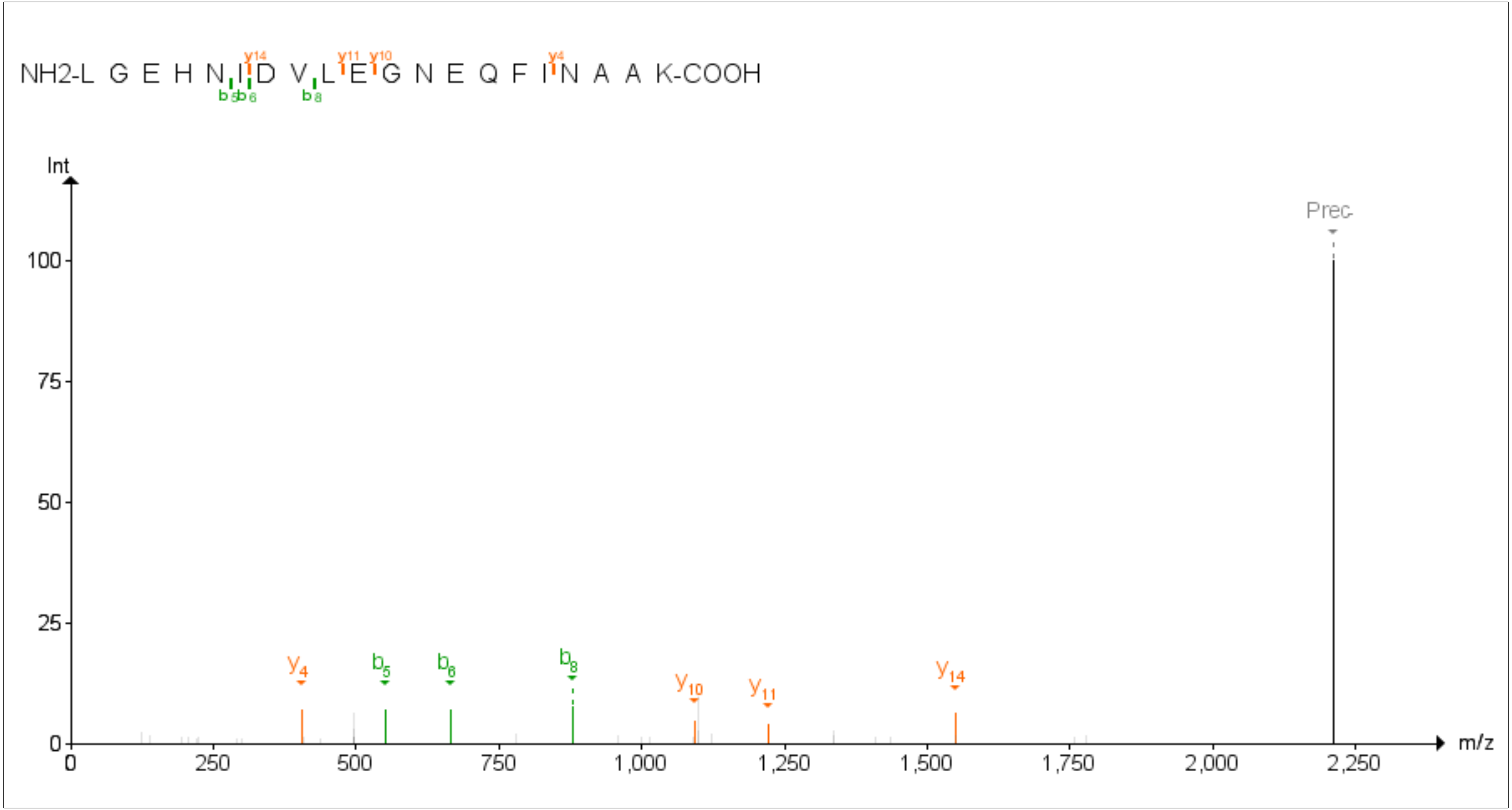
NH2-L G E H N I D V L E G N E Q F I N A A K-COOH
y14 y11 y10 y4
b5 b6 b8
Int
100
75
50
25
0
Prec
y4
b5
b6
b8
y10
y11
y14
0 250 500 750 1,000 1,250 1,500 1,750 2,000 2,250
m/z

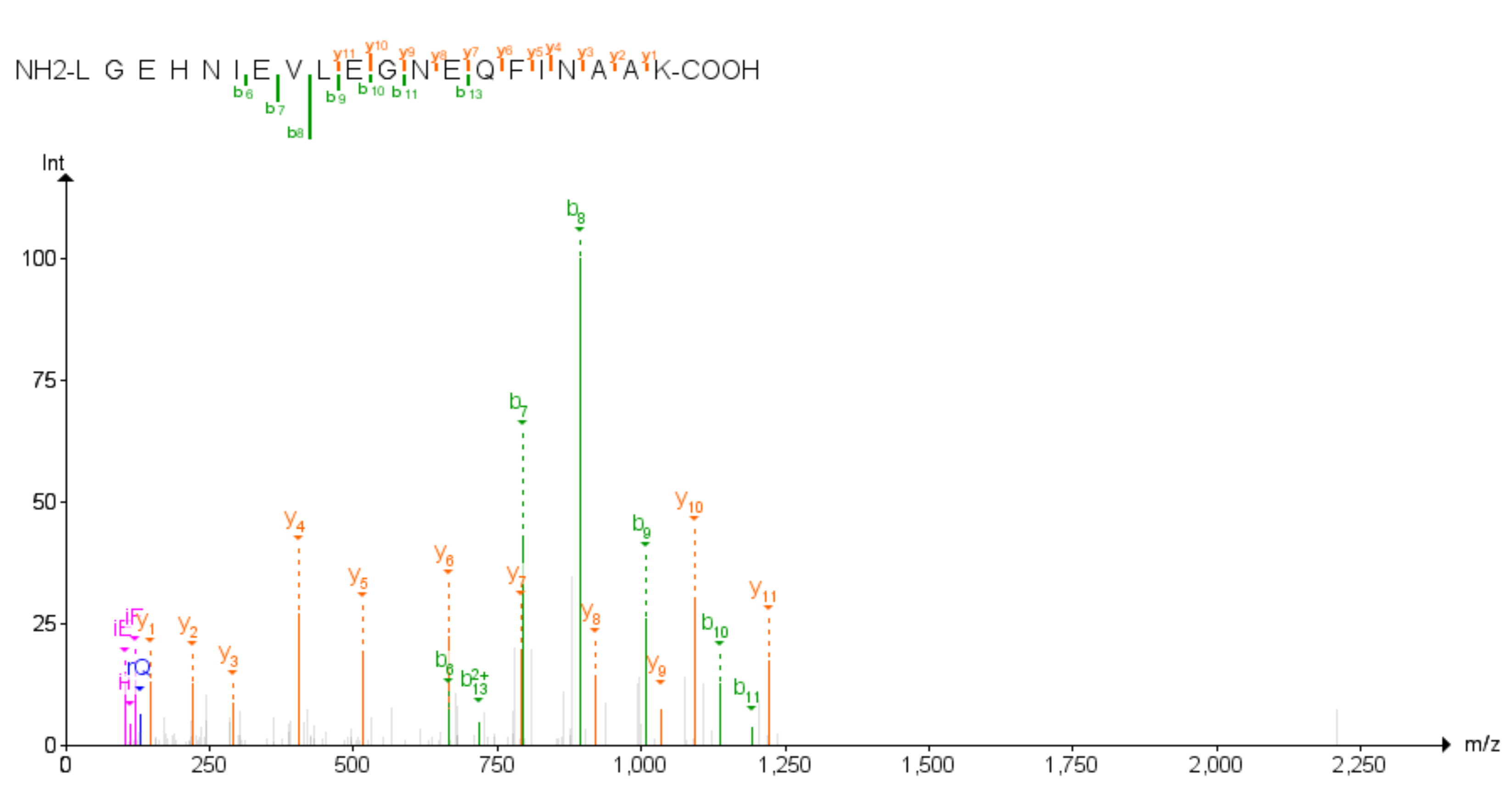

NH2-L G E H N I E V L E G N E Q F I N A A K-COOH
y11 y10 y9 y8 y7 y6 y5 y4 y3 y2 y1
b6 b7 b8 b9 b10 b11 b13
Int
100
75
50
25
0
m/z
0 250 500 750 1,000 1,250 1,500 1,750 2,000 2,250
b8 b7 y10 b9 y4 y6 y5 y7 y11 b10 y8 y9 b11 b6 b13 2+ y1 y2 y3 iF iE iH iQ

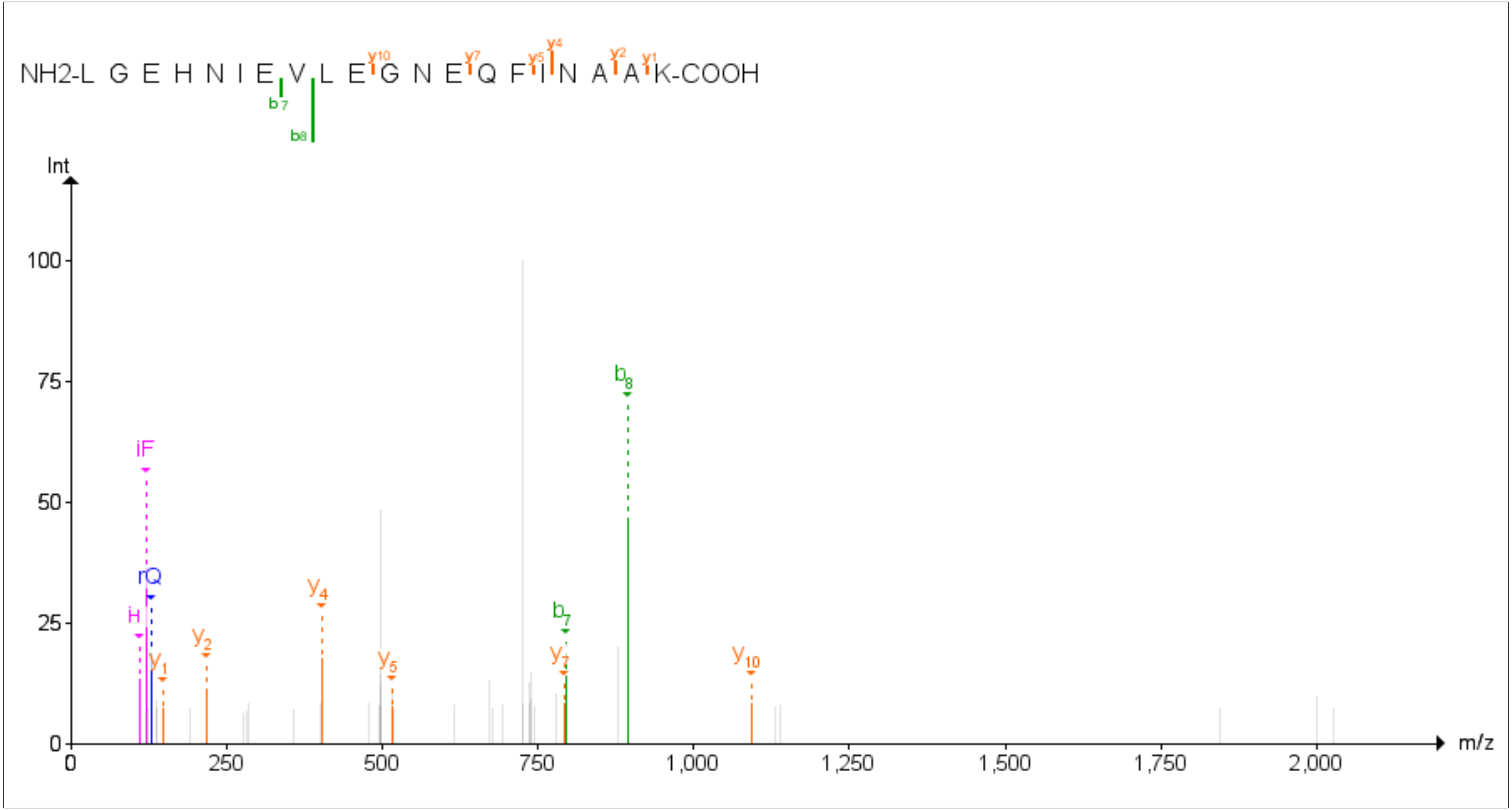

NH2-L G E H N I E V L E G N E Q F I N A A K-COOH
y10
y7
y5
y4
y2
y1
b7
b8
Int
100
75
50
25
0
iF
rQ
iH
y1
y2
y4
y5
b7
y7
b8
y10
0
250
500
750
1,000
1,250
1,500
1,750
2,000
m/z

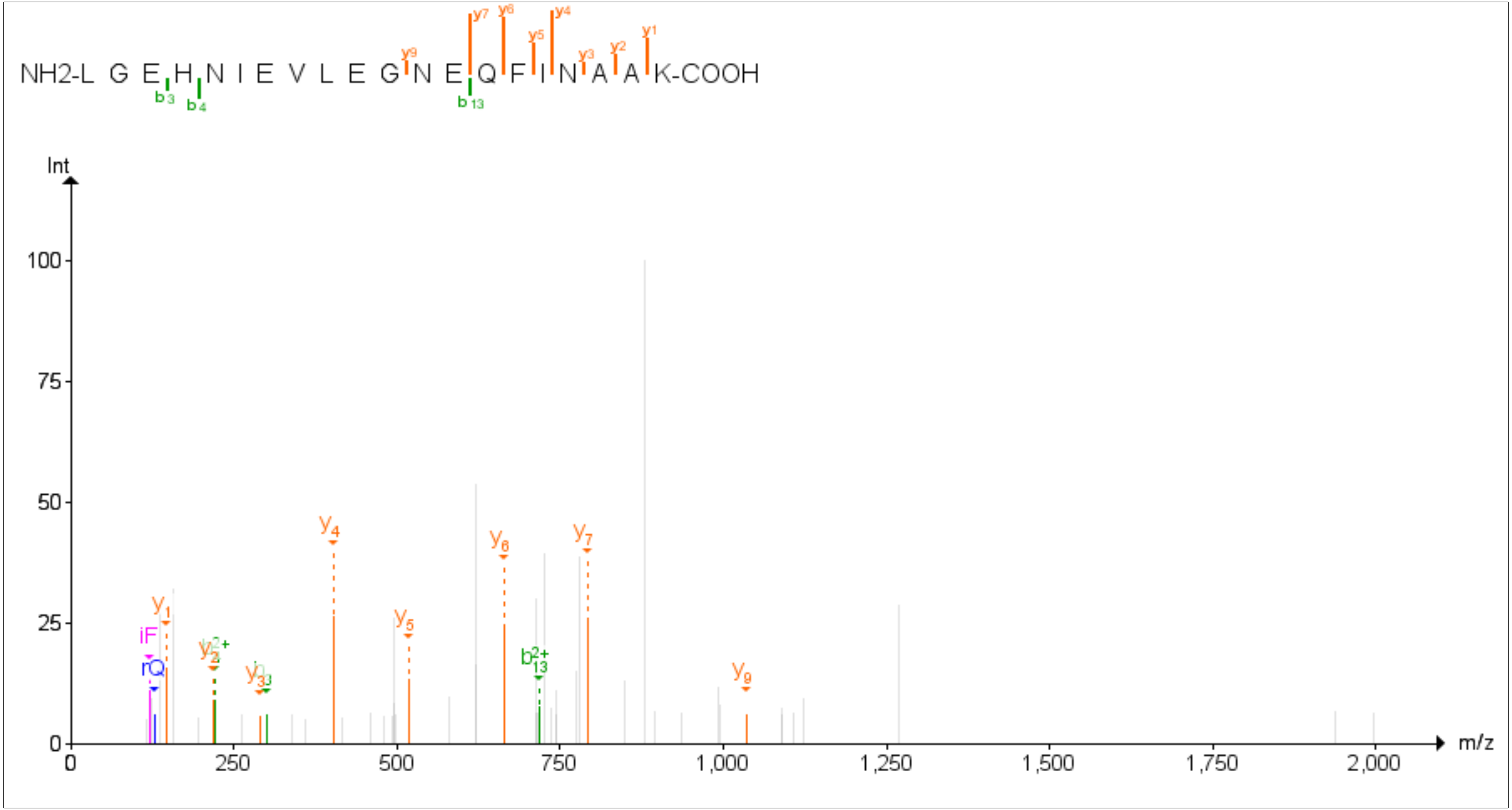

NH2-L G E H N I E V L E G N E Q F I N A A K-COOH
y9 y7 y6 y5 y4 y3 y2 y1
b3 b4 b13
Int
100
75
50
25
0
y4
y6
y7
y1
iF
rQ
y5
y2
y3
b2+ 13
y9
0
250
500
750
1,000
1,250
1,500
1,750
2,000
m/z

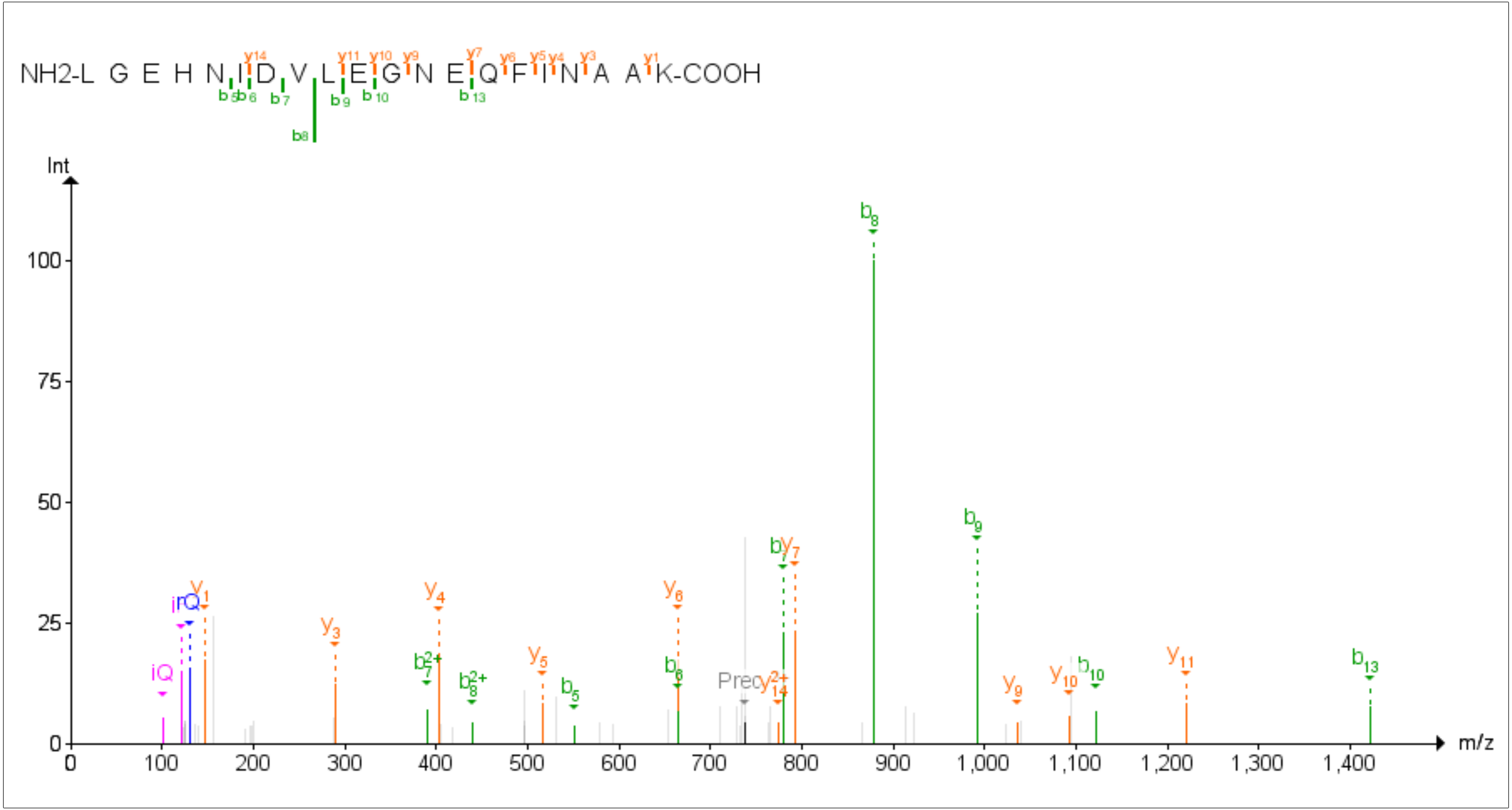

NH2-L G E H N I D V L E G N E Q F I N A A K-COOH
Int
m/z
0
25
50
75
100
100
200
300
400
500
600
700
800
900
1,000
1,100
1,200
1,300
1,400

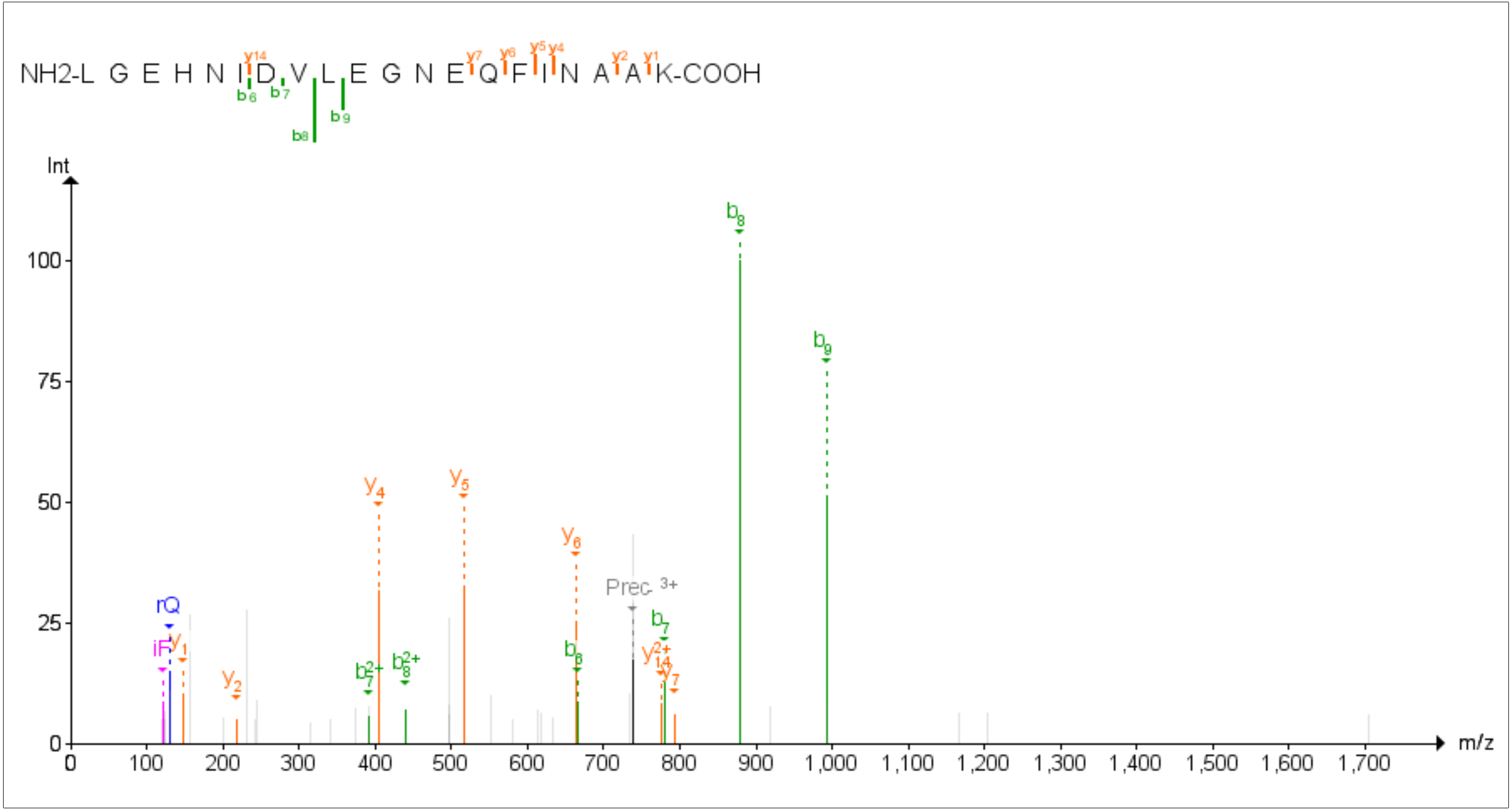

NH2-L G E H N I D V L E G N E Q F I N A A K-COOH
y14 y7 y6 y5 y4 y2 y1
b6 b7 b8 b9
Int
100
75
50
25
0
b8
b9
y4
y5
y6
Prec 3+
rQ
iF
y1
y2
b7 2+
b8 2+
b6
b7
y14 2+
y7
m/z
0 100 200 300 400 500 600 700 800 900 1,000 1,100 1,200 1,300 1,400 1,500 1,600 1,700

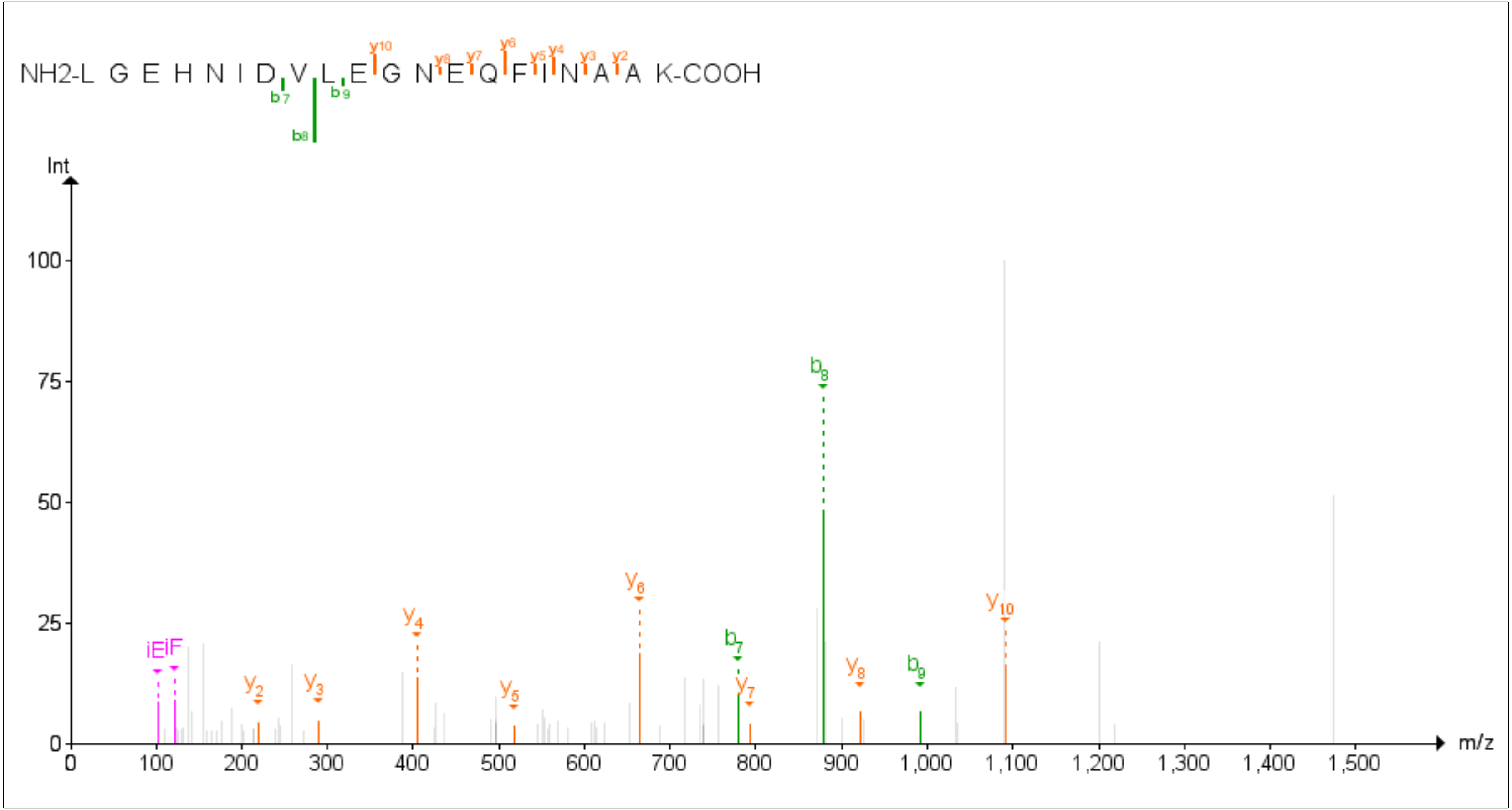

NH2-L G E H N I D V L E G N E Q F I N A A K-COOH
y10 y8 y7 y6 y5 y4 y3 y2
b7 b8 b9
Int
100
75
50
25
0
iE iF y2 y3 y4 y5 y6 b7 y7 b8 y8 b9 y10
0 100 200 300 400 500 600 700 800 900 1,000 1,100 1,200 1,300 1,400 1,500
m/z

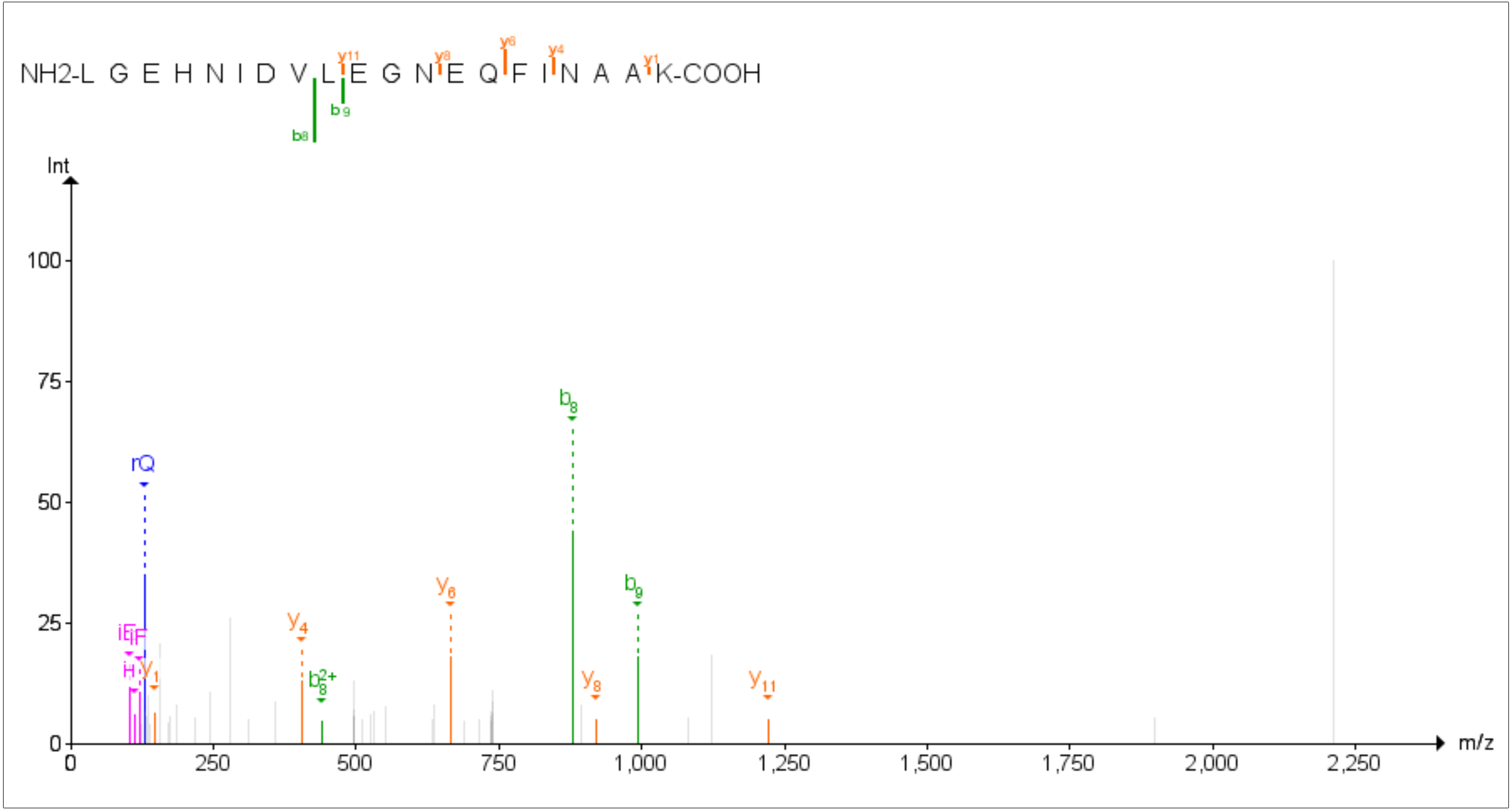
NH2-L G E H N I D V L I E G N E Q F I N A A K-COOH
y11
y8
y6
y4
y1
b8
b9
Int
100
75
50
25
0
rQ
b8
b9
y4
y6
y8
y11
y1
iH
iF
m/z
0
250
500
750
1,000
1,250
1,500
1,750
2,000
2,250

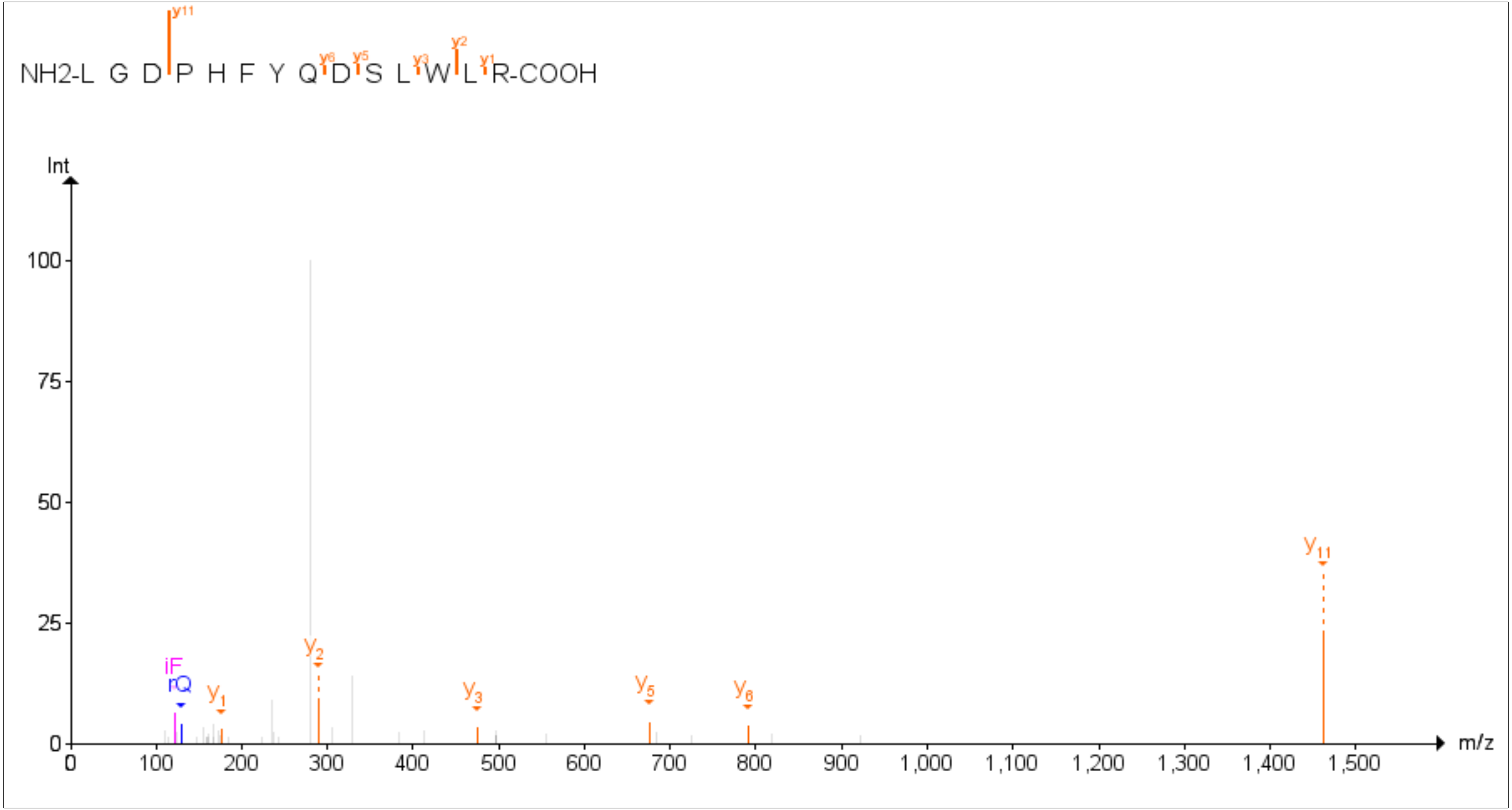

NH2-L G D P H F Y Q D S L W L R-COOH
y11
y6
y5
y3
y2
y1
Int
100
75
50
25
0
iF
rQ
y1
y2
y3
y5
y6
y11
0
100
200
300
400
500
600
700
800
900
1,000
1,100
1,200
1,300
1,400
1,500
m/z

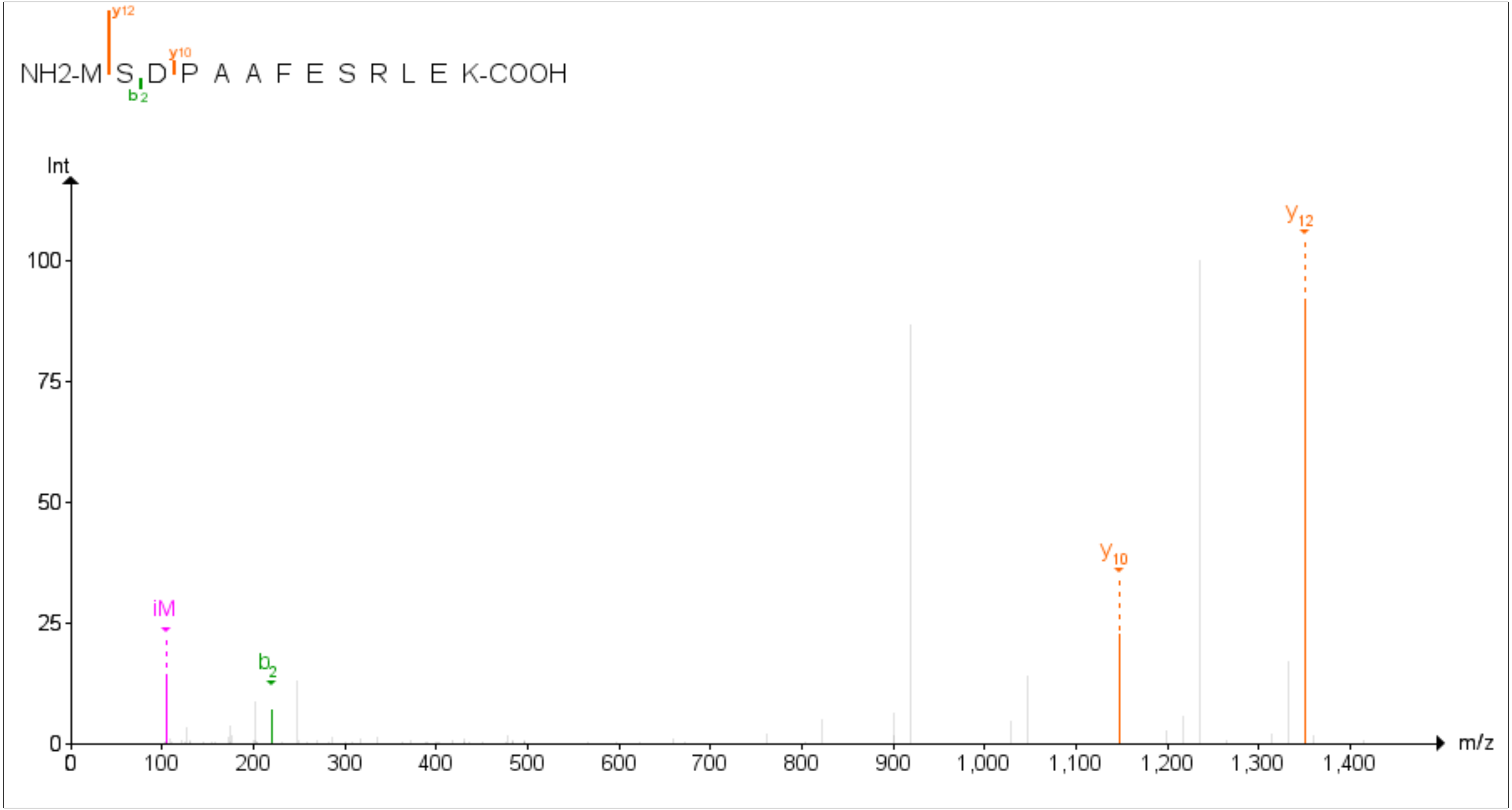
NH2-M S D P A A F E S R L E K-COOH
y12
y10
b2
Int
100
75
50
25
0
iM
b2
y10
y12
0
100
200
300
400
500
600
700
800
900
1,000
1,100
1,200
1,300
1,400
m/z

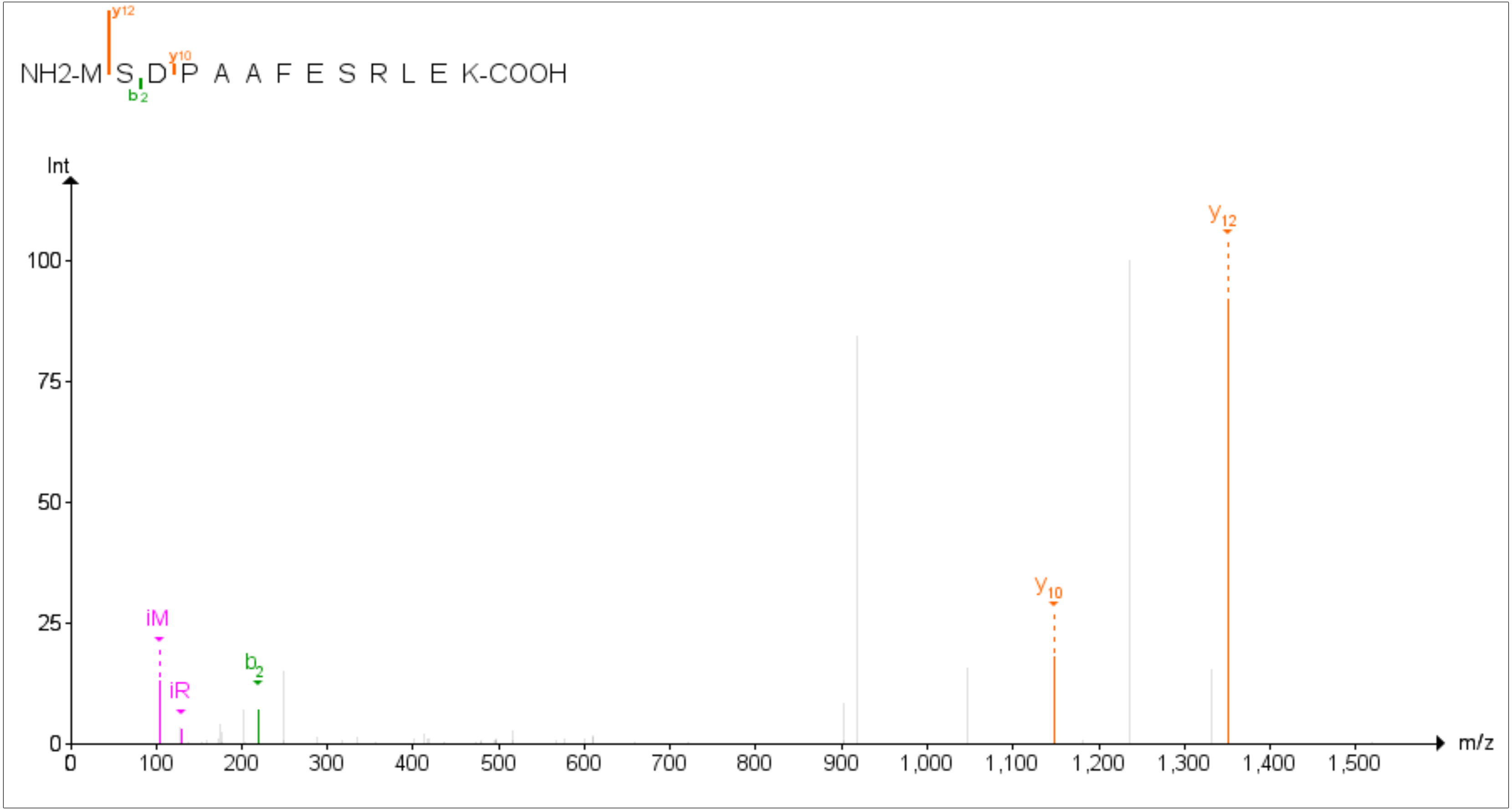

NH2-M S D P A A F E S R L E K-COOH
y12
y10
b2
Int
100
75
50
25
0
iM
iR
b2
y10
y12
0
100
200
300
400
500
600
700
800
900
1,000
1,100
1,200
1,300
1,400
1,500
m/z

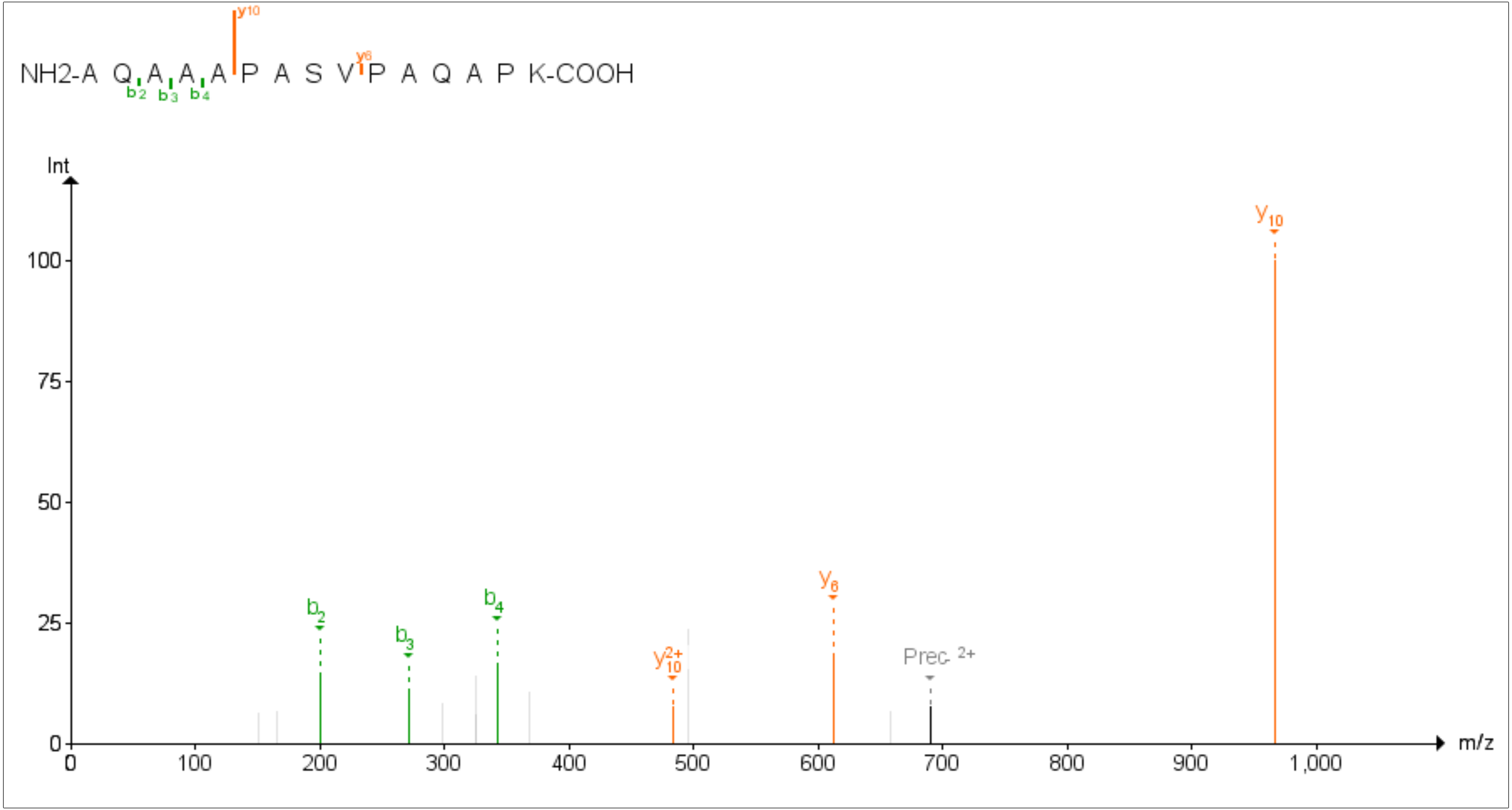

NH2-A Q A A A P A S V P A Q A P K-COOH
y10
y6
b2
b3
b4
Int
100
75
50
25
0
y10
y6
b2
b3
b4
y10 2+
Prec 2+
0
100
200
300
400
500
600
700
800
900
1,000
m/z

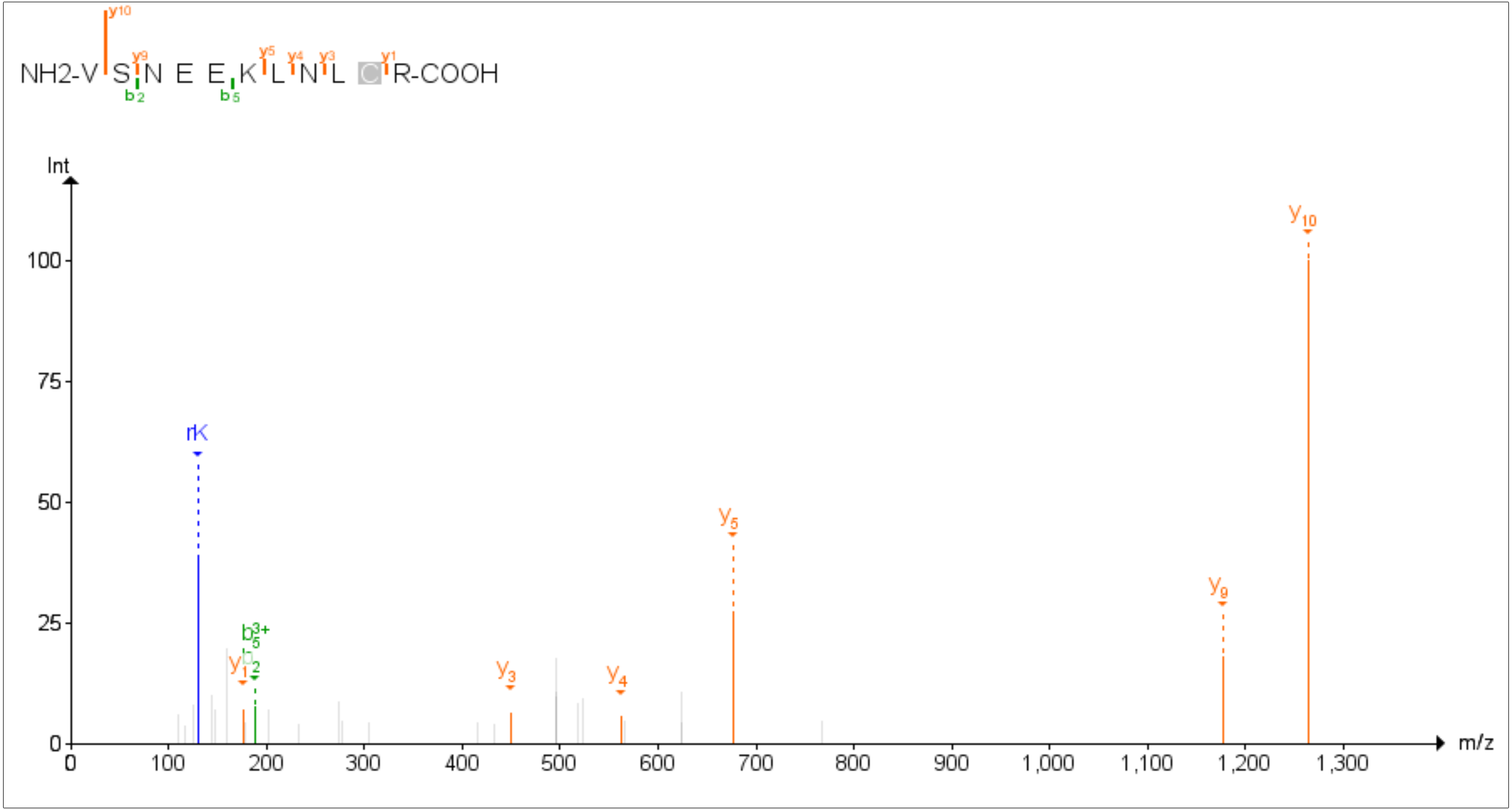

NH2-V S N E E K L N L C R-COOH
y10
y9
y5
y4
y3
y1
b2
b5
Int
100
75
50
25
0
rK
b_5^{3+}
y_1
b_2
y_3
y_4
y_5
y_9
y_{10}
m/z
0
100
200
300
400
500
600
700
800
900
1,000
1,100
1,200
1,300

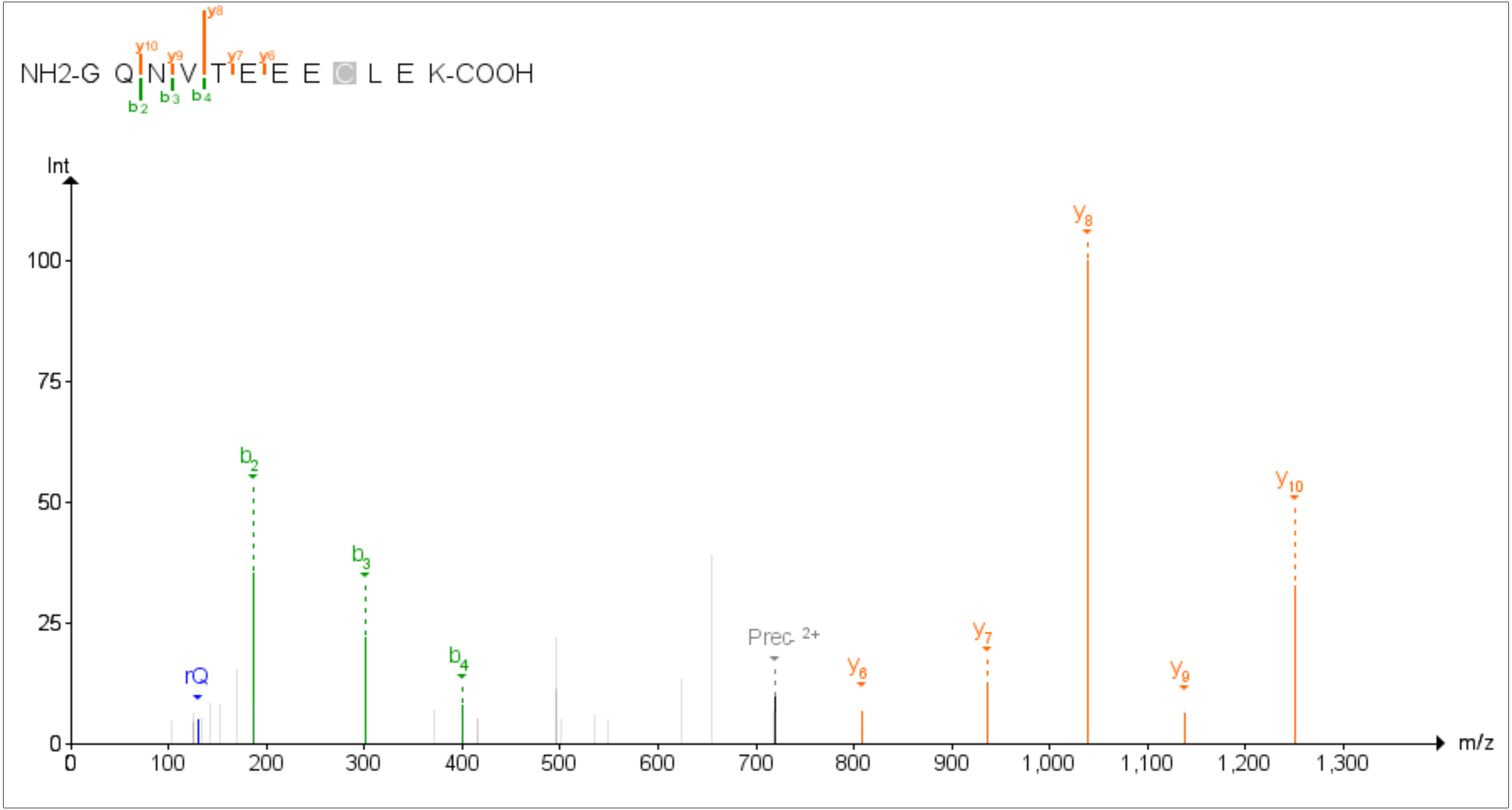

NH2-G Q N V T E E E C L E K-COOH
y10 y9 y8 y7 y6
b2 b3 b4
Int
100
75
50
25
0
rQ
b2
b3
b4
Prec 2+
y6
y7
y8
y9
y10
0 100 200 300 400 500 600 700 800 900 1,000 1,100 1,200 1,300
m/z

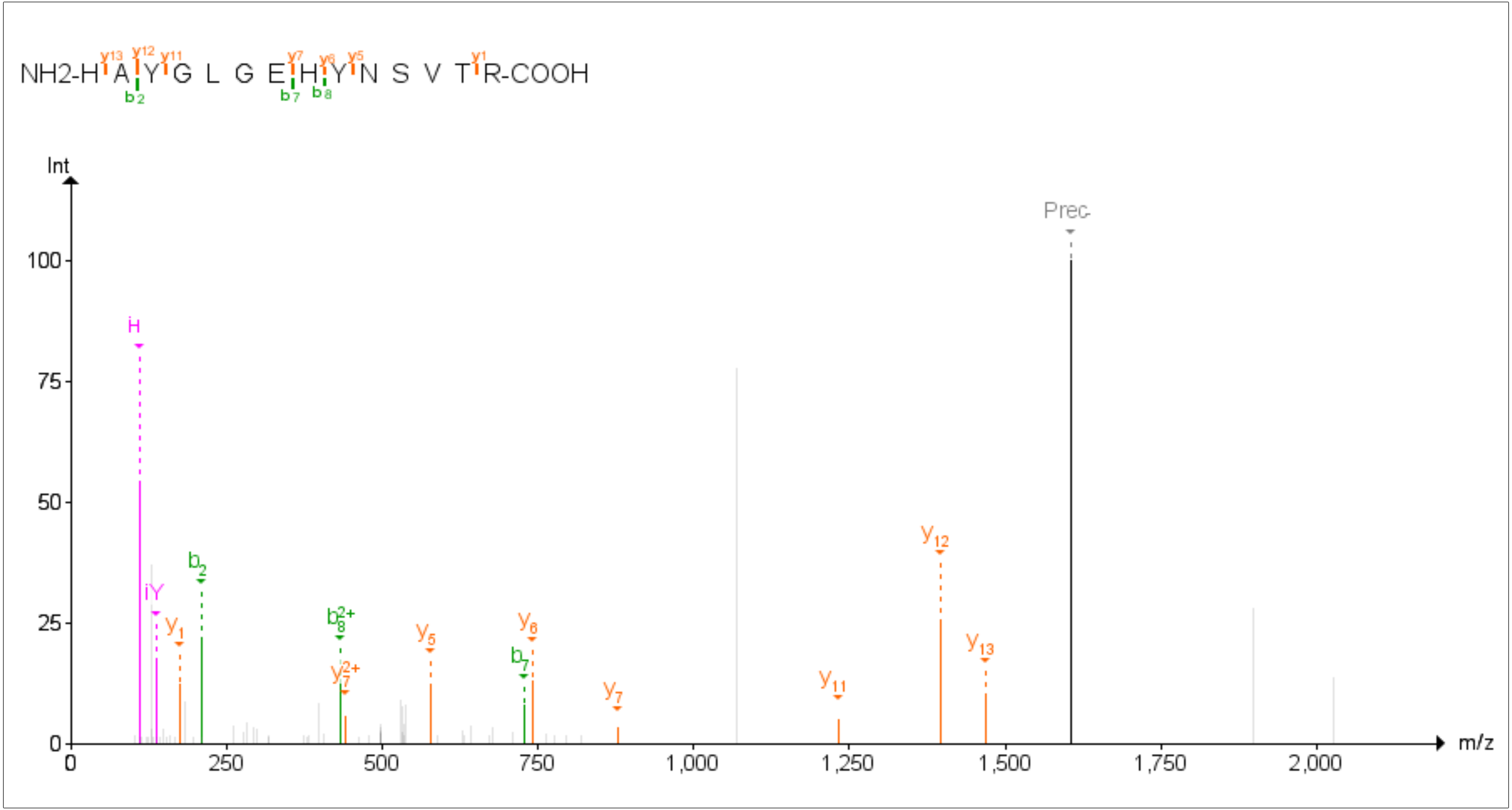

NH2-H A Y G L G E H Y N S V T R-COOH
y13 y12 y11 y7 y6 y5 y1
b2 b7 b8
Int
m/z
Prec
H
iY
y1
b2
b8 2+
y7 2+
y5
y6
b7
y7
y11
y12
y13
0
25
50
75
100
250
500
750
1,000
1,250
1,500
1,750
2,000

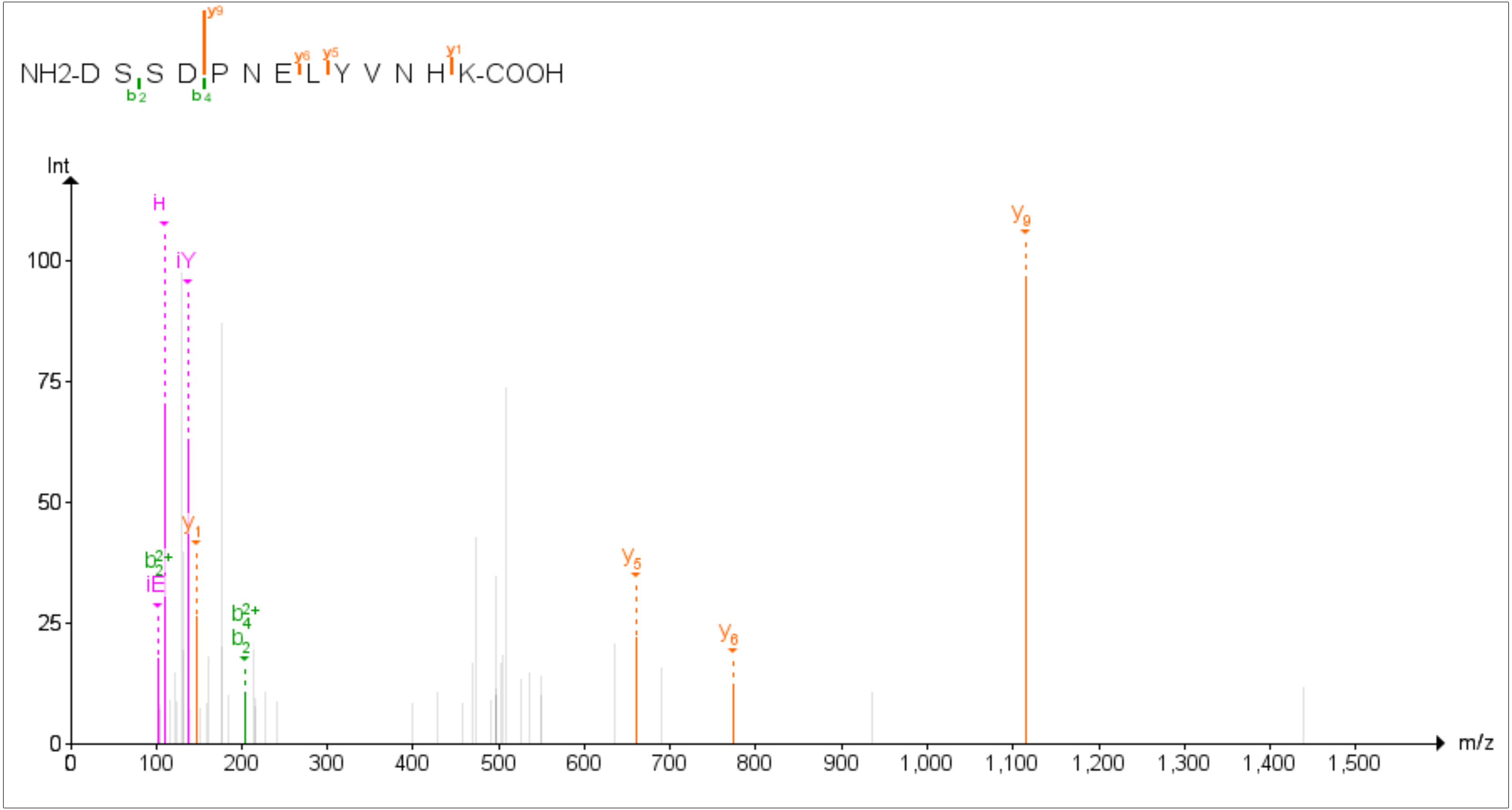
NH2-D S S D P N E L Y V N H K-COOH
y9
y6
y5
y1
b2
b4
Int
iH
iY
y1
iE
y5
y6
y9
100
75
50
25
0
0
100
200
300
400
500
600
700
800
900
1,000
1,100
1,200
1,300
1,400
1,500
m/z

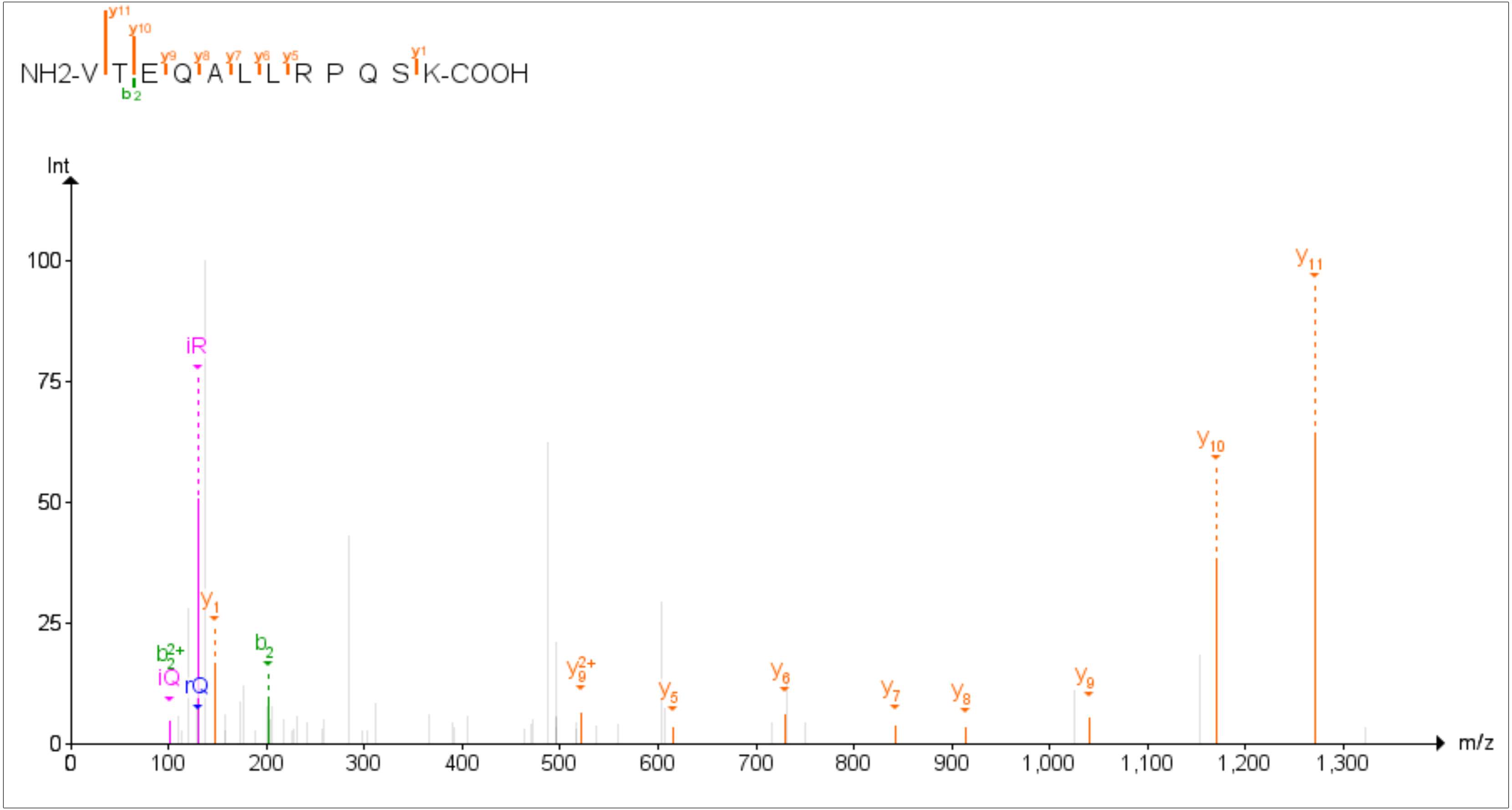

NH2-V T E Q A L L R P Q S K-COOH
y11
y10
y9
y8
y7
y6
y5
y1
b2
Int
100
75
50
25
0
m/z
iR
iQ
rQ
y1
b2
y11
y10
y9
y8
y7
y6
y5
0
100
200
300
400
500
600
700
800
900
1,000
1,100
1,200
1,300

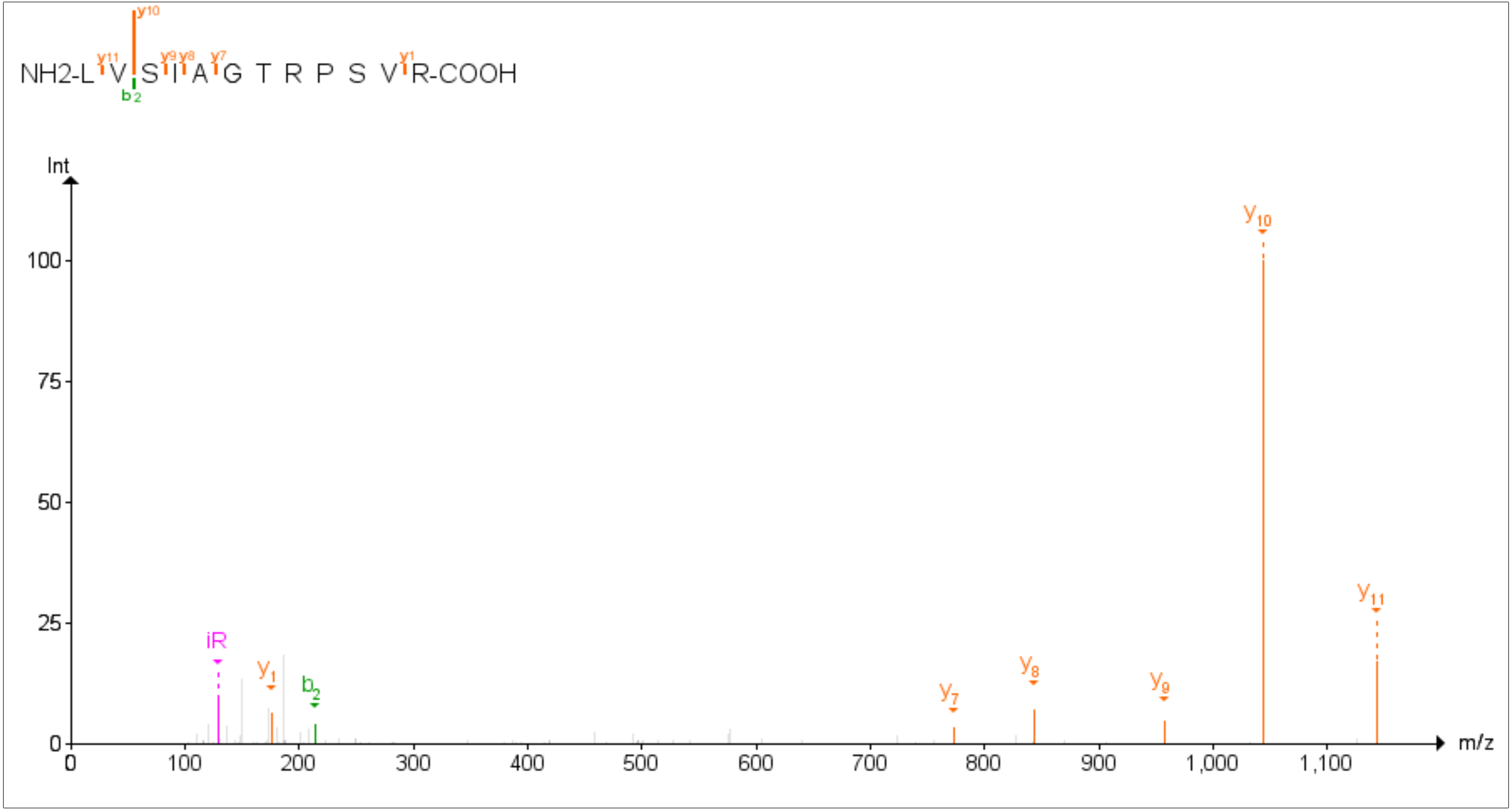

NH2-L V S I A G T R P S V R-COOH
y10
y11
y9 y8
y7
y1
b2
Int
100
75
50
25
0
m/z
0
100
200
300
400
500
600
700
800
900
1,000
1,100
iR
y1
b2
y7
y8
y9
y10
y11

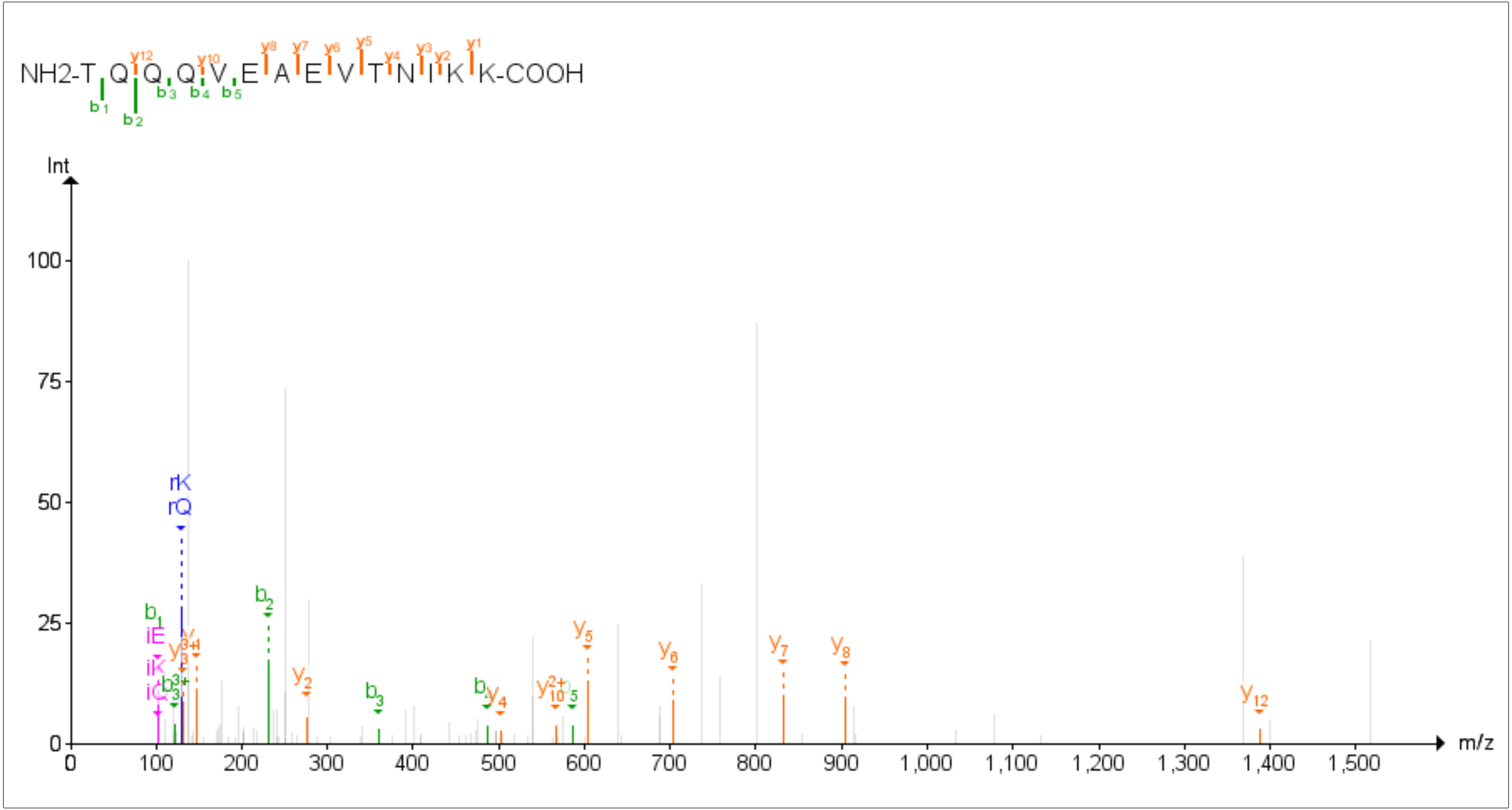

NH2-T Q Q Q V E A E V T N I K K-COOH
Int
m/z

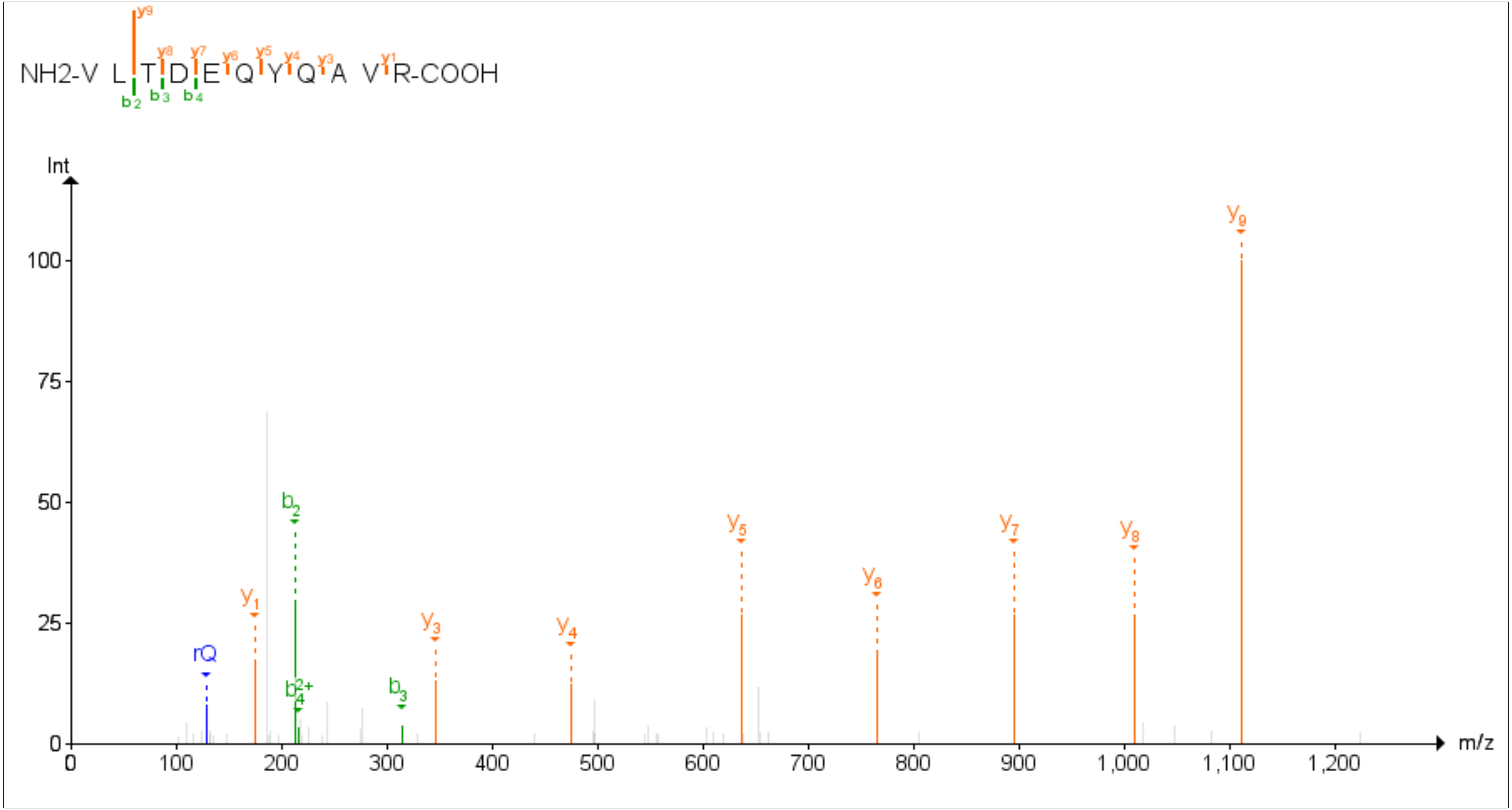

NH2-V L T D E Q Y Q A V R-COOH
y9 y8 y7 y6 y5 y4 y3 y1
b2 b3 b4
Int
100
75
50
25
0
rQ
y1
b2
b4 2+
b3
y3
y4
y5
y6
y7
y8
y9
0 100 200 300 400 500 600 700 800 900 1,000 1,100 1,200
m/z

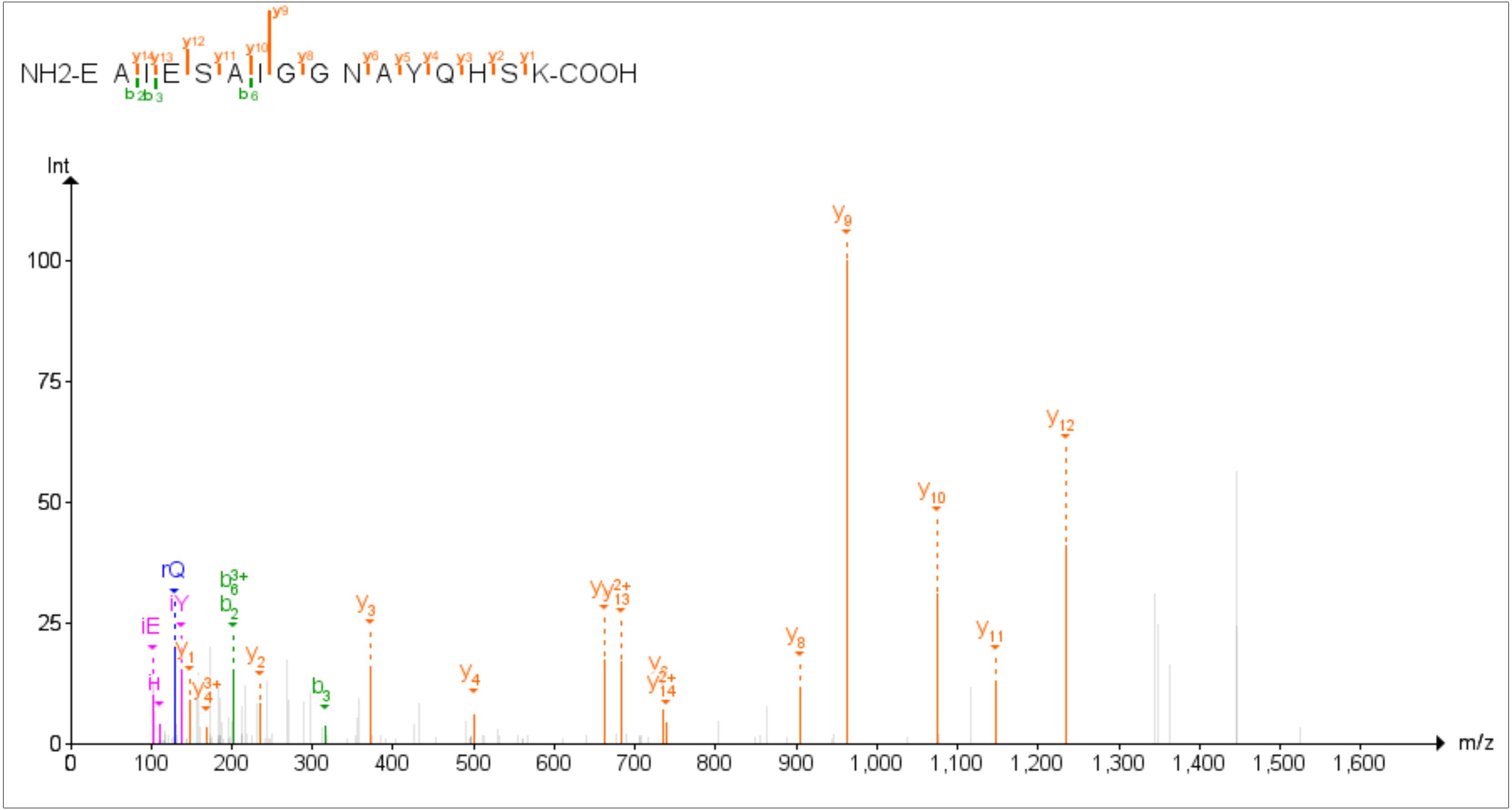
NH2-E A I I E S A I G G N A Y Q H S K-COOH
Int
m/z
y9
y12
y10
y3
y4
y8
y11
y13 2+
y14 2+
y1
y2
b3
b2
b6 3+
rQ
iY
iE
iH
y4 3+

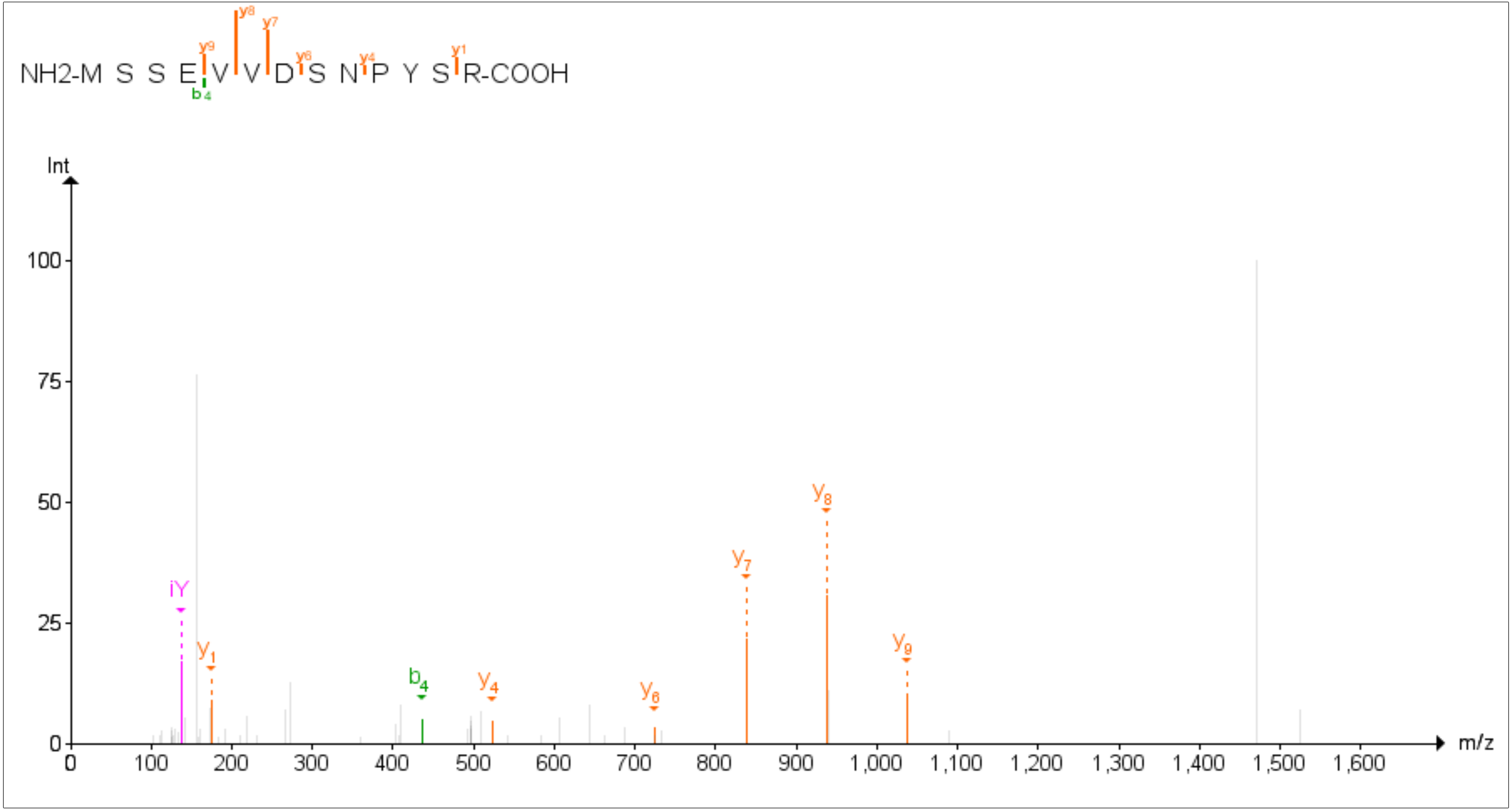

NH2-M S S E V V D S N P Y S R-COOH
y9
y8
y7
y6
y4
y1
b4
Int
100
75
50
25
0
iY
y1
b4
y4
y6
y7
y8
y9
0
100
200
300
400
500
600
700
800
900
1,000
1,100
1,200
1,300
1,400
1,500
1,600
m/z

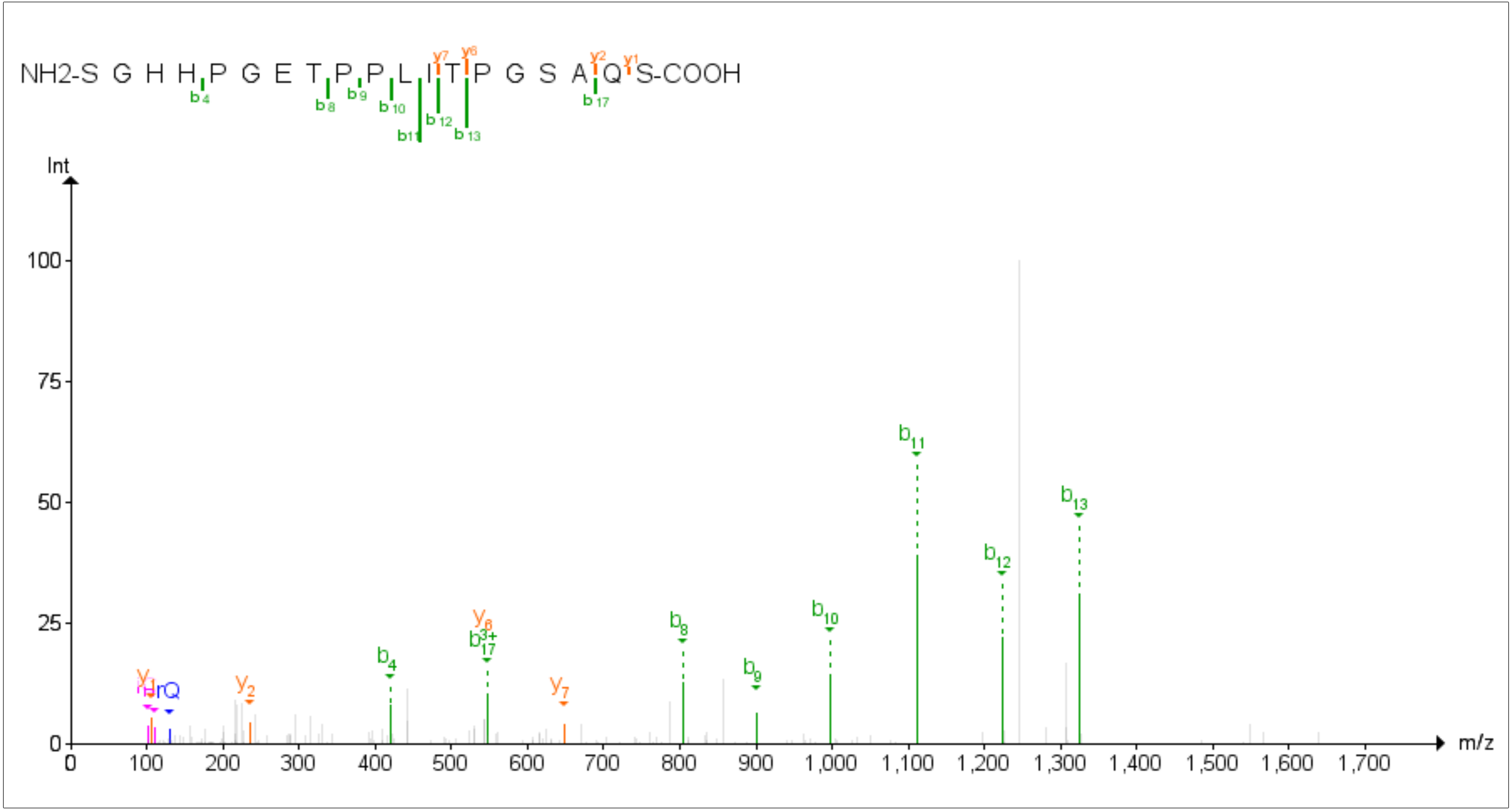

NH2-S G H H P G E T P P L I T P G S A Q S-COOH
y7
y6
y2
y1
b4
b8
b9
b10
b11
b12
b13
b17
Int
100
75
50
25
0
m/z
0
100
200
300
400
500
600
700
800
900
1,000
1,100
1,200
1,300
1,400
1,500
1,600
1,700
y2
b4
y6
b17 3+
y7
b8
b9
b10
b11
b12
b13
rQ

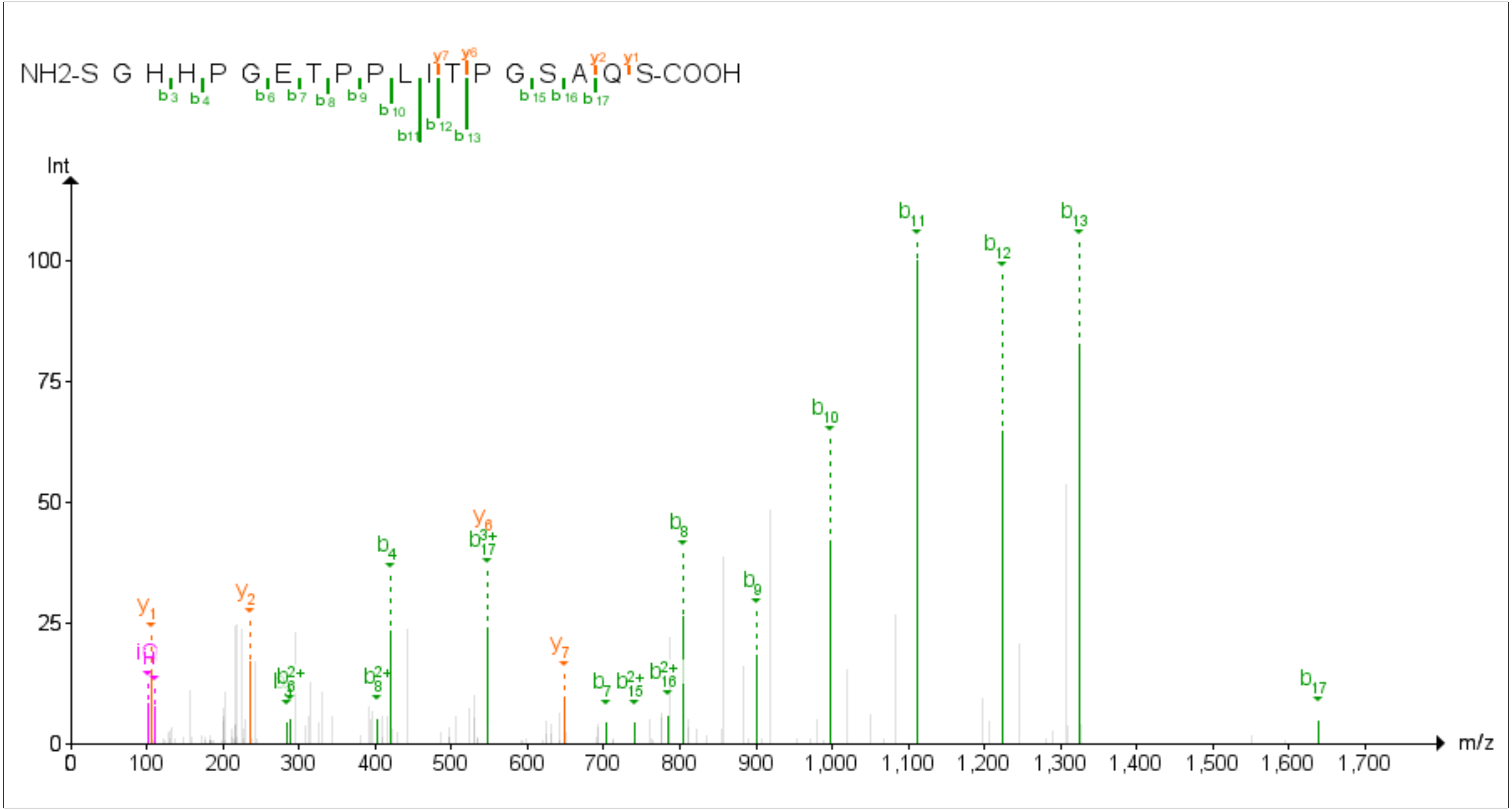

NH2-S G H H P G E T P P L I T P G S A Q S-COOH
b3 b4 b6 b7 b8 b9 b10 b11 b12 b13 b15 b16 b17
y7 y6 y2 y1
Int
100
75
50
25
0
m/z
0 100 200 300 400 500 600 700 800 900 1,000 1,100 1,200 1,300 1,400 1,500 1,600 1,700

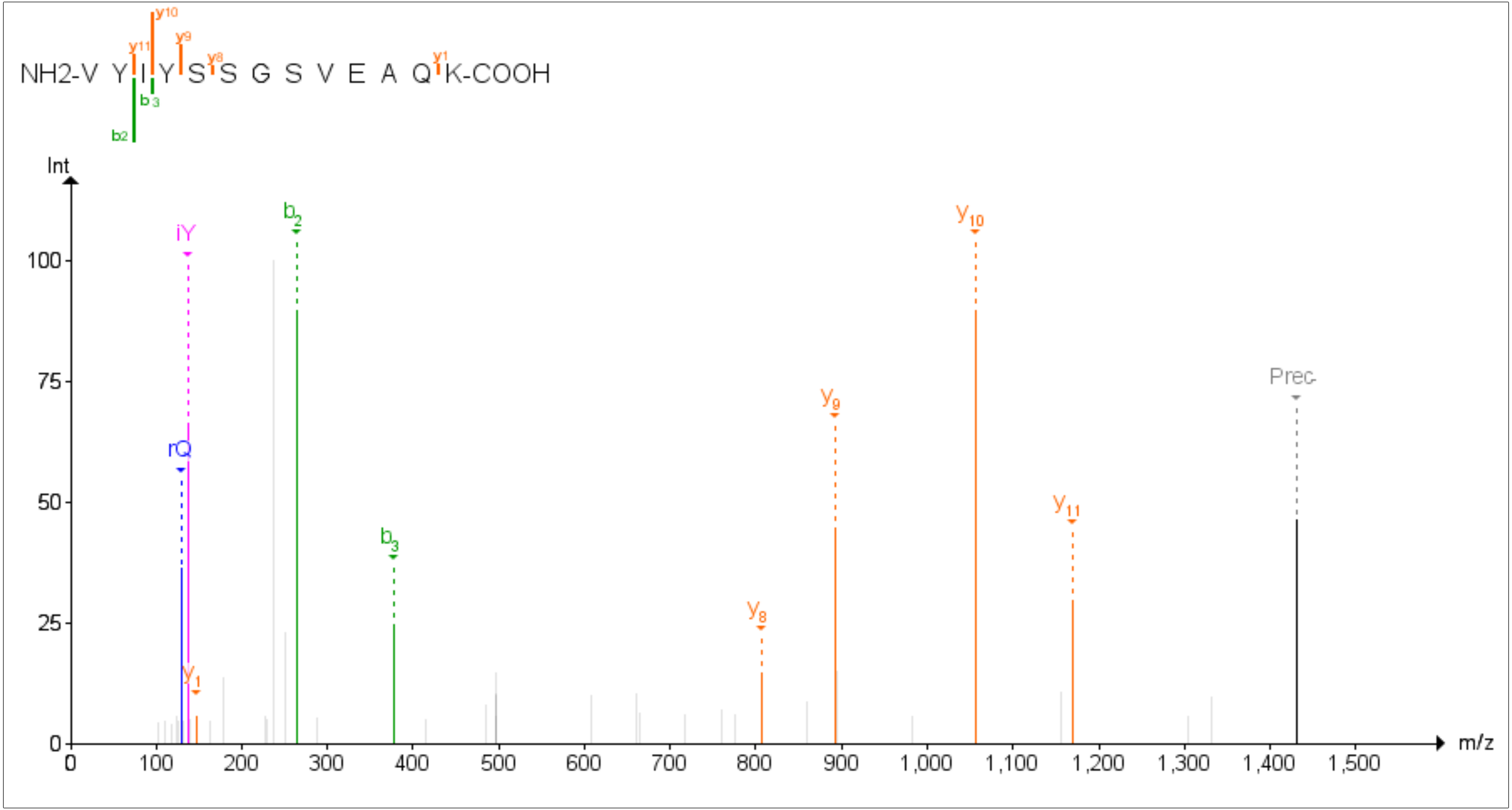

NH2-V Y I Y S S G S V E A Q K-COOH
y10
y11
y9
y8
y1
b2
b 3
Int
m/z
iY
b2
rQ
b3
y1
y10
y9
y8
y11
Prec
0
25
50
75
100
0
100
200
300
400
500
600
700
800
900
1,000
1,100
1,200
1,300
1,400
1,500

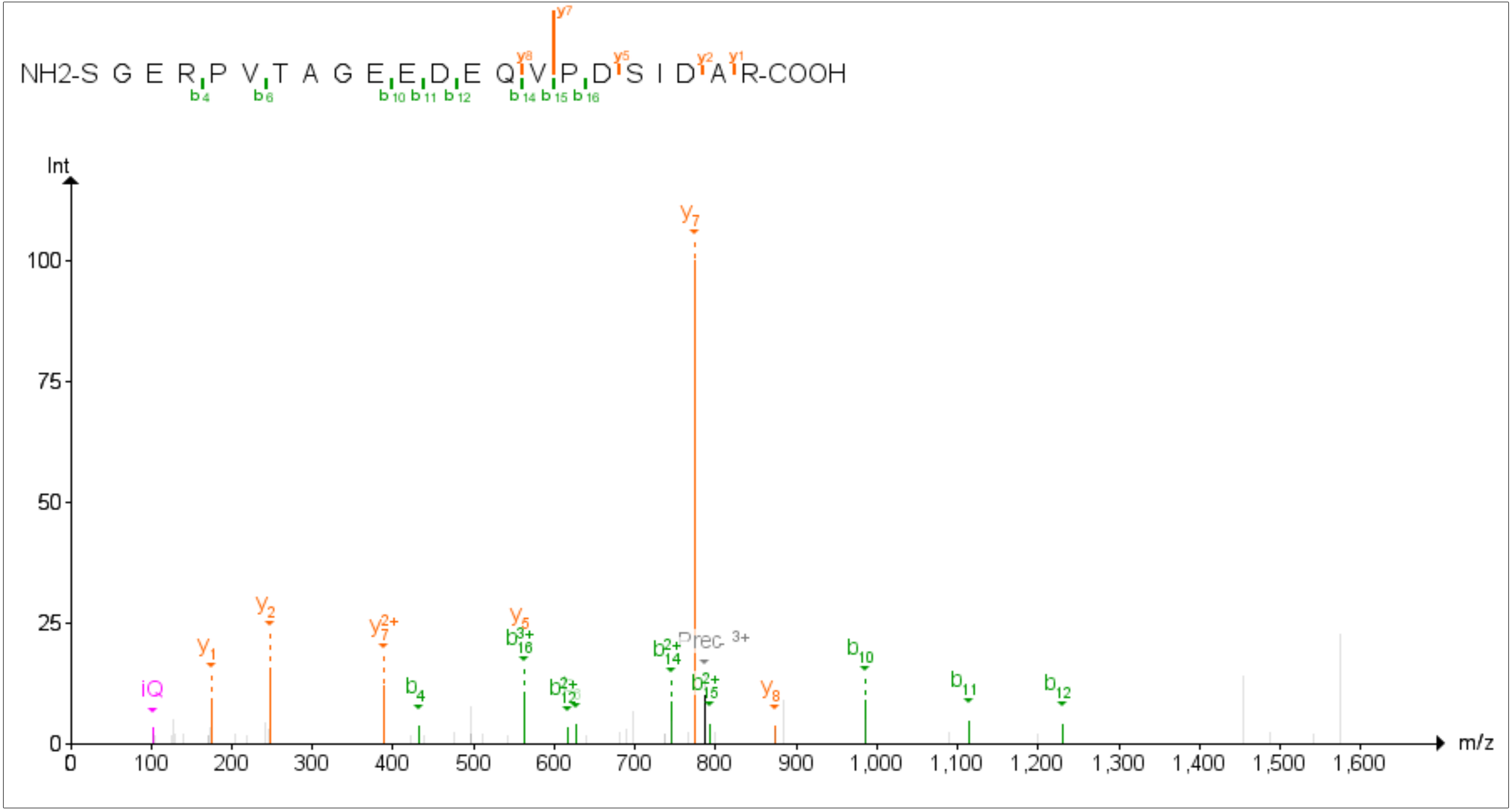

NH2-S G E R P V T A G E E D E Q V P D S I D A R-COOH
y7
y8
y5
y2
y1
b4
b6
b10
b11
b12
b14
b15
b16
Int
100
75
50
25
0
iQ
y1
y2
y7 2+
y5
b16 3+
b4
b12 2+
b14 2+
Prec. 3+
b15 2+
y8
b10
b11
b12
y7
0
100
200
300
400
500
600
700
800
900
1,000
1,100
1,200
1,300
1,400
1,500
1,600
m/z

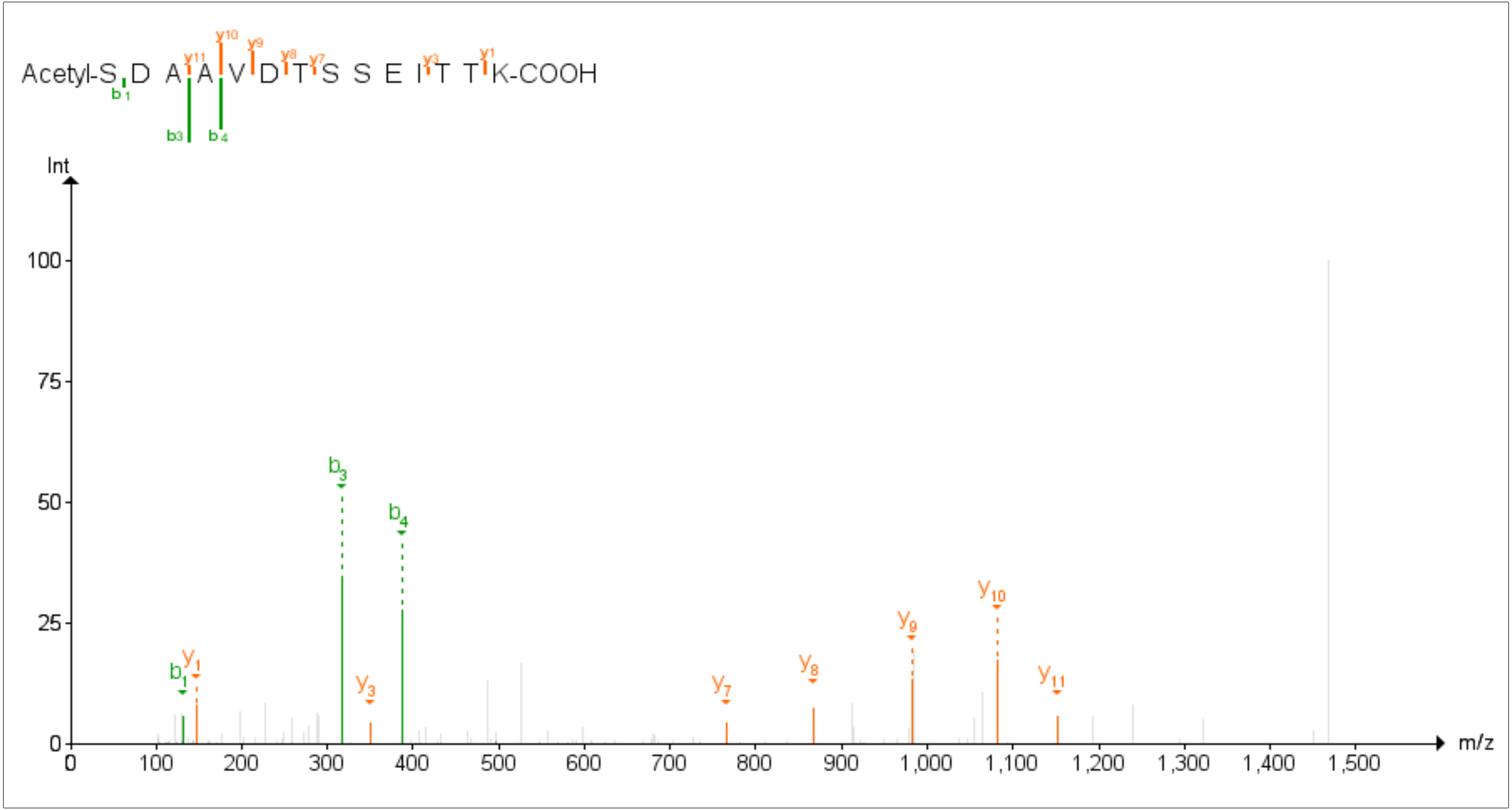

Acetyl-S D A A V D T S S E I T T K-COOH
b1 b3 b4
y11 y10 y9 y8 y7 y3 y1
Int
100
75
50
25
0
m/z
0 100 200 300 400 500 600 700 800 900 1,000 1,100 1,200 1,300 1,400 1,500
b1 y1 b3 y3 b4 y7 y8 y9 y10 y11

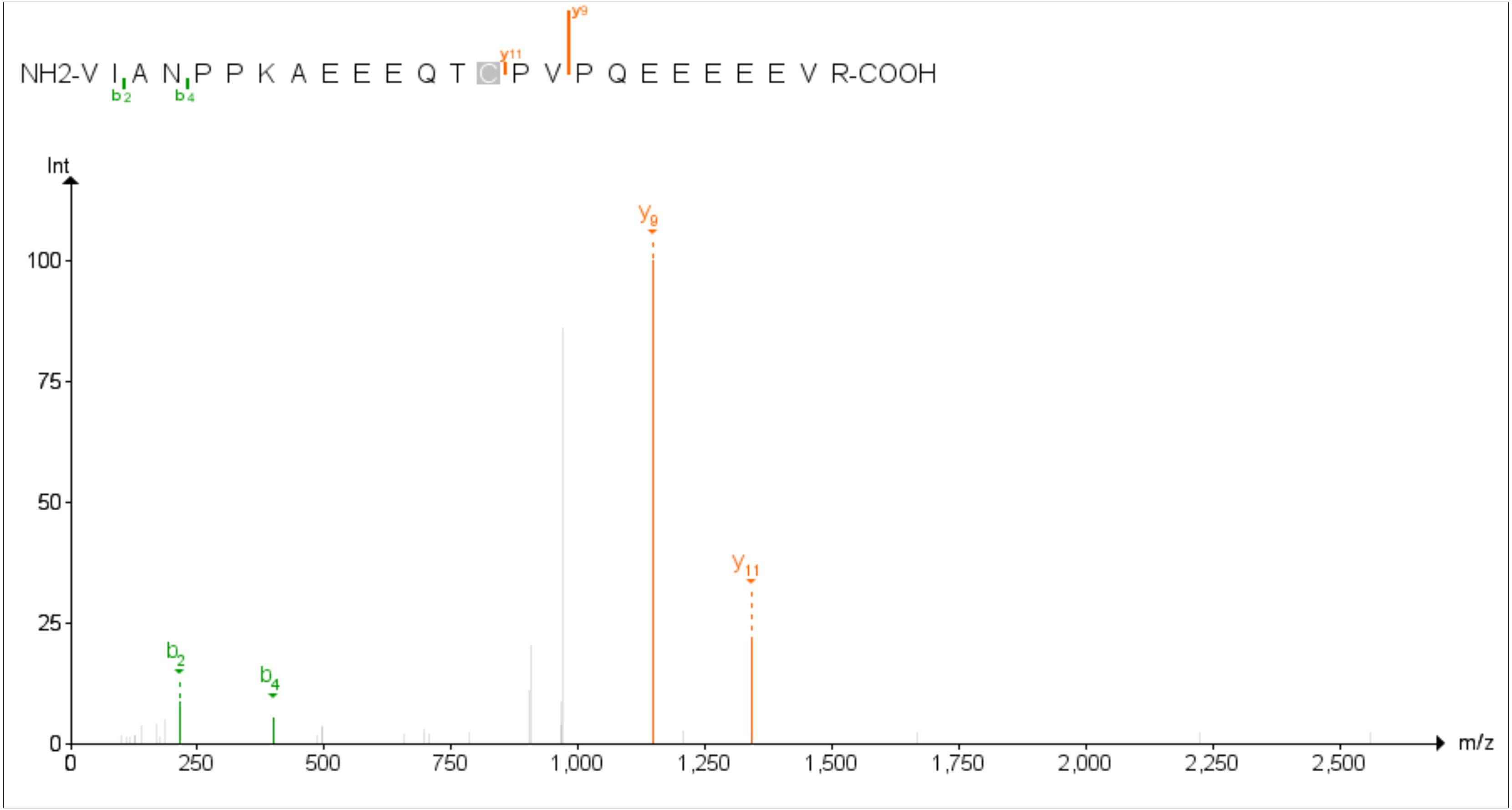

NH2-V I A N P P K A E E E Q T C P V P Q E E E E E V R-COOH
b2
b4
y11
y9
Int
100
75
50
25
0
0
250
500
750
1,000
1,250
1,500
1,750
2,000
2,250
2,500
m/z

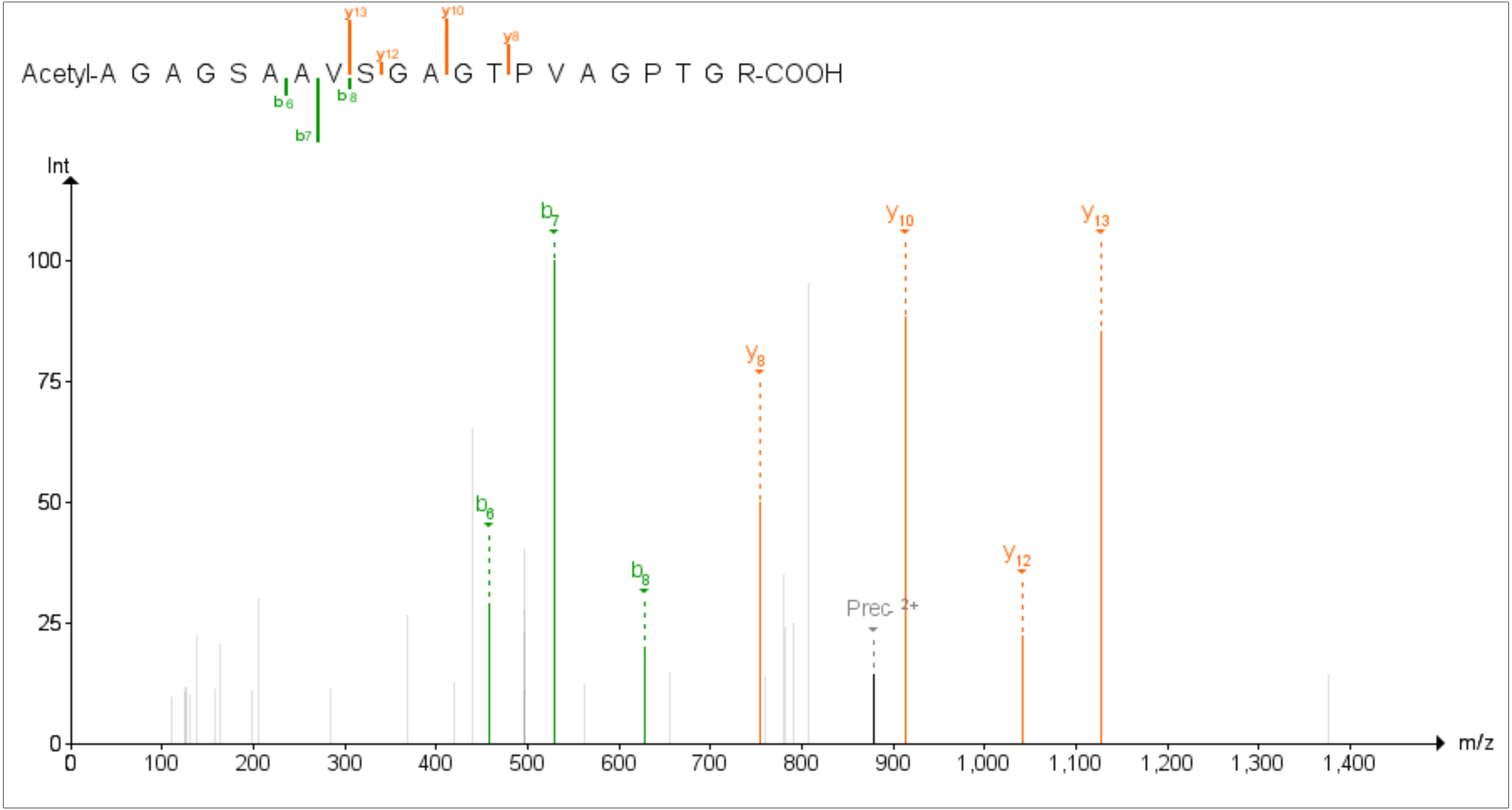
Acetyl-A G A G S A A V S G A G T P V A G P T G R-COOH
y13
y10
y12
y8
b6
b8
b7
Int
100
75
50
25
0
b7
y10
y13
y8
b6
b8
y12
Prec. 2+
0
100
200
300
400
500
600
700
800
900
1,000
1,100
1,200
1,300
1,400
m/z

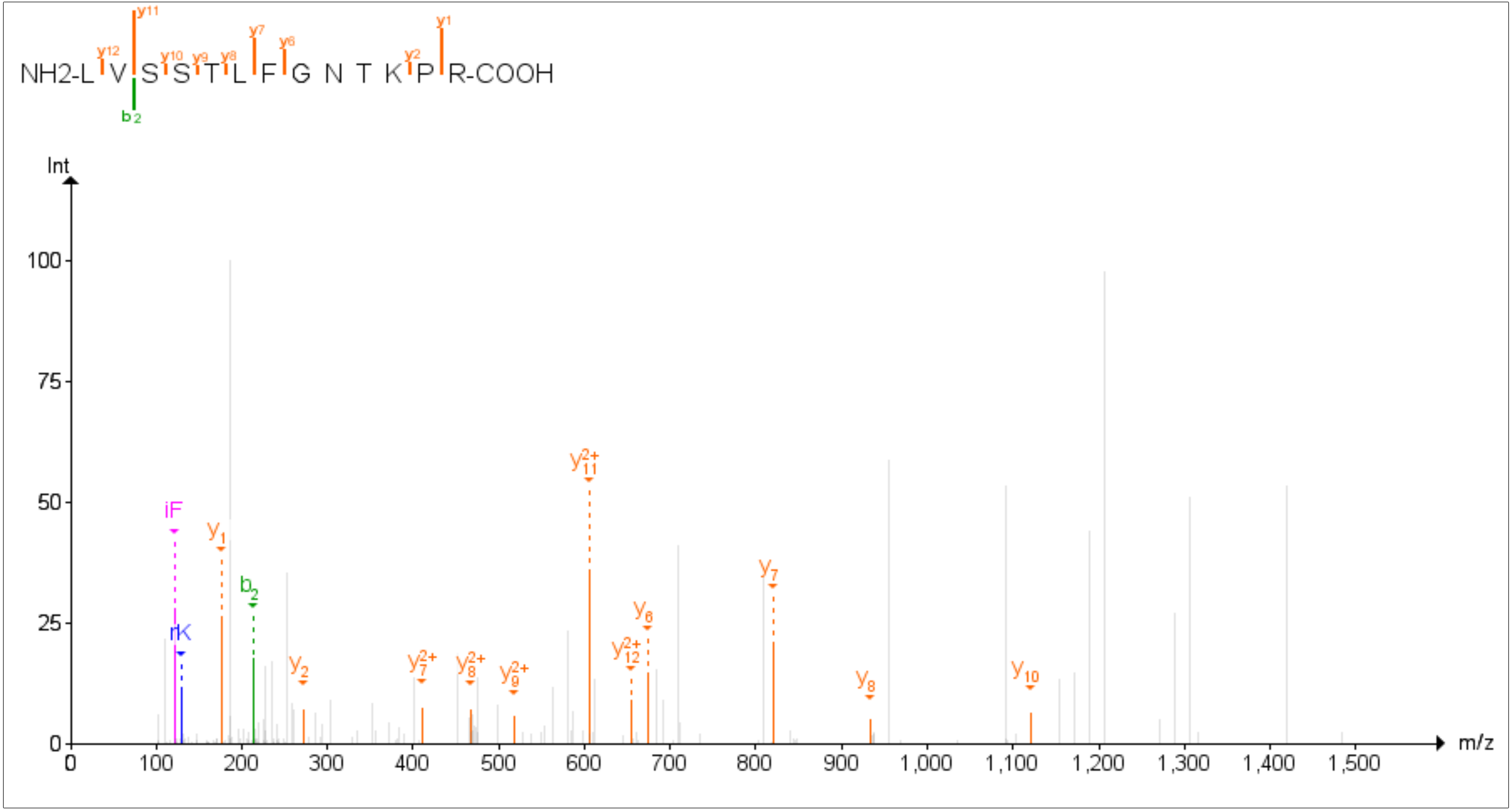

NH2-L V S S T L F G N T K P R-COOH
y12 y11 y10 y9 y8 y7 y6 y2 y1
b2
Int
m/z
iF
rK
y1
b2
y2
y7 2+
y8 2+
y9 2+
y11 2+
y12 2+
y6
y7
y8
y10

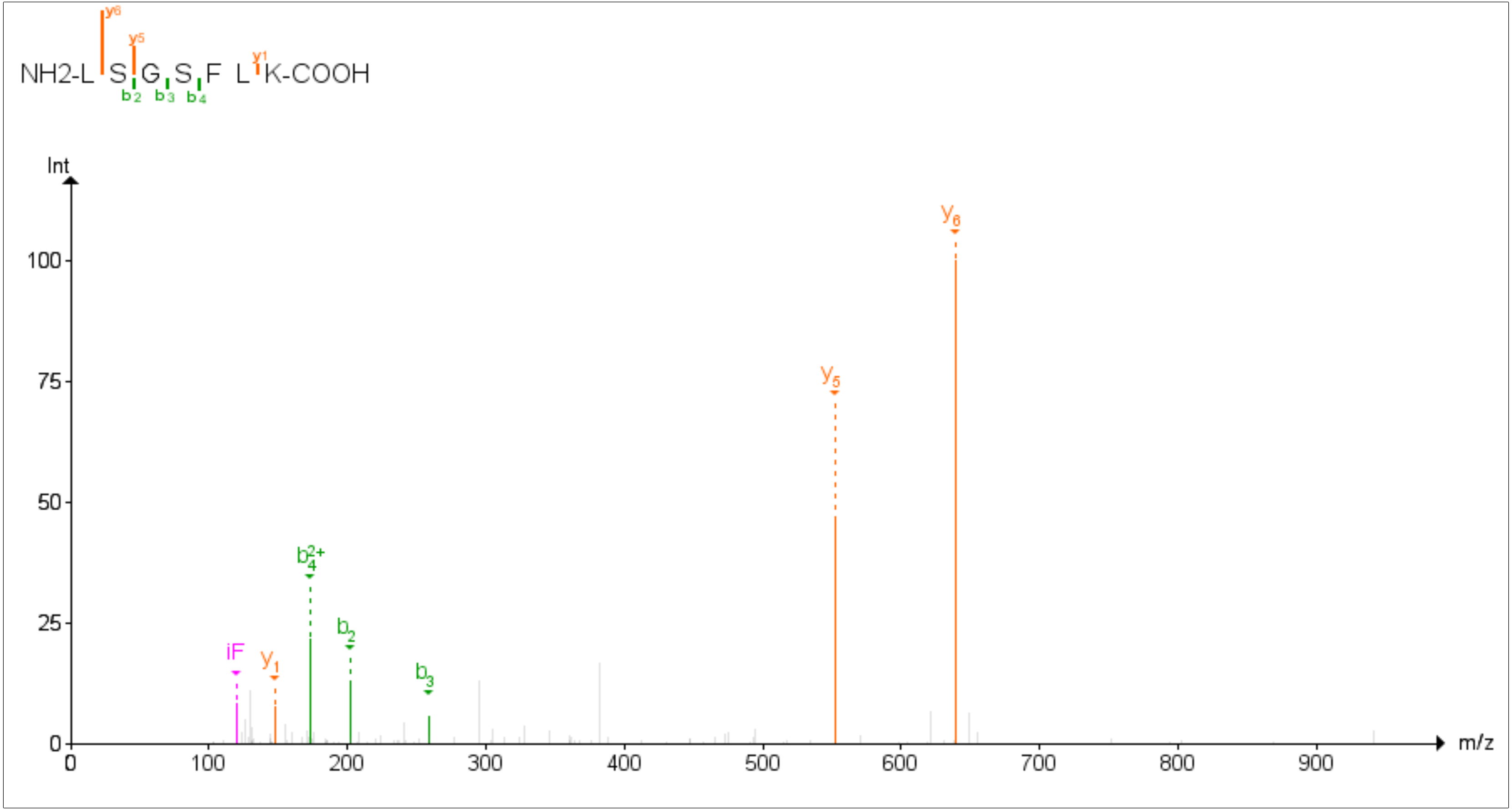

NH2-L S G S F L K-COOH
y6
y5
y1
b2
b3
b4
Int
100
75
50
25
0
y6
y5
b4 2+
b2
b3
iF
y1
0
100
200
300
400
500
600
700
800
900
m/z

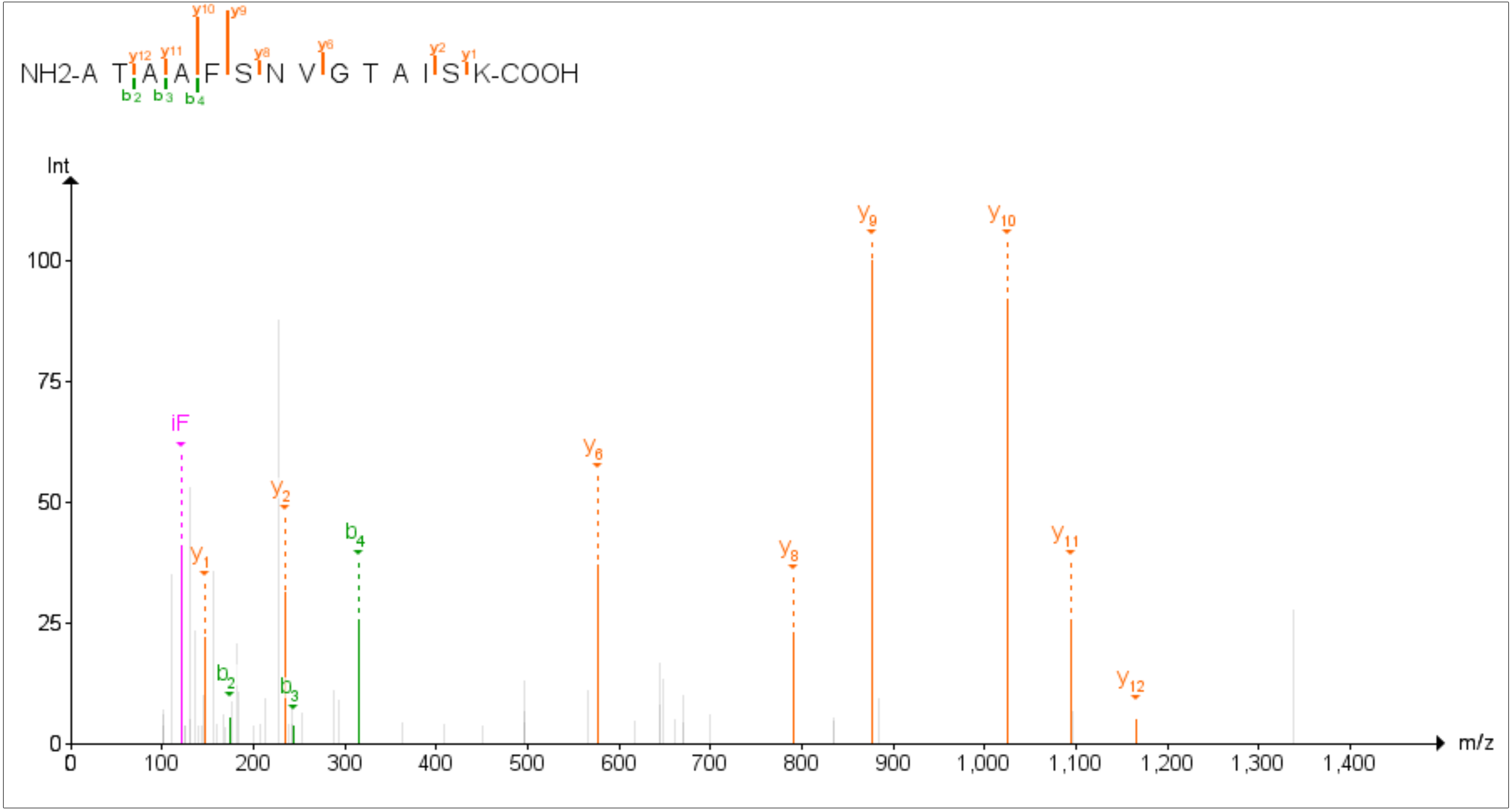
NH2-A T A A F S N V G T A I S K-COOH
y12 y11 y10 y9 y8 y6 y2 y1
b2 b3 b4
Int
100
75
50
25
0
iF
y1
y2
b2
b3
b4
y6
y8
y9
y10
y11
y12
0 100 200 300 400 500 600 700 800 900 1,000 1,100 1,200 1,300 1,400
m/z

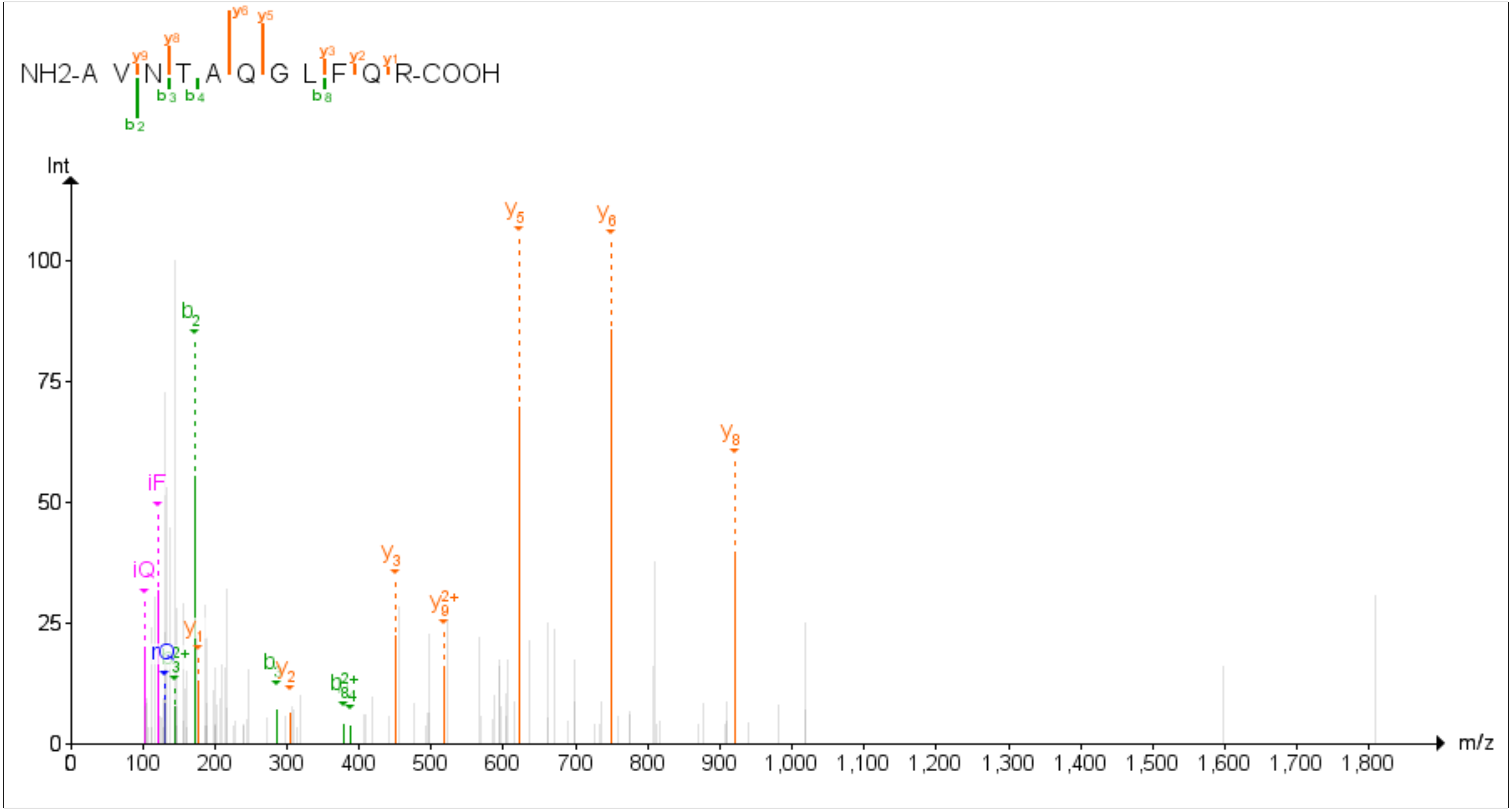

NH2-A V N T A Q G L F Q R-COOH
y9 y8 y6 y5 y3 y2 y1
b2 b3 b4 b8
Int
m/z
y5
y6
y8
b2
iF
iQ
y3
y1
y9 2+
b3
y2
b8 2+

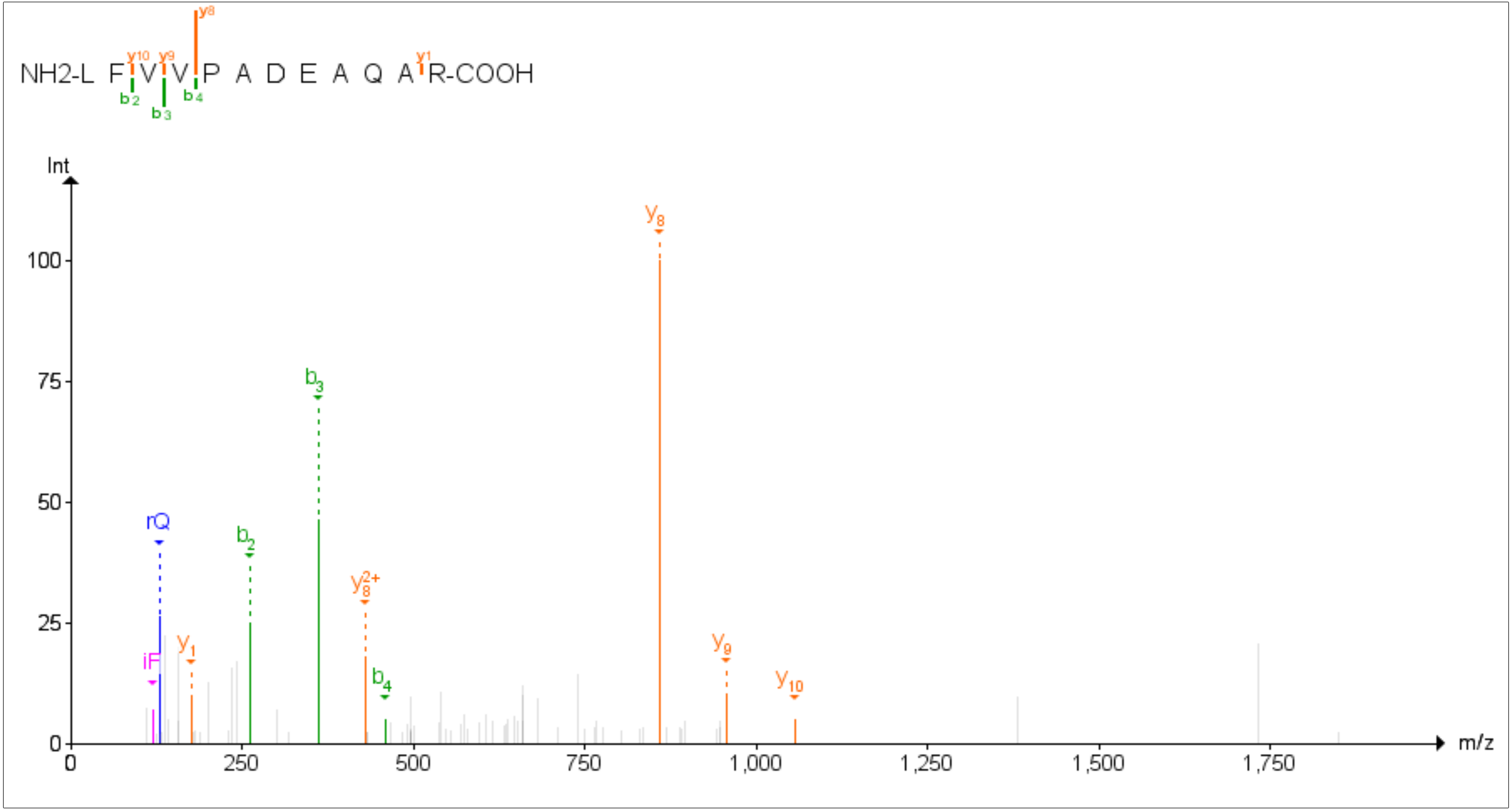

NH2-L F V V P A D E A Q A R-COOH
y10
y9
y8
y1
b2
b3
b4
Int
100
75
50
25
0
rQ
iF
y1
b2
b3
y8 2+
b4
y8
y9
y10
0
250
500
750
1,000
1,250
1,500
1,750
m/z

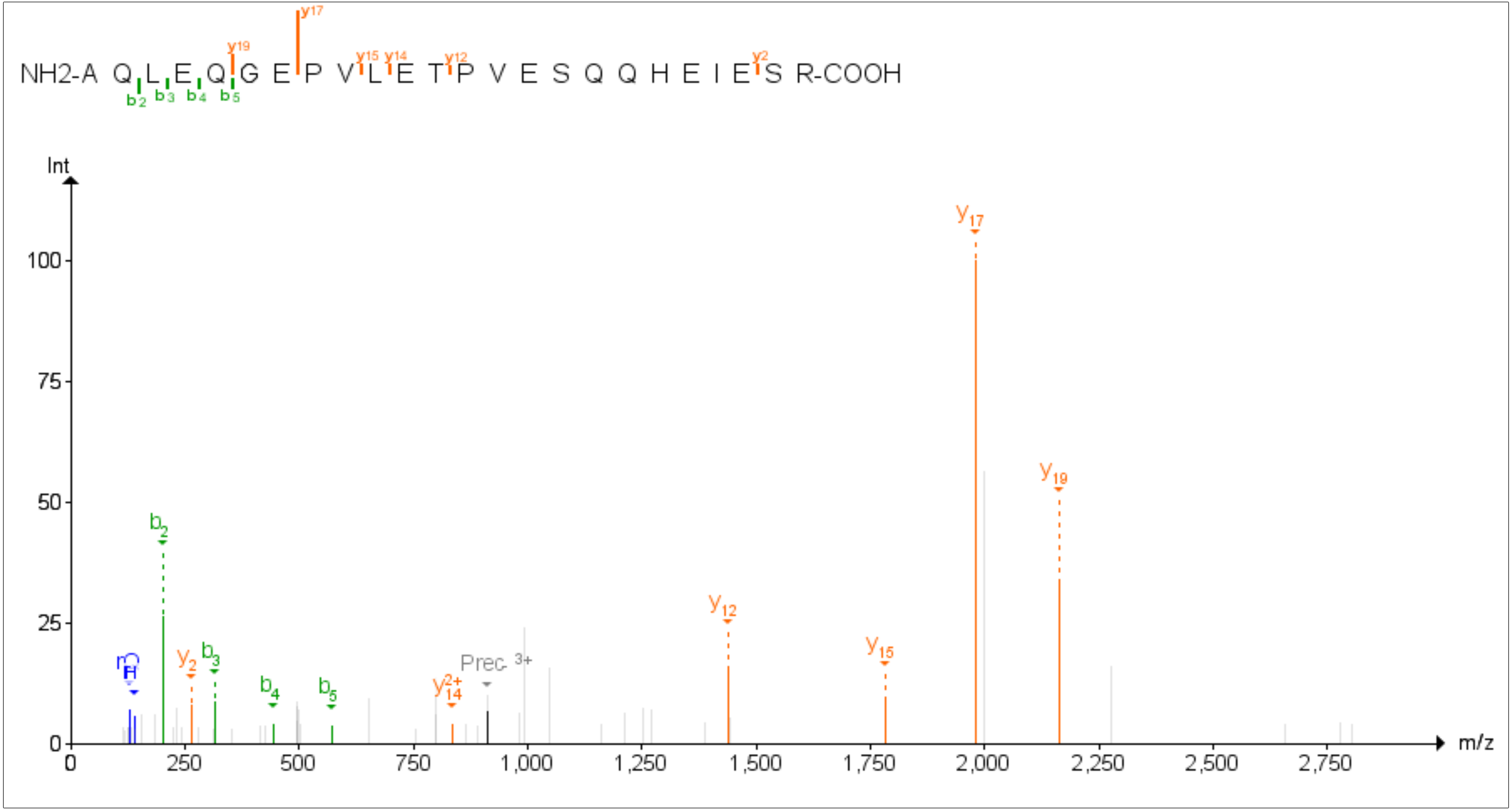

NH2-A Q L E Q G E P V L E T P V E S Q Q H E I E S R-COOH
y17
y19
y15 y14
y12
y2
b2 b3 b4 b5
Int
100
75
50
25
0
y17
y19
b2
y12
y15
y2
b3
b4
b5
Prec 3+
y14 2+
m/z
0
250
500
750
1,000
1,250
1,500
1,750
2,000
2,250
2,500
2,750

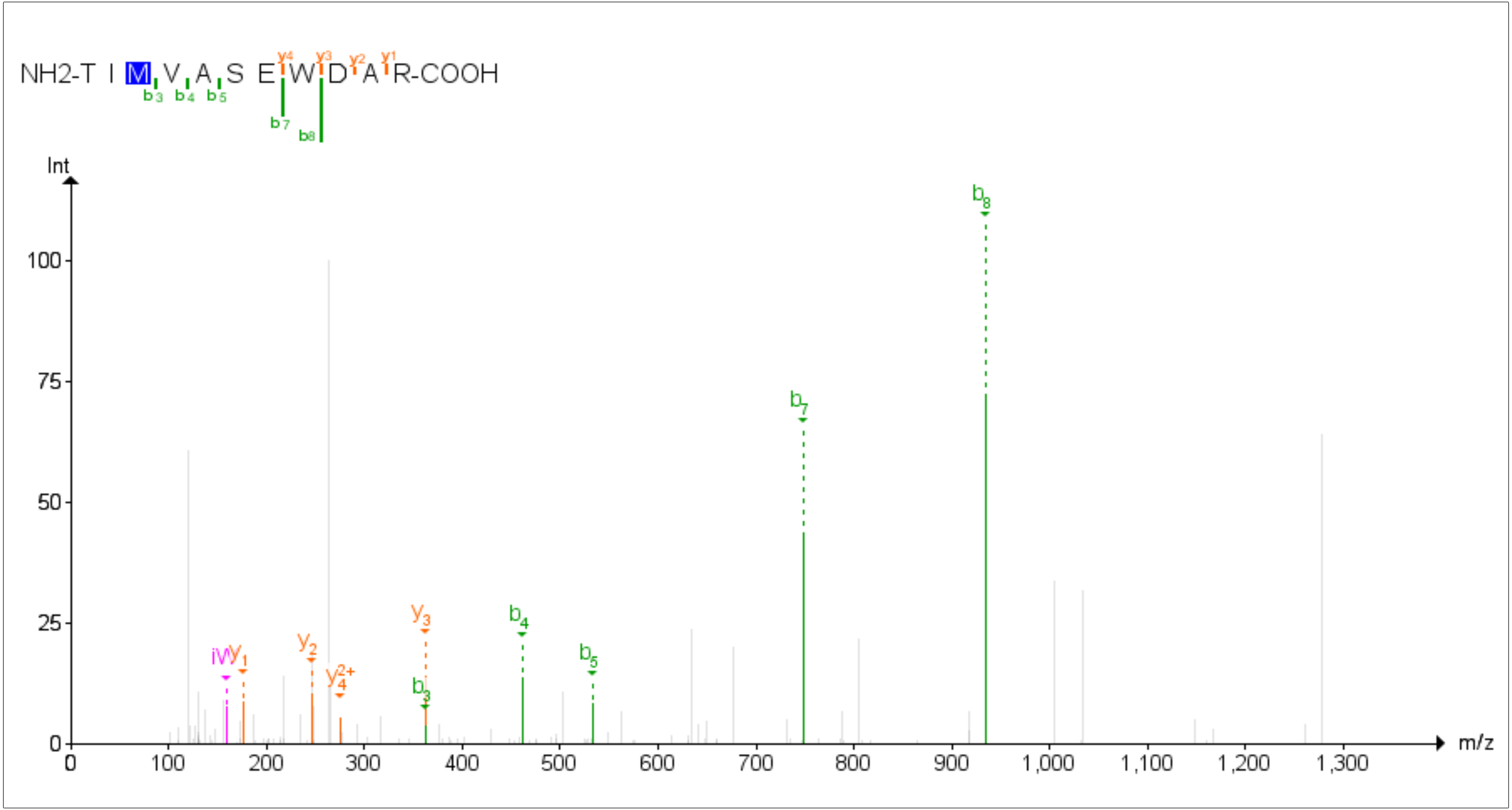
NH2-T I M V A S E W D A R-COOH
b3 b4 b5 b7 b8
y4 y3 y2 y1
Int
100
75
50
25
0
b8
b7
b4
b5
b3
y3
y2
y1
iW
y4 2+
0 100 200 300 400 500 600 700 800 900 1,000 1,100 1,200 1,300
m/z

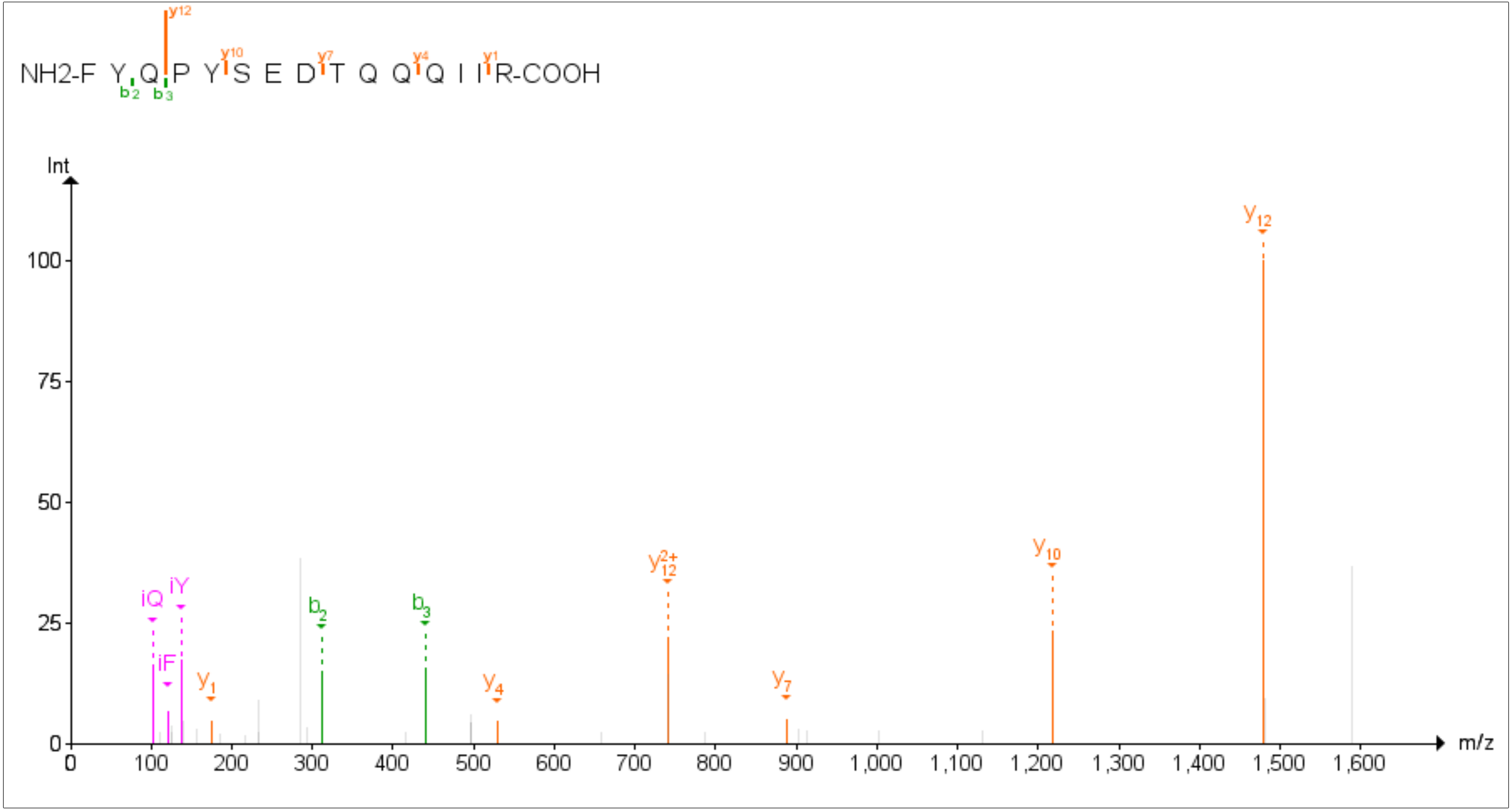
NH2-F Y Q P Y S E D T Q Q Q I I R-COOH
y12
y10
y7
y4
y1
b2
b3
Int
100
75
50
25
0
m/z
0
100
200
300
400
500
600
700
800
900
1,000
1,100
1,200
1,300
1,400
1,500
1,600
iQ
iY
iF
y1
b2
b3
y4
y12 2+
y7
y10
y12

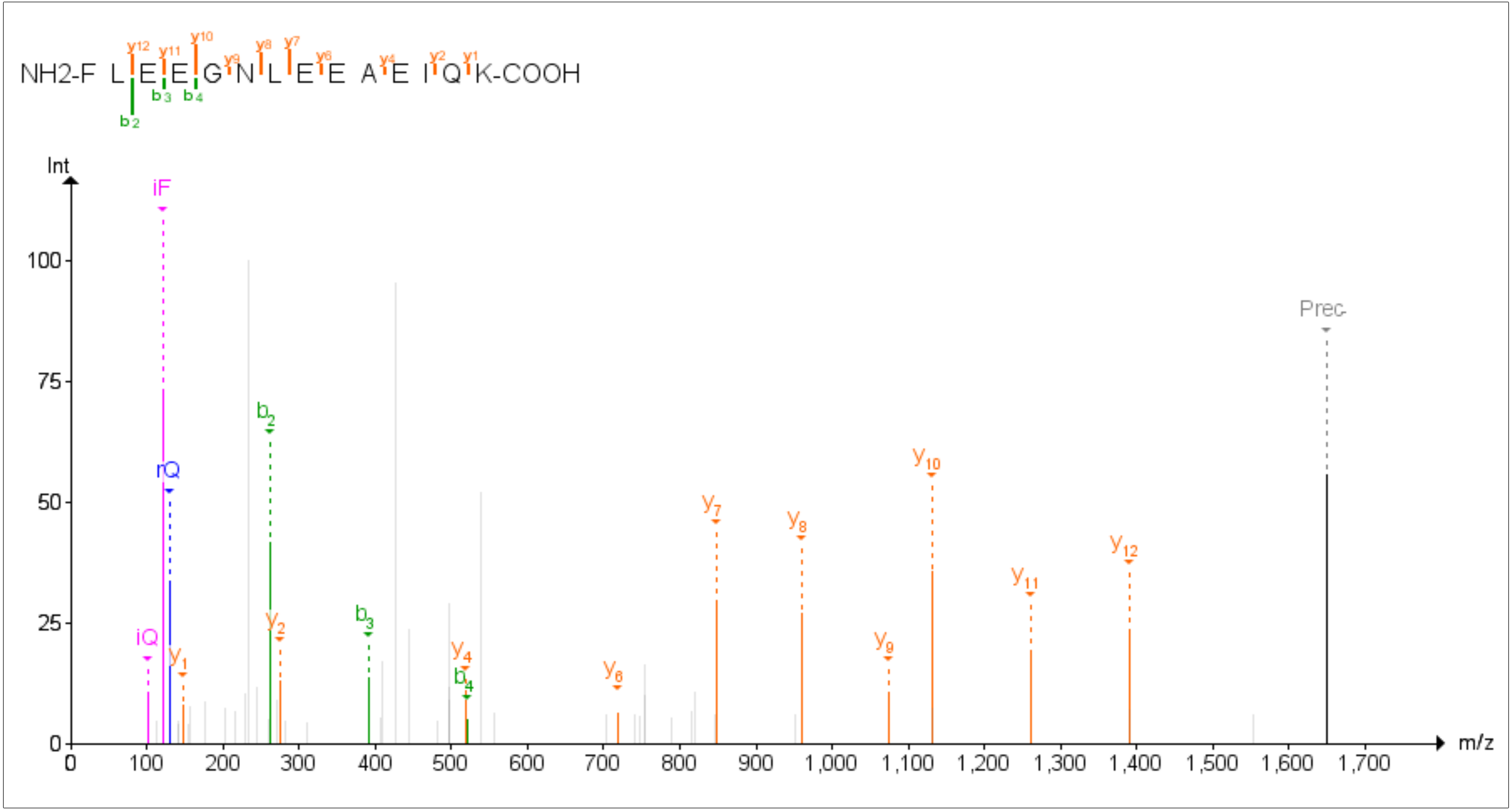
NH2-F L E E G N L E E A E I Q K-COOH
y12 y11 y10 y9 y8 y7 y6 y4 y2 y1
b2 b3 b4
Int
m/z
iF
iQ
rQ
y1
y2
b2
b3
y4
b4
y6
y7
y8
y9
y10
y11
y12
Prec.
0
25
50
75
100
0 100 200 300 400 500 600 700 800 900 1,000 1,100 1,200 1,300 1,400 1,500 1,600 1,700

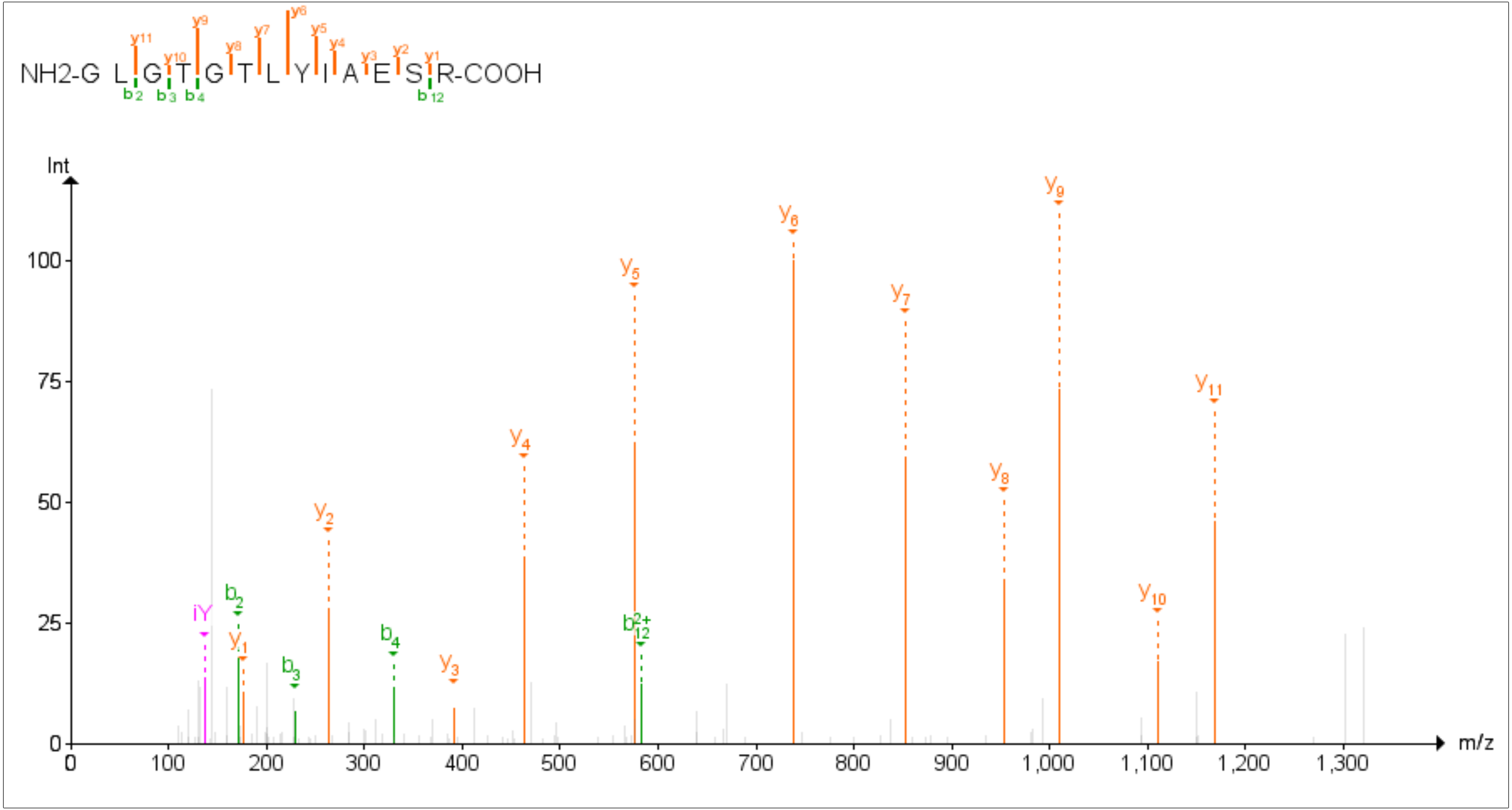

NH2-G L G T G T L Y I A E S R-COOH
y11 y10 y9 y8 y7 y6 y5 y4 y3 y2 y1
b2 b3 b4 b12
Int
100
75
50
25
0
m/z
0 100 200 300 400 500 600 700 800 900 1,000 1,100 1,200 1,300
iY
b2
y1
b3
y2
b4
y3
y4
y5
b12 2+
y6
y7
y8
y9
y10
y11

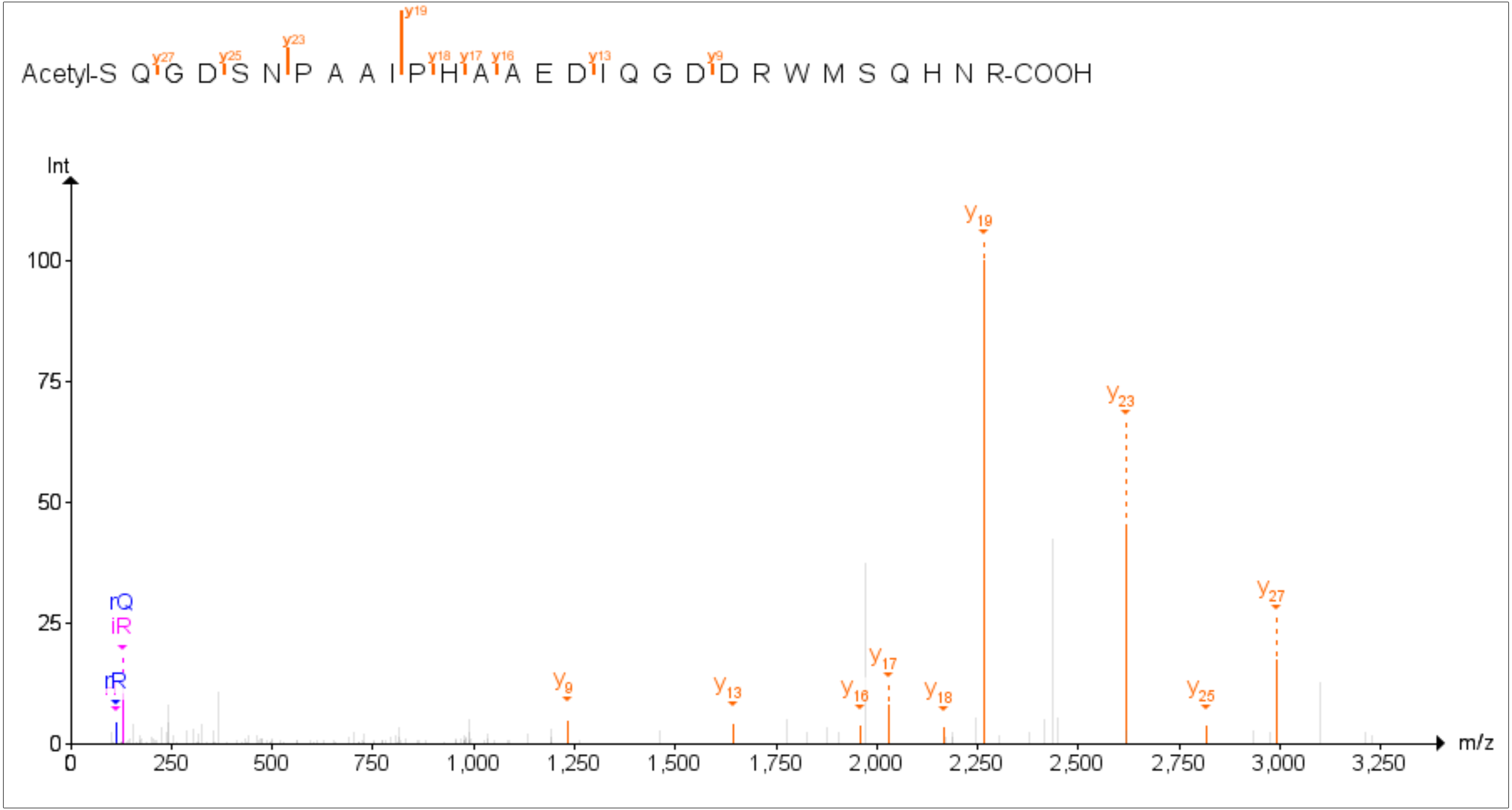

Acetyl-S Q G D S N P A A I P H A A E D I Q G D D R W M S Q H N R-COOH
y27
y25
y23
y19
y18
y17
y16
y13
y9
Int
100
75
50
25
0
rQ
iR
rR
y19
y23
y27
y9
y13
y16
y17
y18
y25
0
250
500
750
1,000
1,250
1,500
1,750
2,000
2,250
2,500
2,750
3,000
3,250
m/z

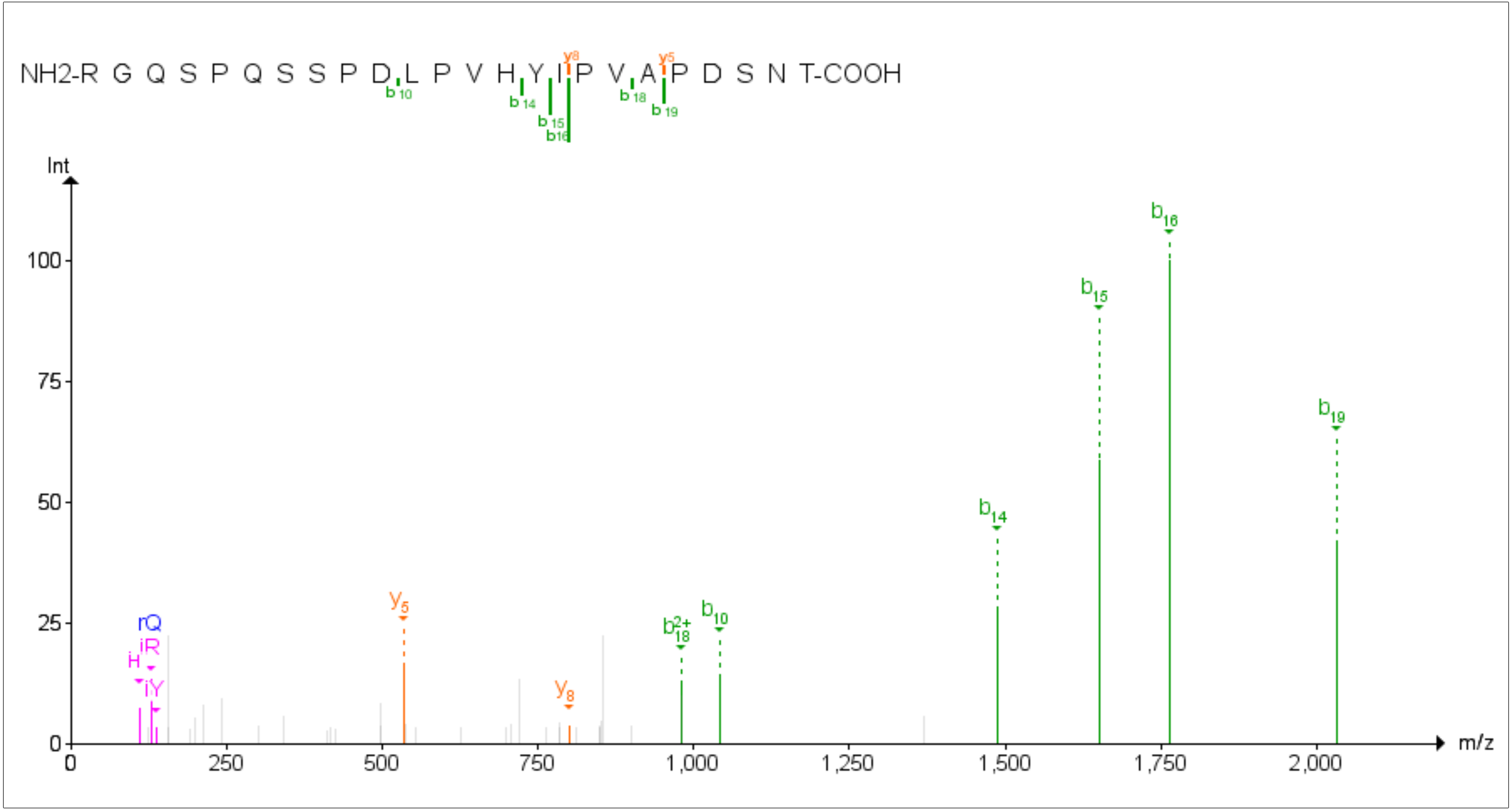

NH2-R G Q S P Q S S P D L P V H Y I P V A P D S N T-COOH
y8
y5
b 10
b 14
b 15
b 16
b 18
b 19
Int
100
75
50
25
0
b16
b15
b19
b14
y5
b2+ 18
b10
y8
rQ
iR
H
iY
0
250
500
750
1,000
1,250
1,500
1,750
2,000
m/z

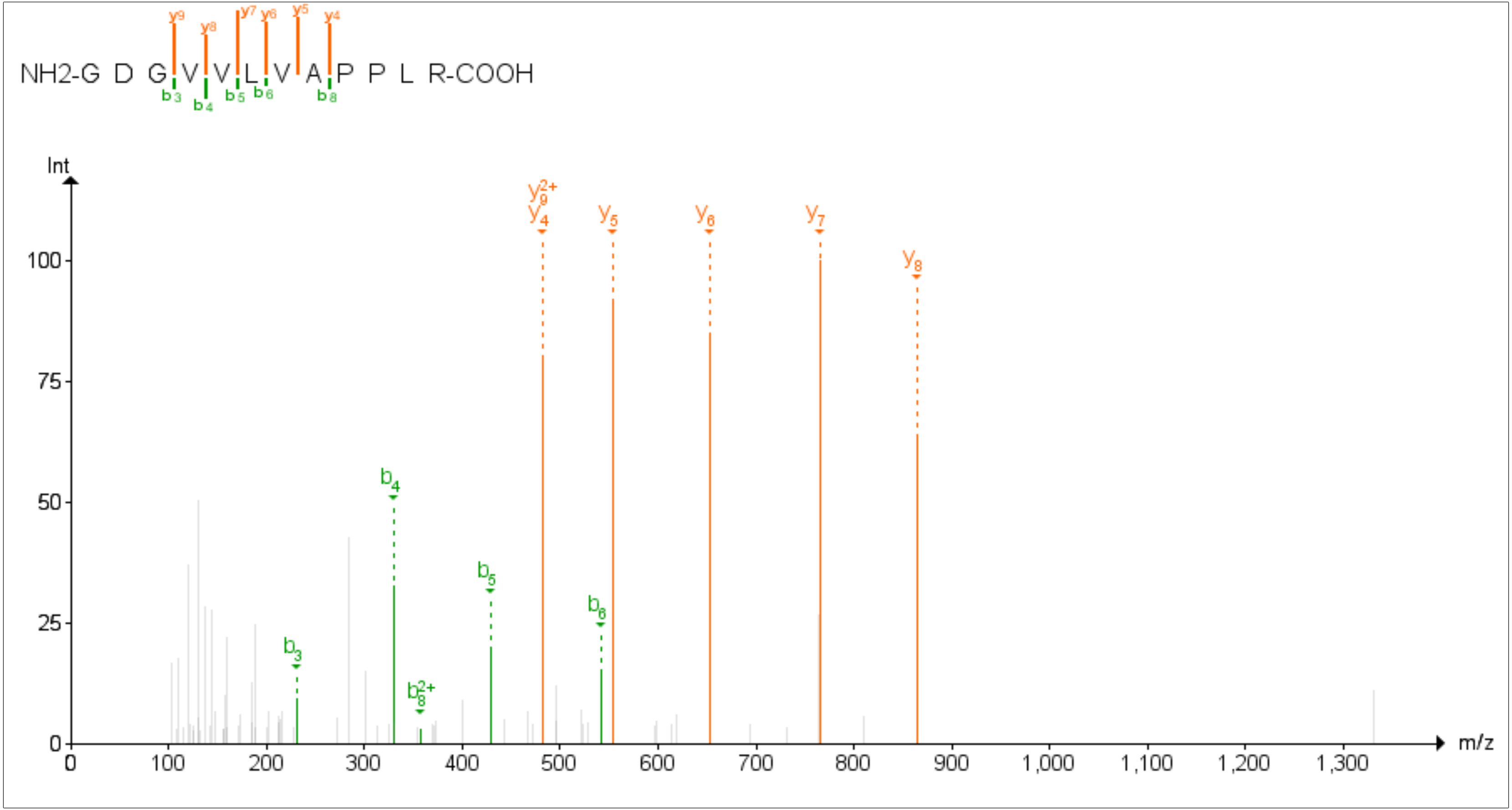

NH2-G D G V V L V A P P L R-COOH
y9 y8 y7 y6 y5 y4
b3 b4 b5 b6 b8
Int
m/z
y4
y9 2+
y5
y6
y7
y8
b3
b4
b8 2+
b5
b6
0
25
50
75
100
0
100
200
300
400
500
600
700
800
900
1,000
1,100
1,200
1,300

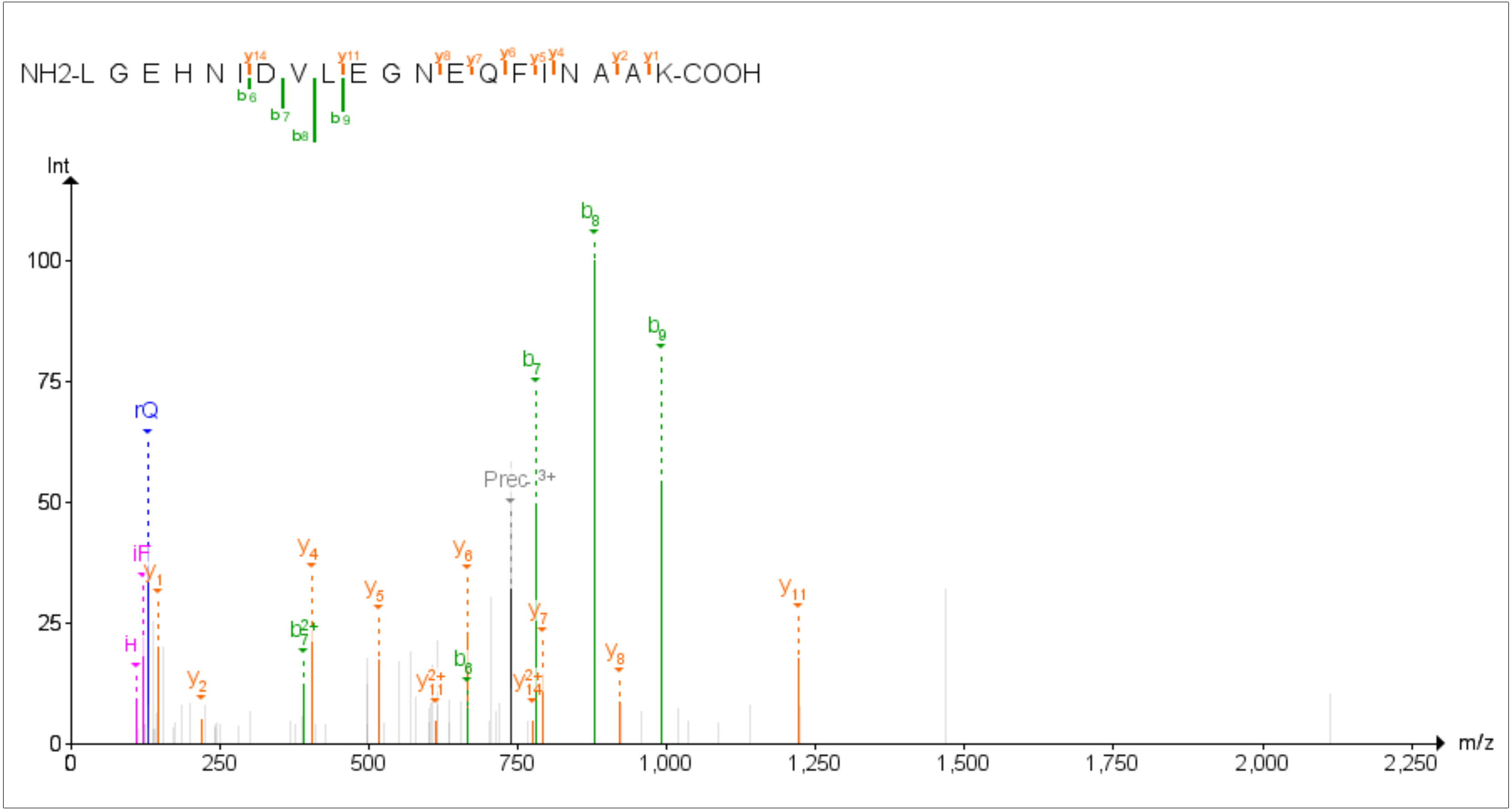
NH2-L G E H N I D V L E G N E Q F I N A A K-COOH
y14 y11 y8 y7 y6 y5 y4 y2 y1
b6 b7 b8 b9
Int
100
75
50
25
0
rQ
iF
iH
Prec 3+
b8
b9
b7
y1
y2
y4
y5
y6
y7
y8
y11
b6
b7 2+
y11 2+
y14 2+
0
250
500
750
1,000
1,250
1,500
1,750
2,000
2,250
m/z

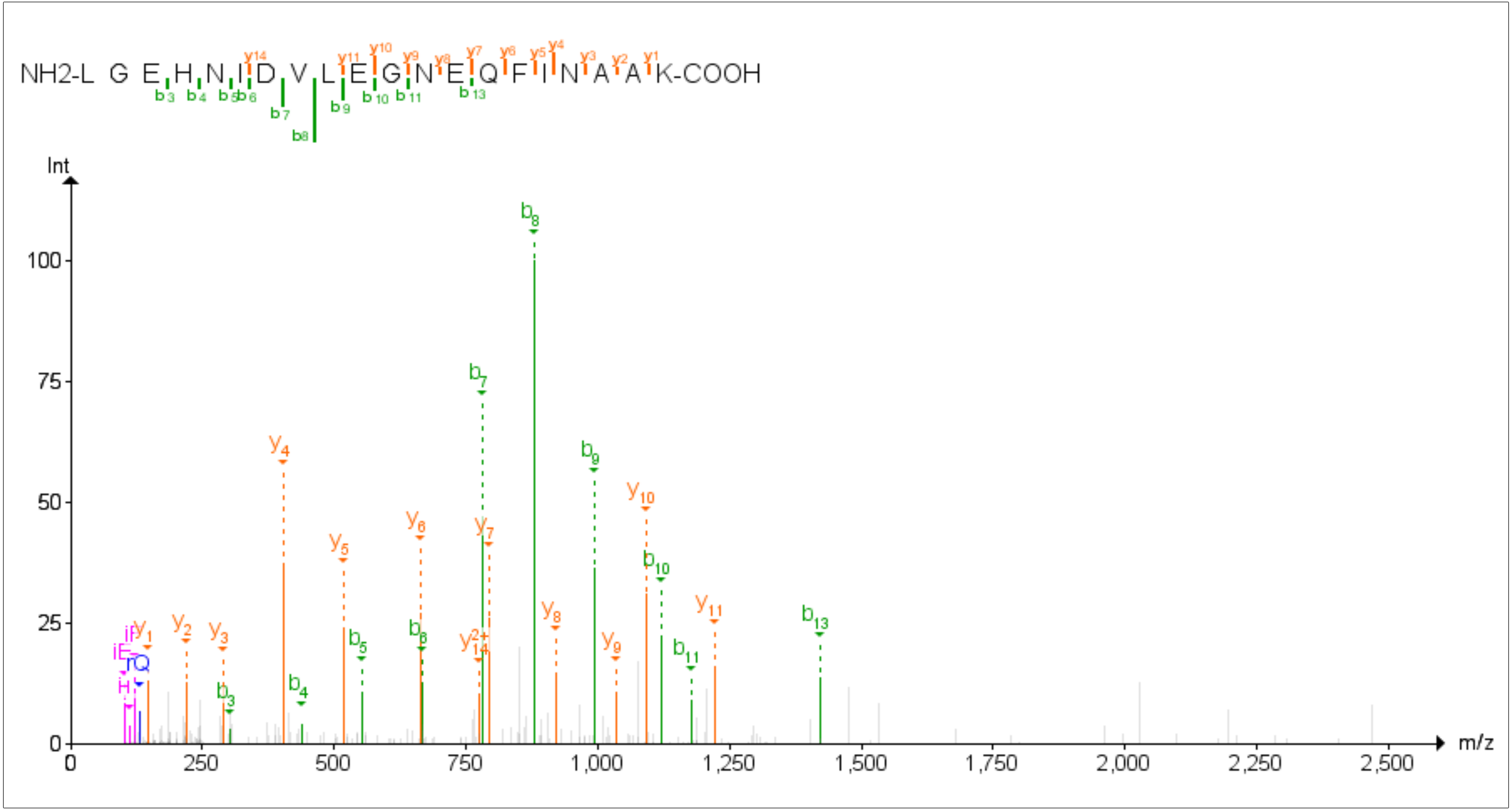
NH2-L G E H N I D V L E G N E Q F I N A A K-COOH
Int
m/z
0
25
50
75
100
250
500
750
1,000
1,250
1,500
1,750
2,000
2,250
2,500

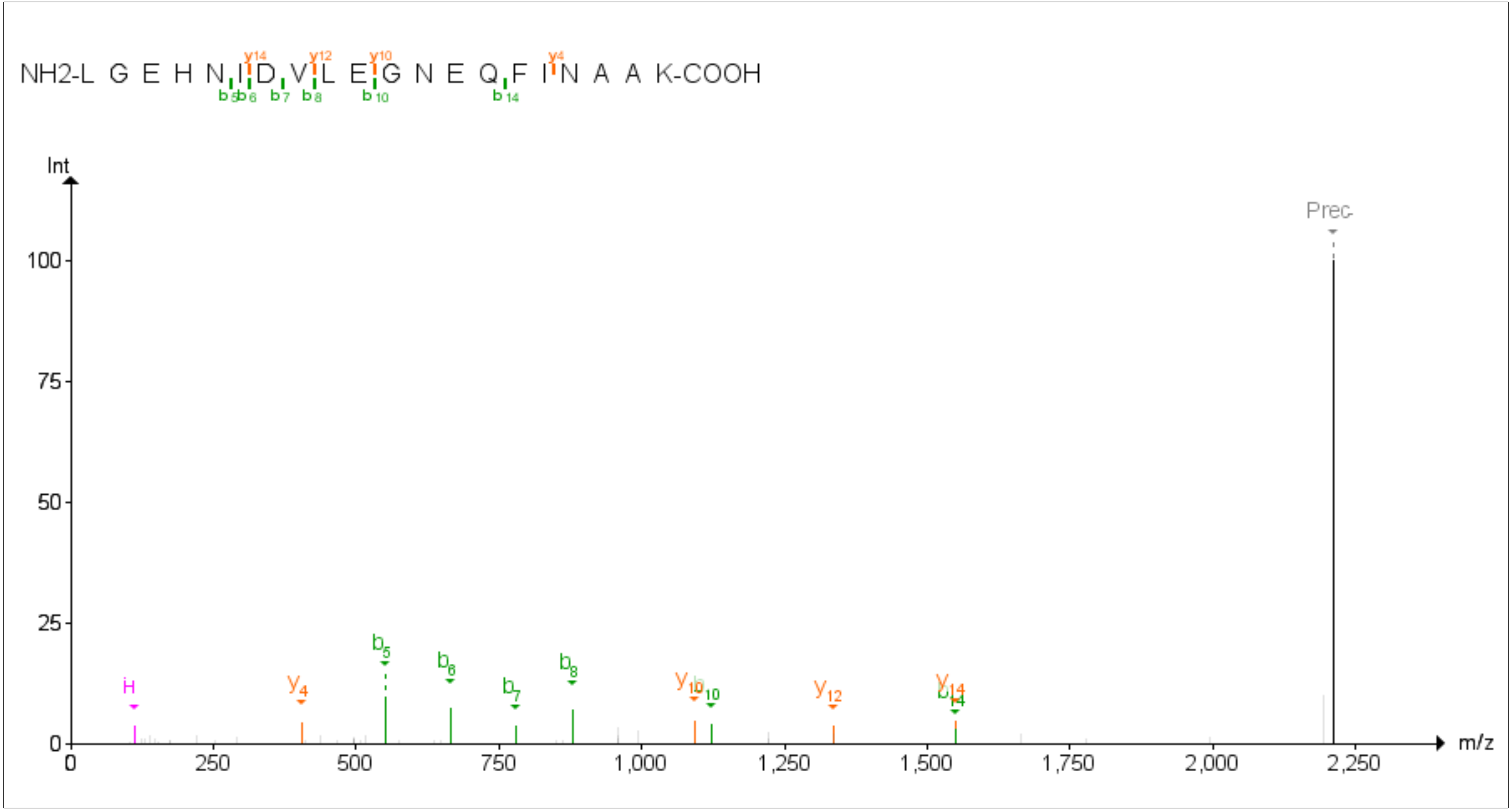

NH2-L G E H N I D V L E G N E Q F I N A A K-COOH
y14 y12 y10 y4
b5 b6 b7 b8 b10 b14
Int
Prec
100
75
50
25
0
H
y4
b5
b6
b7
b8
y10
b10
y12
y14
b14
0
250
500
750
1,000
1,250
1,500
1,750
2,000
2,250
m/z

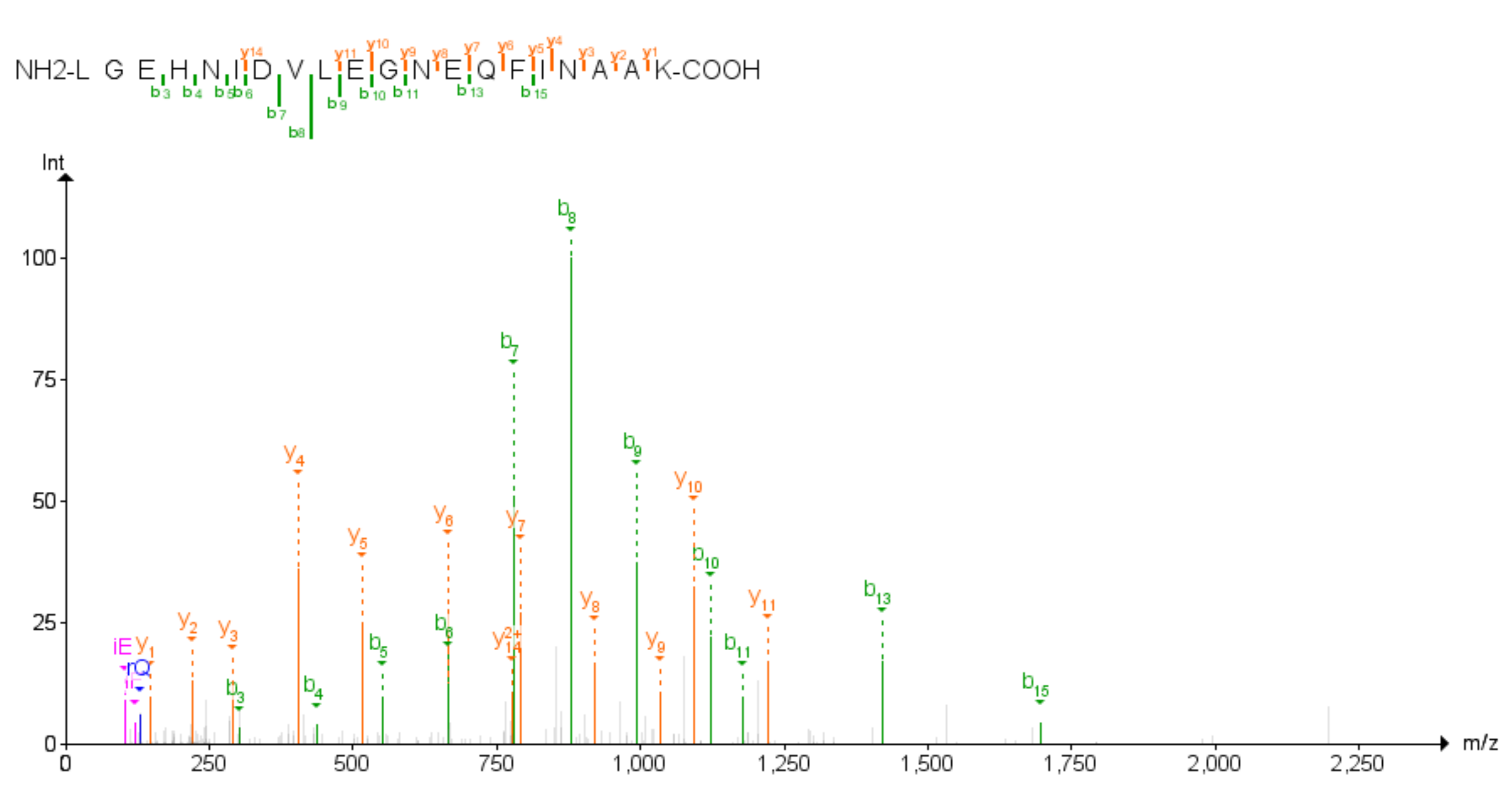

NH2-L G E H N I D V L E G N E Q F I N A A K-COOH
Int
m/z
0
25
50
75
100
250
500
750
1,000
1,250
1,500
1,750
2,000
2,250
b8
b7
b9
y4
y10
b10
y5
y6
y7
y8
y11
b13
y2
y3
b5
b6
y14 2+
y9
b11
iE
y1
rQ
b3
b4
b15

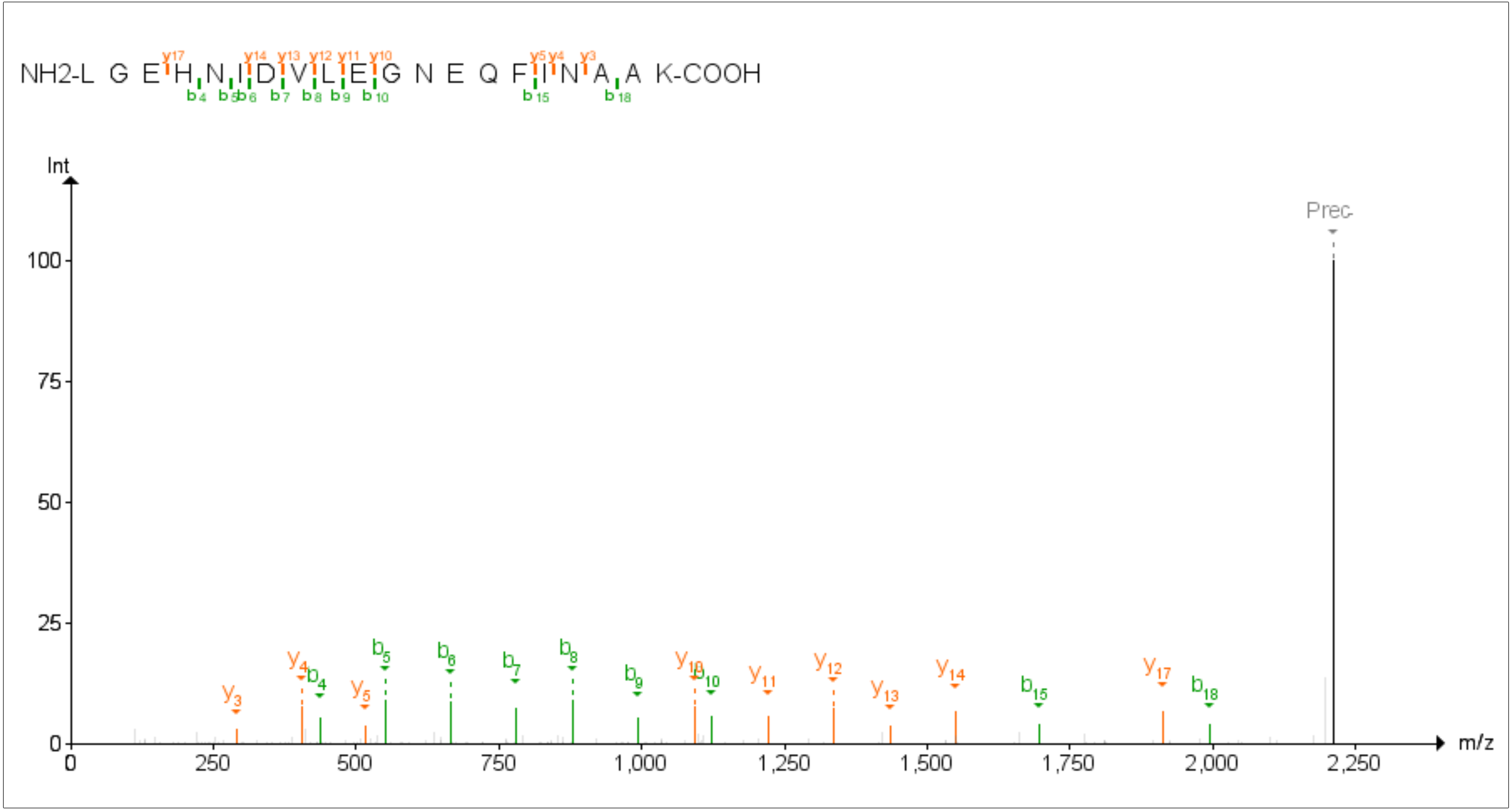

NH2-L G E H N I D V L E G N E Q F I N A A K-COOH
y17 y14 y13 y12 y11 y10 y5 y4 y3
b4 b5 b6 b7 b8 b9 b10 b15 b18
Int
100
75
50
25
0
Prec
y3 y4 b4 y5 b5 b6 b7 b8 b9 y10 b10 y11 y12 y13 y14 b15 y17 b18
0
250
500
750
1,000
1,250
1,500
1,750
2,000
2,250
m/z

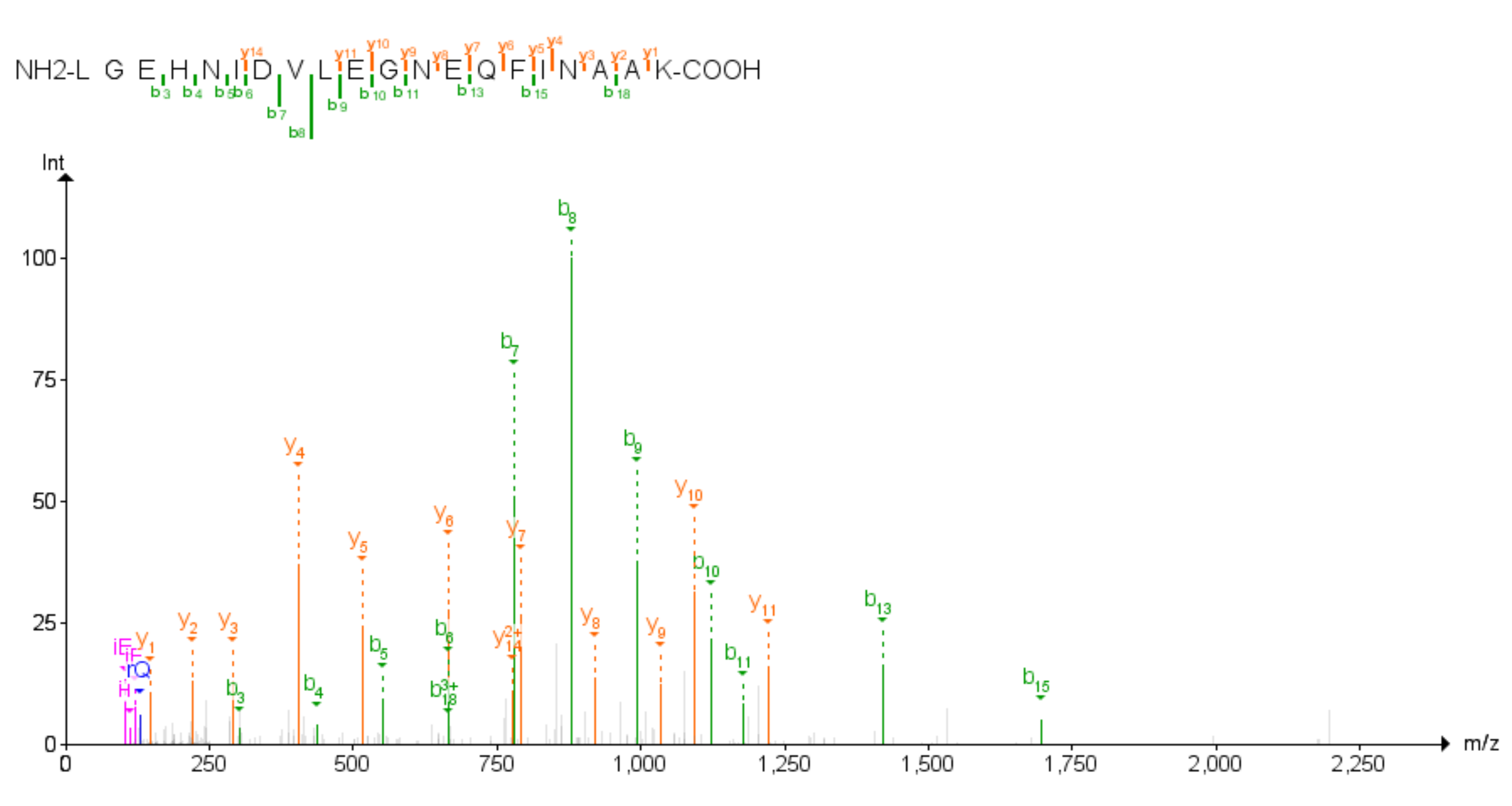

NH2-L G E H N I D V L E G N E Q F I N A A K-COOH
Int
m/z
0
25
50
75
100
250
500
750
1,000
1,250
1,500
1,750
2,000
2,250

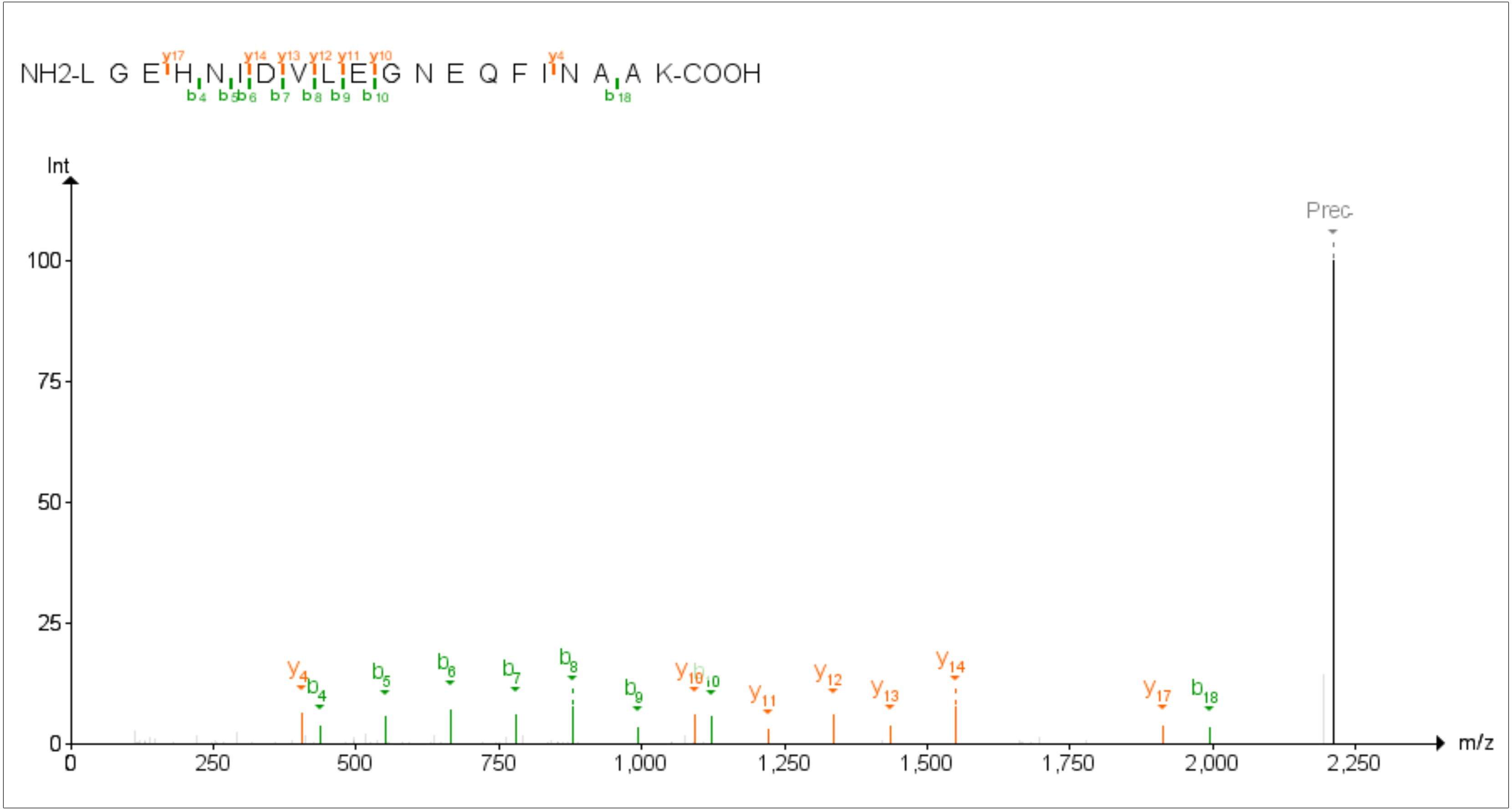

NH2-L G E H N I D V L E G N E Q F I N A A K-COOH
Int
Prec.
100
75
50
25
0
250
500
750
1,000
1,250
1,500
1,750
2,000
2,250
m/z

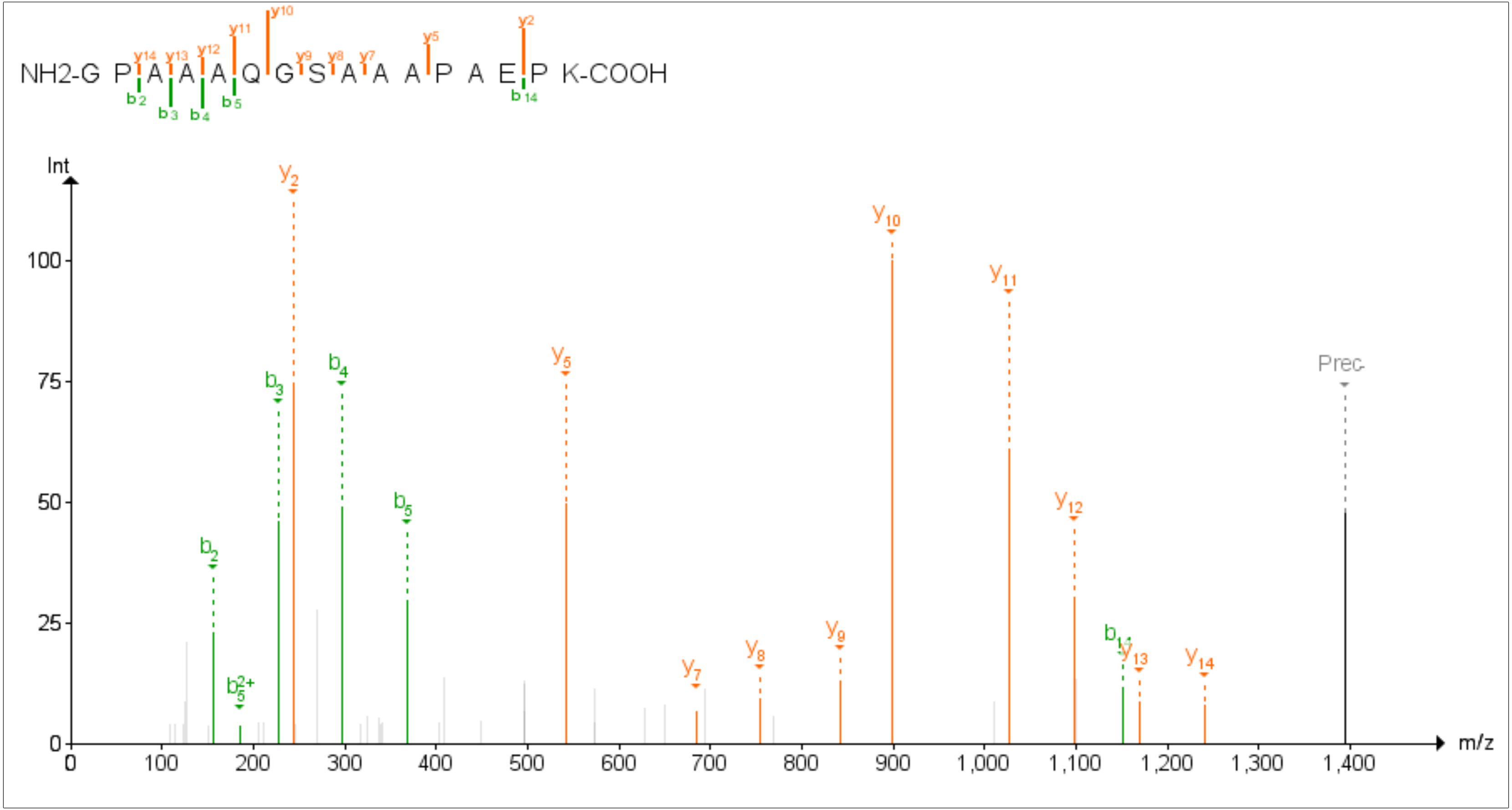

NH2-G P A A A Q G S A A A P A E P K-COOH
y14 y13 y12 y11 y10 y9 y8 y7 y5 y2
b2 b3 b4 b5 b14
Int
m/z
y2
y10
y11
y5
b3
b4
b5
b2
b_5^{2+}
y12
b14
y13
y14
y7
y8
y9
Prec
0
25
50
75
100
0
100
200
300
400
500
600
700
800
900
1,000
1,100
1,200
1,300
1,400

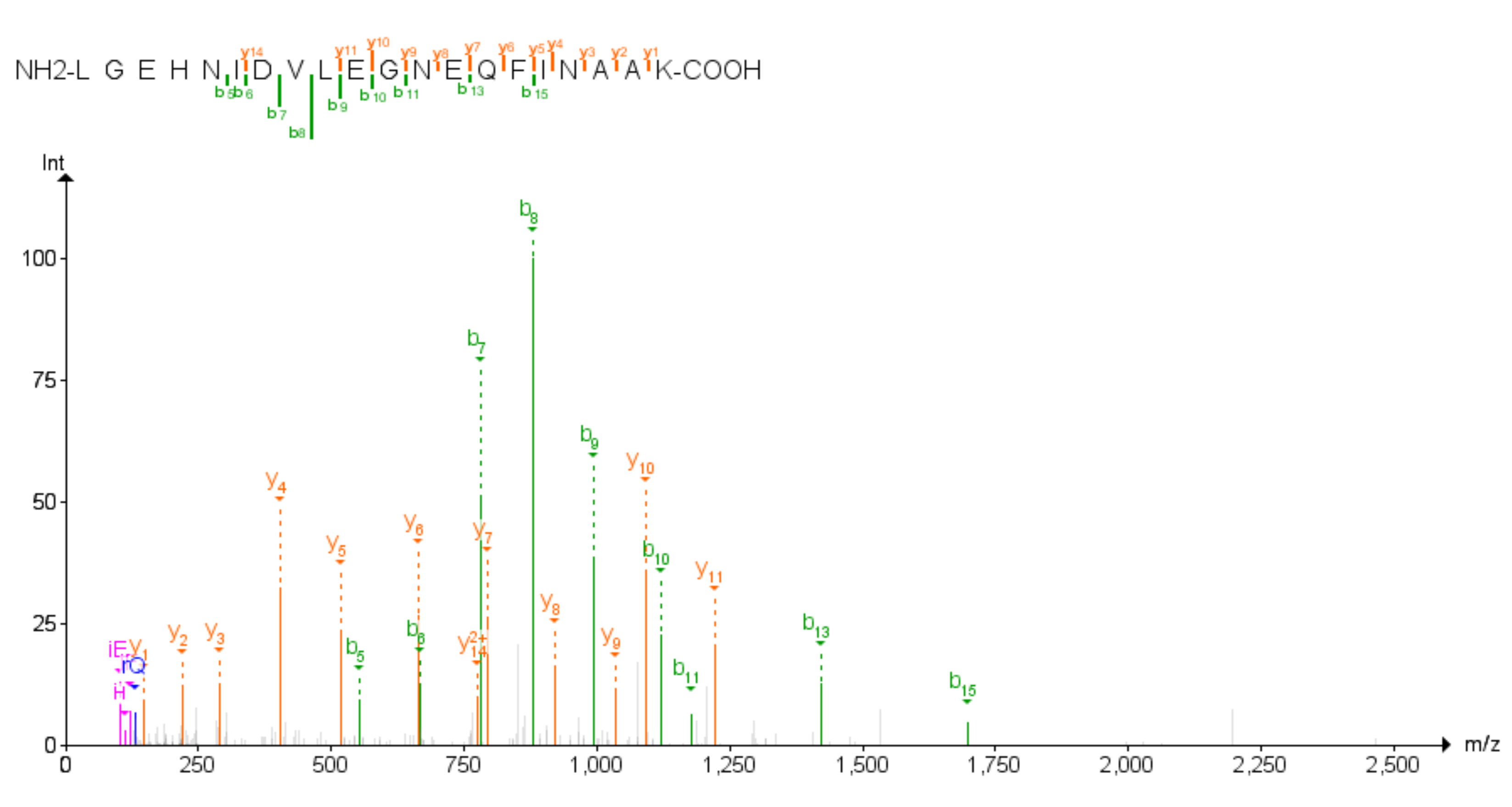
NH2-L G E H N I D V L E G N E Q F I N A A K-COOH
Int
m/z
0
25
50
75
100
0
250
500
750
1,000
1,250
1,500
1,750
2,000
2,250
2,500

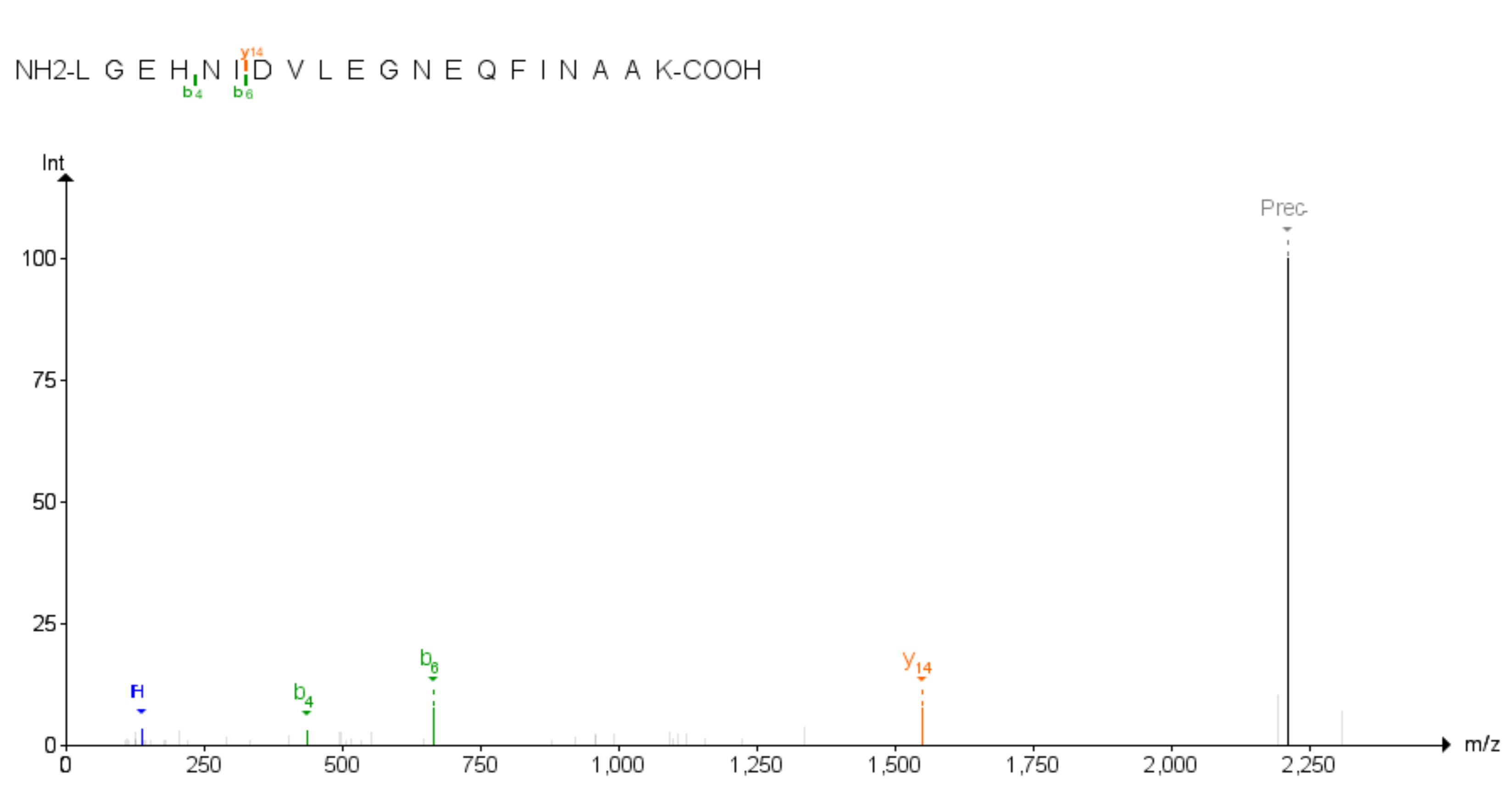
NH2-L G E H N I D V L E G N E Q F I N A A K-COOH
y14
b4
b6
Int
Prec
100
75
50
25
0
H
b4
b6
y14
0
250
500
750
1,000
1,250
1,500
1,750
2,000
2,250
m/z

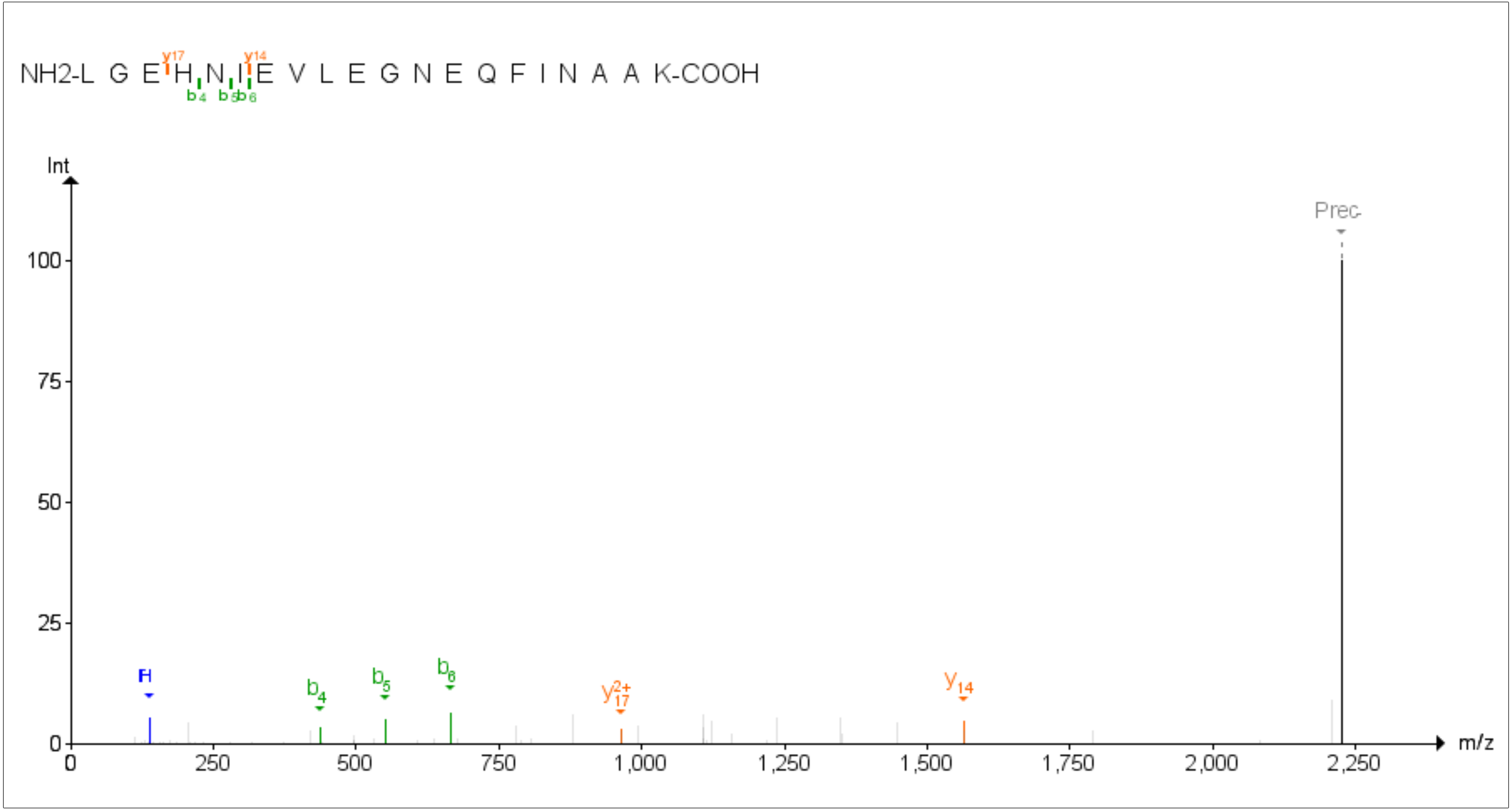
NH2-L G E H N I E V L E G N E Q F I N A A K-COOH
y17
y14
b4
b5
b6
Int
Prec.
100
75
50
25
0
m/z
0
250
500
750
1,000
1,250
1,500
1,750
2,000
2,250
H
b4
b5
b6
y17 2+
y14

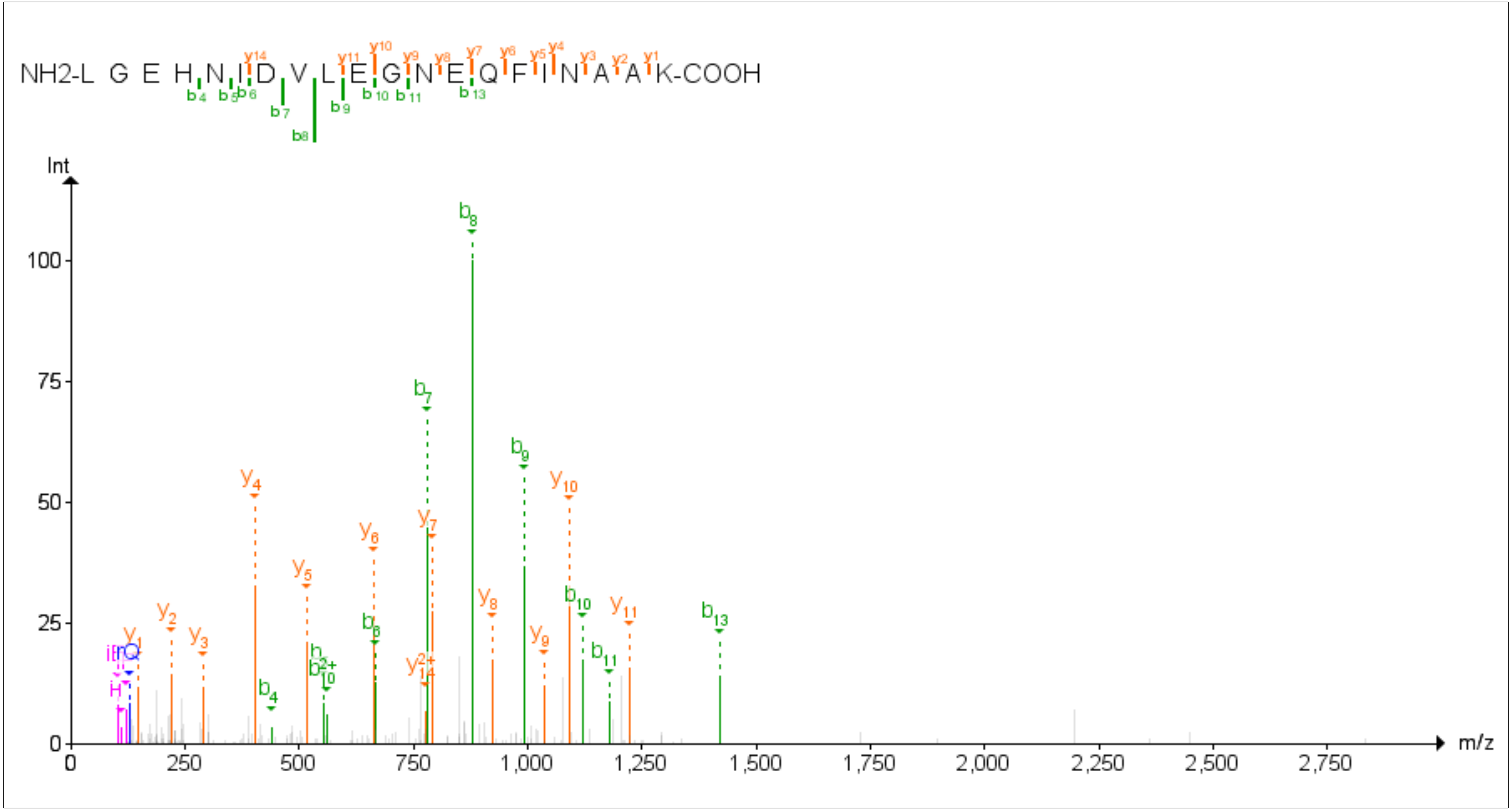

NH2-L G E H N I D V L E G N E Q F I N A A K-COOH
Int
m/z

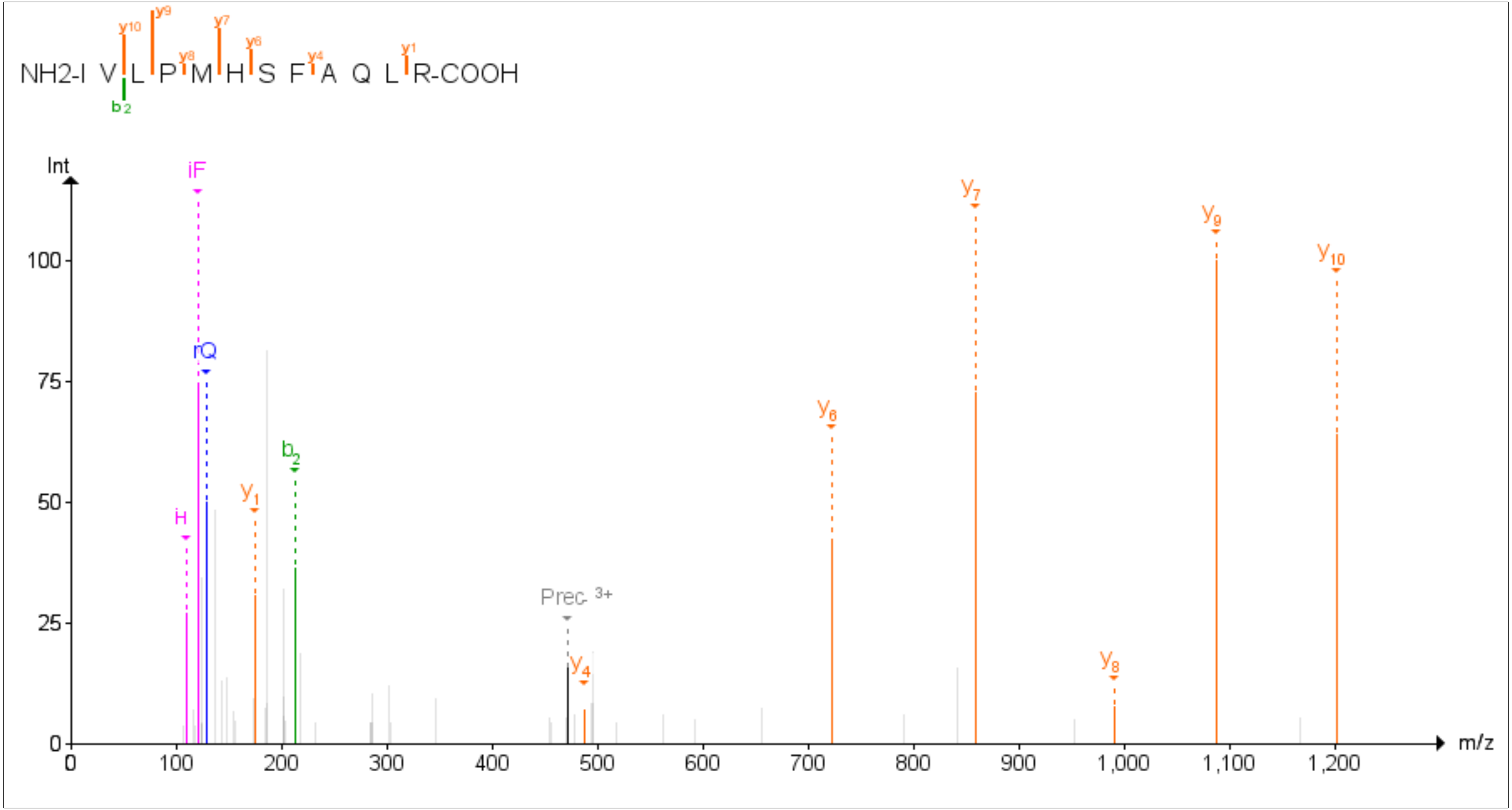

NH2-I V L P M H S F A Q L R-COOH
y10
y9
y8
y7
y6
y4
y1
b2
Int
iF
iH
rQ
y1
b2
Prec 3+
y4
y6
y7
y8
y9
y10
100
75
50
25
0
0
100
200
300
400
500
600
700
800
900
1,000
1,100
1,200
m/z

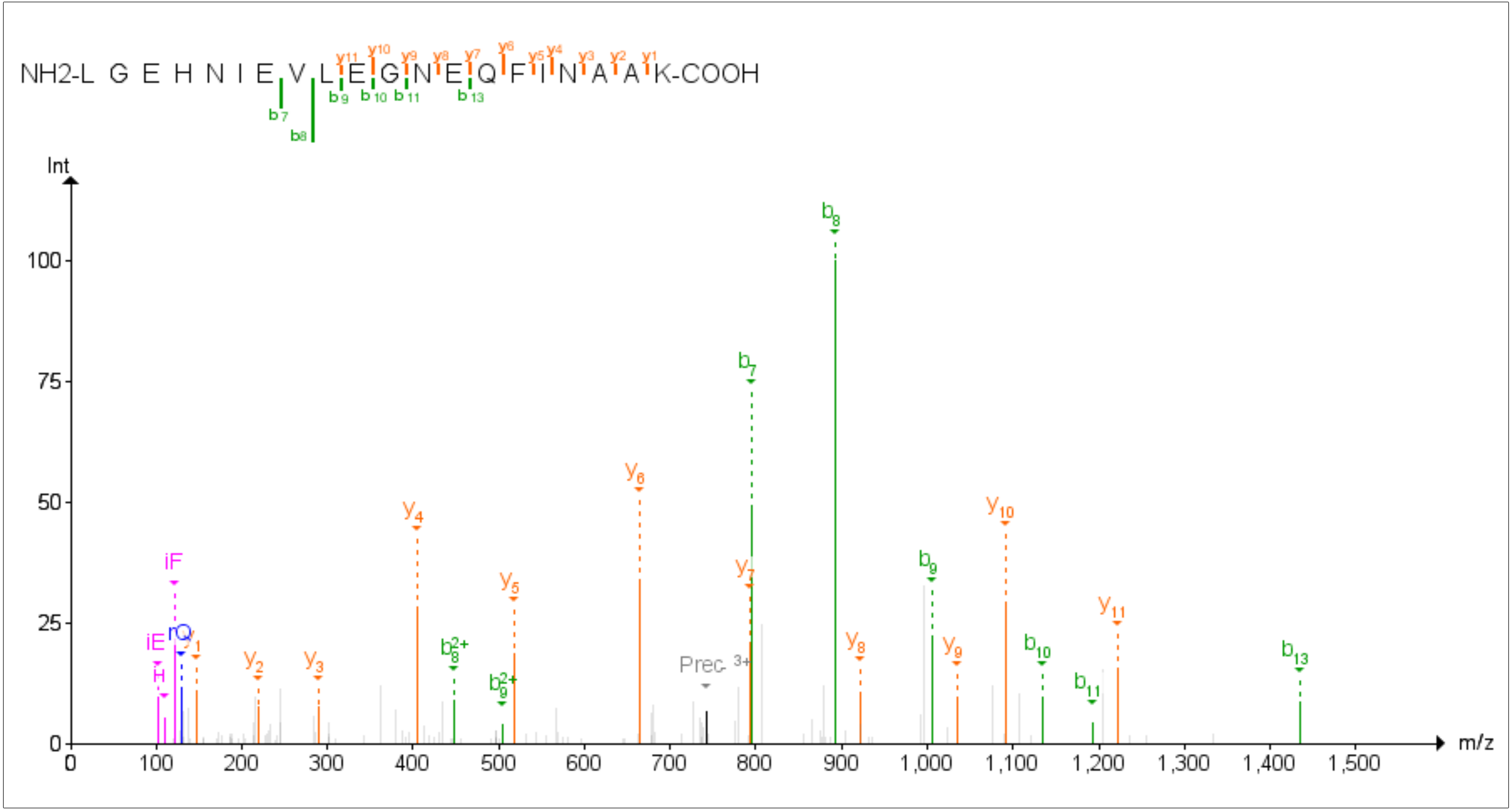
NH2-L G E H N I E V L E G N E Q F I N A A K-COOH
Int
m/z
b8
b7
y10
b9
y4
y6
y7
y5
y11
b10
b13
b11
y8
y9
Prec 3+
iF
iE
iH
rQ
y1
y2
y3
b8 2+
b9 2+
0
25
50
75
100
0
100
200
300
400
500
600
700
800
900
1,000
1,100
1,200
1,300
1,400
1,500

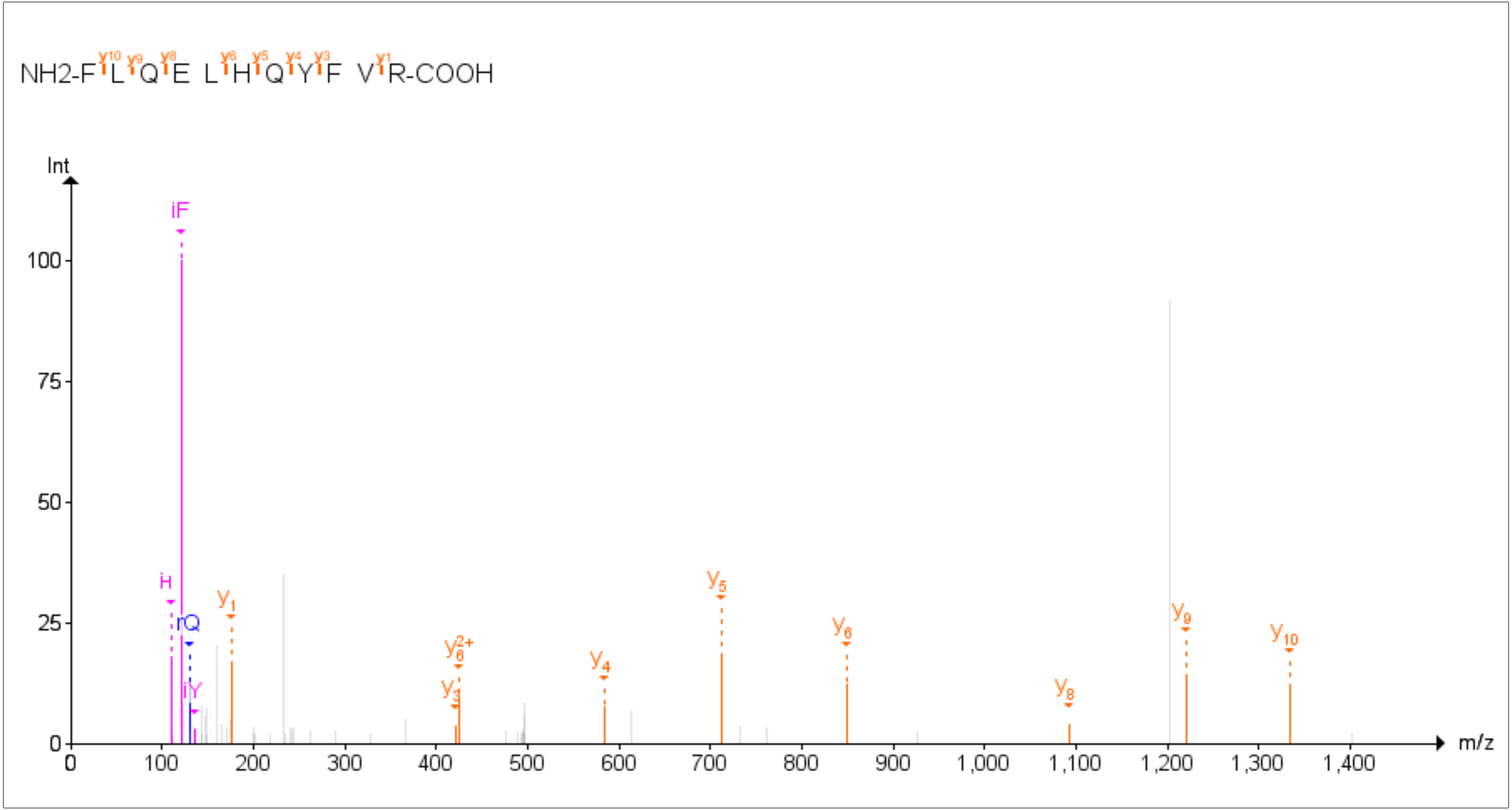

NH2-F L Q E L H Q Y F V R-COOH
y10 y9 y8 y6 y5 y4 y3 y1
Int
100
75
50
25
0
iF
iH
rQ
iY
y1
y3
y6 2+
y4
y5
y6
y8
y9
y10
0
100
200
300
400
500
600
700
800
900
1,000
1,100
1,200
1,300
1,400
m/z

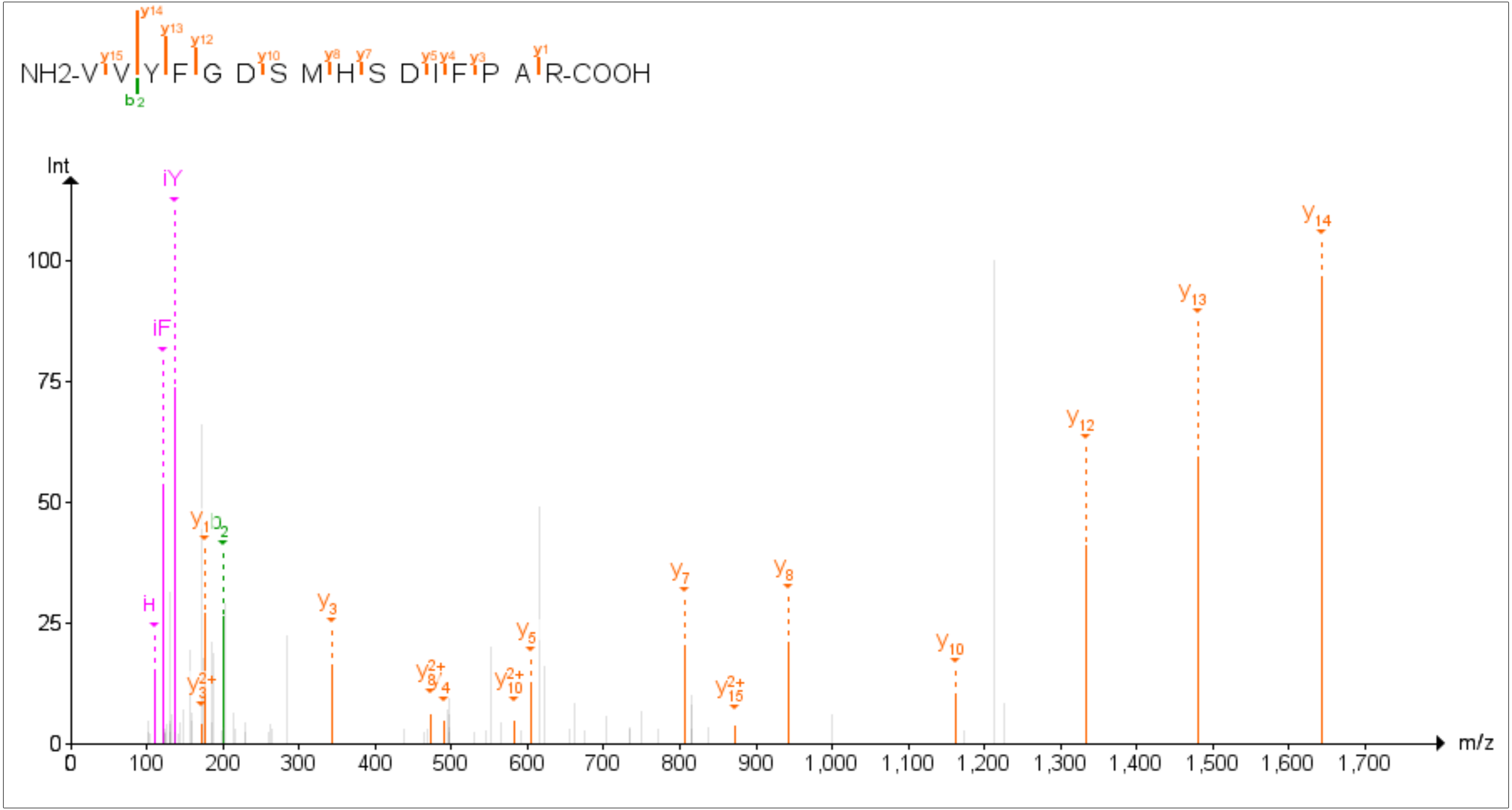

NH2-V V Y F G D S M H S D I F P A R-COOH
y15 y14 y13 y12 y10 y8 y7 y5 y4 y3 y1
b2
Int
m/z
iY
iF
iH
y1 b2
y3
y7
y8
y5
y10
y12
y13
y14
y3 2+
y8 2+
y4
y10 2+
y15 2+
0
25
50
75
100
0
100
200
300
400
500
600
700
800
900
1,000
1,100
1,200
1,300
1,400
1,500
1,600
1,700

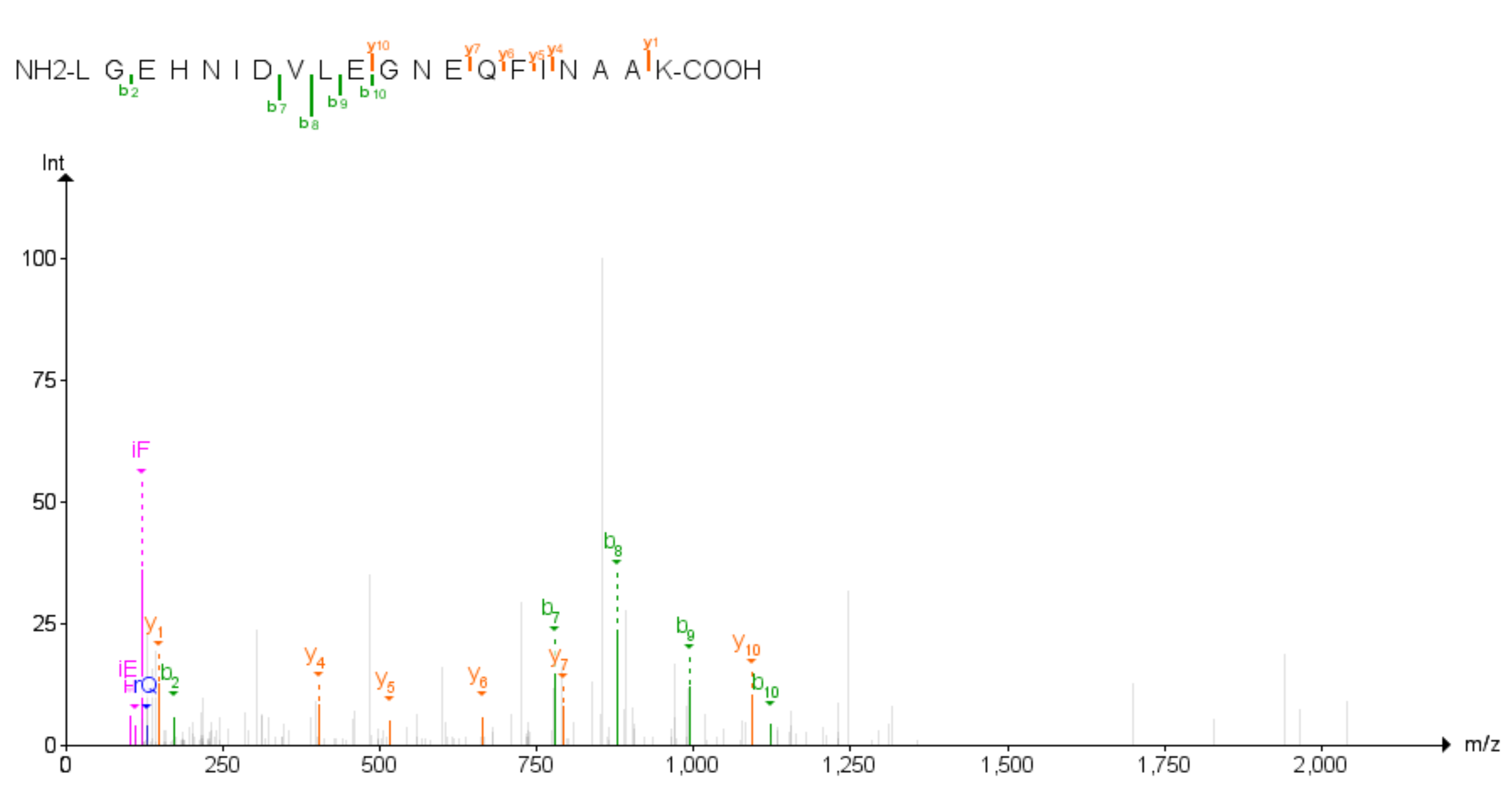

NH2-L G E H N I D V L E G N E Q F I N A A K-COOH
b2
b7
b8
b9
b10
y10
y7
y6
y5
y4
y1
Int
100
75
50
25
0
iF
iE
H
rQ
y1
b2
y4
y5
y6
b7
y7
b8
b9
y10
b10
0
250
500
750
1,000
1,250
1,500
1,750
2,000
m/z

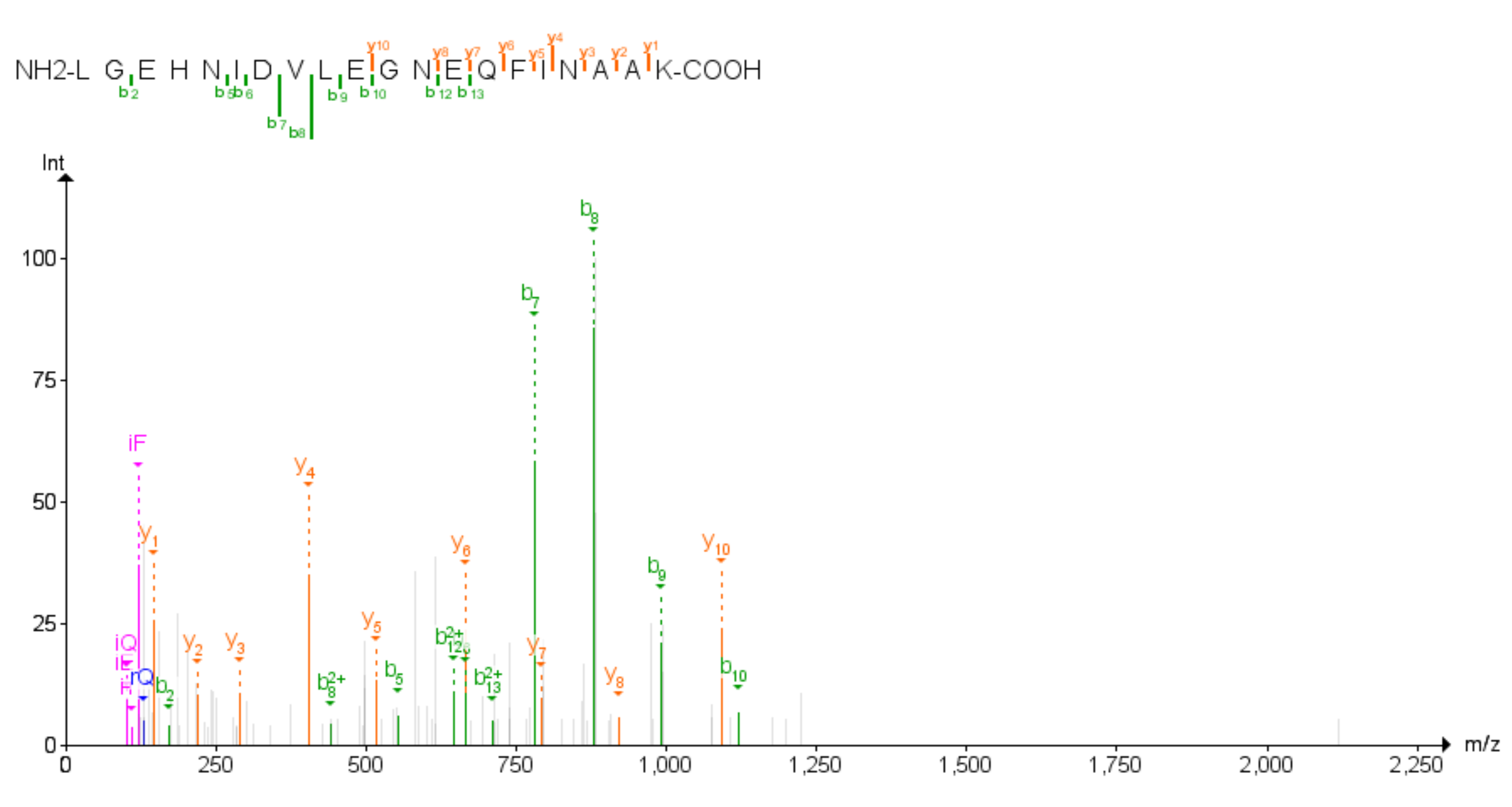

NH2-L G E H N I D V L E G N E Q F I N A A K-COOH
b2 b5 b6 b7 b8 b9 b10 b12 b13
y10 y8 y7 y6 y5 y4 y3 y2 y1
Int
100
75
50
25
0
m/z
0
250
500
750
1,000
1,250
1,500
1,750
2,000
2,250
b8
b7
iF
y4
y1
y6
y10
b9
y5
iQ
y2
y3
rQ
b2
b5
y7
y8
b10

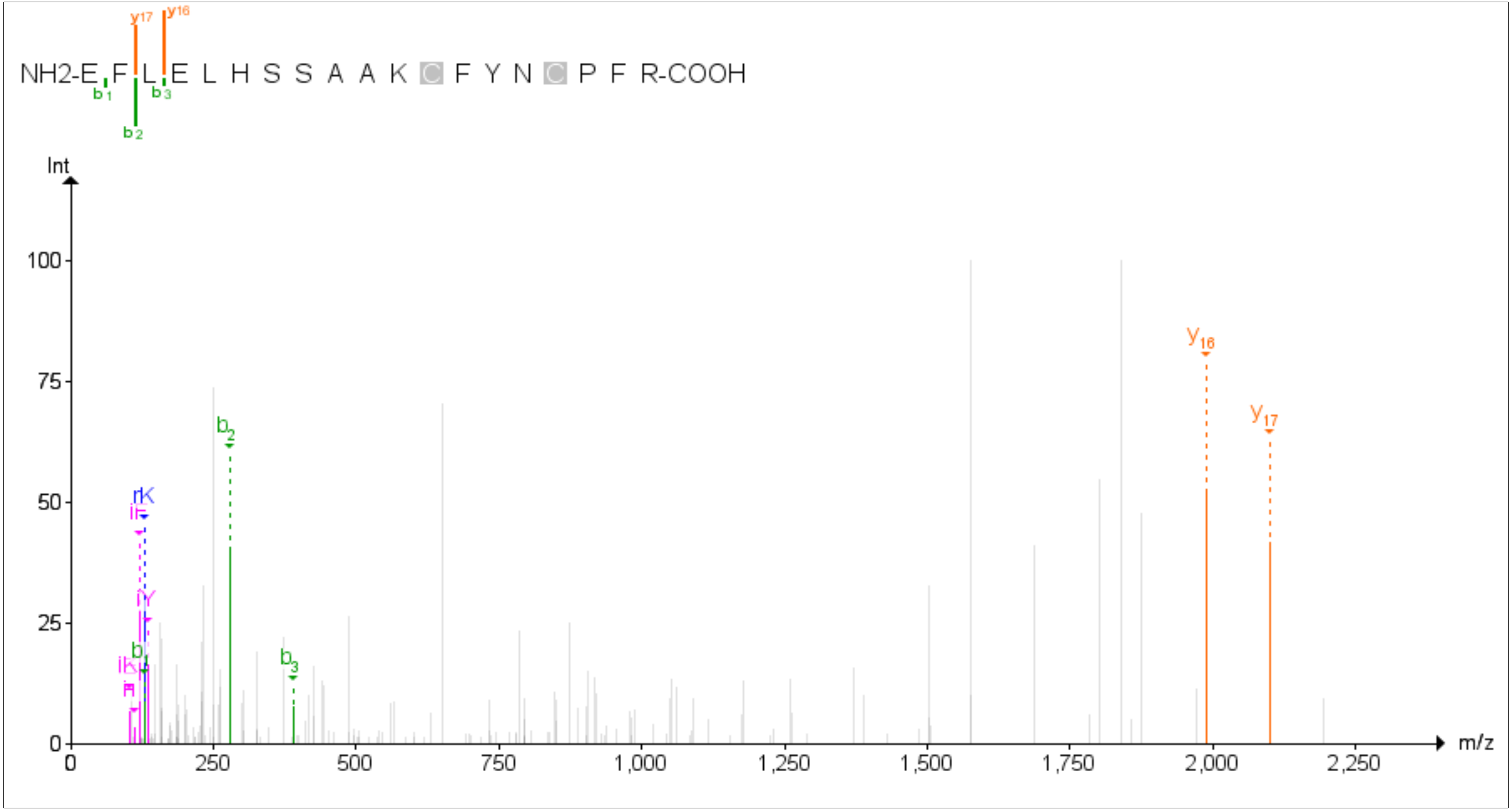

NH2-E F L E L H S S A A K C F Y N C P F R-COOH
y17
y16
b1
b2
b3
Int
100
75
50
25
0
m/z
0
250
500
750
1,000
1,250
1,500
1,750
2,000
2,250
y16
y17
b2
b3
rK
iF
iY
b
iK

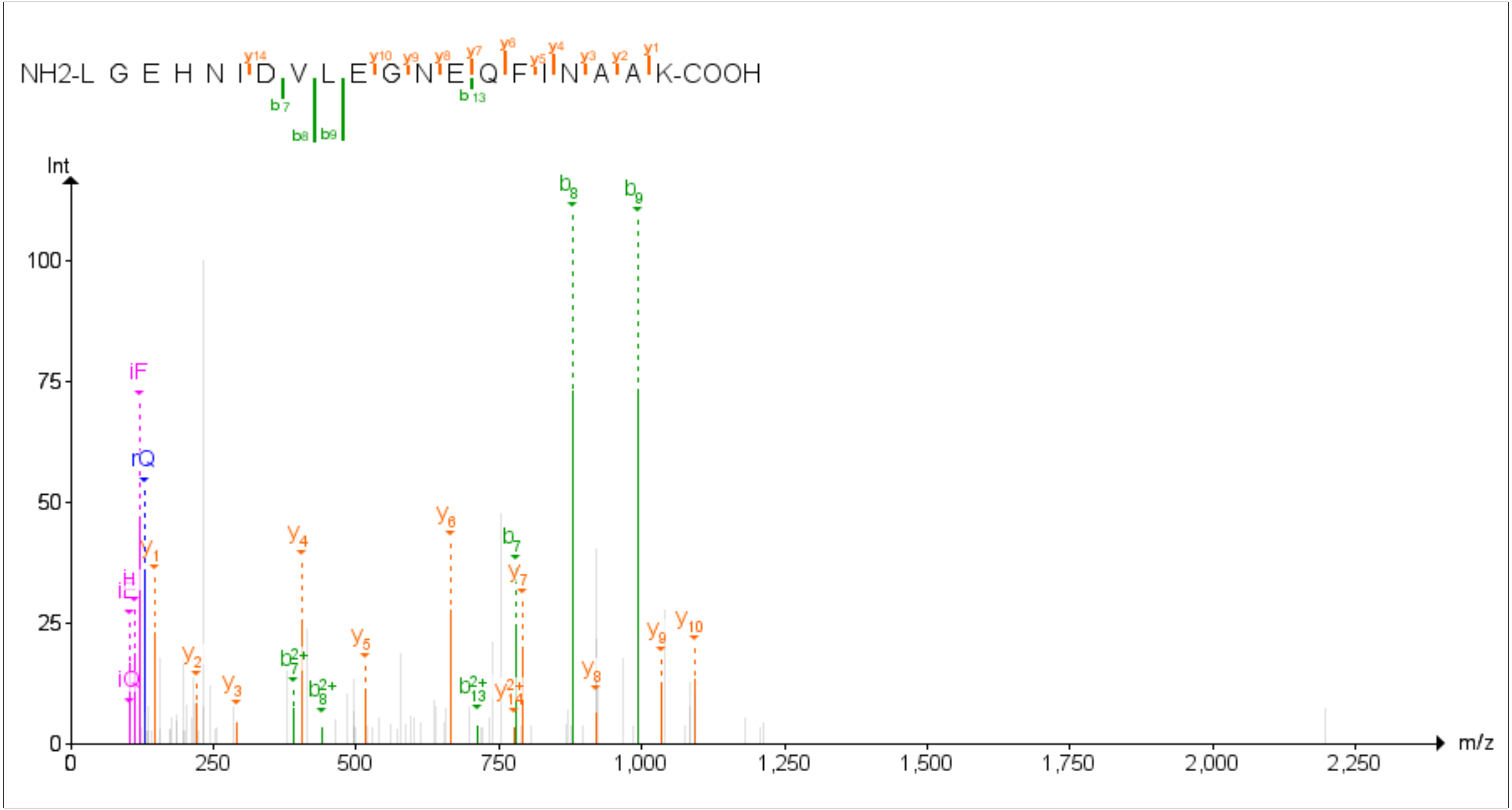

NH2-L G E H N I D V L E G N E Q F I N A A K-COOH
y14 y10 y9 y8 y7 y6 y5 y4 y3 y2 y1
b7 b8 b9 b13
Int
100
75
50
25
0
iF
rQ
iH
iL
iQ
y1 y2 y3 y4 y5 y6 y7 y8 y9 y10
b8 b9 b7
b7 2+ b8 2+ b13 2+ y14 2+
0 250 500 750 1,000 1,250 1,500 1,750 2,000 2,250
m/z

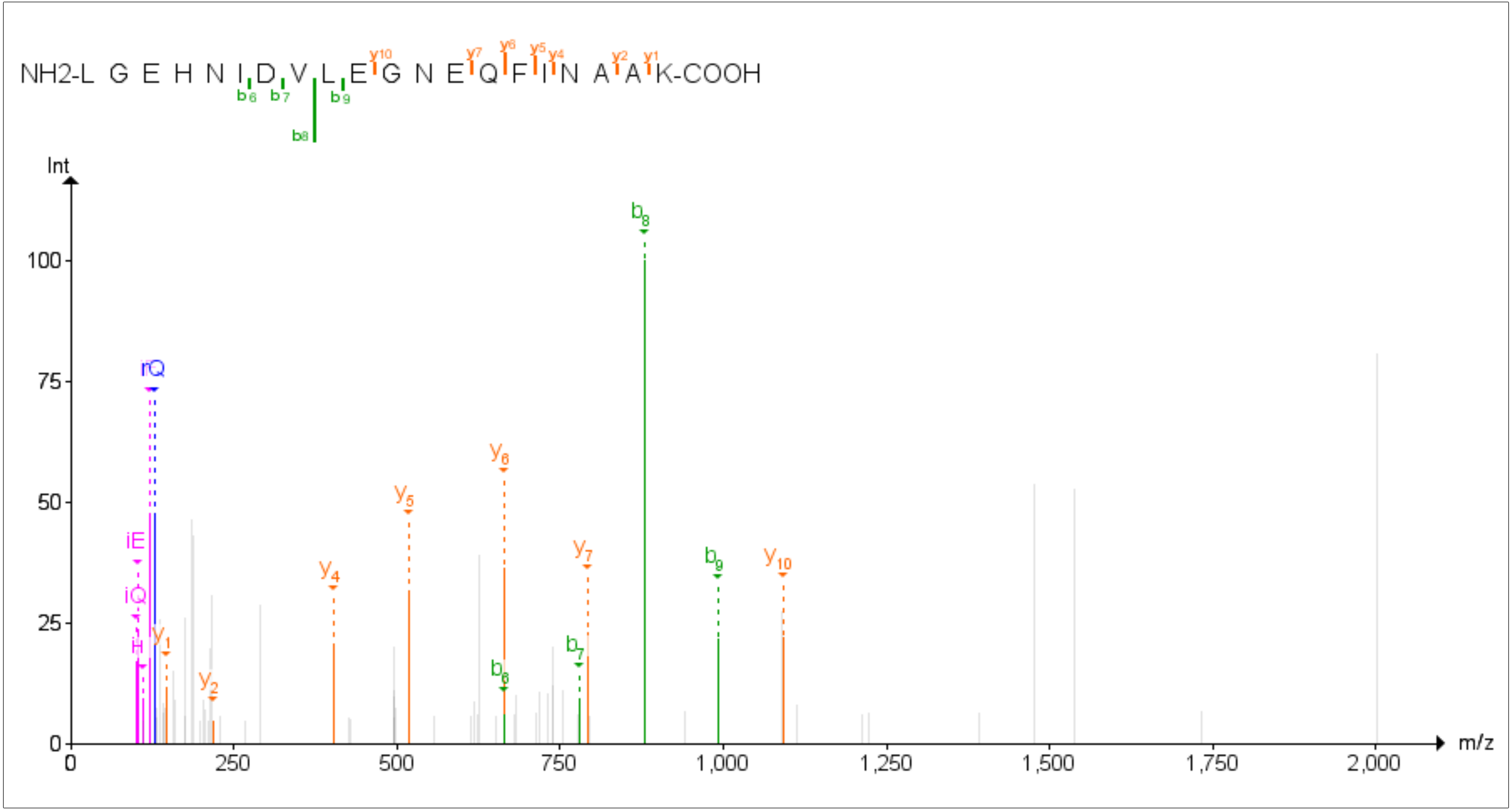

NH2-L G E H N I D V L E G N E Q F I N A A K-COOH
b6 b7 b8 b9
y10 y7 y6 y5 y4 y2 y1
Int
m/z
0
25
50
75
100
0
250
500
750
1,000
1,250
1,500
1,750
2,000
rQ
iE
iQ
iH
y1
y2
y4
y5
y6
y7
y10
b6
b7
b8
b9

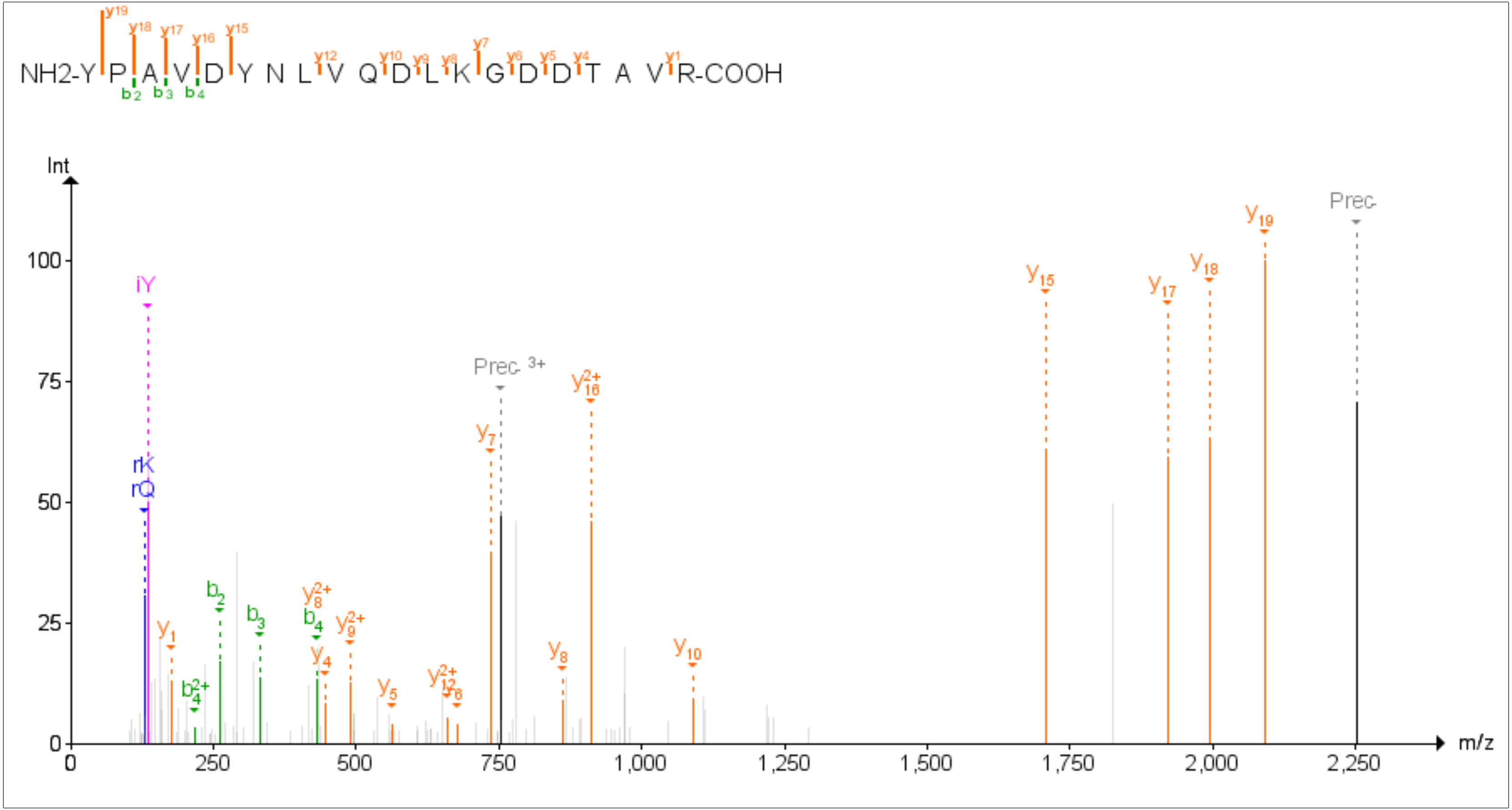

NH2-Y P A V D Y N L V Q D L K G D D T A V R-COOH
y19 y18 y17 y16 y15 y12 y10 y9 y8 y7 y6 y5 y4 y1
b2 b3 b4
Int
m/z
iY
rK
rQ
y1
b2
b3
b4
b4 2+
y8 2+
y9 2+
y4
y5
y12 2+
y6
y7
Prec 3+
y16 2+
y8
y10
y15
y17
y18
y19
Prec
0
25
50
75
100
0
250
500
750
1,000
1,250
1,500
1,750
2,000
2,250

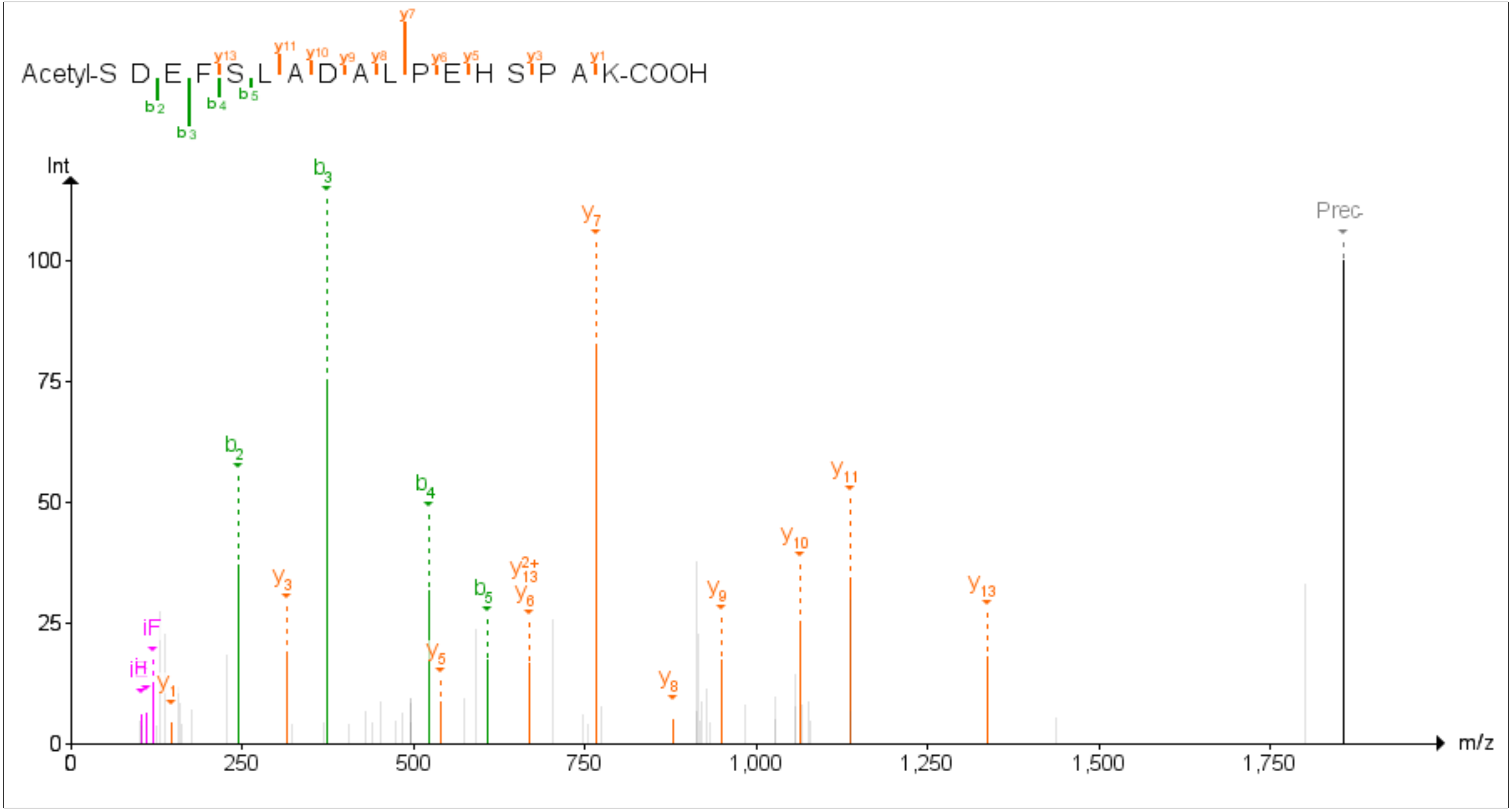

Acetyl-S D E F S L A D A L P E H S P A K-COOH
b2
b3
b4
b5
y13
y11
y10
y9
y8
y7
y6
y5
y3
y1
Int
100
75
50
25
0
m/z
250
500
750
1,000
1,250
1,500
1,750
Prec.
iF
iH
y2+13

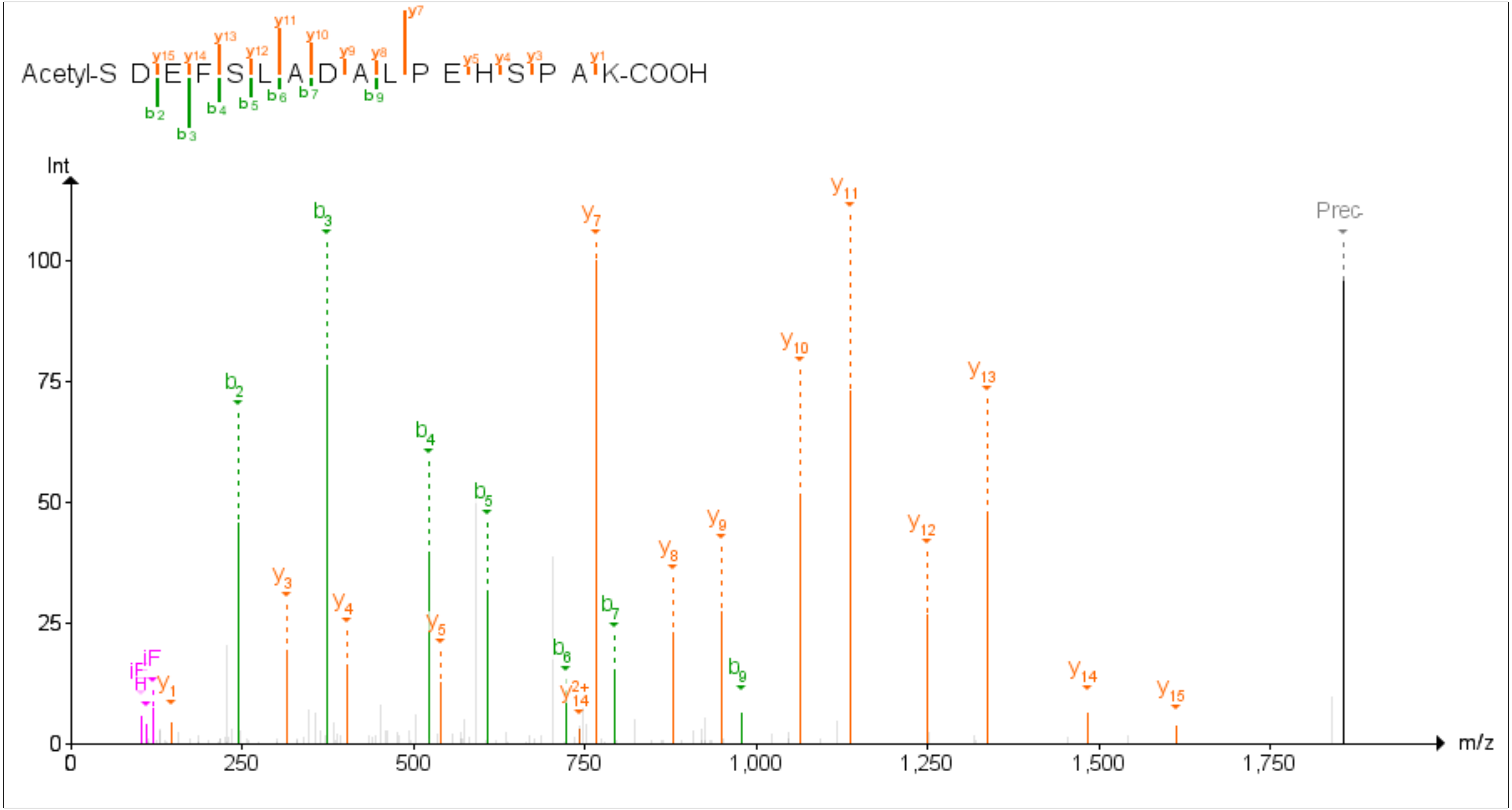

Acetyl-S D E F S L A D A L P E H S P A K-COOH
Int
m/z
Prec.
0
25
50
75
100
250
500
750
1,000
1,250
1,500
1,750

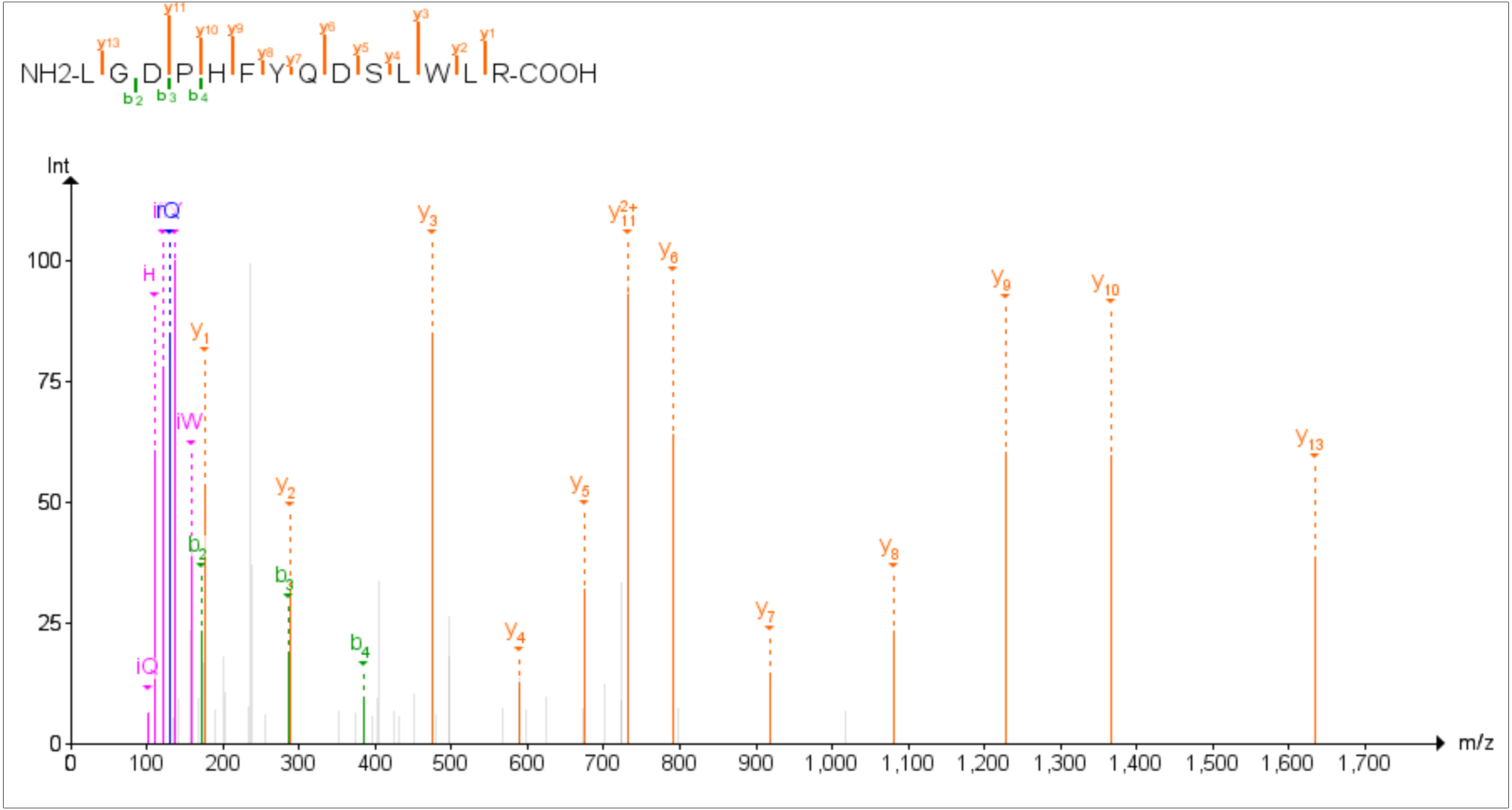

NH2-L G D P H F Y Q D S L W L R-COOH
y13 y11 y10 y9 y8 y7 y6 y5 y4 y3 y2 y1
b2 b3 b4
Int
100
75
50
25
0
iH
iW
iQ
y1
y2
y3
y4
y5
y6
y7
y8
y9
y10
y11 2+
y13
b2
b3
b4
0 100 200 300 400 500 600 700 800 900 1,000 1,100 1,200 1,300 1,400 1,500 1,600 1,700
m/z

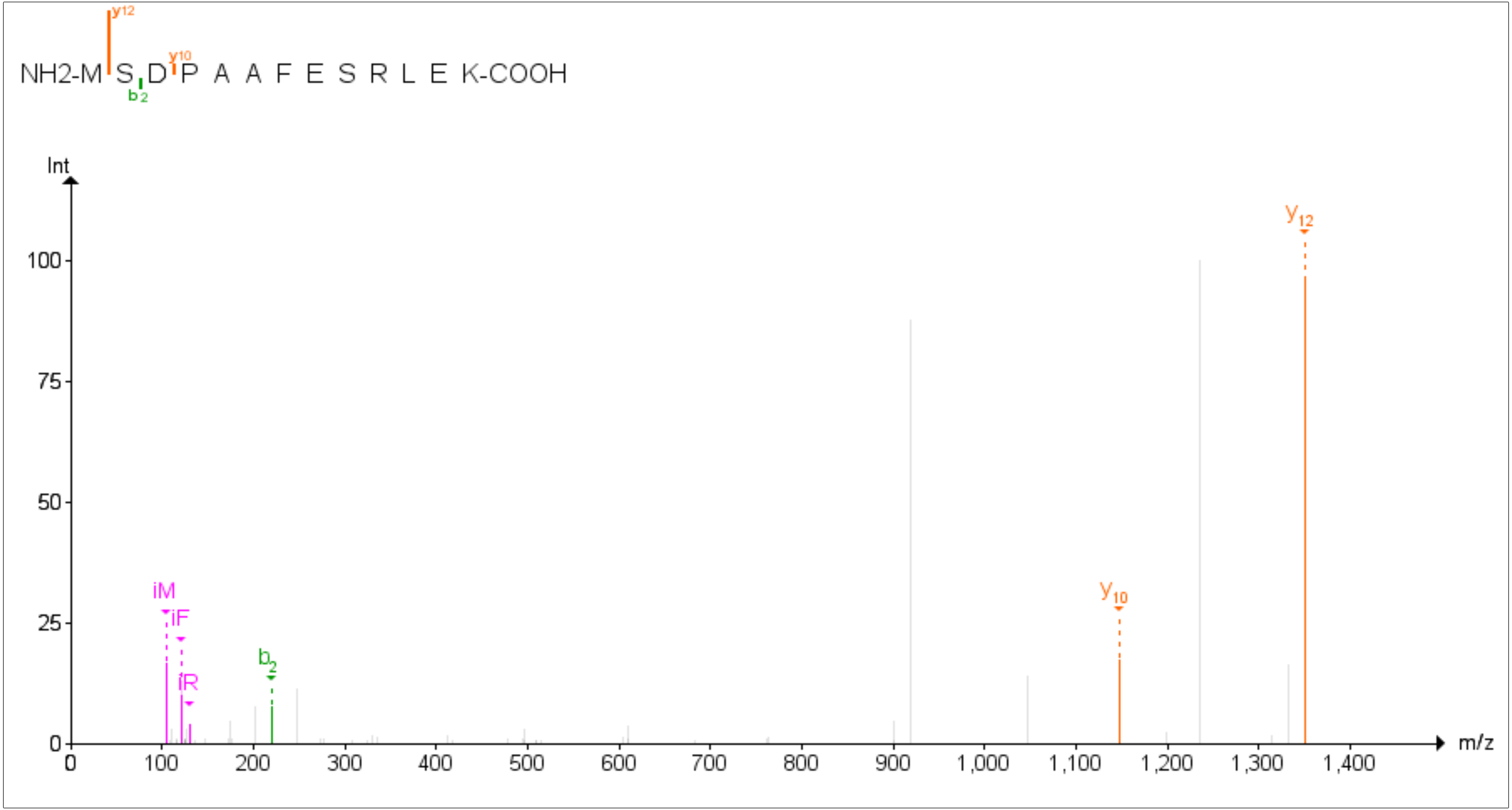

y12
y10
NH2-M S D P A A F E S R L E K-COOH
b2
Int
100
75
50
25
0
iM
iF
iR
b2
y10
y12
0
100
200
300
400
500
600
700
800
900
1,000
1,100
1,200
1,300
1,400
m/z

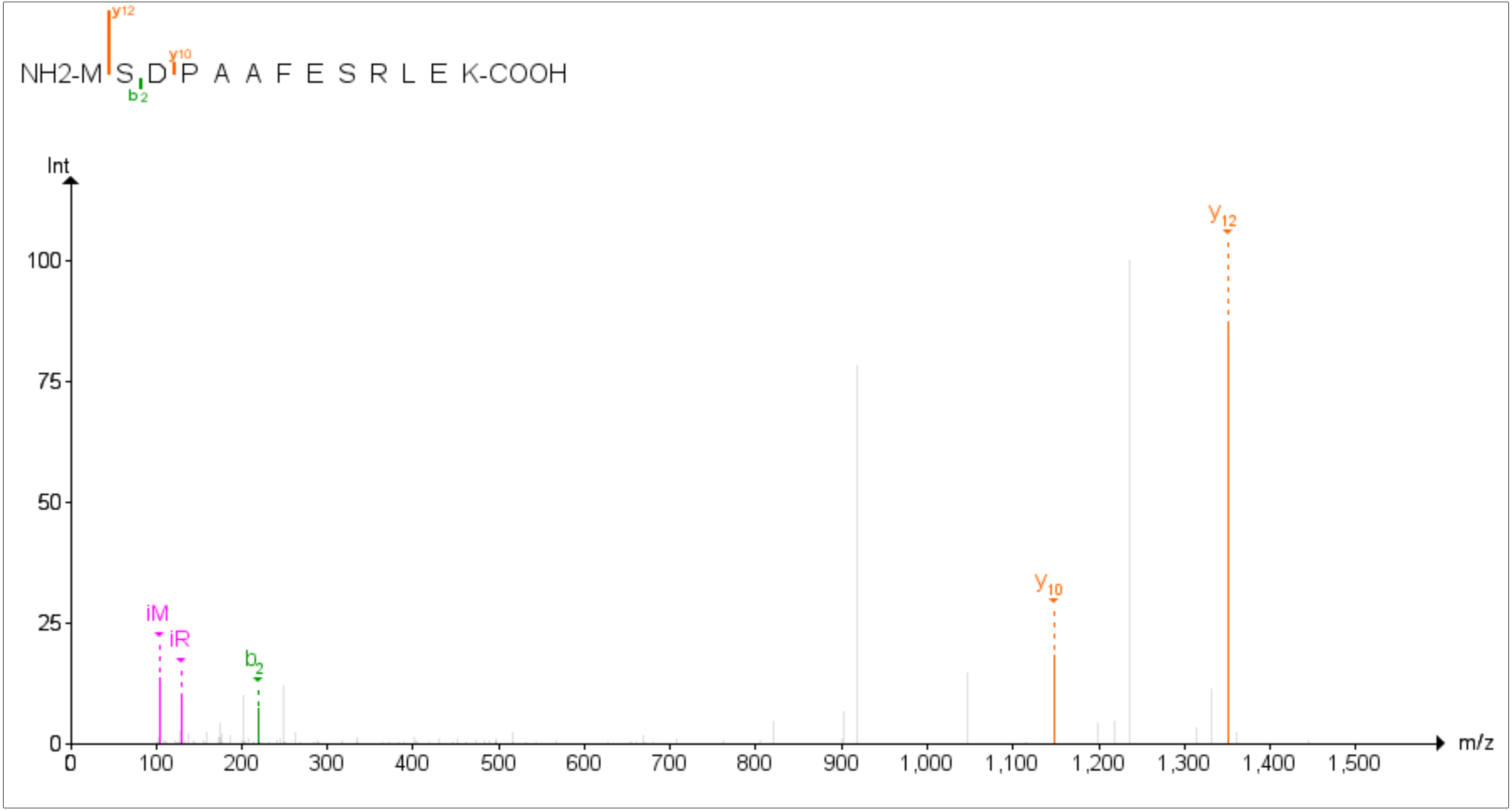

NH2-M S D P A A F E S R L E K-COOH
y12
y10
b2
Int
100
75
50
25
0
iM
iR
b2
y10
y12
0
100
200
300
400
500
600
700
800
900
1,000
1,100
1,200
1,300
1,400
1,500
m/z

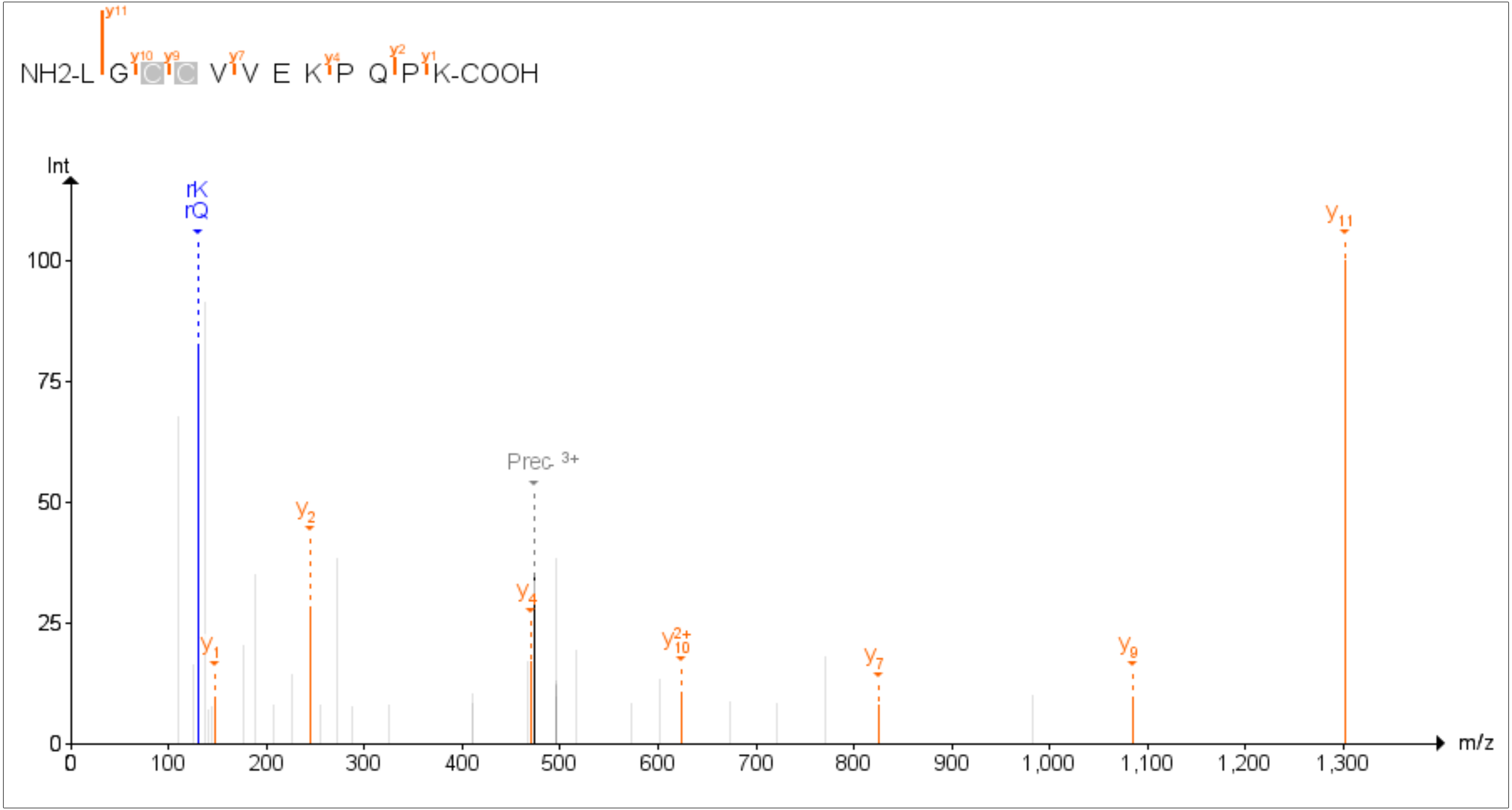
NH2-L G C C V V E K P Q P K-COOH
y11
y10
y9
y7
y4
y2
y1
Int
rK
rQ
Prec 3+
y2
y1
y4
y10 2+
y7
y9
y11
0
25
50
75
100
0
100
200
300
400
500
600
700
800
900
1,000
1,100
1,200
1,300
m/z

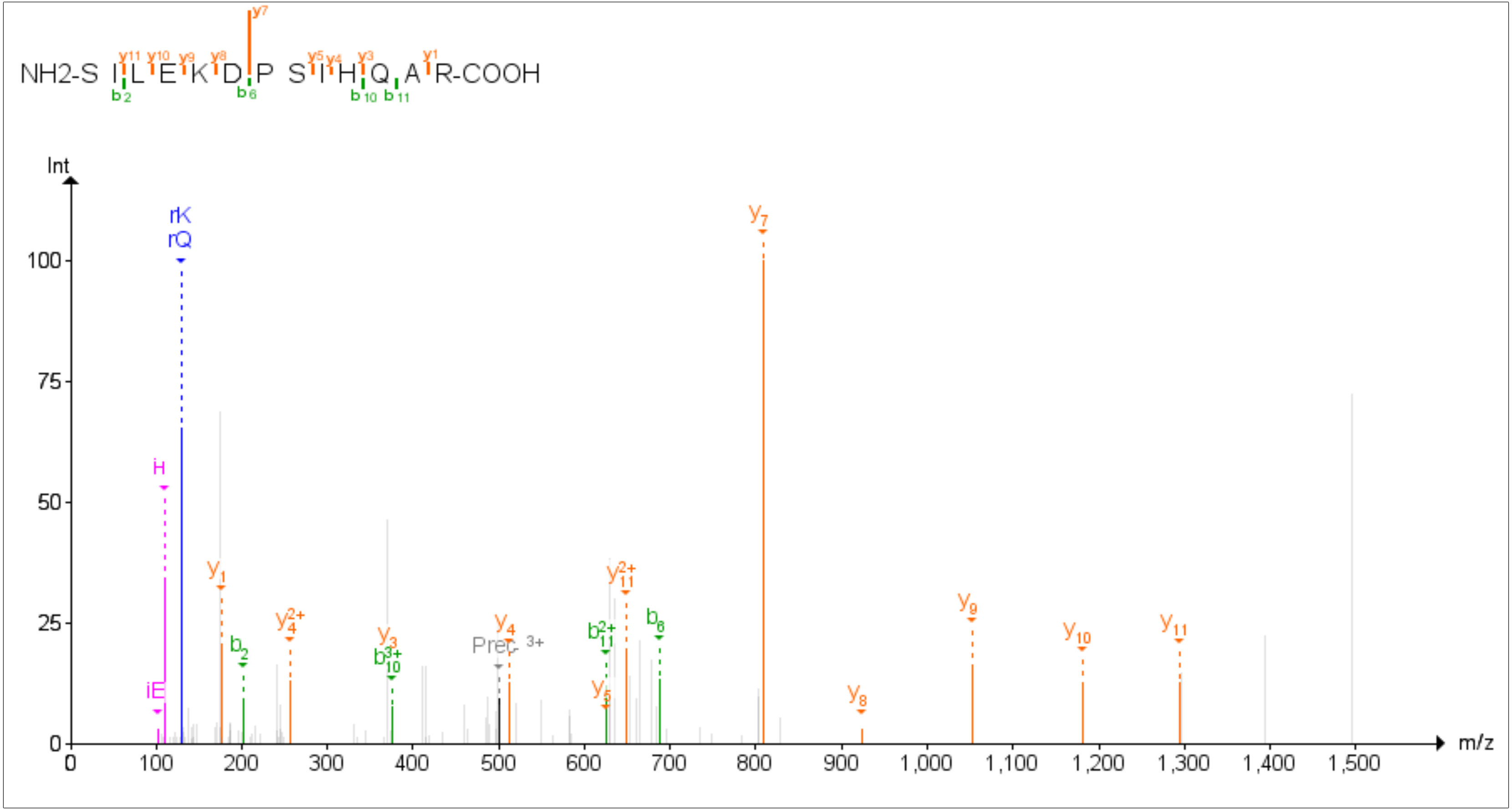
NH2-S I L E K D P S I H Q A R-COOH
y11 y10 y9 y8 y7 y5 y4 y3 y1
b2 b6 b10 b11
Int
100
75
50
25
0
rK
rQ
H
iE
y7
y1
b2
y4 2+
y3
b10 3+
Prec- 3+
y4
b11 2+
y11 2+
y5
b6
y8
y9
y10
y11
m/z
0
100
200
300
400
500
600
700
800
900
1,000
1,100
1,200
1,300
1,400
1,500

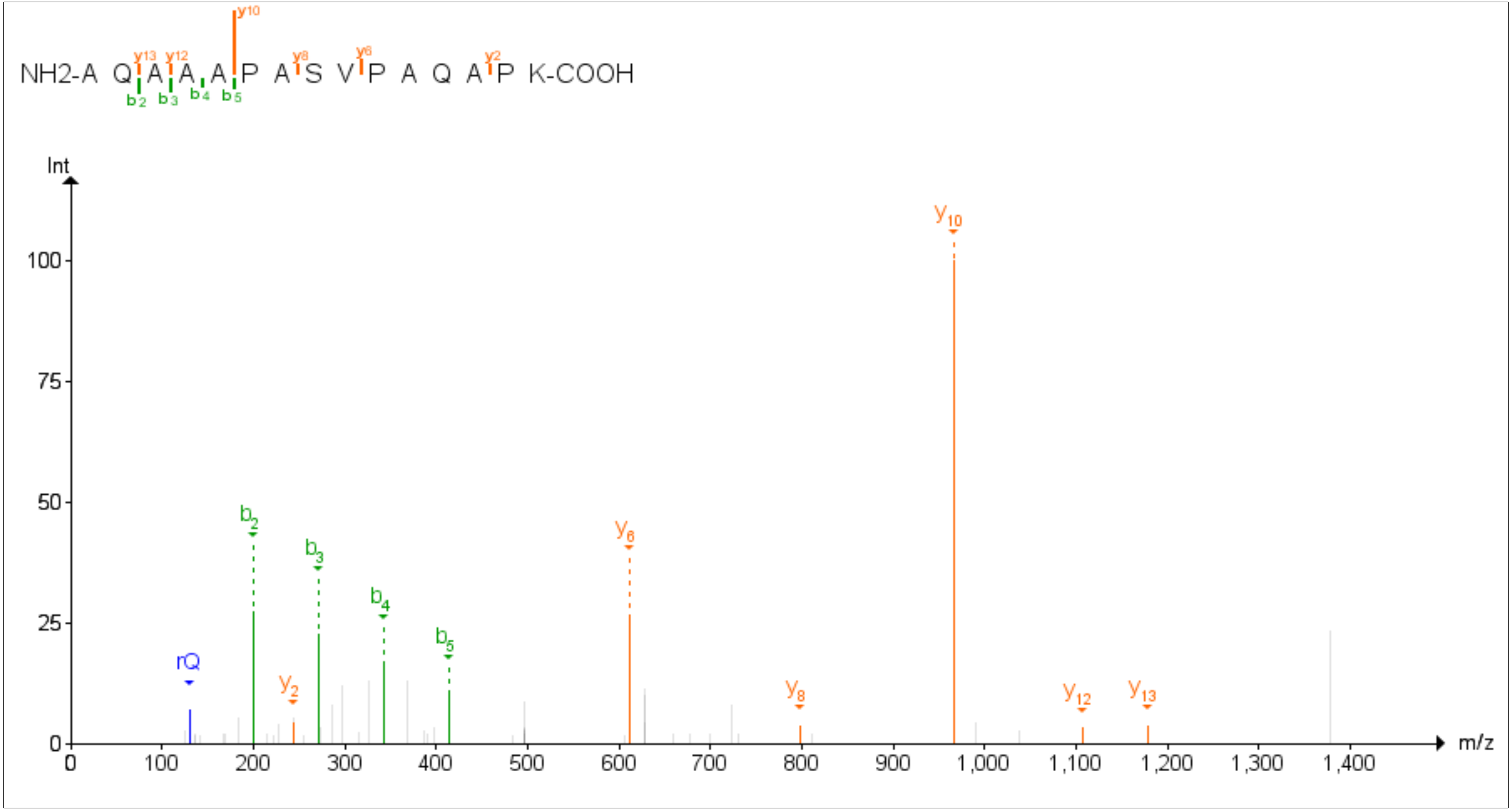

NH2-A Q A A A P A S V P A Q A P K-COOH
y10
y13 y12 y8 y6 y2
b2 b3 b4 b5
Int
100
75
50
25
0
rQ
y2
b2
b3
b4
b5
y6
y8
y10
y12
y13
0 100 200 300 400 500 600 700 800 900 1,000 1,100 1,200 1,300 1,400
m/z

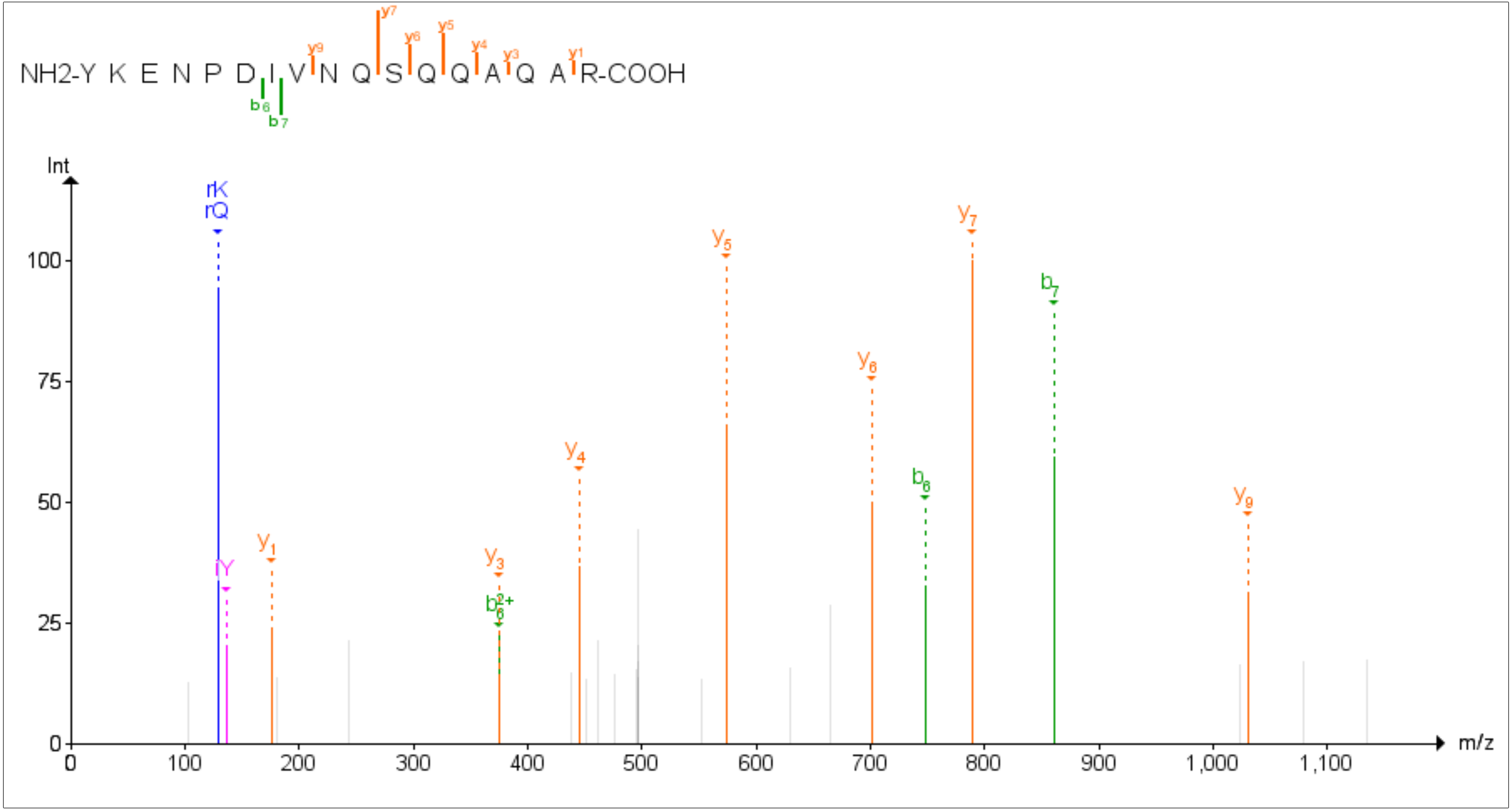

NH2-Y K E N P D I V N Q S Q Q A Q A R-COOH
y9 y7 y6 y5 y4 y3 y1
b6 b7
Int
rK
rQ
iY
y1 y3 y4 y5 y6 y7 y9
b6 b7
0
25
50
75
100
0 100 200 300 400 500 600 700 800 900 1,000 1,100
m/z

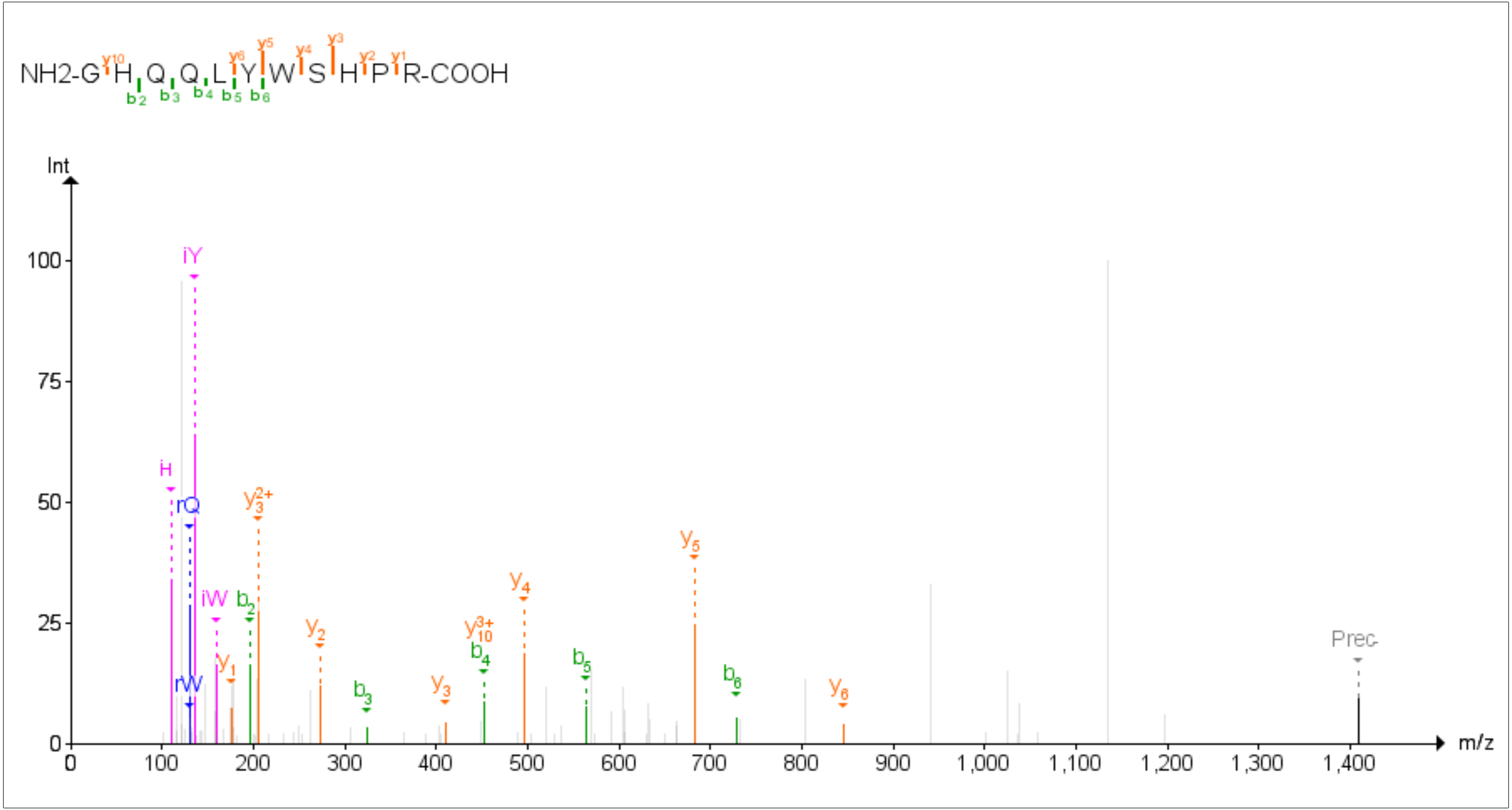

NH2-G H Q Q L Y W S H P R-COOH
y10 y6 y5 y4 y3 y2 y1
b2 b3 b4 b5 b6
Int
100
75
50
25
0
iY
iH
rQ
iW
rW
b2
y3 2+
y1
y2
b3
y3
y10 3+
b4
y4
b5
y5
b6
y6
Prec
0
100
200
300
400
500
600
700
800
900
1,000
1,100
1,200
1,300
1,400
m/z

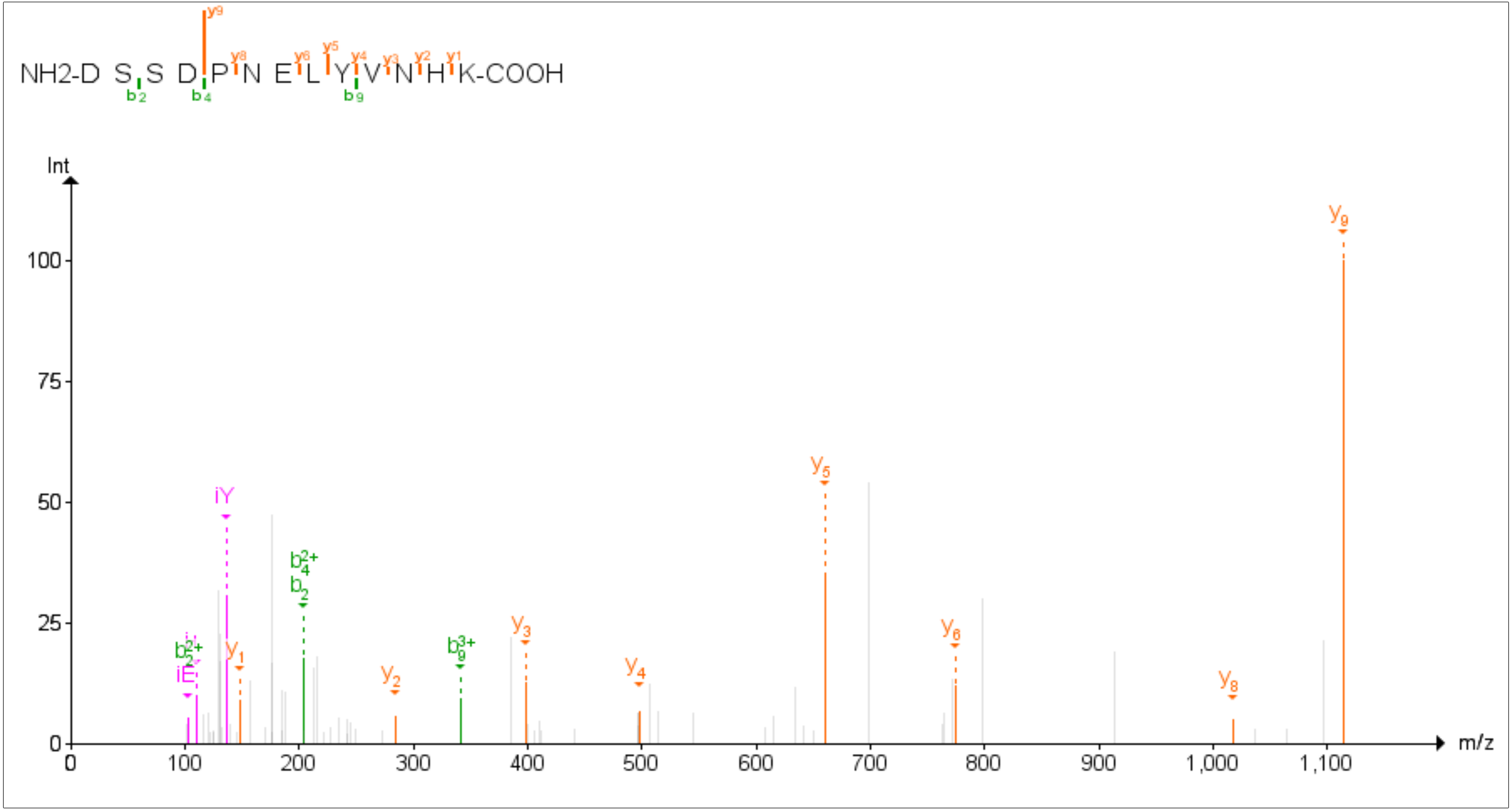

NH2-D S S D P N E L Y V N H K-COOH
y9 y8 y6 y5 y4 y3 y2 y1
b2 b4 b9
Int
m/z
0
25
50
75
100
0 100 200 300 400 500 600 700 800 900 1,000 1,100
iY
iE
b_4^{2+}
b_2
b_2^{2+}
b_9^{3+}
y_1
y_2
y_3
y_4
y_5
y_6
y_8
y_9

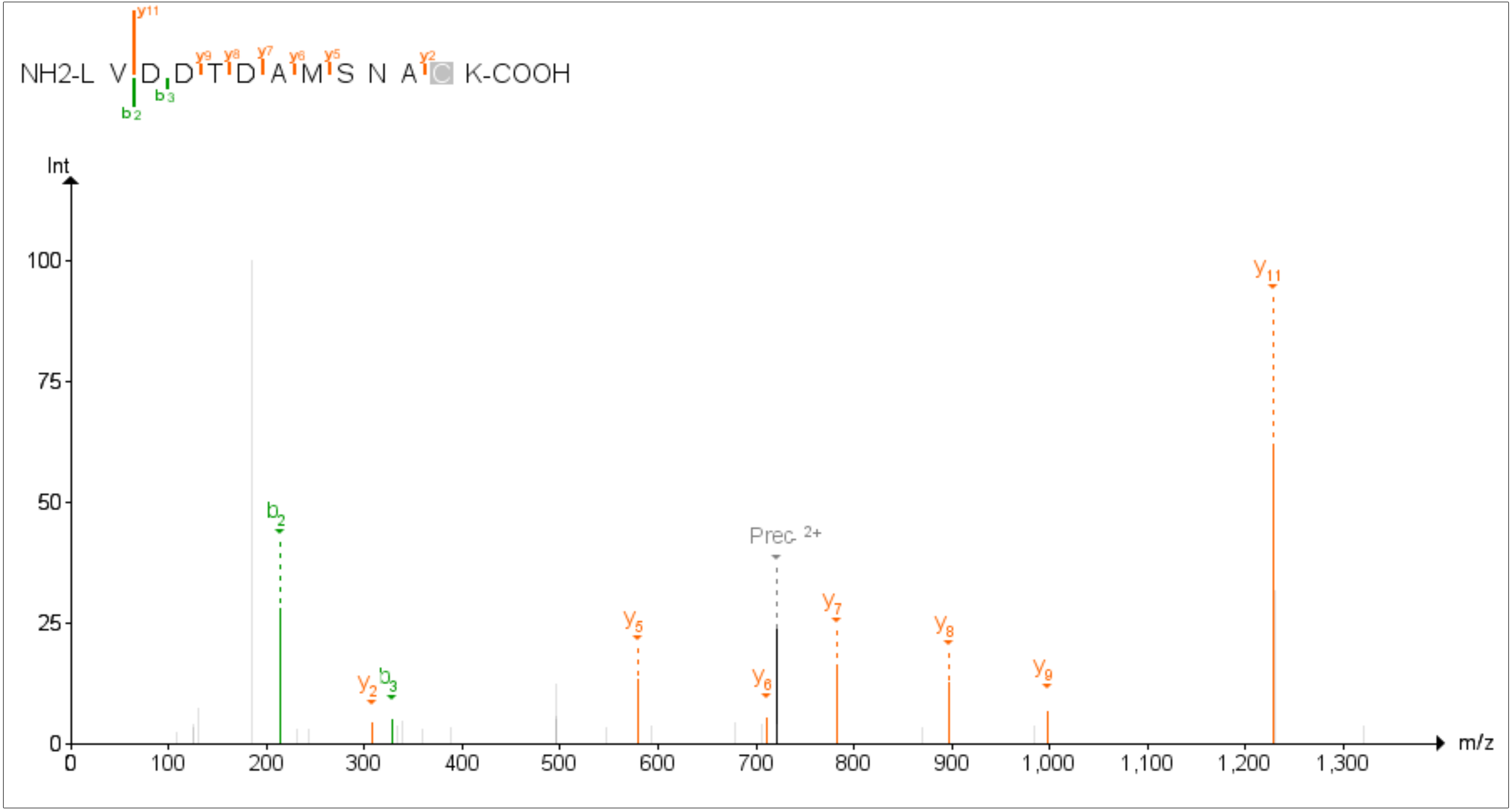

NH2-L V D D T D A M S N A C K-COOH
y11
y9
y8
y7
y6
y5
y2
b2
b3
Int
100
75
50
25
0
y11
b2
Prec 2+
y5
y7
y8
y9
y6
y2
b3
0
100
200
300
400
500
600
700
800
900
1,000
1,100
1,200
1,300
m/z

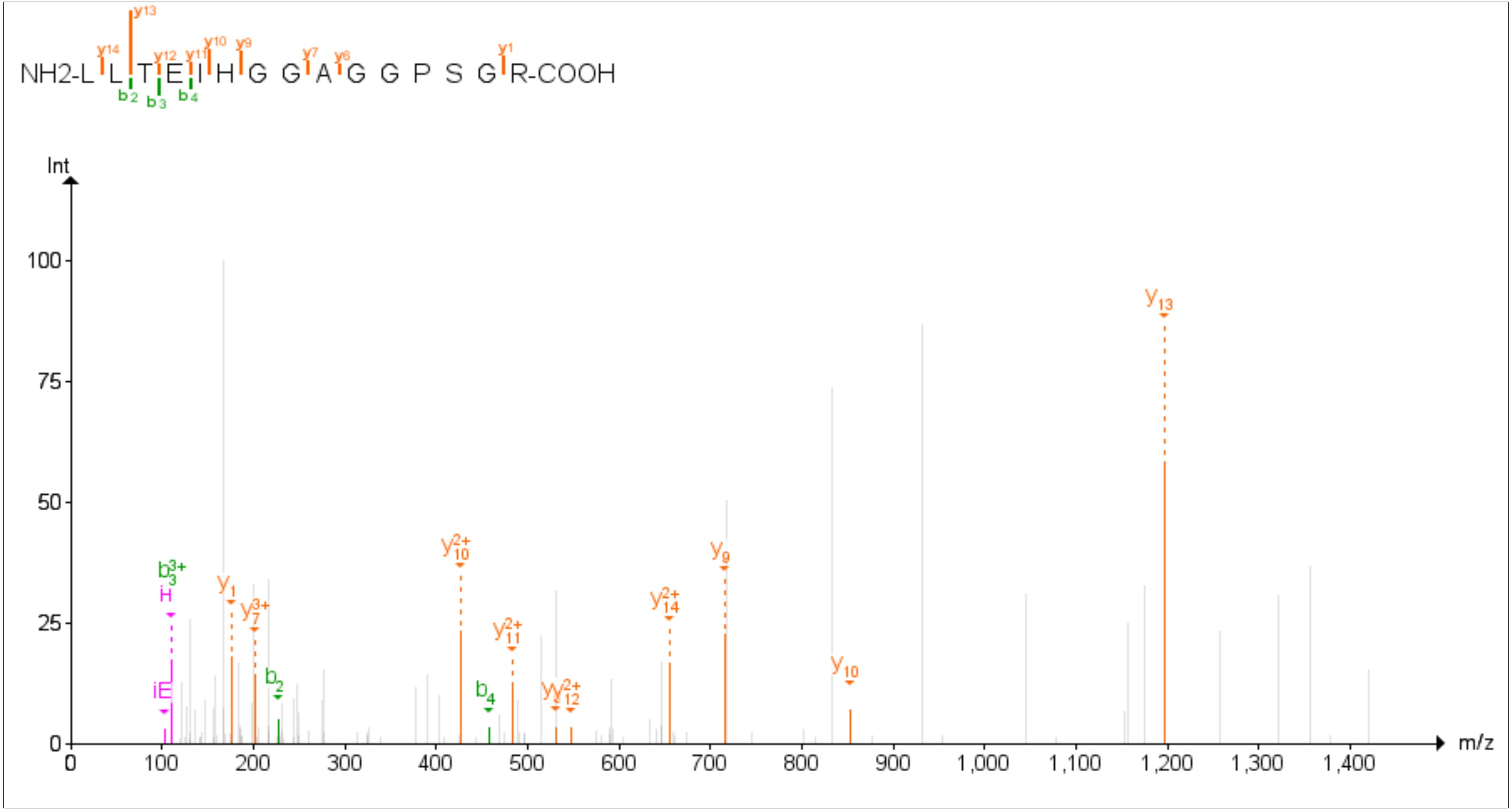

NH2-L L T E I H G G A G G P S G R-COOH
Int
m/z
y13
y14
y12
y11
y10
y9
y7
y6
y1
b2
b3
b4
b_3^{3+}
iH
iE
y_1
y_7^{3+}
b_2
y_{10}^{2+}
b_4
y_{11}^{2+}
y_{12}^{2+}
y_{14}^{2+}
y_9
y_{10}
y_{13}
0
25
50
75
100
0
100
200
300
400
500
600
700
800
900
1,000
1,100
1,200
1,300
1,400

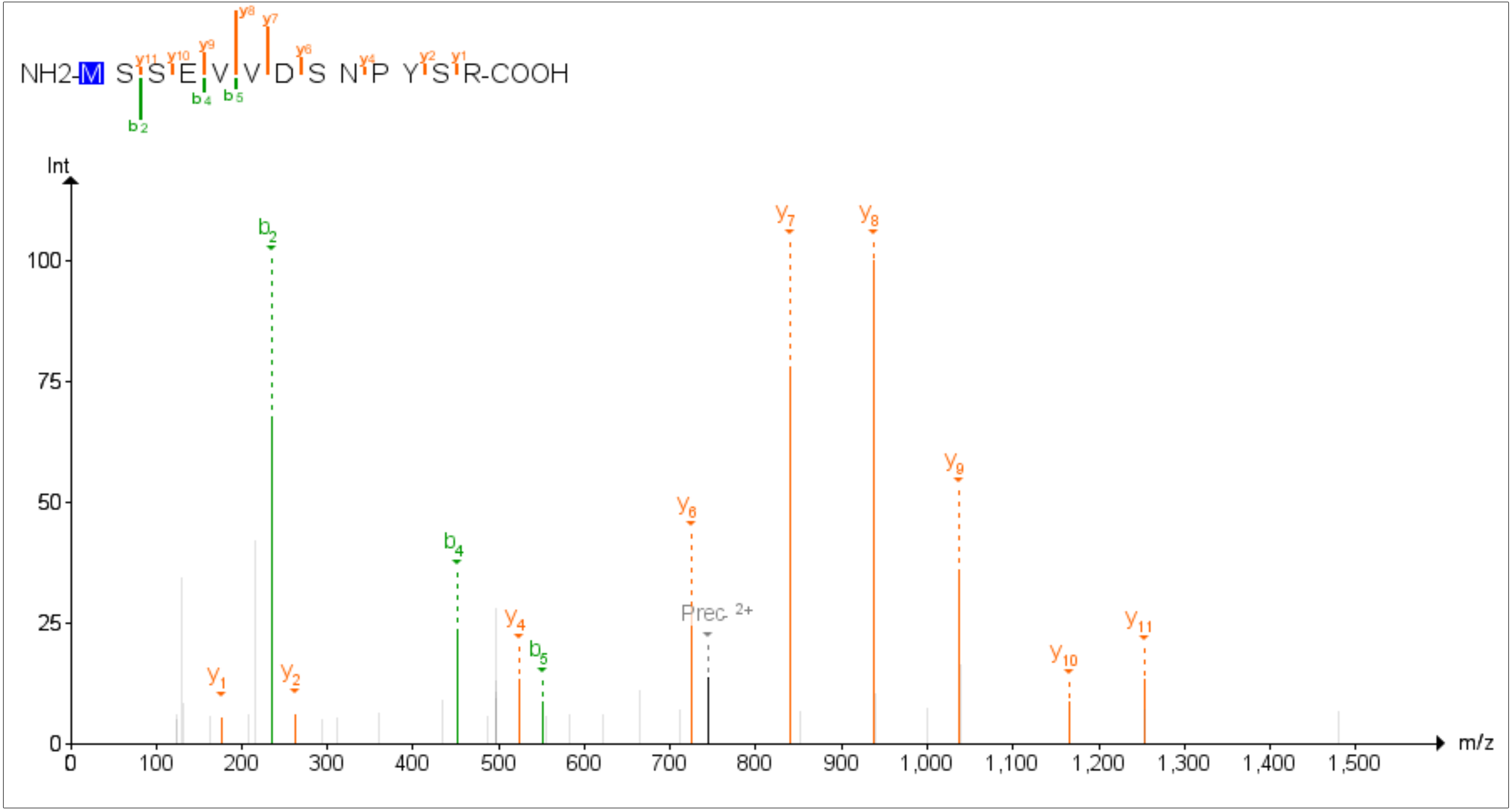
NH2-M S S E V V D S N P Y S R-COOH
Int
m/z
0
25
50
75
100
0
100
200
300
400
500
600
700
800
900
1,000
1,100
1,200
1,300
1,400
1,500
b2
b4
b5
y1
y2
y4
y6
y7
y8
y9
y10
y11
Prec 2+

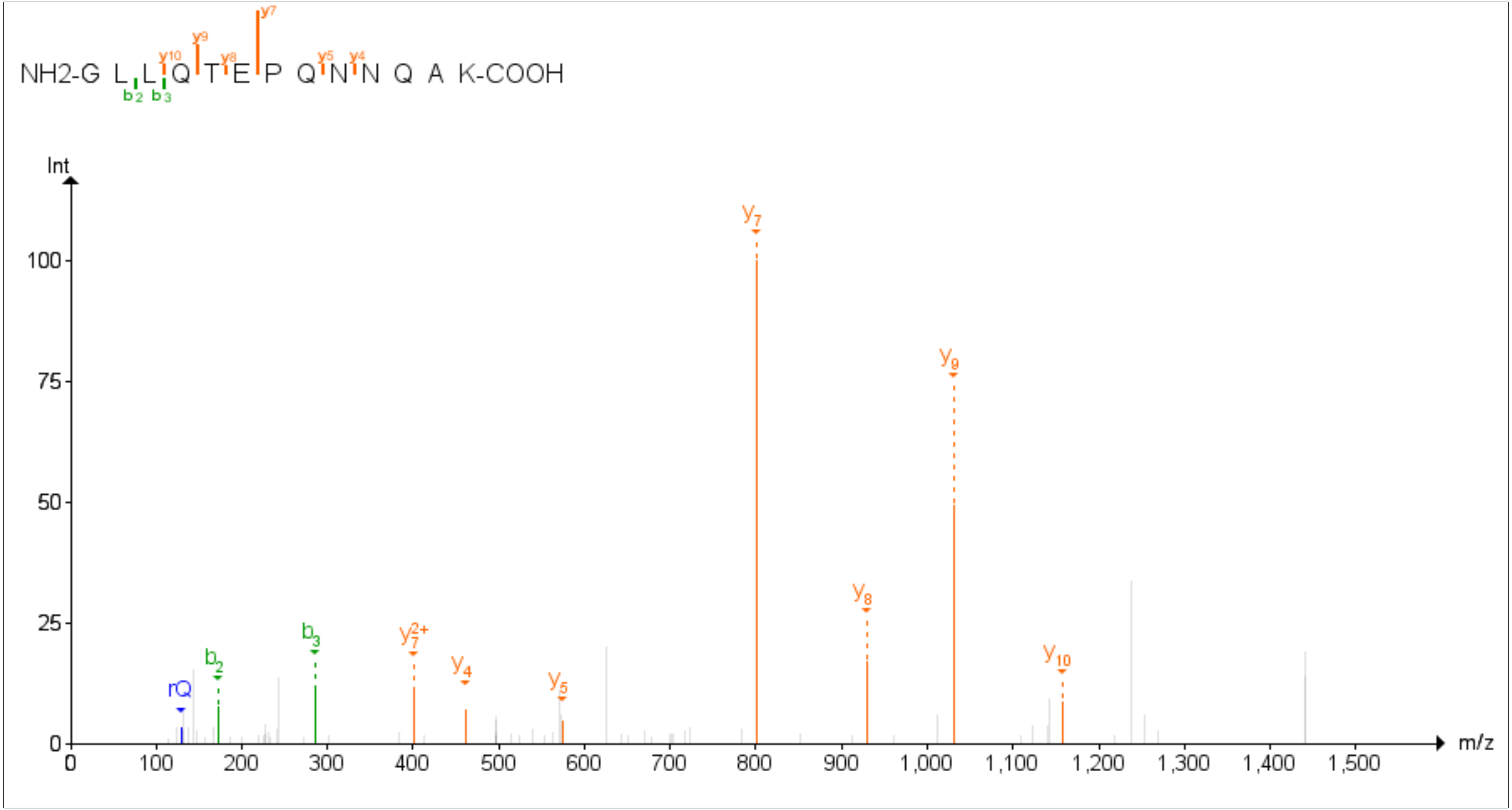

NH2-G L L Q T E P Q N N Q A K-COOH
b2 b3
y10 y9 y8 y7 y5 y4
Int
100
75
50
25
0
rQ
b2
b3
y7 2+
y4
y5
y7
y8
y9
y10
m/z
0
100
200
300
400
500
600
700
800
900
1,000
1,100
1,200
1,300
1,400
1,500

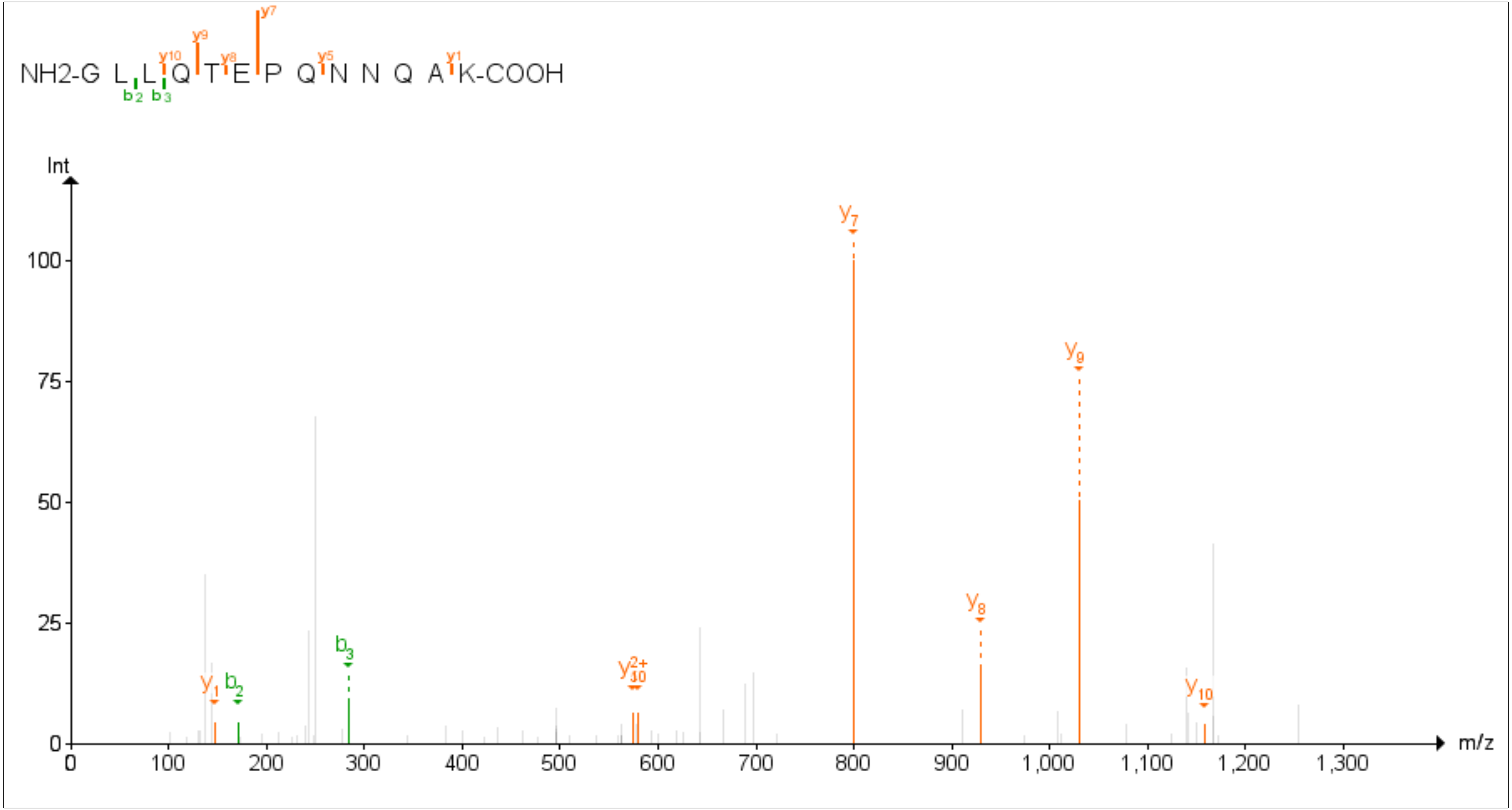
NH2-G L L Q T E P Q N N Q A K-COOH
y7
y9
y10
y8
y5
y1
b2
b3
Int
100
75
50
25
0
y7
y9
y8
b3
y1
b2
y10 2+
y10
0
100
200
300
400
500
600
700
800
900
1,000
1,100
1,200
1,300
m/z

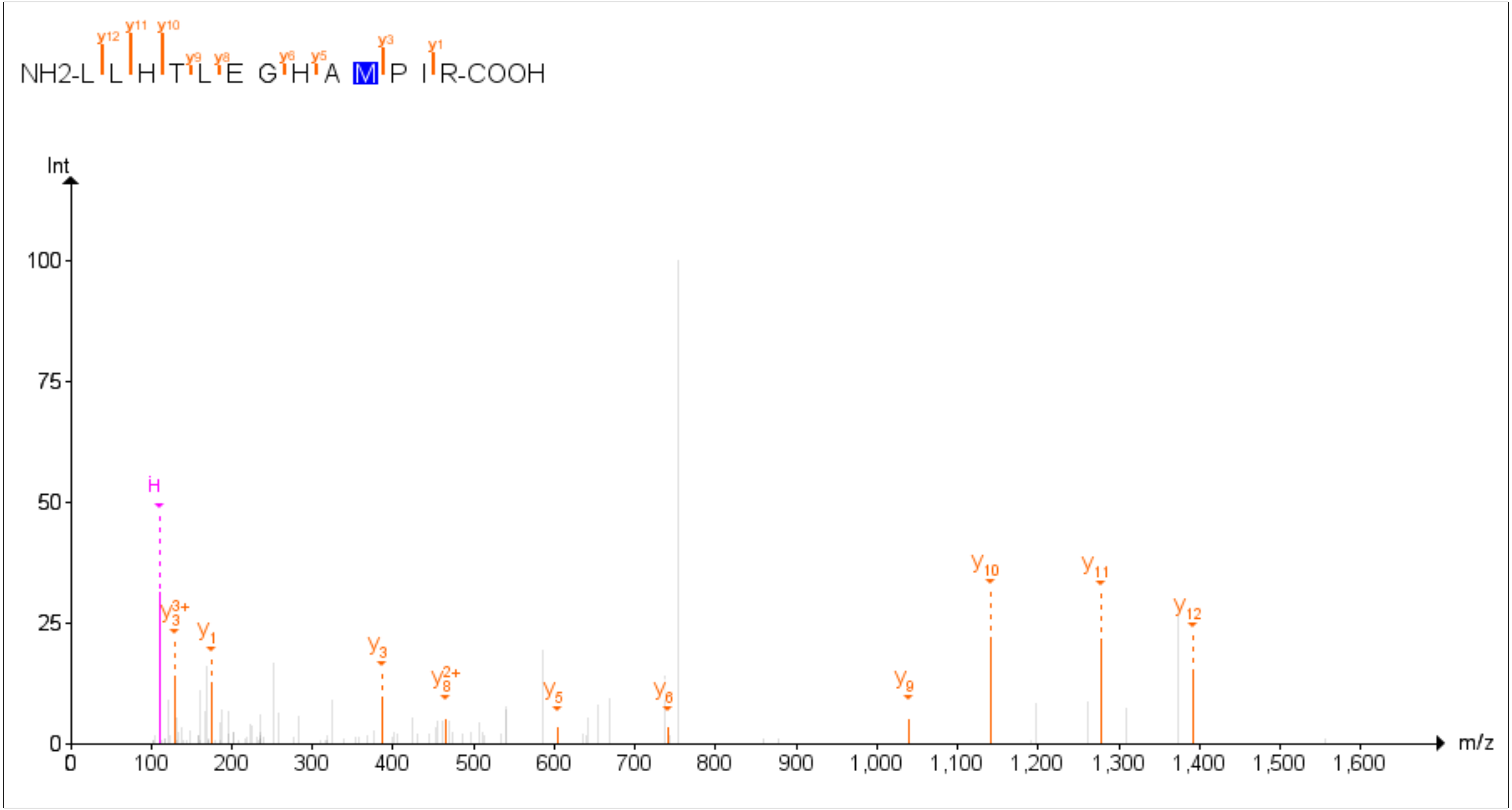

NH2-L L H T L E G H A M P I R-COOH
y12 y11 y10 y9 y8 y6 y5 y3 y1
Int
100
75
50
25
0
m/z
0 100 200 300 400 500 600 700 800 900 1,000 1,100 1,200 1,300 1,400 1,500 1,600
H
y3 3+
y1
y3
y8 2+
y5
y6
y9
y10
y11
y12

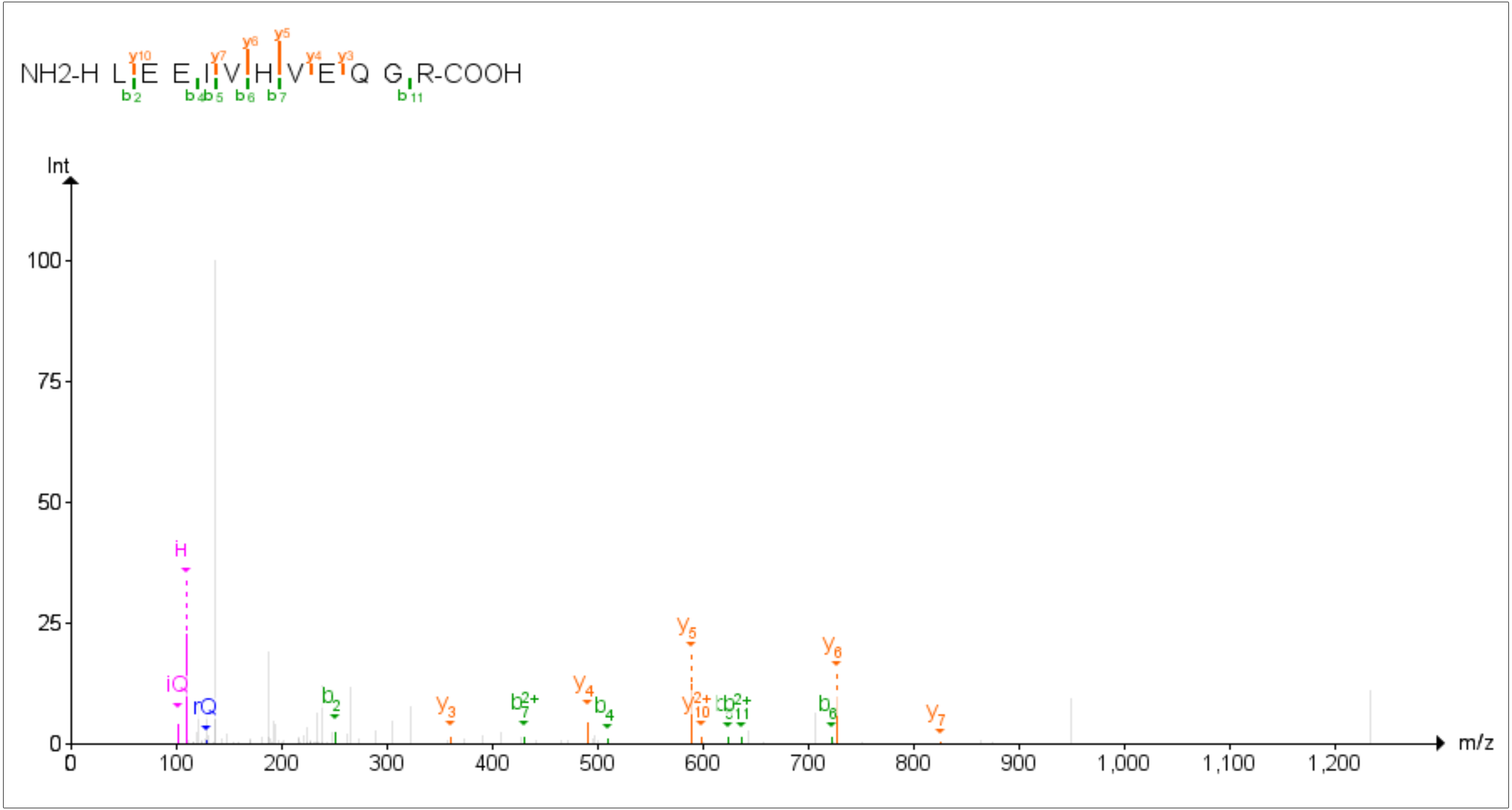

NH2-H L E E I V H V E Q G R-COOH
y10
y7
y6
y5
y4
y3
b2
b4
b5
b6
b7
b11
Int
100
75
50
25
0
iH
iQ
rQ
b2
y3
b7 2+
y4
b4
y5
y10 2+
b9 2+
b11 2+
b6
y6
y7
0
100
200
300
400
500
600
700
800
900
1,000
1,100
1,200
m/z

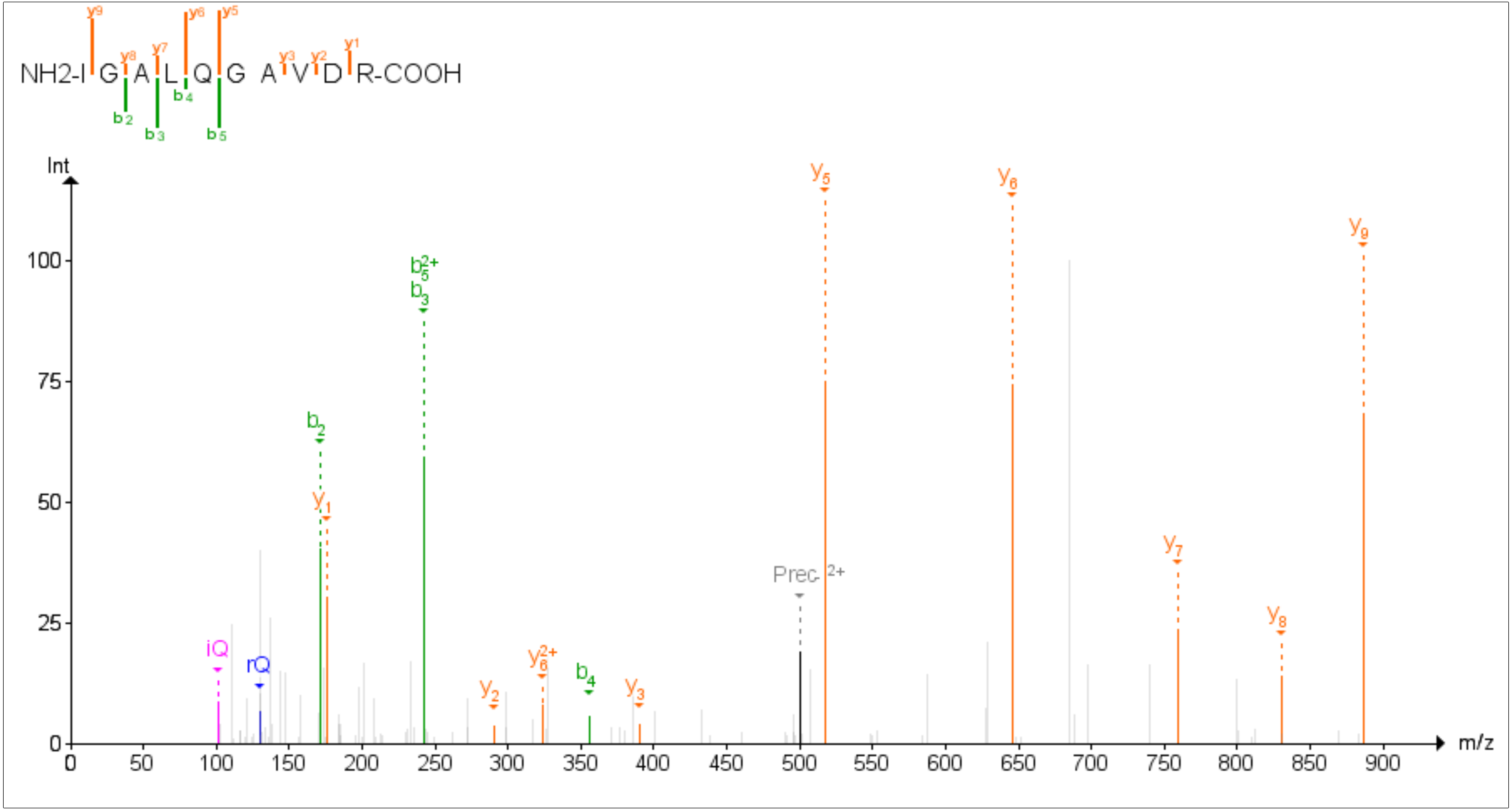

NH2-I G A L Q G A V D R-COOH
y9 y8 y7 y6 y5 y3 y2 y1
b2 b3 b4 b5
Int
100
75
50
25
0
b5 2+
b3
b2
y1
iQ
rQ
y2
y6 2+
b4
y3
Prec 2+
y5
y6
y7
y8
y9
0 50 100 150 200 250 300 350 400 450 500 550 600 650 700 750 800 850 900
m/z

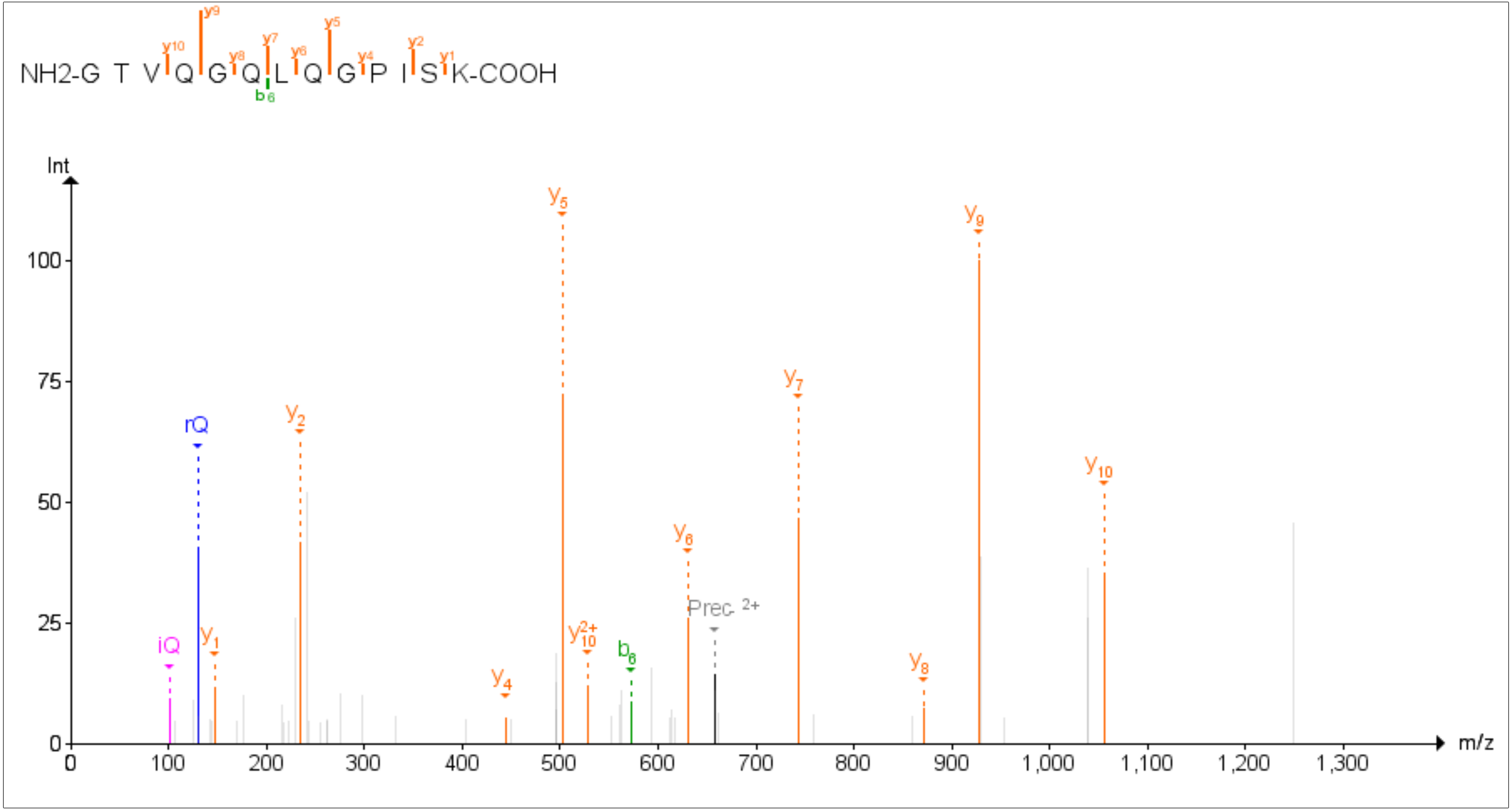

NH2-G T V Q G Q L Q G P I S K-COOH
y10 y9 y8 y7 y6 y5 y4 y2 y1
b6
Int
100
75
50
25
0
y5
y9
y7
rQ
y2
y10
y6
Prec 2+
iQ
y1
y4
y10 2+
b6
y8
0 100 200 300 400 500 600 700 800 900 1,000 1,100 1,200 1,300
m/z

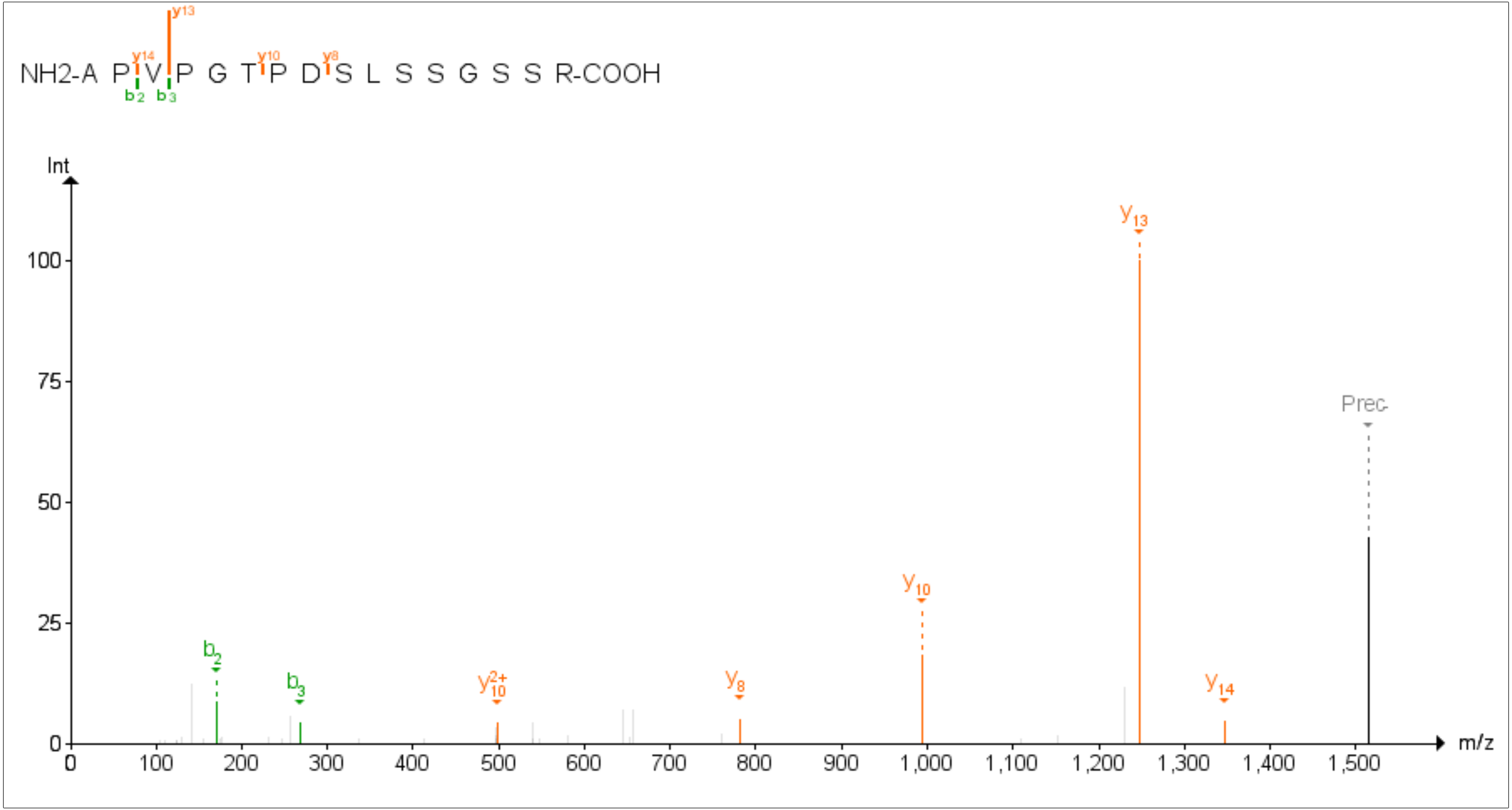
NH2-A P V P G T P D S L S S G S S R-COOH
y14
y13
y10
y8
b2
b3
Int
100
75
50
25
0
y13
Prec.
y10
b2
b3
y10 2+
y8
y14
0
100
200
300
400
500
600
700
800
900
1,000
1,100
1,200
1,300
1,400
1,500
m/z

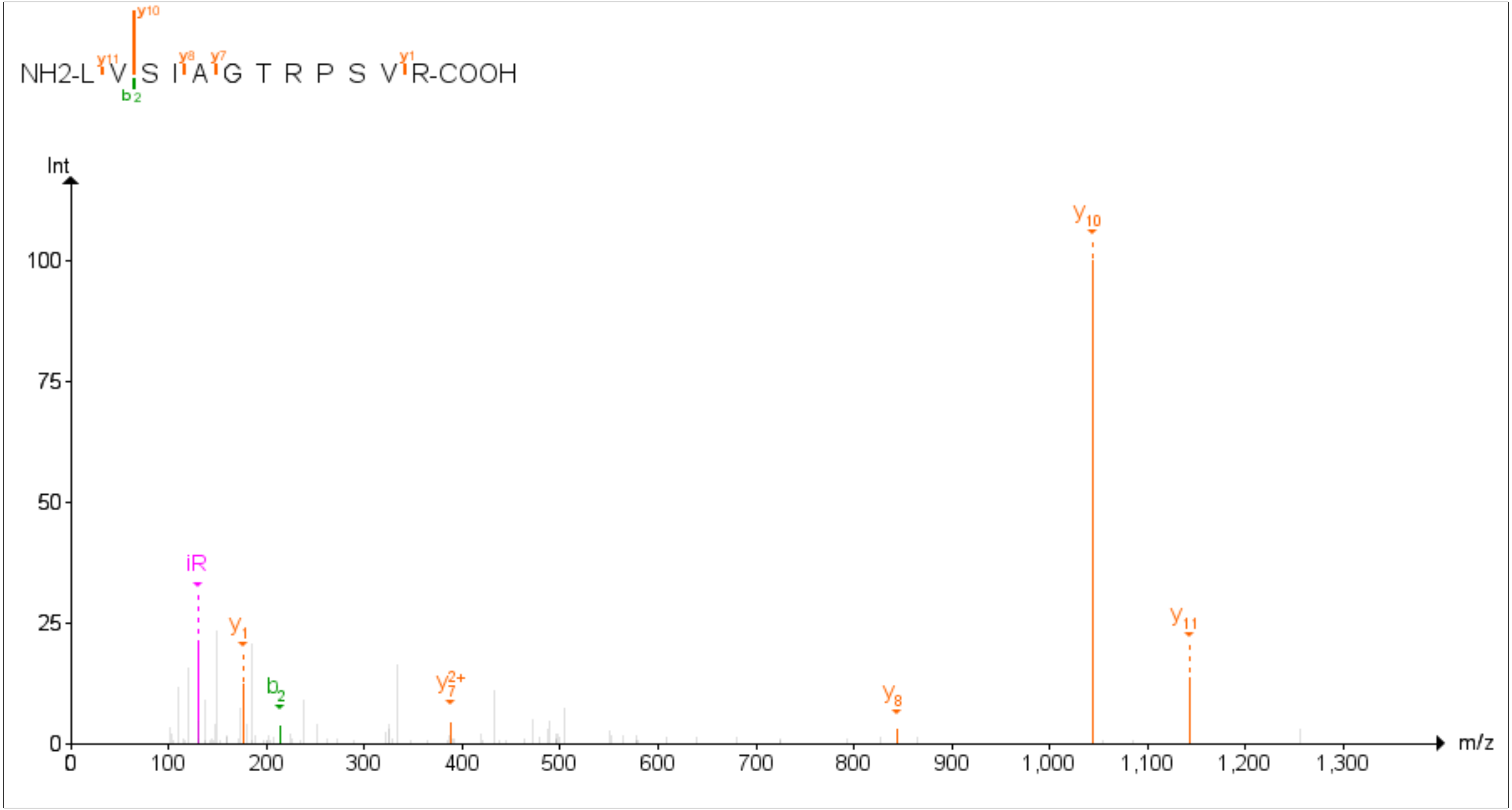
NH2-L V S I A G T R P S V R-COOH
y11
y10
b2
y8
y7
y1
Int
100
75
50
25
0
iR
y1
b2
y7 2+
y8
y10
y11
0
100
200
300
400
500
600
700
800
900
1,000
1,100
1,200
1,300
m/z

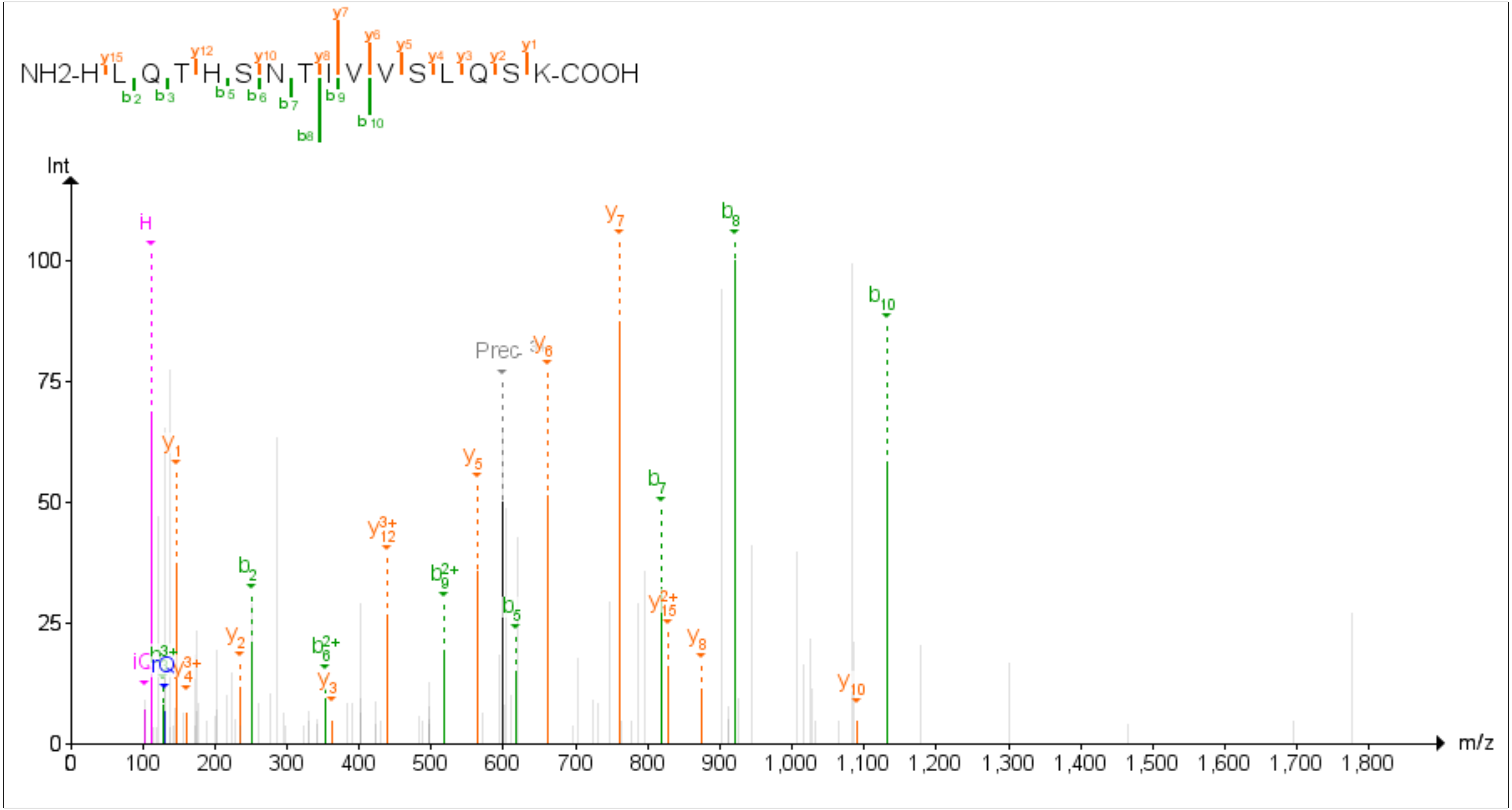

NH2-H L Q T H S N T I V V S L Q S K-COOH
y15 y12 y10 y8 y7 y6 y5 y4 y3 y2 y1
b2 b3 b5 b6 b7 b8 b9 b10
Int
m/z
Prec

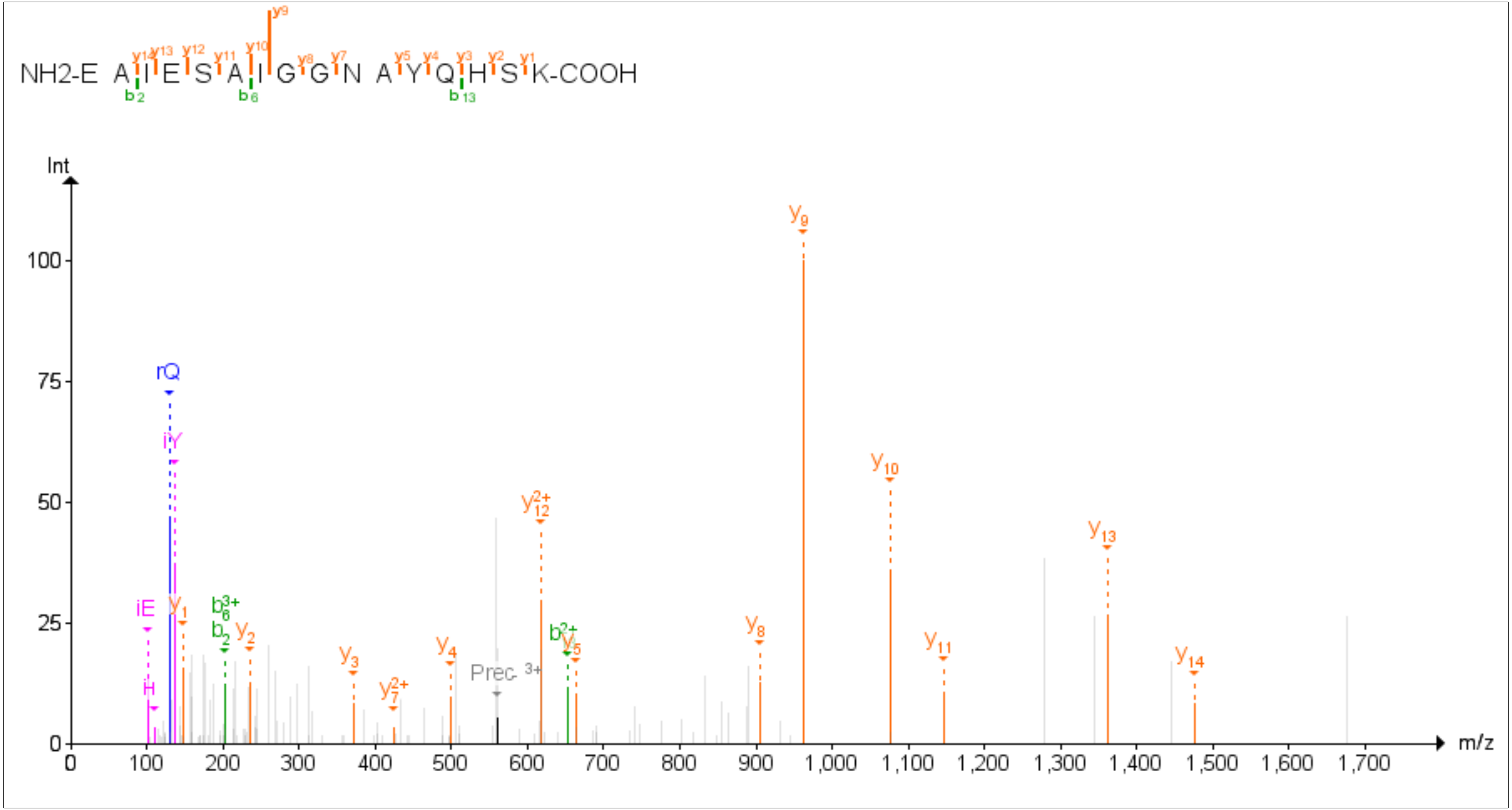
NH2-E A I I E S A I G G N A Y Q H S K-COOH
y14 y13 y12 y11 y10 y9 y8 y7 y5 y4 y3 y2 y1
b2 b6 b13
Int
m/z
100
75
50
25
0
rQ
iY
iE
iH
y1
b_6^{3+}
b_2
y_2
y_3
y_7^{2+}
y_4
Prec- 3+
y_{12}^{2+}
b_{13}^{2+}
y_5
y_8
y_9
y_{10}
y_{11}
y_{13}
y_{14}
0
100
200
300
400
500
600
700
800
900
1,000
1,100
1,200
1,300
1,400
1,500
1,600
1,700

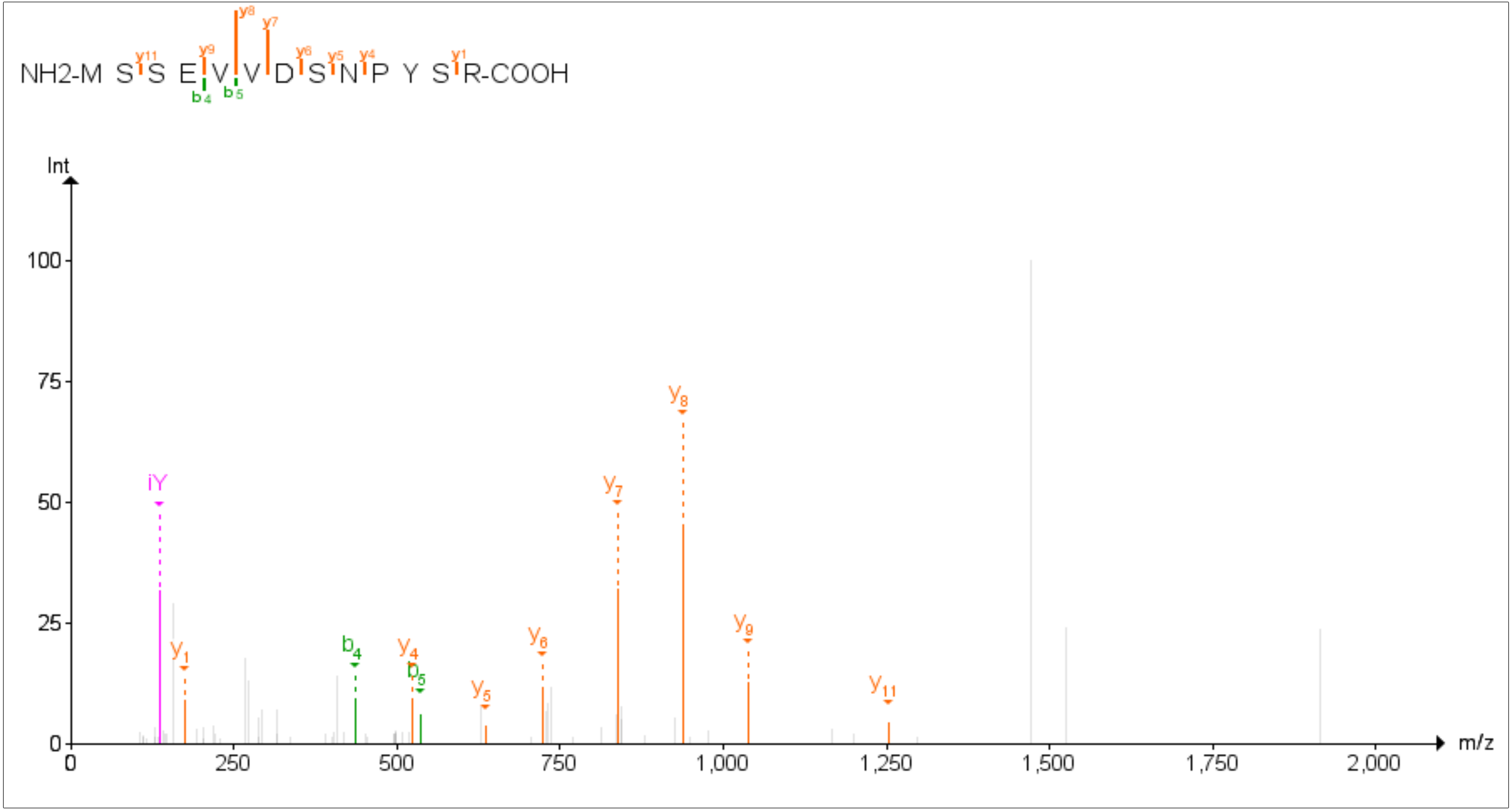
NH2-M S S E V V D S N P Y S R-COOH
y11 y9 y8 y7 y6 y5 y4 y1
b4 b5
Int
100
75
50
25
0
iY
y1
b4
y4
b5
y5
y6
y7
y8
y9
y11
0
250
500
750
1,000
1,250
1,500
1,750
2,000
m/z

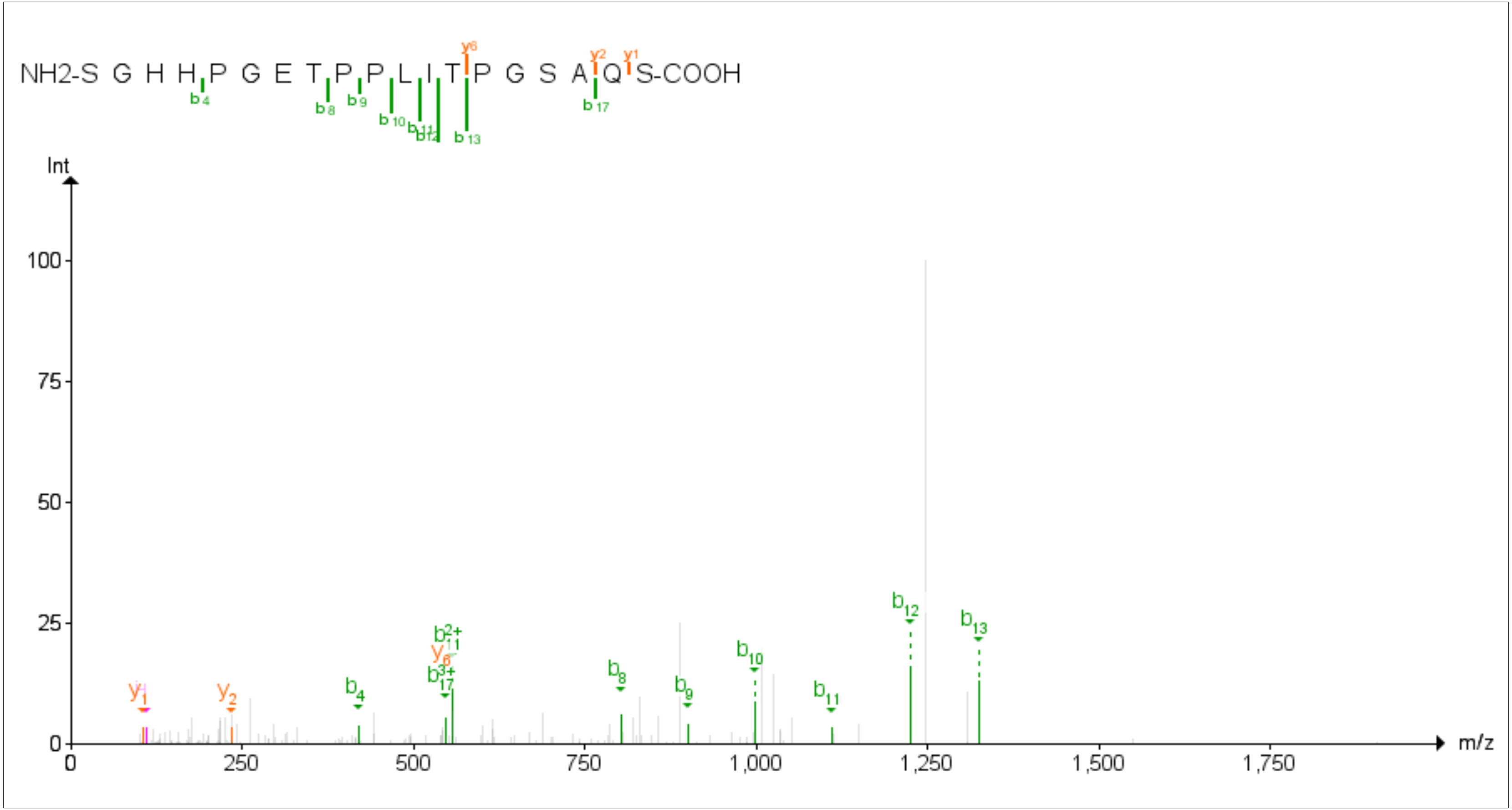

NH2-S G H H P G E T P P L I T P G S A Q S-COOH
Int
m/z

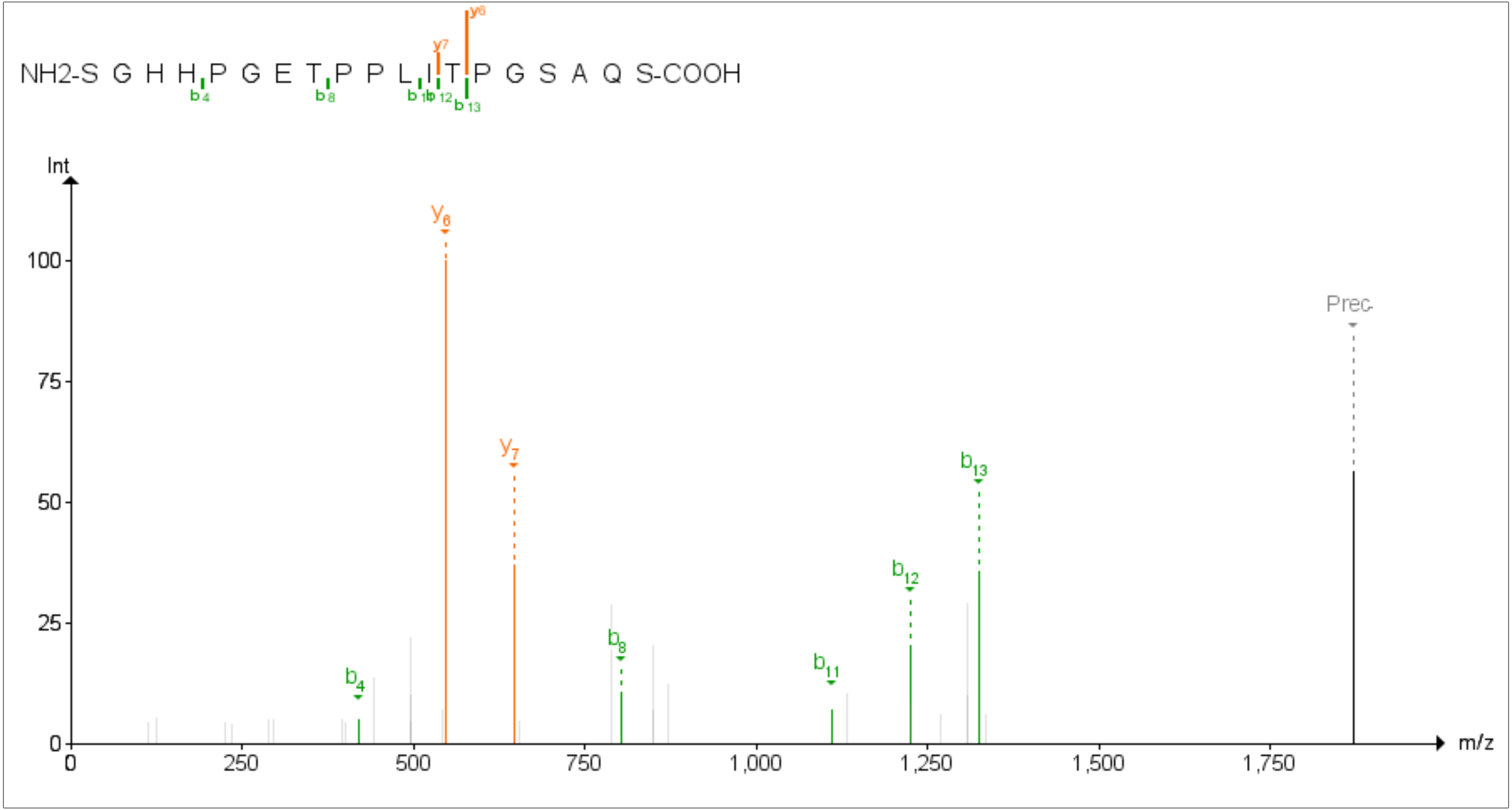

NH2-S G H H P G E T P P L I T P G S A Q S-COOH
b4
b8
b11
b12
b13
y7
y6
Int
100
75
50
25
0
y6
y7
b4
b8
b11
b12
b13
Prec
0
250
500
750
1,000
1,250
1,500
1,750
m/z

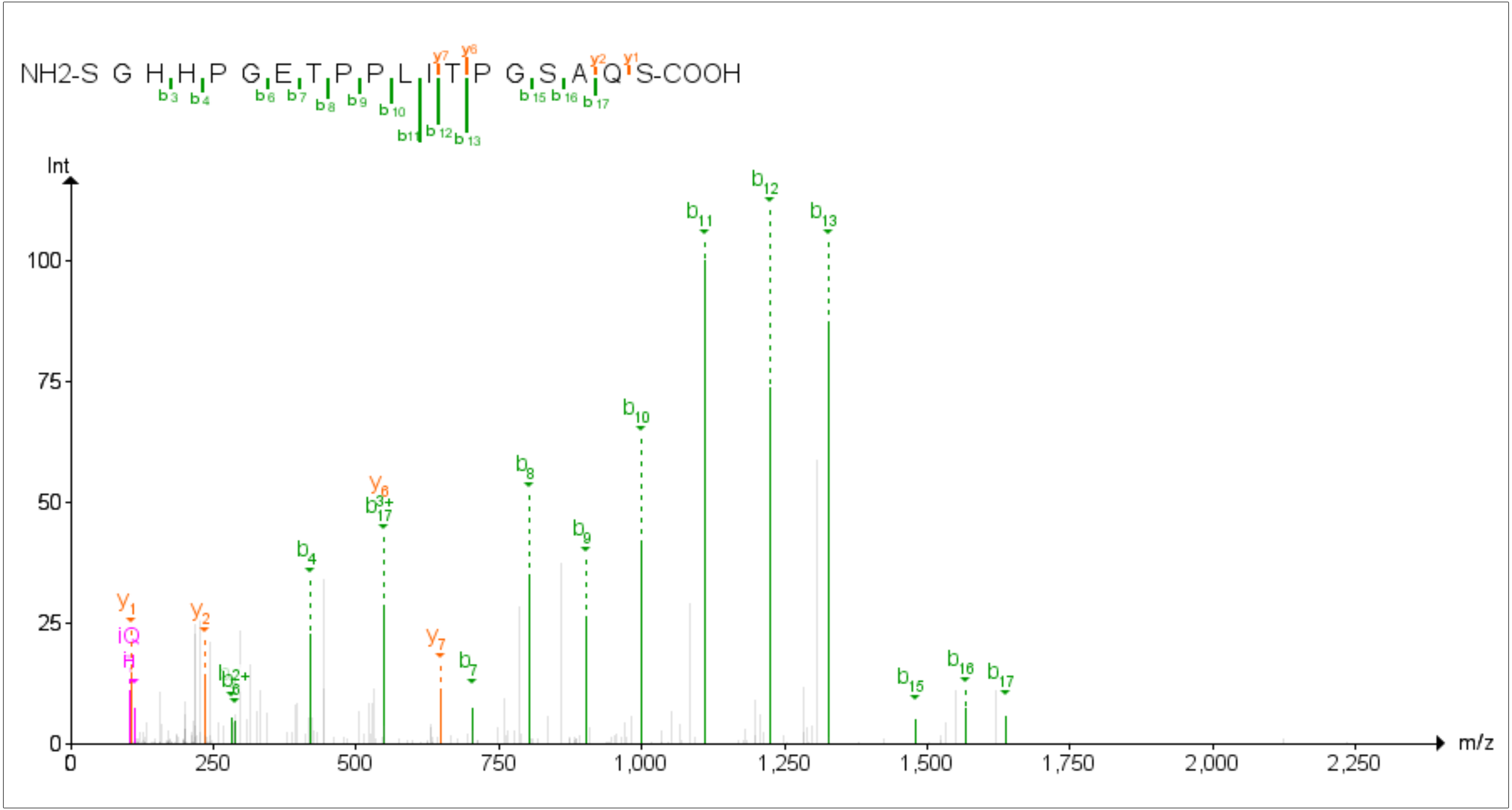

NH2-S G H H P G E T P P L I T P G S A Q S-COOH
Int
m/z
0
25
50
75
100
250
500
750
1,000
1,250
1,500
1,750
2,000
2,250

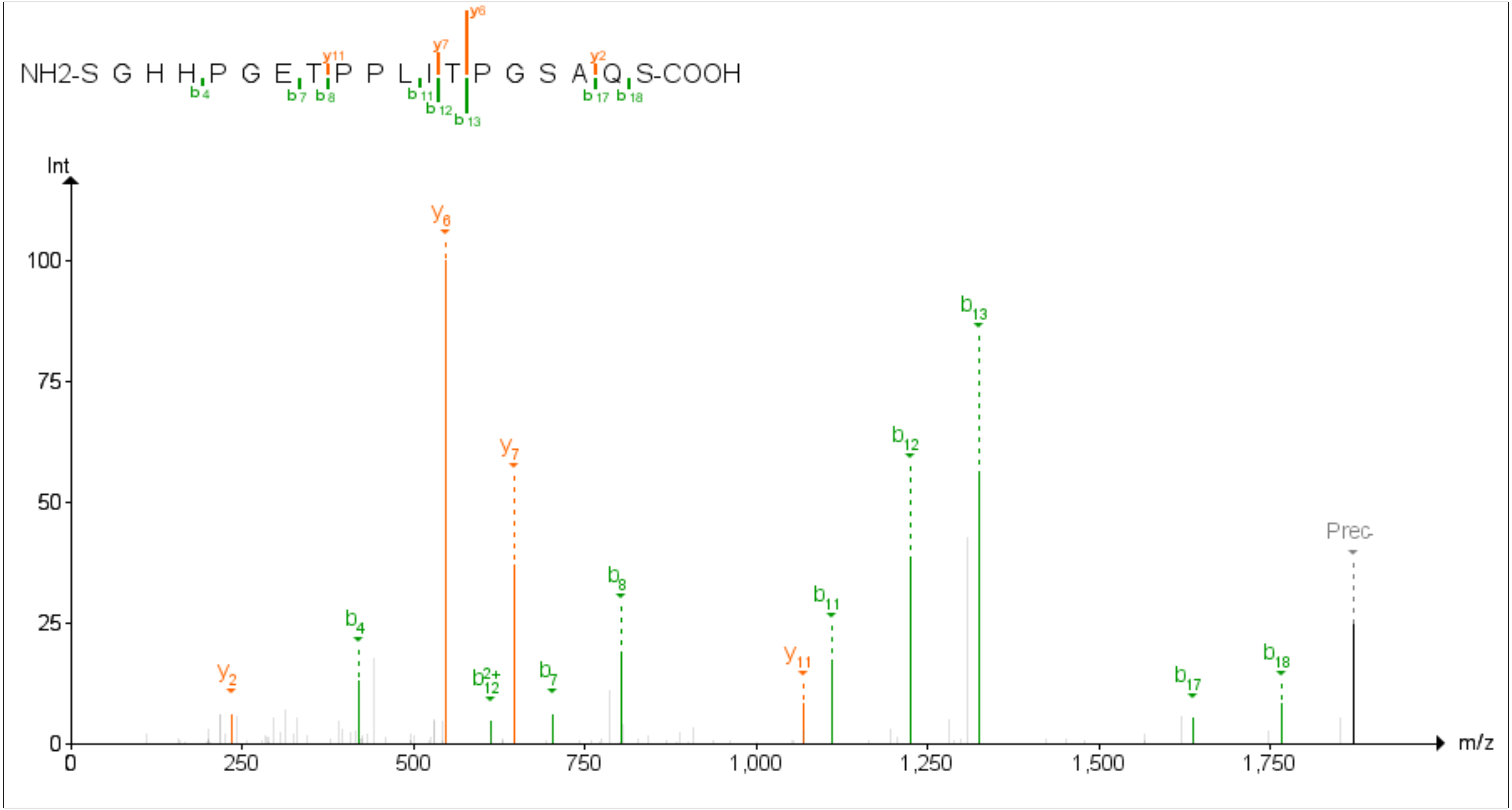
NH2-S G H H P G E T P P L I T P G S A Q S-COOH
y11 y7 y6 y2
b4 b7 b8 b11 b12 b13 b17 b18
Int
m/z
0
25
50
75
100
0
250
500
750
1,000
1,250
1,500
1,750
y6
y7
y2
y11
b4
b12 2+
b7
b8
b11
b12
b13
b17
b18
Prec

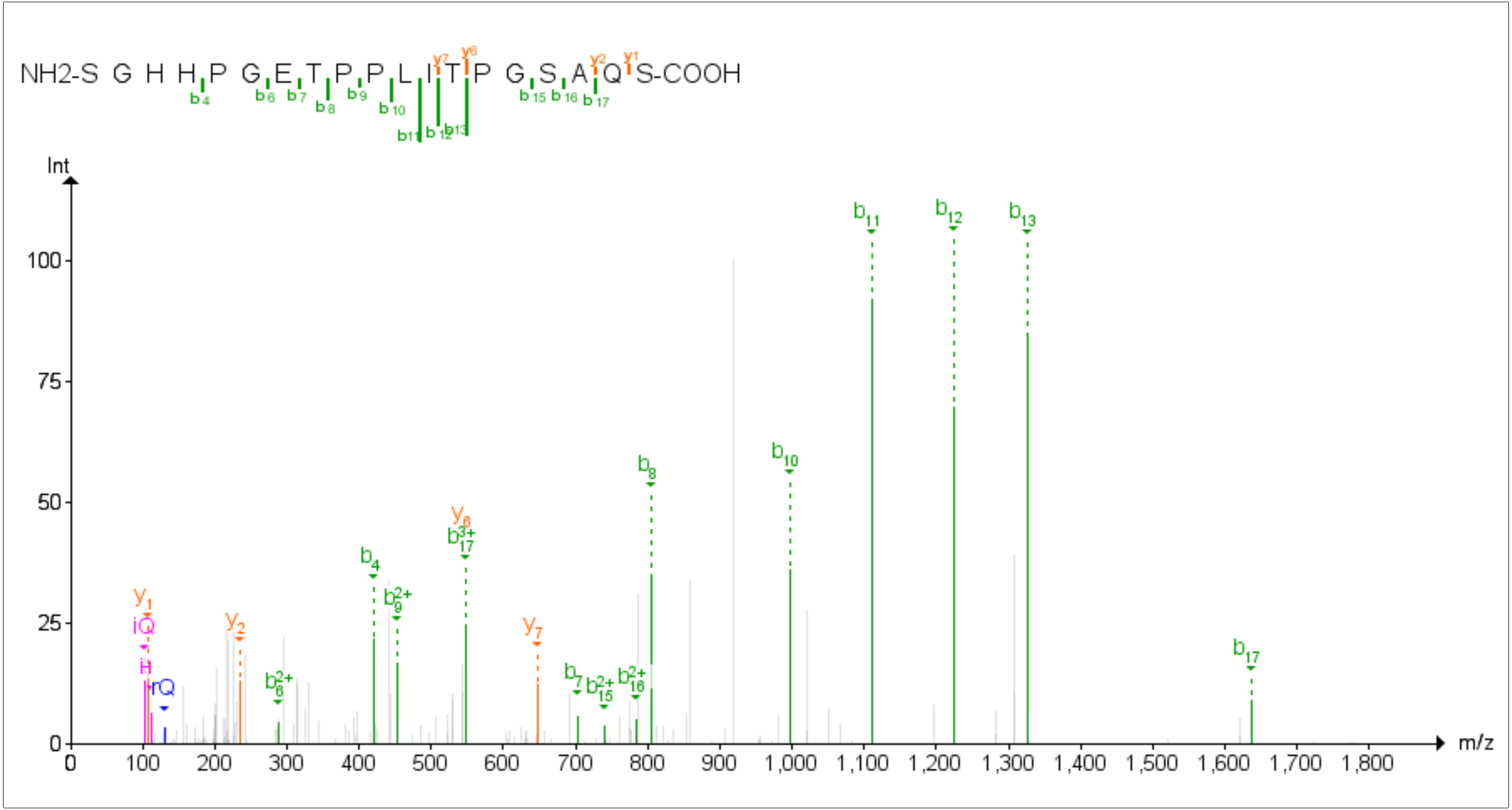

NH2-S G H H P G E T P P L I T P G S A Q S-COOH
Int
m/z
b11
b12
b13
b10
b8
y6
b4
y1
iQ
y2
y7
b17

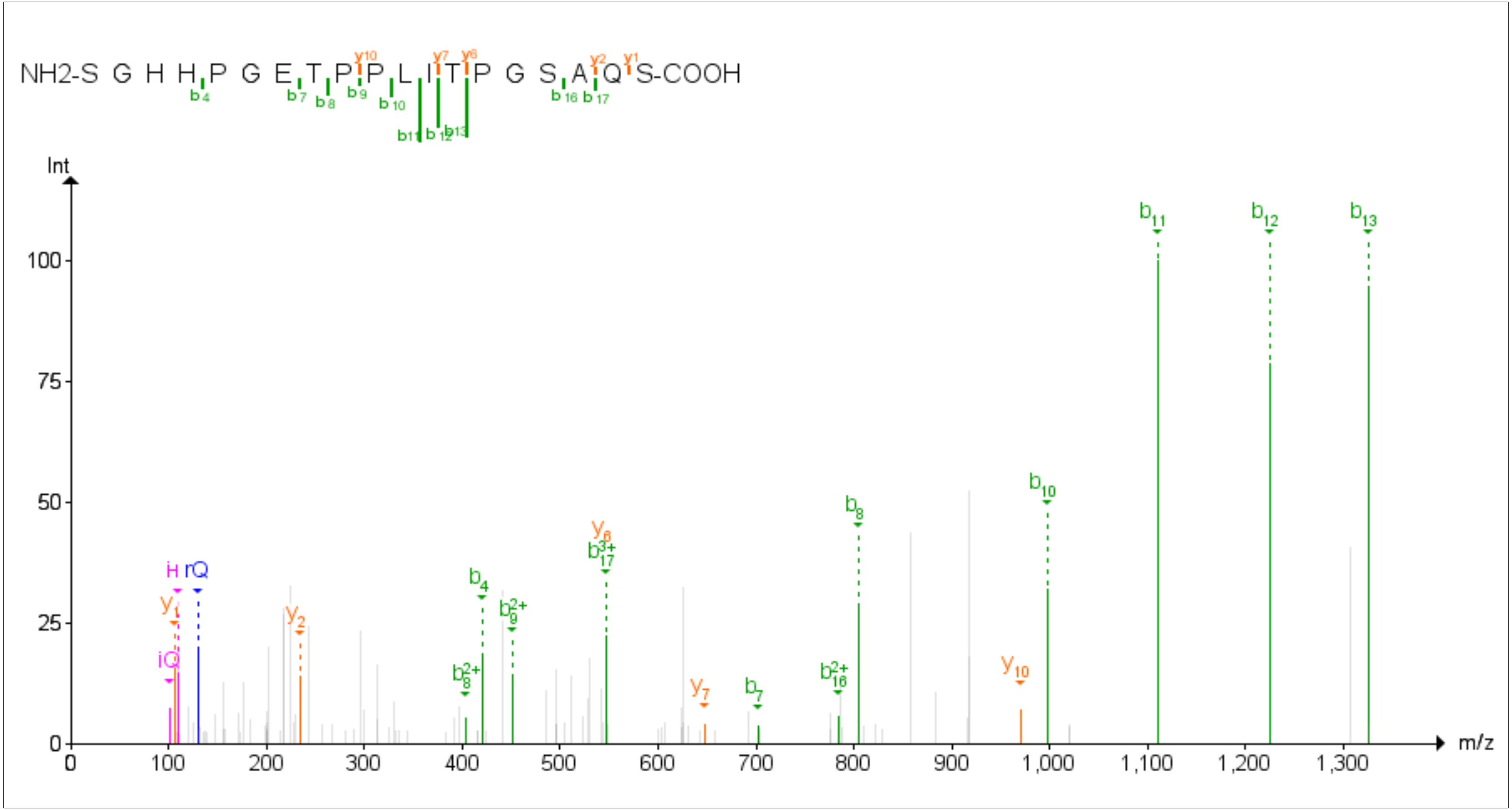

NH2-S G H H P G E T P P L I T P G S A Q S-COOH
Int
m/z
b11
b12
b13
b10
b8
y6
b17 3+
b4
b9 2+
b8 2+
y7
b7
b16 2+
y10
y1
y2
iH
rQ
iQ

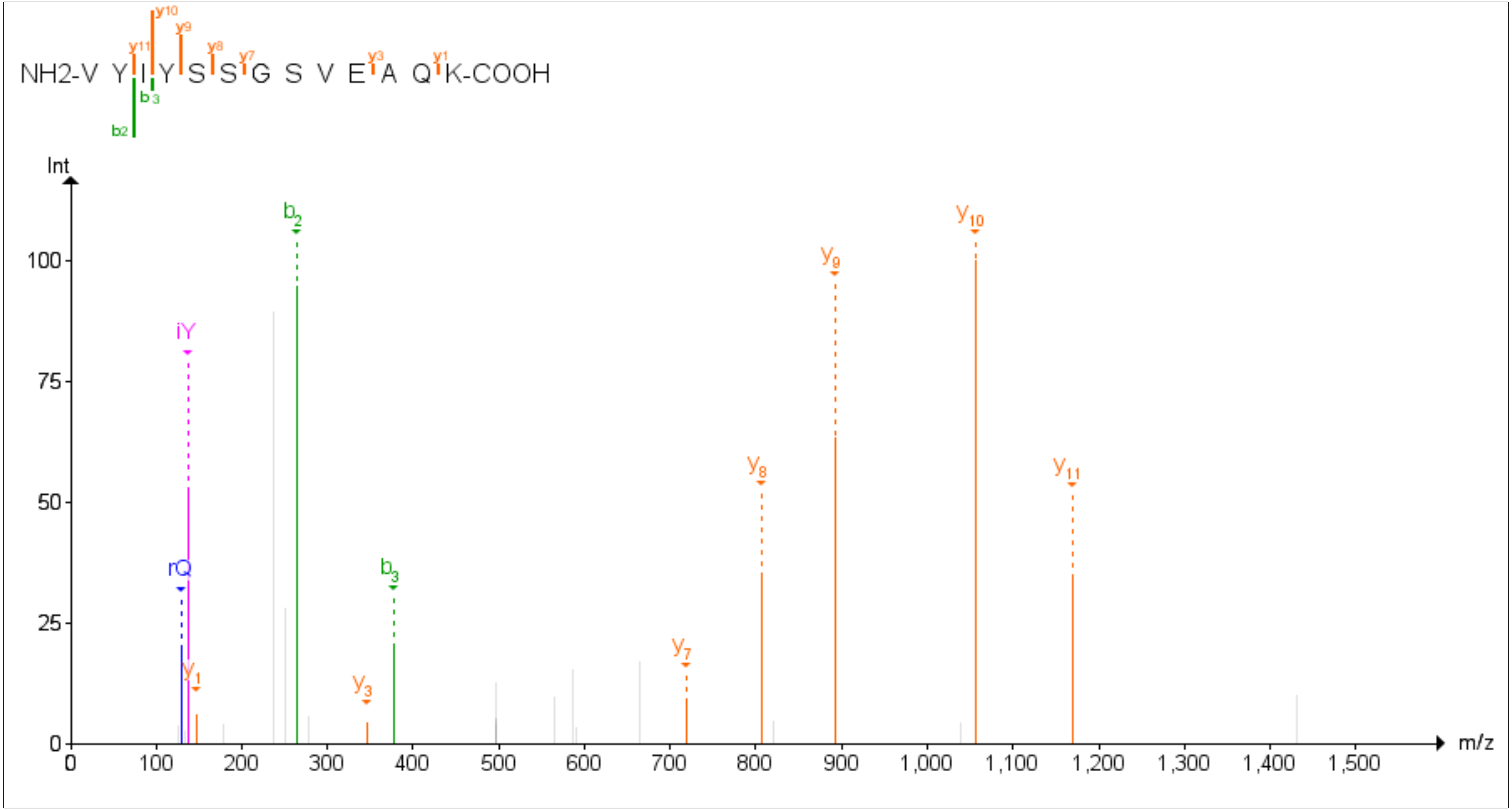

NH2-V Y I Y S S G S V E A Q K-COOH
y11 y10 y9 y8 y7 y3 y1
b2 b3
Int
100
75
50
25
0
b2
iY
rQ
y1
y3
b3
y7
y8
y9
y10
y11
0
100
200
300
400
500
600
700
800
900
1,000
1,100
1,200
1,300
1,400
1,500
m/z

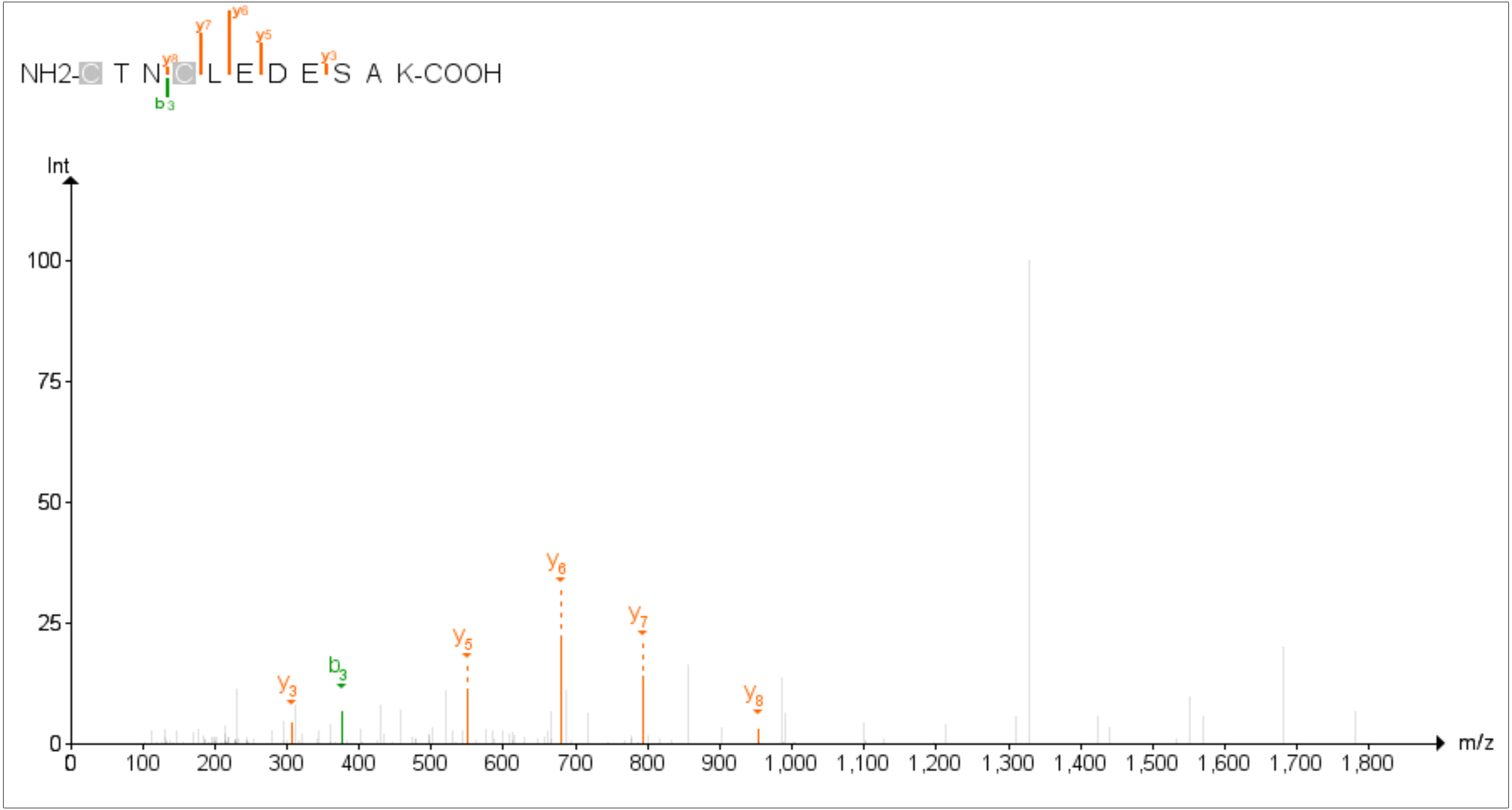
NH2-C T N C L E D E S A K-COOH
y8
y7
y6
y5
y3
b3
Int
100
75
50
25
0
y3
b3
y5
y6
y7
y8
0
100
200
300
400
500
600
700
800
900
1,000
1,100
1,200
1,300
1,400
1,500
1,600
1,700
1,800
m/z

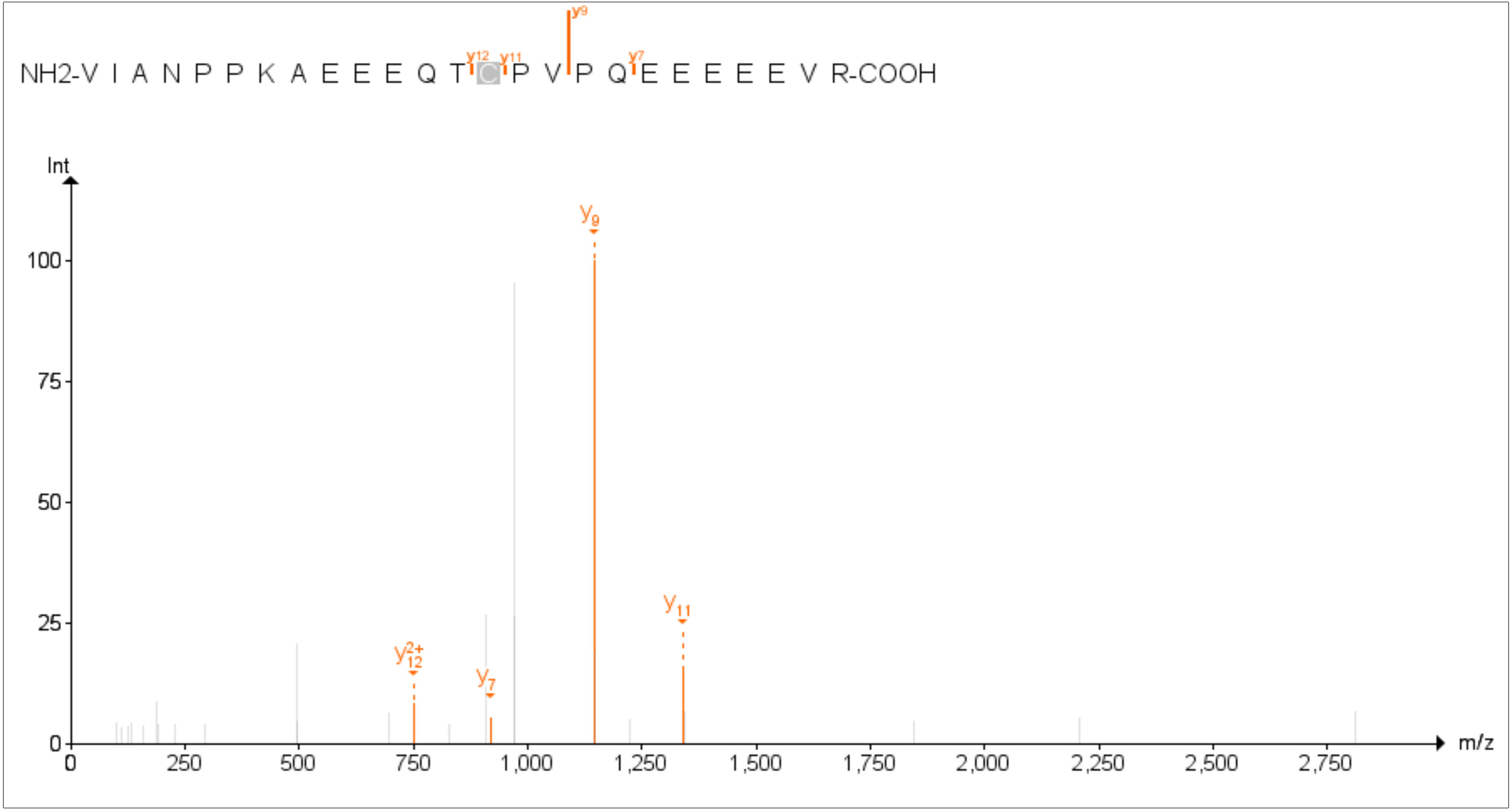
NH2-V I A N P P K A E E E Q T C P V P Q E E E E E V R-COOH
y12
y11
y9
y7
Int
100
75
50
25
0
y_9
y_{11}
y_{12}^{2+}
y_7
0
250
500
750
1,000
1,250
1,500
1,750
2,000
2,250
2,500
2,750
m/z

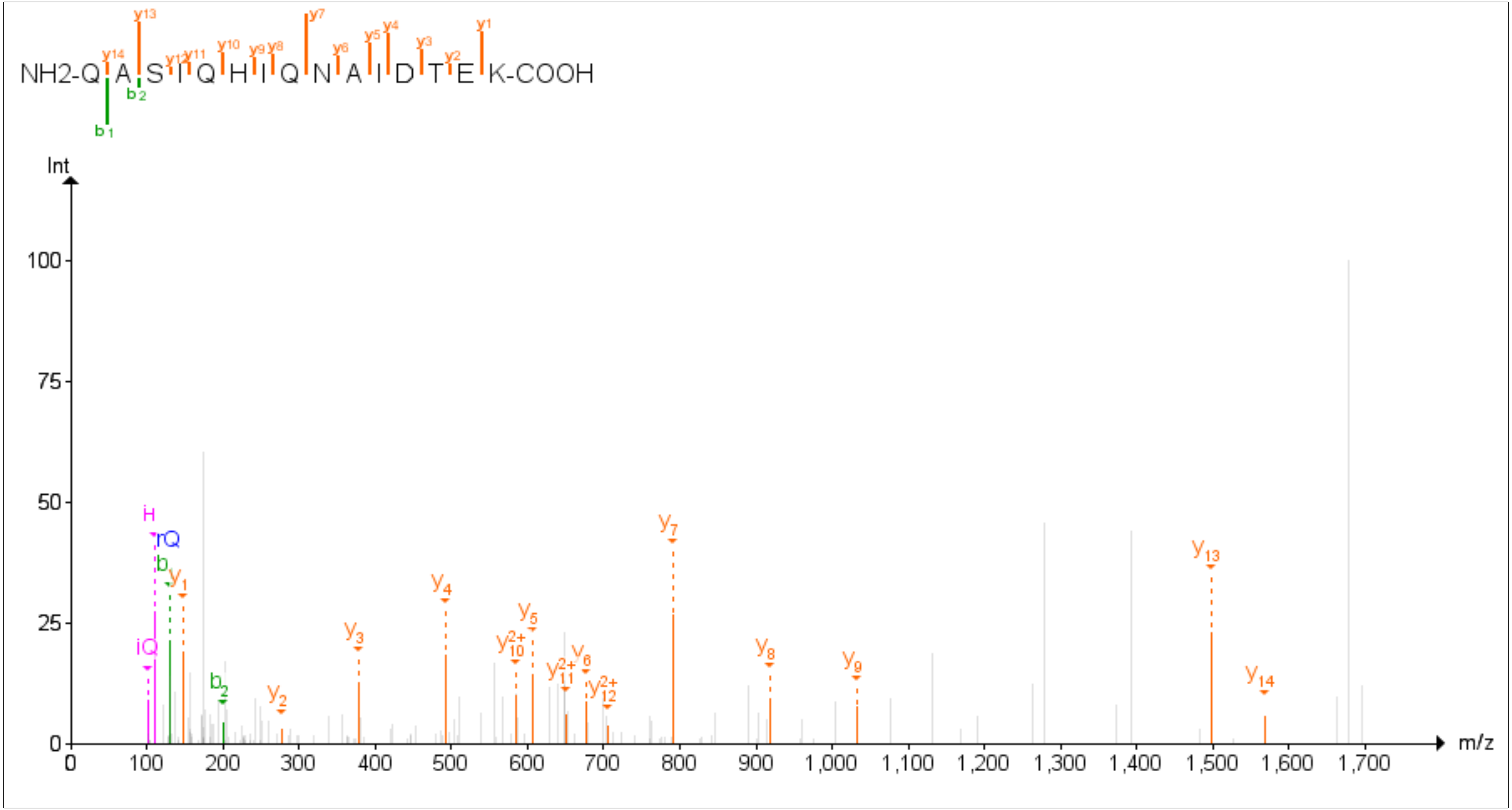

NH2-Q A S I Q H I Q N A I D T E K-COOH
y14 y13 y12 y11 y10 y9 y8 y7 y6 y5 y4 y3 y2 y1
b1 b2
Int
m/z
100
75
50
25
0
0 100 200 300 400 500 600 700 800 900 1,000 1,100 1,200 1,300 1,400 1,500 1,600 1,700
iH
rQ
iQ
b
y1
b2
y2
y3
y4
y5
y7
y8
y9
y13
y14

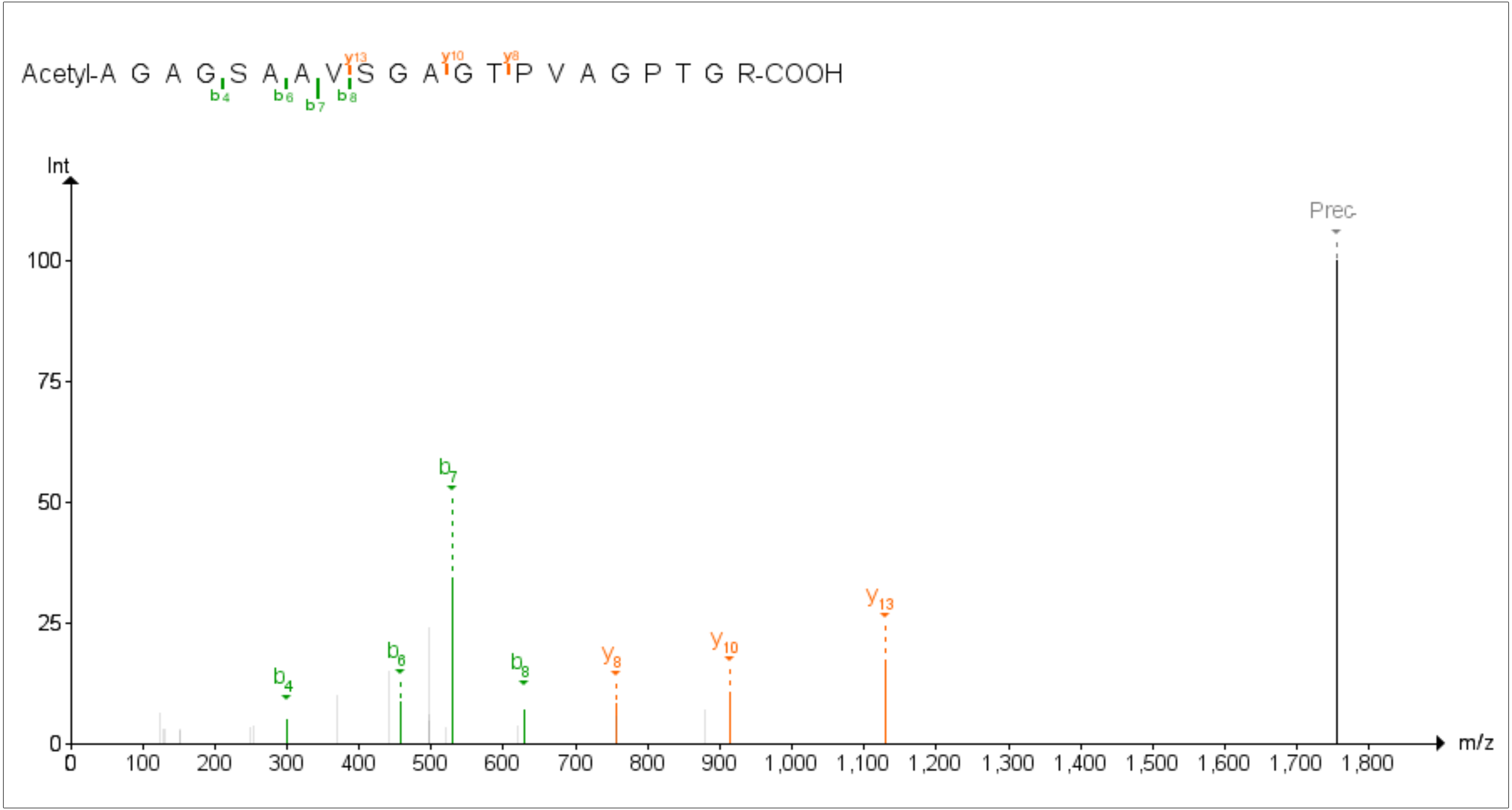

Acetyl-A G A G S A A V S G A G T P V A G P T G R-COOH
b4
b6
b7
b8
y13
y10
y8
Int
100
75
50
25
0
Prec
m/z
0
100
200
300
400
500
600
700
800
900
1,000
1,100
1,200
1,300
1,400
1,500
1,600
1,700
1,800

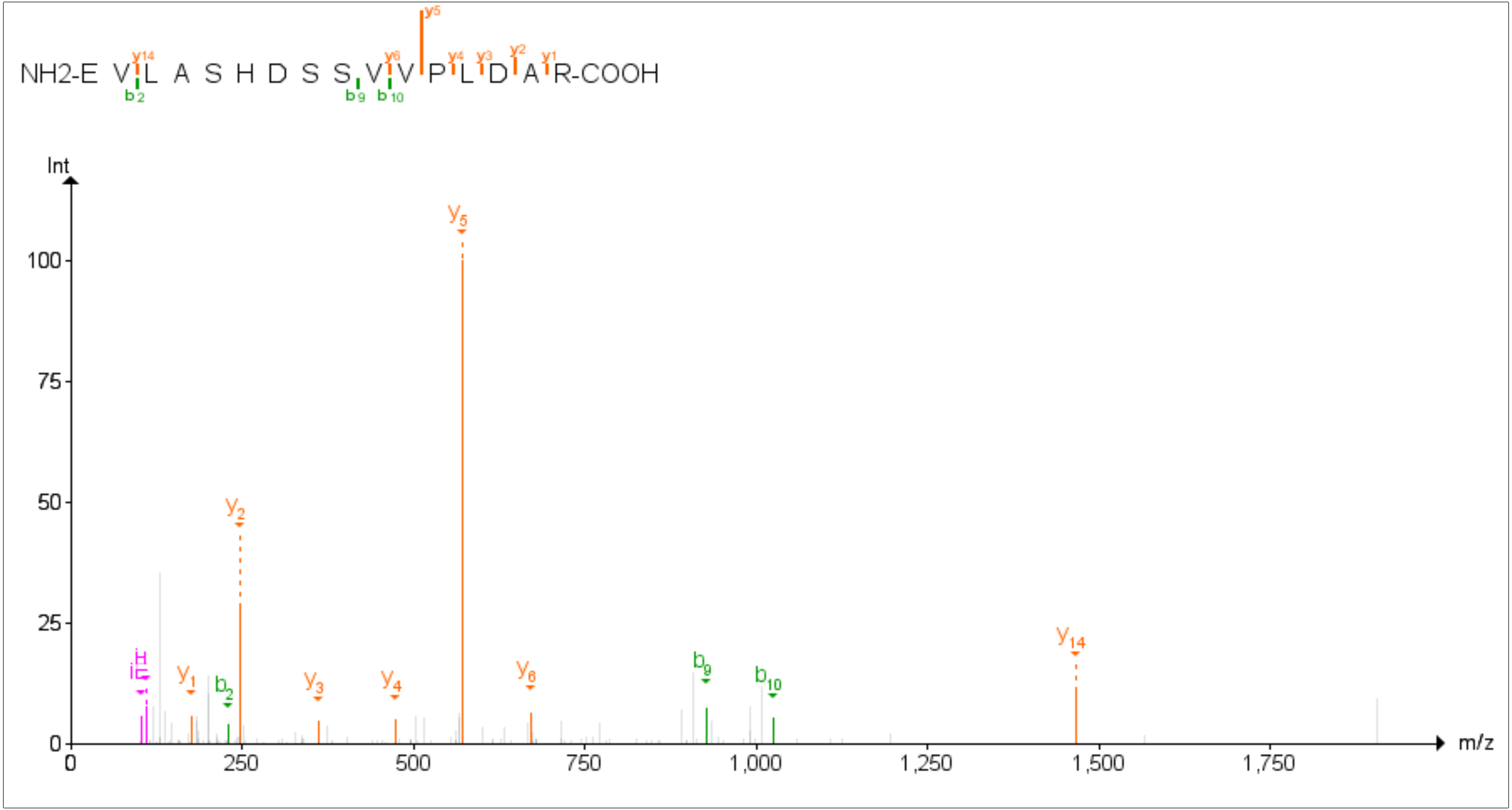

NH2-E V L A S H D S S V V P L D A R-COOH
y14
y6
y5
y4
y3
y2
y1
b2
b9
b10
Int
100
75
50
25
0
y5
y2
y1
b2
y3
y4
y6
b9
b10
y14
m/z
0
250
500
750
1,000
1,250
1,500
1,750

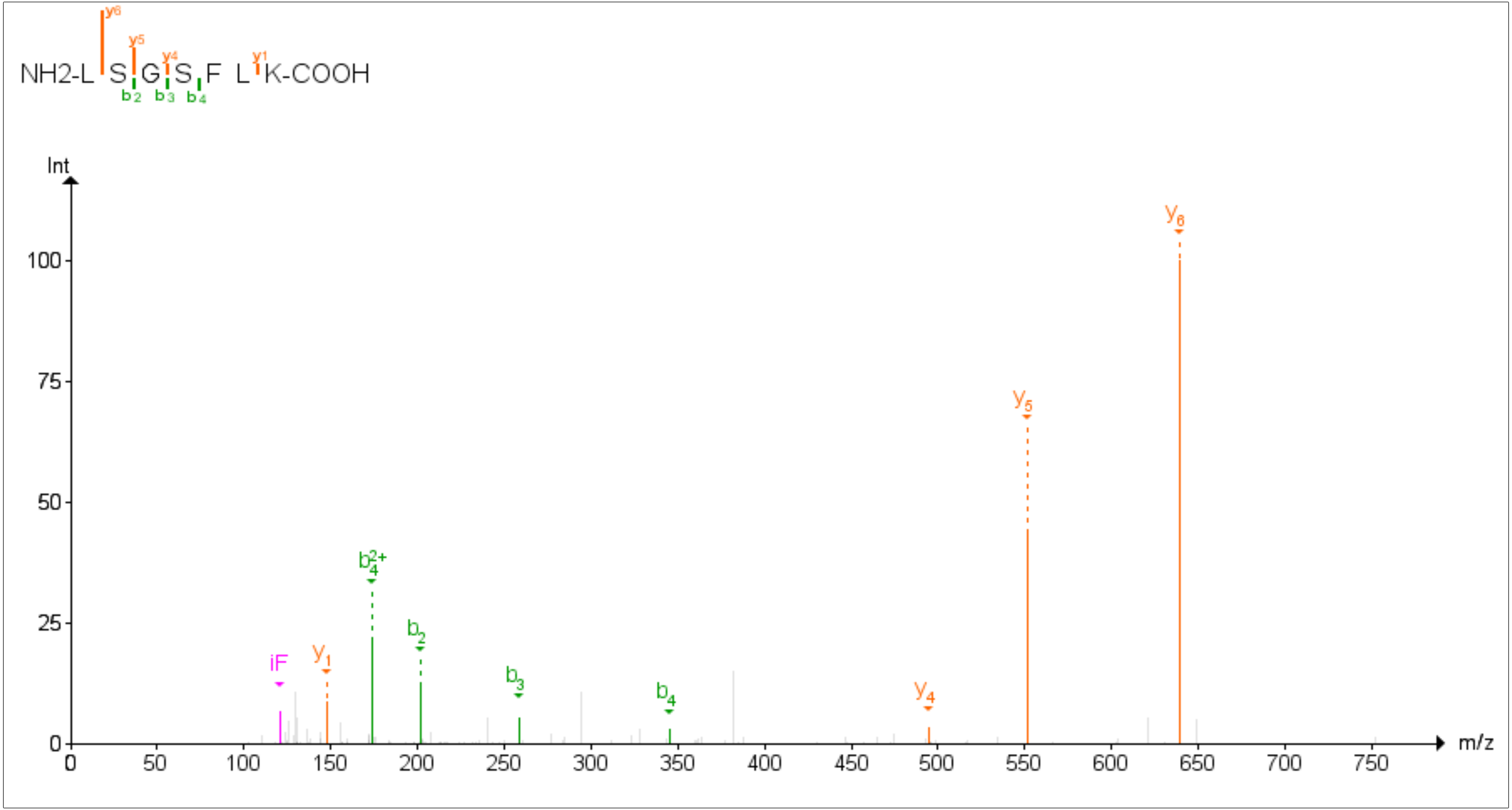

NH2-L S G S F L K-COOH
y6
y5
y4
y1
b2
b3
b4
Int
100
75
50
25
0
iF
y1
b4 2+
b2
b3
b4
y4
y5
y6
0
50
100
150
200
250
300
350
400
450
500
550
600
650
700
750
m/z

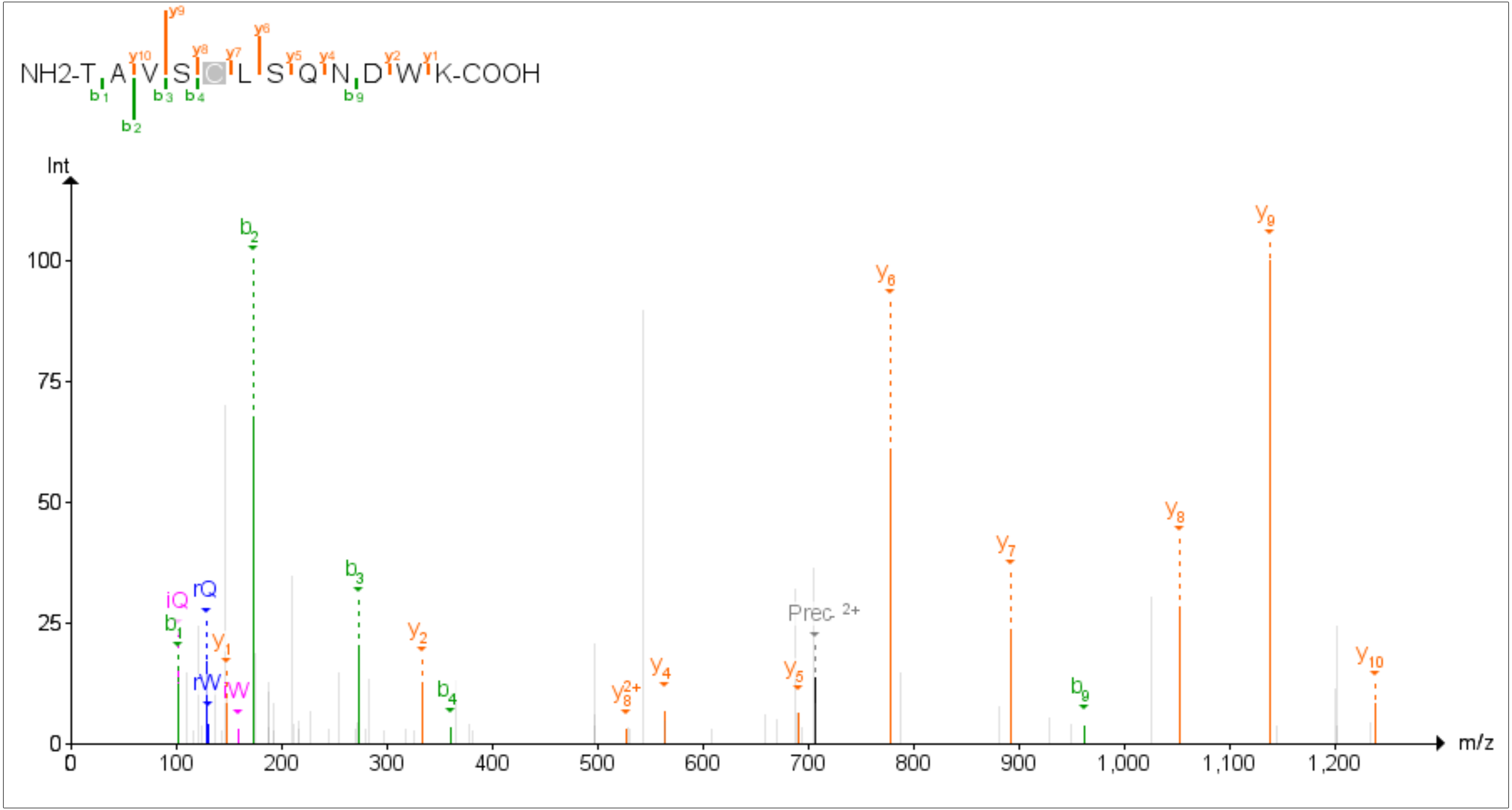

NH2-T A V S C L S Q N D W K-COOH
y9 y6 y10 y8 y7 y5 y4 y2 y1
b1 b3 b4 b9 b2
Int
m/z
0
25
50
75
100
0
100
200
300
400
500
600
700
800
900
1,000
1,100
1,200
b2
y9
y6
y8
y7
b3
rQ
iQ
b1
y1
y2
rW
iW
b4
y8 2+
y4
y5
Prec- 2+
b9
y10

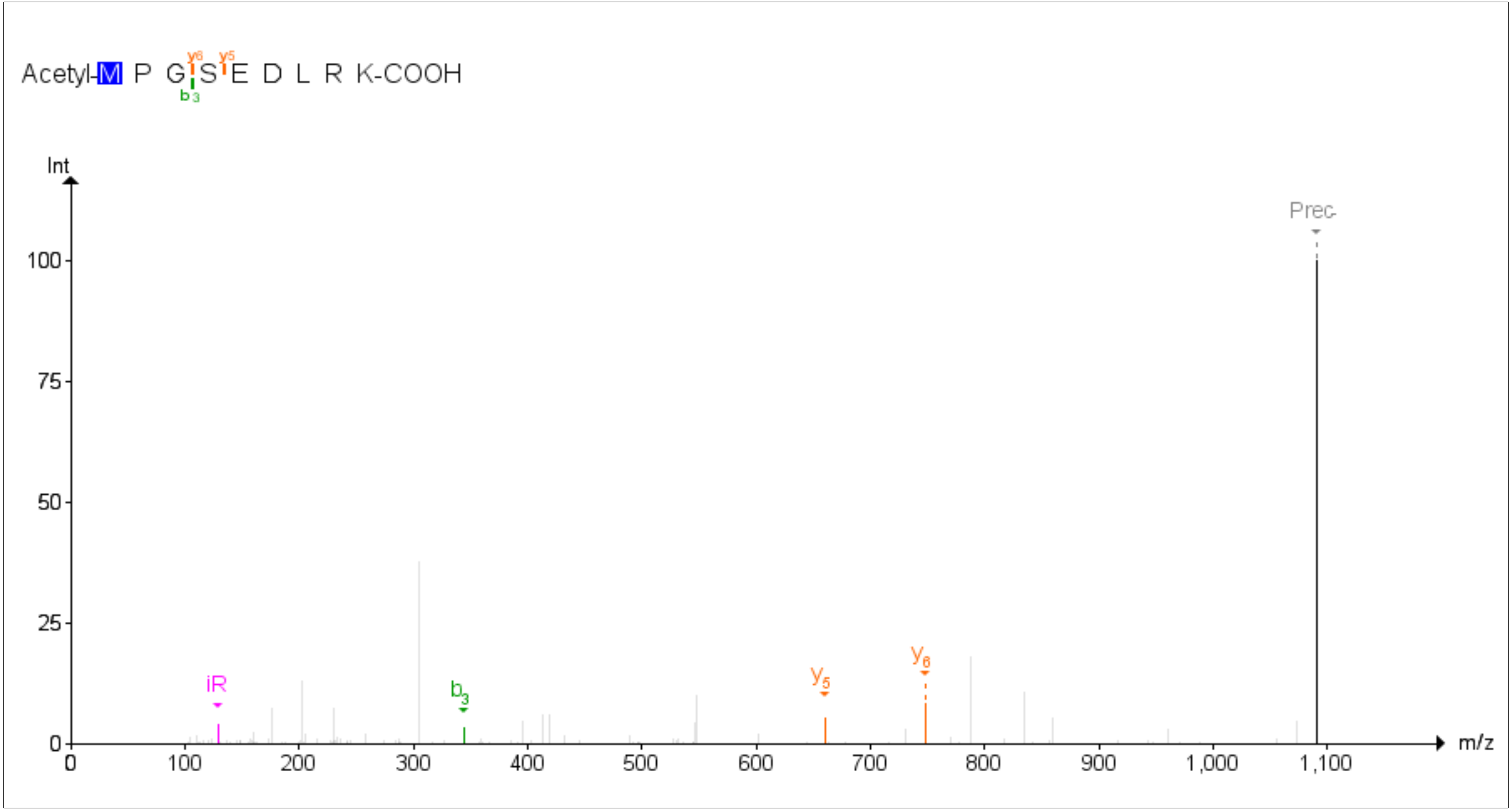
Acetyl-M P G S E D L R K-COOH
y6
y5
b3
Int
100
75
50
25
0
Prec.
iR
b3
y5
y6
0
100
200
300
400
500
600
700
800
900
1,000
1,100
m/z

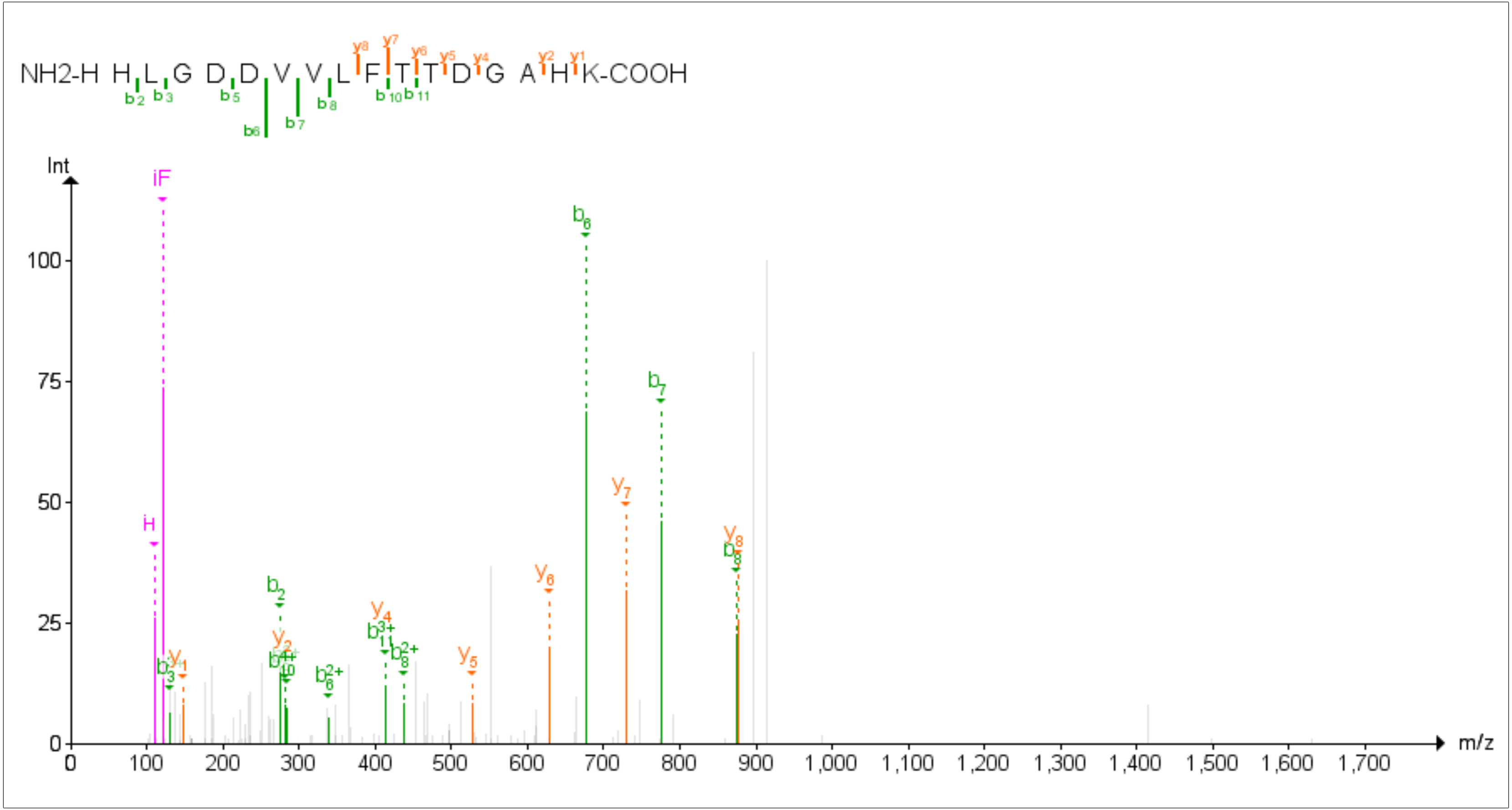
NH2-H H L G D D V V L F T T D G A H K-COOH
b2 b3 b5 b6 b7 b8 b10 b11
y8 y7 y6 y5 y4 y2 y1
Int
iF
iH
b6
b7
y7
y8
b8
y6
b2
y4
y5
0
25
50
75
100
0
100
200
300
400
500
600
700
800
900
1,000
1,100
1,200
1,300
1,400
1,500
1,600
1,700
m/z

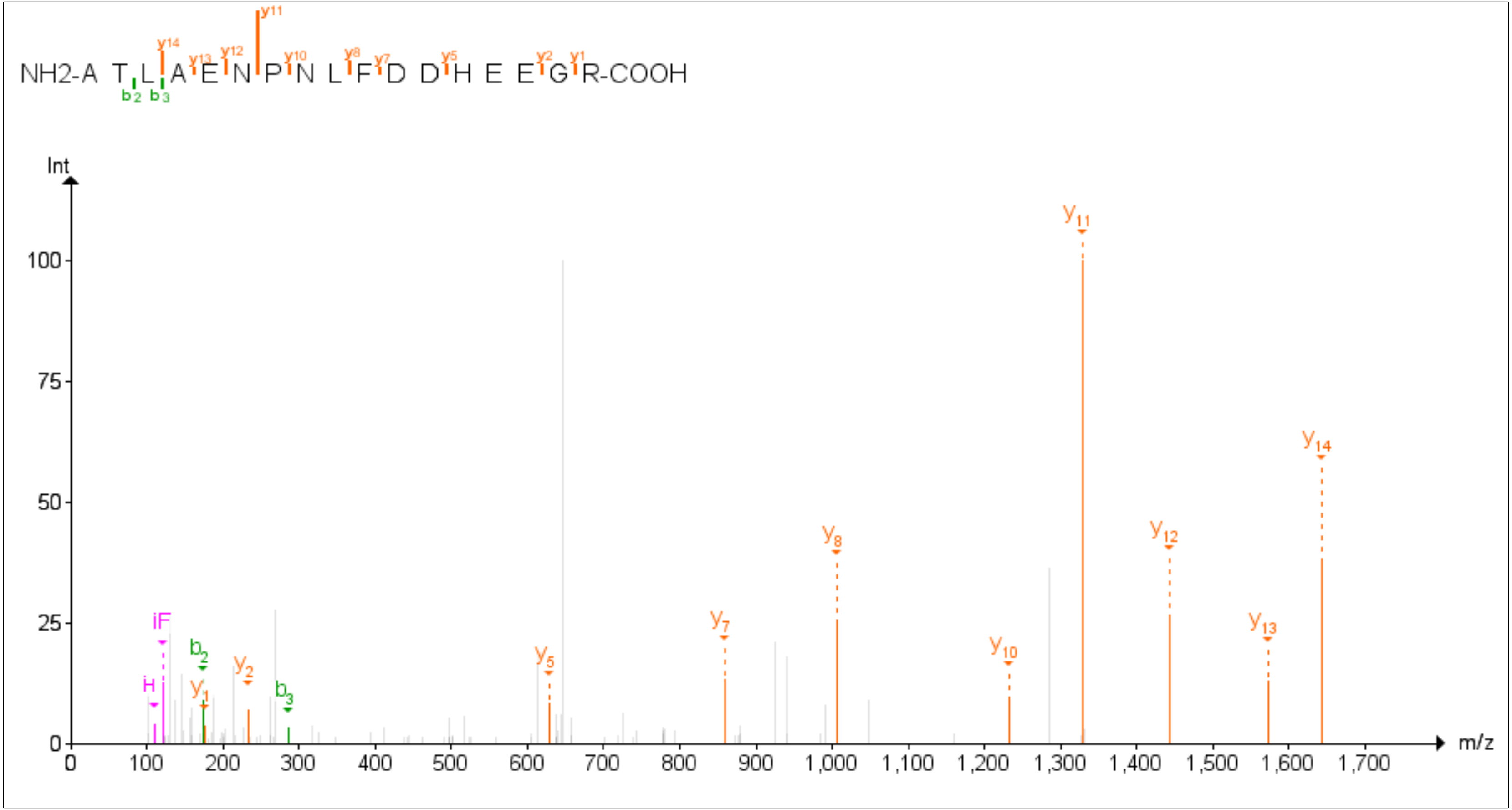
NH2-A T L A E N P N L F D D H E E G R-COOH
b2
b3
y14
y13
y12
y11
y10
y8
y7
y5
y2
y1
Int
100
75
50
25
0
m/z
0
100
200
300
400
500
600
700
800
900
1,000
1,100
1,200
1,300
1,400
1,500
1,600
1,700
iF
iH
y1
y2
y5
y7
y8
y10
y11
y12
y13
y14

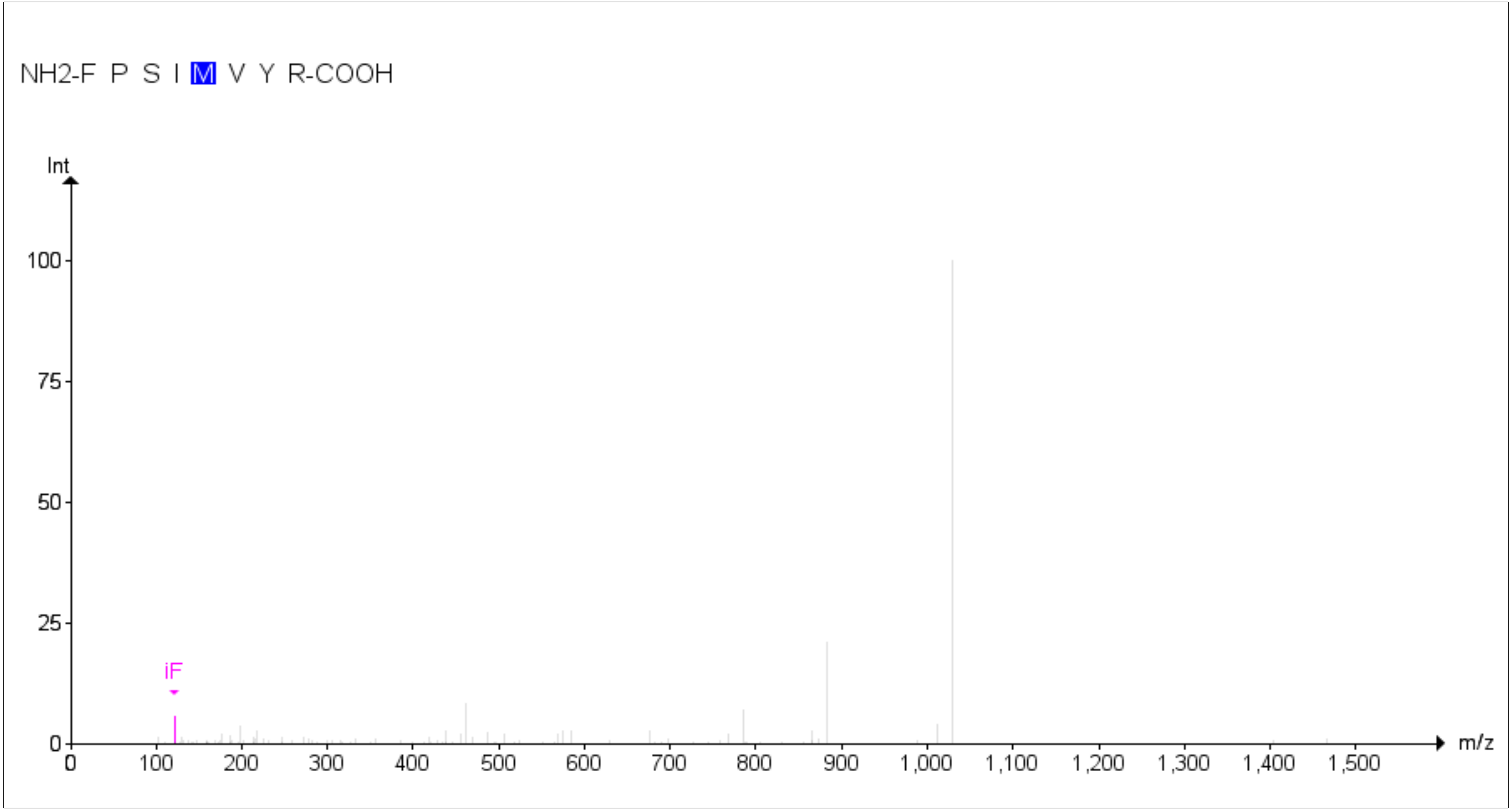

NH2-F P S I M V Y R-COOH
Int
100
75
50
25
0
iF
0
100
200
300
400
500
600
700
800
900
1,000
1,100
1,200
1,300
1,400
1,500
m/z

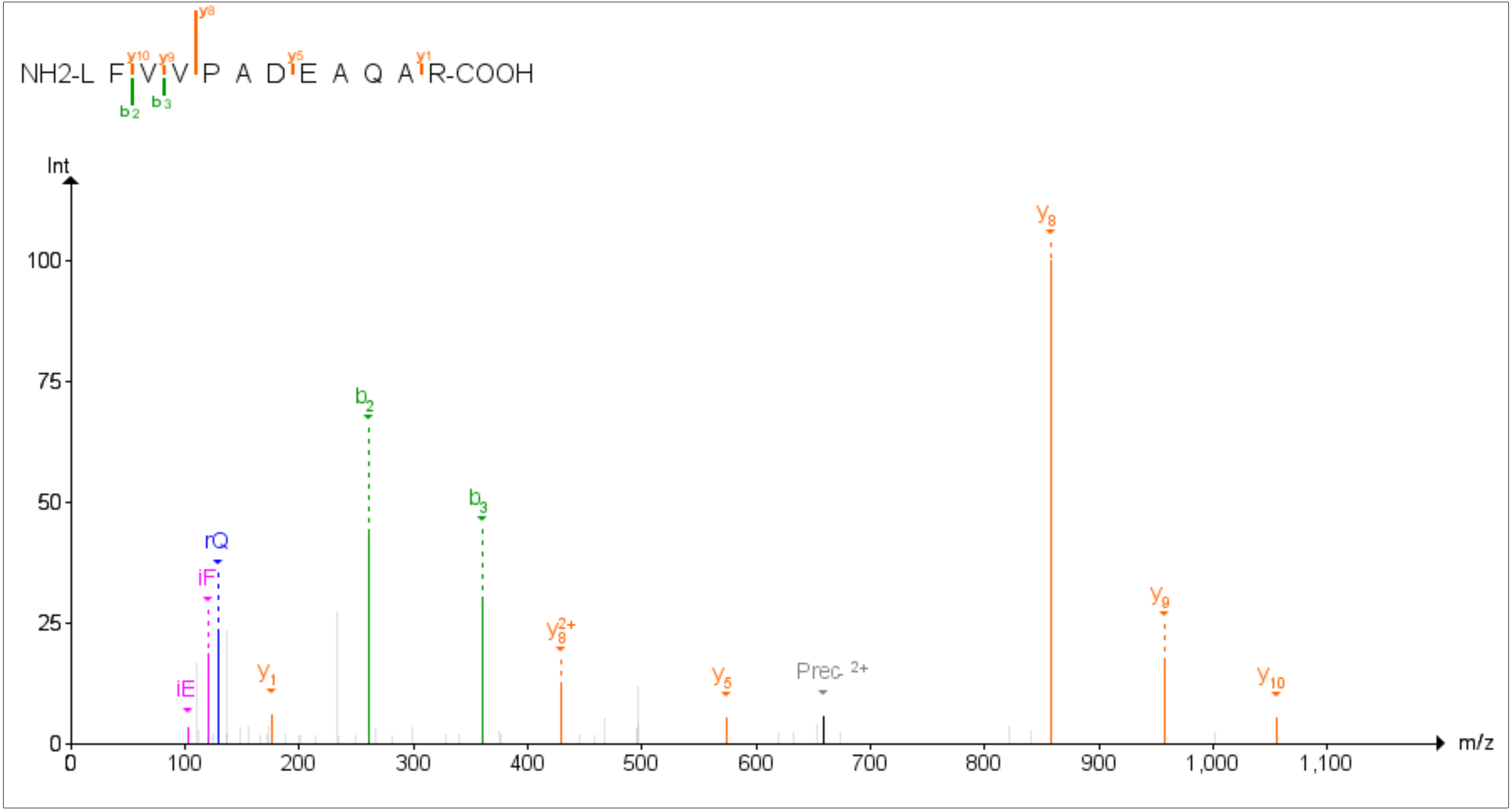

NH2-L F V V P A D E A Q A R-COOH
y10 y9 y8 y5 y1
b2 b3
Int
100
75
50
25
0
rQ
iF
iE
y1
b2
b3
y8 2+
y5
Prec- 2+
y8
y9
y10
0 100 200 300 400 500 600 700 800 900 1,000 1,100
m/z

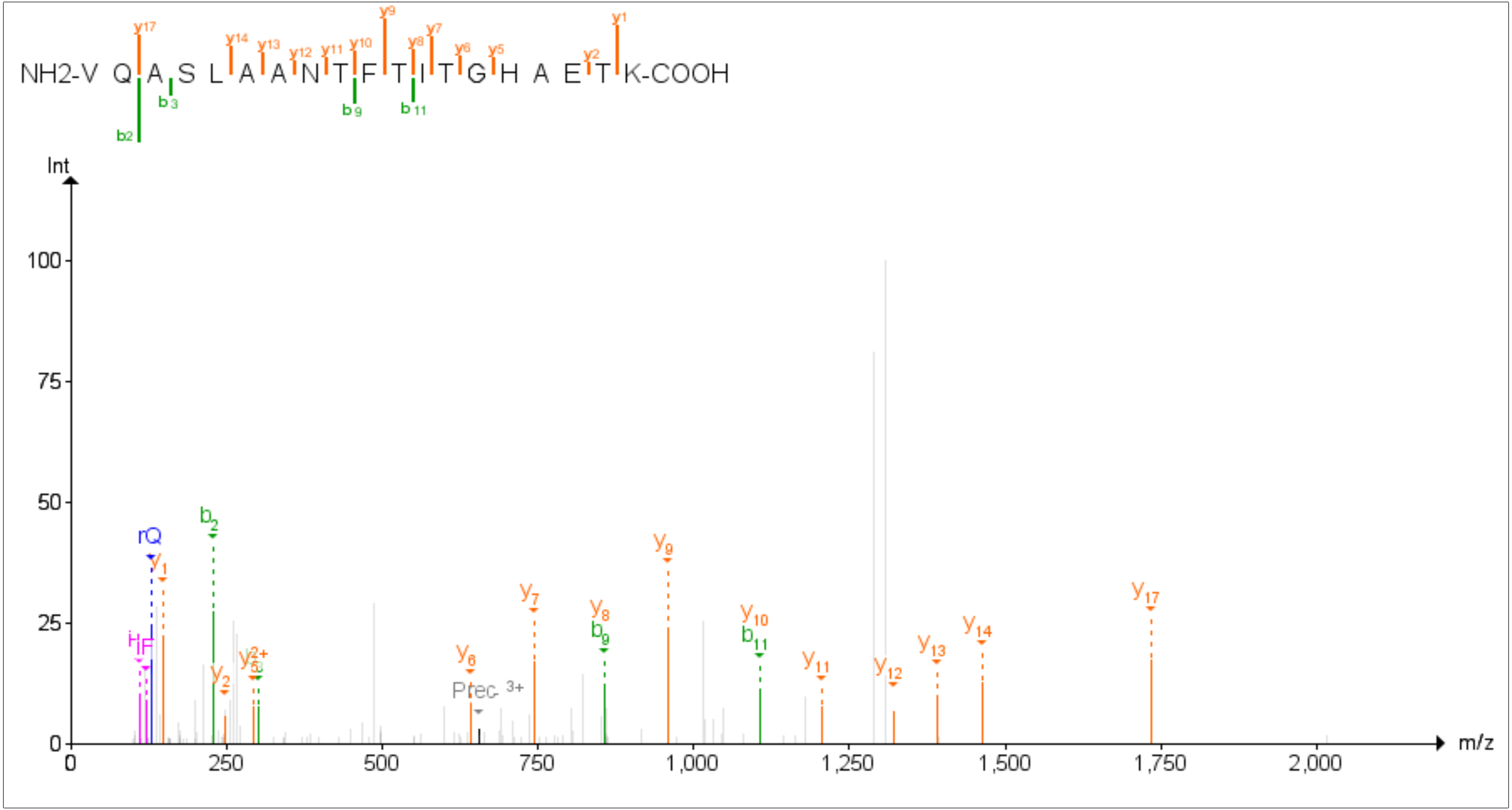

NH2-V Q A S L A A N T F T I T G H A E T K-COOH
y17 y14 y13 y12 y11 y10 y9 y8 y7 y6 y5 y2 y1
b2 b3 b9 b11
Int
100
75
50
25
0
rQ
b2
y1
y9
y7
y8
b9
y10
b11
y17
y14
y13
y11
y12
y6
y2
Prec 3+
0
250
500
750
1,000
1,250
1,500
1,750
2,000
m/z

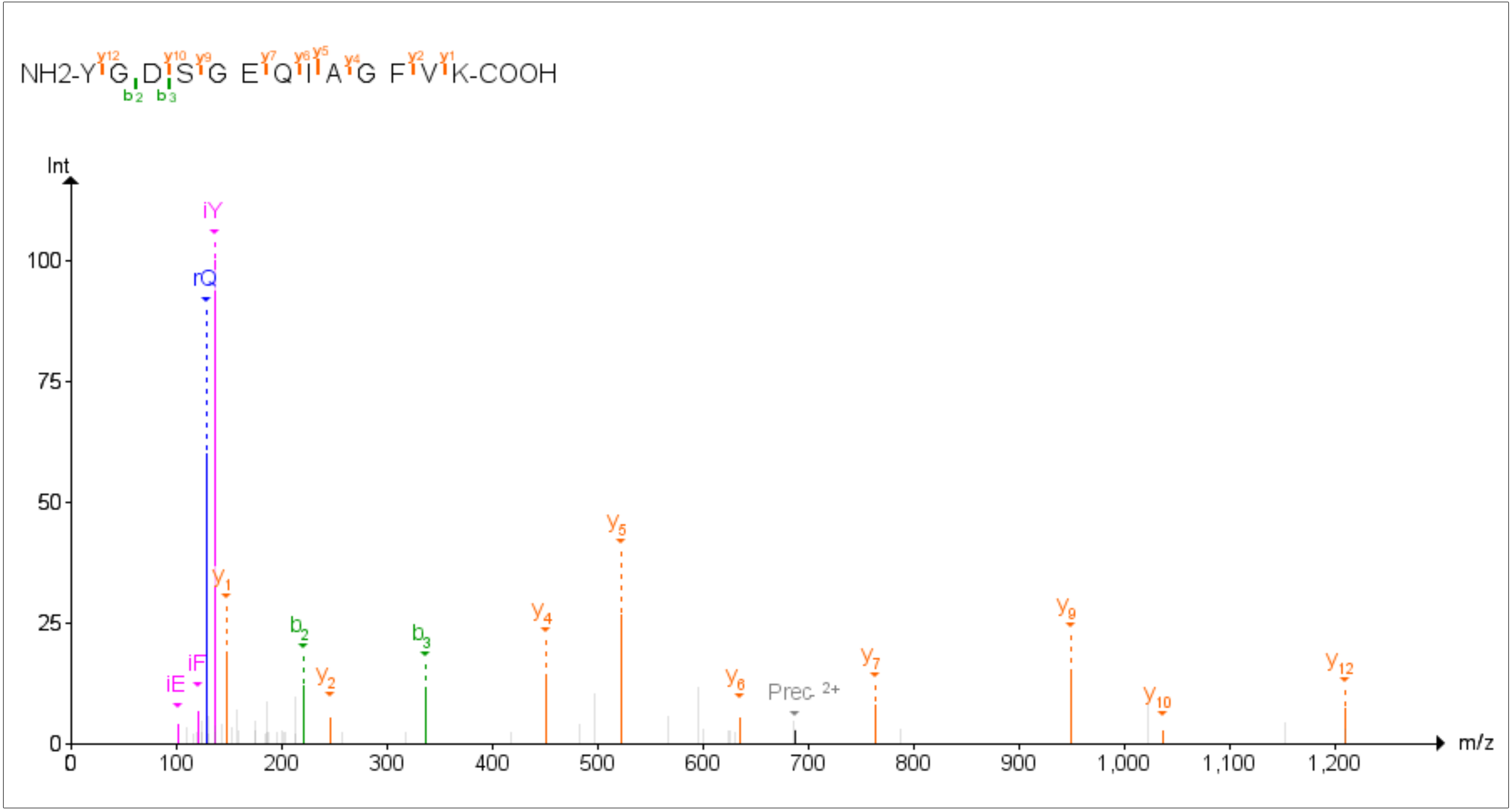
NH2-YGDISGEQIAGFVK-COOH
y12 y10 y9 y7 y6 y5 y4 y2 y1
b2 b3
Int
100
75
50
25
0
iY
rQ
y1
b2
b3
y4
y5
y9
iF
iE
y2
y6
Prec 2+
y7
y10
y12
0
100
200
300
400
500
600
700
800
900
1,000
1,100
1,200
m/z

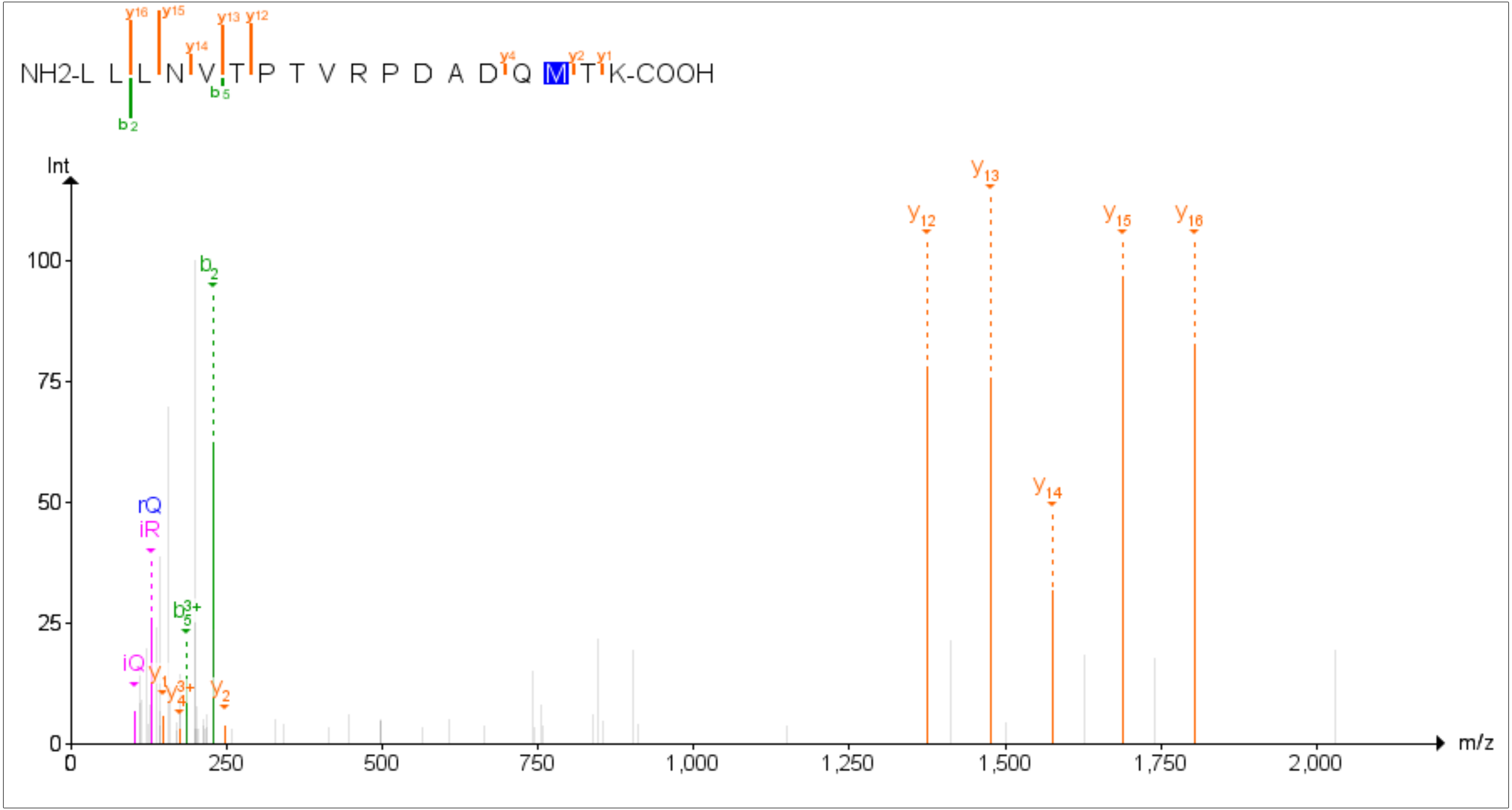

NH2-L L L N V T P T V R P D A D Q M T K-COOH
y16 y15 y14 y13 y12 y4 y2 y1
b2 b5
Int
100
75
50
25
0
0 250 500 750 1,000 1,250 1,500 1,750 2,000
m/z
b2
rQ
iR
b5 3+
iQ
y1
y4 3+
y2
y12
y13
y14
y15
y16

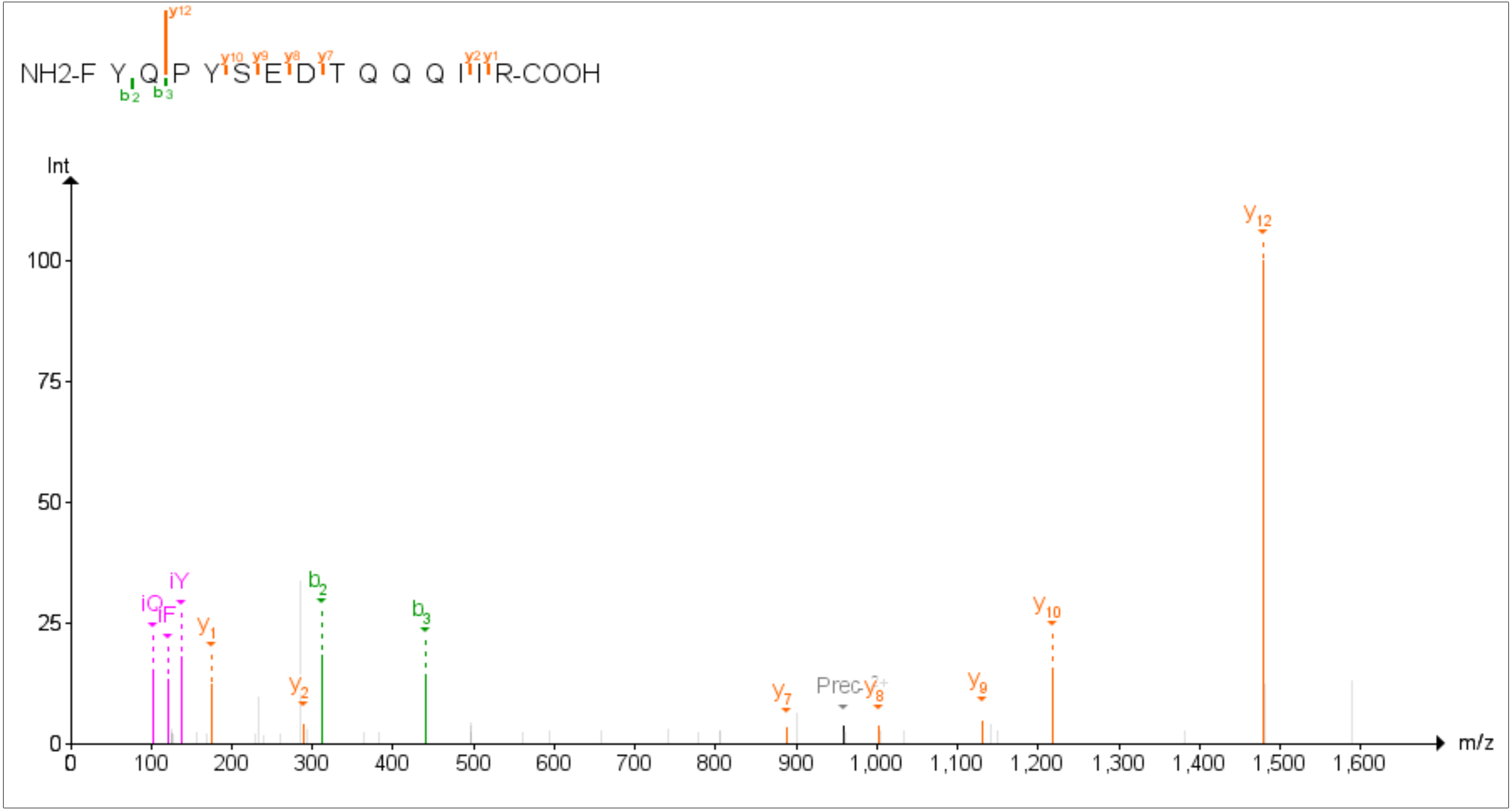
NH2-F Y Q P Y S E D T Q Q Q I I R-COOH
y12
y10 y9 y8 y7
y2 y1
b2 b3
Int
100
75
50
25
0
m/z
0 100 200 300 400 500 600 700 800 900 1,000 1,100 1,200 1,300 1,400 1,500 1,600
y12
iY
iQ
iF
y1
b2
b3
y2
y7
Prec
y8
y9
y10

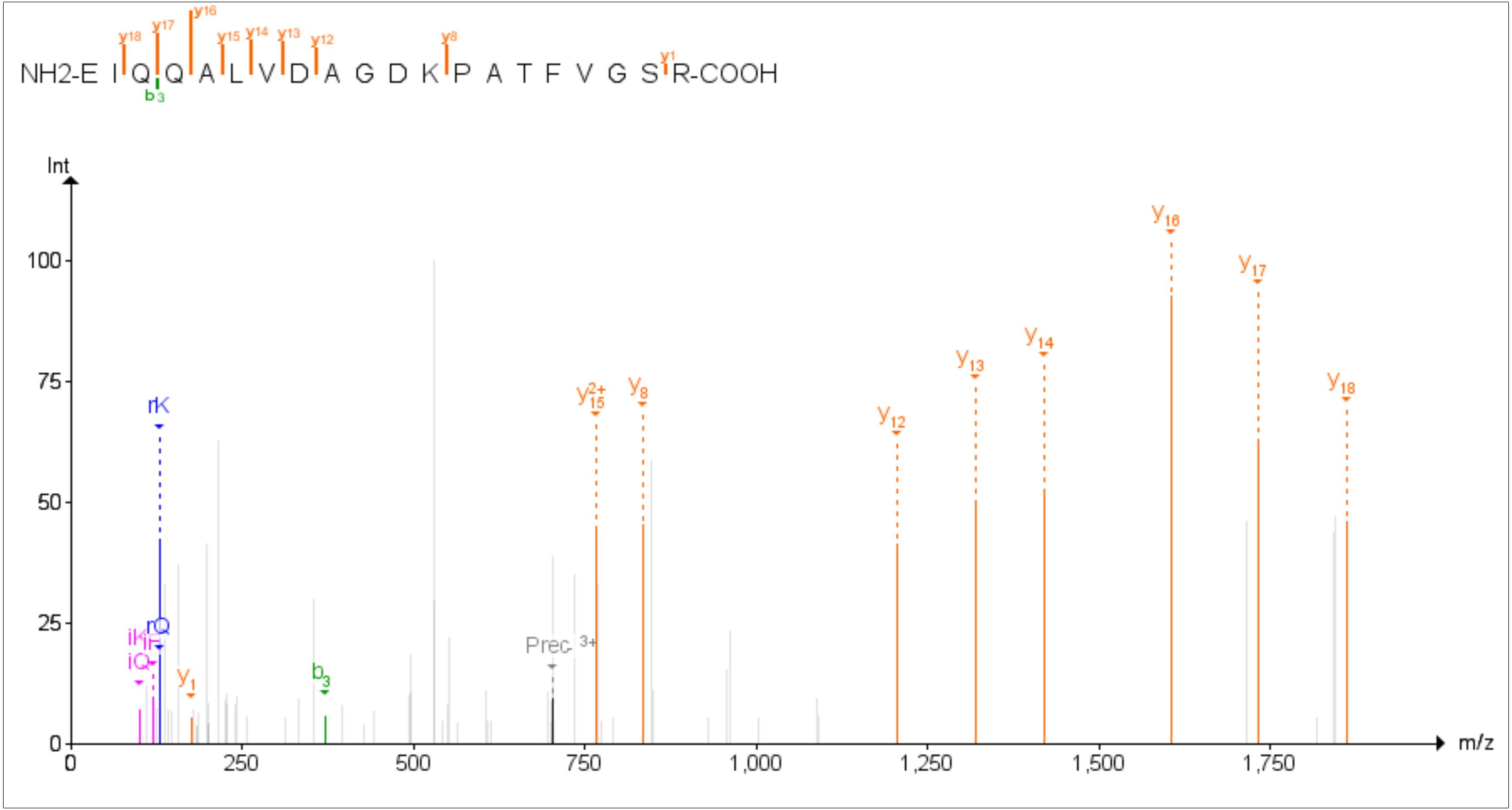

NH2-E I Q Q A L V D A G D K P A T F V G S R-COOH
y18 y17 y16 y15 y14 y13 y12 y8 y1
b3
Int
100
75
50
25
0
rK
rQ
iK
iF
iQ
y1
b3
Prec 3+
y15 2+
y8
y12
y13
y14
y16
y17
y18
0 250 500 750 1,000 1,250 1,500 1,750
m/z

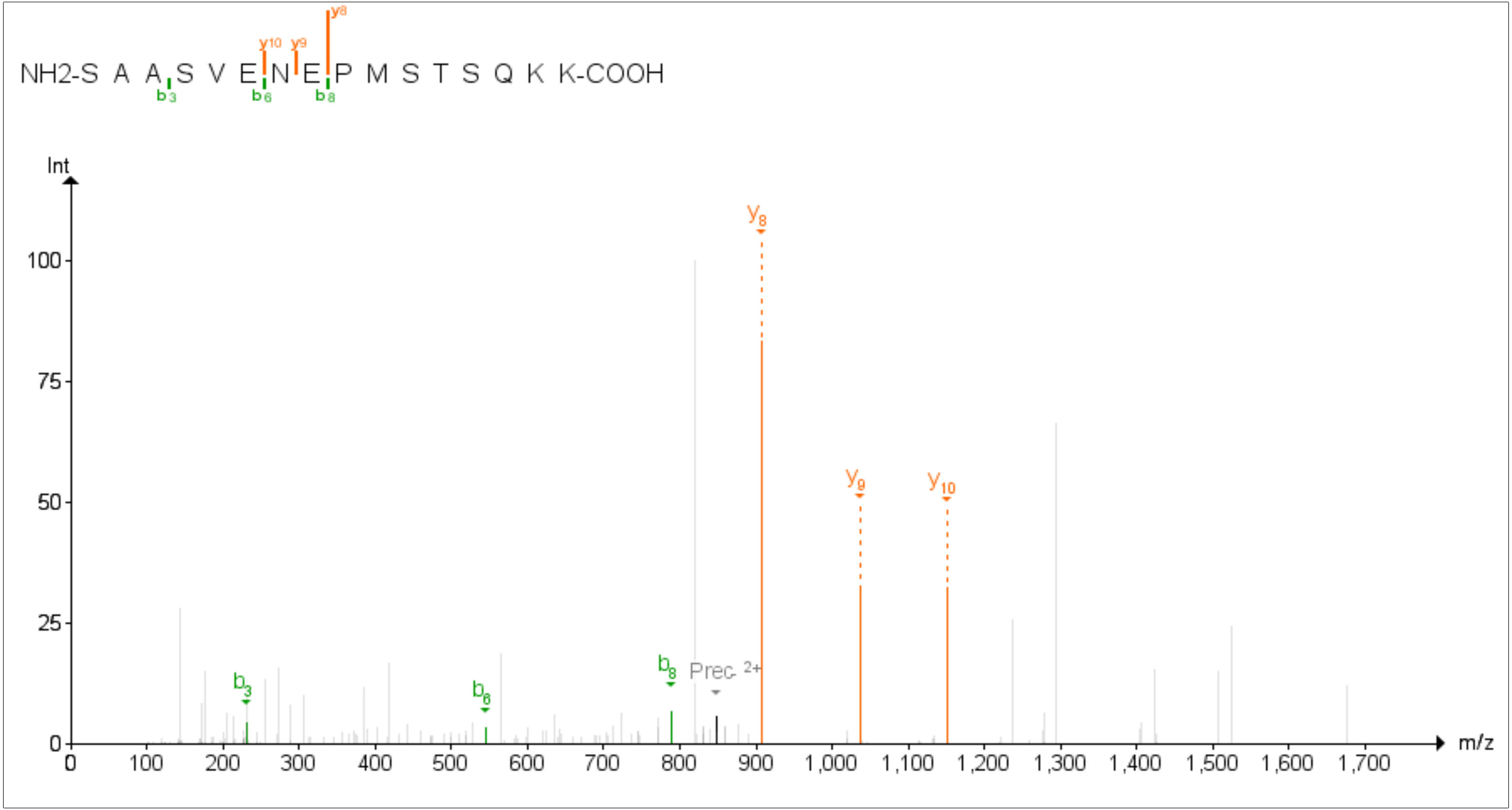
NH2-S A A S V E N E P M S T S Q K K-COOH
y10 y9 y8
b3 b6 b8
Int
m/z
y8
y9
y10
b3
b6
b8
Prec 2+
0
25
50
75
100
0
100
200
300
400
500
600
700
800
900
1,000
1,100
1,200
1,300
1,400
1,500
1,600
1,700

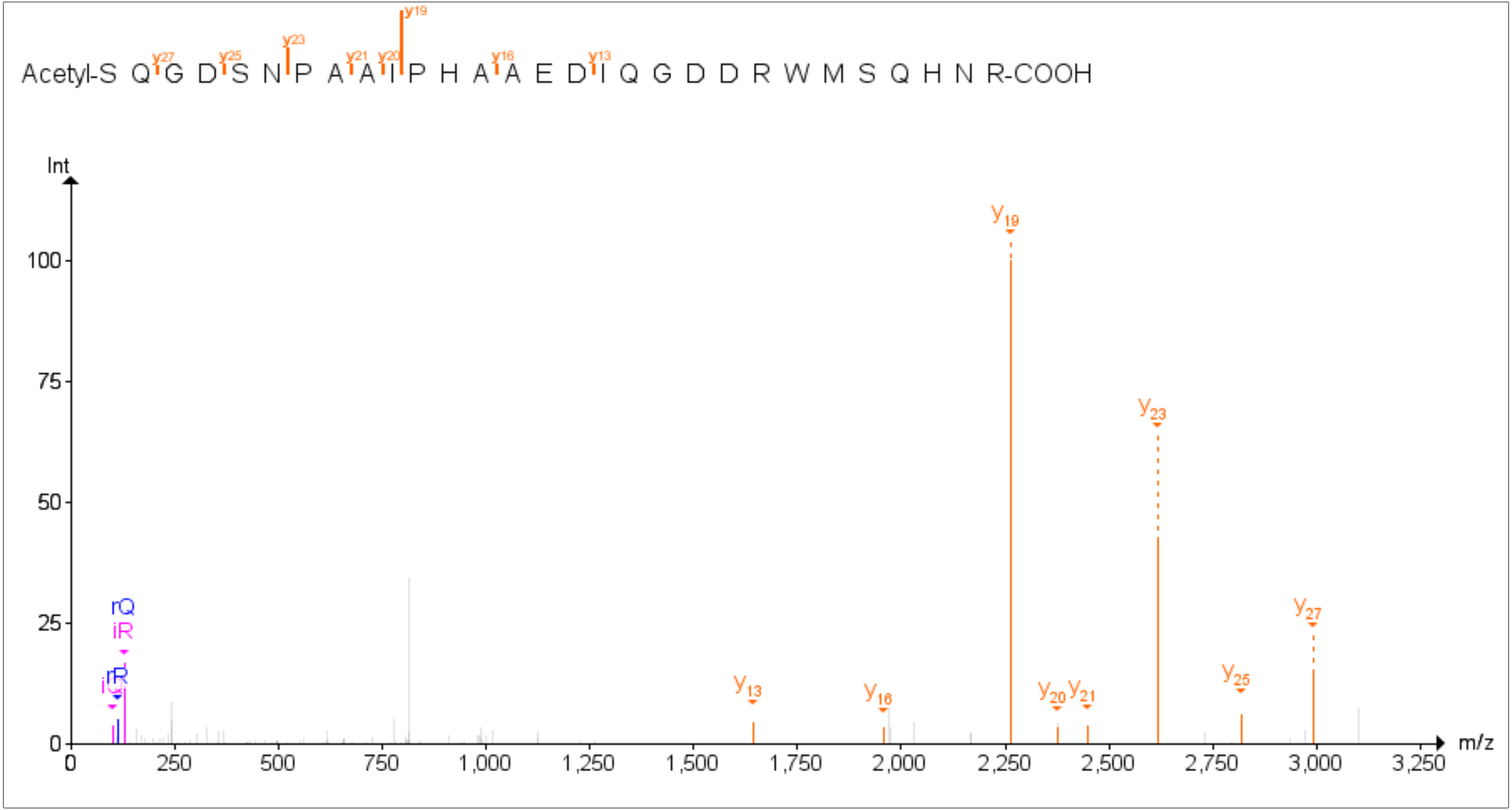
Acetyl-S Q G D S N P A A I P H A A E D I Q G D D R W M S Q H N R-COOH
y27
y25
y23
y21
y20
y19
y16
y13
Int
100
75
50
25
0
rQ
iR
rR
iQ
y13
y16
y19
y20
y21
y23
y25
y27
0
250
500
750
1,000
1,250
1,500
1,750
2,000
2,250
2,500
2,750
3,000
3,250
m/z

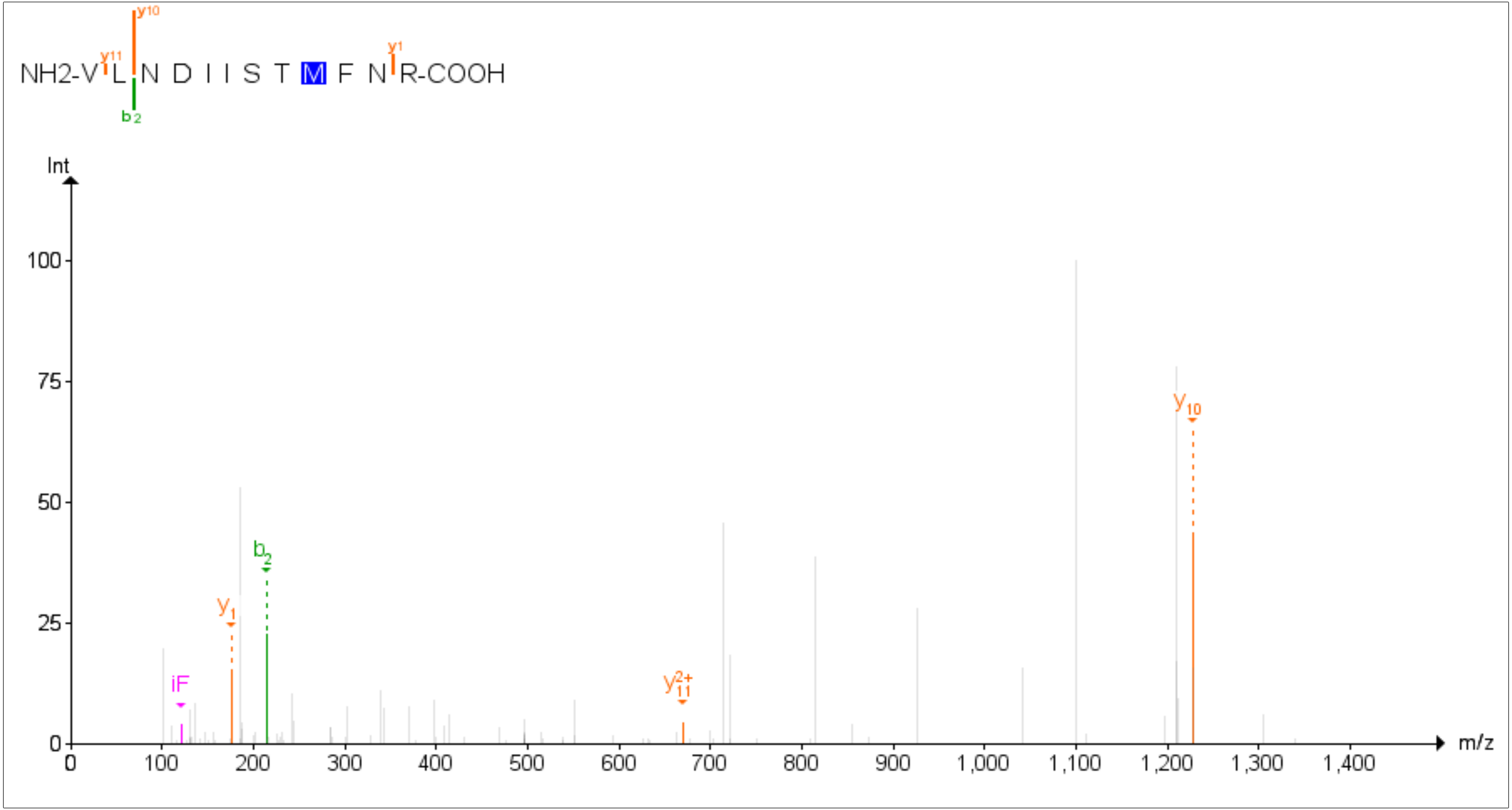

NH2-V L N D I I S T M F N R-COOH
y10
y11
y1
b2
Int
100
75
50
25
0
b2
y1
iF
y10
y11 2+
0
100
200
300
400
500
600
700
800
900
1,000
1,100
1,200
1,300
1,400
m/z

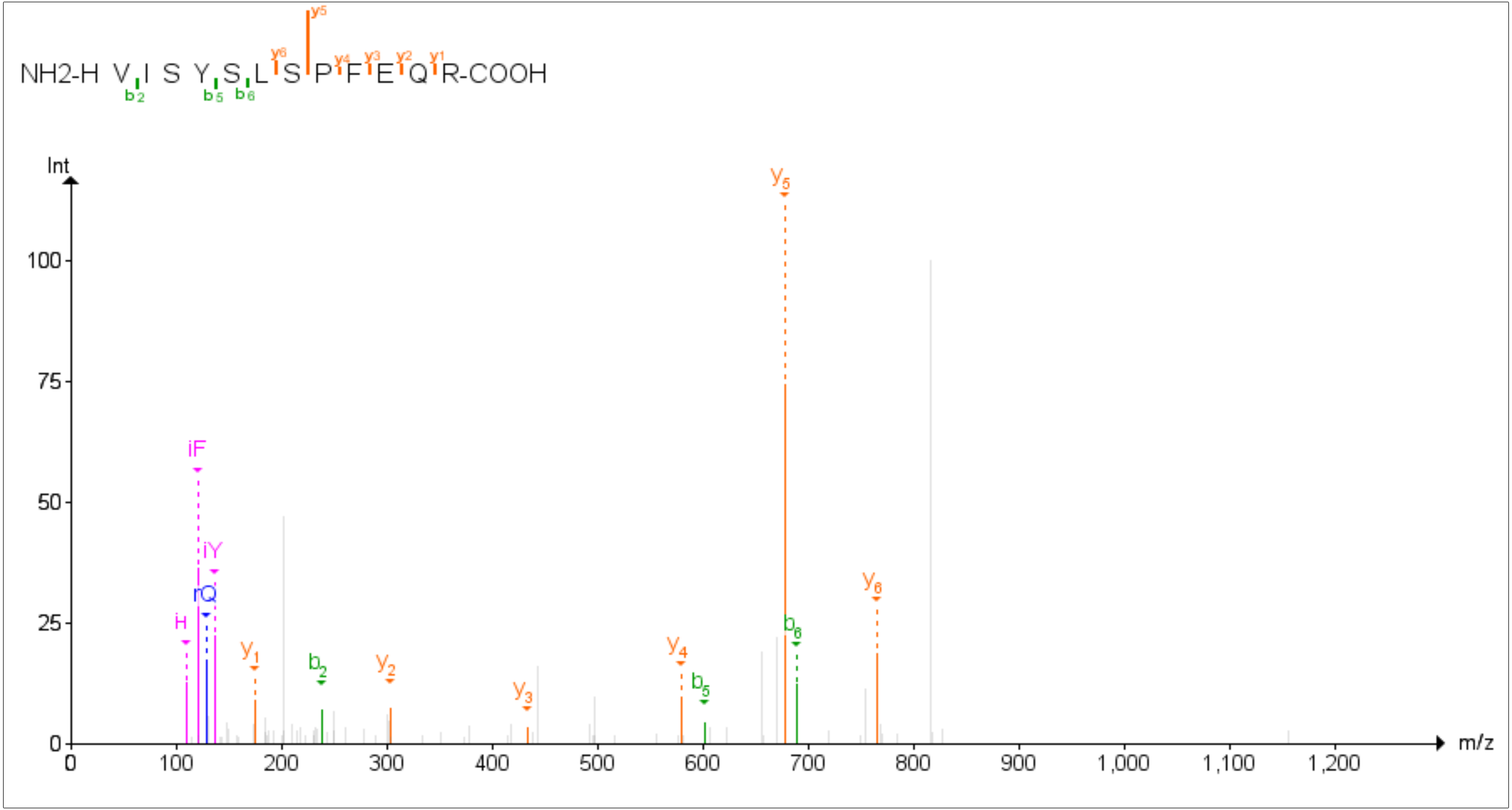

NH2-H V I S Y S L S P F E Q R-COOH
b2
b5
b6
y6
y5
y4
y3
y2
y1
Int
m/z
iF
iY
rQ
iH
y1
b2
y2
y3
y4
b5
y5
b6
y6

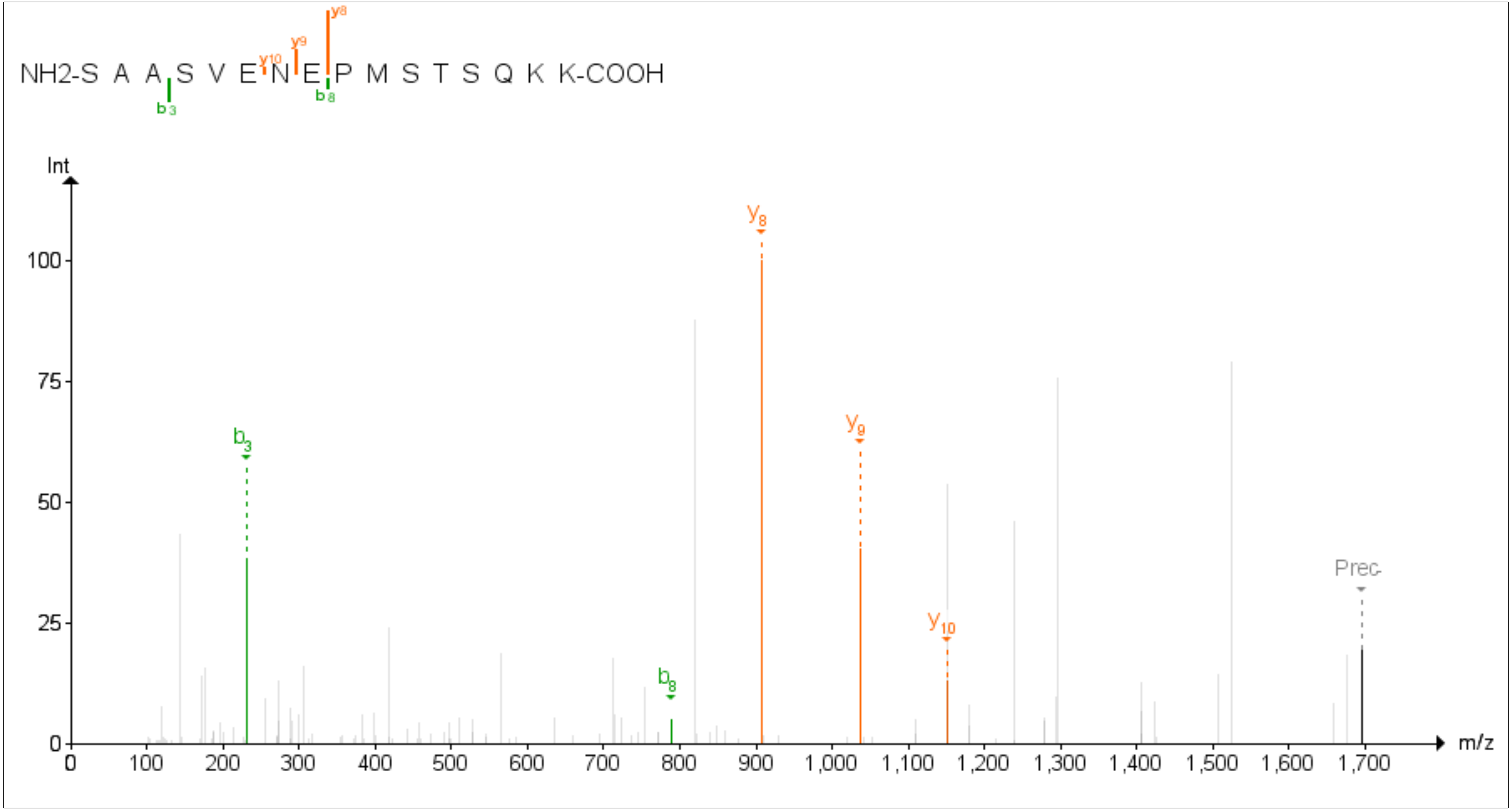

NH2-S A A S V E N E P M S T S Q K K-COOH
y8
y9
y10
b3
b8
Int
100
75
50
25
0
y8
b3
y9
Prec
y10
b8
m/z
0
100
200
300
400
500
600
700
800
900
1,000
1,100
1,200
1,300
1,400
1,500
1,600
1,700

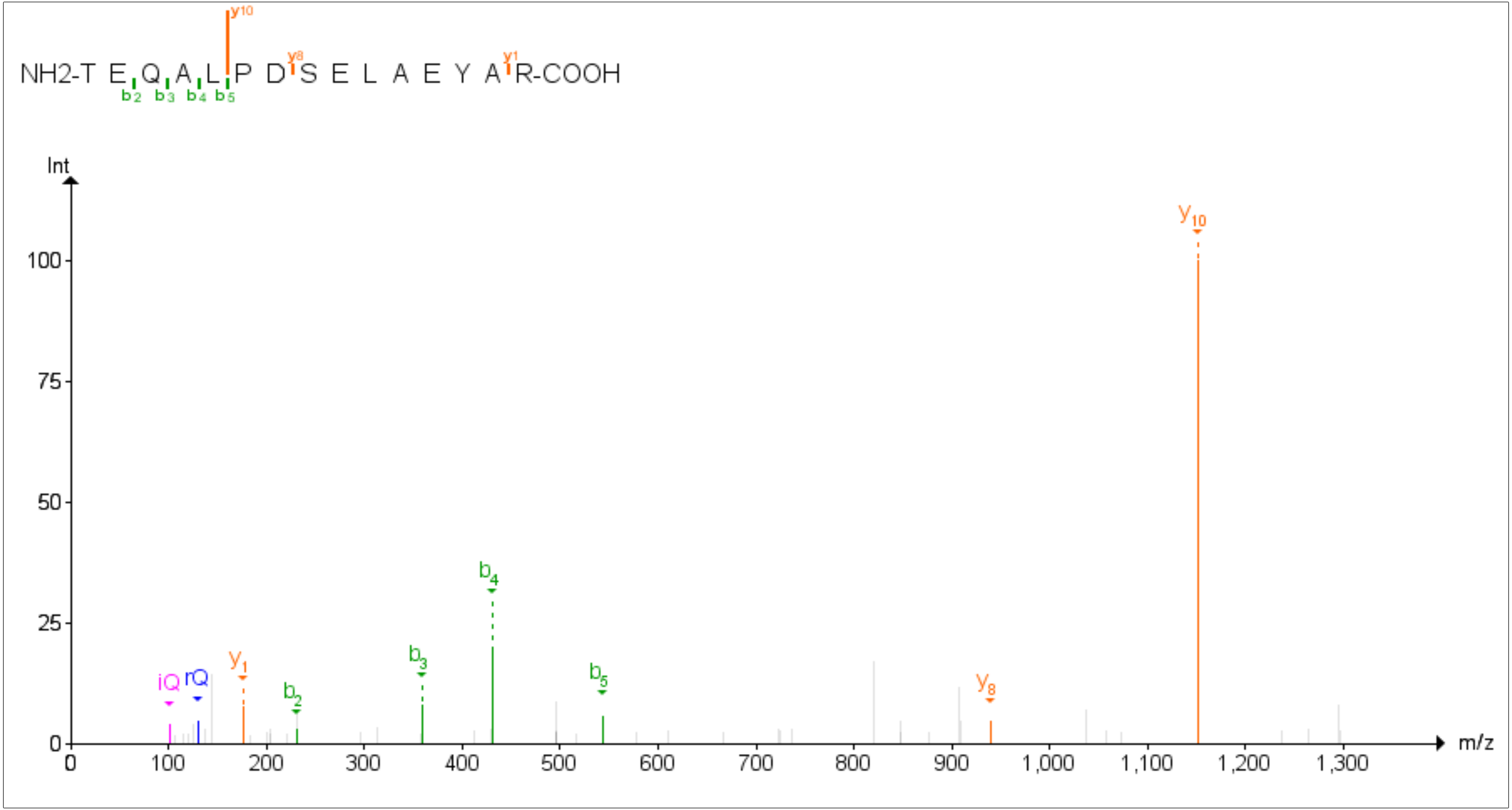

NH2-T E Q A L P D S E L A E Y A R-COOH
y10
y8
y1
b2 b3 b4 b5
Int
100
75
50
25
0
y10
b4
b3
b5
y8
y1
iQ
rQ
b2
0
100
200
300
400
500
600
700
800
900
1,000
1,100
1,200
1,300
m/z

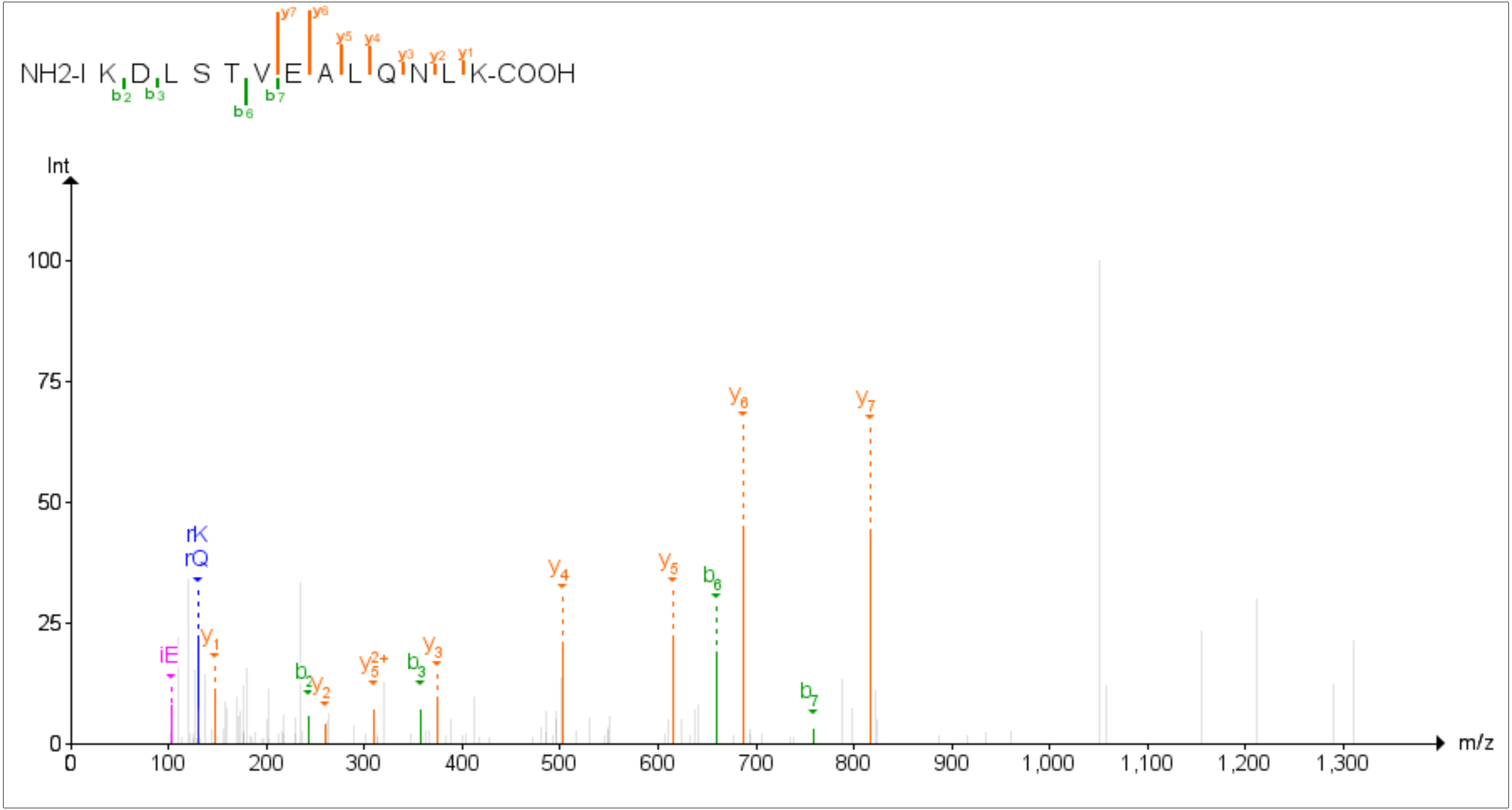

NH2-I K D L S T V E A L Q N L K-COOH
b2 b3 b6 b7
y7 y6 y5 y4 y3 y2 y1
Int
100
75
50
25
0
m/z
0 100 200 300 400 500 600 700 800 900 1,000 1,100 1,200 1,300
rK
rQ
iE
y1
b2
y2
y5 2+
b3
y3
y4
y5
b6
y6
y7
b7

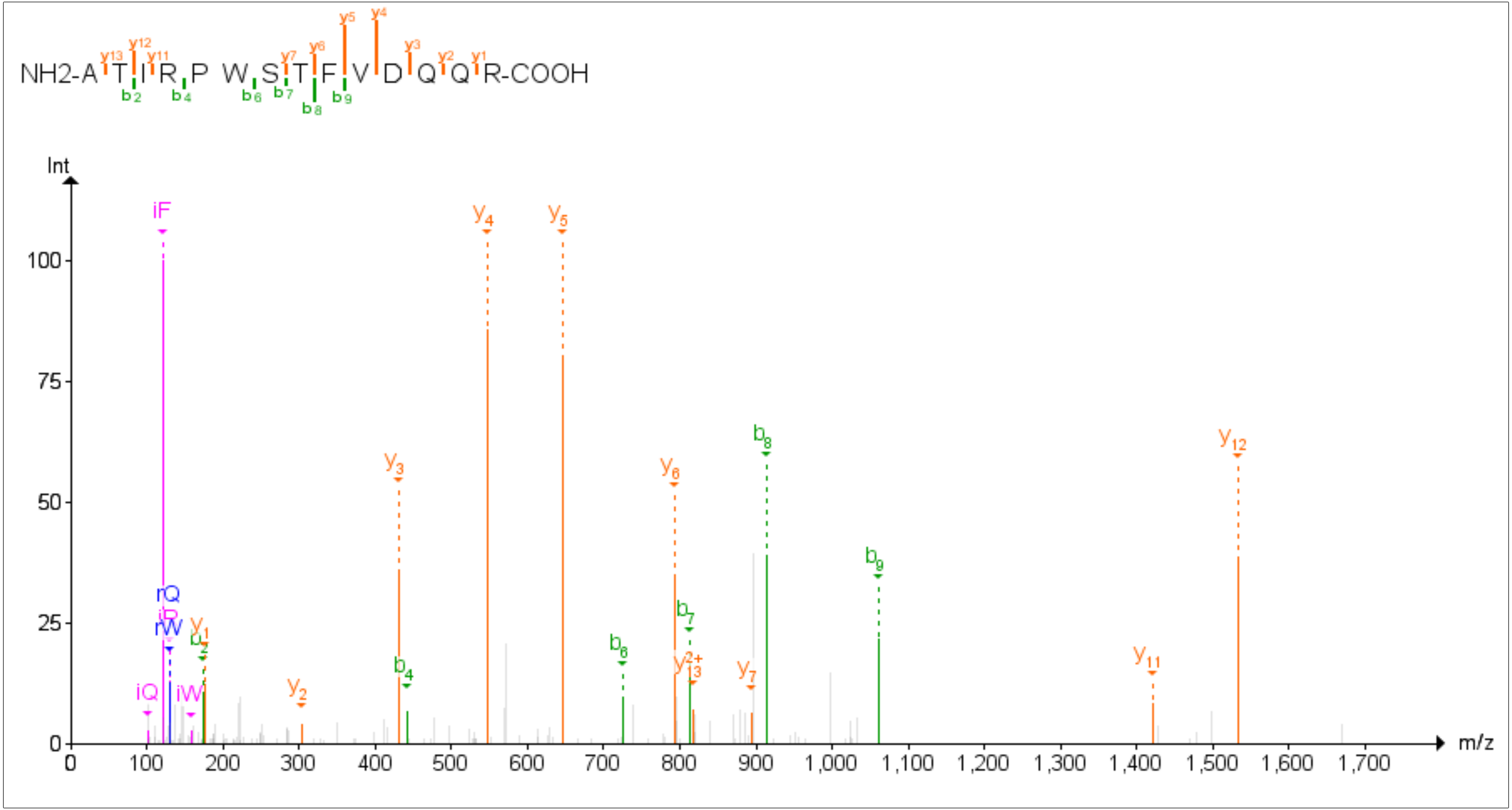

NH2-A T I R P W S T F V D Q Q R-COOH
y13 y12 y11 y7 y6 y5 y4 y3 y2 y1
b2 b4 b6 b7 b8 b9
Int
m/z
iF
y4
y5
y3
y6
b8
y12
b9
rQ
iP
rW
y1
b2
iQ
iW
y2
b4
b6
b7
y13 2+
y7
y11
0
25
50
75
100
0
100
200
300
400
500
600
700
800
900
1,000
1,100
1,200
1,300
1,400
1,500
1,600
1,700

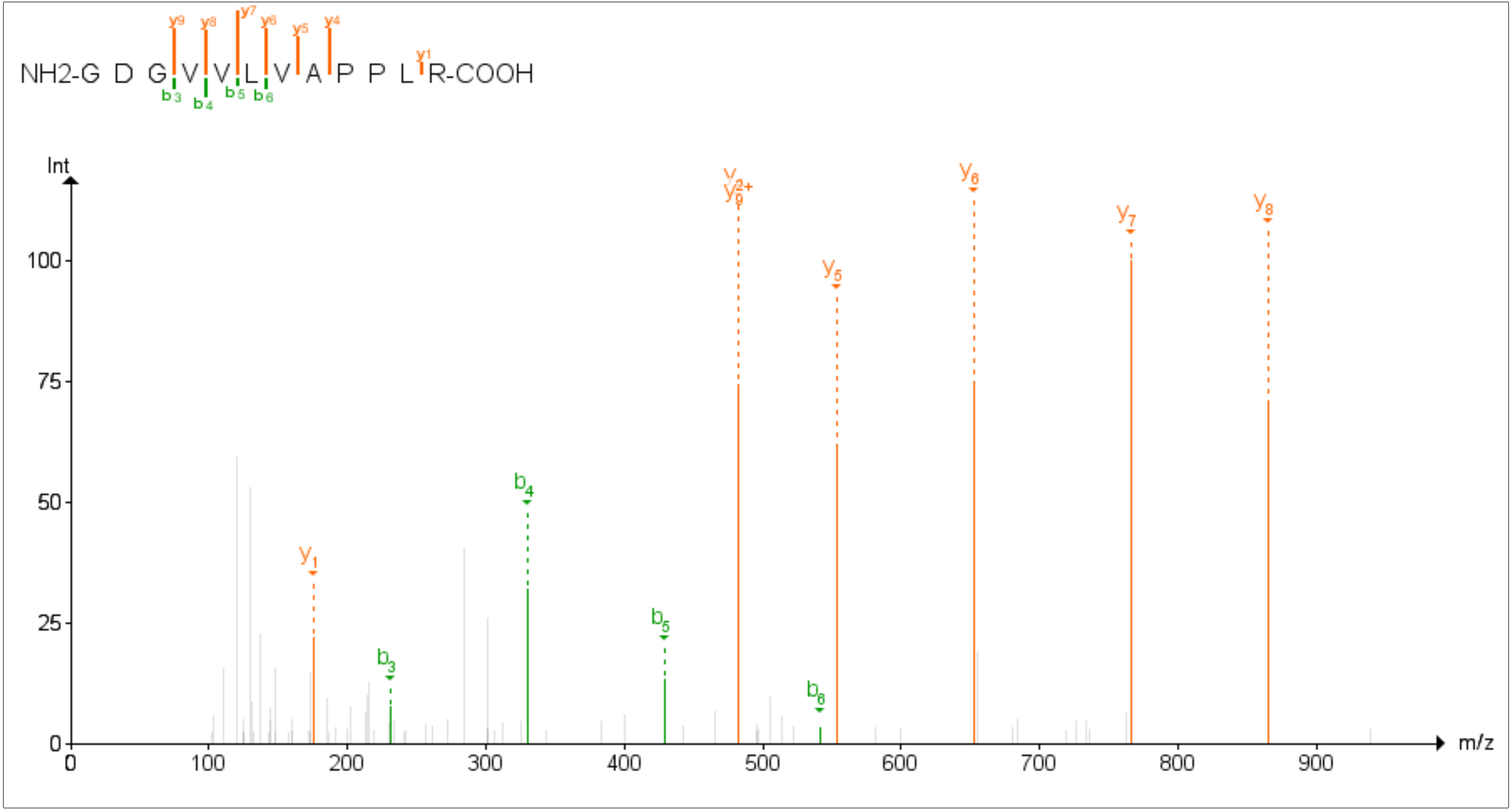

NH2-G D G V V L V A P P L R-COOH
y9 y8 y7 y6 y5 y4 y1
b3 b4 b5 b6
Int
m/z
0
25
50
75
100
100
200
300
400
500
600
700
800
900
y1
b3
b4
b5
y9 2+
y5
b6
y6
y7
y8

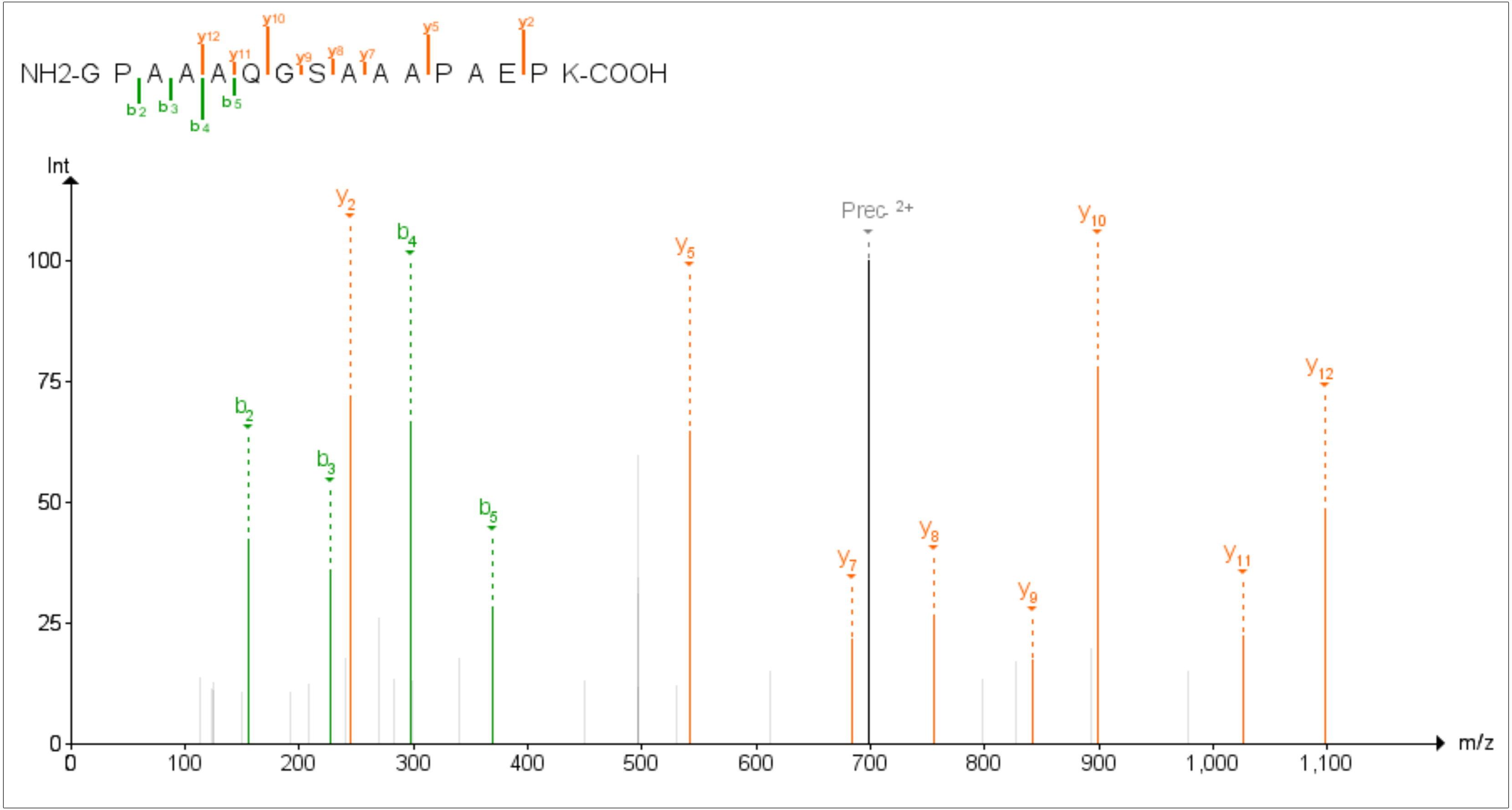
NH2-G P A A A Q G S A A A P A E P K-COOH
y12
y10
y11
y9
y8
y7
y5
y2
b2
b3
b4
b5
Int
100
75
50
25
0
y2
b4
y5
Prec. 2+
y10
b2
b3
b5
y12
y7
y8
y9
y11
0
100
200
300
400
500
600
700
800
900
1,000
1,100
m/z

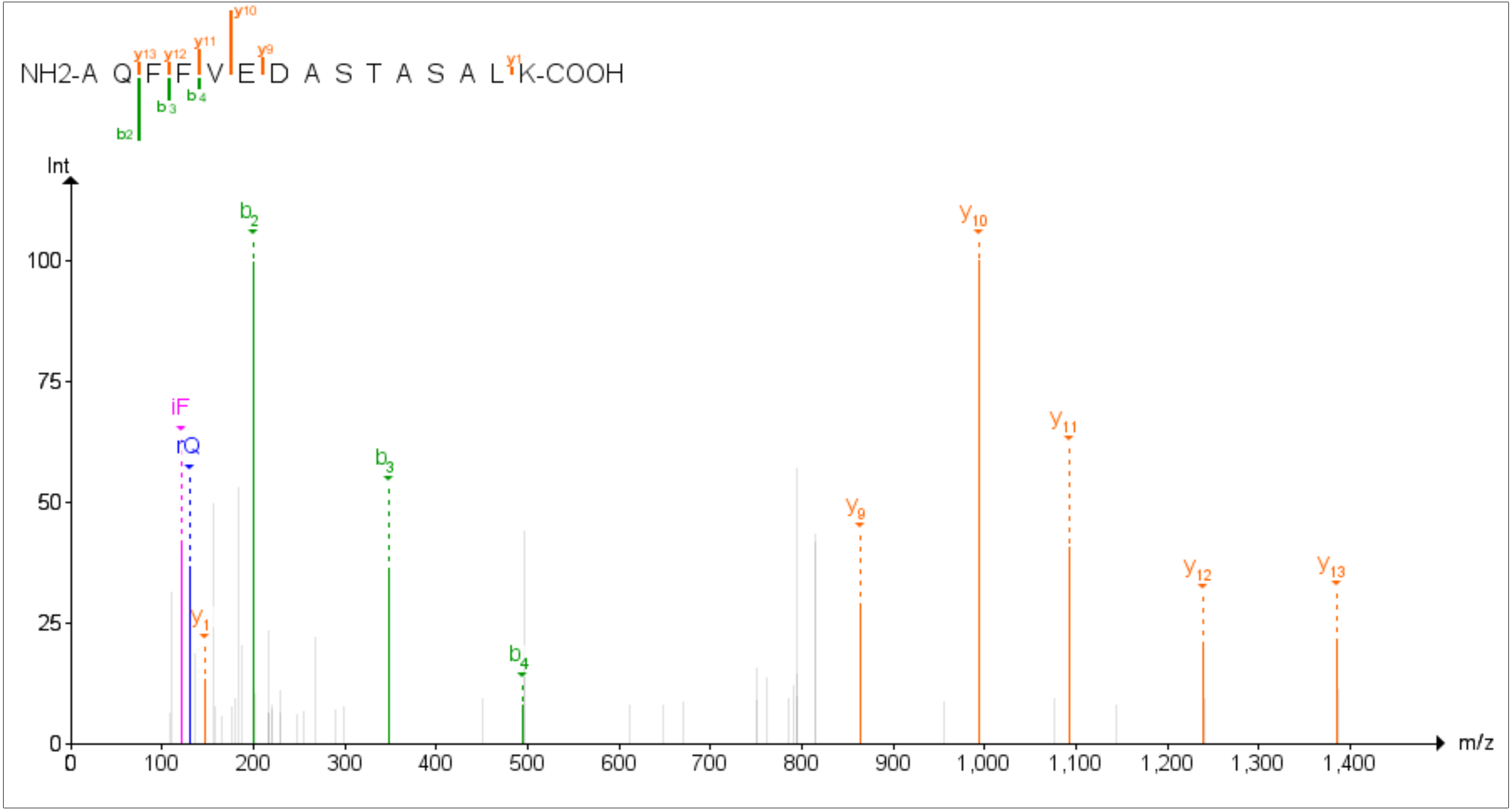

NH2-A Q F F V E D A S T A S A L K-COOH
y13
y12
y11
y10
y9
y1
b2
b3
b4
Int
m/z
b2
y10
iF
rQ
b3
y11
y9
y1
b4
y12
y13
0
25
50
75
100
0
100
200
300
400
500
600
700
800
900
1,000
1,100
1,200
1,300
1,400

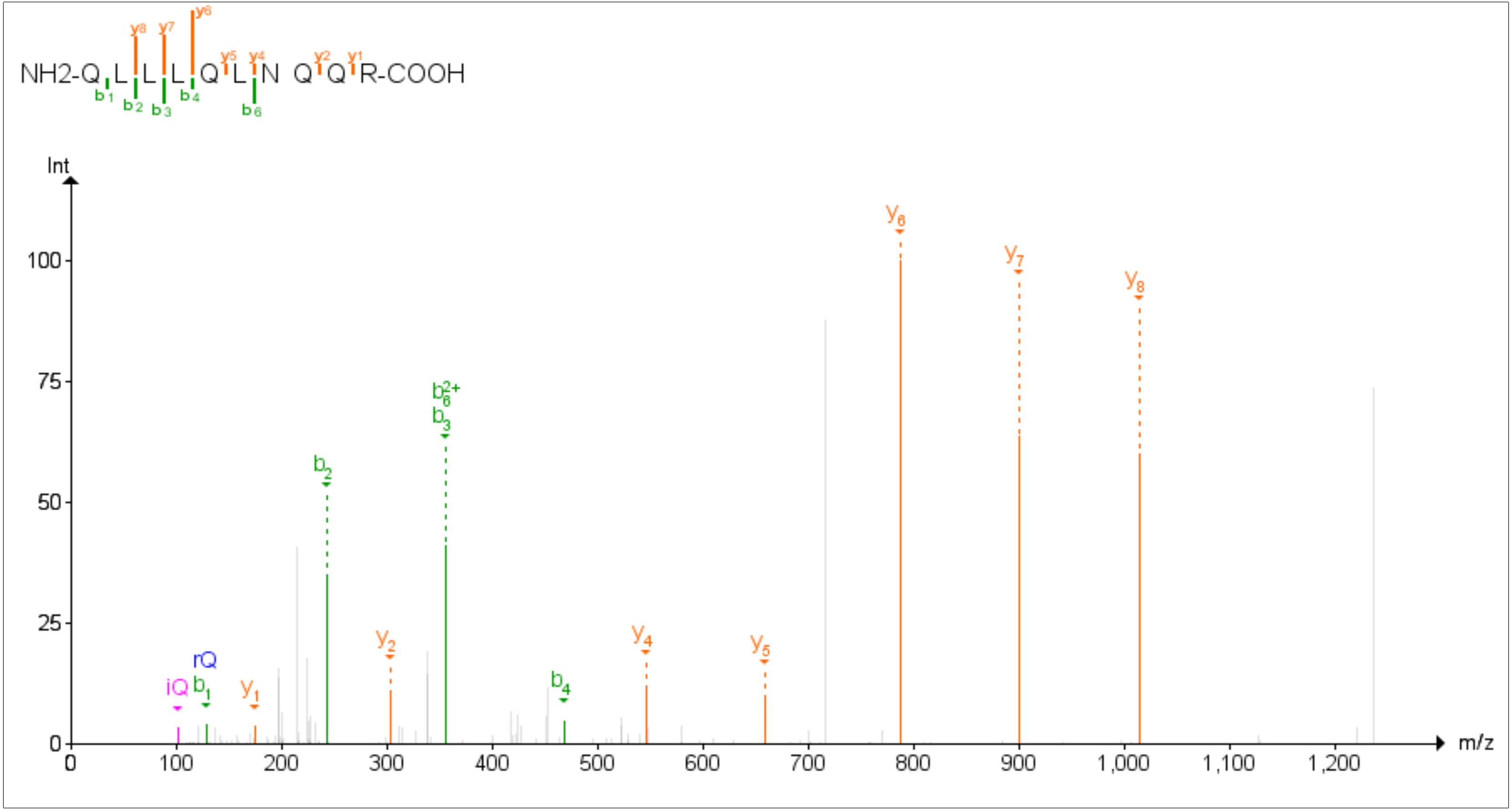

NH2-Q L L L Q L N Q Q R-COOH
y8 y7 y6 y5 y4 y2 y1
b1 b2 b3 b4 b6
Int
100
75
50
25
0
m/z
0 100 200 300 400 500 600 700 800 900 1,000 1,100 1,200
iQ
rQ
b1
y1
b2
b3
b6 2+
y2
b4
y4
y5
y6
y7
y8

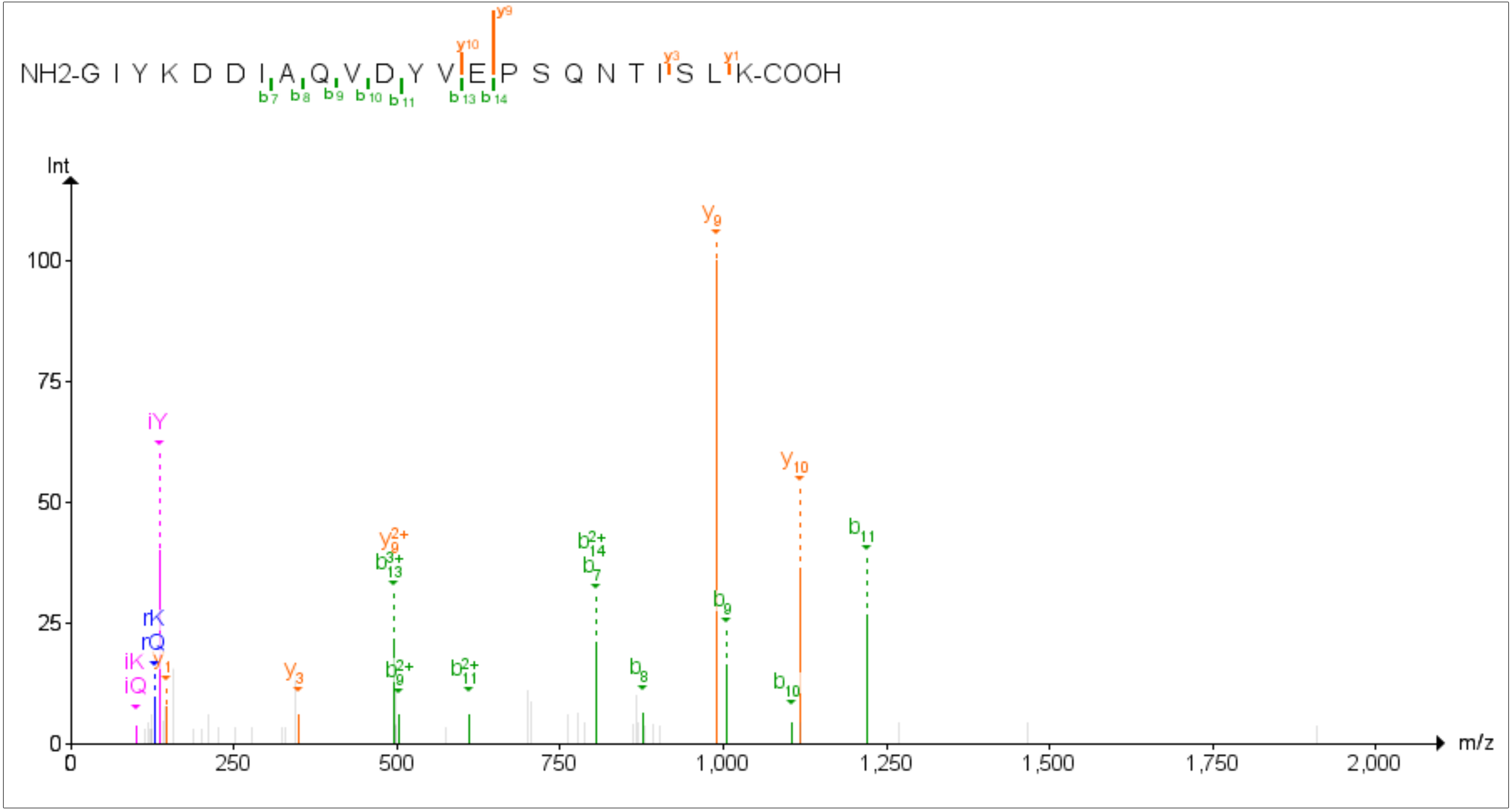
NH2-G I Y K D D I A Q V D Y V E P S Q N T I S L K-COOH
Int
m/z
iY
rK
rQ
iK
iQ
y1
y3
y9
y10
b11
b9
b8
b10
b7
b14 2+
b13 3+
y9 2+
b9 2+
b11 2+
0
25
50
75
100
250
500
750
1,000
1,250
1,500
1,750
2,000

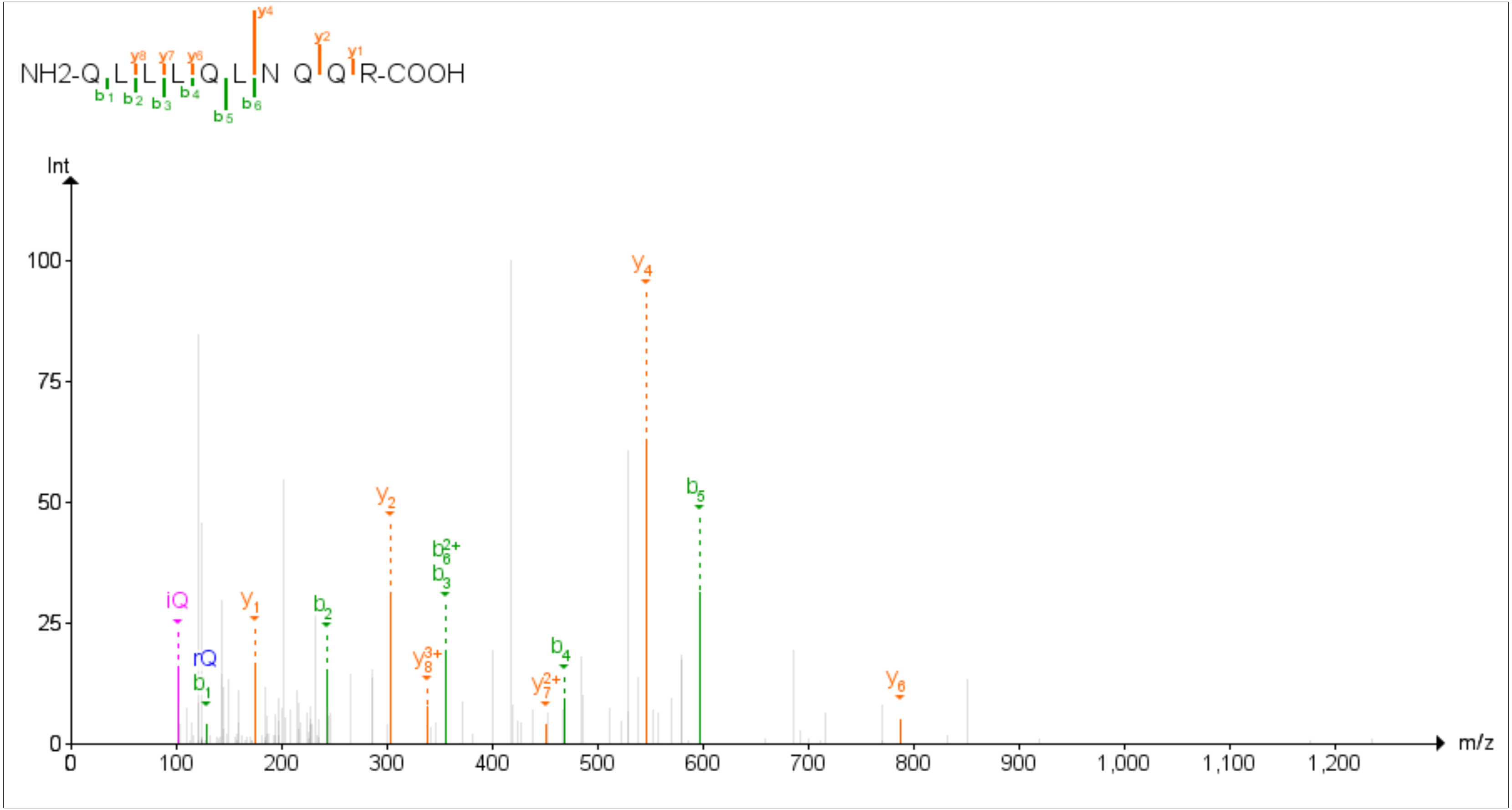

NH2-Q L L L Q L N Q Q R-COOH
y8 y7 y6 y4 y2 y1
b1 b2 b3 b4 b5 b6
Int
100
75
50
25
0
iQ
rQ
b1
y1
b2
y2
y8 3+
b6 2+
b3
y7 2+
b4
y4
b5
y6
0
100
200
300
400
500
600
700
800
900
1,000
1,100
1,200
m/z

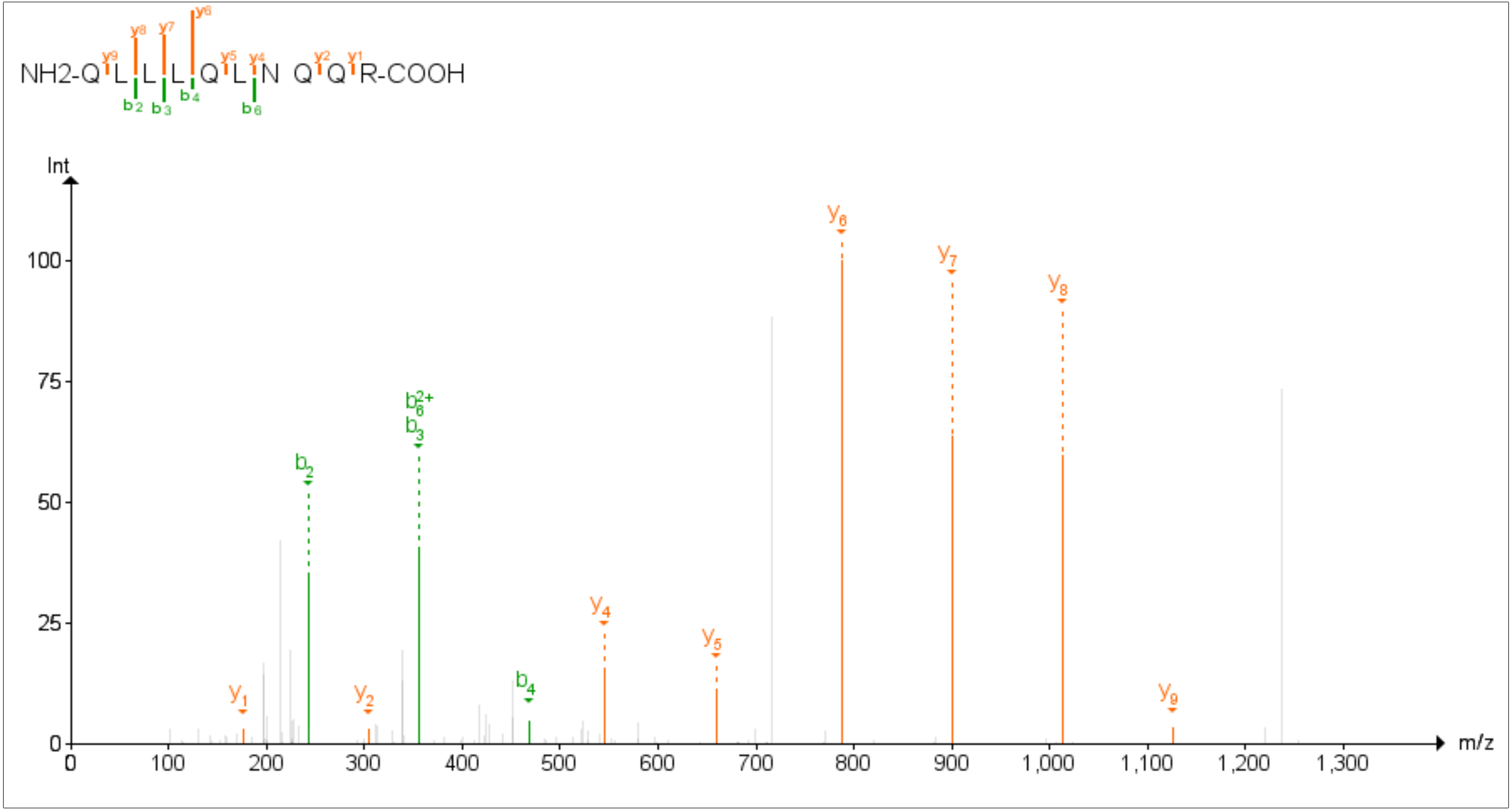

NH2-Q L L L Q L N Q Q R-COOH
y9 y8 y7 y6 y5 y4 y2 y1
b2 b3 b4 b6
Int
100
75
50
25
0
b2
b6 2+
b3
b4
y1
y2
y4
y5
y6
y7
y8
y9
0 100 200 300 400 500 600 700 800 900 1,000 1,100 1,200 1,300
m/z

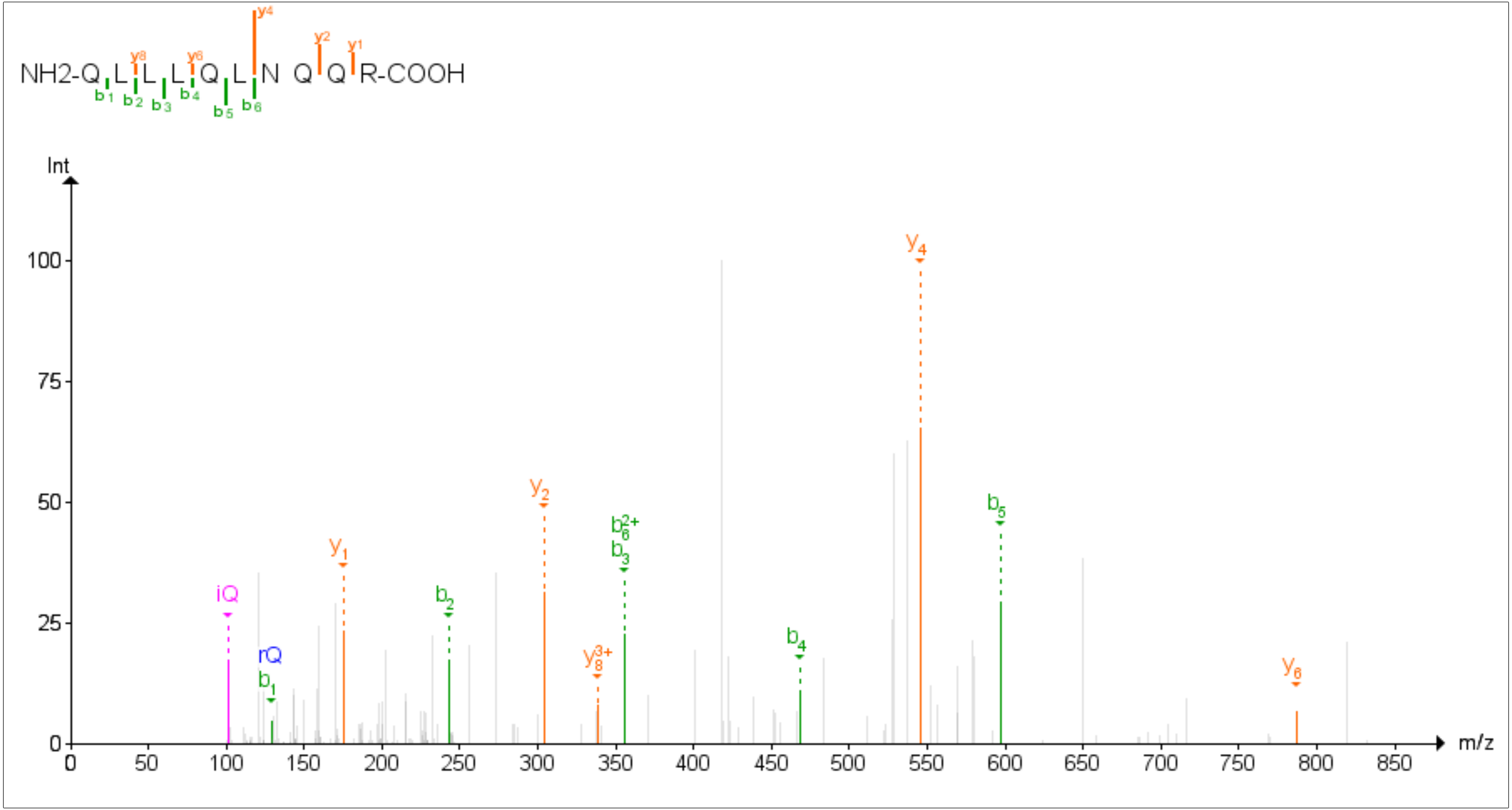

NH2-Q L L L Q L N Q Q R-COOH
y8 y6 y4 y2 y1
b1 b2 b3 b4 b5 b6
Int
100
75
50
25
0
iQ
rQ
b1
y1
b2
y2
y8 3+
b6 2+
b3
b4
y4
b5
y6
0 50 100 150 200 250 300 350 400 450 500 550 600 650 700 750 800 850
m/z

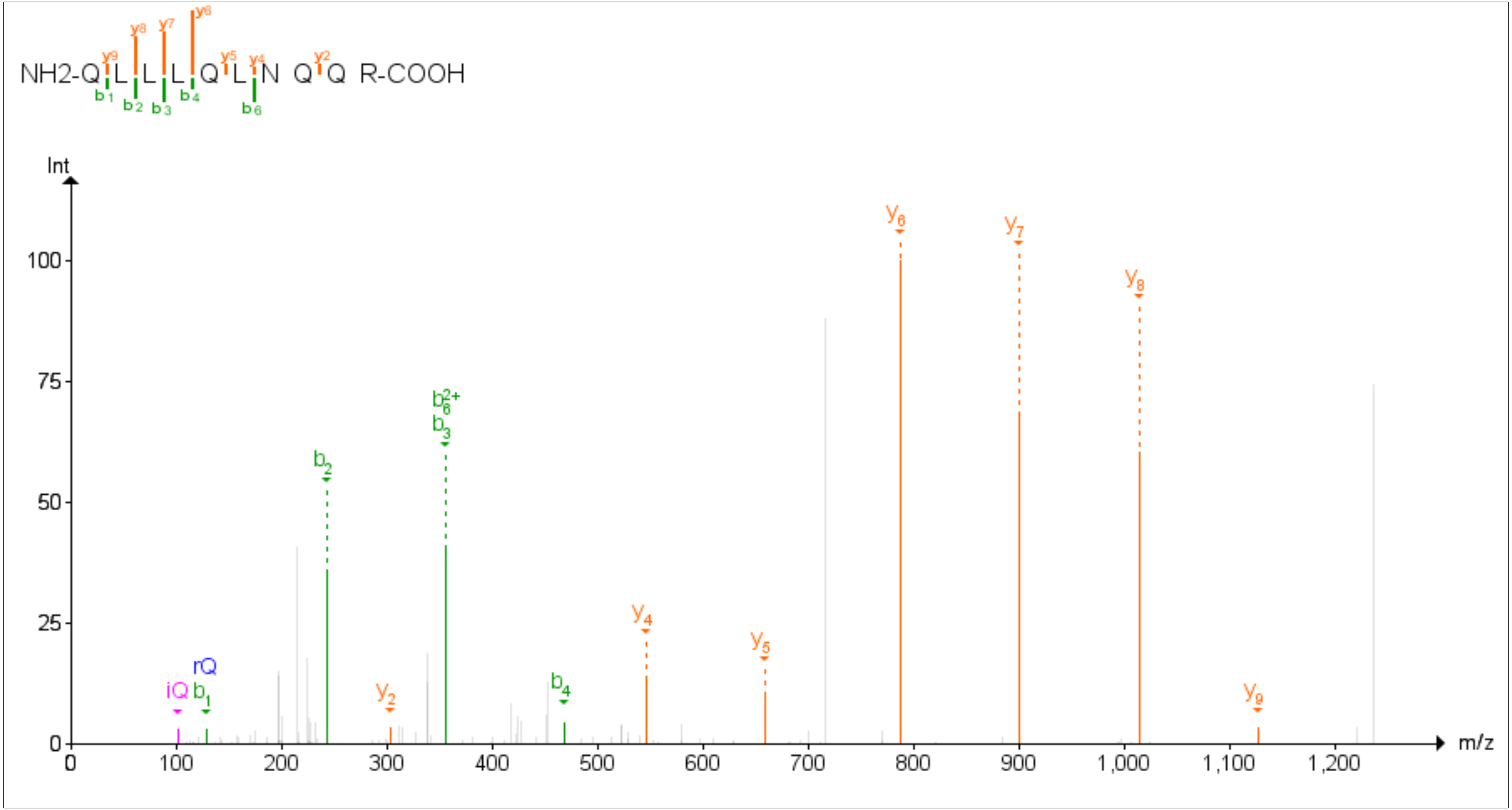

NH2-Q L L L Q L N Q Q R-COOH
y9 y8 y7 y6 y5 y4 y2
b1 b2 b3 b4 b6
Int
100
75
50
25
0
y6
y7
y8
b6 2+
b3
b2
y4
y5
rQ
iQ
b1
y2
b4
y9
0 100 200 300 400 500 600 700 800 900 1,000 1,100 1,200
m/z

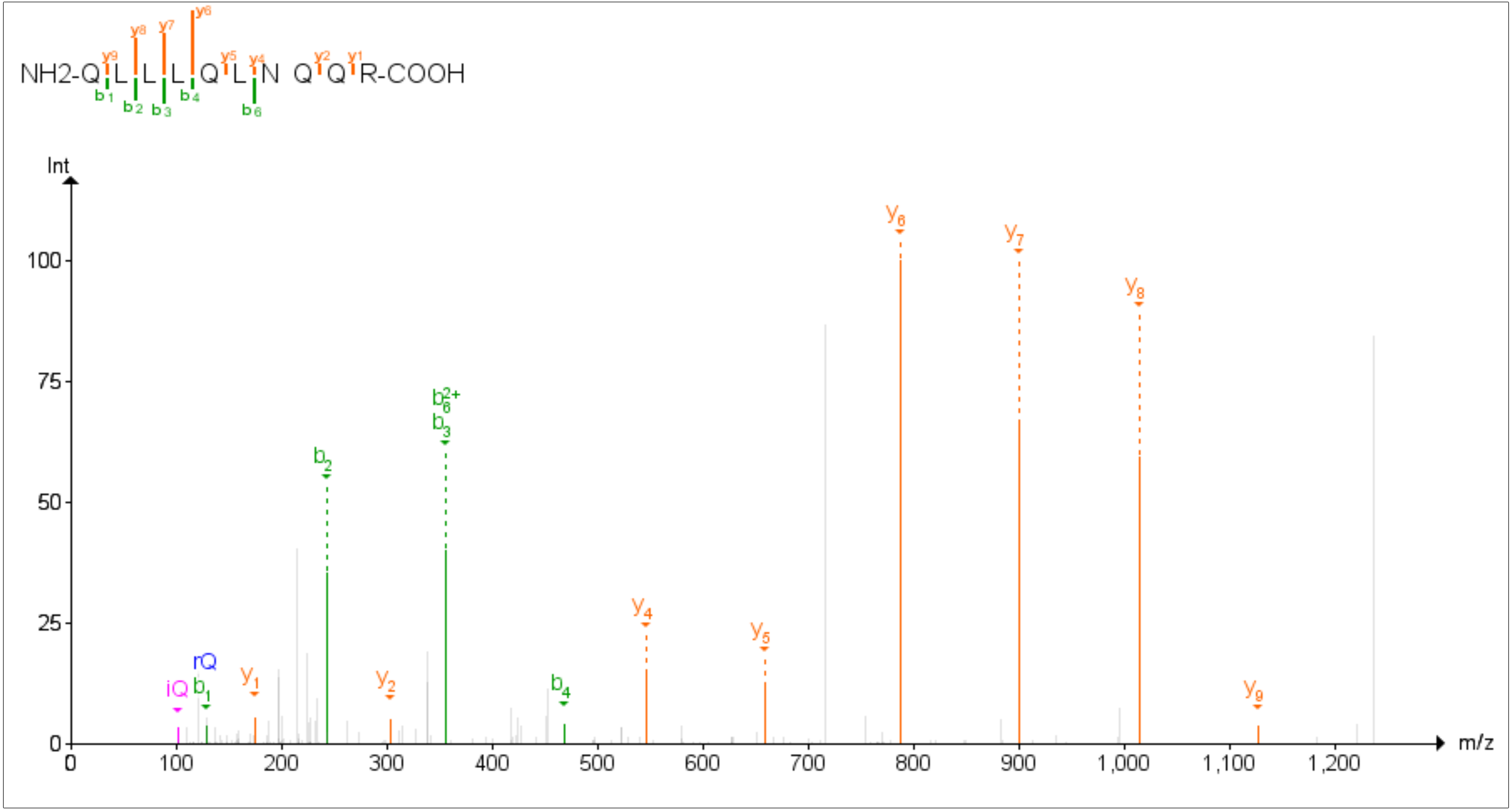
NH2-Q L L L Q L N Q Q R-COOH
y9 y8 y7 y6 y5 y4 y2 y1
b1 b2 b3 b4 b6
Int
m/z
y6
y7
y8
b6 2+
b3
b2
y4
y5
iQ
rQ
b1
y1
y2
b4
y9
0
25
50
75
100
0
100
200
300
400
500
600
700
800
900
1,000
1,100
1,200

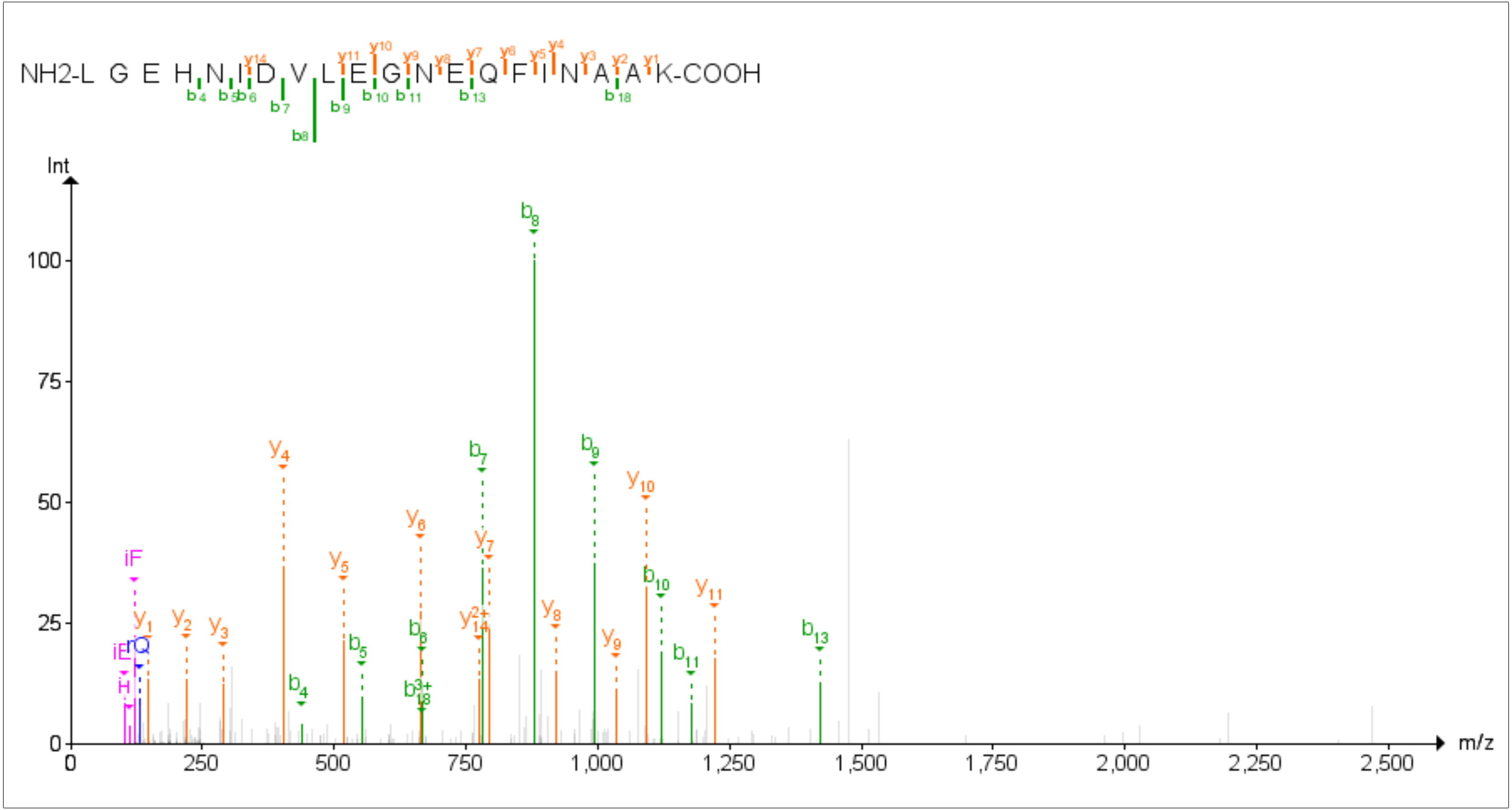
NH2-L G E H N I D V L E G N E Q F I N A A K-COOH
y14 y11 y10 y9 y8 y7 y6 y5 y4 y3 y2 y1
b4 b5 b6 b7 b8 b9 b10 b11 b13 b18
Int
m/z
0
25
50
75
100
250
500
750
1,000
1,250
1,500
1,750
2,000
2,250
2,500

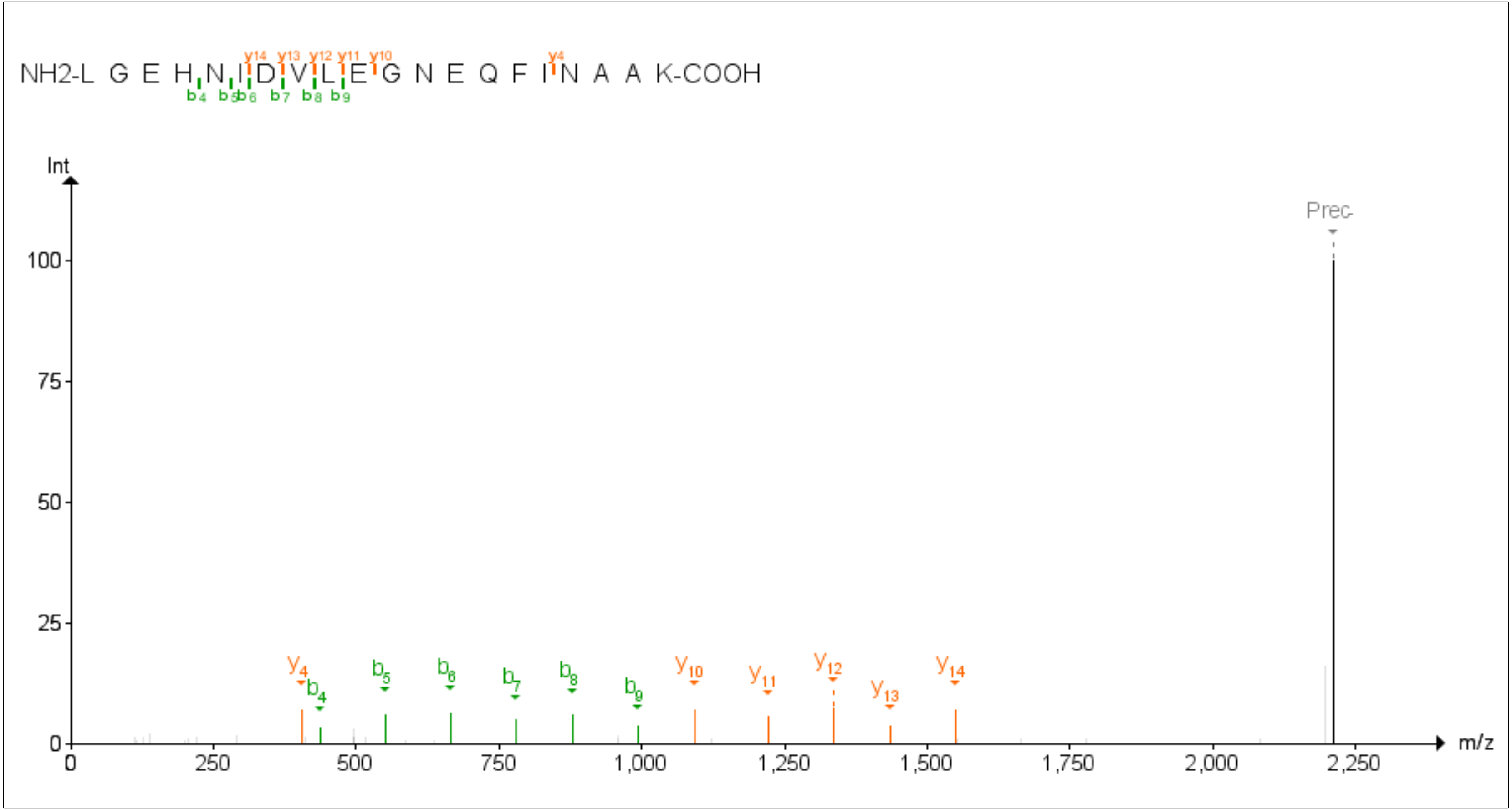

NH2-L G E H N I D V L E G N E Q F I N A A K-COOH
y14 y13 y12 y11 y10 y4
b4 b5 b6 b7 b8 b9
Int
100
75
50
25
0
Prec.
y4
b4
b5
b6
b7
b8
b9
y10
y11
y12
y13
y14
0
250
500
750
1,000
1,250
1,500
1,750
2,000
2,250
m/z

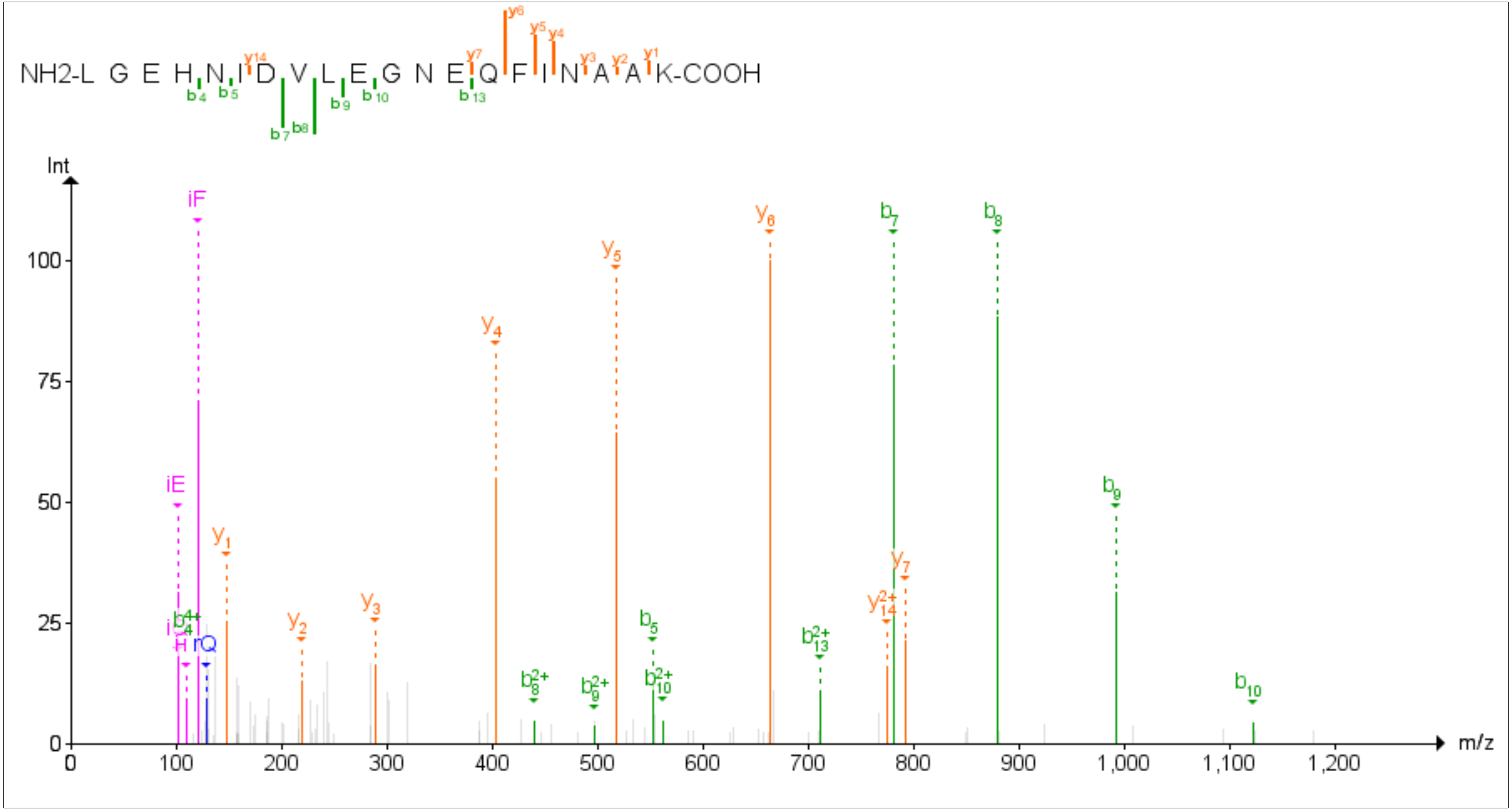

NH2-L G E H N I D V L E G N E Q F I N A A K-COOH
y14 y7 y6 y5 y4 y3 y2 y1
b4 b5 b7 b8 b9 b10 b13
Int
m/z
iF
iE
y1
y2
y3
y4
y5
y6
y7
b5
b7
b8
b9
b10
rQ
0
25
50
75
100
200
300
400
500
600
700
800
900
1,000
1,100
1,200

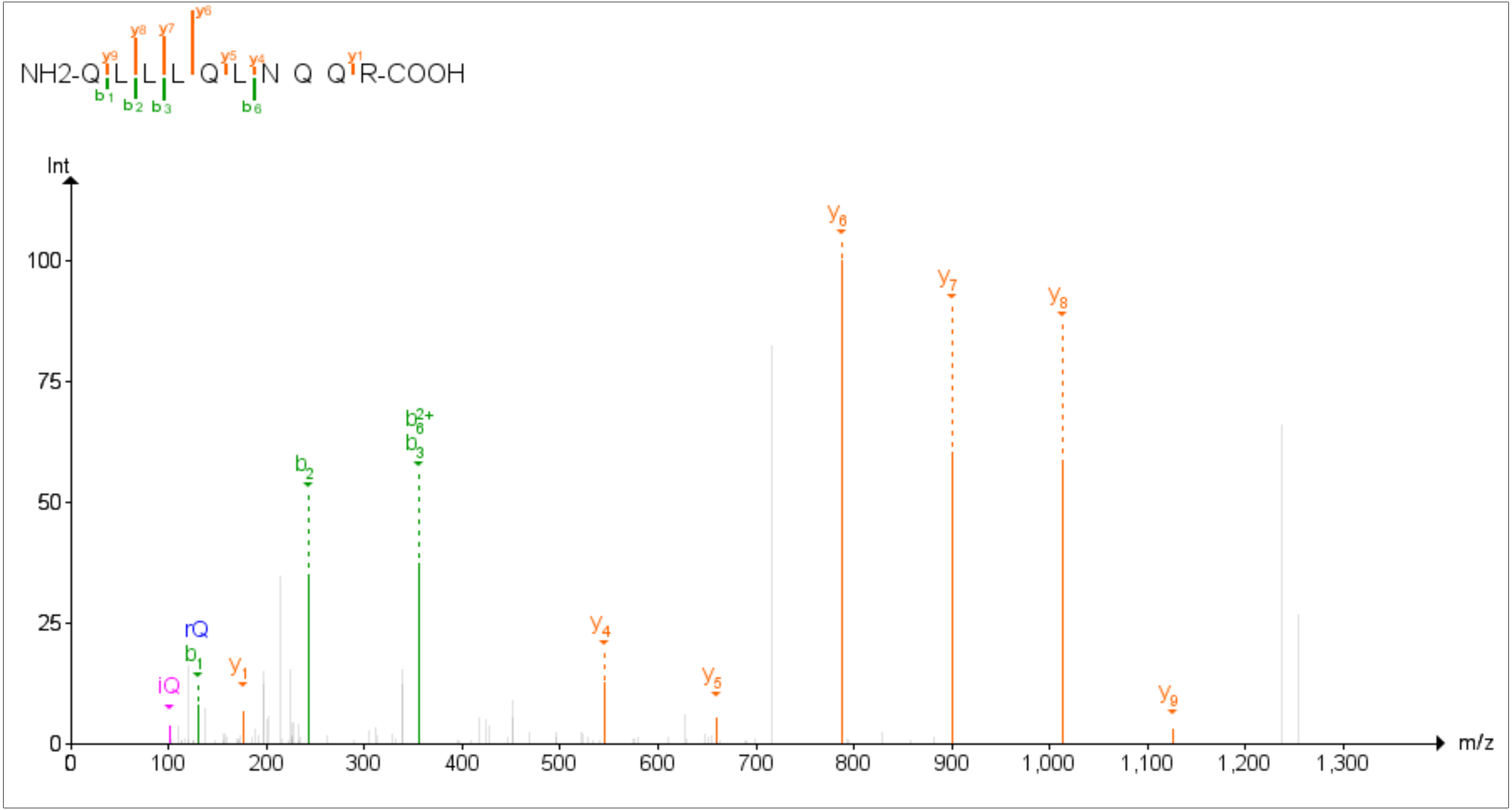

NH2-Q L L L Q L N Q Q R-COOH
Int
m/z
y6
y7
y8
b2
b6 2+
b3
rQ
b1
iQ
y1
y4
y5
y9

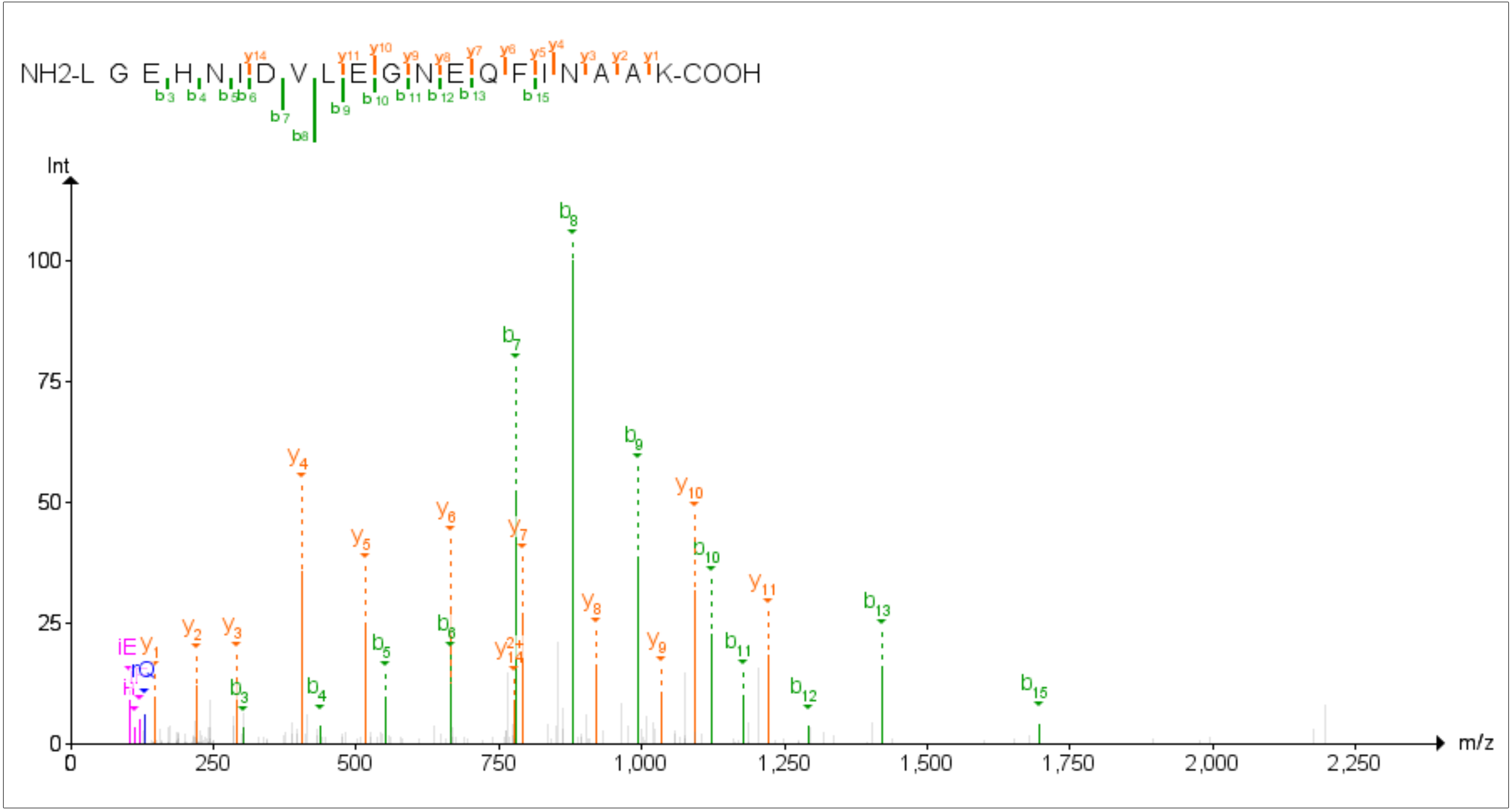
NH2-L G E H N I D V L E G N E Q F I N A A K-COOH
Int
m/z
b8
b7
b9
y4
y10
y6
y5
y7
b10
y11
b13
y8
b6
y3
y2
y1
iE
rQ
b5
b3
b4
y9
b11
b12
b15
0
25
50
75
100
0
250
500
750
1,000
1,250
1,500
1,750
2,000
2,250

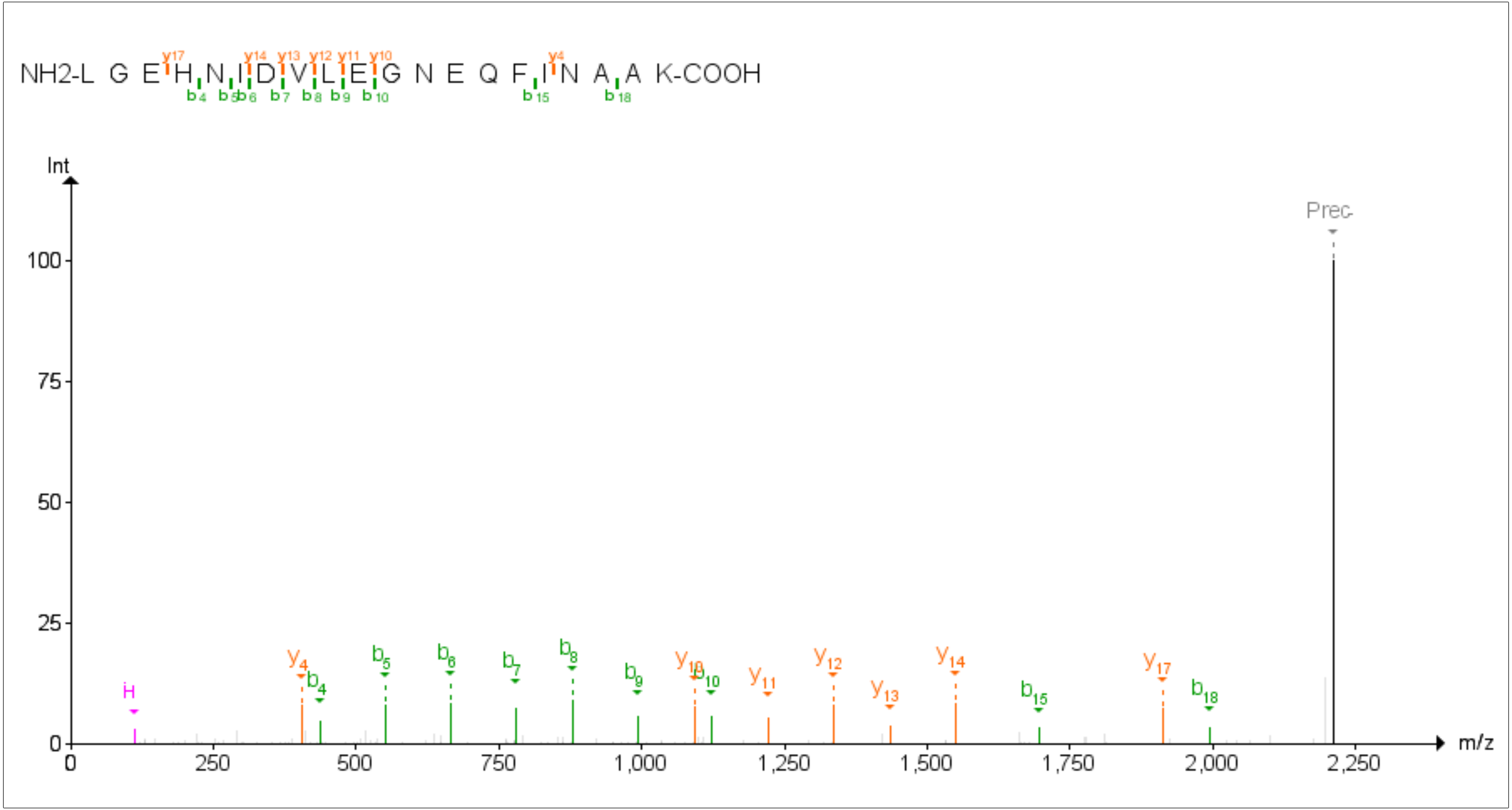
NH2-L G E H N I D V L E G N E Q F I N A A K-COOH
y17 y14 y13 y12 y11 y10 y4
b4 b5 b6 b7 b8 b9 b10 b15 b18
Int
Prec
100
75
50
25
0
H
y4 b4 b5 b6 b7 b8 b9 y10 b10 y11 y12 y13 y14 b15 y17 b18
0 250 500 750 1,000 1,250 1,500 1,750 2,000 2,250
m/z

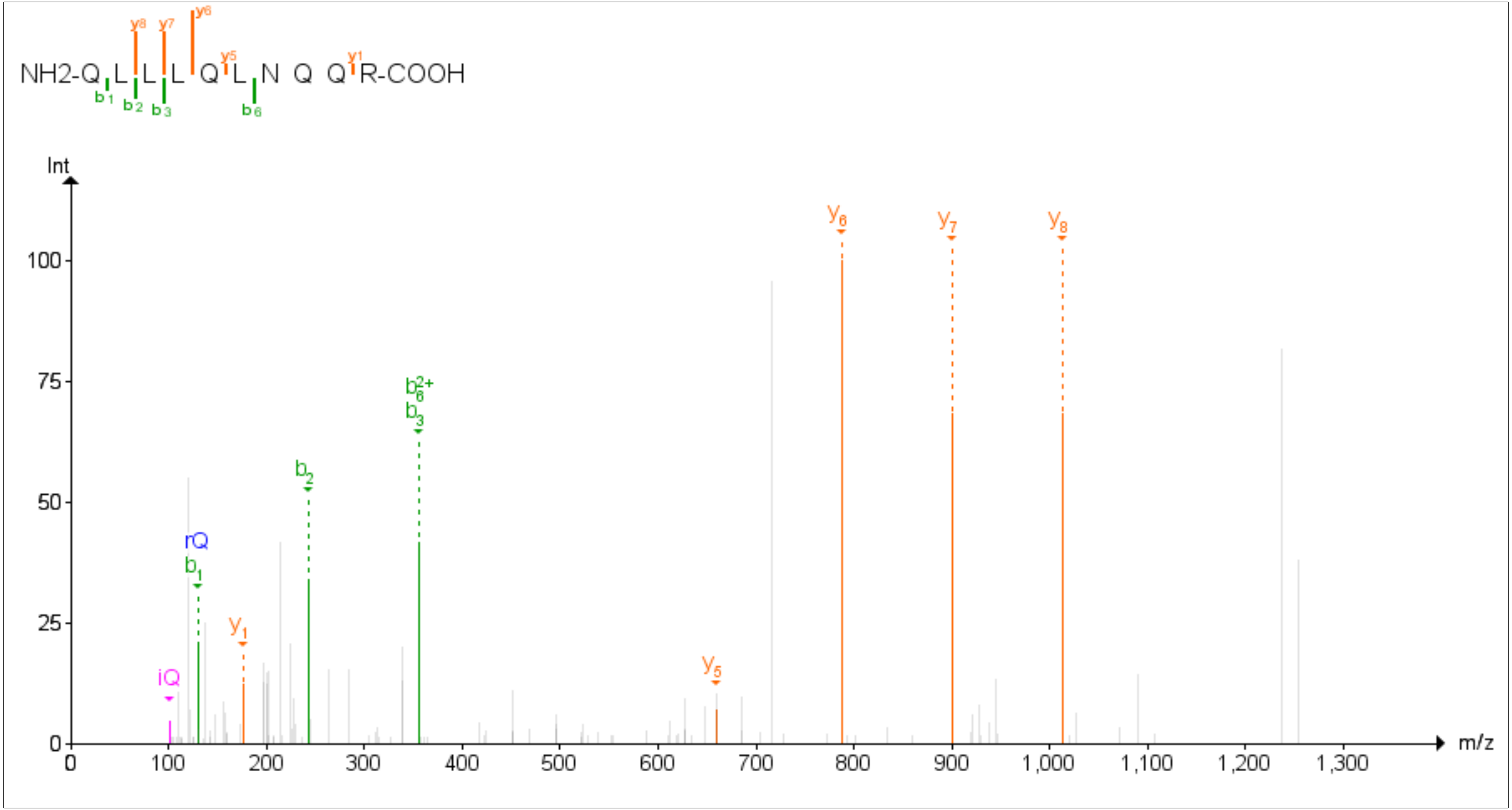

NH2-Q L L L L Q L N Q Q R-COOH
y8 y7 y6 y5 y1
b1 b2 b3 b6
Int
100
75
50
25
0
m/z
0 100 200 300 400 500 600 700 800 900 1,000 1,100 1,200 1,300
iQ
rQ
b1
y1
b2
b6 2+
b3
y5
y6
y7
y8

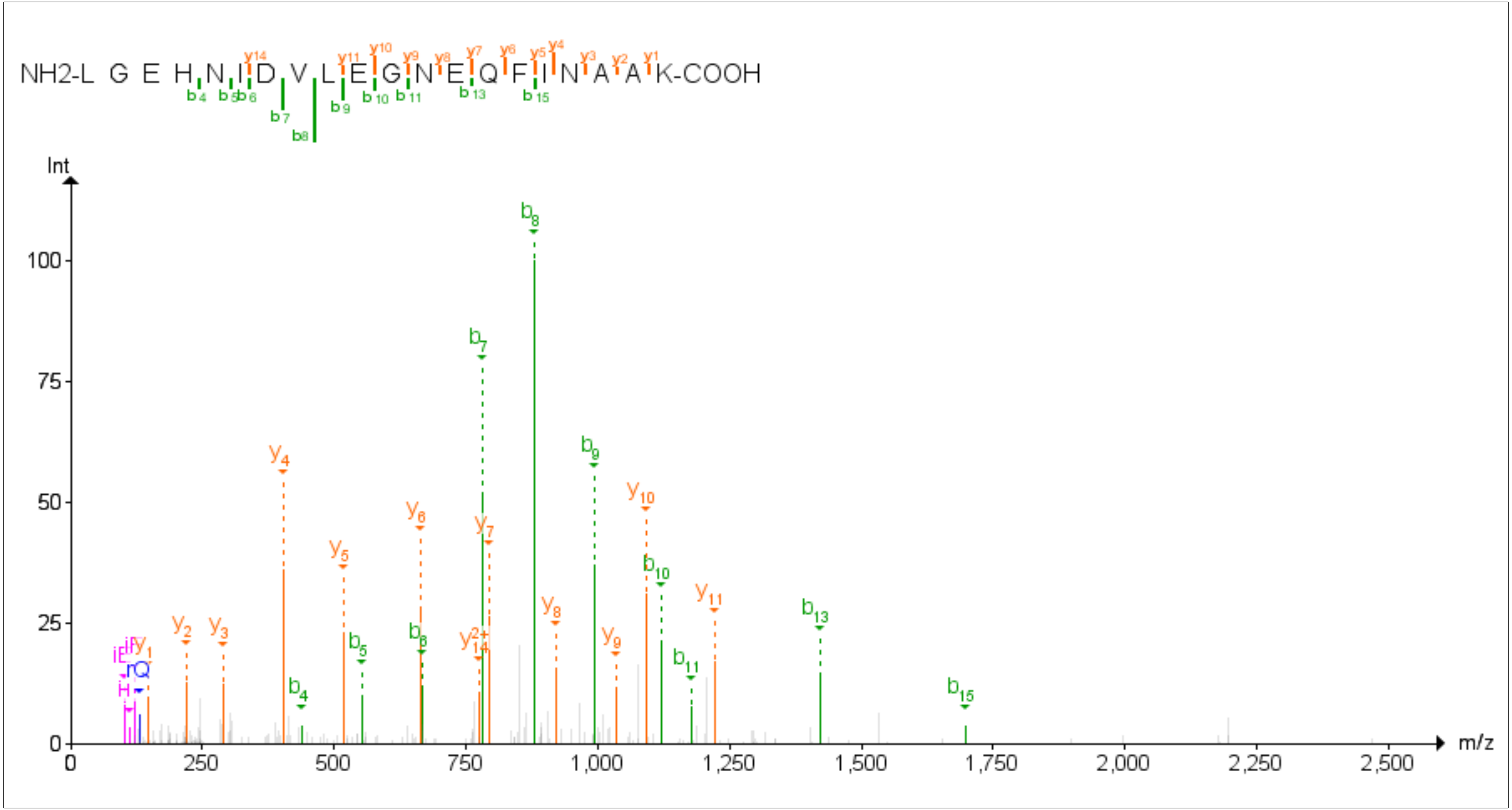

NH2-L G E H N I D V L E G N E Q F I N A A K-COOH
Int
m/z
0
25
50
75
100
250
500
750
1,000
1,250
1,500
1,750
2,000
2,250
2,500

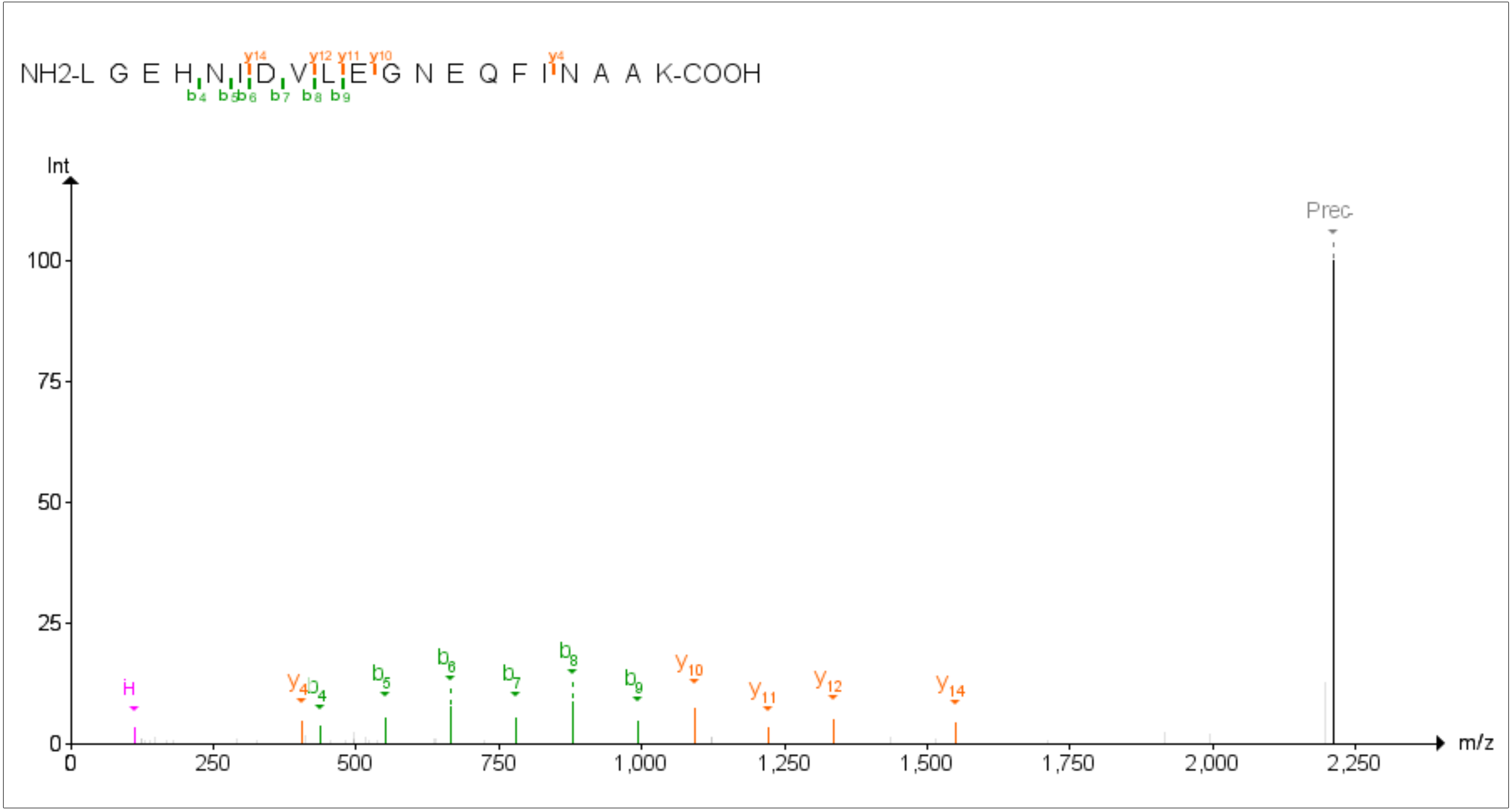

NH2-L G E H N I D V L E G N E Q F I N A A K-COOH
y14 y12 y11 y10 y4
b4 b5 b6 b7 b8 b9
Int
100
75
50
25
0
Prec
H
y4 b4 b5 b6 b7 b8 b9 y10 y11 y12 y14
0 250 500 750 1,000 1,250 1,500 1,750 2,000 2,250
m/z

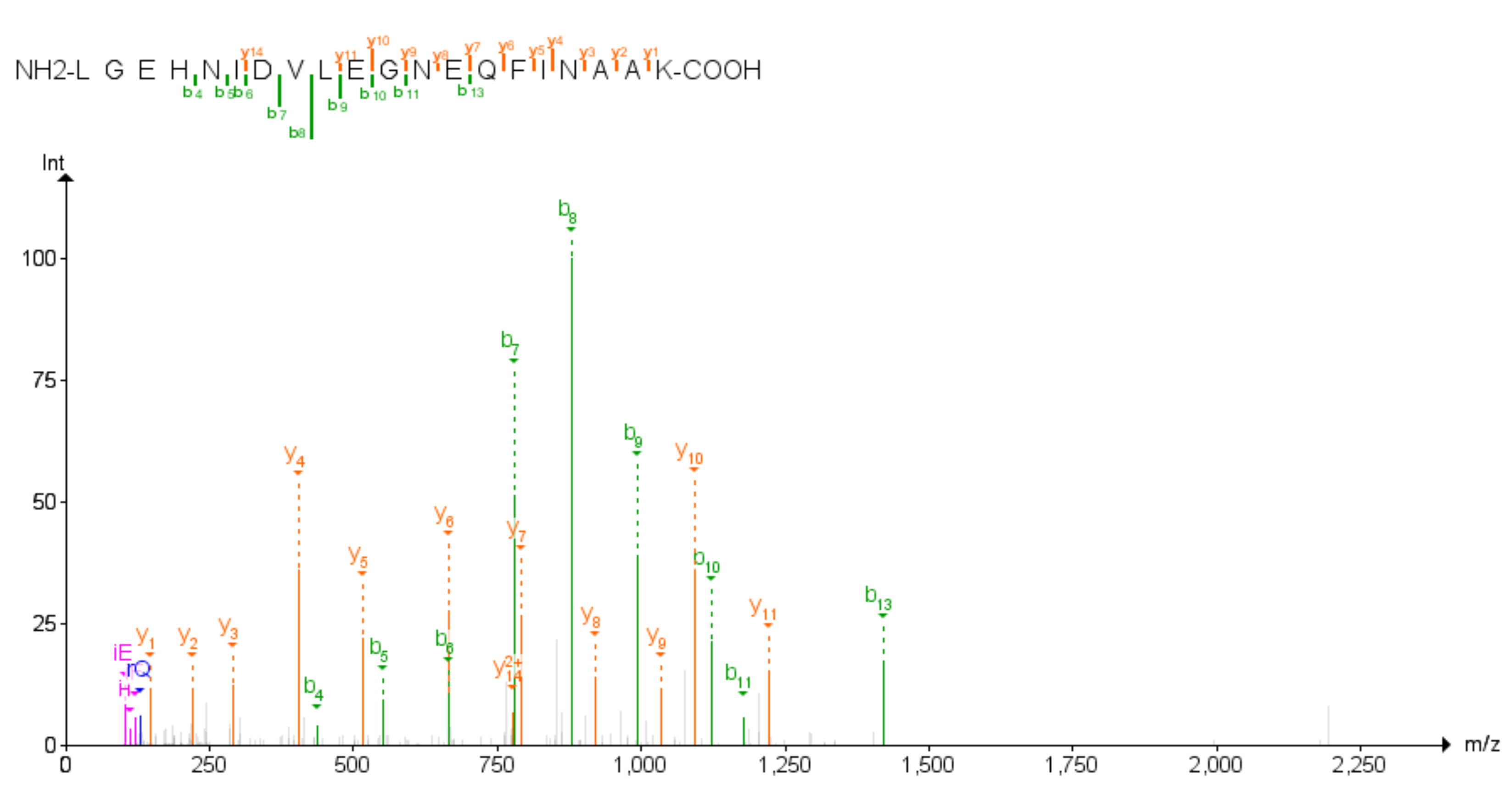

NH2-L G E H N I D V L E G N E Q F I N A A K-COOH
Int
m/z
0
25
50
75
100
250
500
750
1,000
1,250
1,500
1,750
2,000
2,250

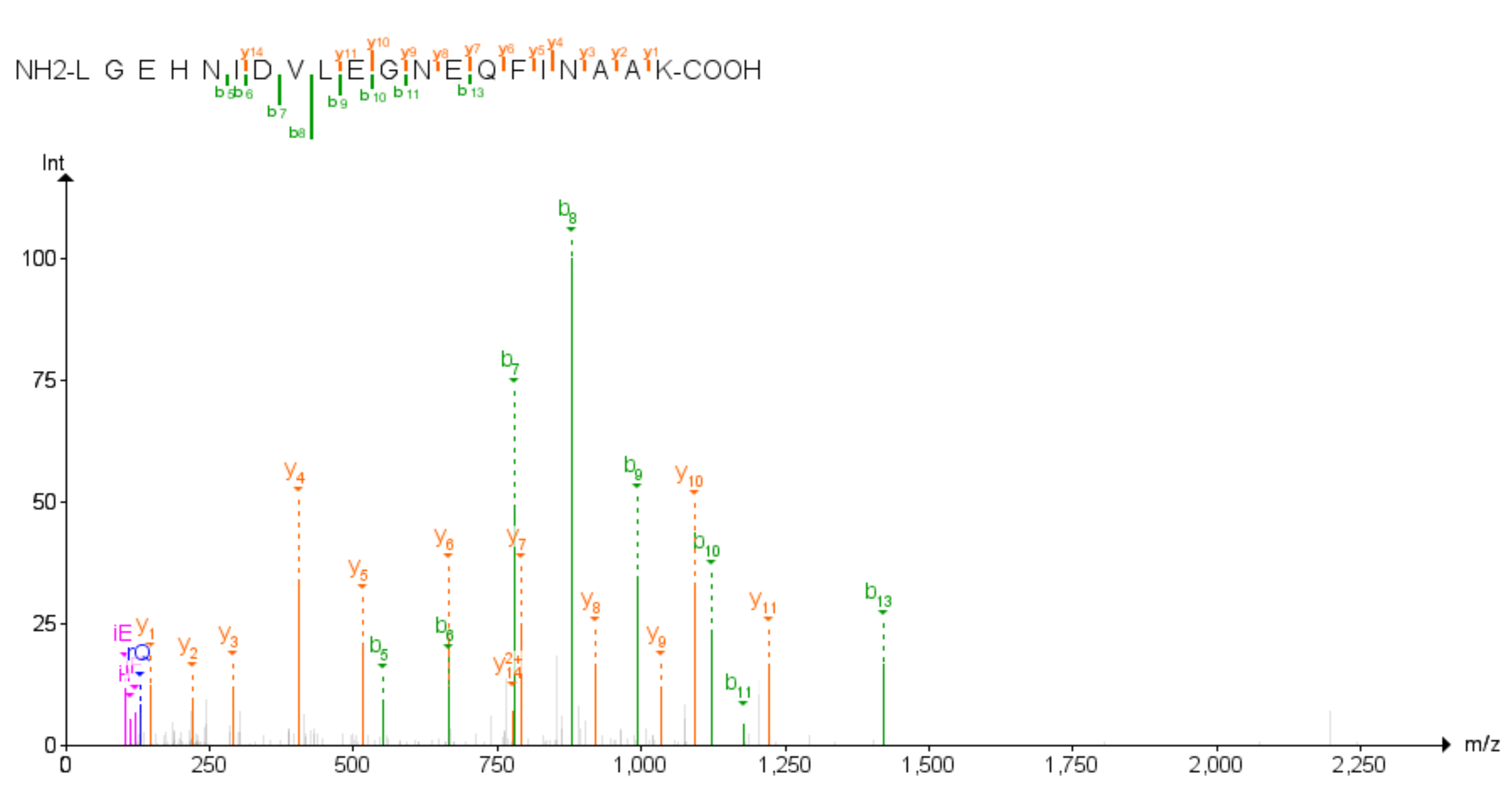

NH2-L G E H N I D V L E G N E Q F I N A A K-COOH
Int
m/z
0
25
50
75
100
250
500
750
1,000
1,250
1,500
1,750
2,000
2,250

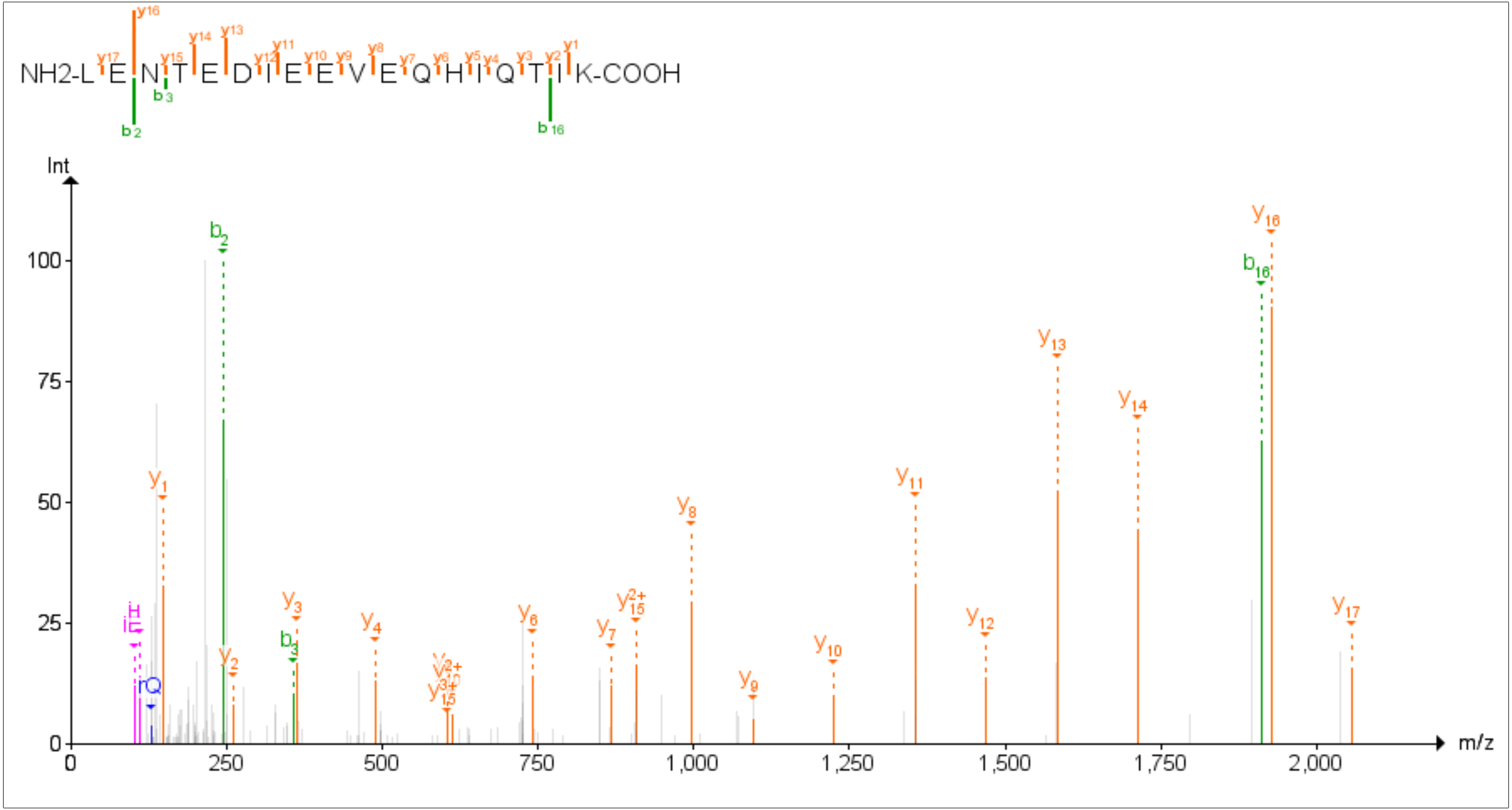

NH2-LENTEDIEEVEQHIQTIK-COOH
Int
m/z
0
25
50
75
100
250
500
750
1,000
1,250
1,500
1,750
2,000

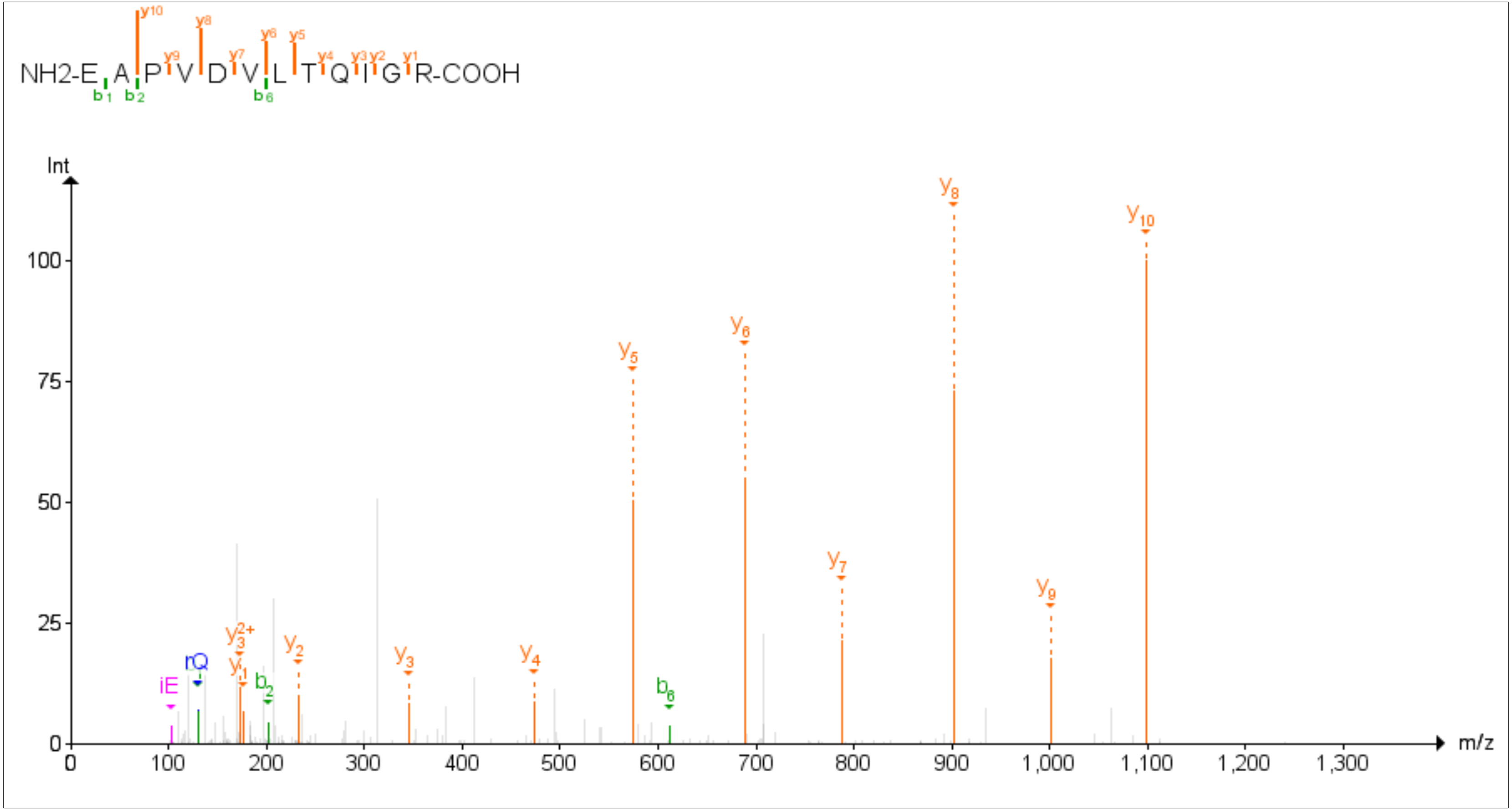
NH2-E A P V D V L T Q I G R-COOH
b1 b2 b6
y10 y9 y8 y7 y6 y5 y4 y3 y2 y1
Int
100
75
50
25
0
m/z
0
100
200
300
400
500
600
700
800
900
1,000
1,100
1,200
1,300
y8
y10
y6
y5
y7
y9
y3 2+
y2
y1
rQ
iE
b2
y3
y4
b6

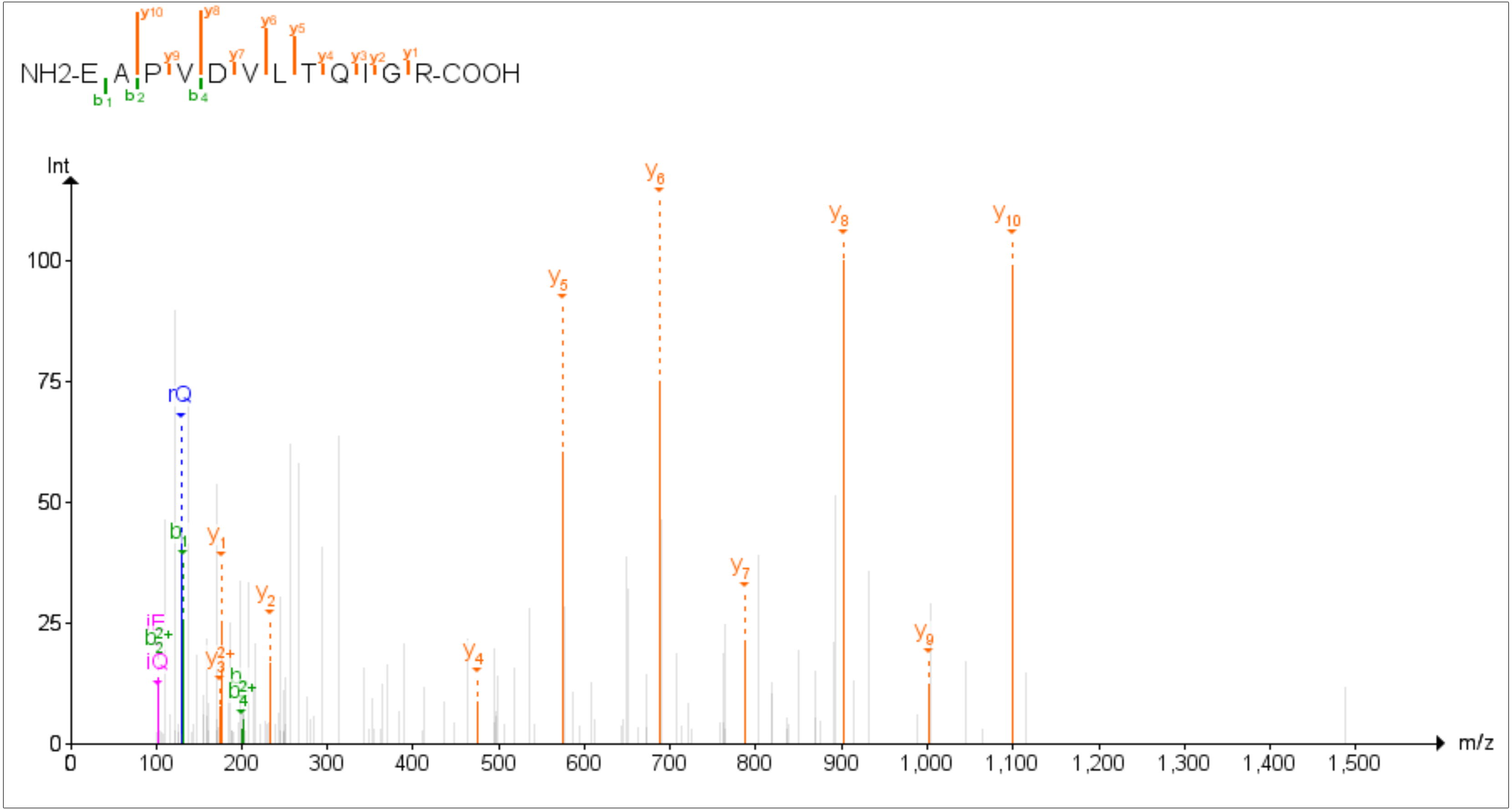
NH2-E A P V D V L T Q I G R-COOH
y10 y9 y8 y7 y6 y5 y4 y3 y2 y1
b1 b2 b4
Int
m/z
0
25
50
75
100
0 100 200 300 400 500 600 700 800 900 1,000 1,100 1,200 1,300 1,400 1,500
rQ
b1
y1
y2
iF
b2 2+
iQ
y3 2+
b4 2+
y4
y5
y6
y7
y8
y9
y10

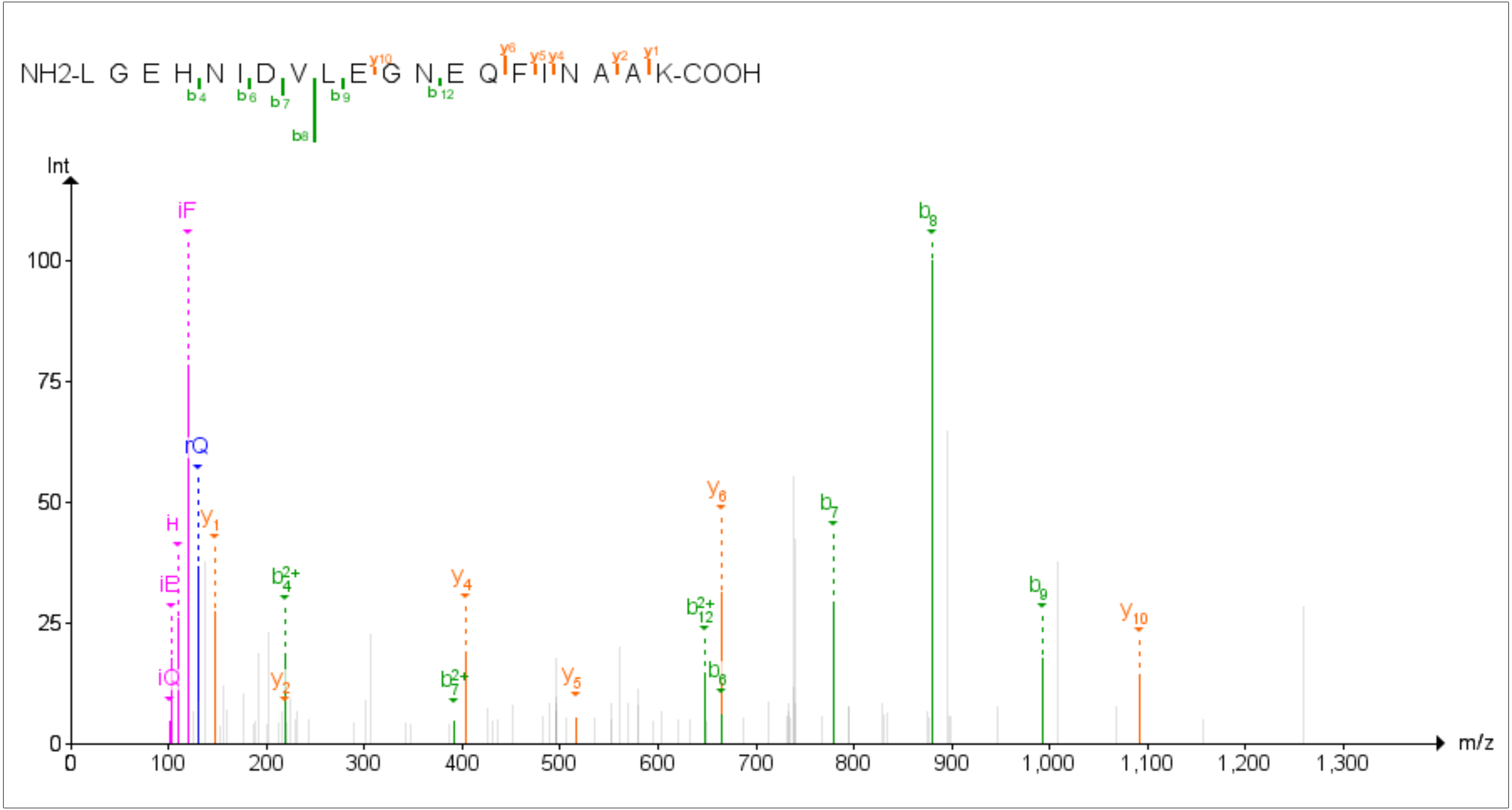

NH2-L G E H N I D V L E G N E Q F I N A A K-COOH
b4 b6 b7 b8 b9 b12
y10 y6 y5 y4 y2 y1
Int
m/z
iF
rQ
iH
iE
iQ
y1
y2
y4
y5
y6
y10
b8
b7
b9
b6
b4 2+
b7 2+
b12 2+
0
25
50
75
100
0
100
200
300
400
500
600
700
800
900
1,000
1,100
1,200
1,300

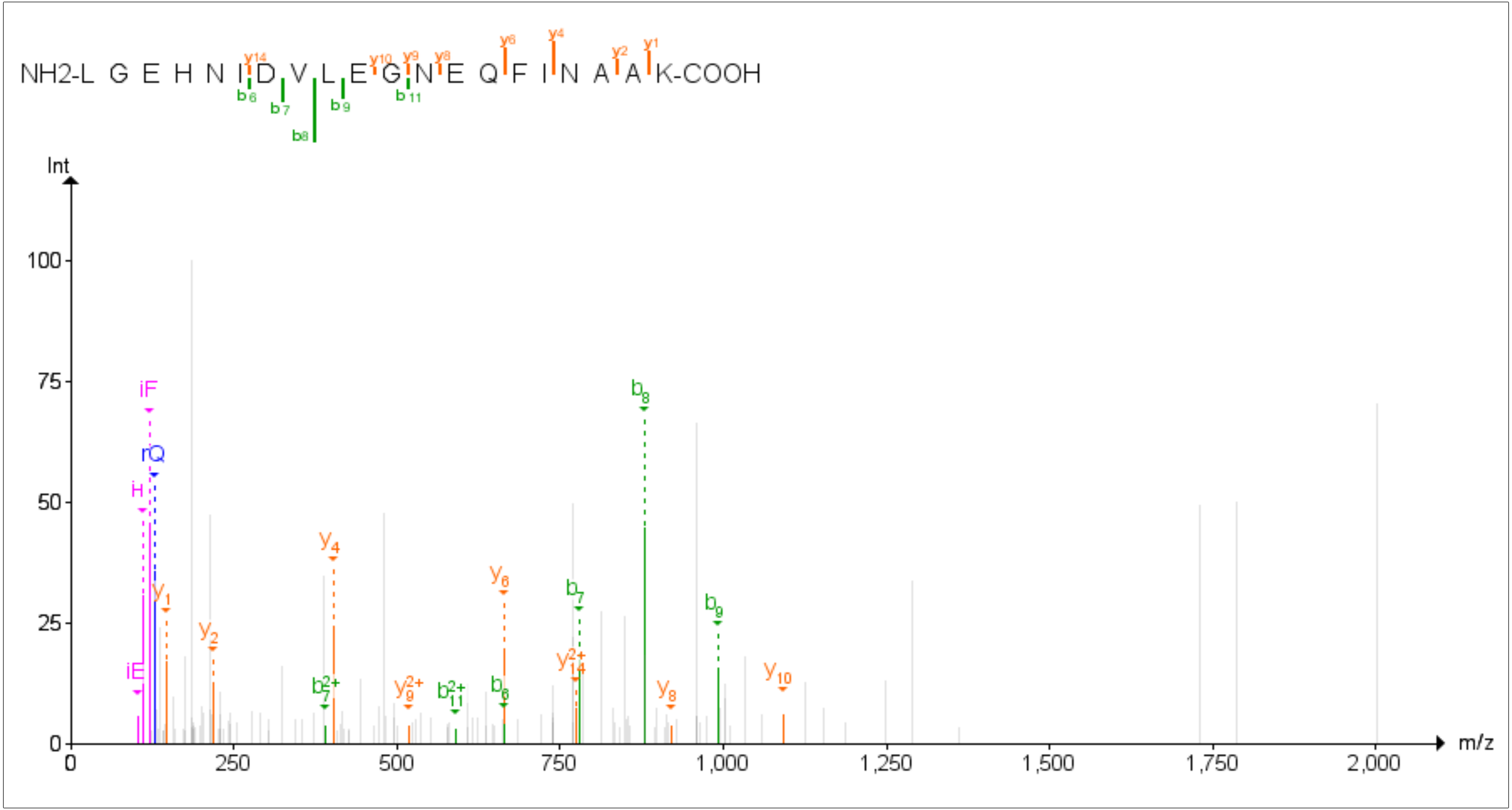

NH2-L G E H N I D V L E G N E Q F I N A A K-COOH
y14 y10 y9 y8 y6 y4 y2 y1
b6 b7 b8 b9 b11
Int
m/z
iF
rQ
iH
iE
y1 y2 y4 y6 y8 y10
y_9^{2+} y_{14}^{2+}
b_7^{2+} b_{11}^{2+}
b6 b7 b8 b9
0 25 50 75 100
0 250 500 750 1,000 1,250 1,500 1,750 2,000

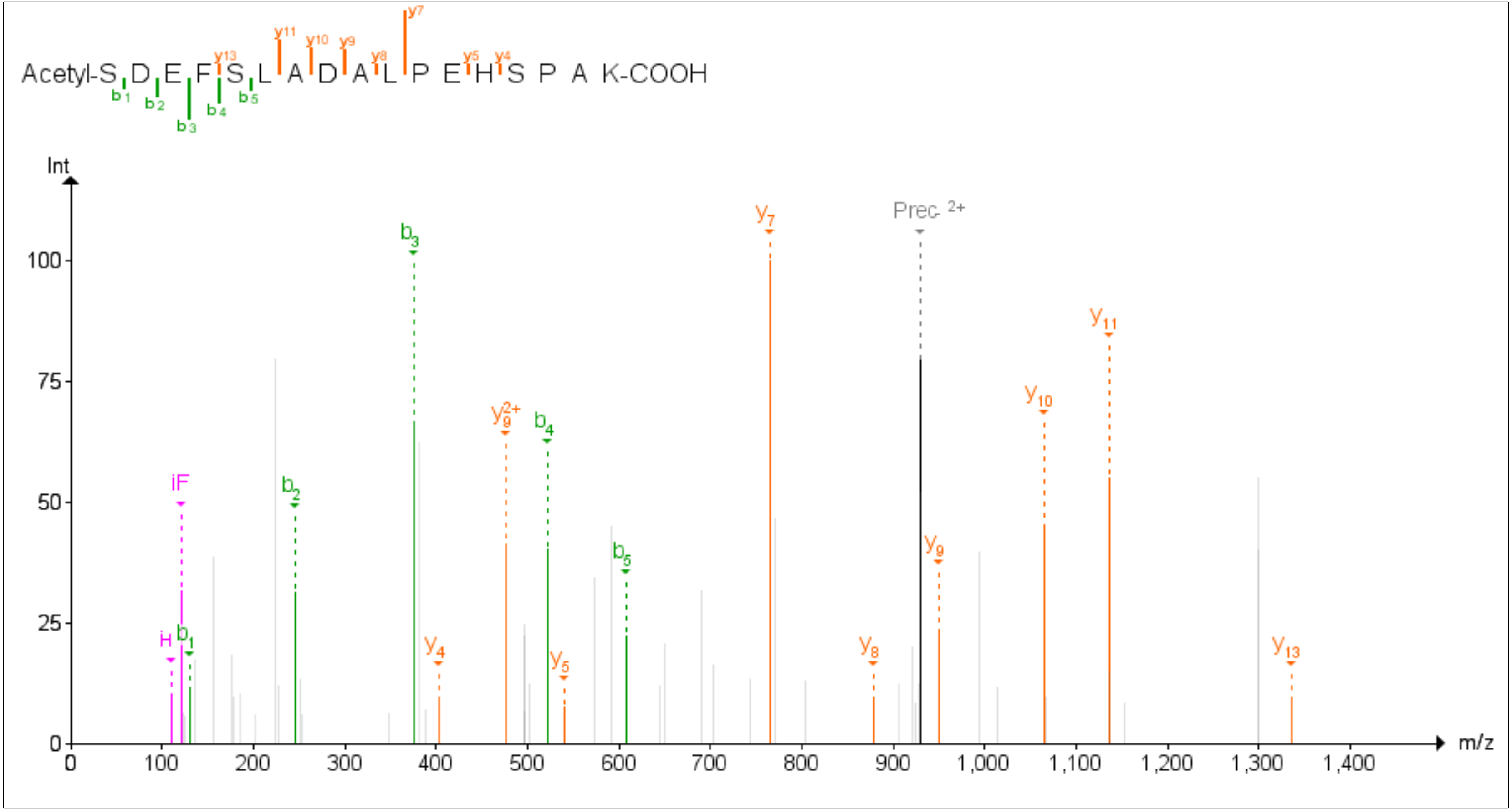
Acetyl-S D E F S L A D A L P E H S P A K-COOH
b1 b2 b3 b4 b5
y13 y11 y10 y9 y8 y7 y5 y4
Int
100
75
50
25
0
m/z
0
100
200
300
400
500
600
700
800
900
1,000
1,100
1,200
1,300
1,400
iH
iF
b1
b2
b3
b4
b5
y4
y5
y7
y8
y9
y9 2+
y10
y11
y13
Prec 2+

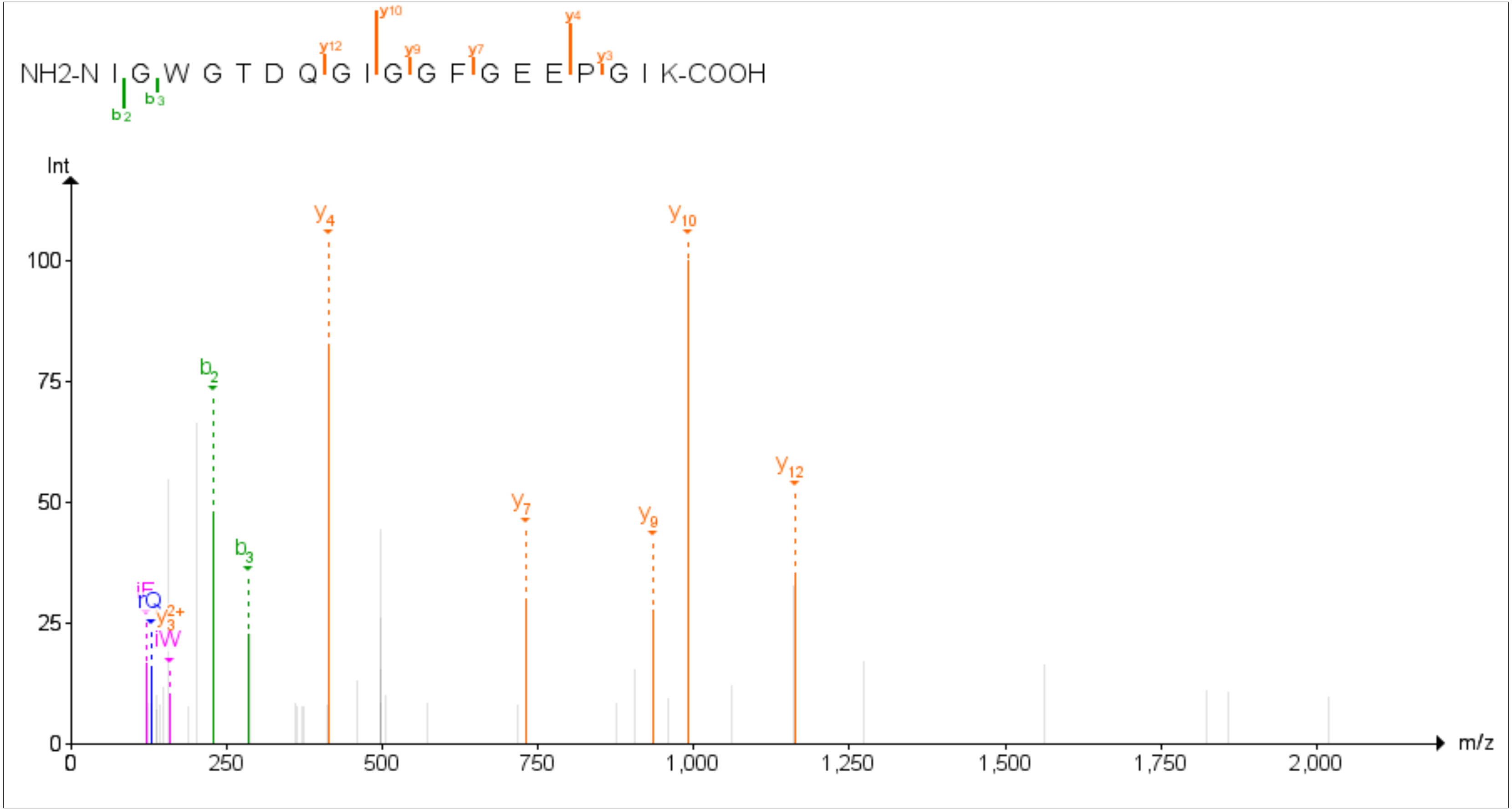

NH2-N I G W G T D Q G I G G F G E E P G I K-COOH
y10
y12
y9
y7
y4
y3
b2
b3
Int
100
75
50
25
0
y4
y10
b2
b3
y7
y9
y12
iF
rQ
y3 2+
iW
0
250
500
750
1,000
1,250
1,500
1,750
2,000
m/z

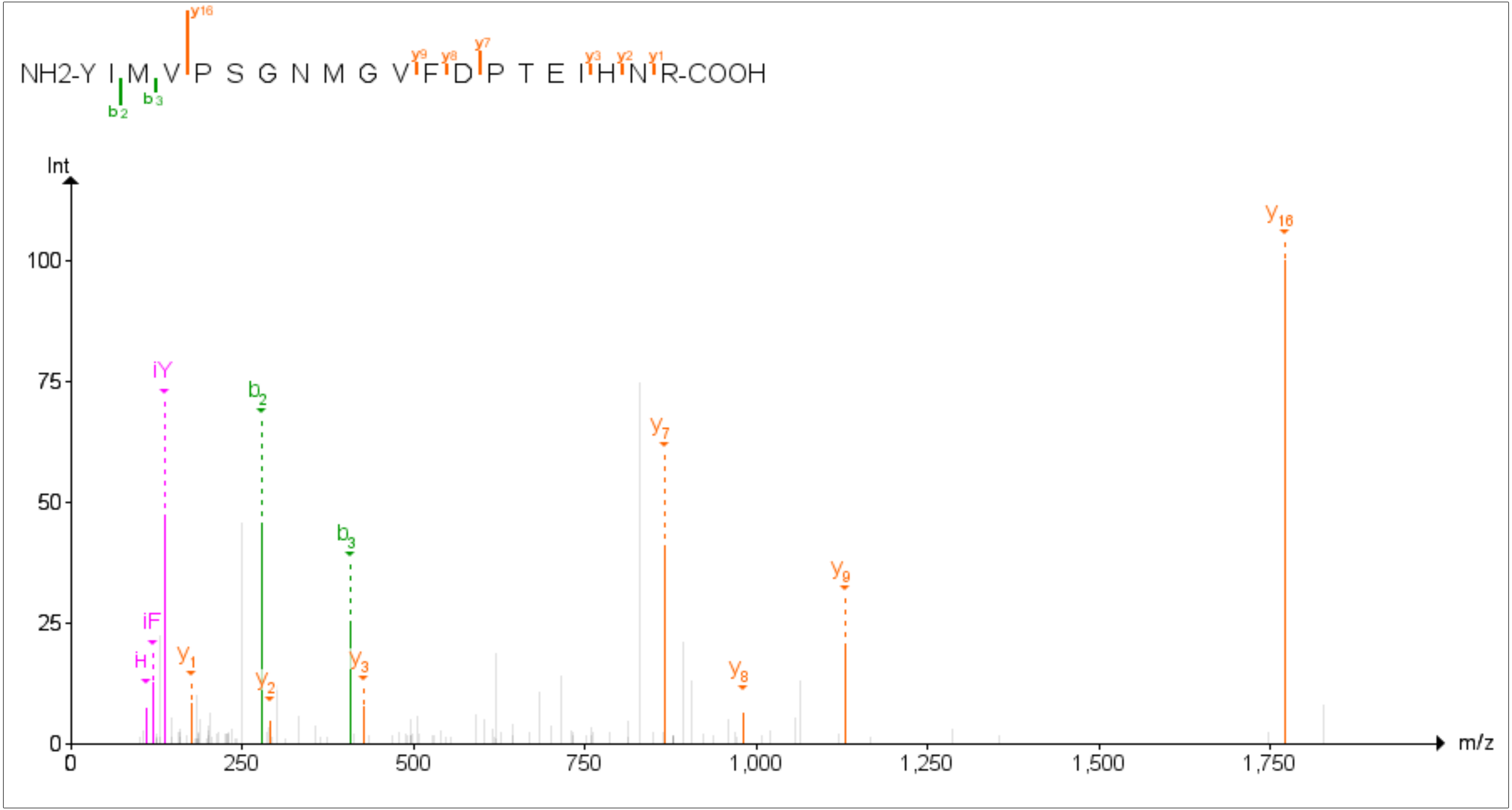

NH2-Y I M V P S G N M G V F D P T E I H N R-COOH
y16
y9
y8
y7
y3
y2
y1
b2
b3
Int
100
75
50
25
0
iY
b2
y7
b3
y16
y9
iF
iH
y1
y2
y3
y8
m/z
0
250
500
750
1,000
1,250
1,500
1,750

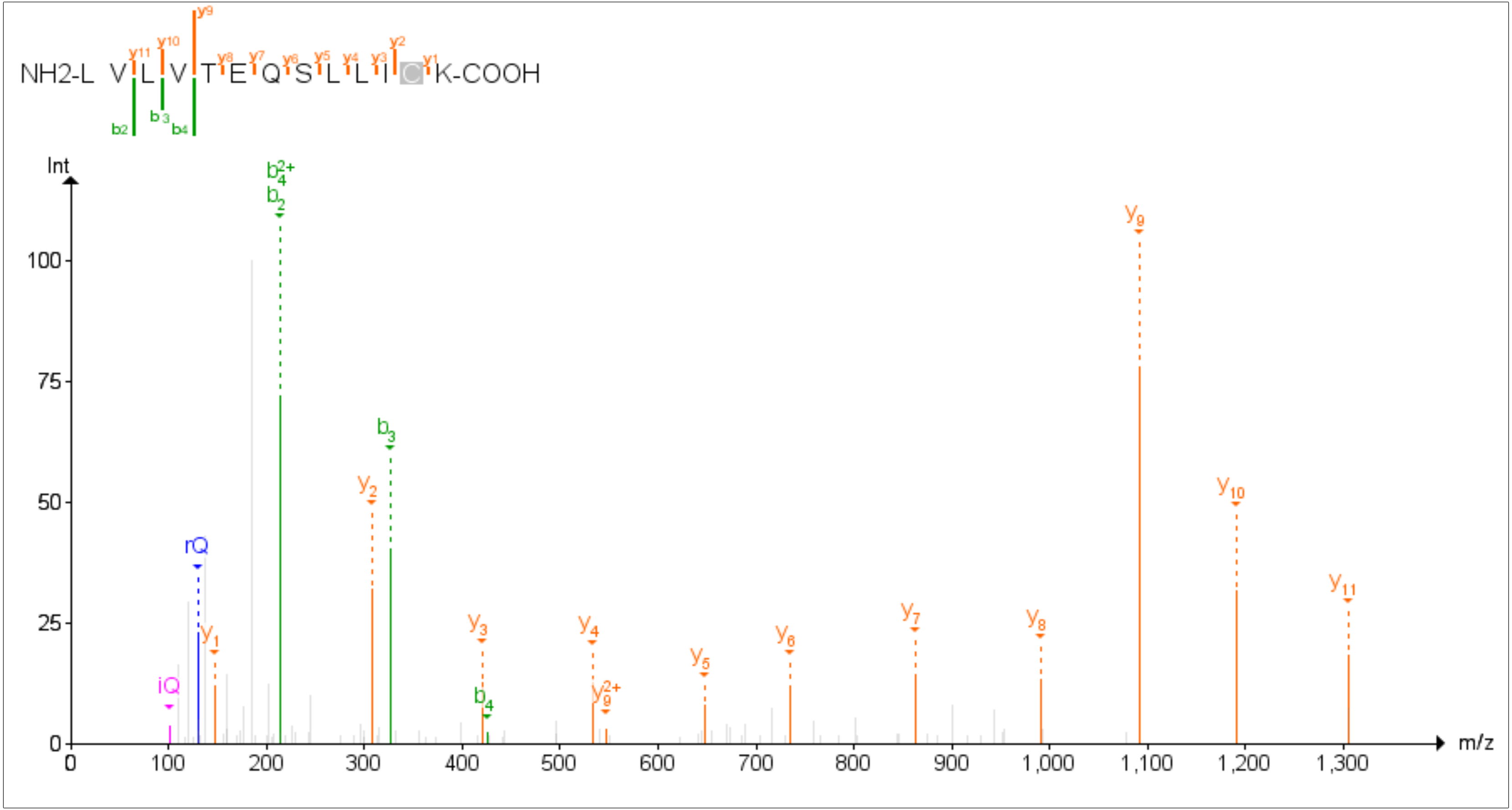
NH2-L V L V T E Q S L L I C K-COOH
y11 y10 y9 y8 y7 y6 y5 y4 y3 y2 y1
b2 b3 b4
Int
m/z
0 25 50 75 100
0 100 200 300 400 500 600 700 800 900 1,000 1,100 1,200 1,300
b_4^{2+}
b_2
b_3
b_4
rQ
iQ
y_1 y_2 y_3 y_4 y_5 y_6 y_7 y_8 y_9 y_{10} y_{11}
y_9^{2+}

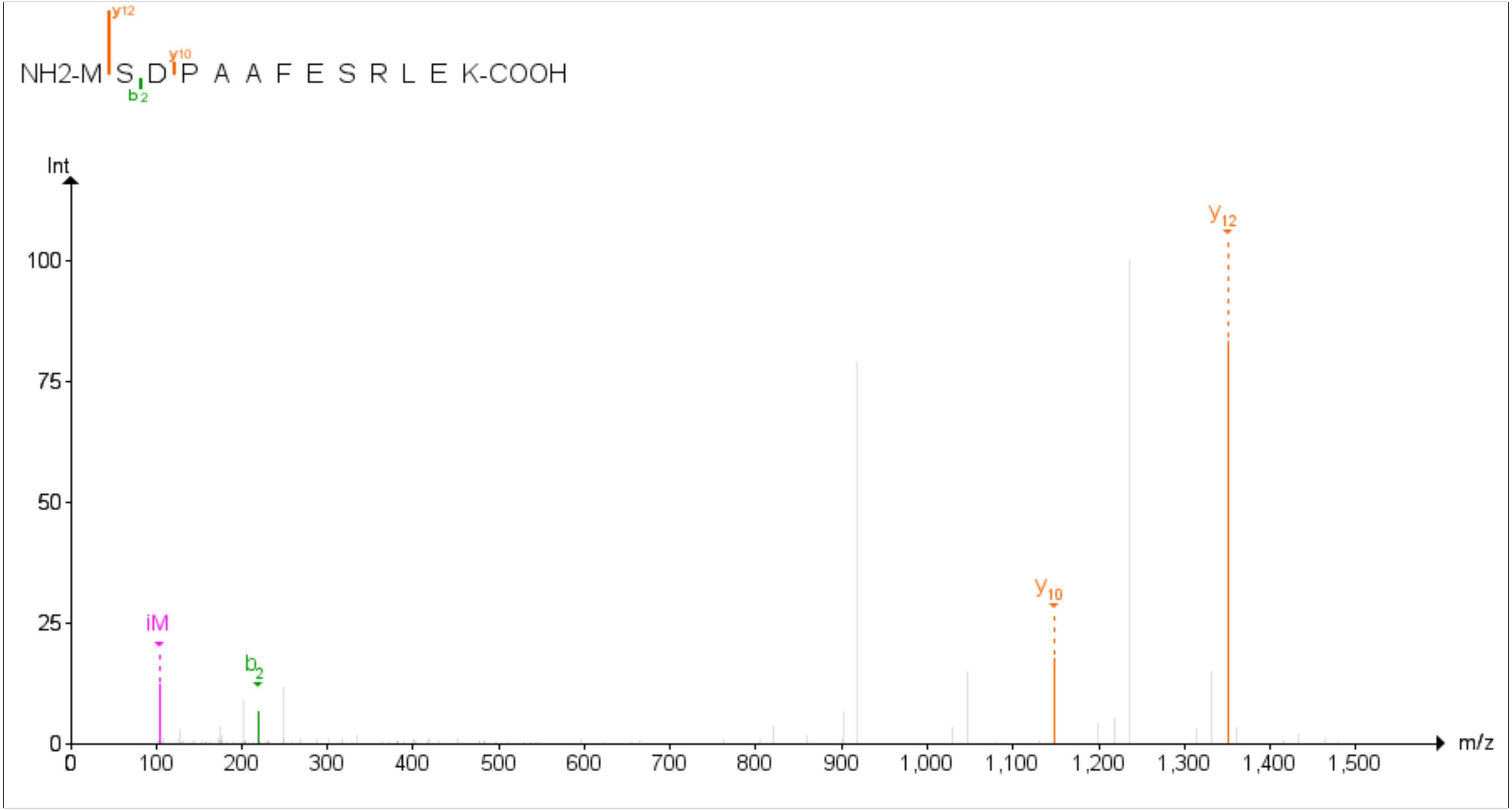
NH2-M S D P A A F E S R L E K-COOH
y12
y10
b2
Int
100
75
50
25
0
iM
b2
y10
y12
0
100
200
300
400
500
600
700
800
900
1,000
1,100
1,200
1,300
1,400
1,500
m/z

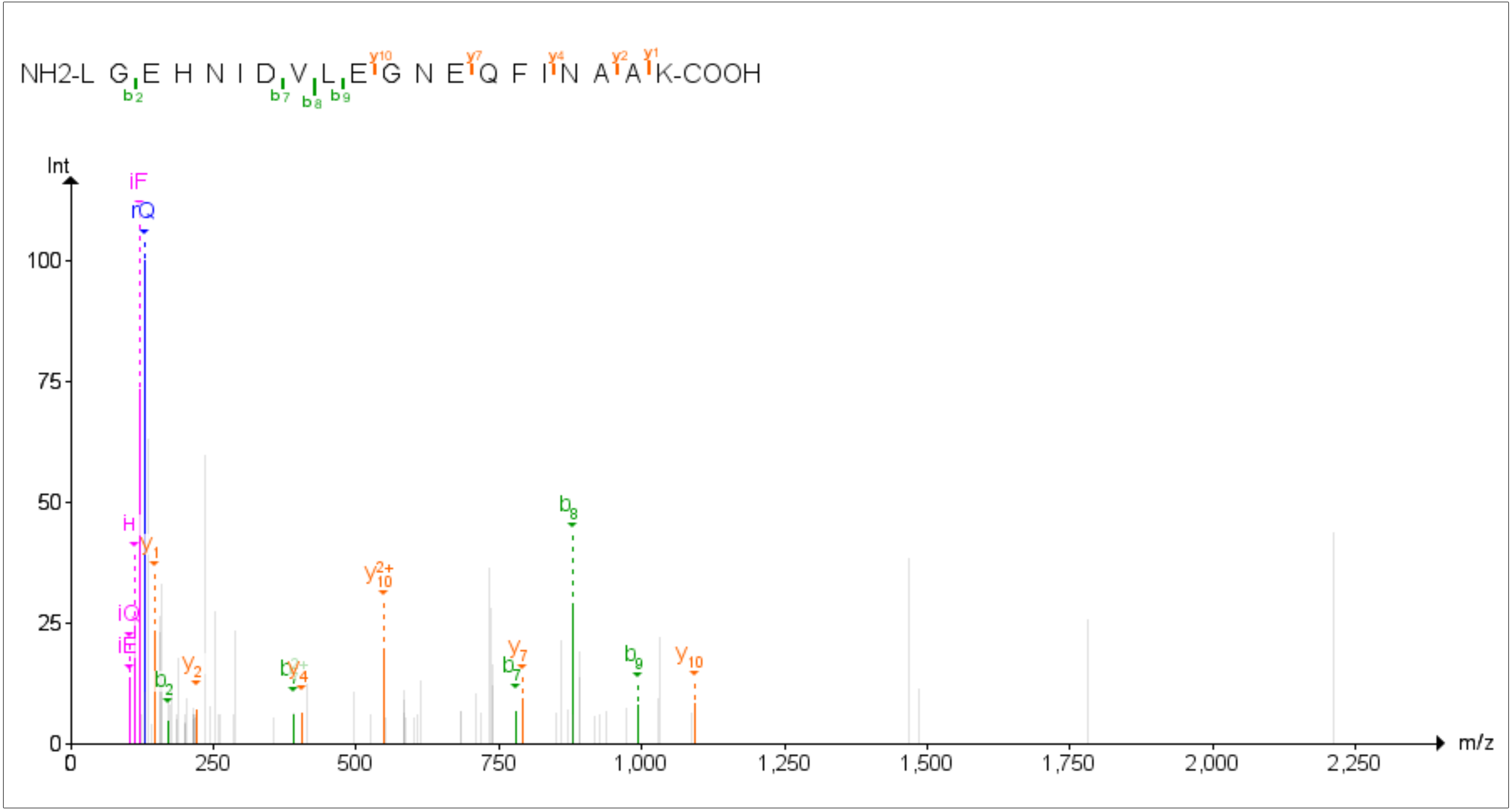
NH2-L G E H N I D V L E G N E Q F I N A A K-COOH
b2
b7
b8
b9
y10
y7
y4
y2
y1
Int
iF
rQ
iH
iQ
iE
y1
b2
y2
y10 2+
b8
b7
y7
b9
y10
100
75
50
25
0
250
500
750
1,000
1,250
1,500
1,750
2,000
2,250
m/z

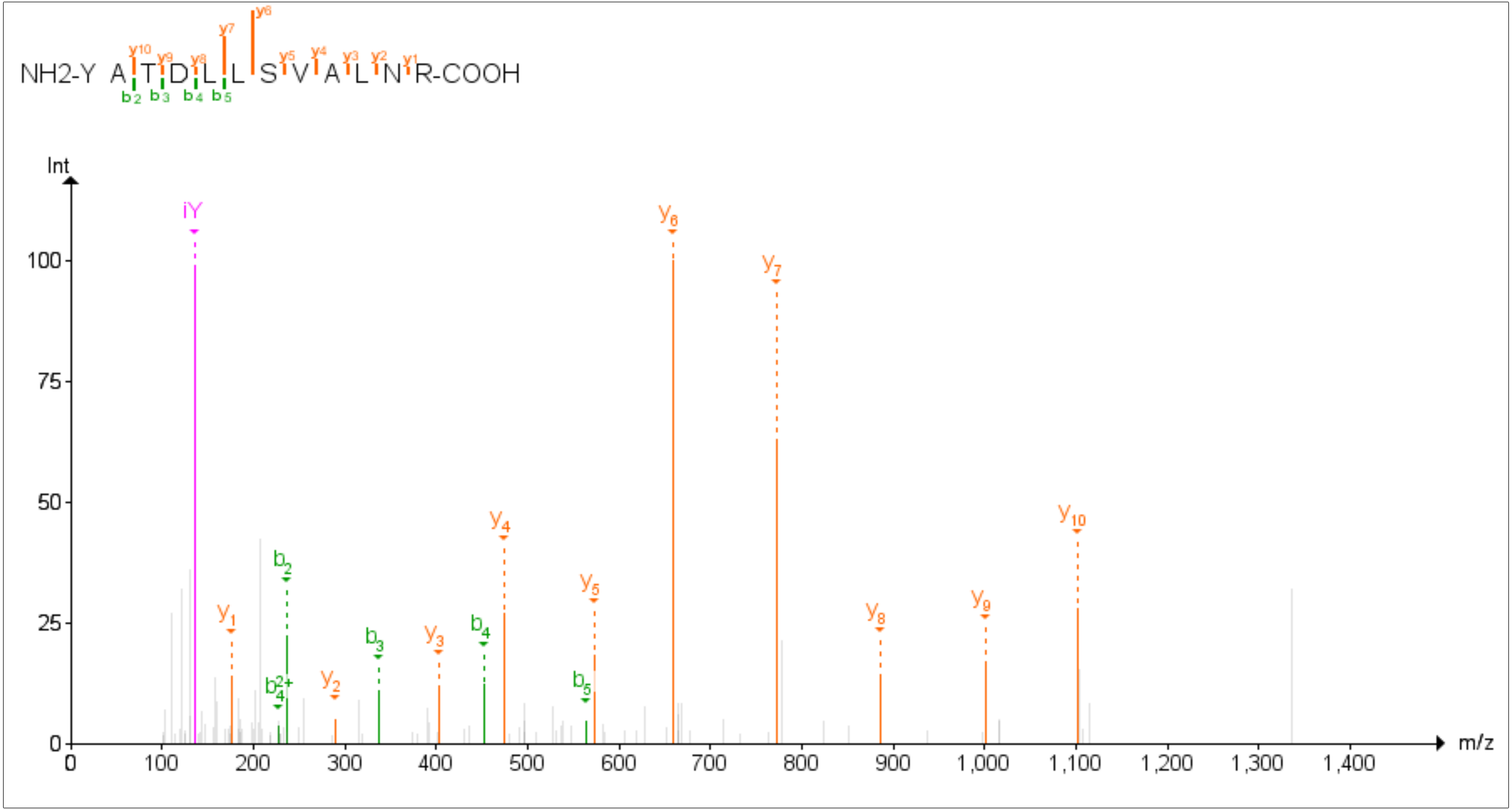

NH2-Y A T D L L S V A L N R-COOH
y10 y9 y8 y7 y6 y5 y4 y3 y2 y1
b2 b3 b4 b5
Int
iY
y6
y7
y4
y10
b2
y5
y1
b3
y3
b4
y8
y9
b4 2+
y2
b5
100
75
50
25
0
0
100
200
300
400
500
600
700
800
900
1,000
1,100
1,200
1,300
1,400
m/z

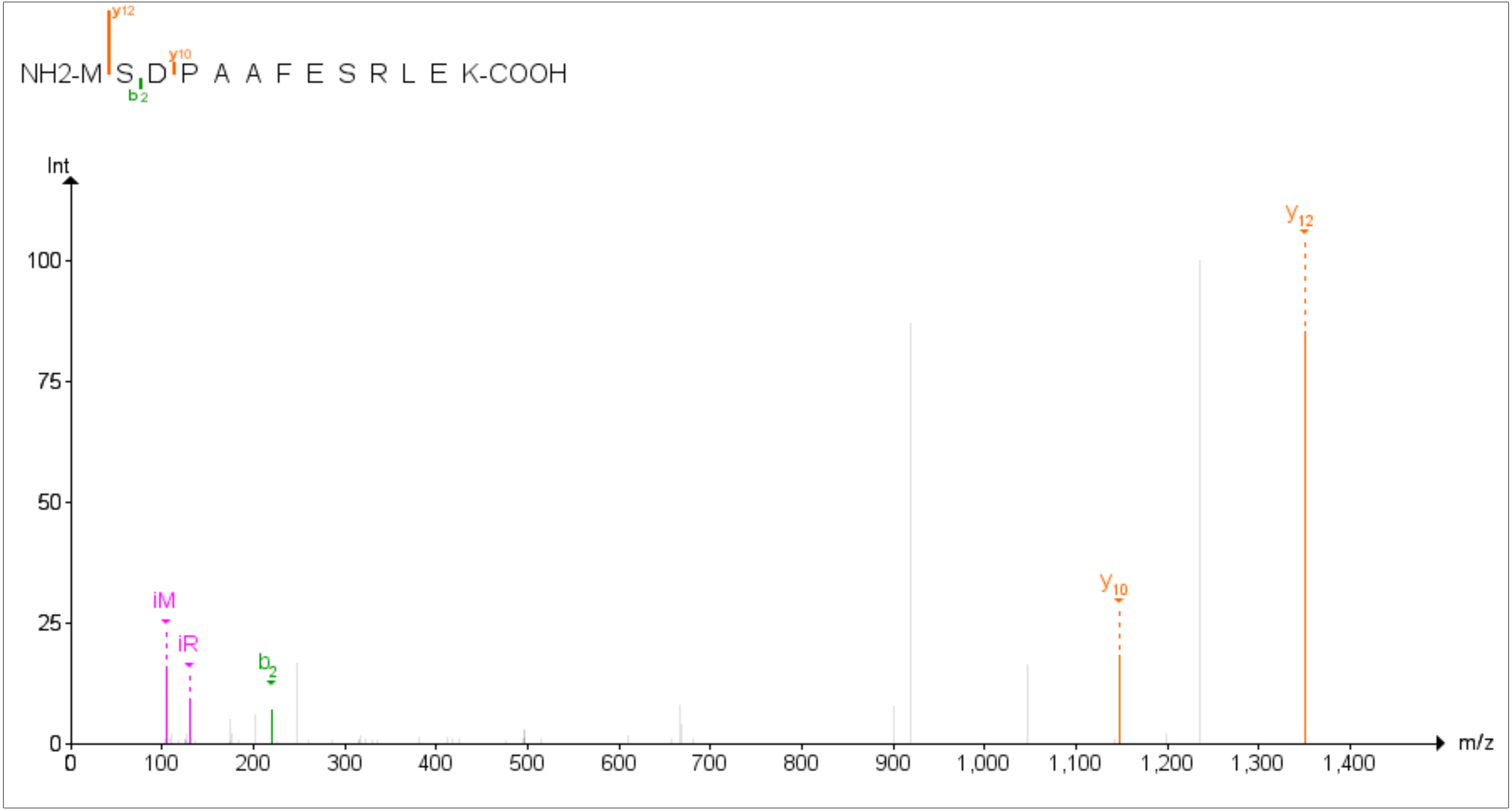

NH2-M S D P A A F E S R L E K-COOH
y12
y10
b2
Int
100
75
50
25
0
iM
iR
m/z
0
100
200
300
400
500
600
700
800
900
1,000
1,100
1,200
1,300
1,400

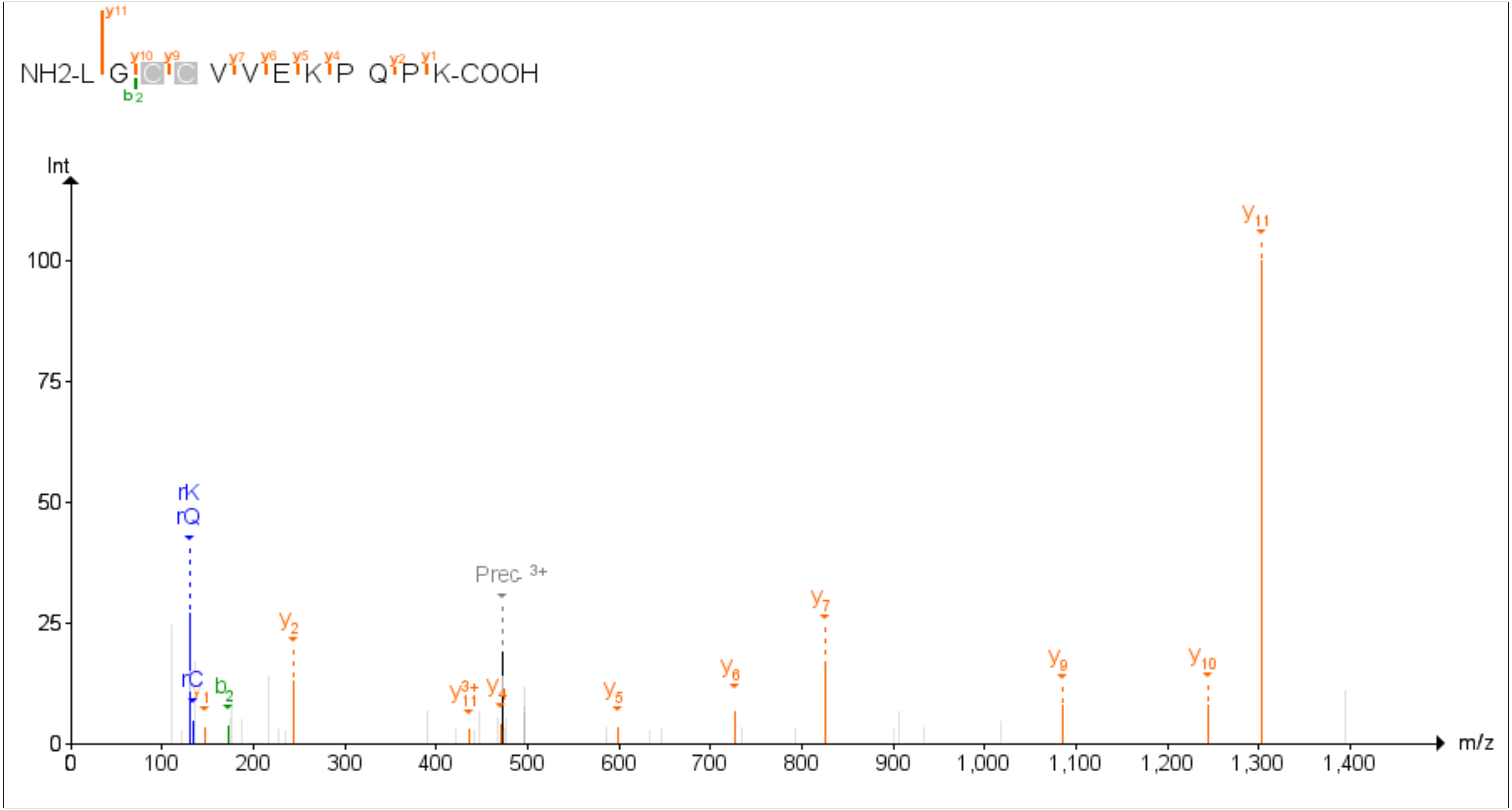

NH2-L G C C V V E K P Q P K-COOH
y11 y10 y9 y7 y6 y5 y4 y2 y1
b2
Int
100
75
50
25
0
rK
rQ
rC
y1
b2
y2
Prec 3+
y11 3+
y4
y5
y6
y7
y9
y10
y11
0 100 200 300 400 500 600 700 800 900 1,000 1,100 1,200 1,300 1,400
m/z

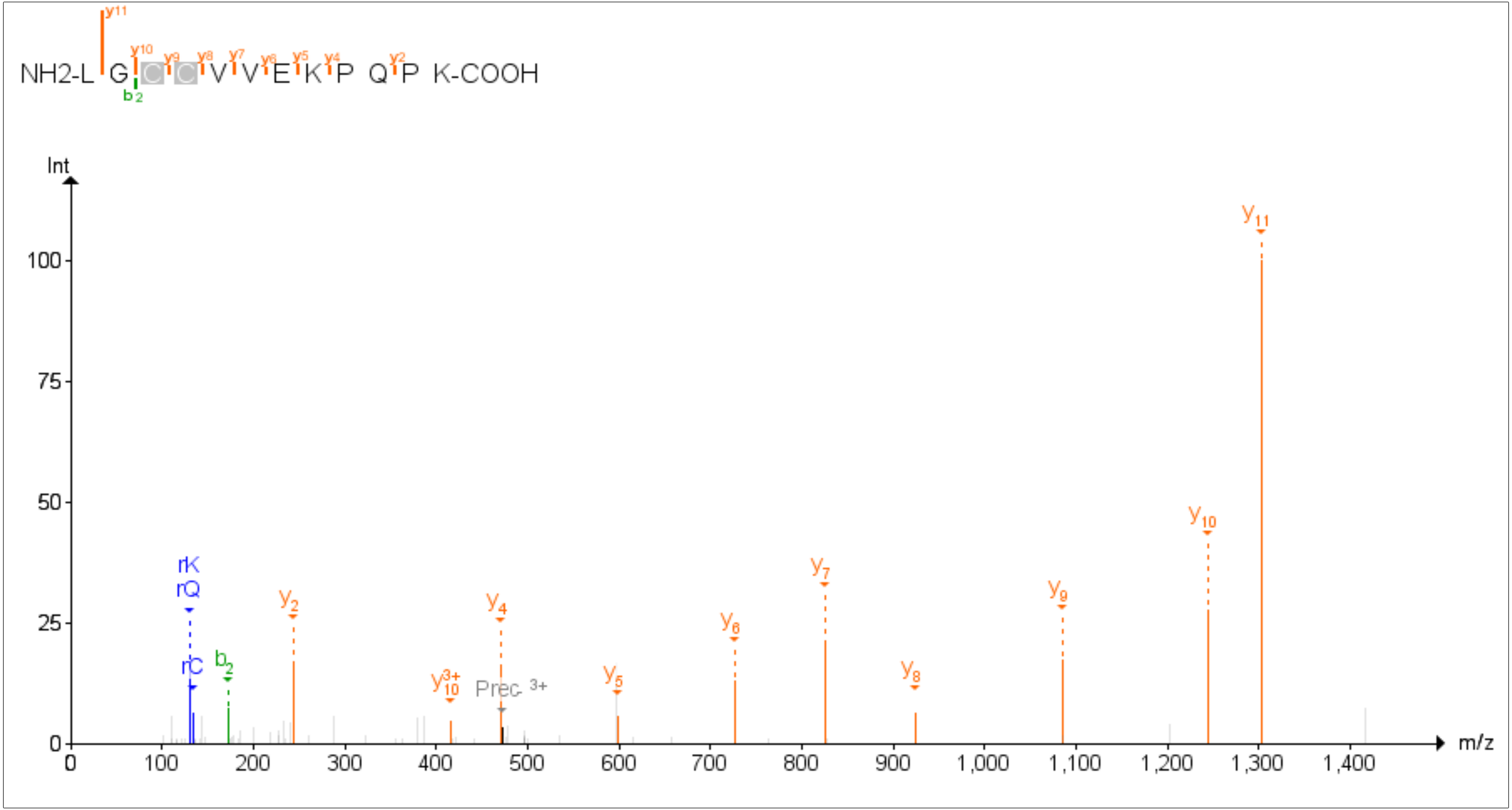

NH2-L G C C V V E K P Q P K-COOH
y11
y10
y9
y8
y7
y6
y5
y4
y2
b2
Int
100
75
50
25
0
rK
rQ
rC
b2
y2
y10 3+
Prec 3+
y4
y5
y6
y7
y8
y9
y10
y11
0
100
200
300
400
500
600
700
800
900
1,000
1,100
1,200
1,300
1,400
m/z

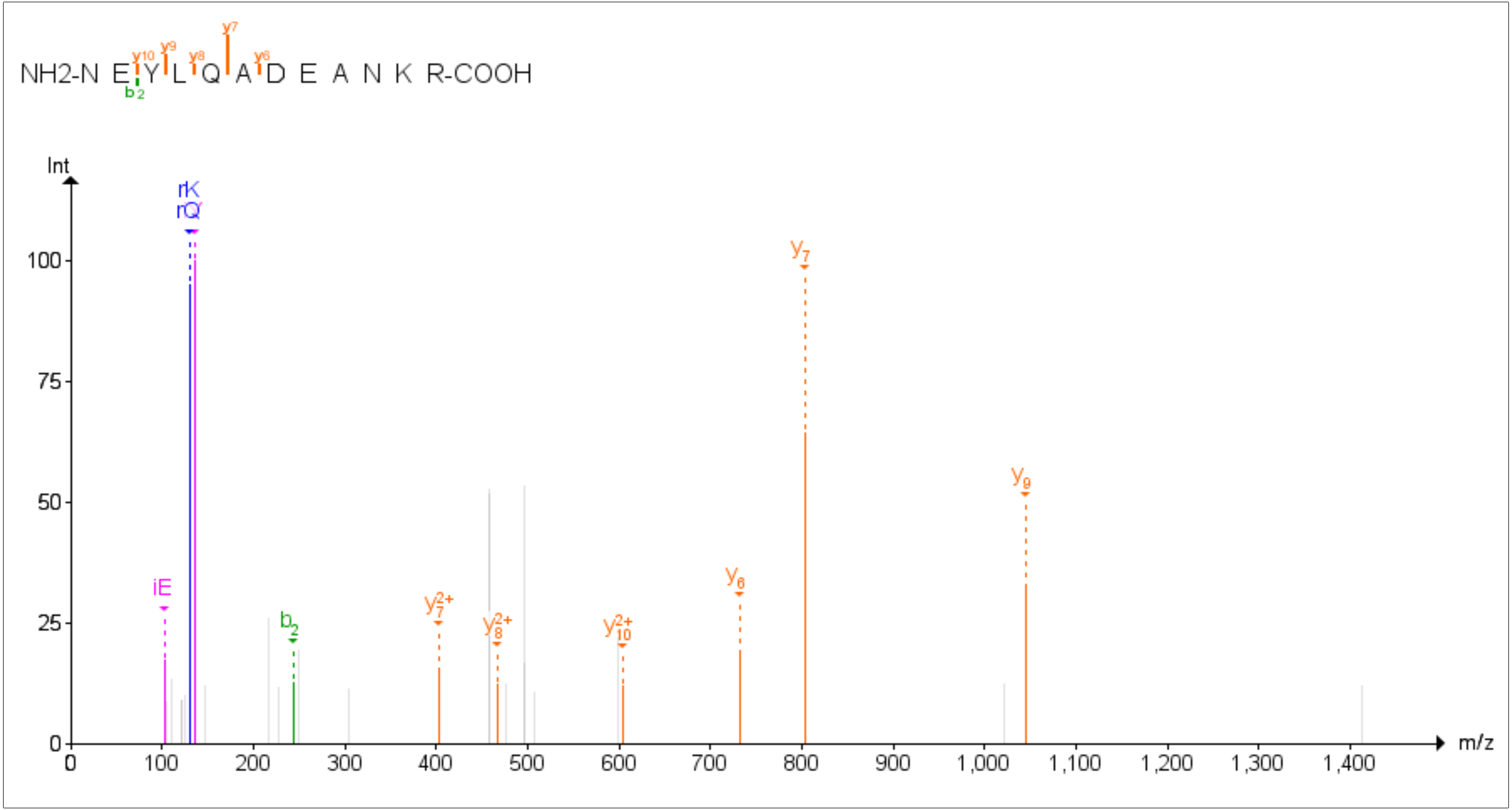

NH2-N E Y L Q A D E A N K R-COOH
y10
y9
y8
y7
y6
b2
Int
rK
rQ
iE
b2
y7 2+
y8 2+
y10 2+
y6
y7
y9
m/z
100
75
50
25
0
0
100
200
300
400
500
600
700
800
900
1,000
1,100
1,200
1,300
1,400

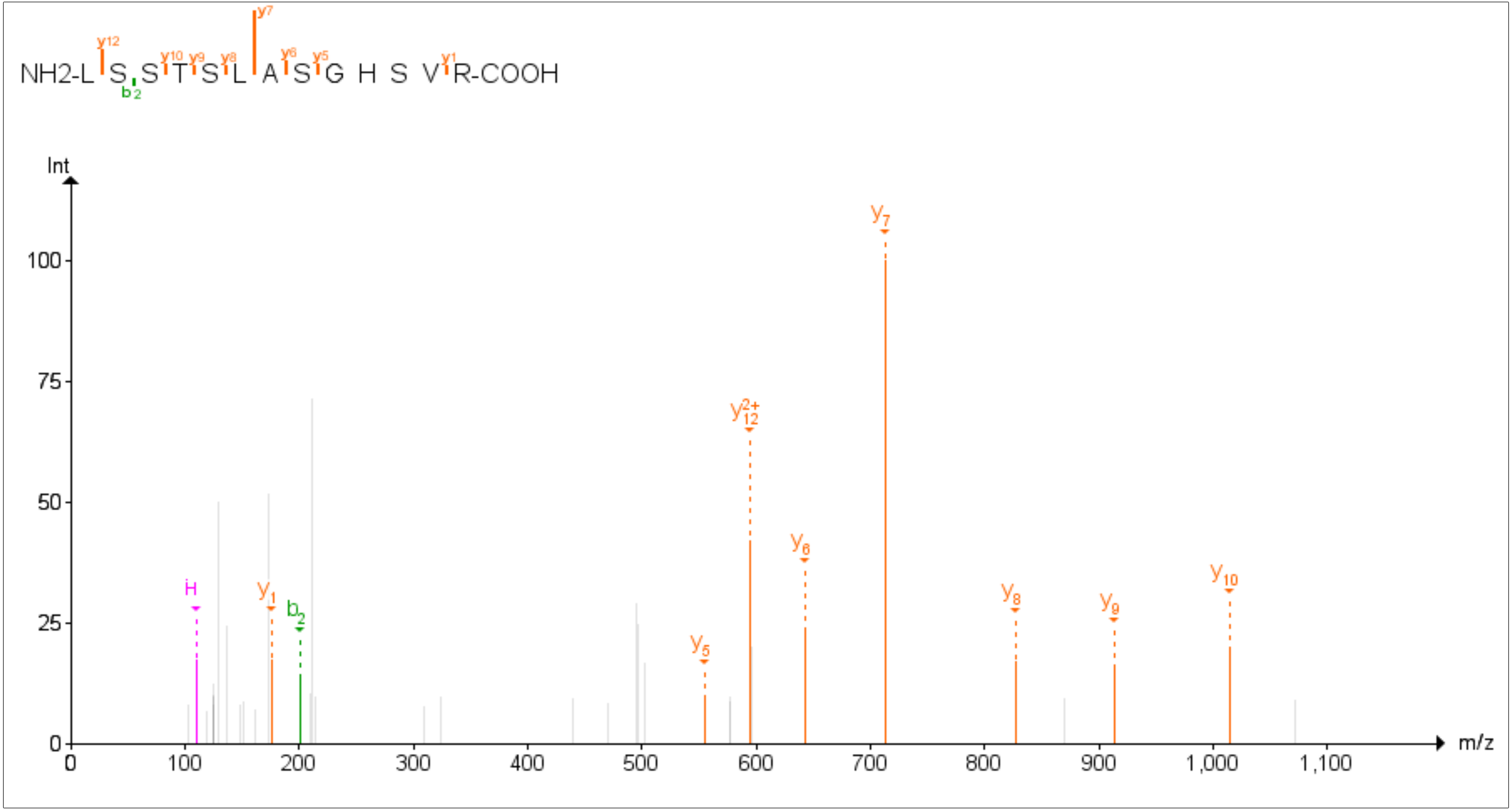
NH2-L S S T S L A S G H S V R-COOH
y12
y10
y9
y8
y7
y6
y5
y1
b2
Int
m/z
0
25
50
75
100
0
100
200
300
400
500
600
700
800
900
1,000
1,100
H
y1
b2
y5
y12 2+
y6
y7
y8
y9
y10

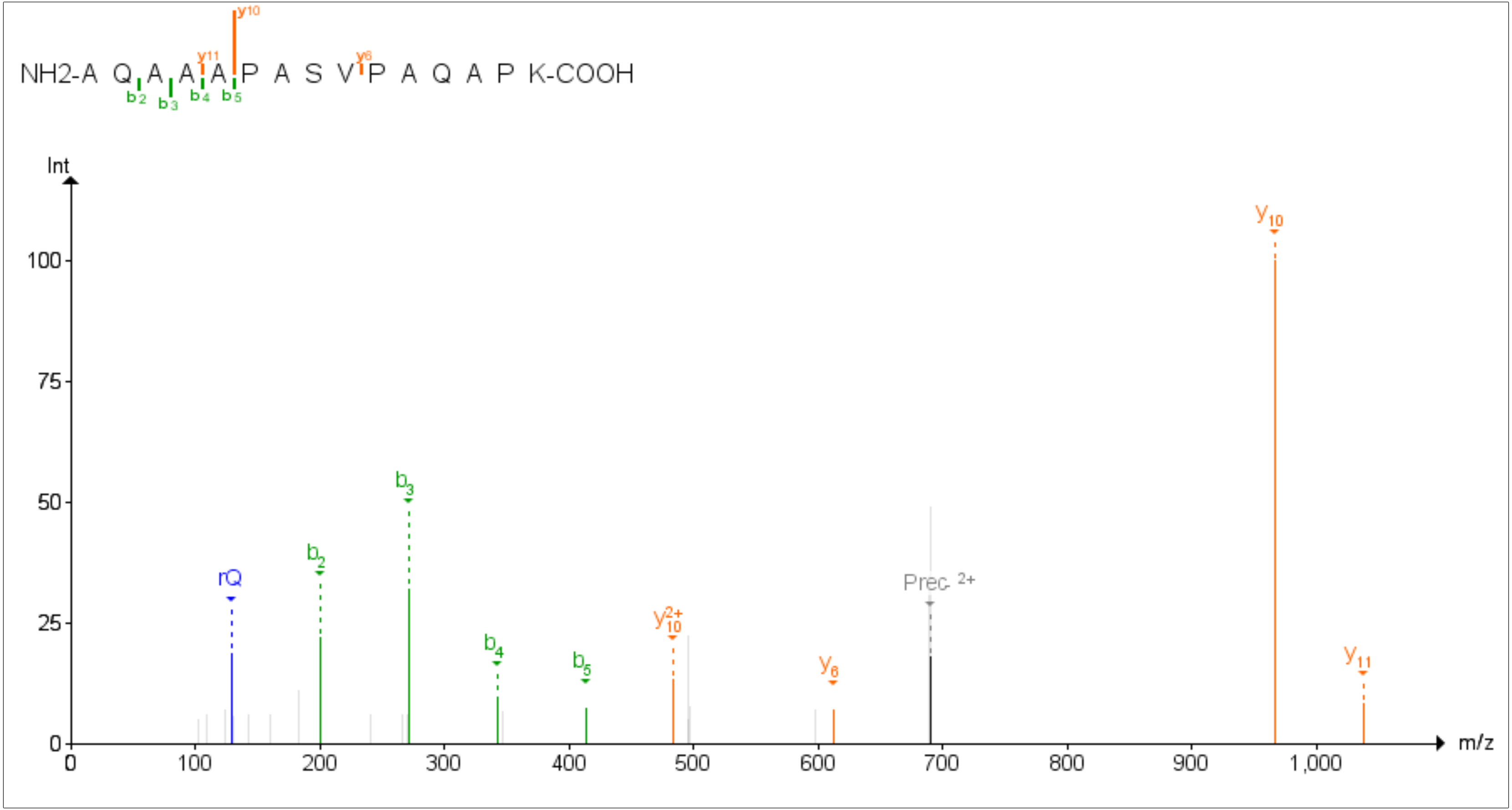

NH2-A Q A A A P A S V P A Q A P K-COOH
y10
y11
y6
b2
b3
b4
b5
Int
100
75
50
25
0
rQ
b2
b3
b4
b5
y10 2+
y6
Prec- 2+
y10
y11
0
100
200
300
400
500
600
700
800
900
1,000
m/z

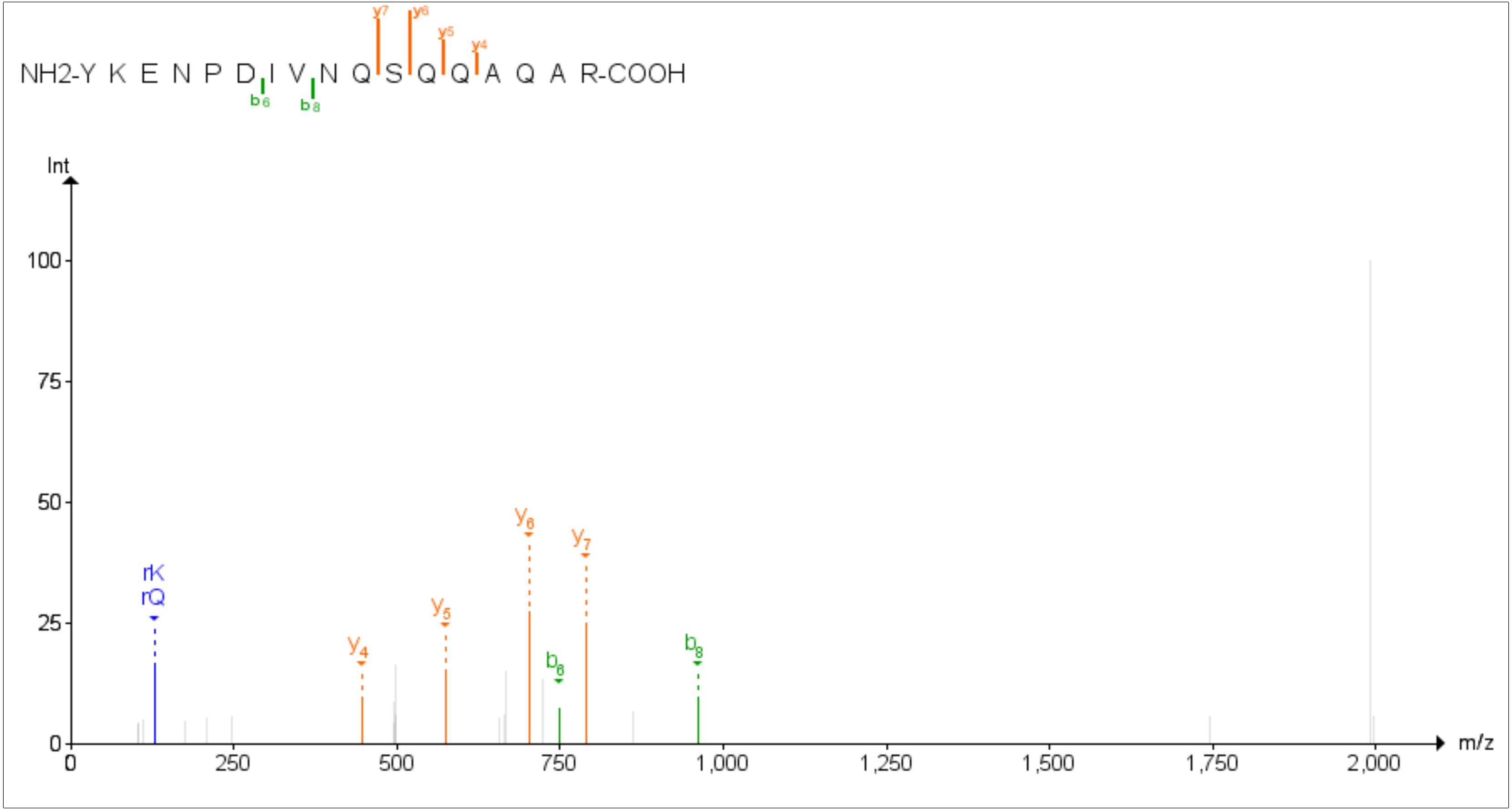

NH2-Y K E N P D I V N Q S Q Q A Q A R-COOH
y7
y6
y5
y4
b6
b8
Int
100
75
50
25
0
rK
rQ
y4
y5
y6
y7
b6
b8
0
250
500
750
1,000
1,250
1,500
1,750
2,000
m/z

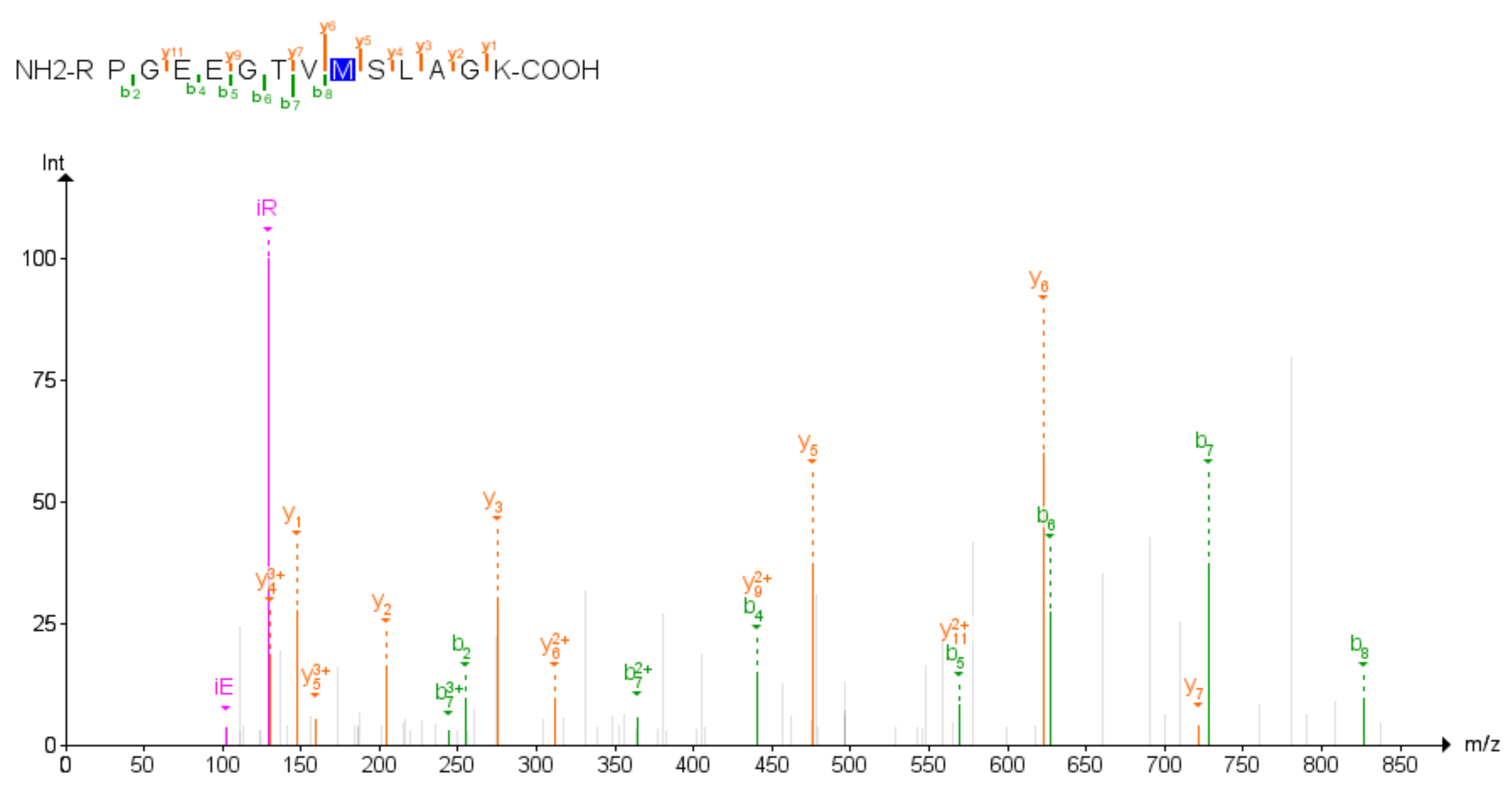
NH2-R P G E E G T V M S L A G K-COOH
y11 y9 y7 y6 y5 y4 y3 y2 y1
b2 b4 b5 b6 b7 b8
Int
m/z
iR
iE
y6
y5
b7
b8
y3
y1
y4 3+
y2
y5 3+
b2
b7 3+
y6 2+
b7 2+
y9 2+
b4
y11 2+
b5
y7
b8
0 25 50 75 100
0 50 100 150 200 250 300 350 400 450 500 550 600 650 700 750 800 850

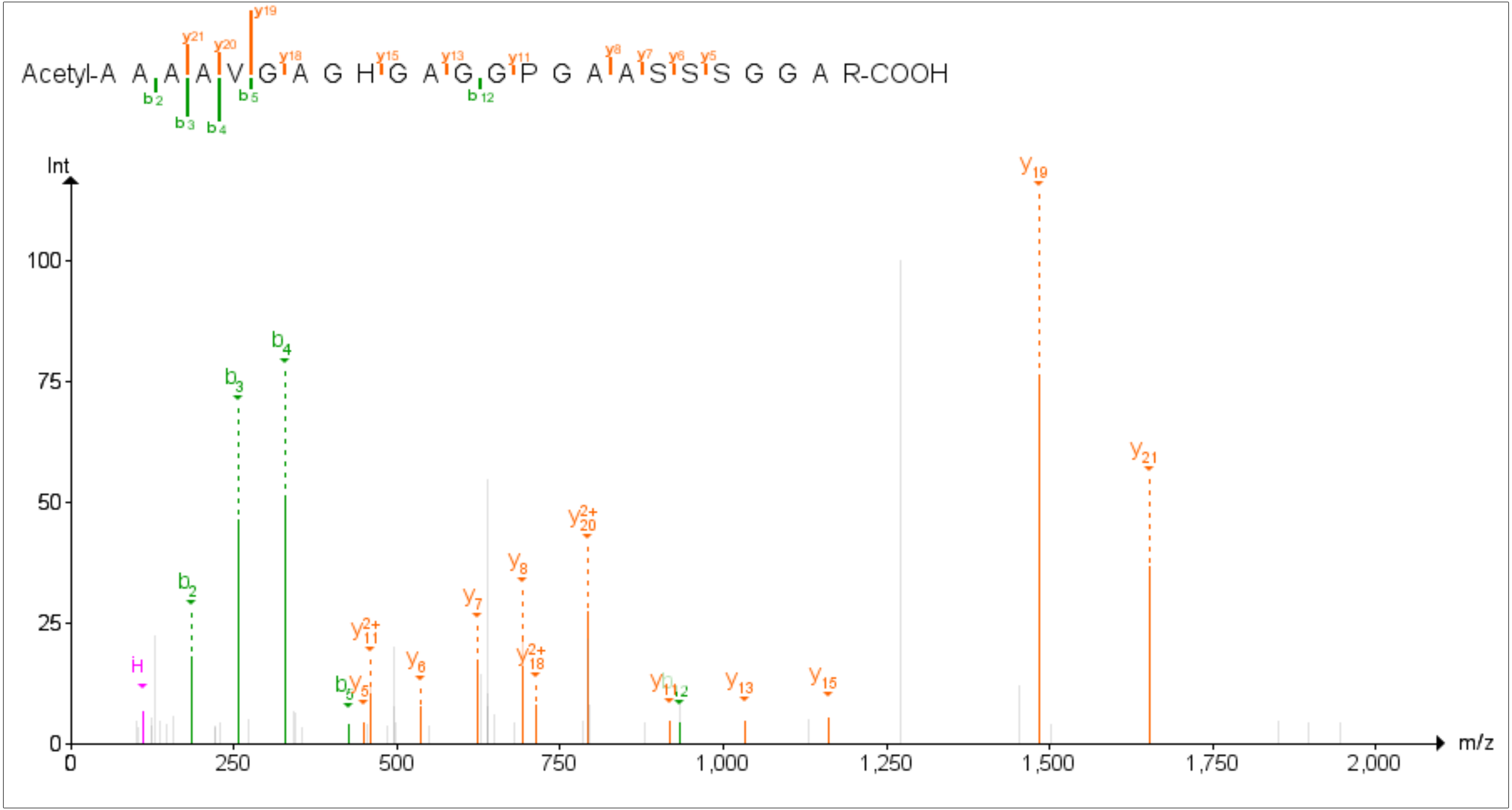
Acetyl-A A A A V G A G H G A G G P G A A S S S G G A R-COOH
Int
m/z
0
25
50
75
100
250
500
750
1,000
1,250
1,500
1,750
2,000
y19
y21
b4
b3
y20 2+
y8
y7
b2
y11 2+
y6
y18 2+
H
b5
y5
y13
y15
y12
