# Supplementary material for: Surface Proteomics of Cancer-Derived Extracellular Vesicles Reveals Universal Heat Shock Proteins and Cancer Signatures
Source: Mol Cell Proteomics. 2026 Aug 22;25(10):101649. doi: 10.1016/j.mcpro.2026.101649 (PMC13634791; doi:10.1016/j.mcpro.2026.101649)
Supplement: Supplementary file 4 [file mmc5.pdf]

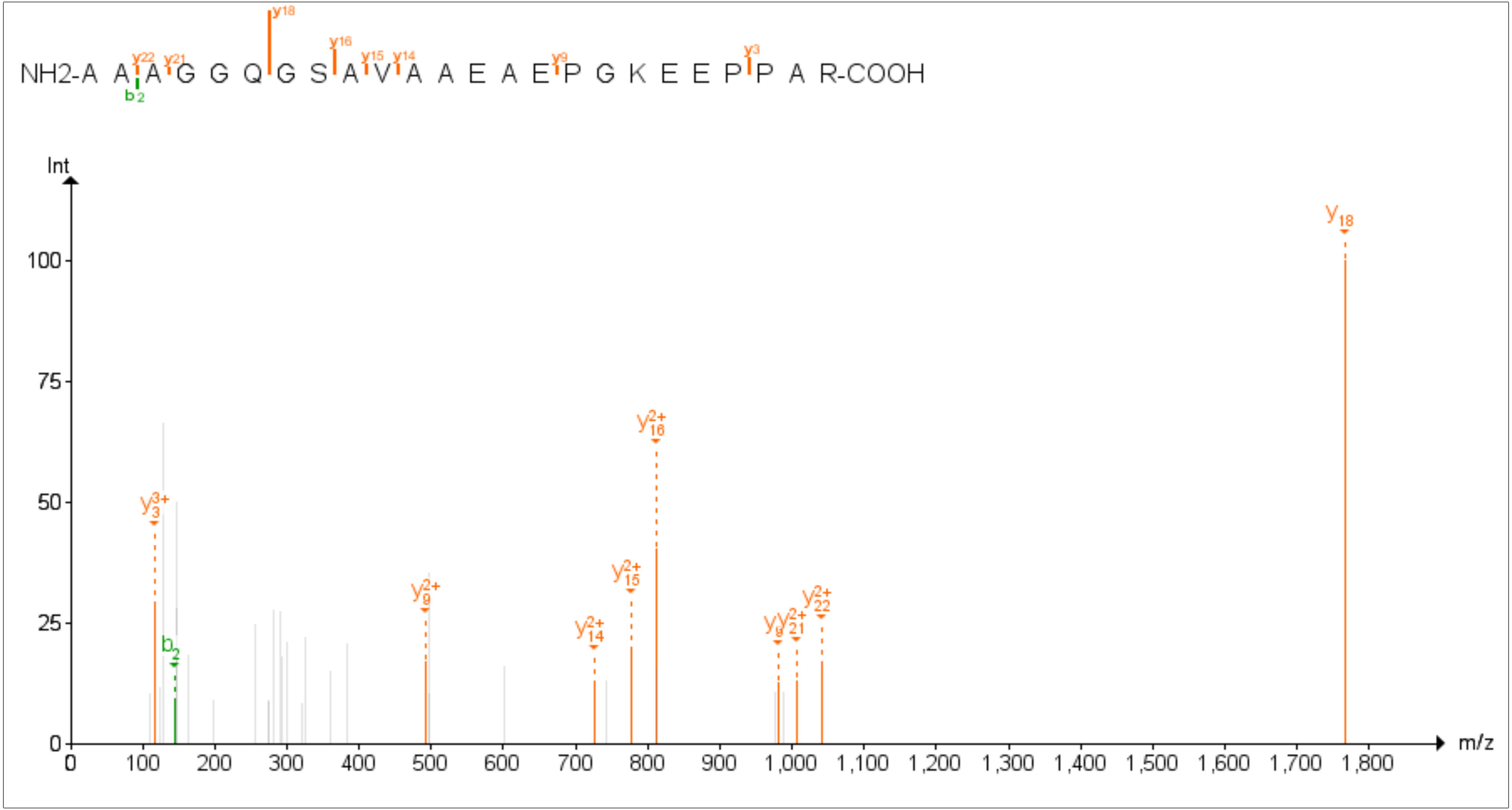

NH2-A A A G G Q G S A V A A E A E P G K E E P P A R-COOH
y22
y21
y18
y16
y15
y14
y9
y3
b2
Int
100
75
50
25
0
m/z
0
100
200
300
400
500
600
700
800
900
1,000
1,100
1,200
1,300
1,400
1,500
1,600
1,700
1,800
y18
y16 2+
y3 3+
y9 2+
y15 2+
y14 2+
y22 2+
y9
y21 2+
b2

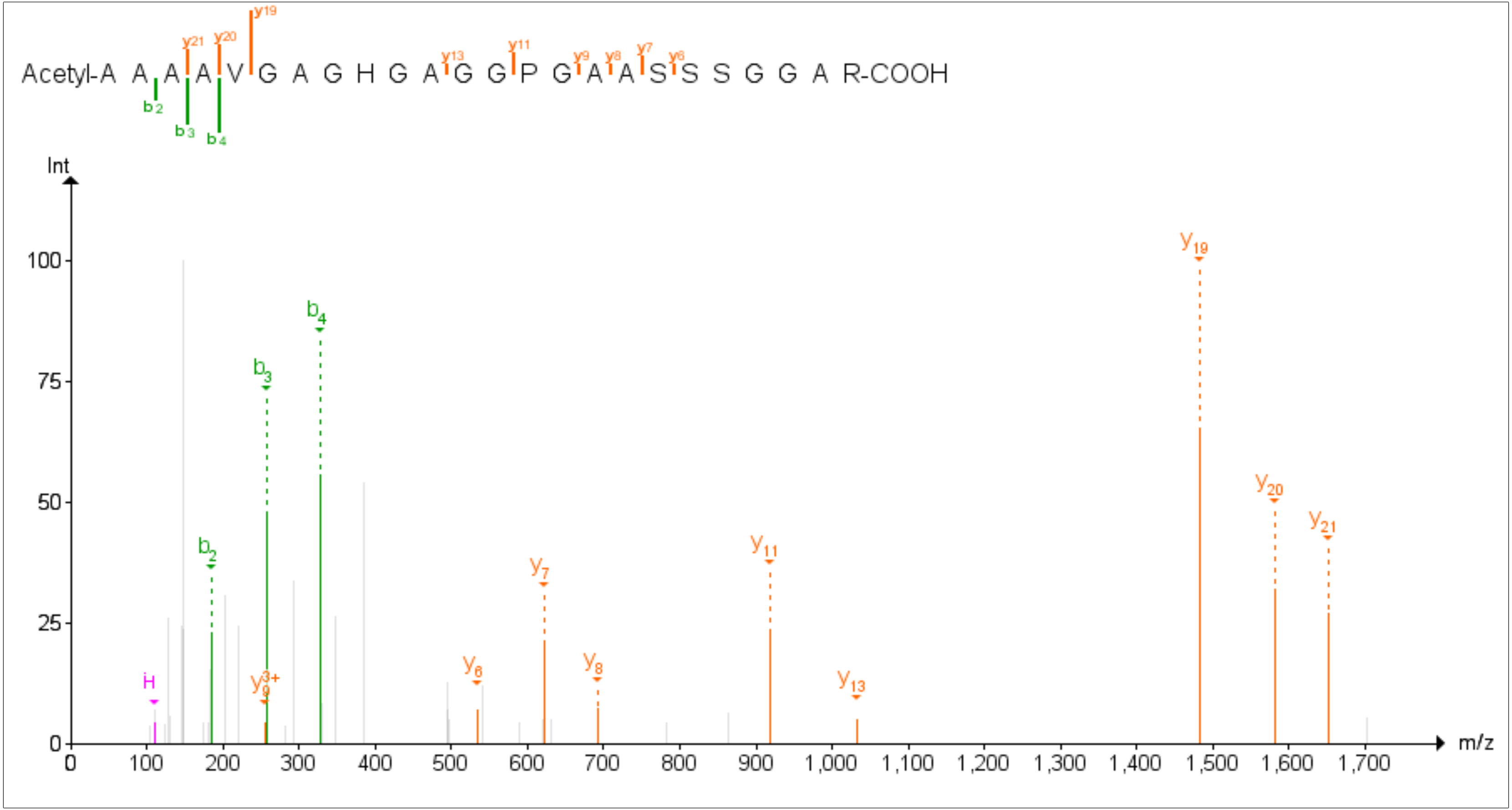

Acetyl-A A A A V G A G H G A G G P G A A S S S G G A R-COOH
b2
b3
b4
y21
y20
y19
y13
y11
y9
y8
y7
y6
Int
100
75
50
25
0
m/z
0
100
200
300
400
500
600
700
800
900
1,000
1,100
1,200
1,300
1,400
1,500
1,600
1,700

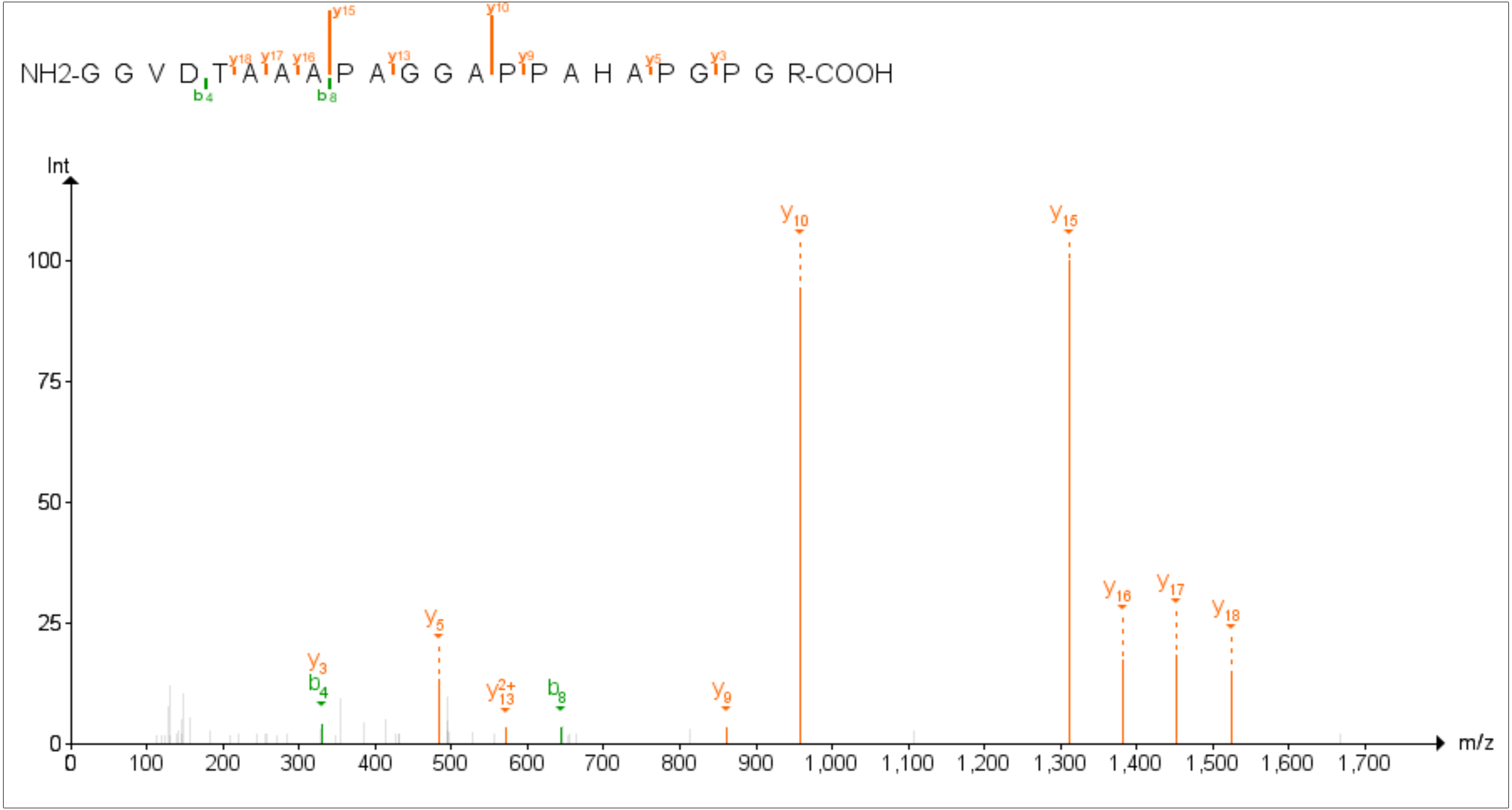

NH2-G G V D T A A A P A G G A P P A H A P G P G R-COOH
b4
b8
y18
y17
y16
y15
y13
y10
y9
y5
y3
Int
m/z
0
25
50
75
100
0
100
200
300
400
500
600
700
800
900
1,000
1,100
1,200
1,300
1,400
1,500
1,600
1,700
y3
b4
y5
y13 2+
b8
y9
y10
y15
y16
y17
y18

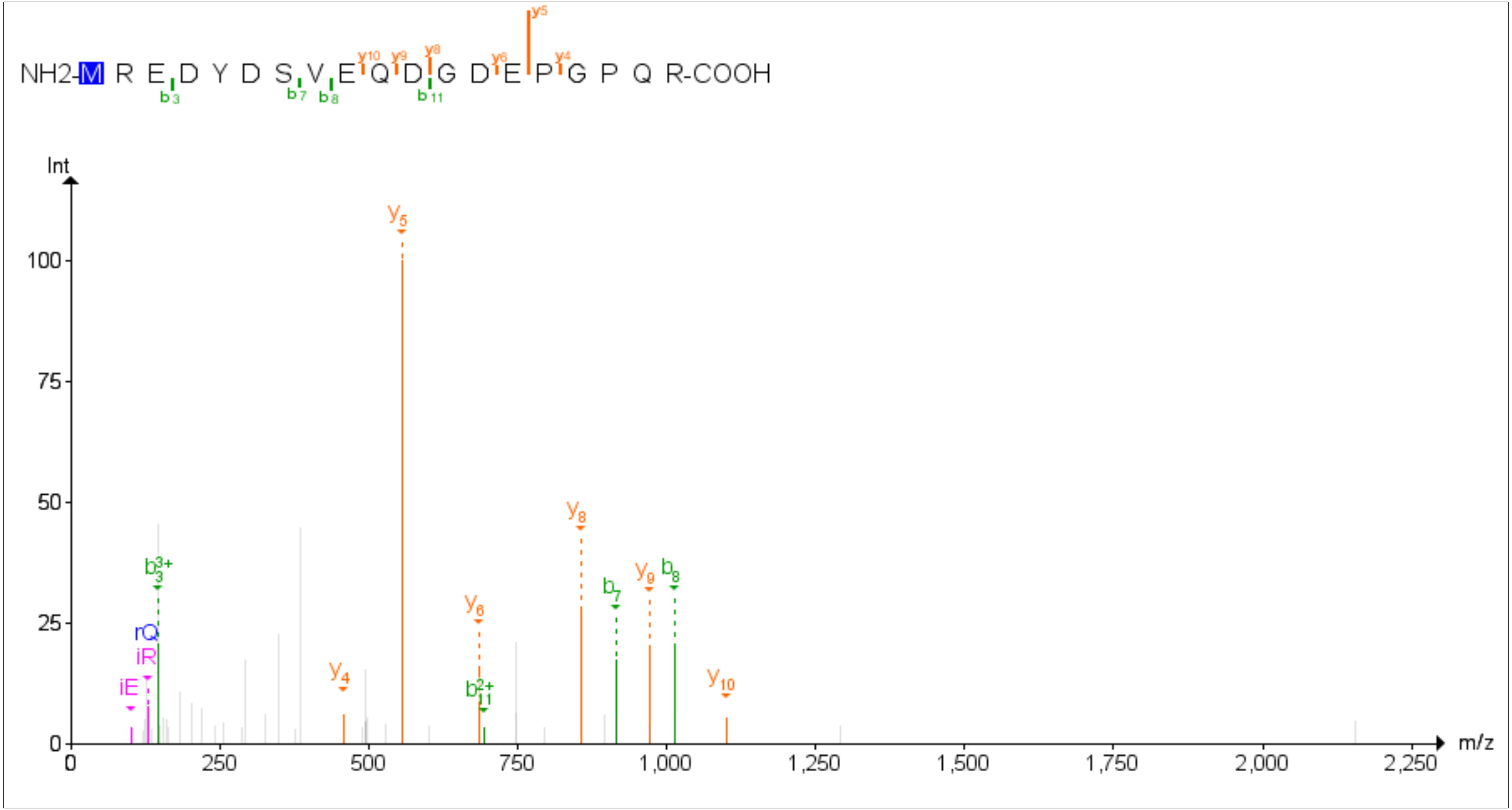
NH2-M R E D Y D S V E Q D G D E P G P Q R-COOH
b3 b7 b8 b11
y10 y9 y8 y6 y5 y4
Int
100
75
50
25
0
y5
y8
y6
y9
b7
b8
b3 3+
rQ
iR
iE
y4
b11 2+
y10
0 250 500 750 1,000 1,250 1,500 1,750 2,000 2,250
m/z

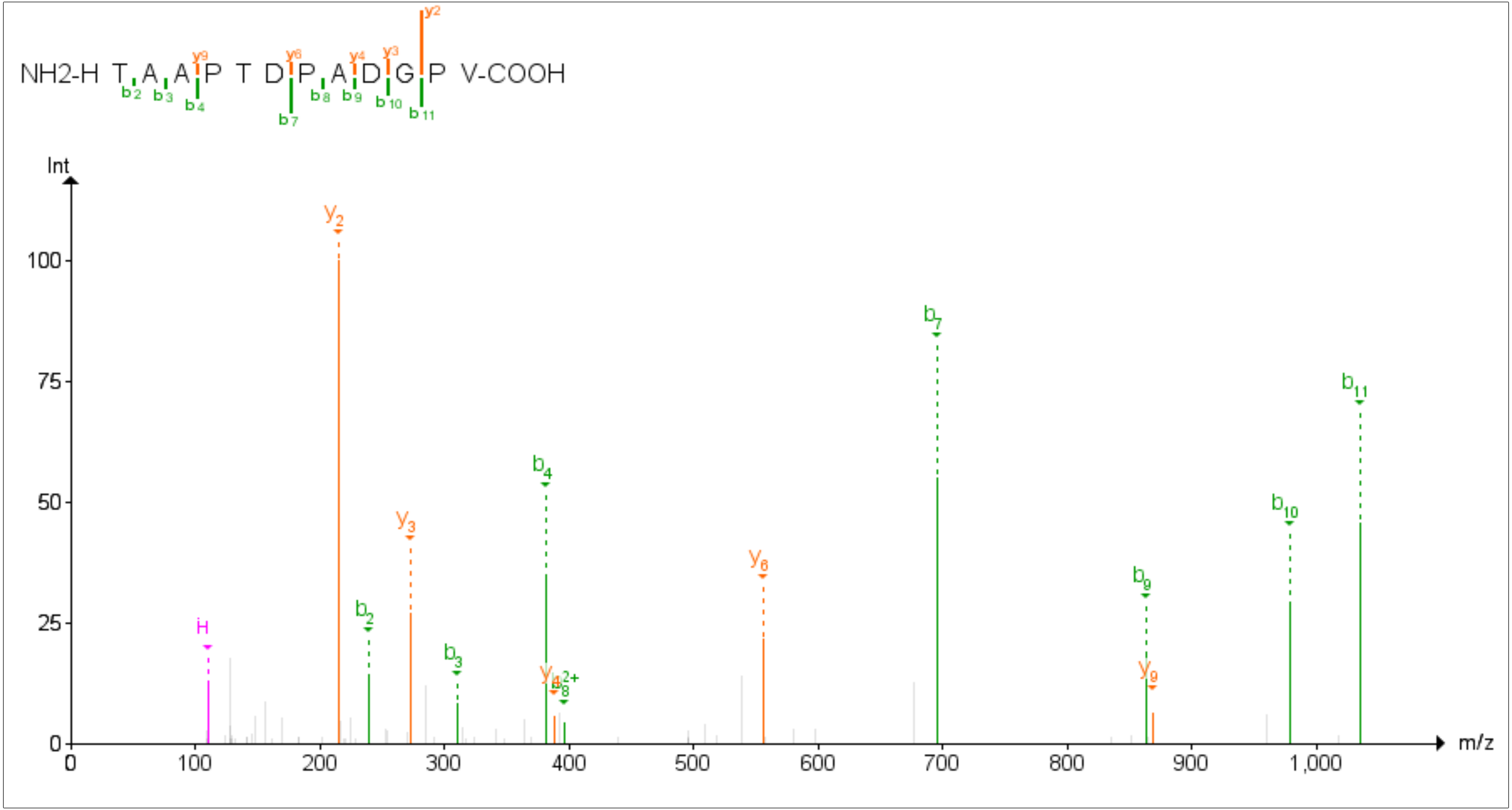
NH2-H T A A P T D P A D G P V-COOH
Int
m/z
y2
y3
y4
y6
y9
b2
b3
b4
b7
b9
b10
b11
H
0
25
50
75
100
0
100
200
300
400
500
600
700
800
900
1,000

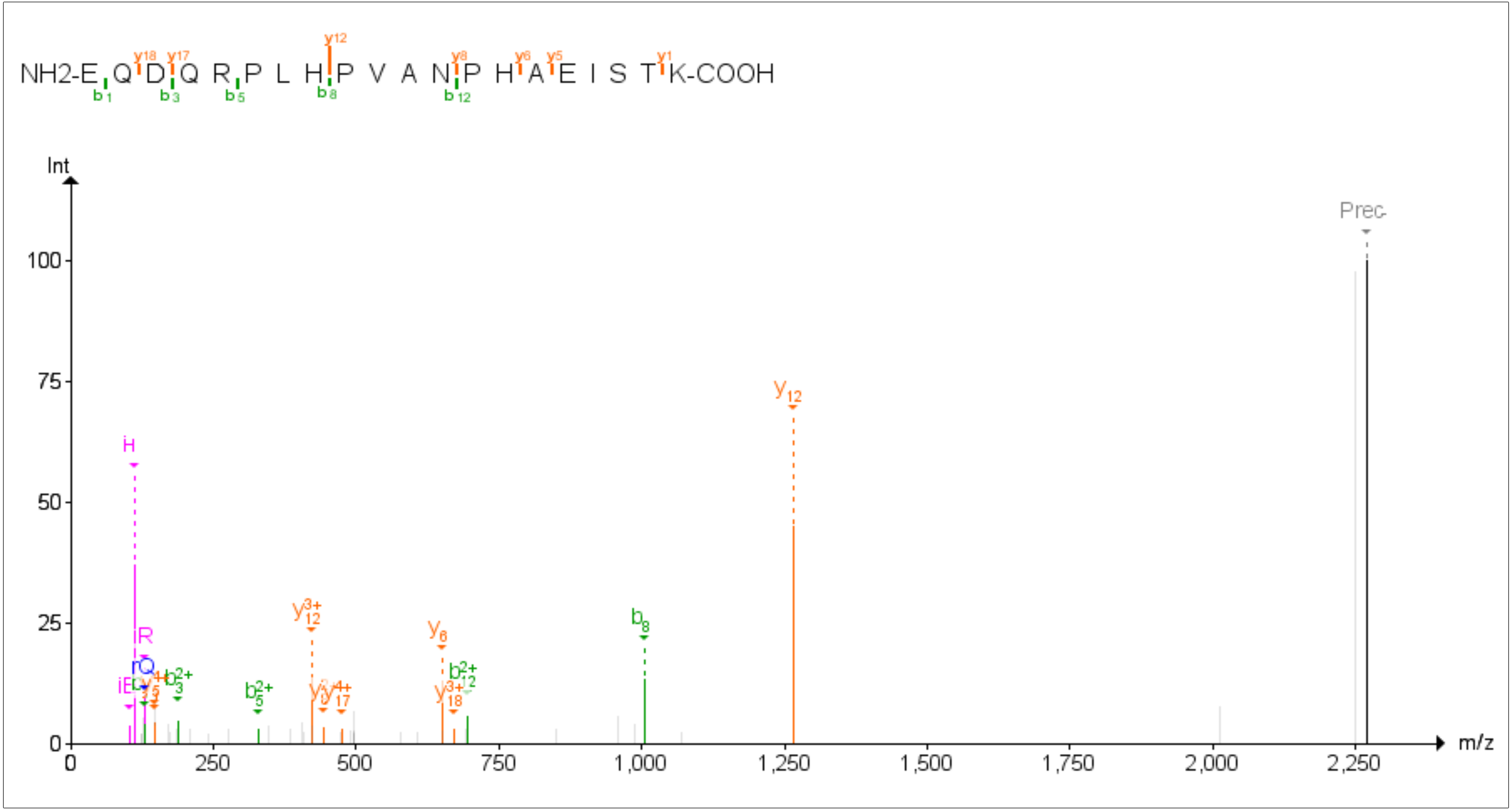

NH2-E Q D Q R P L H P V A N P H A E I S T K-COOH
y18 y17 y12 y8 y6 y5 y1
b1 b3 b5 b8 b12
Int
m/z
Prec.
y12
b8
y6
iH
iR
rQ

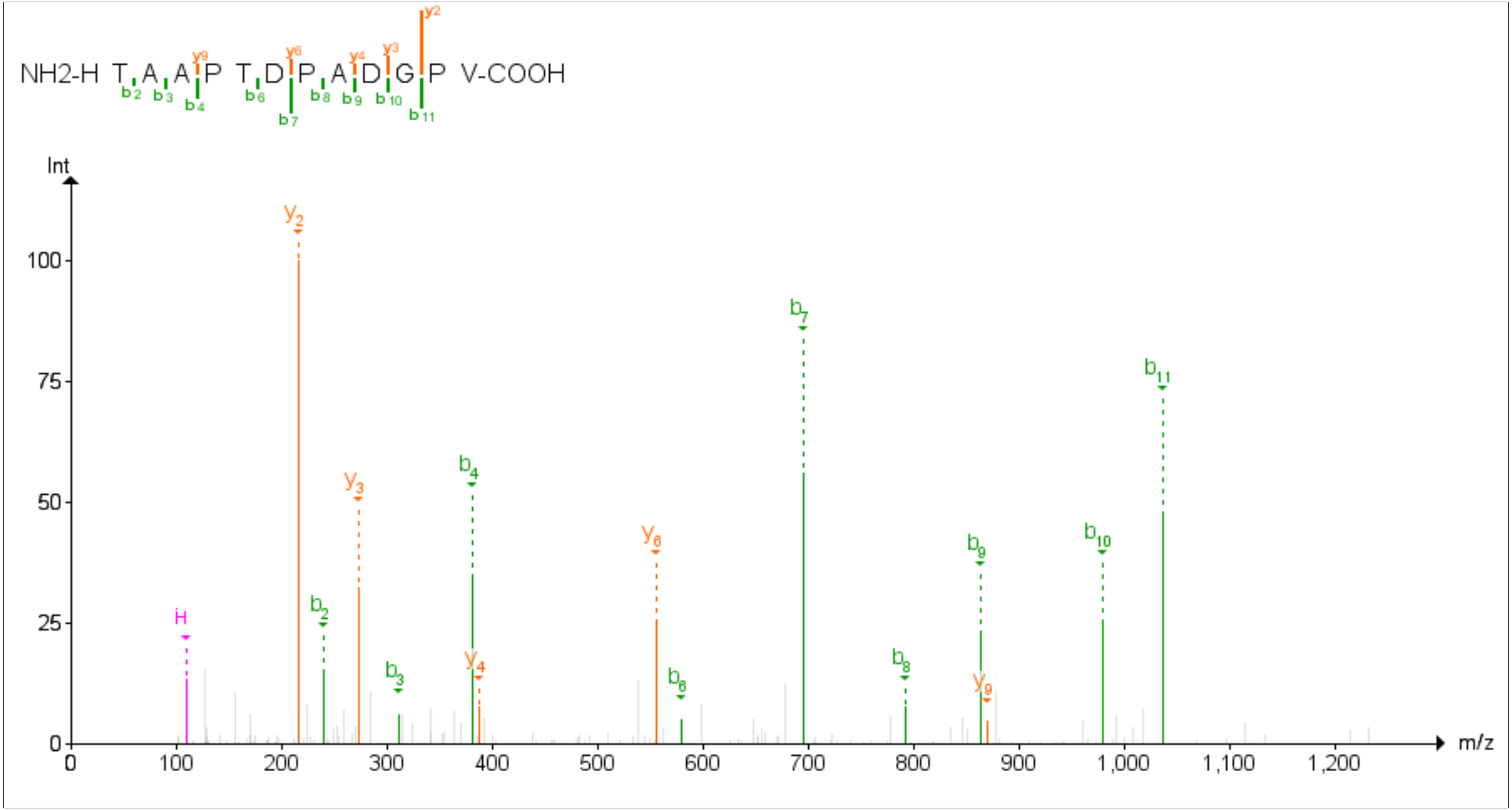

NH2-H T A A P T D P A D G P V-COOH
y9 y6 y4 y3 y2
b2 b3 b4 b6 b7 b8 b9 b10 b11
Int
100
75
50
25
0
m/z
0 100 200 300 400 500 600 700 800 900 1,000 1,100 1,200
H
y2 y3 y4 y6 y9
b2 b3 b4 b6 b7 b8 b9 b10 b11

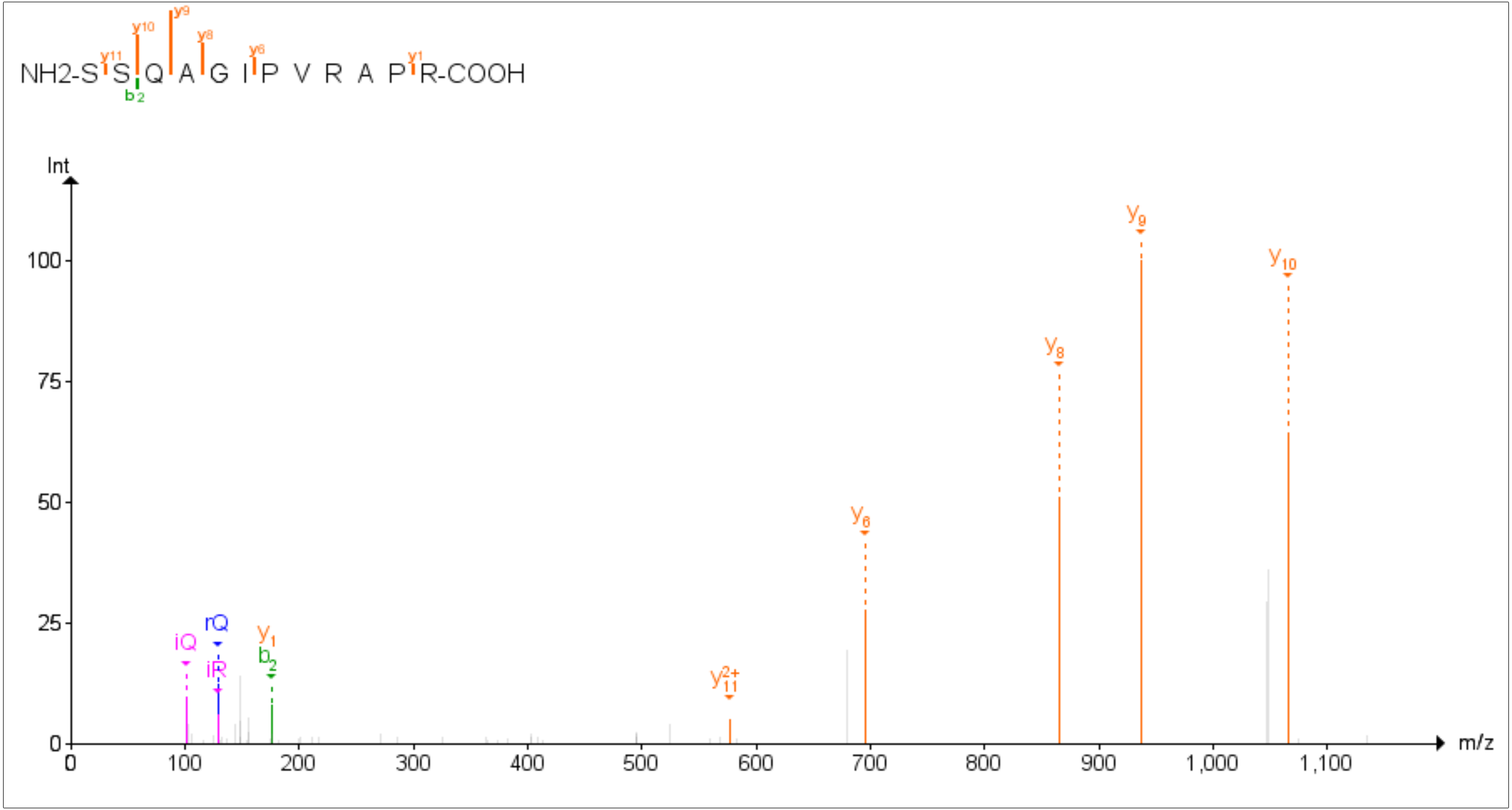
NH2-S S Q A G I P V R A P R-COOH
y11
y10
y9
y8
y6
y1
b2
Int
100
75
50
25
0
m/z
0
100
200
300
400
500
600
700
800
900
1,000
1,100
iQ
rQ
iR
y1
b2
y11 2+
y6
y8
y9
y10

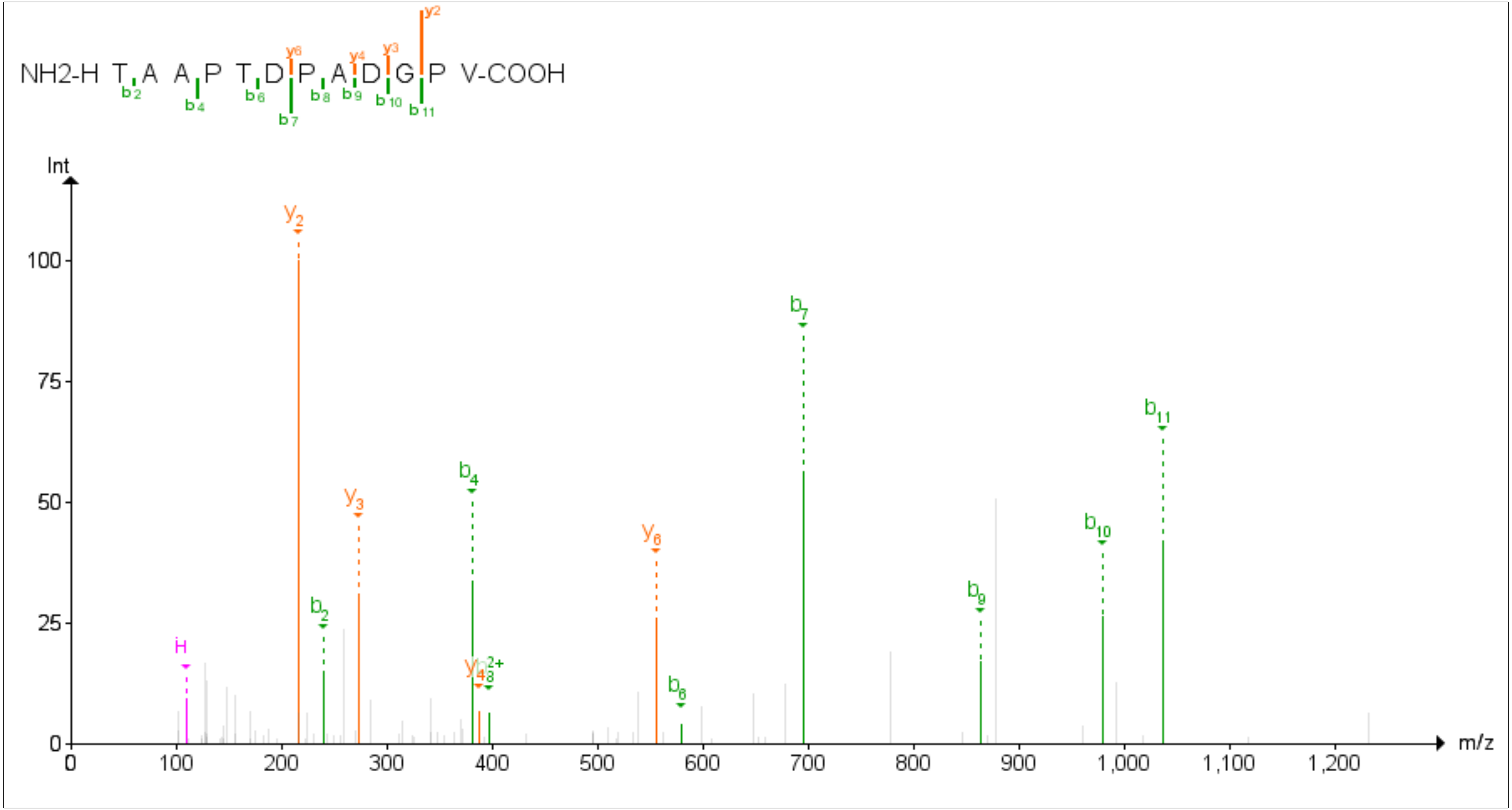
NH2-H T A A P T D P A D G P V-COOH
y6 y4 y3 y2
b2 b4 b6 b7 b8 b9 b10 b11
Int
m/z
0
25
50
75
100
0 100 200 300 400 500 600 700 800 900 1,000 1,100 1,200
H
y2
y3
b2
b4
y4
b8 2+
y6
b6
b7
b9
b10
b11

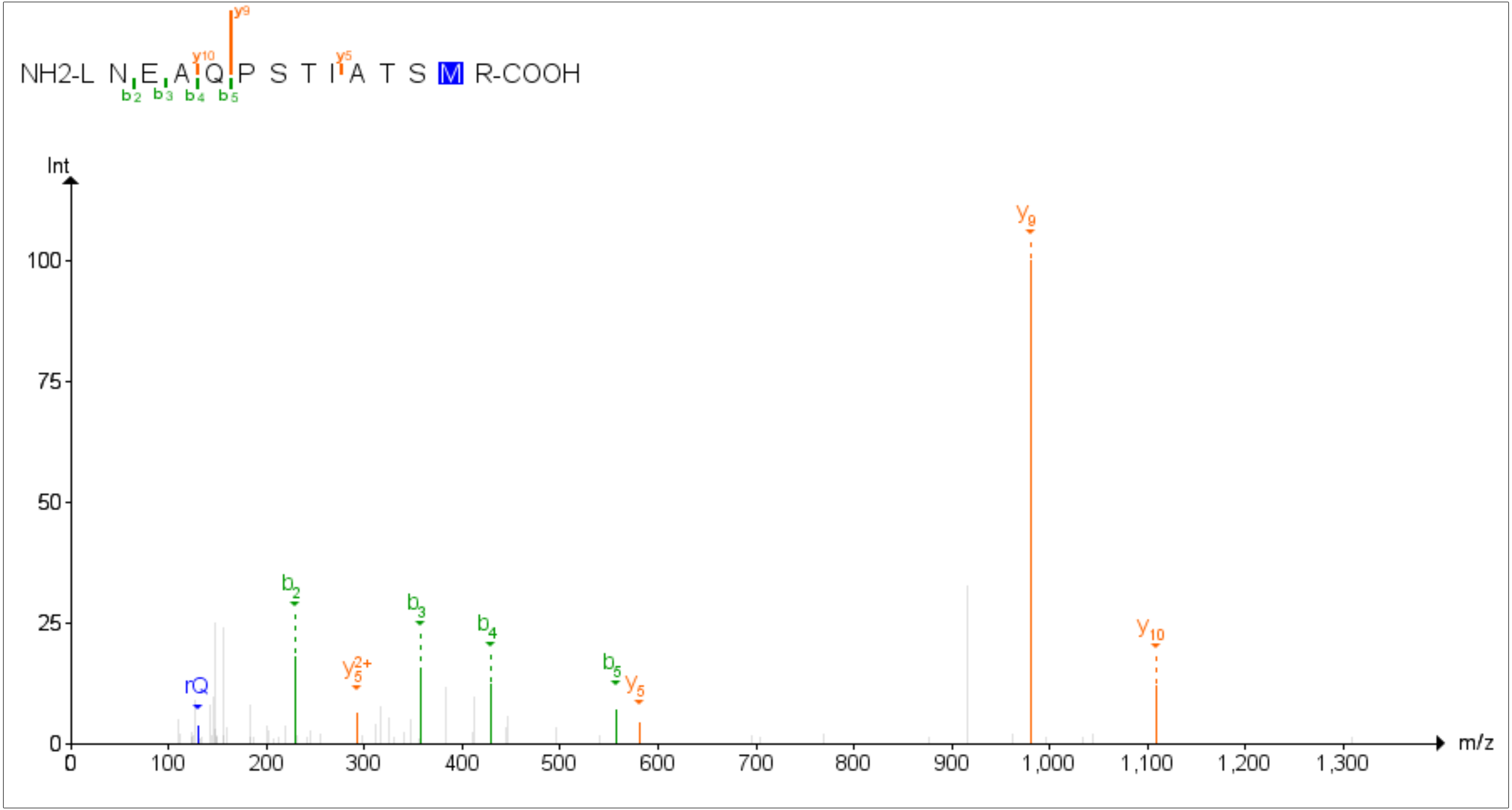
NH2-L N E A Q P S T I A T S M R-COOH
b2 b3 b4 b5
y10 y9 y5
Int
100
75
50
25
0
m/z
0 100 200 300 400 500 600 700 800 900 1,000 1,100 1,200 1,300
rQ
b2
y5 2+
b3
b4
b5
y5
y9
y10

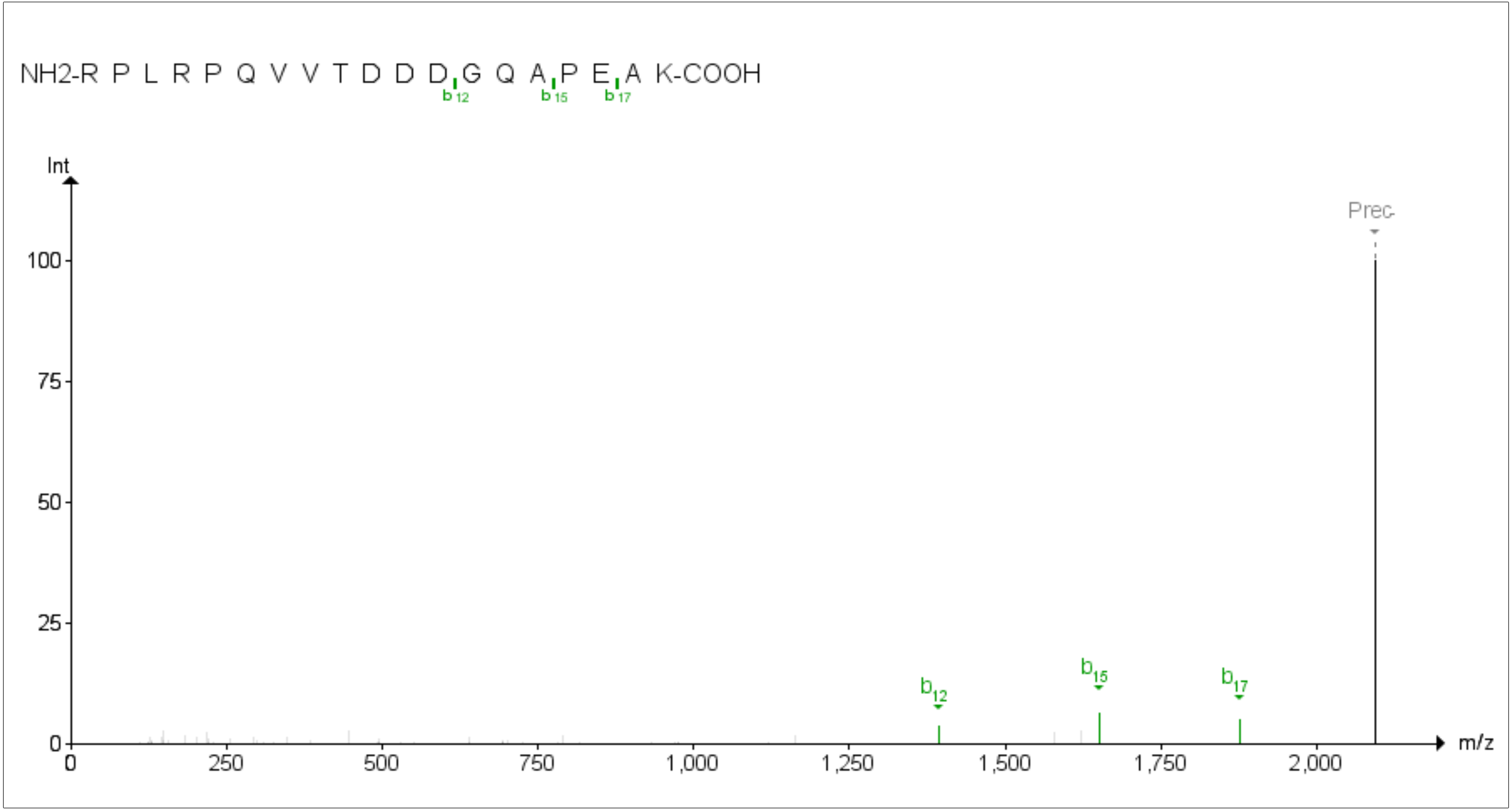
NH2-R P L R P Q V V T D D D G Q A P E A K-COOH
b12
b15
b17
Int
100
75
50
25
0
Prec
b12
b15
b17
0
250
500
750
1,000
1,250
1,500
1,750
2,000
m/z

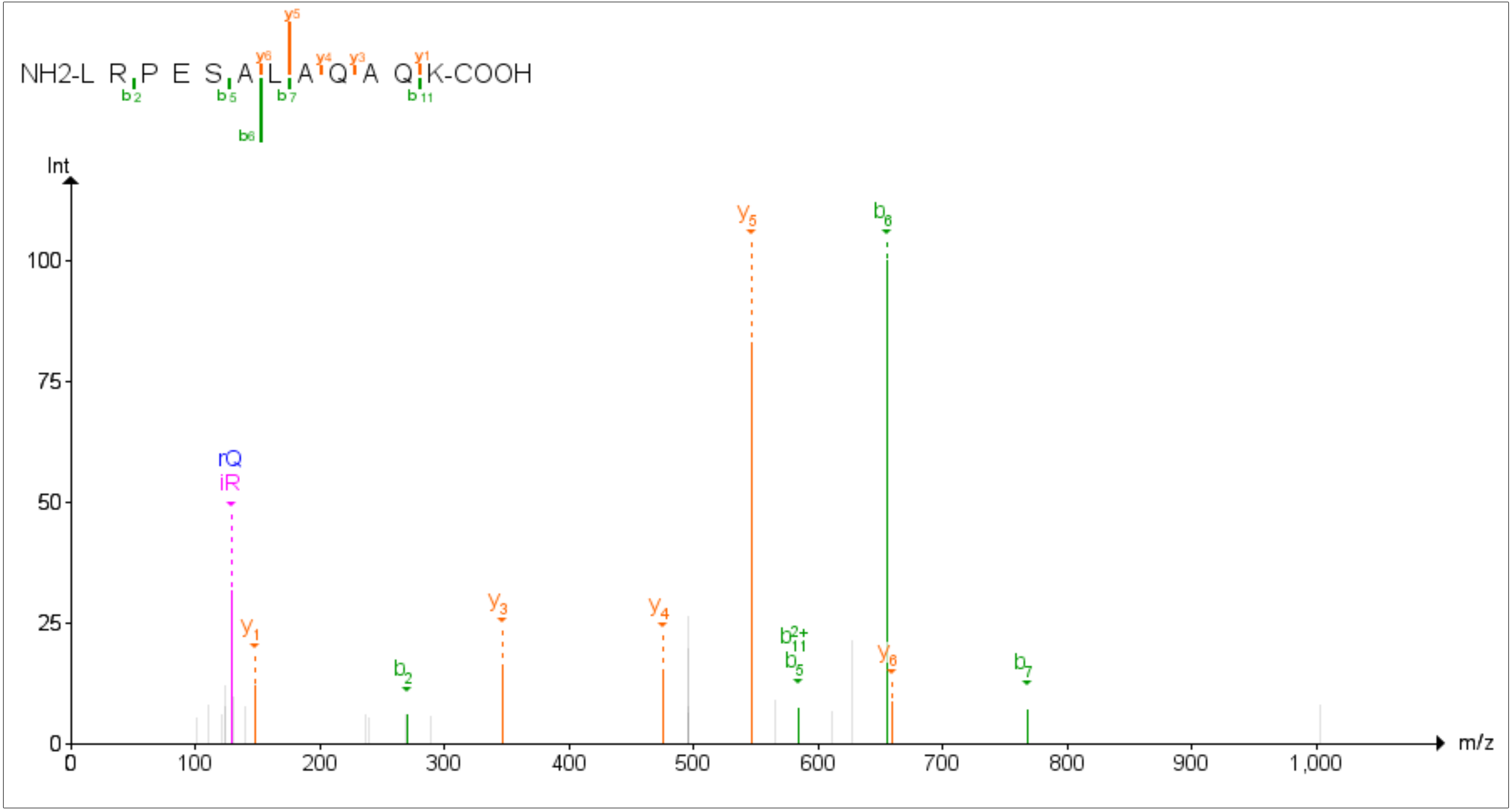
NH2-L R P E S A L A Q A Q K-COOH
b2 b5 b6 b7 b11
y6 y5 y4 y3 y1
Int
100
75
50
25
0
rQ
iR
y1
b2
y3
y4
y5
b6
b11 2+
b5
y6
b7
0
100
200
300
400
500
600
700
800
900
1,000
m/z

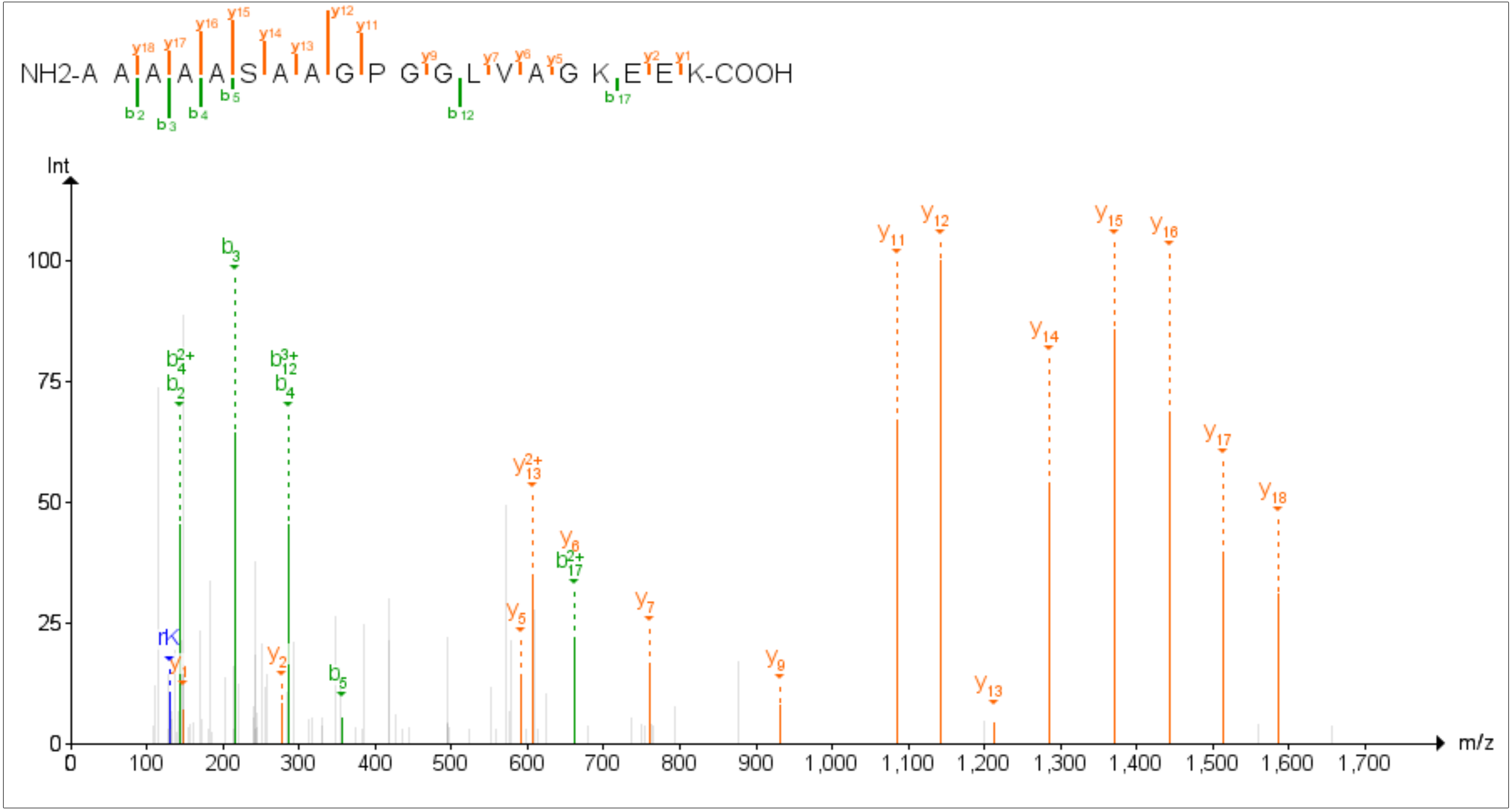

NH2-A A A A A S A A G P G G L V A G K E E K-COOH
y18 y17 y16 y15 y14 y13 y12 y11 y9 y7 y6 y5 y2 y1
b2 b3 b4 b5 b12 b17
Int
100
75
50
25
0
m/z
0 100 200 300 400 500 600 700 800 900 1,000 1,100 1,200 1,300 1,400 1,500 1,600 1,700
b3
b4 2+
b2
b12 3+
b4
rK
y1
y2
b5
y5
y13 2+
y6
b17 2+
y7
y9
y11
y12
y13
y14
y15
y16
y17
y18

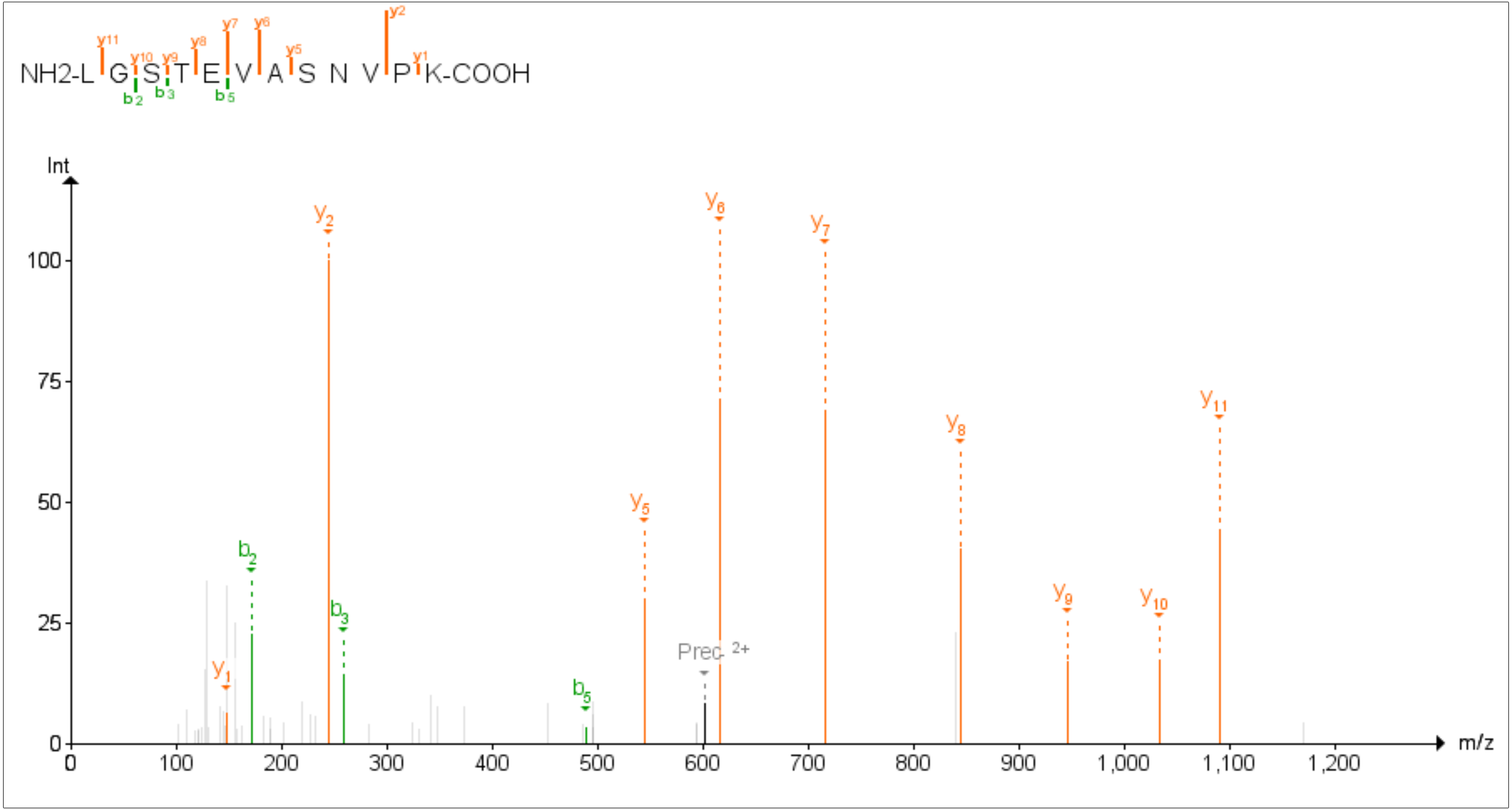

NH2-L G S T E V A S N V P K-COOH
y11
y10
y9
y8
y7
y6
y5
y2
y1
b2
b3
b5
Int
100
75
50
25
0
y2
y6
y7
y8
y11
y5
b2
b3
y9
y10
Prec 2+
y1
b5
0
100
200
300
400
500
600
700
800
900
1,000
1,100
1,200
m/z

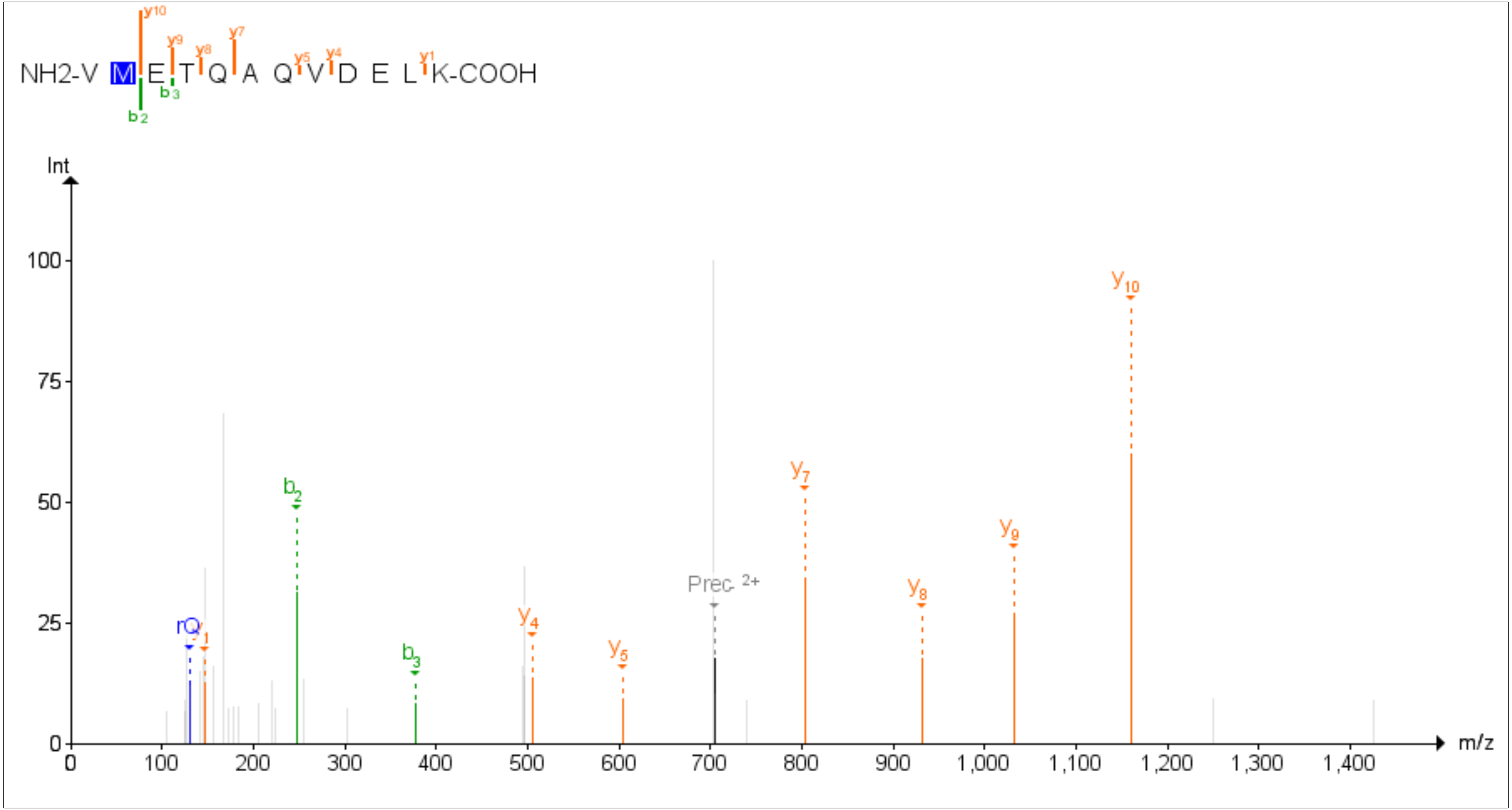

NH2-V M E T Q A Q V D E L K-COOH
y10
y9
y8
y7
y5
y4
y1
b2
b3
Int
100
75
50
25
0
b2
b3
rQ
y1
y4
y5
Prec 2+
y7
y8
y9
y10
0
100
200
300
400
500
600
700
800
900
1,000
1,100
1,200
1,300
1,400
m/z

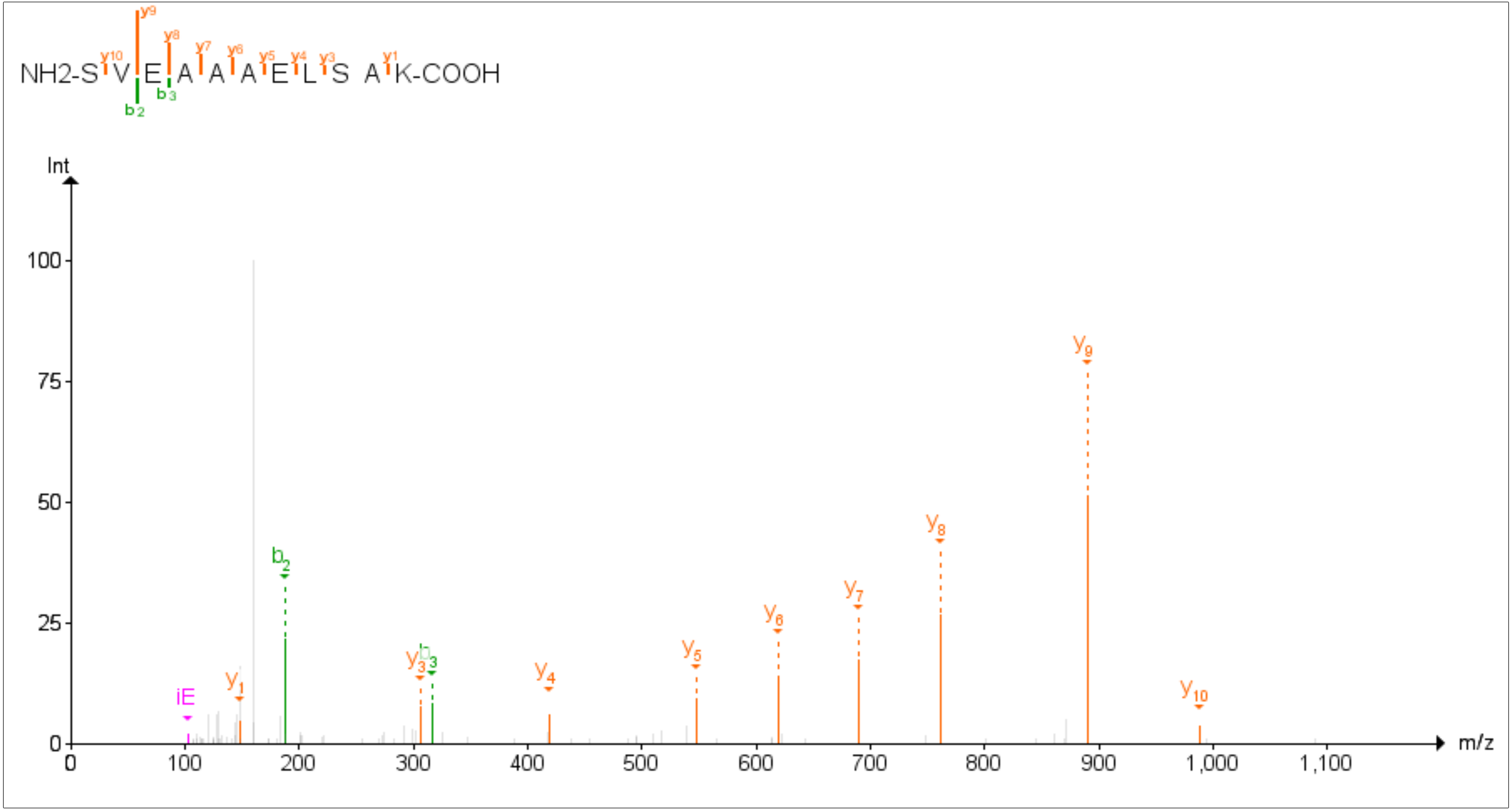

NH2-S V E A A A E L S A K-COOH
y10 y9 y8 y7 y6 y5 y4 y3 y1
b2 b3
Int
100
75
50
25
0
m/z
0 100 200 300 400 500 600 700 800 900 1,000 1,100
iE
y1
b2
y3
b3
y4
y5
y6
y7
y8
y9
y10

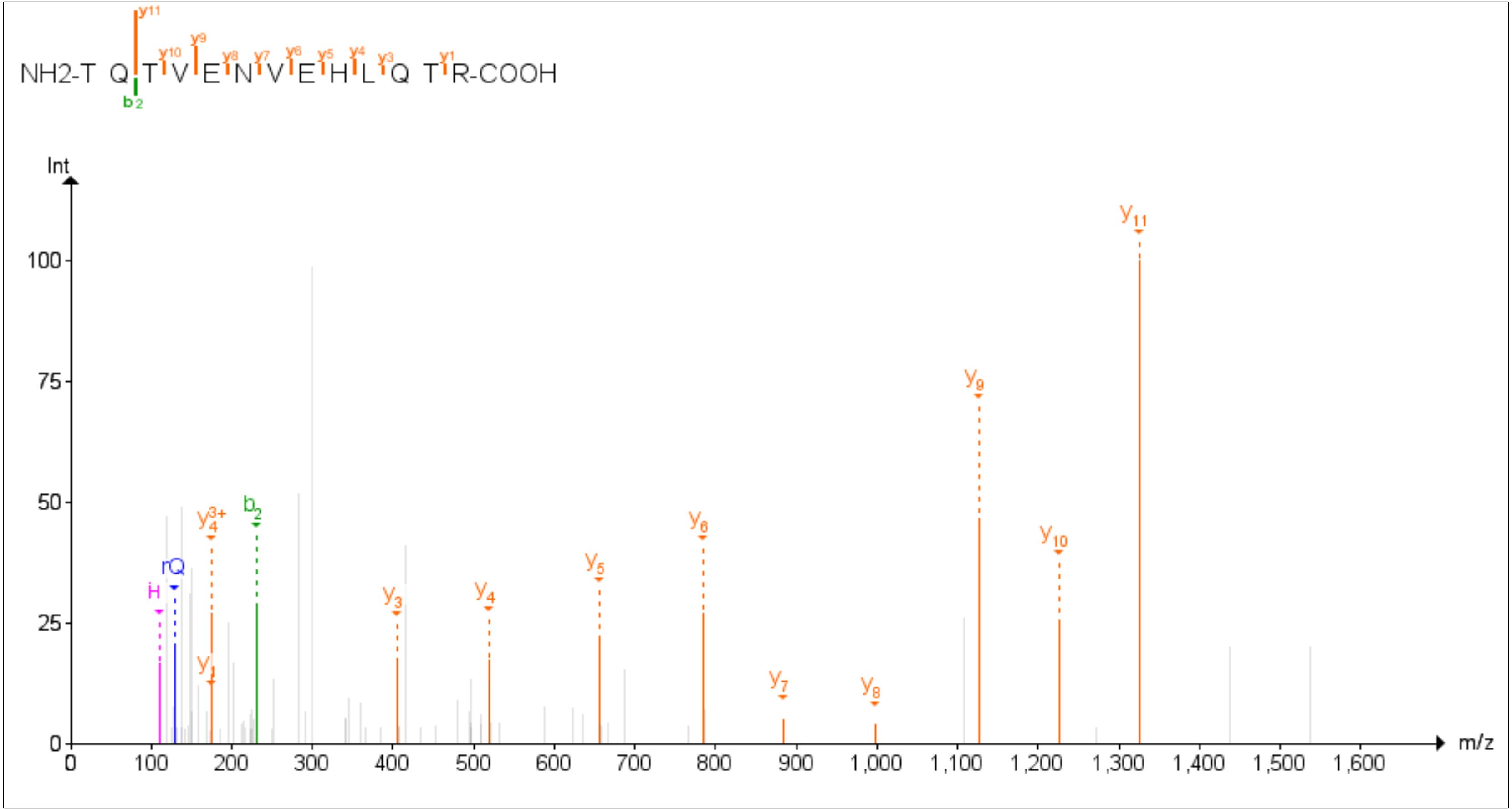

NH2-T Q T V E N V E H L Q T R-COOH
y11
y10
y9
y8
y7
y6
y5
y4
y3
y1
b2
Int
100
75
50
25
0
m/z
0
100
200
300
400
500
600
700
800
900
1,000
1,100
1,200
1,300
1,400
1,500
1,600
H
rQ
y4 3+
b2
y1
y3
y4
y5
y6
y7
y8
y9
y10
y11

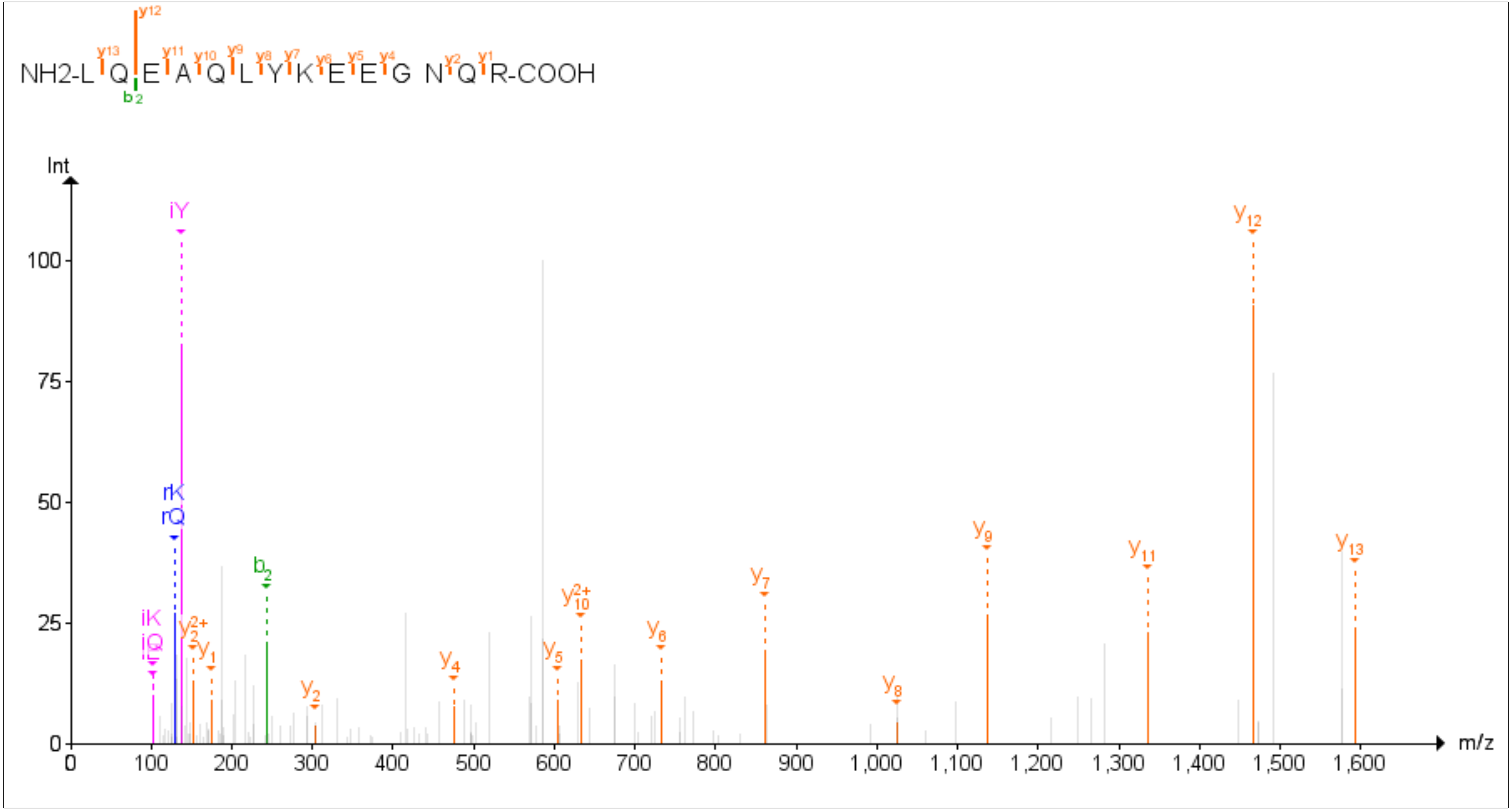

NH2-L Q E A Q L Y K E E G N Q R-COOH
y12
y13
y11
y10
y9
y8
y7
y6
y5
y4
y2
y1
b2
Int
m/z
iY
rK
rQ
iK
iQ
b2
y_2^{2+}
y_1
y_2
y_4
y_5
y_{10}^{2+}
y_6
y_7
y_8
y_9
y_{11}
y_{12}
y_{13}
0
25
50
75
100
0
100
200
300
400
500
600
700
800
900
1,000
1,100
1,200
1,300
1,400
1,500
1,600

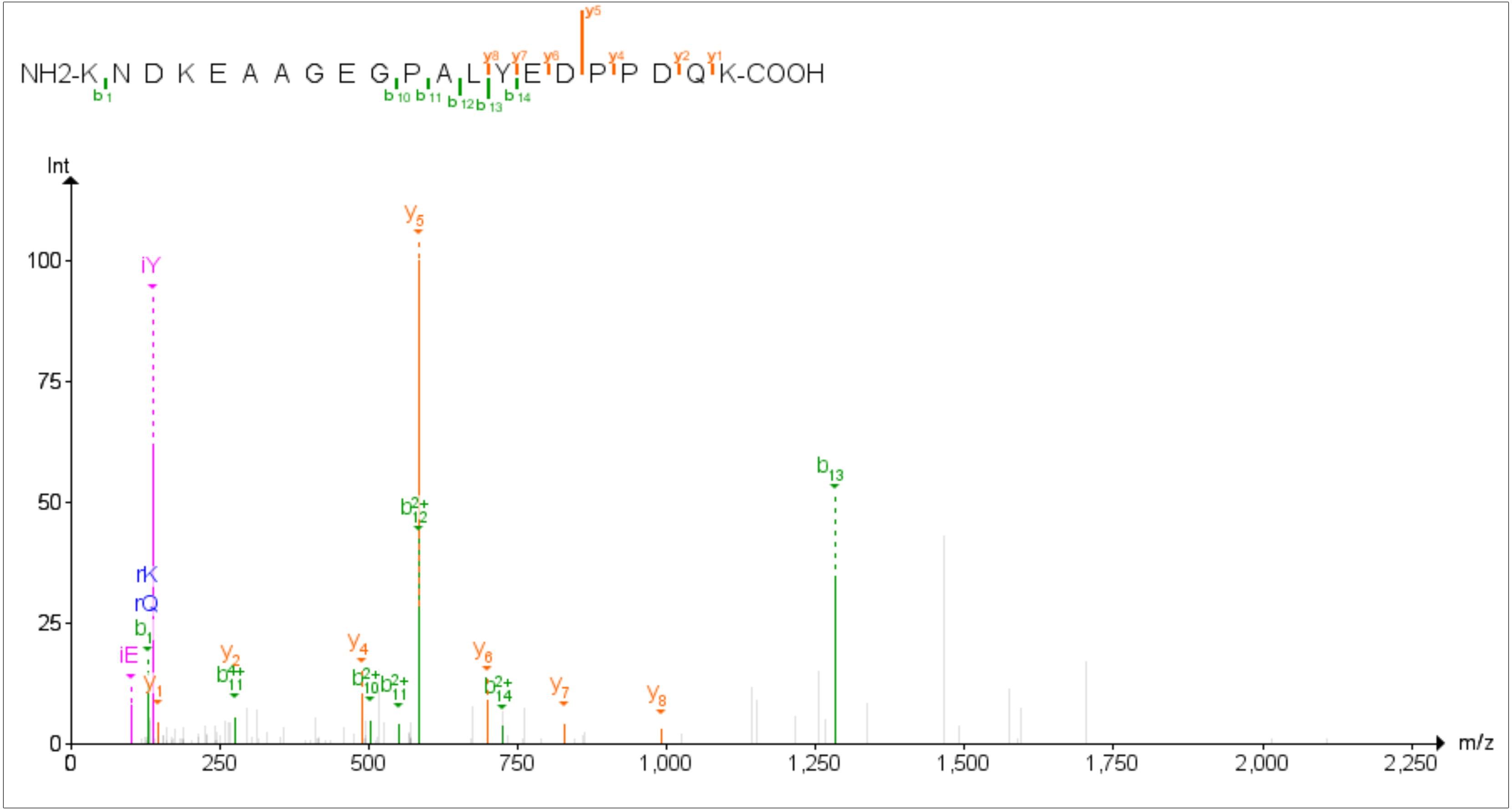

NH2-K N D K E A A G E G P A L Y E D P P D Q K-COOH
y5
y8 y7 y6 y4 y2 y1
b 1 b 10 b 11 b 12 b 13 b 14
Int
100
75
50
25
0
0 250 500 750 1,000 1,250 1,500 1,750 2,000 2,250
m/z
iY
rK
rQ
iE

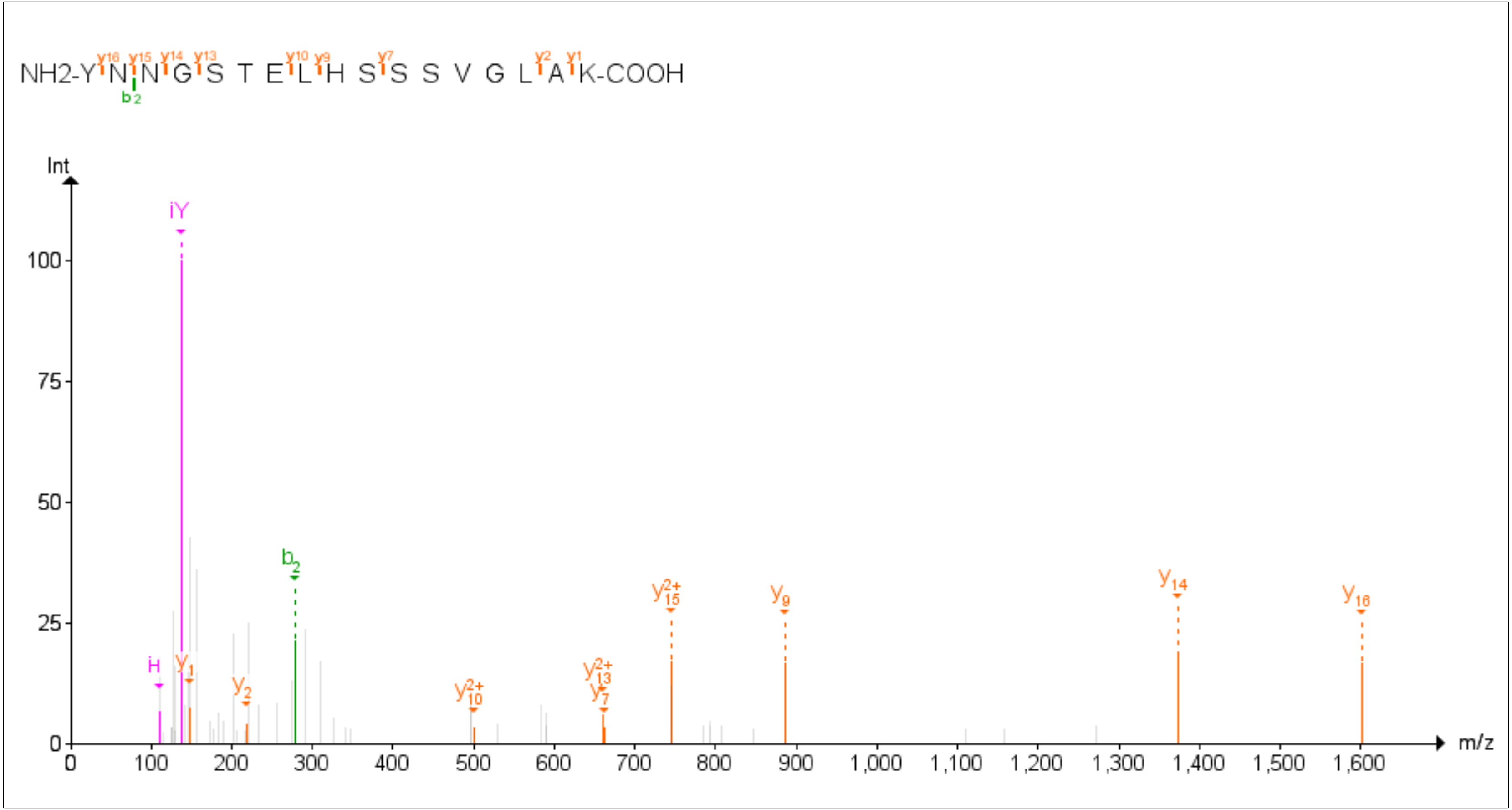

NH2-Y N N G S T E L H S S S V G L A K-COOH
y16 y15 y14 y13 y10 y9 y7 y2 y1
b2
Int
m/z
iY
iH
y1
y2
b2
y10 2+
y13 2+
y7
y15 2+
y9
y14
y16
0
25
50
75
100
0
100
200
300
400
500
600
700
800
900
1,000
1,100
1,200
1,300
1,400
1,500
1,600

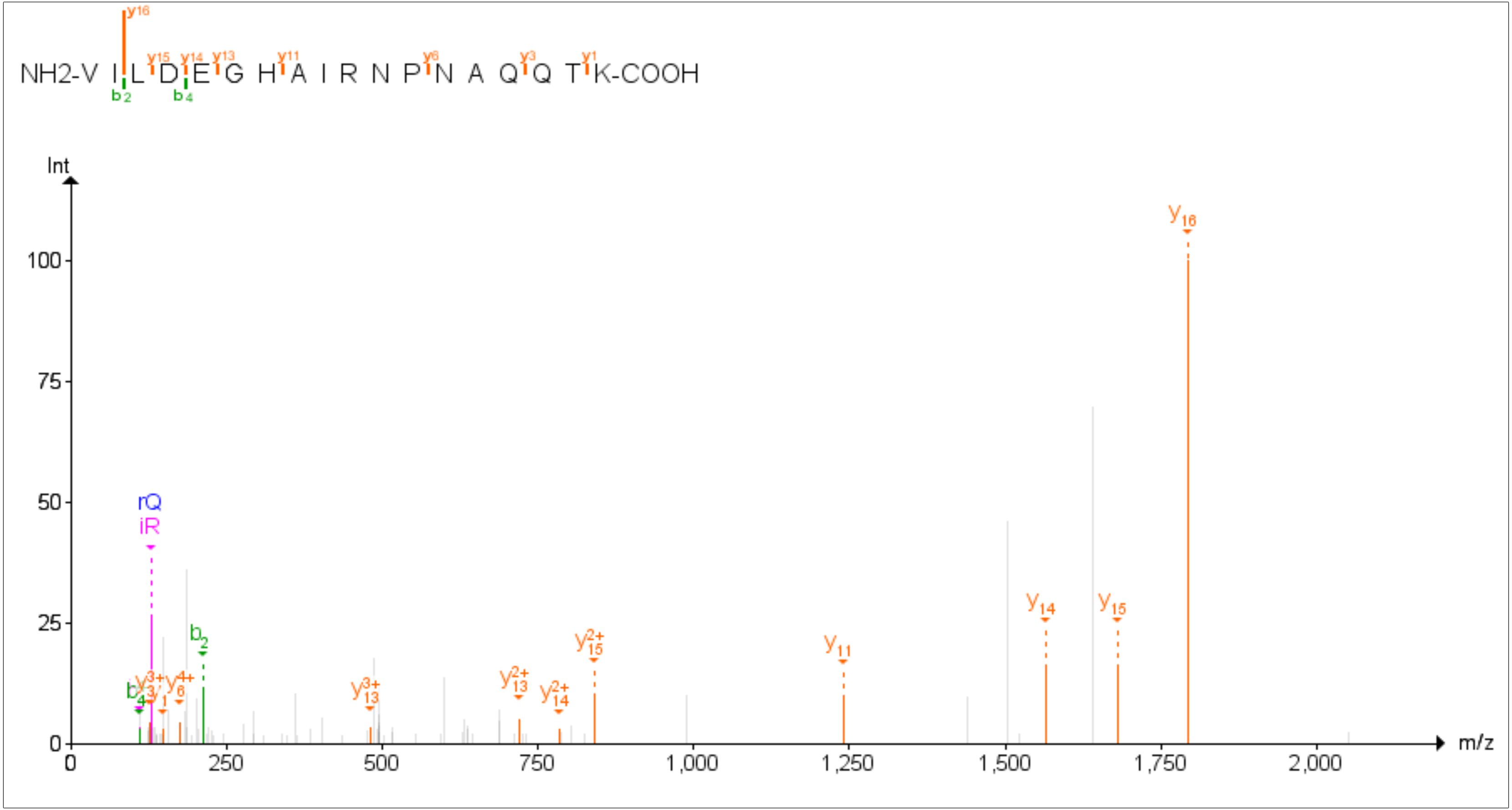

NH2-V I L D E G H A I R N P N A Q Q T K-COOH
y16
y15
y14
y13
y11
y6
y3
y1
b2
b4
Int
100
75
50
25
0
rQ
iR
m/z
250
500
750
1,000
1,250
1,500
1,750
2,000

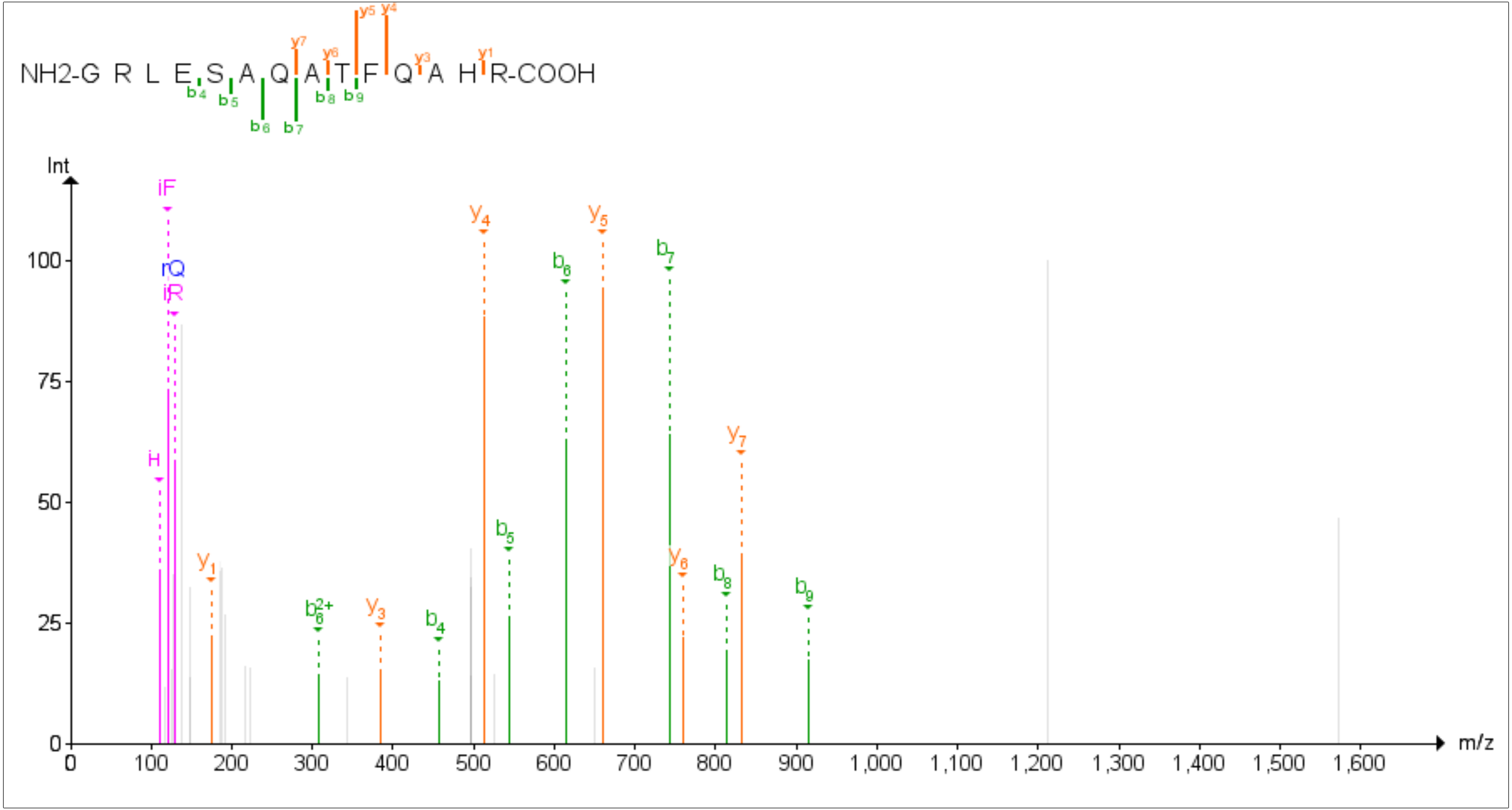

NH2-G R L E S A Q A T F Q A H R-COOH
b4 b5 b6 b7 b8 b9
y7 y6 y5 y4 y3 y1
Int
100
75
50
25
0
iF
rQ
iR
iH
y1
b_6^{2+}
y3
b4
y4
b6
b5
y5
b7
y6
b8
y7
b9
0 100 200 300 400 500 600 700 800 900 1,000 1,100 1,200 1,300 1,400 1,500 1,600
m/z

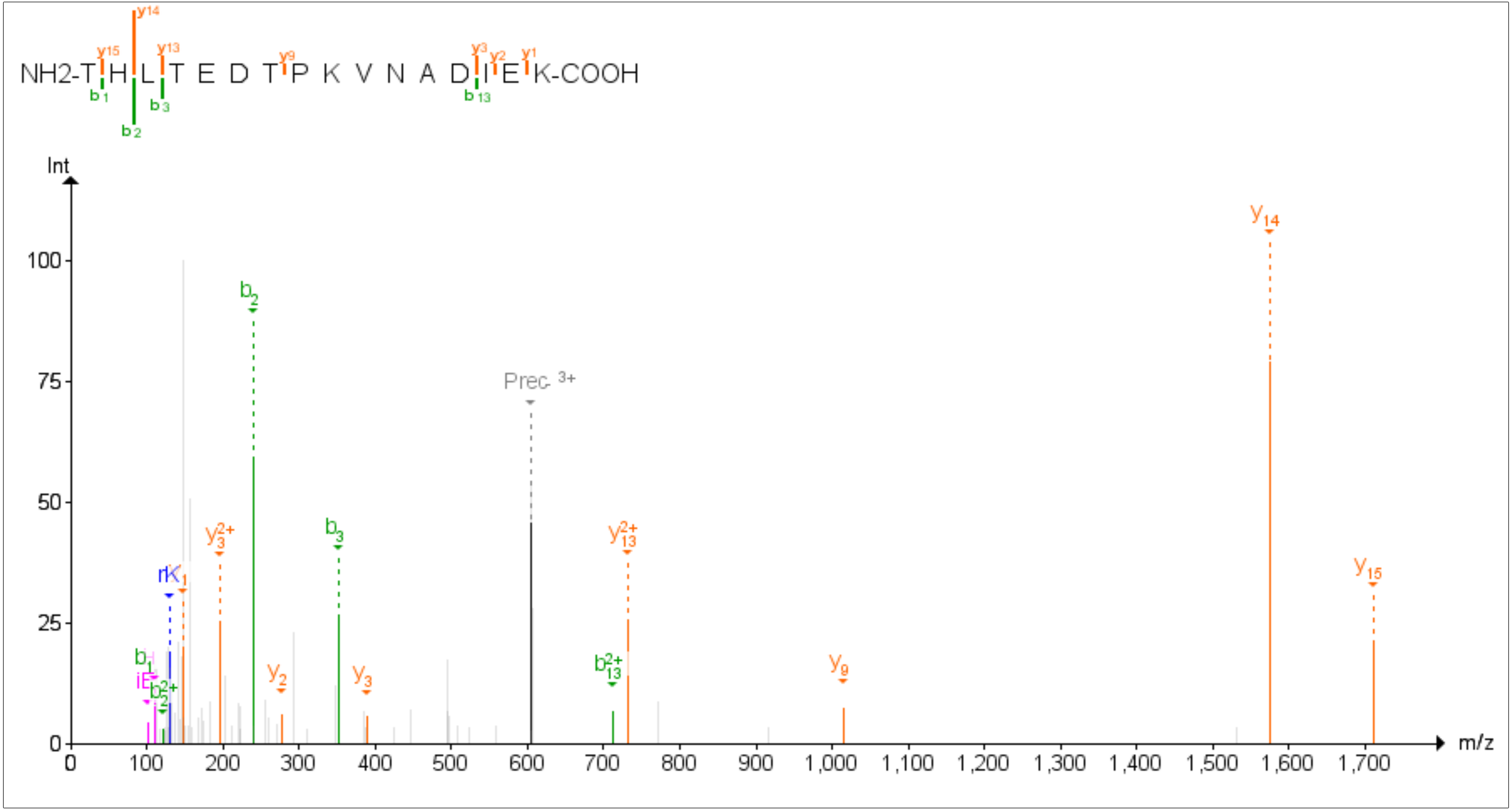

NH2-T H L T E D T P K V N A D I E K-COOH
y14
y15
y13
y9
y3
y2
y1
b1
b2
b3
b13
Int
Prec 3+
m/z

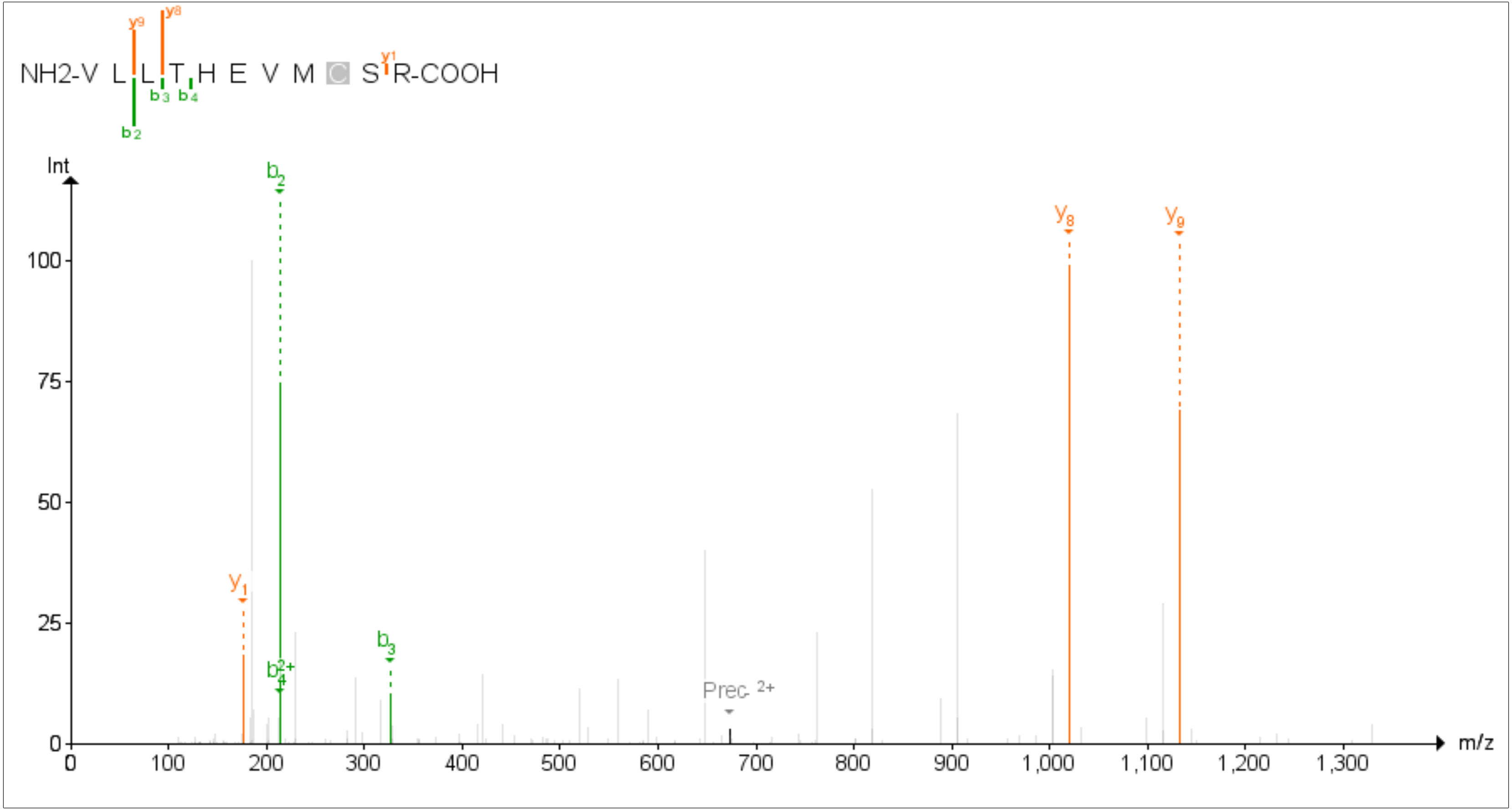

NH2-V L L T H E V M C S R-COOH
y9
y8
y1
b2
b3
b4
Int
b2
y8
y9
y1
b4 2+
b3
Prec 2+
100
75
50
25
0
0
100
200
300
400
500
600
700
800
900
1,000
1,100
1,200
1,300
m/z

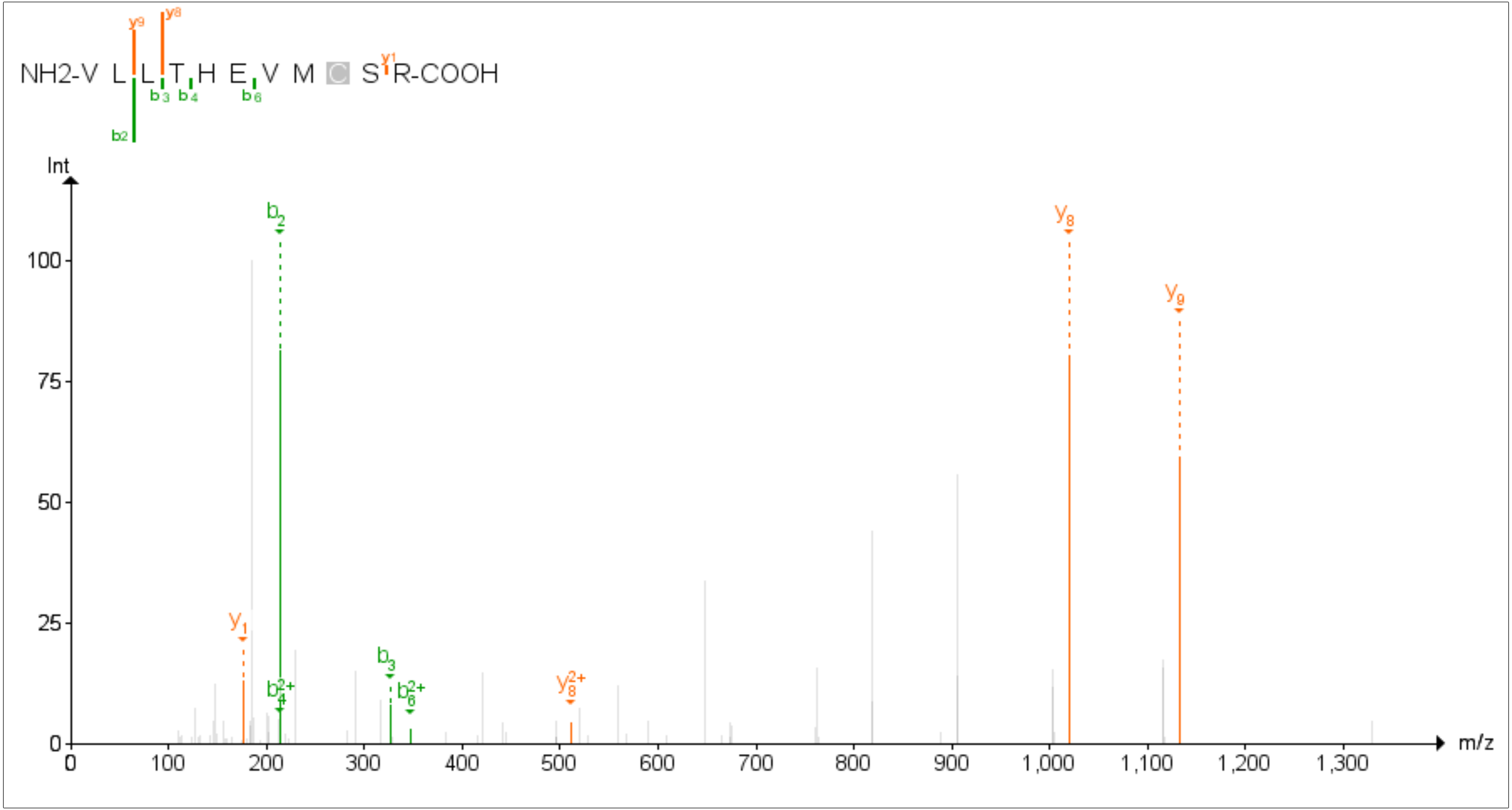

NH2-V L L T H E V M C S R-COOH
y9
y8
y1
b2
b3
b4
b6
Int
100
75
50
25
0
b2
y8
y9
y1
b4 2+
b3
b6 2+
y8 2+
0
100
200
300
400
500
600
700
800
900
1,000
1,100
1,200
1,300
m/z

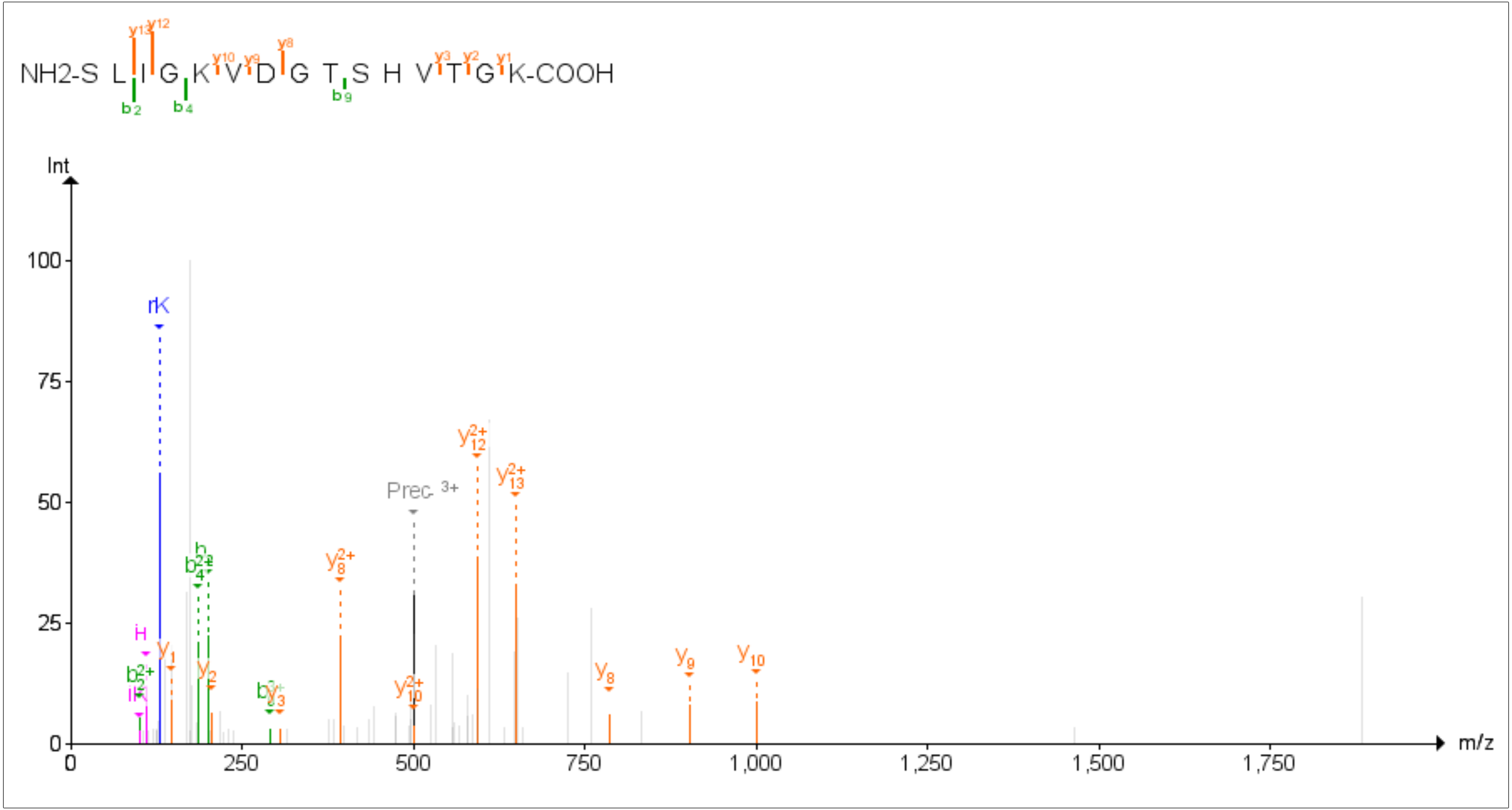

NH2-S L I G K V D G T S H V T G K-COOH
y13 y12 y10 y9 y8 y3 y2 y1
b2 b4 b9
Int
100
75
50
25
0
rK
Prec 3+
m/z
0
250
500
750
1,000
1,250
1,500
1,750

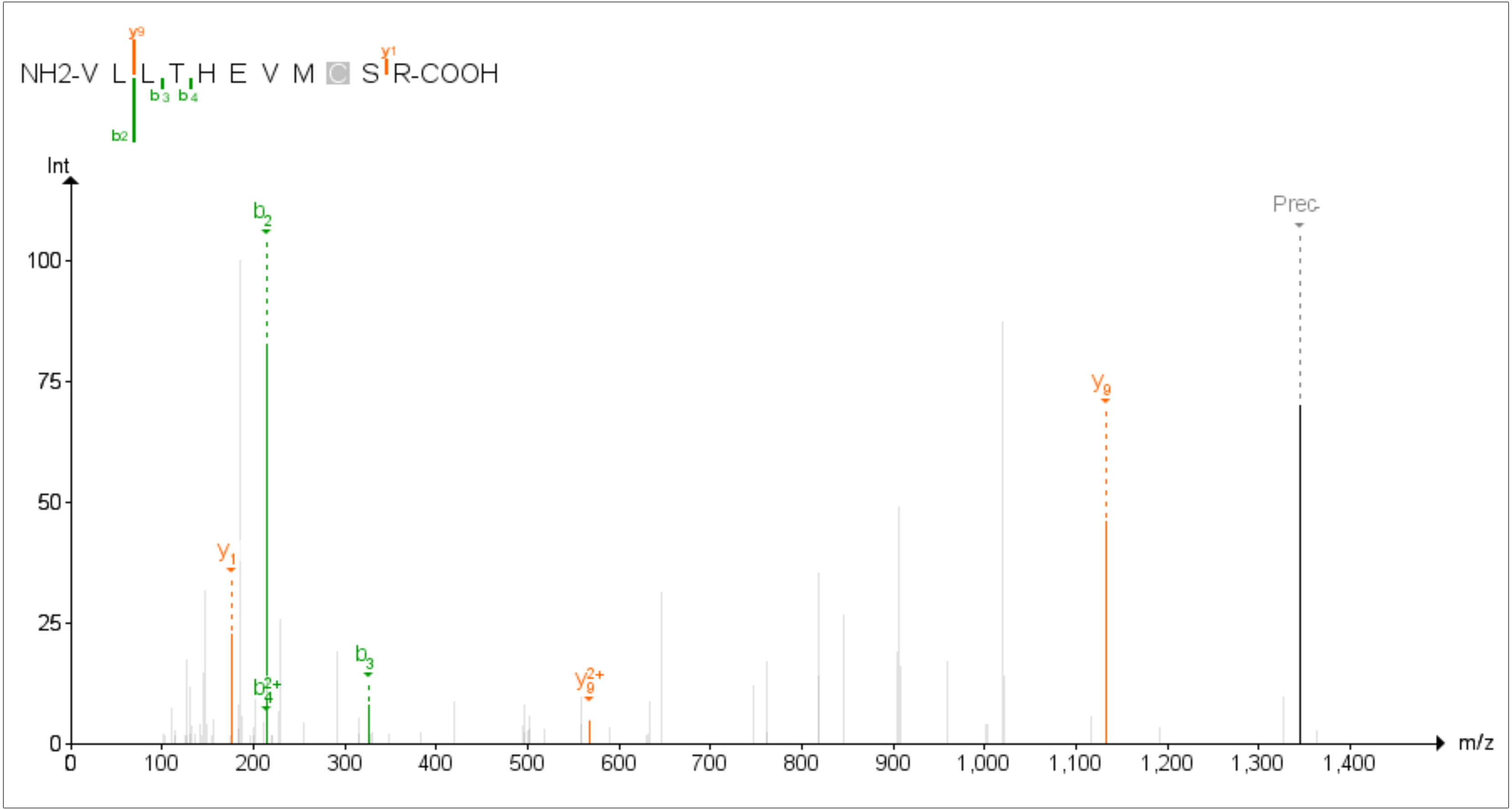

NH2-V L L T H E V M C S R-COOH
y9
y1
b2
b3
b4
Int
m/z
Prec.
b2
b3
b_4^{2+}
y1
y9
y_9^{2+}
100
75
50
25
0
0
100
200
300
400
500
600
700
800
900
1,000
1,100
1,200
1,300
1,400

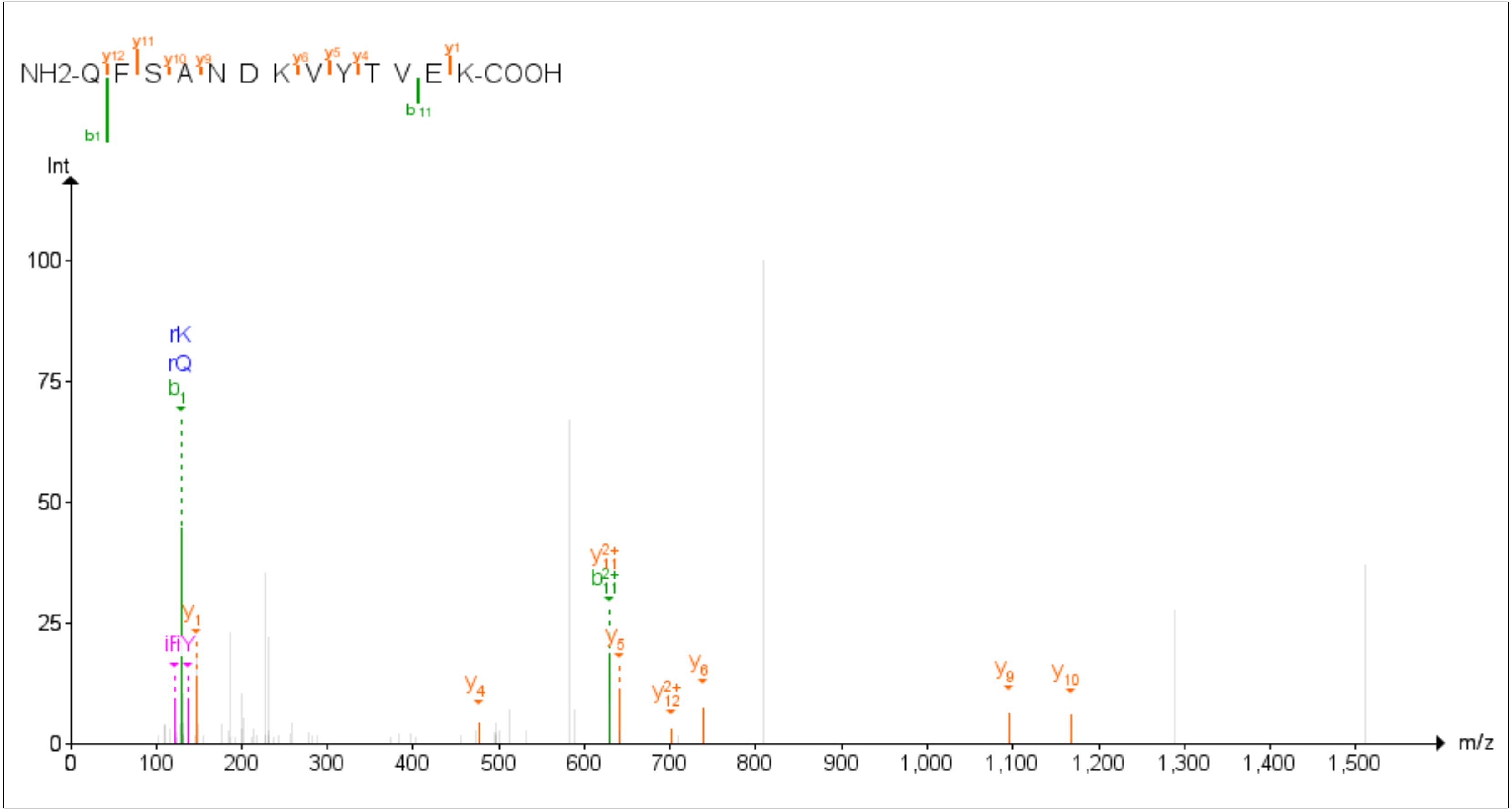

NH2-Q F S A N D K V Y T V E K-COOH
y12 y11 y10 y9 y6 y5 y4 y1
b1 b11
Int
m/z
rK
rQ
b1
y1
iFiY
y4
y11 2+
b11 2+
y5
y12 2+
y6
y9
y10

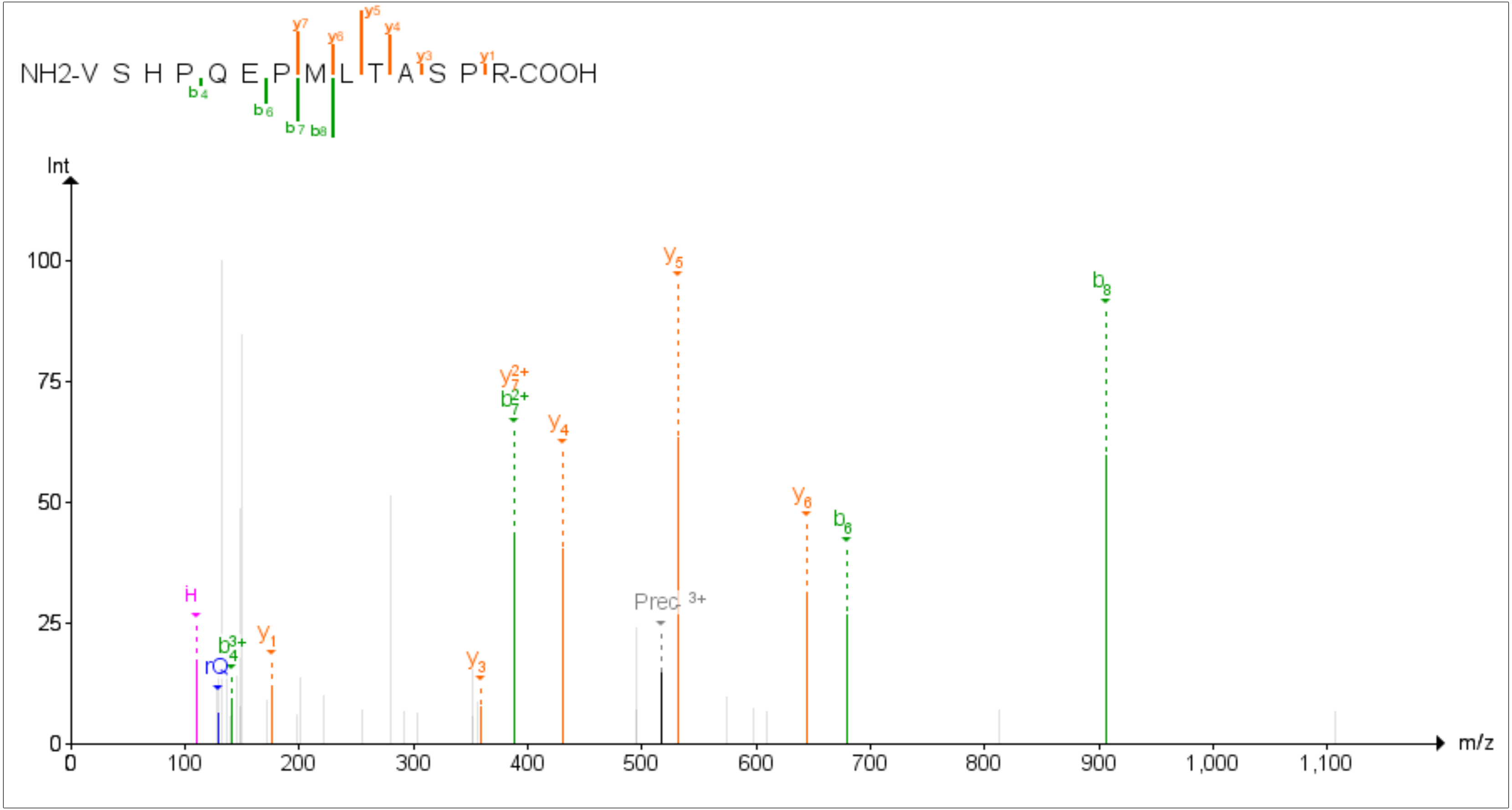
NH2-V S H P Q E P M L T A S P R-COOH
y7 y6 y5 y4 y3 y1
b4 b6 b7 b8
Int
m/z
0
25
50
75
100
200
300
400
500
600
700
800
900
1,000
1,100
Prec 3+

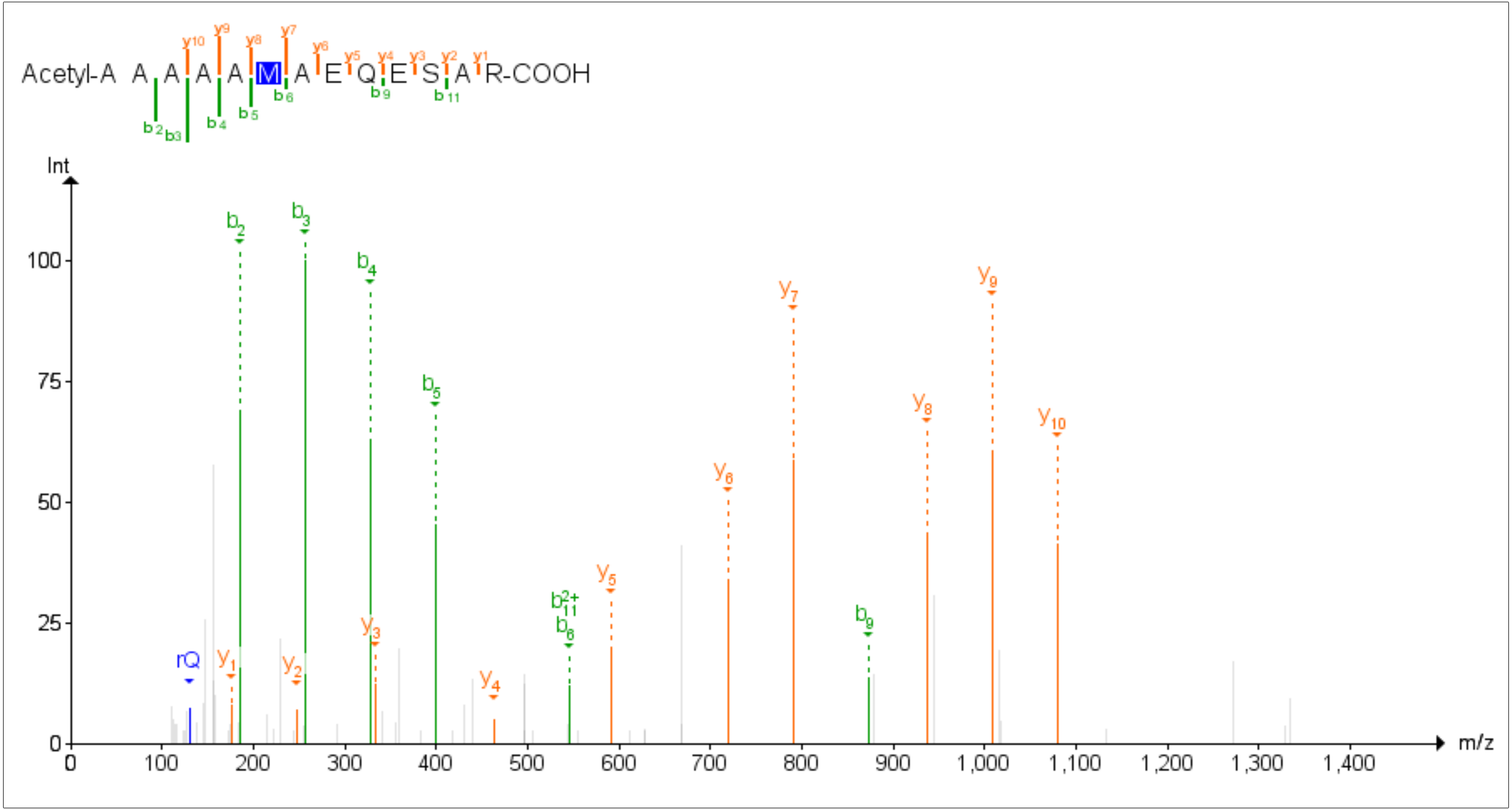

Acetyl-A A A A A M A E Q E S A R-COOH
y10 y9 y8 y7 y6 y5 y4 y3 y2 y1
b2 b3 b4 b5 b6 b9 b11
Int
m/z
0
25
50
75
100
0 100 200 300 400 500 600 700 800 900 1,000 1,100 1,200 1,300 1,400
rQ
y1 y2 y3 y4 y5 y6 y7 y8 y9 y10
b2 b3 b4 b5 b6 b9
b11 2+

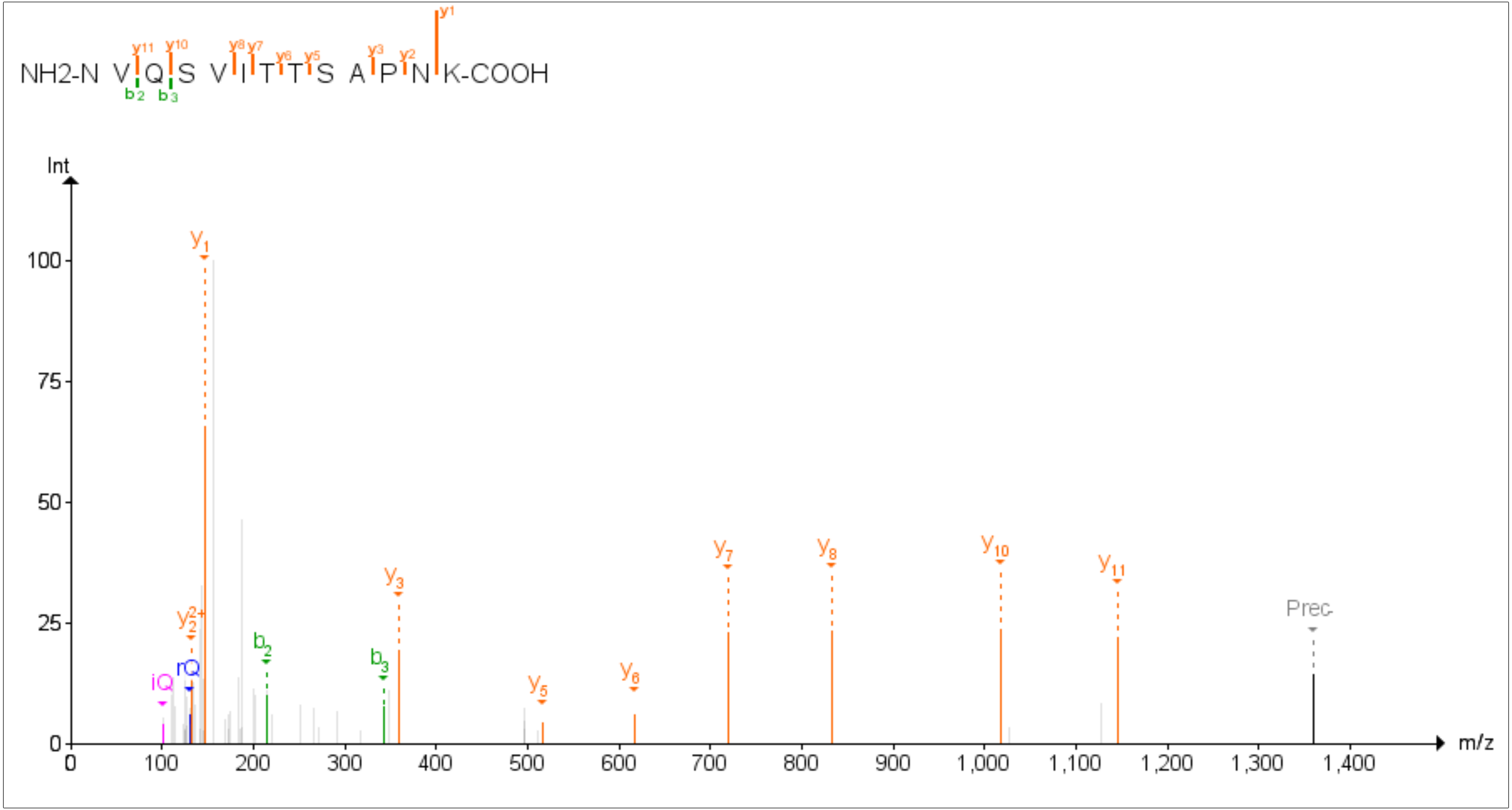

NH2-N V Q S V I T T S A P N K-COOH
y11 y10 y8 y7 y6 y5 y3 y2 y1
b2 b3
Int
m/z
y1
y2 2+
iQ
rQ
b2
b3
y3
y5
y6
y7
y8
y10
y11
Prec
0
25
50
75
100
0
100
200
300
400
500
600
700
800
900
1,000
1,100
1,200
1,300
1,400

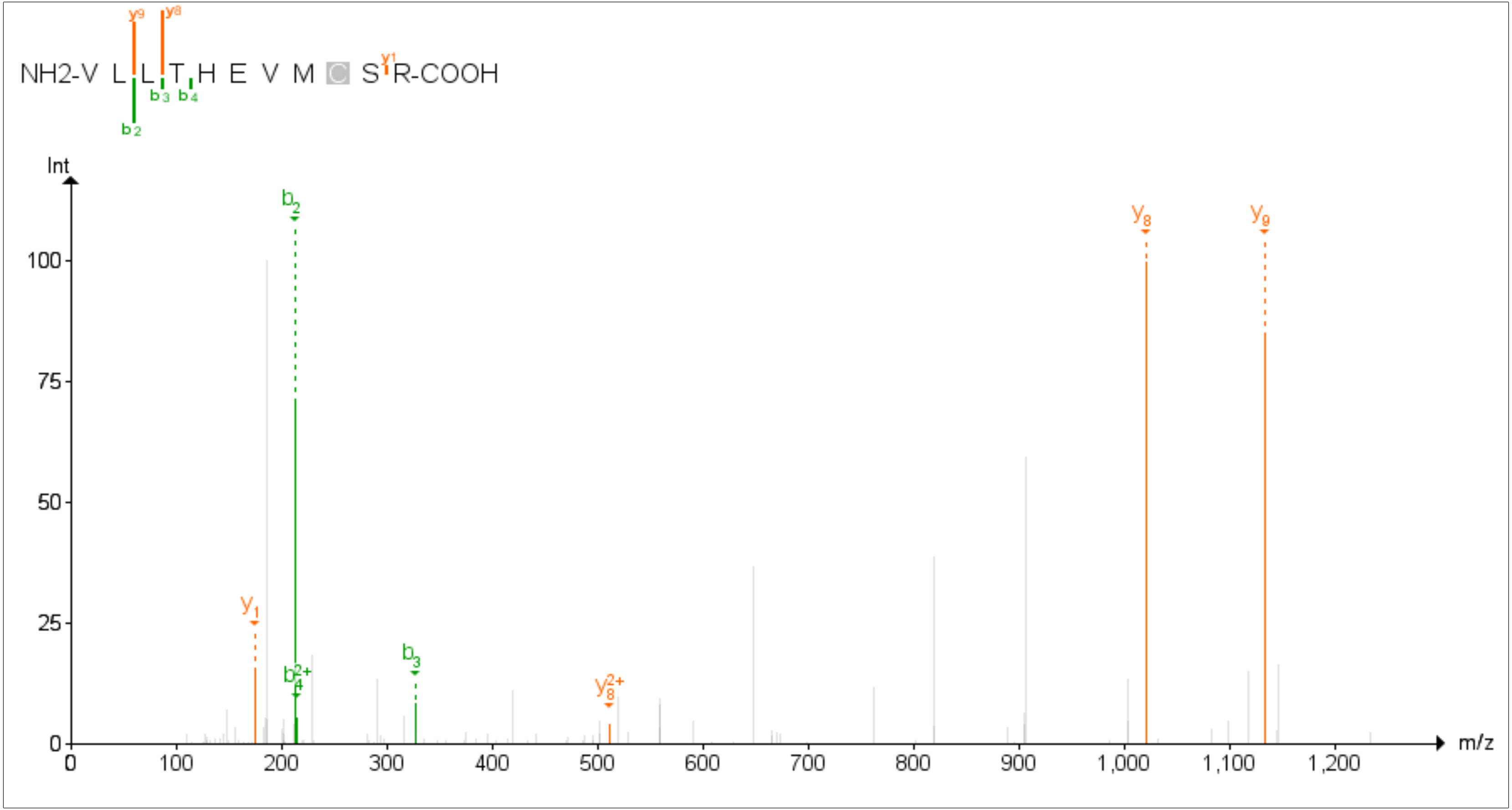

NH2-V L L T H E V M C S R-COOH
y9
y8
y1
b2
b3
b4
Int
100
75
50
25
0
b2
y8
y9
y1
b4 2+
b3
y8 2+
0
100
200
300
400
500
600
700
800
900
1,000
1,100
1,200
m/z

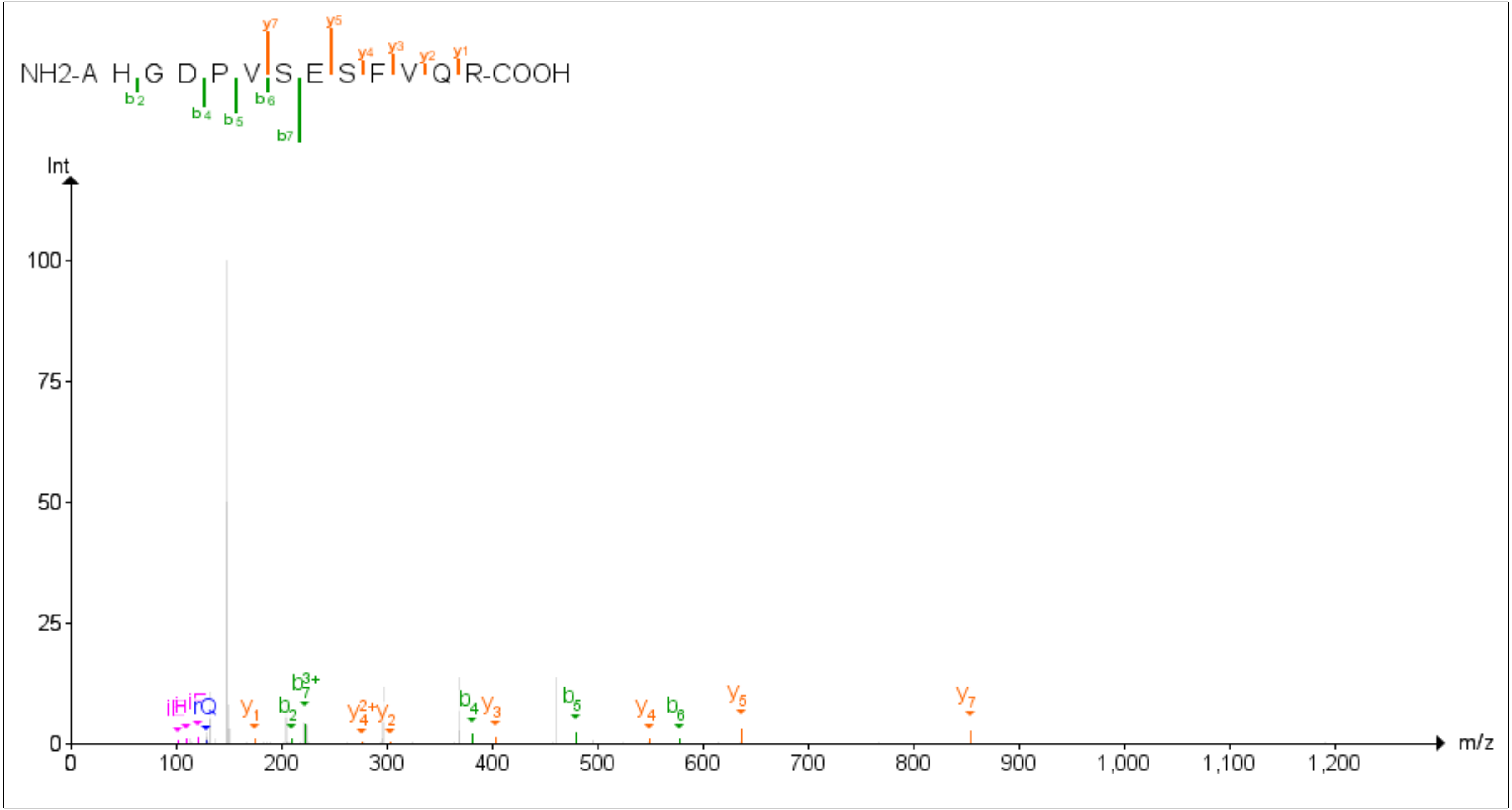

NH2-A H G D P V S E S F V Q R-COOH
Int
m/z
100
75
50
25
0
0
100
200
300
400
500
600
700
800
900
1,000
1,100
1,200

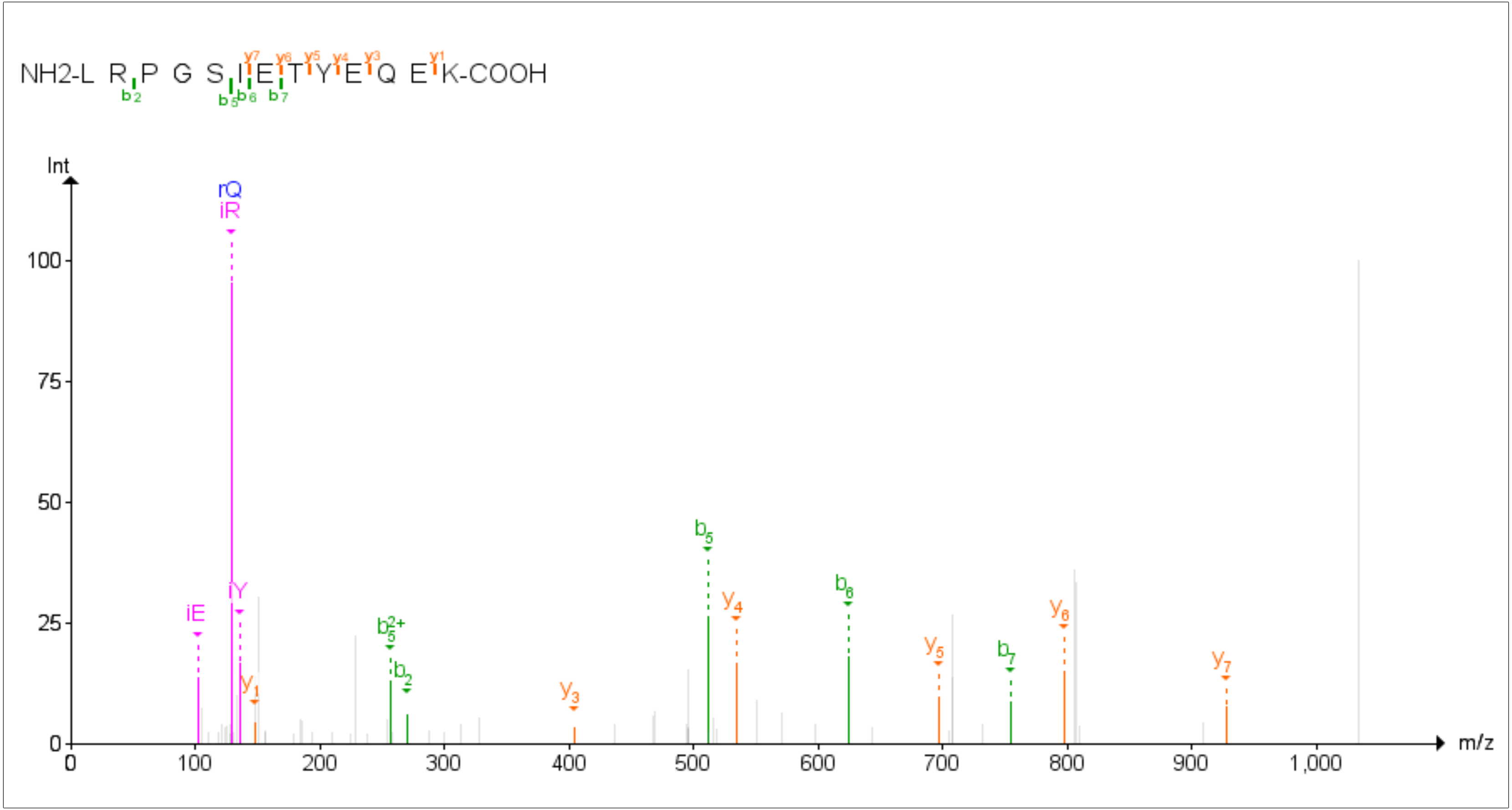

NH2-L R P G S I E T Y E Q E K-COOH
b2
b5
b6
b7
y7
y6
y5
y4
y3
y1
Int
rQ
iR
iE
iY
y1
b5 2+
b2
y3
b5
y4
b6
y5
b7
y6
y7
100
75
50
25
0
0
100
200
300
400
500
600
700
800
900
1,000
m/z

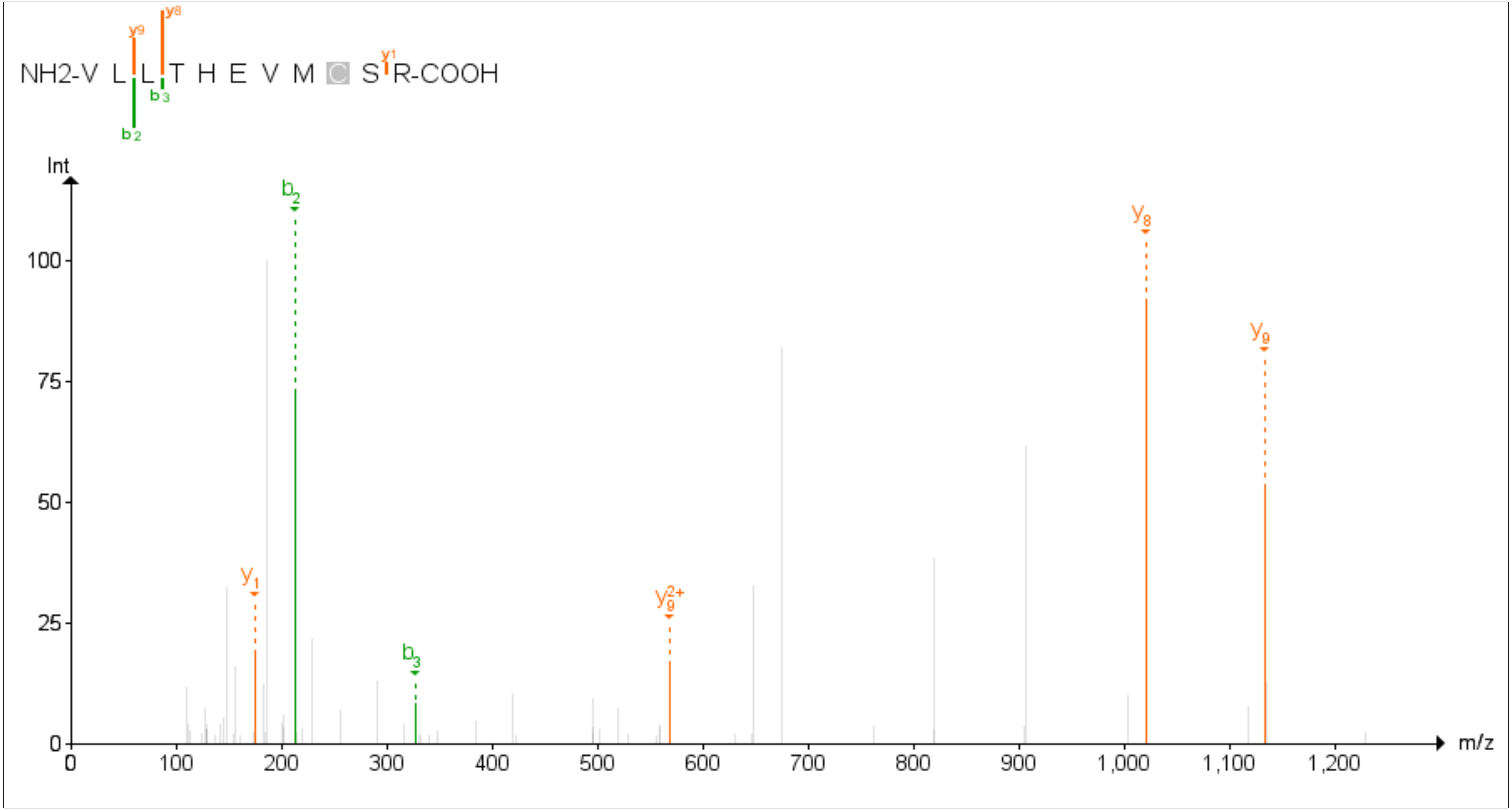

NH2-V L L T H E V M C S R-COOH
y9
y8
y1
b2
b3
Int
100
75
50
25
0
b_2
y_8
y_9
y_1
y_9^{2+}
b_3
0
100
200
300
400
500
600
700
800
900
1,000
1,100
1,200
m/z

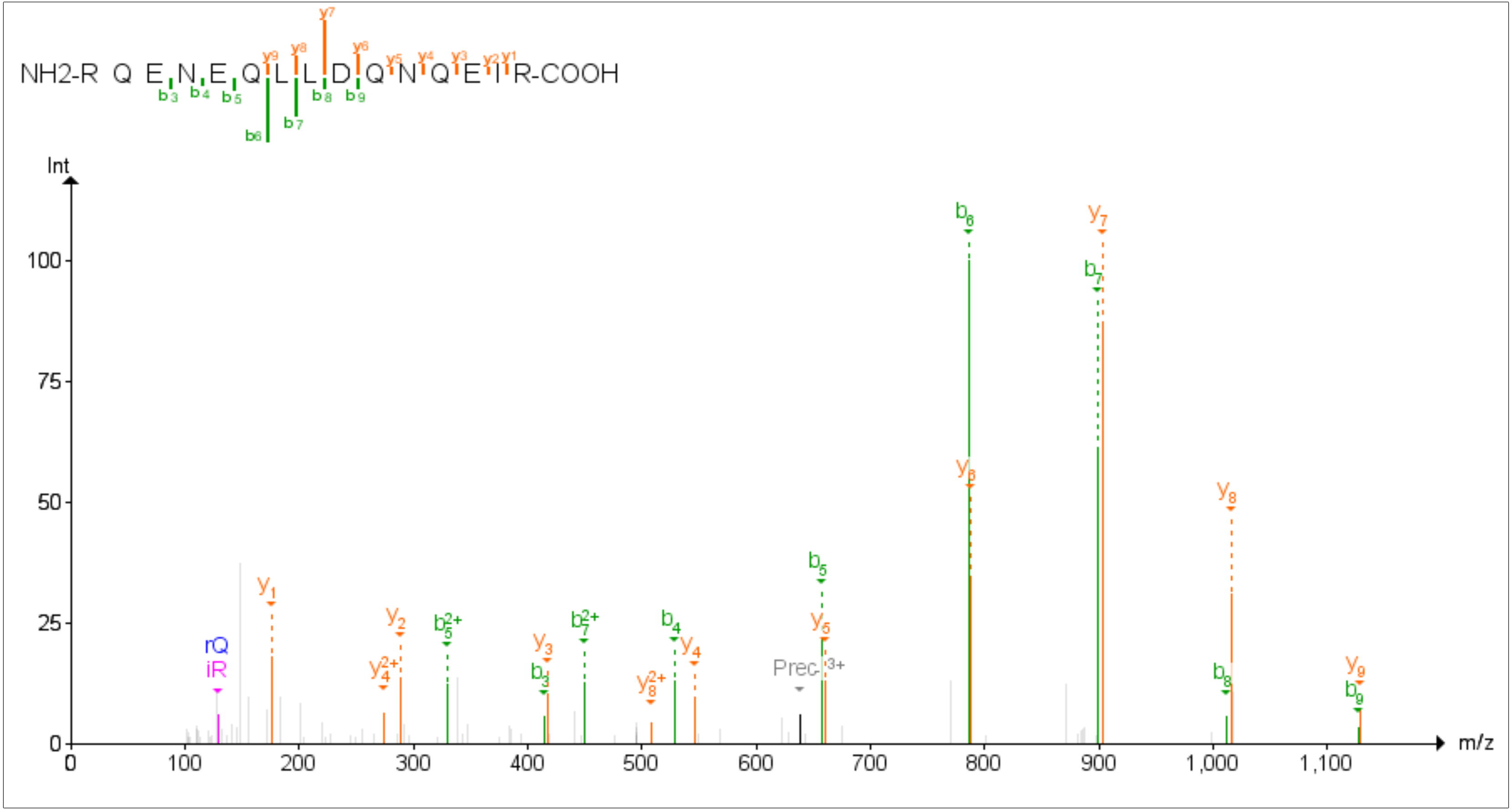

NH2-R Q E N E Q L L D Q N Q E I R-COOH
Int
m/z
Prec 3+
rQ
iR

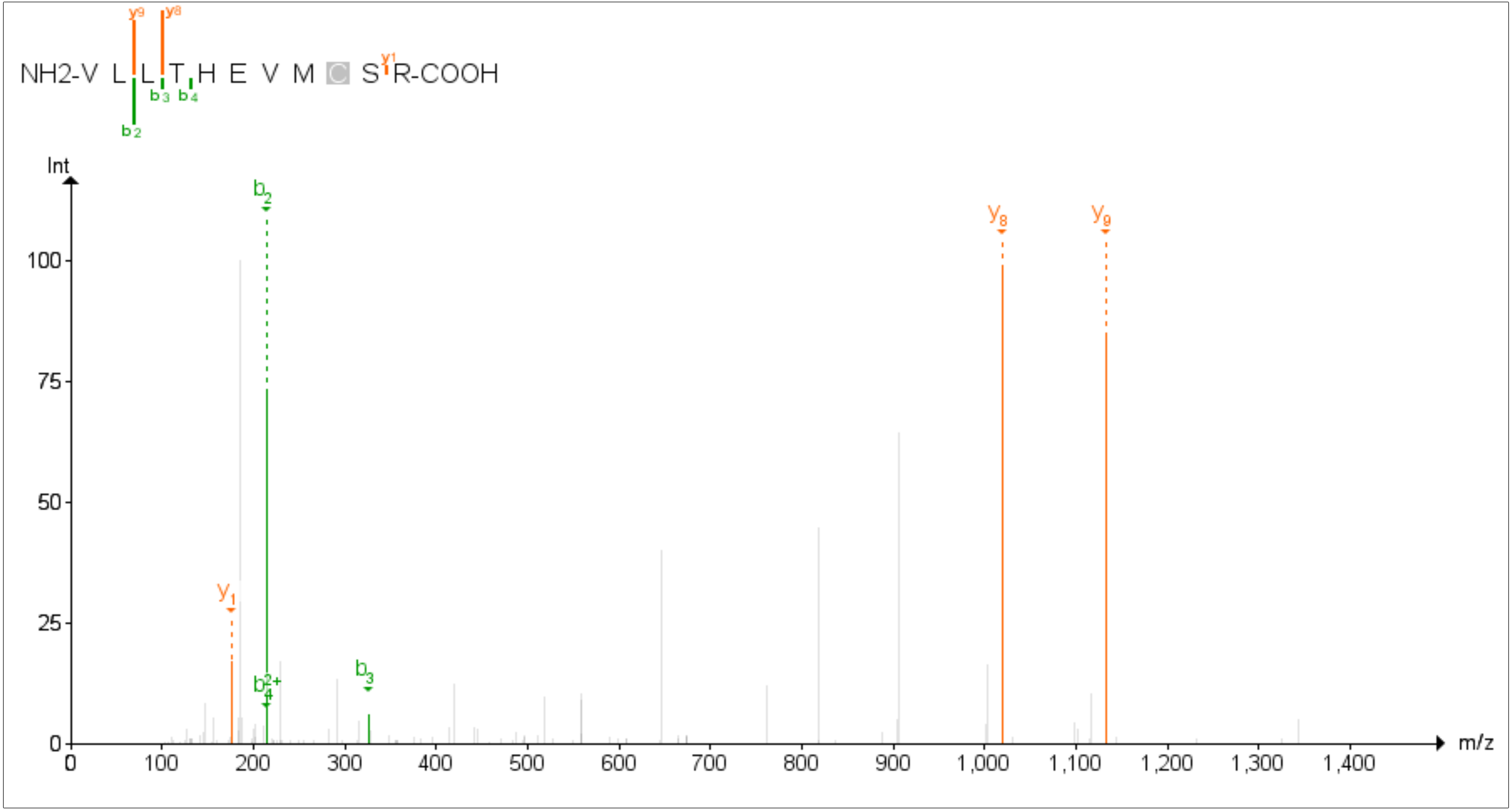
NH2-V L L T H E V M C S R-COOH
y9
y8
y1
b2
b3
b4
Int
100
75
50
25
0
b2
y8
y9
y1
b4 2+
b3
0
100
200
300
400
500
600
700
800
900
1,000
1,100
1,200
1,300
1,400
m/z

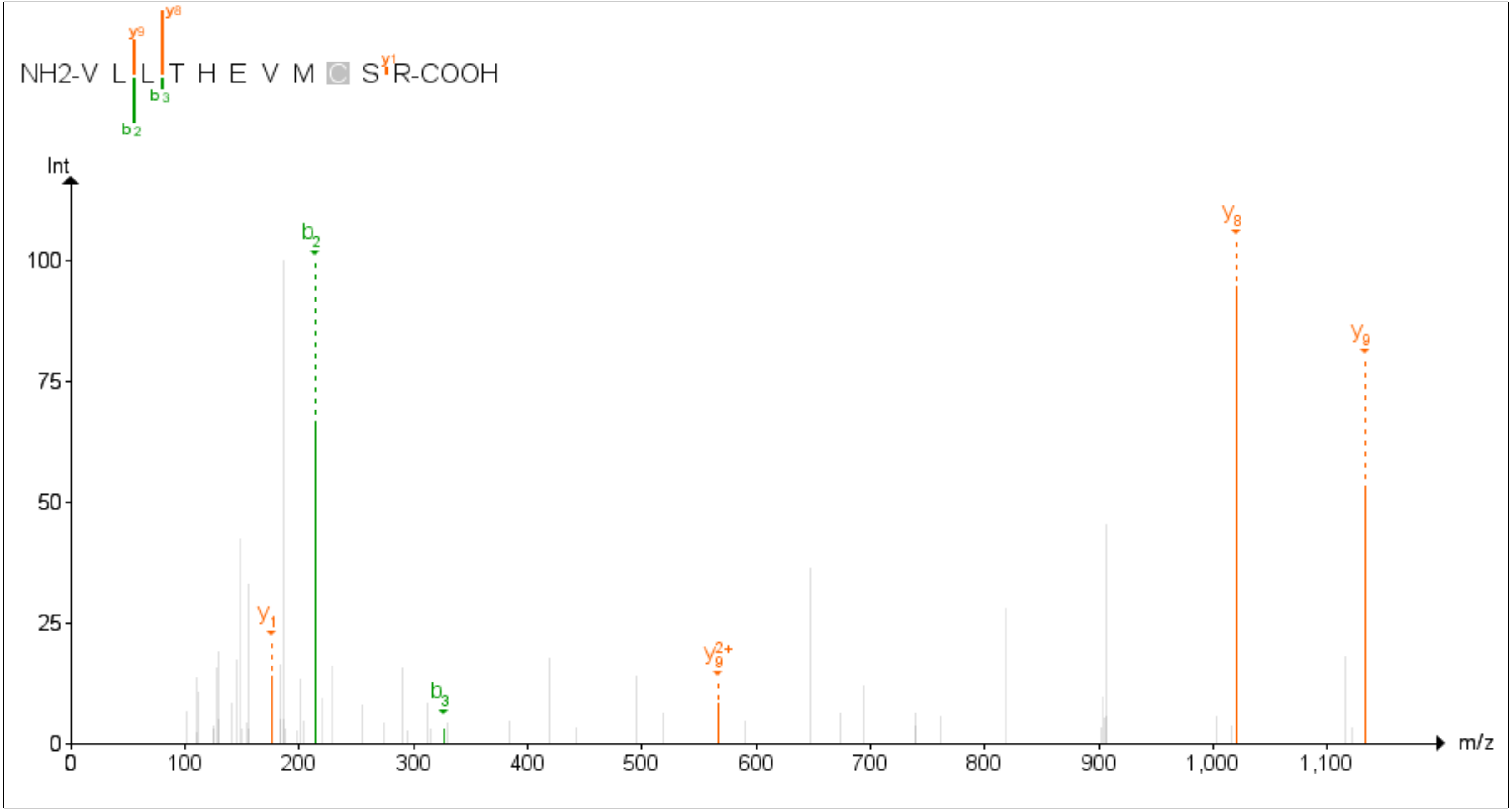

NH2-V L L T H E V M C S R-COOH
y9
y8
y1
b2
b3
Int
100
75
50
25
0
b2
b3
y1
y9 2+
y8
y9
m/z
0
100
200
300
400
500
600
700
800
900
1,000
1,100

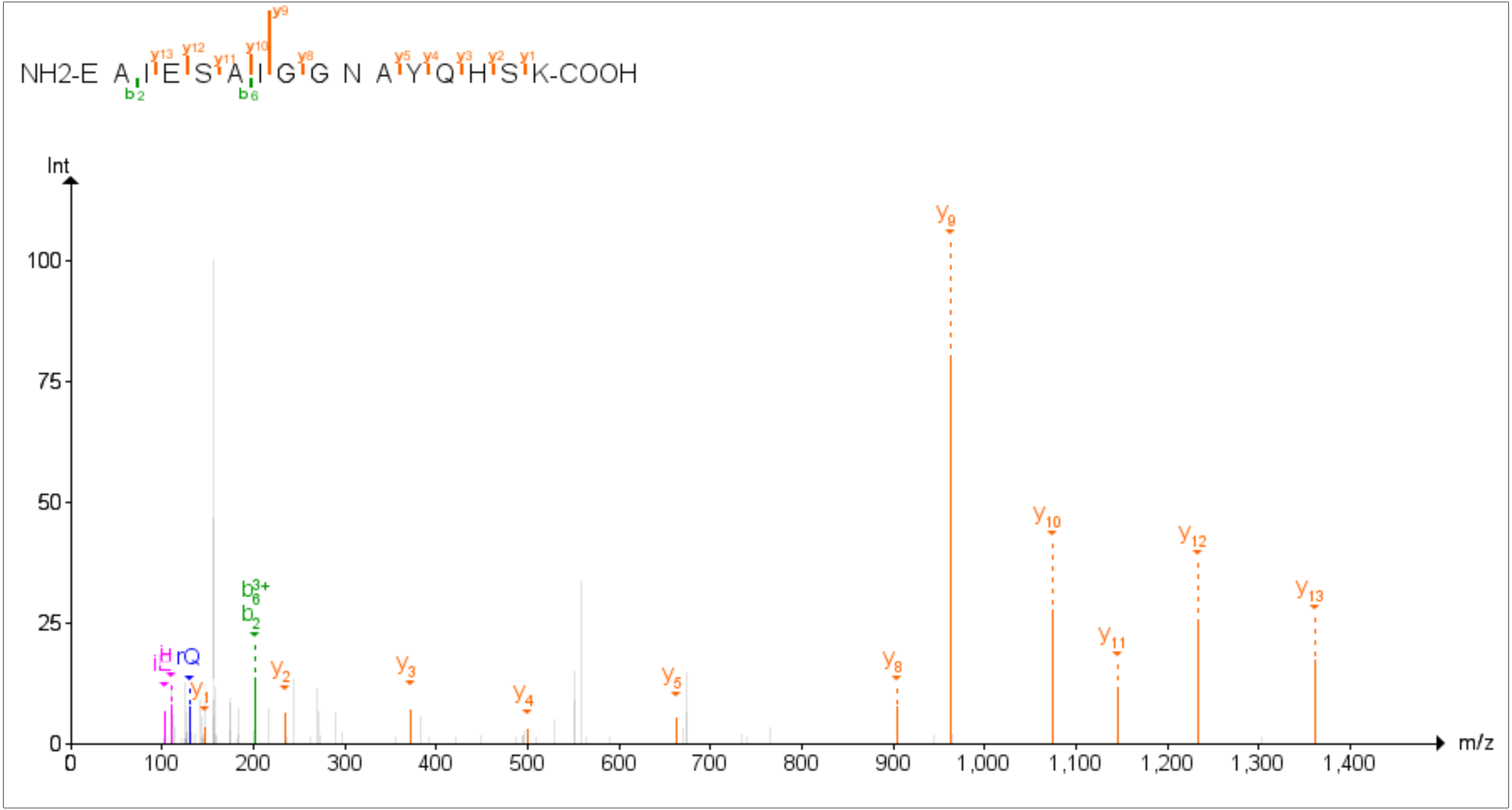

NH2-E A I E S A I G G N A Y Q H S K-COOH
y13 y12 y11 y10 y9 y8 y5 y4 y3 y2 y1
b2 b6
Int
100
75
50
25
0
m/z
0 100 200 300 400 500 600 700 800 900 1,000 1,100 1,200 1,300 1,400
y9 y10 y11 y12 y13 y8 y5 y4 y3 y2 y1
b6 3+
b2
iL iH rQ

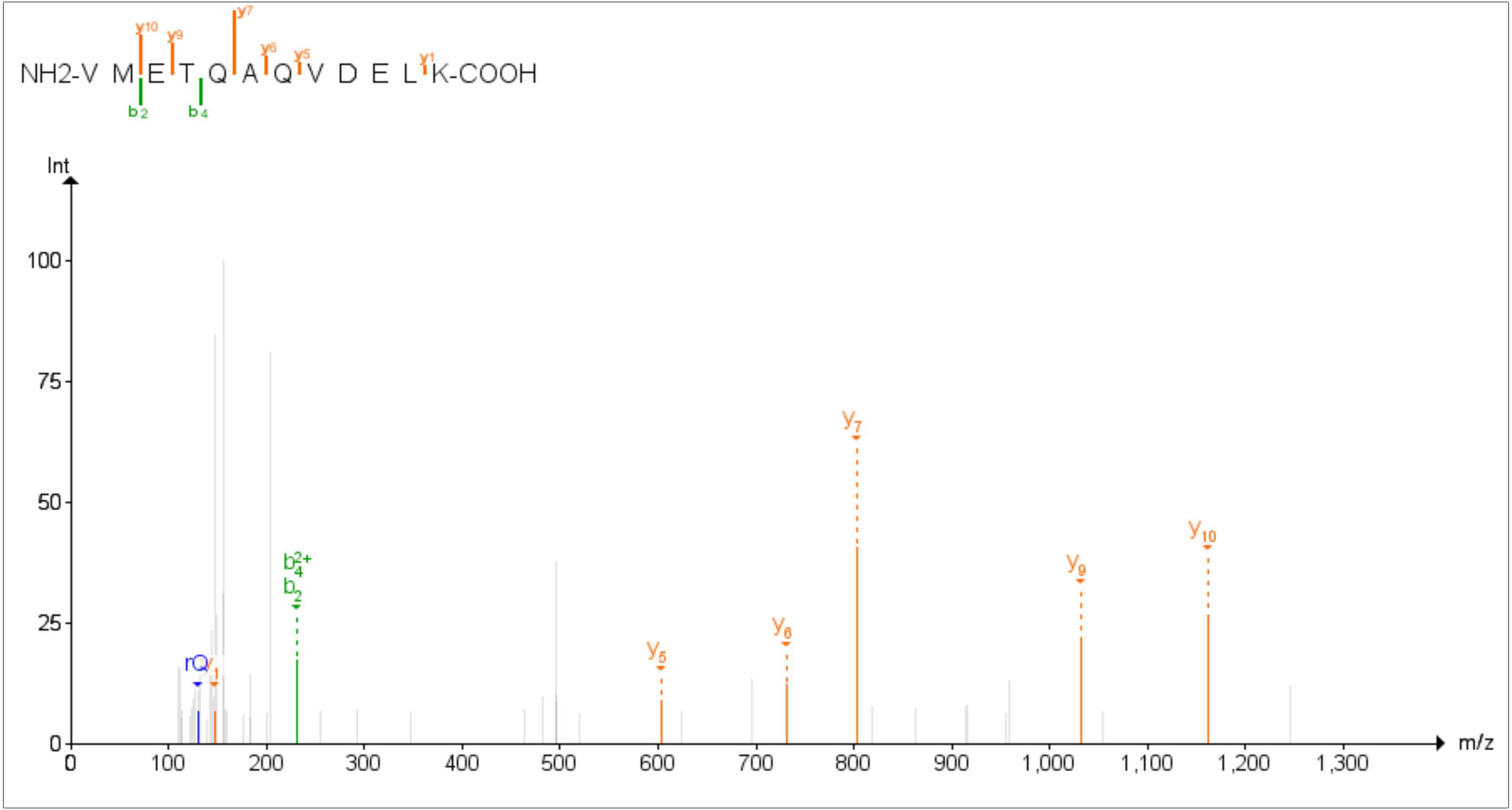

NH2-V M E T Q A Q V D E L K-COOH
y10
y9
y7
y6
y5
y1
b2
b4
Int
100
75
50
25
0
rQ
y1
b2+4
b2
y5
y6
y7
y9
y10
0
100
200
300
400
500
600
700
800
900
1,000
1,100
1,200
1,300
m/z

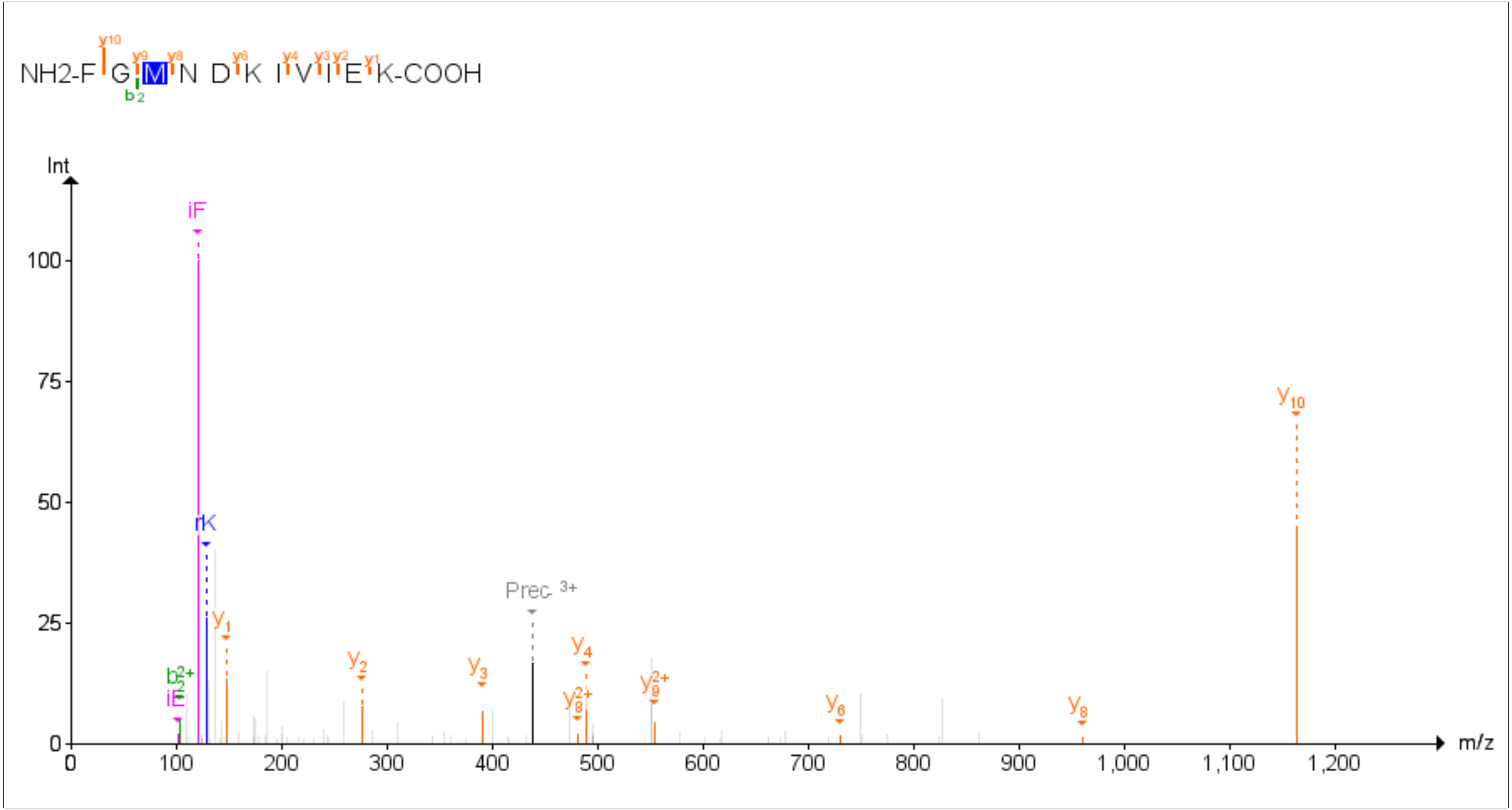

NH2-F G M N D K I V I E K-COOH
y10 y9 y8 y6 y4 y3 y2 y1
b2
Int
iF
rK
y1
Prec 3+
y2
y3
y4
y8 2+
y9 2+
y6
y8
y10
b2 2+
iE
m/z
0
25
50
75
100
100
200
300
400
500
600
700
800
900
1,000
1,100
1,200

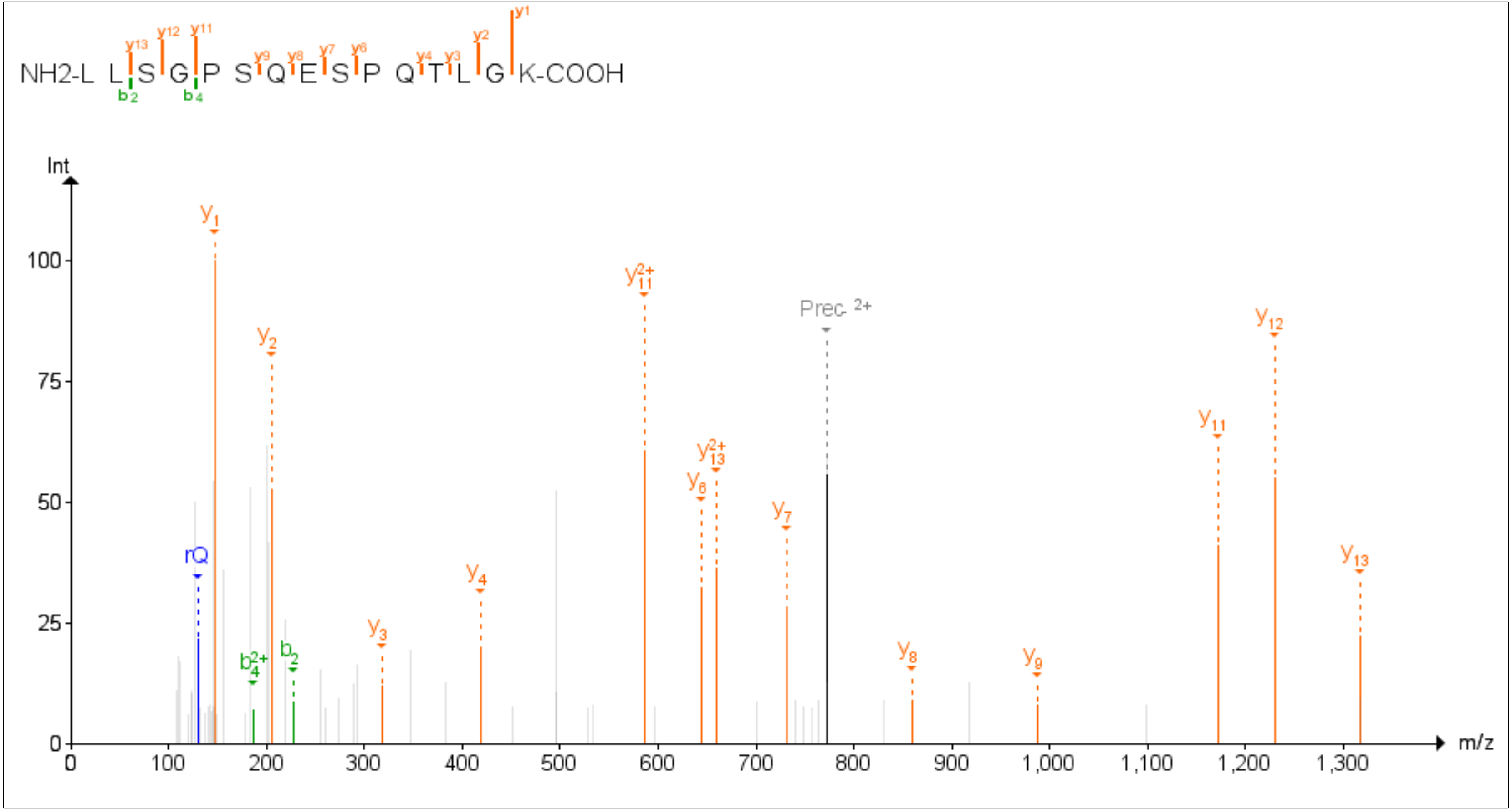
NH2-L L S G P S Q E S P Q T L G K-COOH
y13 y12 y11 y9 y8 y7 y6 y4 y3 y2 y1
b2 b4
Int
100
75
50
25
0
m/z
0 100 200 300 400 500 600 700 800 900 1,000 1,100 1,200 1,300
y1
y2
rQ
b4 2+
b2
y3
y4
y11 2+
Prec. 2+
y6
y13 2+
y7
y8
y9
y11
y12
y13

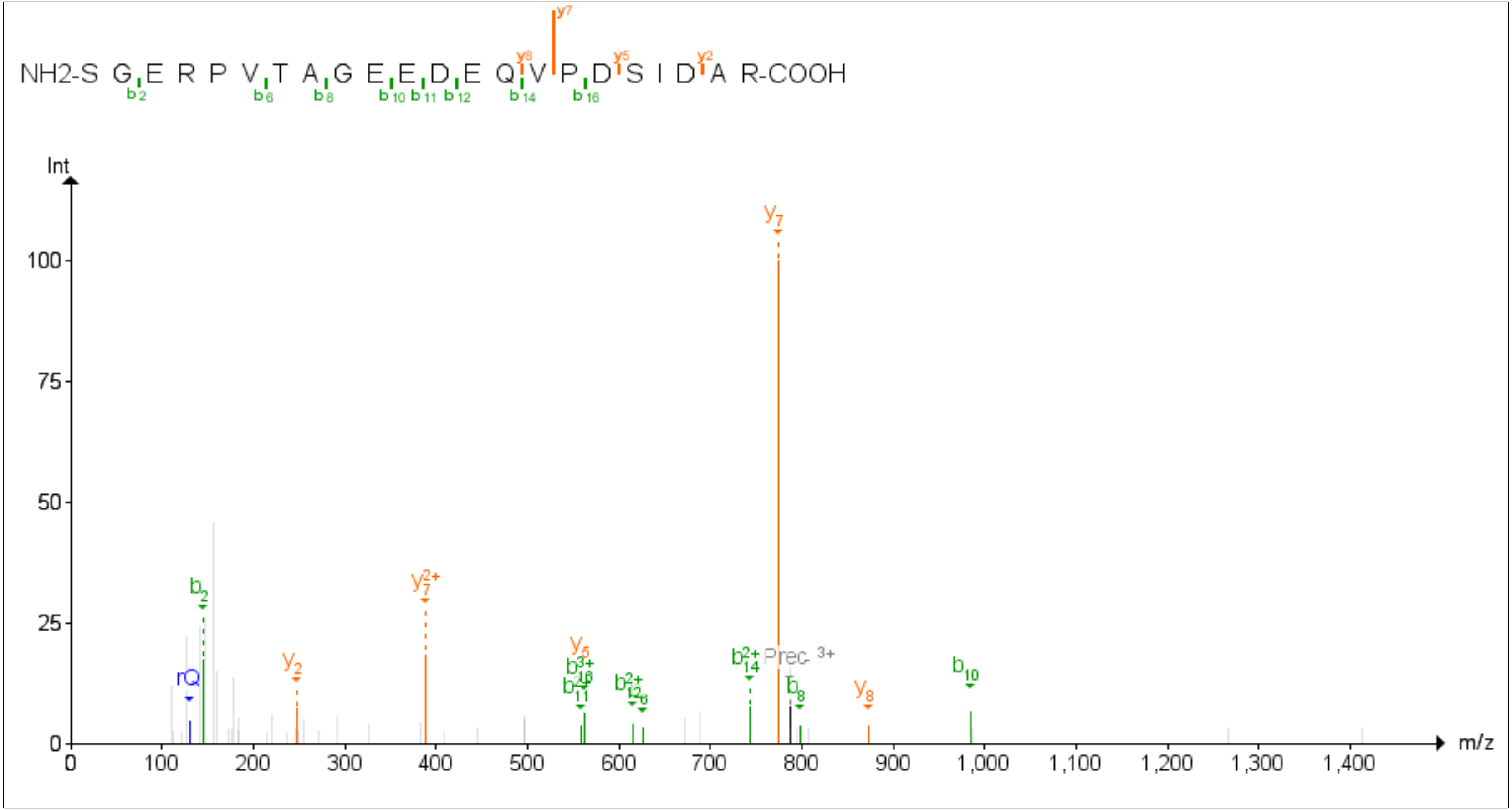

NH2-S G E R P V T A G E E D E Q V P D S I D A R-COOH
y7
y8
y5
y2
b2
b6
b8
b10
b11
b12
b14
b16
Int
100
75
50
25
0
y7
b2
y7 2+
rQ
y2
y5
b16 3+
b11
b12 2+
b14 2+
Prec- 3+
b8
y8
b10
0
100
200
300
400
500
600
700
800
900
1,000
1,100
1,200
1,300
1,400
m/z

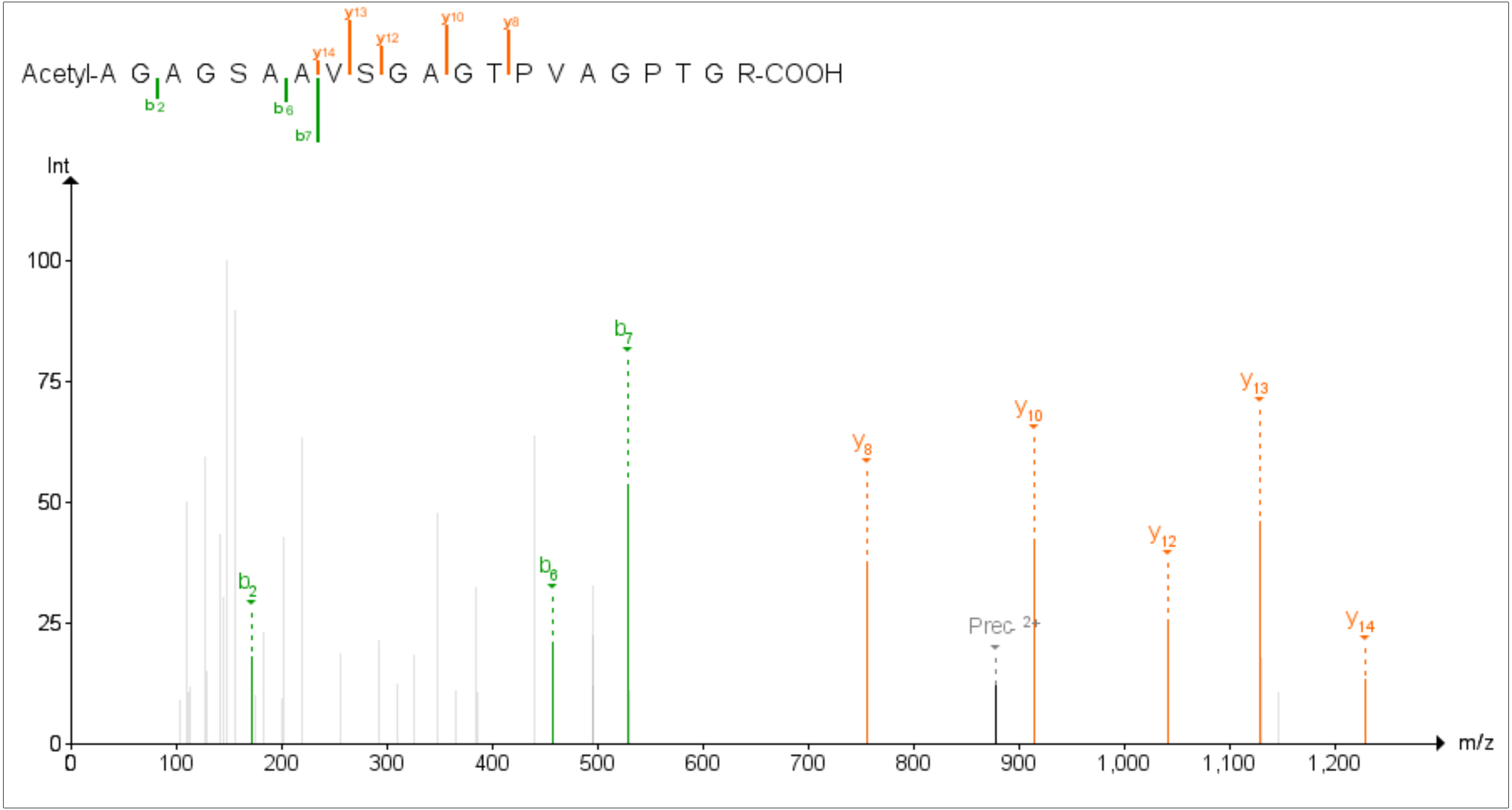
Acetyl-A G A G S A A V S G A G T P V A G P T G R-COOH
b2
b6
b7
y14
y13
y12
y10
y8
Int
100
75
50
25
0
m/z
0
100
200
300
400
500
600
700
800
900
1,000
1,100
1,200
Prec. 2+

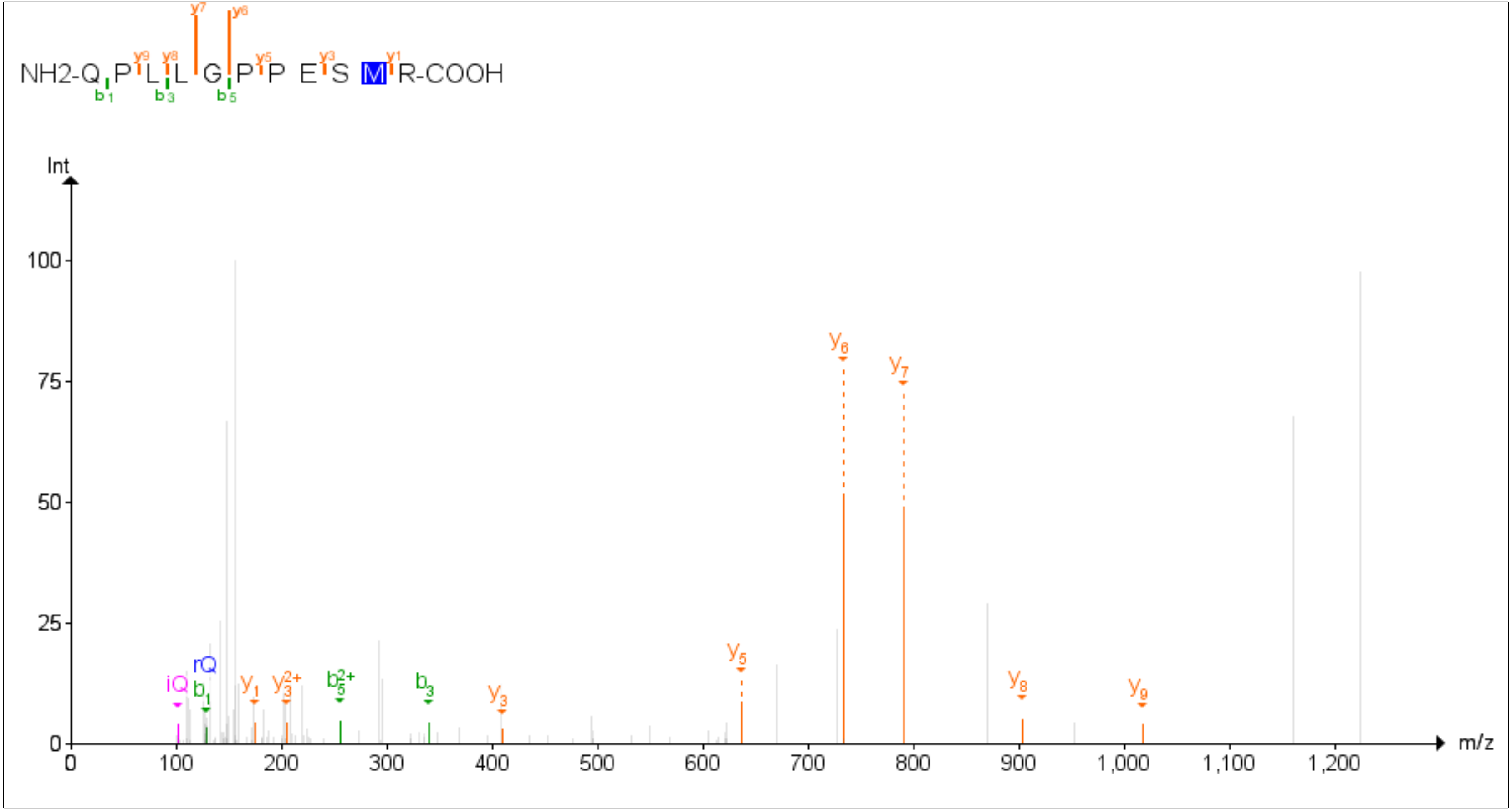

NH2-Q P L L G P P E S M R-COOH
y7
y6
y9
y8
y5
y3
y1
b1
b3
b5
Int
100
75
50
25
0
y6
y7
y5
y8
y9
iQ
rQ
b1
y1
y3 2+
b5 2+
b3
y3
m/z
0
100
200
300
400
500
600
700
800
900
1,000
1,100
1,200

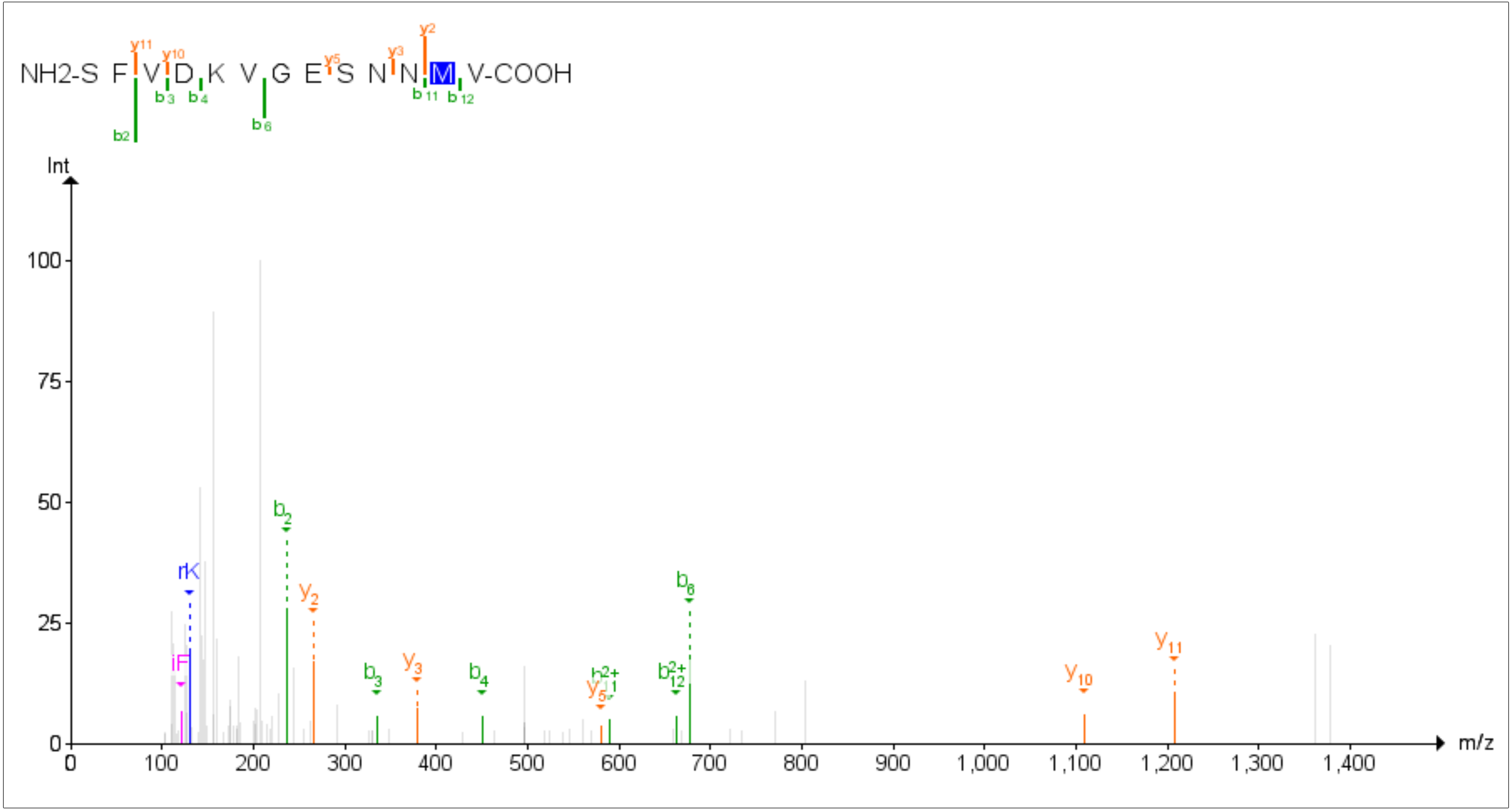

NH2-S F V D K V G E S N N M V-COOH
y11
y10
y5
y3
y2
b2
b3
b4
b6
b11
b12
Int
100
75
50
25
0
b_2
rK
y_2
iF
b_3
y_3
b_4
y_5
b_6
b^{2+}_{12}
y_{10}
y_{11}
0
100
200
300
400
500
600
700
800
900
1,000
1,100
1,200
1,300
1,400
m/z

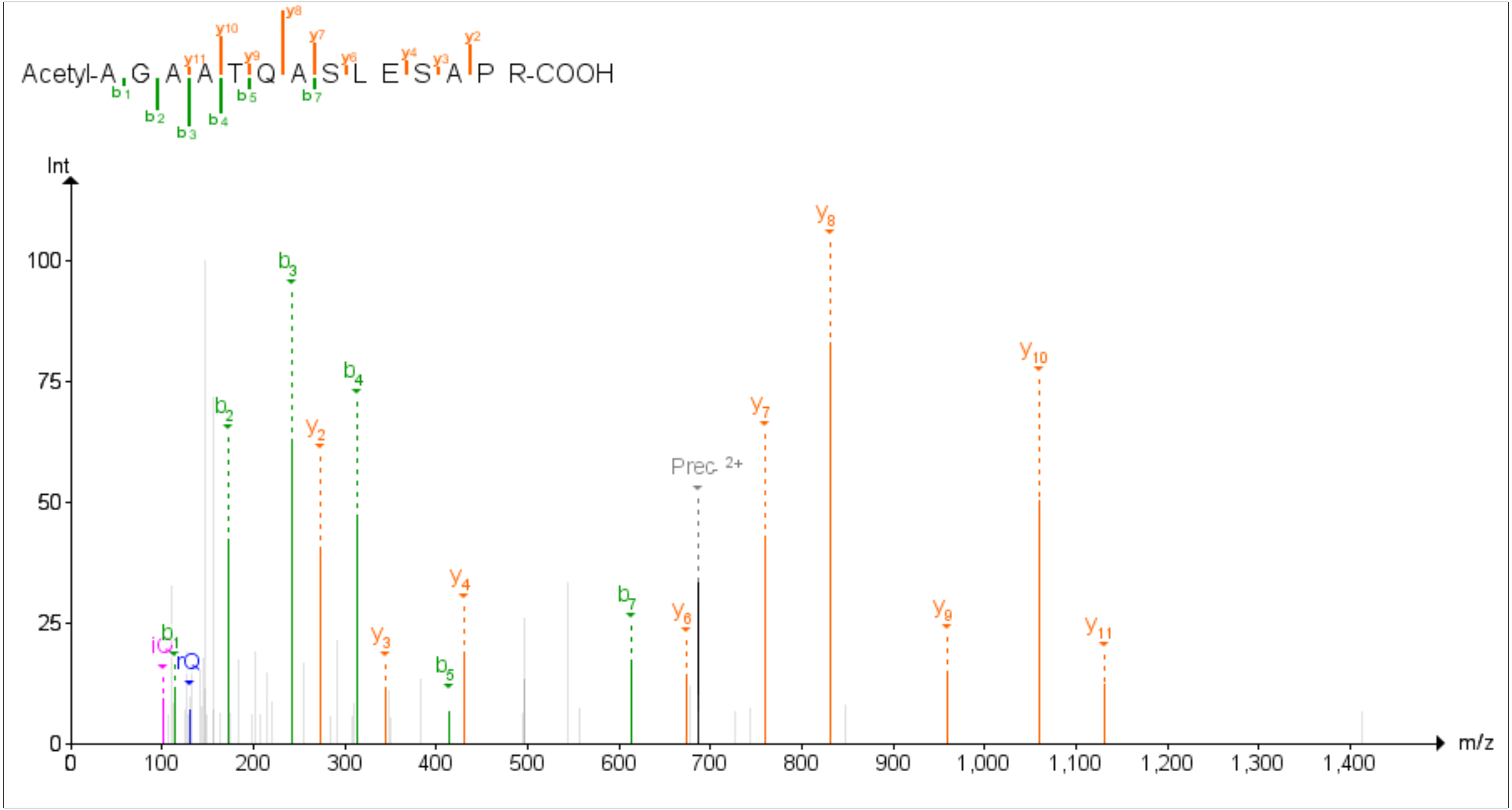

Acetyl-A G A A T Q A S L E S A P R-COOH
b1 b2 b3 b4 b5 b7
y11 y10 y9 y8 y7 y6 y4 y3 y2
Int
100
75
50
25
0
iQ
rQ
Prec. 2+
m/z
0 100 200 300 400 500 600 700 800 900 1,000 1,100 1,200 1,300 1,400

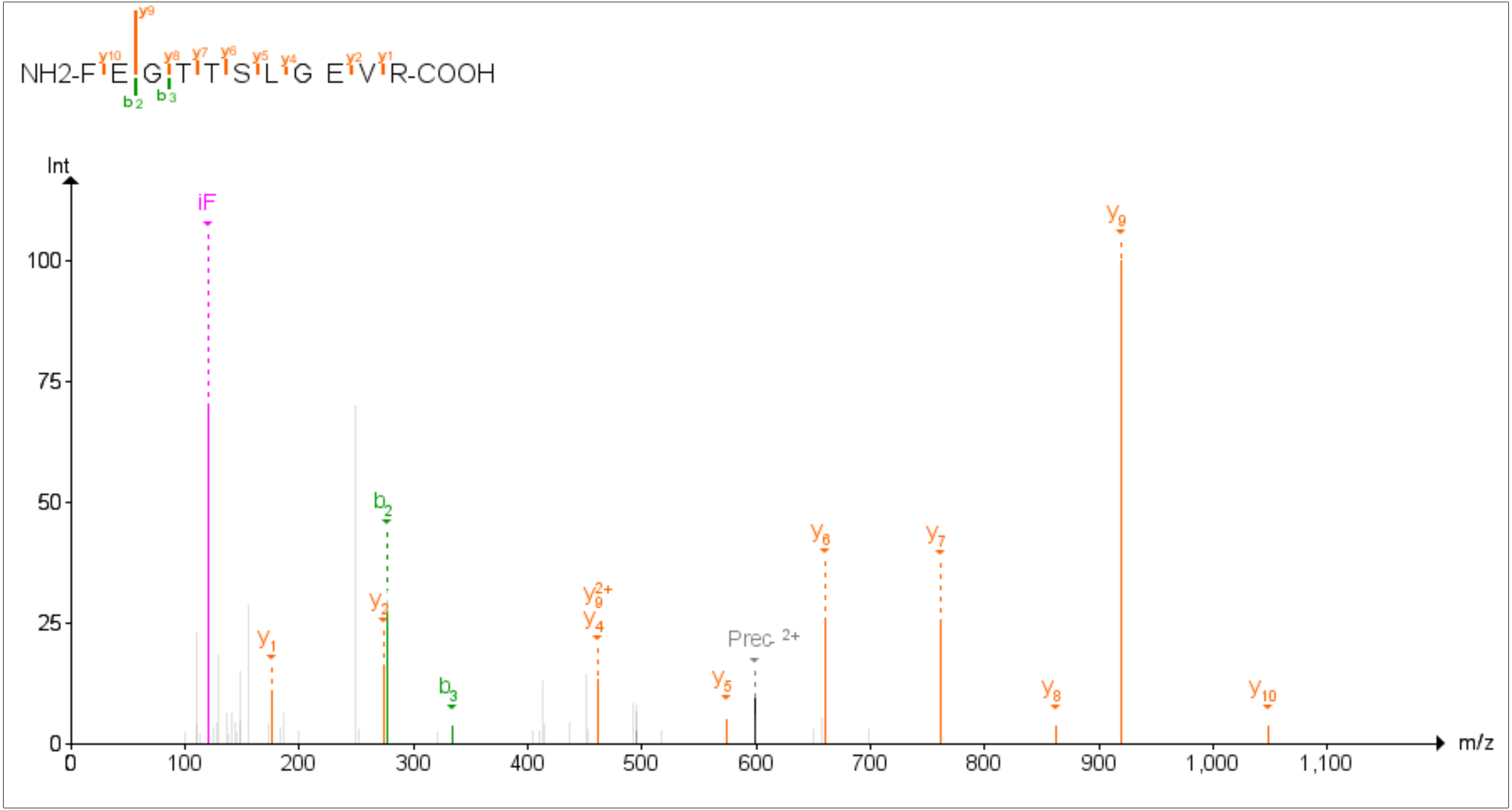

NH2-F E G T T S L G E V R-COOH
y9
y10
y8
y7
y6
y5
y4
y2
y1
b2
b3
Int
iF
y9
b2
y6
y7
y2
y9 2+
y4
y1
b3
Prec. 2+
y5
y8
y10
m/z
0
25
50
75
100
0
100
200
300
400
500
600
700
800
900
1,000
1,100

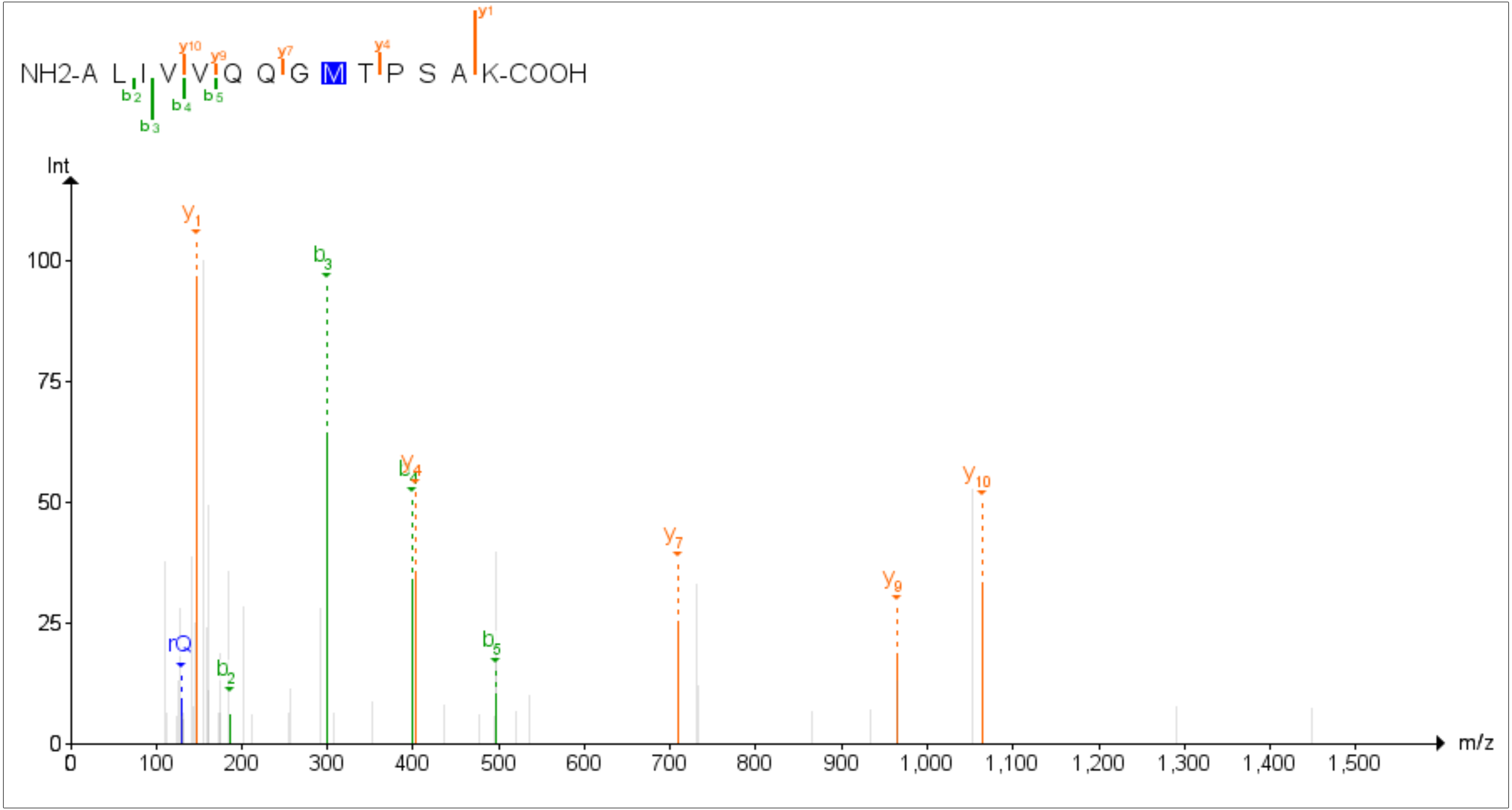

NH2-A L I V V Q Q G M T P S A K-COOH
y1
y4
y7
y9
y10
b2
b3
b4
b5
Int
m/z
rQ
0
25
50
75
100
0
100
200
300
400
500
600
700
800
900
1,000
1,100
1,200
1,300
1,400
1,500

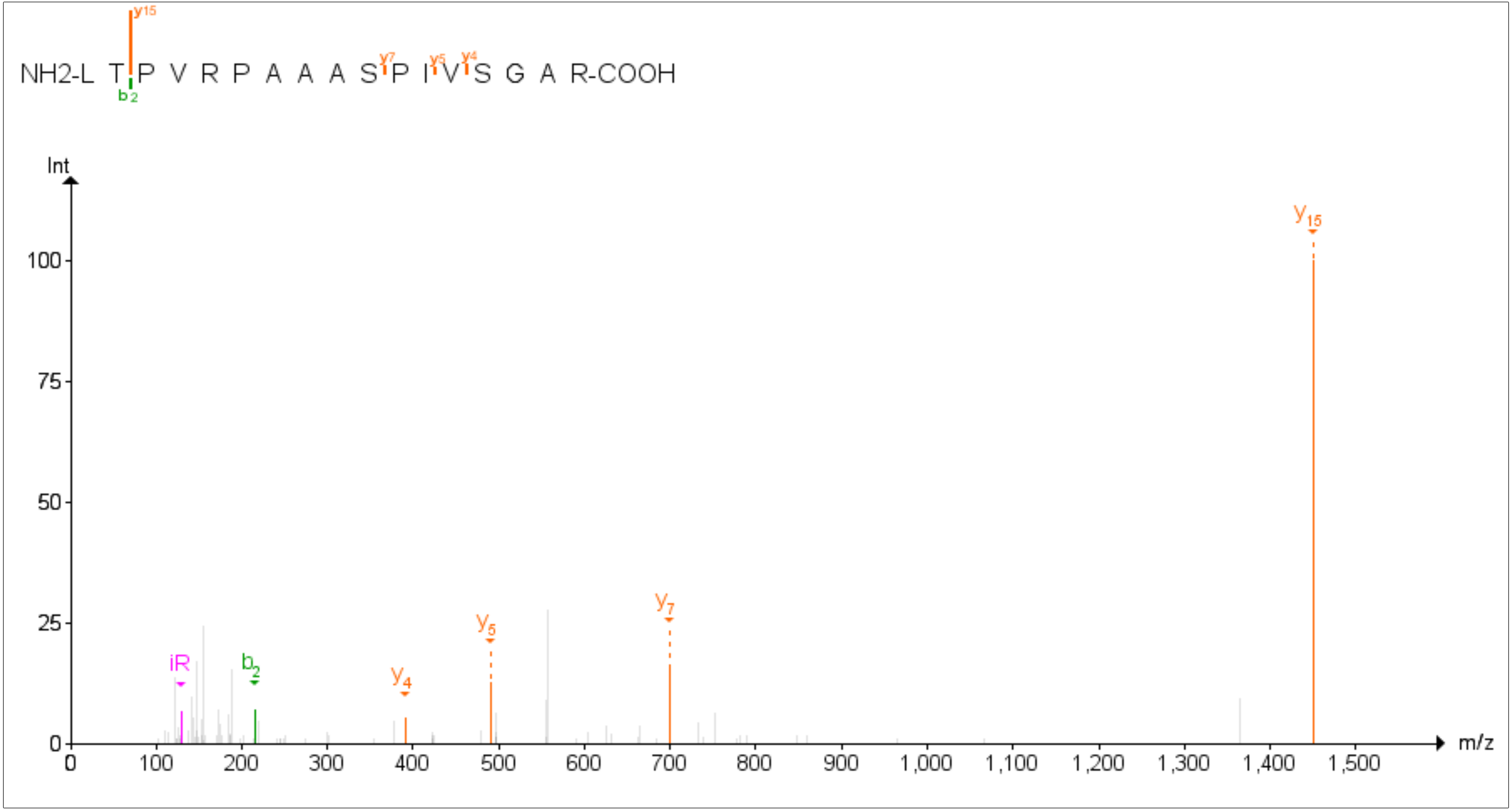

NH2-L T P V R P A A A S P I V S G A R-COOH
y15
b2
y7
y5
y4
Int
100
75
50
25
0
iR
b2
y4
y5
y7
y15
0
100
200
300
400
500
600
700
800
900
1,000
1,100
1,200
1,300
1,400
1,500
m/z

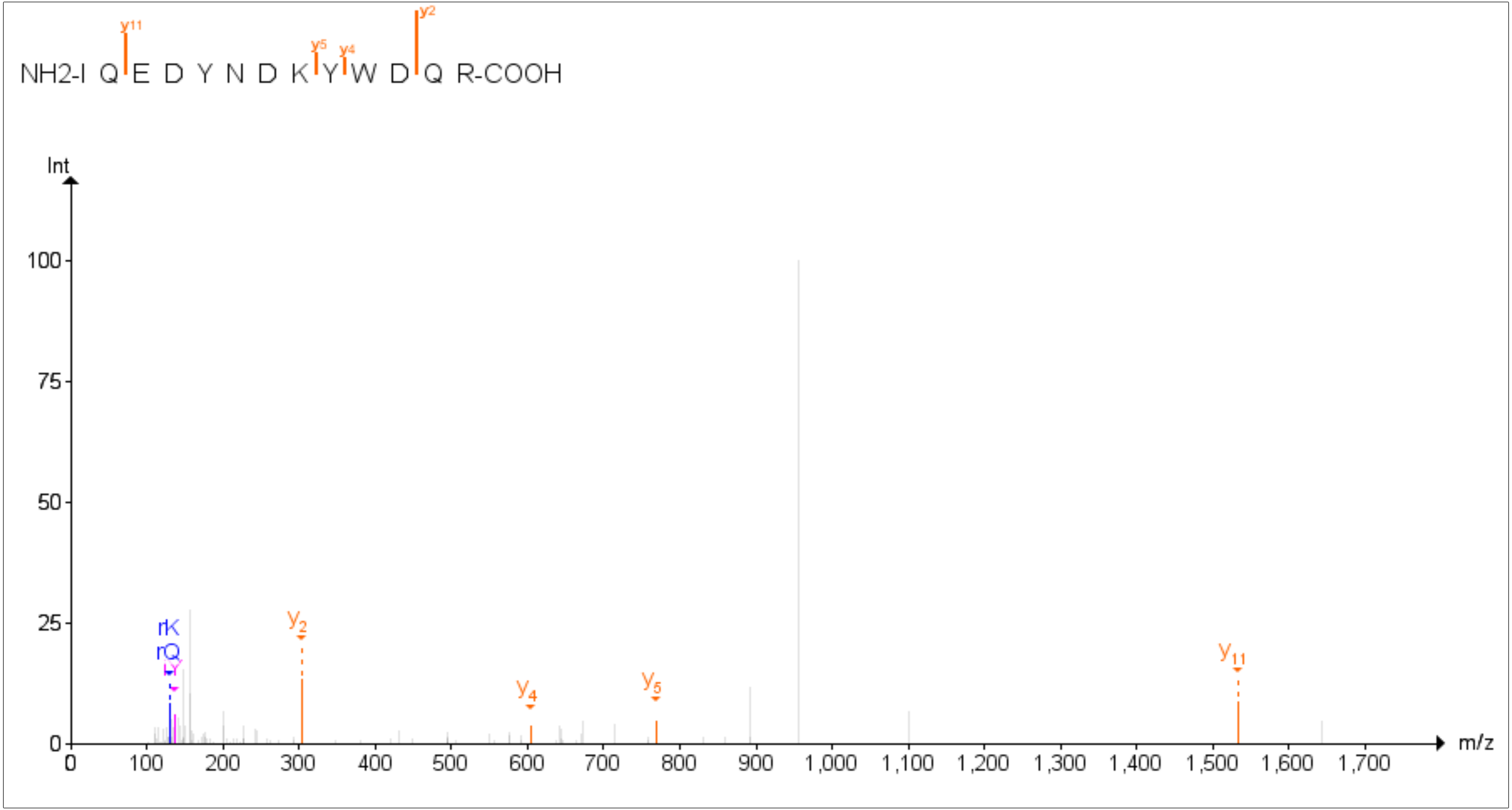

NH2-I Q E D Y N D K Y W D Q R-COOH
y11
y5
y4
y2
Int
100
75
50
25
0
rK
rQ
y2
y4
y5
y11
0
100
200
300
400
500
600
700
800
900
1,000
1,100
1,200
1,300
1,400
1,500
1,600
1,700
m/z

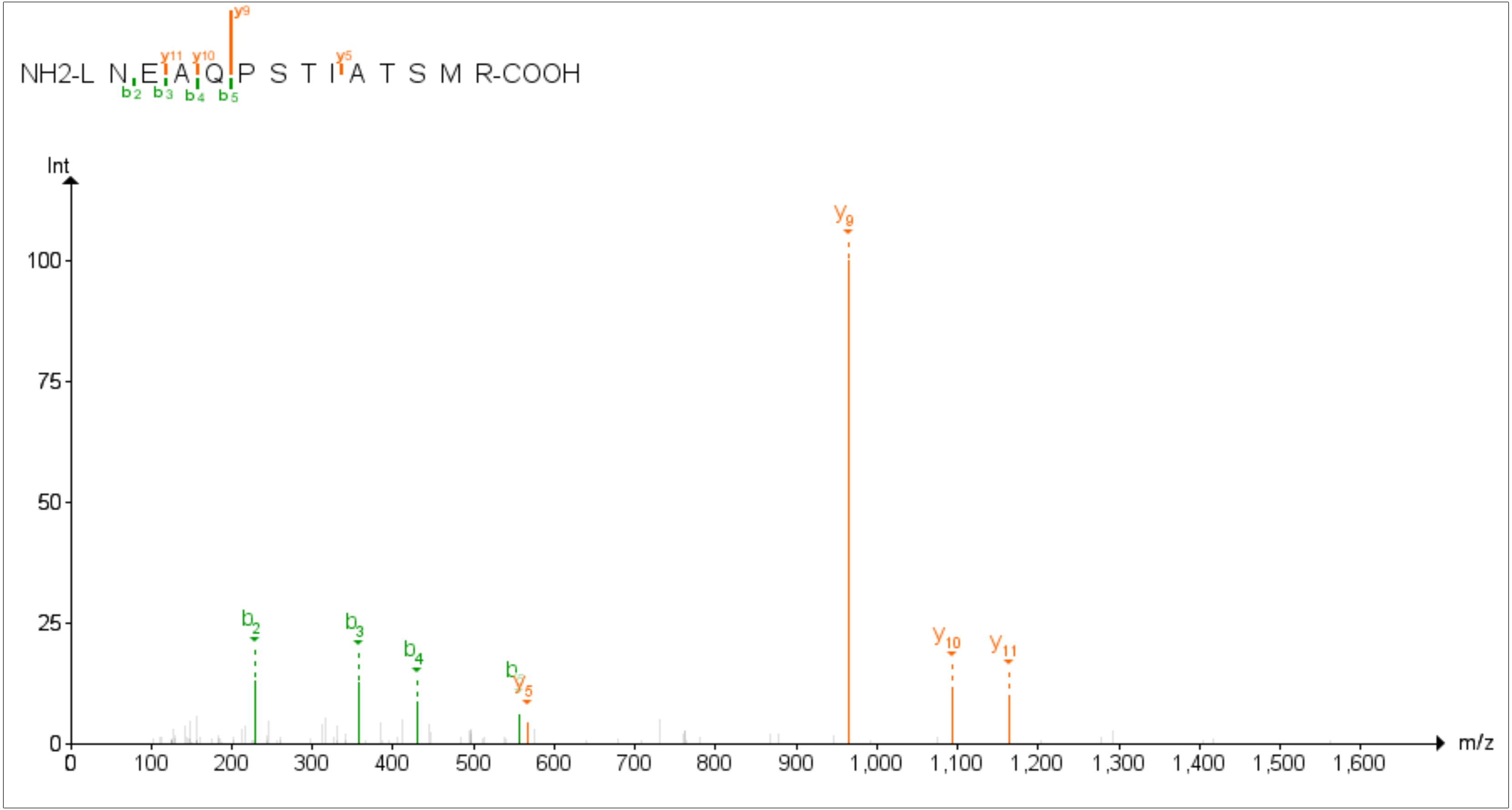

NH2-L N E A Q P S T I A T S M R-COOH
y9
y11
y10
y5
b2
b3
b4
b5
Int
100
75
50
25
0
m/z
0
100
200
300
400
500
600
700
800
900
1,000
1,100
1,200
1,300
1,400
1,500
1,600

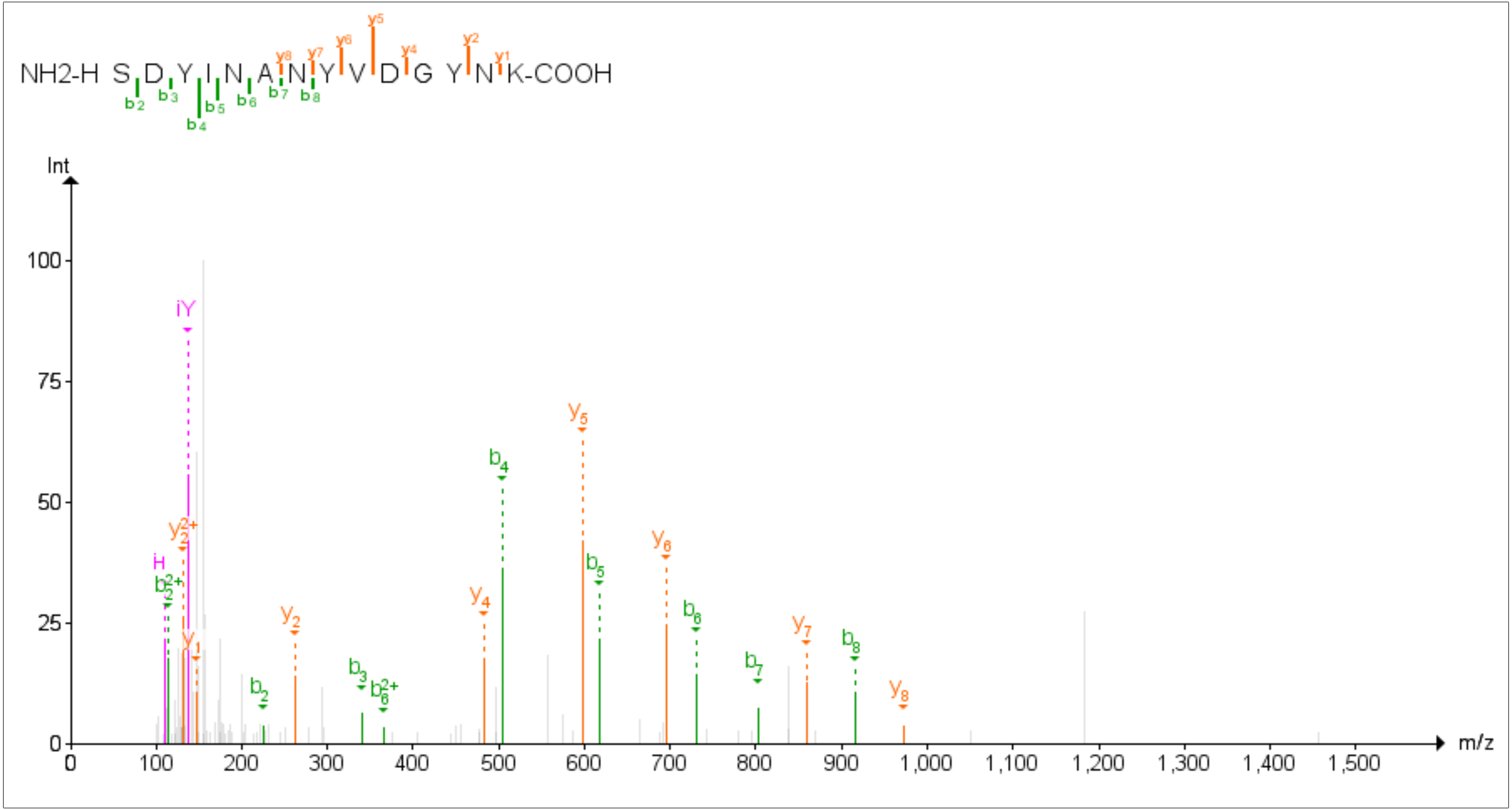

NH2-H S D Y I N A N Y V D G Y N K-COOH
Int
m/z
iY
iH

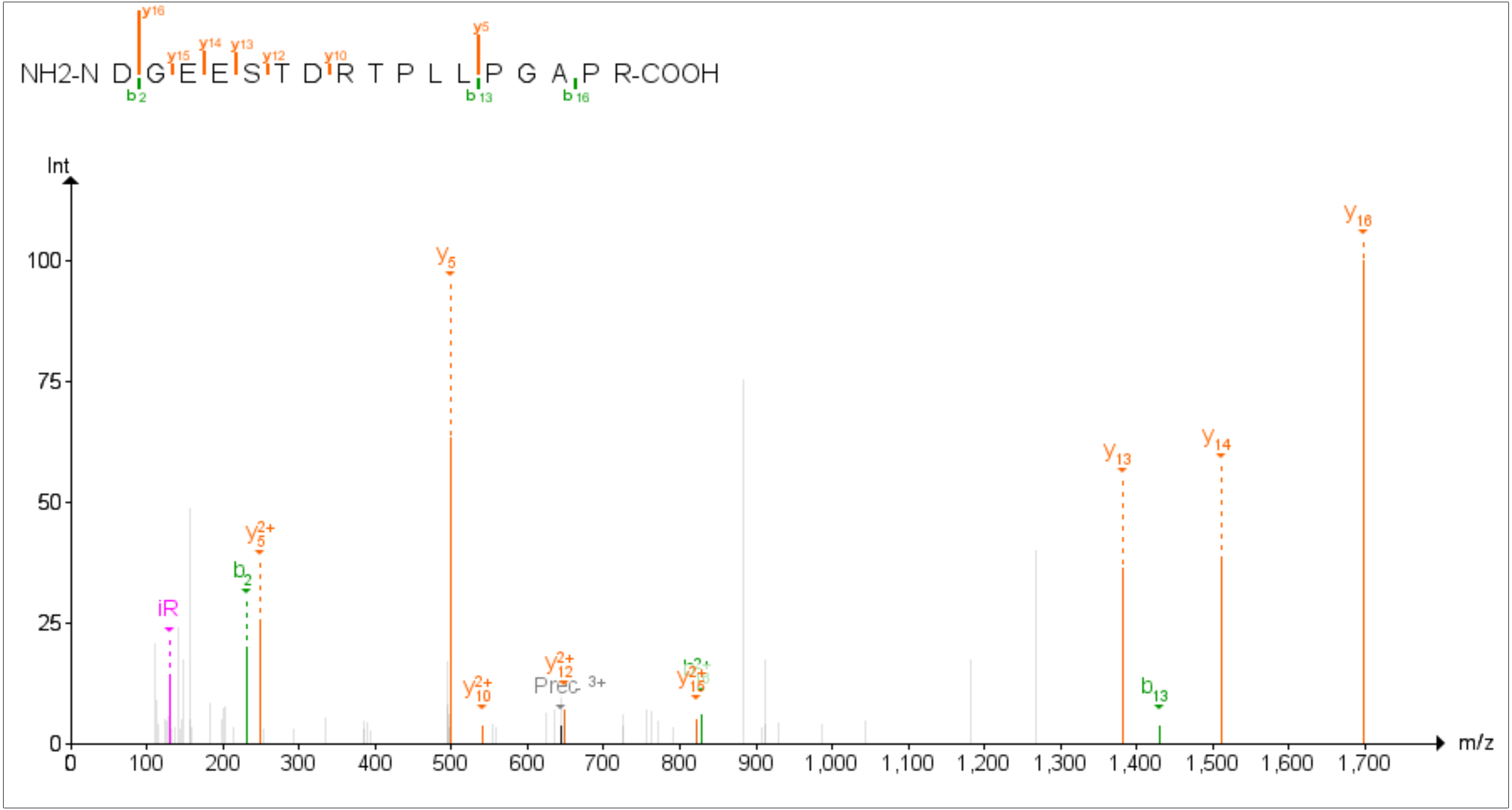
NH2-N D G E E S T D R T P L L P G A P R-COOH
y16
y15
y14
y13
y12
y10
y5
b2
b13
b16
Int
100
75
50
25
0
m/z
0
100
200
300
400
500
600
700
800
900
1,000
1,100
1,200
1,300
1,400
1,500
1,600
1,700
iR
b2
y5 2+
y5
y10 2+
y12 2+
Prec- 3+
y15 2+
y13
y14
b13
y16

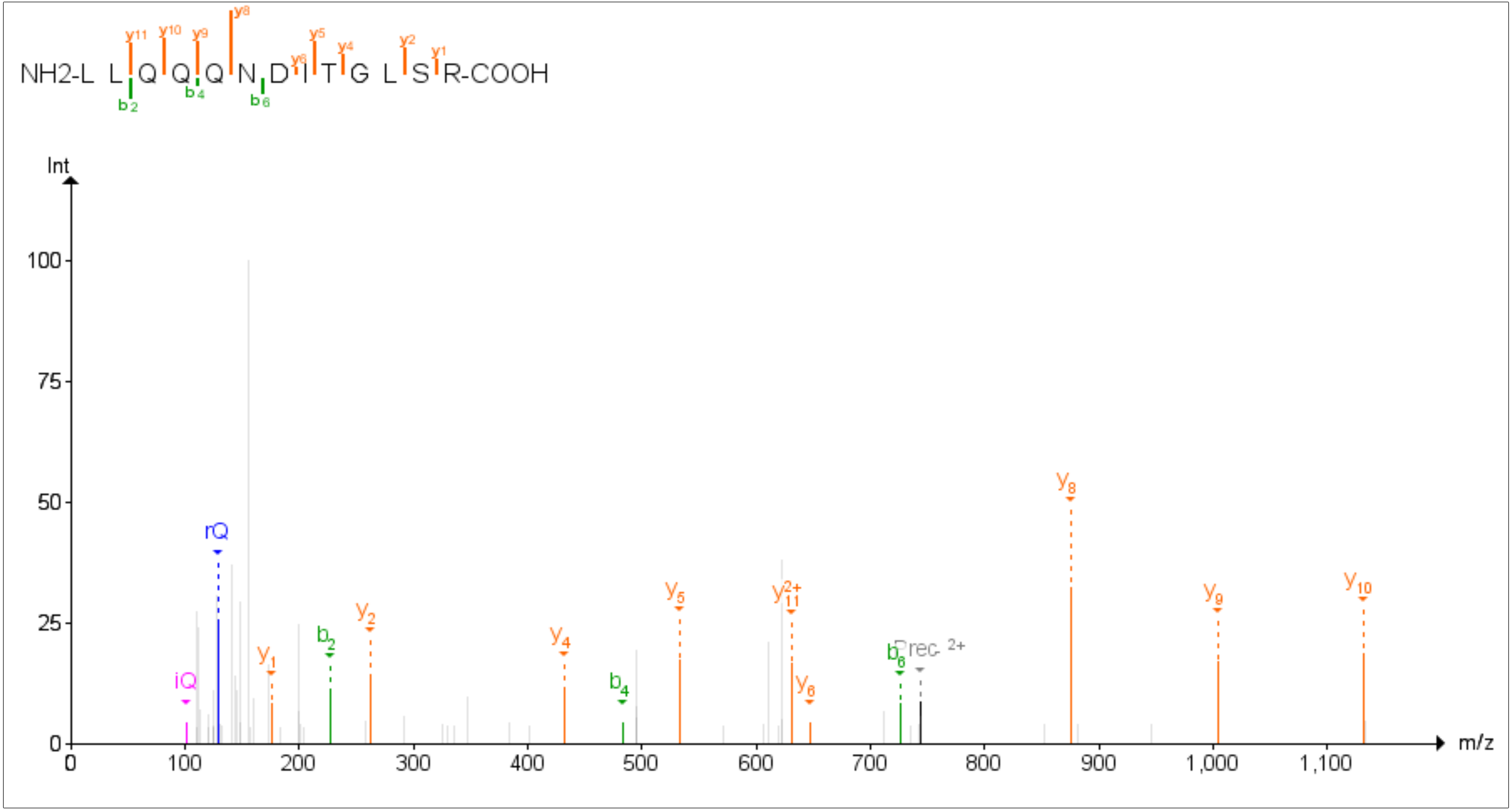

NH2-L L Q Q Q N D I T G L S R-COOH
y11 y10 y9 y8 y6 y5 y4 y2 y1
b2 b4 b6
Int
100
75
50
25
0
m/z
0 100 200 300 400 500 600 700 800 900 1,000 1,100
rQ
iQ
y1
b2
y2
y4
b4
y5
y11 2+
y6
b6
Prec. 2+
y8
y9
y10

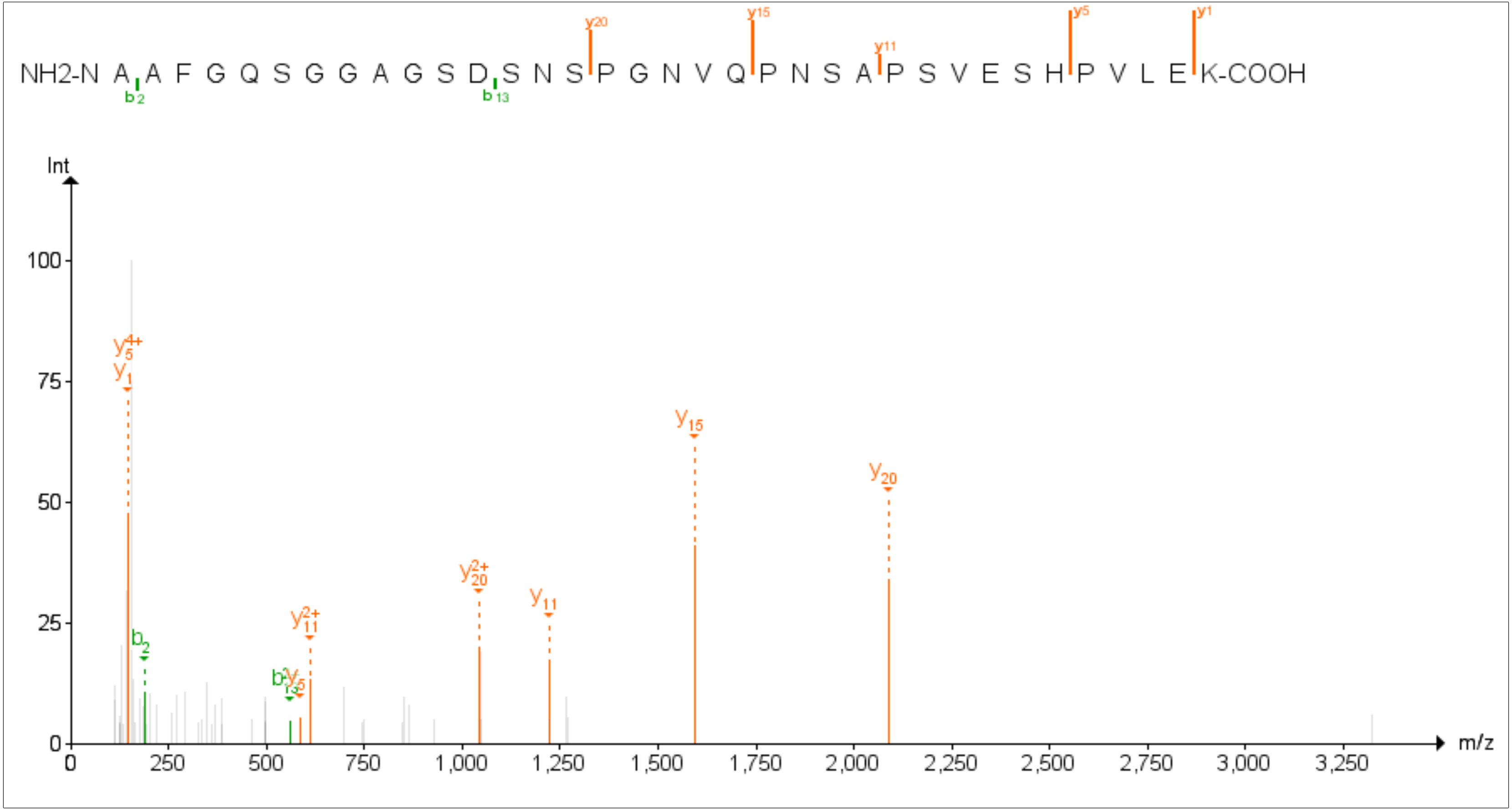

NH2-N A A F G Q S G G A G S D S N S P G N V Q P N S A P S V E S H P V L E K-COOH
b2
b13
y20
y15
y11
y5
y1
Int
m/z
100
75
50
25
0
250
500
750
1,000
1,250
1,500
1,750
2,000
2,250
2,500
2,750
3,000
3,250

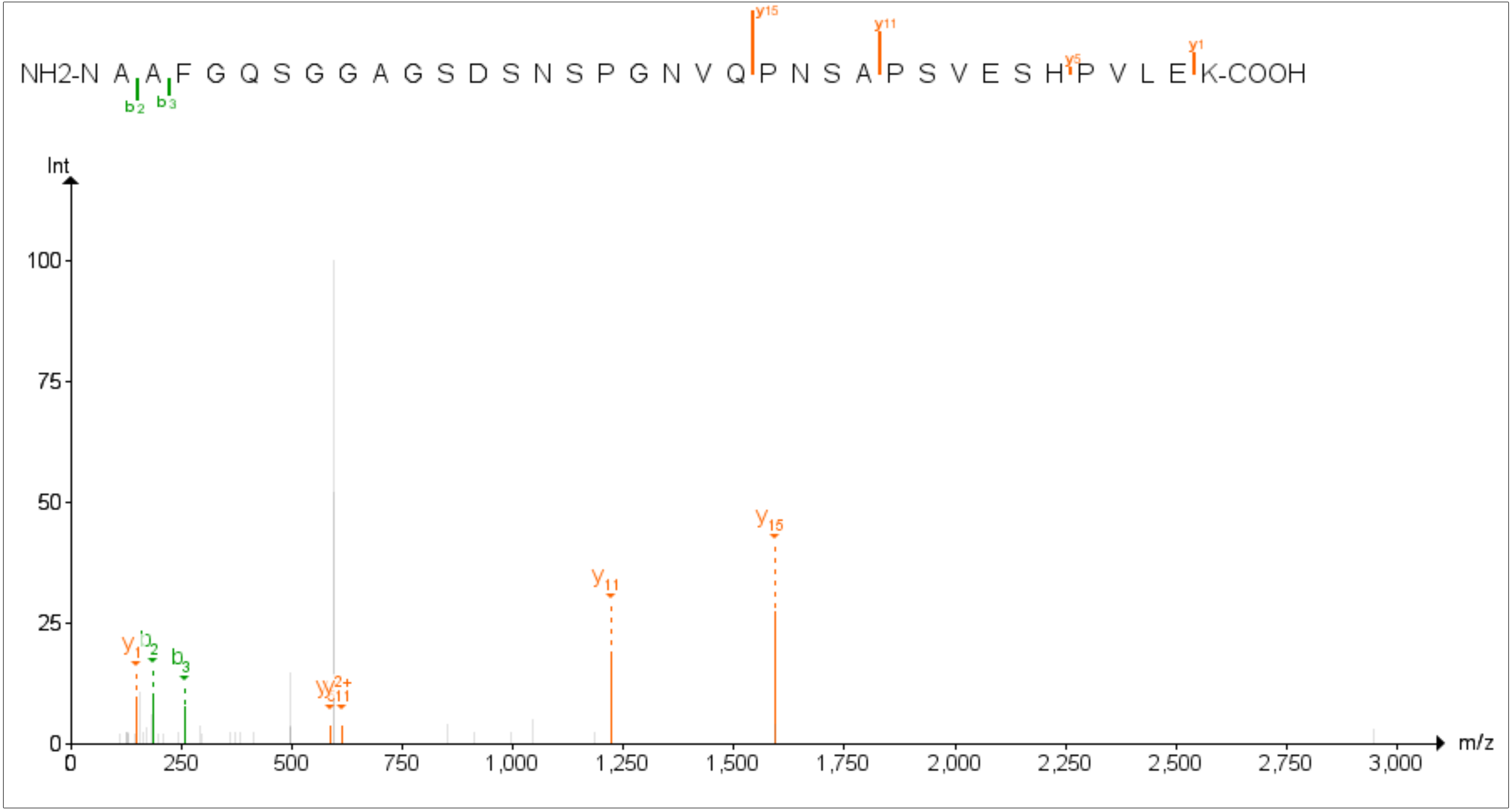

NH2-N A A F G Q S G G A G S D S N S P G N V Q P N S A P S V E S H P V L E K-COOH
Int
m/z

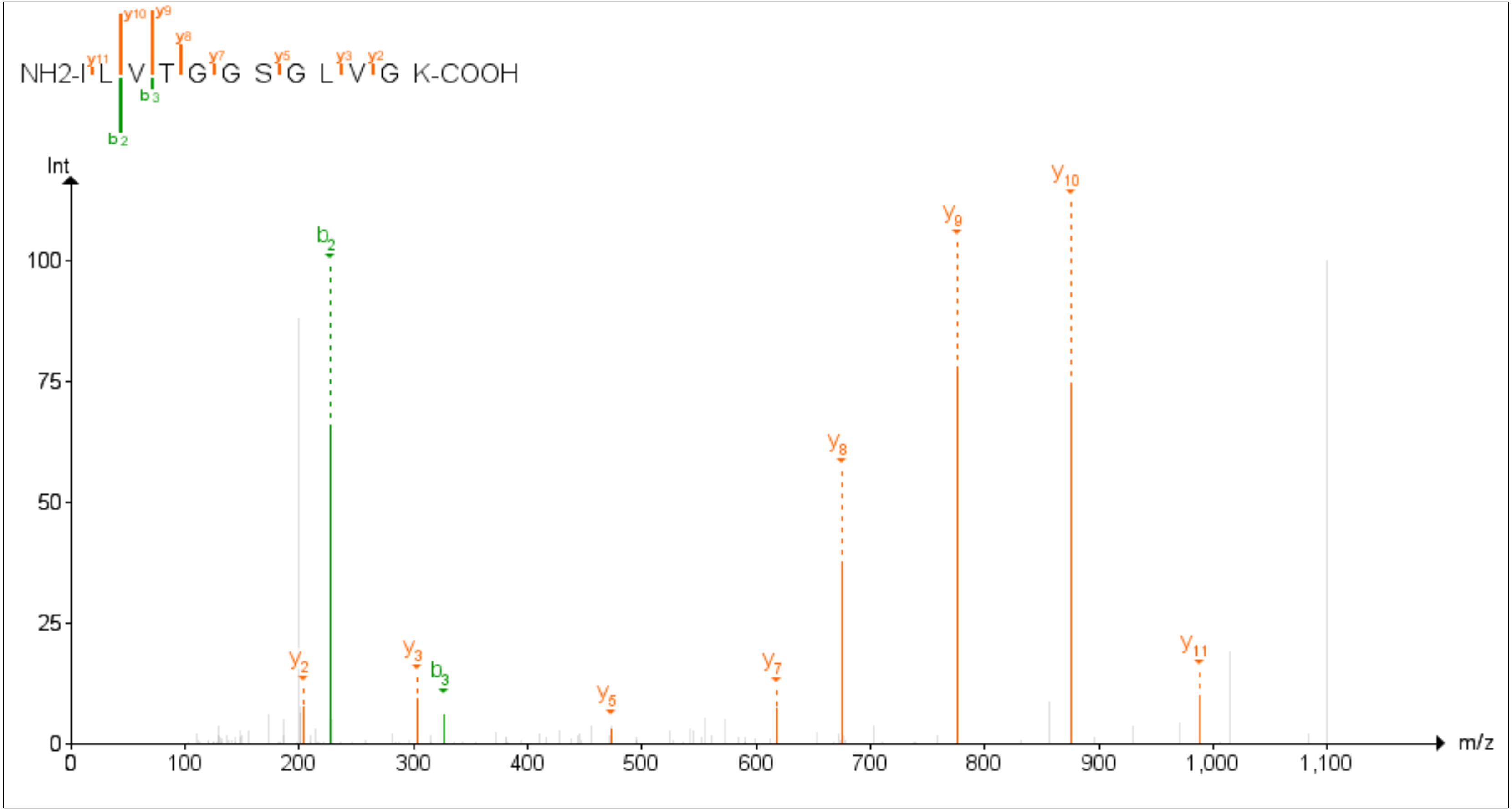

NH2-I L V T G G S G L V G K-COOH
y11 y10 y9 y8 y7 y5 y3 y2
b2 b3
Int
100
75
50
25
0
b2
y9
y10
y8
y2
y3
b3
y5
y7
y11
0 100 200 300 400 500 600 700 800 900 1,000 1,100
m/z

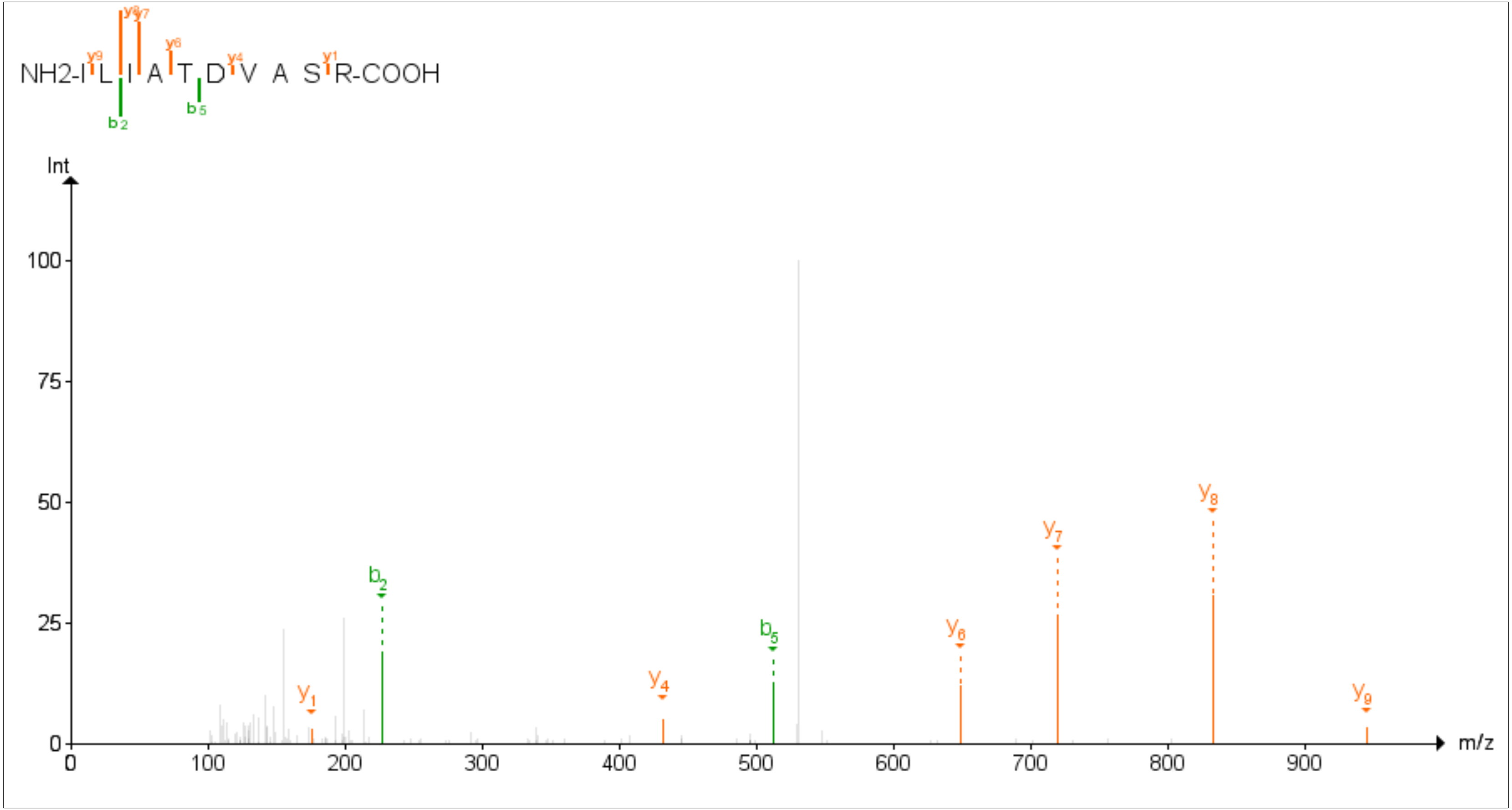
NH2-I L I A T D V A S R-COOH
y9
y8
y7
y6
y4
y1
b2
b5
Int
100
75
50
25
0
0
100
200
300
400
500
600
700
800
900
m/z
b2
b5
y1
y4
y6
y7
y8
y9

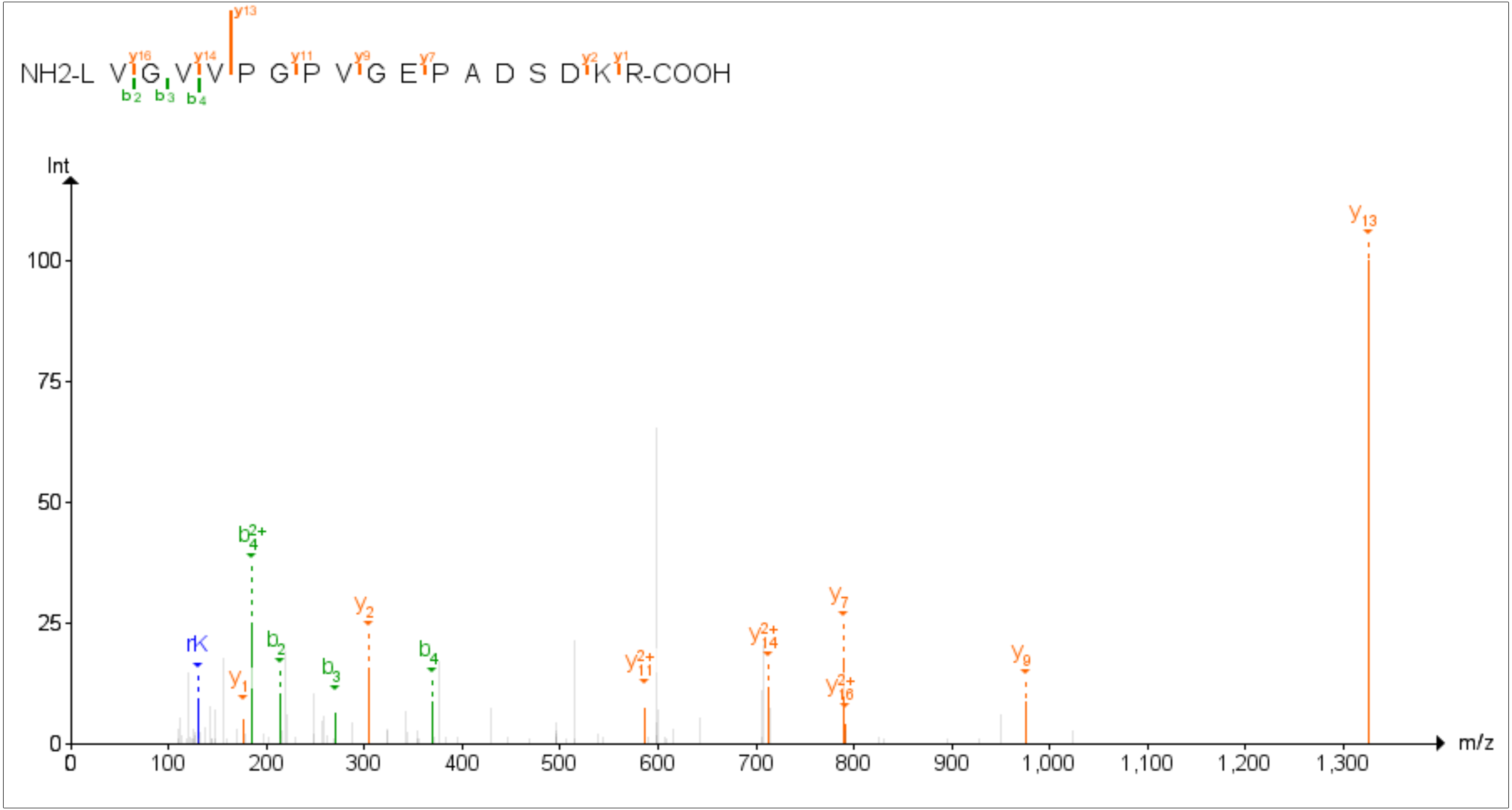

NH2-L V G V V P G P V G E P A D S D K R-COOH
y16 y14 y13 y11 y9 y7 y2 y1
b2 b3 b4
Int
m/z
y13
b4 2+
y2
y7
y14 2+
y11 2+
y16 2+
y9
rK
y1
b2
b3
b4

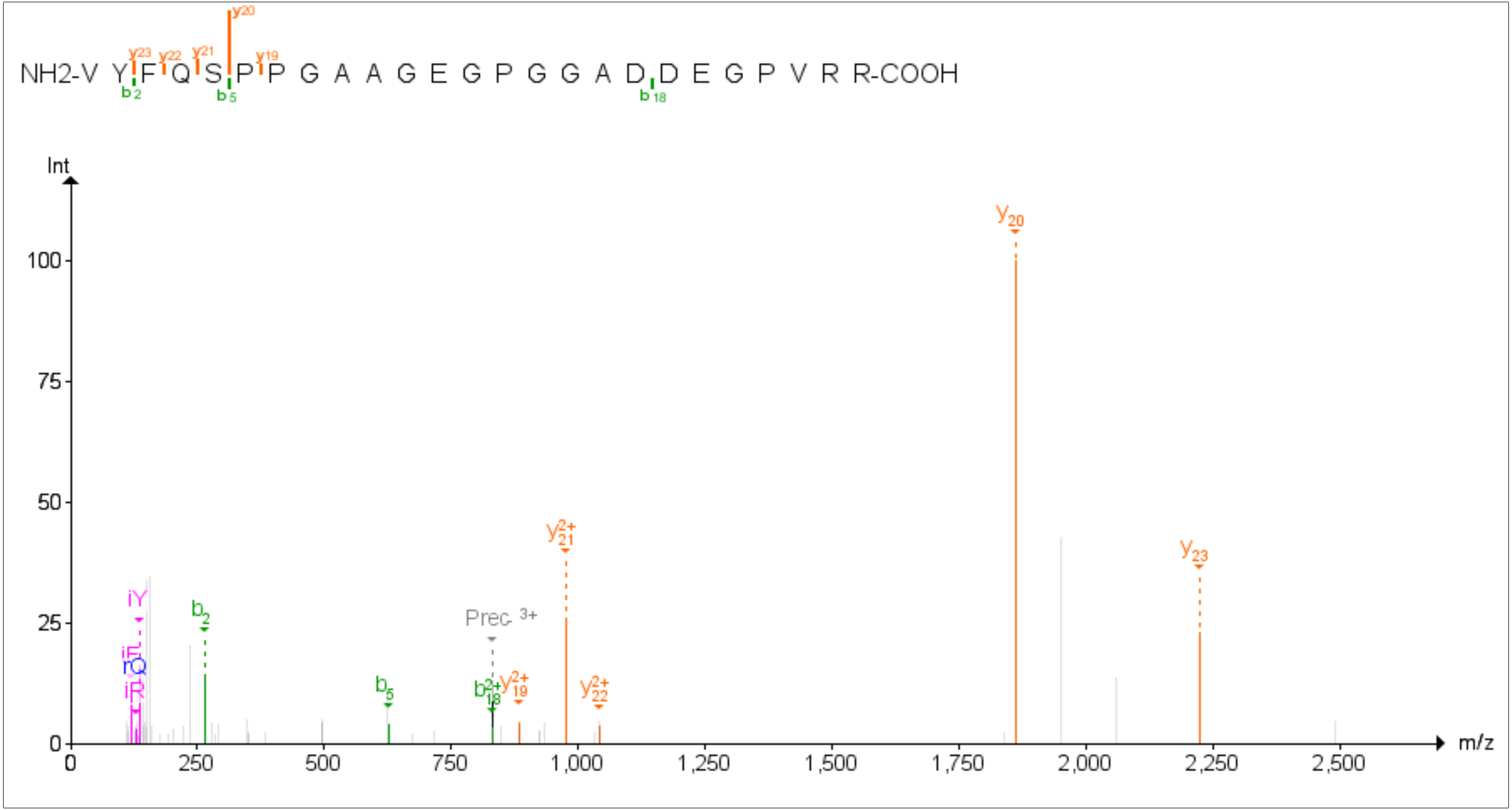

NH2-V Y F Q S P P G A A G E G P G G A D D E G P V R R-COOH
y23
y22
y21
y20
y19
b2
b5
b18
Int
100
75
50
25
0
y20
y21 2+
y23
iY
b2
Prec 3+
iF
rQ
iR
b5
b18 2+
y19 2+
y22 2+
0
250
500
750
1,000
1,250
1,500
1,750
2,000
2,250
2,500
m/z

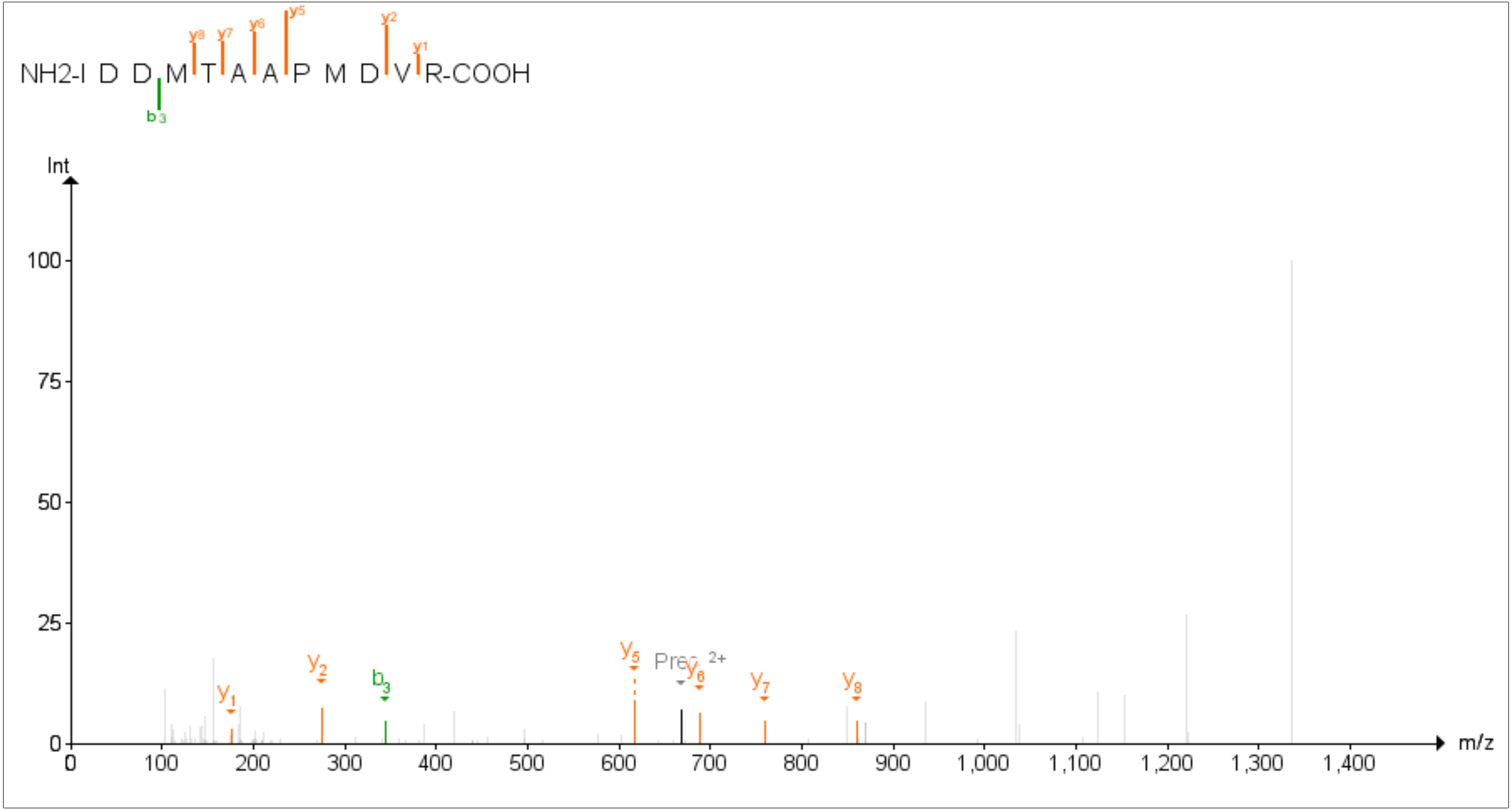

NH2-I D D M T A A P M D V R-COOH
y8 y7 y6 y5 y2 y1
b3
Int
100
75
50
25
0
y1 y2 b3 y5 Prec 2+ y6 y7 y8
0 100 200 300 400 500 600 700 800 900 1,000 1,100 1,200 1,300 1,400
m/z

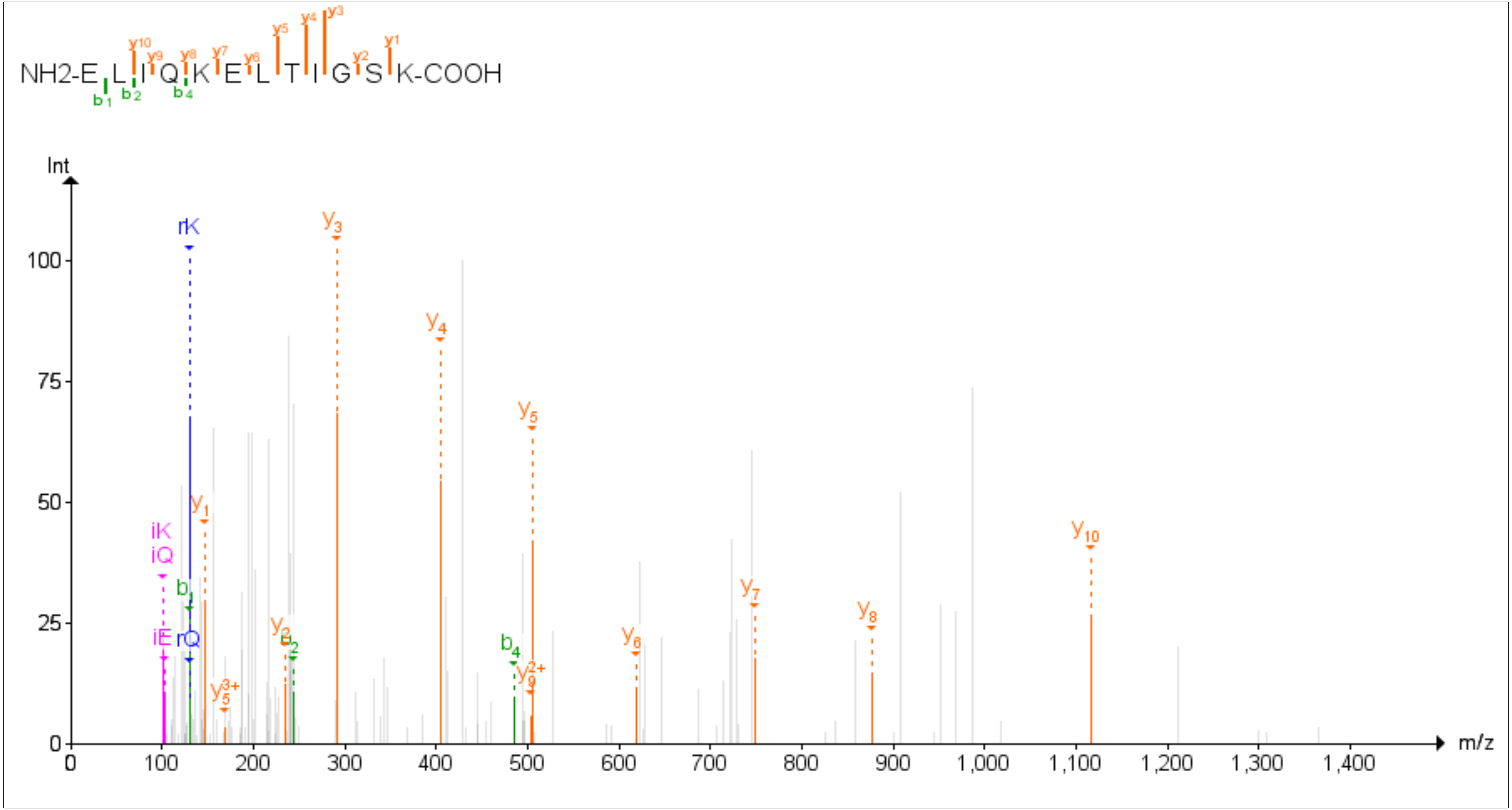

NH2-E L I Q K E L T I G S K-COOH
b1 b2 b4
y10 y9 y8 y7 y6 y5 y4 y3 y2 y1
Int
m/z
rK
iK
iQ
iE
rQ
b1
b2
b4
y1
y2
y3
y4
y5
y6
y7
y8
y10
y5 3+
y9 2+
0
25
50
75
100
0
100
200
300
400
500
600
700
800
900
1,000
1,100
1,200
1,300
1,400

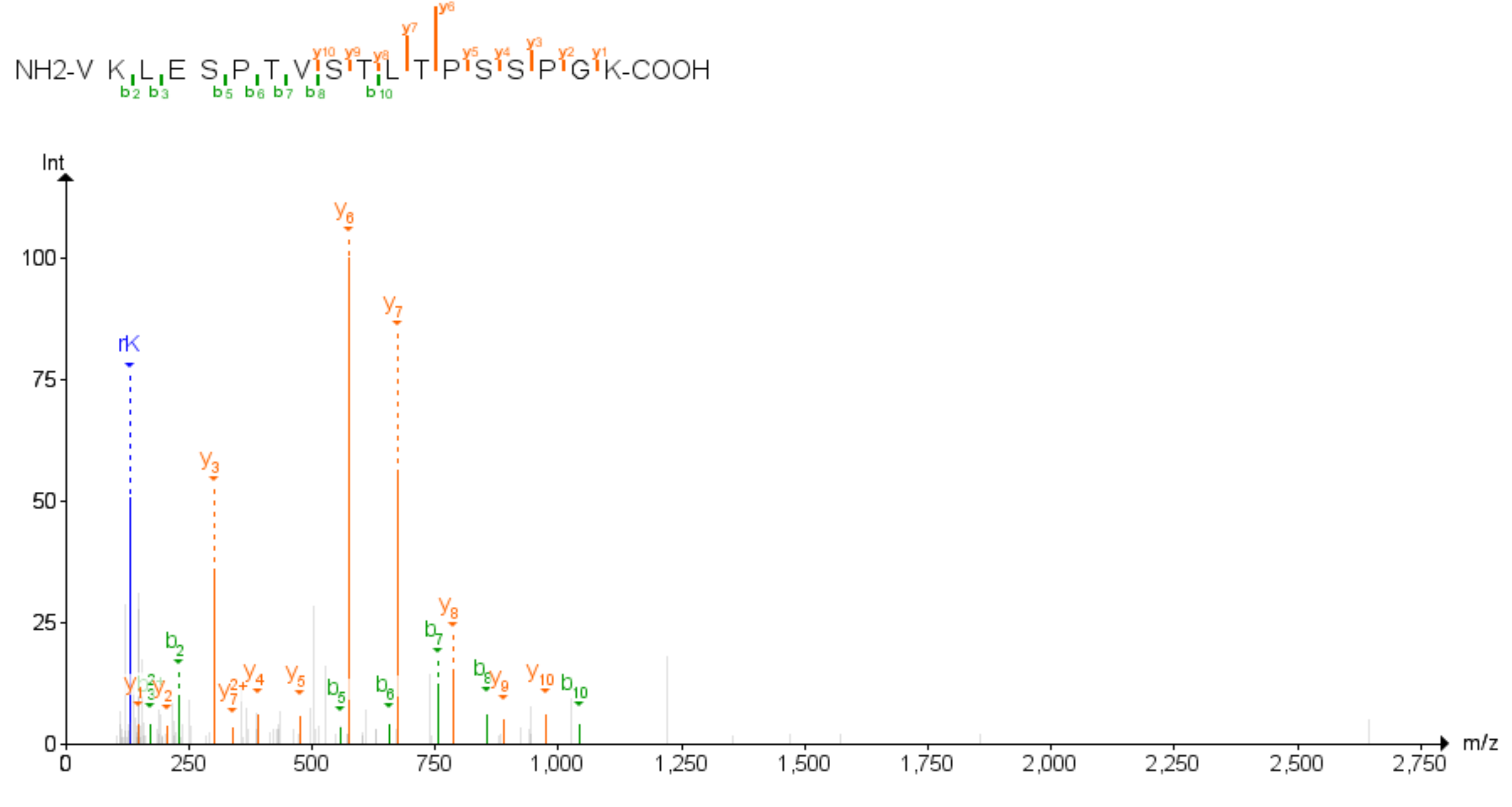
NH2-V K L E S P T V S T L T P S S P G K-COOH
Int
m/z
y6
y7
y3
rK
y8
b7
b2
b8
y9
y10
b10
y4
y5
b5
b6
y2
0
25
50
75
100
250
500
750
1,000
1,250
1,500
1,750
2,000
2,250
2,500
2,750

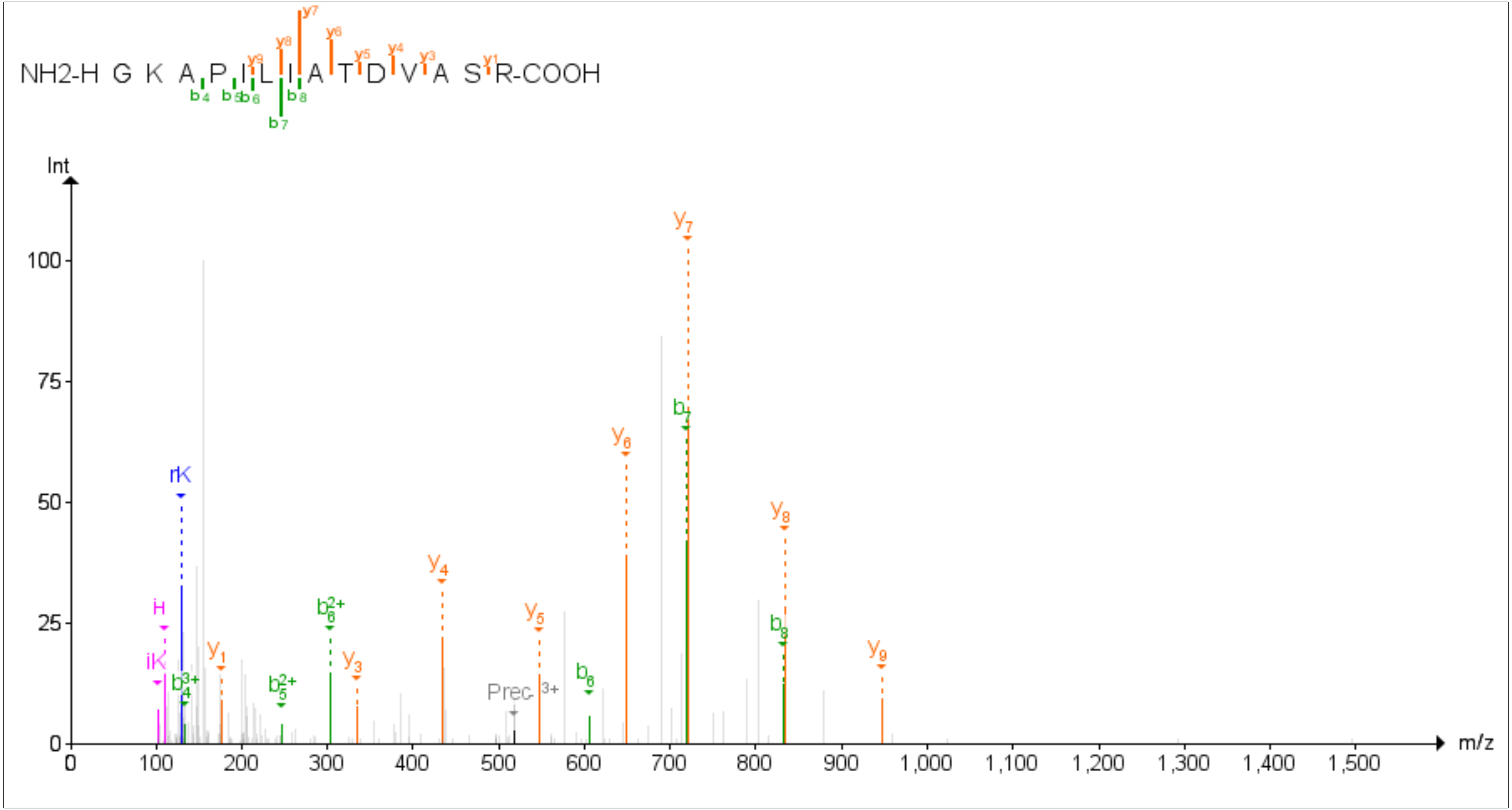

NH2-H G K A P I L I A T D V A S R-COOH
Int
m/z

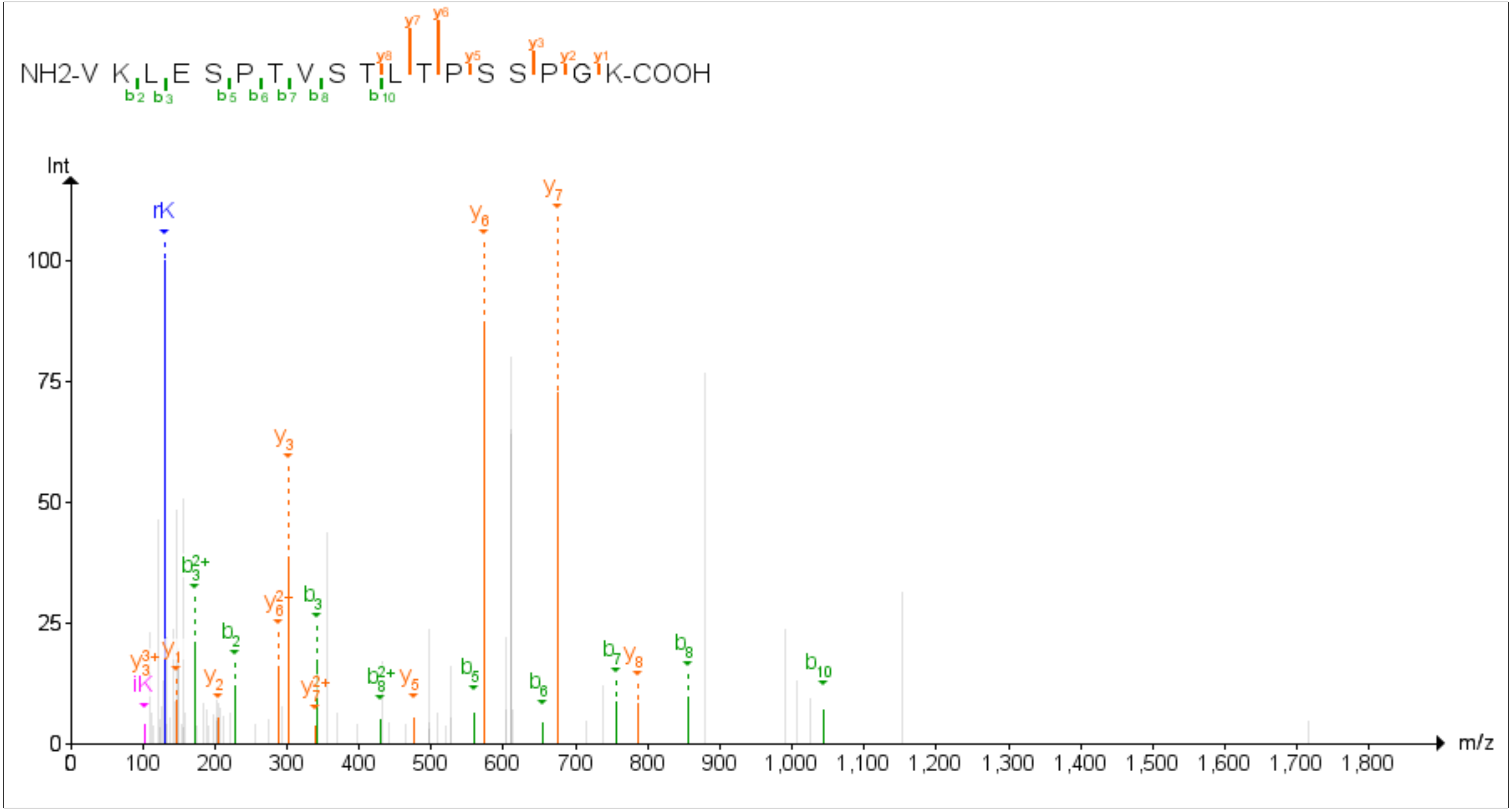

NH2-V K L E S P T V S T L T P S S P G K-COOH
b2 b3 b5 b6 b7 b8 b10
y8 y7 y6 y5 y3 y2 y1
Int
m/z
rK
iK
0
25
50
75
100
0
100
200
300
400
500
600
700
800
900
1,000
1,100
1,200
1,300
1,400
1,500
1,600
1,700
1,800

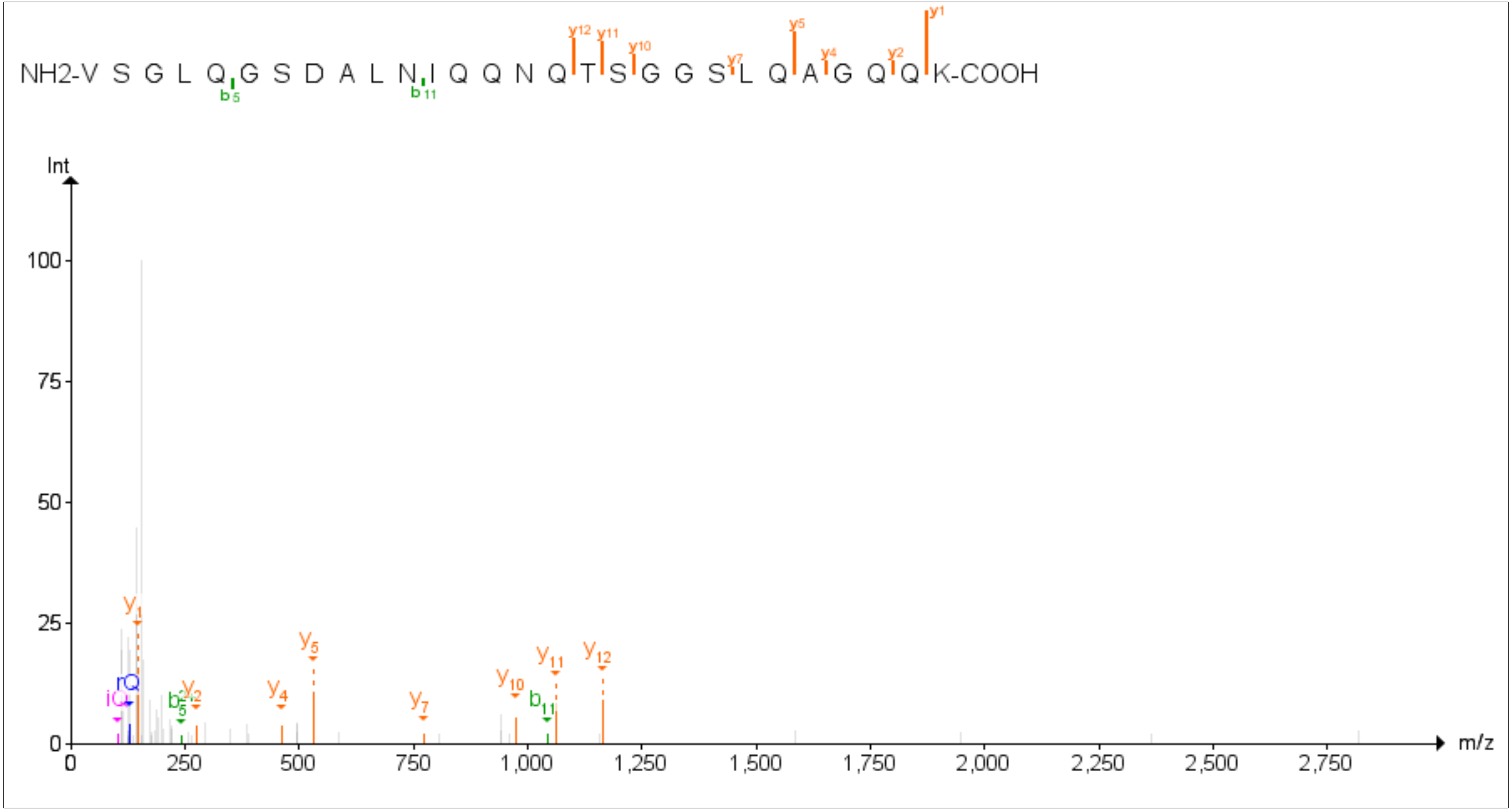

NH2-V S G L Q G S D A L N I Q Q N Q T S G G S L Q A G Q Q K-COOH
b5
b11
y12
y11
y10
y7
y5
y4
y2
y1
Int
100
75
50
25
0
m/z
0
250
500
750
1,000
1,250
1,500
1,750
2,000
2,250
2,500
2,750
y1
rQ
iQ
b5
y2
y4
y5
y7
y10
y11
b11
y12

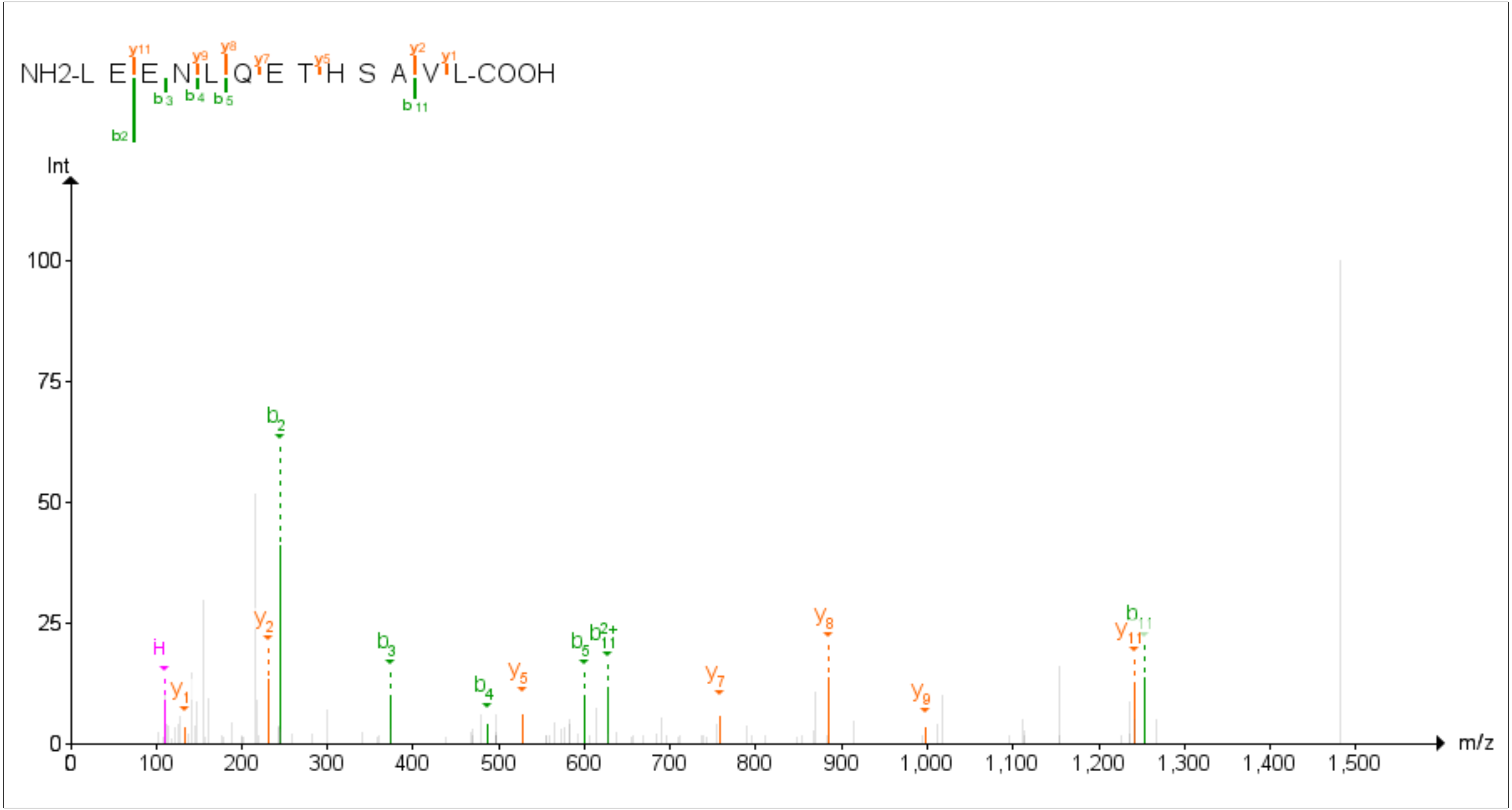

NH2-L E E N L Q E T H S A V L-COOH
y11 y9 y8 y7 y5 y2 y1
b2 b3 b4 b5 b11
Int
100
75
50
25
0
H
y1
y2
b2
b3
b4
y5
b5
b11 2+
y7
y8
y9
y11
b11
0 100 200 300 400 500 600 700 800 900 1,000 1,100 1,200 1,300 1,400 1,500
m/z

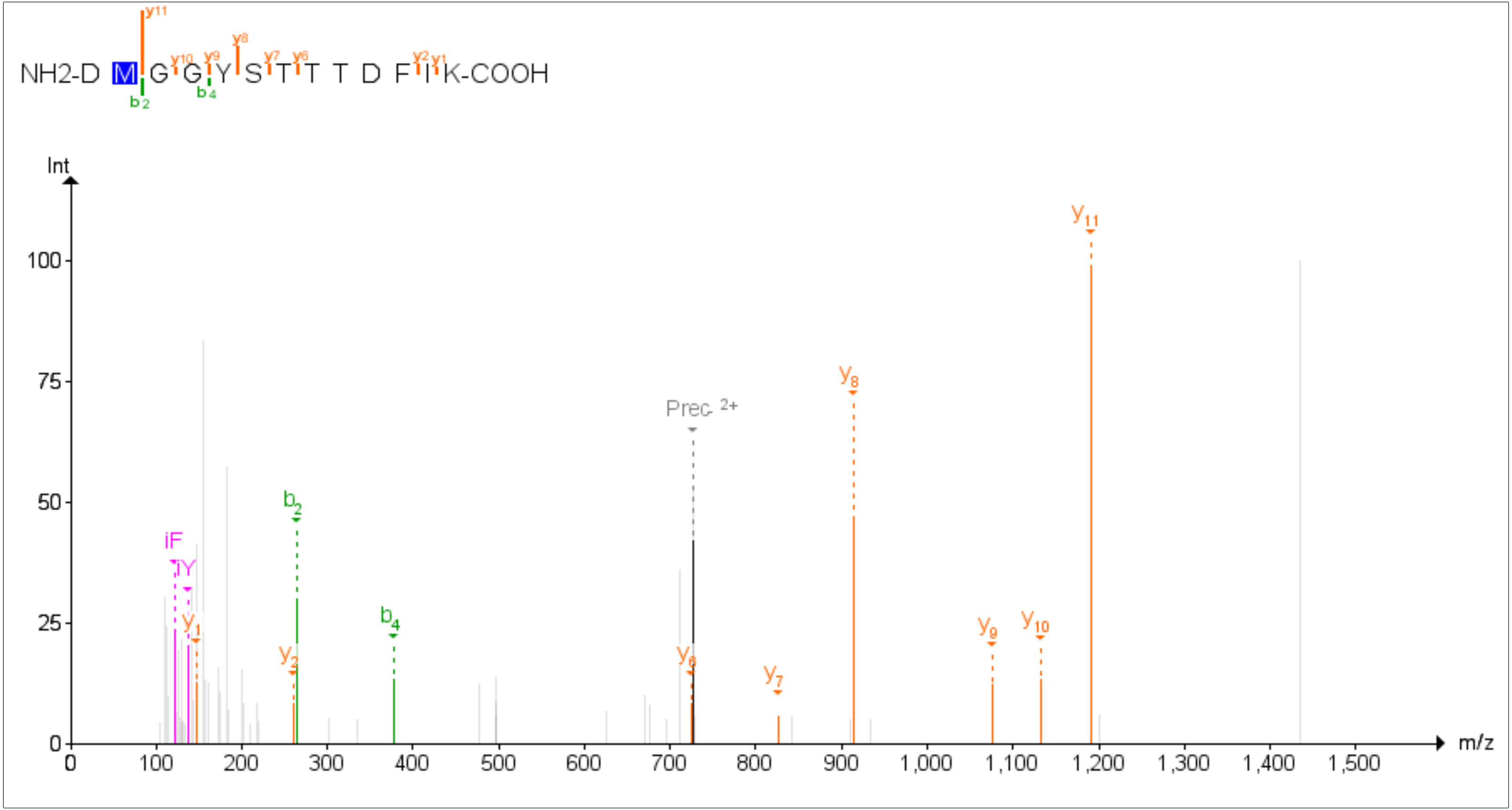

NH2-D M G G Y S T T T D F I K-COOH
y11
y10
y9
y8
y7
y6
y2
y1
b2
b4
Int
100
75
50
25
0
Prec 2+
iF
iY
m/z
0
100
200
300
400
500
600
700
800
900
1,000
1,100
1,200
1,300
1,400
1,500

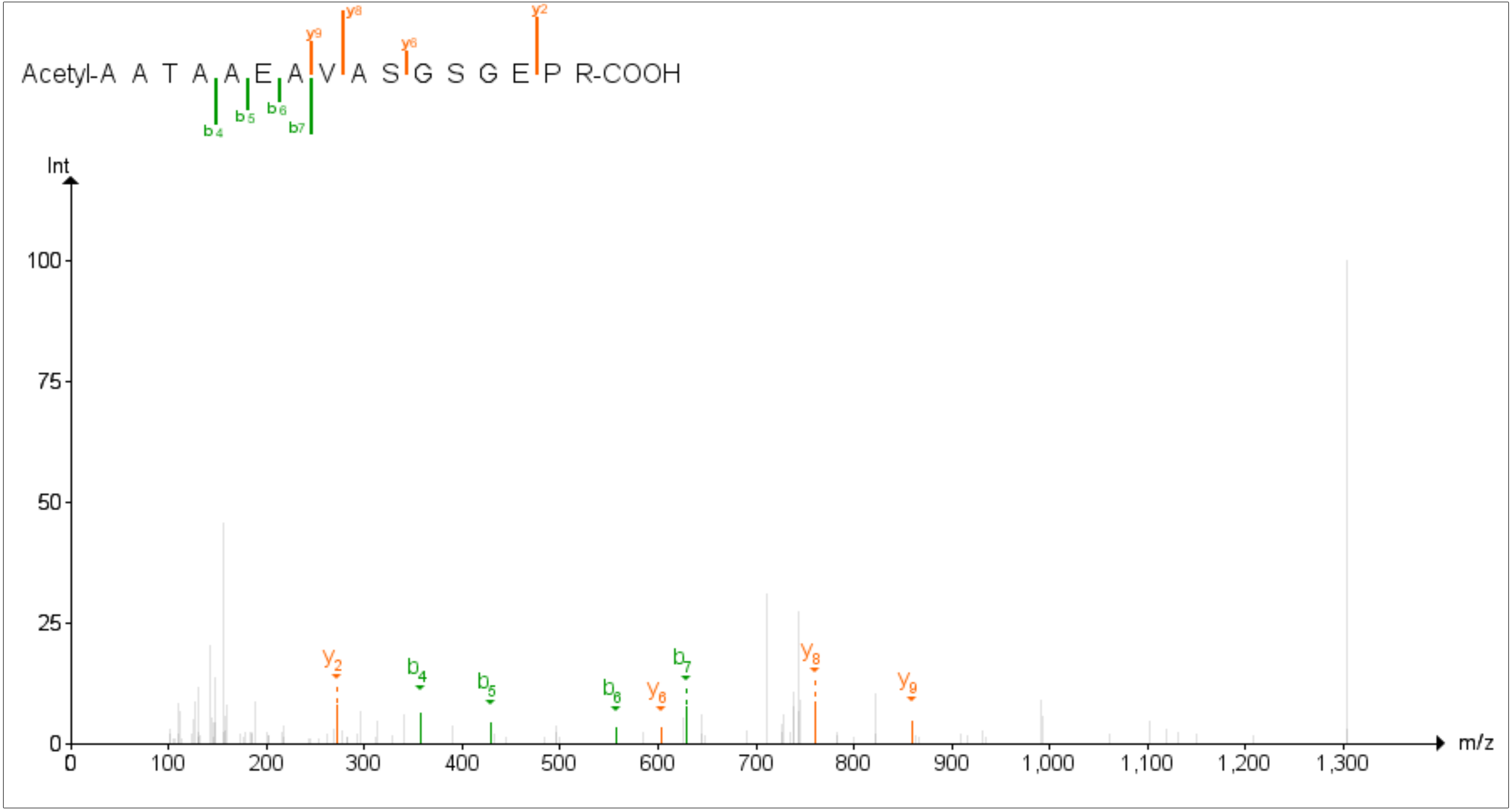

Acetyl-A A T A A E A V A S G S G E P R-COOH
y9 y8 y6 y2
b4 b5 b6 b7
Int
100
75
50
25
0
y2
b4
b5
b6
y6
b7
y8
y9
0 100 200 300 400 500 600 700 800 900 1,000 1,100 1,200 1,300
m/z

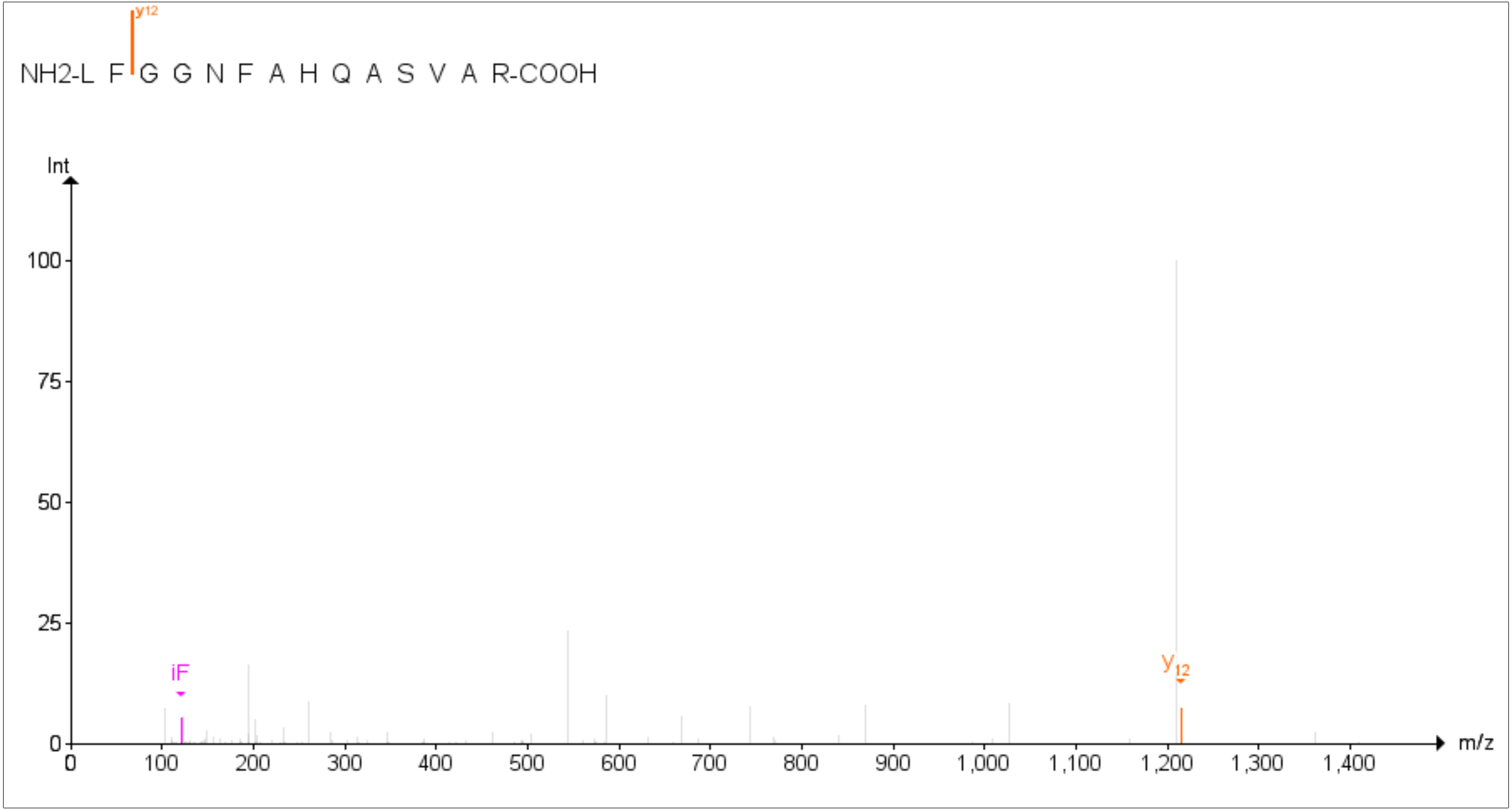

y12
NH2-L F G G N F A H Q A S V A R-COOH
Int
100
75
50
25
0
iF
y12
0
100
200
300
400
500
600
700
800
900
1,000
1,100
1,200
1,300
1,400
m/z

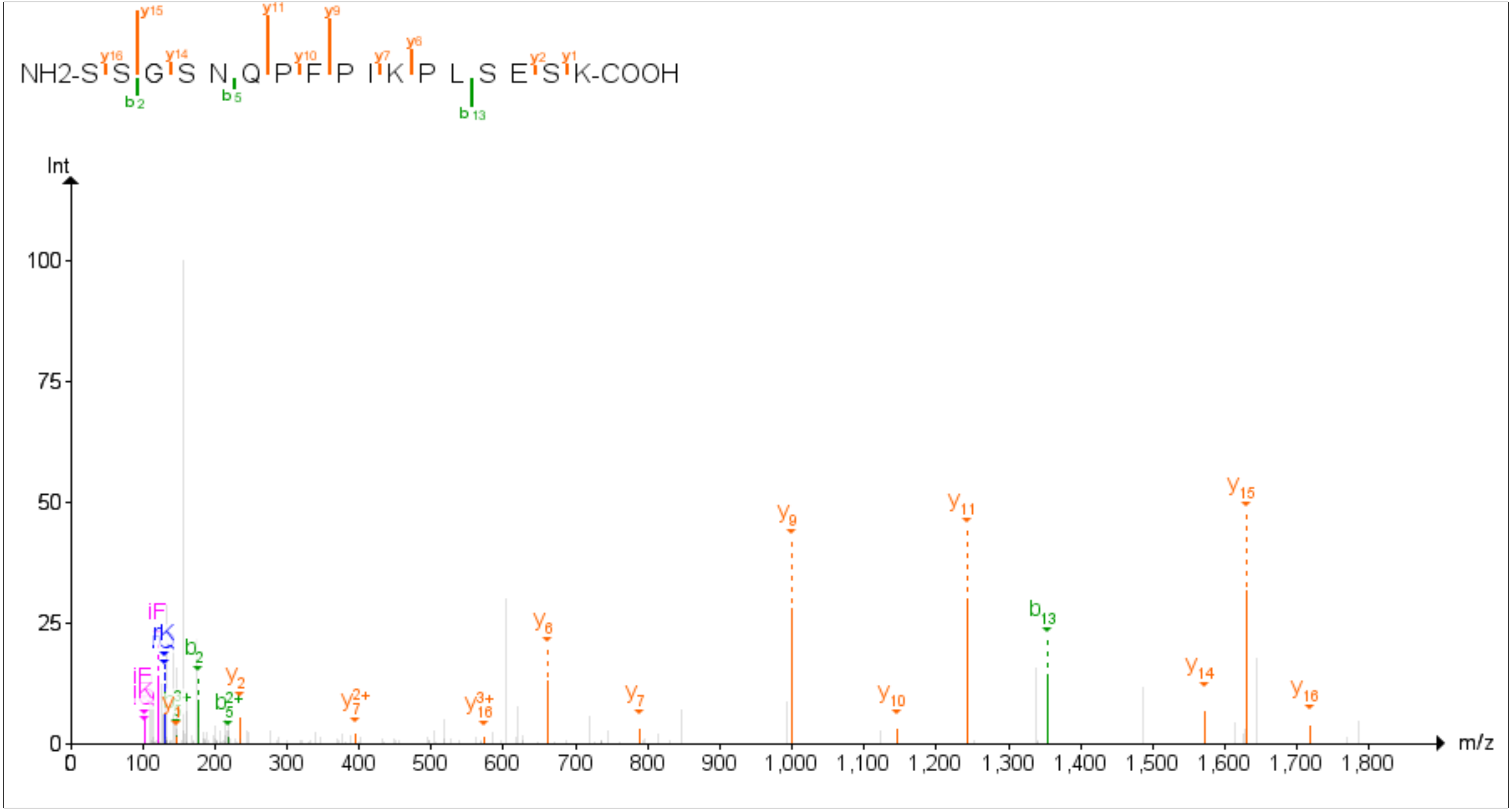
NH2-S S G S N Q P F P I K P L S E S K-COOH
Int
m/z
y15
y11
y9
y6
y14
y10
y7
y16
y2
y1
b2
b5
b13
iF
iK
rK
y2+7
y3+16
b2+5
y3+
100
75
50
25
0
0
100
200
300
400
500
600
700
800
900
1,000
1,100
1,200
1,300
1,400
1,500
1,600
1,700
1,800

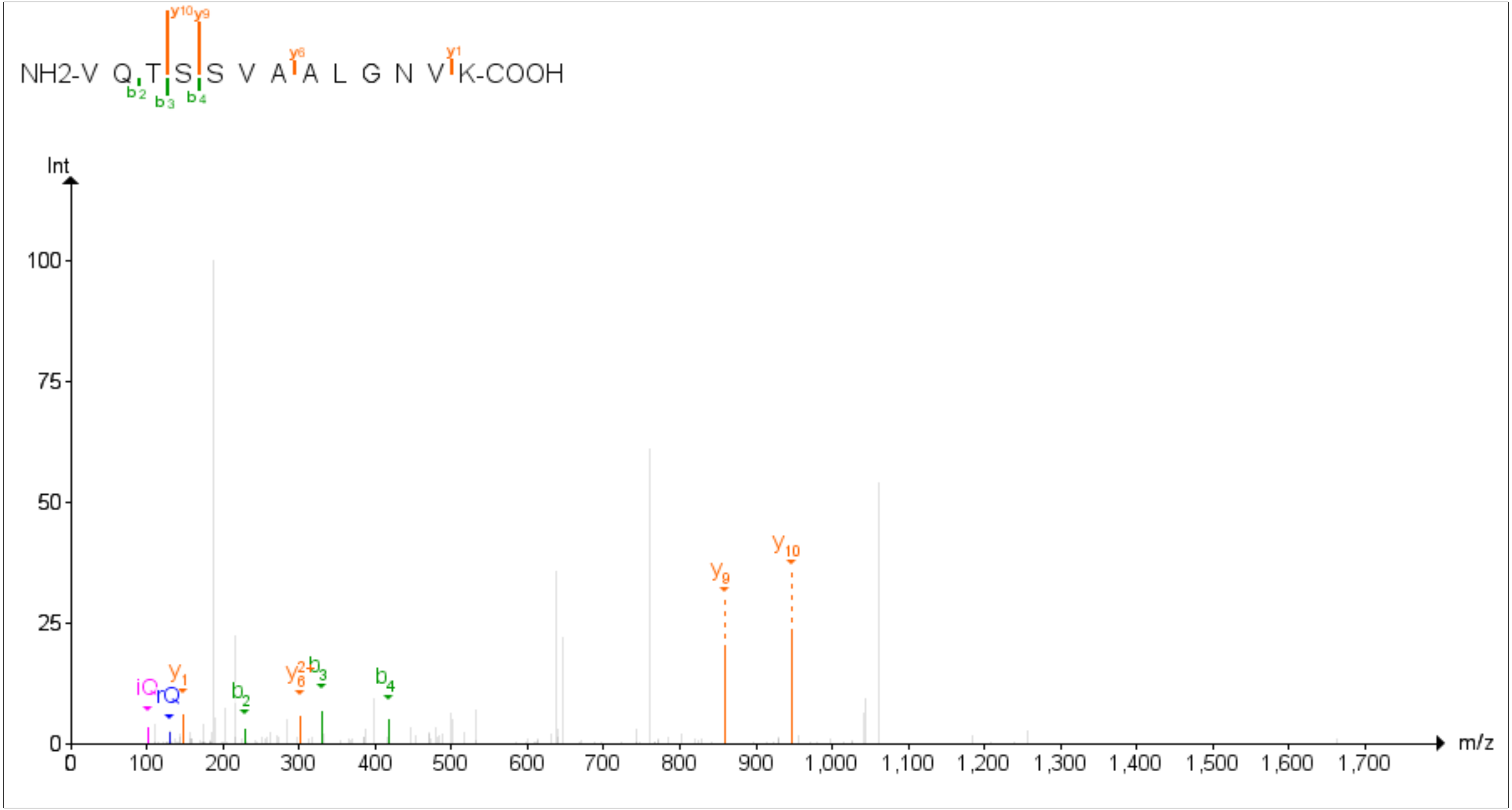

NH2-V Q T S S V A A L G N V K-COOH
y10 y9 y6 y1
b2 b3 b4
Int
100
75
50
25
0
m/z
0 100 200 300 400 500 600 700 800 900 1,000 1,100 1,200 1,300 1,400 1,500 1,600 1,700
iQ
rQ
y1
b2
y6 2+
b3
b4
y9
y10

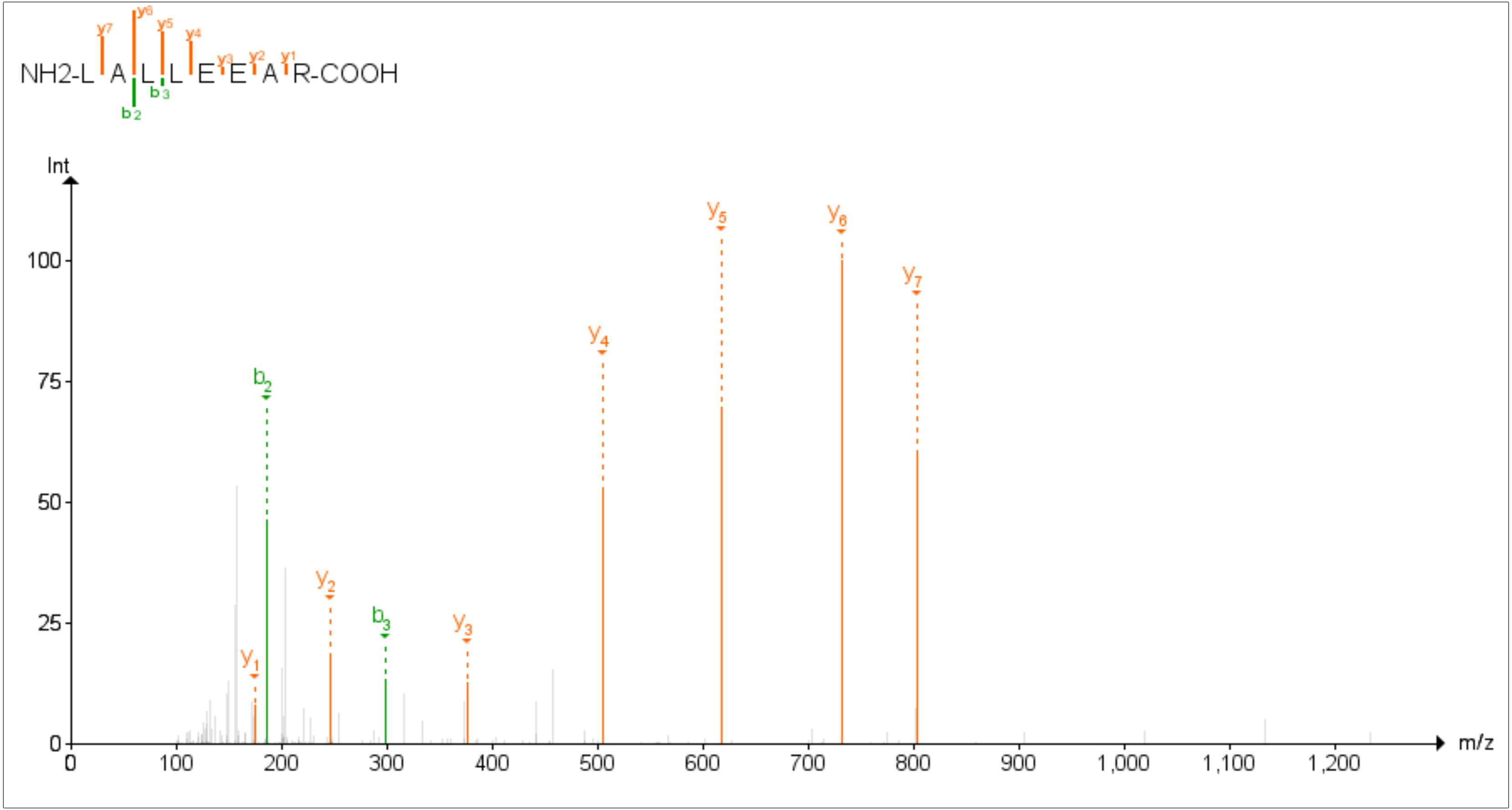

NH2-L A L L E E A R-COOH
y7 y6 y5 y4 y3 y2 y1
b2 b3
Int
100
75
50
25
0
m/z
0 100 200 300 400 500 600 700 800 900 1,000 1,100 1,200
y1 b2 y2 b3 y3 y4 y5 y6 y7

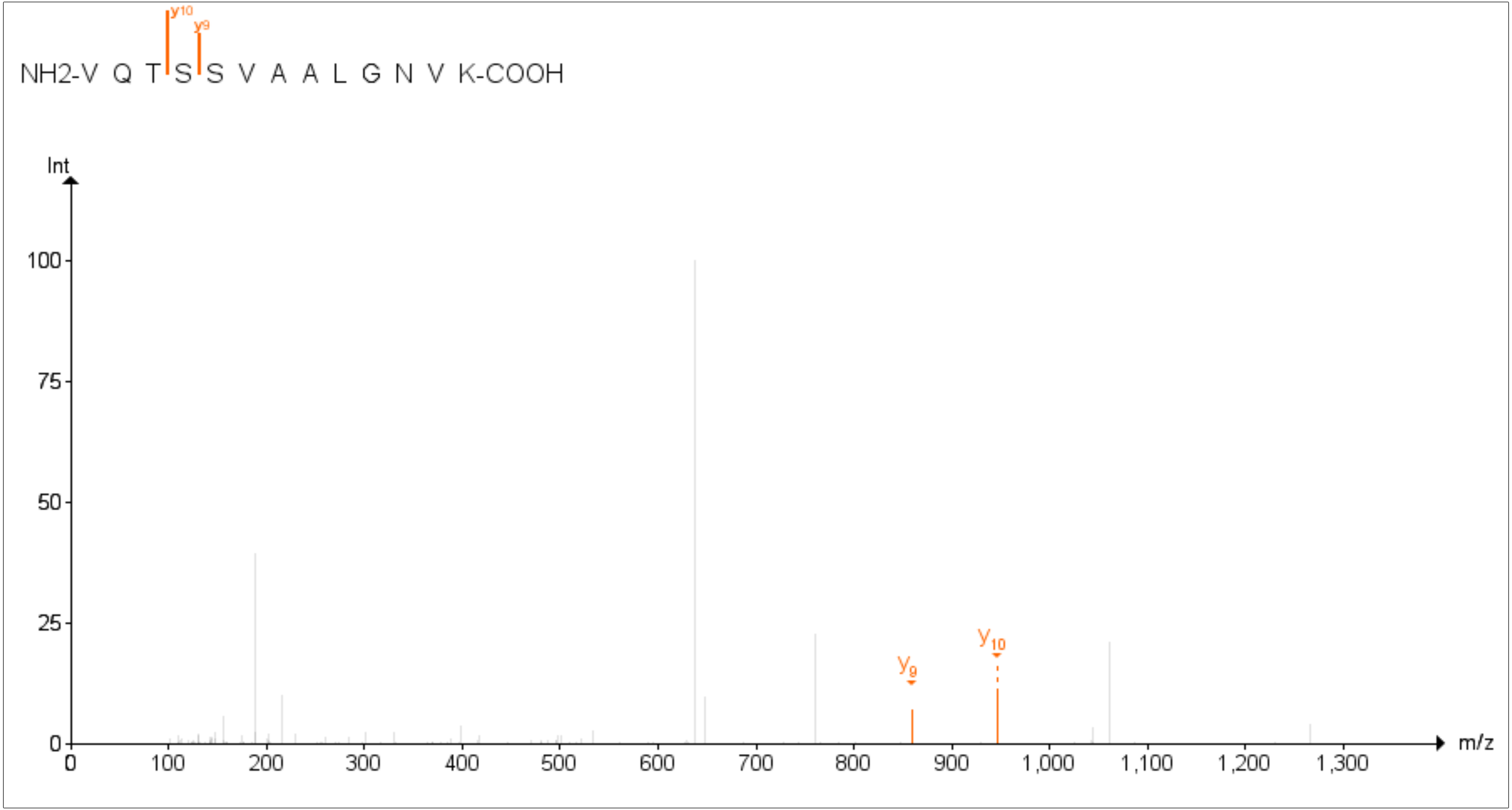
NH2-V Q T S S V A A L G N V K-COOH
y10
y9
Int
100
75
50
25
0
m/z
0
100
200
300
400
500
600
700
800
900
1,000
1,100
1,200
1,300
y9
y10

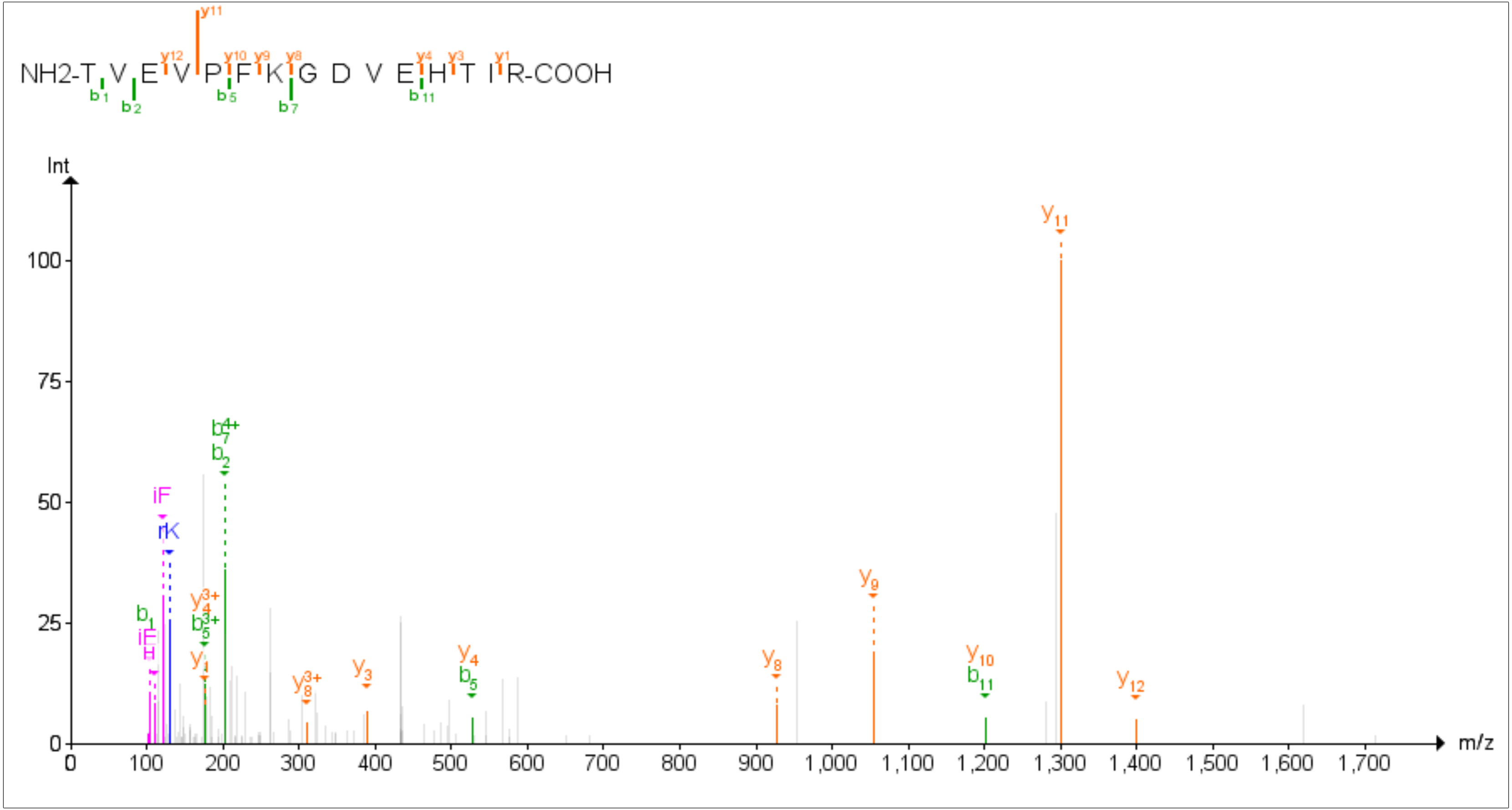

NH2-T V E V P F K G D V E H T I R-COOH
y11
y12
y10
y9
y8
y4
y3
y1
b1
b2
b5
b7
b11
Int
m/z
0
25
50
75
100
0
100
200
300
400
500
600
700
800
900
1,000
1,100
1,200
1,300
1,400
1,500
1,600
1,700

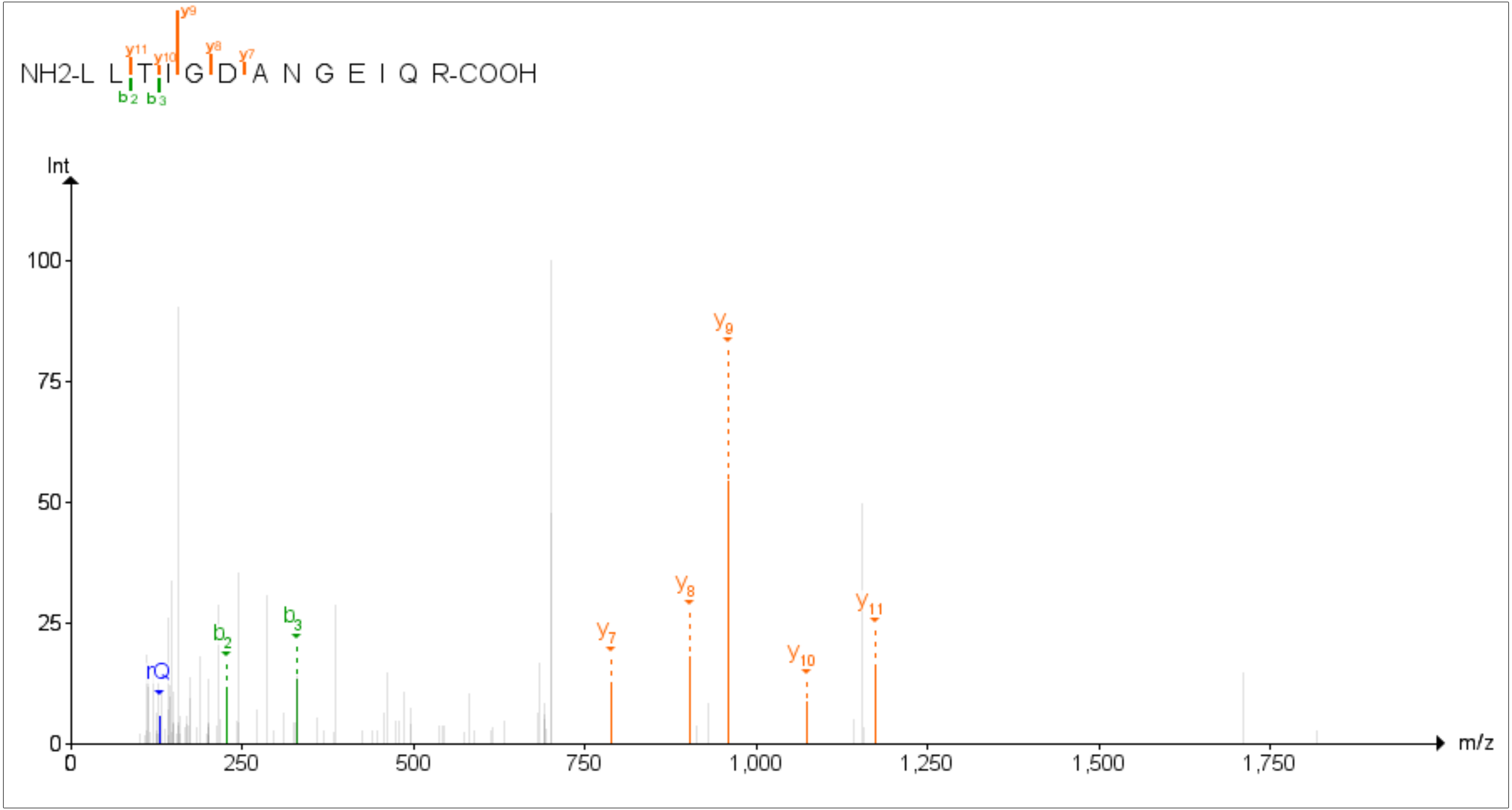
NH2-L L T I G D A N G E I Q R-COOH
y9
y11
y10
y8
y7
b2
b3
Int
100
75
50
25
0
rQ
b2
b3
y7
y8
y9
y10
y11
0
250
500
750
1,000
1,250
1,500
1,750
m/z

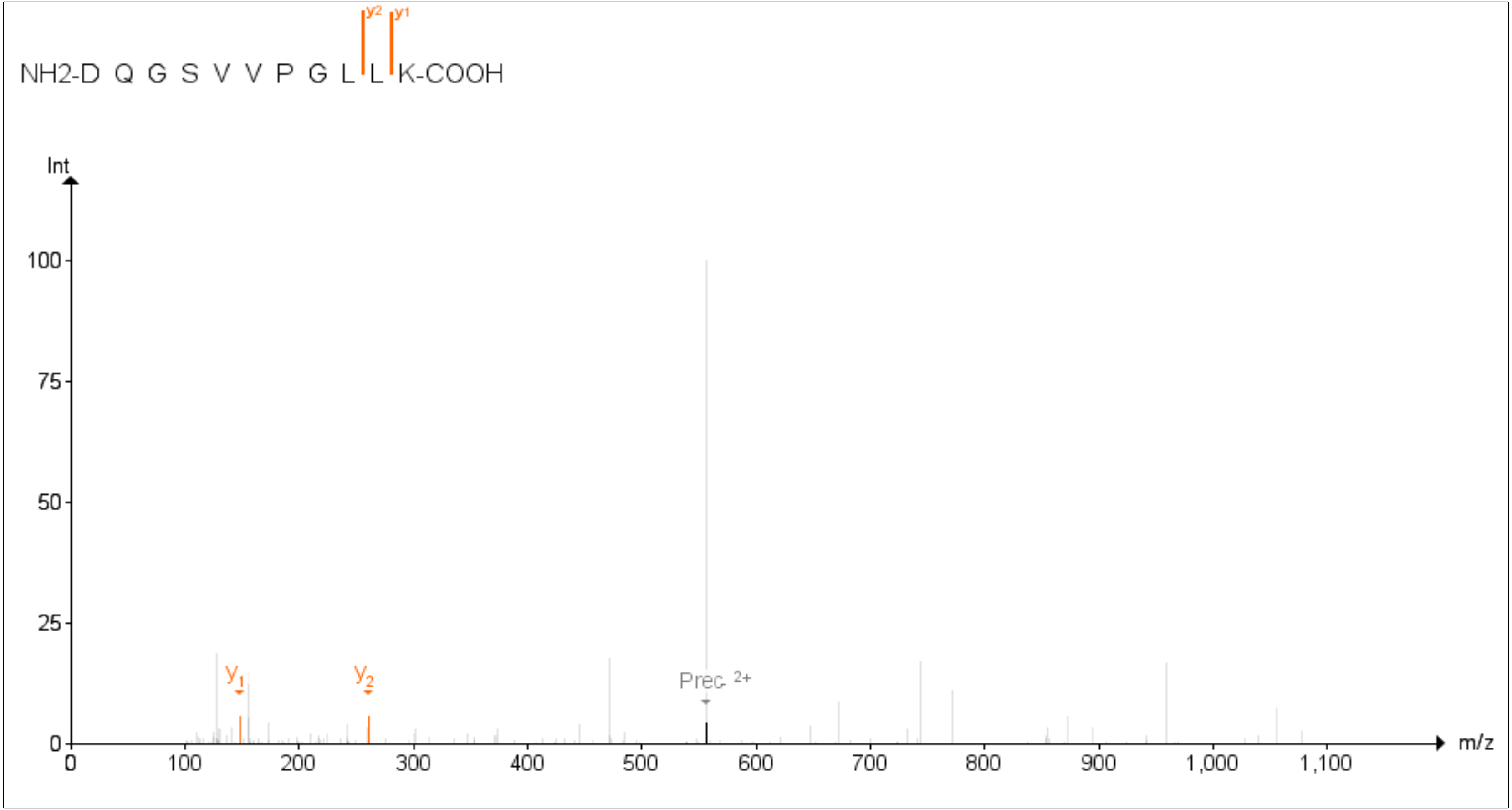

y2
y1
NH2-D Q G S V V P G L L K-COOH
Int
100
75
50
25
0
y1
y2
Prec 2+
0
100
200
300
400
500
600
700
800
900
1,000
1,100
m/z

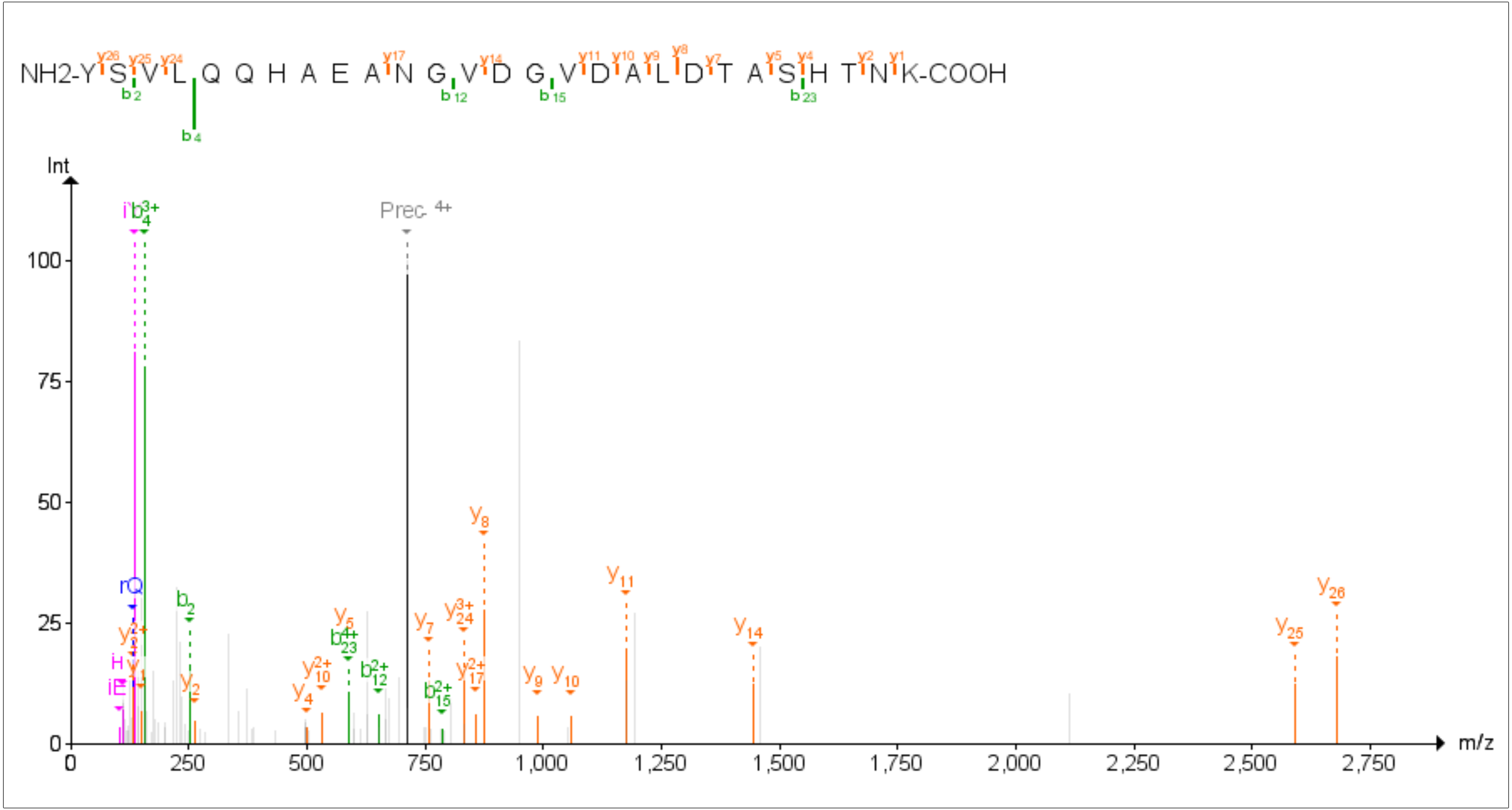

NH2-Y S V L Q Q H A E A N G V D G V D A L D T A S H T N K-COOH
Int
m/z
Prec 4+

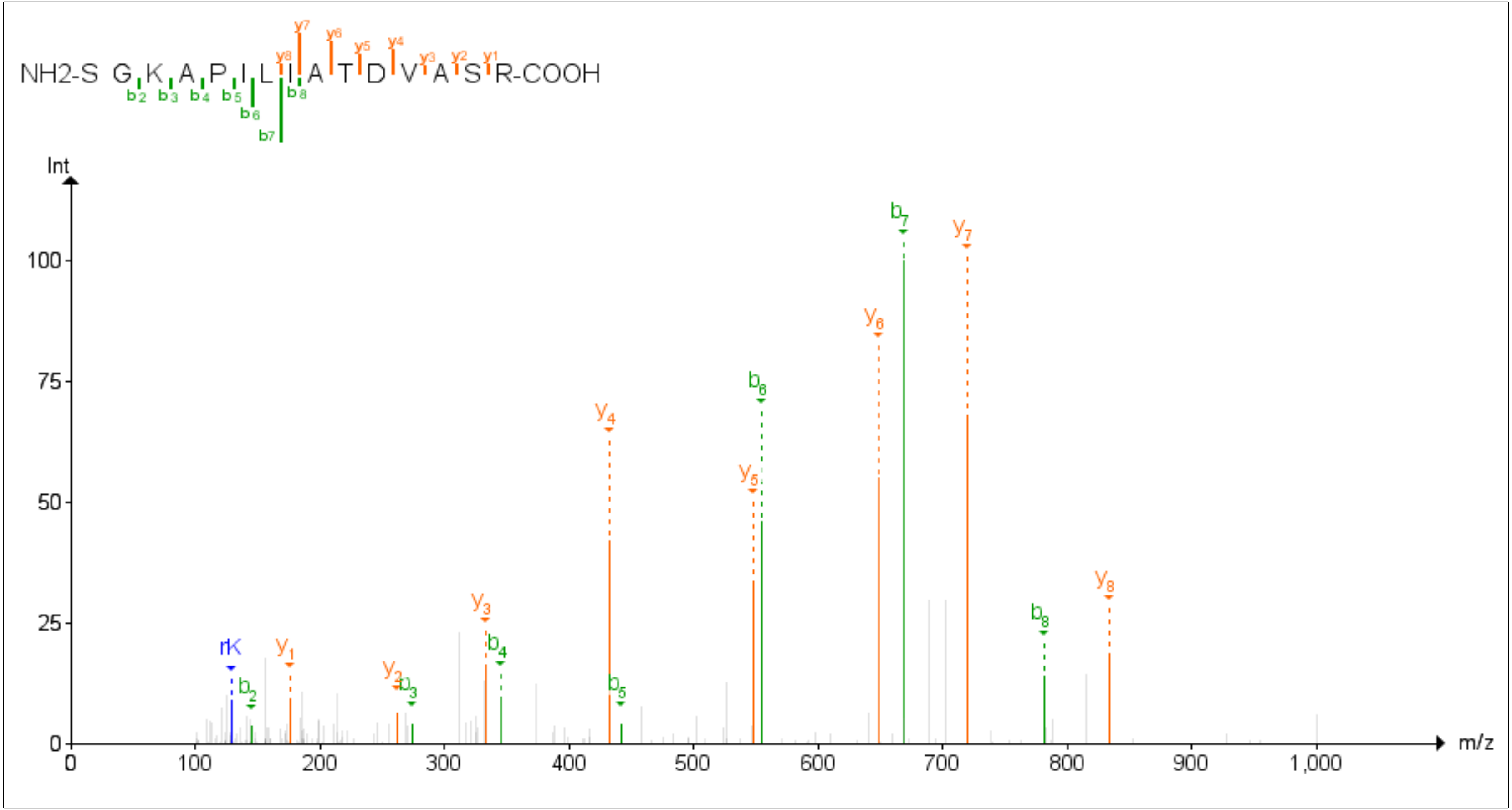
NH2-S G K A P I L I A T D V A S R-COOH
b2 b3 b4 b5 b6 b7 b8
y8 y7 y6 y5 y4 y3 y2 y1
Int
m/z
0
25
50
75
100
0
100
200
300
400
500
600
700
800
900
1,000
rK
y1
y2
y3
y4
y5
y6
y7
y8
b2
b3
b4
b5
b6
b7
b8

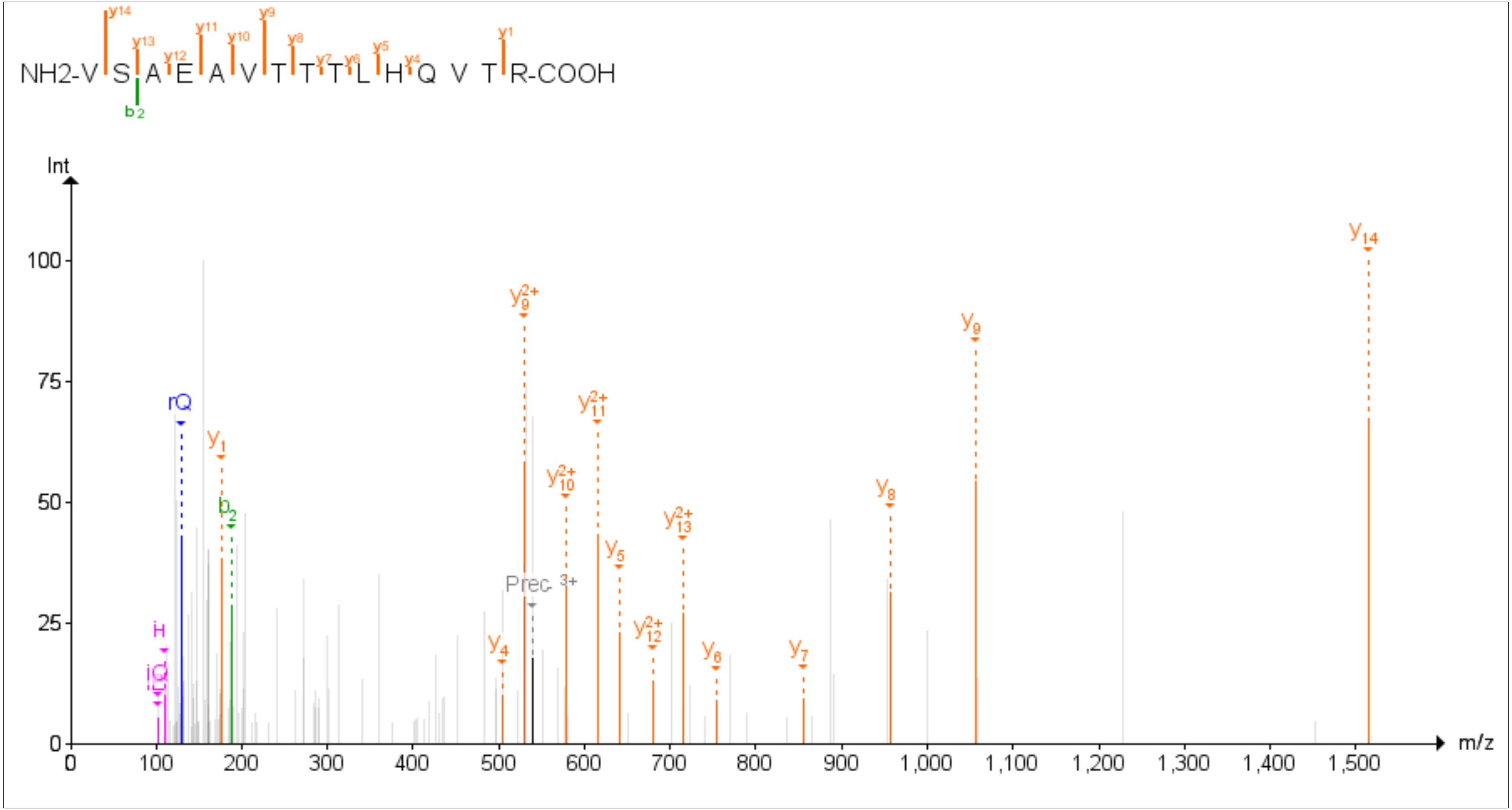

NH2-V S A E A V T T T L H Q V T R-COOH
y14
y13
y12
y11
y10
y9
y8
y7
y6
y5
y4
y1
b2
Int
m/z
Prec 3+
rQ
iQ
H

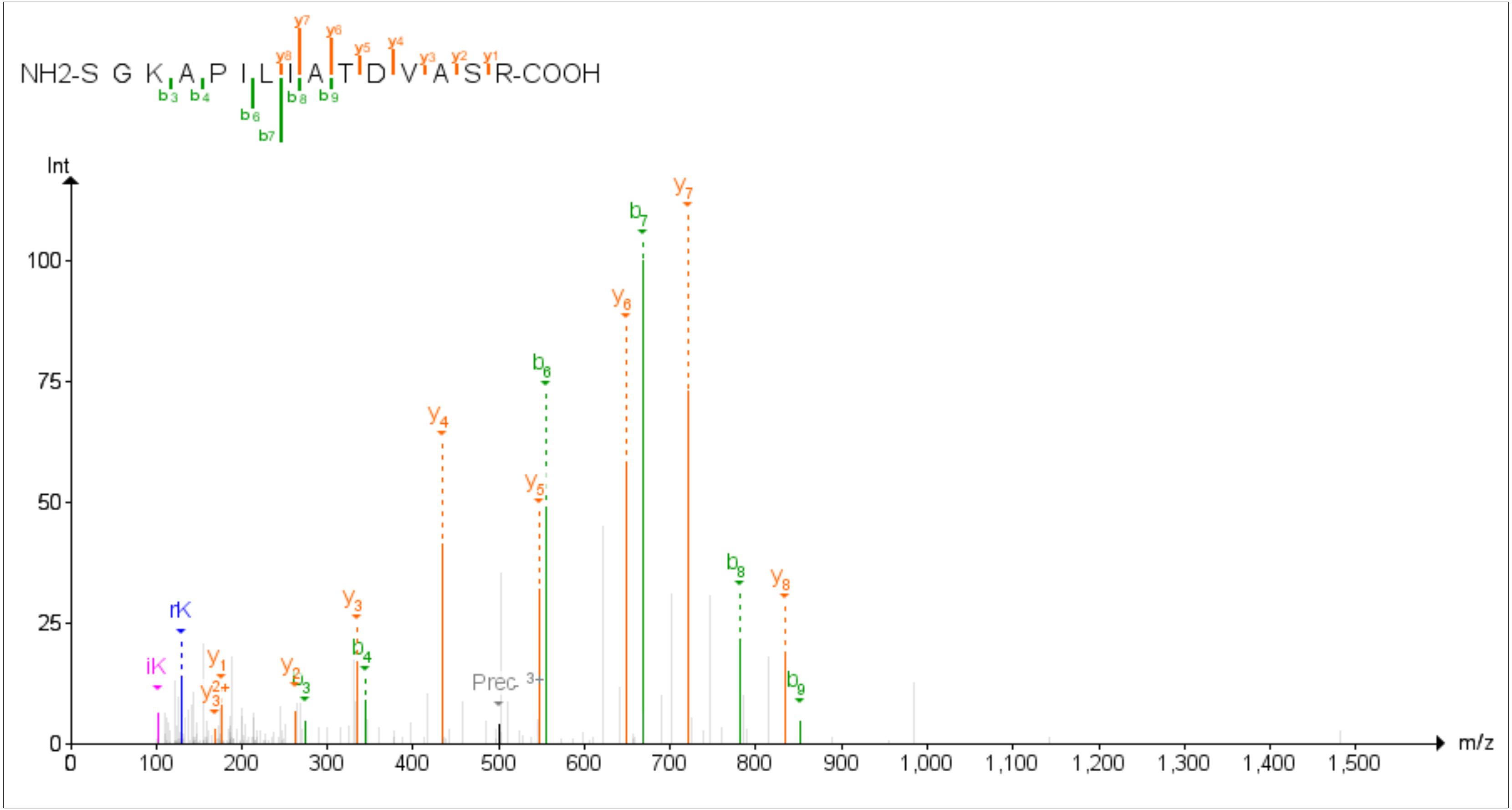

NH2-S G K A P I L I A T D V A S R-COOH
y7 y6 y5 y4 y3 y2 y1 y8
b3 b4 b6 b7 b8 b9
Int
m/z
iK rK y1 y3 2+ y2 b3 y3 b4 Prec 3+ y4 y5 b6 y6 b7 y7 b8 y8 b9
0 25 50 75 100
0 100 200 300 400 500 600 700 800 900 1,000 1,100 1,200 1,300 1,400 1,500

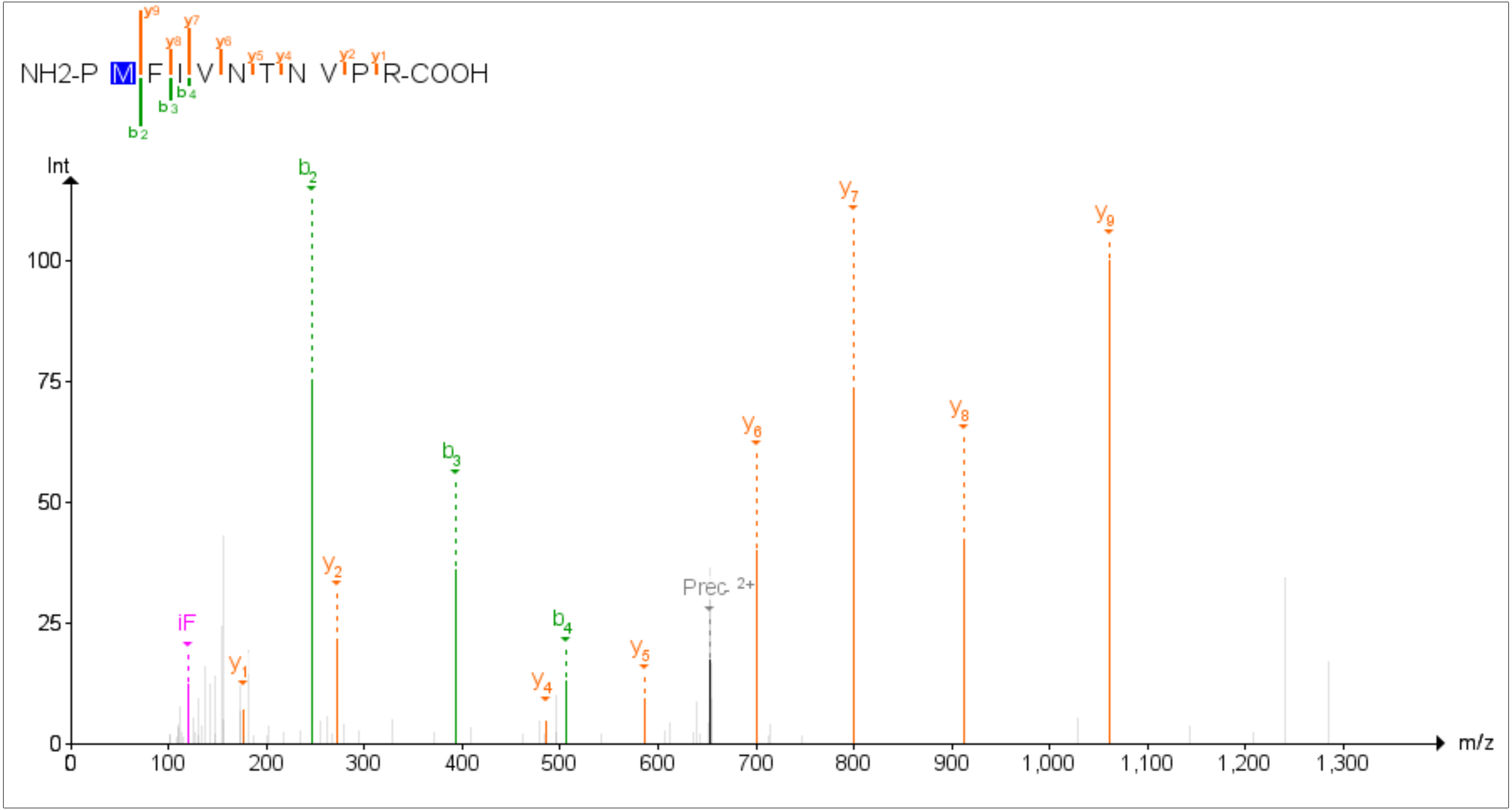

NH2-P M F I V N T N V P R-COOH
y9 y8 y7 y6 y5 y4 y2 y1
b2 b3 b4
Int
100
75
50
25
0
b2
y7
y9
y8
y6
b3
y2
Prec 2+
iF
b4
y5
y1
y4
0 100 200 300 400 500 600 700 800 900 1,000 1,100 1,200 1,300
m/z

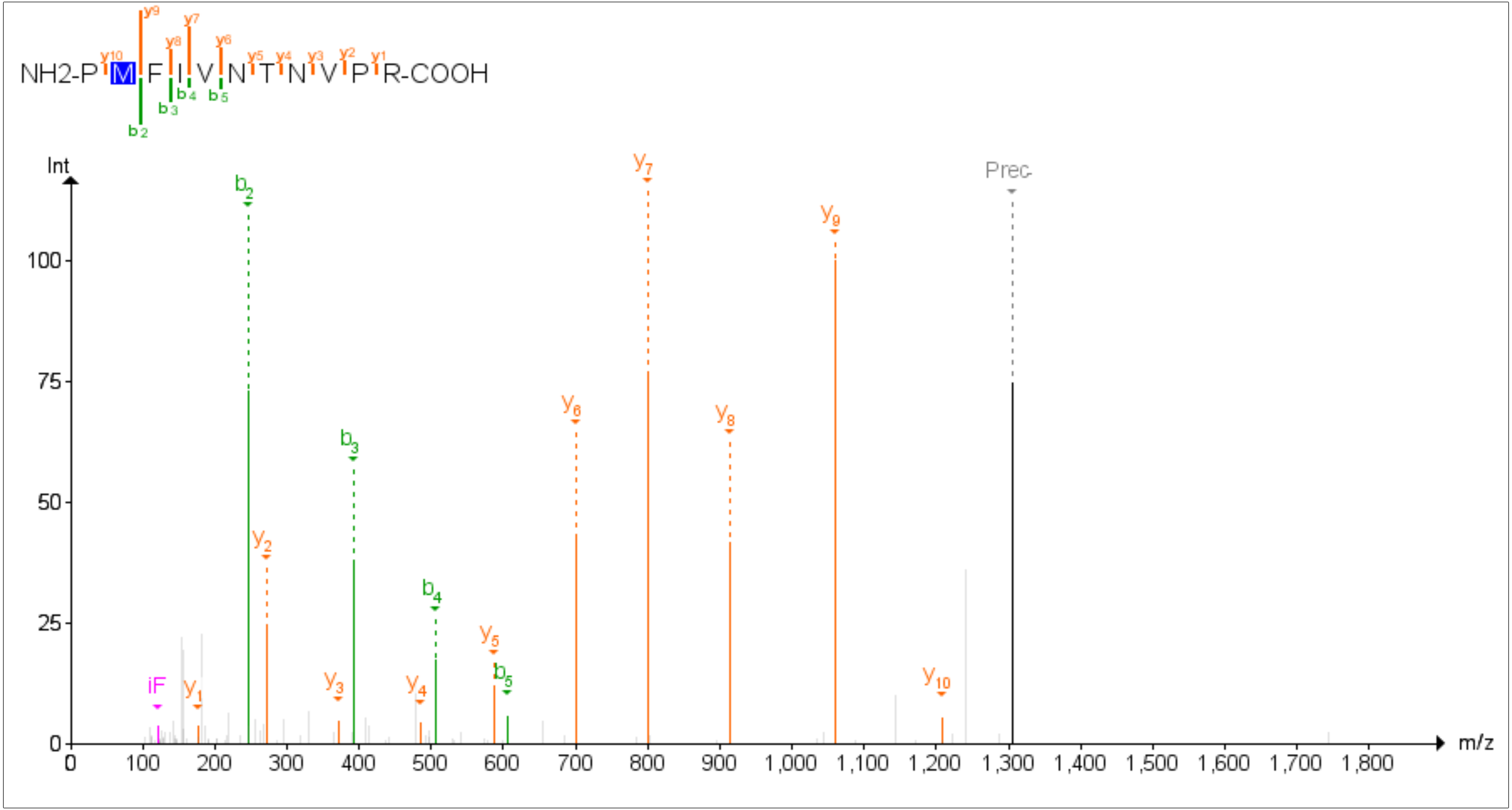

NH2-P M F I V N T N V P R-COOH
y10 y9 y8 y7 y6 y5 y4 y3 y2 y1
b2 b3 b4 b5
Int
m/z
Prec
0
25
50
75
100
0 100 200 300 400 500 600 700 800 900 1,000 1,100 1,200 1,300 1,400 1,500 1,600 1,700 1,800

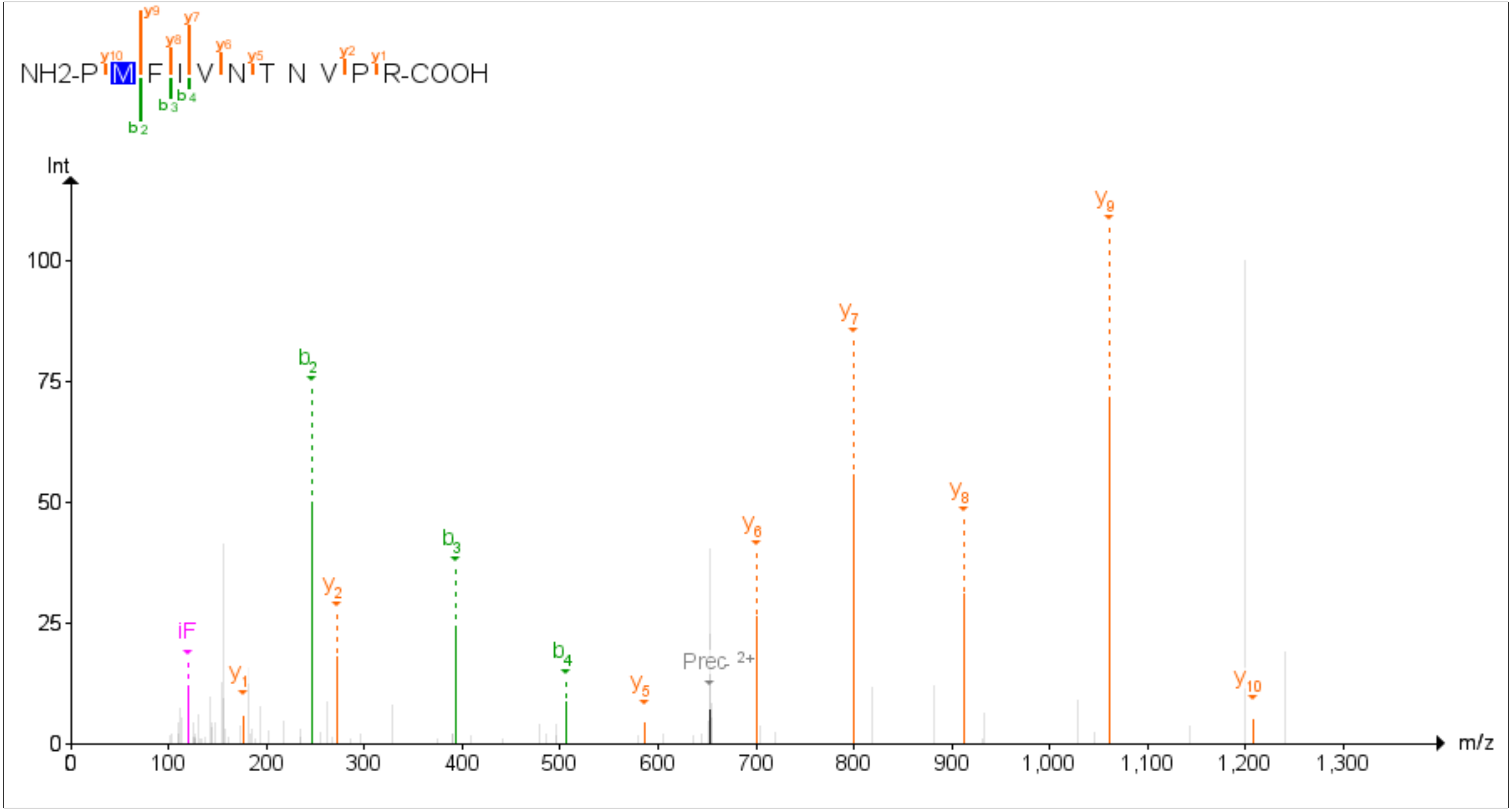

NH2-P M F I V N T N V P R-COOH
y10 y9 y8 y7 y6 y5 y2 y1
b2 b3 b4
Int
m/z
iF
y1 y2 y5 y6 y7 y8 y9 y10
b2 b3 b4
Prec 2+
0
25
50
75
100
0
100
200
300
400
500
600
700
800
900
1,000
1,100
1,200
1,300

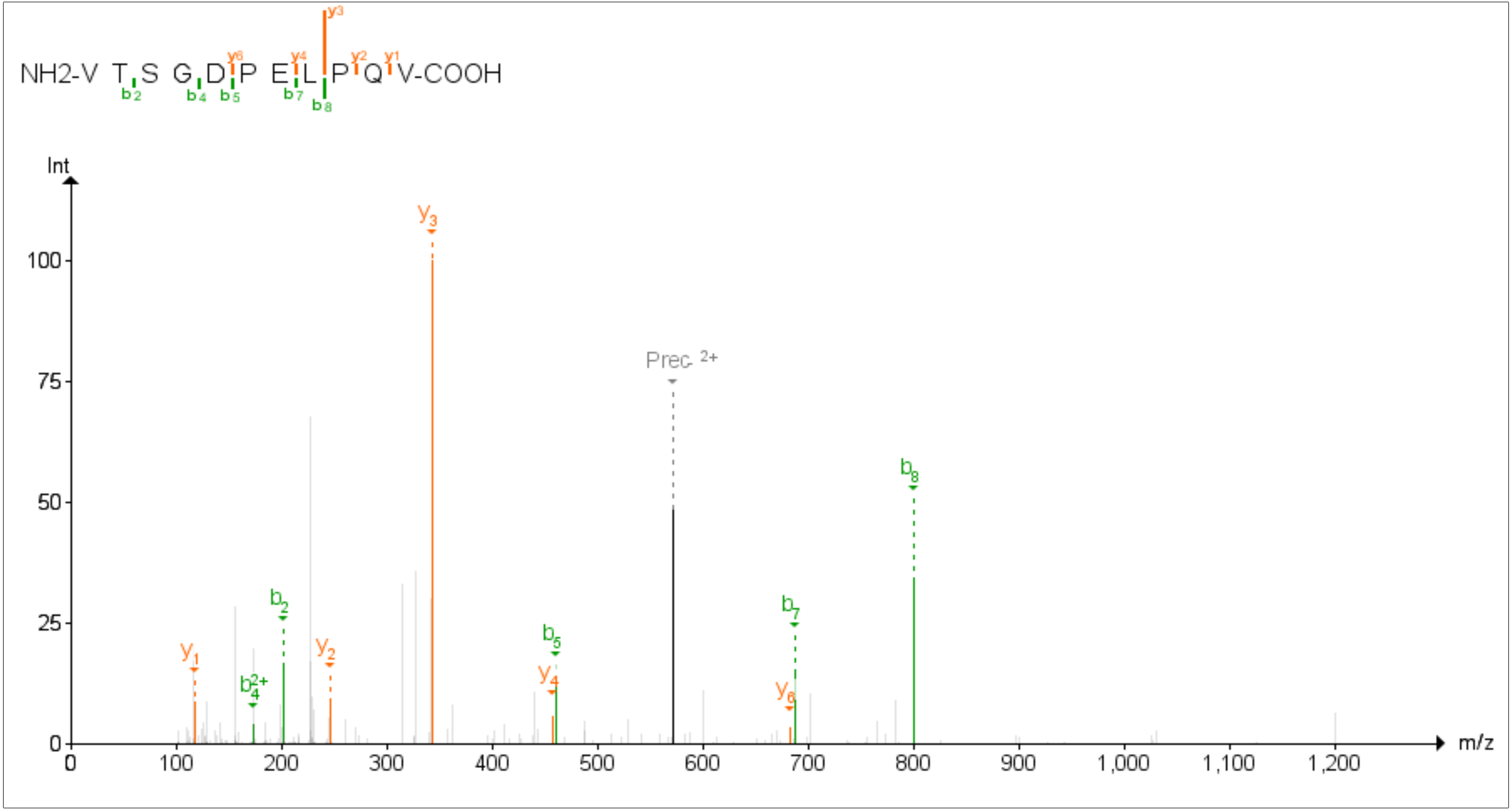

NH2-V T S G D P E L P Q V-COOH
b2 b4 b5 b7 b8
y6 y4 y3 y2 y1
Int
m/z
0
25
50
75
100
0
100
200
300
400
500
600
700
800
900
1,000
1,100
1,200
Prec. 2+

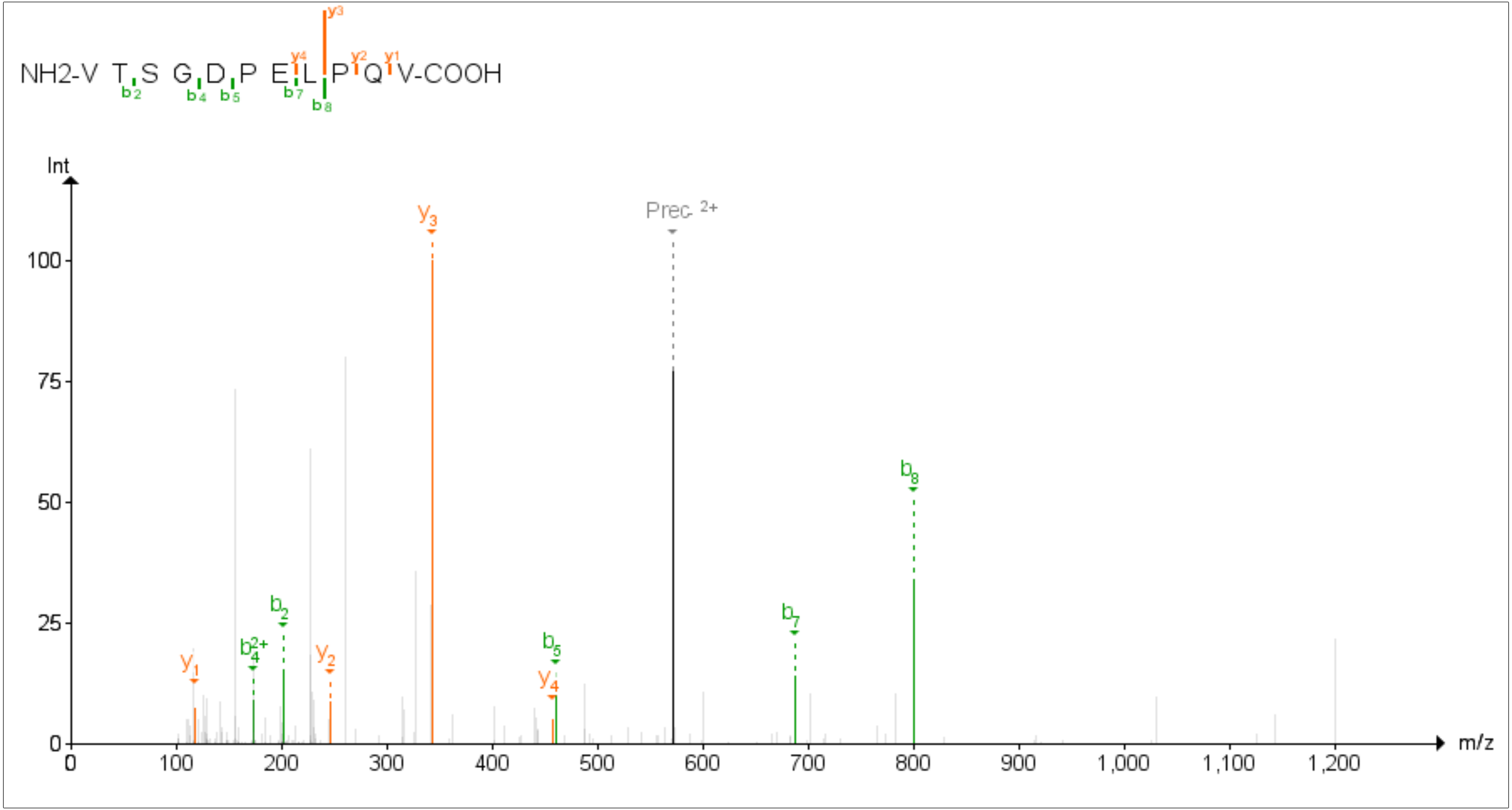

NH2-V T S G D P E L P Q V-COOH
b2 b4 b5 b7 b8
y4 y3 y2 y1
Int
m/z
Prec 2+
y3
y1
b4 2+
b2
y2
b5
y4
b7
b8
0
25
50
75
100
0
100
200
300
400
500
600
700
800
900
1,000
1,100
1,200

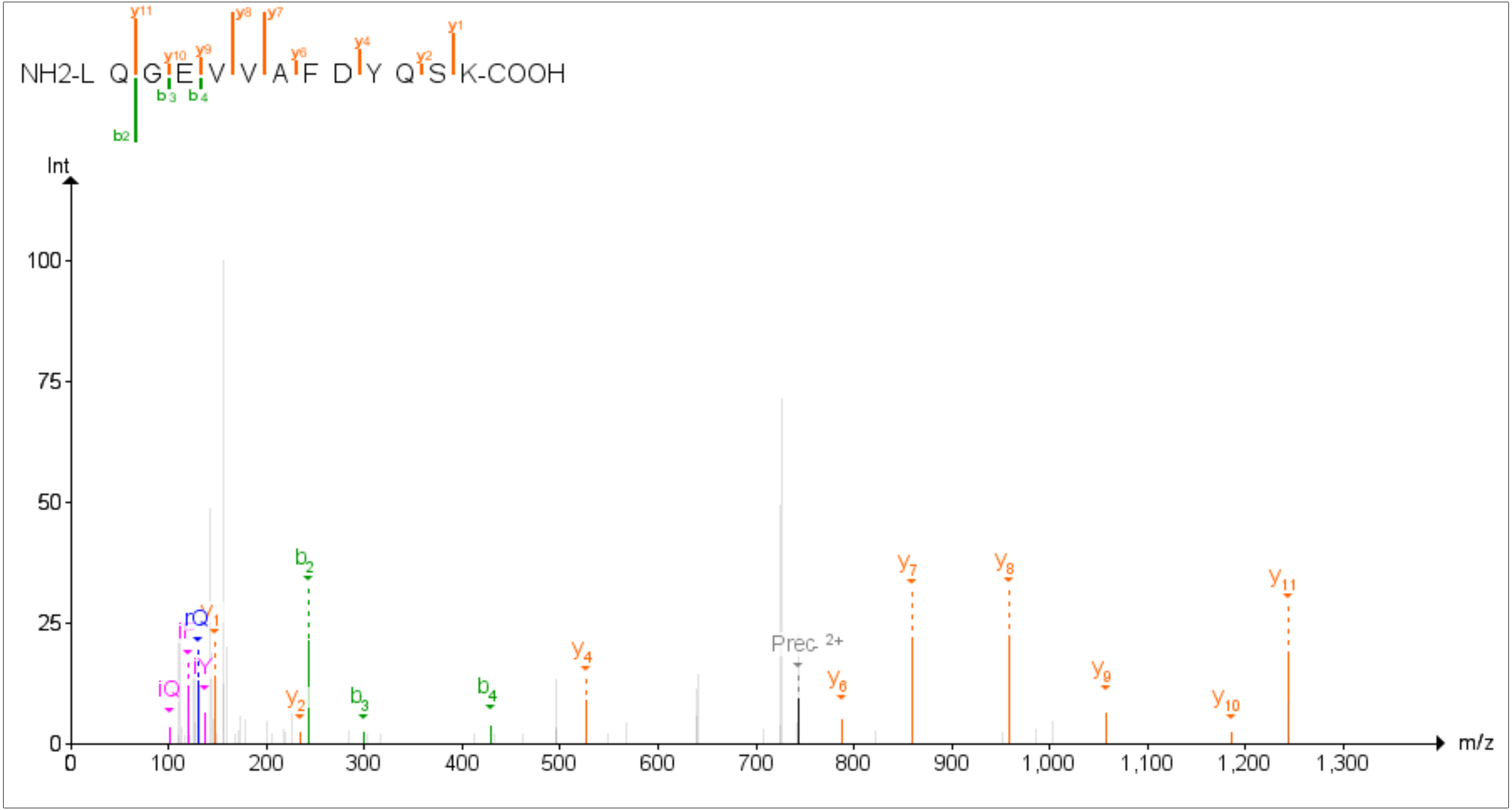

NH2-L Q G E V V A F D Y Q S K-COOH
y11
y10
y9
y8
y7
y6
y4
y2
y1
b2
b3
b4
Int
100
75
50
25
0
m/z
0
100
200
300
400
500
600
700
800
900
1,000
1,100
1,200
1,300
Prec 2+
iQ
iF
rQ
iY

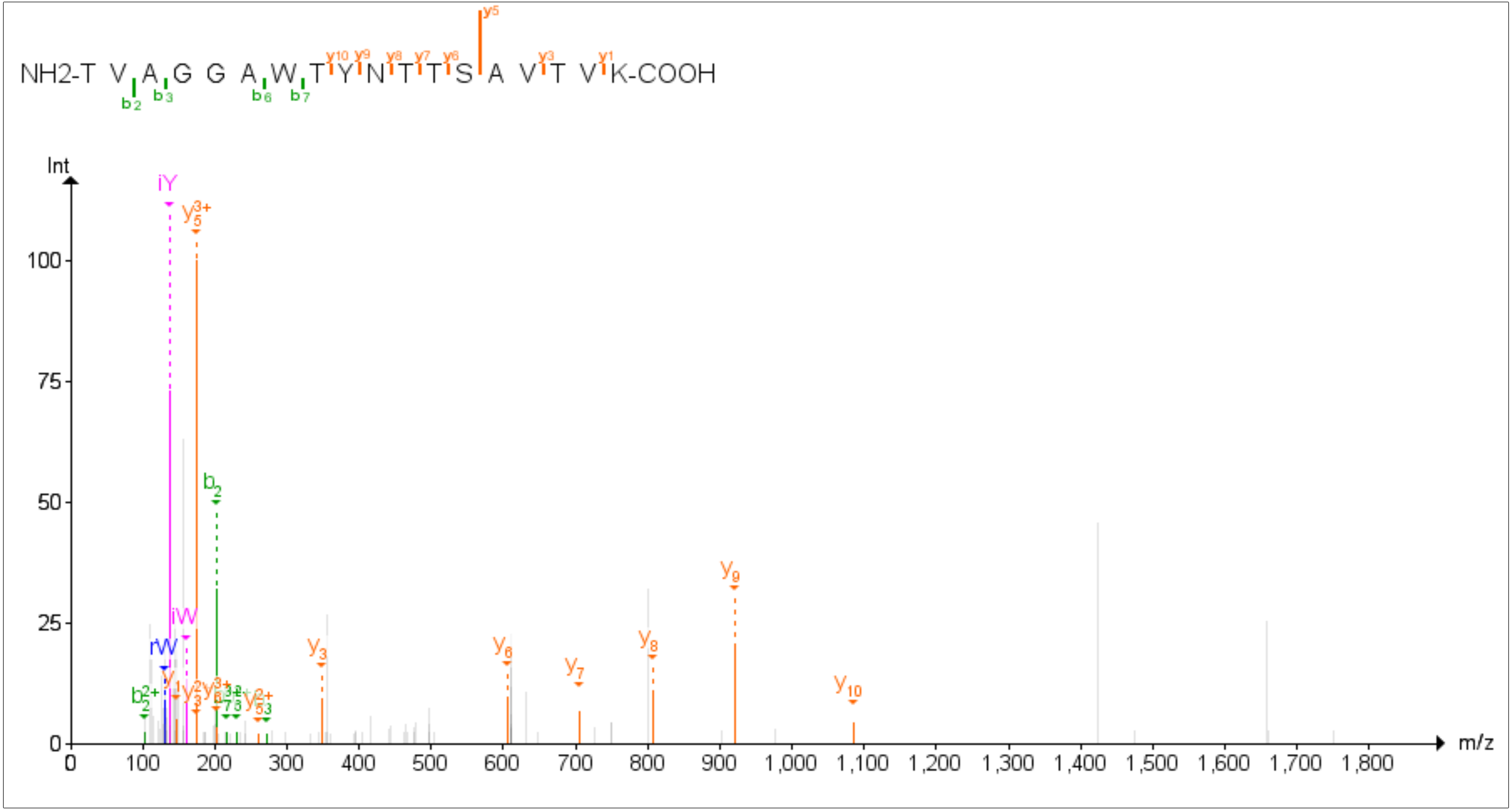

NH2-T V A G G A W T Y N T T S A V T V K-COOH
b2 b3 b6 b7
y10 y9 y8 y7 y6 y5 y3 y1
Int
m/z
iY
iW
rW
b2
y3
y6
y7
y8
y9
y10
0
25
50
75
100
0
100
200
300
400
500
600
700
800
900
1,000
1,100
1,200
1,300
1,400
1,500
1,600
1,700
1,800

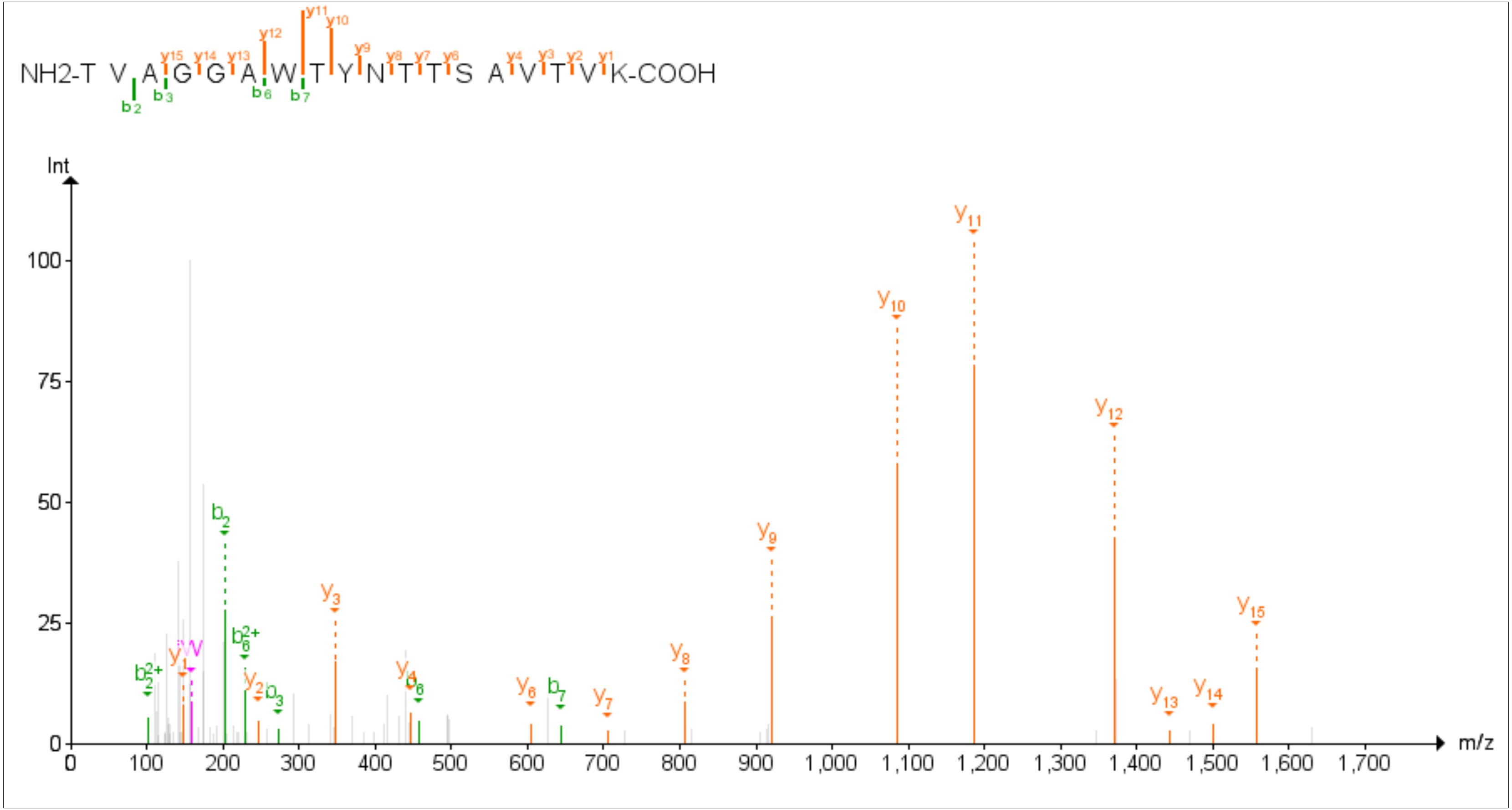

NH2-T V A G G A W T Y N T T S A V T V K-COOH
Int
m/z
y11
y10
y12
y9
y3
y15
b2
y8

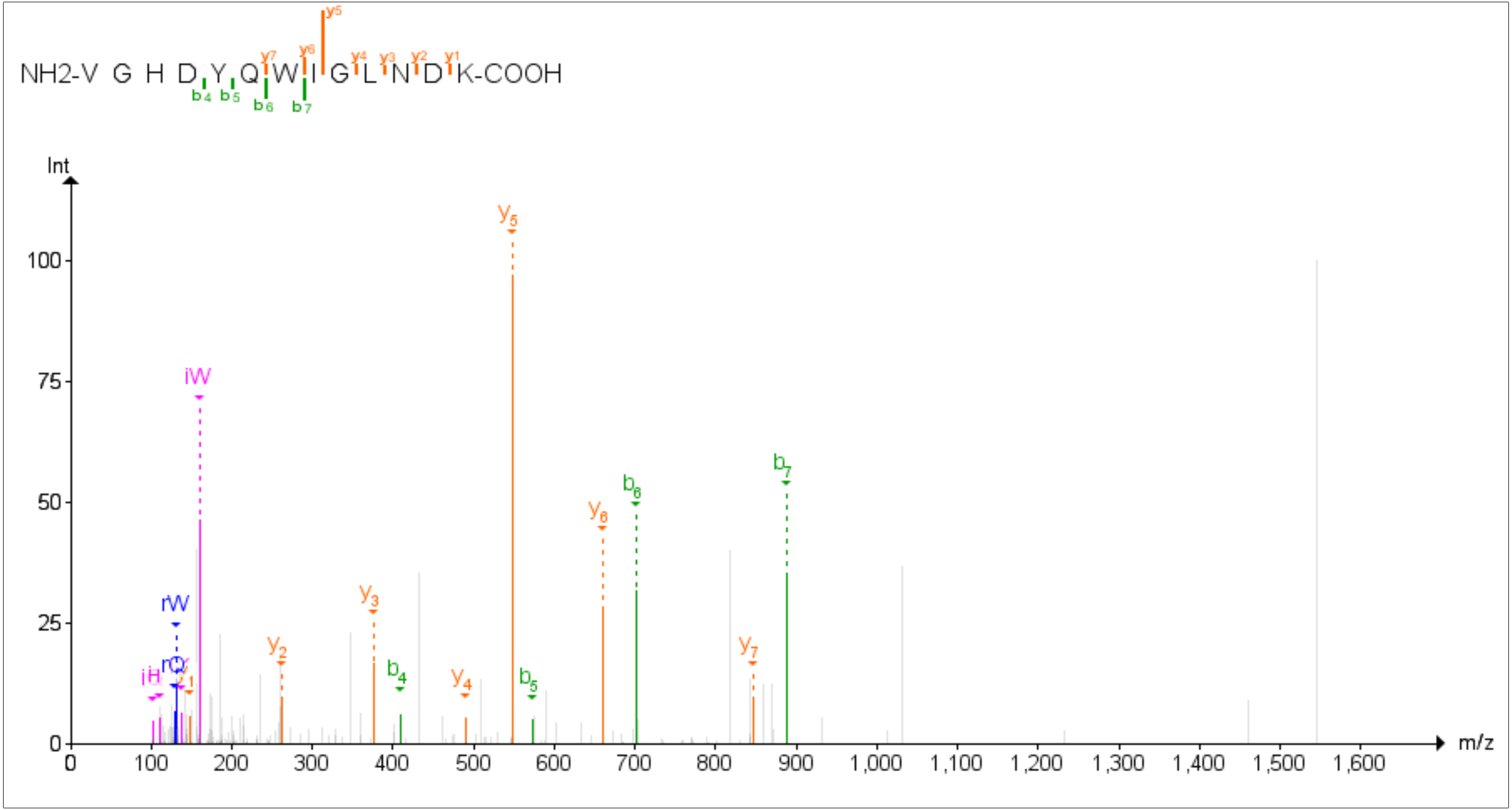

NH2-V G H D Y Q W I G L N D K-COOH
y7 y6 y5 y4 y3 y2 y1
b4 b5 b6 b7
Int
m/z
0
25
50
75
100
0
100
200
300
400
500
600
700
800
900
1,000
1,100
1,200
1,300
1,400
1,500
1,600
iW
rW
rQ
iH
y5
y6
y3
y2
y4
y7
b4
b5
b6
b7

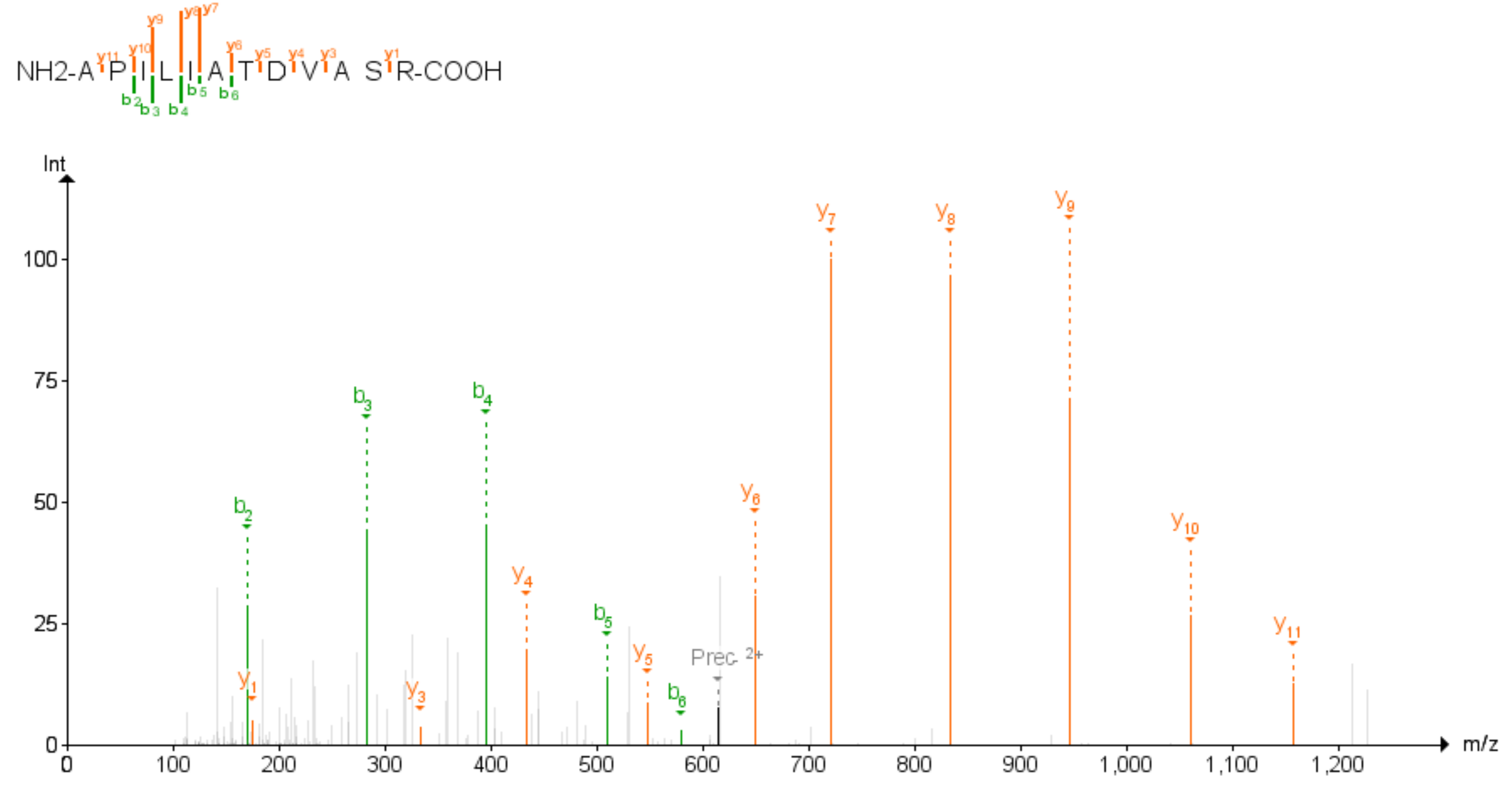
NH2-A P I L I A T D V A S R-COOH
Int
m/z
0
25
50
75
100
0
100
200
300
400
500
600
700
800
900
1,000
1,100
1,200
Prec 2+

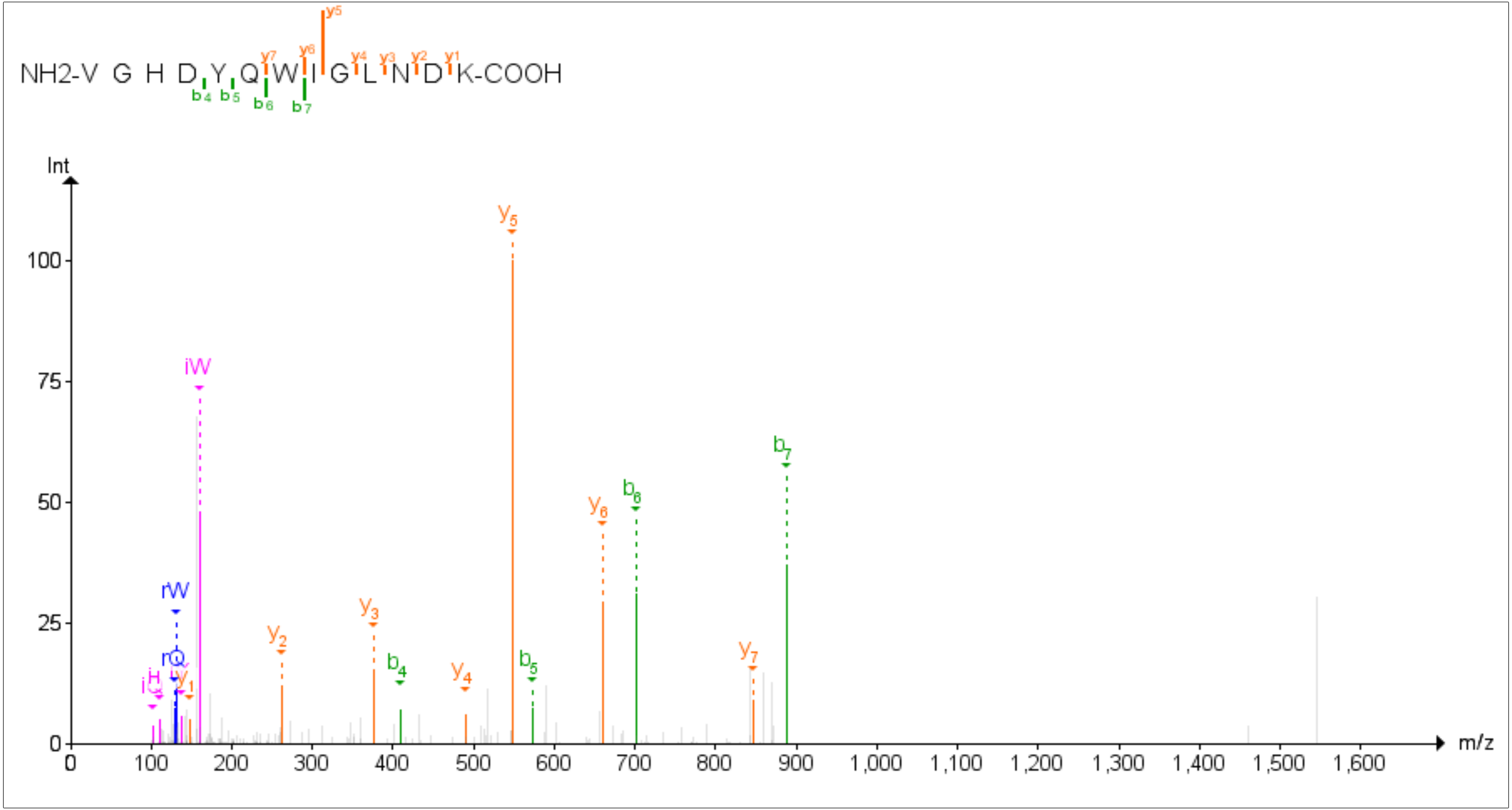

NH2-V G H D Y Q W I G L N D K-COOH
y5
y7
y6
y4
y3
y2
y1
b4
b5
b6
b7
Int
100
75
50
25
0
iW
rW
rQ
iH
iQ
y1
y2
y3
b4
y4
y5
b5
y6
b6
y7
b7
0
100
200
300
400
500
600
700
800
900
1,000
1,100
1,200
1,300
1,400
1,500
1,600
m/z

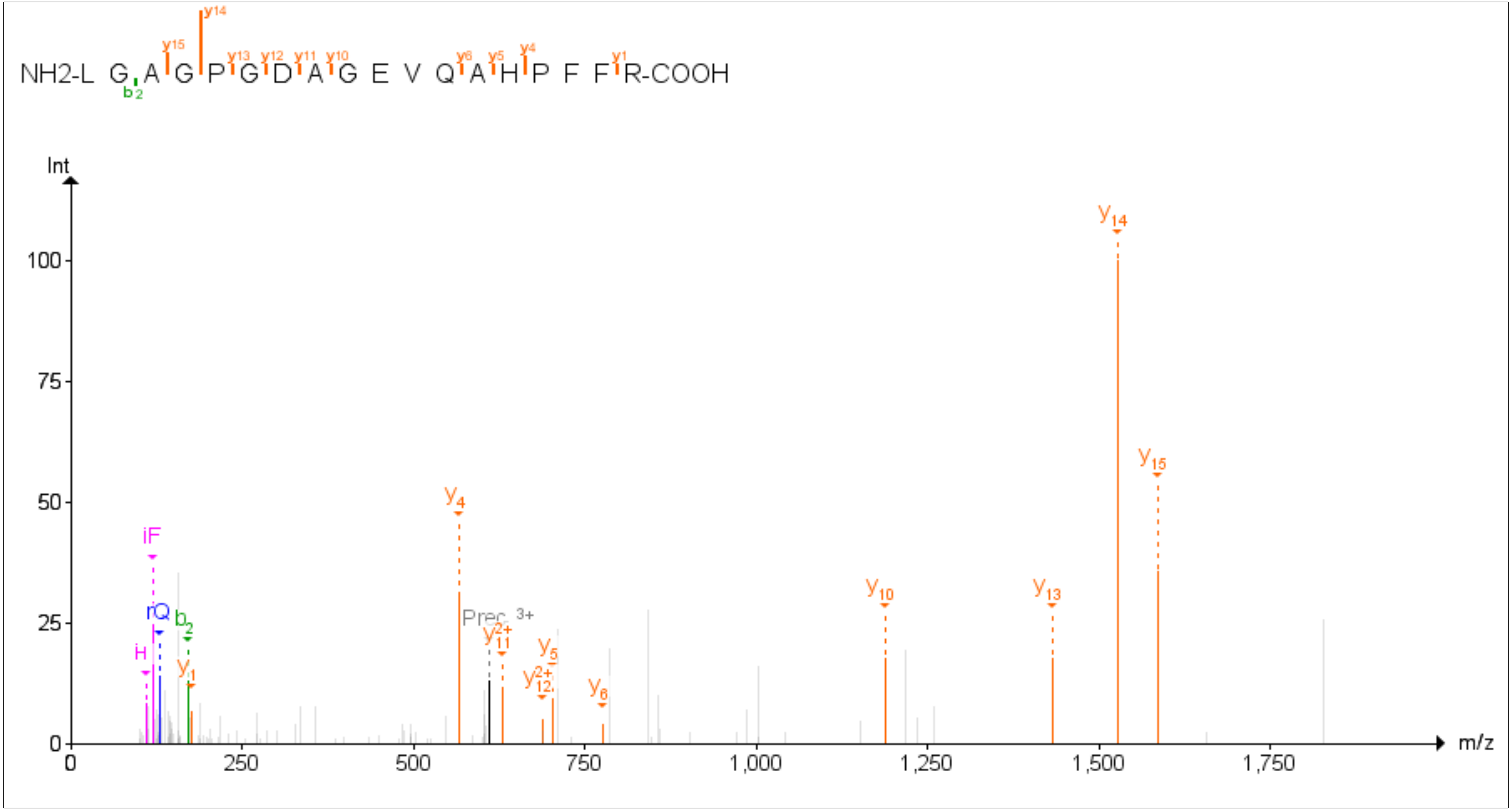

NH2-L G A G P G D A G E V Q A H P F F R-COOH
b2
y15 y14 y13 y12 y11 y10 y6 y5 y4 y1
Int
100
75
50
25
0
m/z
0
250
500
750
1,000
1,250
1,500
1,750
iF
rQ
b2
iH
y1
y4
Prec. 3+
y11 2+
y5
y12 2+
y6
y10
y13
y14
y15

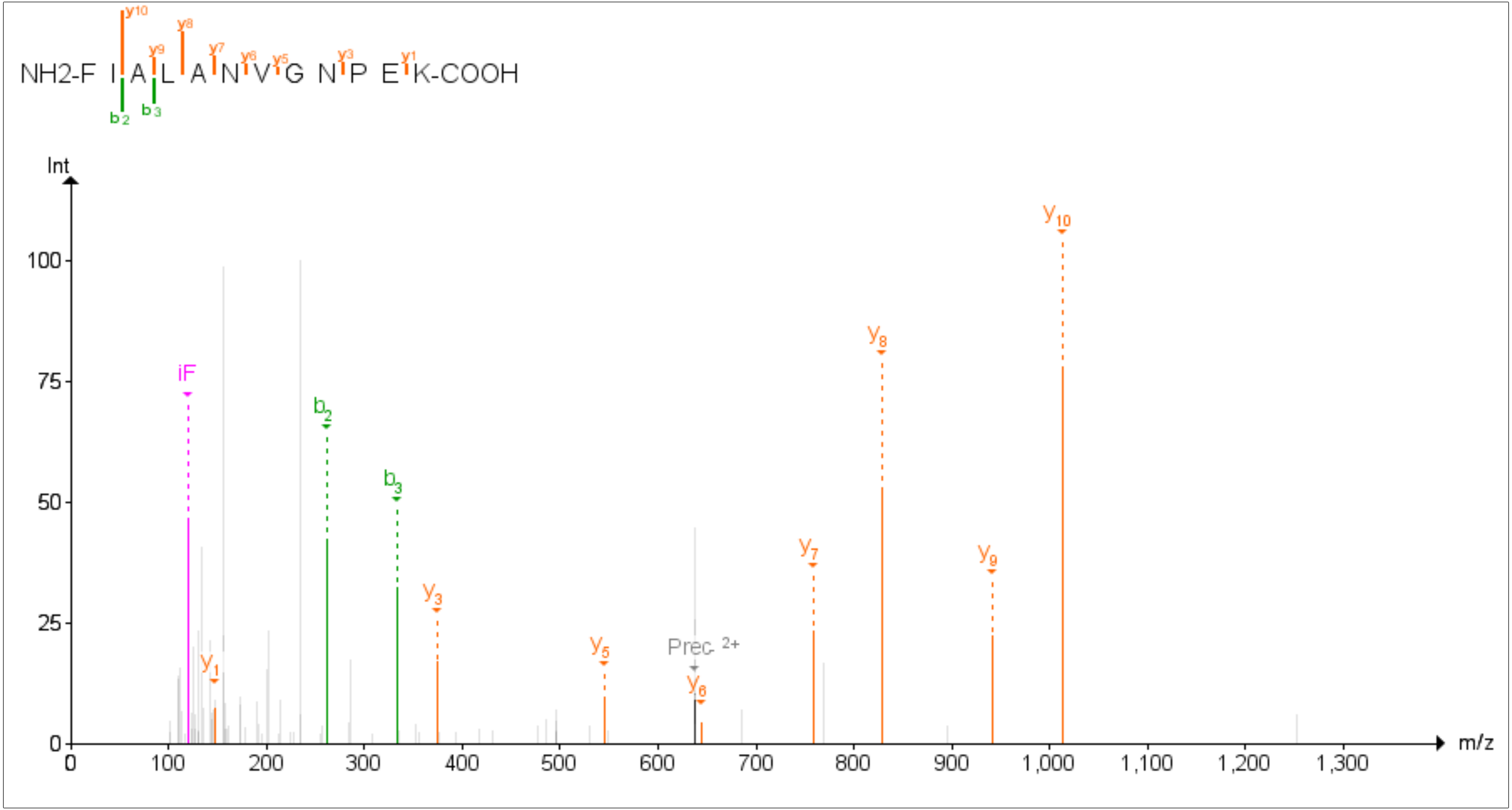

NH2-F I A L A N V G N P E K-COOH
y10
y9
y8
y7
y6
y5
y3
y1
b2
b3
Int
m/z
iF
Prec 2+

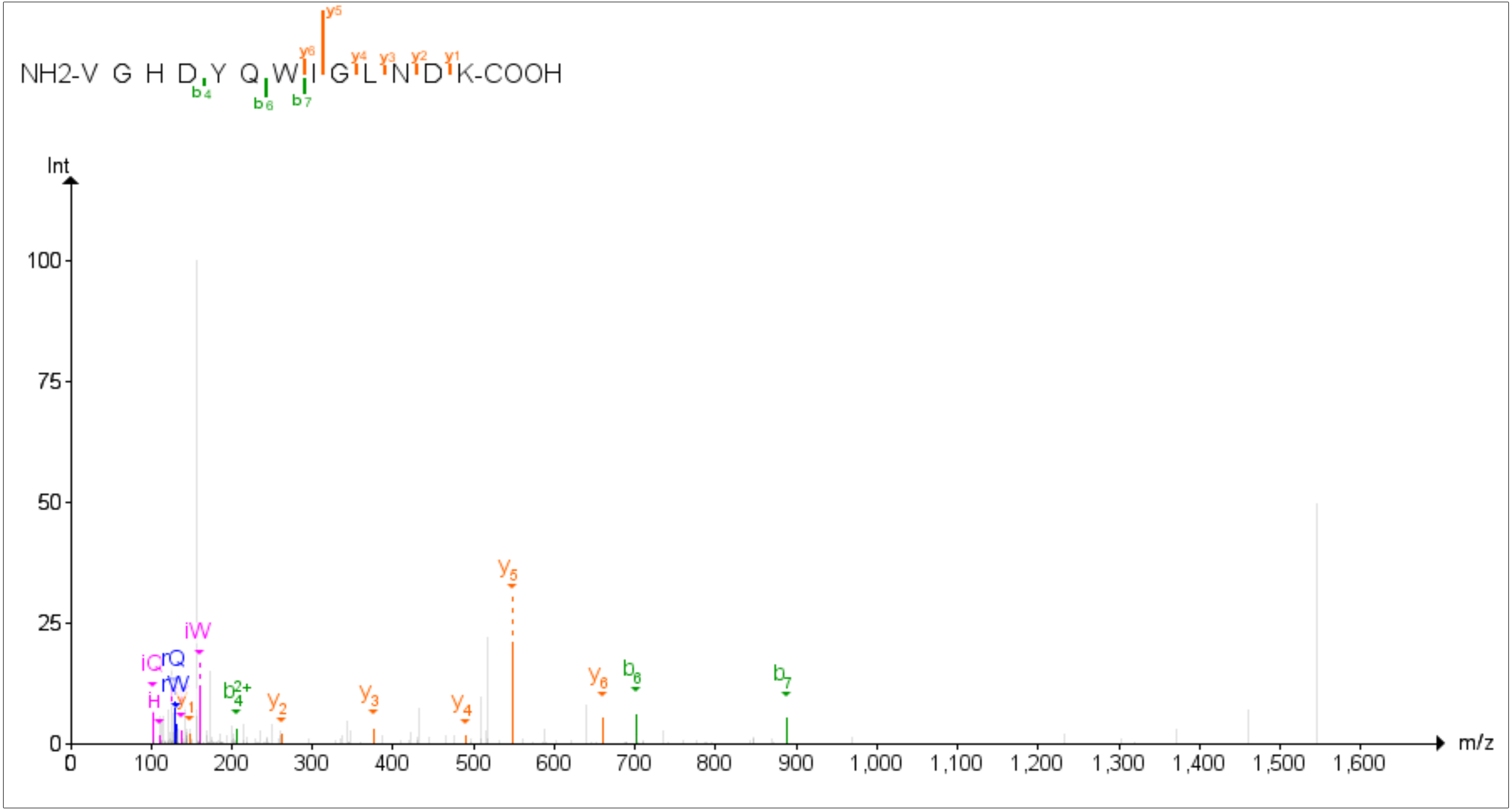

NH2-V G H D Y Q W I G L N D K-COOH
y5
y6
y4
y3
y2
y1
b4
b6
b7
Int
100
75
50
25
0
y5
iW
iQ
rQ
rW
H
y1
b_4^{2+}
y2
y3
y4
y6
b6
b7
0
100
200
300
400
500
600
700
800
900
1,000
1,100
1,200
1,300
1,400
1,500
1,600
m/z

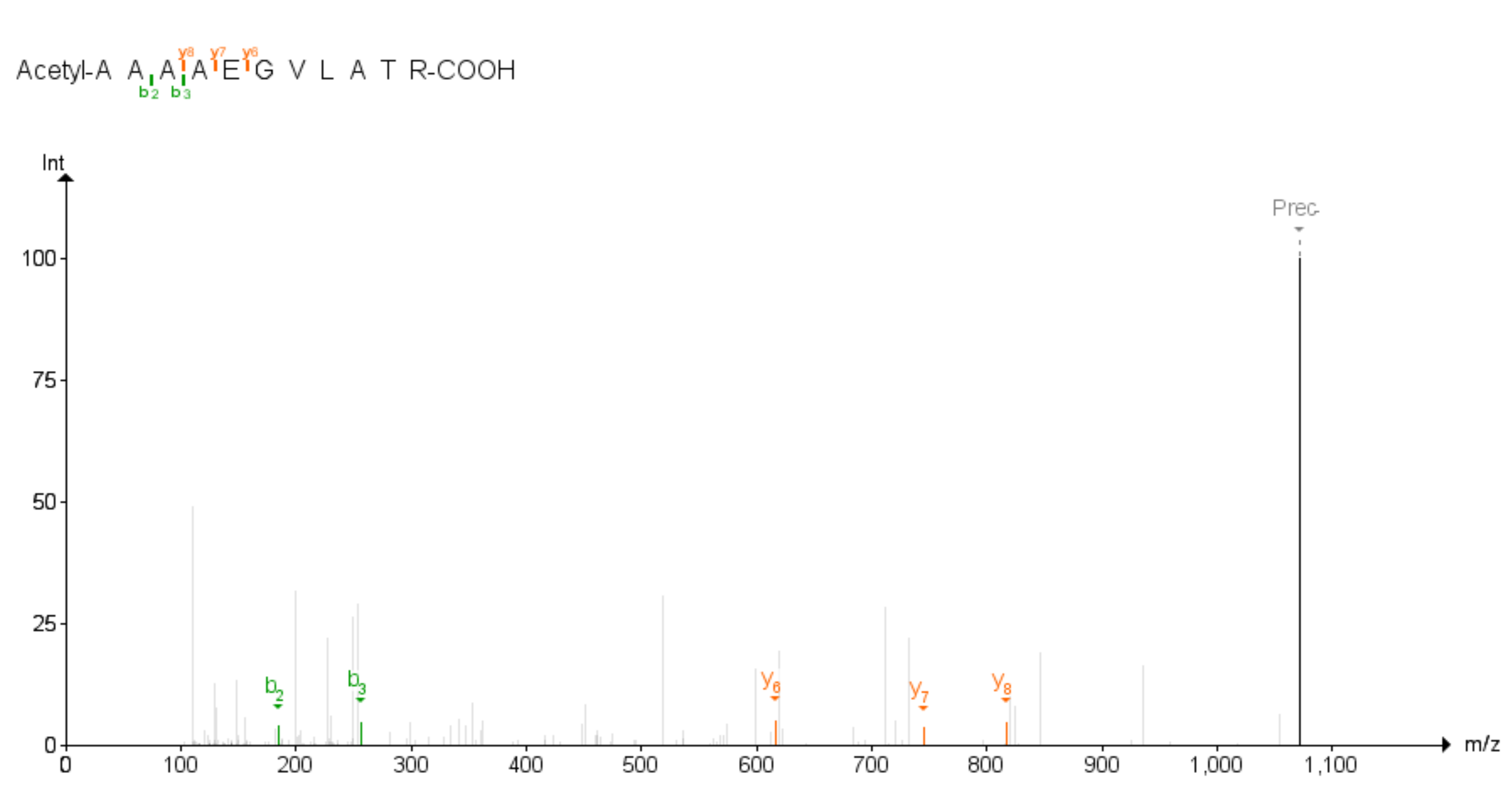

Acetyl-A A A A E G V L A T R-COOH
y8 y7 y6
b2 b3
Int
Prec
100
75
50
25
0
b2
b3
y6
y7
y8
0
100
200
300
400
500
600
700
800
900
1,000
1,100
m/z

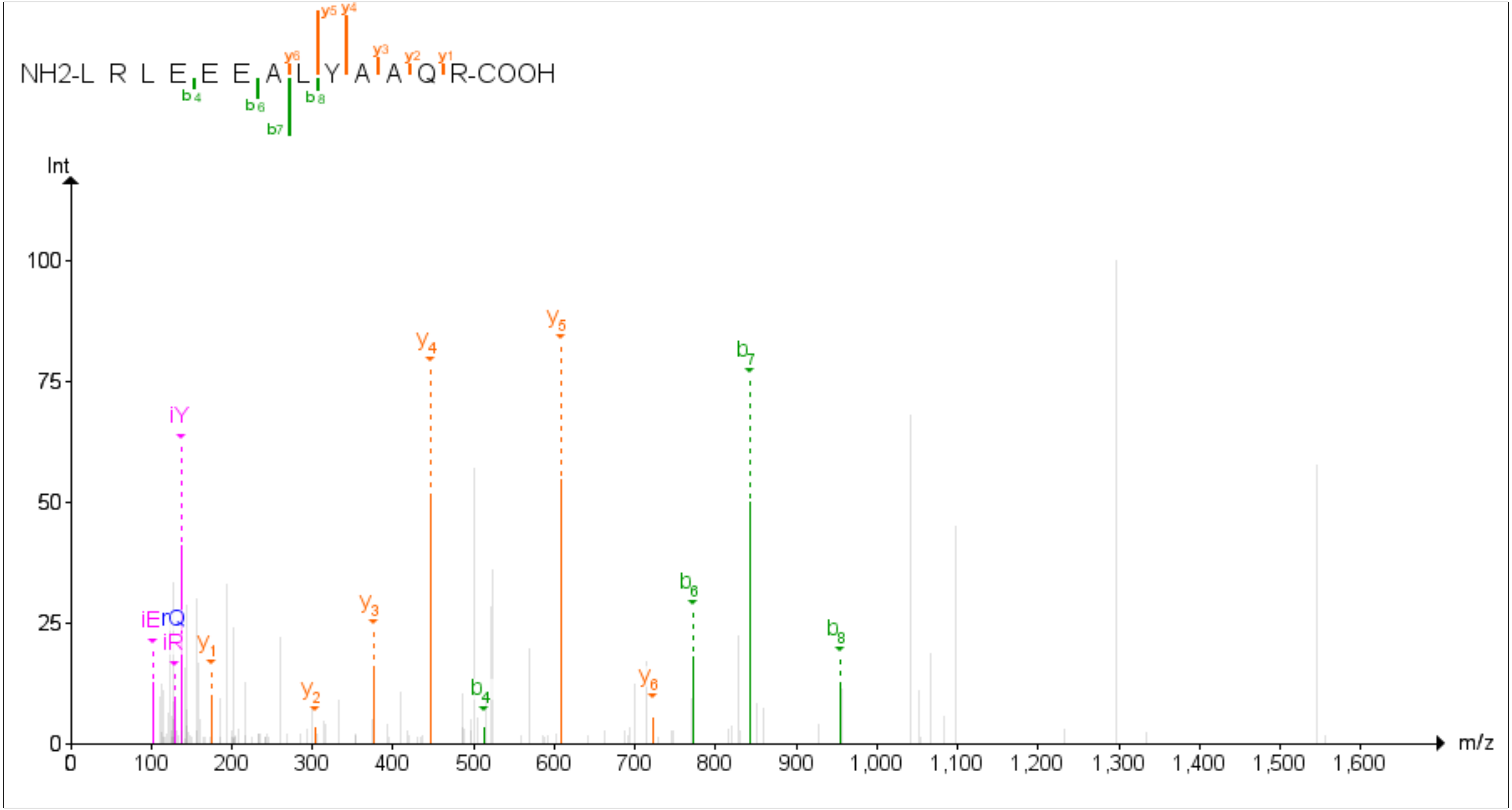

NH2-L R L E E E A L Y A A Q R-COOH
y5 y4 y6 y3 y2 y1
b4 b6 b7 b8
Int
m/z
iY
y4
y5
b7
iE rQ
iR
y1
y3
y2
b4
y6
b6
b8
0
25
50
75
100
0
100
200
300
400
500
600
700
800
900
1,000
1,100
1,200
1,300
1,400
1,500
1,600

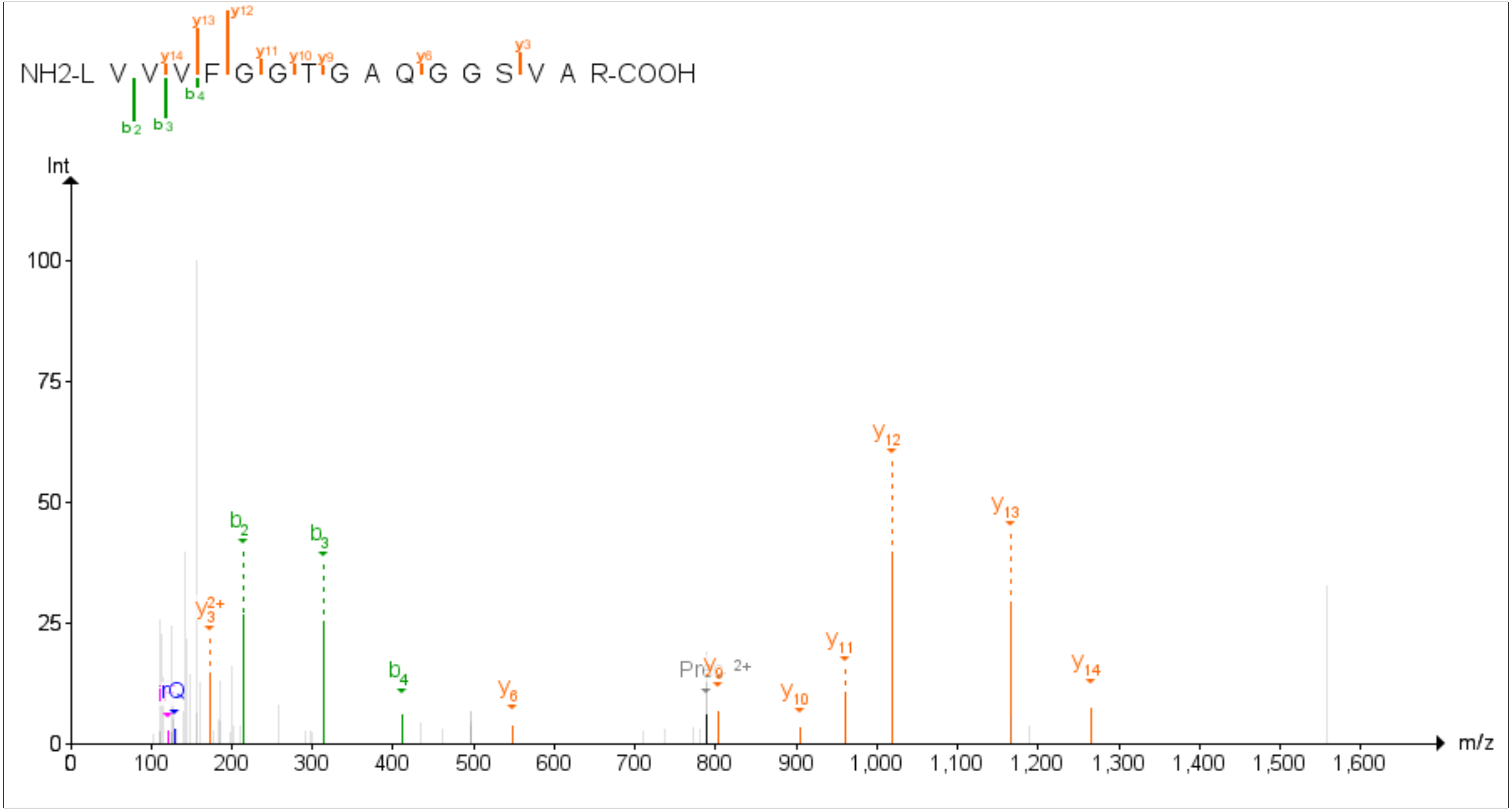
NH2-L V V V F G G T G A Q G G S V A R-COOH
y13 y12 y14 y11 y10 y9 y6 y3
b2 b3 b4
Int
100
75
50
25
0
m/z
0 100 200 300 400 500 600 700 800 900 1,000 1,100 1,200 1,300 1,400 1,500 1,600
b2 b3 b4 y3 2+ y6 y9 y10 y11 y12 y13 y14
irQ
Prec 2+

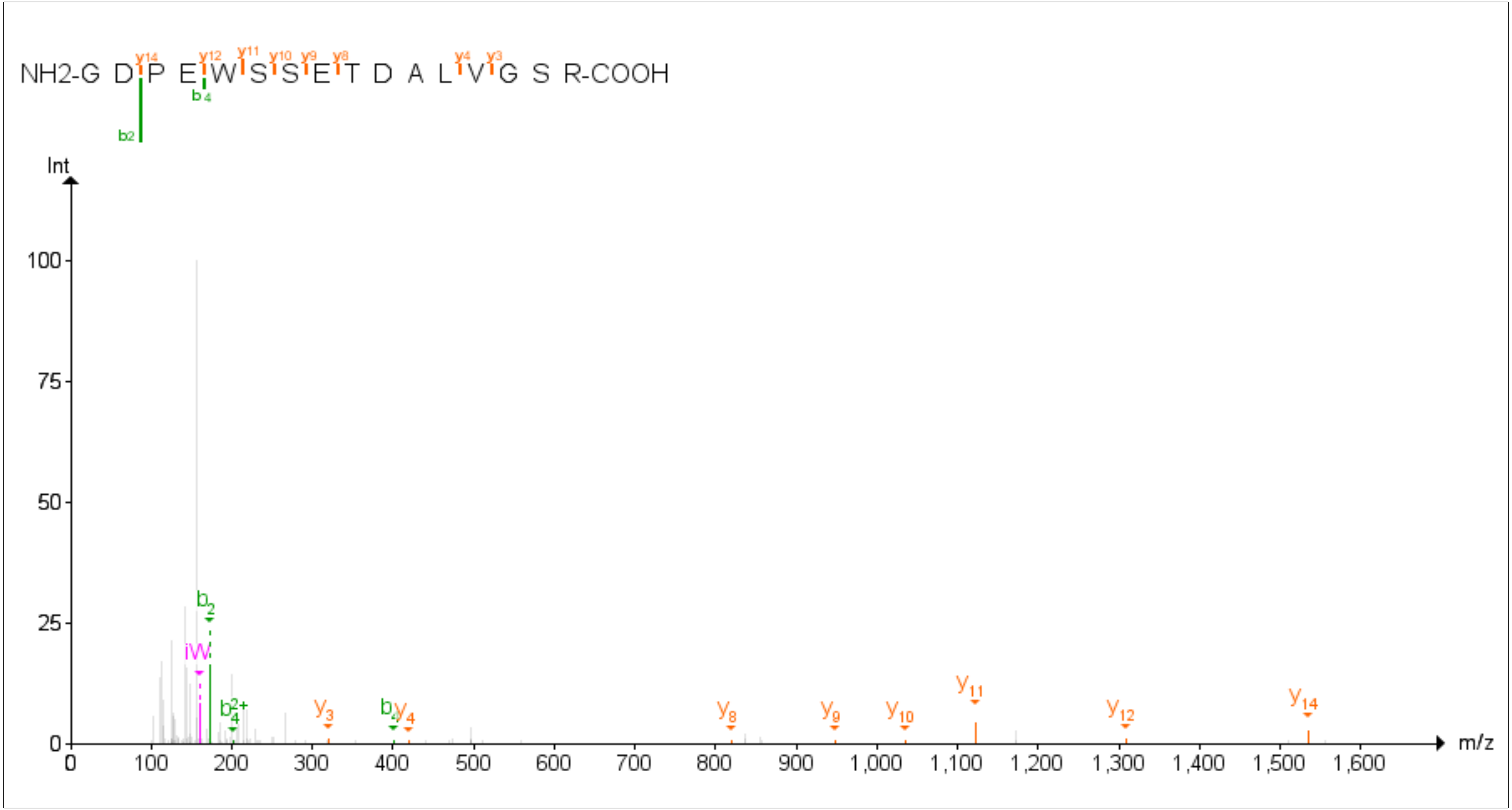
NH2-G D P E W S S E T D A L V G S R-COOH
y14
y12
y11
y10
y9
y8
y4
y3
b4
b2
Int
100
75
50
25
0
iW
m/z
0
100
200
300
400
500
600
700
800
900
1,000
1,100
1,200
1,300
1,400
1,500
1,600

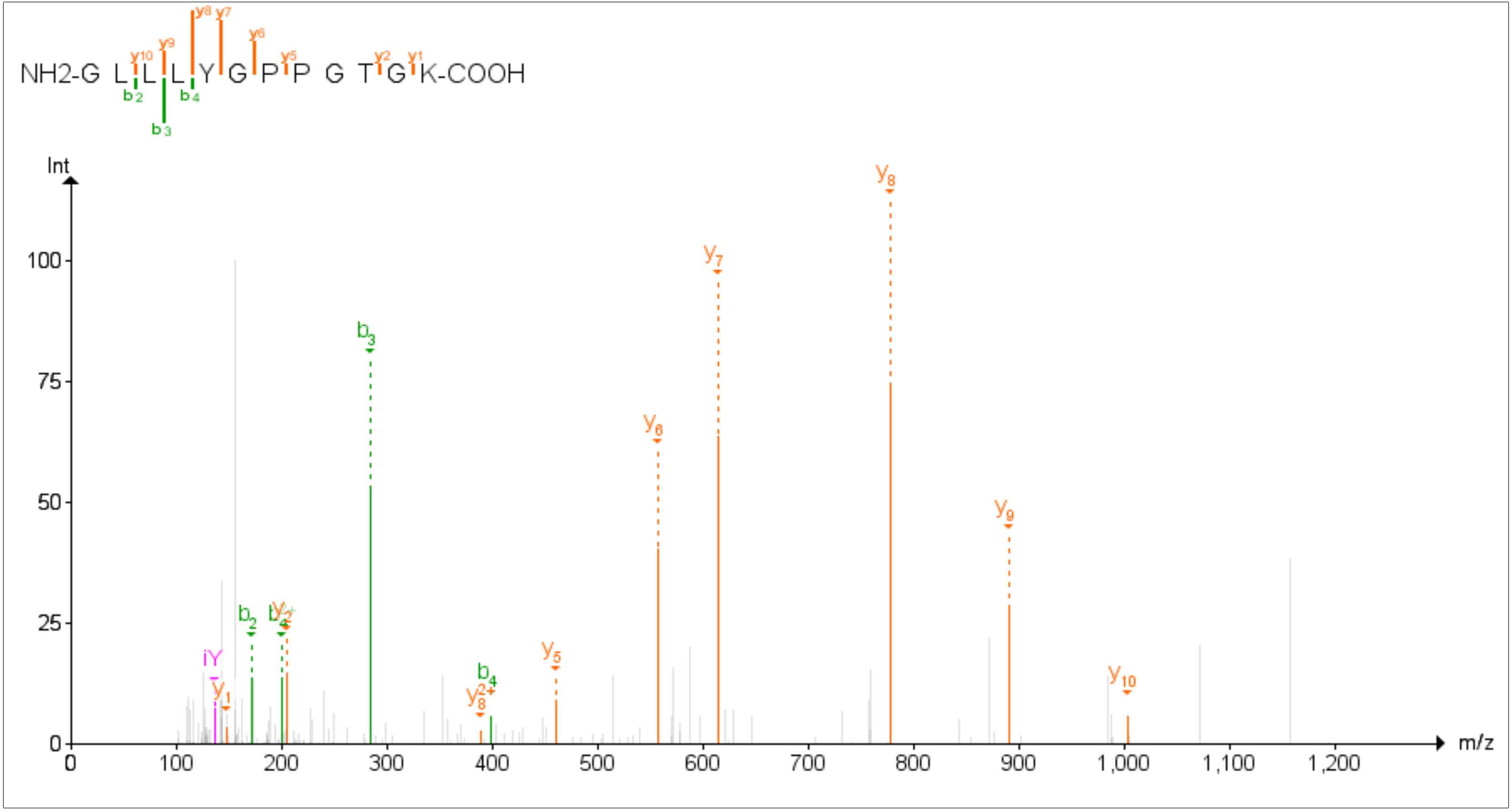
NH2-G L L L Y G P P G T G K-COOH
Int
m/z
y8
y7
y6
y9
y10
y5
y2
y1
b2
b3
b4
iY
y1
0
25
50
75
100
0
100
200
300
400
500
600
700
800
900
1,000
1,100
1,200

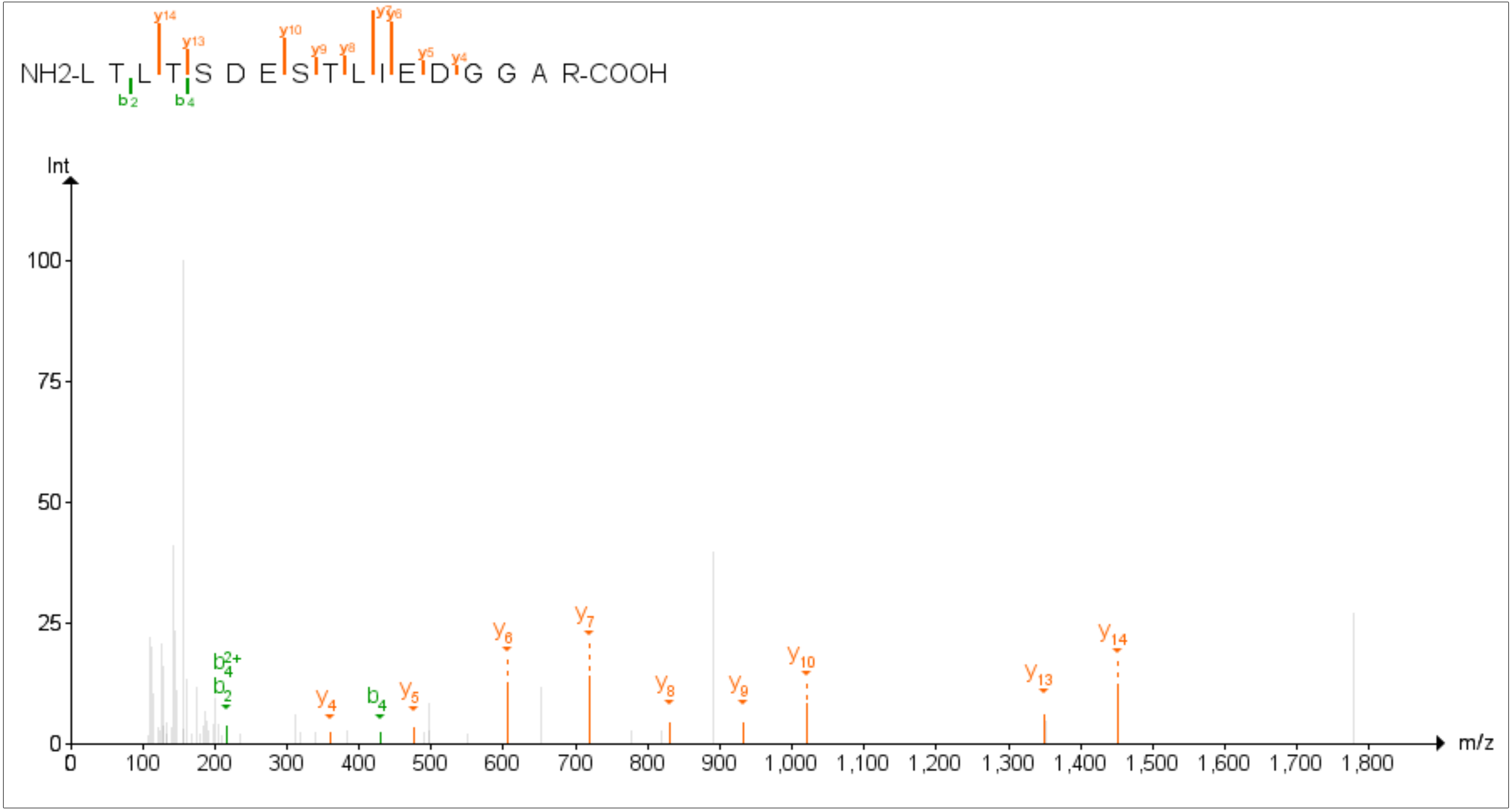

NH2-L T L T S D E S T L I E D G G A R-COOH
y14 y13 y10 y9 y8 y7 y6 y5 y4
b2 b4
Int
100
75
50
25
0
b4 2+
b2
y4
b4
y5
y6
y7
y8
y9
y10
y13
y14
0 100 200 300 400 500 600 700 800 900 1,000 1,100 1,200 1,300 1,400 1,500 1,600 1,700 1,800
m/z

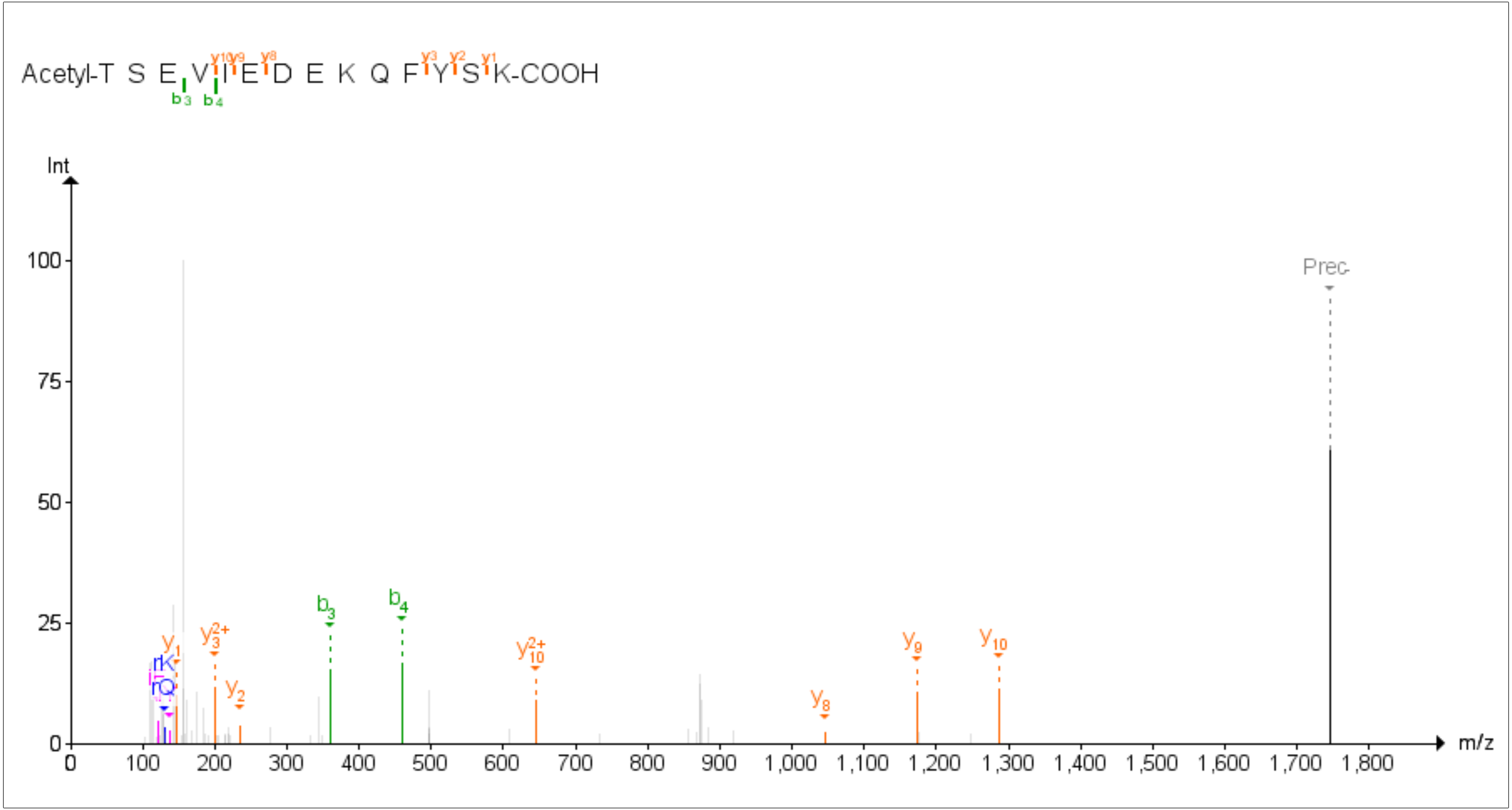
Acetyl-T S E V I E D E K Q F Y S K-COOH
b3 b4
y10 y9 y8 y3 y2 y1
Int
100
75
50
25
0
Prec
b3
b4
y1
y2
y3 2+
y10 2+
y8
y9
y10
rK
rQ
m/z
0 100 200 300 400 500 600 700 800 900 1,000 1,100 1,200 1,300 1,400 1,500 1,600 1,700 1,800

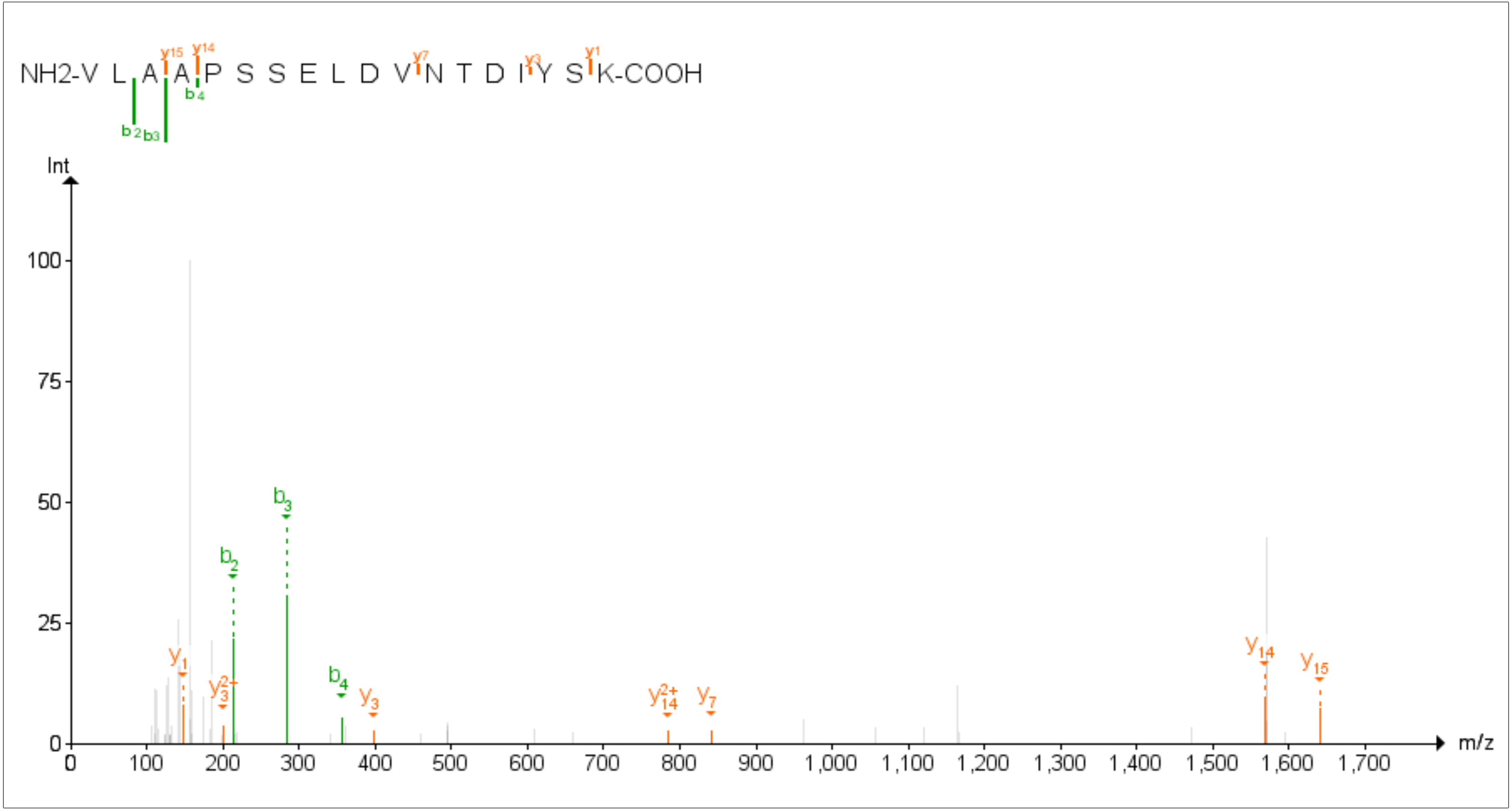

NH2-V L A A P S S E L D V N T D I Y S K-COOH
y15
y14
y7
y3
y1
b2
b3
b4
Int
100
75
50
25
0
b3
b2
y1
y_3^{2+}
b4
y3
y_{14}^{2+}
y7
y14
y15
0
100
200
300
400
500
600
700
800
900
1,000
1,100
1,200
1,300
1,400
1,500
1,600
1,700
m/z

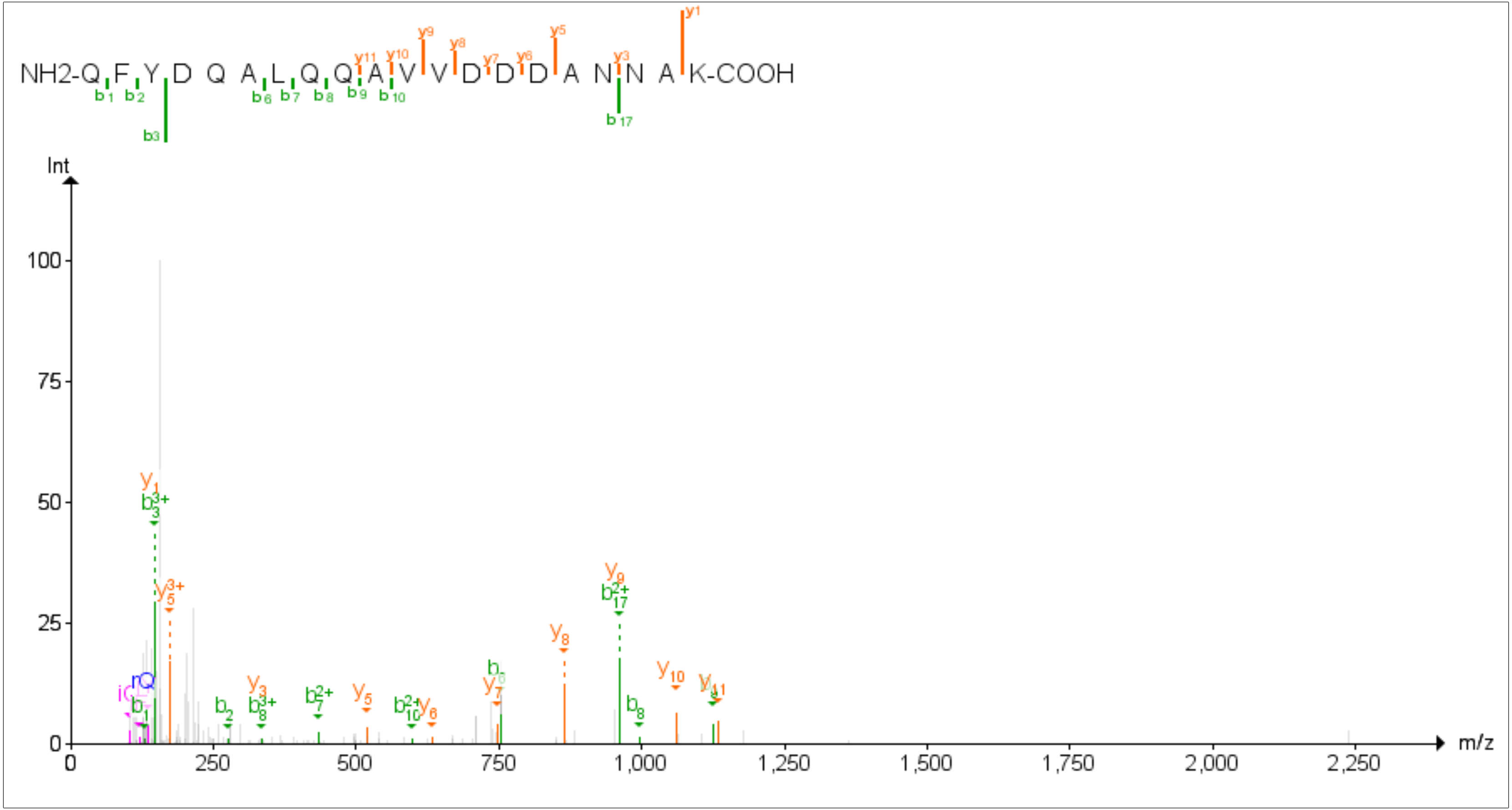

NH2-Q F Y D Q A L Q Q A V V D D D A N N A K-COOH
Int
100
75
50
25
0
m/z
0
250
500
750
1,000
1,250
1,500
1,750
2,000
2,250

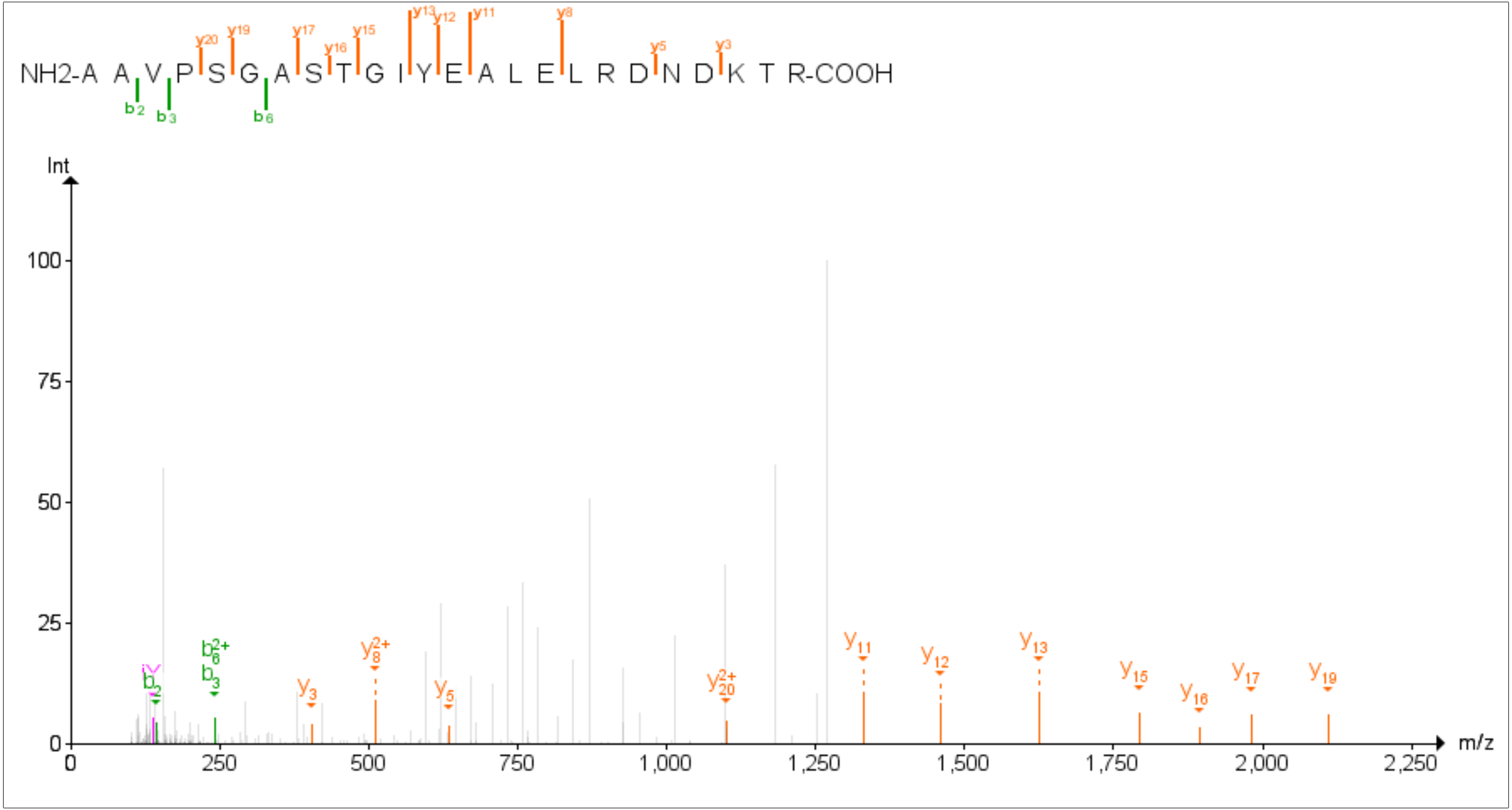

NH2-A A V P S G A S T G I Y E A L E L R D N D K T R-COOH
y20 y19 y17 y16 y15 y13 y12 y11 y8 y5 y3
b2 b3 b6
Int
100
75
50
25
0
0 250 500 750 1,000 1,250 1,500 1,750 2,000 2,250
m/z
iY
b2
b6 2+
b3
y3
y8 2+
y5
y20 2+
y11
y12
y13
y15
y16
y17
y19

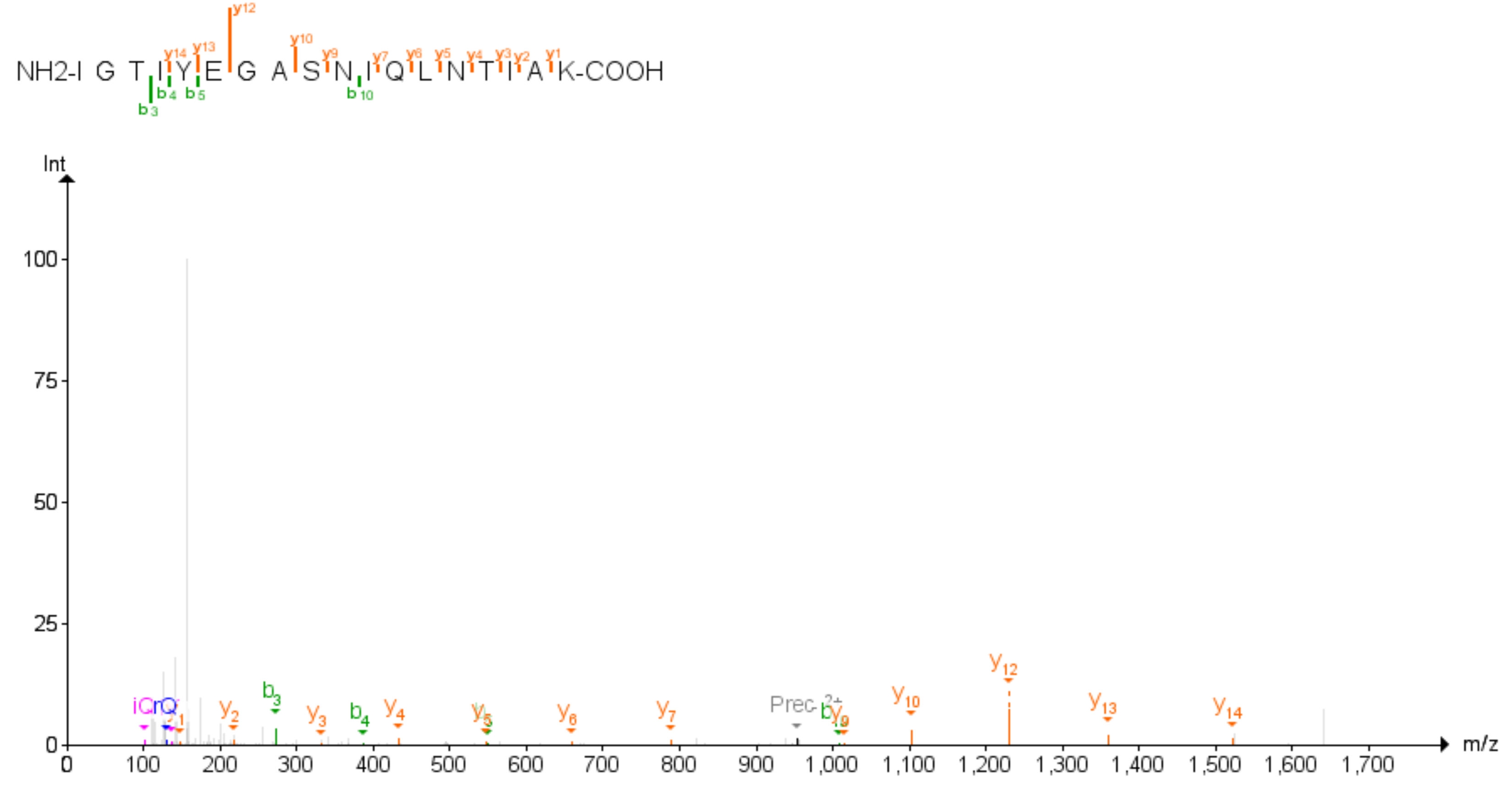
NH2-I G T I Y E G A S N I Q L N T I A K-COOH
y12 y14 y13 y10 y9 y7 y6 y5 y4 y3 y2 y1
b3 b4 b5 b10
Int
100
75
50
25
0
m/z
0 100 200 300 400 500 600 700 800 900 1,000 1,100 1,200 1,300 1,400 1,500 1,600 1,700
iCnQ y2 b3 y3 b4 y4 y5 y6 y7 Prec 2+ y9 y10 y12 y13 y14

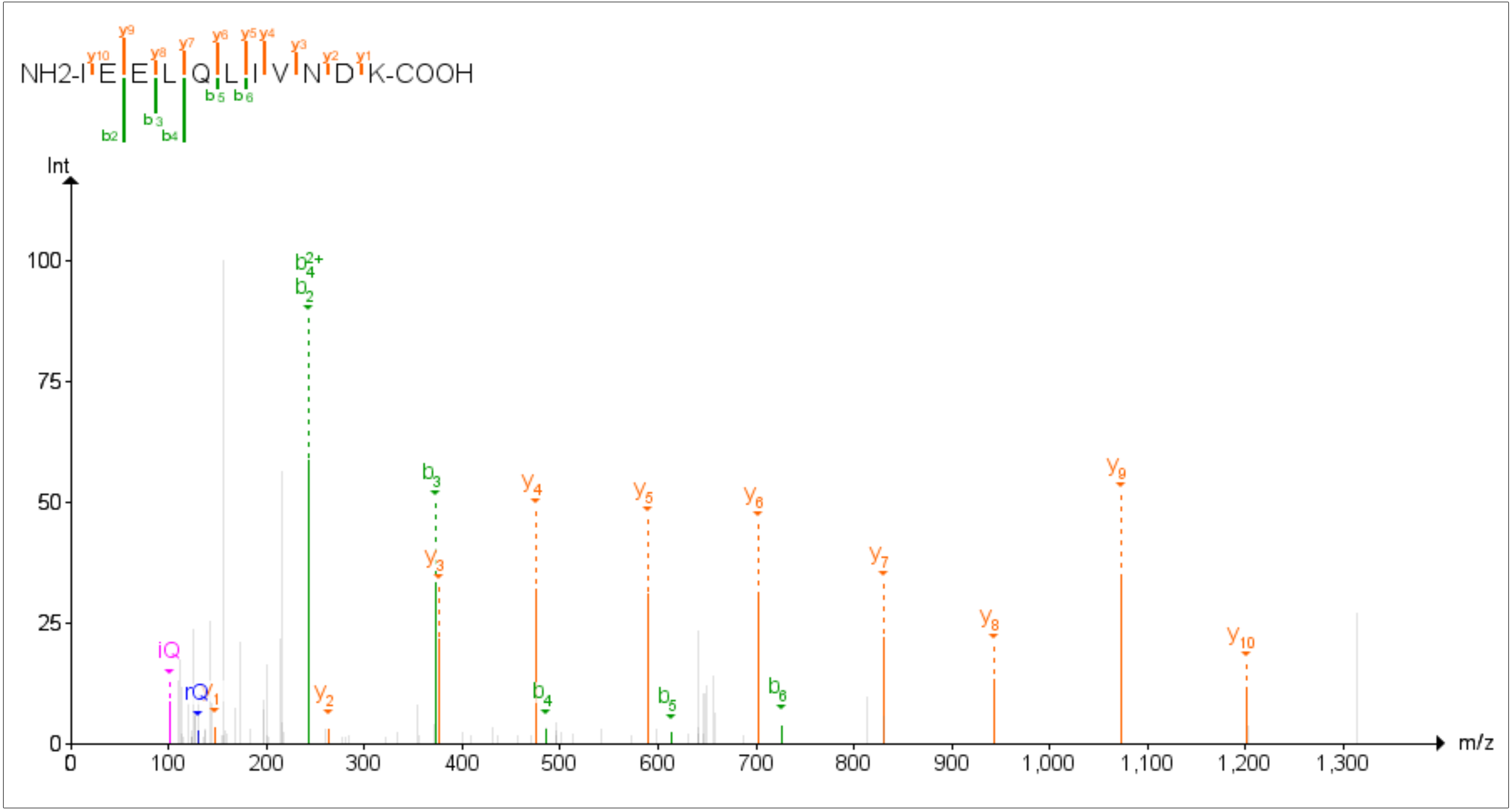

NH2-I E E L Q L I V N D K-COOH
y10 y9 y8 y7 y6 y5 y4 y3 y2 y1
b2 b3 b4 b5 b6
Int
m/z
0 25 50 75 100
0 100 200 300 400 500 600 700 800 900 1,000 1,100 1,200 1,300
b_4^{2+}
b_2
b_3
y_3
y_4
y_5
y_6
y_7
y_8
y_9
y_{10}
b_4
b_5
b_6
y_2
y_1
iQ
rQ

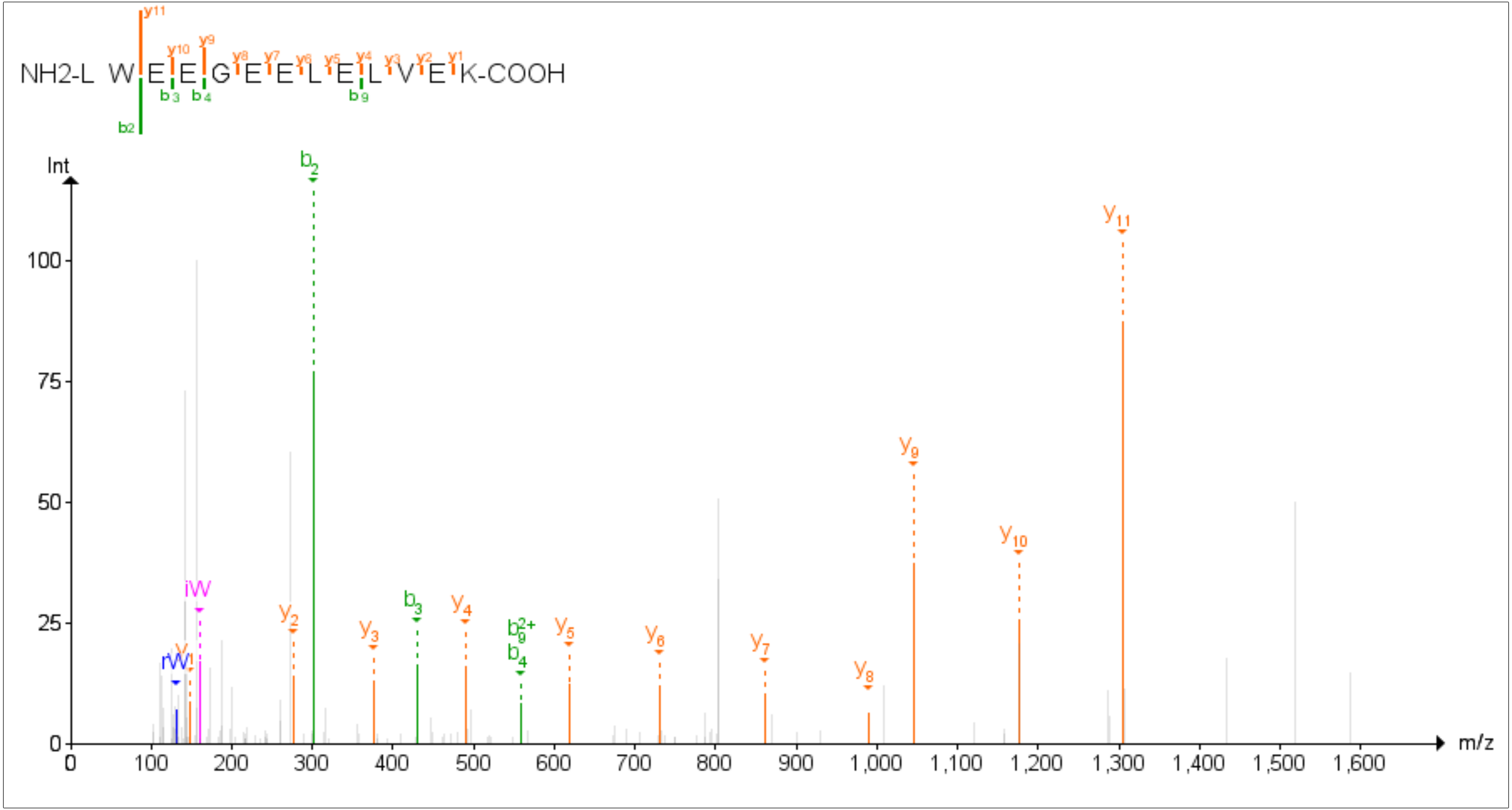

NH2-L W E E G E E L E L V E K-COOH
y11
y10
y9
y8
y7
y6
y5
y4
y3
y2
y1
b2
b3
b4
b9
Int
100
75
50
25
0
b2
y11
y9
y10
iW
rW
y1
y2
y3
b3
y4
b9 2+
b4
y5
y6
y7
y8
0
100
200
300
400
500
600
700
800
900
1,000
1,100
1,200
1,300
1,400
1,500
1,600
m/z

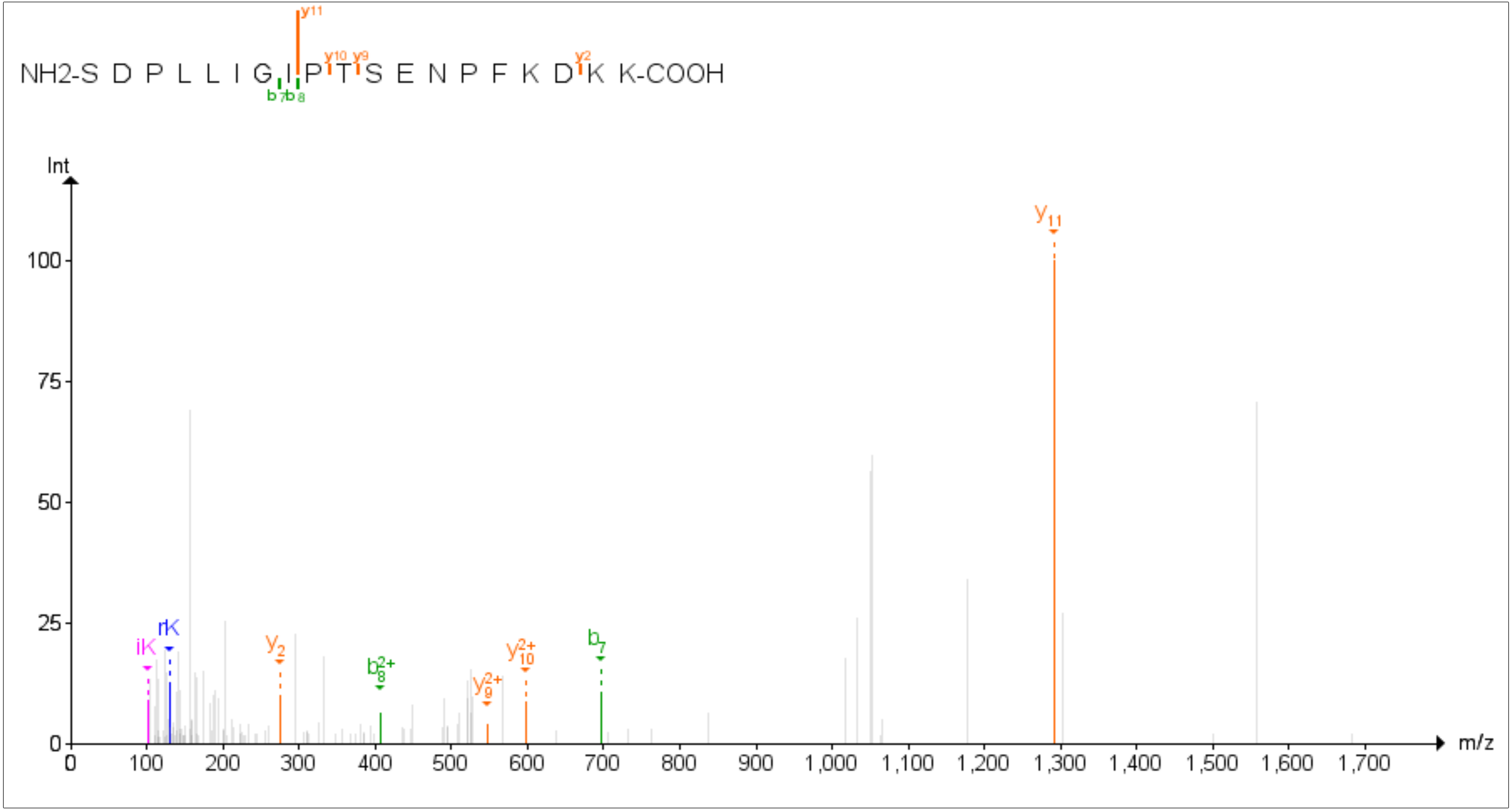

NH2-S D P L L I G I P T S E N P F K D K K-COOH
y11
y10
y9
y2
b7
b8
Int
100
75
50
25
0
iK
rK
y2
b8 2+
y9 2+
y10 2+
b7
y11
m/z
0
100
200
300
400
500
600
700
800
900
1,000
1,100
1,200
1,300
1,400
1,500
1,600
1,700

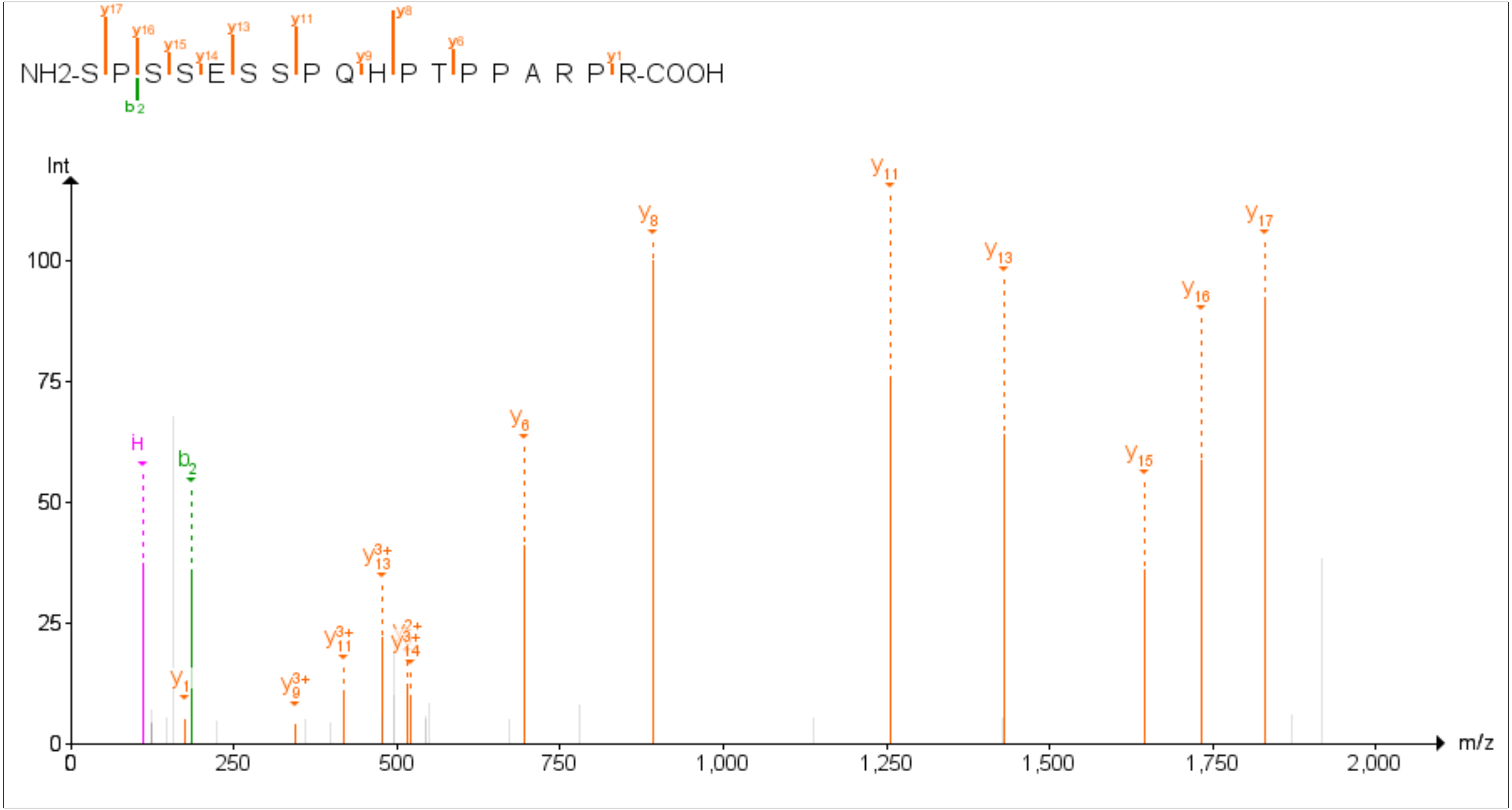
NH2-S P S S E S S P Q H P T P P A R P R-COOH
y17 y16 y15 y14 y13 y11 y9 y8 y6 y1
b2
Int
m/z
y11
y8
y17
y13
y16
y15
y6
b2
y1
y9 3+
y11 3+
y13 3+
y14 3+
0
25
50
75
100
0
250
500
750
1,000
1,250
1,500
1,750
2,000

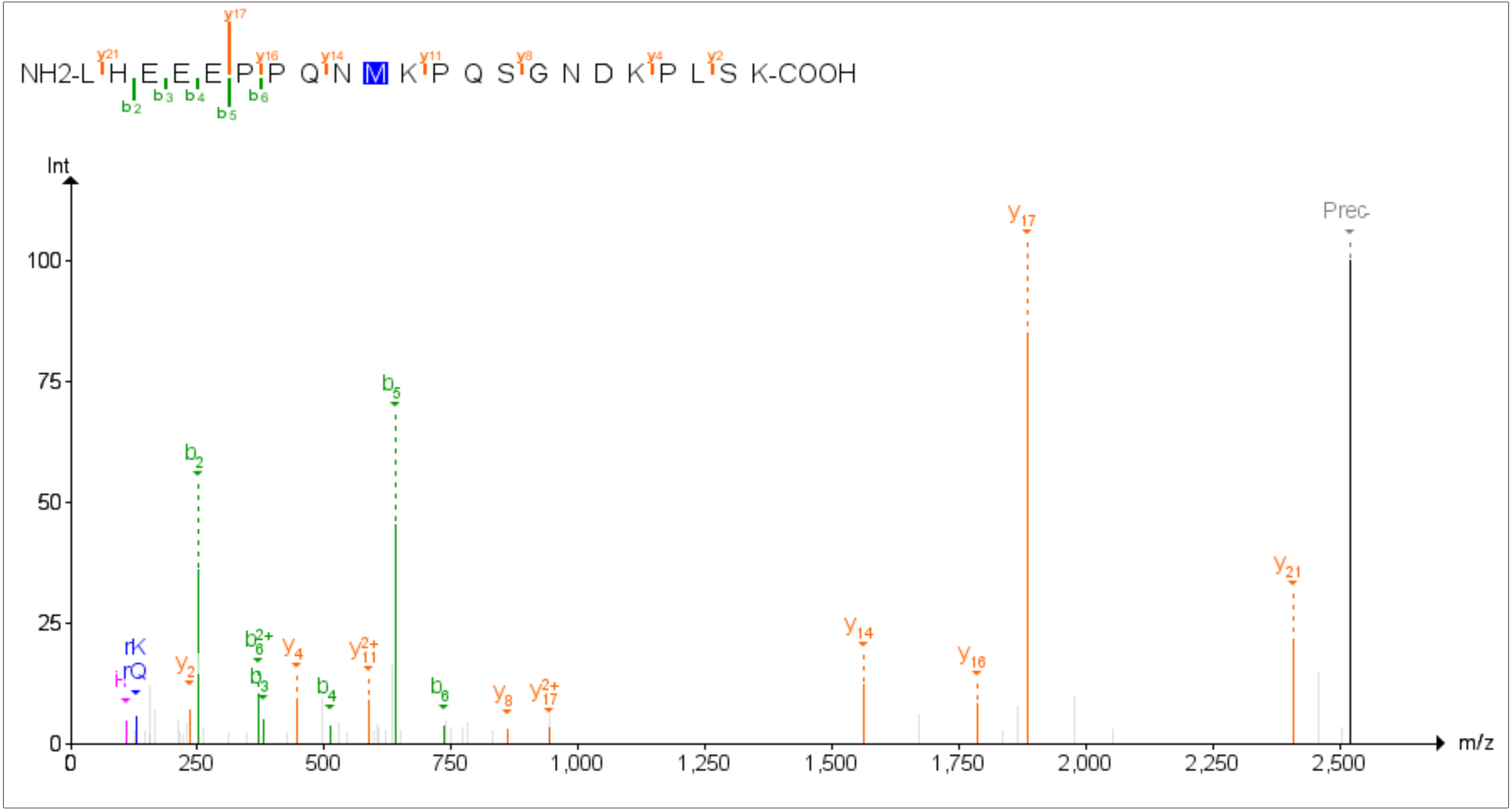

NH2-L H E E E P P Q N M K P Q S G N D K P L S K-COOH
y21 y17 y16 y14 y11 y8 y4 y2
b2 b3 b4 b5 b6
Int
m/z
Prec.
y17
b5
b2
y21
y14
y16
rK
rQ
y2
b6 2+
b3
y4
y11 2+
b4
b6
y8
y17 2+
0
25
50
75
100
250
500
750
1,000
1,250
1,500
1,750
2,000
2,250
2,500

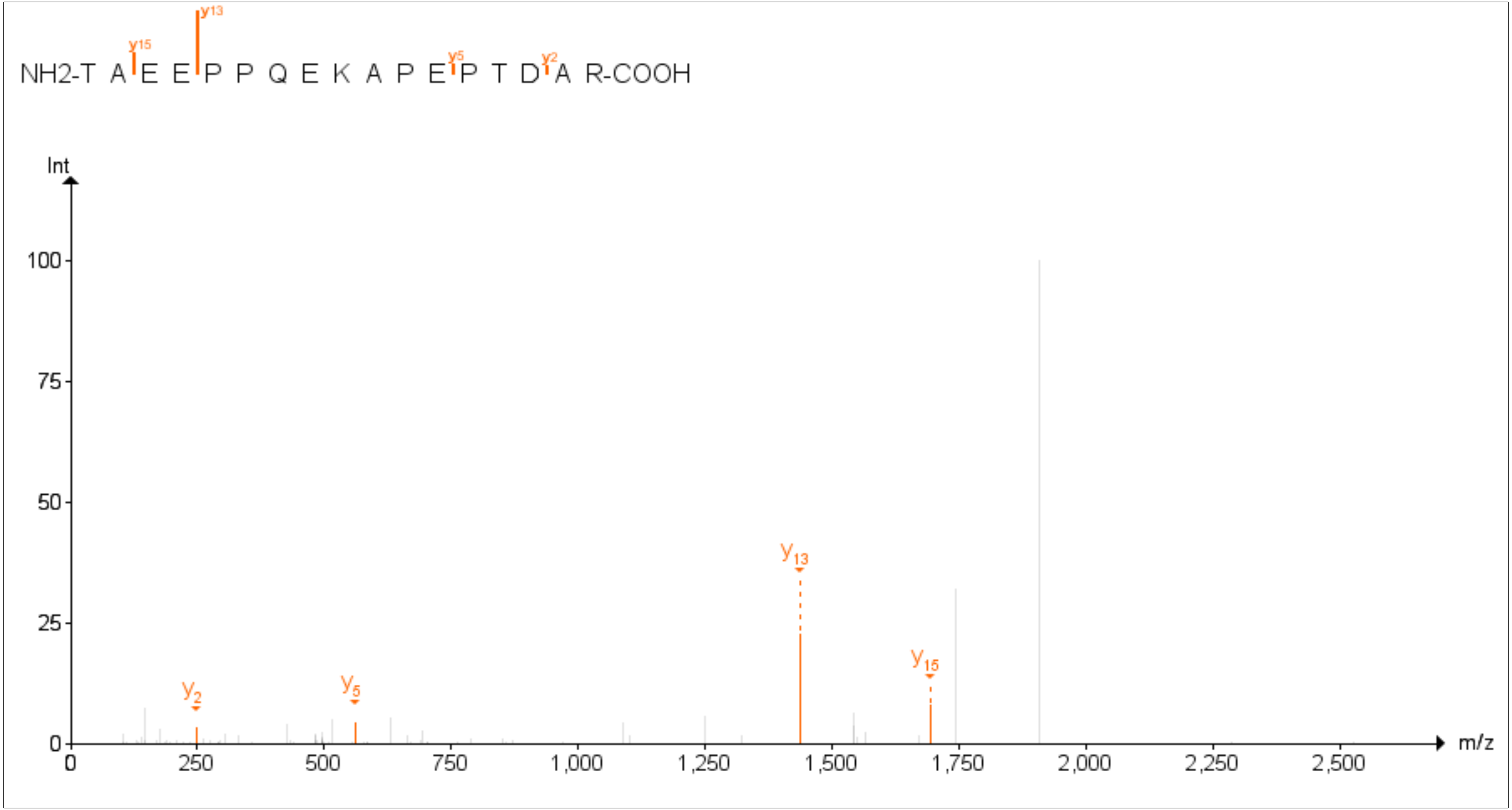

NH2-T A E E P P Q E K A P E P T D A R-COOH
y15
y13
y5
y2
Int
100
75
50
25
0
y13
y15
y2
y5
0
250
500
750
1,000
1,250
1,500
1,750
2,000
2,250
2,500
m/z

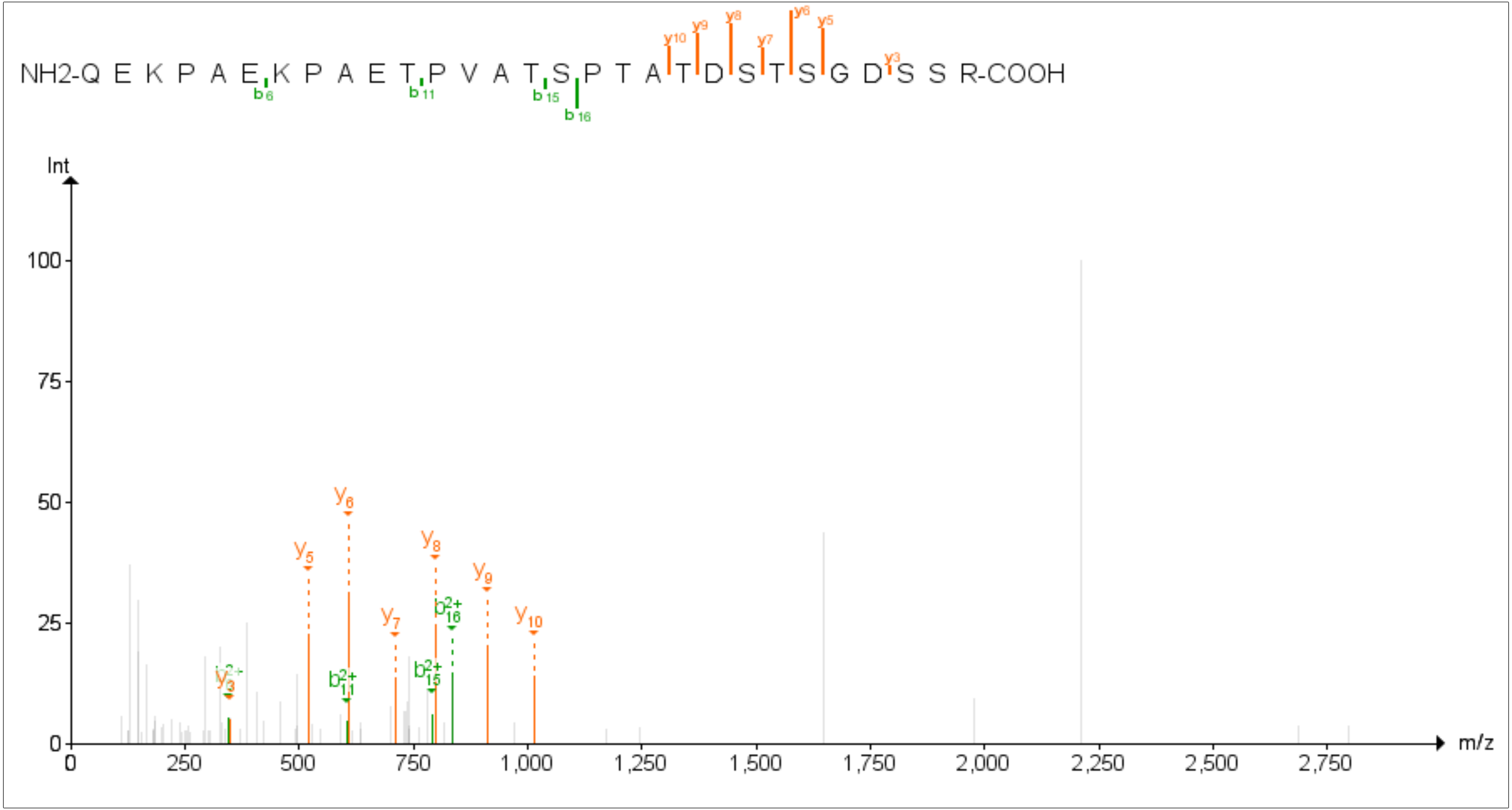
NH2-Q E K P A E K P A E T P V A T S P T A T D S T S G D S S R-COOH
Int
m/z
100
75
50
25
0
0
250
500
750
1,000
1,250
1,500
1,750
2,000
2,250
2,500
2,750
y5
y6
y7
y8
y9
y10

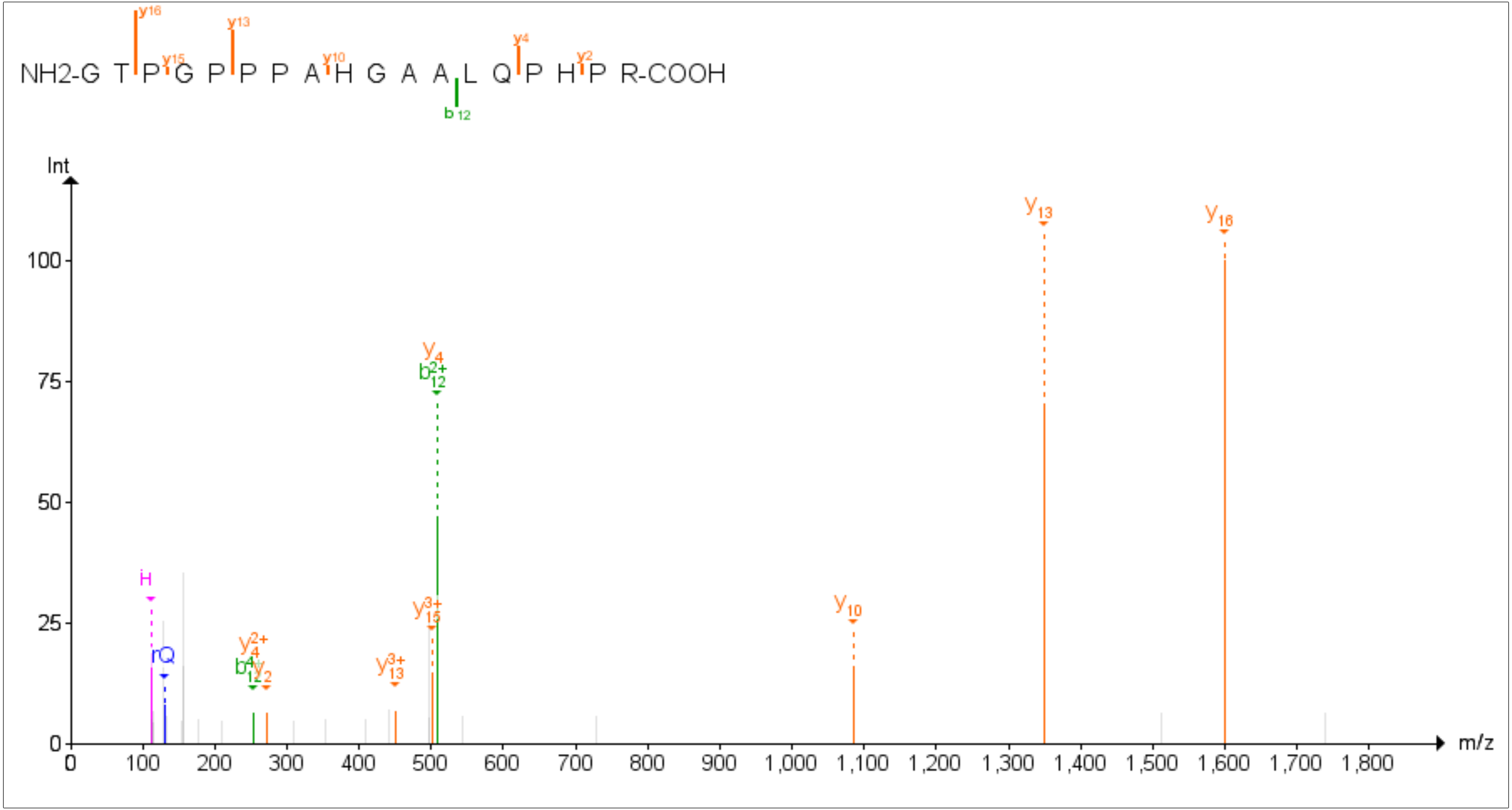

NH2-G T P G P P P A H G A A L Q P H P R-COOH
y16
y15
y13
y10
y4
y2
b 12
Int
100
75
50
25
0
m/z
0
100
200
300
400
500
600
700
800
900
1,000
1,100
1,200
1,300
1,400
1,500
1,600
1,700
1,800
y13
y16
y4
b12 2+
y10
y15 3+
y13 3+
y4 2+
b12 4+
y2
iH
rQ

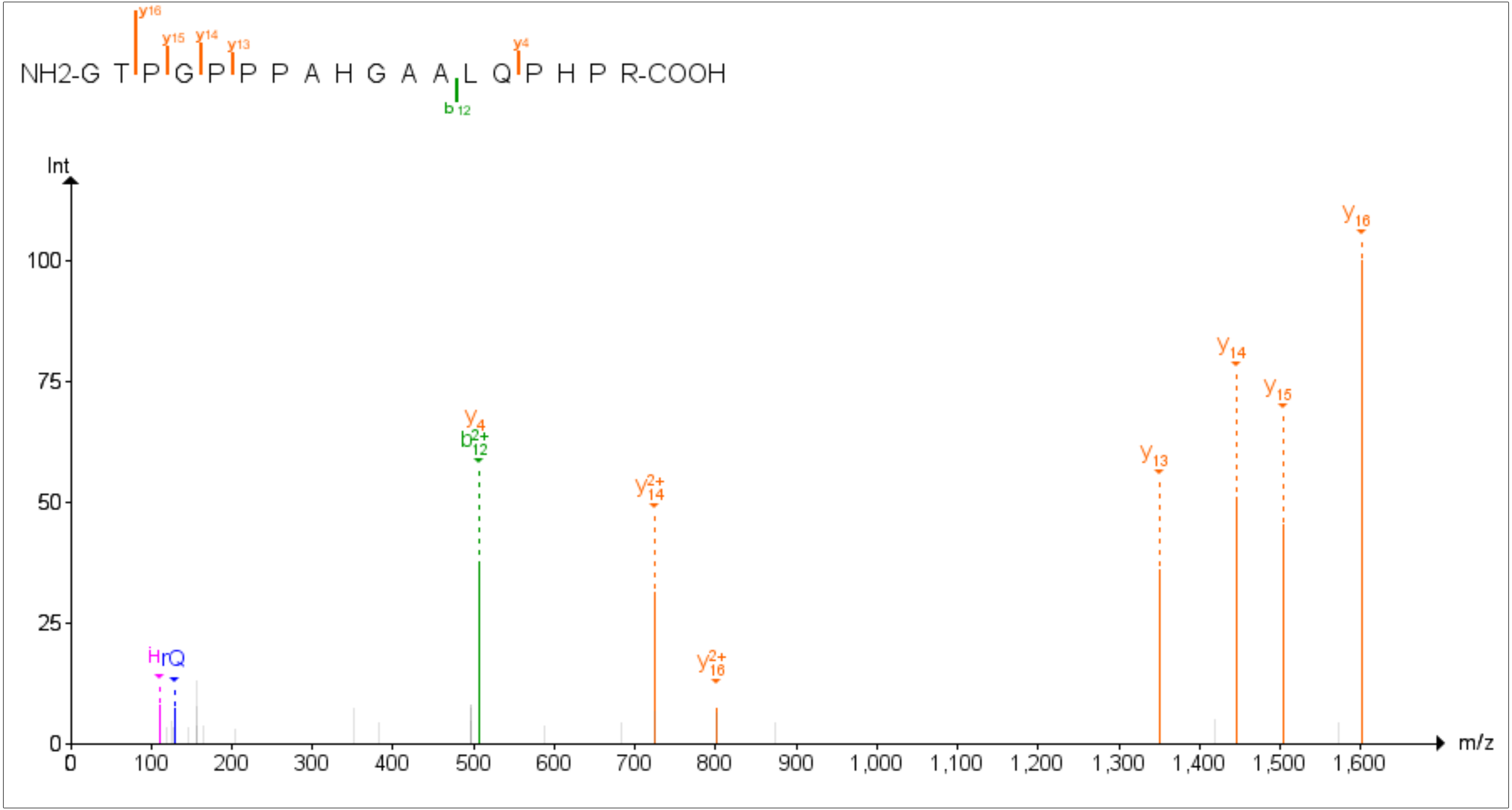

NH2-G T P G P P P A H G A A L Q P H P R-COOH
y16
y15
y14
y13
y4
b12
Int
100
75
50
25
0
y16
y14
y15
y13
y4
b12 2+
y14 2+
y16 2+
iH
rQ
0
100
200
300
400
500
600
700
800
900
1,000
1,100
1,200
1,300
1,400
1,500
1,600
m/z

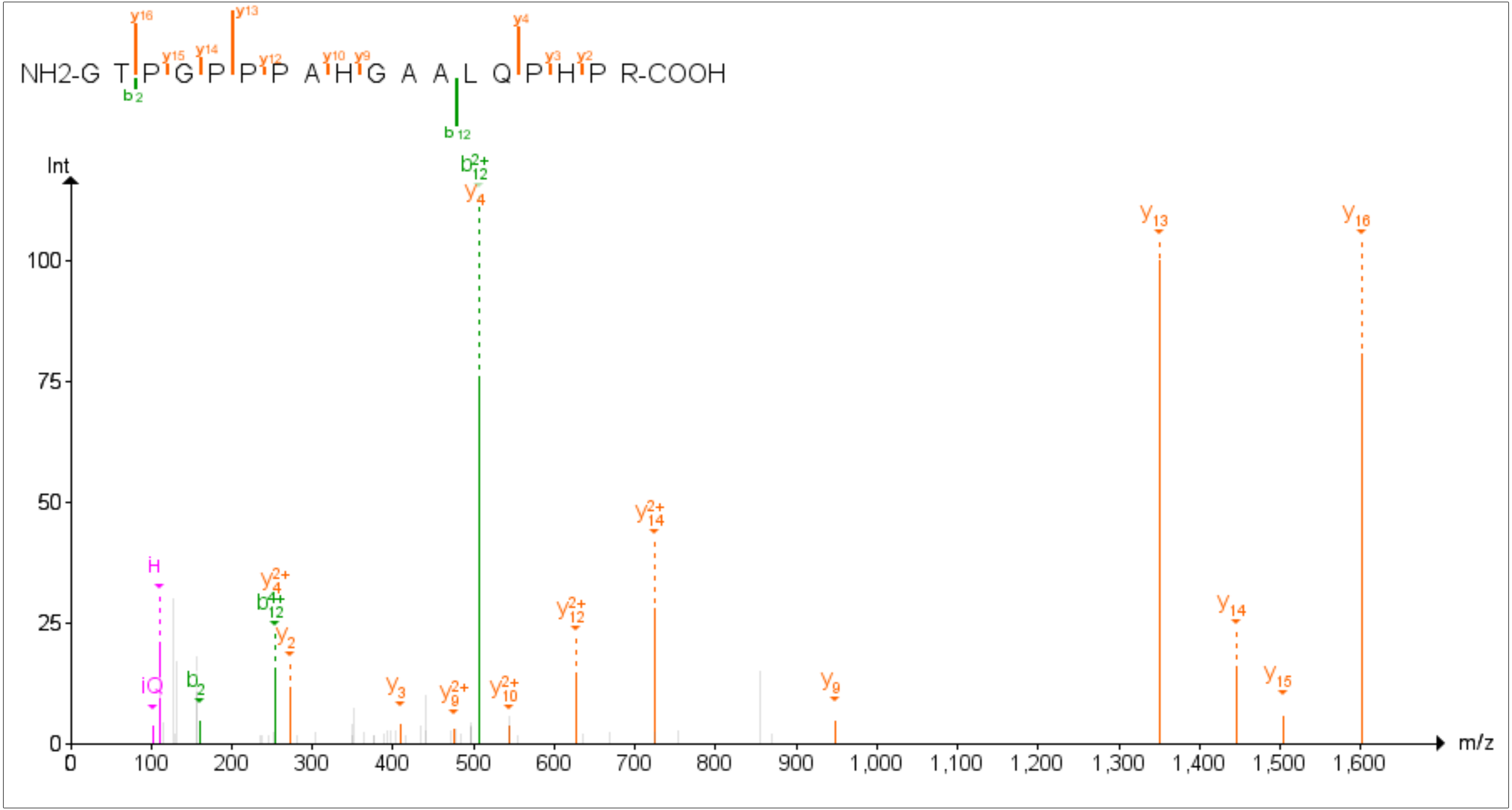

NH2-G T P G P P P A H G A A L Q P H P R-COOH
Int
m/z

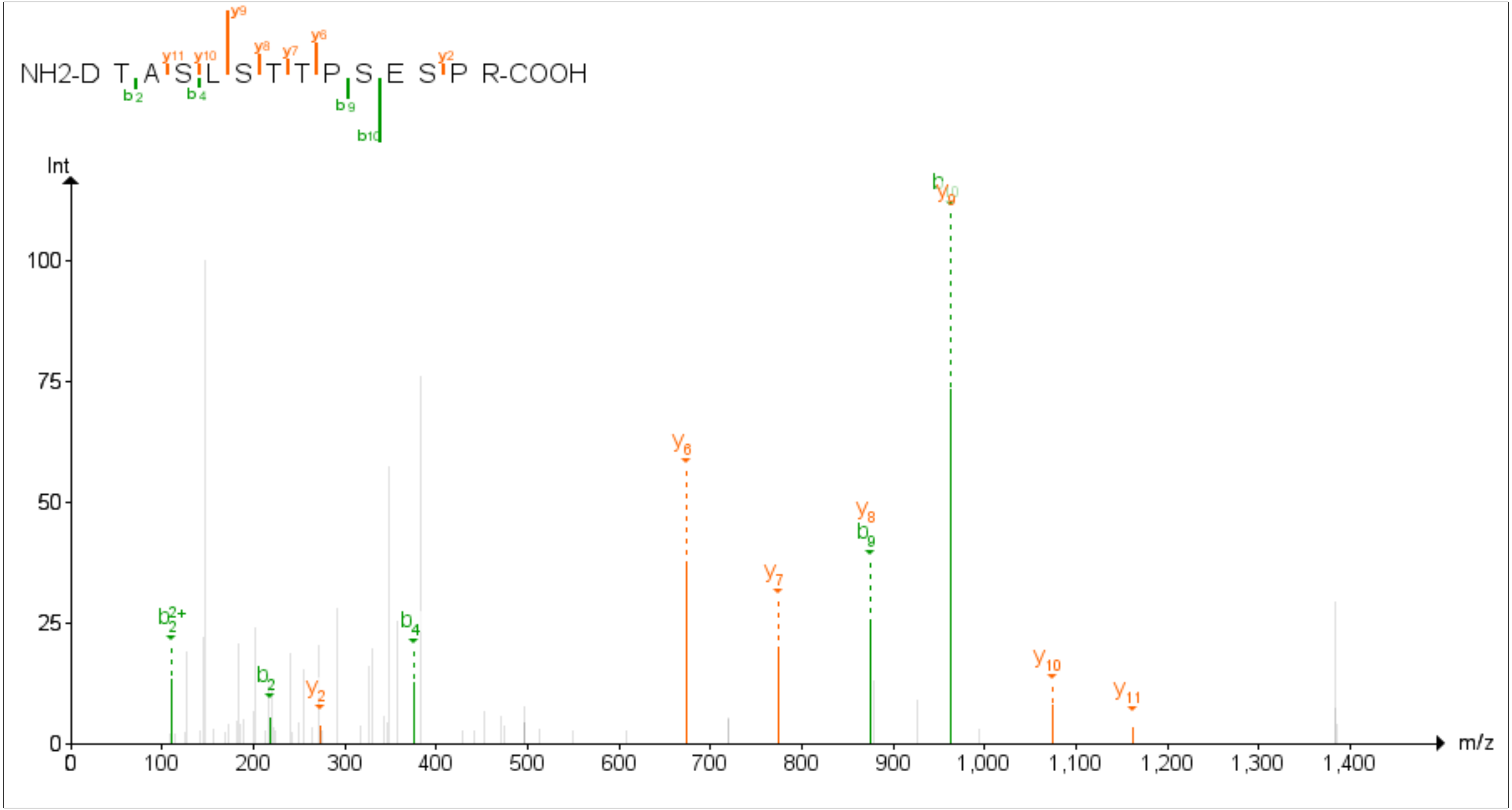

NH2-D T A S L S T T P S E S P R-COOH
y11 y10 y9 y8 y7 y6 y2
b2 b4 b9 b10
Int
100
75
50
25
0
m/z
0 100 200 300 400 500 600 700 800 900 1,000 1,100 1,200 1,300 1,400
b_2^{2+}
b_2
y_2
b_4
y_6
y_7
y_8
b_9
b_{10}
y_9
y_{10}
y_{11}

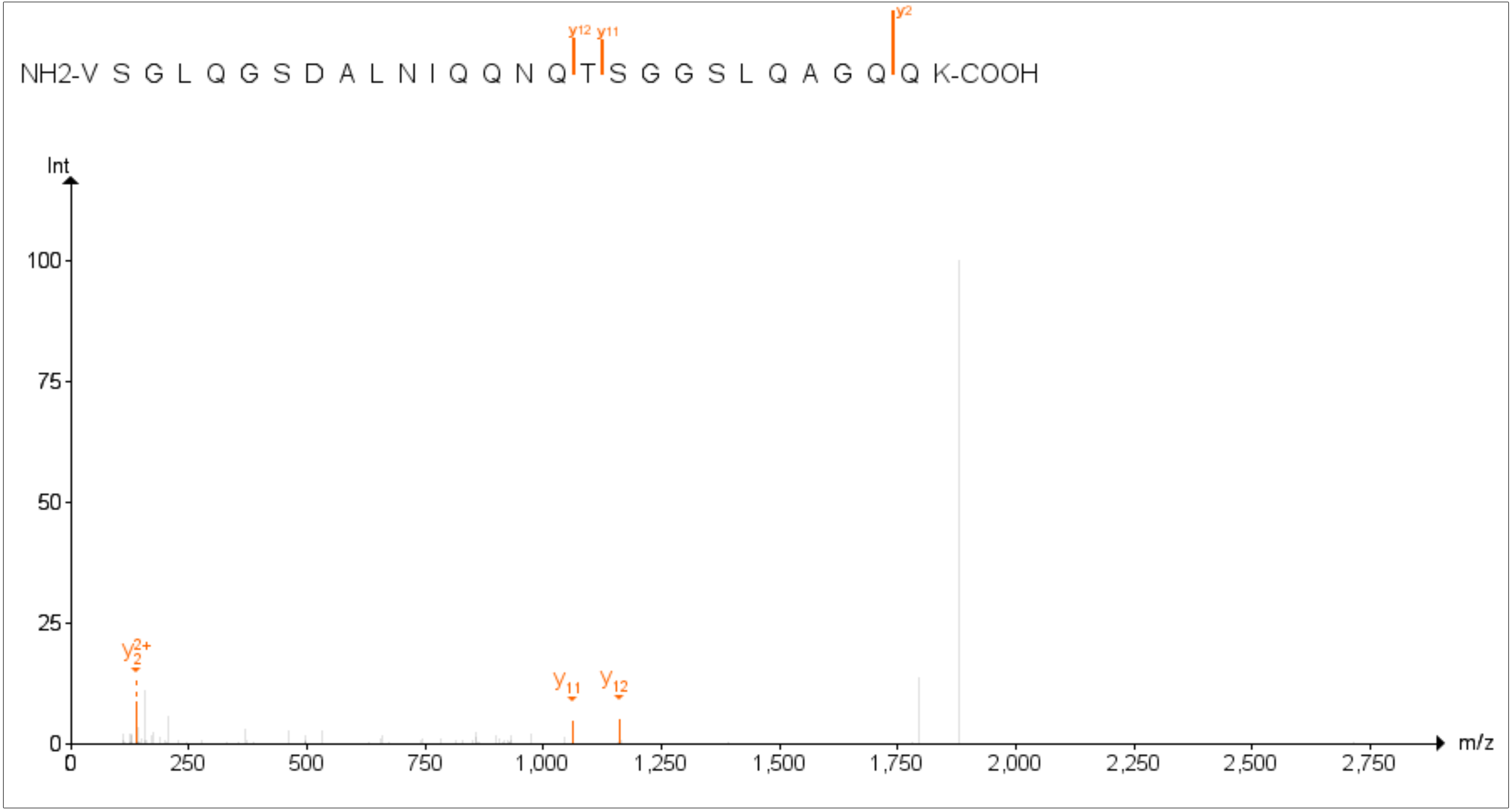

NH2-V S G L Q G S D A L N I Q Q N Q T S G G S L Q A G Q Q K-COOH
y12
y11
y2
Int
100
75
50
25
0
y2 2+
y11
y12
0
250
500
750
1,000
1,250
1,500
1,750
2,000
2,250
2,500
2,750
m/z

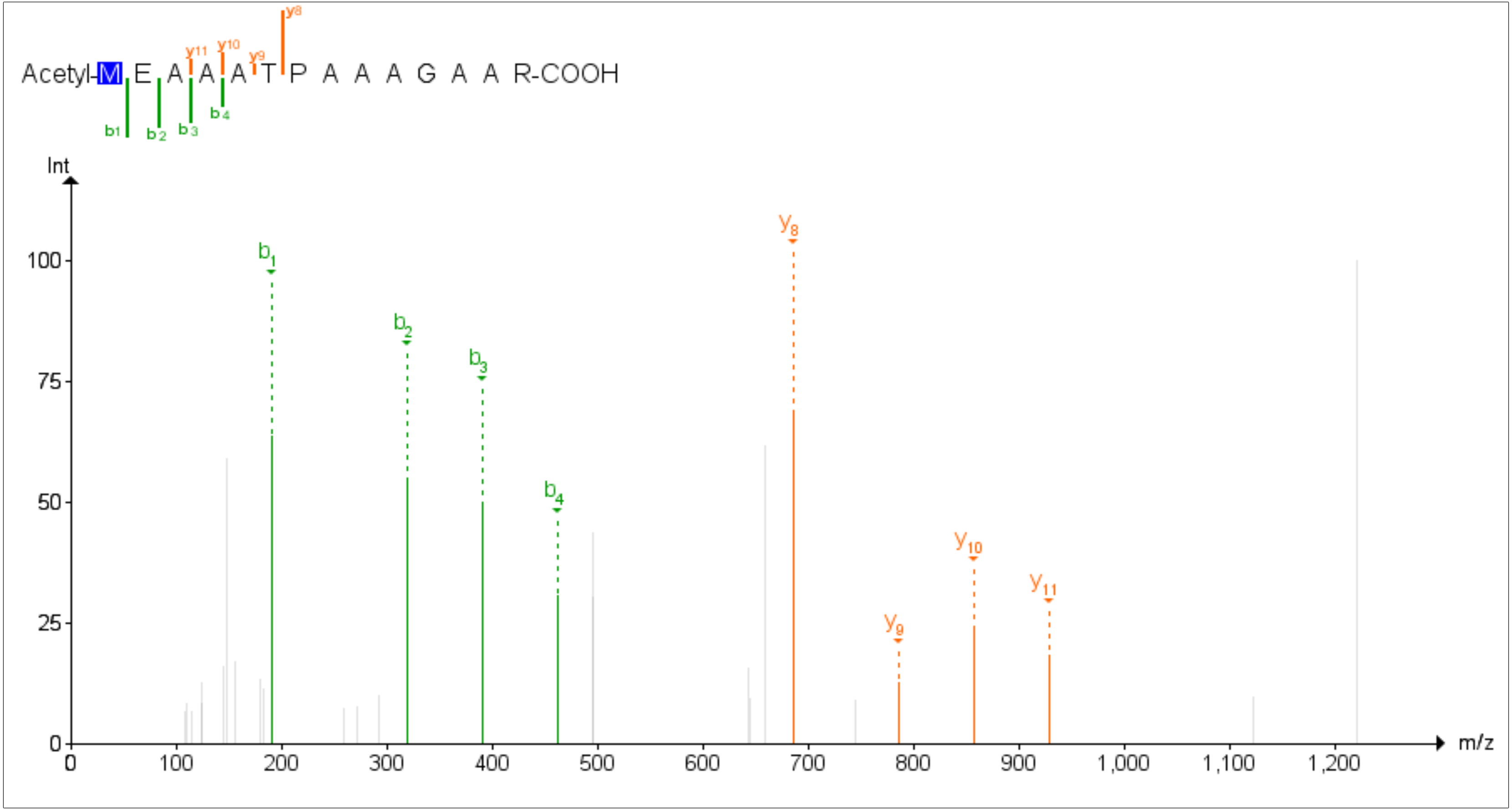

Acetyl-M E A A A T P A A A G A A R-COOH
y8
y11
y10
y9
b1
b2
b3
b4
Int
100
75
50
25
0
m/z
0
100
200
300
400
500
600
700
800
900
1,000
1,100
1,200

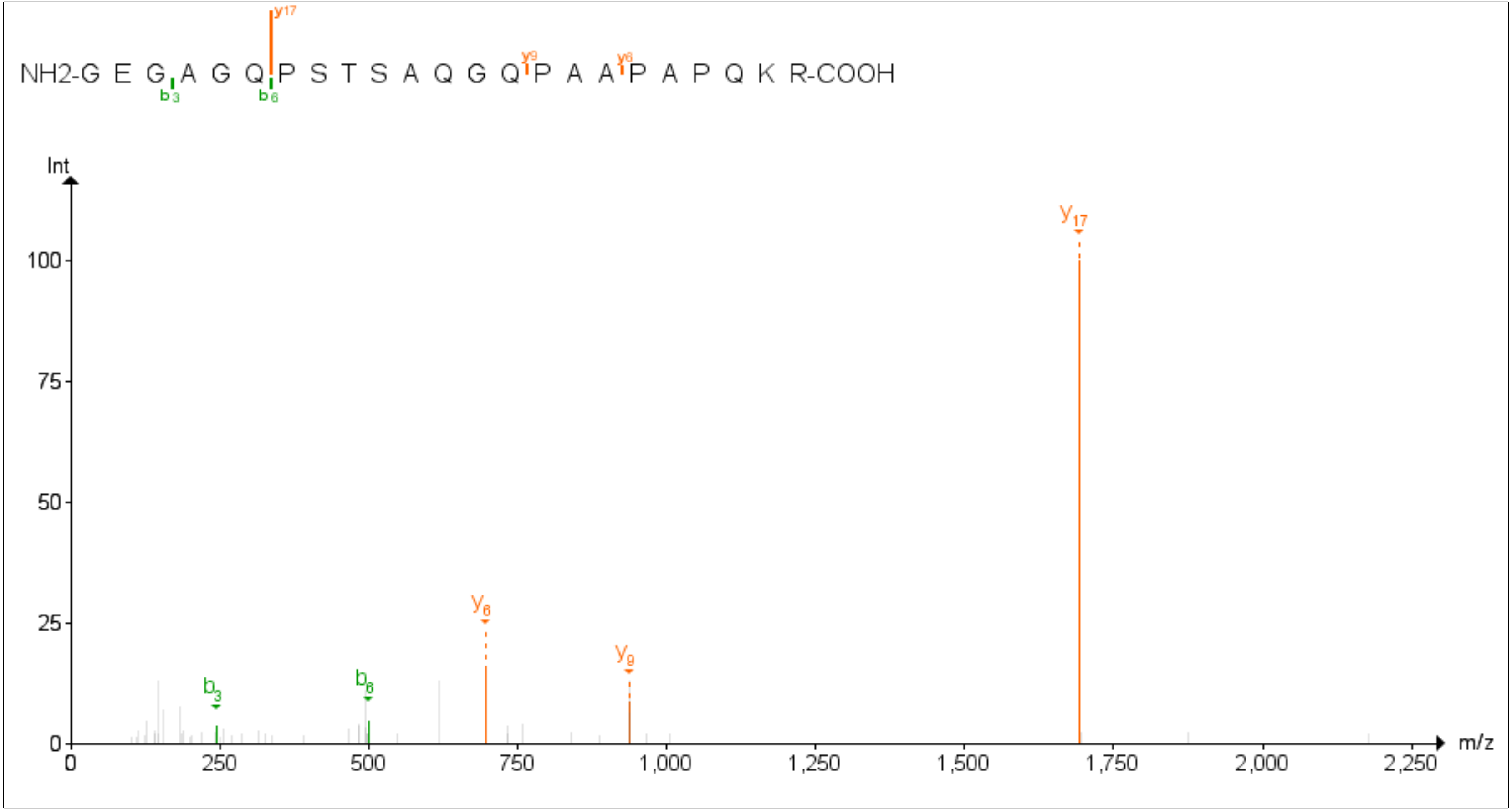

NH2-G E G A G Q P S T S A Q G G Q P A A P A P Q K R-COOH
y17
y9
y6
b3
b6
Int
100
75
50
25
0
y17
y6
y9
b3
b6
0
250
500
750
1,000
1,250
1,500
1,750
2,000
2,250
m/z

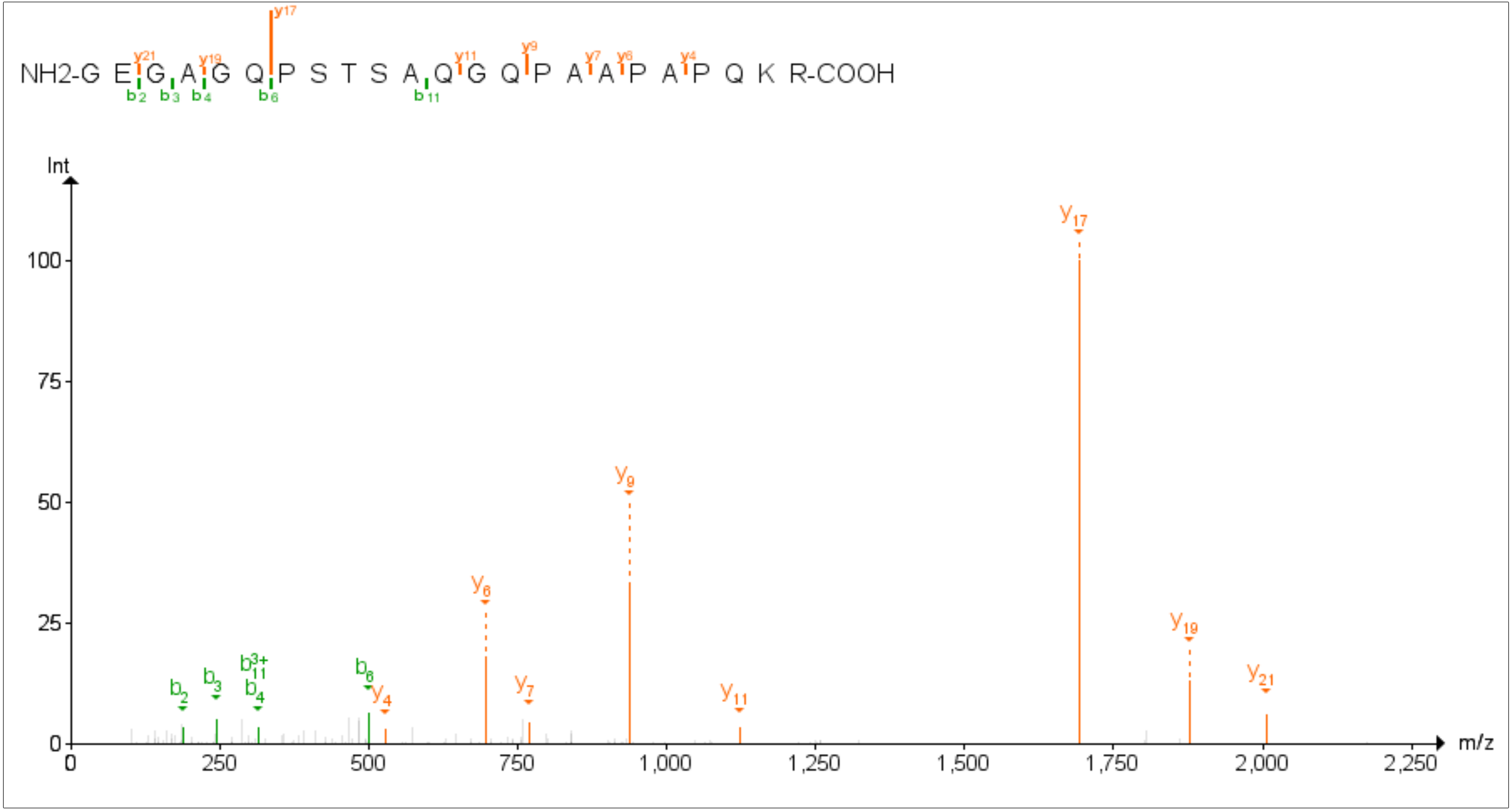

NH2-G E G A G Q P S T S A Q G Q P A A P A P Q K R-COOH
y21 y19 y17 y11 y9 y7 y6 y4
b2 b3 b4 b6 b11
Int
100
75
50
25
0
y17
y9
y6
y19
y21
y7
y11
y4
b2
b3
b11 3+
b4
b6
0
250
500
750
1,000
1,250
1,500
1,750
2,000
2,250
m/z

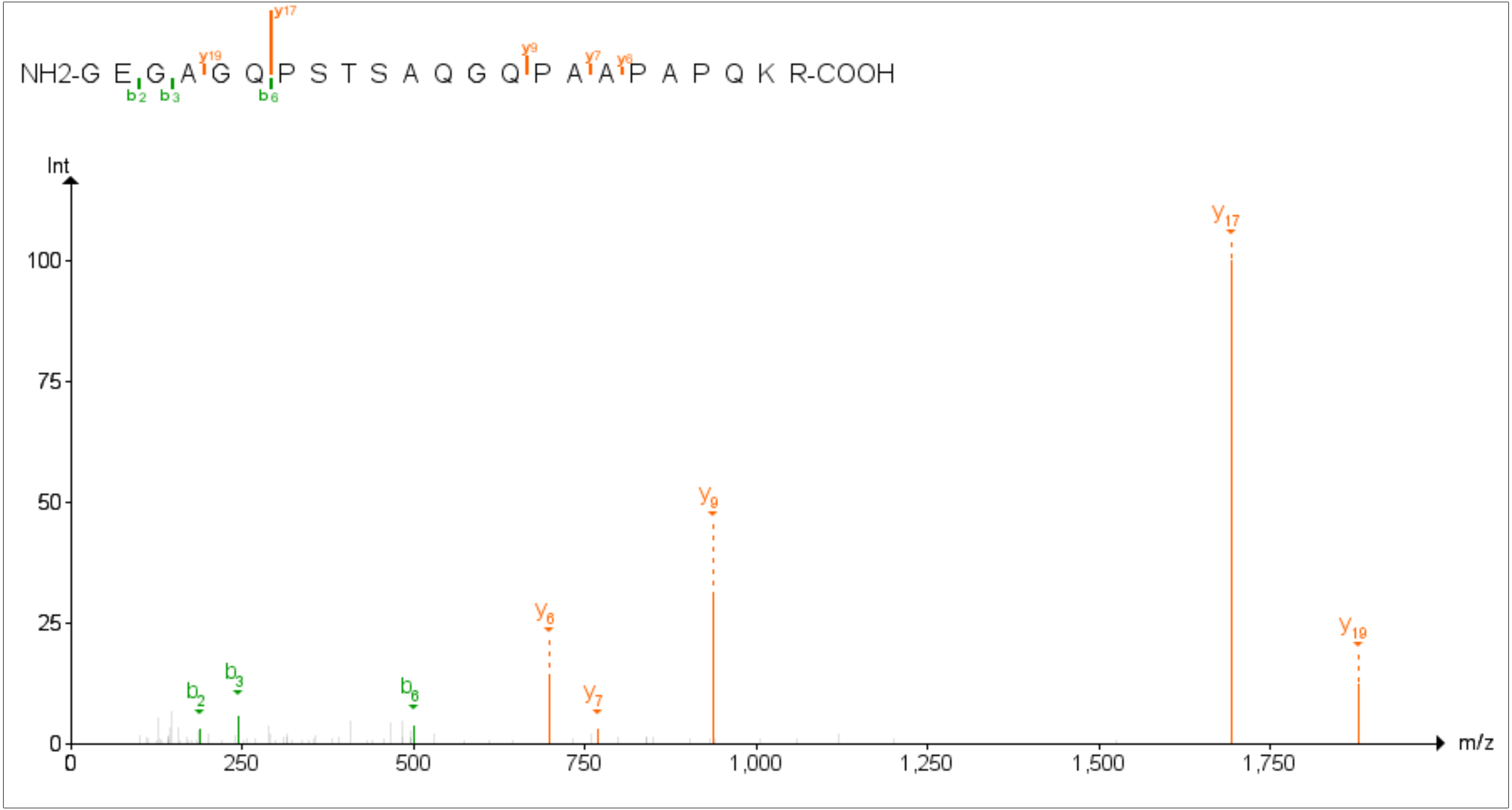
NH2-G E G A G Q P S T S A Q G G Q P A A P A P Q K R-COOH
y17
y19
y9
y7
y6
b2
b3
b6
Int
100
75
50
25
0
y17
y9
y6
y7
y19
b2
b3
b6
0
250
500
750
1,000
1,250
1,500
1,750
m/z

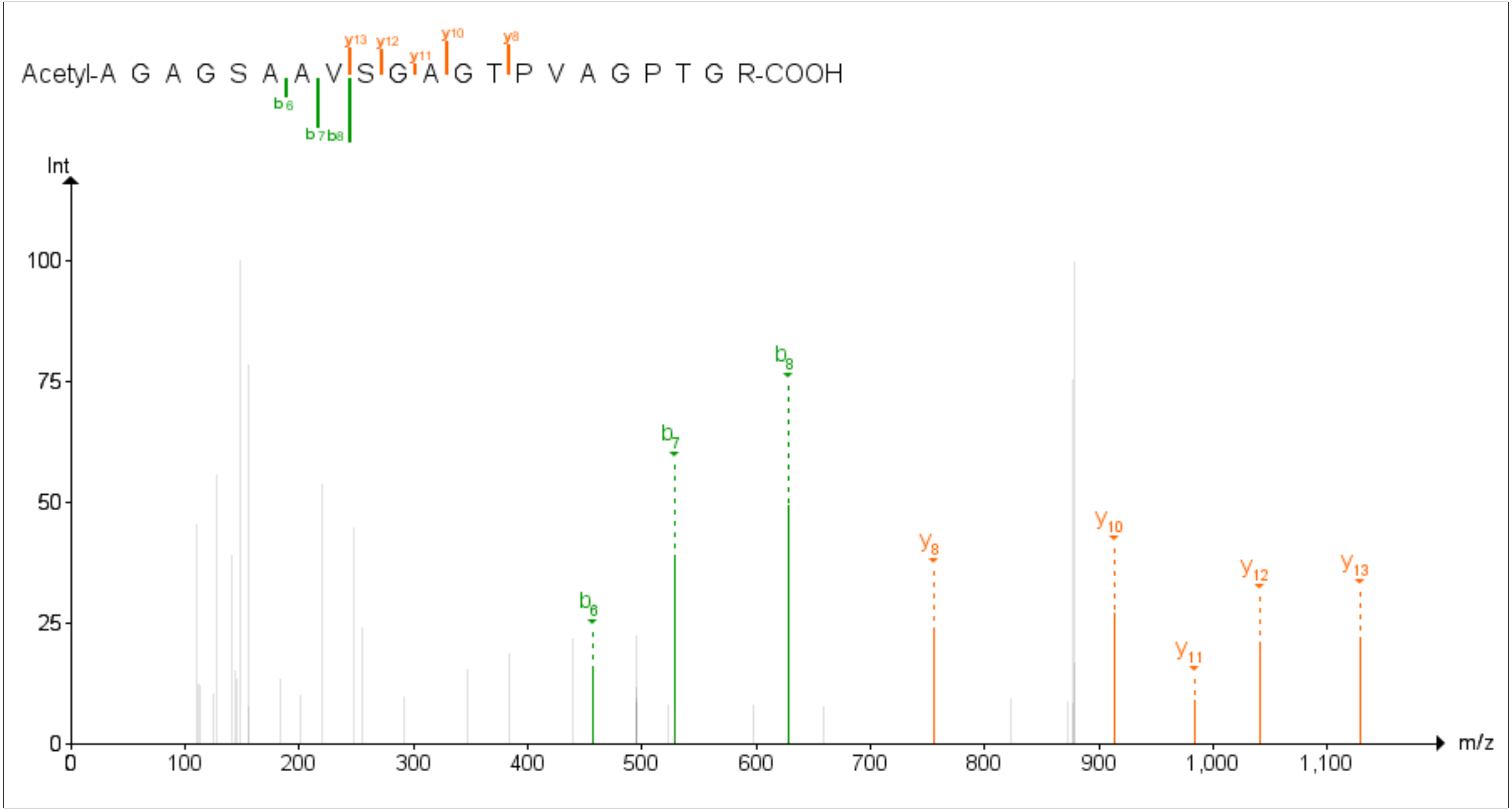

Acetyl-A G A G S A A V S G A G T P V A G P T G R-COOH
b6 b7 b8
y13 y12 y11 y10 y8
Int
100
75
50
25
0
m/z
0 100 200 300 400 500 600 700 800 900 1,000 1,100
b6 b7 b8 y8 y10 y11 y12 y13

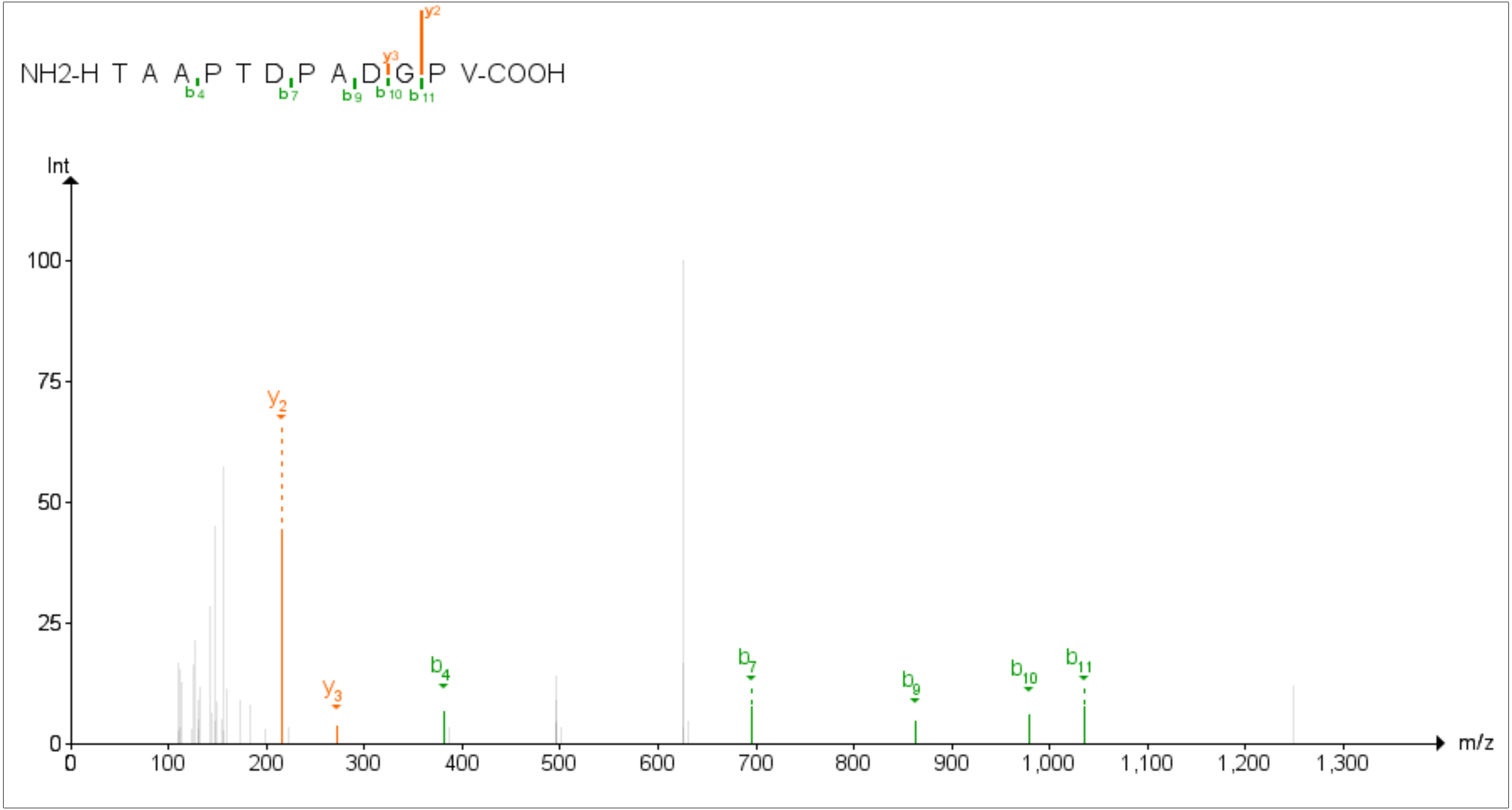
NH2-H T A A P T D P A D G P V-COOH
b4
b7
b9
b10
b11
y3
y2
Int
100
75
50
25
0
y2
y3
b4
b7
b9
b10
b11
0
100
200
300
400
500
600
700
800
900
1,000
1,100
1,200
1,300
m/z

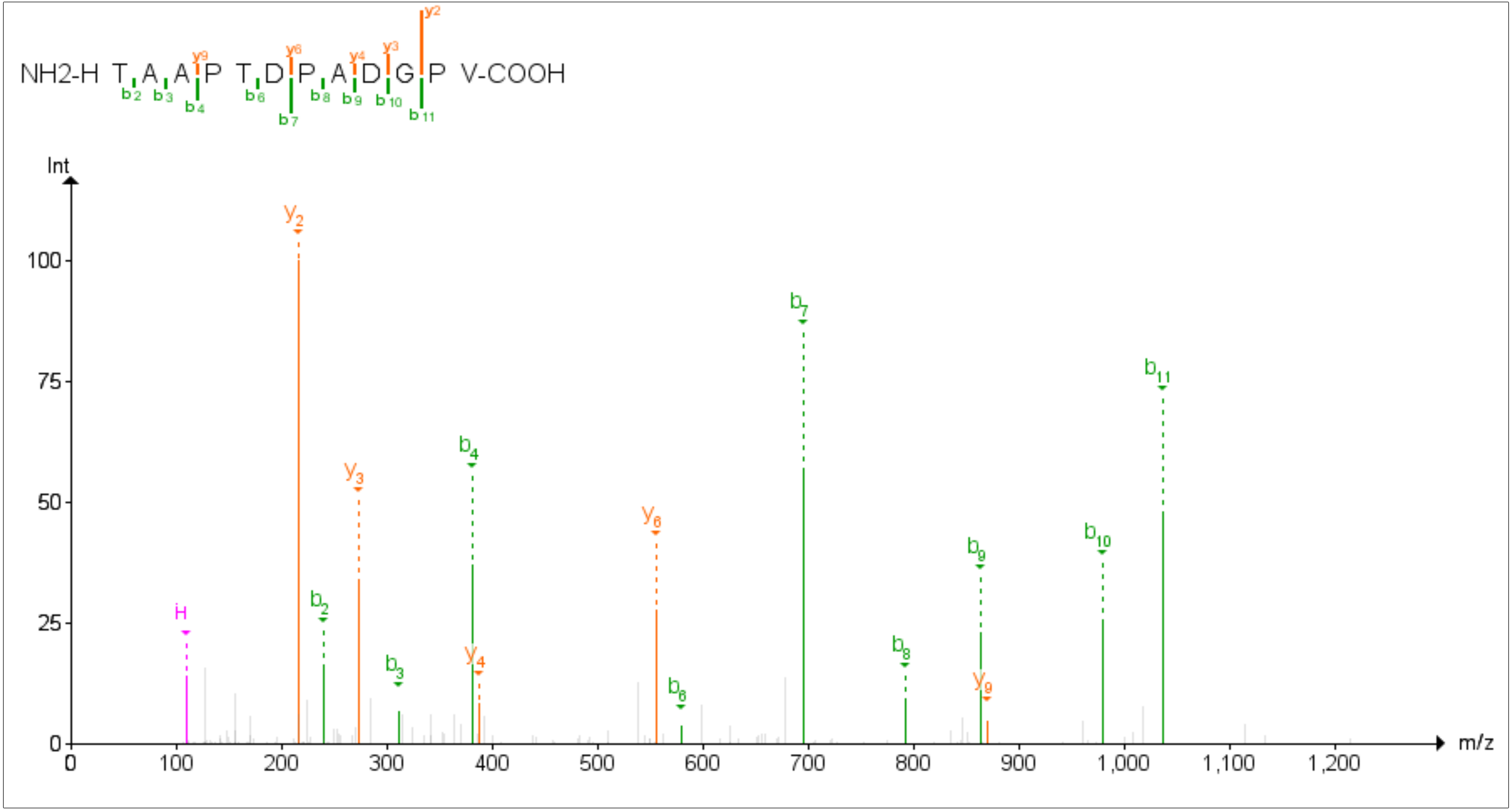

NH2-H T A A P T D P A D G P V-COOH
y9 y6 y4 y3 y2
b2 b3 b4 b6 b7 b8 b9 b10 b11
Int
100
75
50
25
0
m/z
0 100 200 300 400 500 600 700 800 900 1,000 1,100 1,200
y2 y3 y4 y6 y9
b2 b3 b4 b6 b7 b8 b9 b10 b11
H

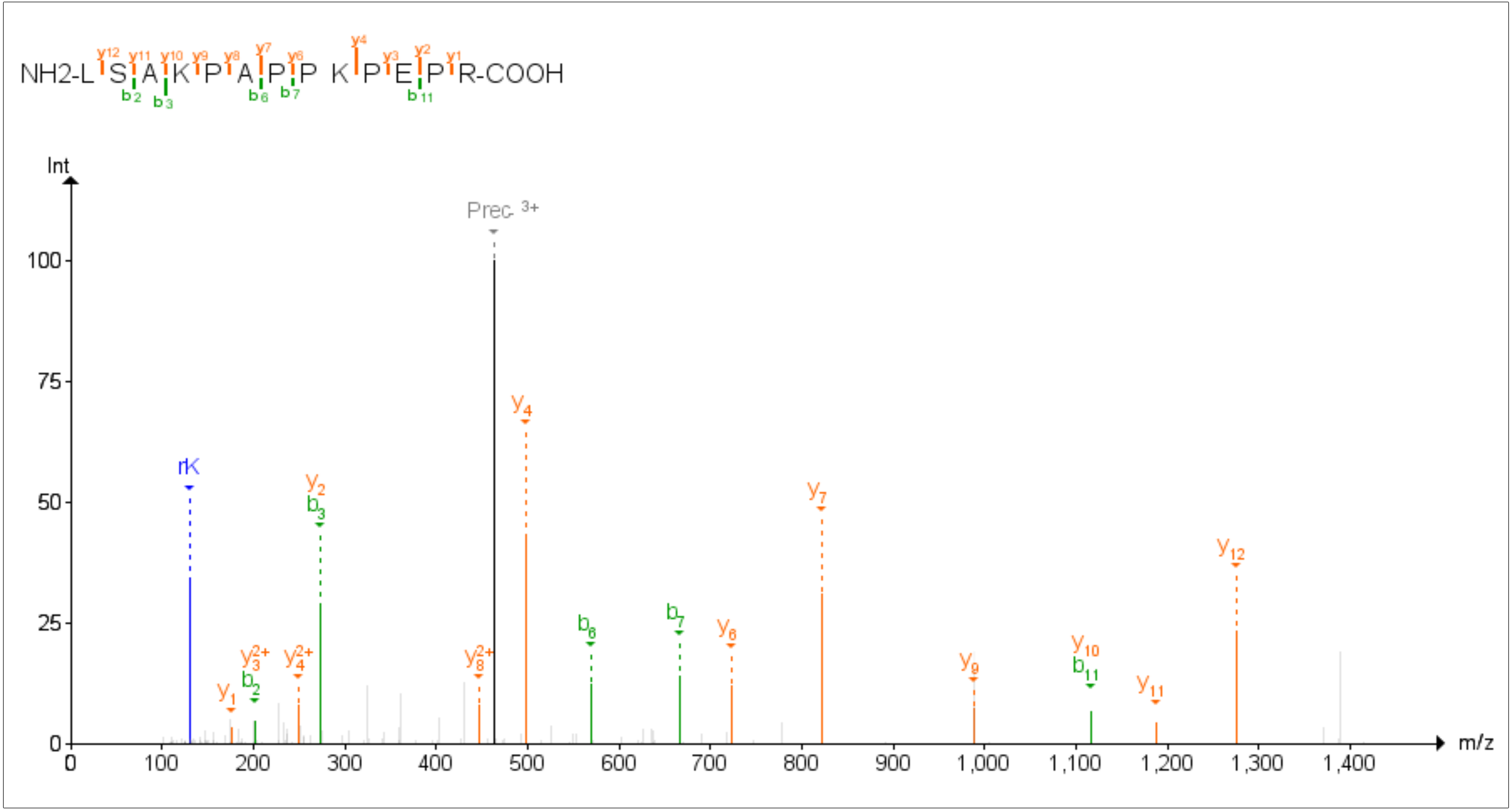
NH2-LSAKPAPP KPEPR-COOH
y12 y11 y10 y9 y8 y7 y6 y4 y3 y2 y1
b2 b3 b6 b7 b11
Int
Prec 3+
rK
y4
y2
b3
y7
y12
b6
b7
y6
y1
b2
y3 2+
y4 2+
y8 2+
y9
y10
b11
y11
0
25
50
75
100
0
100
200
300
400
500
600
700
800
900
1,000
1,100
1,200
1,300
1,400
m/z

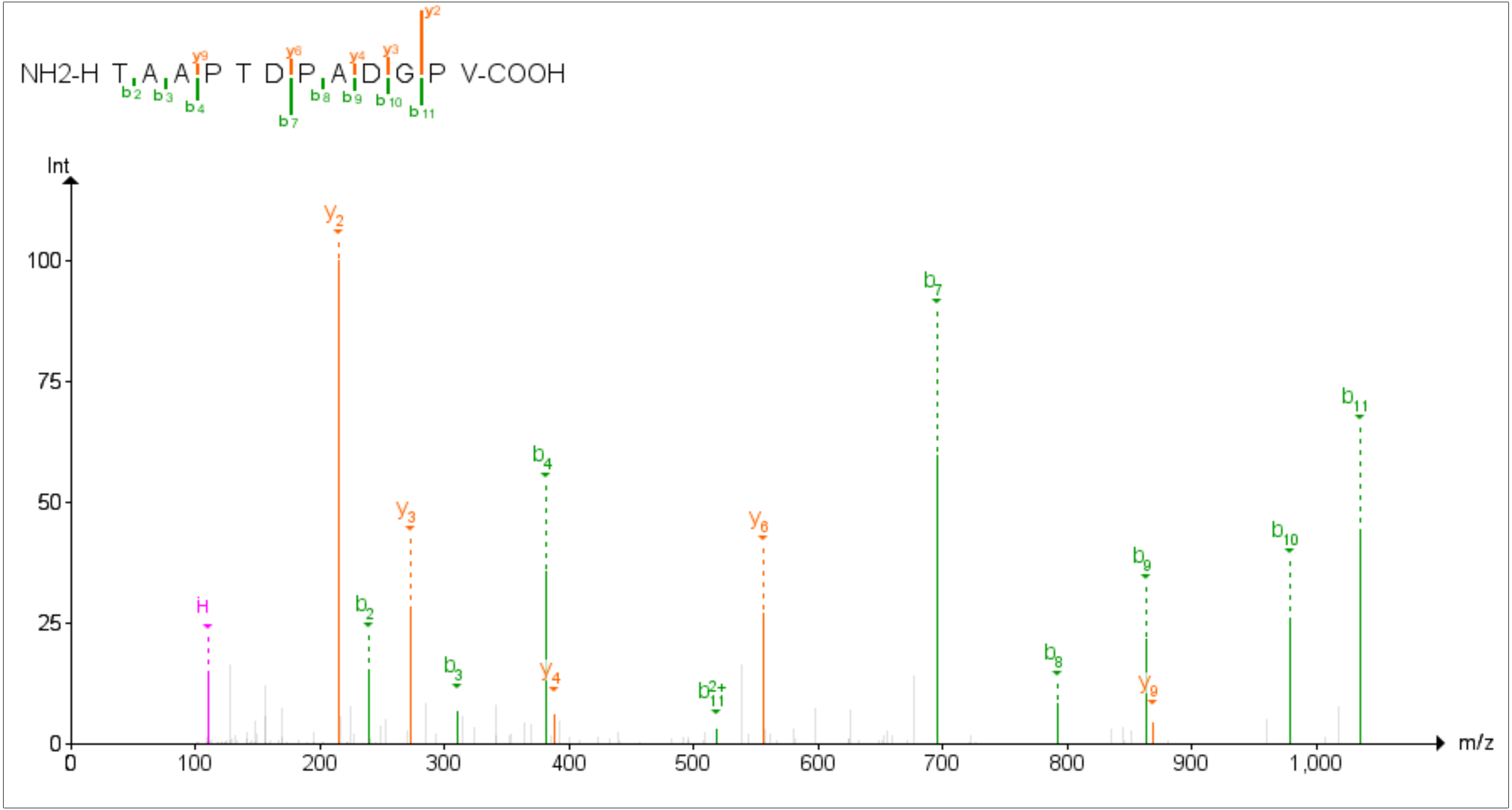

NH2-H T A A P T D P A D G P V-COOH
y9 y6 y4 y3 y2
b2 b3 b4 b7 b8 b9 b10 b11
Int
100
75
50
25
0
m/z
0
100
200
300
400
500
600
700
800
900
1,000
H
y2
b2
y3
b3
b4
y4
b11 2+
y6
b7
b8
b9
y9
b10
b11

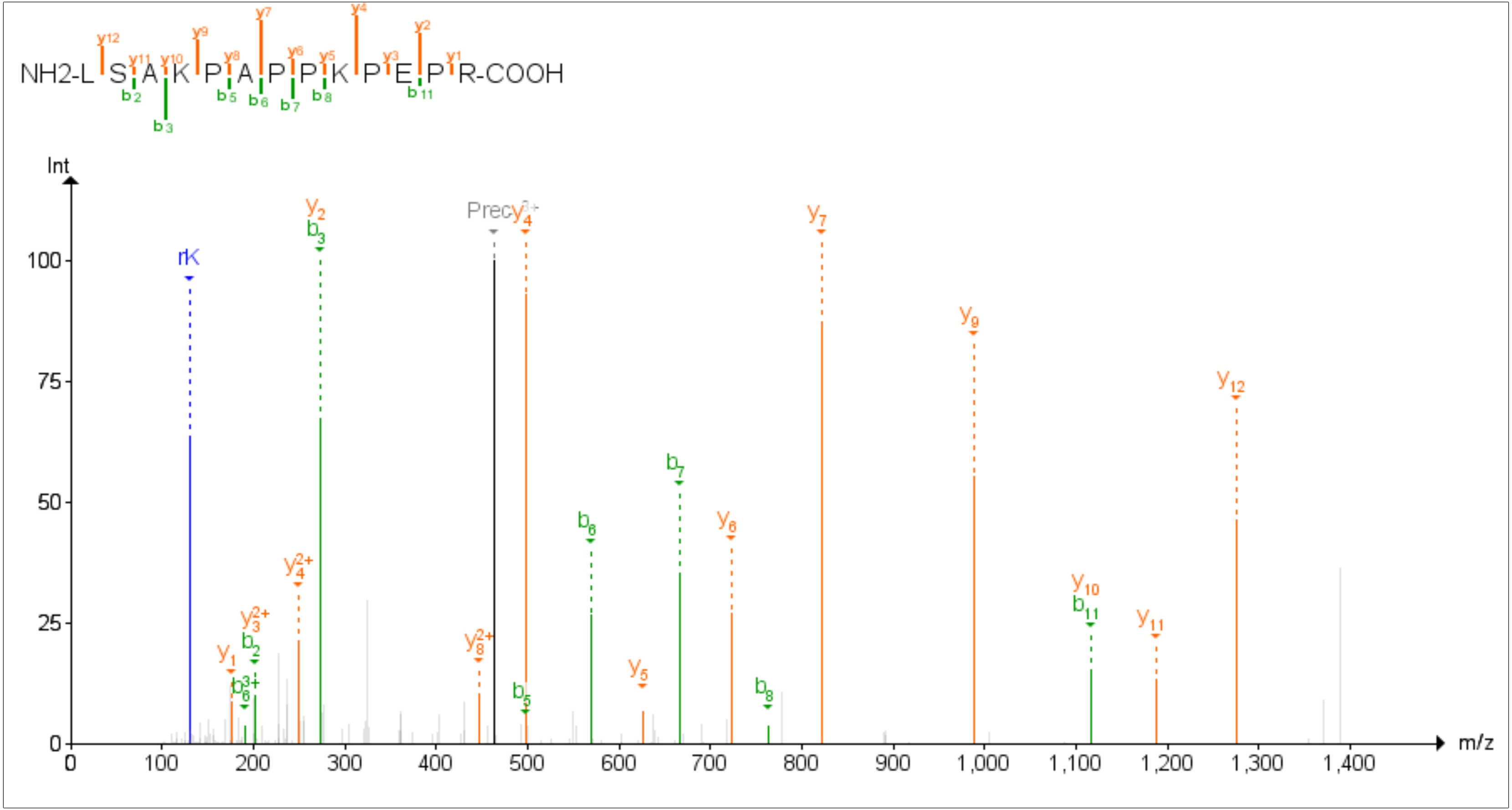

NH2-L S A K P A P P K P E P R-COOH
Int
m/z
rK
Prec

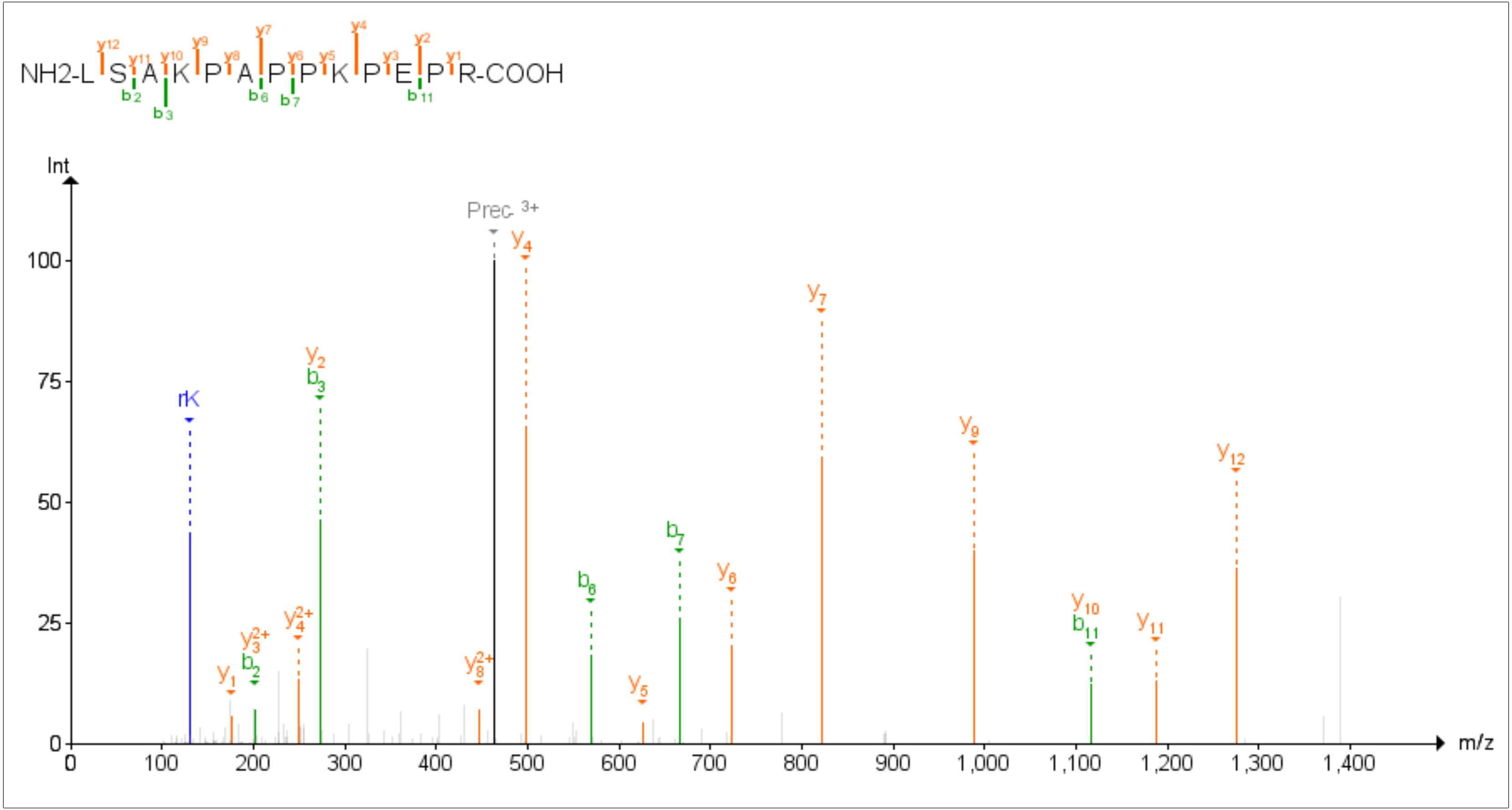

NH2-L S A K P A P P K P E P R-COOH
y12 y11 y10 y9 y8 y7 y6 y5 y4 y3 y2 y1
b2 b3 b6 b7 b11
Int
Prec 3+
rK
y1
b2
y3 2+
y4 2+
y2
b3
y8 2+
y4
b6
y5
b7
y6
y7
y9
y10
b11
y11
y12
0
25
50
75
100
0
100
200
300
400
500
600
700
800
900
1,000
1,100
1,200
1,300
1,400
m/z

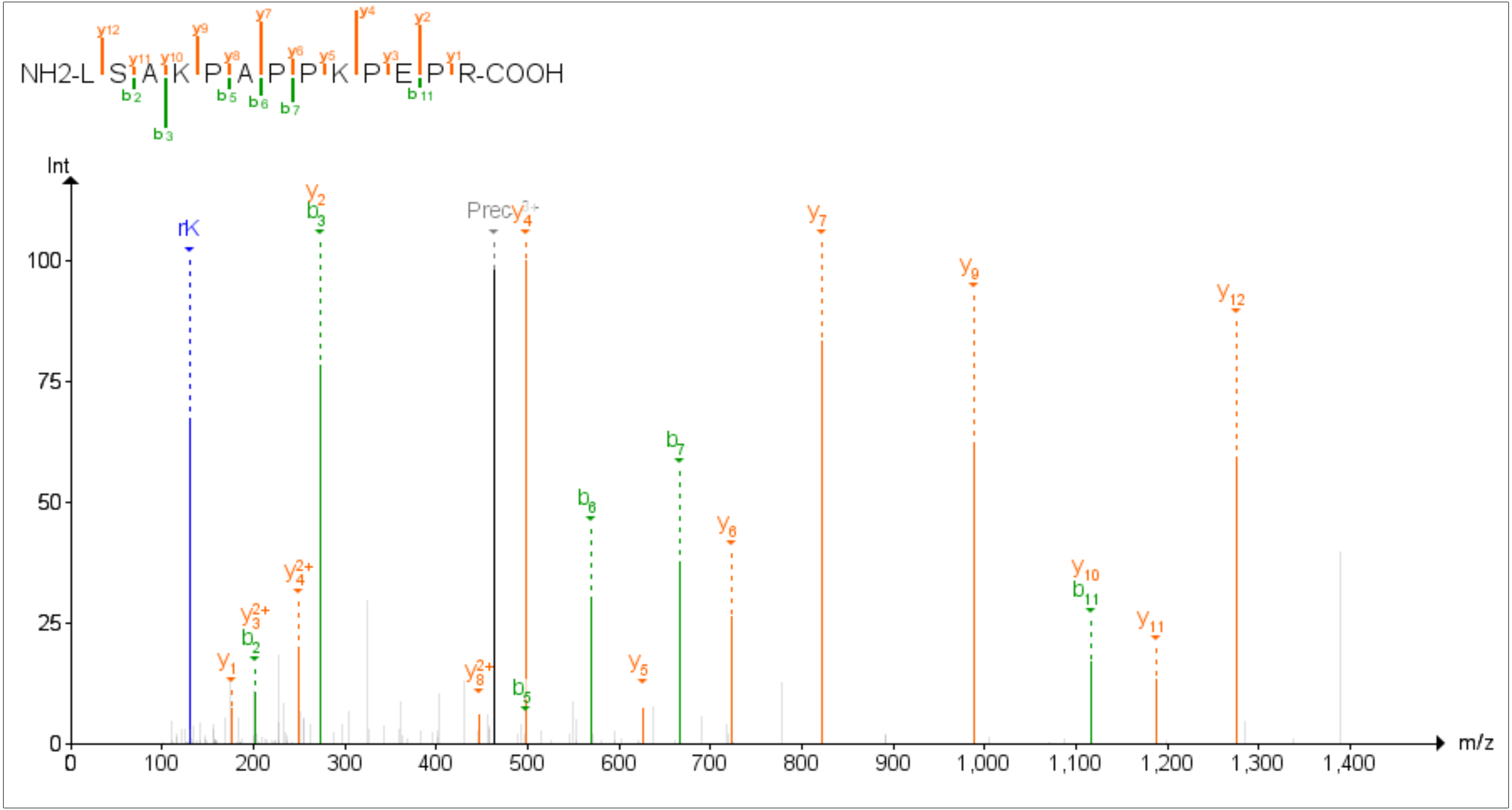

NH2-LSAKPAPPKPEPR-COOH
y12 y11 y10 y9 y8 y7 y6 y5 y4 y3 y2 y1
b2 b3 b5 b6 b7 b11
Int
100
75
50
25
0
rK
y2
b3
Prec
y4
y7
y9
y12
b7
b6
y6
y10
b11
y11
y4 2+
y3 2+
b2
y1
y8 2+
b5
y5
0
100
200
300
400
500
600
700
800
900
1,000
1,100
1,200
1,300
1,400
m/z

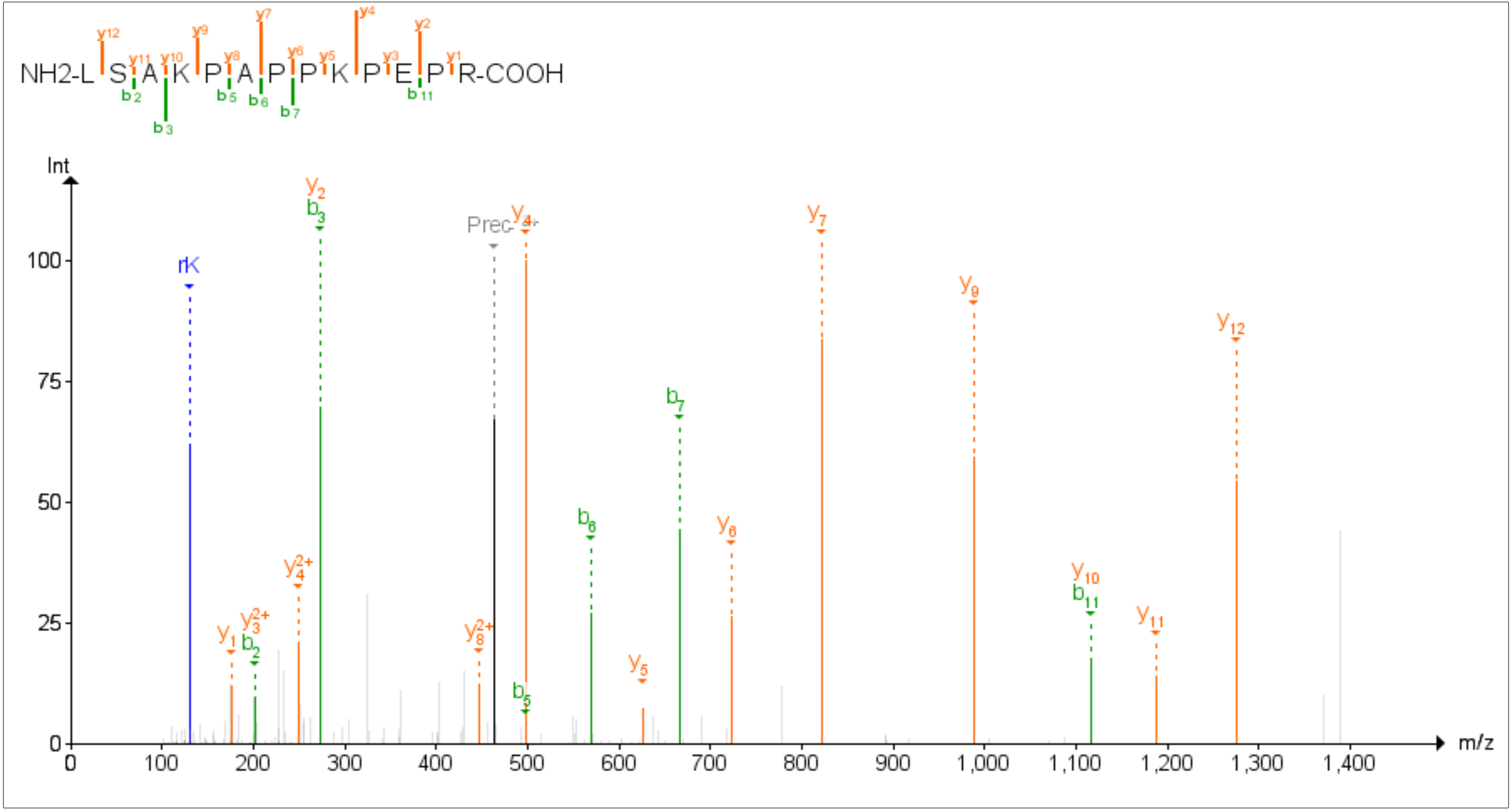

NH2-L S A K P A P P K P E P R-COOH
Int
m/z
Prec 4+
rK

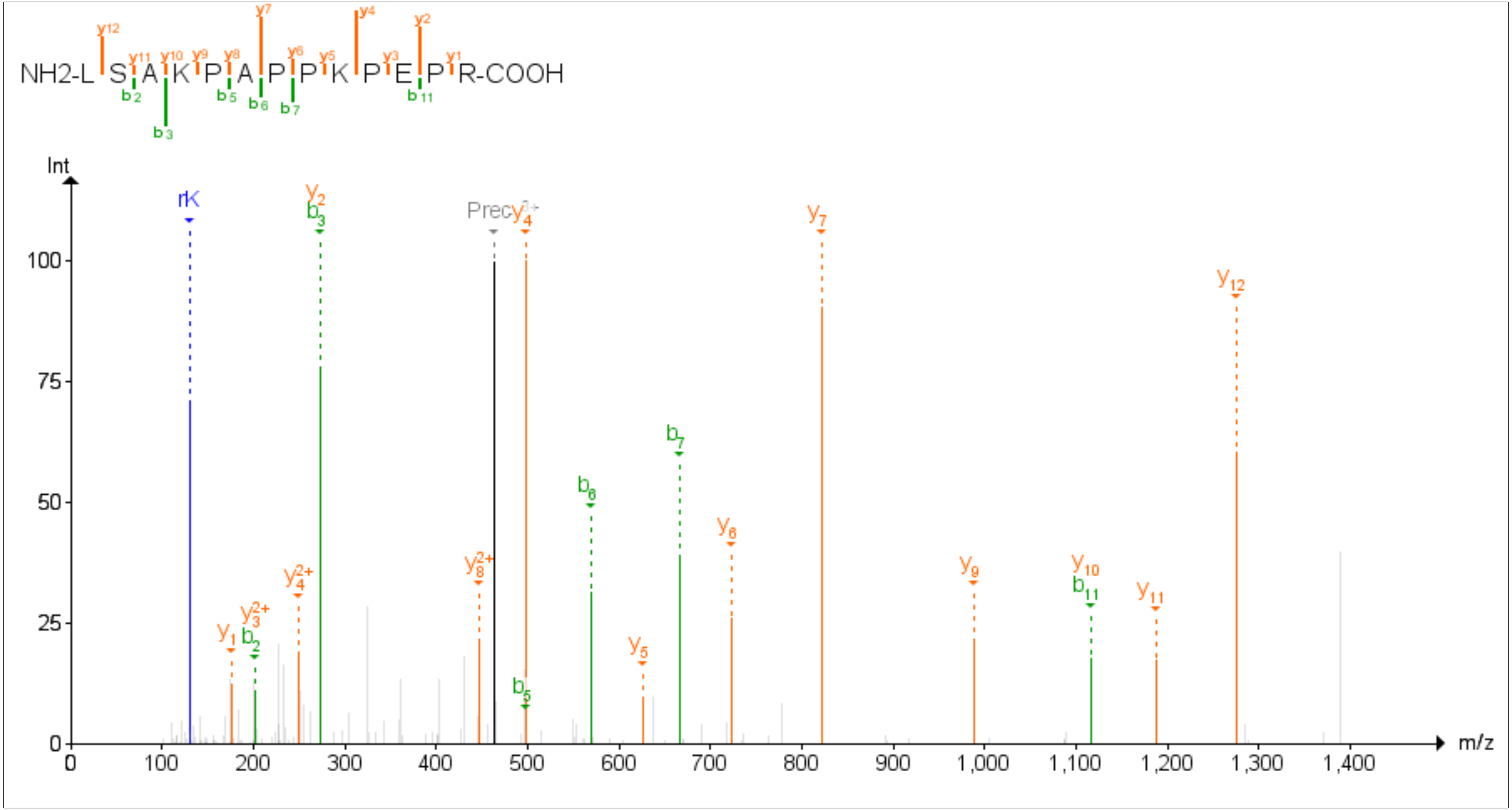

NH2-LSAKPAPPKPEPR-COOH
Int
m/z
rK
Prec

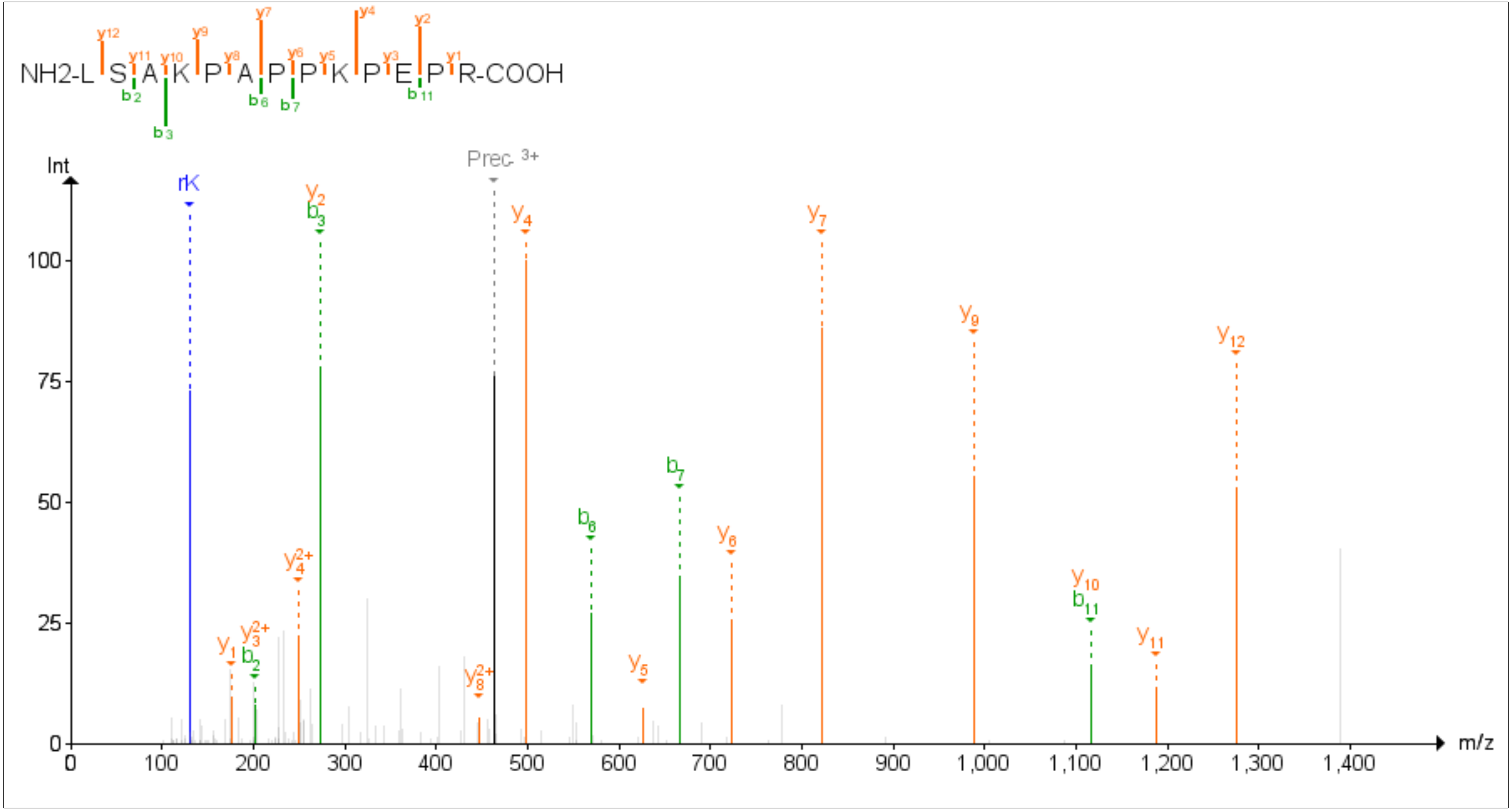

NH2-LSAKPAPPKPEPR-COOH
y12 y11 y10 y9 y8 y7 y6 y5 y4 y3 y2 y1
b2 b3 b6 b7 b11
Int
Prec 3+
rK
m/z
0
25
50
75
100
100
200
300
400
500
600
700
800
900
1,000
1,100
1,200
1,300
1,400

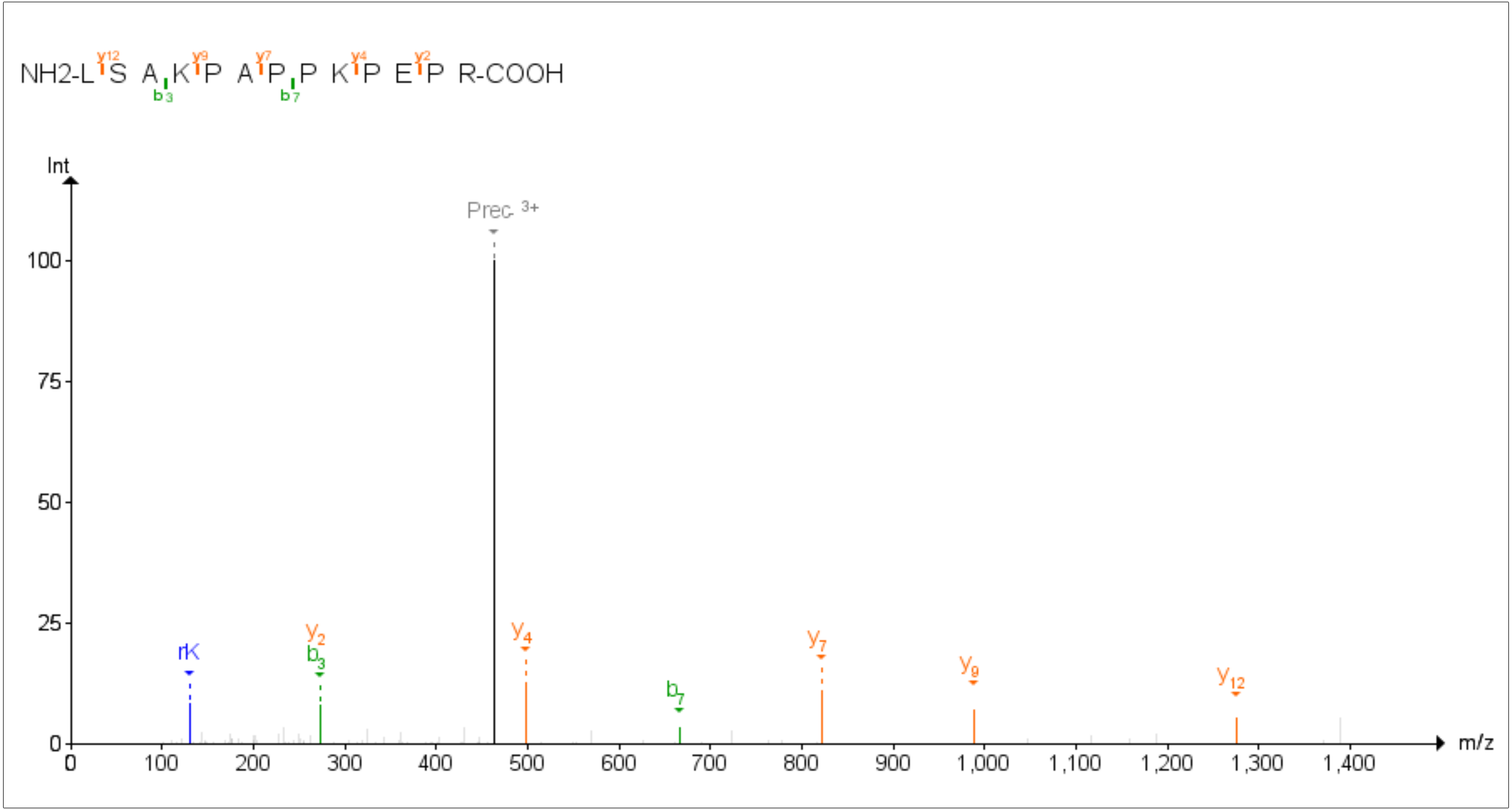

NH2-L S A K P A P P K P E P R-COOH
y12 y9 y7 y4 y2
b3 b7
Int
Prec 3+
100
75
50
25
0
rK
y2
b3
y4
b7
y7
y9
y12
0 100 200 300 400 500 600 700 800 900 1,000 1,100 1,200 1,300 1,400
m/z

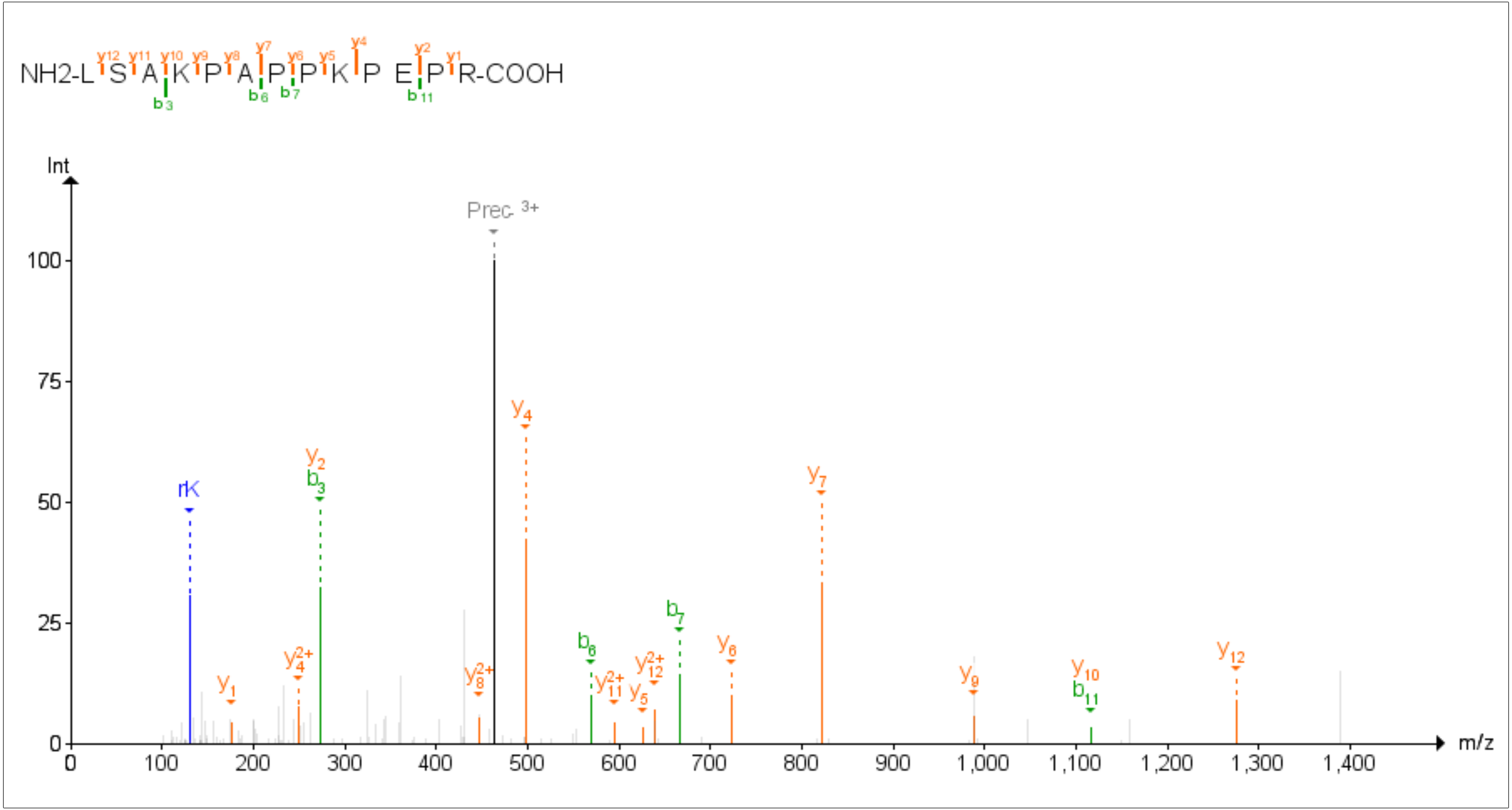

NH2-L S A K P A P P K P E P R-COOH
y12 y11 y10 y9 y8 y7 y6 y5 y4 y2 y1
b3 b6 b7 b11
Int
Prec 3+
rK
y2
b3
y4
y7
b6
b7
y6
y1
y4 2+
y8 2+
y11 2+
y12 2+
y5
y9
y10
b11
y12
m/z

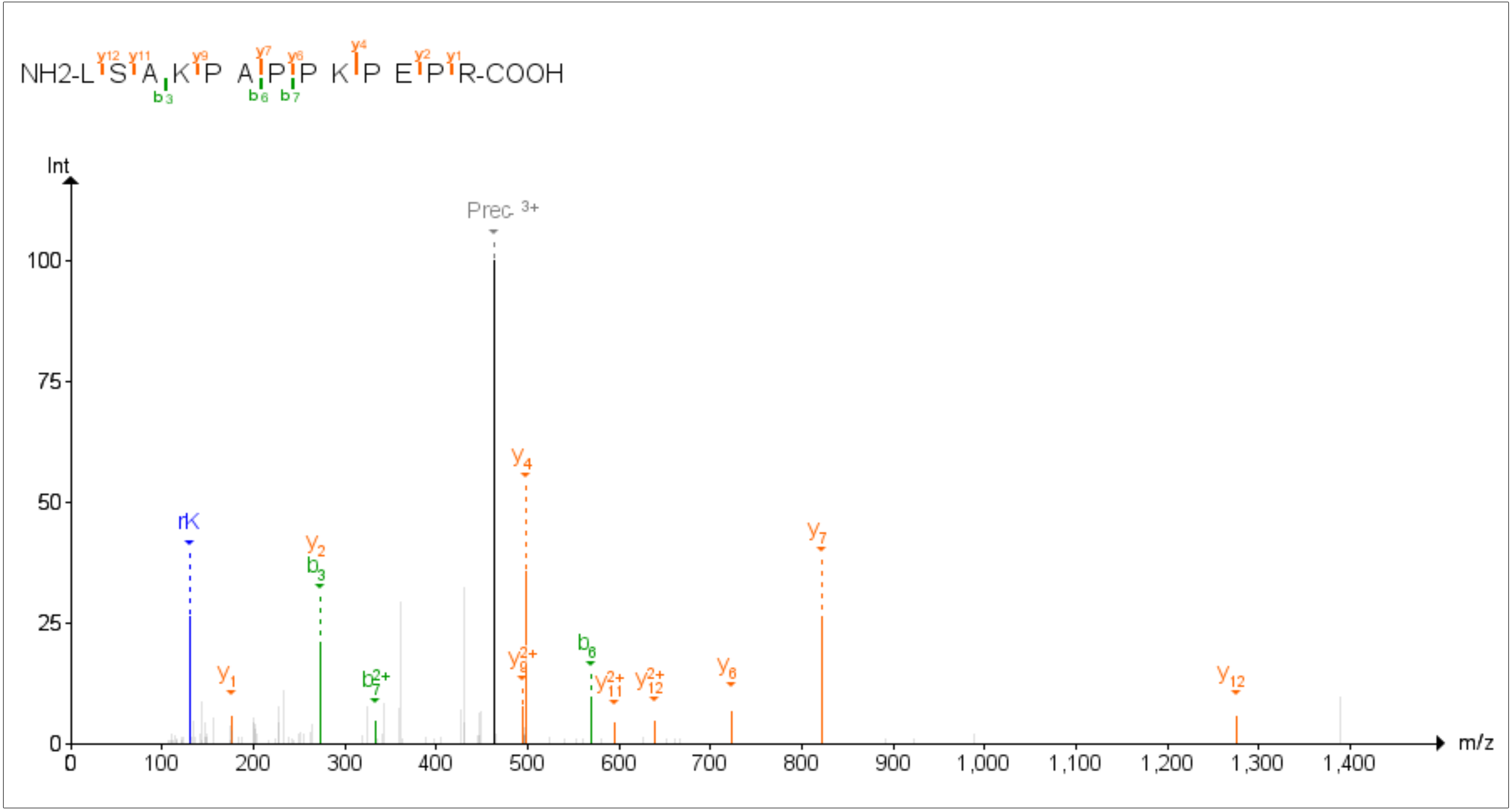

NH2-L S A K P A P P K P E P R-COOH
y12 y11 y9 y7 y6 y4 y2 y1
b3 b6 b7
Int
Prec 3+
100
75
50
25
0
rK
y1
y2
b3
b7 2+
y4
y9 2+
b6
y11 2+
y12 2+
y6
y7
y12
0
100
200
300
400
500
600
700
800
900
1,000
1,100
1,200
1,300
1,400
m/z

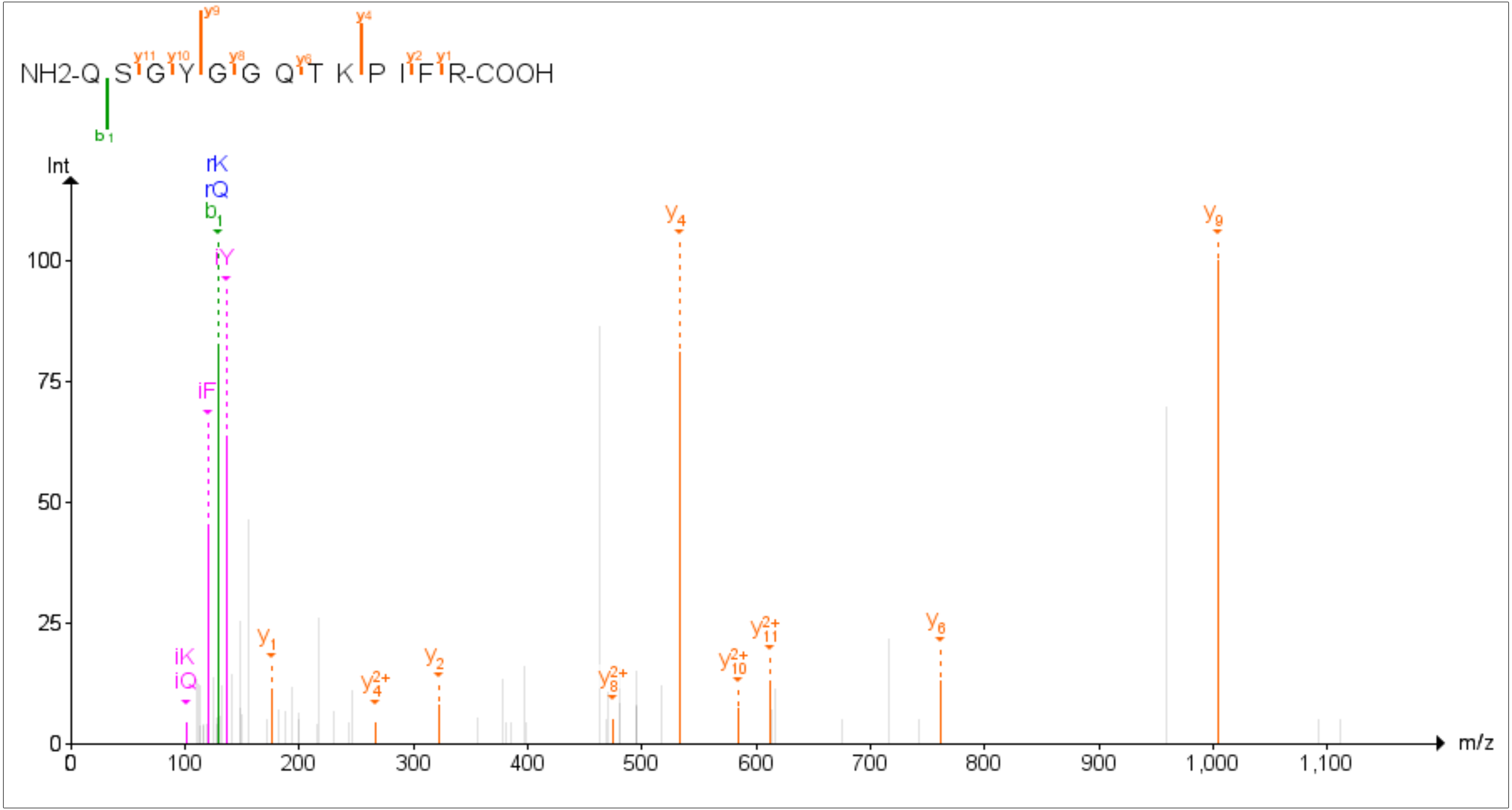

NH2-Q S G Y G G Q T K P I F R-COOH
b1
y11 y10 y9 y8 y6 y4 y2 y1
Int
m/z
rK
rQ
b1
iY
iF
iK
iQ
y1
y2
y4
y9
y6
0
25
50
75
100
0
100
200
300
400
500
600
700
800
900
1,000
1,100

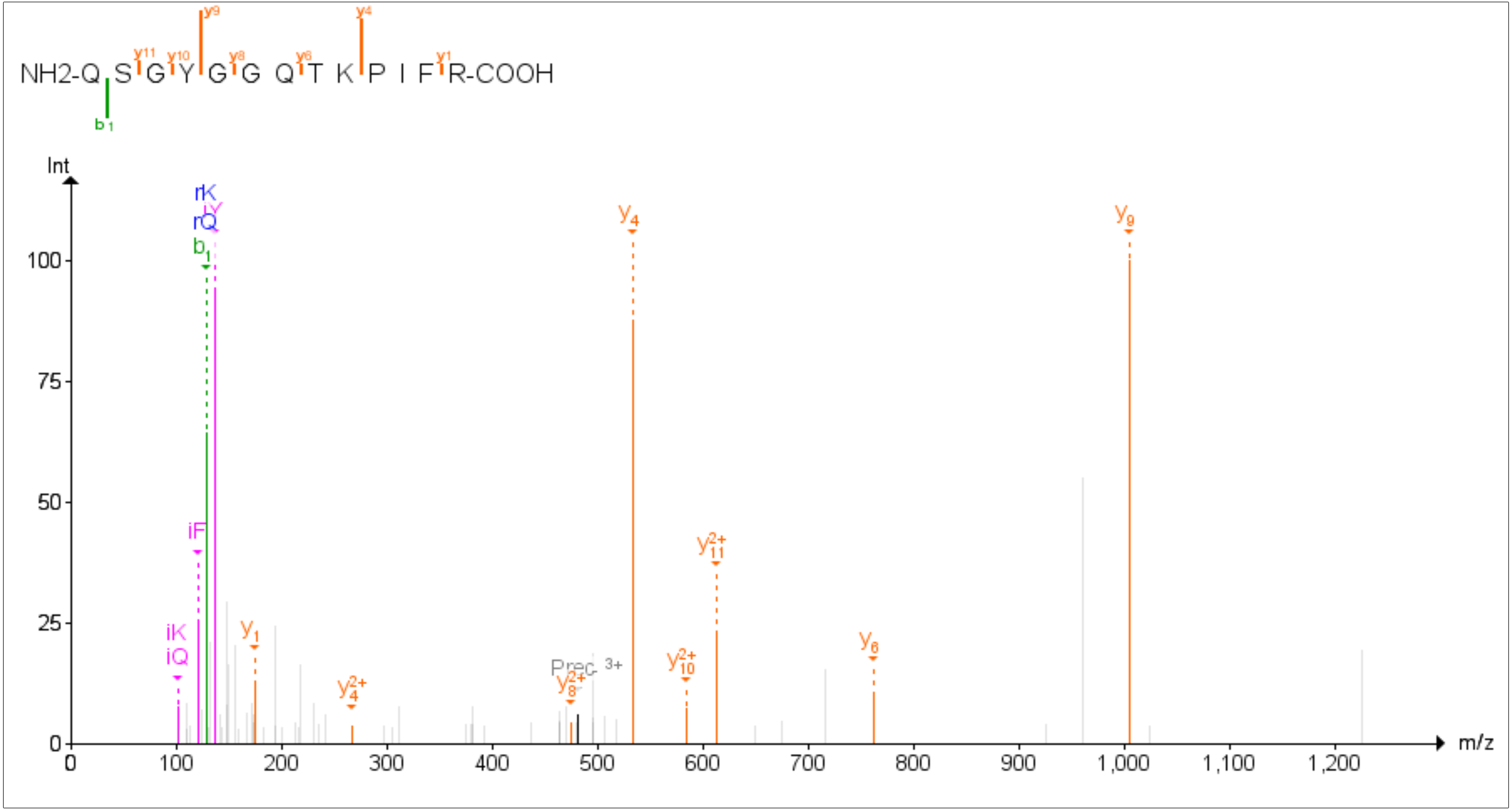
NH2-Q S G Y G G Q T K P I F R-COOH
y11 y10 y9 y8 y6 y4 y1
b1
Int
100
75
50
25
0
rK
iY
rQ
b1
iF
iK
iQ
y1
y4
y9
y4 2+
Prec 3+
y8 2+
y10 2+
y11 2+
y6
0
100
200
300
400
500
600
700
800
900
1,000
1,100
1,200
m/z

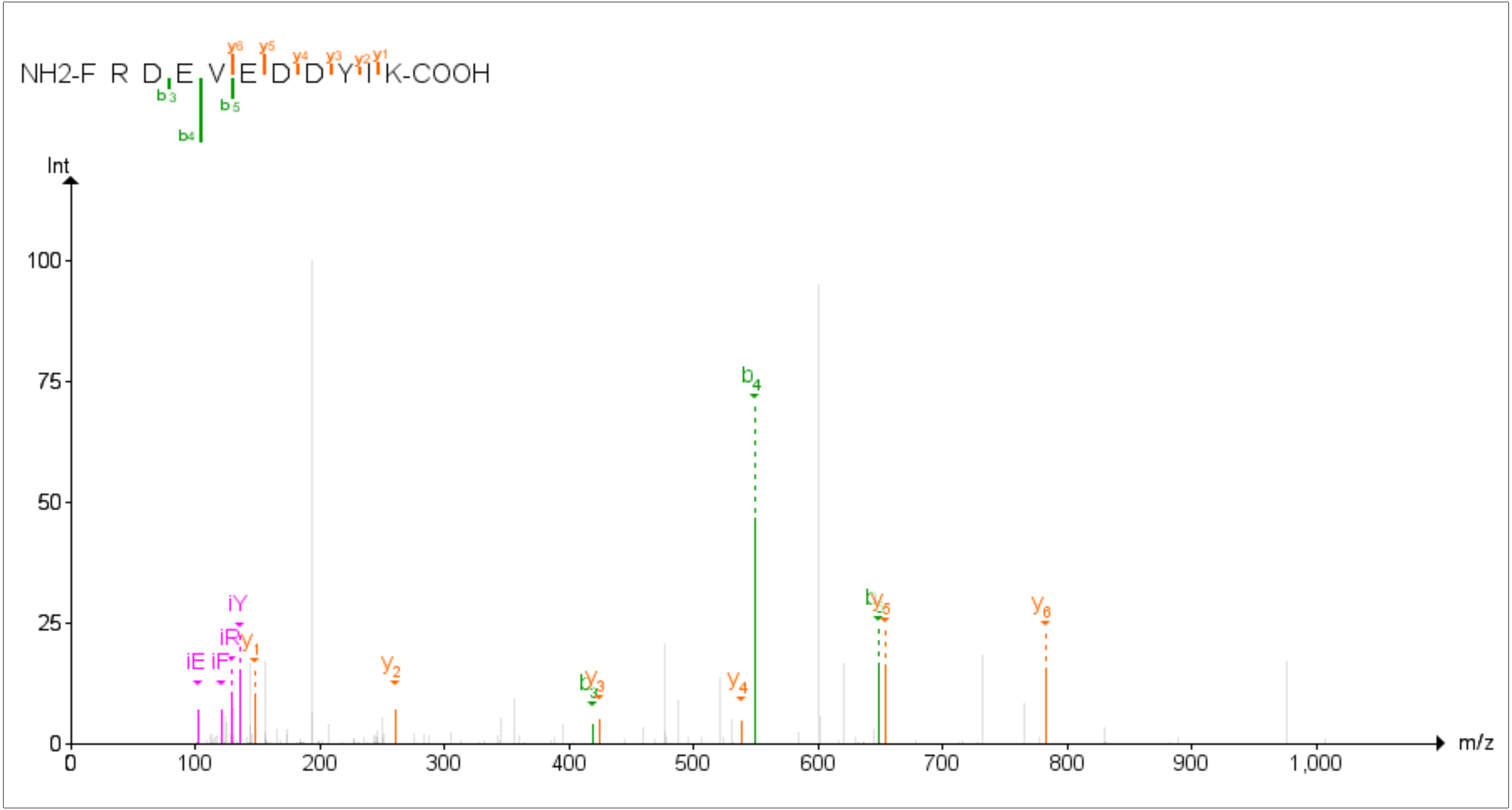

NH2-F R D E V E D D Y I K-COOH
y6 y5 y4 y3 y2 y1
b3 b5 b4
Int
m/z
b4
y6
y5
b5
y4
y3
b3
y2
y1
iY
iR
iE
iF

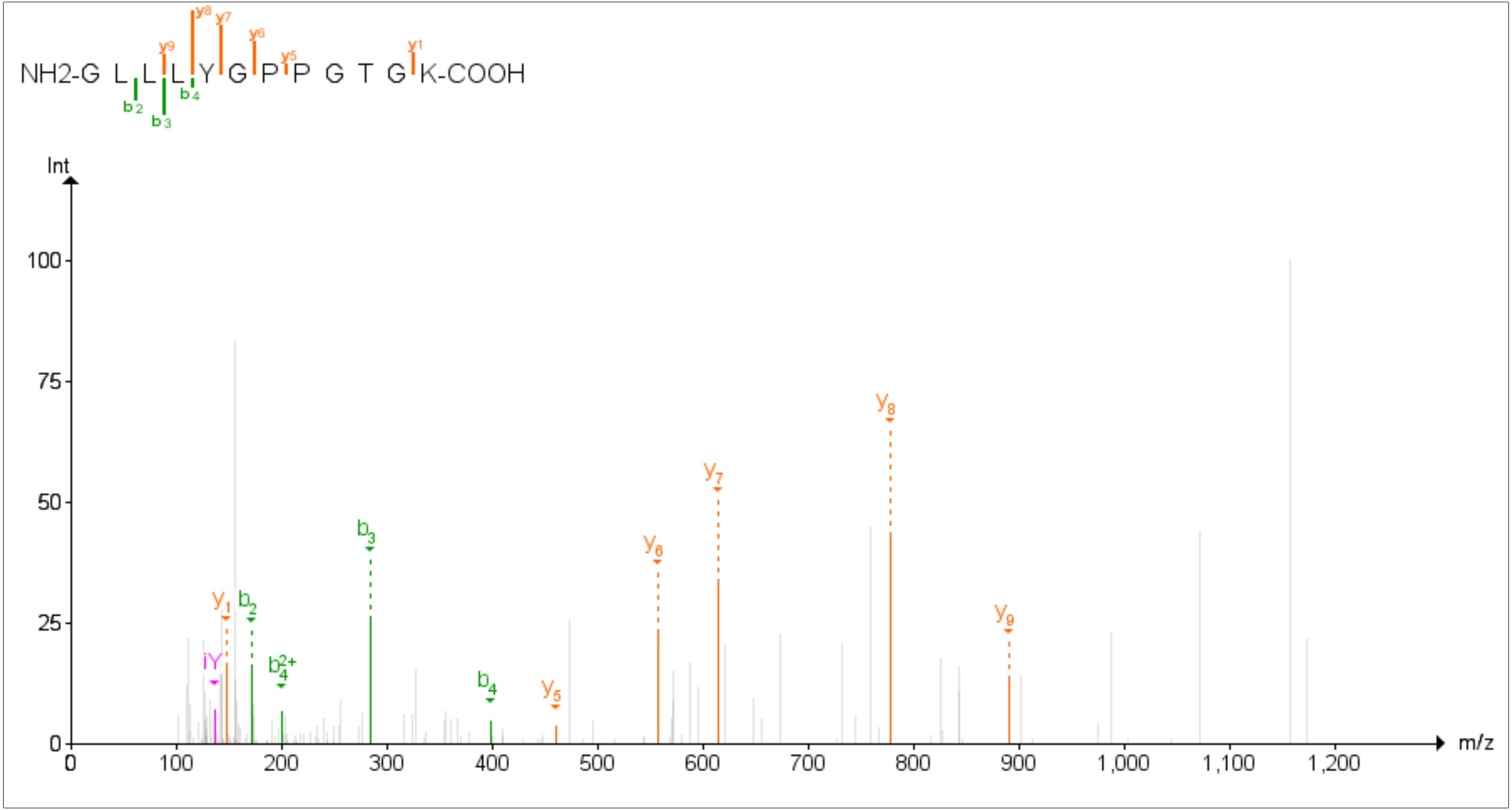
NH2-G L L L Y G P P G T G K-COOH
y9 y8 y7 y6 y5 y1
b2 b3 b4
Int
100
75
50
25
0
m/z
0 100 200 300 400 500 600 700 800 900 1,000 1,100 1,200
iY
y1
b2
b4 2+
b3
b4
y5
y6
y7
y8
y9

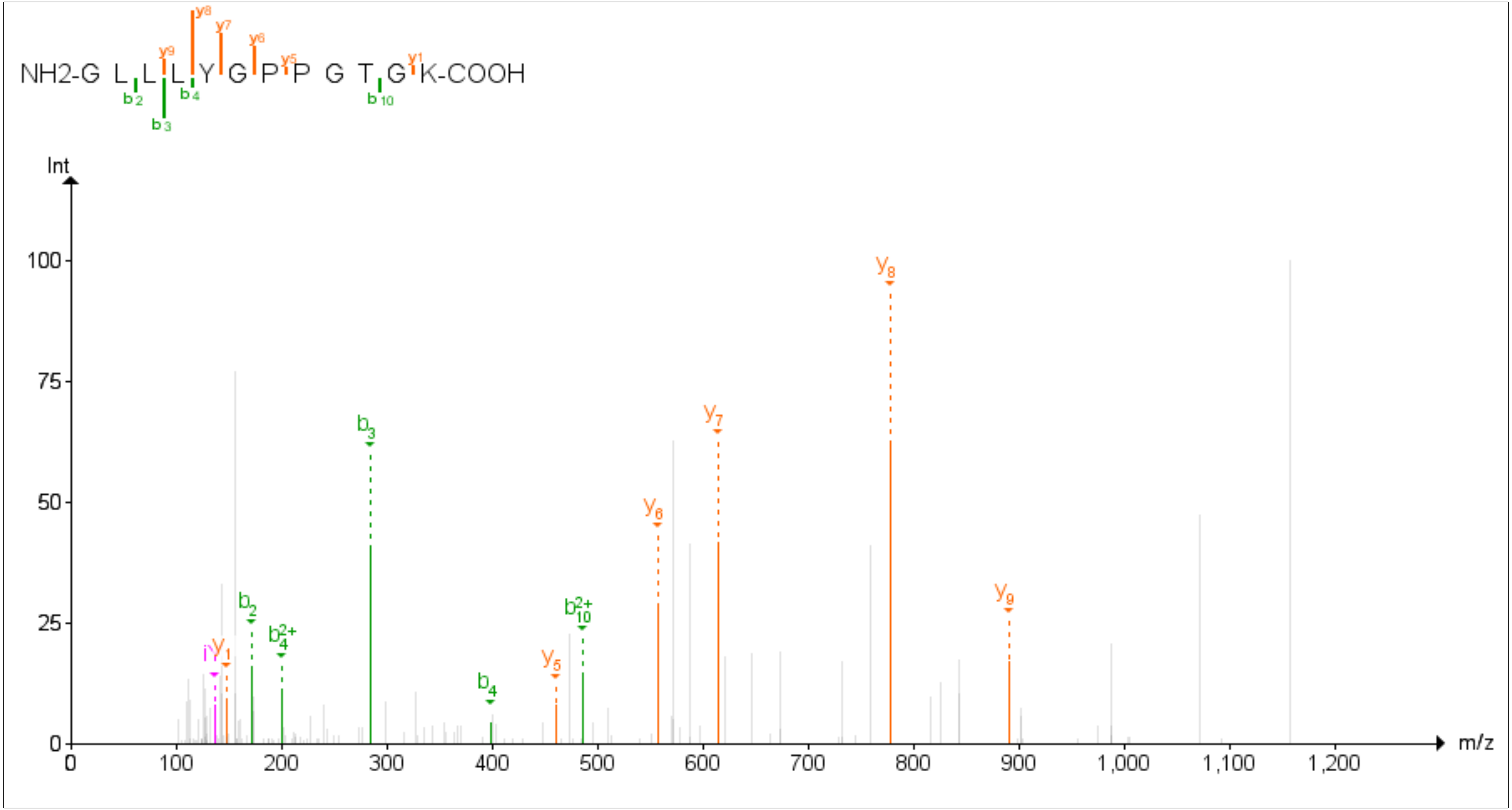
NH2-G L L L Y G P P G T G K-COOH
y9 y8 y7 y6 y5 y1
b2 b3 b4 b10
Int
100
75
50
25
0
m/z
0 100 200 300 400 500 600 700 800 900 1,000 1,100 1,200
y8
y7
b3
y6
y9
b2
b_4^{2+}
b_{10}^{2+}
y5
b4
y1

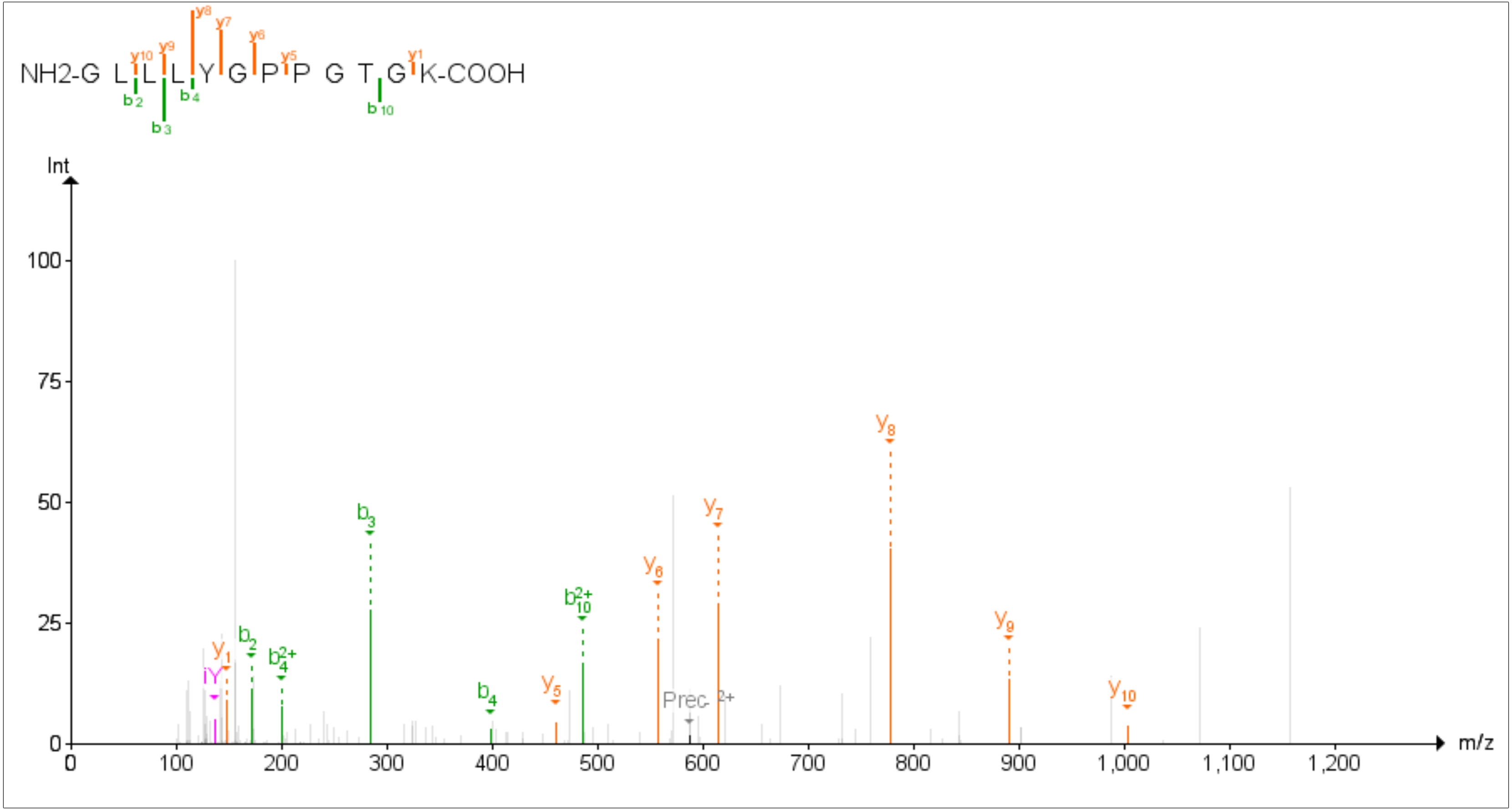

NH2-G L L L Y G P P G T G K-COOH
y10 y9 y8 y7 y6 y5 y1
b2 b3 b4 b10
Int
100
75
50
25
0
iY
y1
b2
b4 2+
b3
b4
y5
b10 2+
y6
Prec 2+
y7
y8
y9
y10
0
100
200
300
400
500
600
700
800
900
1,000
1,100
1,200
m/z

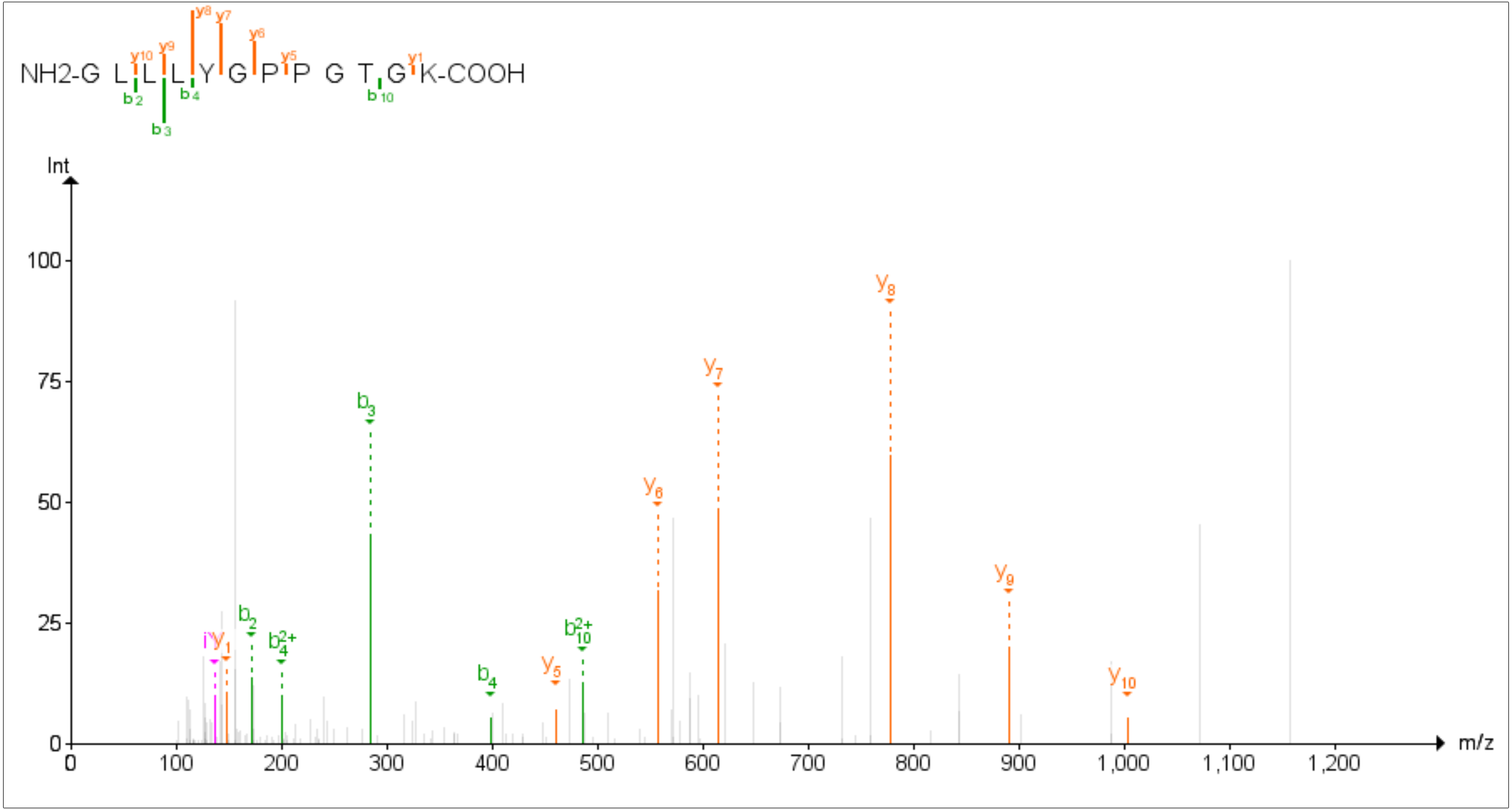

NH2-G L L L Y G P P G T G K-COOH
y10 y9 y8 y7 y6 y5 y1
b2 b3 b4 b10
Int
m/z
b3
y8
y7
y6
y9
y10
y5
b4
b2
y1

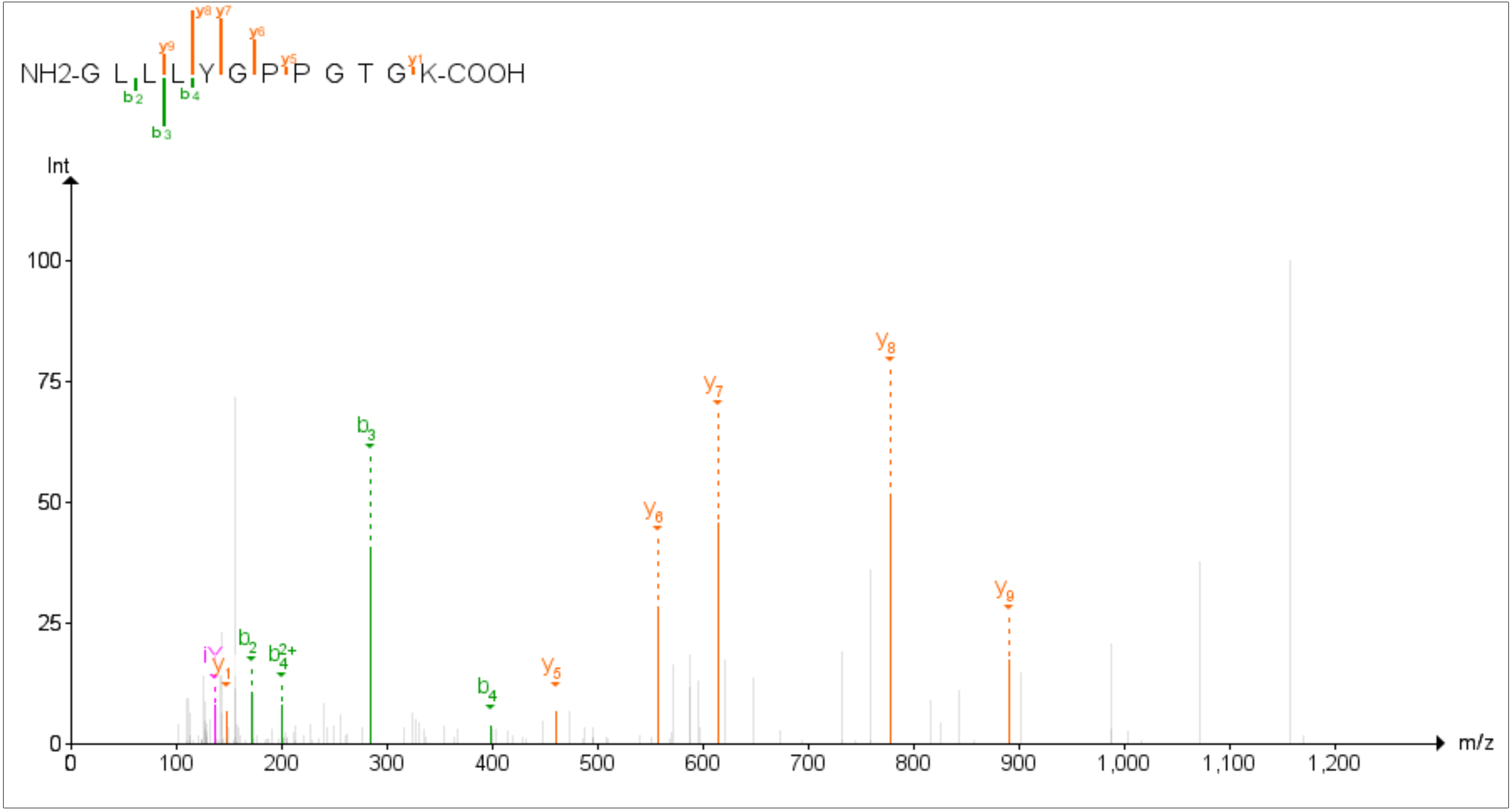

NH2-G L L L Y G P P G T G K-COOH
y9 y8 y7 y6 y5 y1
b2 b3 b4
Int
100
75
50
25
0
m/z
0
100
200
300
400
500
600
700
800
900
1,000
1,100
1,200
b3
y8
y7
y6
y9
b2
b4 2+
b4
y5
y1
iY

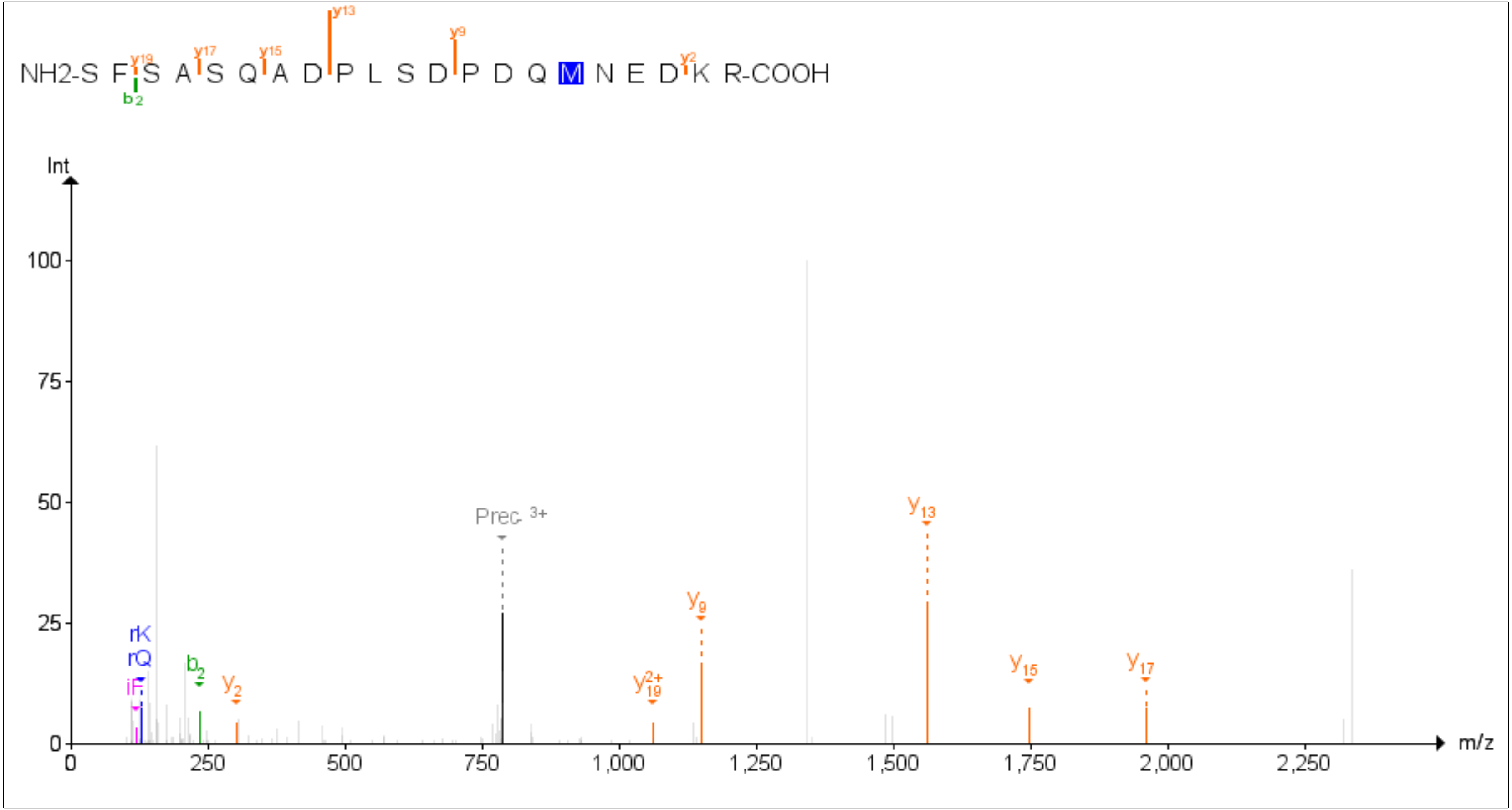

NH2-S F S A S Q A D P L S D P D Q M N E D K R-COOH
y19 y17 y15 y13 y9 y2
b2
Int
100
75
50
25
0
Prec 3+
rK
rQ
iF
b_2
y_2
y_{19}^{2+}
y_9
y_{13}
y_{15}
y_{17}
0 250 500 750 1,000 1,250 1,500 1,750 2,000 2,250
m/z

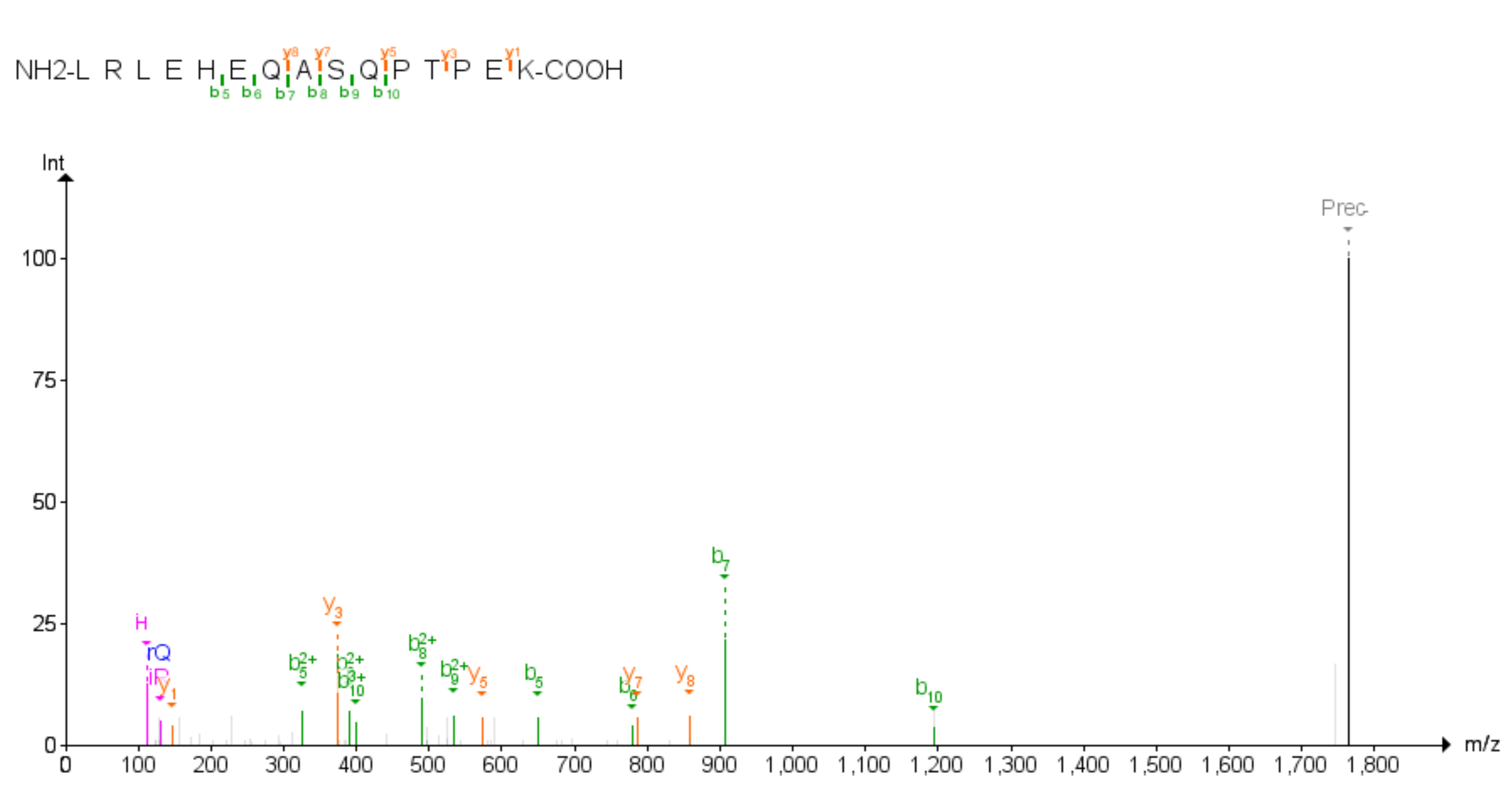

NH2-L R L E H E Q A S Q P T P E K-COOH
y8 y7 y5 y3 y1
b5 b6 b7 b8 b9 b10
Int
100
75
50
25
0
Prec
b7
y3
H
rQ
iP
y1
b5 2+
b5 2+
b10 3+
b8 2+
b9 2+
y5
b5
y7
b6
y8
b10
m/z
0
100
200
300
400
500
600
700
800
900
1,000
1,100
1,200
1,300
1,400
1,500
1,600
1,700
1,800

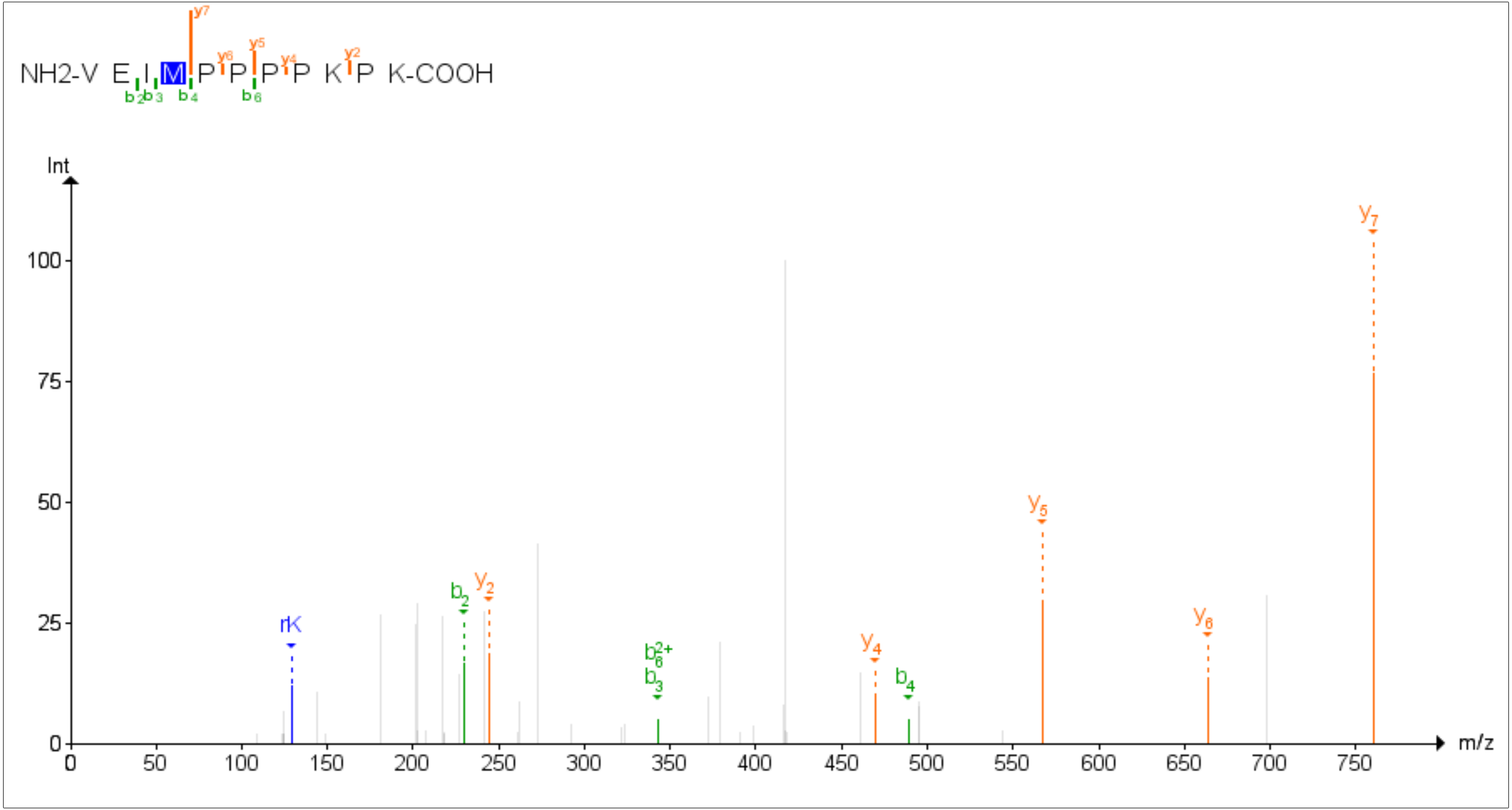

NH2-V E I M P P P P K P K-COOH
y7
y6
y5
y4
y2
b2
b3
b4
b6
Int
m/z
rK
b2
y2
b6 2+
b3
y4
b4
y5
y6
y7

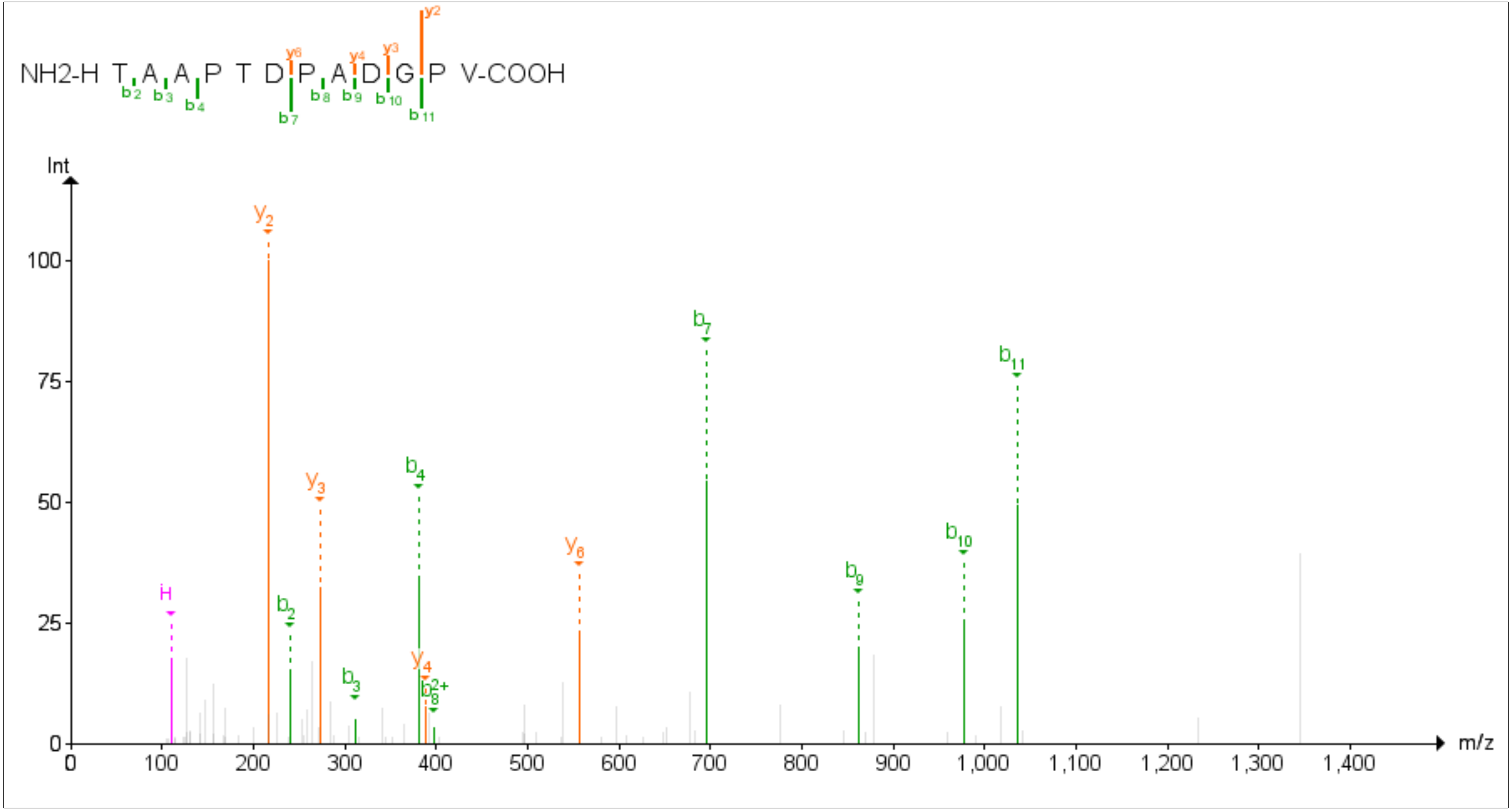

NH2-H T A A P T D P A D G P V-COOH
b2 b3 b4 b7 b8 b9 b10 b11
y6 y4 y3 y2
Int
100
75
50
25
0
y2
y3
b2
b3
b4
y4
b8 2+
y6
b7
b9
b10
b11
H
m/z
0 100 200 300 400 500 600 700 800 900 1,000 1,100 1,200 1,300 1,400

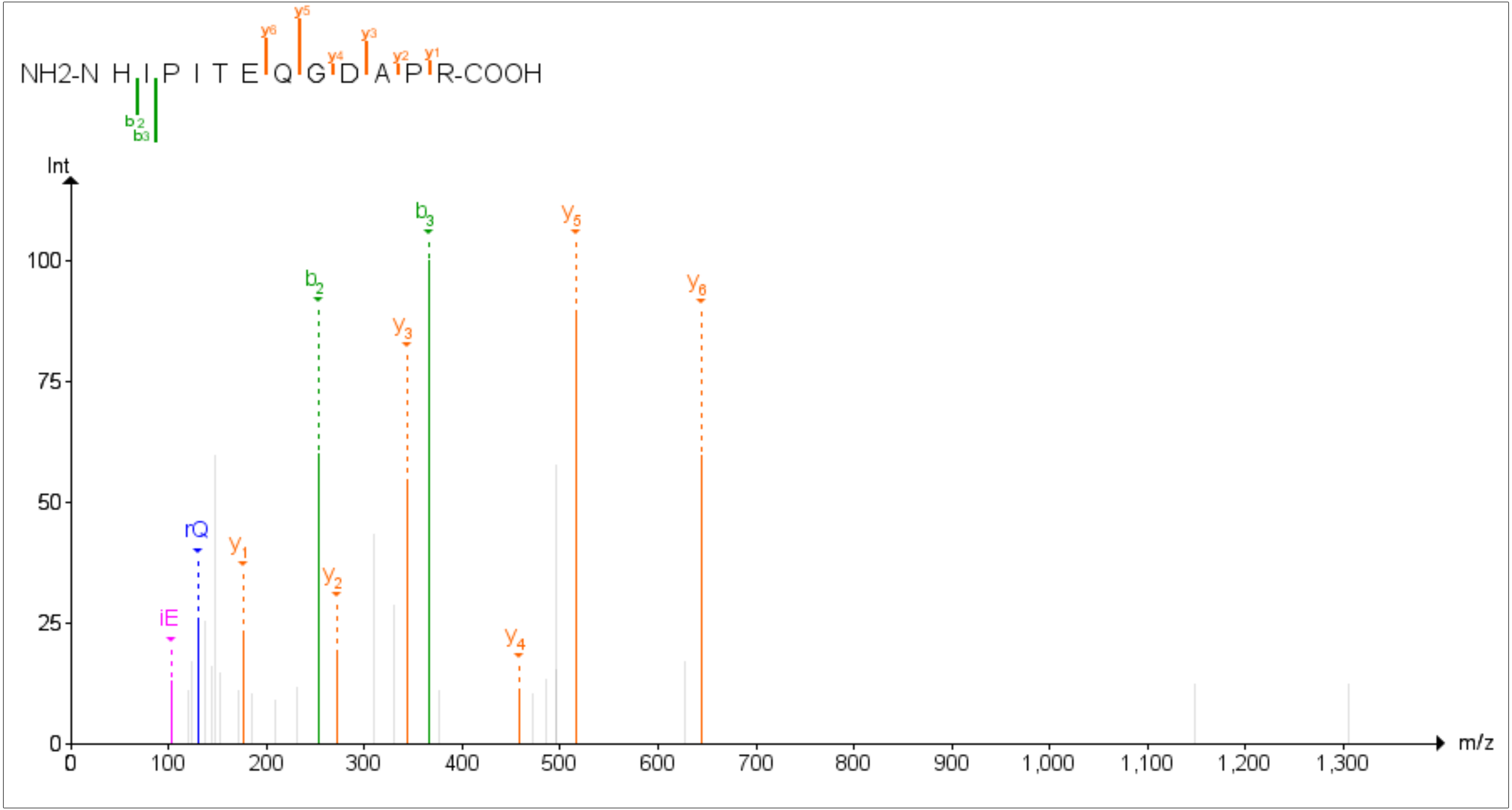

NH2-N H I P I T E Q G D A P R-COOH
b2 b3
y6 y5 y4 y3 y2 y1
Int
m/z
0
25
50
75
100
0
100
200
300
400
500
600
700
800
900
1,000
1,100
1,200
1,300
iE
rQ
y1
b2
y2
y3
b3
y4
y5
y6

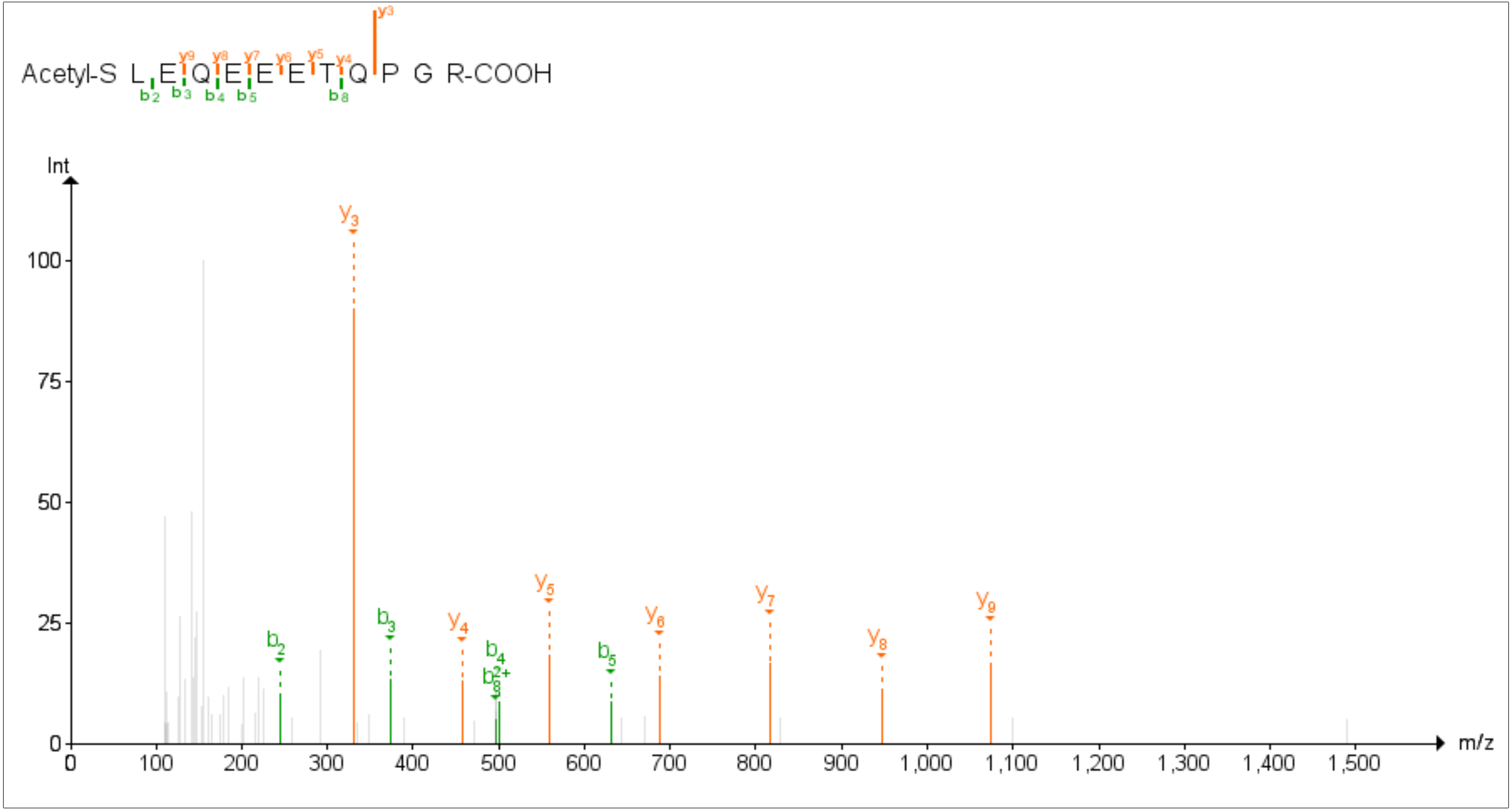

Acetyl-S L E Q E E E T Q P G R-COOH
Int
m/z

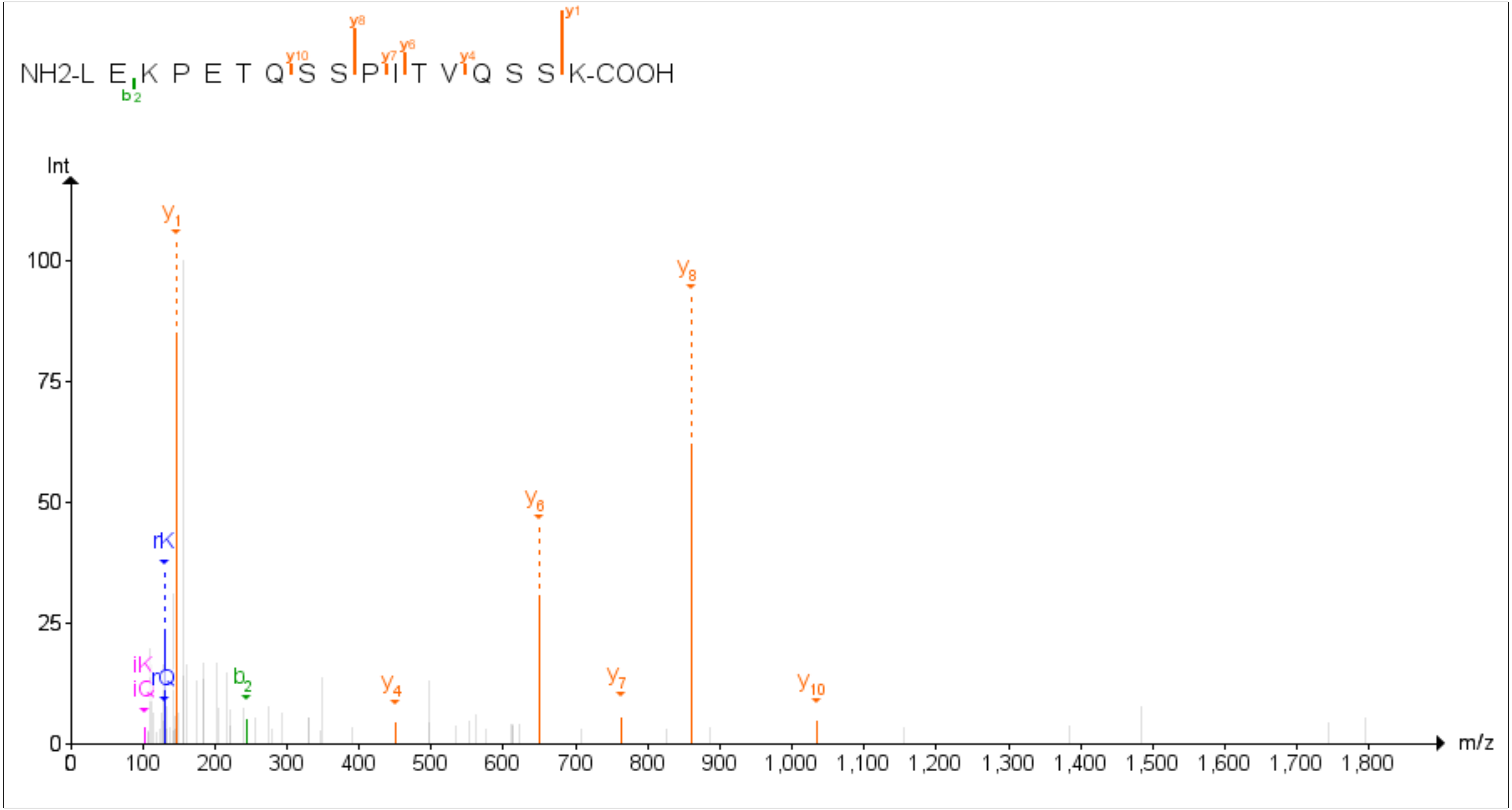

NH2-L E K P E T Q S S P I T V Q S S K-COOH
b2
y10
y8
y7
y6
y4
y1
Int
m/z
rK
iK
iQ
rQ
b2
y4
y6
y7
y8
y10

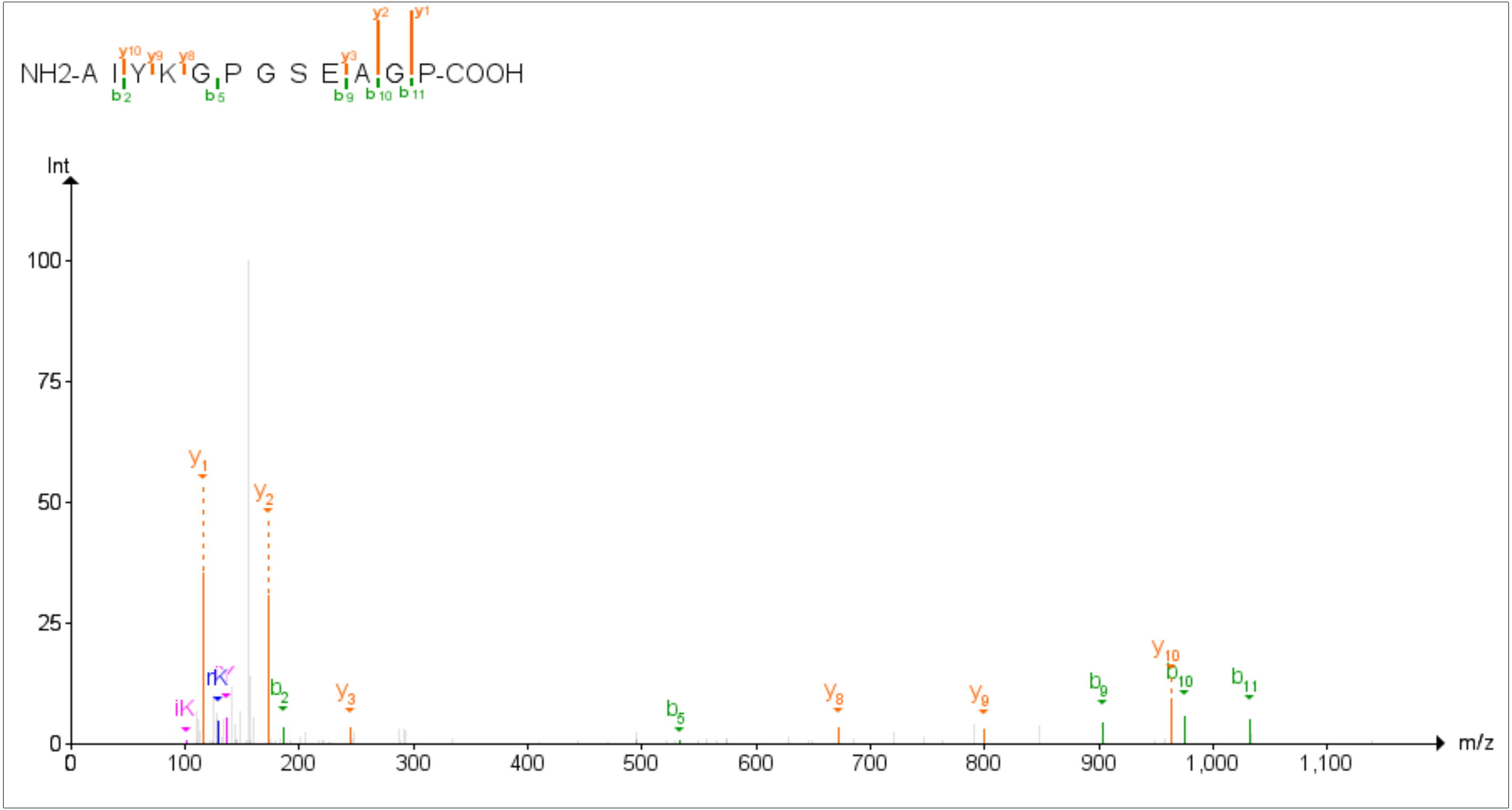

NH2-A I Y K G P G S E A G P-COOH
Int
m/z
y1
y2
y3
y8
y9
y10
b2
b5
b9
b10
b11
iK
rK

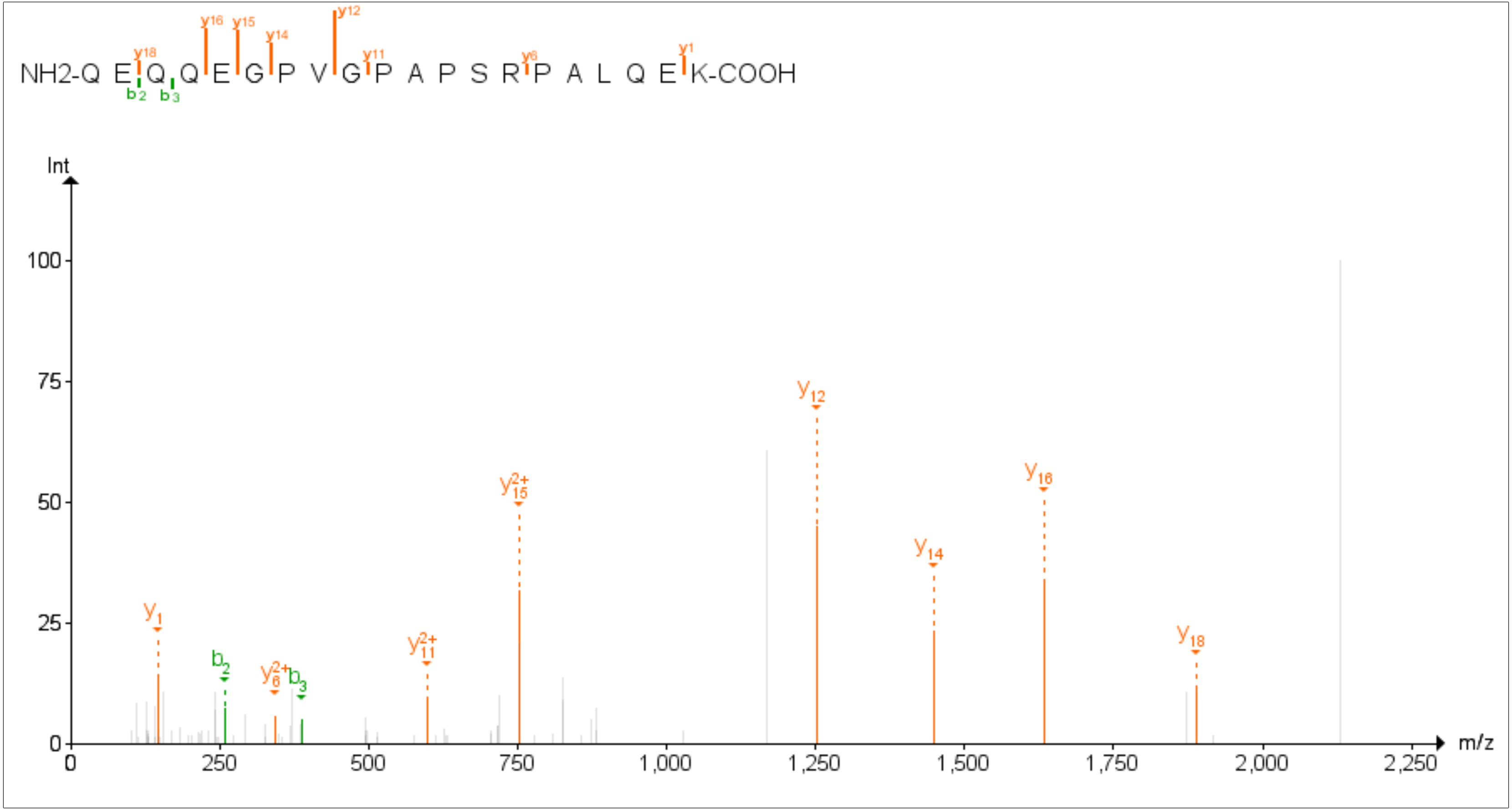
NH2-Q E Q Q E G P V G P A P S R P A L Q E K-COOH
y18 y16 y15 y14 y12 y11 y6 y1
b2 b3
Int
100
75
50
25
0
y1
b2
y6 2+
b3
y11 2+
y15 2+
y12
y14
y16
y18
0 250 500 750 1,000 1,250 1,500 1,750 2,000 2,250
m/z

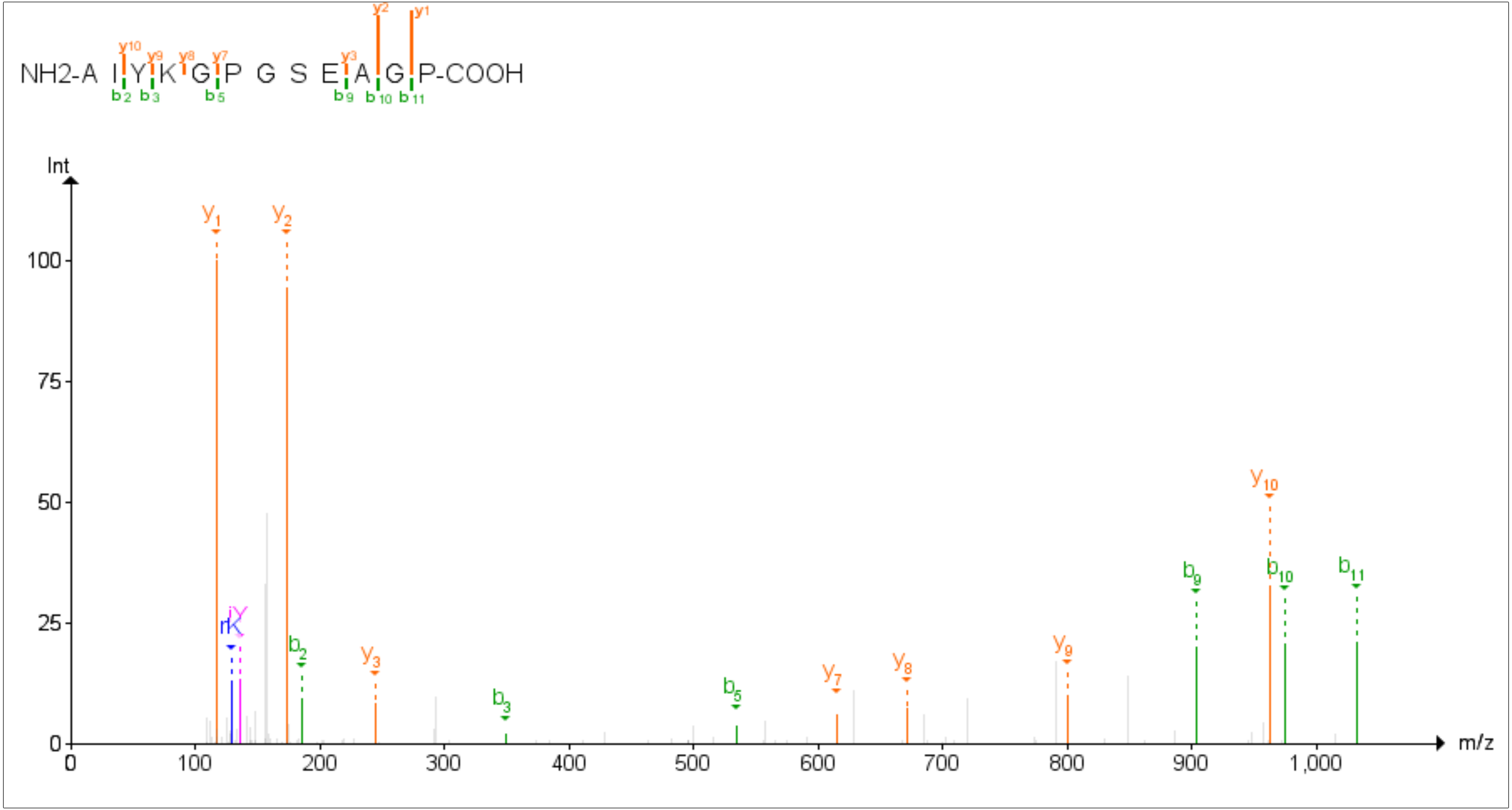

NH2-A I Y K G P G S E A G P-COOH
y10 y9 y8 y7 y3 y2 y1
b2 b3 b5 b9 b10 b11
Int
100
75
50
25
0
y1 y2 rK iY b2 y3 b3 b5 y7 y8 y9 b9 y10 b10 b11
0 100 200 300 400 500 600 700 800 900 1,000
m/z

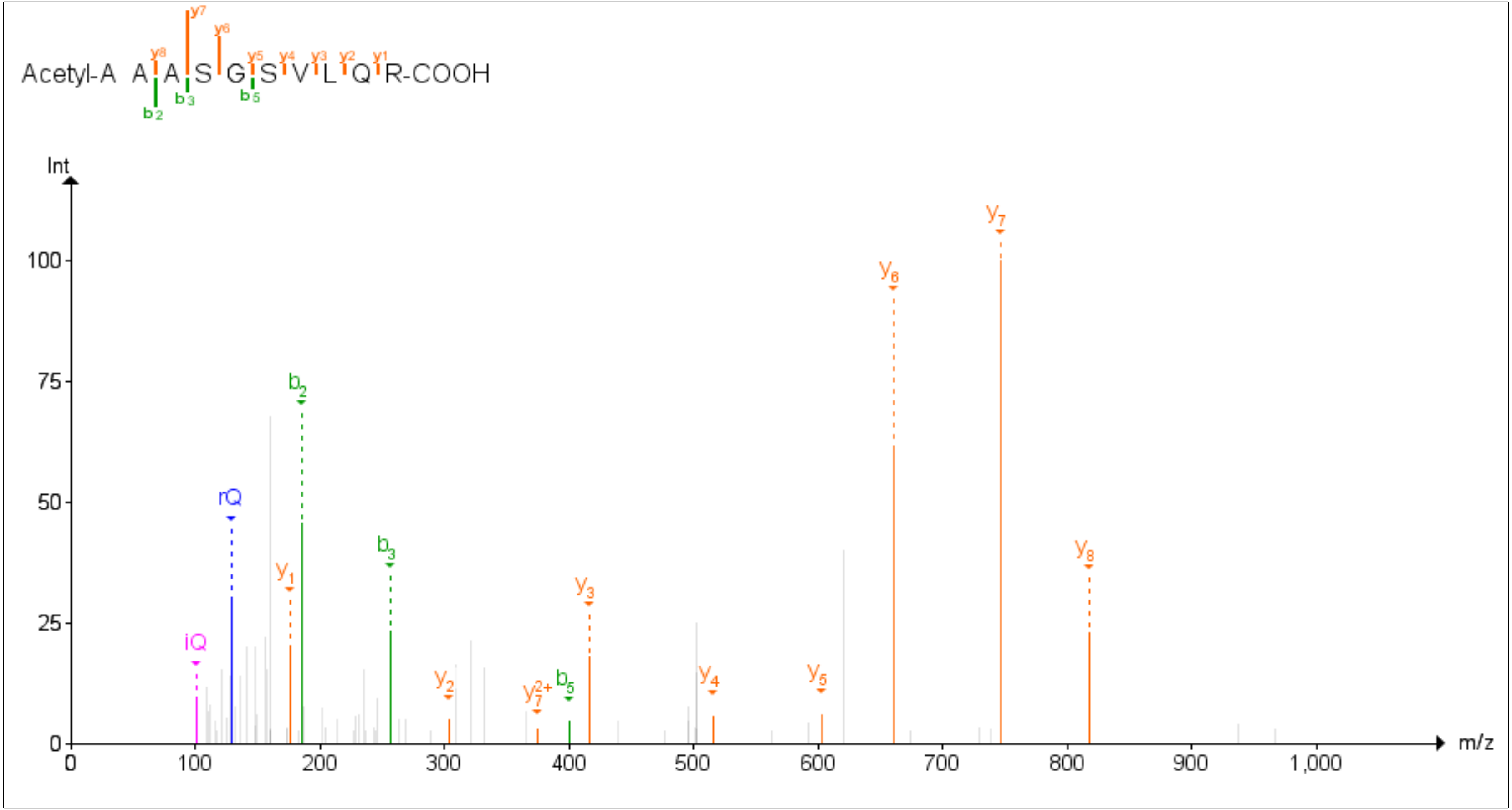

Acetyl-A A A S G S V L Q R-COOH
y8 y7 y6 y5 y4 y3 y2 y1
b2 b3 b5
Int
m/z
iQ
rQ
y1
b2
b3
y2
y7 2+
b5
y3
y4
y5
y6
y7
y8
0
25
50
75
100
0
100
200
300
400
500
600
700
800
900
1,000

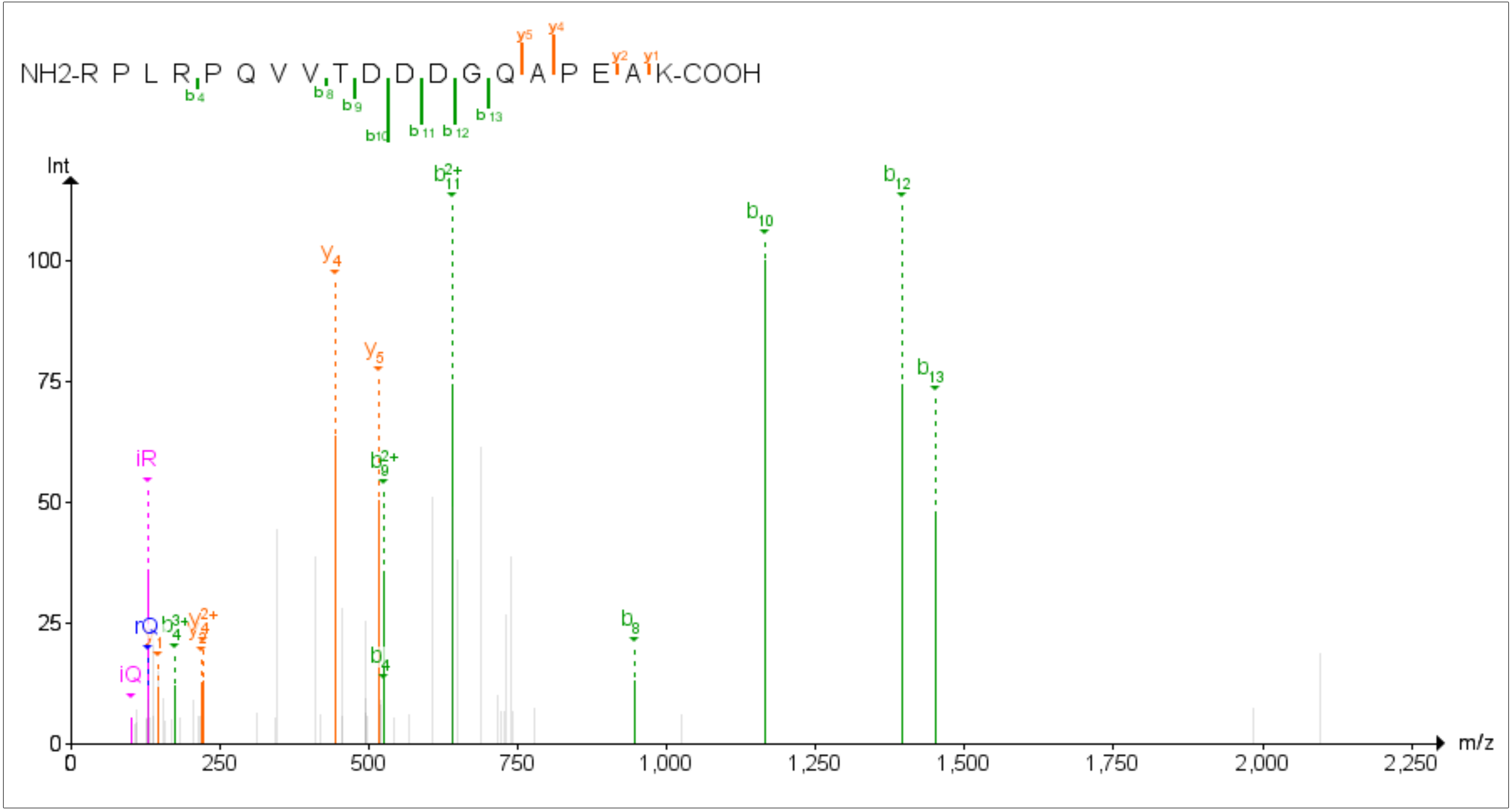

NH2-R P L R P Q V V T D D D G Q A P E A K-COOH
b4
b8
b9
b10
b11
b12
b13
y5
y4
y2
y1
Int
100
75
50
25
0
0
250
500
750
1,000
1,250
1,500
1,750
2,000
2,250
m/z
b11 2+
b12
b10
y4
y5
b13
b9 2+
iR
rQ
b4 3+
y4 2+
iQ
b4
b8

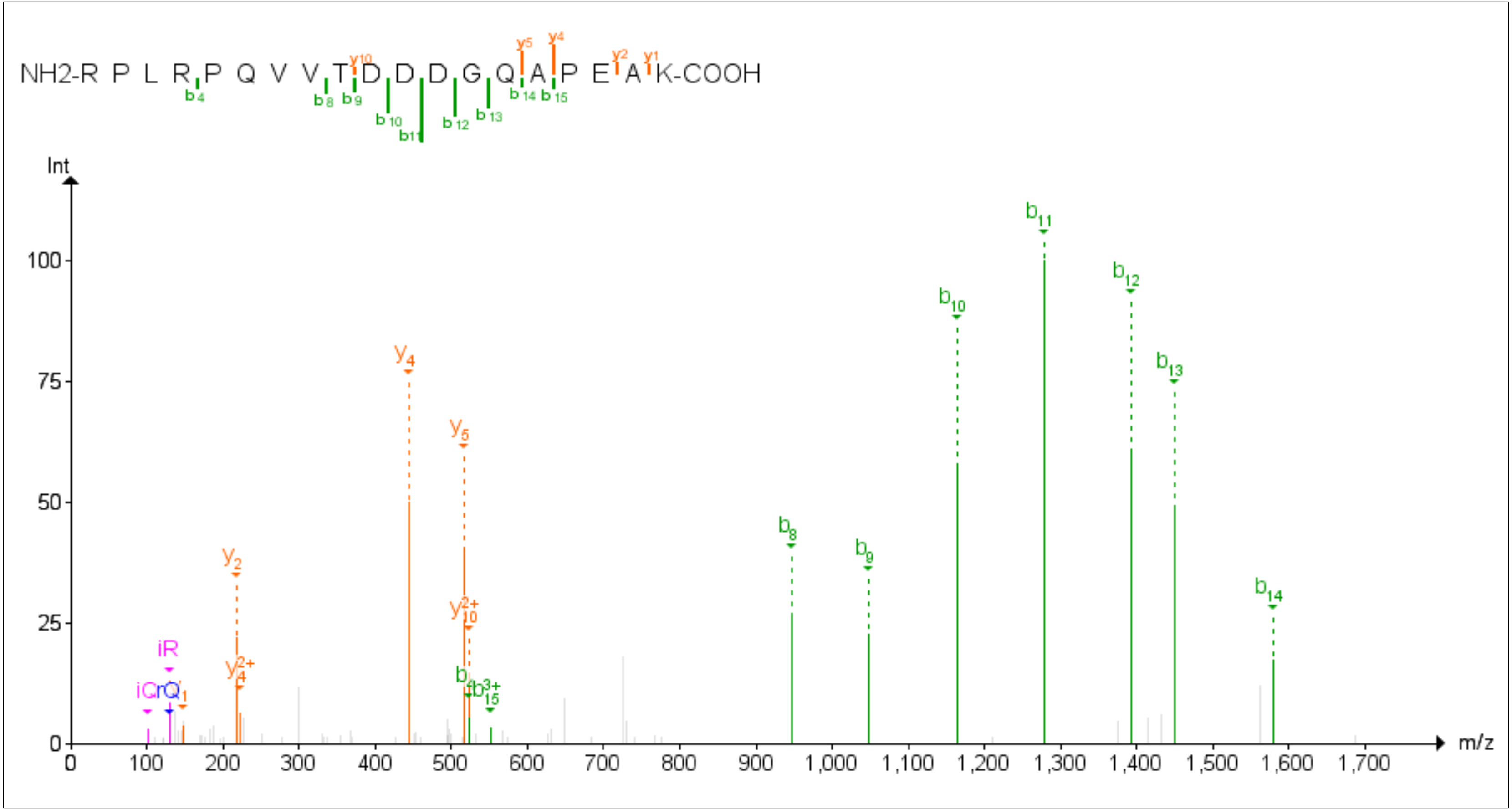
NH2-R P L R P Q V V T D D D G Q A P E A K-COOH
y10 y5 y4 y2 y1
b4 b8 b9 b10 b11 b12 b13 b14 b15
Int
m/z
0
25
50
75
100
0 100 200 300 400 500 600 700 800 900 1,000 1,100 1,200 1,300 1,400 1,500 1,600 1,700
iR
iQ
iQ
y1
y2
y4 2+
y4
y5
y10 2+
b4
b15 3+
b8
b9
b10
b11
b12
b13
b14

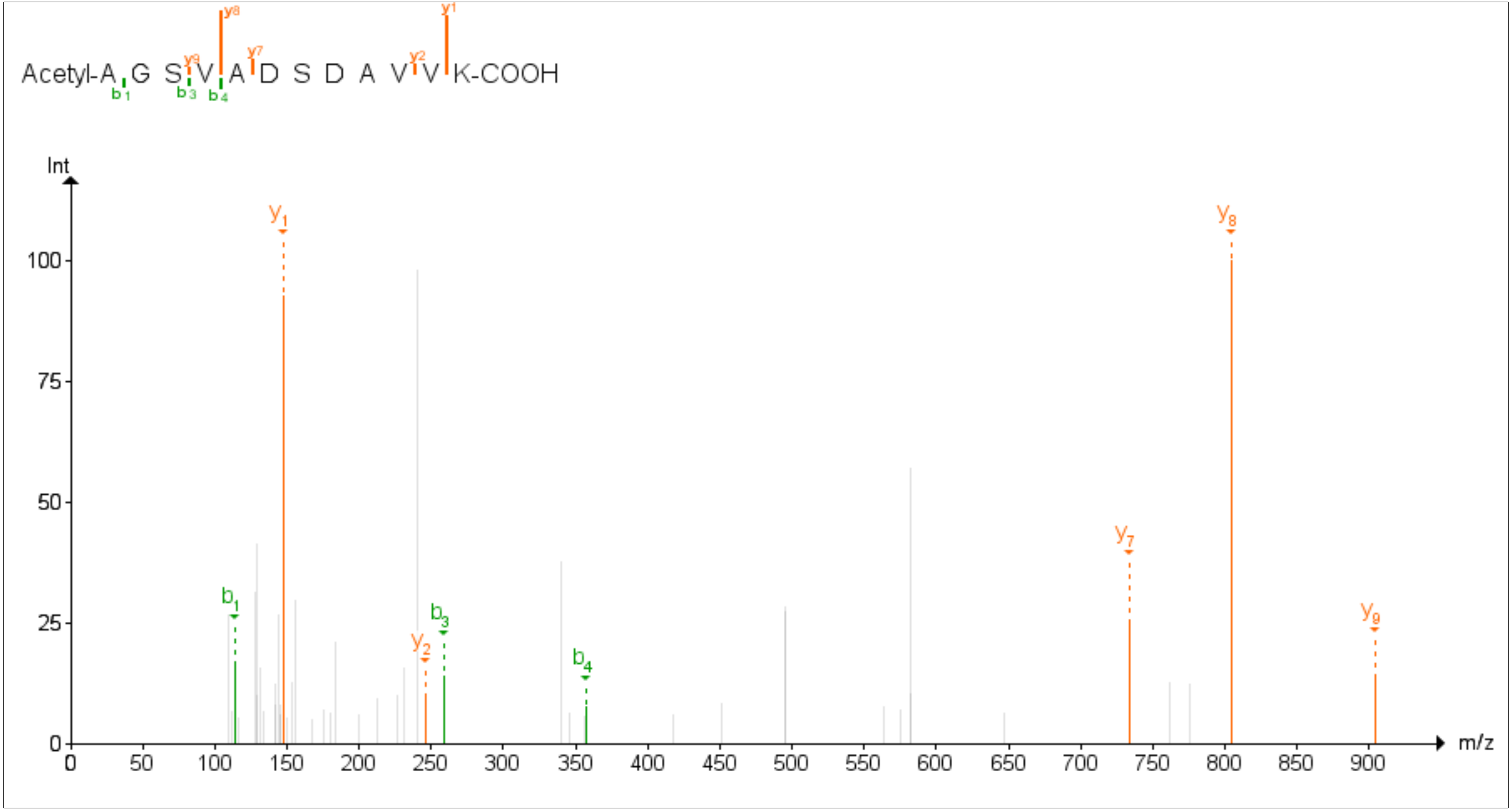

Acetyl-A G S V A D S D A V V K-COOH
y8
y9
y7
y2
y1
b1
b3
b4
Int
100
75
50
25
0
y1
y8
y7
y9
b1
b3
b4
y2
0
50
100
150
200
250
300
350
400
450
500
550
600
650
700
750
800
850
900
m/z

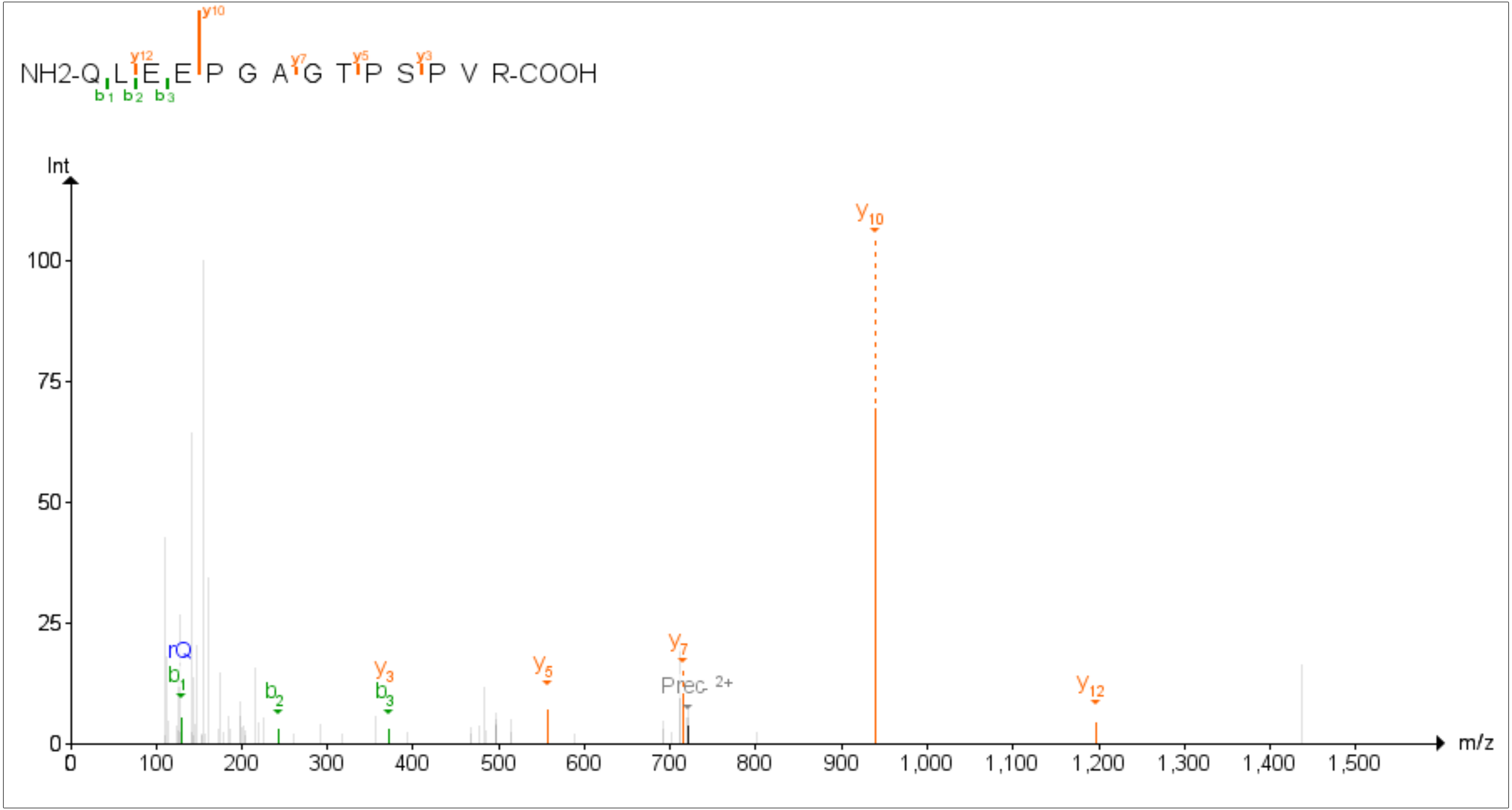
NH2-Q L E E P G A G T P S P V R-COOH
y10
y12
y7
y5
y3
b1
b2
b3
Int
100
75
50
25
0
rQ
b1
b2
y3
b3
y5
y7
Prec 2+
y10
y12
m/z
0
100
200
300
400
500
600
700
800
900
1,000
1,100
1,200
1,300
1,400
1,500

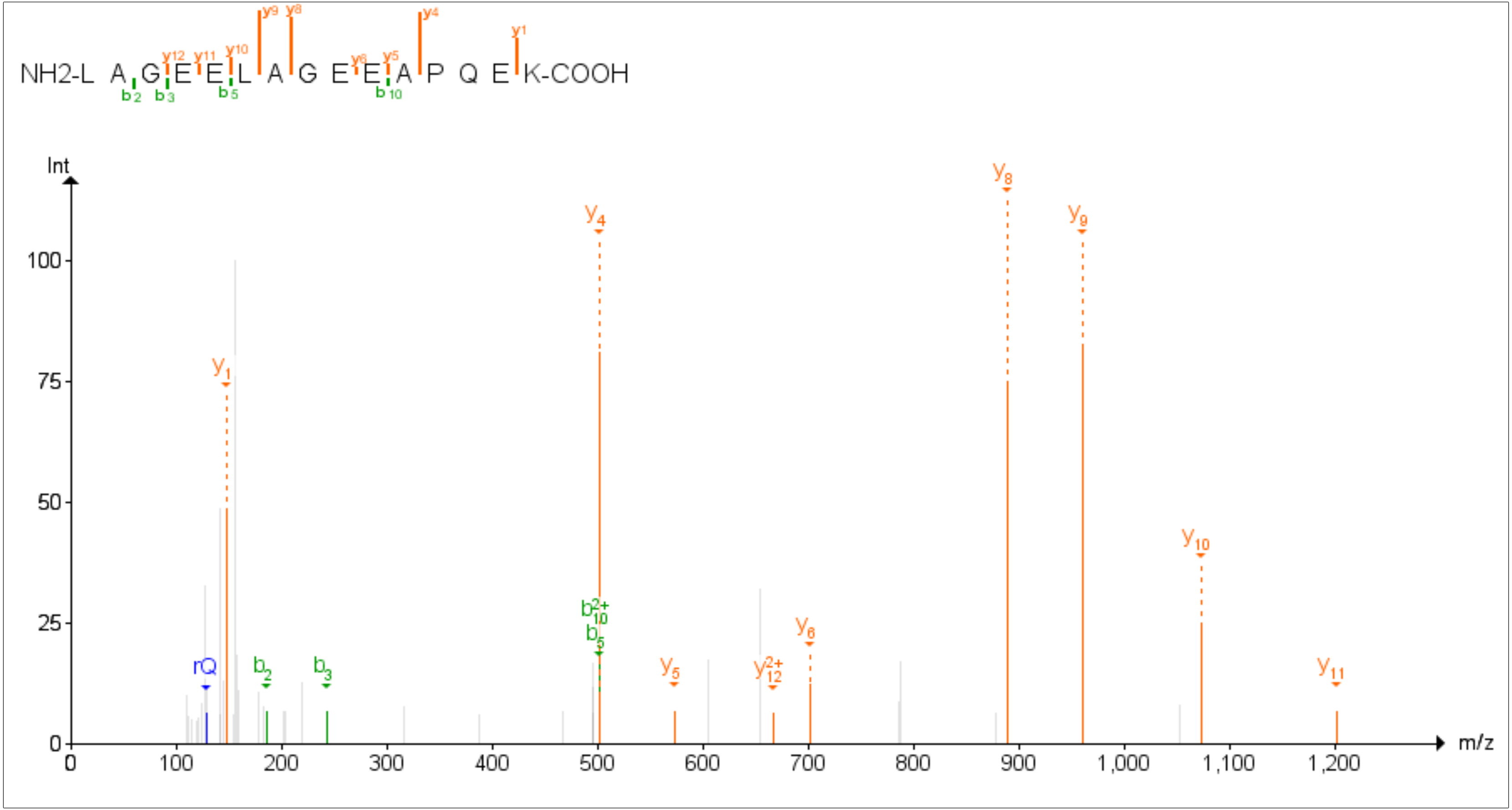

NH2-L A G E E L A G E E A P Q E K-COOH
y12 y11 y10 y9 y8 y6 y5 y4 y1
b2 b3 b5 b10
Int
m/z
rQ
b2
b3
y1
y4
y5
y6
y8
y9
y10
y11
y12 2+
b10 2+
b5

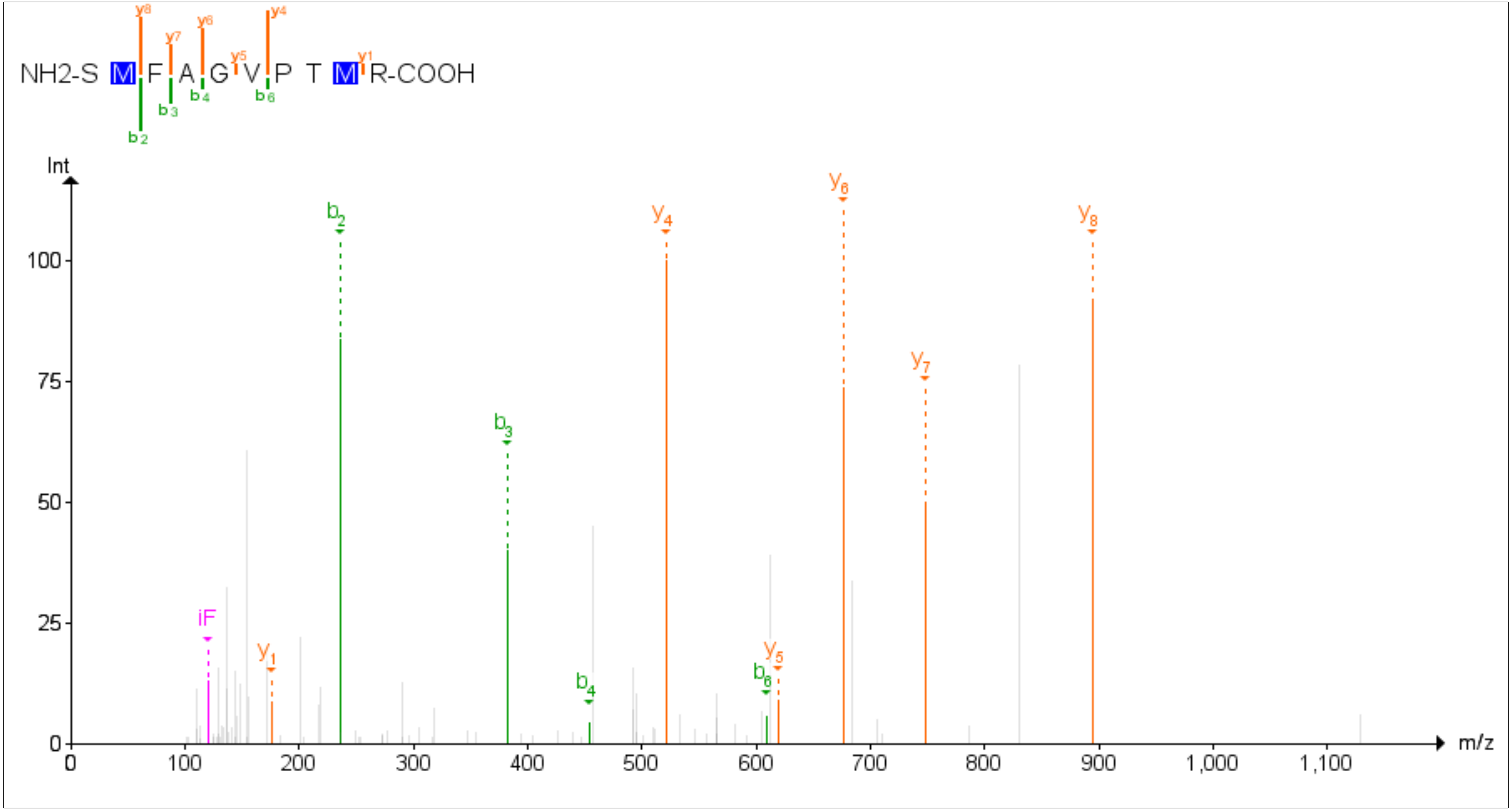

NH2-S M F A G V P T M R-COOH
y8
y7
y6
y5
y4
y1
b2
b3
b4
b6
Int
iF
100
75
50
25
0
m/z
0
100
200
300
400
500
600
700
800
900
1,000
1,100

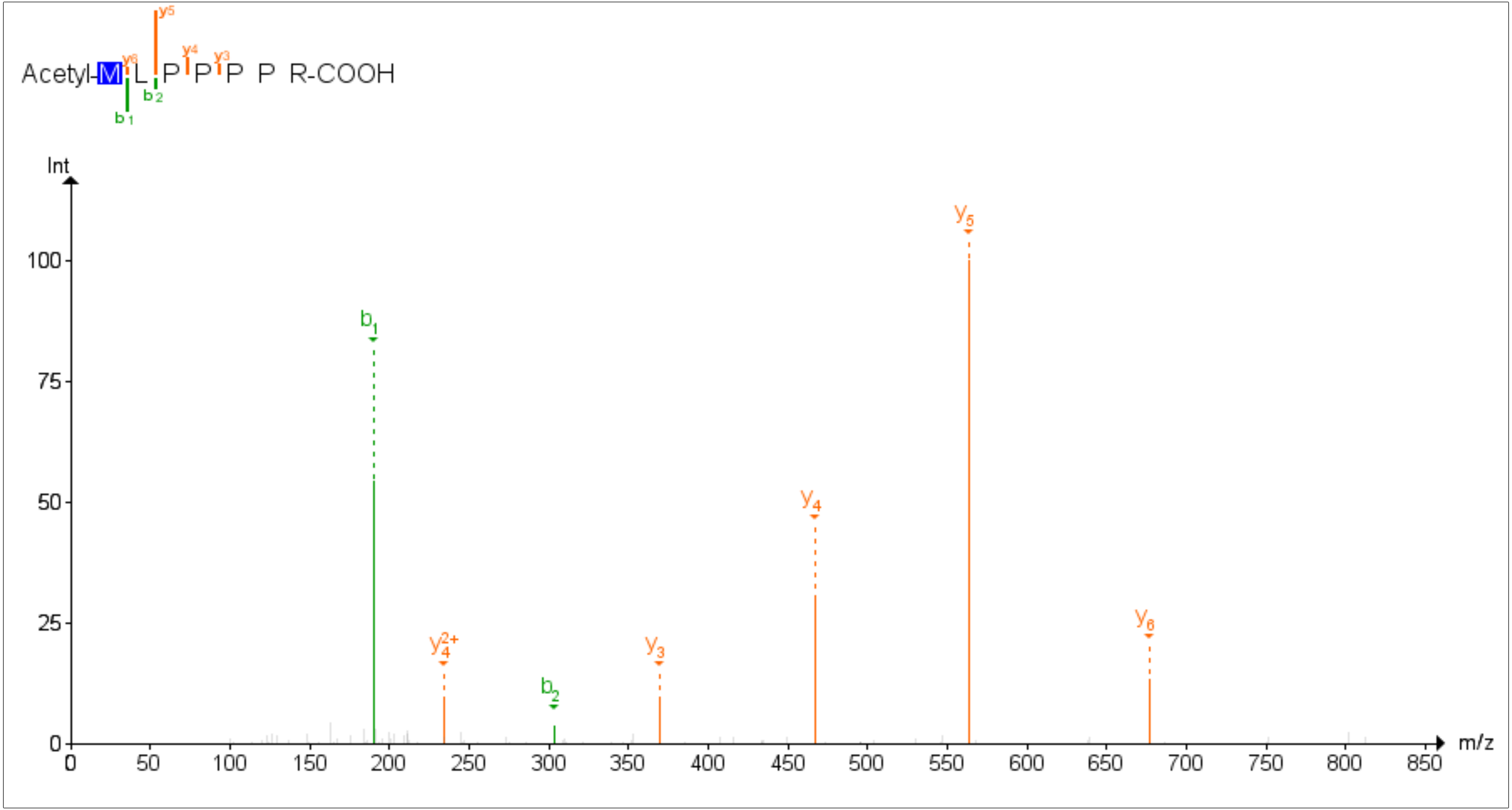

Acetyl-M L P P P P R-COOH
y5
y6
y4
y3
b 1
b 2
Int
m/z
b1
y4 2+
b2
y3
y4
y5
y6
0
25
50
75
100
0
50
100
150
200
250
300
350
400
450
500
550
600
650
700
750
800
850

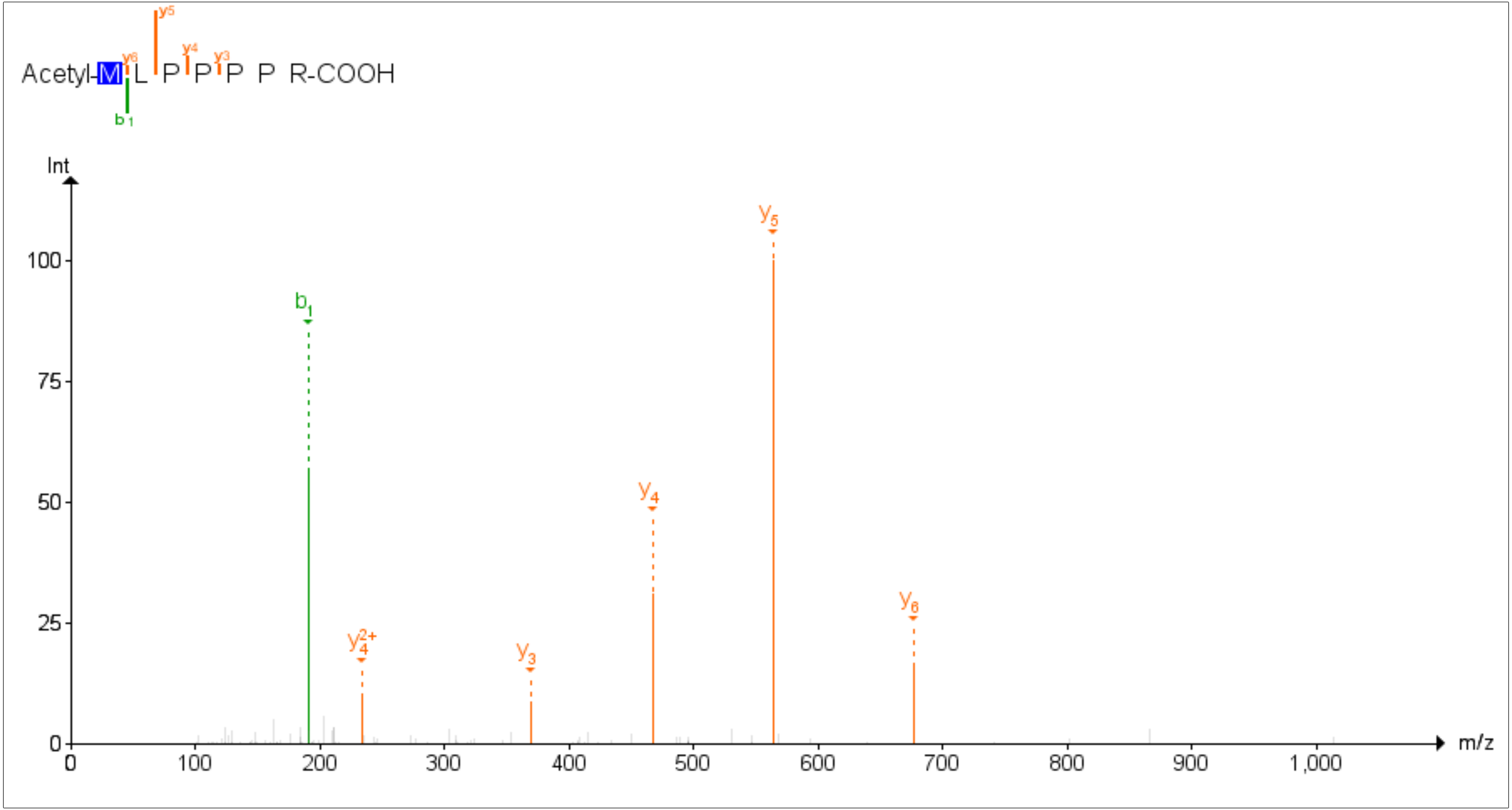

Acetyl-M L P P P P R-COOH
y5
y6
y4
y3
b1
Int
100
75
50
25
0
b1
y5
y4
y6
y4 2+
y3
0
100
200
300
400
500
600
700
800
900
1,000
m/z

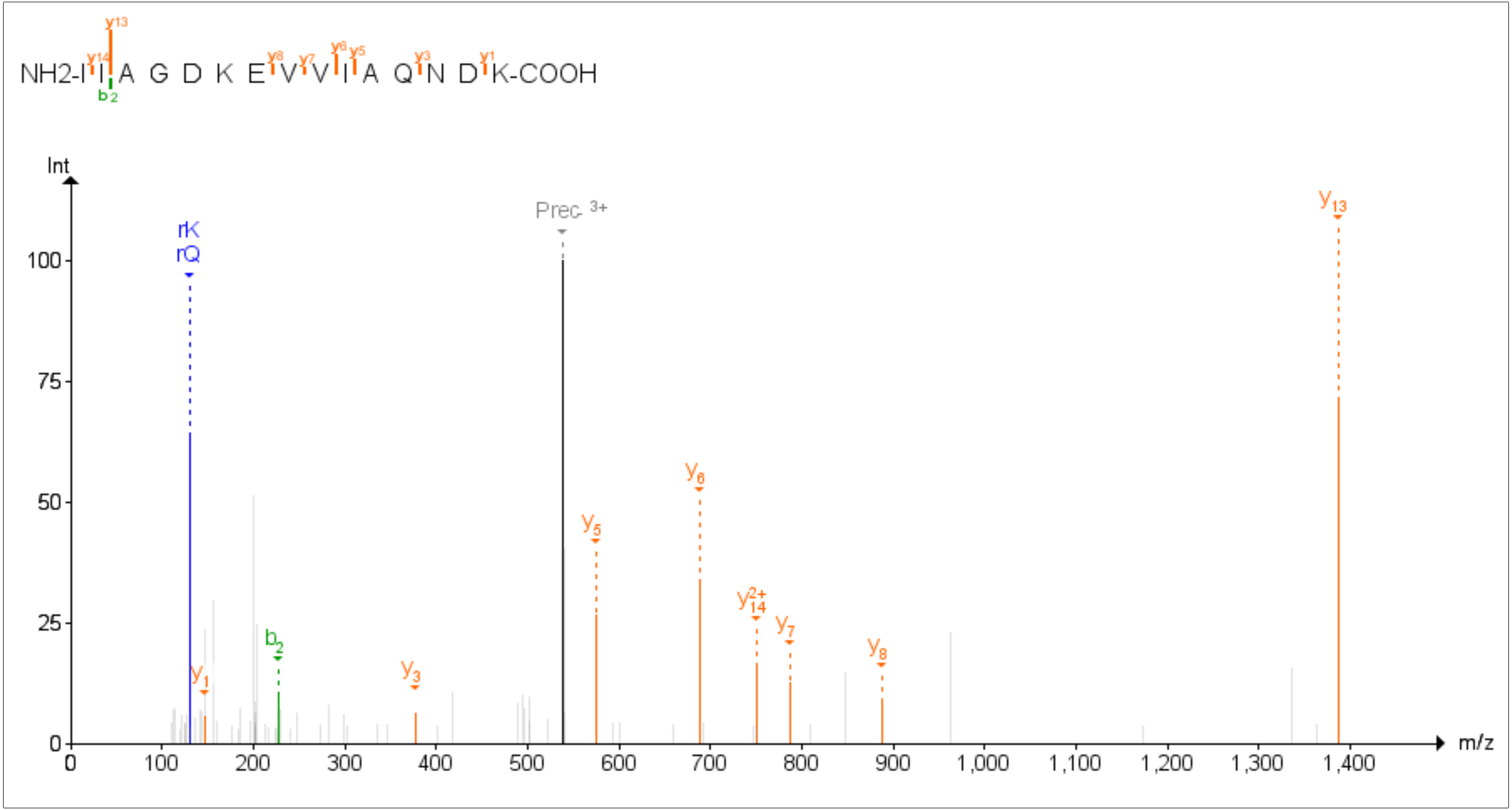

NH2-I I A G D K E V V I A Q N D K-COOH
y13
y14
b2
y8
y7
y6
y5
y3
y1
Int
100
75
50
25
0
rK
rQ
Prec 3+
y13
y6
y5
y14 2+
y7
y8
b2
y1
y3
0
100
200
300
400
500
600
700
800
900
1,000
1,100
1,200
1,300
1,400
m/z

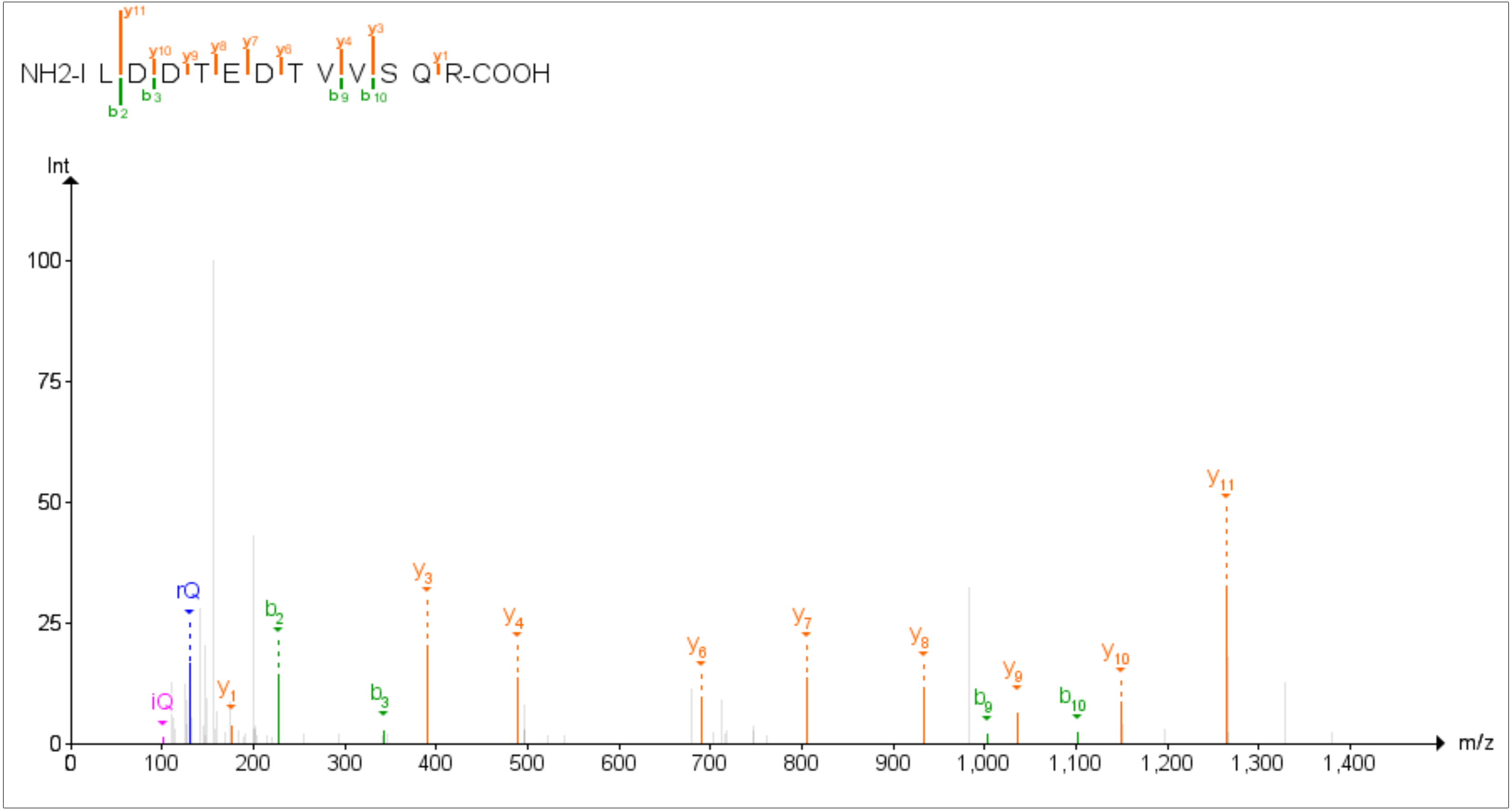

NH2-I L D D T E D T V V S Q R-COOH
y11 y10 y9 y8 y7 y6 y4 y3 y1
b2 b3 b9 b10
Int
100
75
50
25
0
m/z
0 100 200 300 400 500 600 700 800 900 1,000 1,100 1,200 1,300 1,400
iQ
rQ
y1
b2
b3
y3
y4
y6
y7
y8
b9
y9
b10
y10
y11

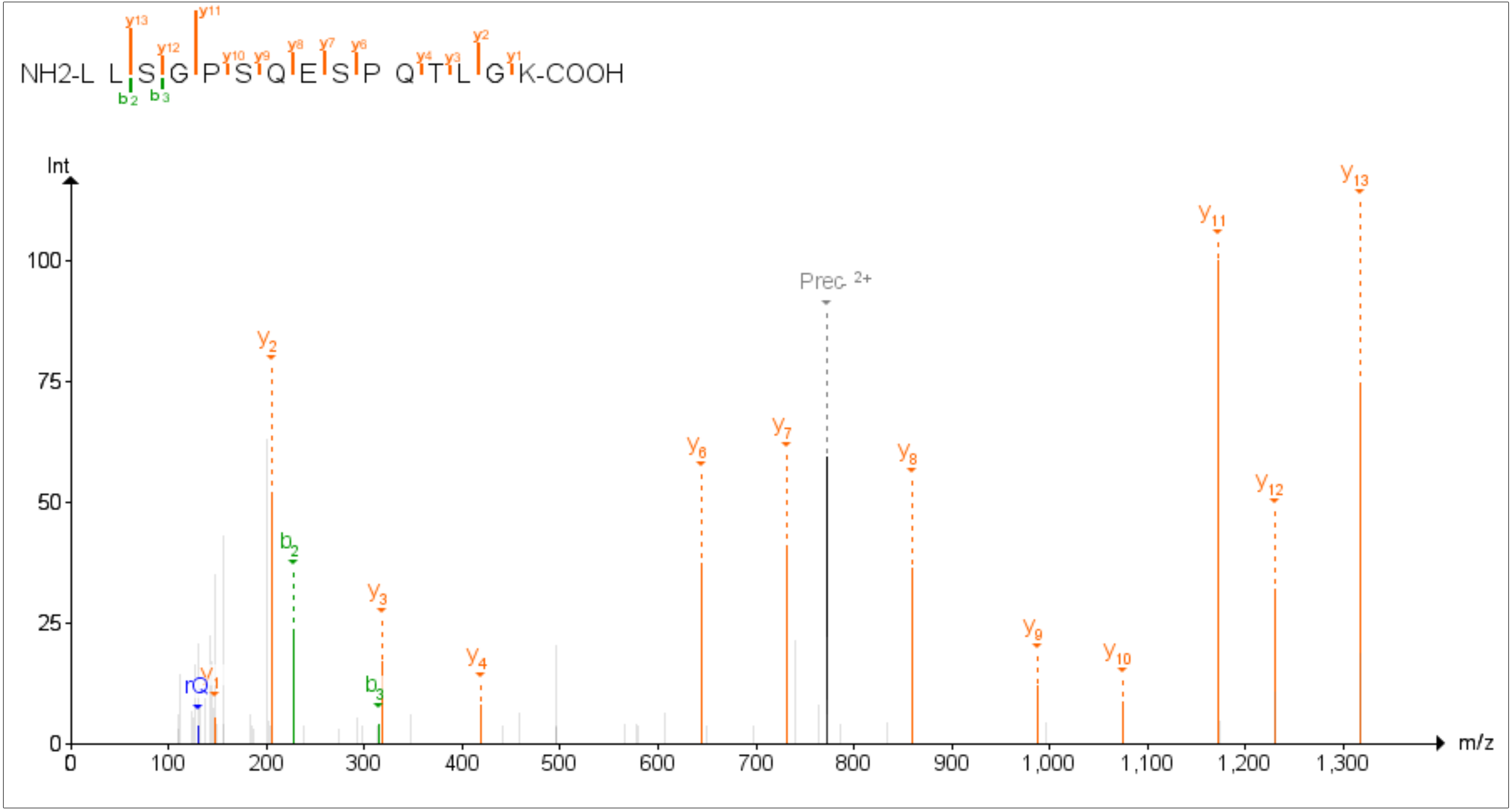

NH2-L L S G P S Q E S P Q T L G K-COOH
y13 y12 y11 y10 y9 y8 y7 y6 y4 y3 y2 y1
b2 b3
Int
m/z
Prec 2+
rQ
y1 y2 y3 y4 y6 y7 y8 y9 y10 y11 y12 y13
b2 b3
0
25
50
75
100
0
100
200
300
400
500
600
700
800
900
1,000
1,100
1,200
1,300

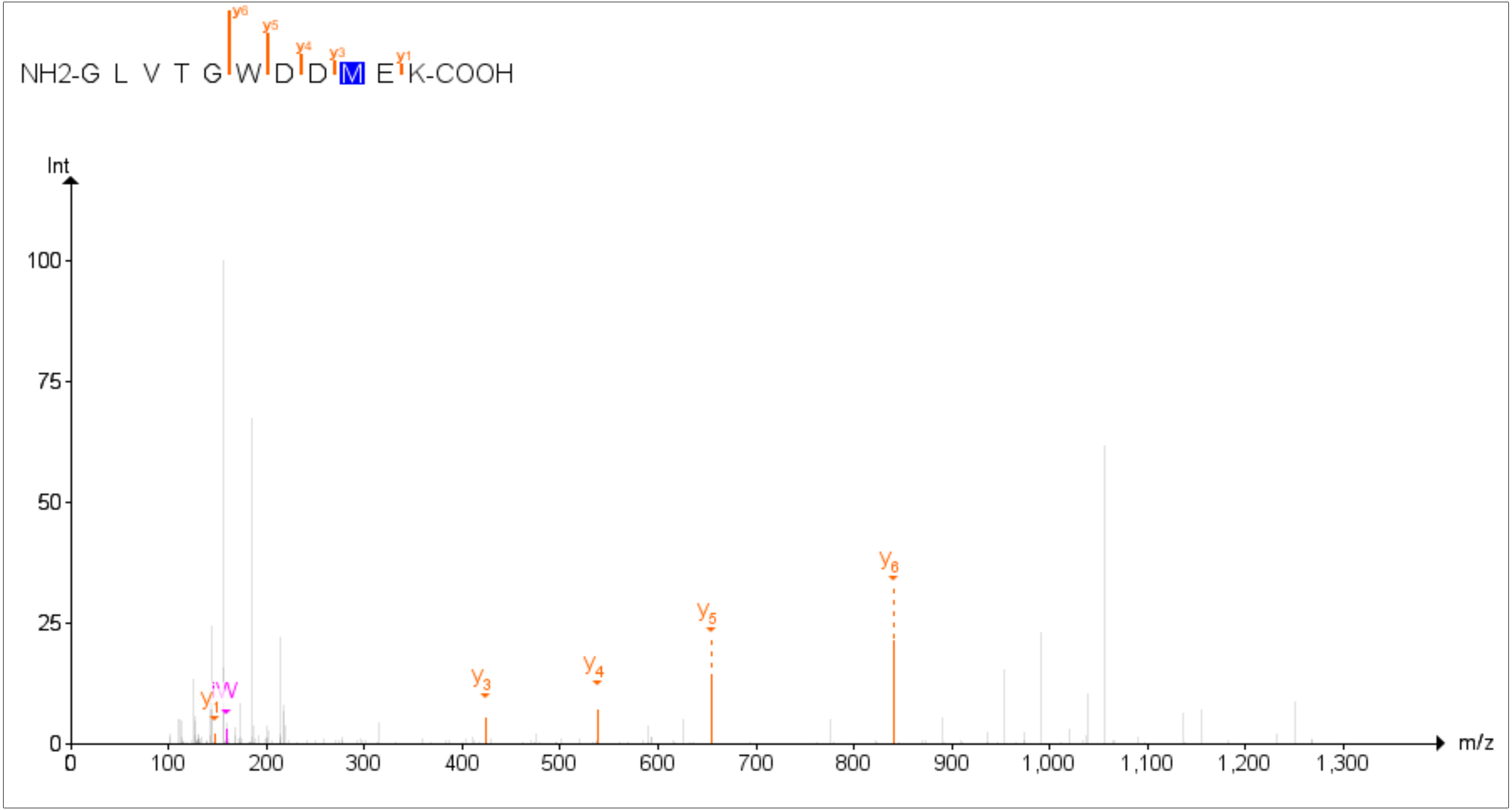

NH2-G L V T G W D D M E K-COOH
y6
y5
y4
y3
y1
Int
100
75
50
25
0
m/z
0
100
200
300
400
500
600
700
800
900
1,000
1,100
1,200
1,300
y1
iW
y3
y4
y5
y6

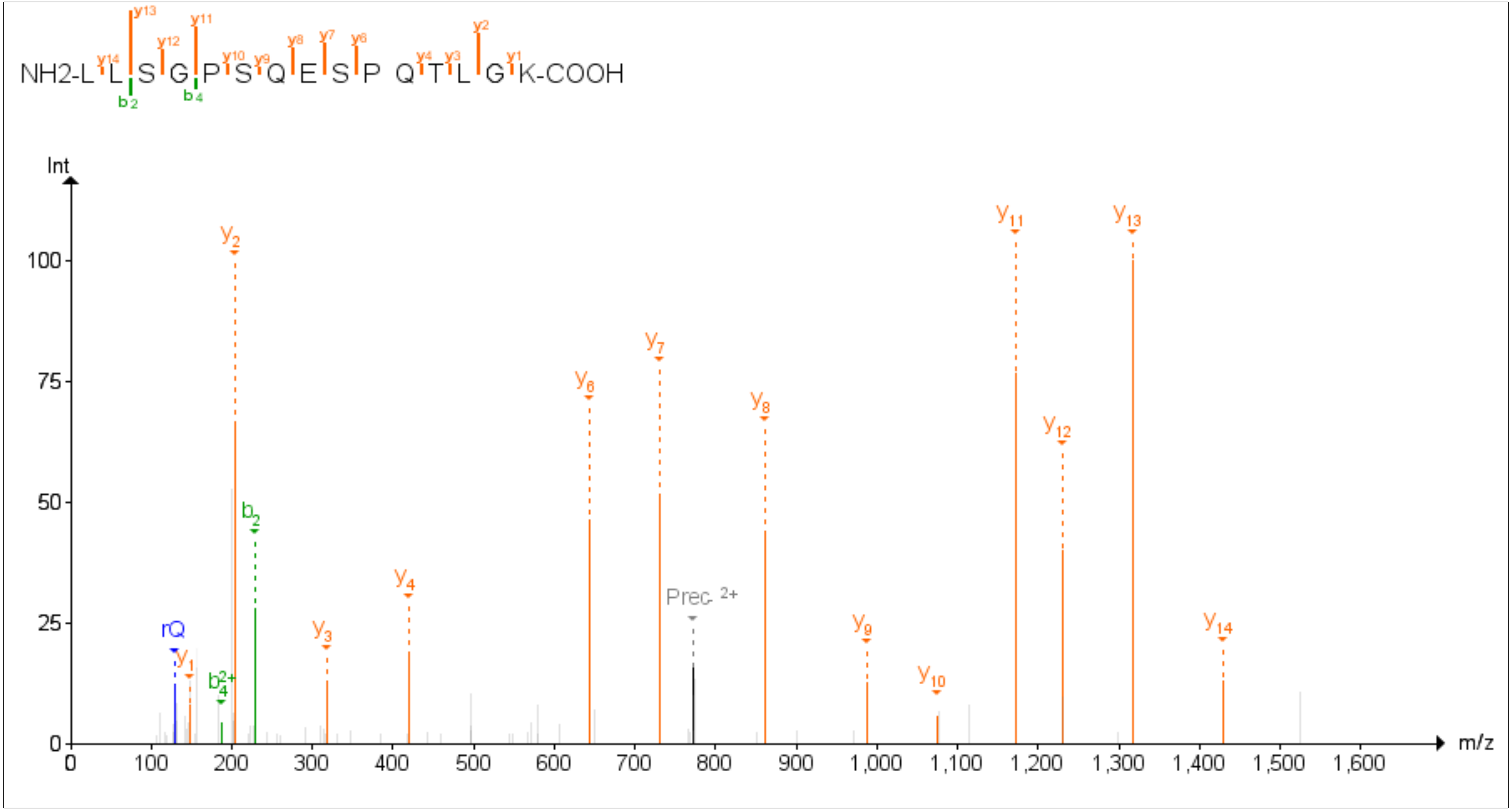

NH2-L L S G P S Q E S P Q T L G K-COOH
y14 y13 y12 y11 y10 y9 y8 y7 y6 y4 y3 y2 y1
b2 b4
Int
m/z
rQ
Prec 2+

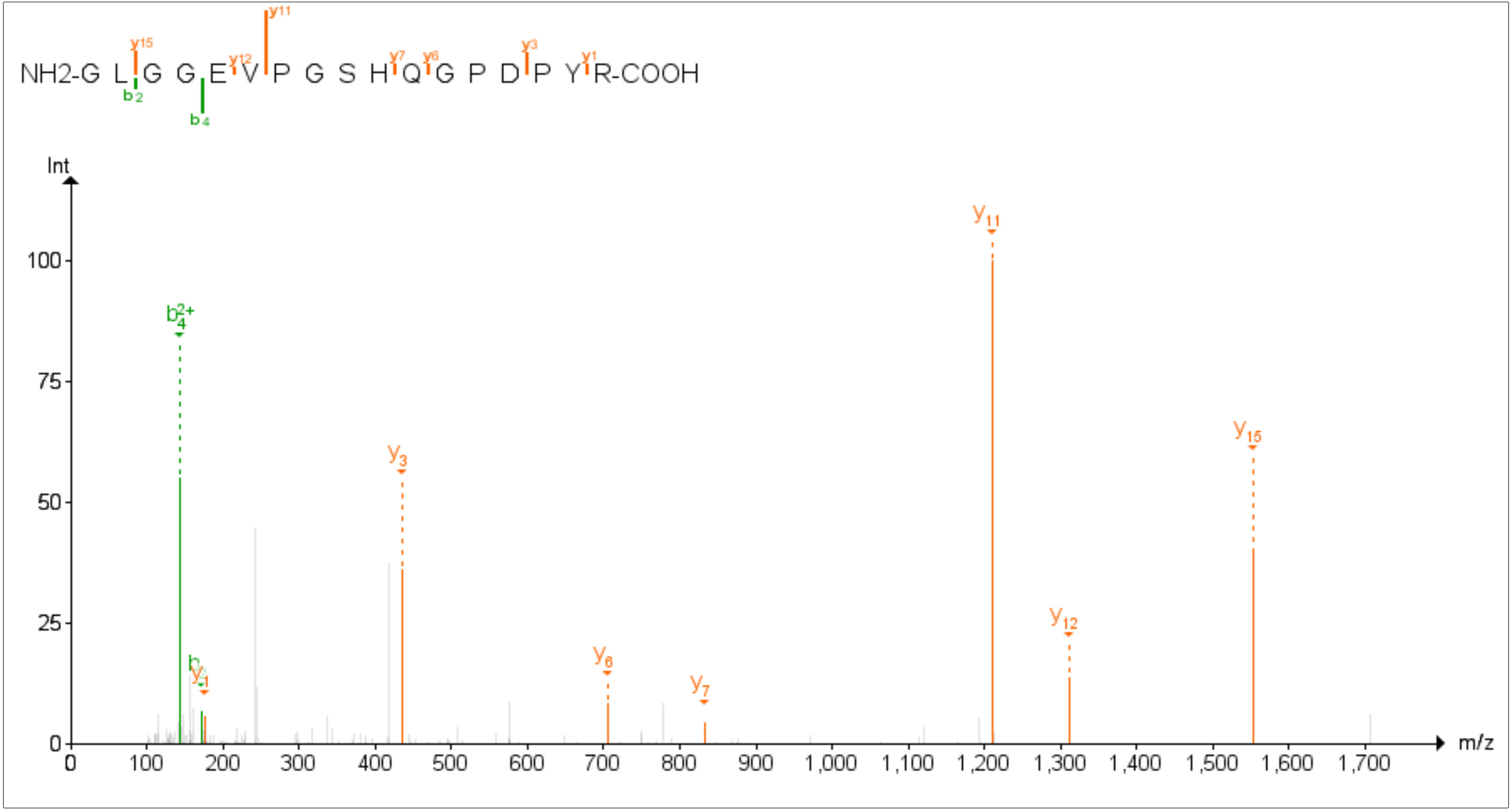

NH2-G L G G E V P G S H Q G P D P Y R-COOH
y15
y12
y11
y7
y6
y3
y1
b2
b4
Int
m/z
y11
b4 2+
y3
y15
y12
y6
y7
y1
0
25
50
75
100
0
100
200
300
400
500
600
700
800
900
1,000
1,100
1,200
1,300
1,400
1,500
1,600
1,700

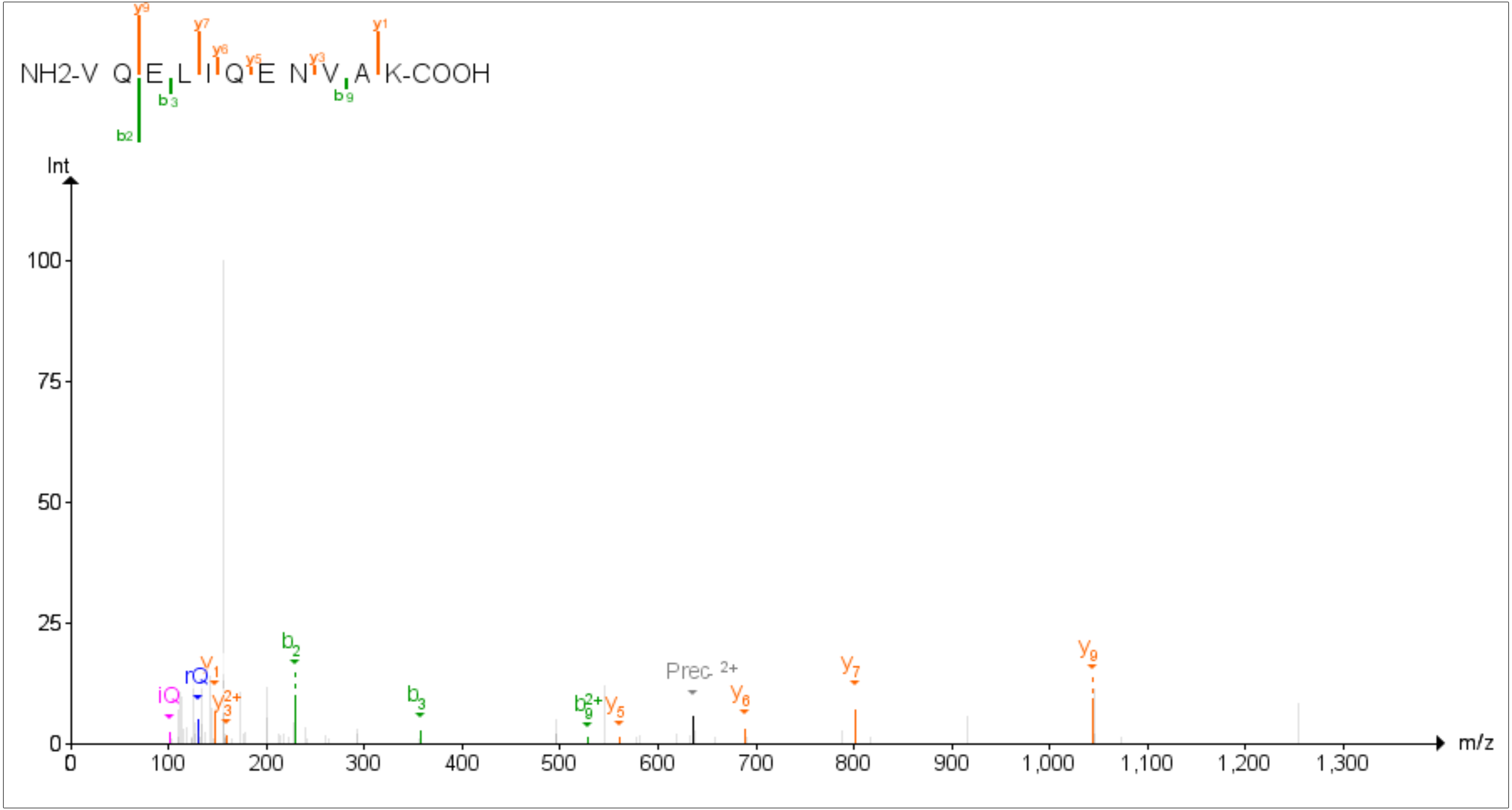

NH2-V Q E L I Q E N V A K-COOH
y9
y7
y6
y5
y3
y1
b2
b3
b9
Int
100
75
50
25
0
iQ
rQ
y1
y3 2+
b2
b3
b9 2+
y5
Prec 2+
y6
y7
y9
m/z
0
100
200
300
400
500
600
700
800
900
1,000
1,100
1,200
1,300

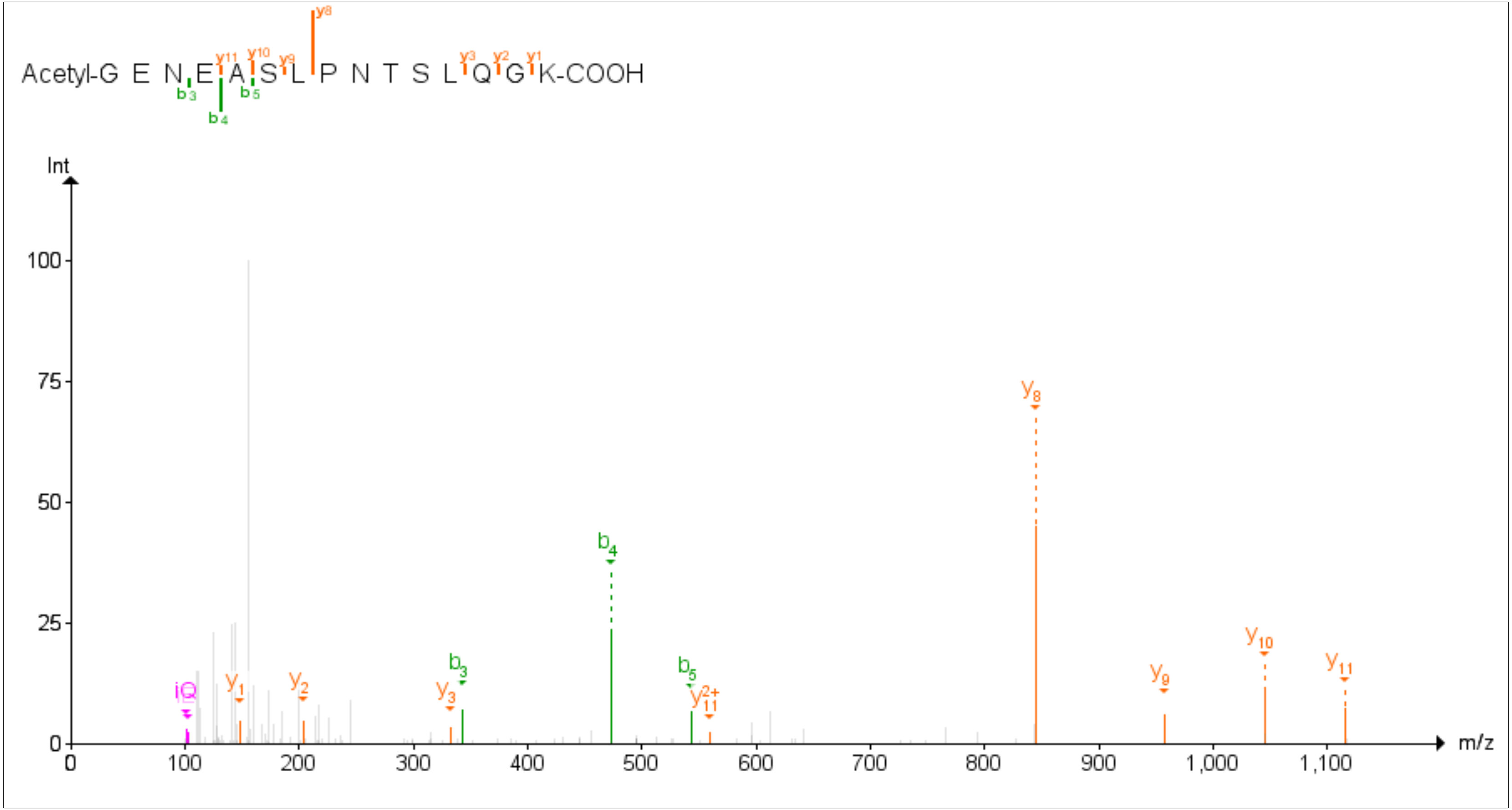
Acetyl-G E N E A S L P N T S L Q G K-COOH
Int
100
75
50
25
0
m/z
0
100
200
300
400
500
600
700
800
900
1,000
1,100
y8
b4
y9
y10
y11
b3
b5
y3
y1
y2
iQ

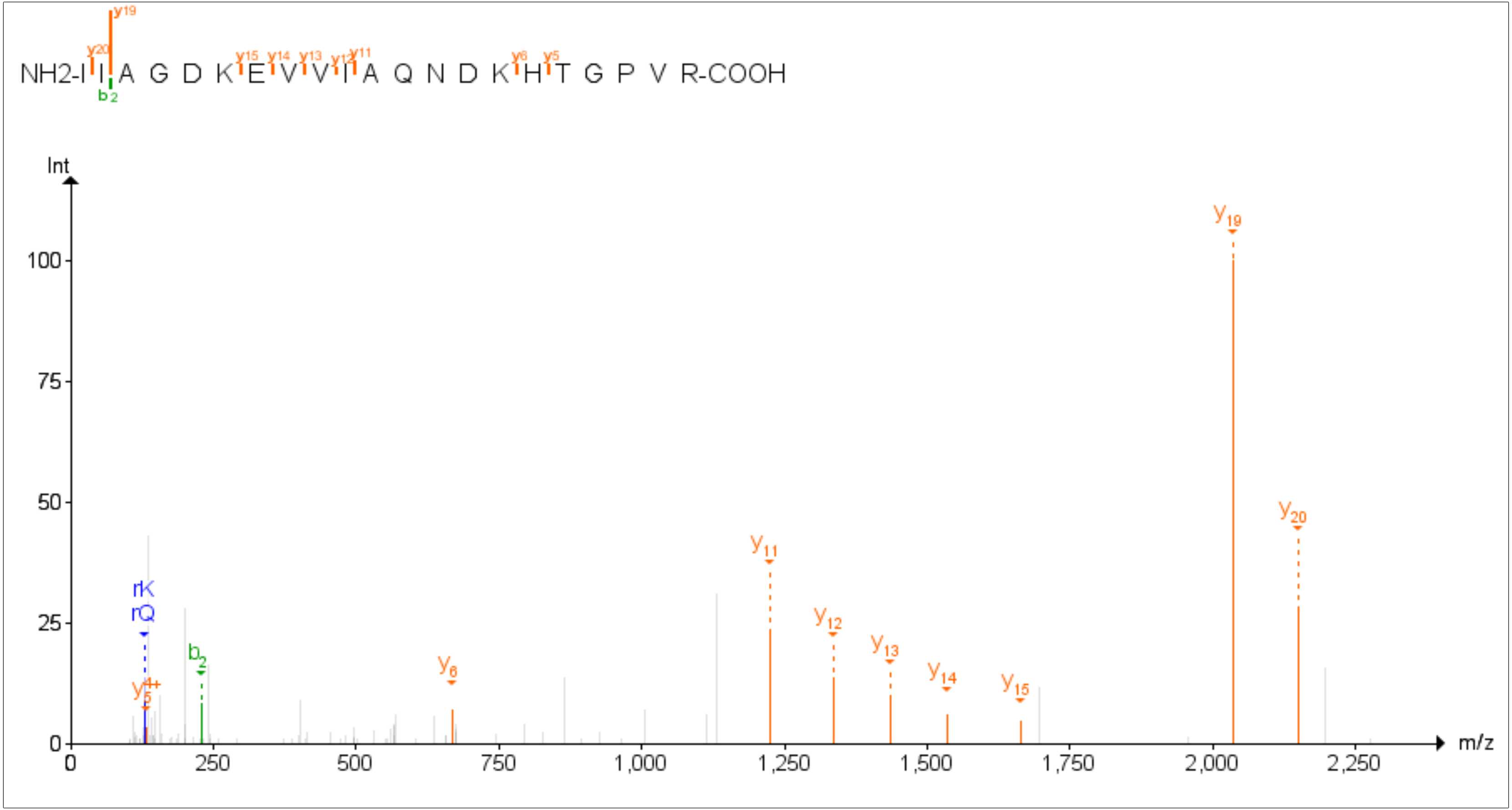
NH2-I I A G D K E V V I A Q N D K H T G P V R-COOH
y19
y20
b2
y15 y14 y13 y12 y11
y6 y5
Int
100
75
50
25
0
m/z
0
250
500
750
1,000
1,250
1,500
1,750
2,000
2,250
rK
rQ
y5++
b2
y6
y11
y12
y13
y14
y15
y19
y20

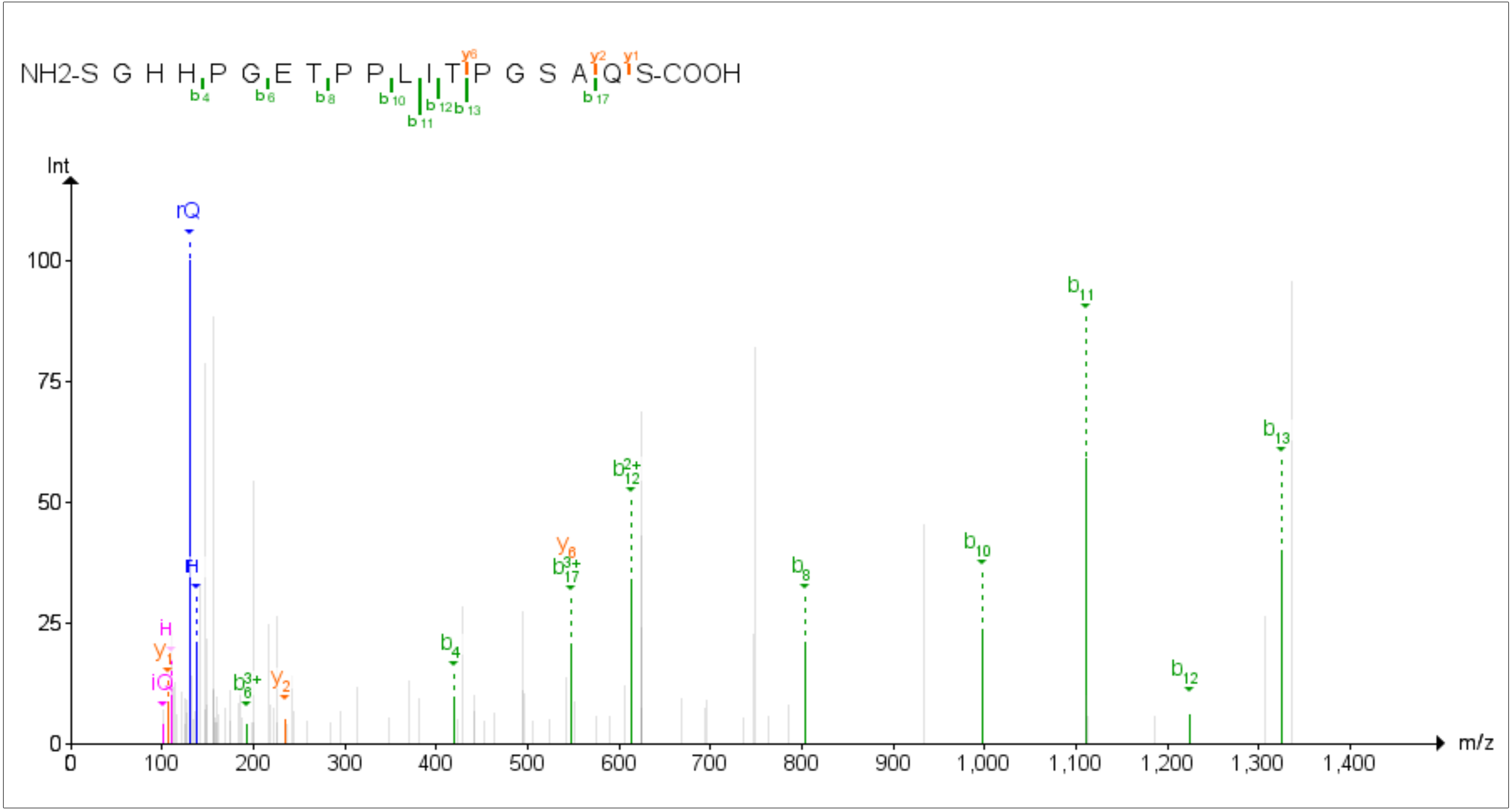

NH2-S G H H P G E T P P L I T P G S A Q S-COOH
b4 b6 b8 b10 b11 b12 b13 b17
y6 y2 y1
Int
rQ
iH
y1
iQ
b6 3+
y2
b4
y6
b17 3+
b12 2+
b8
b10
b11
b12
b13
0
25
50
75
100
0
100
200
300
400
500
600
700
800
900
1,000
1,100
1,200
1,300
1,400
m/z

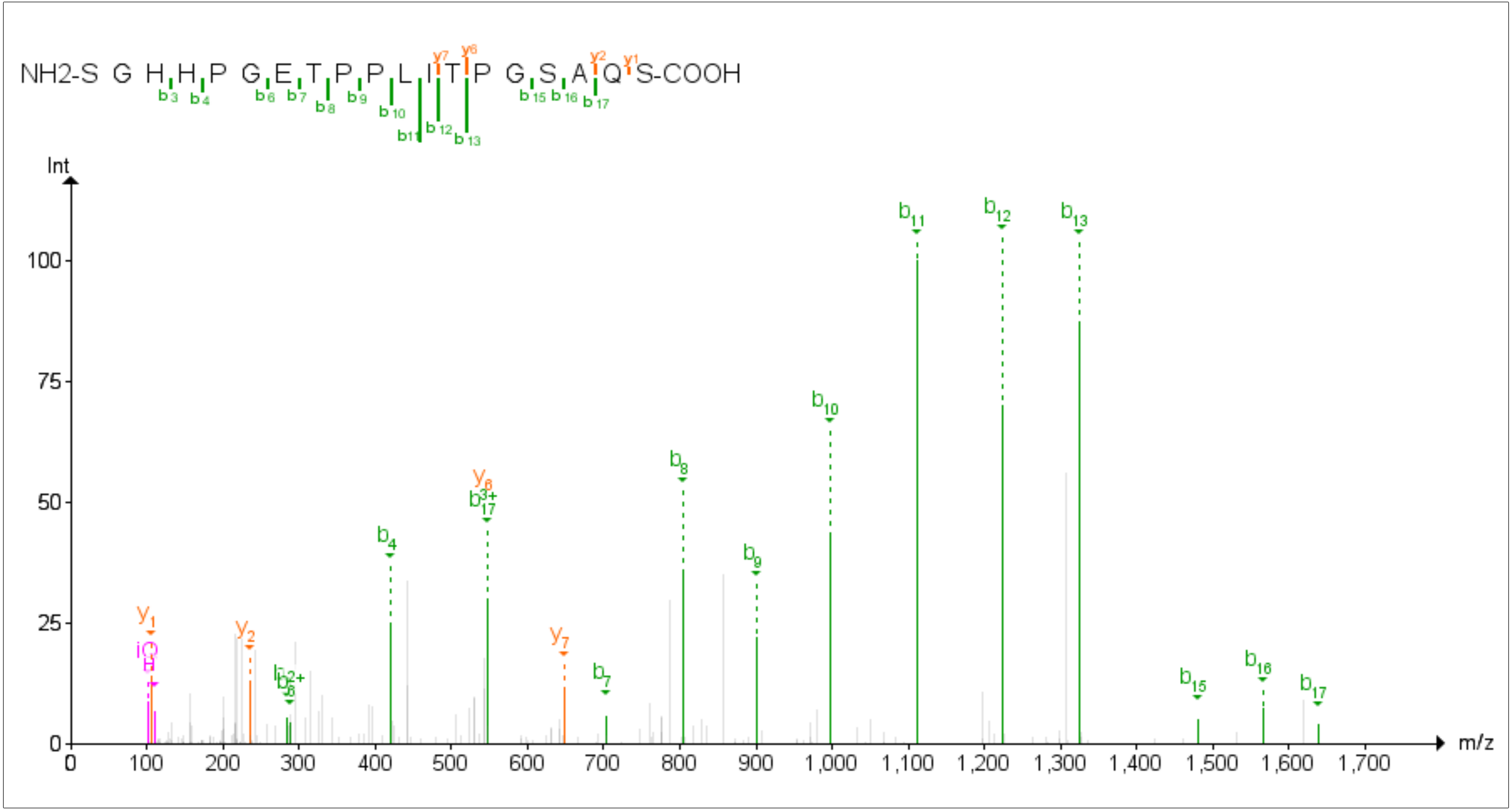

NH2-S G H H P G E T P P L I T P G S A Q S-COOH
Int
m/z
b11
b12
b13
b10
b8
b9
b4
y6
y7
y1
y2
b7
b15
b16
b17

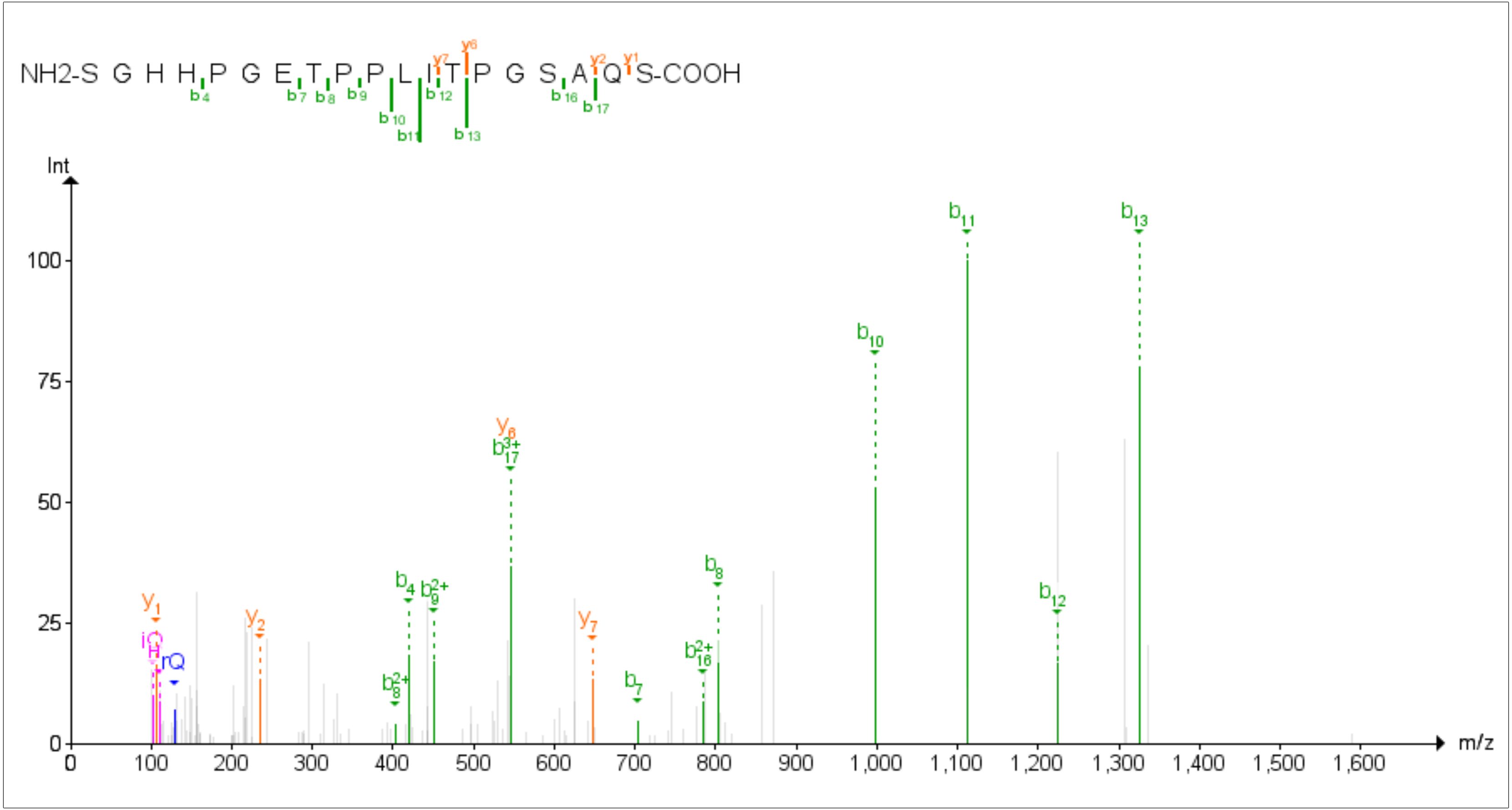

NH2-S G H H P G E T P P L I T P G S A Q S-COOH
b4 b7 b8 b9 b10 b11 b12 b13 b16 b17
y7 y6 y2 y1
Int
m/z
0
25
50
75
100
0
100
200
300
400
500
600
700
800
900
1,000
1,100
1,200
1,300
1,400
1,500
1,600
b11
b13
b10
y6
b17 3+
y1
y2
b4
b9 2+
b8 2+
y7
b7
b16 2+
b8
b12
rQ

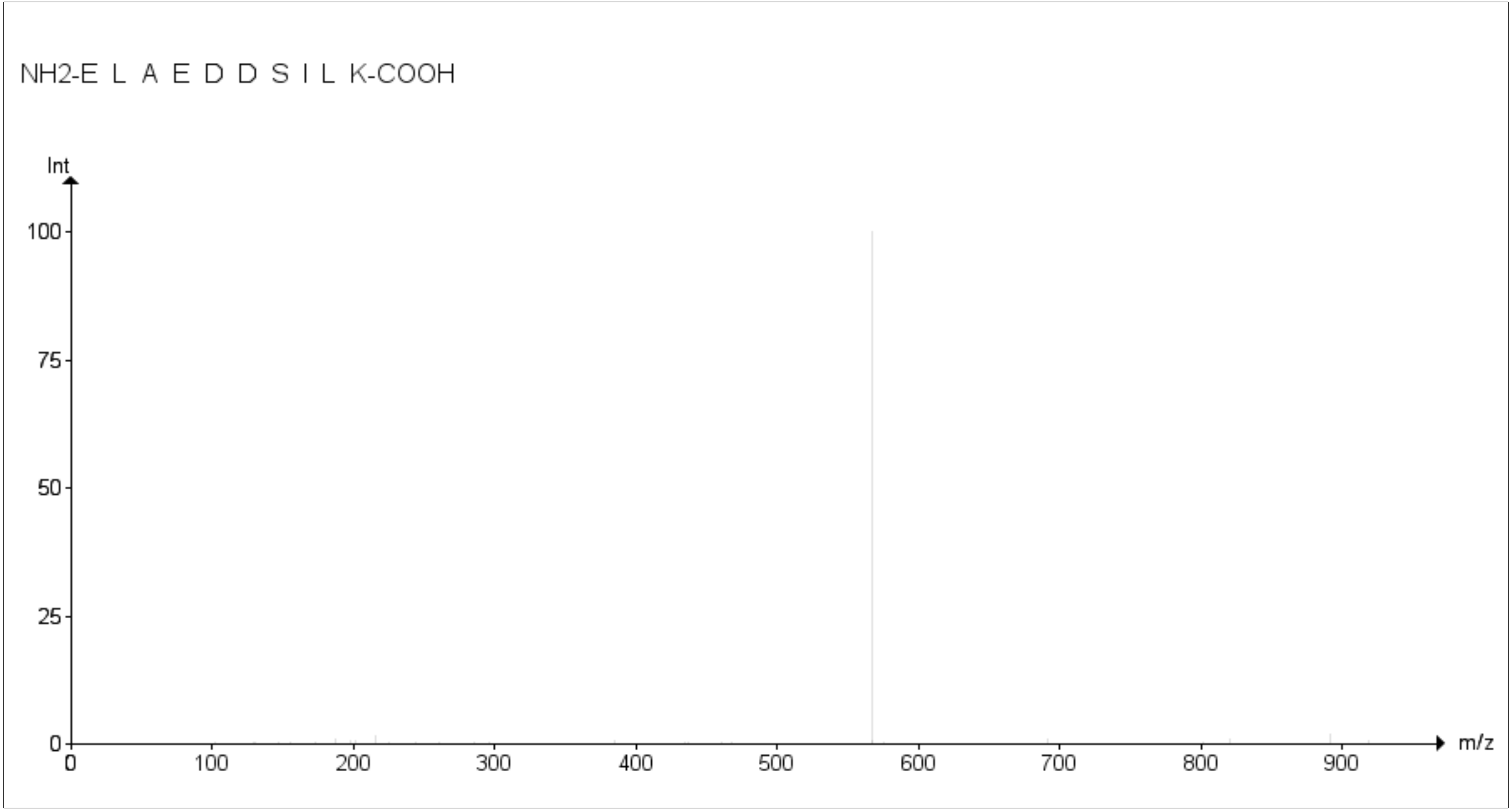
NH2-E L A E D D S I L K-COOH
Int
100
75
50
25
0
0
100
200
300
400
500
600
700
800
900
m/z

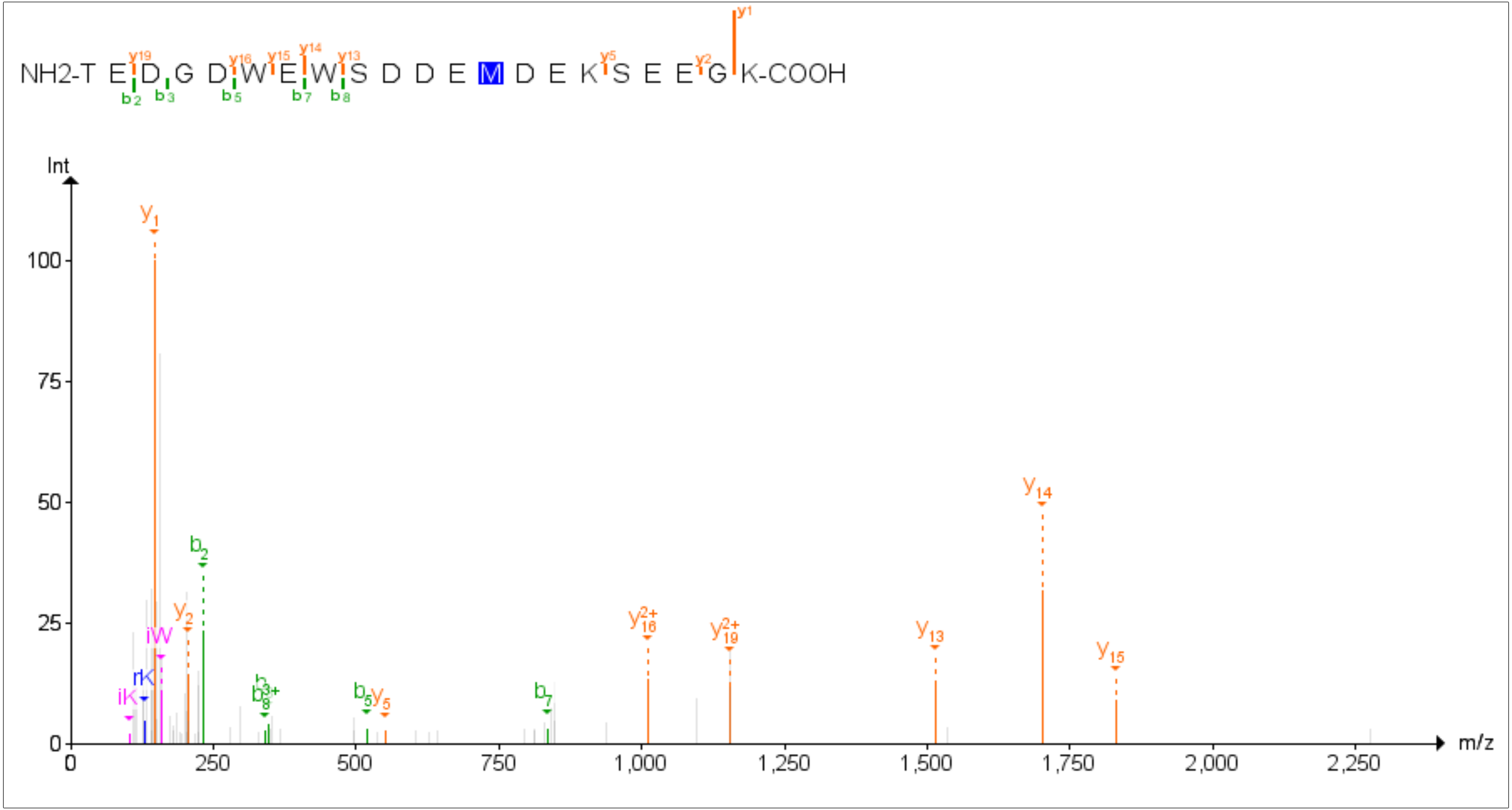
NH2-T E D G D W E W S D D E M D E K S E E G K-COOH
y19 y16 y15 y14 y13 y5 y2 y1
b2 b3 b5 b7 b8
Int
m/z
y1
b2
y2
iW
rK
iK
b3 b8 3+
b5 y5
b7
y16 2+
y19 2+
y13
y14
y15
0
25
50
75
100
0
250
500
750
1,000
1,250
1,500
1,750
2,000
2,250

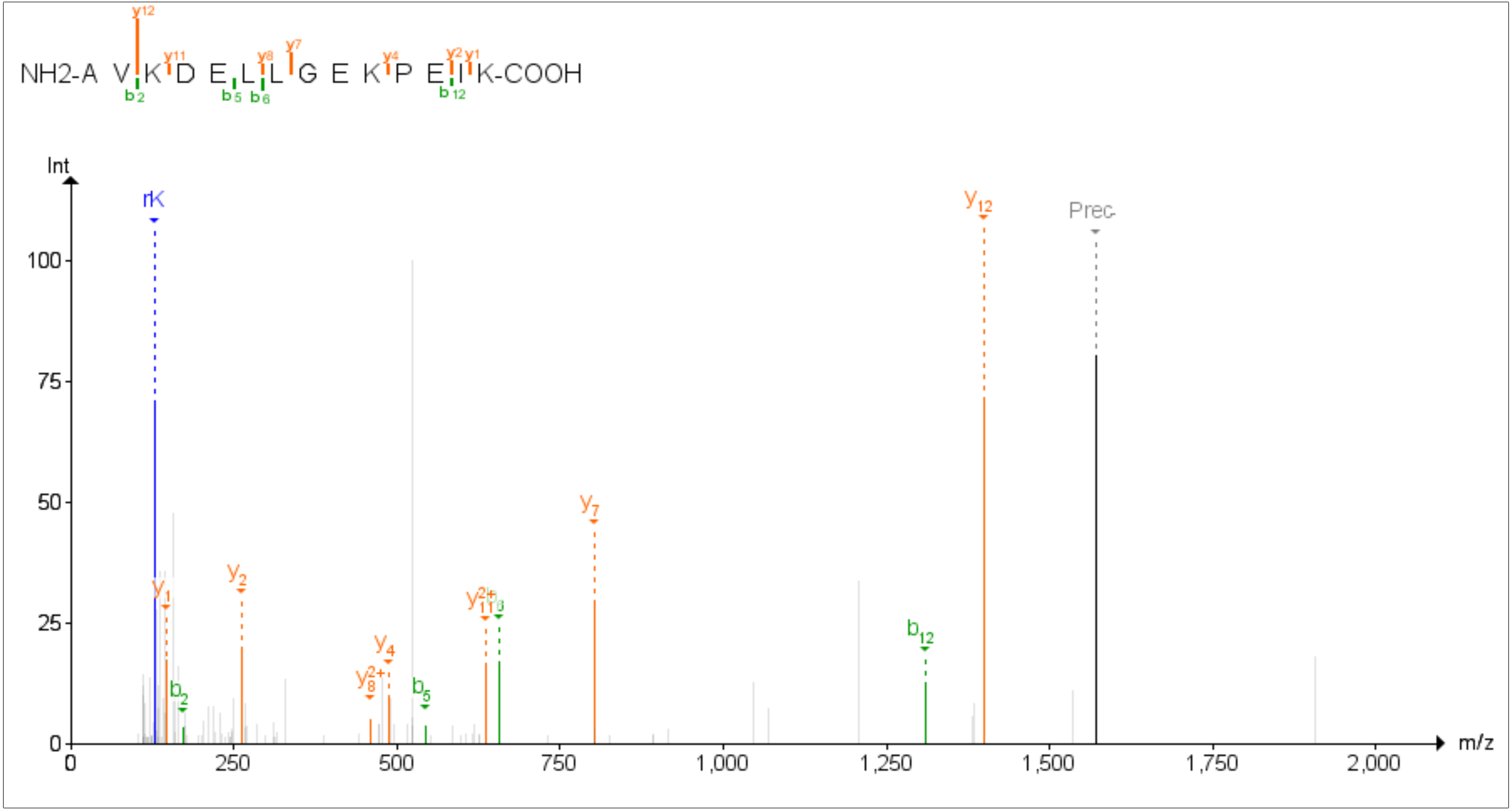

NH2-A V K D E L L L G E K P E I I K-COOH
Int
m/z
rK
y12
Prec
y7
y2
y1
b2
y4
b5
b12
0
25
50
75
100
250
500
750
1,000
1,250
1,500
1,750
2,000

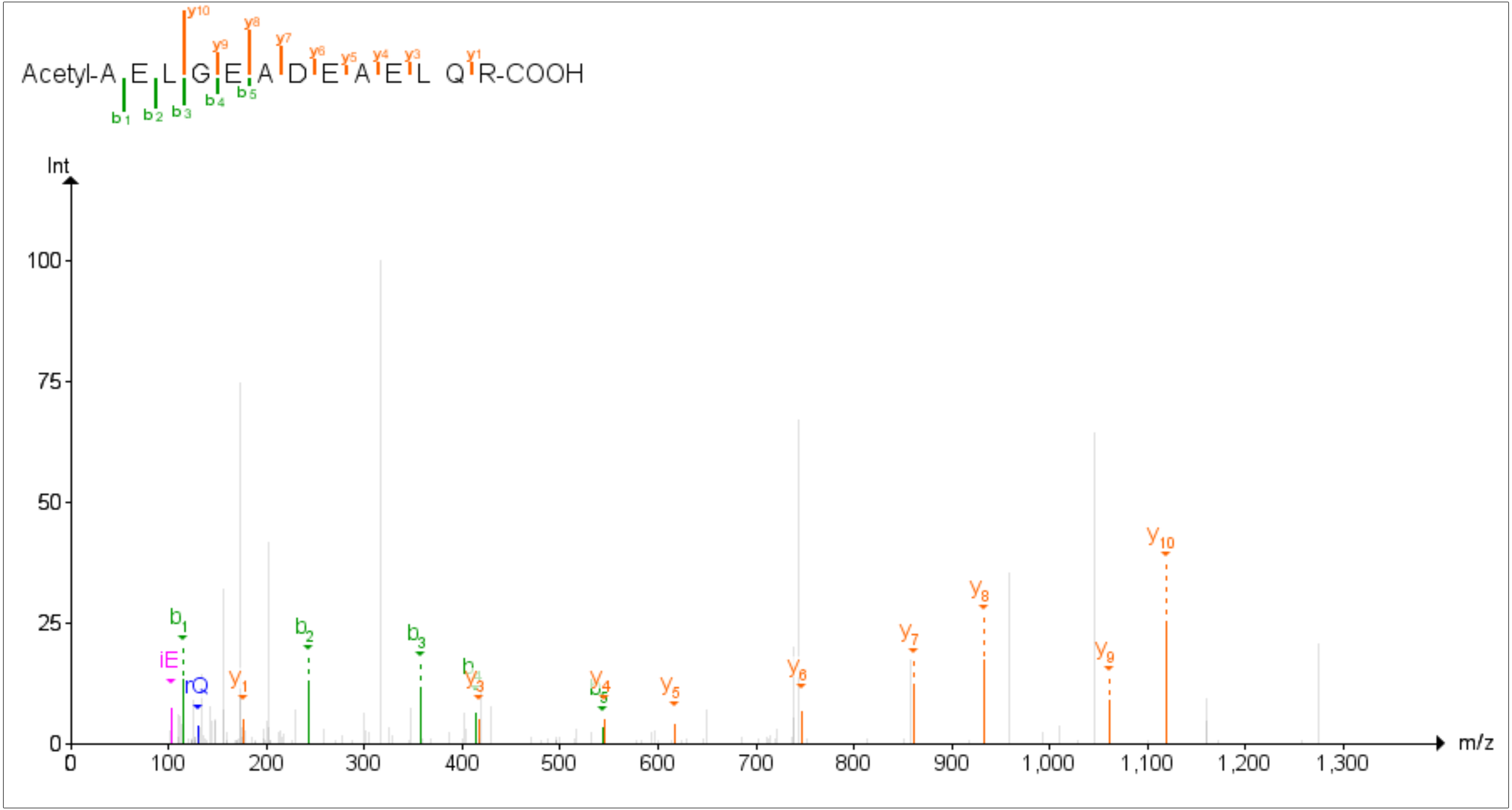

Acetyl-A E L G E A D E A E L Q R-COOH
b1 b2 b3 b4 b5
y10 y9 y8 y7 y6 y5 y4 y3 y1
Int
100
75
50
25
0
m/z
0
100
200
300
400
500
600
700
800
900
1,000
1,100
1,200
1,300
iE
rQ

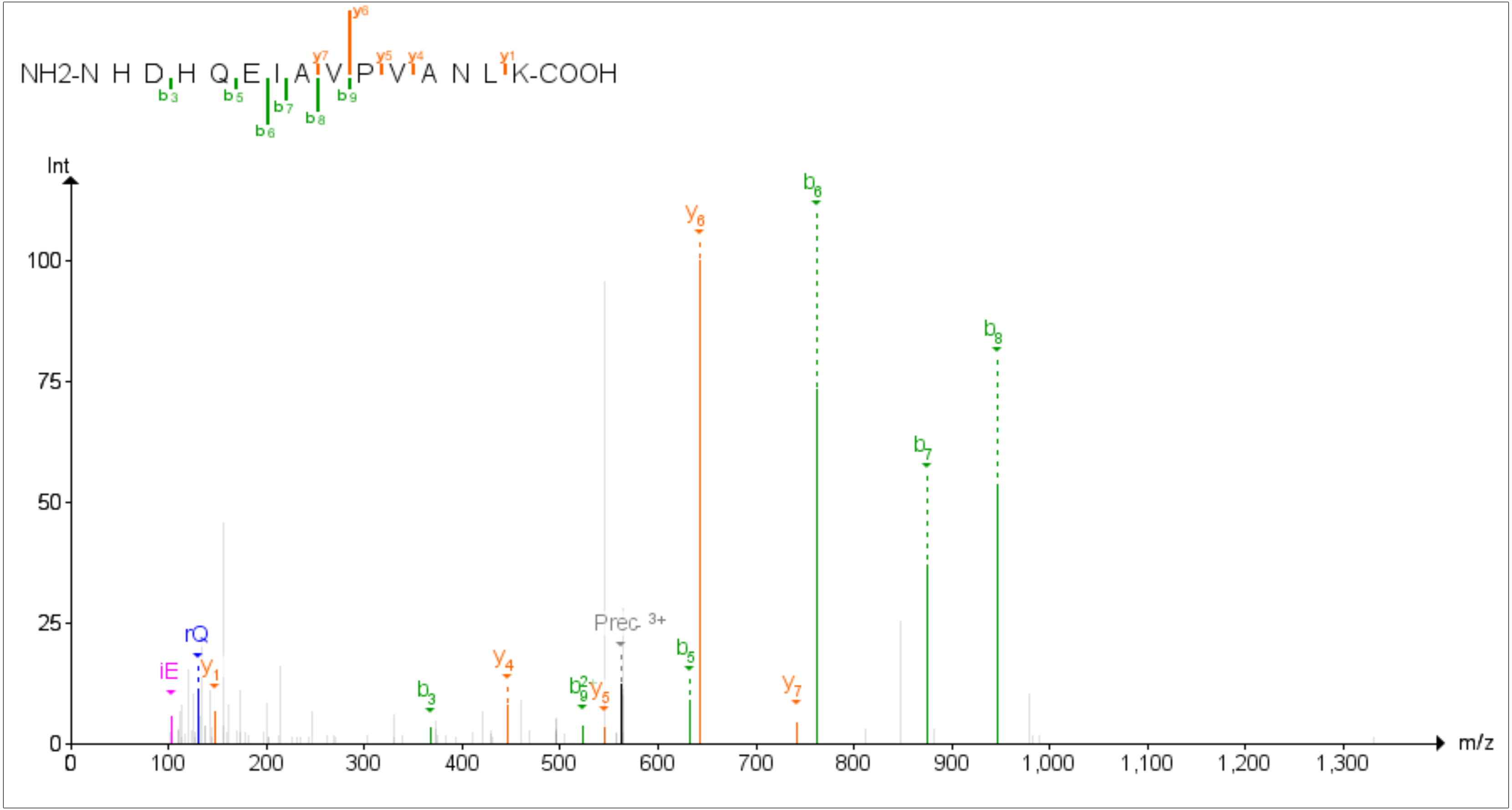

NH2-N H D H Q E I A V P V A N L K-COOH
b3 b5 b6 b7 b8 b9
y8 y7 y5 y4 y1
Int
100
75
50
25
0
m/z
0 100 200 300 400 500 600 700 800 900 1,000 1,100 1,200 1,300
iE
rQ
y1
b3
y4
b9 2+
y5
Prec- 3+
b5
y6
y7
b6
b7
b8

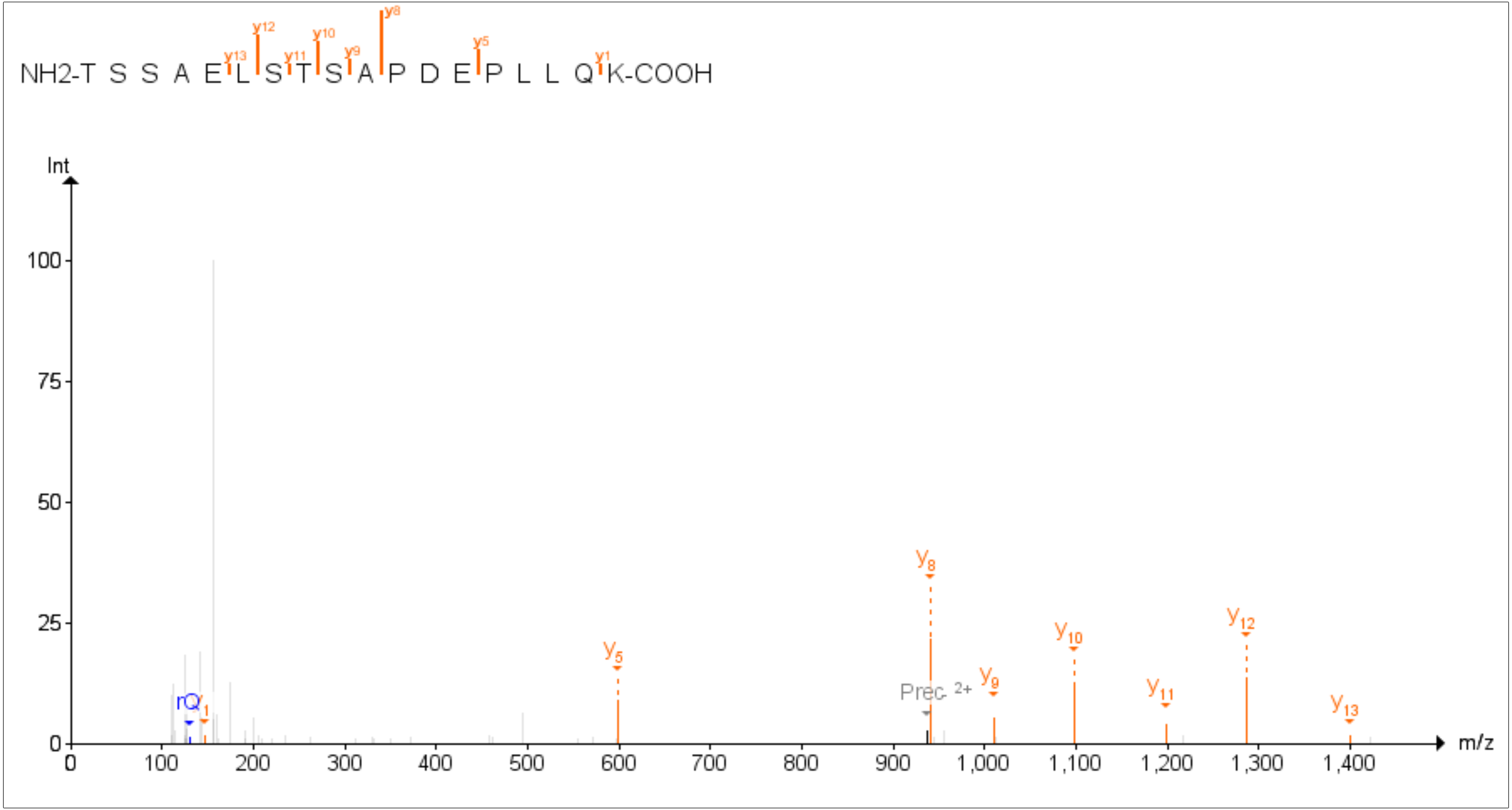

NH2-T S S A E L S T S A P D E P L L Q K-COOH
y13 y12 y11 y10 y9 y8 y5 y1
Int
100
75
50
25
0
rQ
y1
y5
Prec 2+
y8
y9
y10
y11
y12
y13
0 100 200 300 400 500 600 700 800 900 1,000 1,100 1,200 1,300 1,400
m/z

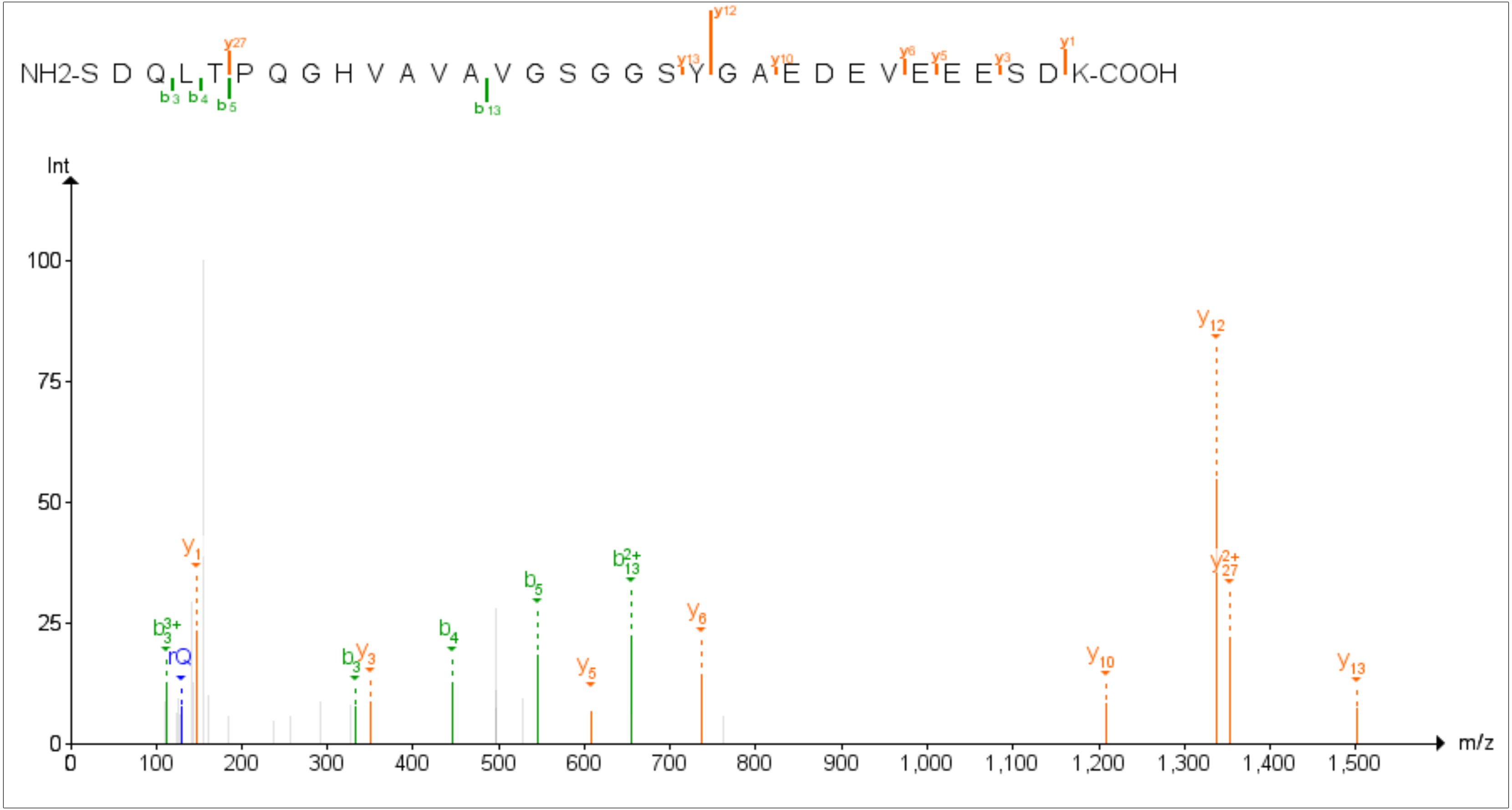

NH2-S D Q L T P Q G H V A V A V G S G G S Y G A E D E V E E E S D K-COOH
Int
m/z
y1
b3 3+
rQ
b3
y3
b4
b5
y5
b13 2+
y6
y10
y12
y27 2+
y13

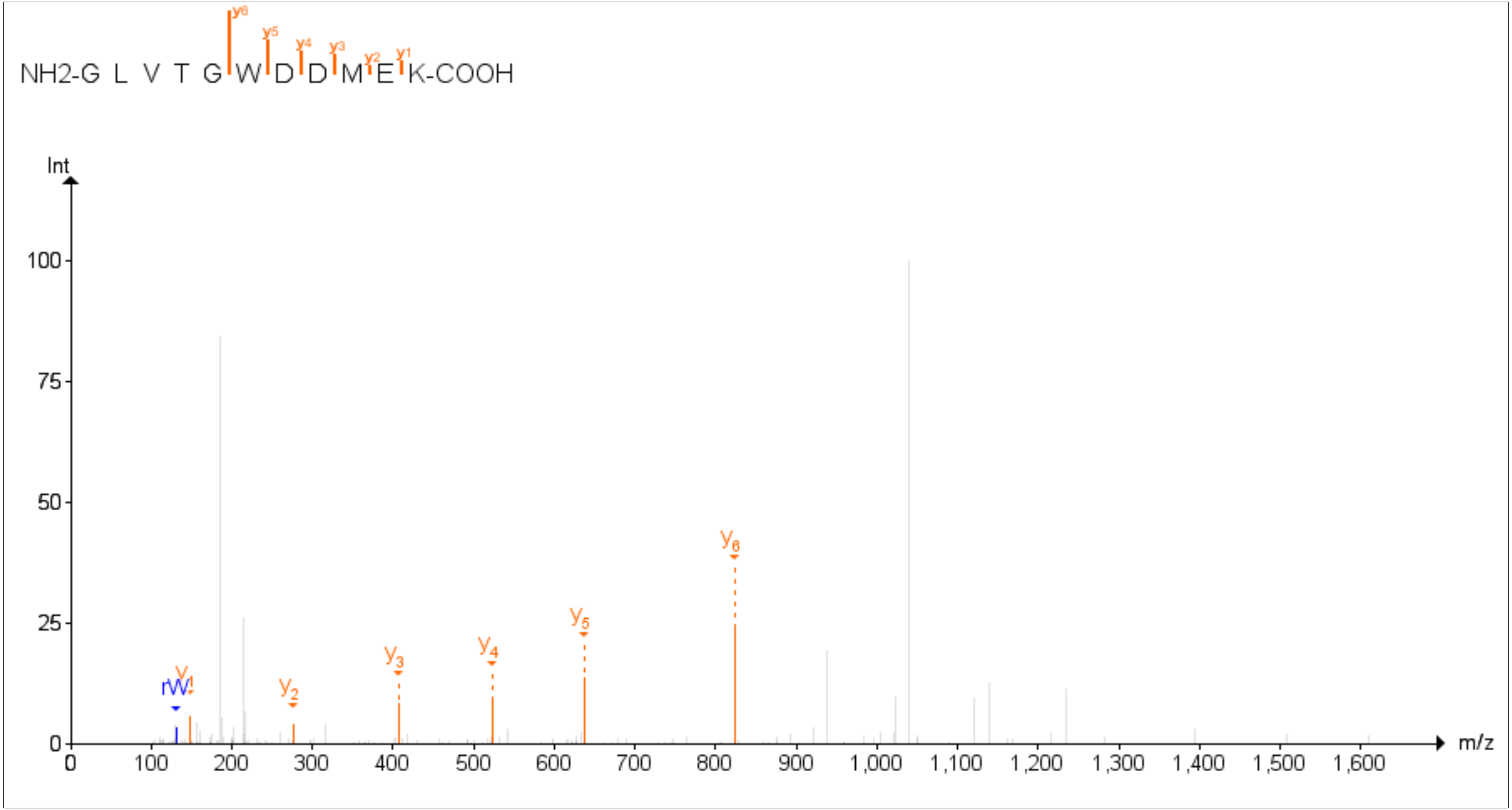

NH2-G L V T G W D D M E K-COOH
y6
y5
y4
y3
y2
y1
Int
100
75
50
25
0
0
100
200
300
400
500
600
700
800
900
1,000
1,100
1,200
1,300
1,400
1,500
1,600
m/z

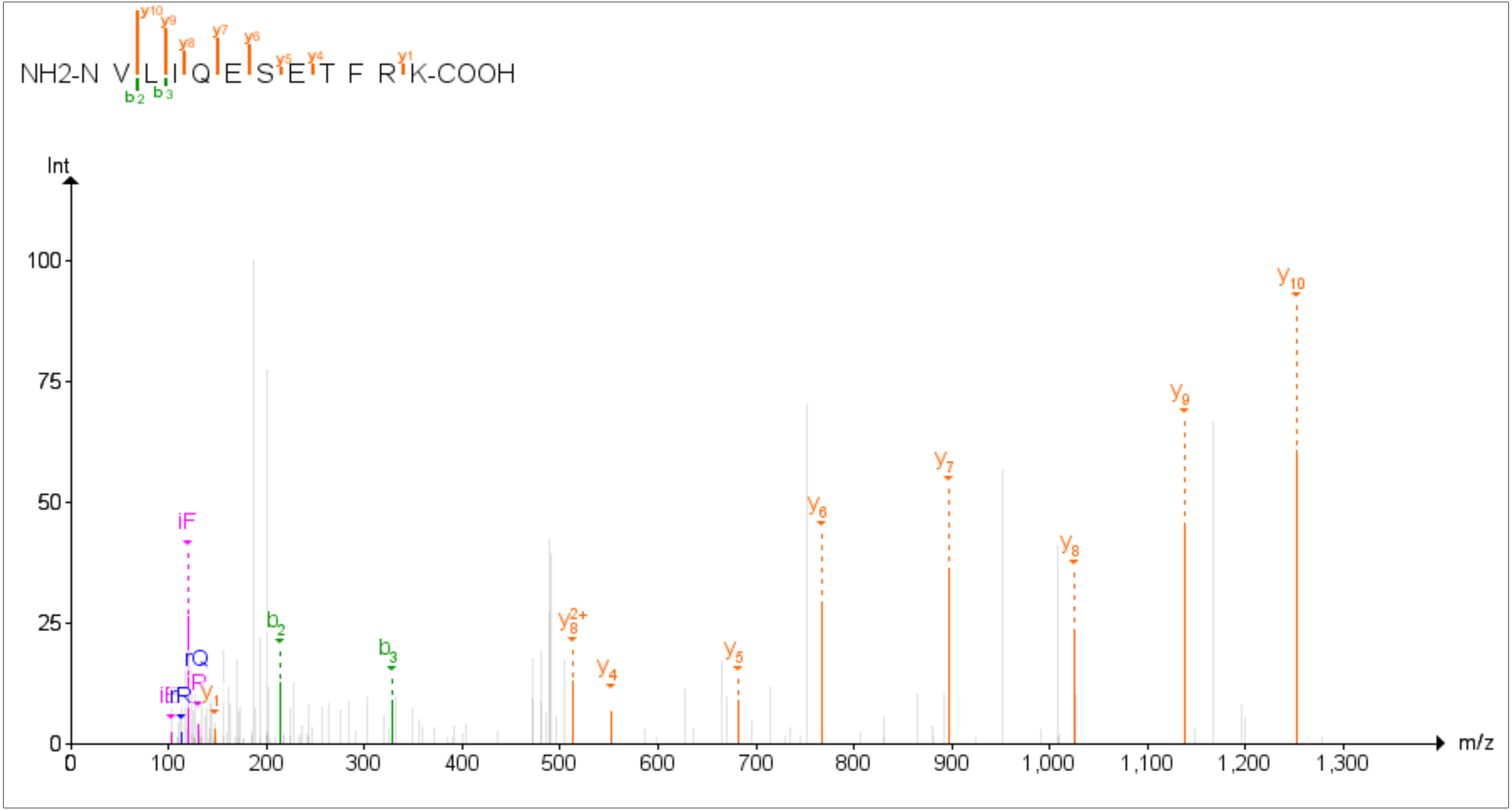
NH2-N V L I Q E S E T F R K-COOH
y10 y9 y8 y7 y6 y5 y4 y1
b2 b3
Int
100
75
50
25
0
m/z
0 100 200 300 400 500 600 700 800 900 1,000 1,100 1,200 1,300
iF
rQ
iR
rR
y1
b2
b3
y8 2+
y4
y5
y6
y7
y8
y9
y10

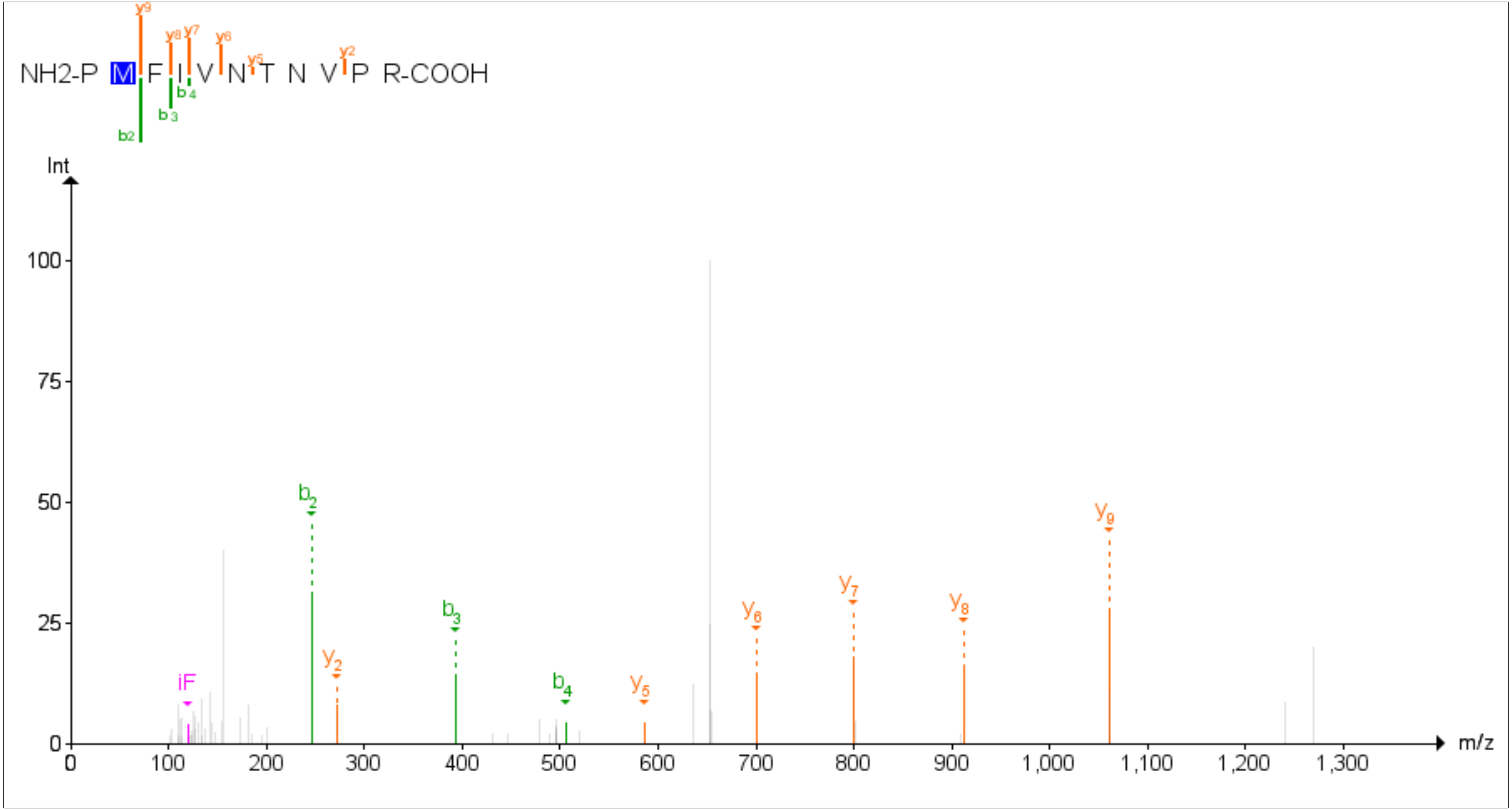
NH2-P M F I V N T N V P R-COOH
y9 y8 y7 y6 y5 y2
b2 b3 b4
Int
100
75
50
25
0
iF
b2
y2
b3
b4
y5
y6
y7
y8
y9
0 100 200 300 400 500 600 700 800 900 1,000 1,100 1,200 1,300
m/z

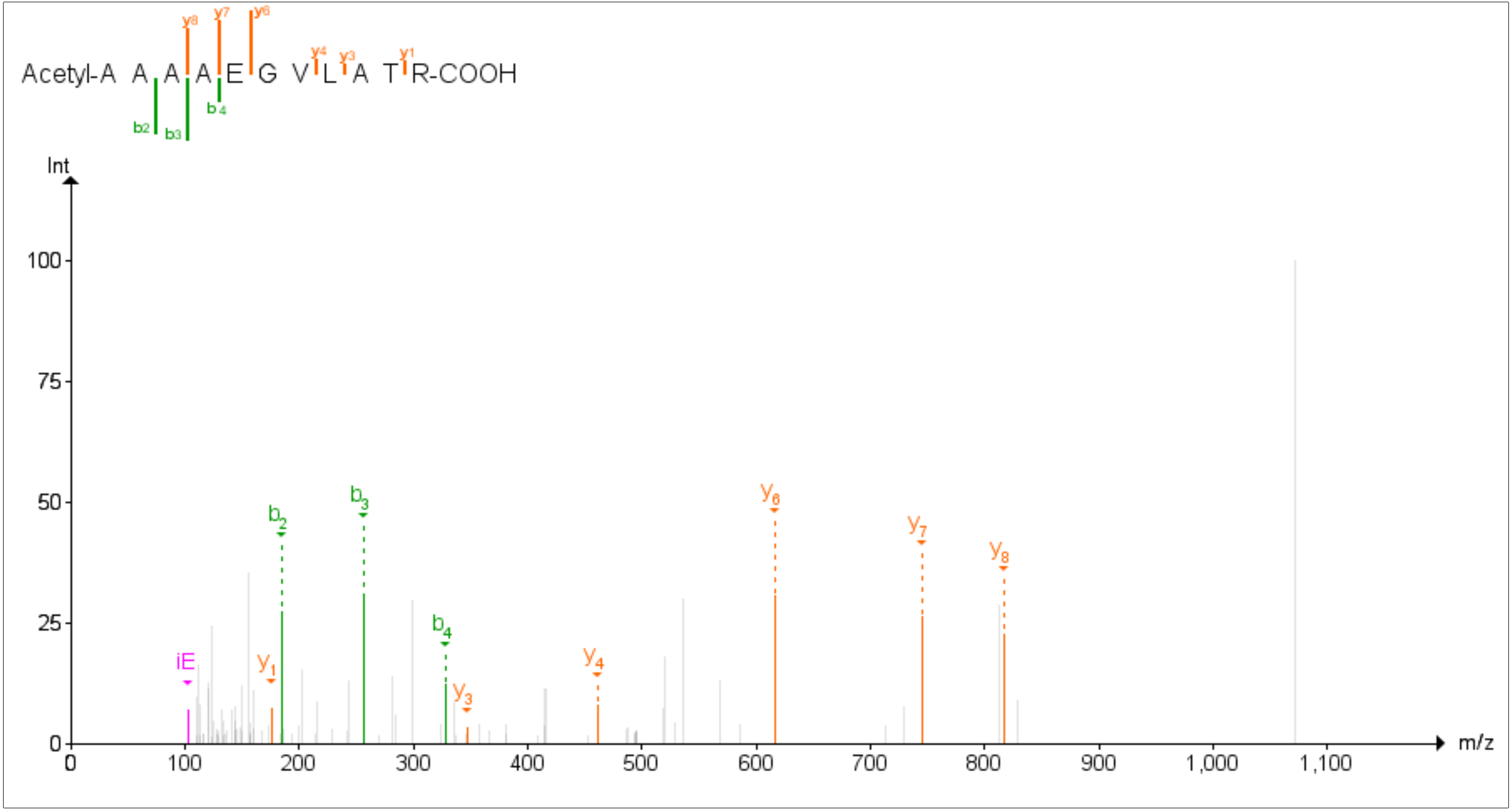

Acetyl-A A A A E G V L A T R-COOH
y8
y7
y6
y4
y3
y1
b2
b3
b4
Int
100
75
50
25
0
b2
b3
b4
iE
y1
y3
y4
y6
y7
y8
m/z
0
100
200
300
400
500
600
700
800
900
1,000
1,100

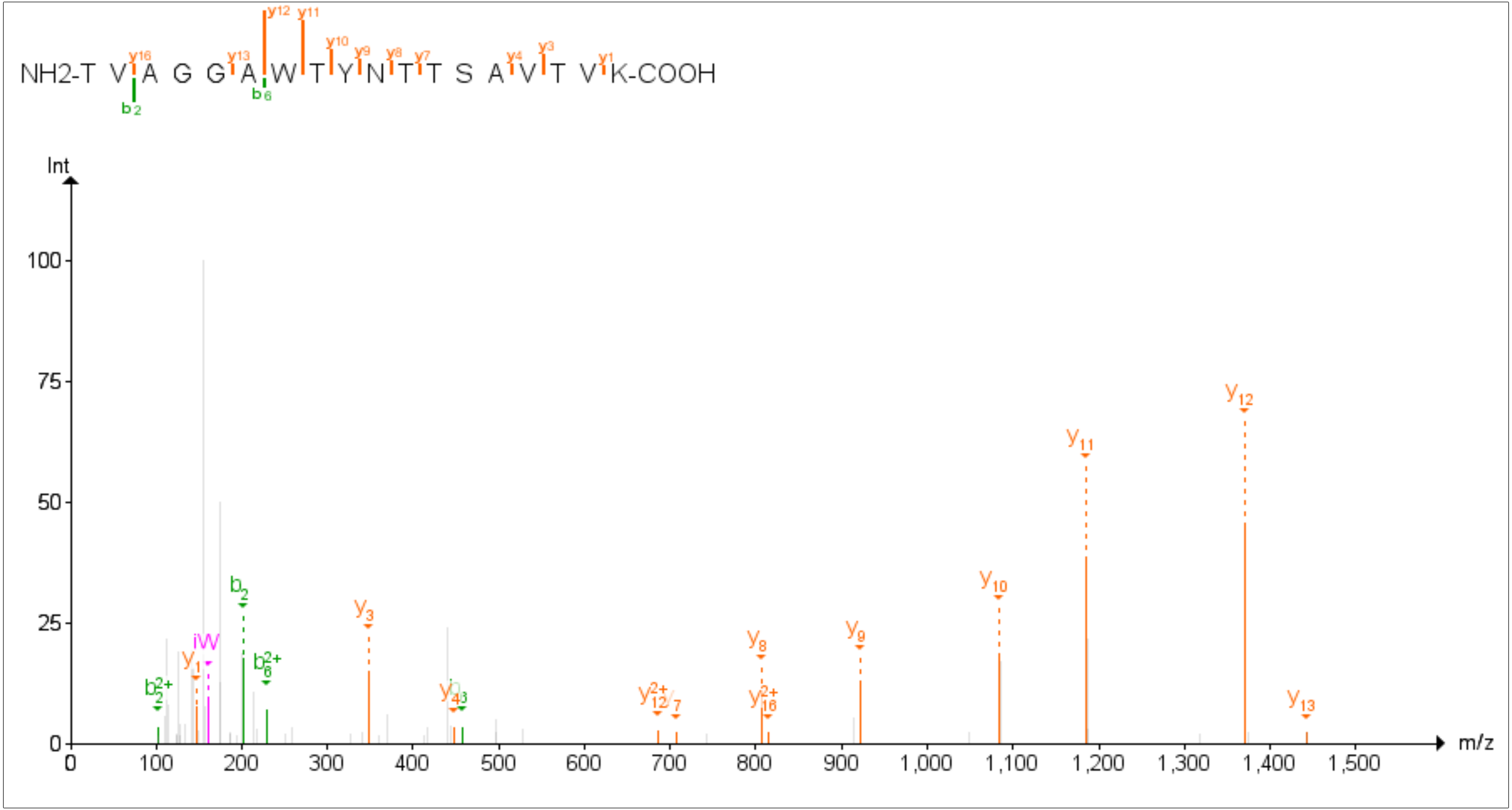

NH2-T V A G G A W T Y N T T S A V T V K-COOH
Int
m/z
y16 y13 y12 y11 y10 y9 y8 y7 y4 y3 y1
b2 b6
iW

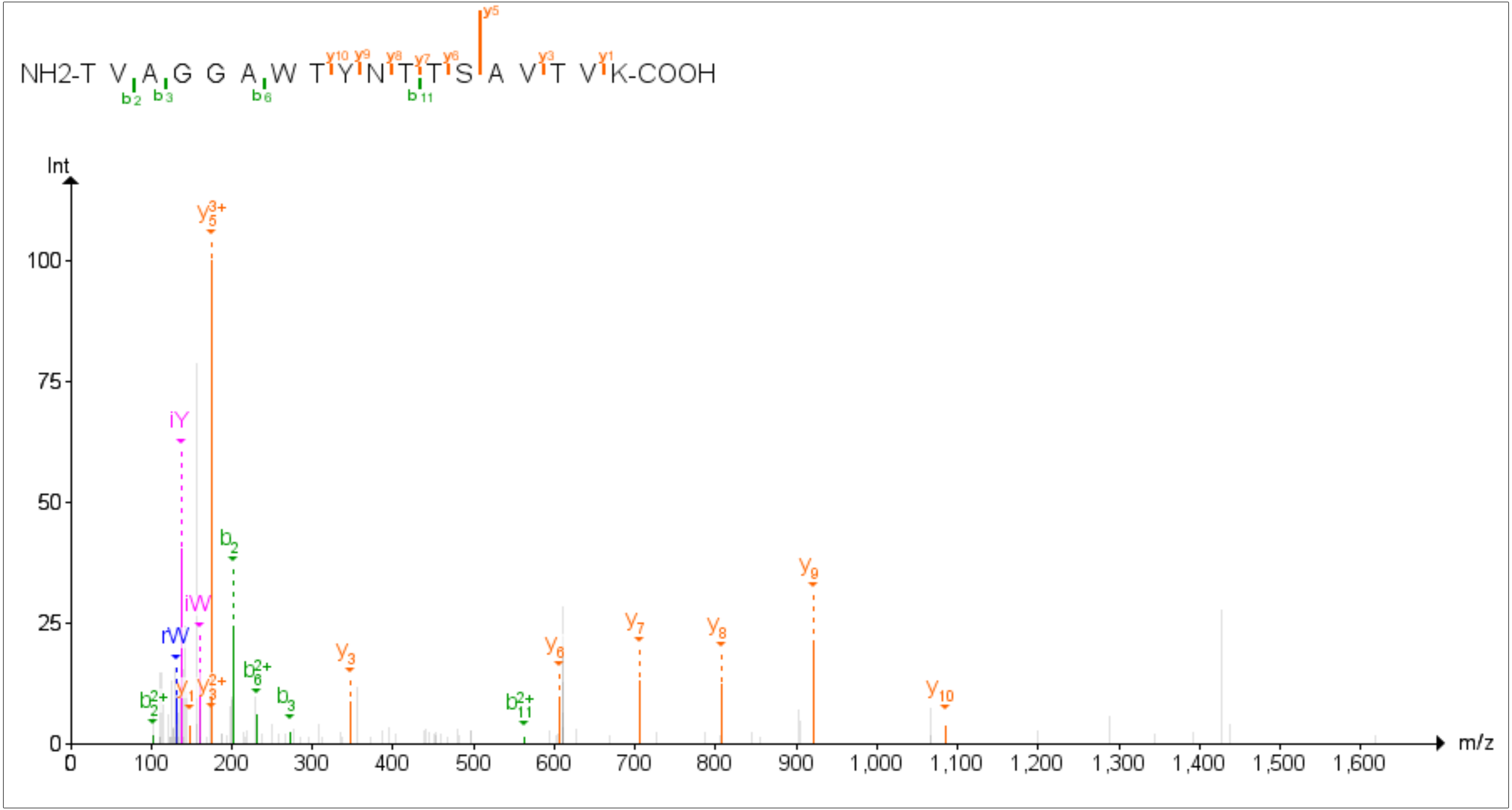

NH2-T V A G G A W T Y N T T T S A V T V K-COOH
b2 b3 b6 b11
y10 y9 y8 y7 y6 y5 y3 y1
Int
m/z
0
25
50
75
100
0
100
200
300
400
500
600
700
800
900
1,000
1,100
1,200
1,300
1,400
1,500
1,600
y5 3+
iY
b2
iW
rW
y9
y7
y8
y6
y3
b6 2+
y3 2+
y1
b2 2+
b3
b11 2+
y10

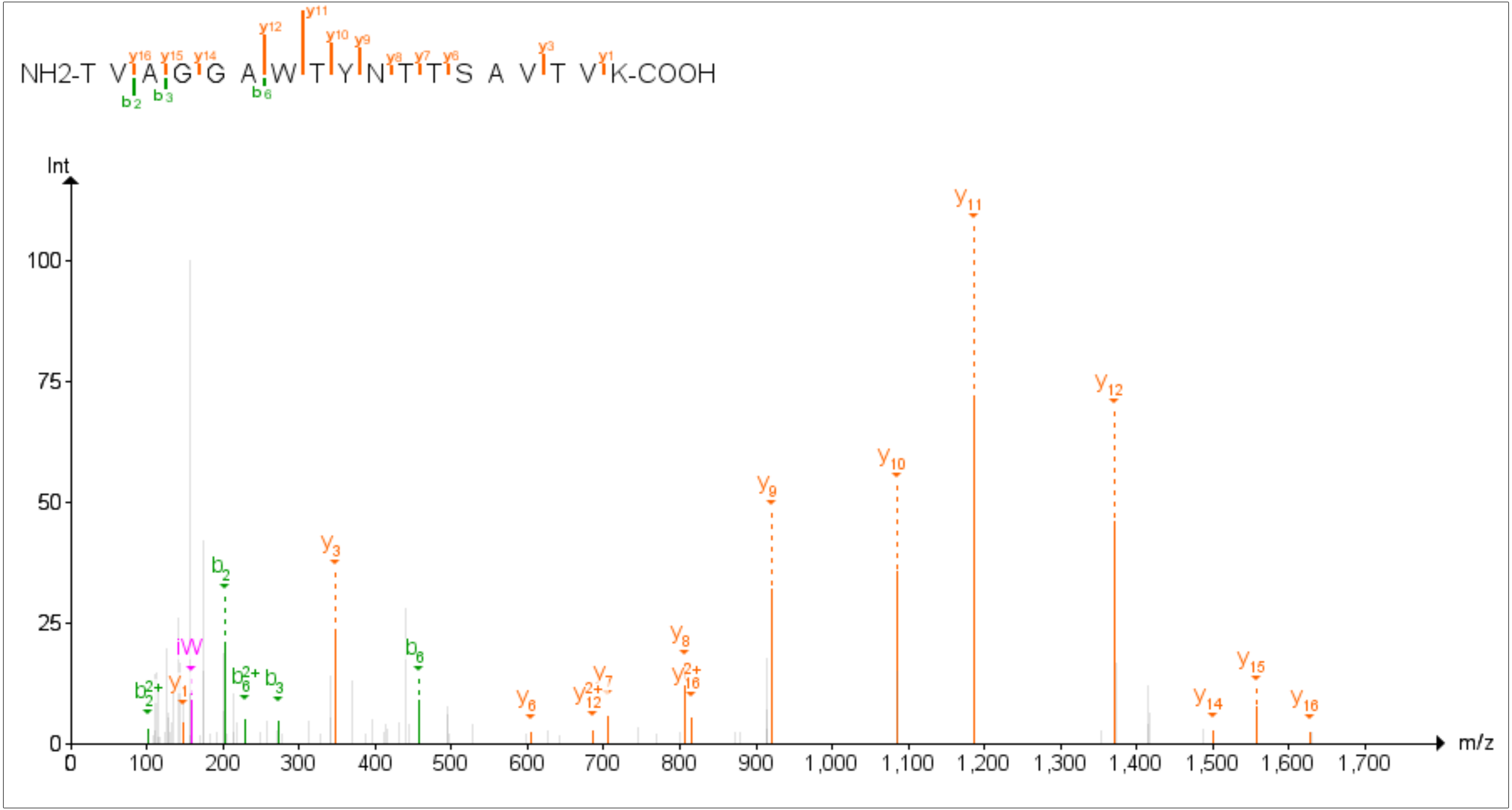
NH2-T V A G G A W T Y N T T S A V T V K-COOH
Int
m/z
y11
y12
y10
y9
y3
y8
y7
y6
y14
y15
y16
y1
b2
b3
b6
iW
0
25
50
75
100
0
100
200
300
400
500
600
700
800
900
1,000
1,100
1,200
1,300
1,400
1,500
1,600
1,700

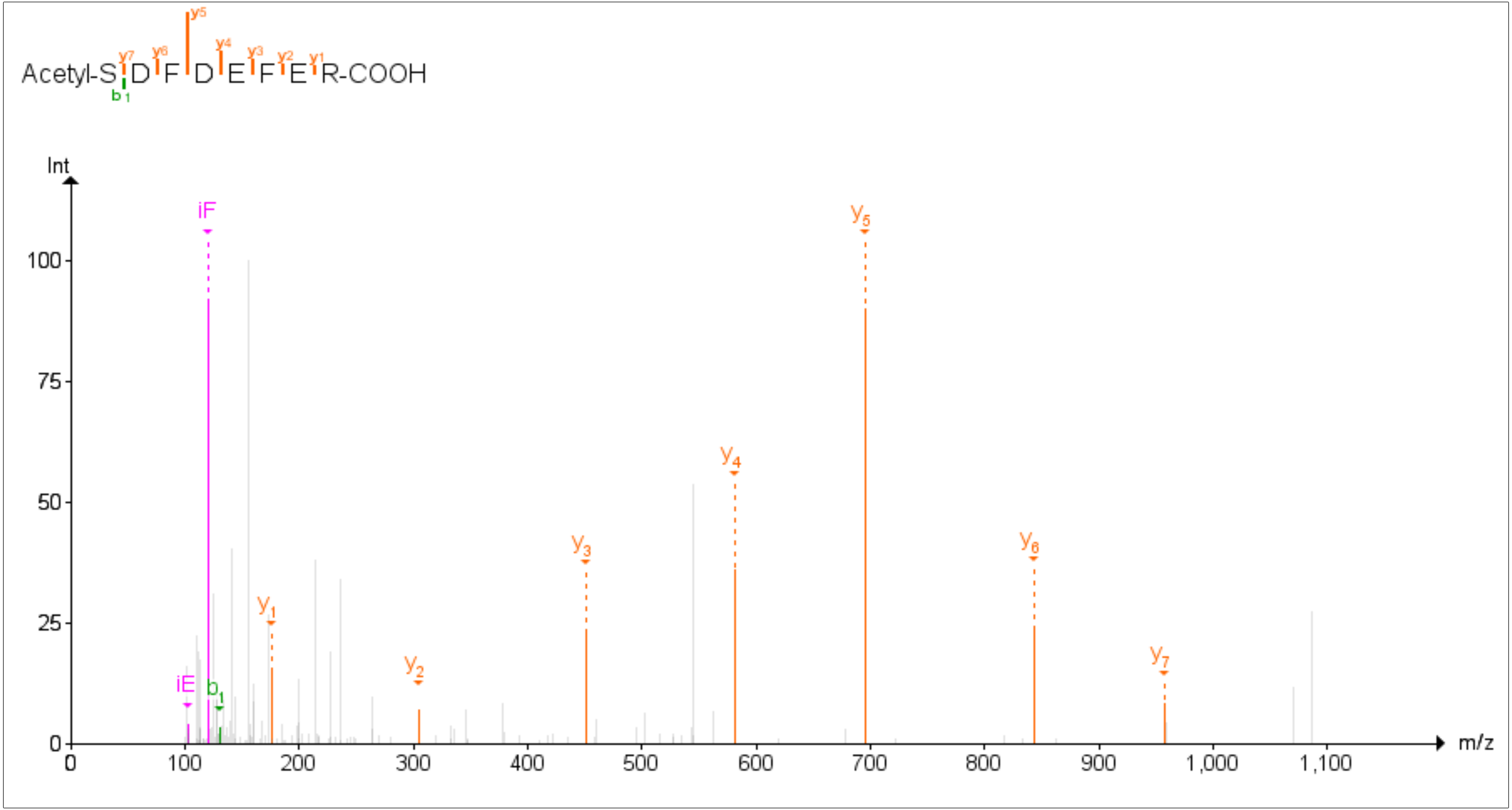

Acetyl-S D F D E F E R-COOH
y7 y6 y5 y4 y3 y2 y1
b1
Int
m/z
0
25
50
75
100
100
200
300
400
500
600
700
800
900
1,000
1,100
iF
iE
b1
y1
y2
y3
y4
y5
y6
y7

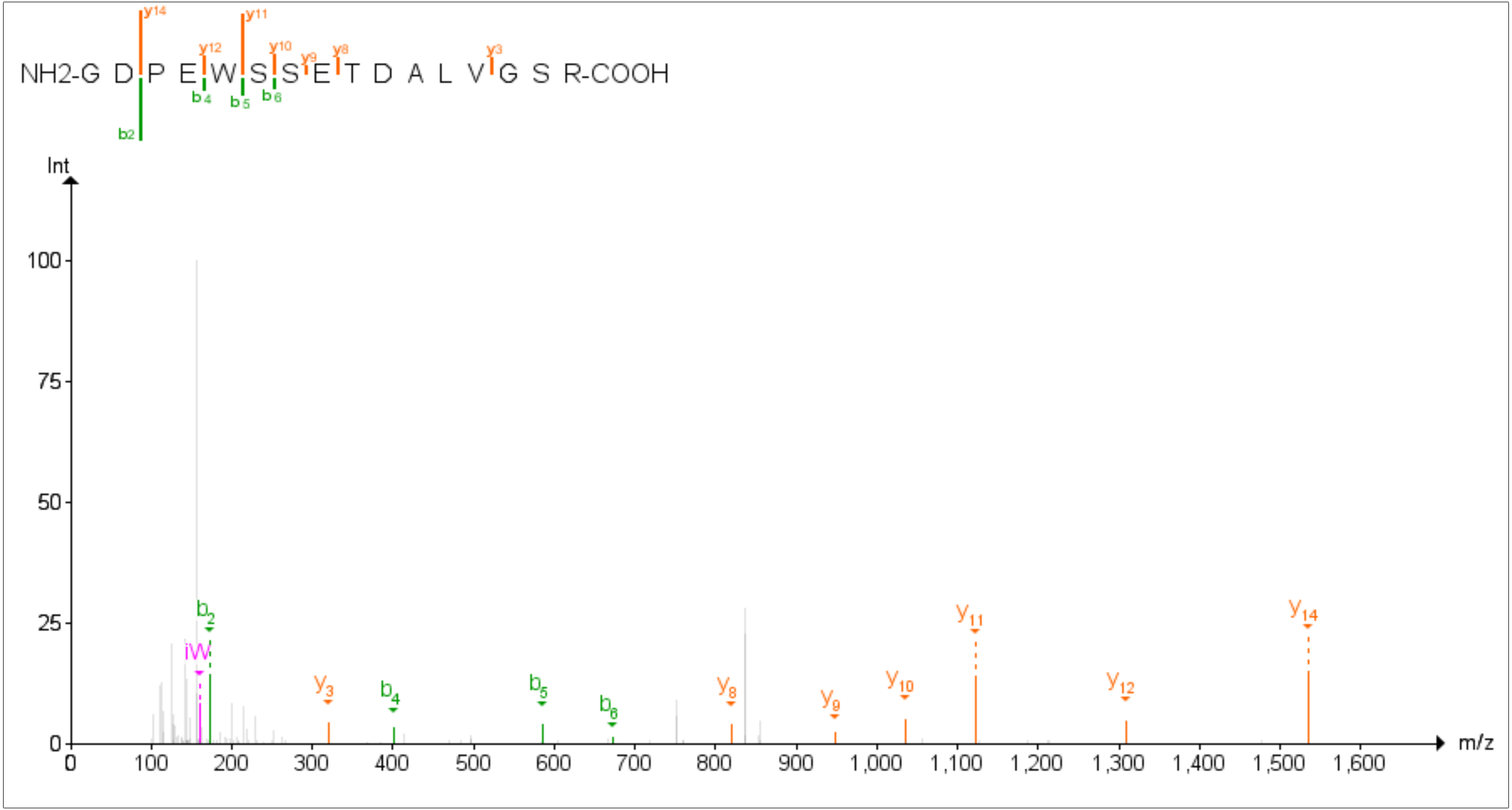
NH2-G D P E W S S E T D A L V G S R-COOH
y14
y12
y11
y10
y9
y8
y3
b2
b4
b5
b6
Int
100
75
50
25
0
iW
b2
y3
b4
b5
b6
y8
y9
y10
y11
y12
y14
0
100
200
300
400
500
600
700
800
900
1,000
1,100
1,200
1,300
1,400
1,500
1,600
m/z

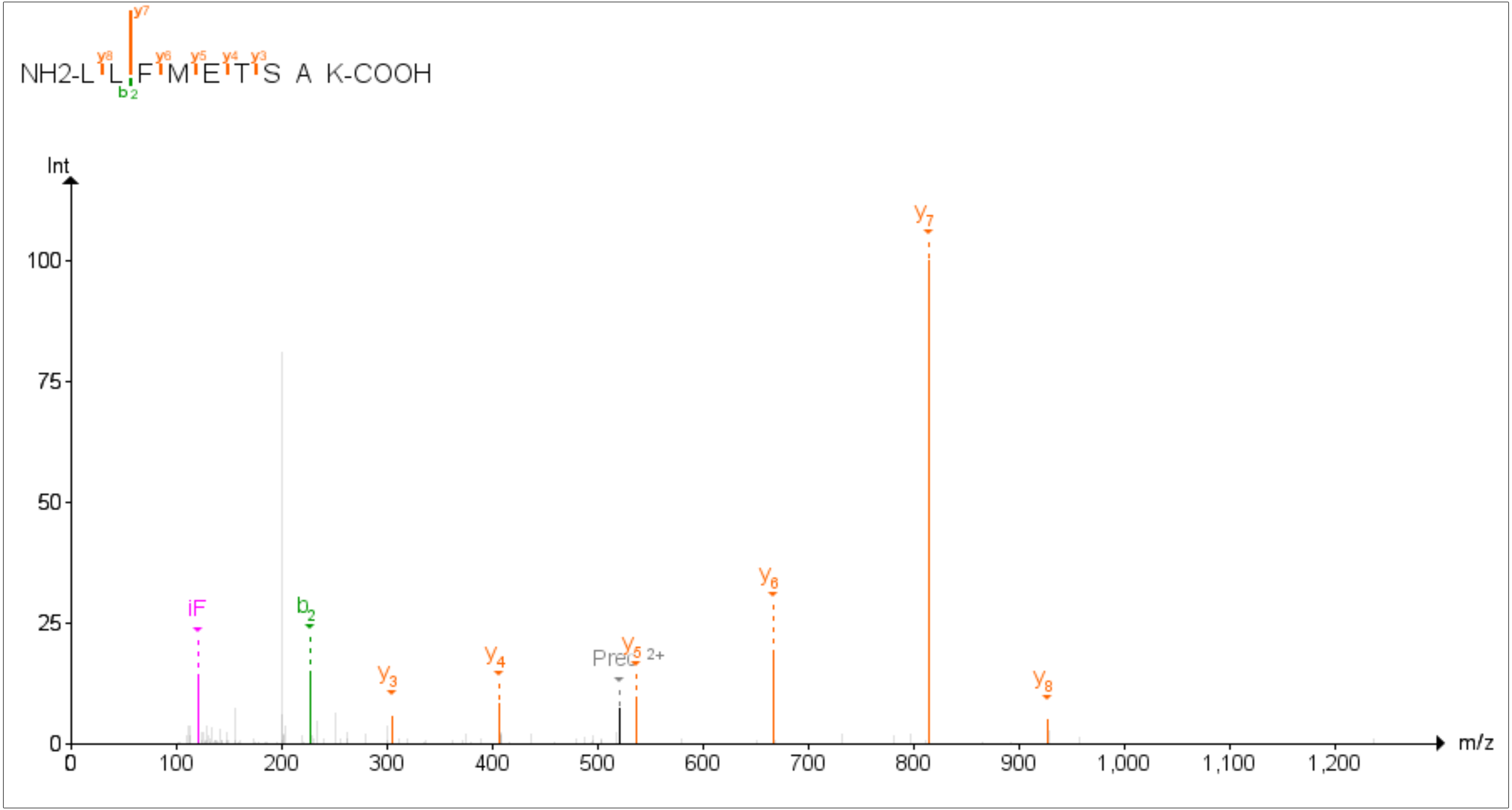
NH2-L L F M E T S A K-COOH
y8 y7 y6 y5 y4 y3
b2
Int
100
75
50
25
0
iF
b2
y3
y4
Prec 2+
y5
y6
y7
y8
0
100
200
300
400
500
600
700
800
900
1,000
1,100
1,200
m/z

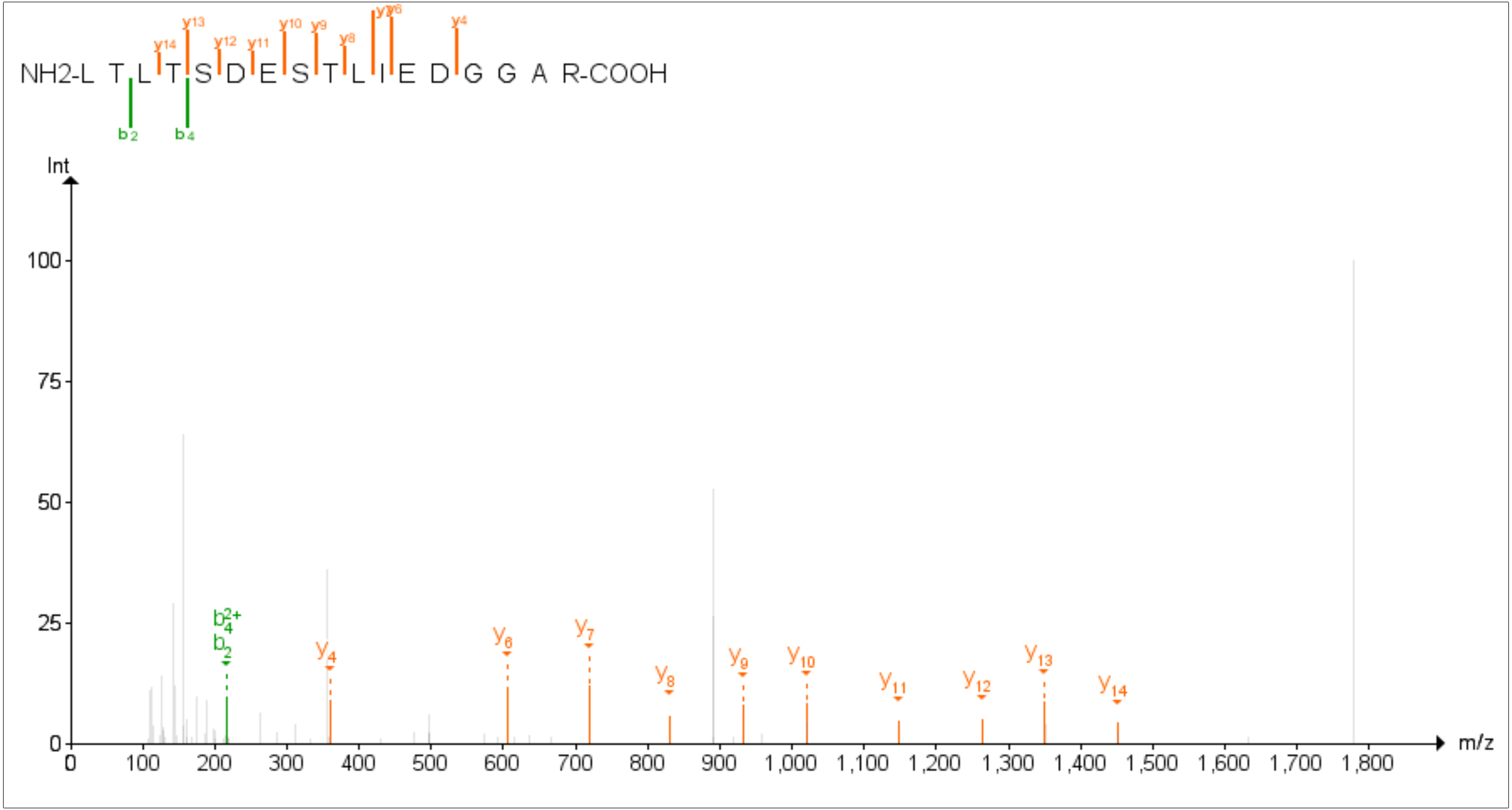

NH2-L T L T S D E S T L I E D G G A R-COOH
y14 y13 y12 y11 y10 y9 y8 y7 y6 y4
b2 b4
Int
m/z
100
75
50
25
0
0 100 200 300 400 500 600 700 800 900 1,000 1,100 1,200 1,300 1,400 1,500 1,600 1,700 1,800
b_4^{2+}
b_2
y_4 y_6 y_7 y_8 y_9 y_{10} y_{11} y_{12} y_{13} y_{14}

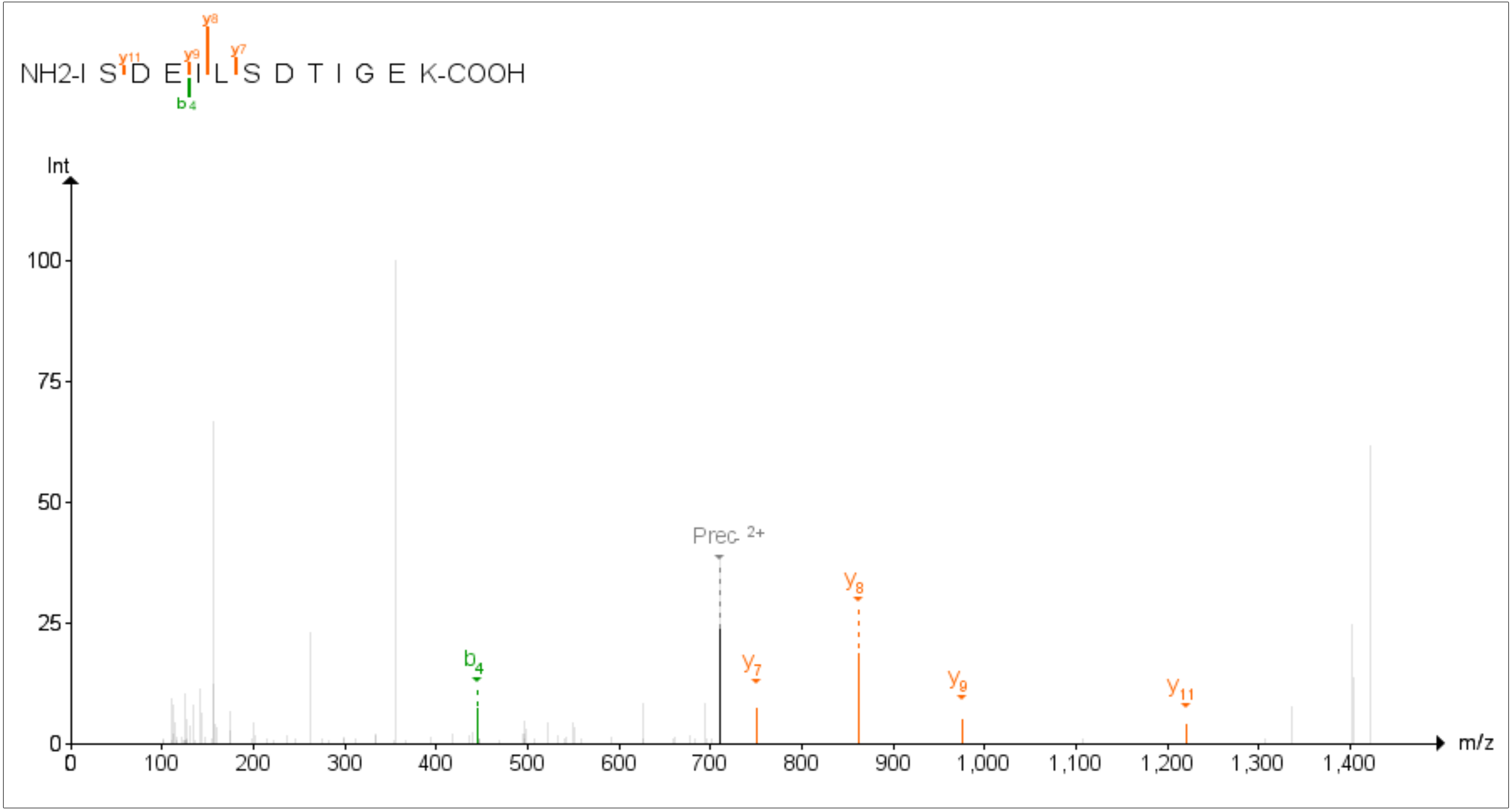

NH2-I S D E I L S D T I G E K-COOH
y11
y9
y8
y7
b4
Int
100
75
50
25
0
Prec 2+
b4
y7
y8
y9
y11
0
100
200
300
400
500
600
700
800
900
1,000
1,100
1,200
1,300
1,400
m/z

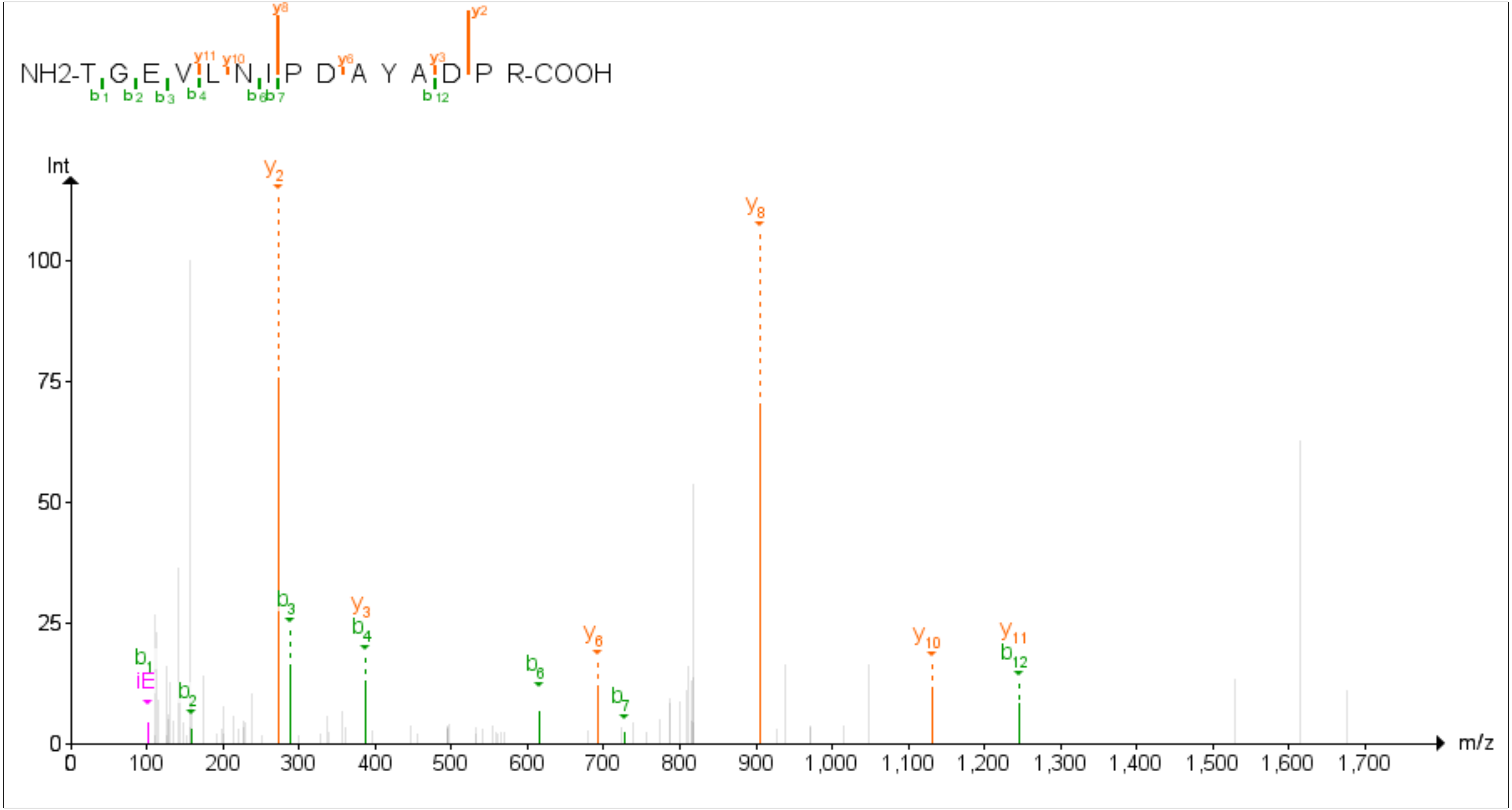
NH2-T G E V L N I P D A Y A D P R-COOH
Int
m/z
0
25
50
75
100
0
100
200
300
400
500
600
700
800
900
1,000
1,100
1,200
1,300
1,400
1,500
1,600
1,700
y2
y8
y3
y6
y10
y11
b1
iE
b2
b3
b4
b6
b7
b12

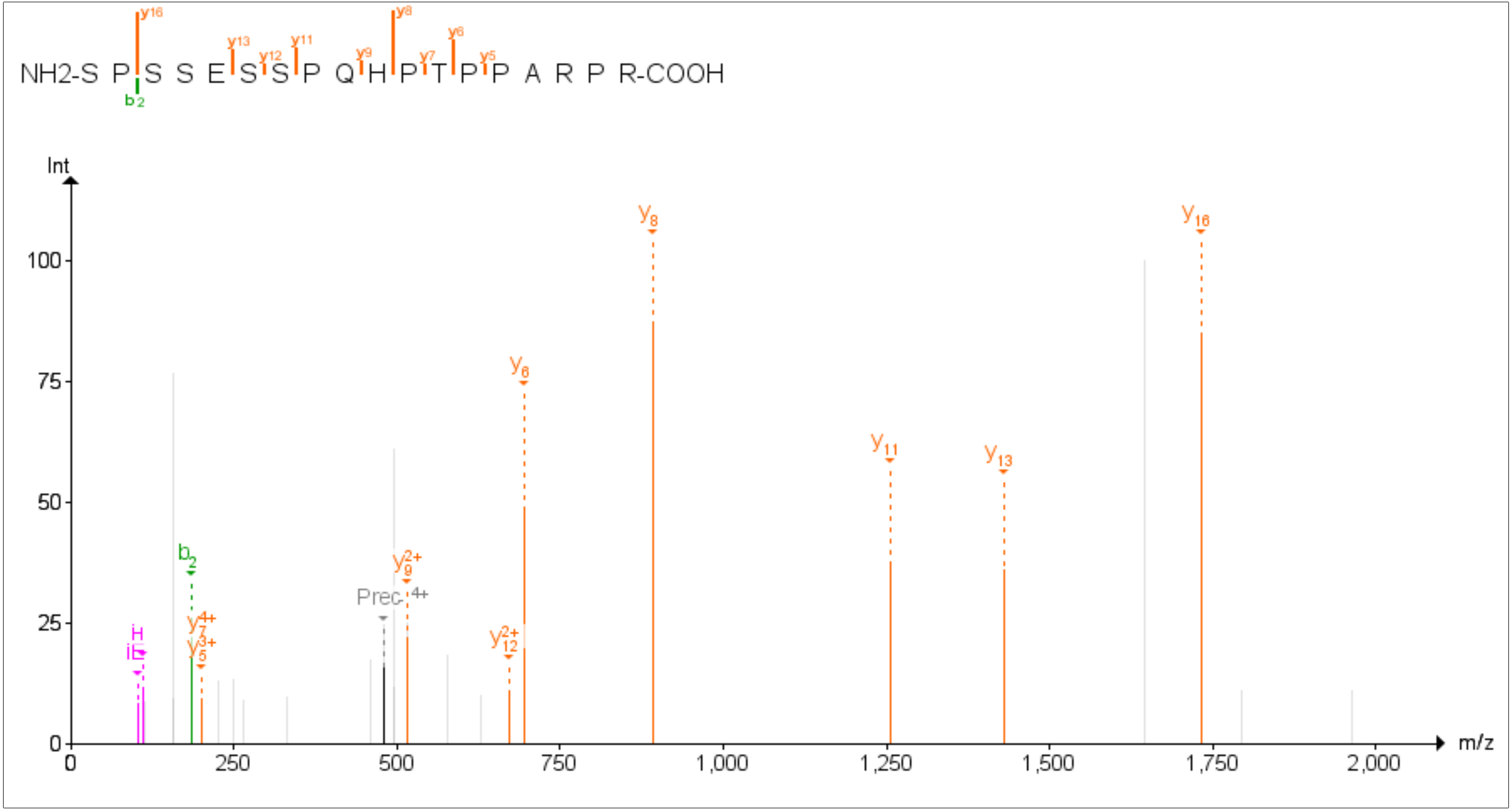

NH2-S P S S E S S P Q H P T P P A R P R-COOH
y16 y13 y12 y11 y9 y8 y7 y6 y5
b2
Int
m/z
y8
y16
y6
y11
y13
b2
y9 2+
Prec 4+
y7 4+
y5 3+
y12 2+
iH
iE
0
25
50
75
100
0
250
500
750
1,000
1,250
1,500
1,750
2,000

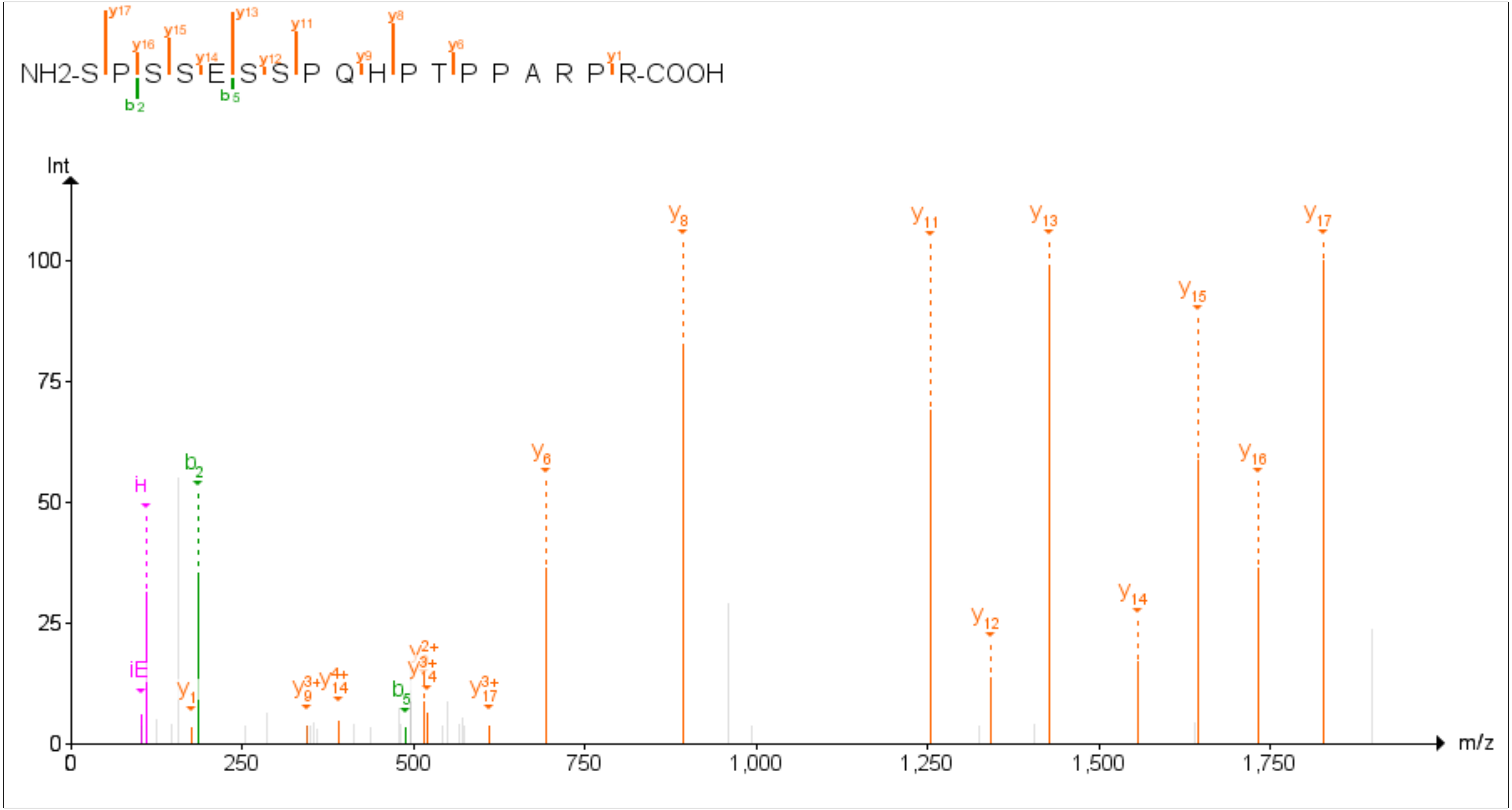
NH2-S P S S E S S P Q H P T P P A R P R-COOH
y17 y16 y15 y14 y13 y12 y11 y9 y8 y6 y1
b2 b5
Int
100
75
50
25
0
m/z
0
250
500
750
1,000
1,250
1,500
1,750
y8
y11
y13
y17
y15
y6
y16
b2
iH
y14
y12
iE
y1
y9 3+
y14 4+
b5
y14 2+
y14 3+
y17 3+

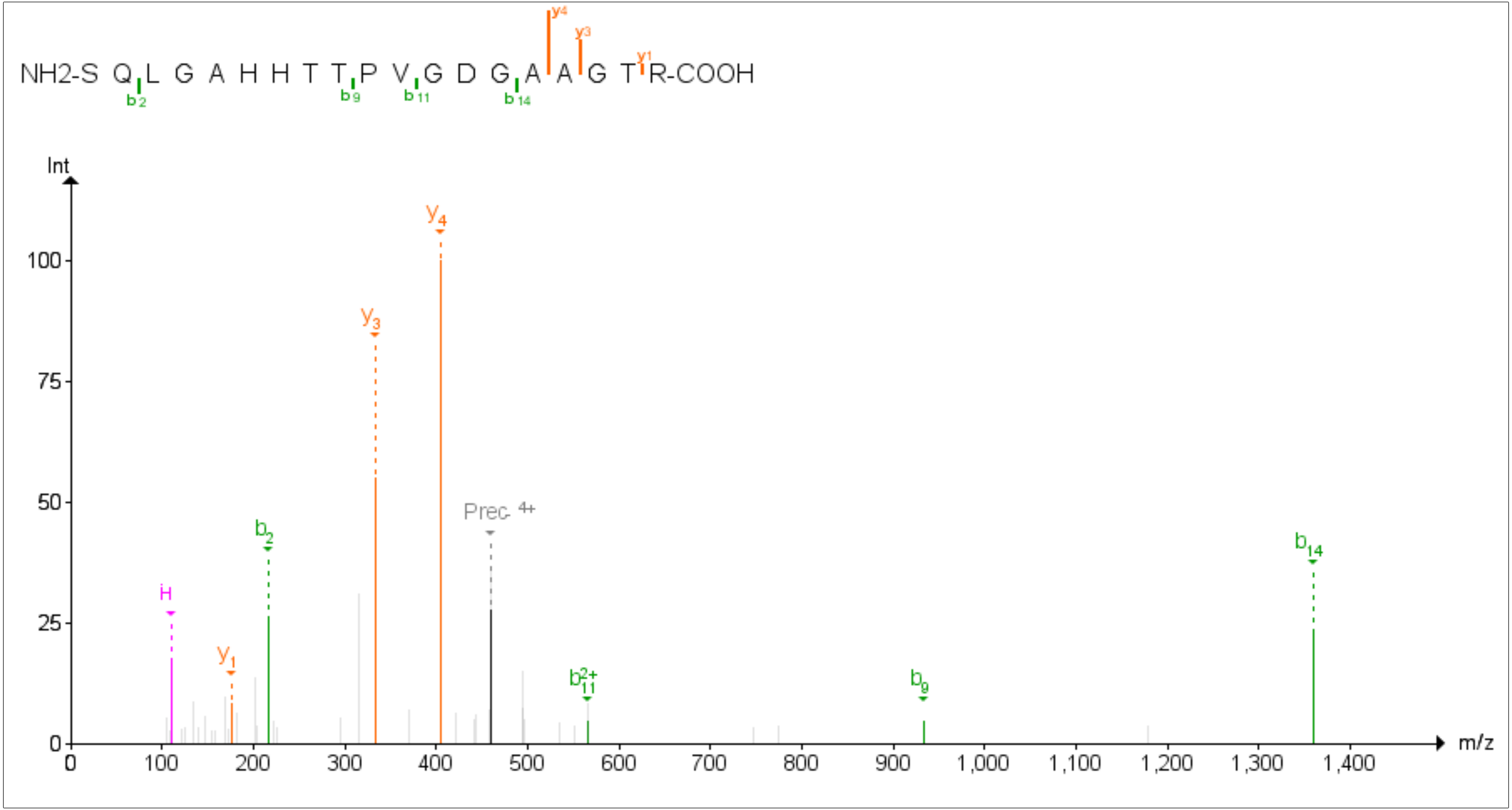
NH2-S Q L G A H H T T P V G D G A A G T R-COOH
b2
b9
b11
b14
y4
y3
y1
Int
100
75
50
25
0
y4
y3
Prec 4+
b2
b14
H
y1
b_{11}^{2+}
b9
0
100
200
300
400
500
600
700
800
900
1,000
1,100
1,200
1,300
1,400
m/z

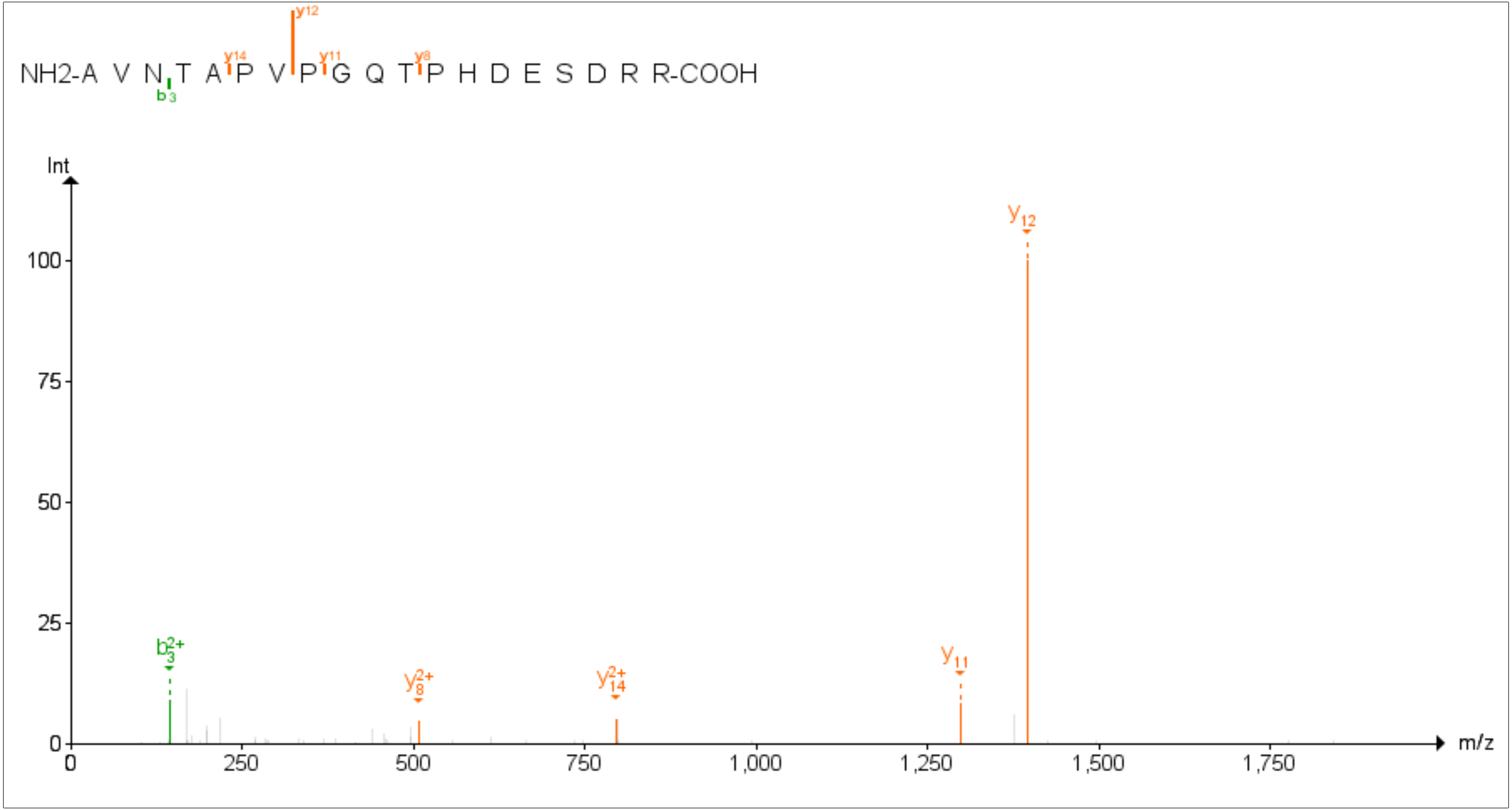

NH2-A V N T A P V P G Q T P H D E S D R R-COOH
b3
y14
y12
y11
y8
Int
100
75
50
25
0
m/z
0
250
500
750
1,000
1,250
1,500
1,750
b_3^{2+}
y_8^{2+}
y_{14}^{2+}
y_{11}
y_{12}

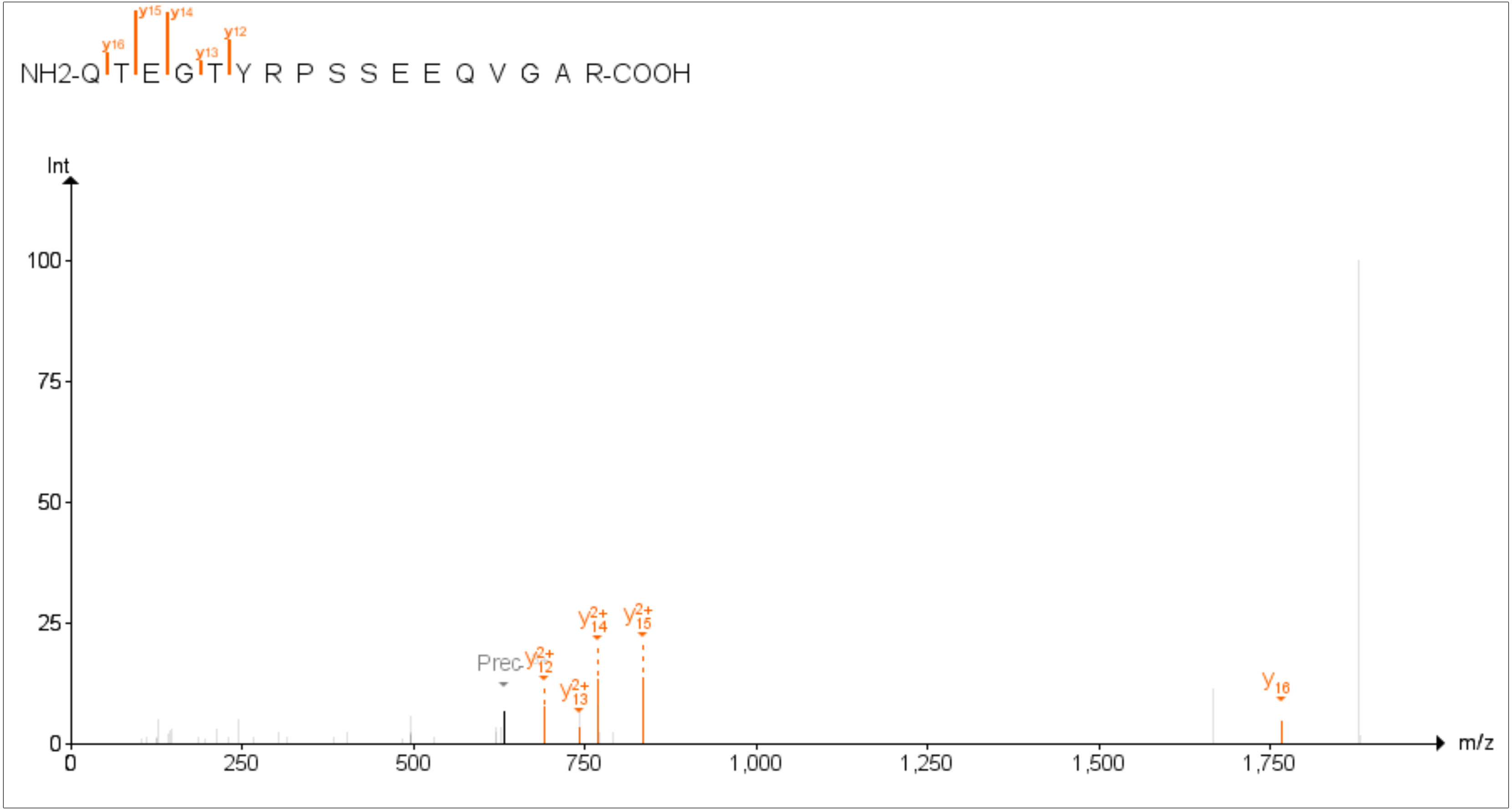

y15 y14 y12
y16 y13
NH2-Q T E G T Y R P S S E E Q V G A R-COOH
Int
100
75
50
25
0
Prec
y^{2+}_{12}
y^{2+}_{13}
y^{2+}_{14}
y^{2+}_{15}
y_{16}
0 250 500 750 1,000 1,250 1,500 1,750
m/z

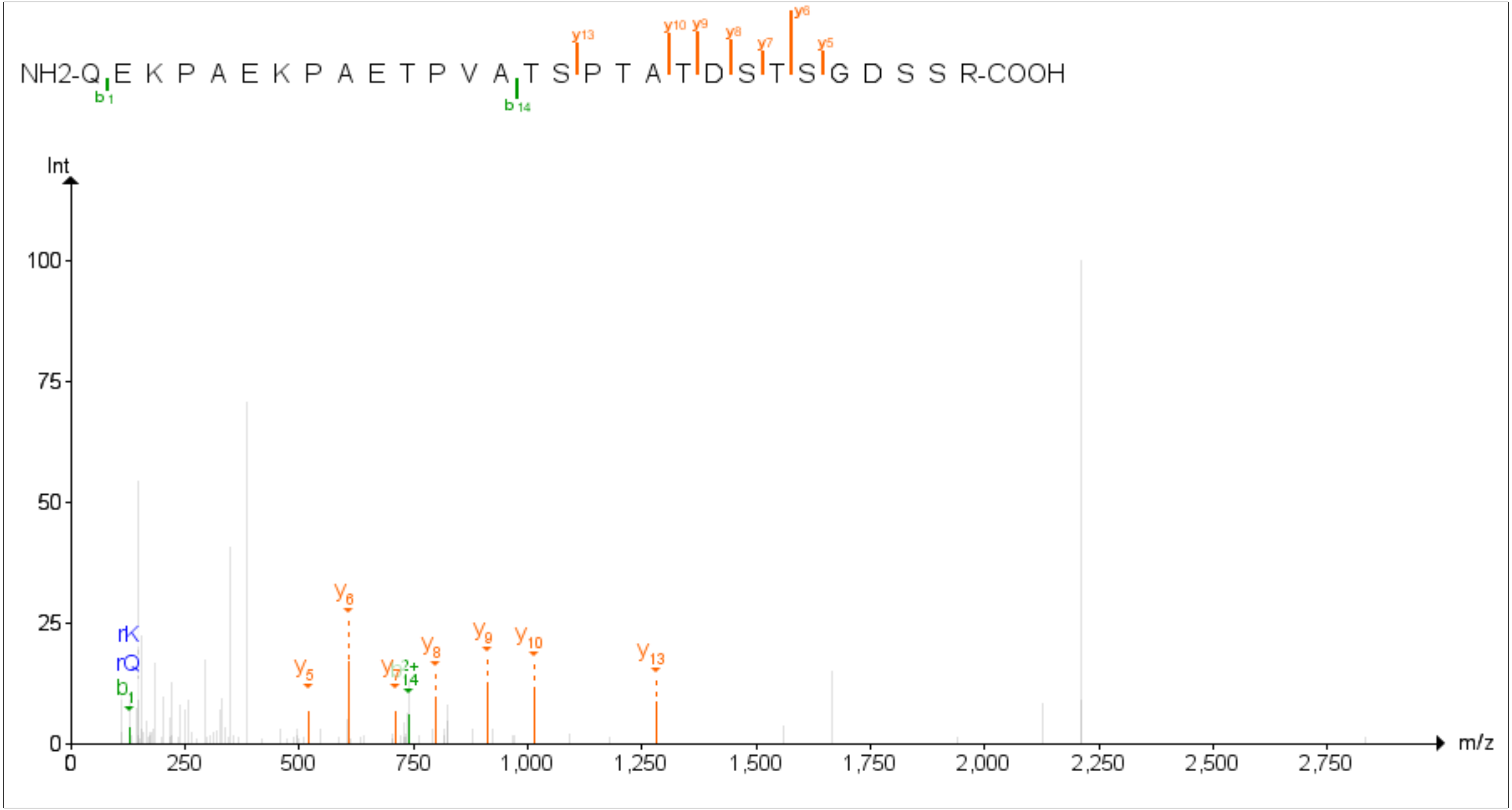

NH2-Q E K P A E K P A E T P V A T S P T A T D S T S G D S S R-COOH
b1
b14
y13
y10
y9
y8
y7
y6
y5
Int
100
75
50
25
0
rK
rQ
b1
y5
y6
y7
b14 2+
y8
y9
y10
y13
0
250
500
750
1,000
1,250
1,500
1,750
2,000
2,250
2,500
2,750
m/z

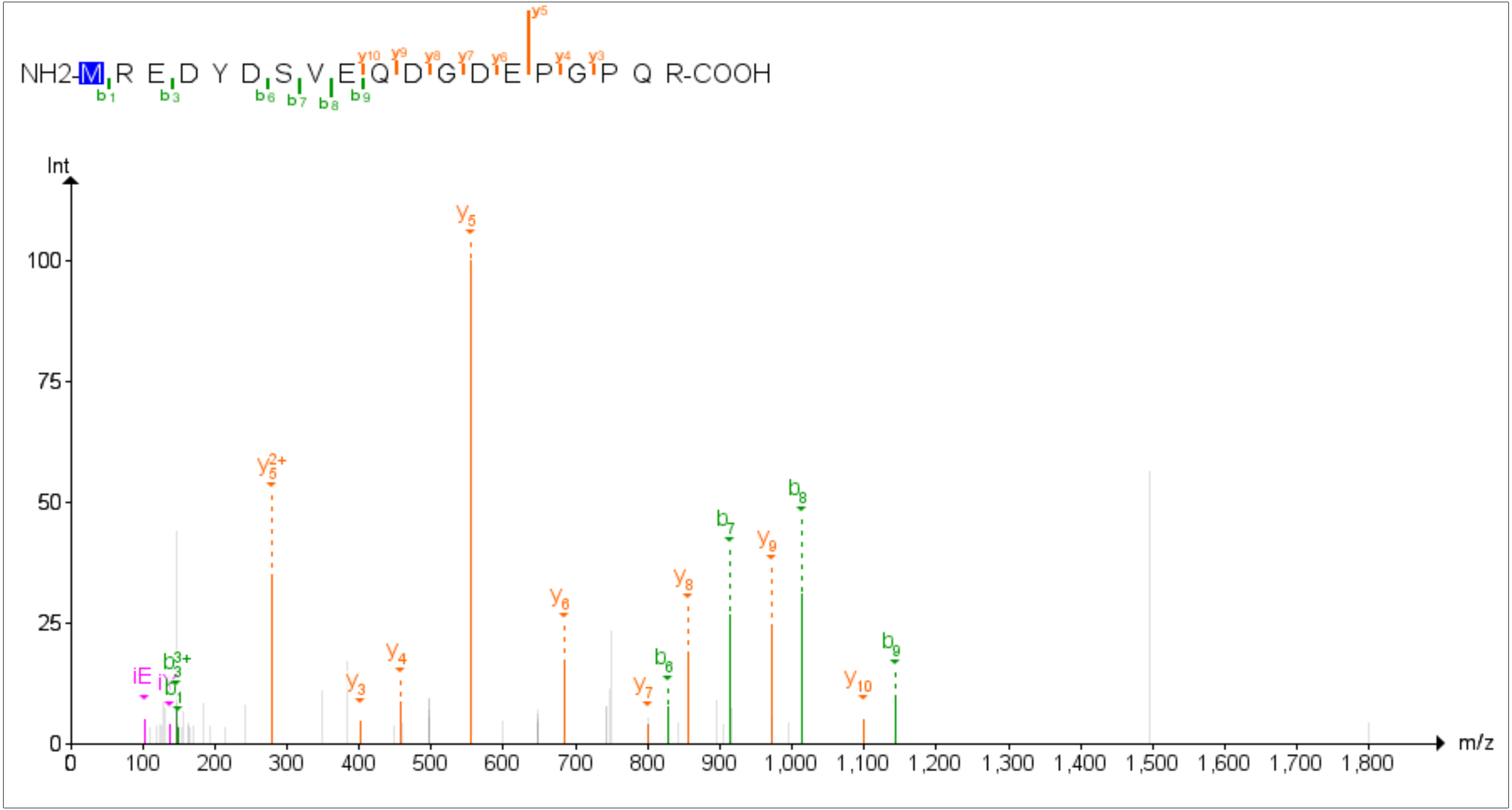

NH2-M R E D Y D S V E Q D G D E P G P Q R-COOH
b1 b3 b6 b7 b8 b9
y10 y9 y8 y7 y6 y5 y4 y3
Int
100
75
50
25
0
m/z
0 100 200 300 400 500 600 700 800 900 1,000 1,100 1,200 1,300 1,400 1,500 1,600 1,700 1,800
y5
y5 2+
b8
b7
y9
y8
y6
b6
b9
y4
y3
y7
y10
b3 3+
iE
b1

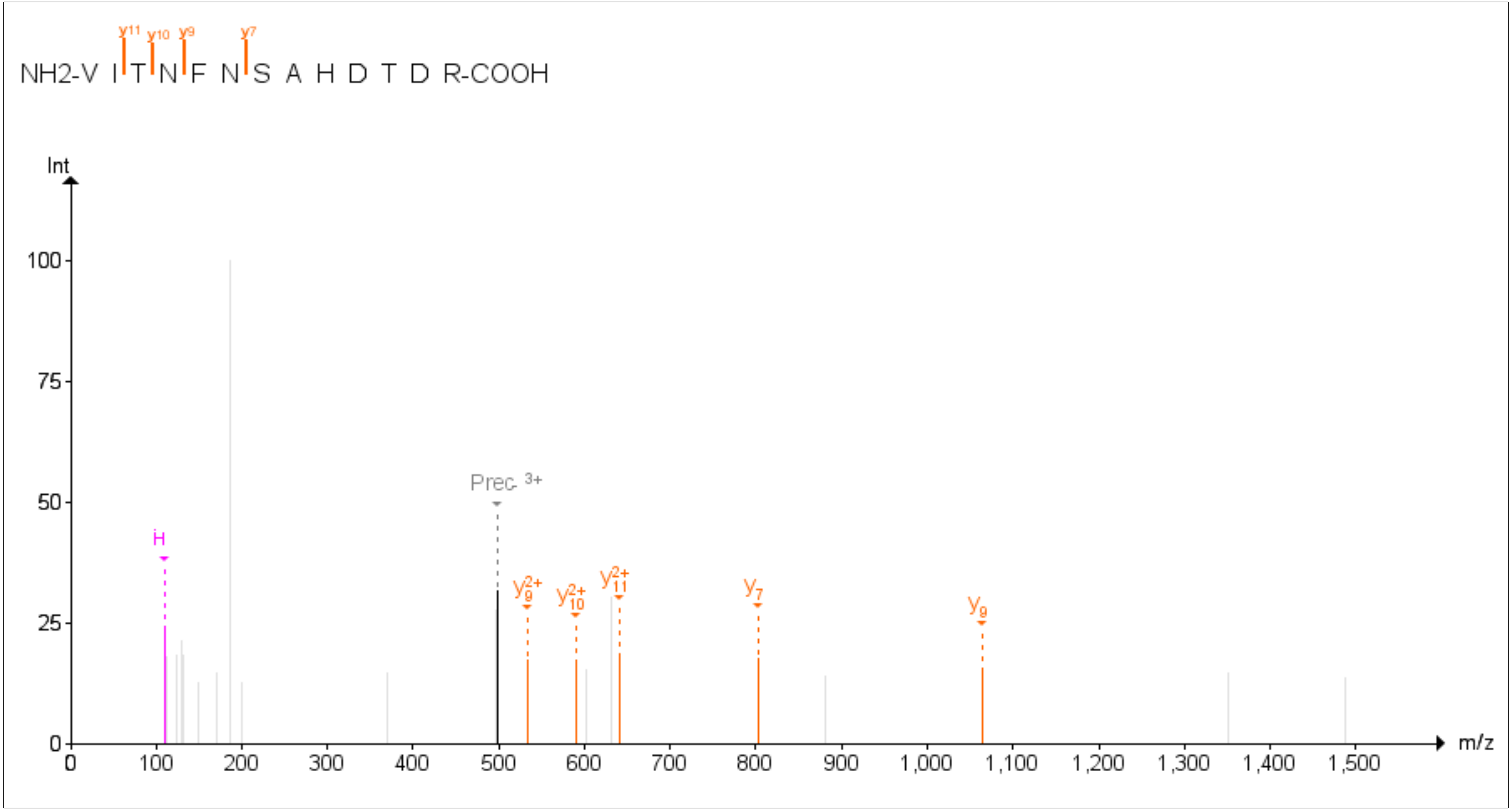

NH2-V I T N F N S A H D T D R-COOH
y11 y10 y9 y7
Int
100
75
50
25
0
H
Prec 3+
y_9^{2+}
y_{10}^{2+}
y_{11}^{2+}
y_7
y_9
0 100 200 300 400 500 600 700 800 900 1,000 1,100 1,200 1,300 1,400 1,500
m/z

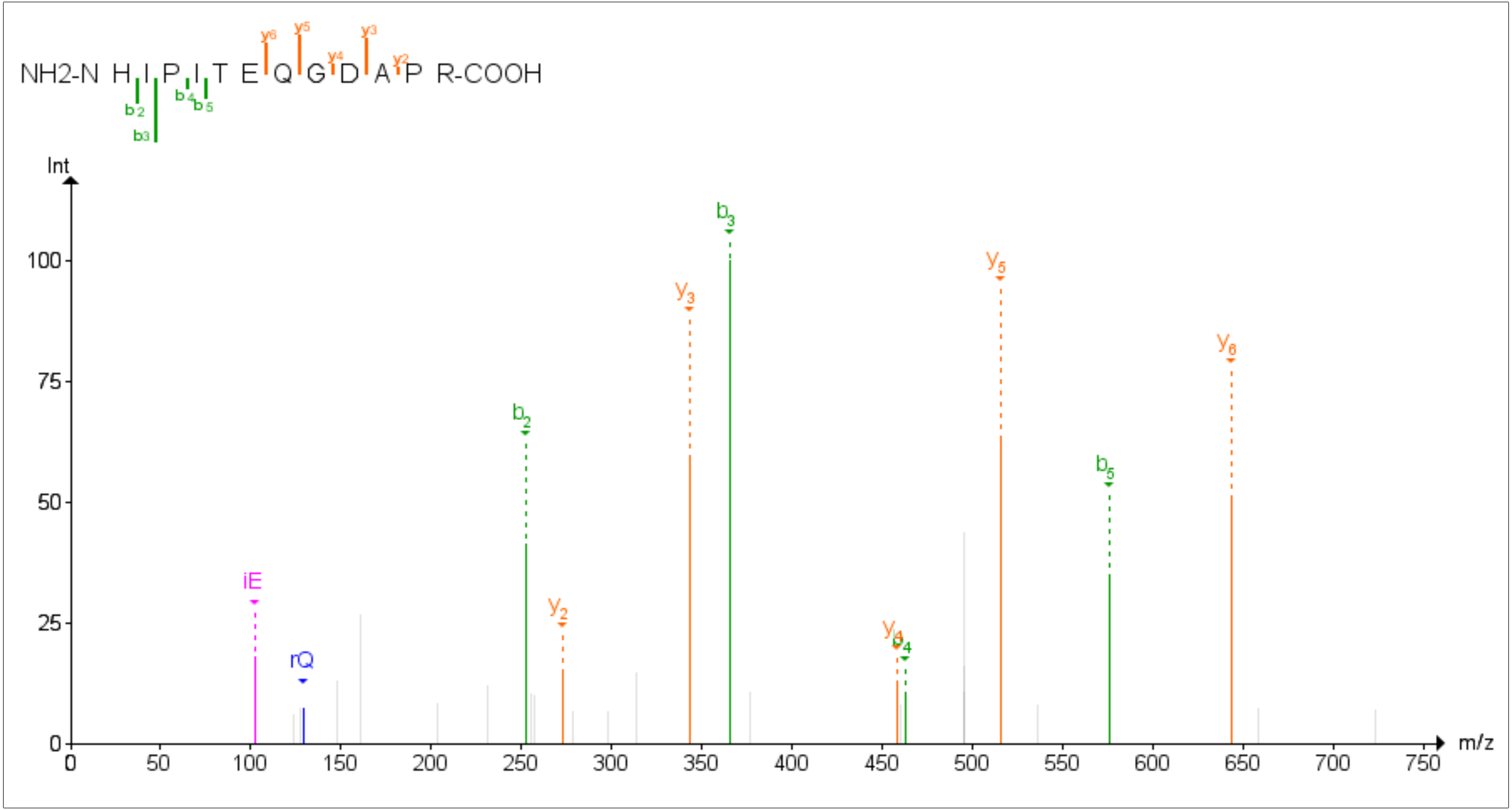

NH2-N H I P I T E Q G D A P R-COOH
y6
y5
y4
y3
y2
b2
b3
b4
b5
Int
m/z
100
75
50
25
0
0
50
100
150
200
250
300
350
400
450
500
550
600
650
700
750
iE
rQ
b2
y2
y3
b3
y4
b4
y5
b5
y6

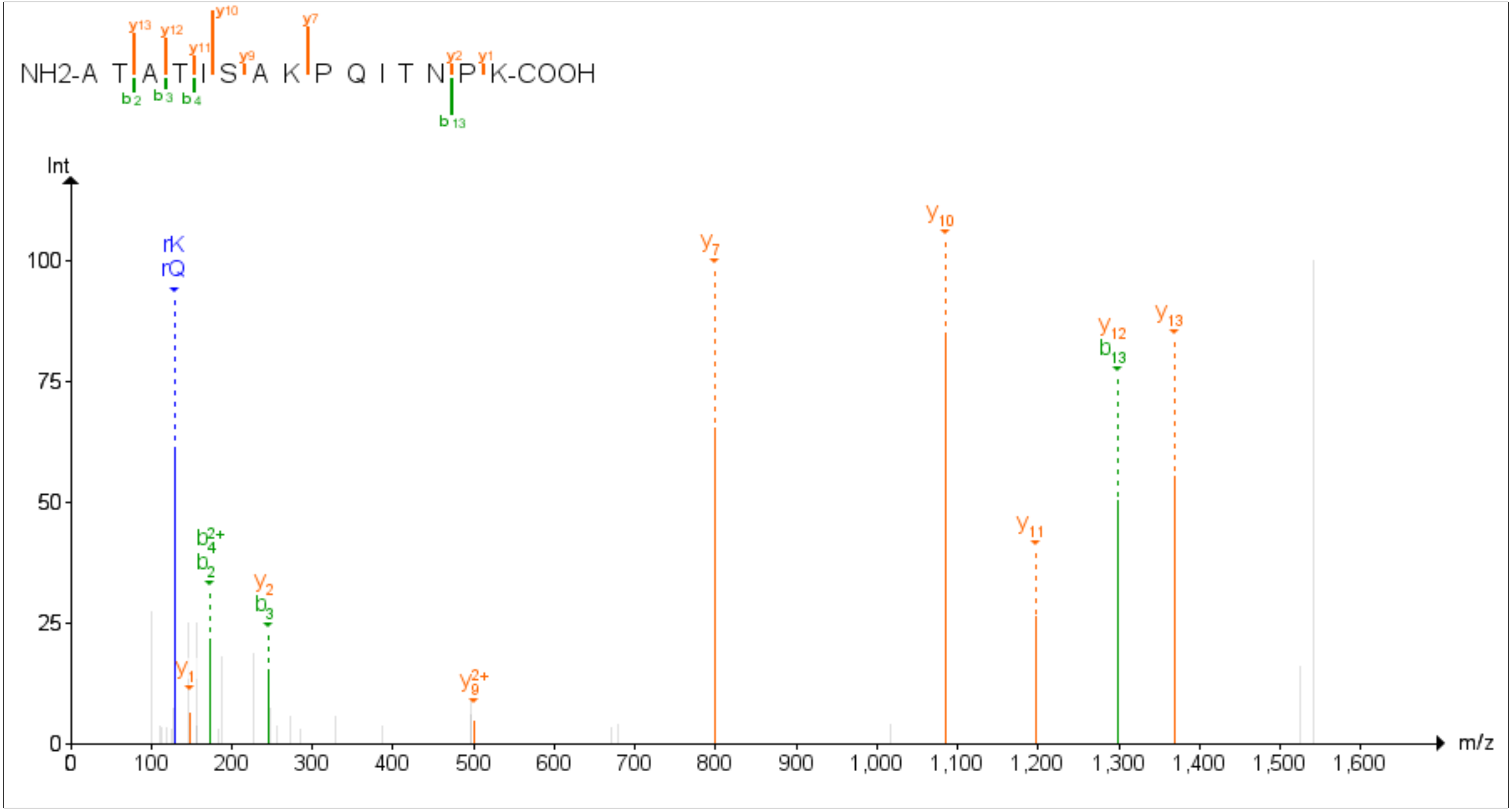
NH2-A T A T I S A K P Q I T N P K-COOH
y13 y12 y11 y10 y9 y7 y2 y1
b2 b3 b4 b13
Int
rK
rQ
y7
y10
y12
b13
y13
y11
b4 2+
b2
y2
b3
y1
y9 2+
0
25
50
75
100
0 100 200 300 400 500 600 700 800 900 1,000 1,100 1,200 1,300 1,400 1,500 1,600
m/z

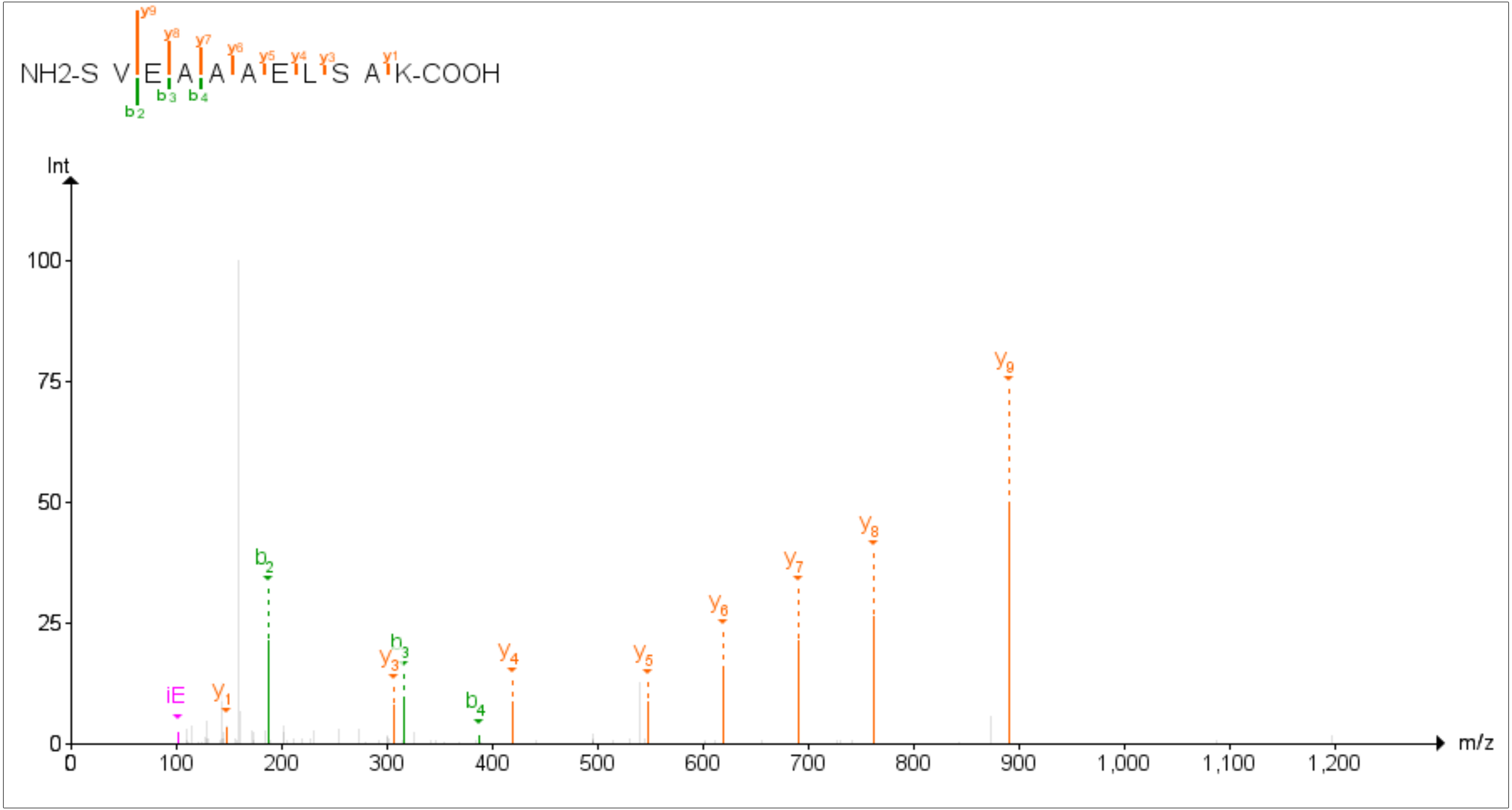

NH2-S V E A A A E L S A K-COOH
y9 y8 y7 y6 y5 y4 y3 y1
b2 b3 b4
Int
100
75
50
25
0
m/z
0 100 200 300 400 500 600 700 800 900 1,000 1,100 1,200
iE
y1
b2
y3
b3
b4
y4
y5
y6
y7
y8
y9

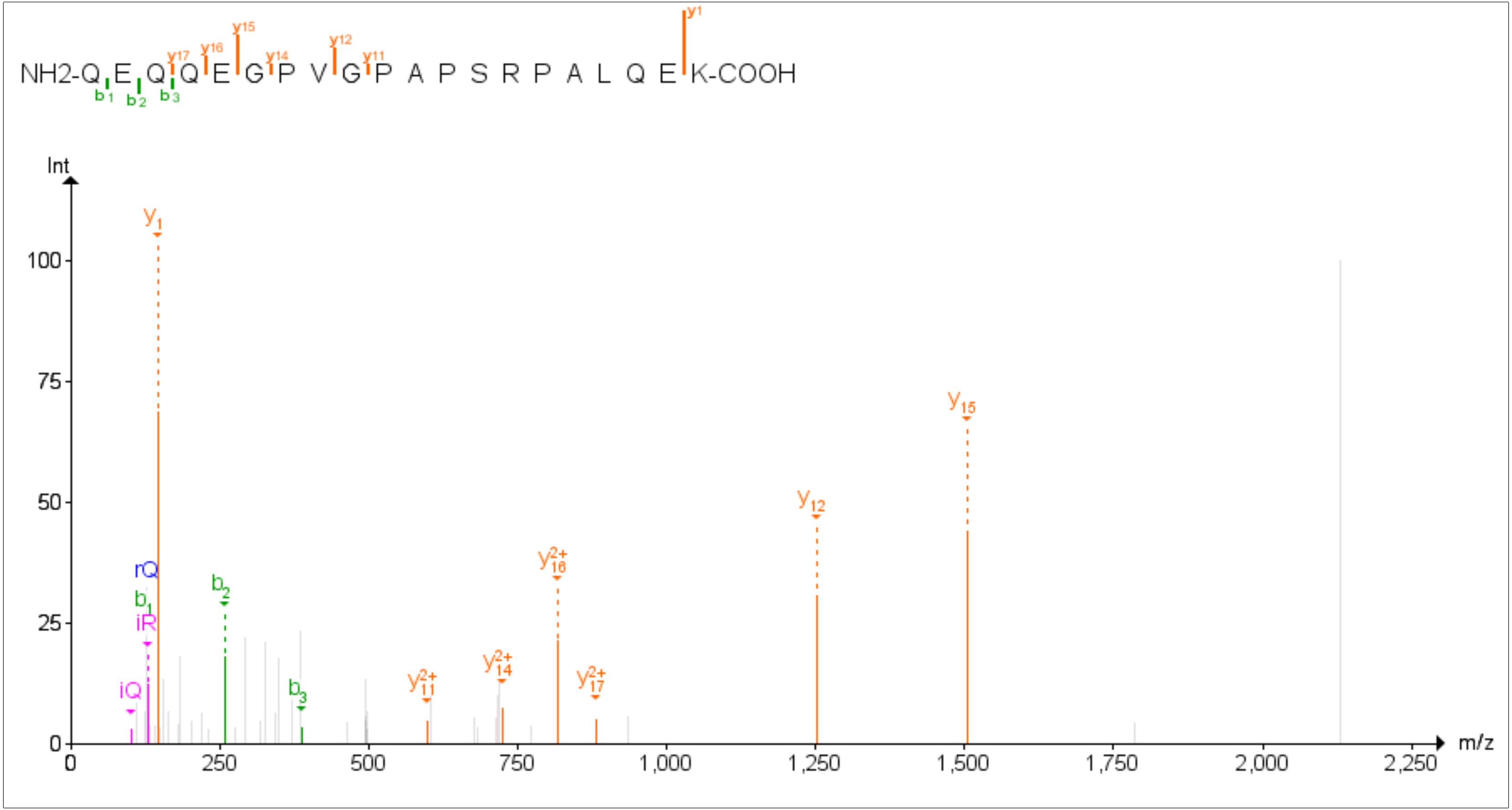

NH2-Q E Q Q E G P V G P A P S R P A L Q E K-COOH
y1 y11 y12 y14 y15 y16 y17
b1 b2 b3
Int
m/z
0 25 50 75 100
0 250 500 750 1,000 1,250 1,500 1,750 2,000 2,250
y1
y15
y12
y16 2+
y14 2+
y11 2+
y17 2+
rQ
b1
iR
iQ
b2
b3

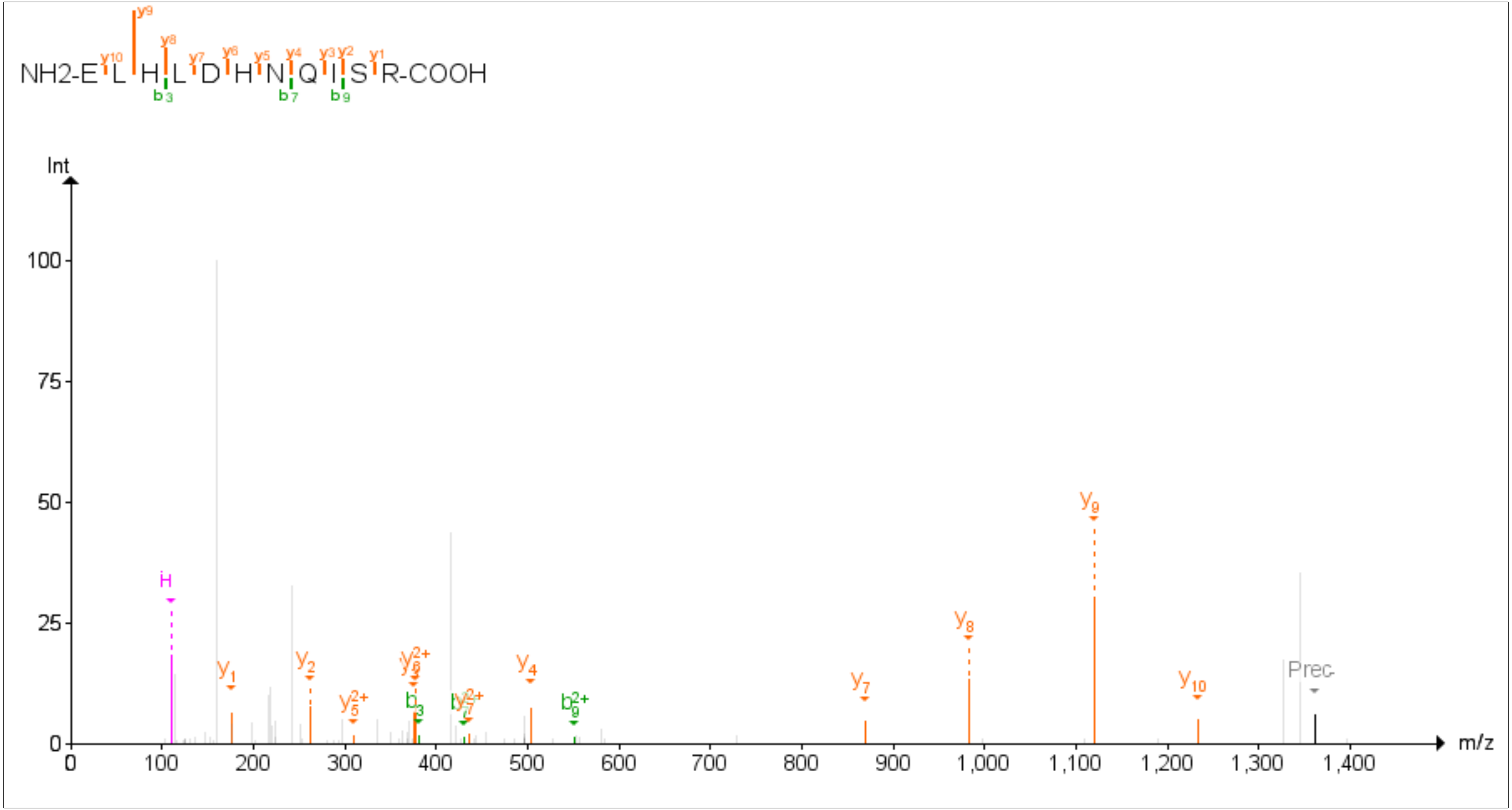

NH2-E L H L D H N Q I S R-COOH
y10 y9 y8 y7 y6 y5 y4 y3 y2 y1
b3 b7 b9
Int
100
75
50
25
0
m/z
0 100 200 300 400 500 600 700 800 900 1,000 1,100 1,200 1,300 1,400
iH
y1
y2
y5 2+
y3
b3
y7 2+
y4
b9 2+
y7
y8
y9
y10
Prec

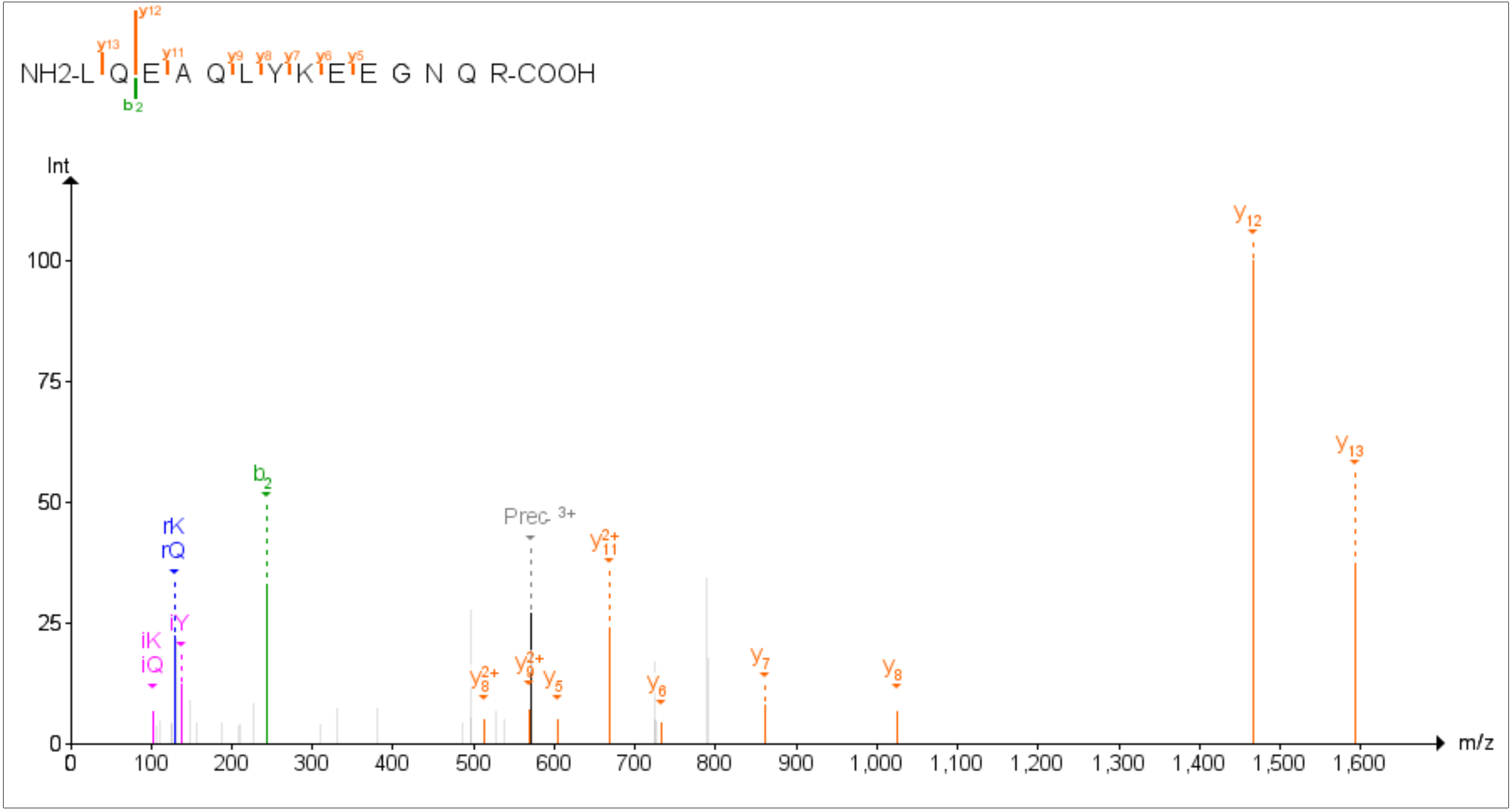

NH2-L Q E A Q L Y K E E G N Q R-COOH
y13 y12 y11 y9 y8 y7 y6 y5
b2
Int
100
75
50
25
0
y12
y13
b2
rK
rQ
Prec 3+
y11 2+
iK
iQ
iY
y8 2+
y9 2+
y5
y6
y7
y8
0 100 200 300 400 500 600 700 800 900 1,000 1,100 1,200 1,300 1,400 1,500 1,600
m/z

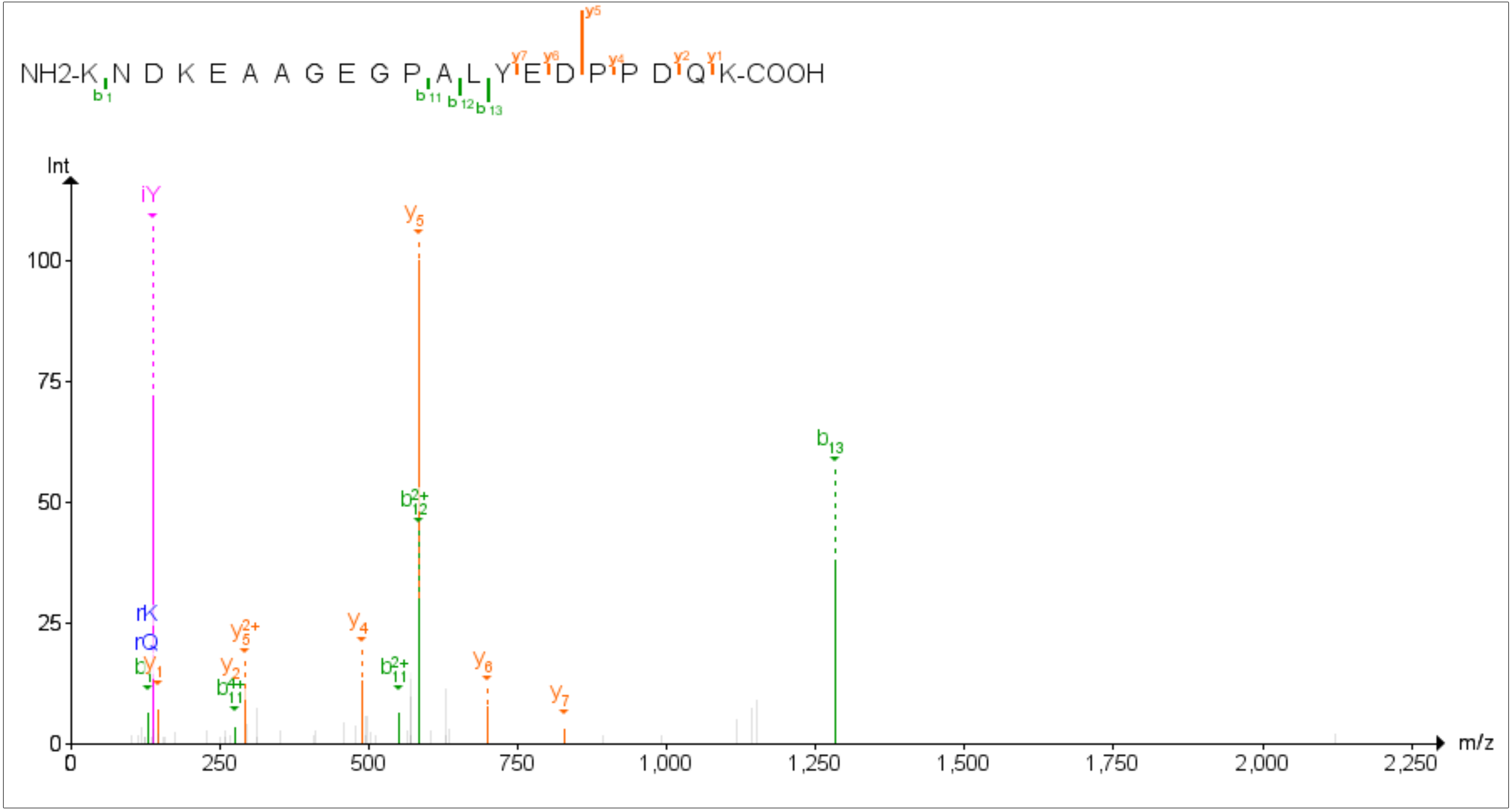

NH2-K N D K E A A G E G P A L Y E D P P D Q K-COOH
b1
b11
b12
b13
y7
y6
y5
y4
y2
y1
Int
iY
y5
100
75
50
25
0
b13
b12 2+
rK
rQ
b y1
y5 2+
y2
b11 4+
y4
b11 2+
y6
y7
0
250
500
750
1,000
1,250
1,500
1,750
2,000
2,250
m/z

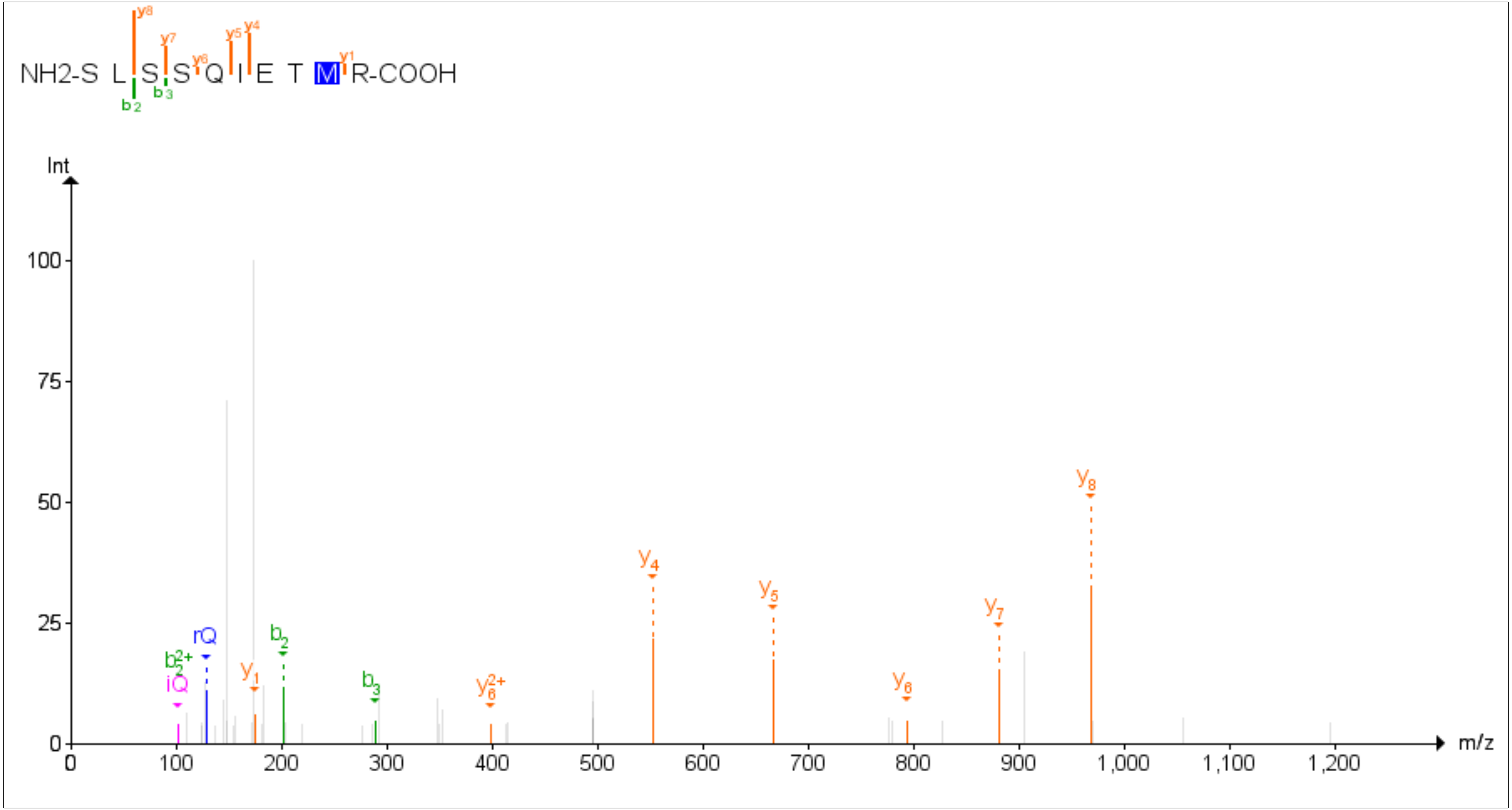

NH2-S L S S Q I E T M R-COOH
y8
y7
y6
y5
y4
y1
b2
b3
Int
100
75
50
25
0
m/z
0
100
200
300
400
500
600
700
800
900
1,000
1,100
1,200
b_2^{2+}
iQ
rQ
y_1
b_2
b_3
y_6^{2+}
y_4
y_5
y_6
y_7
y_8

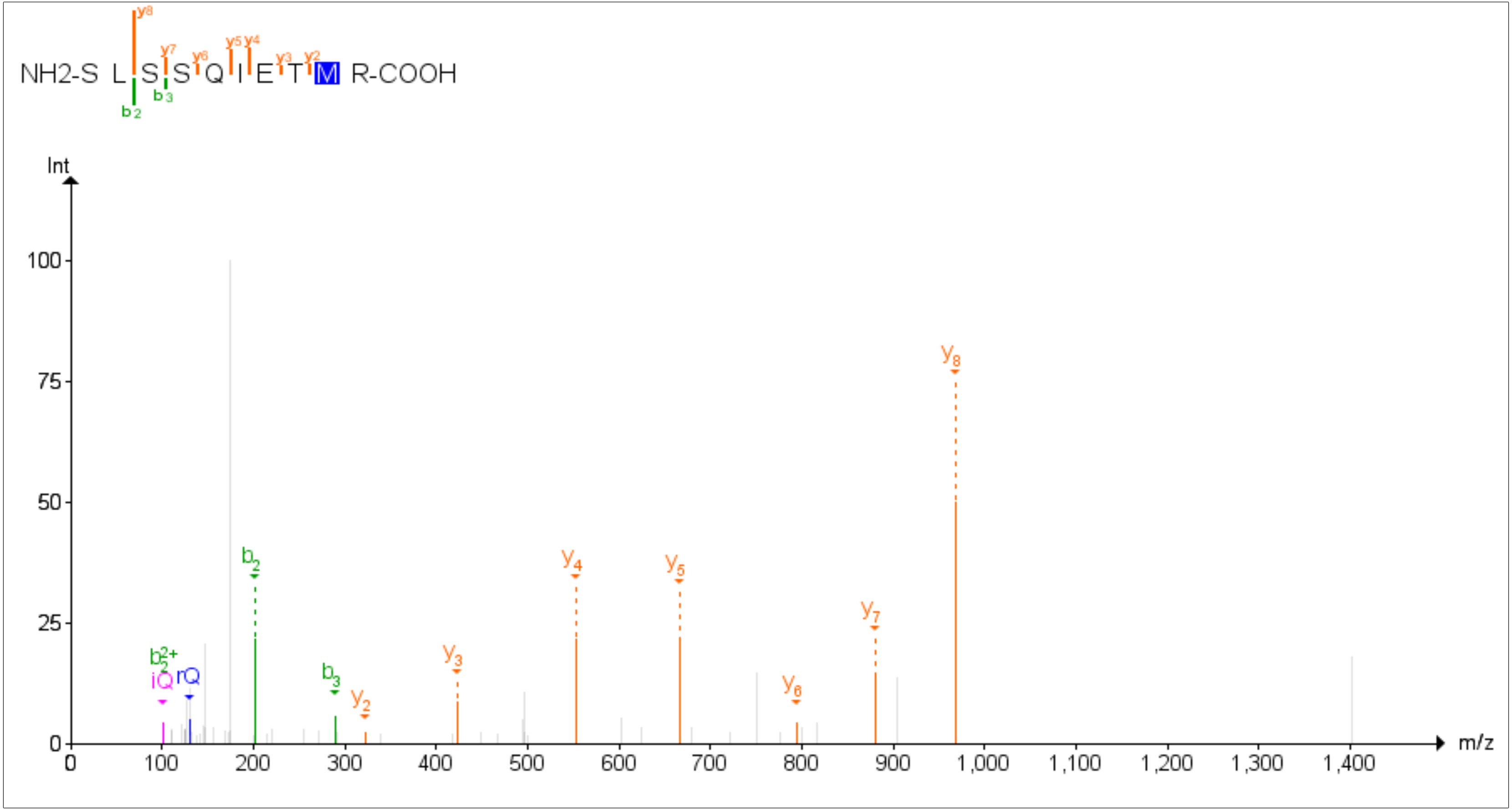

NH2-S L S S Q I E T M R-COOH
y8
y7
y6
y5
y4
y3
y2
b2
b3
Int
m/z
100
75
50
25
0
0
100
200
300
400
500
600
700
800
900
1,000
1,100
1,200
1,300
1,400
y8
b2
y4
y5
y7
y3
b2 2+
iQ
rQ
b3
y2
y6

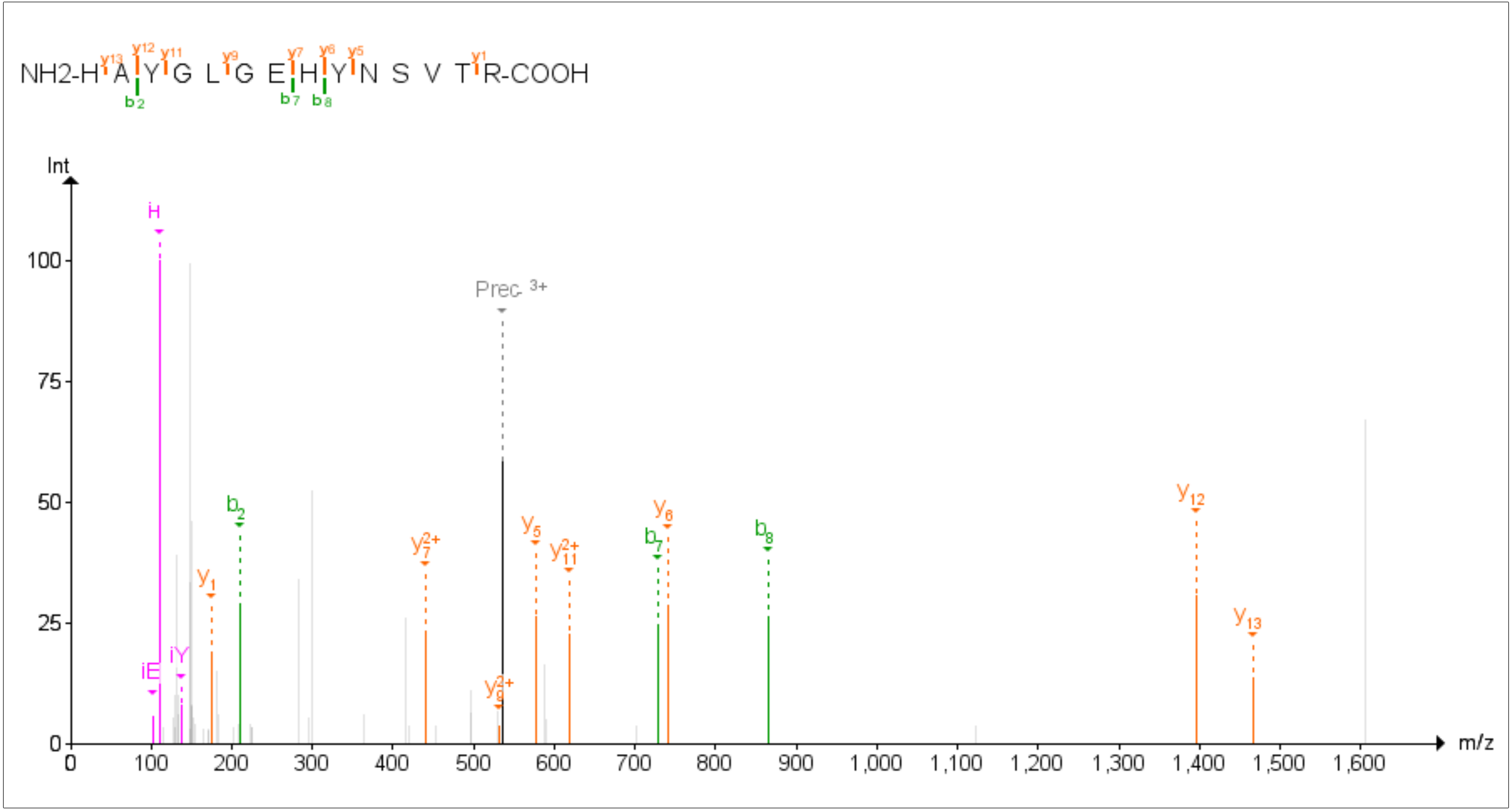

NH2-H A Y G L G E H Y N S V T R-COOH
y13 y12 y11 y9 y7 y6 y5 y1
b2 b7 b8
Int
100
75
50
25
0
iH
iE
iY
y1
b2
y7 2+
Prec 3+
y9 2+
y5
y11 2+
b7
y6
b8
y12
y13
0 100 200 300 400 500 600 700 800 900 1,000 1,100 1,200 1,300 1,400 1,500 1,600
m/z

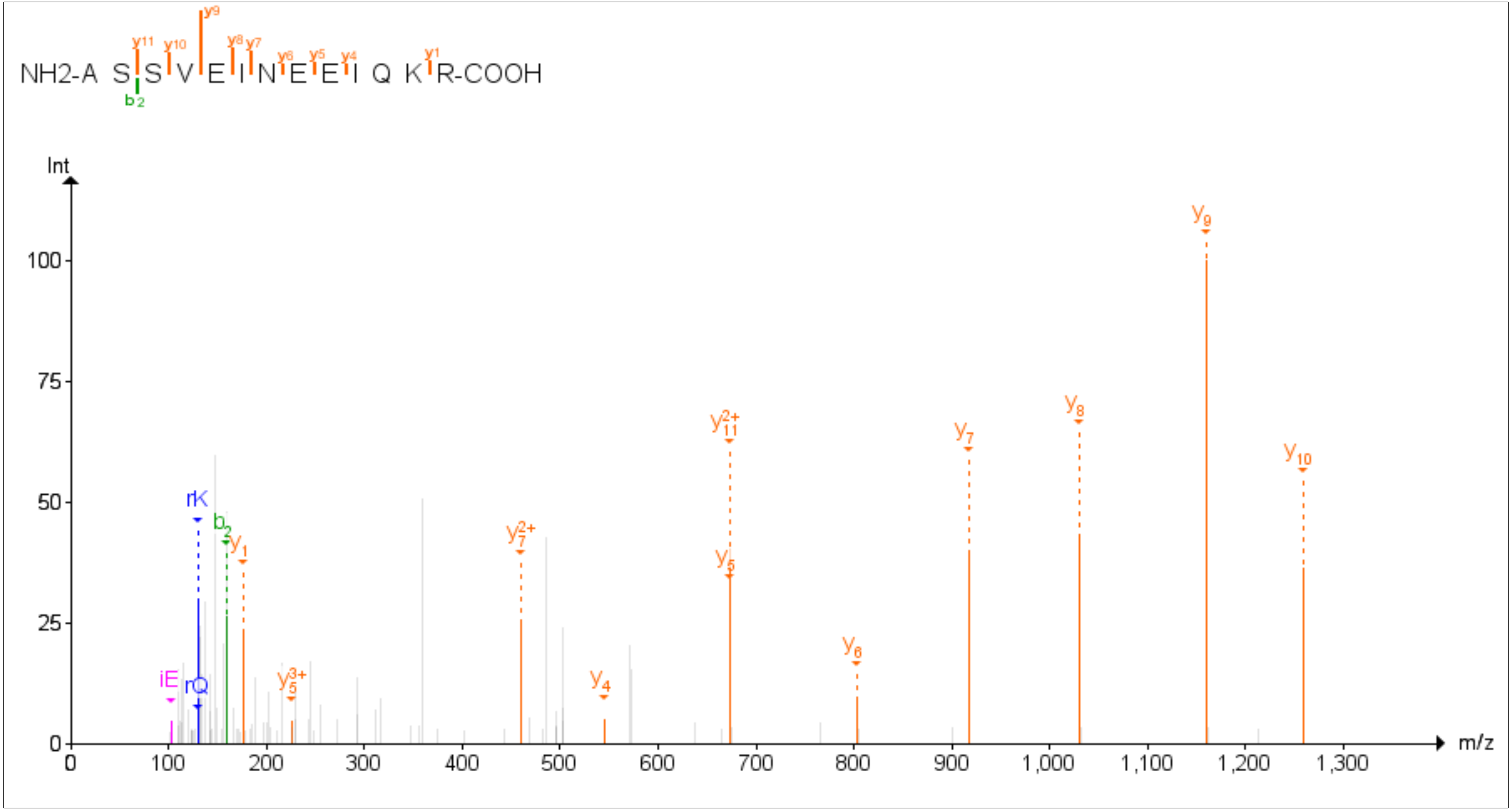

NH2-A S S V E I N E E I Q K R-COOH
y11 y10 y9 y8 y7 y6 y5 y4 y1
b2
Int
m/z
0
25
50
75
100
0
100
200
300
400
500
600
700
800
900
1,000
1,100
1,200
1,300
iE
rK
rQ
b2
y1
y5 3+
y7 2+
y4
y11 2+
y5
y6
y7
y8
y9
y10

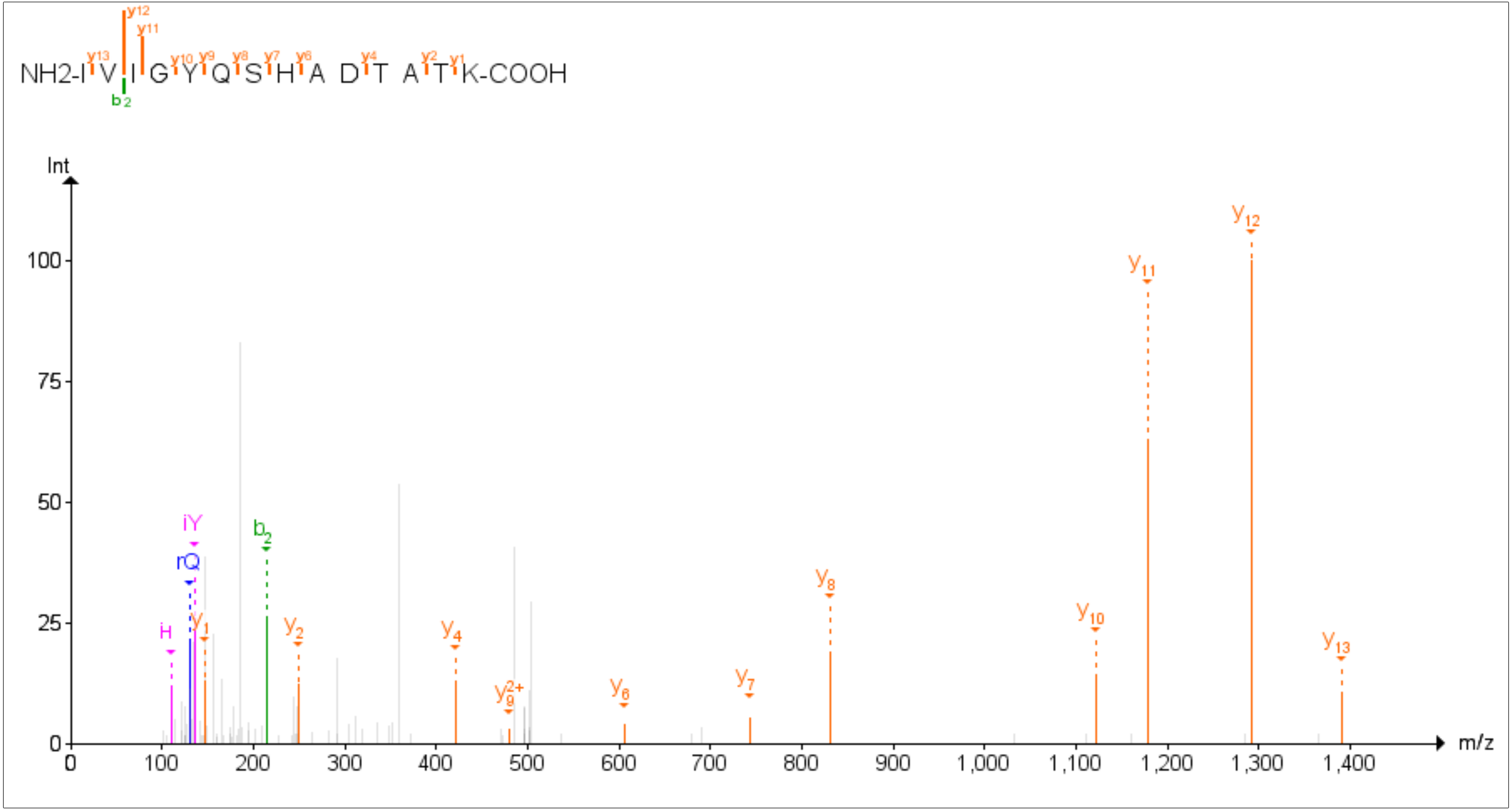

NH2-I V I G Y Q S H A D T A T K-COOH
y13
y12
y11
y10
y9
y8
y7
y6
y4
y2
y1
b2
Int
100
75
50
25
0
iY
rQ
iH
b2
y1
y2
y4
y9 2+
y6
y7
y8
y10
y11
y12
y13
0
100
200
300
400
500
600
700
800
900
1,000
1,100
1,200
1,300
1,400
m/z

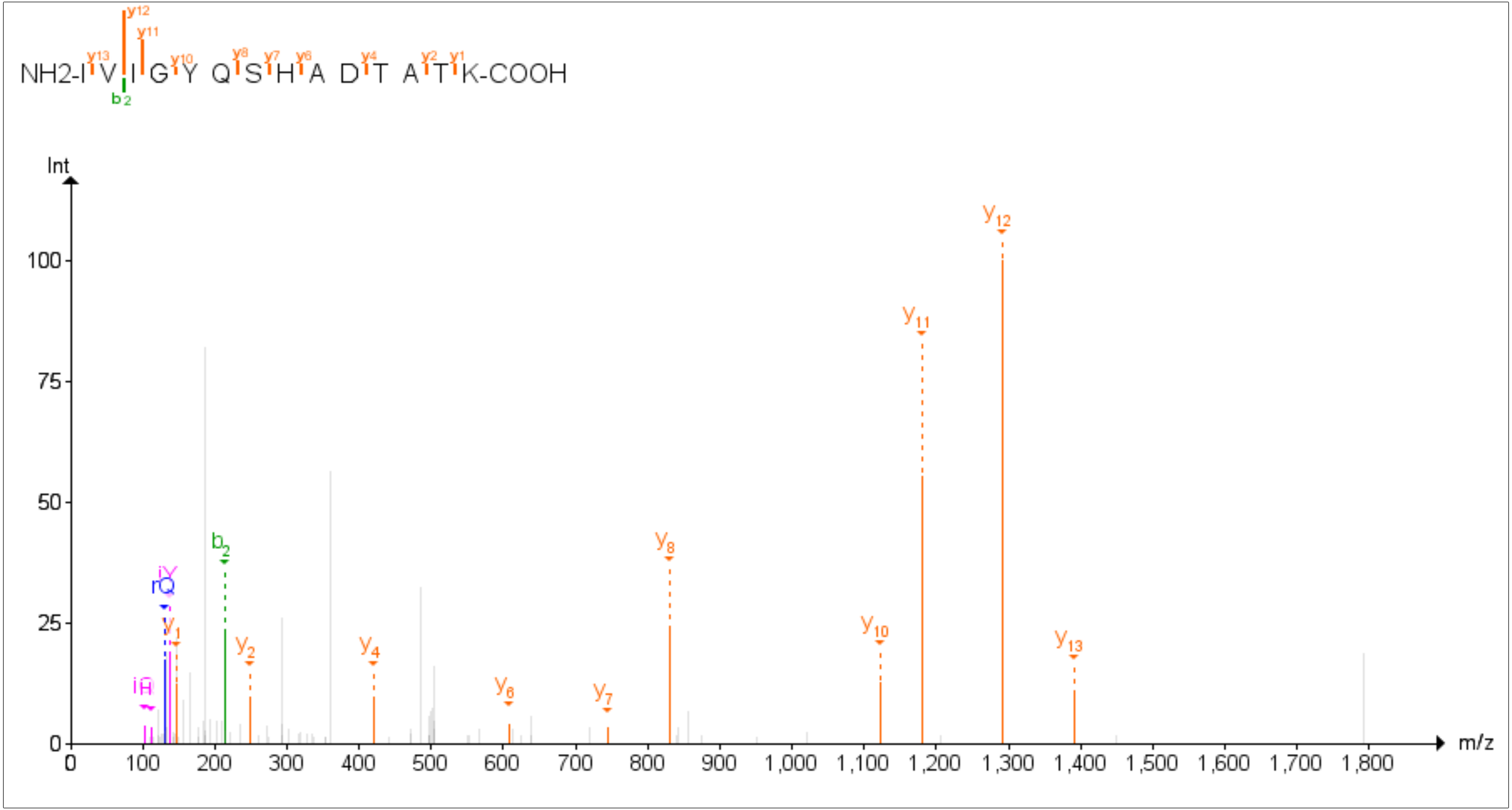

NH2-IVIGYQSHADTATK-COOH
y13
y12
y11
y10
y8
y7
y6
y4
y2
y1
b2
Int
100
75
50
25
0
iH
rQ
iY
m/z
0
100
200
300
400
500
600
700
800
900
1,000
1,100
1,200
1,300
1,400
1,500
1,600
1,700
1,800

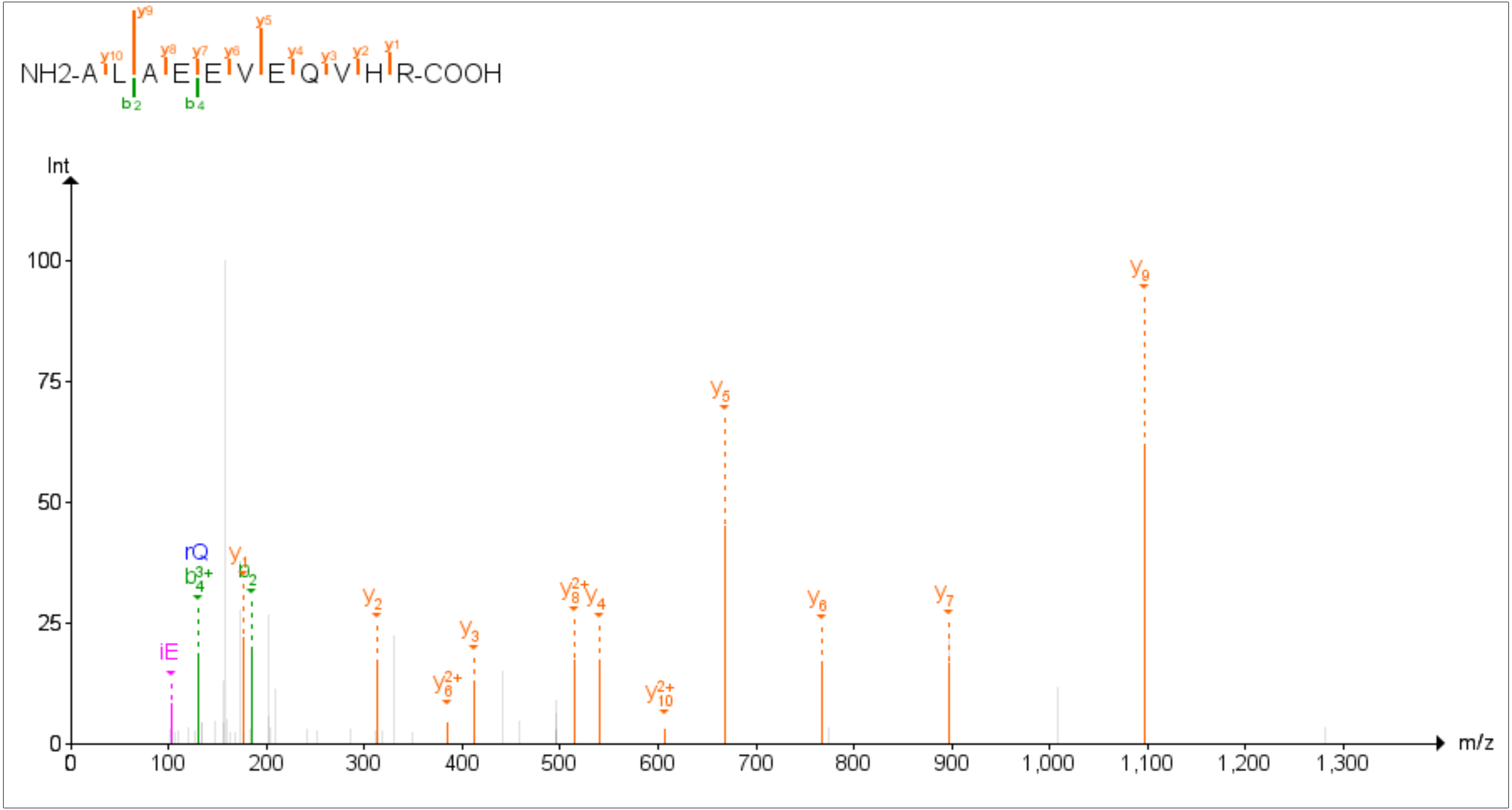

NH2-A L A E E V E Q V H R-COOH
y10 y9 y8 y7 y6 y5 y4 y3 y2 y1
b2 b4
Int
m/z
y9
y5
rQ
b_4^{3+}
y_1
b_2
iE
y_2
y_6^{2+}
y_3
y_8^{2+}
y_4
y_{10}^{2+}
y_6
y_7

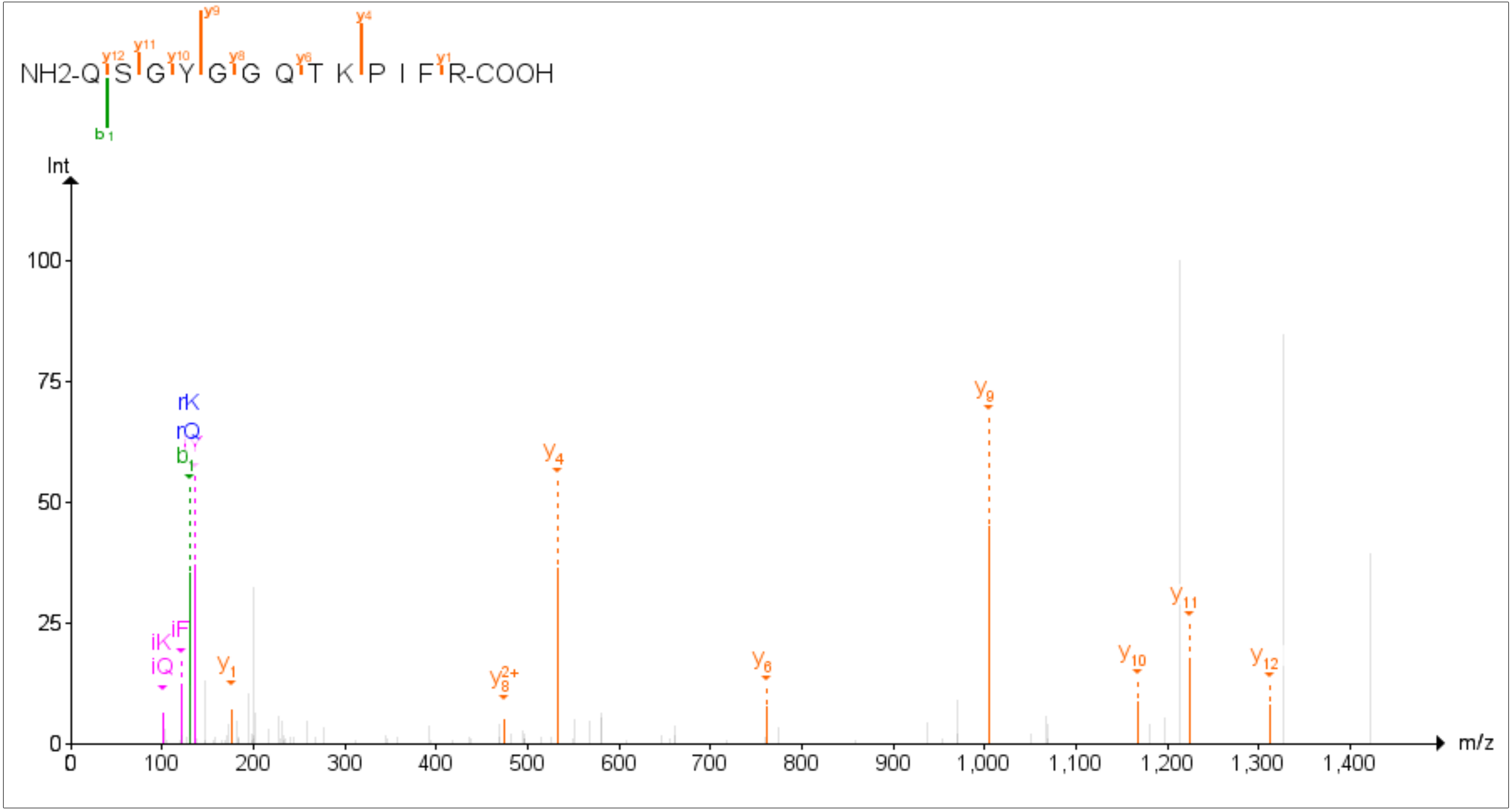

NH2-Q S G Y G G Q T K P I F R-COOH
y12 y11 y10 y9 y8 y6 y4 y1
b1
Int
100
75
50
25
0
rK
rQ
b1
iK
iF
iQ
y1
y8 2+
y4
y6
y9
y10
y11
y12
0 100 200 300 400 500 600 700 800 900 1,000 1,100 1,200 1,300 1,400
m/z

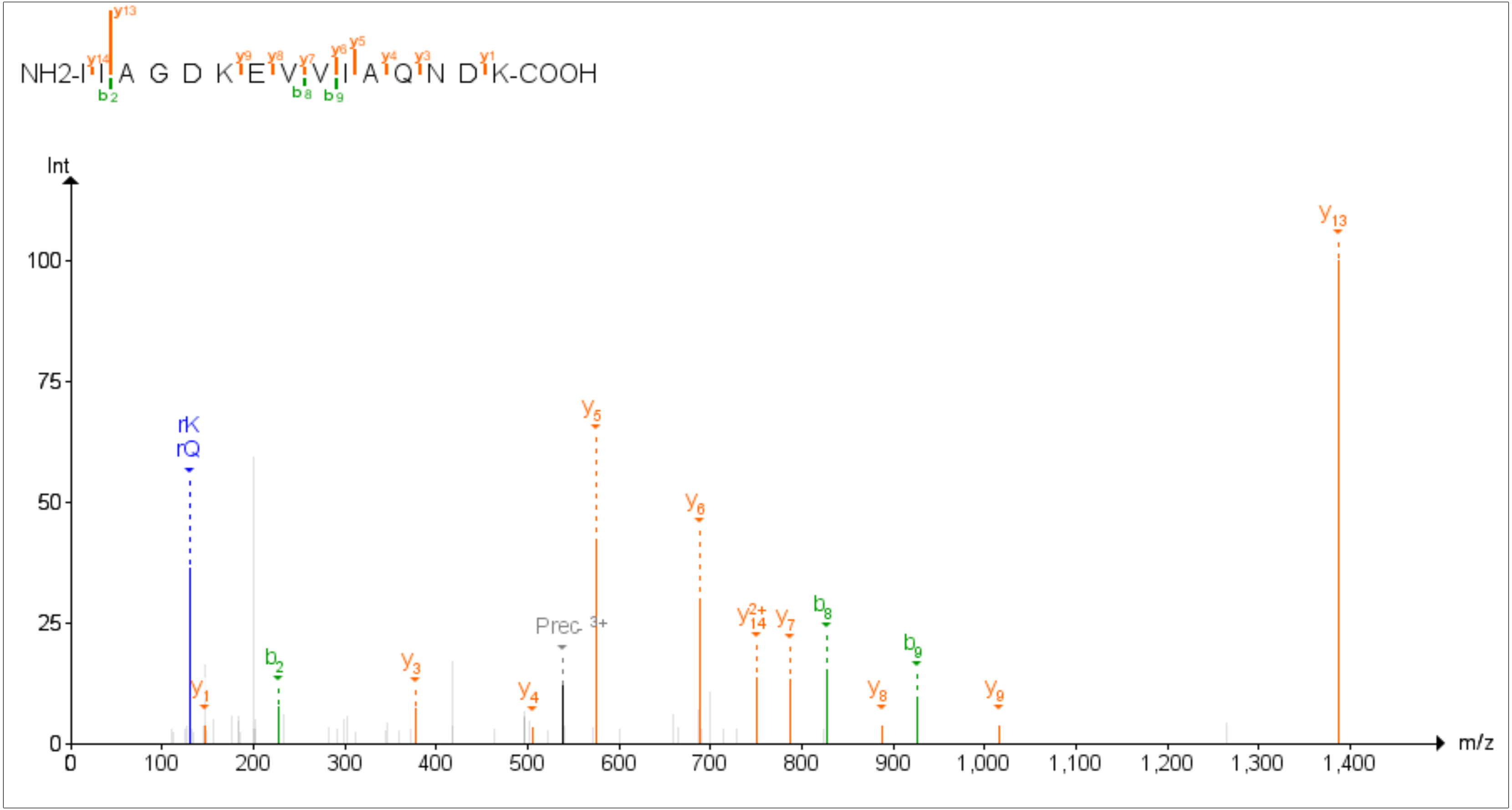

NH2-I I A G D K E V V I A Q N D K-COOH
y13
y14
y9
y8
y7
y6
y5
y4
y3
y1
b2
b8
b9
Int
100
75
50
25
0
rK
rQ
y5
y6
y13
y1
b2
y3
Prec 3+
y4
y14 2+
y7
b8
b9
y8
y9
0
100
200
300
400
500
600
700
800
900
1,000
1,100
1,200
1,300
1,400
m/z

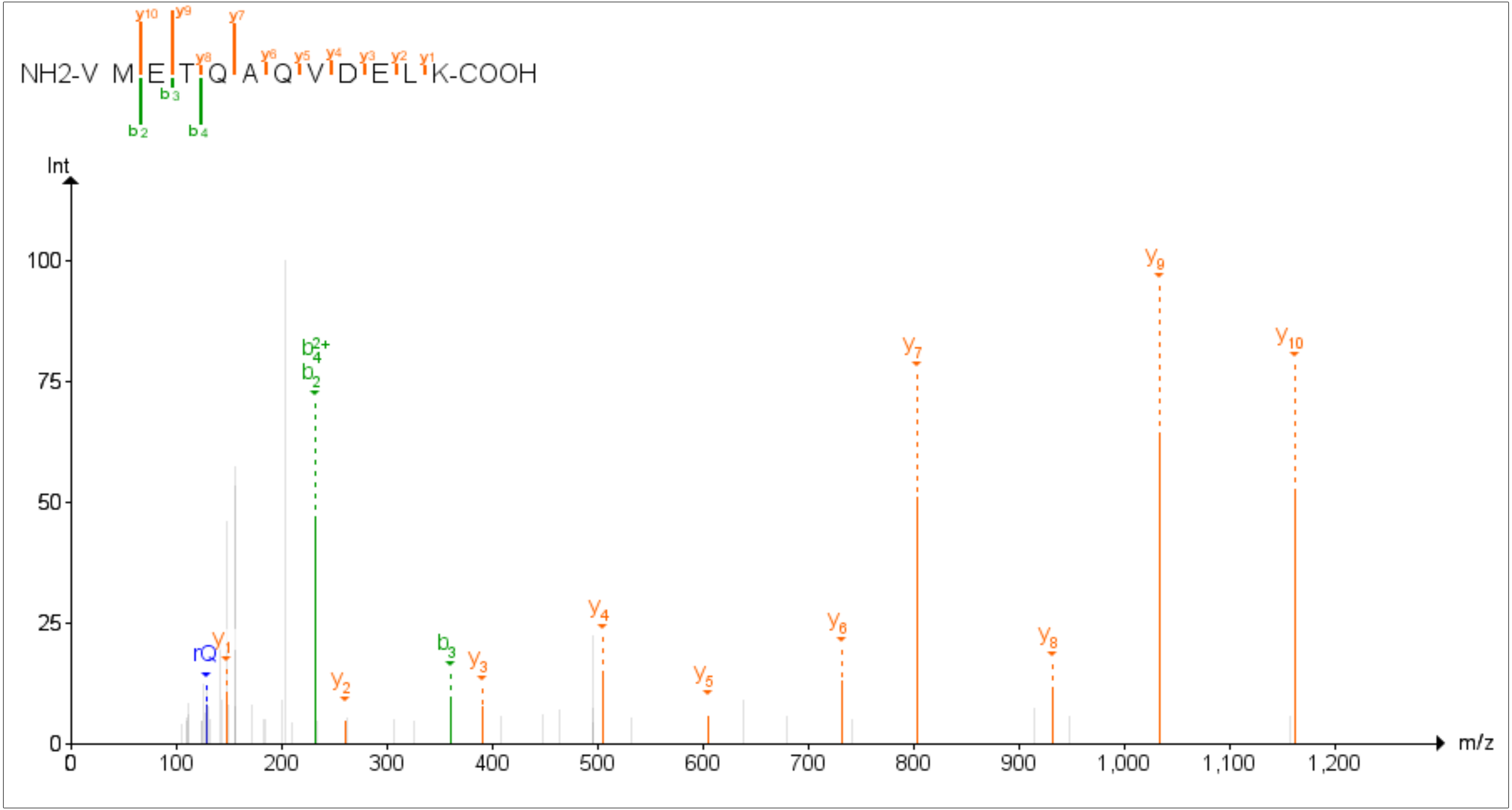

NH2-V M E T Q A Q V D E L K-COOH
y10 y9 y8 y7 y6 y5 y4 y3 y2 y1
b2 b3 b4
Int
m/z
0 25 50 75 100
0 100 200 300 400 500 600 700 800 900 1,000 1,100 1,200
rQ y1 y2 b3 y3 y4 y5 y6 y7 y8 y9 y10
b4 2+
b2

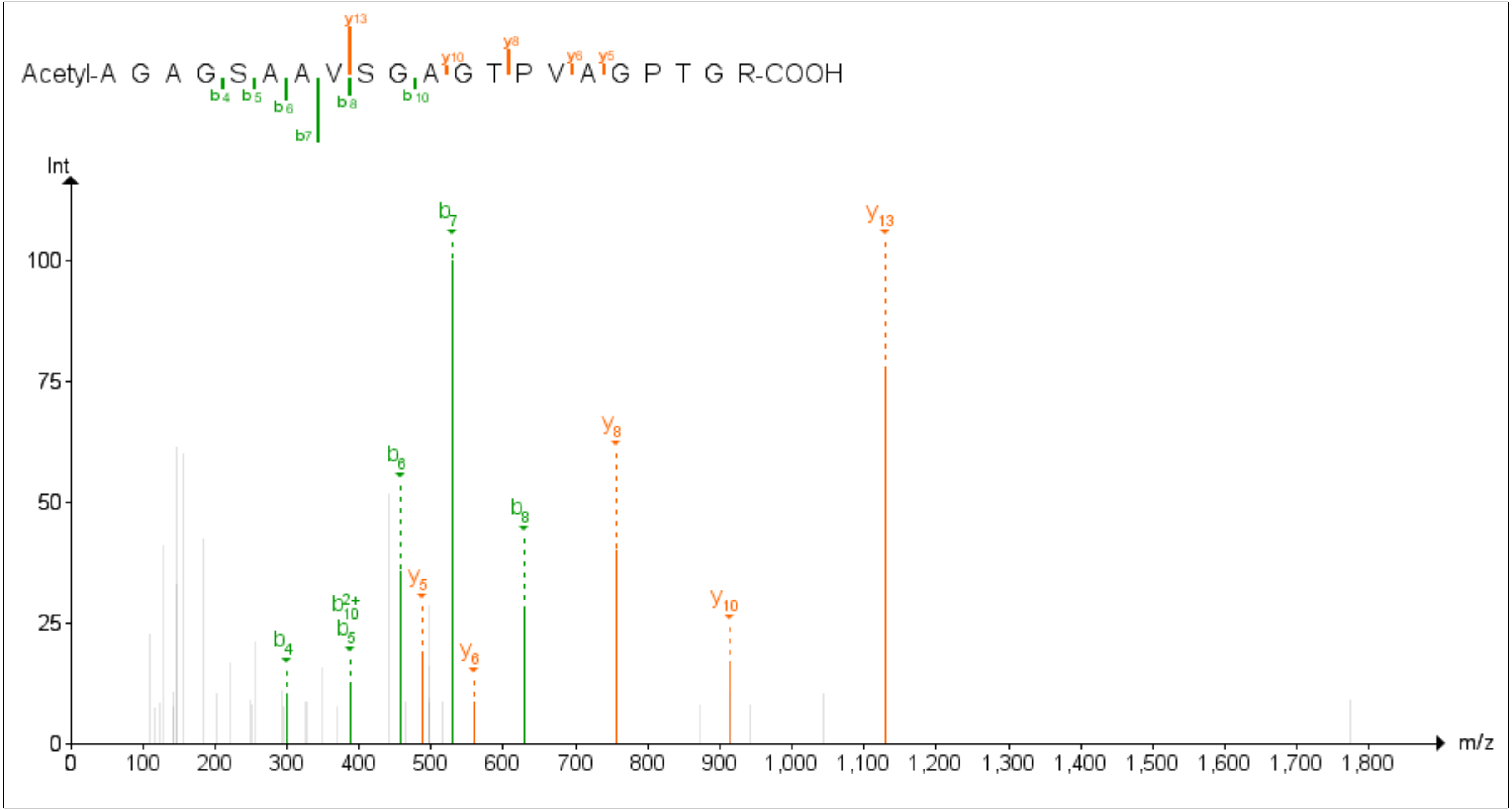

Acetyl-A G A G S A A V S G A G T P V A G P T G R-COOH
b4 b5 b6 b7 b8 b10
y13 y10 y8 y6 y5
Int
m/z
0
25
50
75
100
0
100
200
300
400
500
600
700
800
900
1,000
1,100
1,200
1,300
1,400
1,500
1,600
1,700
1,800
b4
b10 2+
b5
b6
y5
b7
y6
b8
y8
y10
y13

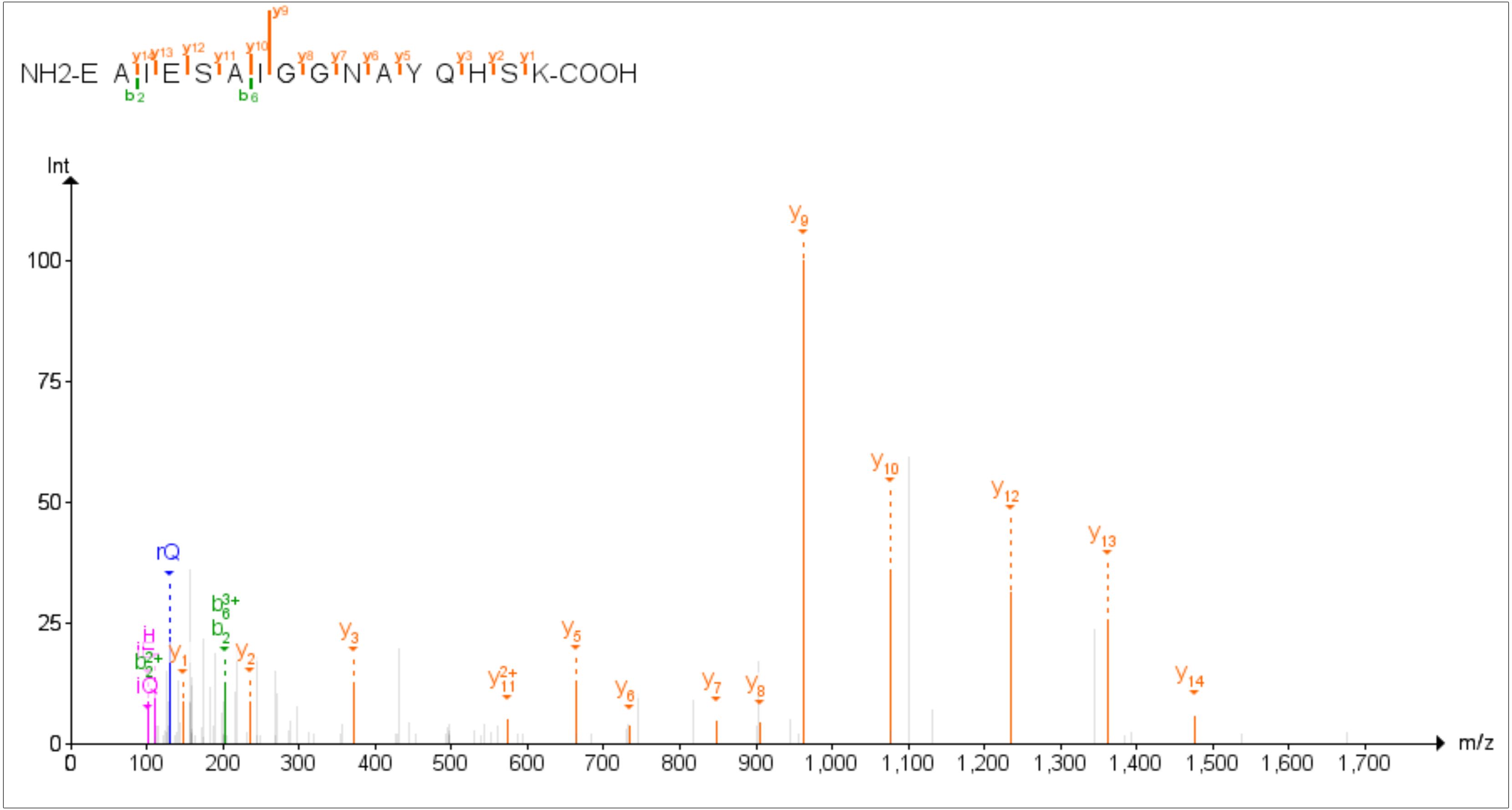

NH2-E A I E S A I G G N A Y Q H S K-COOH
y14 y13 y12 y11 y10 y9 y8 y7 y6 y5 y3 y2 y1
b2 b6
Int
m/z
rQ
iH
iQ
b_2^{2+}
b_6^{3+}
b_2
y_1
y_2
y_3
y_{11}^{2+}
y_5
y_6
y_7
y_8
y_9
y_{10}
y_{12}
y_{13}
y_{14}

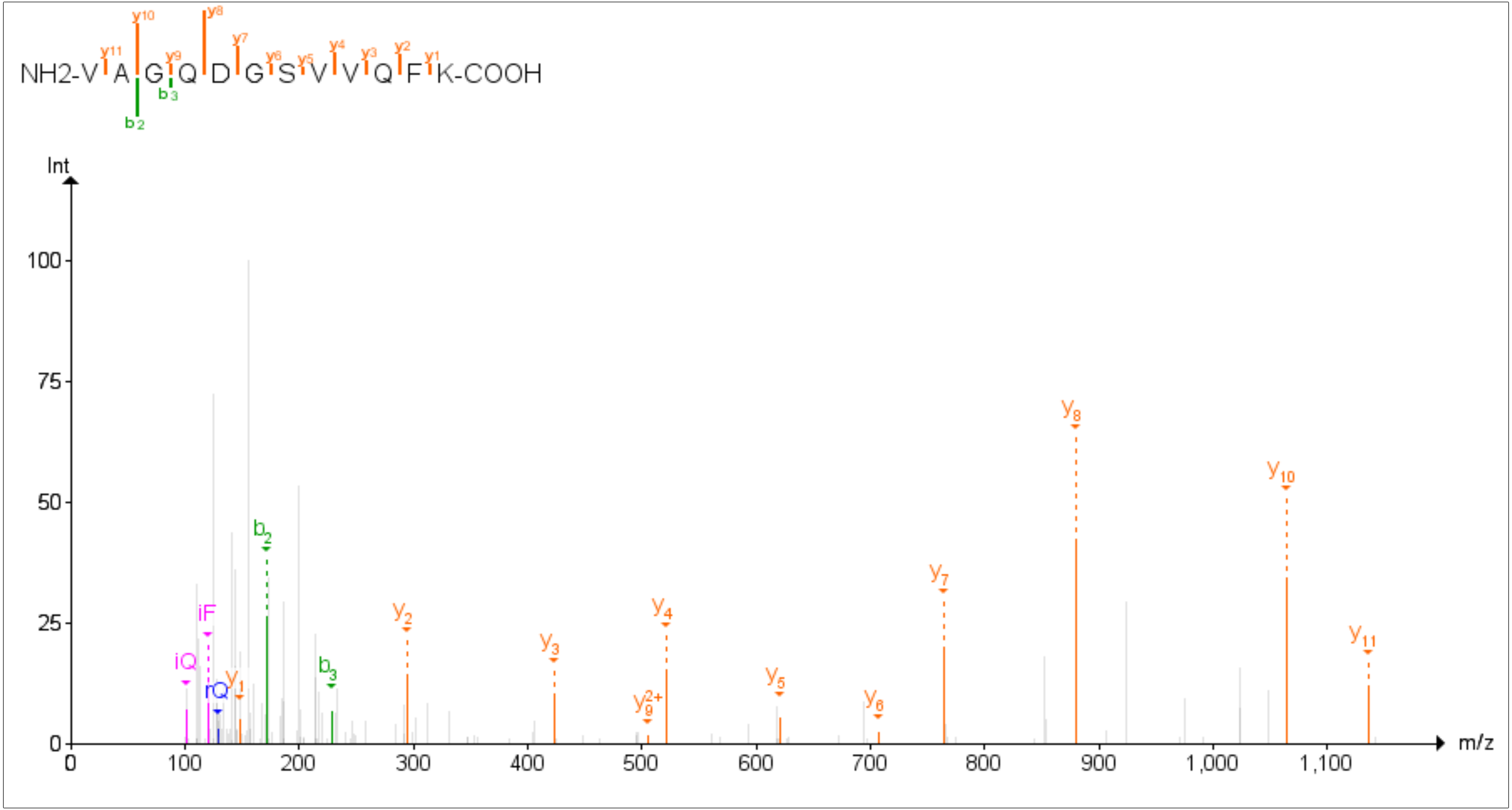
NH2-V A G Q D G S V V Q F K-COOH
y11 y10 y9 y8 y7 y6 y5 y4 y3 y2 y1
b2 b3
Int
100
75
50
25
0
m/z
0 100 200 300 400 500 600 700 800 900 1,000 1,100
iQ iF rQ y1 b2 b3 y2 y3 y9 2+ y4 y5 y6 y7 y8 y10 y11

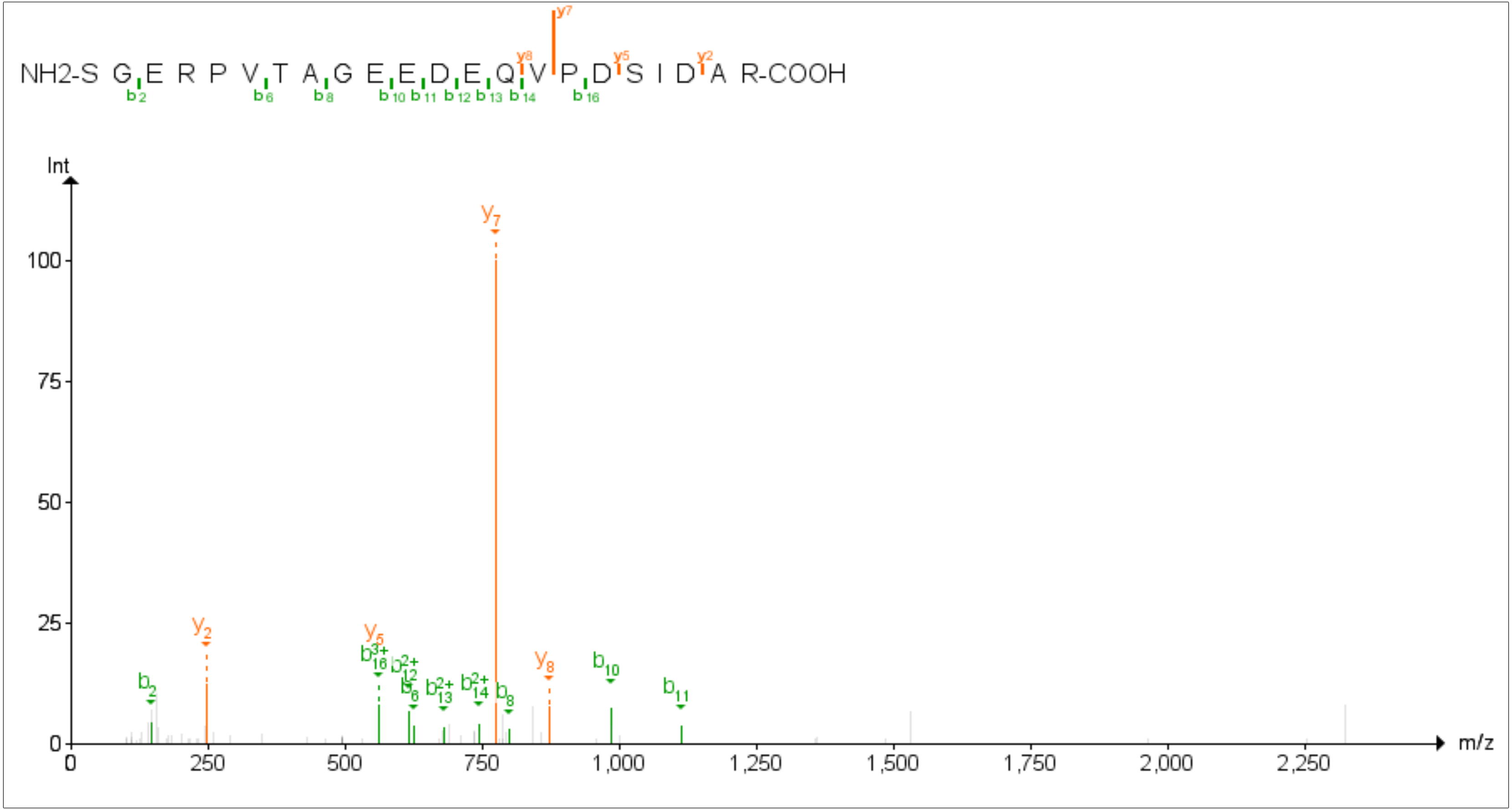

NH2-S G E R P V T A G E E D E Q V P D S I D A R-COOH
y8 y7 y5 y2
b2 b6 b8 b10 b11 b12 b13 b14 b16
Int
100
75
50
25
0
y7
y2
y5
b2
b3+16
b2+12
b6
b2+13
b2+14
b8
y8
b10
b11
0
250
500
750
1,000
1,250
1,500
1,750
2,000
2,250
m/z

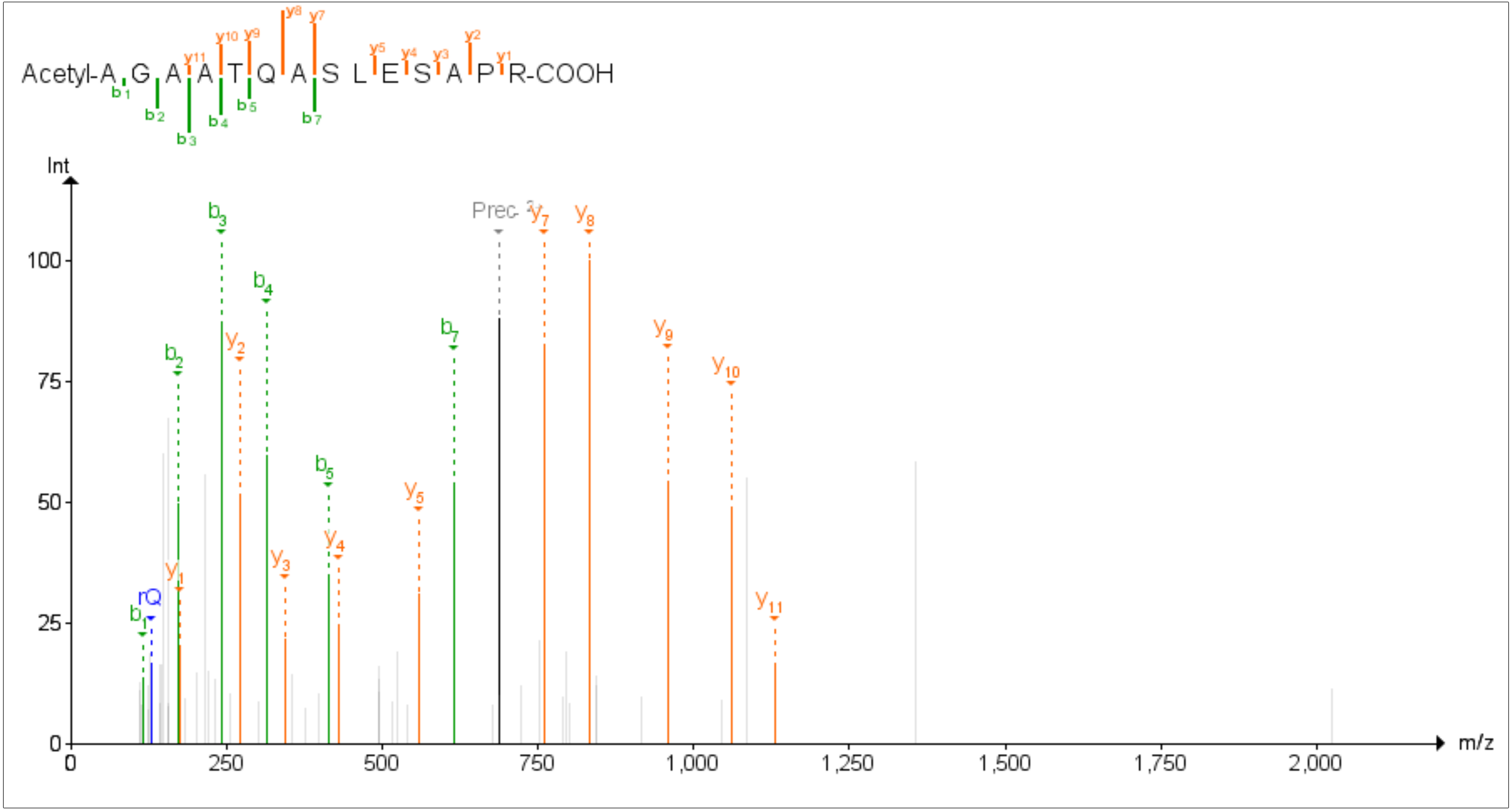
Acetyl-A G A A T Q A S L E S A P R-COOH
Int
m/z
Prec 2+
rQ
0
25
50
75
100
250
500
750
1,000
1,250
1,500
1,750
2,000

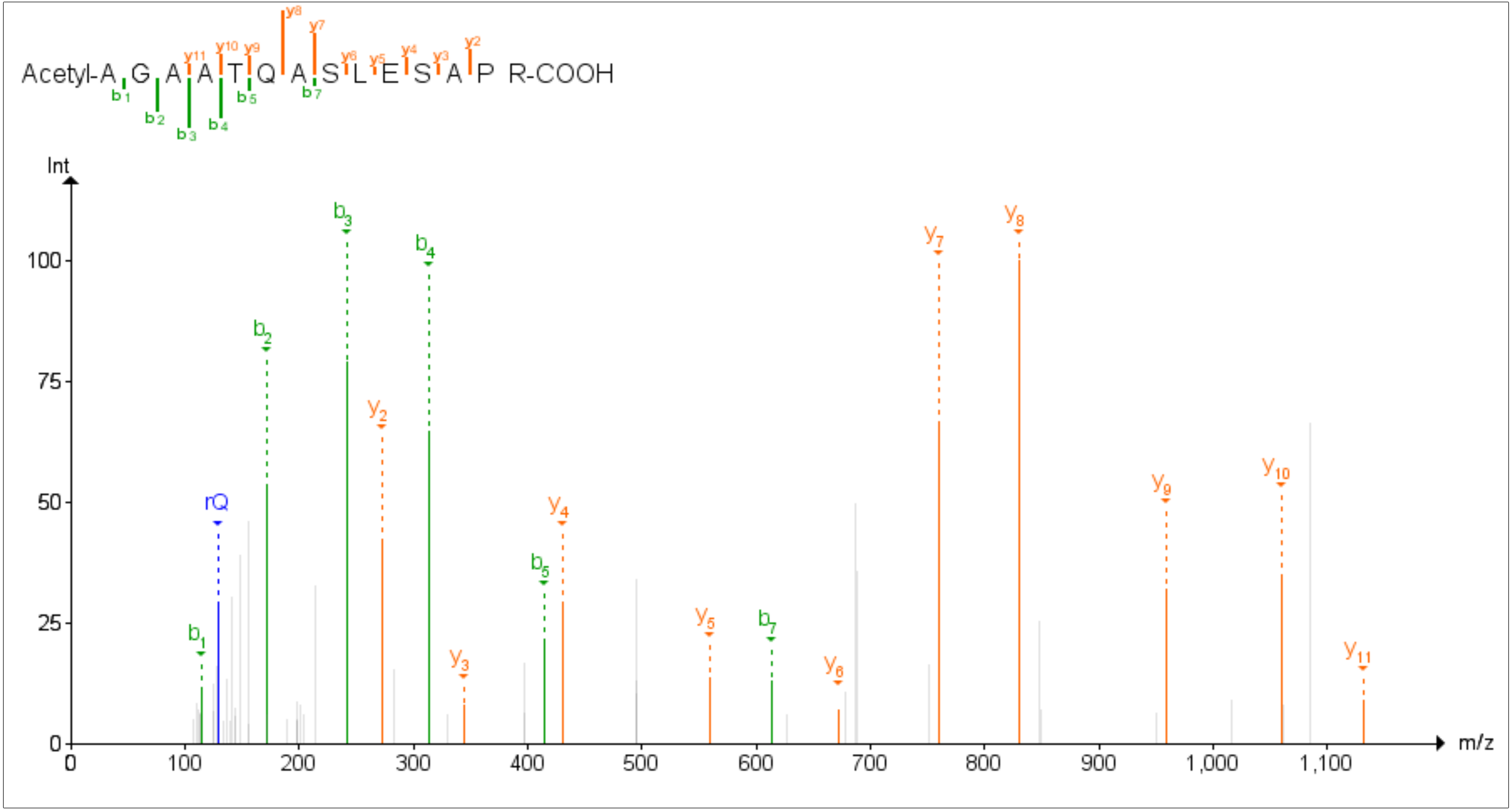
Acetyl-A G A A T Q A S L E S A P R-COOH
b1 b2 b3 b4 b5 b7
y11 y10 y9 y8 y7 y6 y5 y4 y3 y2
Int
m/z
rQ
0
25
50
75
100
0
100
200
300
400
500
600
700
800
900
1,000
1,100

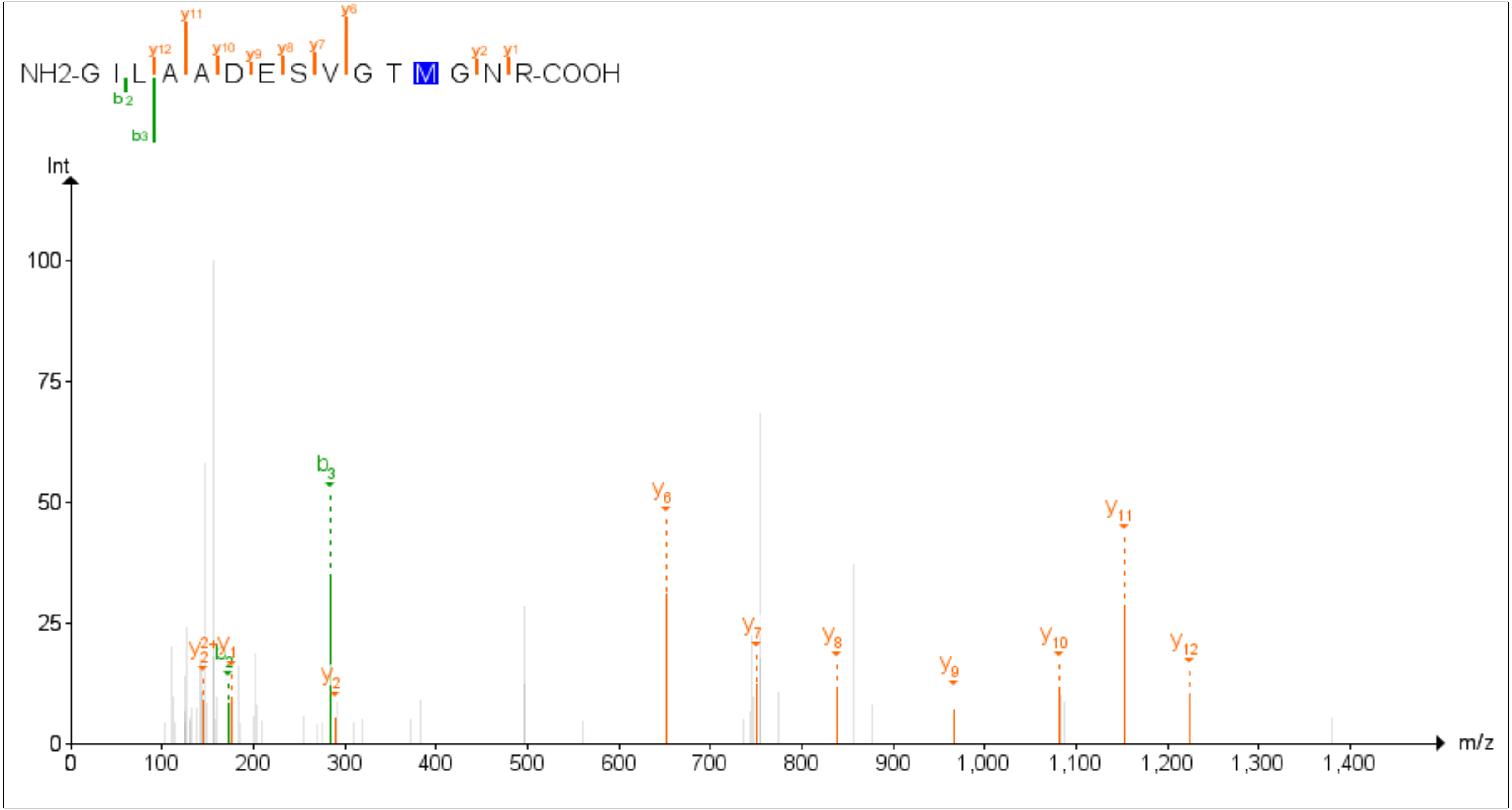

NH2-G I L A A D E S V G T M G N R-COOH
b2
b3
y12 y11 y10 y9 y8 y7 y6 y2 y1
Int
m/z
b3
y6
y11
y7
y8
y9
y10
y12
y2

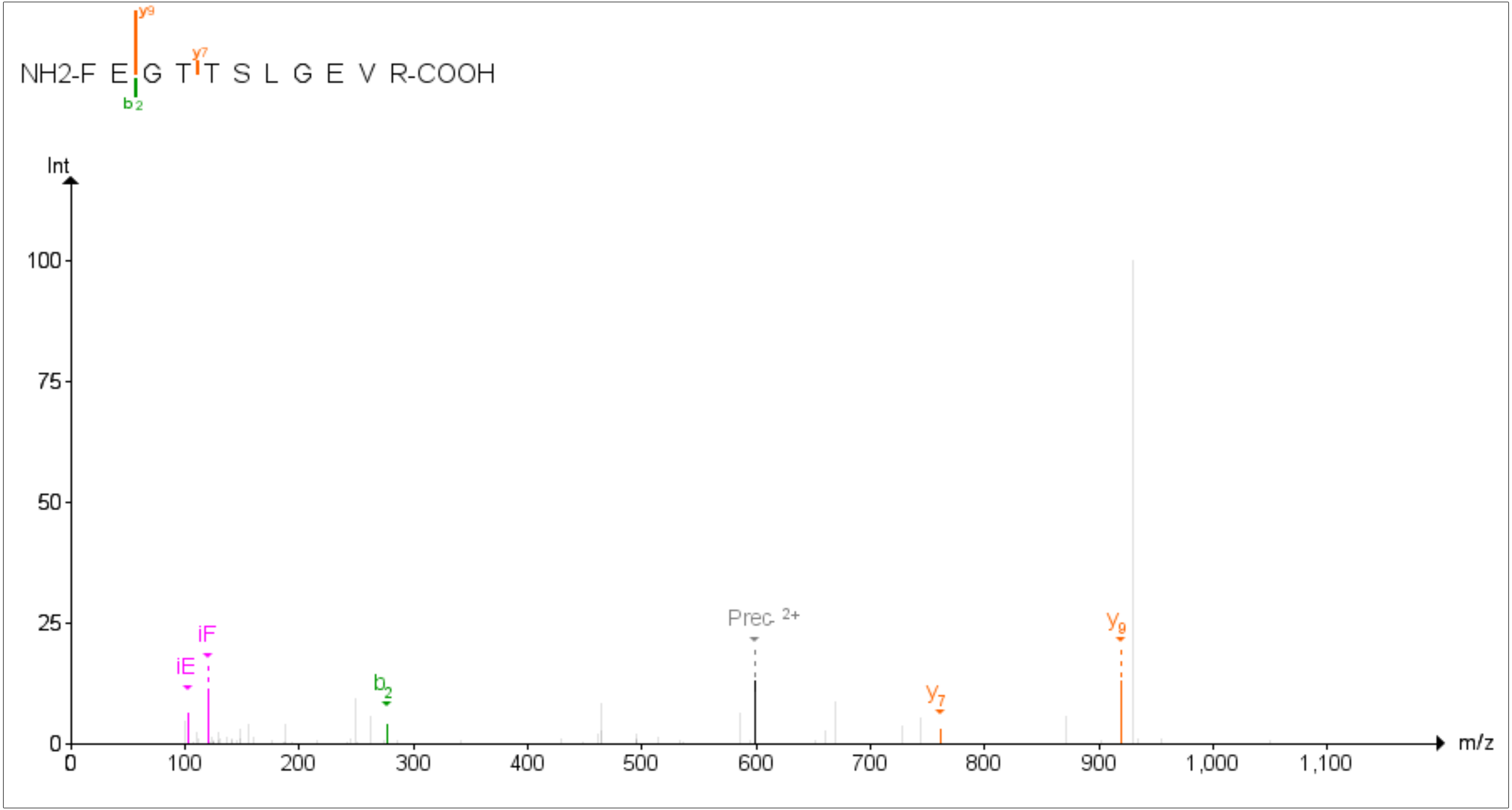

NH2-F E G T T S L G E V R-COOH
y9
y7
b2
Int
100
75
50
25
0
iE
iF
b2
Prec 2+
y7
y9
0
100
200
300
400
500
600
700
800
900
1,000
1,100
m/z

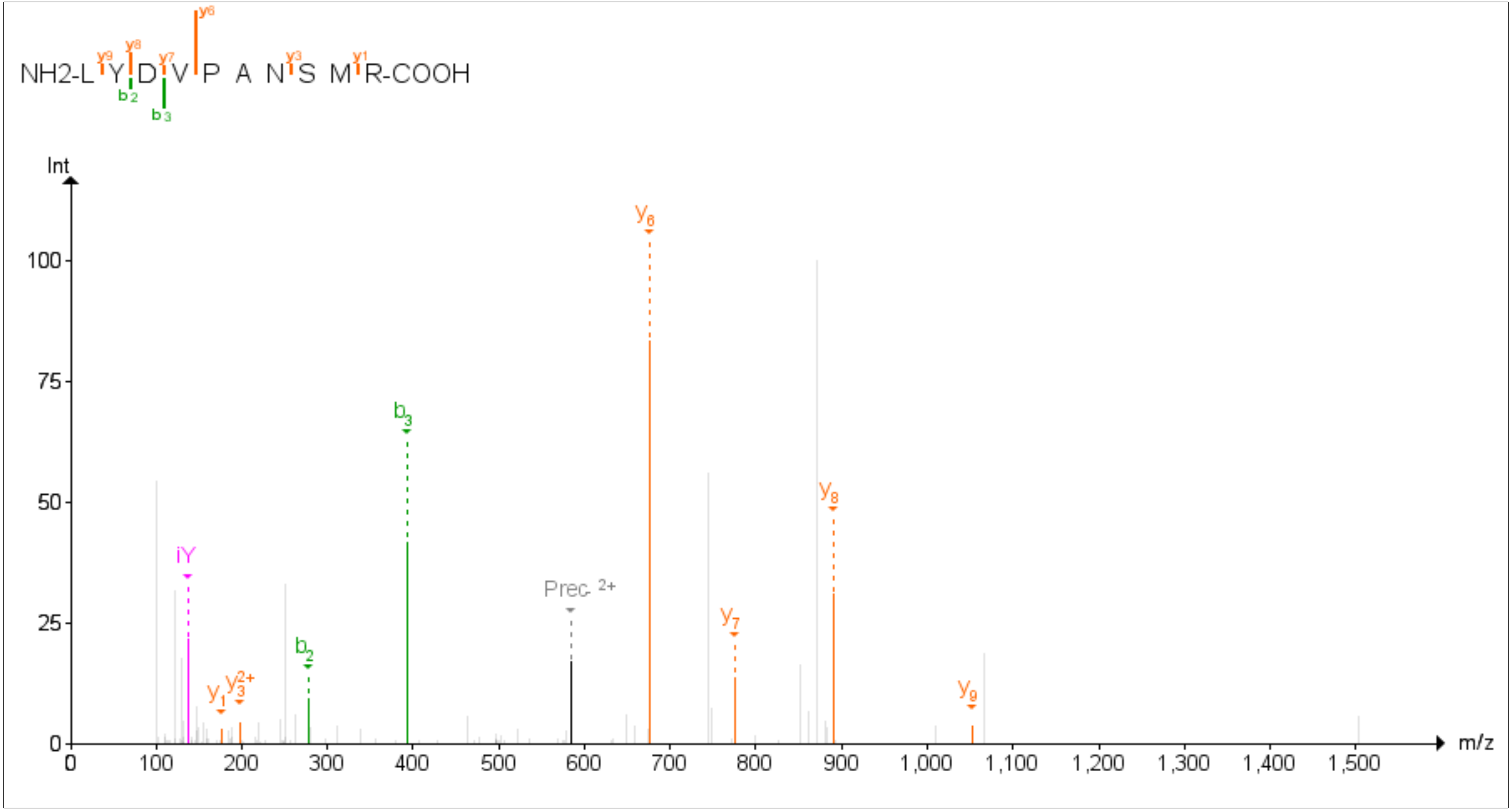

NH2-L Y D V P A N S M R-COOH
y9
y8
y7
y6
y3
y1
b2
b3
Int
100
75
50
25
0
y6
b3
y8
iY
Prec 2+
y7
y1
y3 2+
b2
y9
m/z
0
100
200
300
400
500
600
700
800
900
1,000
1,100
1,200
1,300
1,400
1,500

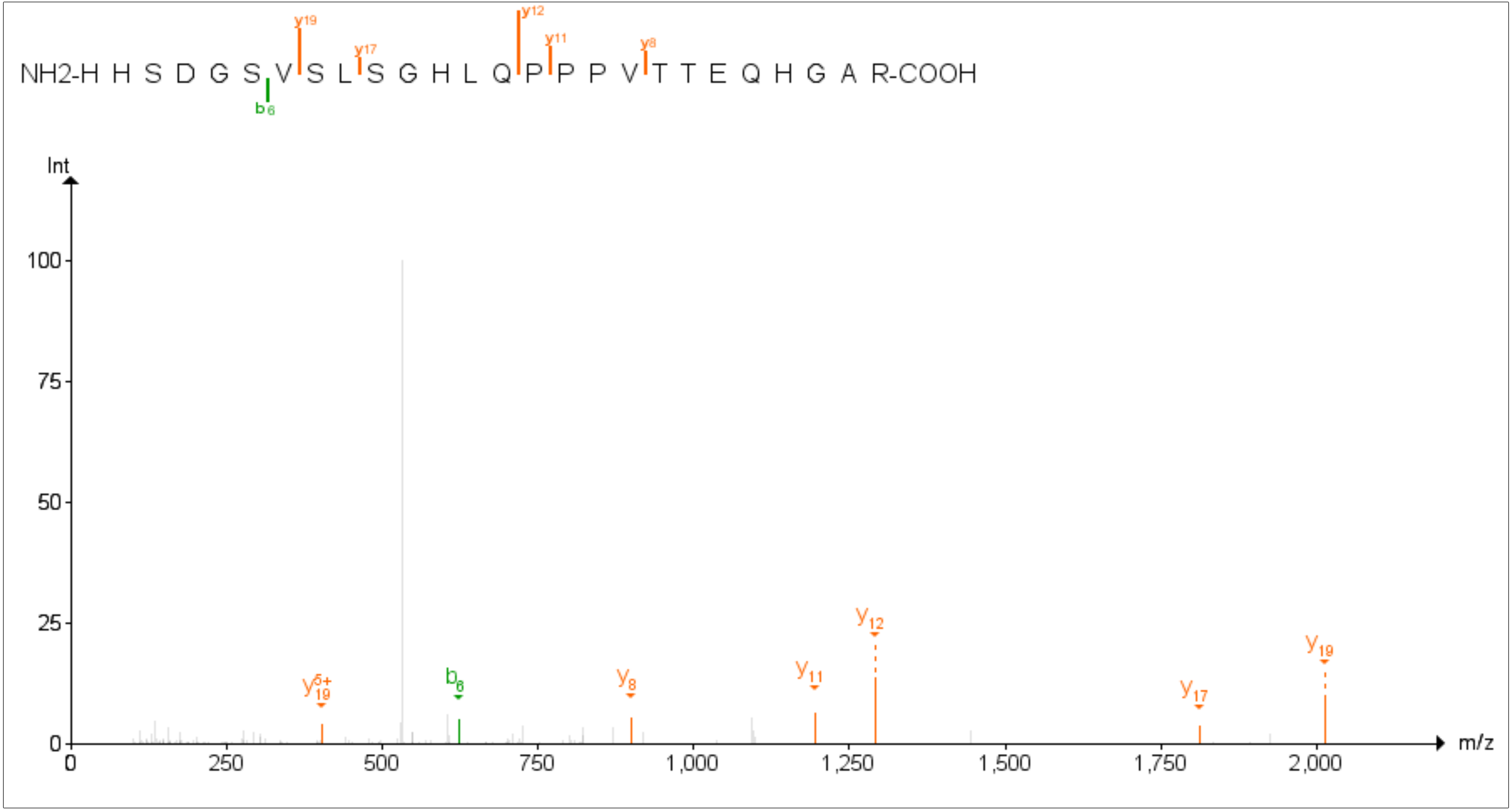

NH2-H H S D G S V S L S G H L Q P P P V T T E Q H G A R-COOH
y19
y17
y12
y11
y8
b6
Int
100
75
50
25
0
0
250
500
750
1,000
1,250
1,500
1,750
2,000
m/z
y19 5+
b6
y8
y11
y12
y17
y19

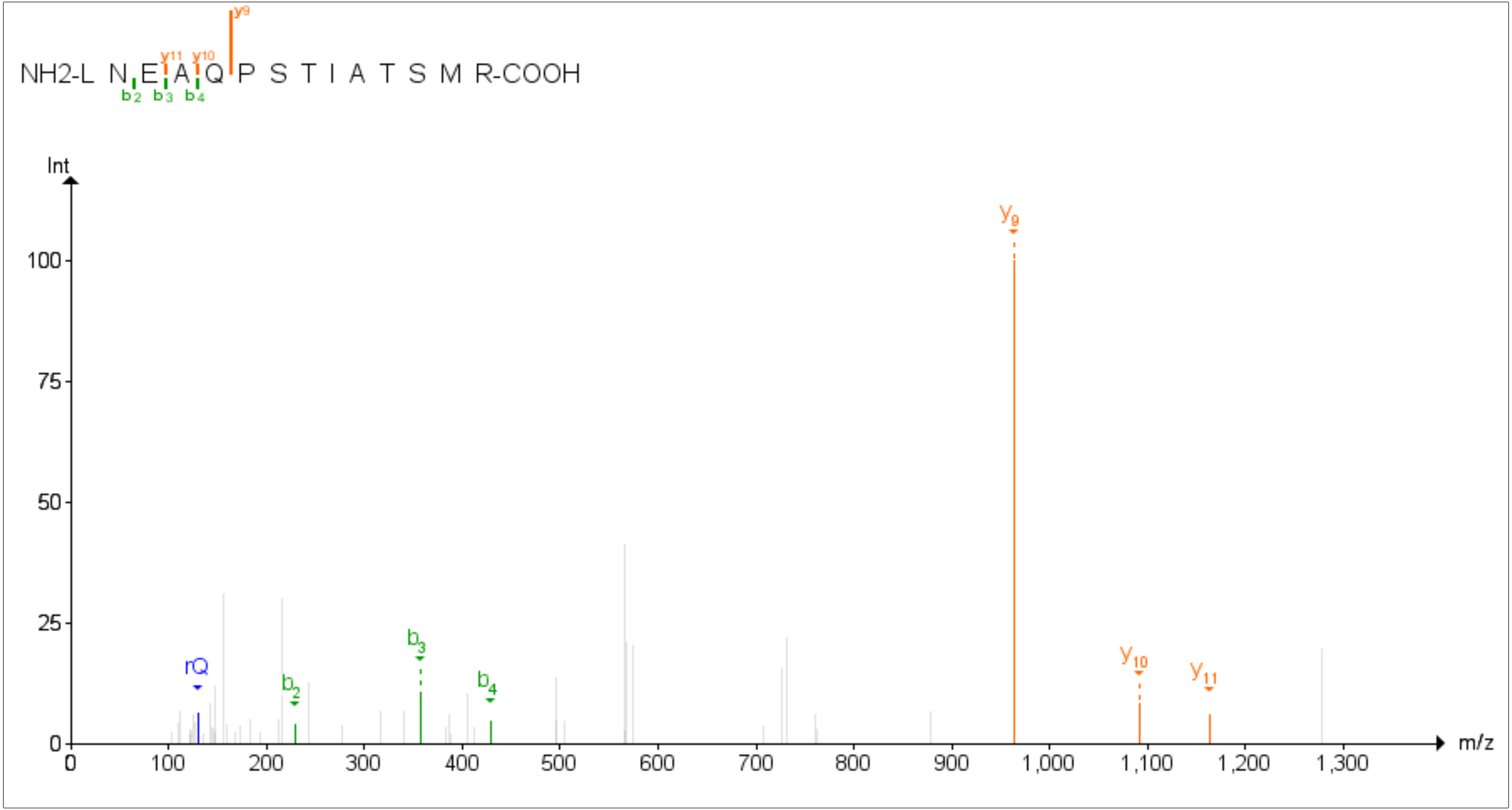
NH2-L N E A Q P S T I A T S M R-COOH
y9
y11
y10
b2
b3
b4
Int
100
75
50
25
0
rQ
b_2
b_3
b_4
y_9
y_{10}
y_{11}
0
100
200
300
400
500
600
700
800
900
1,000
1,100
1,200
1,300
m/z

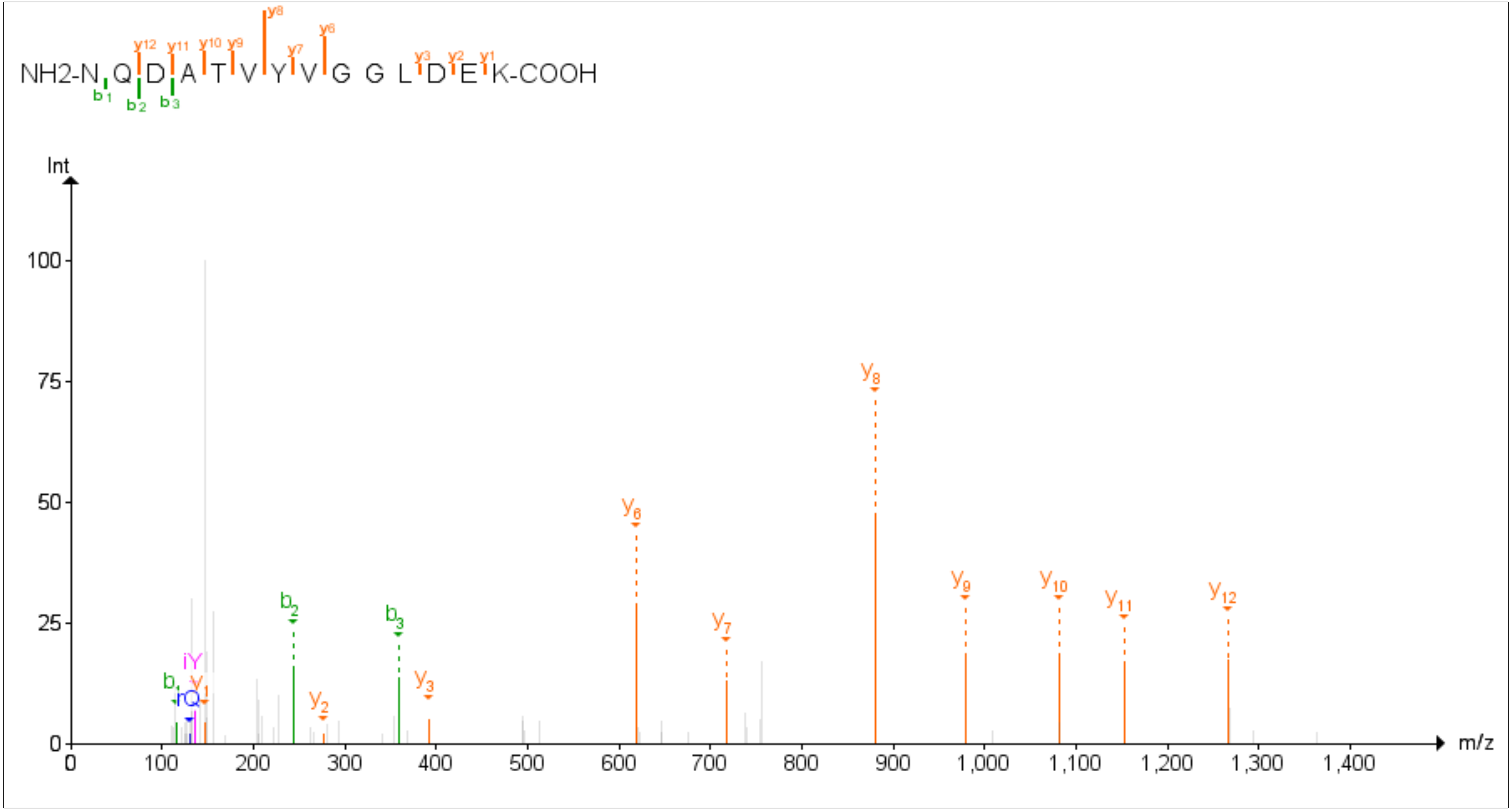

NH2-N Q D A T V Y V G G L D E K-COOH
y12 y11 y10 y9 y8 y7 y6 y3 y2 y1
b1 b2 b3
Int
100
75
50
25
0
y8
y6
y9
y10
y11
y12
y7
b2
b3
y3
y2
iY
b1
y1
rQ
0 100 200 300 400 500 600 700 800 900 1,000 1,100 1,200 1,300 1,400
m/z

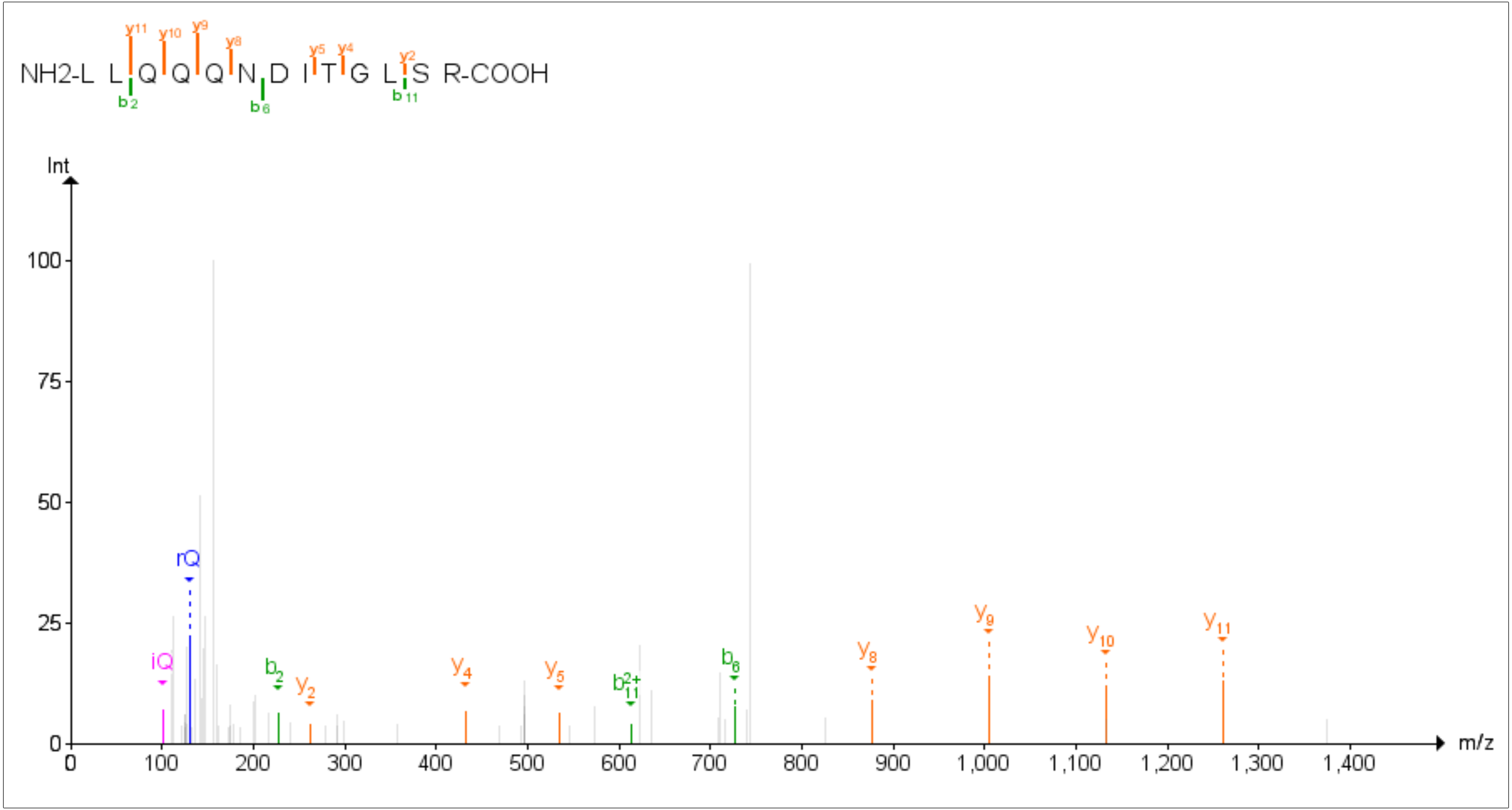
NH2-L L Q Q Q N D I T G L S R-COOH
y11 y10 y9 y8 y5 y4 y2
b2 b6 b11
Int
100
75
50
25
0
rQ
iQ
b2
y2
y4
y5
b11 2+
b6
y8
y9
y10
y11
0 100 200 300 400 500 600 700 800 900 1,000 1,100 1,200 1,300 1,400
m/z

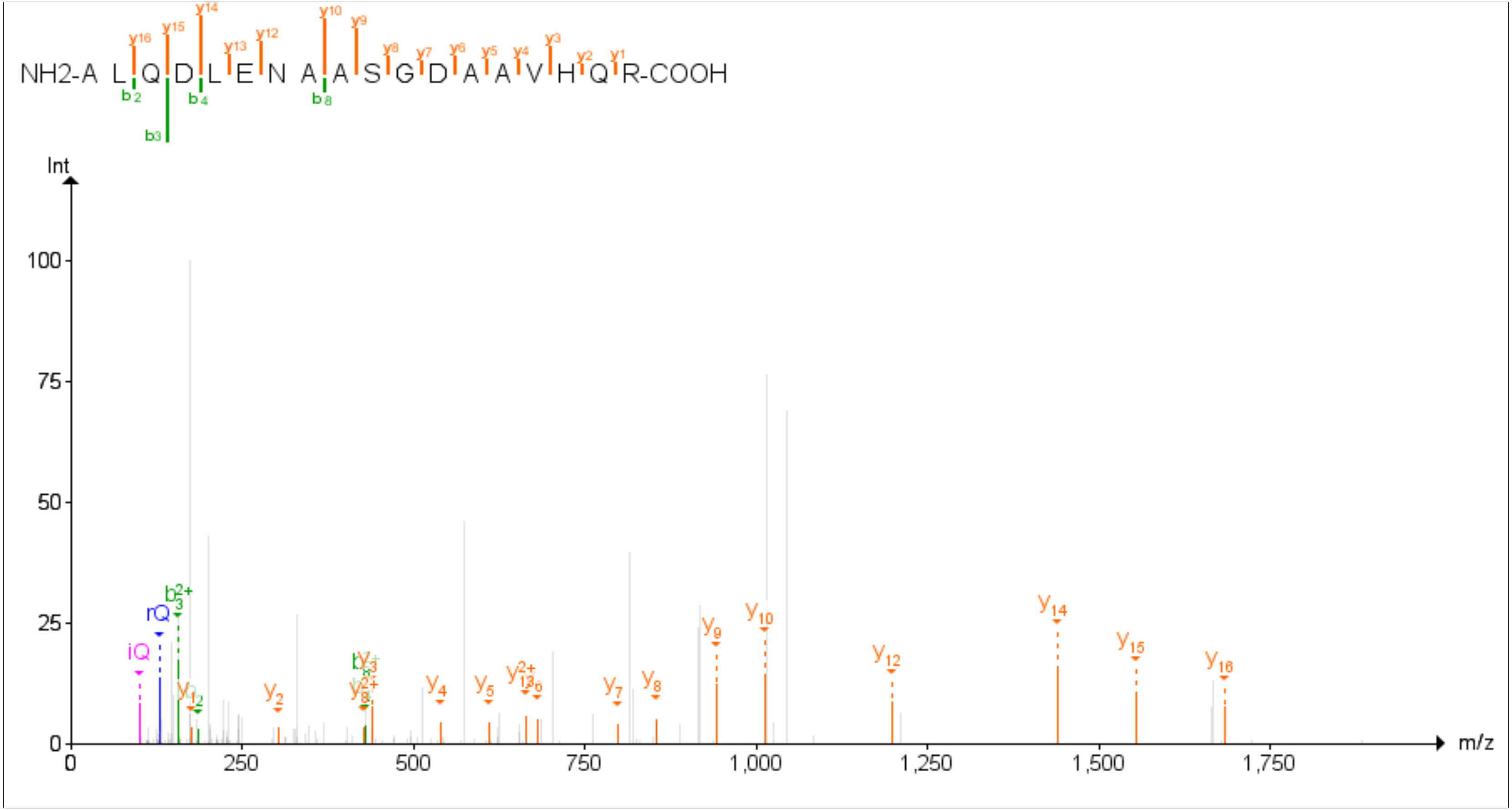
NH2-A L Q D L E N A A S G D A A V H Q R-COOH
y16 y15 y14 y13 y12 y10 y9 y8 y7 y6 y5 y4 y3 y2 y1
b2 b3 b4 b8
Int
100
75
50
25
0
iQ
rQ
y2
y4
y5
y7
y8
y9
y10
y12
y14
y15
y16
0
250
500
750
1,000
1,250
1,500
1,750
m/z

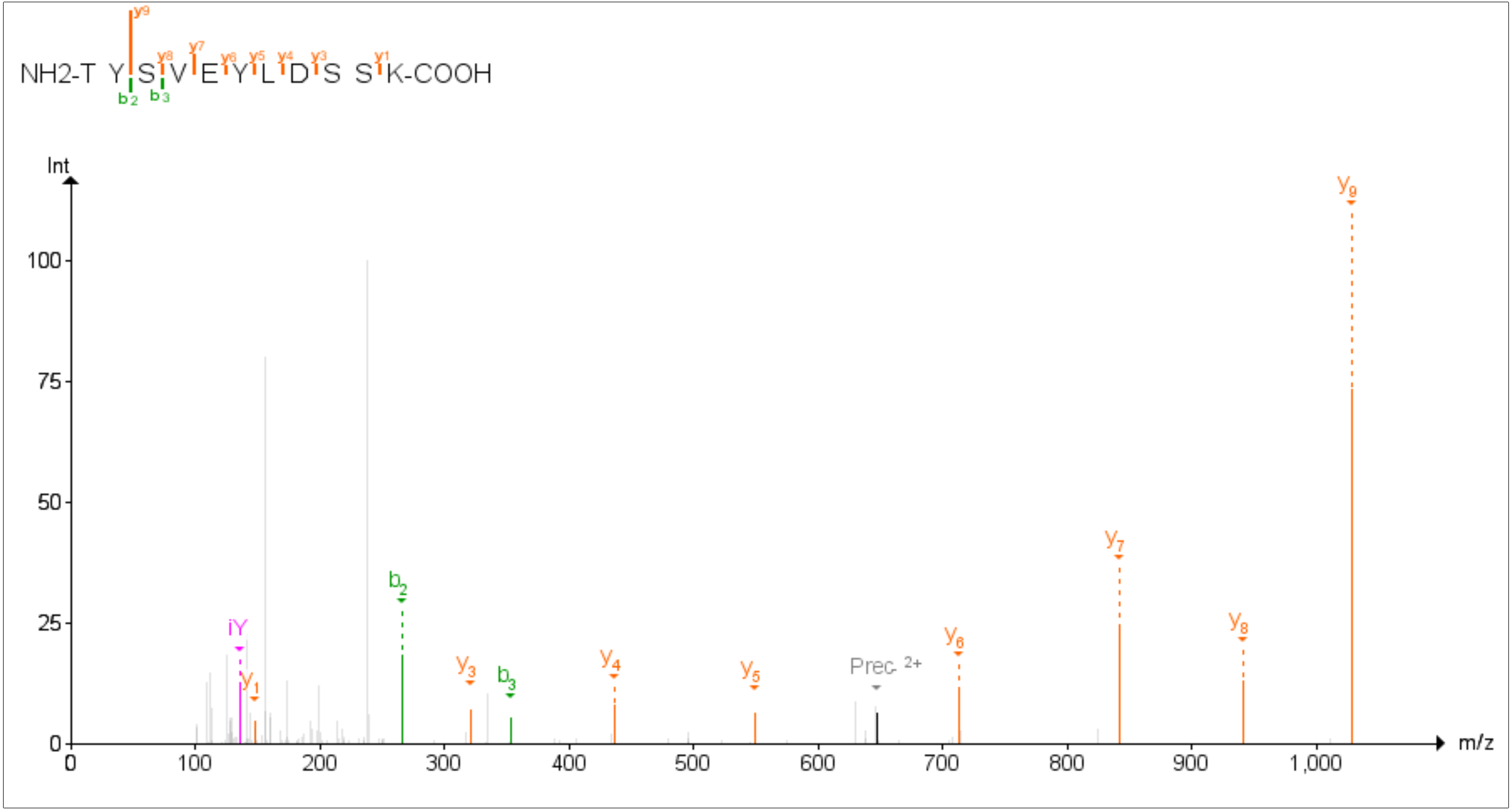

NH2-T Y S V E Y L D S S K-COOH
y9
y8
y7
y6
y5
y4
y3
y1
b2
b3
Int
100
75
50
25
0
y9
y7
y8
y6
b2
iY
y1
y3
b3
y4
y5
Prec 2+
0
100
200
300
400
500
600
700
800
900
1,000
m/z

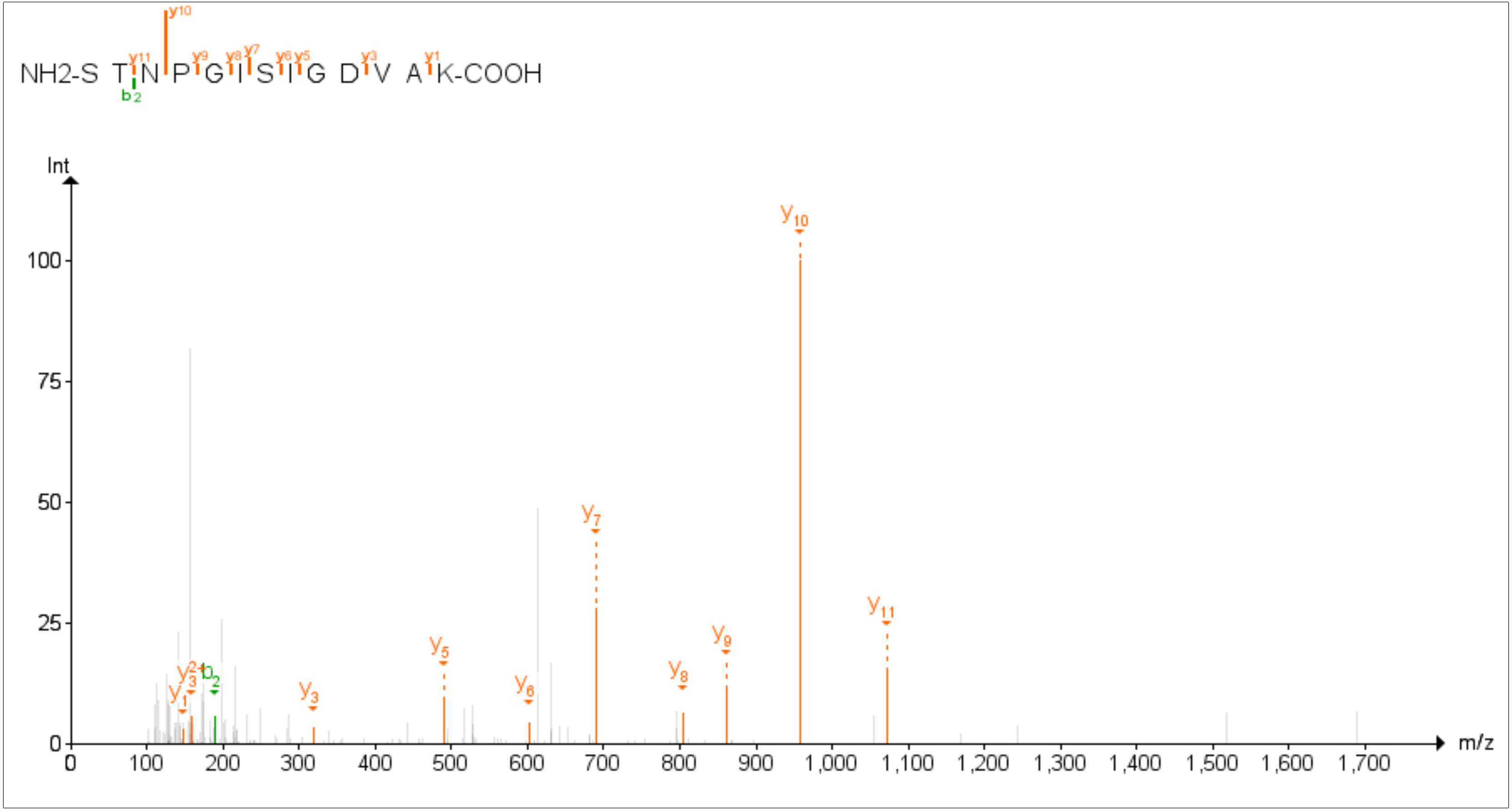

NH2-S T N P G I S I G D V A K-COOH
y11 y10 y9 y8 y7 y6 y5 y3 y1
b2
Int
100
75
50
25
0
m/z
0 100 200 300 400 500 600 700 800 900 1,000 1,100 1,200 1,300 1,400 1,500 1,600 1,700
y10
y7
y11
y9
y8
y6
y5
y3
y1
b2

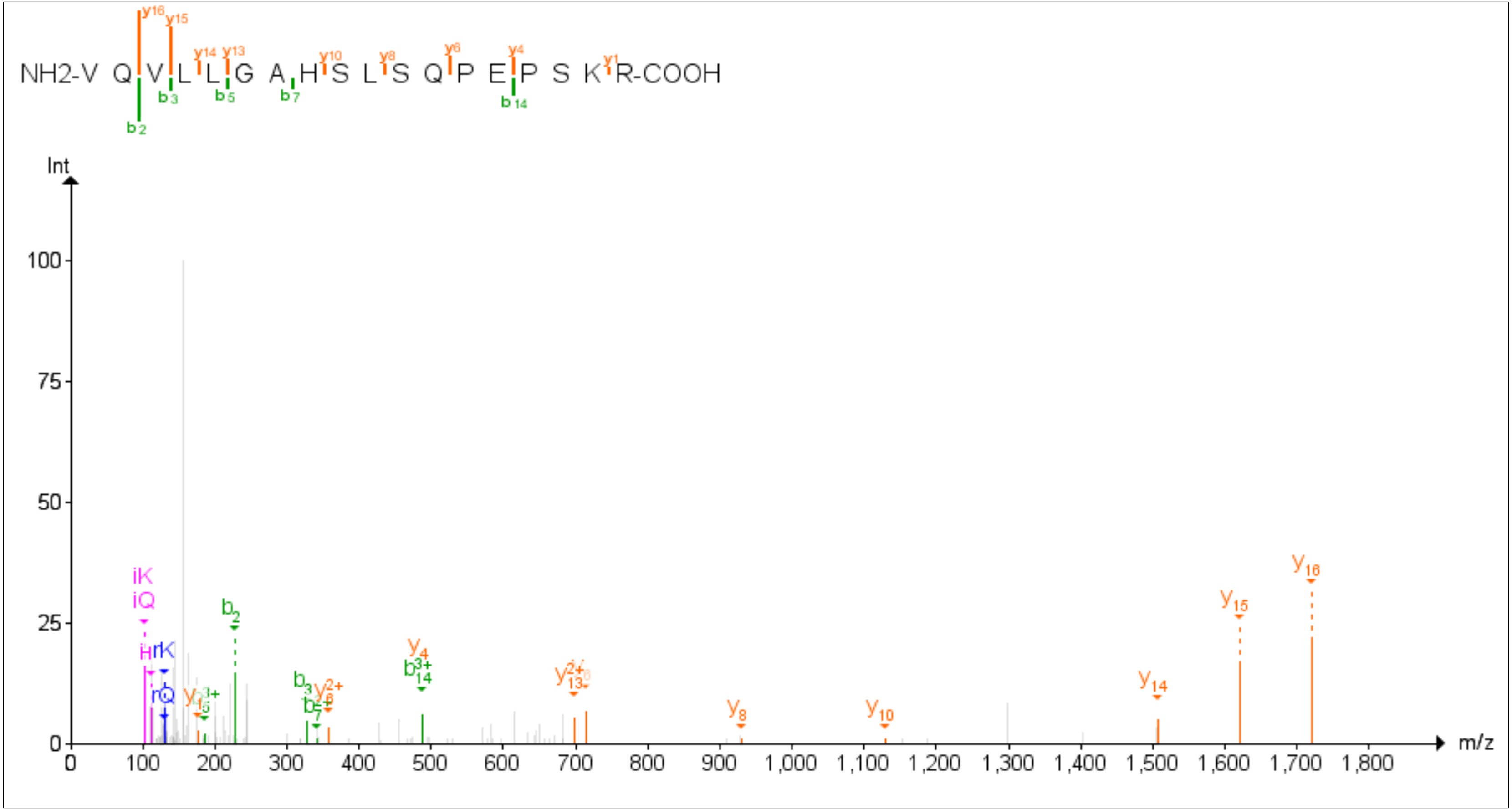

NH2-V Q V L L G A H S L S Q P E P S K R-COOH
y16 y15 y14 y13 y10 y8 y6 y4 y1
b2 b3 b5 b7 b14
Int
100
75
50
25
0
m/z
0 100 200 300 400 500 600 700 800 900 1,000 1,100 1,200 1,300 1,400 1,500 1,600 1,700 1,800
iK
iQ
iH
rK
rQ
b2
b3
y4
y8
y10
y14
y15
y16

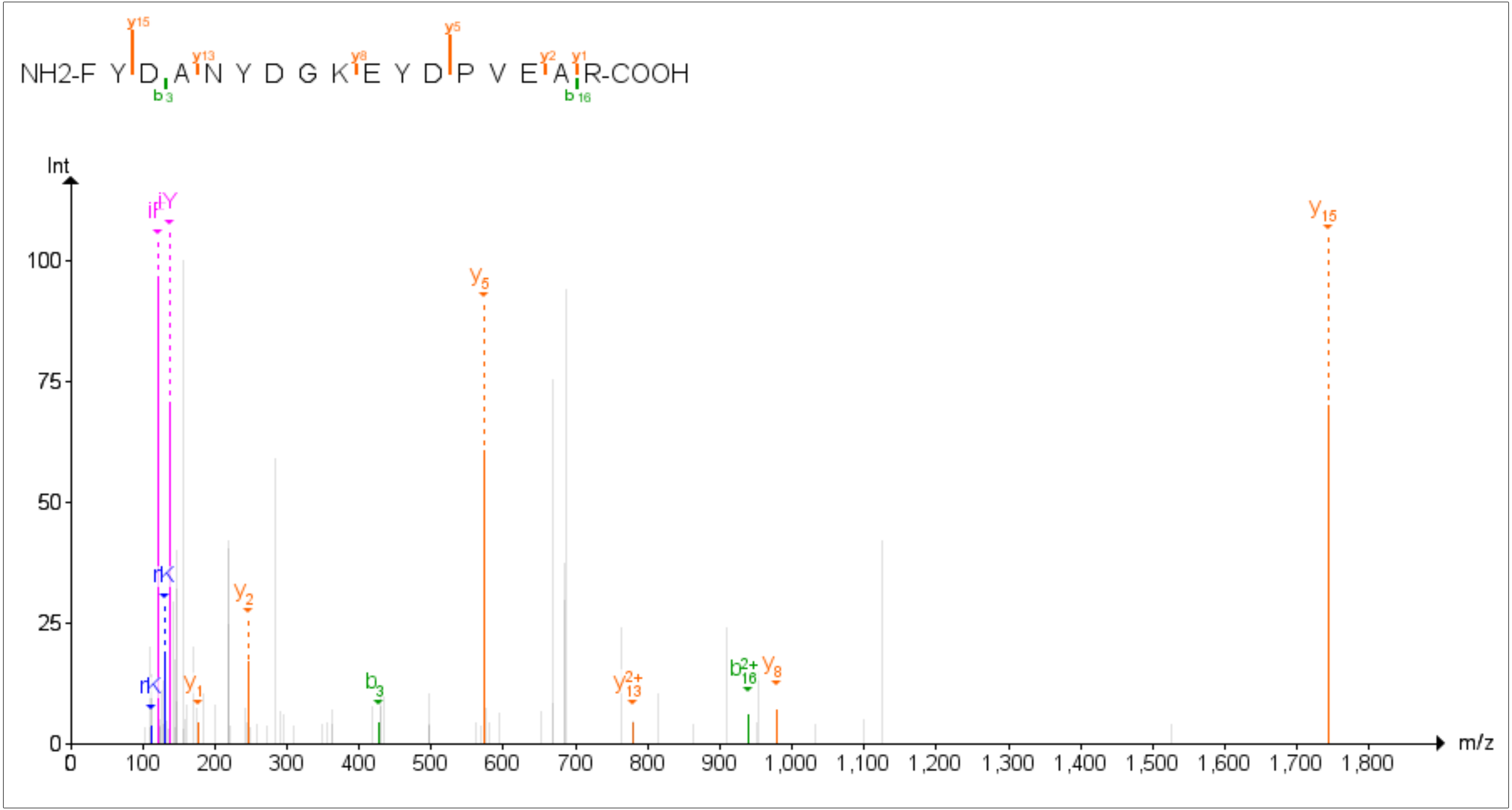

NH2-F Y D A N Y D G K E Y D P V E A R-COOH
y15
y13
y8
y5
y2
y1
b3
b 16
Int
m/z
iF
iY
rK
y1
y2
b3
y5
y13 2+
b16 2+
y8
y15
0
25
50
75
100
0
100
200
300
400
500
600
700
800
900
1,000
1,100
1,200
1,300
1,400
1,500
1,600
1,700
1,800

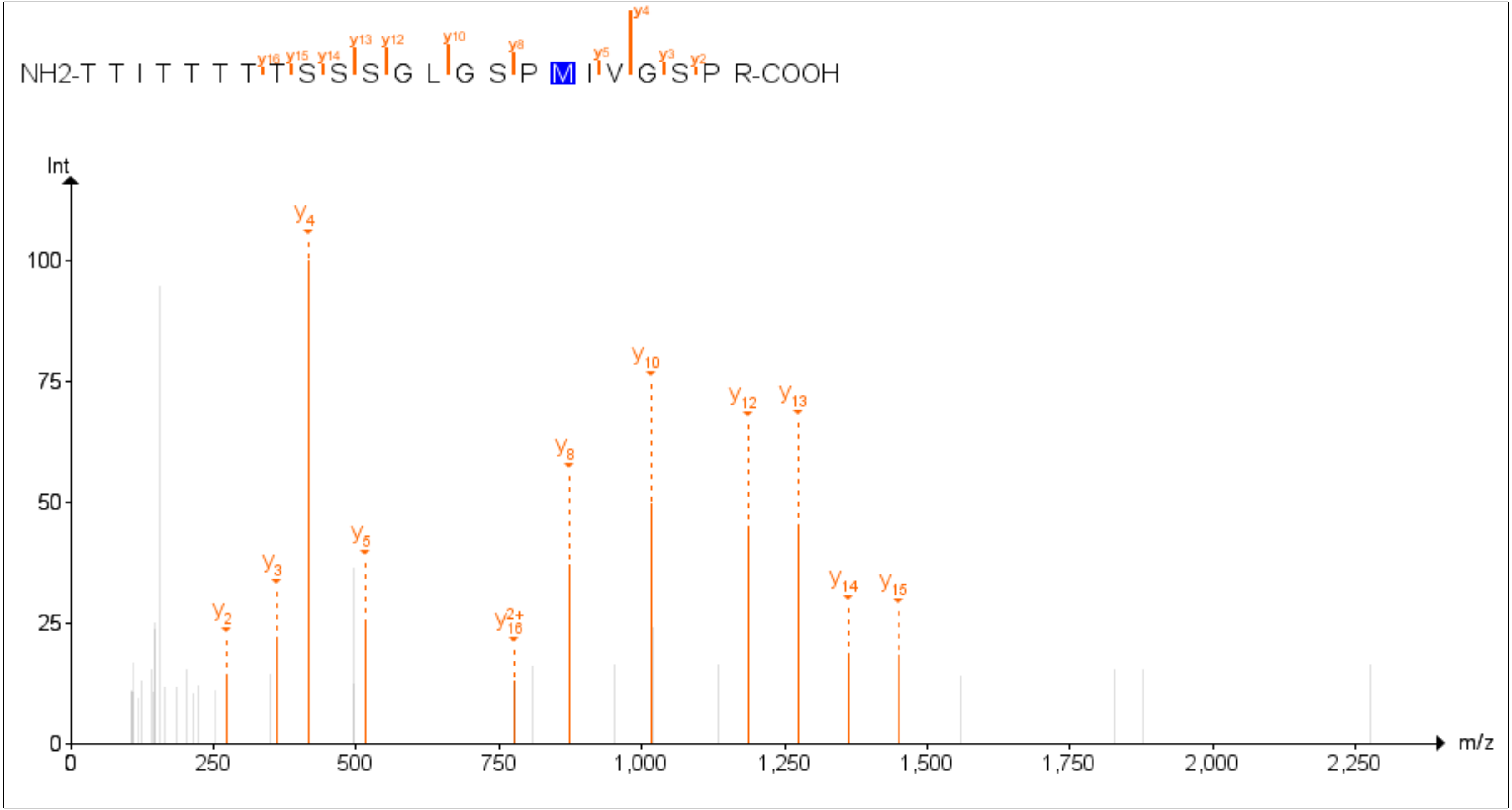

NH2-T T I T T T T T S S S G L G S P M I V G S P R-COOH
y16 y15 y14 y13 y12 y10 y8 y5 y4 y3 y2
Int
100
75
50
25
0
y4
y10
y8
y12
y13
y5
y3
y2
y16 2+
y14
y15
0
250
500
750
1,000
1,250
1,500
1,750
2,000
2,250
m/z

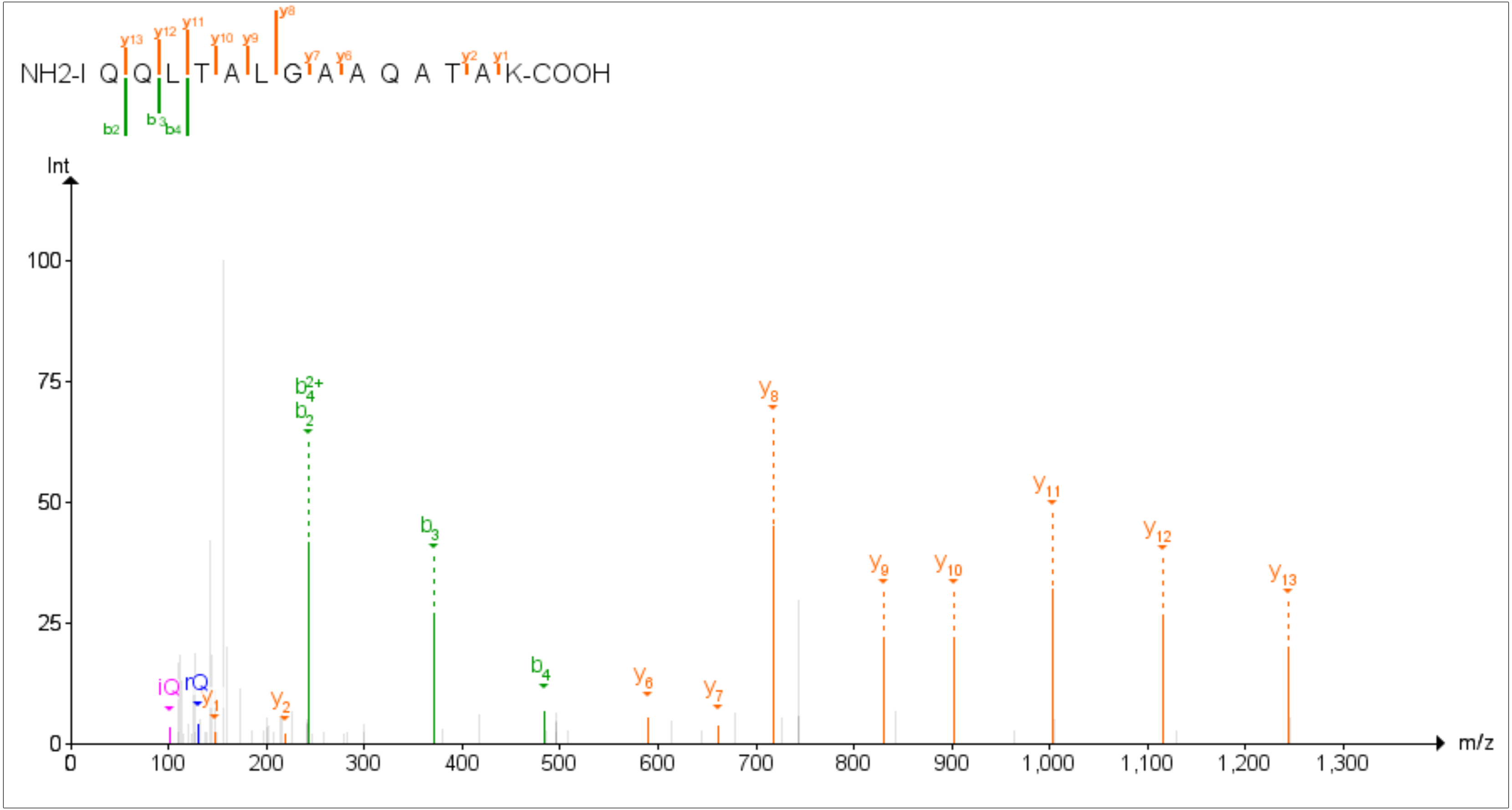

NH2-I Q Q L T A L G A A Q A T A K-COOH
y13 y12 y11 y10 y9 y8 y7 y6 y2 y1
b2 b3 b4
Int
m/z
iQ
rQ
y1
y2
b4 2+
b2
b3
b4
y6
y7
y8
y9
y10
y11
y12
y13

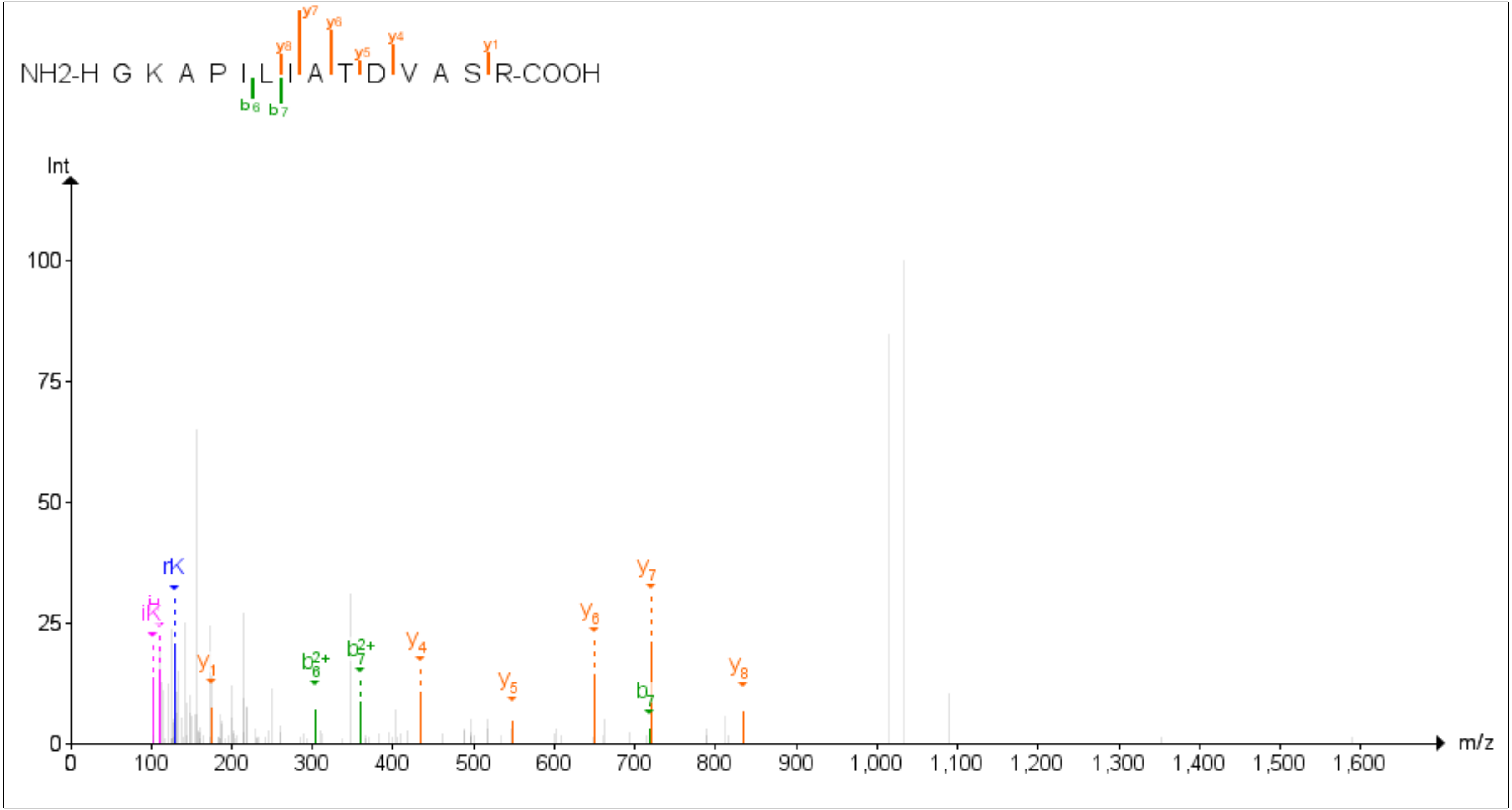

NH2-H G K A P I L I A T D V A S R-COOH
y7
y6
y8
y5
y4
y1
b6
b7
Int
100
75
50
25
0
rK
iK
y1
b6 2+
b7 2+
y4
y5
y6
y7
b7
y8
0
100
200
300
400
500
600
700
800
900
1,000
1,100
1,200
1,300
1,400
1,500
1,600
m/z

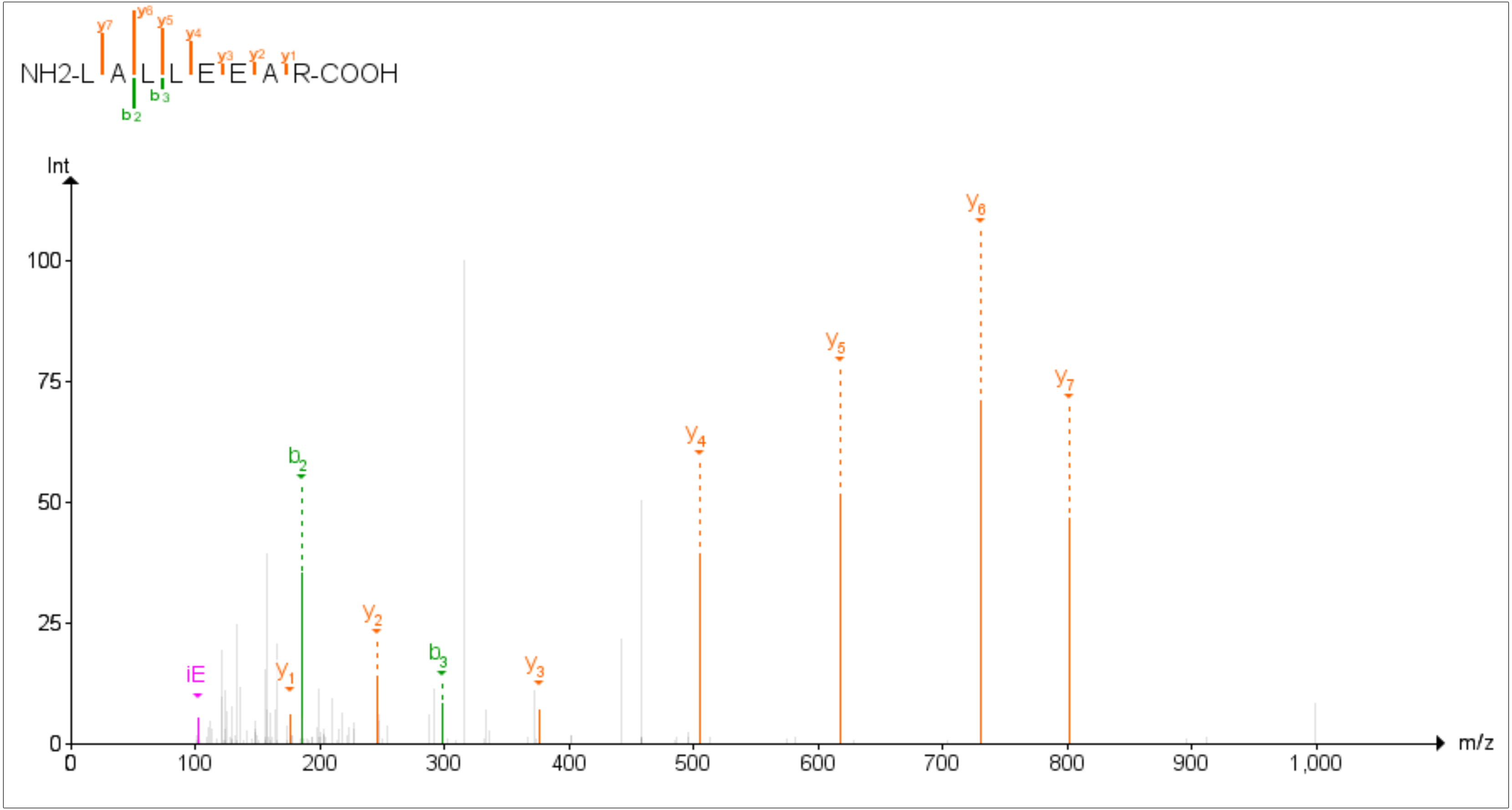

NH2-L A L L E E A R-COOH
y7
y6
y5
y4
y3
y2
y1
b2
b3
Int
100
75
50
25
0
m/z
0
100
200
300
400
500
600
700
800
900
1,000
iE
y1
b2
y2
b3
y3
y4
y5
y6
y7

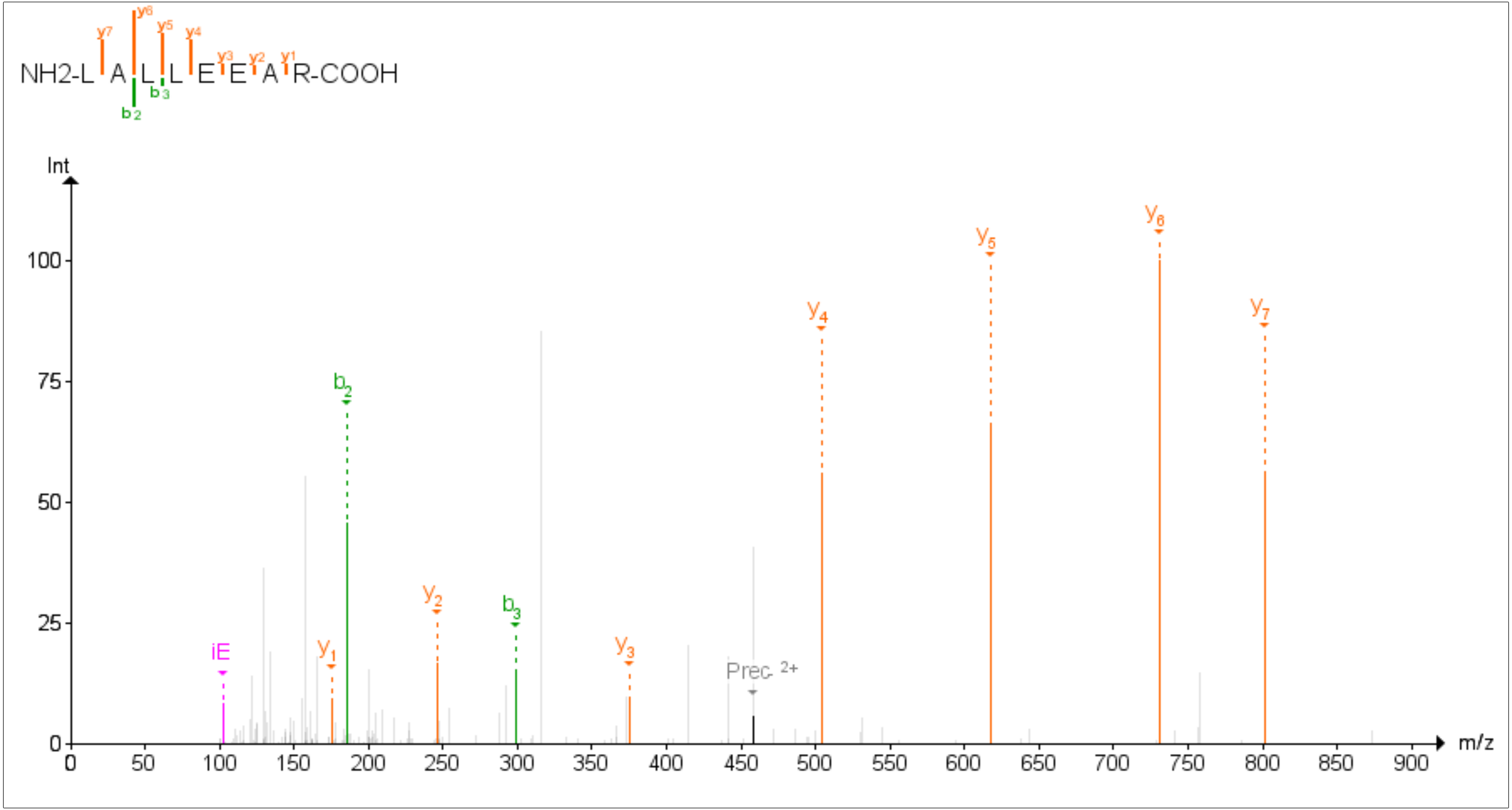

NH2-L A L L E E A R-COOH
y7 y6 y5 y4 y3 y2 y1
b2 b3
Int
100
75
50
25
0
m/z
0 50 100 150 200 250 300 350 400 450 500 550 600 650 700 750 800 850 900
iE
y1
b2
y2
b3
y3
Prec 2+
y4
y5
y6
y7

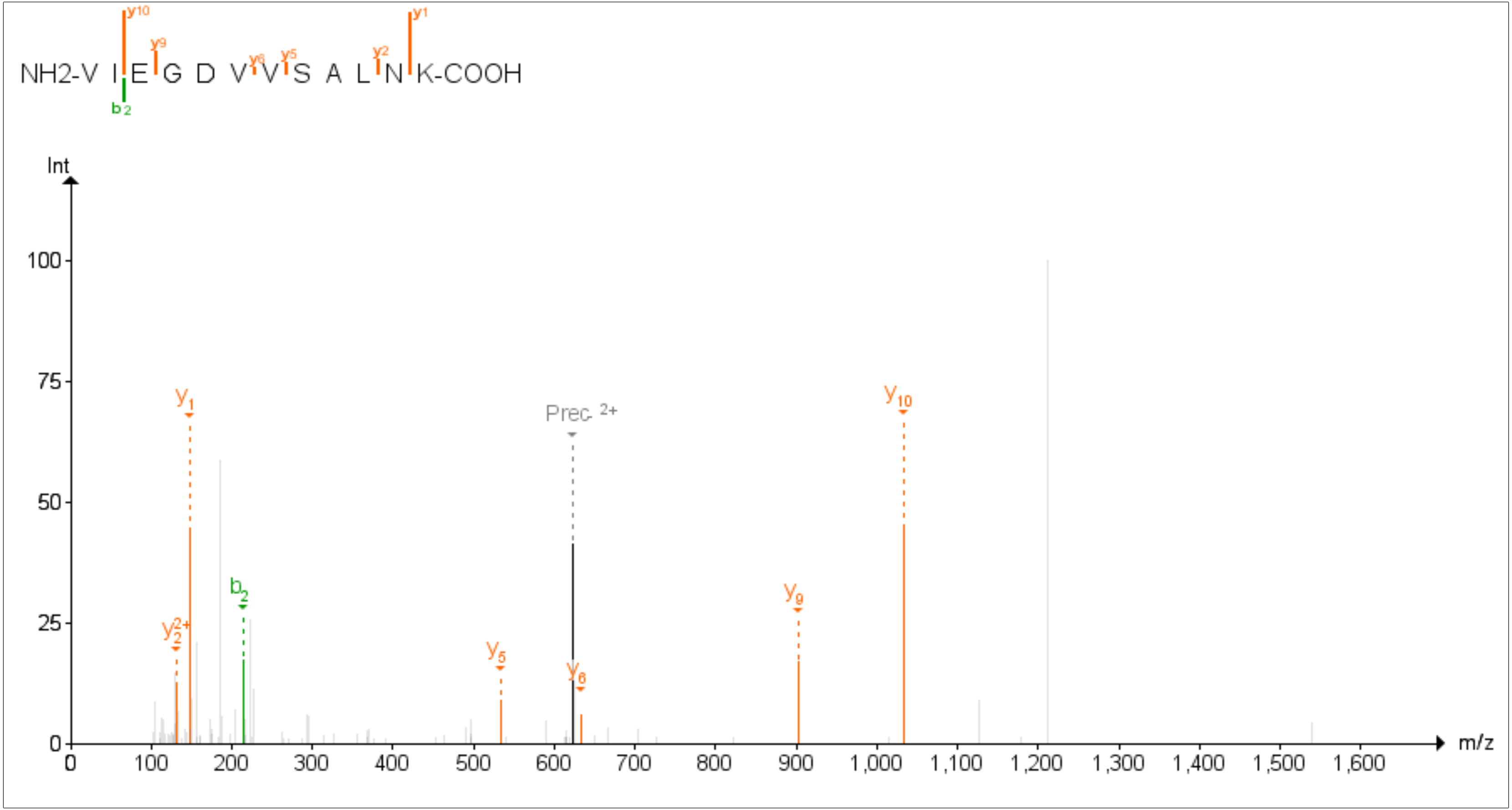

NH2-V I E G D V V S A L N K-COOH
y10
y9
y6
y5
y2
y1
b2
Int
m/z
100
75
50
25
0
y1
Prec 2+
y10
b2
y2 2+
y9
y5
y6
0
100
200
300
400
500
600
700
800
900
1,000
1,100
1,200
1,300
1,400
1,500
1,600

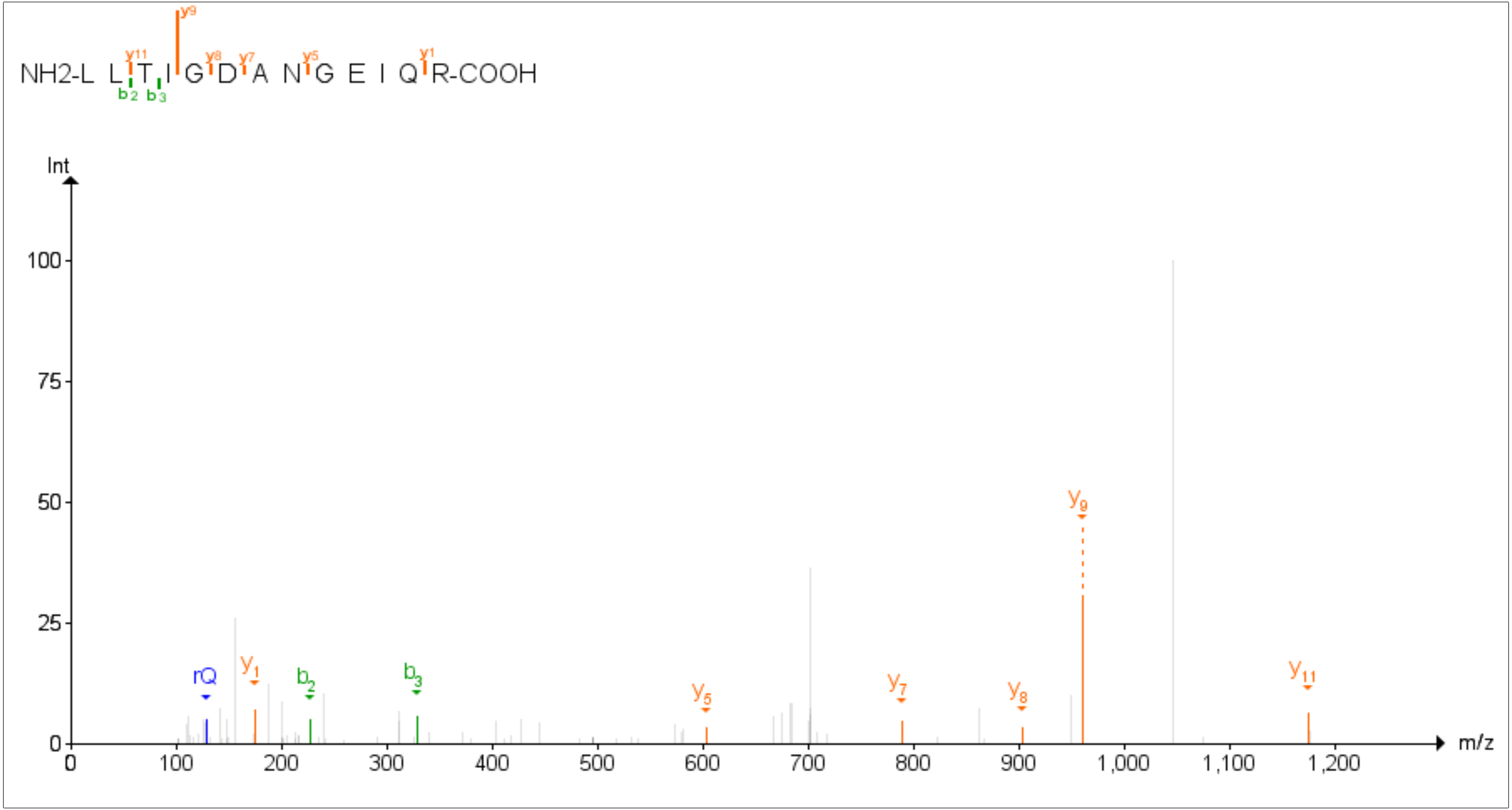

NH2-L L T I G D A N G E I Q R-COOH
y11 y9 y8 y7 y5 y1
b2 b3
Int
100
75
50
25
0
rQ
y1
b2
b3
y5
y7
y8
y9
y11
0 100 200 300 400 500 600 700 800 900 1,000 1,100 1,200
m/z

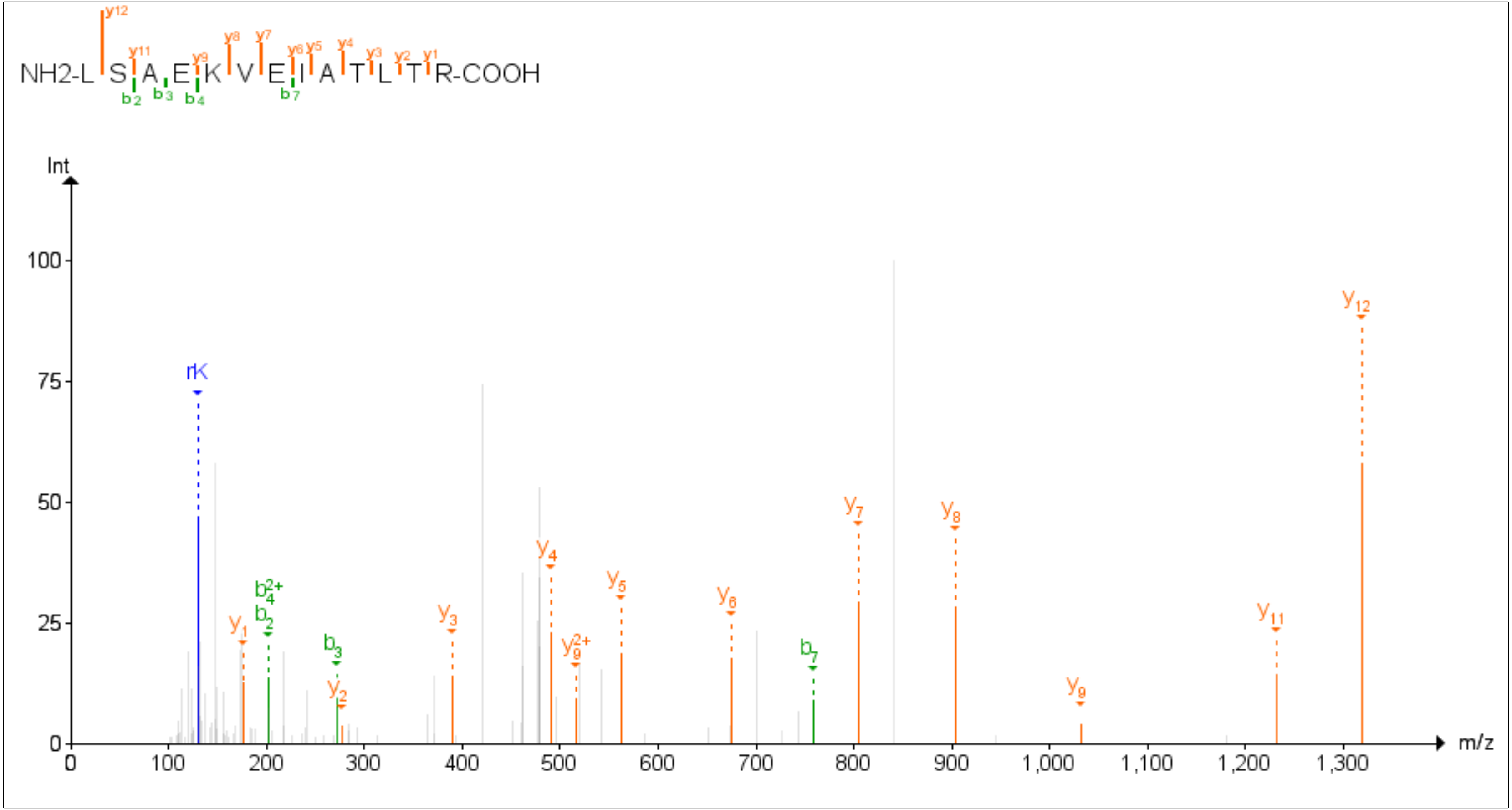
NH2-LSAEKVEIATLTR-COOH
y12
y11
y9
y8
y7
y6
y5
y4
y3
y2
y1
b2
b3
b4
b7
Int
100
75
50
25
0
rK
b4 2+
b2
y1
b3
y2
y3
y4
y9 2+
y5
y6
b7
y7
y8
y9
y11
y12
0
100
200
300
400
500
600
700
800
900
1,000
1,100
1,200
1,300
m/z

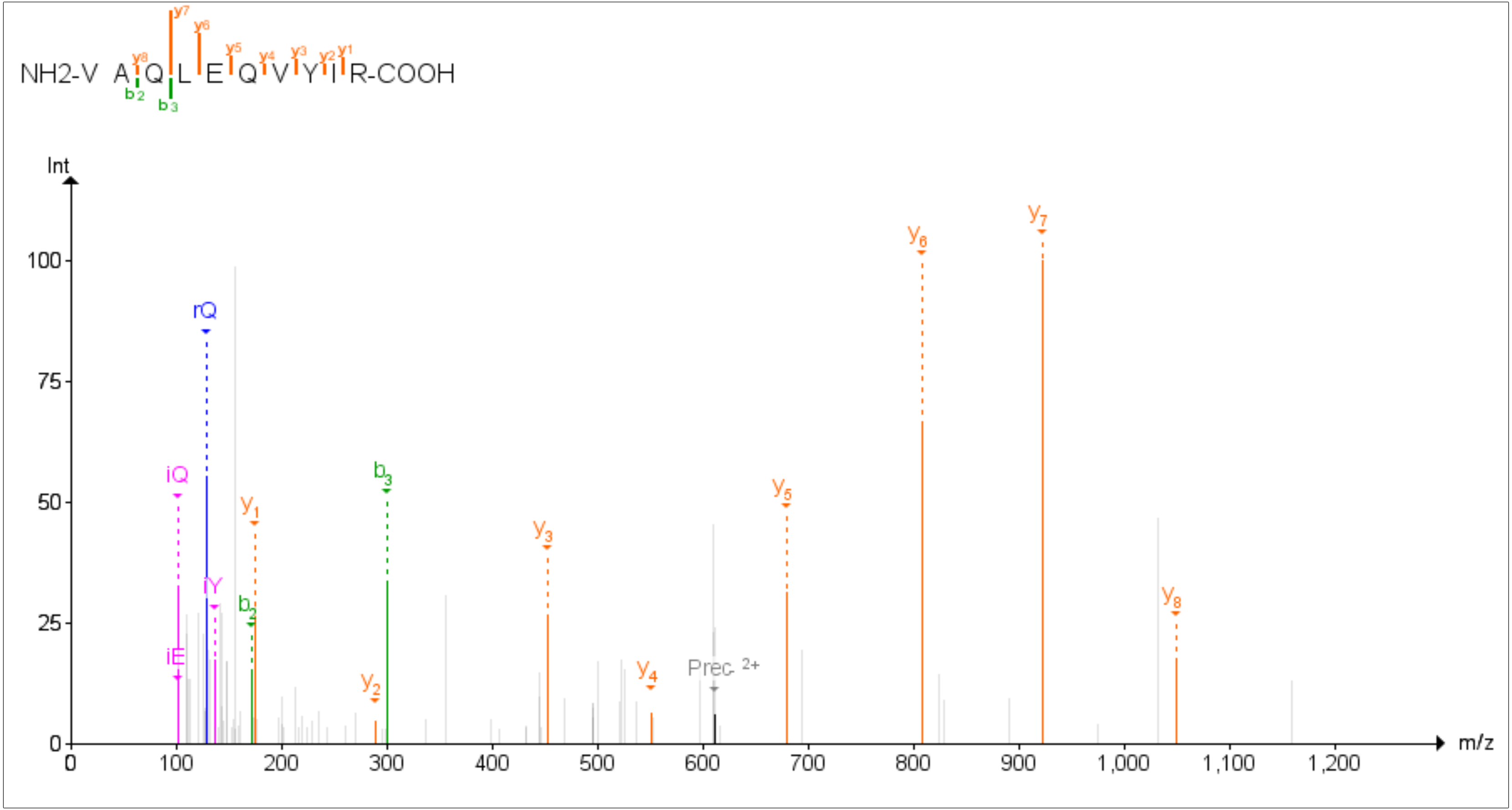

NH2-V A Q L E Q V Y I R-COOH
y8 y7 y6 y5 y4 y3 y2 y1
b2 b3
Int
m/z
rQ
iQ
iY
iE
y1
b2
b3
y2
y3
y4
Prec 2+
y5
y6
y7
y8

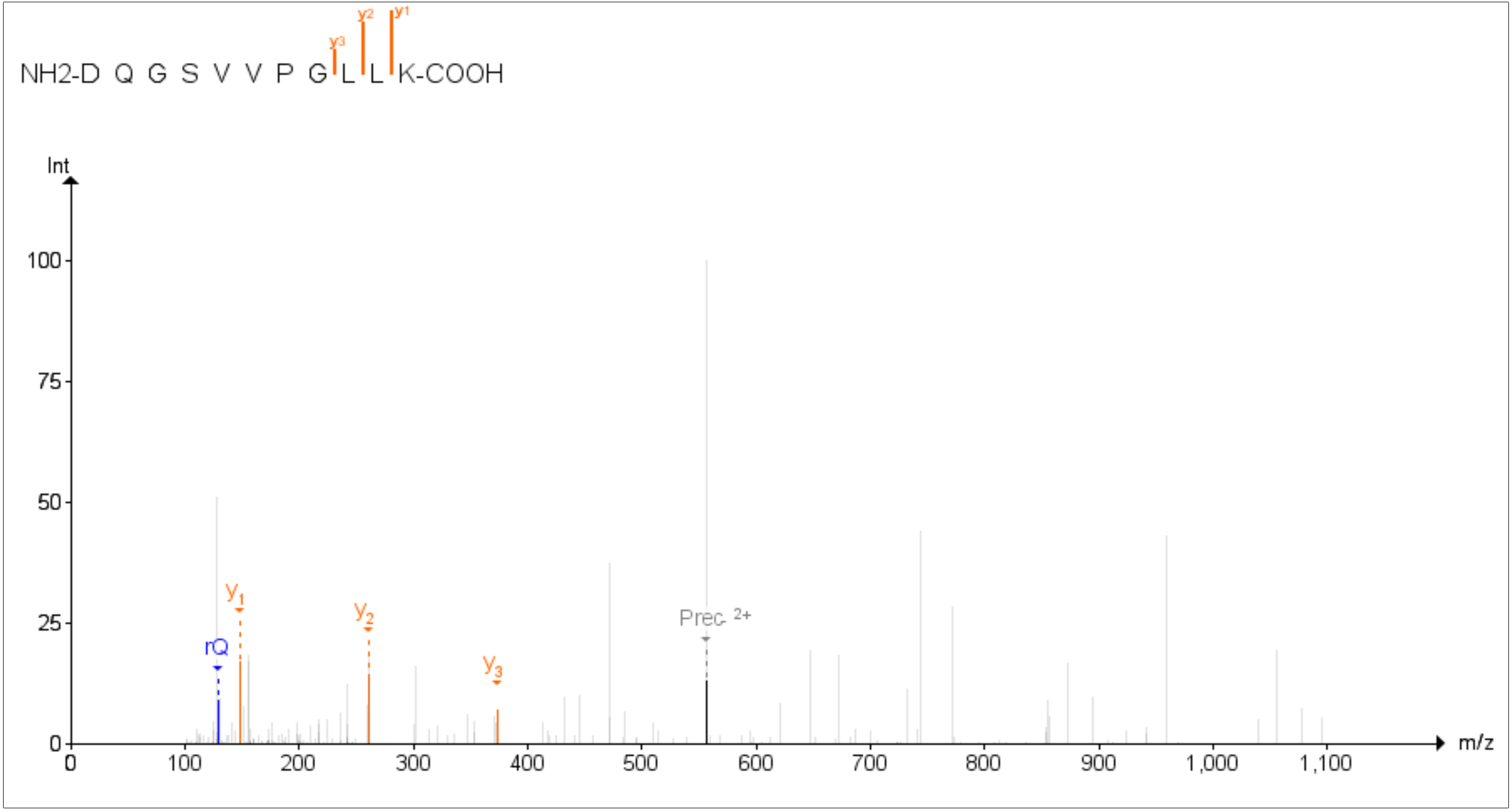

y3
y2
y1
NH2-D Q G S V V P G L L K-COOH
Int
100
75
50
25
0
y1
y2
y3
rQ
Prec 2+
0
100
200
300
400
500
600
700
800
900
1,000
1,100
m/z

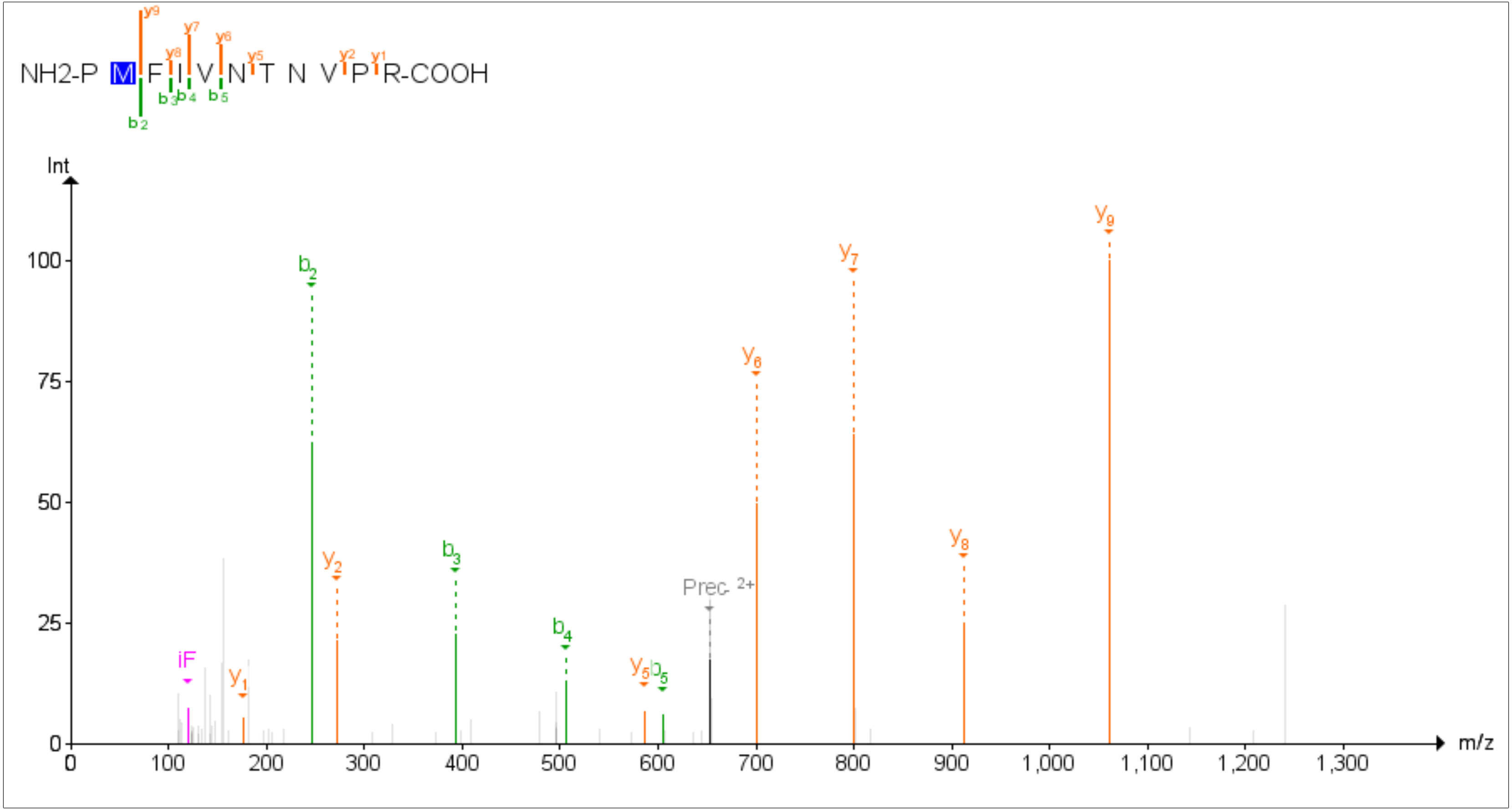

NH2-P M F I V N T N V P R-COOH
y9
y8
y7
y6
y5
y2
y1
b2
b3
b4
b5
Int
100
75
50
25
0
iF
Prec 2+
m/z
0
100
200
300
400
500
600
700
800
900
1,000
1,100
1,200
1,300

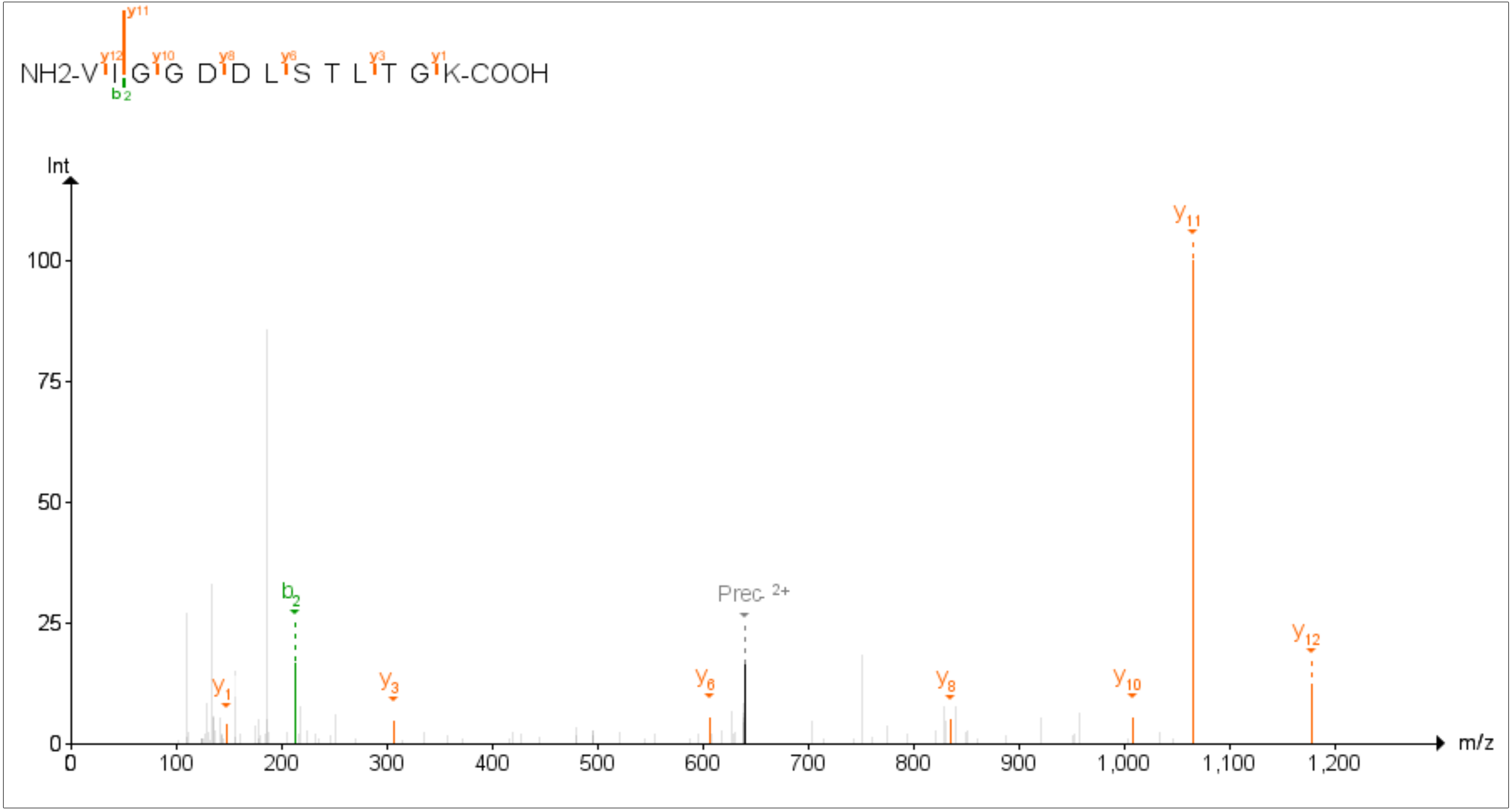

NH2-V I G G D D L S T L T G K-COOH
y12
y11
y10
y8
y6
y3
y1
b2
Int
100
75
50
25
0
y11
b2
Prec 2+
y12
y1
y3
y6
y8
y10
0
100
200
300
400
500
600
700
800
900
1,000
1,100
1,200
m/z

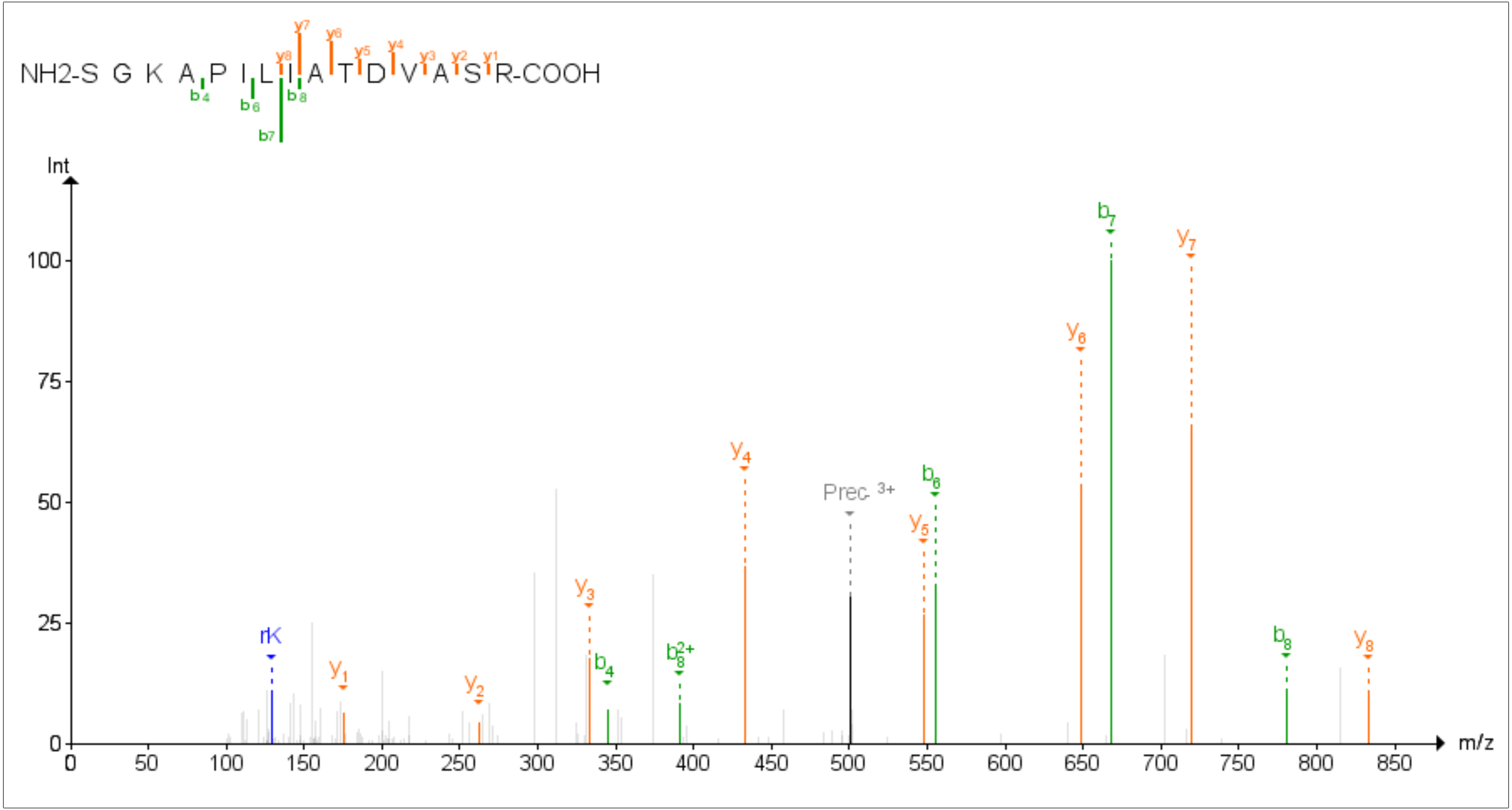
NH2-S G K A P I L I A T D V A S R-COOH
y7 y6 y8 y5 y4 y3 y2 y1
b4 b6 b8 b7
Int
m/z
Prec 3+
rK

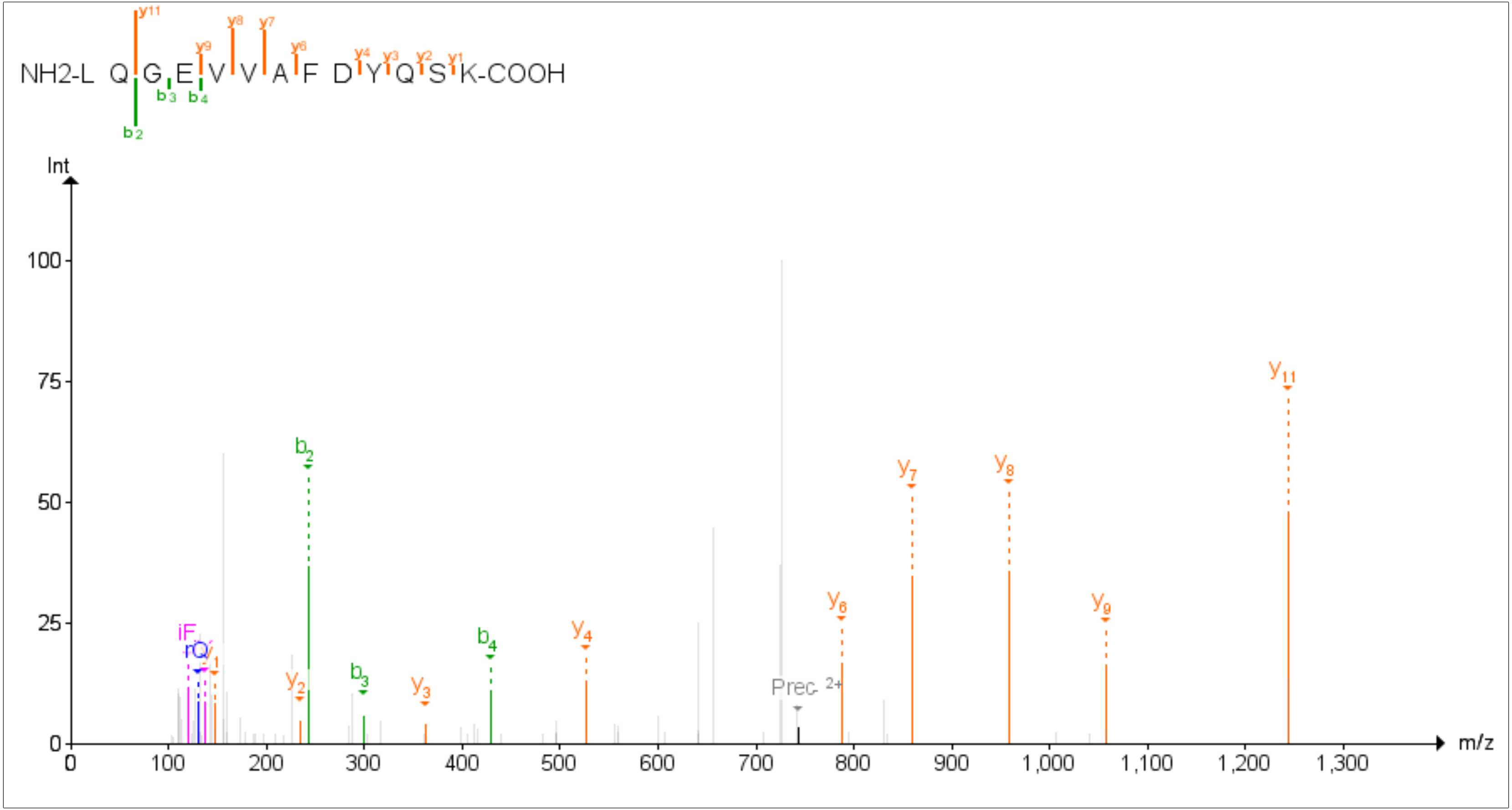

NH2-L Q G E V V A F D Y Q S K-COOH
Int
m/z
Prec 2+

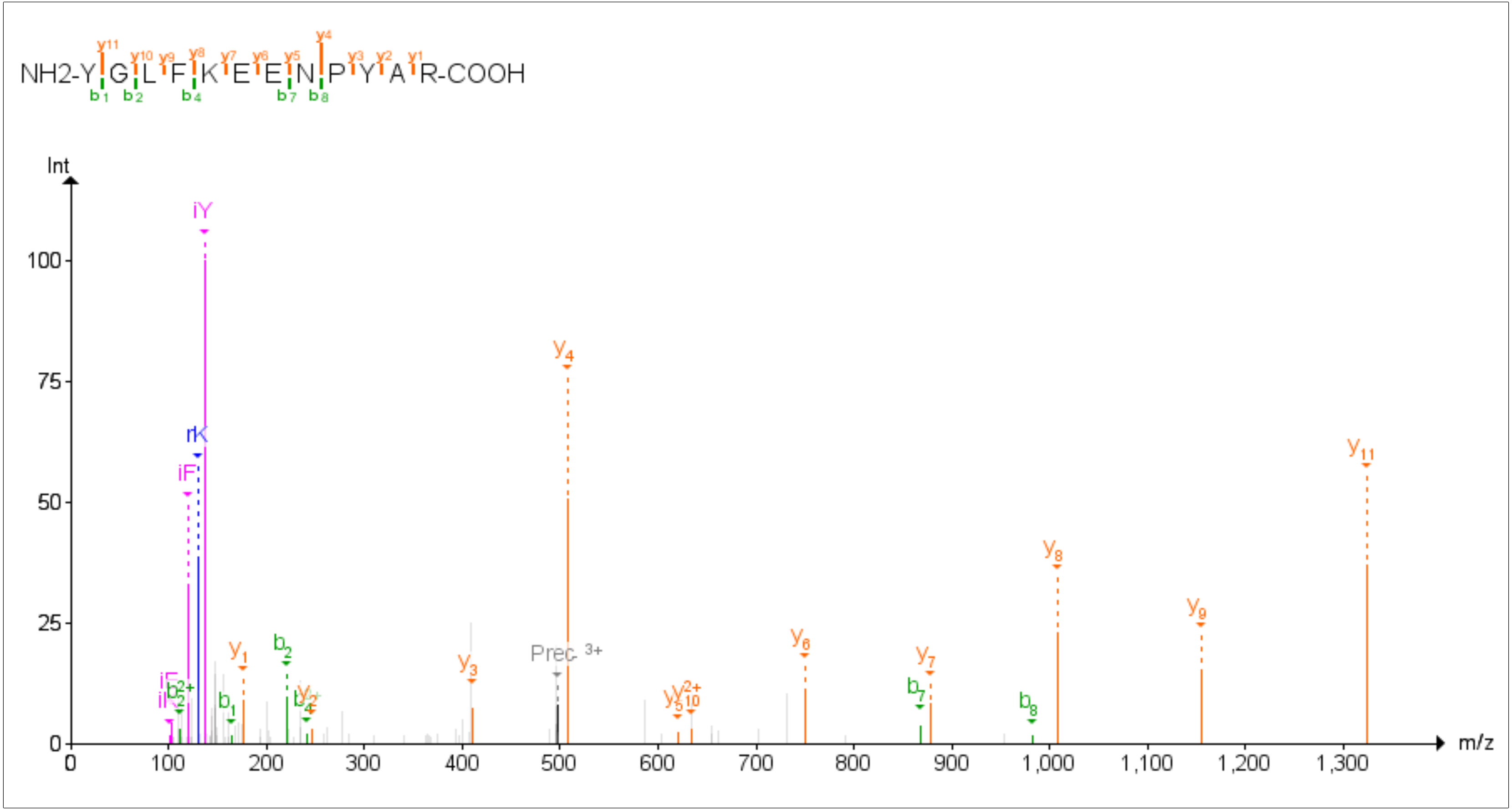
NH2-Y G L F K E E N P Y A R-COOH
y11 y10 y9 y8 y7 y6 y5 y4 y3 y2 y1
b1 b2 b4 b7 b8
Int
100
75
50
25
0
iY
y4
y11
rK
iF
y8
y9
y1
b2
y3
Prec 3+
y6
y7
b7
b8
m/z
0
100
200
300
400
500
600
700
800
900
1,000
1,100
1,200
1,300

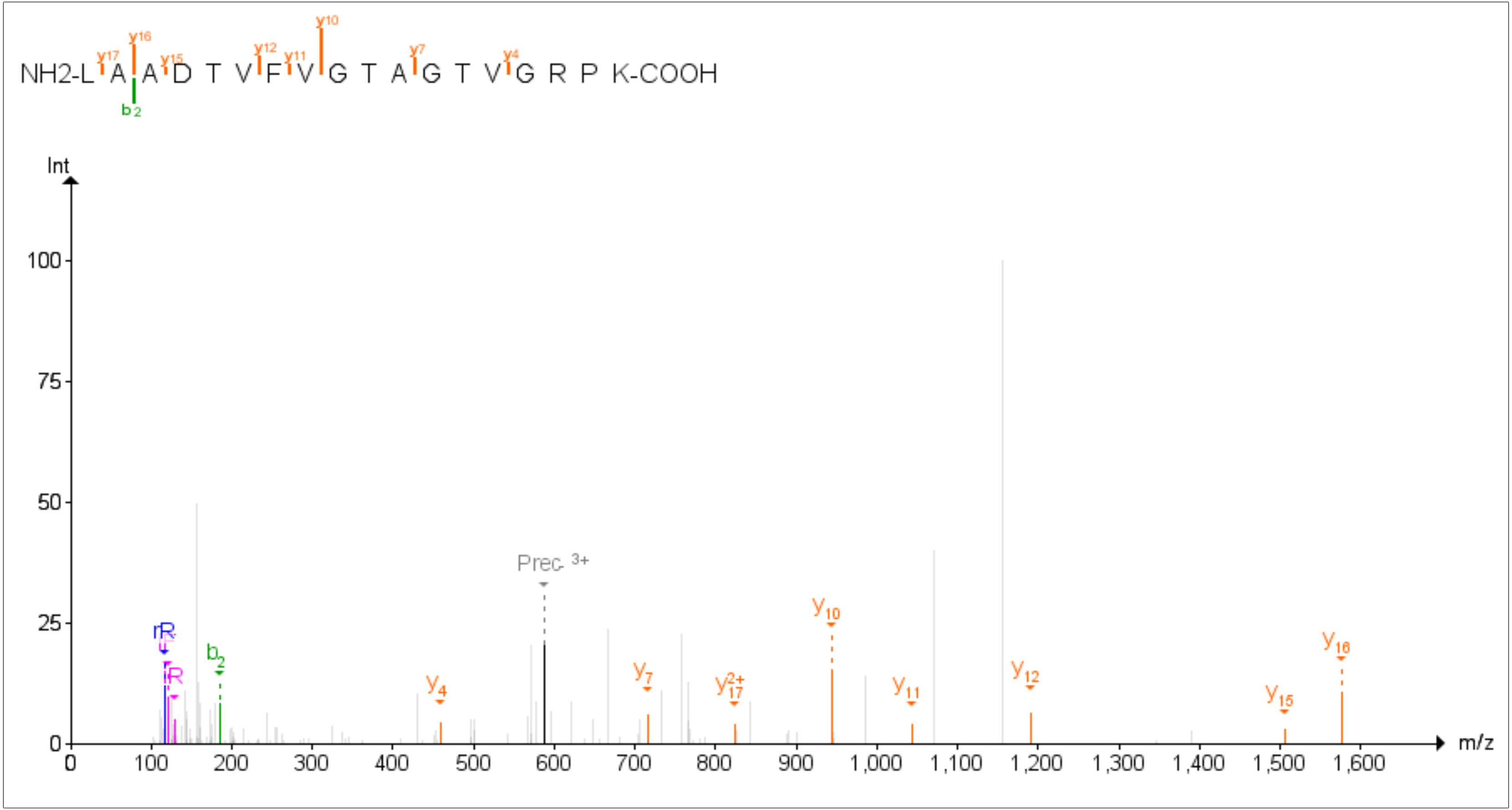

NH2-L A A D T V F V G T A G T V G R P K-COOH
y17 y16 y15 y12 y11 y10 y7 y4
b2
Int
100
75
50
25
0
Prec 3+
y10
y16
y12
y7
y17 2+
y11
y15
y4
b2
m/z
0 100 200 300 400 500 600 700 800 900 1,000 1,100 1,200 1,300 1,400 1,500 1,600

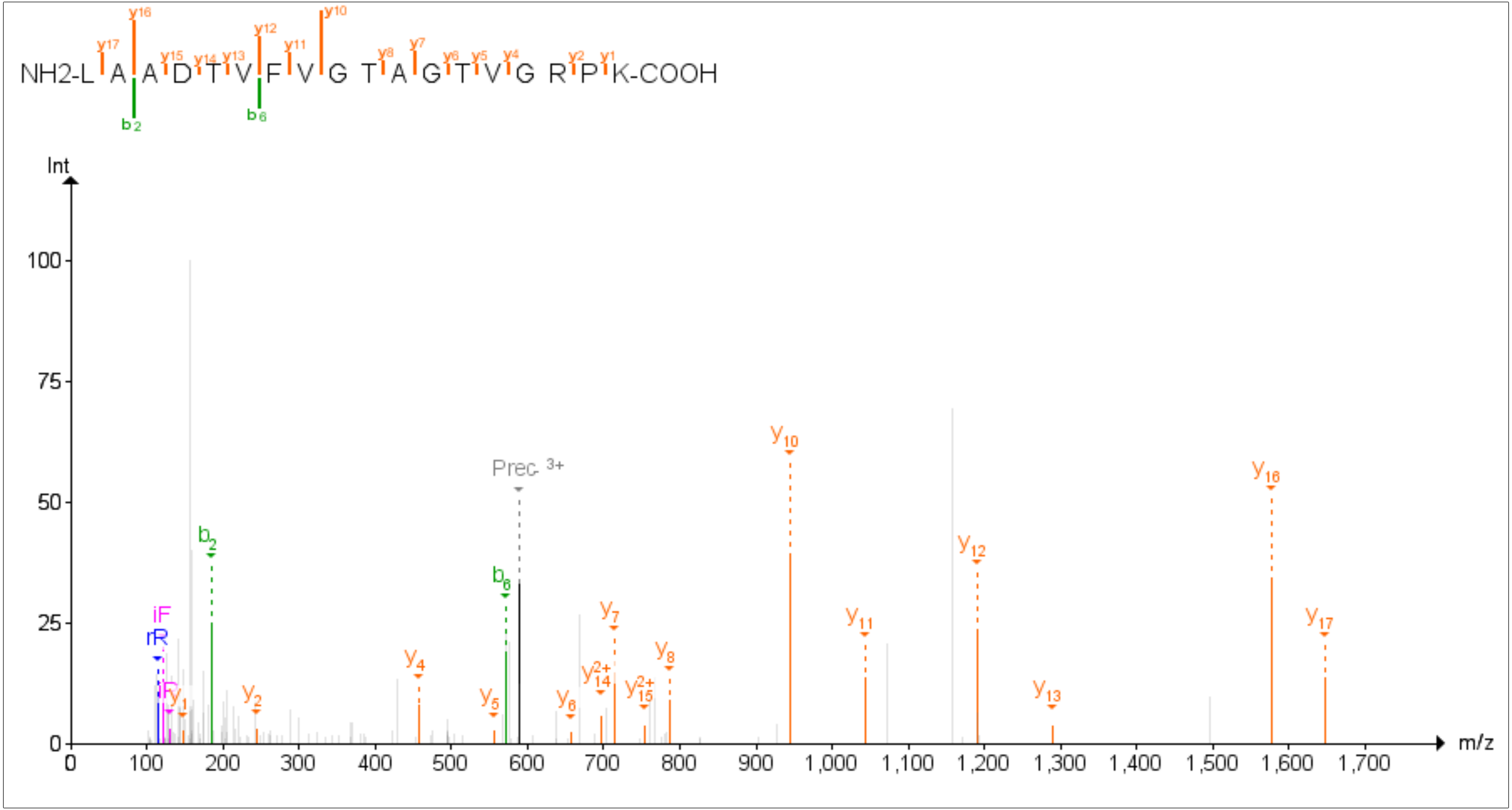
NH2-L A A D T V F V G T A G T V G R P K-COOH
y17 y16 y15 y14 y13 y12 y11 y10 y8 y7 y6 y5 y4 y2 y1
b2 b6
Int
m/z
Prec 3+
y10
y16
y12
b2
b6
y7
y11
y17
iF
rR
y4
y8
y14 2+
y15 2+
y2
y1
y5
y6
y13
0
25
50
75
100
0 100 200 300 400 500 600 700 800 900 1,000 1,100 1,200 1,300 1,400 1,500 1,600 1,700

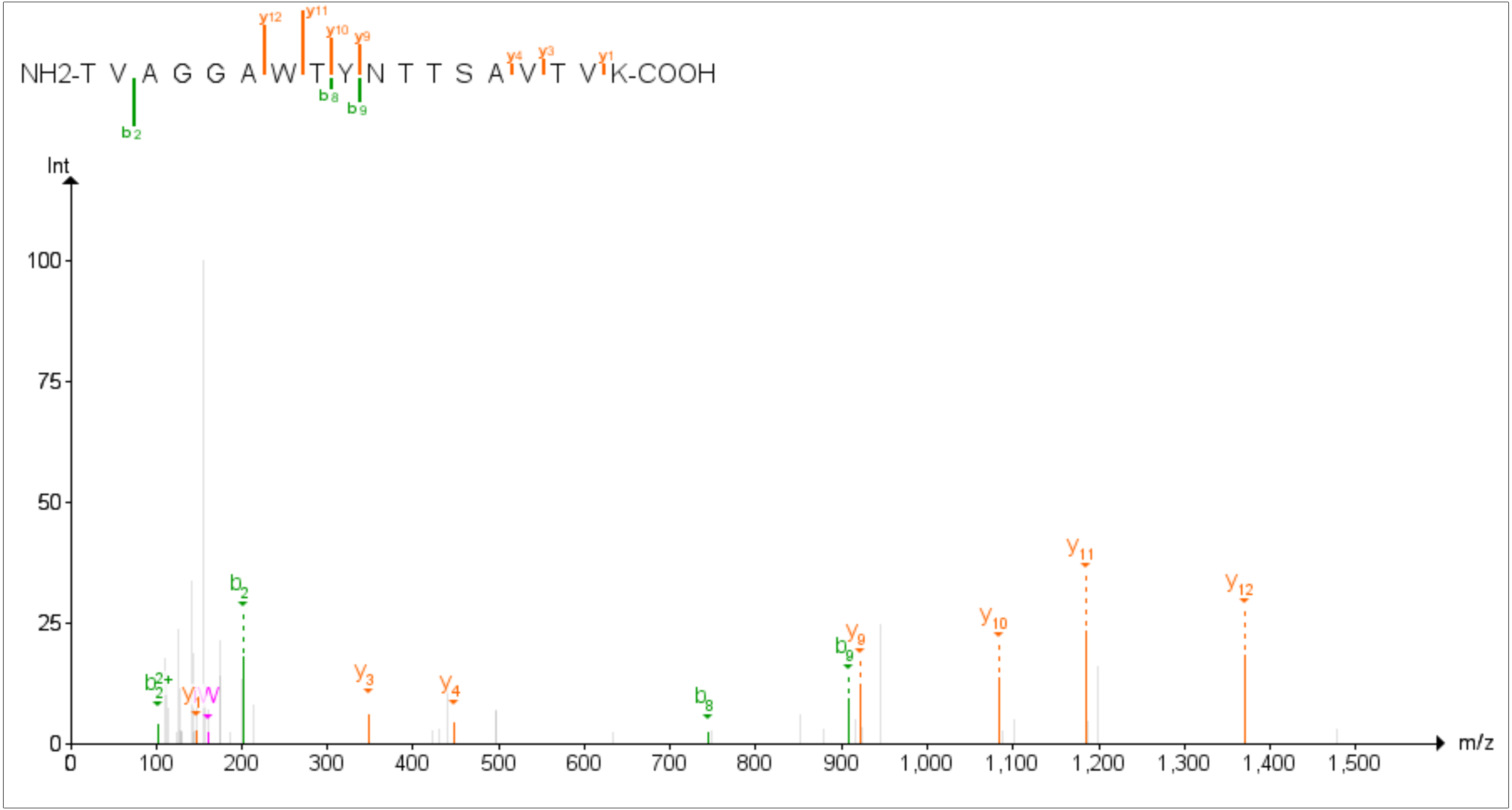

NH2-T V A G G A W T Y N T T S A V T V K-COOH
y12
y11
y10
y9
y4
y3
y1
b2
b8
b9
Int
100
75
50
25
0
m/z
0
100
200
300
400
500
600
700
800
900
1,000
1,100
1,200
1,300
1,400
1,500
b2 2+
y1
W
b2
y3
y4
b8
b9
y9
y10
y11
y12

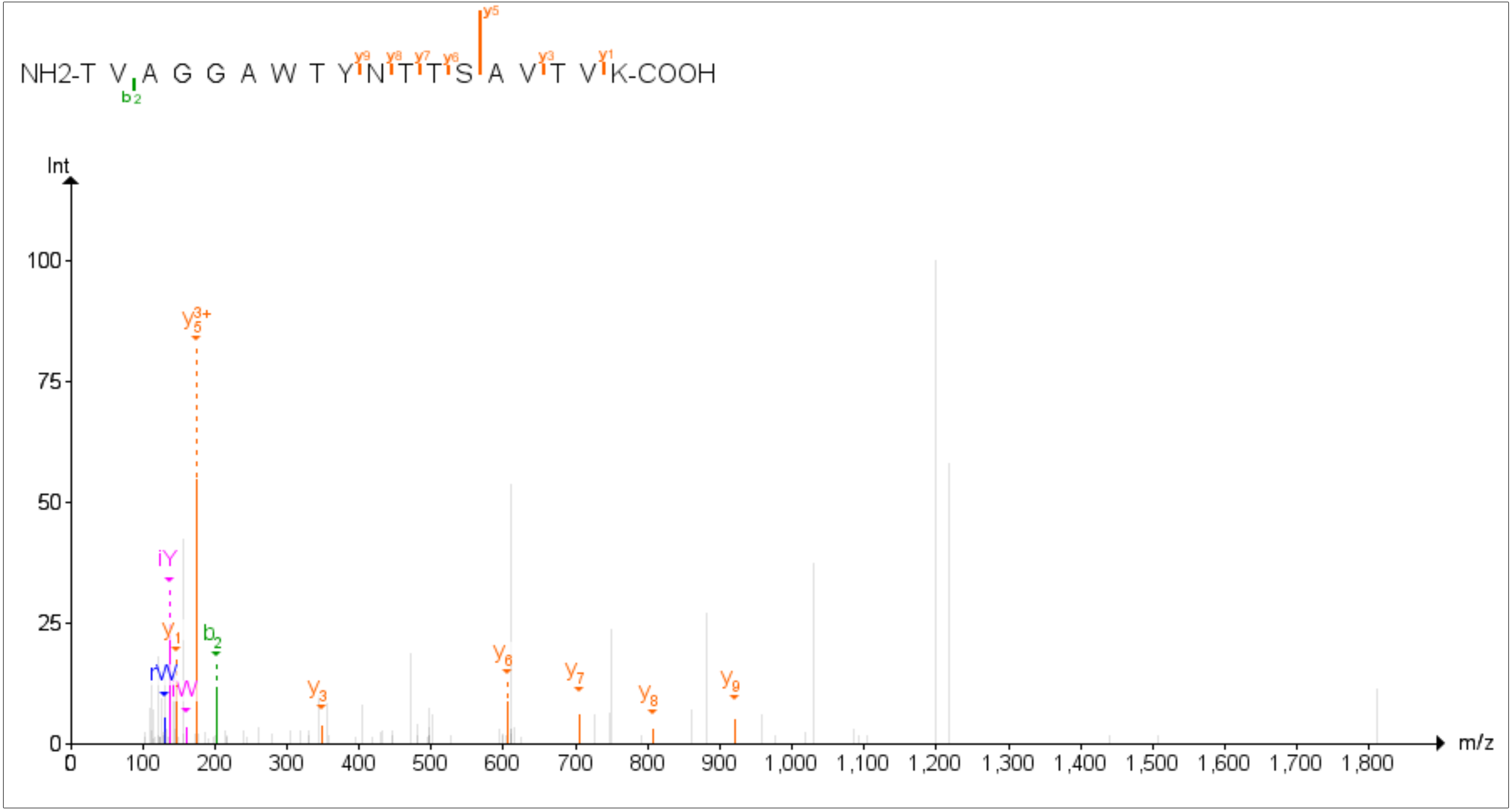
NH2-T V A G G A W T Y N T T S A V T V K-COOH
b2
y9 y8 y7 y6 y5 y3 y1
Int
m/z
100
75
50
25
0
y_5^{3+}
iY
y_1
b_2
rW
iW
y_3
y_6
y_7
y_8
y_9
0 100 200 300 400 500 600 700 800 900 1,000 1,100 1,200 1,300 1,400 1,500 1,600 1,700 1,800

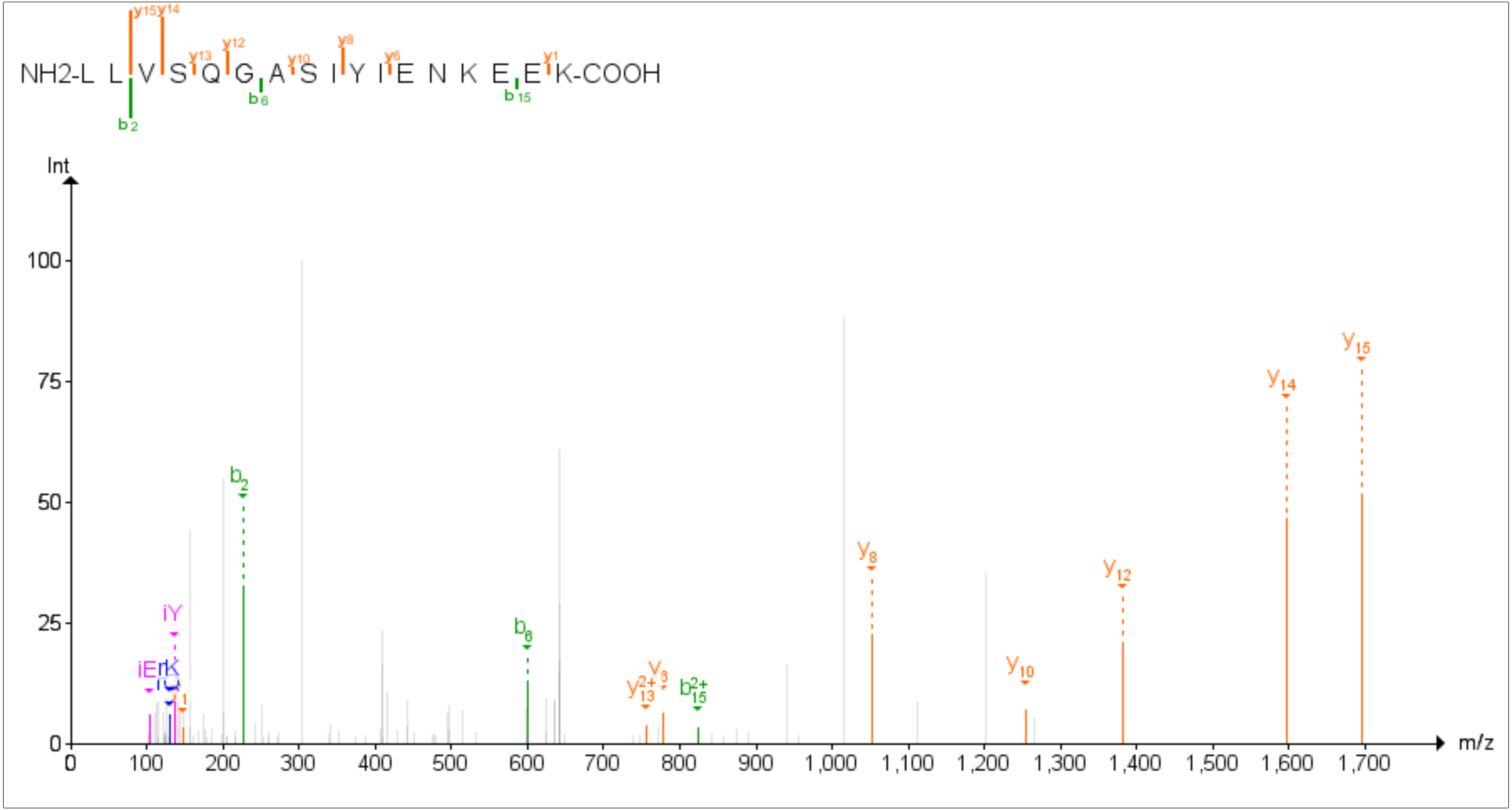
NH2-L L V S Q G A S I Y I E N K E E K-COOH
y15 y14 y13 y12 y10 y8 y6 y1
b2 b6 b15
Int
m/z
b2
b6
iY
iE
y8
y12
y10
y14
y15
y13 2+
b15 2+
0
25
50
75
100
0
100
200
300
400
500
600
700
800
900
1,000
1,100
1,200
1,300
1,400
1,500
1,600
1,700

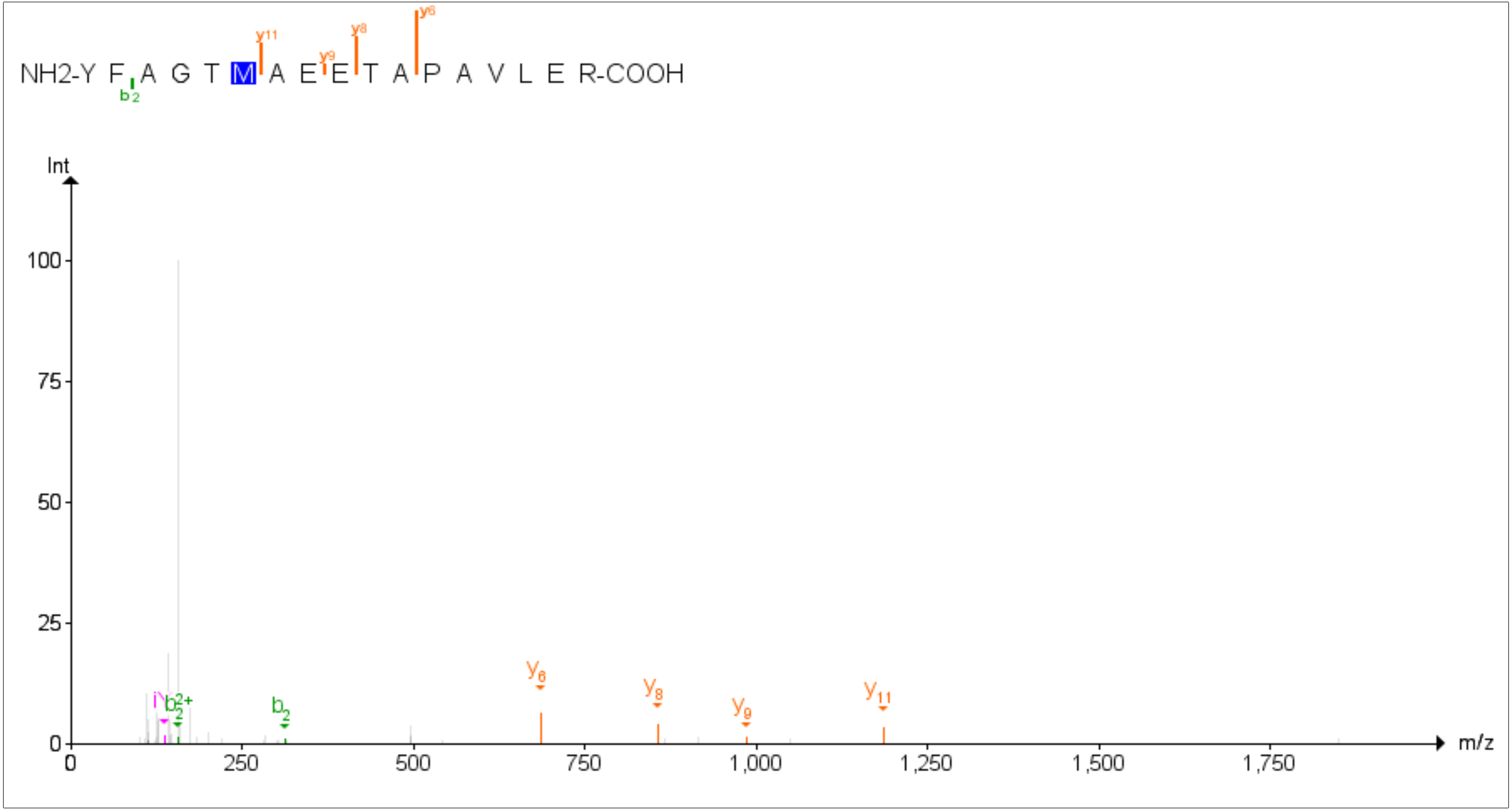

NH2-Y F A G T M A E E T A P A V L E R-COOH
b2
y11
y9
y8
y6
Int
m/z
100
75
50
25
0
250
500
750
1,000
1,250
1,500
1,750
b2
y6
y8
y9
y11

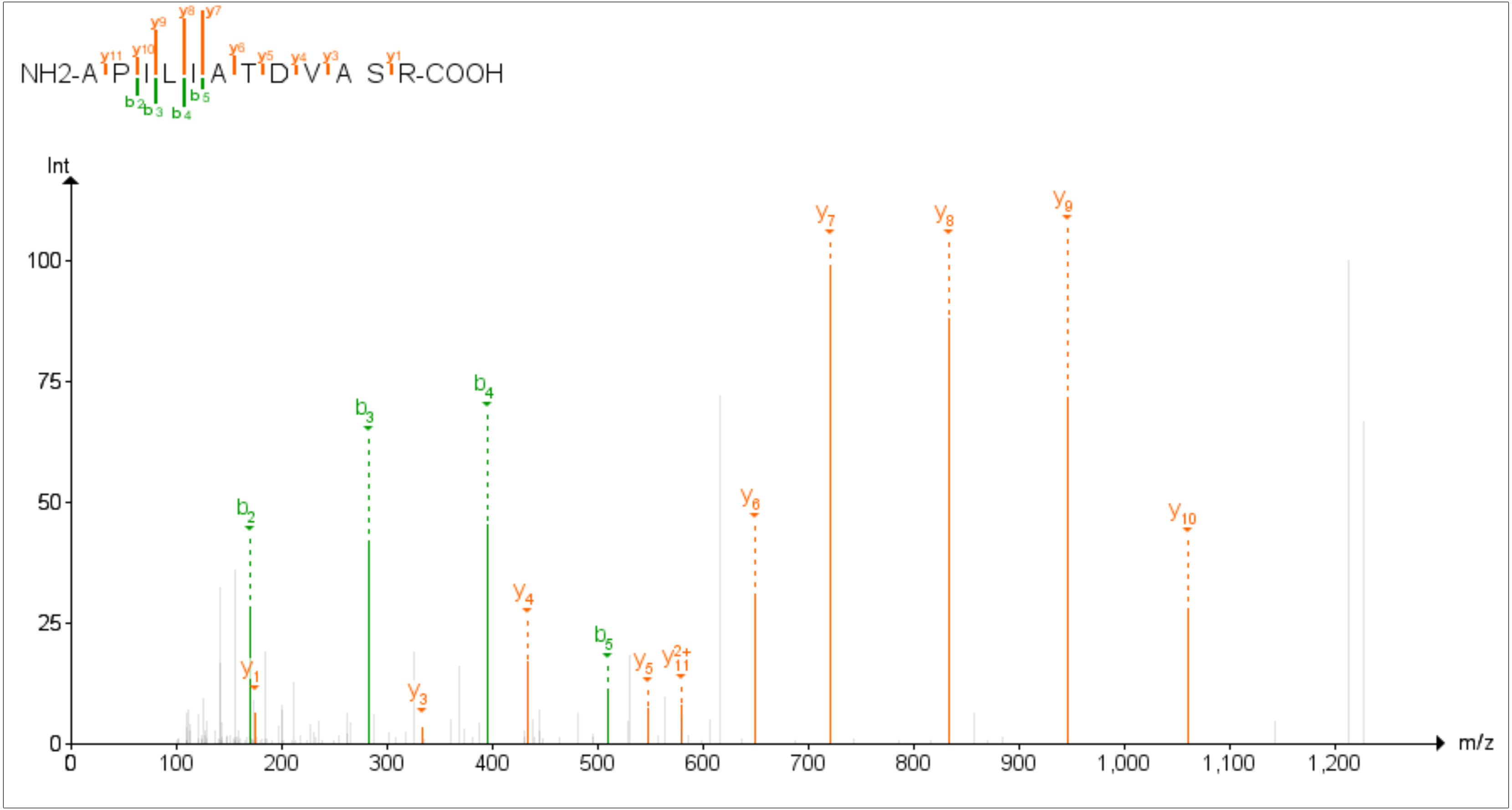

NH2-A P I L I A T D V A S R-COOH
y11 y10 y9 y8 y7 y6 y5 y4 y3 y1
b2 b3 b4 b5
Int
100
75
50
25
0
0 100 200 300 400 500 600 700 800 900 1,000 1,100 1,200
m/z
y_7 y_8 y_9 b_4 b_3 b_2 y_6 y_{10} y_4 b_5 y_5 y_{11}^{2+} y_1 y_3

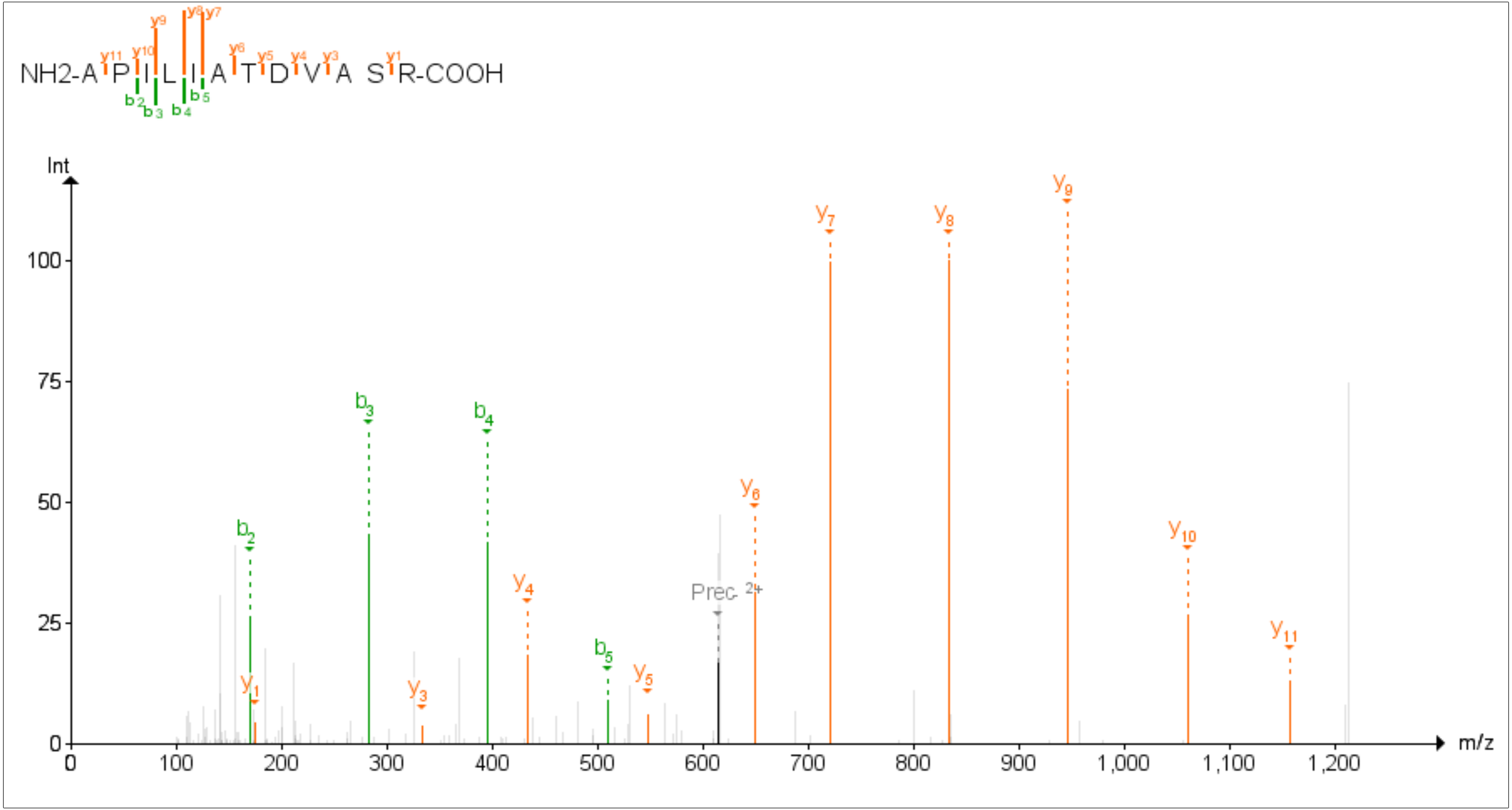

NH2-A P I L I I A T D V A S R-COOH
y11 y10 y9 y8 y7 y6 y5 y4 y3 y1
b2 b3 b4 b5
Int
100
75
50
25
0
m/z
0 100 200 300 400 500 600 700 800 900 1,000 1,100 1,200
b2 b3 b4 b5
y1 y3 y4 y5 y6 y7 y8 y9 y10 y11
Prec- 2+

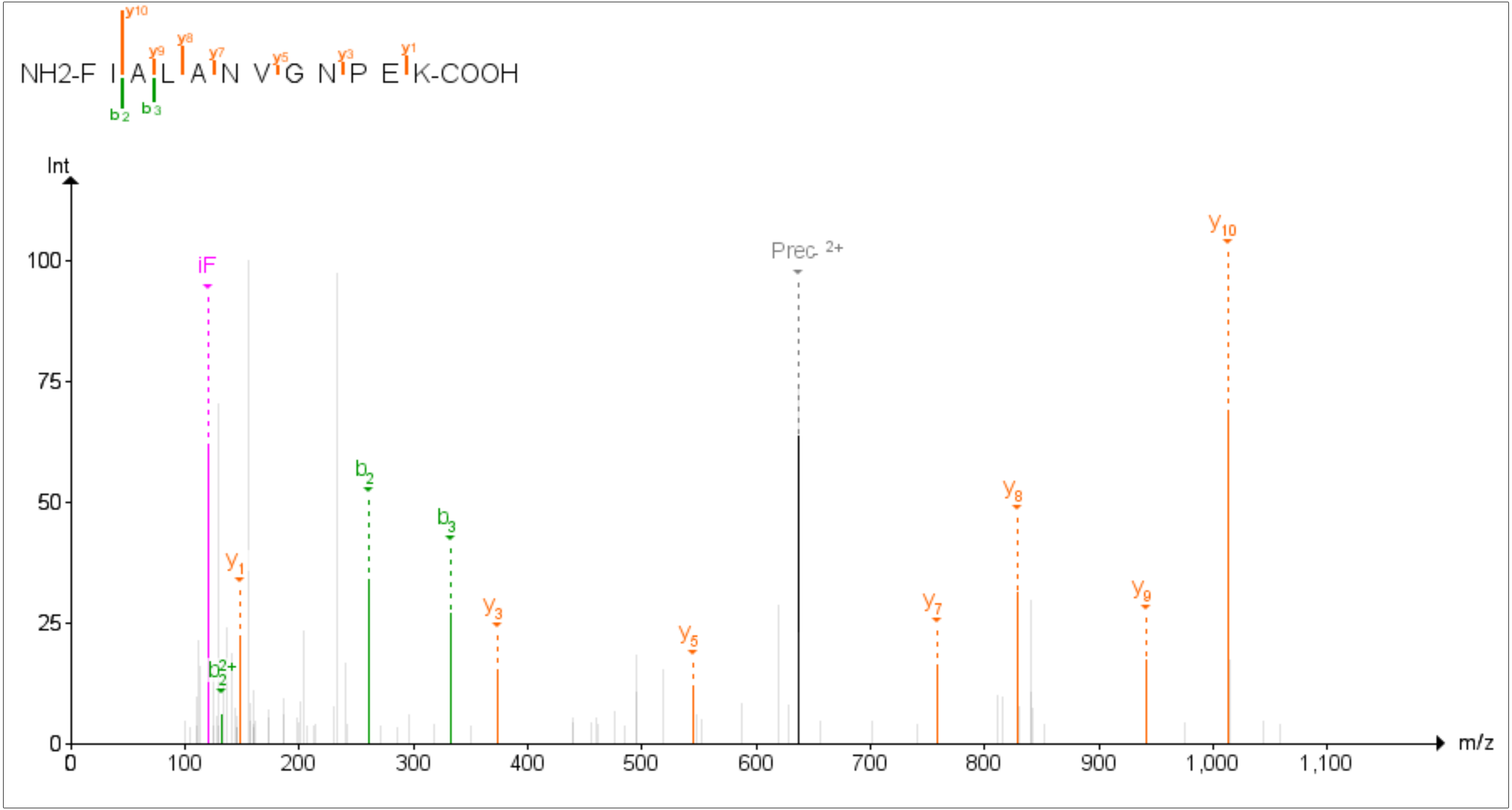

NH2-F I A L A N V G N P E K-COOH
y10
y9
y8
y7
y5
y3
y1
b2
b3
Int
100
75
50
25
0
iF
Prec 2+
b2
b3
y1
y3
y5
y7
y8
y9
y10
b2 2+
0
100
200
300
400
500
600
700
800
900
1,000
1,100
m/z

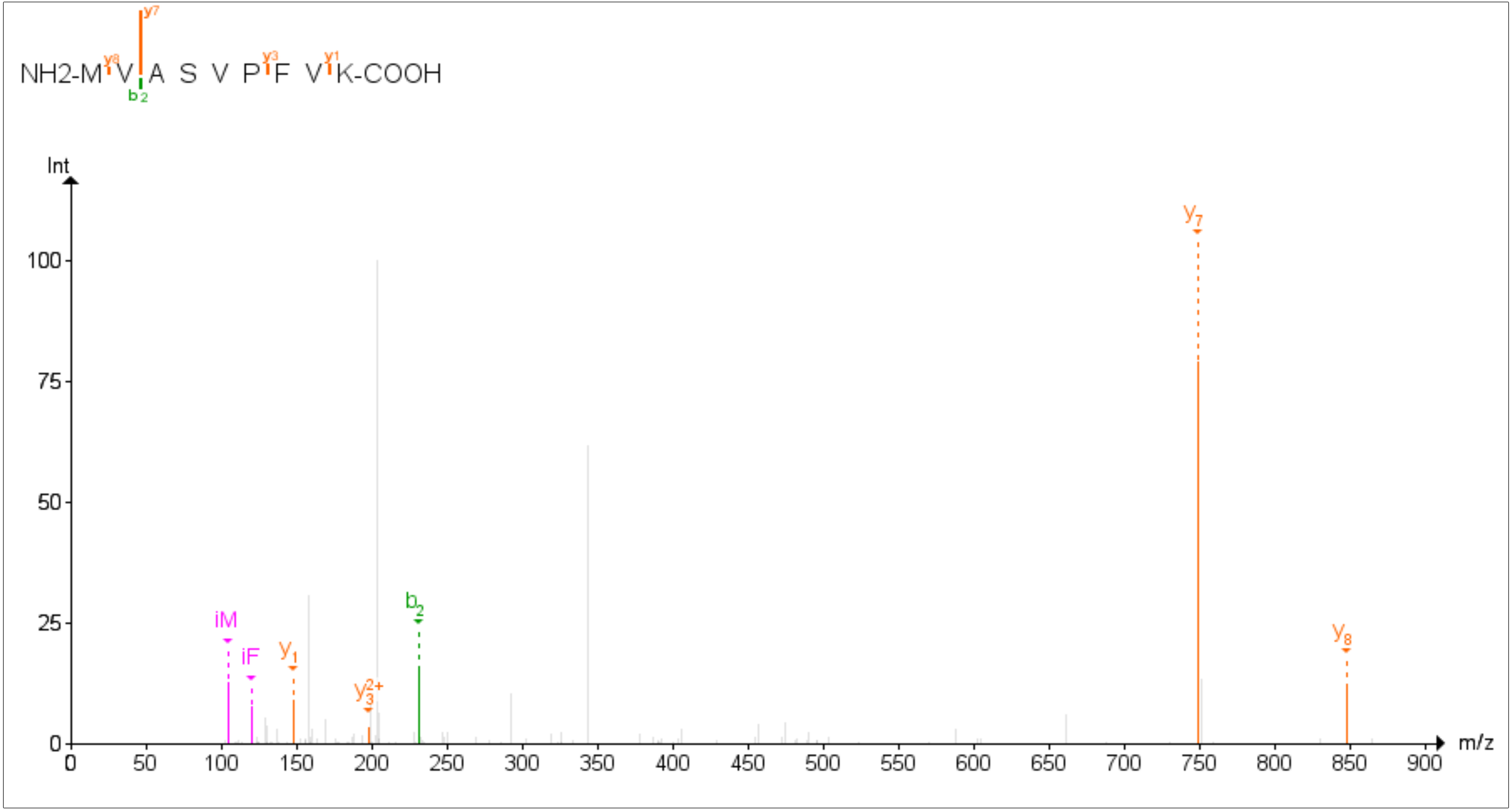
NH2-M V A S V P F V K-COOH
y8
y7
b2
y3
y1
Int
100
75
50
25
0
iM
iF
y1
y3 2+
b2
y7
y8
0
50
100
150
200
250
300
350
400
450
500
550
600
650
700
750
800
850
900
m/z

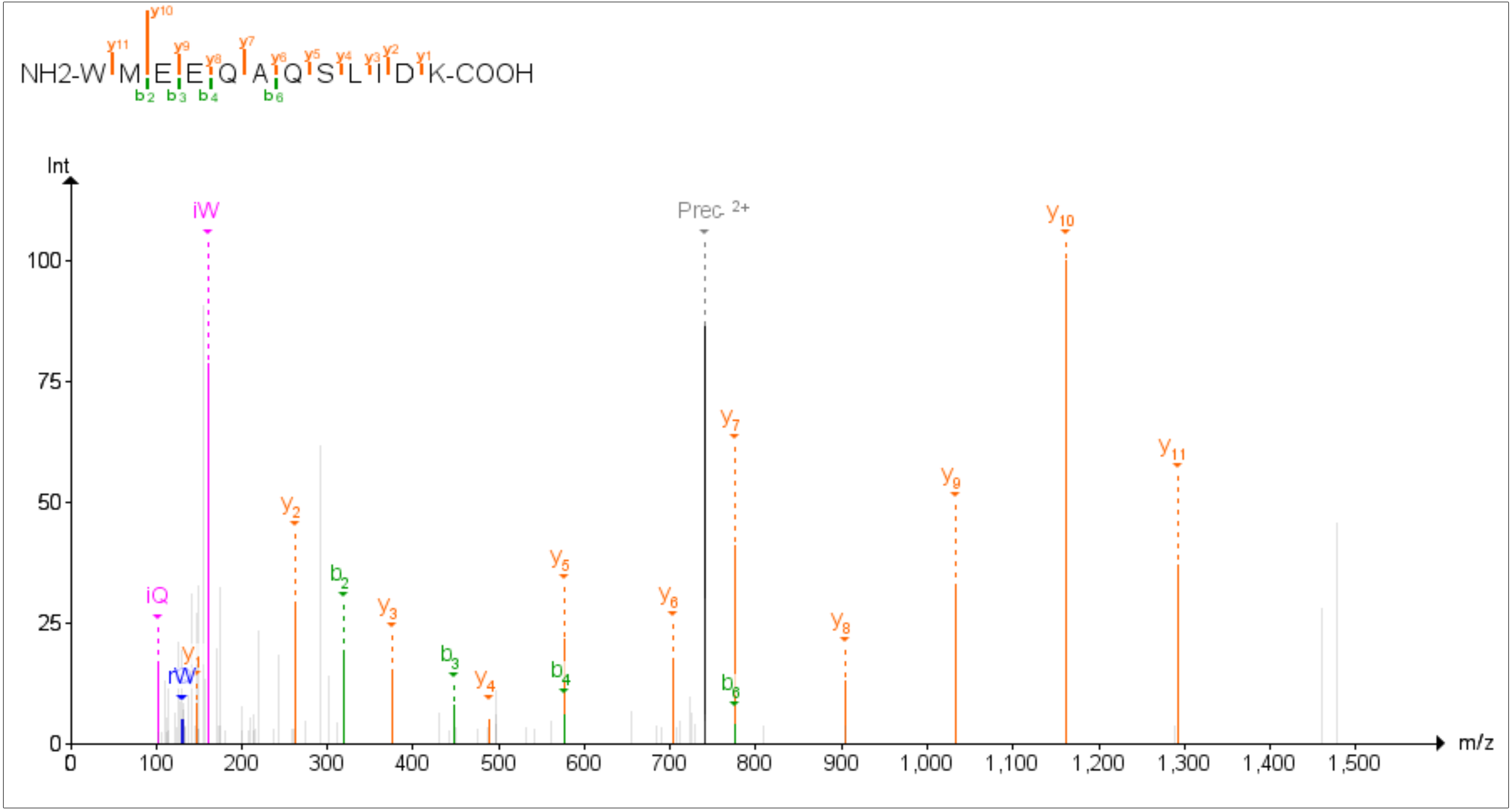

NH2-WMEEQAQSLIDK-COOH
y11 y10 y9 y8 y7 y6 y5 y4 y3 y2 y1
b2 b3 b4 b6
Int
m/z
iW
Prec. 2+
y10
y7
y11
y9
y2
b2
y5
y6
iQ
y3
b3
y8
y1
rW
y4
b4
b6
0
25
50
75
100
0
100
200
300
400
500
600
700
800
900
1,000
1,100
1,200
1,300
1,400
1,500

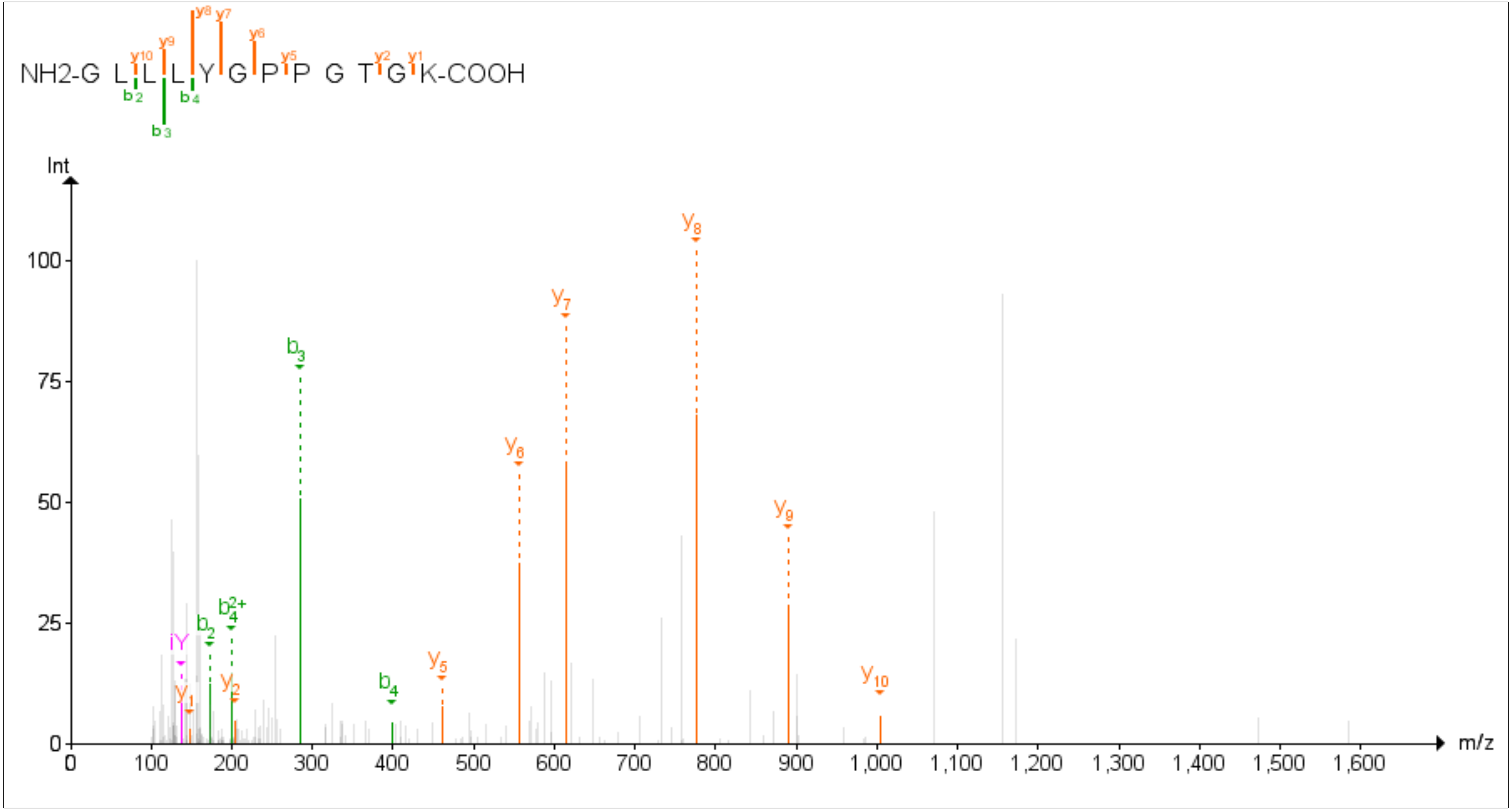
NH2-G L L L Y G P P G T G K-COOH
y10 y9 y8 y7 y6 y5 y2 y1
b2 b3 b4
Int
100
75
50
25
0
b3
y8
y7
y6
y9
y10
y5
b4
iY
b2
b4 2+
y2
y1
0 100 200 300 400 500 600 700 800 900 1,000 1,100 1,200 1,300 1,400 1,500 1,600
m/z

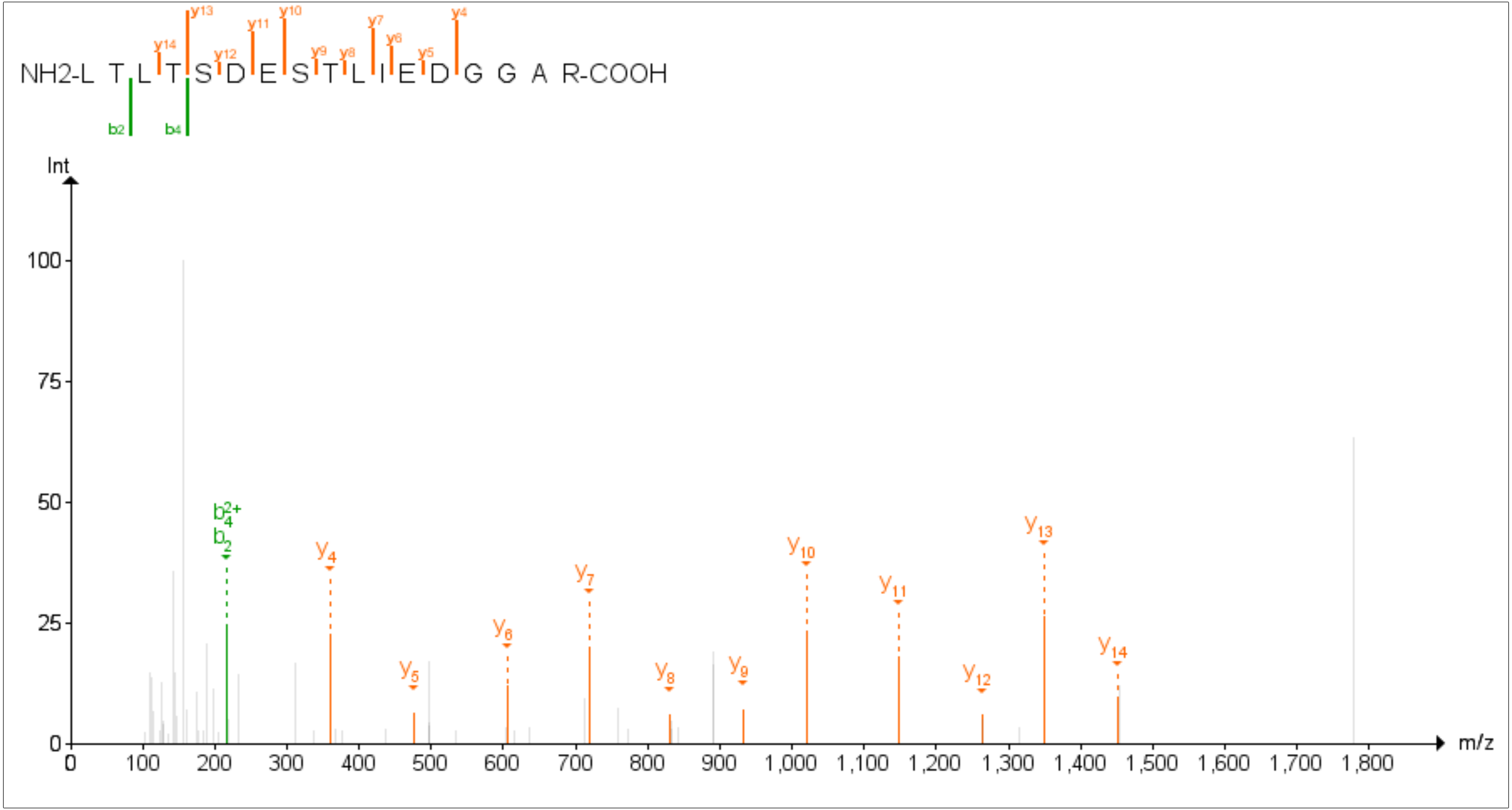

NH2-L T L T S D E S T L I E D G G A R-COOH
y14
y13
y12
y11
y10
y9
y8
y7
y6
y5
y4
b2
b4
Int
m/z
b4 2+
b2

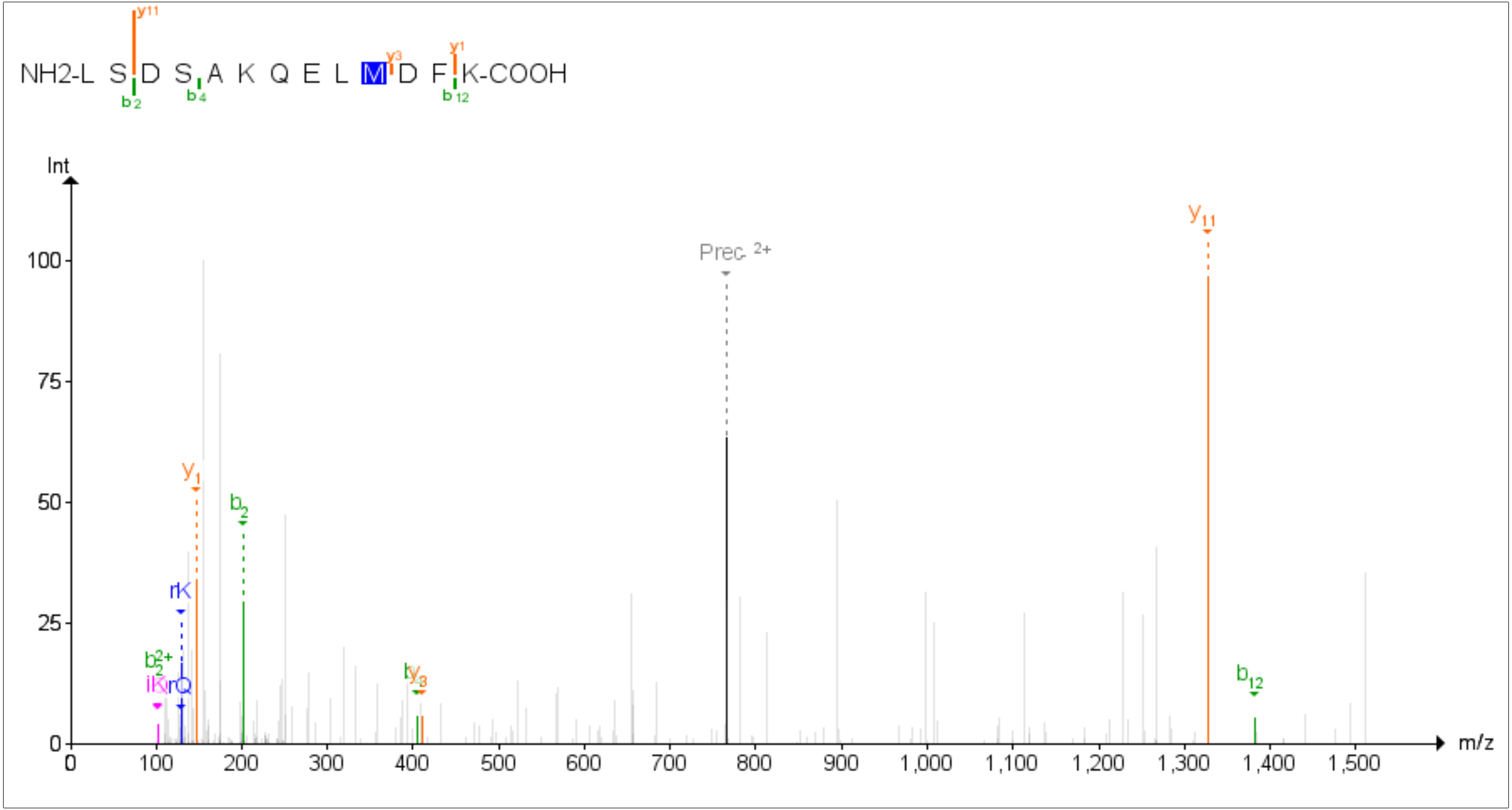

NH2-L S D S A K Q E L M D F K-COOH
Int
m/z
Prec 2+
y11
y1
b2
rK
b2 2+
iK
rQ
y3
b12
0
25
50
75
100
0
100
200
300
400
500
600
700
800
900
1,000
1,100
1,200
1,300
1,400
1,500

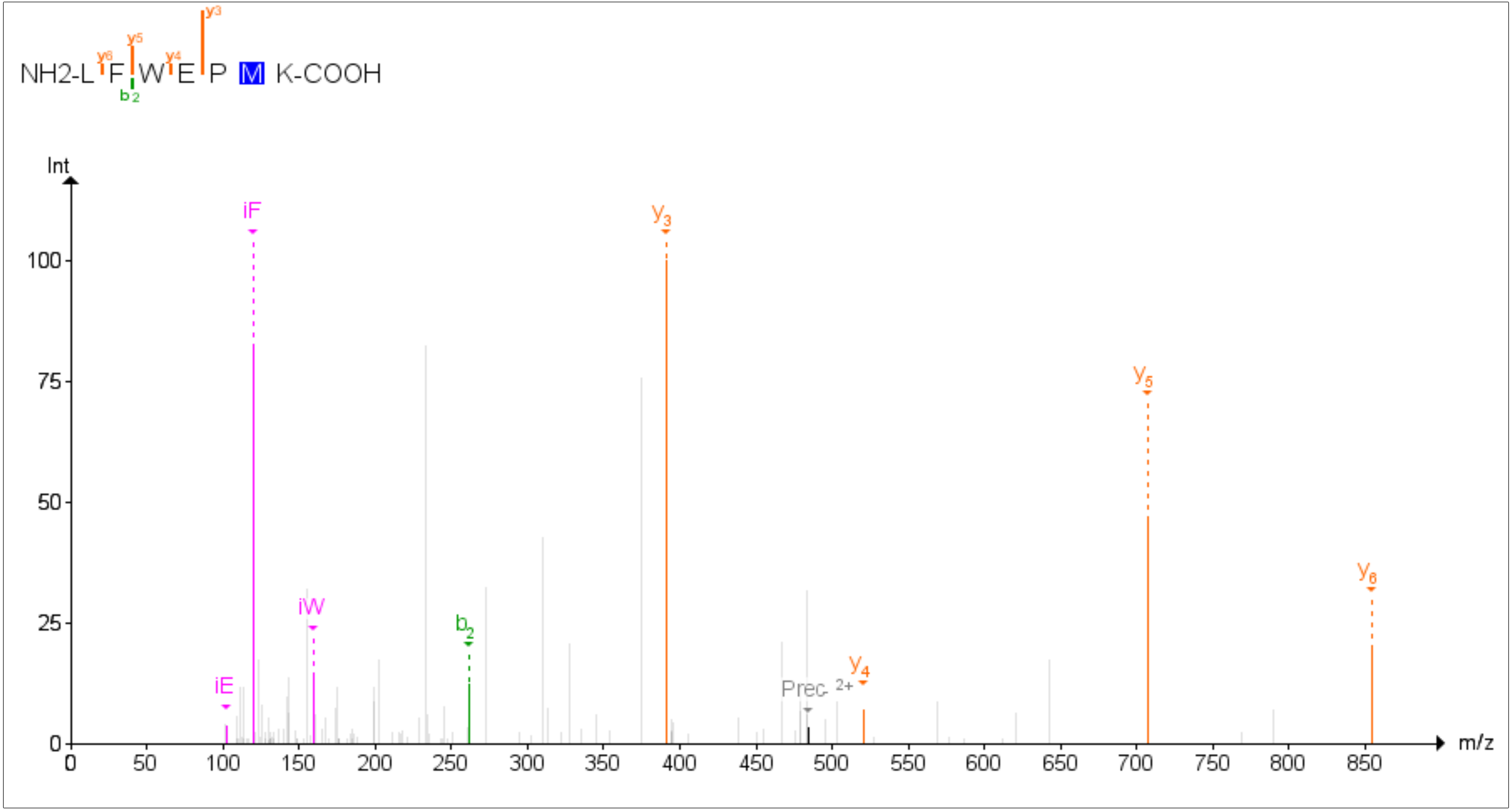

NH2-L F W E P M K-COOH
y6 y5 y4 y3 b2
Int
m/z
iF
iE
iW
b2
y3
y4
y5
y6
Prec 2+
0 25 50 75 100
0 50 100 150 200 250 300 350 400 450 500 550 600 650 700 750 800 850

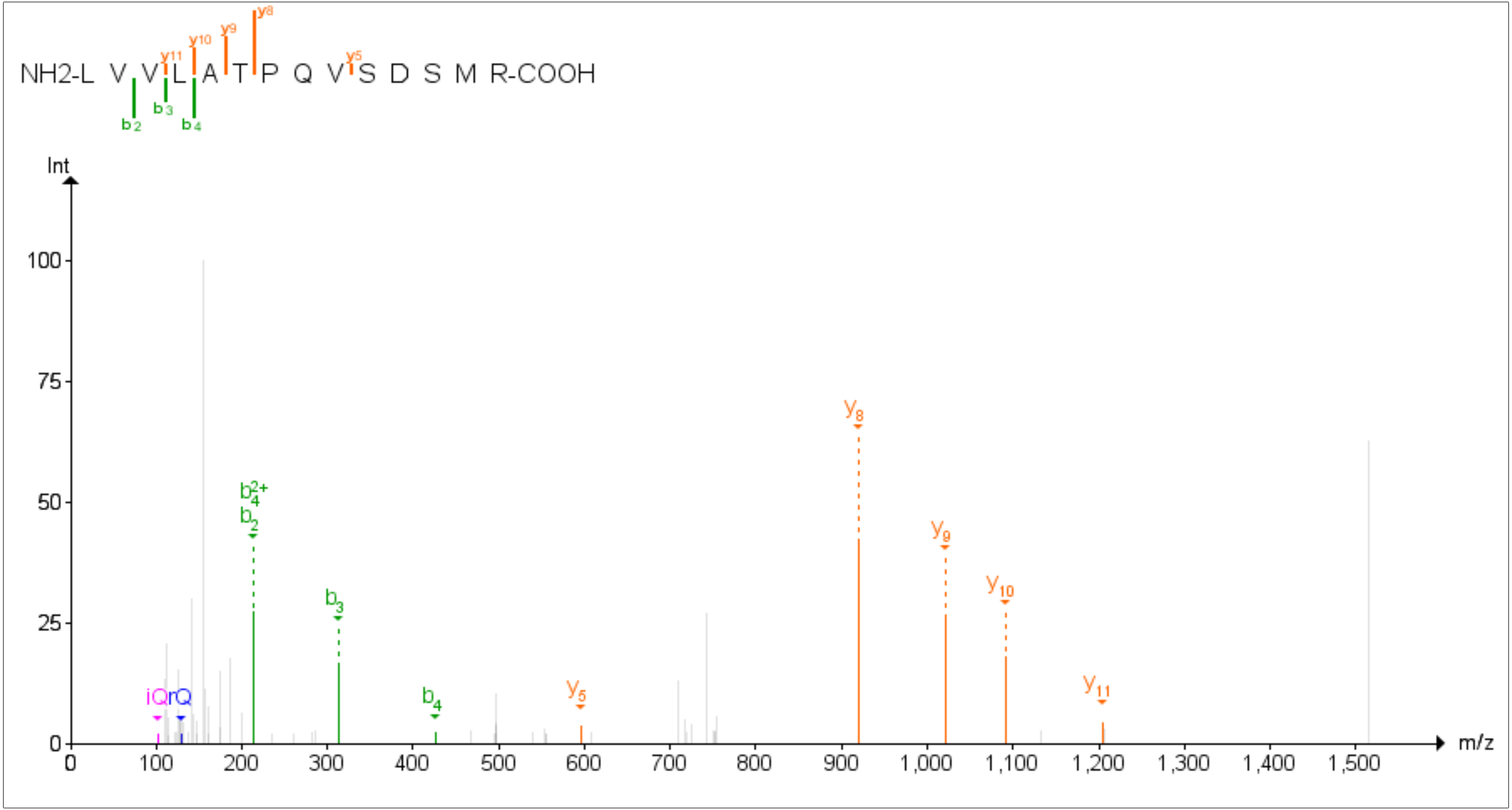

NH2-L V V L A T P Q V S D S M R-COOH
y11 y10 y9 y8 y5
b2 b3 b4
Int
100
75
50
25
0
b_4^{2+}
b_2
b_3
b_4
iQ rQ
y_5
y_8
y_9
y_{10}
y_{11}
0 100 200 300 400 500 600 700 800 900 1,000 1,100 1,200 1,300 1,400 1,500
m/z

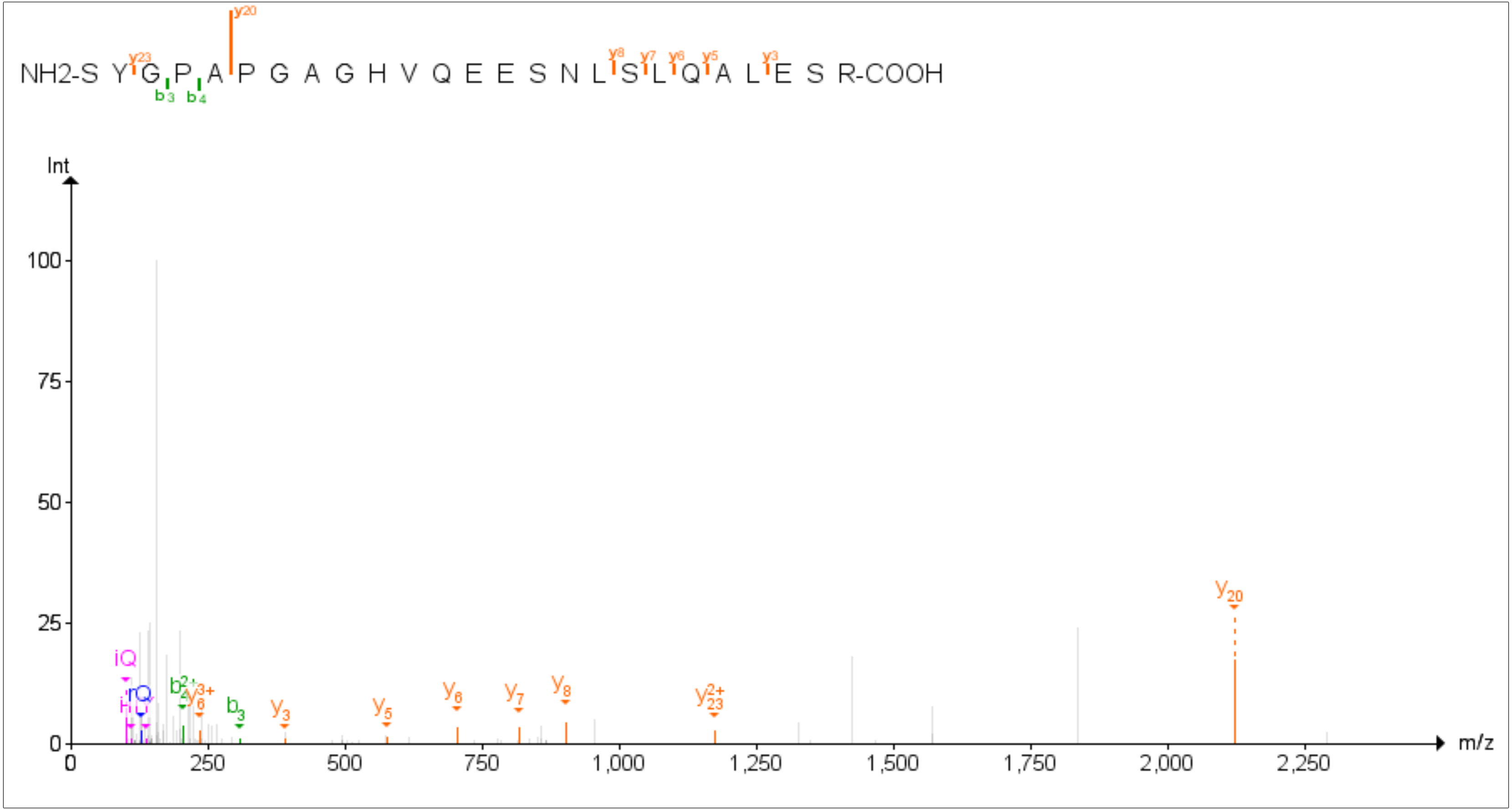

NH2-S Y G P A P G A G H V Q E E S N L S L Q A L E S R-COOH
y23
y20
b3
b4
y8
y7
y6
y5
y3
Int
100
75
50
25
0
m/z
0
250
500
750
1,000
1,250
1,500
1,750
2,000
2,250
iQ
rQ
b3
y3
y5
y6
y7
y8
y20

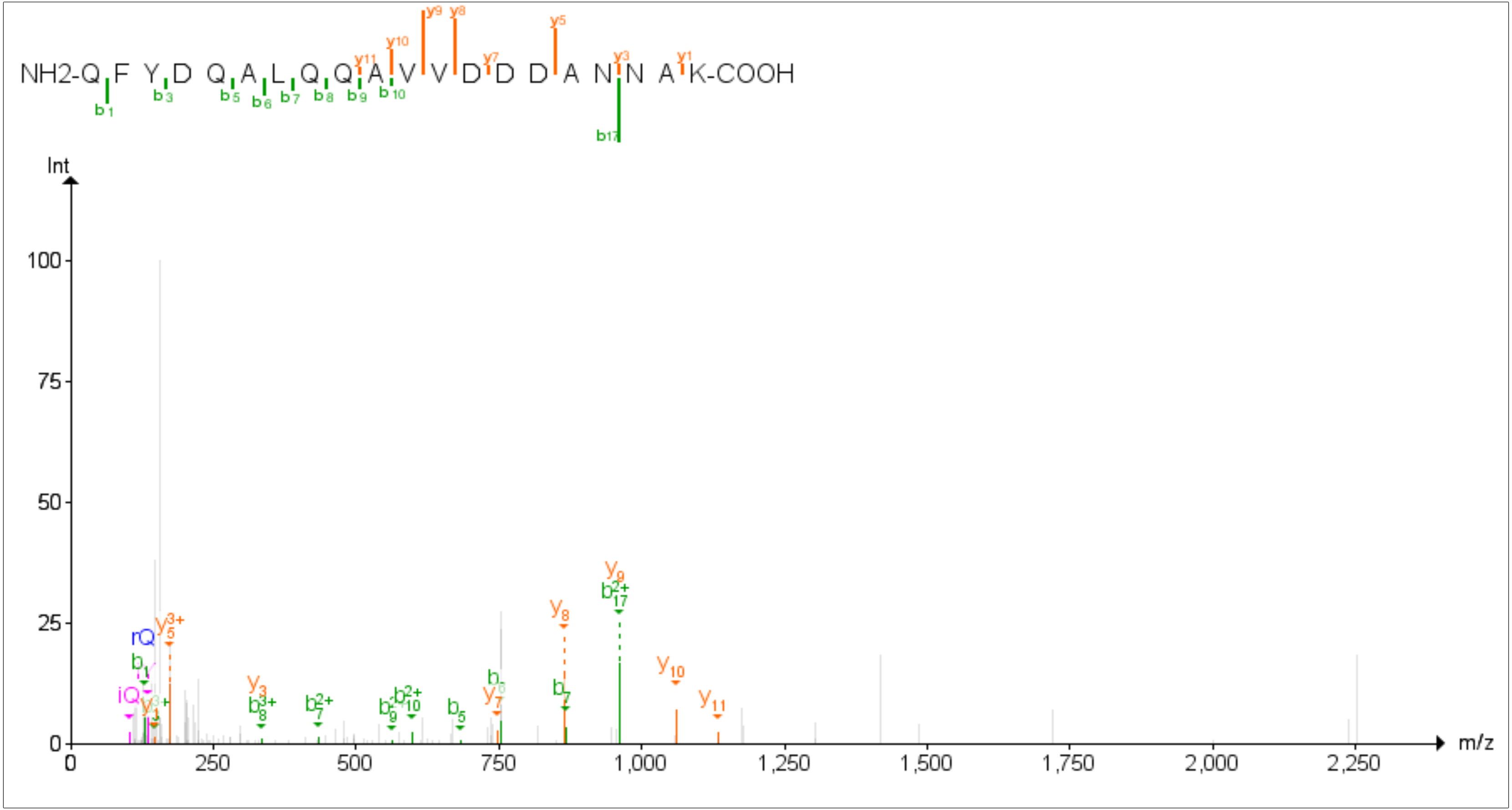
NH2-Q F Y D Q A L Q Q A V V D D D A N N A K-COOH
Int
m/z
0
25
50
75
100
250
500
750
1,000
1,250
1,500
1,750
2,000
2,250

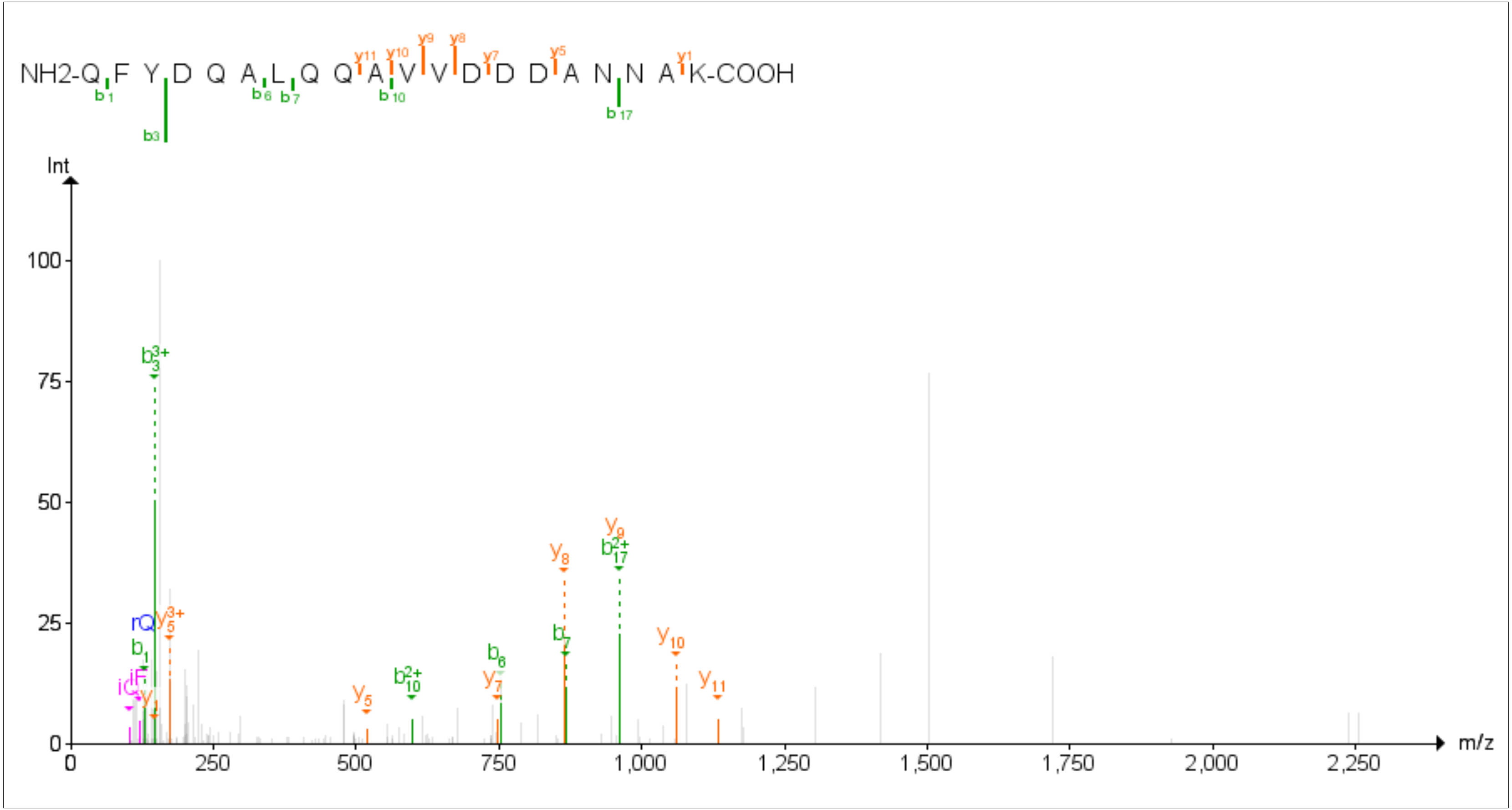

NH2-Q F Y D Q A L Q Q A V V D D D A N N A K-COOH
Int
m/z

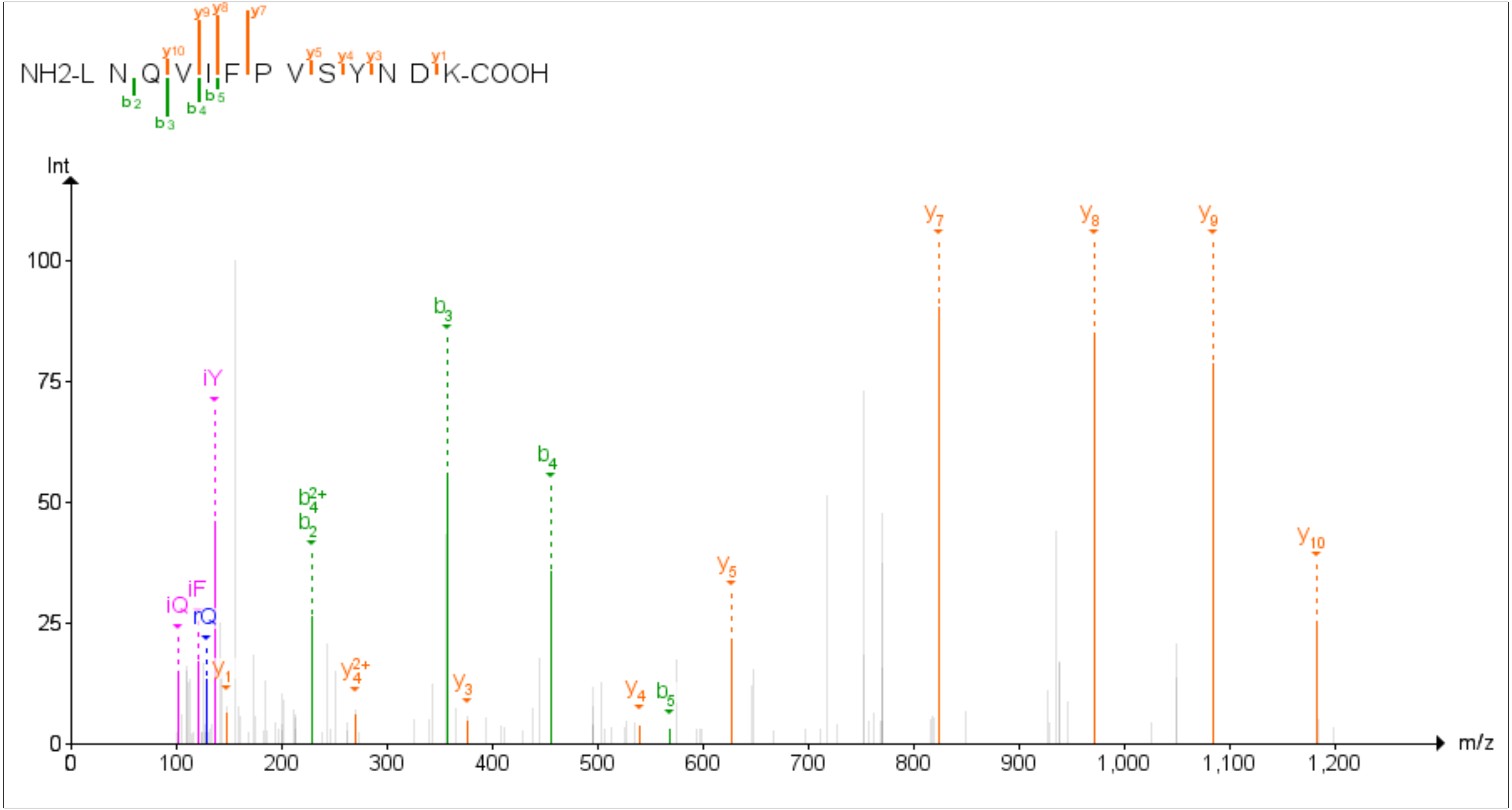

NH2-L N Q V I F P V S Y N D K-COOH
y10 y9 y8 y7 y5 y4 y3 y1
b2 b3 b4 b5
Int
m/z
y7 y8 y9 y10 y5 y4 y3 y1
b3 b4 b4 2+ b2 b5
y4 2+
iY iQ iF rQ

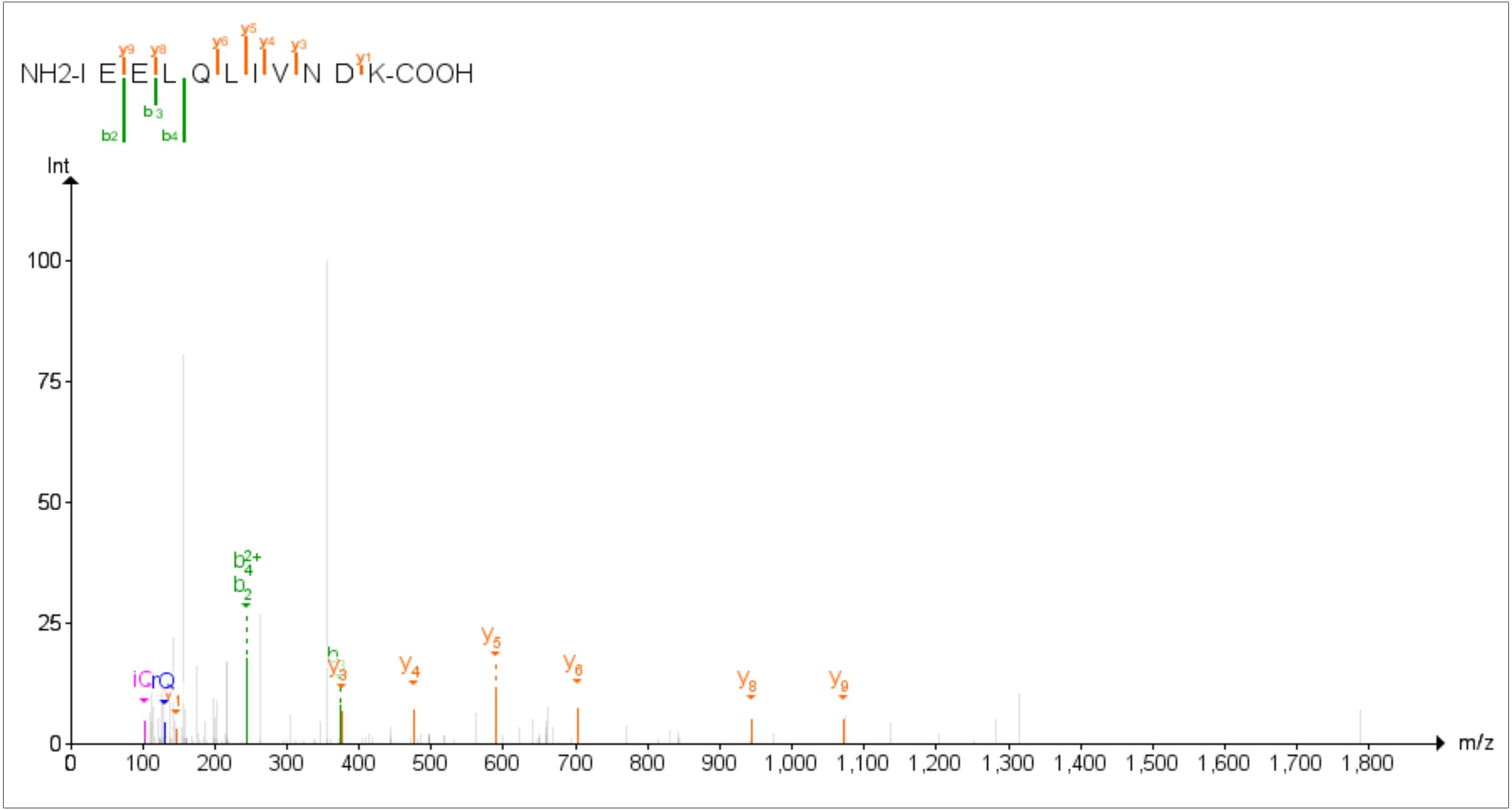

NH2-I E E L Q L I V N D K-COOH
y9
y8
y6
y5
y4
y3
y1
b2
b3
b4
Int
100
75
50
25
0
b_4^{2+}
b_2
y_3
y_4
y_5
y_6
y_8
y_9
iC
rQ
m/z
0
100
200
300
400
500
600
700
800
900
1,000
1,100
1,200
1,300
1,400
1,500
1,600
1,700
1,800

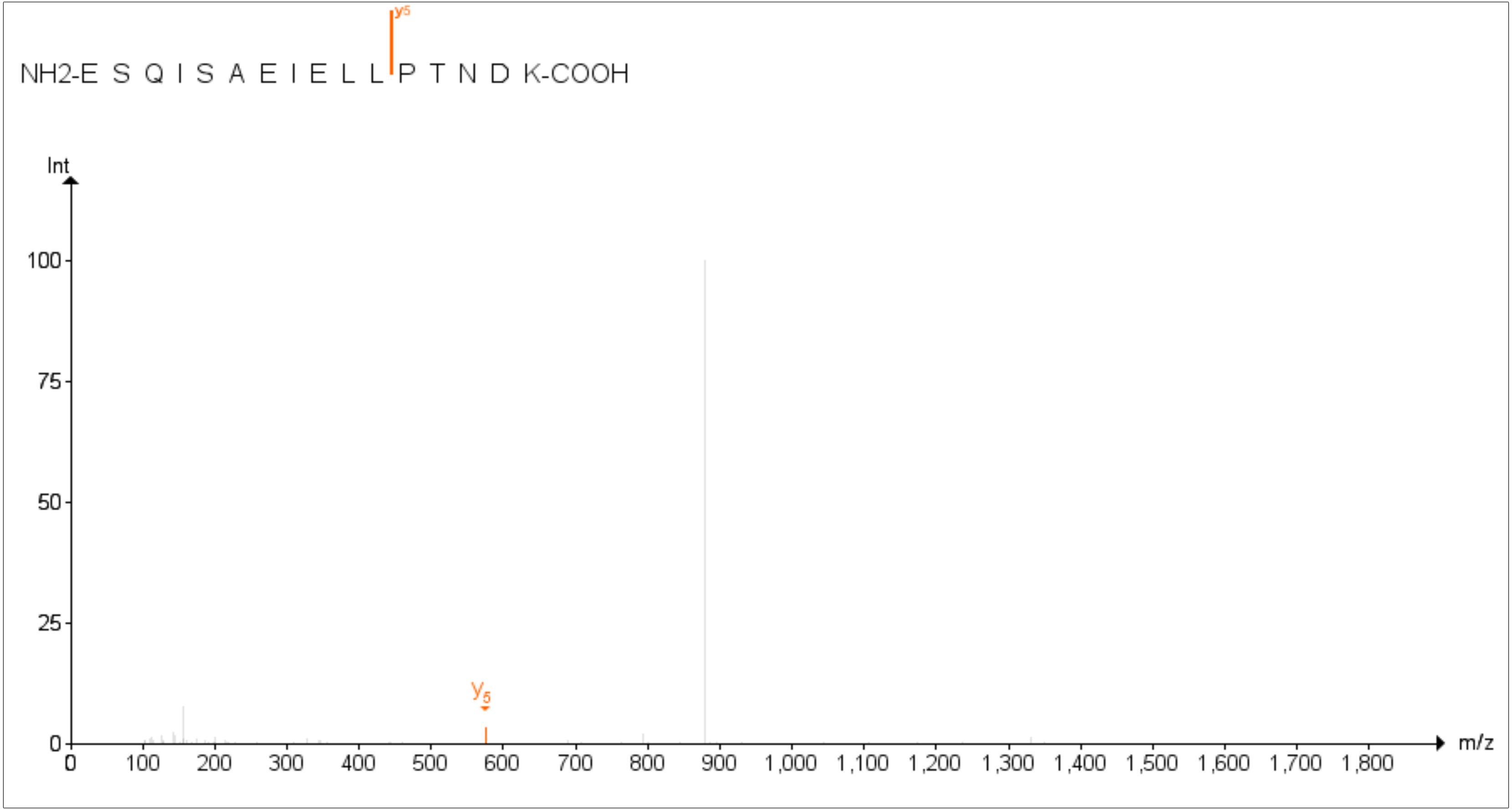

y5
NH2-E S Q I S A E I E L L P T N D K-COOH
Int
100
75
50
25
0
y5
0
100
200
300
400
500
600
700
800
900
1,000
1,100
1,200
1,300
1,400
1,500
1,600
1,700
1,800
m/z

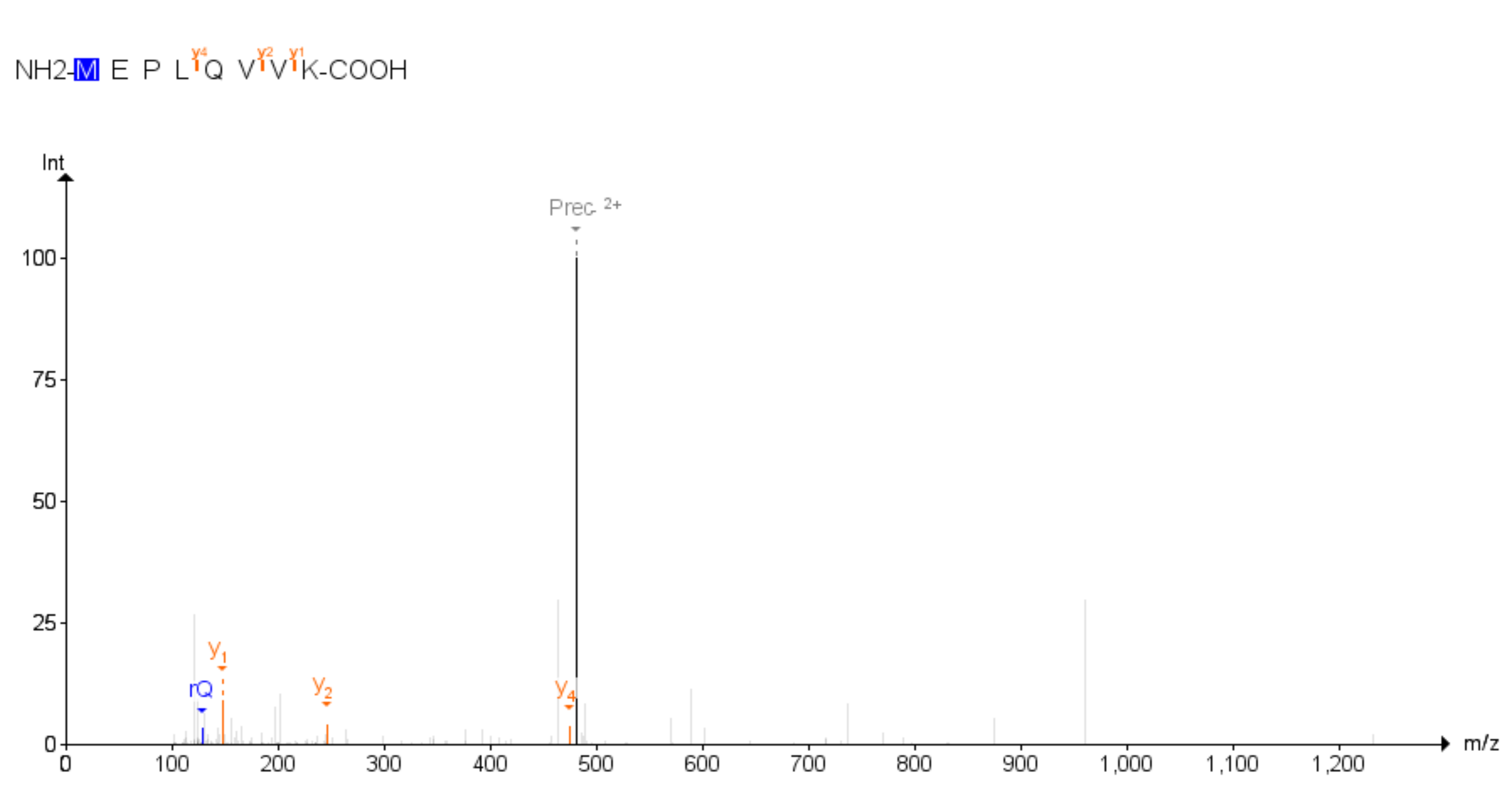
NH2-M E P L Q V V K-COOH
y4 y2 y1
Int
Prec 2+
100
75
50
25
0
y1
rQ
y2
y4
0 100 200 300 400 500 600 700 800 900 1,000 1,100 1,200
m/z

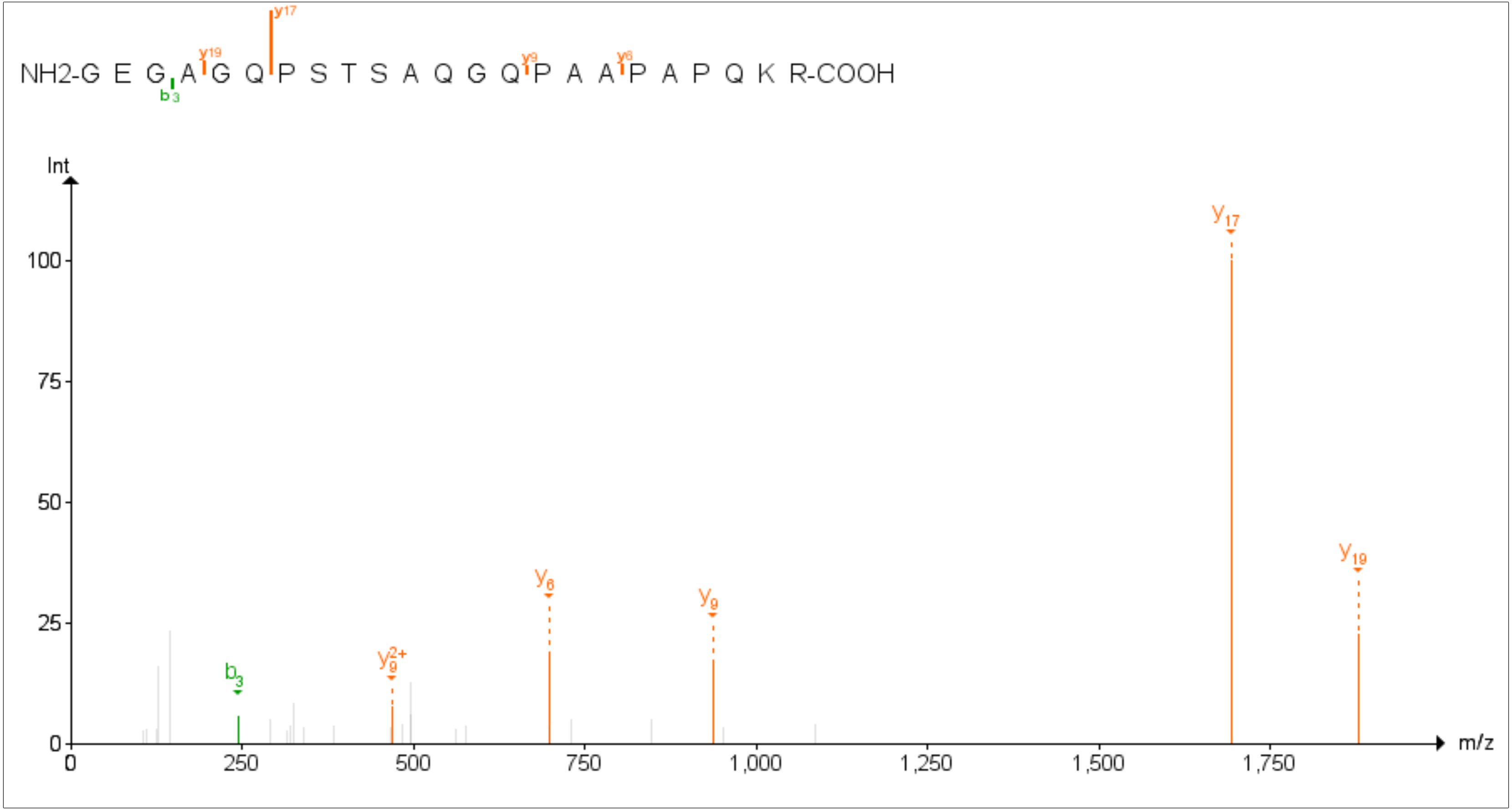

NH2-G E G A G Q P S T S A Q G G Q P A A P A P Q K R-COOH
y17
y19
y9
y6
b3
Int
100
75
50
25
0
y17
y19
y6
y9
y9 2+
b3
0
250
500
750
1,000
1,250
1,500
1,750
m/z

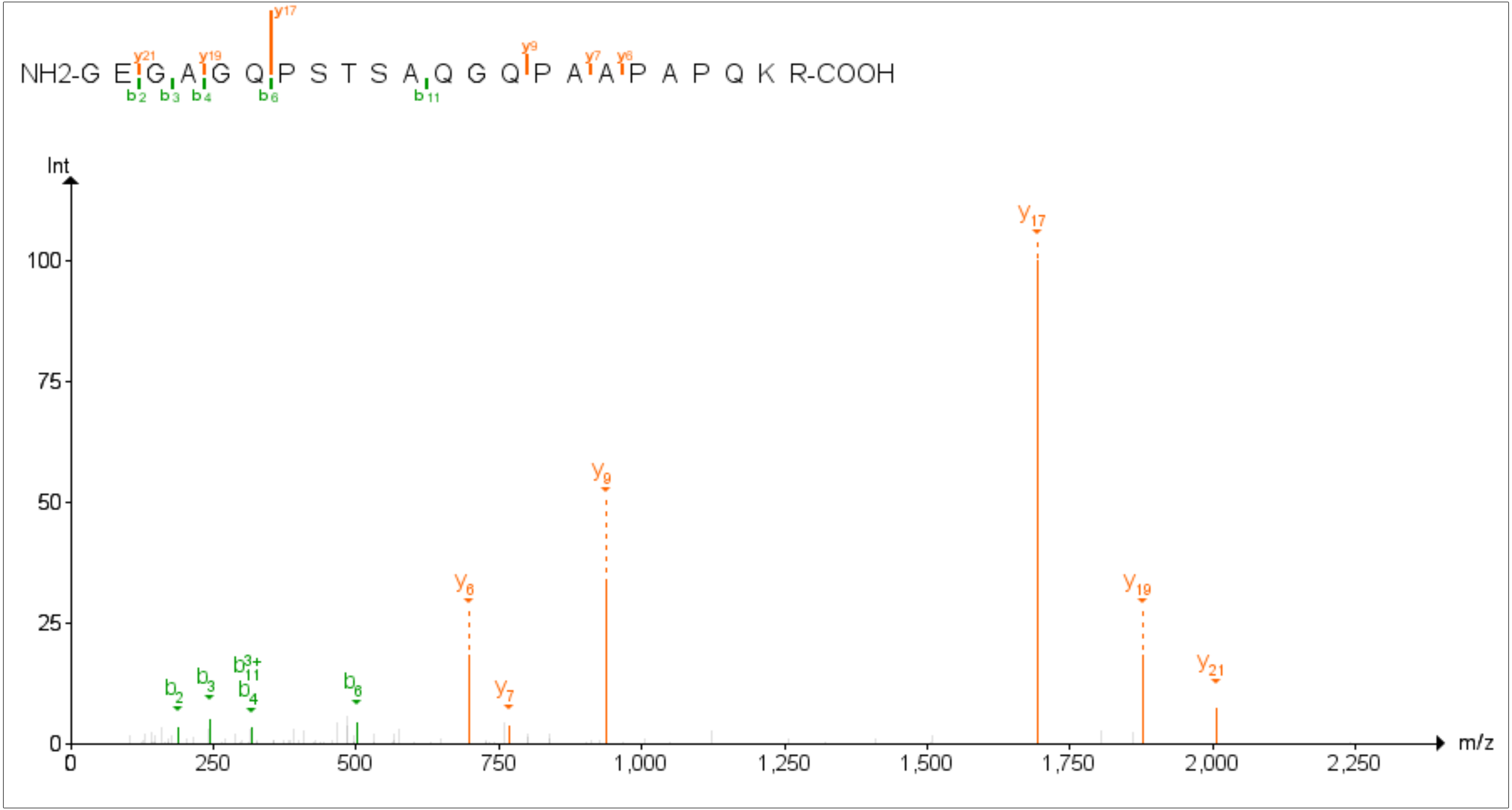
NH2-G E G A G Q P S T S A Q G G Q P A A P A P Q K R-COOH
y21 y19 y17 y9 y7 y6
b2 b3 b4 b6 b11
Int
100
75
50
25
0
m/z
0 250 500 750 1,000 1,250 1,500 1,750 2,000 2,250
y17
y9
y6
y7
y19
y21
b2
b3
b11 3+
b4
b6

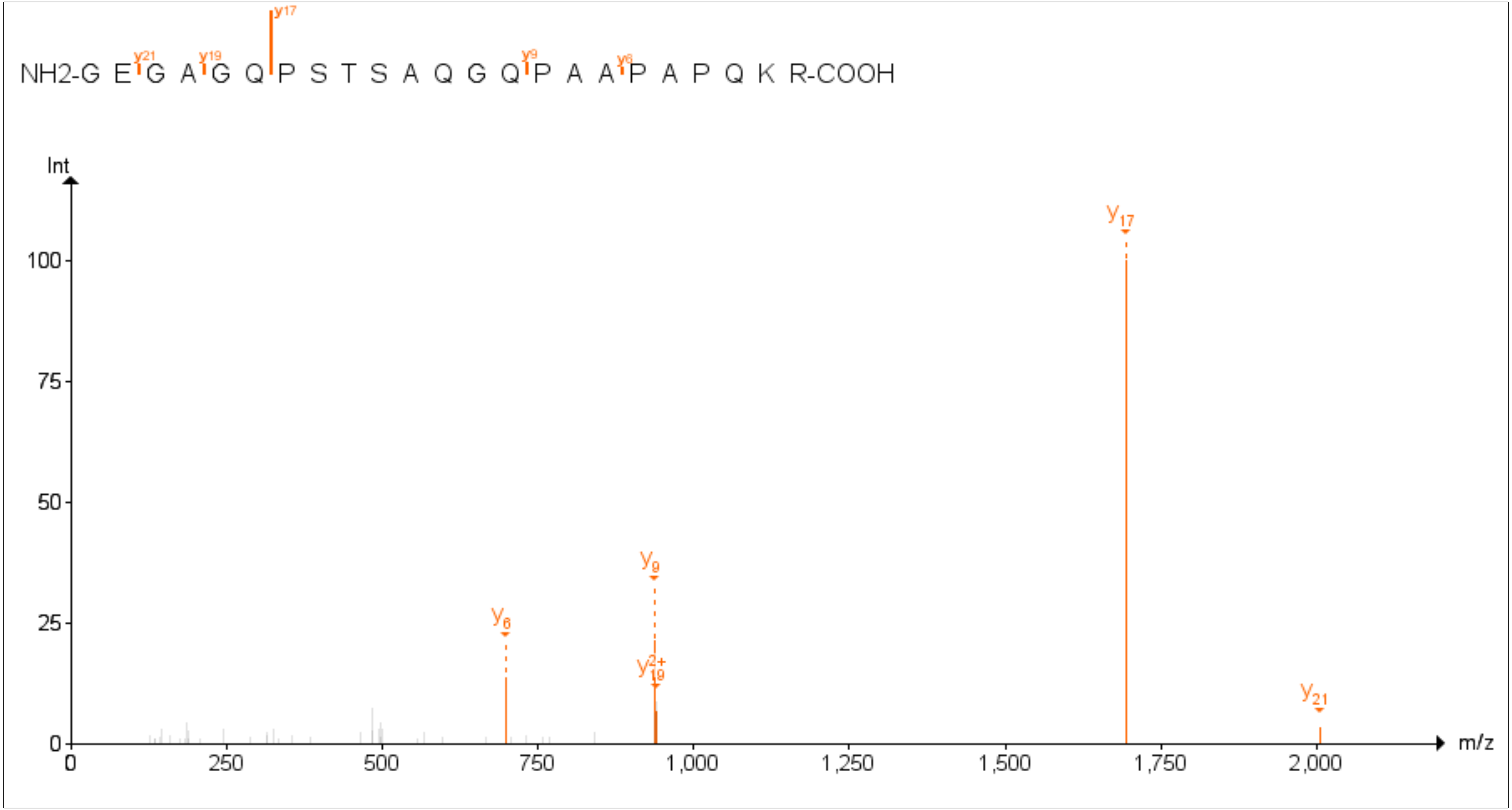
NH2-G E G A G Q P S T S A Q G G Q P A A P A P Q K R-COOH
y21
y19
y17
y9
y6
Int
100
75
50
25
0
m/z
0
250
500
750
1,000
1,250
1,500
1,750
2,000
y6
y9
y19 2+
y17
y21

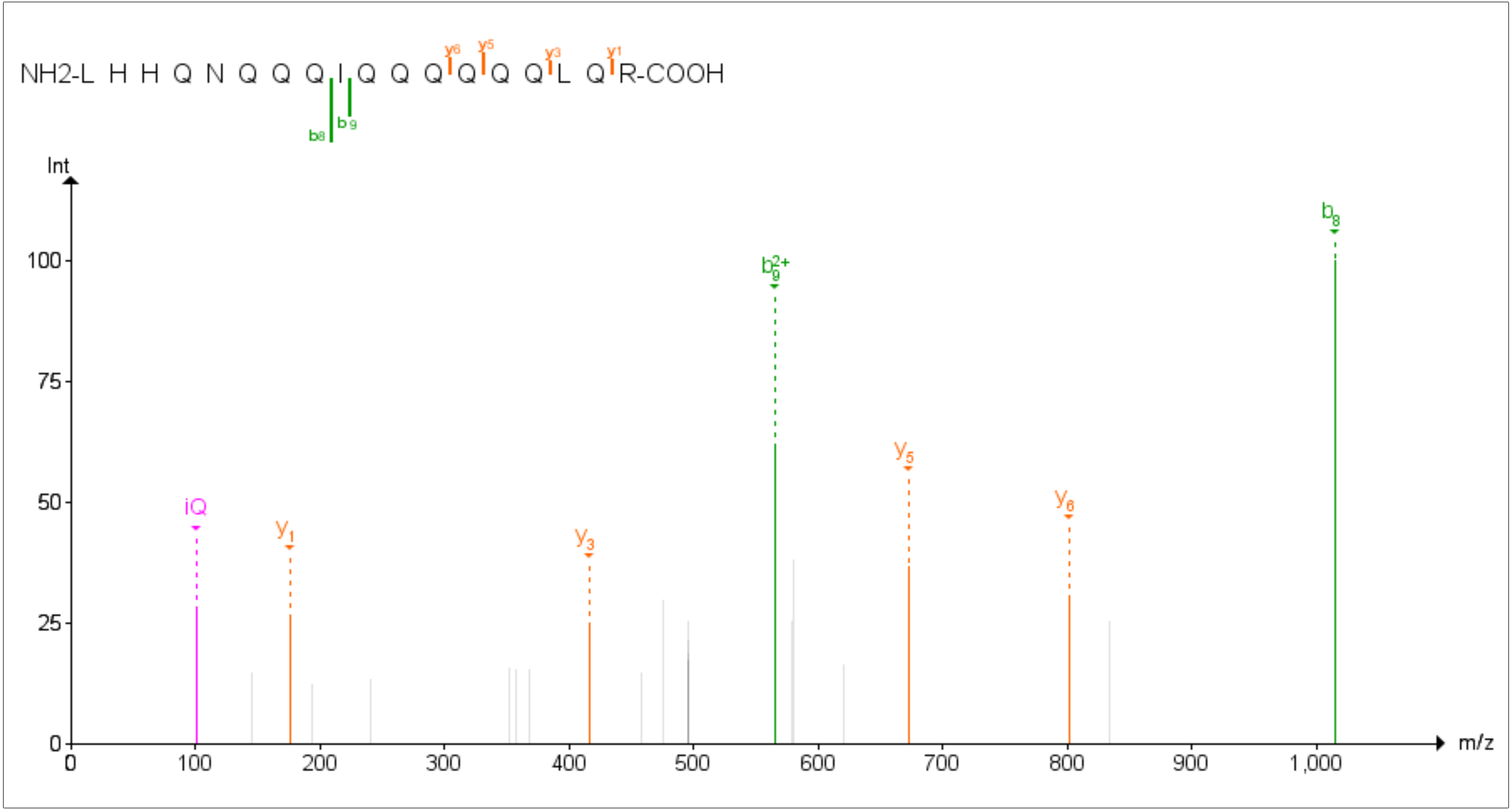
NH2-L H H Q N Q Q Q I Q Q Q Q Q Q L Q R-COOH
y6
y5
y3
y1
b8
b9
Int
100
75
50
25
0
iQ
y1
y3
b9 2+
y5
y6
b8
0
100
200
300
400
500
600
700
800
900
1,000
m/z

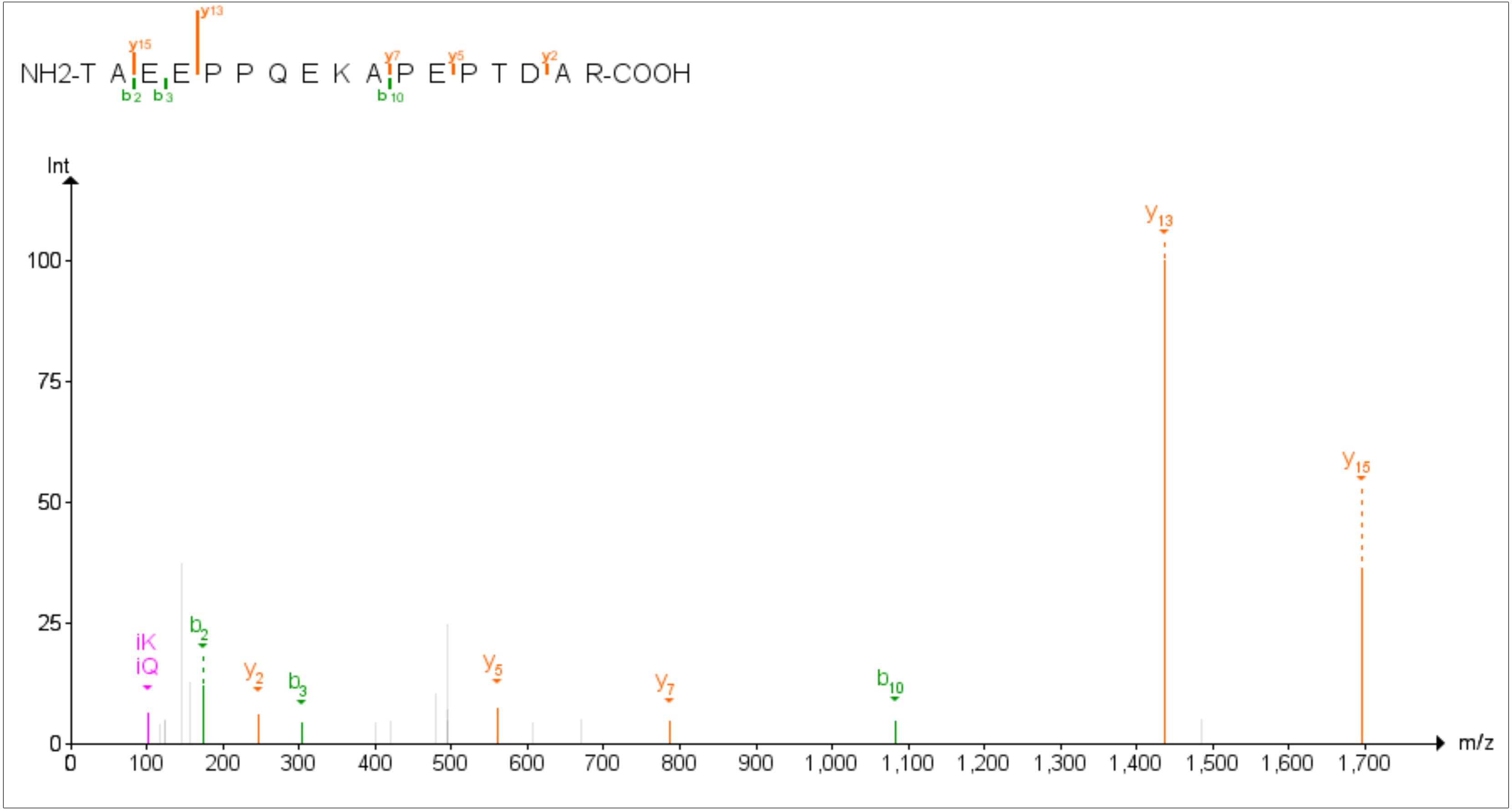

NH2-T A E E P P Q E K A P E P T D A R-COOH
y15
y13
y7
y5
y2
b2
b3
b10
Int
100
75
50
25
0
iK
iQ
b2
y2
b3
y5
y7
b10
y13
y15
m/z
0
100
200
300
400
500
600
700
800
900
1,000
1,100
1,200
1,300
1,400
1,500
1,600
1,700

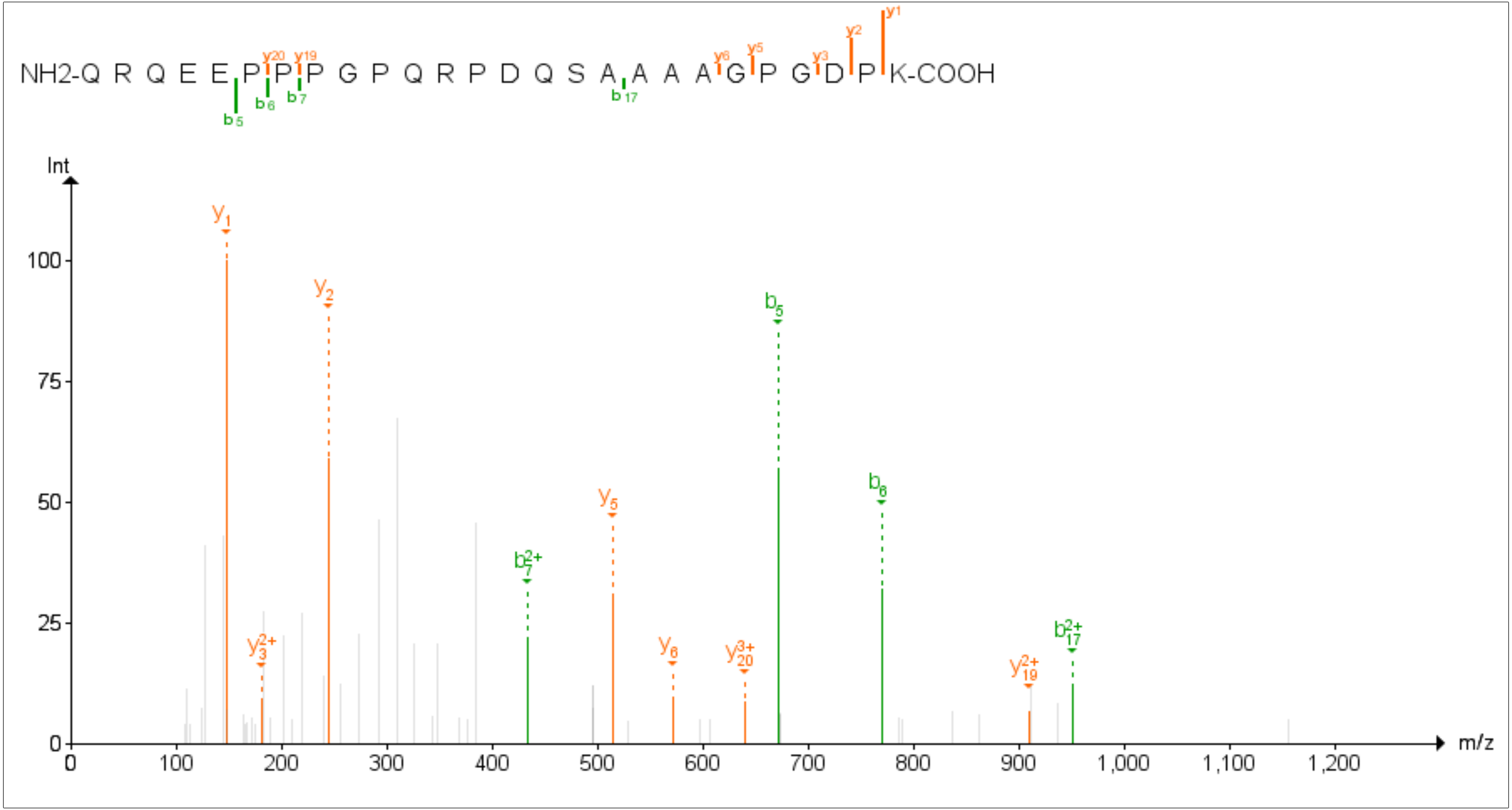

NH2-Q R Q E E P P P G P Q R P D Q S A A A A G P G D P K-COOH
Int
m/z
y1
y2
y3 2+
y5
y6
y19 2+
y20 3+
b5
b6
b7 2+
b17 2+

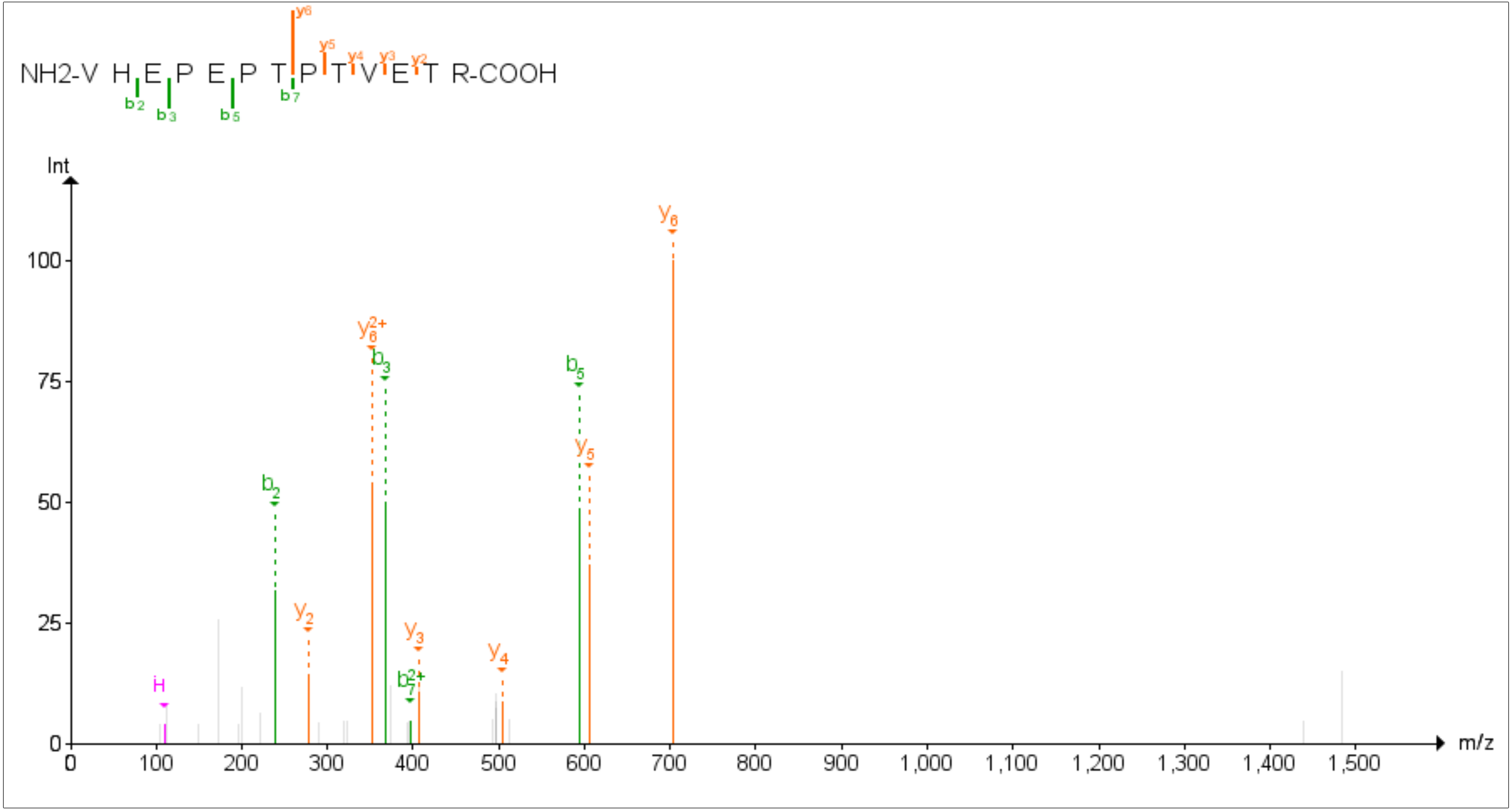

NH2-V H E P E P T P T V E T R-COOH
y6
y5
y4
y3
y2
b2
b3
b5
b7
Int
100
75
50
25
0
m/z
0
100
200
300
400
500
600
700
800
900
1,000
1,100
1,200
1,300
1,400
1,500
y6
y6 2+
b3
b5
y5
b2
y2
y3
b7 2+
y4
H

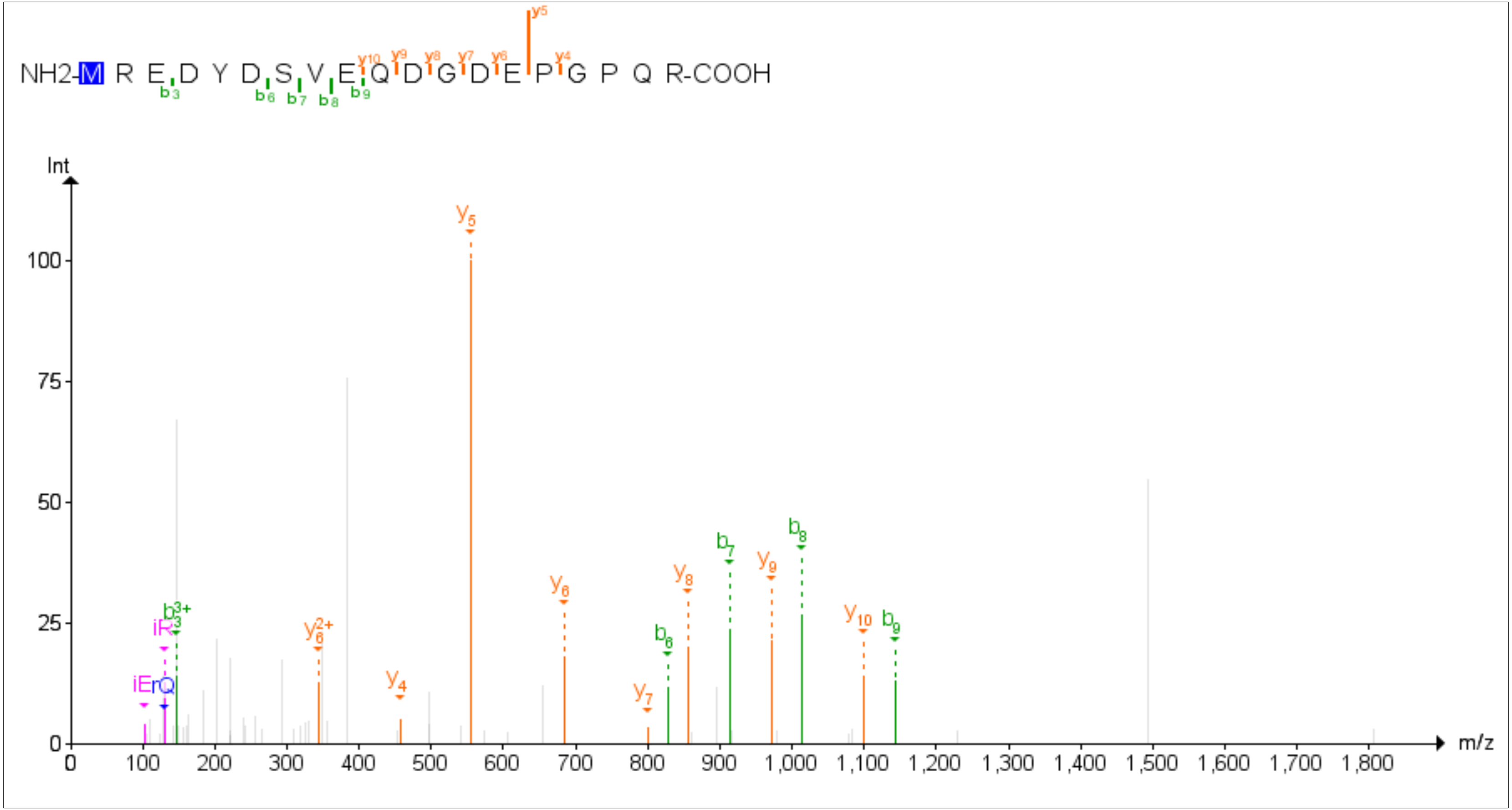

NH2-M R E D Y D S V E Q D G D E P G P Q R-COOH
y5 y10 y9 y8 y7 y6 y4
b3 b6 b7 b8 b9
Int
m/z
0 25 50 75 100
0 100 200 300 400 500 600 700 800 900 1,000 1,100 1,200 1,300 1,400 1,500 1,600 1,700 1,800
iErQ
iR
b3 3+
y6 2+
y4
y5
y6
y7
b6
y8
b7
y9
b8
y10
b9

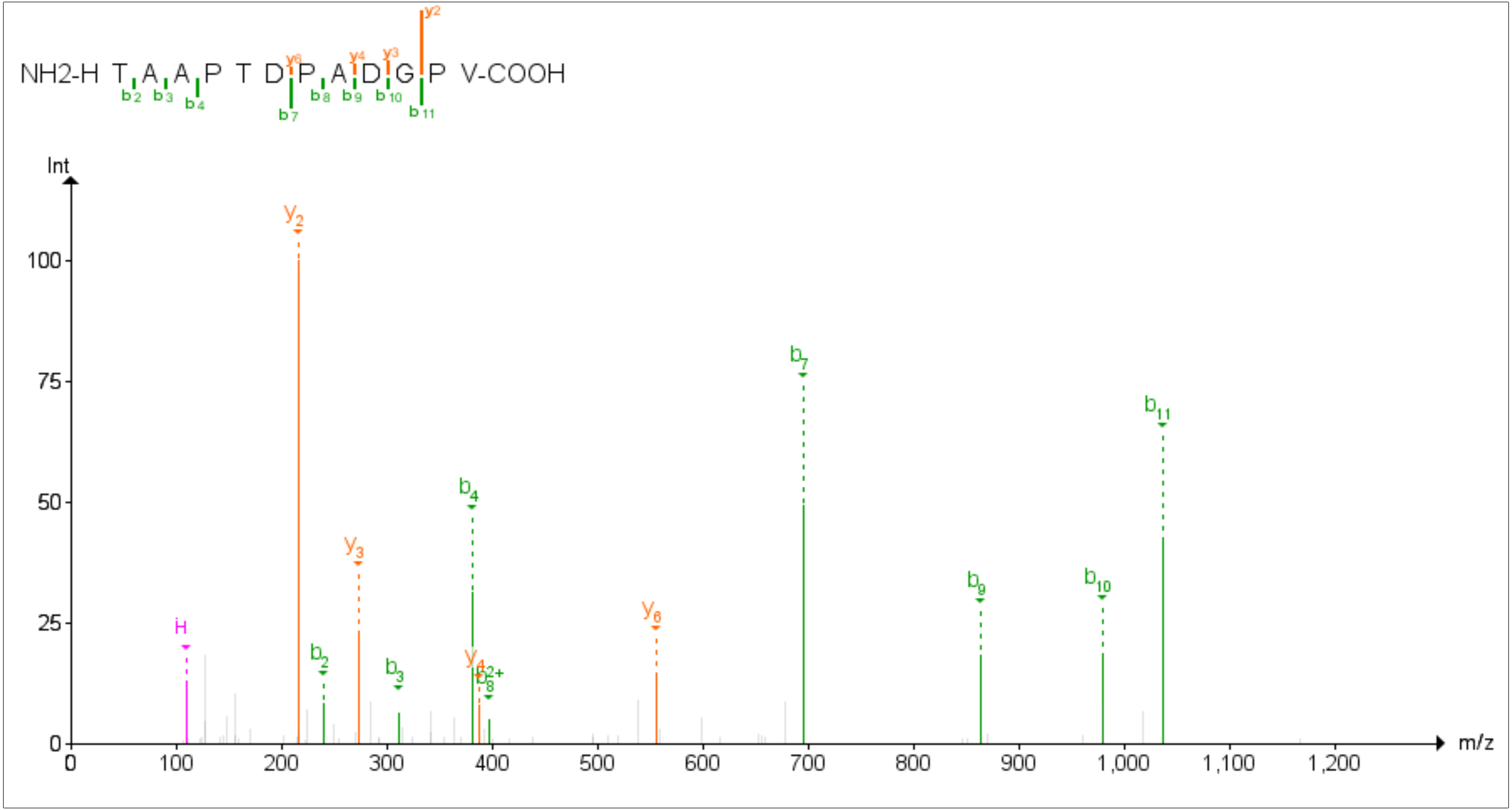
NH2-H T A A P T D P A D G P V-COOH
b2 b3 b4 b7 b8 b9 b10 b11
y6 y4 y3 y2
Int
m/z
0
25
50
75
100
0 100 200 300 400 500 600 700 800 900 1,000 1,100 1,200
y2
y3
y4
y6
b2
b3
b4
b7
b8
b9
b10
b11
H

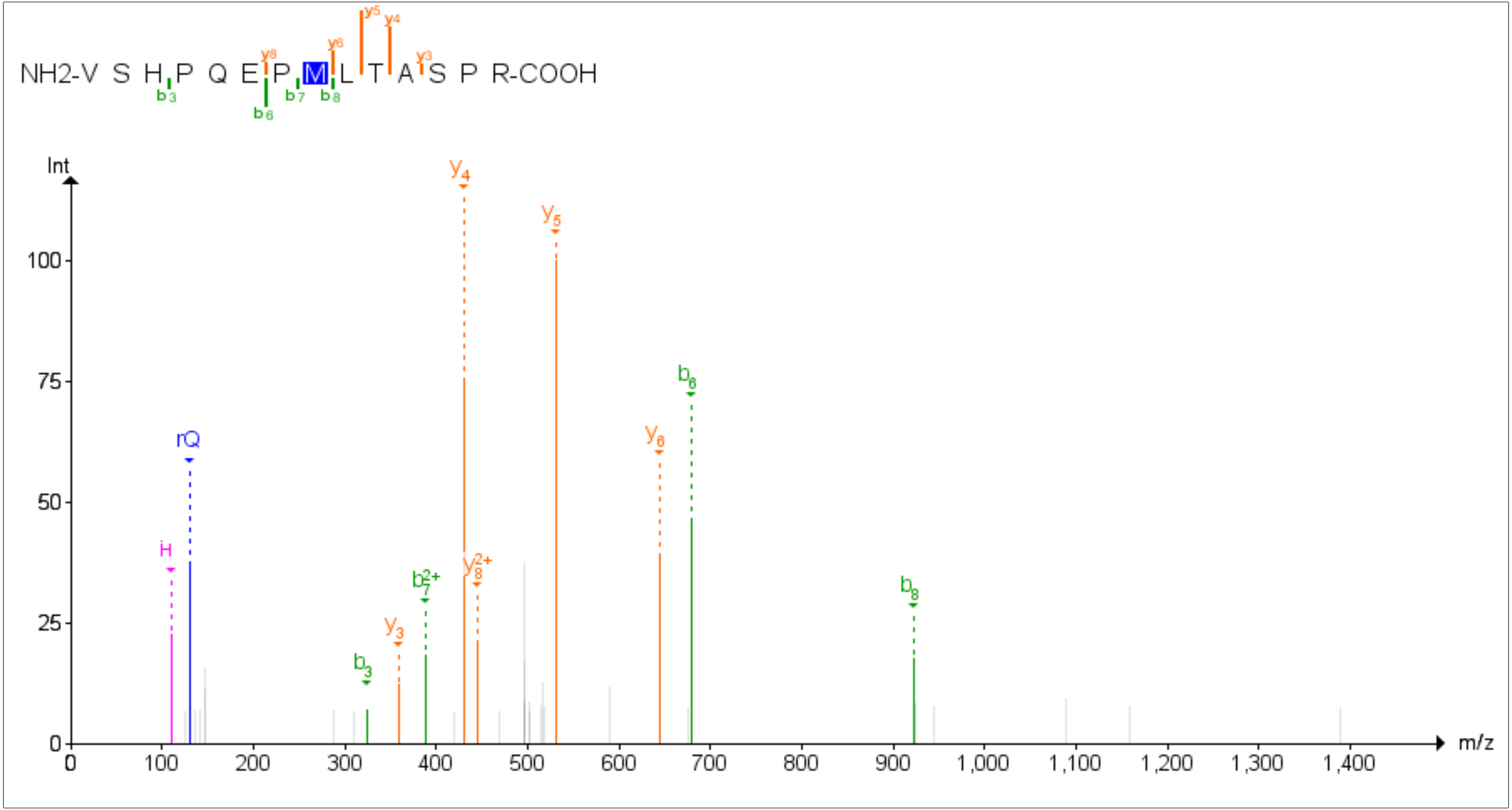

NH2-V S H P Q E P M L T A S P R-COOH
y5 y4 y8 y6 y3
b3 b6 b7 b8
Int
m/z
y4
y5
rQ
H
b3
y3
b7 2+
y8 2+
y6
b6
b8

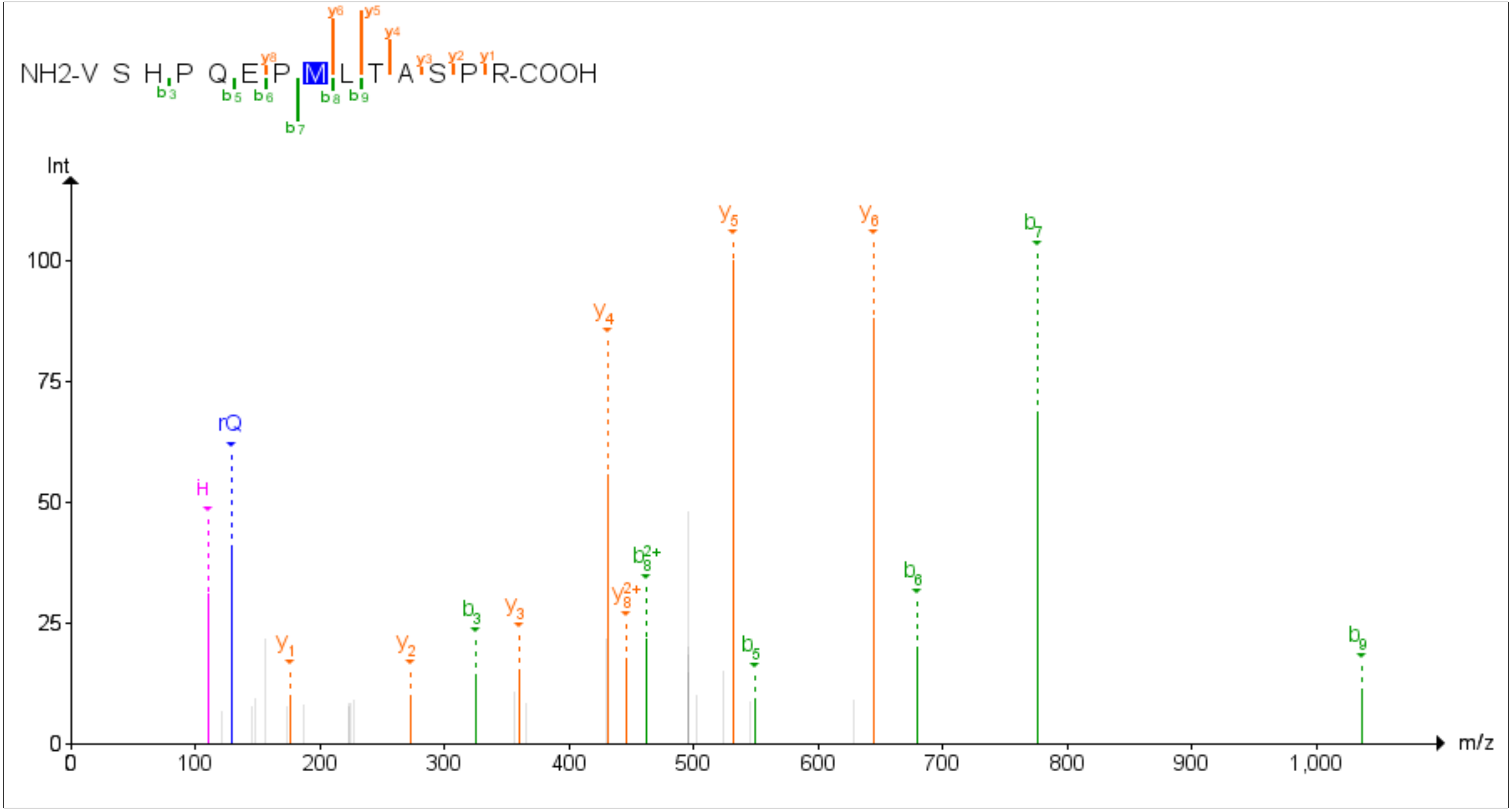

NH2-V S H P Q E P M L T A S P R-COOH
b3 b5 b6 b7 b8 b9
y8 y6 y5 y4 y3 y2 y1
Int
100
75
50
25
0
m/z
0
100
200
300
400
500
600
700
800
900
1,000
H
rQ
y1
y2
b3
y3
y4
y8 2+
b8 2+
y5
b5
y6
b6
b7
b9

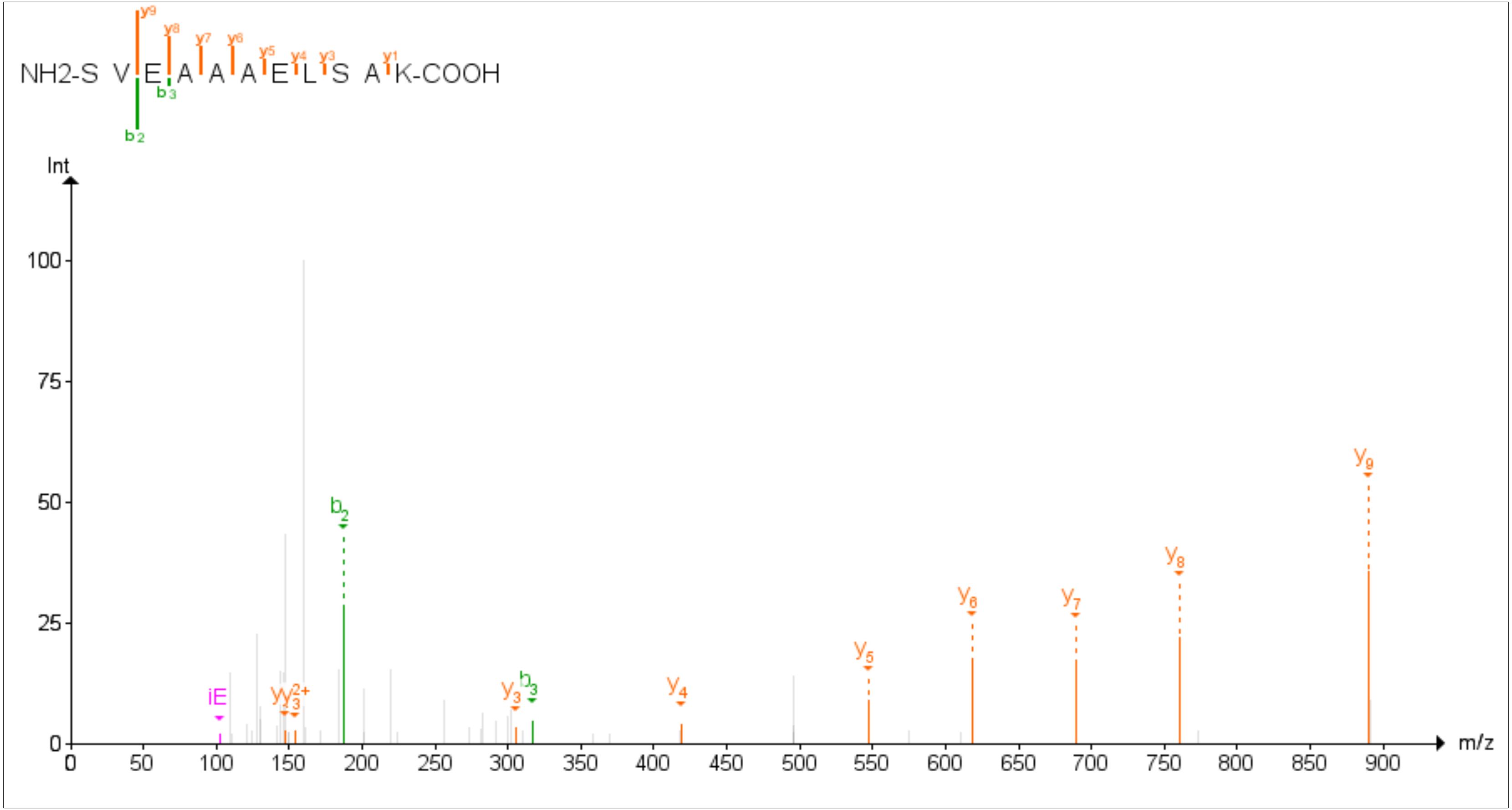

NH2-S V E A A A E L S A K-COOH
y9
y8
y7
y6
y5
y4
y3
y1
b2
b3
Int
100
75
50
25
0
m/z
0
50
100
150
200
250
300
350
400
450
500
550
600
650
700
750
800
850
900
iE
y
y3 2+
b2
y3
b3
y4
y5
y6
y7
y8
y9

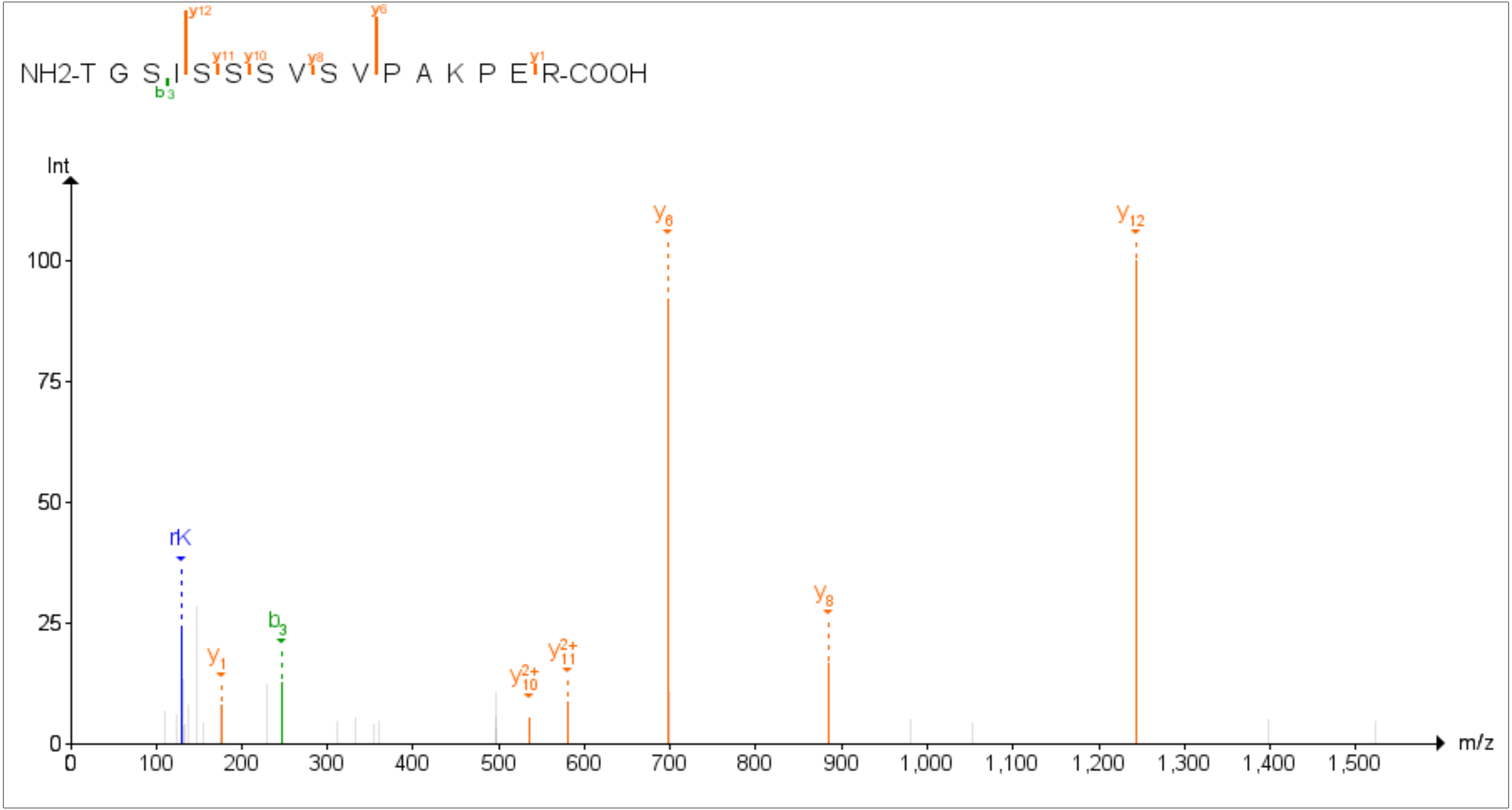

NH2-T G S I S S S V S V P A K P E R-COOH
y12
y11
y10
y8
y6
y1
b3
Int
100
75
50
25
0
rK
y1
b3
y10 2+
y11 2+
y6
y8
y12
0
100
200
300
400
500
600
700
800
900
1,000
1,100
1,200
1,300
1,400
1,500
m/z

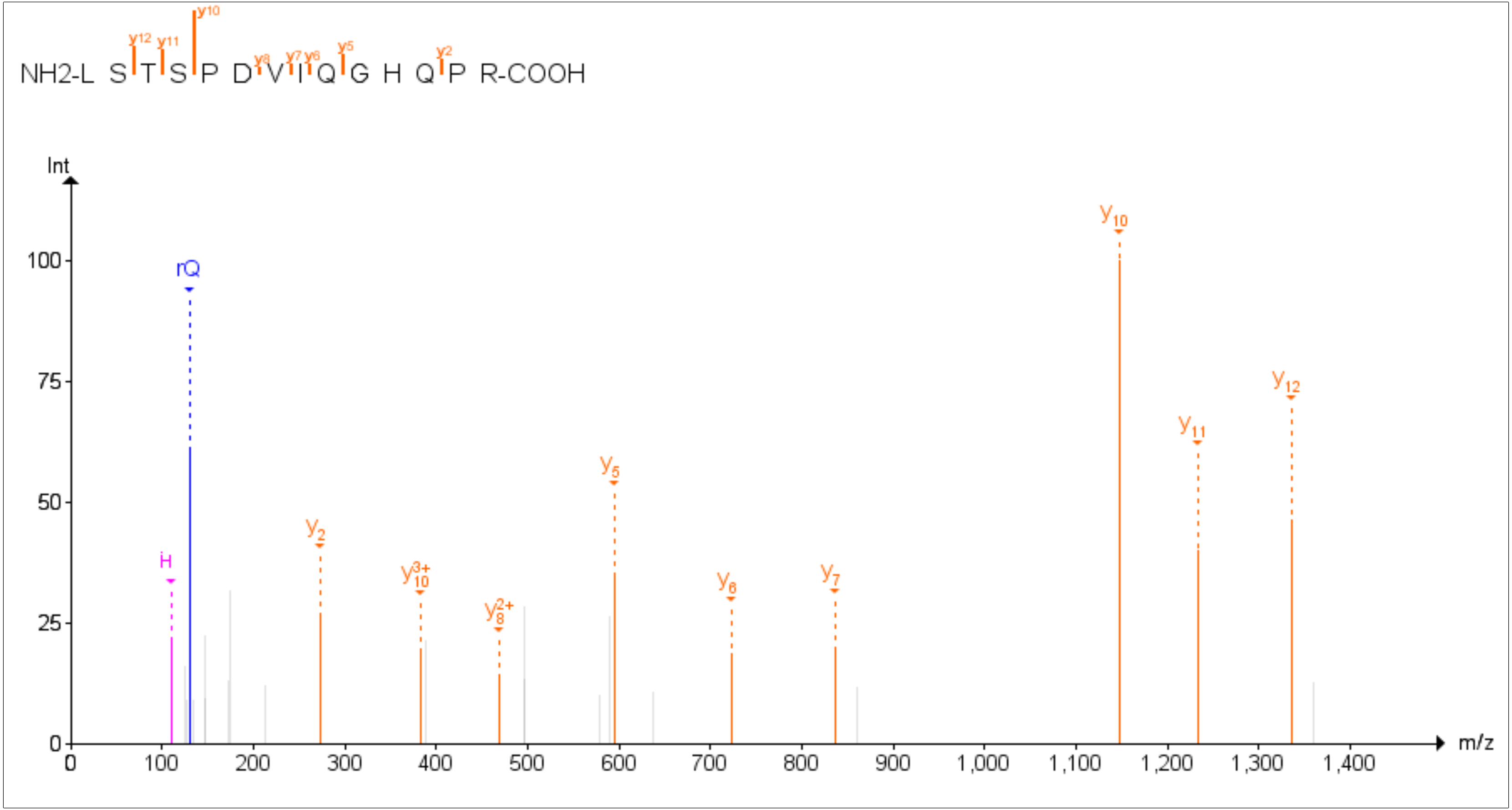
NH2-L S T S P D V I Q G H Q P R-COOH
y12 y11 y10 y8 y7 y6 y5 y2
Int
100
75
50
25
0
rQ
H
y2
y5
y6
y7
y10
y11
y12
0
100
200
300
400
500
600
700
800
900
1,000
1,100
1,200
1,300
1,400
m/z

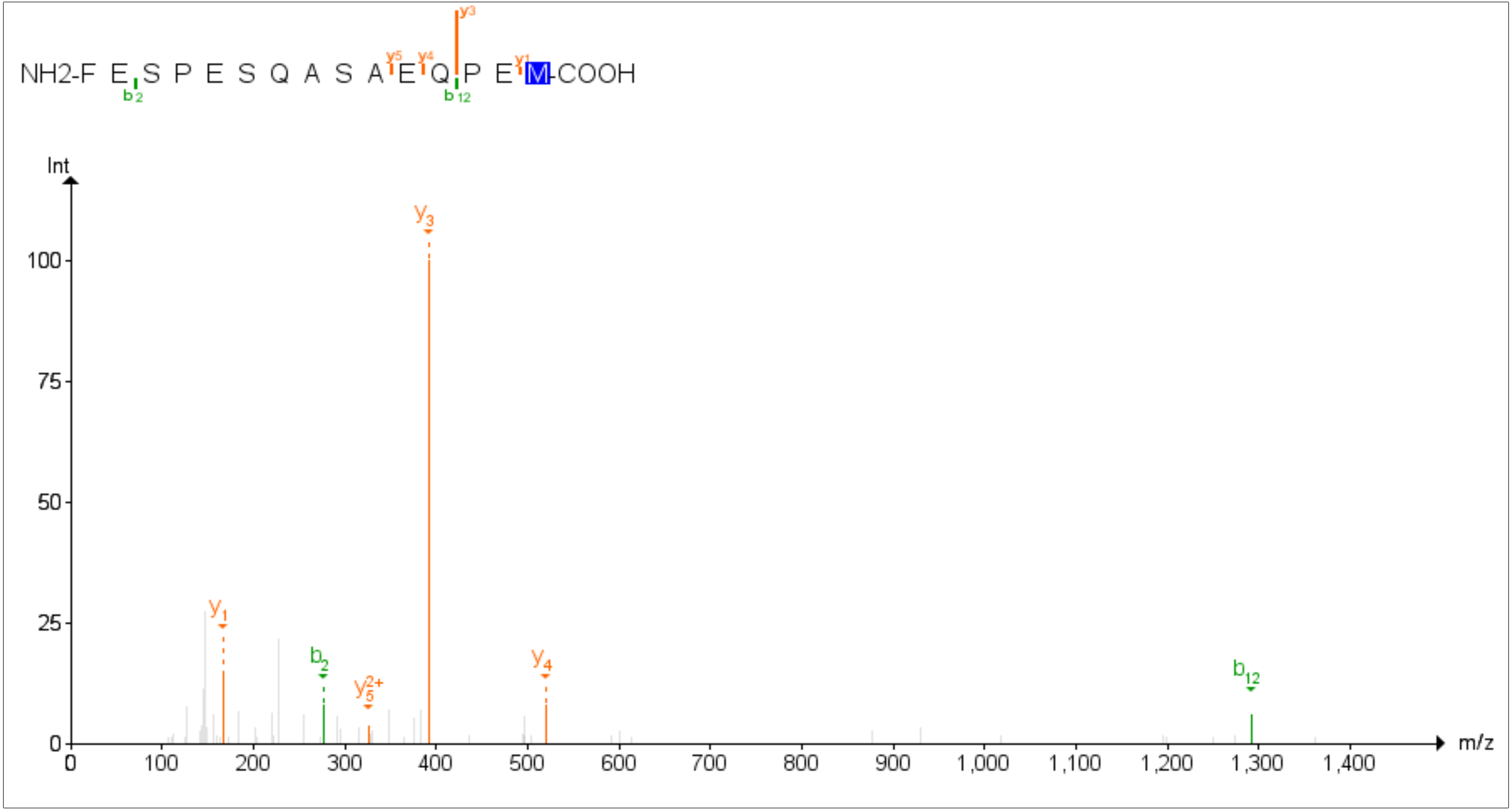

NH2-F E S P E S Q A S A E Q P E M-COOH
b2
b12
y5
y4
y3
y1
Int
100
75
50
25
0
y3
y1
b2
y5 2+
y4
b12
0
100
200
300
400
500
600
700
800
900
1,000
1,100
1,200
1,300
1,400
m/z

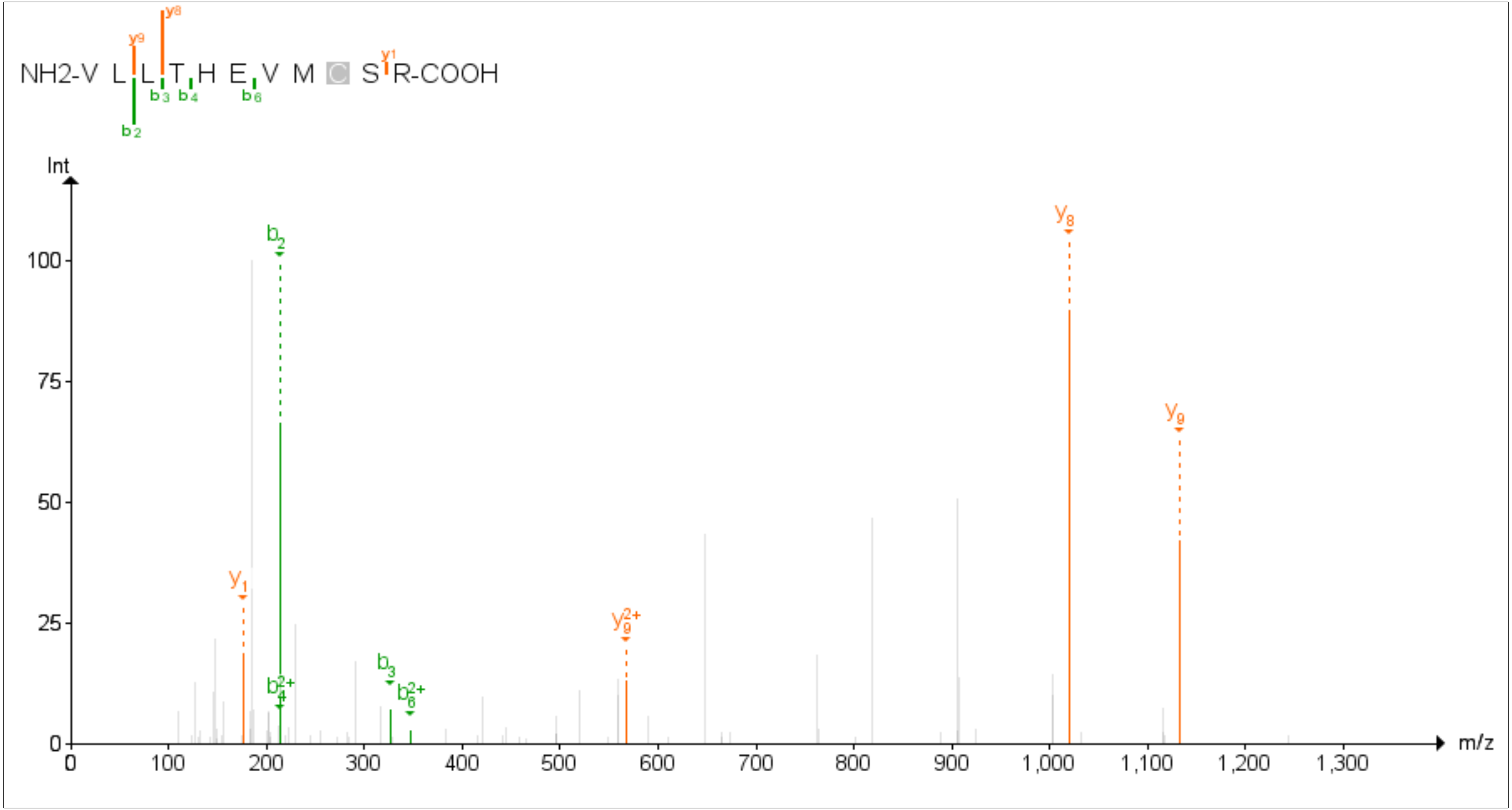

NH2-V L L T H E V M C S R-COOH
y9
y8
y1
b2
b3
b4
b6
Int
m/z
0
25
50
75
100
0
100
200
300
400
500
600
700
800
900
1,000
1,100
1,200
1,300
y1
b2
b4 2+
b3
b6 2+
y9 2+
y8
y9

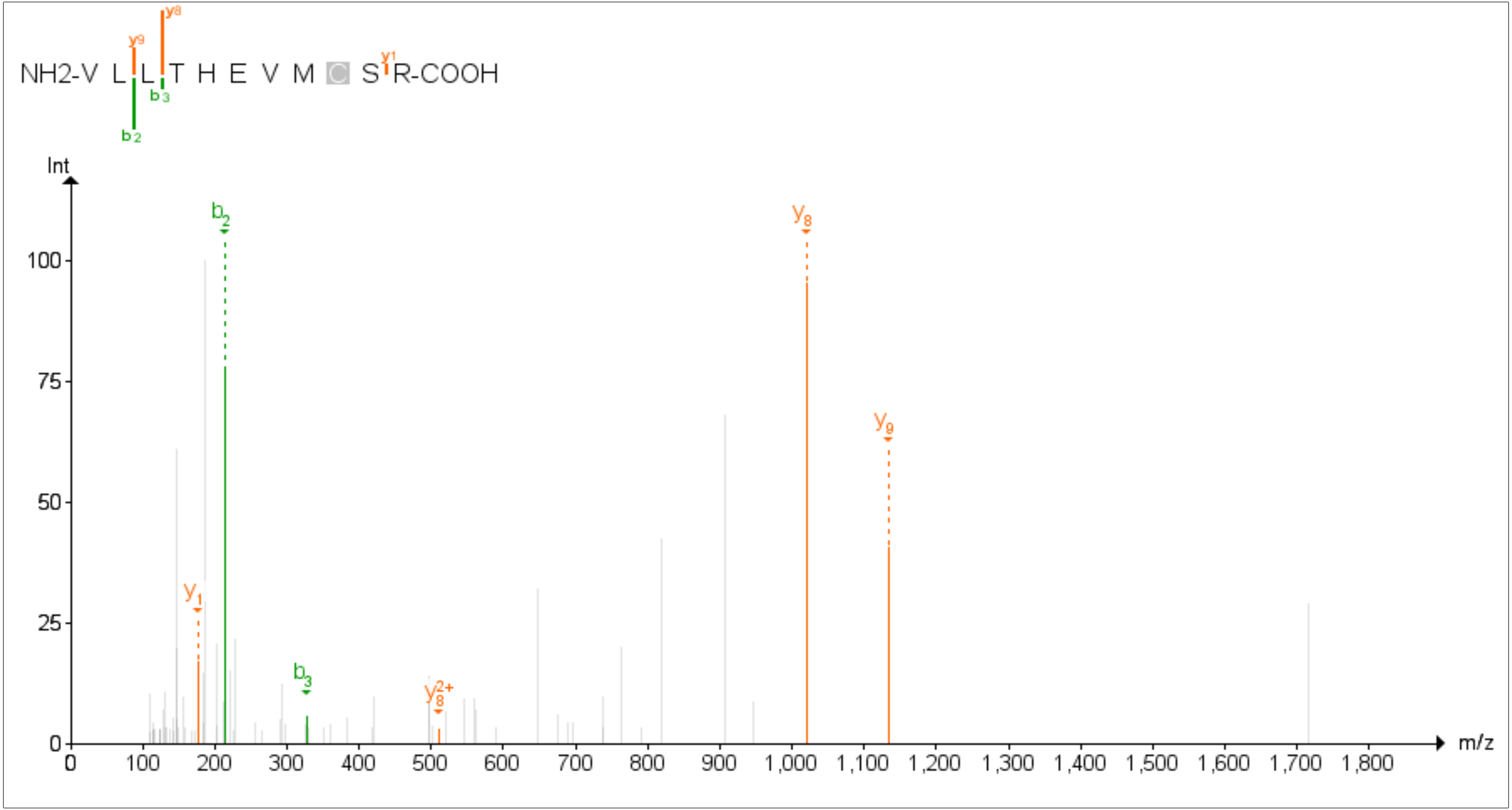

NH2-V L L T H E V M C S R-COOH
y9
y8
y1
b2
b3
Int
100
75
50
25
0
m/z
0
100
200
300
400
500
600
700
800
900
1,000
1,100
1,200
1,300
1,400
1,500
1,600
1,700
1,800
b_2
y_8
y_9
y_1
b_3
y_8^{2+}

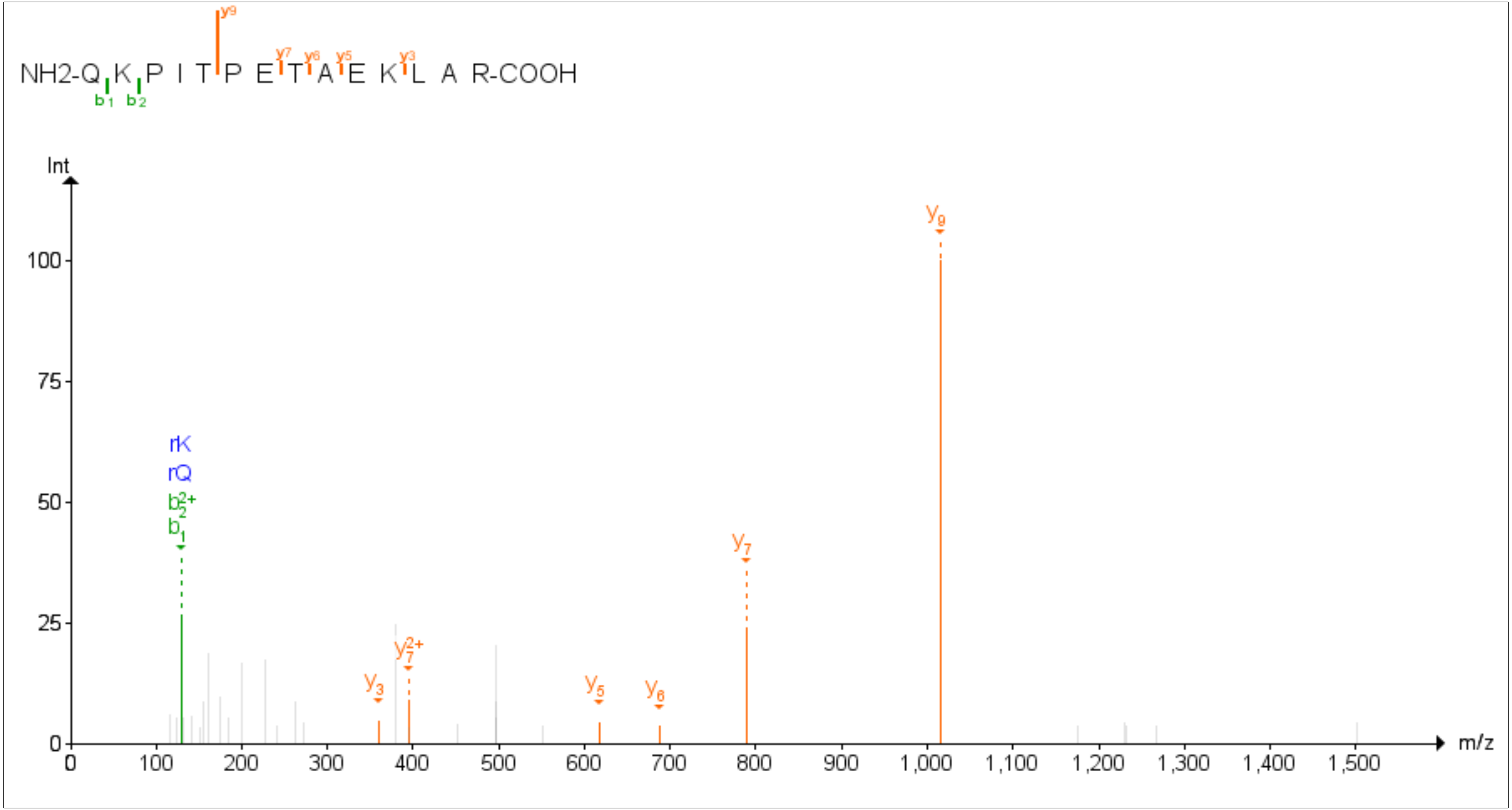
NH2-Q K P I T P E T A E K L A R-COOH
y9
y7
y6
y5
y3
b1
b2
Int
m/z
y9
y7
y7 2+
y3
y5
y6
rK
rQ
b2 2+
b1
0
25
50
75
100
0
100
200
300
400
500
600
700
800
900
1,000
1,100
1,200
1,300
1,400
1,500

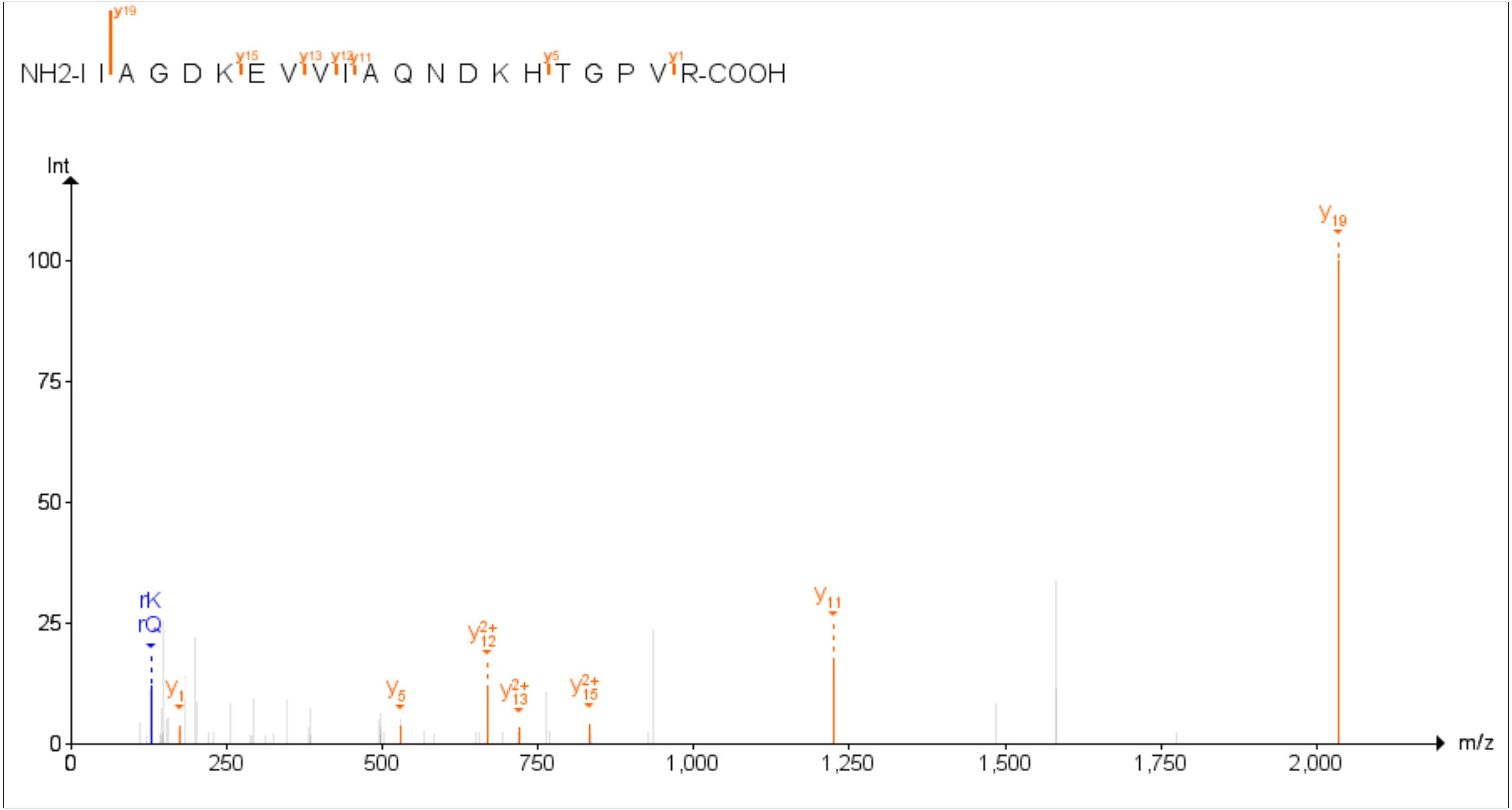

NH2-I I A G D K E V V I A Q N D K H T G P V R-COOH
y19
y15
y13
y12
y11
y5
y1
Int
100
75
50
25
0
rK
rQ
y1
y5
y12 2+
y13 2+
y15 2+
y11
y19
0
250
500
750
1,000
1,250
1,500
1,750
2,000
m/z

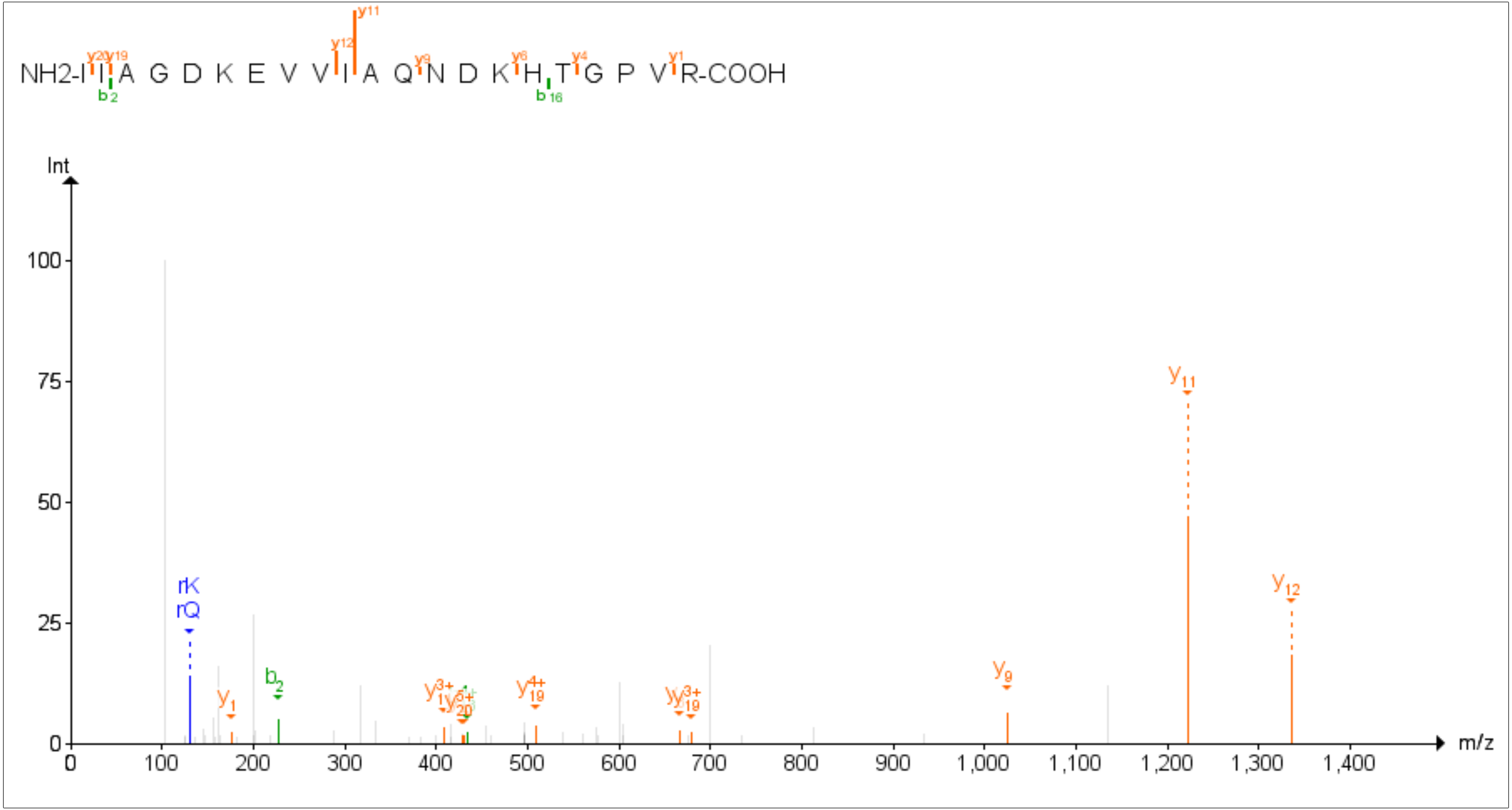
NH2-I I I A G D K E V V I A Q N D K H T G P V R-COOH
y20 y19 y12 y11 y9 y6 y4 y1
b2 b16
Int
m/z
rK
rQ
y1
b2
y11
y12
y9
0
25
50
75
100
0
100
200
300
400
500
600
700
800
900
1,000
1,100
1,200
1,300
1,400

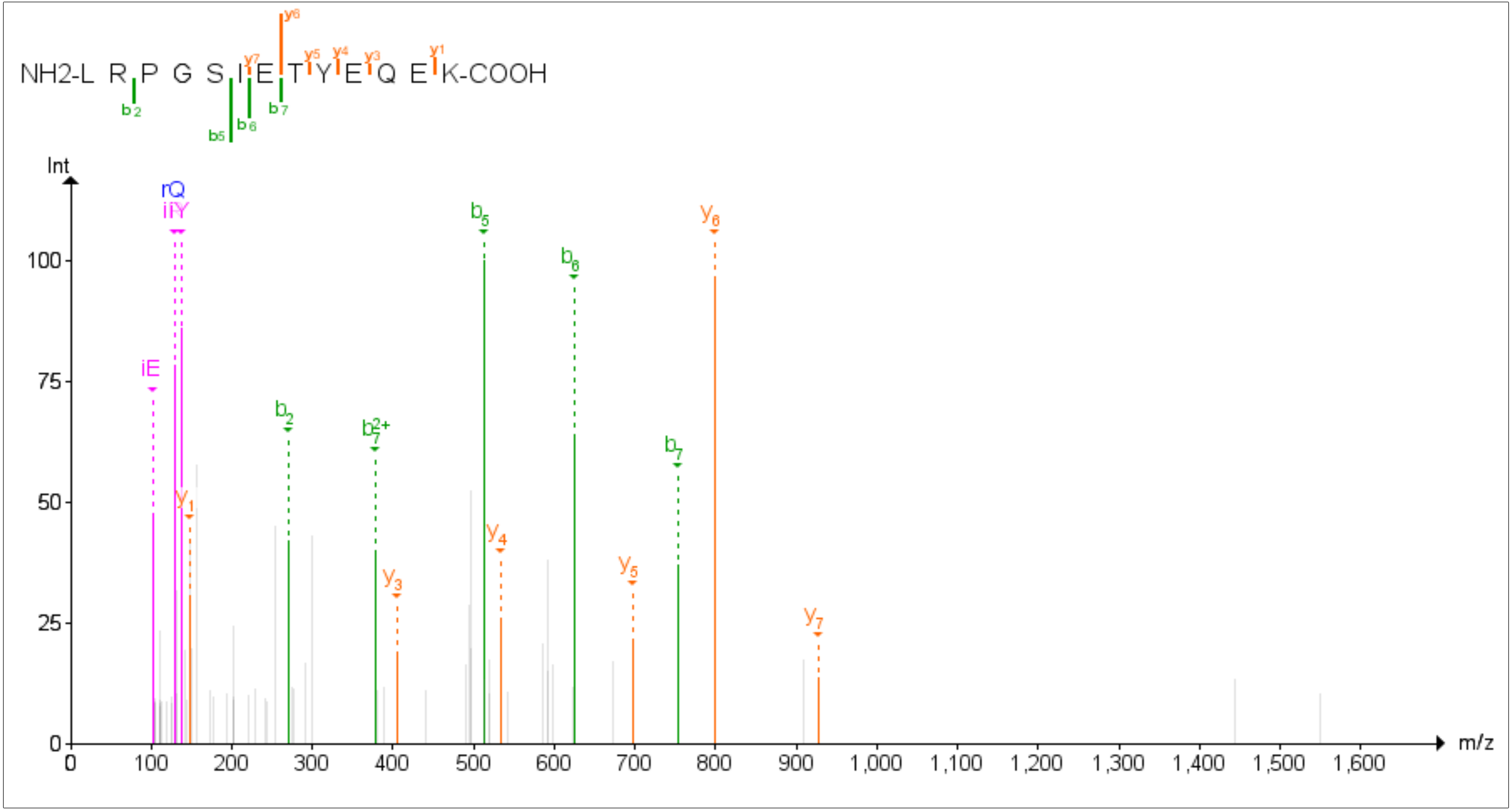

NH2-L R P G S I E T Y E Q E K-COOH
b2
b5
b6
b7
y7
y6
y5
y4
y3
y1
Int
rQ
iiY
iE
y1
b2
b7 2+
b5
y4
y3
b6
y5
b7
y6
y7
100
75
50
25
0
0
100
200
300
400
500
600
700
800
900
1,000
1,100
1,200
1,300
1,400
1,500
1,600
m/z

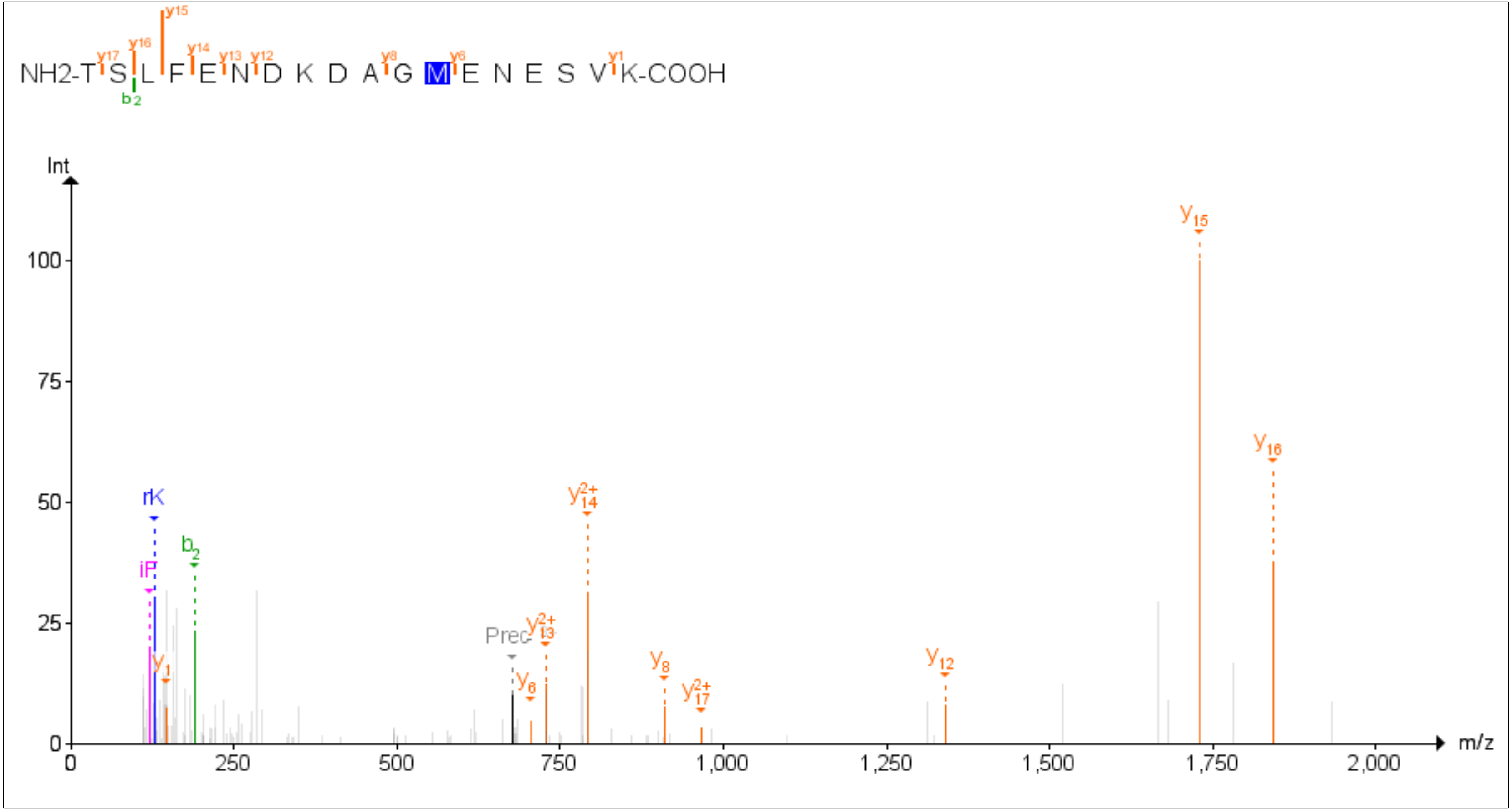

NH2-T S L F E N D K D A G M E N E S V K-COOH
y17 y16 y15 y14 y13 y12 y8 y6 y1
b2
Int
m/z
0
25
50
75
100
250
500
750
1,000
1,250
1,500
1,750
2,000
y15
y16
y14 2+
rK
b2
iP
y1
Prec
y13 2+
y6
y8
y17 2+
y12

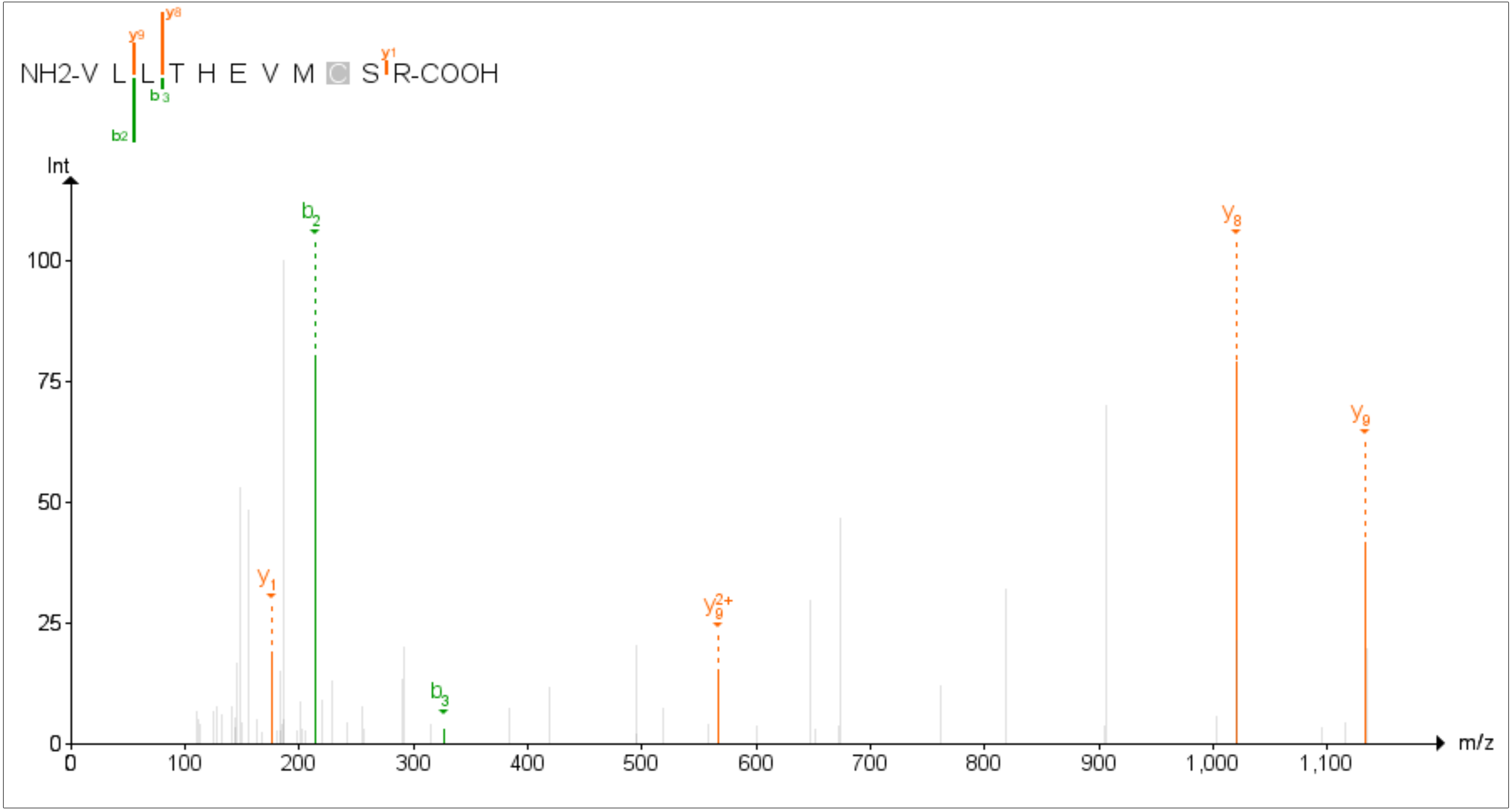

NH2-V L L T H E V M C S R-COOH
y9
y8
y1
b2
b3
Int
100
75
50
25
0
m/z
0
100
200
300
400
500
600
700
800
900
1,000
1,100
b2
y1
b3
y9 2+
y8
y9

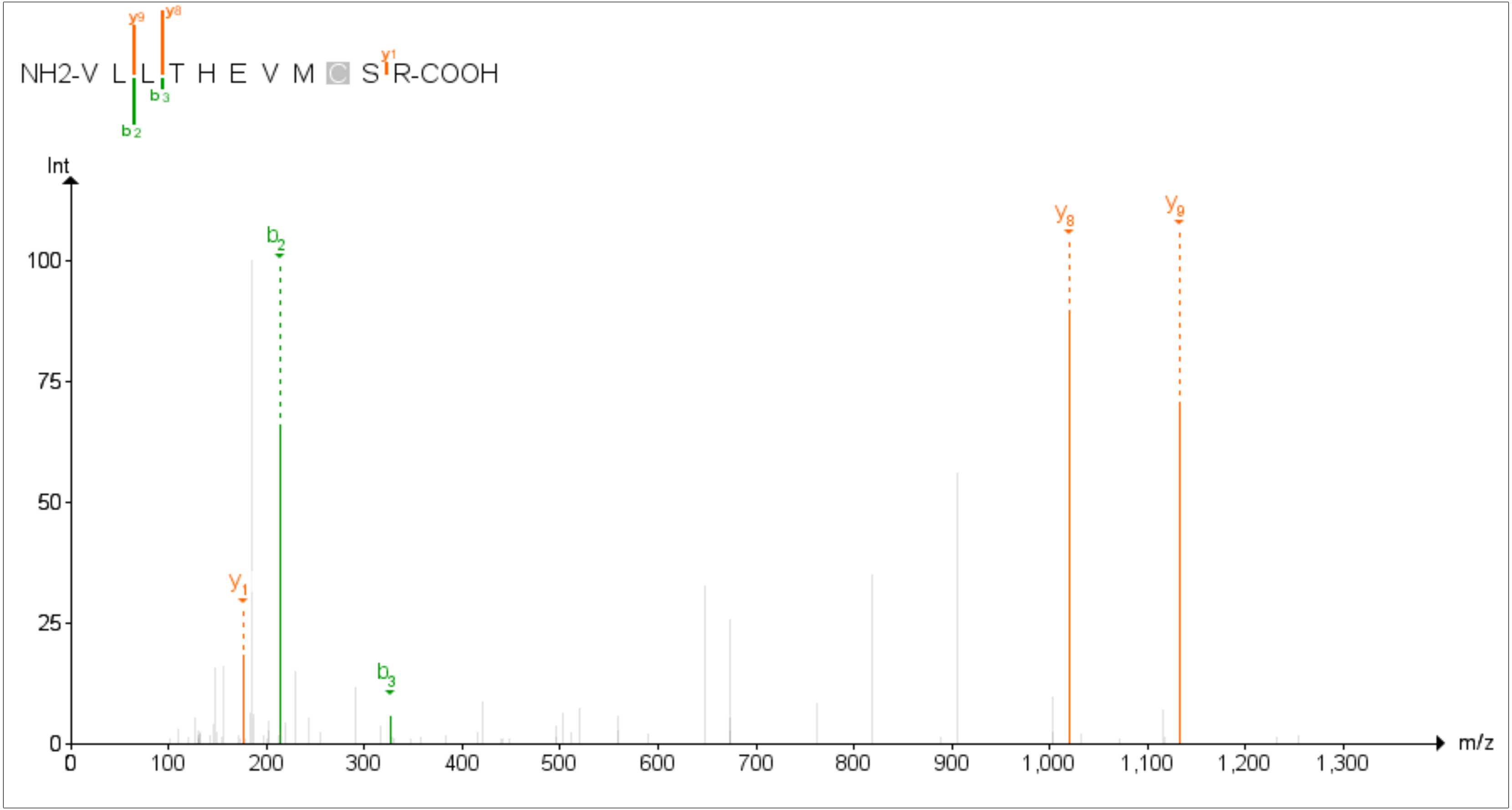

NH2-V L L T H E V M C S R-COOH
y9
y8
y1
b2
b3
Int
100
75
50
25
0
b2
y8
y9
y1
b3
m/z
0
100
200
300
400
500
600
700
800
900
1,000
1,100
1,200
1,300

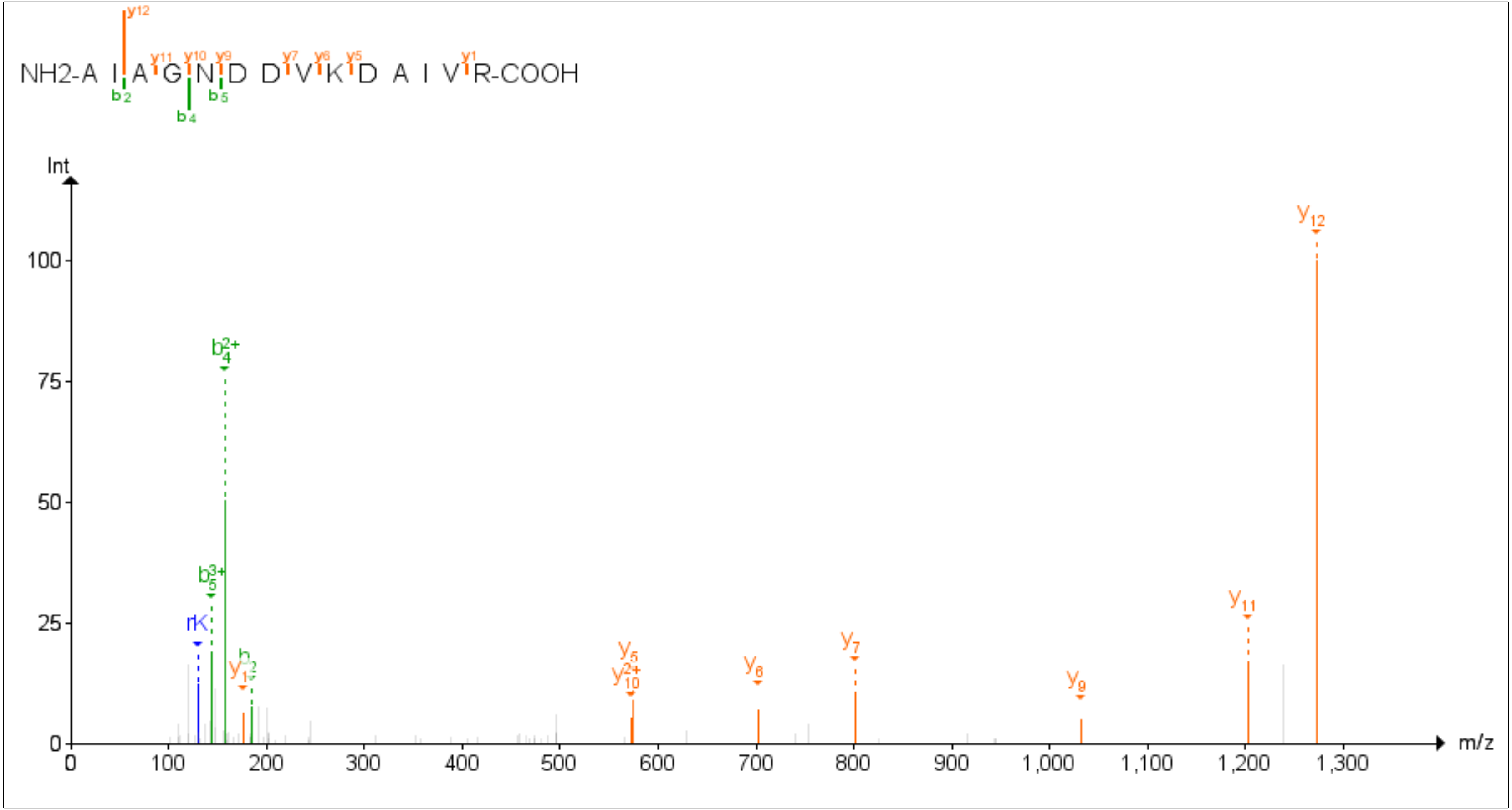

NH2-A I A G N D D V K D A I V R-COOH
y12
y11
y10
y9
y7
y6
y5
y1
b2
b4
b5
Int
100
75
50
25
0
m/z
0
100
200
300
400
500
600
700
800
900
1,000
1,100
1,200
1,300
y12
b4 2+
b5 3+
rK
b2
y1
y5
y10 2+
y6
y7
y9
y11

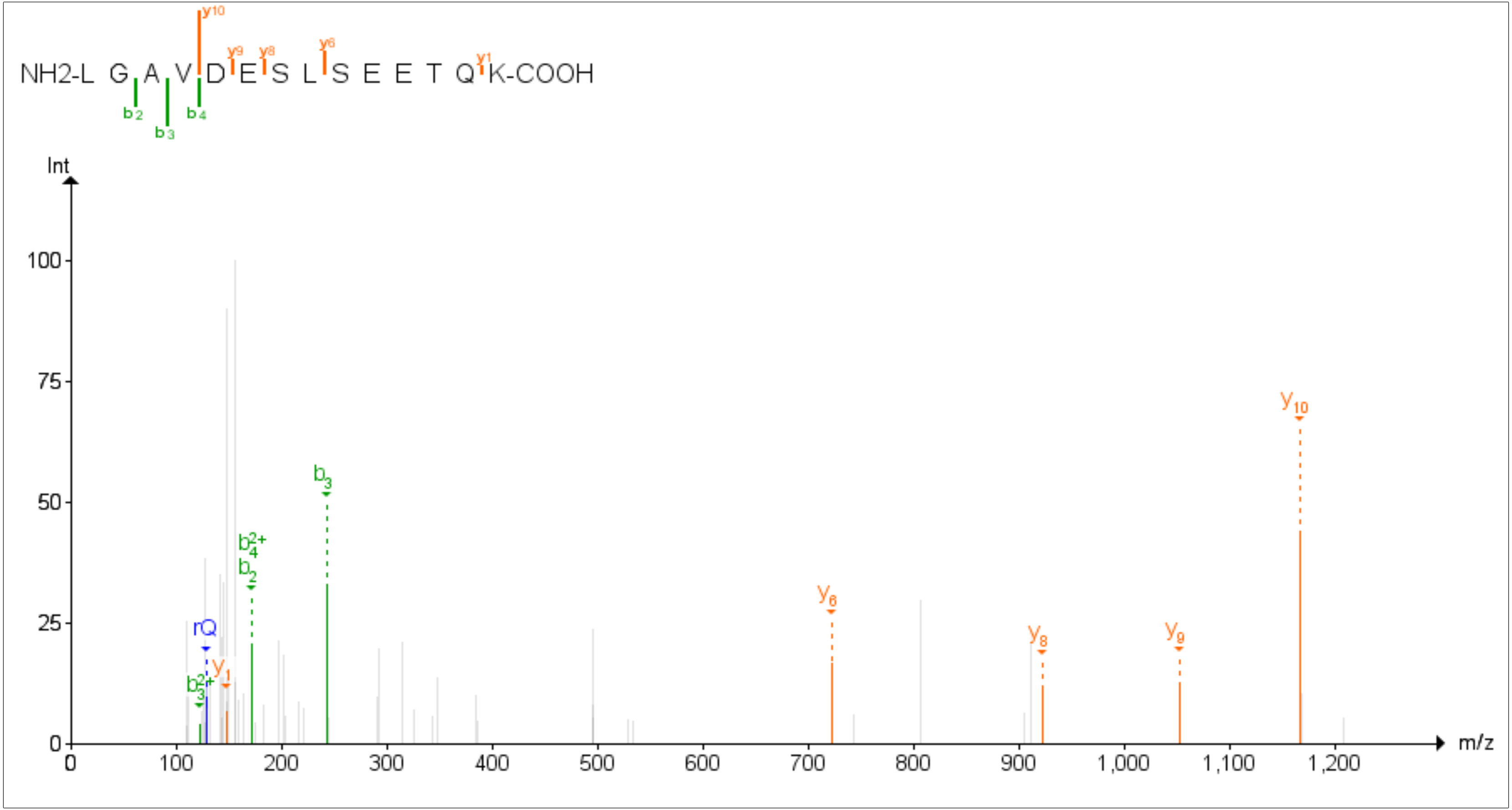
NH2-L G A V D E S L S E E T Q K-COOH
y10
y9
y8
y6
y1
b2
b3
b4
Int
100
75
50
25
0
b3
b4 2+
b2
rQ
y1
b3 2+
y6
y8
y9
y10
m/z
0
100
200
300
400
500
600
700
800
900
1,000
1,100
1,200

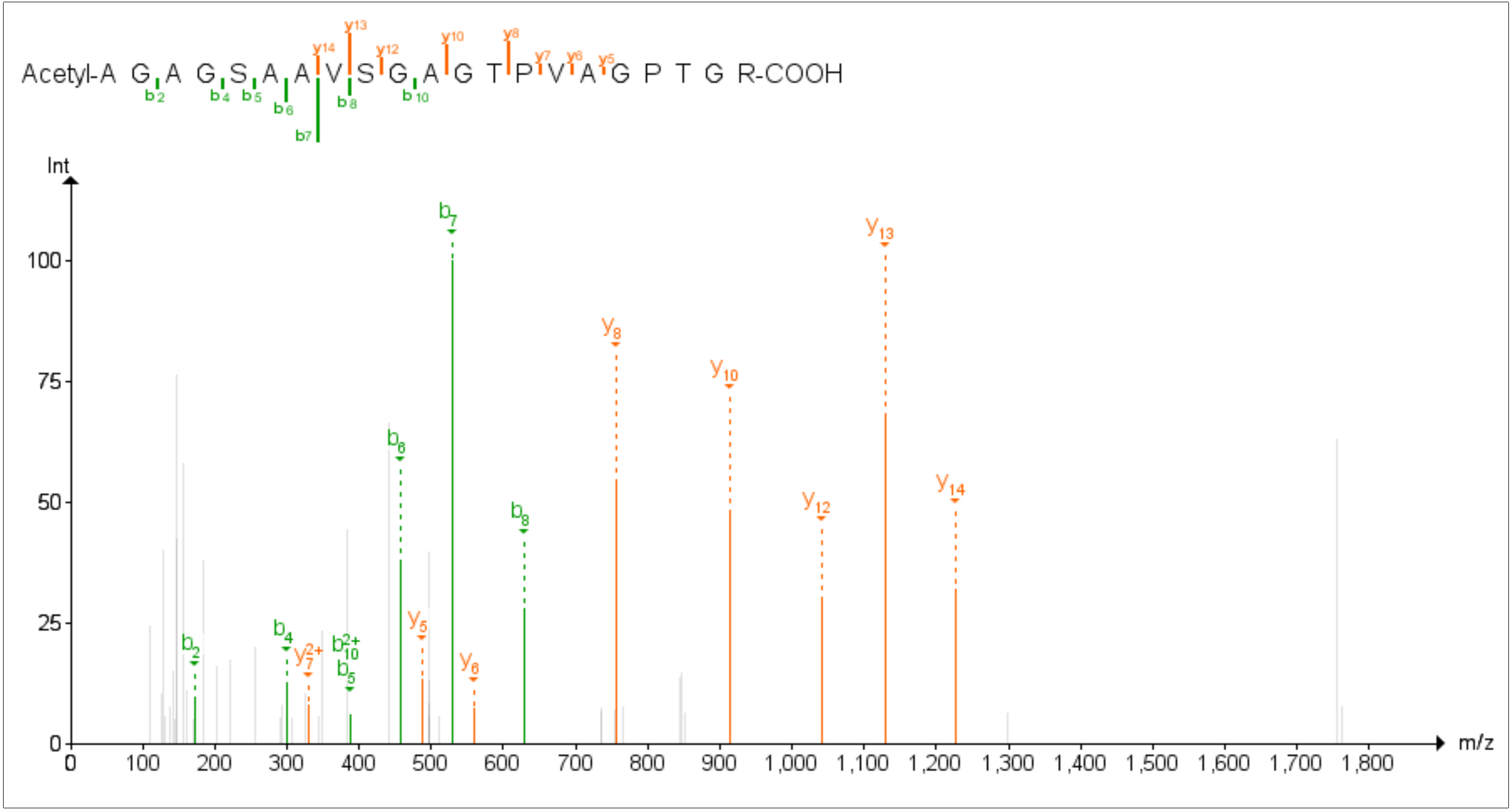
Acetyl-A G A G S A A V S G A G T P V A G P T G R-COOH
y13 y14 y12 y10 y8 y7 y6 y5
b2 b4 b5 b6 b7 b8 b10
Int
m/z
b7
y13
y8
y10
b6
b8
y12
y14
y5
b4
b2
y7 2+
b10 2+
b5
y6
0
25
50
75
100
0 100 200 300 400 500 600 700 800 900 1,000 1,100 1,200 1,300 1,400 1,500 1,600 1,700 1,800

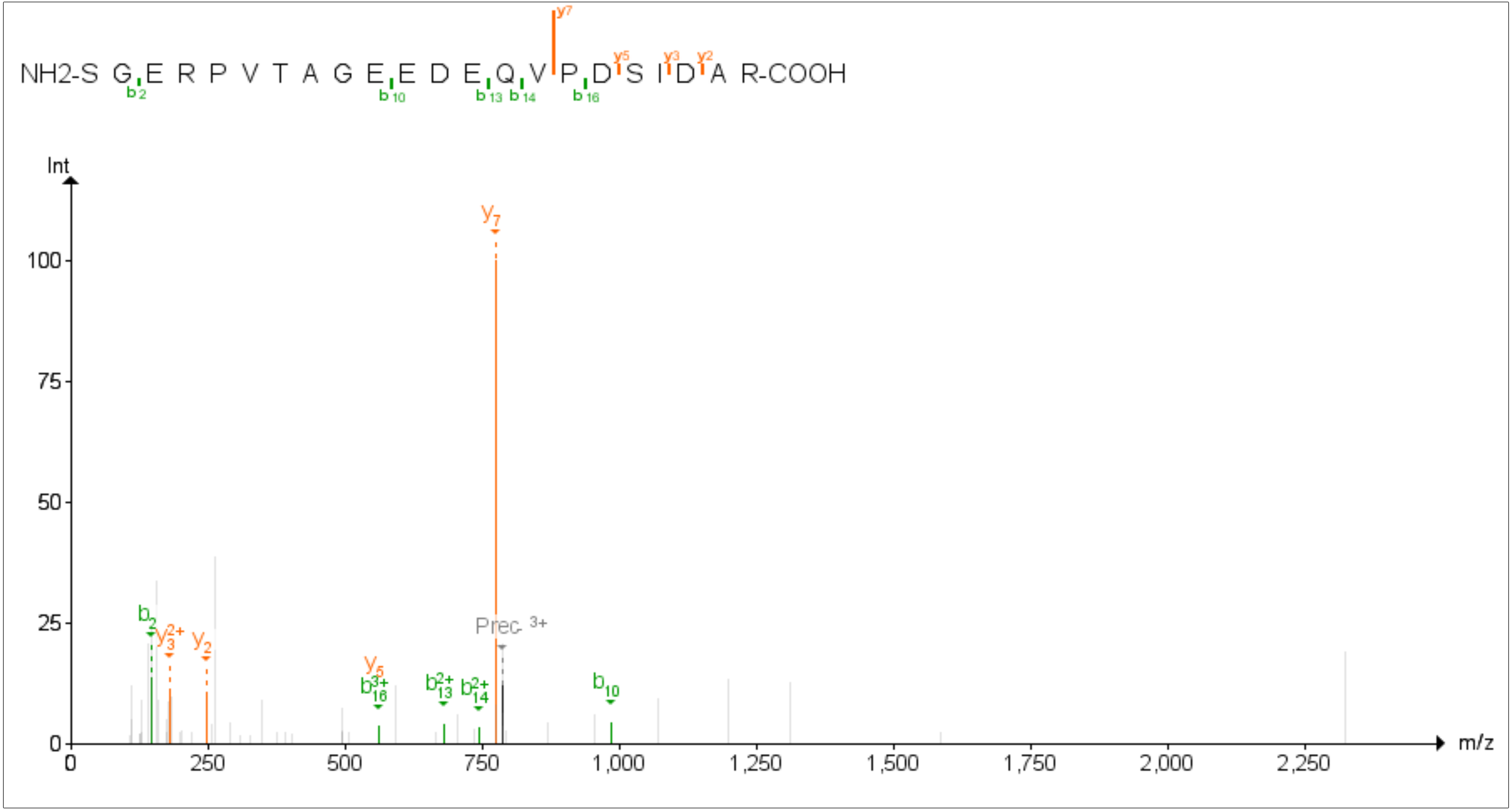

NH2-S G E R P V T A G E E D E Q V P D S I D A R-COOH
b2 b10 b13 b14 b16
y7 y5 y3 y2
Int
100
75
50
25
0
y7
b2
y3 2+
y2
y5
b16 3+
b13 2+
b14 2+
Prec- 3+
b10
0
250
500
750
1,000
1,250
1,500
1,750
2,000
2,250
m/z

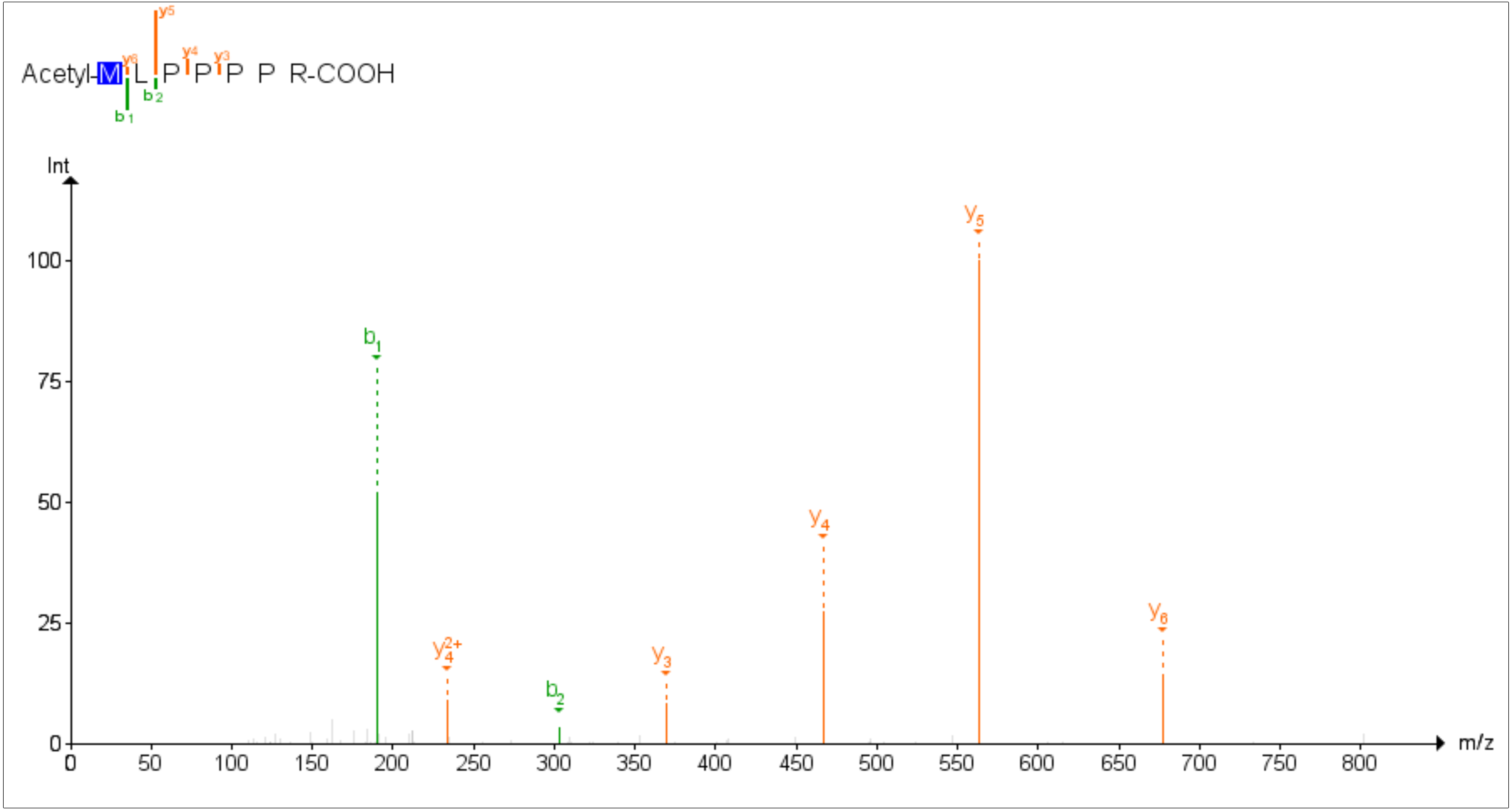

Acetyl-M L P P P P R-COOH
y5
y6
y4
y3
b1
b2
Int
100
75
50
25
0
m/z
0
50
100
150
200
250
300
350
400
450
500
550
600
650
700
750
800
b1
y4 2+
b2
y3
y4
y5
y6

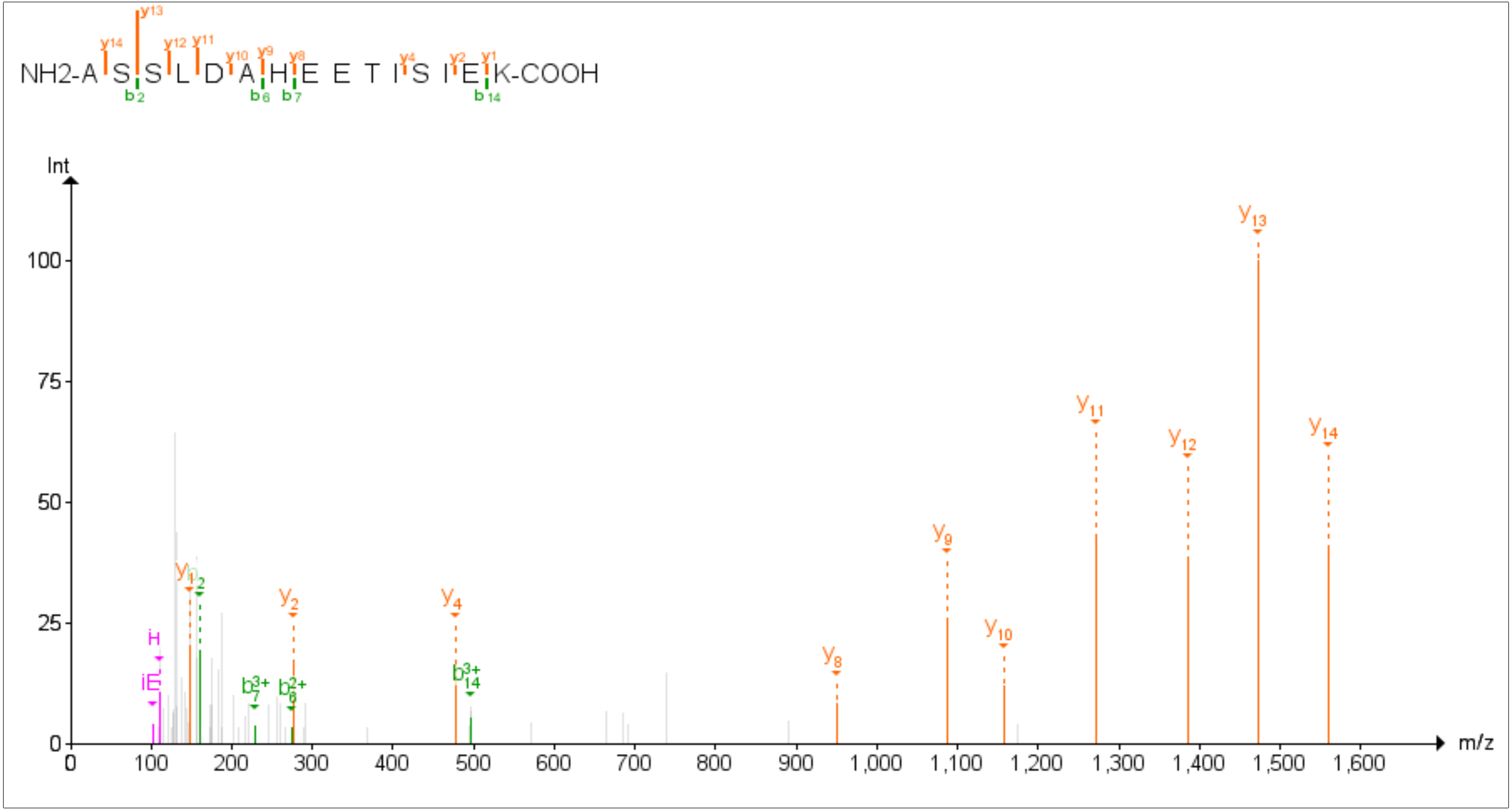

NH2-A S S L D A H E E T I S I E K-COOH
y14 y13 y12 y11 y10 y9 y8 y4 y2 y1
b2 b6 b7 b14
Int
m/z
y13
y11
y12
y14
y9
y10
y8
y4
y2
y1
b2
b7 3+
b6 2+
b14 3+
iH
iE
0
25
50
75
100
0
100
200
300
400
500
600
700
800
900
1,000
1,100
1,200
1,300
1,400
1,500
1,600

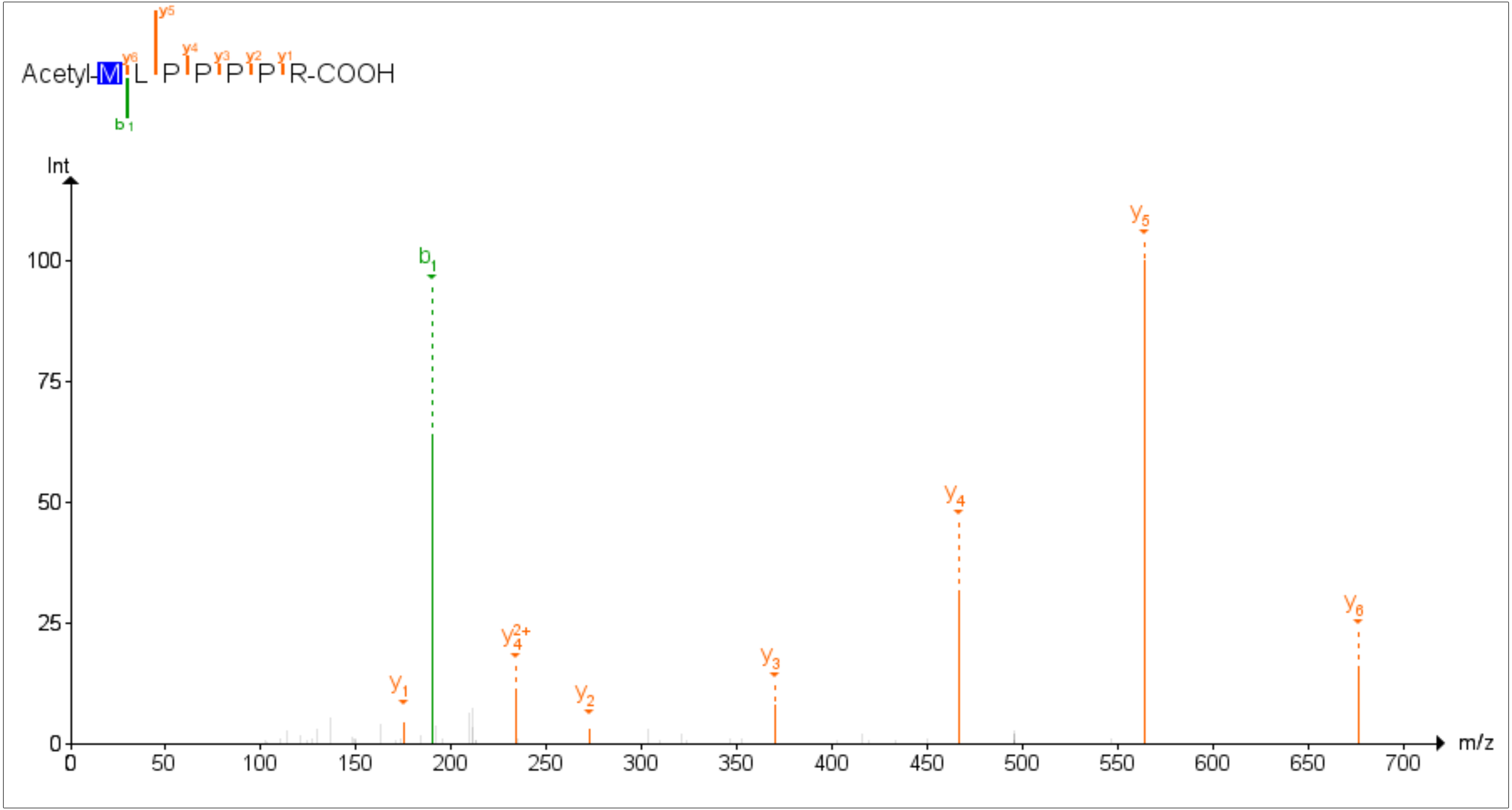

Acetyl-M L P P P P R-COOH
y6 y5 y4 y3 y2 y1
b 1
Int
0
25
50
75
100
m/z
0 50 100 150 200 250 300 350 400 450 500 550 600 650 700
b1
y1
y4 2+
y2
y3
y4
y5
y6

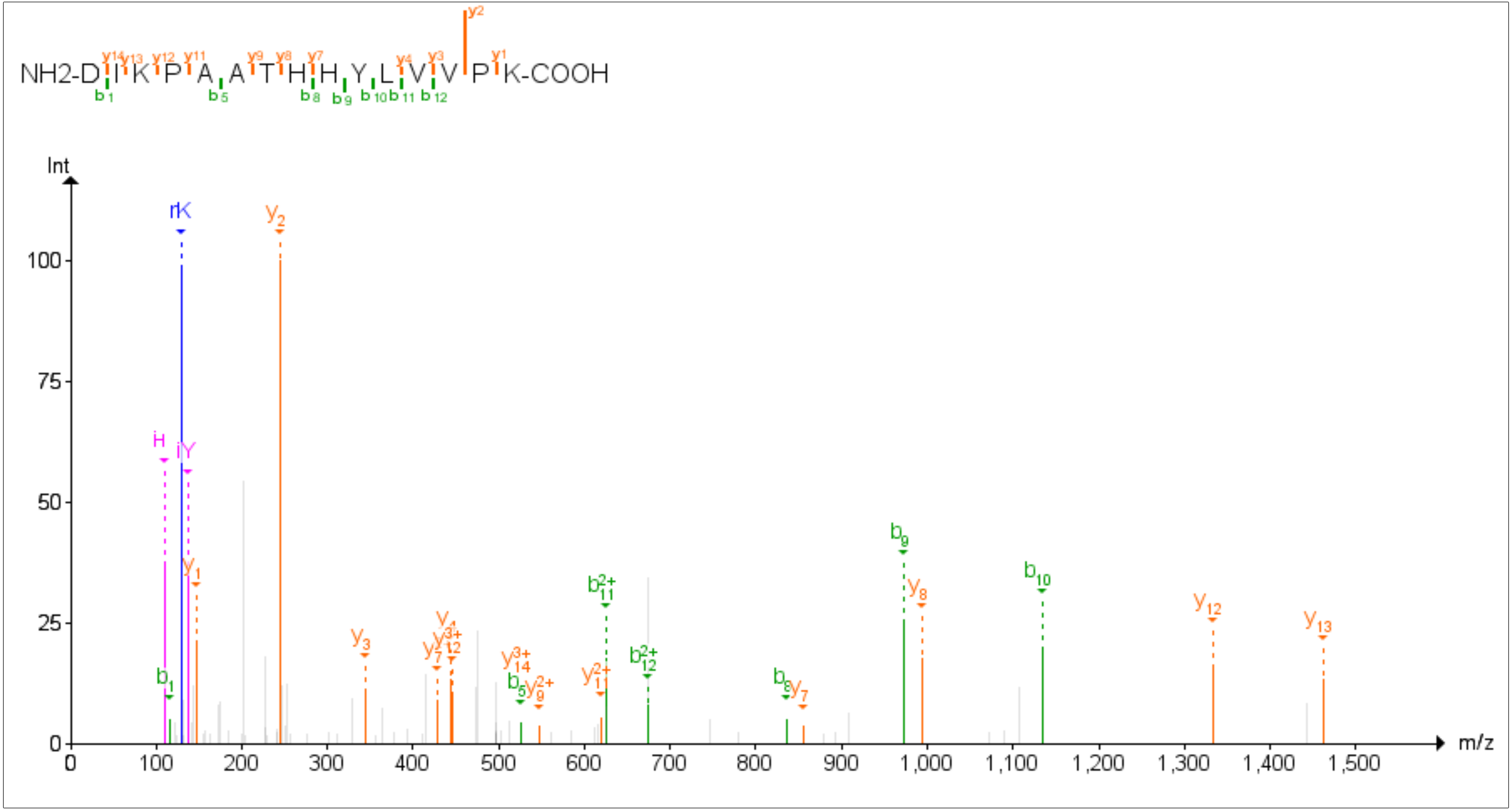

NH2-DIKPAATHHYLVVPK-COOH
Int
m/z
rK
y2
iH
iY
y1
b1
y3
y4
y7
b5
b8
b9
b10
y8
y12
y13

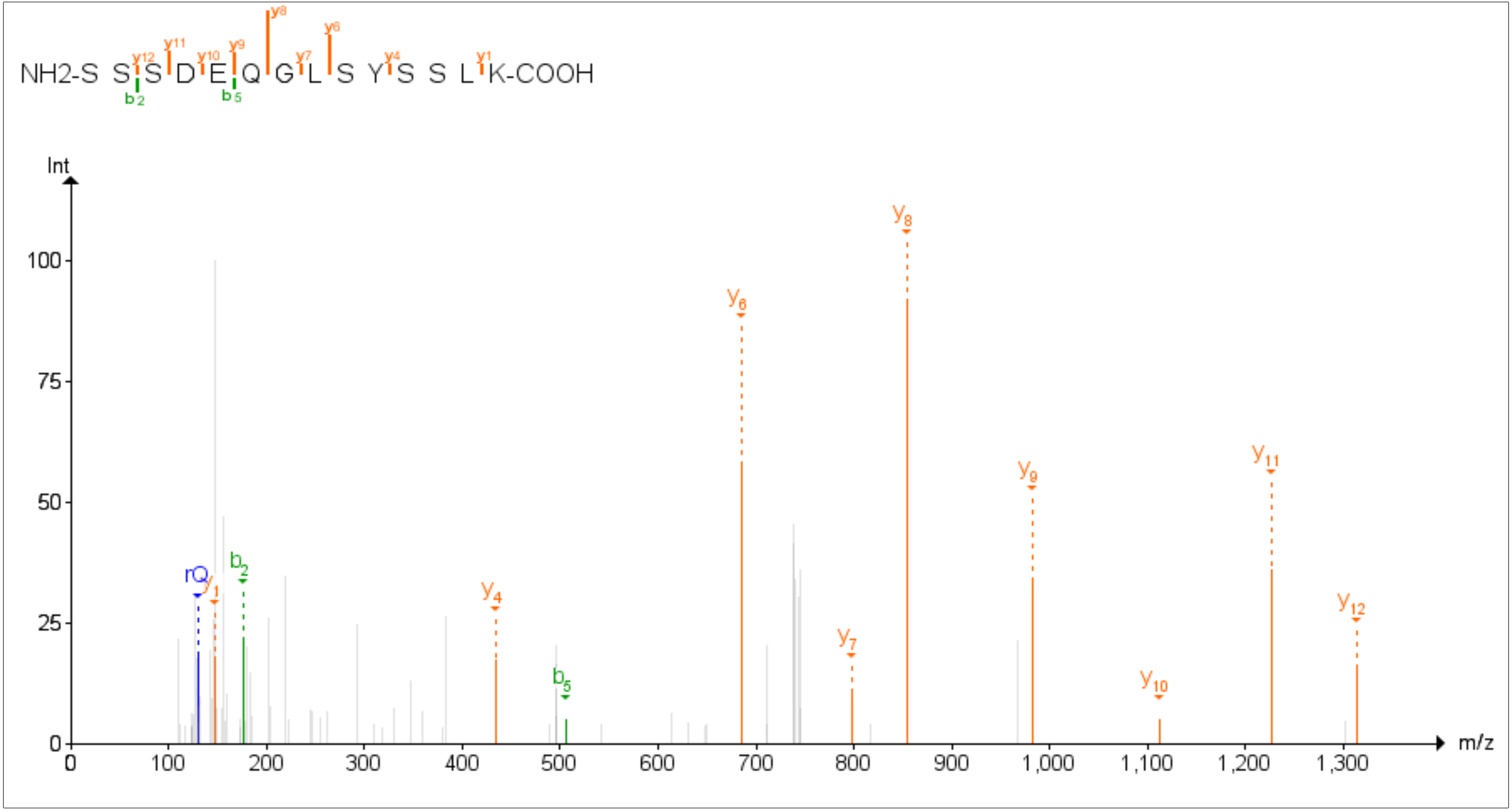

NH2-S S S D E Q G L S Y S S L K-COOH
y12 y11 y10 y9 y8 y7 y6 y4 y1
b2 b5
Int
m/z
rQ
y1 b2 y4 b5 y6 y7 y8 y9 y10 y11 y12
0 25 50 75 100
0 100 200 300 400 500 600 700 800 900 1,000 1,100 1,200 1,300

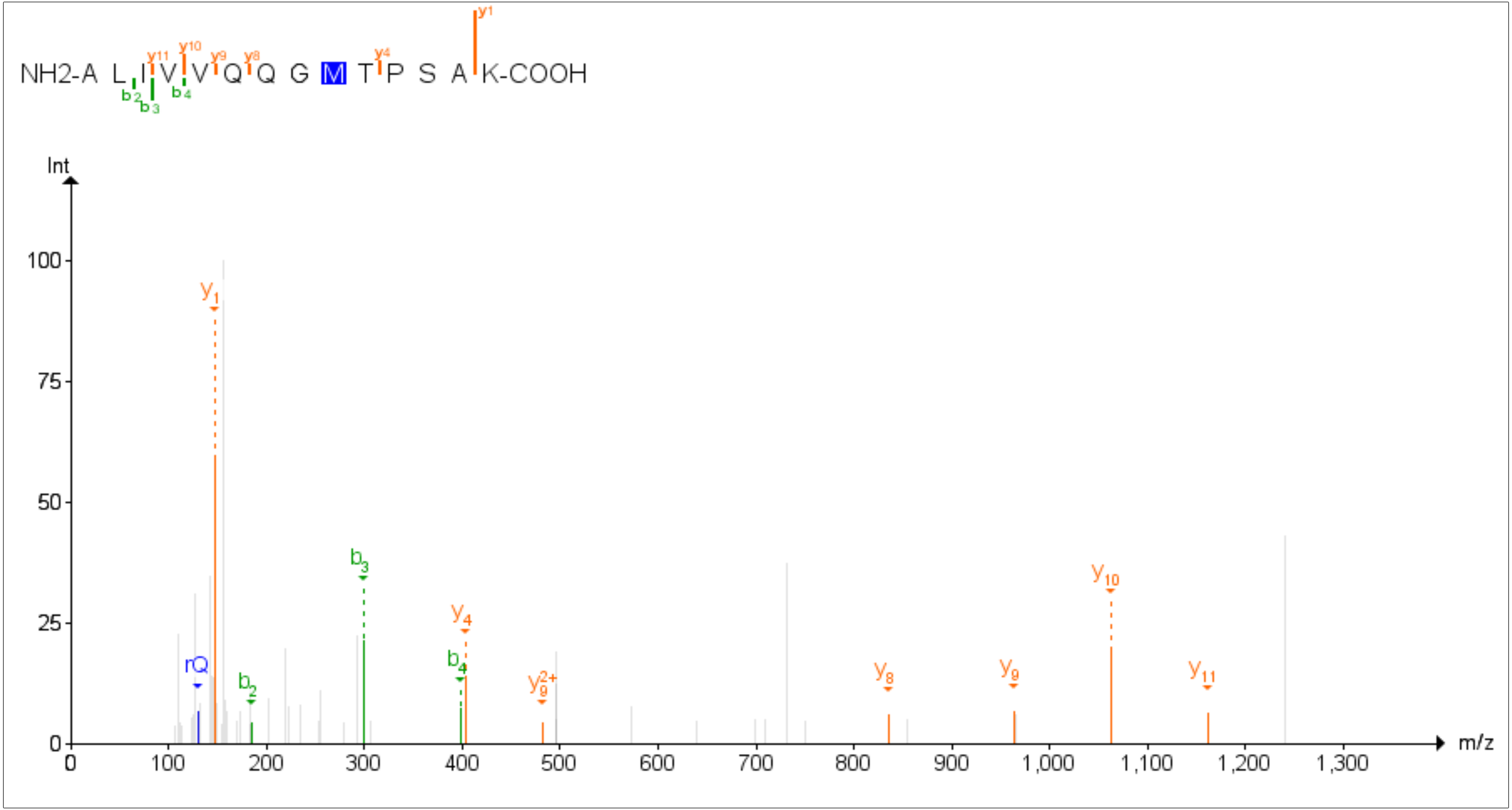

NH2-A L I V V Q Q G M T P S A K-COOH
y11 y10 y9 y8 y4 y1
b2 b3 b4
Int
m/z
y1
b3
y4
b4
y10
rQ
b2
y9 2+
y8
y9
y11
0
25
50
75
100
0
100
200
300
400
500
600
700
800
900
1,000
1,100
1,200
1,300

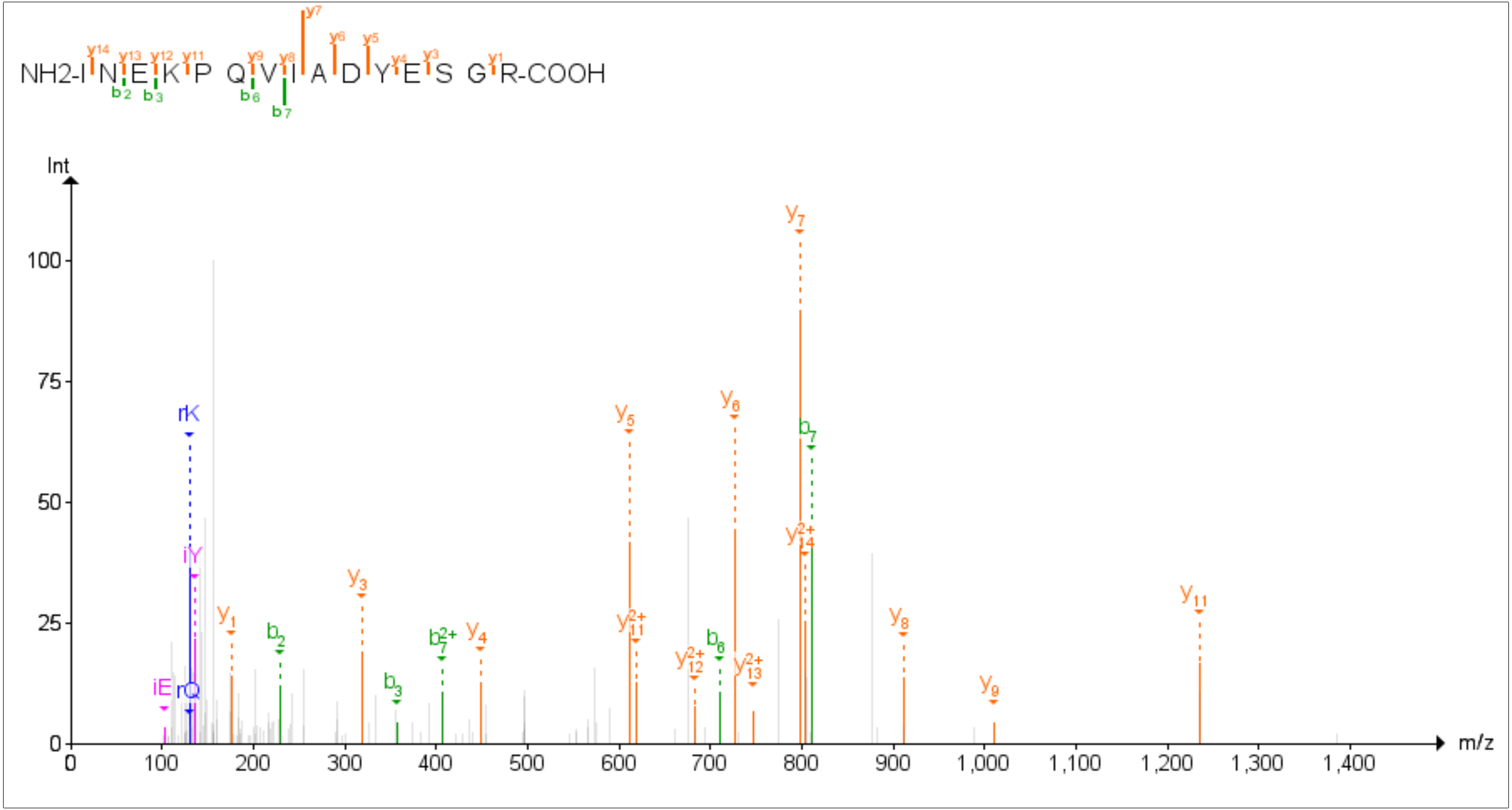

NH2-I N E K P Q V I A D Y E S G R-COOH
y14 y13 y12 y11 y9 y8 y7 y6 y5 y4 y3 y1
b2 b3 b6 b7
Int
100
75
50
25
0
m/z
0 100 200 300 400 500 600 700 800 900 1,000 1,100 1,200 1,300 1,400
rK
iY
iE
rQ
y1
b2
y3
b3
b7 2+
y4
y5
y11 2+
y12 2+
b6
y6
y13 2+
y7
b7
y14 2+
y8
y9
y11

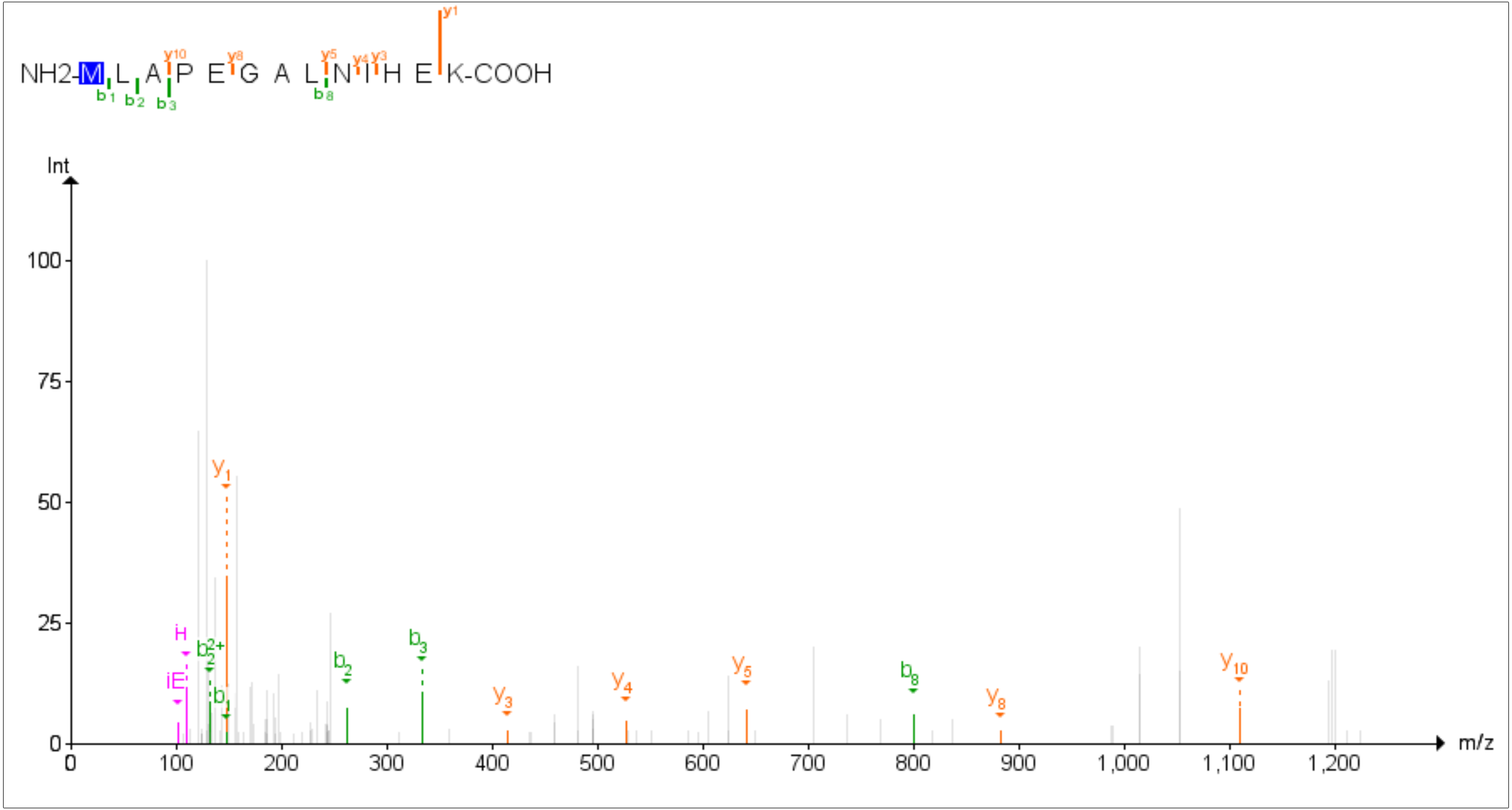

NH2-M L A P E G A L N I H E K-COOH
y1
y10
y8
y5
y4
y3
b1
b2
b3
b8
Int
100
75
50
25
0
m/z
0
100
200
300
400
500
600
700
800
900
1,000
1,100
1,200

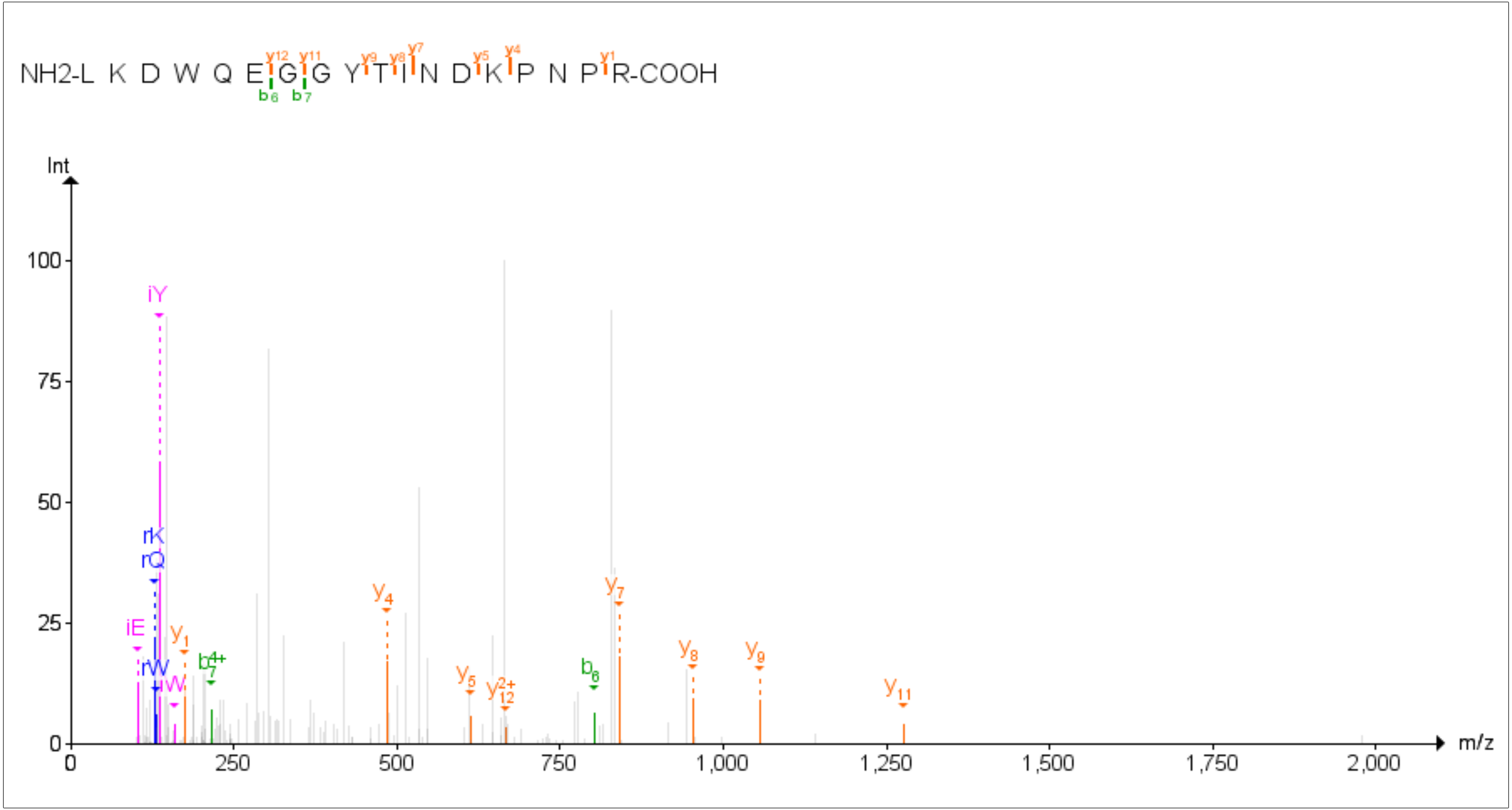
NH2-L K D W Q E G G Y T I N D K P N P R-COOH
y12
y11
y9
y8
y7
y5
y4
y1
b6
b7
Int
m/z
100
75
50
25
0
0
250
500
750
1,000
1,250
1,500
1,750
2,000
iY
rK
rQ
iE
y1
rW
iW
b7 4+
y4
y5
y12 2+
b6
y7
y8
y9
y11

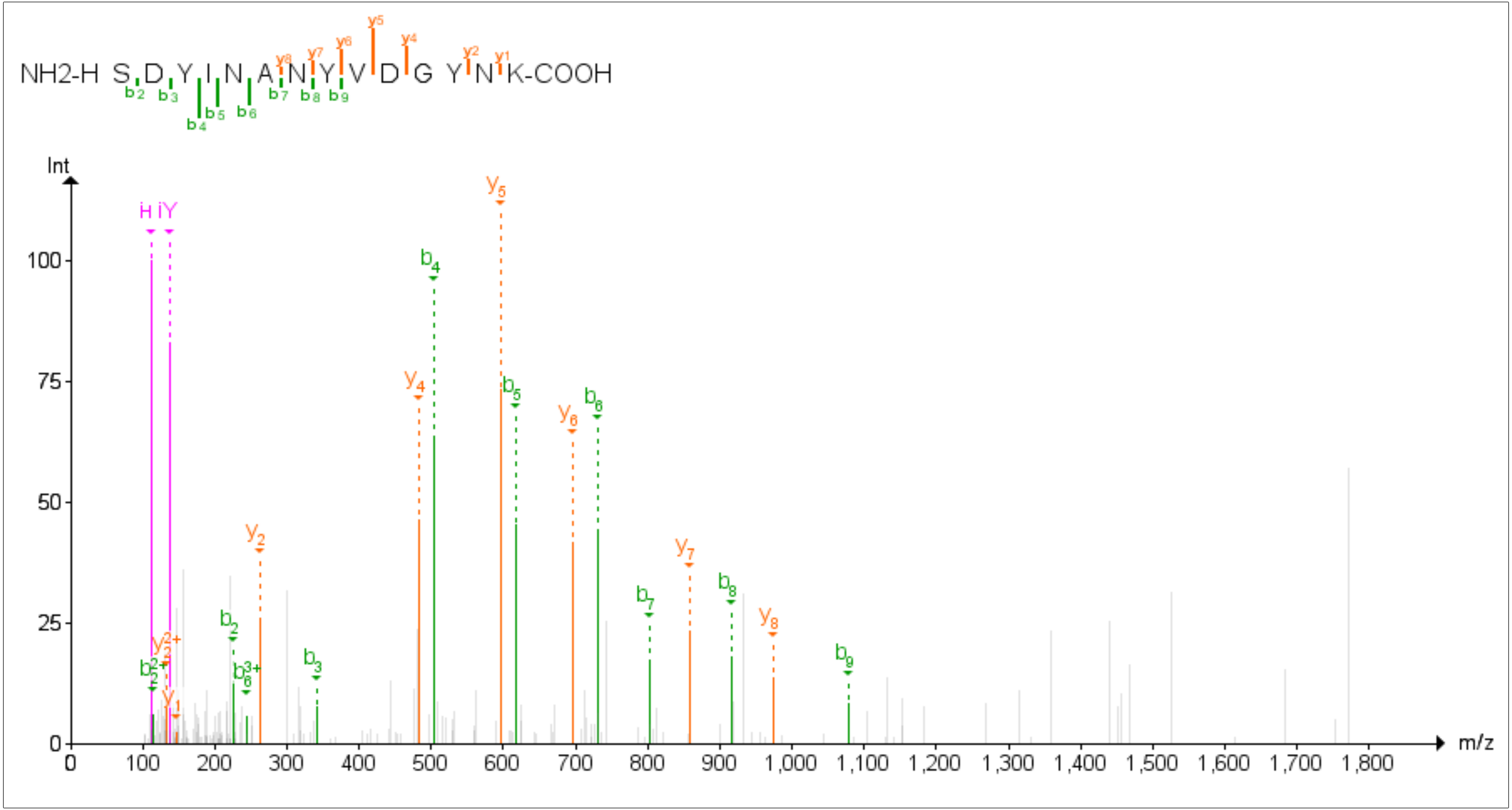

NH2-H S D Y I N A N Y V D G Y N K-COOH
Int
100
75
50
25
0
m/z
0
100
200
300
400
500
600
700
800
900
1,000
1,100
1,200
1,300
1,400
1,500
1,600
1,700
1,800
H
iY

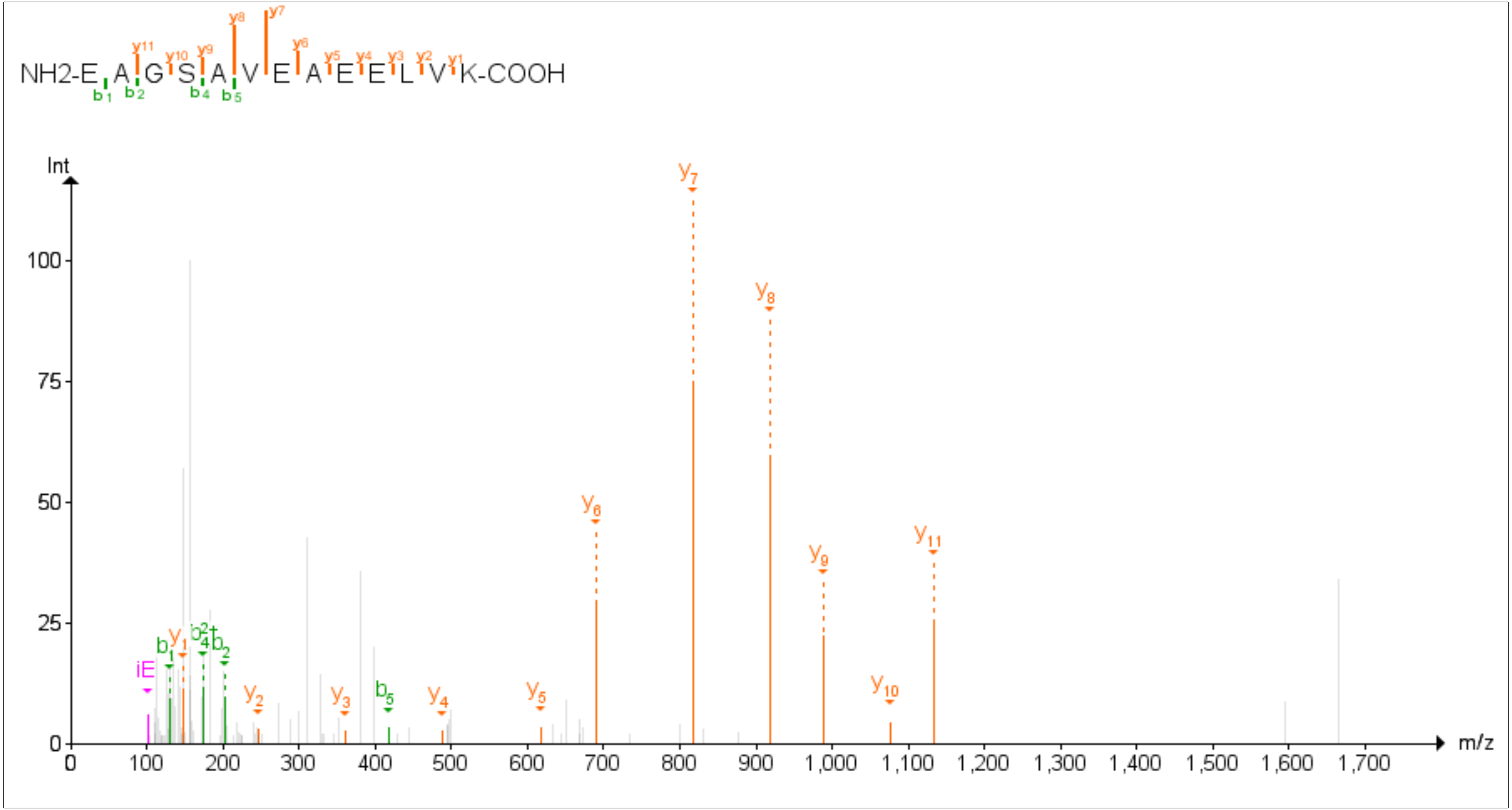
NH2-E A G S A V E A E E L V K-COOH
y11 y10 y9 y8 y7 y6 y5 y4 y3 y2 y1
b1 b2 b4 b5
Int
100
75
50
25
0
iE
b1
y1
b4 2+
b2
y2
y3
b5
y4
y5
y6
y7
y8
y9
y10
y11
0 100 200 300 400 500 600 700 800 900 1,000 1,100 1,200 1,300 1,400 1,500 1,600 1,700
m/z

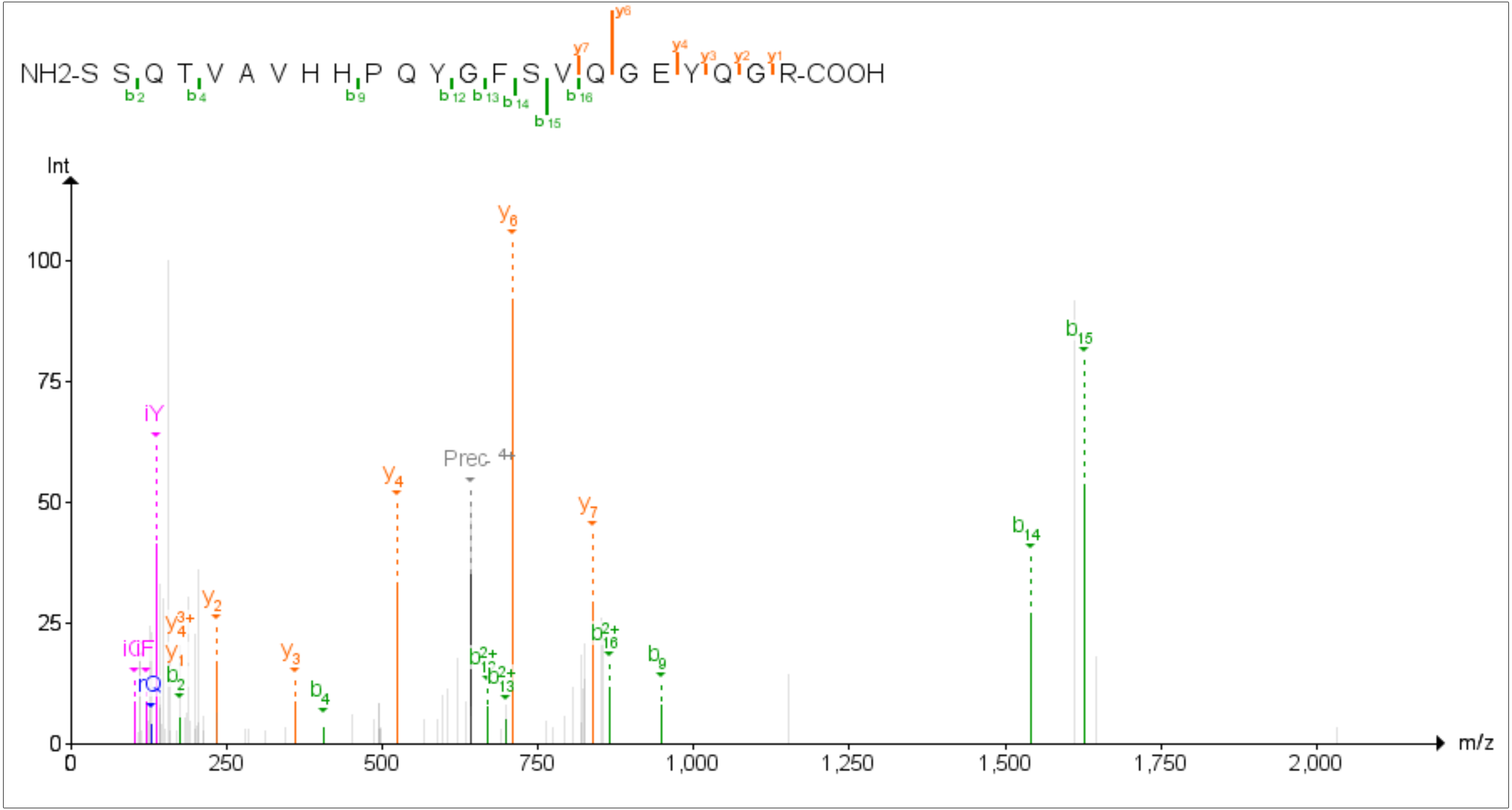

NH2-S S Q T V A V H H P Q Y G F S V Q G E Y Q G R-COOH
b2 b4 b9 b12 b13 b14 b15 b16
y7 y6 y4 y3 y2 y1
Int
100
75
50
25
0
y6
b15
iY
Prec- 4+
y4
y7
b14
y2
y4 3+
iGF
y1
rQ
b2
y3
b4
b16 2+
b12 2+
b13 2+
b9
0
250
500
750
1,000
1,250
1,500
1,750
2,000
m/z

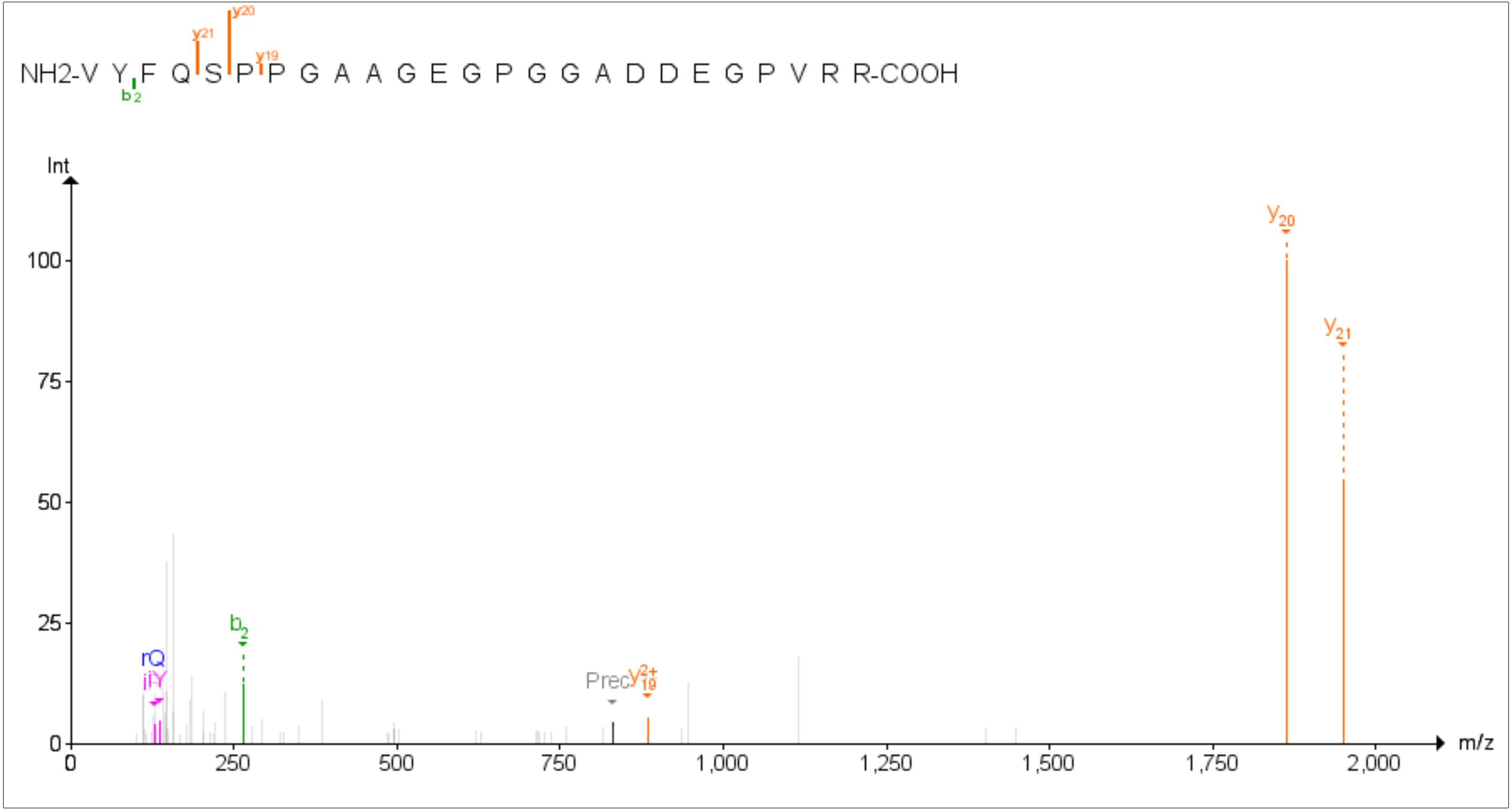

NH2-V Y F Q S P P G A A G E G P G G A D D E G P V R R-COOH
y20
y21
y19
b2
Int
100
75
50
25
0
rQ
iY
b2
Prec
y19 2+
y20
y21
0
250
500
750
1,000
1,250
1,500
1,750
2,000
m/z

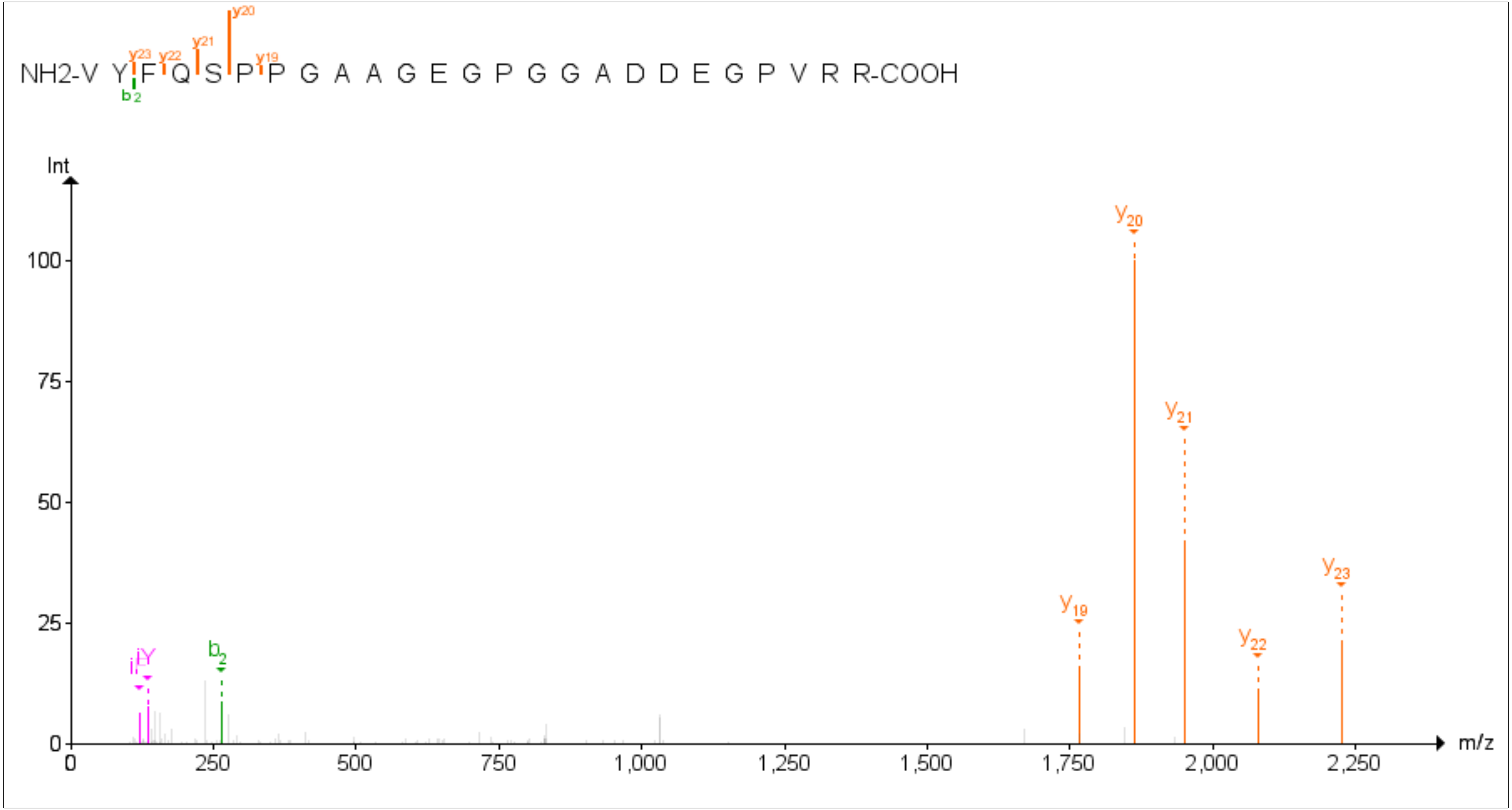

NH2-V Y F Q S P P G A A G E G P G G A D D E G P V R R-COOH
b2
y23
y22
y21
y20
y19
Int
100
75
50
25
0
m/z
0
250
500
750
1,000
1,250
1,500
1,750
2,000
2,250
iY
b2
y19
y20
y21
y22
y23

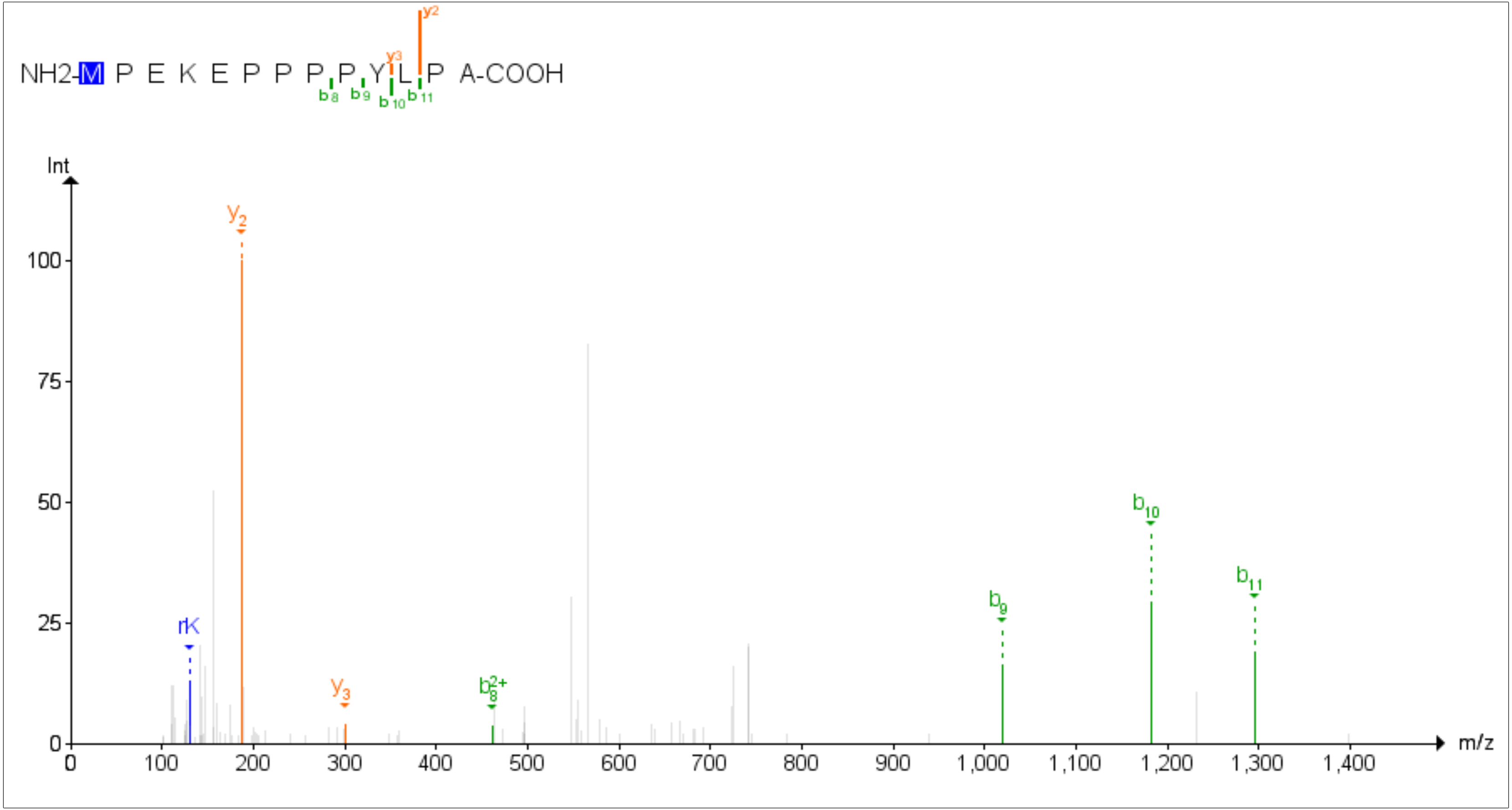

NH2-M P E K E P P P P Y L P A-COOH
b8 b9 b10 b11
y3 y2
Int
m/z
y2
y3
rK
b8 2+
b9
b10
b11

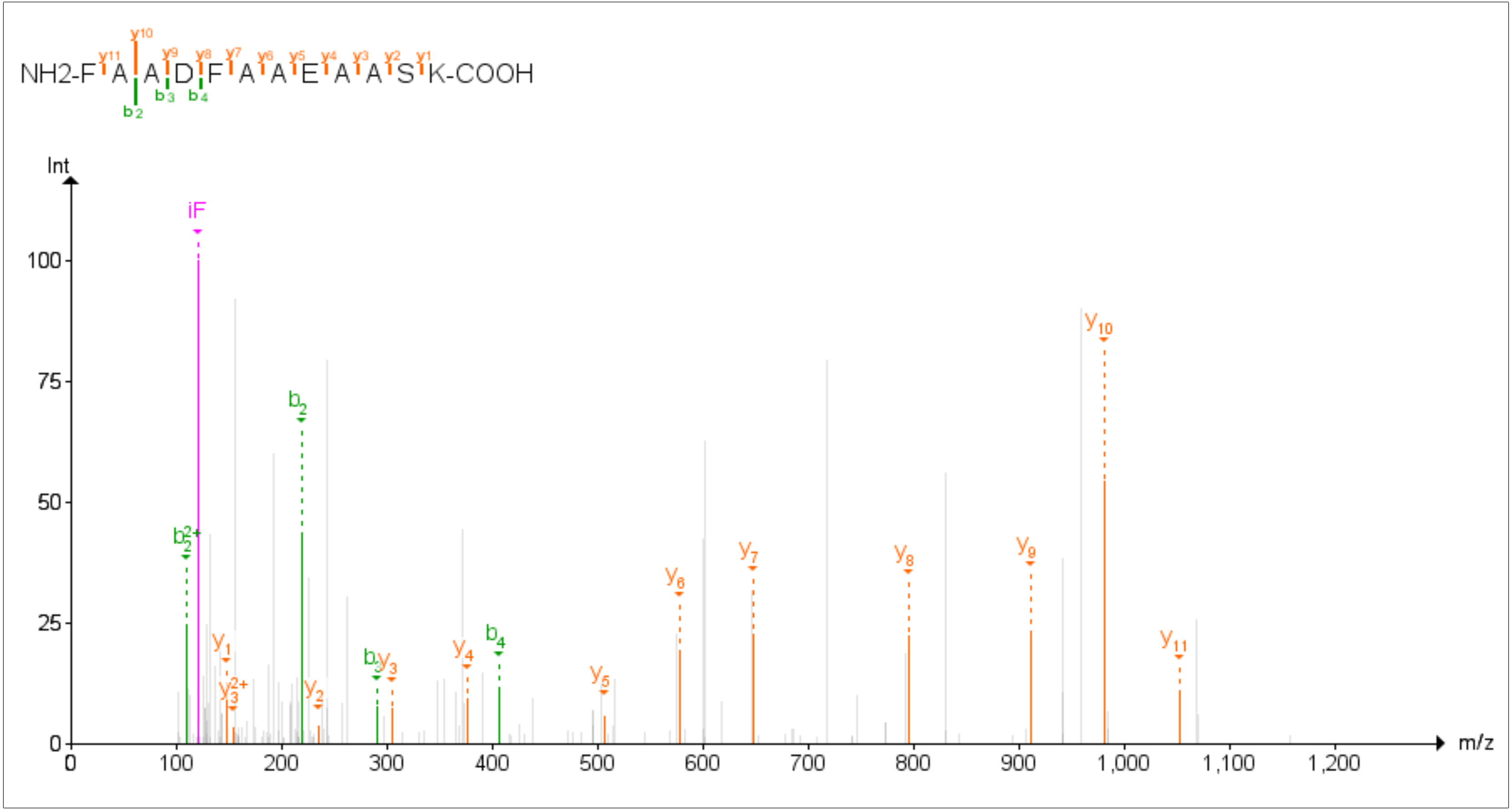

NH2-F A A D F A A E A A S K-COOH
y11 y10 y9 y8 y7 y6 y5 y4 y3 y2 y1
b2 b3 b4
Int
iF
100
75
50
25
0
b2
b2 2+
y1
y3 2+
y2
b3
y3
y4
b4
y5
y6
y7
y8
y9
y10
y11
0 100 200 300 400 500 600 700 800 900 1,000 1,100 1,200
m/z

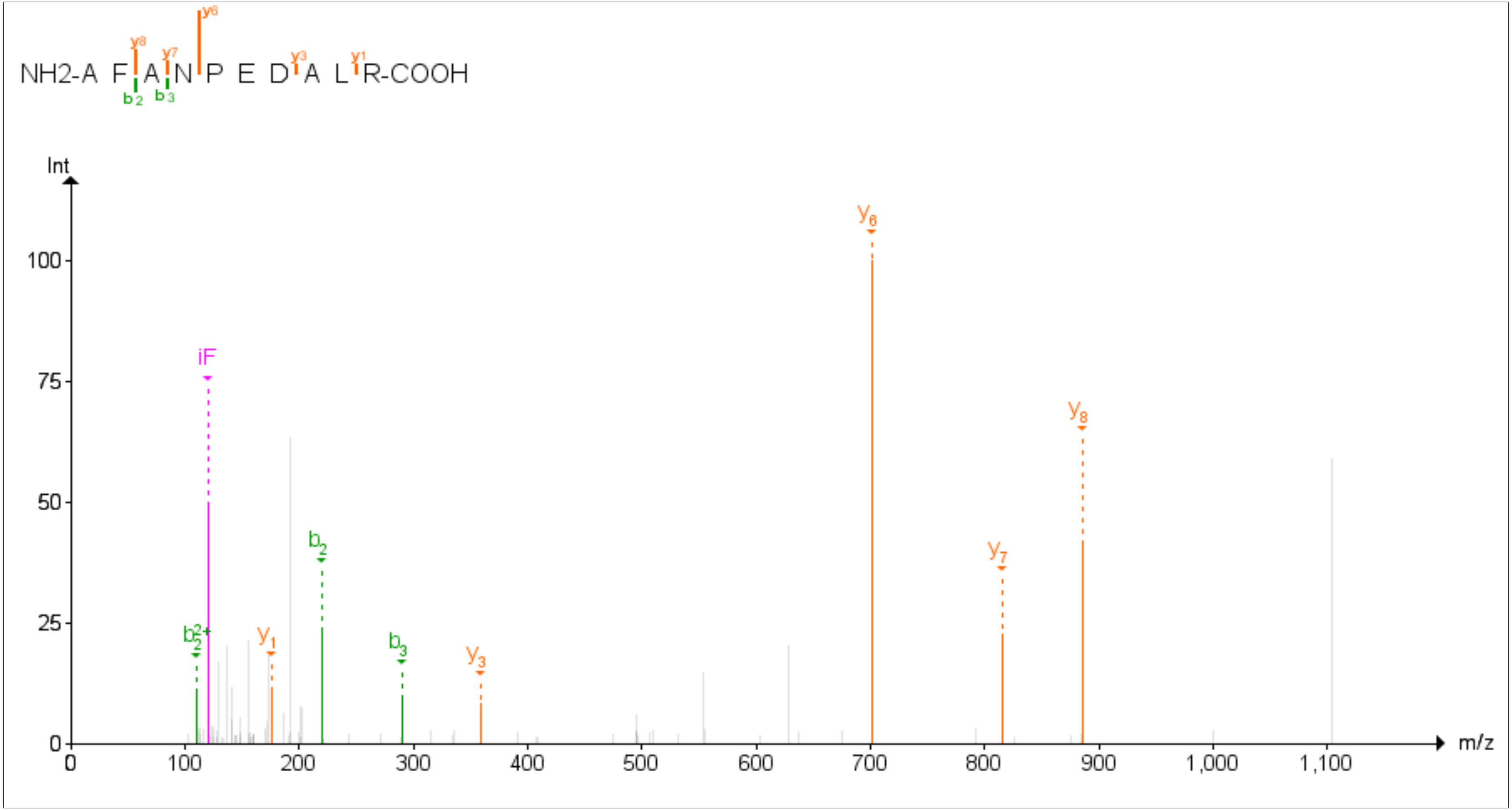
NH2-A F A N P E D A L R-COOH
y8 y7 y6 y3 y1
b2 b3
Int
m/z
iF
b2 y1 b3 y3 y6 y7 y8
0
25
50
75
100
100
200
300
400
500
600
700
800
900
1,000
1,100

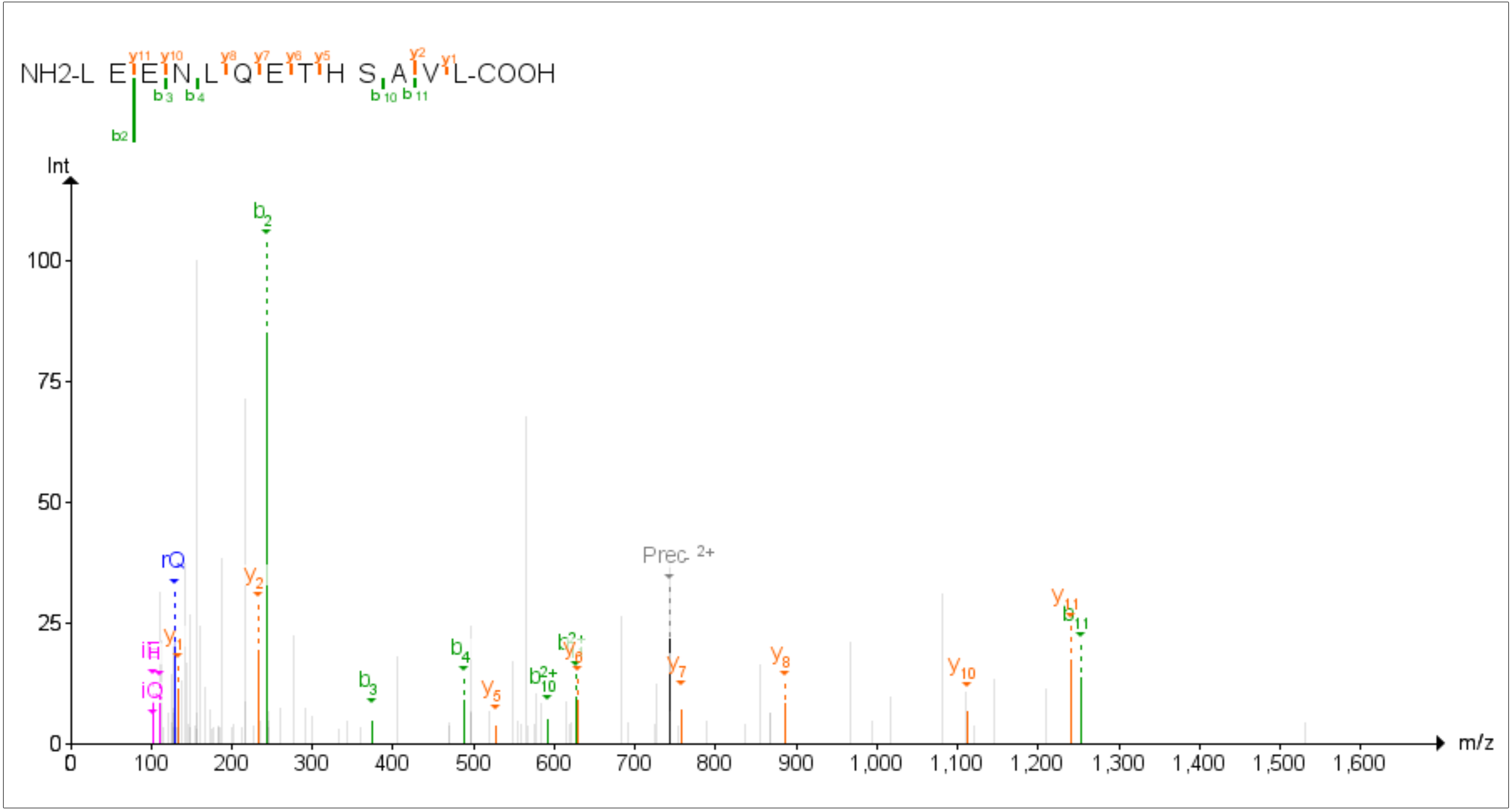

NH2-L E E N L Q E T H S A V L-COOH
y11 y10 y8 y7 y6 y5 y2 y1
b2 b3 b4 b10 b11
Int
m/z
b2
rQ
y2
iH
y1
iQ
b3
b4
y5
b10 2+
b6 2+
y6
Prec 2+
y7
y8
y10
y11
b11
0
25
50
75
100
0
100
200
300
400
500
600
700
800
900
1,000
1,100
1,200
1,300
1,400
1,500
1,600

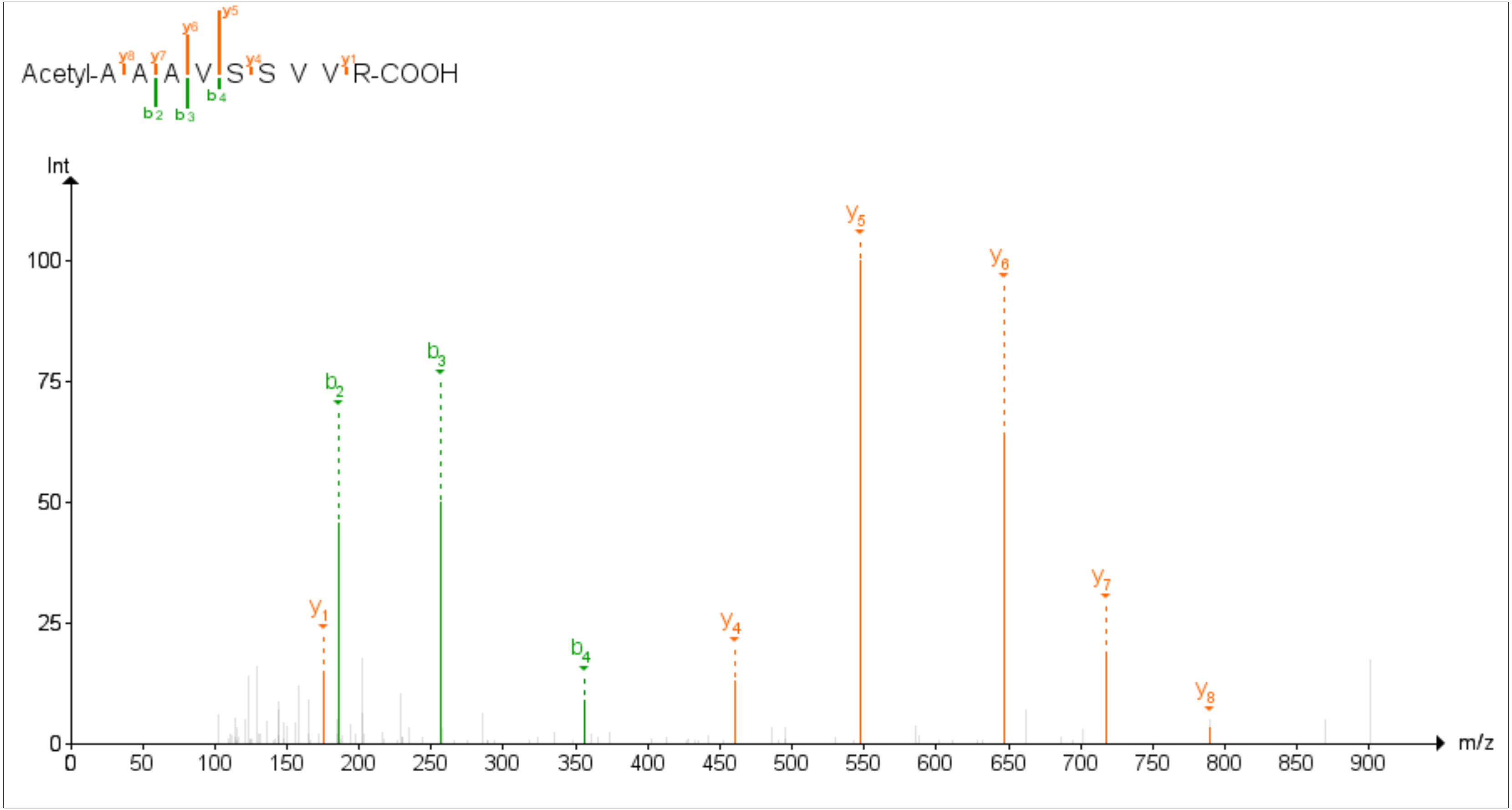

Acetyl-A A A V S S V V R-COOH
y8 y7 y6 y5 y4 y1
b2 b3 b4
Int
100
75
50
25
0
y5
y6
b3
b2
y1
b4
y4
y7
y8
0 50 100 150 200 250 300 350 400 450 500 550 600 650 700 750 800 850 900
m/z

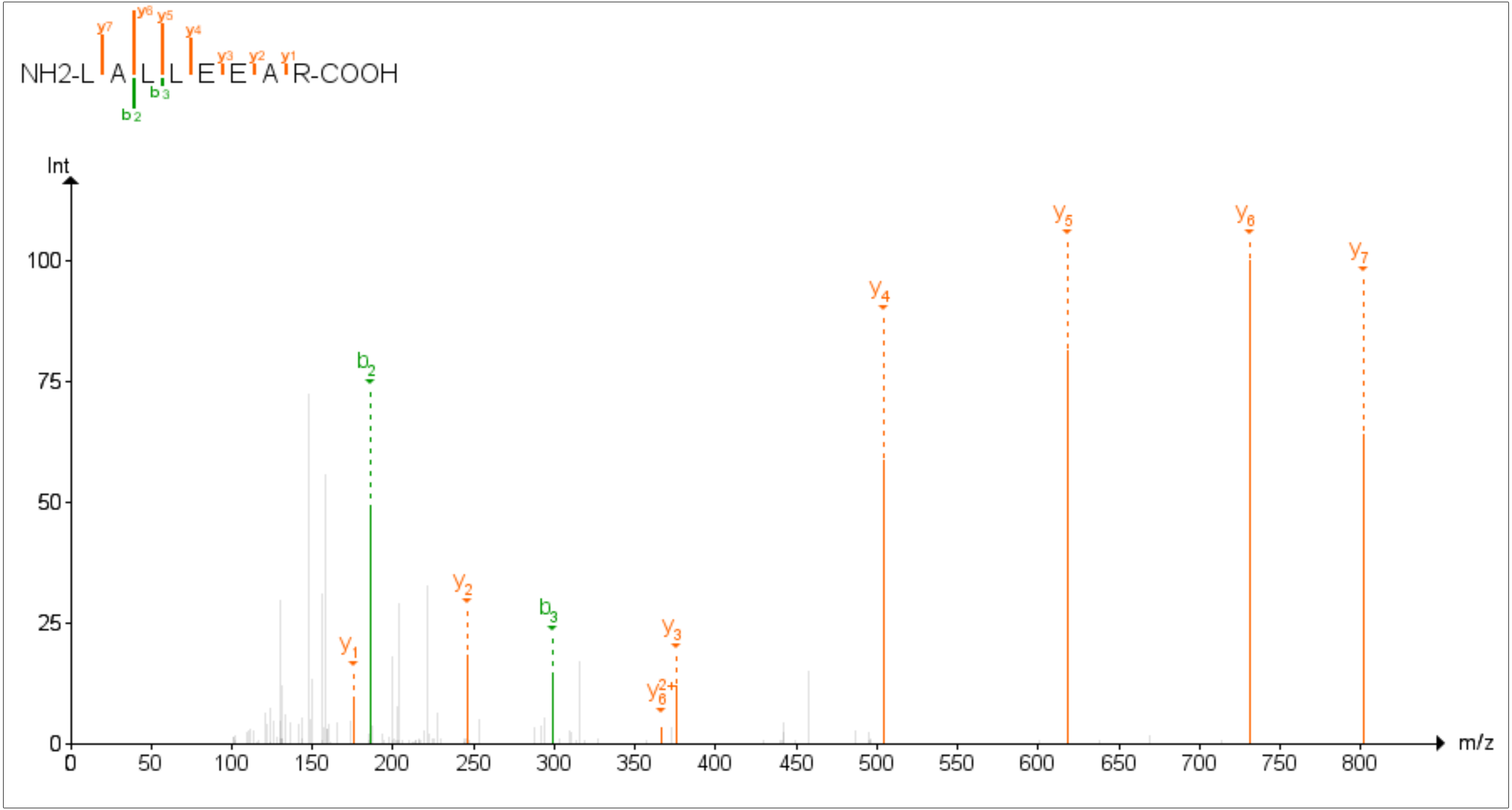

NH2-L A L L E E A R-COOH
y7 y6 y5 y4 y3 y2 y1
b2 b3
Int
100
75
50
25
0
0 50 100 150 200 250 300 350 400 450 500 550 600 650 700 750 800
m/z
b2
y1
y2
b3
y3
y6 2+
y4
y5
y6
y7

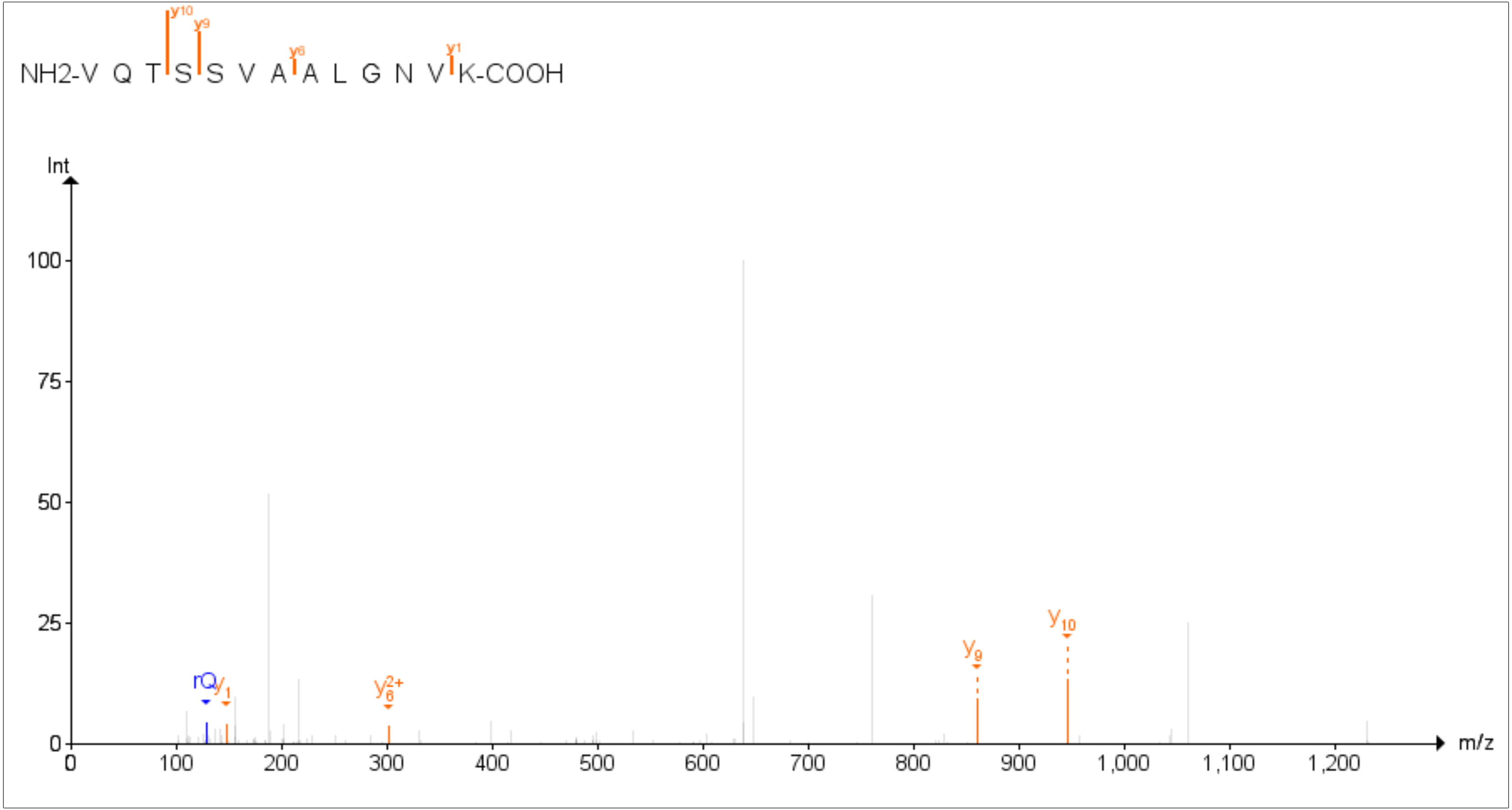
NH2-V Q T S S V A A L G N V K-COOH
y10
y9
y6
y1
Int
100
75
50
25
0
rQ
y1
y6 2+
y9
y10
0
100
200
300
400
500
600
700
800
900
1,000
1,100
1,200
m/z

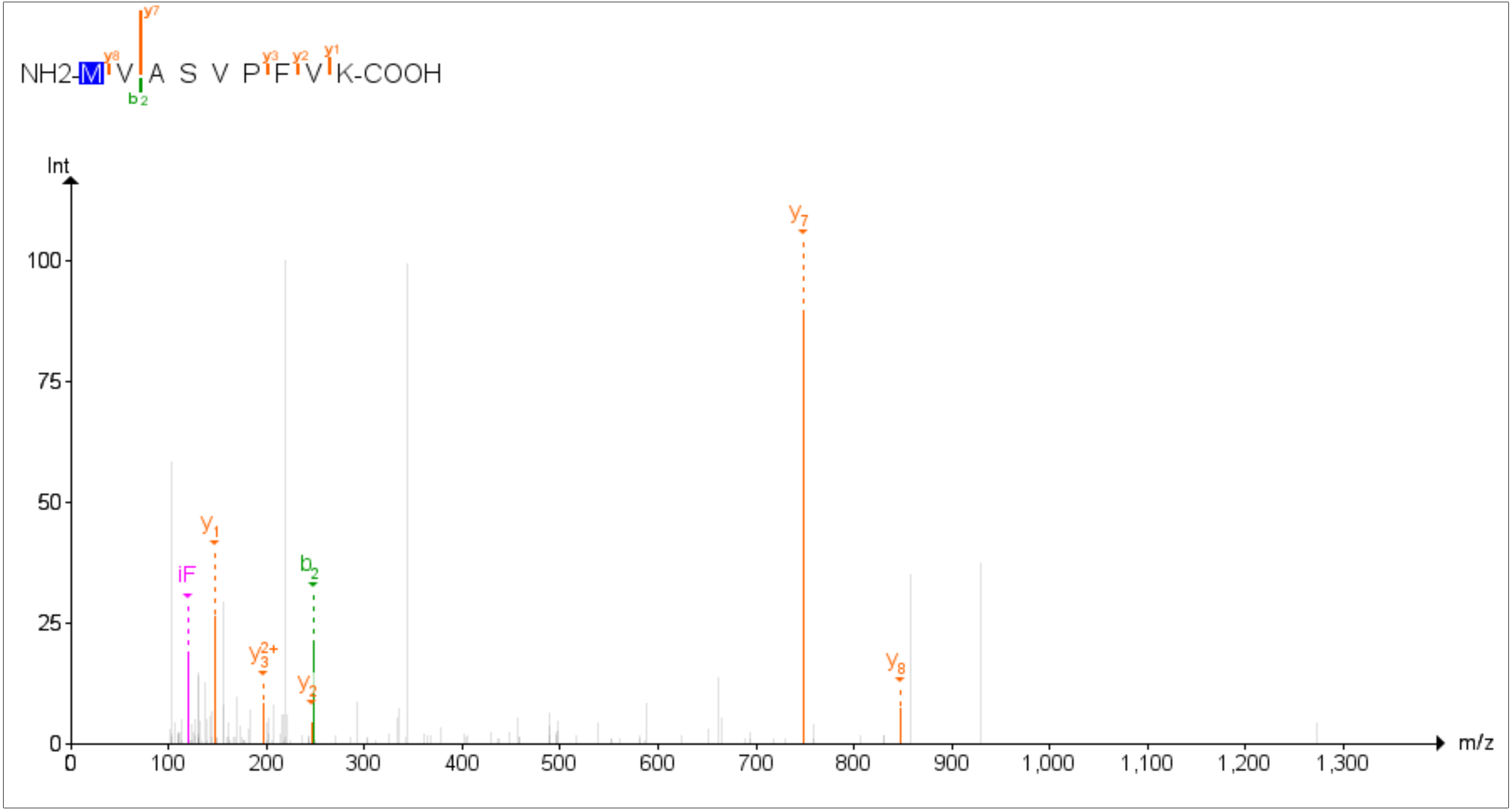

NH2-M V A S V P F V K-COOH
y7
y8
y3
y2
y1
b2
Int
y7
y1
iF
b2
y3 2+
y2
y8
100
75
50
25
0
0
100
200
300
400
500
600
700
800
900
1,000
1,100
1,200
1,300
m/z

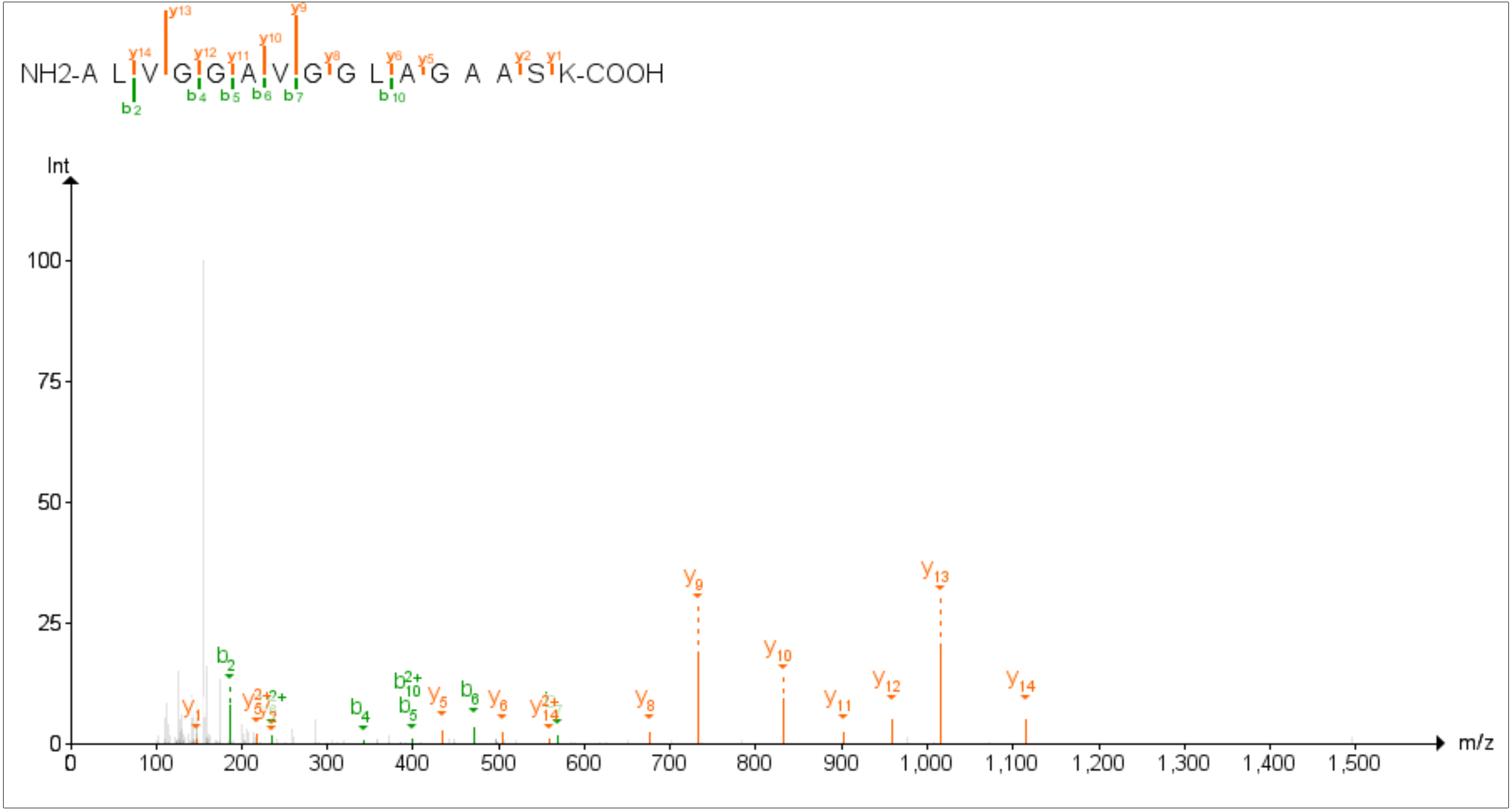

NH2-A L V G G A V G G L A G A A S K-COOH
y13
y9
y14
y12
y11
y10
y8
y6
y5
y2
y1
b2
b4
b5
b6
b7
b10
Int
100
75
50
25
0
m/z
0
100
200
300
400
500
600
700
800
900
1,000
1,100
1,200
1,300
1,400
1,500

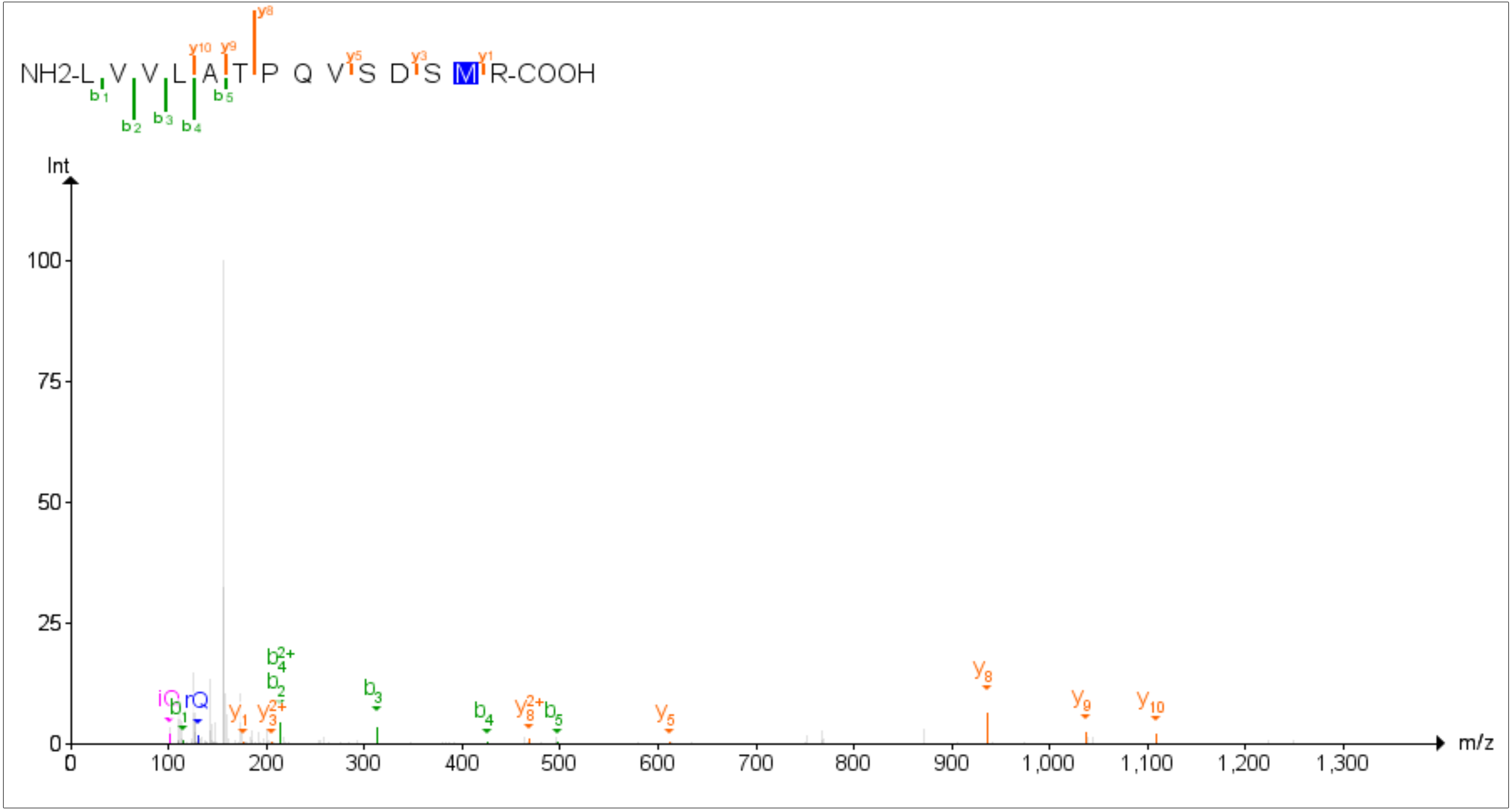

NH2-L V V L A T P Q V S D S M R-COOH
b1 b2 b3 b4 b5
y10 y9 y8 y5 y3 y1
Int
100
75
50
25
0
iQ
b1
rQ
y1
y3 2+
b4 2+
b2
b3
b4
y8 2+
b5
y5
y8
y9
y10
0 100 200 300 400 500 600 700 800 900 1,000 1,100 1,200 1,300
m/z

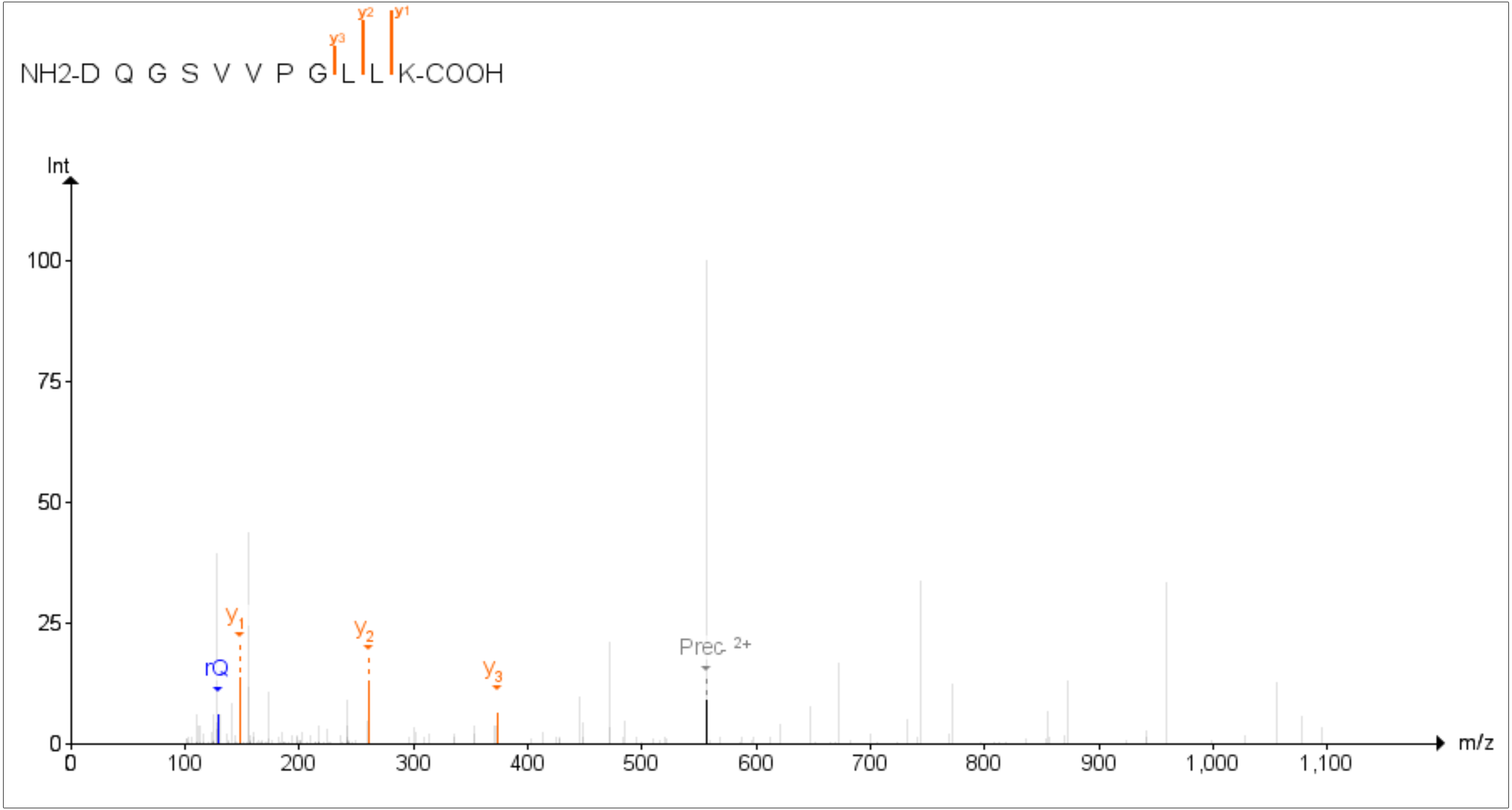

y3
y2
y1
NH2-D Q G S V V P G L L K-COOH
Int
100
75
50
25
0
rQ
y_1
y_2
y_3
Prec 2+
0
100
200
300
400
500
600
700
800
900
1,000
1,100
m/z

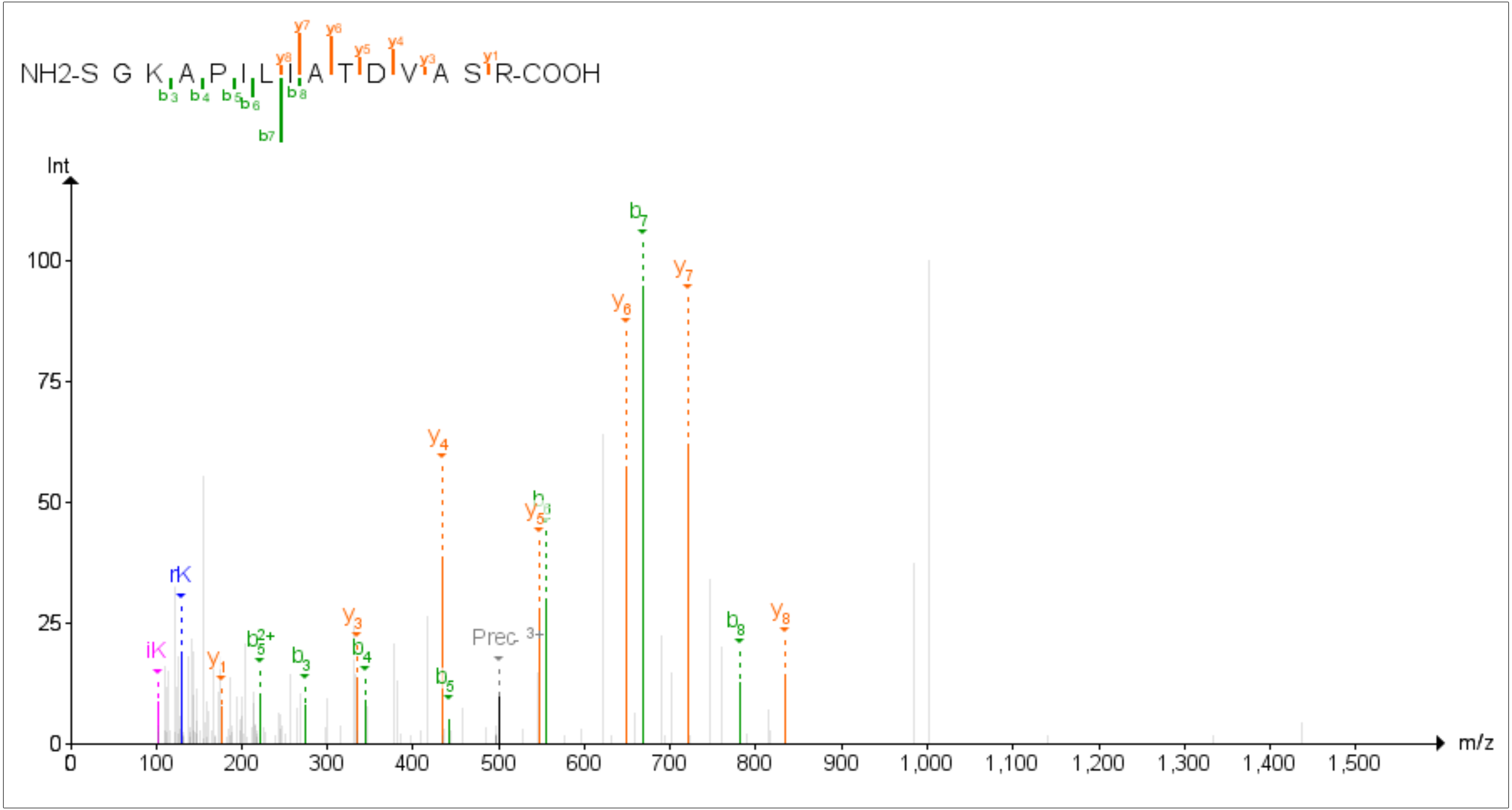

NH2-S G K A P I L I A T D V A S R-COOH
y7 y6 y5 y4 y3 y1 y8
b3 b4 b5 b6 b7 b8
Int
m/z
iK
rK
Prec 3+

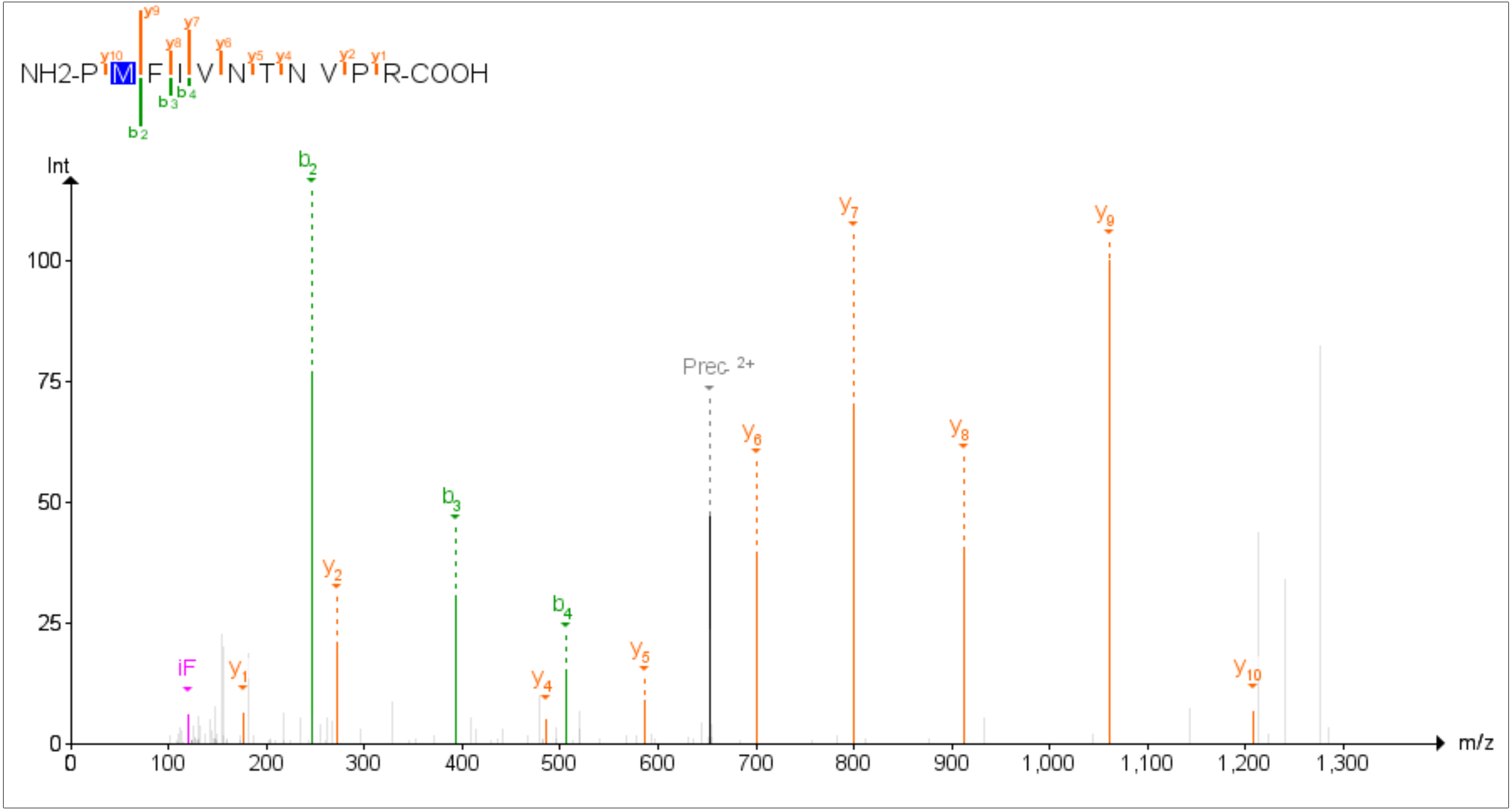

NH2-P M F I V N T N V P R-COOH
y10 y9 y8 y7 y6 y5 y4 y2 y1
b2 b3 b4
Int
100
75
50
25
0
b2
y7
y9
Prec 2+
y6
y8
b3
y2
b4
iF
y1
y4
y5
y10
0 100 200 300 400 500 600 700 800 900 1,000 1,100 1,200 1,300
m/z

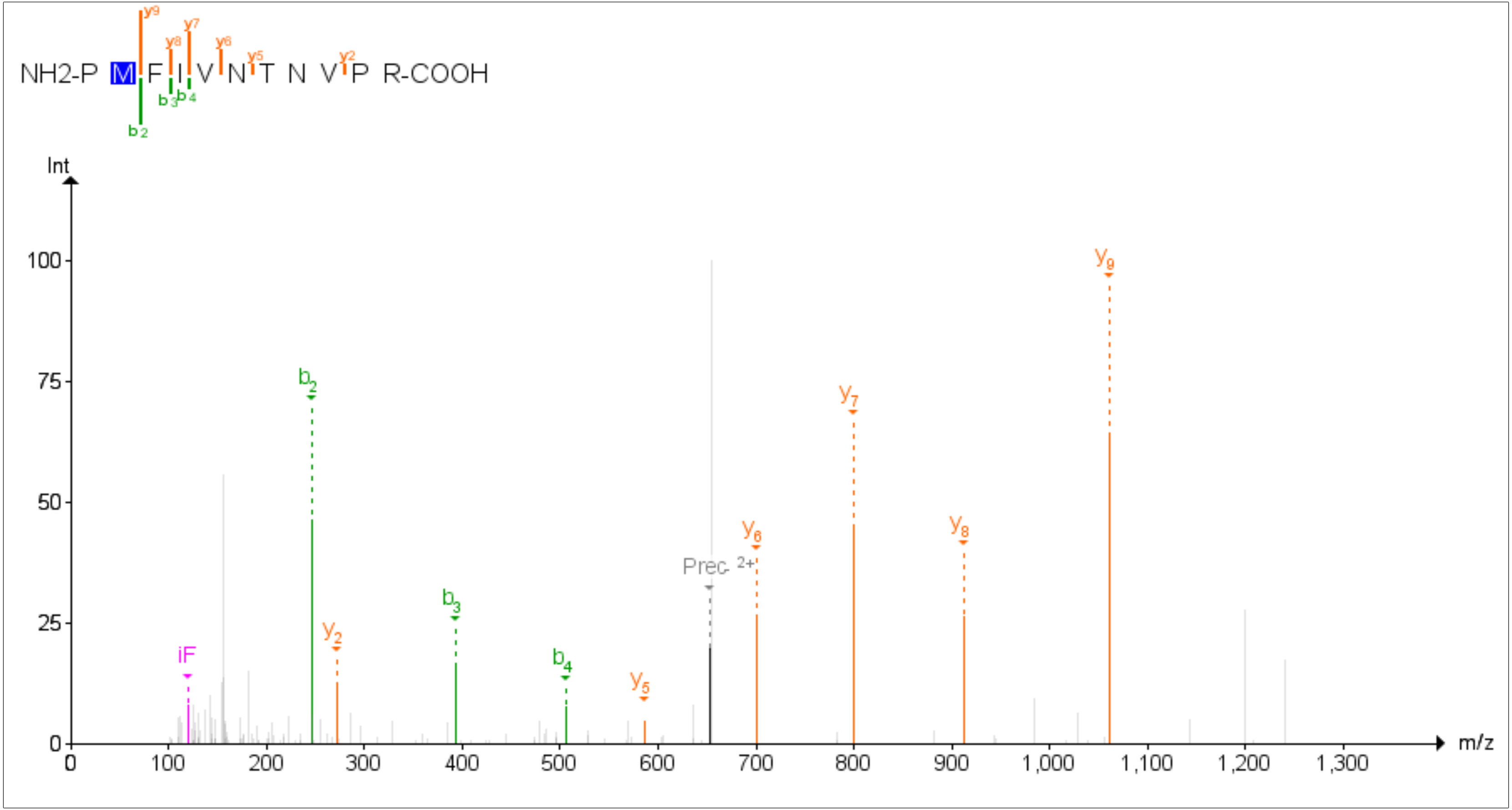
NH2-P M F I V N T N V P R-COOH
y9 y8 y7 y6 y5 y2
b2 b3 b4
Int
100
75
50
25
0
b2
y2
iF
b3
b4
y5
Prec 2+
y6
y7
y8
y9
0 100 200 300 400 500 600 700 800 900 1,000 1,100 1,200 1,300
m/z

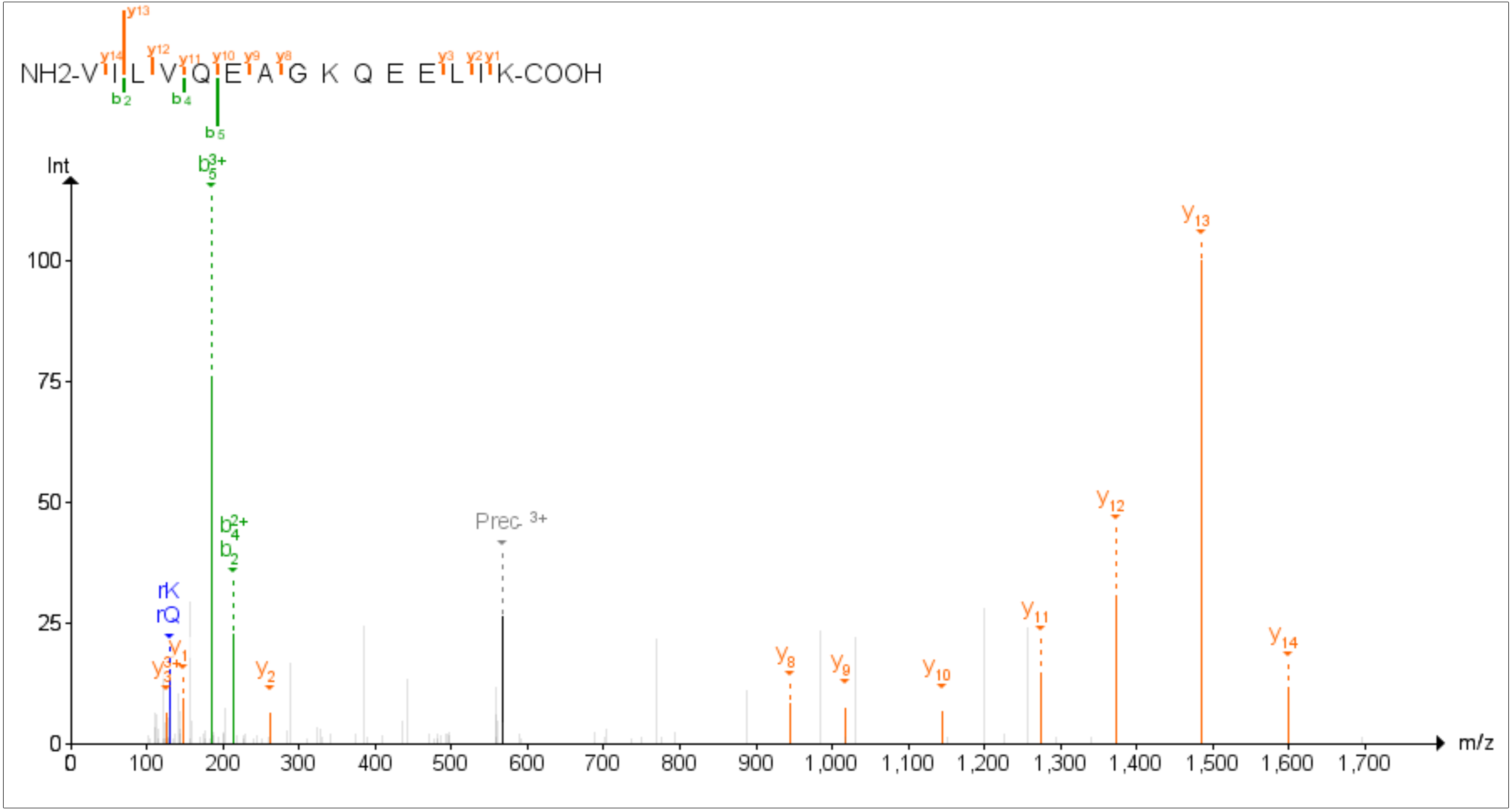

NH2-V I L V Q E A G K Q E E L I K-COOH
y14 y13 y12 y11 y10 y9 y8 y3 y2 y1
b2 b4 b5
Int
100
75
50
25
0
m/z
0 100 200 300 400 500 600 700 800 900 1,000 1,100 1,200 1,300 1,400 1,500 1,600 1,700
b5 3+
b4 2+
b2
Prec 3+
rK
rQ
y1
y3 3+
y2
y8
y9
y10
y11
y12
y13
y14

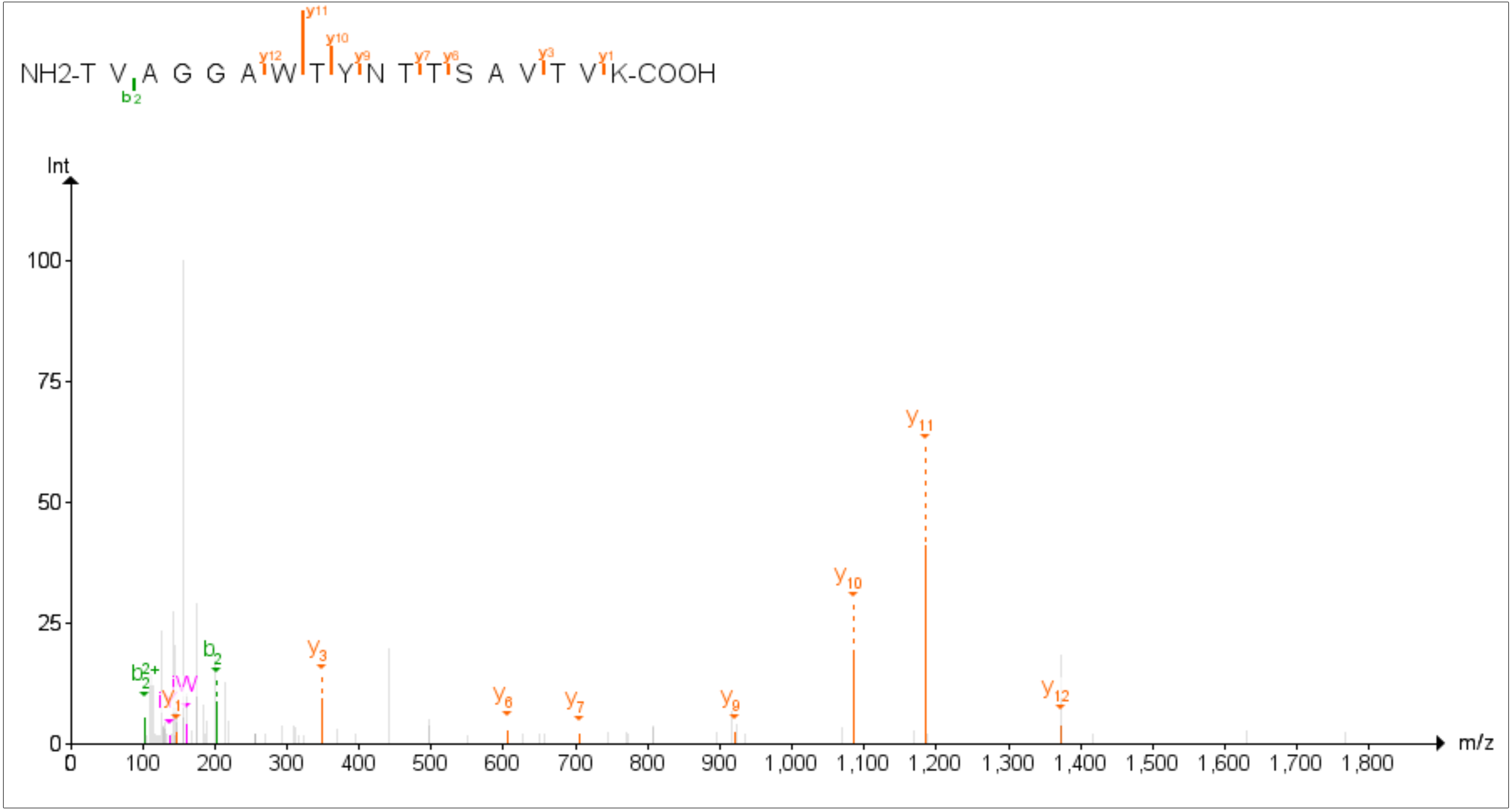

NH2-T V A G G A W T Y N T T S A V T V K-COOH
b2
y12
y11
y10
y9
y7
y6
y3
y1
Int
100
75
50
25
0
b2 2+
b2
iY
iW
y1
y3
y6
y7
y9
y10
y11
y12
0
100
200
300
400
500
600
700
800
900
1,000
1,100
1,200
1,300
1,400
1,500
1,600
1,700
1,800
m/z

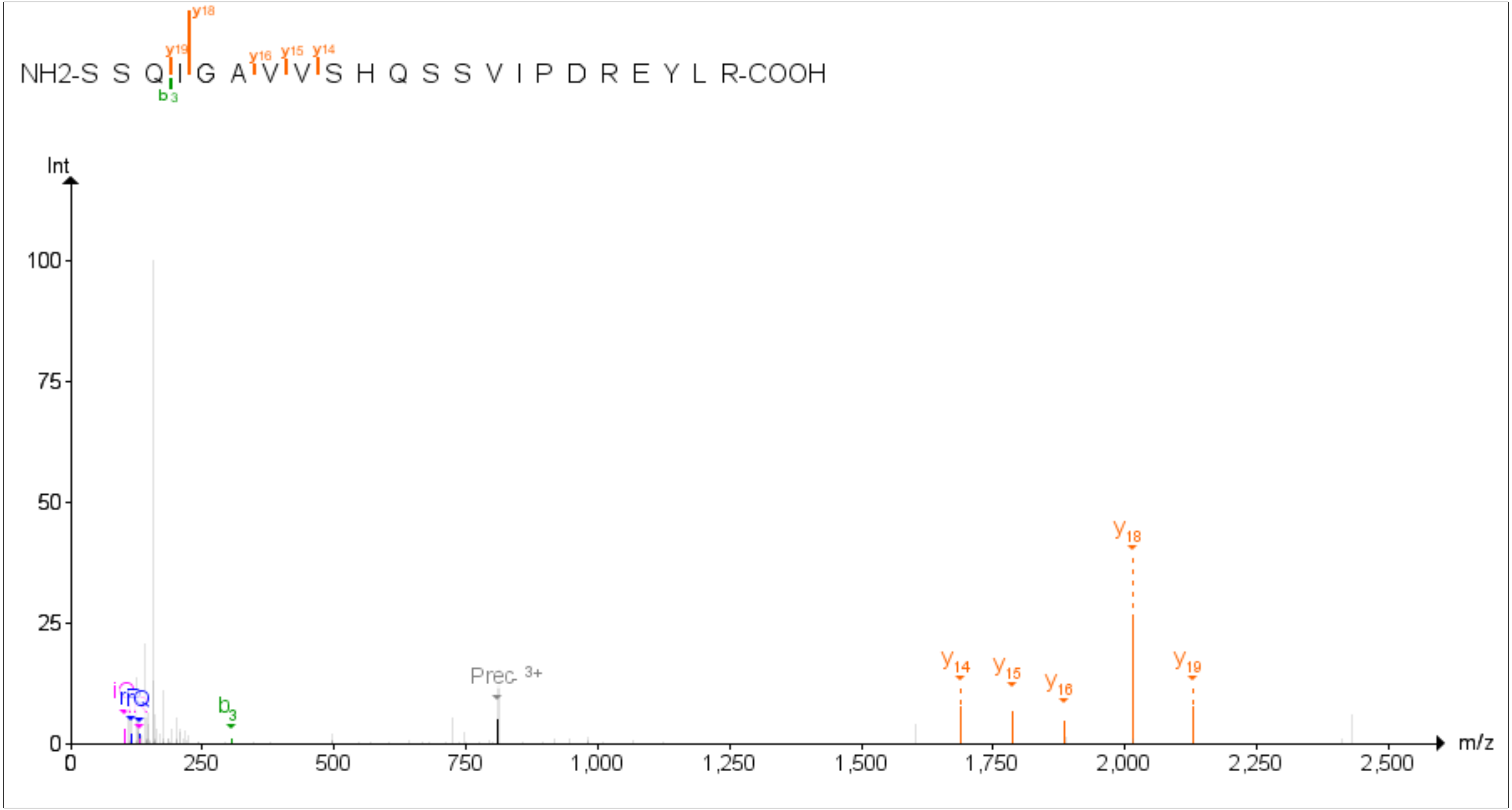

NH2-S S Q I G A V V S H Q S S V I P D R E Y L R-COOH
y18
y19
y16
y15
y14
b3
Int
100
75
50
25
0
Prec- 3+
m/z
0
250
500
750
1,000
1,250
1,500
1,750
2,000
2,250
2,500

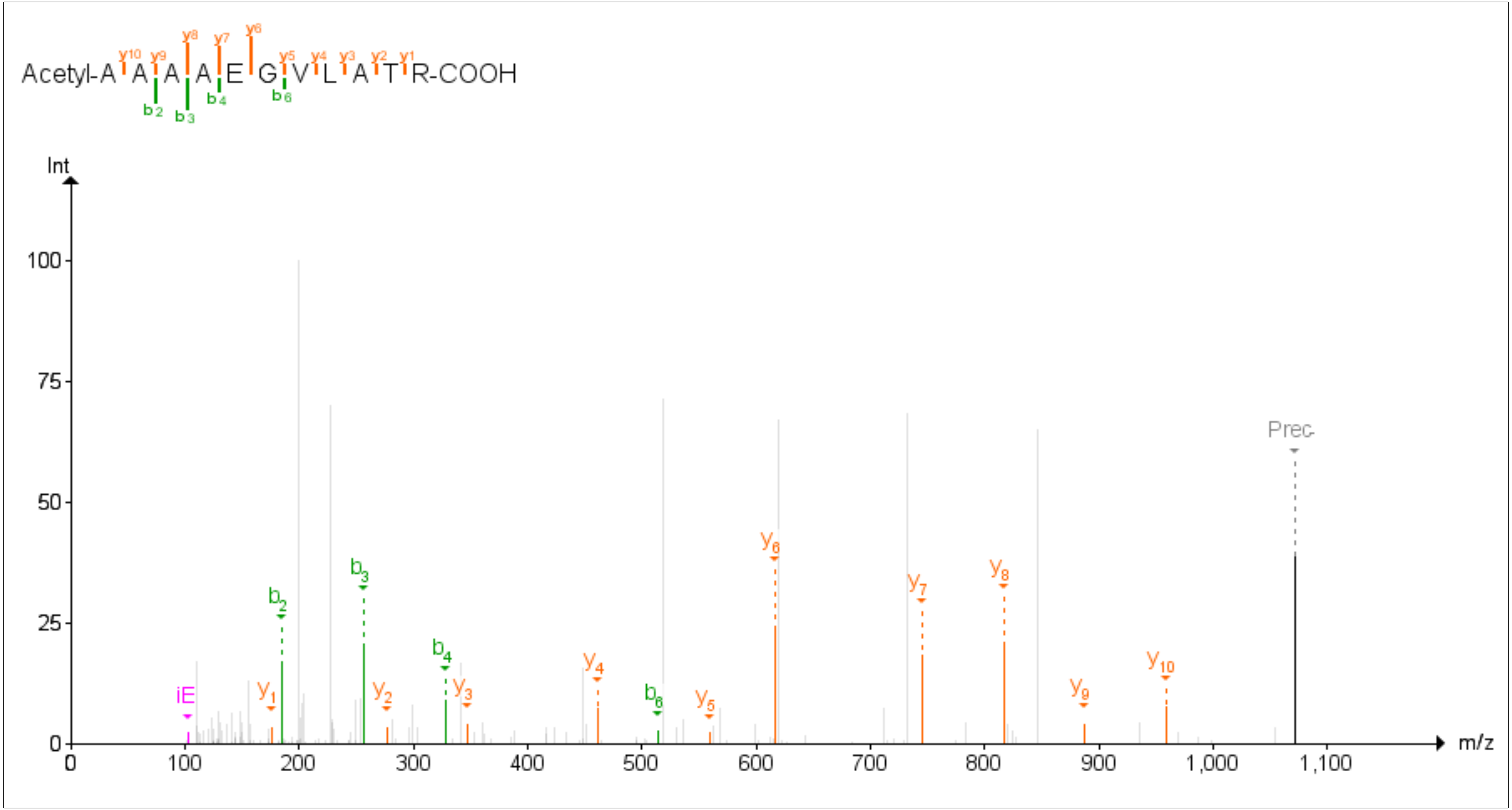

Acetyl-A A A A E G V L A T R-COOH
y10 y9 y8 y7 y6 y5 y4 y3 y2 y1
b2 b3 b4 b6
Int
100
75
50
25
0
iE
y1
b2
y2
b3
b4
y3
y4
b6
y5
y6
y7
y8
y9
y10
Prec
0
100
200
300
400
500
600
700
800
900
1,000
1,100
m/z

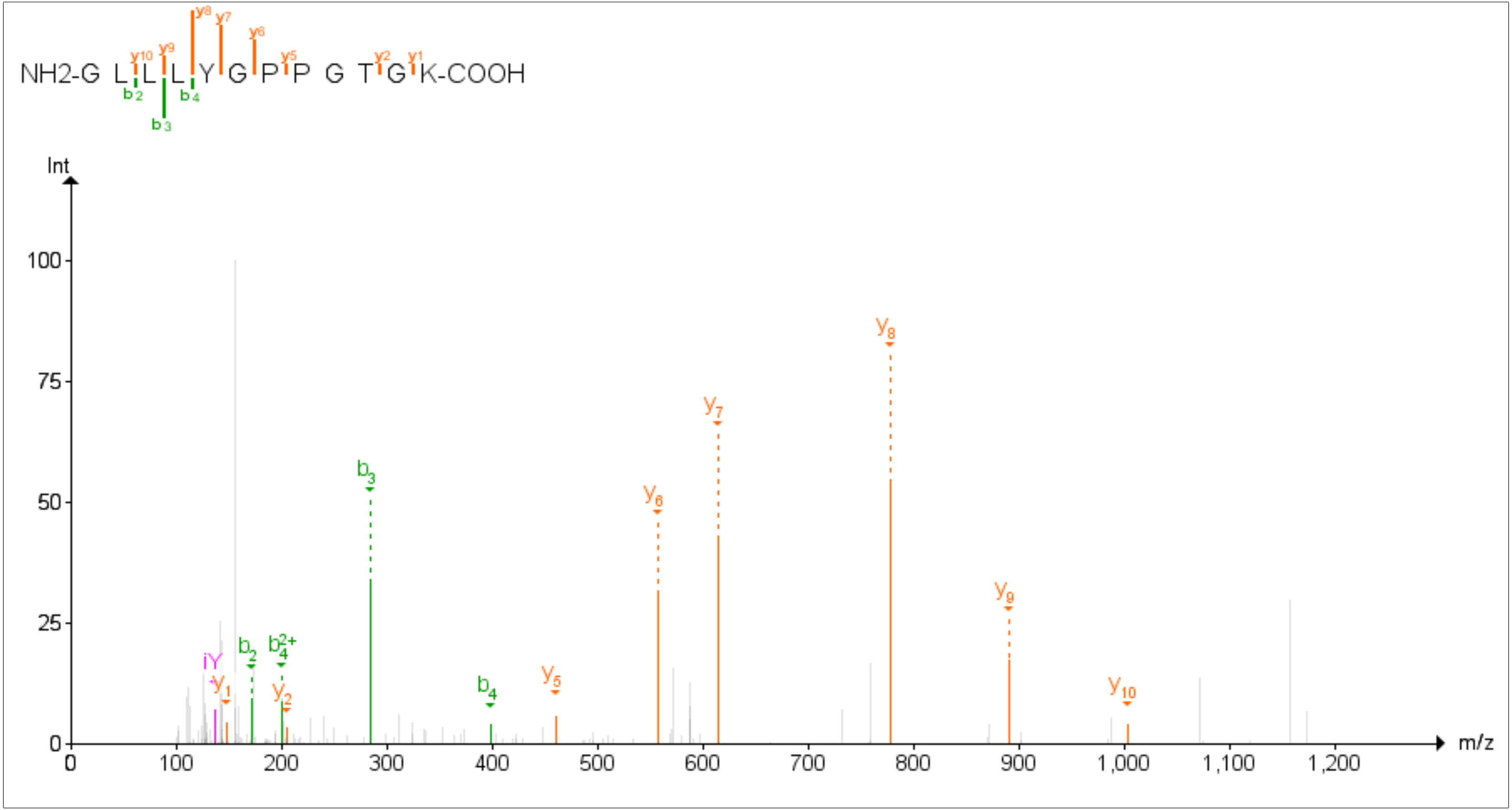
NH2-G L L L Y G P P G T G K-COOH
y10 y9 y8 y7 y6 y5 y2 y1
b2 b3 b4
Int
100
75
50
25
0
y8
y7
b3
y6
y9
iY
y1
b2
b4 2+
y2
b4
y5
y10
0 100 200 300 400 500 600 700 800 900 1,000 1,100 1,200
m/z

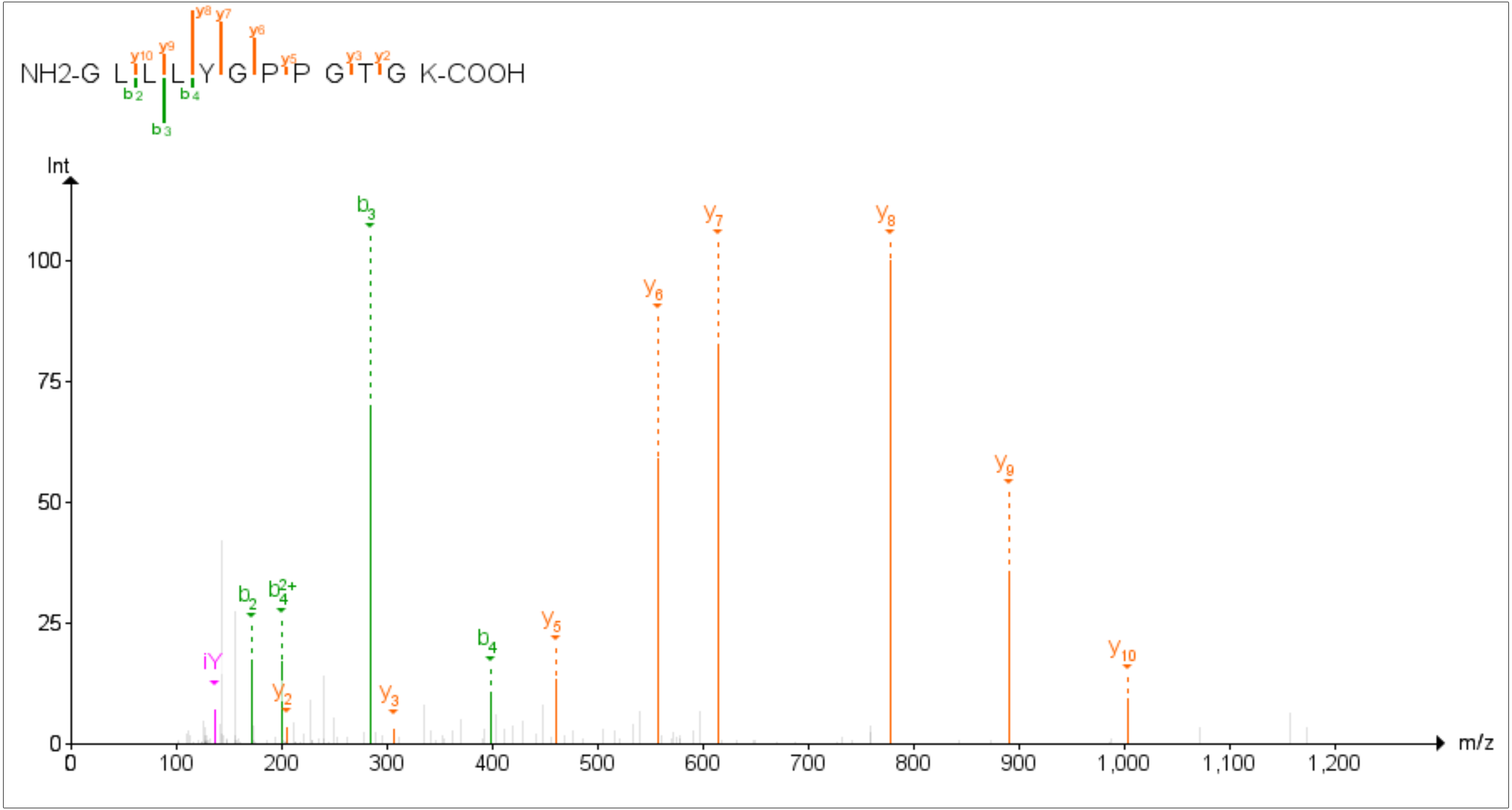

NH2-G L L L Y G P P G T G K-COOH
y10 y9 y8 y7 y6 y5 y3 y2
b2 b3 b4
Int
100
75
50
25
0
b3
y7
y8
y6
y9
b2
b4 2+
iY
y2
y3
b4
y5
y10
0
100
200
300
400
500
600
700
800
900
1,000
1,100
1,200
m/z

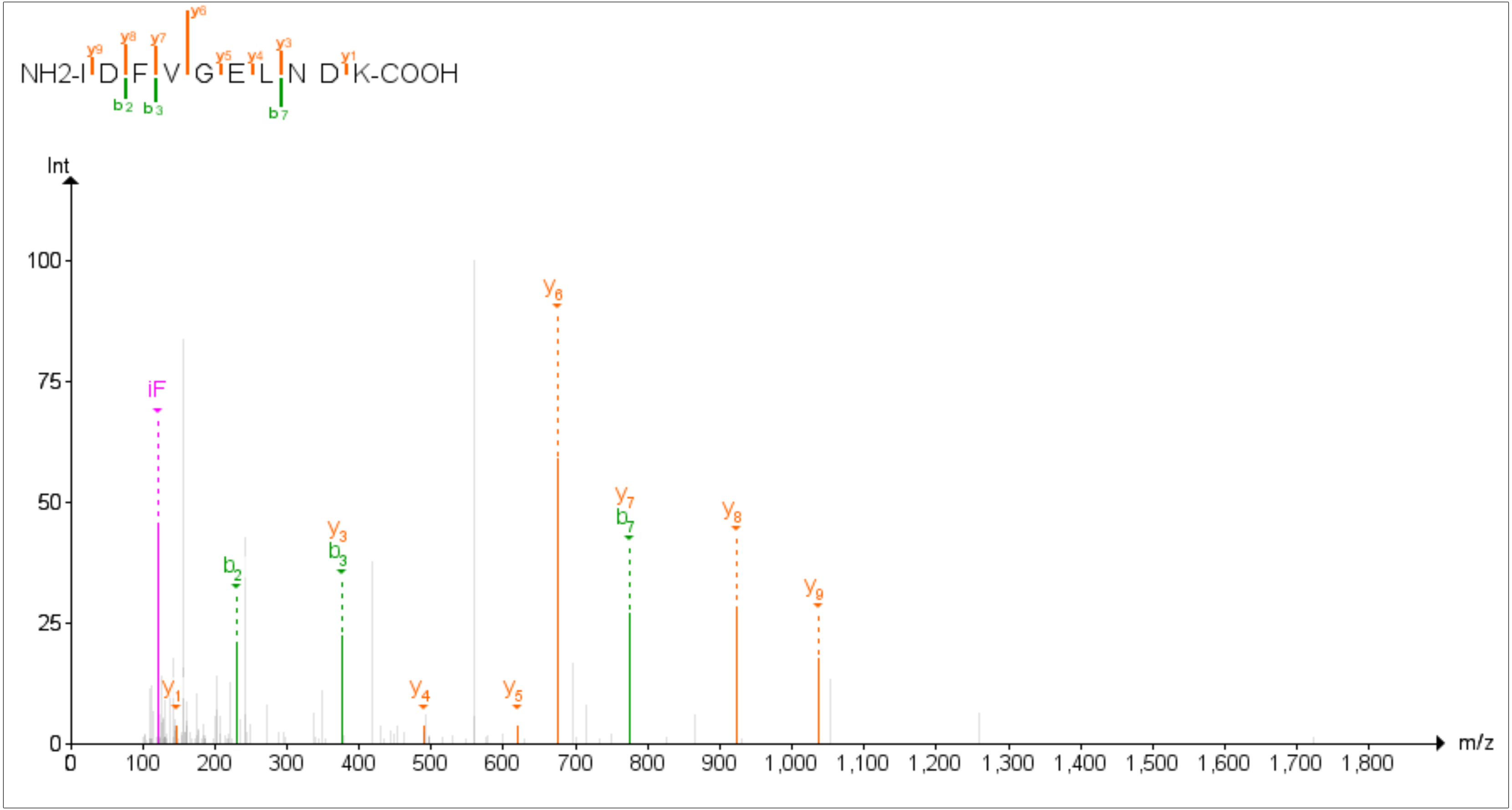
NH2-I D F V G E L N D K-COOH
y9 y8 y7 y6 y5 y4 y3 y1
b2 b3 b7
Int
100
75
50
25
0
iF
y1
b2
y3
b3
y4
y5
y6
y7
b7
y8
y9
0 100 200 300 400 500 600 700 800 900 1,000 1,100 1,200 1,300 1,400 1,500 1,600 1,700 1,800
m/z

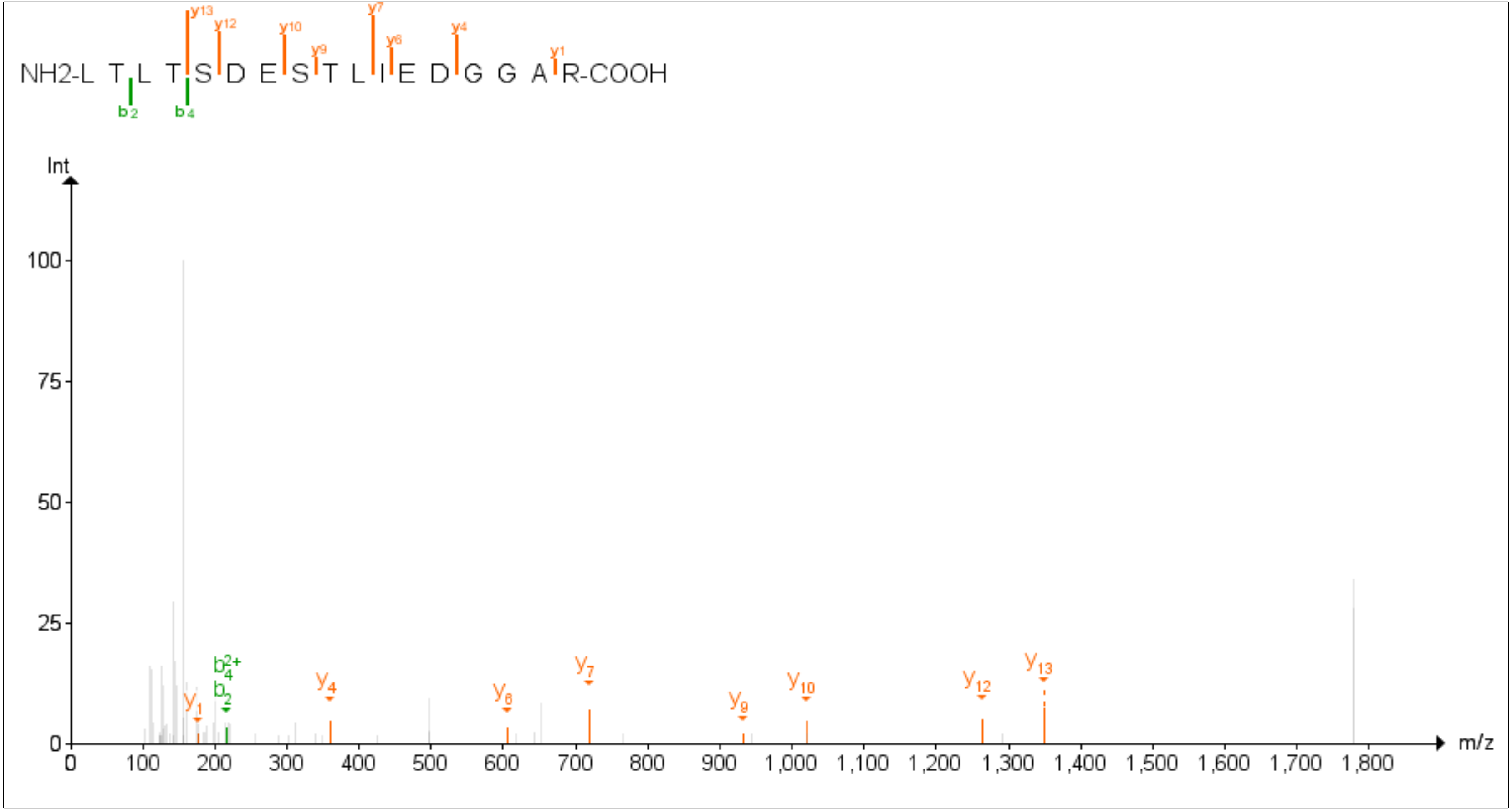

NH2-L T L T S D E S T L I E D G G A R-COOH
y13
y12
y10
y9
y7
y6
y4
y1
b2
b4
Int
100
75
50
25
0
b$_4^{2+}$
b$_2$
y$_1$
y$_4$
y$_6$
y$_7$
y$_9$
y$_{10}$
y$_{12}$
y$_{13}$
0
100
200
300
400
500
600
700
800
900
1,000
1,100
1,200
1,300
1,400
1,500
1,600
1,700
1,800
m/z

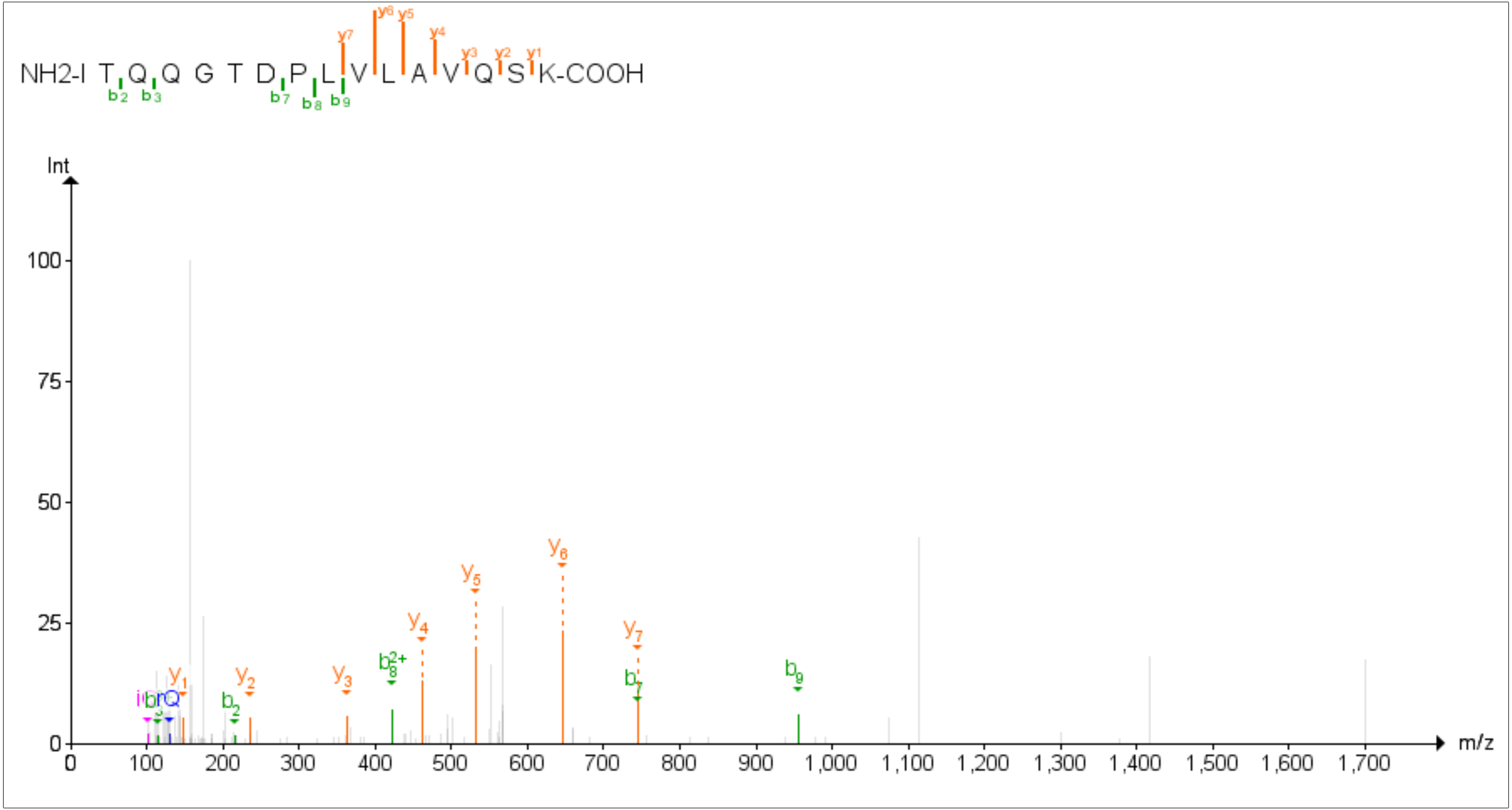

NH2-I T Q Q G T D P L V L A V Q S K-COOH
b2 b3 b7 b8 b9
y7 y6 y5 y4 y3 y2 y1
Int
100
75
50
25
0
y1 y2 y3 y4 y5 y6 y7
b2 b7 b9
m/z
0 100 200 300 400 500 600 700 800 900 1,000 1,100 1,200 1,300 1,400 1,500 1,600 1,700

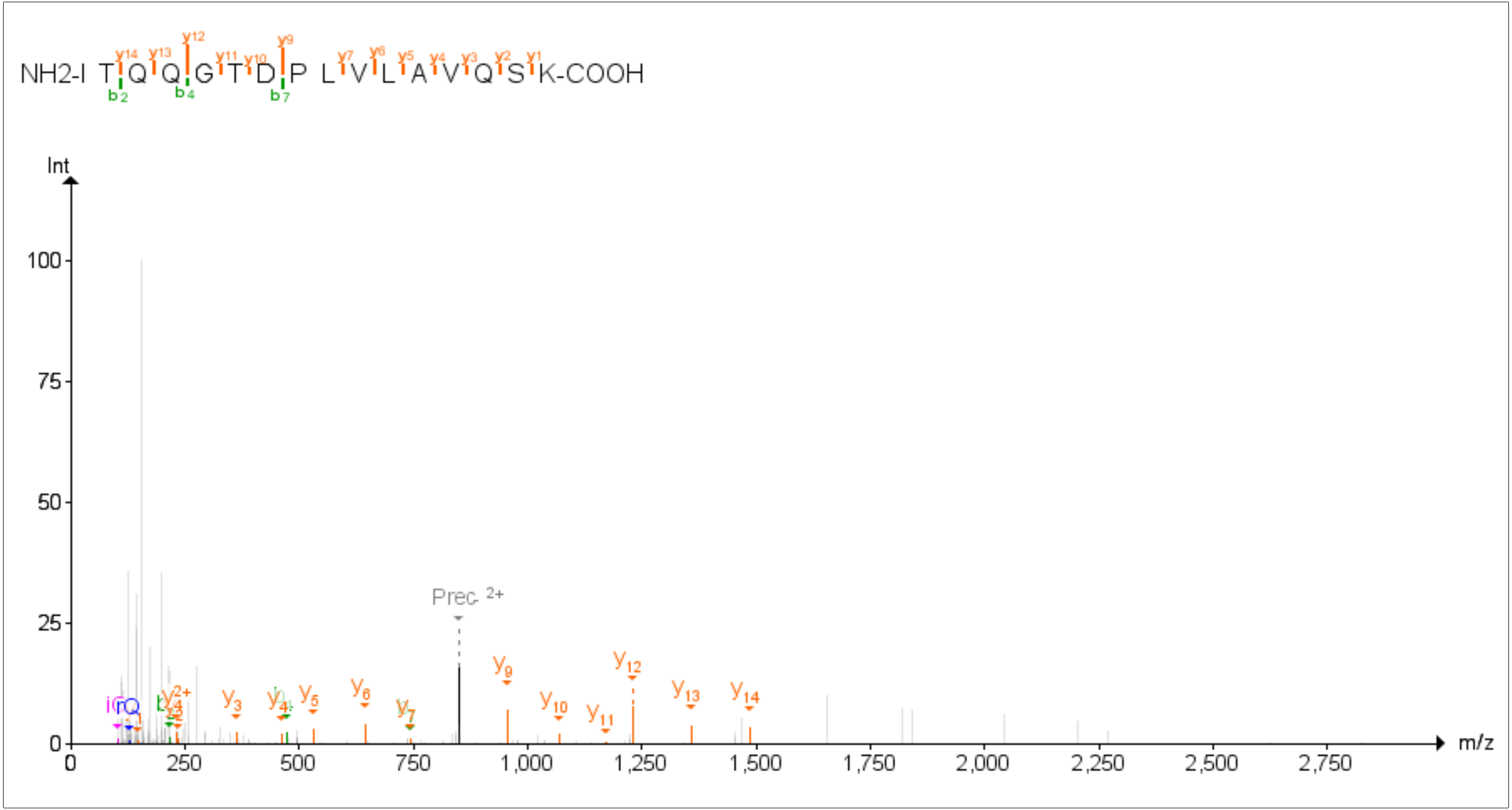
NH2-I T Q Q G T D P L V L A V Q S K-COOH
Int
m/z
Prec 2+
y3
y5
y6
y7
y9
y10
y11
y12
y13
y14

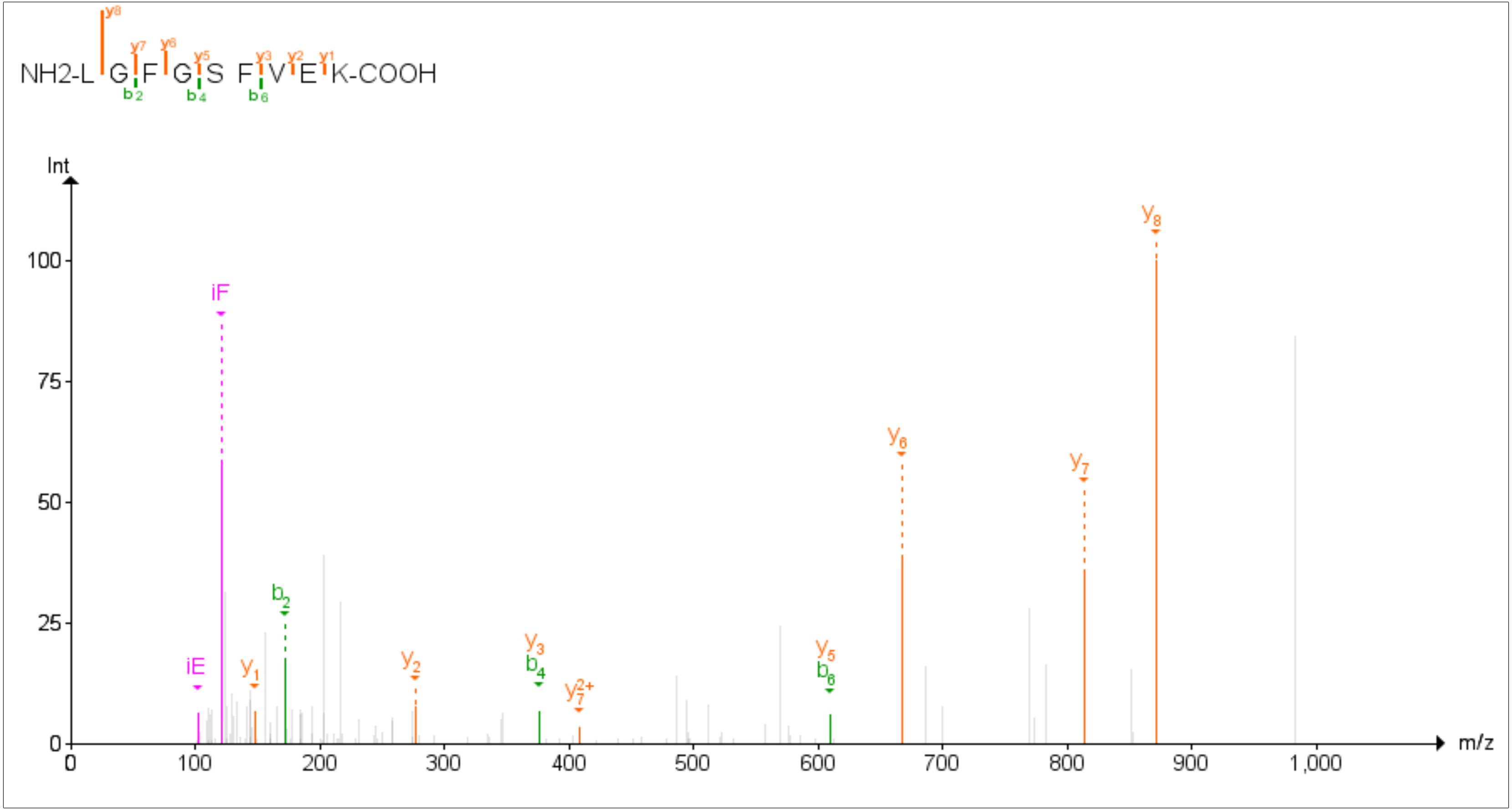
NH2-L G F G S F V E K-COOH
y8 y7 y6 y5 y3 y2 y1
b2 b4 b6
Int
m/z
iF
iE
y1
b2
y2
y3
b4
y7 2+
y5
b6
y6
y7
y8
0
25
50
75
100
0
100
200
300
400
500
600
700
800
900
1,000

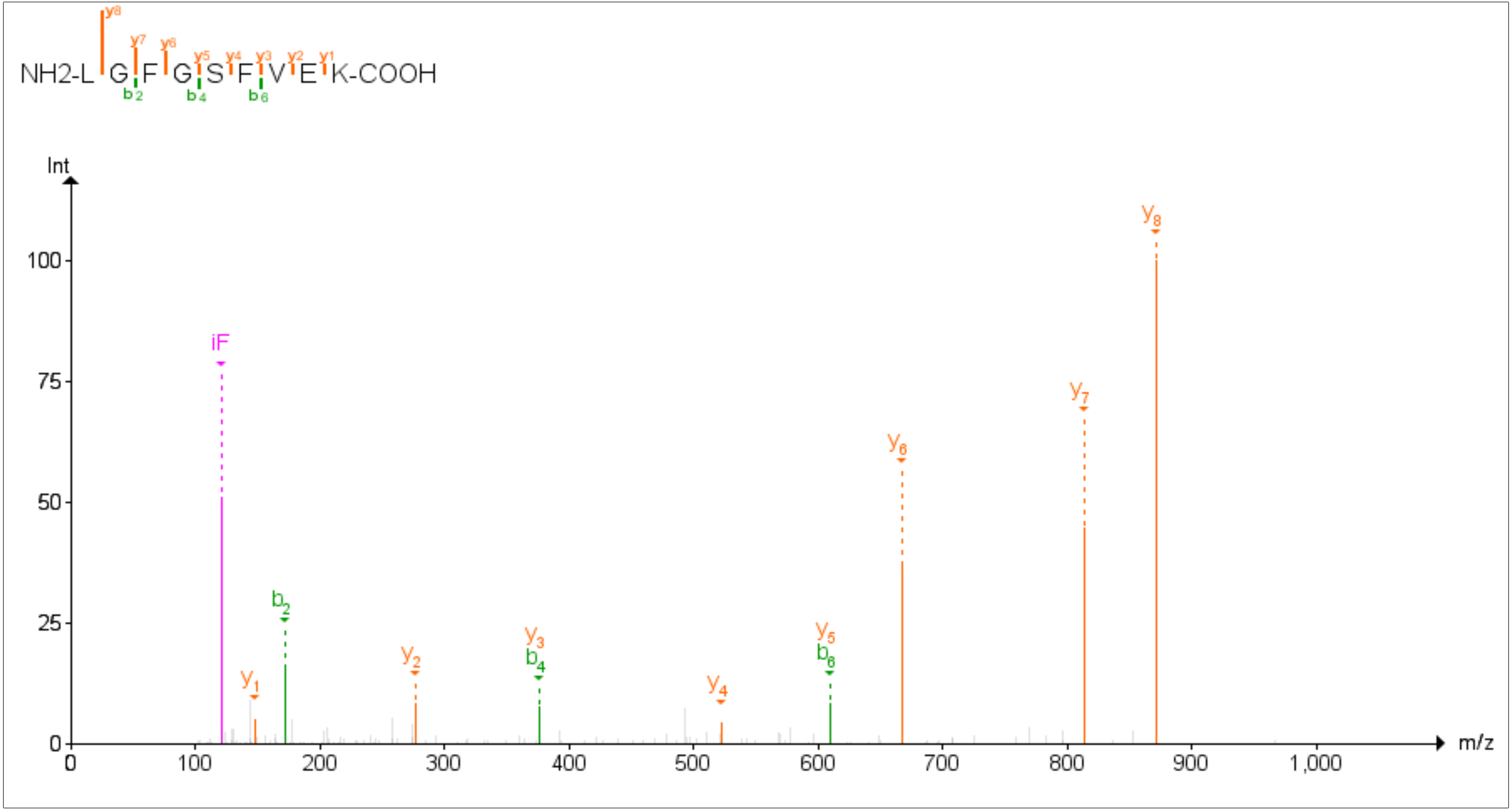

NH2-L G F G S F V E K-COOH
y8
y7
y6
y5
y4
y3
y2
y1
b2
b4
b6
Int
100
75
50
25
0
iF
y1
b2
y2
y3
b4
y4
y5
b6
y6
y7
y8
0
100
200
300
400
500
600
700
800
900
1,000
m/z

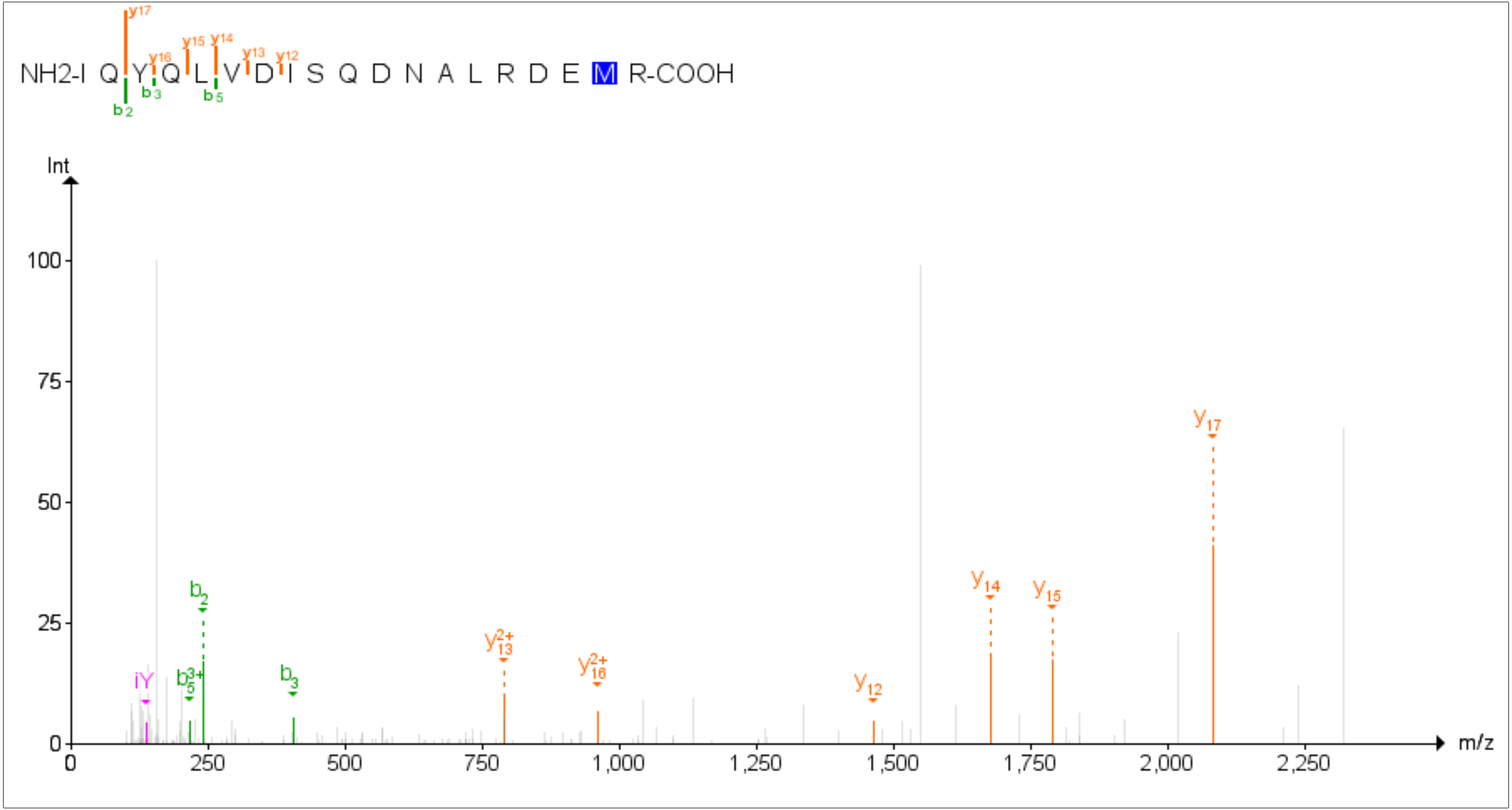
NH2-I Q Y Q L V D I S Q D N A L R D E M R-COOH
y17
y16
y15
y14
y13
y12
b2
b3
b5
Int
100
75
50
25
0
m/z
0
250
500
750
1,000
1,250
1,500
1,750
2,000
2,250
iY
b5 3+
b2
b3
y13 2+
y16 2+
y12
y14
y15
y17

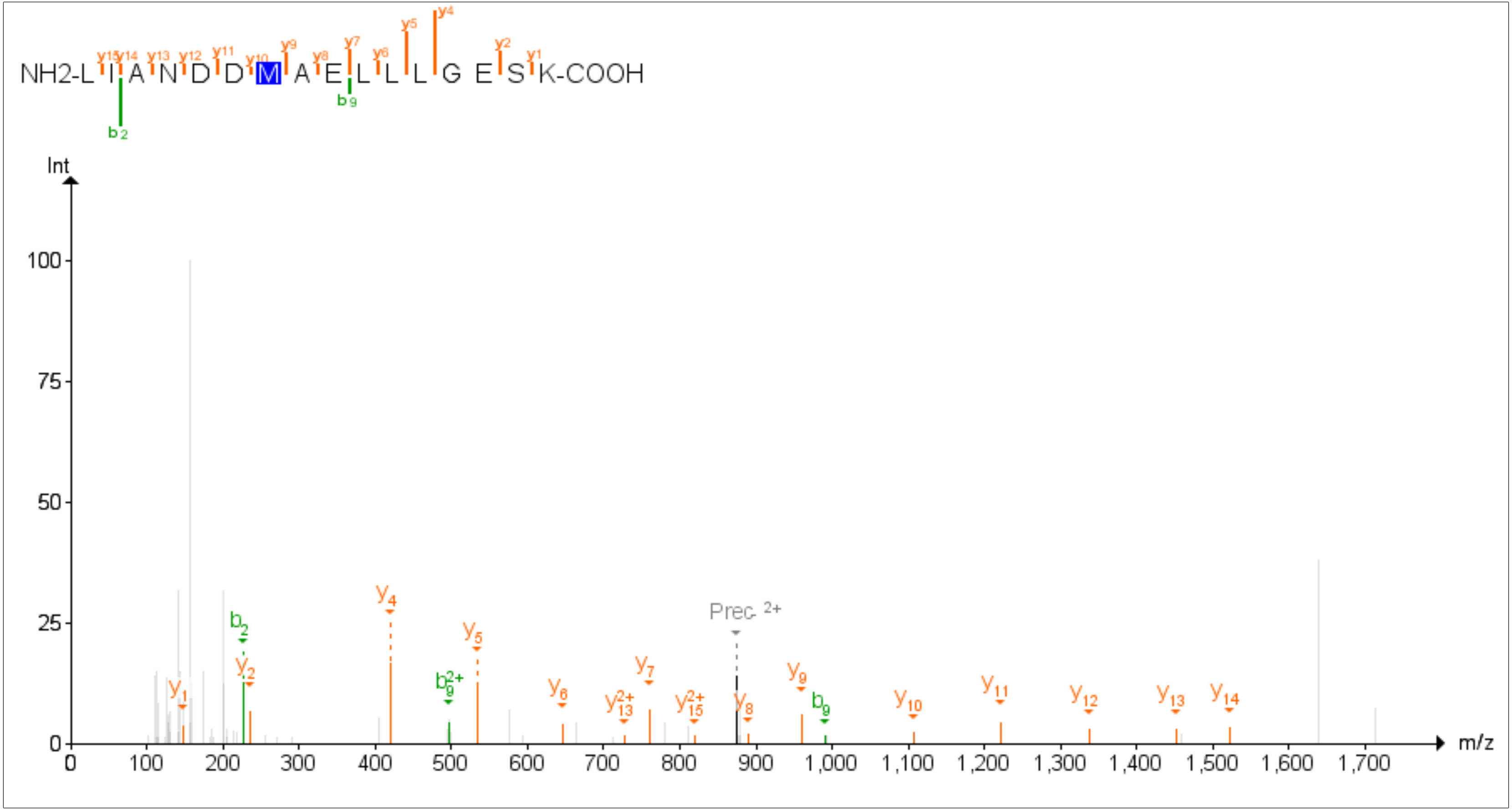
NH2-L I A N D D M A E L L L G E S K-COOH
Int
100
75
50
25
0
m/z
0
100
200
300
400
500
600
700
800
900
1,000
1,100
1,200
1,300
1,400
1,500
1,600
1,700
Prec 2+

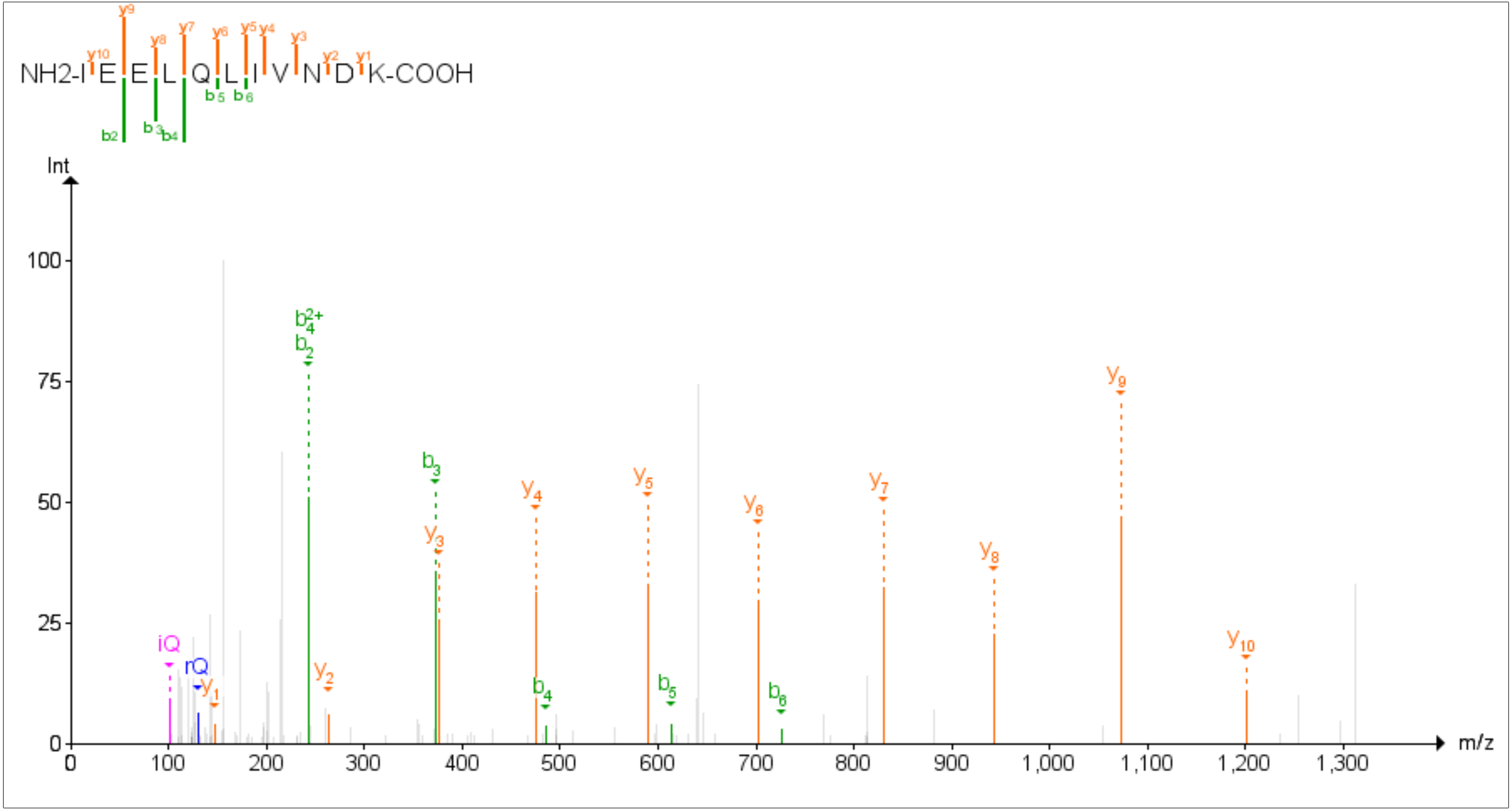

NH2-I E E L Q L I V N D K-COOH
y10 y9 y8 y7 y6 y5 y4 y3 y2 y1
b2 b3 b4 b5 b6
Int
100
75
50
25
0
m/z
0 100 200 300 400 500 600 700 800 900 1,000 1,100 1,200 1,300
iQ
rQ
y1
y2
b4 2+
b2
b3
y3
y4
b4
y5
b5
y6
b6
y7
y8
y9
y10

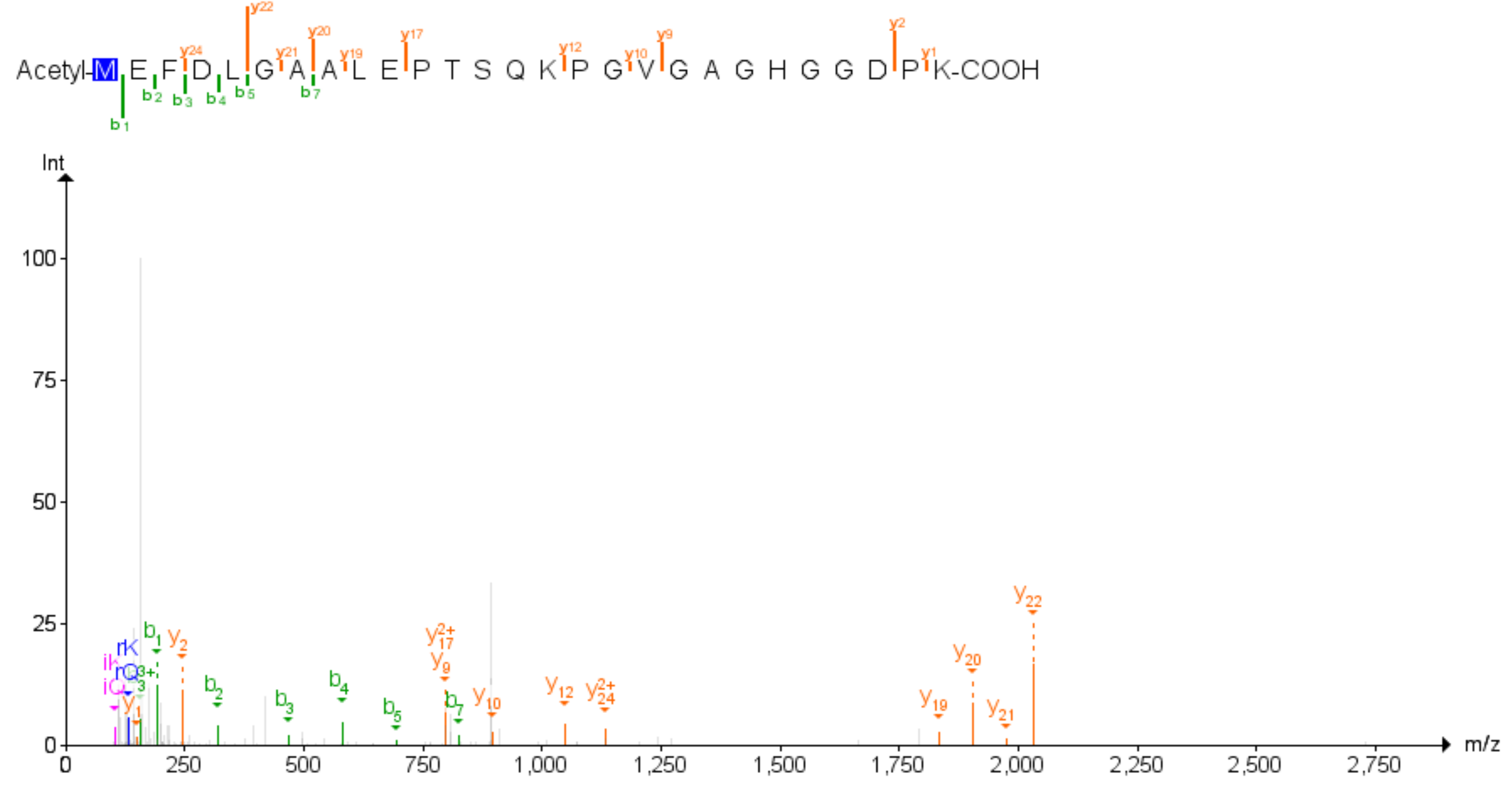

Acetyl-M E F D L G A A L E P T S Q K P G V G A G H G G D P K-COOH
Int
m/z
100
75
50
25
0
0
250
500
750
1,000
1,250
1,500
1,750
2,000
2,250
2,500
2,750
y22
y20
y21
y19
y12
y10
y9
y17
y24
y2
y1
y2+17
y2+24
b1
b2
b3
b4
b5
b7

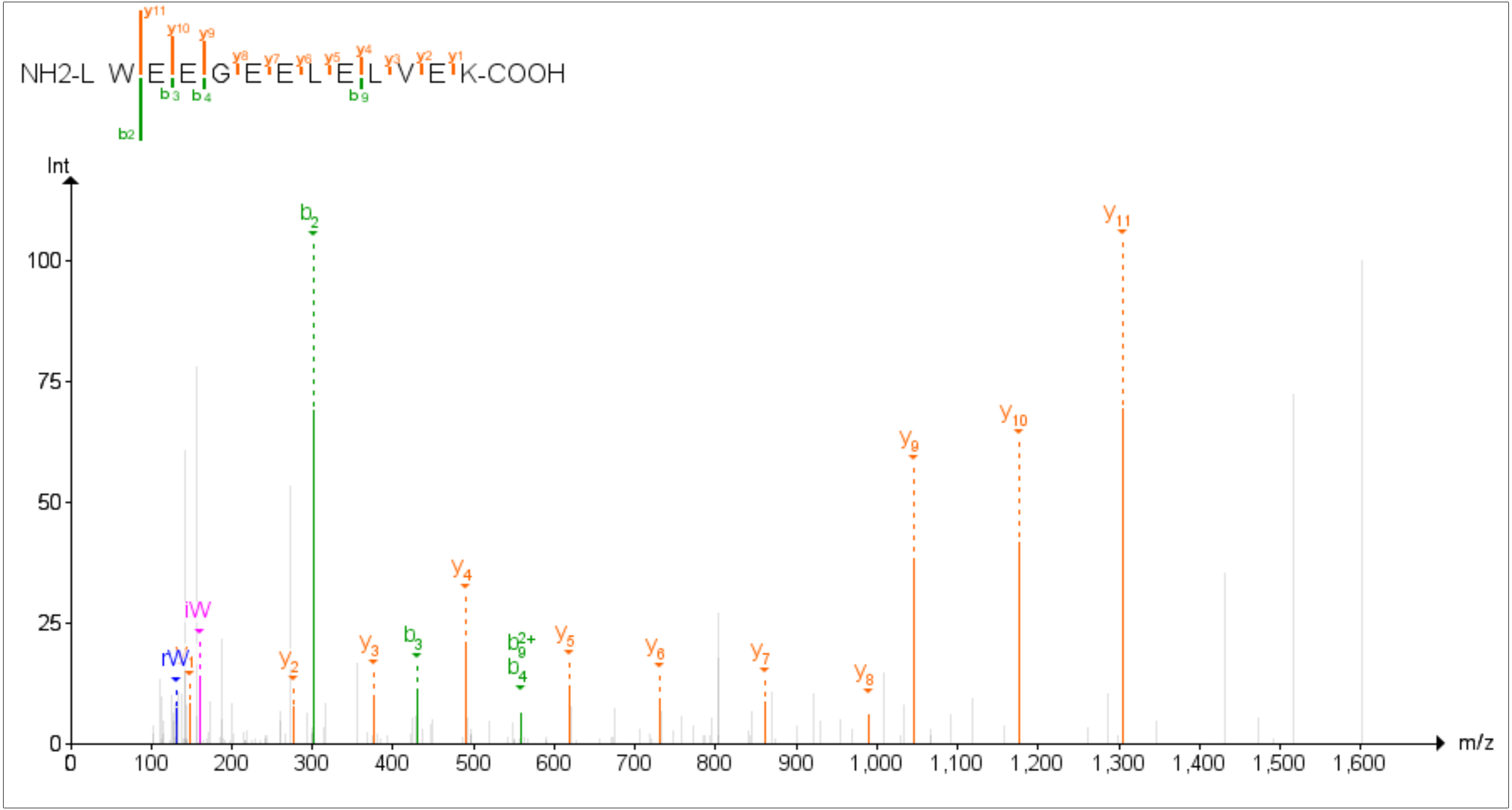
NH2-L W E E G E E L E L V E K-COOH
y11 y10 y9 y8 y7 y6 y5 y4 y3 y2 y1
b2 b3 b4 b9
Int
m/z
0 25 50 75 100
0 100 200 300 400 500 600 700 800 900 1,000 1,100 1,200 1,300 1,400 1,500 1,600
rW iW y_1 y_2 b_2 y_3 b_3 y_4 b_9^{2+} b_4 y_5 y_6 y_7 y_8 y_9 y_{10} y_{11}

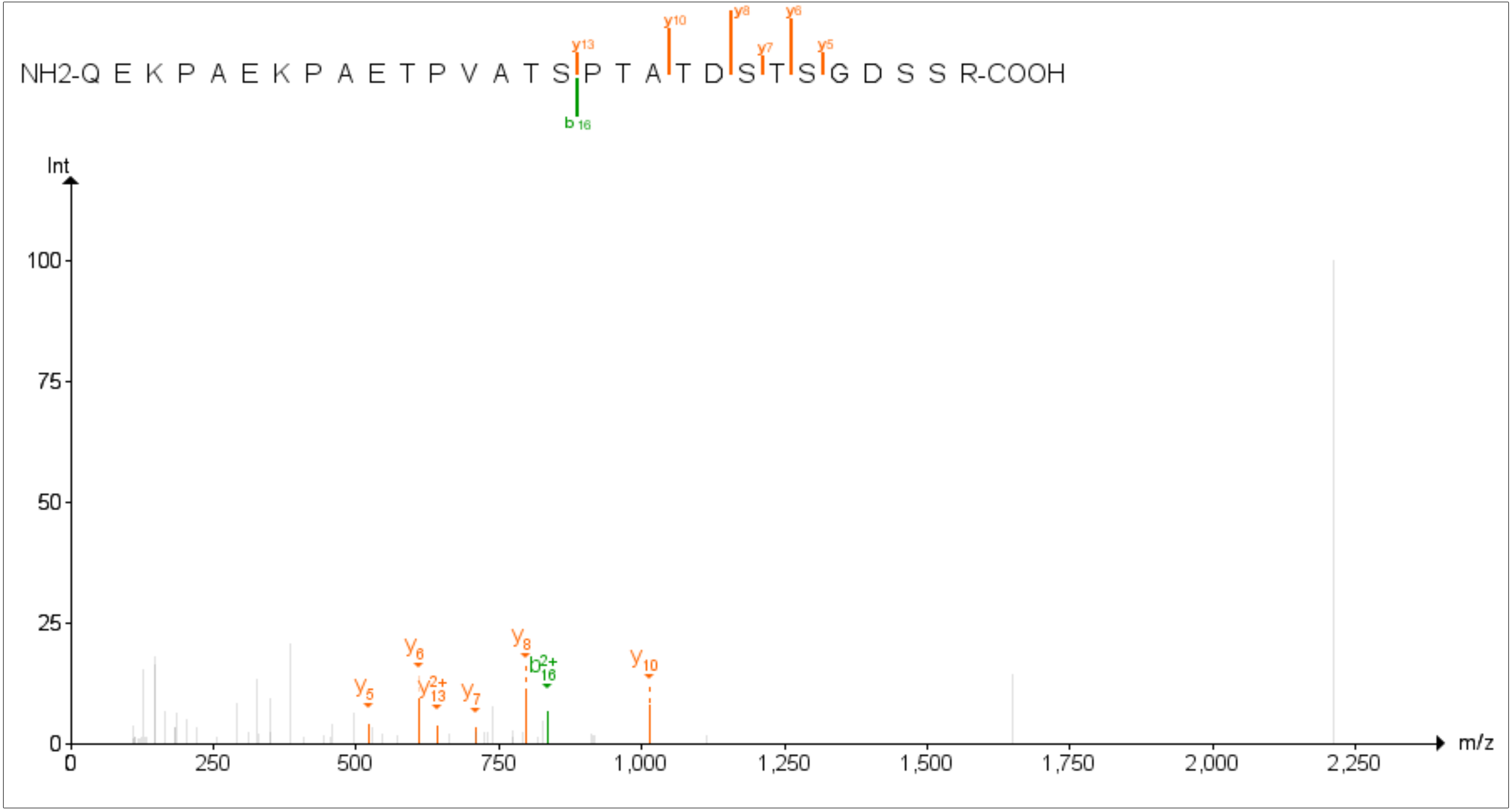

NH2-Q E K P A E K P A E T P V A T S P T A T D S T S G D S S R-COOH
y13
y10
y8
y7
y6
y5
b16
Int
100
75
50
25
0
y5
y6
y13 2+
y7
y8
b16 2+
y10
0
250
500
750
1,000
1,250
1,500
1,750
2,000
2,250
m/z

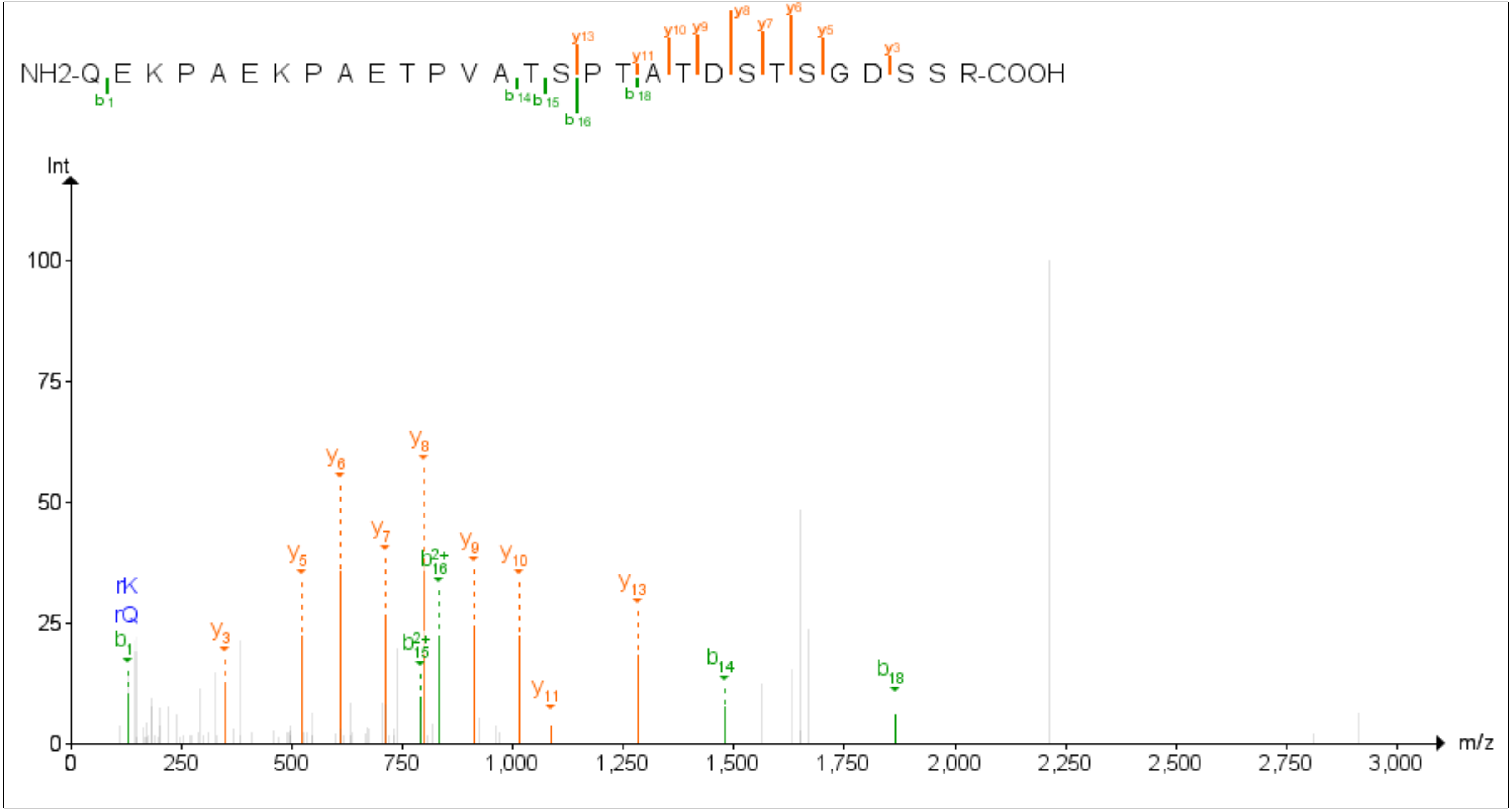
NH2-Q E K P A E K P A E T P V A T S P T A T D S T S G D S S R-COOH
Int
m/z
y3
y5
y6
y7
y8
y9
y10
y11
y13
b1
b14
b15
b16
b18
rK
rQ

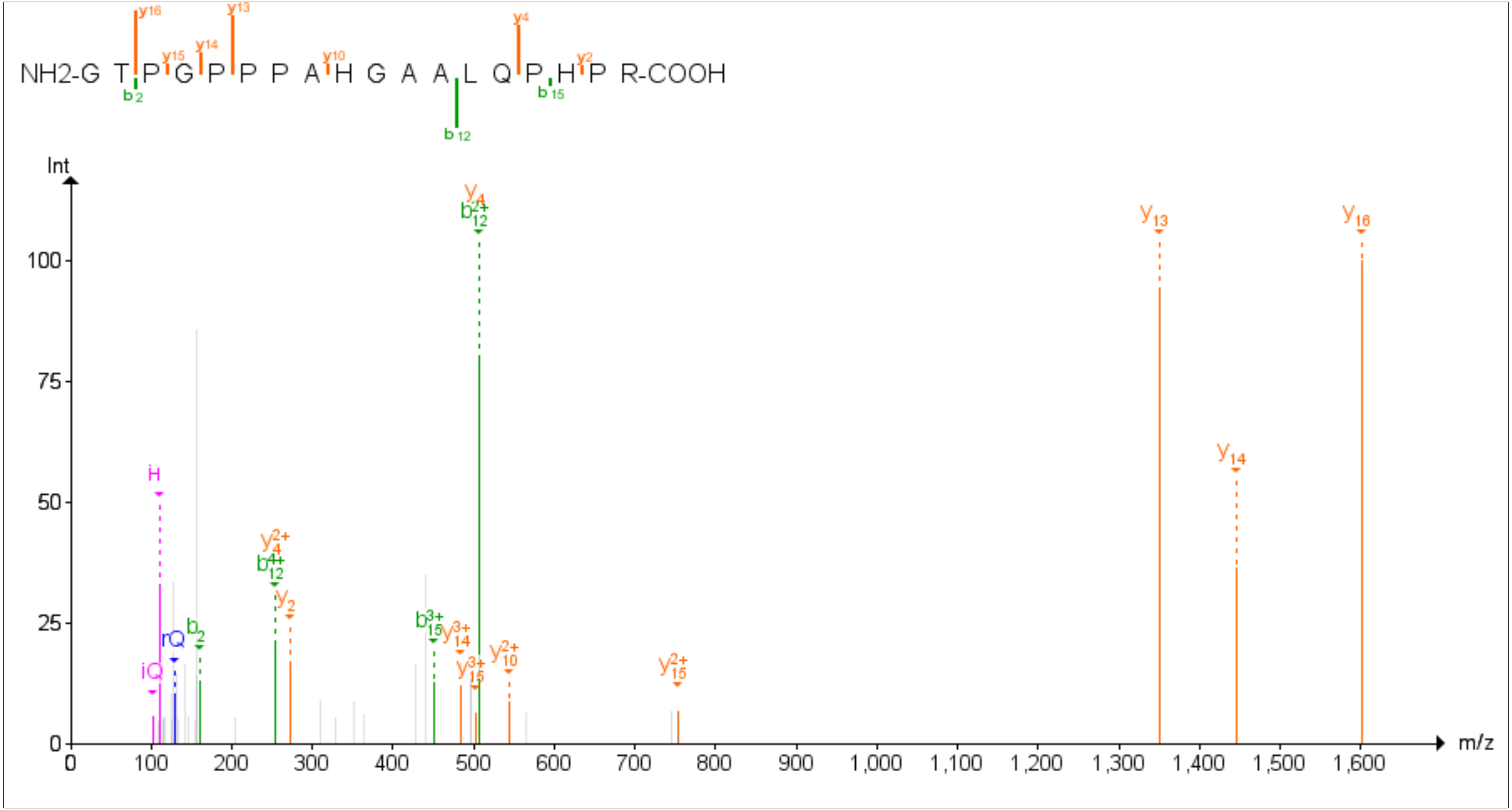

NH2-G T P G P P P A H G A A L Q P H P R-COOH
y16 y15 y14 y13 y10 y4 y2
b2 b12 b15
Int
m/z
0
25
50
75
100
0 100 200 300 400 500 600 700 800 900 1,000 1,100 1,200 1,300 1,400 1,500 1,600
iH
iQ
rQ
b2
y4 2+
b12 4+
y2
b15 3+
y14 3+
y15 3+
y4
b12 2+
y10 2+
y15 2+
y13
y14
y16

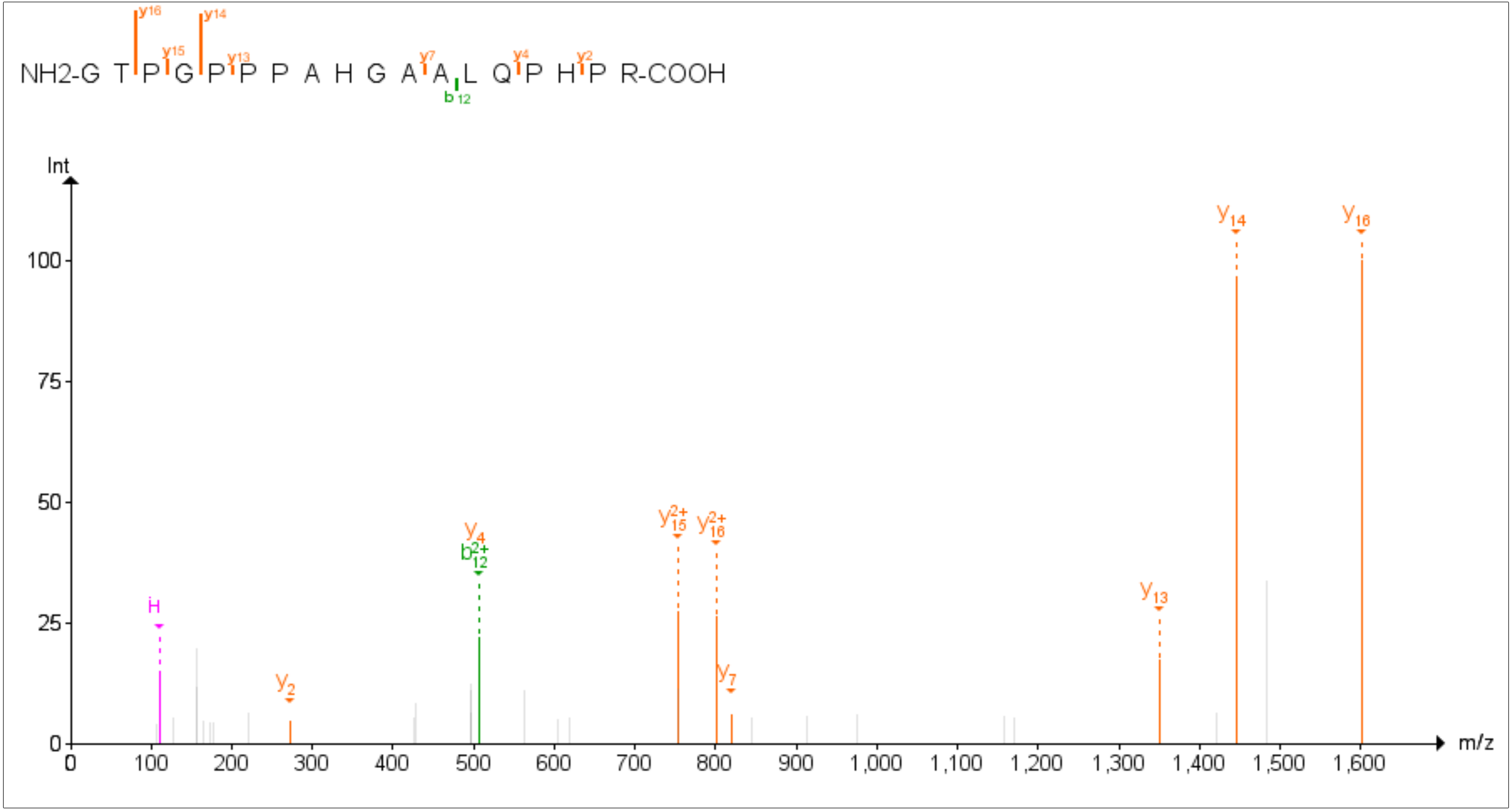
NH2-G T P G P P P A H G A A L Q P H P R-COOH
y16
y15
y14
y13
y7
y4
y2
b12
Int
100
75
50
25
0
H
y2
y4
b12 2+
y15 2+
y16 2+
y7
y13
y14
y16
m/z
0
100
200
300
400
500
600
700
800
900
1,000
1,100
1,200
1,300
1,400
1,500
1,600

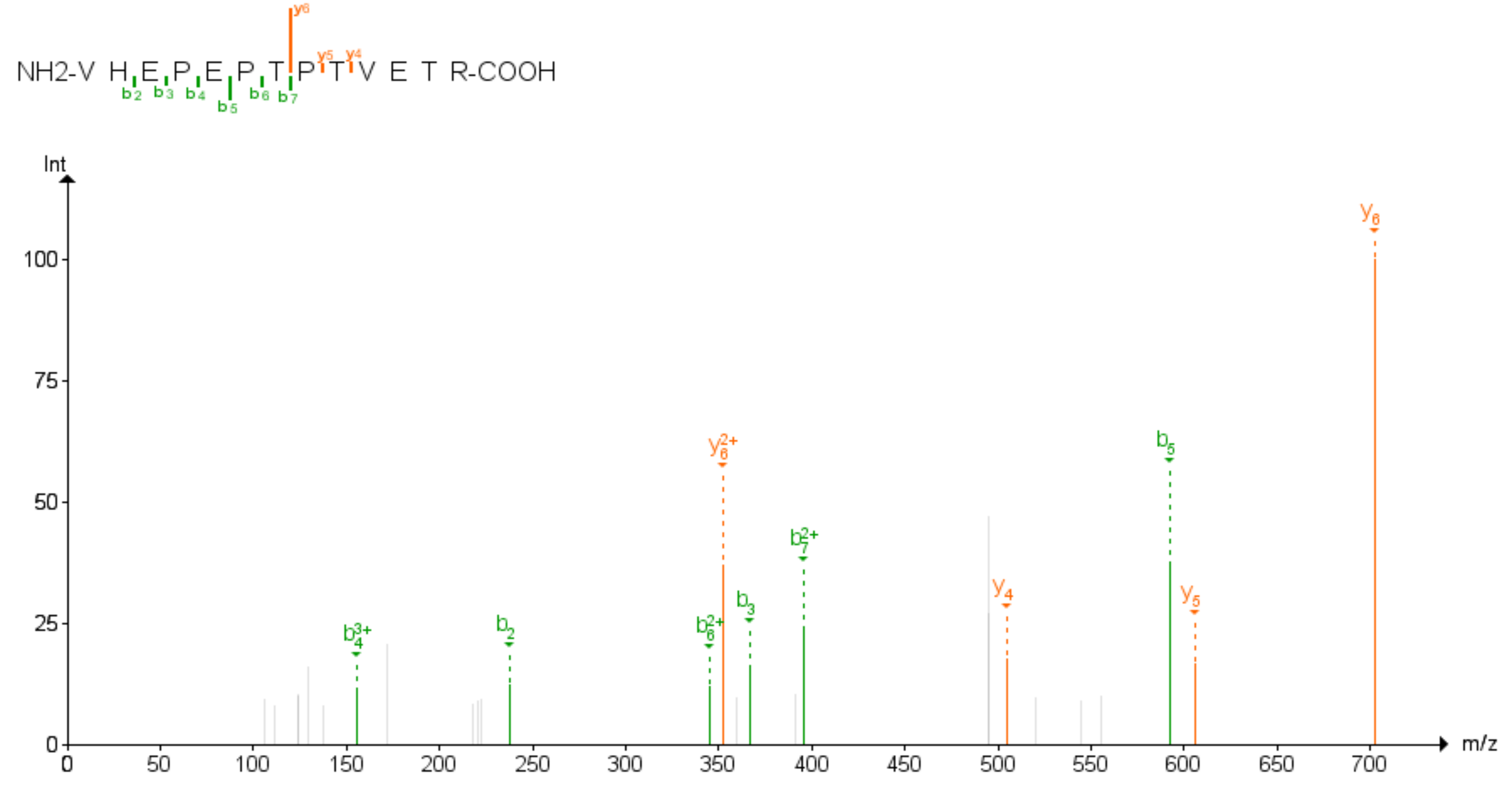
NH2-V H E P E P T P T V E T R-COOH
b2 b3 b4 b5 b6 b7
y6 y5 y4
Int
m/z
0
25
50
75
100
0 50 100 150 200 250 300 350 400 450 500 550 600 650 700

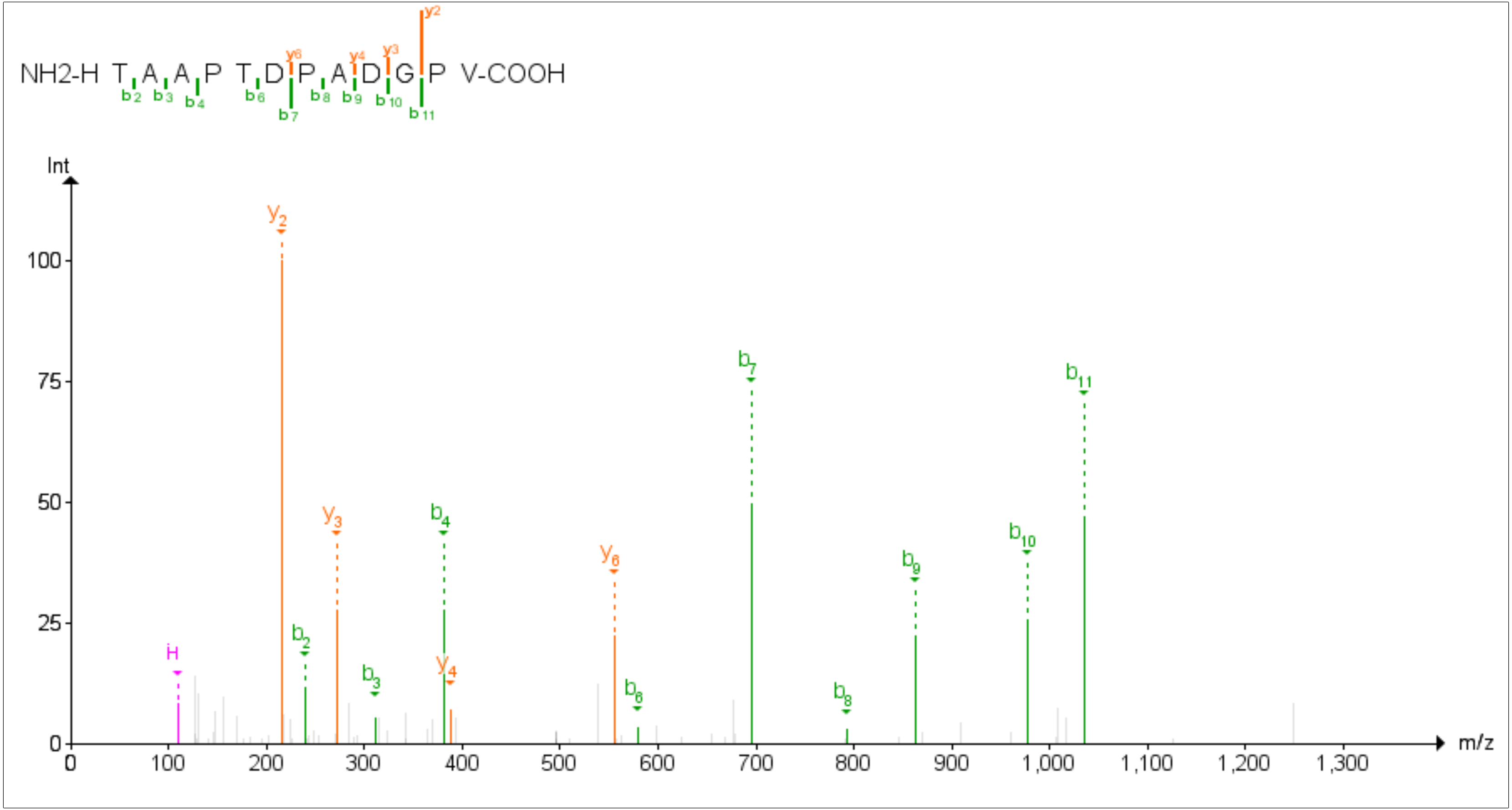

NH2-H T A A P T D P A D G P V-COOH
b2 b3 b4 b6 b7 b8 b9 b10 b11
y6 y4 y3 y2
Int
100
75
50
25
0
y2
y3
b4
y4
b2
b3
H
y6
b6
b7
b8
b9
b10
b11
0 100 200 300 400 500 600 700 800 900 1,000 1,100 1,200 1,300
m/z

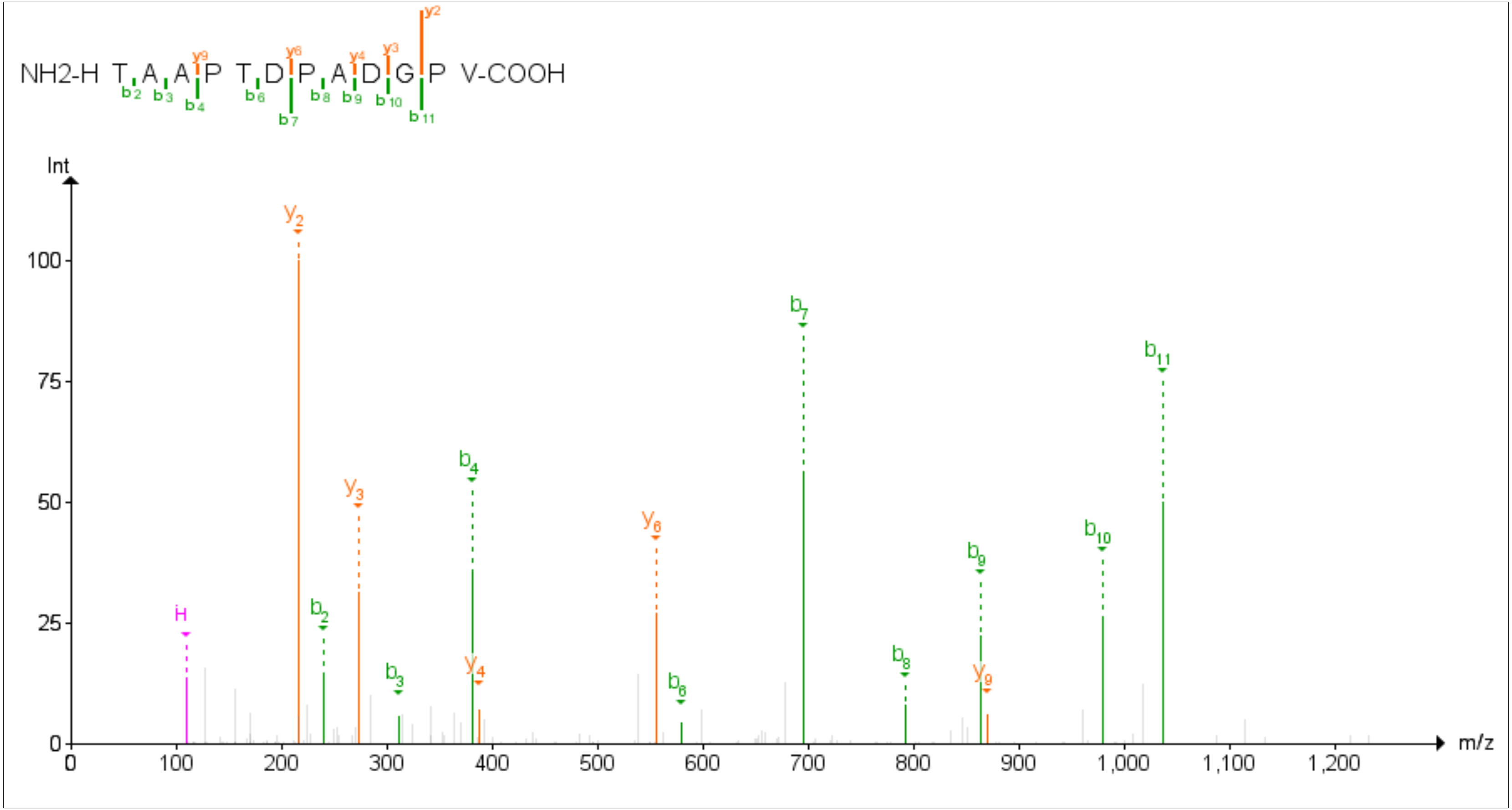

NH2-H T A A P T D P A D G P V-COOH
y9 y6 y4 y3 y2
b2 b3 b4 b6 b7 b8 b9 b10 b11
Int
100
75
50
25
0
H
y2
y3
y4
y6
y9
b2
b3
b4
b6
b7
b8
b9
b10
b11
0 100 200 300 400 500 600 700 800 900 1,000 1,100 1,200
m/z

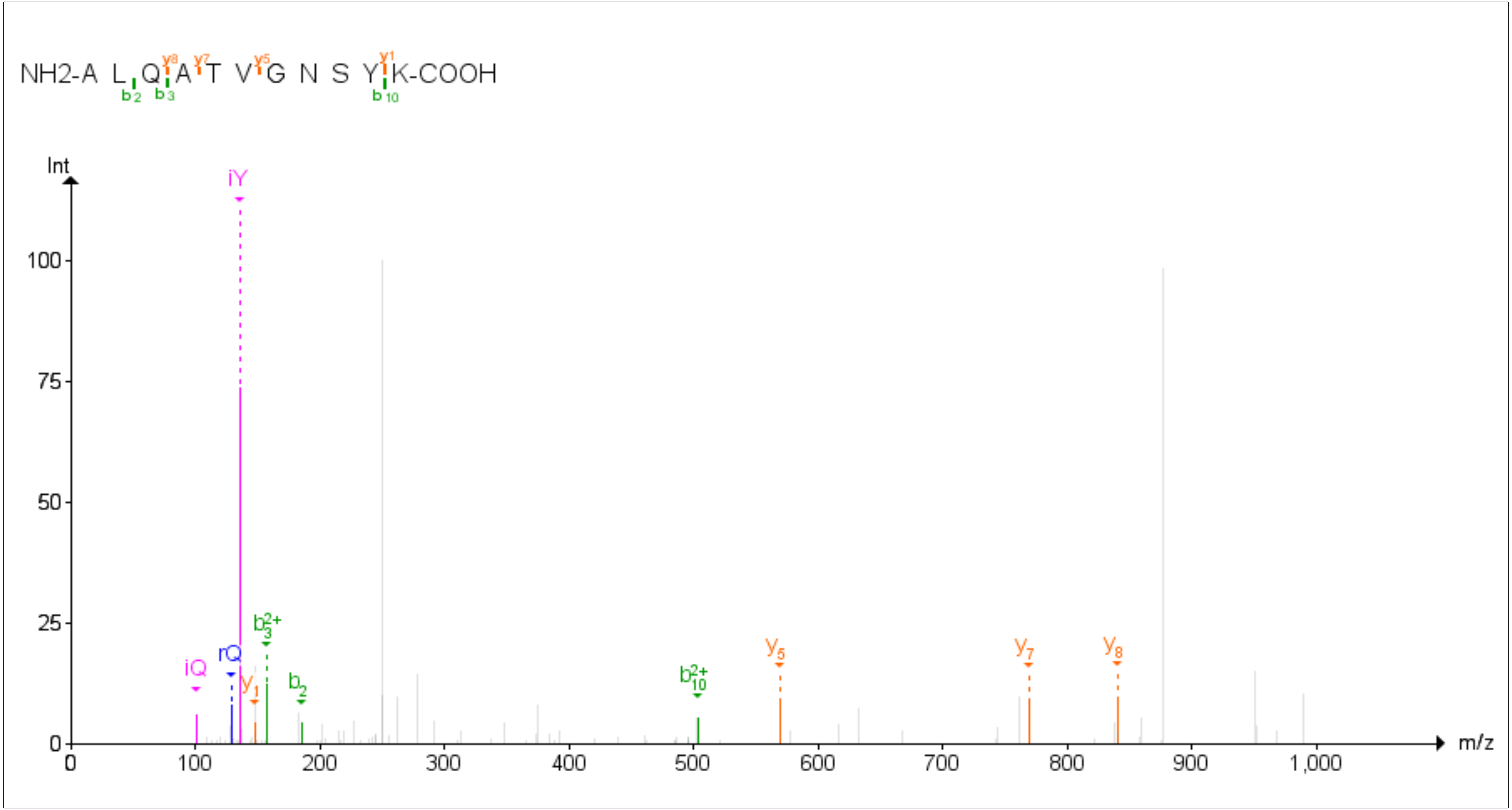
NH2-A L Q A T V G N S Y K-COOH
y8 y7 y5 y1
b2 b3 b10
Int
iY
100
75
50
25
0
iQ
rQ
y1
b3 2+
b2
b10 2+
y5
y7
y8
0 100 200 300 400 500 600 700 800 900 1,000
m/z

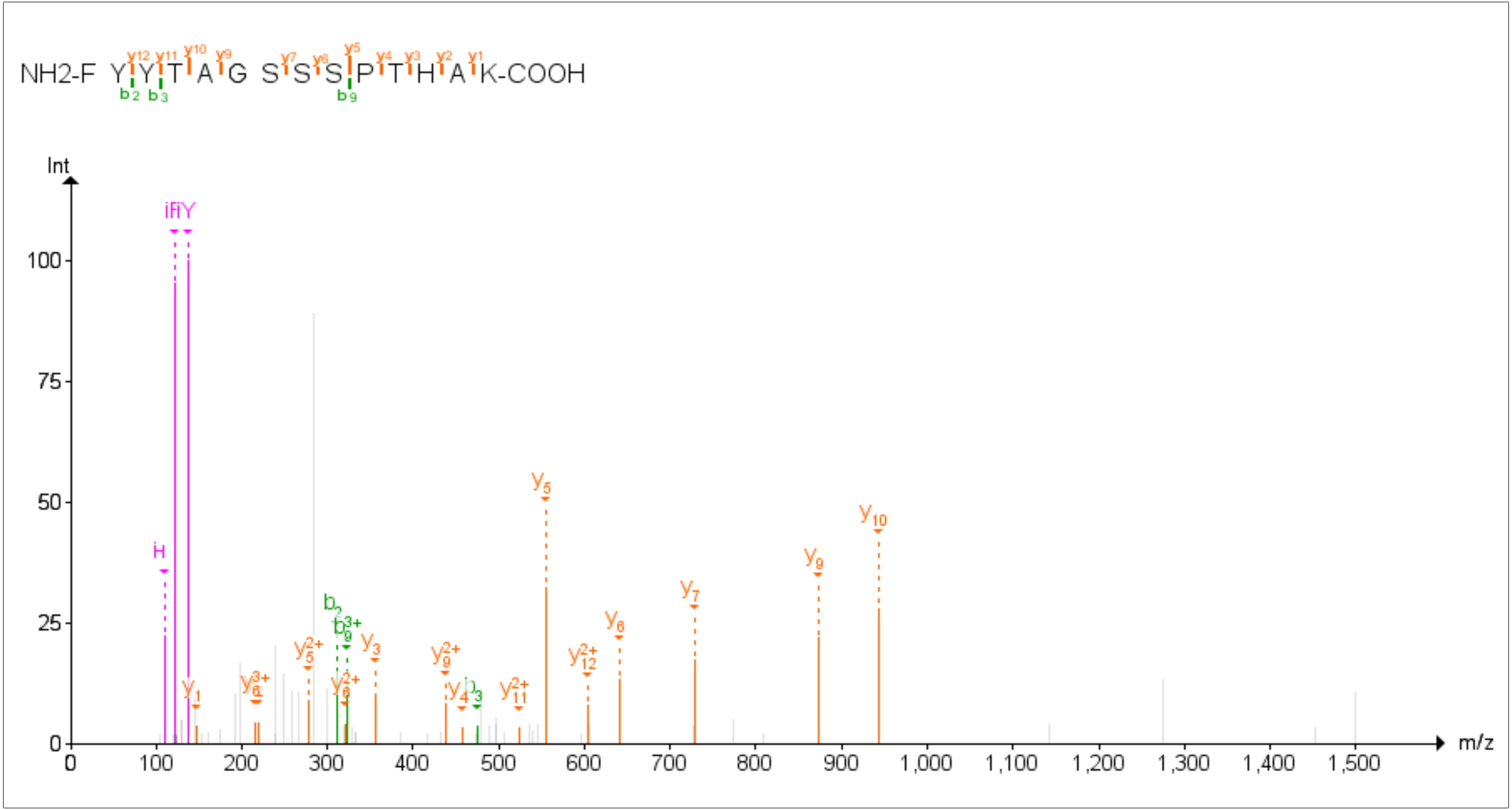

NH2-F Y Y T A G S S S P T H A K-COOH
y12 y11 y10 y9 y7 y6 y5 y4 y3 y2 y1
b2 b3 b9
Int
100
75
50
25
0
iFiY
iH
y1
y5
y10
y9
y7
y6
y12
y11
y4
y3
b3
b2
b9
m/z
0 100 200 300 400 500 600 700 800 900 1,000 1,100 1,200 1,300 1,400 1,500

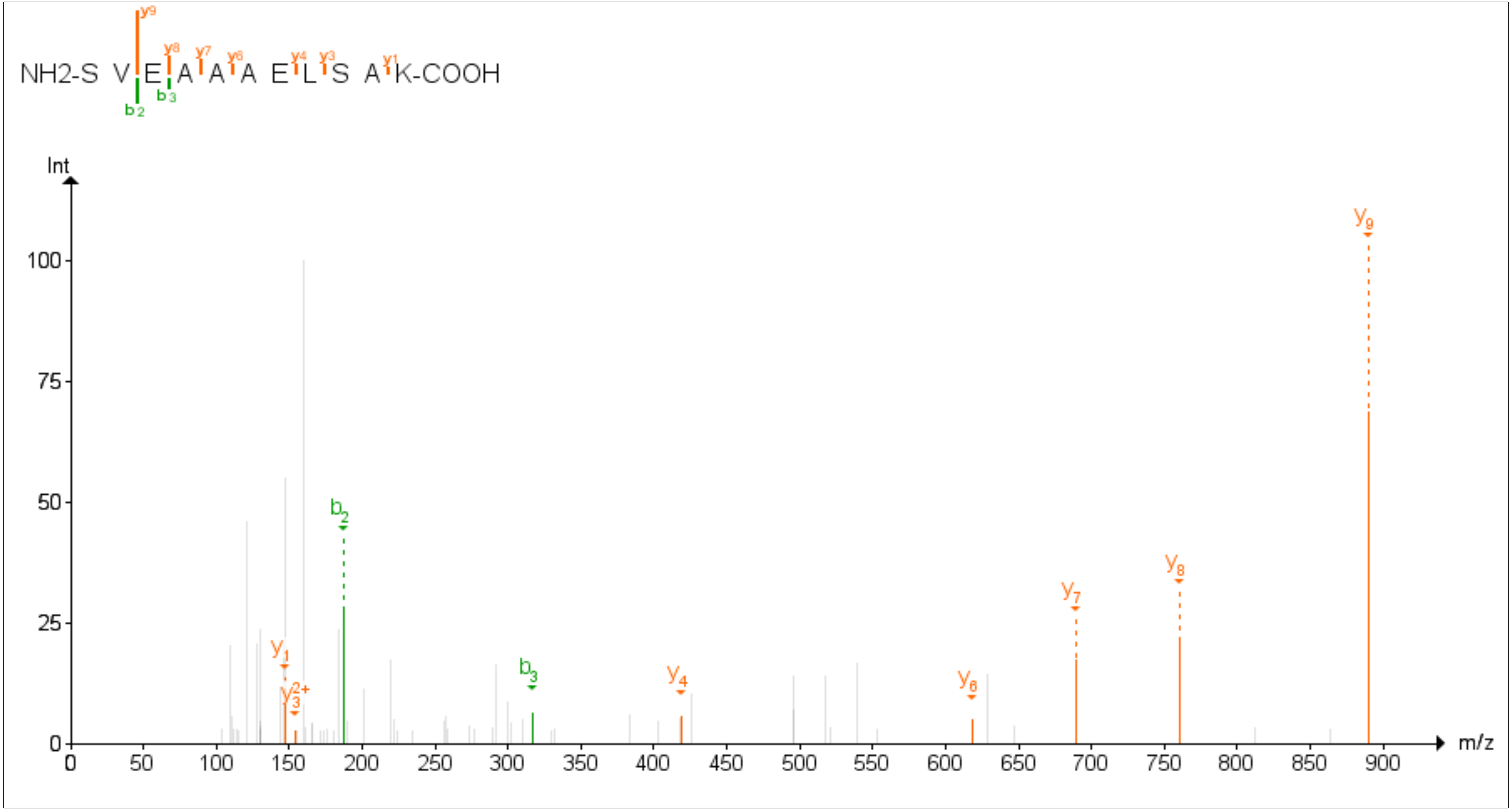

NH2-S V E A A A E L S A K-COOH
y9
y8
y7
y6
y4
y3
y1
b2
b3
Int
m/z
0
25
50
75
100
0
50
100
150
200
250
300
350
400
450
500
550
600
650
700
750
800
850
900

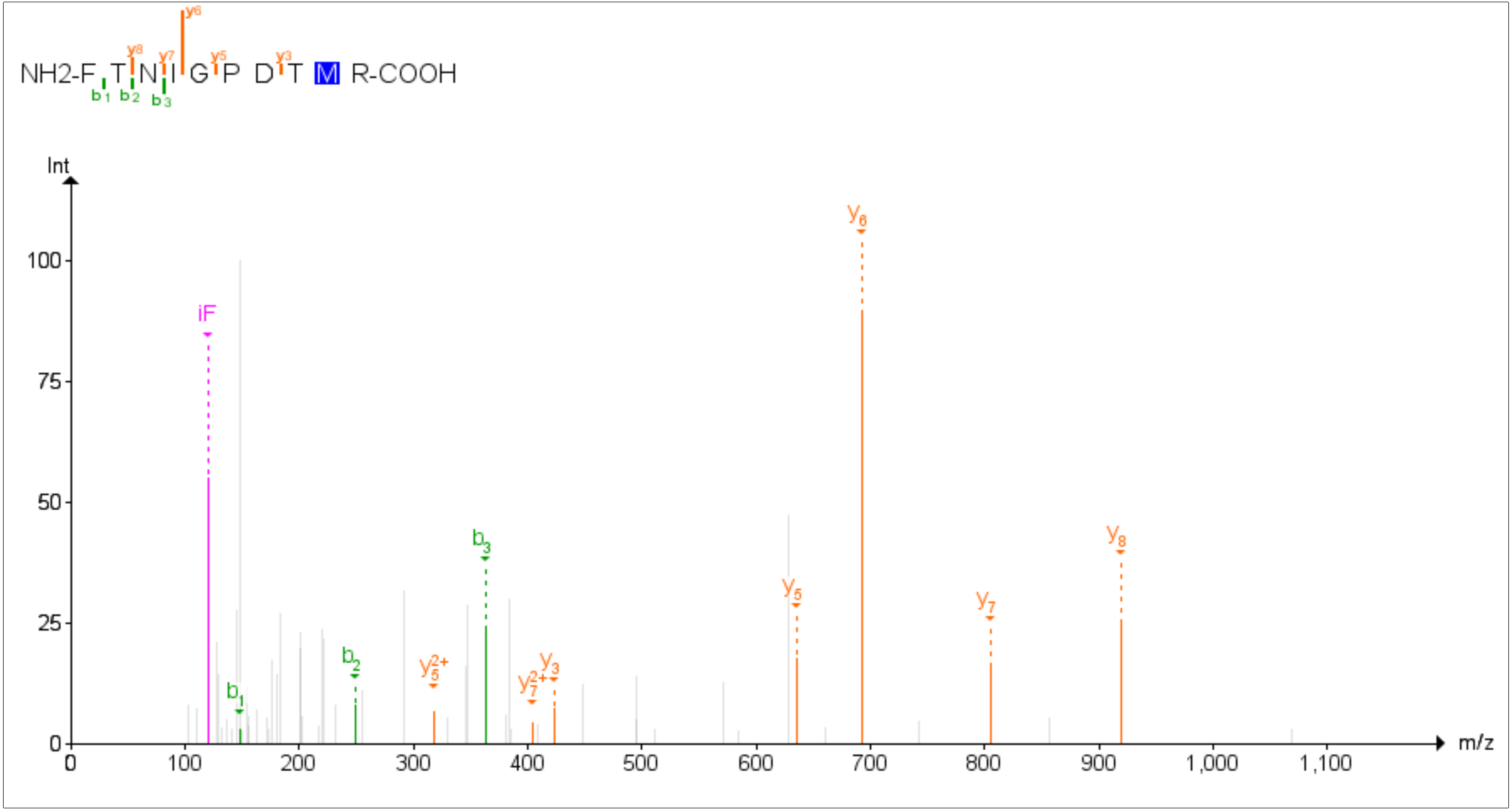

NH2-F T N I G P D T M R-COOH
b1 b2 b3
y8 y7 y6 y5 y3
Int
m/z
iF
b1 b2 b3
y5 y6 y7 y8 y3
y5 2+
y7 2+
0
25
50
75
100
0
100
200
300
400
500
600
700
800
900
1,000
1,100

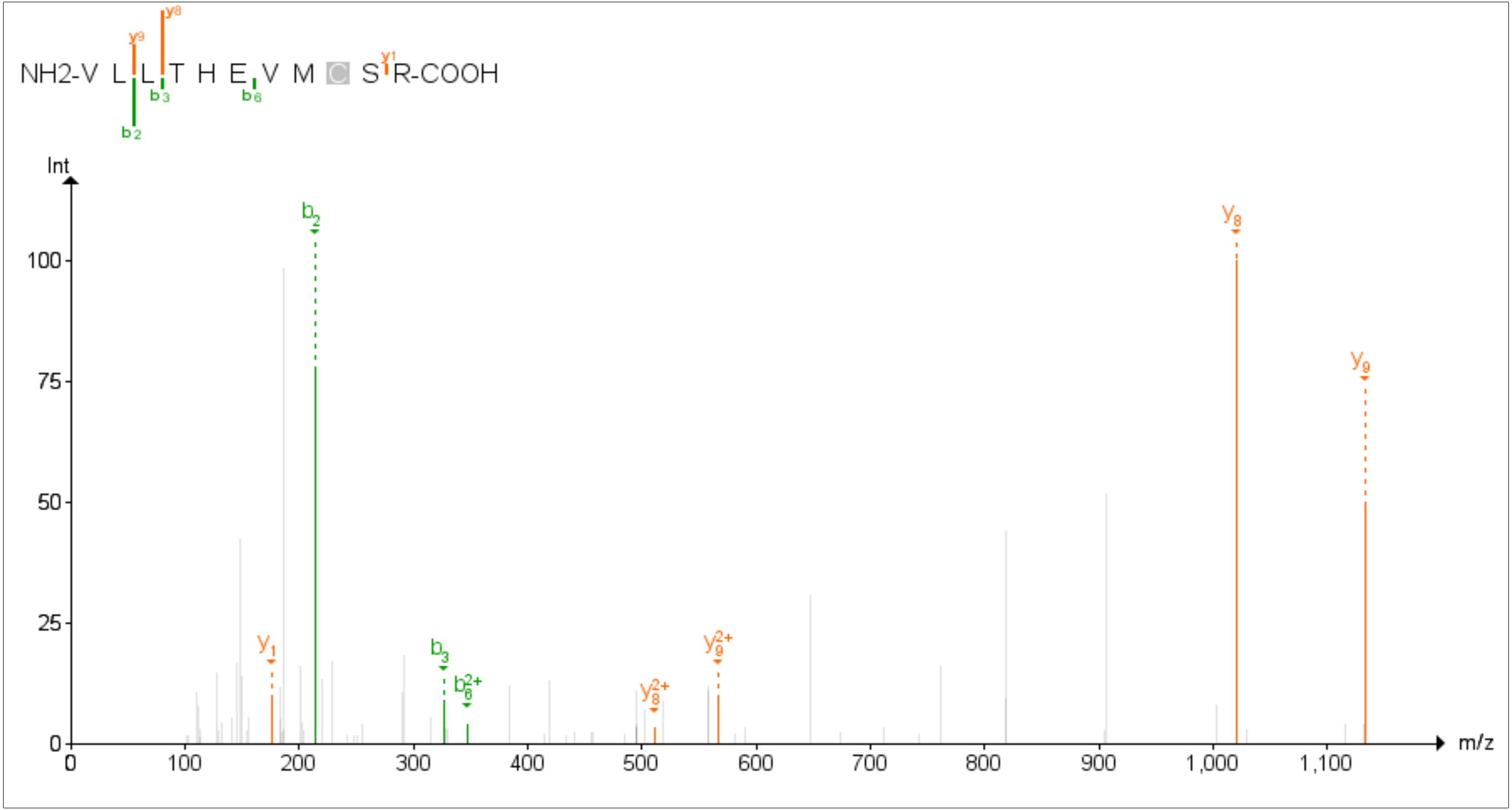
NH2-V L L T H E V M C S R-COOH
y9
y8
y1
b2
b3
b6
Int
100
75
50
25
0
m/z
0
100
200
300
400
500
600
700
800
900
1,000
1,100
b2
y8
y9
y1
b3
b6 2+
y8 2+
y9 2+

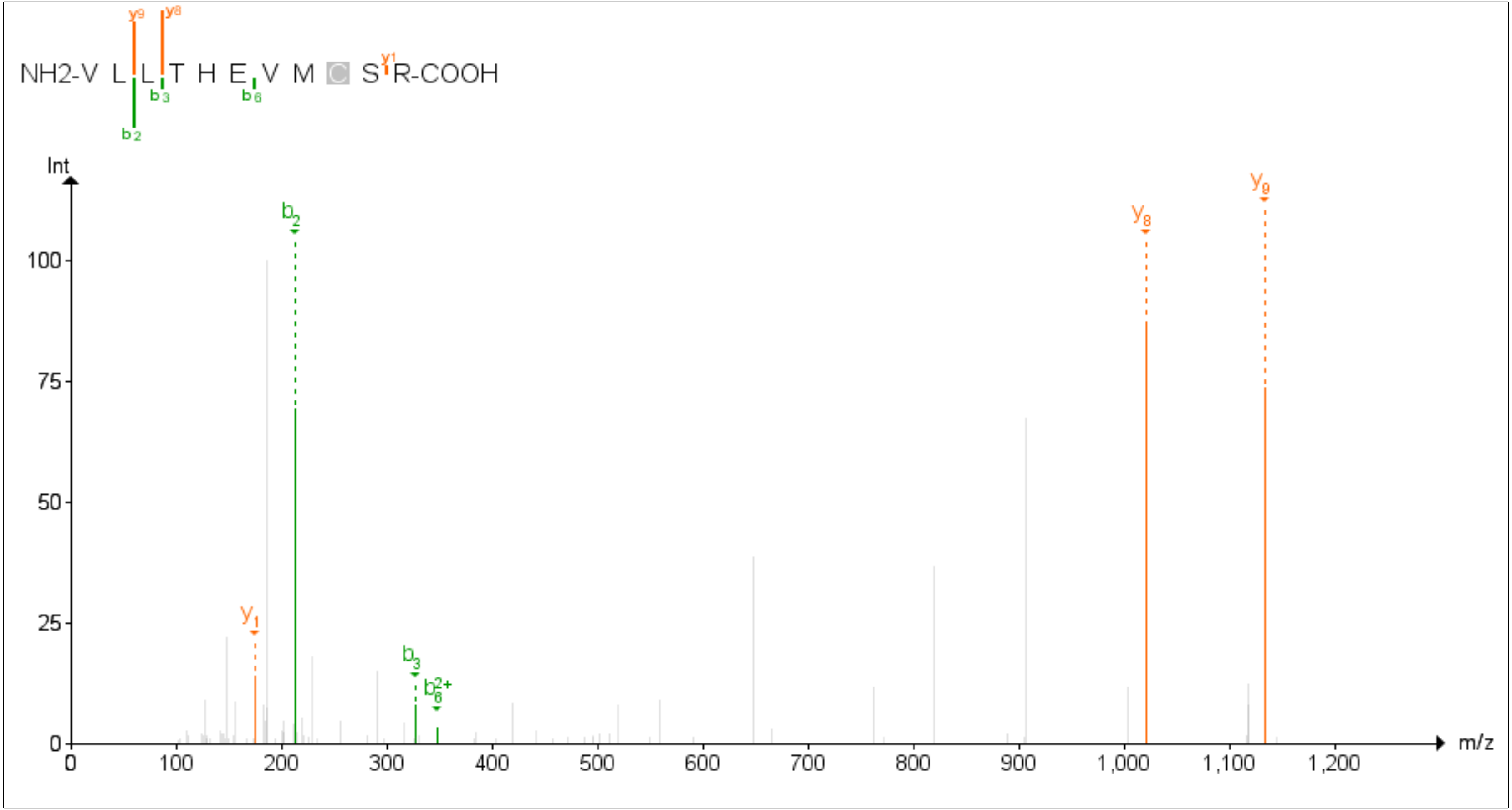

NH2-V L L T H E V M C S R-COOH
y9
y8
y1
b2
b3
b6
Int
100
75
50
25
0
m/z
0
100
200
300
400
500
600
700
800
900
1,000
1,100
1,200
y1
b2
b3
b6 2+
y8
y9

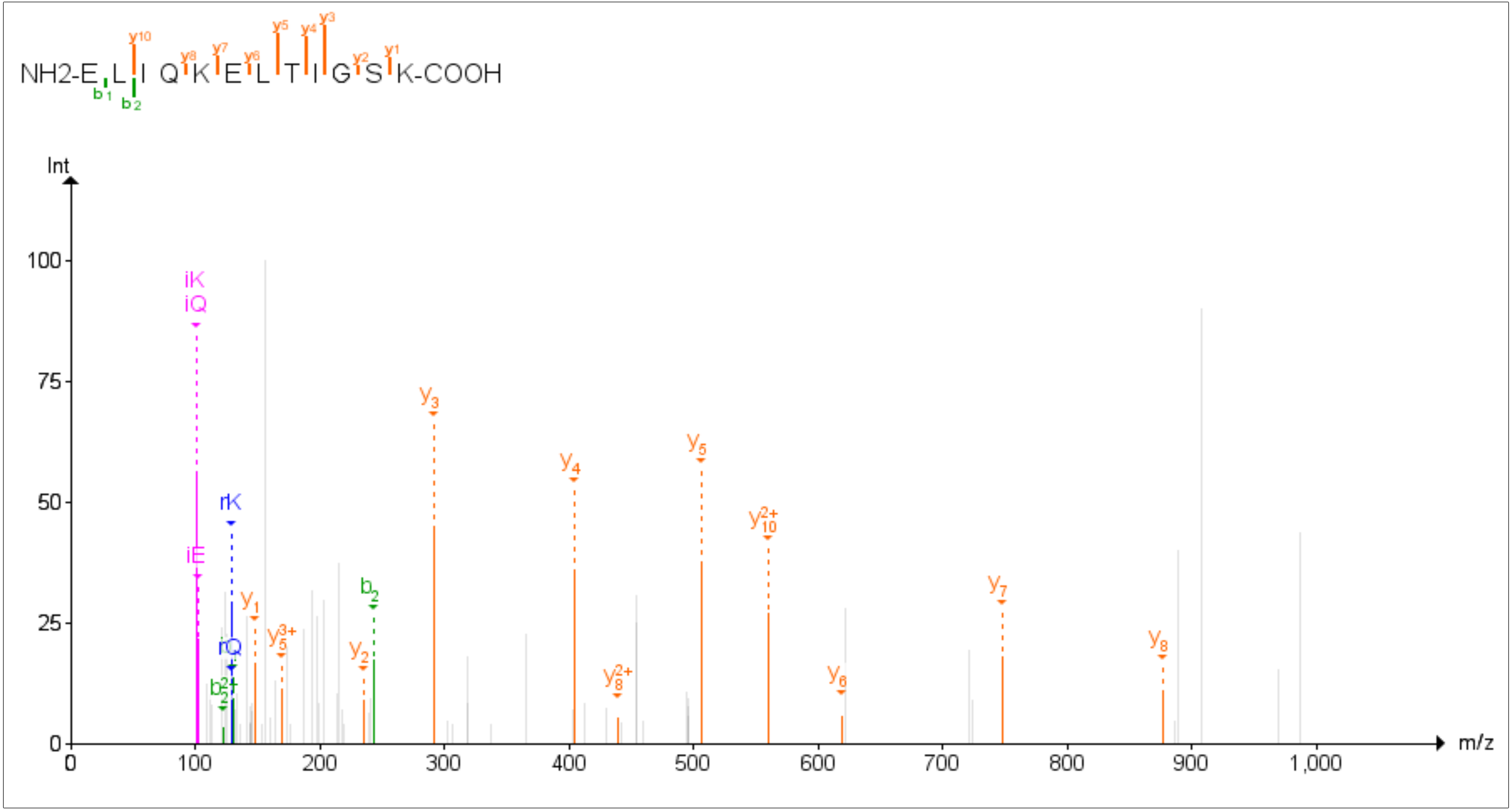
NH2-E L I Q K E L T I G S K-COOH
y10 y8 y7 y6 y5 y4 y3 y2 y1
b1 b2
Int
100
75
50
25
0
m/z
0 100 200 300 400 500 600 700 800 900 1,000
iK
iQ
iE
rK
rQ
y1
y3+5
y2
b2
b2+2
y3
y4
y5
y2+8
y2+10
y6
y7
y8

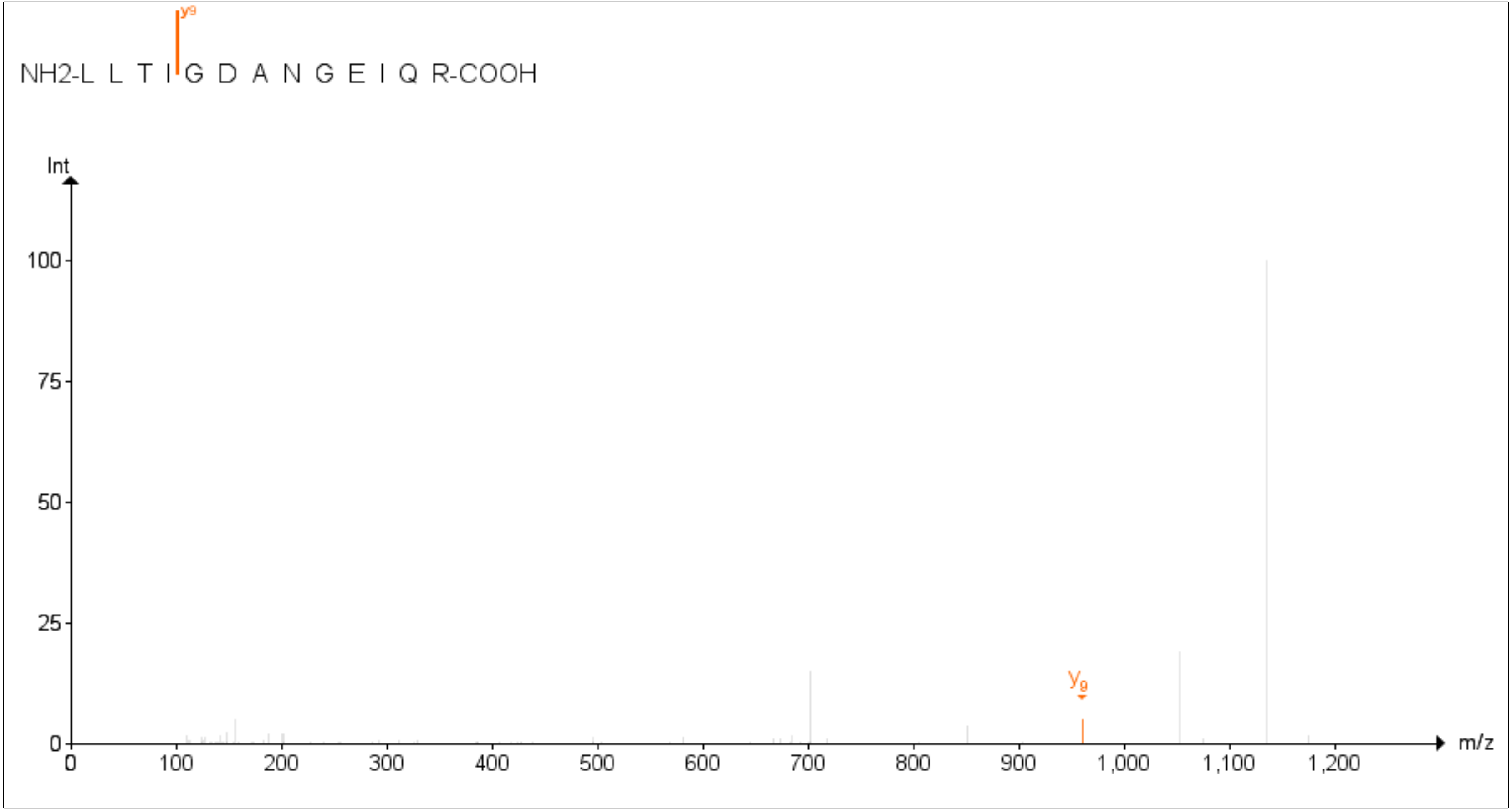

y9
NH2-L L T I G D A N G E I Q R-COOH
Int
100
75
50
25
0
y9
0
100
200
300
400
500
600
700
800
900
1,000
1,100
1,200
m/z

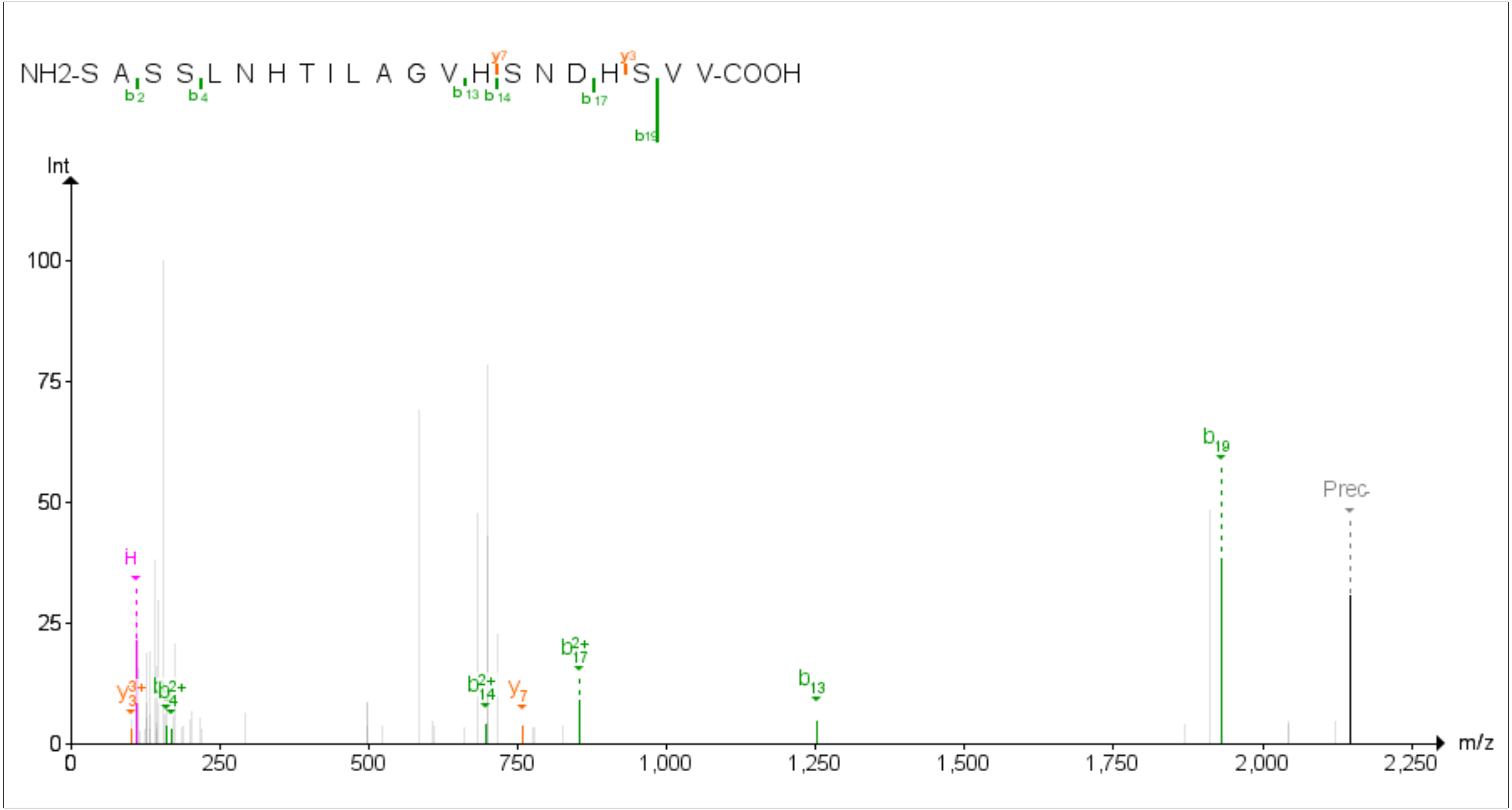

NH2-S A S S L N H T I L A G V H S N D H S V V-COOH
b2
b4
b13
b14
b17
b19
y7
y3
Int
100
75
50
25
0
m/z
0
250
500
750
1,000
1,250
1,500
1,750
2,000
2,250
H
y3 3+
b4 2+
b14 2+
y7
b17 2+
b13
b19
Prec.

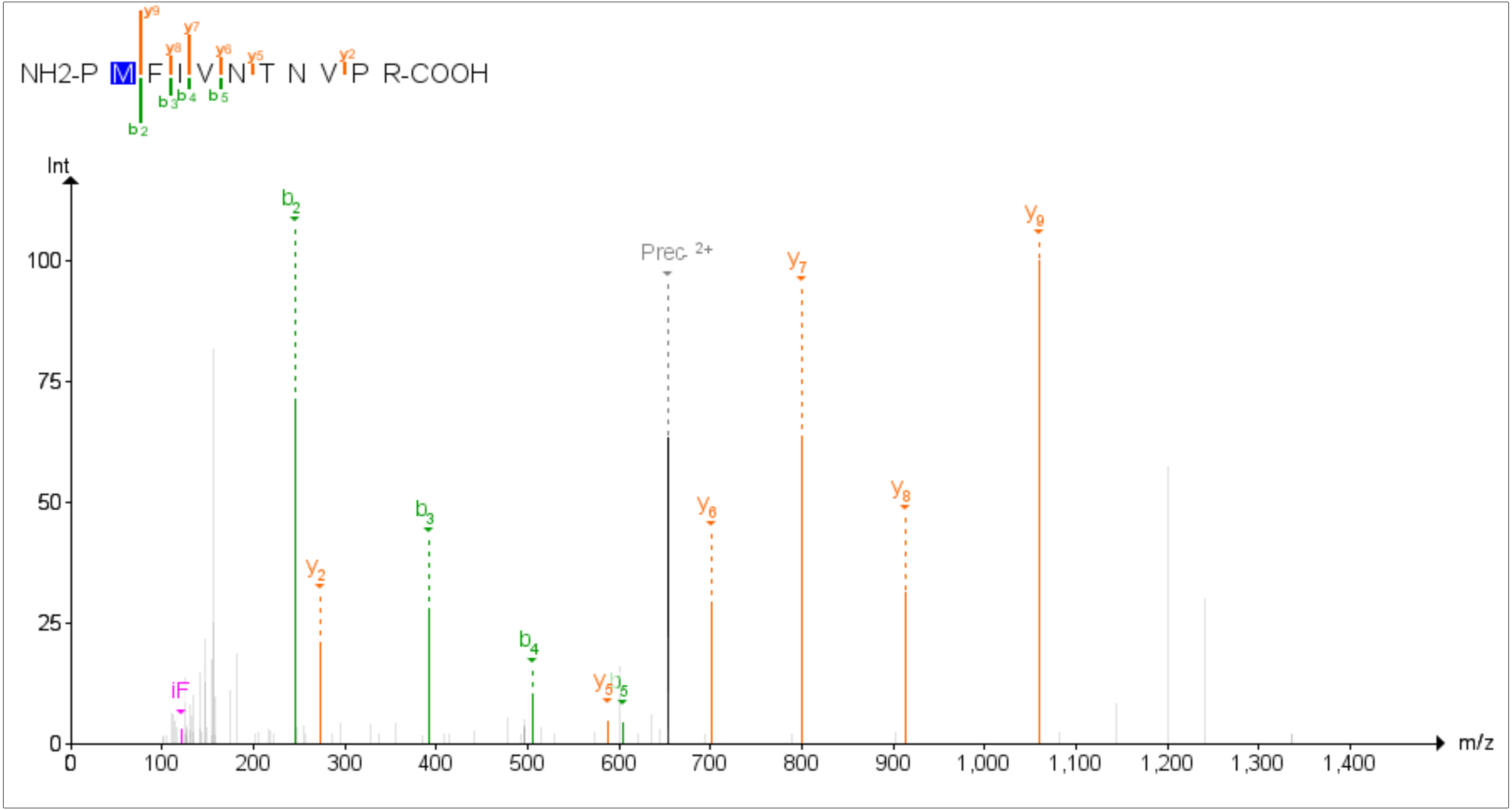
NH2-P M F I V N T N V P R-COOH
y9 y8 y7 y6 y5 y2
b2 b3 b4 b5
Int
m/z
Prec 2+
b2 b3 b4 b5 y2 y5 y6 y7 y8 y9 iF
0 25 50 75 100
0 100 200 300 400 500 600 700 800 900 1,000 1,100 1,200 1,300 1,400

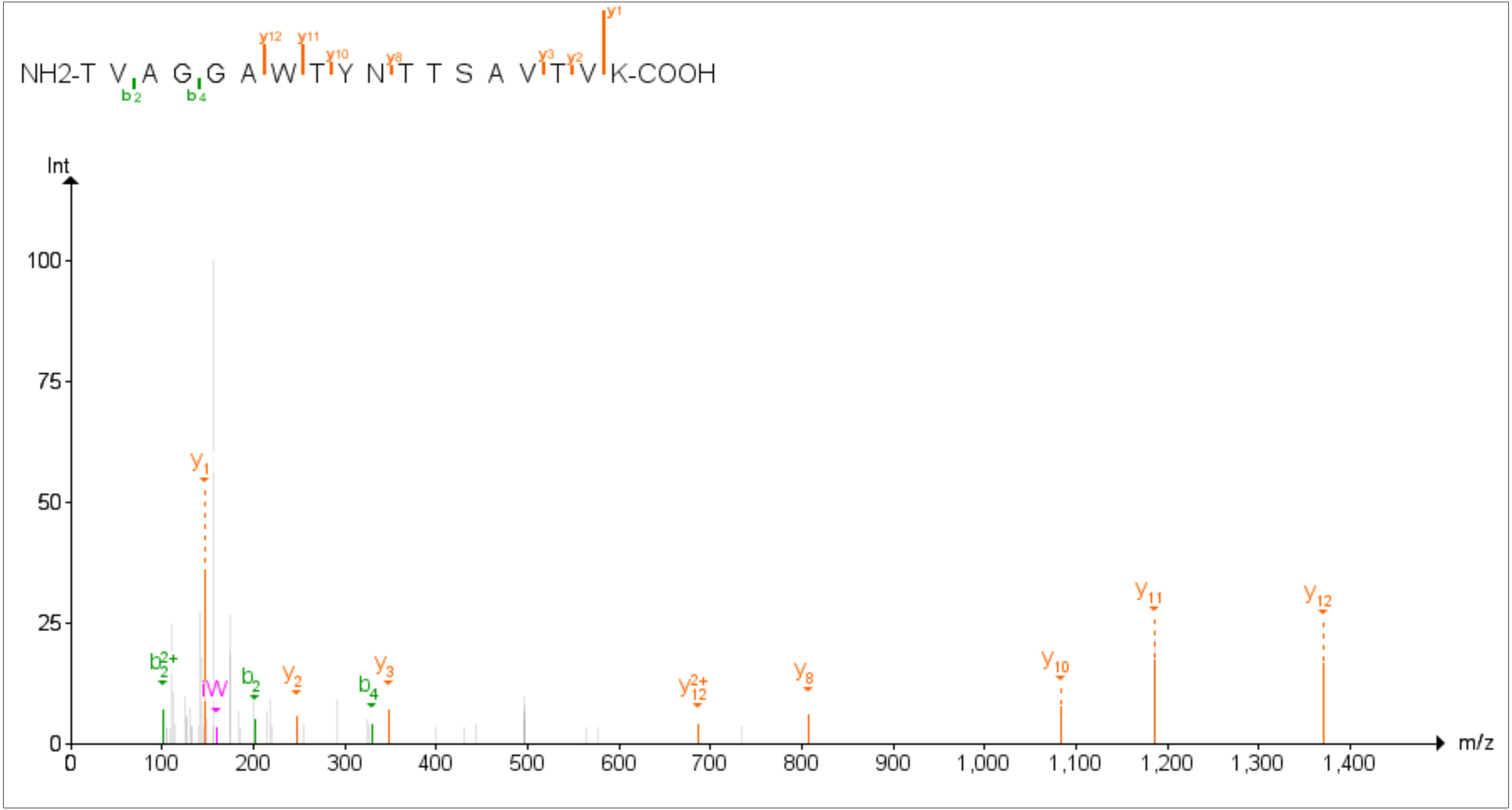
NH2-T V A G G A W T Y N T T S A V T V K-COOH
b2
b4
y12
y11
y10
y8
y3
y2
y1
Int
100
75
50
25
0
m/z
0
100
200
300
400
500
600
700
800
900
1,000
1,100
1,200
1,300
1,400

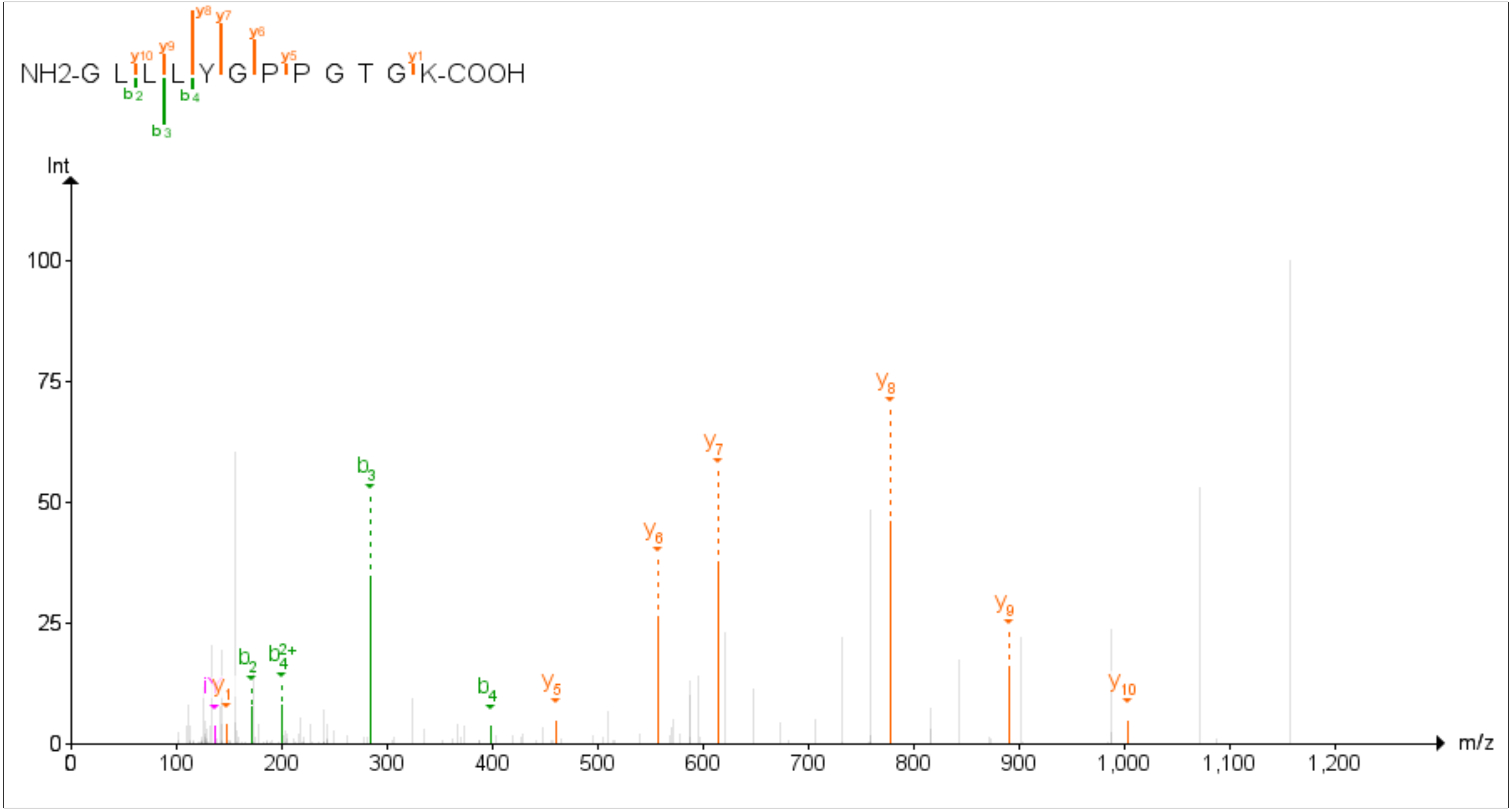
NH2-G L L L Y G P P G T G K-COOH
Int
m/z
b3
y8
y7
y6
y9
y10
y5
b4
b2
y1
0
25
50
75
100
200
300
400
500
600
700
800
900
1,000
1,100
1,200

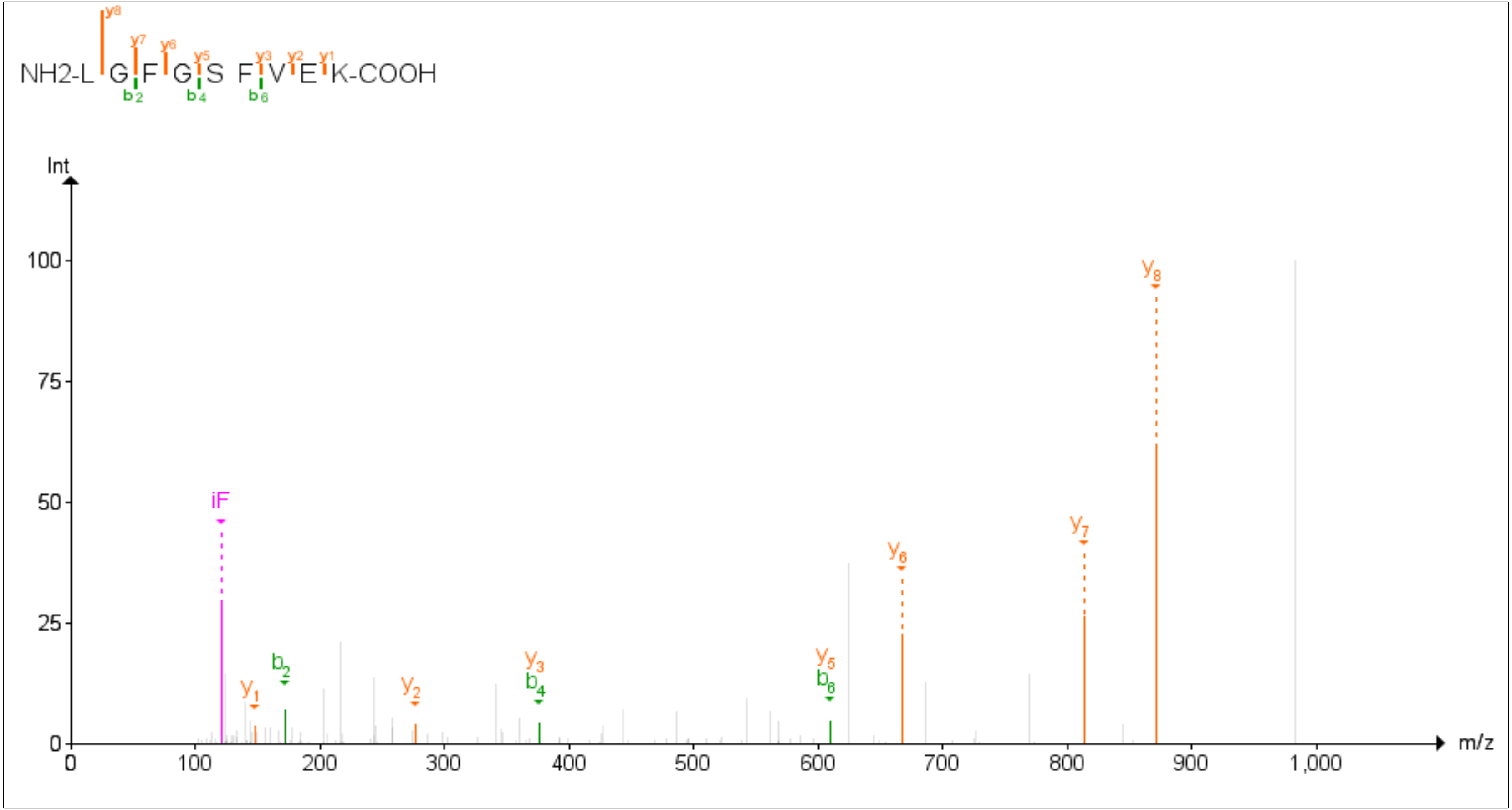

NH2-L G F G S F V E K-COOH
y8
y7
y6
y5
y3
y2
y1
b2
b4
b6
Int
100
75
50
25
0
iF
y1
b2
y2
y3
b4
y5
b6
y6
y7
y8
0
100
200
300
400
500
600
700
800
900
1,000
m/z

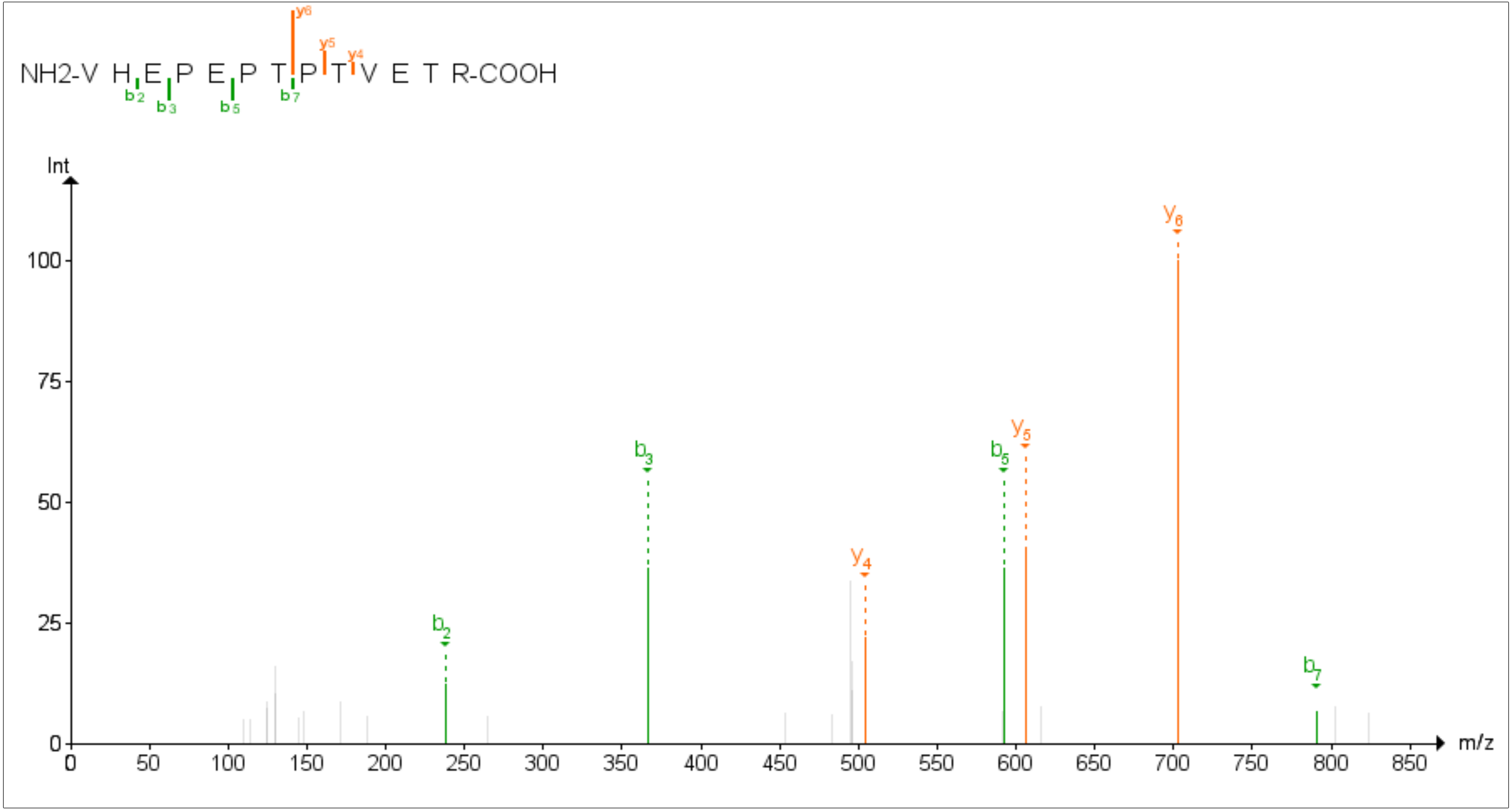
NH2-V H E P E P T P T V E T R-COOH
b2
b3
b5
b7
y6
y5
y4
Int
m/z
0
25
50
75
100
0
50
100
150
200
250
300
350
400
450
500
550
600
650
700
750
800
850

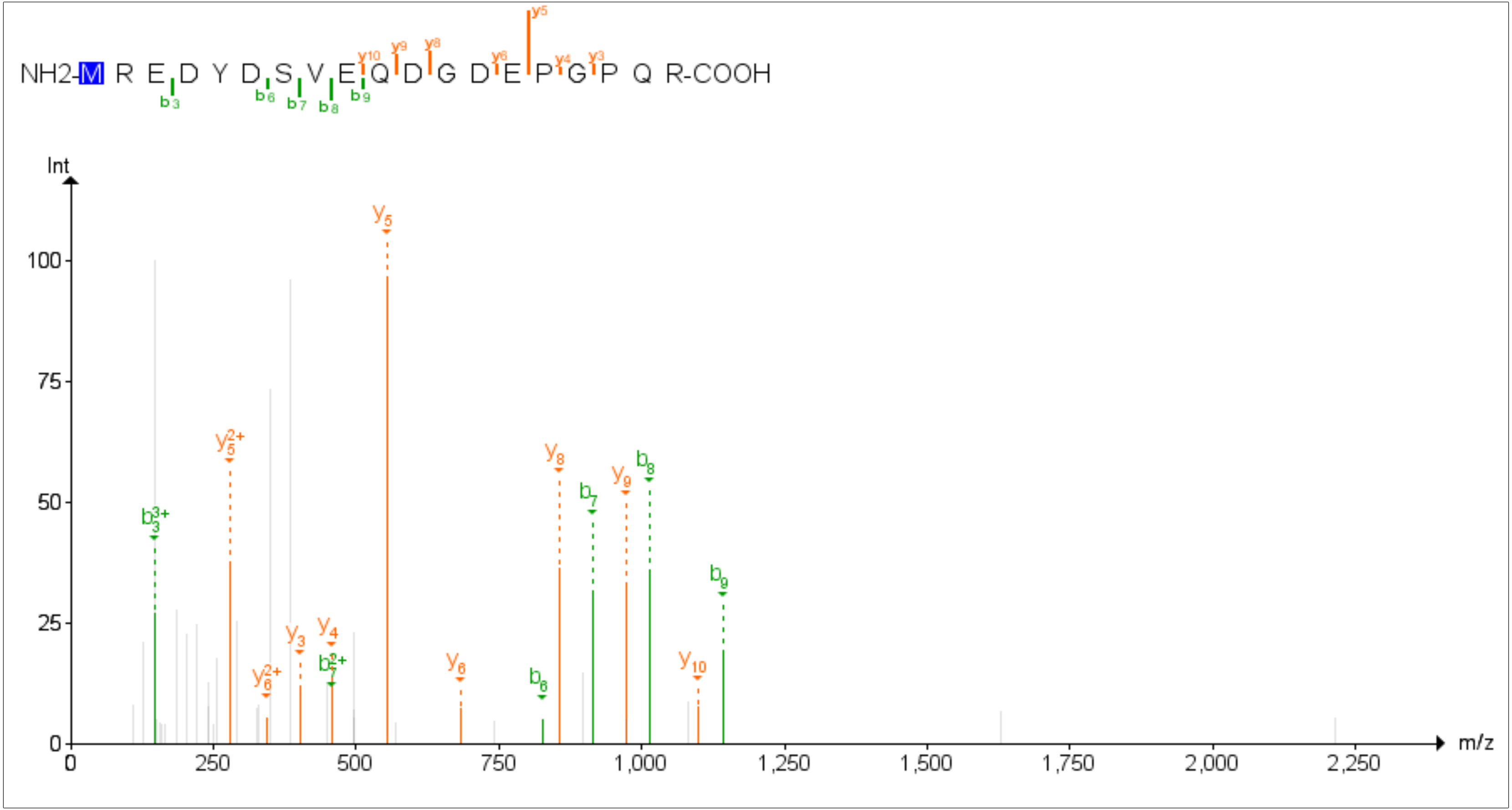
NH2-M R E D Y D S V E Q D G D E P G P Q R-COOH
b3 b6 b7 b8 b9
y10 y9 y8 y6 y5 y4 y3
Int
m/z
100
75
50
25
0
0
250
500
750
1,000
1,250
1,500
1,750
2,000
2,250

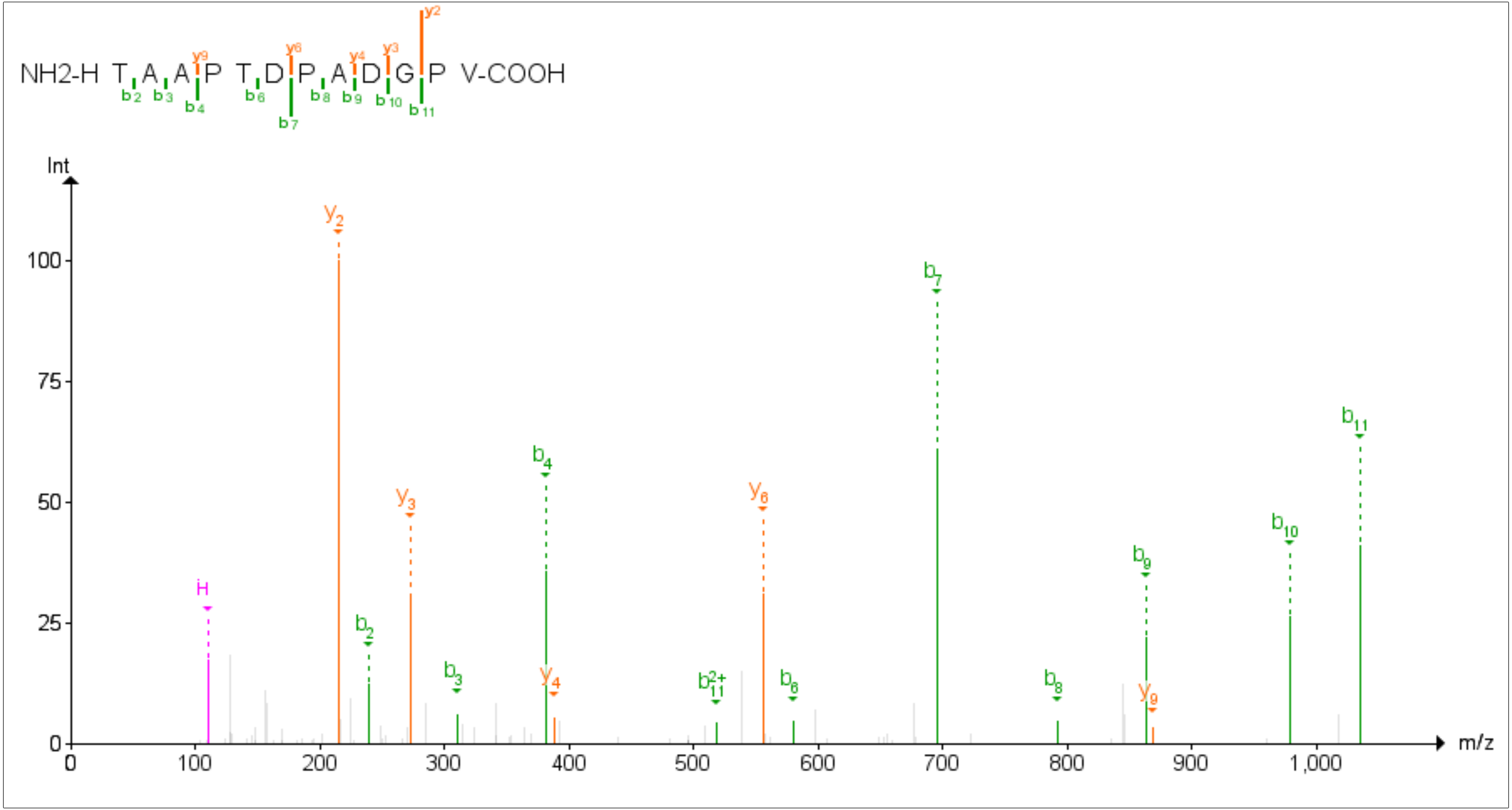
NH2-H T A A P T D P A D G P V-COOH
Int
m/z
y2
y3
y4
y6
y9
b2
b3
b4
b7
b8
b9
b10
b11
b2+11
H
0
25
50
75
100
100
200
300
400
500
600
700
800
900
1,000

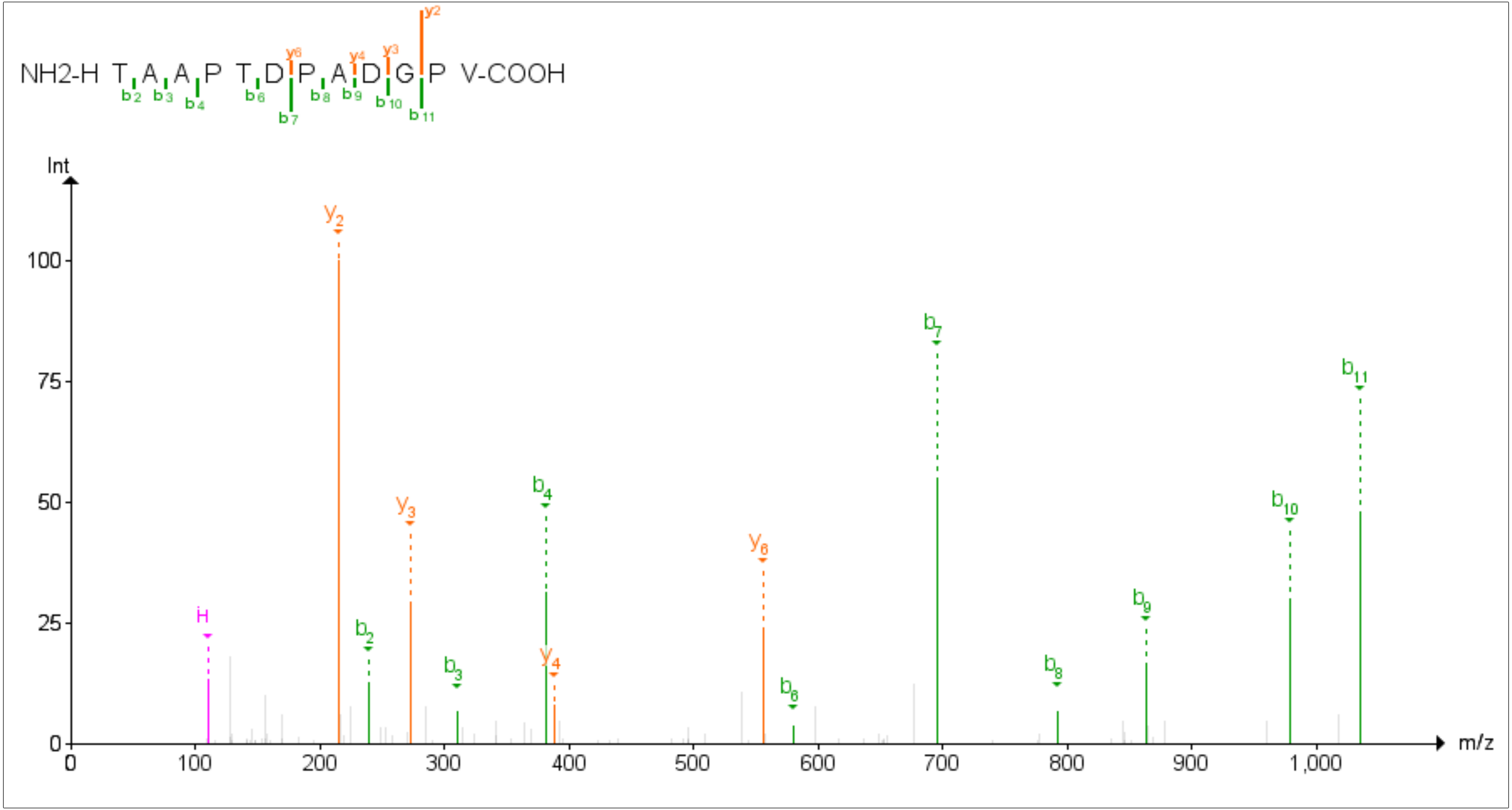

NH2-H T A A P T D P A D G P V-COOH
y6 y4 y3 y2
b2 b3 b4 b6 b7 b8 b9 b10 b11
Int
m/z
0
25
50
75
100
0
100
200
300
400
500
600
700
800
900
1,000
y2
y3
y4
y6
b2
b3
b4
b6
b7
b8
b9
b10
b11
H

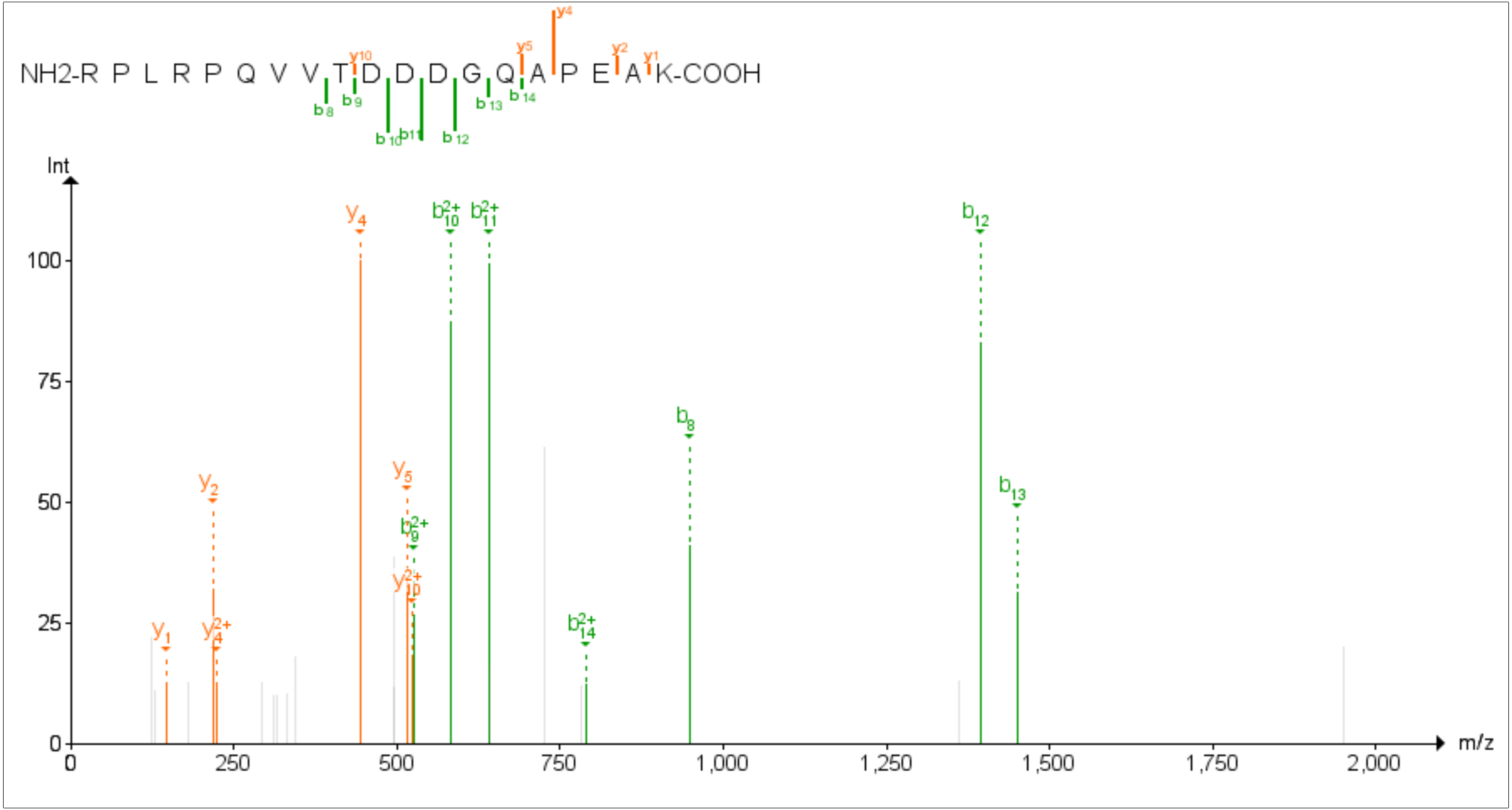
NH2-R P L R P Q V V T D D D G Q A P E A K-COOH
y10
y5
y4
y2
y1
b8
b9
b10
b11
b12
b13
b14
Int
100
75
50
25
0
y4
b10 2+
b11 2+
b12
b8
b13
y5
y2
b9 2+
y10 2+
y1
y4 2+
b14 2+
0
250
500
750
1,000
1,250
1,500
1,750
2,000
m/z

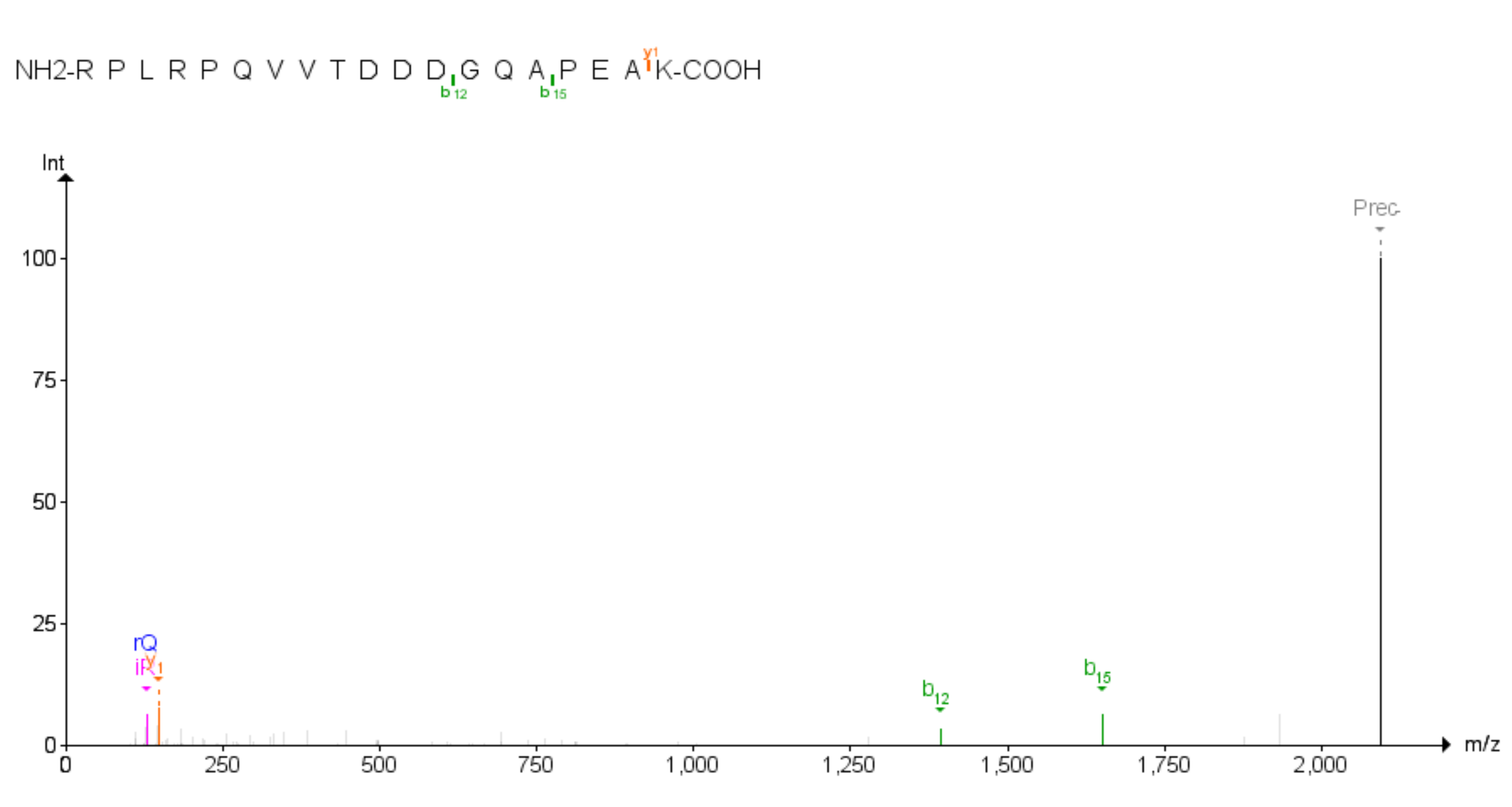

NH2-R P L R P Q V V T D D D G Q A P E A K-COOH
b12
b15
y1
Int
100
75
50
25
0
Prec
rQ
iK
y1
b12
b15
0
250
500
750
1,000
1,250
1,500
1,750
2,000
m/z

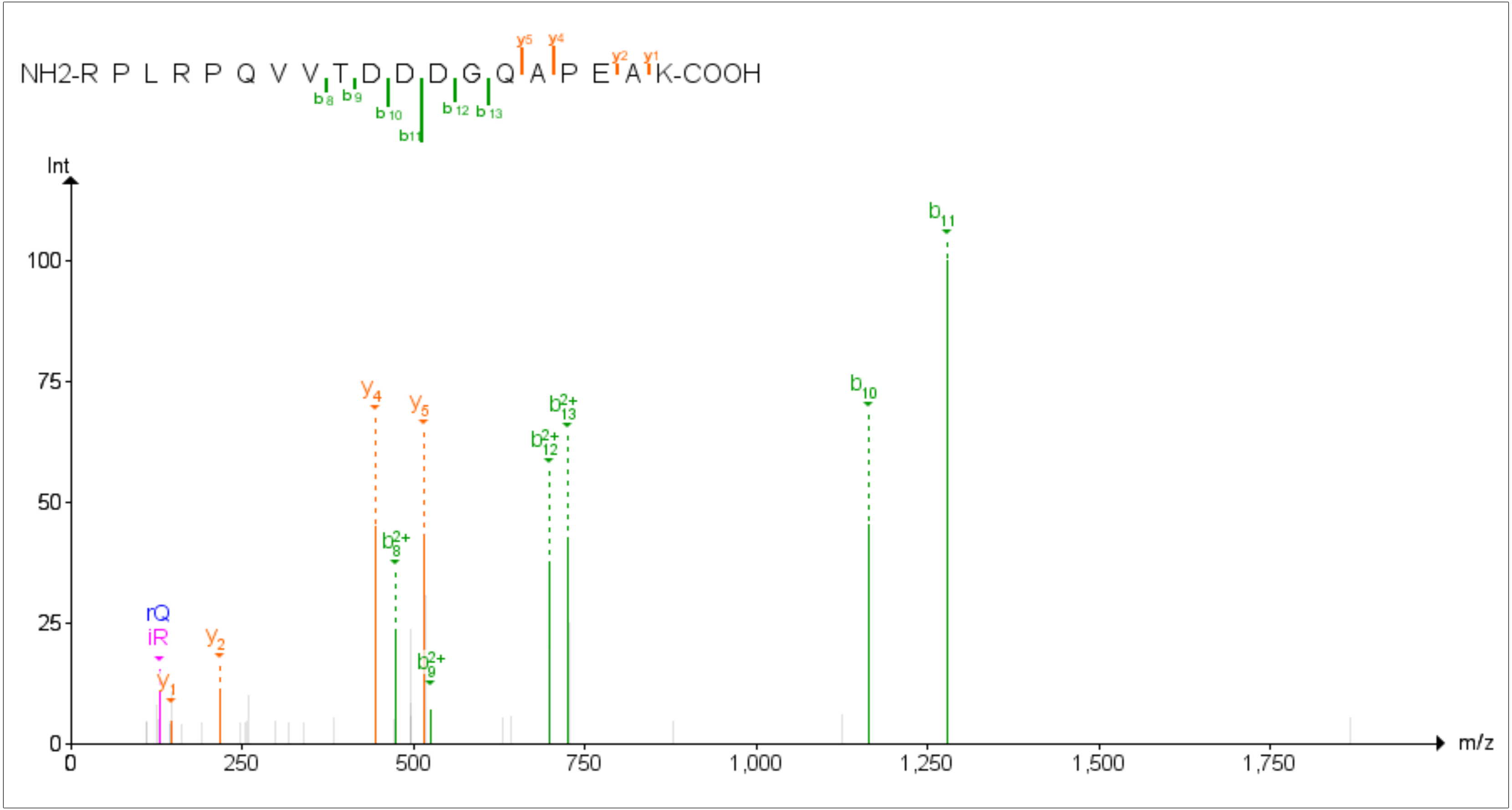

NH2-R P L R P Q V V T D D D G Q A P E A K-COOH
y5 y4 y2 y1
b8 b9 b10 b11 b12 b13
Int
100
75
50
25
0
b11
b10
y4
y5
b13 2+
b12 2+
b8 2+
b9 2+
rQ
iR
y2
y1
0
250
500
750
1,000
1,250
1,500
1,750
m/z

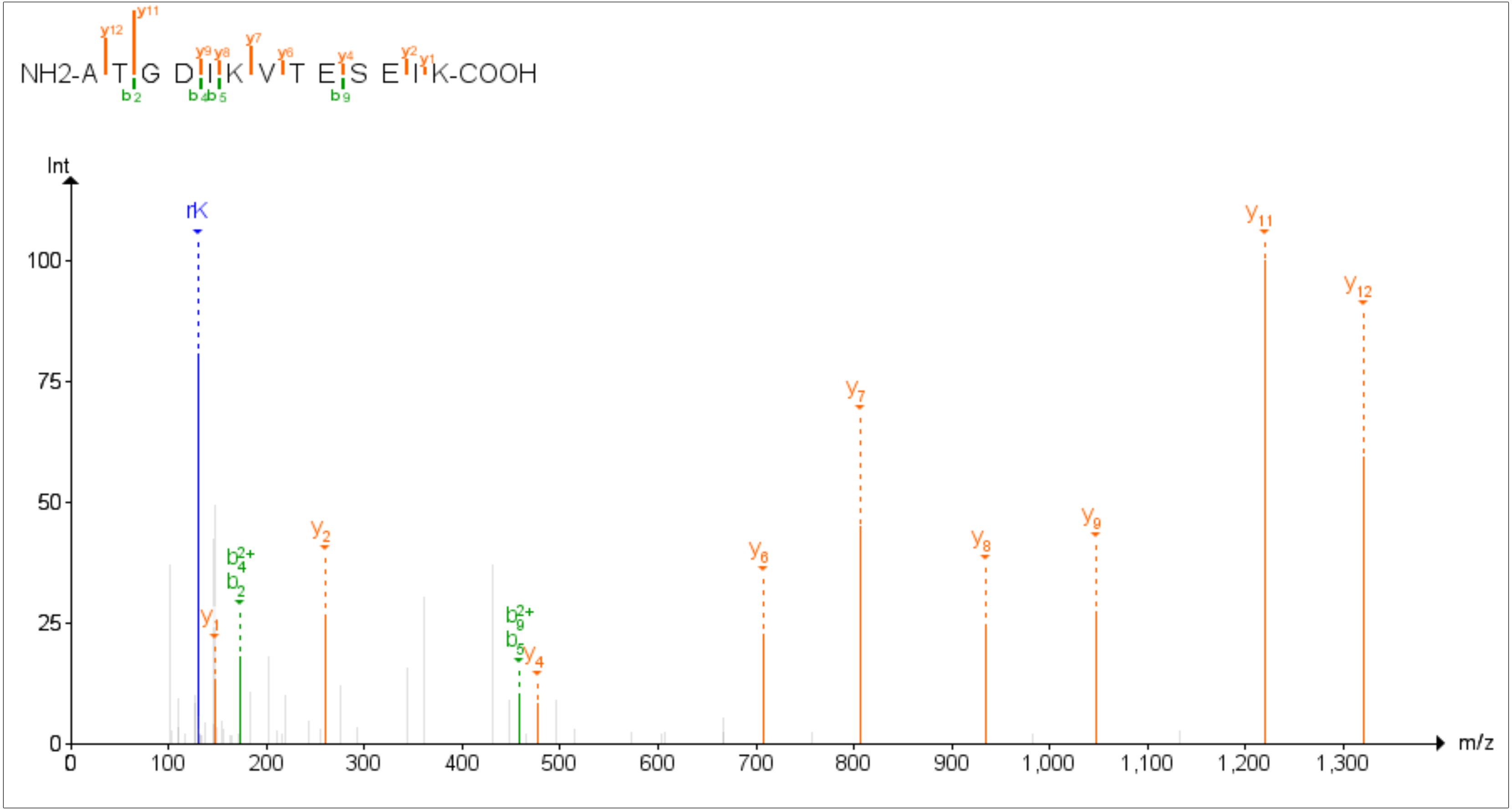

NH2-A T G D I I K V T E S E I K-COOH
y12 y11 y9 y8 y7 y6 y4 y2 y1
b2 b4 b5 b9
Int
rK
y11
y12
y7
y2
b4 2+
b2
y1
y6
y8
y9
b9 2+
b5
y4
0
25
50
75
100
0
100
200
300
400
500
600
700
800
900
1,000
1,100
1,200
1,300
m/z

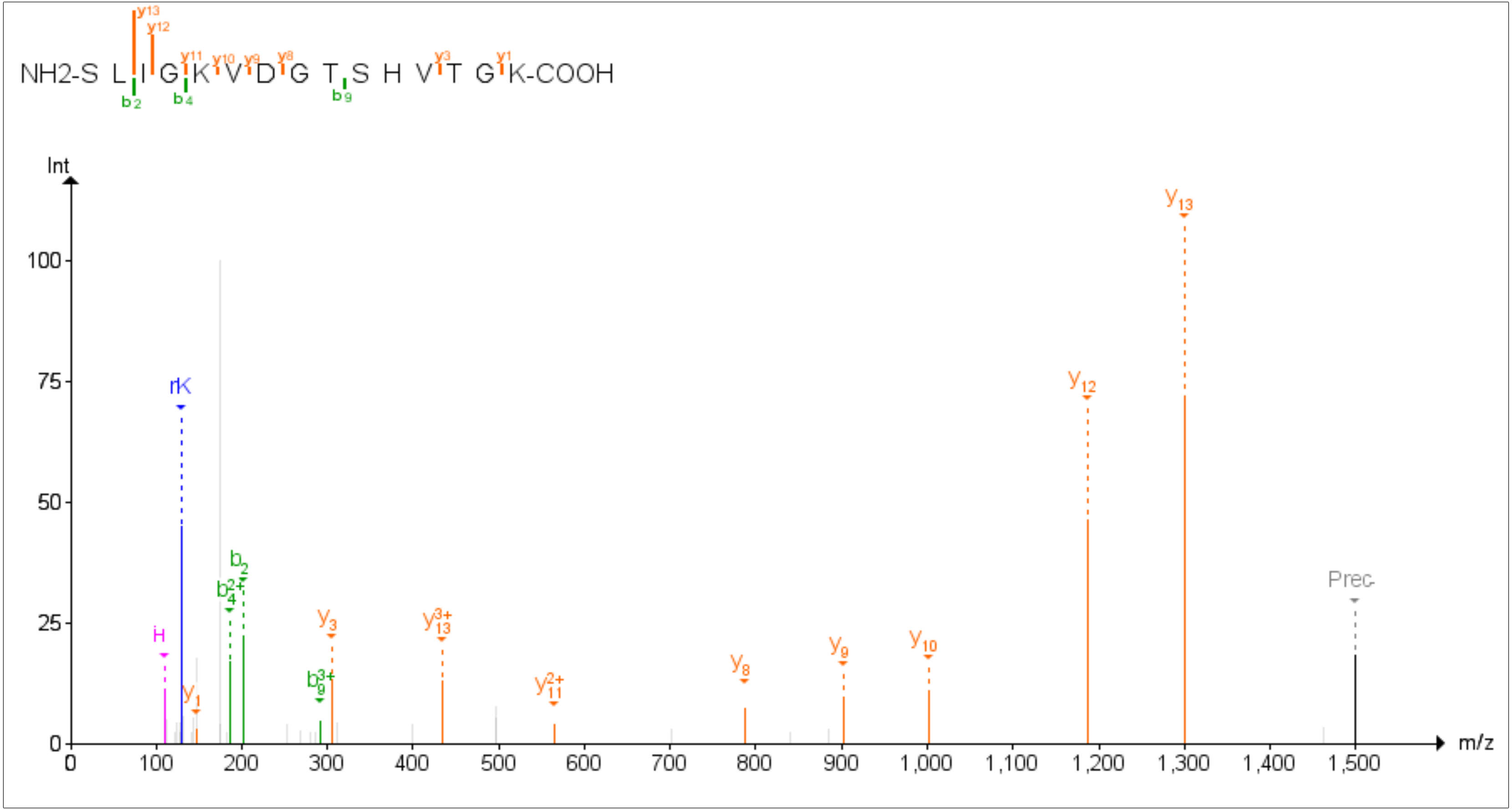

NH2-S L I G K V D G T S H V T G K-COOH
y13
y12
y11
y10
y9
y8
y3
y1
b2
b4
b9
Int
100
75
50
25
0
m/z
0
100
200
300
400
500
600
700
800
900
1,000
1,100
1,200
1,300
1,400
1,500
rK
iH
b2
b4 2+
y1
b9 3+
y3
y13 3+
y11 2+
y8
y9
y10
y12
y13
Prec

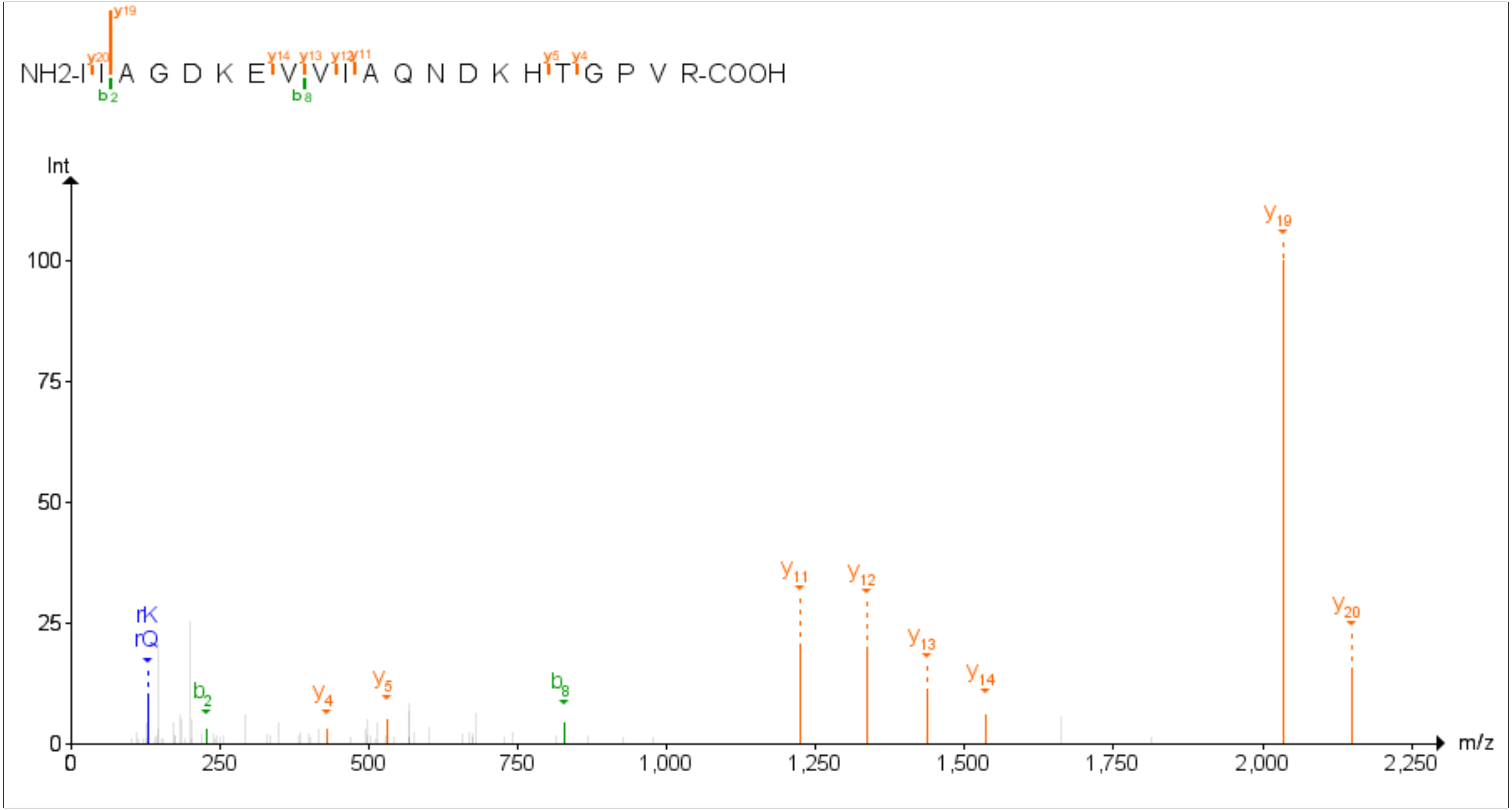

NH2-I I A G D K E V V I A Q N D K H T G P V R-COOH
y19
y20
y14
y13
y12
y11
y5
y4
b 2
b 8
Int
100
75
50
25
0
rK
rQ
b2
y4
y5
b8
y11
y12
y13
y14
y19
y20
0
250
500
750
1,000
1,250
1,500
1,750
2,000
2,250
m/z

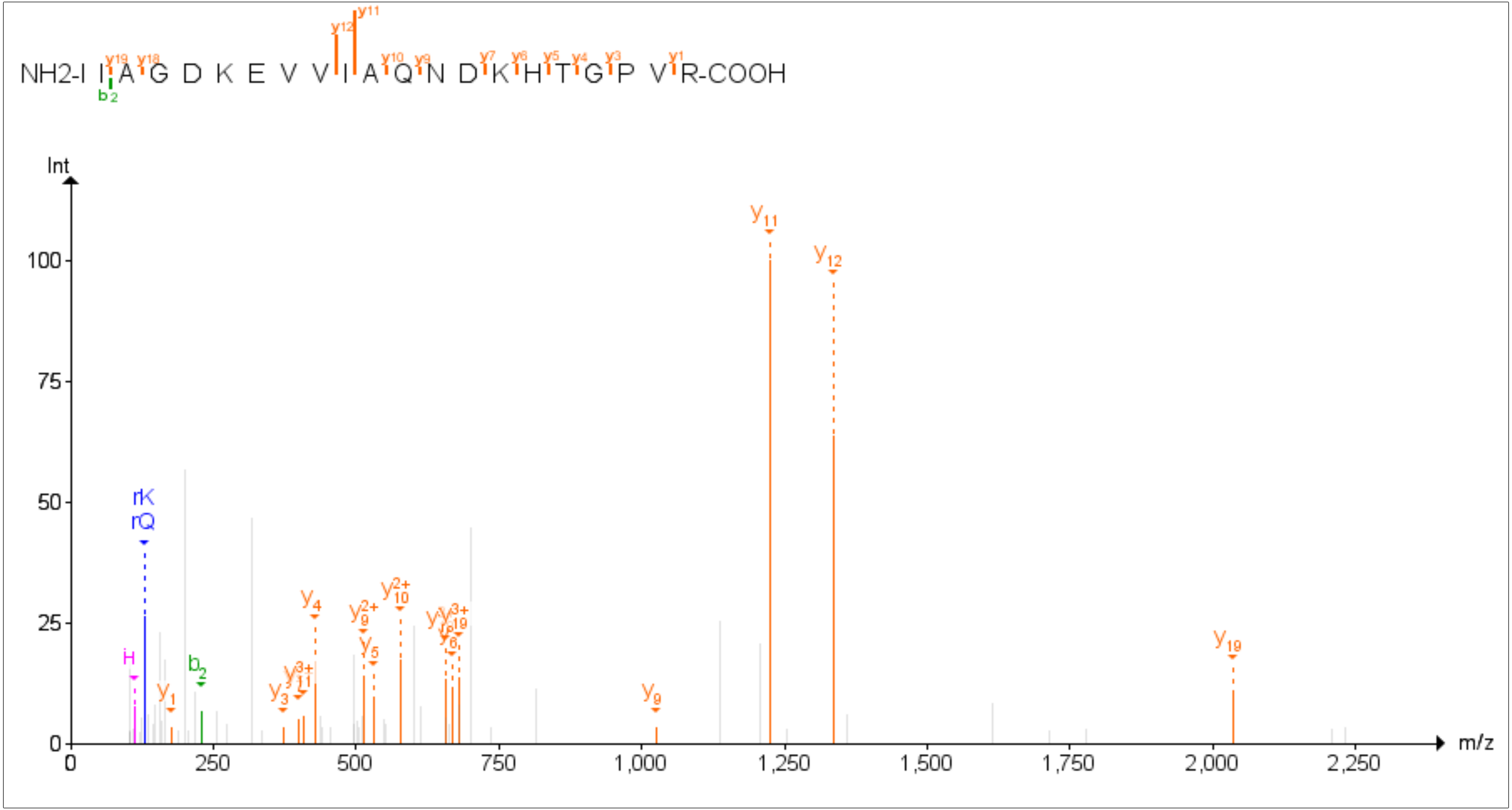

NH2-I I A G D K E V V I A Q N D K H T G P V R-COOH
y19 y18 y12 y11 y10 y9 y7 y6 y5 y4 y3 y1
b2
Int
100
75
50
25
0
rK
rQ
iH
b2
y1
y3
y4
y9 2+
y5
y10 2+
y19 3+
y9
y11
y12
y19
0
250
500
750
1,000
1,250
1,500
1,750
2,000
2,250
m/z

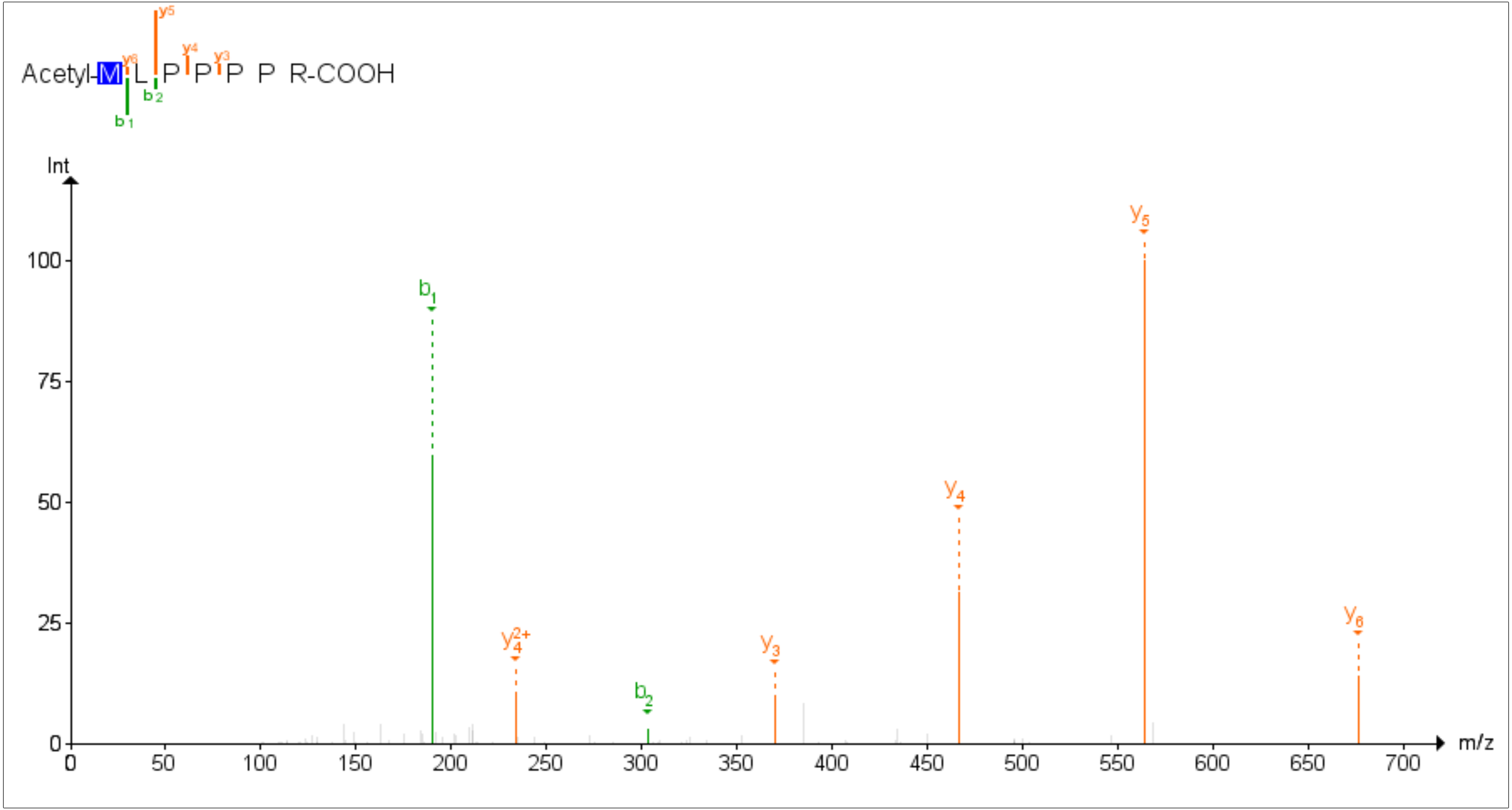

Acetyl-M L P P P P R-COOH
y5
y4
y3
y6
b1
b2
Int
100
75
50
25
0
b1
y4 2+
b2
y3
y4
y5
y6
0
50
100
150
200
250
300
350
400
450
500
550
600
650
700
m/z

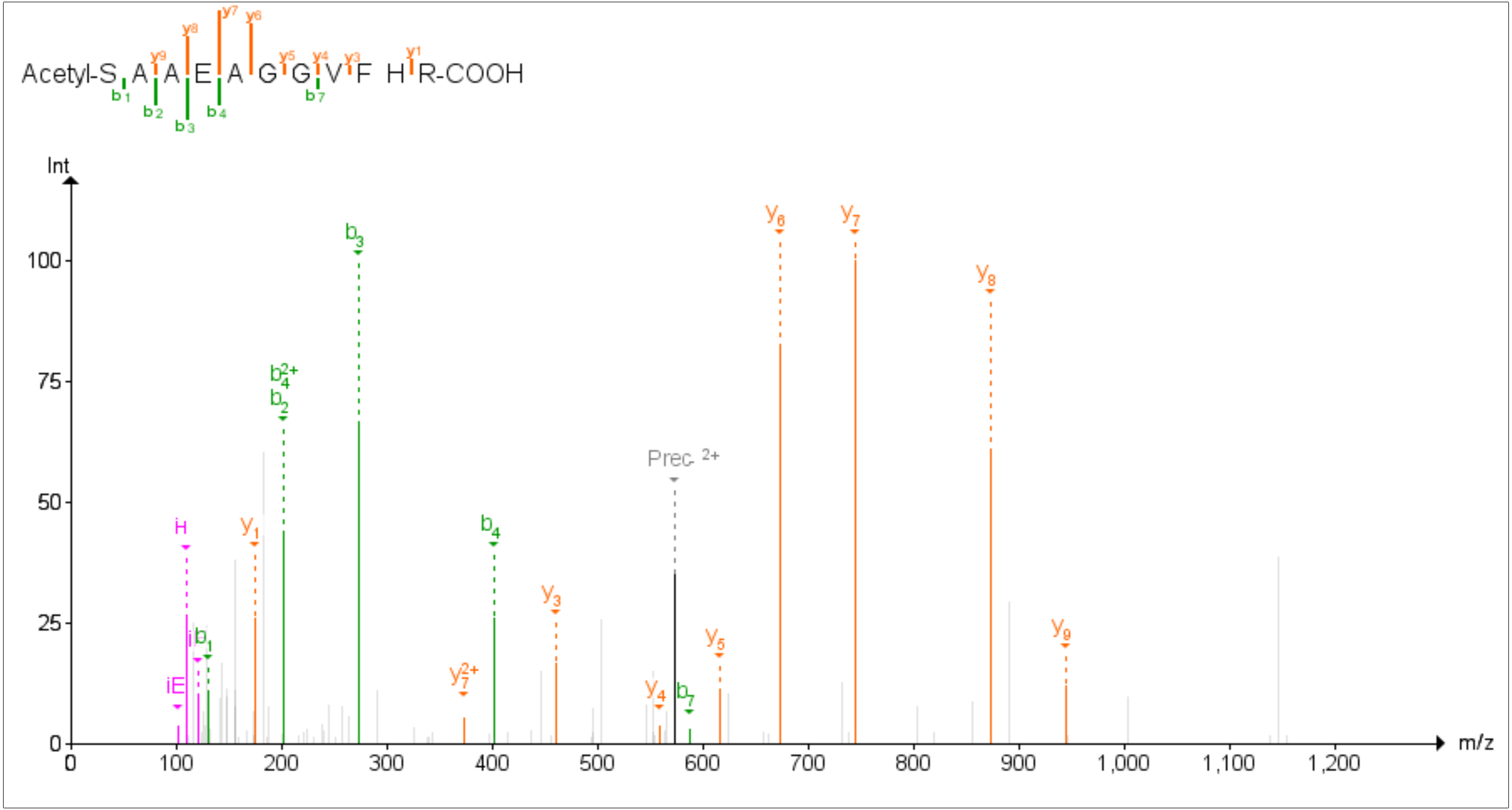

Acetyl-S A A E A G G V F H R-COOH
Int
m/z
100
75
50
25
0
0
100
200
300
400
500
600
700
800
900
1,000
1,100
1,200
Prec. 2+

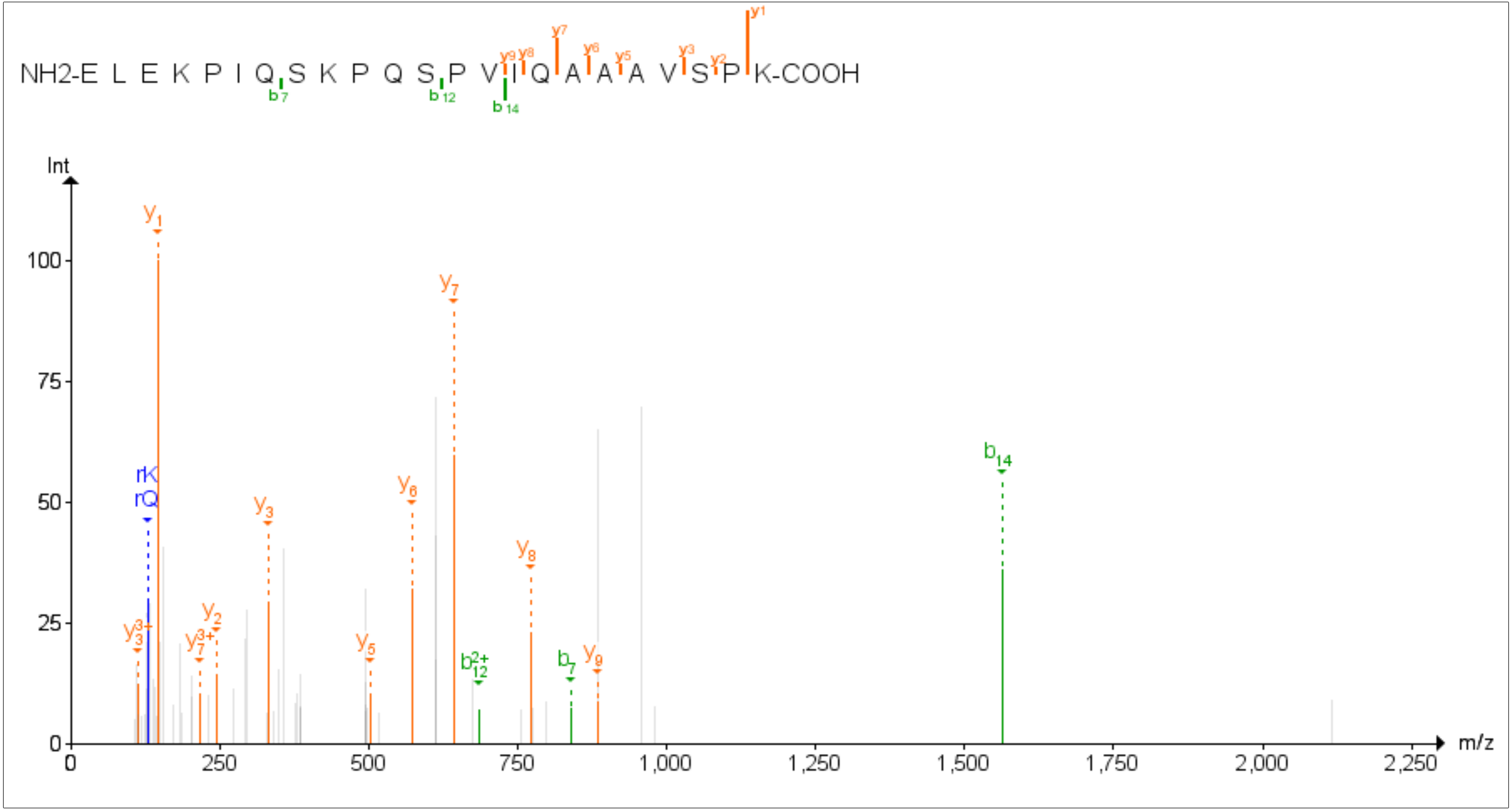

NH2-E L E K P I Q S K P Q S P V I Q A A A V S P K-COOH
b7
b12
b14
y1
y2
y3
y5
y6
y7
y8
y9
Int
100
75
50
25
0
m/z
0
250
500
750
1,000
1,250
1,500
1,750
2,000
2,250
rK
rQ
b14
b12 2+
b7
y3 3+
y7 3+

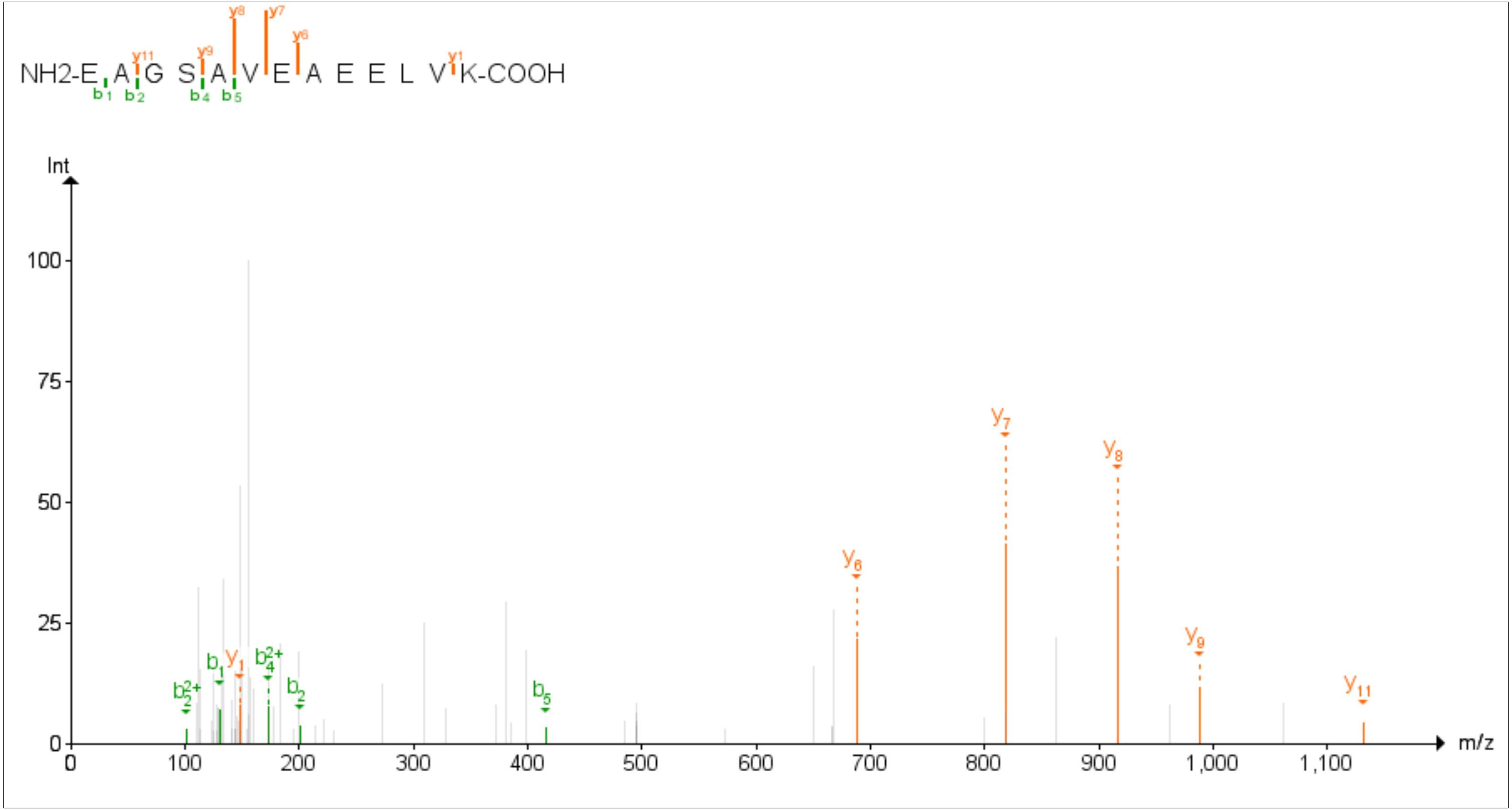
NH2-E A G S A V E A E E L V K-COOH
b1 b2 b4 b5
y11 y9 y8 y7 y6 y1
Int
100
75
50
25
0
b2 2+
b1
y1
b4 2+
b2
b5
y6
y7
y8
y9
y11
0 100 200 300 400 500 600 700 800 900 1,000 1,100
m/z

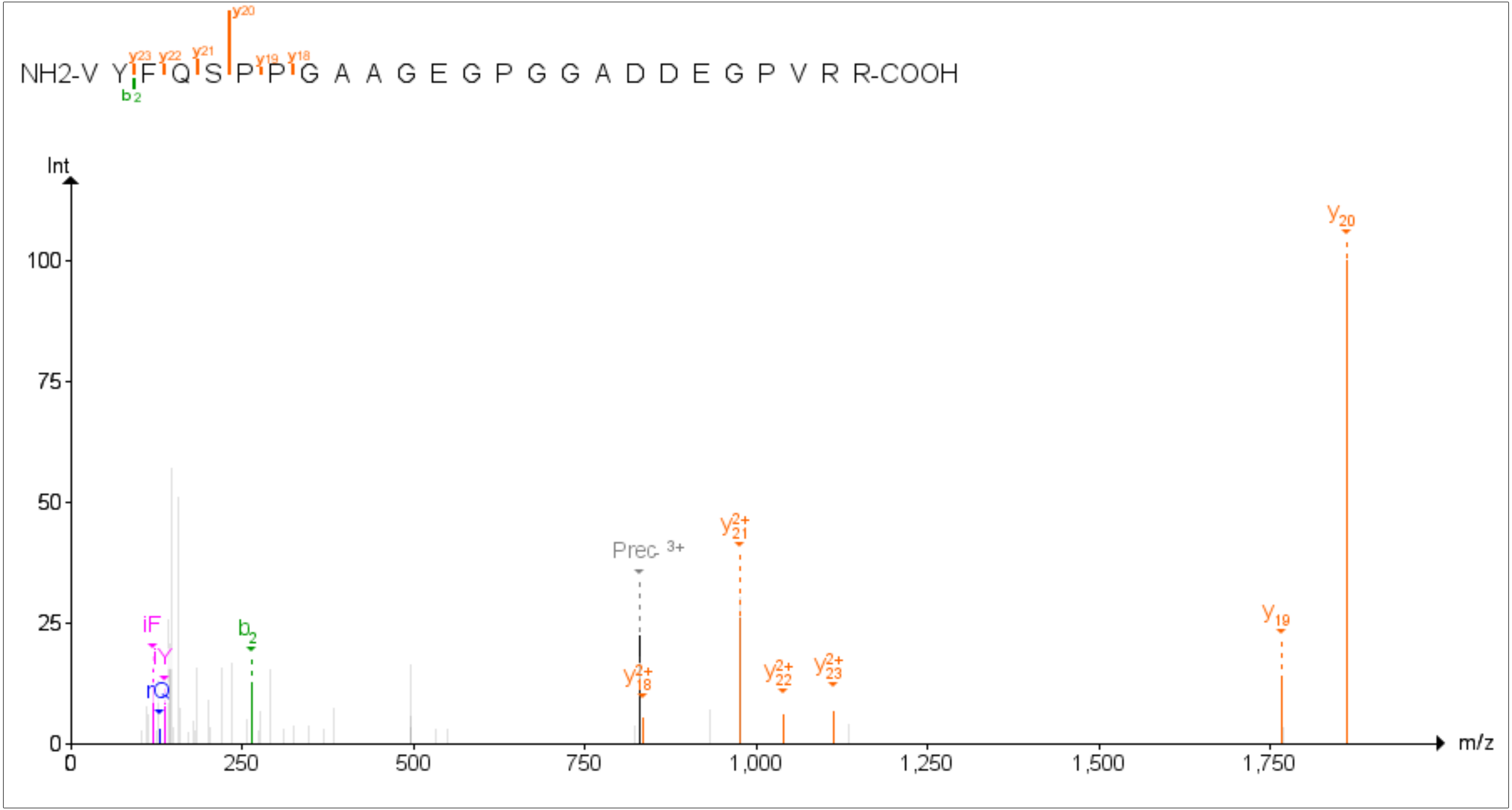

NH2-V Y F Q S P P G A A G E G P G G A D D E G P V R R-COOH
y23 y22 y21 y20 y19 y18
b2
Int
100
75
50
25
0
m/z
0
250
500
750
1,000
1,250
1,500
1,750
y20
y19
Prec 3+
y21 2+
y18 2+
y22 2+
y23 2+
iF
iY
rQ
b2

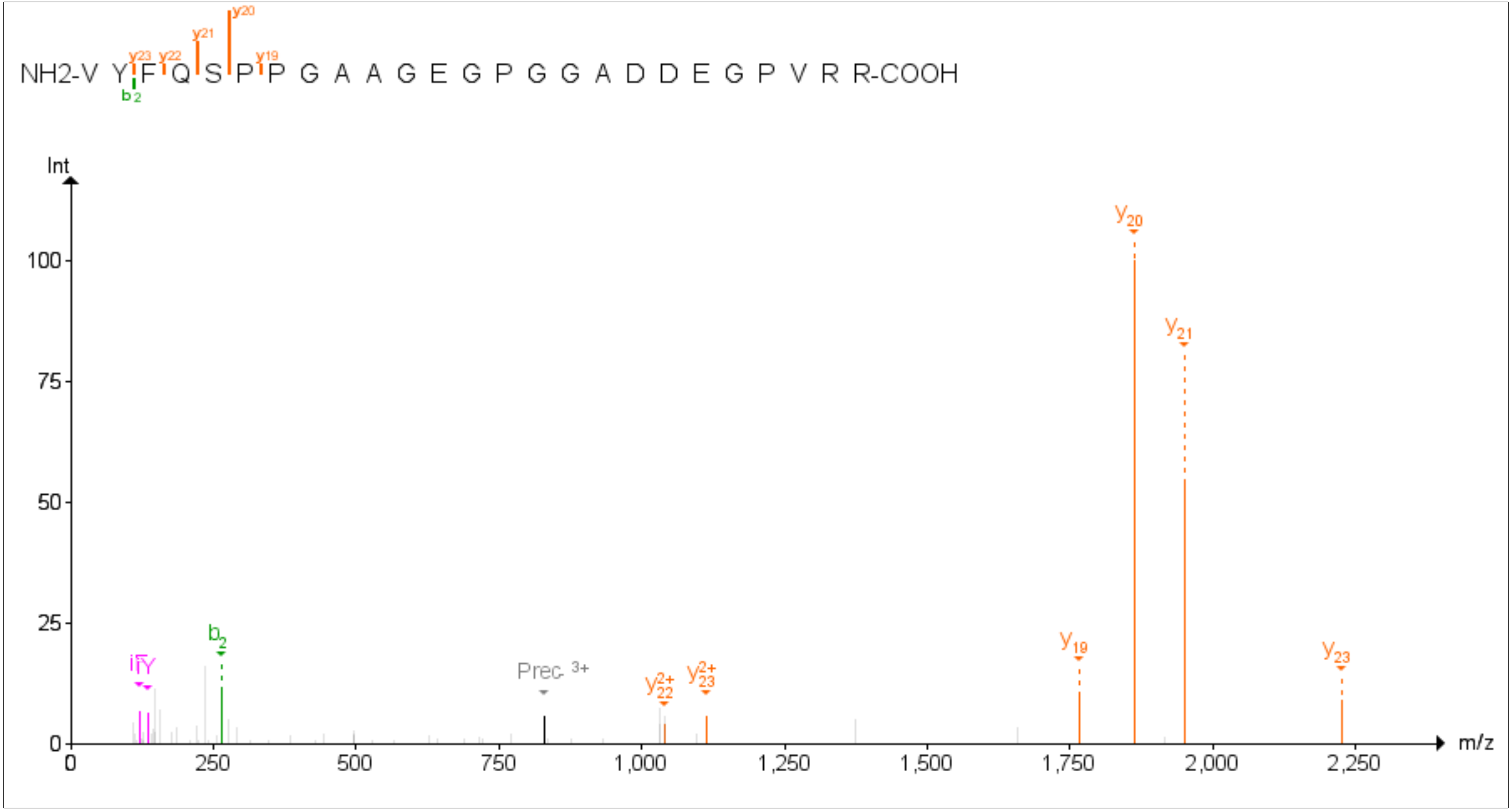

NH2-V Y F Q S P P G A A G E G P G G A D D E G P V R R-COOH
Int
m/z
y20
y21
y19
y23
y22
b2
iY
Prec. 3+

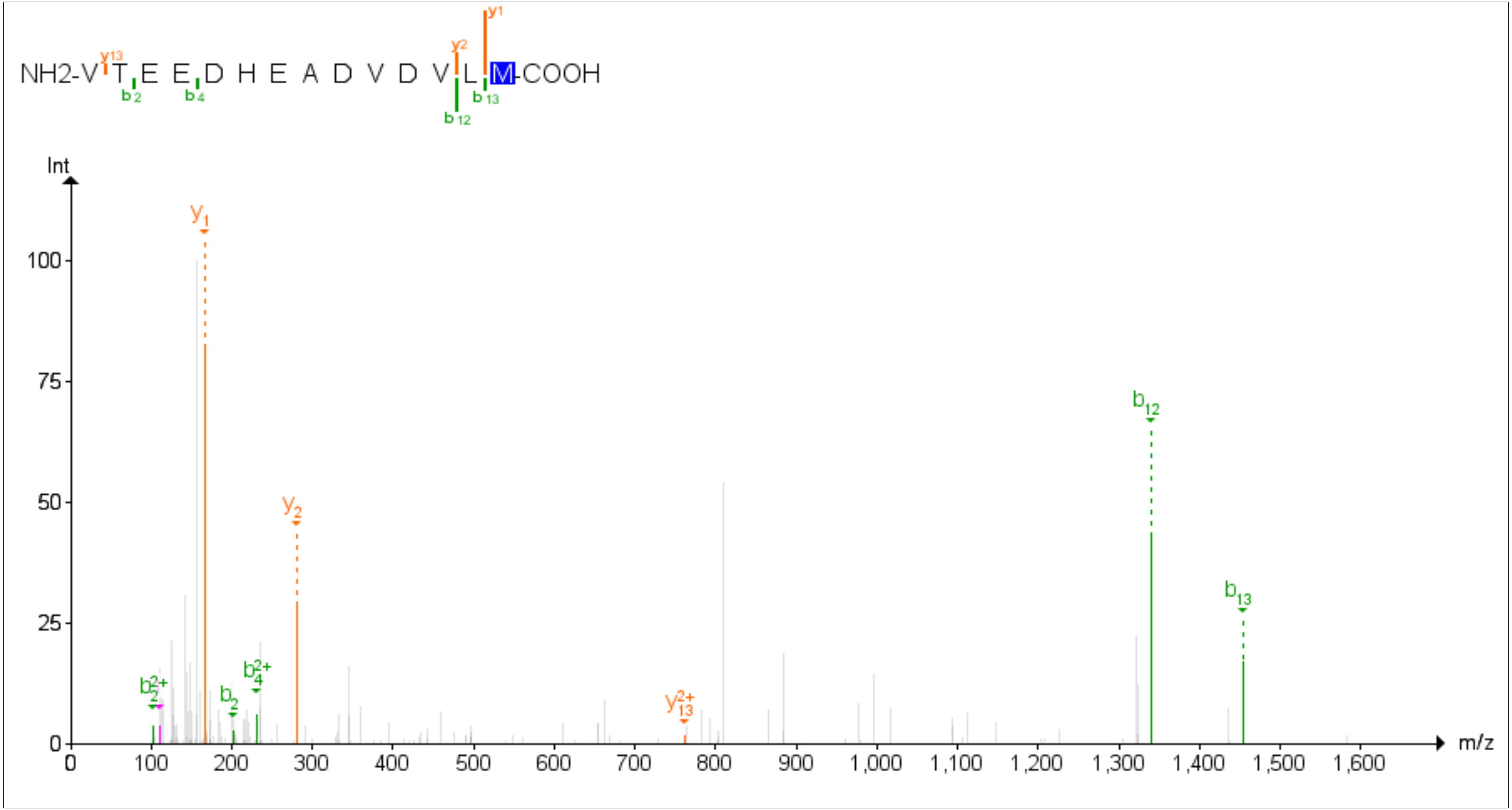

NH2-V T E E D H E A D V D V L M-COOH
y13
b2
b4
y2
y1
b12
b13
Int
m/z
y1
y2
b12
b13
b2 2+
b2
b4 2+
y13 2+
0
25
50
75
100
0
100
200
300
400
500
600
700
800
900
1,000
1,100
1,200
1,300
1,400
1,500
1,600

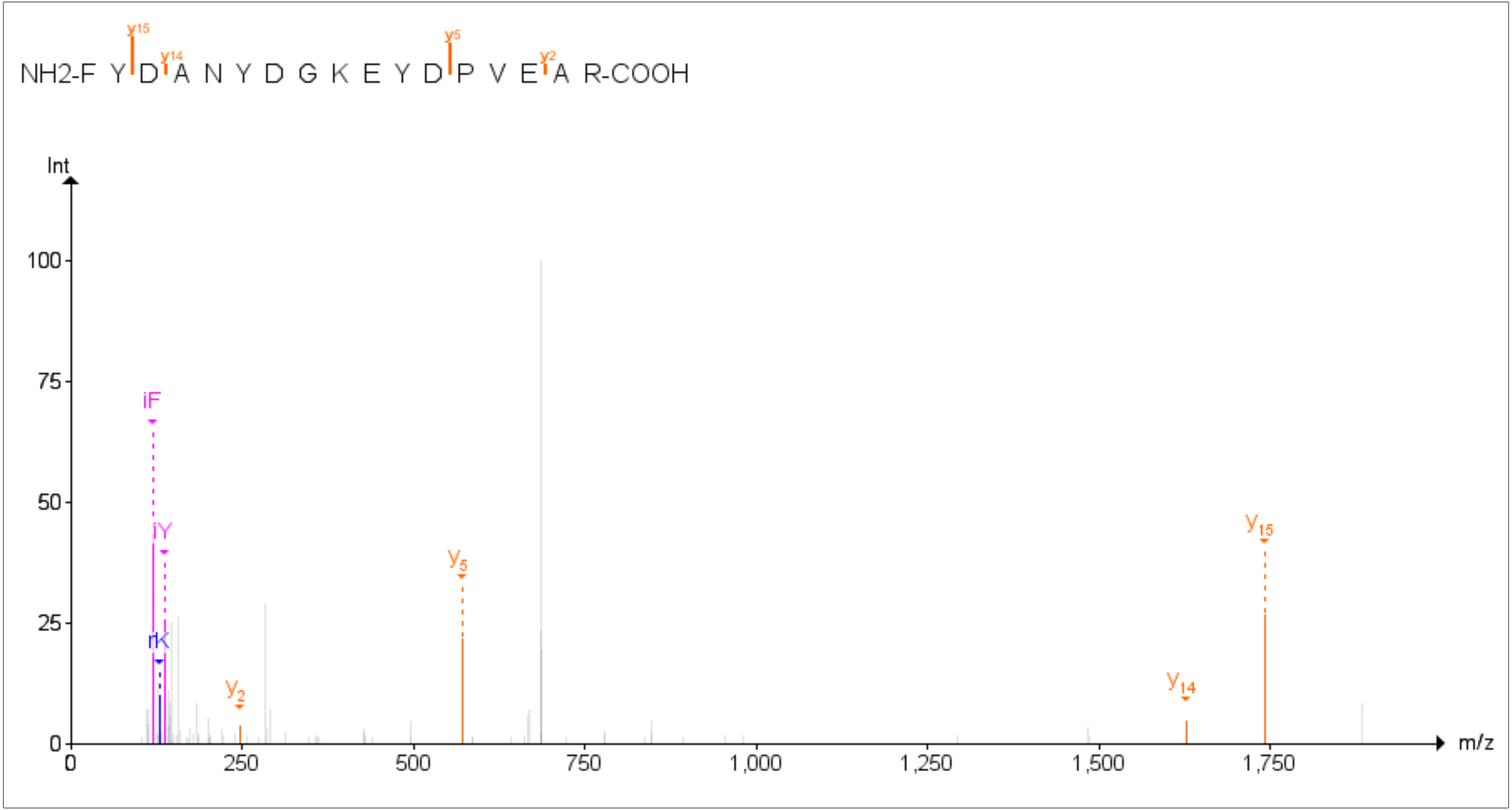

NH2-F Y D A N Y D G K E Y D P V E A R-COOH
y15
y14
y5
y2
Int
100
75
50
25
0
iF
iY
rK
y2
y5
y14
y15
0
250
500
750
1,000
1,250
1,500
1,750
m/z

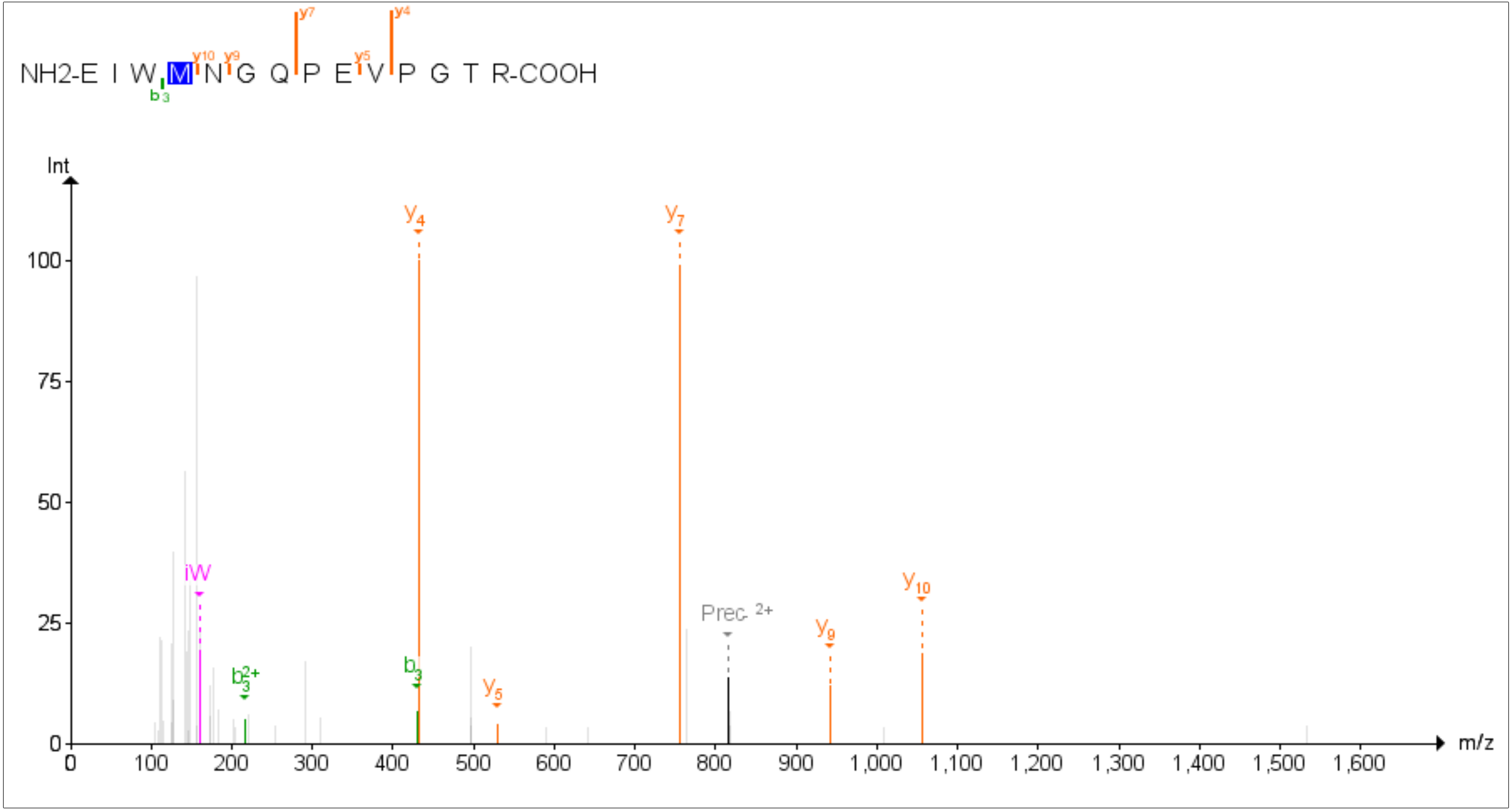

NH2-E I W M N G Q P E V P G T R-COOH
b3
y10 y9 y7 y5 y4
Int
m/z
y4
y7
iW
b3 2+
b3
y5
Prec 2+
y9
y10

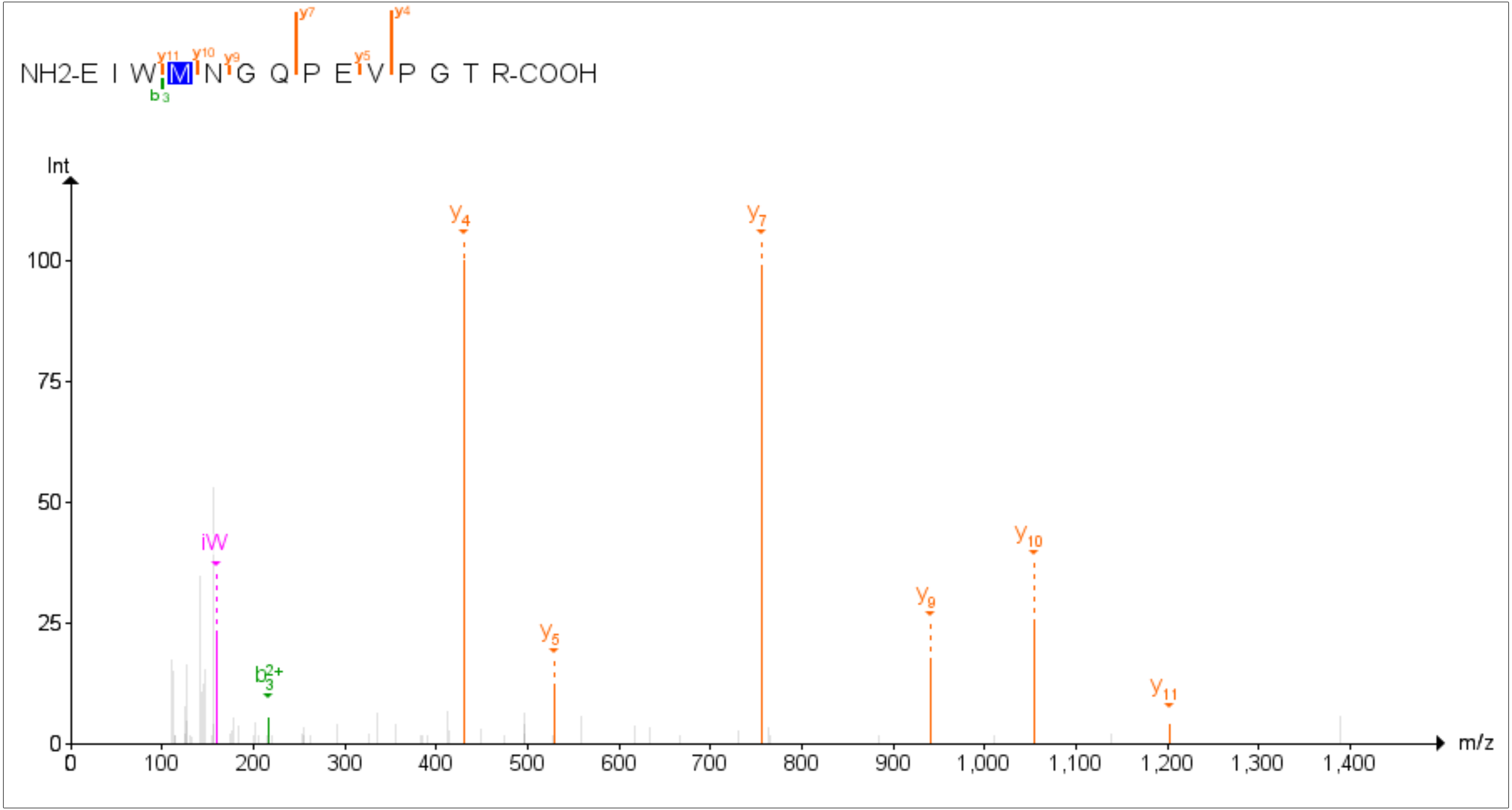

NH2-E I W M N G Q P E V P G T R-COOH
y11 y10 y9 y7 y5 y4
b3
Int
m/z
y4
y7
iW
b3 2+
y5
y9
y10
y11
0 25 50 75 100
0 100 200 300 400 500 600 700 800 900 1,000 1,100 1,200 1,300 1,400

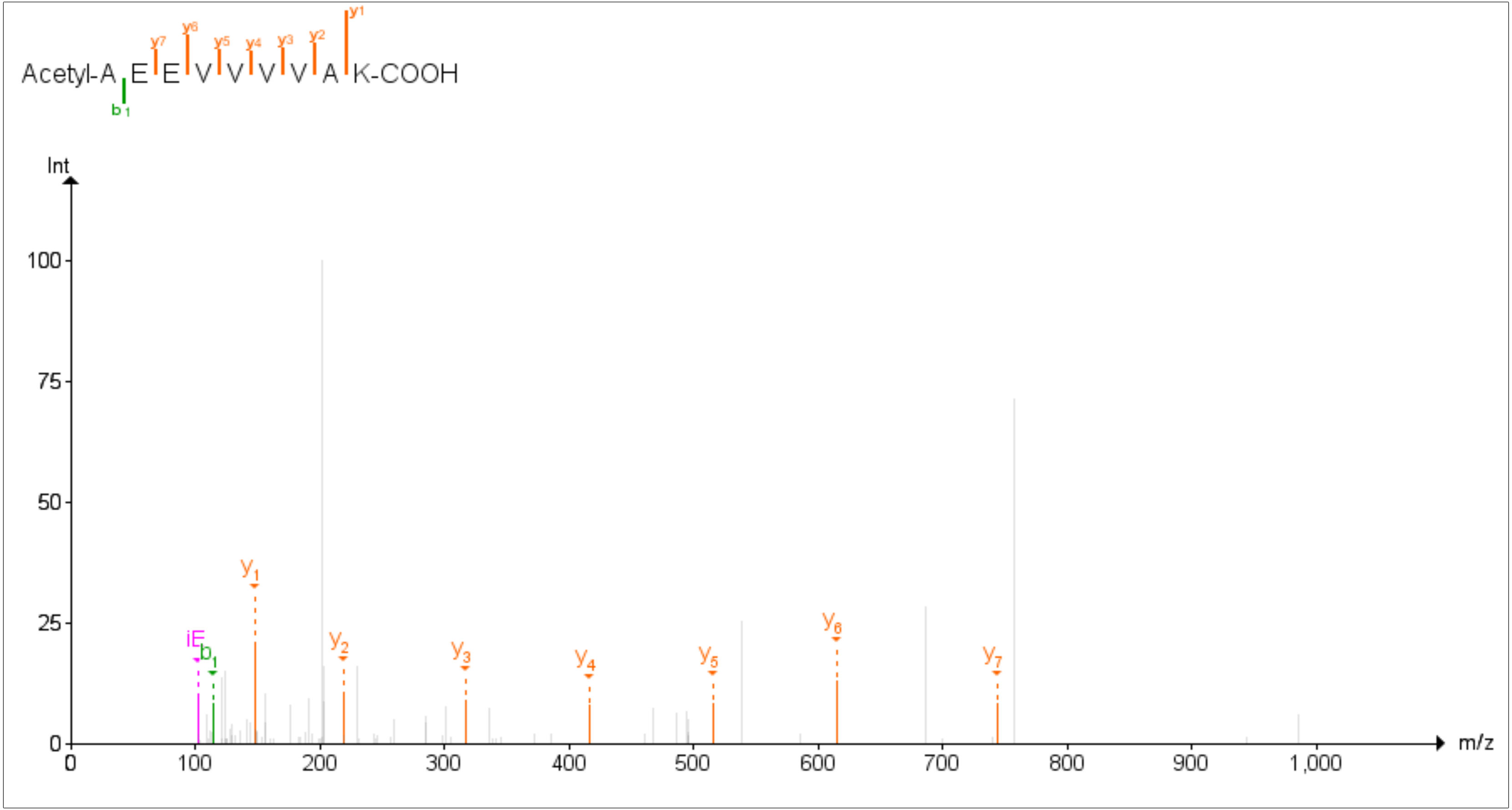

Acetyl-A E E V V V V A K-COOH
y7 y6 y5 y4 y3 y2 y1
b1
Int
100
75
50
25
0
iE
b1
y1
y2
y3
y4
y5
y6
y7
0 100 200 300 400 500 600 700 800 900 1,000
m/z

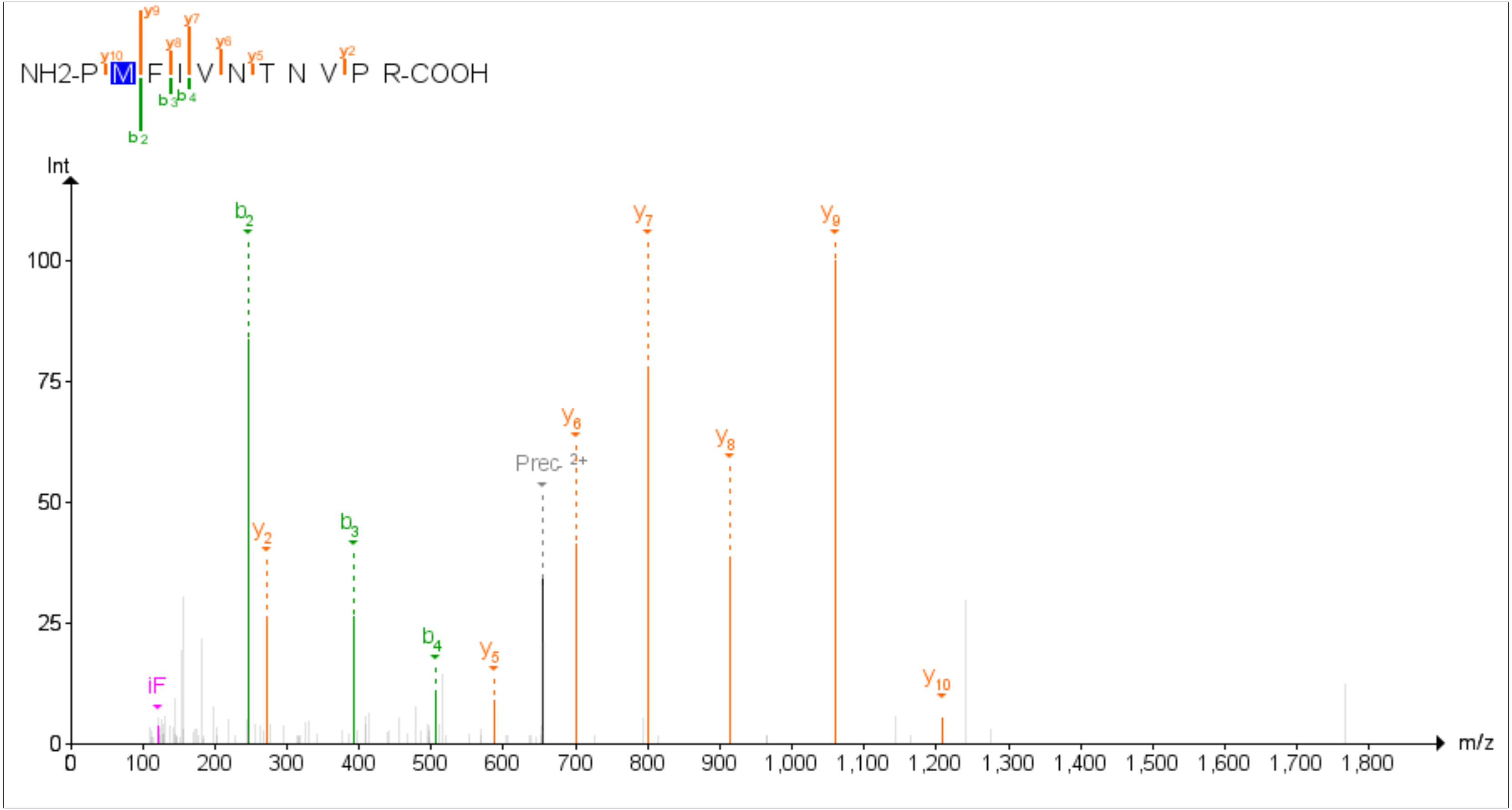

NH2-P M F I V N T N V P R-COOH
y10 y9 y8 y7 y6 y5 y2
b2 b3 b4
Int
m/z
b2
y7
y9
y6
y8
Prec 2+
y2
b3
b4
y5
y10
iF
0
25
50
75
100
0 100 200 300 400 500 600 700 800 900 1,000 1,100 1,200 1,300 1,400 1,500 1,600 1,700 1,800

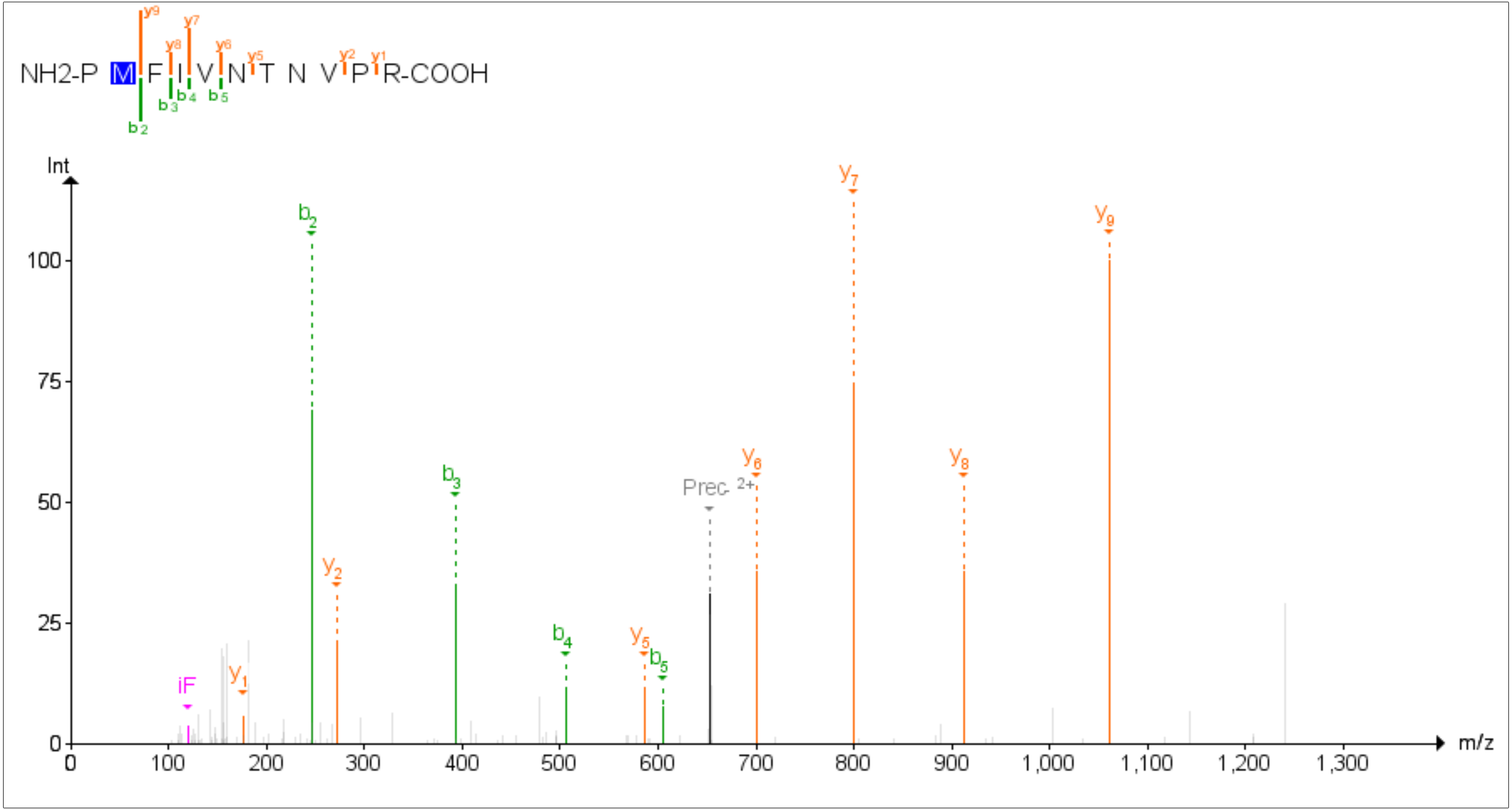

NH2-P M F I V N T N V P R-COOH
y9 y8 y7 y6 y5 y2 y1
b2 b3 b4 b5
Int
100
75
50
25
0
b2
y7
y9
b3
y6
y8
Prec 2+
y2
b4
y5
b5
iF
y1
0 100 200 300 400 500 600 700 800 900 1,000 1,100 1,200 1,300
m/z

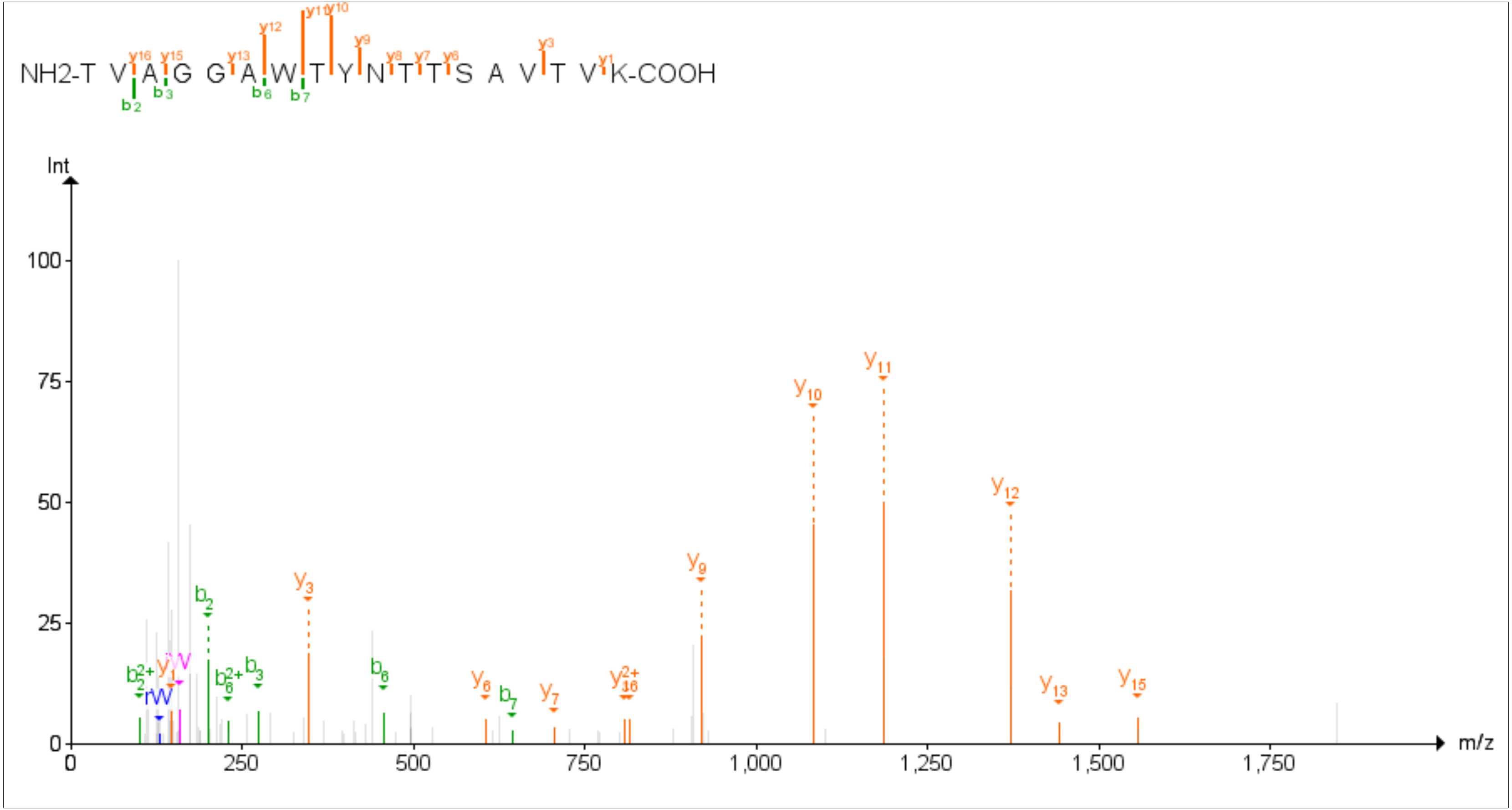

NH2-T V A G G A W T Y N T T S A V T V K-COOH
Int
m/z
0
25
50
75
100
250
500
750
1,000
1,250
1,500
1,750

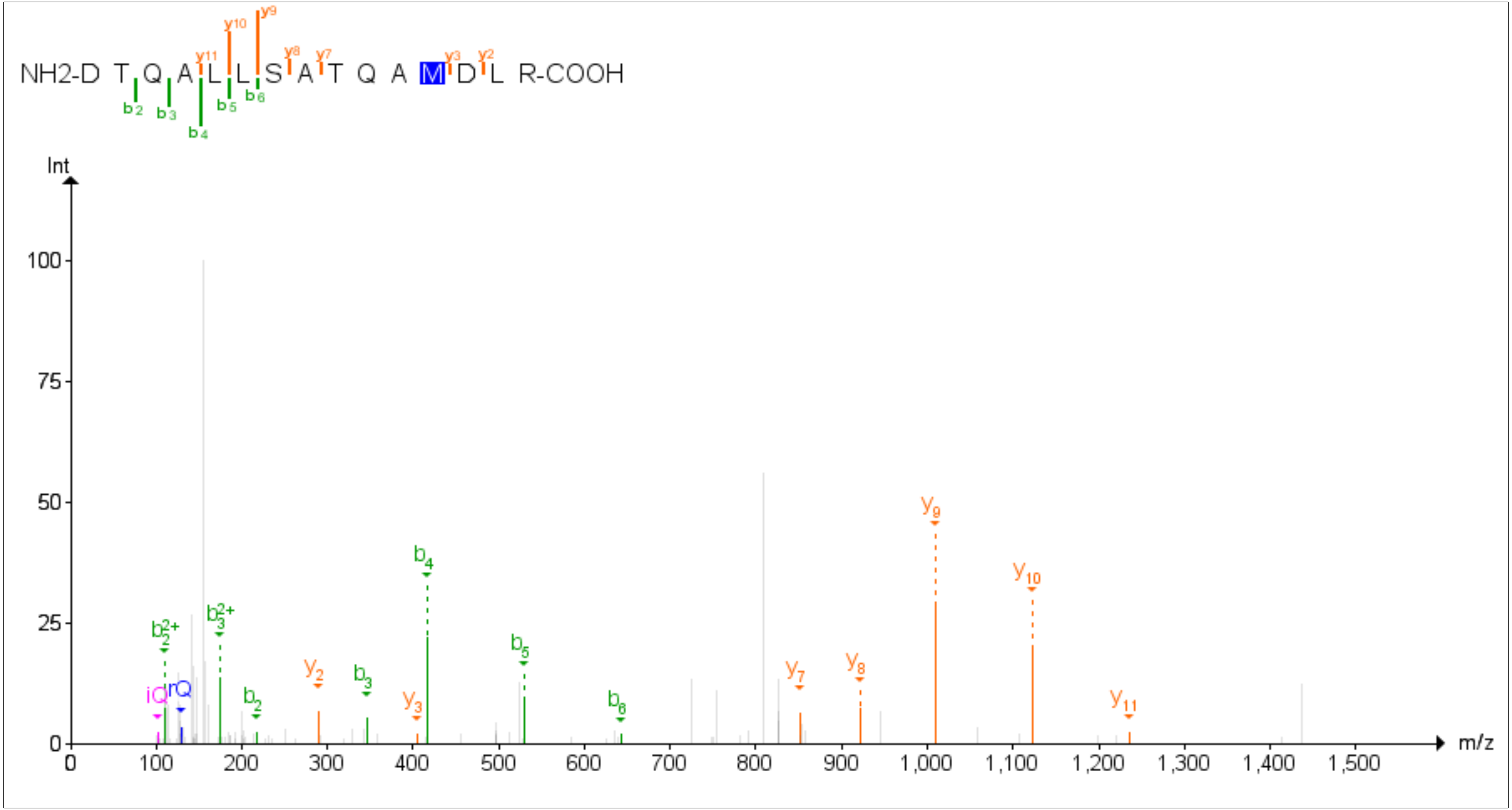

NH2-D T Q A L L S A T Q A M D L R-COOH
Int
m/z

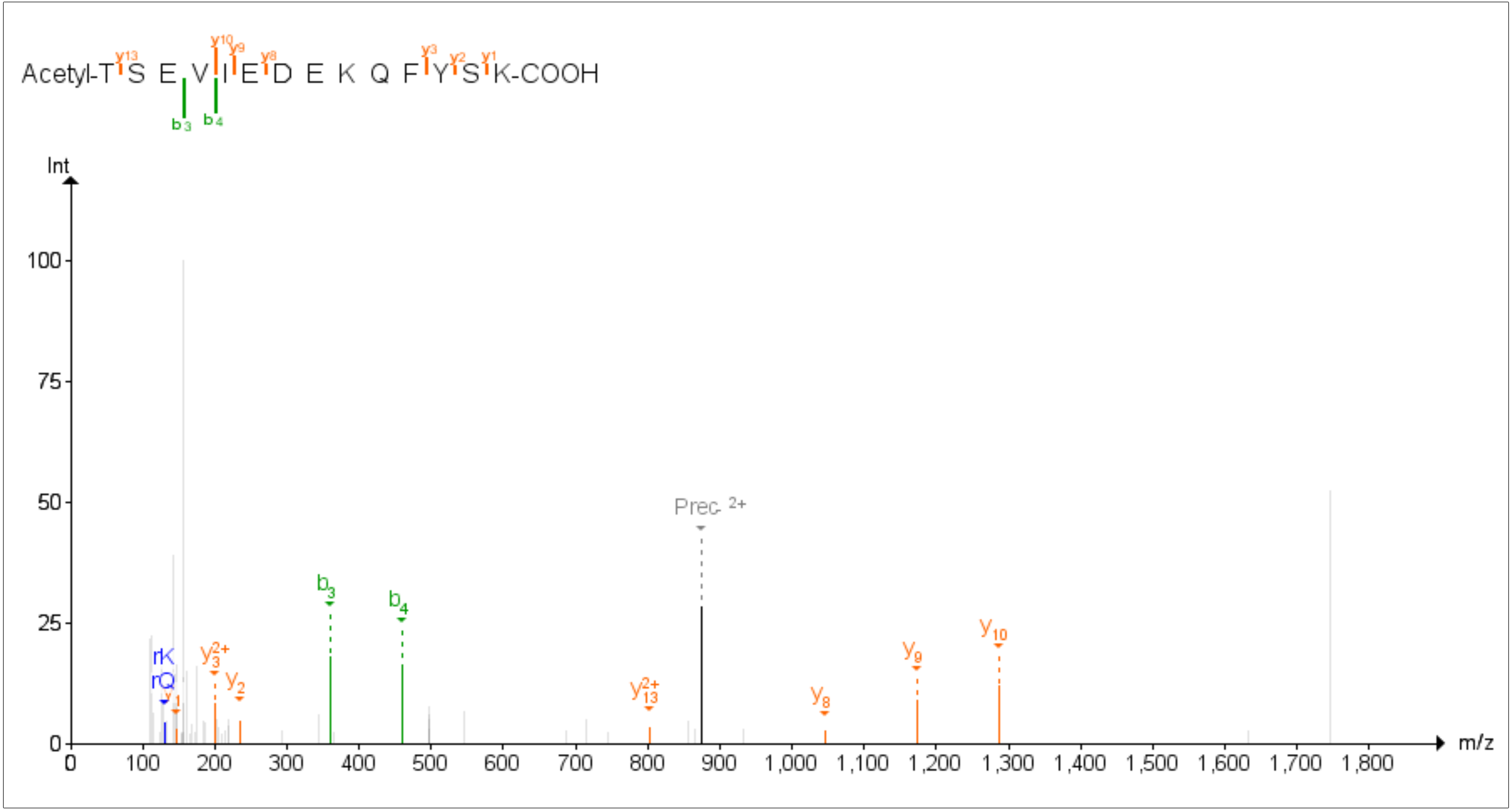

Acetyl-T S E V I E D E K Q F Y S K-COOH
y13 y10 y9 y8 y3 y2 y1
b3 b4
Int
m/z
Prec. 2+
b3
b4
rK
rQ
y1
y3 2+
y2
y13 2+
y8
y9
y10

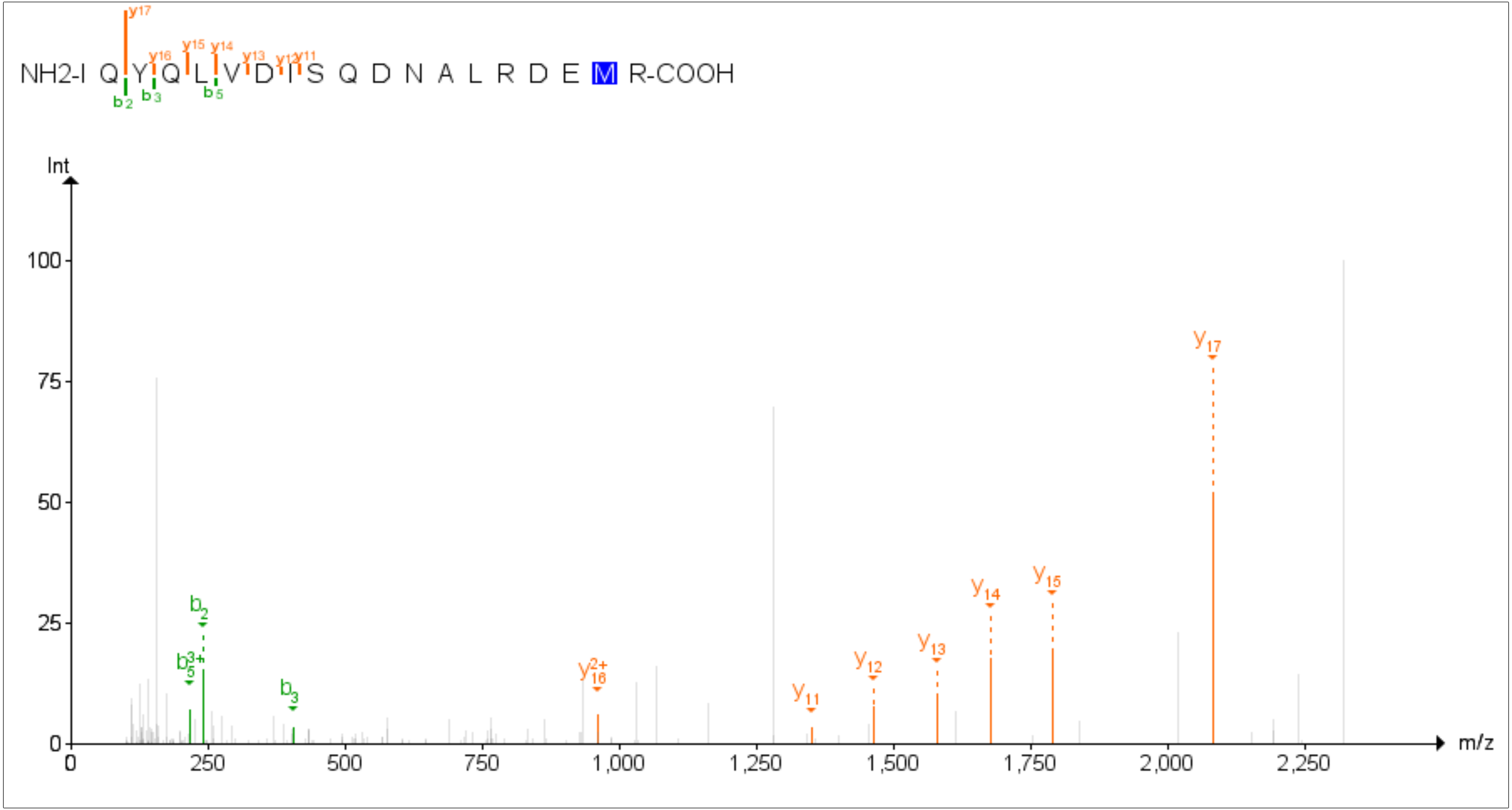

NH2-I Q Y Q L V D I S Q D N A L R D E M R-COOH
y17 y16 y15 y14 y13 y12 y11
b2 b3 b5
Int
100
75
50
25
0
0
250
500
750
1,000
1,250
1,500
1,750
2,000
2,250
m/z
b2
b5 3+
b3
y16 2+
y11
y12
y13
y14
y15
y17

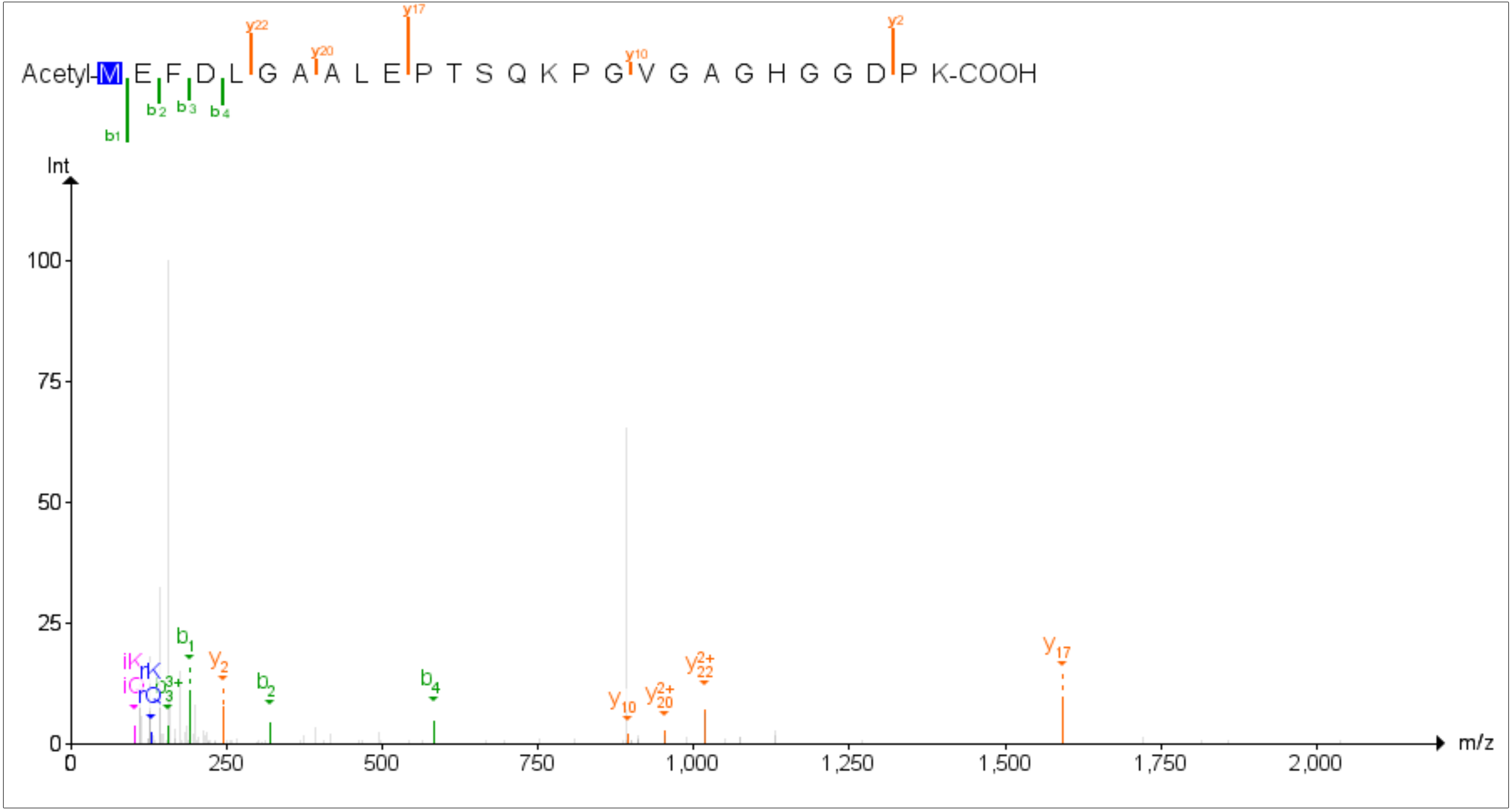

Acetyl-M E F D L G A A L E P T S Q K P G V G A G H G G D P K-COOH
y22
y20
y17
y10
y2
b1
b2
b3
b4
Int
100
75
50
25
0
iK
rK
iC
rQ
b3+
3
b1
y2
b2
b4
y10
y2+
20
y2+
22
y17
0
250
500
750
1,000
1,250
1,500
1,750
2,000
m/z

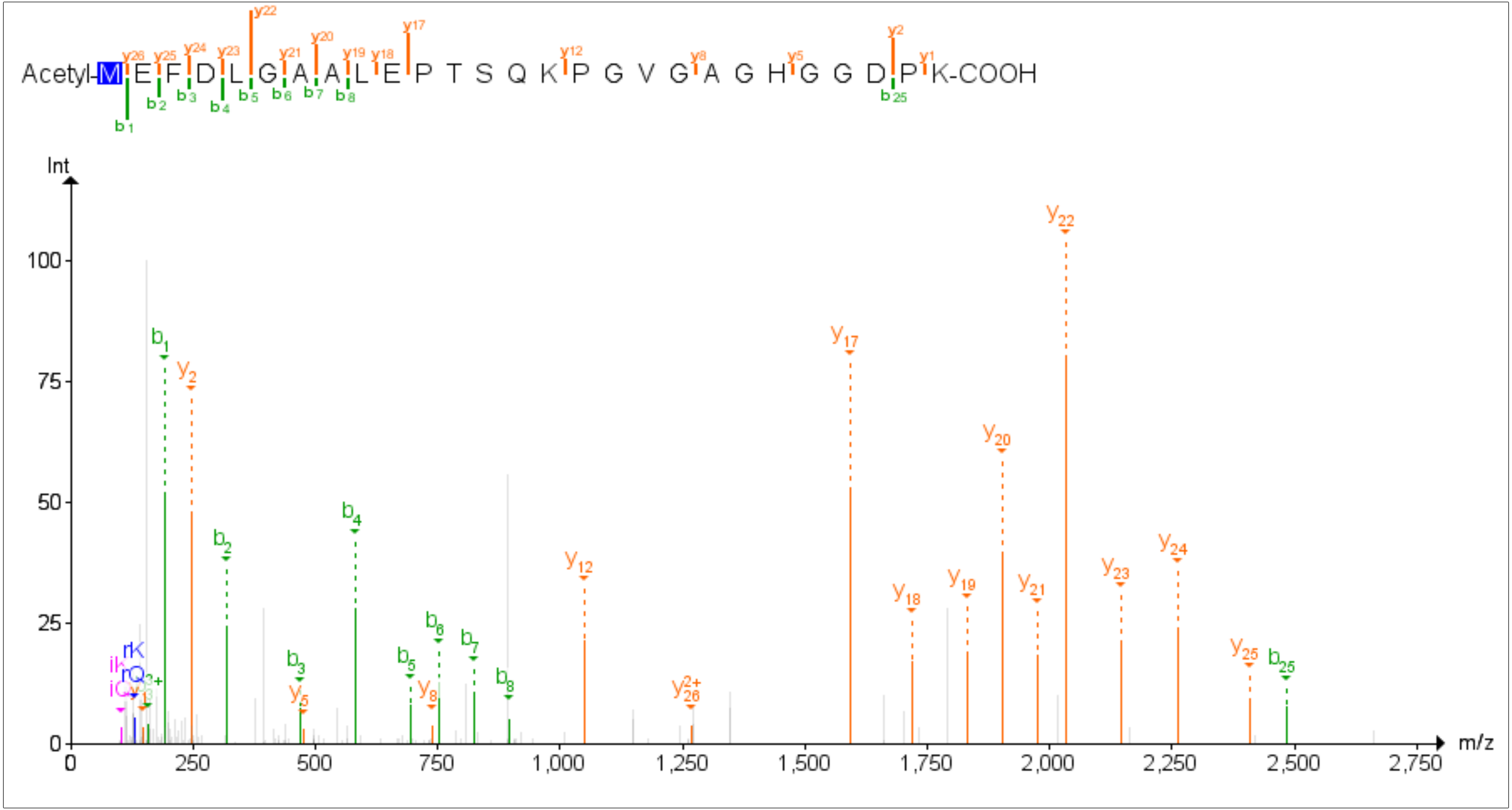

Acetyl-M E F D L G A A L E P T S Q K P G V G A G H G G D P K-COOH
Int
m/z
0
25
50
75
100
250
500
750
1,000
1,250
1,500
1,750
2,000
2,250
2,500
2,750

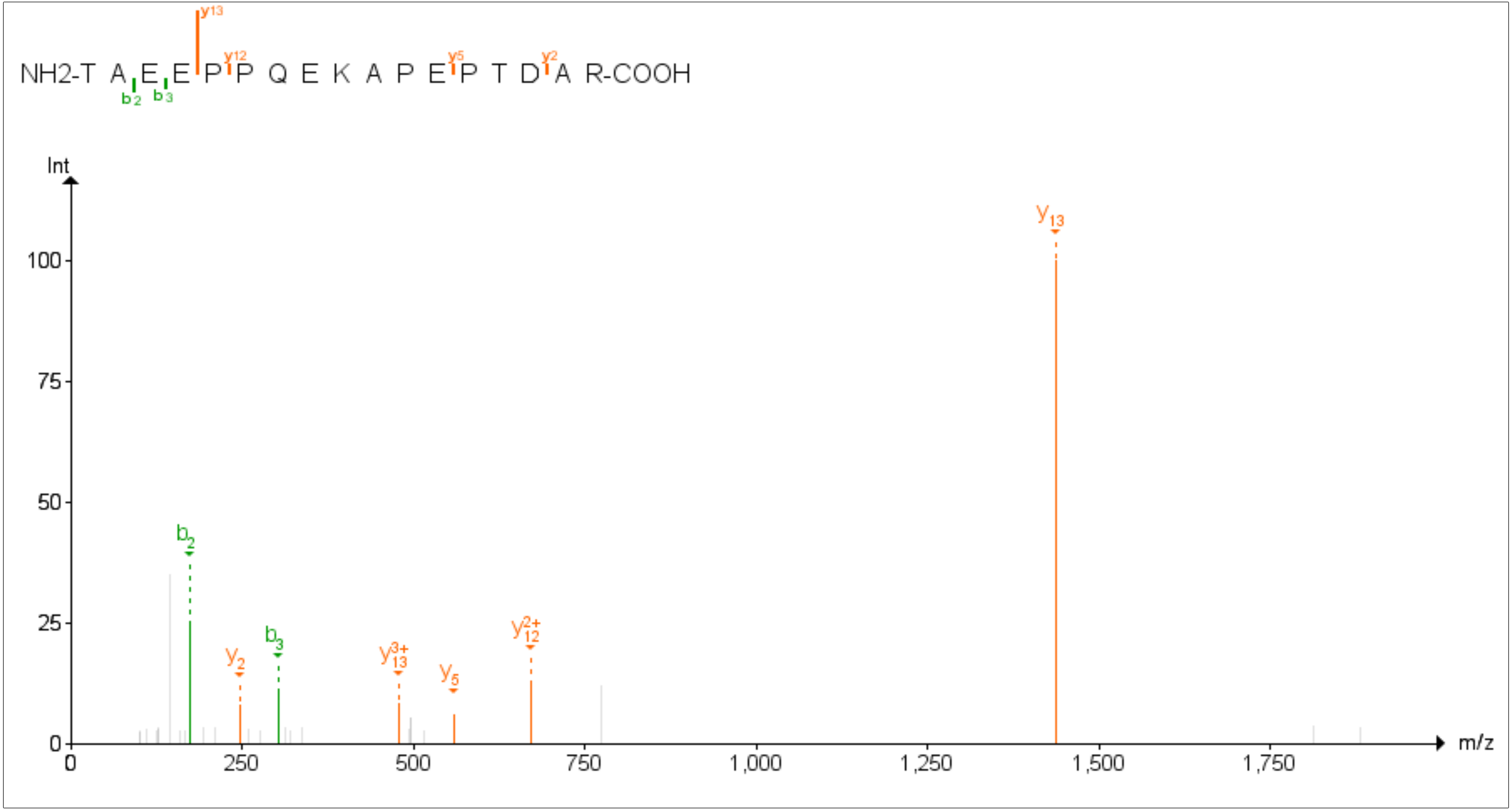

NH2-T A E E P P Q E K A P E P T D A R-COOH
y13
y12
y5
y2
b2
b3
Int
100
75
50
25
0
y13
b2
y2
b3
y13 3+
y5
y12 2+
0
250
500
750
1,000
1,250
1,500
1,750
m/z

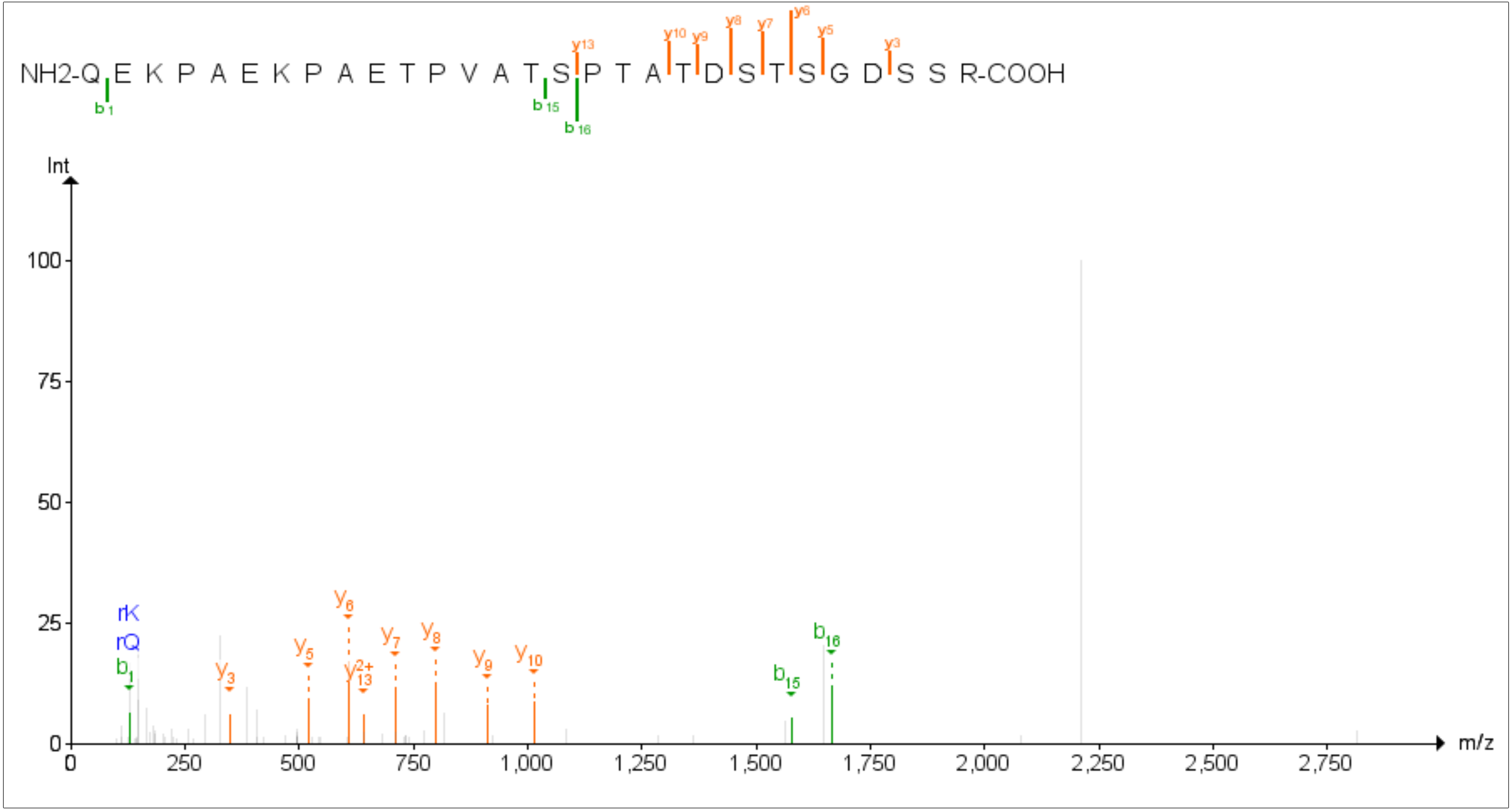

NH2-Q E K P A E K P A E T P V A T S P T A T D S T S G D S S R-COOH
b1
b15
b16
y13
y10
y9
y8
y7
y6
y5
y3
Int
100
75
50
25
0
rK
rQ
b1
y3
y5
y6
y13 2+
y7
y8
y9
y10
b15
b16
0
250
500
750
1,000
1,250
1,500
1,750
2,000
2,250
2,500
2,750
m/z

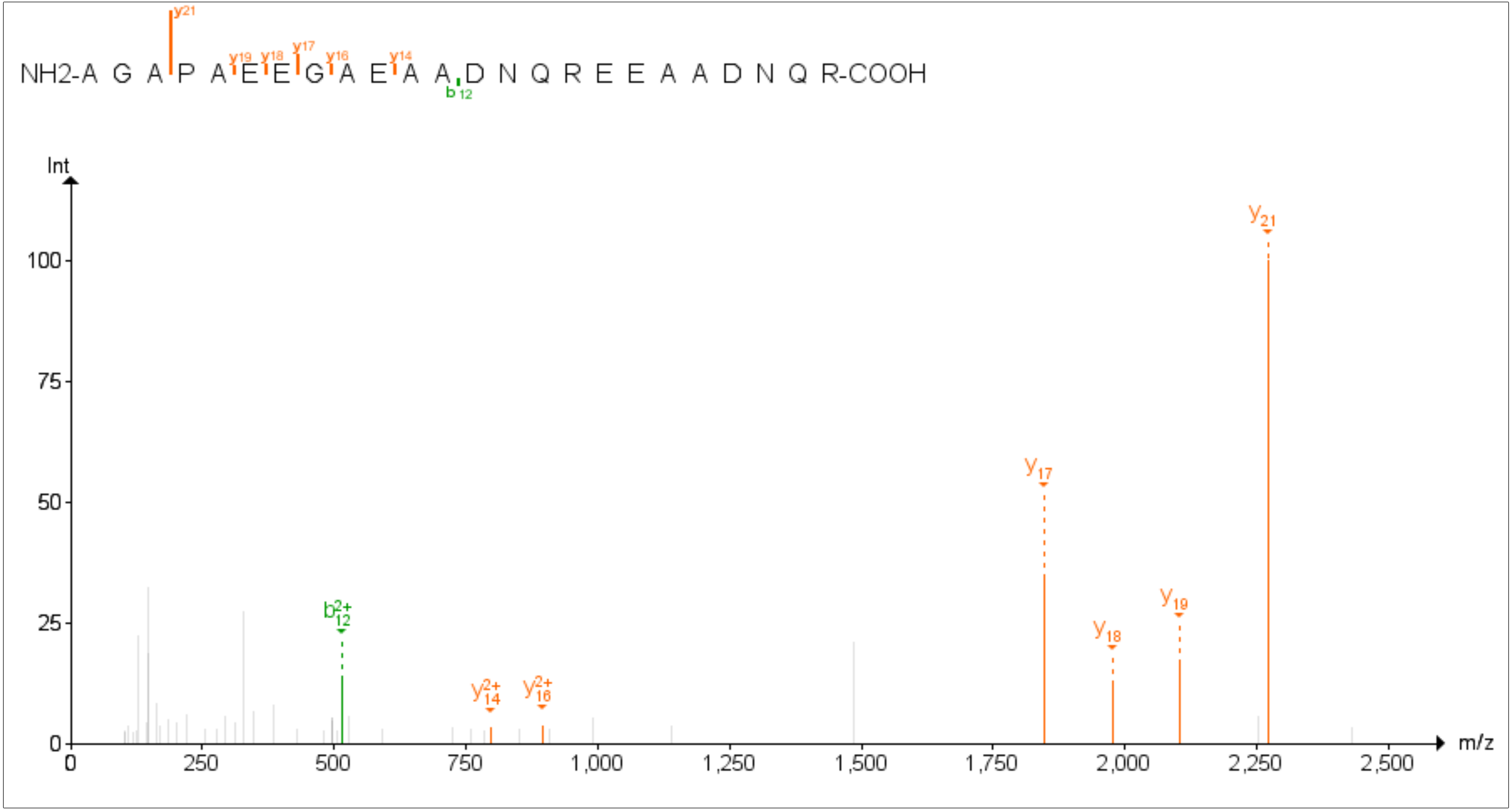

NH2-A G A P A E E G A E A A D N Q R E E A A D N Q R-COOH
y21
y19
y18
y17
y16
y14
b12
Int
100
75
50
25
0
y21
y17
y18
y19
b$^{2+}_{12}$
y$^{2+}_{14}$
y$^{2+}_{16}$
0
250
500
750
1,000
1,250
1,500
1,750
2,000
2,250
2,500
m/z

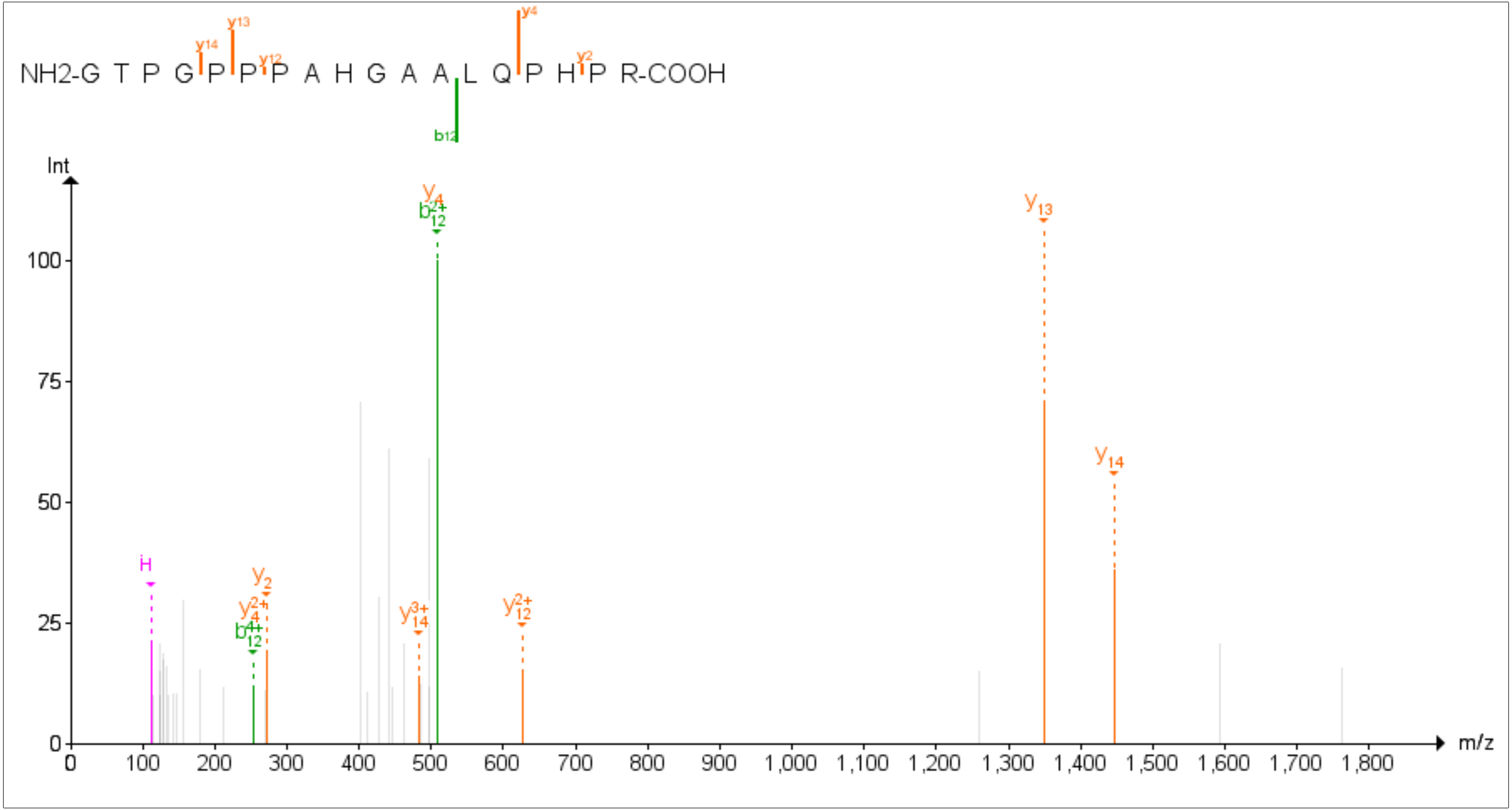

NH2-G T P G P P P A H G A A L Q P H P R-COOH
y14
y13
y12
y4
y2
b12
Int
100
75
50
25
0
m/z
0
100
200
300
400
500
600
700
800
900
1,000
1,100
1,200
1,300
1,400
1,500
1,600
1,700
1,800
H
y_2
y_4^{2+}
b_{12}^{4+}
y_{14}^{3+}
y_4
b_{12}^{2+}
y_{12}^{2+}
y_{13}
y_{14}

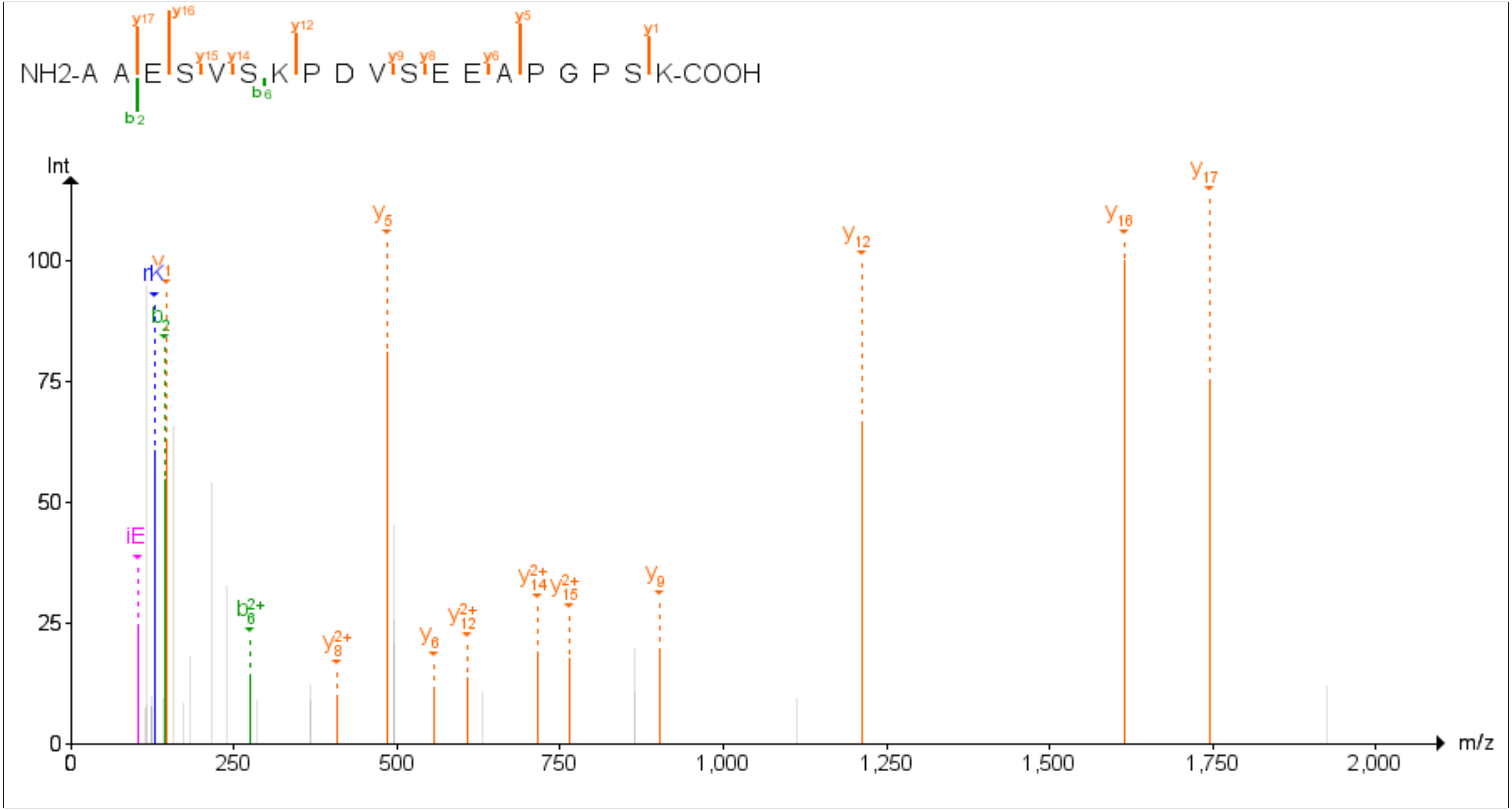

NH2-A A E S V S K P D V S E E A P G P S K-COOH
y17 y16 y15 y14 y12 y9 y8 y6 y5 y1
b2 b6
Int
100
75
50
25
0
y5
y12
y16
y17
y1
rK
b2
iE
b6 2+
y8 2+
y6
y12 2+
y14 2+
y15 2+
y9
0
250
500
750
1,000
1,250
1,500
1,750
2,000
m/z

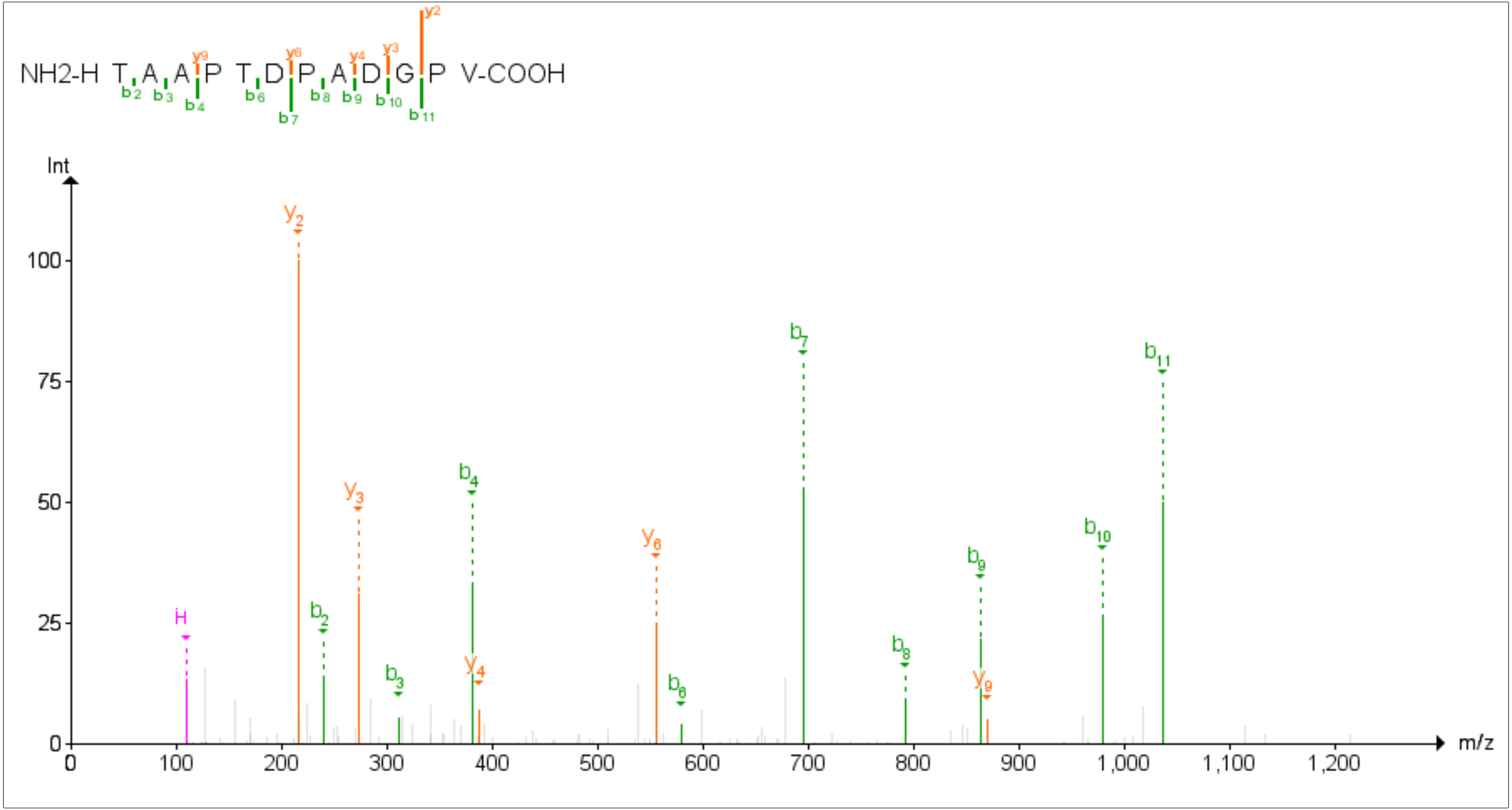
NH2-H T A A P T D P A D G P V-COOH
Int
m/z
y2
y3
y4
y6
y9
b2
b3
b4
b6
b7
b8
b9
b10
b11
H
0
25
50
75
100
0
100
200
300
400
500
600
700
800
900
1,000
1,100
1,200

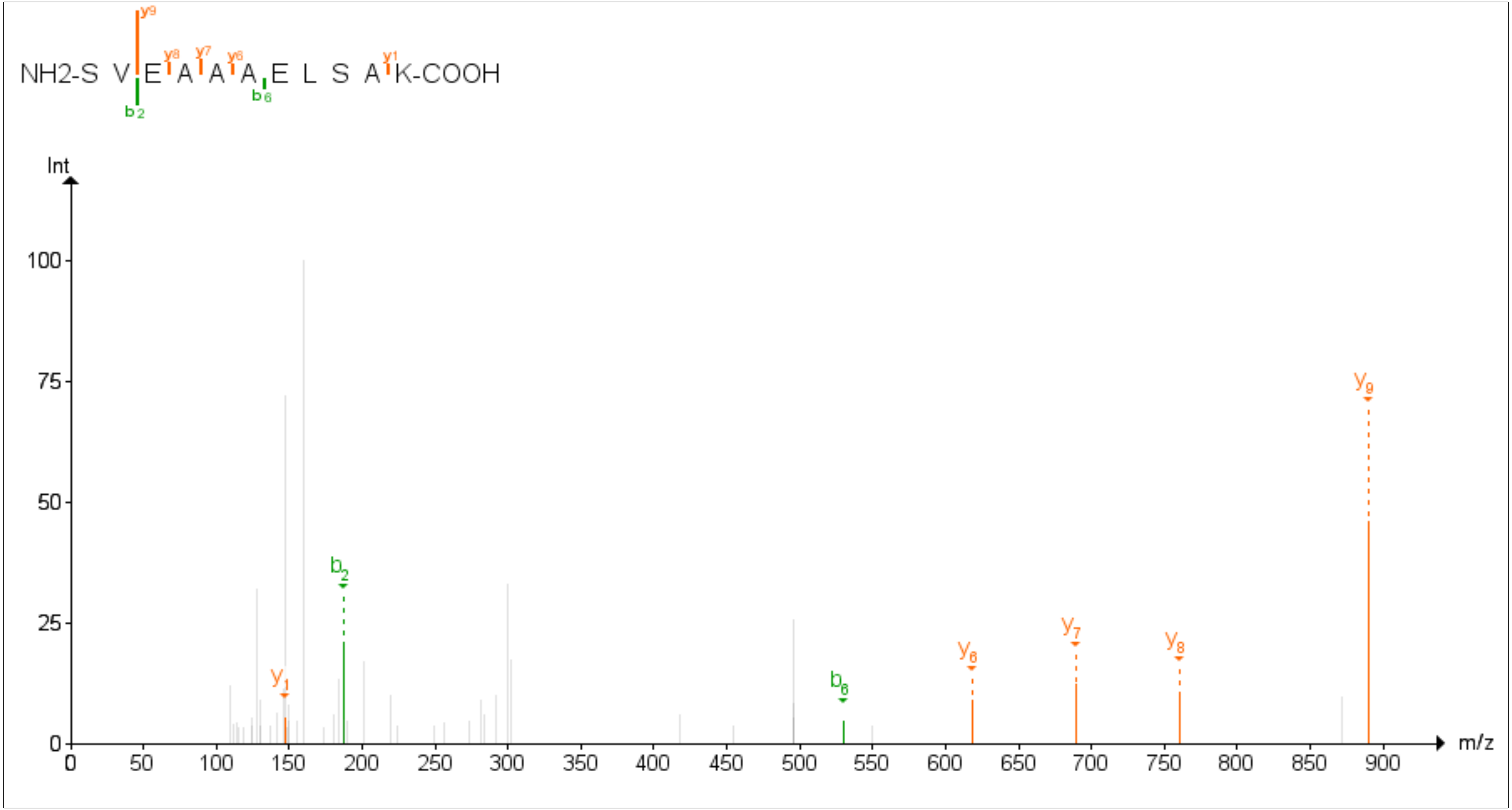

NH2-S V E A A A E L S A K-COOH
y9
y8
y7
y6
y1
b2
b6
Int
100
75
50
25
0
m/z
0 50 100 150 200 250 300 350 400 450 500 550 600 650 700 750 800 850 900
y_9
b_2
y_1
b_6
y_6
y_7
y_8

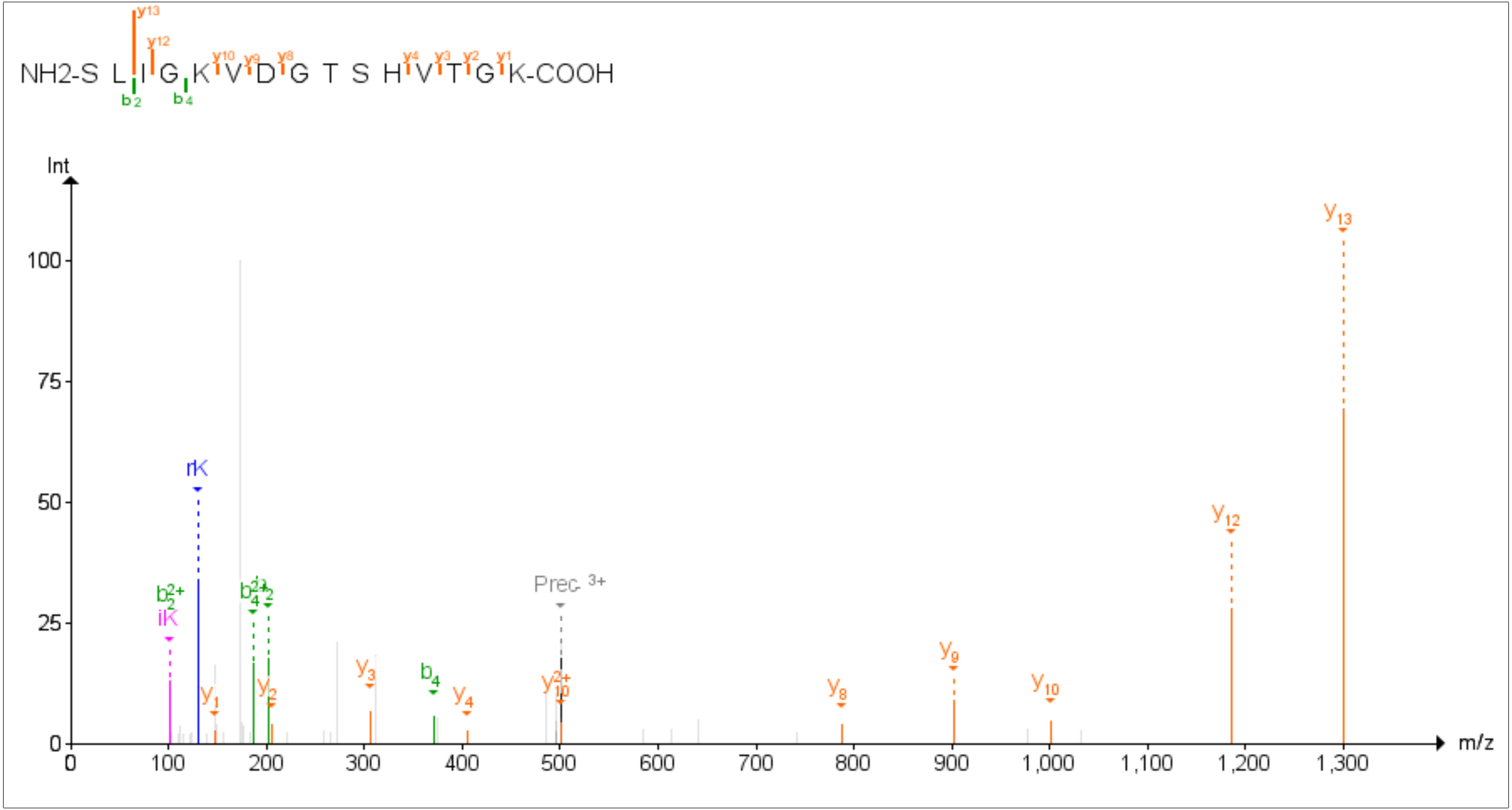

NH2-S L I G K V D G T S H V T G K-COOH
y13
y12
y10
y9
y8
y4
y3
y2
y1
b2
b4
Int
100
75
50
25
0
rK
b2 2+
iK
b4 2+
y2
Prec 3+
y1
y3
b4
y4
y10 2+
y8
y9
y10
y12
y13
m/z
0
100
200
300
400
500
600
700
800
900
1,000
1,100
1,200
1,300

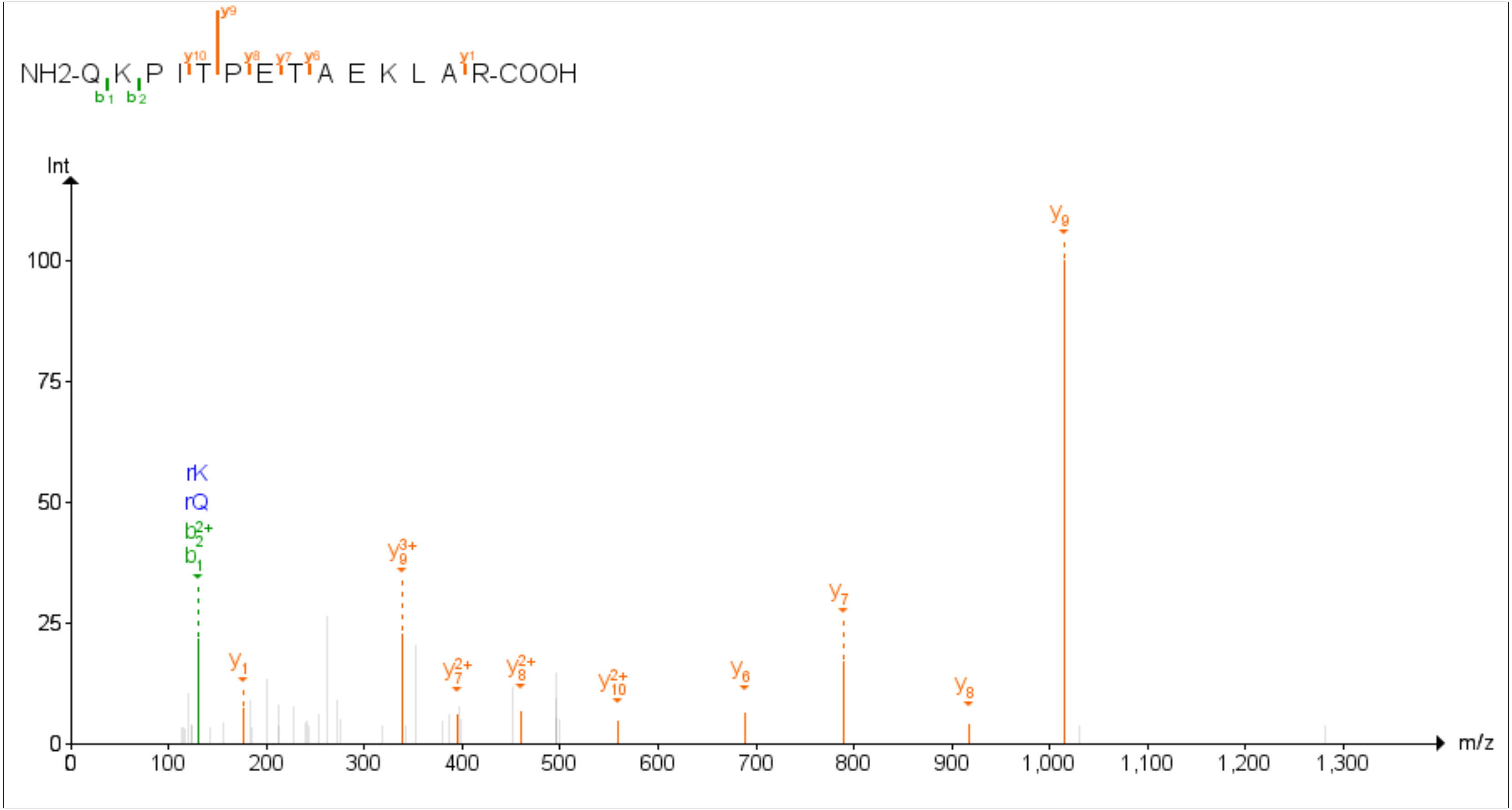

NH2-Q K P I T P E T A E K L A R-COOH
b1 b2
y10 y9 y8 y7 y6 y1
Int
100
75
50
25
0
rK
rQ
b2 2+
b1
y9 3+
y1
y7 2+
y8 2+
y10 2+
y6
y7
y8
y9
0
100
200
300
400
500
600
700
800
900
1,000
1,100
1,200
1,300
m/z

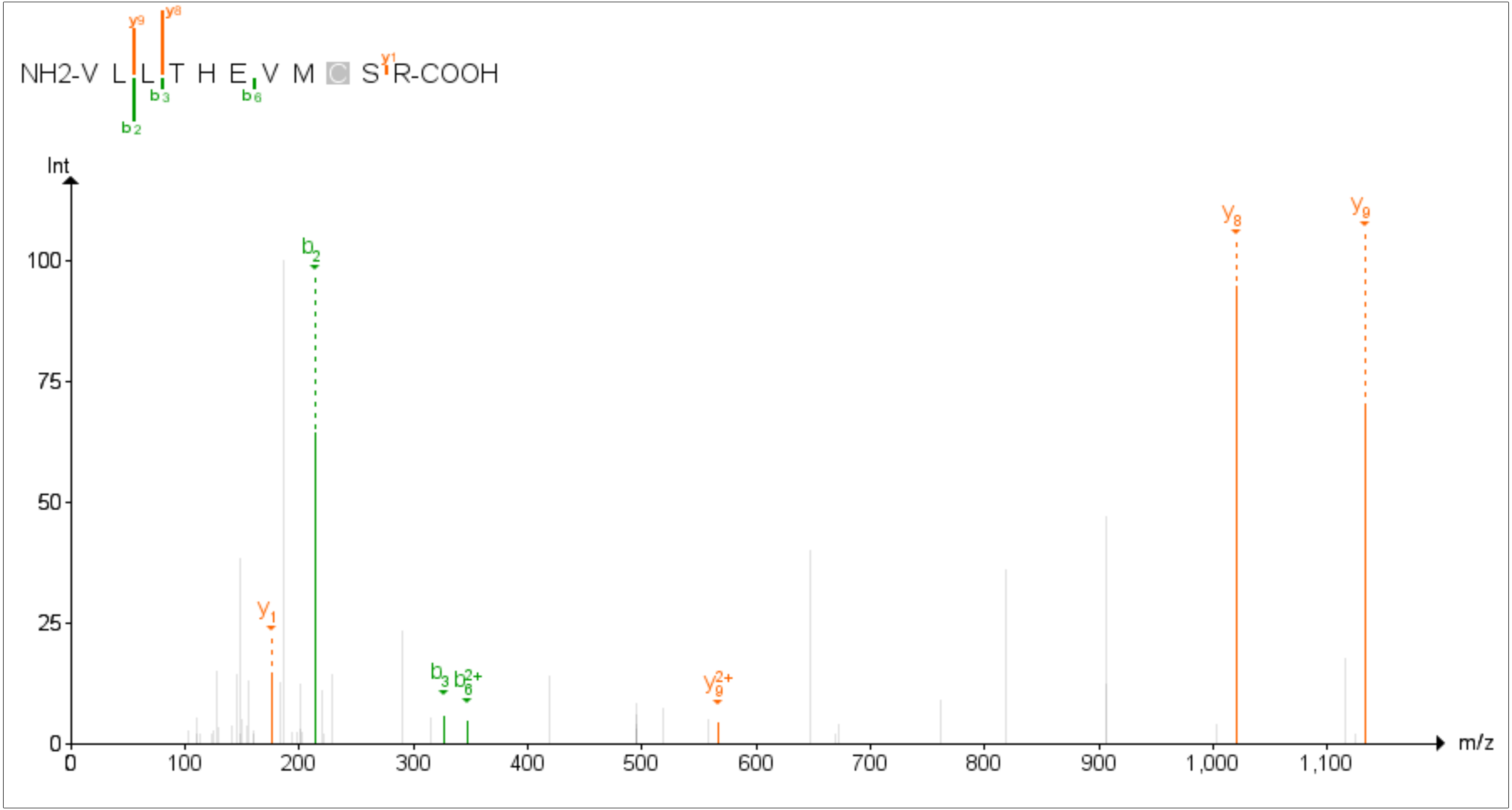

NH2-V L L T H E V M C S R-COOH
y9
y8
y1
b2
b3
b6
Int
100
75
50
25
0
b2
y8
y9
y1
b3
b6 2+
y9 2+
0
100
200
300
400
500
600
700
800
900
1,000
1,100
m/z

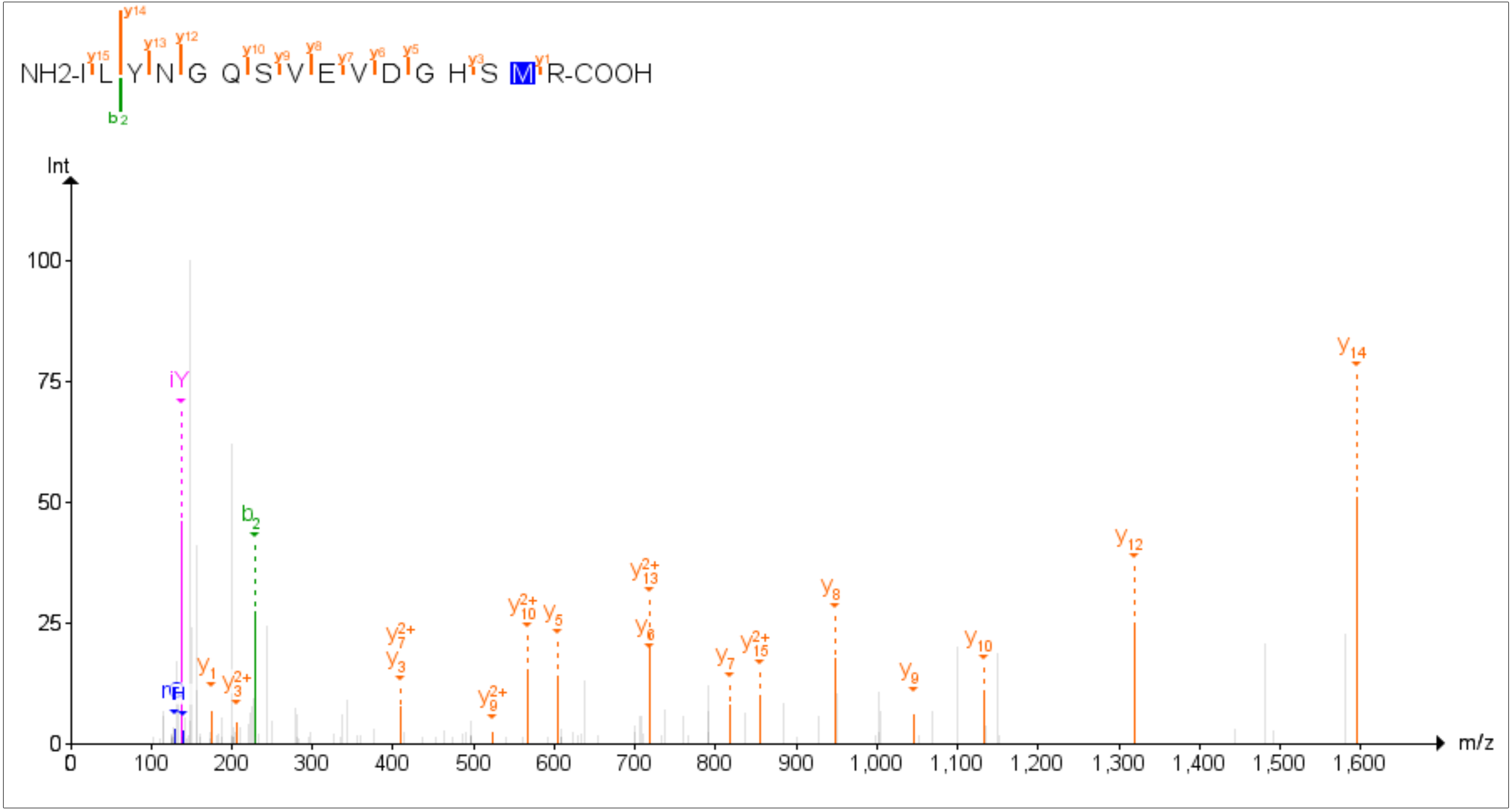

NH2-I L Y N G Q S V E V D G H S M R-COOH
Int
m/z
iY
b2
y1
y3
y5
y6
y7
y8
y9
y10
y12
y14

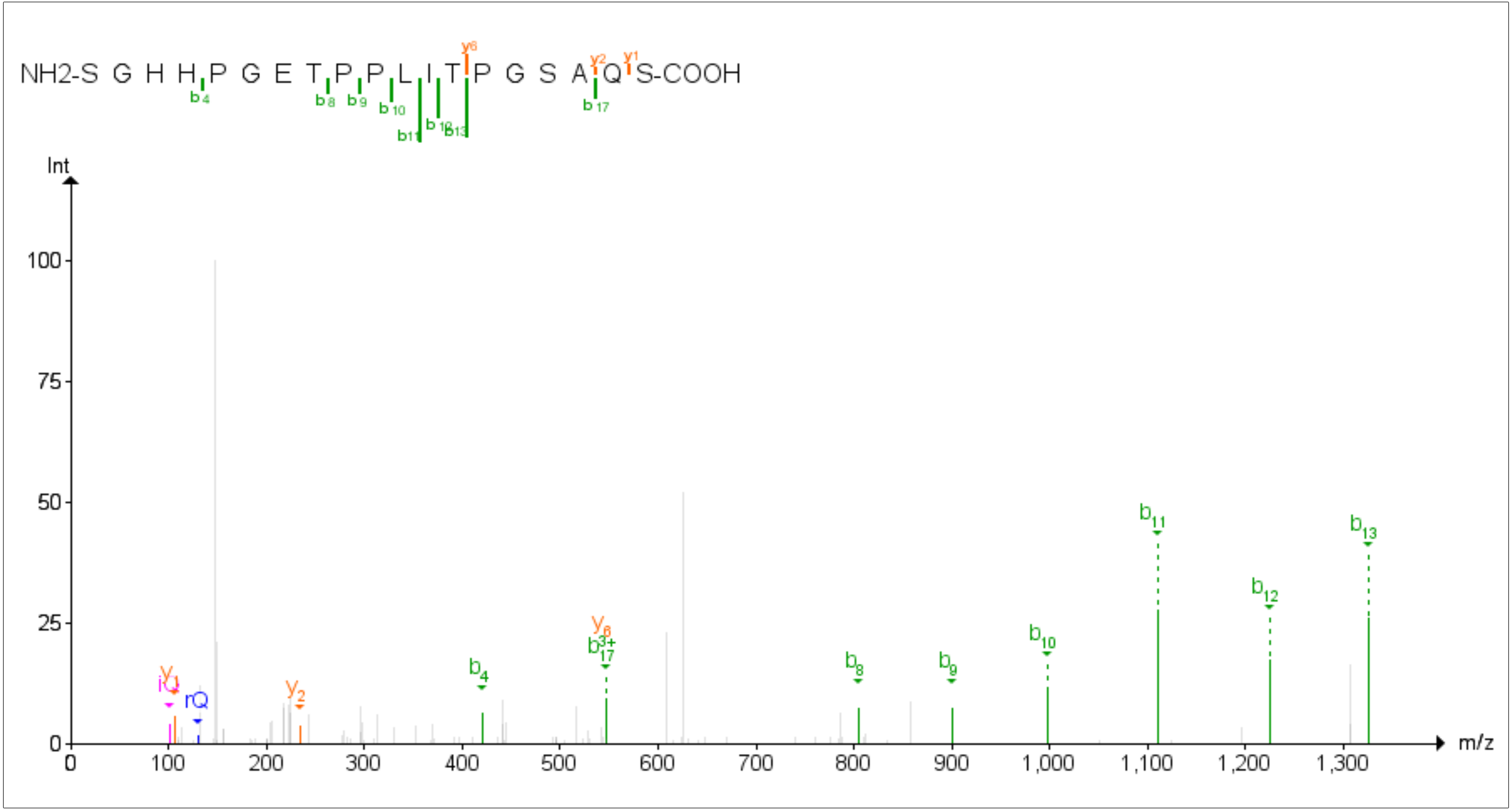

NH2-S G H H P G E T P P L I T P G S A Q S-COOH
Int
m/z

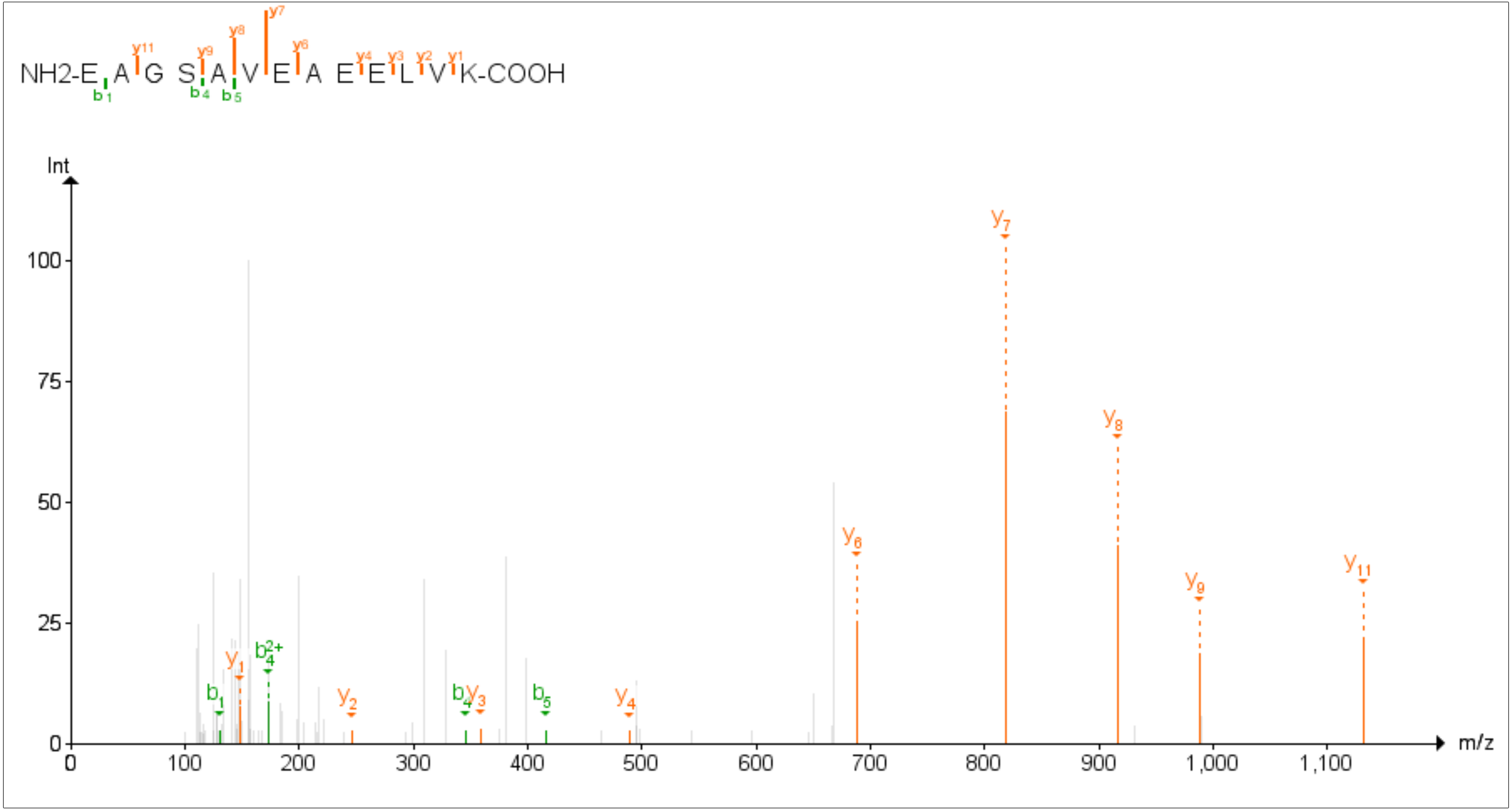
NH2-E A G S A V E A E E L V K-COOH
y11 y9 y8 y7 y6 y4 y3 y2 y1
b1 b4 b5
Int
100
75
50
25
0
m/z
0
100
200
300
400
500
600
700
800
900
1,000
1,100
y7
y8
y6
y9
y11
y1
b4 2+
b1
y2
b4
y3
b5
y4

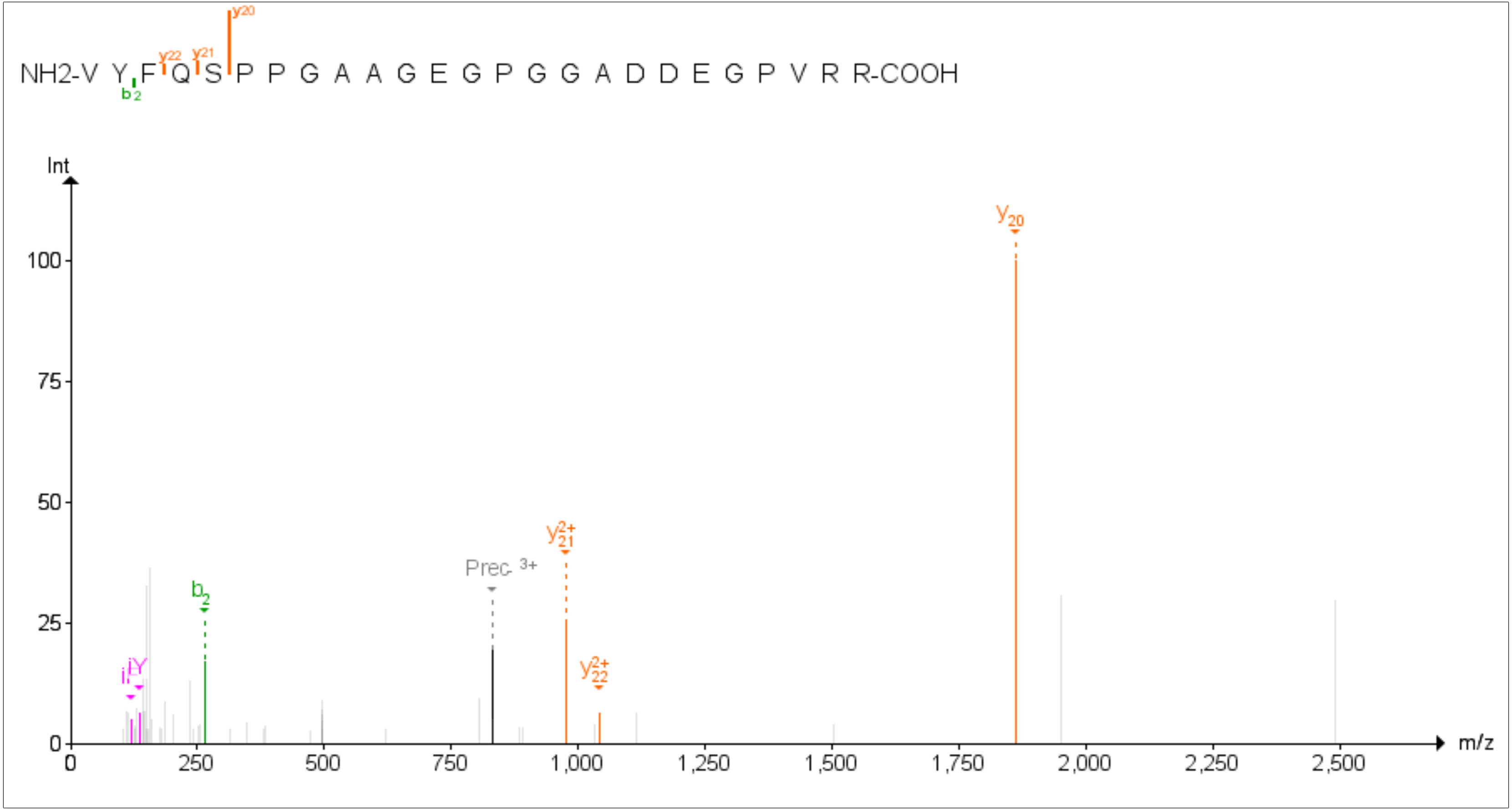

NH2-V Y F Q S P P G A A G E G P G G A D D E G P V R R-COOH
y20
y22
y21
b2
Int
m/z
Prec. 3+
iY

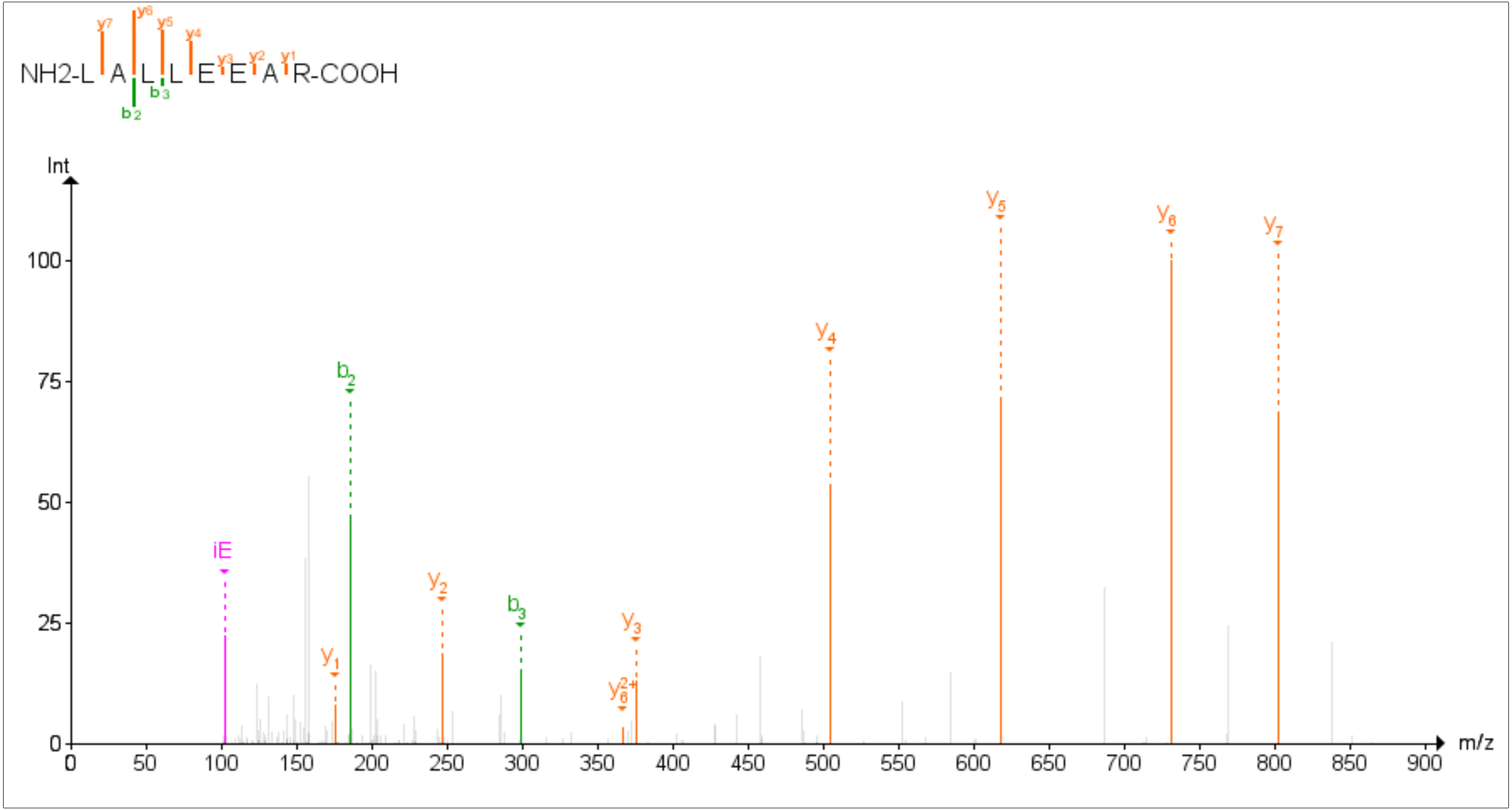

NH2-L A L L E E A R-COOH
y7
y6
y5
y4
y3
y2
y1
b2
b3
Int
100
75
50
25
0
iE
y1
b2
y2
b3
y6 2+
y3
y4
y5
y6
y7
0
50
100
150
200
250
300
350
400
450
500
550
600
650
700
750
800
850
900
m/z

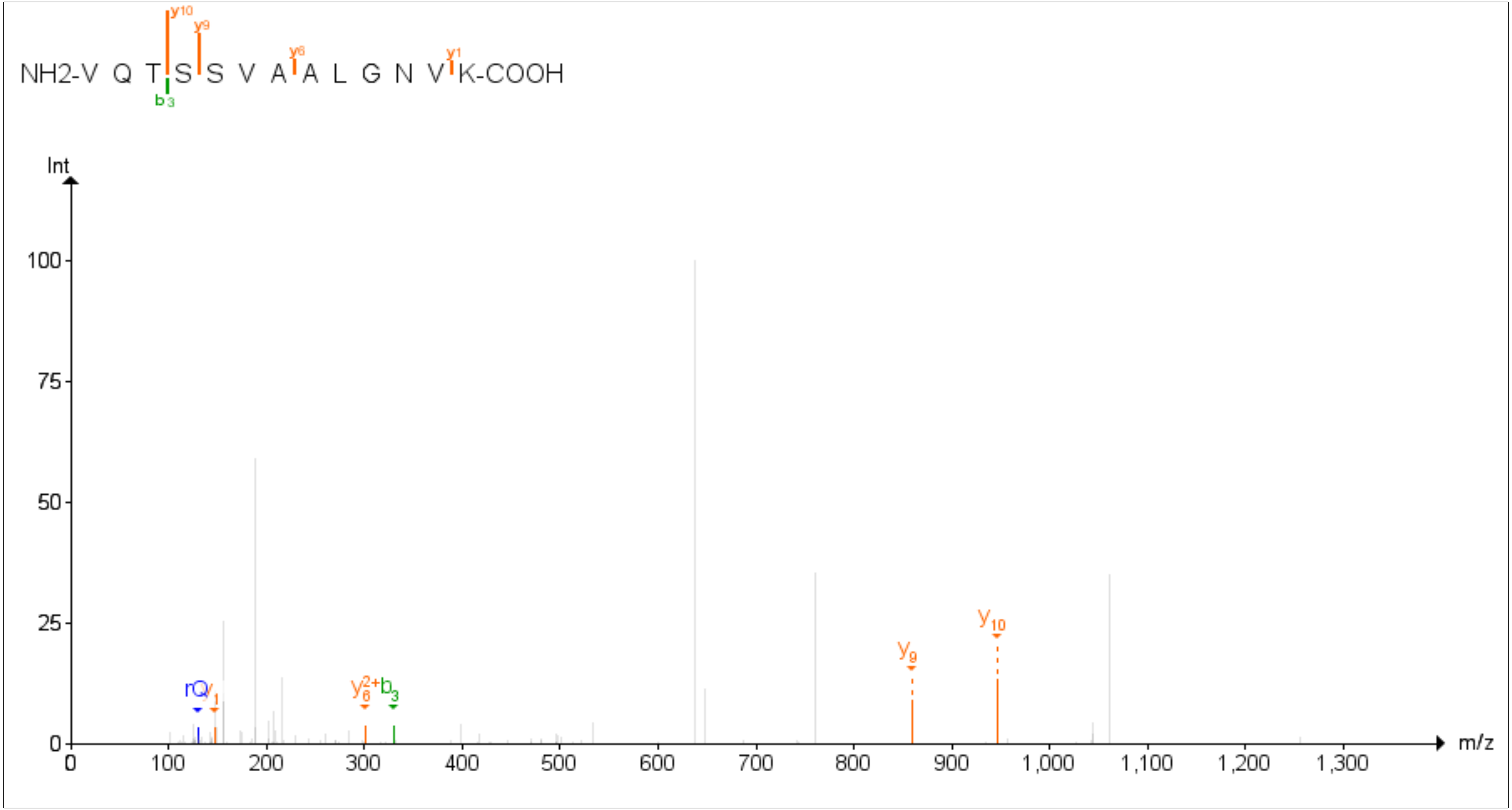

NH2-V Q T S S V A A L G N V K-COOH
y10
y9
y6
y1
b3
Int
100
75
50
25
0
rQ
y1
y6 2+
b3
y9
y10
0
100
200
300
400
500
600
700
800
900
1,000
1,100
1,200
1,300
m/z

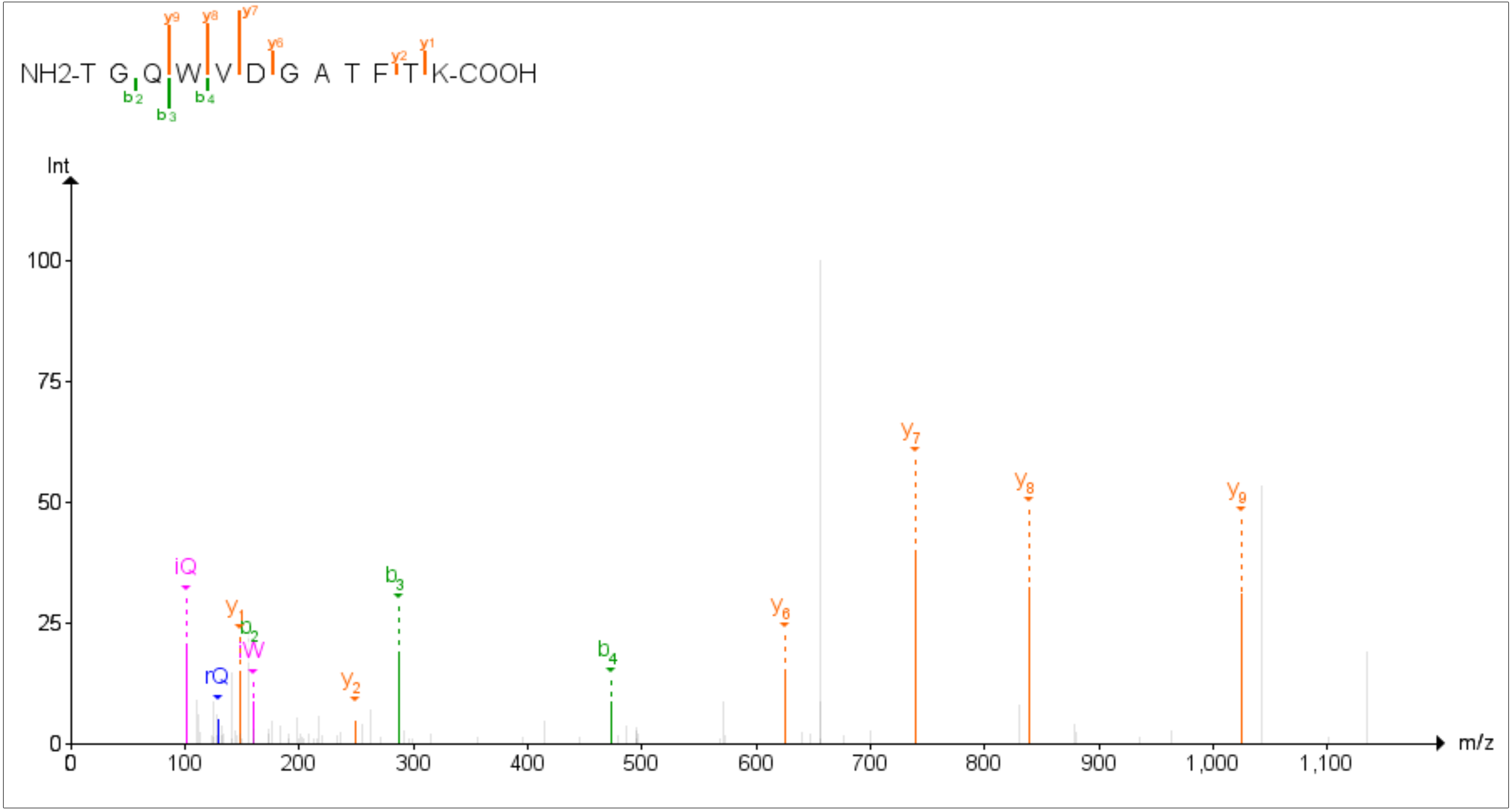

NH2-T G Q W V D G A T F T K-COOH
y9 y8 y7 y6 y2 y1
b2 b3 b4
Int
m/z
0
25
50
75
100
0
100
200
300
400
500
600
700
800
900
1,000
1,100
iQ
rQ
y1
b2
iW
y2
b3
b4
y6
y7
y8
y9

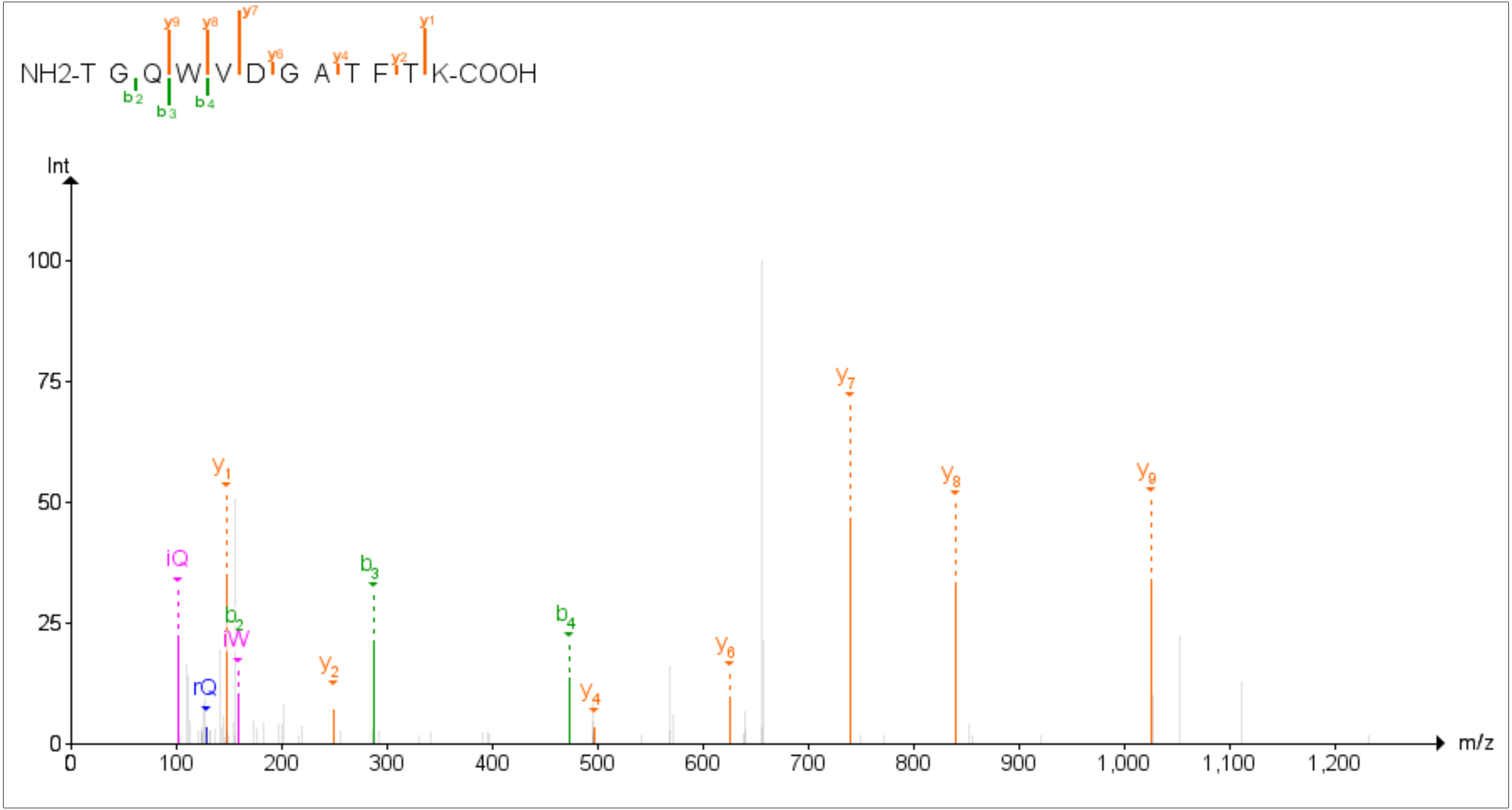
NH2-T G Q W V D G A T F T K-COOH
y9 y8 y7 y6 y4 y2 y1
b2 b3 b4
Int
100
75
50
25
0
m/z
0 100 200 300 400 500 600 700 800 900 1,000 1,100 1,200
y1
iQ
b3
b2
iW
rQ
y2
b4
y4
y6
y7
y8
y9

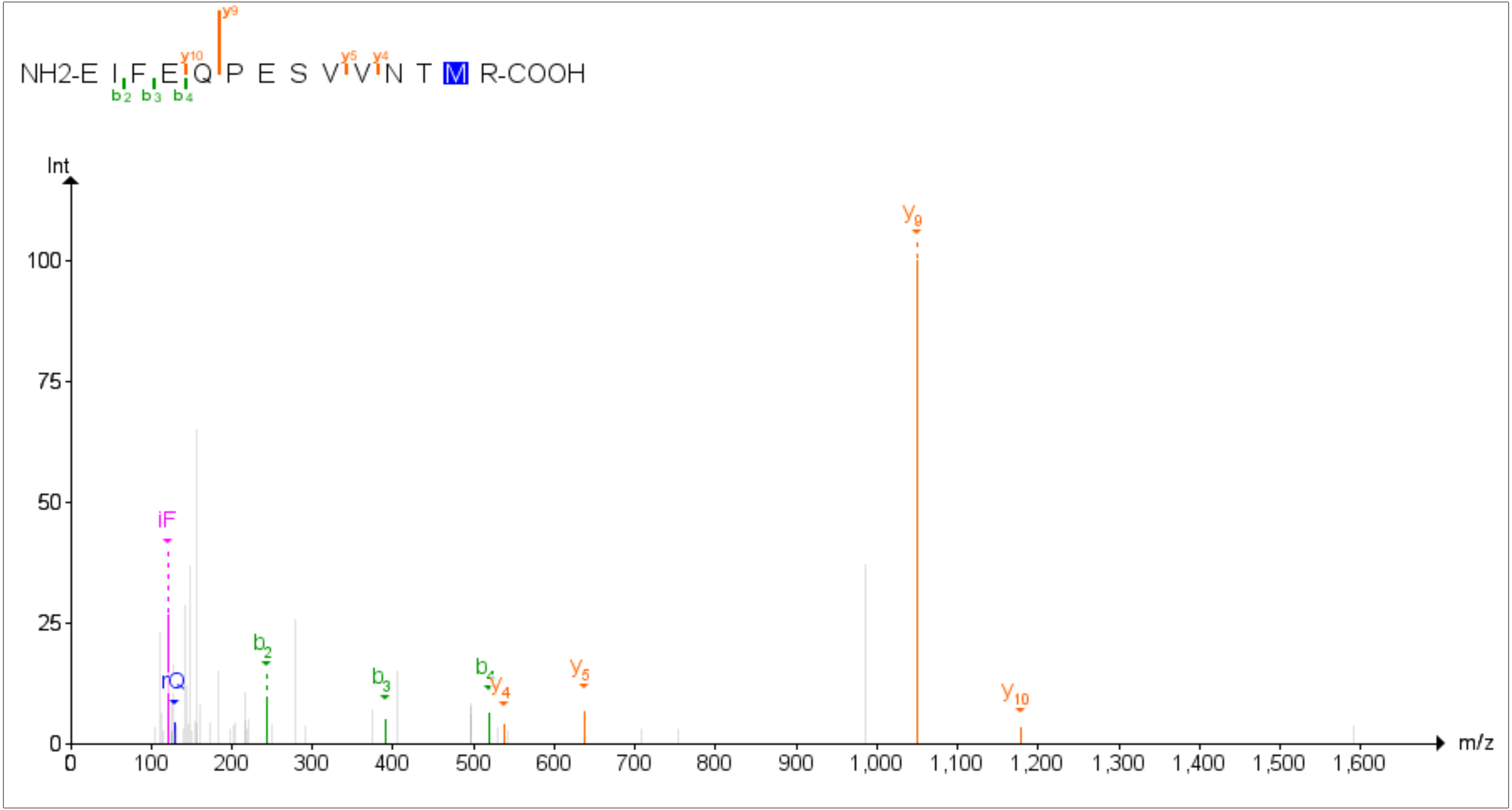

NH2-E I F E Q P E S V V N T M R-COOH
y9
y10
y5
y4
b2
b3
b4
Int
100
75
50
25
0
iF
rQ
b2
b3
b4
y4
y5
y9
y10
m/z
0
100
200
300
400
500
600
700
800
900
1,000
1,100
1,200
1,300
1,400
1,500
1,600

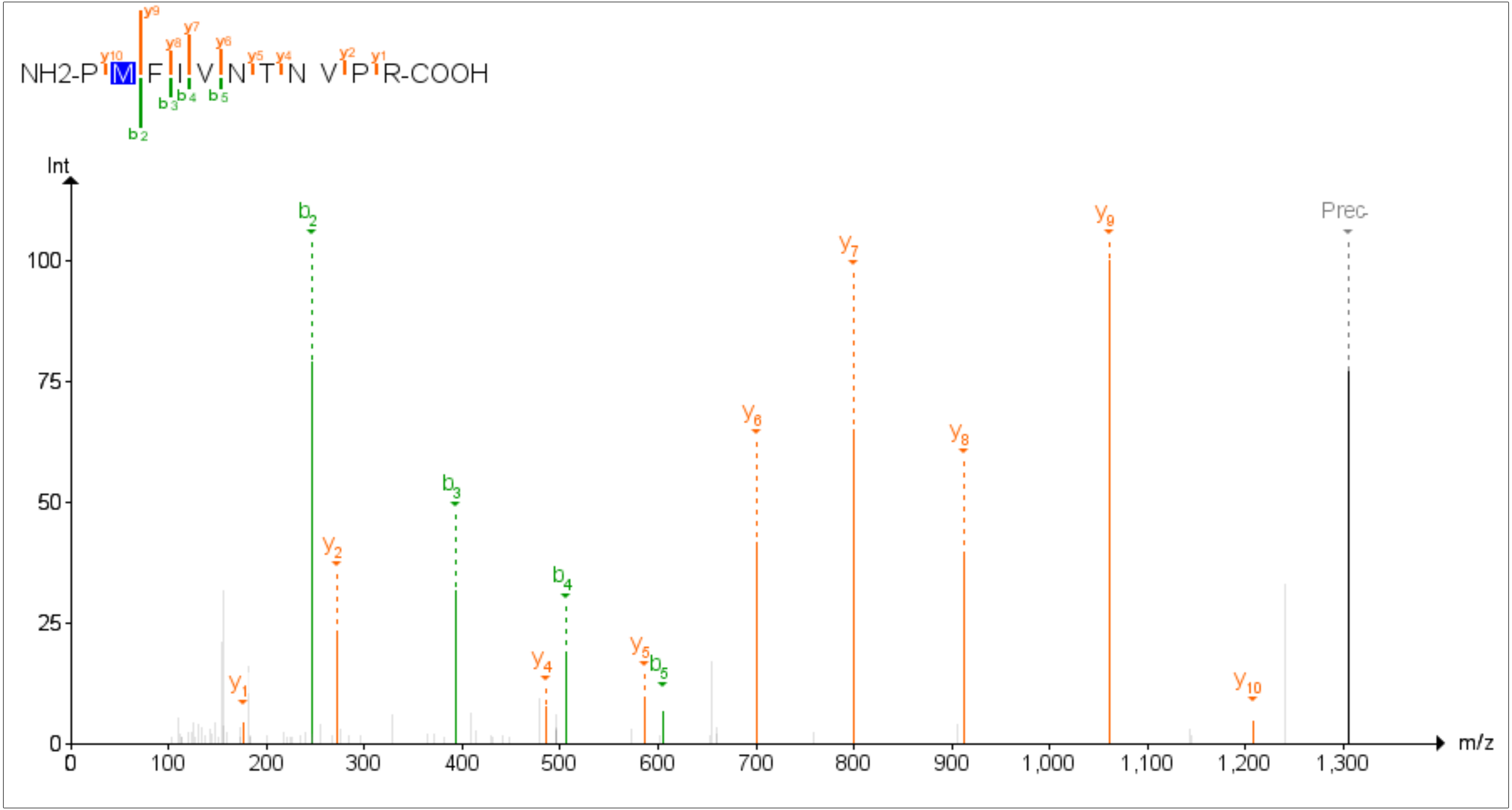
NH2-PMFIVNTNVPR-COOH
y10 y9 y8 y7 y6 y5 y4 y2 y1
b2 b3 b4 b5
Int
Prec.
m/z
0 25 50 75 100
0 100 200 300 400 500 600 700 800 900 1,000 1,100 1,200 1,300

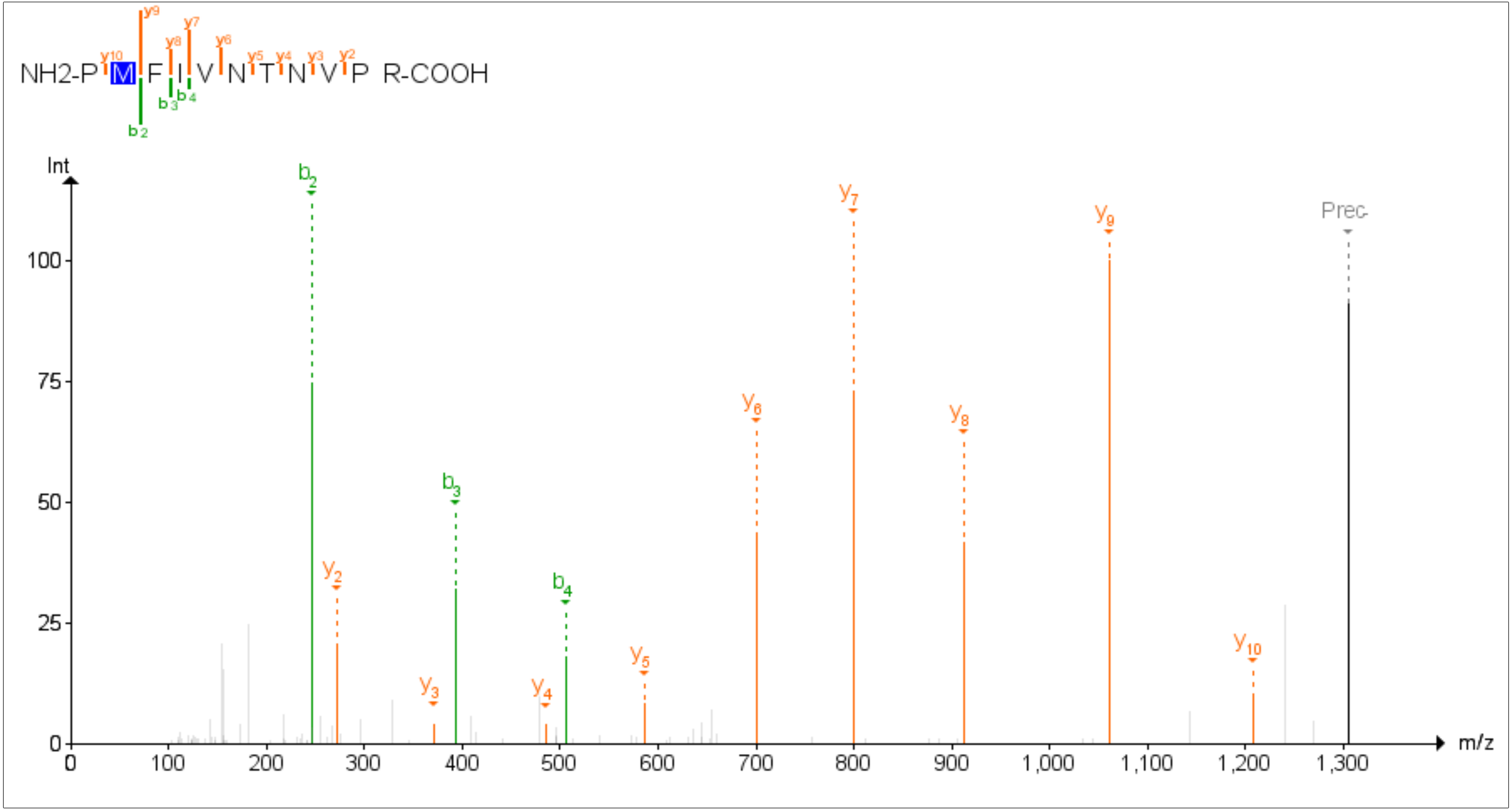
NH2-P M F I V N T N V P R-COOH
y10 y9 y8 y7 y6 y5 y4 y3 y2
b2 b3 b4
Int
m/z
Prec.
b2 b3 b4 y2 y3 y4 y5 y6 y7 y8 y9 y10
0 25 50 75 100
0 100 200 300 400 500 600 700 800 900 1,000 1,100 1,200 1,300

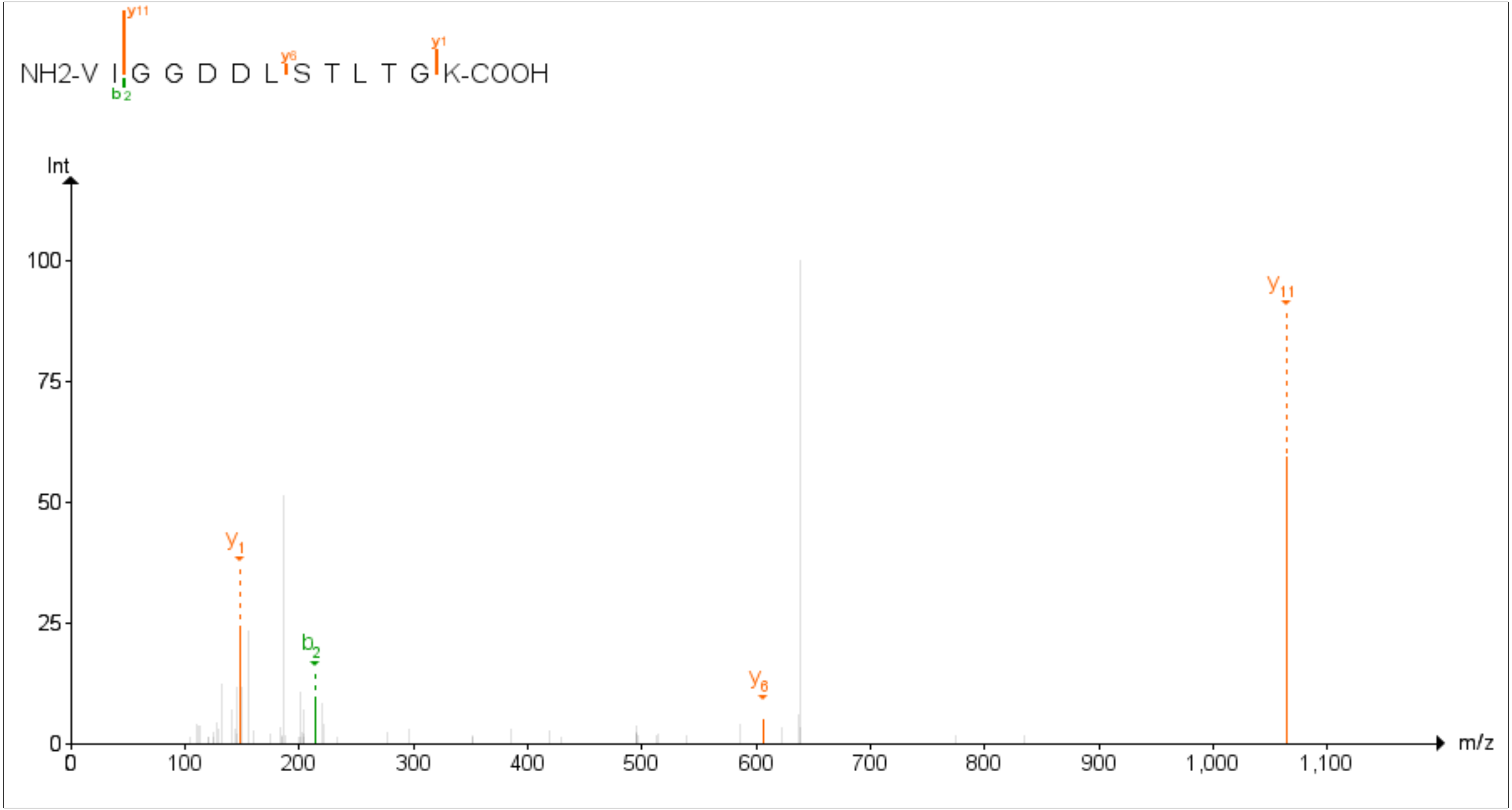

NH2-V I G G D D L S T L T G K-COOH
y11
y6
y1
b2
Int
100
75
50
25
0
m/z
0
100
200
300
400
500
600
700
800
900
1,000
1,100
y11
y1
b2
y6

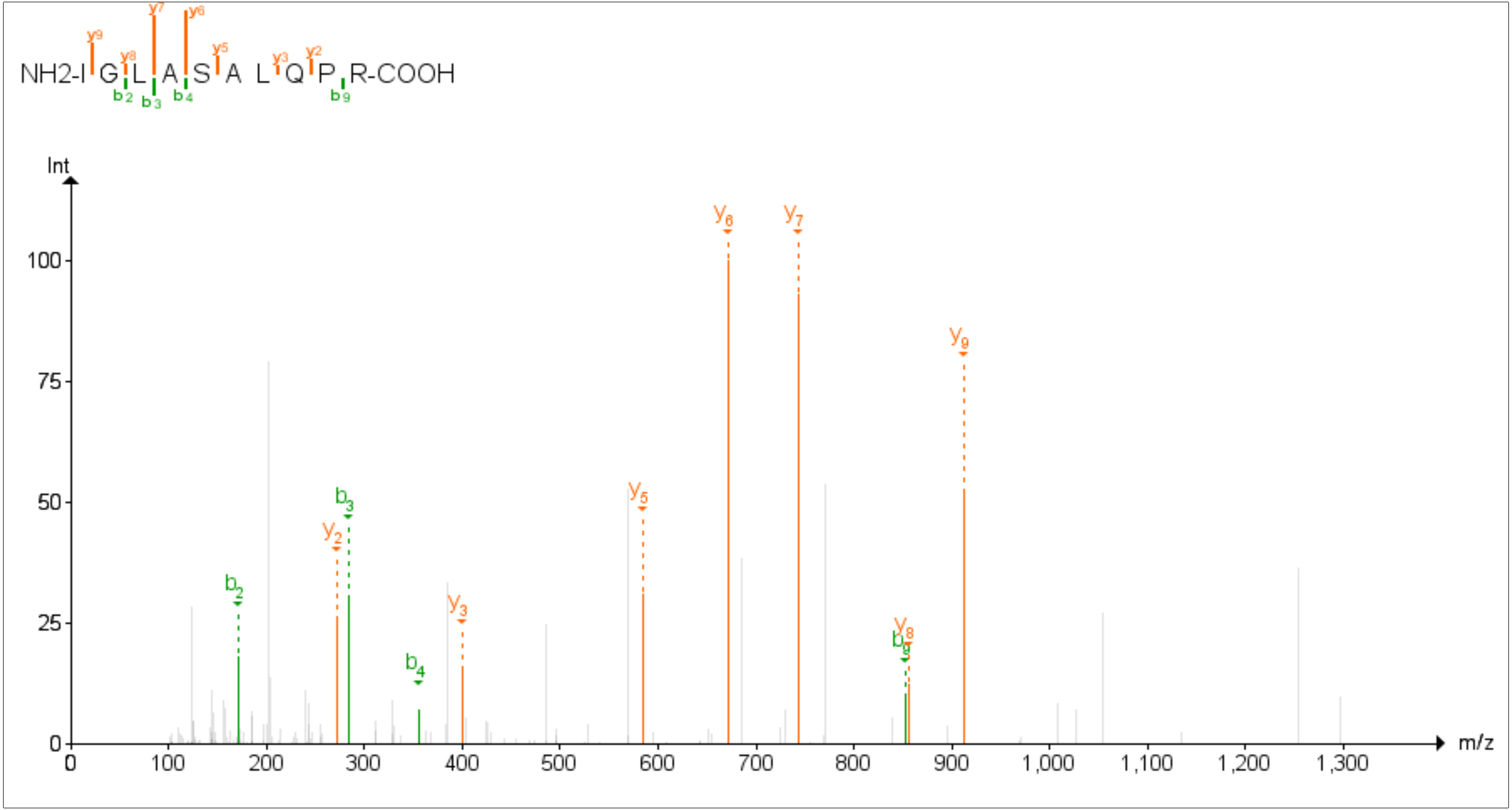

NH2-I G L A S A L Q P R-COOH
y9
y8
y7
y6
y5
y3
y2
b2
b3
b4
b9
Int
100
75
50
25
0
m/z
0
100
200
300
400
500
600
700
800
900
1,000
1,100
1,200
1,300
b2
y2
b3
b4
y3
y5
y6
y7
y8
b9
y9

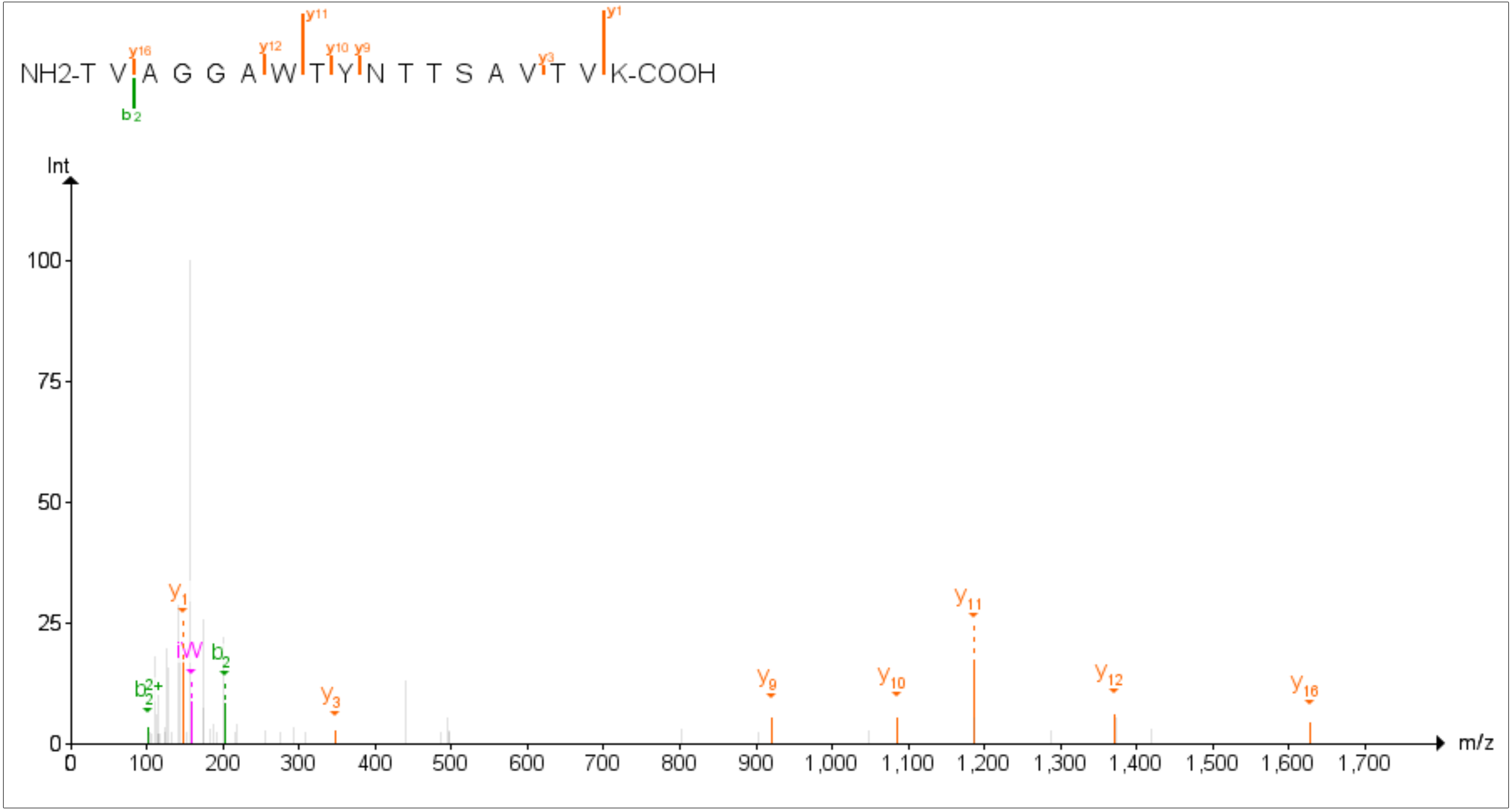

NH2-T V A G G A W T Y N T T S A V T V K-COOH
y16
y12
y11
y10
y9
y3
y1
b2
Int
m/z
y1
iW
b2
b2 2+
y3
y9
y10
y11
y12
y16

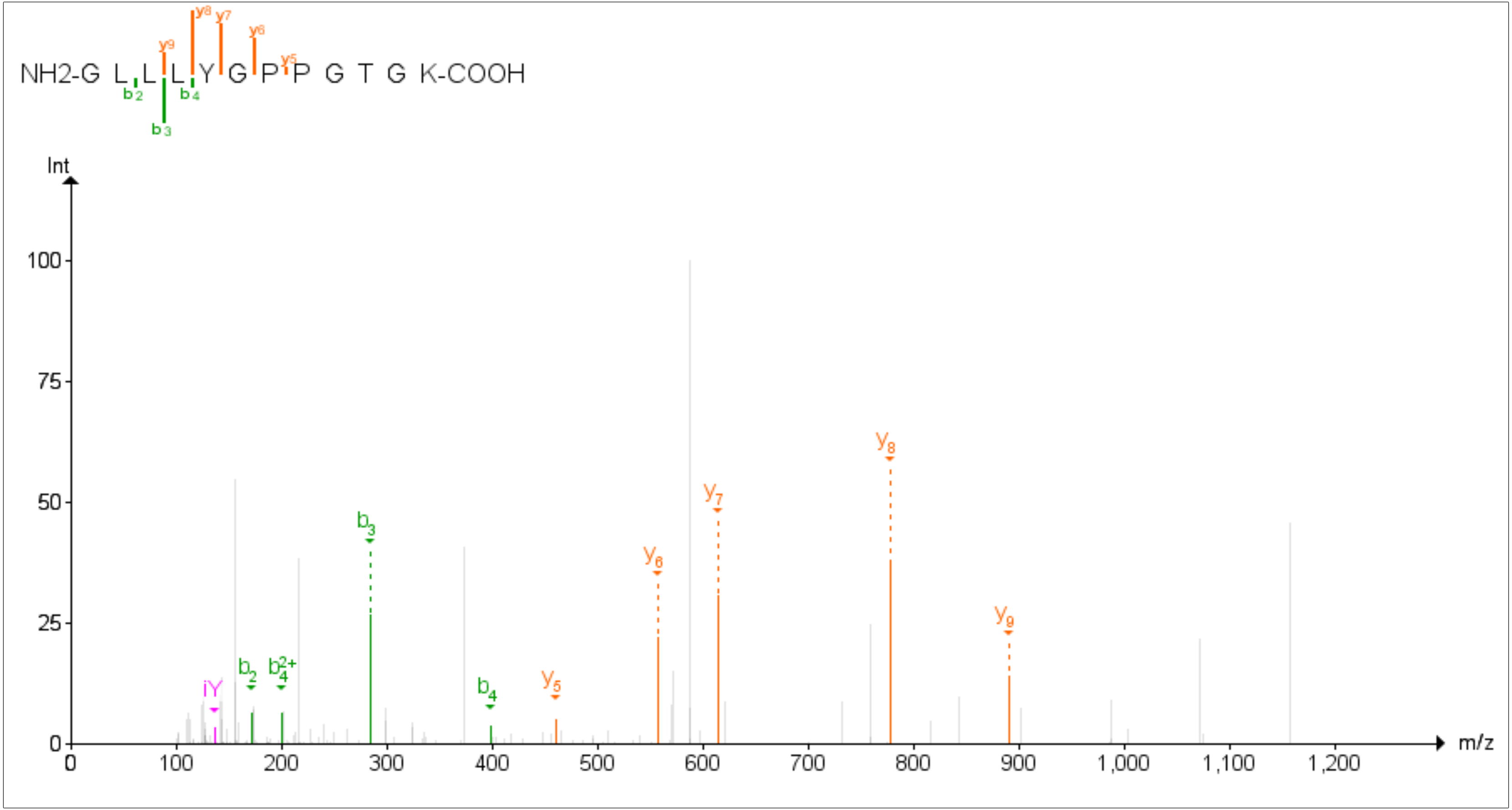

NH2-G L L L Y G P P G T G K-COOH
y9 y8 y7 y6 y5
b2 b3 b4
Int
100
75
50
25
0
iY
b2
b4 2+
b3
b4
y5
y6
y7
y8
y9
0 100 200 300 400 500 600 700 800 900 1,000 1,100 1,200
m/z

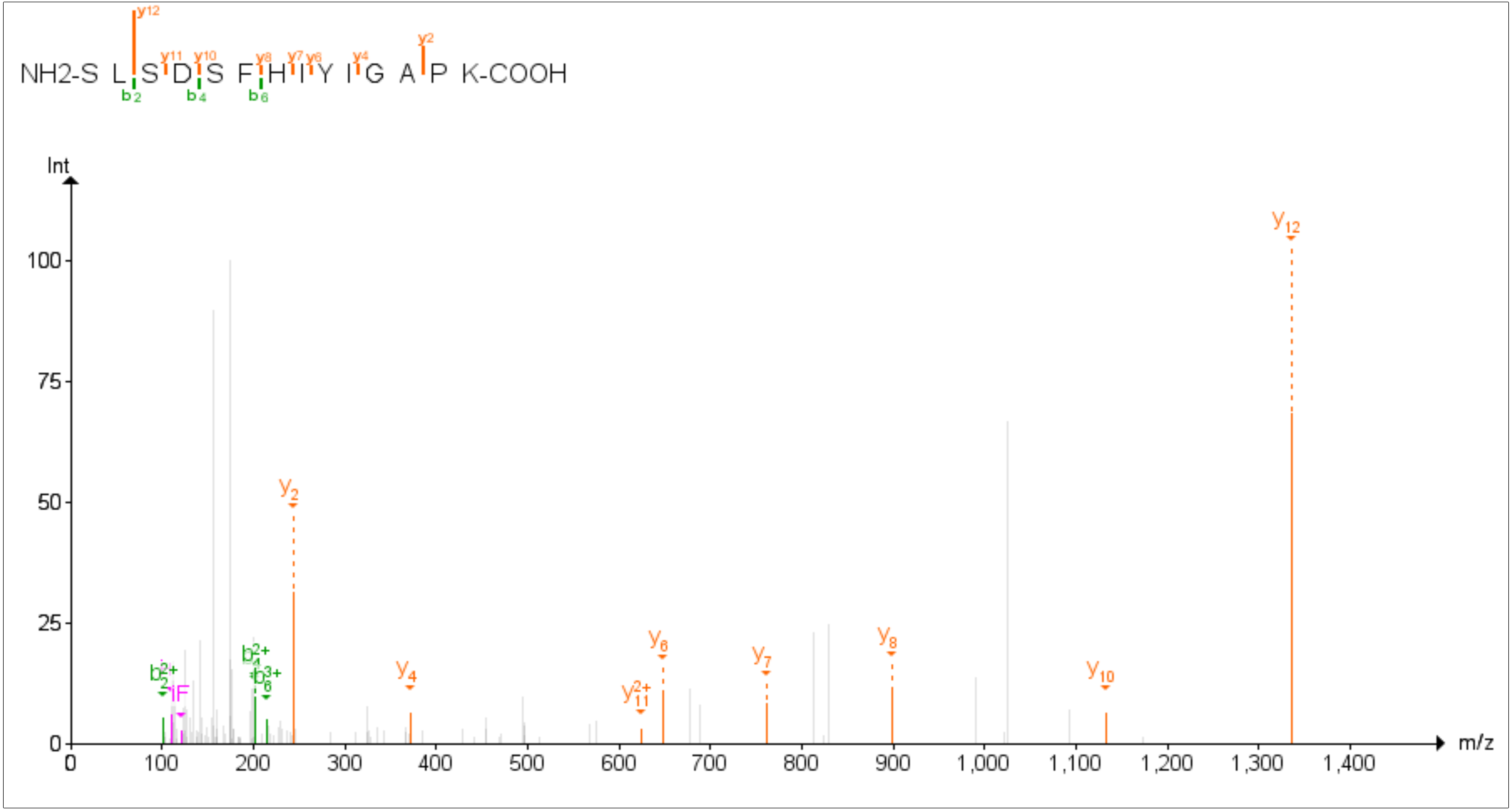

NH2-S L S D S F H I Y I G A P K-COOH
Int
m/z
y12
y2
y4
y6
y7
y8
y10
y11
b2
b4
b6

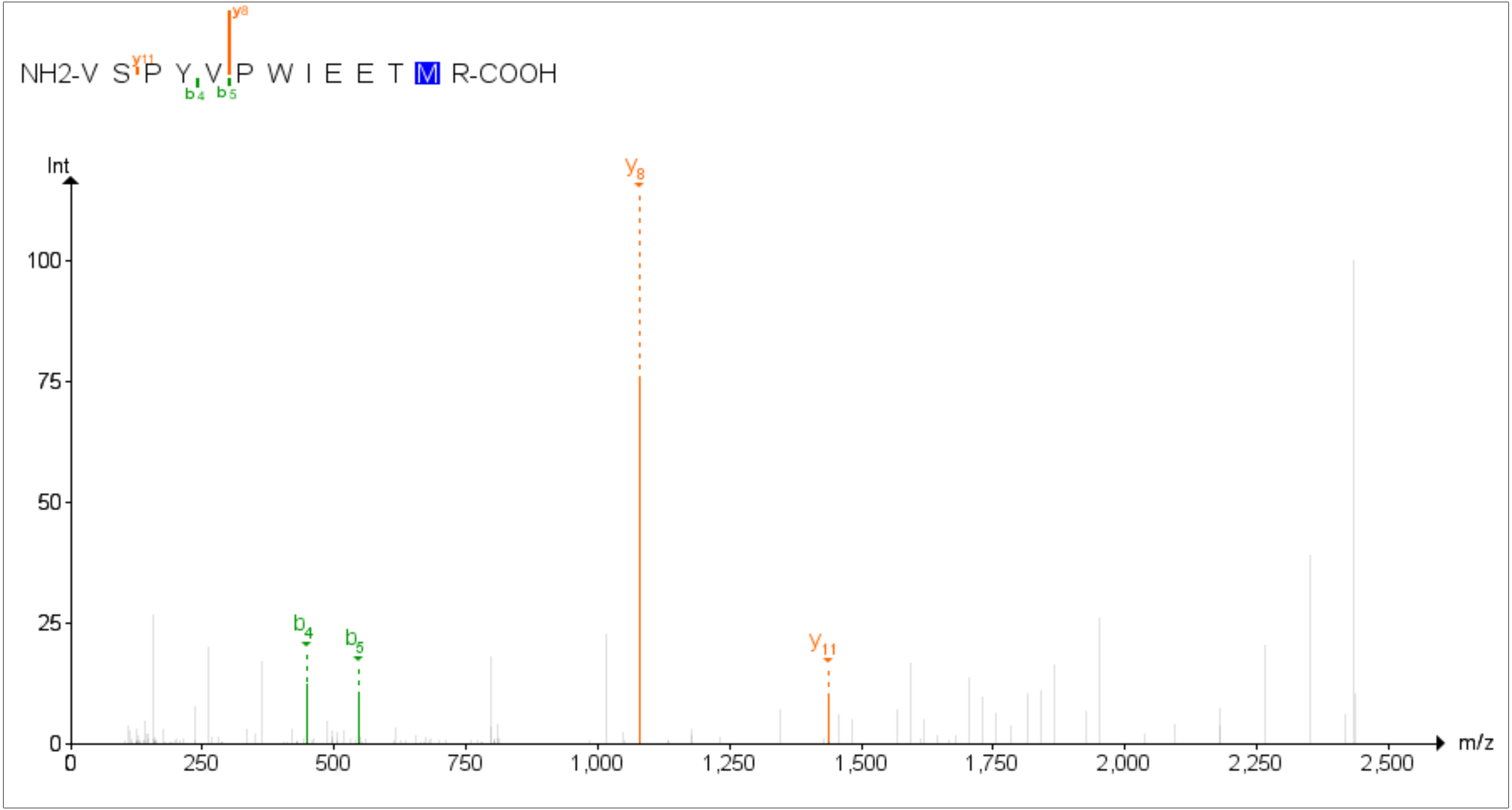

NH2-V S P Y V P W I E E T M R-COOH
y11
y8
b4
b5
Int
100
75
50
25
0
y8
b4
b5
y11
0
250
500
750
1,000
1,250
1,500
1,750
2,000
2,250
2,500
m/z

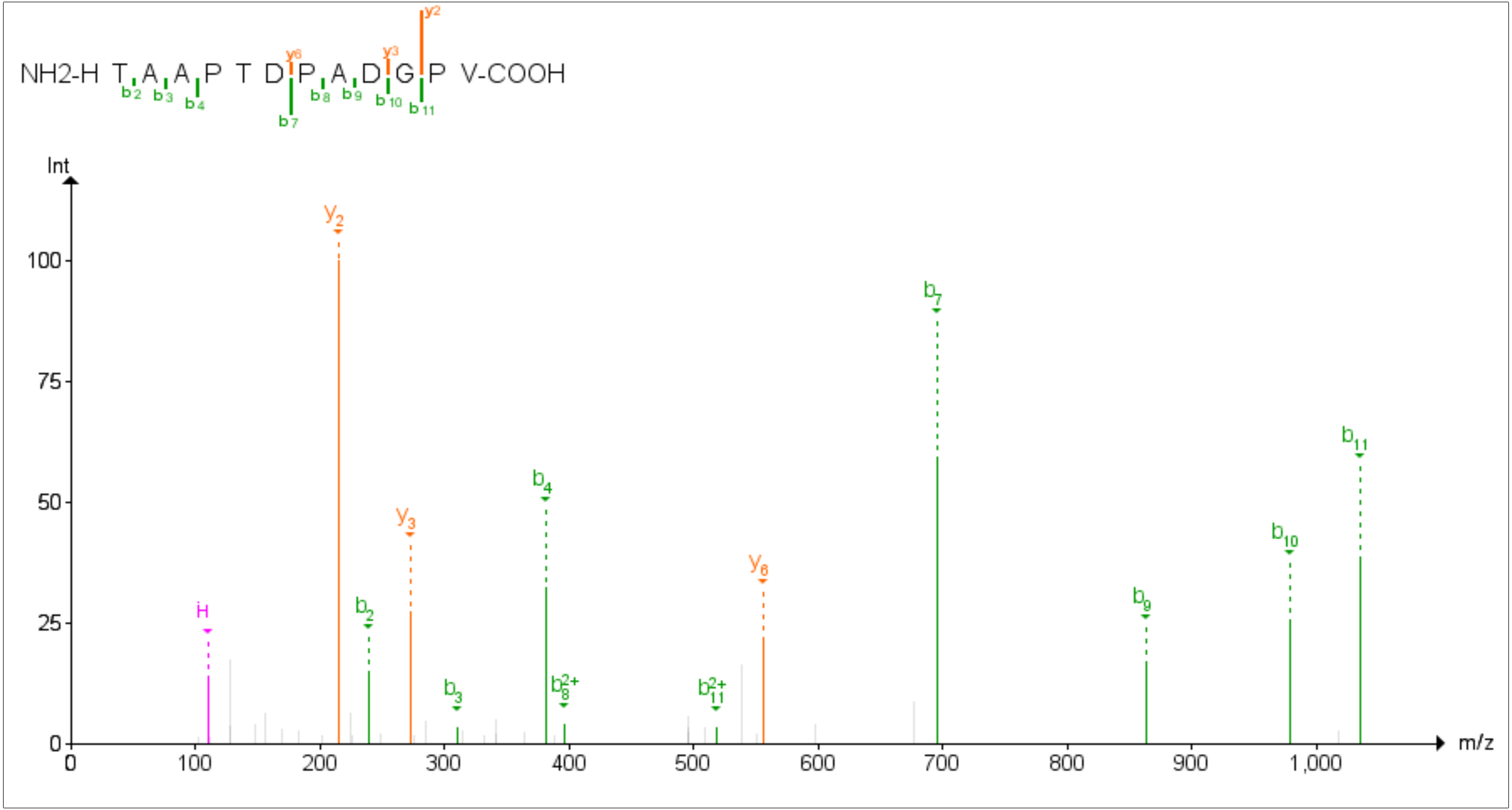

NH2-H T A A P T D P A D G P V-COOH
y6 y3 y2
b2 b3 b4 b7 b8 b9 b10 b11
Int
100
75
50
25
0
y2
b7
b11
b4
y3
b10
y6
b9
H
b2
b3
b8 2+
b11 2+
0 100 200 300 400 500 600 700 800 900 1,000
m/z

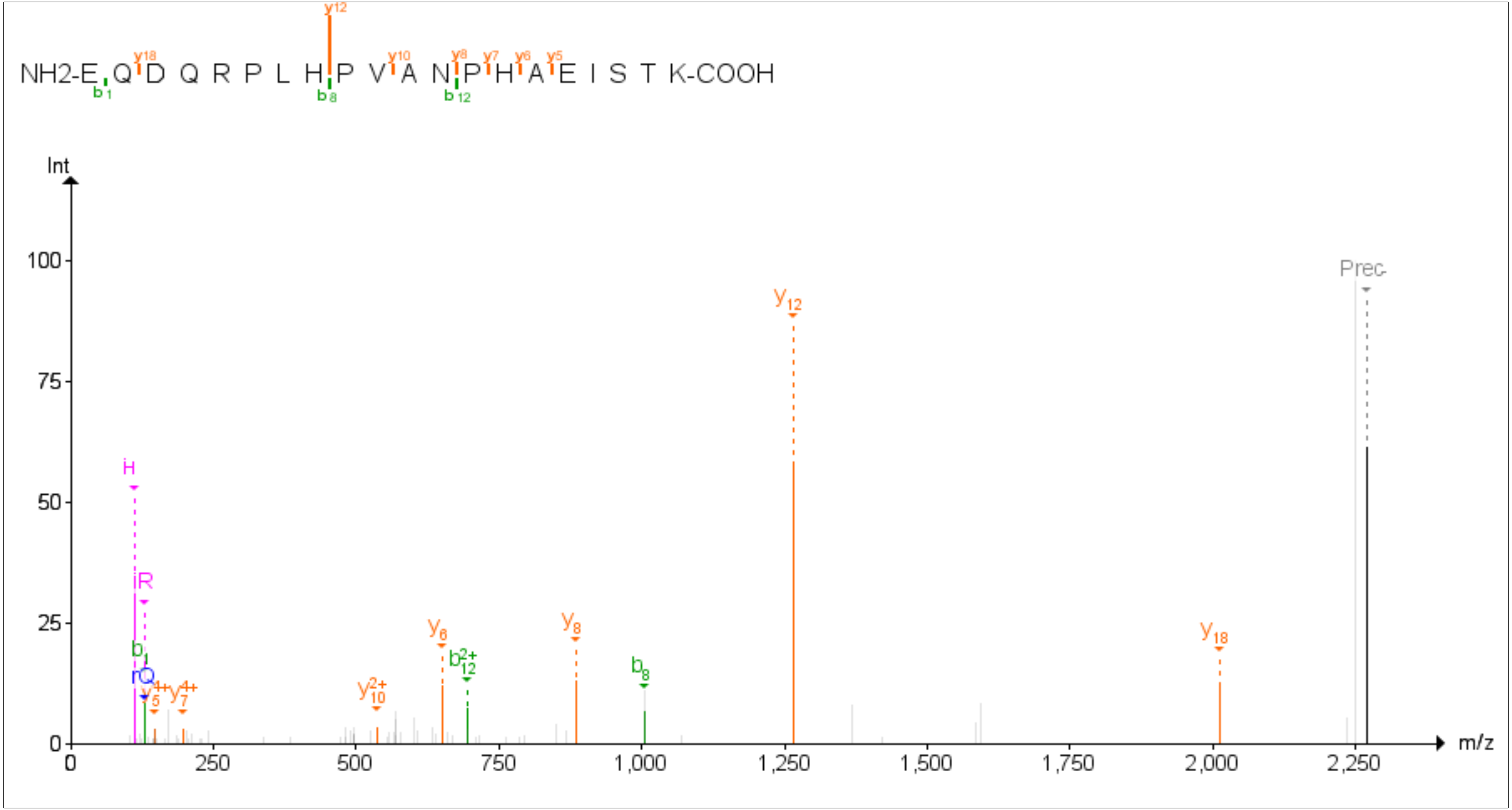

NH2-E Q D Q R P L H P V A N P H A E I S T K-COOH
b1
b8
b12
y18
y12
y10
y8
y7
y6
y5
Int
100
75
50
25
0
m/z
0
250
500
750
1,000
1,250
1,500
1,750
2,000
2,250
Prec
iH
iR
b1
nQ
y_5^{4+}
y_7^{4+}
y_{10}^{2+}
b_{12}^{2+}
b8

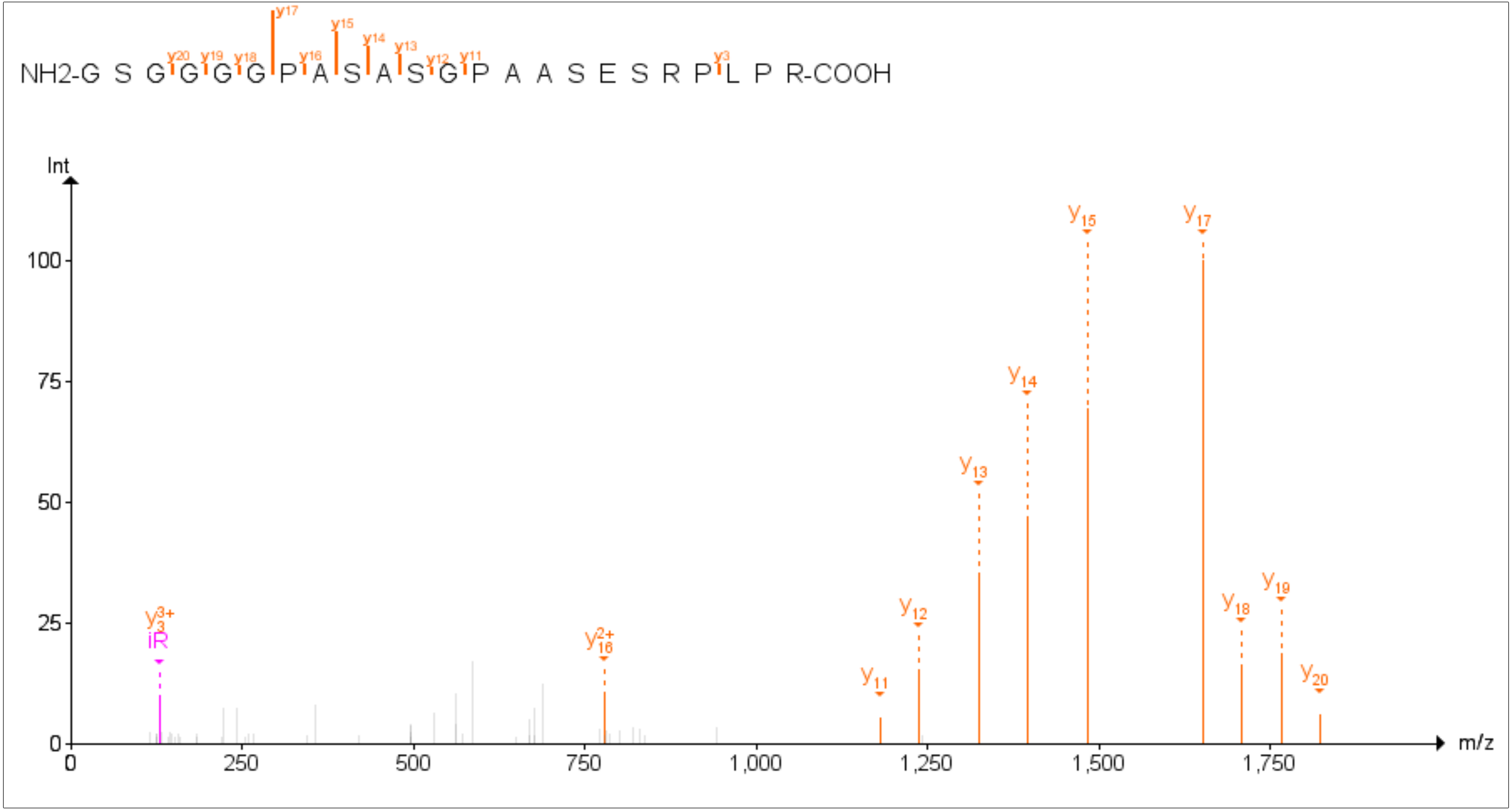

NH2-G S G G G G P A S A S G P A A S E S R P L P R-COOH
y20 y19 y18 y17 y16 y15 y14 y13 y12 y11 y3
Int
100
75
50
25
0
m/z
0
250
500
750
1,000
1,250
1,500
1,750
y15 y17 y14 y13 y19 y18 y12 y20 y11
iR

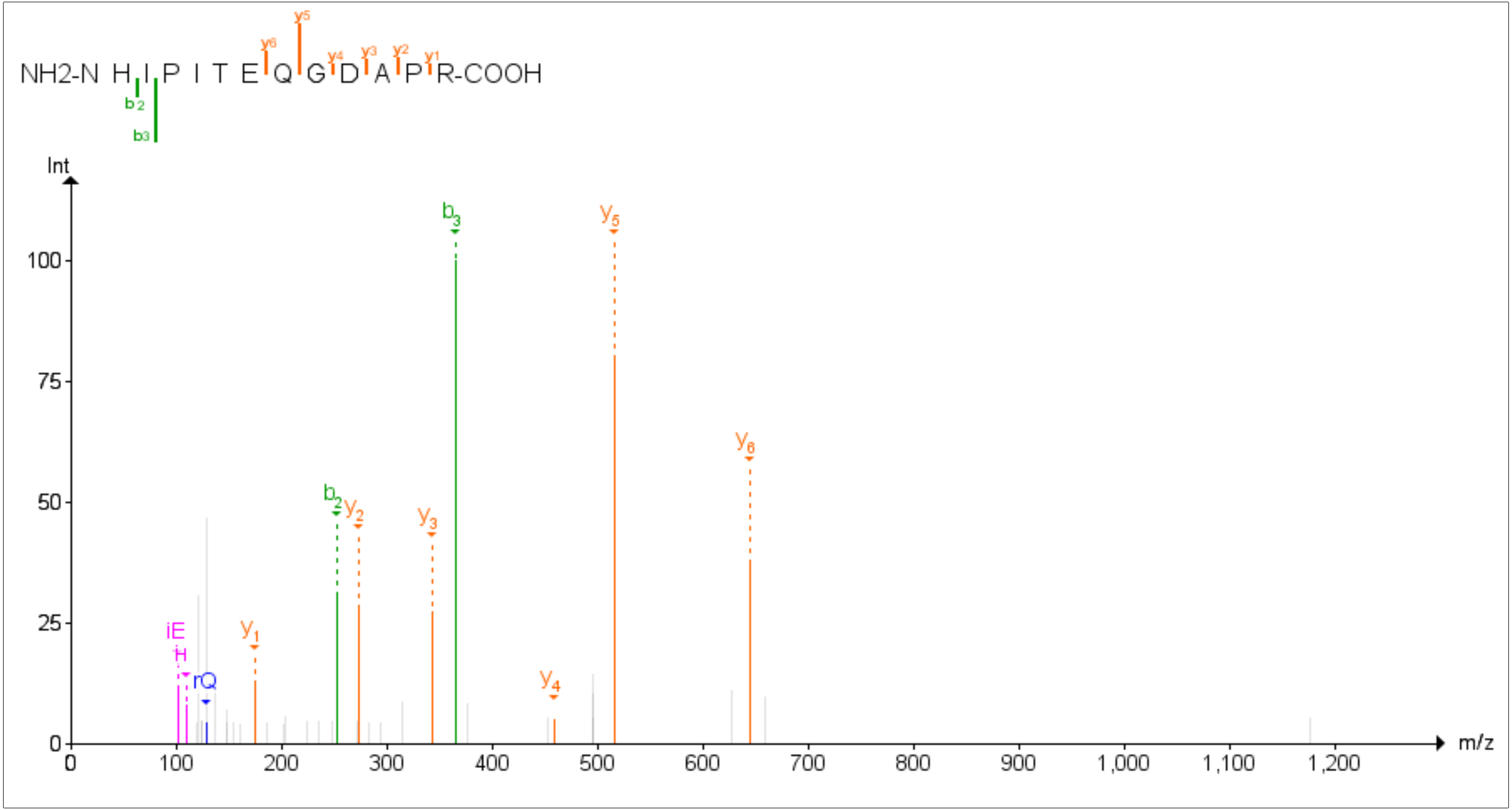

NH2-N H I P I T E Q G D A P R-COOH
b2
b3
y6
y5
y4
y3
y2
y1
Int
100
75
50
25
0
m/z
0
100
200
300
400
500
600
700
800
900
1,000
1,100
1,200
iE
H
rQ
y1
b2
y2
y3
b3
y4
y5
y6

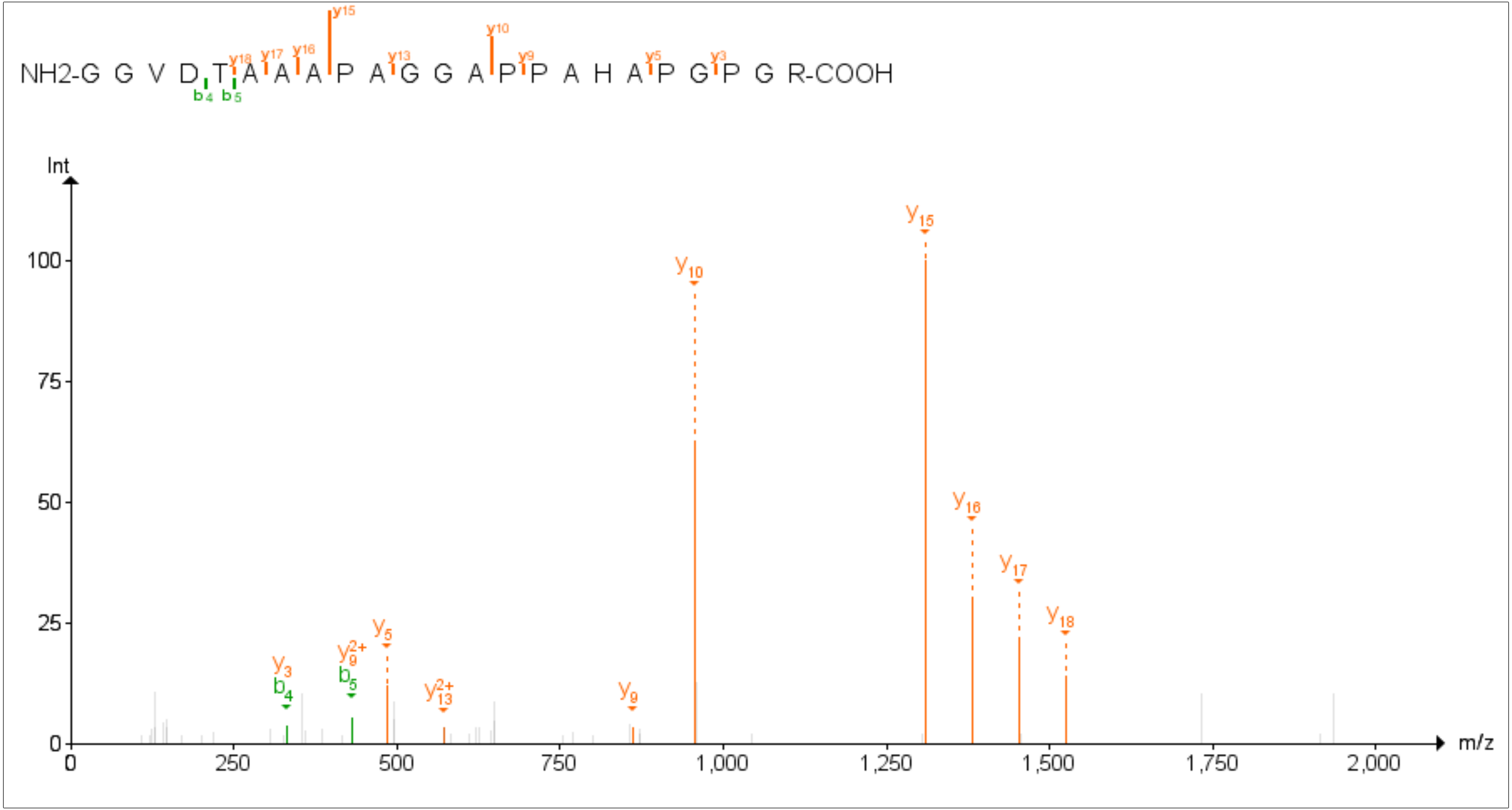

NH2-G G V D T A A A P A G G A P P A H A P G P G R-COOH
y15
y10
y18 y17 y16 y13 y9 y5 y3
b4 b5
Int
100
75
50
25
0
y15
y10
y16
y17
y18
y5
y3
b4
y9 2+
b5
y13 2+
y9
m/z
0
250
500
750
1,000
1,250
1,500
1,750
2,000

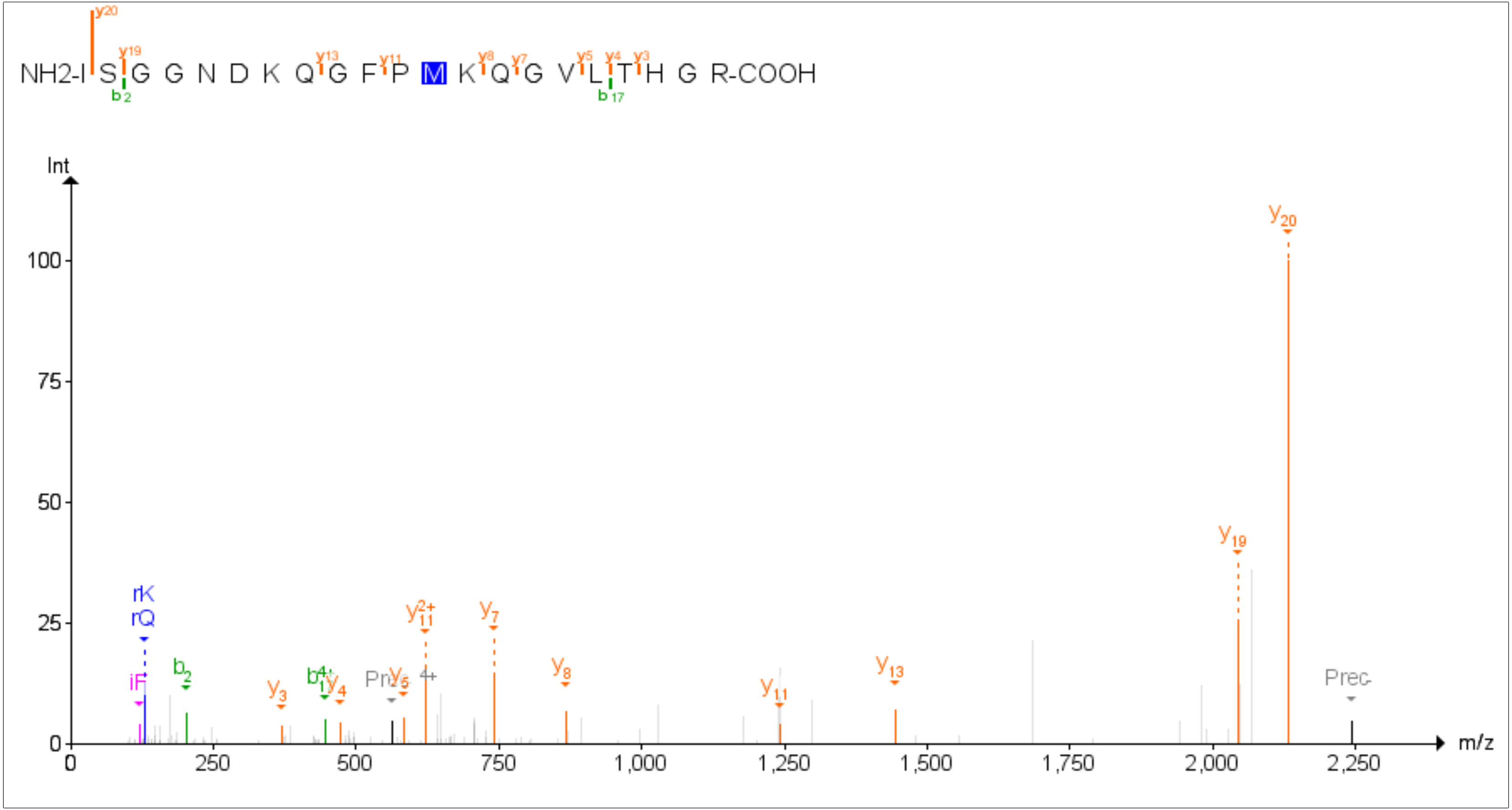

NH2-I S G G N D K Q G F P M K Q G V L T H G R-COOH
Int
m/z
0
25
50
75
100
250
500
750
1,000
1,250
1,500
1,750
2,000
2,250
y20
y19
y13
y11
y8
y7
y5
y4
y3
b2
b17
rK
rQ
iF
Prec

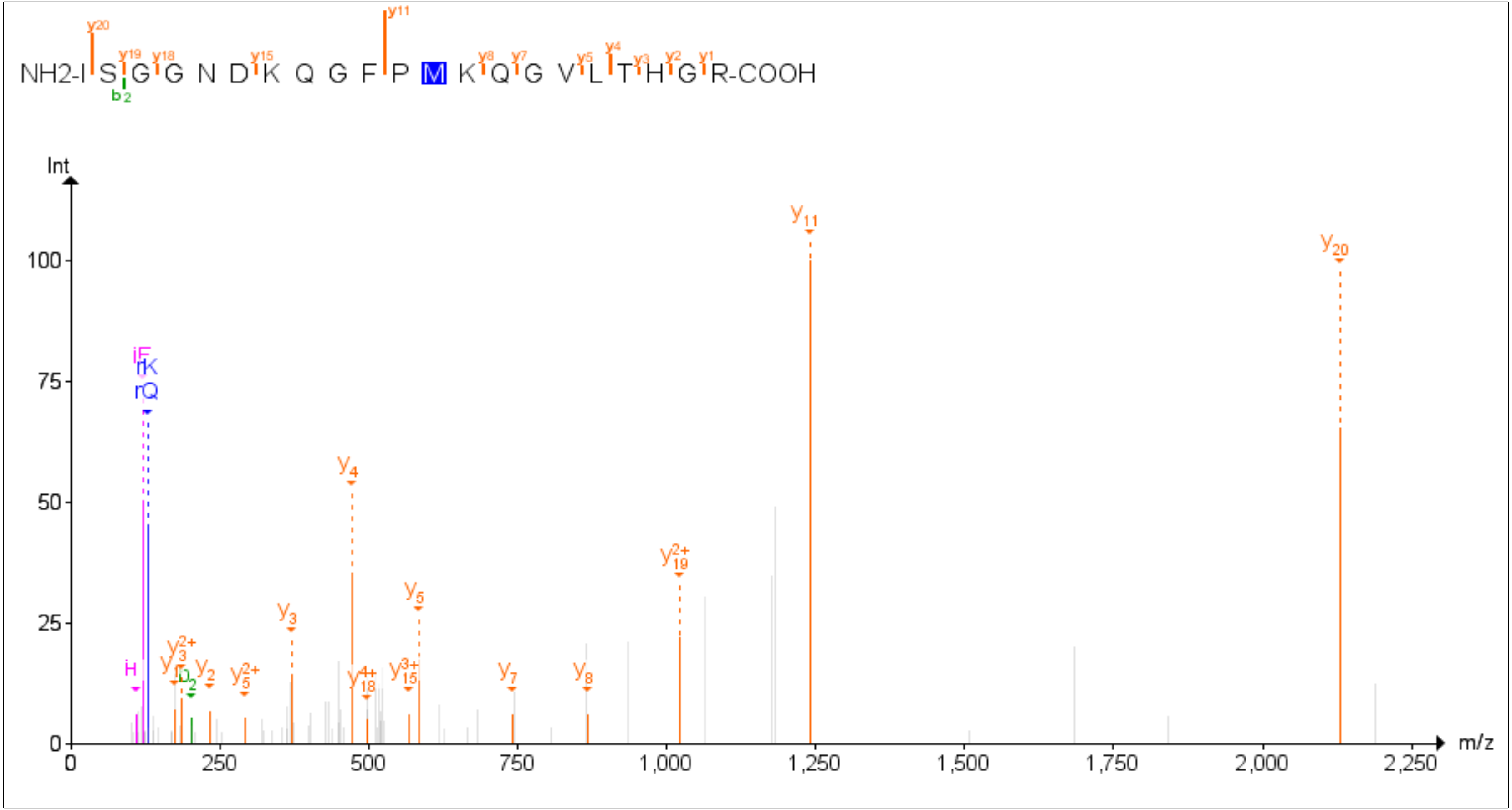

NH2-I S G G N D K Q G F P M K Q G V L T H G R-COOH
Int
m/z

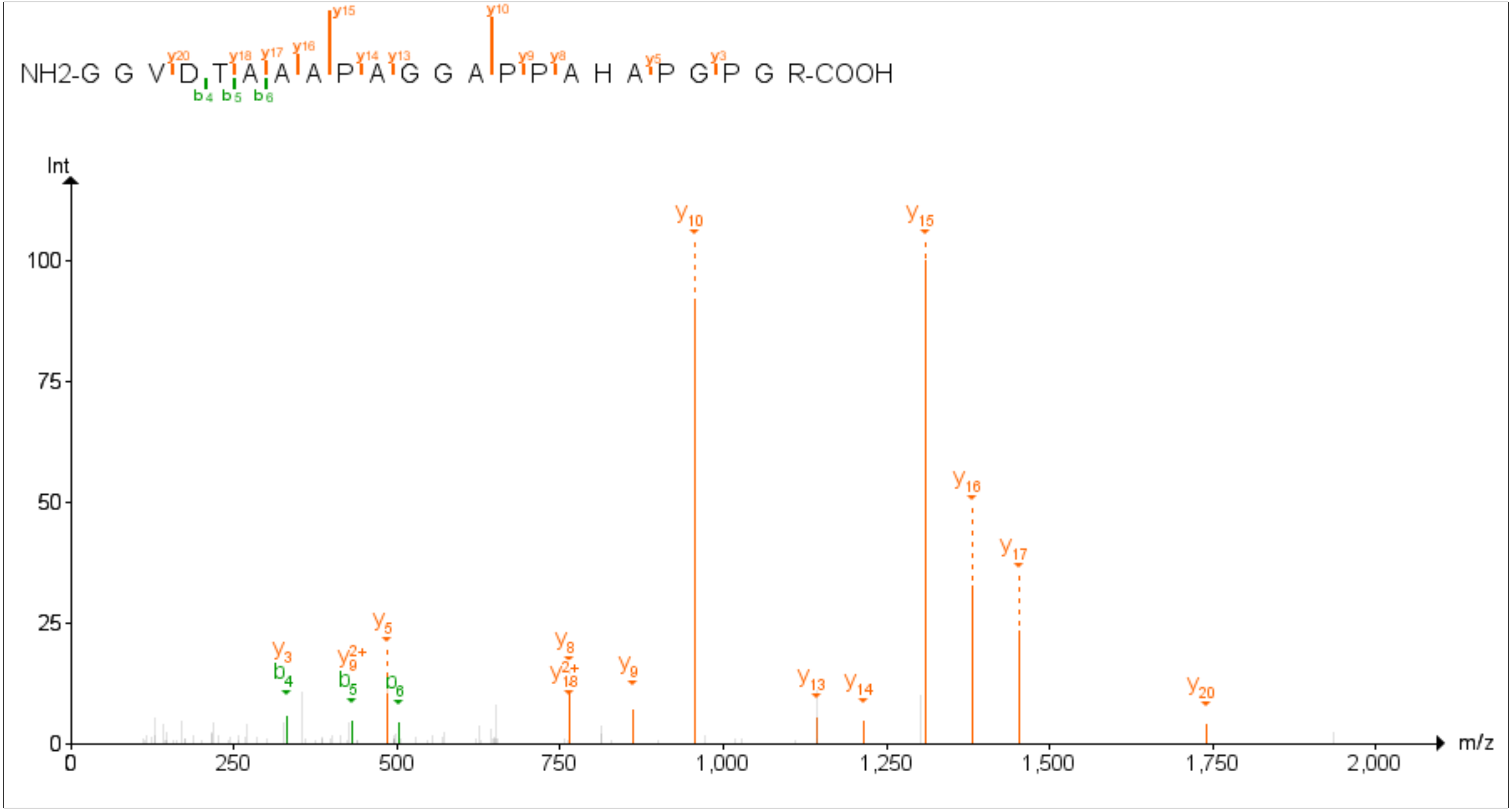

NH2-G G V D T A A A P A G G A P P A H A P G P G R-COOH
y20 y18 y17 y16 y15 y14 y13 y10 y9 y8 y5 y3
b4 b5 b6
Int
100
75
50
25
0
y10
y15
y16
y17
y5
y3
b4
y9 2+
b5
b6
y8
y18 2+
y9
y13
y14
y20
0
250
500
750
1,000
1,250
1,500
1,750
2,000
m/z

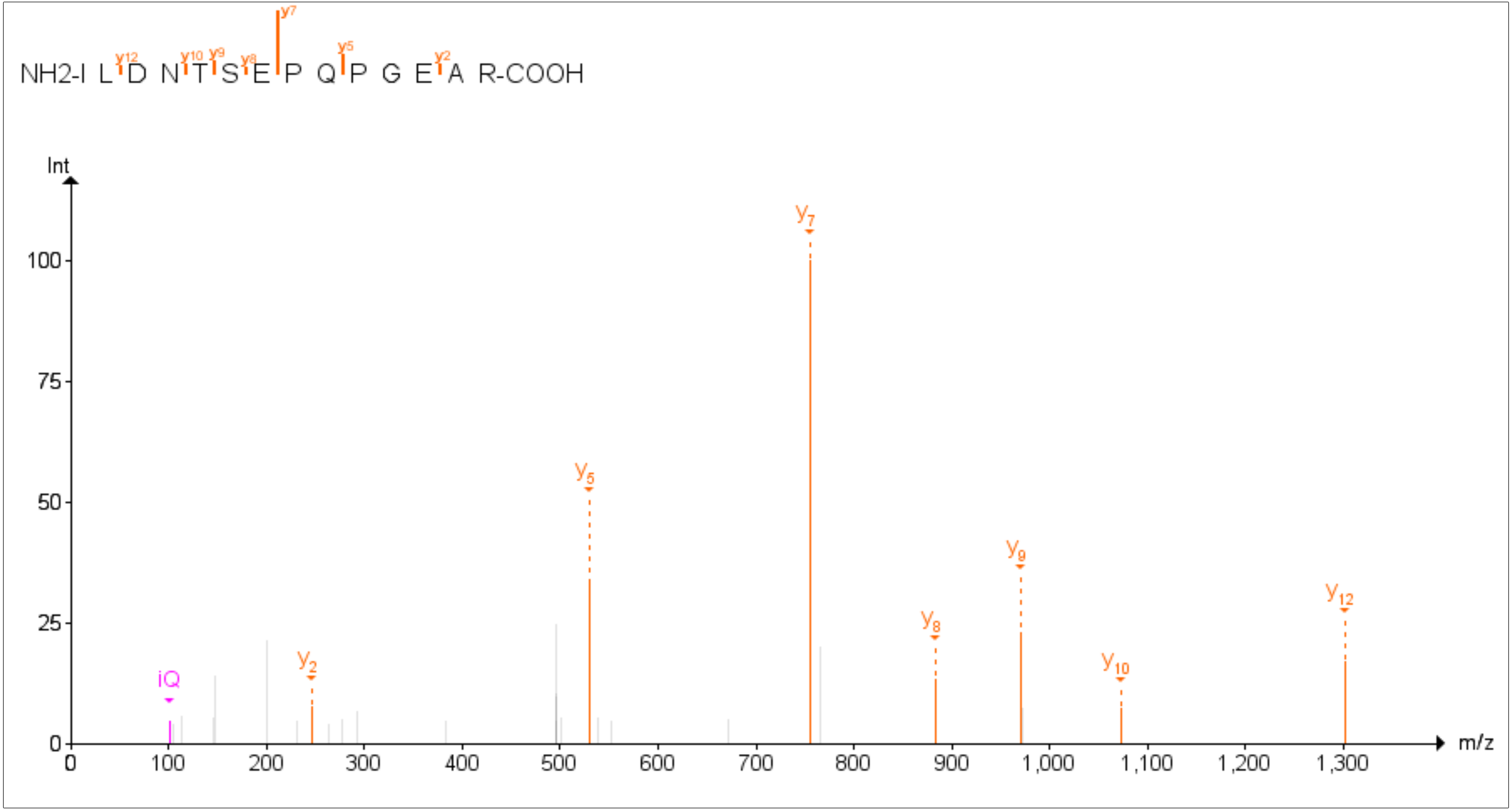

NH2-I L D N T S E P Q P G E A R-COOH
y12
y10
y9
y8
y7
y5
y2
Int
100
75
50
25
0
iQ
y2
y5
y7
y8
y9
y10
y12
0
100
200
300
400
500
600
700
800
900
1,000
1,100
1,200
1,300
m/z

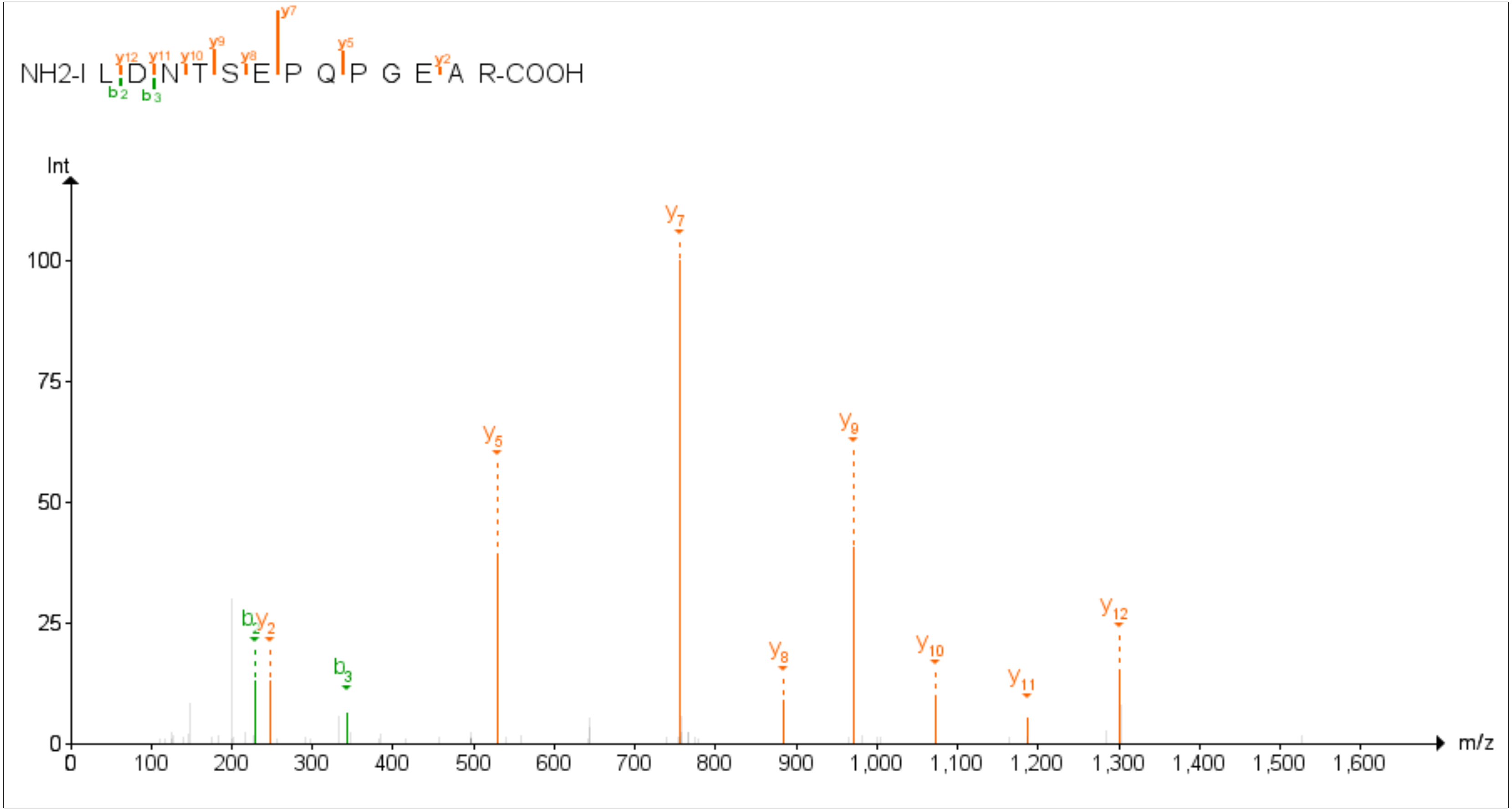
NH2-I L D N T S E P Q P G E A R-COOH
y12 y11 y10 y9 y8 y7 y5 y2
b2 b3
Int
100
75
50
25
0
y_7
y_5
y_9
b_2 y_2
b_3
y_8
y_{10}
y_{11}
y_{12}
0 100 200 300 400 500 600 700 800 900 1,000 1,100 1,200 1,300 1,400 1,500 1,600
m/z

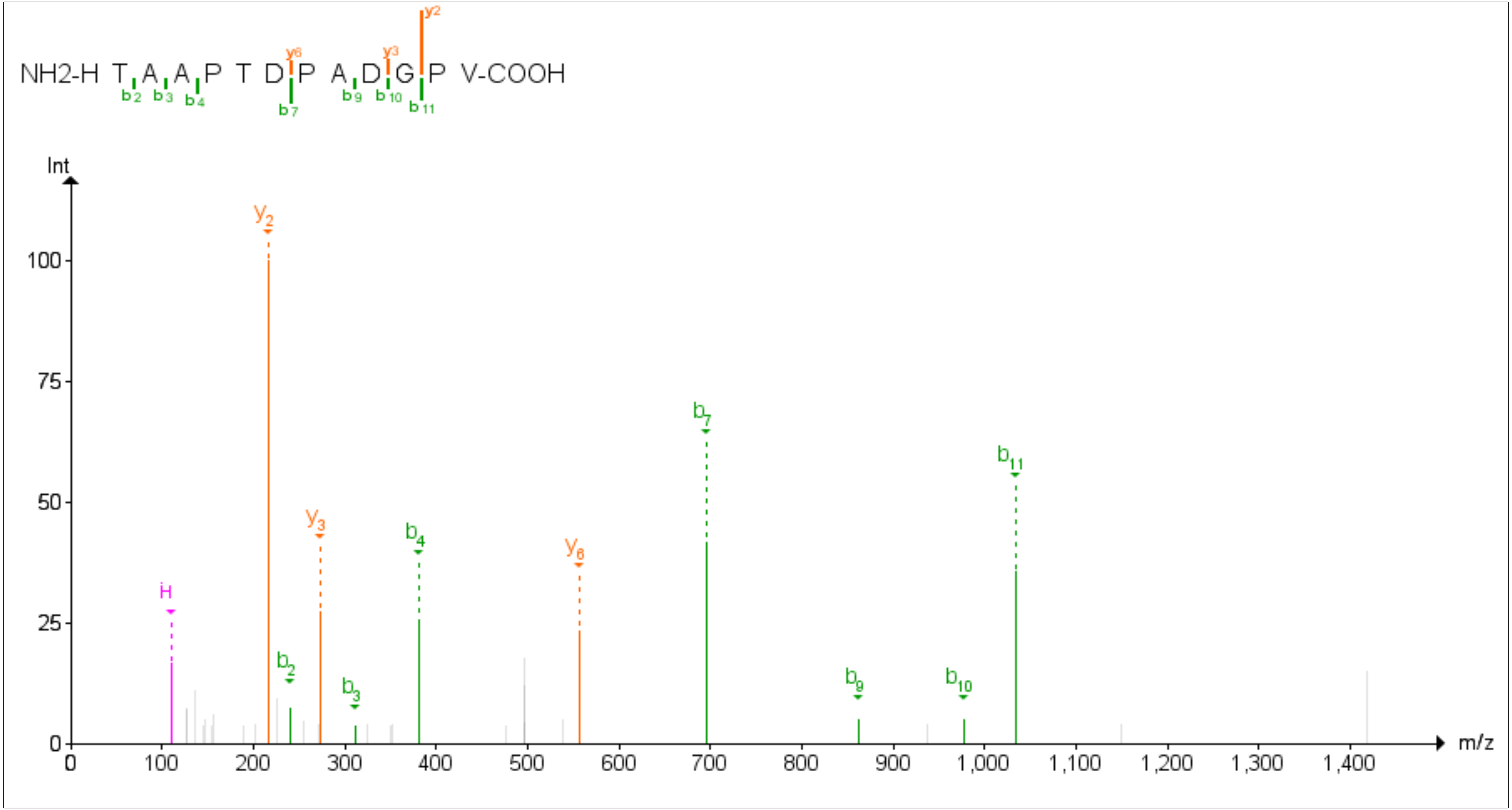

NH2-H T A A P T D P A D G P V-COOH
y2
y6
y3
b2
b3
b4
b7
b9
b10
b11
Int
100
75
50
25
0
y2
y3
b4
y6
b7
b11
b2
b3
b9
b10
H
m/z
0
100
200
300
400
500
600
700
800
900
1,000
1,100
1,200
1,300
1,400

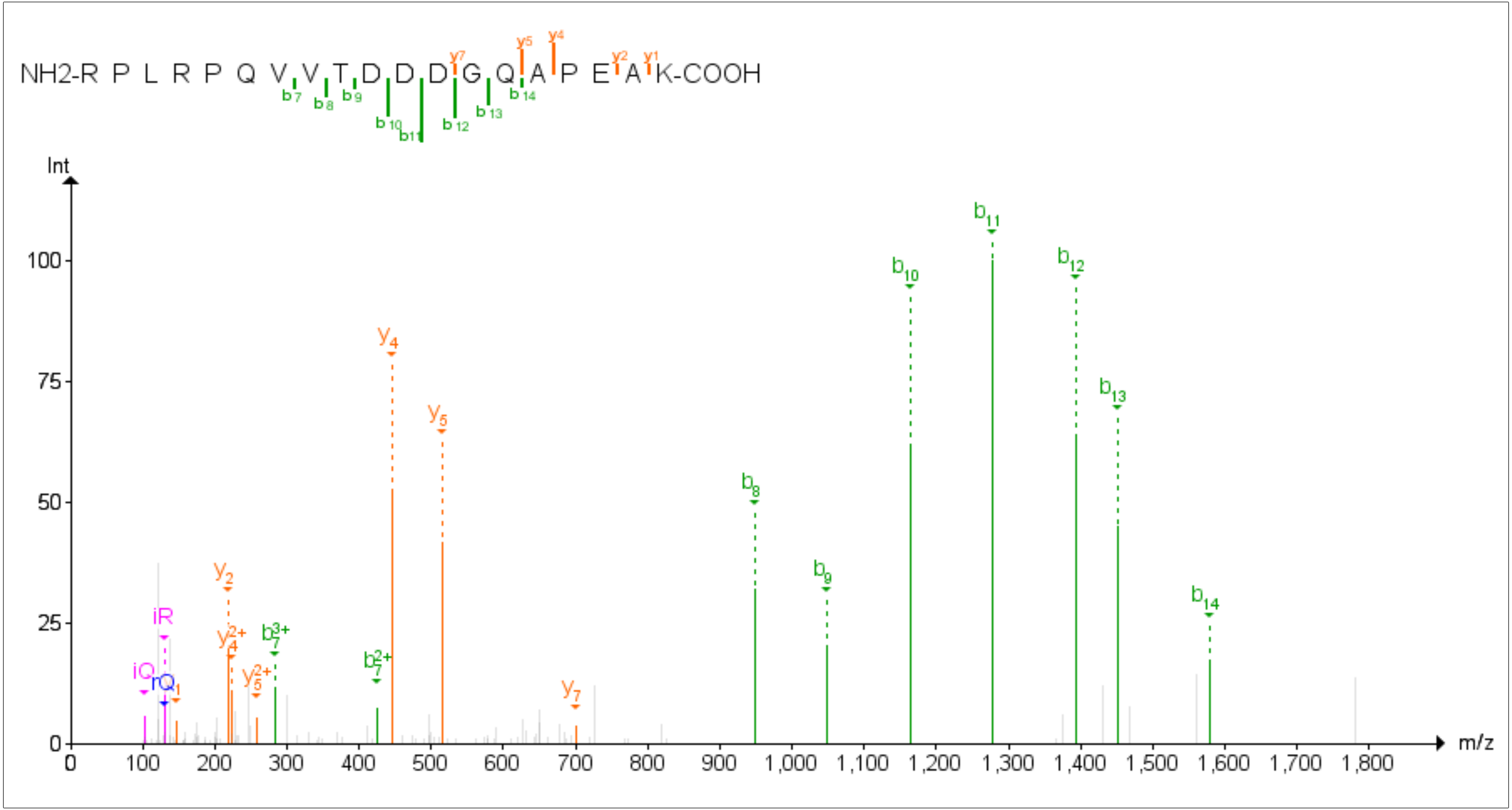

NH2-R P L R P Q V V T D D D G Q A P E A K-COOH
Int
m/z
b11
b10
b12
b13
y4
y5
b8
b9
b14
y2
iR
iQ
rQ
y7
0
25
50
75
100
0
100
200
300
400
500
600
700
800
900
1,000
1,100
1,200
1,300
1,400
1,500
1,600
1,700
1,800

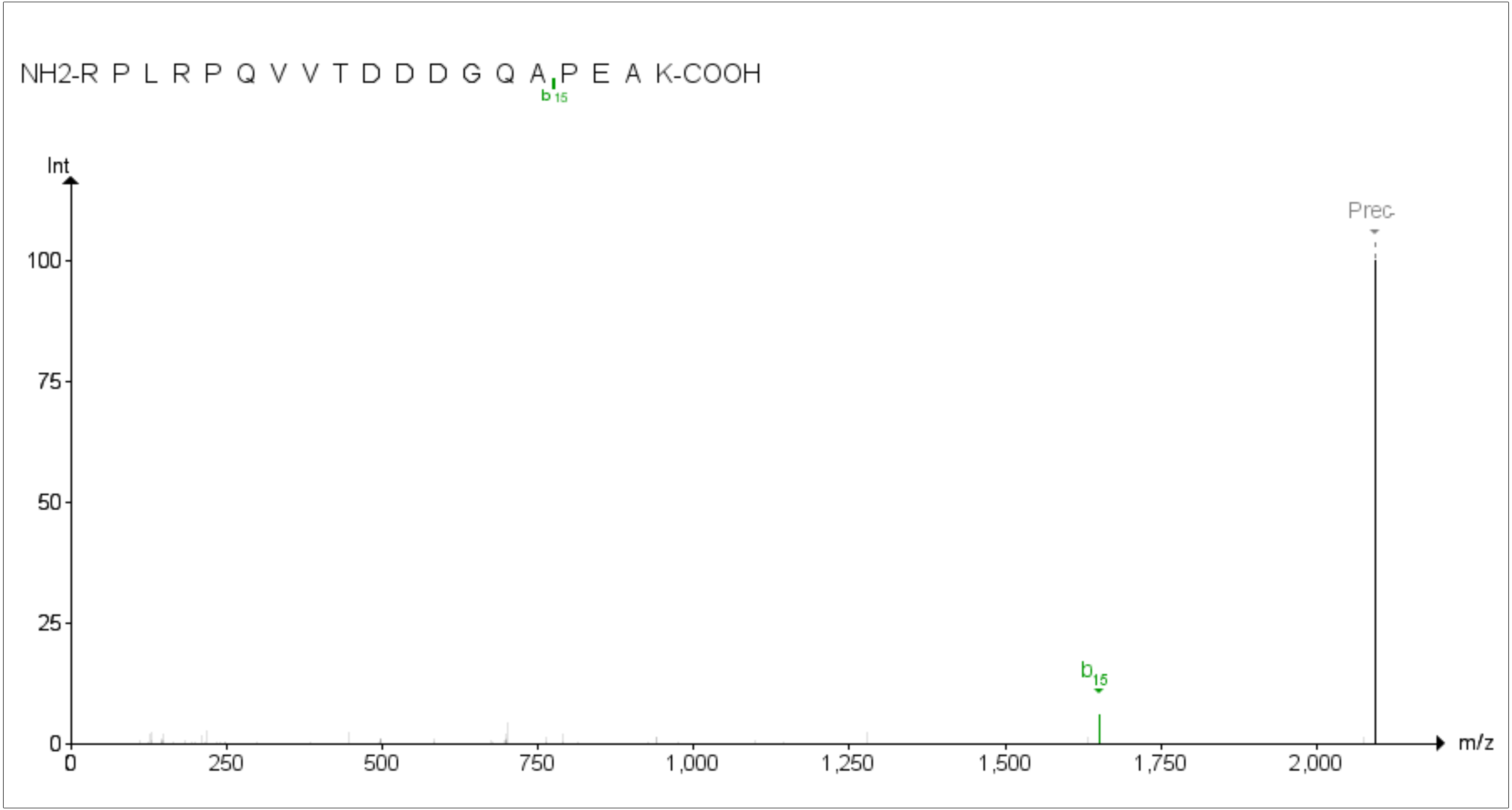

NH2-R P L R P Q V V T D D D G Q A P E A K-COOH
b15
Int
100
75
50
25
0
Prec
b15
0
250
500
750
1,000
1,250
1,500
1,750
2,000
m/z

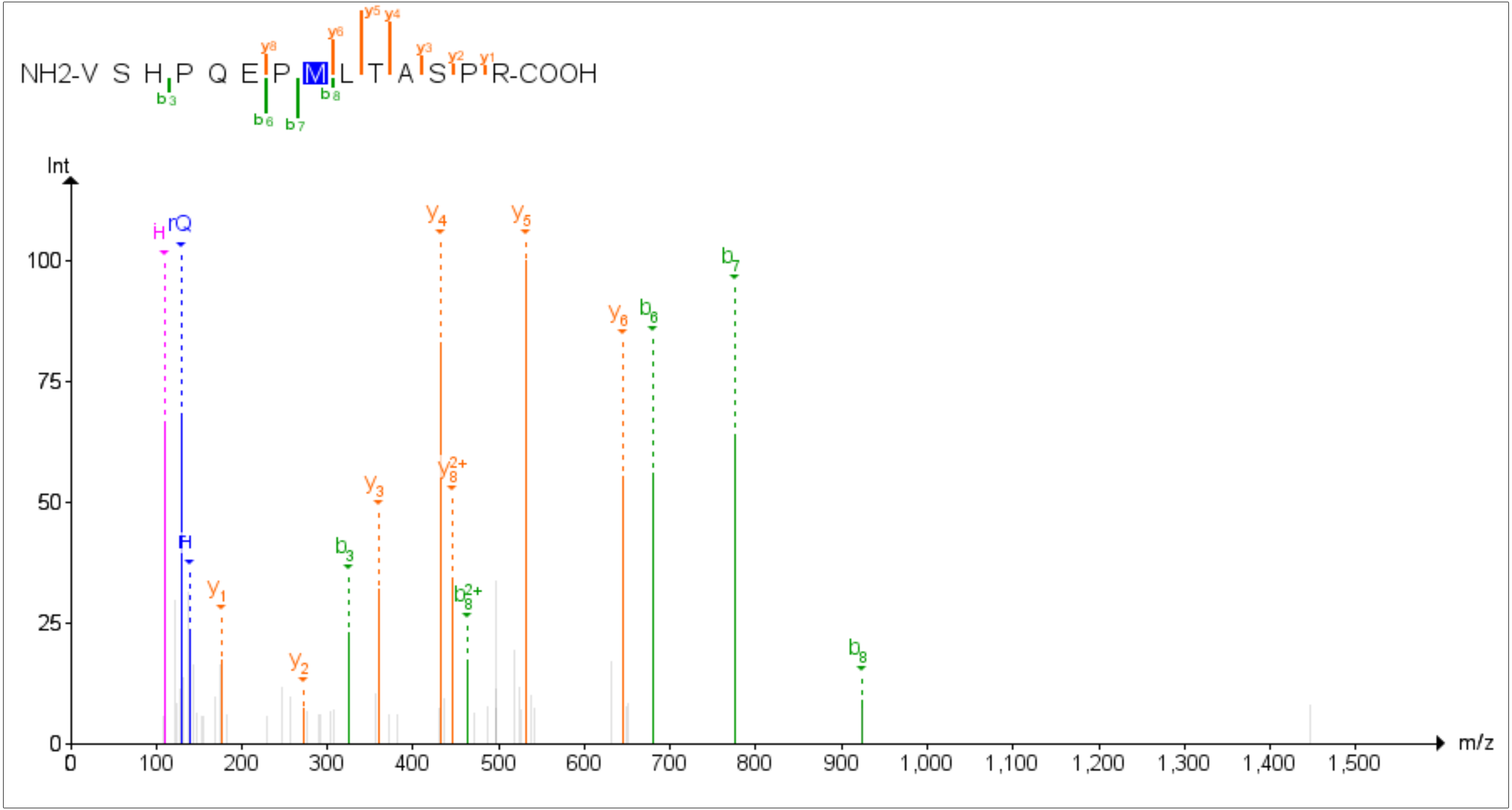

NH2-V S H P Q E P M L T A S P R-COOH
b3 b6 b7 b8
y8 y6 y5 y4 y3 y2 y1
Int
m/z
iH rQ H
y1 y2 y3 y4 y5 y6 y8 2+
b3 b6 b7 b8 b8 2+
0 25 50 75 100
0 100 200 300 400 500 600 700 800 900 1,000 1,100 1,200 1,300 1,400 1,500

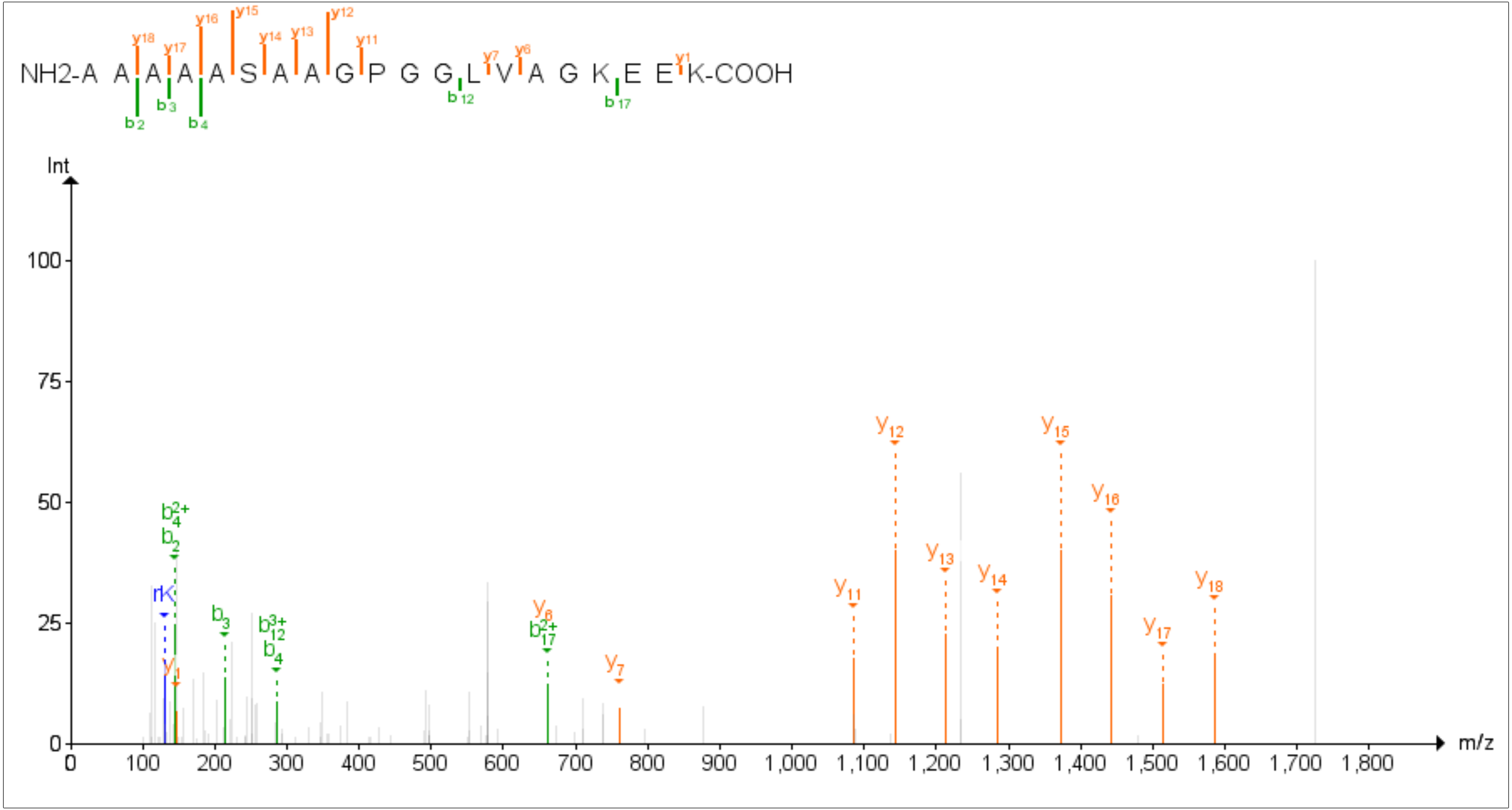

NH2-A A A A A S A A G P G G L V A G K E E K-COOH
y18 y17 y16 y15 y14 y13 y12 y11 y7 y6 y1
b2 b3 b4 b12 b17
Int
100
75
50
25
0
m/z
0 100 200 300 400 500 600 700 800 900 1,000 1,100 1,200 1,300 1,400 1,500 1,600 1,700 1,800
b4 2+
b2
rK
y1
b3
b12 3+
b4
y6
b17 2+
y7
y11
y12
y13
y14
y15
y16
y17
y18

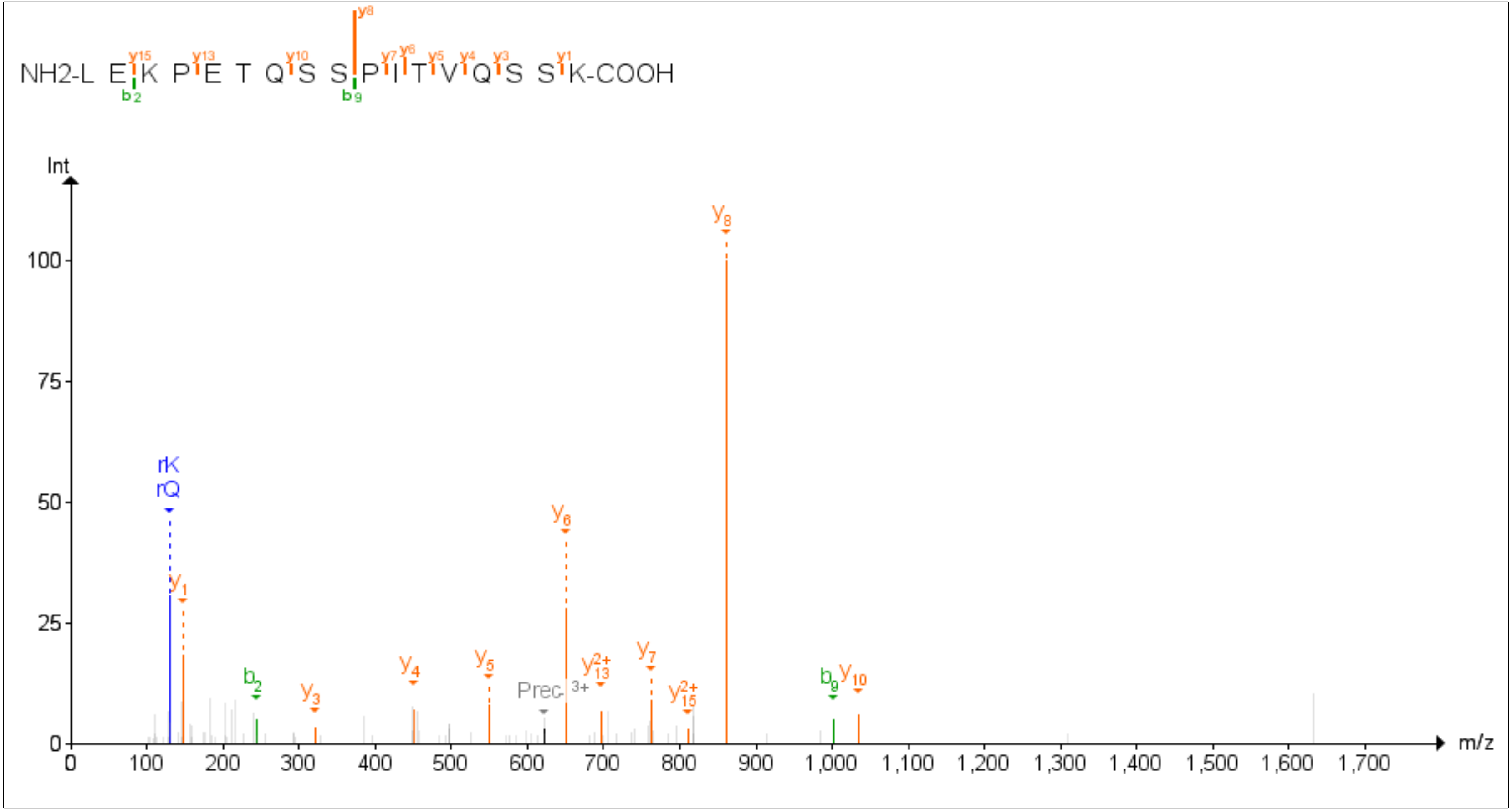

NH2-L E K P E T Q S S P I T V Q S S K-COOH
y15 y13 y10 y8 y7 y6 y5 y4 y3 y1
b2 b9
Int
m/z
rK
rQ
y1
b2
y3
y4
y5
Prec 3+
y6
y13 2+
y7
y15 2+
y8
b9
y10

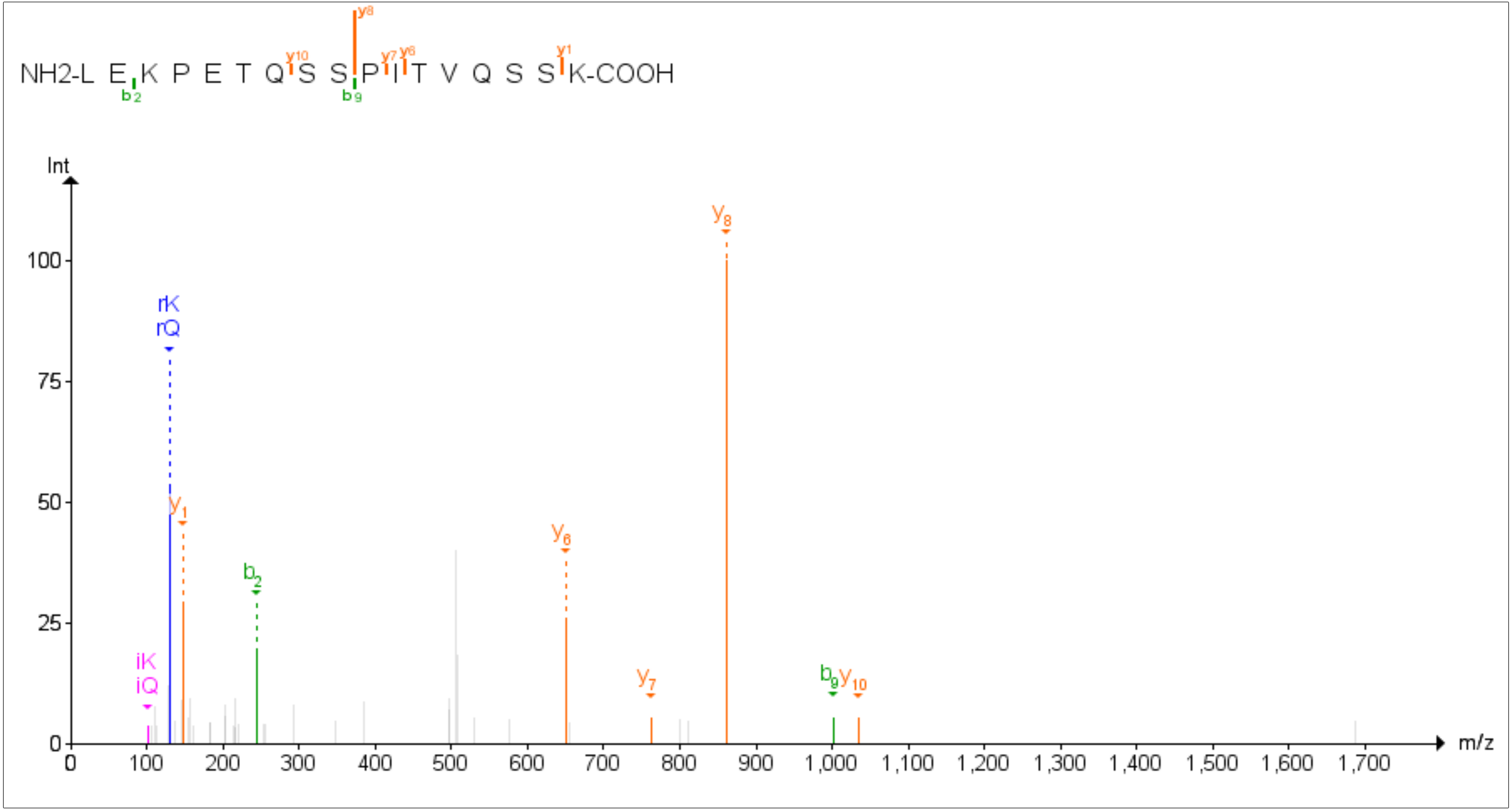
NH2-L E K P E T Q S S P I T V Q S S K-COOH
y8
y10
y7
y6
y1
b2
b9
Int
rK
rQ
y1
b2
iK
iQ
y6
y8
y7
b9
y10
m/z
0
25
50
75
100
0
100
200
300
400
500
600
700
800
900
1,000
1,100
1,200
1,300
1,400
1,500
1,600
1,700

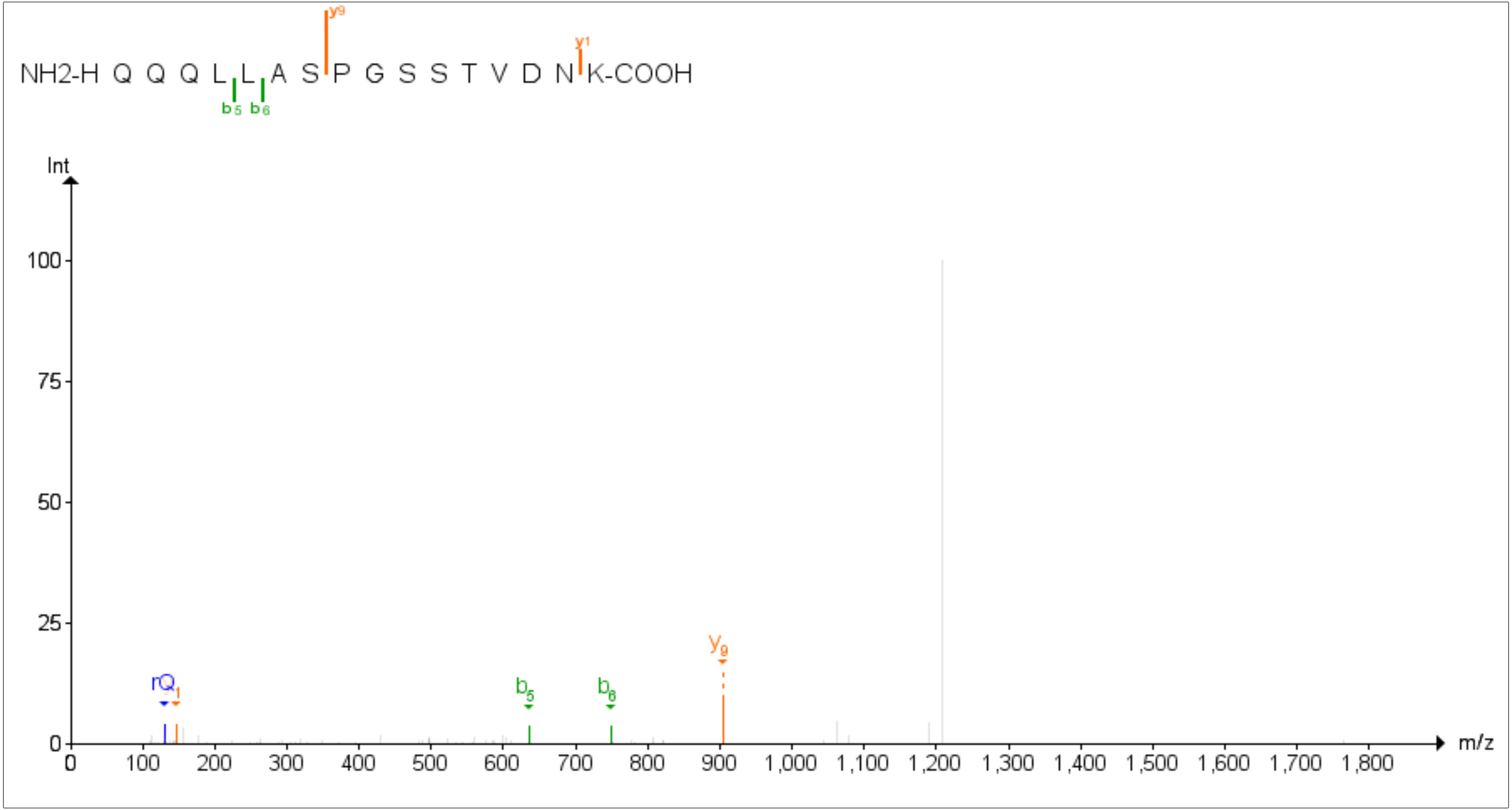

NH2-H Q Q Q L L A S P G S S T V D N K-COOH
y9
y1
b5 b6
Int
100
75
50
25
0
rQ1
b5
b6
y9
m/z
0 100 200 300 400 500 600 700 800 900 1,000 1,100 1,200 1,300 1,400 1,500 1,600 1,700 1,800

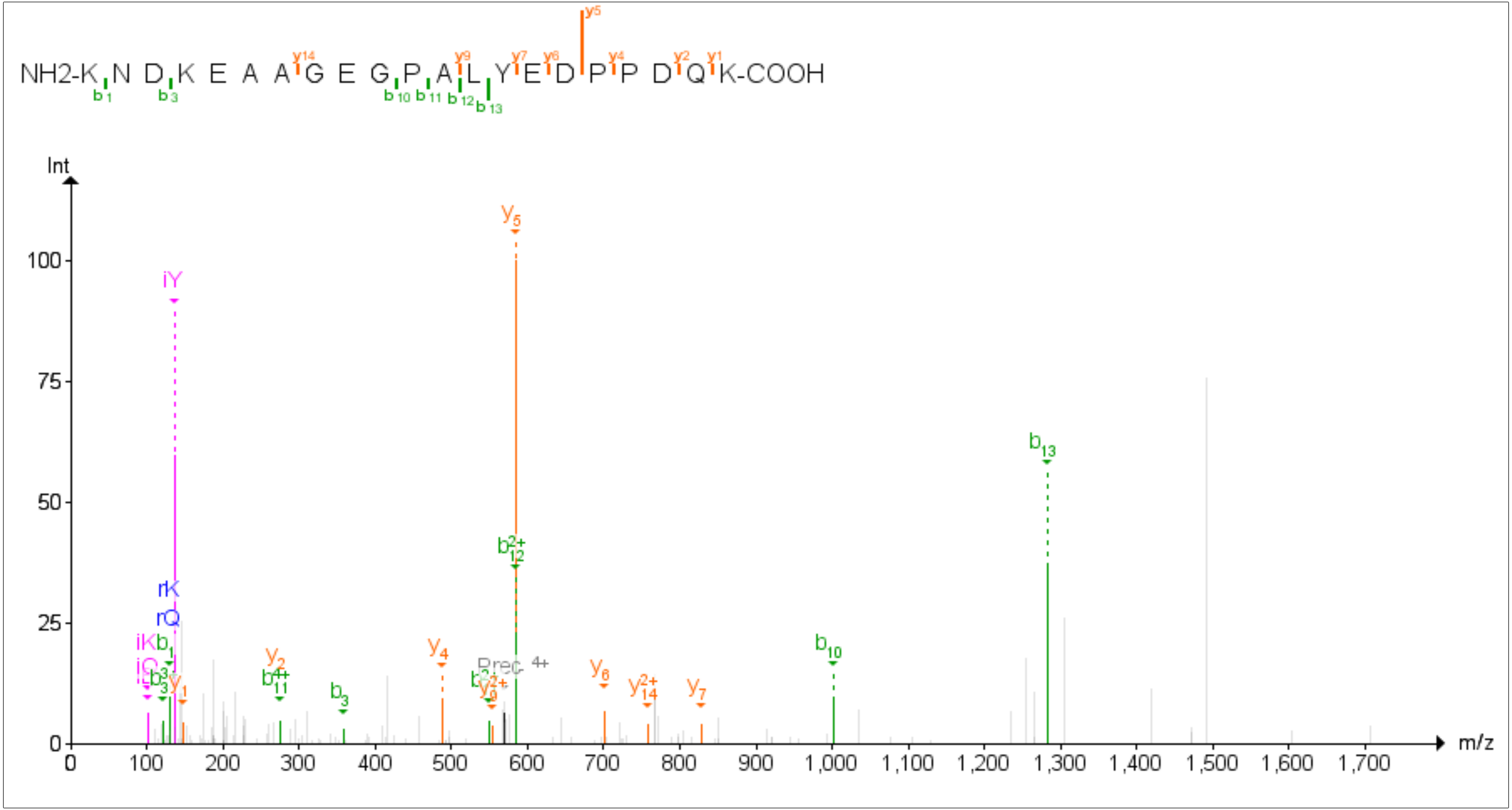
NH2-KNDKEAAGEGPALYEDPPDQK-COOH
Int
m/z
y5
b13
b10
iY
rK
rQ
y4
y6
y7
y2
b3
y1
b1

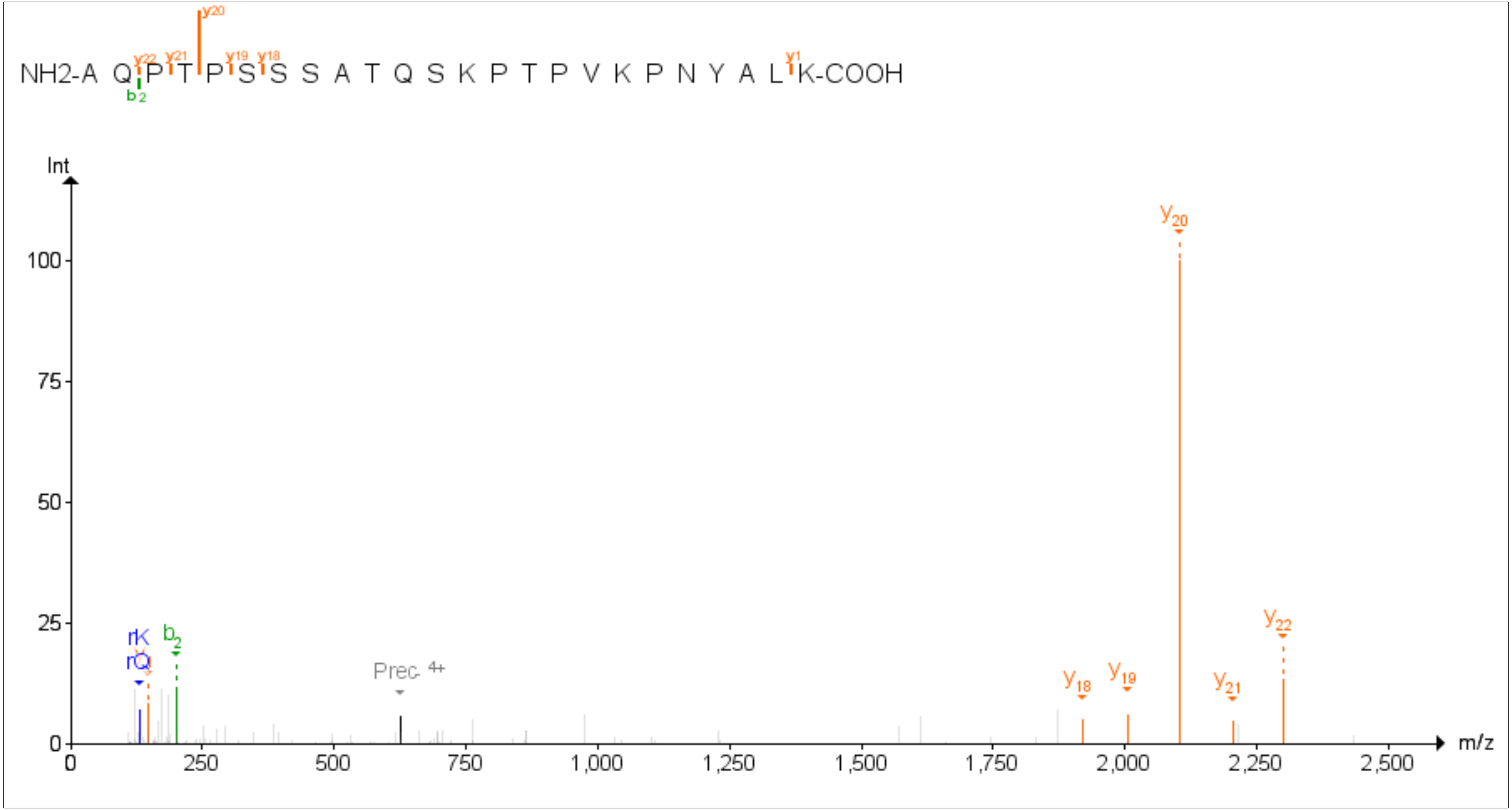
NH2-A Q P T P S S S A T Q S K P T P V K P N Y A L K-COOH
y22 y21 y20 y19 y18 y1
b2
Int
100
75
50
25
0
rK
rQ
b2
Prec 4+
y18
y19
y20
y21
y22
0
250
500
750
1,000
1,250
1,500
1,750
2,000
2,250
2,500
m/z

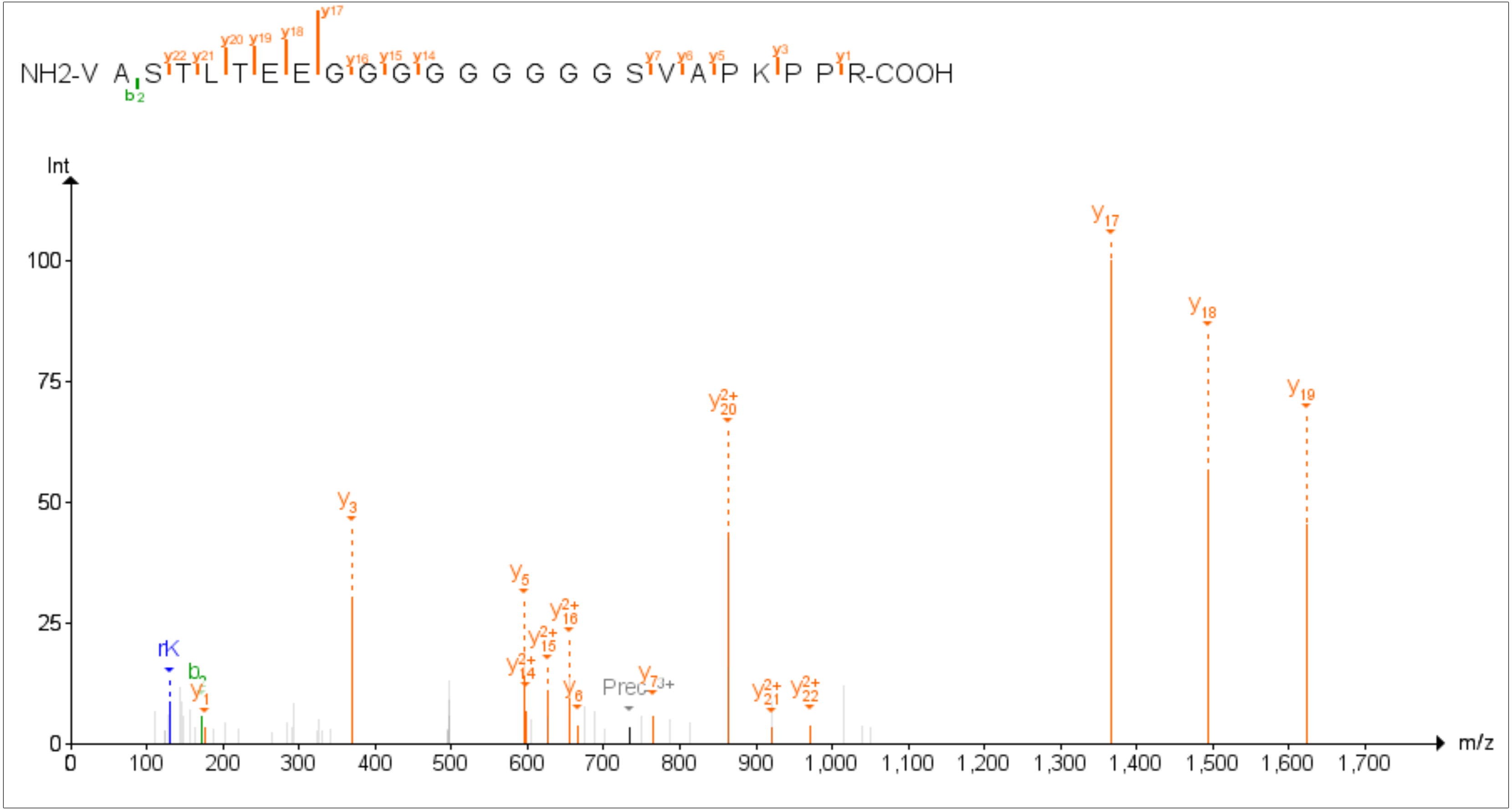

NH2-V A S T L T E E G G G G G G G G G S V A P K P P R-COOH
b2
y22 y21 y20 y19 y18 y17 y16 y15 y14 y7 y6 y5 y3 y1
Int
m/z
0
25
50
75
100
0
100
200
300
400
500
600
700
800
900
1,000
1,100
1,200
1,300
1,400
1,500
1,600
1,700
y17
y18
y19
y20 2+
y3
y5
y16 2+
y15 2+
y14 2+
y6
Prec
y7 3+
y21 2+
y22 2+
rK
b2
y1

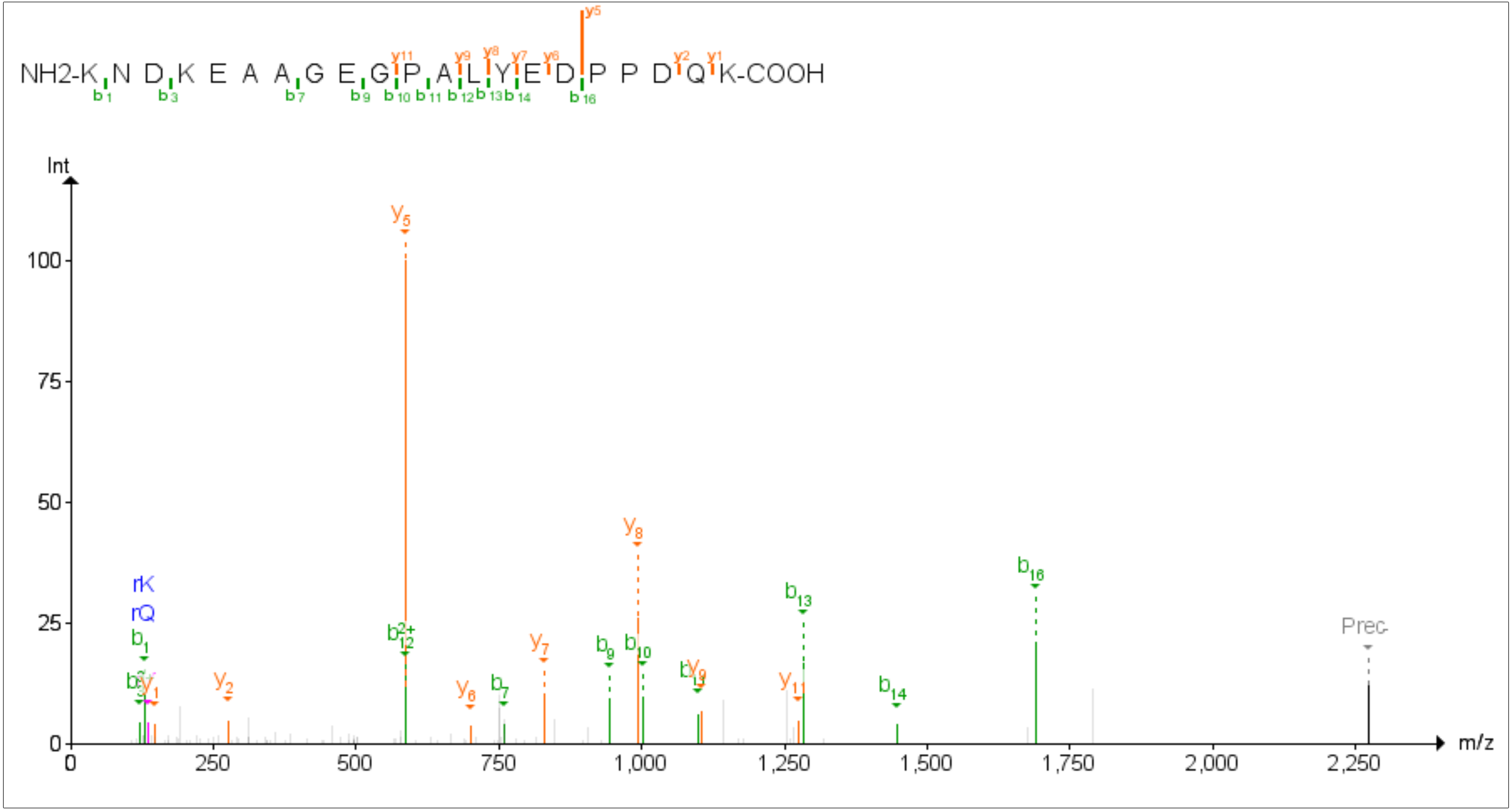
NH2-K N D K E A A G E G P A L Y E D P P D Q K-COOH
Int
m/z
Prec
y5
y8
b16
b13
b14
b12 2+
y7
b9
b10
y6
b7
y2
y11
b1
rK
rQ
0
25
50
75
100
250
500
750
1,000
1,250
1,500
1,750
2,000
2,250

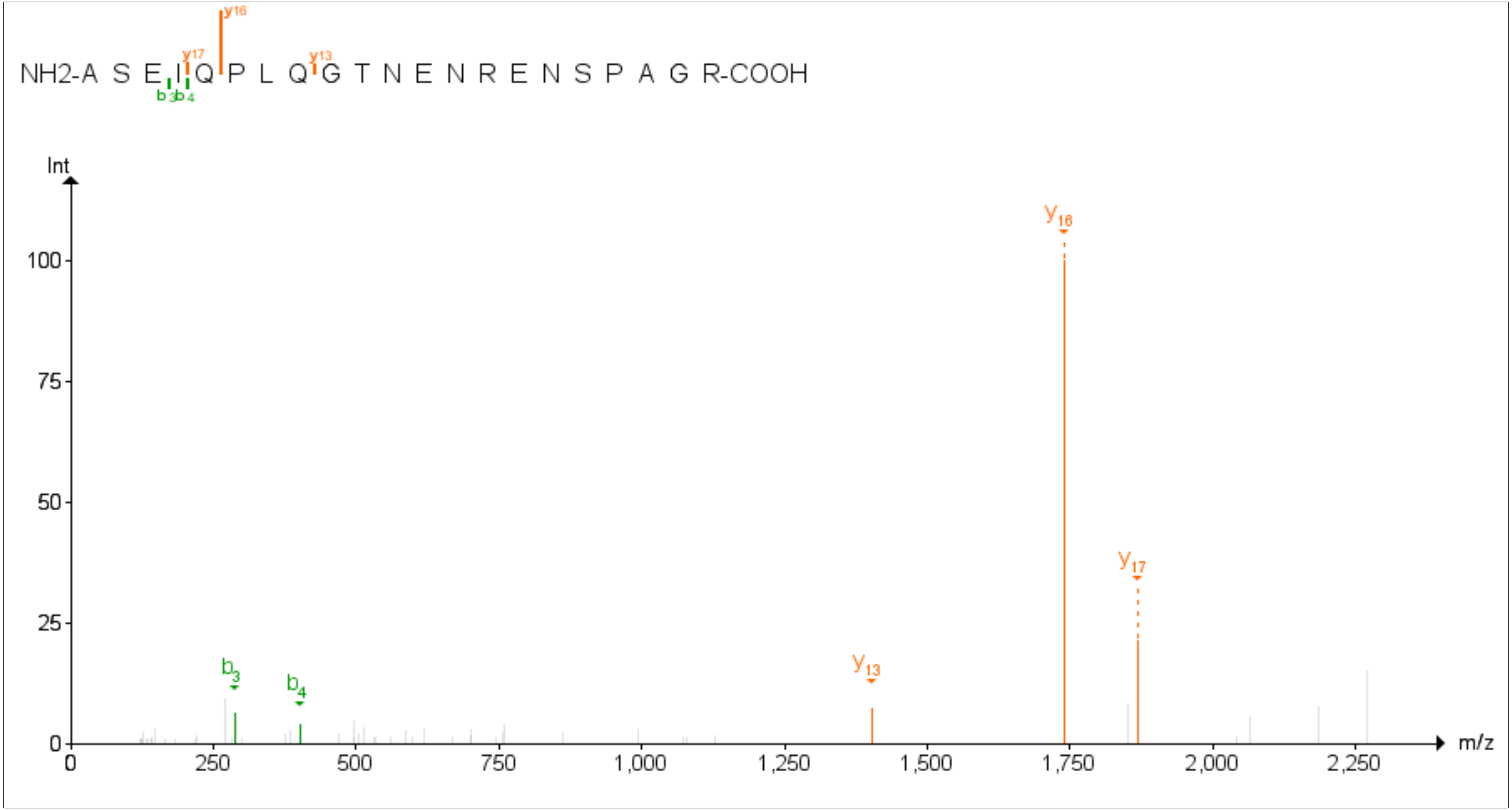

NH2-A S E I Q P L Q G T N E N R E N S P A G R-COOH
y16
y17
y13
b3
b4
Int
100
75
50
25
0
b3
b4
y13
y16
y17
0
250
500
750
1,000
1,250
1,500
1,750
2,000
2,250
m/z

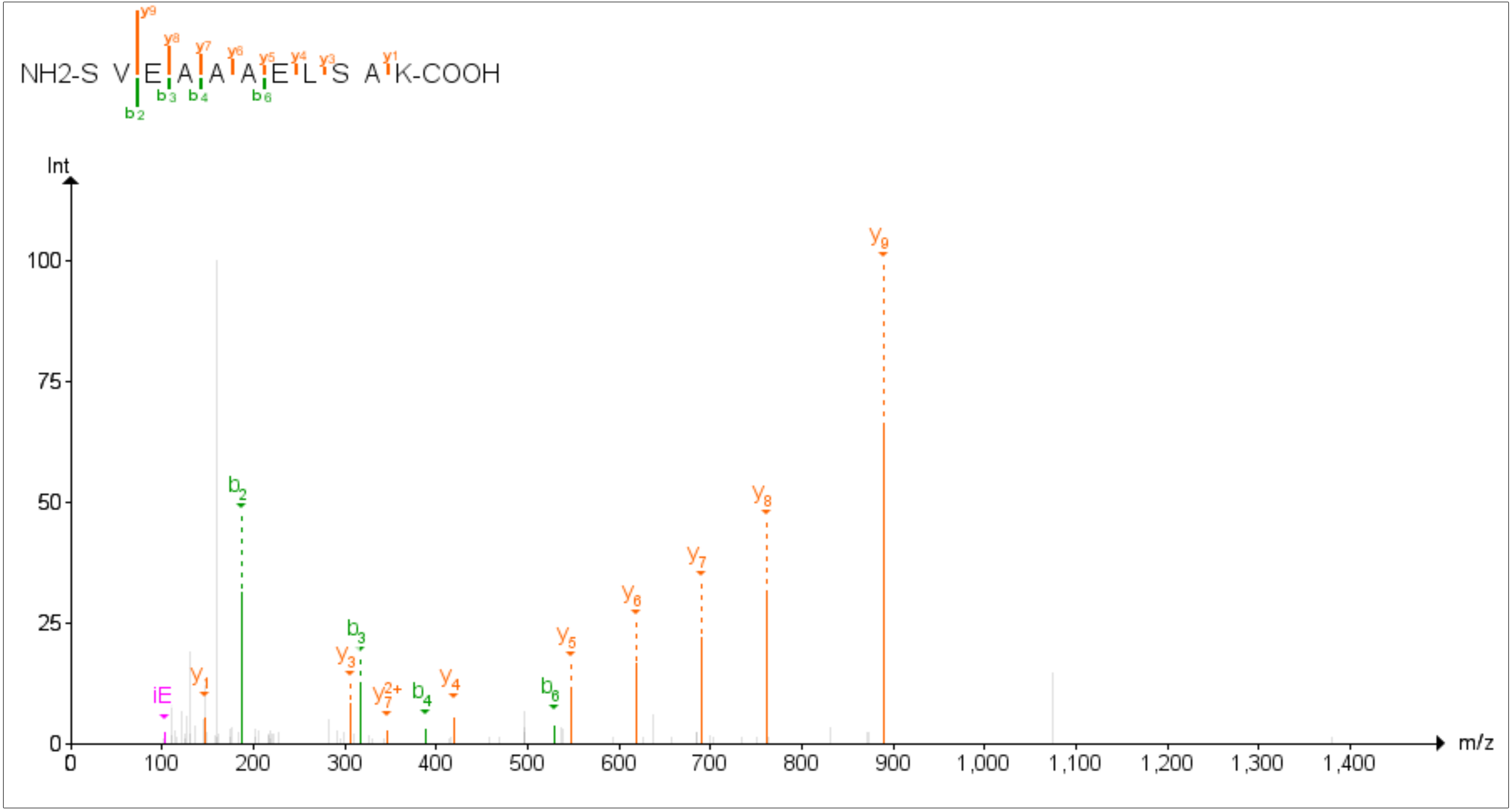

NH2-S V E A A A E L S A K-COOH
y9
y8
y7
y6
y5
y4
y3
y1
b2
b3
b4
b6
Int
100
75
50
25
0
m/z
0
100
200
300
400
500
600
700
800
900
1,000
1,100
1,200
1,300
1,400
iE
y1
b2
y3
b3
y7 2+
b4
y4
b6
y5
y6
y7
y8
y9

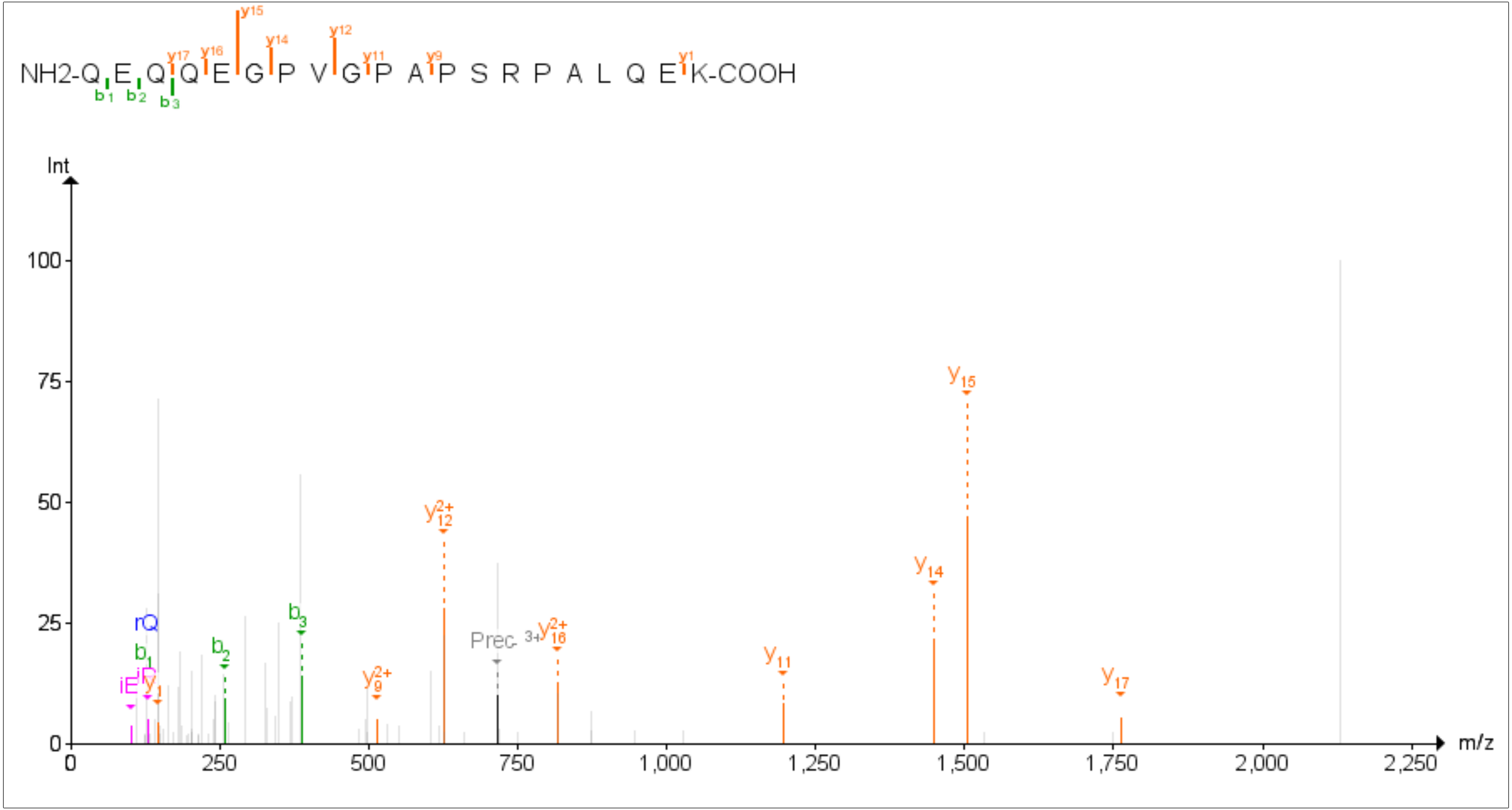

NH2-Q E Q Q E G P V G P A P S R P A L Q E K-COOH
b1 b2 b3
y17 y16 y15 y14 y12 y11 y9 y1
Int
100
75
50
25
0
m/z
0
250
500
750
1,000
1,250
1,500
1,750
2,000
2,250
y15
y14
y12 2+
y16 2+
Prec- 3+
y11
y17
y9 2+
b3
b2
b1
rQ
iE
iP
y1

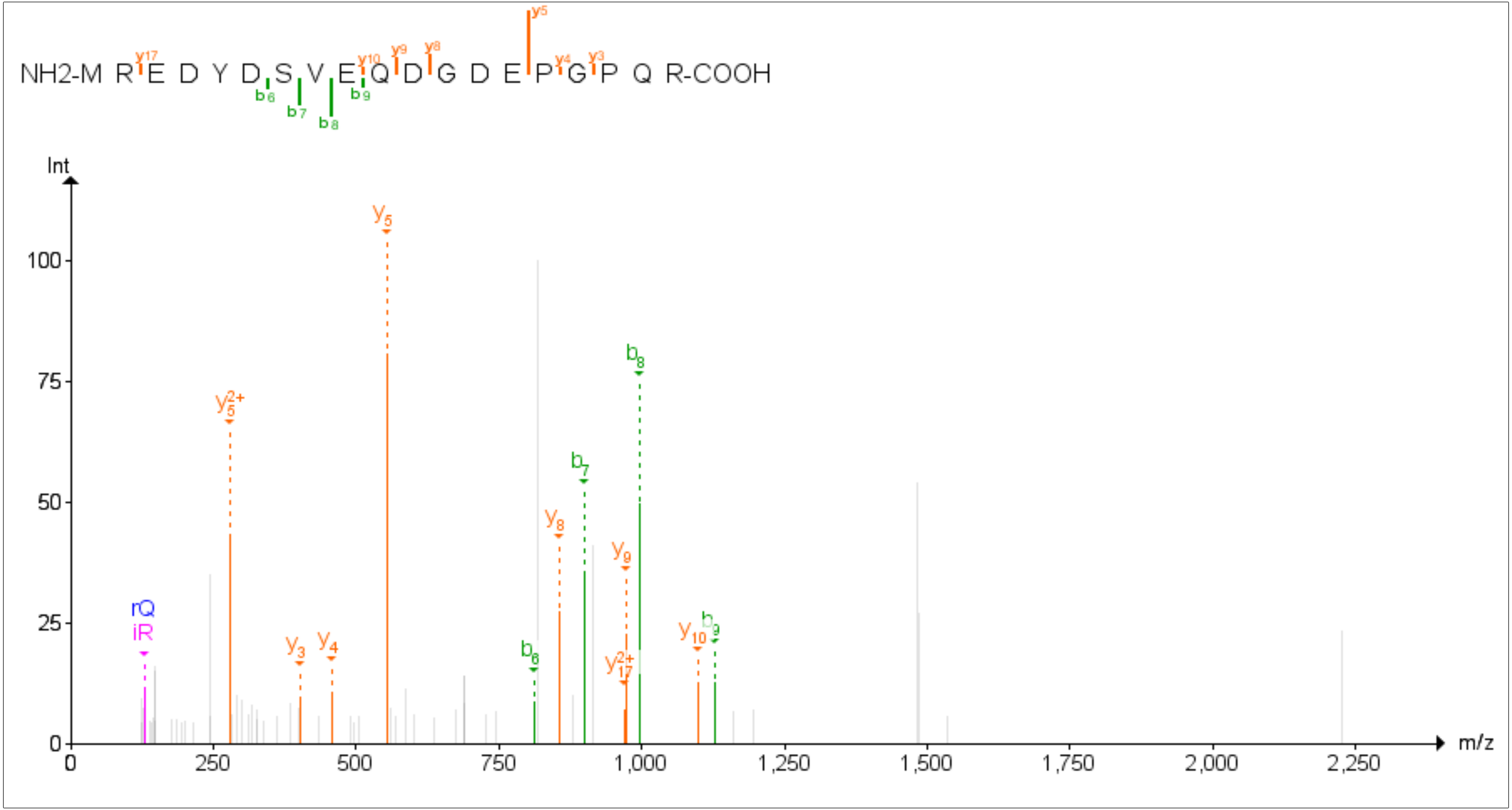

NH2-M R E D Y D S V E Q D G D E P G P Q R-COOH
y17 y10 y9 y8 y5 y4 y3
b6 b7 b8 b9
Int
m/z
100
75
50
25
0
250
500
750
1,000
1,250
1,500
1,750
2,000
2,250
rQ
iR

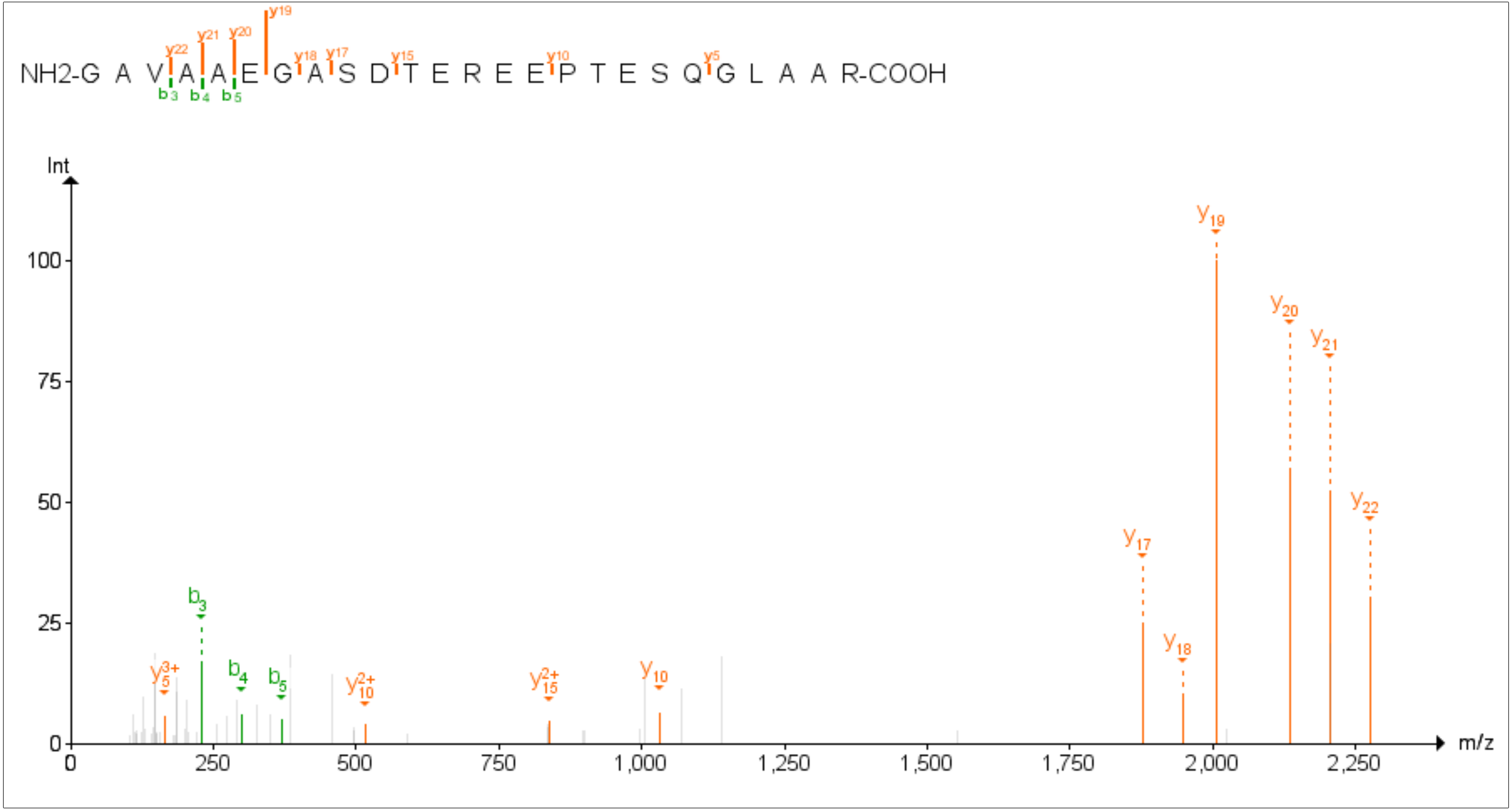

NH2-G A V A A E G A S D T E R E E P T E S Q G L A A R-COOH
y22 y21 y20 y19 y18 y17 y15 y10 y5
b3 b4 b5
Int
100
75
50
25
0
y19
y20
y21
y22
y17
y18
b3
y5 3+
b4
b5
y10 2+
y15 2+
y10
0
250
500
750
1,000
1,250
1,500
1,750
2,000
2,250
m/z

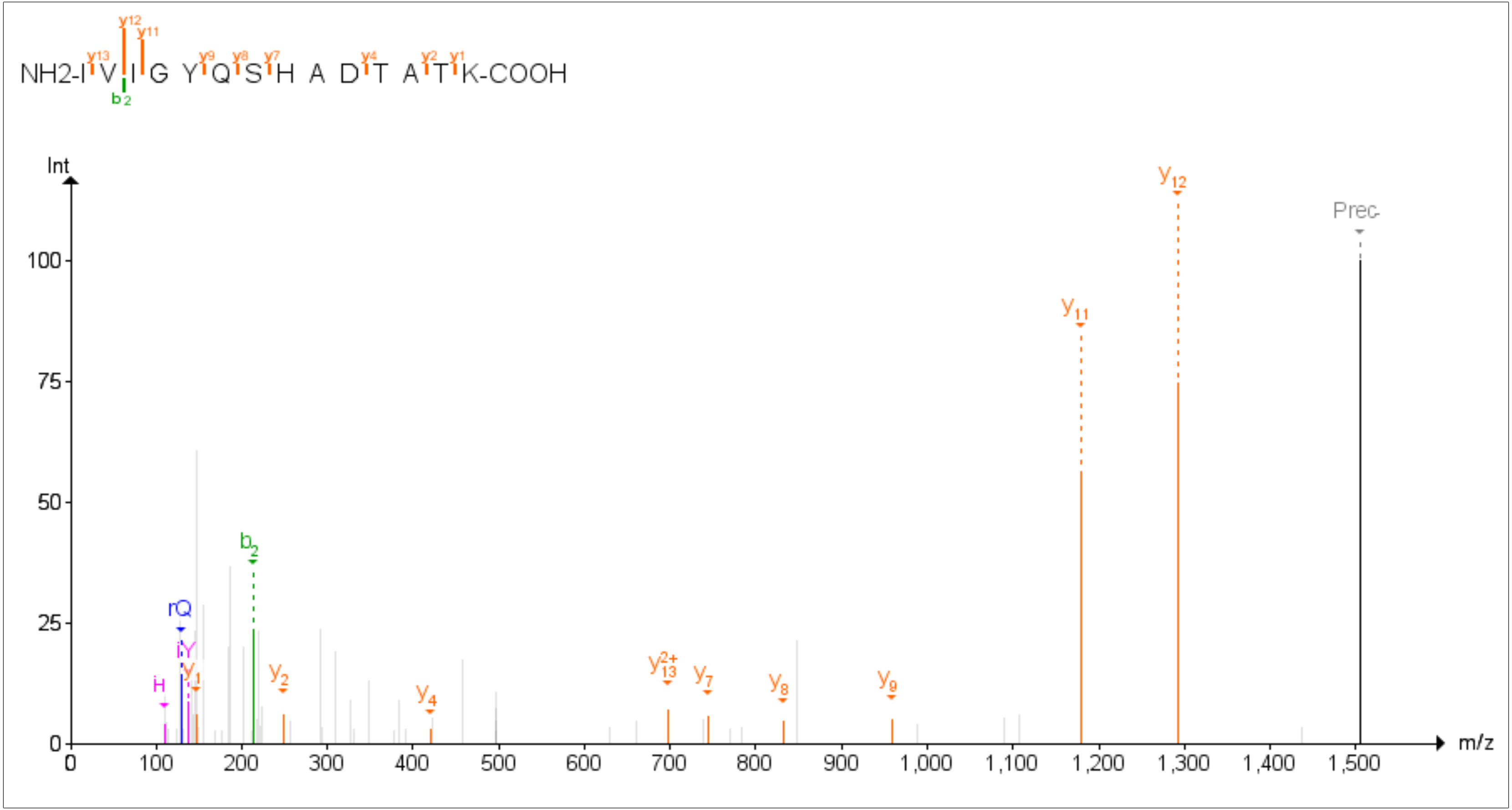

NH2-I V I G Y Q S H A D T A T K-COOH
y13 y12 y11 y9 y8 y7 y4 y2 y1
b2
Int
100
75
50
25
0
m/z
0 100 200 300 400 500 600 700 800 900 1,000 1,100 1,200 1,300 1,400 1,500
Prec
y12
y11
b2
rQ
iY
iH
y1
y2
y4
y13 2+
y7
y8
y9

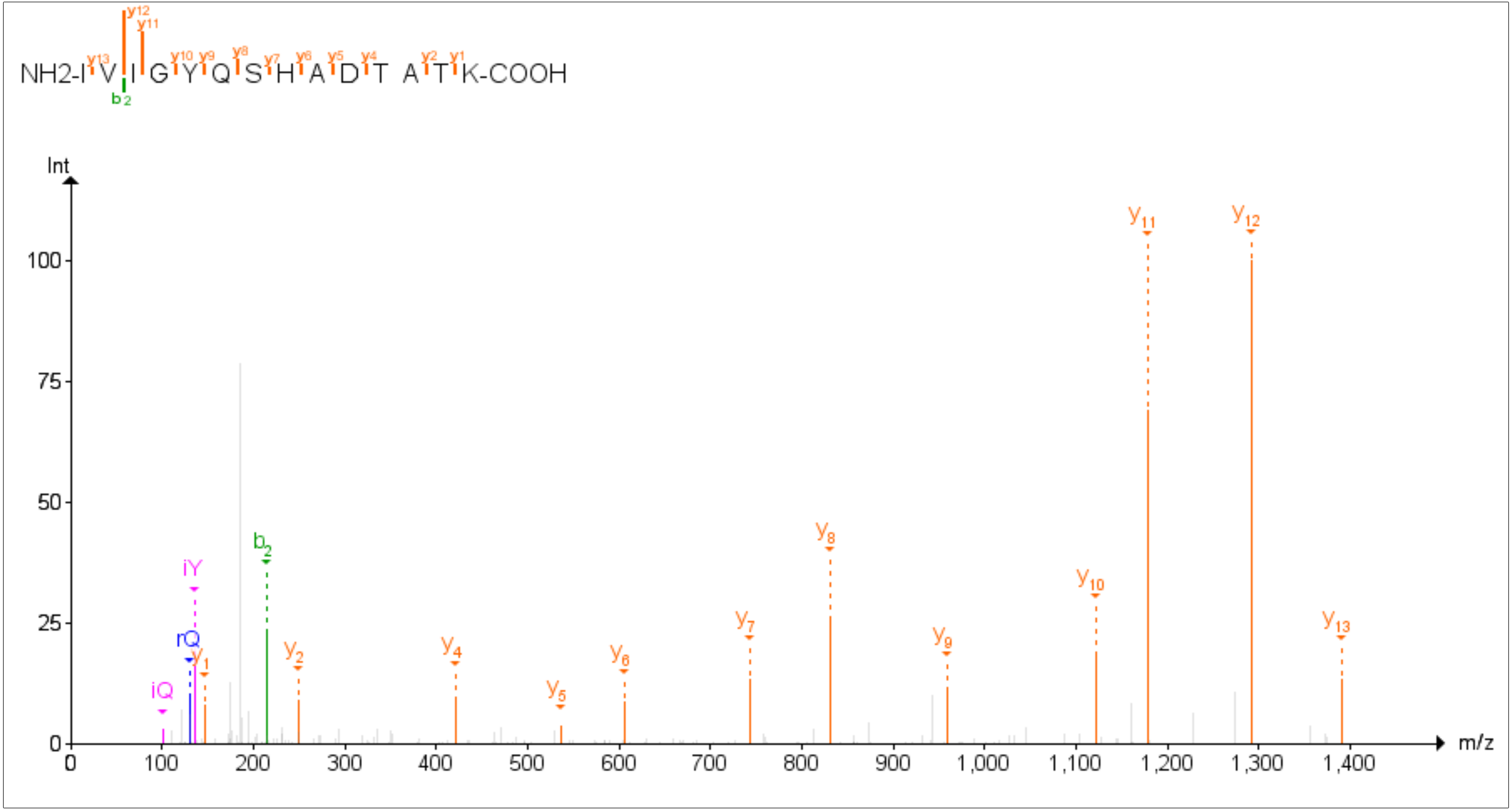

NH2-IVIGYQSHADTATK-COOH
Int
m/z
0
25
50
75
100
100
200
300
400
500
600
700
800
900
1,000
1,100
1,200
1,300
1,400
iQ
rQ
iY
b2
y1
y2
y4
y5
y6
y7
y8
y9
y10
y11
y12
y13

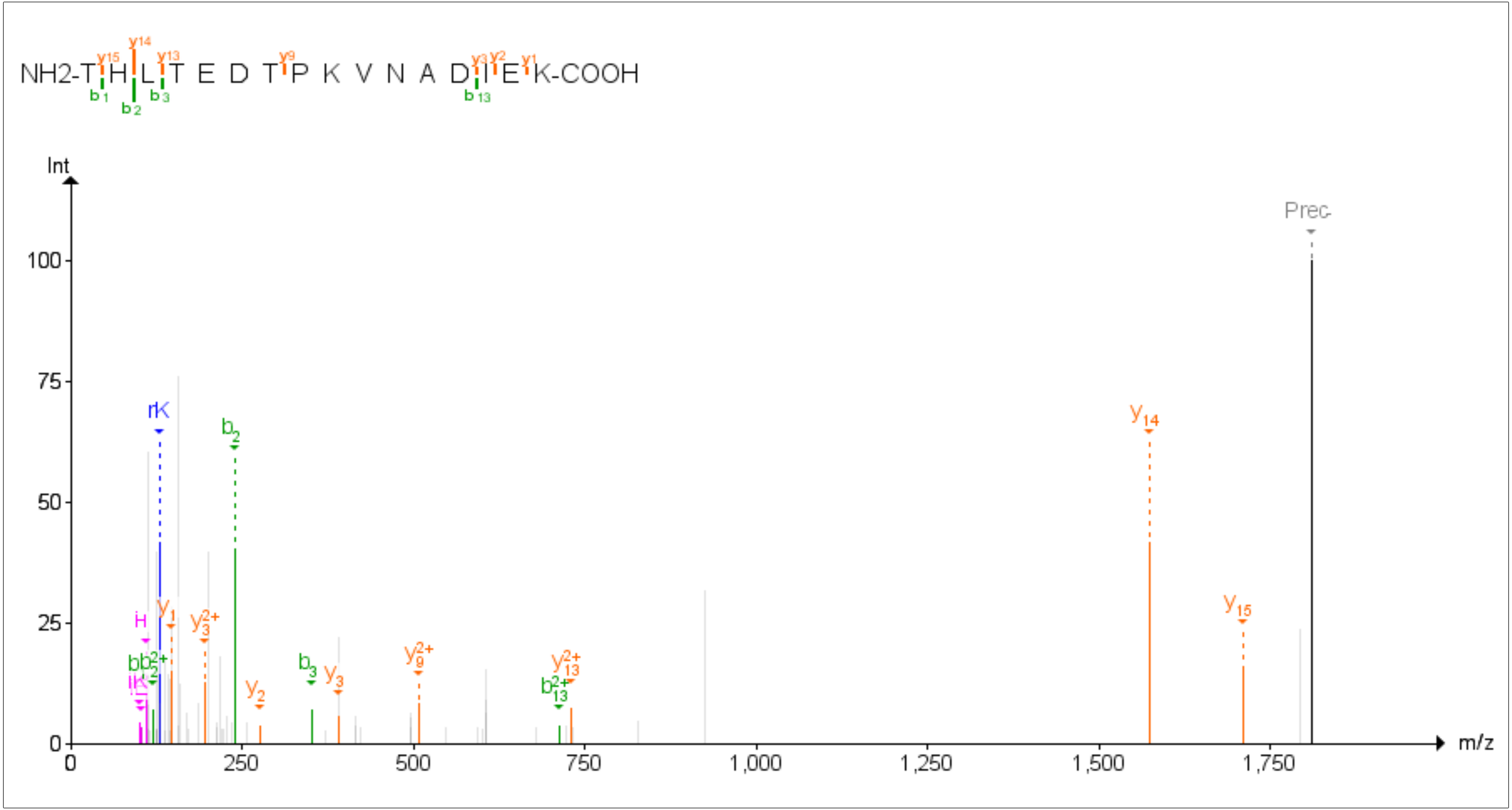
NH2-T H L T E D T P K V N A D I E K-COOH
y15 y14 y13 y9 y3 y2 y1
b1 b2 b3 b13
Int
100
75
50
25
0
Prec
rK
b2
y14
y15
H
y1
y3 2+
bb2 2+
IK
b3
y3
y2
y9 2+
y13 2+
b13 2+
0
250
500
750
1,000
1,250
1,500
1,750
m/z

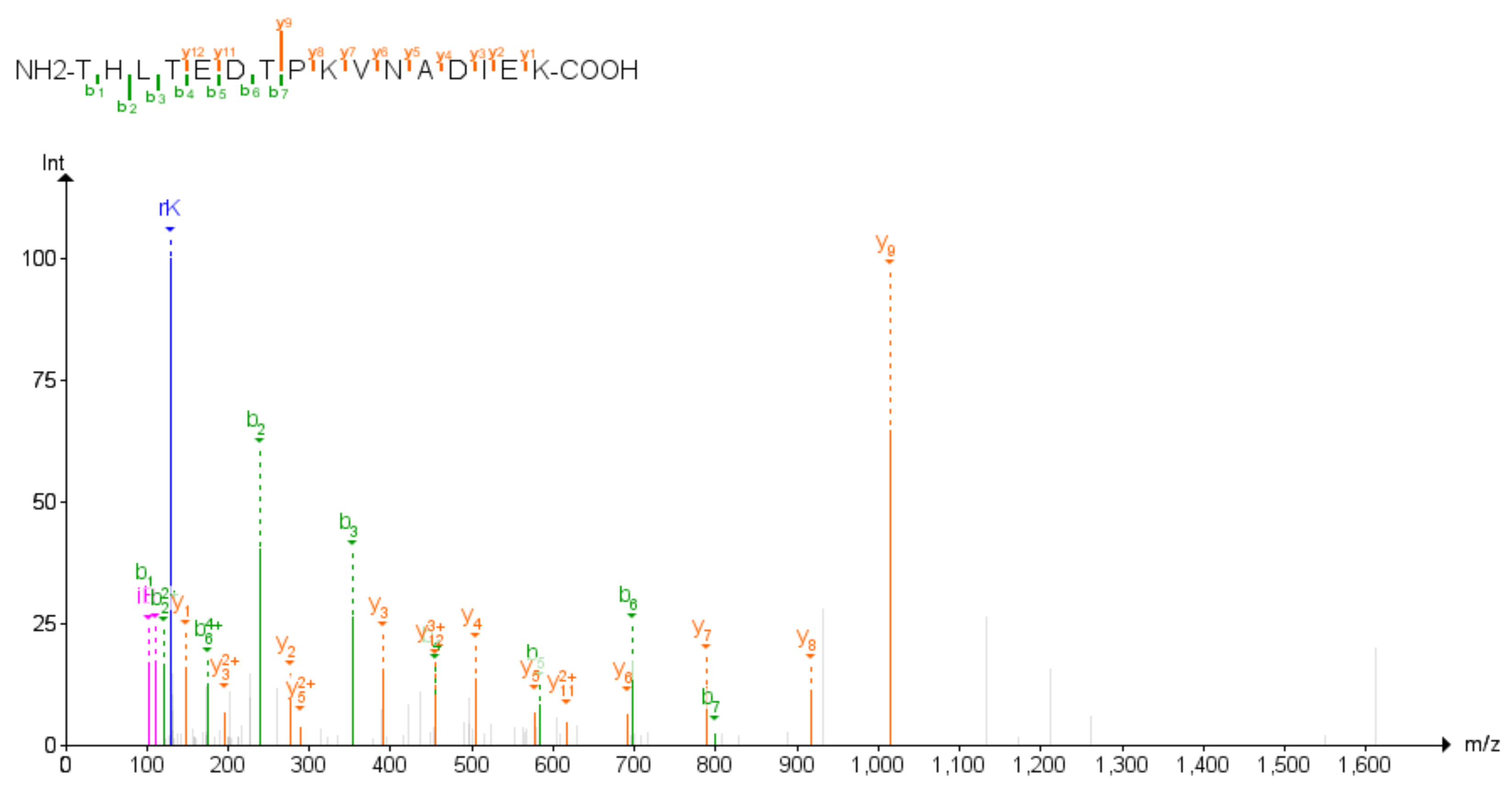
NH2-T H L T E D T P K V N A D I E K-COOH
Int
m/z
rK
y9
b2
b3
b1
y1
y3
y4
b6
y7
y8
y2
y6
b7
0
25
50
75
100
0
100
200
300
400
500
600
700
800
900
1,000
1,100
1,200
1,300
1,400
1,500
1,600

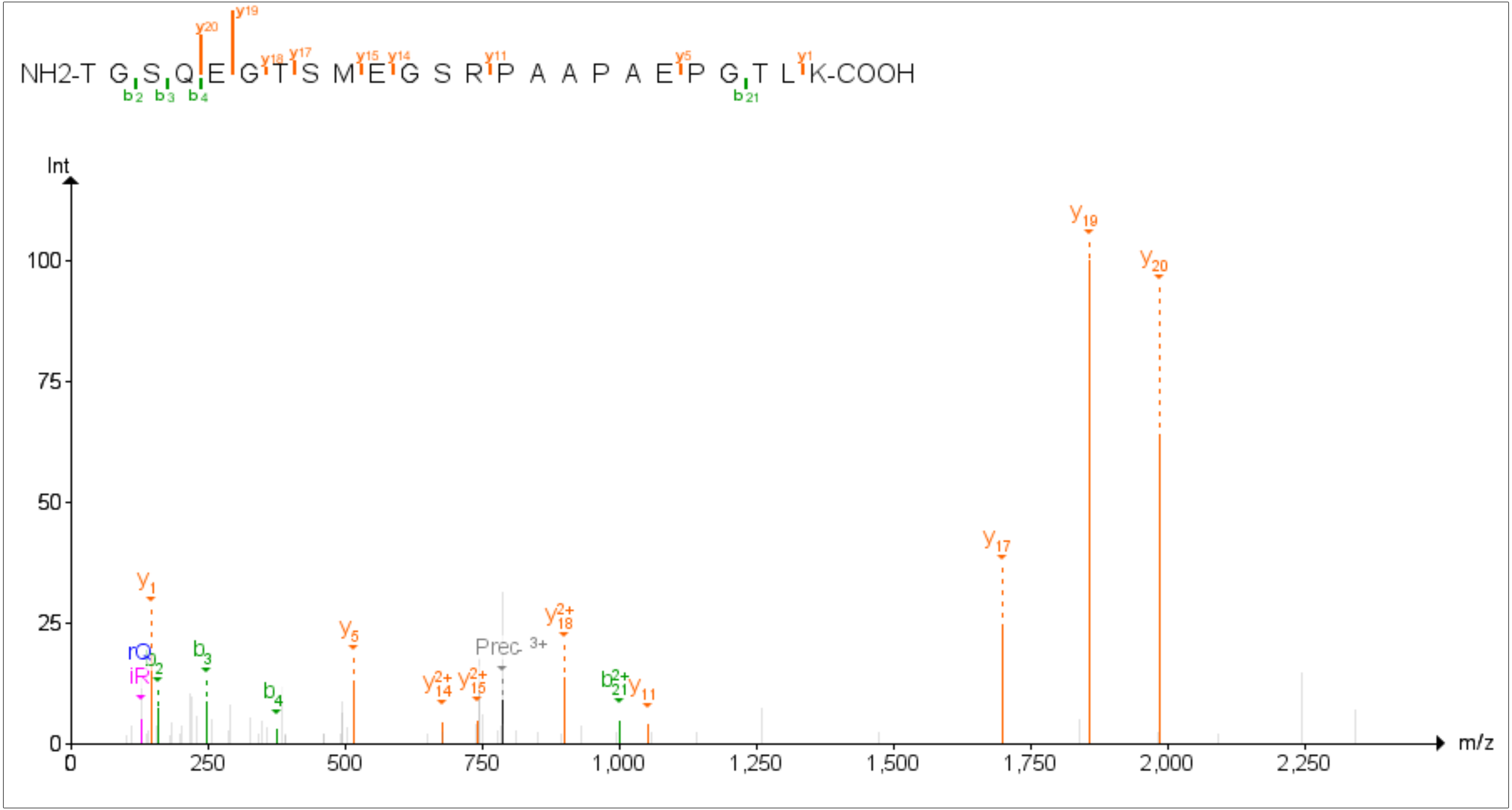

NH2-T G S Q E G T S M E G S R P A A P A E P G T L K-COOH
y20 y19 y18 y17 y15 y14 y11 y5 y1
b2 b3 b4 b21
Int
m/z
0
25
50
75
100
250
500
750
1,000
1,250
1,500
1,750
2,000
2,250
y19
y20
y17
y1
y5
rQ
iR
b2
b3
b4
Prec 3+
y2+ 18
y2+ 14
y2+ 15
b2+ 21
y11

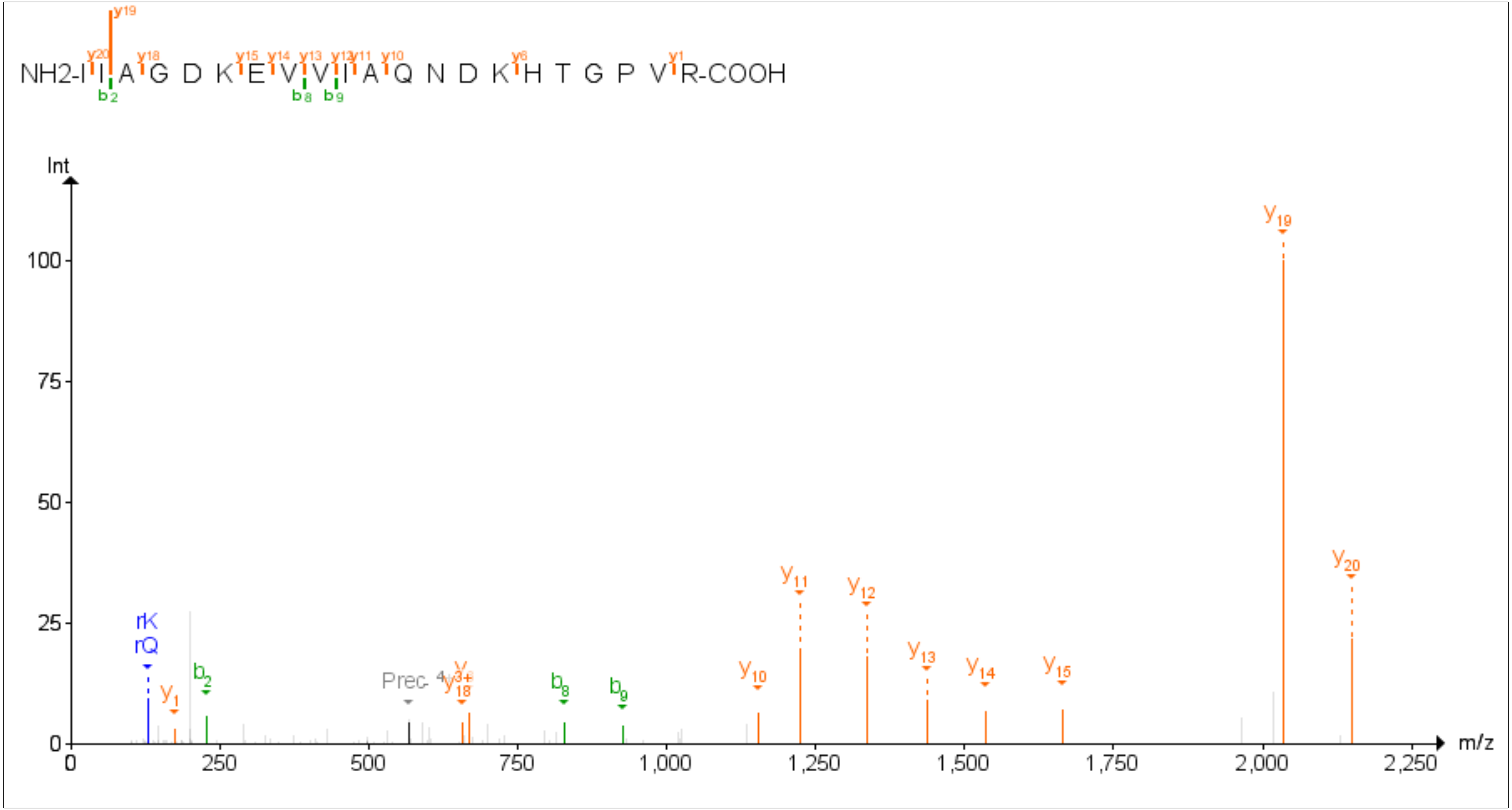

NH2-I I A G D K E V V I A Q N D K H T G P V R-COOH
y19
y20
y18
y15
y14
y13
y12
y11
y10
y6
y1
b2
b8
b9
Int
100
75
50
25
0
m/z
0
250
500
750
1,000
1,250
1,500
1,750
2,000
2,250
rK
rQ
y1
b2
Prec
y18
b8
b9
y10
y11
y12
y13
y14
y15
y19
y20

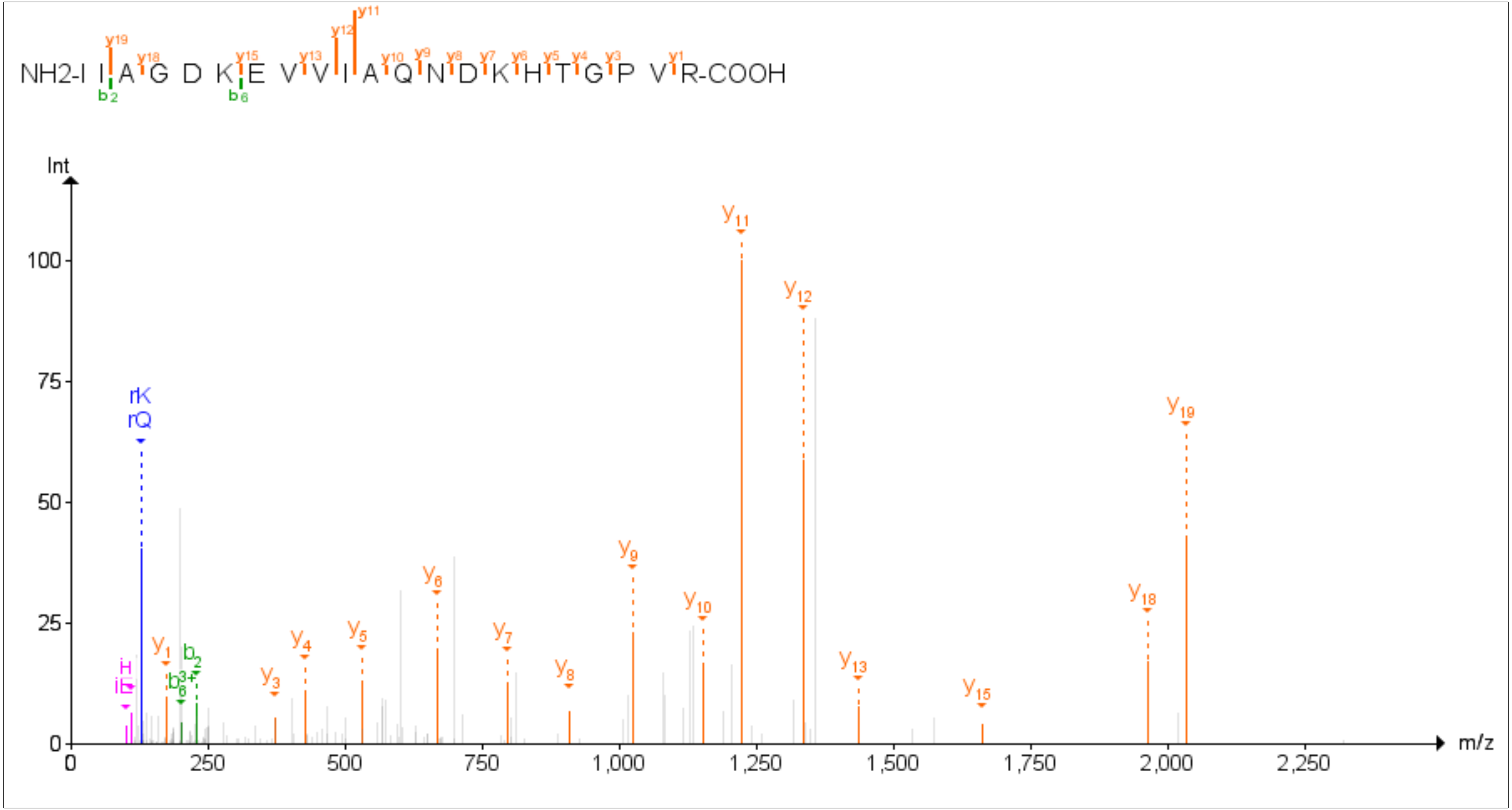

NH2-I I A G D K E V V I A Q N D K H T G P V R-COOH
y19 y18 y15 y13 y12 y11 y10 y9 y8 y7 y6 y5 y4 y3 y1
b2 b6
Int
100
75
50
25
0
m/z
0 250 500 750 1,000 1,250 1,500 1,750 2,000 2,250
rK
rQ
iH
y1 y3 y4 y5 y6 y7 y8 y9 y10 y11 y12 y13 y15 y18 y19
b2

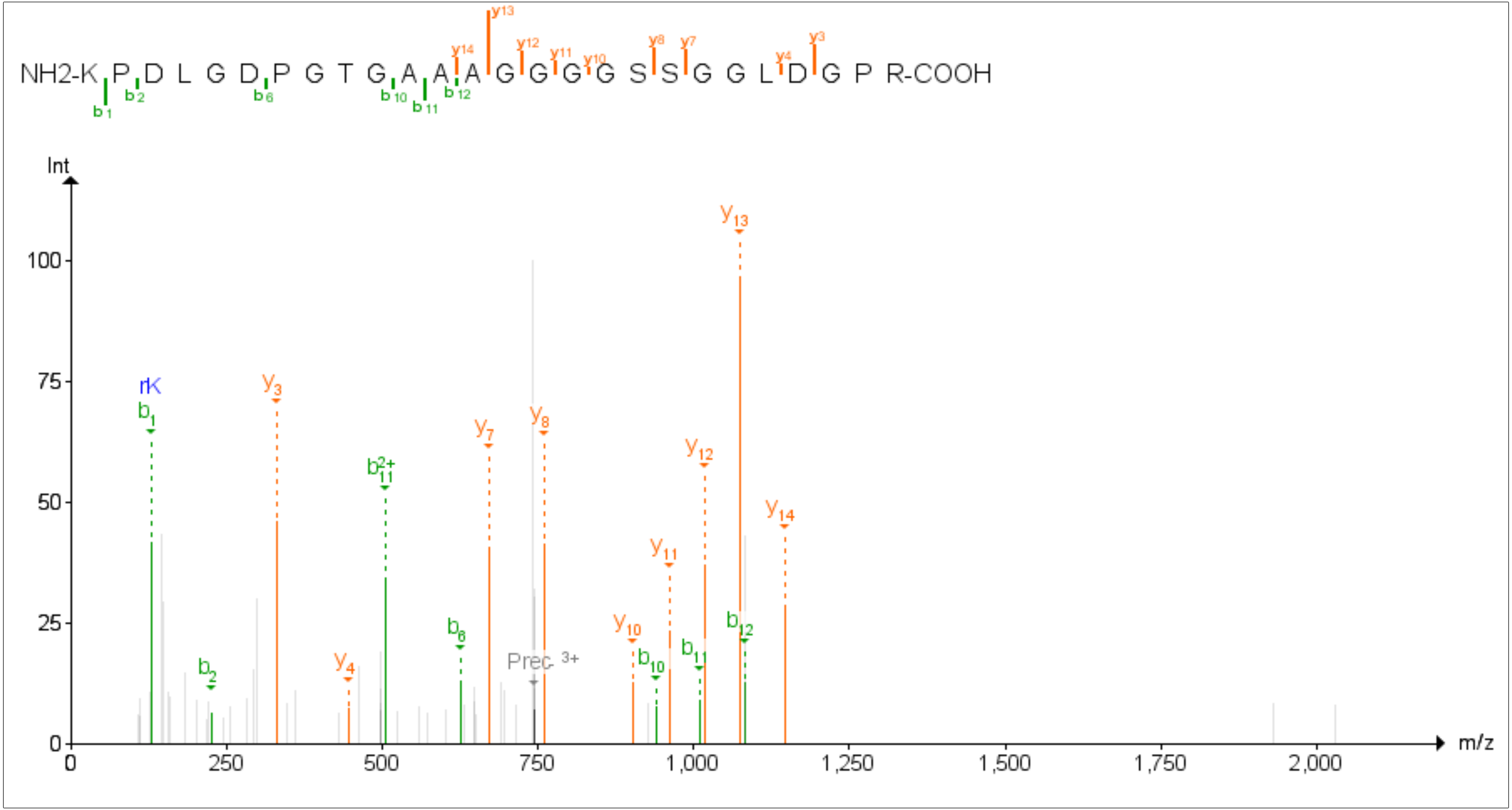
NH2-K P D L G D P G T G A A A G G G G S S G G L D G P R-COOH
b1 b2 b6 b10 b11 b12
y14 y13 y12 y11 y10 y8 y7 y4 y3
Int
100
75
50
25
0
rK
b1
b2
y3
y4
b11 2+
b6
y7
y8
Prec 3+
y10
b10
y11
b11
y12
y13
b12
y14
0
250
500
750
1,000
1,250
1,500
1,750
2,000
m/z

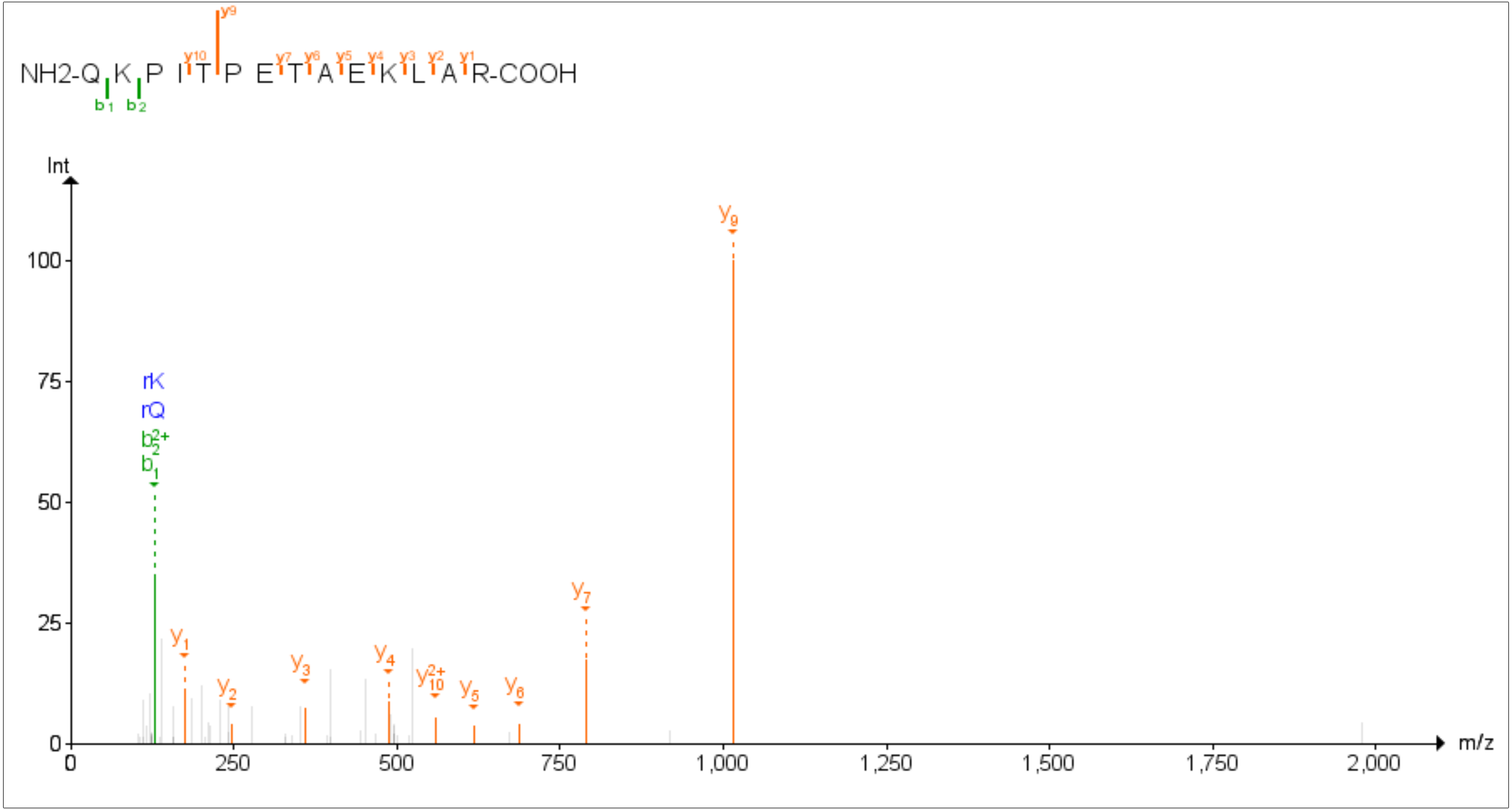

NH2-Q K P I T P E T A E K L A R-COOH
y9
y10 y7 y6 y5 y4 y3 y2 y1
b1 b2
Int
100
75
50
25
0
rK
rQ
b2 2+
b1
y1
y2
y3
y4
y10 2+
y5
y6
y7
y9
0
250
500
750
1,000
1,250
1,500
1,750
2,000
m/z

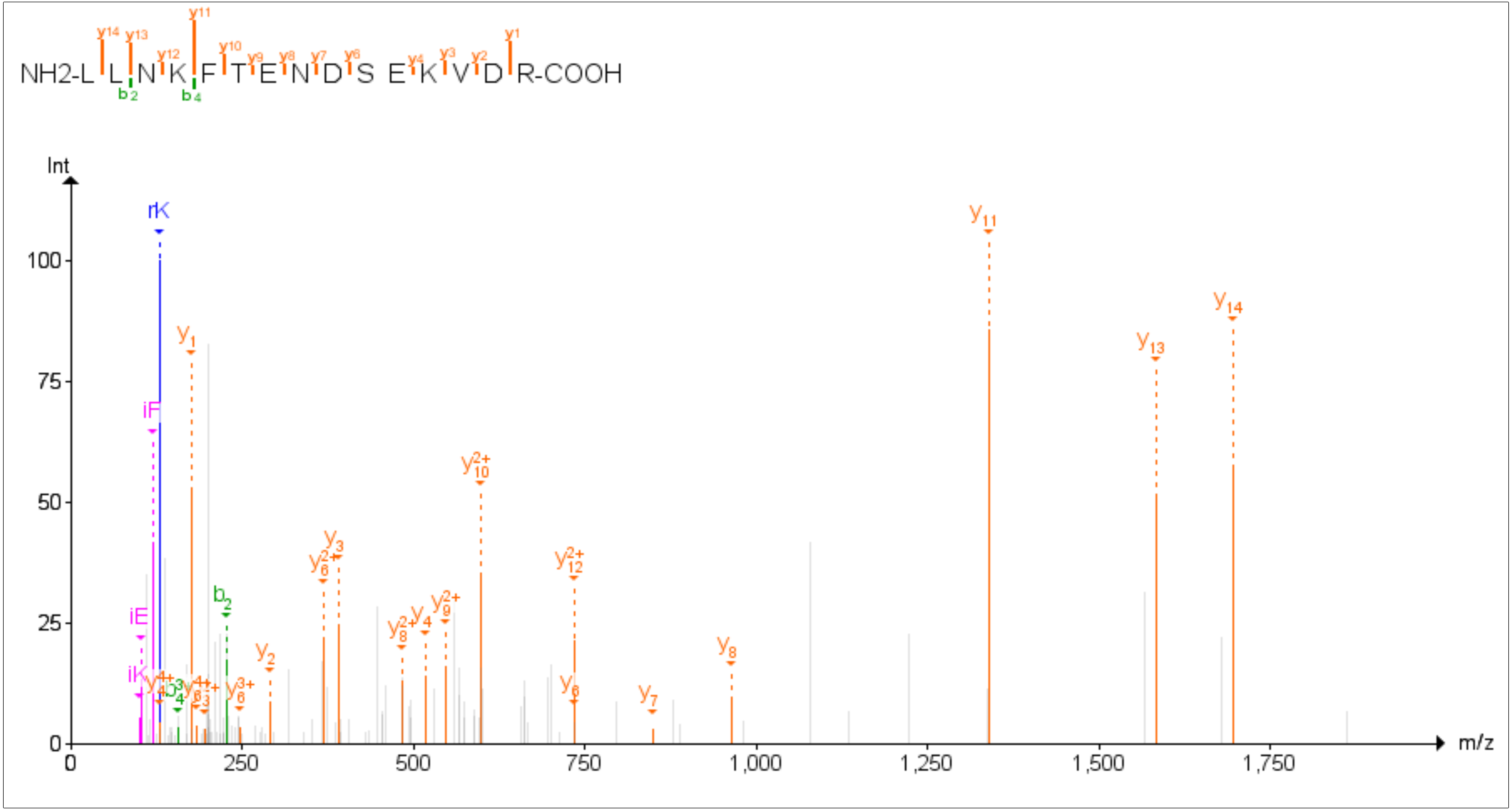
NH2-L L N K F T E N D S E K V D R-COOH
Int
m/z
rK
y11
y1
y14
y13
iF
y10 2+
y3
y6 2+
b2
y12 2+
iE
y4
y9 2+
y8 2+
y2
y8
iK
y6
y7
0
25
50
75
100
250
500
750
1,000
1,250
1,500
1,750

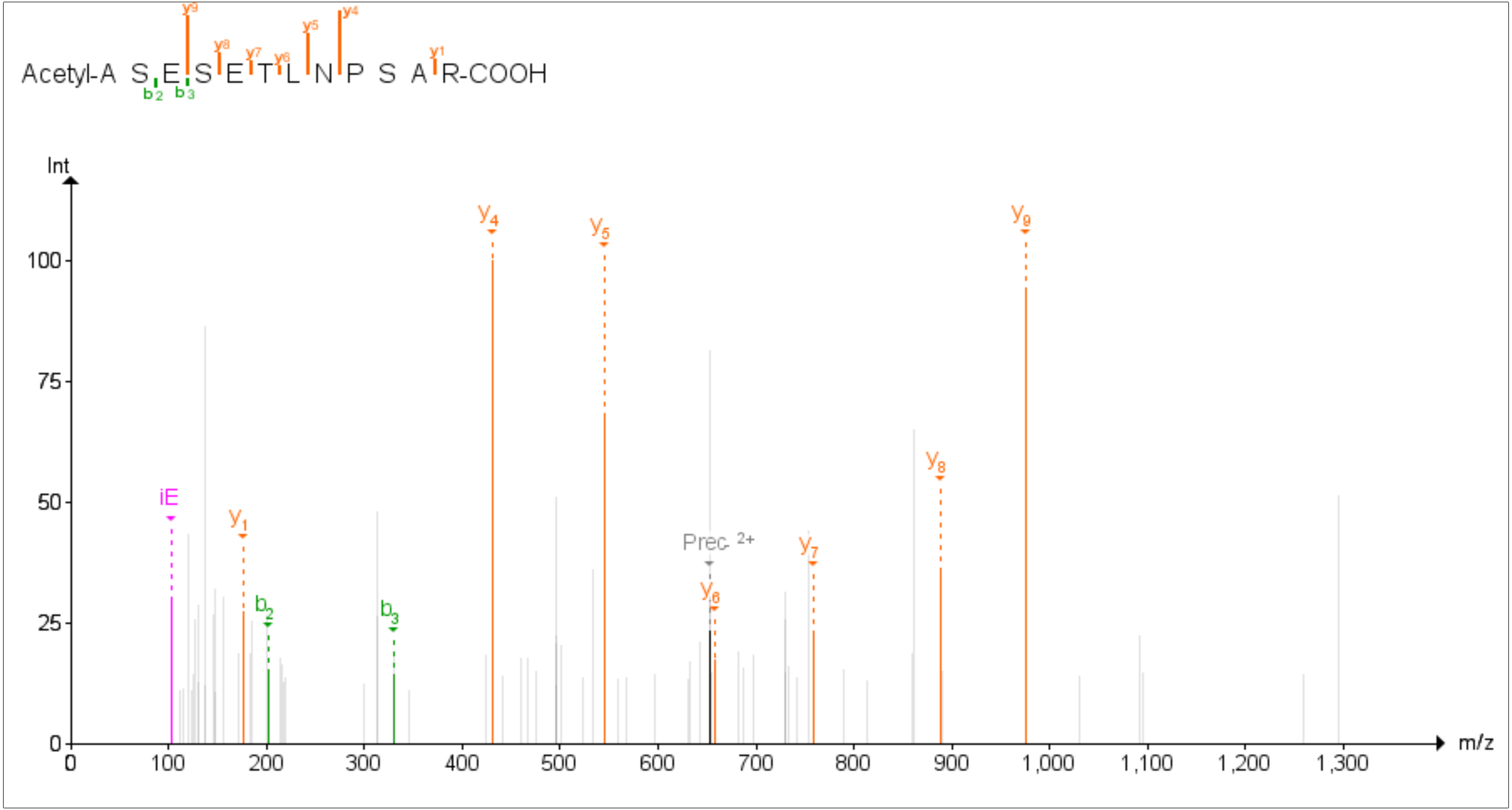

Acetyl-A S E S E T L N P S A R-COOH
b2 b3
y9 y8 y7 y6 y5 y4 y1
Int
m/z
iE
y1
b2
b3
y4
y5
Prec 2+
y6
y7
y8
y9
0 25 50 75 100
0 100 200 300 400 500 600 700 800 900 1,000 1,100 1,200 1,300

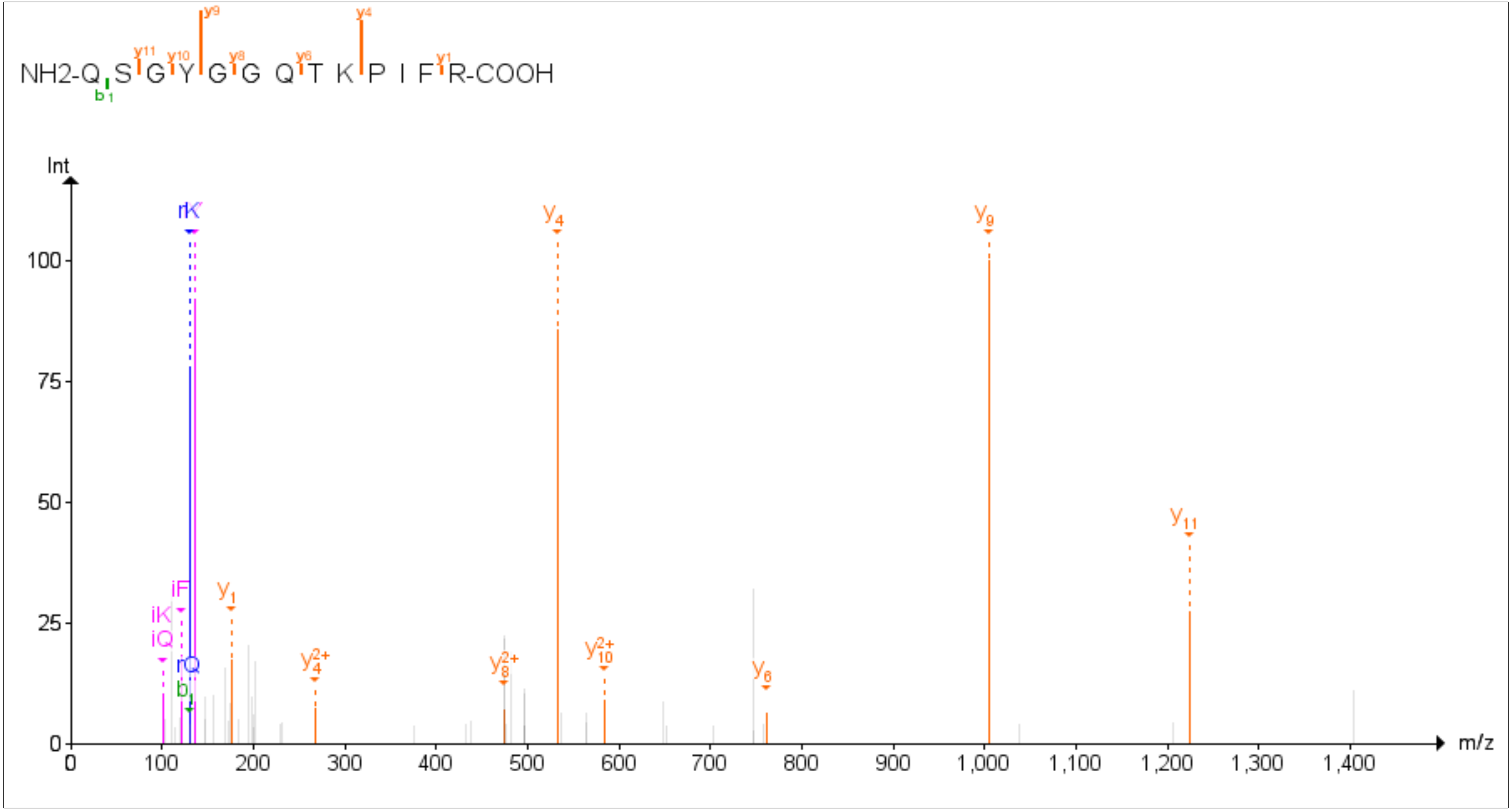

NH2-Q S G Y G G Q T K P I F R-COOH
Int
m/z
0
25
50
75
100
200
300
400
500
600
700
800
900
1,000
1,100
1,200
1,300
1,400
y4
y9
y11
y1
y4 2+
y8 2+
y10 2+
y6
iF
iK
iQ
rQ
b1

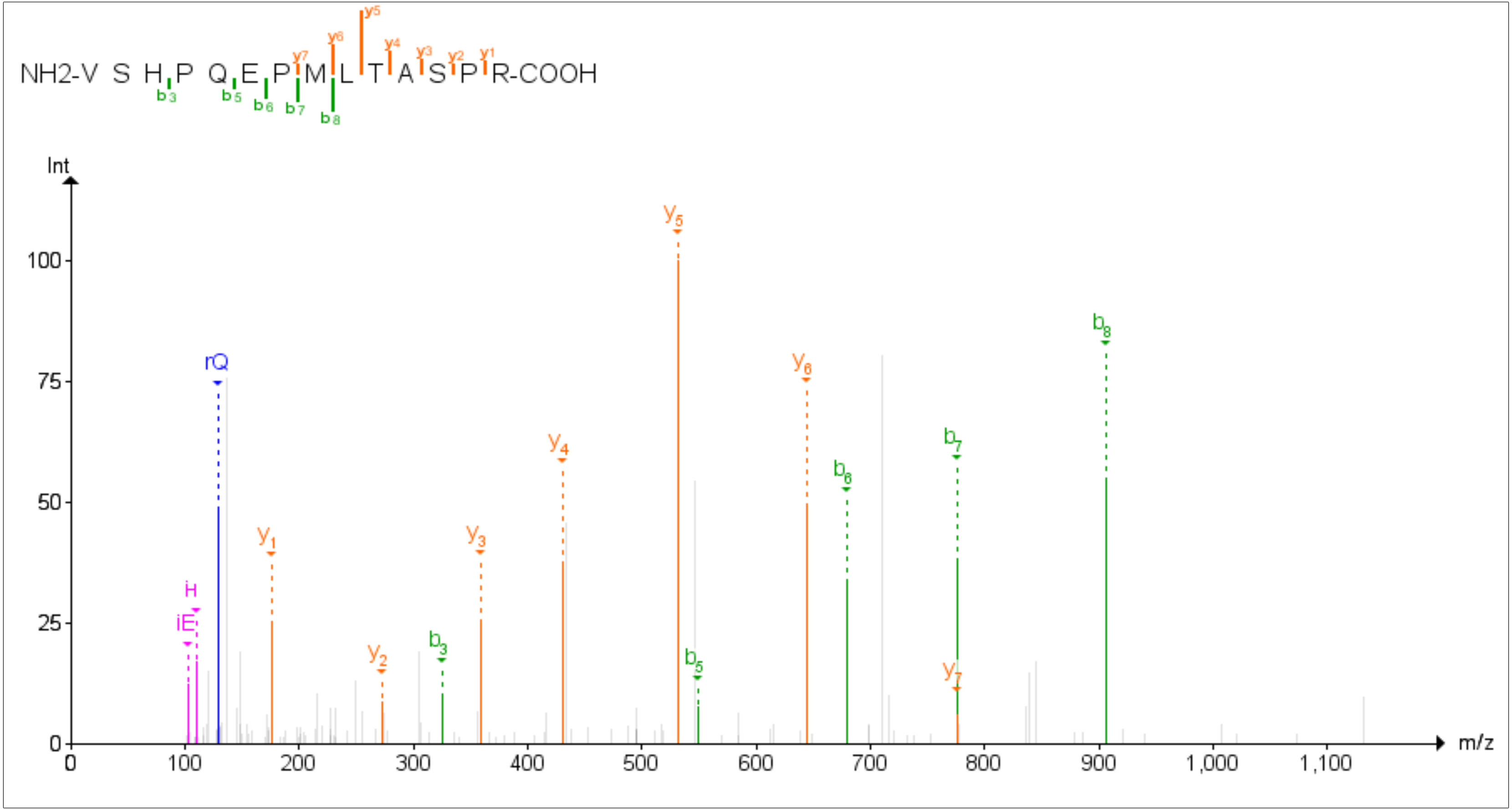

NH2-V S H P Q E P M L T A S P R-COOH
b3 b5 b6 b7 b8
y7 y6 y5 y4 y3 y2 y1
Int
100
75
50
25
0
rQ
iH
iE
y1 y2 y3 y4 y5 y6 y7
b3 b5 b6 b7 b8
0 100 200 300 400 500 600 700 800 900 1,000 1,100
m/z

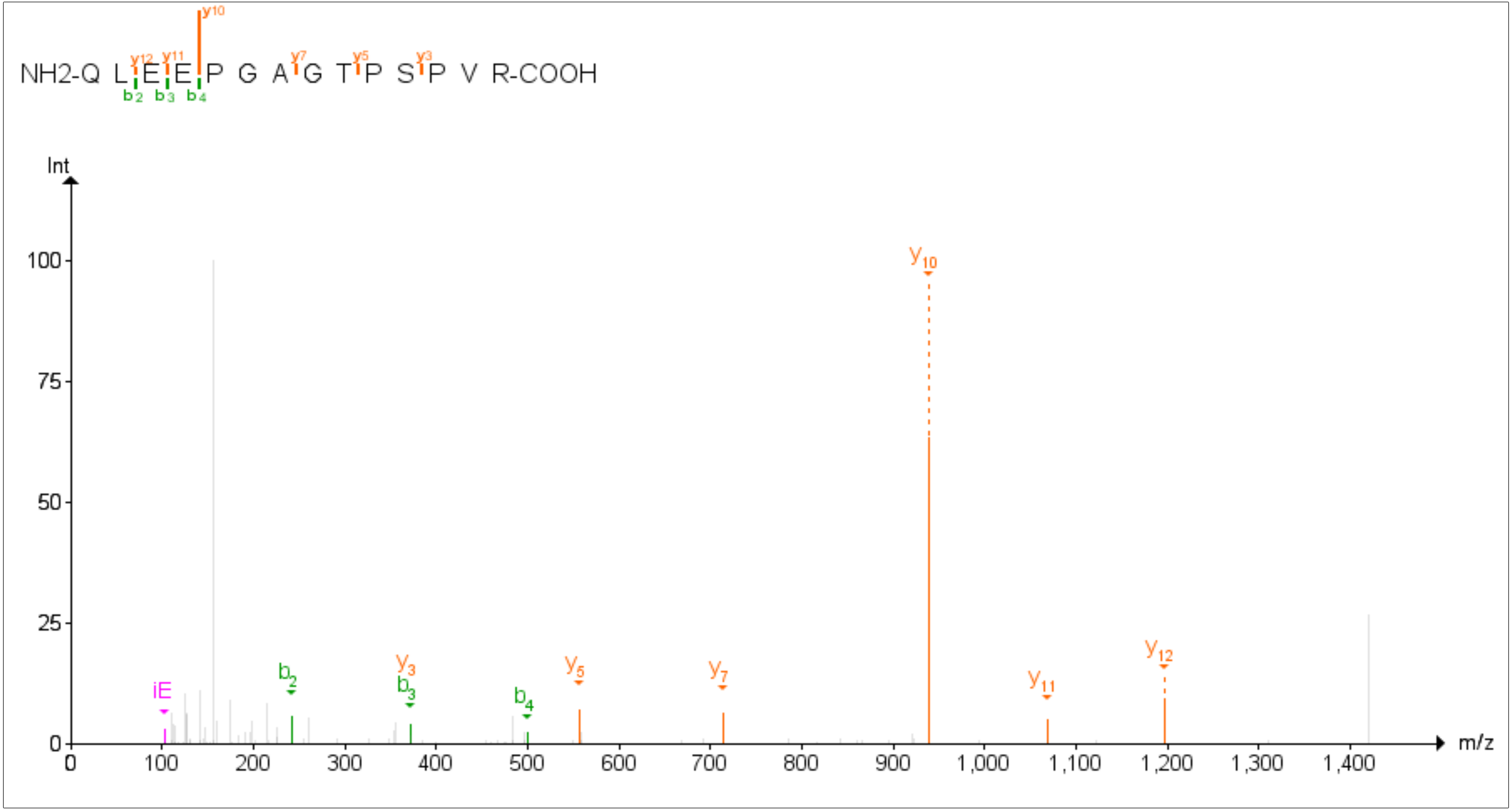

NH2-Q L E E P G A G T P S P V R-COOH
y12 y11 y10 y7 y5 y3
b2 b3 b4
Int
100
75
50
25
0
iE
b2
y3
b3
b4
y5
y7
y10
y11
y12
0 100 200 300 400 500 600 700 800 900 1,000 1,100 1,200 1,300 1,400
m/z

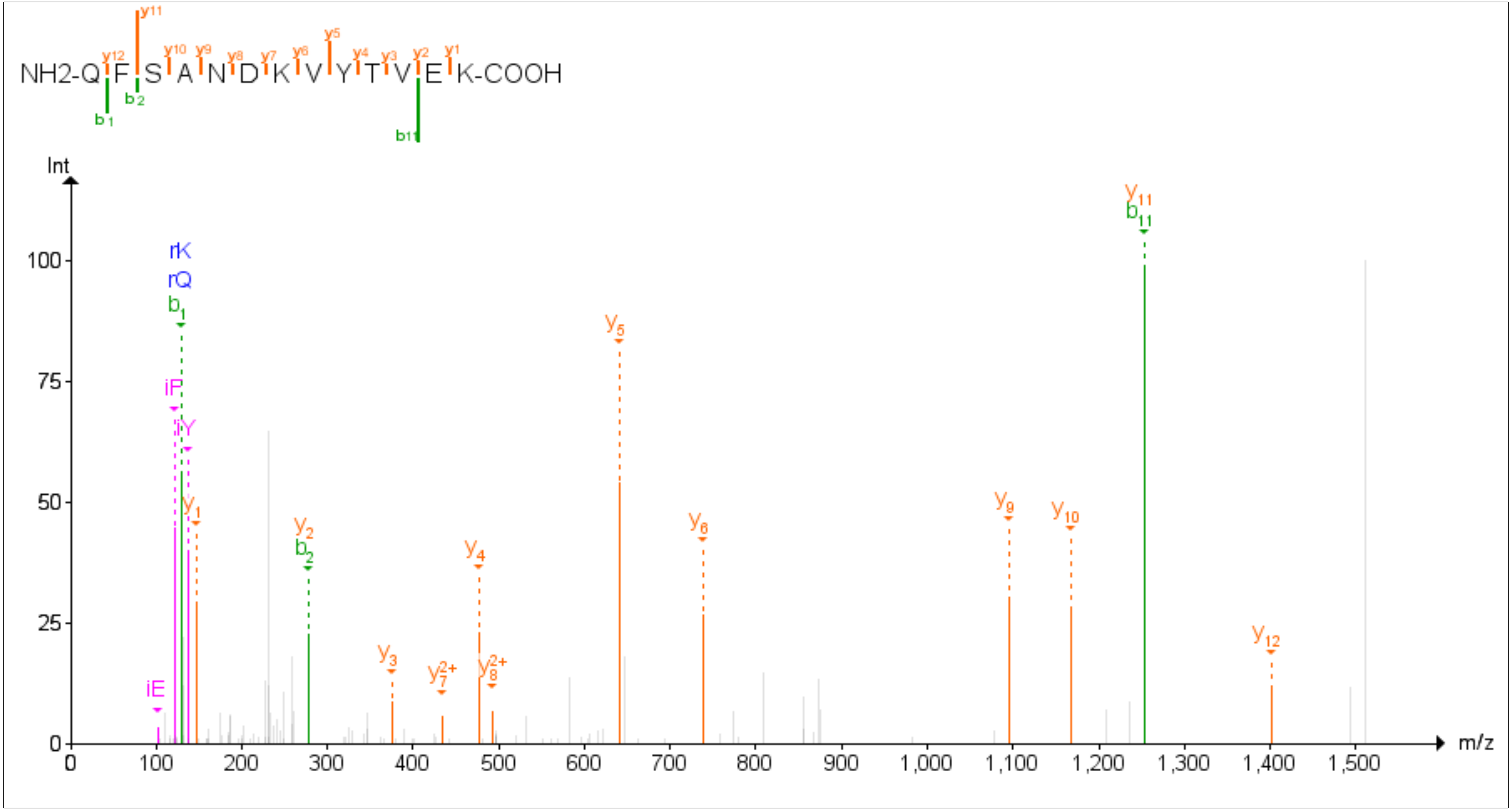
NH2-QFSANDKVYTVEK-COOH
y12 y11 y10 y9 y8 y7 y6 y5 y4 y3 y2 y1
b1 b2 b11
Int
m/z
rK
rQ
b1
iP
iY
y1
iE
y2
b2
y3
y4
y5
y6
y9
y10
y11
b11
y12
0
25
50
75
100
0
100
200
300
400
500
600
700
800
900
1,000
1,100
1,200
1,300
1,400
1,500

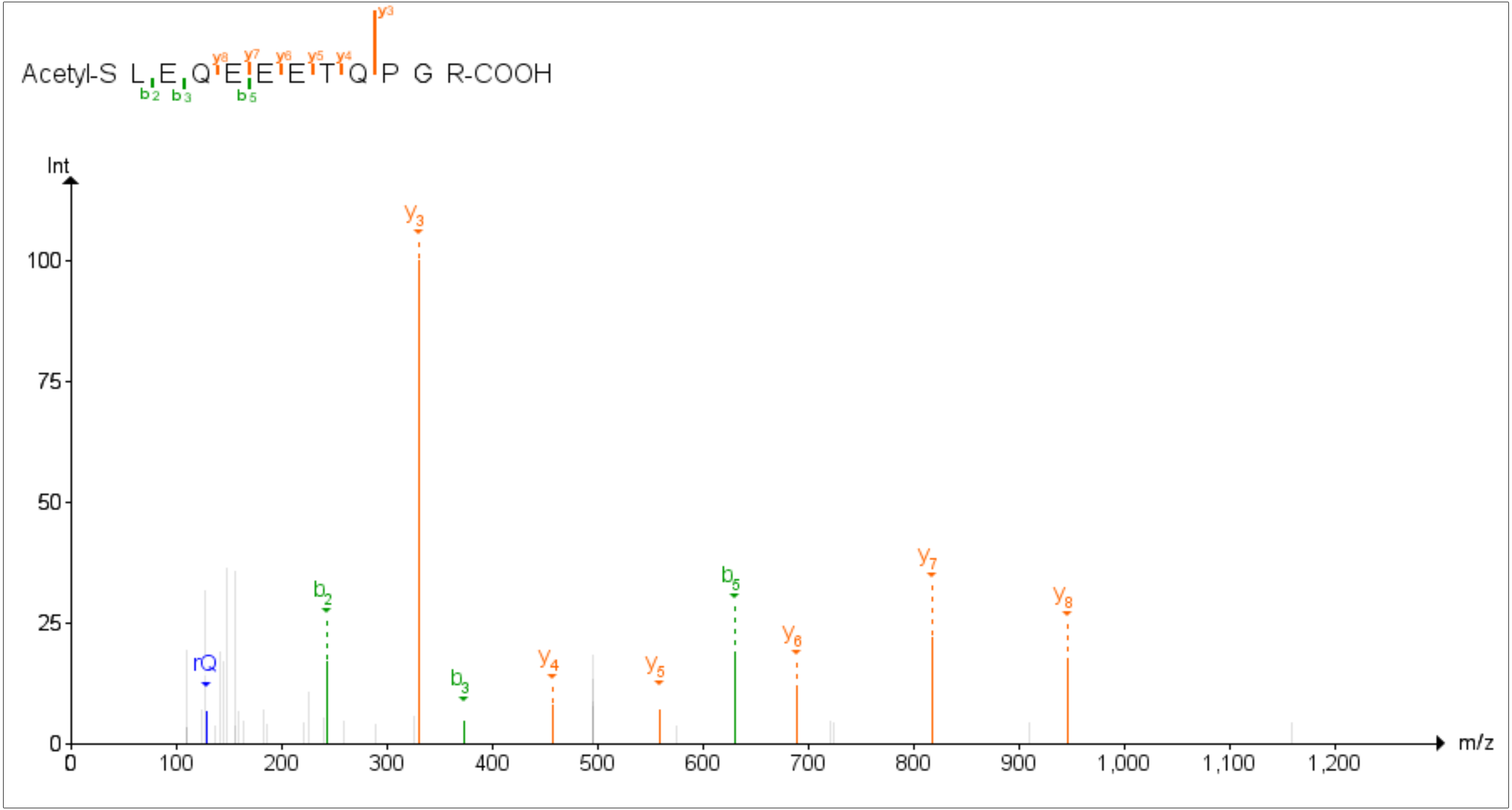

Acetyl-S L E Q E E E T Q P G R-COOH
y3 y4 y5 y6 y7 y8
b2 b3 b5
Int
100
75
50
25
0
rQ
b2
y3
b3
y4
y5
b5
y6
y7
y8
0 100 200 300 400 500 600 700 800 900 1,000 1,100 1,200
m/z

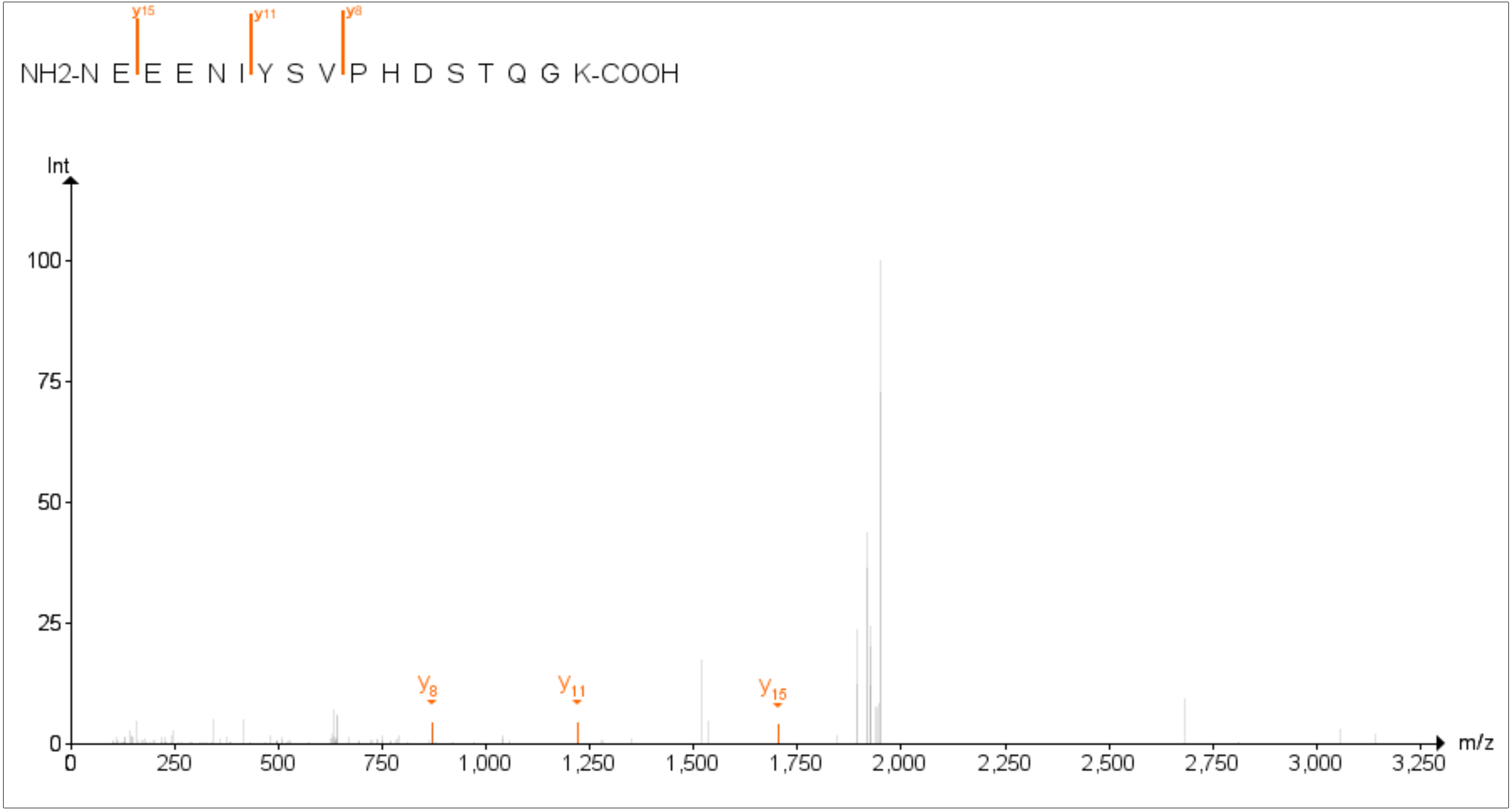
NH2-N E E E N I Y S V P H D S T Q G K-COOH
y15
y11
y8
Int
100
75
50
25
0
y8
y11
y15
0
250
500
750
1,000
1,250
1,500
1,750
2,000
2,250
2,500
2,750
3,000
3,250
m/z

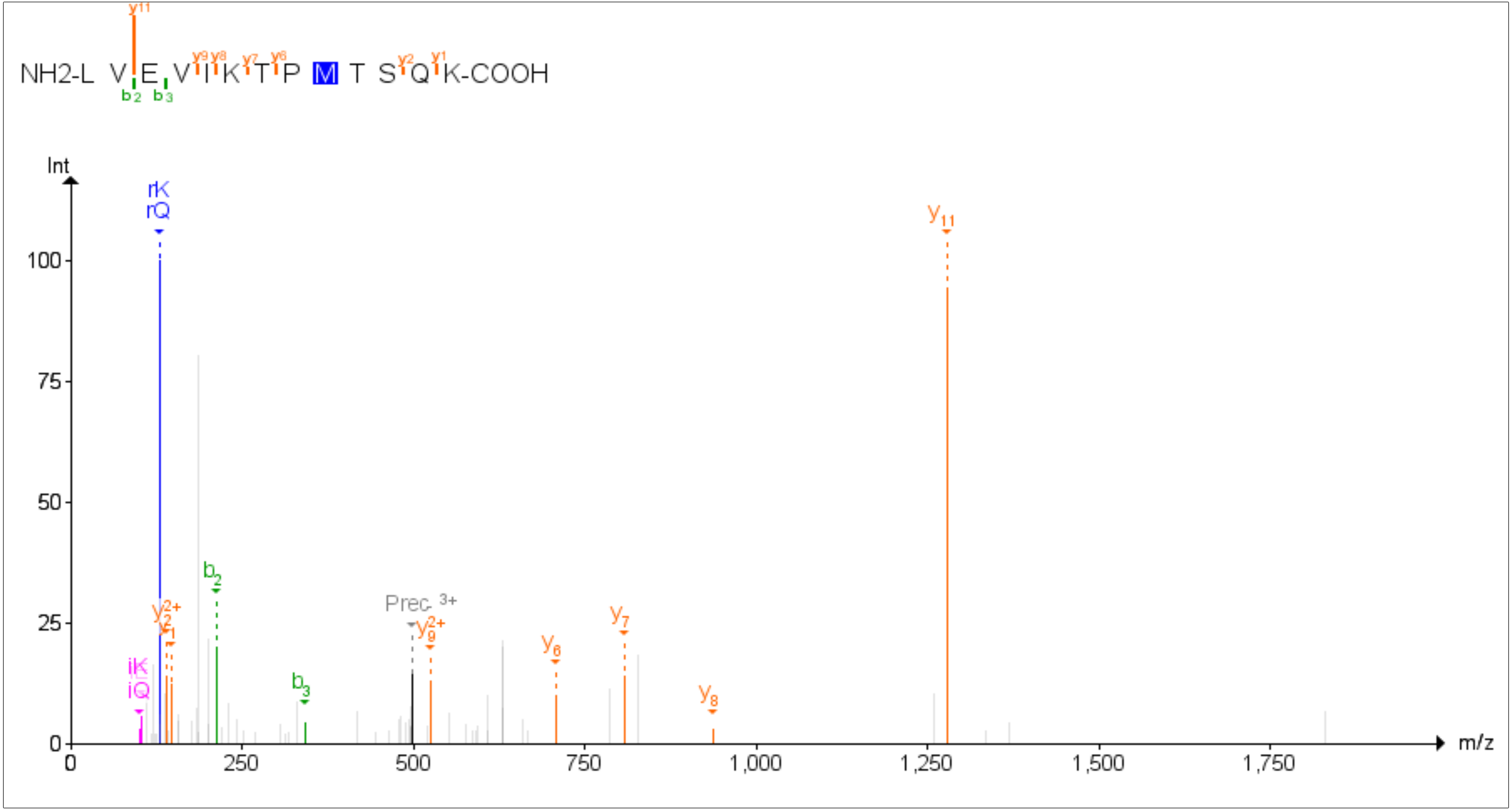

NH2-L V E V I K T P M T S Q K-COOH
y11
y9 y8 y7 y6 y2 y1
b2 b3
Int
rK
rQ
y11
b2
Prec 3+
y2 2+
y1
y9 2+
y6
y7
y8
iK
iQ
b3
100
75
50
25
0
0
250
500
750
1,000
1,250
1,500
1,750
m/z

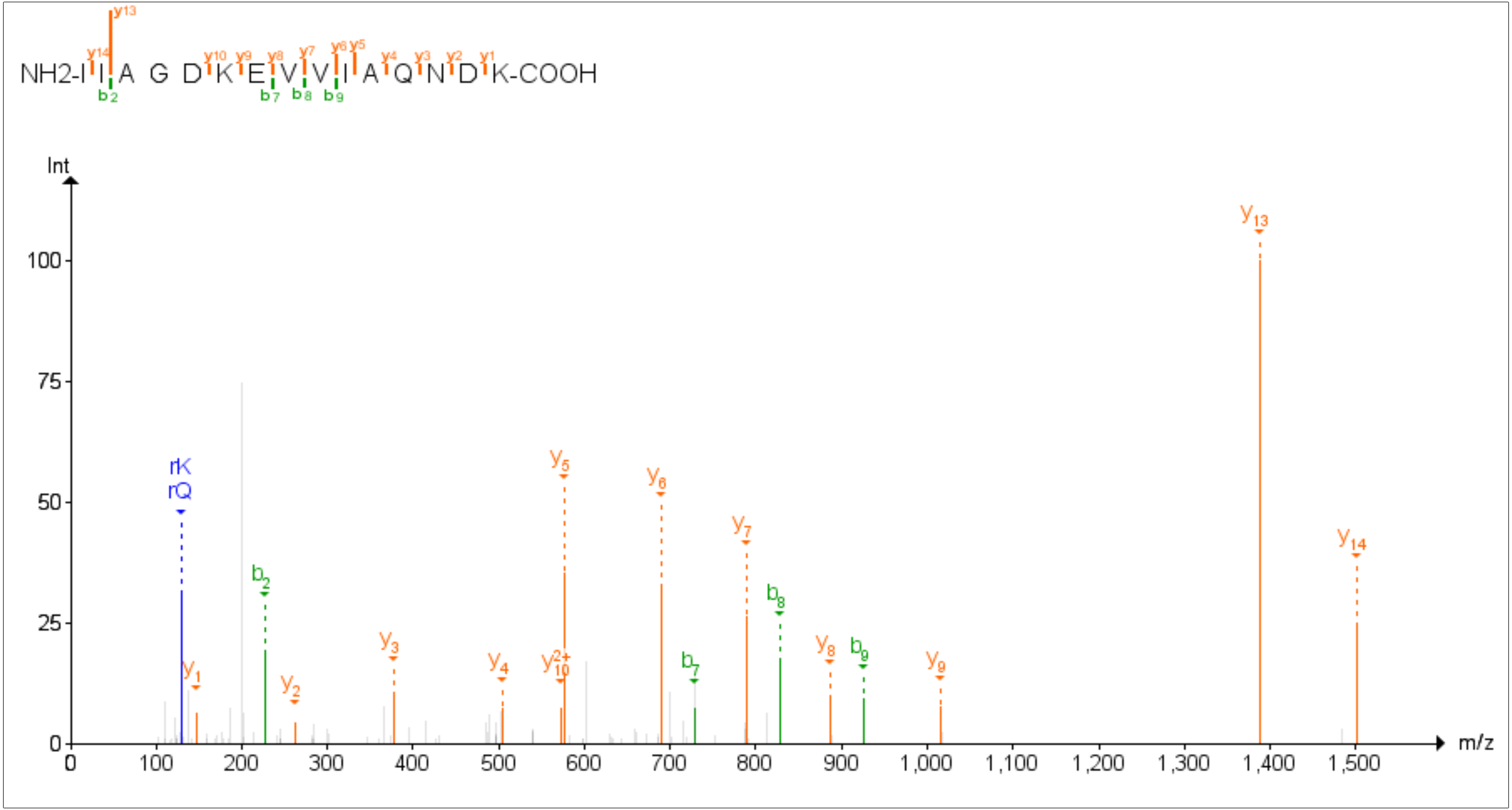

NH2-I I A G D K E V V I A Q N D K-COOH
y13 y14 y10 y9 y8 y7 y6 y5 y4 y3 y2 y1
b2 b7 b8 b9
Int
100
75
50
25
0
rK
rQ
b2
y1
y2
y3
y4
y5
y6
y7
y8
y9
y10 2+
y13
y14
b7
b8
b9
0 100 200 300 400 500 600 700 800 900 1,000 1,100 1,200 1,300 1,400 1,500
m/z

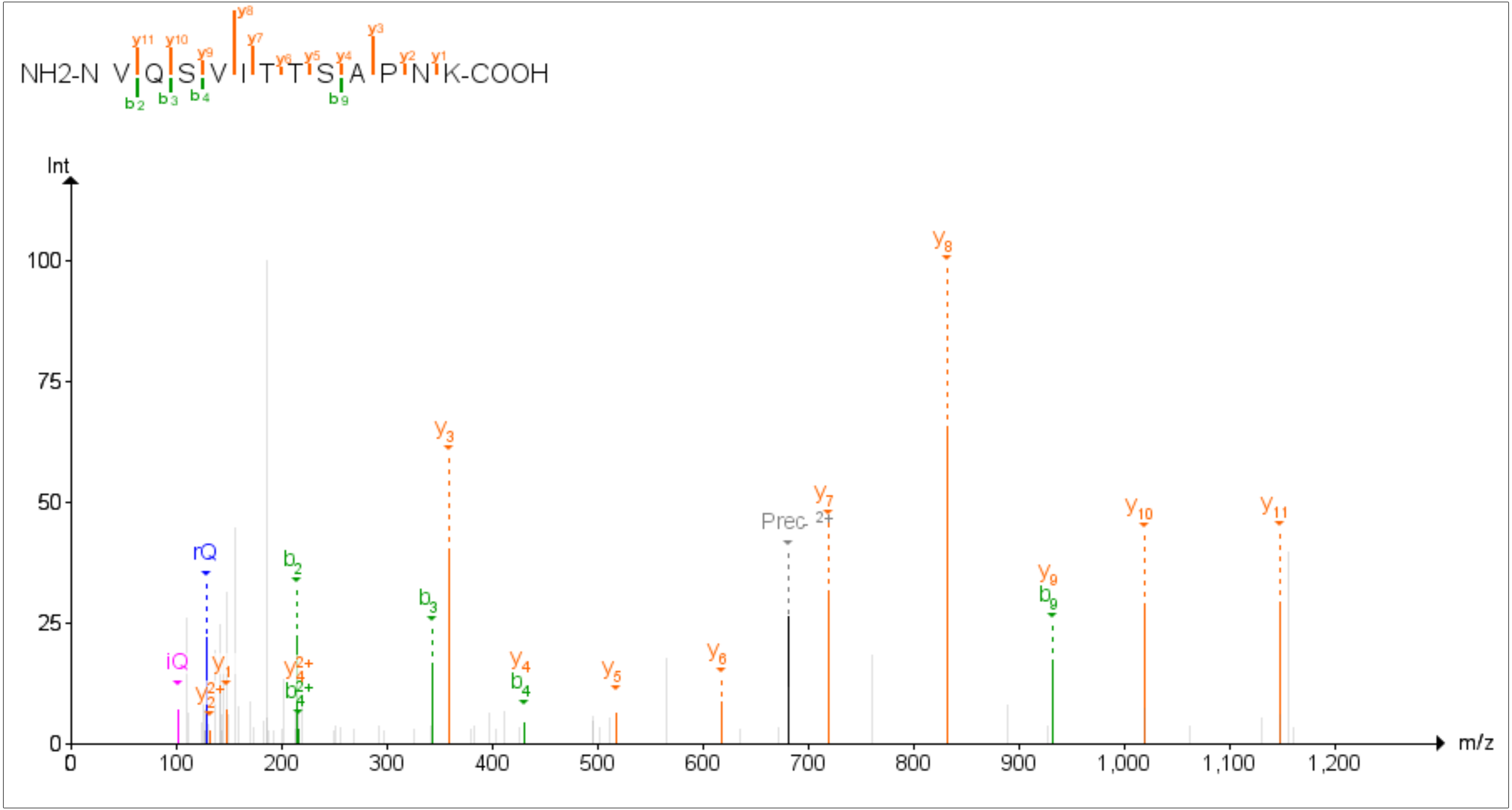

NH2-N V Q S V I T T S A P N K-COOH
Int
m/z
y8
y3
y7
y10
y11
Prec- 2+
y9
b9
rQ
b2
b3
y4
b4
y5
y6
iQ
y1
y2 2+
y4 2+
b4 2+

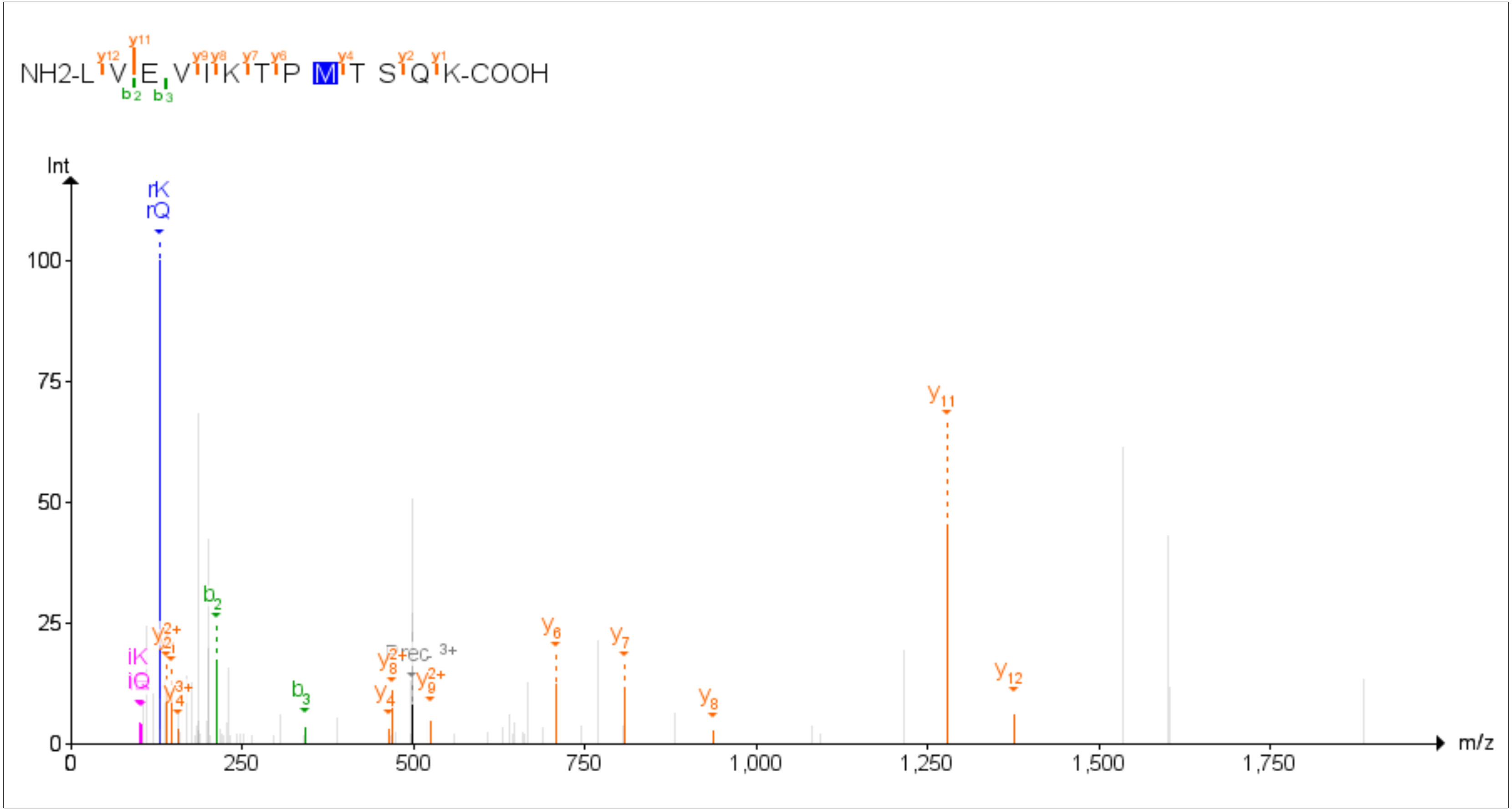

NH2-LVEVIKTPMTSQK-COOH
y12 y11 y9 y8 y7 y6 y4 y2 y1
b2 b3
Int
rK
rQ
100
75
50
25
0
y11
y12
b2
b3
y6
y7
y8
y4
iK
iQ
Prec 3+
m/z
0
250
500
750
1,000
1,250
1,500
1,750

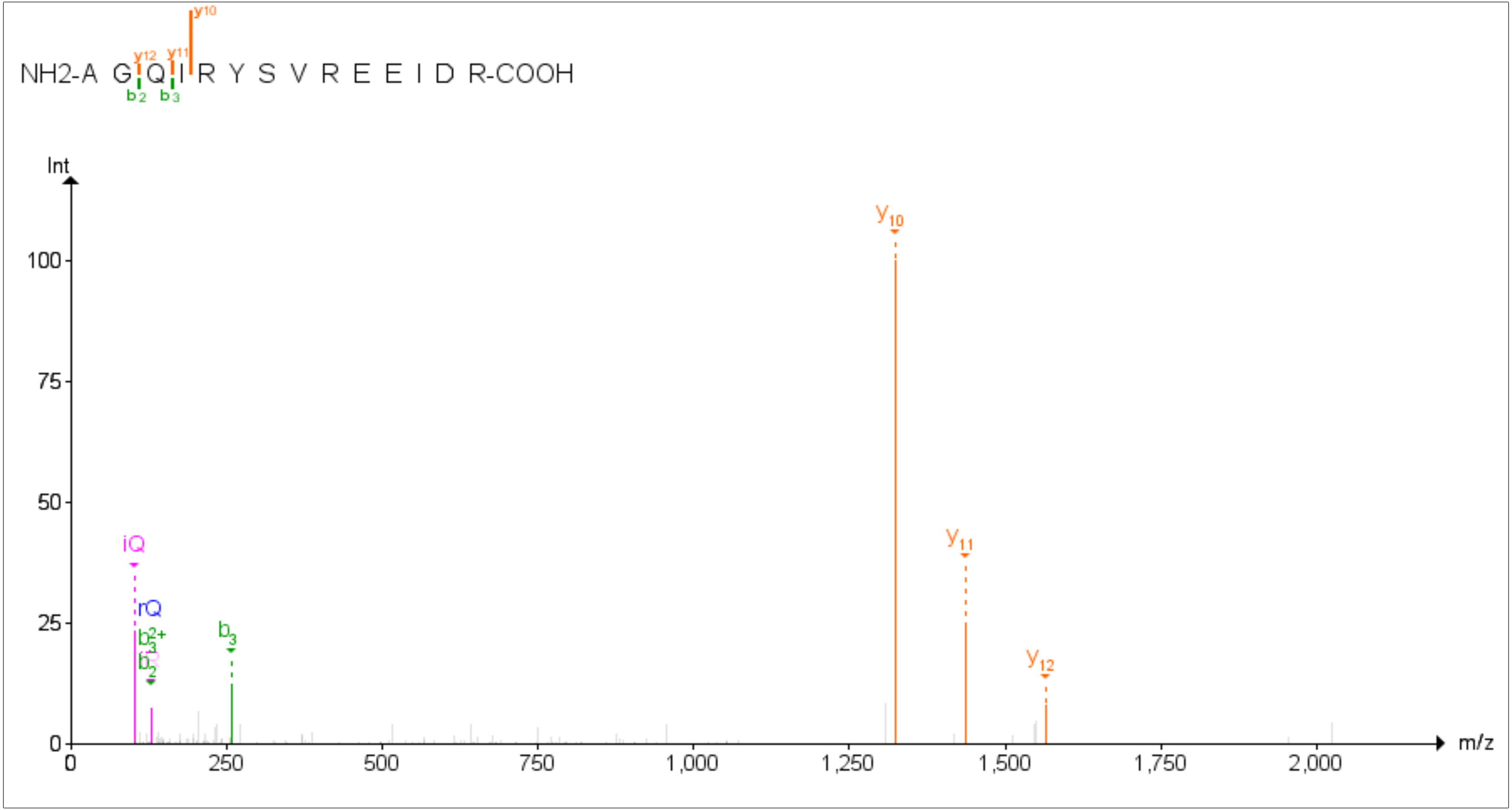

NH2-A G Q I R Y S V R E E I D R-COOH
y10
y12
y11
b2
b3
Int
100
75
50
25
0
y10
y11
y12
iQ
rQ
b3
0
250
500
750
1,000
1,250
1,500
1,750
2,000
m/z

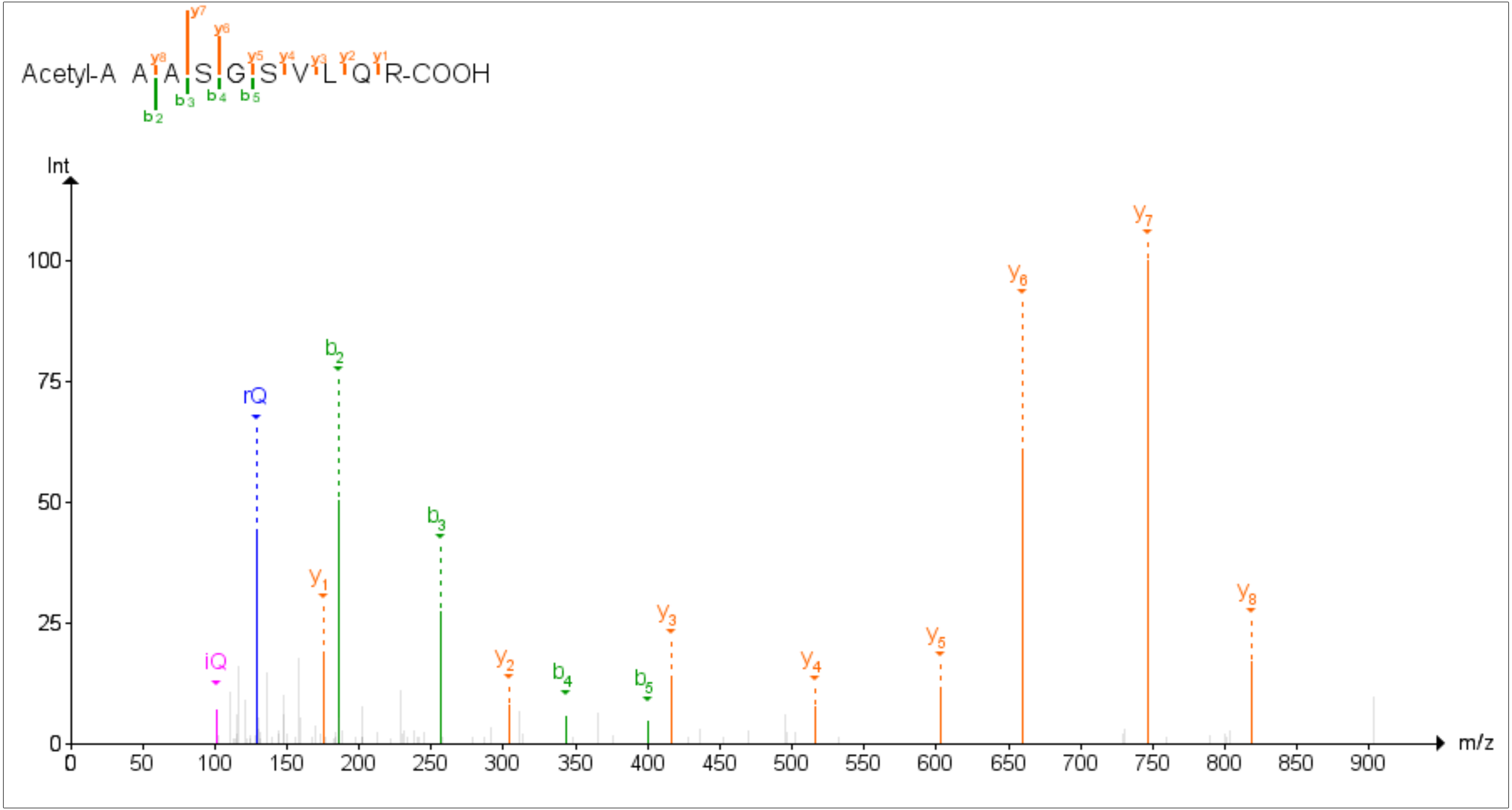

Acetyl-A A A S G S V L Q R-COOH
y8 y7 y6 y5 y4 y3 y2 y1
b2 b3 b4 b5
Int
m/z
100
75
50
25
0
0 50 100 150 200 250 300 350 400 450 500 550 600 650 700 750 800 850 900
iQ
rQ
y1
b2
b3
y2
b4
b5
y3
y4
y5
y6
y7
y8

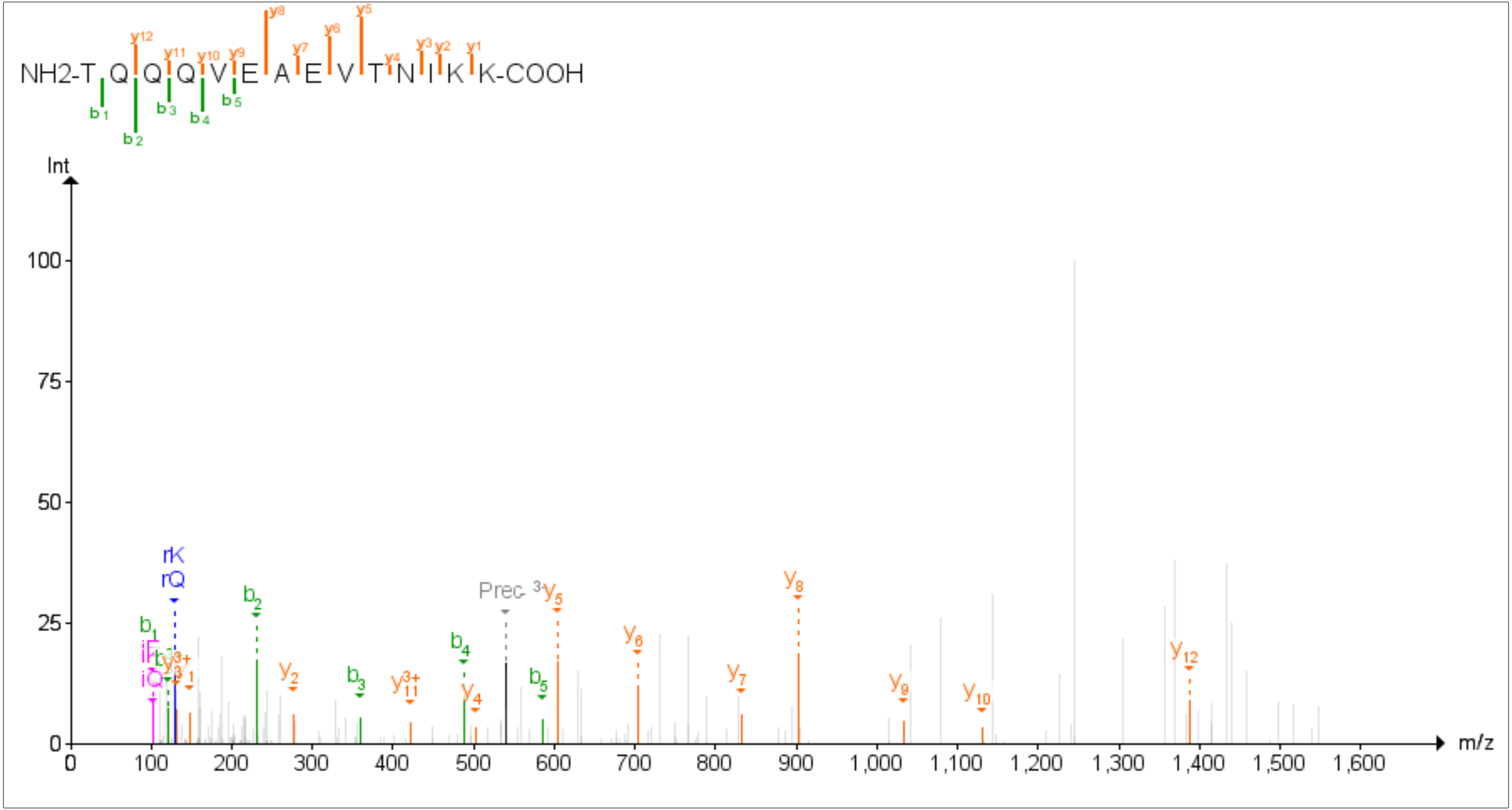

NH2-T Q Q Q V E A E V T N I K K-COOH
Int
m/z
rK
rQ
Prec 3+

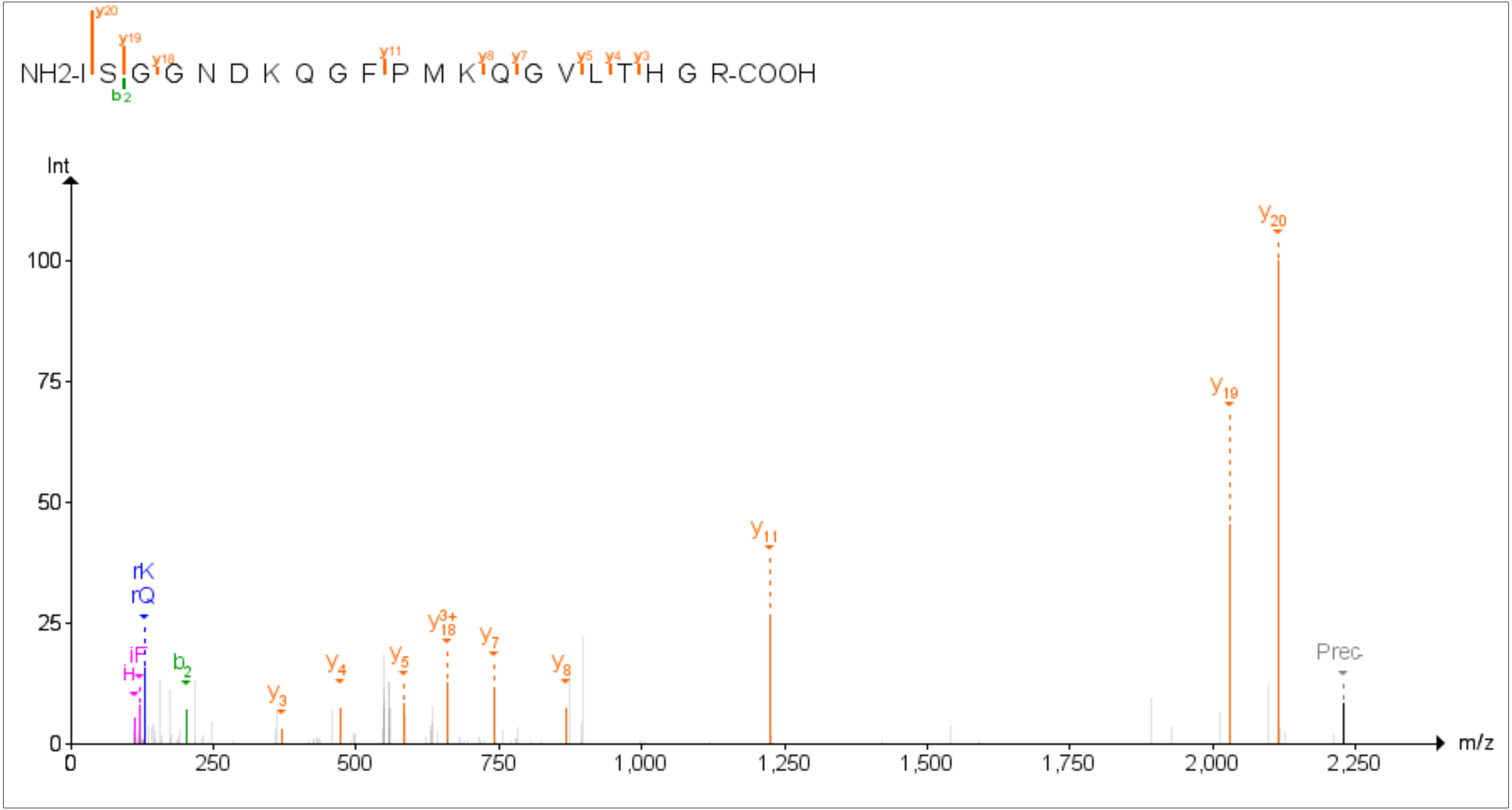
NH2-I S G G N D K Q G F P M K Q G V L T H G R-COOH
y20
y19
y18
y11
y8
y7
y5
y4
y3
b2
Int
100
75
50
25
0
m/z
0
250
500
750
1,000
1,250
1,500
1,750
2,000
2,250
rK
rQ
iF
H
b2
y3
y4
y5
y18 3+
y7
y8
y11
y19
y20
Prec

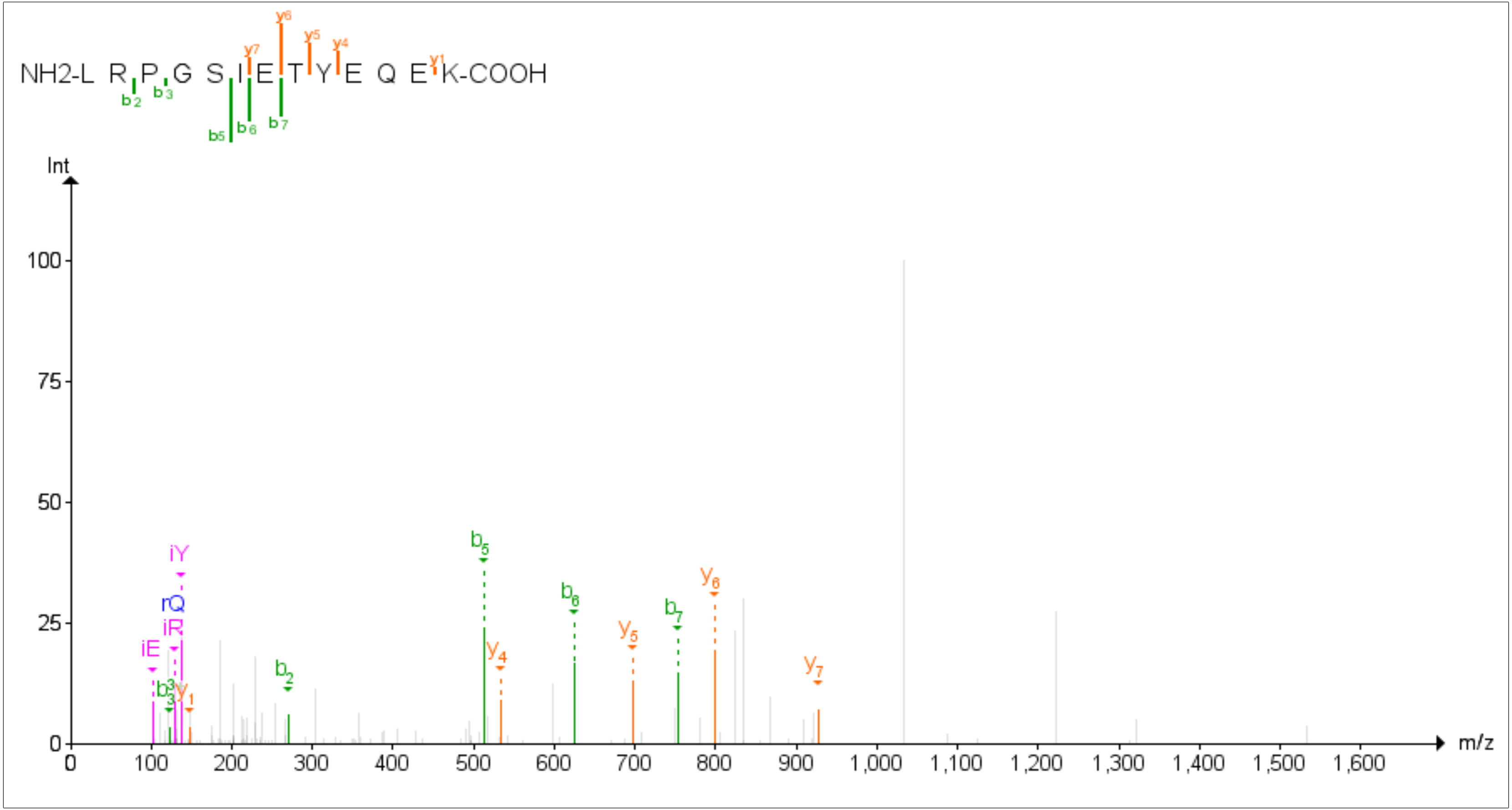

NH2-L R P G S I E T Y E Q E K-COOH
b2 b3 b5 b6 b7
y7 y6 y5 y4 y1
Int
100
75
50
25
0
iY
rQ
iR
iE
b3
y1
b2
b5
y4
b6
y5
b7
y6
y7
0 100 200 300 400 500 600 700 800 900 1,000 1,100 1,200 1,300 1,400 1,500 1,600
m/z

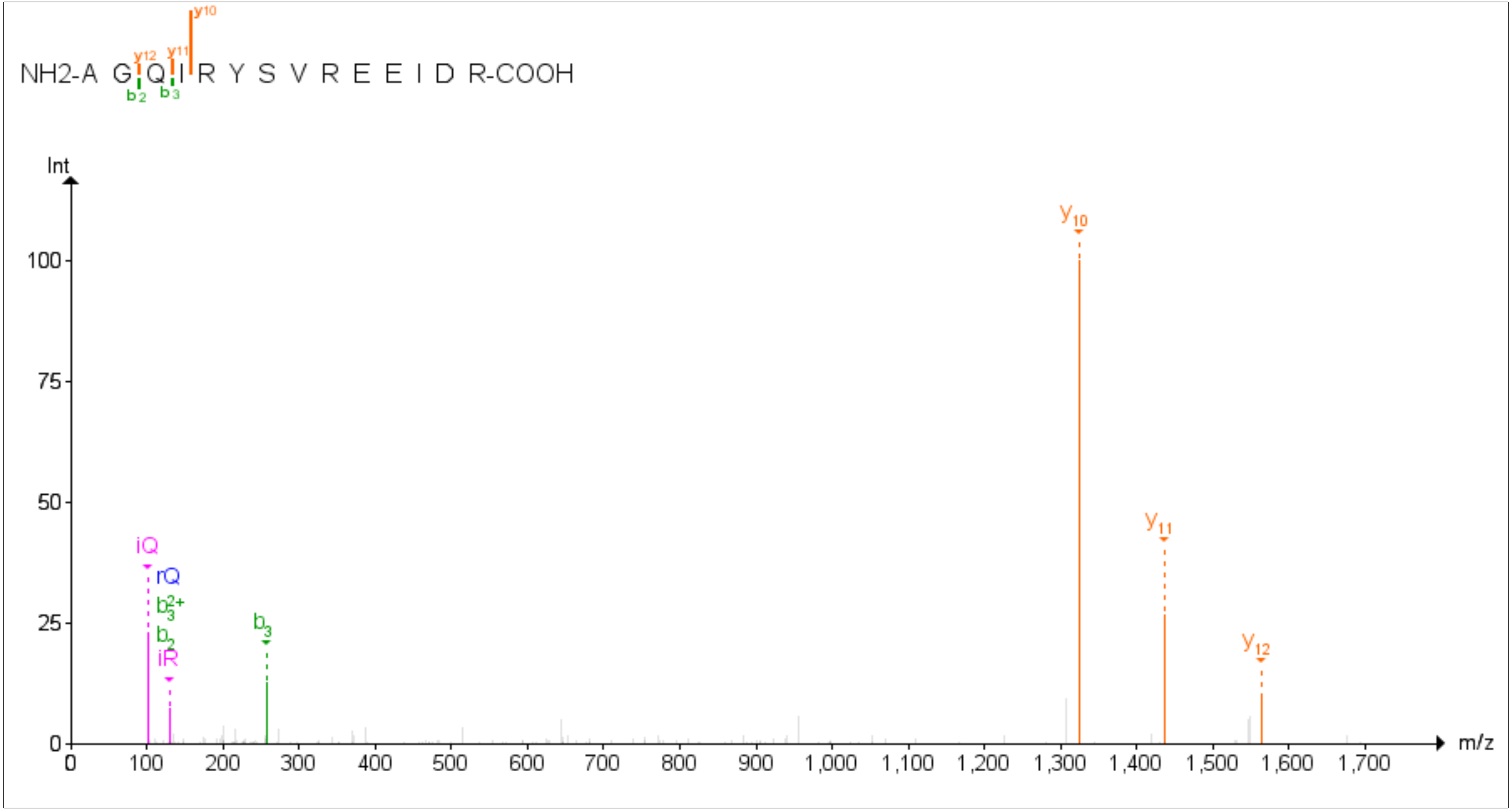
NH2-A G Q I R Y S V R E E I D R-COOH
y10
y11
y12
b2
b3
Int
100
75
50
25
0
y10
y11
y12
iQ
rQ
b3 2+
b2
iR
b3
0
100
200
300
400
500
600
700
800
900
1,000
1,100
1,200
1,300
1,400
1,500
1,600
1,700
m/z

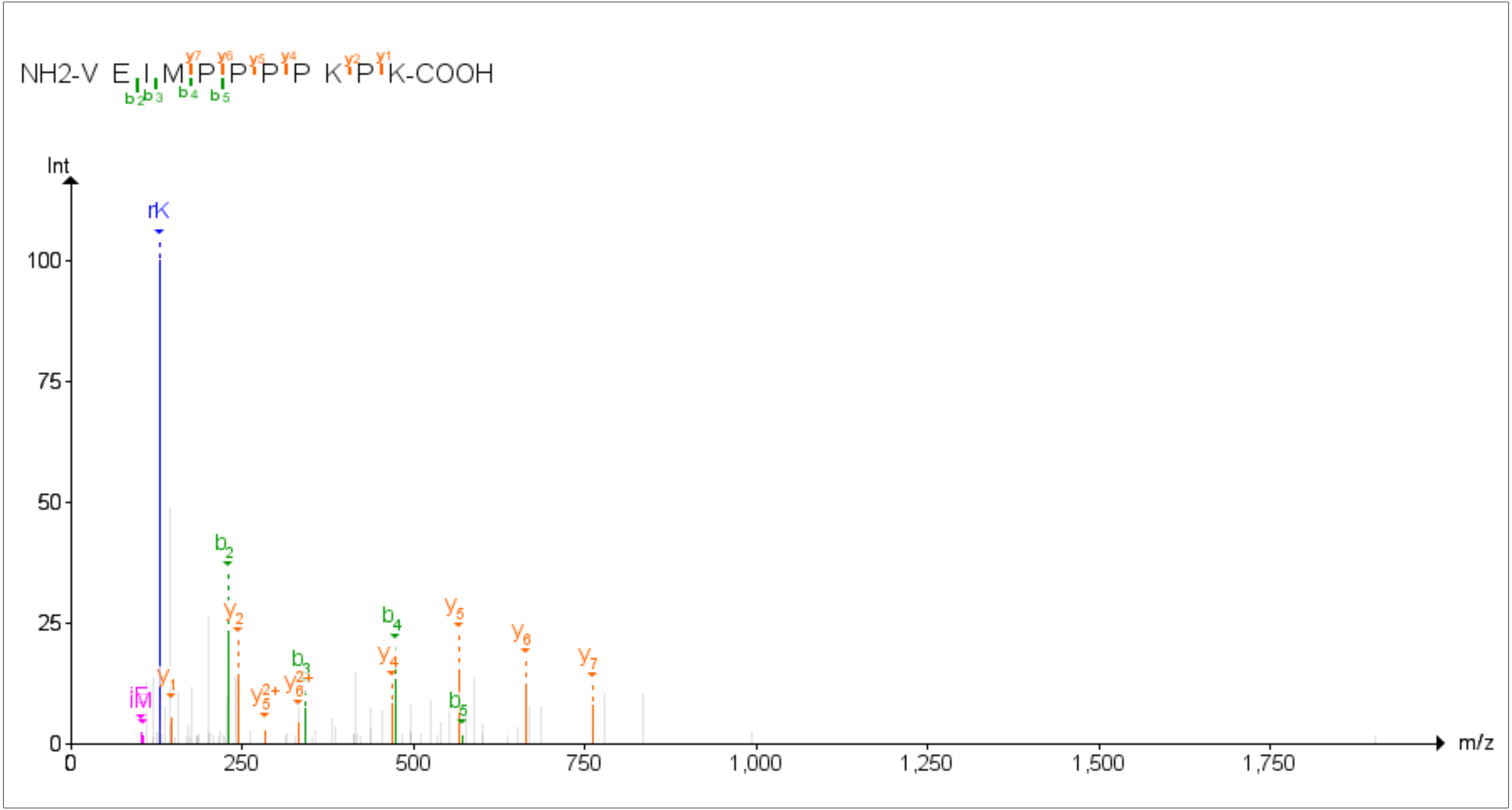

NH2-V E I M P P P P K P K-COOH
y7 y6 y5 y4 y2 y1
b2 b3 b4 b5
Int
rK
100
75
50
25
0
b2
y2
y1
iM
b3
y5 2+
y6 2+
b4
y4
y5
b5
y6
y7
0
250
500
750
1,000
1,250
1,500
1,750
m/z

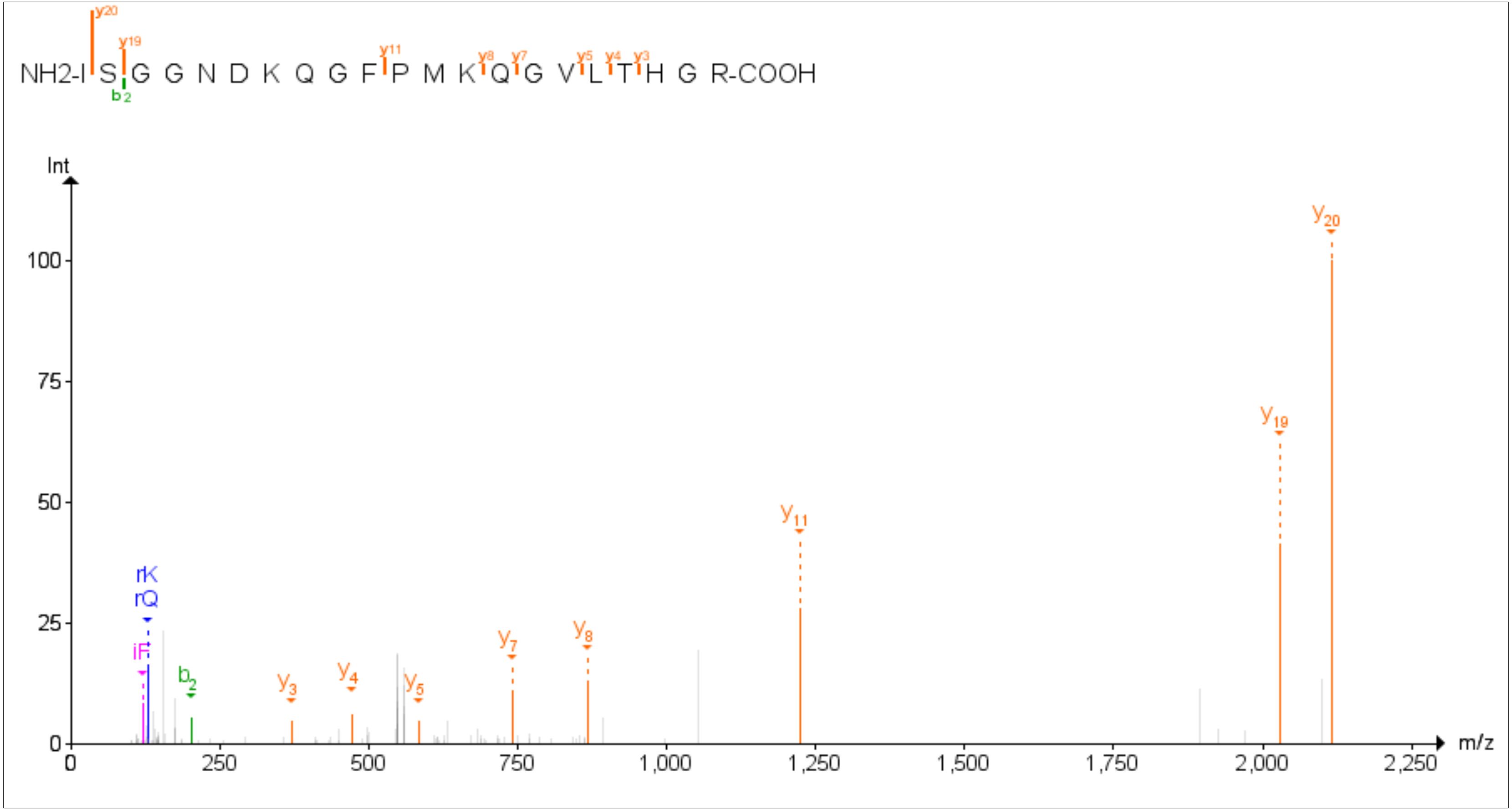

NH2-I S G G N D K Q G F P M K Q G V L T H G R-COOH
y20
y19
y11
y8
y7
y5
y4
y3
b2
Int
100
75
50
25
0
rK
rQ
iF
b2
y3
y4
y5
y7
y8
y11
y19
y20
0
250
500
750
1,000
1,250
1,500
1,750
2,000
2,250
m/z

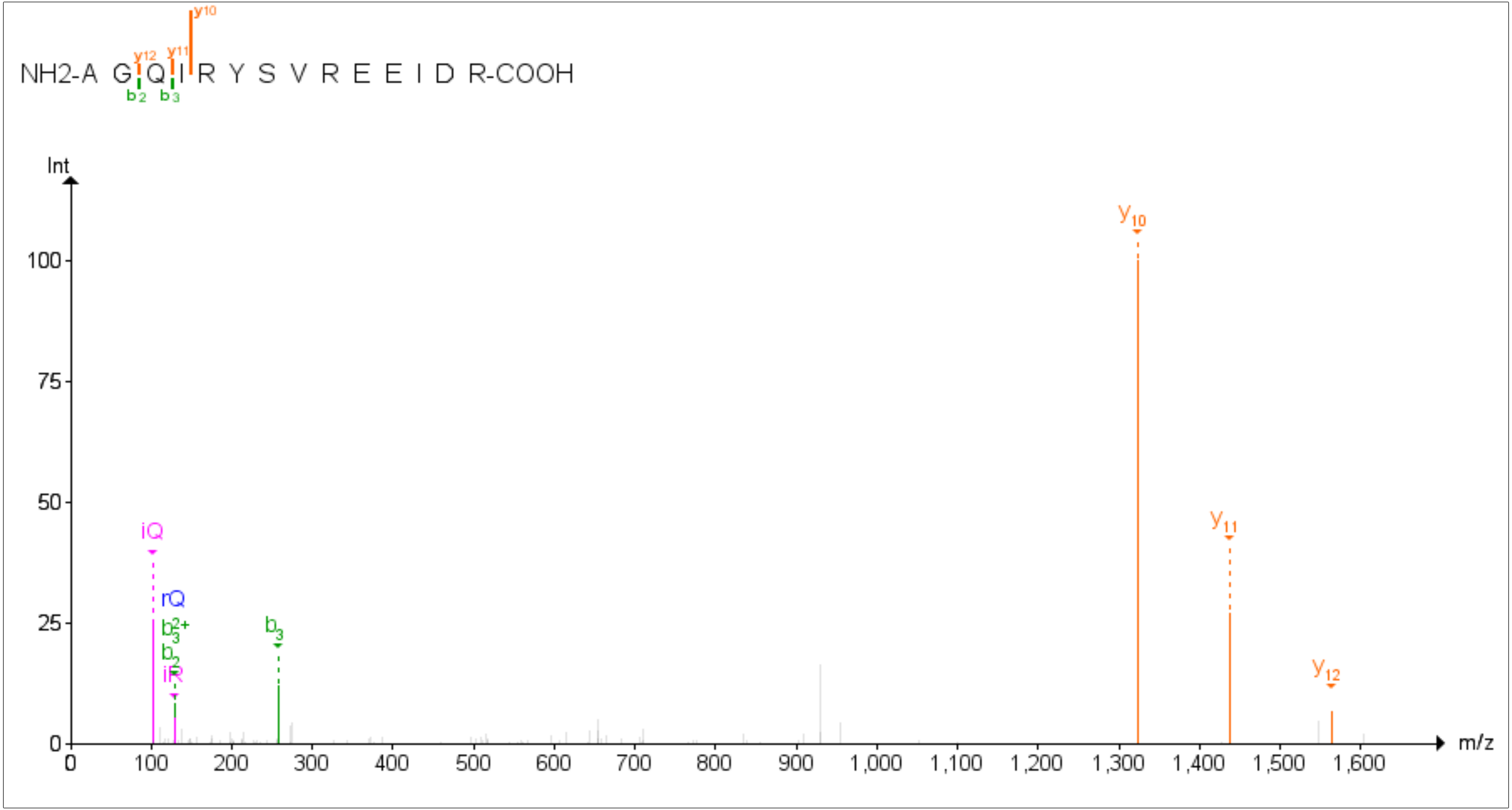

NH2-A G Q I R Y S V R E E I D R-COOH
y10
y12
y11
b2
b3
Int
100
75
50
25
0
iQ
rQ
b3 2+
b2
iR
b3
y10
y11
y12
0
100
200
300
400
500
600
700
800
900
1,000
1,100
1,200
1,300
1,400
1,500
1,600
m/z

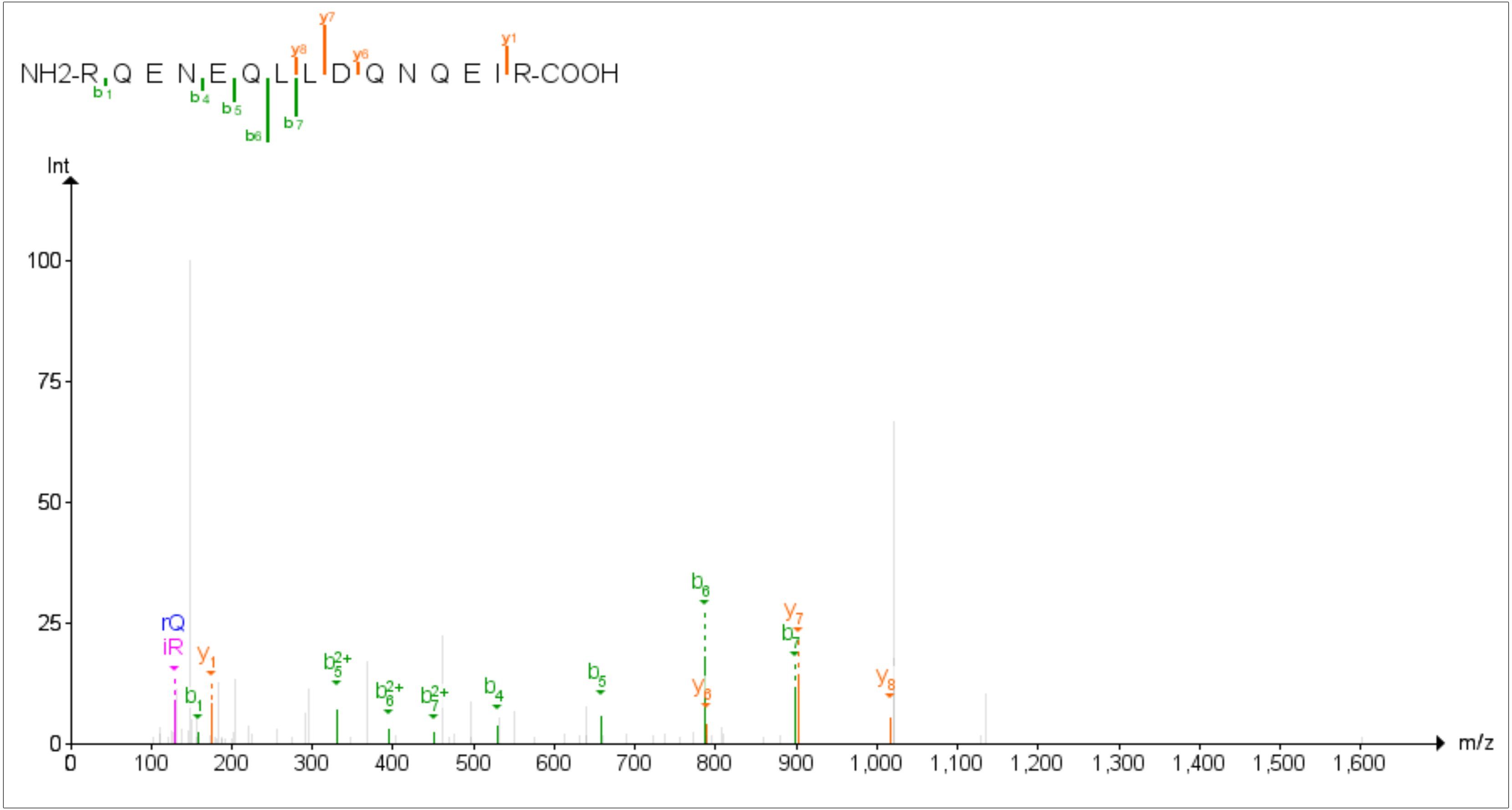

NH2-R Q E N E Q L L D Q N Q E I R-COOH
b1
b4
b5
b6
b7
y8
y7
y6
y1
Int
100
75
50
25
0
rQ
iR
y1
b1
b5 2+
b6 2+
b7 2+
b4
b5
b6
y6
y7
b7
y8
m/z
0
100
200
300
400
500
600
700
800
900
1,000
1,100
1,200
1,300
1,400
1,500
1,600

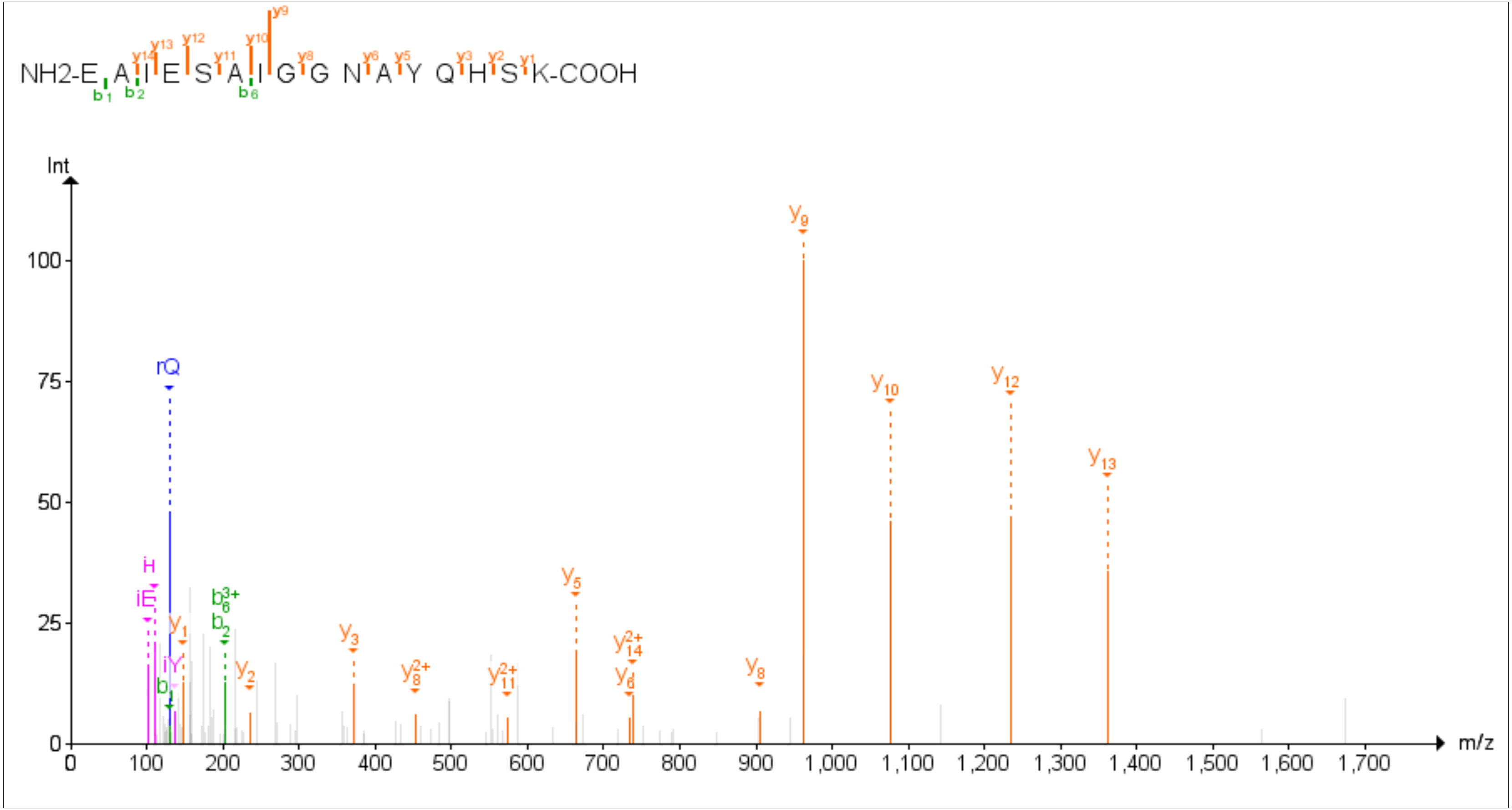

NH2-E A I E S A I G G N A Y Q H S K-COOH
b1 b2 b6
y14 y13 y12 y11 y10 y9 y8 y6 y5 y3 y2 y1
Int
m/z
rQ
iH
iE
iY
y9
y10
y12
y13
y5
y3
y8
y1
y2
b2
b6 3+
y8 2+
y11 2+
y14 2+
y6
0
25
50
75
100
0 100 200 300 400 500 600 700 800 900 1,000 1,100 1,200 1,300 1,400 1,500 1,600 1,700

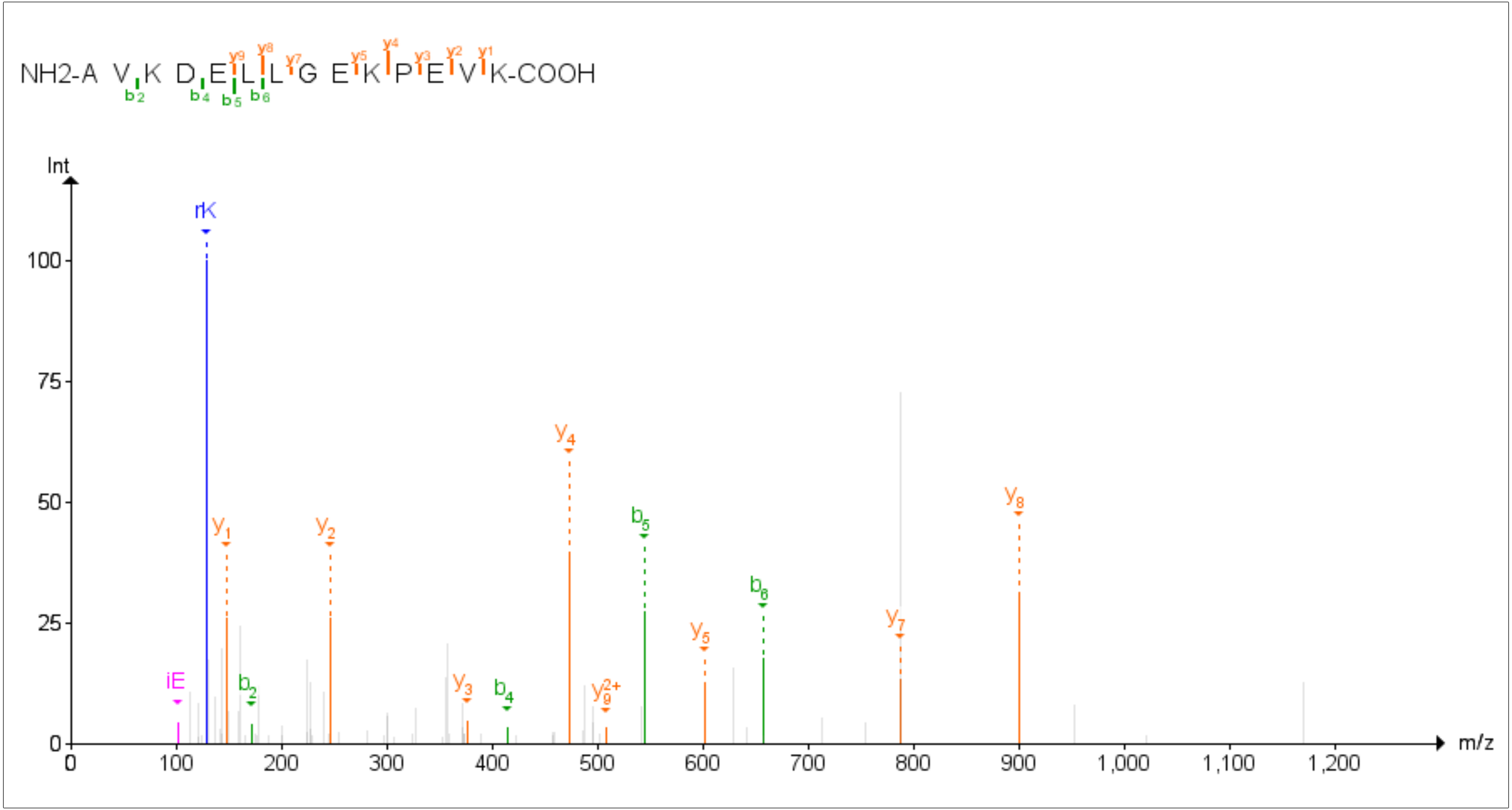

NH2-A V K D E L L G E K P E V K-COOH
b2 b4 b5 b6
y9 y8 y7 y5 y4 y3 y2 y1
Int
m/z
rK
iE
y1 y2 y3 y4 y5 y7 y8 y9 2+
b2 b4 b5 b6
0
25
50
75
100
0 100 200 300 400 500 600 700 800 900 1,000 1,100 1,200

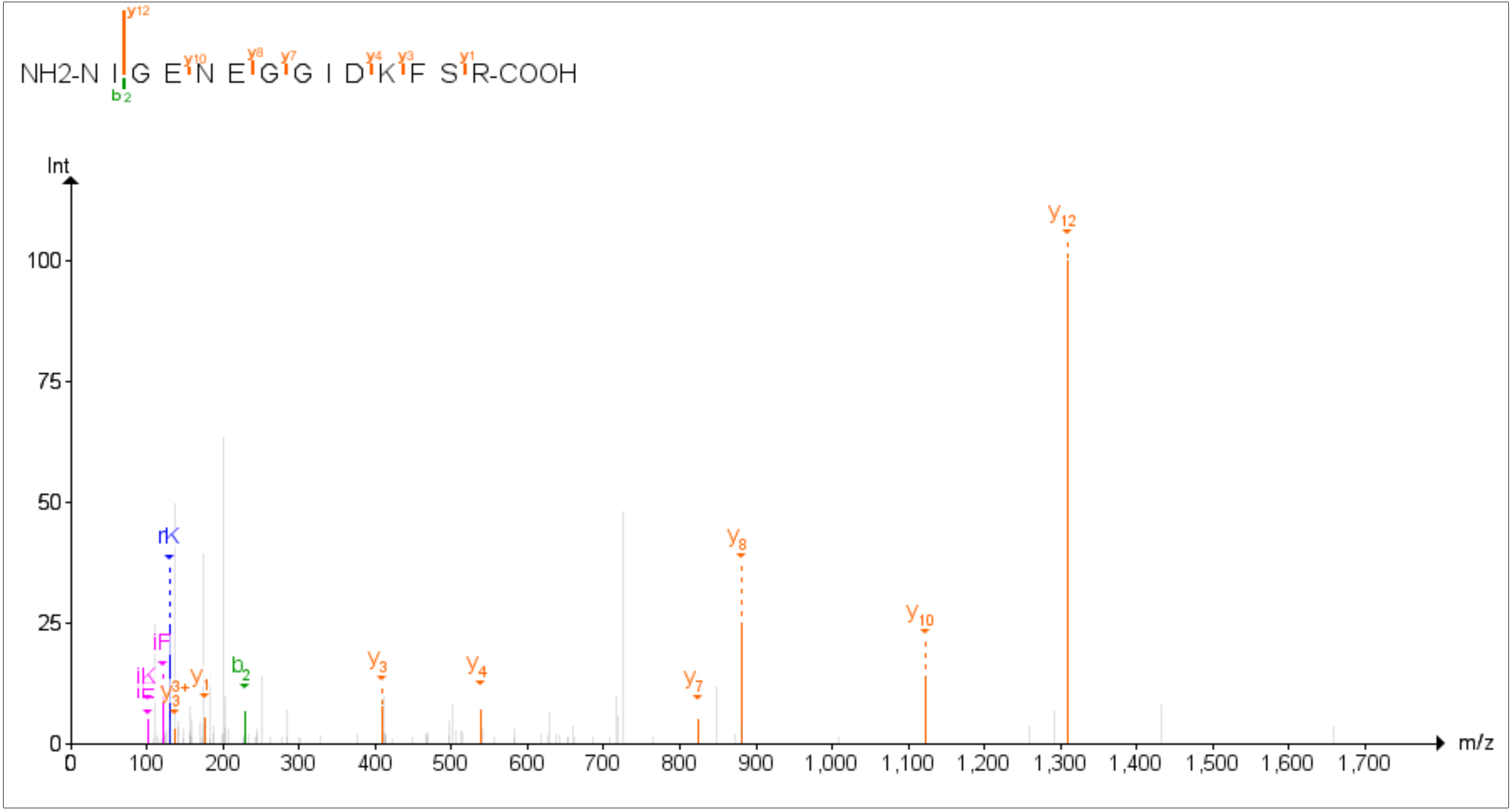

NH2-N I G E N E G G I D K F S R-COOH
y12
y10
y8
y7
y4
y3
y1
b2
Int
100
75
50
25
0
m/z
0
100
200
300
400
500
600
700
800
900
1,000
1,100
1,200
1,300
1,400
1,500
1,600
1,700
y12
rK
y8
y10
iF
iK
iE
y3 3+
y1
b2
y3
y4
y7

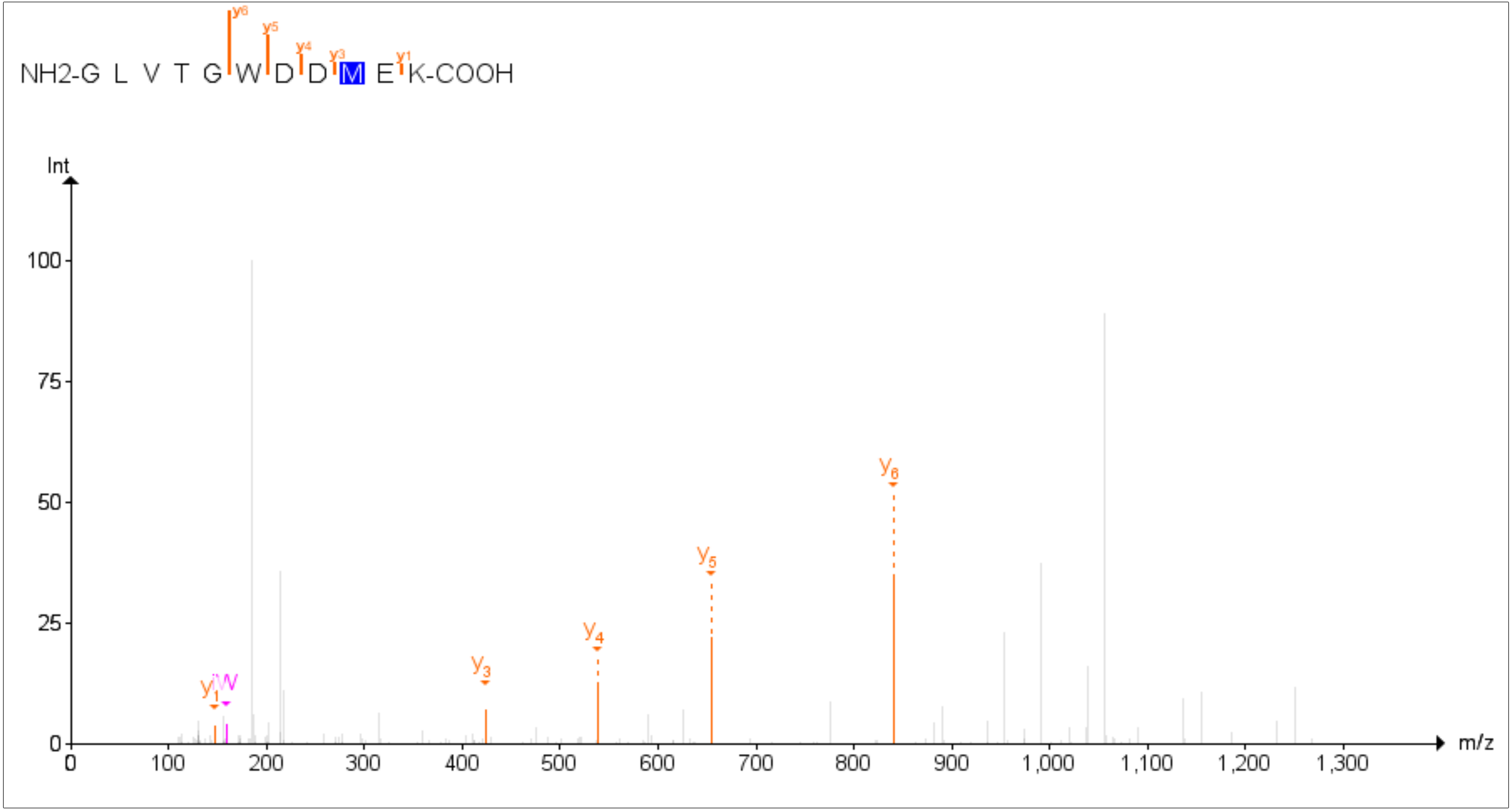

NH2-G L V T G W D D M E K-COOH
y6
y5
y4
y3
y1
Int
100
75
50
25
0
y6
y5
y4
y3
y1
iW
0
100
200
300
400
500
600
700
800
900
1,000
1,100
1,200
1,300
m/z

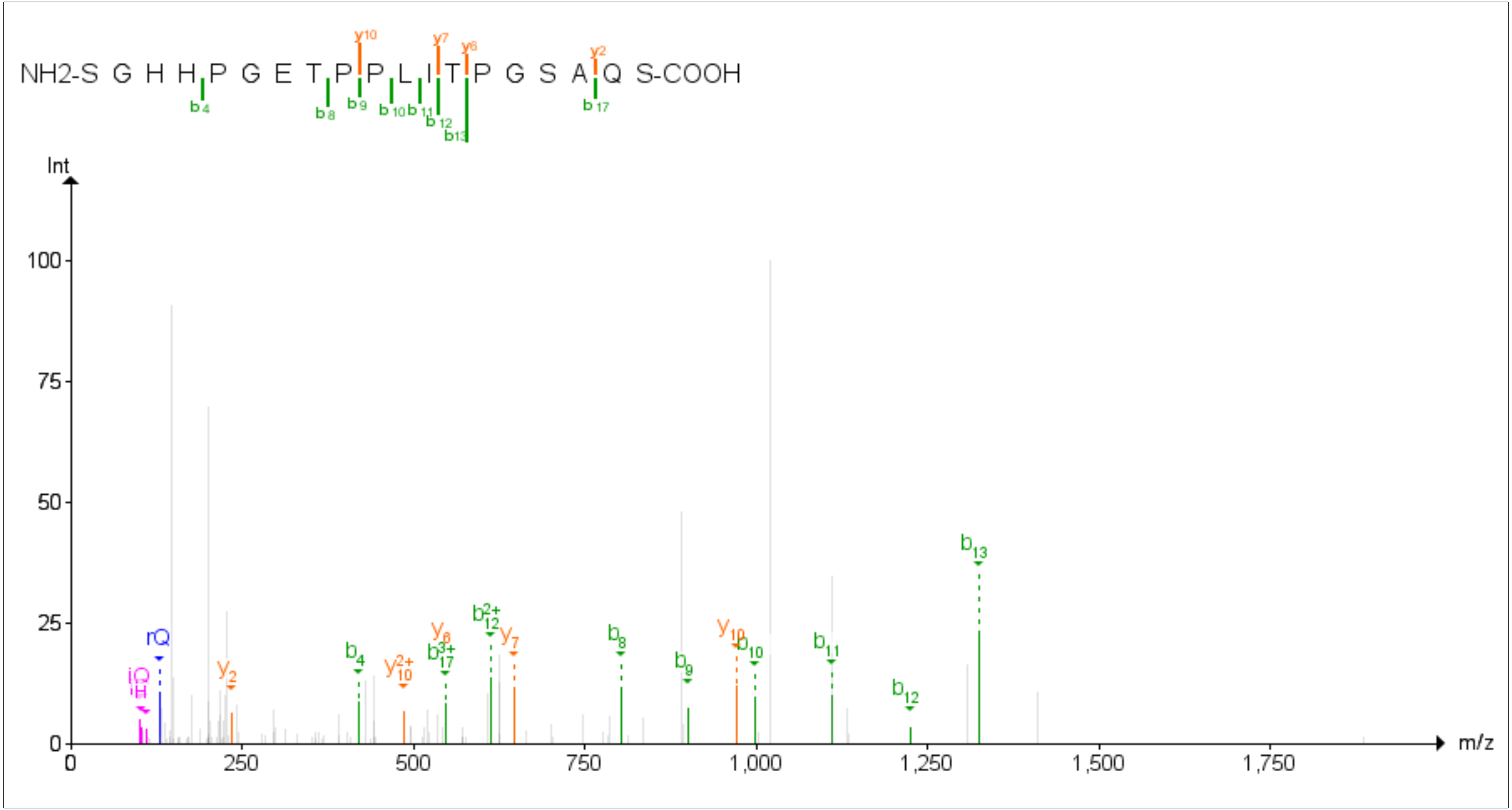

NH2-S G H H P G E T P P L I T P G S A Q S-COOH
y10
y7
y6
y2
b4
b8
b9
b10
b11
b12
b13
b17
Int
100
75
50
25
0
m/z
0
250
500
750
1,000
1,250
1,500
1,750
rQ
iQ
iH
y2
b4
y10 2+
y6
b17 3+
b12 2+
y7
b8
b9
y10
b10
b11
b12
b13

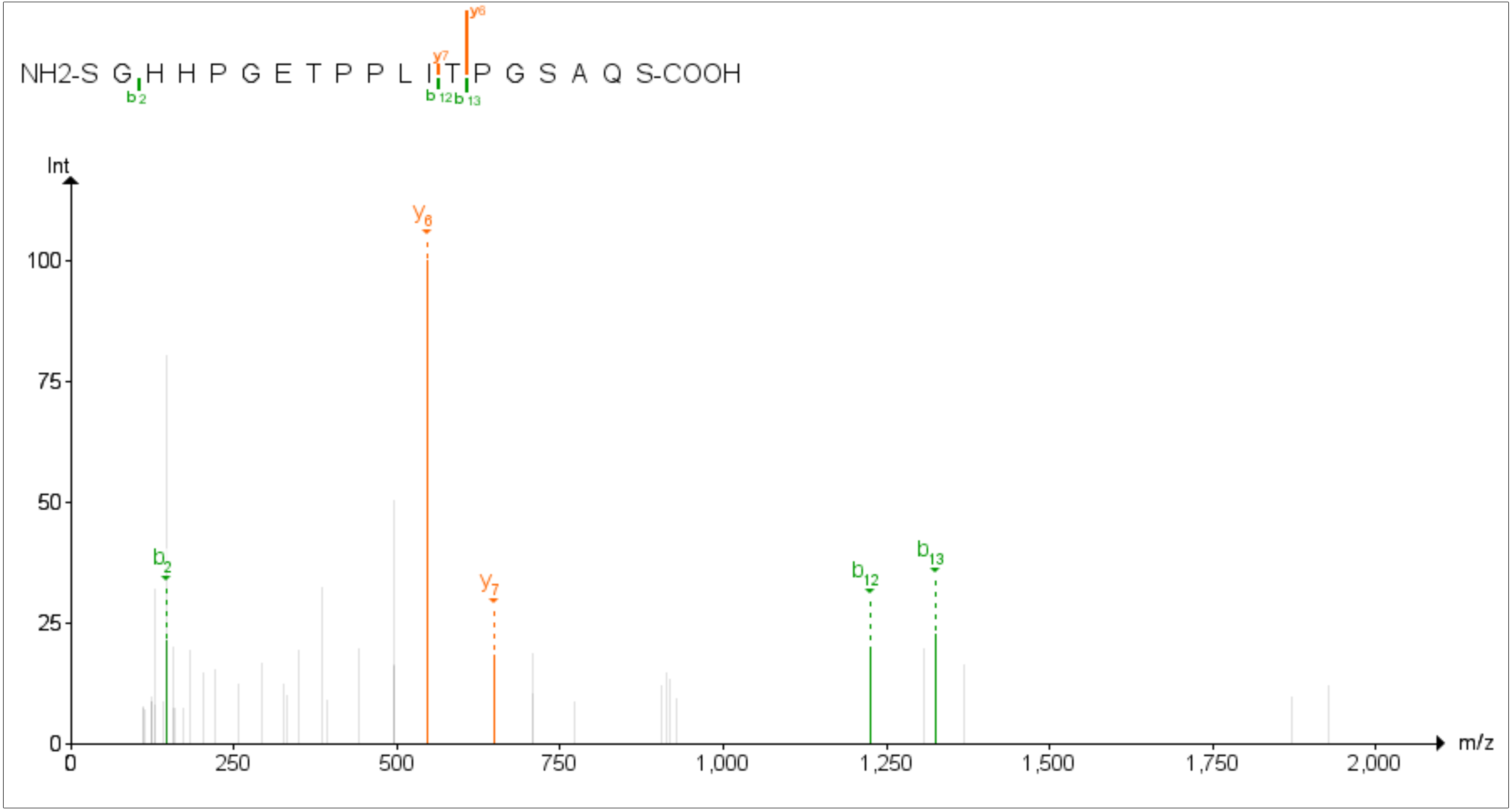

NH2-S G H H P G E T P P L I T P G S A Q S-COOH
b2
b12
b13
y7
y6
Int
100
75
50
25
0
y6
y7
b2
b12
b13
0
250
500
750
1,000
1,250
1,500
1,750
2,000
m/z

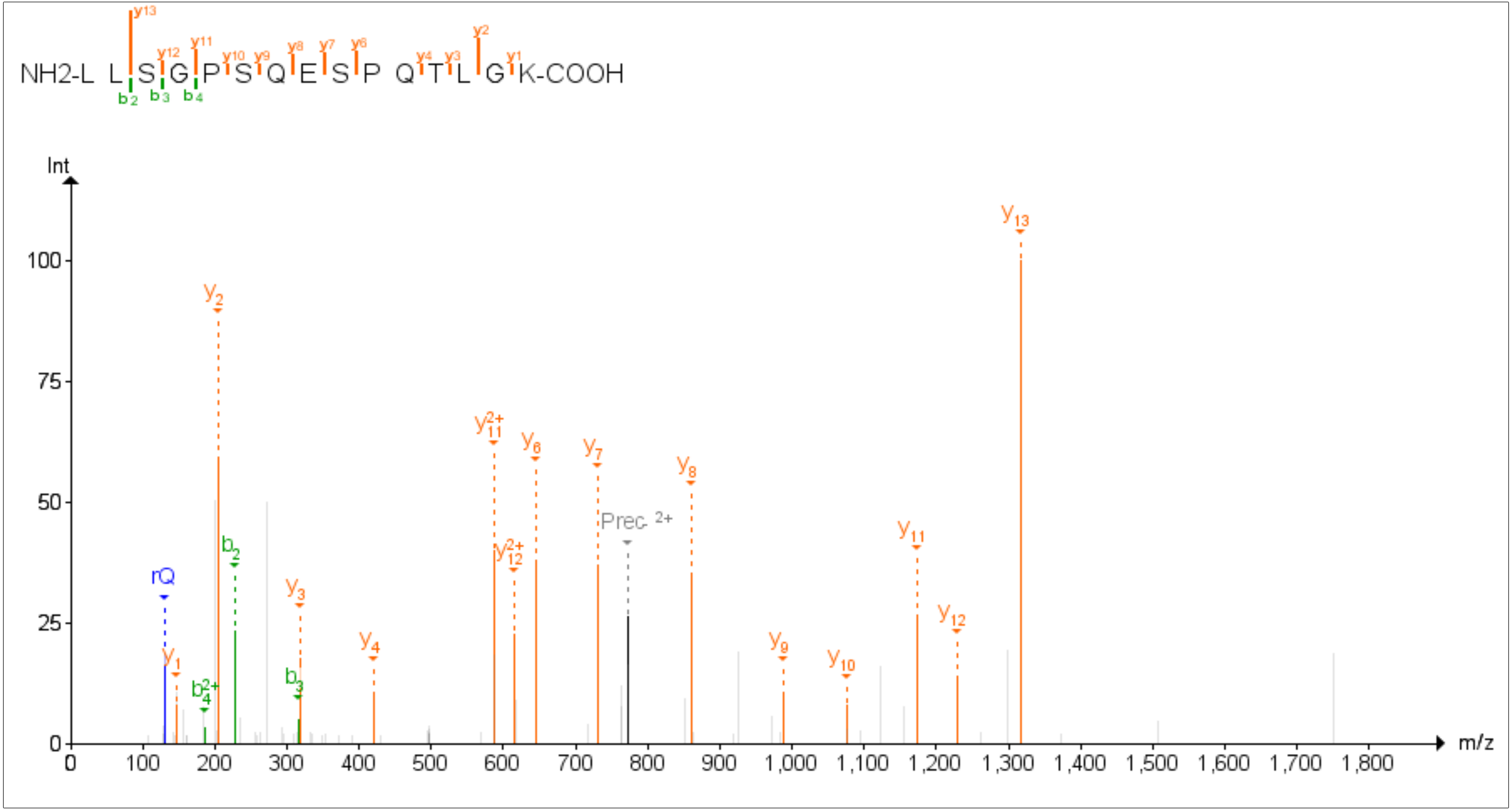
NH2-L L S G P S Q E S P Q T L G K-COOH
y13 y12 y11 y10 y9 y8 y7 y6 y4 y3 y2 y1
b2 b3 b4
Int
m/z
rQ
Prec 2+

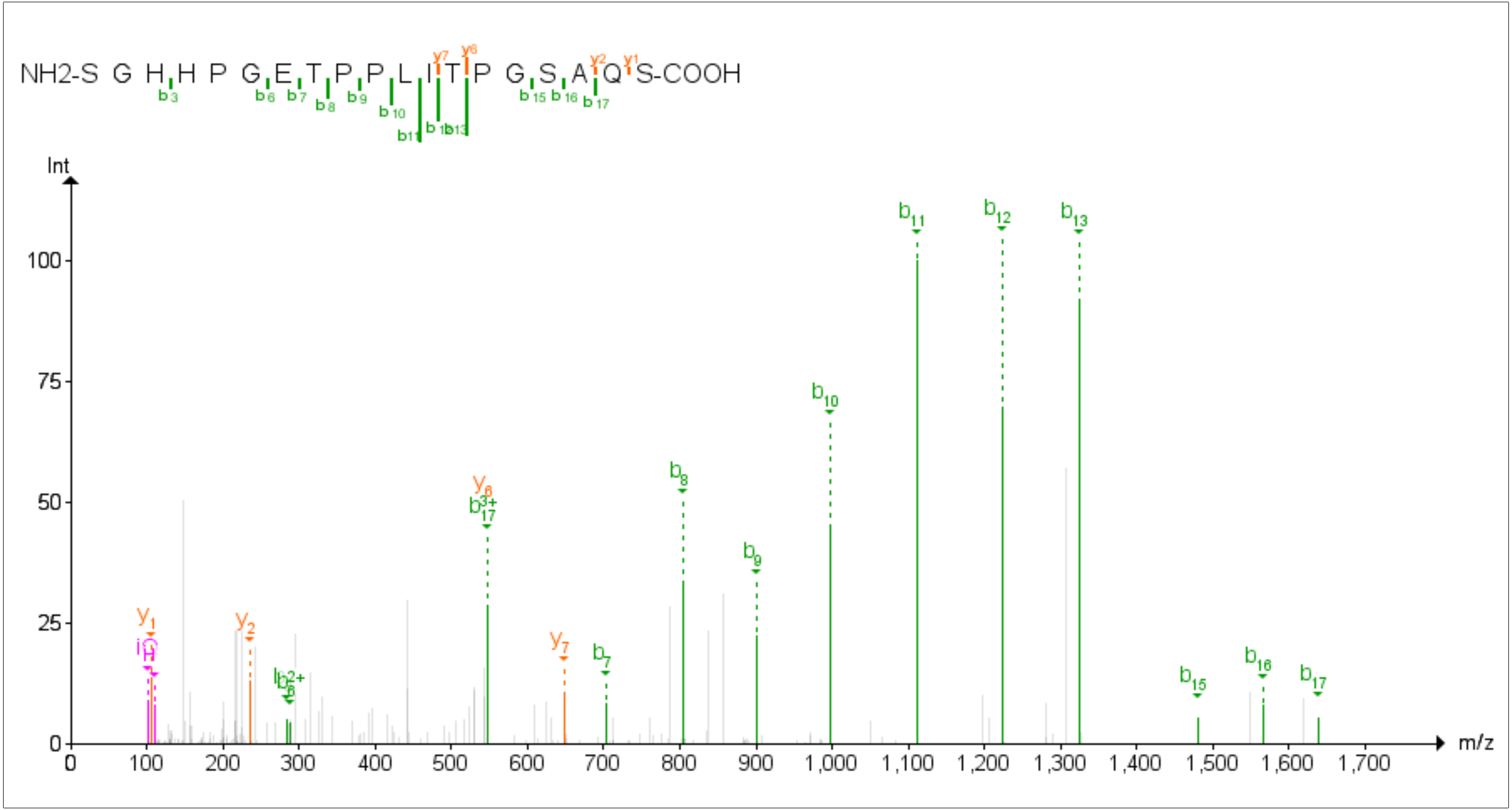

NH2-S G H H P G E T P P L I T P G S A Q S-COOH
Int
m/z
0
25
50
75
100
100
200
300
400
500
600
700
800
900
1,000
1,100
1,200
1,300
1,400
1,500
1,600
1,700

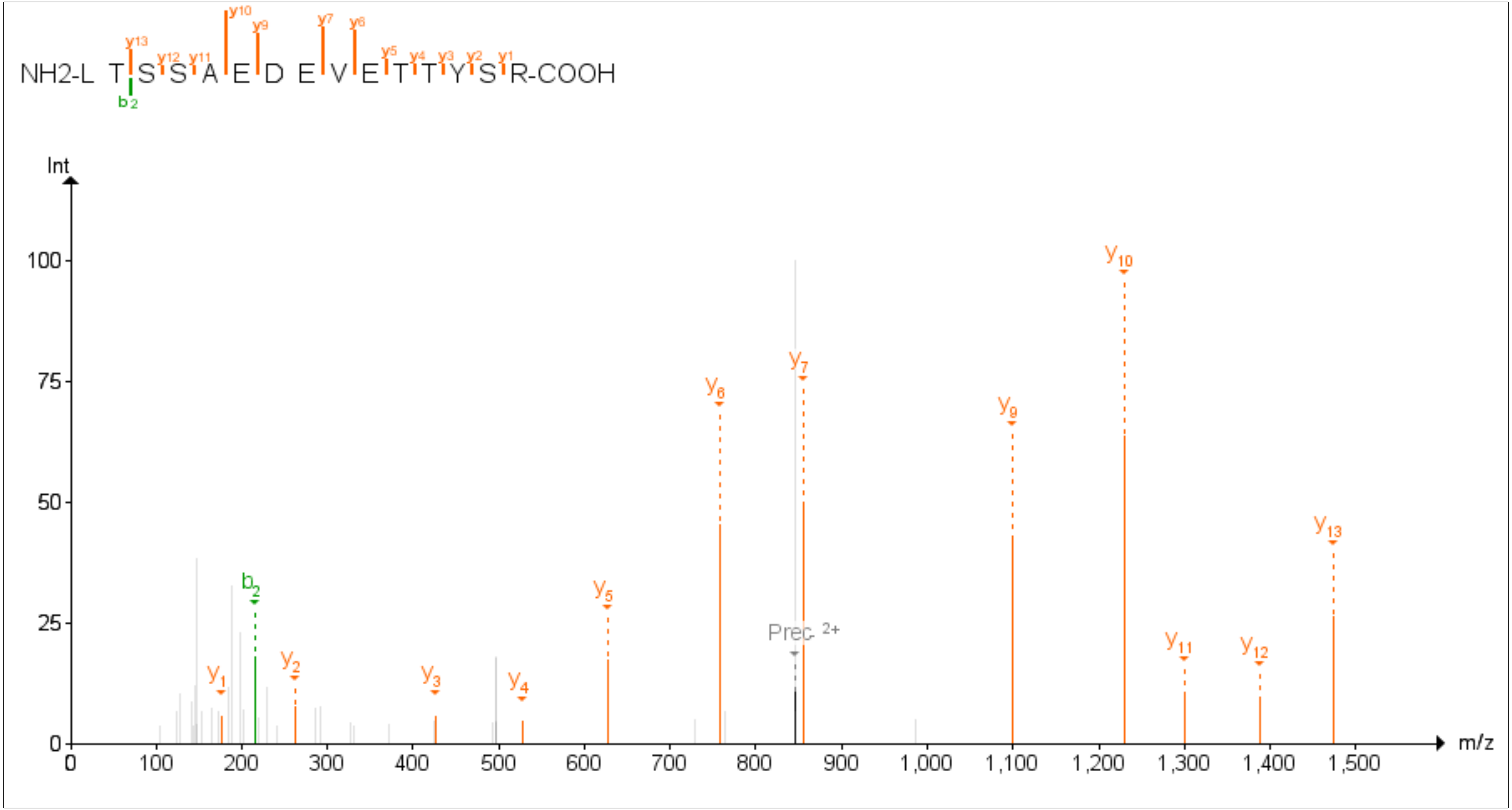

NH2-L T S S A E D E V E T T Y S R-COOH
y13 y12 y11 y10 y9 y7 y6 y5 y4 y3 y2 y1
b2
Int
100
75
50
25
0
m/z
0
100
200
300
400
500
600
700
800
900
1,000
1,100
1,200
1,300
1,400
1,500
b2
y1
y2
y3
y4
y5
y6
Prec 2+
y7
y9
y10
y11
y12
y13

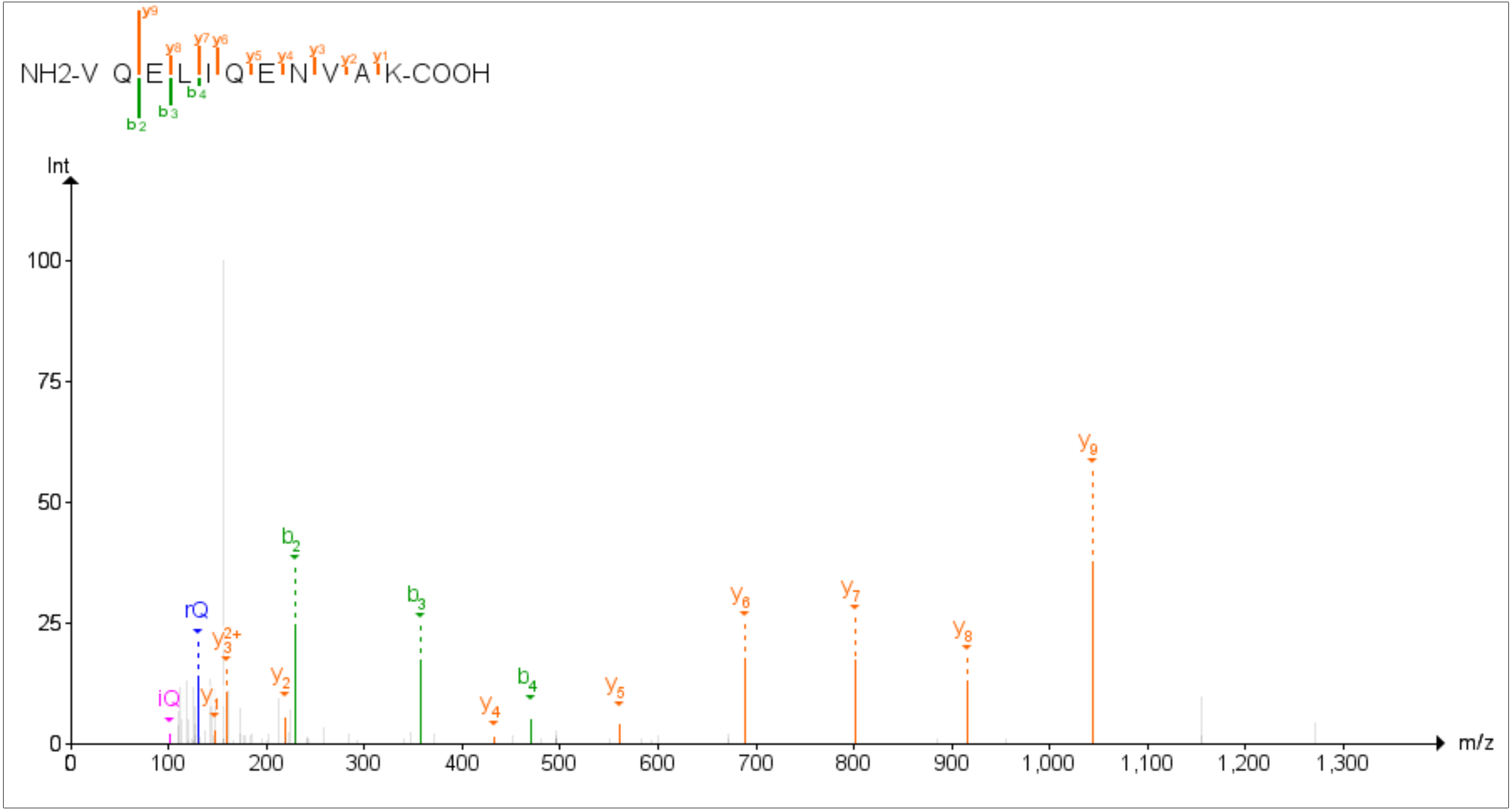
NH2-V Q E L I Q E N V A K-COOH
Int
m/z
b2
b3
b4
y1
y2
y2
y3
y4
y5
y6
y7
y8
y9
rQ
iQ
y3 2+
0
25
50
75
100
0
100
200
300
400
500
600
700
800
900
1,000
1,100
1,200
1,300

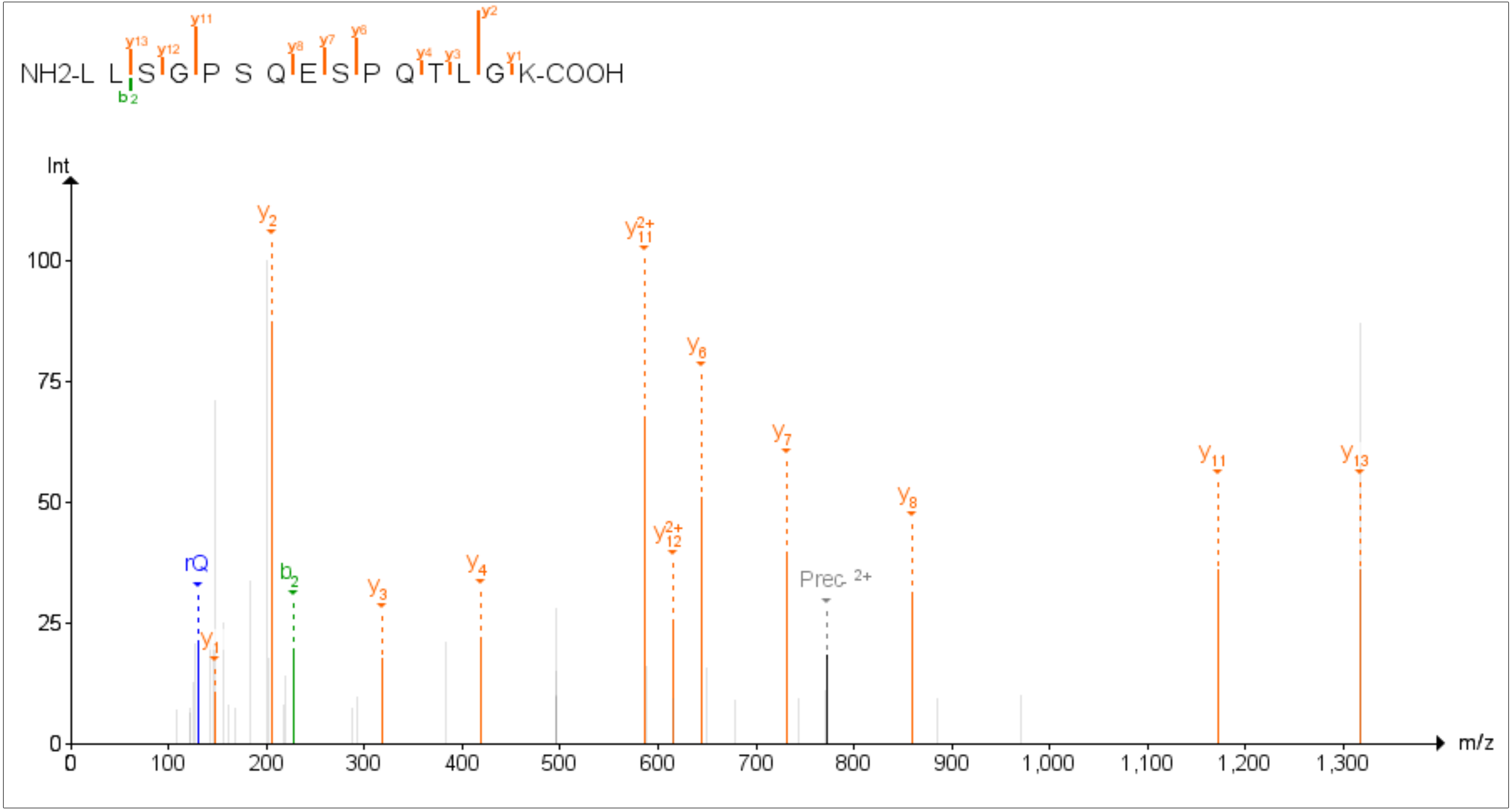
NH2-L L S G P S Q E S P Q T L G K-COOH
y13 y12 y11 y8 y7 y6 y4 y3 y2 y1
b2
Int
100
75
50
25
0
y2
y11 2+
y6
y7
y11
y13
y8
y12 2+
rQ
b2
y3
y4
y1
Prec- 2+
0 100 200 300 400 500 600 700 800 900 1,000 1,100 1,200 1,300
m/z

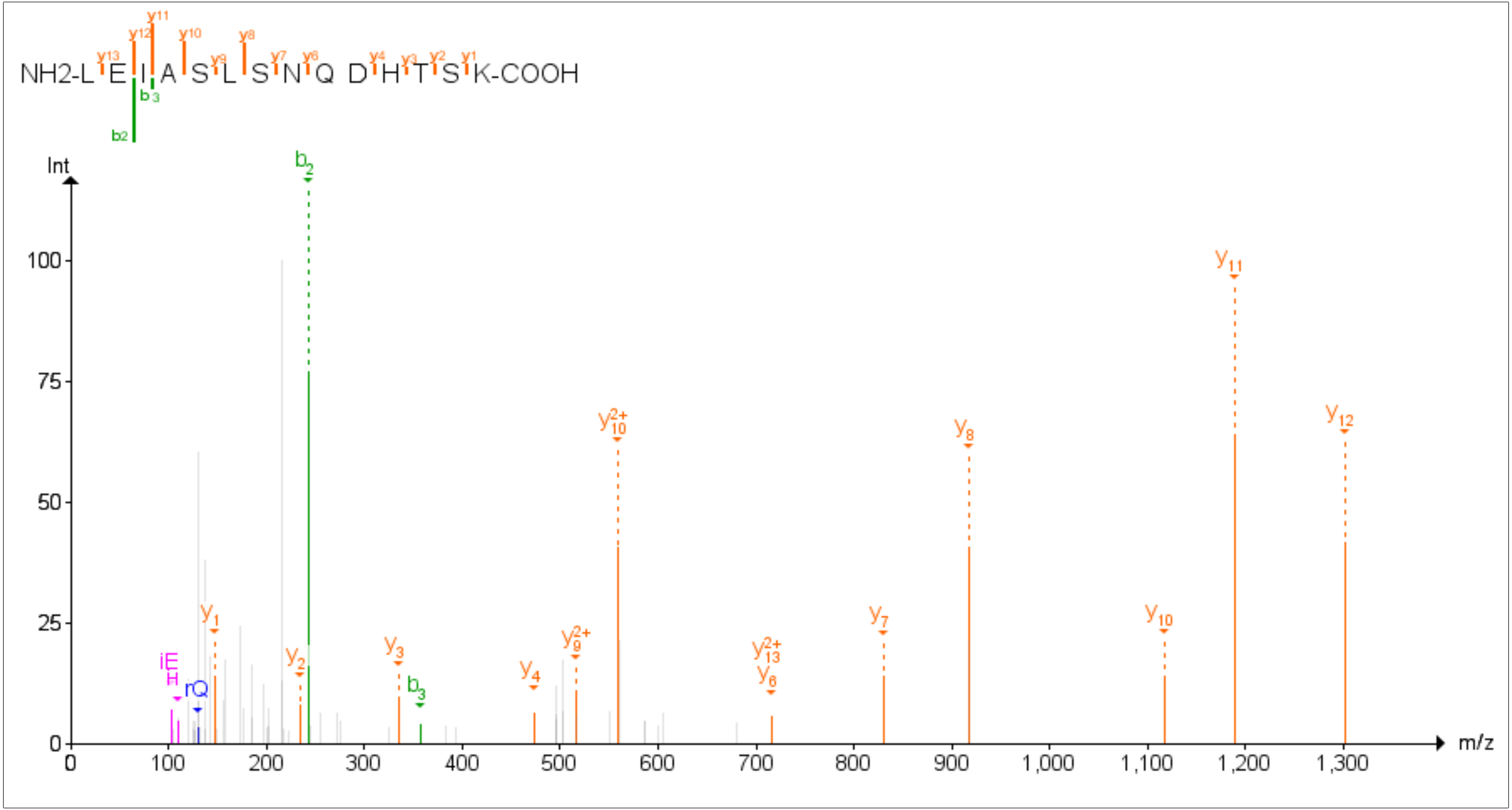
NH2-L E I A S L S N Q D H T S K-COOH
y13 y12 y11 y10 y9 y8 y7 y6 y4 y3 y2 y1
b2 b3
Int
m/z
0
25
50
75
100
0 100 200 300 400 500 600 700 800 900 1,000 1,100 1,200 1,300
b2
y11
y12
y8
y10 2+
y1
y2
y3
b3
y4
y9 2+
y13 2+
y6
y7
y10
iE
H
rQ

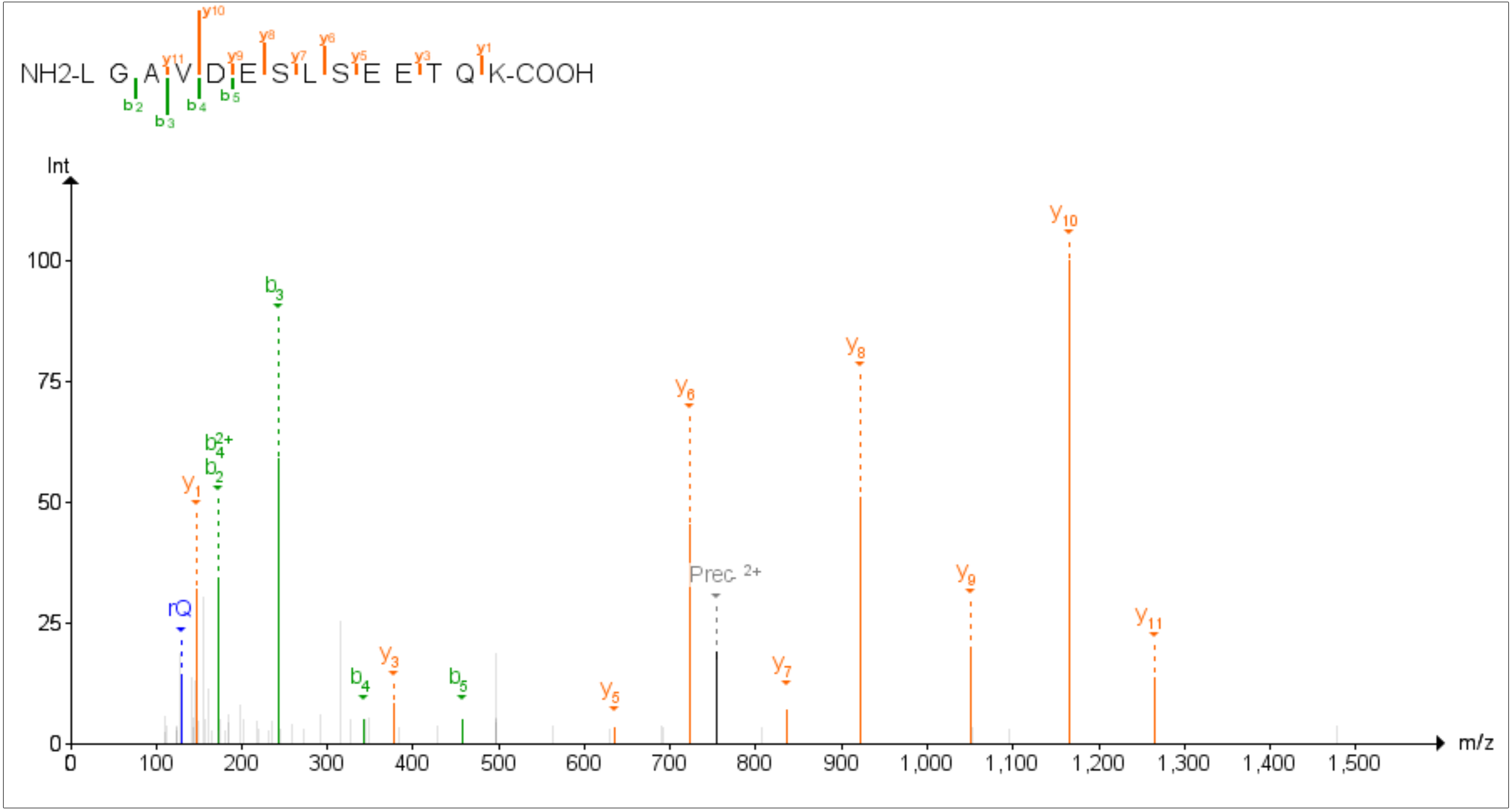

NH2-L G A V D E S L S E E T Q K-COOH
y10
y8
y6
y11
y9
y7
y5
y3
y1
b2
b3
b4
b5
Int
100
75
50
25
0
b3
y10
y8
y6
b4 2+
b2
y1
rQ
Prec. 2+
y9
y11
b4
y3
b5
y5
y7
0
100
200
300
400
500
600
700
800
900
1,000
1,100
1,200
1,300
1,400
1,500
m/z

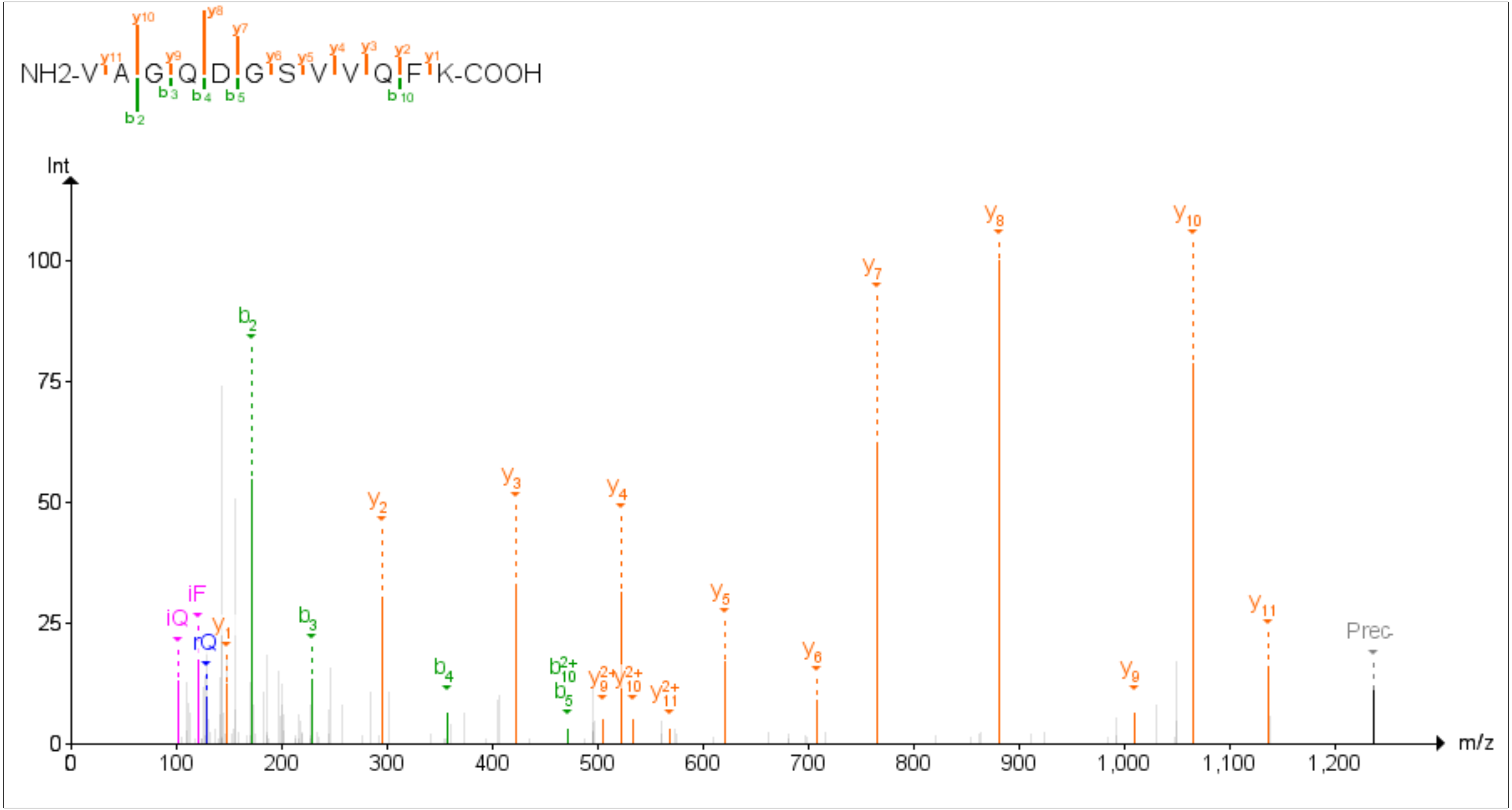

NH2-V A G Q D G S V V Q F K-COOH
y11 y10 y9 y8 y7 y6 y5 y4 y3 y2 y1
b2 b3 b4 b5 b10
Int
100
75
50
25
0
m/z
0
100
200
300
400
500
600
700
800
900
1,000
1,100
1,200
iQ
iF
rQ
y1
b2
b3
b4
y2
y3
y4
b10 2+
b5
y9 2+
y10 2+
y11 2+
y5
y6
y7
y8
y9
y10
y11
Prec

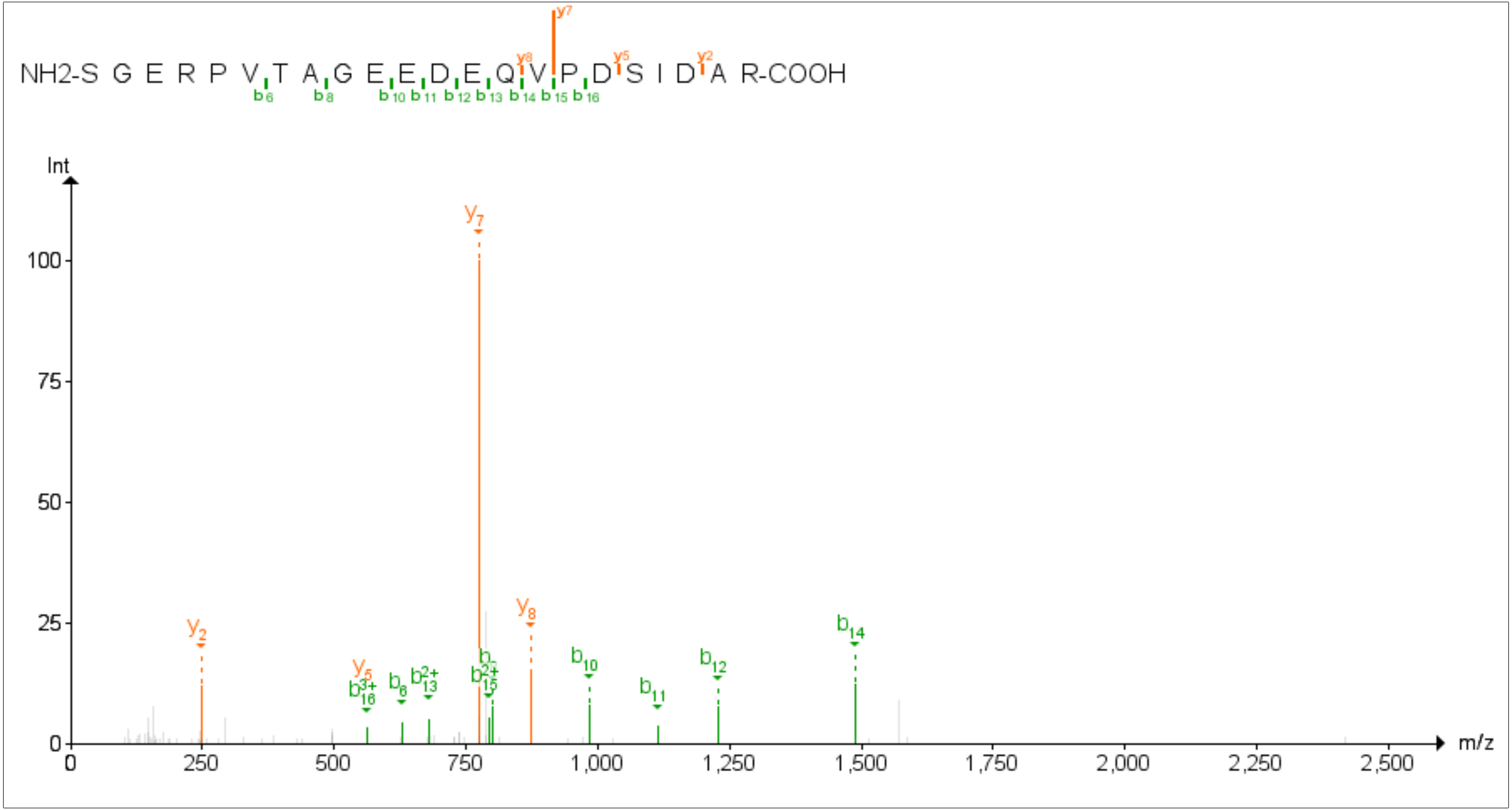

NH2-S G E R P V T A G E E D E Q V P D S I D A R-COOH
y7
y8
y5
y2
b6
b8
b10
b11
b12
b13
b14
b15
b16
Int
100
75
50
25
0
y7
y2
y8
y5
b3+16
b6
b2+13
b2+15
b10
b11
b12
b14
0
250
500
750
1,000
1,250
1,500
1,750
2,000
2,250
2,500
m/z

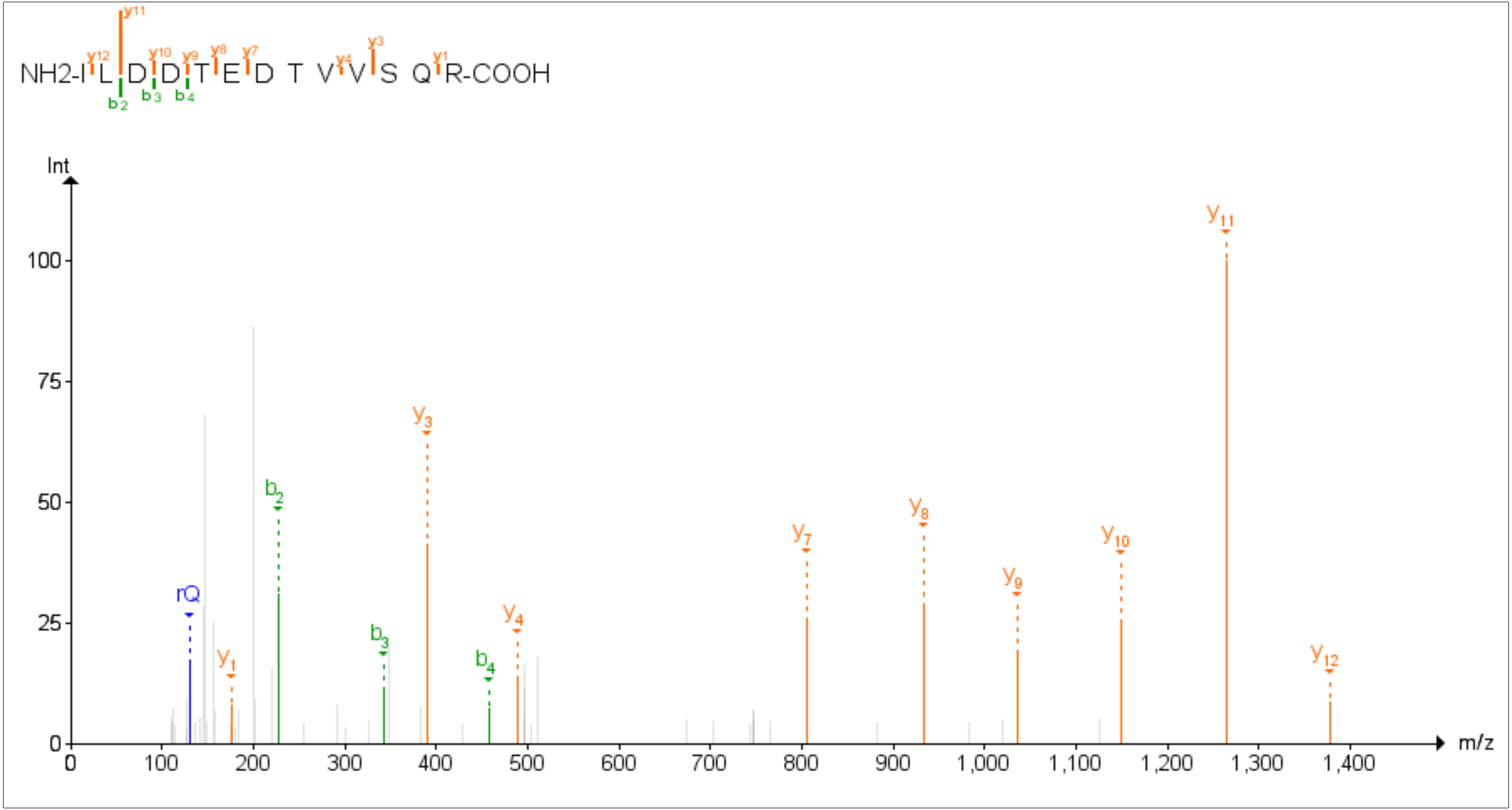

NH2-I L D D T E D T V V S Q R-COOH
y12
y11
y10
y9
y8
y7
y4
y3
y1
b2
b3
b4
Int
100
75
50
25
0
m/z
0
100
200
300
400
500
600
700
800
900
1,000
1,100
1,200
1,300
1,400
rQ
y1
b2
b3
y3
b4
y4
y7
y8
y9
y10
y11
y12

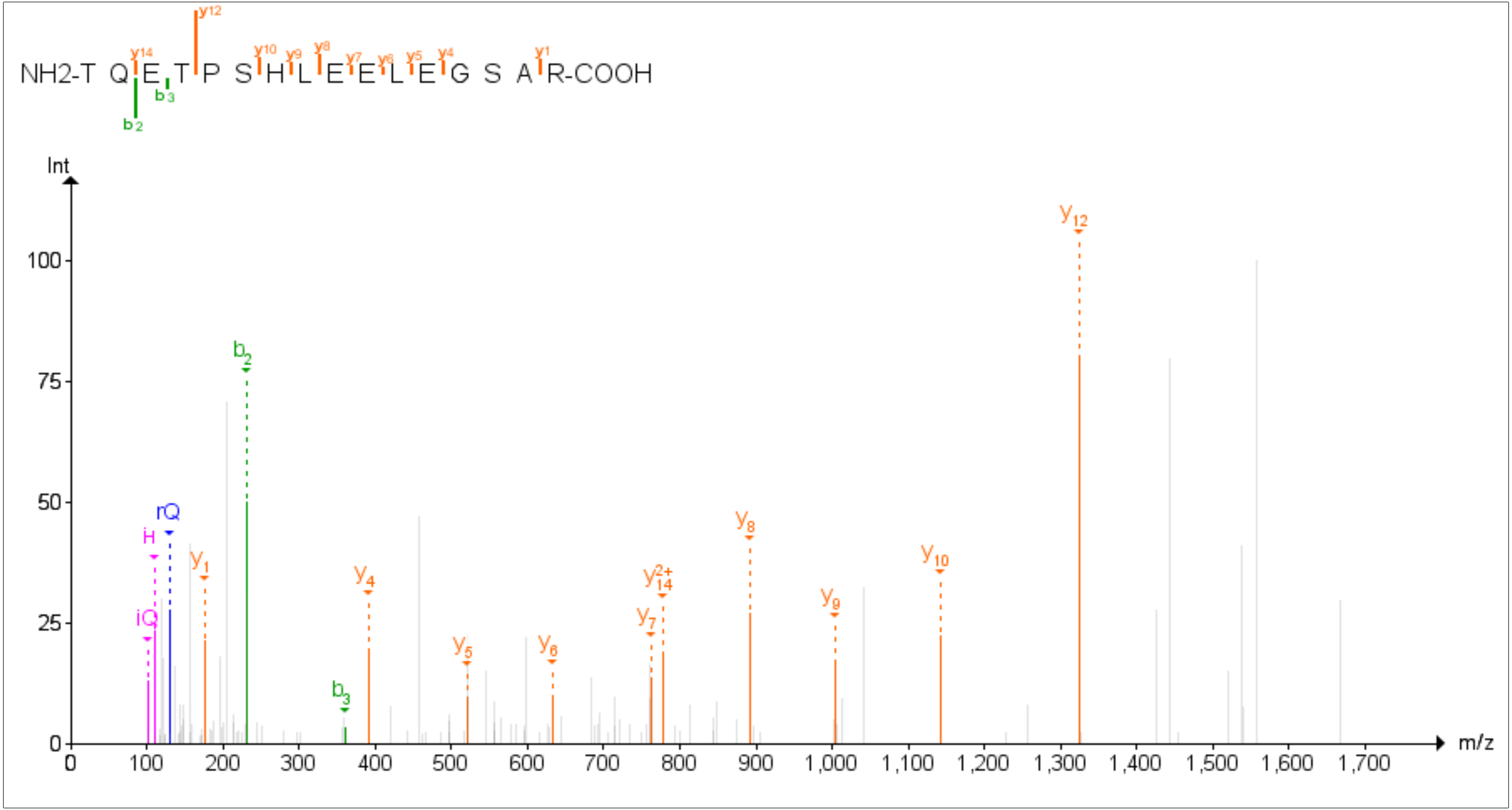

NH2-T Q E T P S H L E E L E G S A R-COOH
y12
y14
y10
y9
y8
y7
y6
y5
y4
y1
b3
b2
Int
100
75
50
25
0
y12
b2
rQ
iH
y1
iQ
y4
y5
y6
y7
y14 2+
y8
y9
y10
b3
m/z
0
100
200
300
400
500
600
700
800
900
1,000
1,100
1,200
1,300
1,400
1,500
1,600
1,700

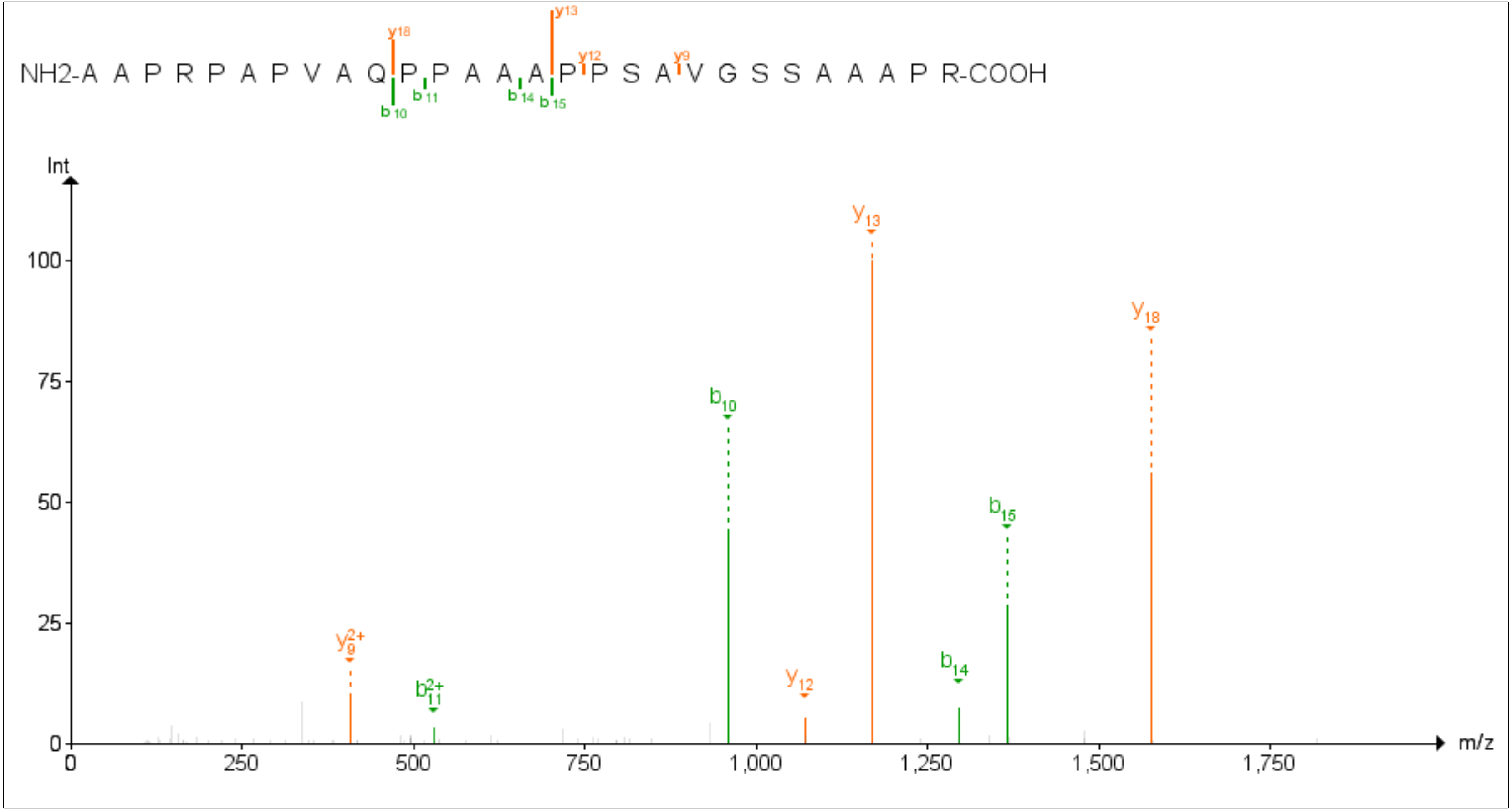

NH2-A A P R P A P V A Q P P A A A P P S A V G S S A A A P R-COOH
y18
y13
y12
y9
b 10
b 11
b 14
b 15
Int
100
75
50
25
0
y13
y18
b10
b15
y9 2+
b11 2+
y12
b14
0
250
500
750
1,000
1,250
1,500
1,750
m/z

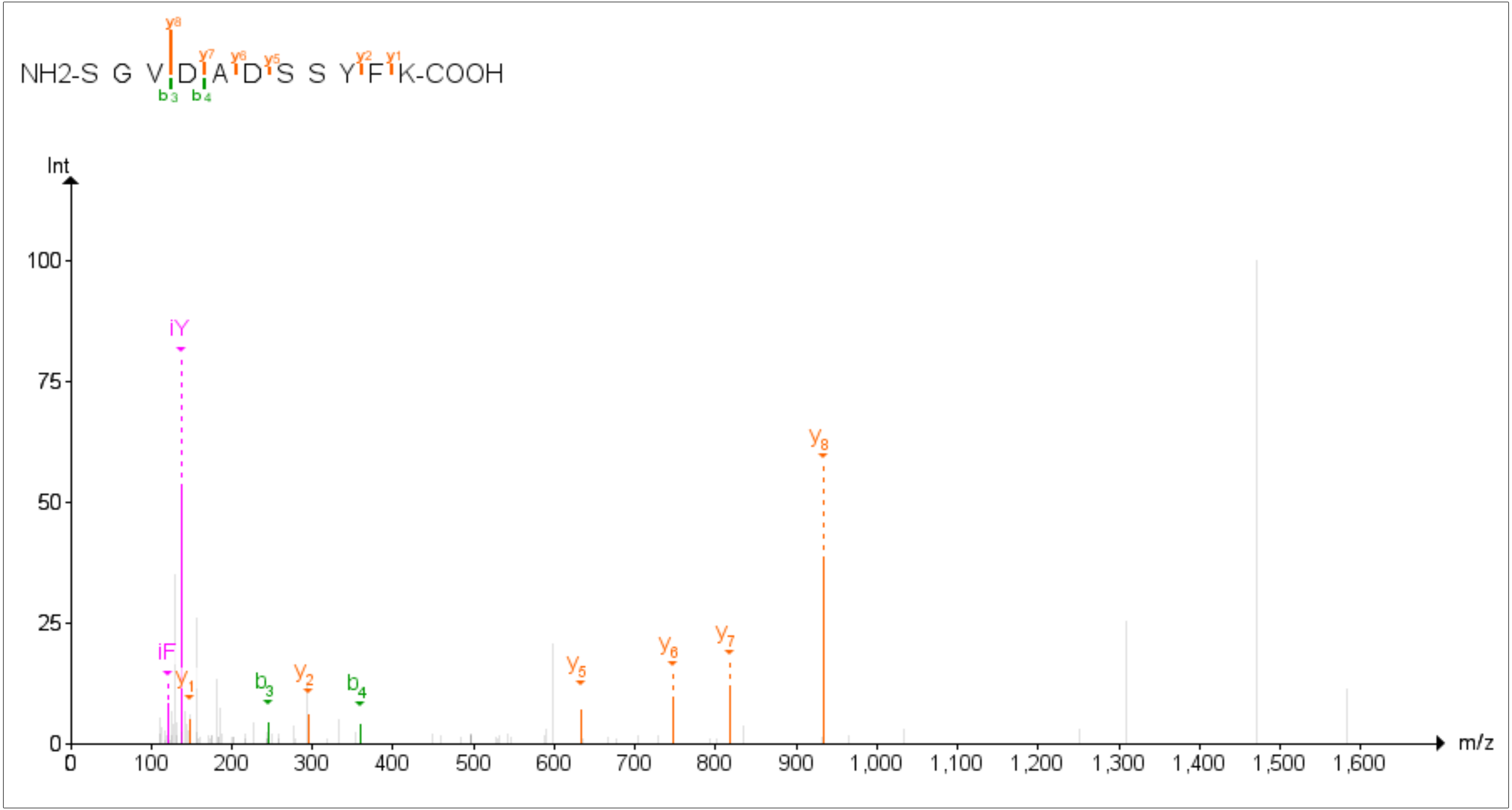

NH2-S G V D A D S S Y F K-COOH
y8
y7
y6
y5
y2
y1
b3
b4
Int
iY
iF
y1
b3
y2
b4
y5
y6
y7
y8
m/z

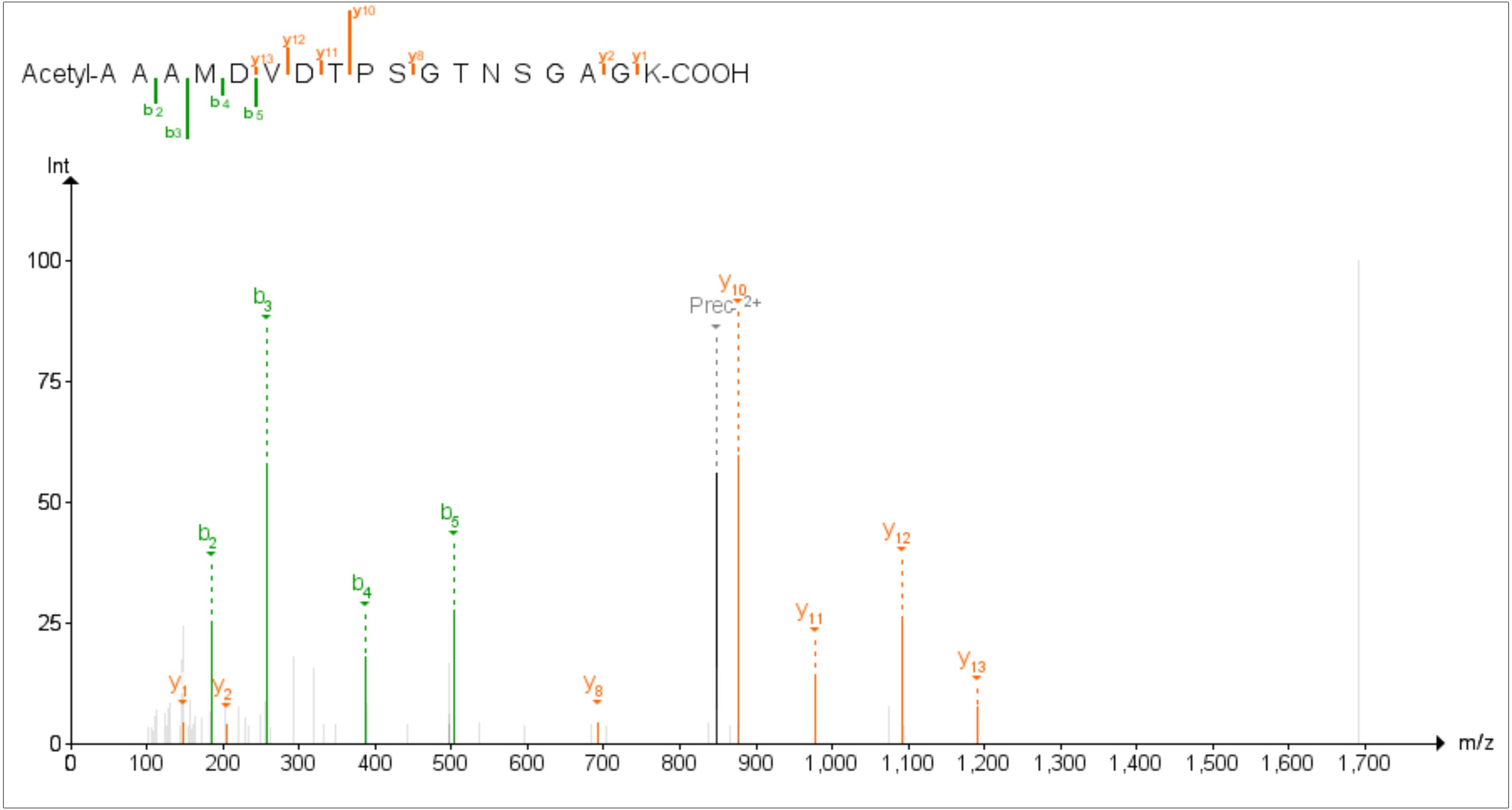

Acetyl-A A A M D V D T P S G T N S G A G K-COOH
b2
b3
b4
b5
y13
y12
y11
y10
y8
y2
y1
Int
100
75
50
25
0
b3
b2
b4
b5
y1
y2
y8
Prec. 2+
y10
y11
y12
y13
0
100
200
300
400
500
600
700
800
900
1,000
1,100
1,200
1,300
1,400
1,500
1,600
1,700
m/z

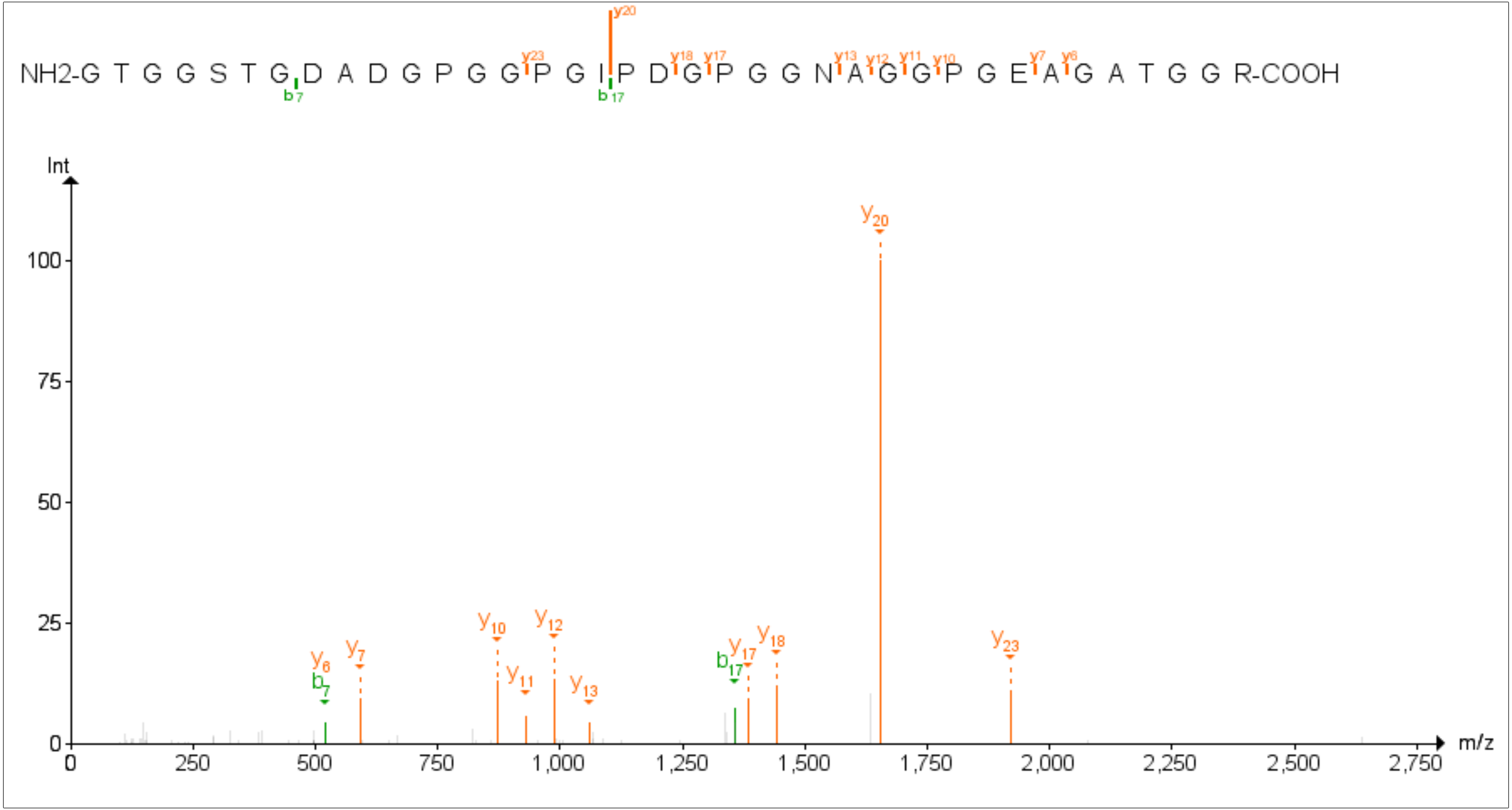

NH2-G T G G S T G D A D G P G G P G I P D G P G G N A G G P G E A G A T G G R-COOH
y20
y23
y18
y17
y13
y12
y11
y10
y7
y6
b7
b17
Int
m/z
100
75
50
25
0
250
500
750
1,000
1,250
1,500
1,750
2,000
2,250
2,500
2,750

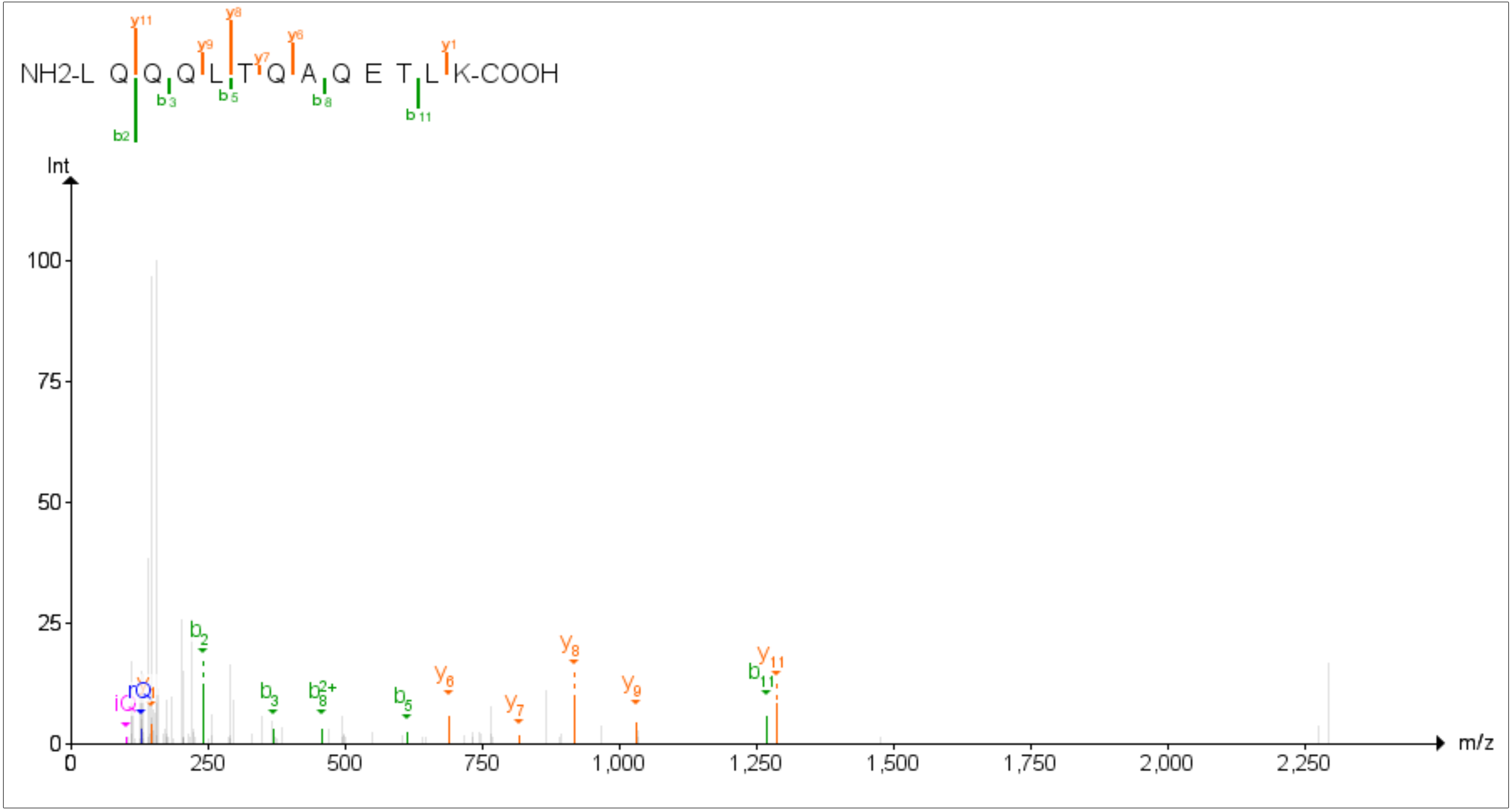

NH2-L Q Q Q L T Q A Q E T L K-COOH
y11
y9
y8
y7
y6
y1
b2
b3
b5
b8
b11
Int
100
75
50
25
0
m/z
0
250
500
750
1,000
1,250
1,500
1,750
2,000
2,250

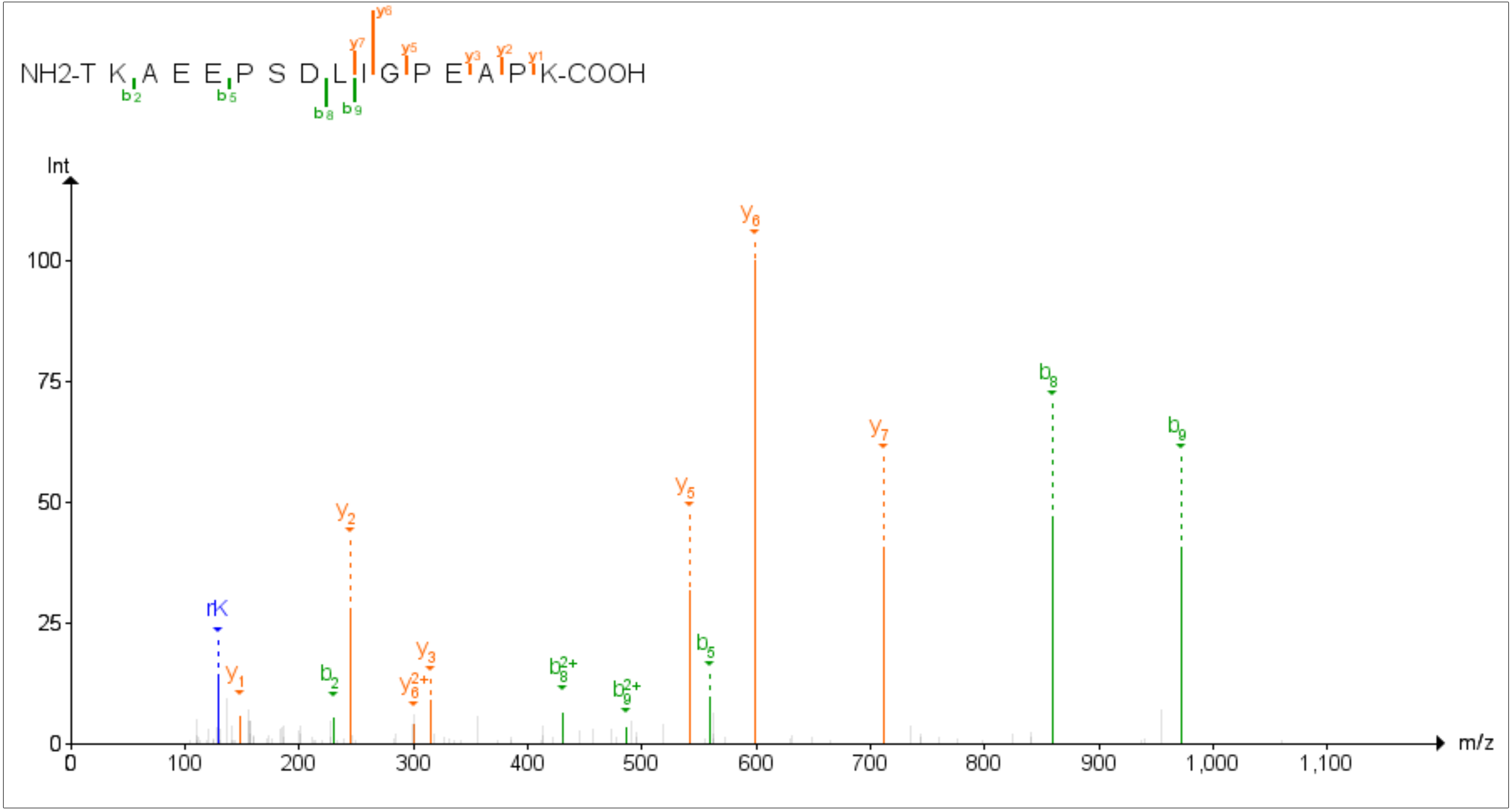

NH2-T K A E E P S D L I G P E A P K-COOH
b2 b5 b8 b9
y7 y6 y5 y3 y2 y1
Int
m/z

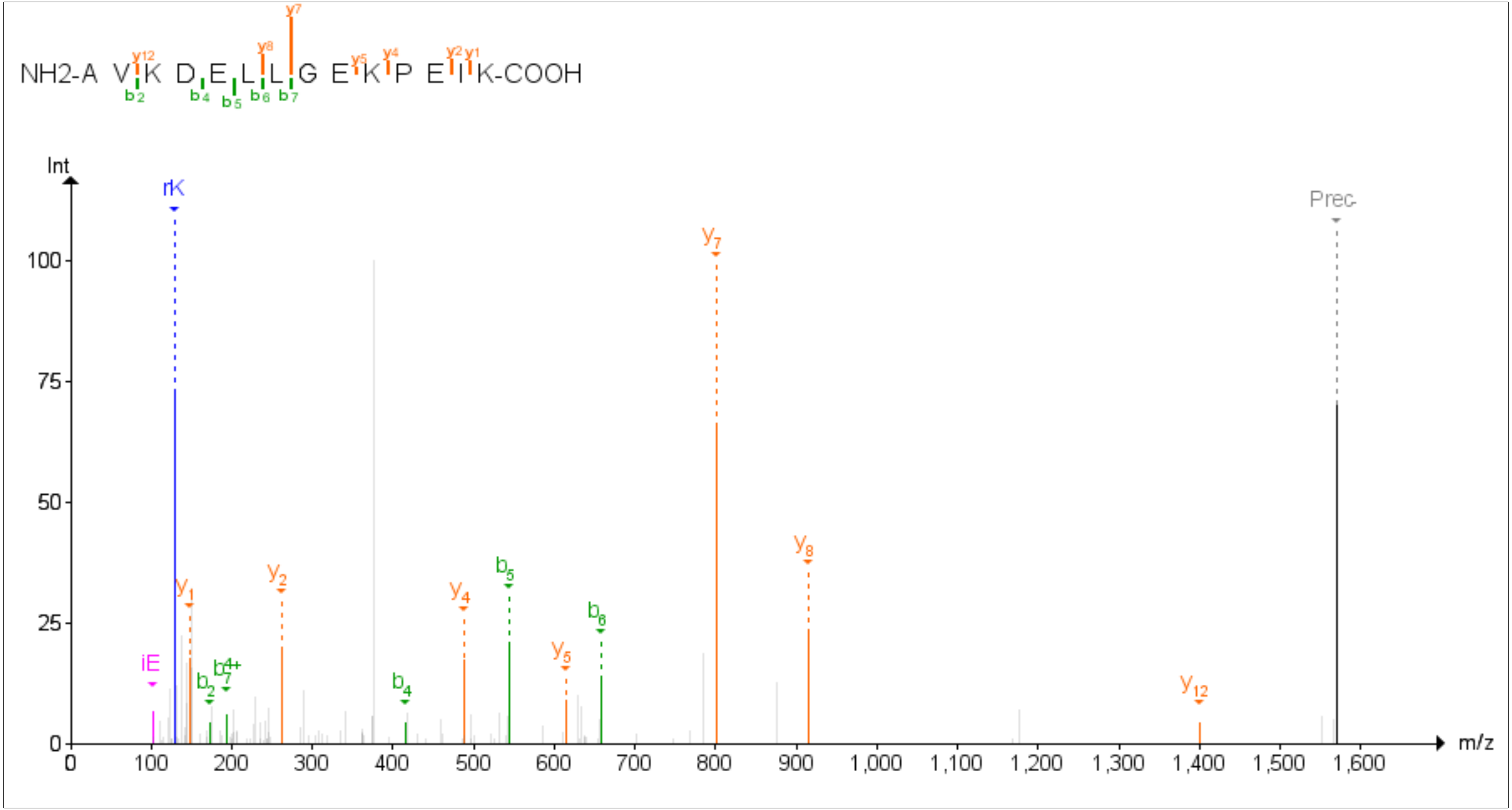

NH2-A V K D E L L G E K P E I K-COOH
y12 y8 y7 y5 y4 y2 y1
b2 b4 b5 b6 b7
Int
m/z
rK
Prec
y7
y8
y1
y2
y4
b5
b6
y5
y12
iE
b2
b7 4+
b4
0
25
50
75
100
0
100
200
300
400
500
600
700
800
900
1,000
1,100
1,200
1,300
1,400
1,500
1,600

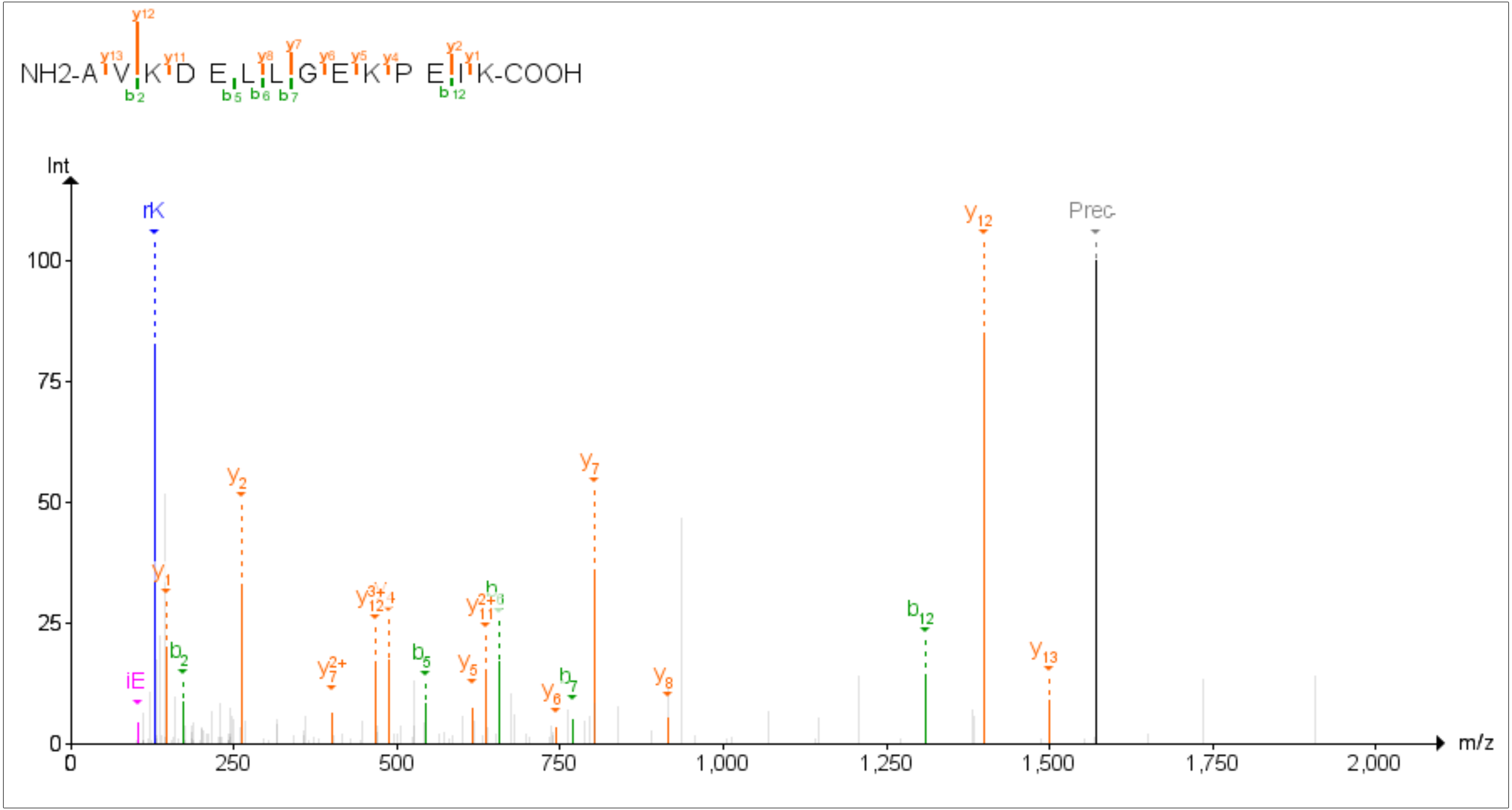

NH2-A V K D E L L G E K P E I K-COOH
y13 y12 y11 y8 y7 y6 y5 y4 y2 y1
b2 b5 b6 b7 b12
Int
m/z
rK
Prec
iE
y1
b2
y2
y7 2+
y12 3+
b5
y5
y11 2+
b6
y6
b7
y7
y8
b12
y12
y13
0
25
50
75
100
0
250
500
750
1,000
1,250
1,500
1,750
2,000

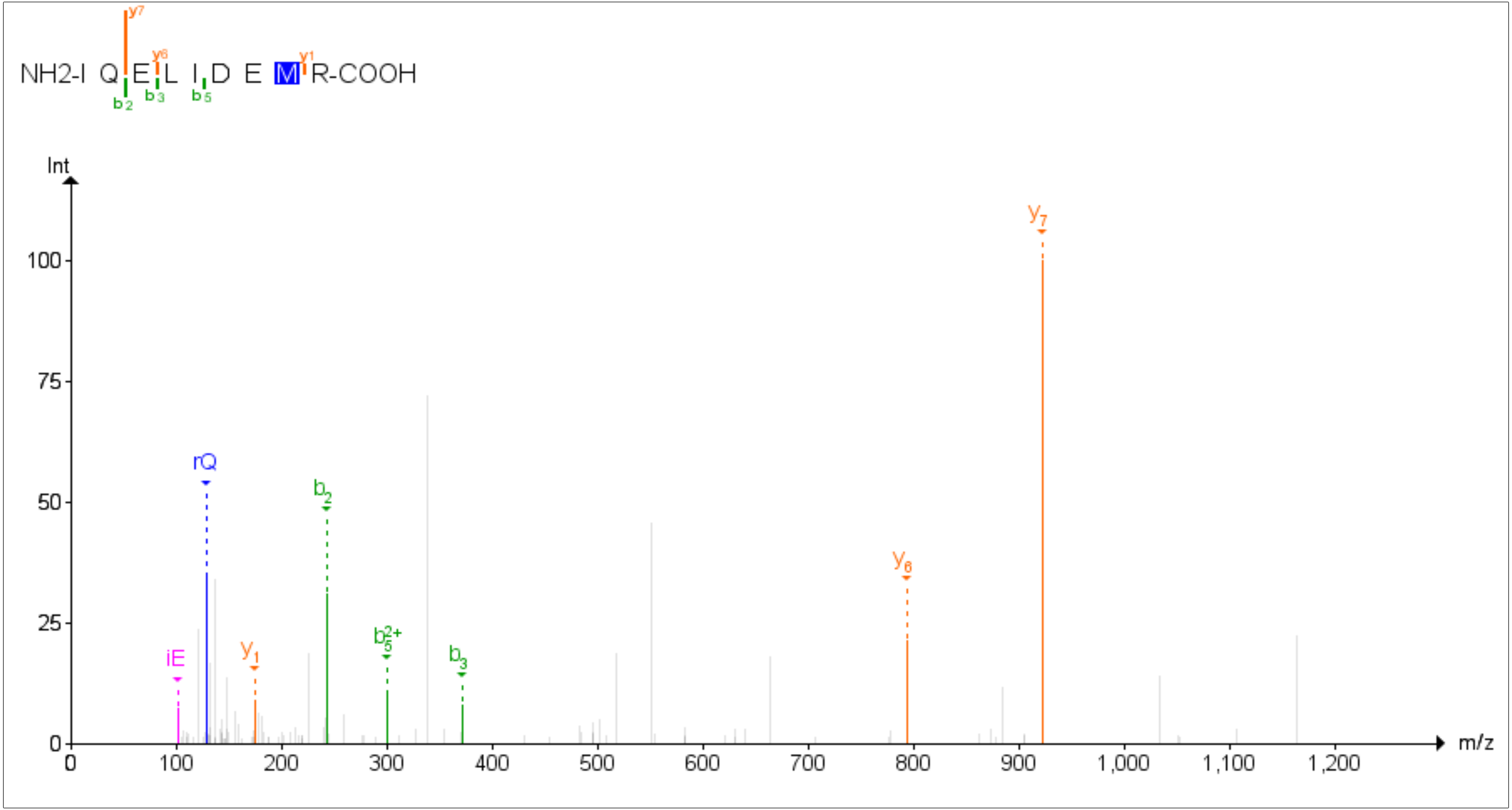

NH2-I Q E L I D E M R-COOH
y7
y6
y1
b2
b3
b5
Int
100
75
50
25
0
rQ
iE
y1
b2
b2+5
b3
y6
y7
0
100
200
300
400
500
600
700
800
900
1,000
1,100
1,200
m/z

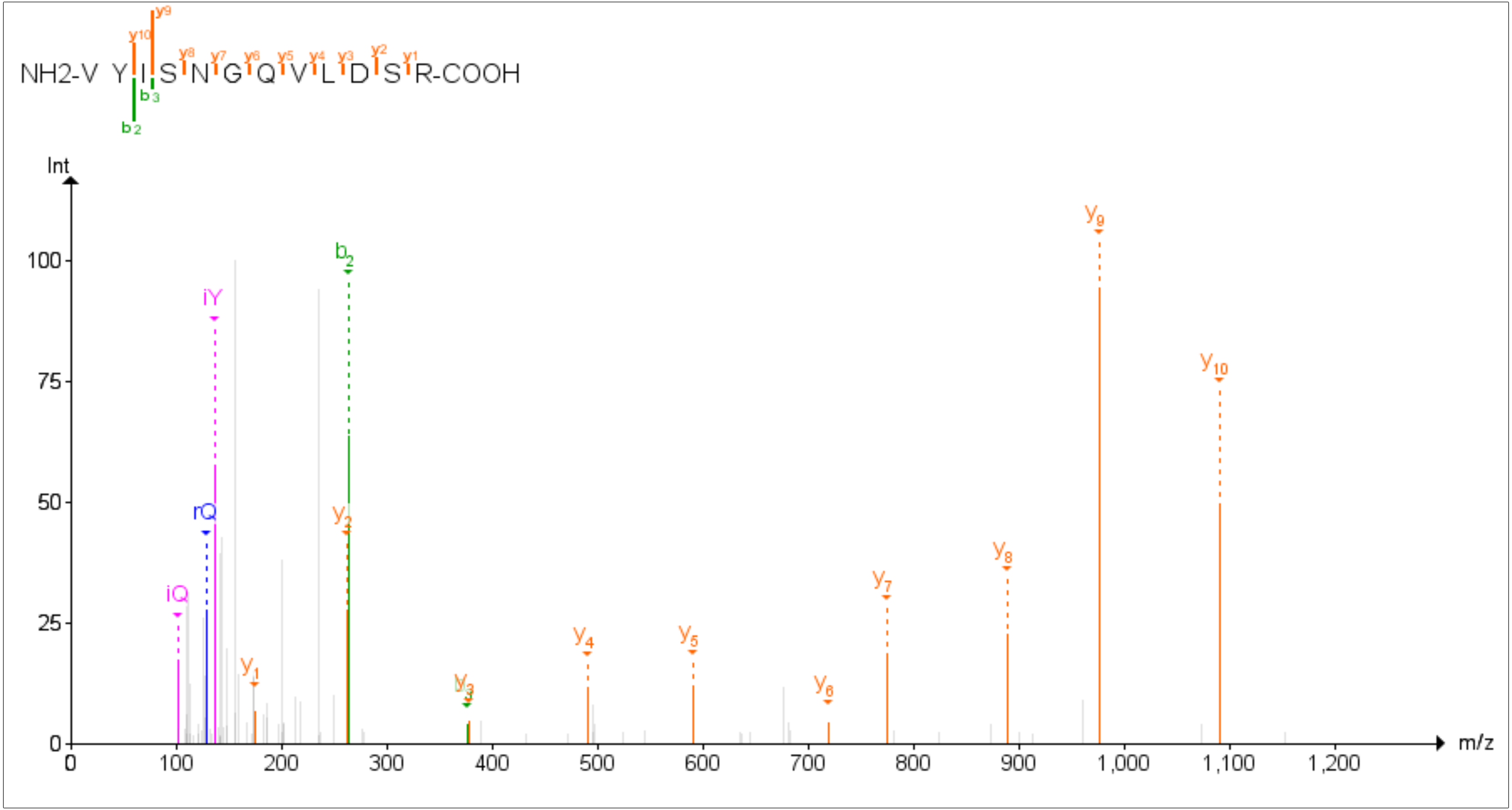

NH2-V Y I S N G Q V L D S R-COOH
y10 y9 y8 y7 y6 y5 y4 y3 y2 y1
b2 b3
Int
m/z
100
75
50
25
0
0 100 200 300 400 500 600 700 800 900 1,000 1,100 1,200
iY
iQ
rQ
b2
y1 y2 y3 y4 y5 y6 y7 y8 y9 y10

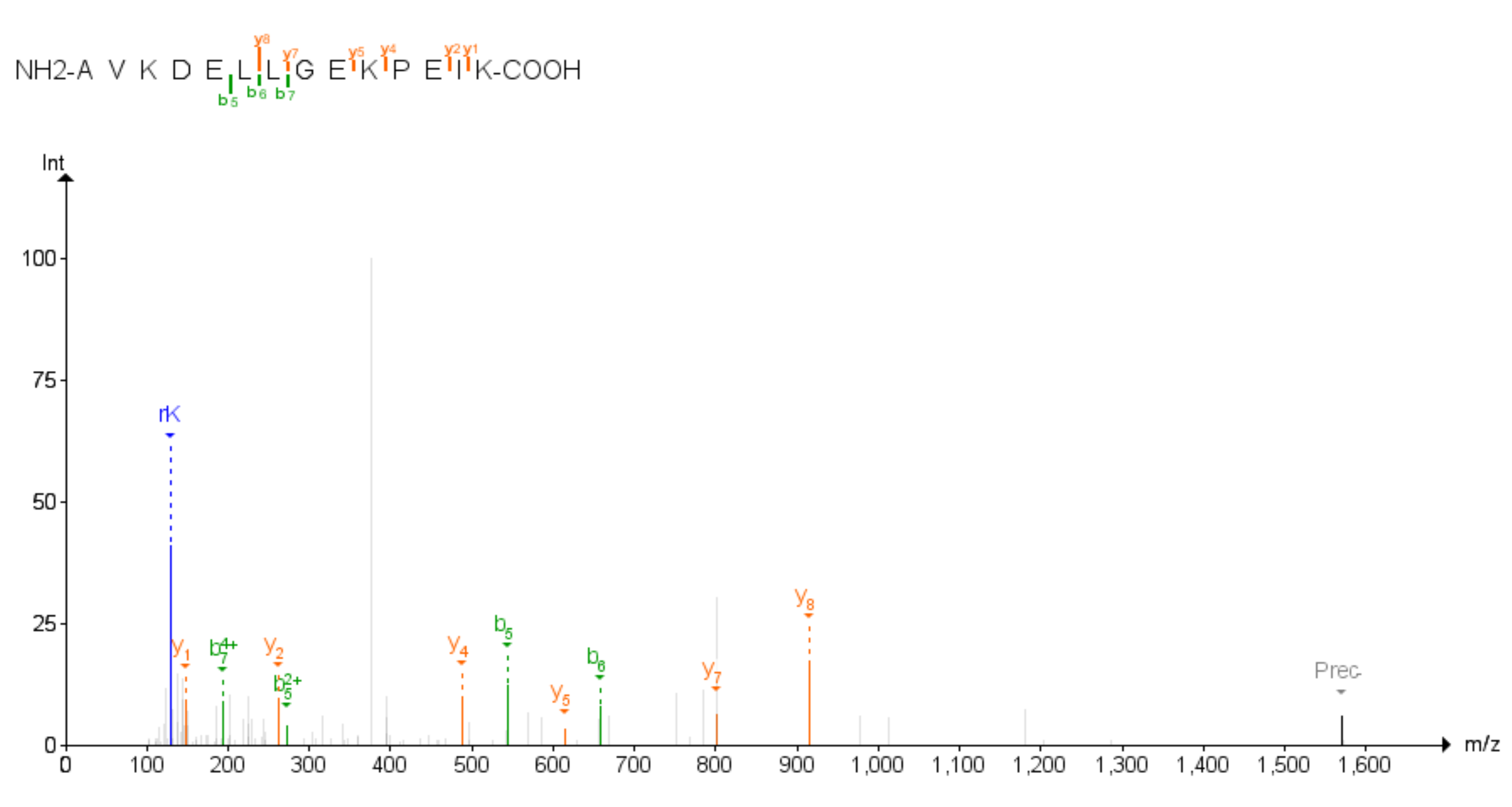

NH2-A V K D E L L G E K P E I K-COOH
Int
m/z
rK
Prec

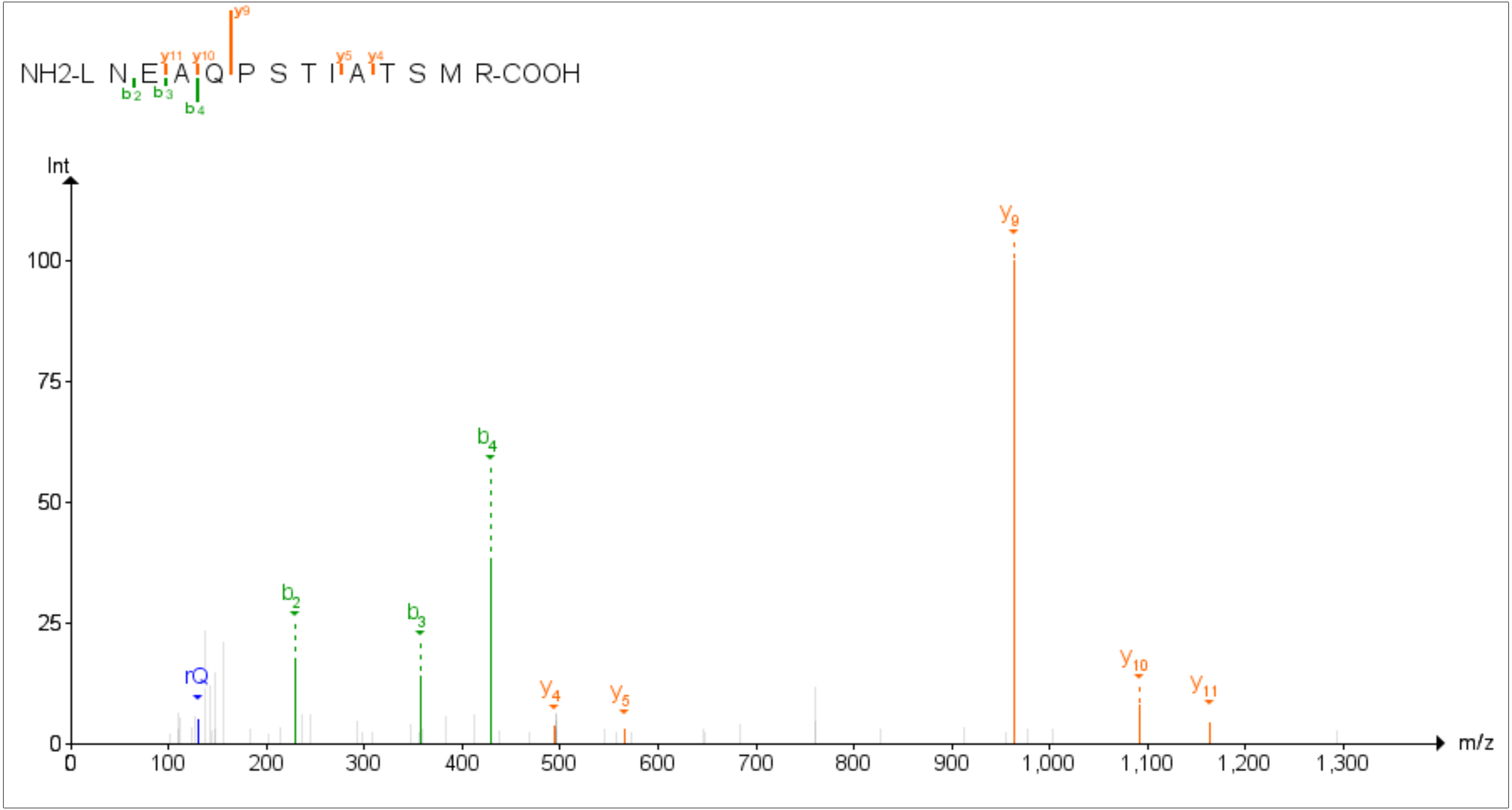

NH2-L N E A Q P S T I A T S M R-COOH
y9
y11 y10 y5 y4
b2 b3 b4
Int
100
75
50
25
0
rQ
b2
b3
b4
y4
y5
y9
y10
y11
0 100 200 300 400 500 600 700 800 900 1,000 1,100 1,200 1,300
m/z

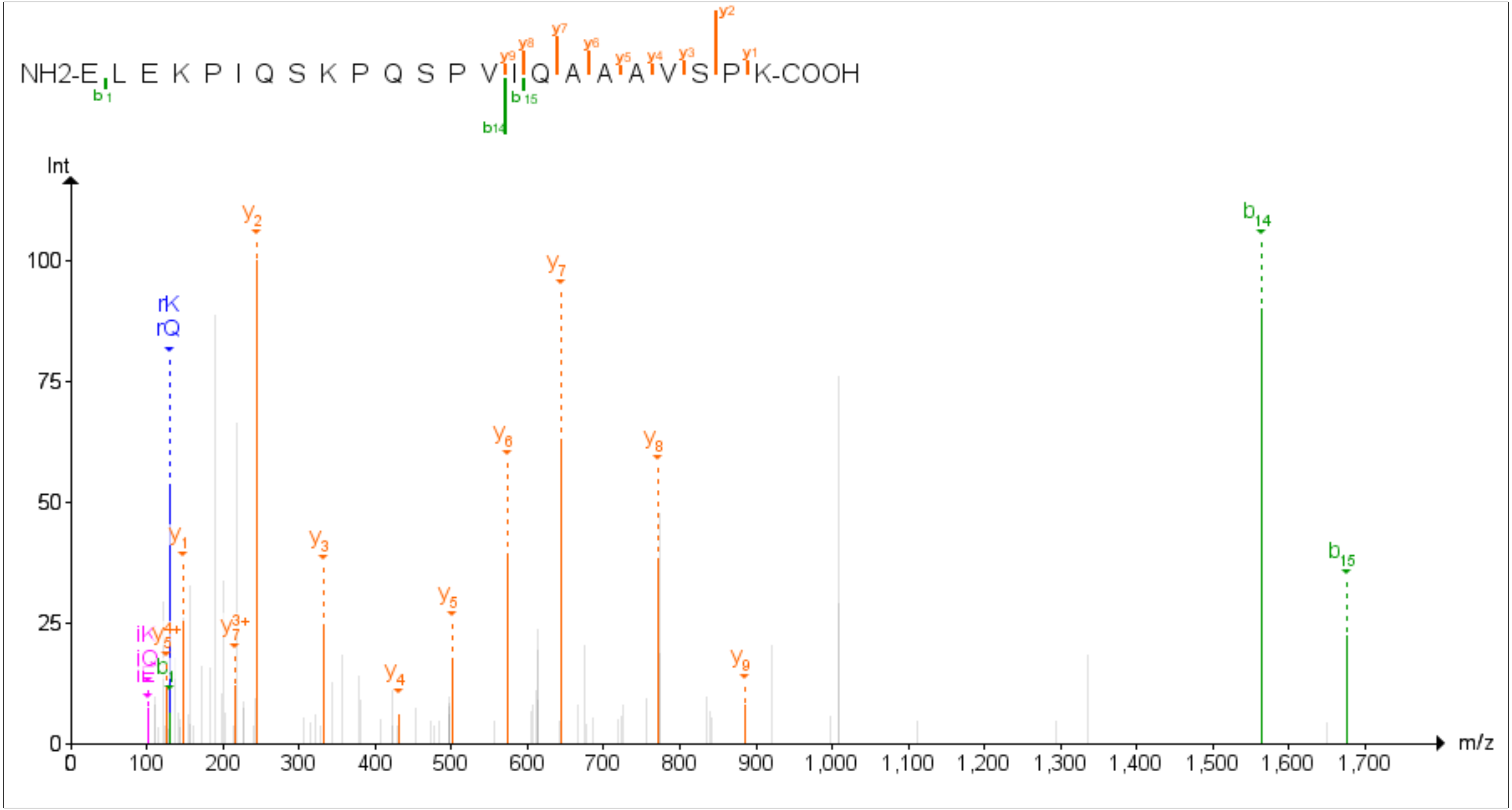

NH2-E L E K P I Q S K P Q S P V I Q A A A V S P K-COOH
b1
b15
b14
y9
y8
y7
y6
y5
y4
y3
y2
y1
Int
rK
rQ
y2
b14
y7
y6
y8
y1
y3
y5
y4
y9
b15
y7 3+
iK
iQ
iE
b1
y5 4+
m/z
0
25
50
75
100
0
100
200
300
400
500
600
700
800
900
1,000
1,100
1,200
1,300
1,400
1,500
1,600
1,700

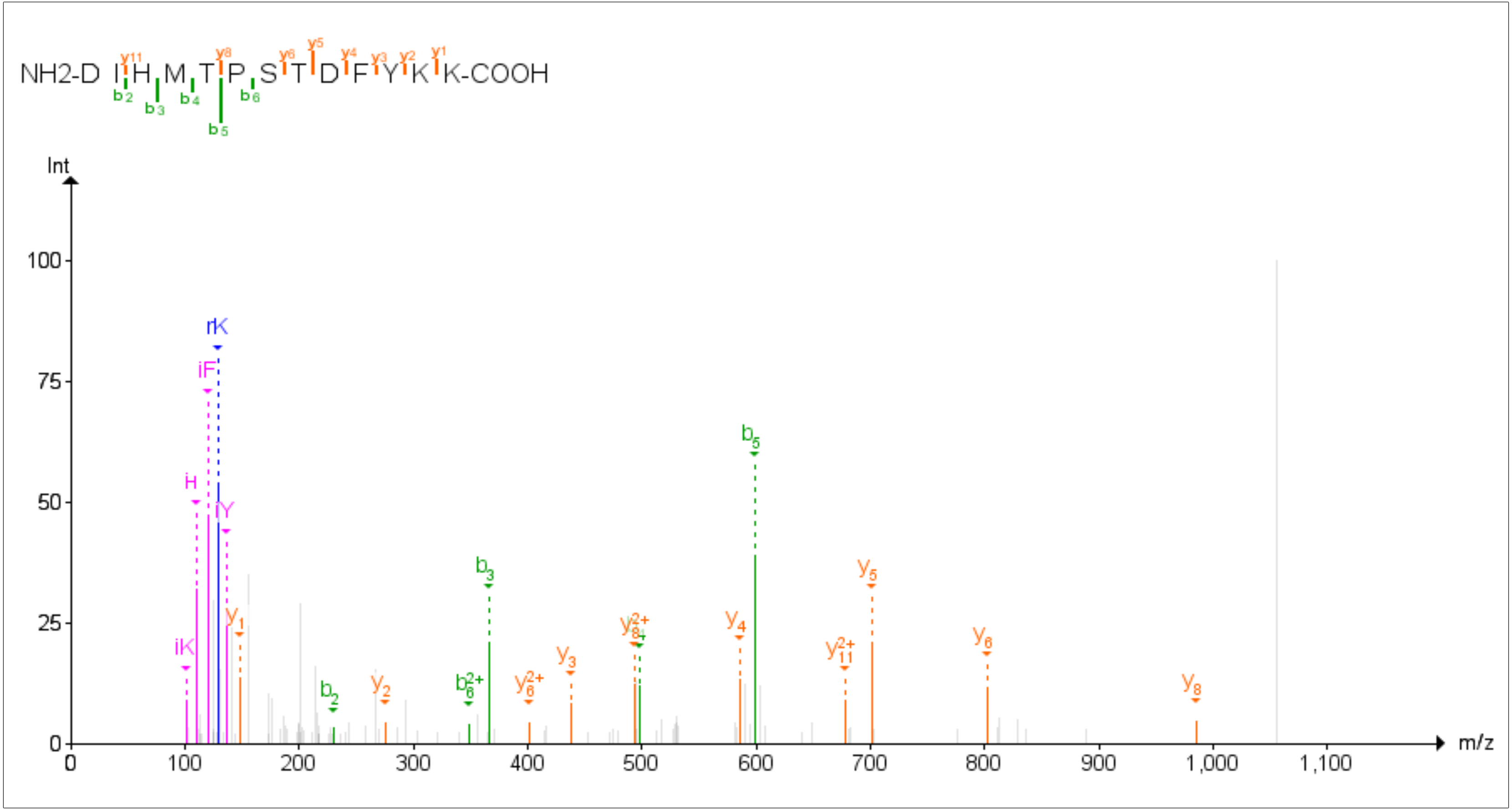

NH2-D I H M T P S T D F Y K K-COOH
Int
m/z
0
25
50
75
100
0
100
200
300
400
500
600
700
800
900
1,000
1,100

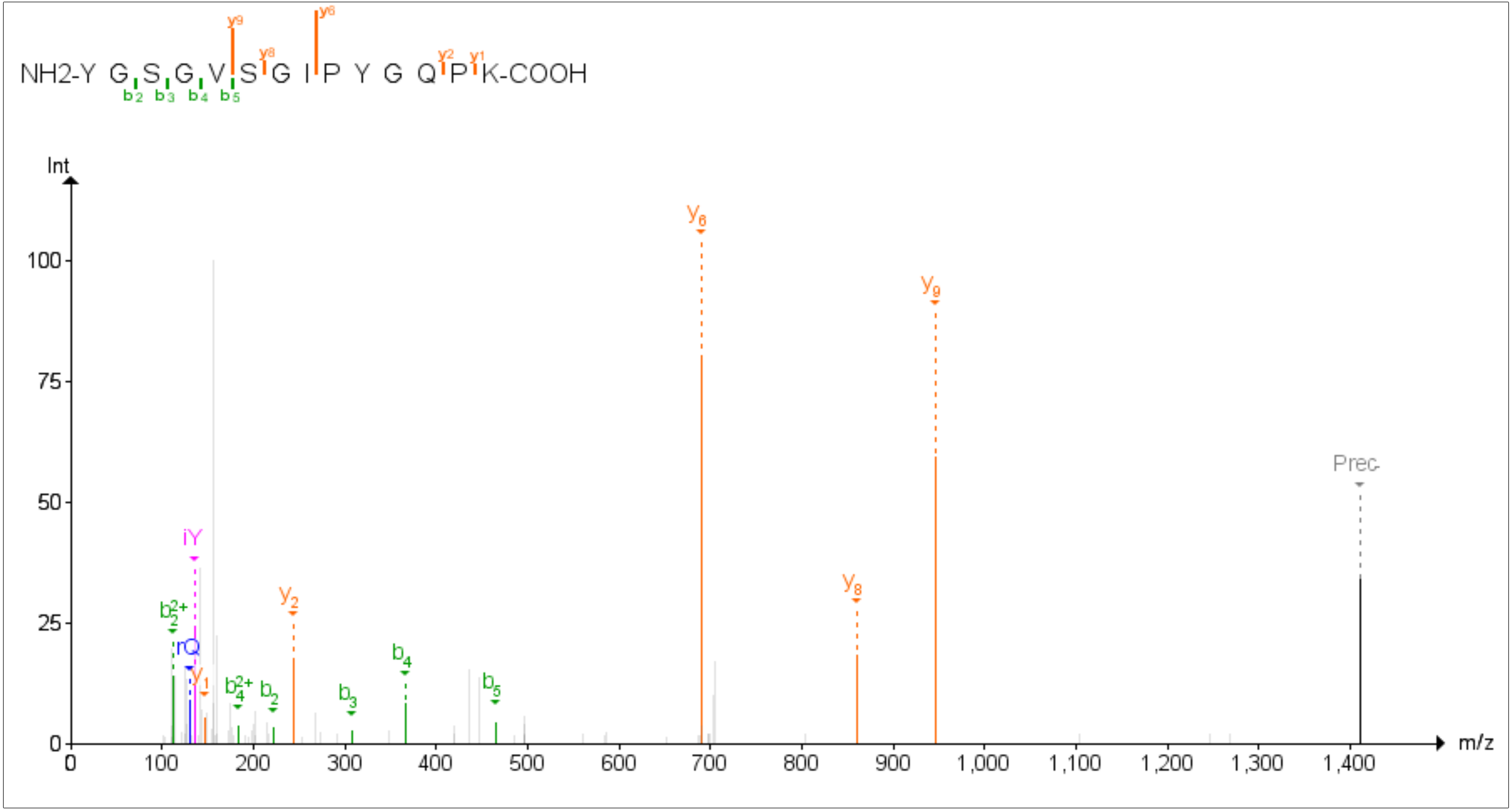

NH2-Y G S G V S G I P Y G Q P K-COOH
b2 b3 b4 b5
y9 y8 y6 y2 y1
Int
m/z
100
75
50
25
0
100
200
300
400
500
600
700
800
900
1,000
1,100
1,200
1,300
1,400
y6
y9
y8
y2
y1
b2 2+
b4 2+
b2
b3
b4
b5
iY
rQ
Prec.

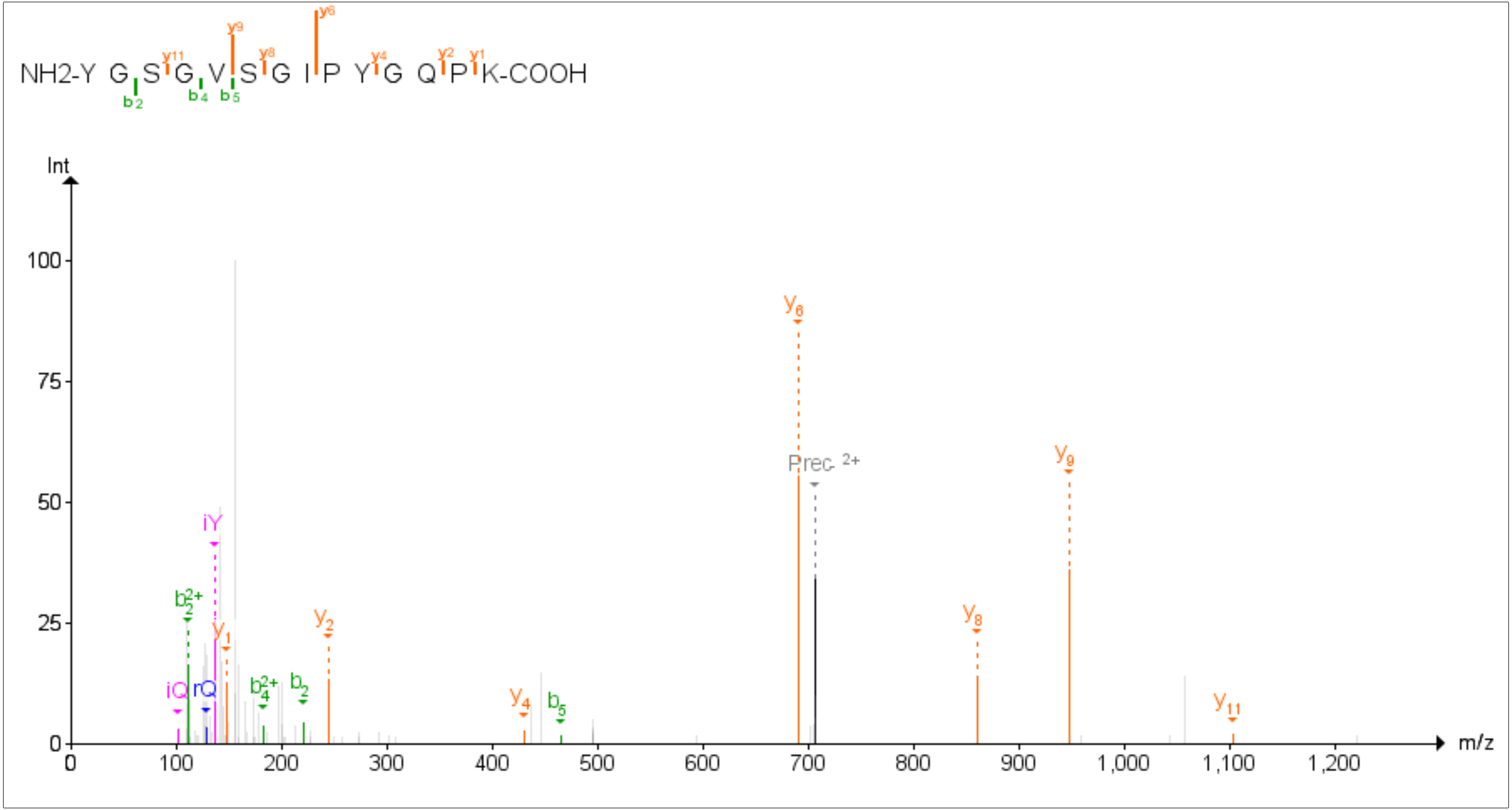

NH2-Y G S G V S G I P Y G Q P K-COOH
y11
y9
y8
y6
y4
y2
y1
b2
b4
b5
Int
100
75
50
25
0
y6
Prec. 2+
y9
y8
y11
y4
b5
y2
b2
b4 2+
y1
iY
b2 2+
iQ
rQ
m/z
0
100
200
300
400
500
600
700
800
900
1,000
1,100
1,200

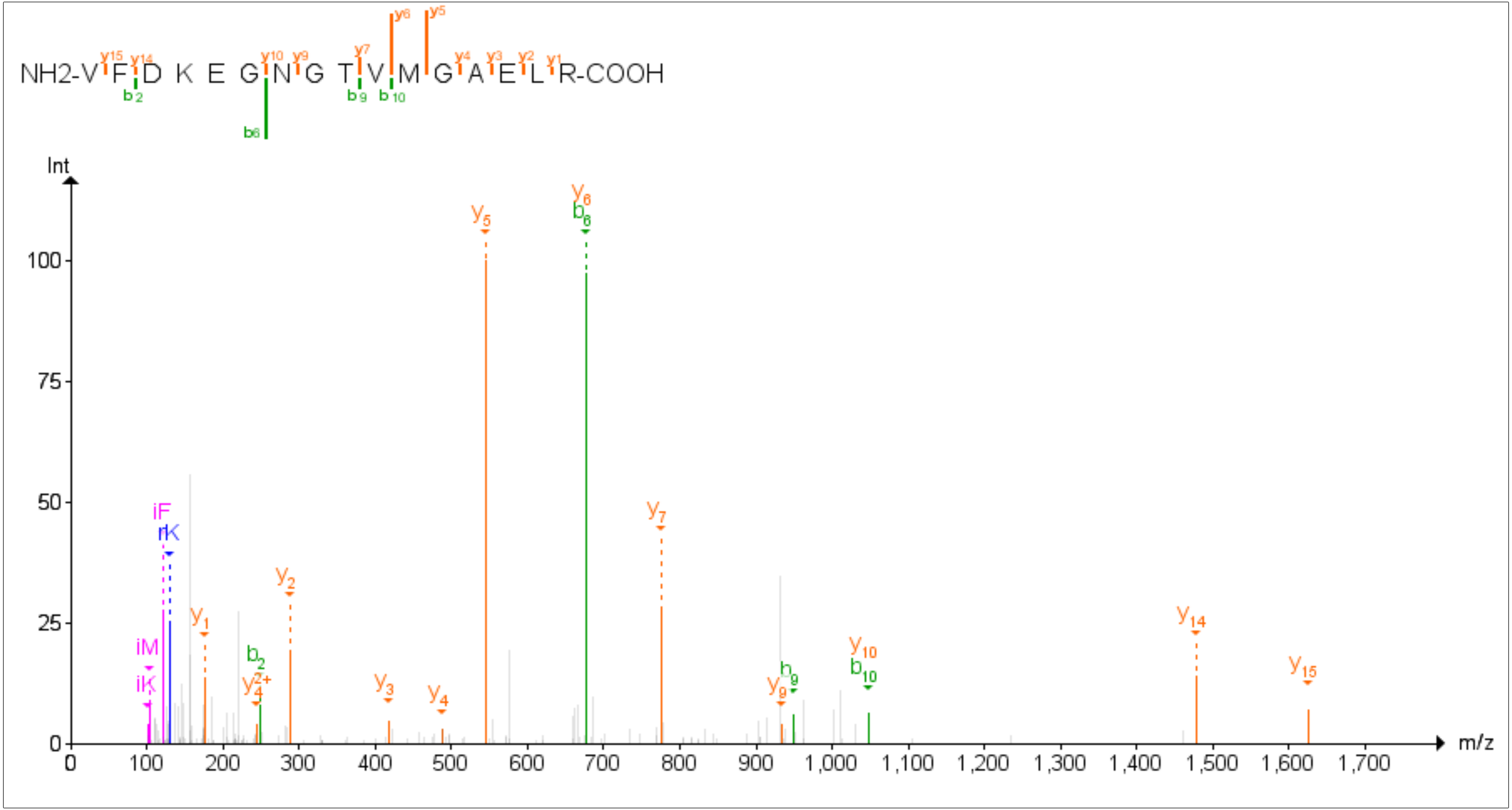
NH2-V F D K E G N G T V M G A E L R-COOH
Int
m/z
y5
y6
b6
y7
y2
y1
y14
y10
b10
y15
b9
y9
y3
y4
b2
iF
rK
iM
iK
0
25
50
75
100
0
100
200
300
400
500
600
700
800
900
1,000
1,100
1,200
1,300
1,400
1,500
1,600
1,700

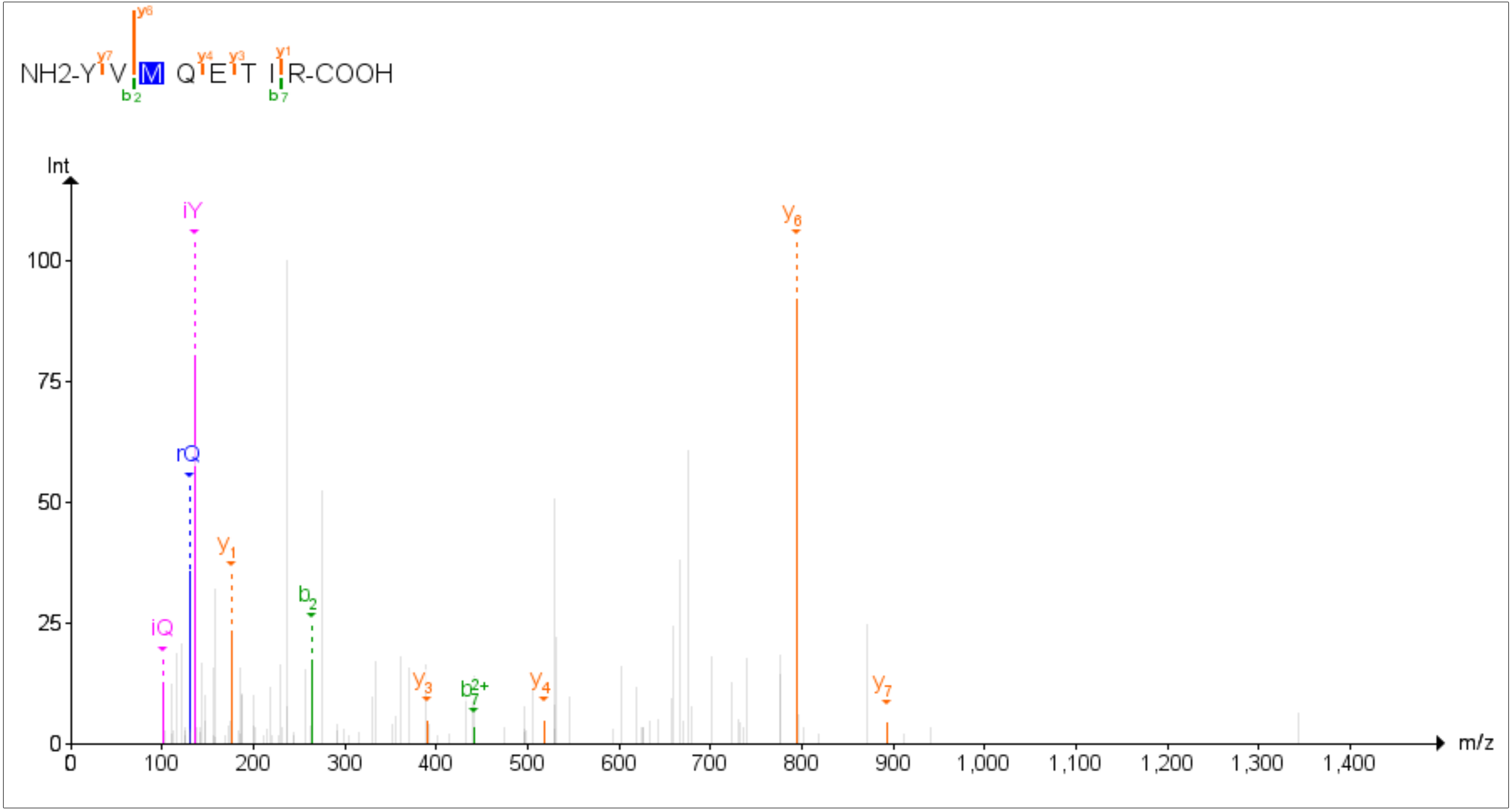

NH2-Y V M Q E T I R-COOH
y7
y6
y4
y3
y1
b2
b7
Int
iY
y6
rQ
y1
b2
iQ
y3
b7 2+
y4
y7
100
75
50
25
0
0
100
200
300
400
500
600
700
800
900
1,000
1,100
1,200
1,300
1,400
m/z

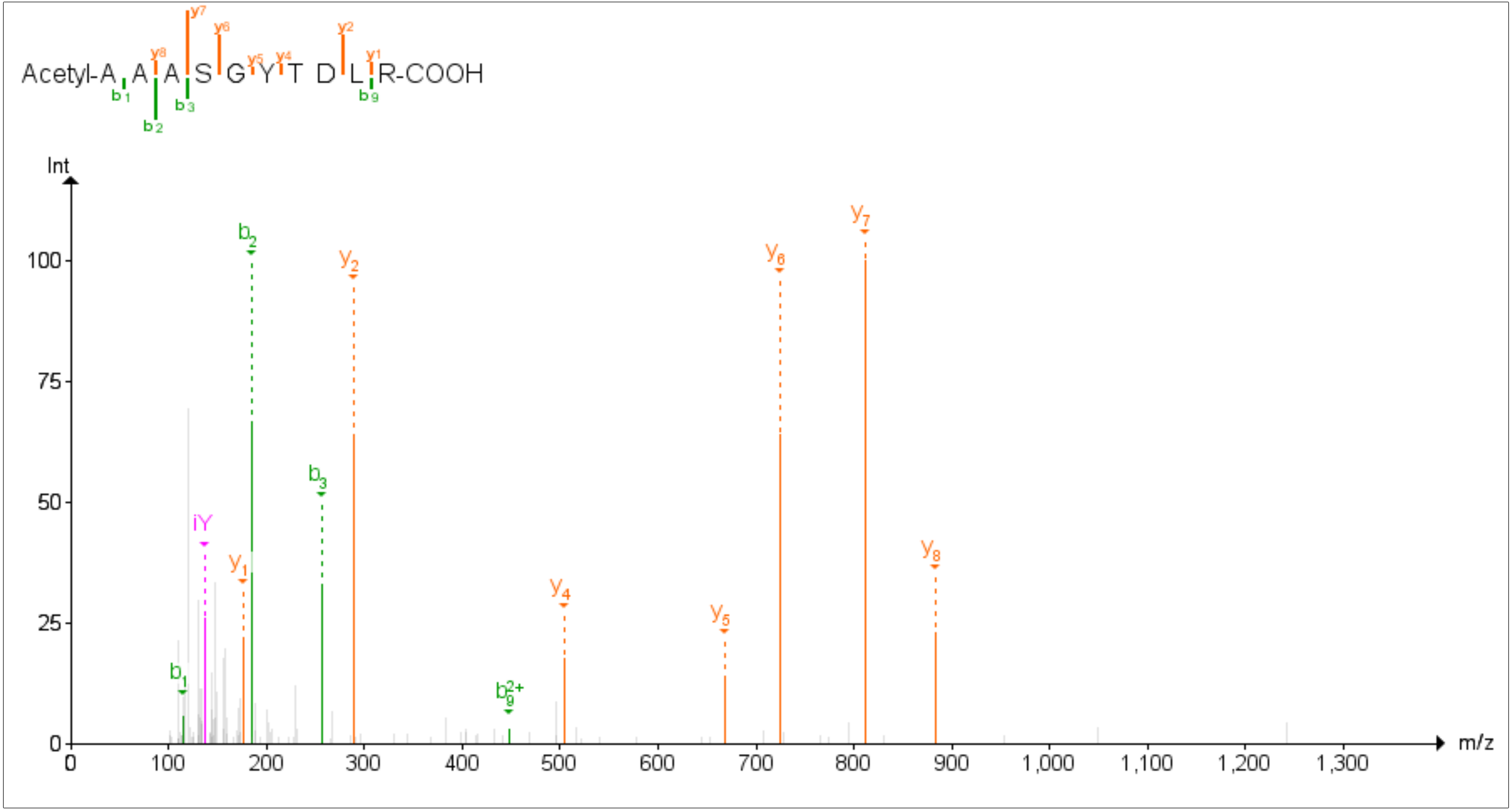

Acetyl-A A A S G Y T D L R-COOH
Int
m/z
b1
b2
b3
b9
y1
y2
y4
y5
y6
y7
y8
iY
b9 2+
0
25
50
75
100
0
100
200
300
400
500
600
700
800
900
1,000
1,100
1,200
1,300

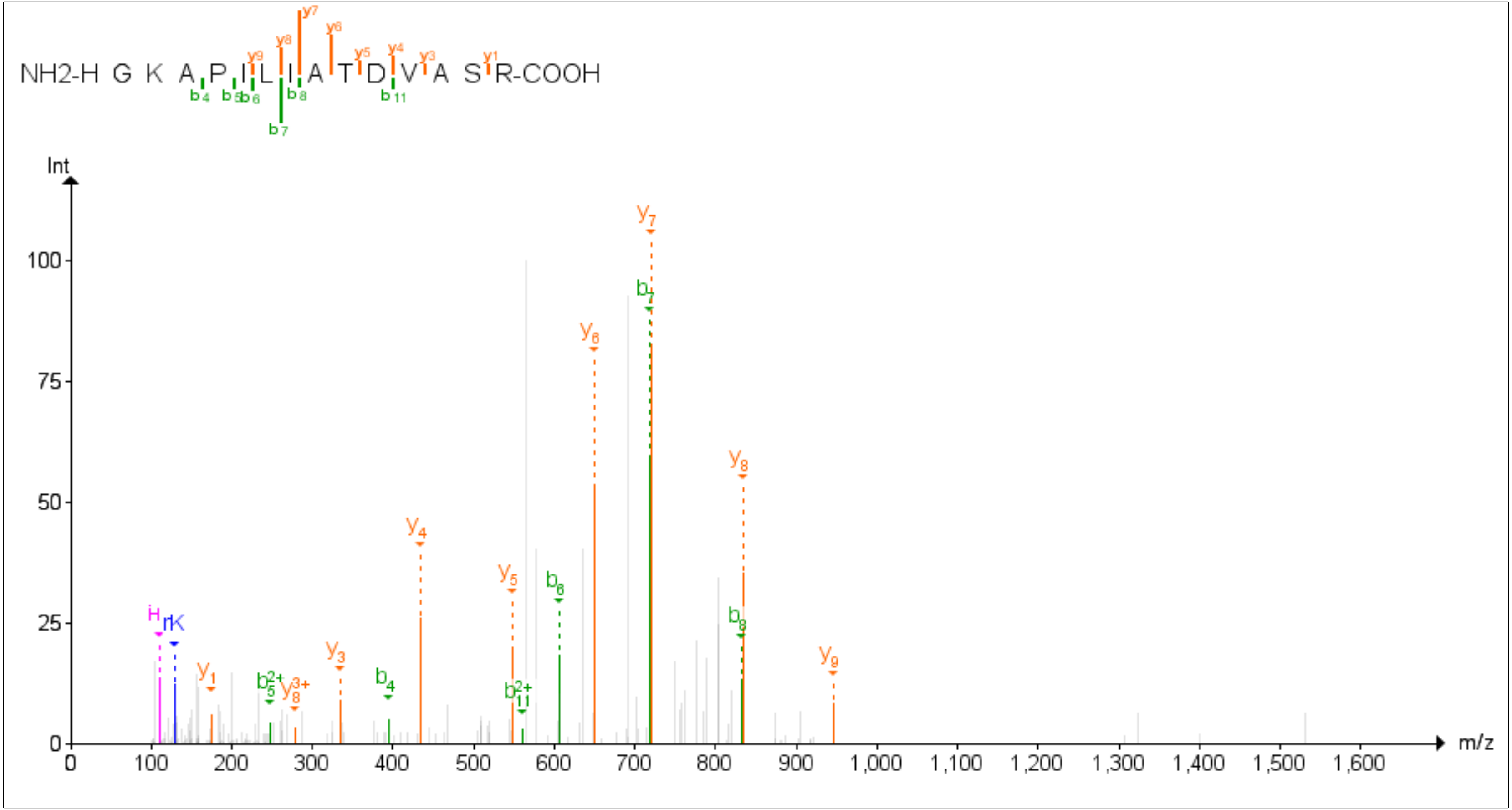
NH2-H G K A P I L I A T D V A S R-COOH
b4 b5 b6 b7 b8 b11
y9 y8 y7 y6 y5 y4 y3 y1
Int
m/z
0
25
50
75
100
0
100
200
300
400
500
600
700
800
900
1,000
1,100
1,200
1,300
1,400
1,500
1,600
iH
iK
y1
b5 2+
y8 3+
y3
b4
y4
y5
b11 2+
b6
y6
y7
b7
y8
b8
y9

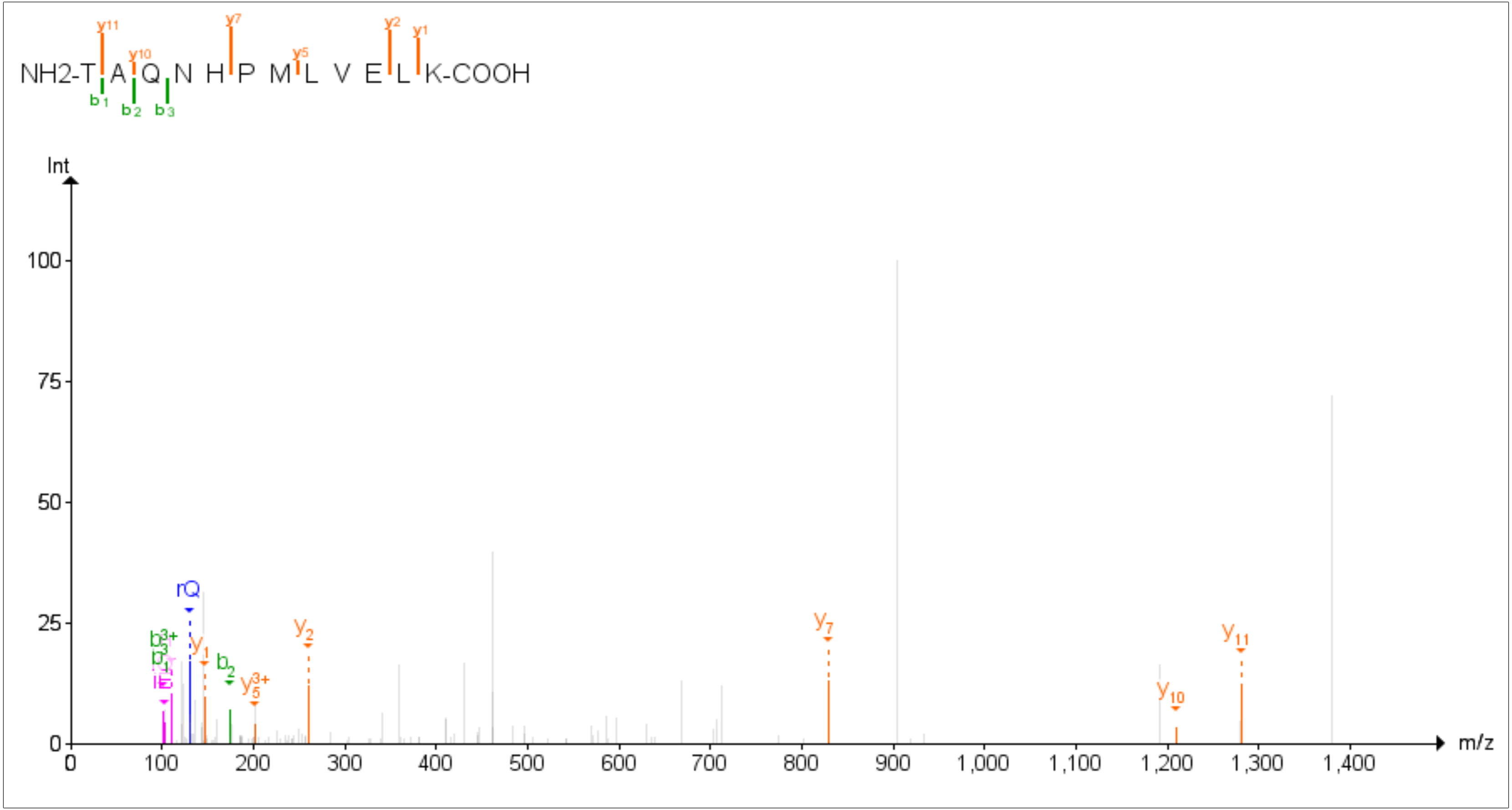

NH2-T A Q N H P M L V E L K-COOH
y11 y10 y7 y5 y2 y1
b1 b2 b3
Int
m/z
rQ
y1
y2
y7
y10
y11
b2

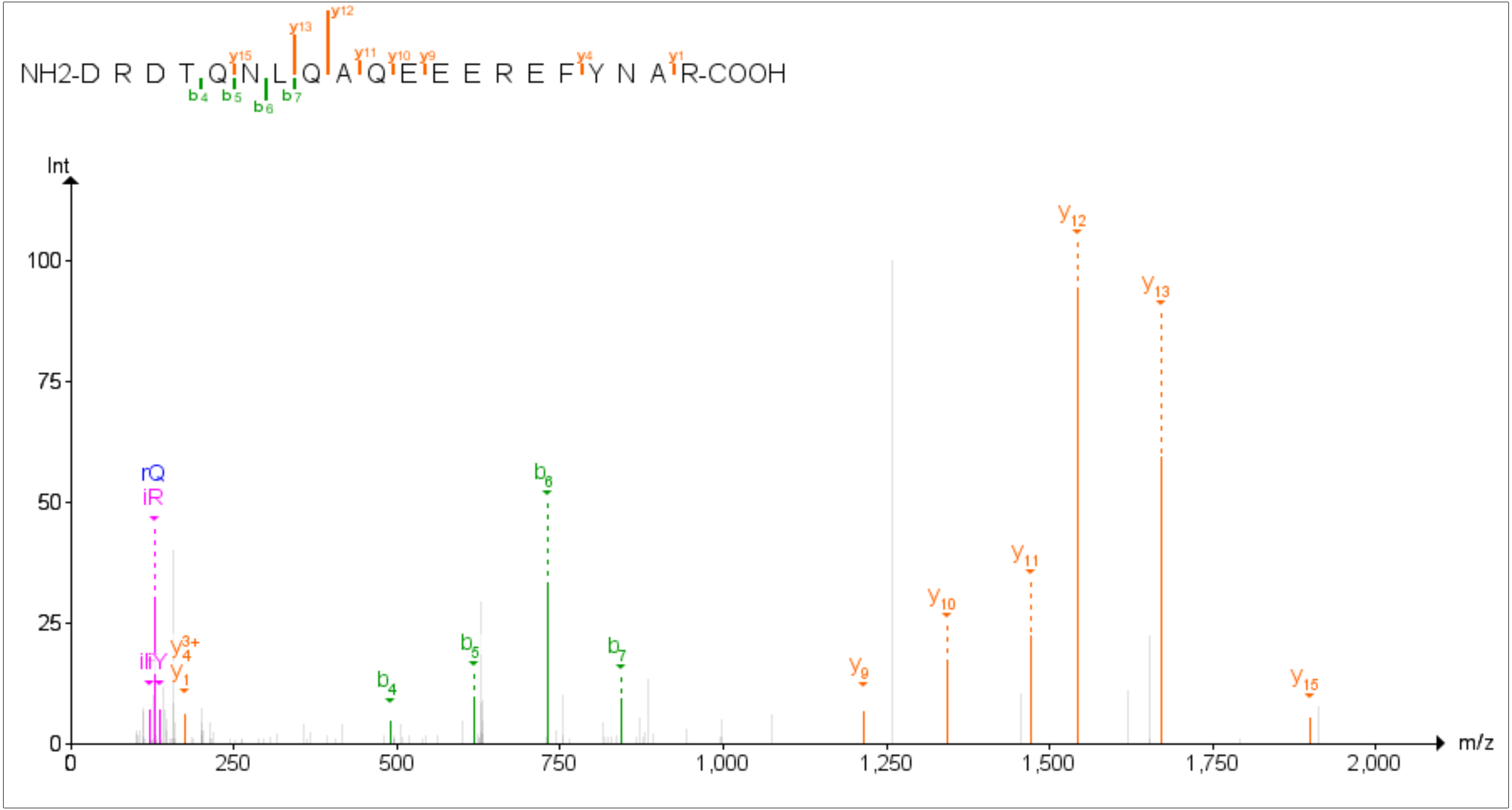

NH2-D R D T Q N L Q A Q E E E R E F Y N A R-COOH
y15 y13 y12 y11 y10 y9 y4 y1
b4 b5 b6 b7
Int
100
75
50
25
0
rQ
iR
iI/Y
y4 3+
y1
b4
b5
b6
b7
y9
y10
y11
y12
y13
y15
0
250
500
750
1,000
1,250
1,500
1,750
2,000
m/z

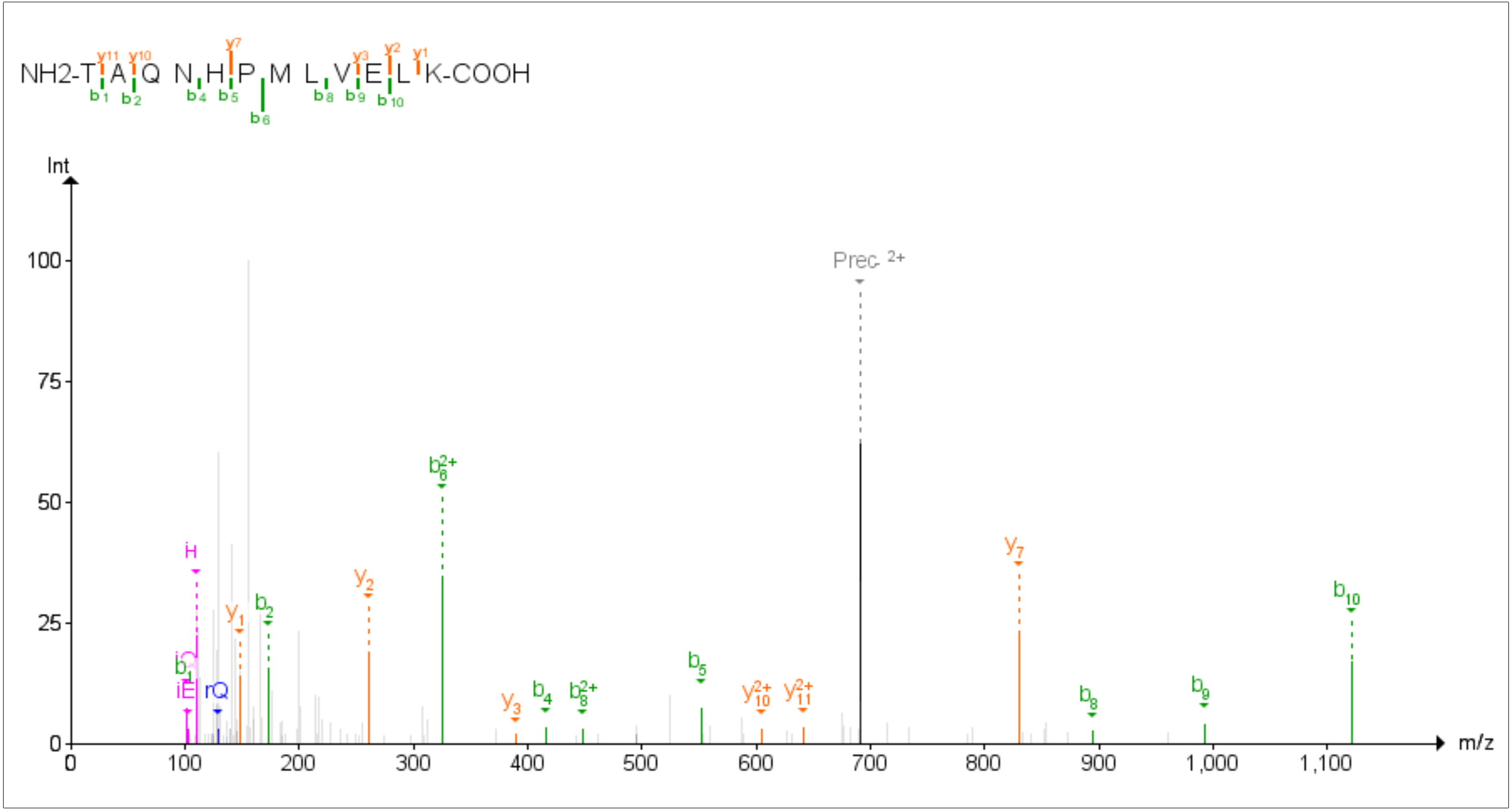

NH2-T A Q N H P M L V E L K-COOH
y11 y10 y7 y3 y2 y1
b1 b2 b4 b5 b6 b8 b9 b10
Int
100
75
50
25
0
Prec. 2+
b6 2+
y7
b10
iH
y2
y1
b2
iQ
b1
iE
rQ
y3
b4
b8 2+
b5
y10 2+
y11 2+
b8
b9
0
100
200
300
400
500
600
700
800
900
1,000
1,100
m/z

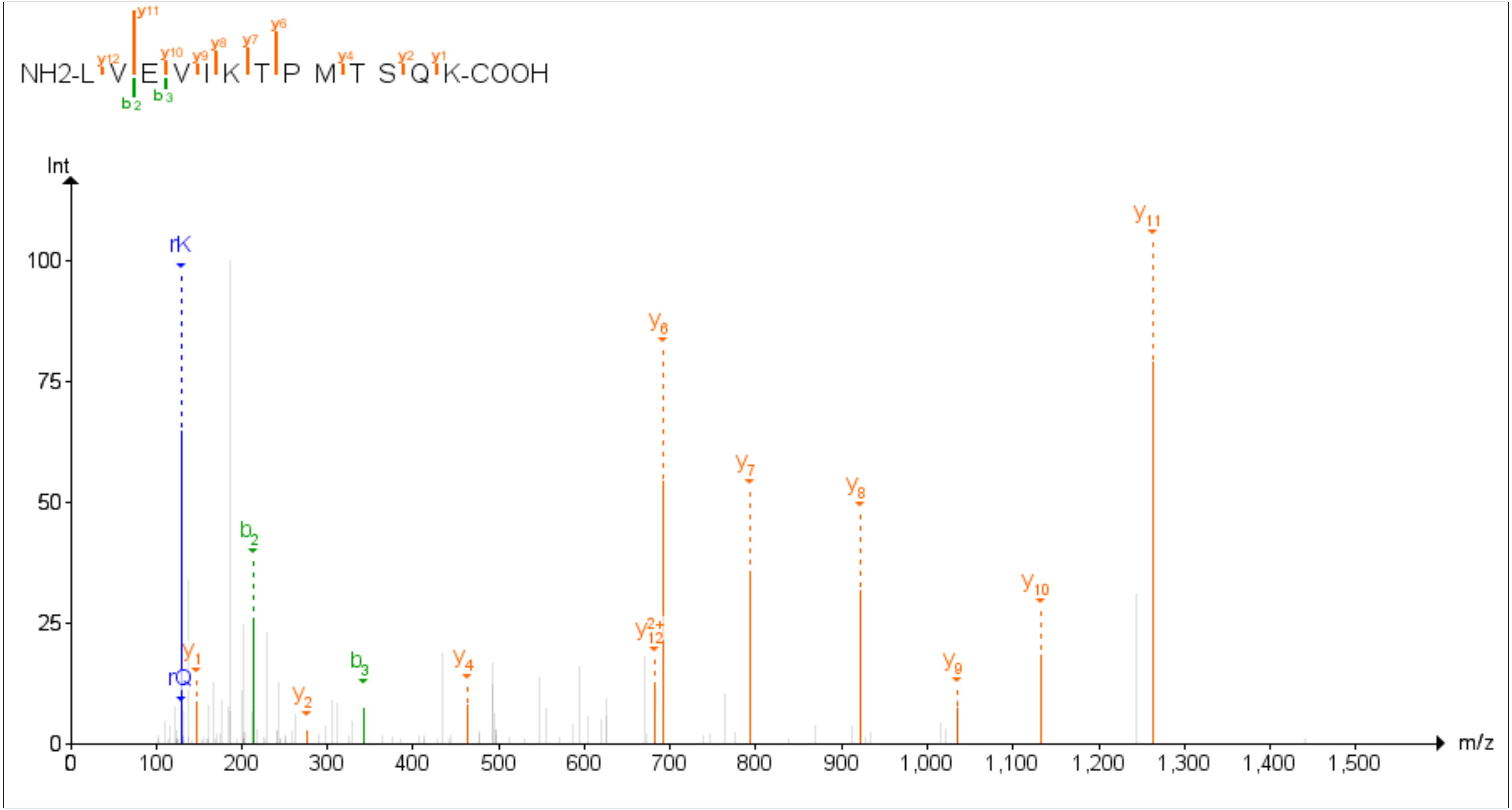
NH2-L V E V I K T P M T S Q K-COOH
y12
y11
y10
y9
y8
y7
y6
y4
y2
y1
b2
b3
Int
m/z
rK
rQ
y1
y2
b2
b3
y4
y12 2+
y6
y7
y8
y9
y10
y11
0
25
50
75
100
0
100
200
300
400
500
600
700
800
900
1,000
1,100
1,200
1,300
1,400
1,500

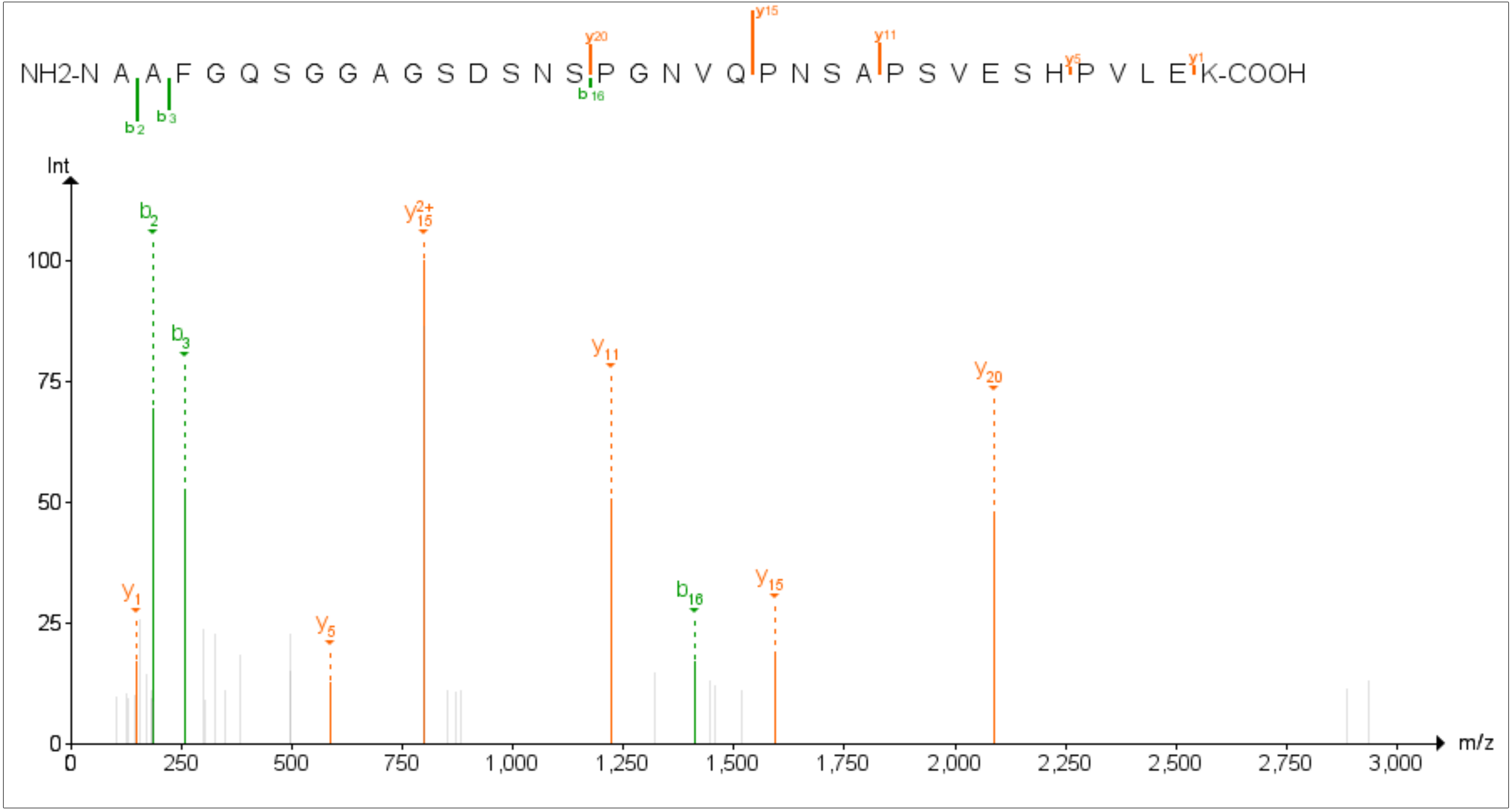

NH2-N A A F G Q S G G A G S D S N S P G N V Q P N S A P S V E S H P V L E K-COOH
y20
y15
y11
y5
y1
b2
b3
b16
Int
m/z
0
25
50
75
100
250
500
750
1,000
1,250
1,500
1,750
2,000
2,250
2,500
2,750
3,000

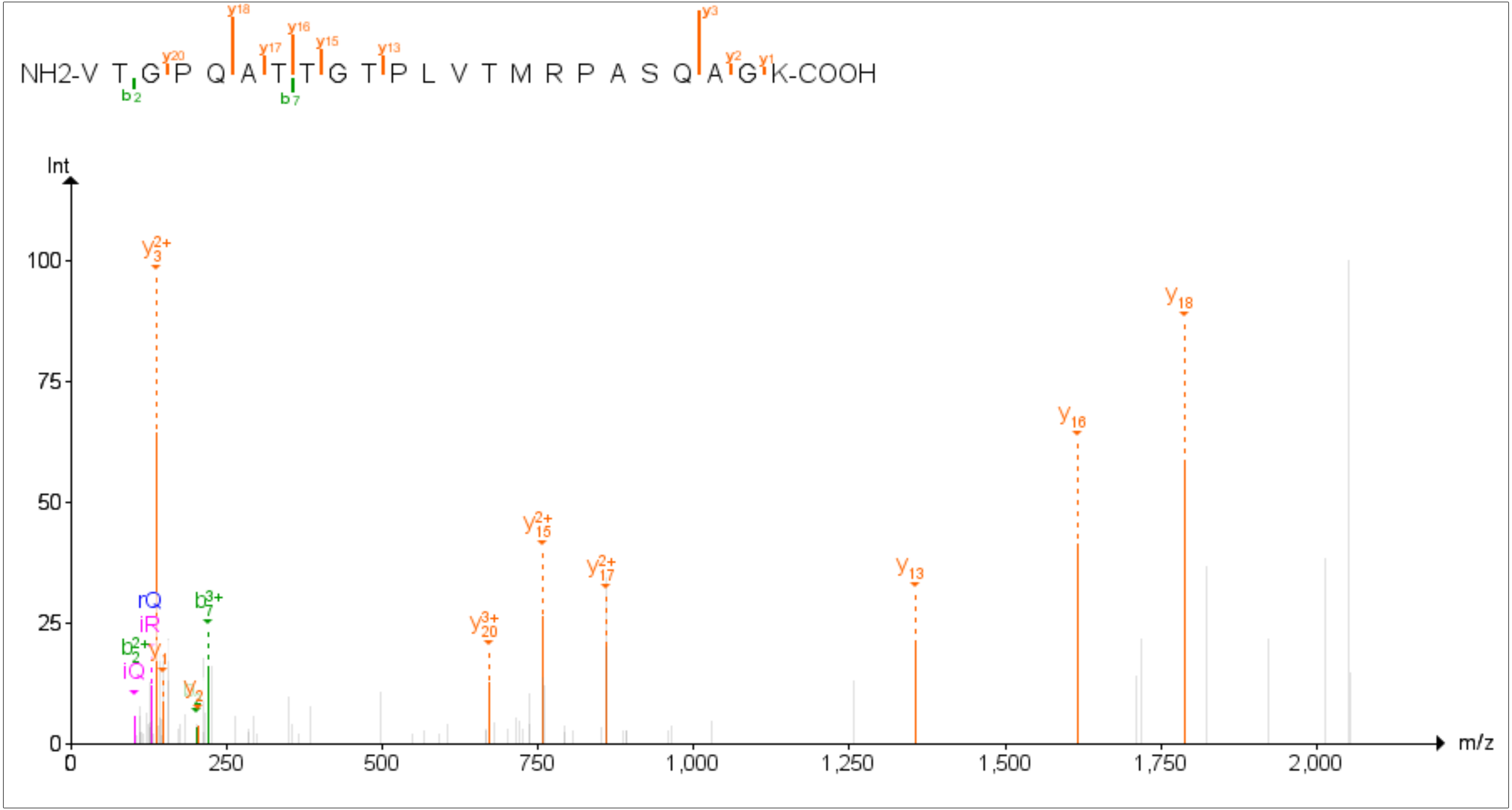
NH2-V T G P Q A T T G T P L V T M R P A S Q A G K-COOH
Int
m/z
100
75
50
25
0
250
500
750
1,000
1,250
1,500
1,750
2,000
y18
y16
y13
y15
y17
y20
y3
y2
y1
rQ
iR
iQ

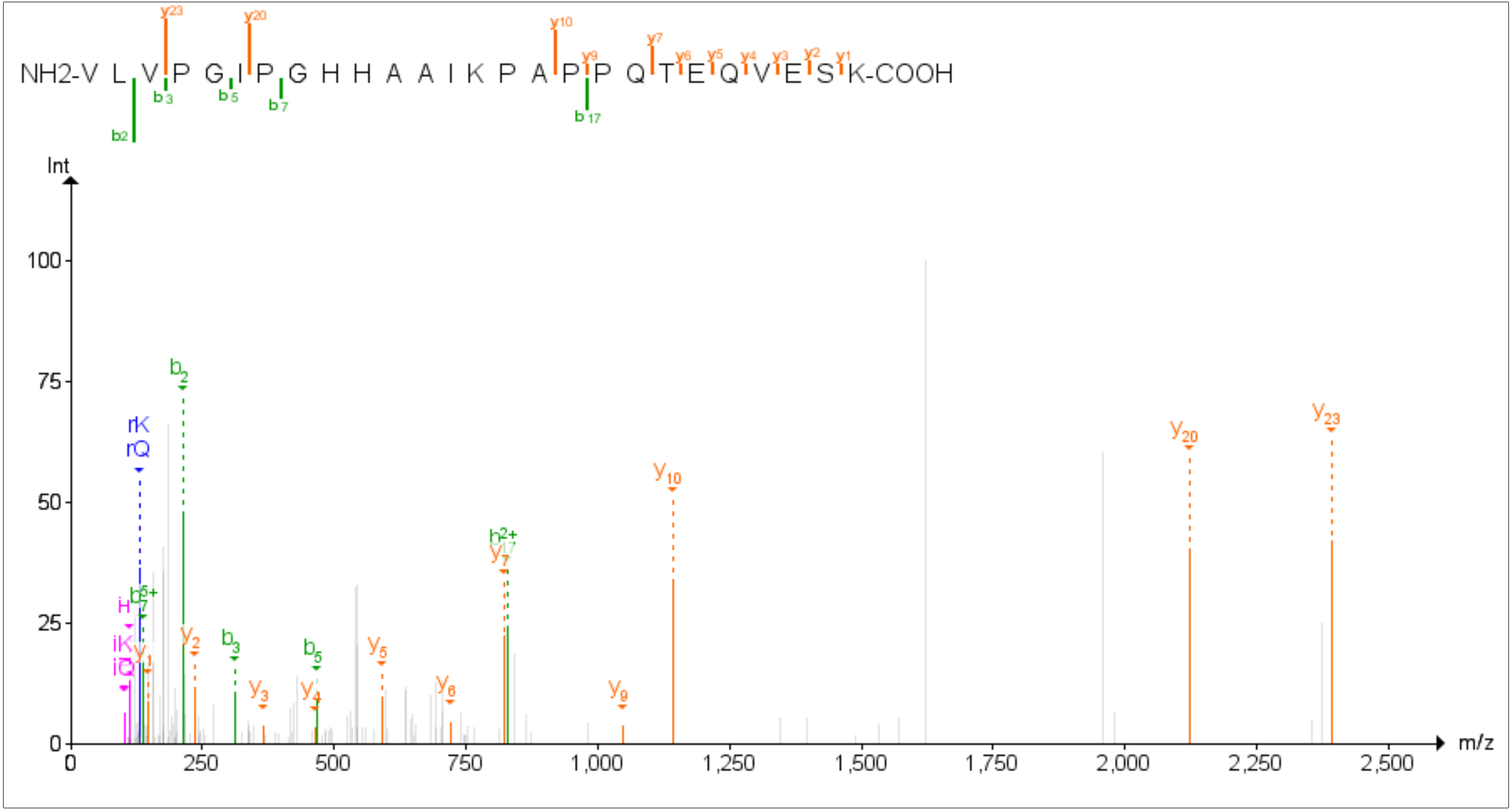
NH2-V L V P G I P G H H A A I K P A P P Q T E Q V E S K-COOH
Int
m/z
0
25
50
75
100
250
500
750
1,000
1,250
1,500
1,750
2,000
2,250
2,500

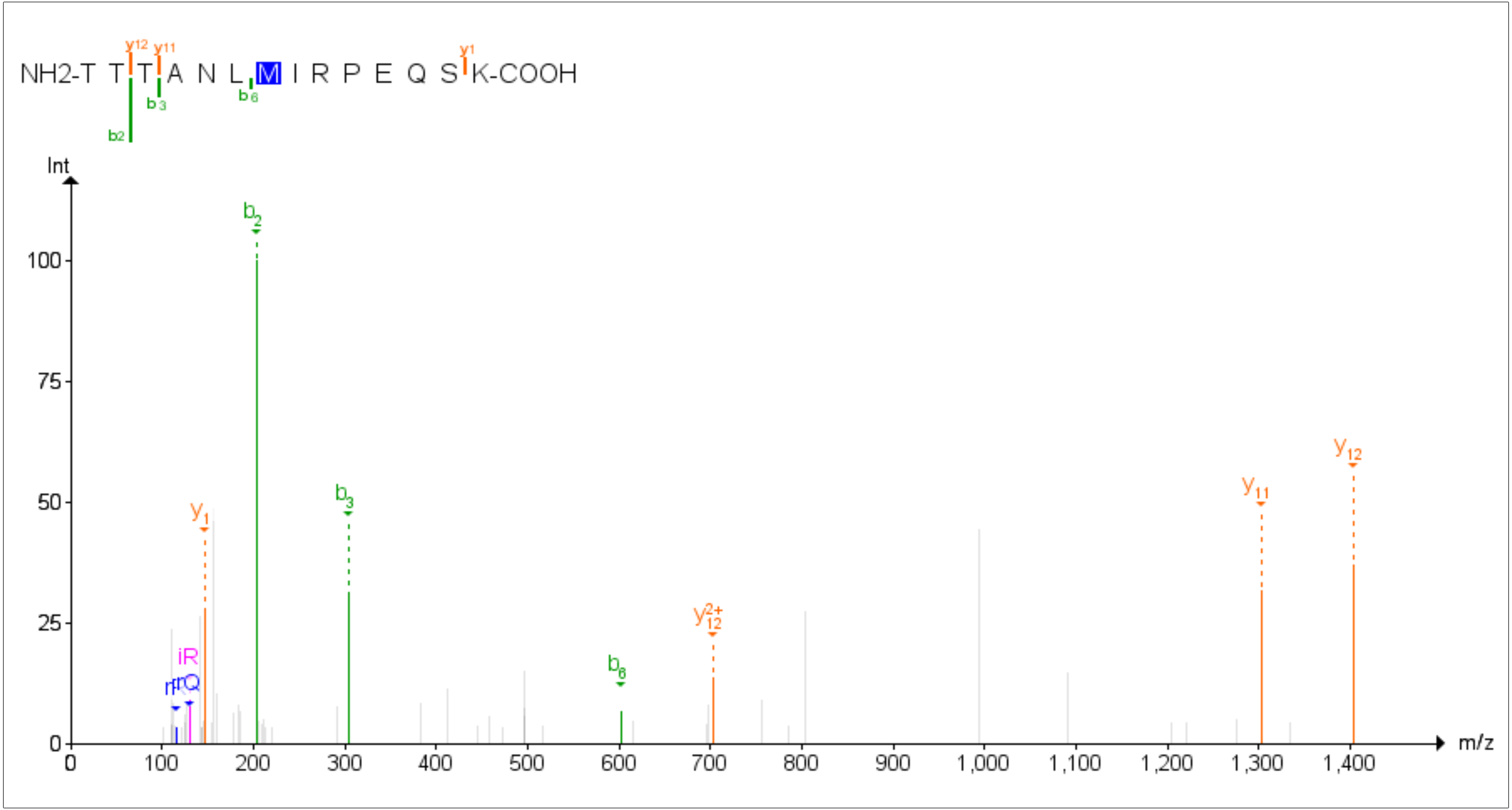

NH2-T T T A N L M I R P E Q S K-COOH
y12
y11
y1
b2
b3
b6
Int
100
75
50
25
0
b2
b3
y1
iR
y2+12
b6
y11
y12
m/z
0
100
200
300
400
500
600
700
800
900
1,000
1,100
1,200
1,300
1,400

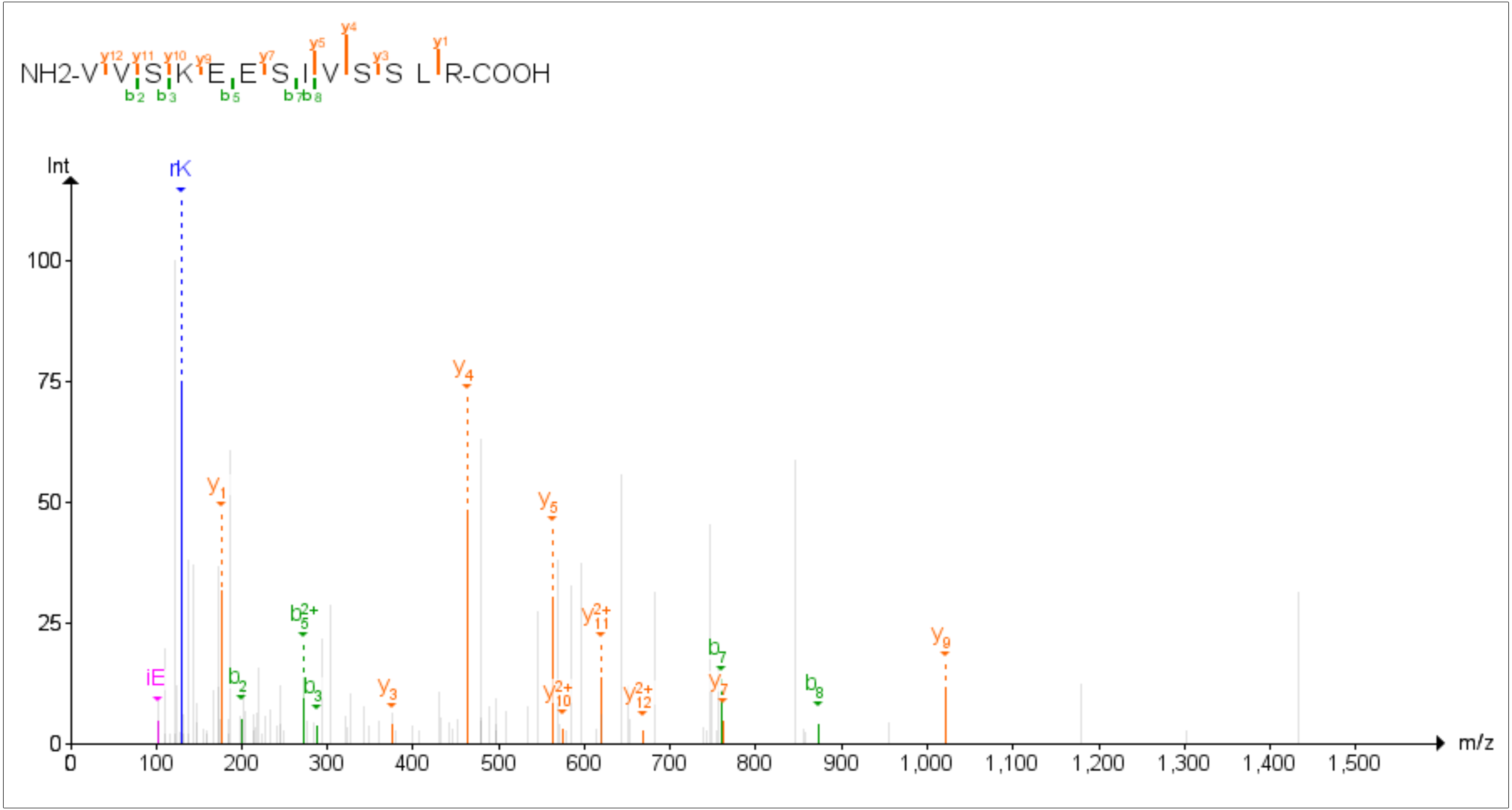

NH2-VVSKEESIVSSLR-COOH
Int
m/z
rK
iE
y1
y3
y4
y5
y7
y9
y2+10
y2+11
y2+12
b2
b3
b2+5
b7
b8

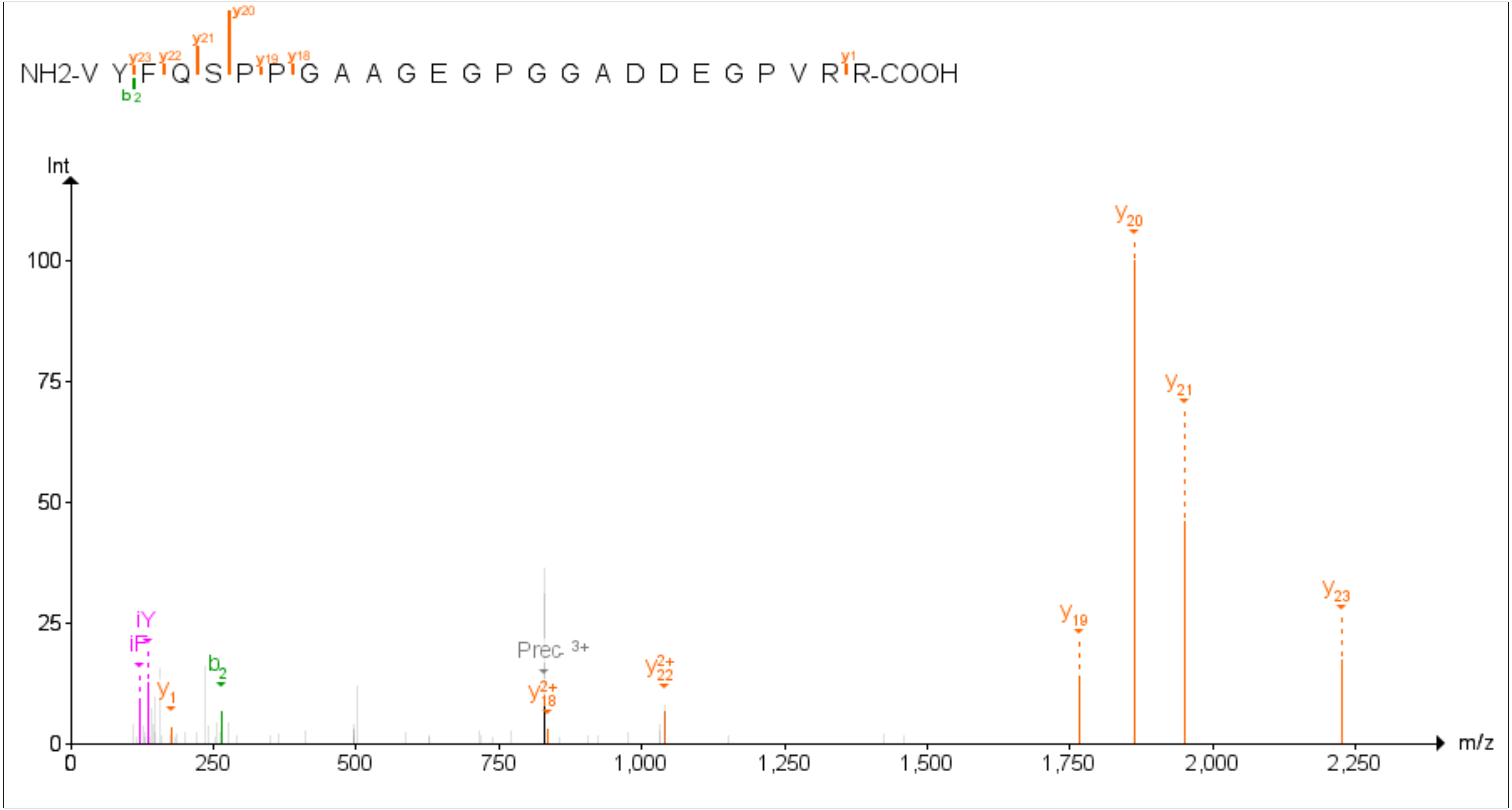
NH2-V Y F Q S P P G A A G E G P G G A D D E G P V R R-COOH
y23
y22
y21
y20
y19
y18
y1
b2
Int
100
75
50
25
0
iY
iF
y1
b2
Prec 3+
y18 2+
y22 2+
y19
y20
y21
y23
0
250
500
750
1,000
1,250
1,500
1,750
2,000
2,250
m/z

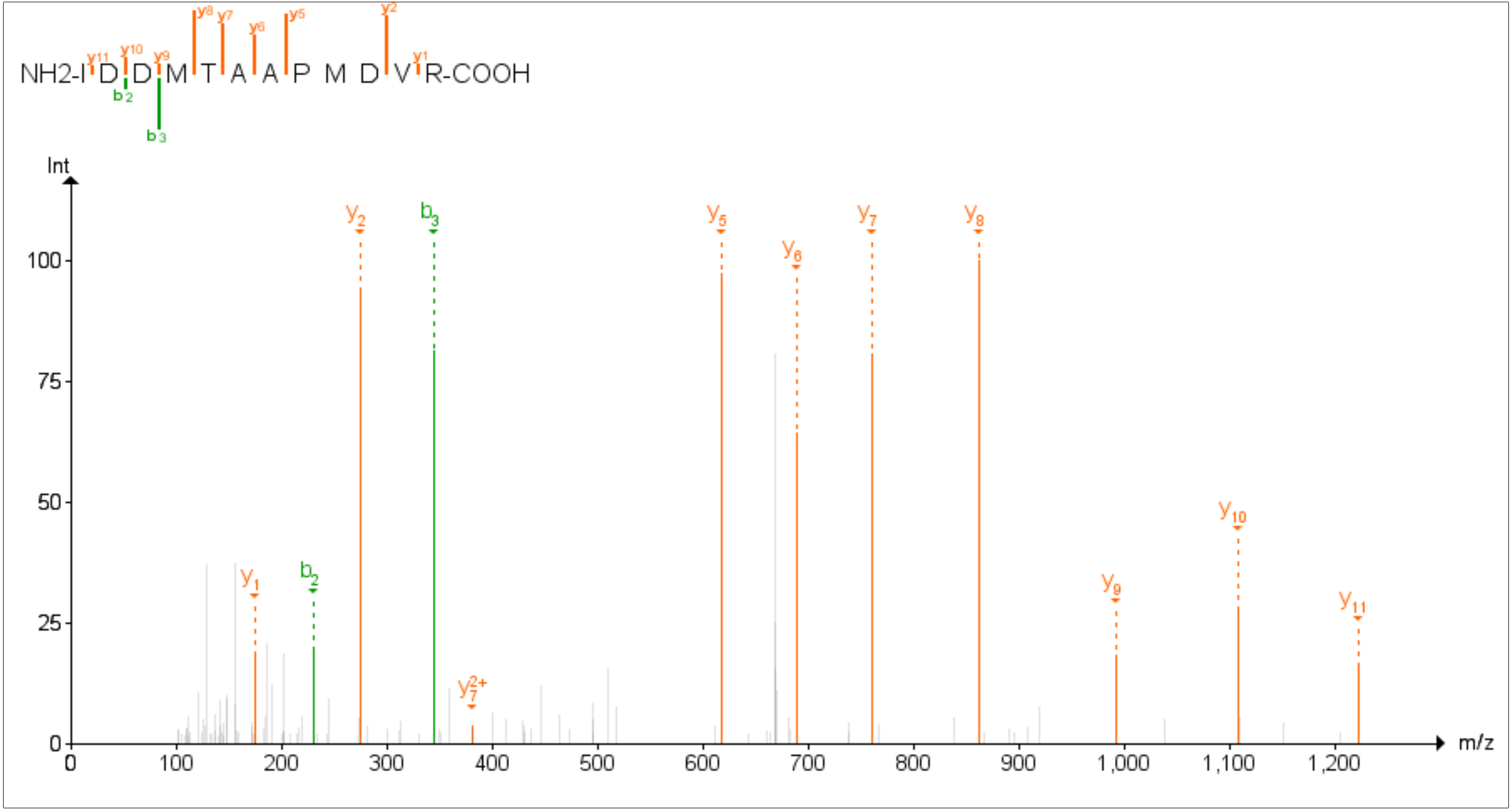

NH2-I D D M T A A P M D V R-COOH
y11 y10 y9 y8 y7 y6 y5 y2 y1
b2 b3
Int
100
75
50
25
0
m/z
0 100 200 300 400 500 600 700 800 900 1,000 1,100 1,200
y1 b2 y2 b3 y7 2+ y5 y6 y7 y8 y9 y10 y11

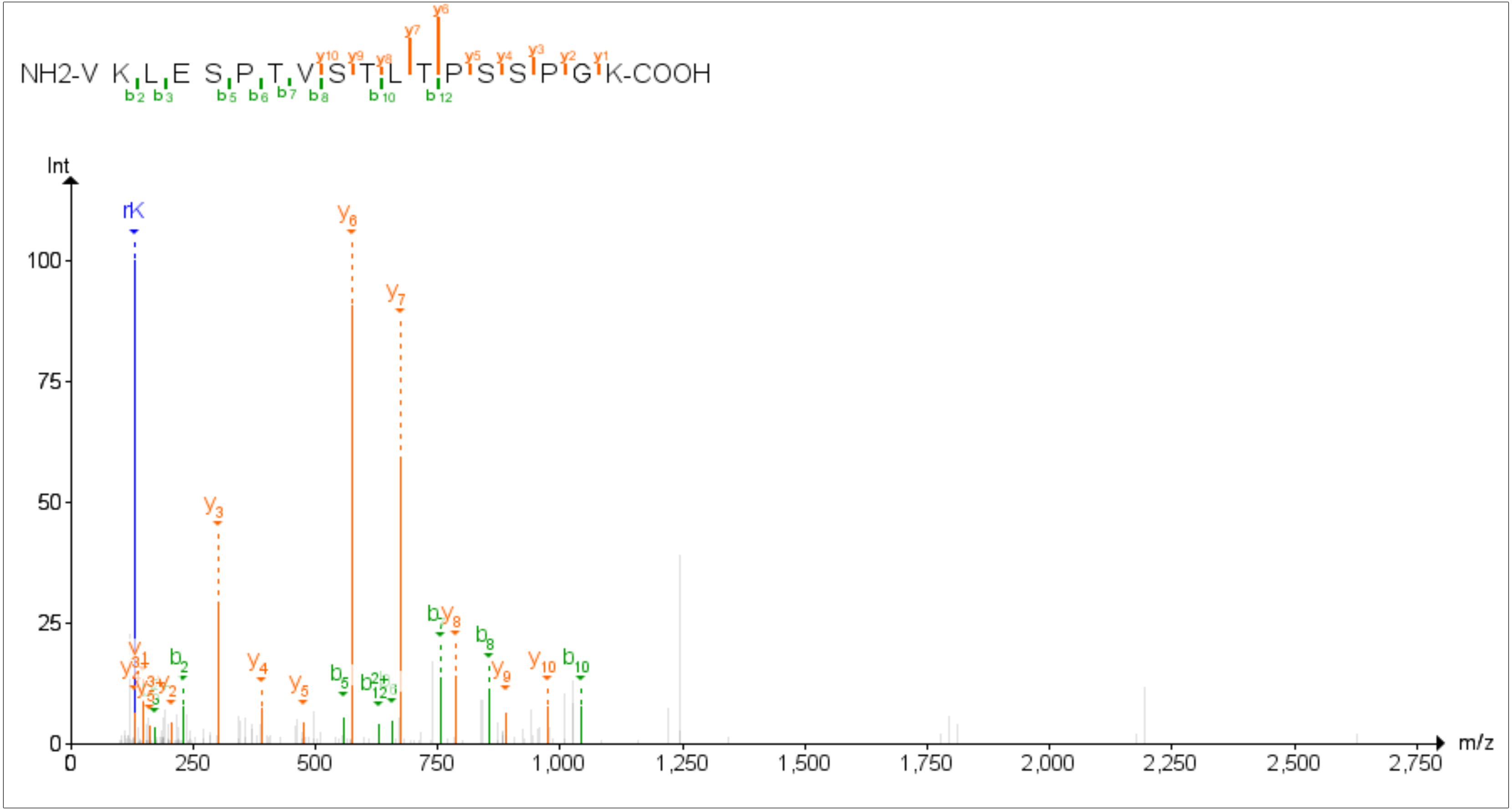

NH2-V K L E S P T V S T L T P S S P G K-COOH
Int
rK
y6
y7
y3
y4
y5
b2
b5
b7
y8
b8
y9
y10
b10
m/z
0
25
50
75
100
250
500
750
1,000
1,250
1,500
1,750
2,000
2,250
2,500
2,750

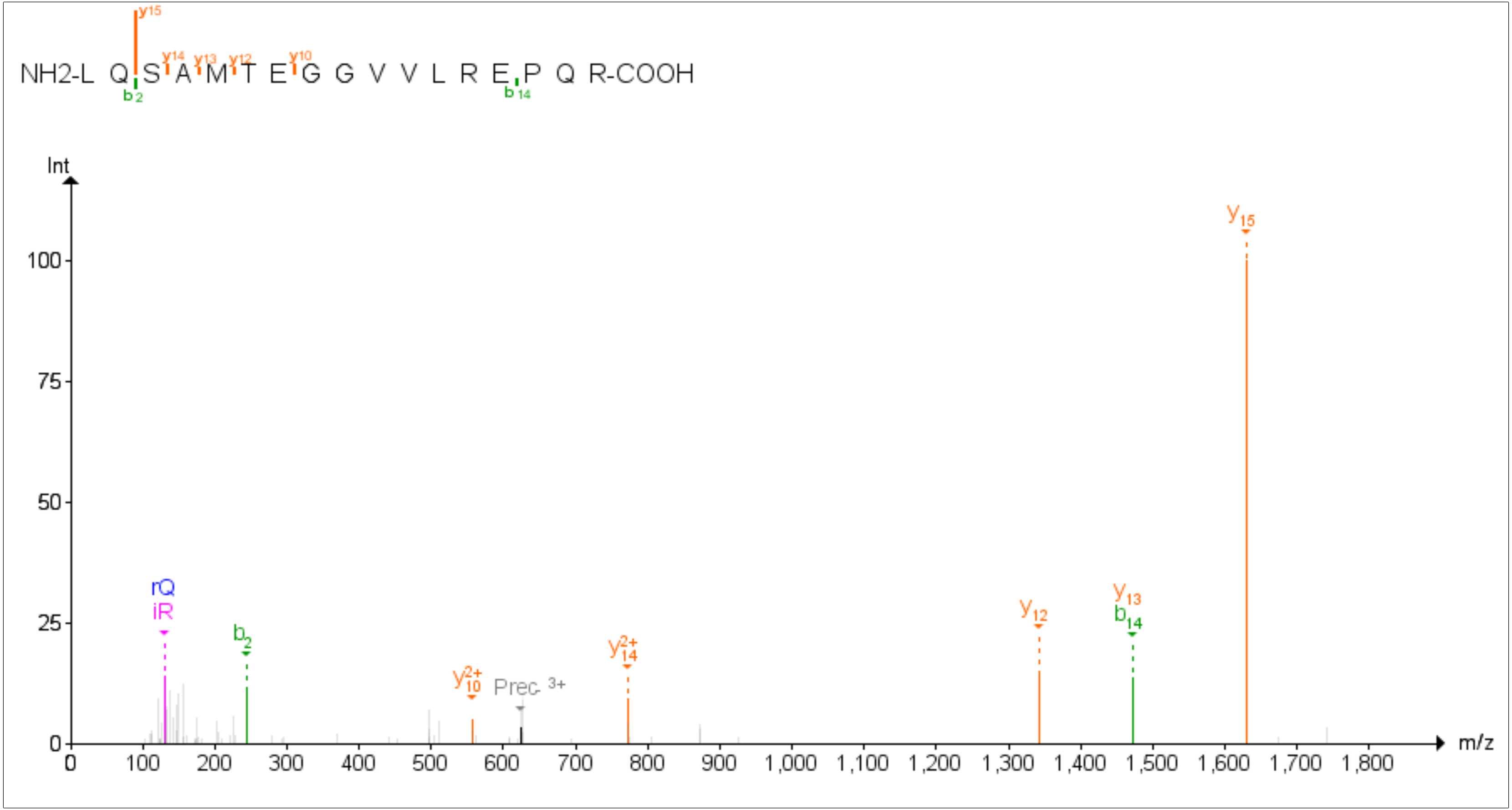
NH2-L Q S A M T E G G V V L R E P Q R-COOH
y15
y14
y13
y12
y10
b2
b14
Int
100
75
50
25
0
rQ
iR
b2
y10 2+
Prec 3+
y14 2+
y12
y13
b14
y15
m/z
0
100
200
300
400
500
600
700
800
900
1,000
1,100
1,200
1,300
1,400
1,500
1,600
1,700
1,800

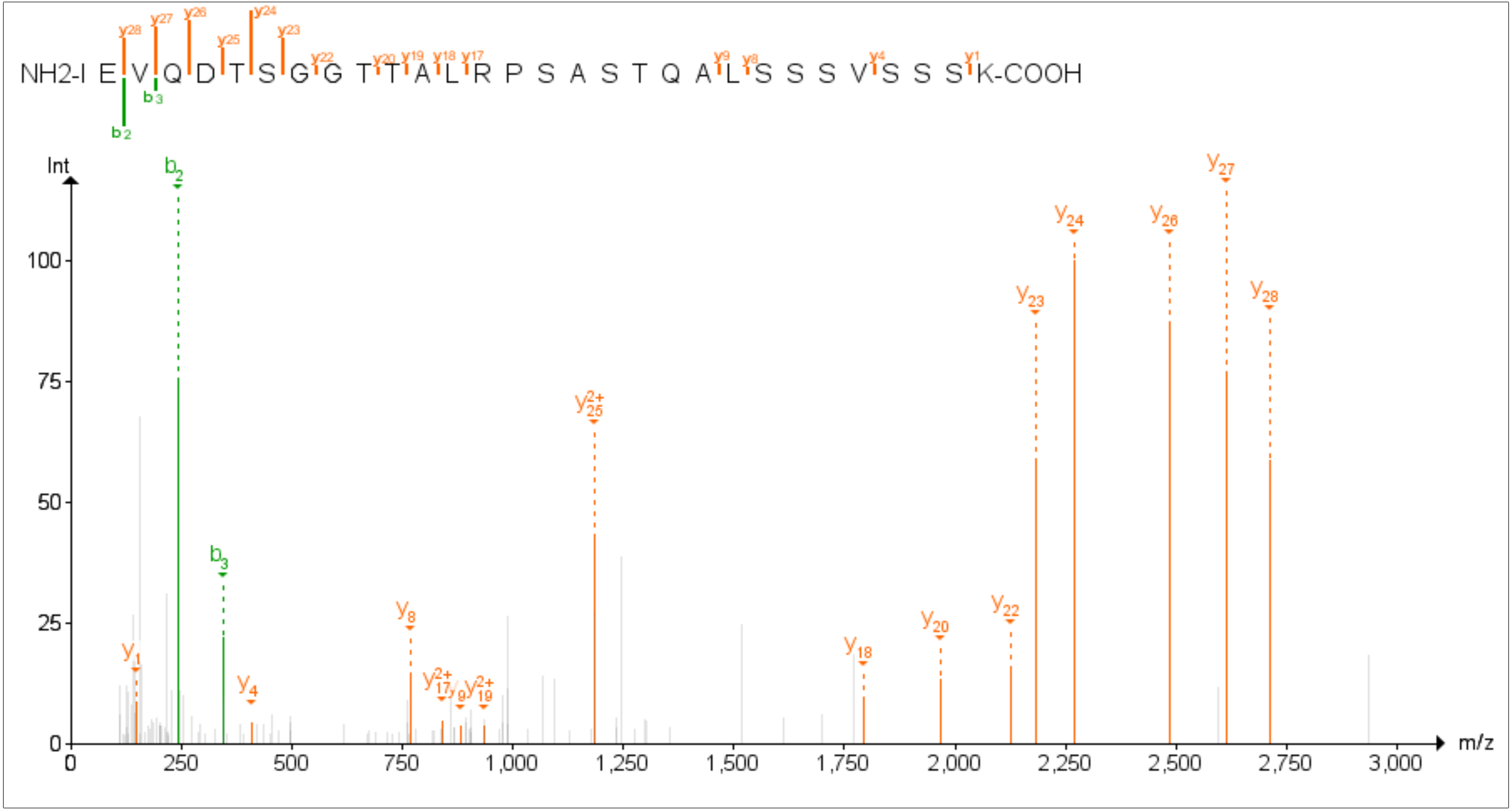

NH2-I E V Q D T S G G T T A L R P S A S T Q A L S S S V S S S K-COOH
Int
m/z
b2
b3
y1
y4
y8
y17 2+
y9
y19 2+
y25 2+
y18
y20
y22
y23
y24
y26
y27
y28
0
25
50
75
100
0
250
500
750
1,000
1,250
1,500
1,750
2,000
2,250
2,500
2,750
3,000

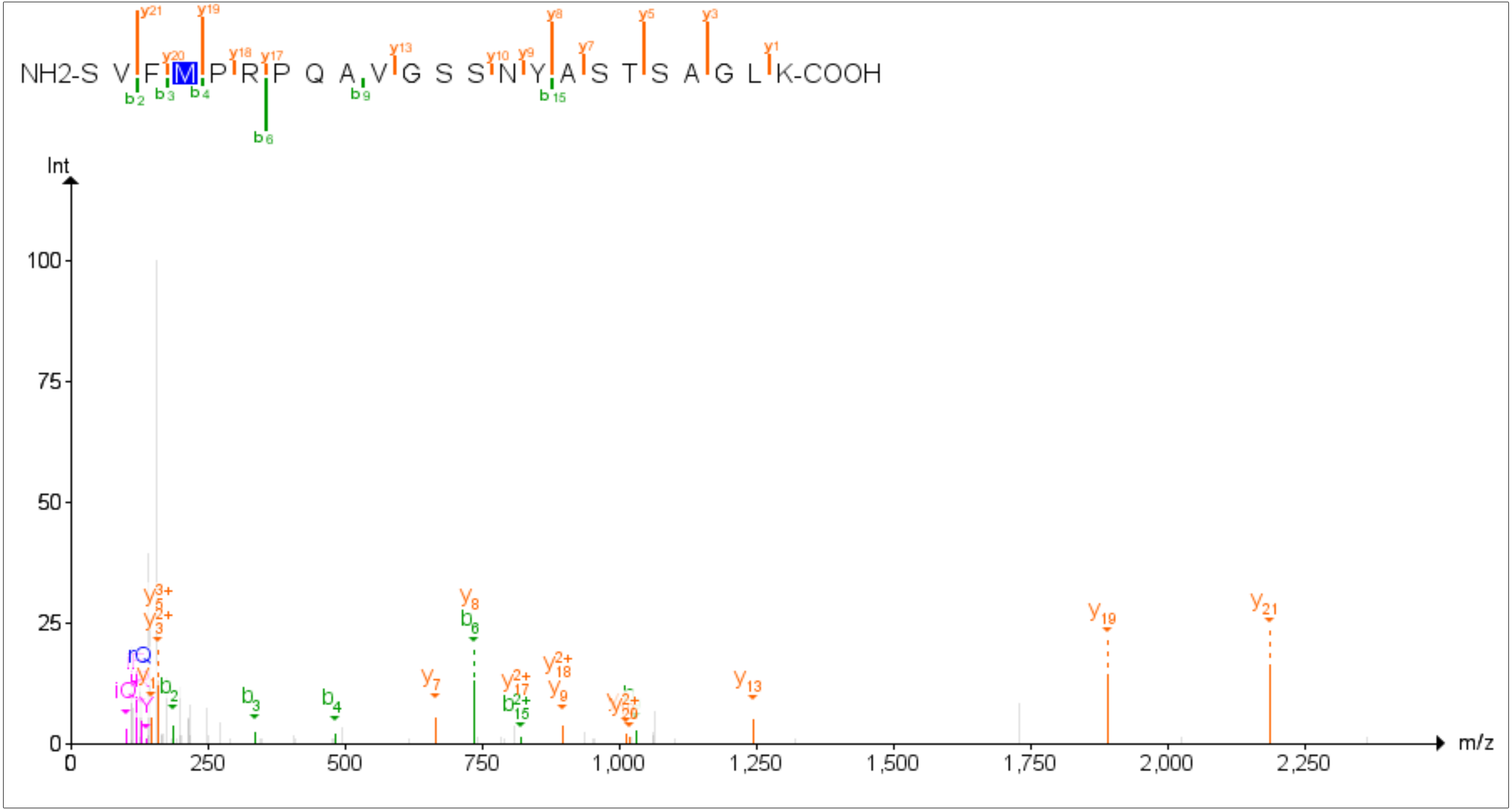

NH2-S V F M P R P Q A V G S S N Y A S T S A G L K-COOH
Int
100
75
50
25
0
0
250
500
750
1,000
1,250
1,500
1,750
2,000
2,250
m/z

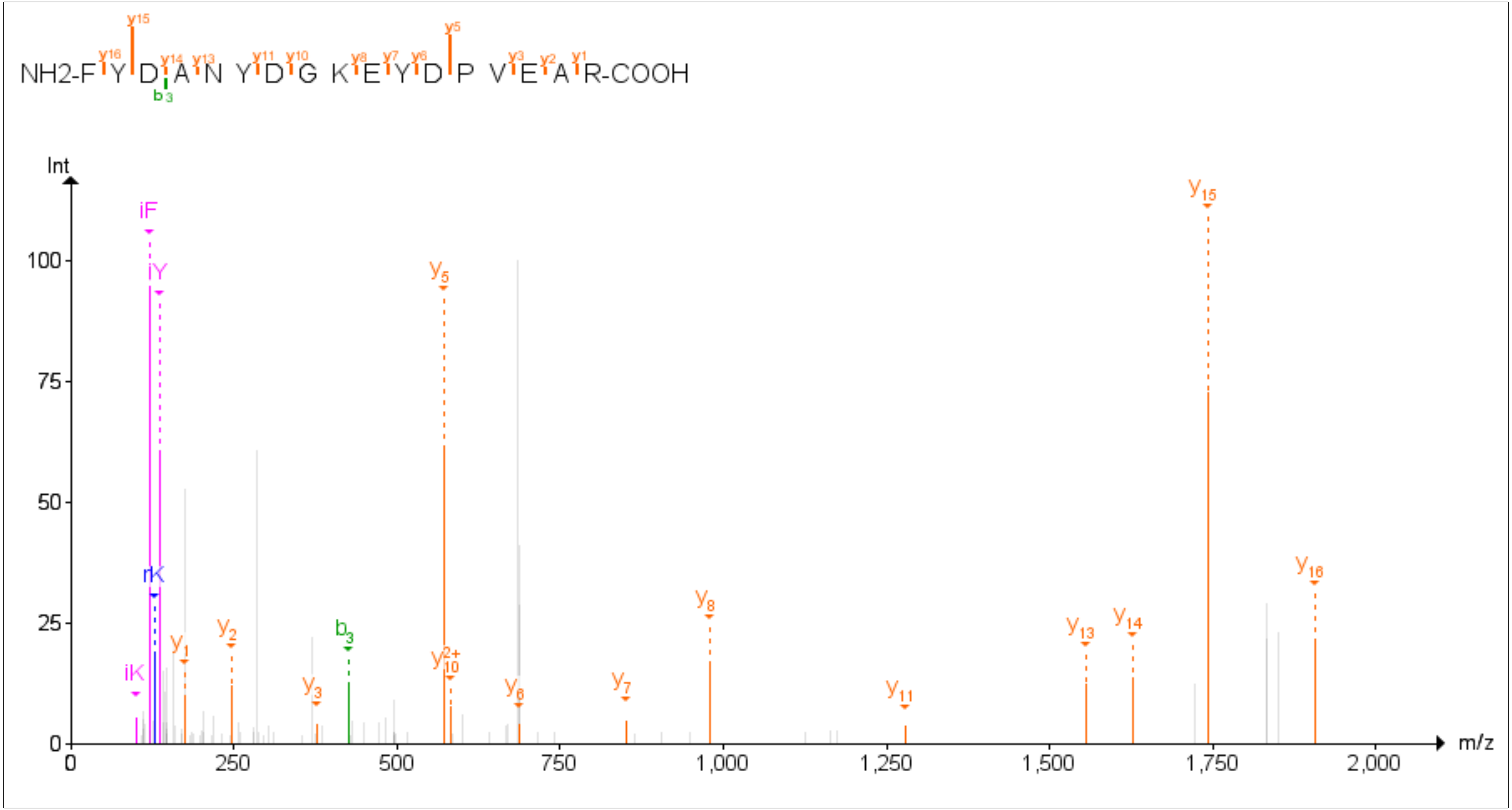
NH2-F Y D A N Y D G K E Y D P V E A R-COOH
y16
y15
y14
y13
y11
y10
y8
y7
y6
y5
y3
y2
y1
b3
Int
100
75
50
25
0
iF
iY
rK
iK
y1
y2
y3
b3
y5
y_{10}^{2+}
y6
y7
y8
y11
y13
y14
y15
y16
0
250
500
750
1,000
1,250
1,500
1,750
2,000
m/z

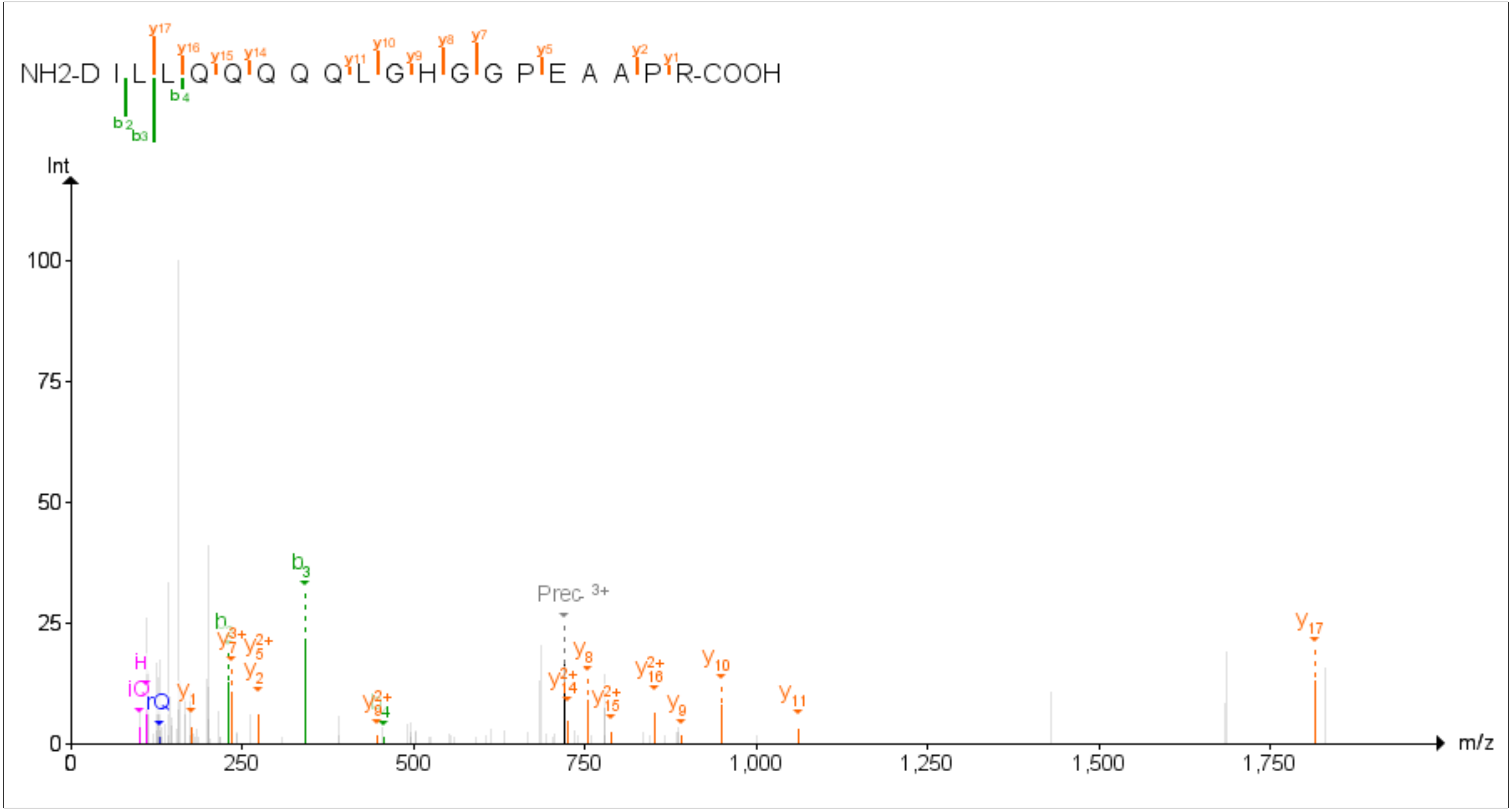

NH2-D I L L Q Q Q Q Q L G H G G P E A A P R-COOH
y17 y16 y15 y14 y11 y10 y9 y8 y7 y5 y2 y1
b2 b3 b4
Int
100
75
50
25
0
m/z
0
250
500
750
1,000
1,250
1,500
1,750
Prec 3+
y17
y8
y10
y11
y9
b3
y2
y1

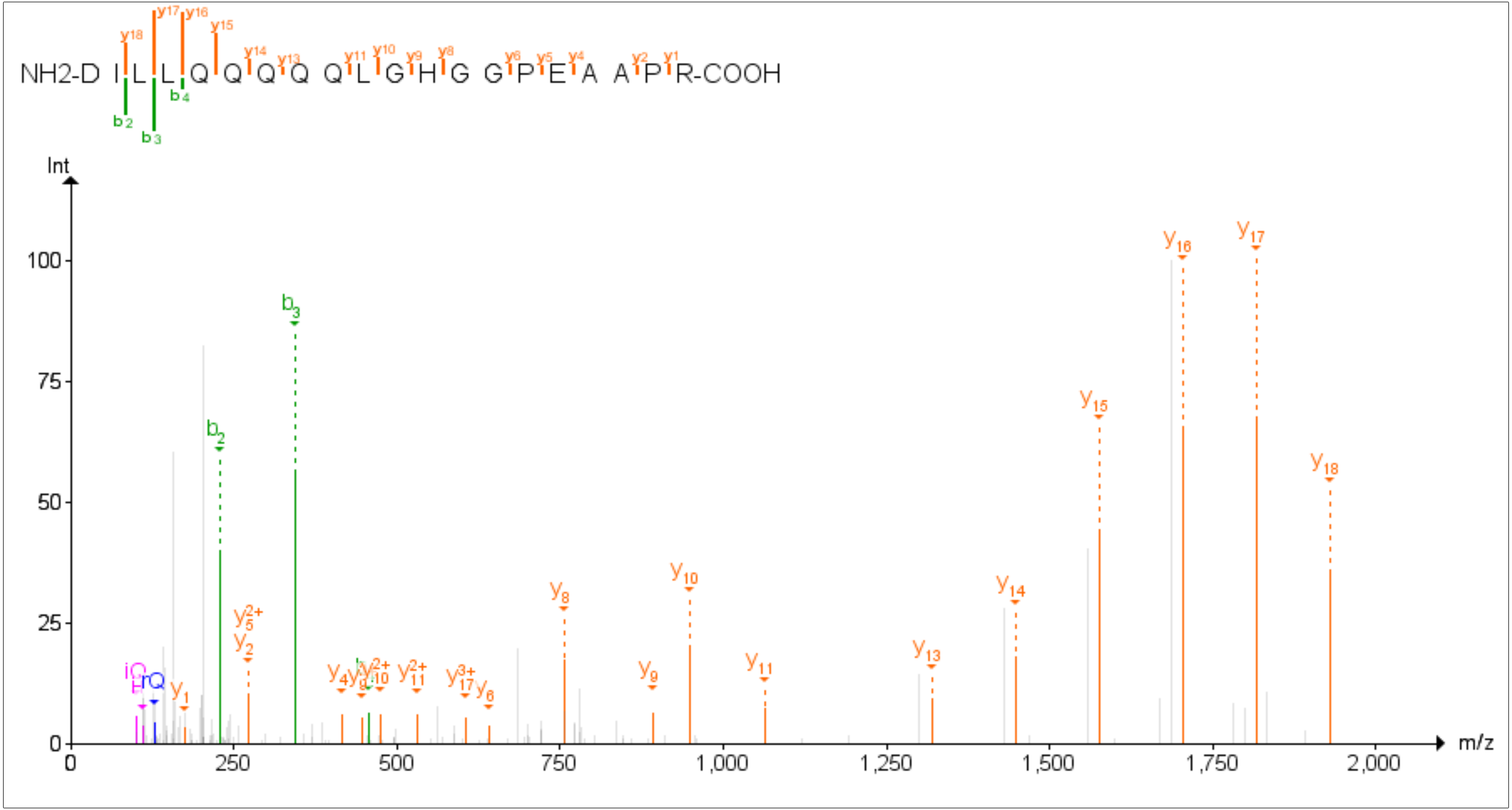

NH2-D I L L Q Q Q Q Q L G H G G P E A A P R-COOH
Int
m/z
0
25
50
75
100
250
500
750
1,000
1,250
1,500
1,750
2,000

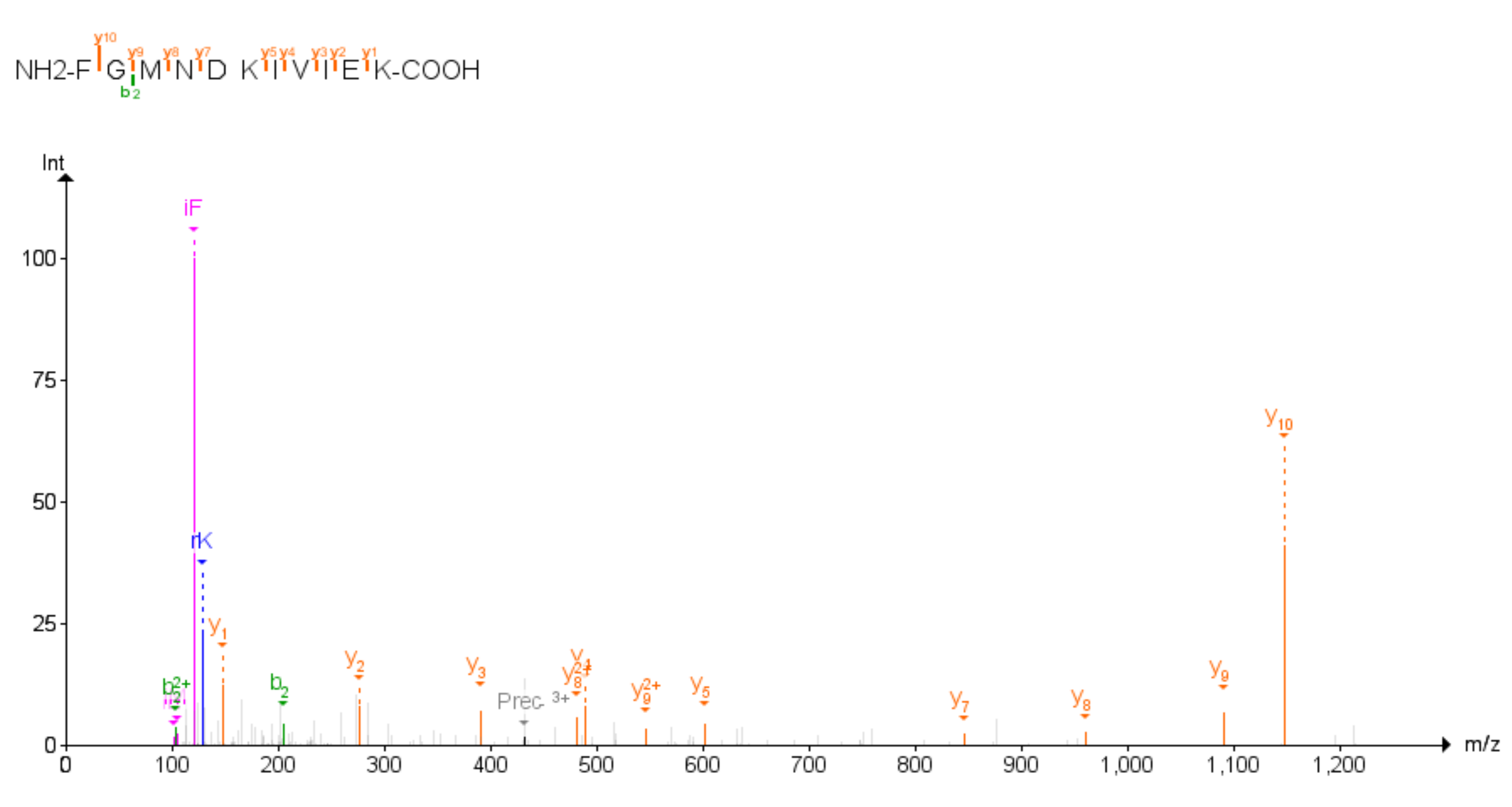

NH2-F G M N D K I V I E K-COOH
y10 y9 y8 y7 y5 y4 y3 y2 y1
b2
Int
iF
rK
y10
y1
y2
y3
Prec- 3+
y9
y5
y7
y8
b2
m/z

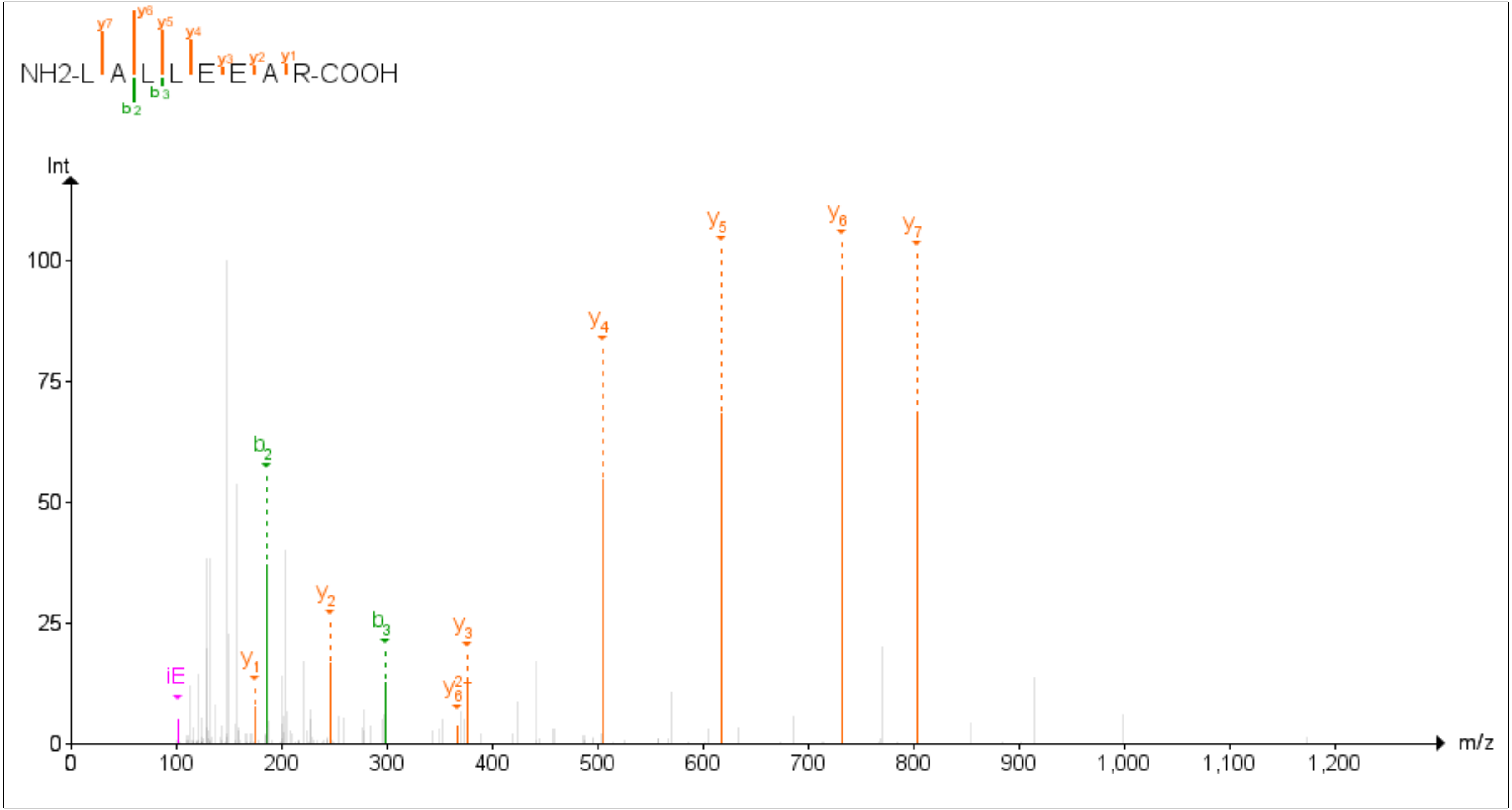

NH2-L A L L E E A R-COOH
y7 y6 y5 y4 y3 y2 y1
b2 b3
Int
m/z
100
75
50
25
0
0 100 200 300 400 500 600 700 800 900 1,000 1,100 1,200
iE
y1
b2
y2
b3
y3
y6 2+
y4
y5
y6
y7

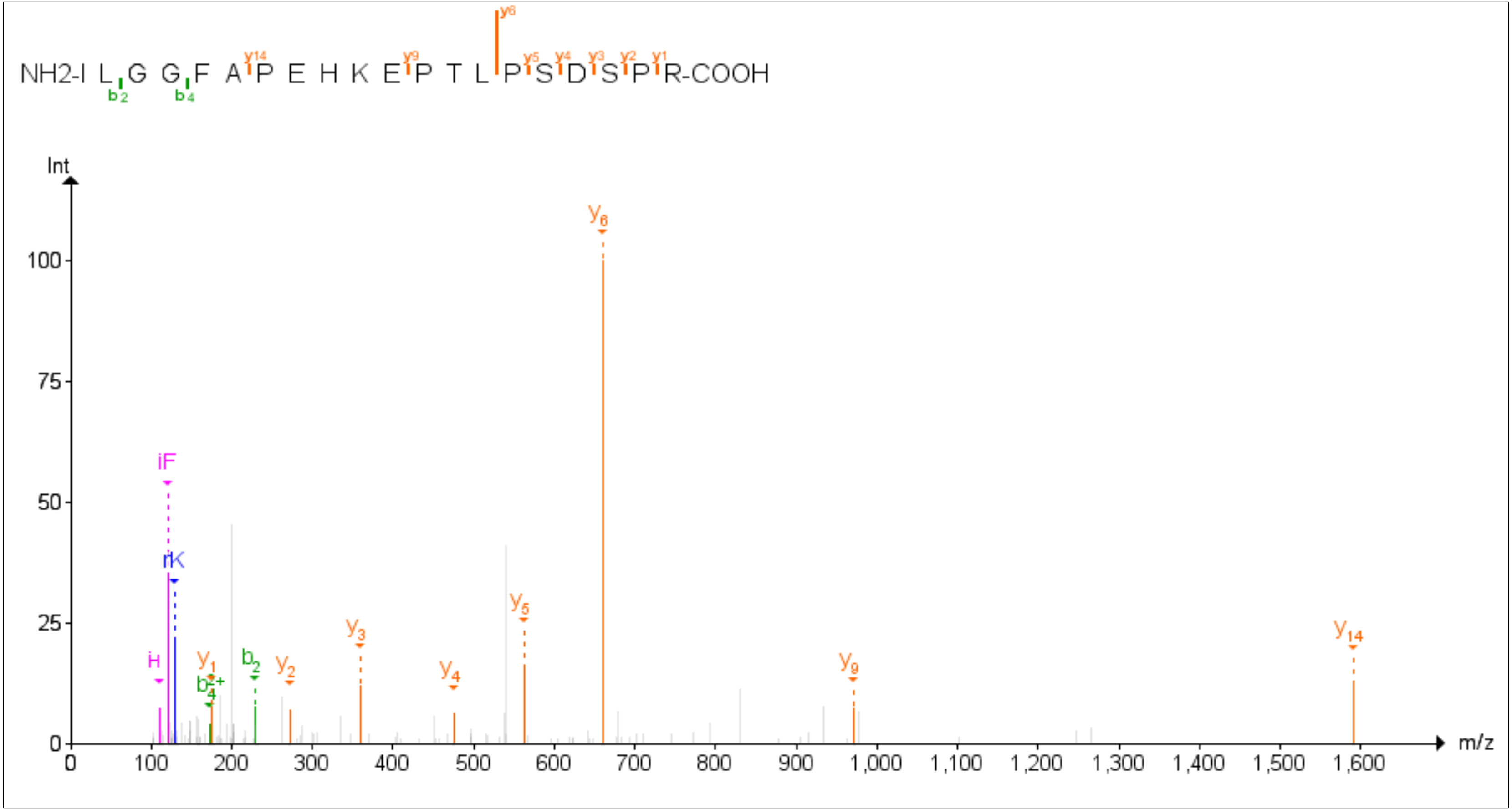
NH2-I L G G F A P E H K E P T L P S D S P R-COOH
b2
b4
y14
y9
y6
y5
y4
y3
y2
y1
Int
m/z
iF
iH
rK
b2
b4 2+
y1
y2
y3
y4
y5
y6
y9
y14
0
25
50
75
100
0
100
200
300
400
500
600
700
800
900
1,000
1,100
1,200
1,300
1,400
1,500
1,600

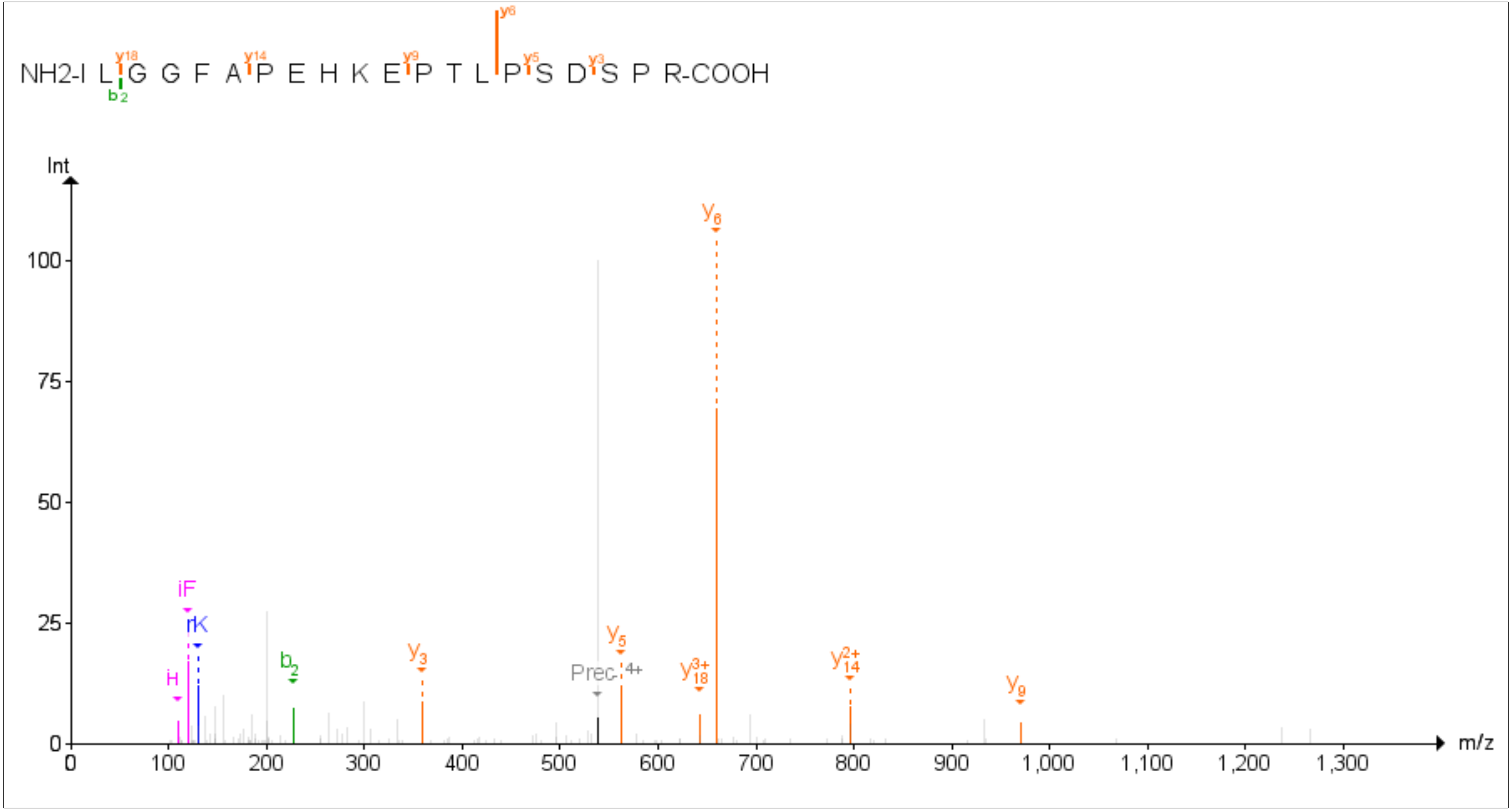

NH2-I L G G F A P E H K E P T L P S D S P R-COOH
y18
y14
y9
y6
y5
y3
b2
Int
100
75
50
25
0
y6
iF
rK
iH
b2
y3
Prec 4+
y5
y18 3+
y14 2+
y9
0
100
200
300
400
500
600
700
800
900
1,000
1,100
1,200
1,300
m/z

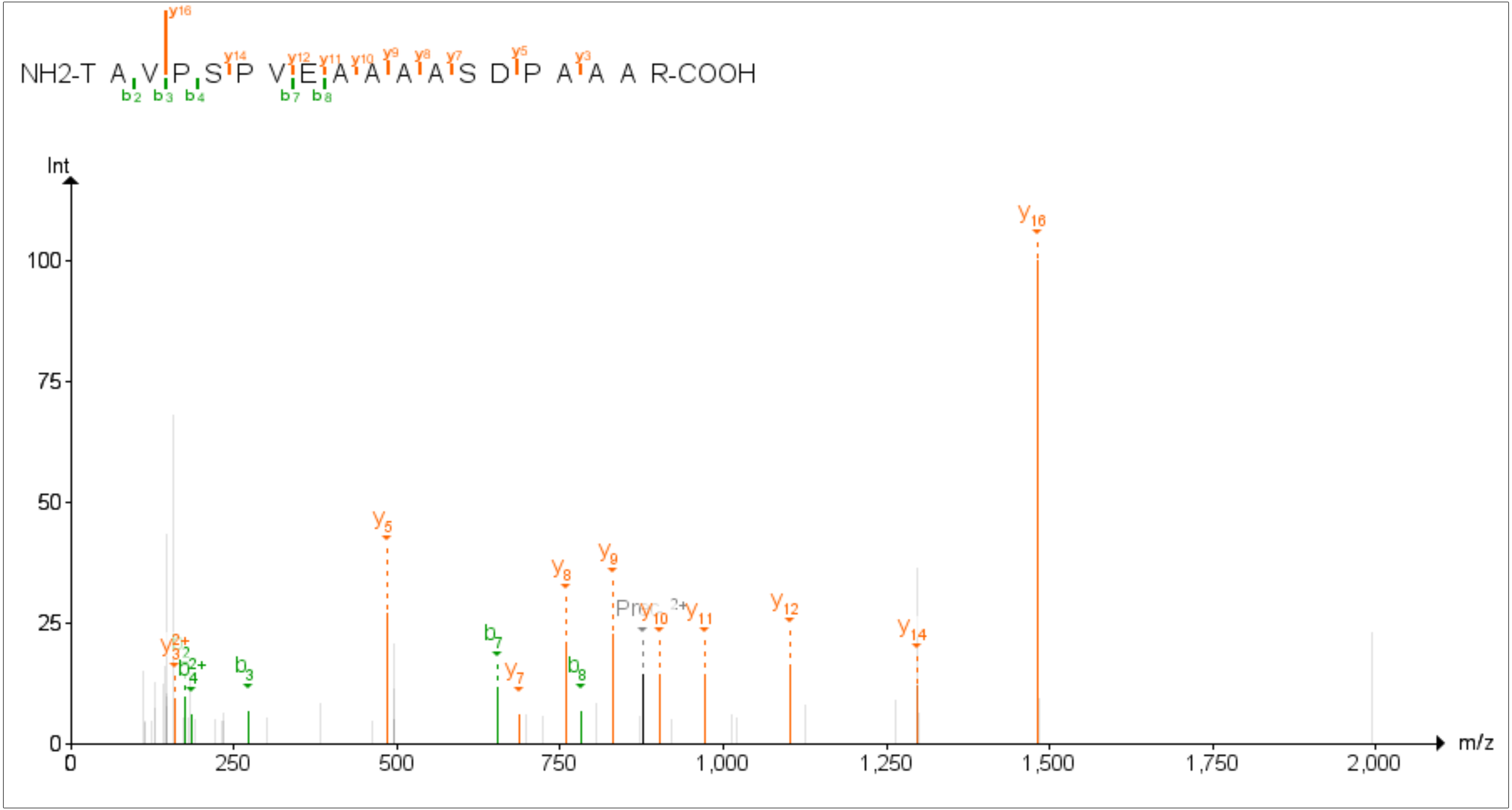

NH2-T A V P S P V E A A A A S D P A A A R-COOH
y16
y14
y12
y11
y10
y9
y8
y7
y5
y3
b2
b3
b4
b7
b8
Int
100
75
50
25
0
y16
y5
y8
y9
y10
y11
y12
y14
y7
b7
b8
b3
y_3^{2+}
b_4^{2+}
0
250
500
750
1,000
1,250
1,500
1,750
2,000
m/z

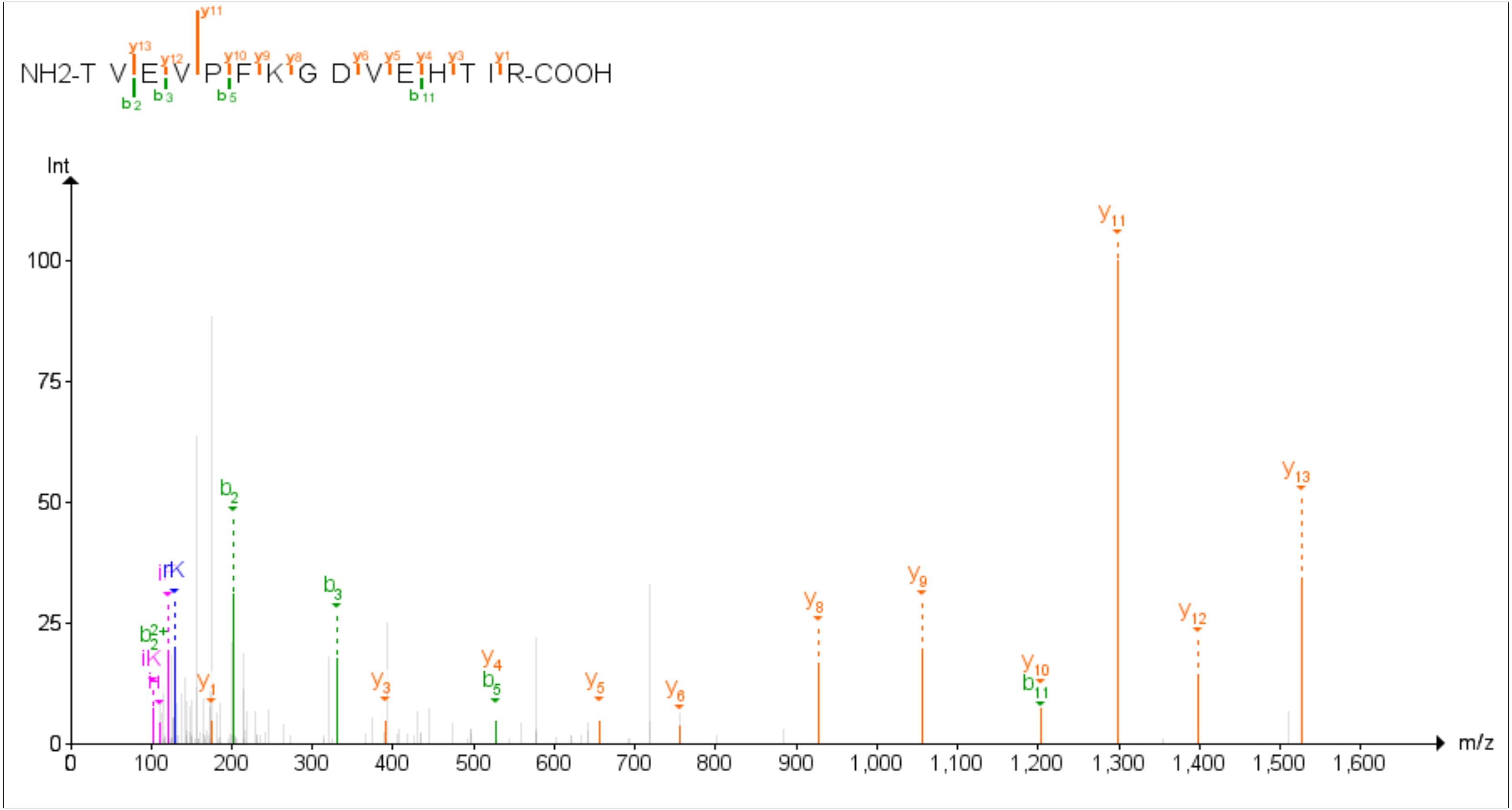

NH2-T V E V P F K G D V E H T I R-COOH
y13 y12 y11 y10 y9 y8 y6 y5 y4 y3 y1
b2 b3 b5 b11
Int
100
75
50
25
0
m/z
0 100 200 300 400 500 600 700 800 900 1,000 1,100 1,200 1,300 1,400 1,500 1,600

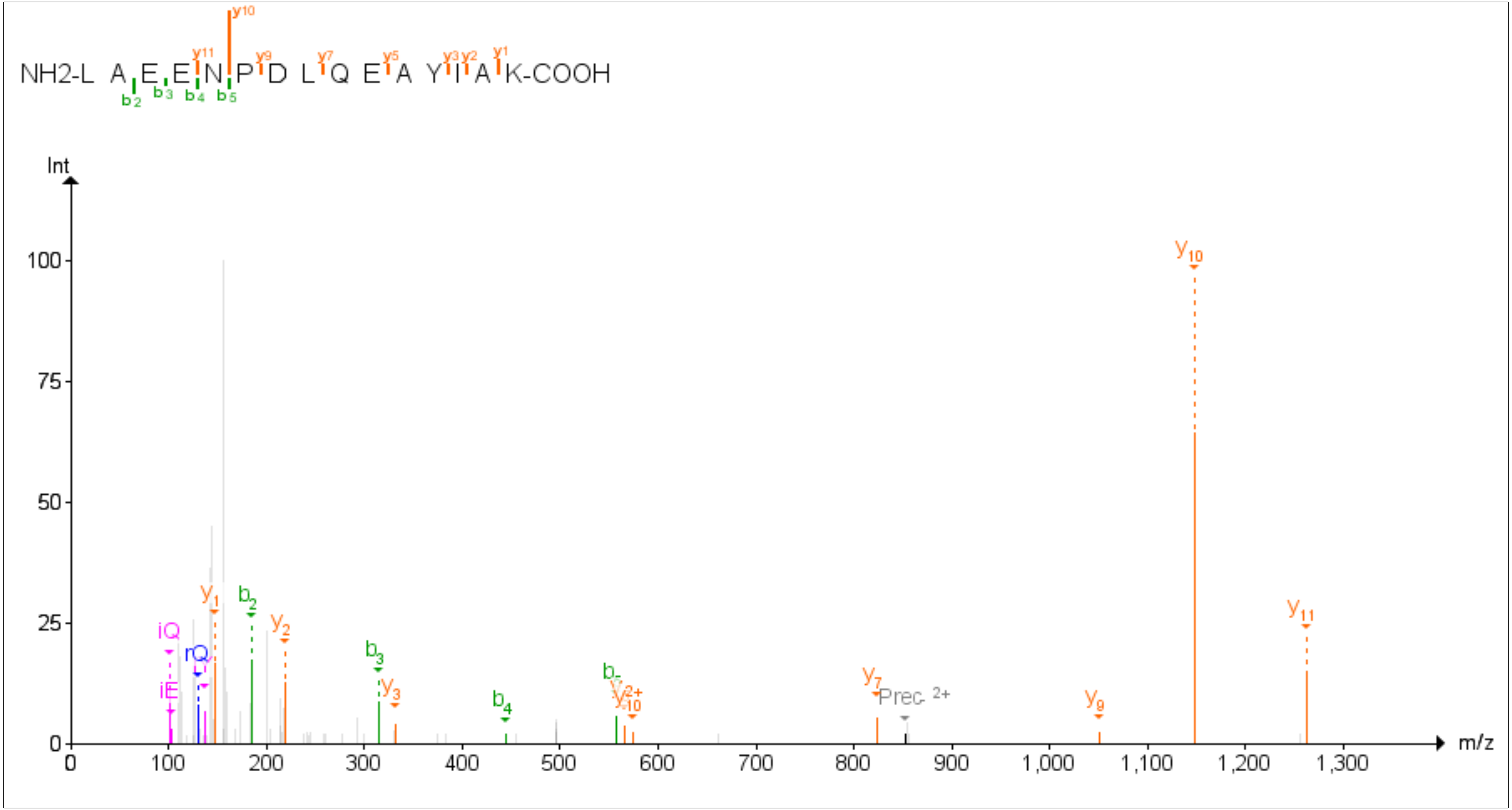

NH2-L A E E N P D L Q E A Y I A K-COOH
Int
m/z
Prec 2+

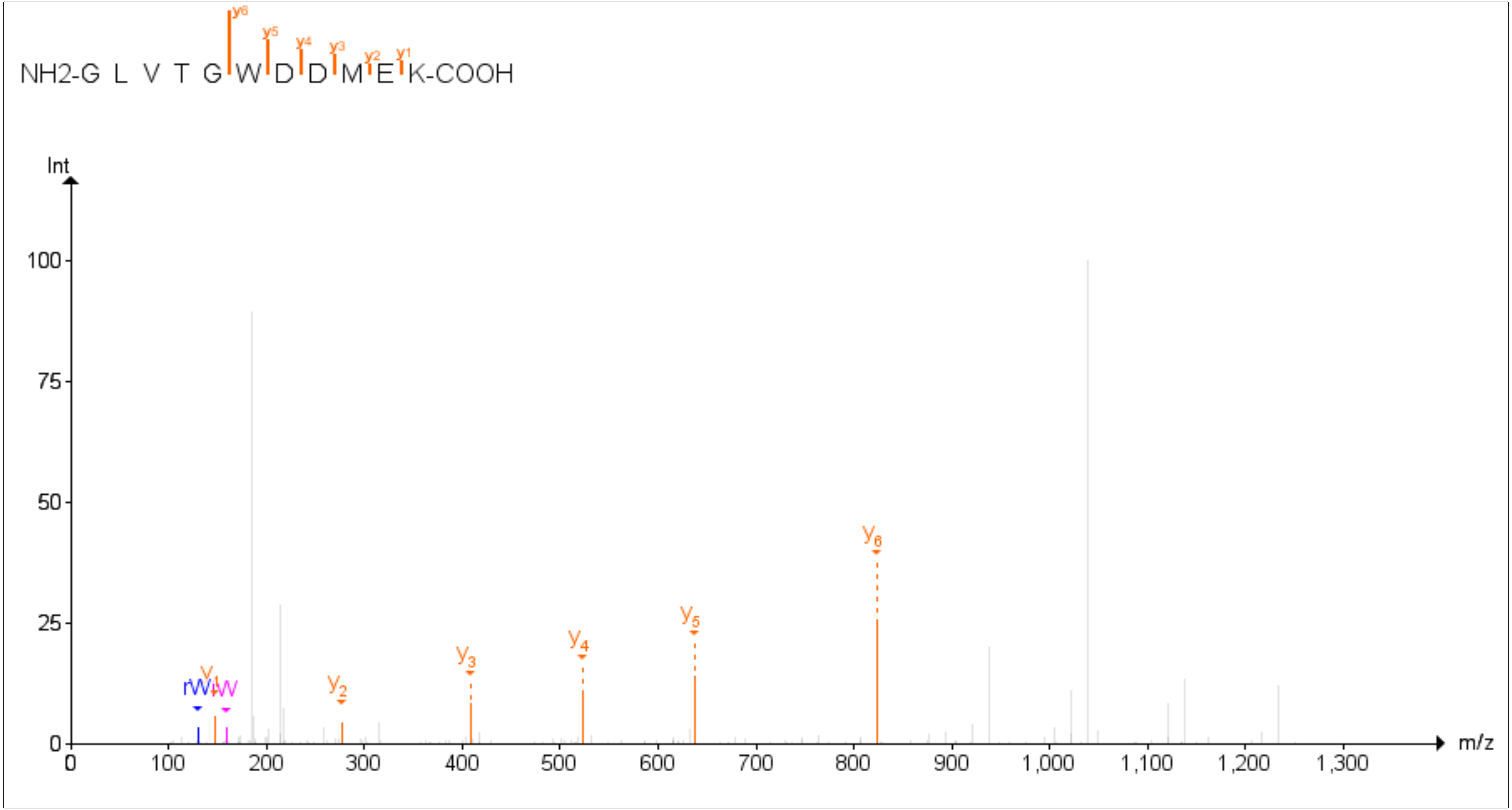

NH2-G L V T G W D D M E K-COOH
y6
y5
y4
y3
y2
y1
Int
100
75
50
25
0
0
100
200
300
400
500
600
700
800
900
1,000
1,100
1,200
1,300
m/z
rW
y1
iW
y2
y3
y4
y5
y6

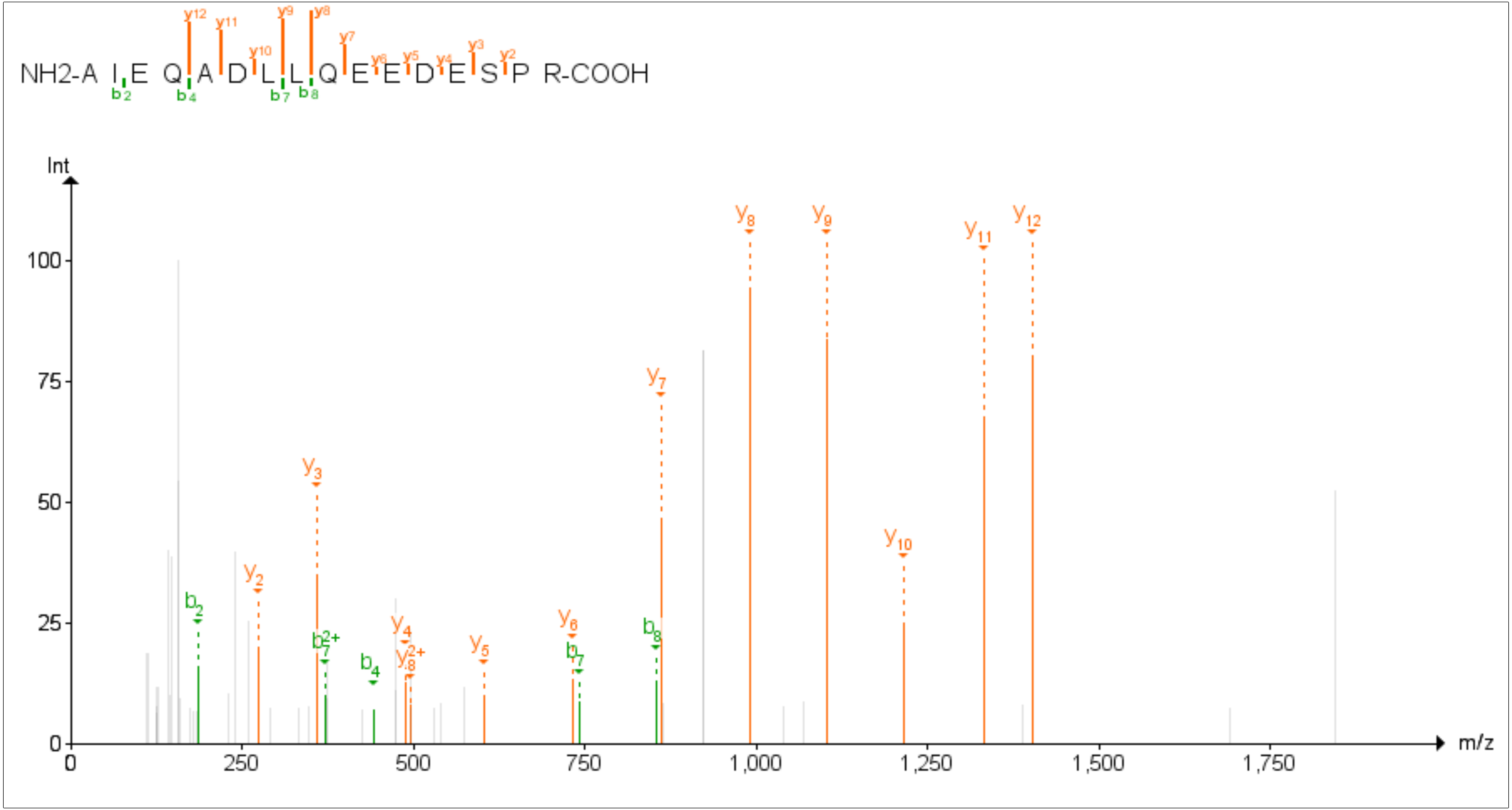
NH2-A I E Q A D L L Q E E D E S P R-COOH
y12 y11 y10 y9 y8 y7 y6 y5 y4 y3 y2
b2 b4 b7 b8
Int
100
75
50
25
0
0 250 500 750 1,000 1,250 1,500 1,750
m/z
y8 y9 y11 y12 y7 y3 y10 y2 b2 b7 2+ y4 y8 2+ b4 y5 y6 b7 b8

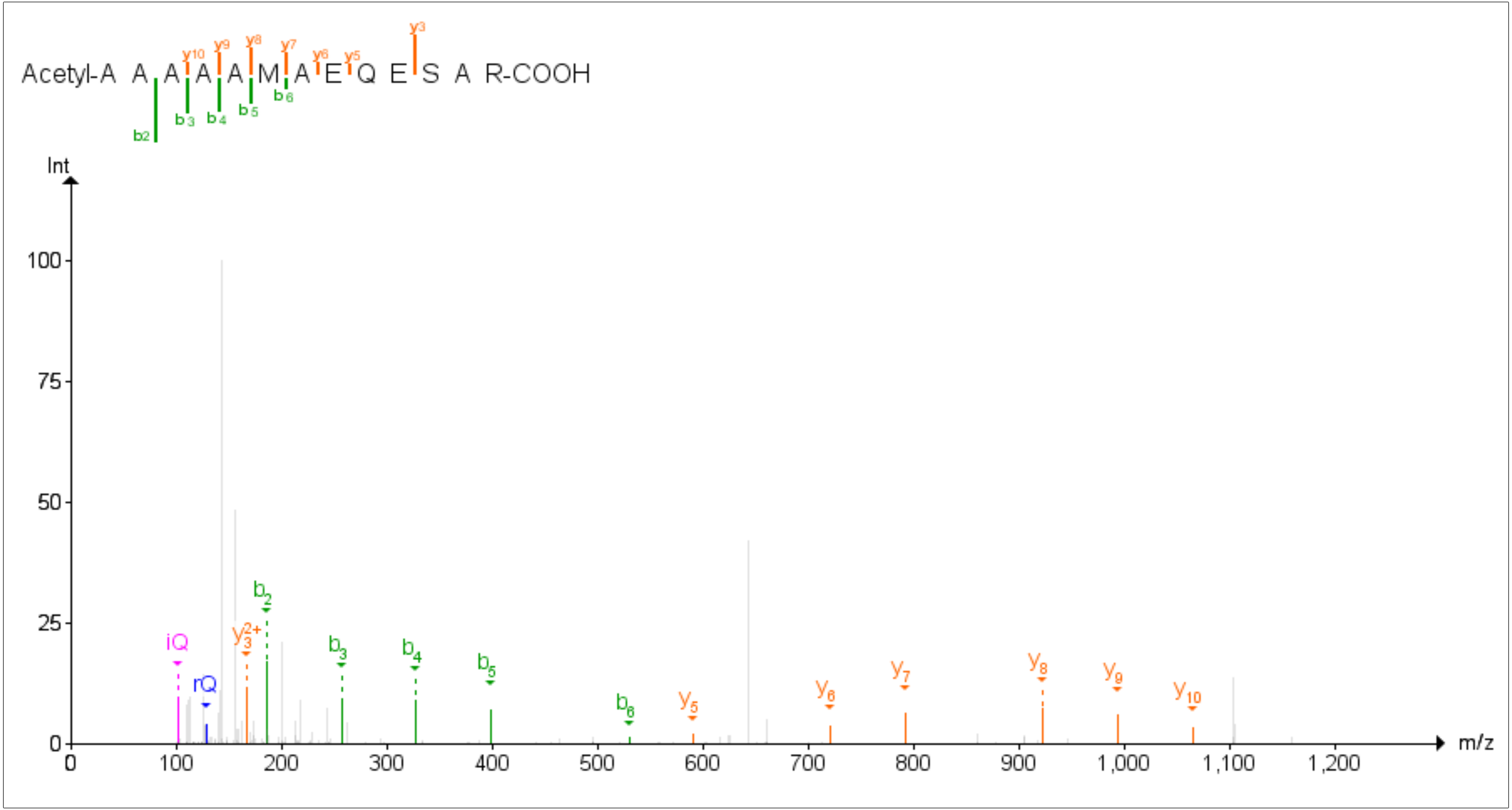

Acetyl-A A A A A M A E Q E S A R-COOH
b2 b3 b4 b5 b6
y10 y9 y8 y7 y6 y5 y3
Int
m/z
iQ
rQ

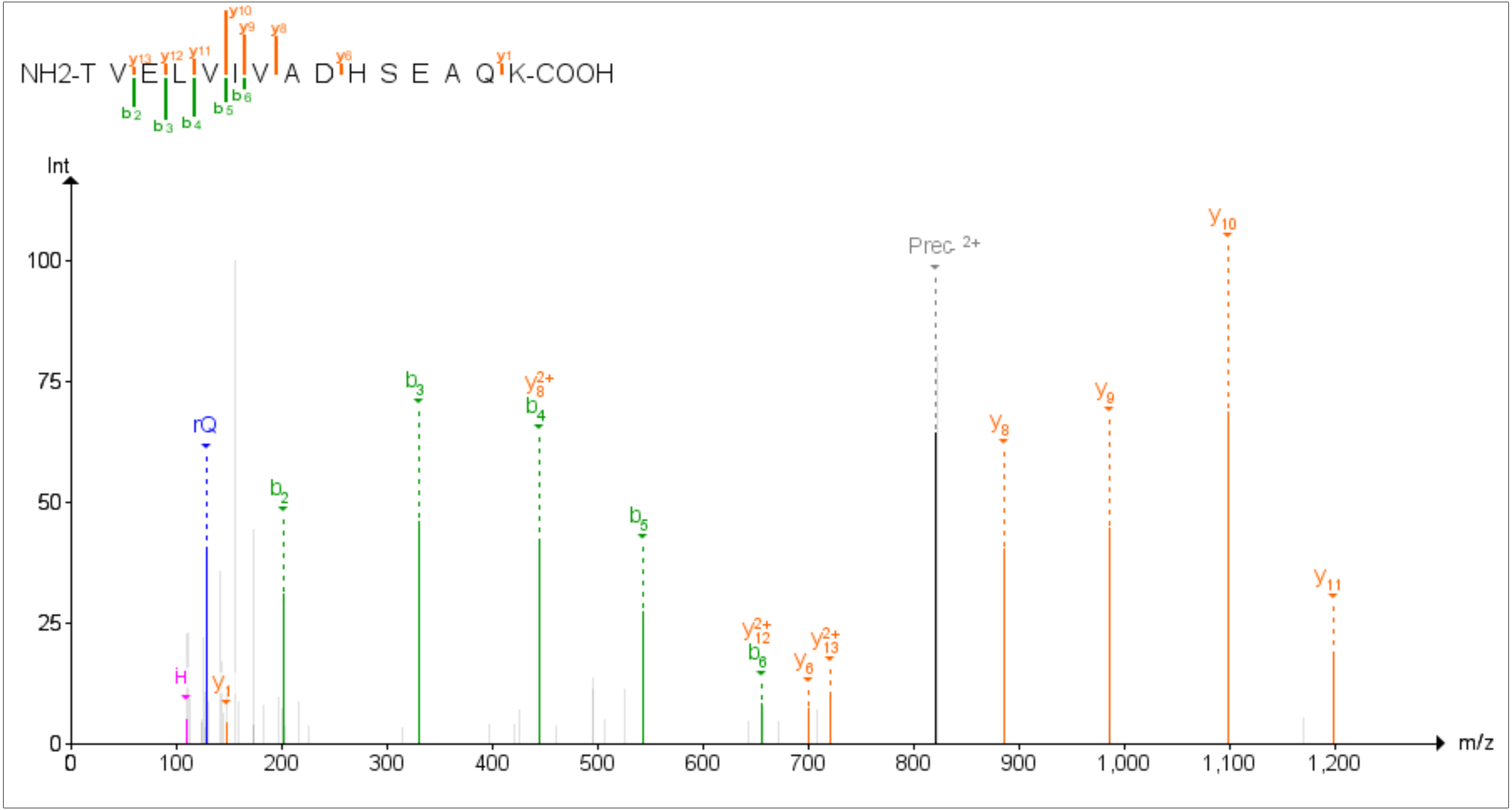

NH2-T V E L V I V A D H S E A Q K-COOH
y13 y12 y11 y10 y9 y8 y6 y1
b2 b3 b4 b5 b6
Int
m/z
Prec 2+
rQ
H
y1
b2
b3
y8 2+
b4
b5
y12 2+
b6
y6
y13 2+
y8
y9
y10
y11
0
25
50
75
100
0
100
200
300
400
500
600
700
800
900
1,000
1,100
1,200

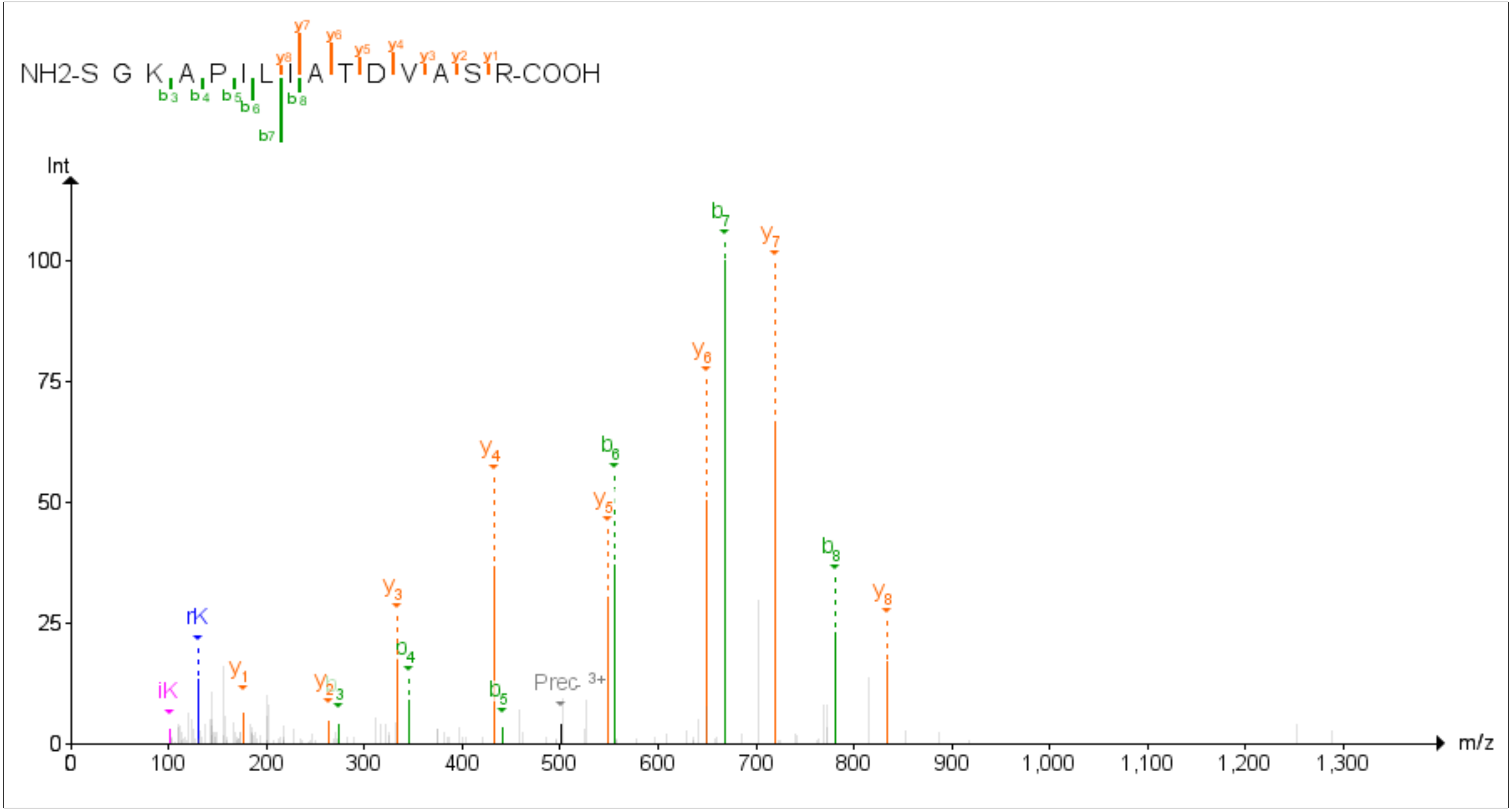

NH2-S G K A P I L I A T D V A S R-COOH
Int
m/z
Prec 3+
iK
rK

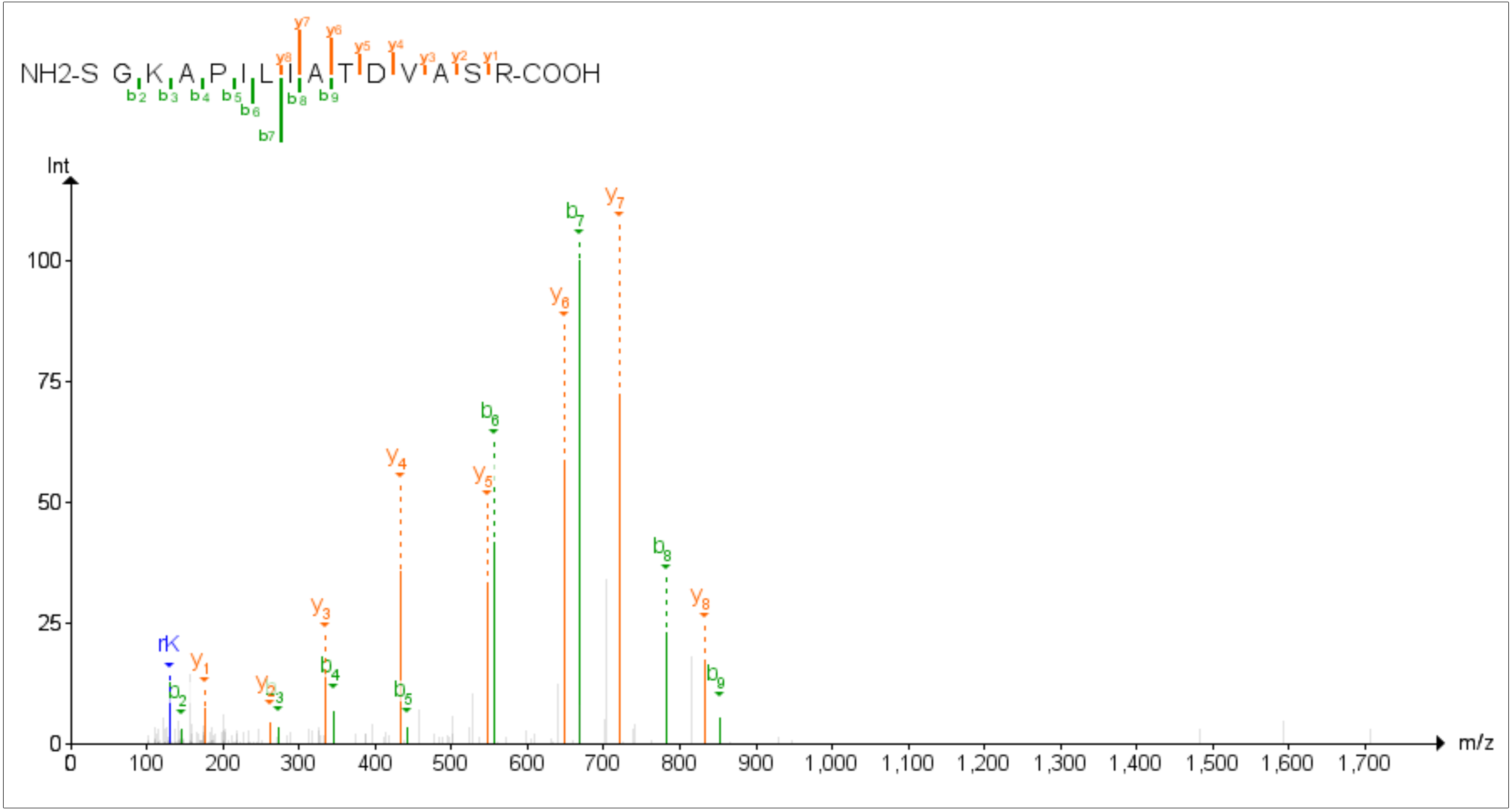

NH2-S G K A P I L I A T D V A S R-COOH
b2 b3 b4 b5 b6 b7 b8 b9
y8 y7 y6 y5 y4 y3 y2 y1
Int
m/z
0
25
50
75
100
0
100
200
300
400
500
600
700
800
900
1,000
1,100
1,200
1,300
1,400
1,500
1,600
1,700
rK

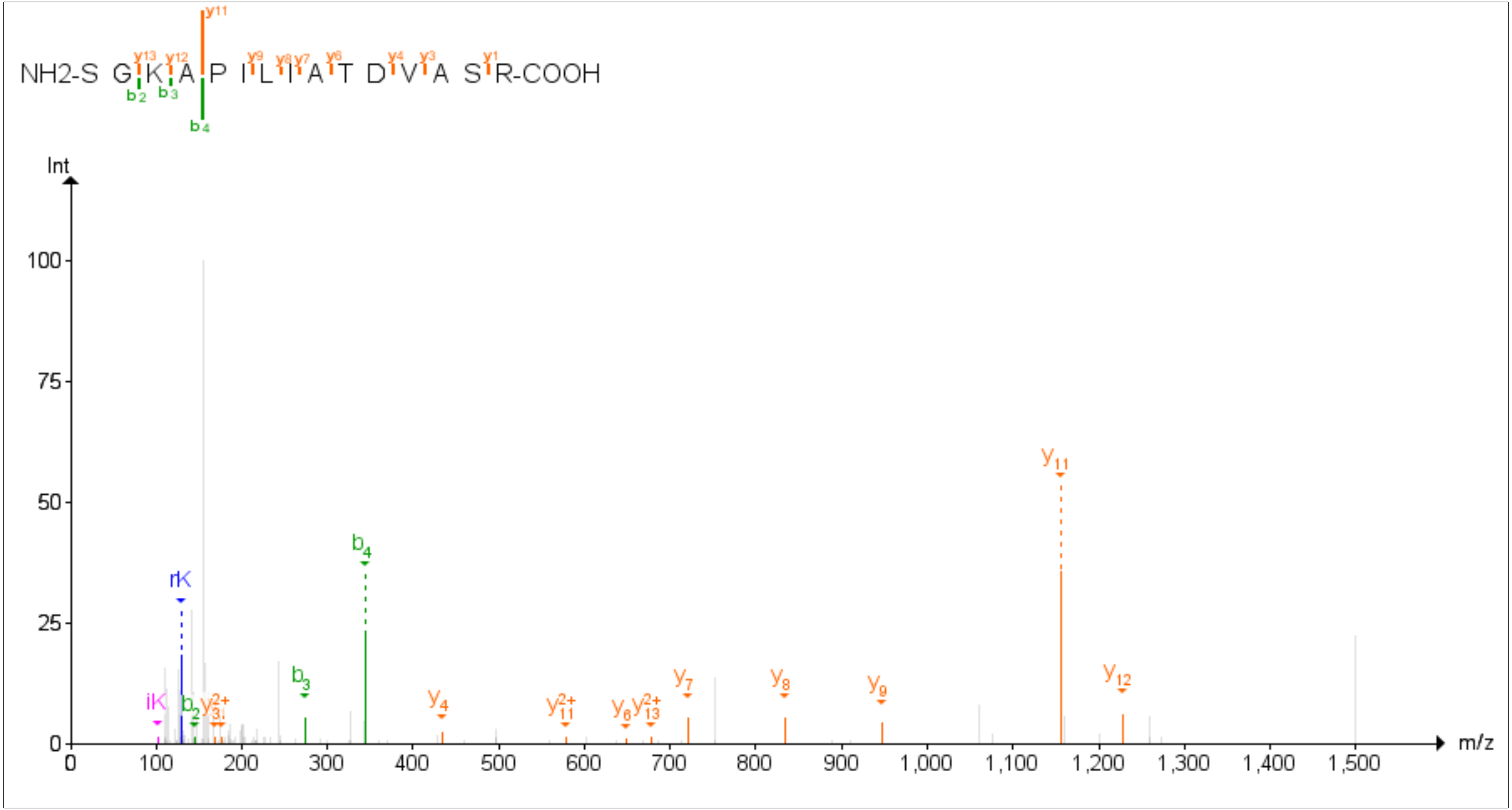

NH2-S G K A P I L I A T D V A S R-COOH
y11
y13
y12
y9
y8
y7
y6
y4
y3
y1
b2
b3
b4
Int
100
75
50
25
0
m/z
0
100
200
300
400
500
600
700
800
900
1,000
1,100
1,200
1,300
1,400
1,500
y11
b4
rK
iK
b2
b3
y4
y7
y8
y9
y12
y6

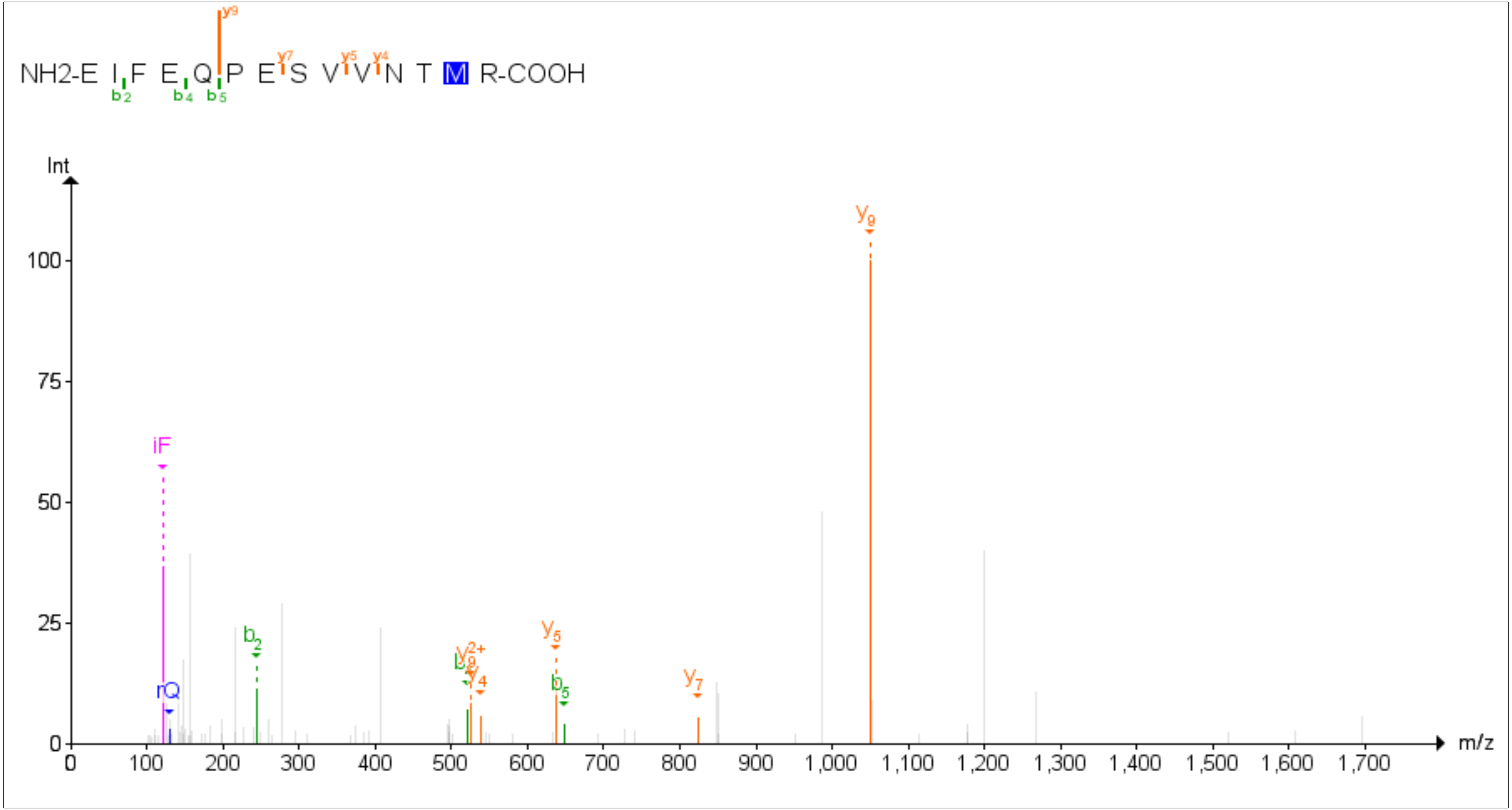

NH2-E I F E Q P E S V V N T M R-COOH
y9
y7
y5
y4
b2
b4
b5
Int
m/z
iF
rQ
b2
y9 2+
b4
y4
y5
b5
y7
y9
0
25
50
75
100
0
100
200
300
400
500
600
700
800
900
1,000
1,100
1,200
1,300
1,400
1,500
1,600
1,700

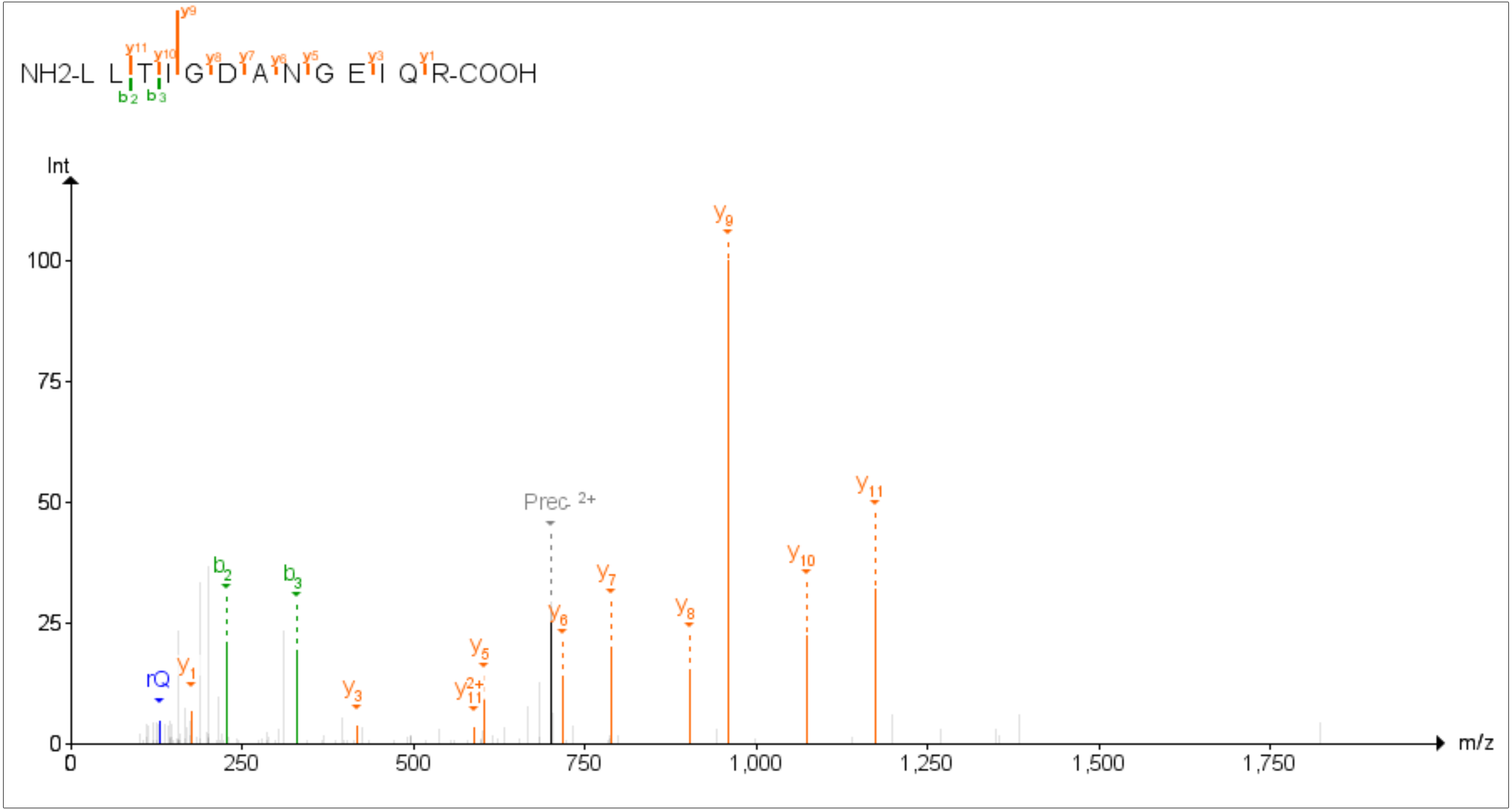

NH2-L L T I G D A N G E I Q R-COOH
y11 y10 y9 y8 y7 y6 y5 y3 y1
b2 b3
Int
100
75
50
25
0
0 250 500 750 1,000 1,250 1,500 1,750
m/z
y9
y11
y10
Prec 2+
b2
b3
y7
y6
y8
y5
rQ
y1
y3
y11 2+

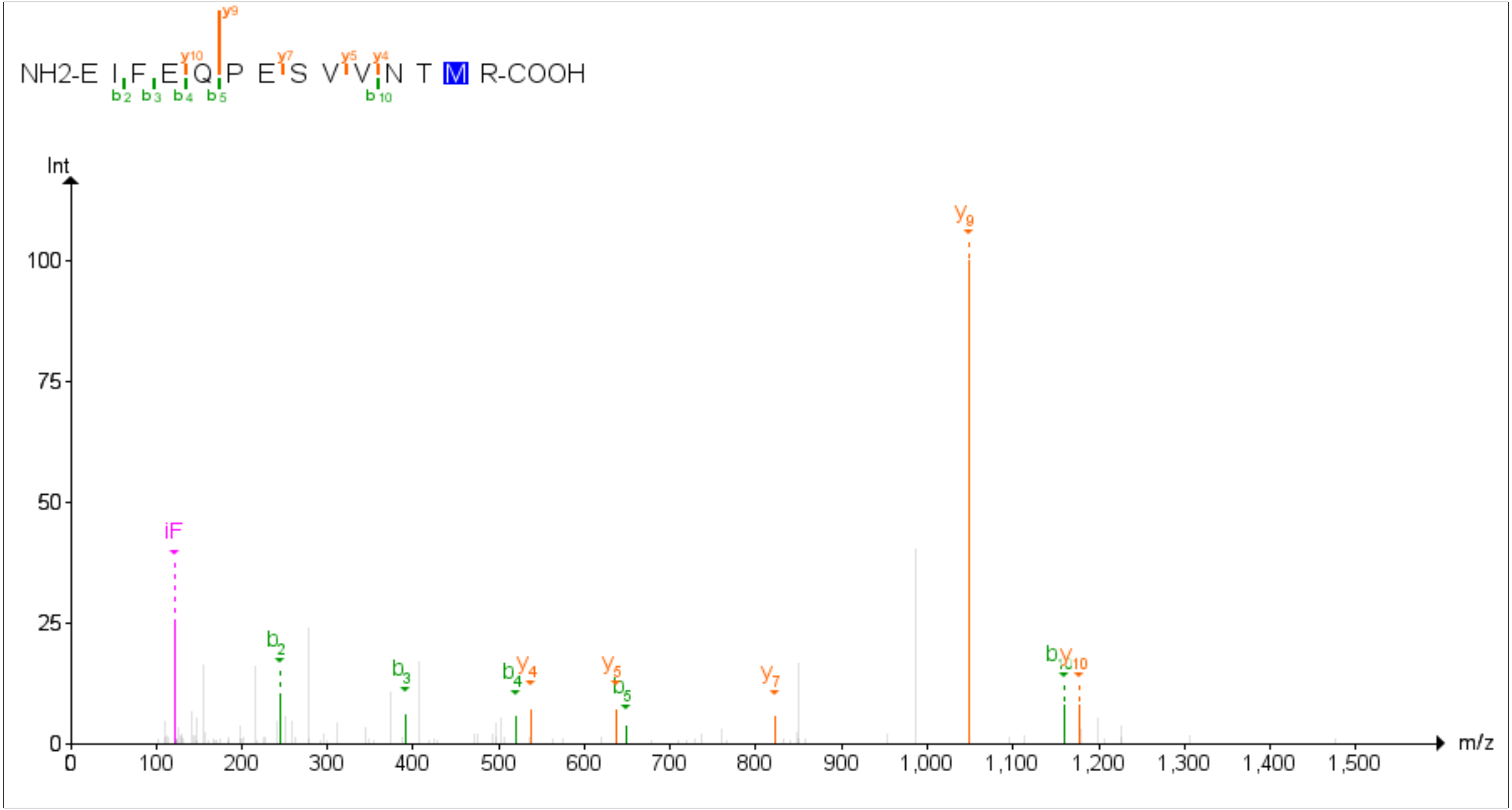

NH2-E I F E Q P E S V V N T M R-COOH
Int
m/z
100
75
50
25
0
y9
iF
b2
b3
b4
y4
y5
b5
y7
b10
y10

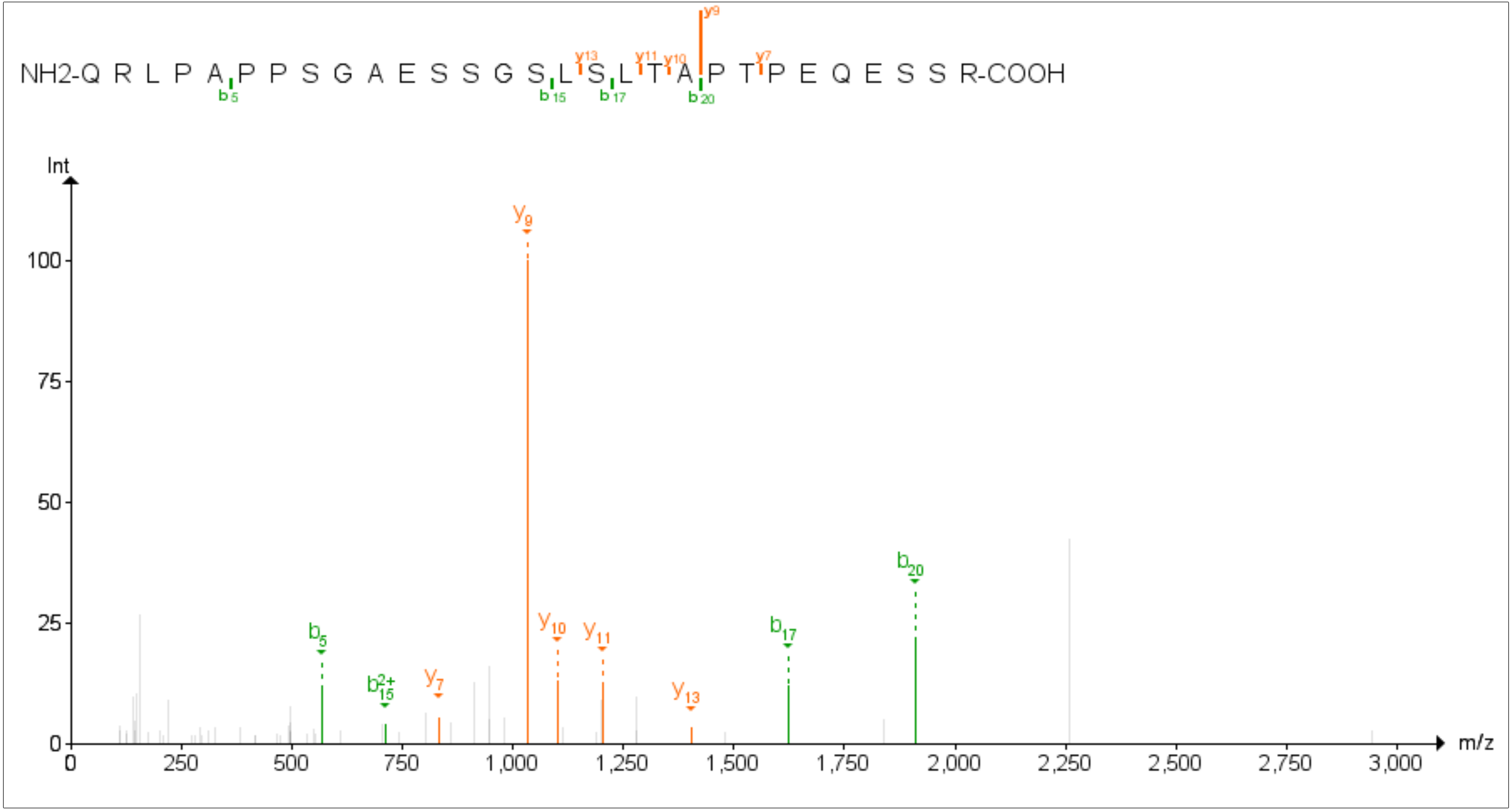

NH2-Q R L P A P P S G A E S S G S L S L T A P T P E Q E S S R-COOH
b5
b15
b17
b20
y13
y11
y10
y9
y7
Int
100
75
50
25
0
m/z
0
250
500
750
1,000
1,250
1,500
1,750
2,000
2,250
2,500
2,750
3,000
y9
b5
b_{15}^{2+}
y7
y10
y11
y13
b17
b20

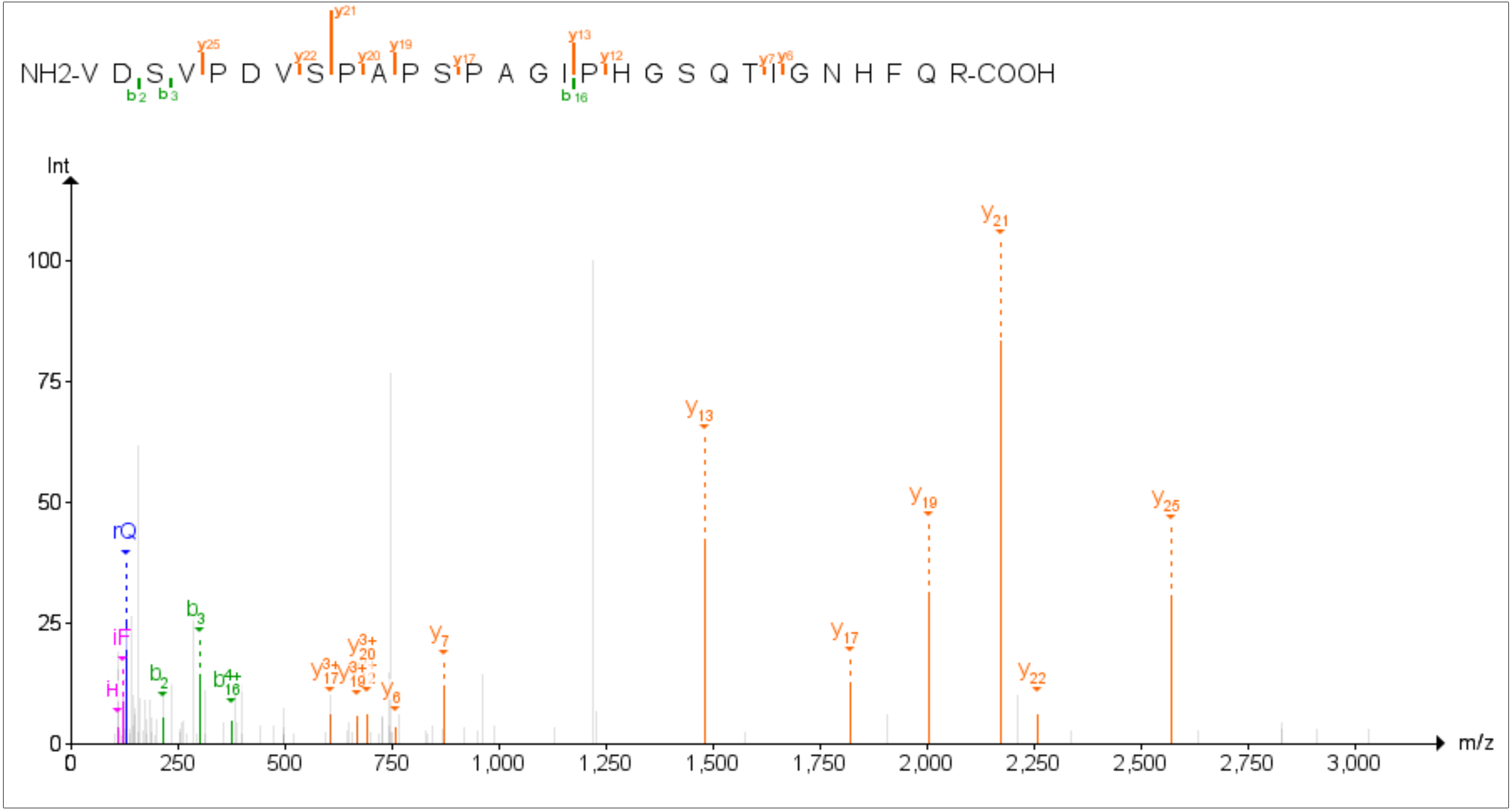

NH2-V D S V P D V S P A P S P A G I P H G S Q T I G N H F Q R-COOH
Int
m/z
0
25
50
75
100
250
500
750
1,000
1,250
1,500
1,750
2,000
2,250
2,500
2,750
3,000
rQ
iF
iH
b2
b3
b16
y21
y13
y19
y25
y17
y22
y7
y6

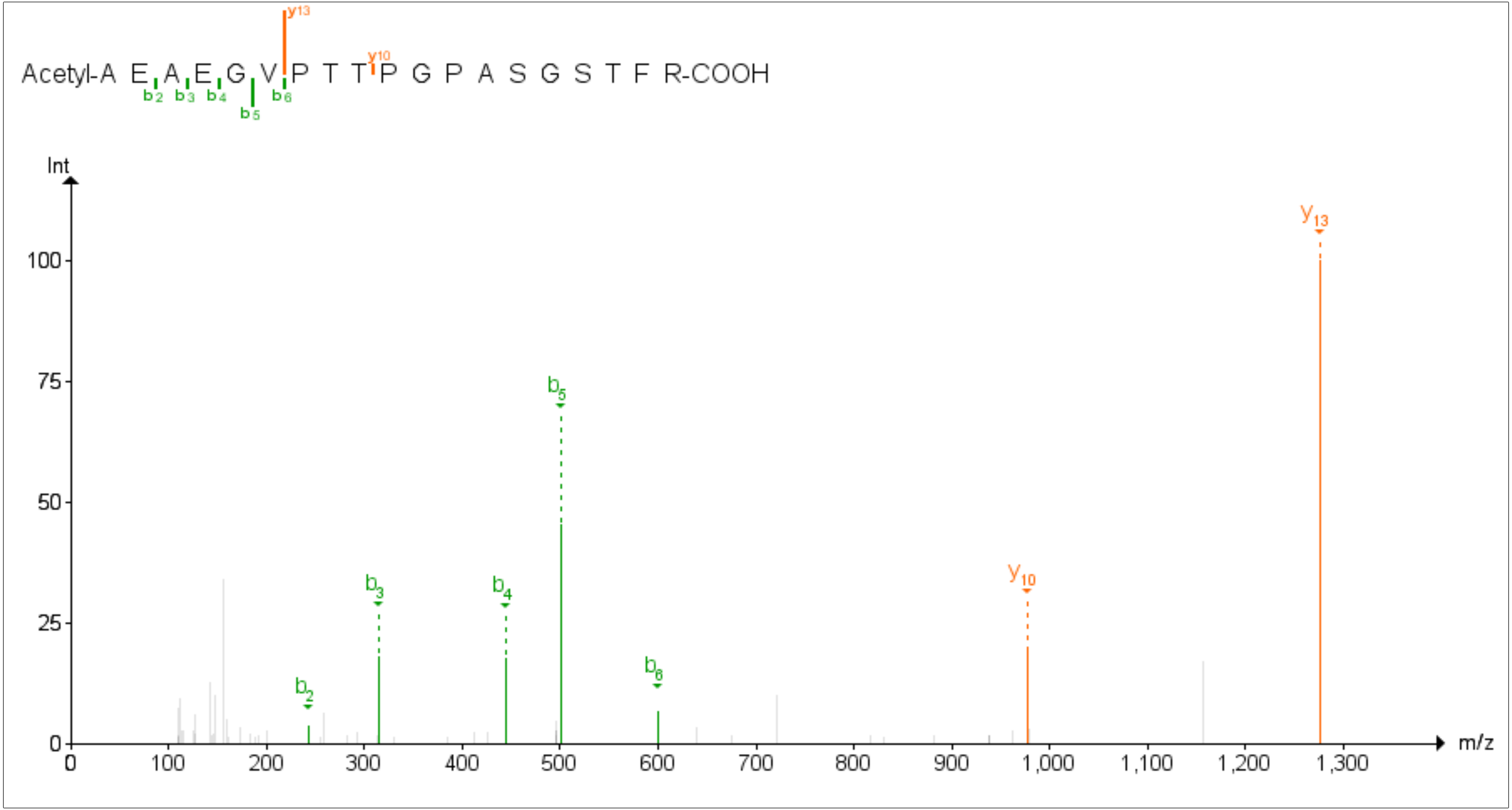

Acetyl-A E A E G V P T T P G P A S G S T F R-COOH
y13
y10
b2
b3
b4
b5
b6
Int
100
75
50
25
0
m/z
0
100
200
300
400
500
600
700
800
900
1,000
1,100
1,200
1,300

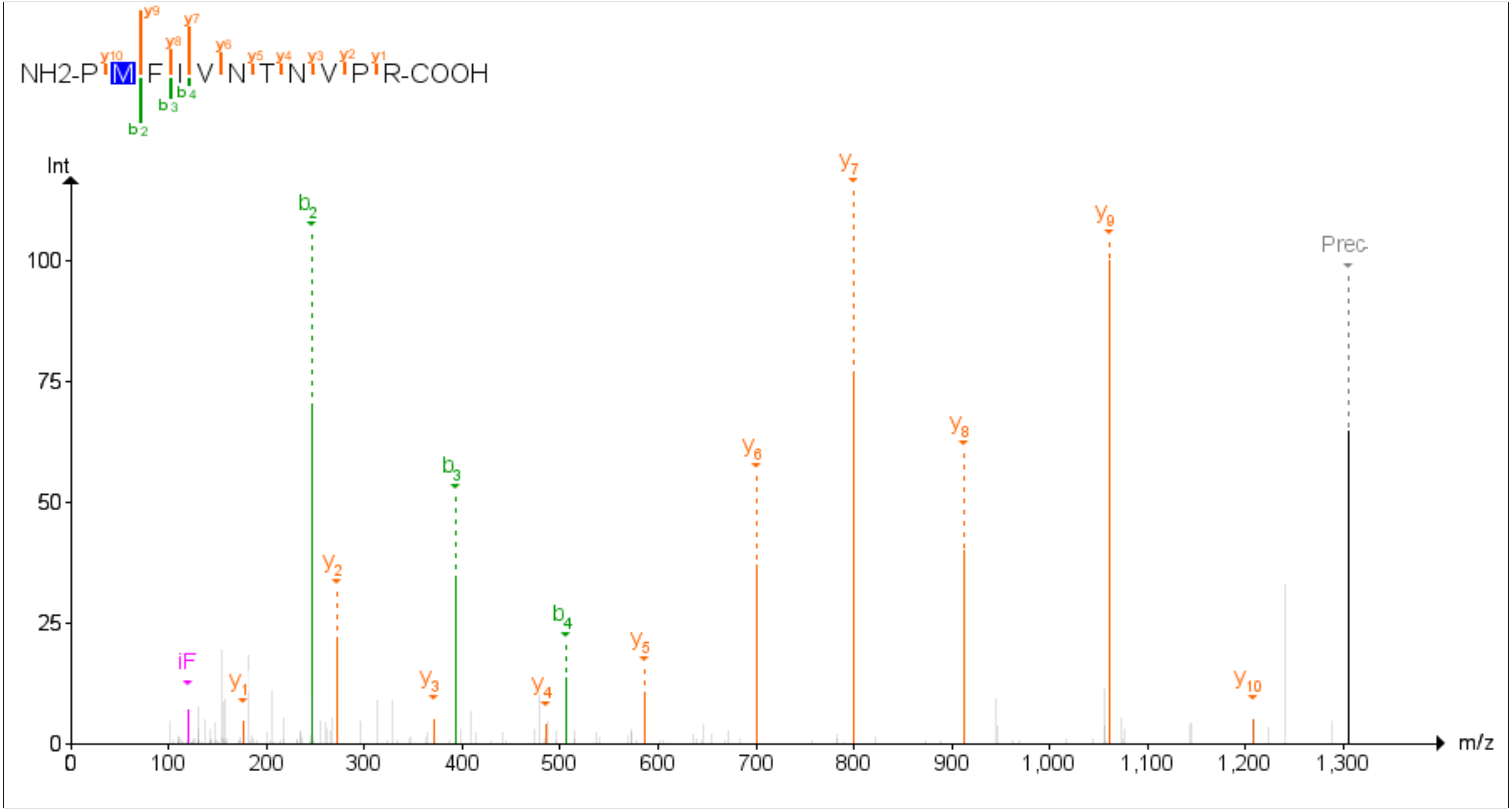

NH2-P M F I V N T N V P R-COOH
y10 y9 y8 y7 y6 y5 y4 y3 y2 y1
b2 b3 b4
Int
m/z
Prec
iF

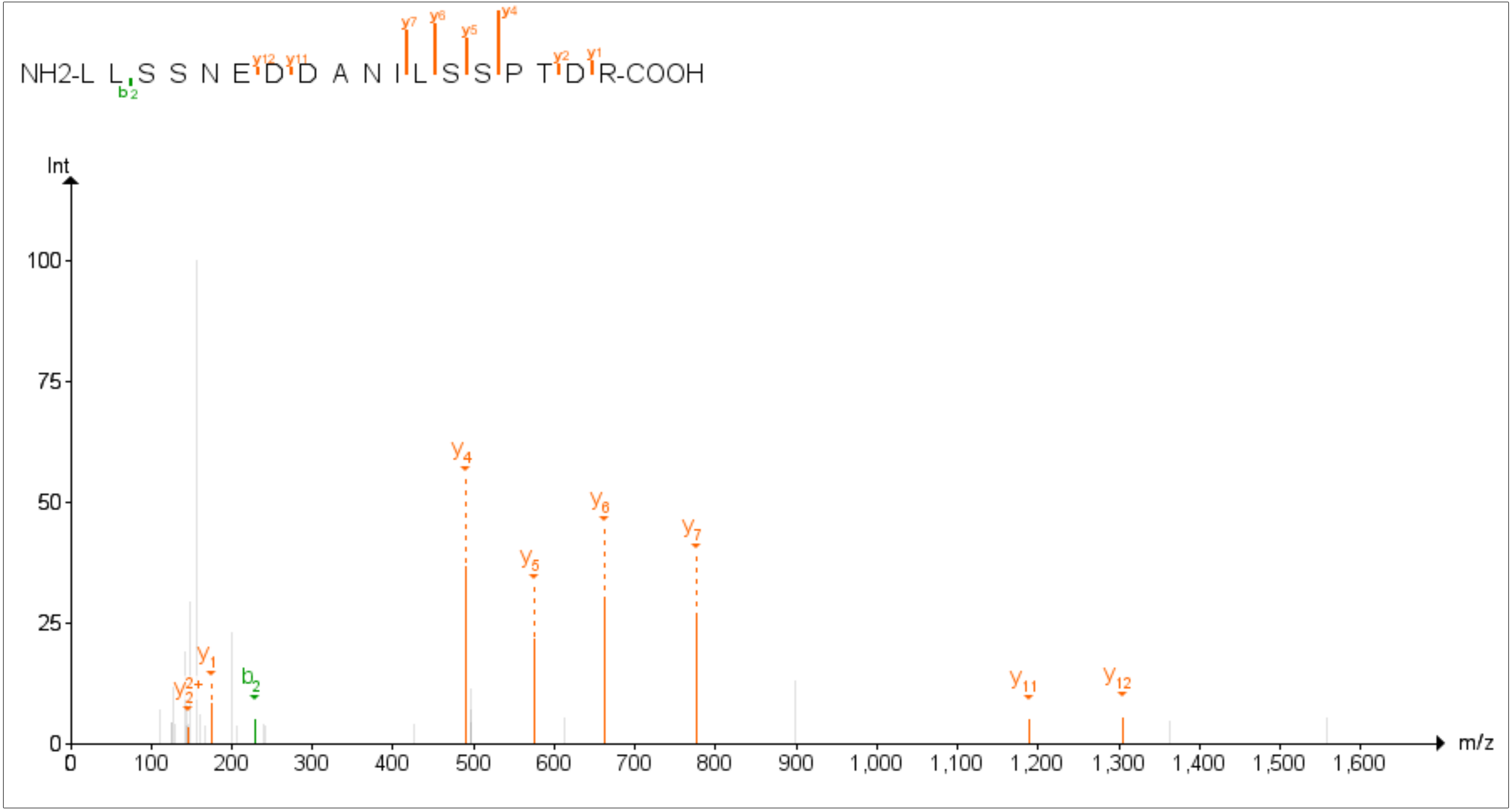
NH2-L L S S N E D D A N I L S S P T D R-COOH
b2
y12 y11 y7 y6 y5 y4 y2 y1
Int
100
75
50
25
0
y4
y6
y7
y5
y1
y_2^{2+}
b2
y11
y12
0 100 200 300 400 500 600 700 800 900 1,000 1,100 1,200 1,300 1,400 1,500 1,600
m/z

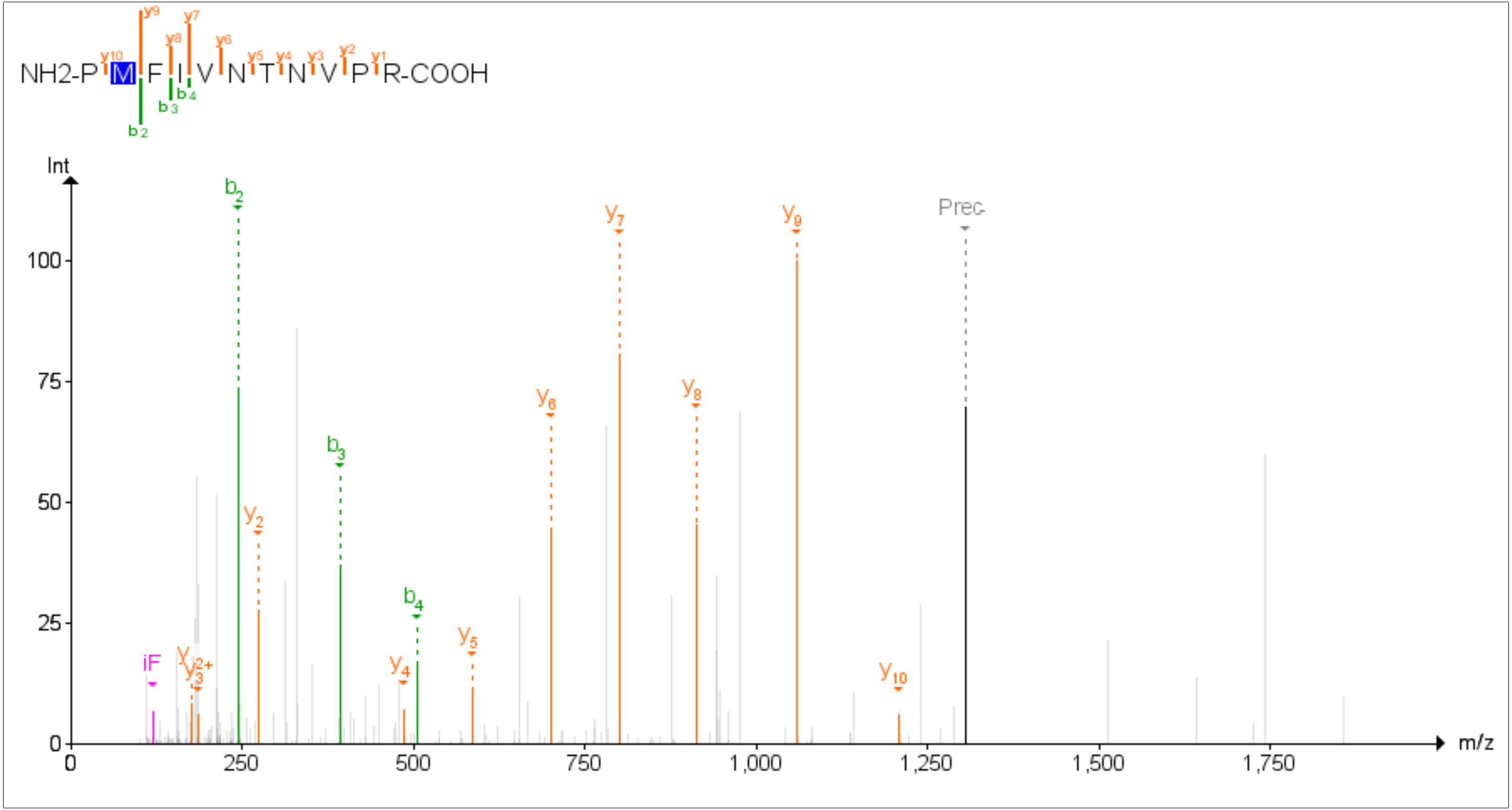

NH2-P M F I V N T N V P R-COOH
y10 y9 y8 y7 y6 y5 y4 y3 y2 y1
b2 b3 b4
Int
m/z
100
75
50
25
0
250
500
750
1,000
1,250
1,500
1,750
b2
b3
b4
y7
y9
y6
y8
y2
y5
y4
y10
Prec
iF
y2+
y3

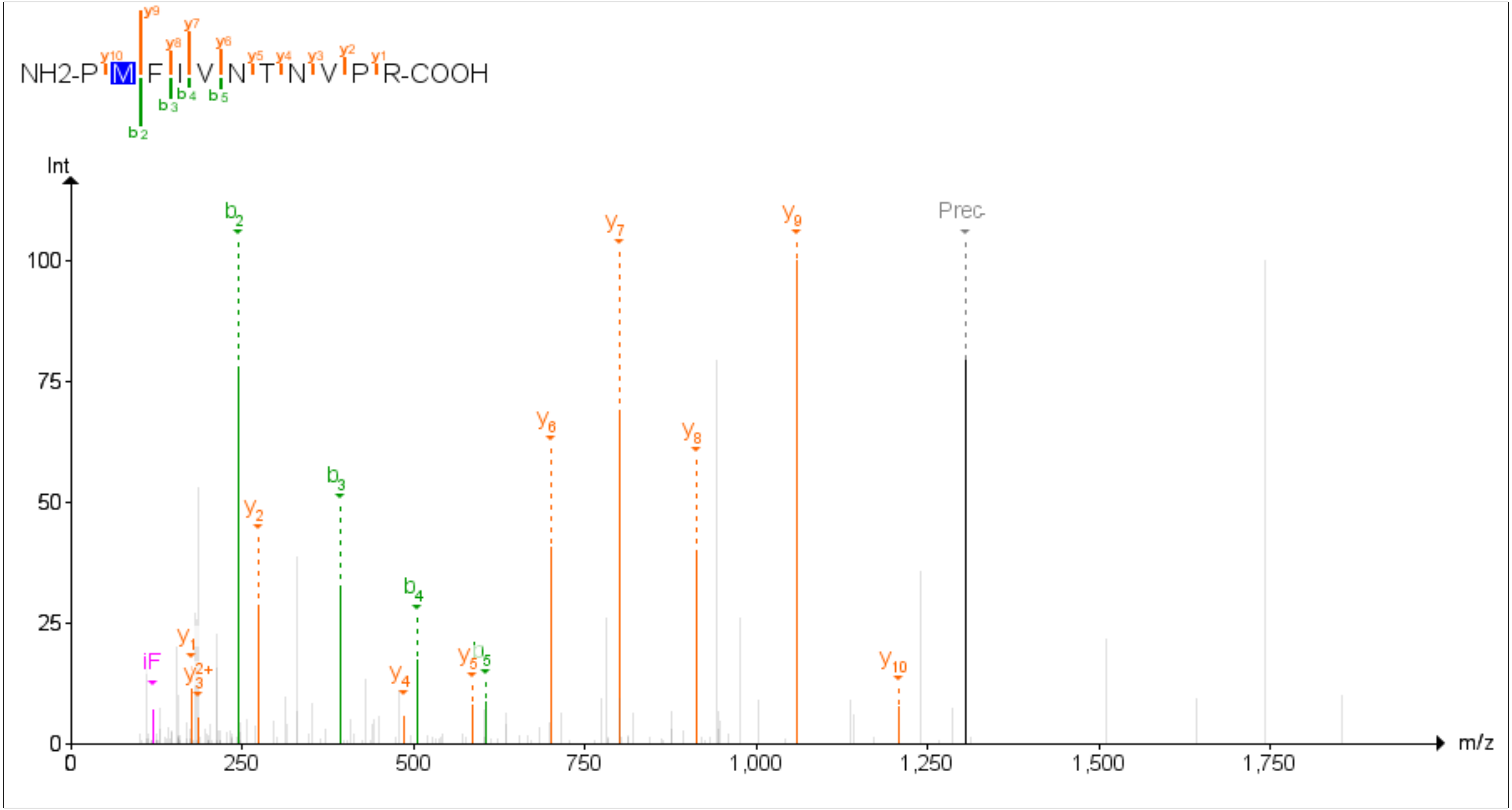

NH2-P M F I V N T N V P R-COOH
y10 y9 y8 y7 y6 y5 y4 y3 y2 y1
b2 b3 b4 b5
Int
m/z
0
25
50
75
100
250
500
750
1,000
1,250
1,500
1,750
b2
y7
y9
Prec.
y6
y8
b3
y2
b4
y1
iF
y3 2+
y4
y5
b5
y10

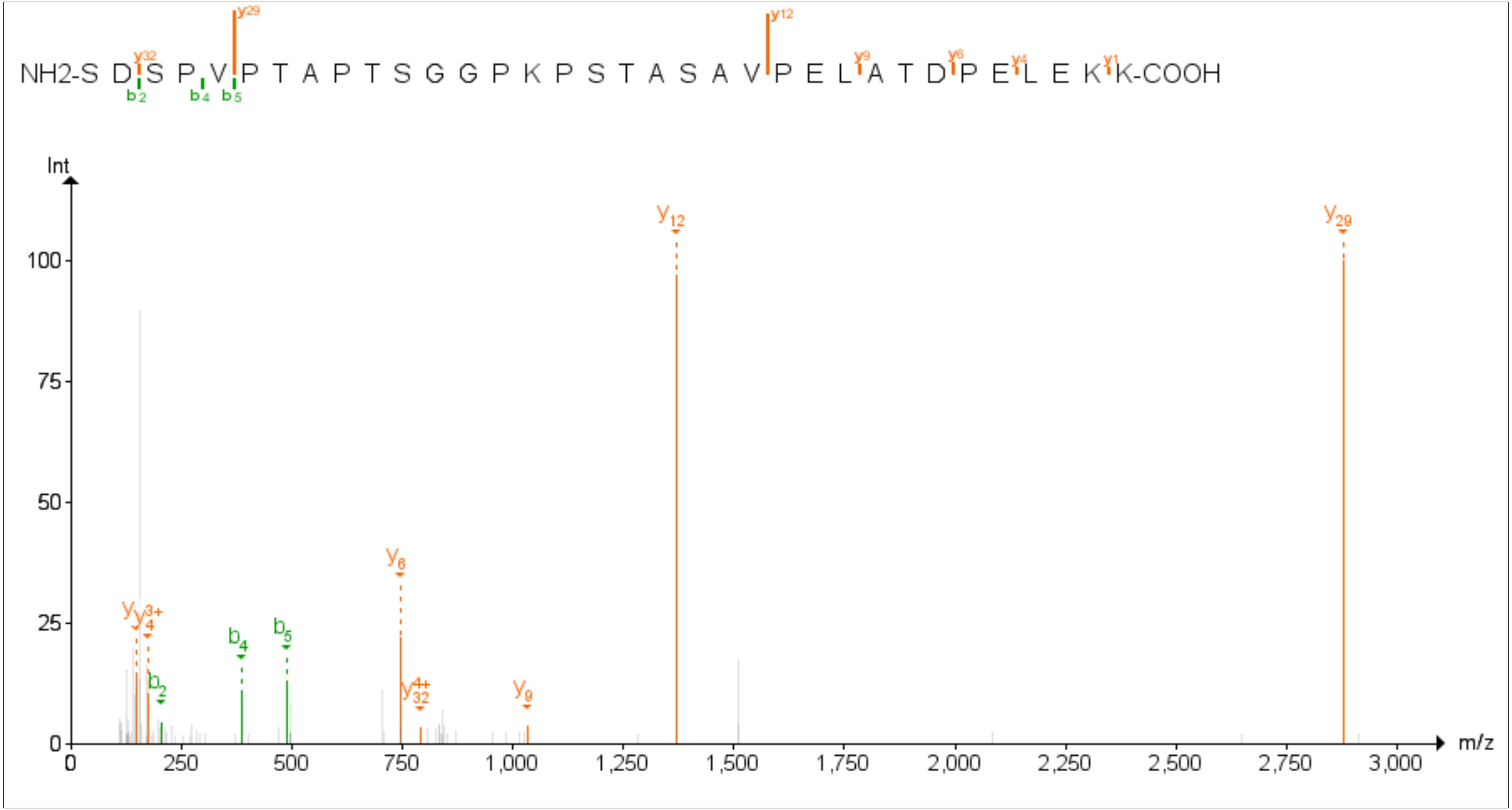
NH2-S D S P V P T A P T S G G P K P S T A S A V P E L A T D P E L E K K-COOH
Int
100
75
50
25
0
m/z
0
250
500
750
1,000
1,250
1,500
1,750
2,000
2,250
2,500
2,750
3,000

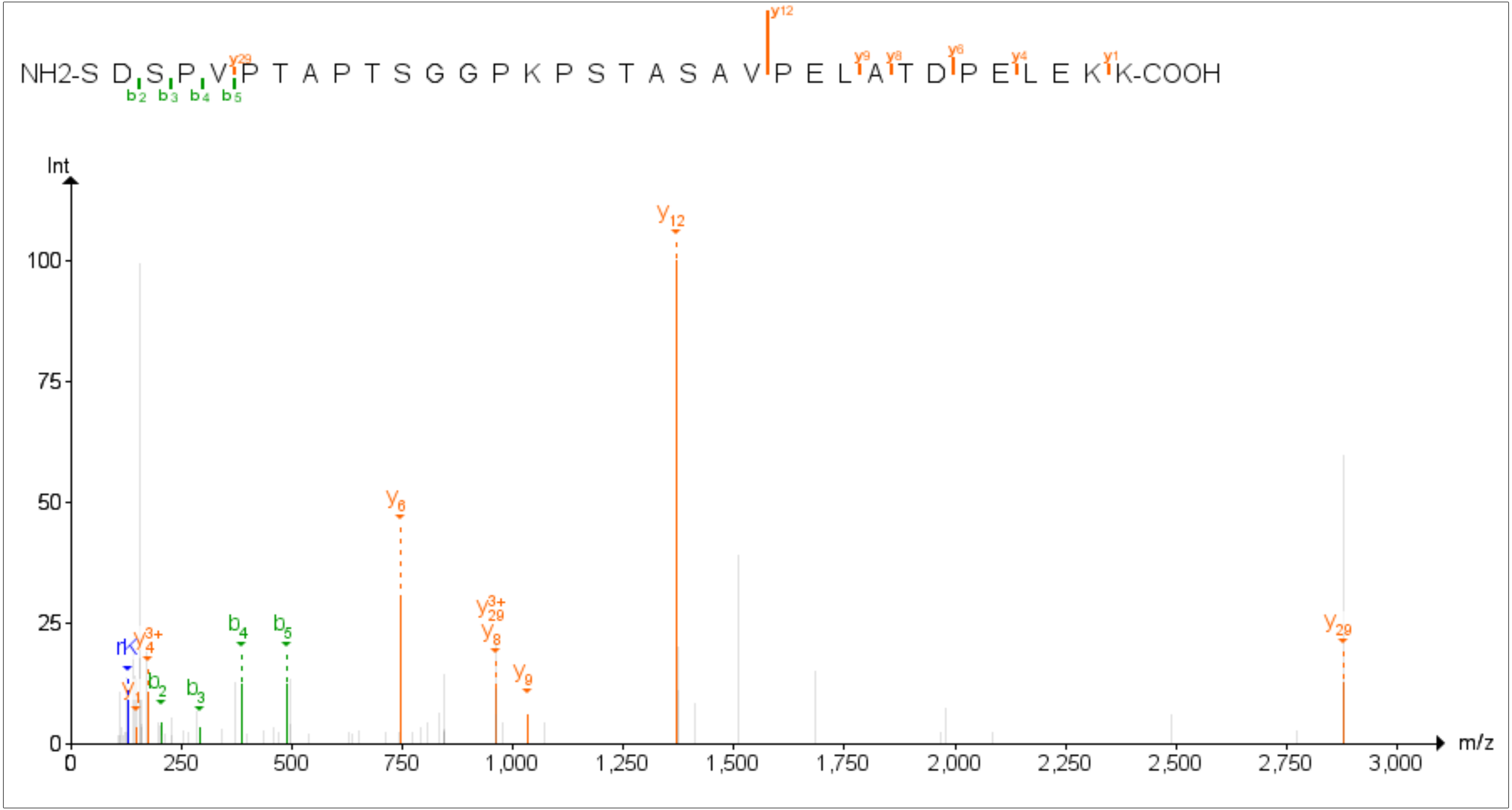

NH2-S D S P V P T A P T S G G P K P S T A S A V P E L A T D P E L E K K-COOH
Int
m/z
y12
y6
y29
y8
y9
b4
b5
b2
b3
y1
rK

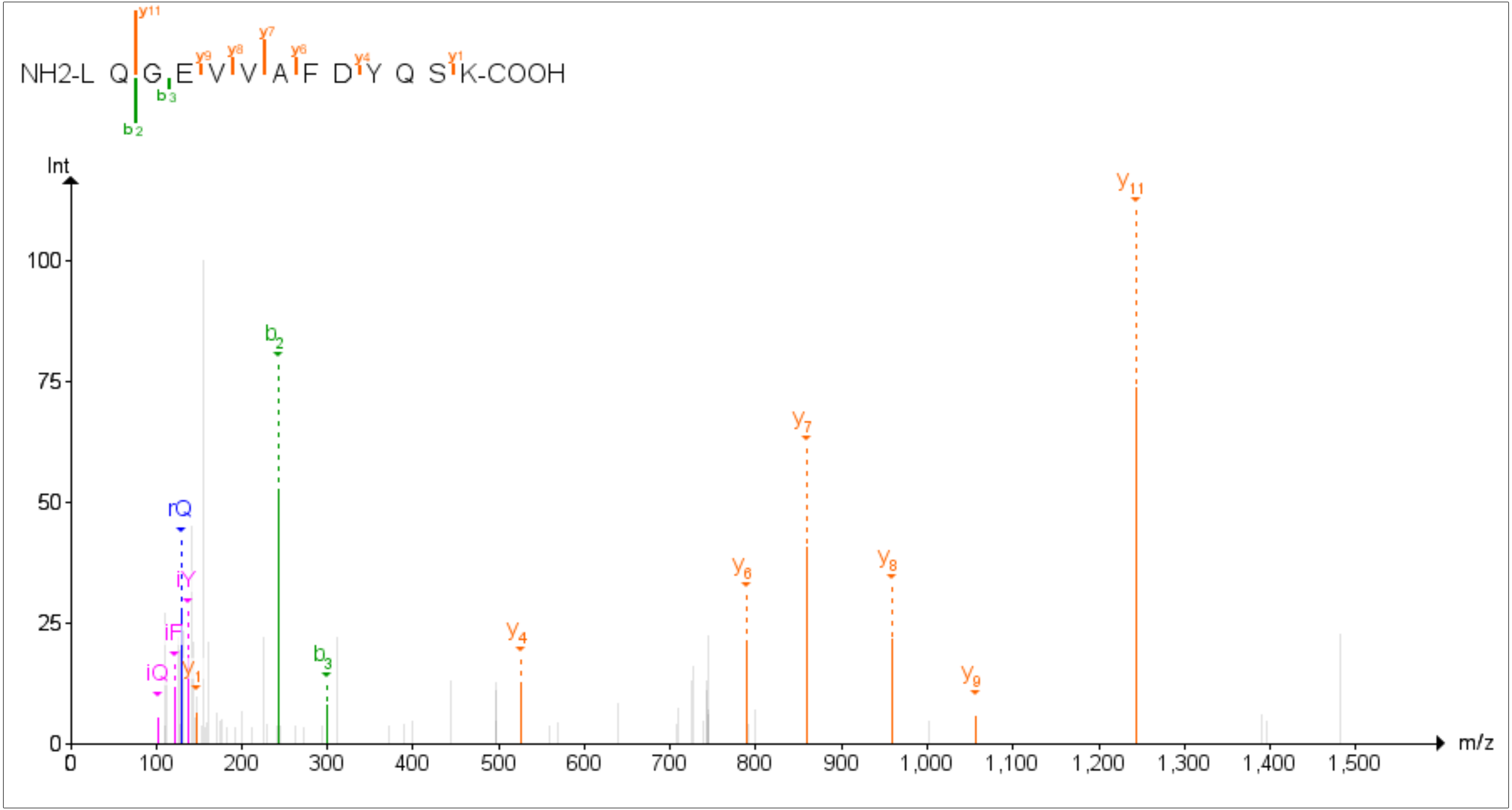

NH2-L Q G E V V A F D Y Q S K-COOH
y11
y9
y8
y7
y6
y4
y1
b2
b3
Int
100
75
50
25
0
rQ
iY
iF
iQ
y1
b2
b3
y4
y6
y7
y8
y9
y11
m/z
0
100
200
300
400
500
600
700
800
900
1,000
1,100
1,200
1,300
1,400
1,500

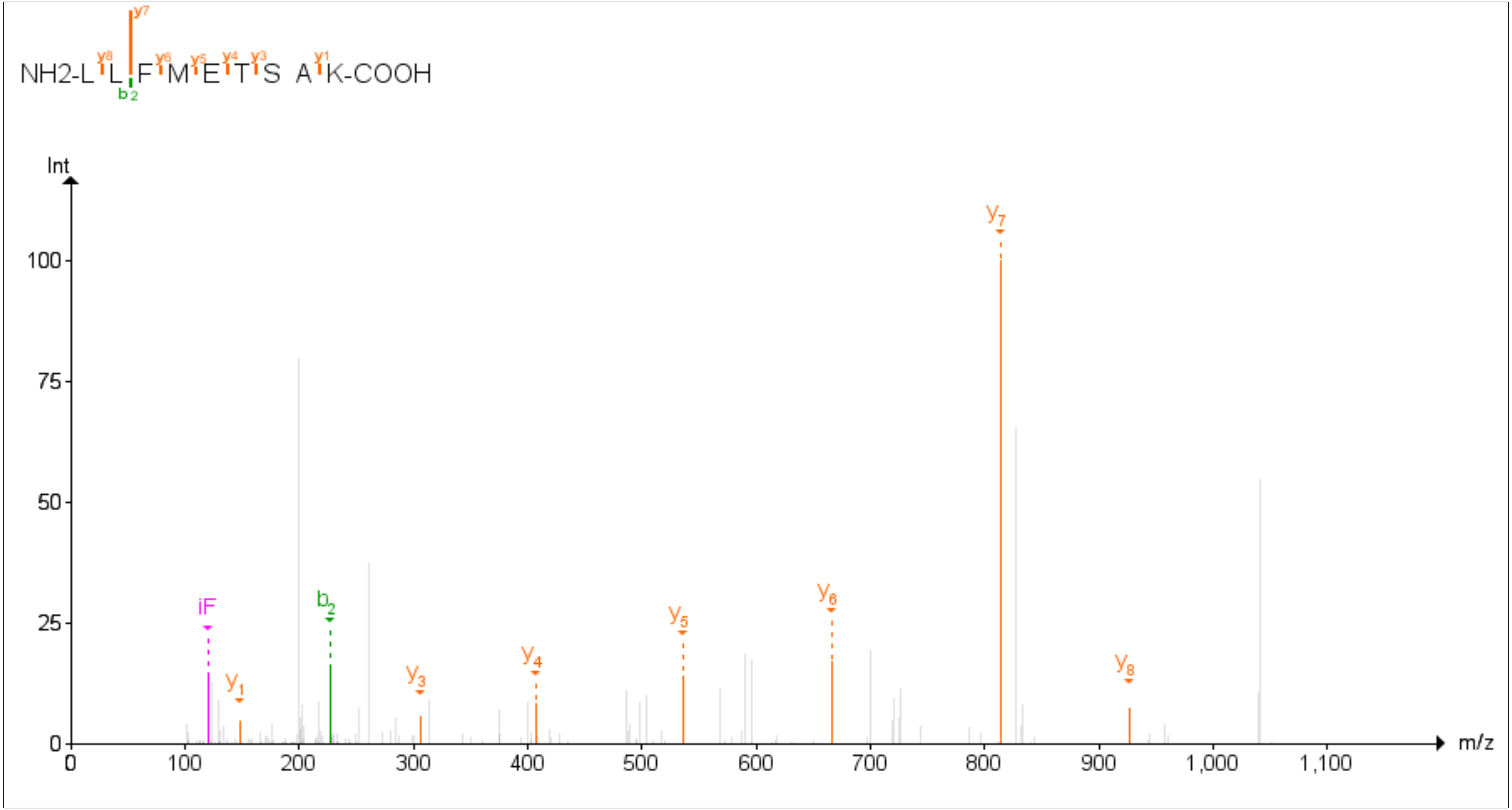
NH2-L L F M E T S A K-COOH
y8
y7
y6
y5
y4
y3
y1
b 2
Int
100
75
50
25
0
iF
y1
b2
y3
y4
y5
y6
y7
y8
0
100
200
300
400
500
600
700
800
900
1,000
1,100
m/z

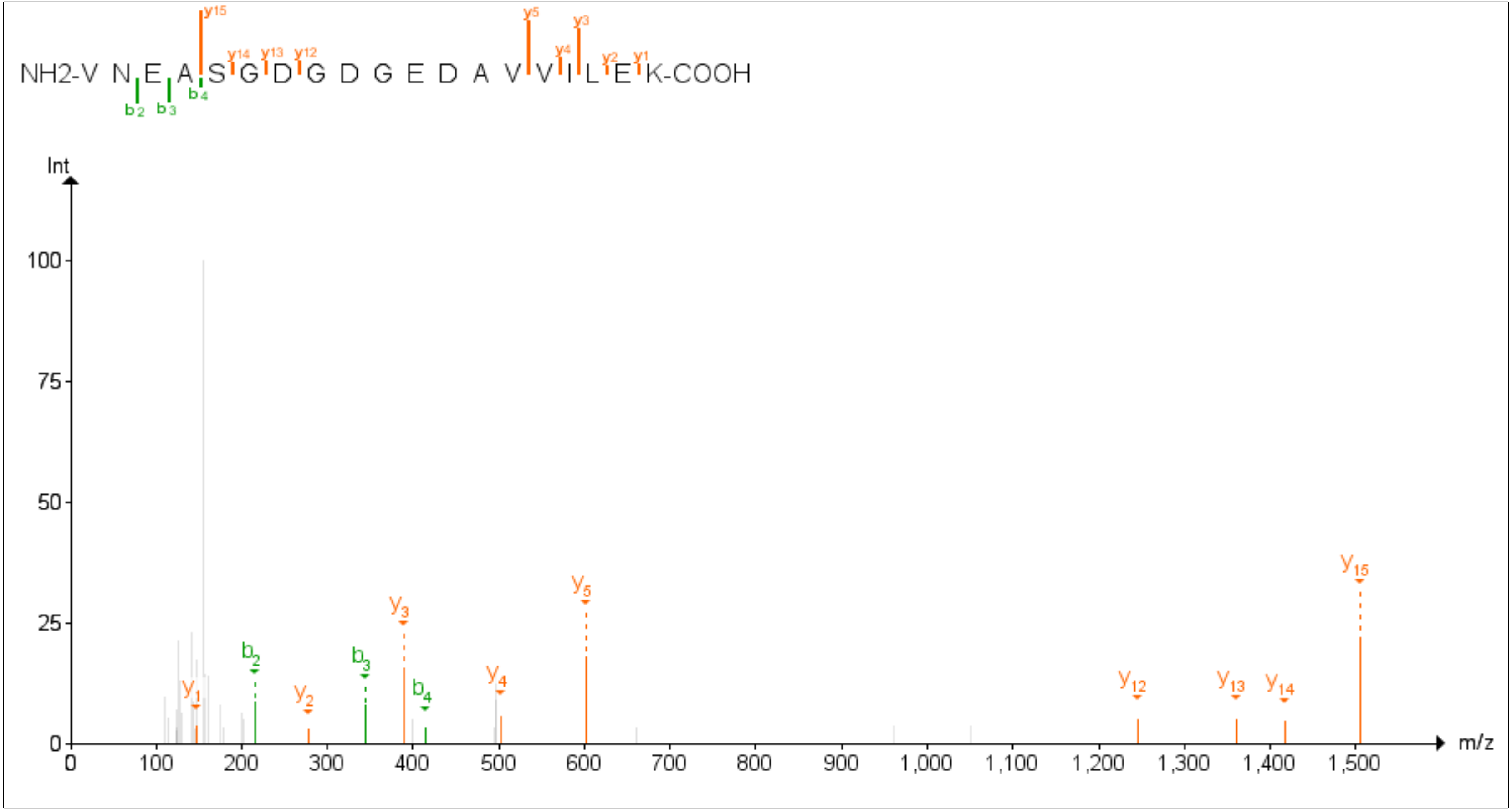

NH2-V N E A S G D G D G E D A V V I L E K-COOH
y15 y14 y13 y12 y5 y4 y3 y2 y1
b2 b3 b4
Int
100
75
50
25
0
m/z
0 100 200 300 400 500 600 700 800 900 1,000 1,100 1,200 1,300 1,400 1,500
y1 b2 y2 b3 y3 b4 y4 y5 y12 y13 y14 y15

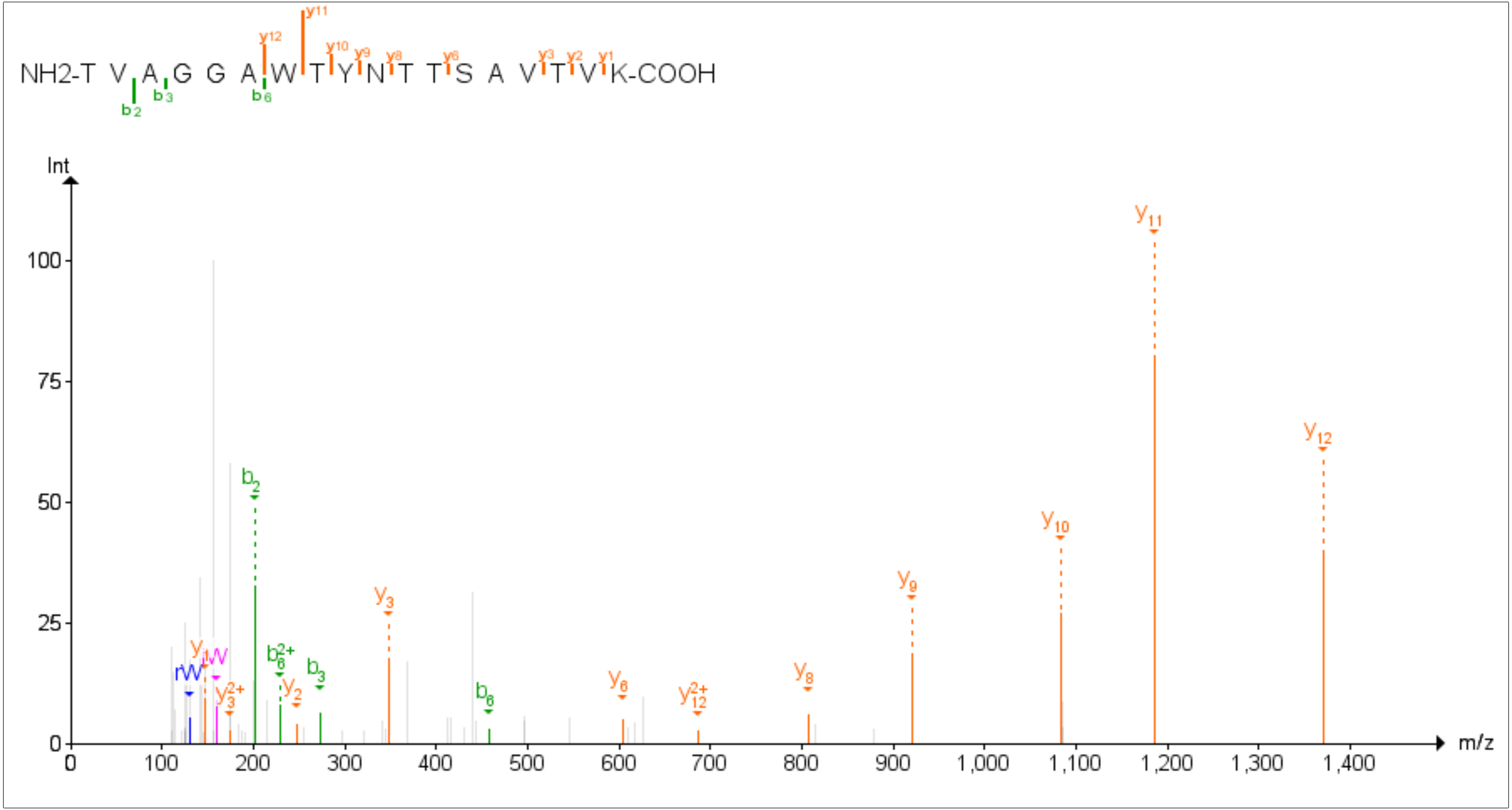
NH2-T V A G G A W T Y N T T S A V T V K-COOH
Int
m/z
y11
y12
y10
y9
y3
y8
y6
y2+12
y2+3
b2
b3
b6
b2+6
rW
iW
y1
y2
0
25
50
75
100
0
100
200
300
400
500
600
700
800
900
1,000
1,100
1,200
1,300
1,400

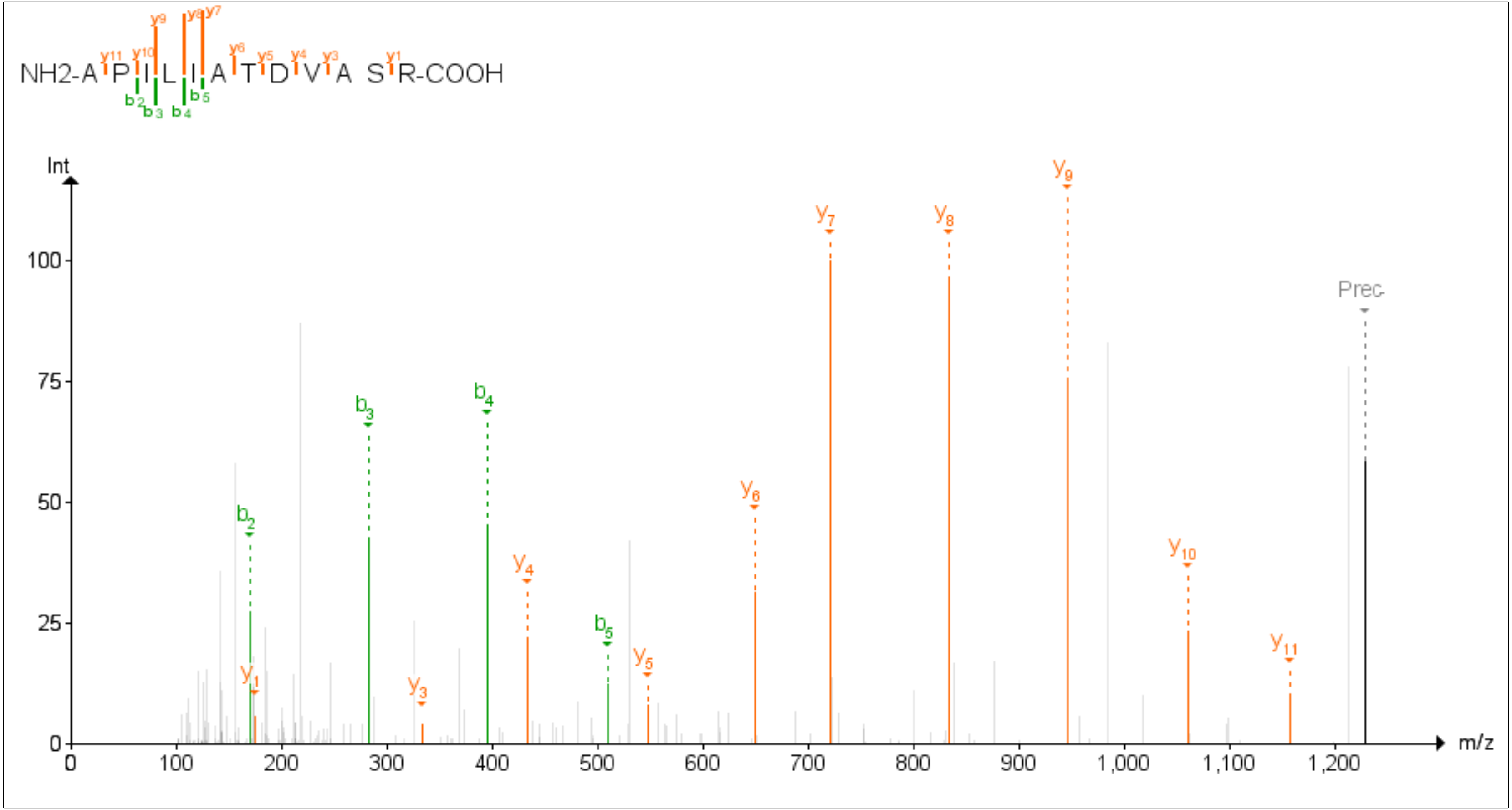

NH2-A P I L I A T D V A S R-COOH
y11 y10 y9 y8 y7 y6 y5 y4 y3 y1
b2 b3 b4 b5
Int
100
75
50
25
0
m/z
0 100 200 300 400 500 600 700 800 900 1,000 1,100 1,200
b2 b3 b4 b5
y1 y3 y4 y5 y6 y7 y8 y9 y10 y11
Prec

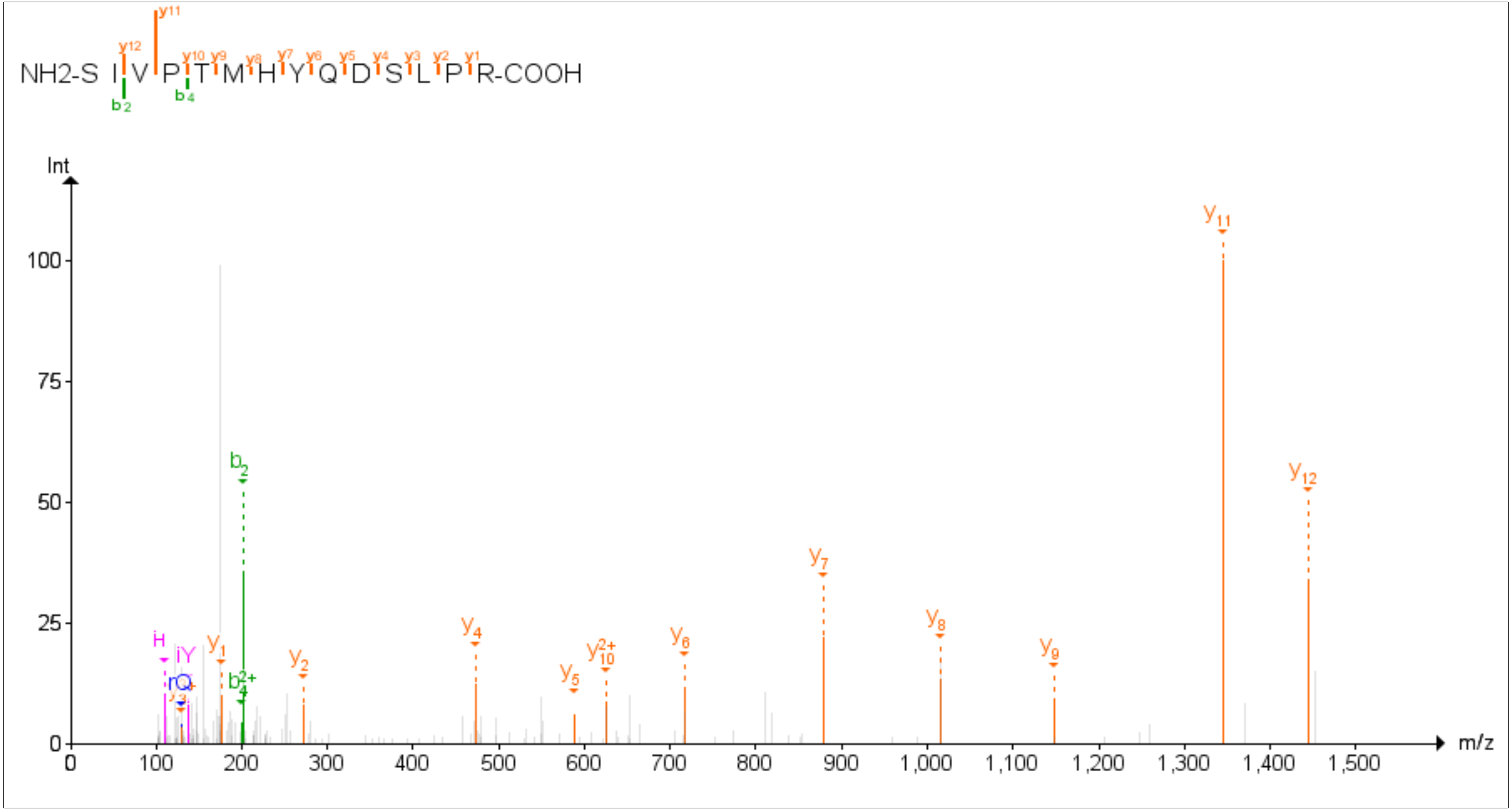

NH2-S I V P I T M H Y Q D S L P R-COOH
y12
y11
y10
y9
y8
y7
y6
y5
y4
y3
y2
y1
b2
b4
Int
100
75
50
25
0
m/z
0
100
200
300
400
500
600
700
800
900
1,000
1,100
1,200
1,300
1,400
1,500
iH
iY
rQ
y1
y2
y4
y5
y10 2+
y6
y7
y8
y9
y11
y12
b2
b4 2+

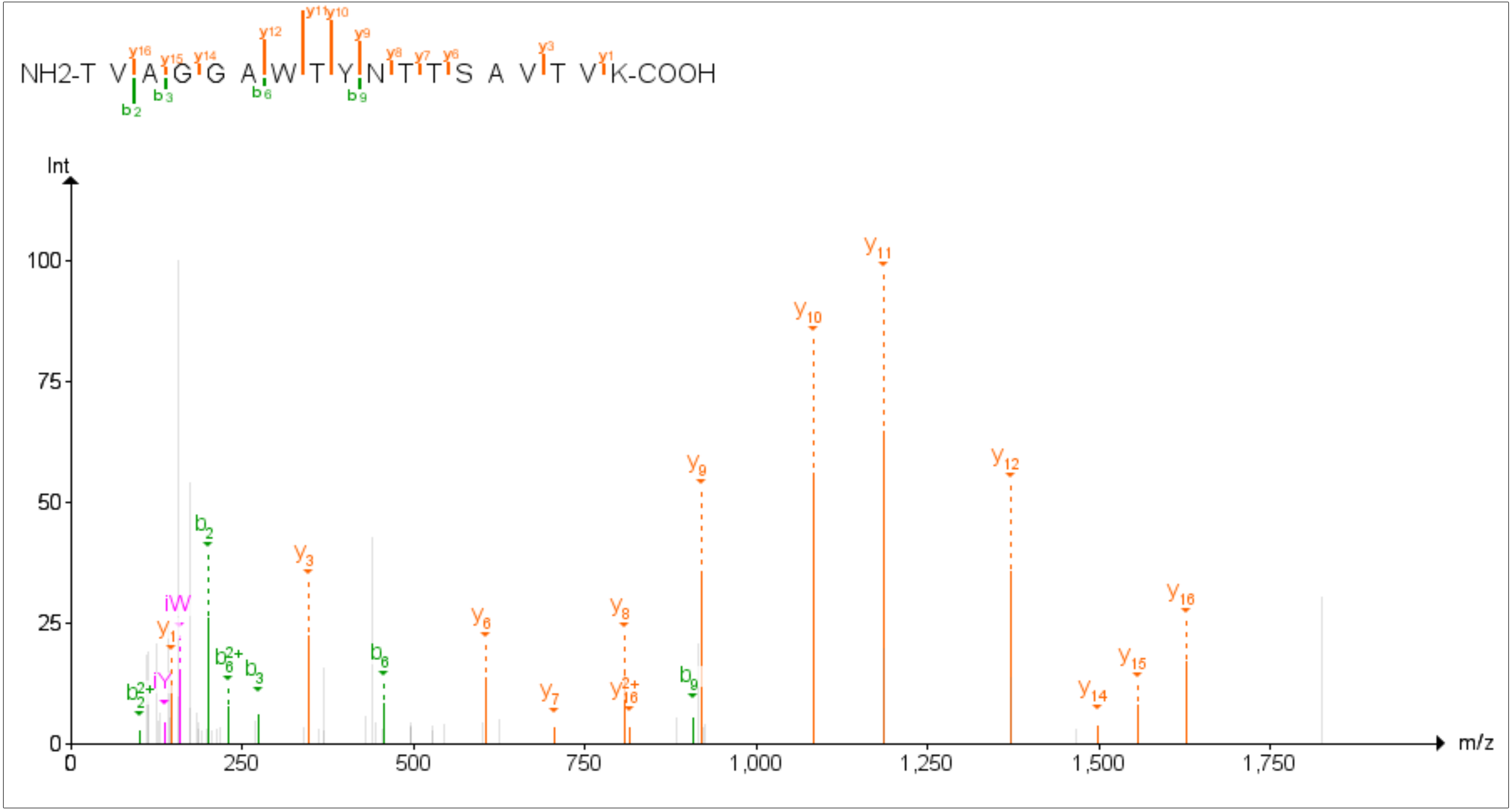

NH2-T V A G G A W T Y N T T S A V T V K-COOH
Int
m/z
0
25
50
75
100
250
500
750
1,000
1,250
1,500
1,750

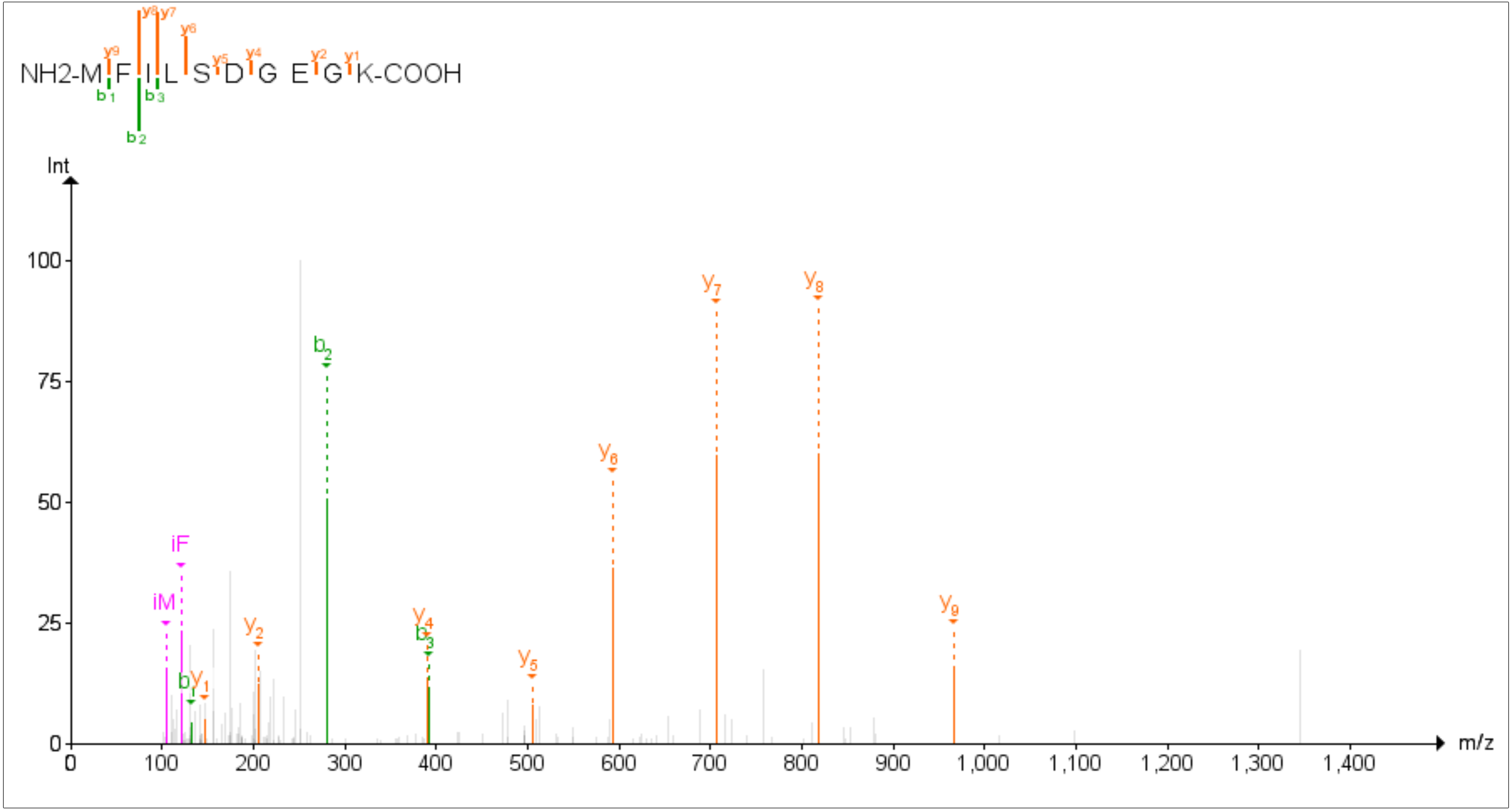

NH2-M F I L S D G E G K-COOH
y9 y8 y7 y6 y5 y4 y2 y1
b1 b2 b3
Int
100
75
50
25
0
b2
y7
y8
y6
iF
iM
y2
y4
b3
y5
y9
b1
y1
0
100
200
300
400
500
600
700
800
900
1,000
1,100
1,200
1,300
1,400
m/z

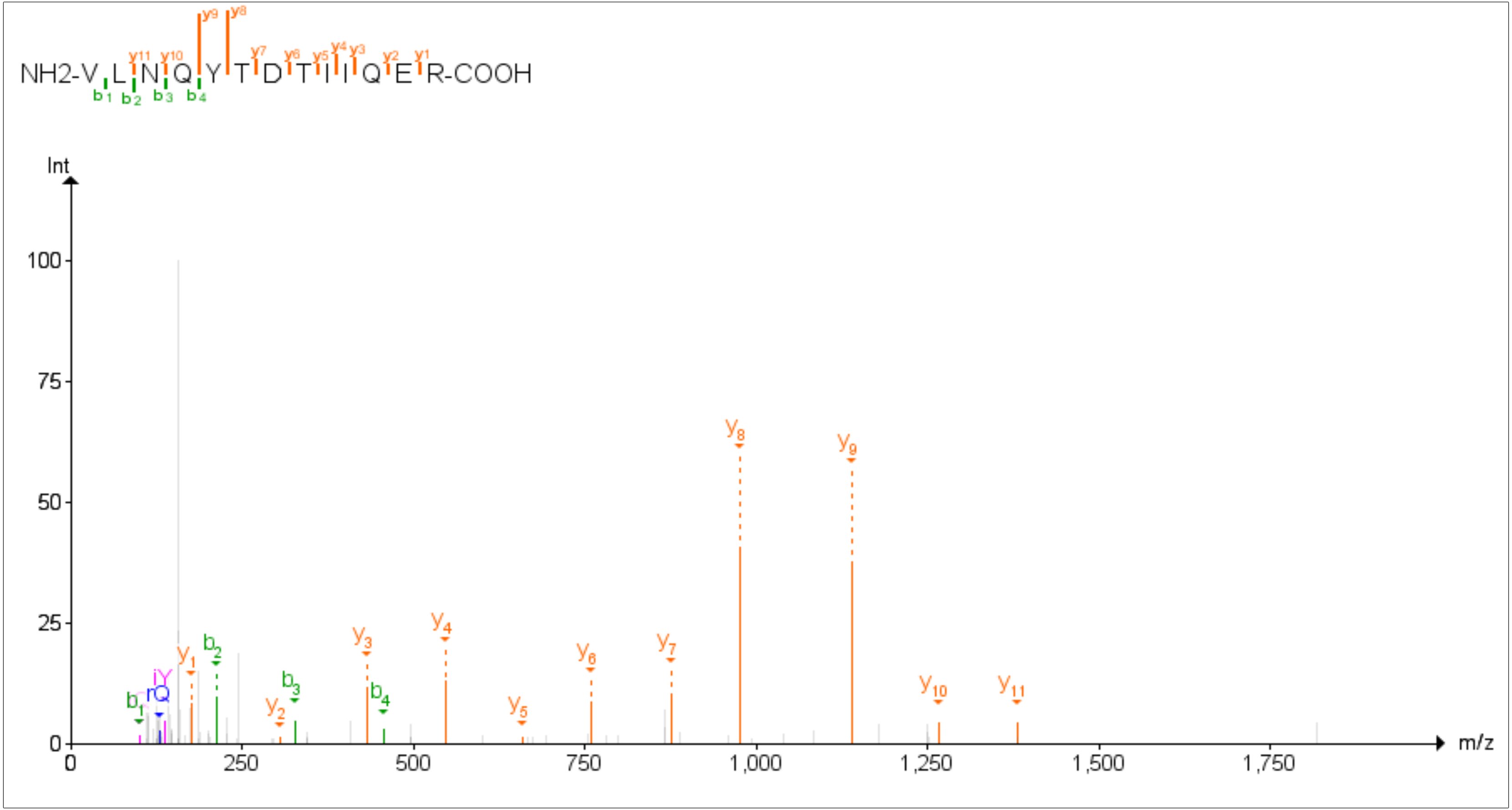

NH2-V L N Q Y T D T I I Q E R-COOH
b1 b2 b3 b4
y11 y10 y9 y8 y7 y6 y5 y4 y3 y2 y1
Int
100
75
50
25
0
0
250
500
750
1,000
1,250
1,500
1,750
m/z
b1
rQ
iY
y1
b2
y2
b3
y3
b4
y4
y5
y6
y7
y8
y9
y10
y11

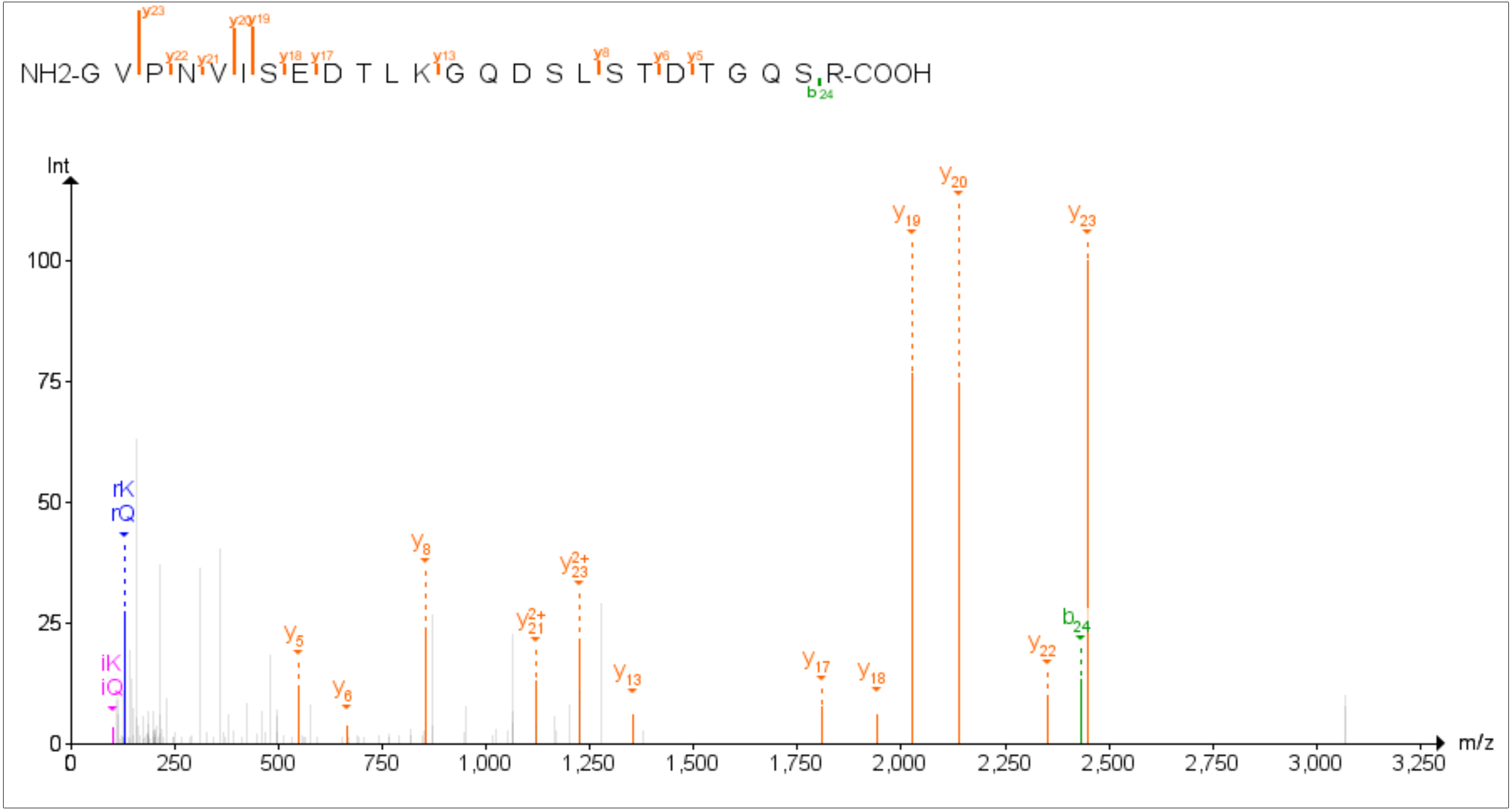

NH2-G V P N V I S E D T L K G Q D S L S T D T G Q S R-COOH
y23 y22 y21 y20 y19 y18 y17 y13 y8 y6 y5 b24
Int
m/z
rK
rQ
iK
iQ
y5 y6 y8 y21 2+ y23 2+ y13 y17 y18 y19 y20 y22 b24 y23
0 25 50 75 100
0 250 500 750 1,000 1,250 1,500 1,750 2,000 2,250 2,500 2,750 3,000 3,250

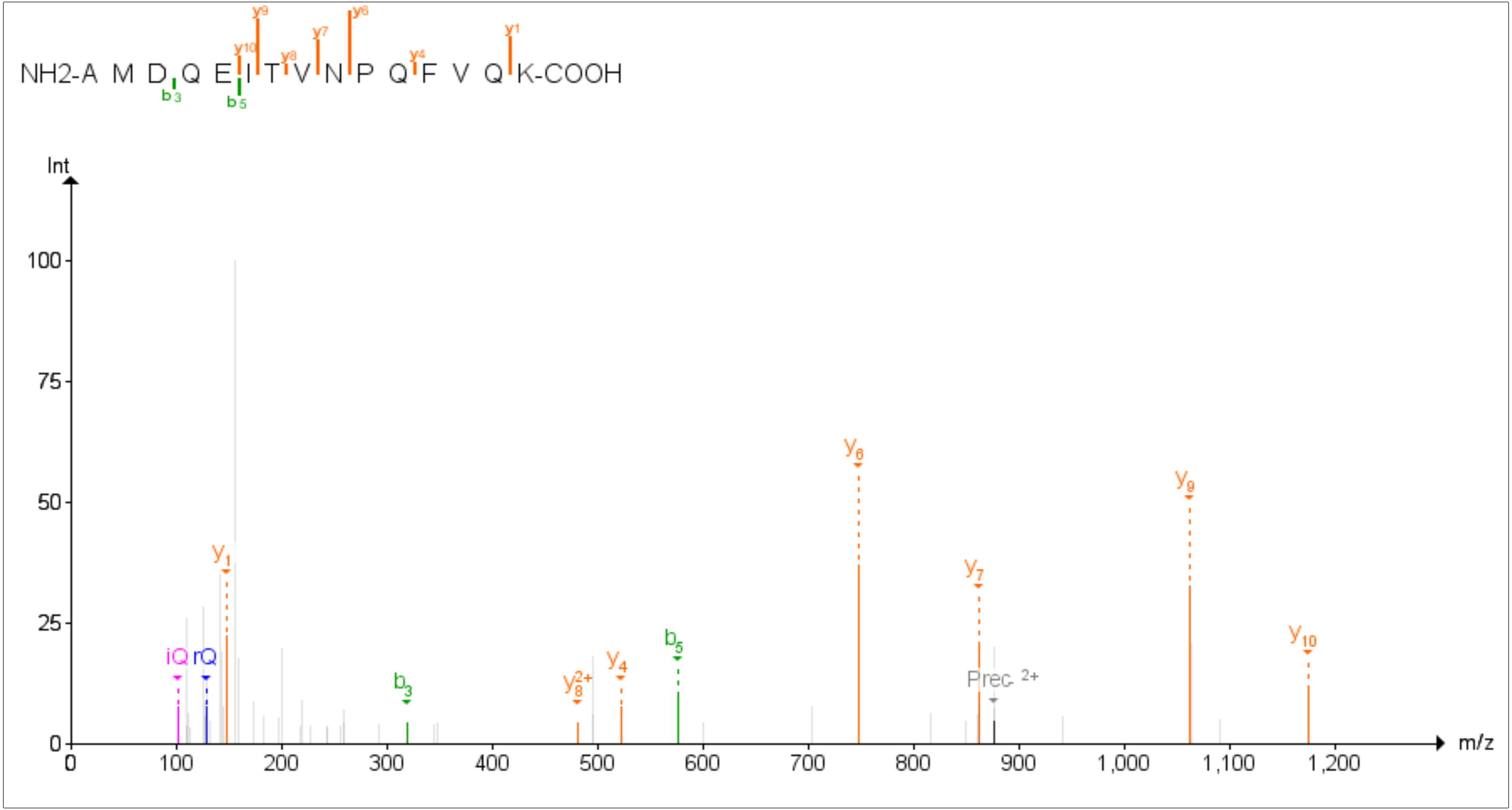

NH2-A M D Q E I T V N P Q F V Q K-COOH
b3
b5
y10
y9
y8
y7
y6
y4
y1
Int
100
75
50
25
0
iQ
rQ
y1
b3
y8 2+
y4
b5
y6
y7
Prec. 2+
y9
y10
0
100
200
300
400
500
600
700
800
900
1,000
1,100
1,200
m/z

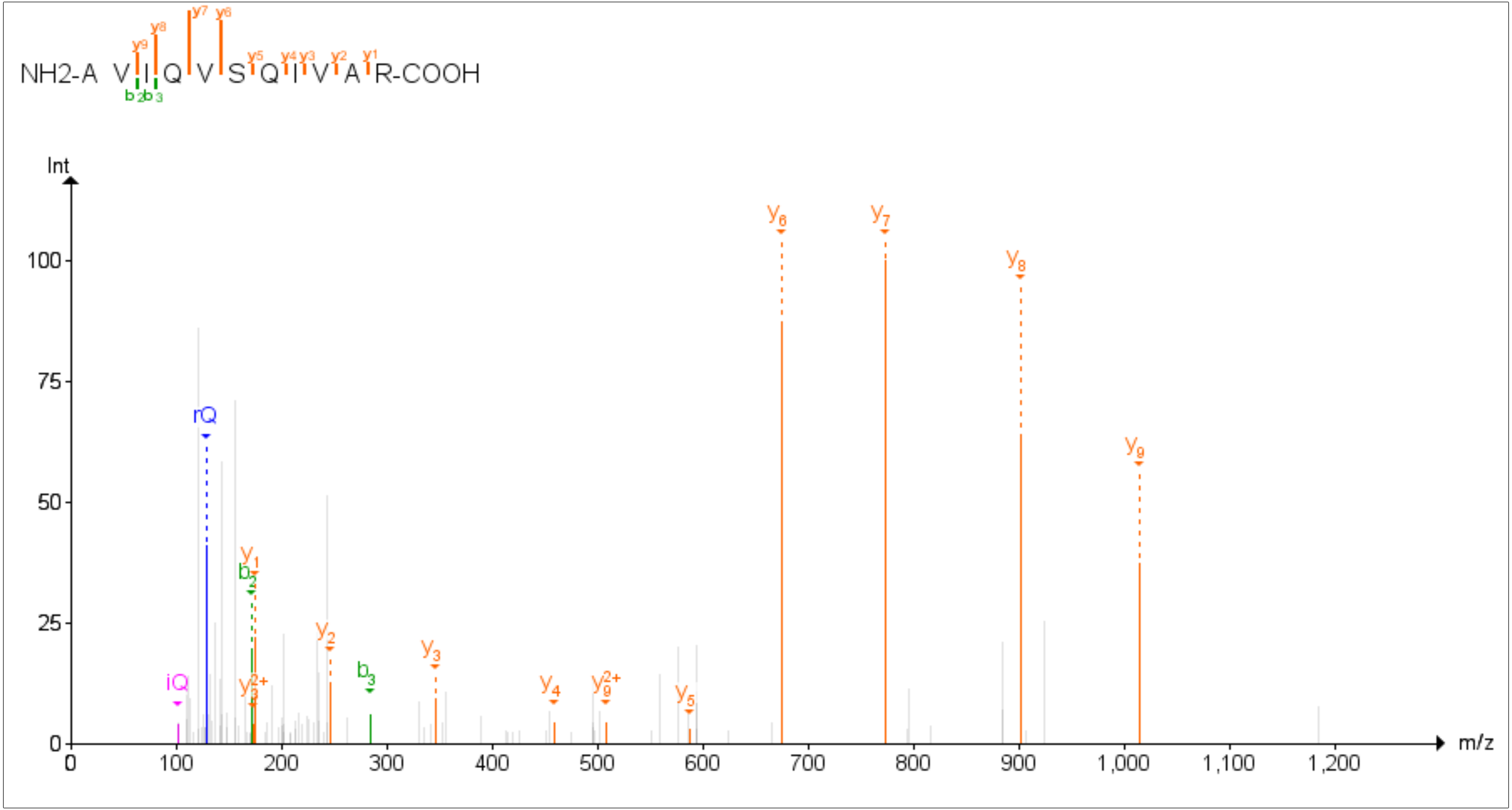

NH2-A V I Q V S Q I V A R-COOH
y9 y8 y7 y6 y5 y4 y3 y2 y1
b2 b3
Int
100
75
50
25
0
rQ
iQ
y6
y7
y8
y9
y1
b2
y2
y3
b3
y4
y5
0 100 200 300 400 500 600 700 800 900 1,000 1,100 1,200
m/z

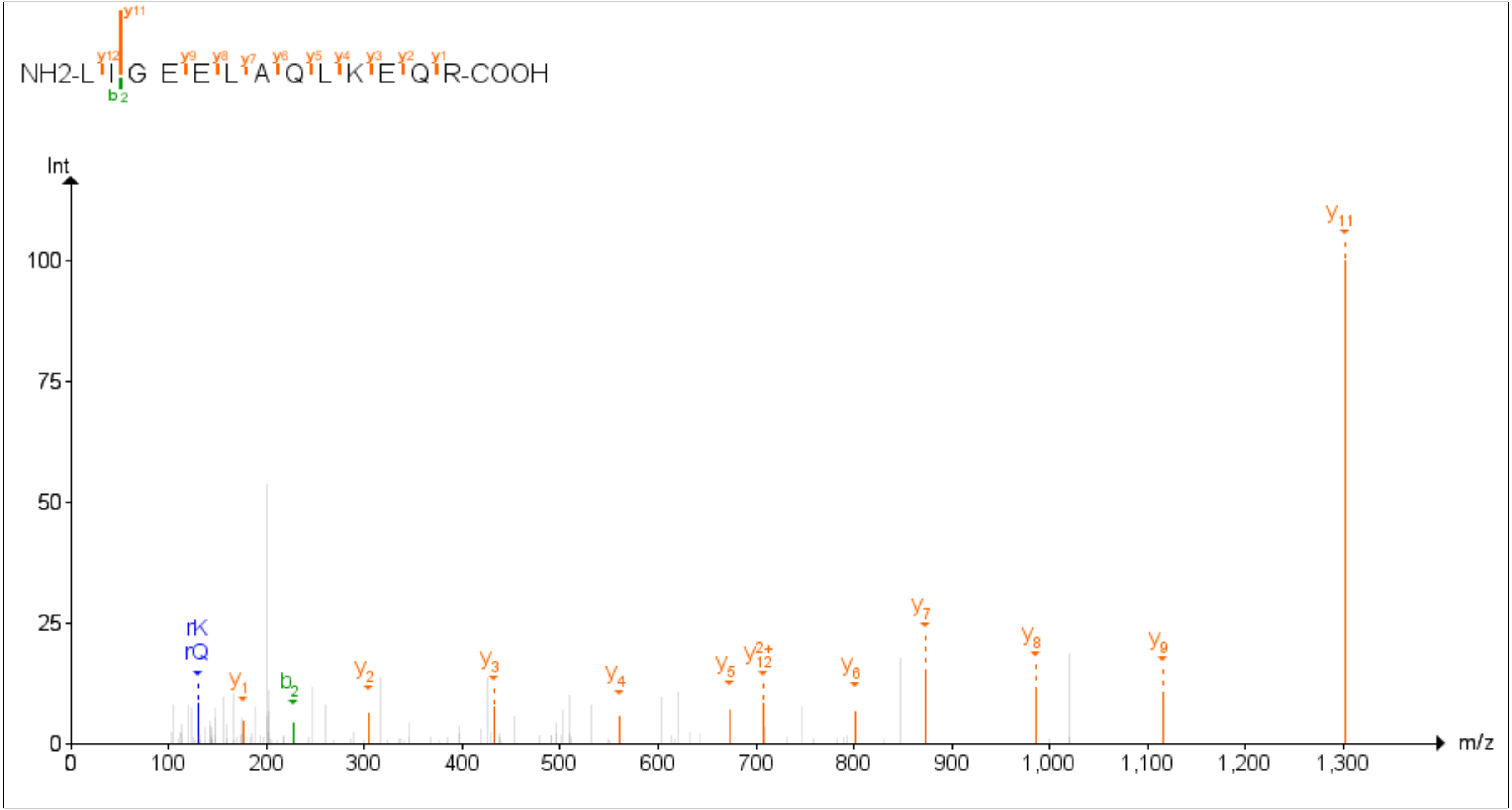

NH2-L I G E E L A Q L K E Q R-COOH
y11
y12 y9 y8 y7 y6 y5 y4 y3 y2 y1
b2
Int
100
75
50
25
0
rK
rQ
y1
b2
y2
y3
y4
y5
y12 2+
y6
y7
y8
y9
y11
0 100 200 300 400 500 600 700 800 900 1,000 1,100 1,200 1,300
m/z

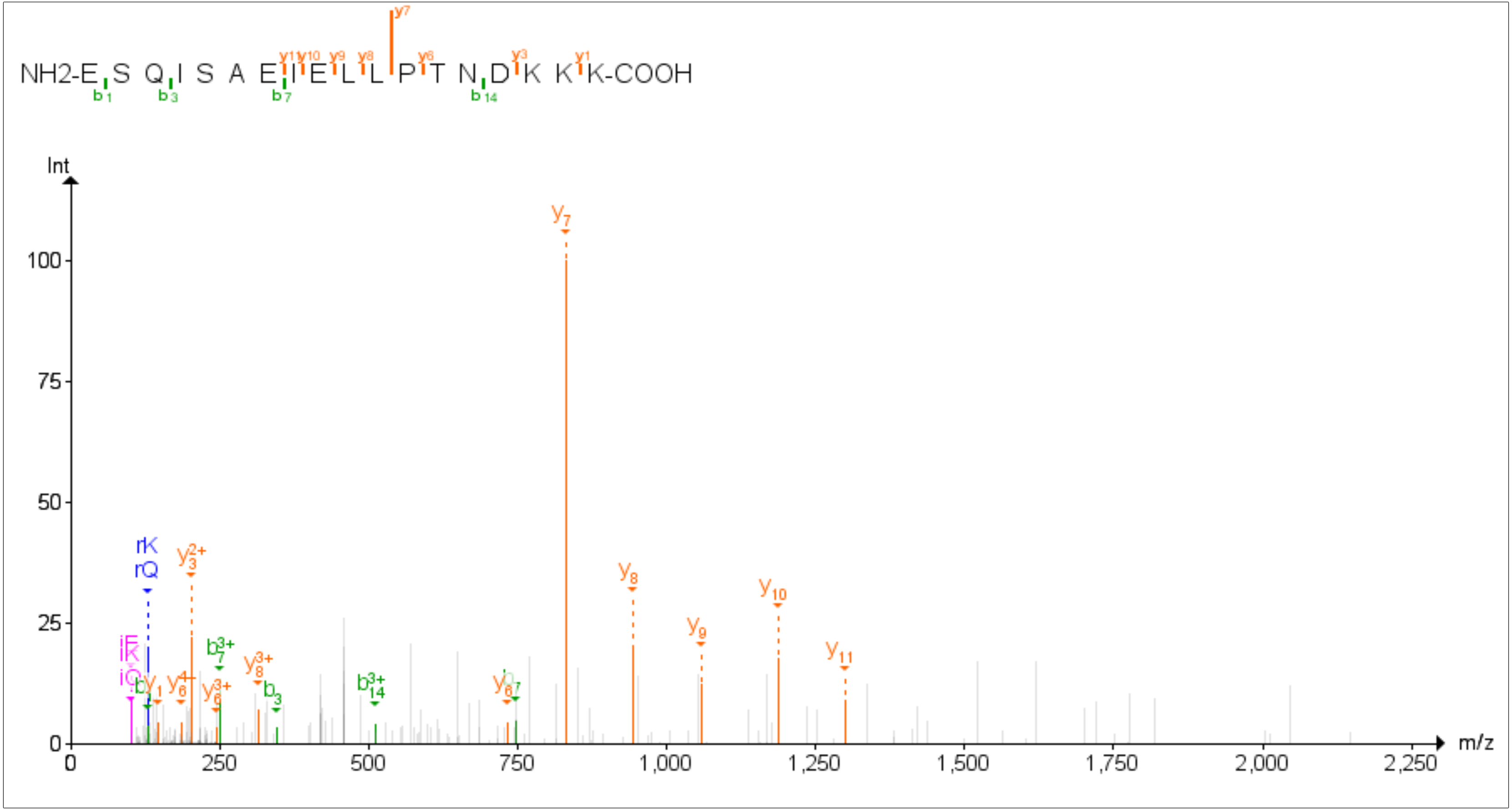

NH2-E S Q I S A E I I E L L P T N D K K K-COOH
Int
m/z
y7
y8
y9
y10
y11
0
25
50
75
100
250
500
750
1,000
1,250
1,500
1,750
2,000
2,250

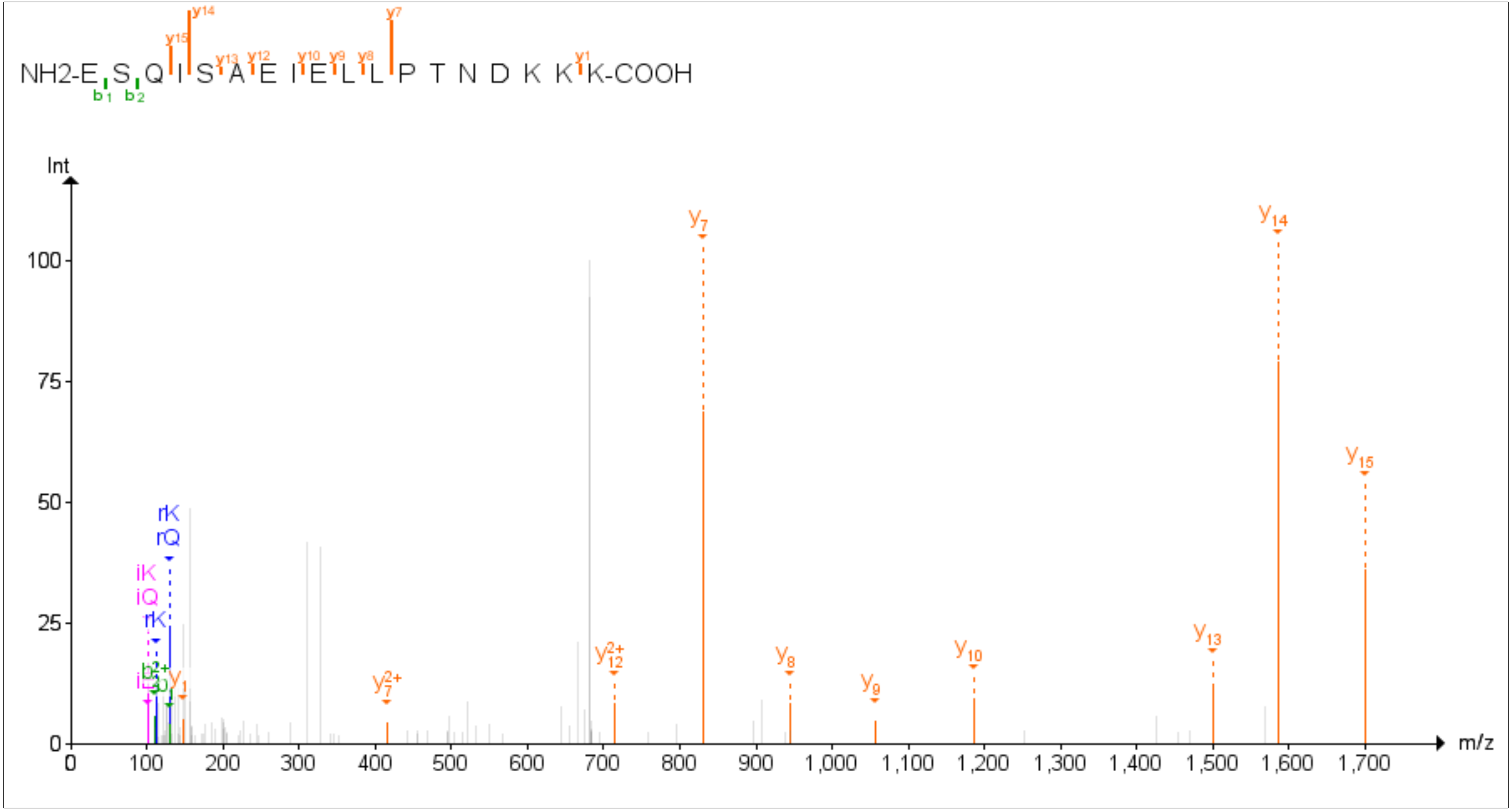

NH2-E S Q I S A E I E L L P T N D K K K-COOH
b1 b2
y15 y14 y13 y12 y10 y9 y8 y7 y1
Int
m/z
y7
y14
y15
y13
y10
y9
y8
y12 2+
y7 2+
y1
rK
rQ
iK
iQ

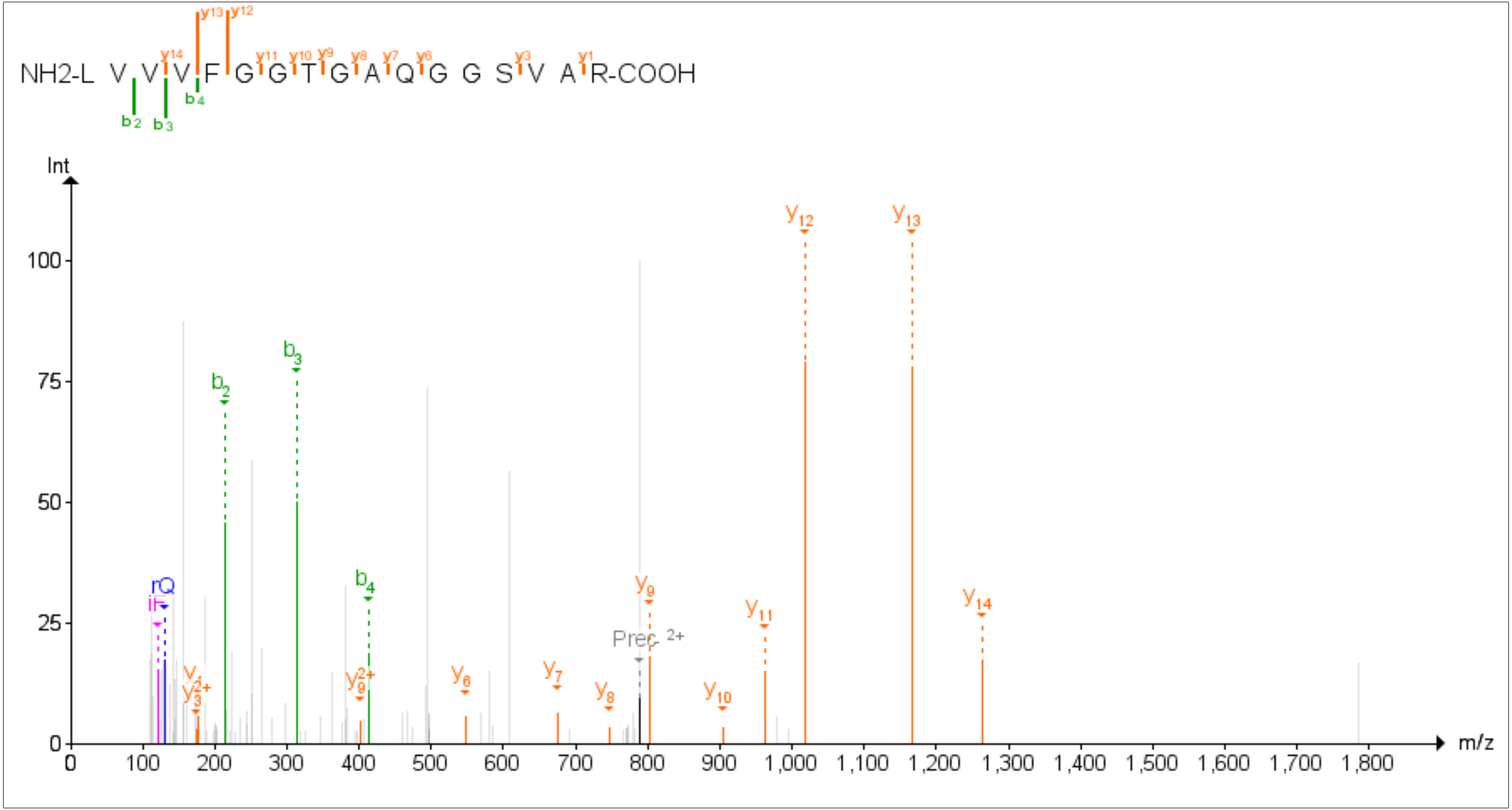

NH2-L V V V F G G T G A Q G G S V A R-COOH
y13 y12 y14 y11 y10 y9 y8 y7 y6 y3 y1
b2 b3 b4
Int
m/z
0 25 50 75 100
0 100 200 300 400 500 600 700 800 900 1,000 1,100 1,200 1,300 1,400 1,500 1,600 1,700 1,800
y12 y13 b3 b2 b4 rQ iF y9 y11 y14 Prec 2+ y3 2+ y9 2+ y6 y7 y8 y10

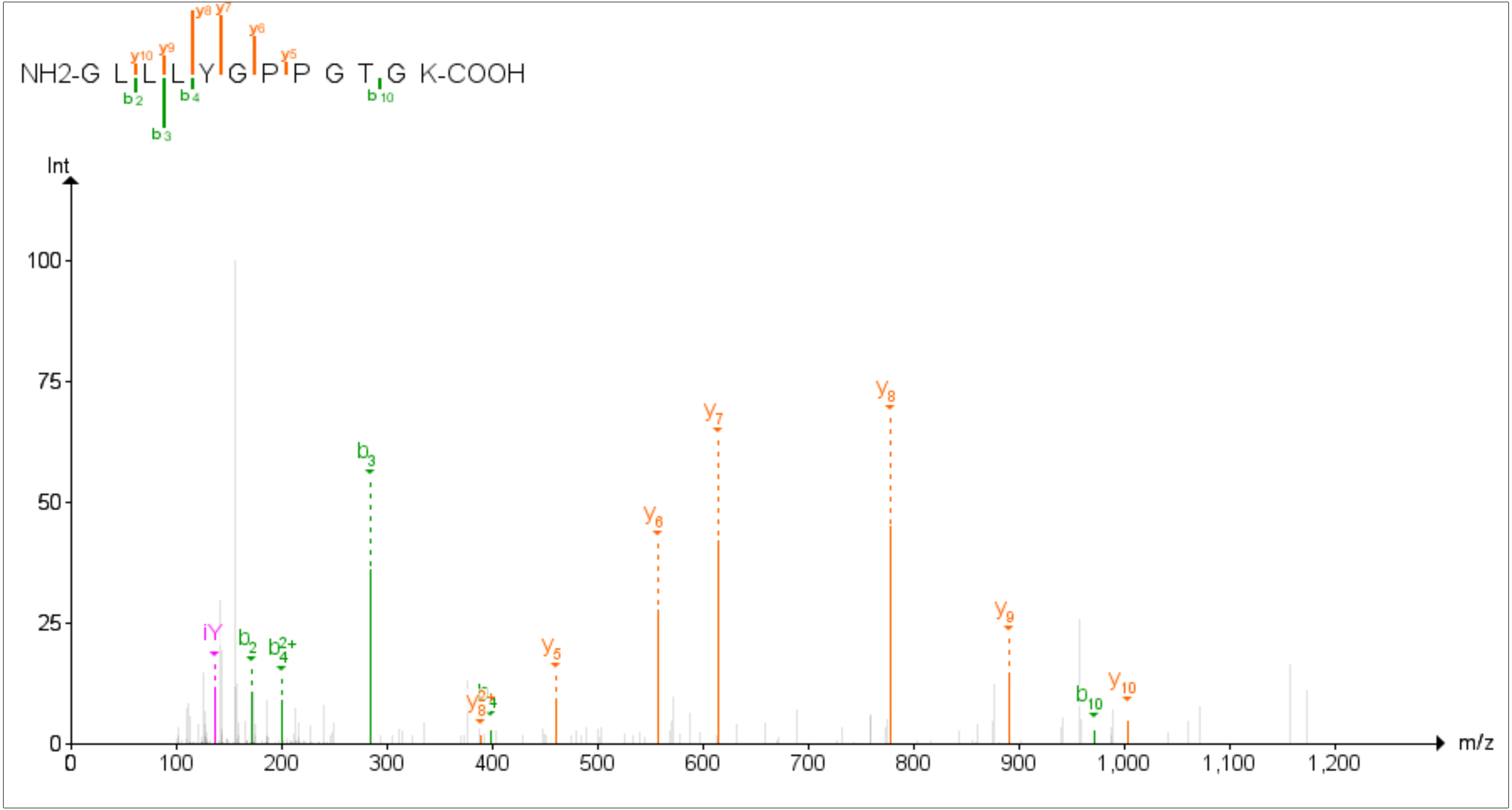
NH2-G L L L Y G P P G T G K-COOH
y10 y9 y8 y7 y6 y5
b2 b3 b4 b10
Int
100
75
50
25
0
m/z
0 100 200 300 400 500 600 700 800 900 1,000 1,100 1,200
iY
b2
b4 2+
b3
y8 2+
y5
y6
y7
y8
y9
b10
y10

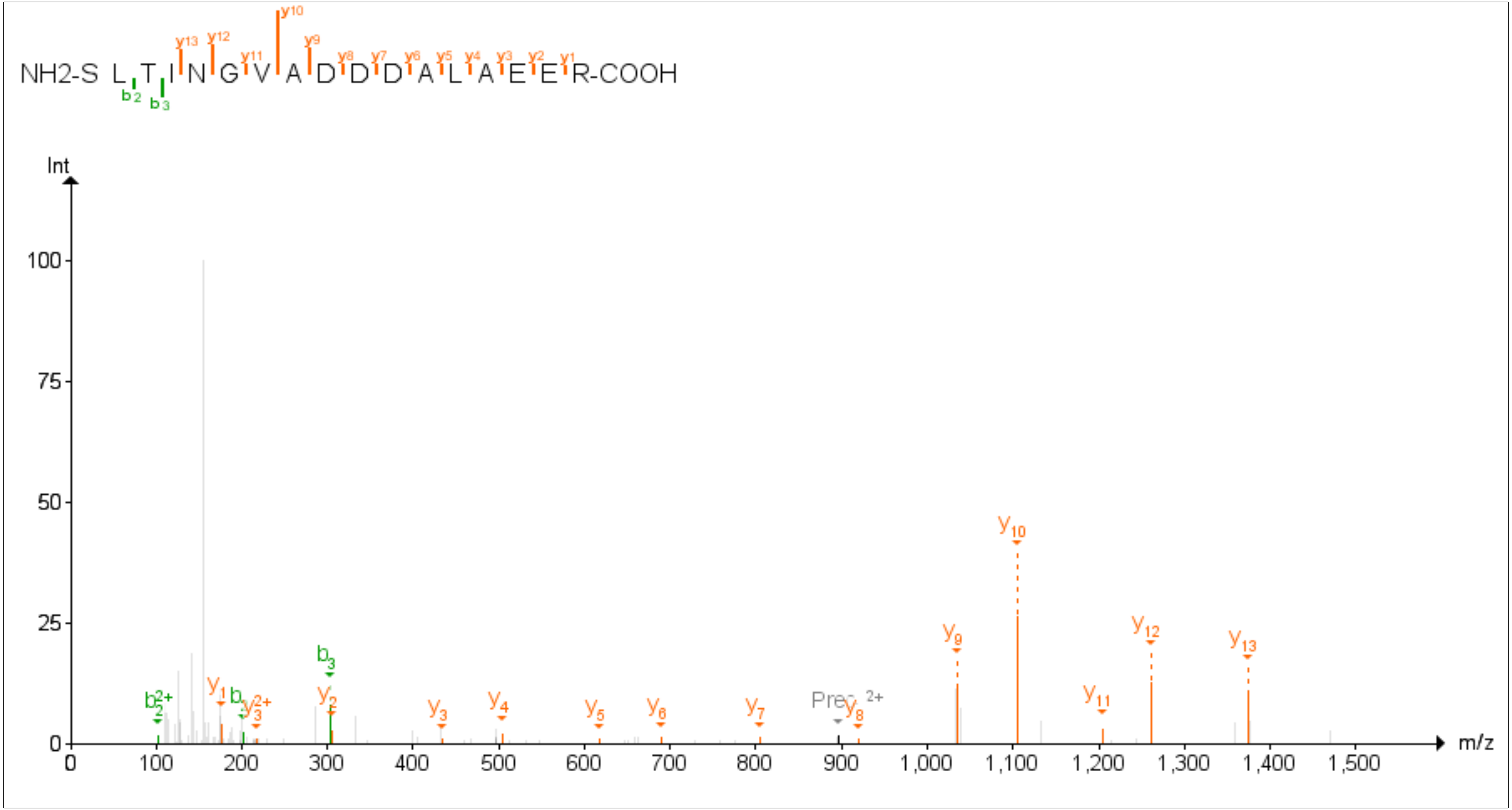

NH2-S L T I N G V A D D D A L A E E R-COOH
b2
b3
y13
y12
y11
y10
y9
y8
y7
y6
y5
y4
y3
y2
y1
Int
100
75
50
25
0
m/z
0
100
200
300
400
500
600
700
800
900
1,000
1,100
1,200
1,300
1,400
1,500
Prec 2+

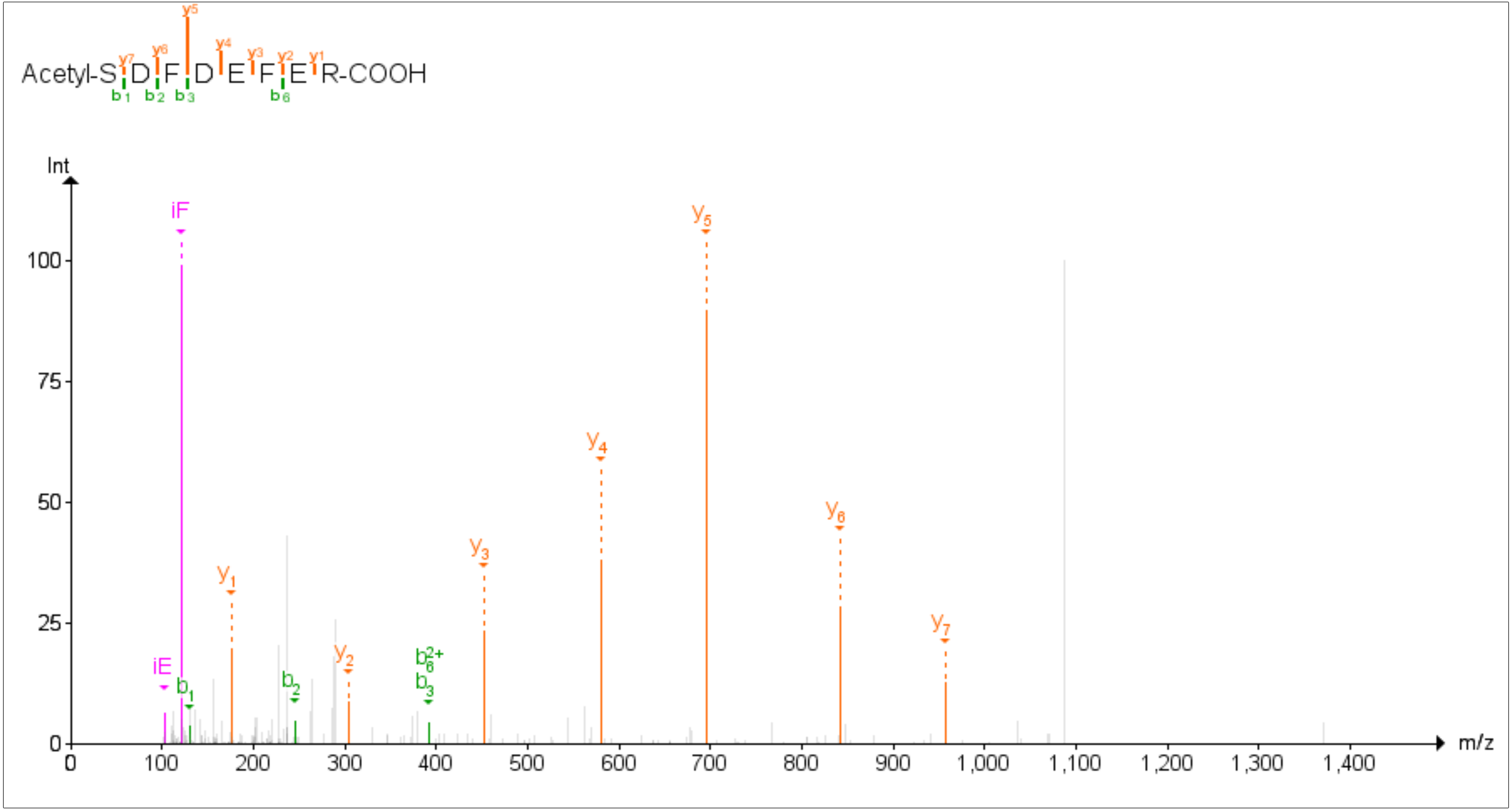
Acetyl-S D F D E F E R-COOH
y7 y6 y5 y4 y3 y2 y1
b1 b2 b3 b6
Int
iF
y5
100
75
50
25
0
y4
y6
y3
y1
y7
y2
iE
b1
b2
b6 2+
b3
0 100 200 300 400 500 600 700 800 900 1,000 1,100 1,200 1,300 1,400
m/z

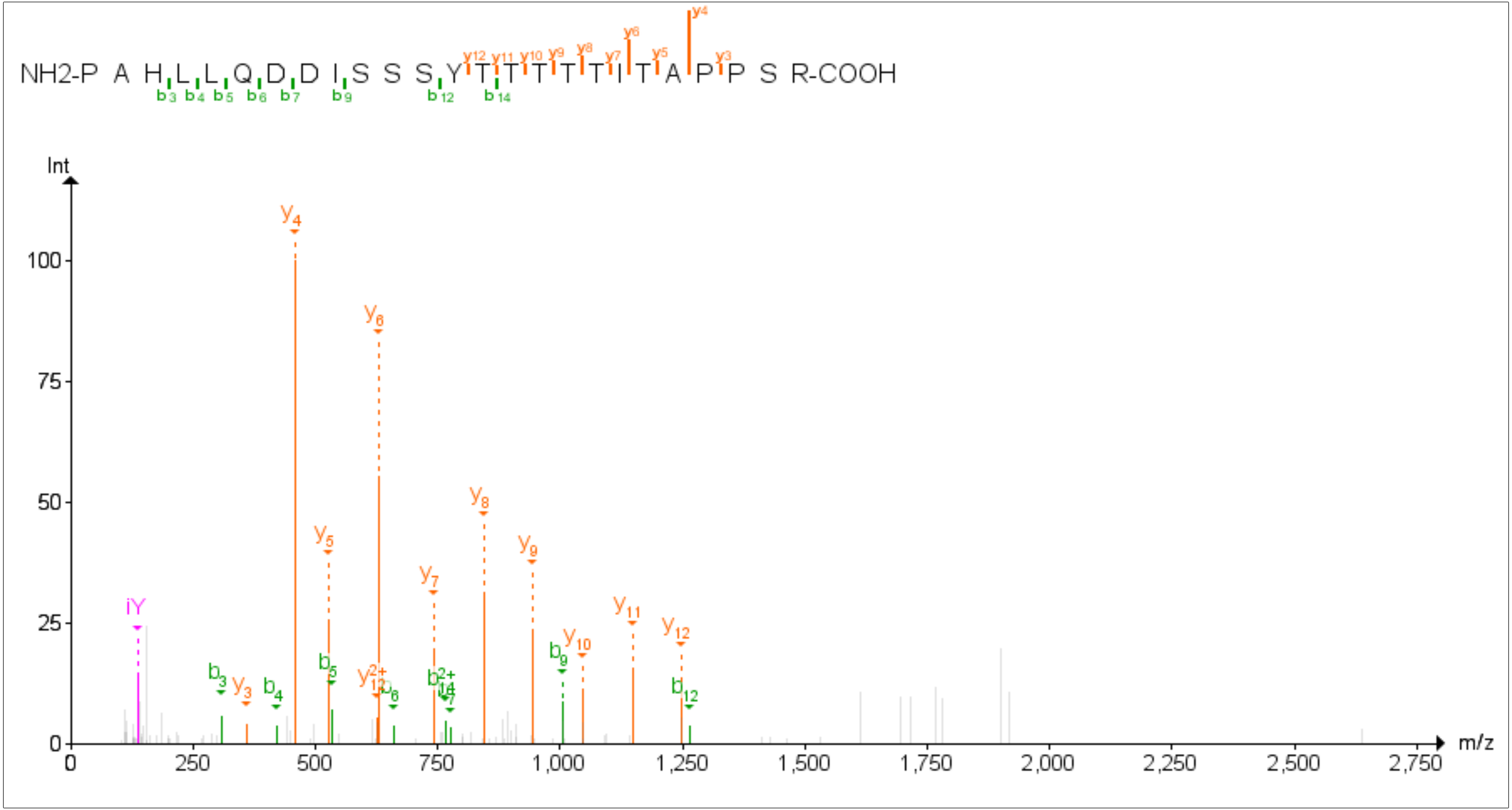

NH2-P A H L L Q D D I S S S Y T T T T T I T A P P S R-COOH
b3 b4 b5 b6 b7 b9 b12 b14
y12 y11 y10 y9 y8 y7 y6 y5 y4 y3
Int
m/z
0
25
50
75
100
250
500
750
1,000
1,250
1,500
1,750
2,000
2,250
2,500
2,750
iY
y4
y6
y5
y8
y9
y7
y11
y12
y10
y3
y12 2+
b3
b4
b5
b6
b9
b12
b14 2+
b7

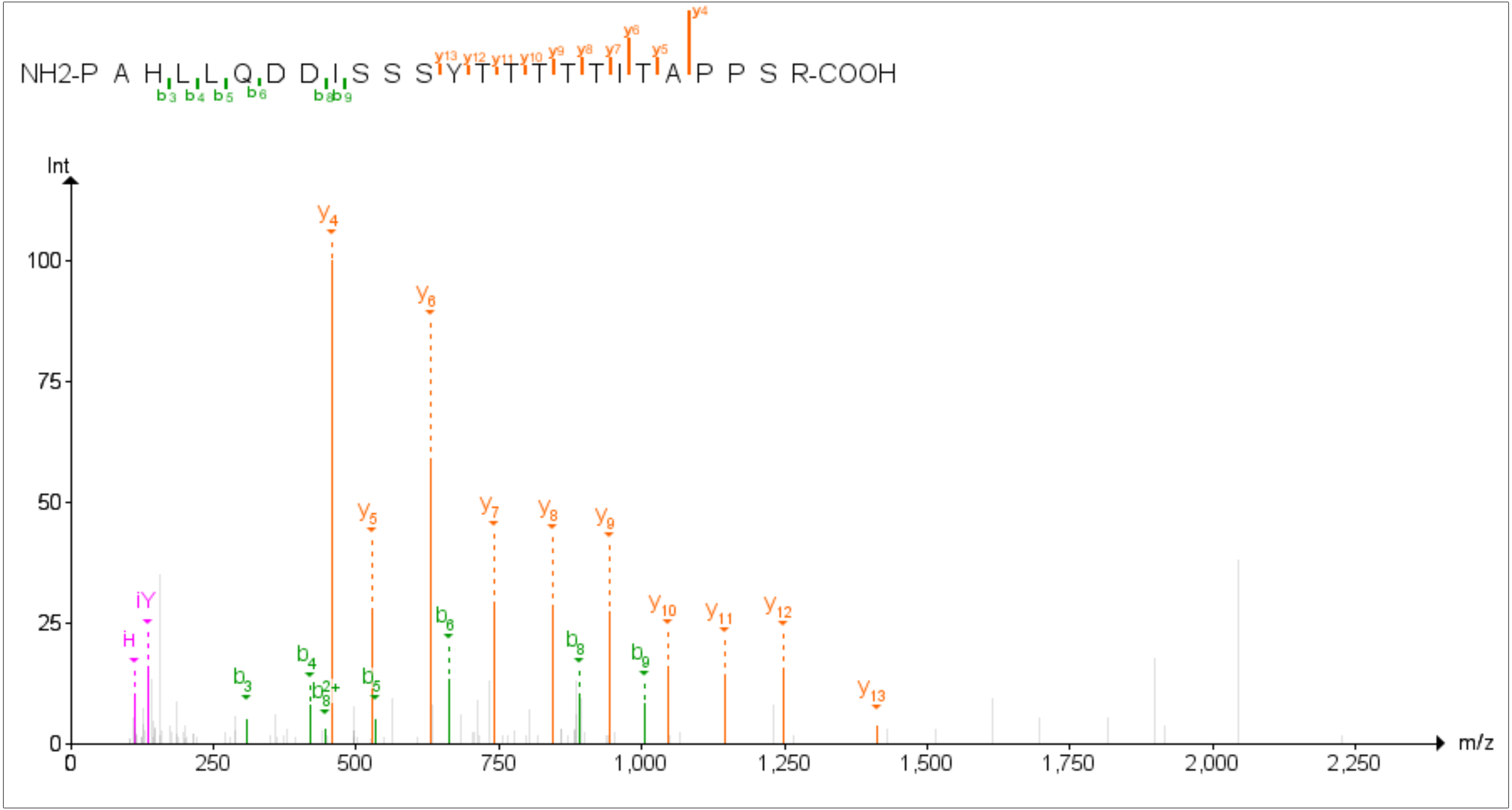
NH2-P A H L L Q D D I S S S Y T T T T T I T A P P S R-COOH
Int
m/z
y4
y6
y5
y7
y8
y9
y10
y11
y12
y13
b3
b4
b5
b6
b8
b9
b8 2+
iY
H
0
25
50
75
100
0
250
500
750
1,000
1,250
1,500
1,750
2,000
2,250

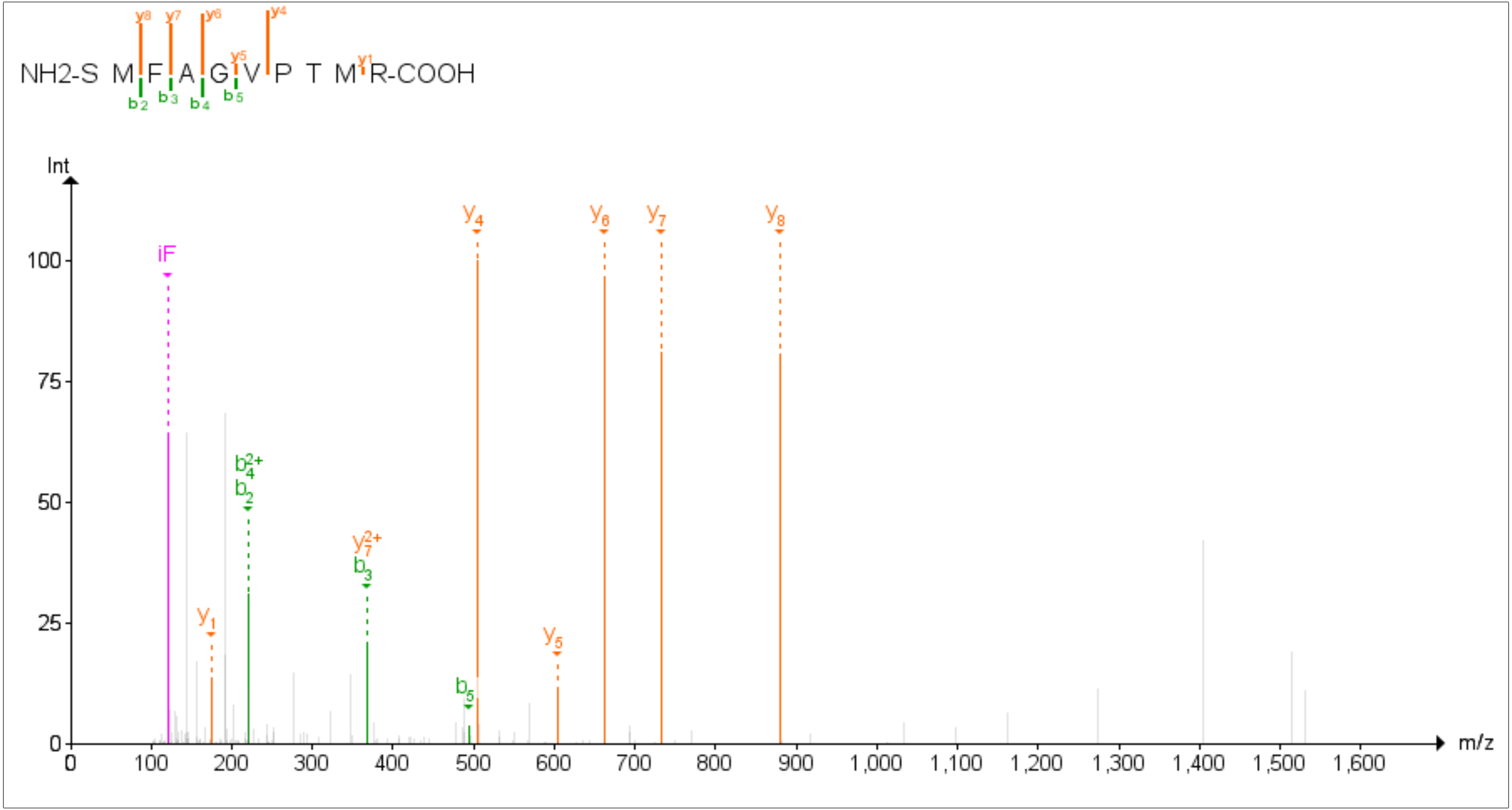

NH2-S M F A G V P T M R-COOH
y8 y7 y6 y5 y4 y1
b2 b3 b4 b5
Int
100
75
50
25
0
iF
y1
b4 2+
b2
y7 2+
b3
b5
y4
y5
y6
y7
y8
0 100 200 300 400 500 600 700 800 900 1,000 1,100 1,200 1,300 1,400 1,500 1,600
m/z

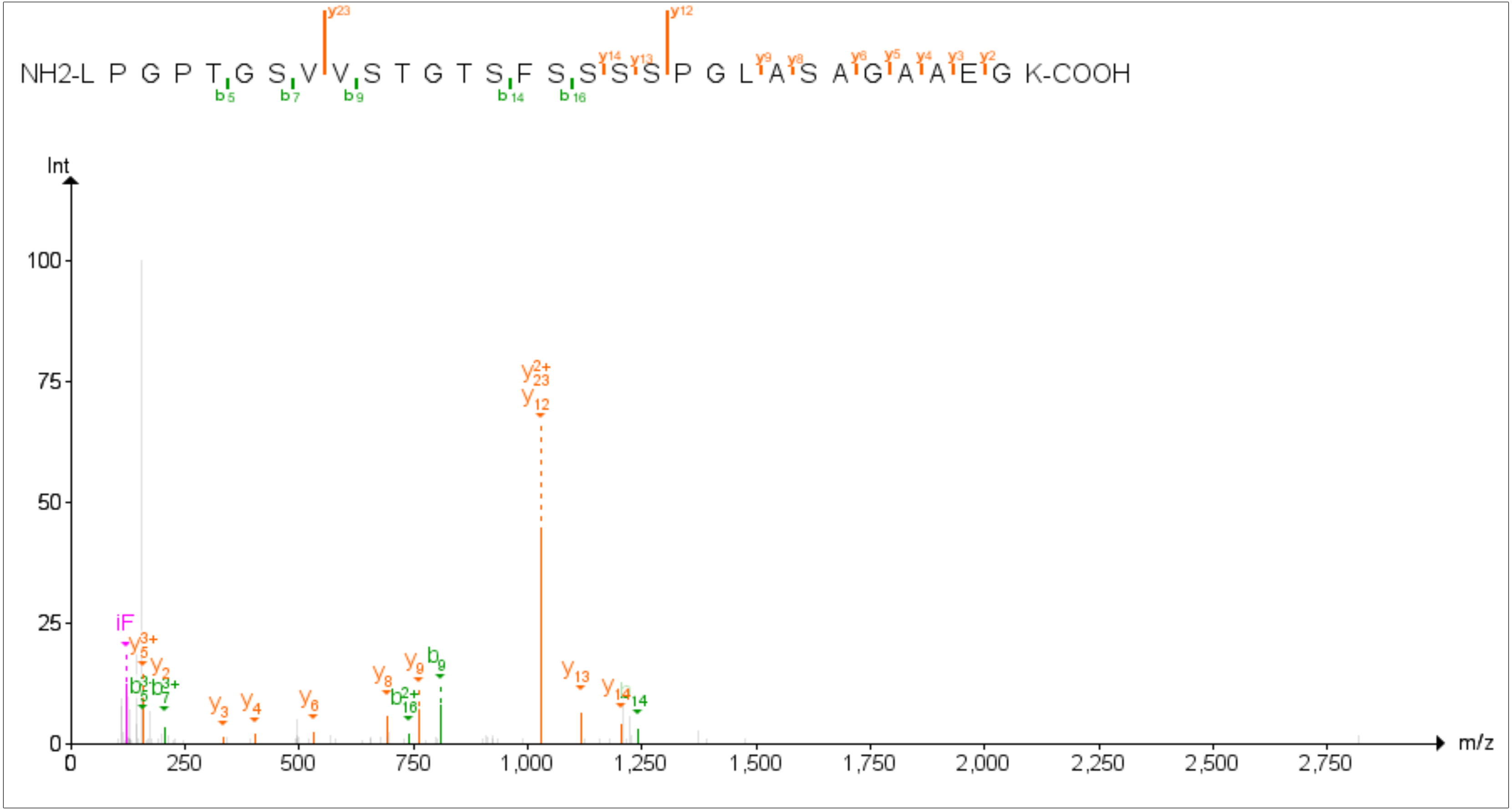

NH2-L P G P T G S V V S T G T S F S S S S P G L A S A G A A E G K-COOH
y23
y12
y14
y13
y9
y8
y6
y5
y4
y3
y2
b5
b7
b9
b14
b16
Int
100
75
50
25
0
iF
y23 2+
y12
y13
y14
y9
y8
y6
y4
y3
y2
b9
b14
m/z
0
250
500
750
1,000
1,250
1,500
1,750
2,000
2,250
2,500
2,750

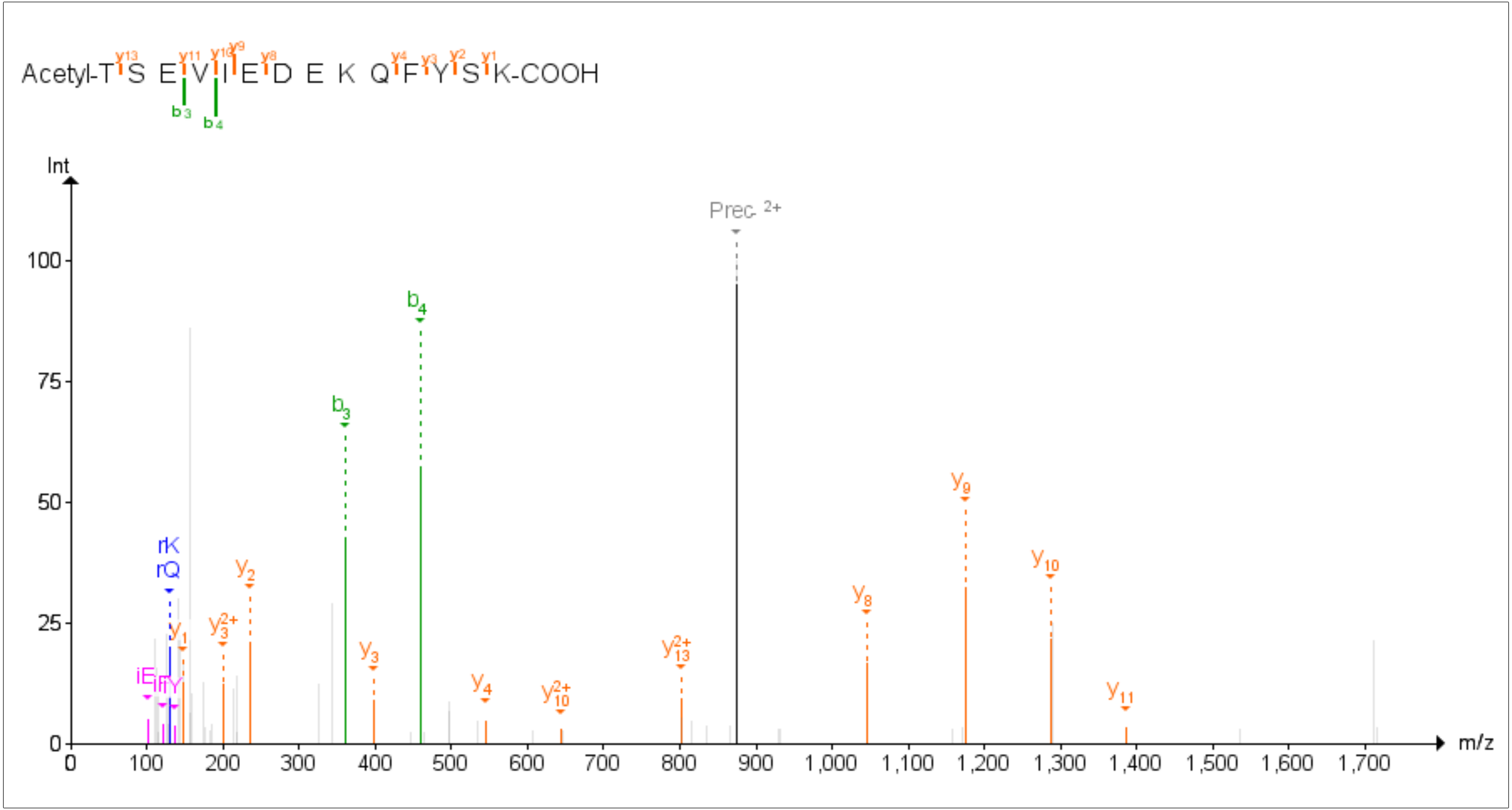

Acetyl-T S E V I E D E K Q F Y S K-COOH
y13 y11 y10 y9 y8 y4 y3 y2 y1
b3 b4
Int
Prec 2+
b4
b3
y9
y10
y8
rK
rQ
y2
y1
y3 2+
y3
y4
y10 2+
y13 2+
y11
iE
iI
iY
100
75
50
25
0
0 100 200 300 400 500 600 700 800 900 1,000 1,100 1,200 1,300 1,400 1,500 1,600 1,700
m/z

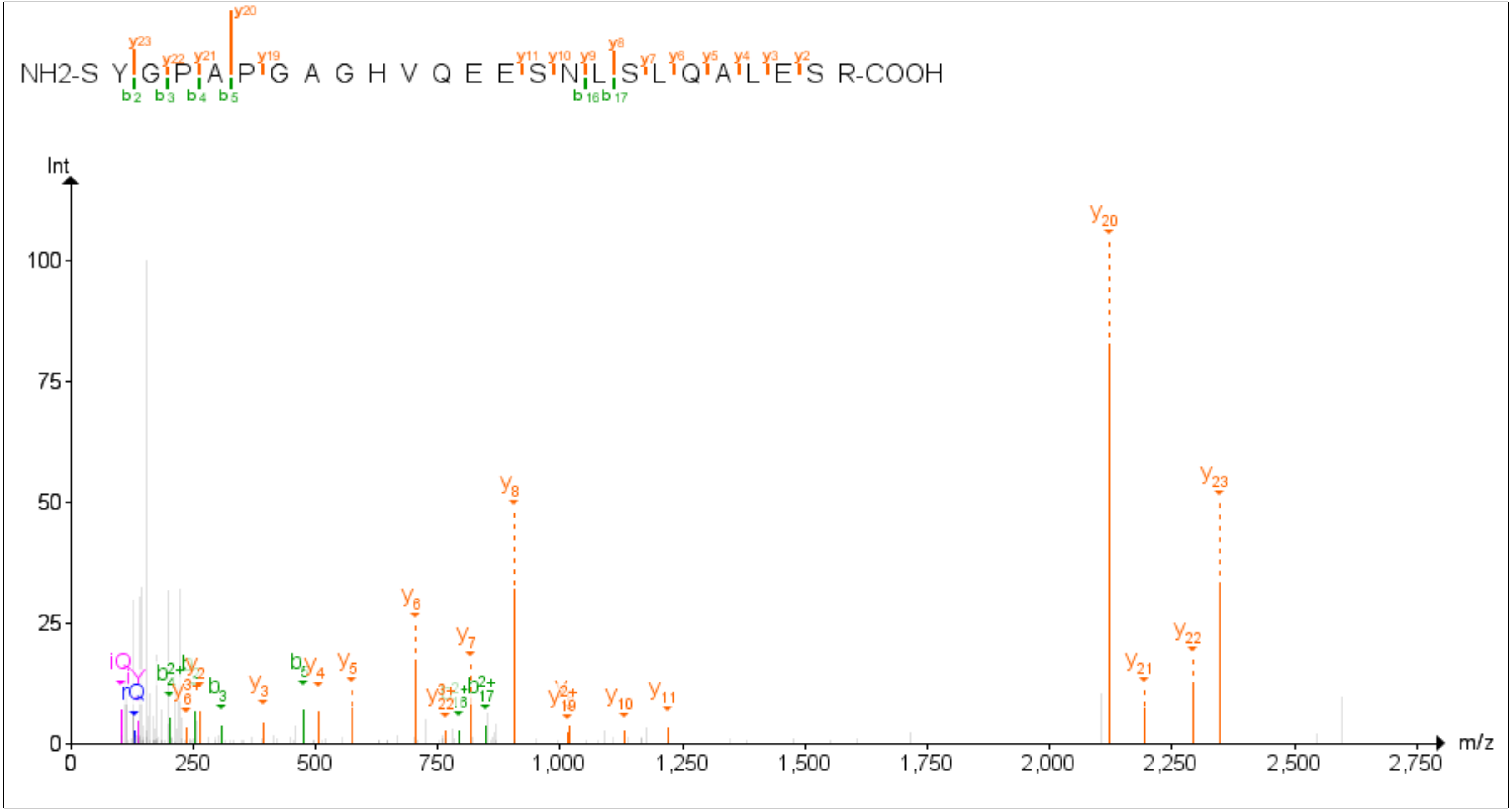
NH2-S Y G P A P G A G H V Q E E S N L S L Q A L E S R-COOH
y23 y22 y21 y20 y19 y11 y10 y9 y8 y7 y6 y5 y4 y3 y2
b2 b3 b4 b5 b16 b17
Int
100
75
50
25
0
m/z
0
250
500
750
1,000
1,250
1,500
1,750
2,000
2,250
2,500
2,750
y20
y23
y8
y6
y7
y22
y21
y5
y4
y3
y2
y10
y11
b3
iQ
rQ

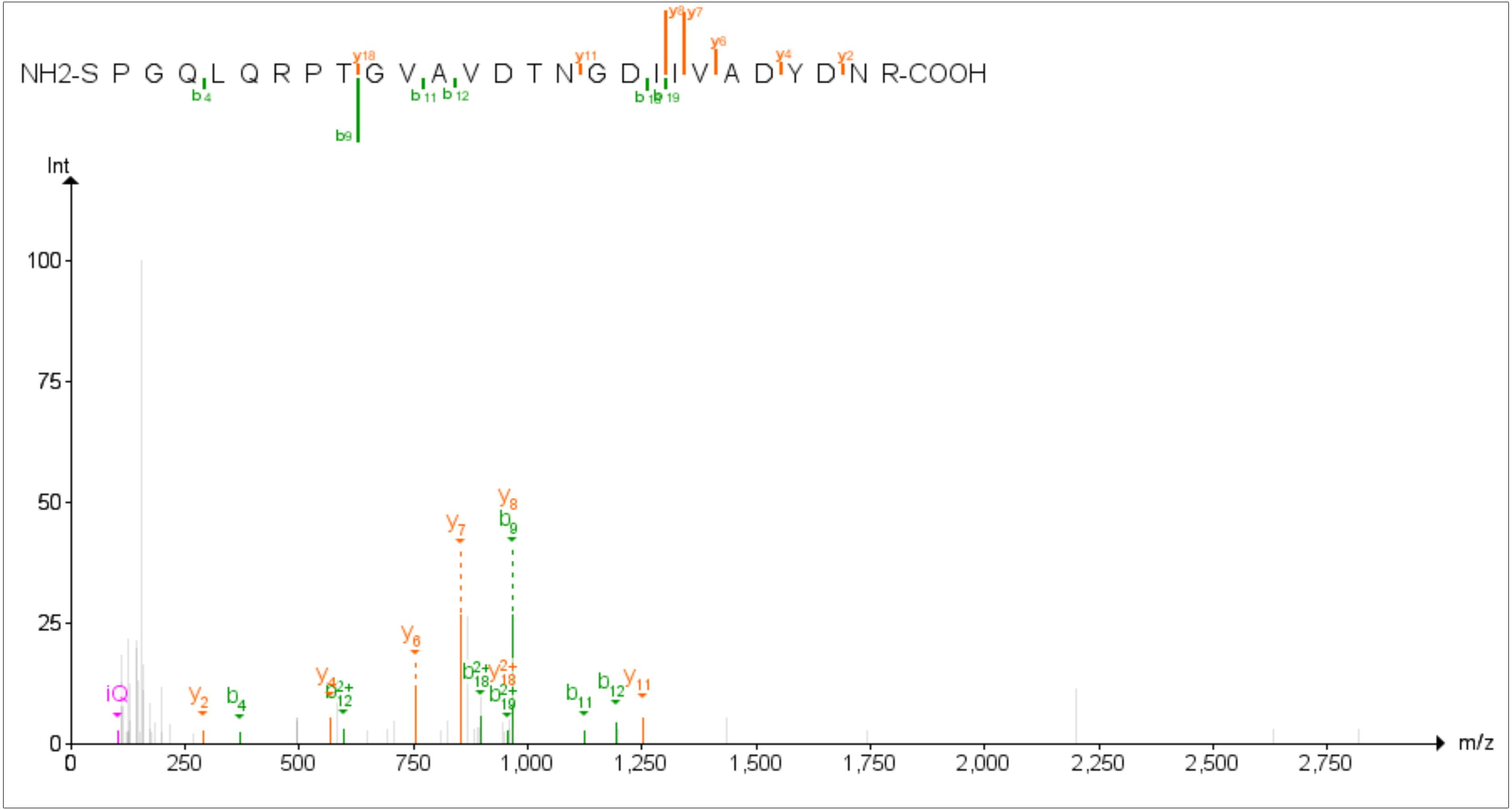
NH2-S P G Q L Q R P T G V A V D T N G D I I V A D Y D N R-COOH
Int
100
75
50
25
0
m/z
0
250
500
750
1,000
1,250
1,500
1,750
2,000
2,250
2,500
2,750
iQ
y2
b4
y4
b12 2+
y6
y7
b18 2+
y18 2+
y8
b9
b19 2+
b11
b12
y11

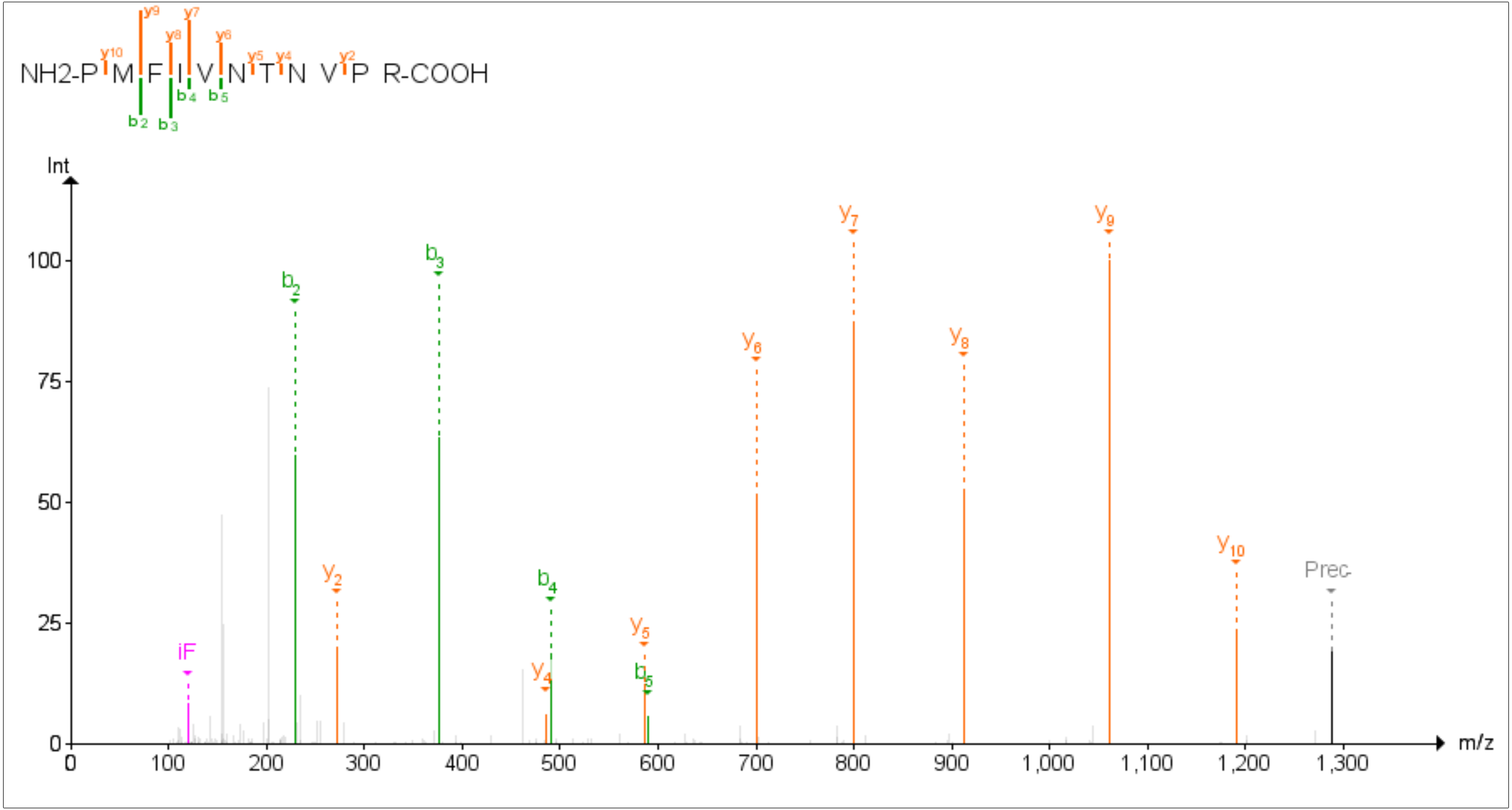

NH2-P M F I V N T N V P R-COOH
y10 y9 y8 y7 y6 y5 y4 y2
b2 b3 b4 b5
Int
m/z
iF
Prec

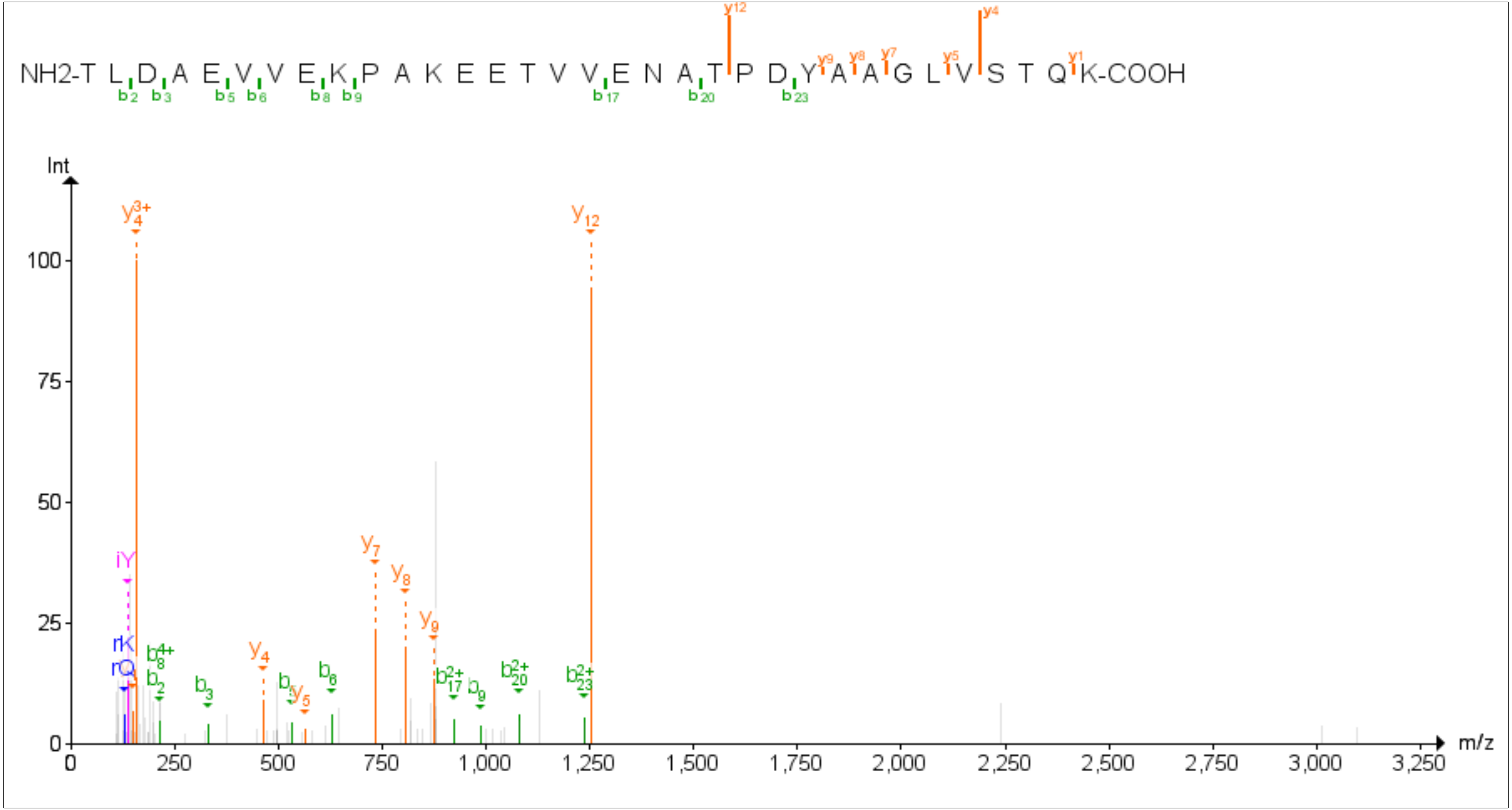

NH2-T L D A E V V E K P A K E E T V V E N A T P D Y A A G L V S T Q K-COOH
Int
m/z

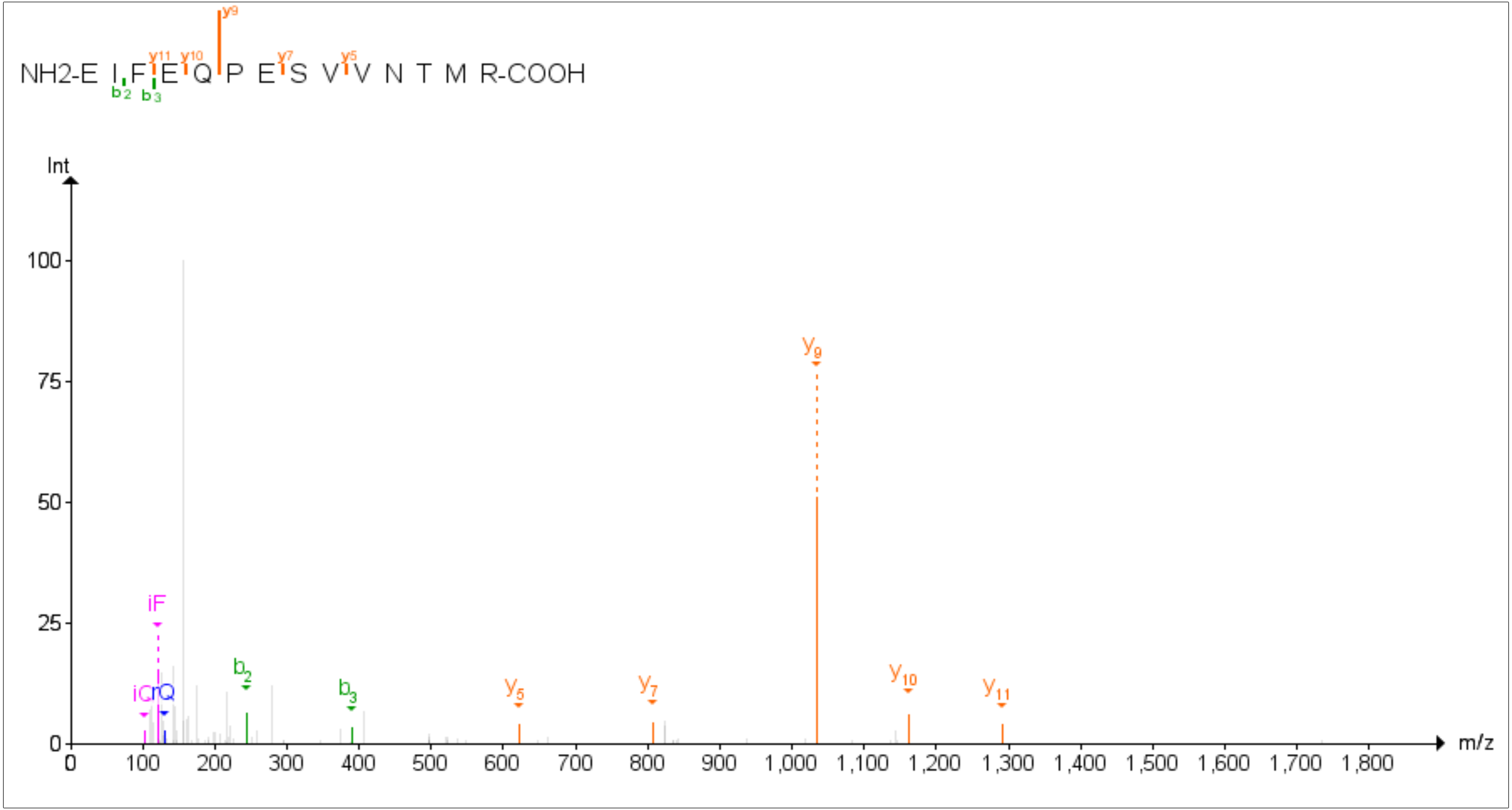
NH2-E I F E Q P E S V V N T M R-COOH
y9
y11 y10 y7 y5
b2 b3
Int
100
75
50
25
0
iF
iC
rQ
b2
b3
y5
y7
y9
y10
y11
0 100 200 300 400 500 600 700 800 900 1,000 1,100 1,200 1,300 1,400 1,500 1,600 1,700 1,800
m/z

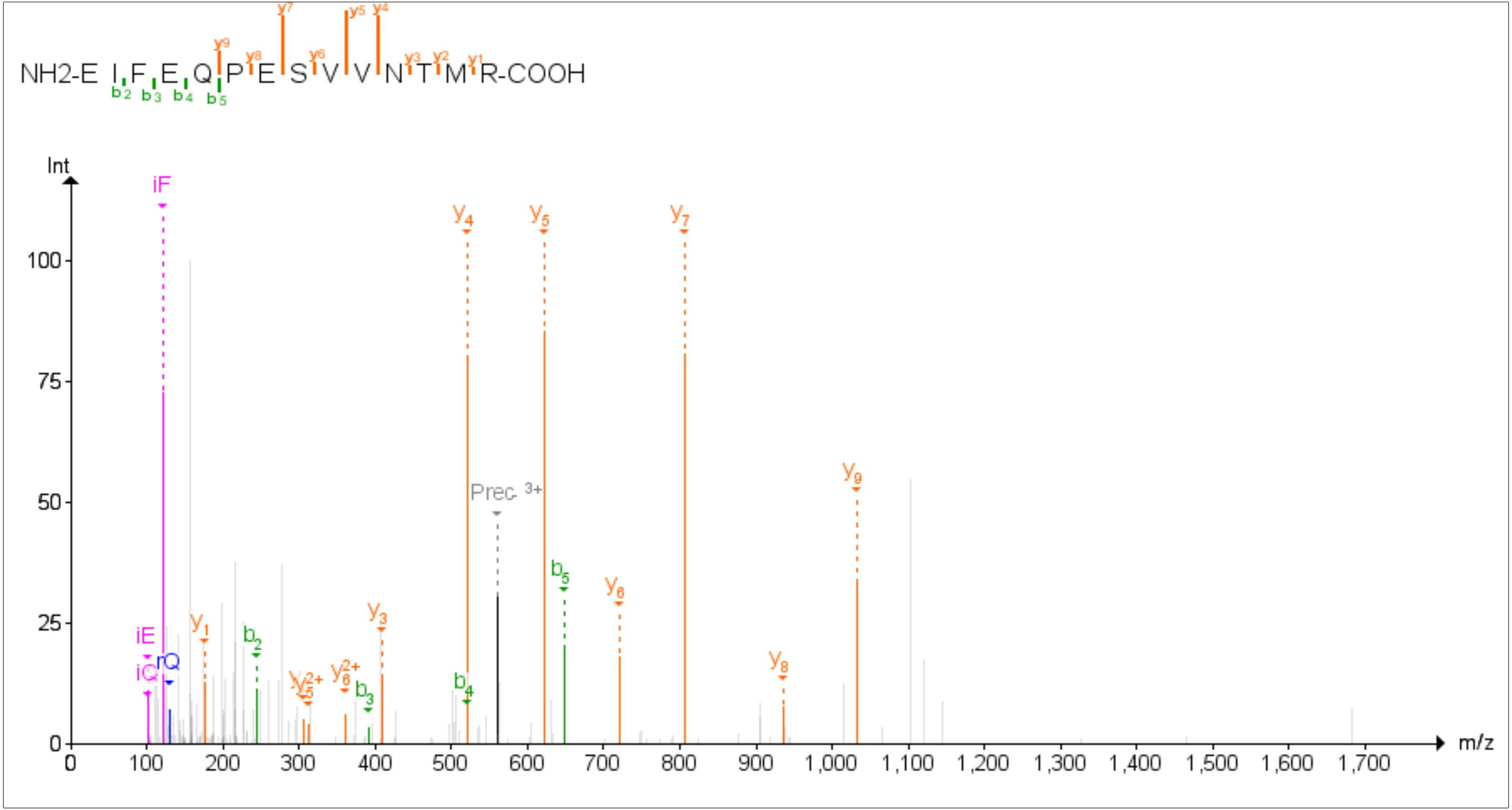

NH2-E I F E Q P E S V V N T M R-COOH
b2
b3
b4
b5
y9
y8
y7
y6
y5
y4
y3
y2
y1
Int
iF
y4
y5
y7
y9
Prec 3+
b5
y6
y3
y1
b2
iE
iQ
rQ
y5 2+
y6 2+
b3
b4
y8
0
25
50
75
100
0
100
200
300
400
500
600
700
800
900
1,000
1,100
1,200
1,300
1,400
1,500
1,600
1,700
m/z

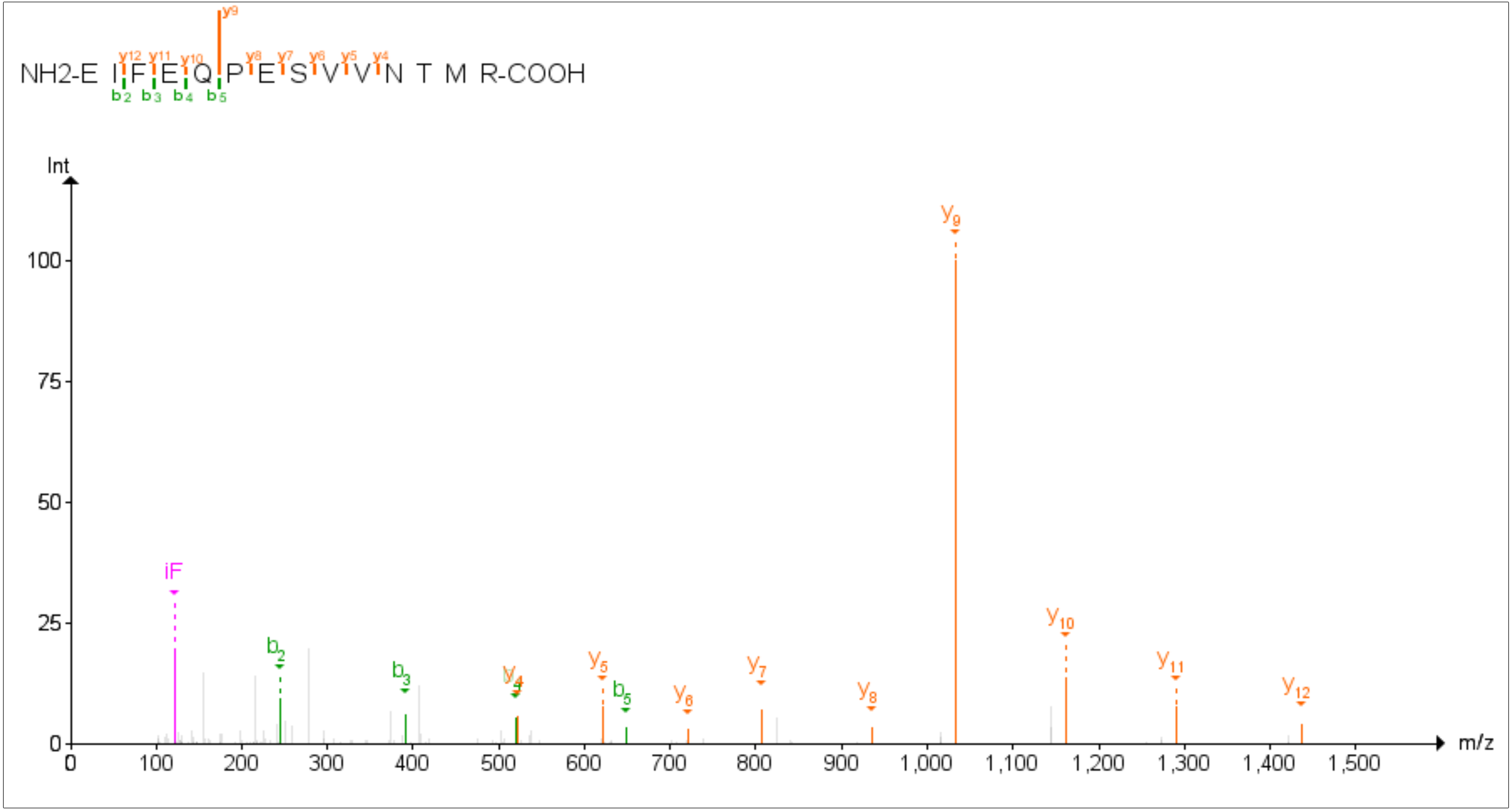
NH2-E I F E Q P E S V V N T M R-COOH
y9
y12 y11 y10 y8 y7 y6 y5 y4
b2 b3 b4 b5
Int
100
75
50
25
0
iF
b2
b3
y4
y5
b5
y6
y7
y8
y9
y10
y11
y12
0 100 200 300 400 500 600 700 800 900 1,000 1,100 1,200 1,300 1,400 1,500
m/z

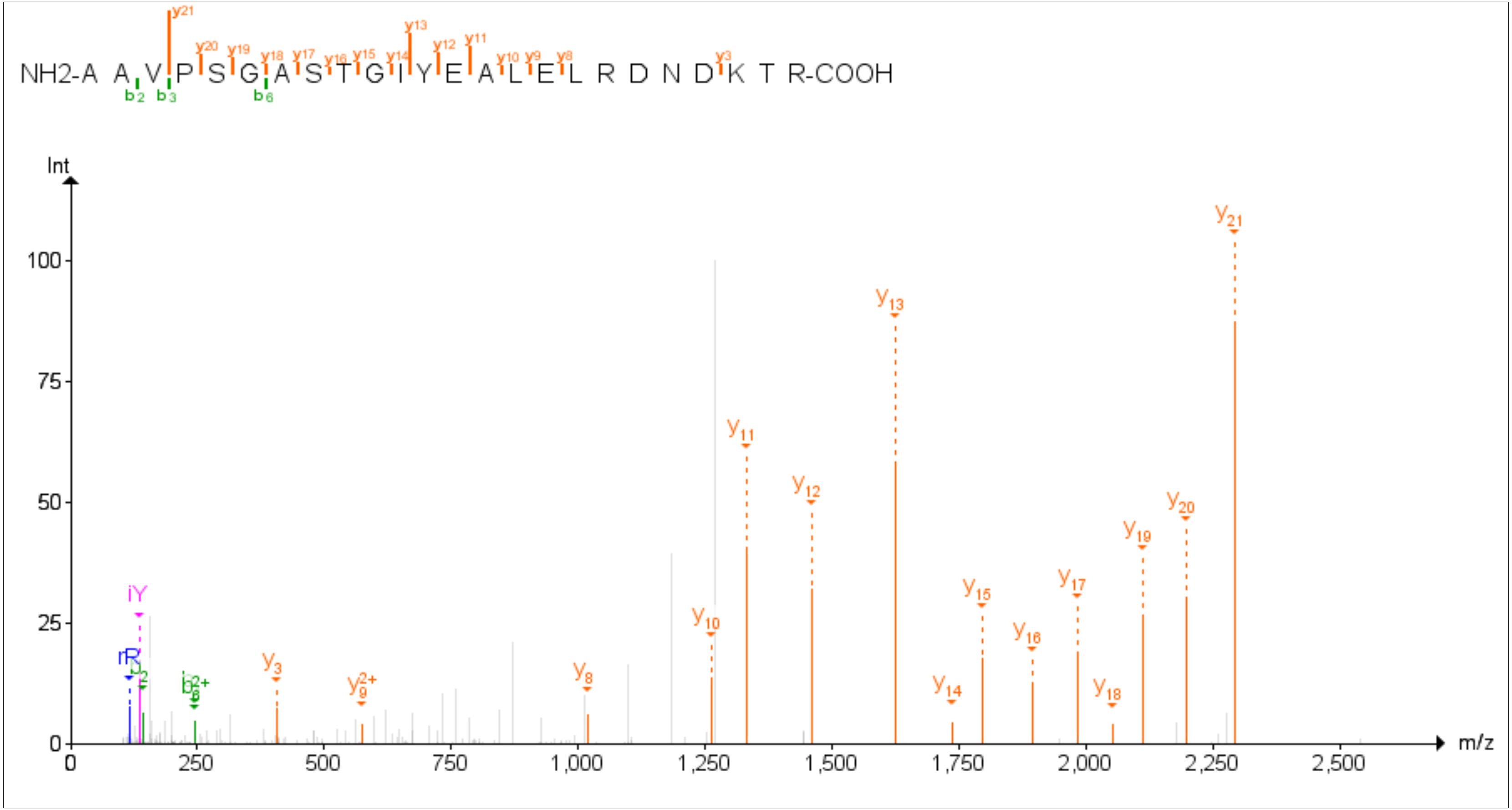

NH2-A A V P S G A S T G I Y E A L E L R D N D K T R-COOH
y21 y20 y19 y18 y17 y16 y15 y14 y13 y12 y11 y10 y9 y8 y3
b2 b3 b6
Int
100
75
50
25
0
m/z
0
250
500
750
1,000
1,250
1,500
1,750
2,000
2,250
2,500
iY
rR
y3
y8
y10
y11
y12
y13
y14
y15
y16
y17
y18
y19
y20
y21

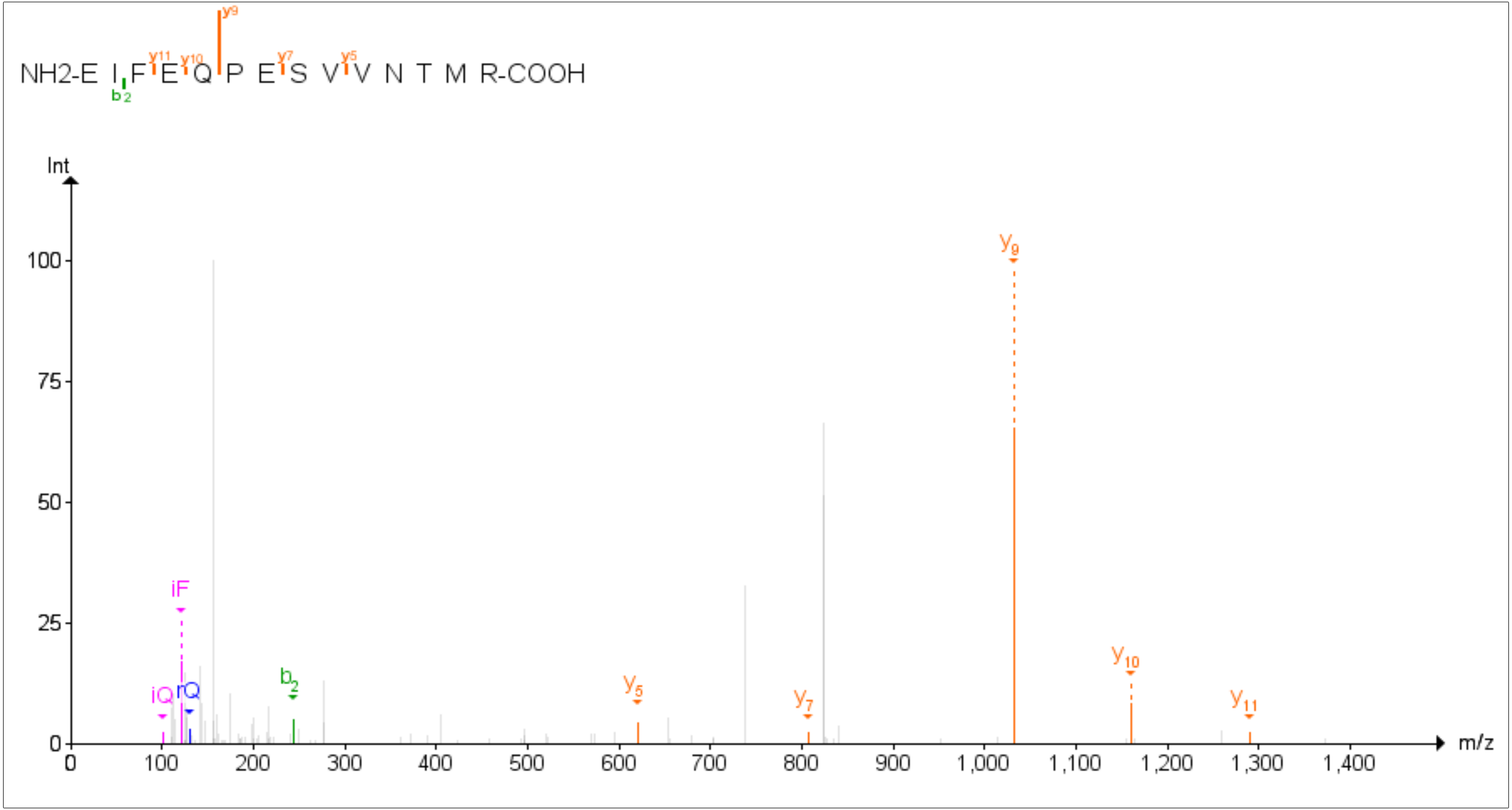

NH2-E I F E Q P E S V V N T M R-COOH
b2
y11
y10
y9
y7
y5
Int
100
75
50
25
0
m/z
0
100
200
300
400
500
600
700
800
900
1,000
1,100
1,200
1,300
1,400
iF
iQ
rQ
b2
y5
y7
y9
y10
y11

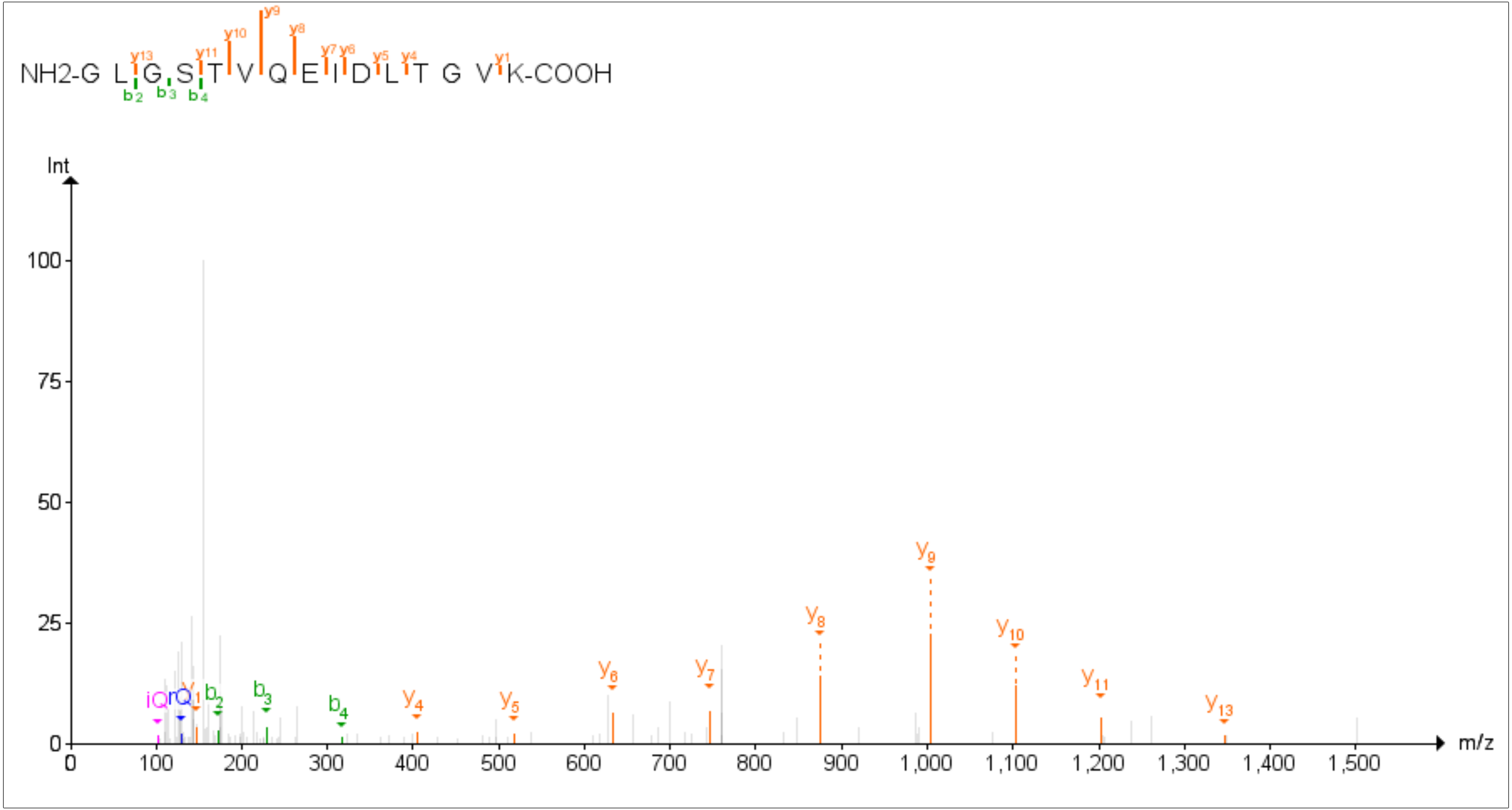
NH2-G L G S T V Q E I D L T G V K-COOH
Int
100
75
50
25
0
m/z
0
100
200
300
400
500
600
700
800
900
1,000
1,100
1,200
1,300
1,400
1,500
iQ
rQ
y1
b2
b3
b4
y4
y5
y6
y7
y8
y9
y10
y11
y13

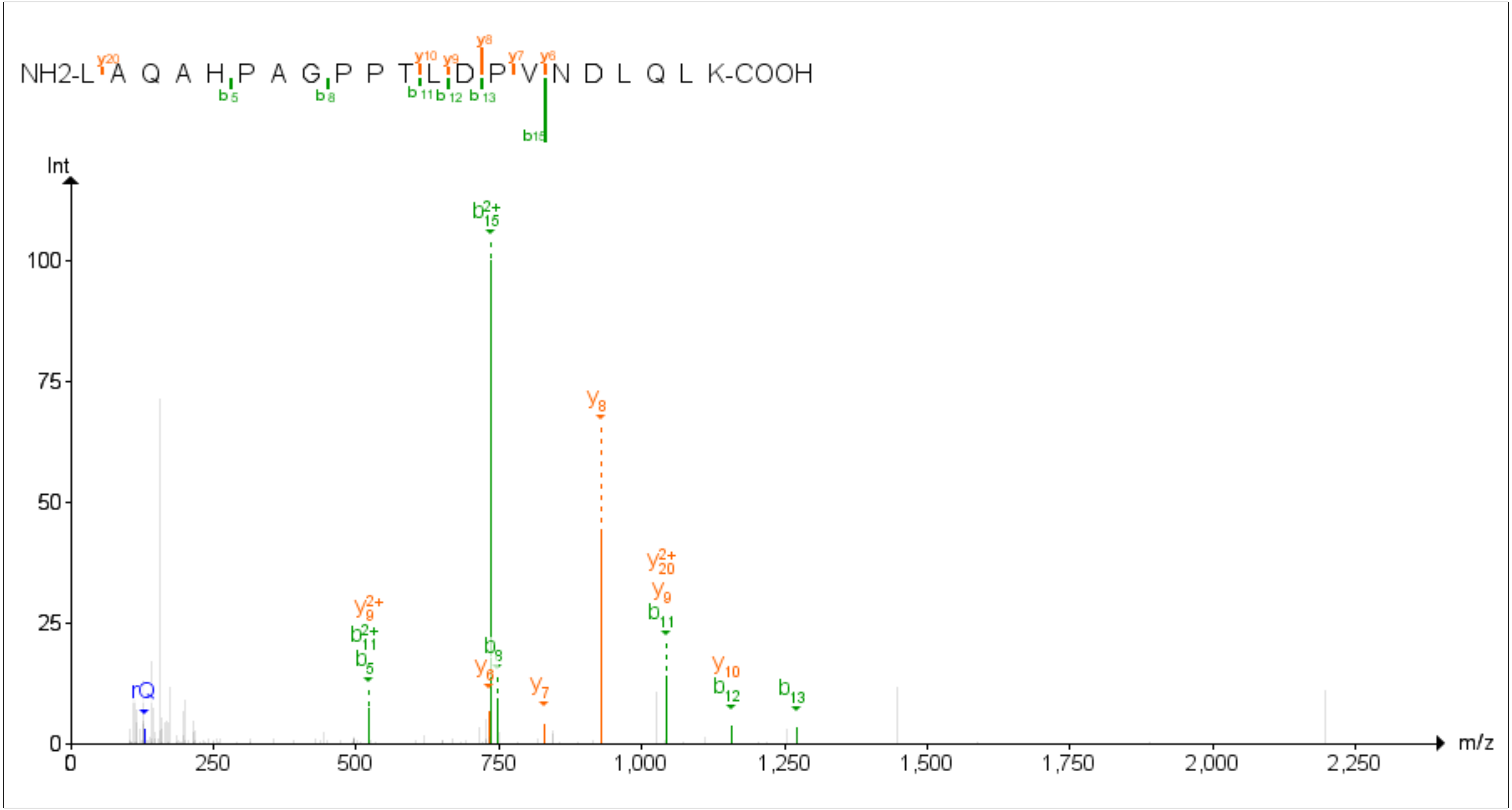

NH2-L A Q A H P A G P P T L D P V N D L Q L K-COOH
Int
m/z
0
25
50
75
100
250
500
750
1,000
1,250
1,500
1,750
2,000
2,250

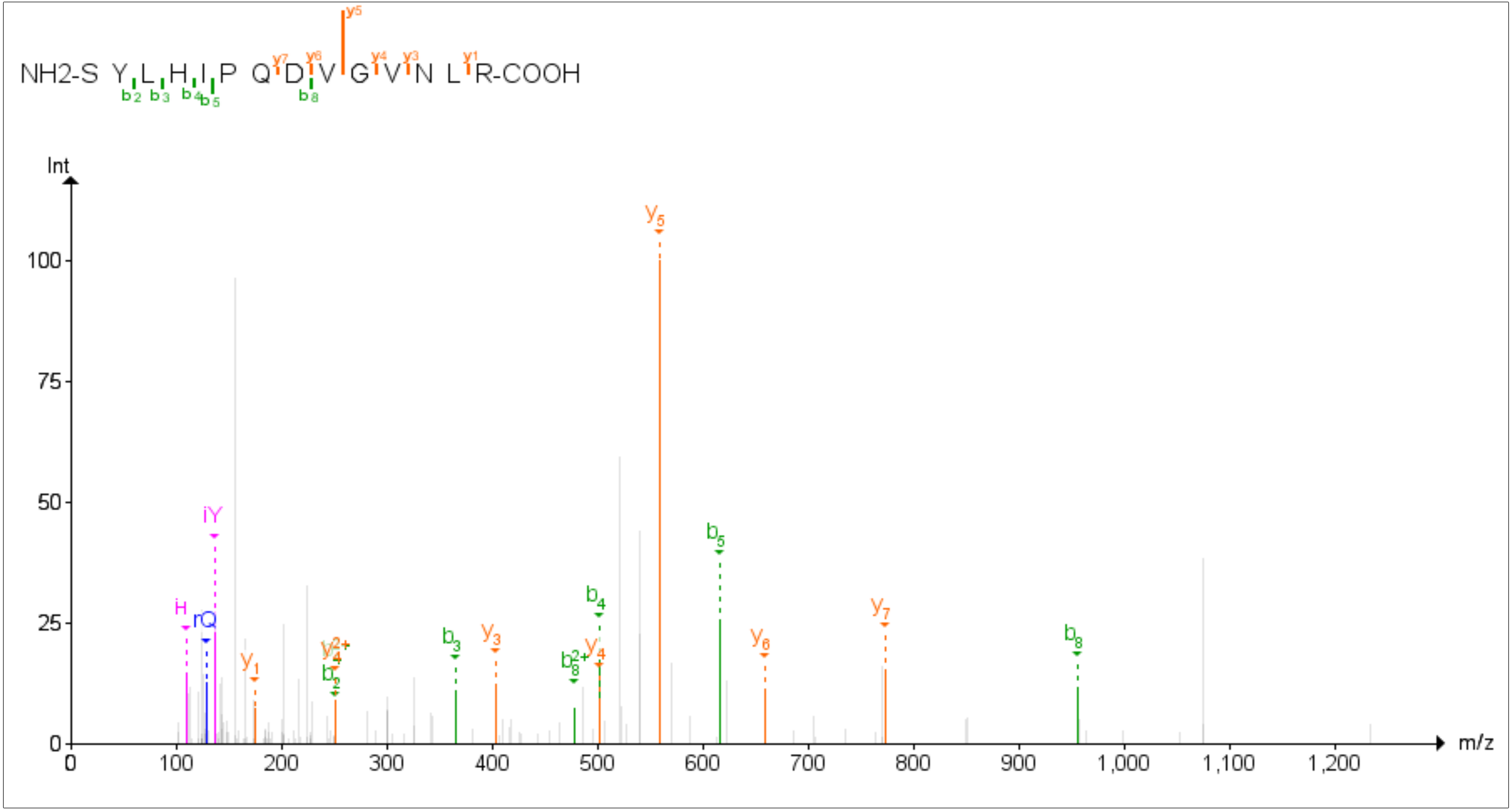

NH2-S Y L H I P Q D V G V N L R-COOH
Int
m/z
y5
b5
y7
b4
y3
b3
y6
b8
y4
y1
iY
iH
rQ

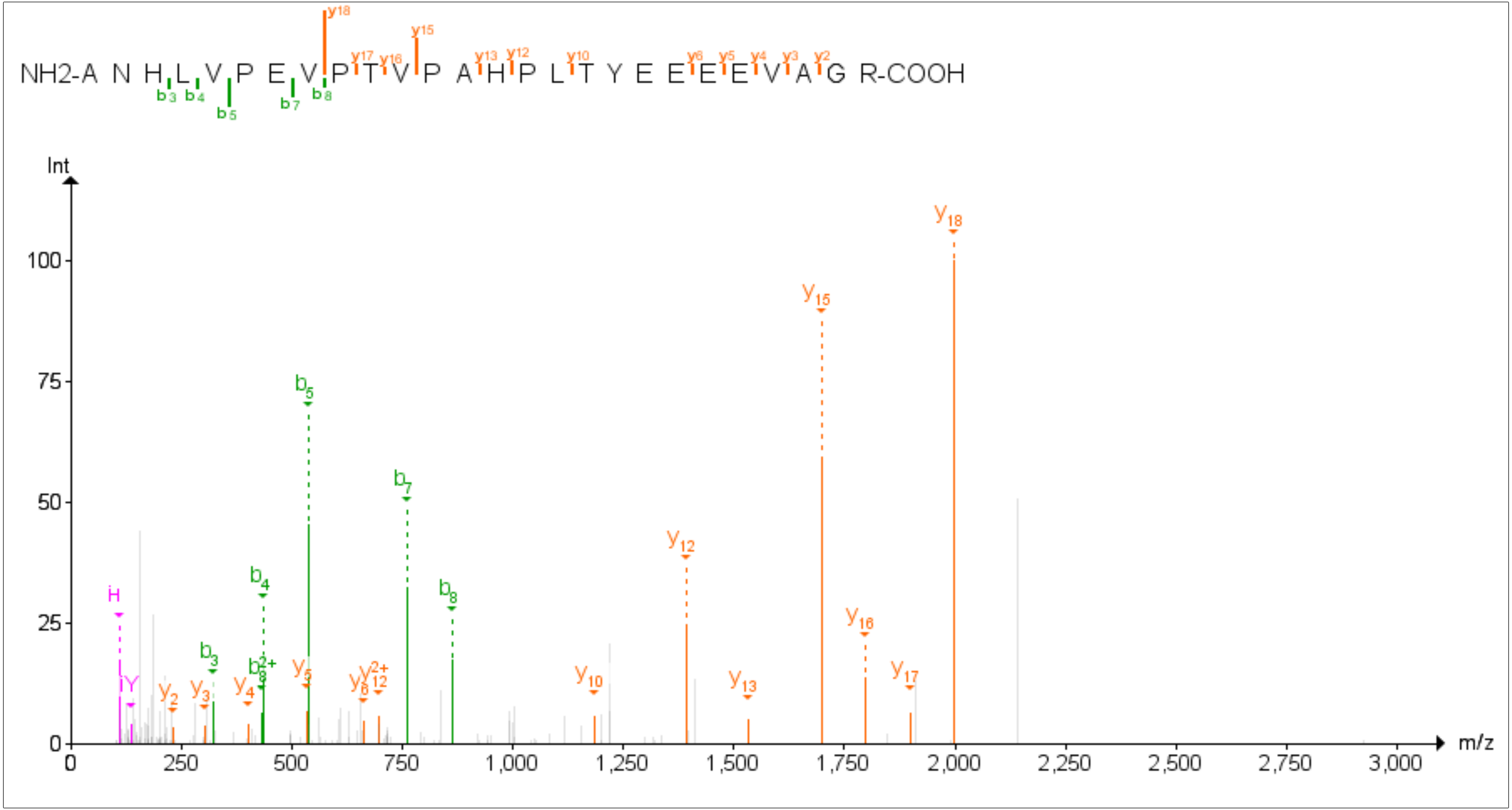

NH2-A N H L V P E V P T V P A H P L T Y E E E E V A G R-COOH
Int
m/z
0
25
50
75
100
250
500
750
1,000
1,250
1,500
1,750
2,000
2,250
2,500
2,750
3,000

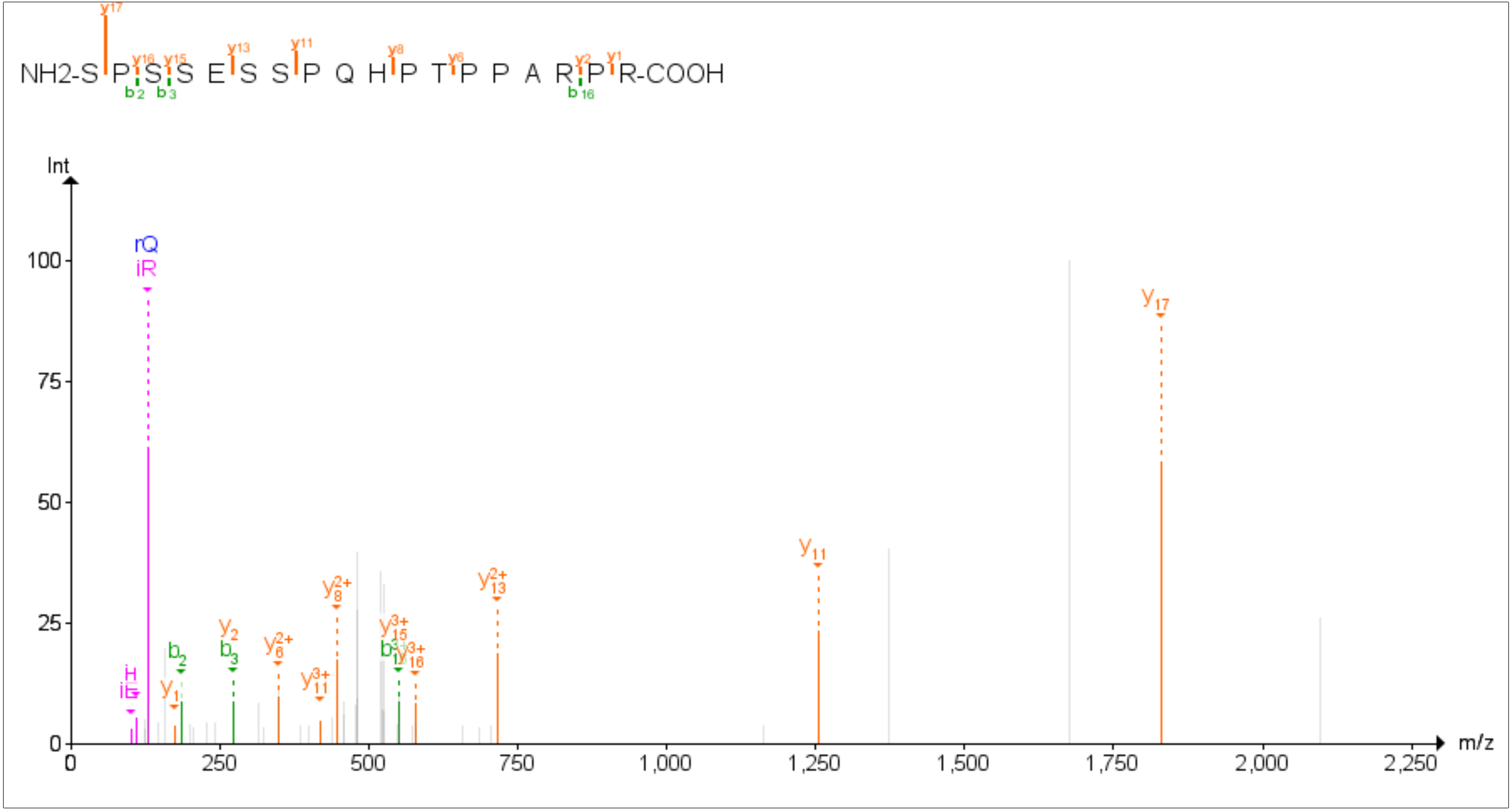

NH2-S P S S E S S P Q H P T P P A R P R-COOH
y17 y16 y15 y13 y11 y8 y6 y2 y1
b2 b3 b16
Int
m/z
0 25 50 75 100
0 250 500 750 1,000 1,250 1,500 1,750 2,000 2,250
rQ
iR
iH
iE
y1
b2
y2
b3
y6 2+
y11 3+
y8 2+
y15 3+
b17 3+
y16 3+
y13 2+
y11
y17

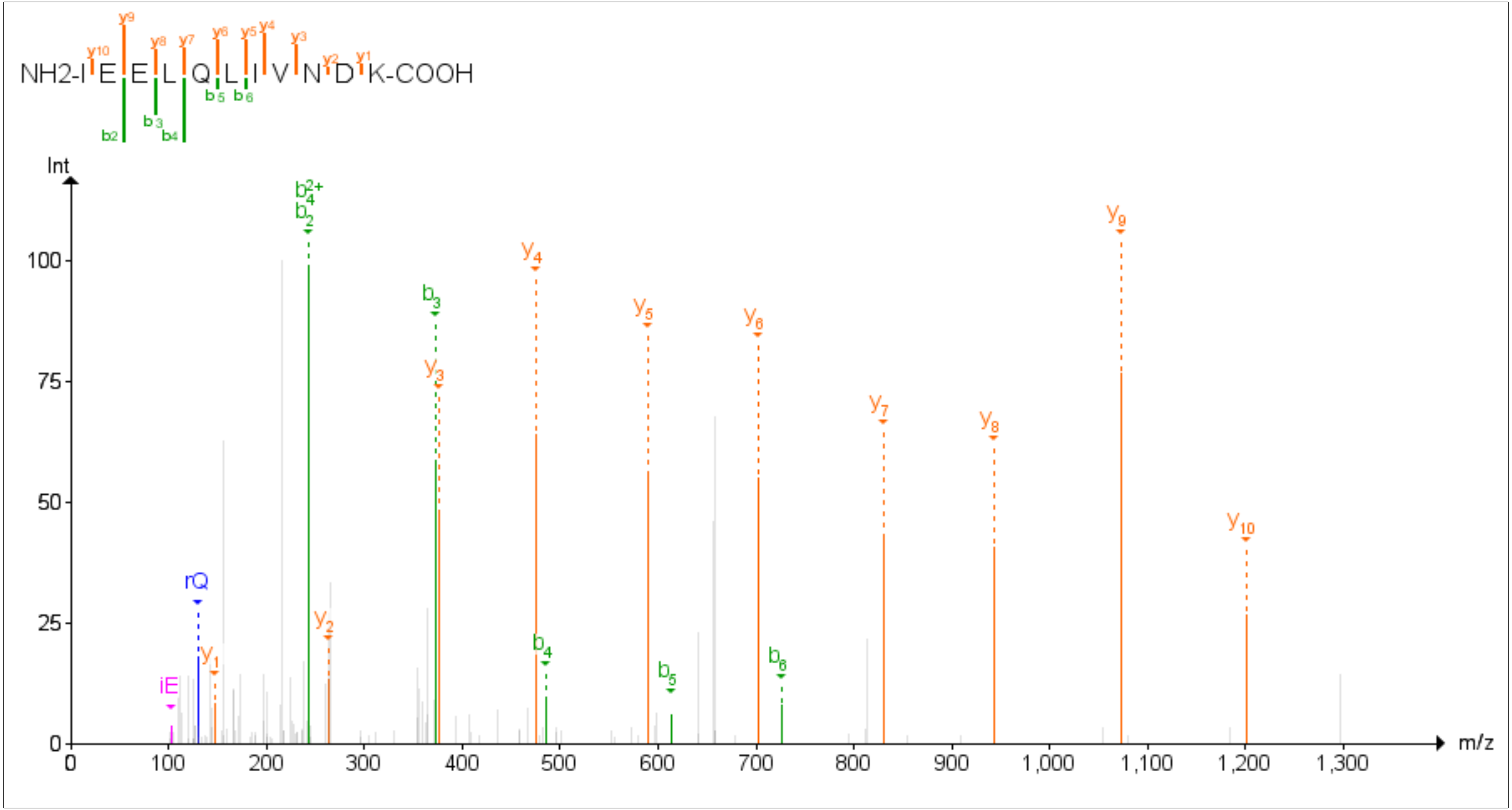

NH2-I E E L Q L I V N D K-COOH
y10 y9 y8 y7 y6 y5 y4 y3 y2 y1
b2 b3 b4 b5 b6
Int
m/z
0
25
50
75
100
0
100
200
300
400
500
600
700
800
900
1,000
1,100
1,200
1,300
iE
rQ
y1
y2
b2
b4 2+
b3
y3
y4
b4
y5
b5
y6
b6
y7
y8
y9
y10

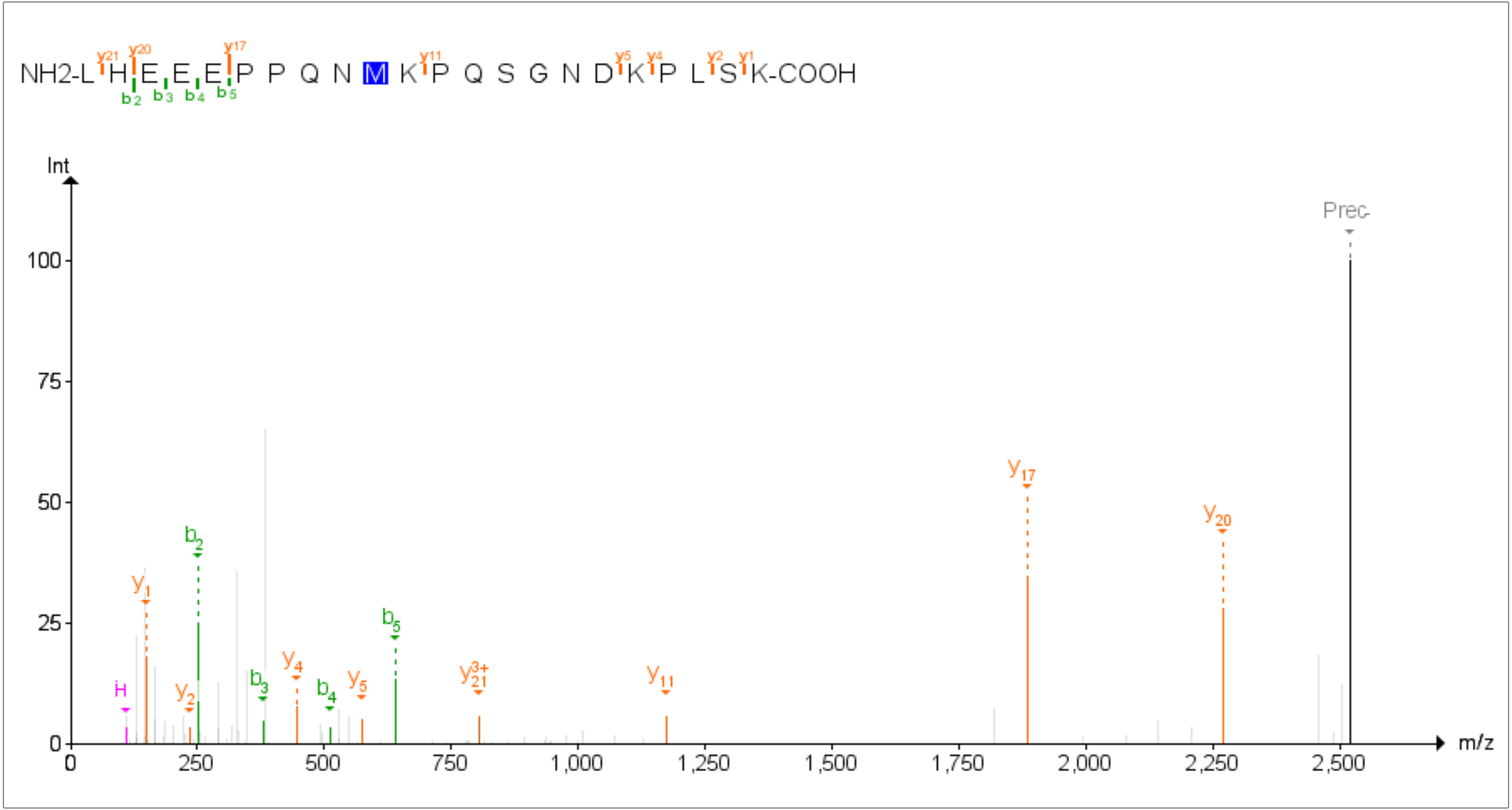

NH2-L H E E E P P Q N M K P Q S G N D K P L S K-COOH
y21 y20 y17 y11 y5 y4 y2 y1
b2 b3 b4 b5
Int
m/z
Prec.
100
75
50
25
0
0
250
500
750
1,000
1,250
1,500
1,750
2,000
2,250
2,500
H
y1
y2
b2
b3
y4
b4
y5
b5
y21 3+
y11
y17
y20

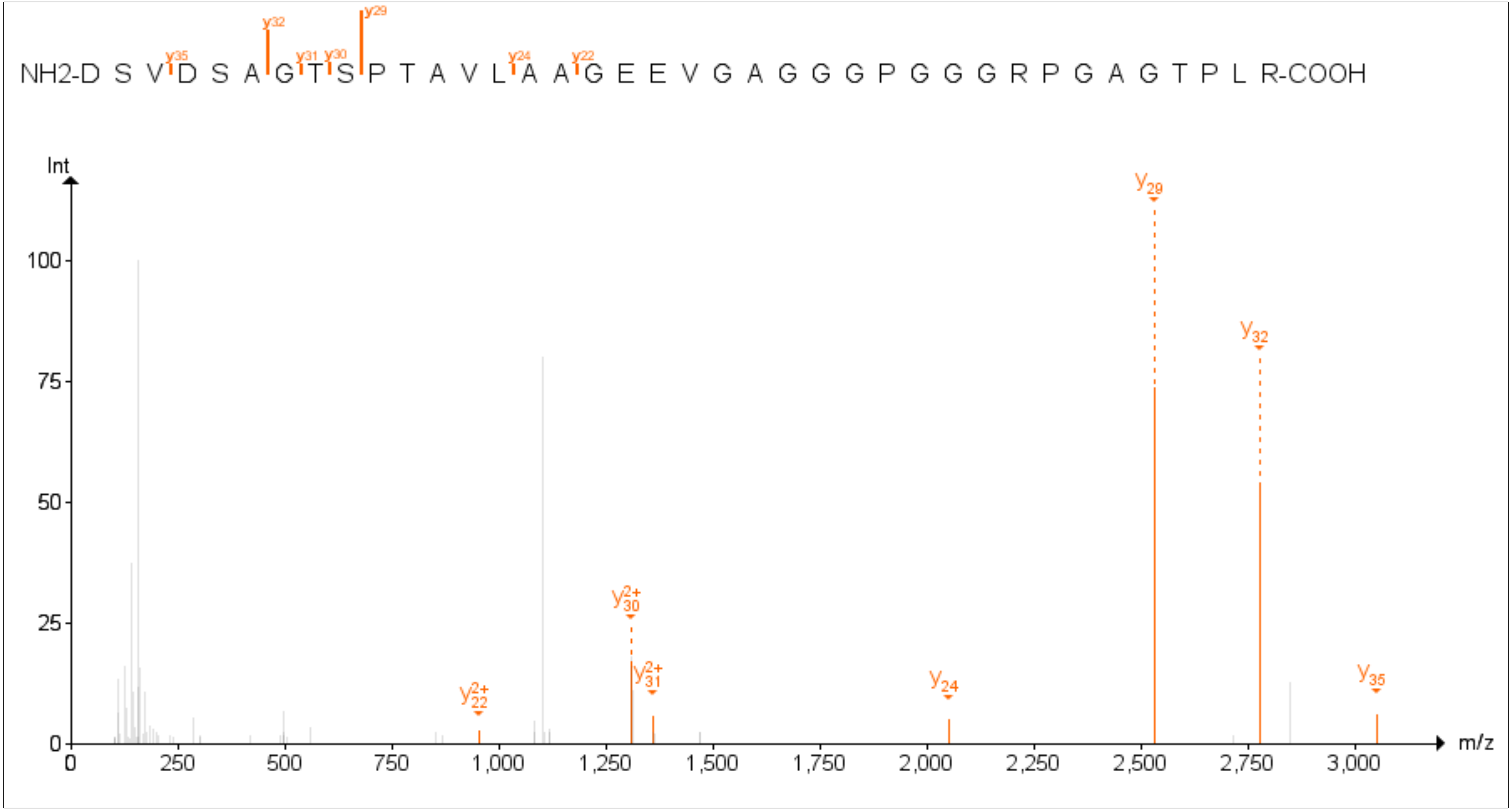
NH2-D S V D S A G T S P T A V L A A G E E V G A G G G P G G G R P G A G T P L R-COOH
y35
y32
y31
y30
y29
y24
y22
Int
100
75
50
25
0
m/z
0
250
500
750
1,000
1,250
1,500
1,750
2,000
2,250
2,500
2,750
3,000
y_{29}
y_{32}
y_{30}^{2+}
y_{31}^{2+}
y_{22}^{2+}
y_{24}
y_{35}

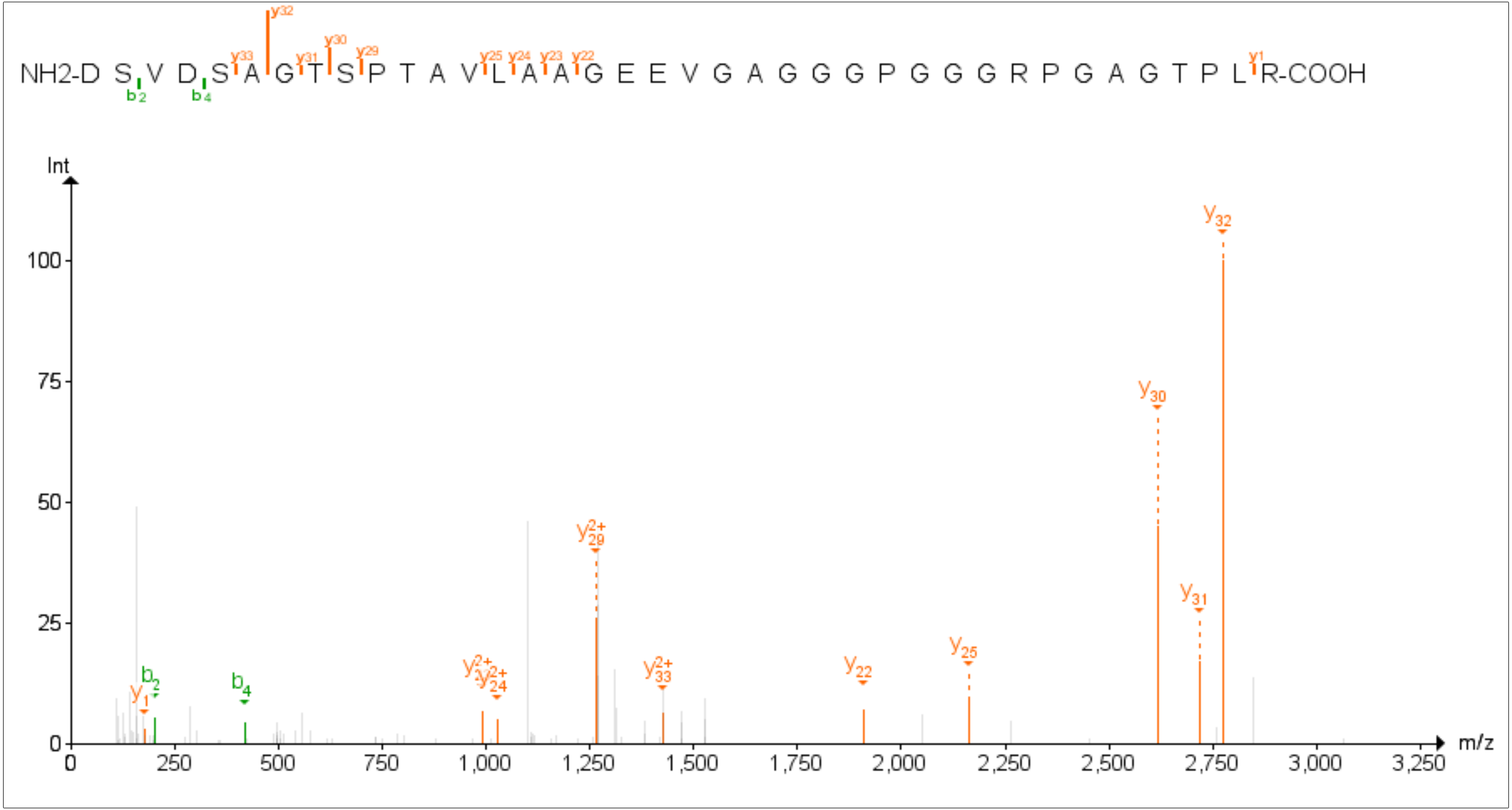

NH2-D S V D S A G T S P T A V L A A G E E V G A G G G P G G G R P G A G T P L R-COOH
Int
m/z
y32
y30
y31
y29
y25
y22
y33
y24
y1
b2
b4

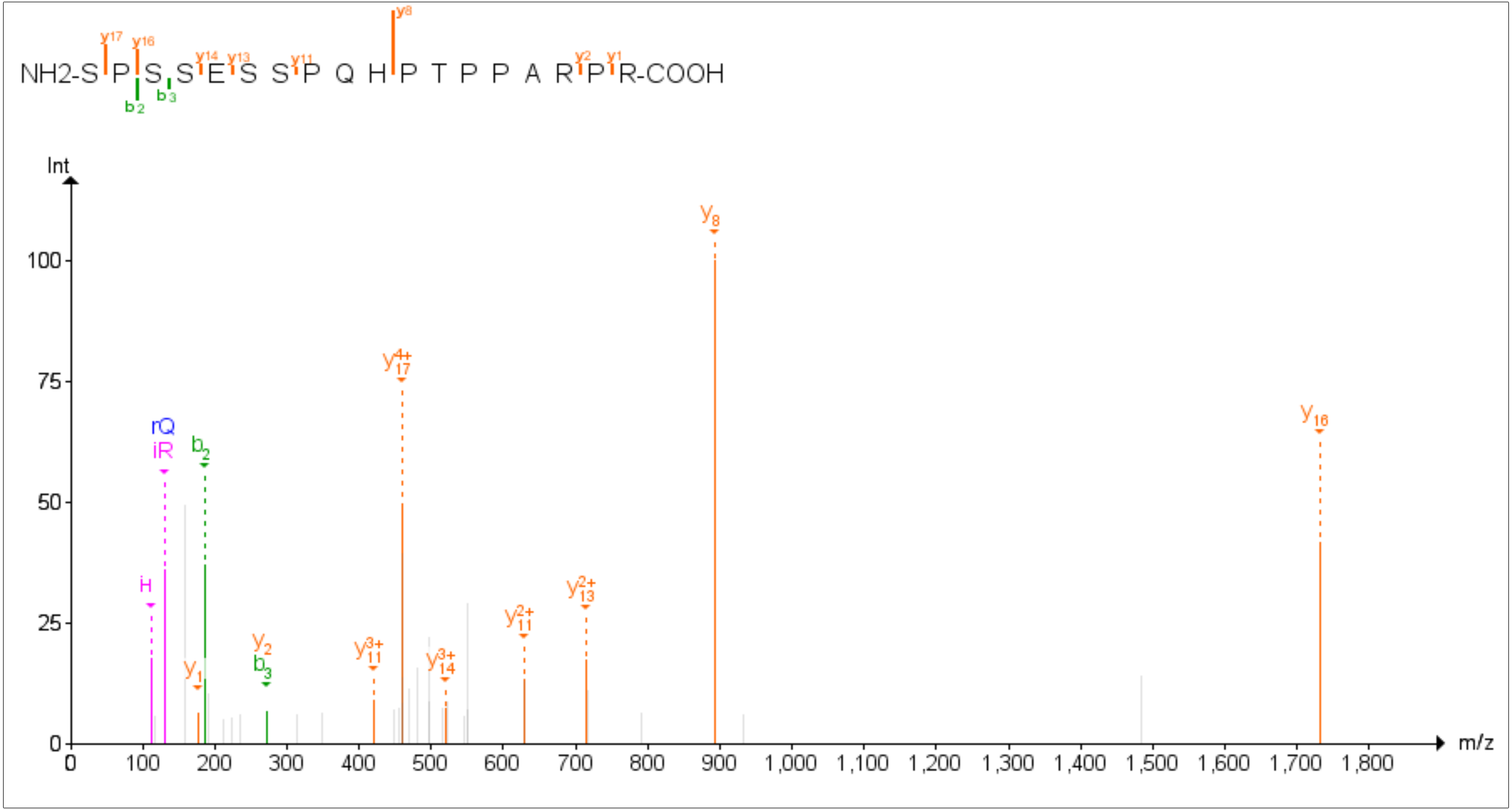

NH2-S P S S E S S P Q H P T P P A R P R-COOH
y17
y16
y14
y13
y11
y8
y2
y1
b2
b3
Int
m/z
y8
y16
y17 4+
rQ
iR
b2
iH
y1
y2
b3
y11 3+
y14 3+
y11 2+
y13 2+
0
25
50
75
100
0
100
200
300
400
500
600
700
800
900
1,000
1,100
1,200
1,300
1,400
1,500
1,600
1,700
1,800

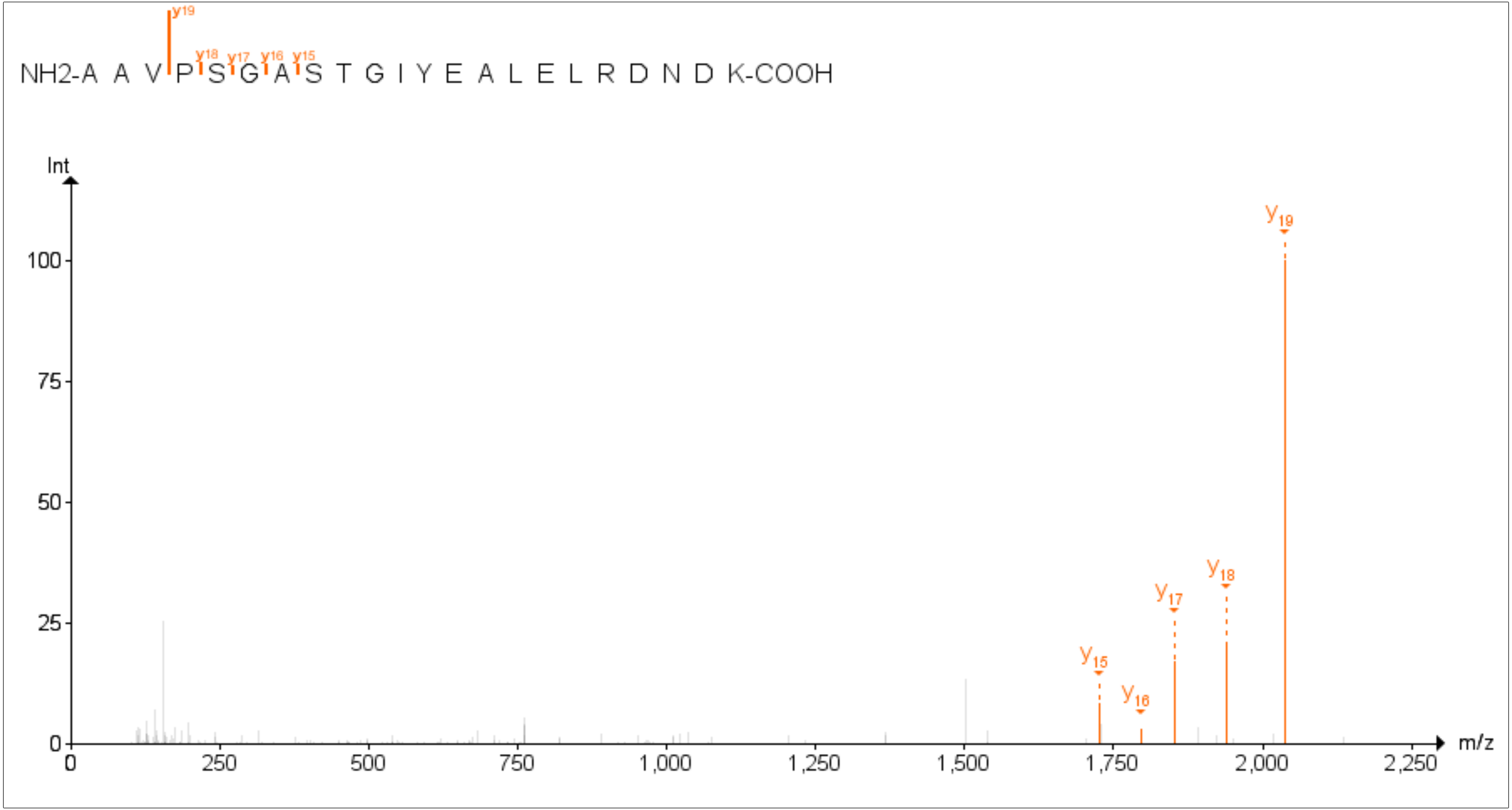

NH2-A A V P S G A S T G I Y E A L E L R D N D K-COOH
y19
y18
y17
y16
y15
Int
100
75
50
25
0
250
500
750
1,000
1,250
1,500
1,750
2,000
2,250
m/z

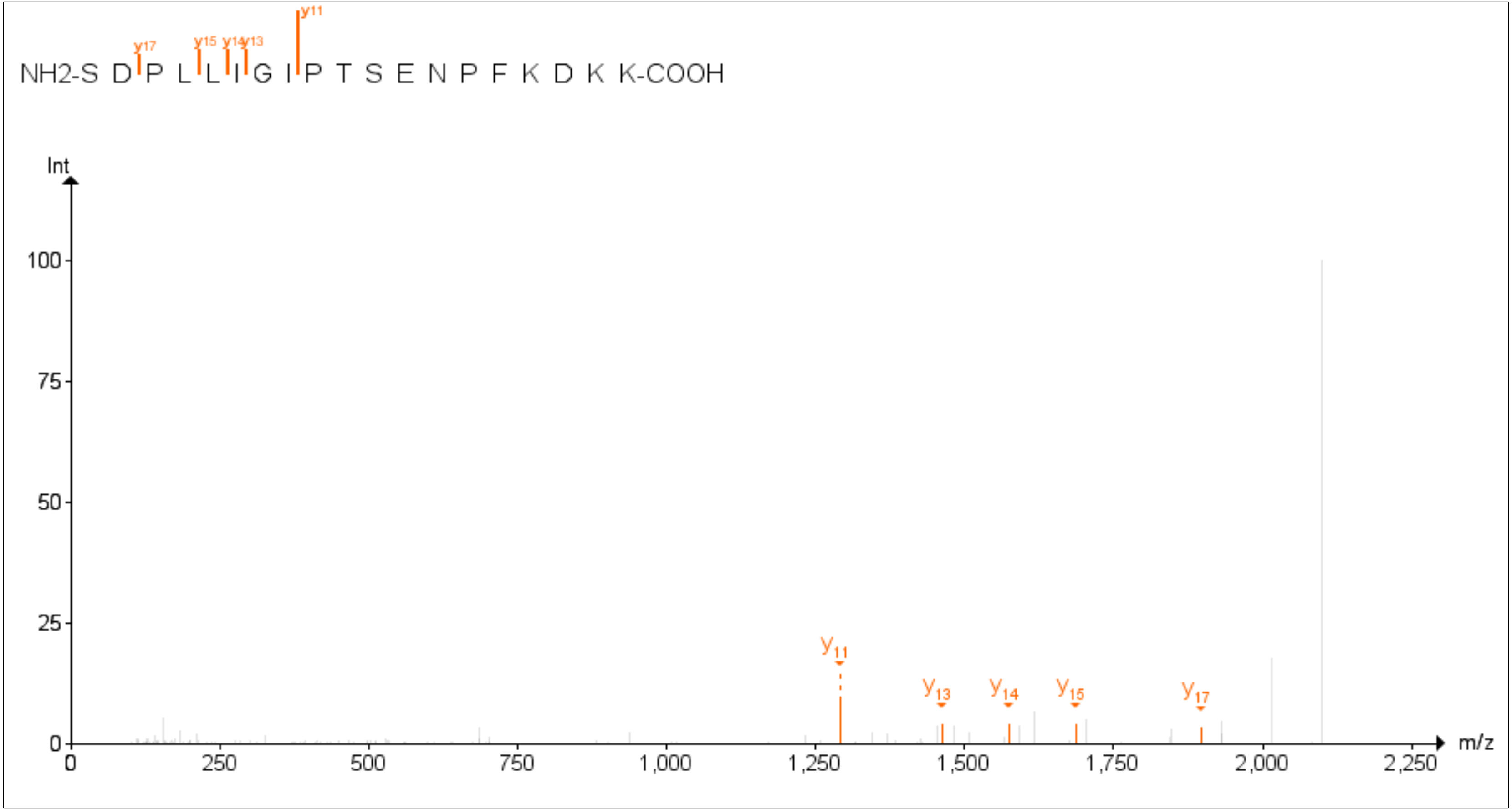

NH2-S D P L L I G I P T S E N P F K D K K-COOH
y17
y15
y14
y13
y11
Int
100
75
50
25
0
y11
y13
y14
y15
y17
0
250
500
750
1,000
1,250
1,500
1,750
2,000
2,250
m/z

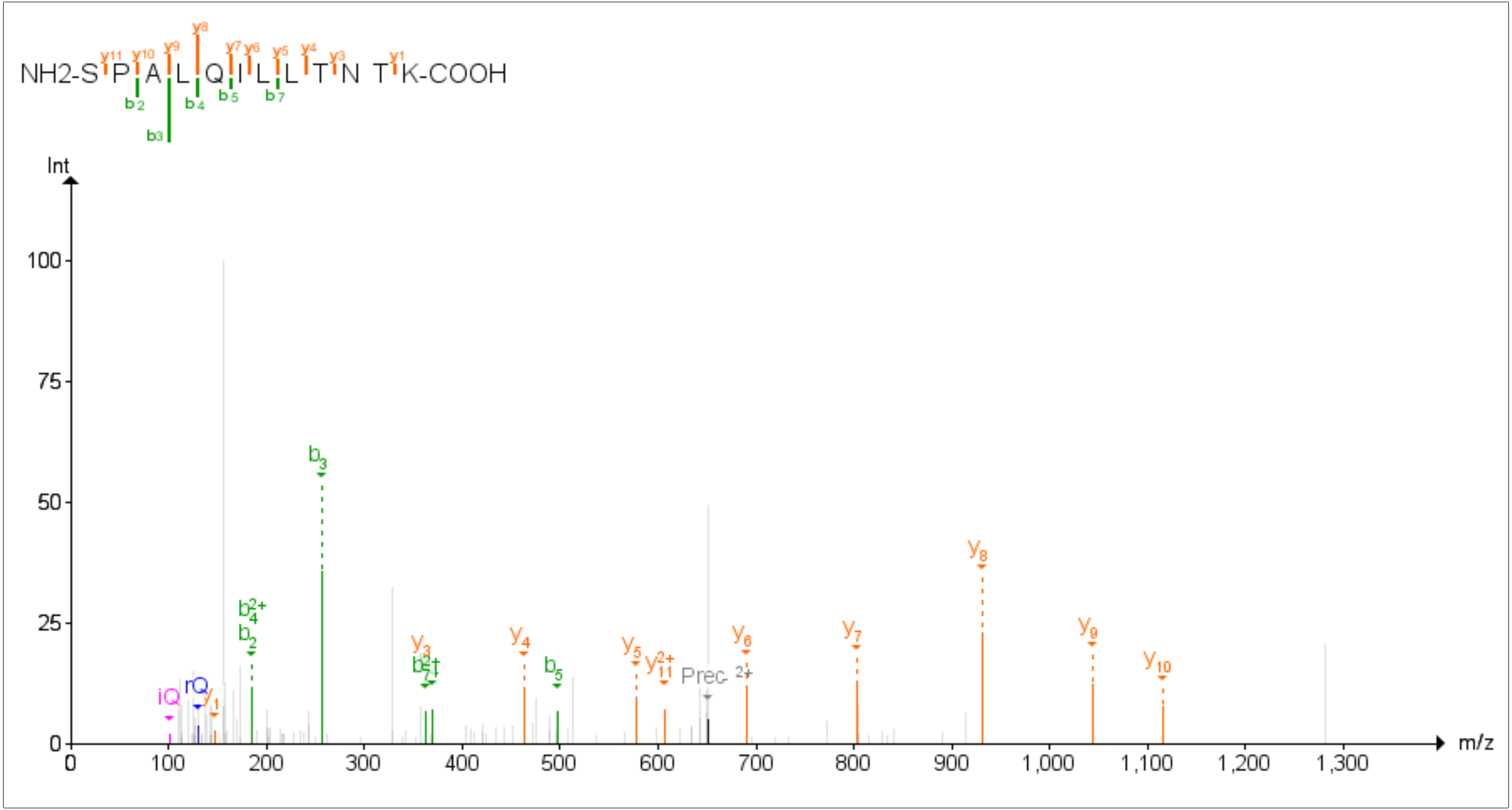

NH2-S P A L Q I L L T N T K-COOH
y11 y10 y9 y8 y7 y6 y5 y4 y3 y1
b2 b3 b4 b5 b7
Int
100
75
50
25
0
m/z
0 100 200 300 400 500 600 700 800 900 1,000 1,100 1,200 1,300
b3
y8
b4 2+
b2
y4
b5
y5
y11 2+
Prec 2+
y6
y7
y9
y10
y3
b7 2+
iQ
rQ
y1

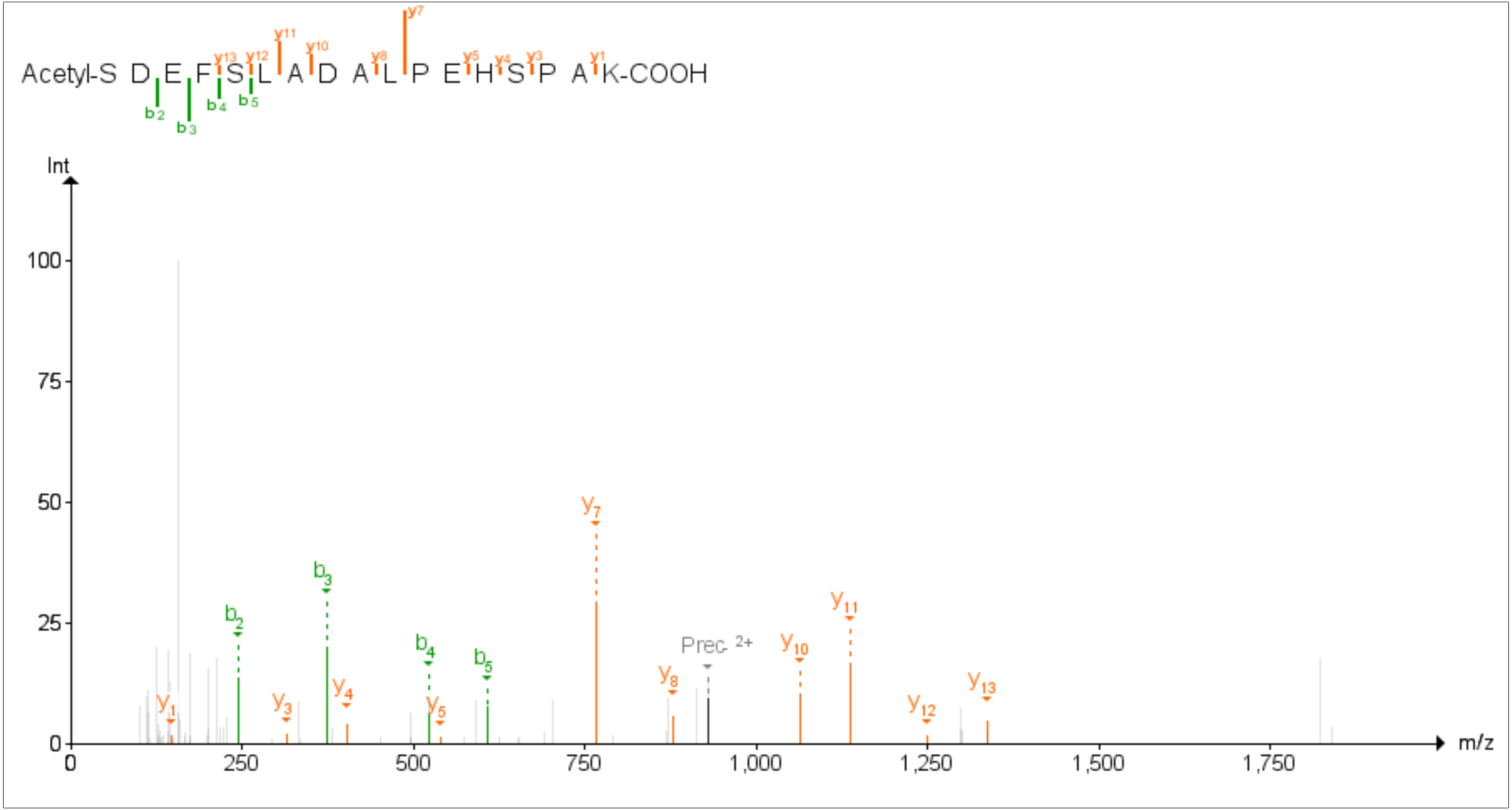

Acetyl-S D E F S L A D A L P E H S P A K-COOH
y13 y12 y11 y10 y8 y7 y5 y4 y3 y1
b2 b3 b4 b5
Int
100
75
50
25
0
0 250 500 750 1,000 1,250 1,500 1,750
m/z
y1 b2 y3 b3 y4 b4 y5 b5 y7 y8 Prec 2+ y10 y11 y12 y13

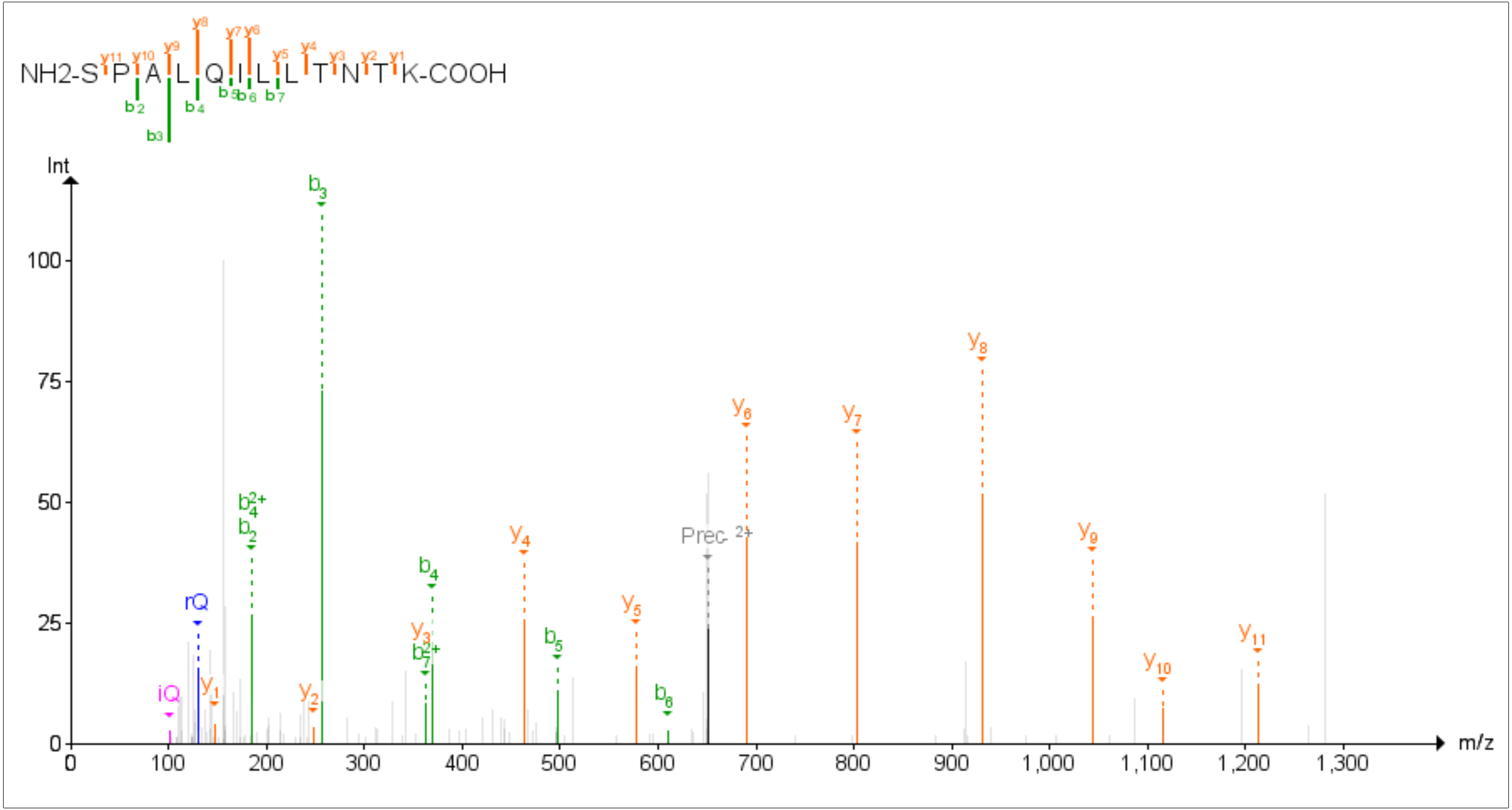

NH2-S P A L Q I L L T N T K-COOH
y11 y10 y9 y8 y7 y6 y5 y4 y3 y2 y1
b2 b3 b4 b5 b6 b7
Int
m/z
b3
b4 2+
b2
rQ
iQ
y1
y2
y3
b7 2+
b4
y4
b5
y5
b6
Prec 2+
y6
y7
y8
y9
y10
y11
0
25
50
75
100
0
100
200
300
400
500
600
700
800
900
1,000
1,100
1,200
1,300

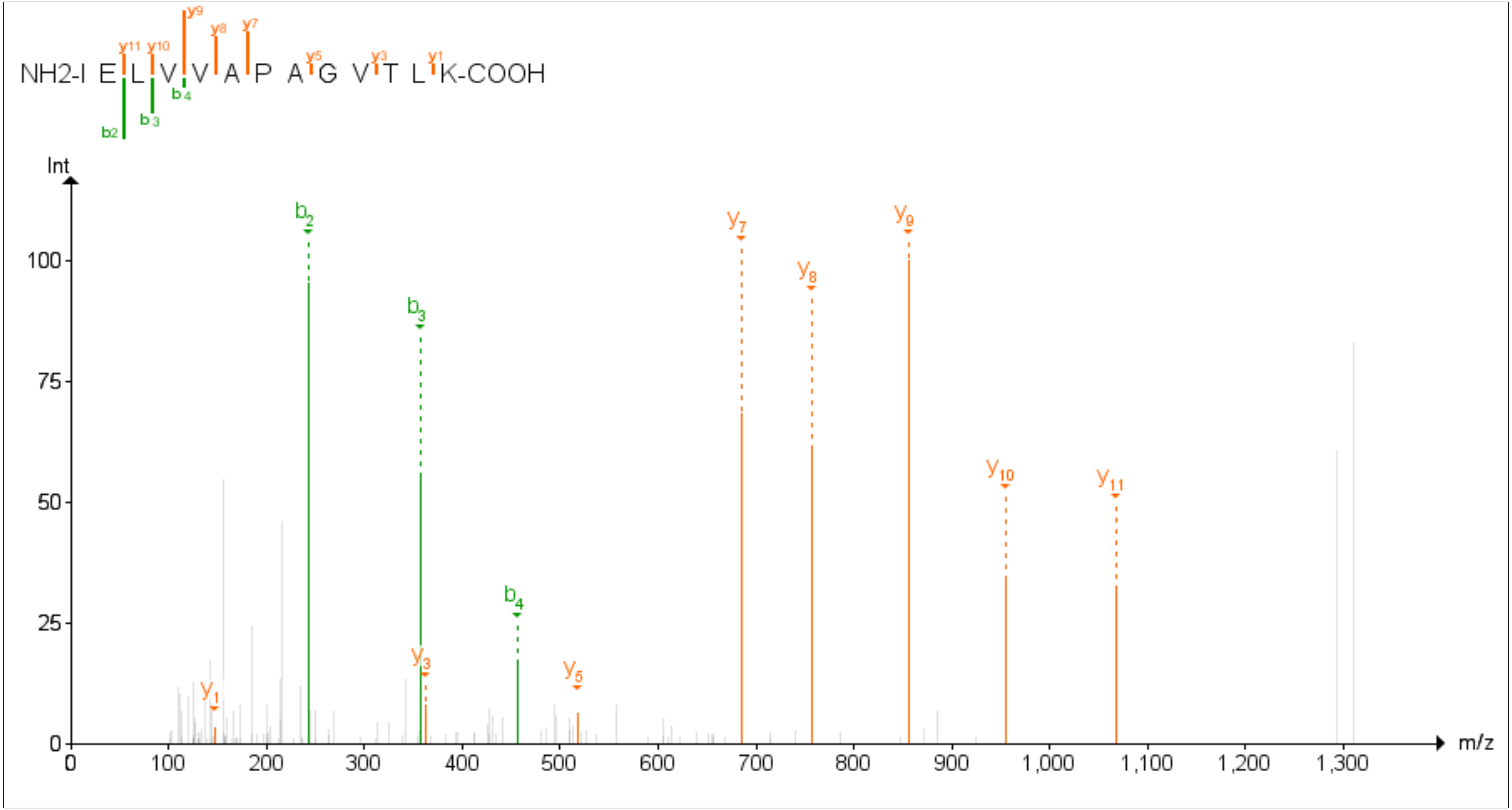

NH2-I E L V V A P A G V T L K-COOH
y11 y10 y9 y8 y7 y5 y3 y1
b2 b3 b4
Int
100
75
50
25
0
b2
b3
b4
y1
y3
y5
y7
y8
y9
y10
y11
0 100 200 300 400 500 600 700 800 900 1,000 1,100 1,200 1,300
m/z

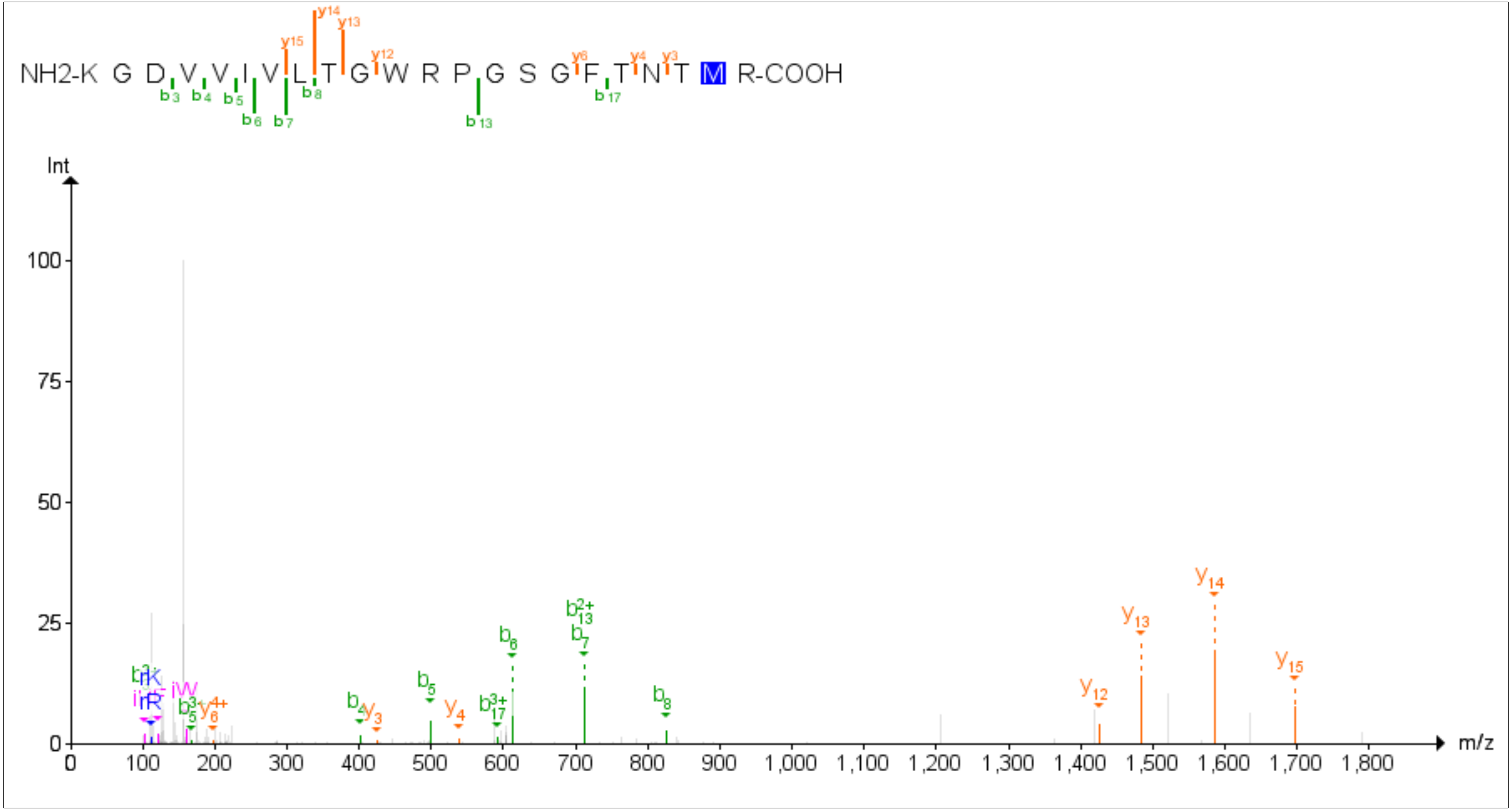
NH2-K G D V V I V L T G W R P G S G F T N T M R-COOH
Int
m/z
100
75
50
25
0
0 100 200 300 400 500 600 700 800 900 1,000 1,100 1,200 1,300 1,400 1,500 1,600 1,700 1,800

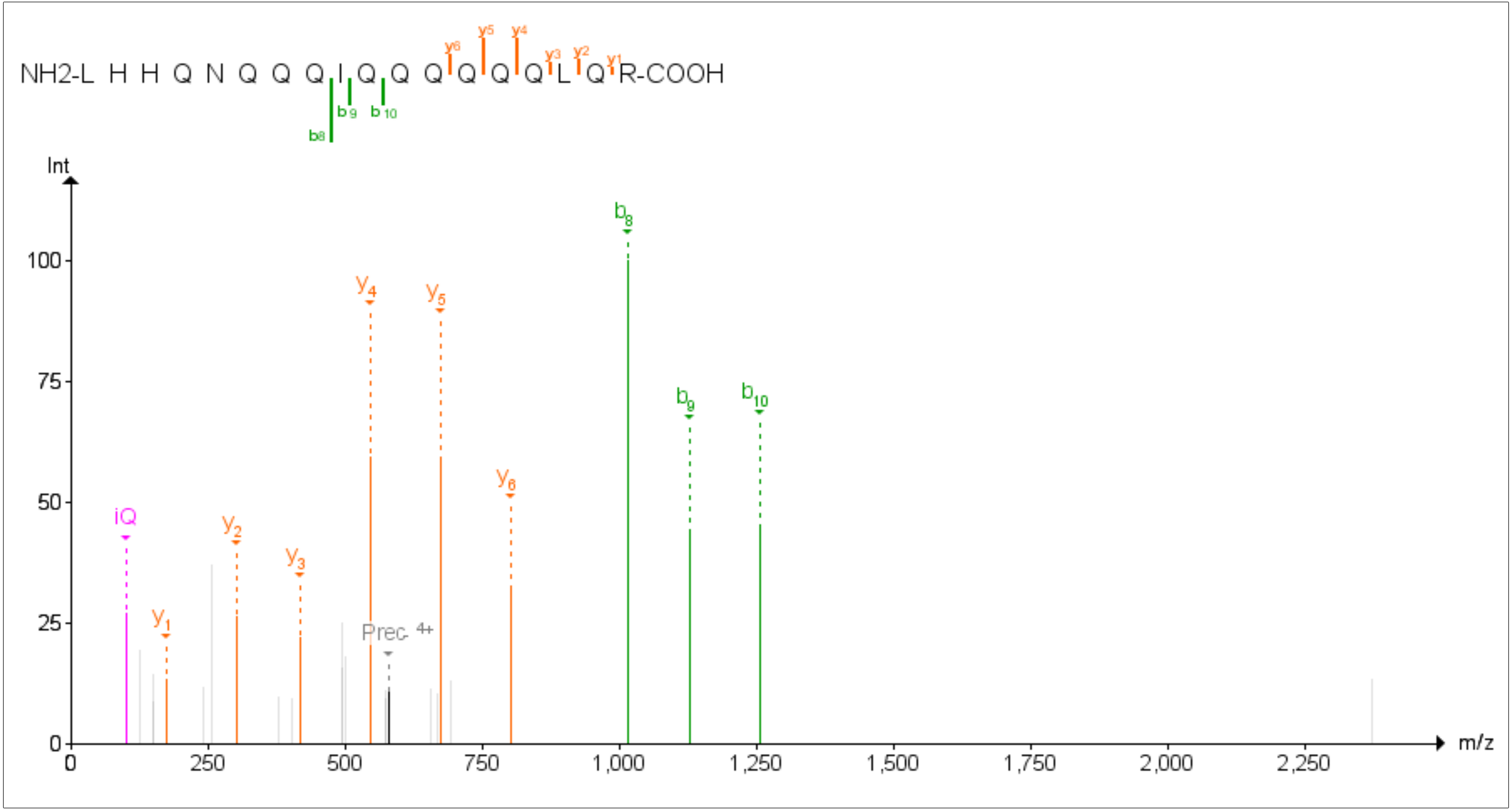
NH2-L H H Q N Q Q Q I Q Q Q Q Q Q L Q R-COOH
y6 y5 y4 y3 y2 y1
b8 b9 b10
Int
m/z
100
75
50
25
0
0 250 500 750 1,000 1,250 1,500 1,750 2,000 2,250
b8
y4
y5
b9
b10
y6
iQ
y2
y3
y1
Prec- 4+

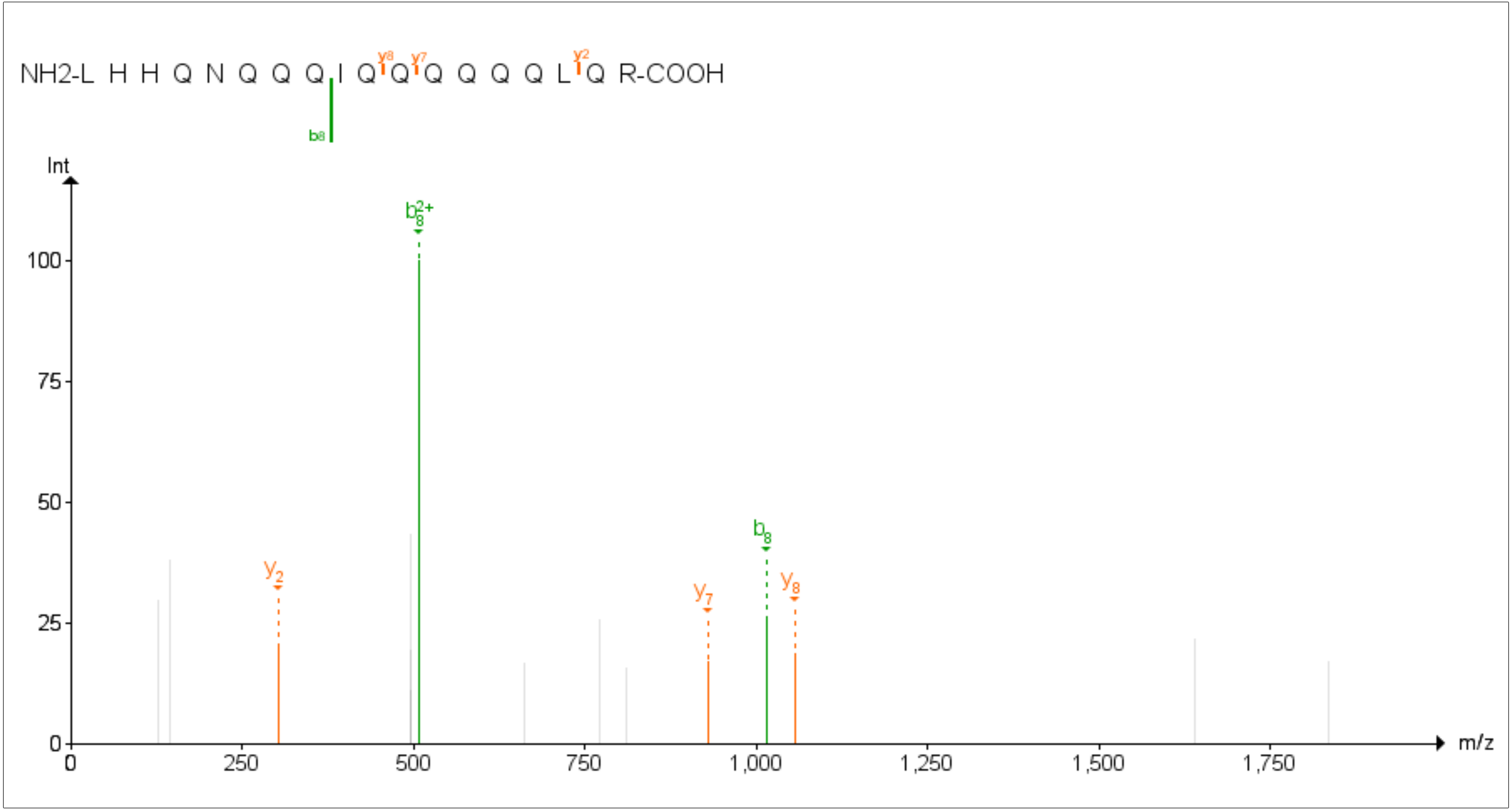

NH2-L H H Q N Q Q Q I Q Q Q Q Q Q L Q R-COOH
b8
y8
y7
y2
Int
100
75
50
25
0
0
250
500
750
1,000
1,250
1,500
1,750
m/z
b_8^{2+}
b_8
y_2
y_7
y_8

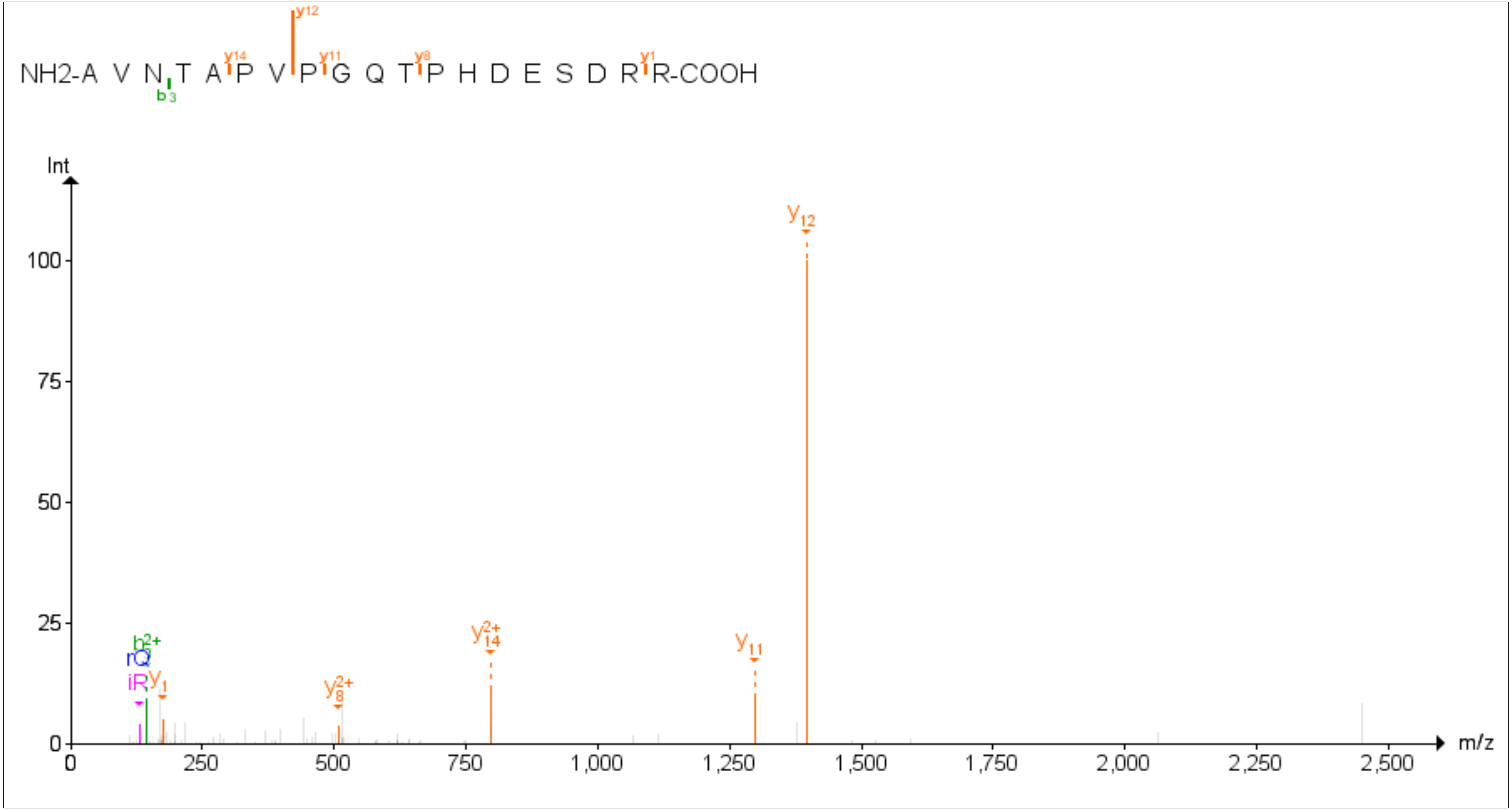

NH2-A V N T A P V P G Q T P H D E S D R R-COOH
y12
y14
y11
y8
y1
b3
Int
100
75
50
25
0
y12
y14 2+
y11
y8 2+
b 2+
rQ
iR
y1
0
250
500
750
1,000
1,250
1,500
1,750
2,000
2,250
2,500
m/z

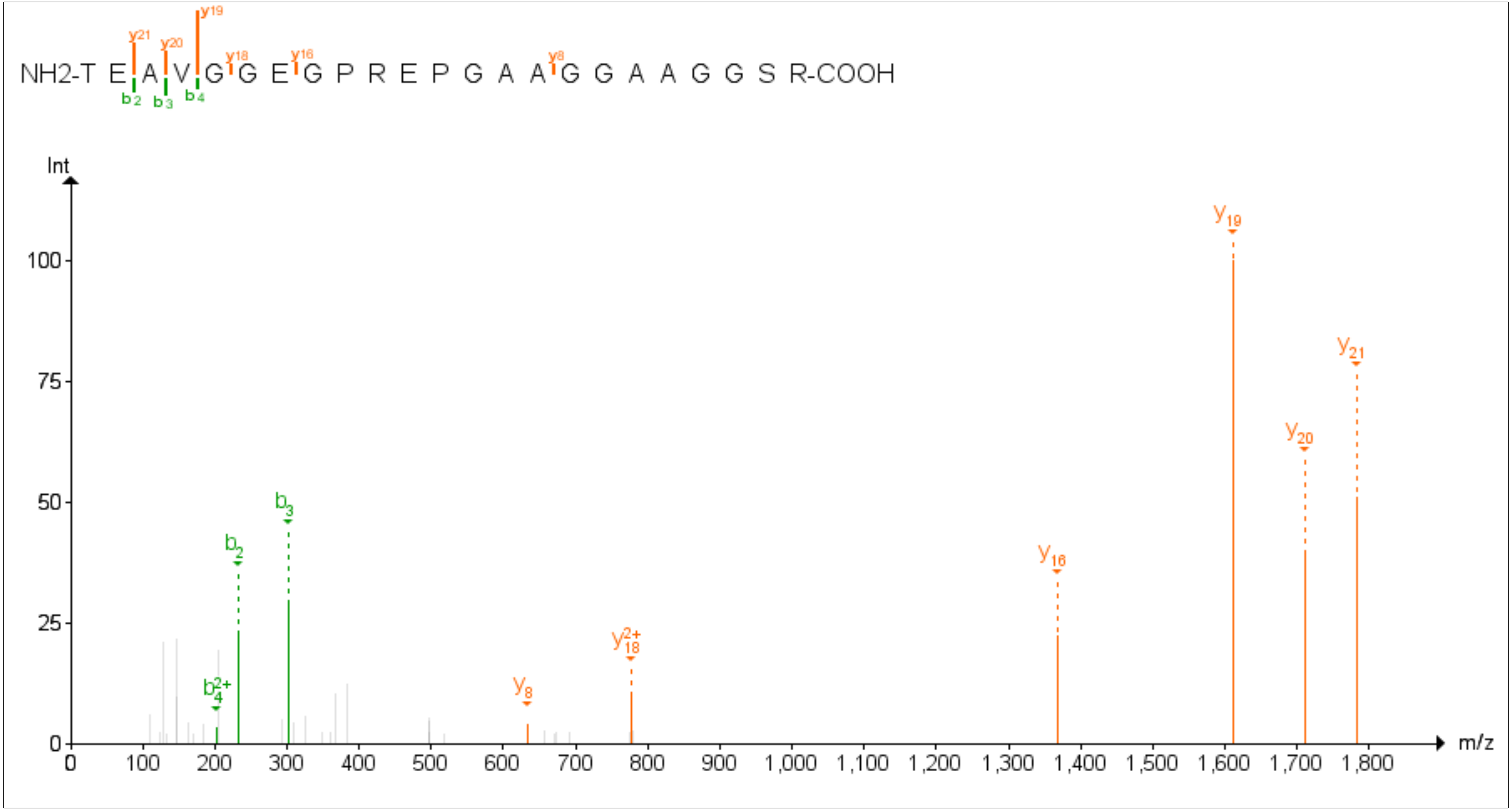

NH2-T E A V G G E G P R E P G A A G G A A G G S R-COOH
y21 y20 y19 y18 y16 y8
b2 b3 b4
Int
100
75
50
25
0
m/z
0 100 200 300 400 500 600 700 800 900 1,000 1,100 1,200 1,300 1,400 1,500 1,600 1,700 1,800
b_4^{2+}
b_2
b_3
y_8
y_{18}^{2+}
y_{16}
y_{19}
y_{20}
y_{21}

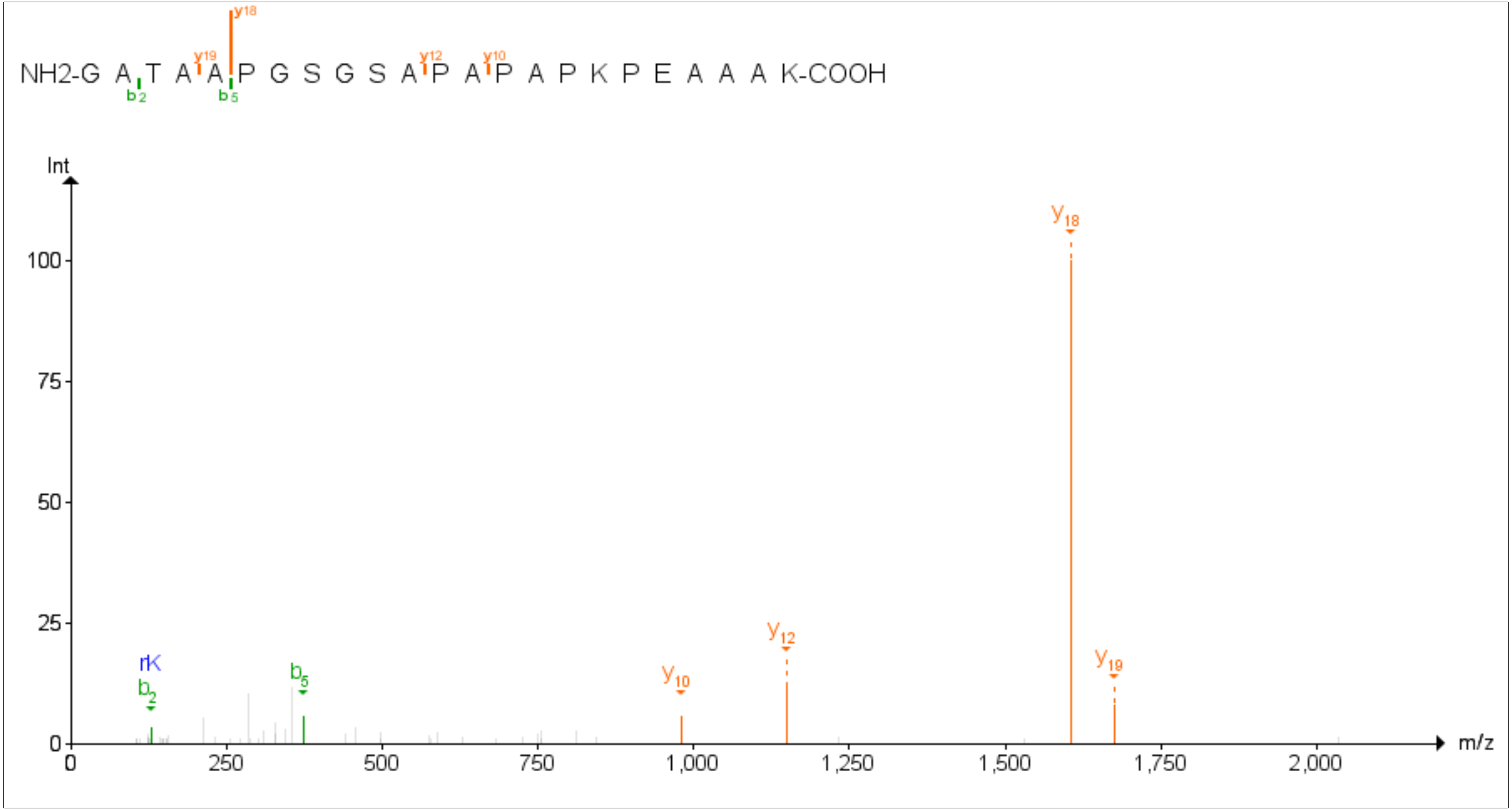

NH2-G A T A A P G S G S A P A P A P K P E A A A K-COOH
y18
y19
y12
y10
b2
b5
Int
100
75
50
25
0
rK
b2
b5
y10
y12
y18
y19
0
250
500
750
1,000
1,250
1,500
1,750
2,000
m/z

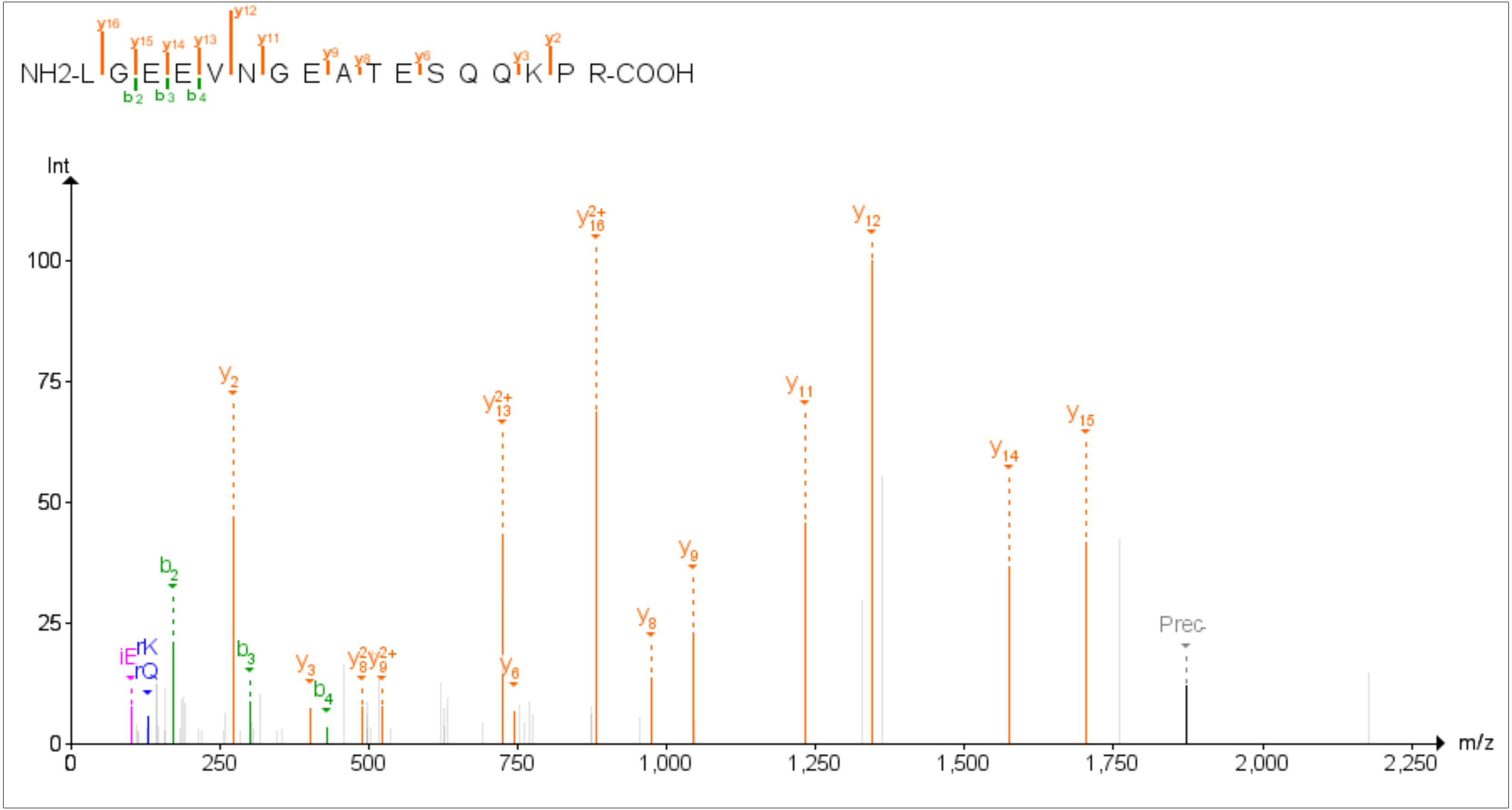

NH2-L G E E V N G E A T E S Q Q K P R-COOH
y16
y15
y14
y13
y12
y11
y9
y8
y6
y3
y2
b2
b3
b4
Int
100
75
50
25
0
m/z
0
250
500
750
1,000
1,250
1,500
1,750
2,000
2,250
y2
y13 2+
y16 2+
y12
y11
y15
y14
y9
y8
y6
y3
y8 2+
y9 2+
b2
b3
b4
iE
rK
rQ
Prec

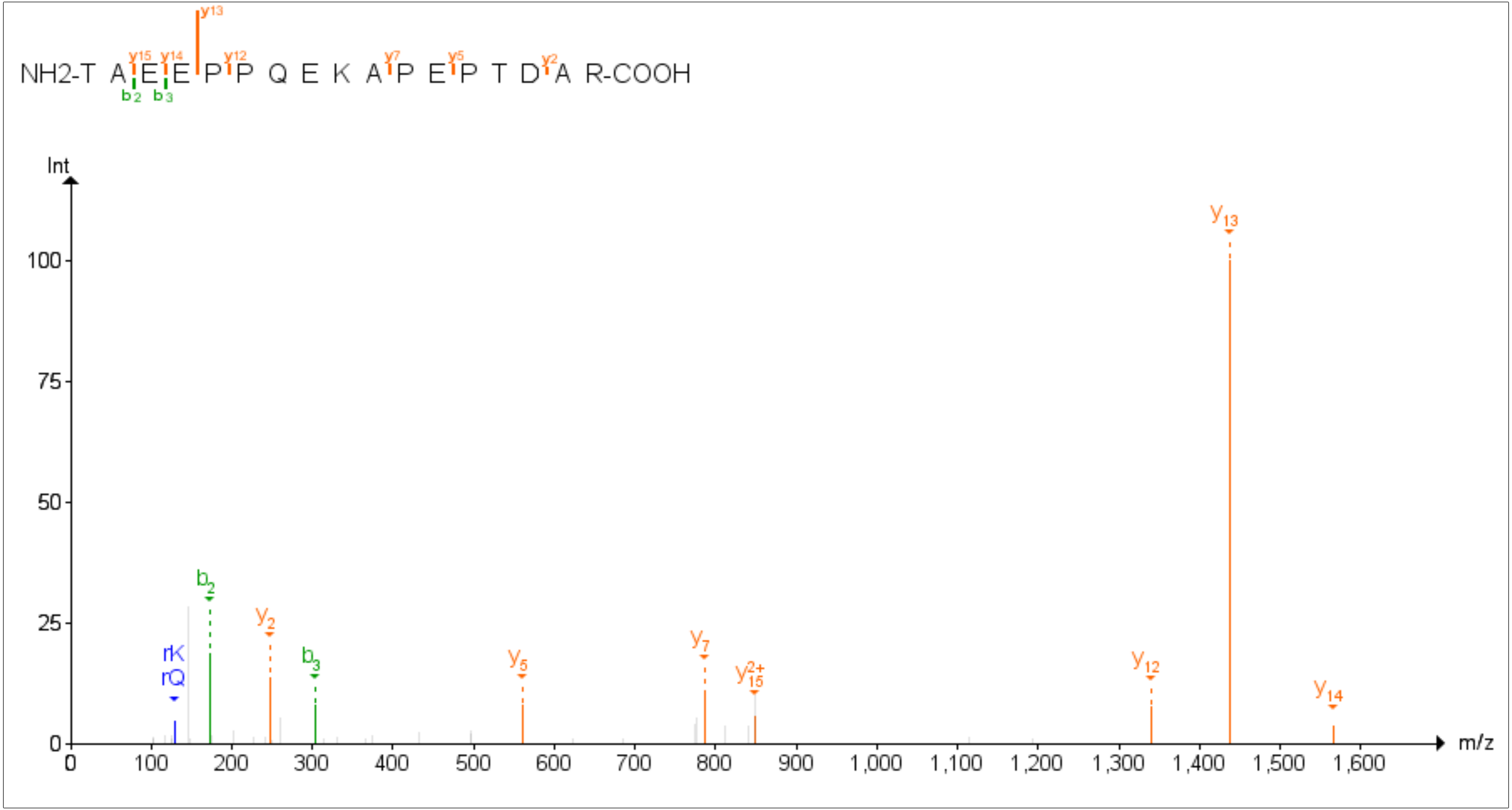

NH2-T A E E P P Q E K A P E P T D A R-COOH
y15 y14 y13 y12 y7 y5 y2
b2 b3
Int
100
75
50
25
0
rK
rQ
b2
y2
b3
y5
y7
y15 2+
y12
y13
y14
0 100 200 300 400 500 600 700 800 900 1,000 1,100 1,200 1,300 1,400 1,500 1,600
m/z

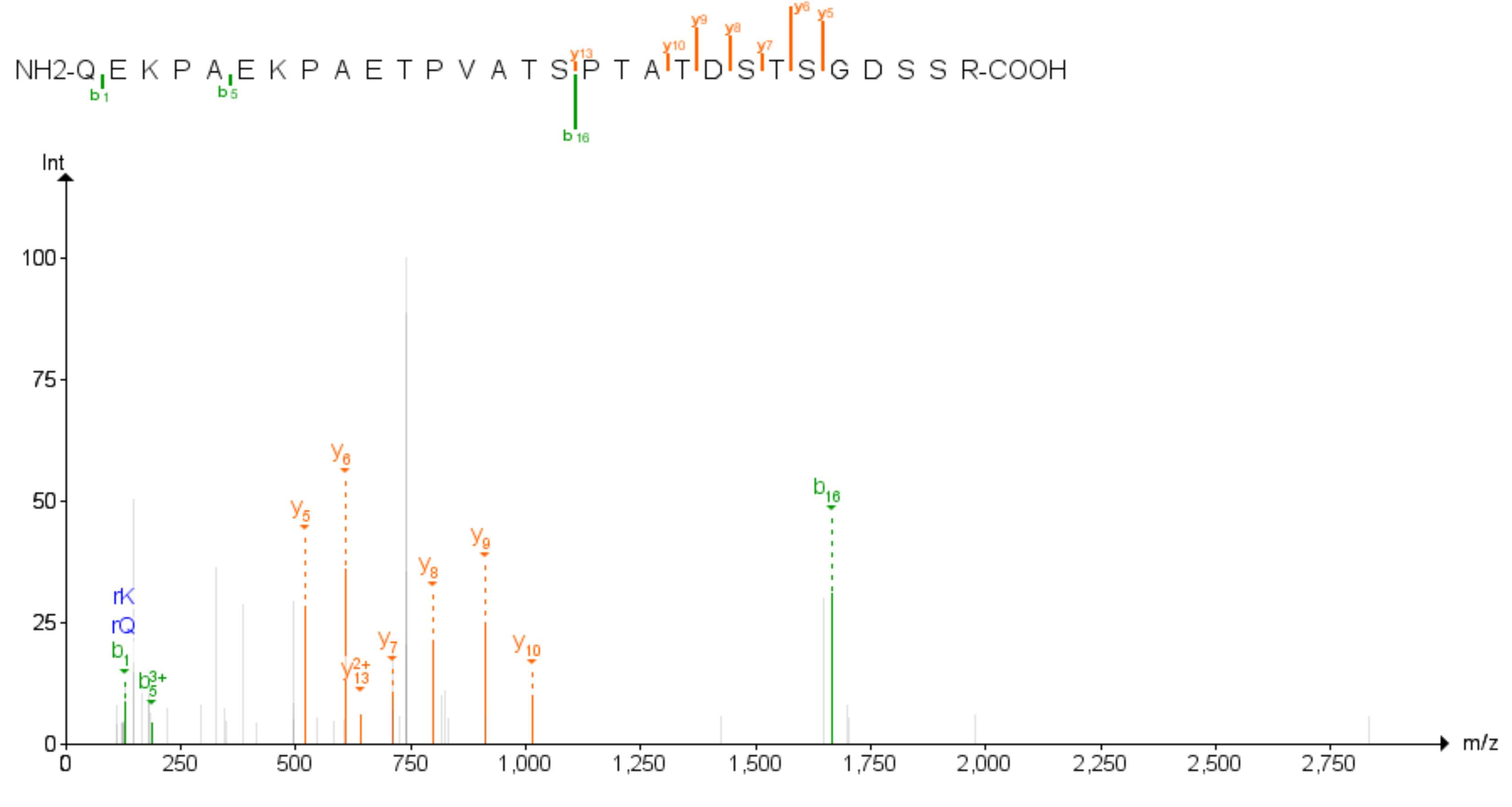

NH2-Q E K P A E K P A E T P V A T S P T A T D S T S G D S S R-COOH
b1
b5
b16
y13
y10
y9
y8
y7
y6
y5
Int
100
75
50
25
0
m/z
0
250
500
750
1,000
1,250
1,500
1,750
2,000
2,250
2,500
2,750
rK
rQ
b1
b5 3+
y5
y6
y13 2+
y7
y8
y9
y10
b16

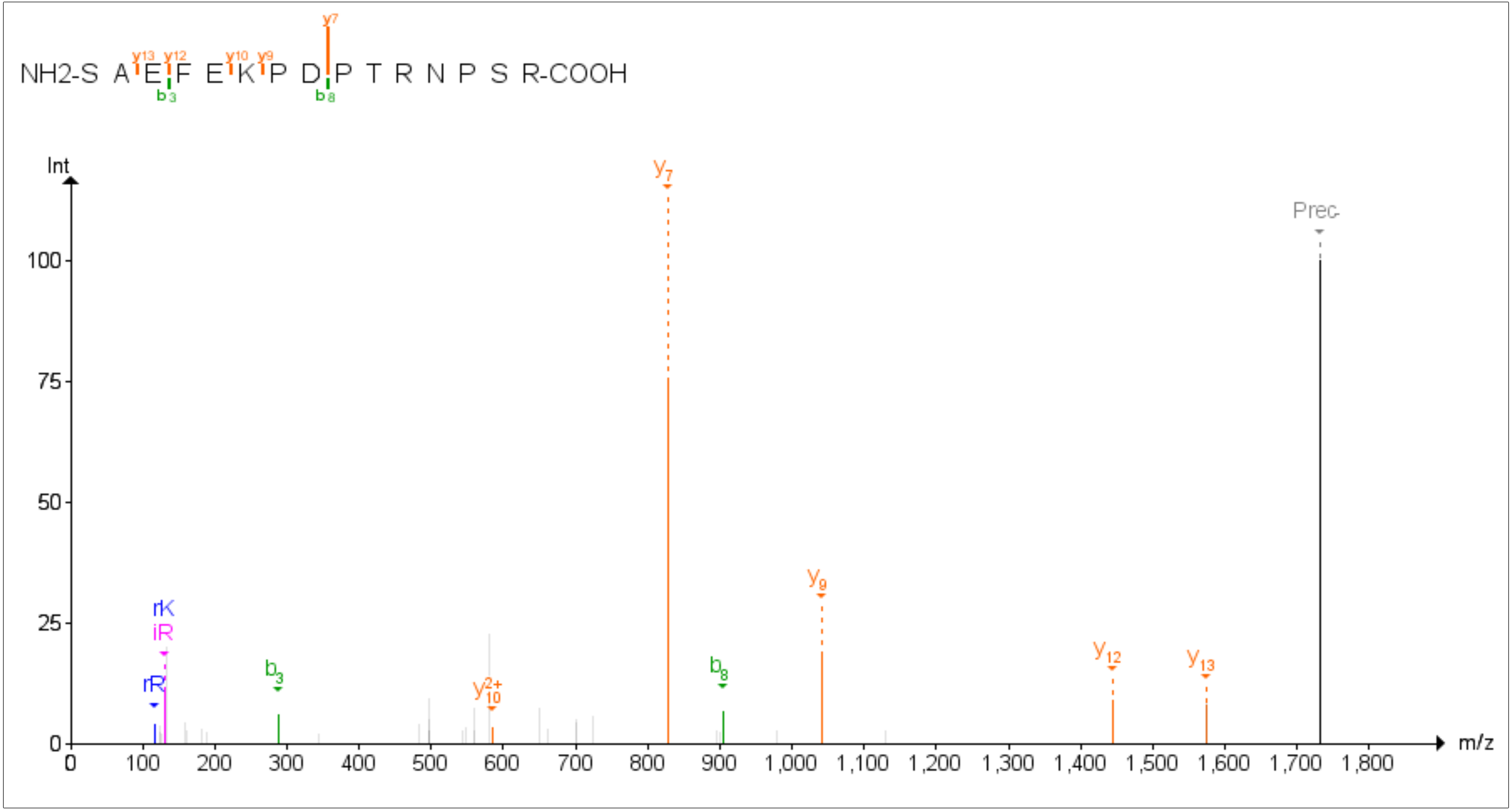

NH2-S A E F E K P D P T R N P S R-COOH
y13 y12 y10 y9 y7
b3 b8
Int
100
75
50
25
0
y7
Prec-
y9
rK
iR
rR
b3
y10 2+
b8
y12
y13
0 100 200 300 400 500 600 700 800 900 1,000 1,100 1,200 1,300 1,400 1,500 1,600 1,700 1,800
m/z

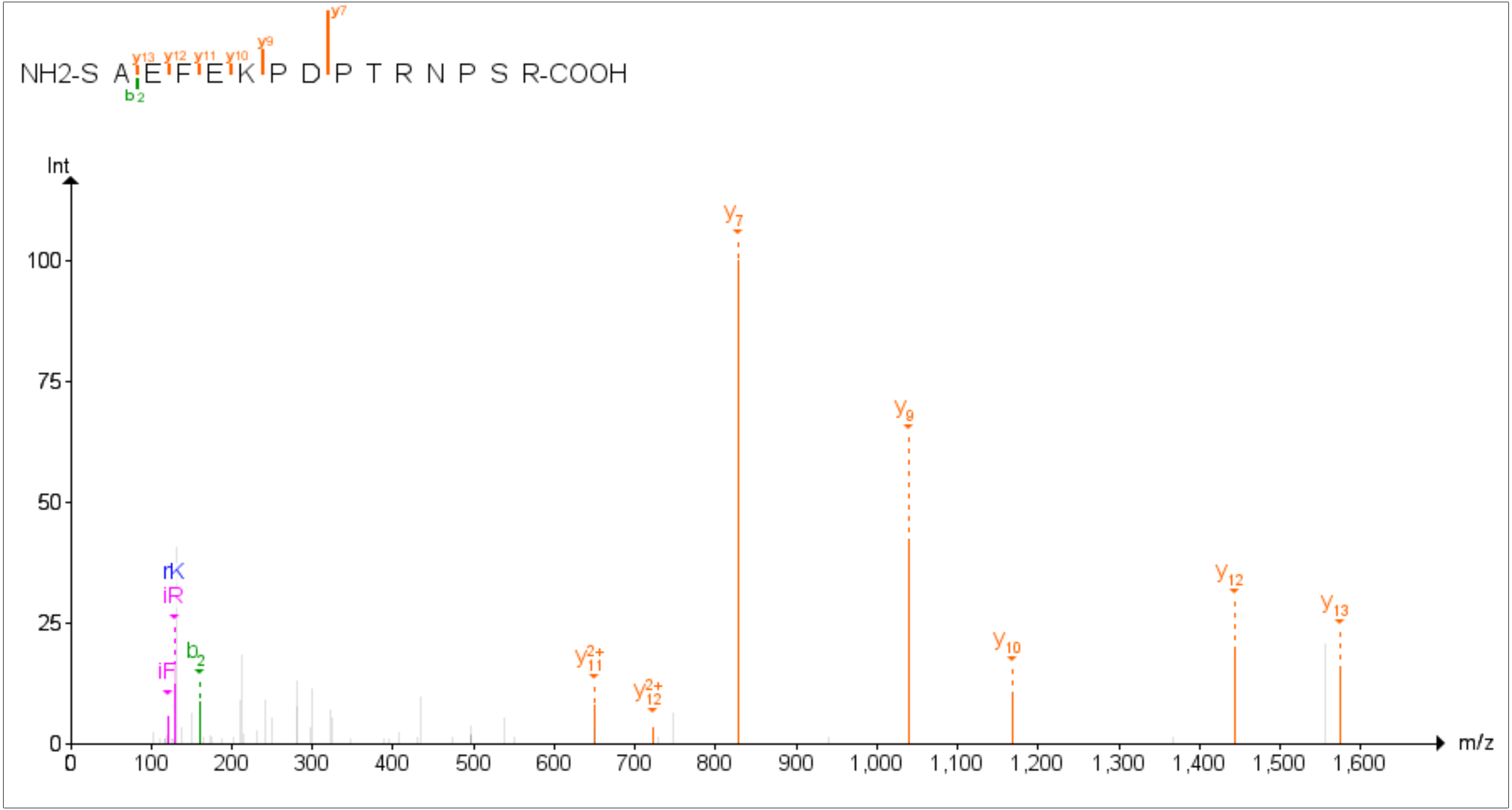

NH2-S A E F E K P D P T R N P S R-COOH
y13 y12 y11 y10 y9 y7
b2
Int
100
75
50
25
0
y7
y9
y12
y13
y10
y11 2+
y12 2+
rK
iR
iF
b2
0 100 200 300 400 500 600 700 800 900 1,000 1,100 1,200 1,300 1,400 1,500 1,600
m/z

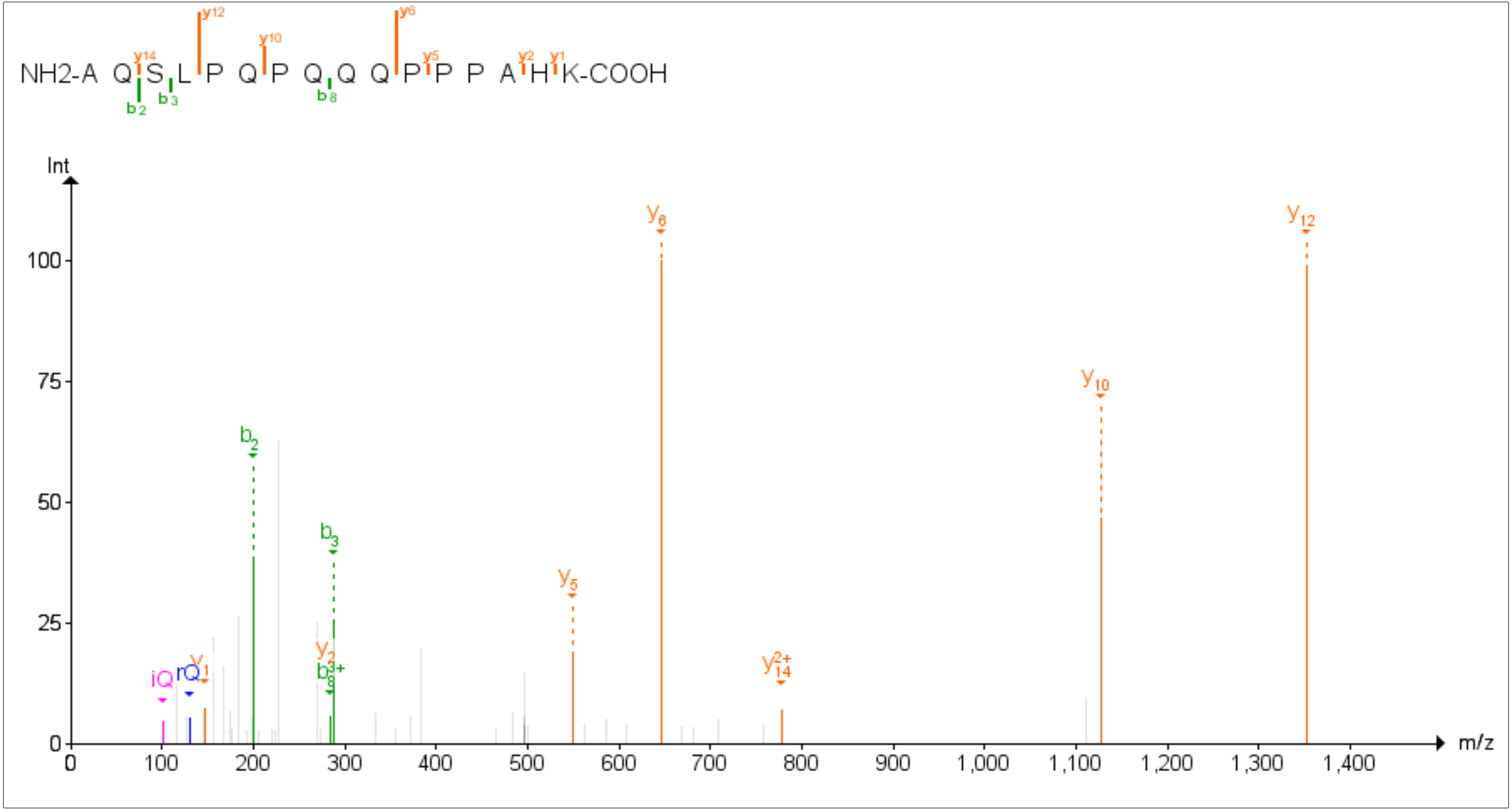

NH2-A Q S L P Q P Q Q Q P P P A H K-COOH
y14 y12 y10 y6 y5 y2 y1
b2 b3 b8
Int
m/z
iQ
rQ
y1
b2
y2
b3
b8 3+
y5
y6
y14 2+
y10
y12
0
25
50
75
100
0
100
200
300
400
500
600
700
800
900
1,000
1,100
1,200
1,300
1,400

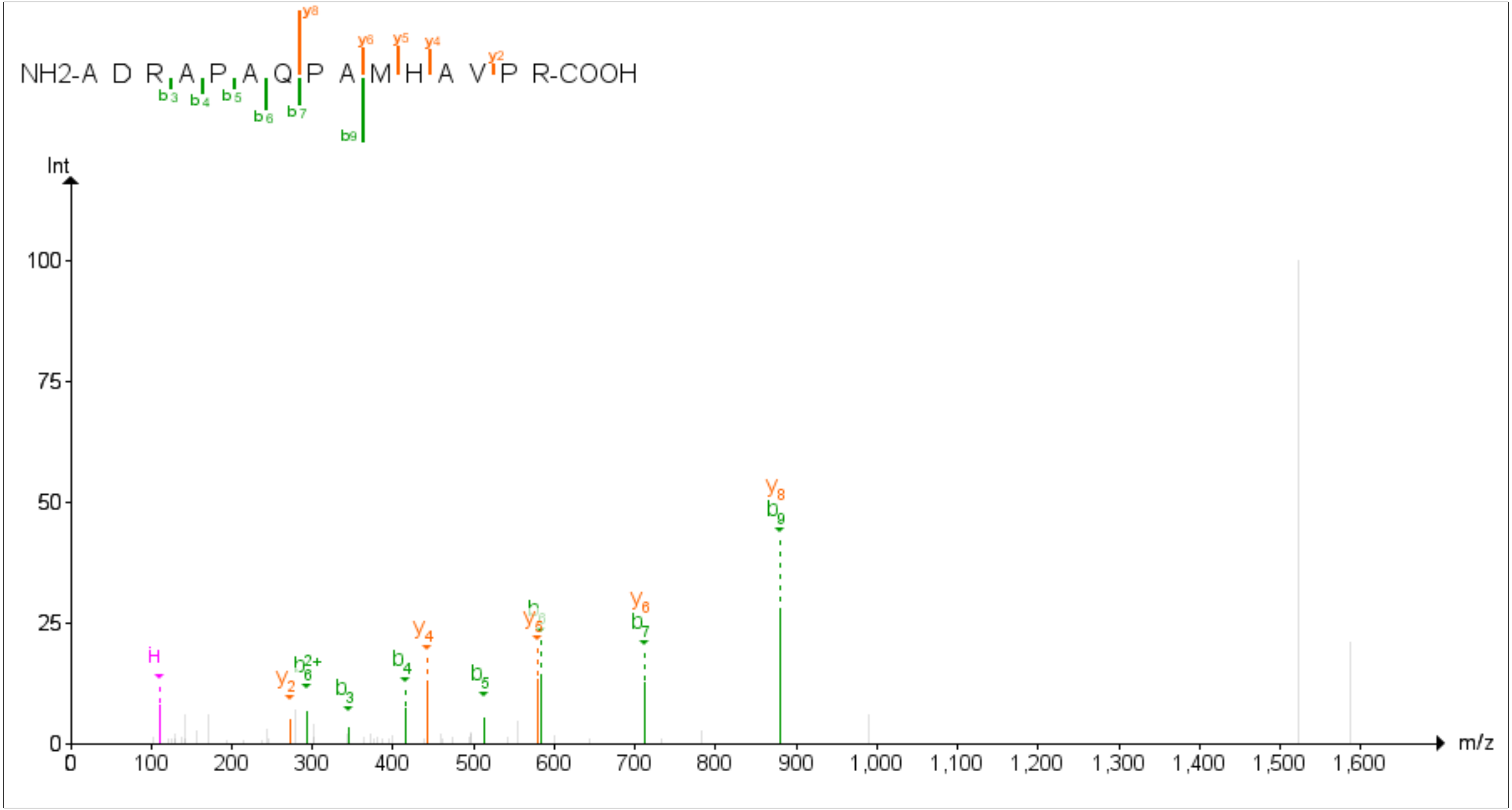

NH2-A D R A P A Q P A M H A V P R-COOH
y8 y6 y5 y4 y2
b3 b4 b5 b6 b7 b9
Int
100
75
50
25
0
m/z
0 100 200 300 400 500 600 700 800 900 1,000 1,100 1,200 1,300 1,400 1,500 1,600
iH
y2
b6 2+
b3
b4
y4
b5
y5
b8
y6
b7
y8
b9

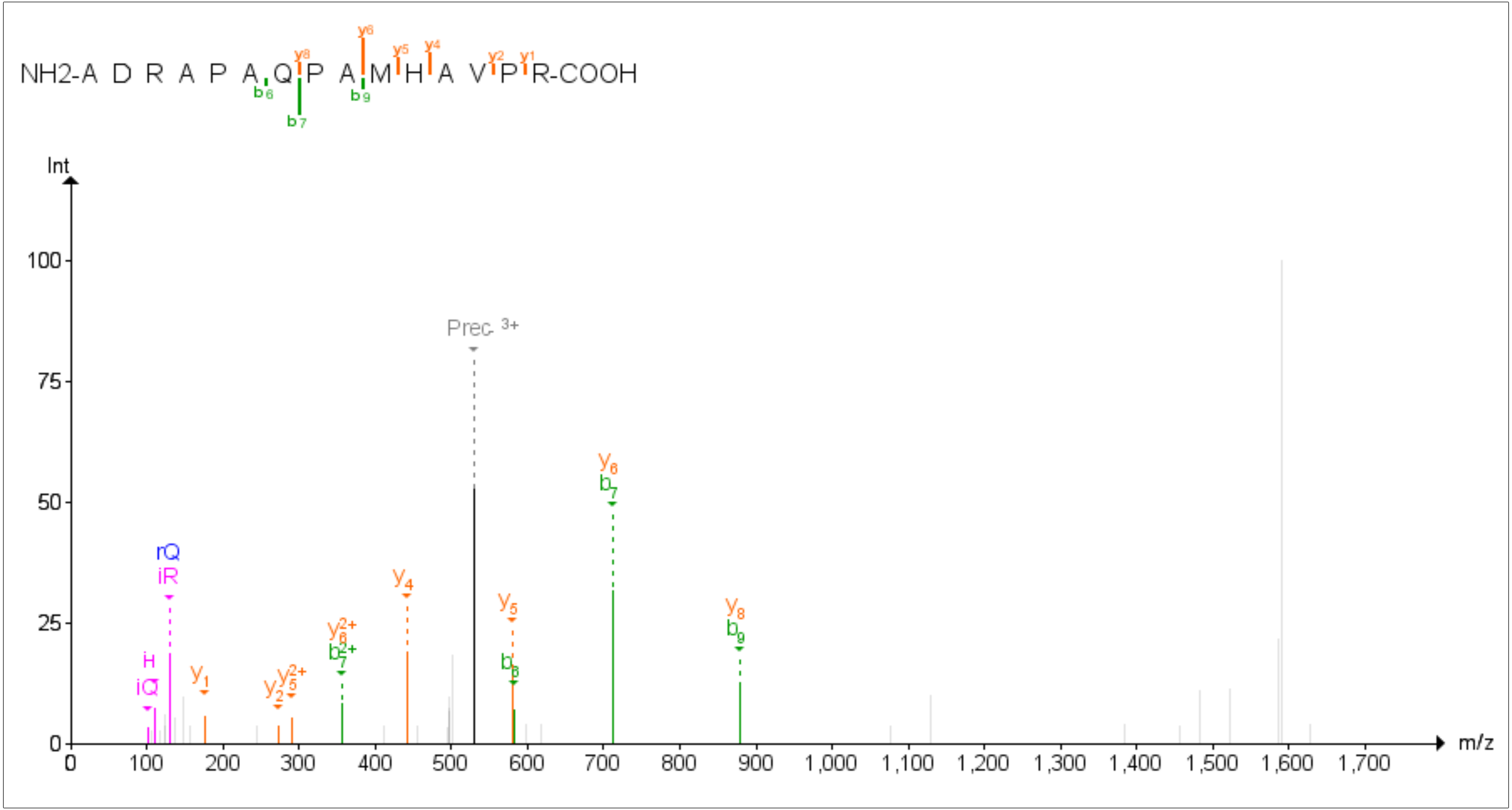
NH2-A D R A P A Q P A M H A V P R-COOH
y8 y6 y5 y4 y2 y1
b6 b7 b9
Int
m/z
Prec 3+
rQ
iR
iH
iQ
y1
y2
y5 2+
y6 2+
b7 2+
y4
y5
b6
y6
b7
y8
b9
0
25
50
75
100
0
100
200
300
400
500
600
700
800
900
1,000
1,100
1,200
1,300
1,400
1,500
1,600
1,700

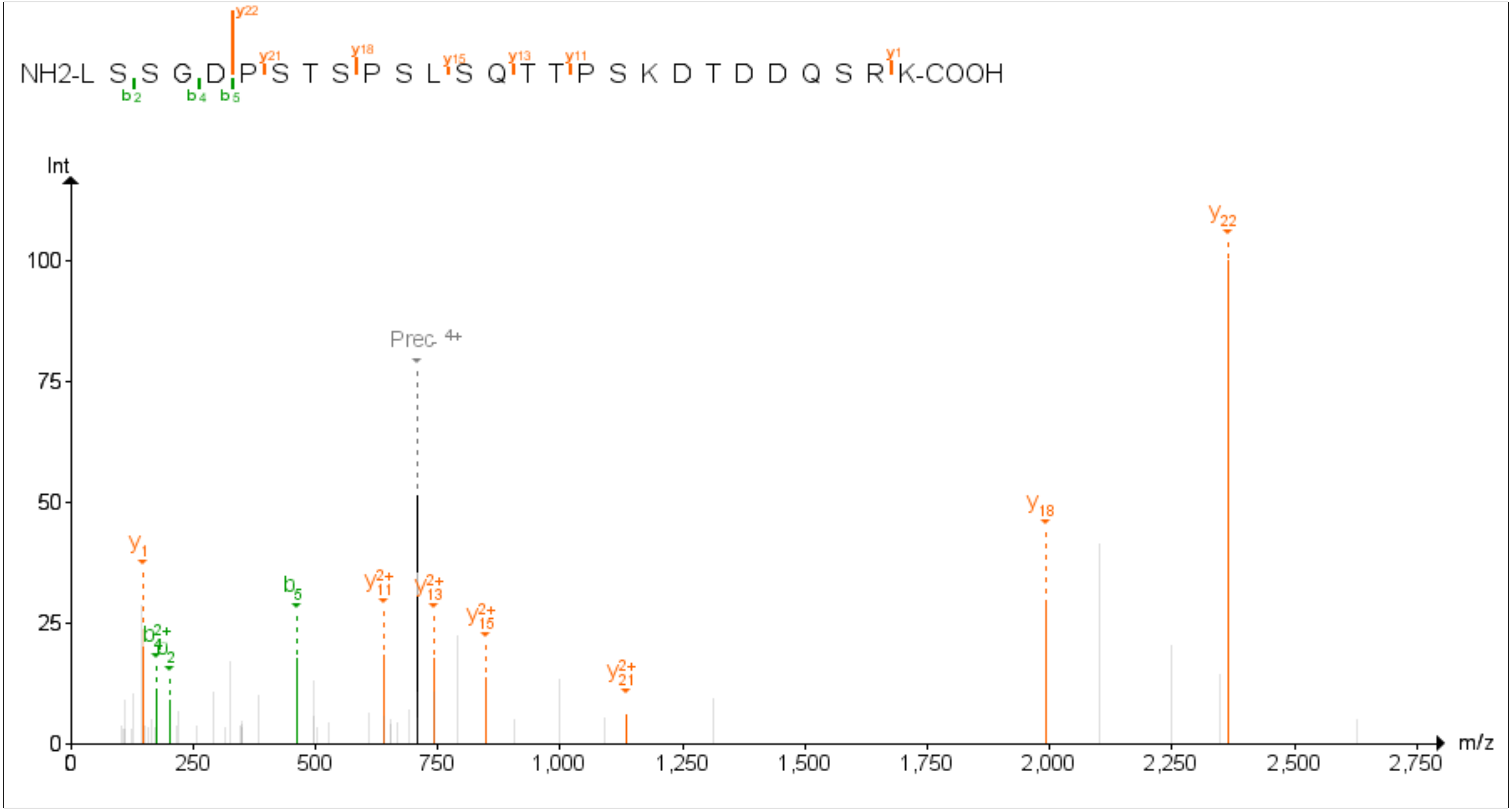
NH2-L S S G D P S T S P S L S Q T T P S K D T D D Q S R K-COOH
Int
m/z
Prec 4+
100
75
50
25
0
250
500
750
1,000
1,250
1,500
1,750
2,000
2,250
2,500
2,750

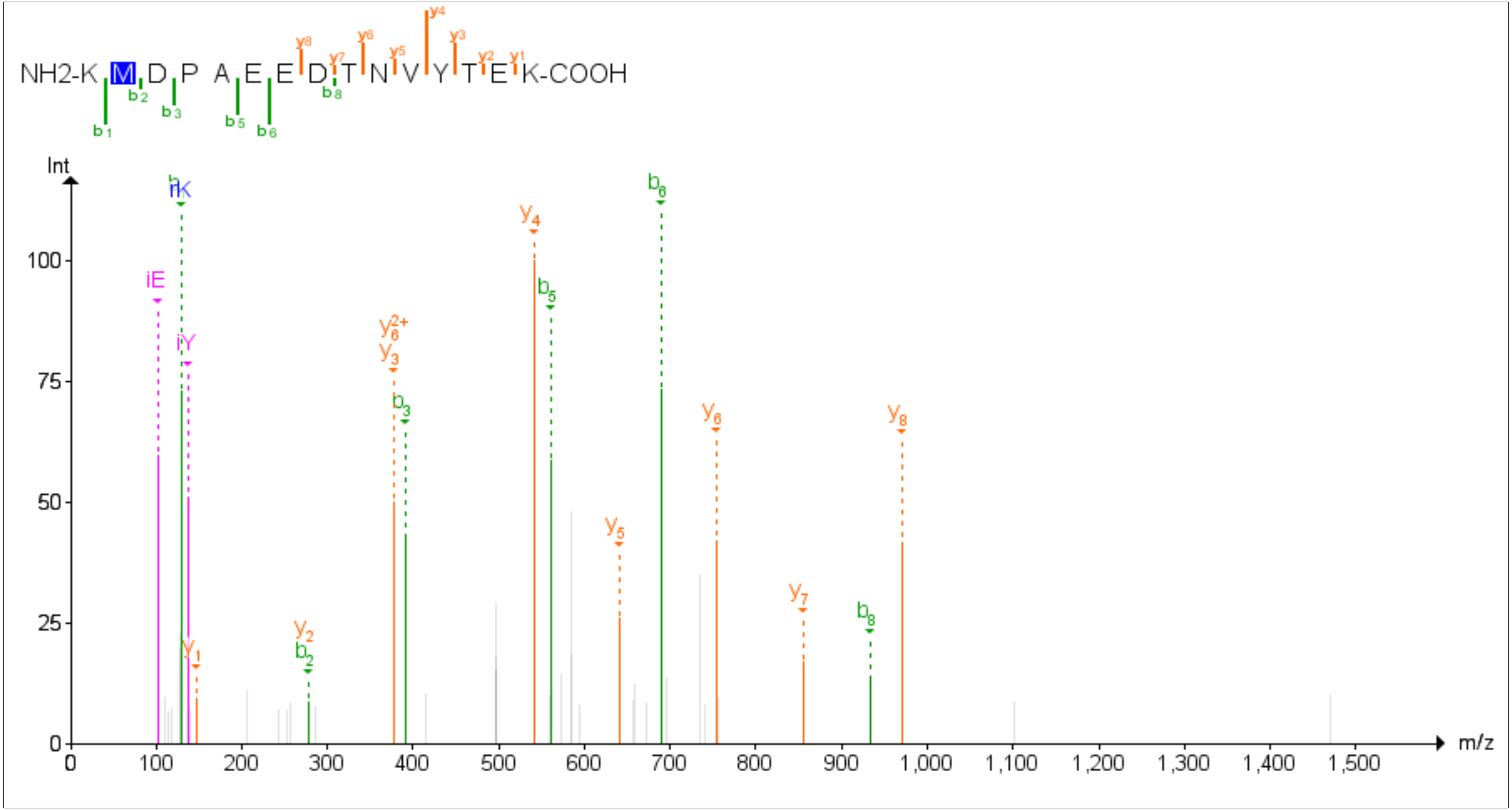

NH2-K M D P A E E D T N V Y T E K-COOH
b1 b2 b3 b5 b6 b8
y8 y7 y6 y5 y4 y3 y2 y1
Int
m/z
iE
iK
iY
y1
y2
b2
y6 2+
y3
b3
y4
b5
y5
b6
y6
y7
b8
y8
0
25
50
75
100
0
100
200
300
400
500
600
700
800
900
1,000
1,100
1,200
1,300
1,400
1,500

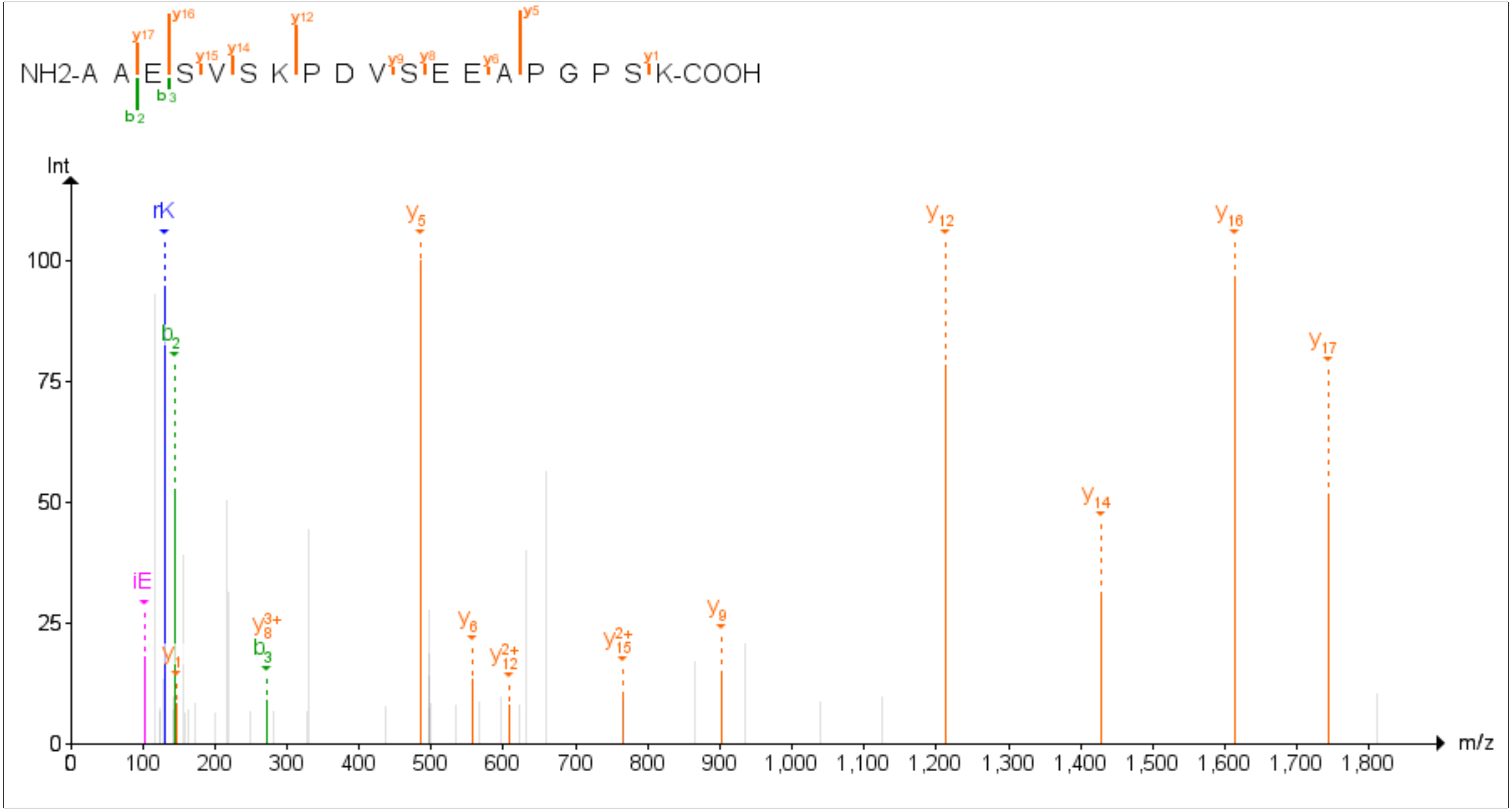

NH2-A A E S V S K P D V S E E A P G P S K-COOH
Int
m/z
rK
iE
b_2
b_3
y_1
y_5
y_6
y_8^{3+}
y_9
y_{12}
y_{12}^{2+}
y_{14}
y_{15}^{2+}
y_{16}
y_{17}

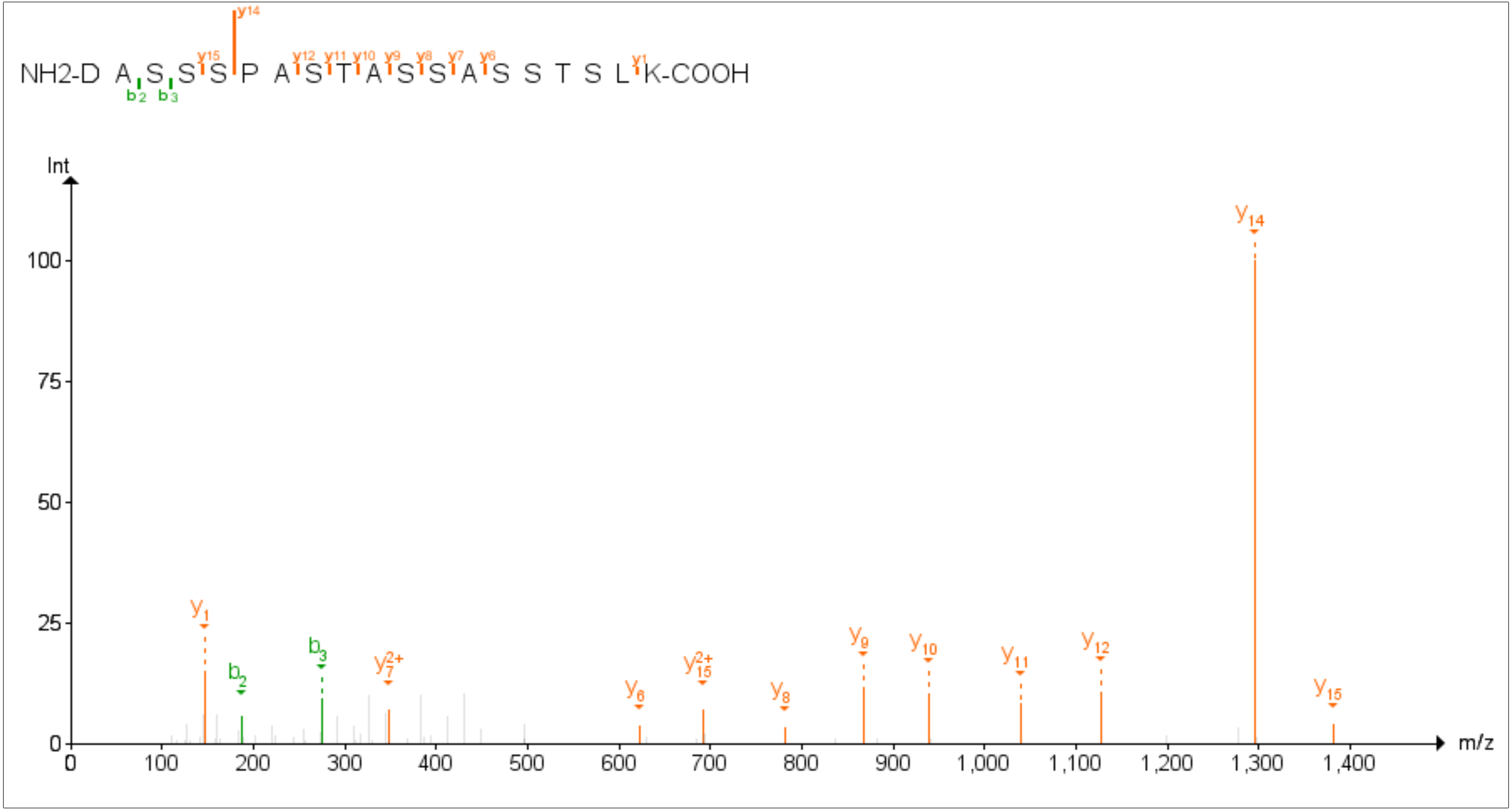

NH2-D A S S S P A S T A S S A S S T S L K-COOH
y14
y15
y12 y11 y10 y9 y8 y7 y6
y1
b2 b3
Int
100
75
50
25
0
m/z
0
100
200
300
400
500
600
700
800
900
1,000
1,100
1,200
1,300
1,400
y14
y1
b2
b3
y7 2+
y6
y15 2+
y8
y9
y10
y11
y12
y15

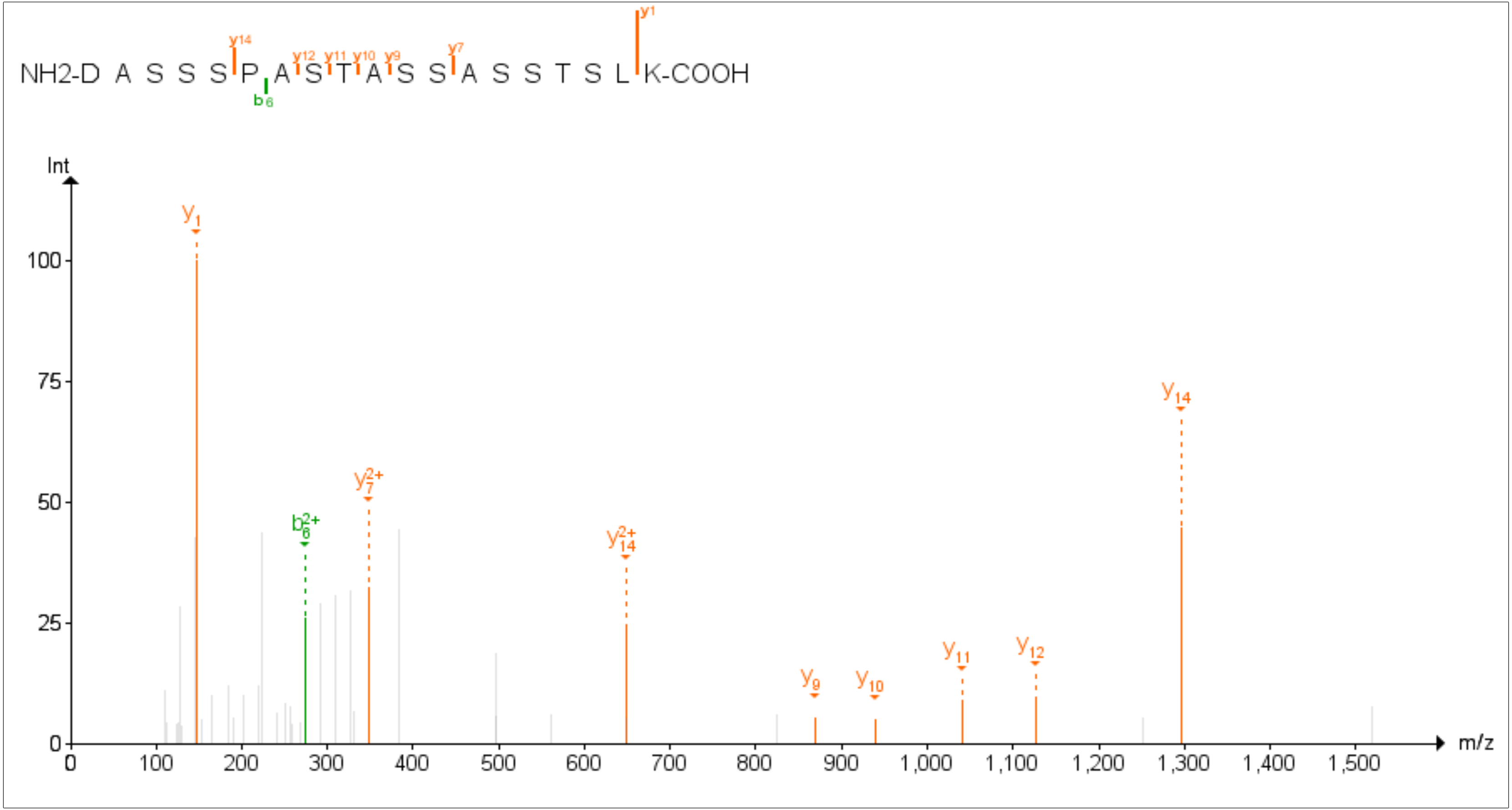

NH2-D A S S S P A S T A S S A S S T S L K-COOH
y14
y12
y11
y10
y9
y7
y1
b6
Int
100
75
50
25
0
y1
y7 2+
b6 2+
y14 2+
y9
y10
y11
y12
y14
0
100
200
300
400
500
600
700
800
900
1,000
1,100
1,200
1,300
1,400
1,500
m/z

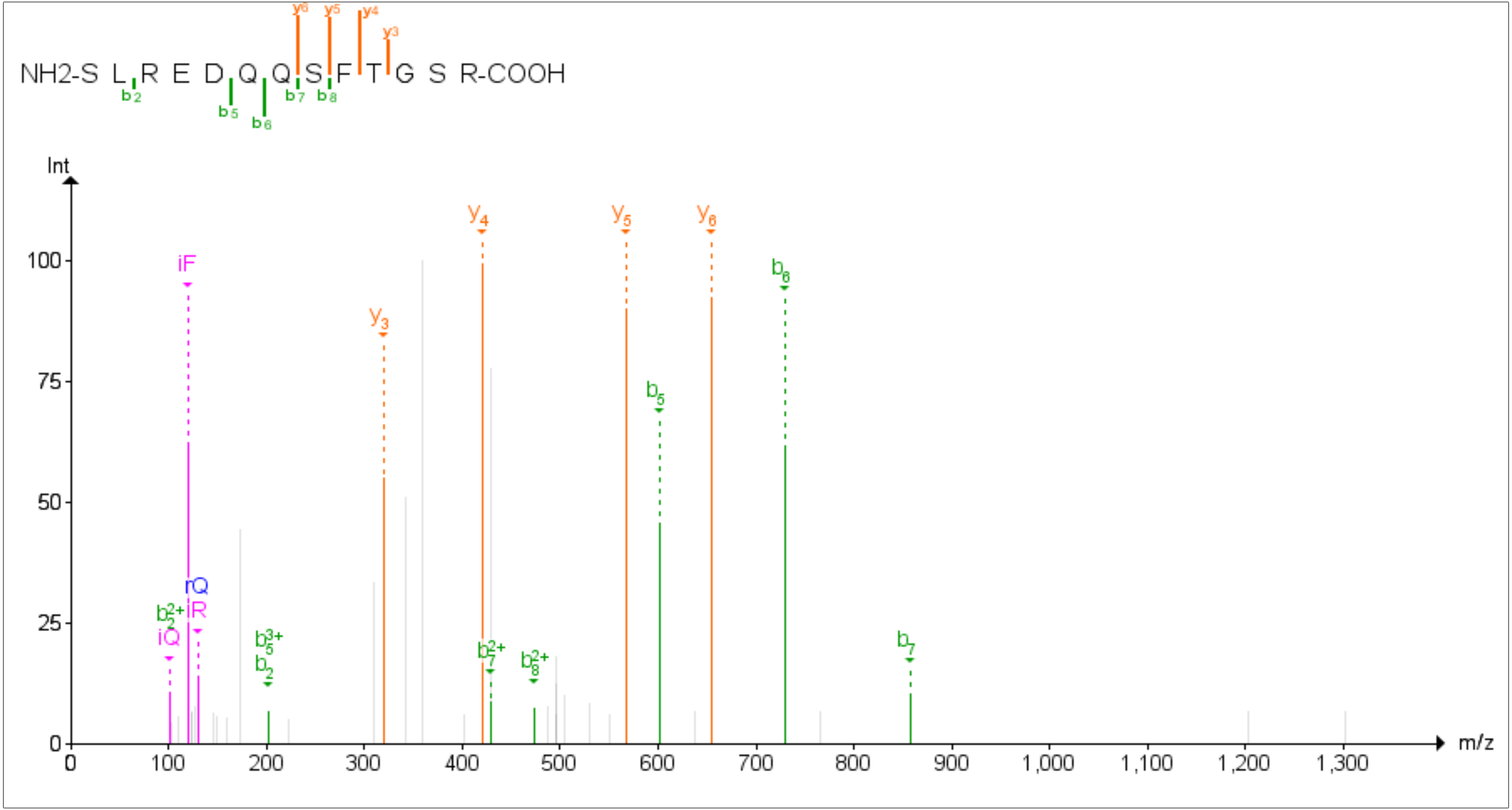

NH2-S L R E D Q Q S F T G S R-COOH
y6 y5 y4 y3
b2 b5 b6 b7 b8
Int
100
75
50
25
0
iF
y3
y4
y5
y6
b6
b5
rQ
iR
iQ
b2
b7
b8
0 100 200 300 400 500 600 700 800 900 1,000 1,100 1,200 1,300
m/z

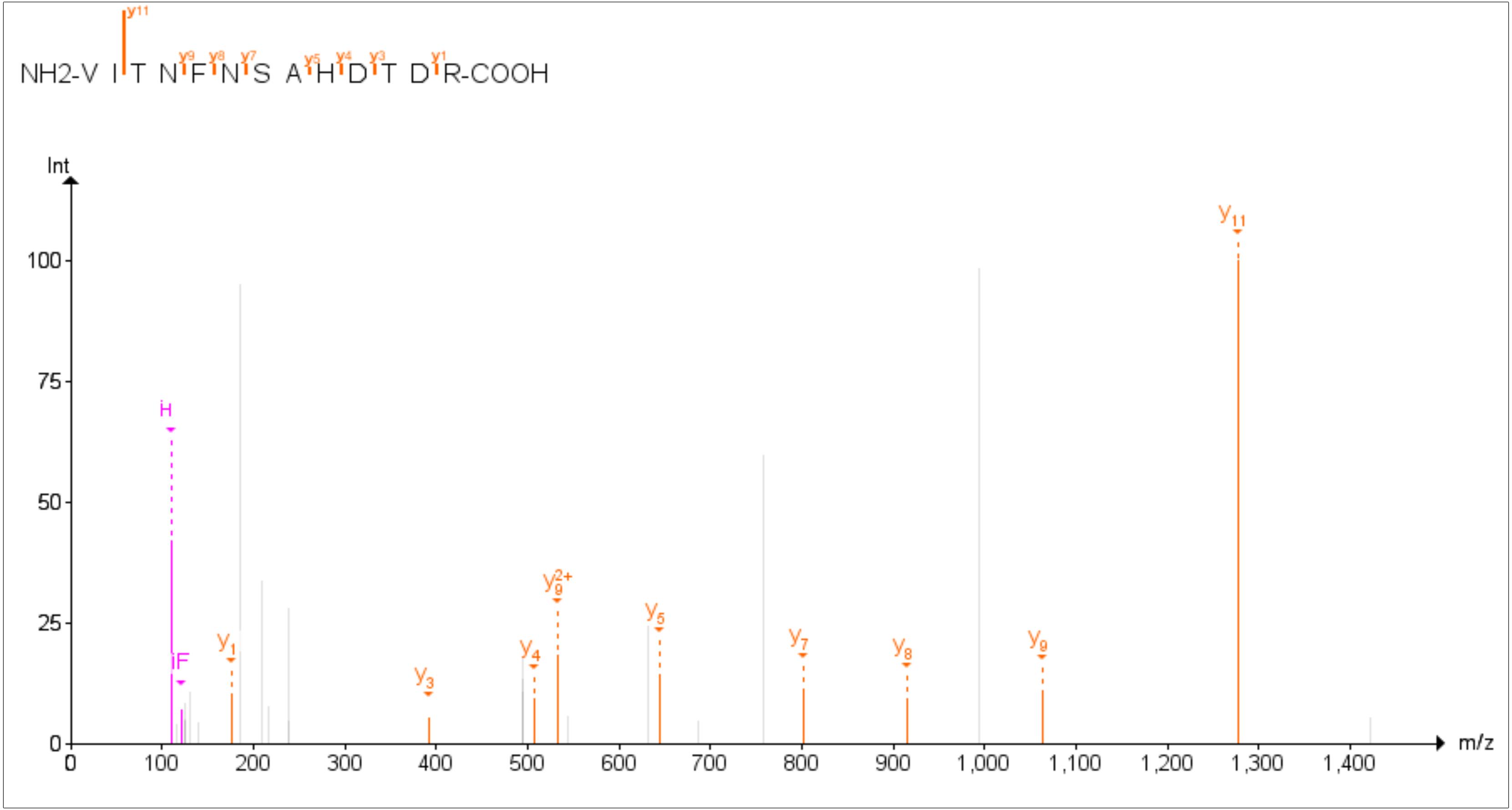

NH2-V I T N F N S A H D T D R-COOH
y11
y9
y8
y7
y5
y4
y3
y1
Int
100
75
50
25
0
m/z
0
100
200
300
400
500
600
700
800
900
1,000
1,100
1,200
1,300
1,400
iH
iF
y1
y3
y4
y9 2+
y5
y7
y8
y9
y11

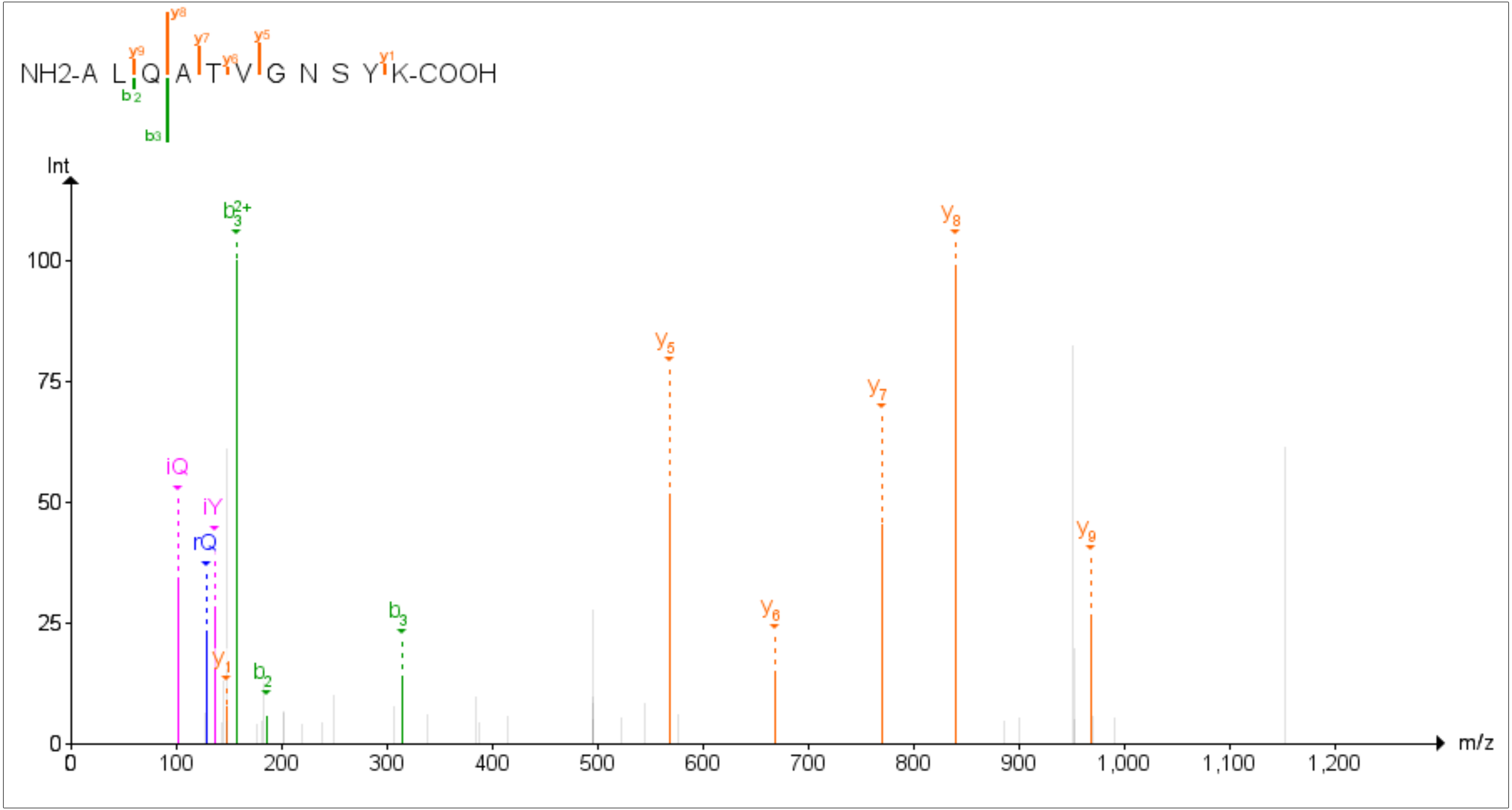

NH2-A L Q A T V G N S Y K-COOH
y9
y8
y7
y6
y5
y1
b2
b3
Int
100
75
50
25
0
iQ
iY
rQ
y1
b2
b3 2+
b3
y5
y6
y7
y8
y9
0
100
200
300
400
500
600
700
800
900
1,000
1,100
1,200
m/z

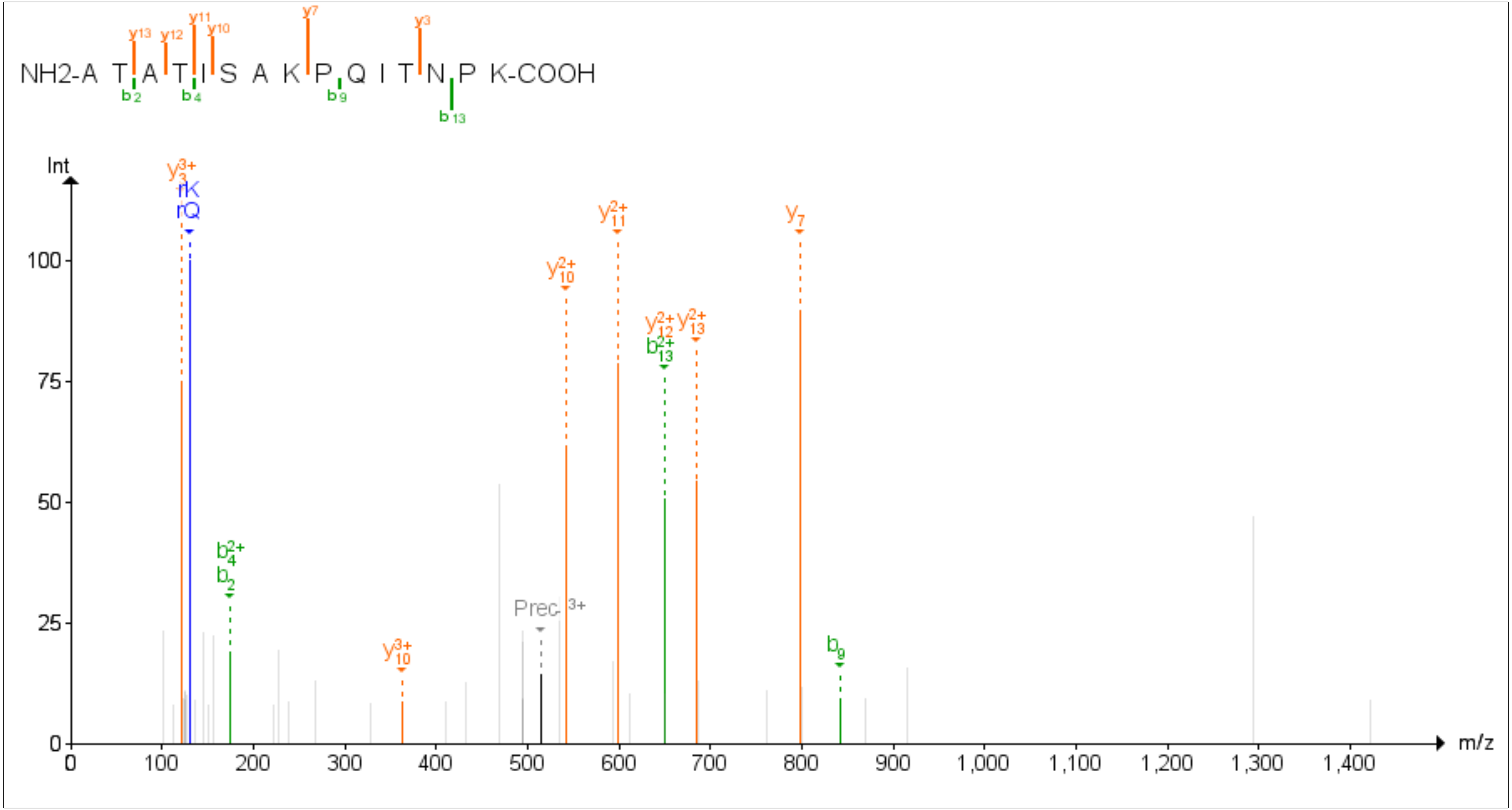

NH2-A T A T I S A K P Q I T N P K-COOH
y13 y12 y11 y10 y7 y3
b2 b4 b9 b13
Int
100
75
50
25
0
y3 3+
rK
rQ
y11 2+
y7
y10 2+
y12 2+ y13 2+
b13 2+
b4 2+
b2
Prec 3+
y10 3+
b9
0 100 200 300 400 500 600 700 800 900 1,000 1,100 1,200 1,300 1,400
m/z

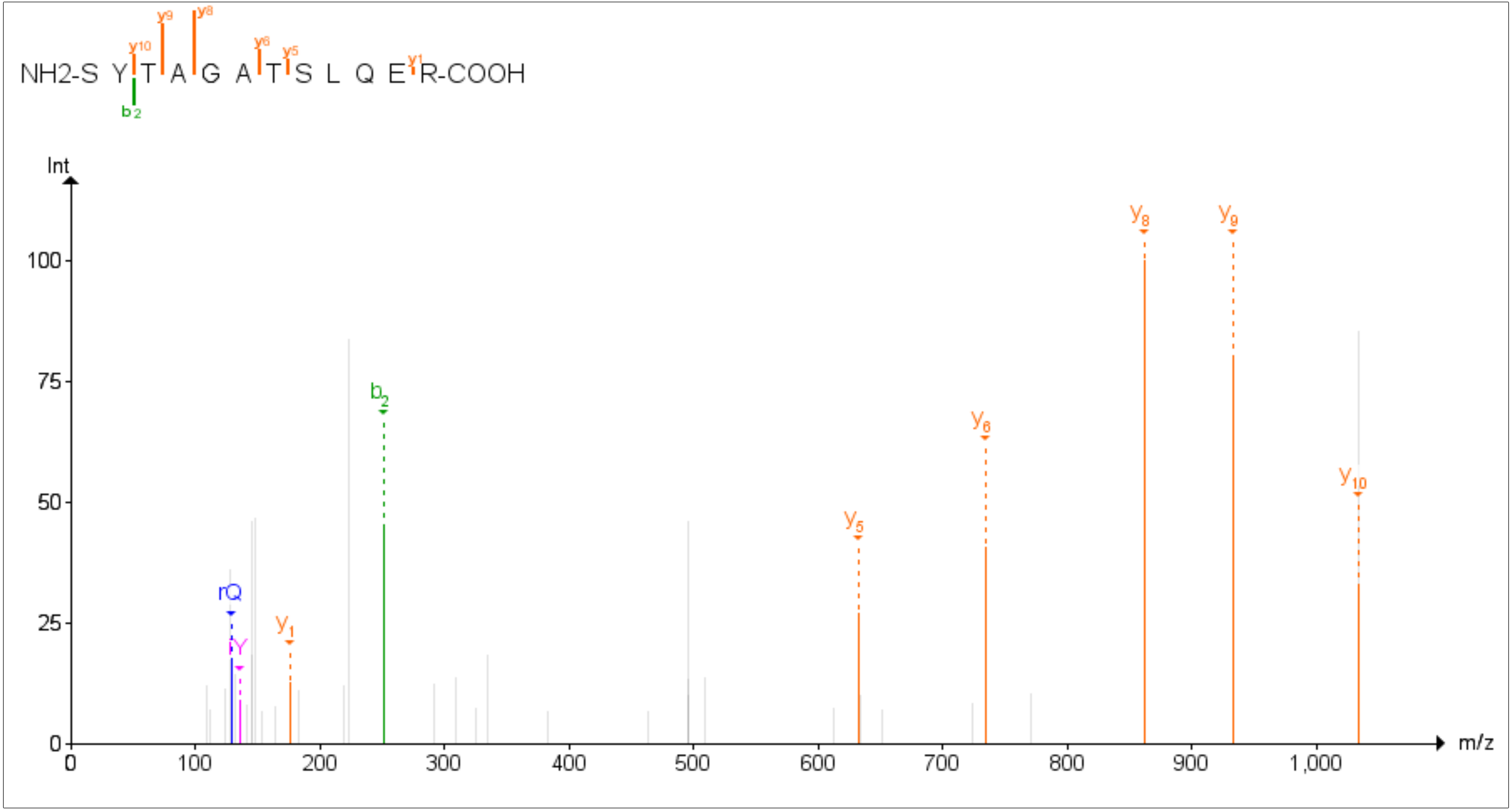

NH2-S Y T A G A T S L Q E R-COOH
y10
y9
y8
y6
y5
y1
b2
Int
100
75
50
25
0
m/z
0
100
200
300
400
500
600
700
800
900
1,000
rQ
iY
b2
y1
y5
y6
y8
y9
y10

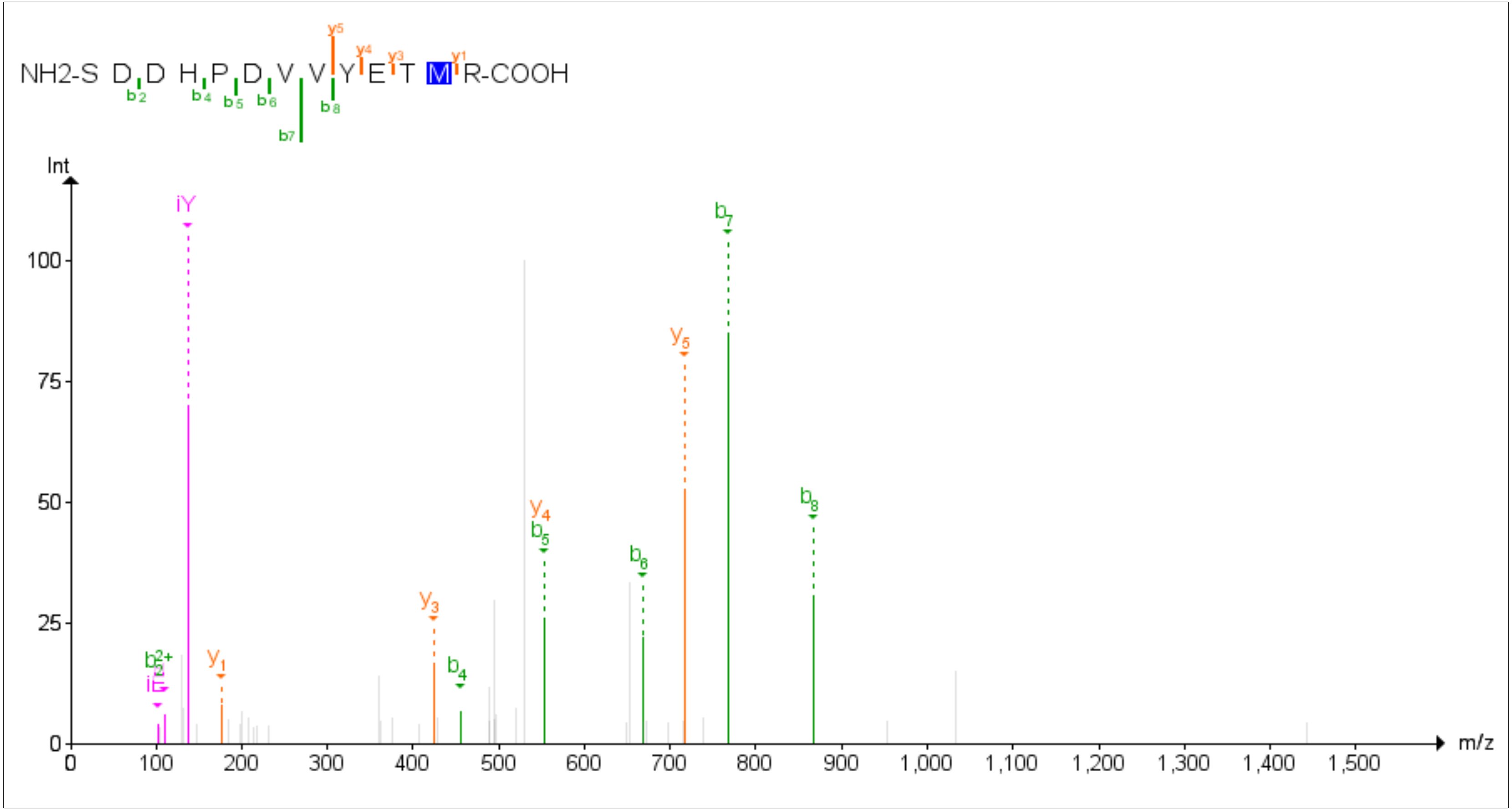

NH2-S D D H P D V V Y E T M R-COOH
y5 y4 y3 y1
b2 b4 b5 b6 b7 b8
Int
iY
b7
100
75
y5
50
y4
b5
b8
b6
25
y3
b2 2+
y1
b4
iE
0
0 100 200 300 400 500 600 700 800 900 1,000 1,100 1,200 1,300 1,400 1,500
m/z

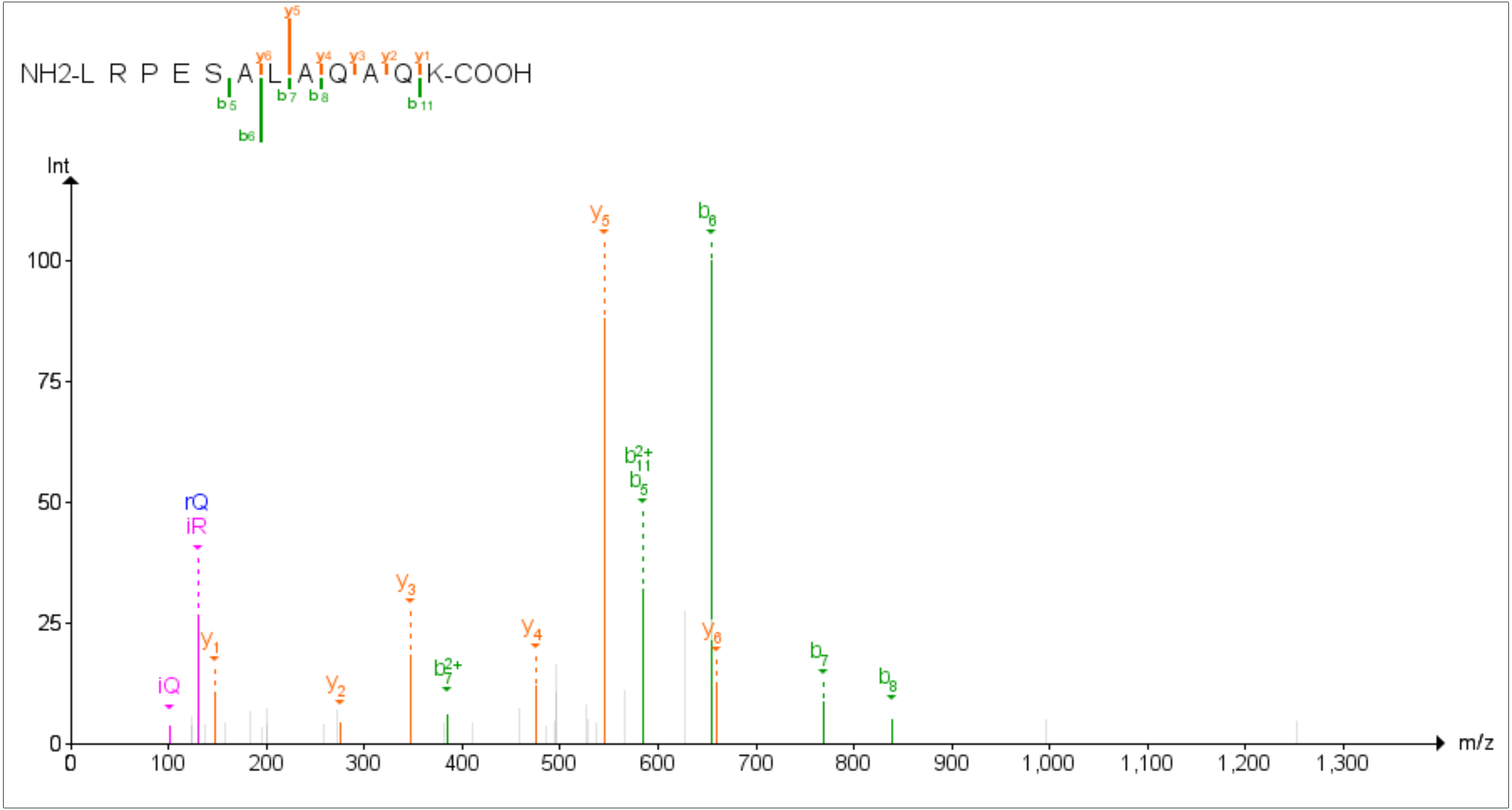
NH2-L R P E S A L A Q A Q K-COOH
y5
y6
y4
y3
y2
y1
b5
b6
b7
b8
b11
Int
m/z
y5
b6
b11 2+
b5
rQ
iR
iQ
y1
y2
y3
b7 2+
y4
y6
b7
b8

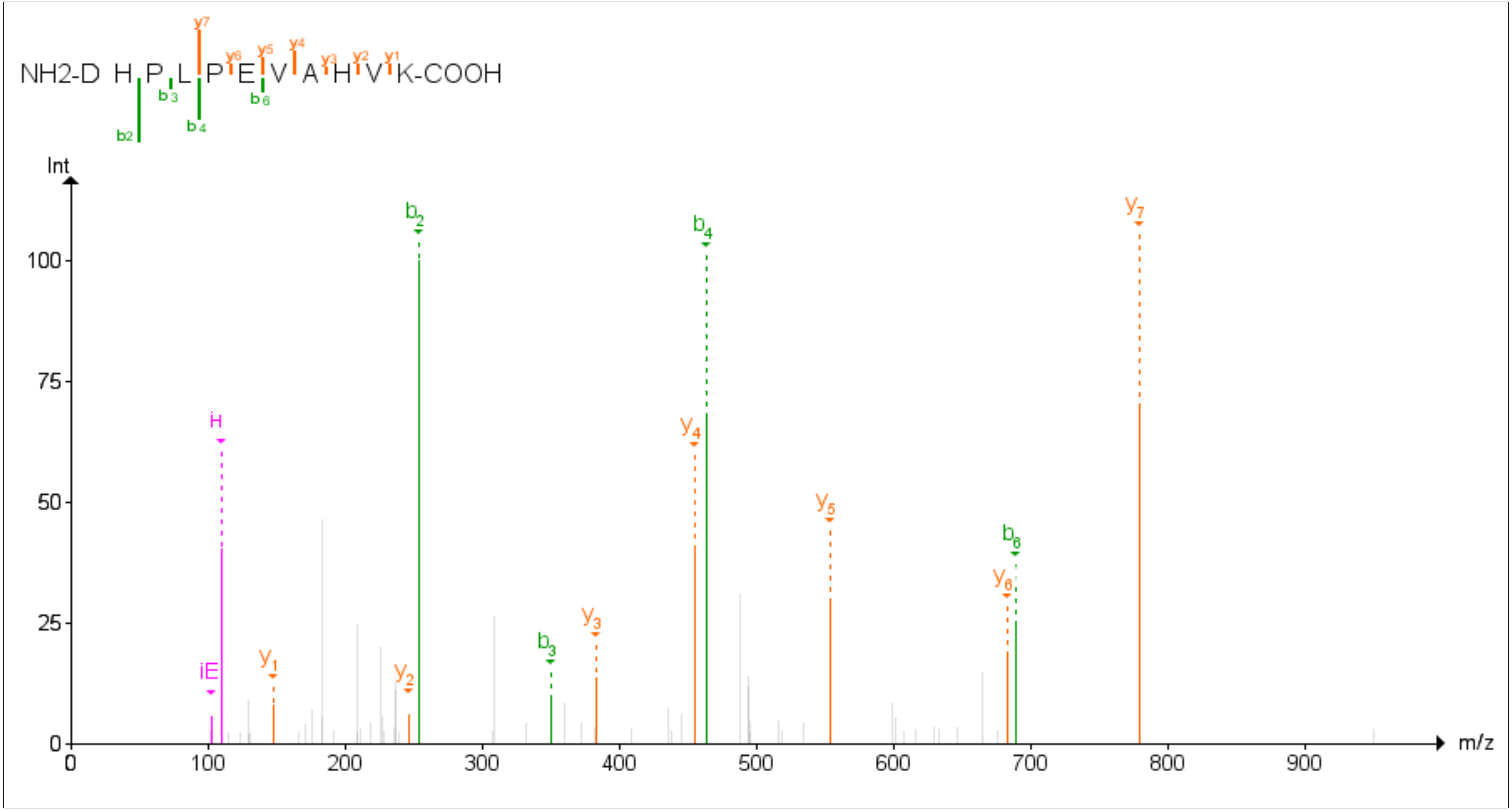

NH2-D H P L P E V A H V K-COOH
y7 y6 y5 y4 y3 y2 y1
b2 b3 b4 b6
Int
100
75
50
25
0
m/z
0 100 200 300 400 500 600 700 800 900
b2
b4
y7
iH
y4
y5
b6
y6
y3
b3
y1
iE
y2

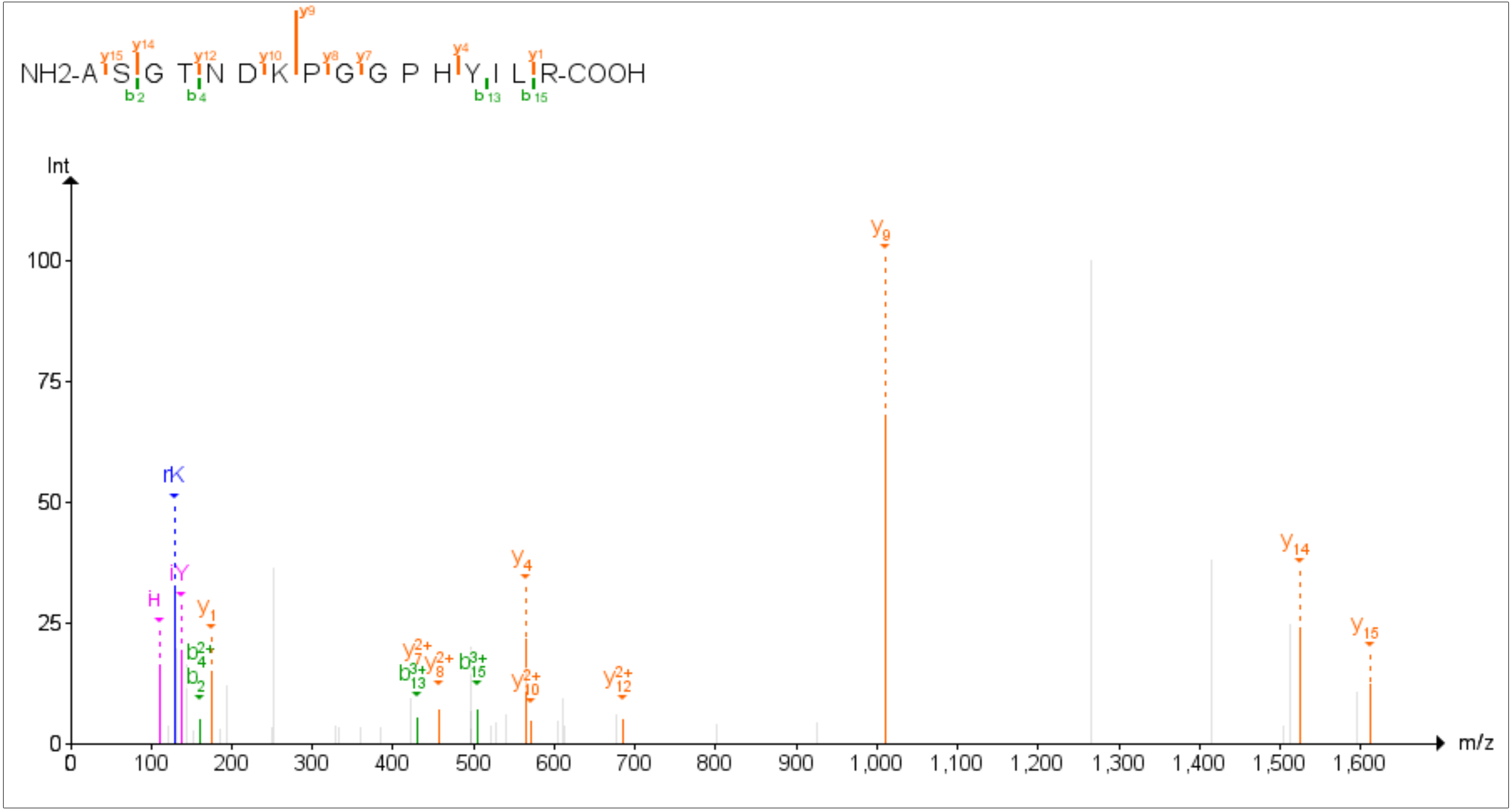

NH2-A S G T N D K P G G P H Y I L R-COOH
y15 y14 y12 y10 y9 y8 y7 y4 y1
b2 b4 b13 b15
Int
m/z
rK
iY
iH
y1
b2
y9
y4
y14
y15
0
25
50
75
100
0
100
200
300
400
500
600
700
800
900
1,000
1,100
1,200
1,300
1,400
1,500
1,600

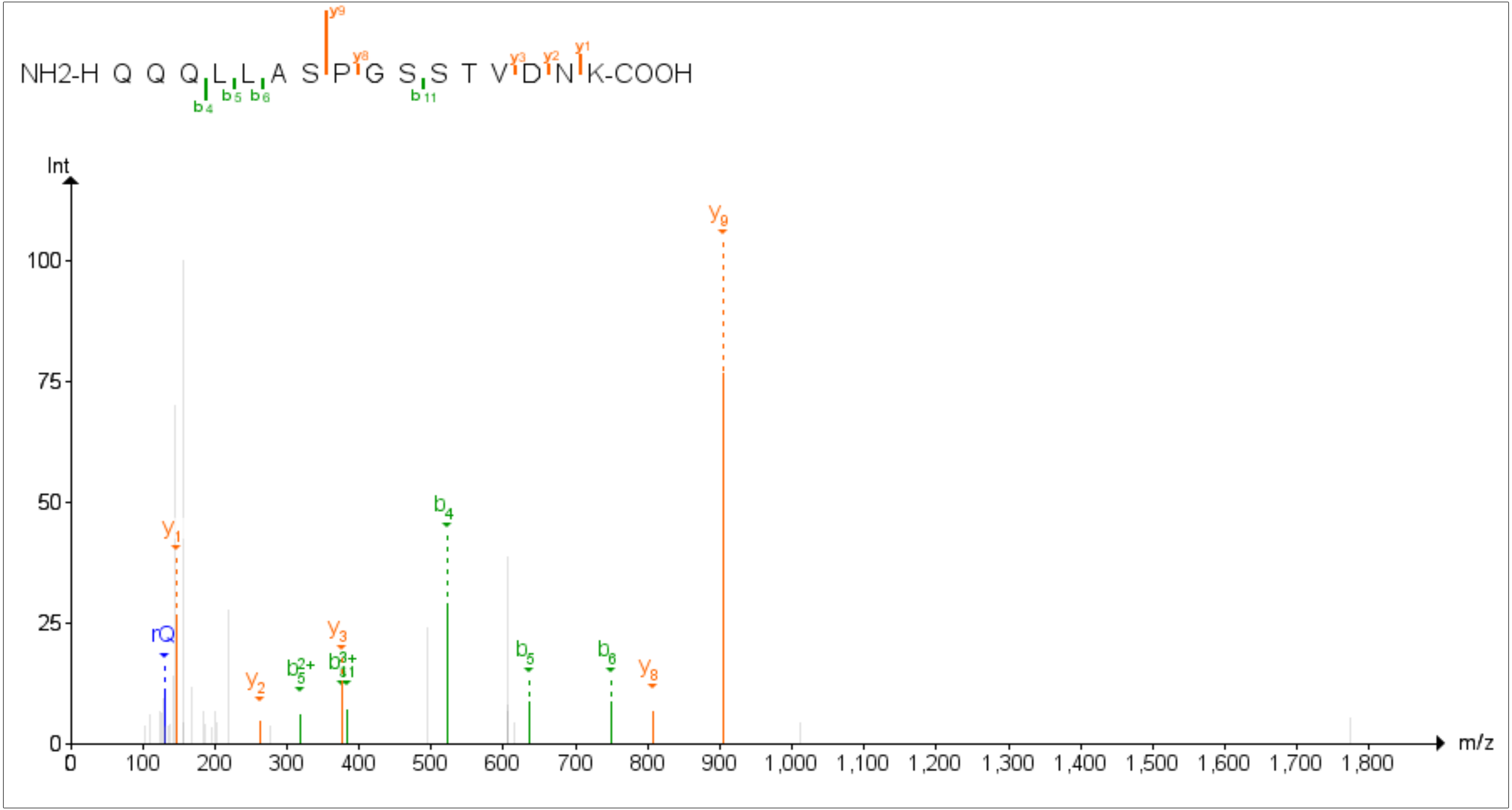
NH2-H Q Q Q L L A S P G S S T V D N K-COOH
y9
y8
y3
y2
y1
b4
b5
b6
b11
Int
100
75
50
25
0
y9
b4
y1
rQ
y3
y2
b5 2+
b11 3+
b5
b6
y8
0
100
200
300
400
500
600
700
800
900
1,000
1,100
1,200
1,300
1,400
1,500
1,600
1,700
1,800
m/z

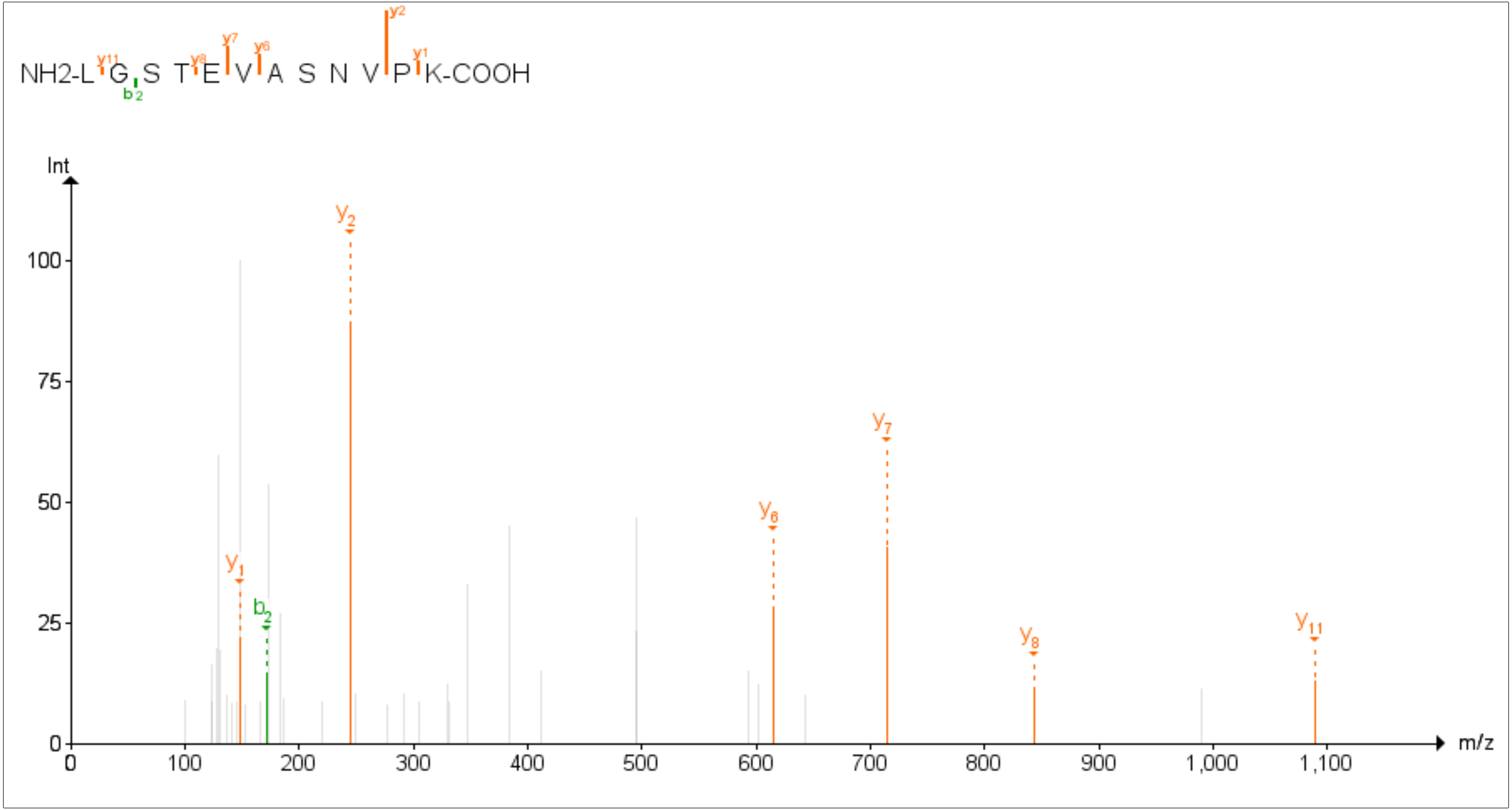

NH2-L G S T E V A S N V P K-COOH
y11
y8
y7
y6
y2
y1
b2
Int
100
75
50
25
0
y2
y7
y6
y1
b2
y8
y11
0
100
200
300
400
500
600
700
800
900
1,000
1,100
m/z

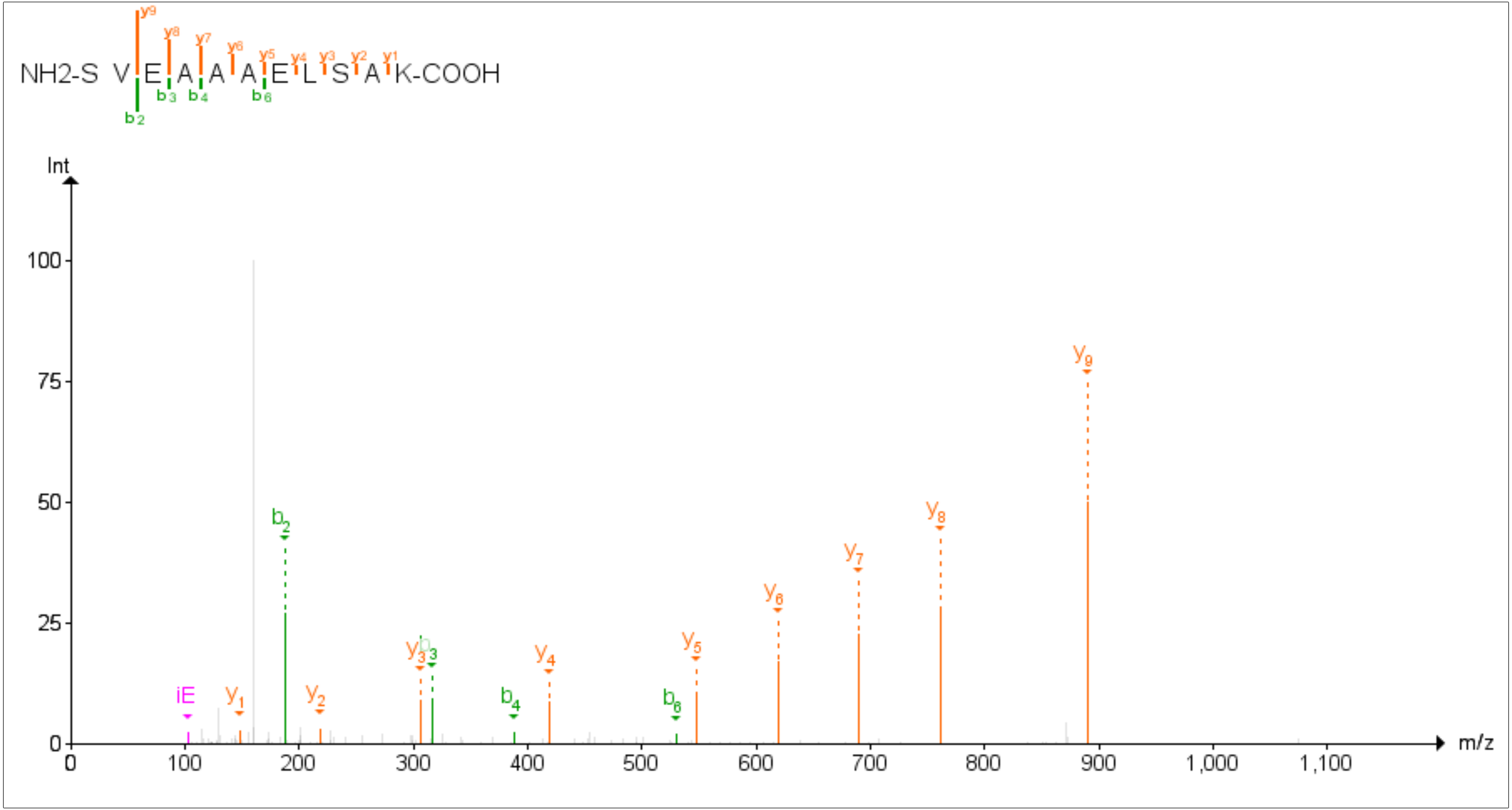

NH2-S V E A A A E L S A K-COOH
y9 y8 y7 y6 y5 y4 y3 y2 y1
b2 b3 b4 b6
Int
100
75
50
25
0
m/z
0
100
200
300
400
500
600
700
800
900
1,000
1,100
iE
y1
b2
y2
y3
b3
b4
y4
b6
y5
y6
y7
y8
y9

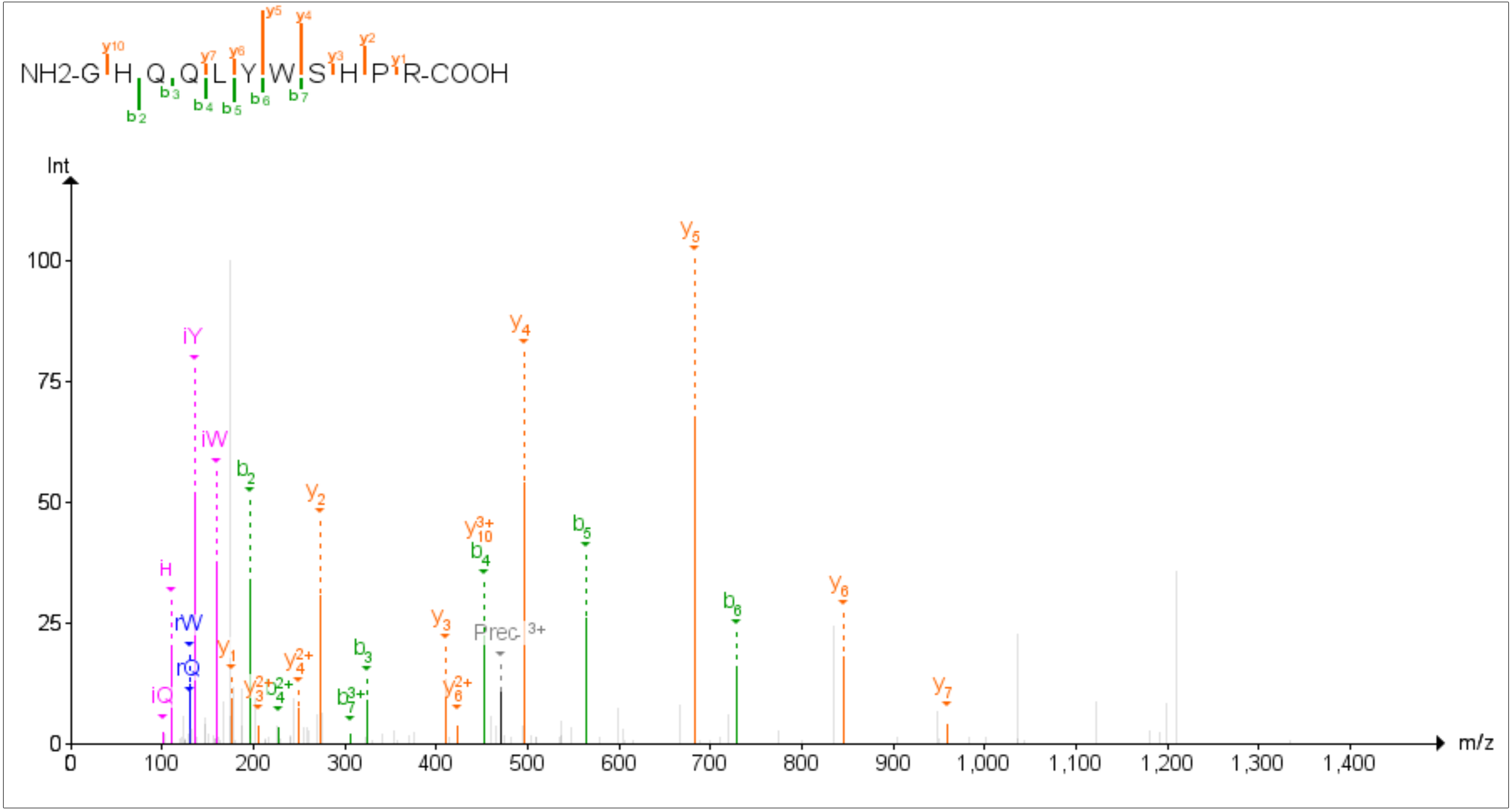

NH2-G H Q Q L Y W S H P R-COOH
y10 y7 y6 y5 y4 y3 y2 y1
b2 b3 b4 b5 b6 b7
Int
m/z
iY
iW
iH
iQ
rW
rQ
y1
y2
y3
y4
y5
y6
y7
b2
b3
b4
b5
b6
Prec 3+

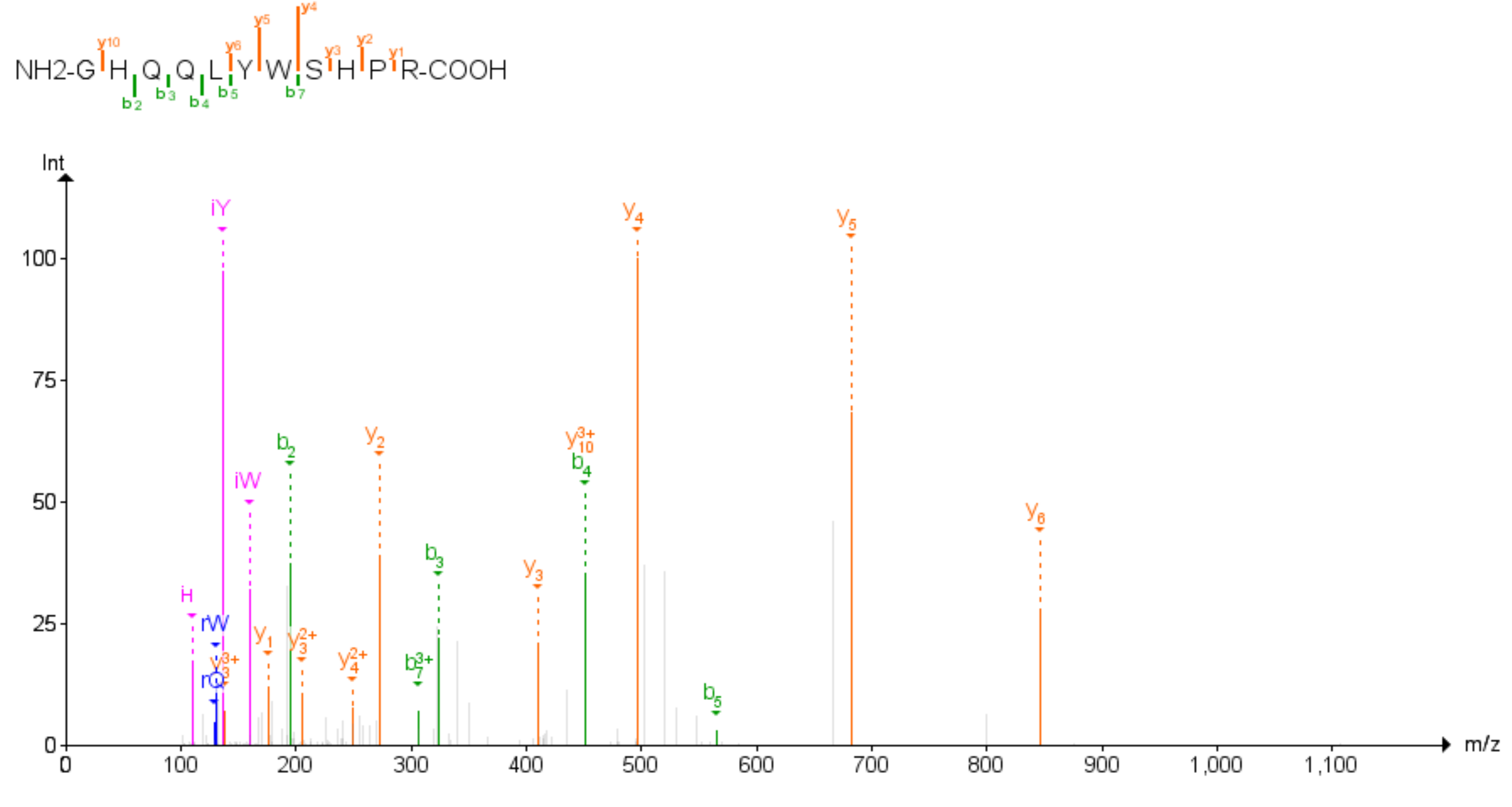

NH2-G H Q Q L Y W S H P R-COOH
y10 y6 y5 y4 y3 y2 y1
b2 b3 b4 b5 b7
Int
m/z
iY
y4
y5
iW
b2
y2
y10 3+
b4
y6
b3
y3
iH
rW
y3 3+
rQ
y1
y3 2+
y4 2+
b7 3+
b5
0
25
50
75
100
0
100
200
300
400
500
600
700
800
900
1,000
1,100

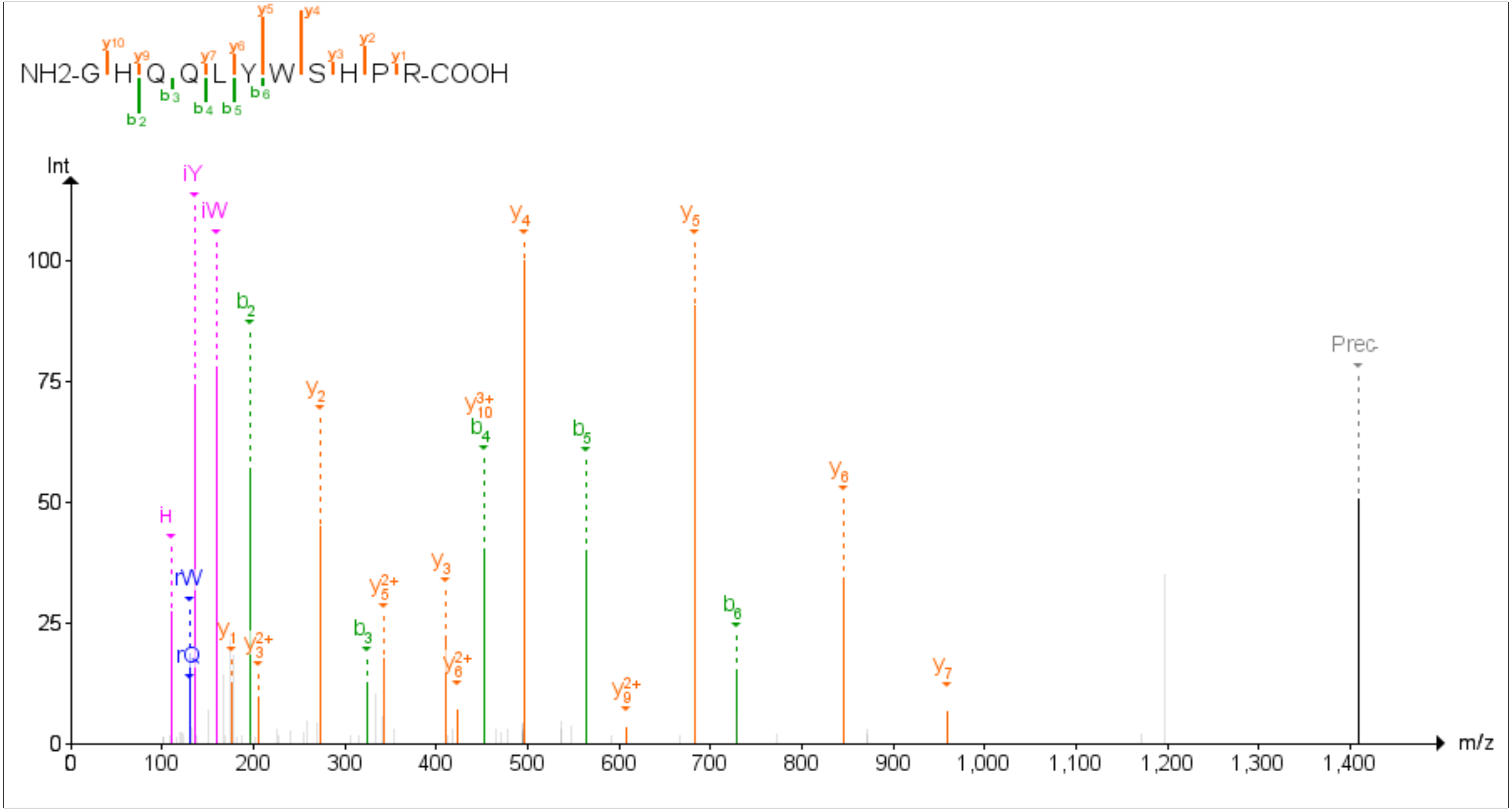
NH2-G H Q Q L Y W S H P R-COOH
y10 y9 y7 y6 y5 y4 y3 y2 y1
b2 b3 b4 b5 b6
Int
m/z
iY
iW
iH
rW
rQ
y1
y2
y3
y4
y5
y6
y7
b2
b3
b4
b5
b6
y3 2+
y5 2+
y6 2+
y9 2+
y10 3+
Prec
0
25
50
75
100
0
100
200
300
400
500
600
700
800
900
1,000
1,100
1,200
1,300
1,400

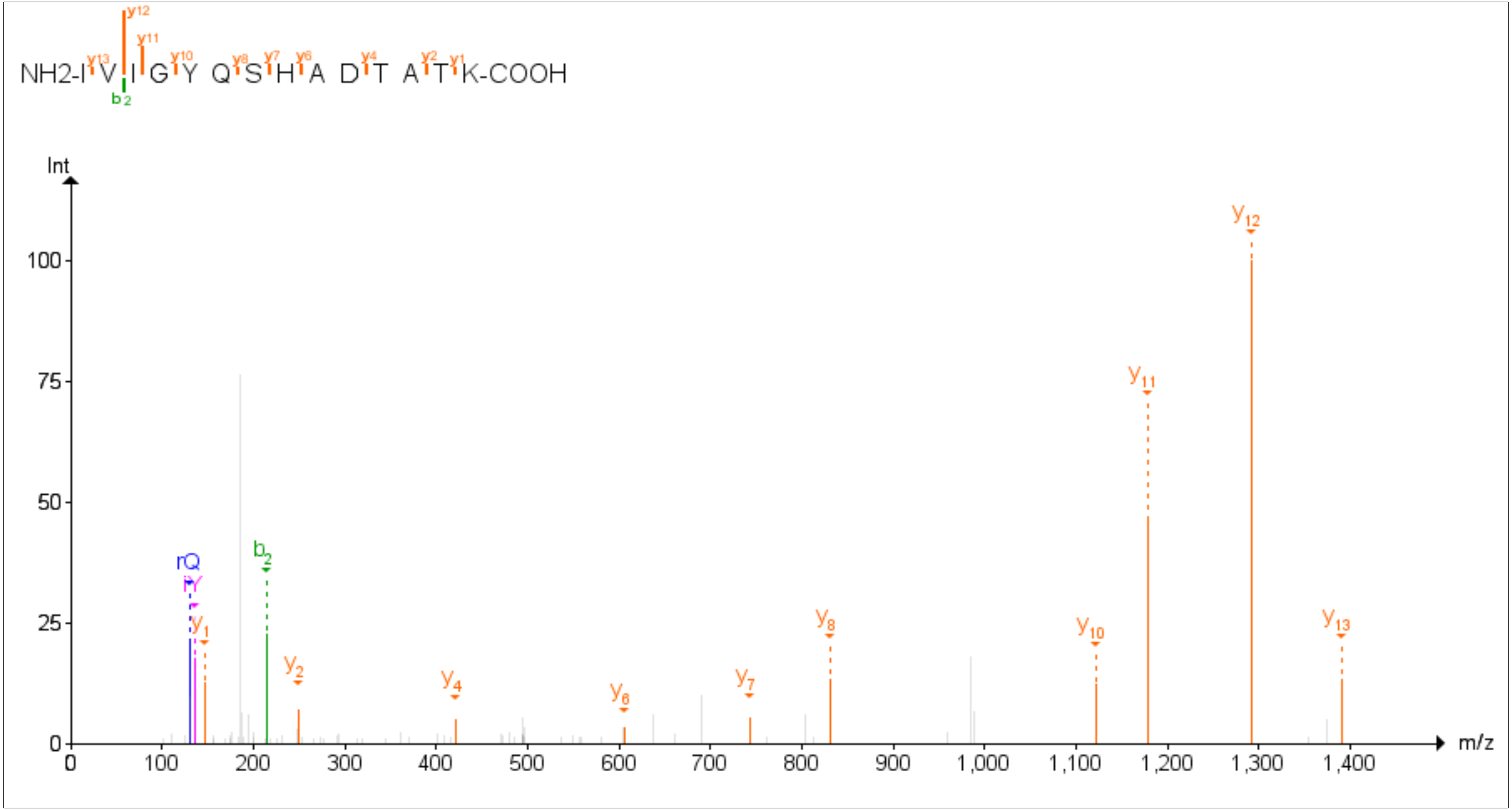

NH2-I V I G Y Q S H A D T A T K-COOH
y13 y12 y11 y10 y8 y7 y6 y4 y2 y1
b2
Int
m/z
0
25
50
75
100
0 100 200 300 400 500 600 700 800 900 1,000 1,100 1,200 1,300 1,400
rQ
iY
y1
b2
y2
y4
y6
y7
y8
y10
y11
y12
y13

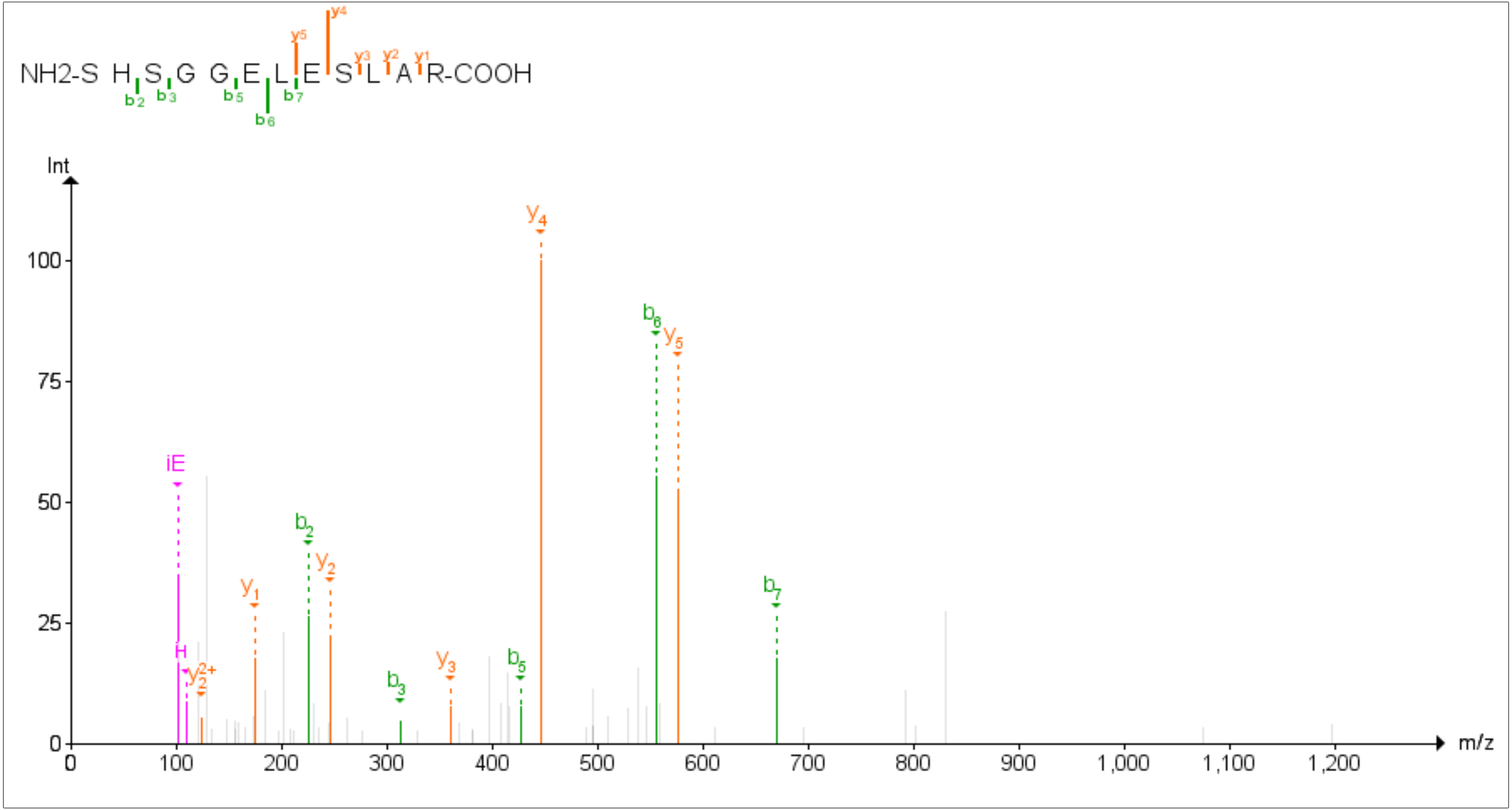

NH2-S H S G G E L E S L A R-COOH
b2
b3
b5
b6
b7
y5
y4
y3
y2
y1
Int
100
75
50
25
0
iE
H
y2 2+
y1
b2
y2
b3
y3
b5
y4
b6
y5
b7
m/z
0
100
200
300
400
500
600
700
800
900
1,000
1,100
1,200

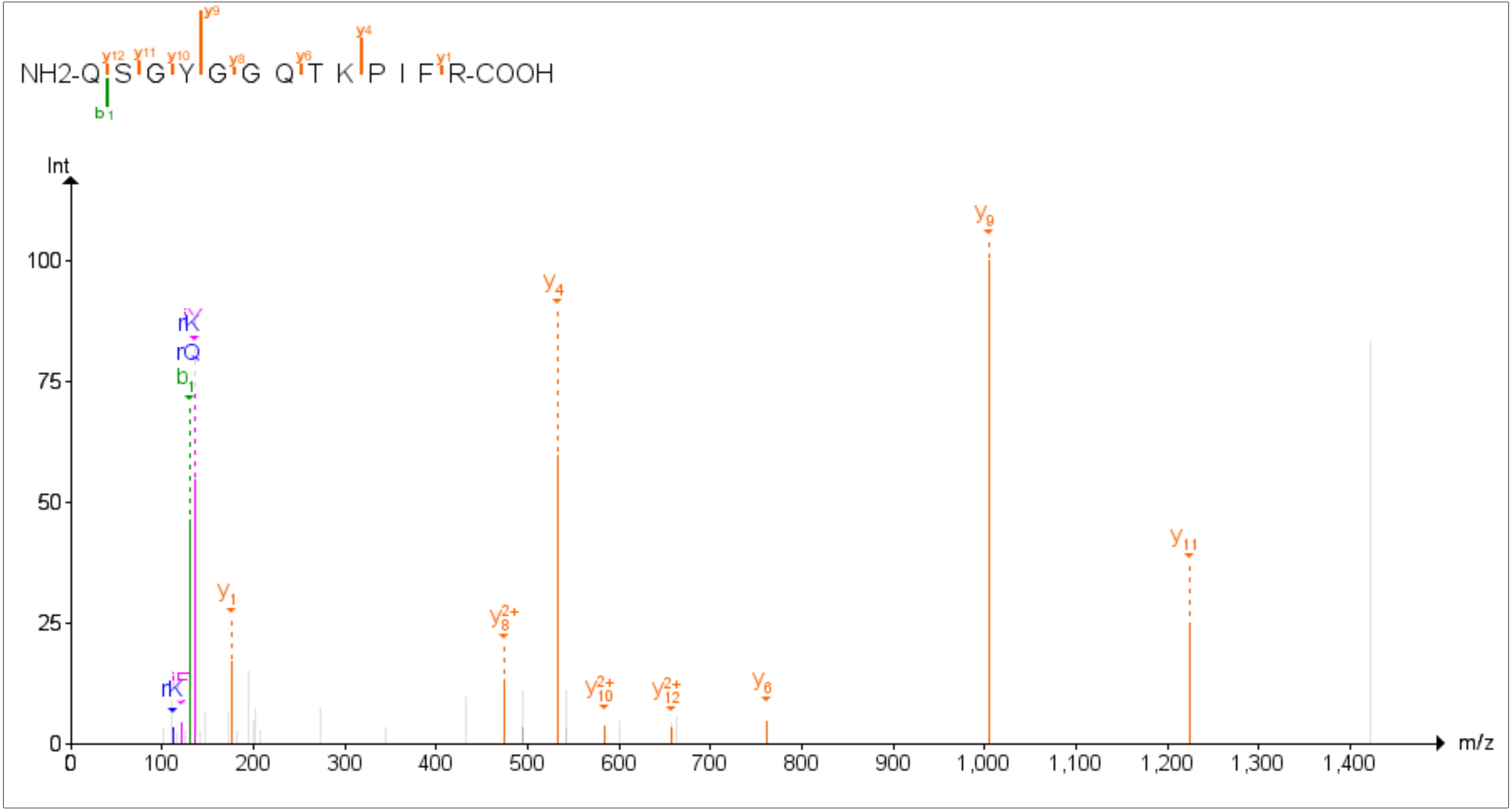

NH2-Q S G Y G G Q T K P I F R-COOH
y12 y11 y10 y9 y8 y6 y4 y1
b1
Int
100
75
50
25
0
iY
rK
rQ
b1
y1
iF
rK
y4
y_8^{2+}
y_{10}^{2+}
y_{12}^{2+}
y6
y9
y11
0
100
200
300
400
500
600
700
800
900
1,000
1,100
1,200
1,300
1,400
m/z

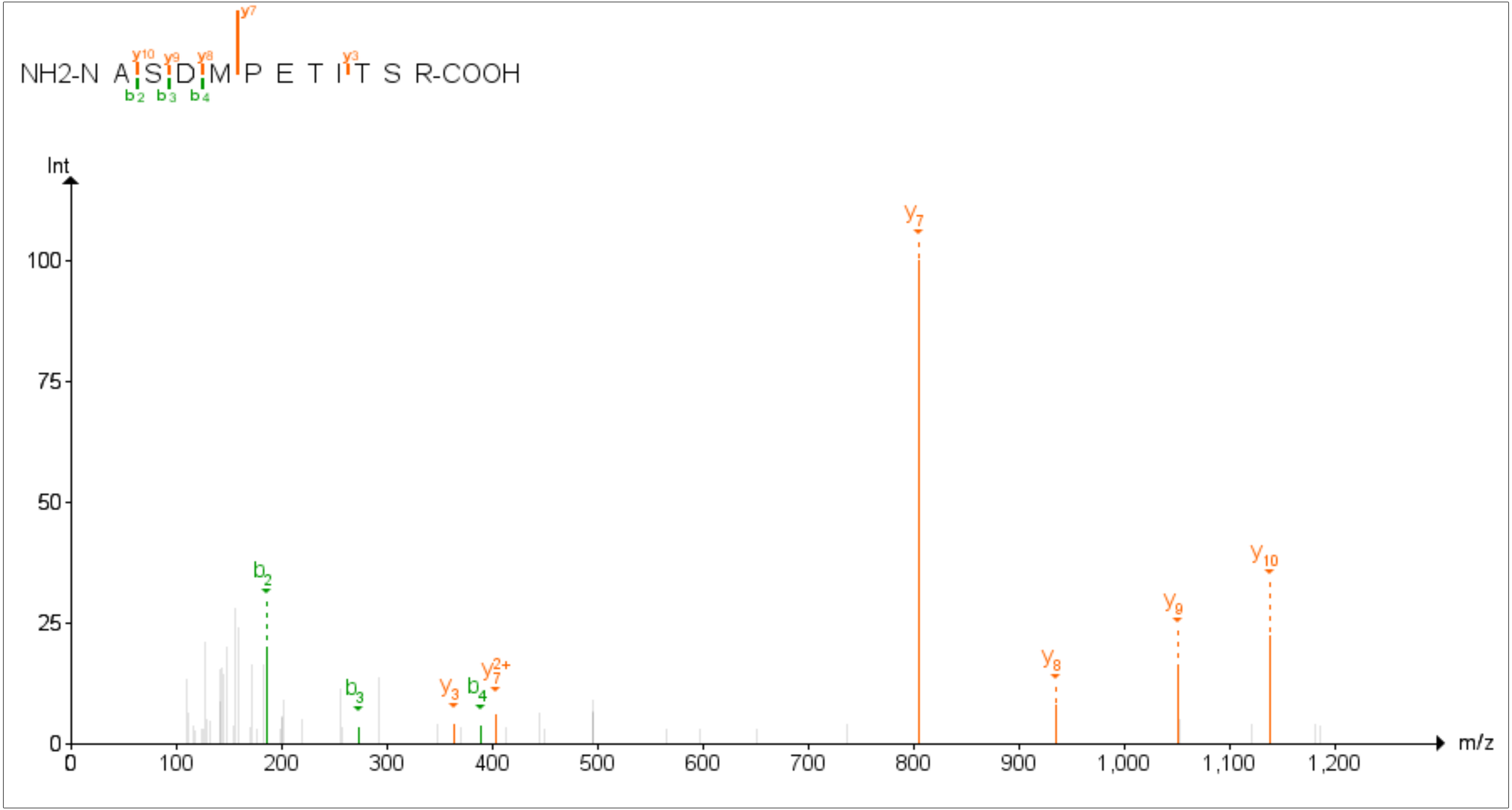

NH2-N A S D M P E T I T S R-COOH
y10 y9 y8 y7 y3
b2 b3 b4
Int
100
75
50
25
0
b2
b3
y3
b4
y7 2+
y7
y8
y9
y10
0 100 200 300 400 500 600 700 800 900 1,000 1,100 1,200
m/z

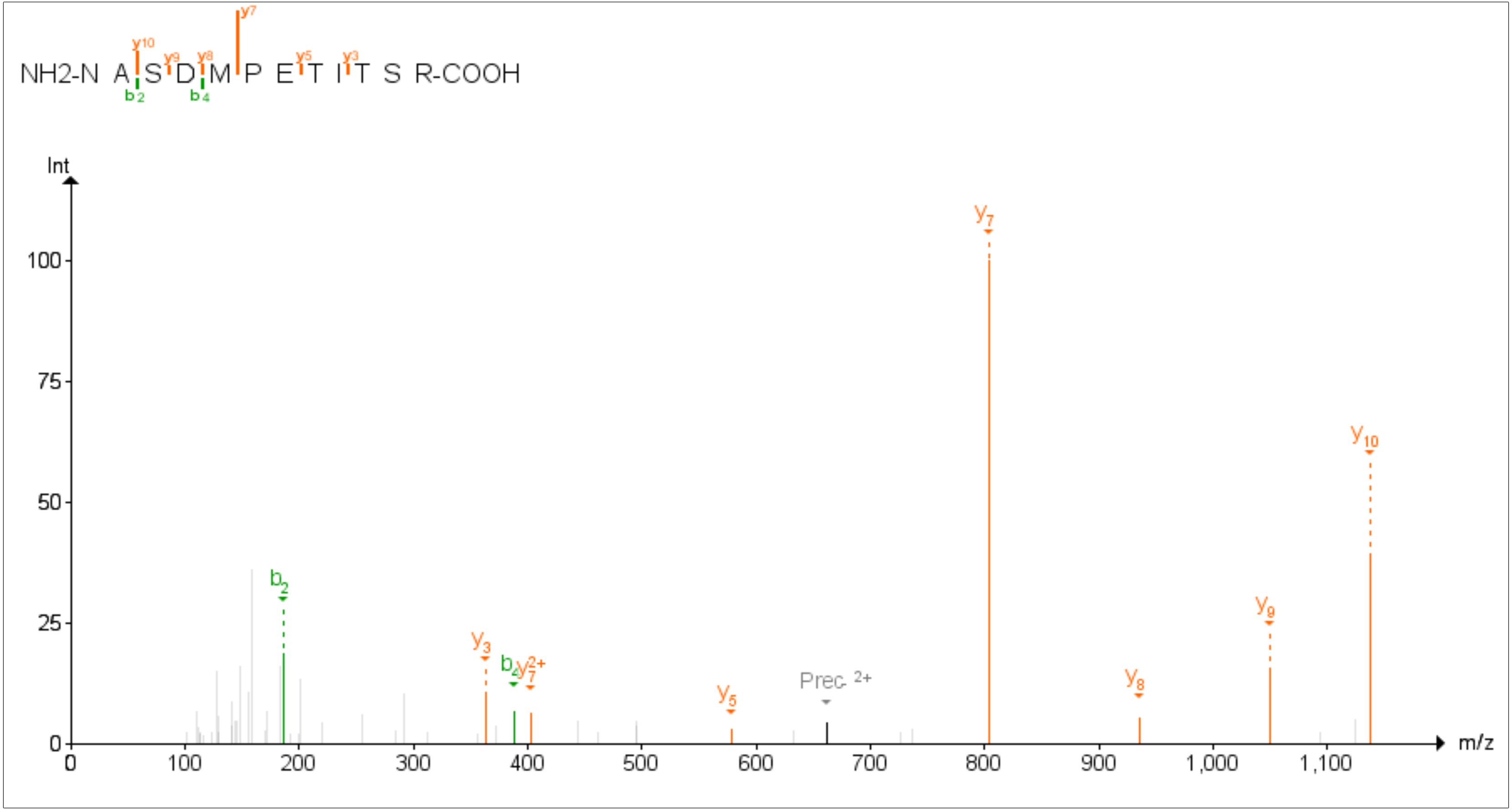
NH2-N A S D M P E T I T S R-COOH
y10 y9 y8 y7 y5 y3
b2 b4
Int
100
75
50
25
0
y7
y10
b2
y3
b4 y7 2+
y5
Prec 2+
y8
y9
0 100 200 300 400 500 600 700 800 900 1,000 1,100
m/z

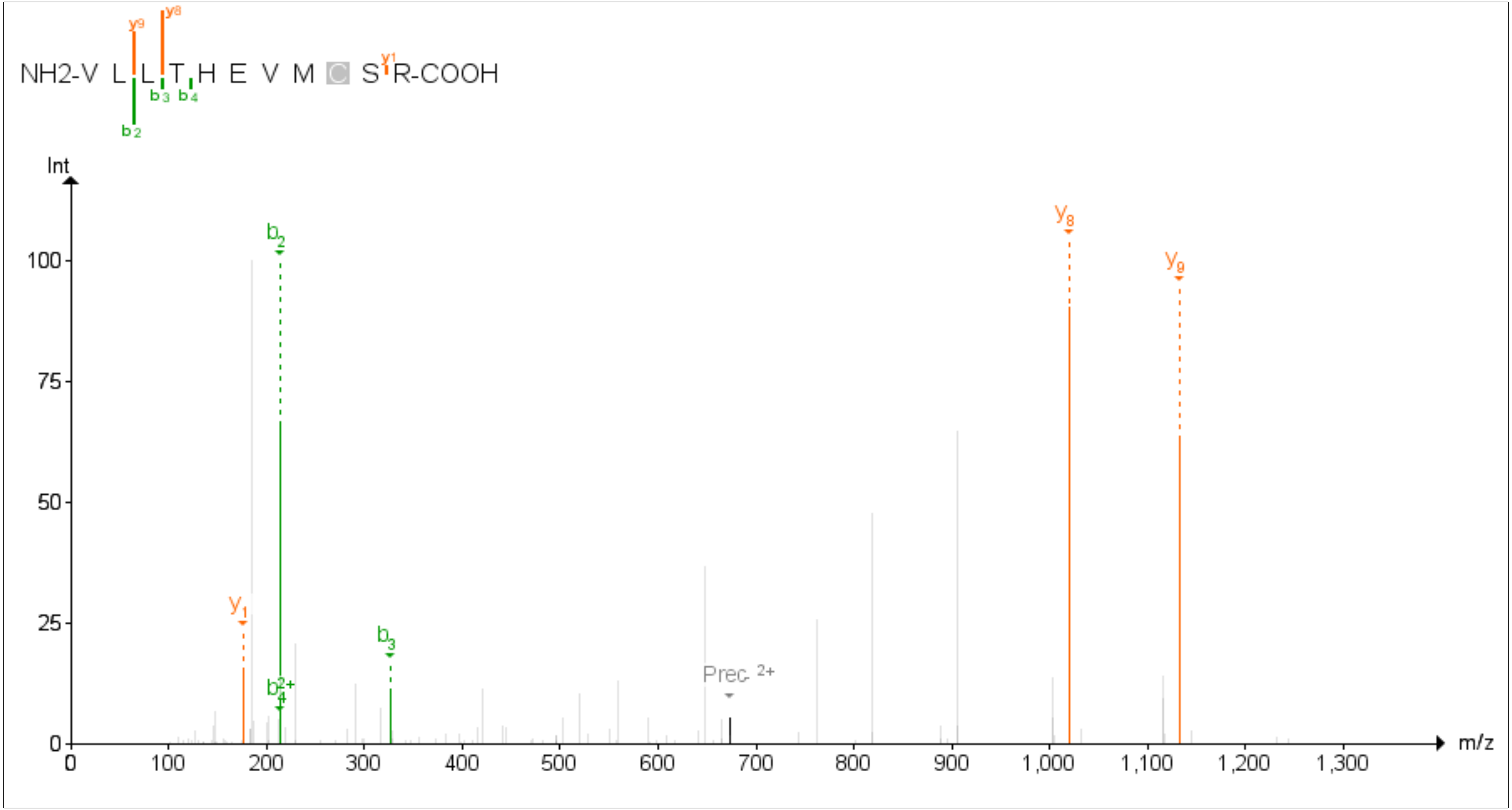
NH2-V L L T H E V M C S R-COOH
y9
y8
y1
b2
b3
b4
Int
100
75
50
25
0
b2
y8
y9
y1
b3
b4 2+
Prec. 2+
0
100
200
300
400
500
600
700
800
900
1,000
1,100
1,200
1,300
m/z

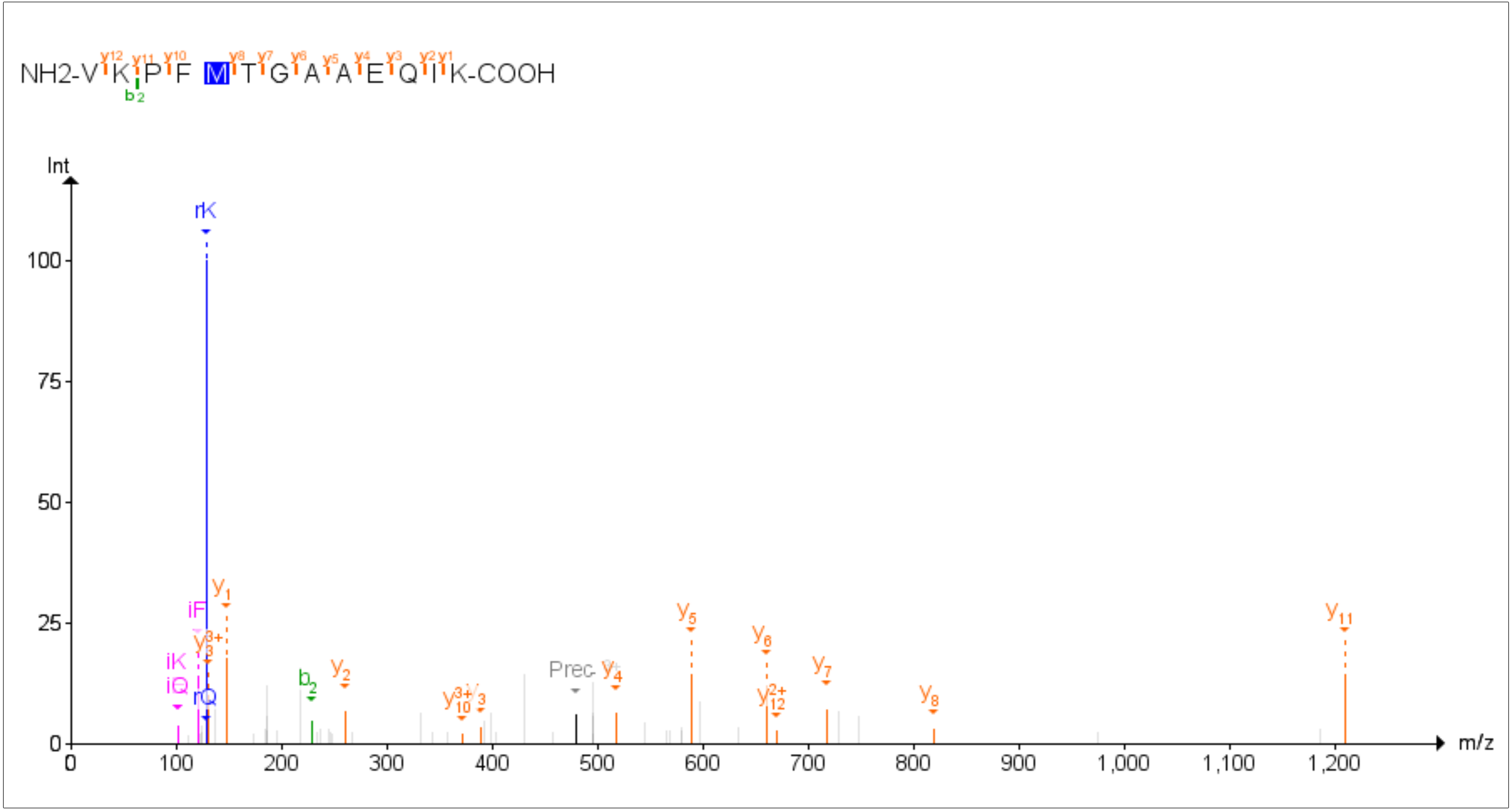

NH2-V K P F M T G A A E Q I K-COOH
y12 y11 y10 y8 y7 y6 y5 y4 y3 y2 y1
b2
Int
100
75
50
25
0
rK
y1
iF
iK
iQ
rQ
b2
y2
Prec
y4
y5
y6
y7
y8
y11
m/z
0
100
200
300
400
500
600
700
800
900
1,000
1,100
1,200

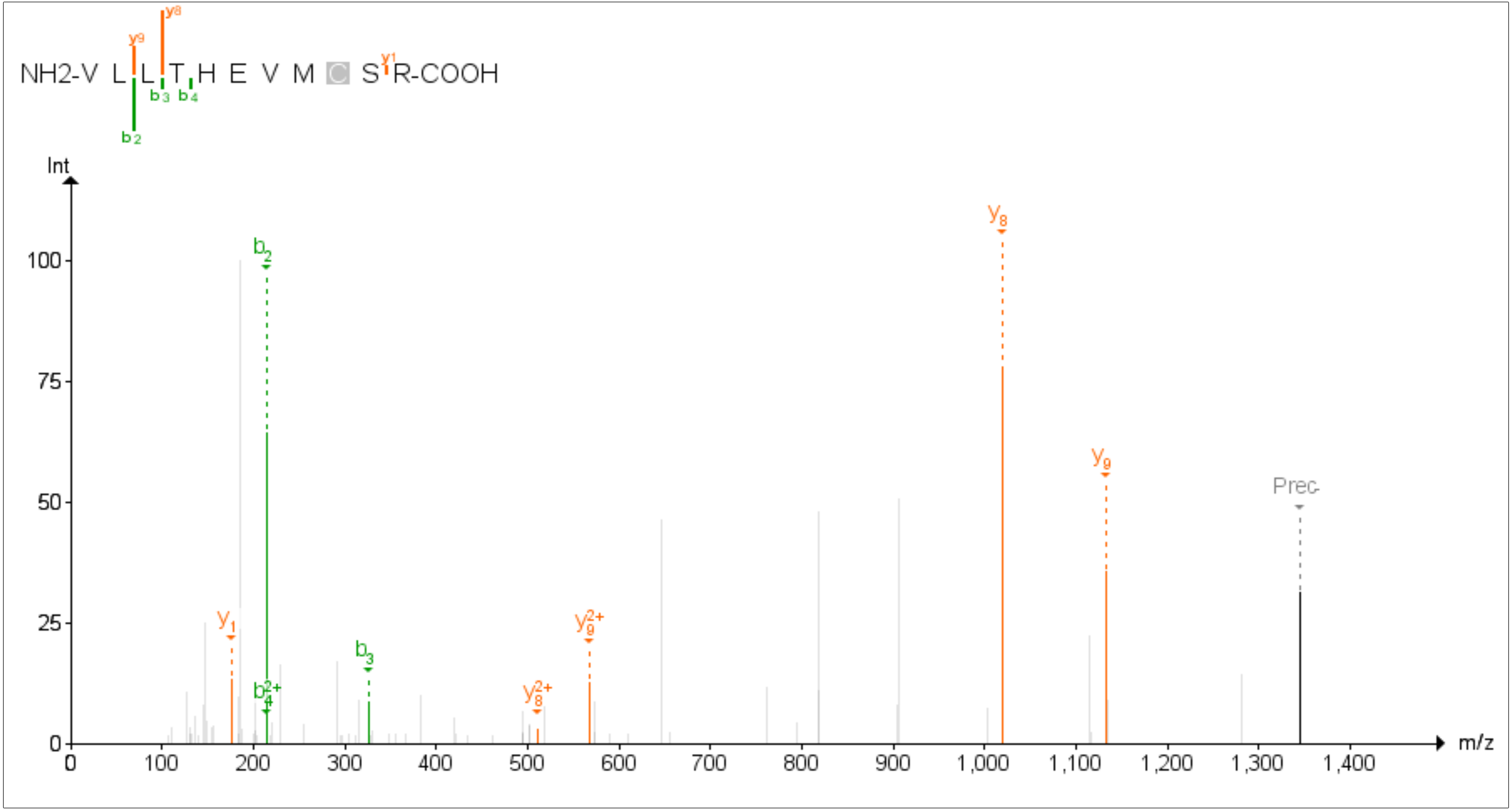

NH2-V L L T H E V M C S R-COOH
y9
y8
y1
b2
b3
b4
Int
m/z
y8
b2
y9
Prec.
y1
b3
b4 2+
y8 2+
y9 2+
0
25
50
75
100
0
100
200
300
400
500
600
700
800
900
1,000
1,100
1,200
1,300
1,400

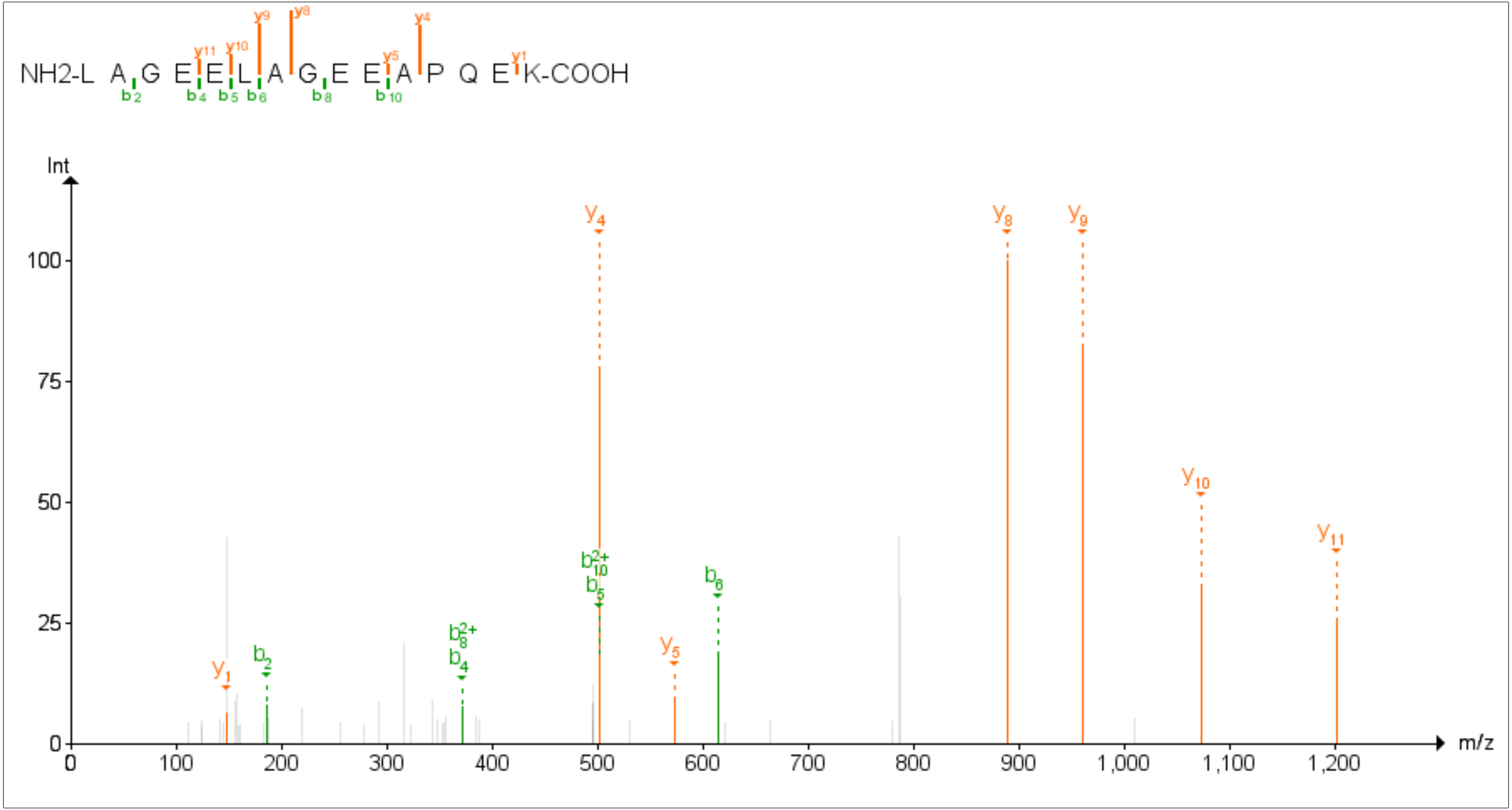

NH2-L A G E E L A G E E A P Q E K-COOH
y11 y10 y9 y8 y5 y4 y1
b2 b4 b5 b6 b8 b10
Int
100
75
50
25
0
y4 y8 y9 y10 y11 y1 b2 b8 2+ b4 b10 2+ b5 y5 b6
0 100 200 300 400 500 600 700 800 900 1,000 1,100 1,200
m/z

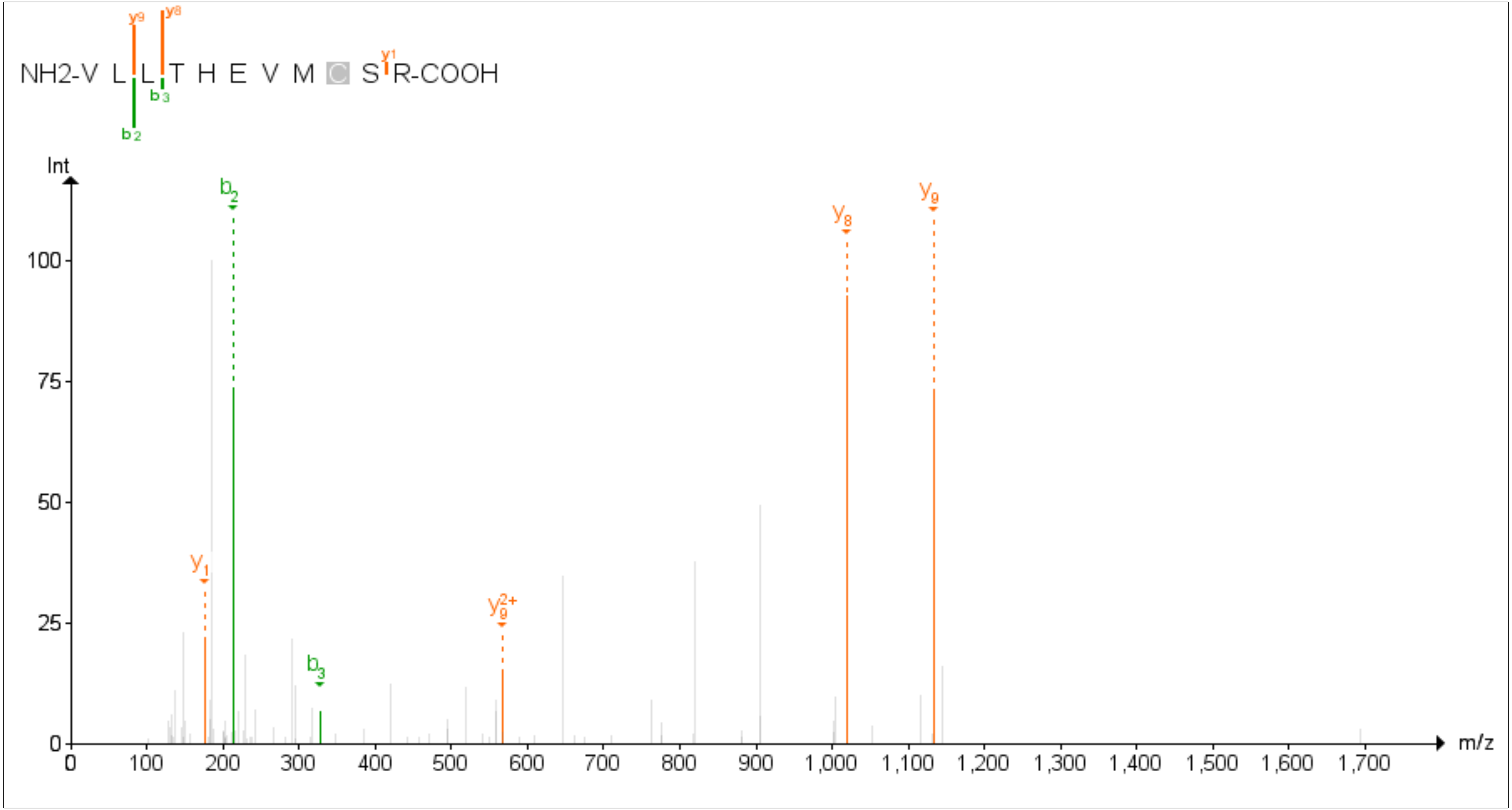

NH2-V L L T H E V M C S R-COOH
y9
y8
y1
b2
b3
Int
100
75
50
25
0
m/z
0
100
200
300
400
500
600
700
800
900
1,000
1,100
1,200
1,300
1,400
1,500
1,600
1,700
b2
y8
y9
y1
b3
y9 2+

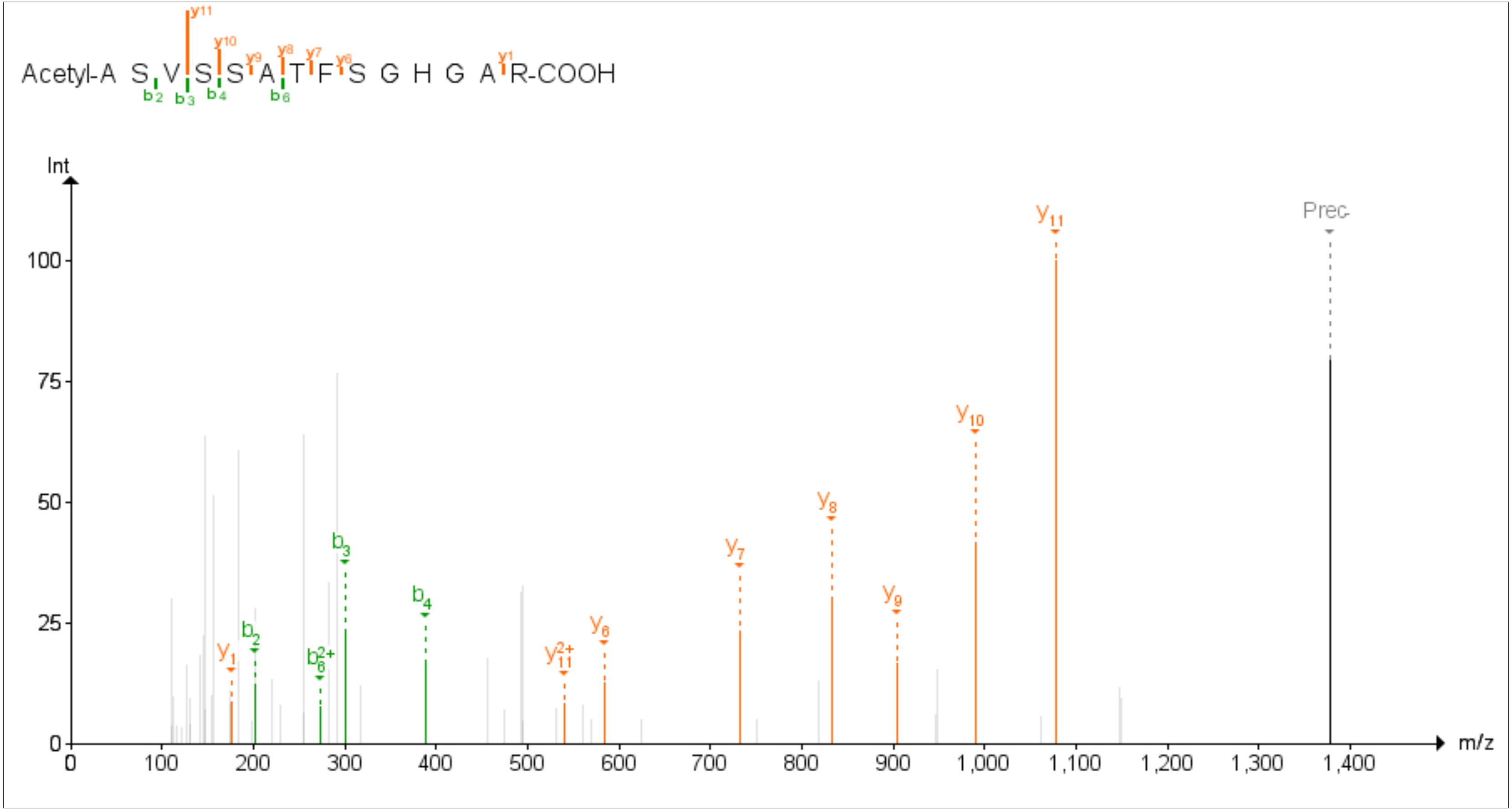

Acetyl-A S V S S A T F S G H G A R-COOH
y11 y10 y9 y8 y7 y6 y1
b2 b3 b4 b6
Int
m/z
Prec
y11
y10
y8
y7
y9
y6
y1
b3
b4
b2
b6 2+
y11 2+
0
25
50
75
100
0
100
200
300
400
500
600
700
800
900
1,000
1,100
1,200
1,300
1,400

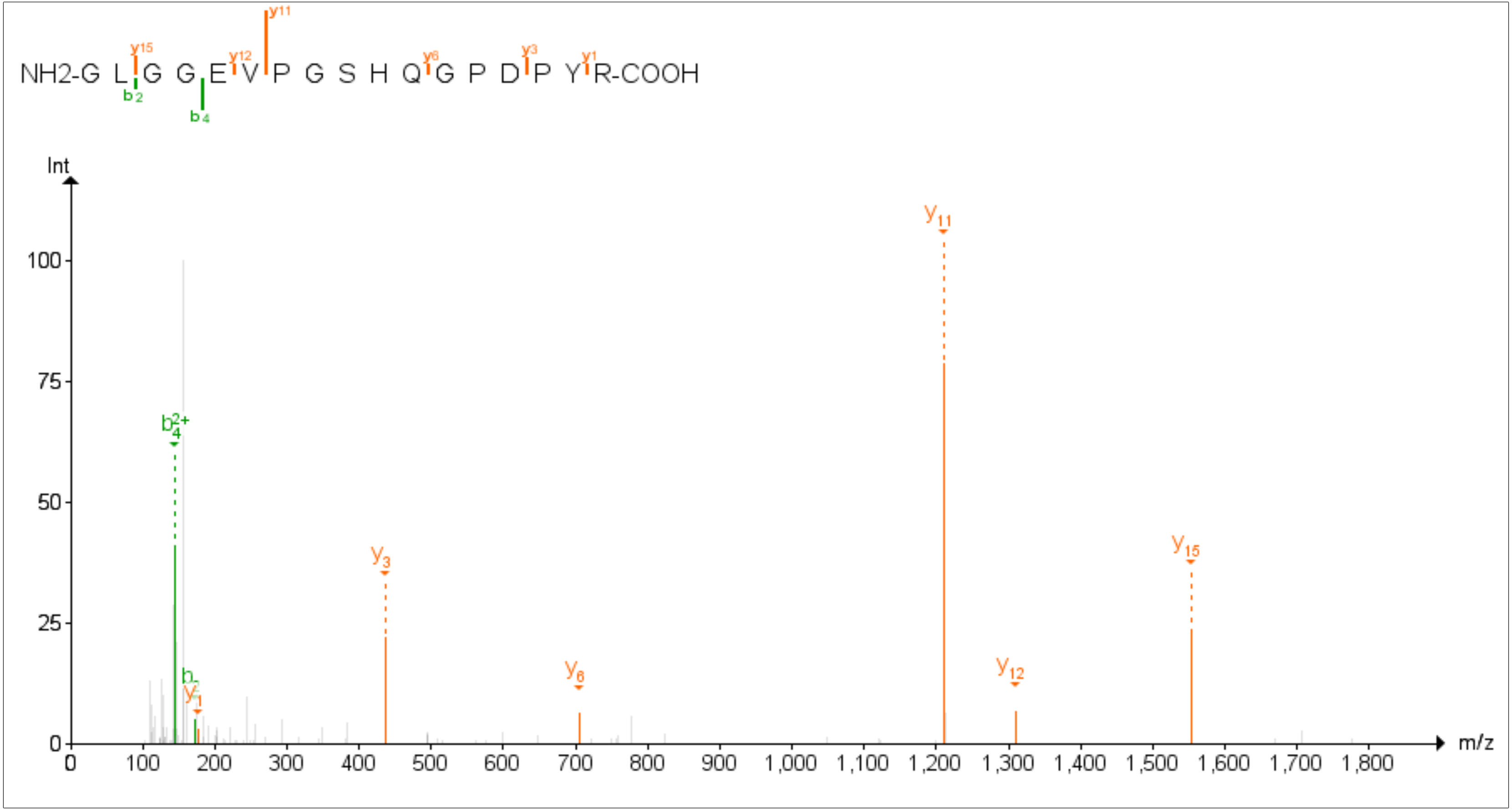

NH2-G L G G E V P G S H Q G P D P Y R-COOH
y15
y12
y11
y6
y3
y1
b2
b4
Int
100
75
50
25
0
b_4^{2+}
b_2
y_1
y_3
y_6
y_{11}
y_{12}
y_{15}
0
100
200
300
400
500
600
700
800
900
1,000
1,100
1,200
1,300
1,400
1,500
1,600
1,700
1,800
m/z

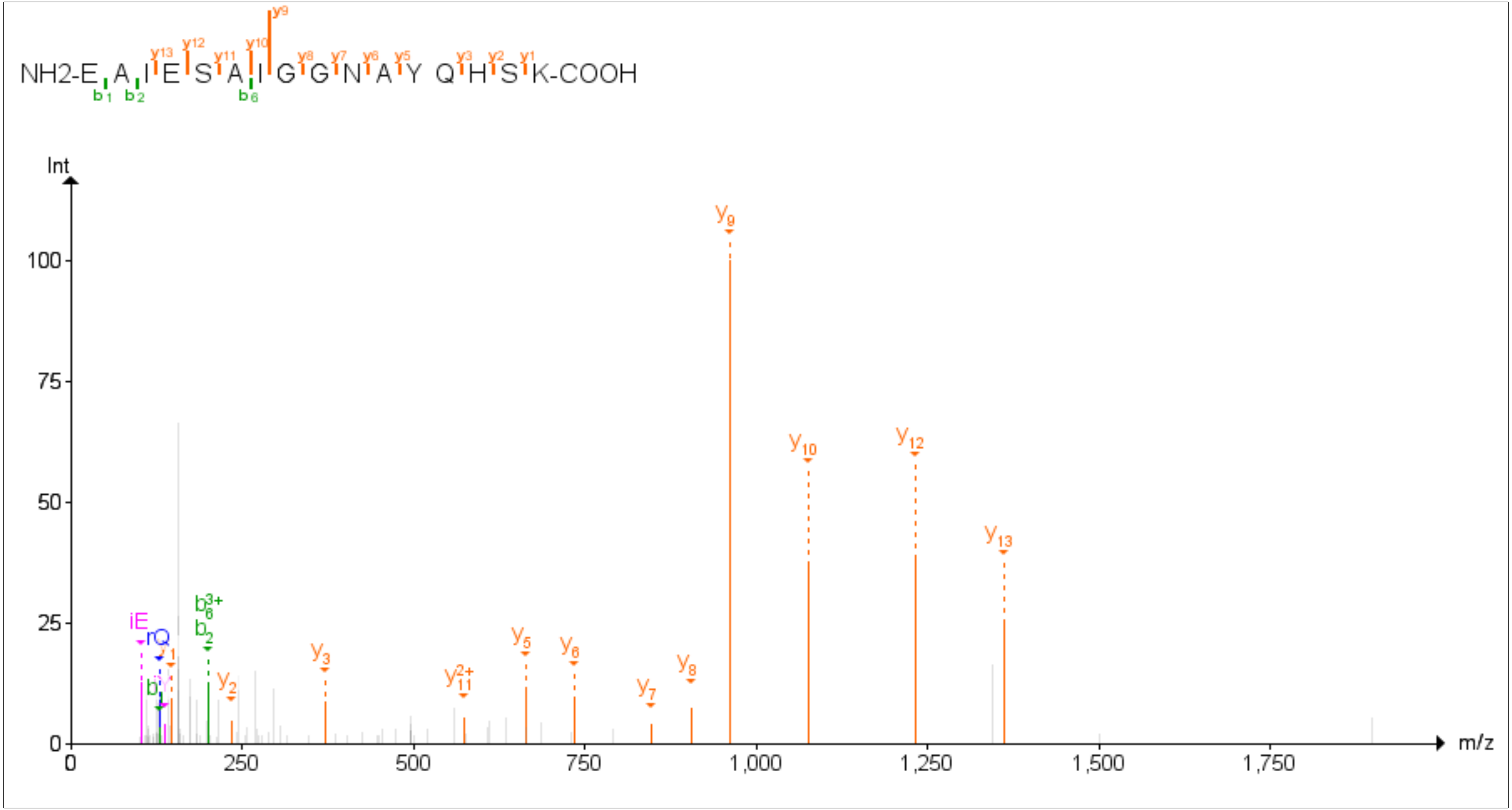

NH2-E A I E S A I G G N A Y Q H S K-COOH
Int
m/z
0
25
50
75
100
250
500
750
1,000
1,250
1,500
1,750
y9
y10
y12
y13
y5
y6
y7
y8
y3
y2
iE
rQ

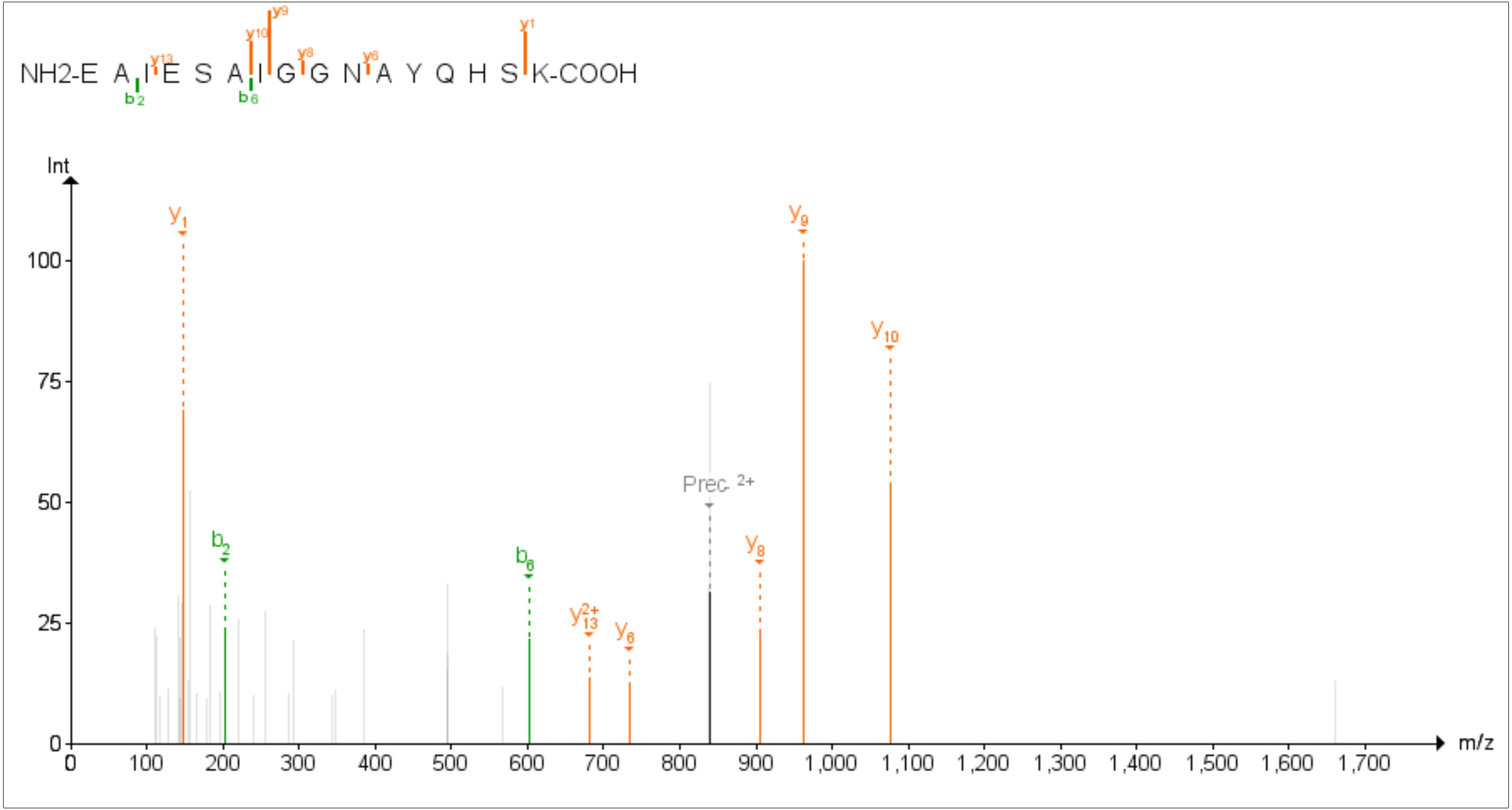
NH2-E A I E S A I G G N A Y Q H S K-COOH
y13 y10 y9 y8 y6 y1
b2 b6
Int
m/z
y1 y9 y10 b2 b6 y13 2+ y6 Prec 2+ y8
0 25 50 75 100
0 100 200 300 400 500 600 700 800 900 1,000 1,100 1,200 1,300 1,400 1,500 1,600 1,700

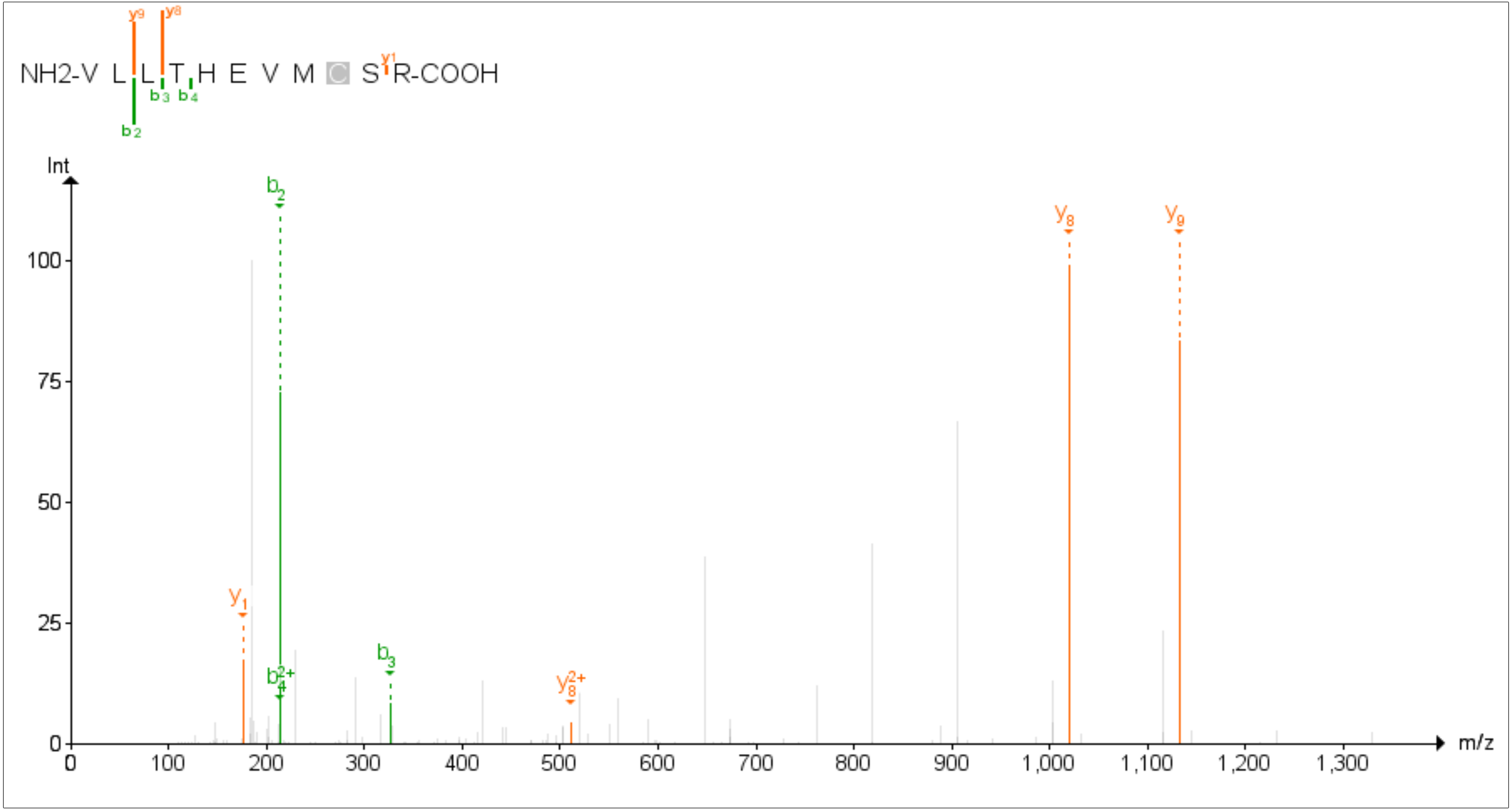

NH2-V L L T H E V M C S R-COOH
y9
y8
y1
b2
b3
b4
Int
m/z
0
25
50
75
100
0
100
200
300
400
500
600
700
800
900
1,000
1,100
1,200
1,300

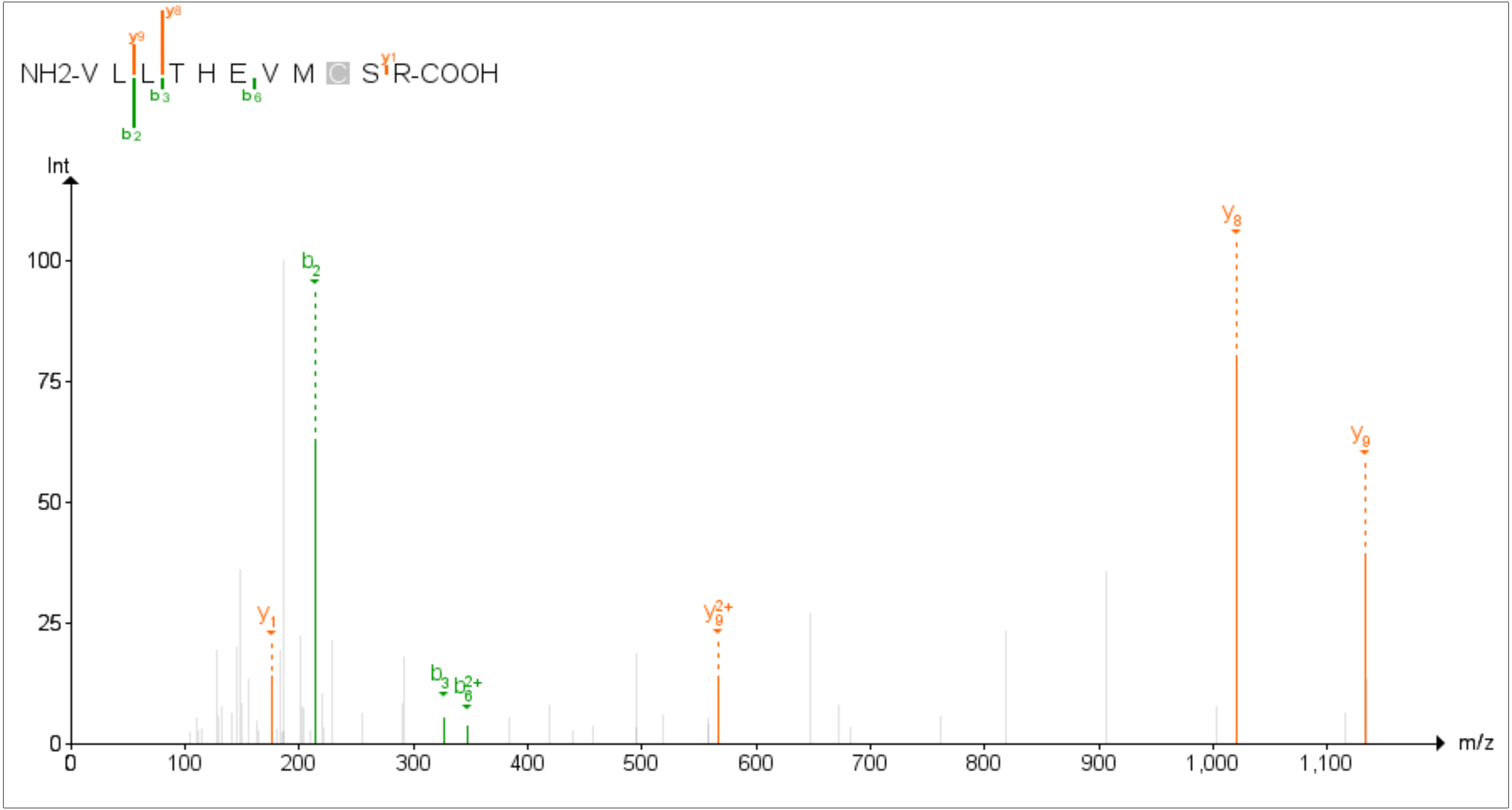
NH2-V L L T H E V M C S R-COOH
y9
y8
y1
b2
b3
b6
Int
100
75
50
25
0
y8
y9
b2
y1
y_9^{2+}
b3
b_6^{2+}
0
100
200
300
400
500
600
700
800
900
1,000
1,100
m/z

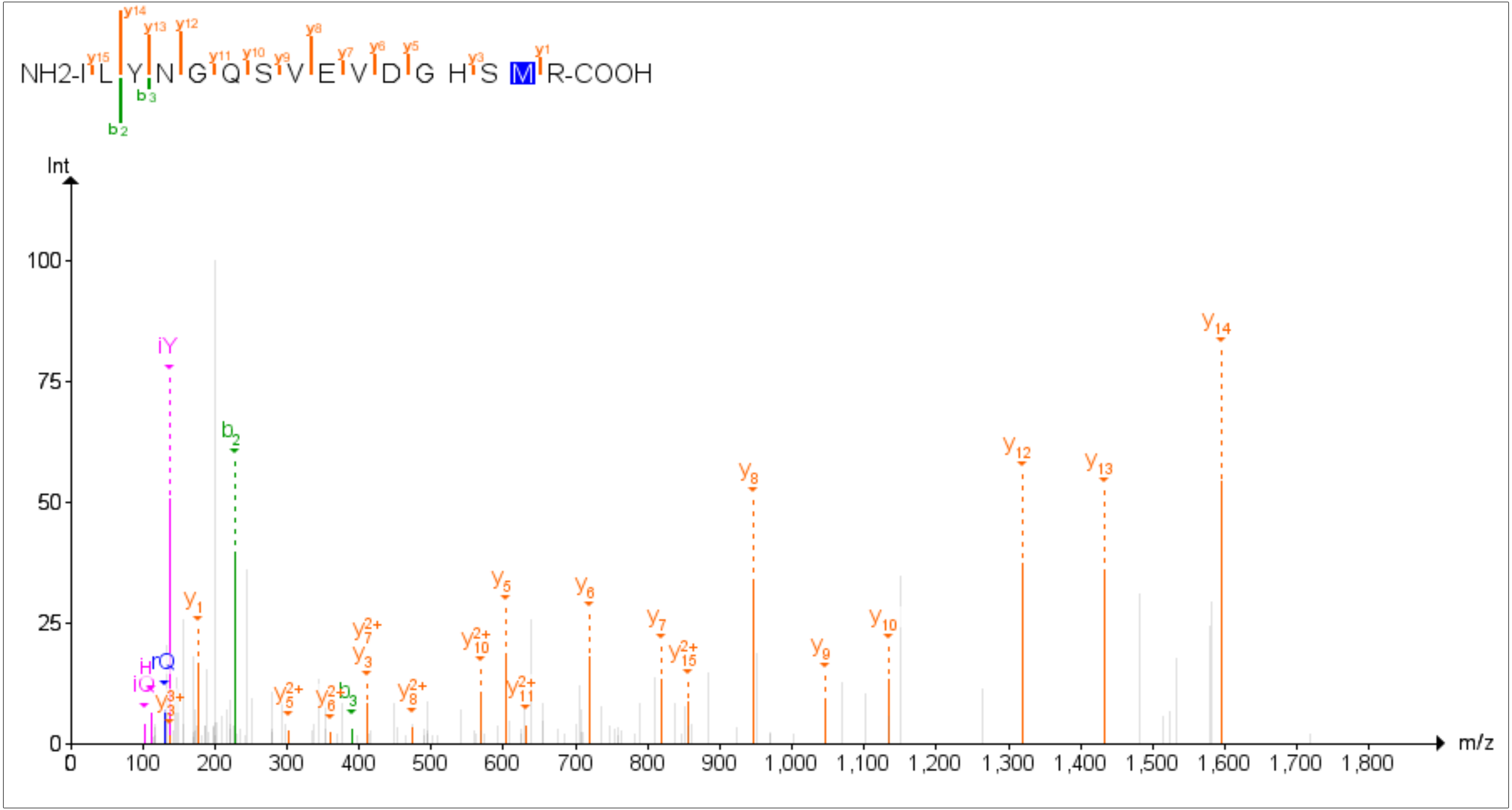

NH2-I L Y N G Q S V E V D G H S M R-COOH
y15 y14 y13 y12 y11 y10 y9 y8 y7 y6 y5 y3 y1
b2 b3
Int
100
75
50
25
0
m/z
0 100 200 300 400 500 600 700 800 900 1,000 1,100 1,200 1,300 1,400 1,500 1,600 1,700 1,800
iY
b2
y1
iH
rQ
iQ
y3+
y2+5
y2+6
b3
y2+7
y3
y2+8
y2+10
y5
y2+11
y6
y7
y2+15
y8
y9
y10
y12
y13
y14

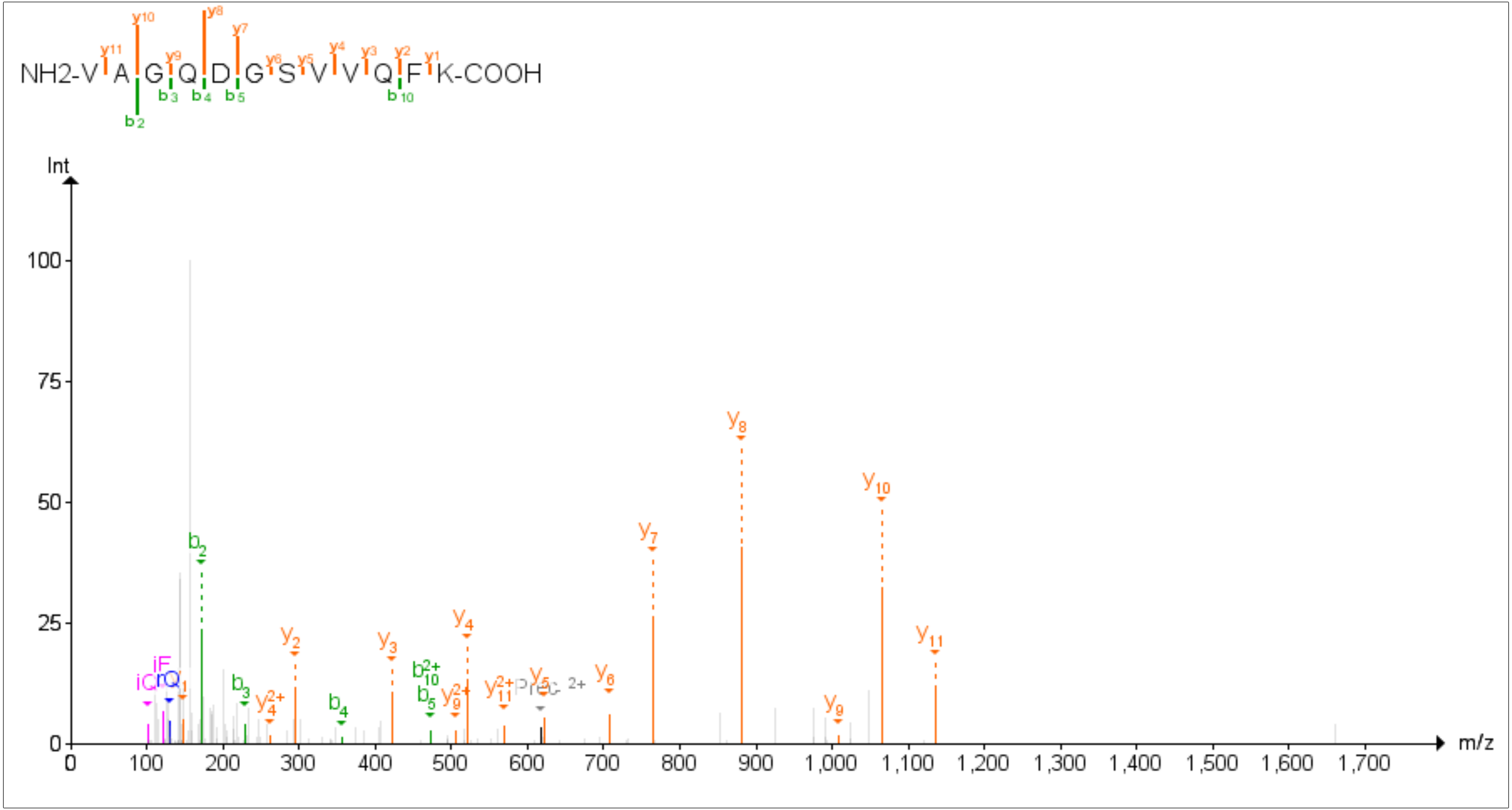
NH2-V A G Q D G S V V Q F K-COOH
Int
m/z
y8
y10
y7
y2
y3
y4
y11
b2
b3
b4
b5
y9
Prec. 2+

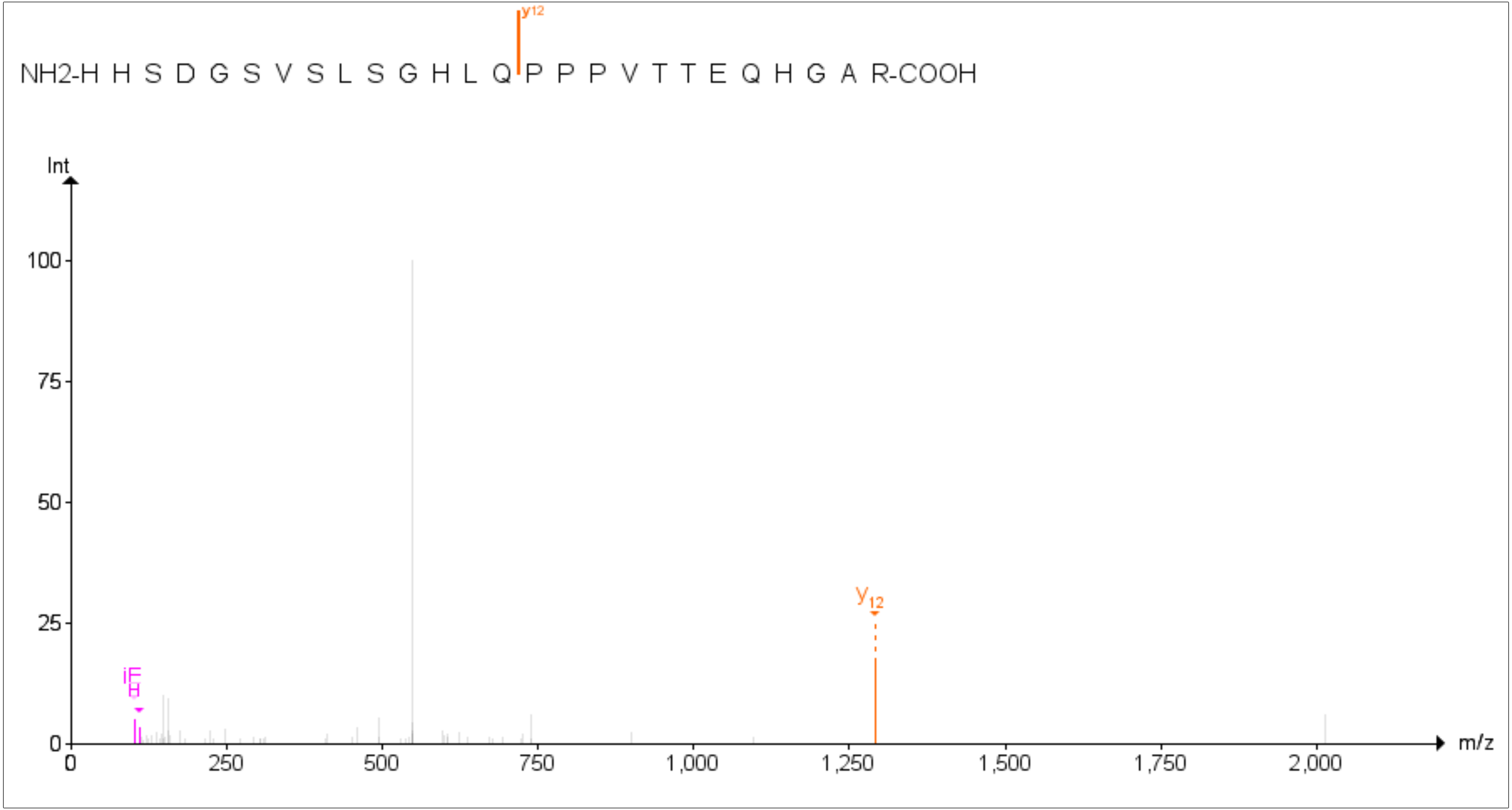

y12
NH2-H H S D G S V S L S G H L Q P P P V T T E Q H G A R-COOH
Int
100
75
50
25
0
iF
H
y12
0
250
500
750
1,000
1,250
1,500
1,750
2,000
m/z

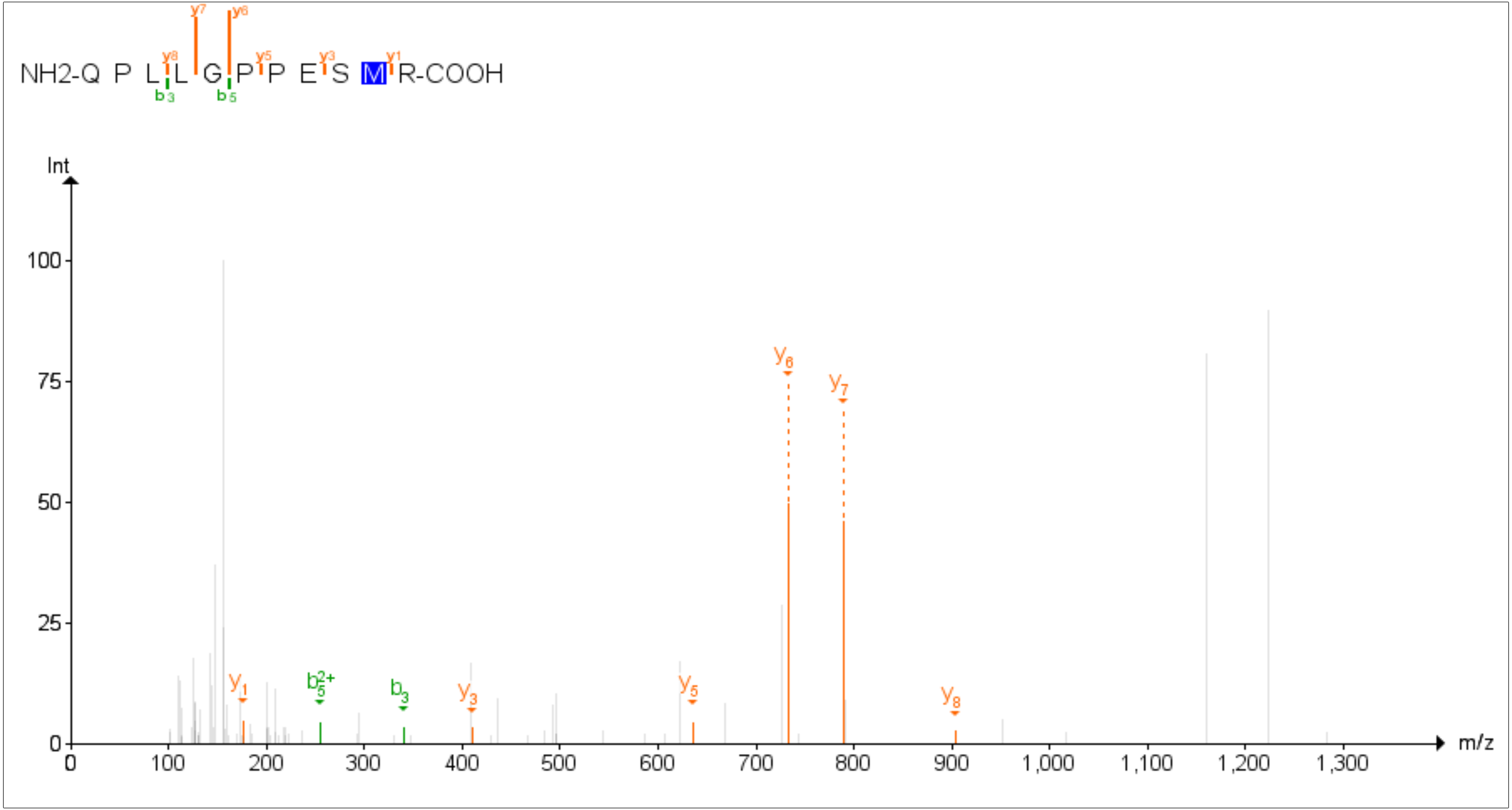
NH2-Q P L L G P P E S M R-COOH
y7
y6
y8
y5
y3
y1
b3
b5
Int
100
75
50
25
0
y6
y7
y1
b5 2+
b3
y3
y5
y8
0
100
200
300
400
500
600
700
800
900
1,000
1,100
1,200
1,300
m/z

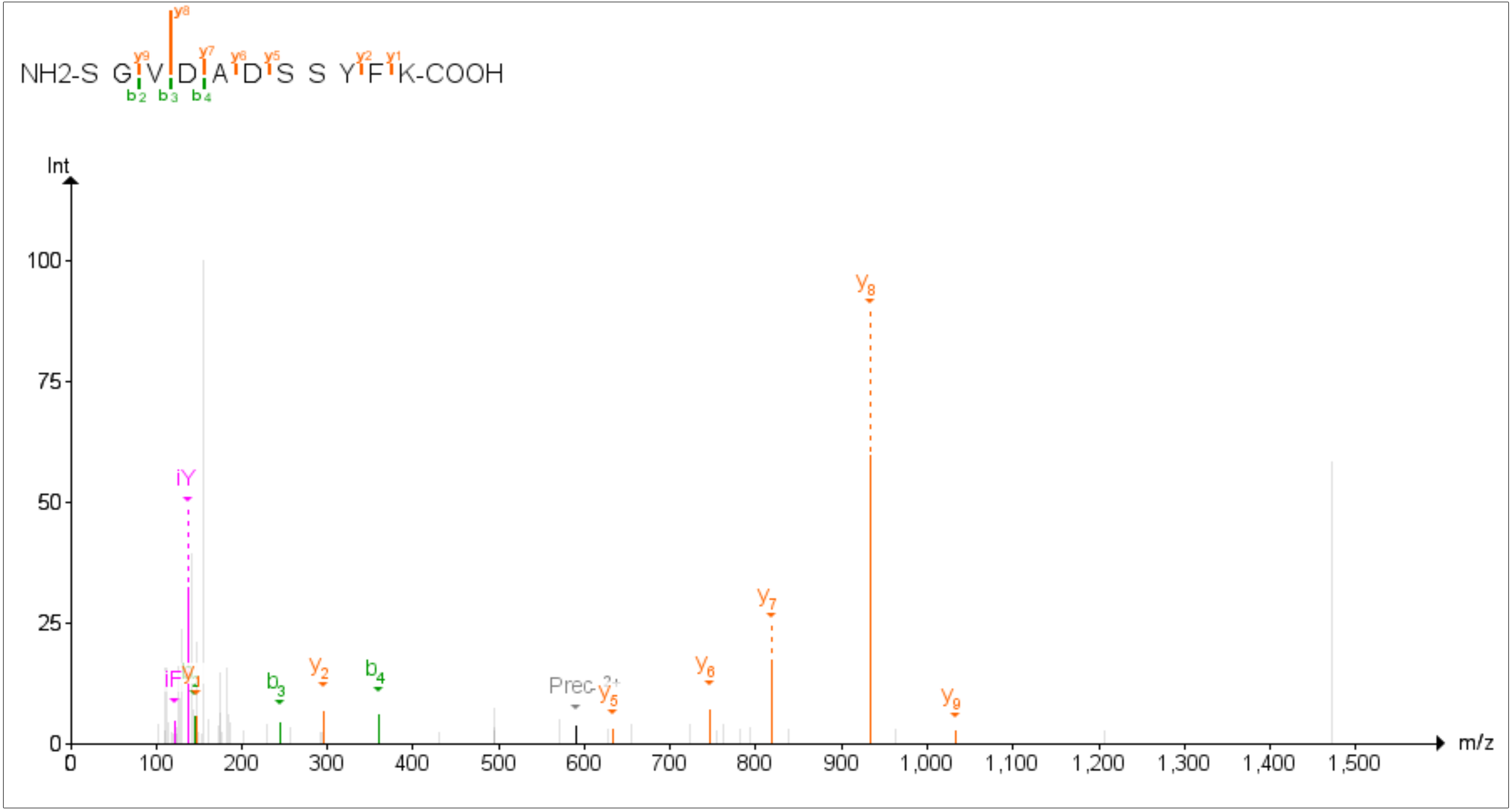

NH2-S G V D A D S S Y F K-COOH
y8 y9 y7 y6 y5 y2 y1
b2 b3 b4
Int
100
75
50
25
0
iY
iF
y1
b3
y2
b4
Prec-2+
y5
y6
y7
y8
y9
0 100 200 300 400 500 600 700 800 900 1,000 1,100 1,200 1,300 1,400 1,500
m/z

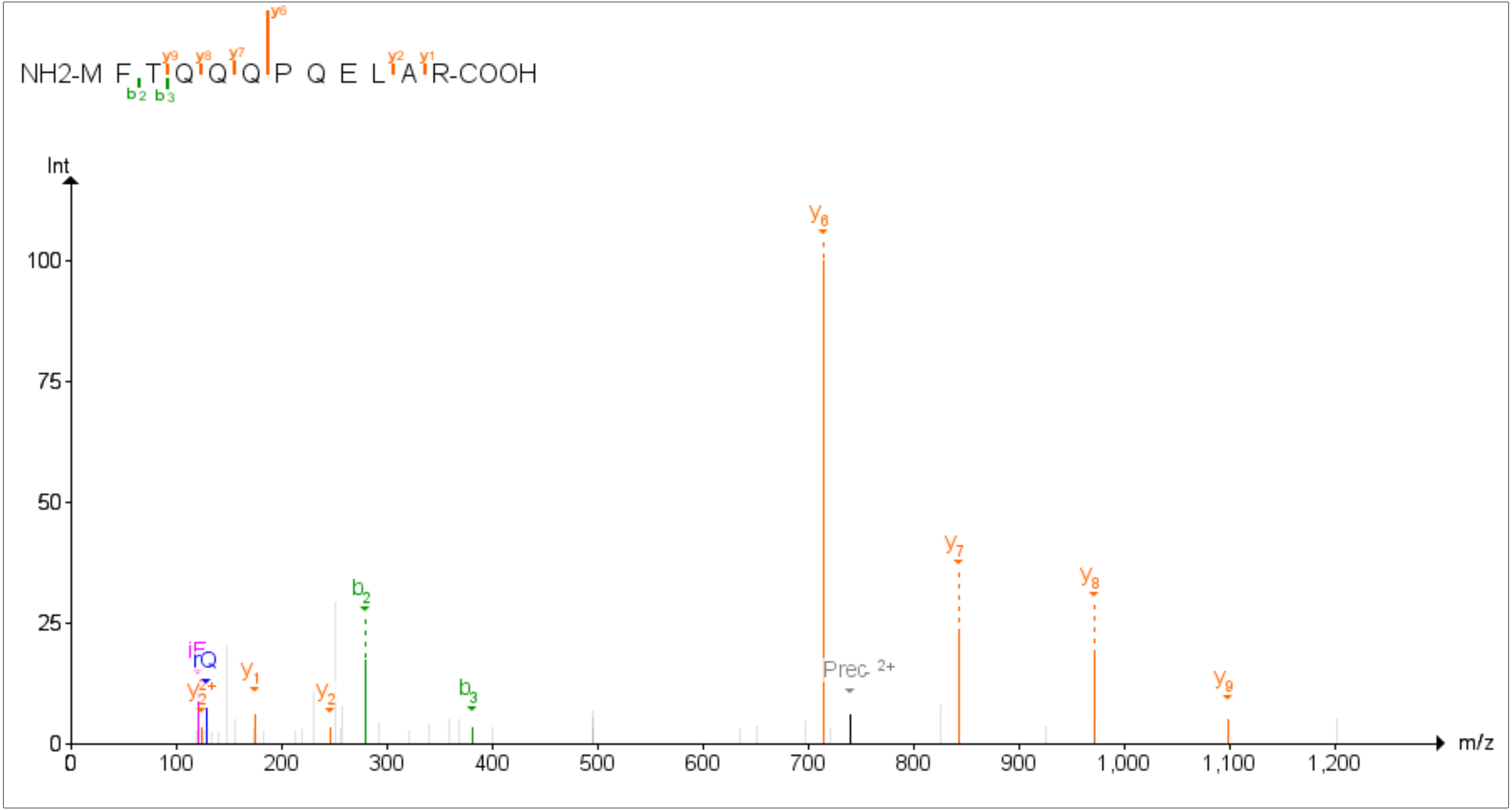

NH2-M F T Q Q Q P Q E L A R-COOH
y9 y8 y7 y6 y2 y1
b2 b3
Int
100
75
50
25
0
y6
y7
y8
b2
iF
rQ
y1
y2 2+
y2
b3
Prec 2+
y9
0 100 200 300 400 500 600 700 800 900 1,000 1,100 1,200
m/z

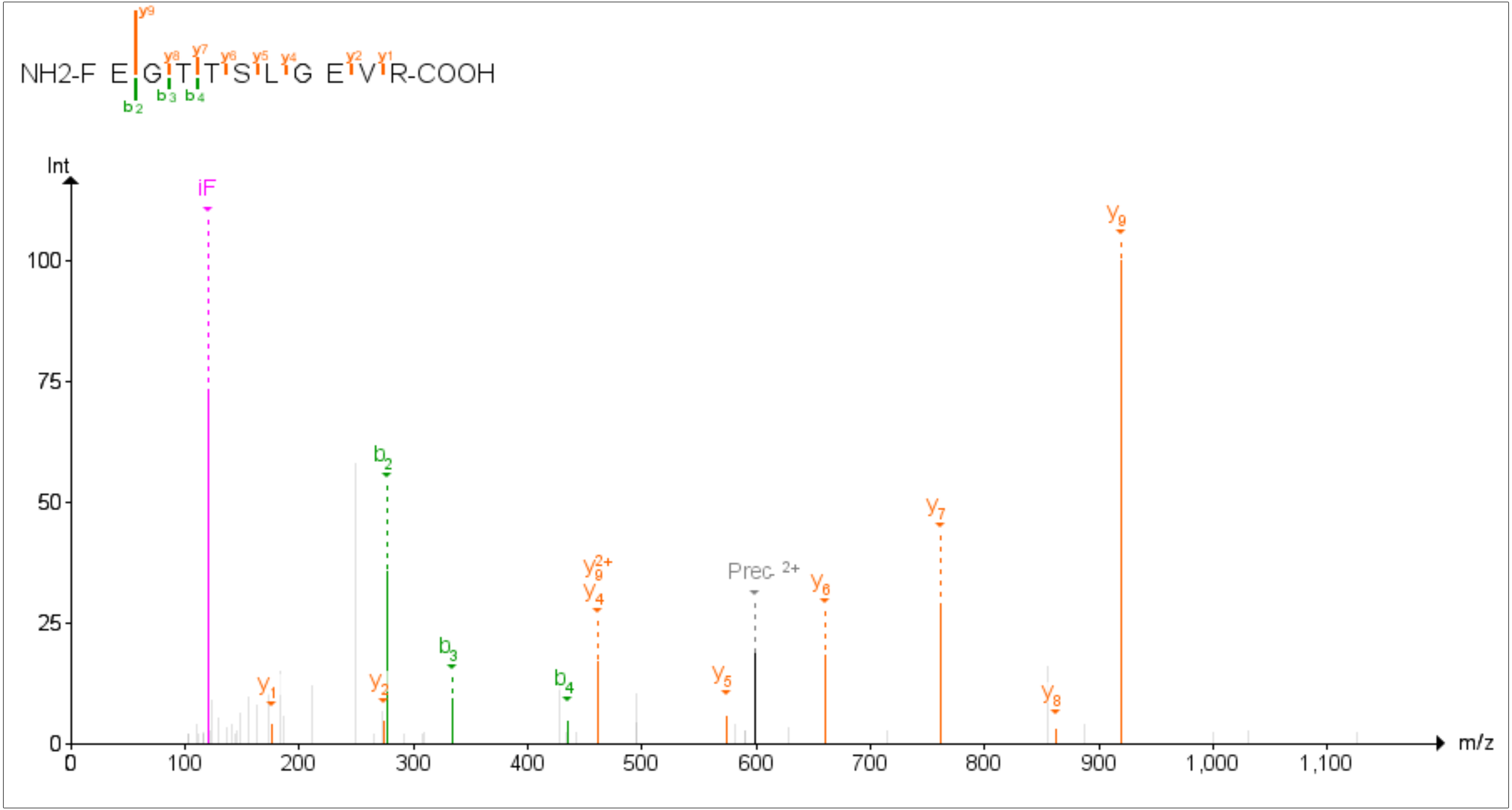

NH2-F E G T T S L G E V R-COOH
y9 y8 y7 y6 y5 y4 y2 y1
b2 b3 b4
Int
iF
b2
b3
b4
y1
y2
y4
y5
y6
y7
y8
y9
y9 2+
Prec 2+
100
75
50
25
0
0
100
200
300
400
500
600
700
800
900
1,000
1,100
m/z

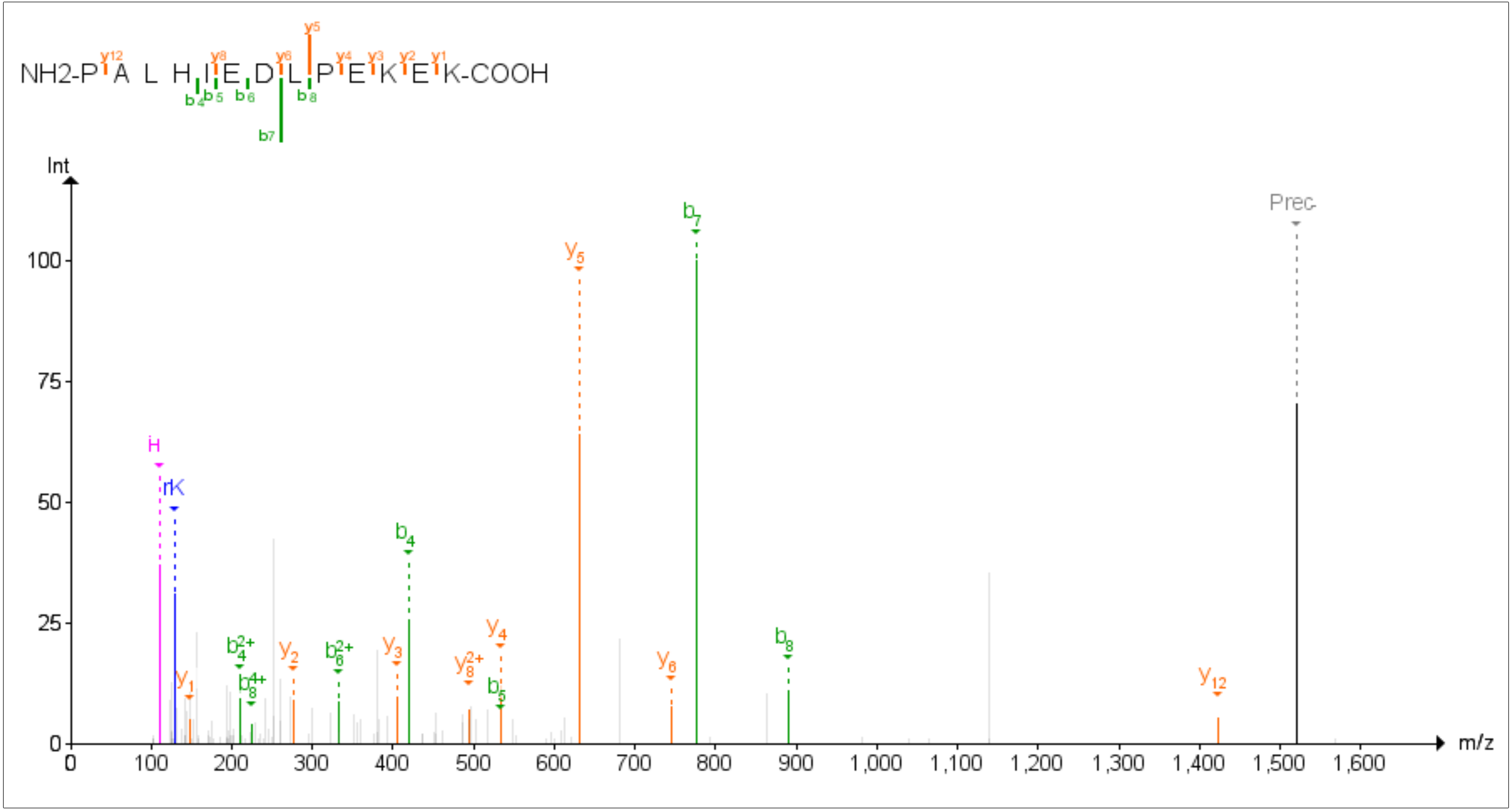

NH2-P A L H I E D L P E K E K-COOH
y12 y8 y6 y5 y4 y3 y2 y1
b4 b5 b6 b7 b8
Int
m/z
Prec
y5
b7
iH
rK
b4
y1
y2
y3
y4
y6
y8
y12
b5
b8
0
25
50
75
100
0
100
200
300
400
500
600
700
800
900
1,000
1,100
1,200
1,300
1,400
1,500
1,600

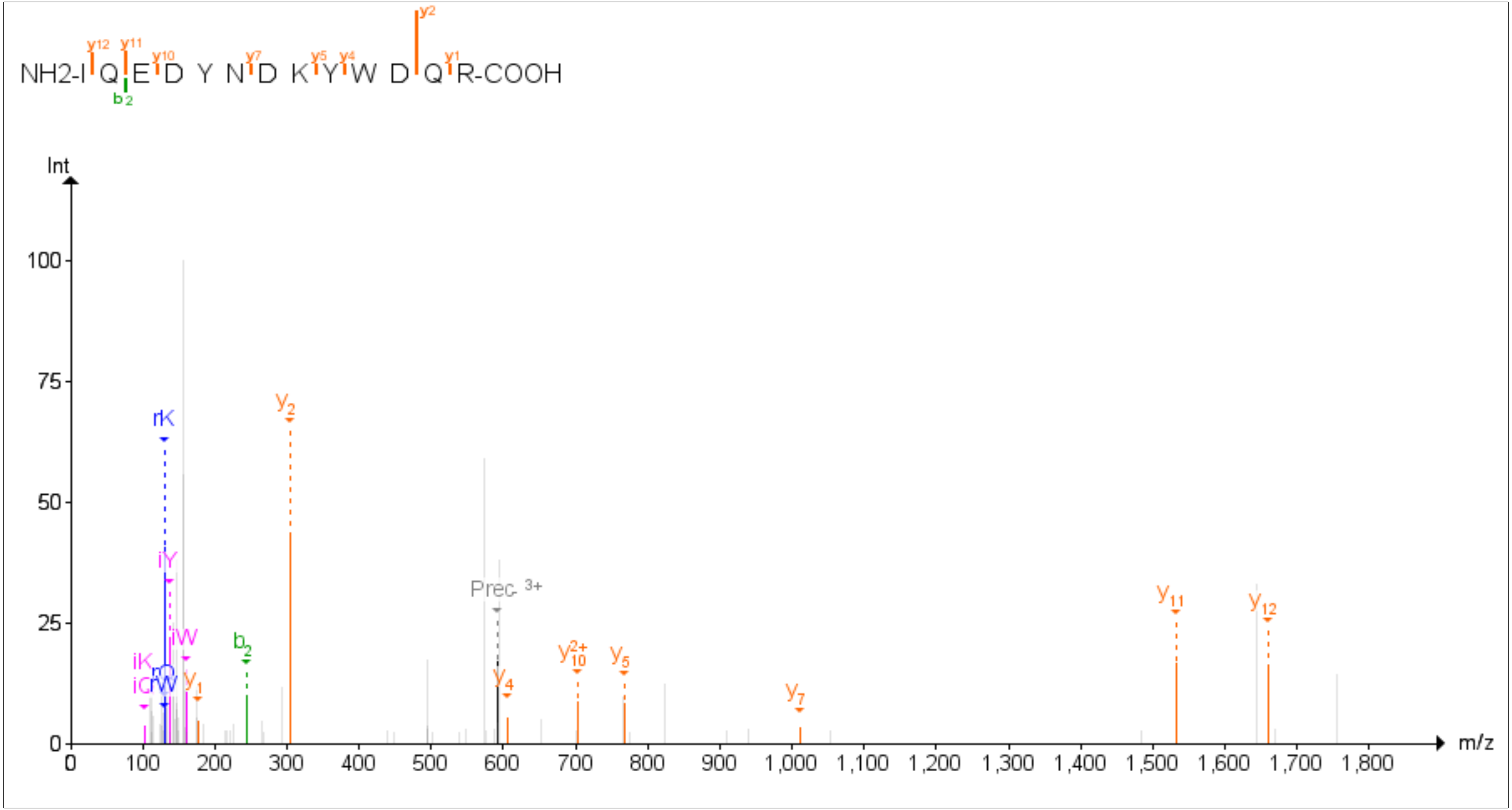

NH2-I Q E D Y N D K Y W D Q R-COOH
y12 y11 y10 y7 y5 y4 y2 y1
b2
Int
100
75
50
25
0
rK
y2
iY
iW
iK
iQ
rQ
rW
y1
b2
Prec 3+
y4
y10 2+
y5
y7
y11
y12
0 100 200 300 400 500 600 700 800 900 1,000 1,100 1,200 1,300 1,400 1,500 1,600 1,700 1,800
m/z

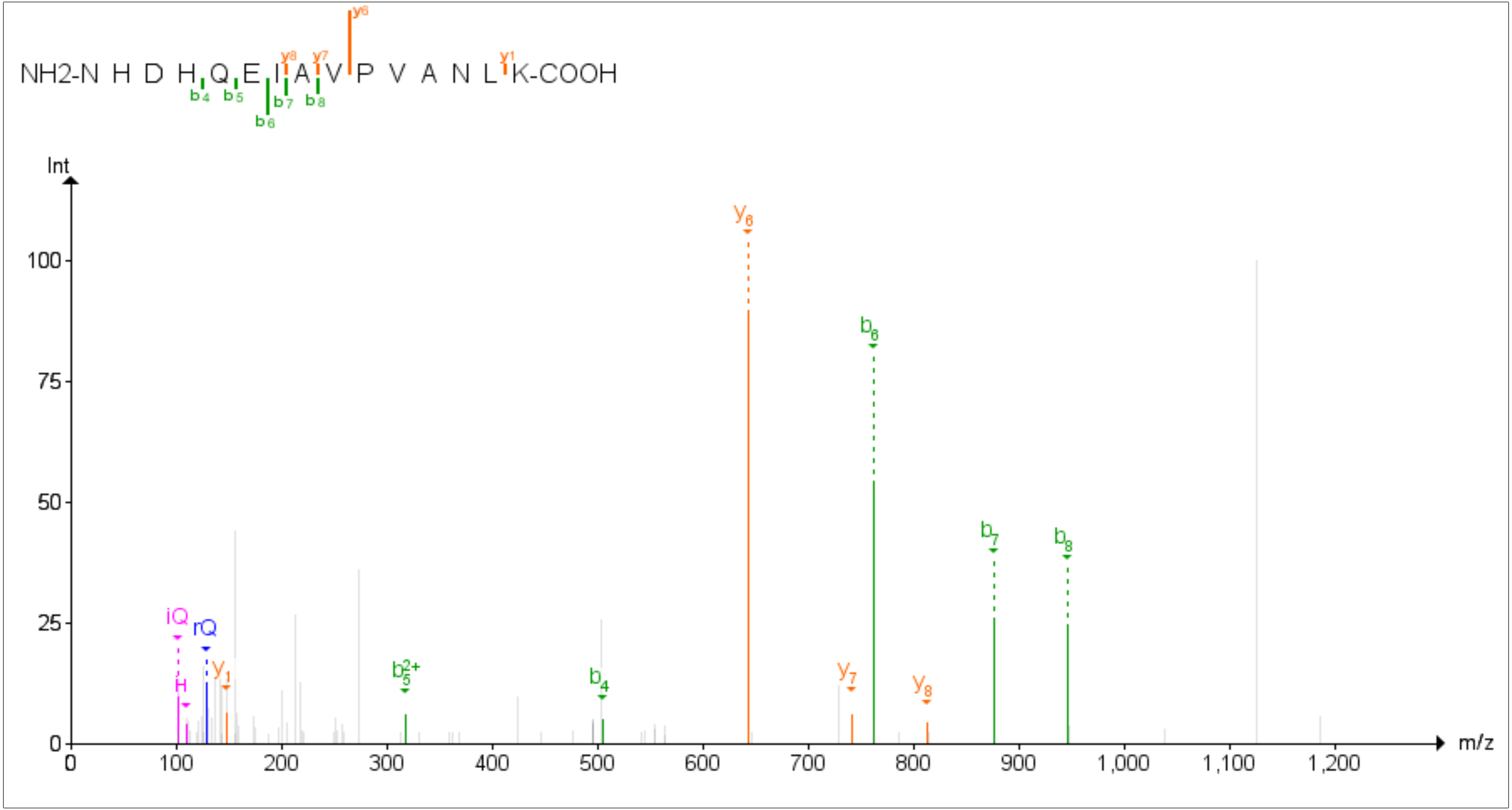

NH2-N H D H Q E I A V P V A N L K-COOH
b4 b5 b6 b7 b8
y8 y7 y6 y1
Int
100
75
50
25
0
y6
b6
b7
b8
iQ
rQ
H
y1
b5 2+
b4
y7
y8
0 100 200 300 400 500 600 700 800 900 1,000 1,100 1,200
m/z

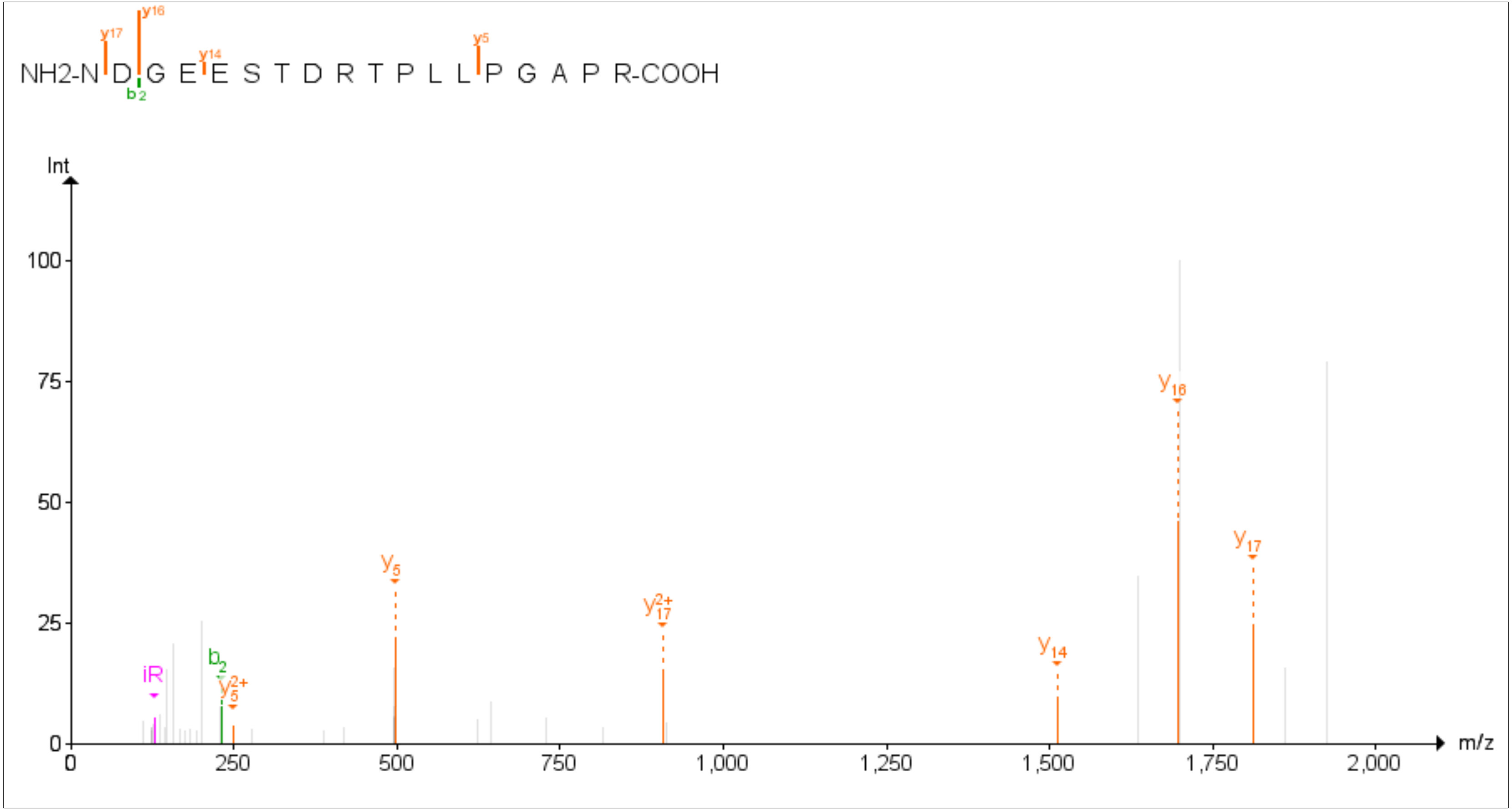

NH2-N D G E E S T D R T P L L P G A P R-COOH
y17
y16
y14
y5
b2
Int
100
75
50
25
0
iR
b2
y5 2+
y5
y17 2+
y14
y16
y17
0
250
500
750
1,000
1,250
1,500
1,750
2,000
m/z

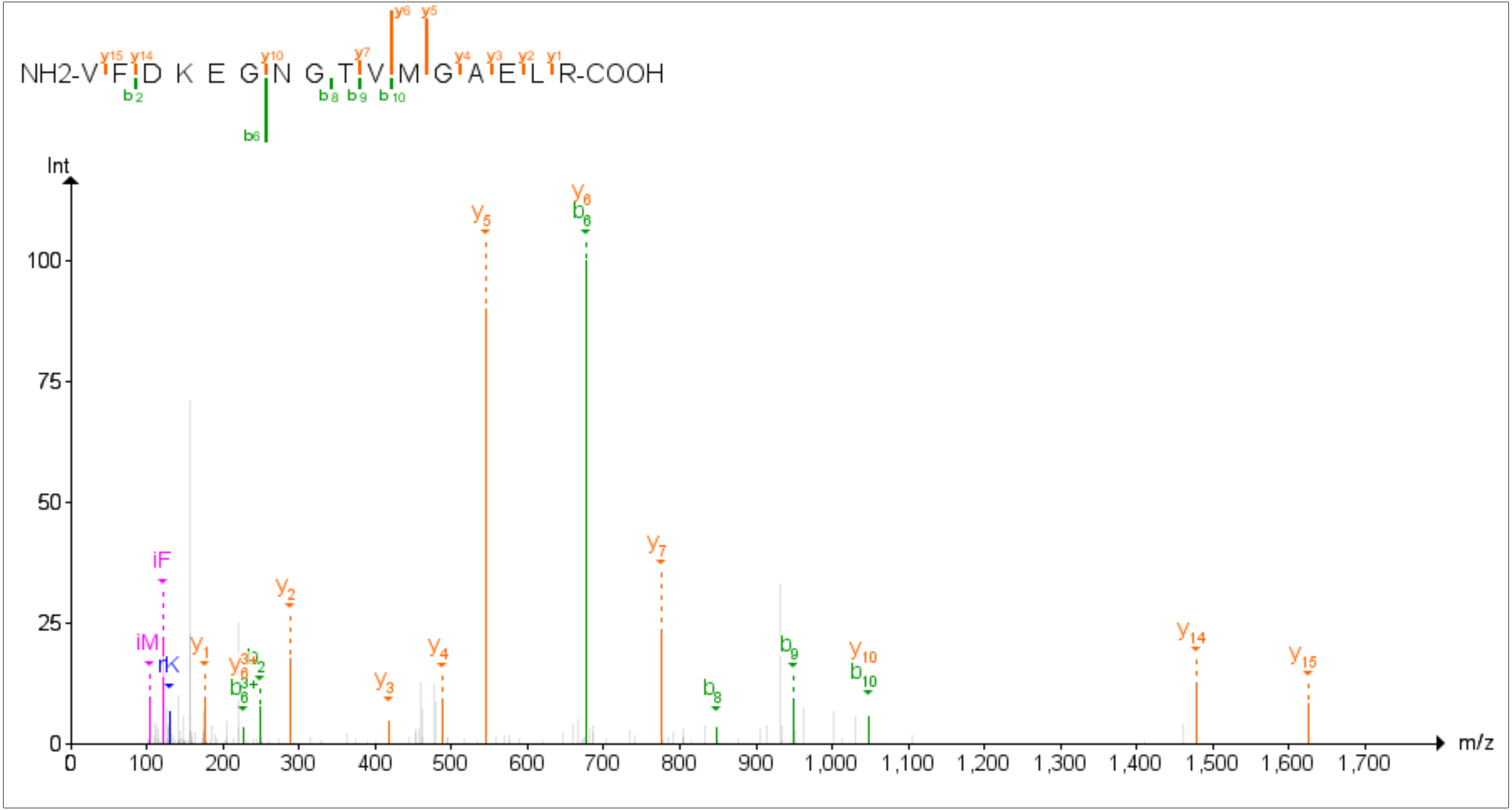

NH2-V F D K E G N G T V M G A E L R-COOH
y15 y14 y10 y7 y6 y5 y4 y3 y2 y1
b2 b6 b8 b9 b10
Int
m/z
iF
iM
rK
y1
y2
y3
y4
y5
y6
b6
y7
b8
b9
y10
b10
y14
y15
0 25 50 75 100
0 100 200 300 400 500 600 700 800 900 1,000 1,100 1,200 1,300 1,400 1,500 1,600 1,700

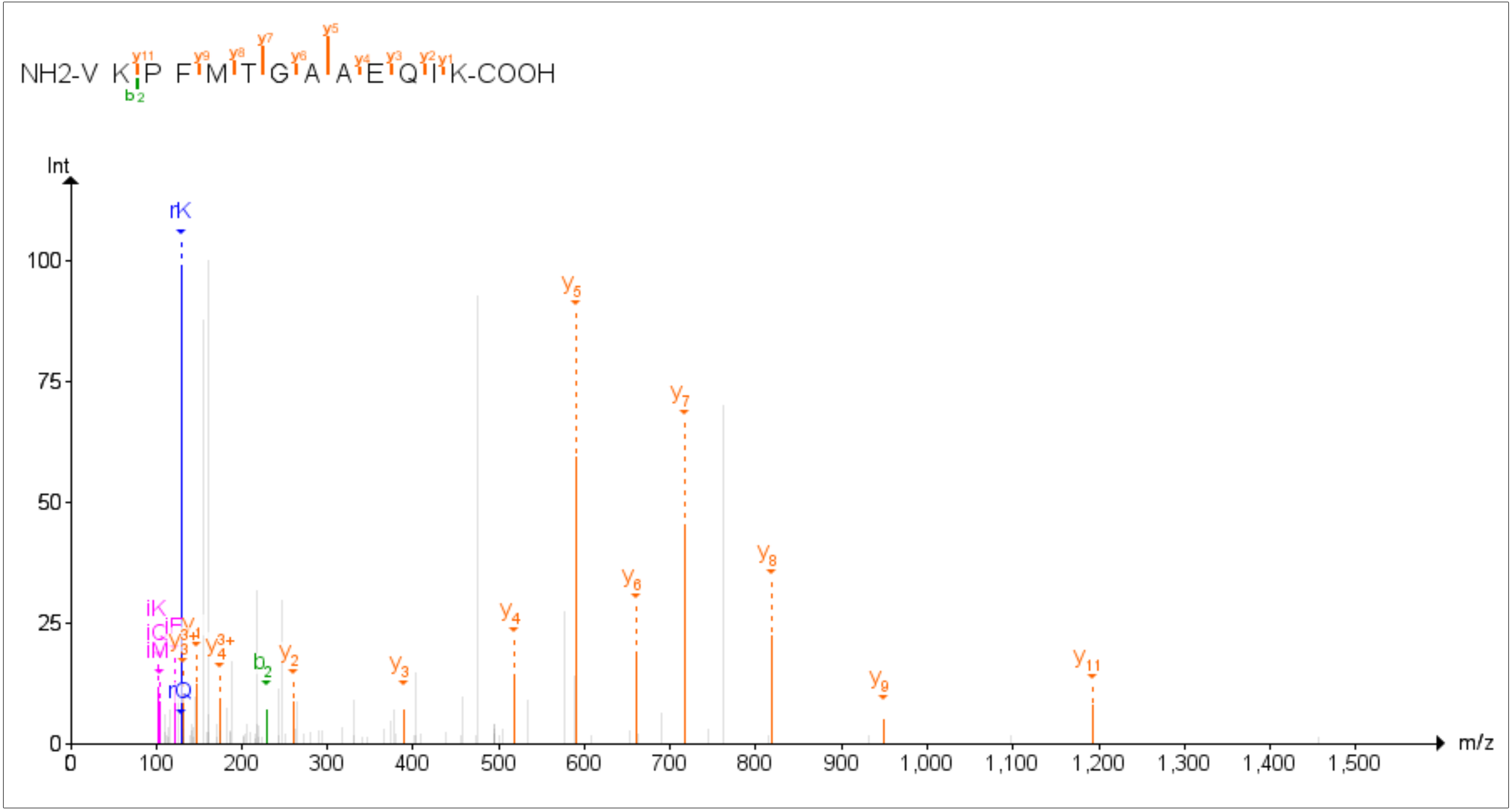
NH2-V K P F M T G A A E Q I K-COOH
y11 y9 y8 y7 y6 y5 y4 y3 y2 y1
b2
Int
m/z
rK
rQ
iK
iM
y5
y7
y8
y6
y4
y2
y3
y9
y11
b2
y4 3+
0
25
50
75
100
0
100
200
300
400
500
600
700
800
900
1,000
1,100
1,200
1,300
1,400
1,500

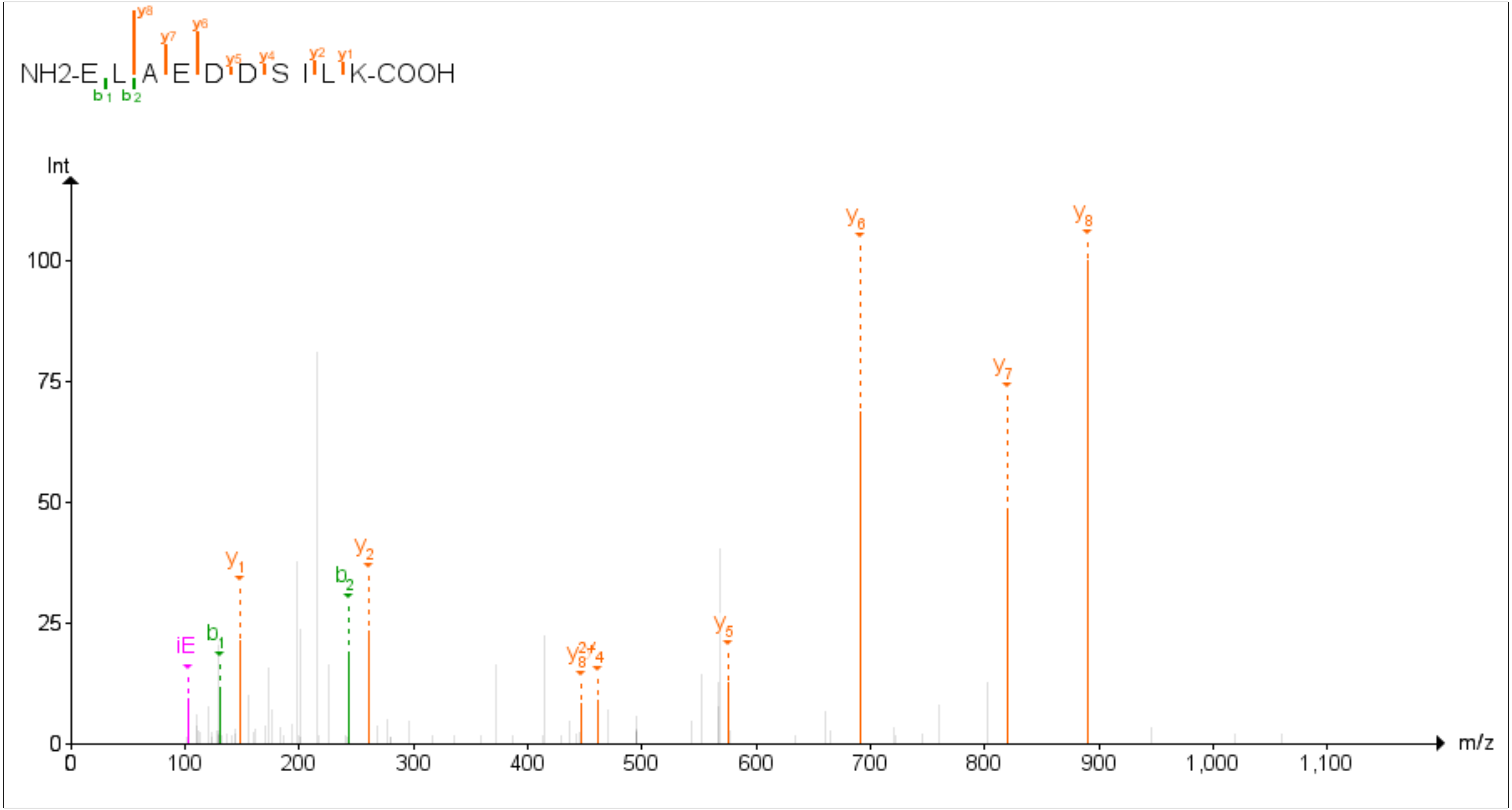

NH2-E L A E D D S I L K-COOH
y8
y7
y6
y5
y4
y2
y1
b1
b2
Int
100
75
50
25
0
m/z
0
100
200
300
400
500
600
700
800
900
1,000
1,100
iE
b1
y1
b2
y2
y8 2+
y4
y5
y6
y7
y8

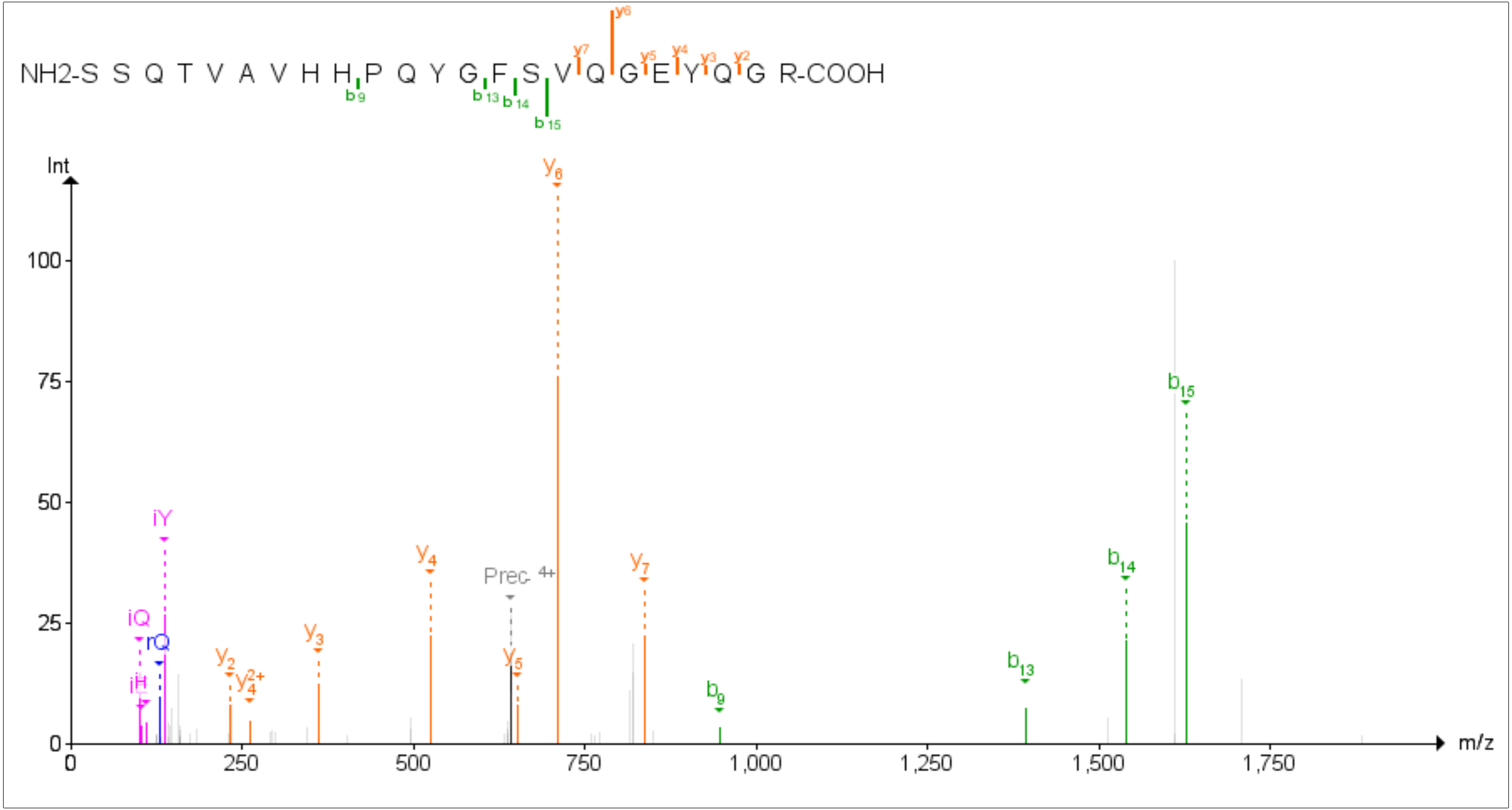
NH2-S S Q T V A V H H P Q Y G F S V Q G E Y Q G R-COOH
y6 y7 y5 y4 y3 y2
b9 b13 b14 b15
Int
100
75
50
25
0
iY
iQ
rQ
iH
y2
y4 2+
y3
y4
Prec 4+
y5
y6
y7
b9
b13
b14
b15
0
250
500
750
1,000
1,250
1,500
1,750
m/z

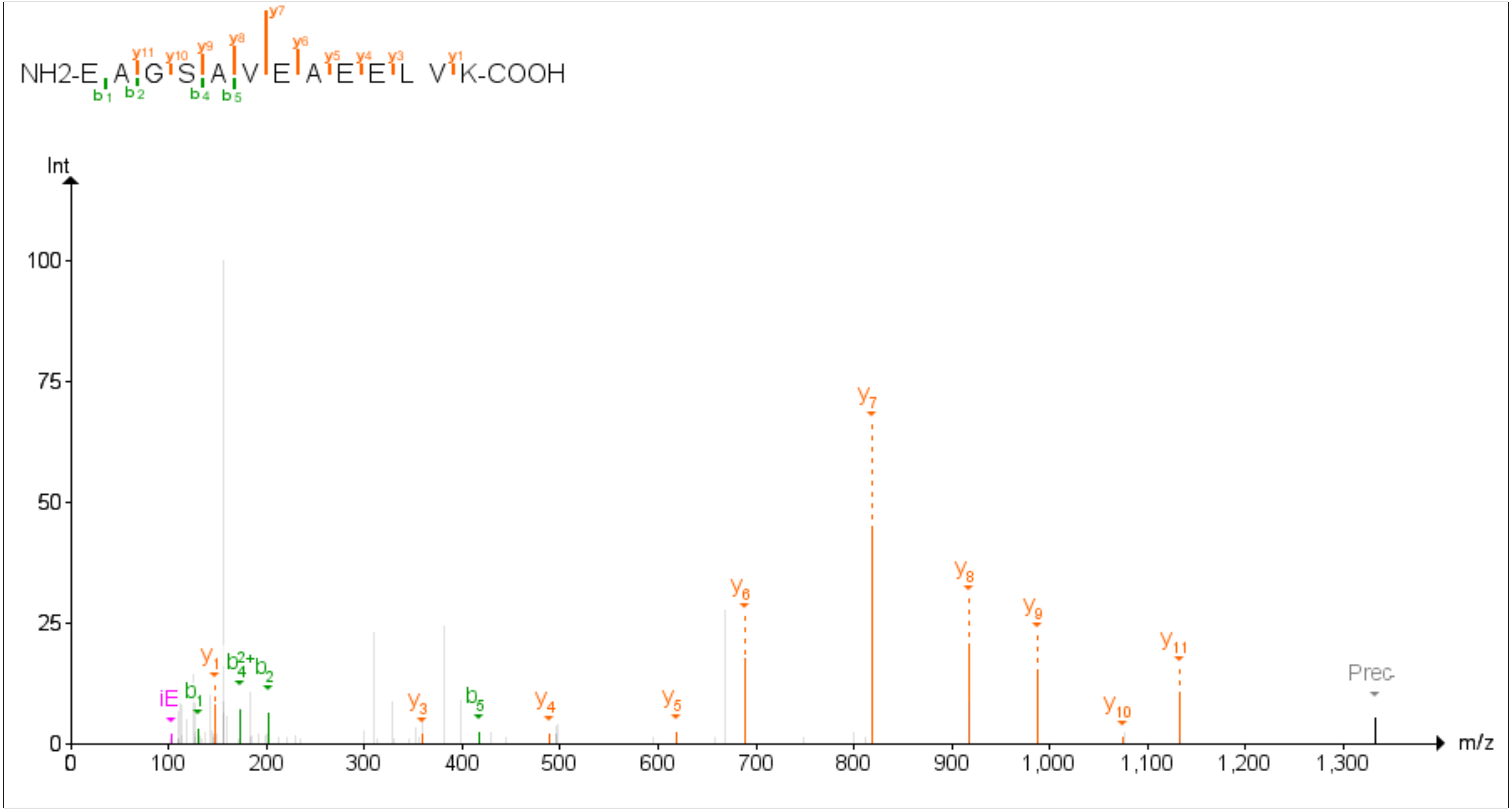
NH2-E A G S A V E A E E L V K-COOH
y11 y10 y9 y8 y7 y6 y5 y4 y3 y1
b1 b2 b4 b5
Int
100
75
50
25
0
y7
y6
y8
y9
y11
y10
Prec.
y1
b4 2+
b2
iE
b1
y3
b5
y4
y5
0 100 200 300 400 500 600 700 800 900 1,000 1,100 1,200 1,300
m/z

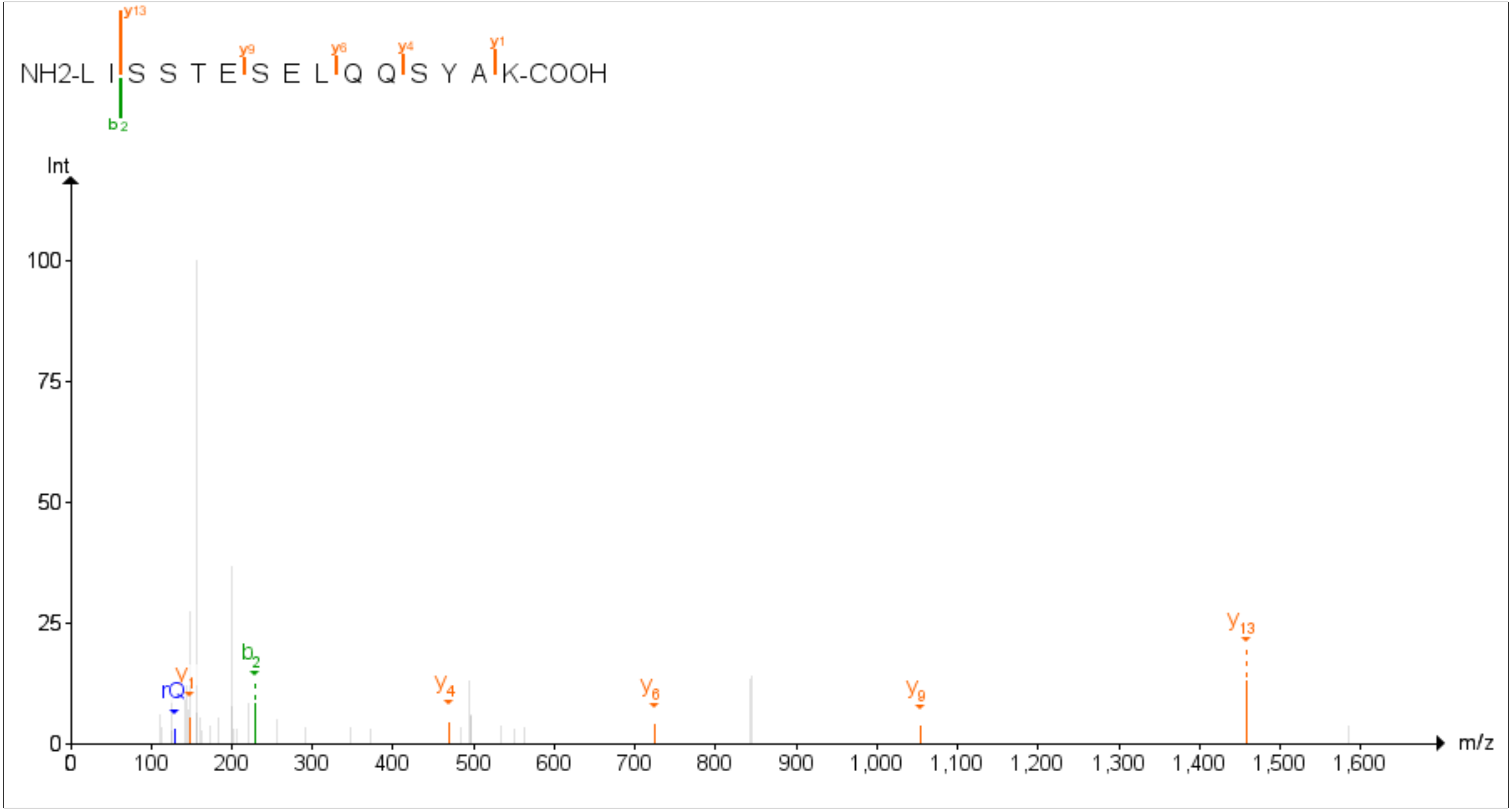

NH2-L I S S T E S E L Q Q S Y A K-COOH
y13
y9
y6
y4
y1
b2
Int
100
75
50
25
0
rQ
y1
b2
y4
y6
y9
y13
0
100
200
300
400
500
600
700
800
900
1,000
1,100
1,200
1,300
1,400
1,500
1,600
m/z

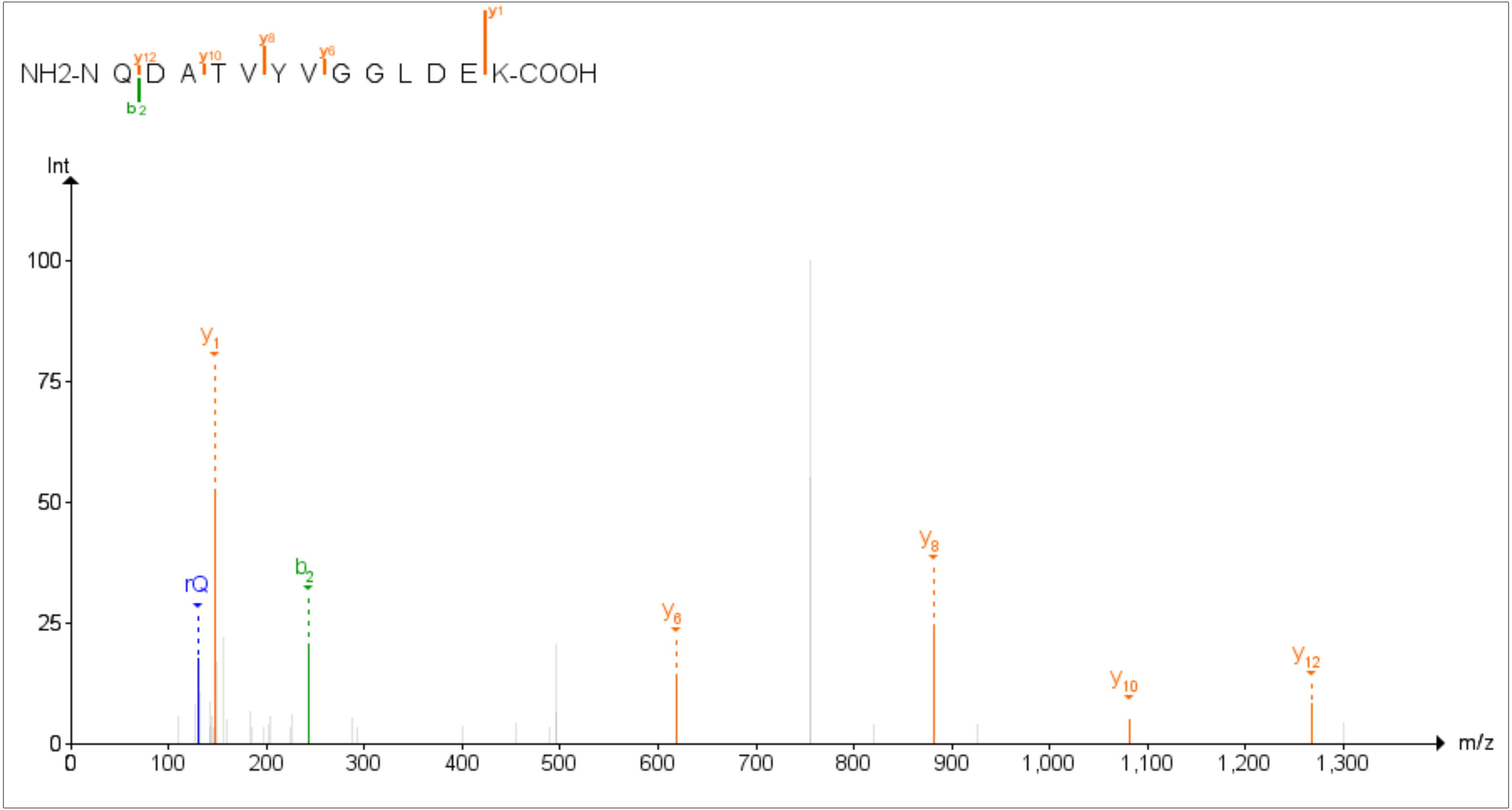
NH2-N Q D A T V Y V G G L D E K-COOH
y1
y12
y10
y8
y6
b2
Int
100
75
50
25
0
y1
rQ
b2
y6
y8
y10
y12
0
100
200
300
400
500
600
700
800
900
1,000
1,100
1,200
1,300
m/z

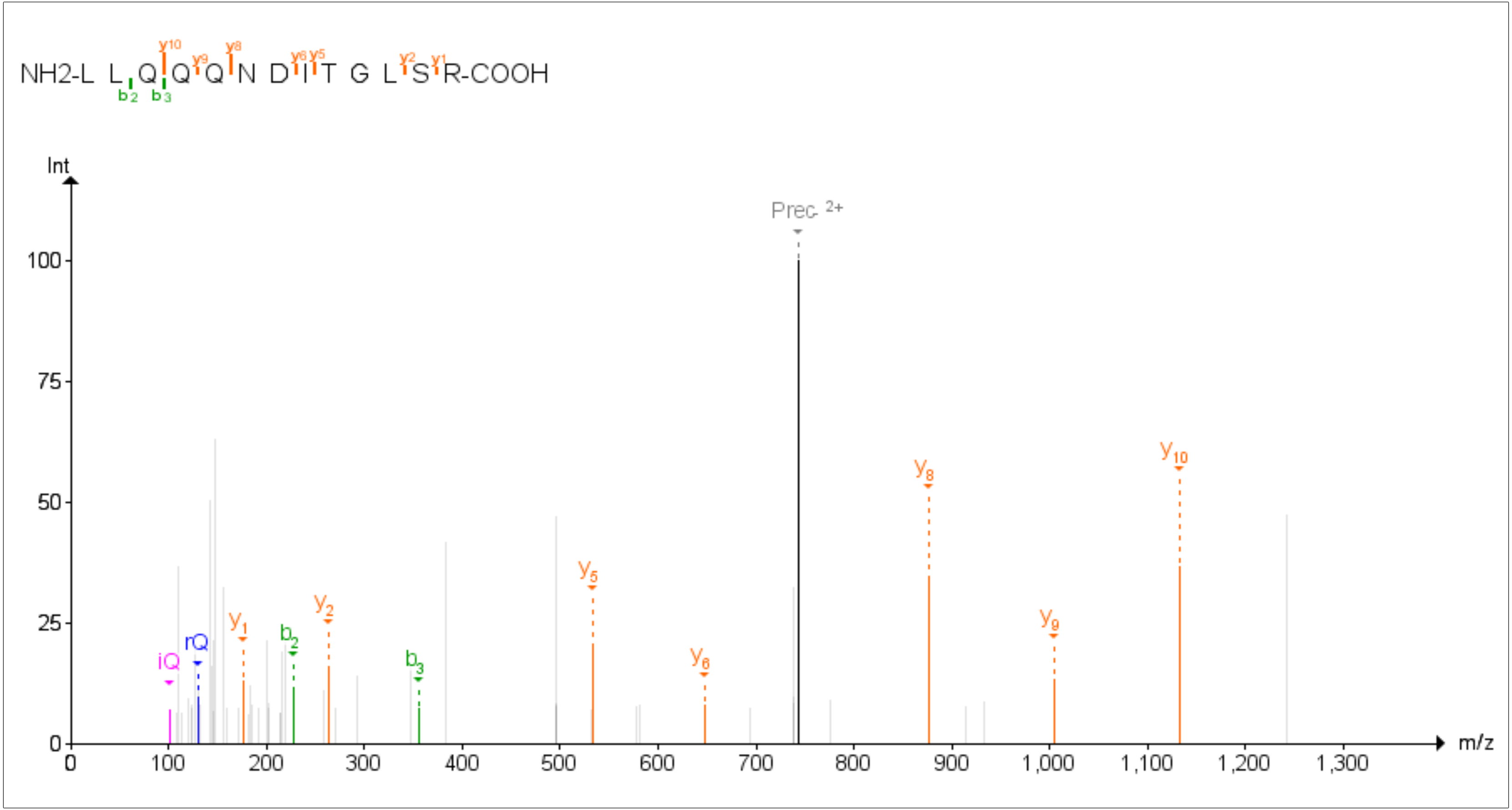

NH2-L L Q Q Q N D I T G L S R-COOH
y10 y9 y8 y6 y5 y2 y1
b2 b3
Int
Prec. 2+
y8
y10
y5
y2
y1
rQ
iQ
b2
b3
y6
y9
0
25
50
75
100
0 100 200 300 400 500 600 700 800 900 1,000 1,100 1,200 1,300
m/z

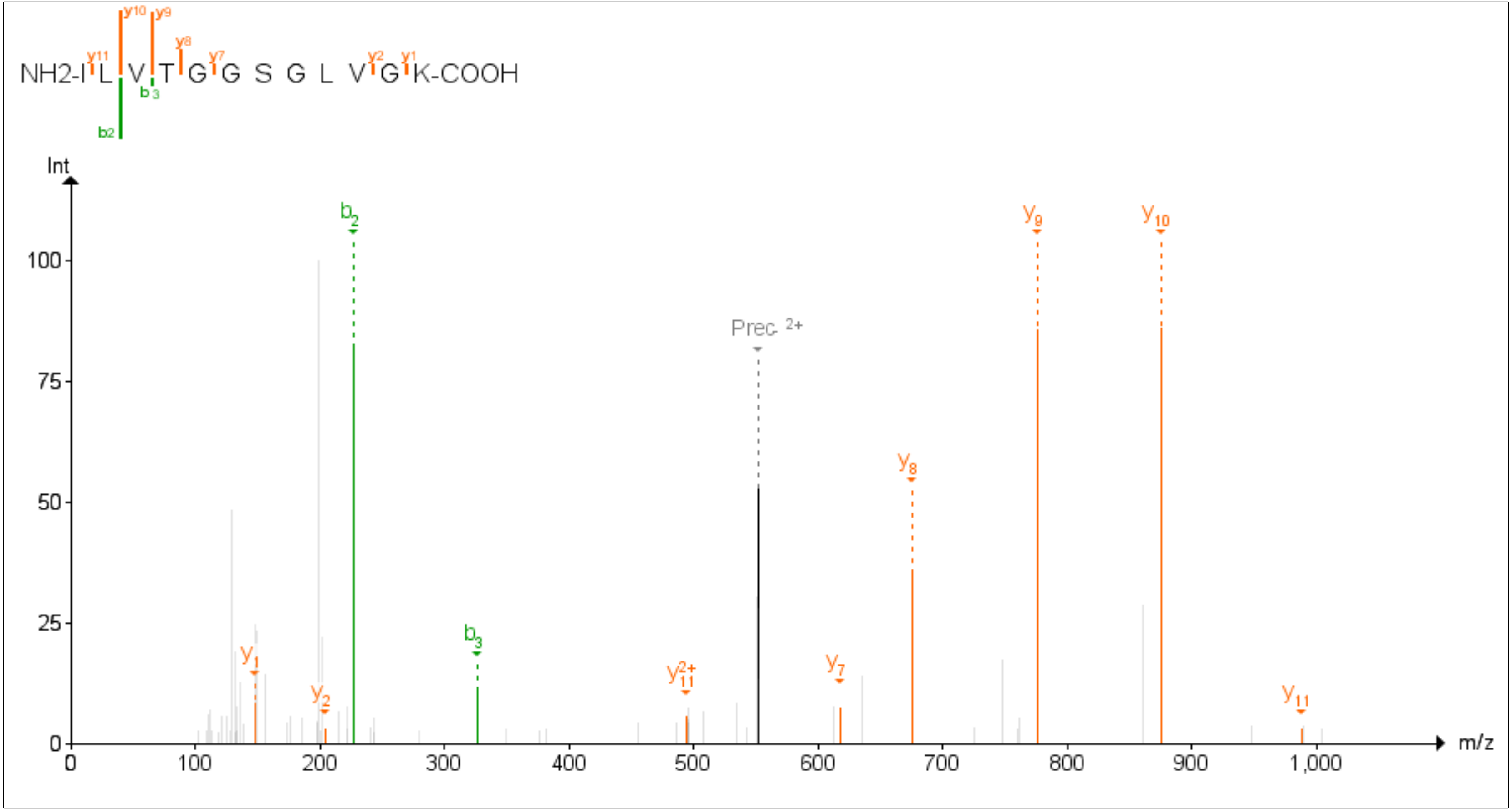
NH2-I L V T G G S G L V G K-COOH
y11
y10
y9
y8
y7
y2
y1
b2
b3
Int
100
75
50
25
0
b2
b3
y9
y10
Prec. 2+
y8
y1
y2
y11 2+
y7
y11
0
100
200
300
400
500
600
700
800
900
1,000
m/z

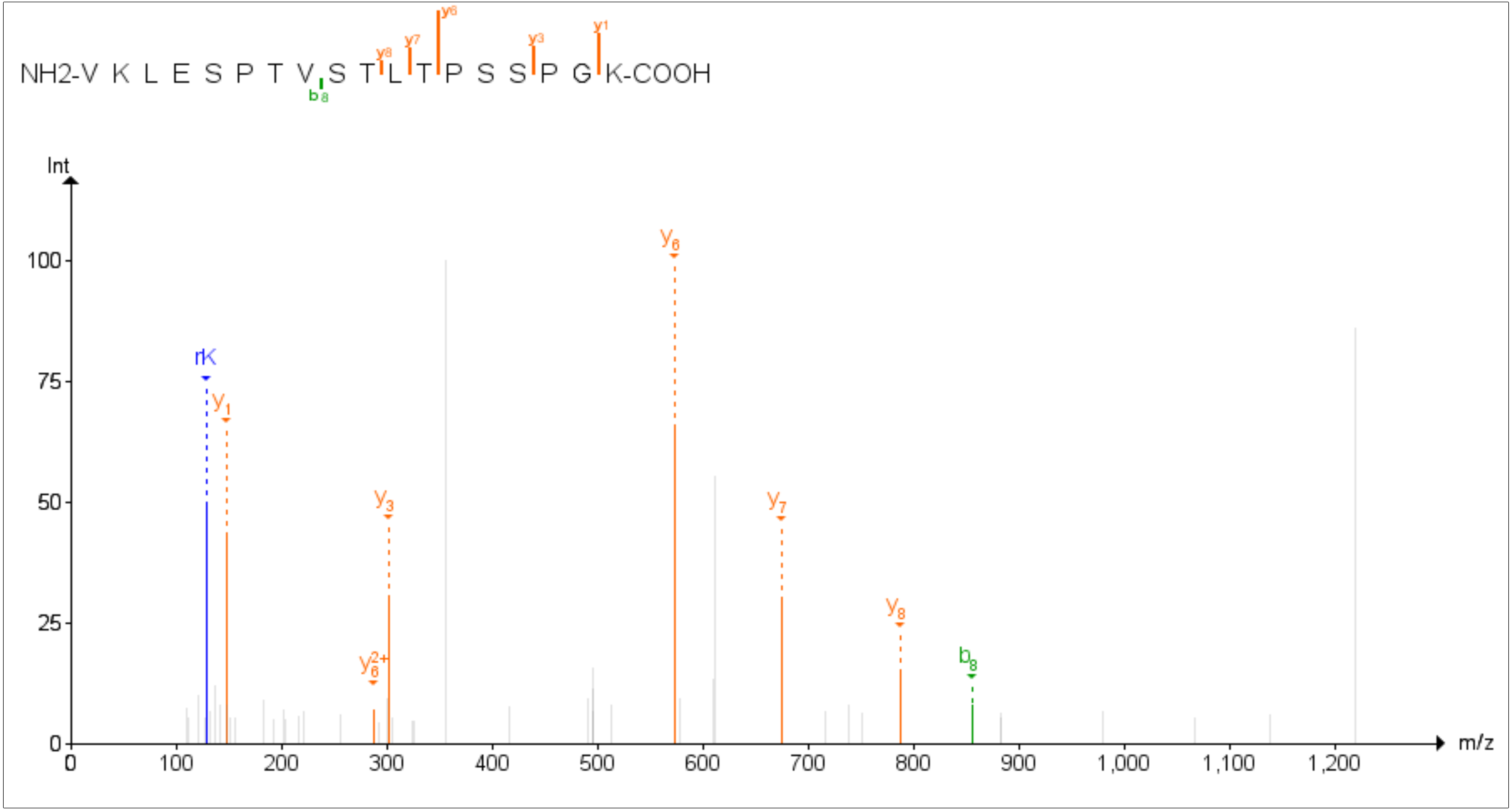

NH2-V K L E S P T V S T L T P S S P G K-COOH
b8
y8
y7
y6
y3
y1
Int
m/z
rK
y1
y3
y6 2+
y6
y7
y8
b8
0
25
50
75
100
0
100
200
300
400
500
600
700
800
900
1,000
1,100
1,200

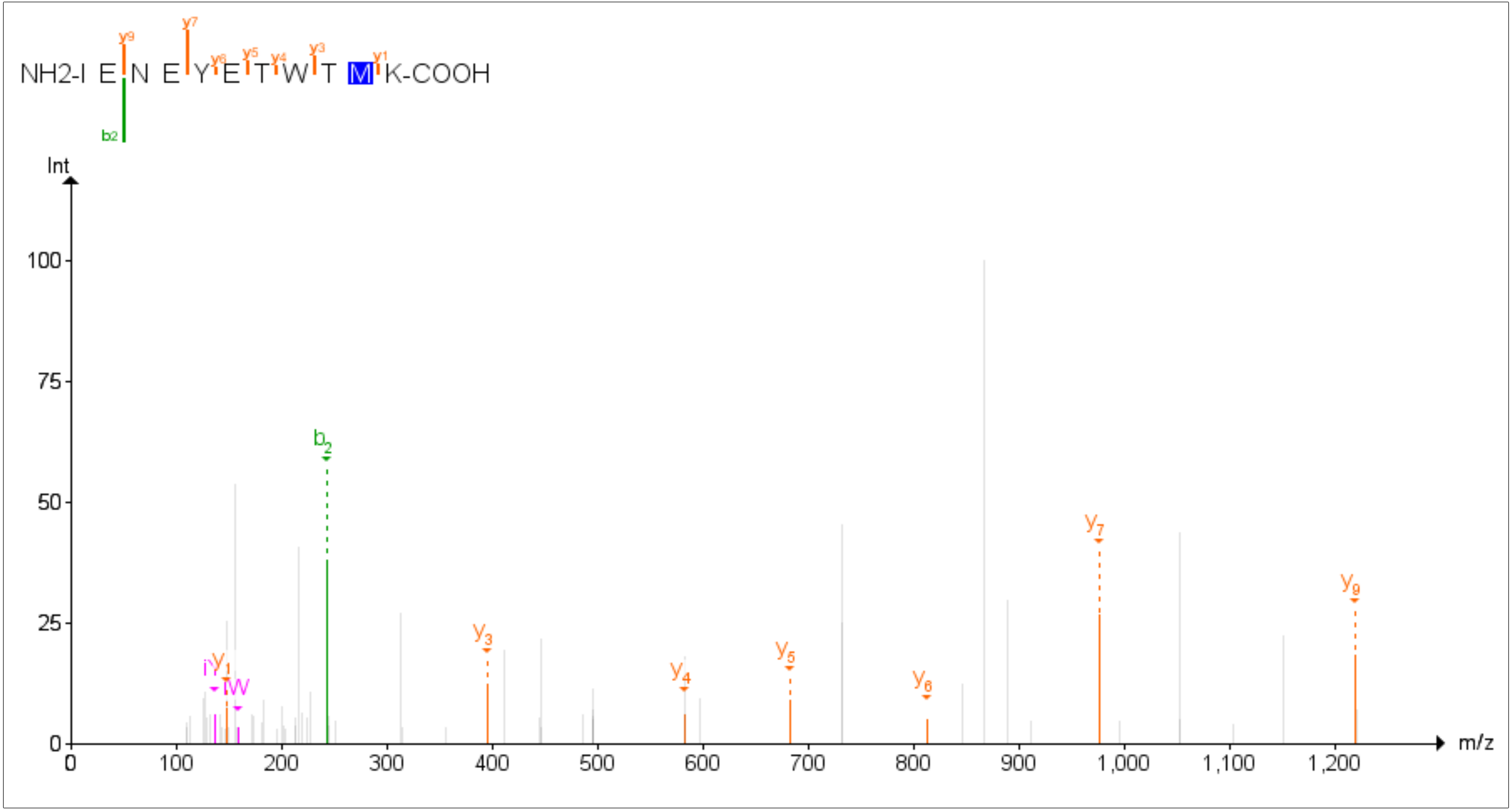

NH2-I E N E Y E T W T M K-COOH
y9 y7 y6 y5 y4 y3 y1
b2
Int
m/z
0
25
50
75
100
0
100
200
300
400
500
600
700
800
900
1,000
1,100
1,200
b2
y1
iY
iW
y3
y4
y5
y6
y7
y9

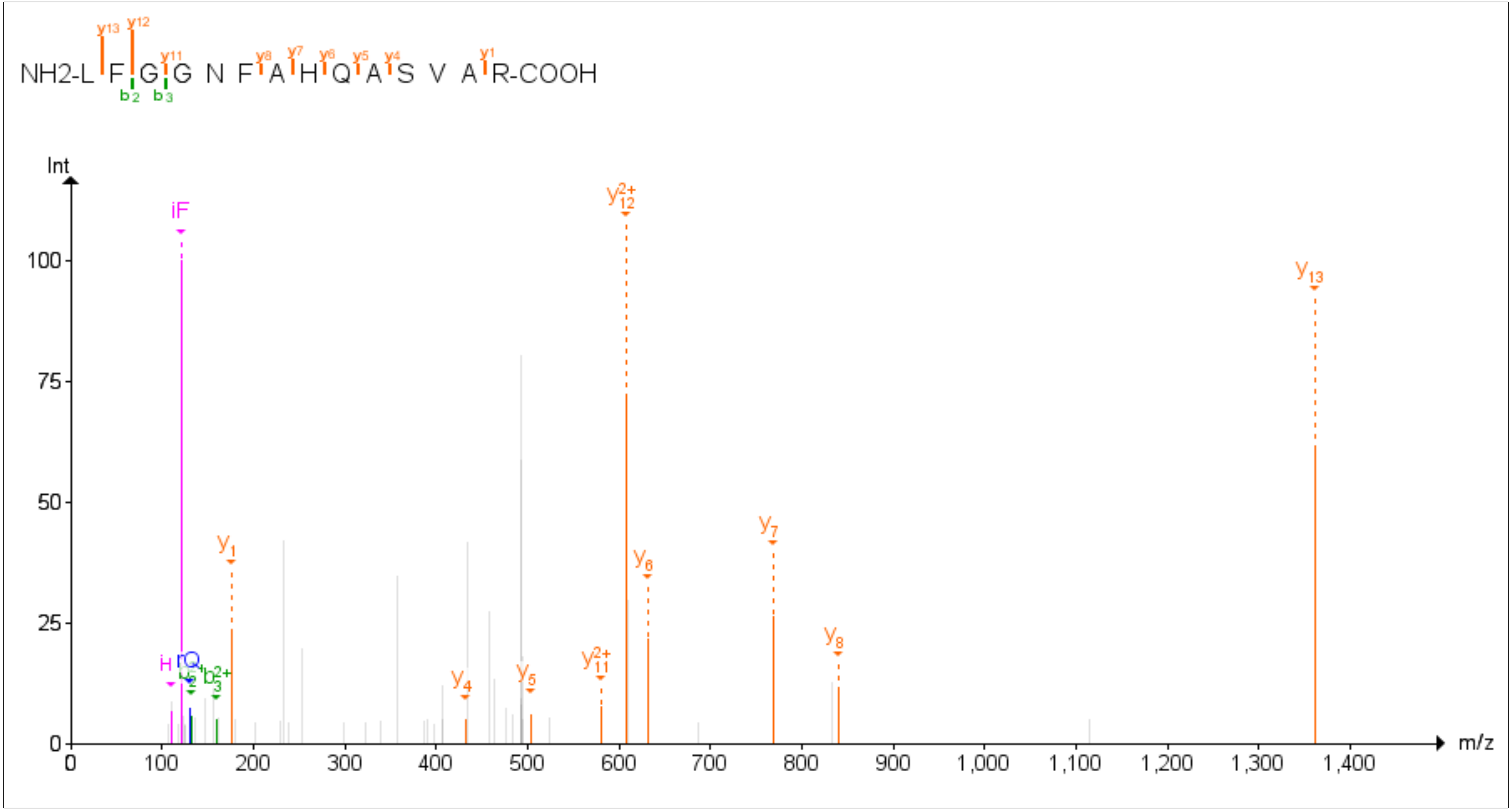

NH2-L F G G N F A H Q A S V A R-COOH
y13 y12 y11 y8 y7 y6 y5 y4 y1
b2 b3
Int
m/z
iF
iH
y1
y4
y5
y6
y7
y8
y13
0
25
50
75
100
0
100
200
300
400
500
600
700
800
900
1,000
1,100
1,200
1,300
1,400

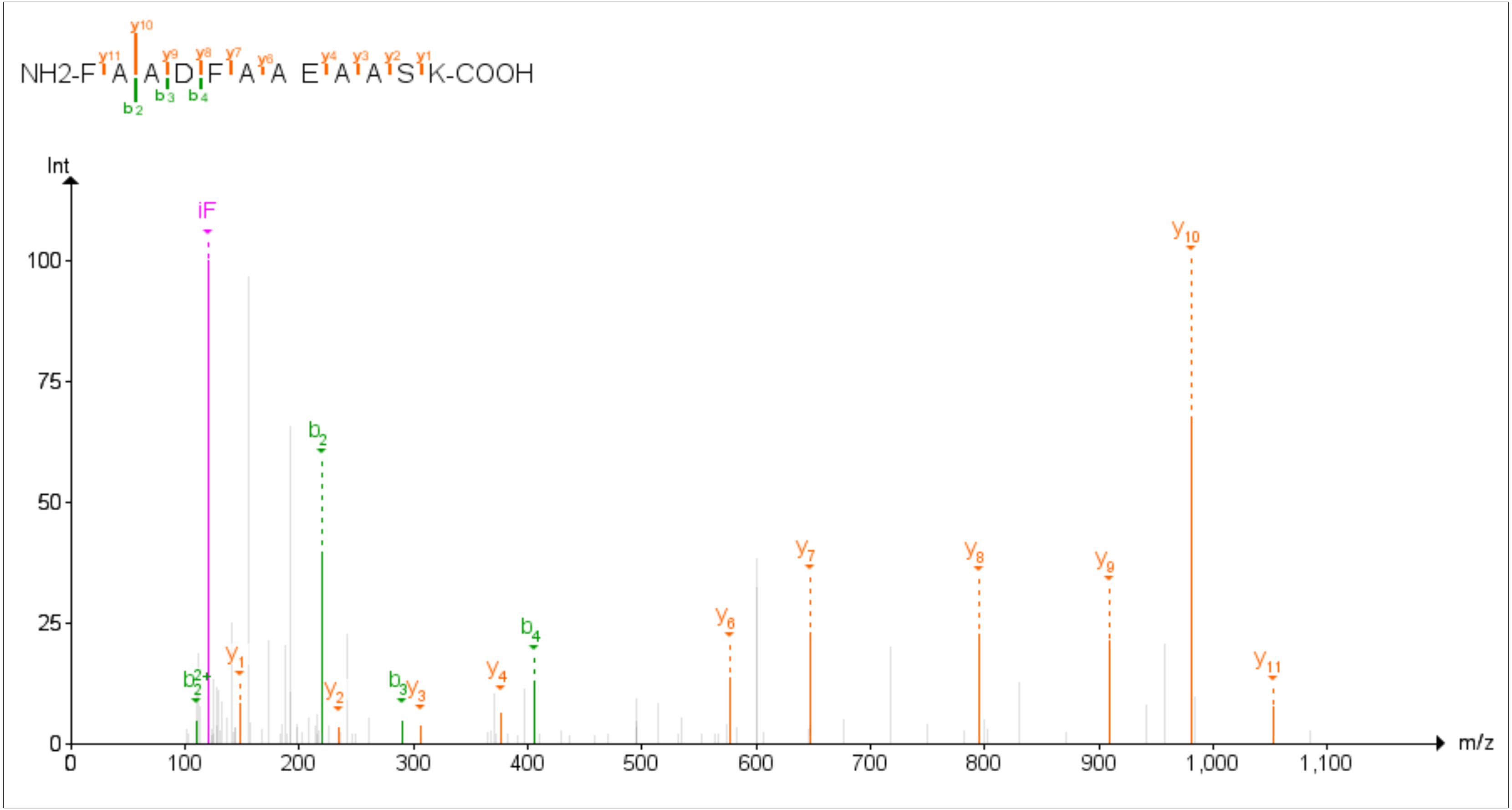

NH2-F A A D F A A E A A S K-COOH
y10 y11 y9 y8 y7 y6 y4 y3 y2 y1
b2 b3 b4
Int
iF
100
75
50
25
0
b2
y10
y7 y8 y9
y6
b4
y1
b2 2+
y2
b3 y3
y4
y11
0 100 200 300 400 500 600 700 800 900 1,000 1,100
m/z

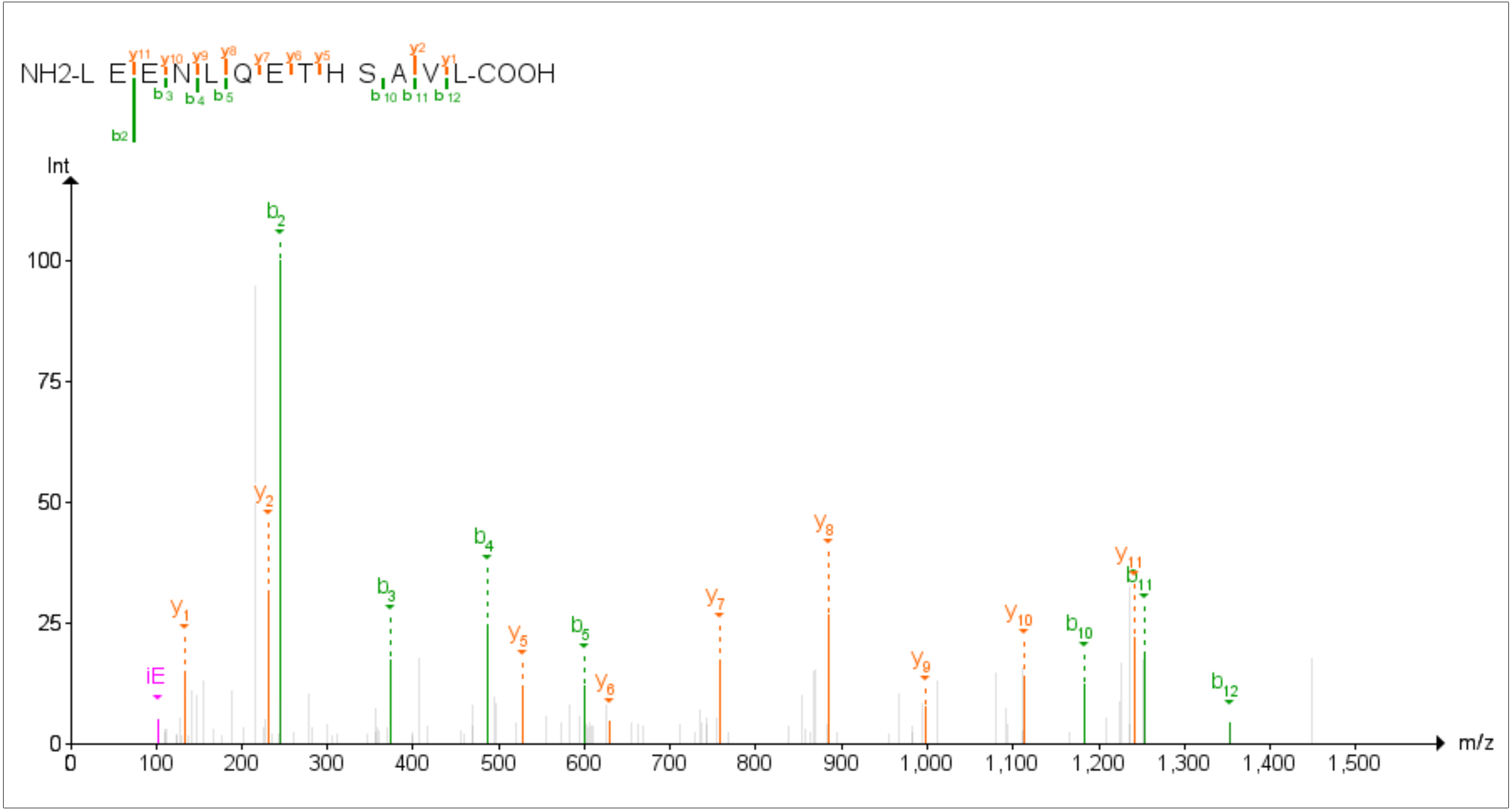
NH2-L E E N L Q E T H S A V L-COOH
y11 y10 y9 y8 y7 y6 y5 y2 y1
b2 b3 b4 b5 b10 b11 b12
Int
m/z
0
25
50
75
100
0
100
200
300
400
500
600
700
800
900
1,000
1,100
1,200
1,300
1,400
1,500
b2
y2
b4
y8
y11
b11
y1
b3
y5
b5
y7
y10
b10
iE
y6
y9
b12

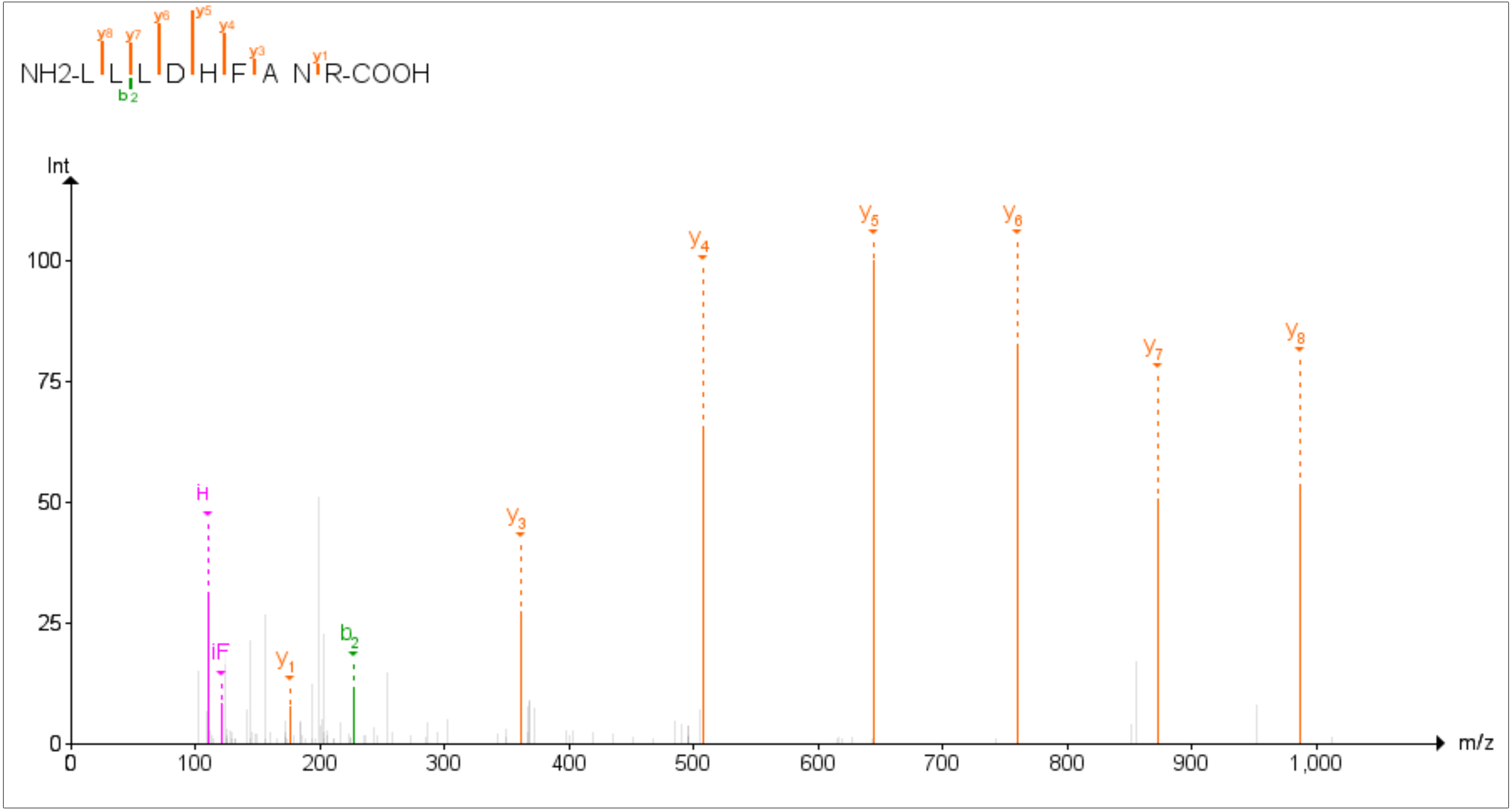

NH2-L L L D H F A N R-COOH
y8 y7 y6 y5 y4 y3 y1
b2
Int
100
75
50
25
0
m/z
0
100
200
300
400
500
600
700
800
900
1,000
iH
iF
y1
b2
y3
y4
y5
y6
y7
y8

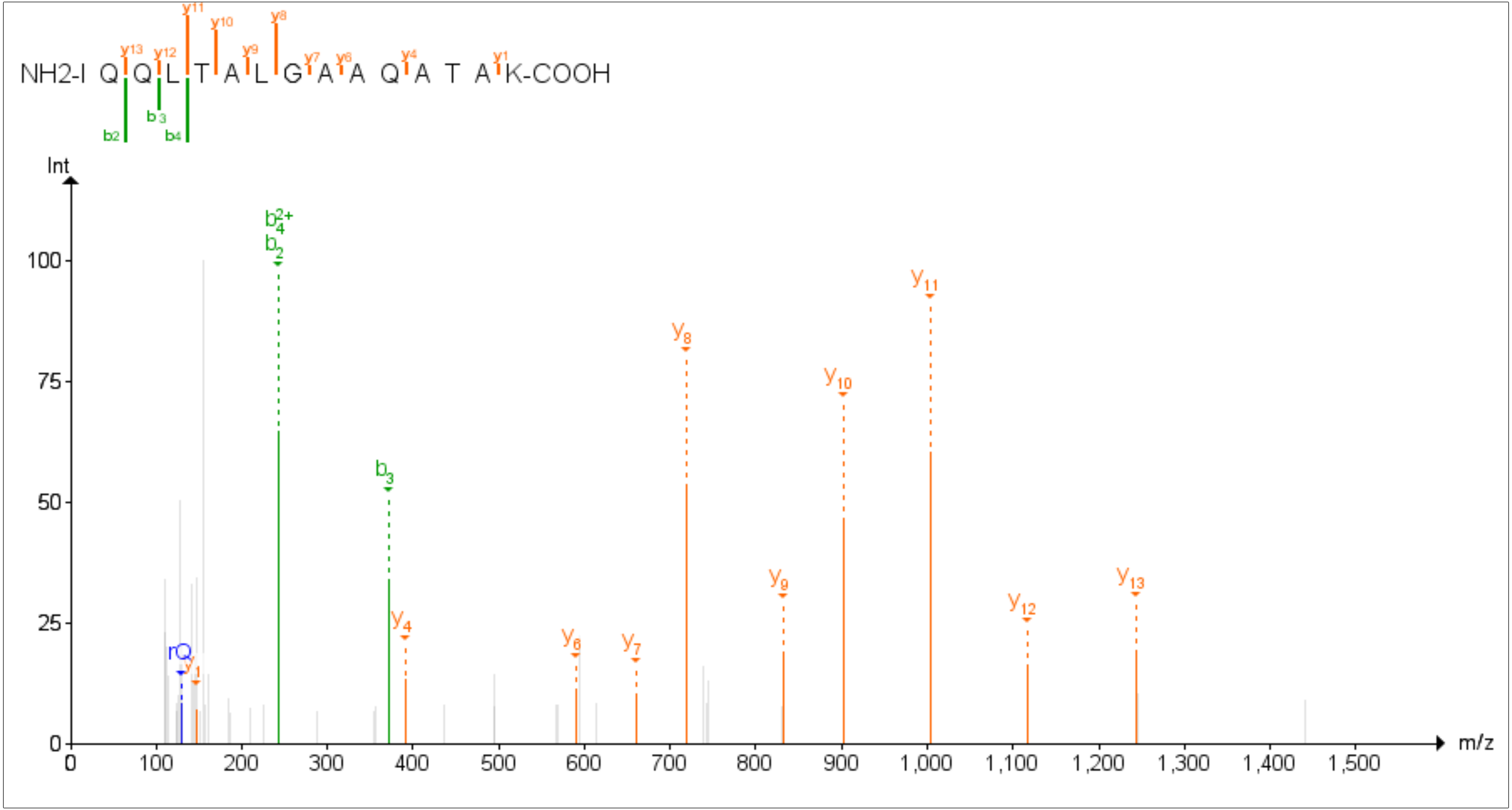

NH2-I Q Q L T A L G A A Q A T A K-COOH
y13 y12 y11 y10 y9 y8 y7 y6 y4 y1
b2 b3 b4
Int
m/z
0
25
50
75
100
0 100 200 300 400 500 600 700 800 900 1,000 1,100 1,200 1,300 1,400 1,500
b2+4
b2
b3
rQ
y1
y4
y6
y7
y8
y9
y10
y11
y12
y13

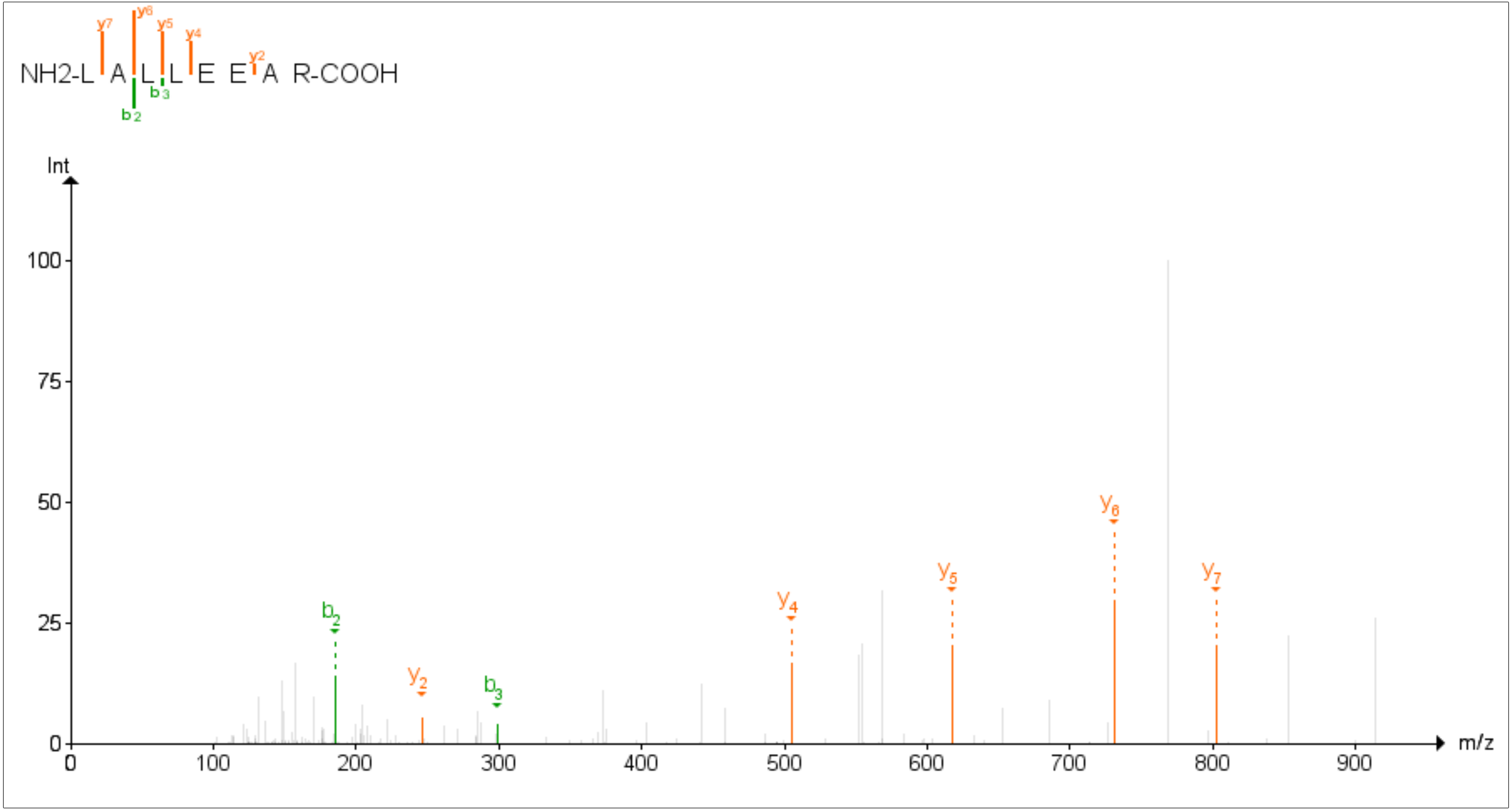

NH2-L A L L E E A R-COOH
y7
y6
y5
y4
y2
b2
b3
Int
100
75
50
25
0
m/z
0
100
200
300
400
500
600
700
800
900
b2
y2
b3
y4
y5
y6
y7

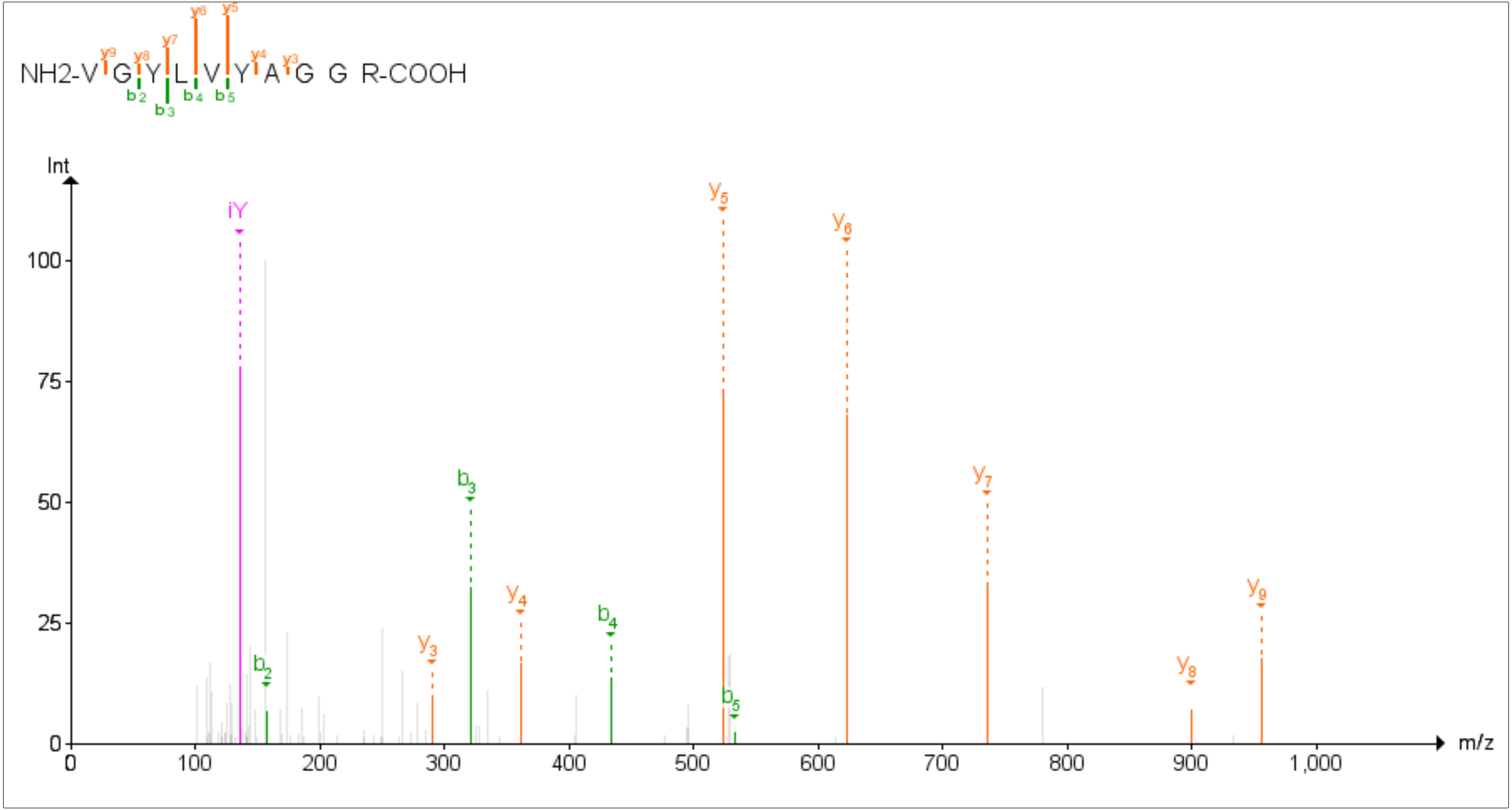

NH2-V G Y L V Y A G G R-COOH
y9 y8 y7 y6 y5 y4 y3
b2 b3 b4 b5
Int
m/z
iY
y5
y6
y7
y8
y9
y3
y4
b2
b3
b4
b5
0
25
50
75
100
0
100
200
300
400
500
600
700
800
900
1,000

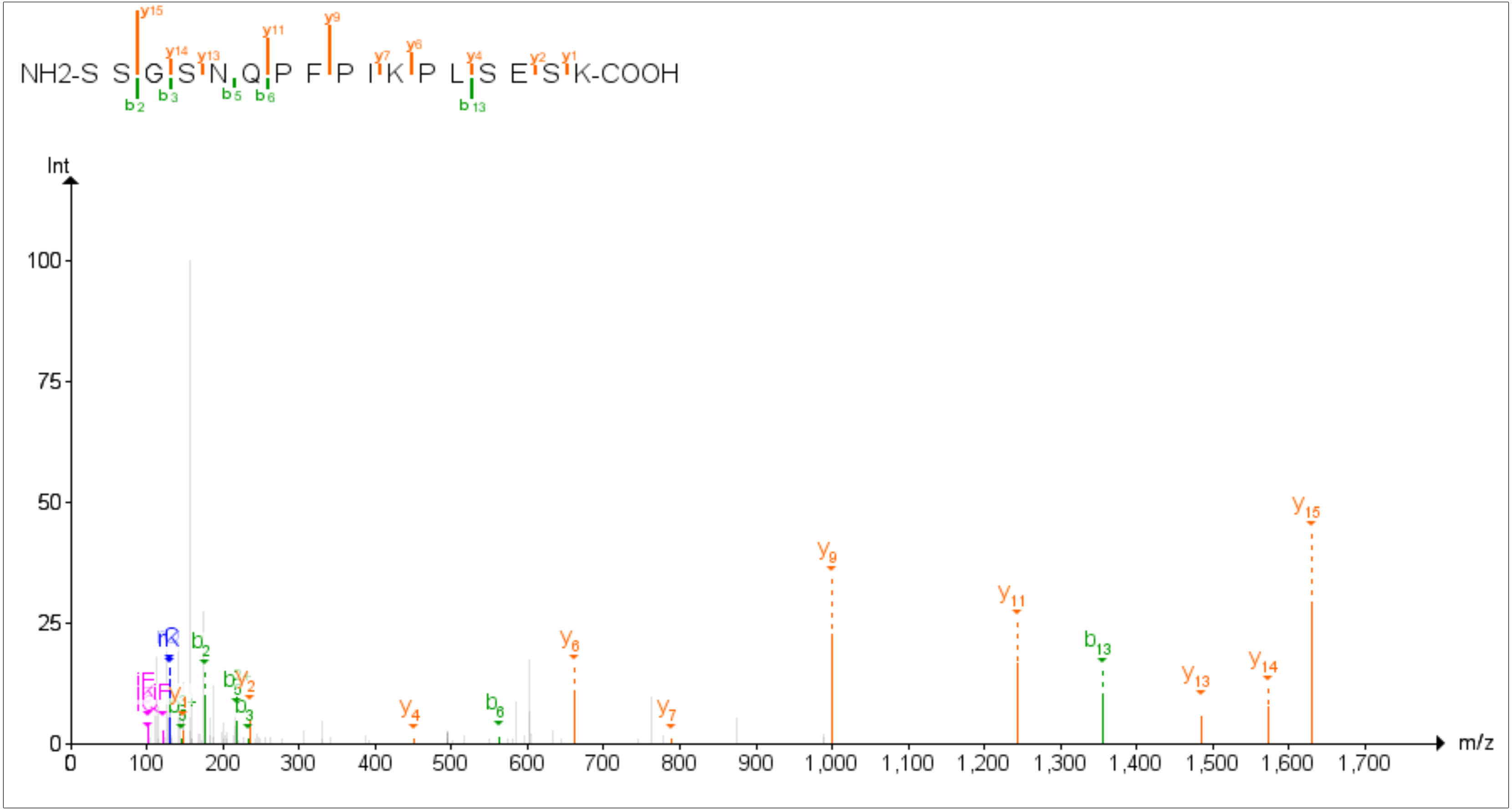

NH2-S S G S N Q P F P I K P L S E S K-COOH
y15 y14 y13 y11 y9 y7 y6 y4 y2 y1
b2 b3 b5 b6 b13
Int
100
75
50
25
0
m/z
0 100 200 300 400 500 600 700 800 900 1,000 1,100 1,200 1,300 1,400 1,500 1,600 1,700
y15
y9
y11
b13
y6
y13
y14
b2
y2
y4
b6
y7
b3

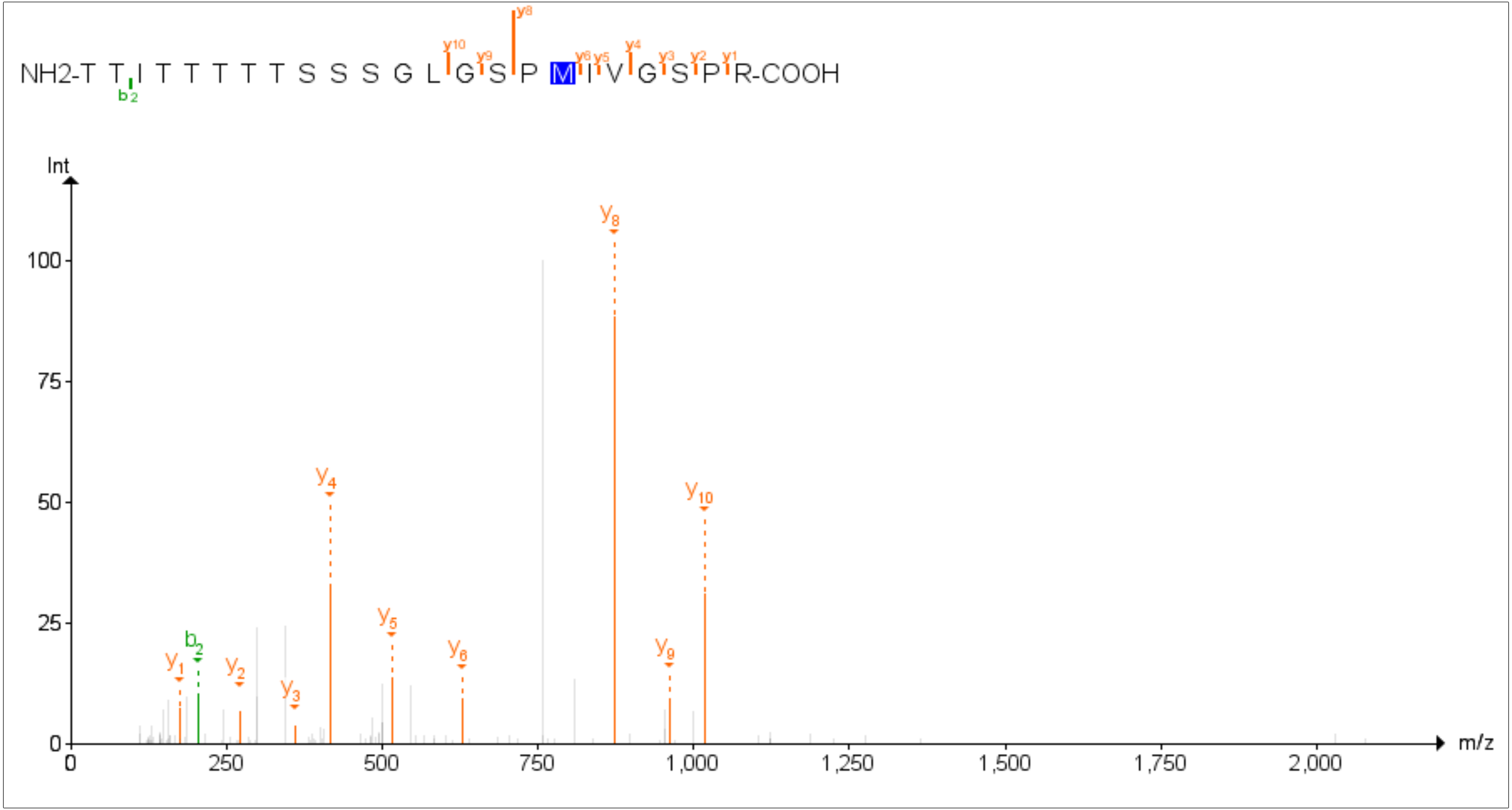
NH2-T T I T T T T T S S S G L G S P M I V G S P R-COOH
b2
y10 y9 y8 y6 y5 y4 y3 y2 y1
Int
100
75
50
25
0
y1
b2
y2
y3
y4
y5
y6
y8
y9
y10
0
250
500
750
1,000
1,250
1,500
1,750
2,000
m/z

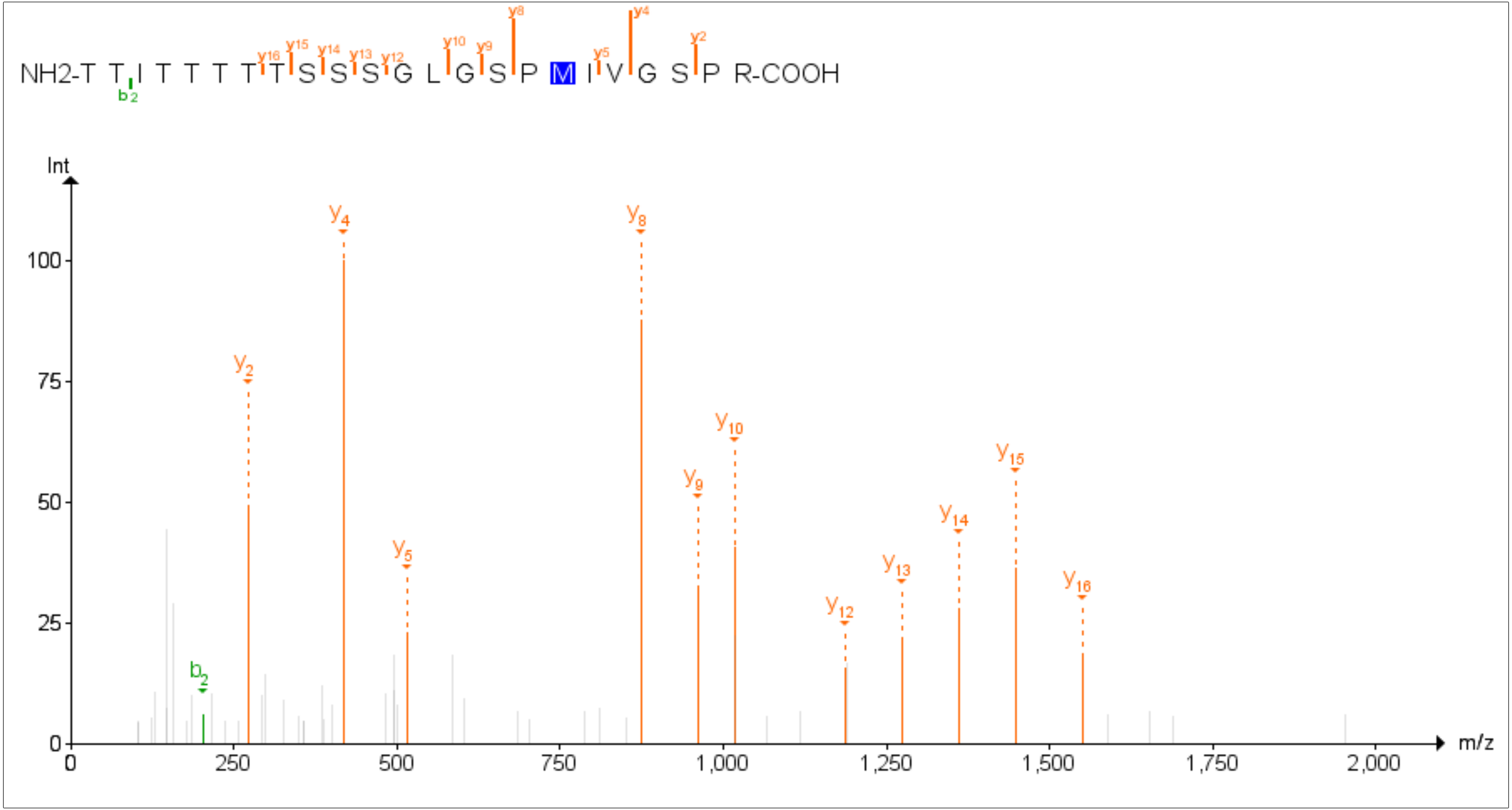
NH2-T T I T T T T T S S S G L G S P M I V G S P R-COOH
b2
y16 y15 y14 y13 y12 y10 y9 y8 y5 y4 y2
Int
100
75
50
25
0
m/z
0
250
500
750
1,000
1,250
1,500
1,750
2,000
b2
y2
y4
y5
y8
y9
y10
y12
y13
y14
y15
y16

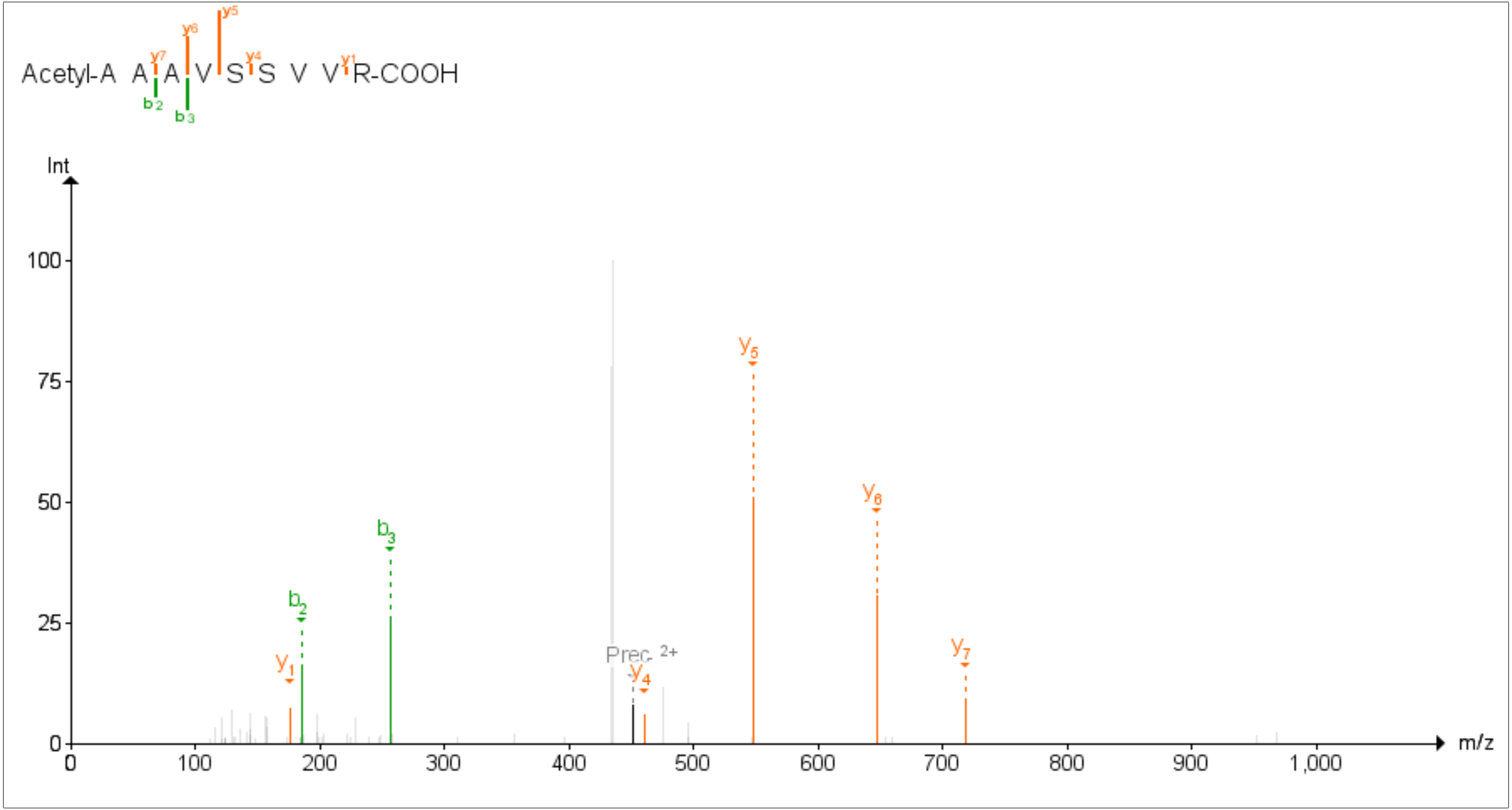

Acetyl-A A A V S S V V R-COOH
y7 y6 y5 y4 y1
b2 b3
Int
100
75
50
25
0
m/z
0
100
200
300
400
500
600
700
800
900
1,000
y1
b2
b3
Prec. 2+
y4
y5
y6
y7

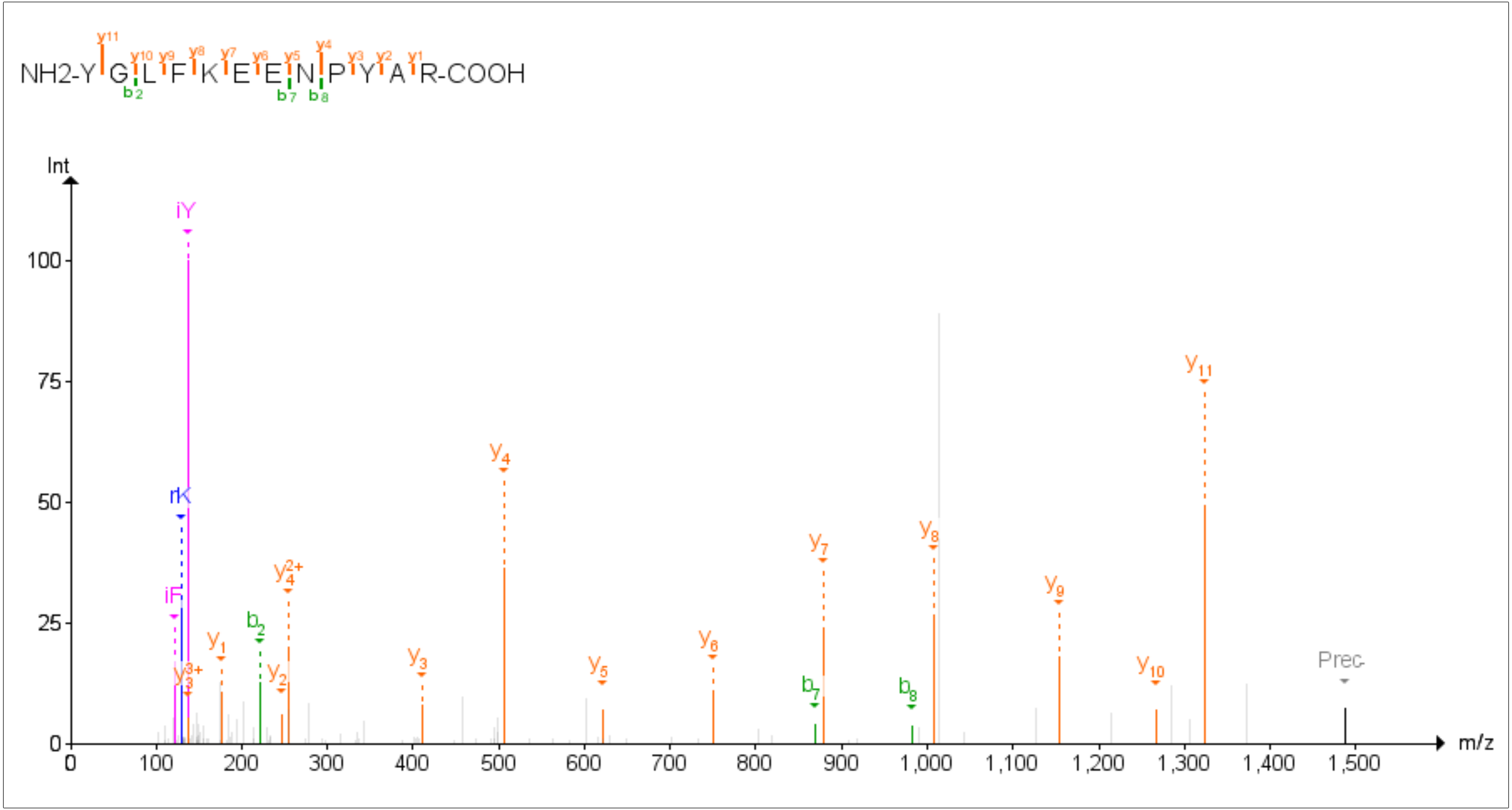
NH2-Y G L F K E E N P Y A R-COOH
y11 y10 y9 y8 y7 y6 y5 y4 y3 y2 y1
b2 b7 b8
Int
100
75
50
25
0
iY
rK
iF
y1
b2
y4 2+
y3 3+
y2
y3
y4
y5
y6
b7
y7
b8
y8
y9
y10
y11
Prec
0 100 200 300 400 500 600 700 800 900 1,000 1,100 1,200 1,300 1,400 1,500
m/z

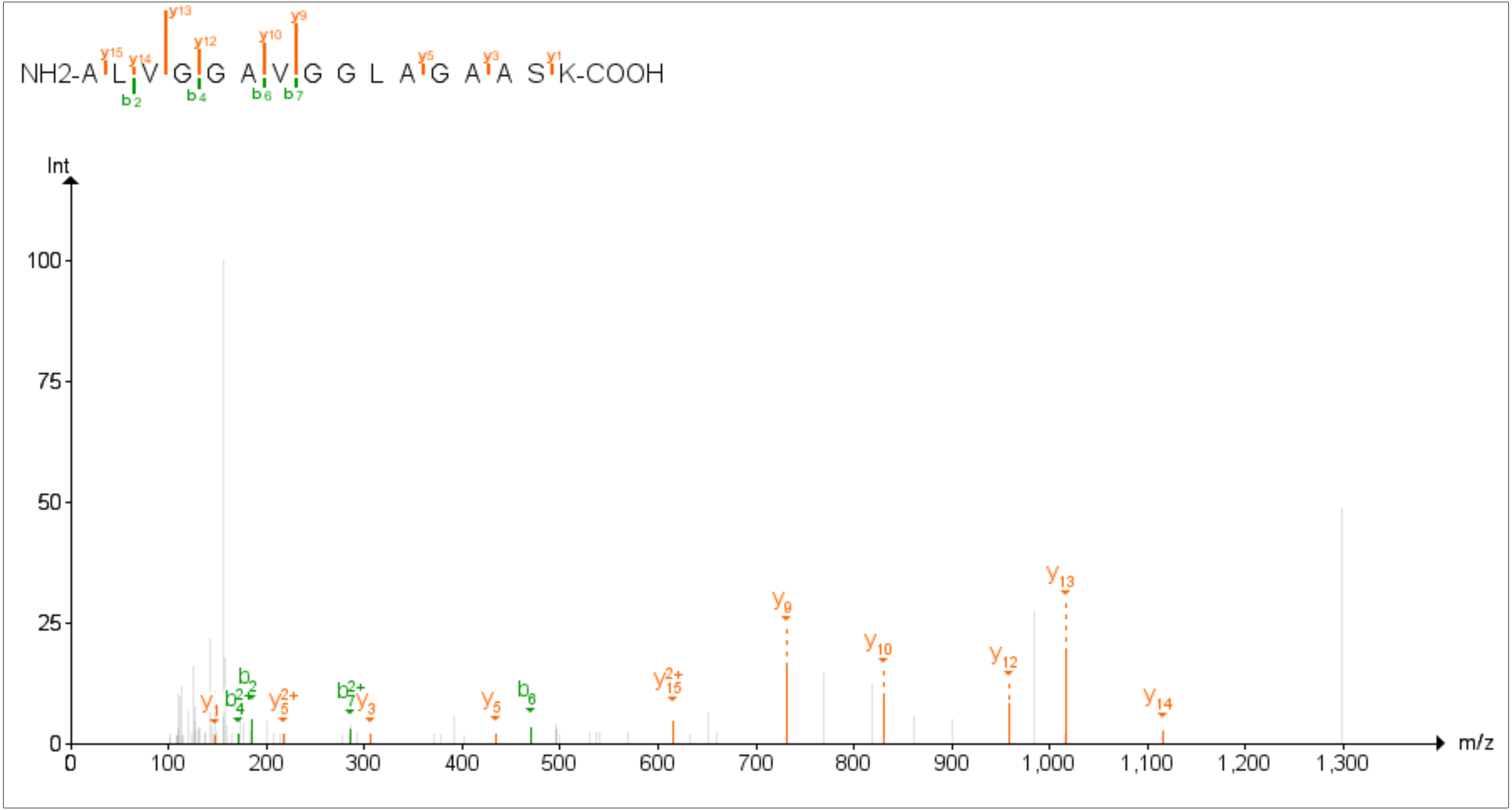

NH2-A L V G G A V G G L A G A A S K-COOH
Int
m/z
y1
y3
y5
y9
y10
y12
y13
y14
b2
b4
b6
b7

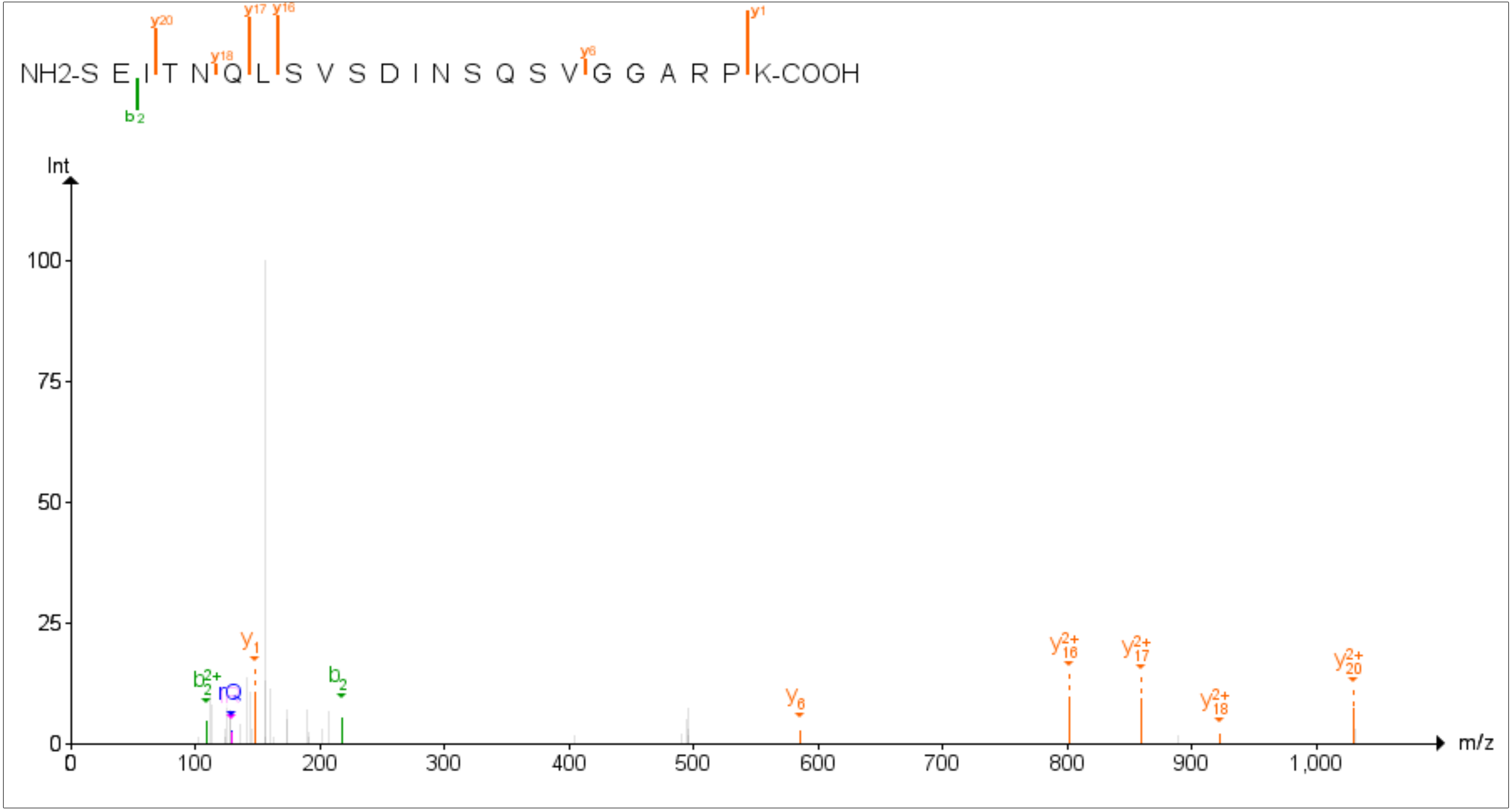
NH2-S E I T N Q L S V S D I N S Q S V G G A R P K-COOH
y20
y18
y17
y16
y6
y1
b2
Int
100
75
50
25
0
m/z
0
100
200
300
400
500
600
700
800
900
1,000
b2 2+
rQ
y1
b2
y6
y16 2+
y17 2+
y18 2+
y20 2+

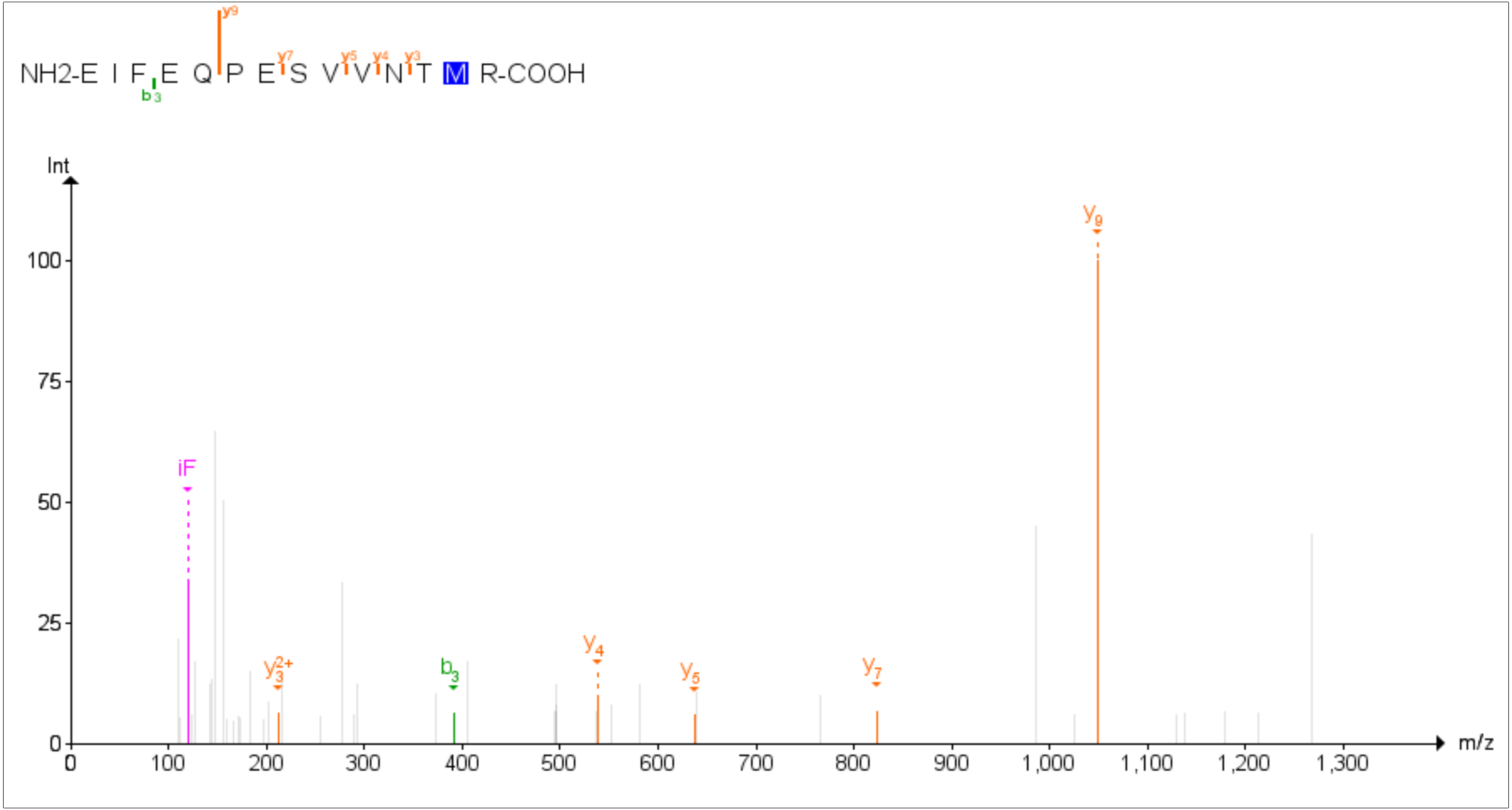

NH2-E I F E Q P E S V V N T M R-COOH
y9
y7
y5
y4
y3
b3
Int
100
75
50
25
0
iF
y3 2+
b3
y4
y5
y7
y9
m/z
0
100
200
300
400
500
600
700
800
900
1,000
1,100
1,200
1,300

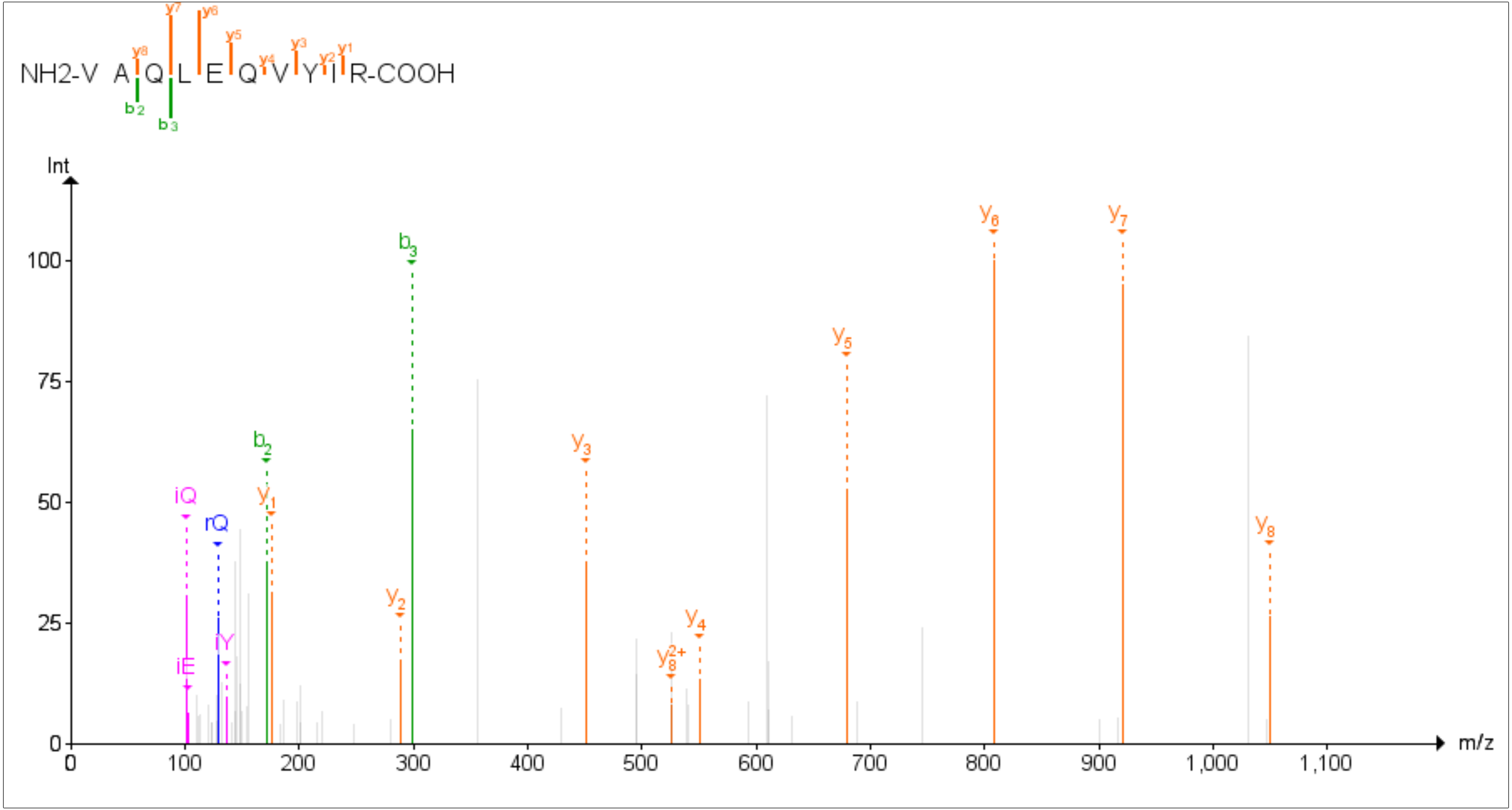

NH2-V A Q L E Q V Y I R-COOH
y7 y6 y5 y8 y4 y3 y2 y1
b2 b3
Int
m/z
0
25
50
75
100
0 100 200 300 400 500 600 700 800 900 1,000 1,100
iQ
rQ
iY
iE
b2
y1
b3
y2
y3
y8 2+
y4
y5
y6
y7
y8

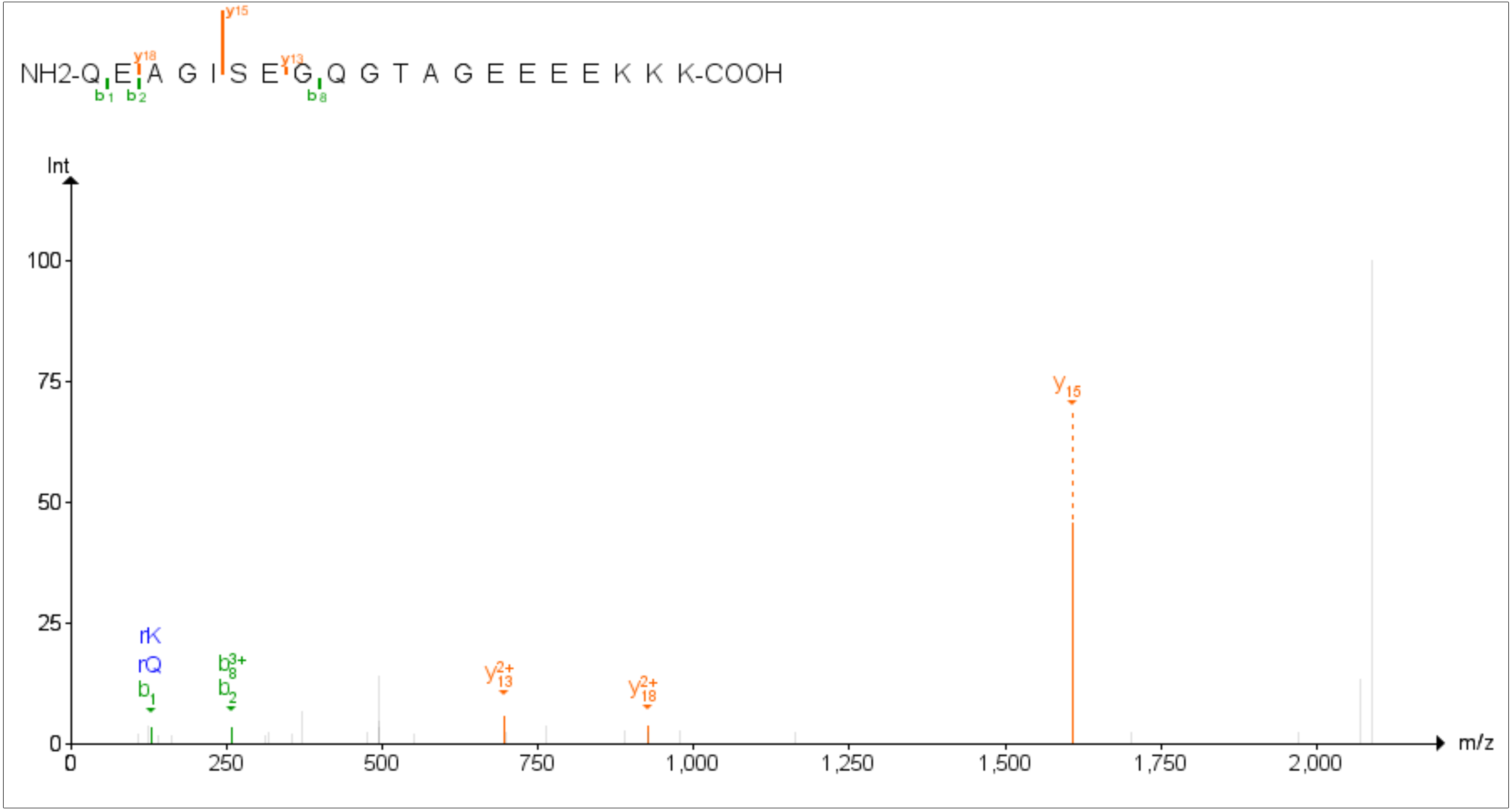

NH2-Q E A G I S E G Q G T A G E E E E K K K-COOH
y15
y18
y13
b1
b2
b8
Int
100
75
50
25
0
y15
rK
rQ
b1
b8 3+
b2
y13 2+
y18 2+
0
250
500
750
1,000
1,250
1,500
1,750
2,000
m/z

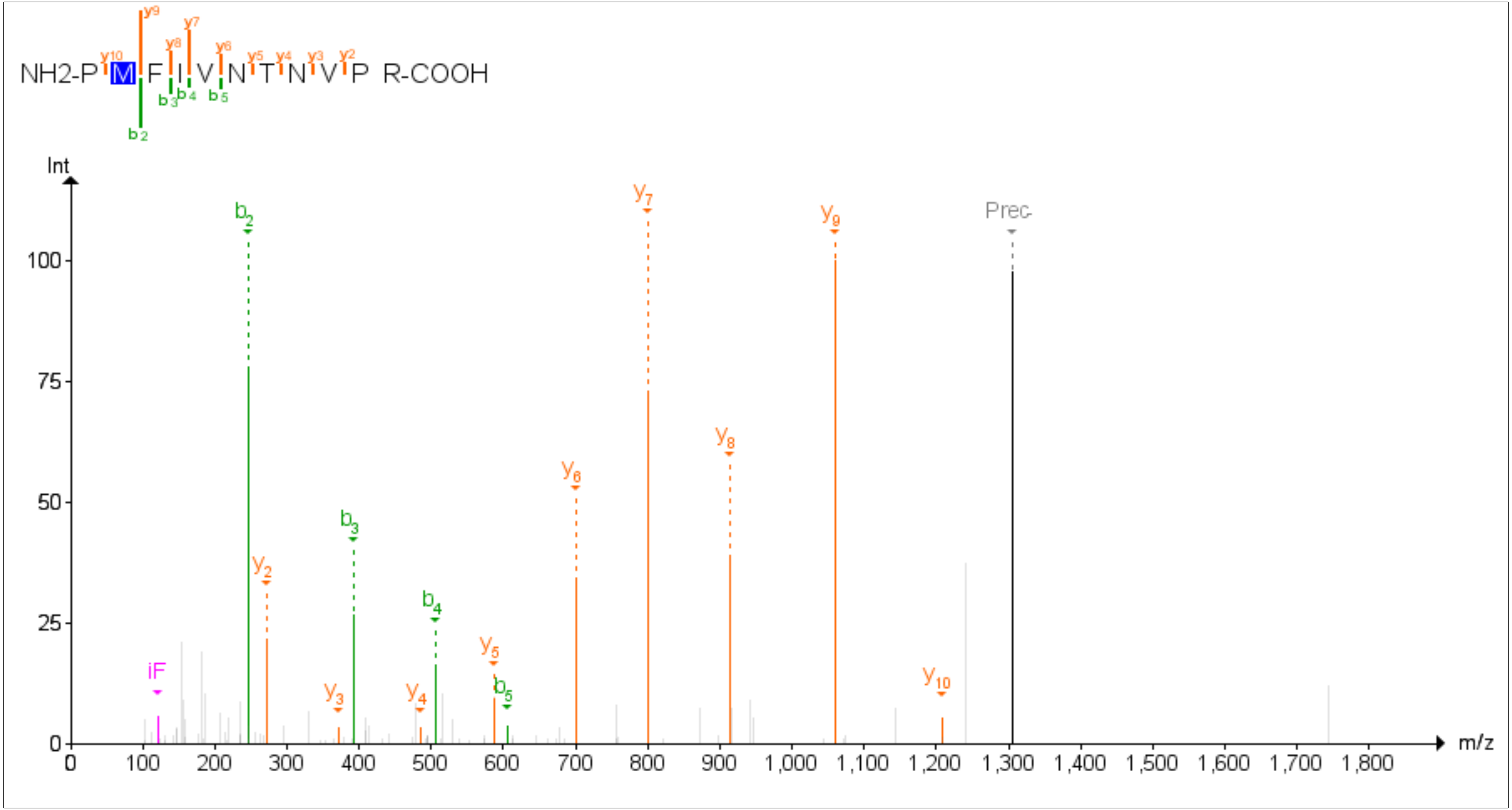

NH2-P M F I V N T N V P R-COOH
y10 y9 y8 y7 y6 y5 y4 y3 y2
b2 b3 b4 b5
Int
100
75
50
25
0
b2
y7
y9
Prec
y8
y6
b3
y2
b4
y5
b5
y3
y4
y10
iF
0 100 200 300 400 500 600 700 800 900 1,000 1,100 1,200 1,300 1,400 1,500 1,600 1,700 1,800
m/z

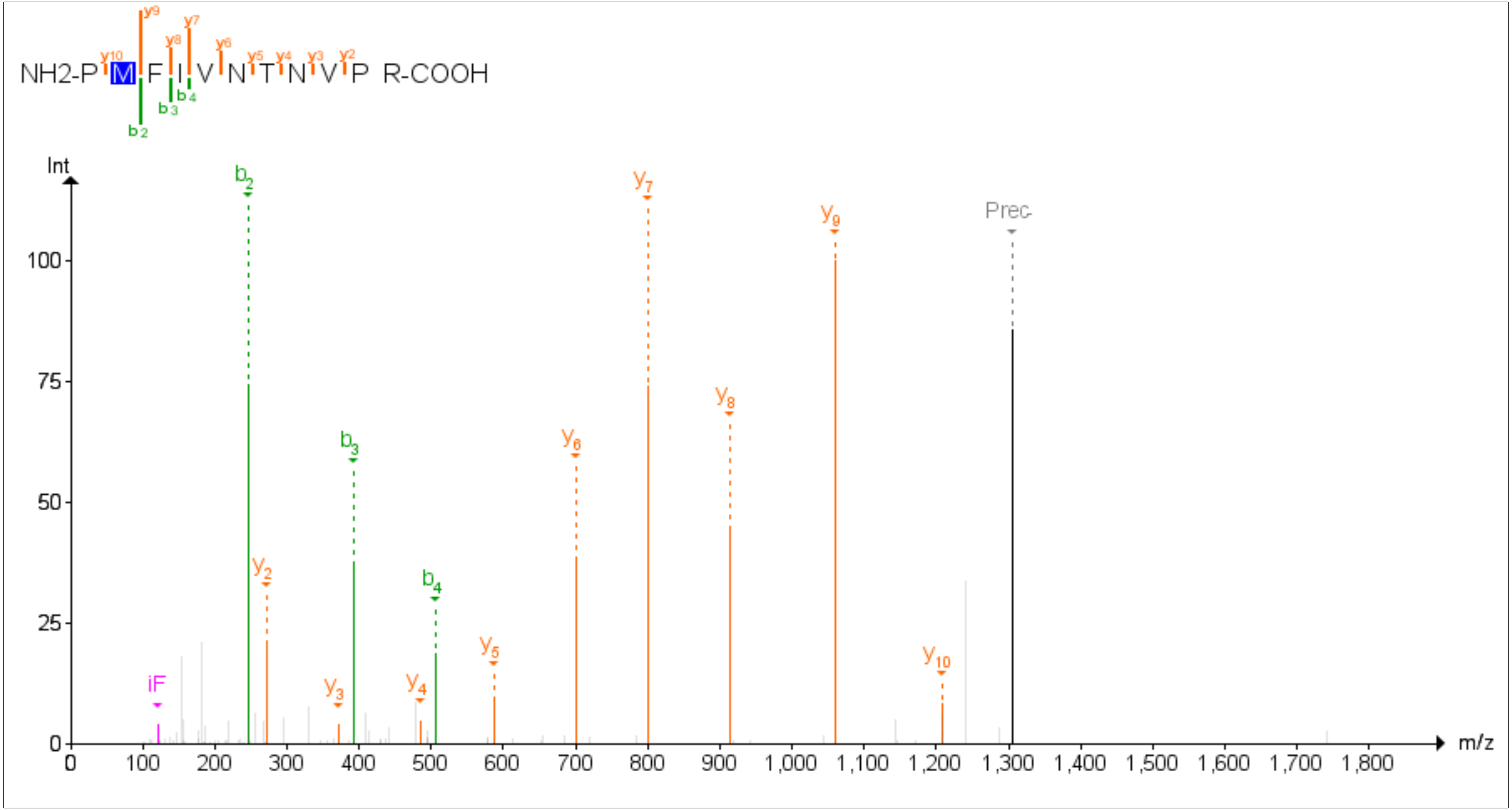
NH2-P M F I V N T N V P R-COOH
y10 y9 y8 y7 y6 y5 y4 y3 y2
b2 b3 b4
Int
m/z
b2
y7
y9
Prec
y8
y6
b3
y2
b4
y5
y10
iF
y3
y4
0
25
50
75
100
0 100 200 300 400 500 600 700 800 900 1,000 1,100 1,200 1,300 1,400 1,500 1,600 1,700 1,800

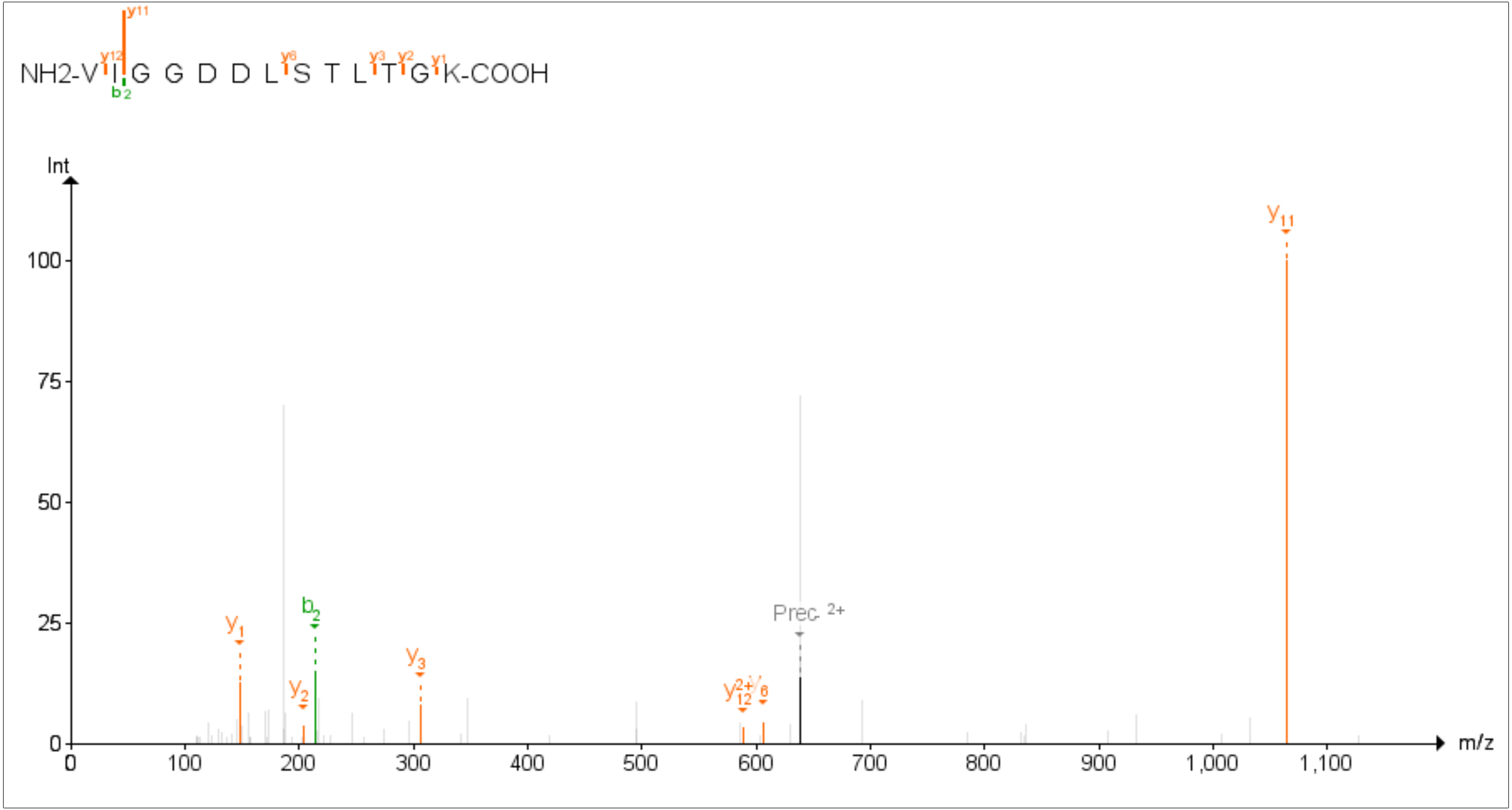

NH2-V I G G D D L S T L T G K-COOH
y11
y12
b2
y6
y3
y2
y1
Int
100
75
50
25
0
y11
y1
b2
y2
y3
Prec 2+
y12 2+
y6
0
100
200
300
400
500
600
700
800
900
1,000
1,100
m/z

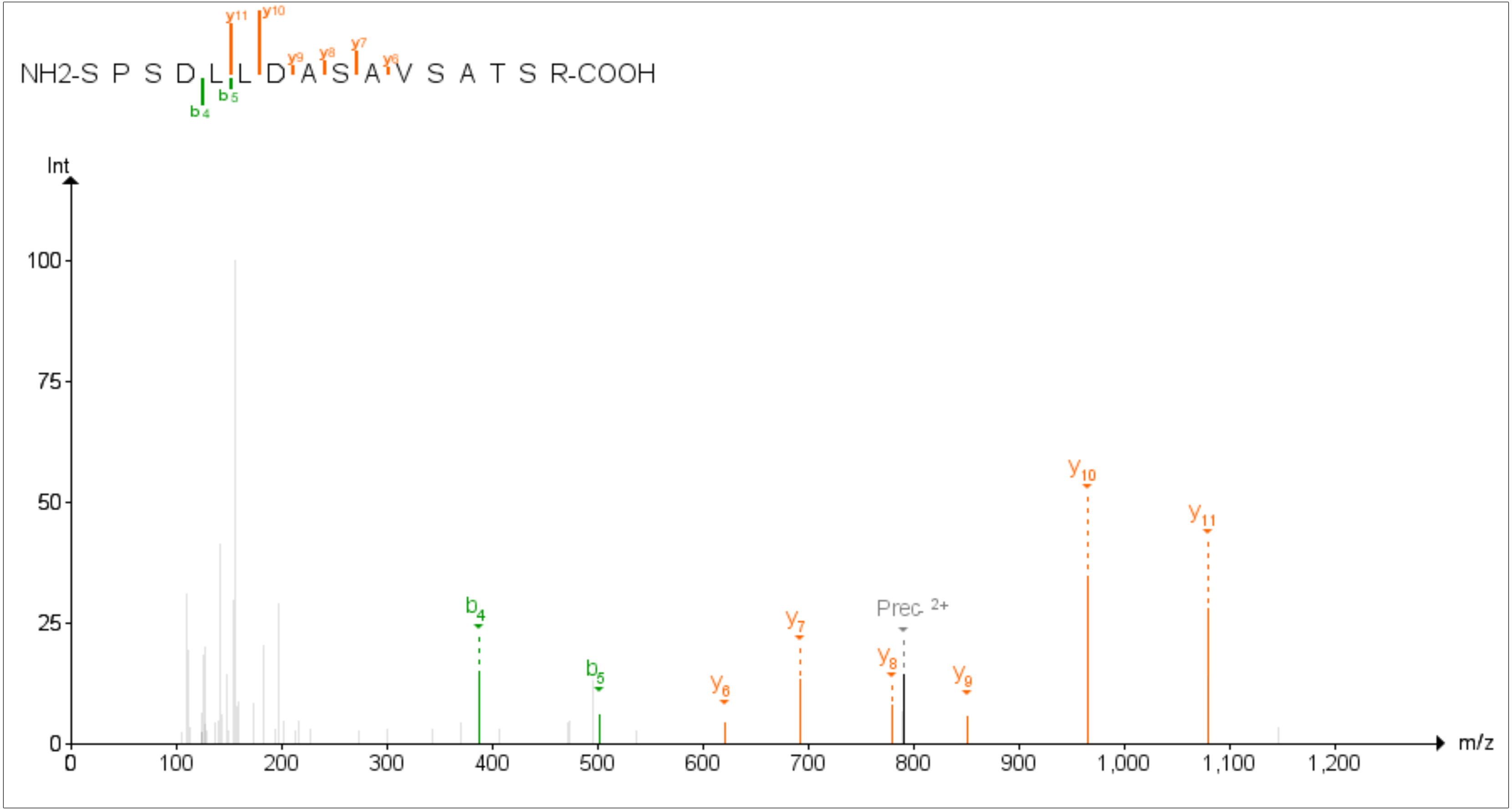

NH2-S P S D L L D A S A V S A T S R-COOH
y11
y10
y9
y8
y7
y6
b4
b5
Int
m/z
Prec. 2+

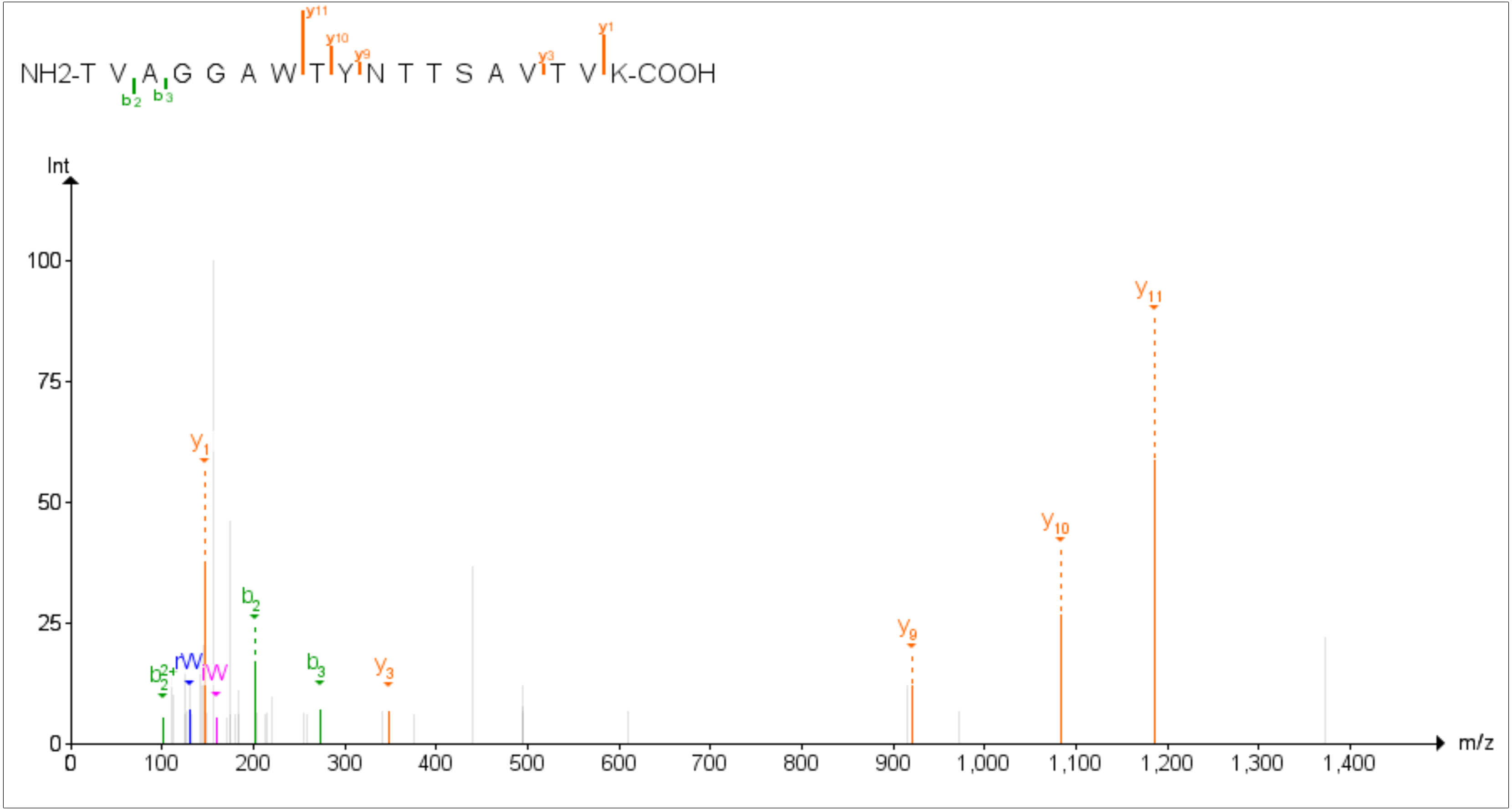

NH2-T V A G G A W T Y N T T S A V T V K-COOH
b2
b3
y11
y10
y9
y3
y1
Int
100
75
50
25
0
y_1
b_2^{2+}
rW
iW
b_2
b_3
y_3
y_9
y_{10}
y_{11}
0
100
200
300
400
500
600
700
800
900
1,000
1,100
1,200
1,300
1,400
m/z

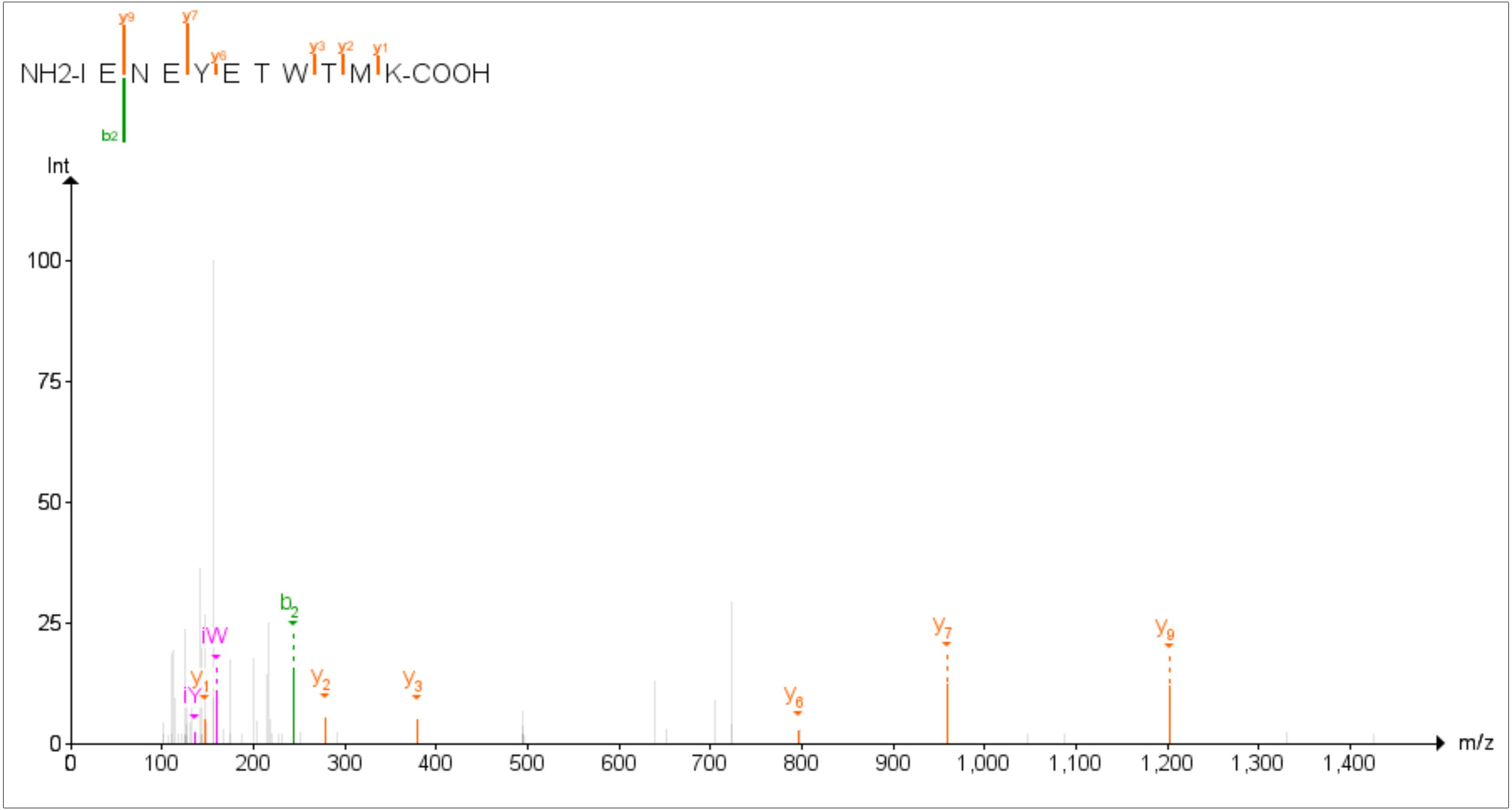

NH2-I E N E Y E T W T M K-COOH
y9
y7
y6
y3
y2
y1
b2
Int
100
75
50
25
0
iW
iY
y1
b2
y2
y3
y6
y7
y9
0
100
200
300
400
500
600
700
800
900
1,000
1,100
1,200
1,300
1,400
m/z

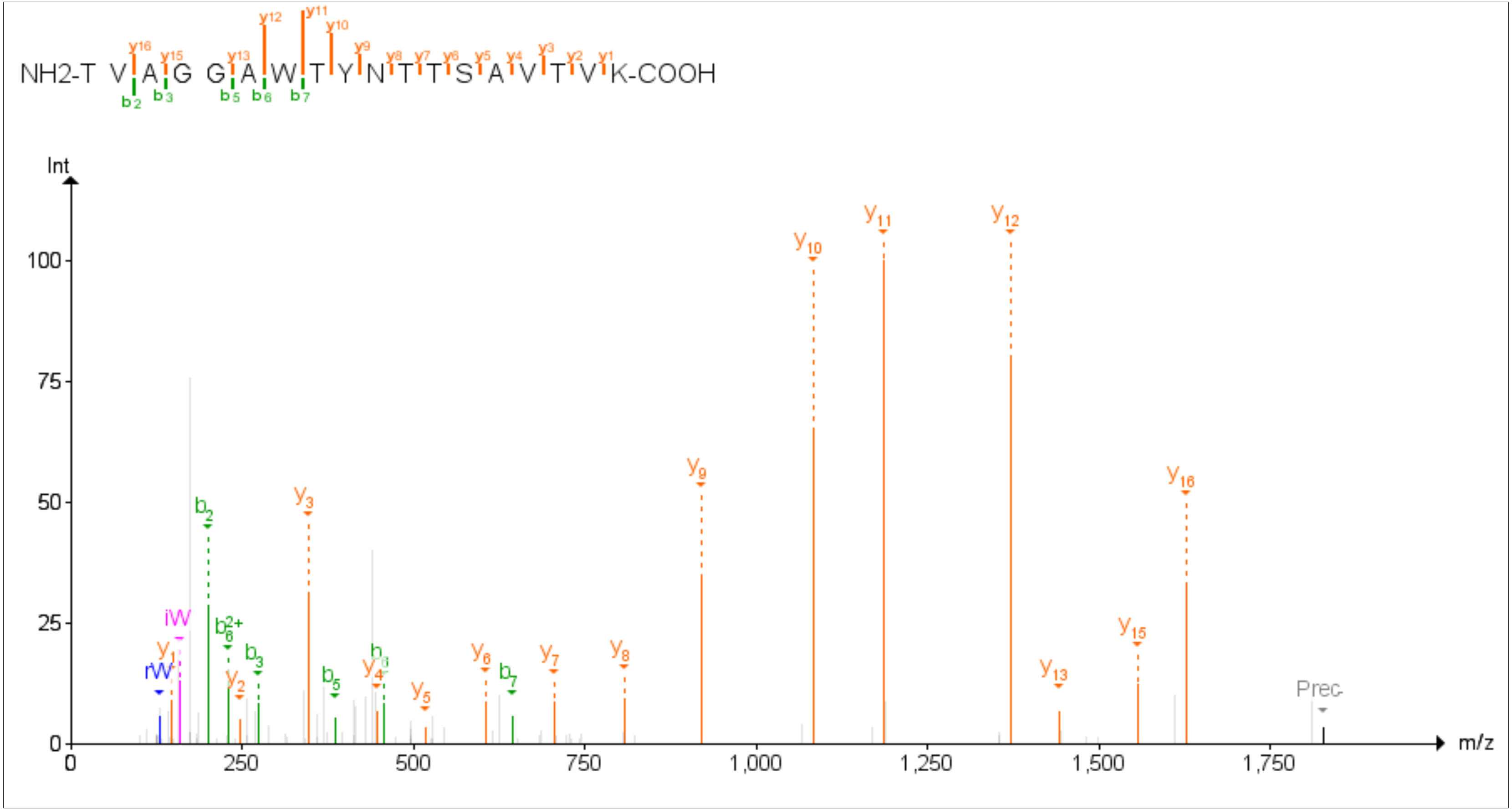

NH2-T V A G G A W T Y N T T S A V T V K-COOH
Int
m/z
Prec

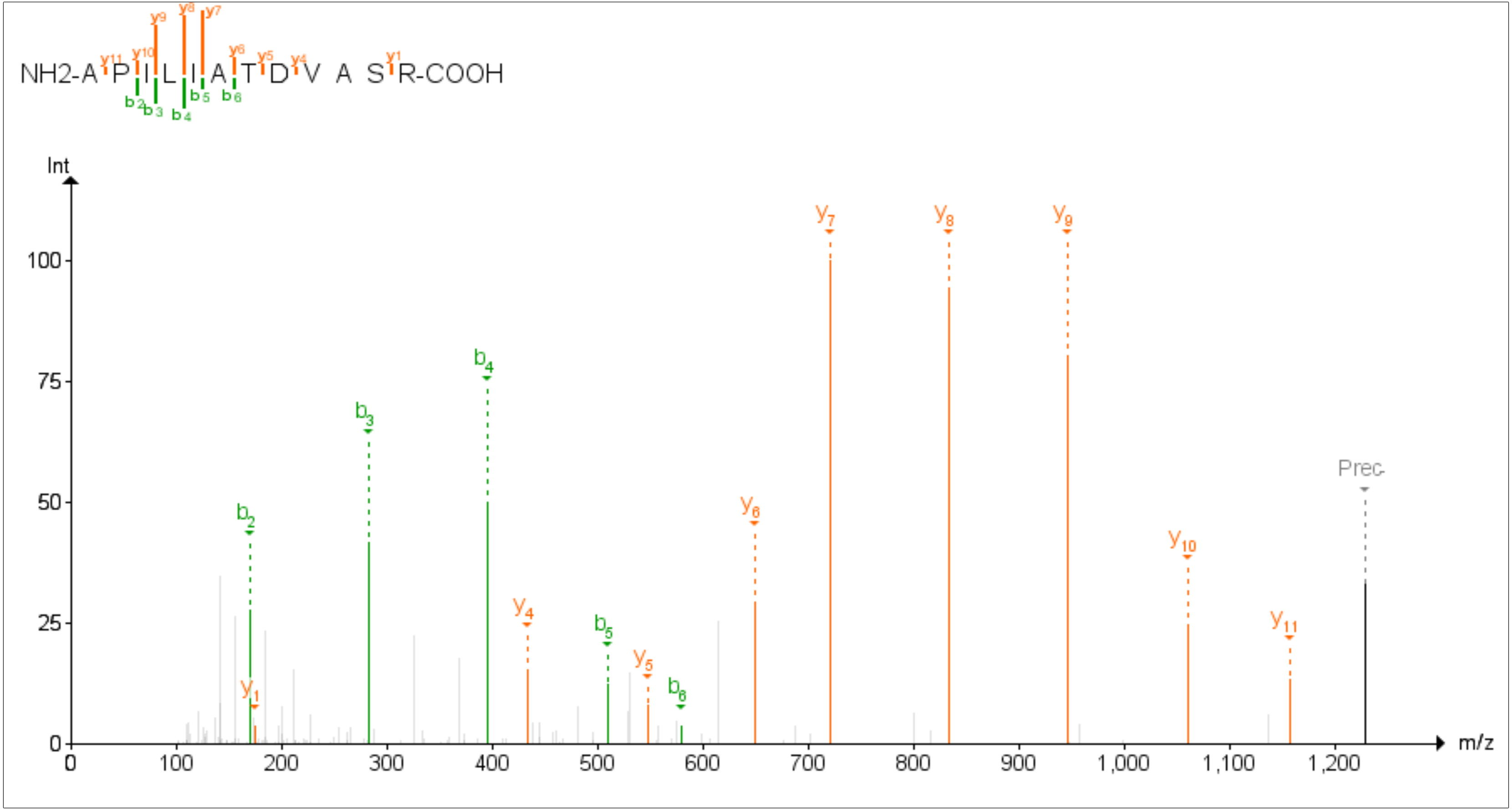

NH2-A P I L I A T D V A S R-COOH
y11 y10 y9 y8 y7 y6 y5 y4 y1
b2 b3 b4 b5 b6
Int
100
75
50
25
0
m/z
0
100
200
300
400
500
600
700
800
900
1,000
1,100
1,200
b2
b3
b4
b5
b6
y1
y4
y5
y6
y7
y8
y9
y10
y11
Prec.

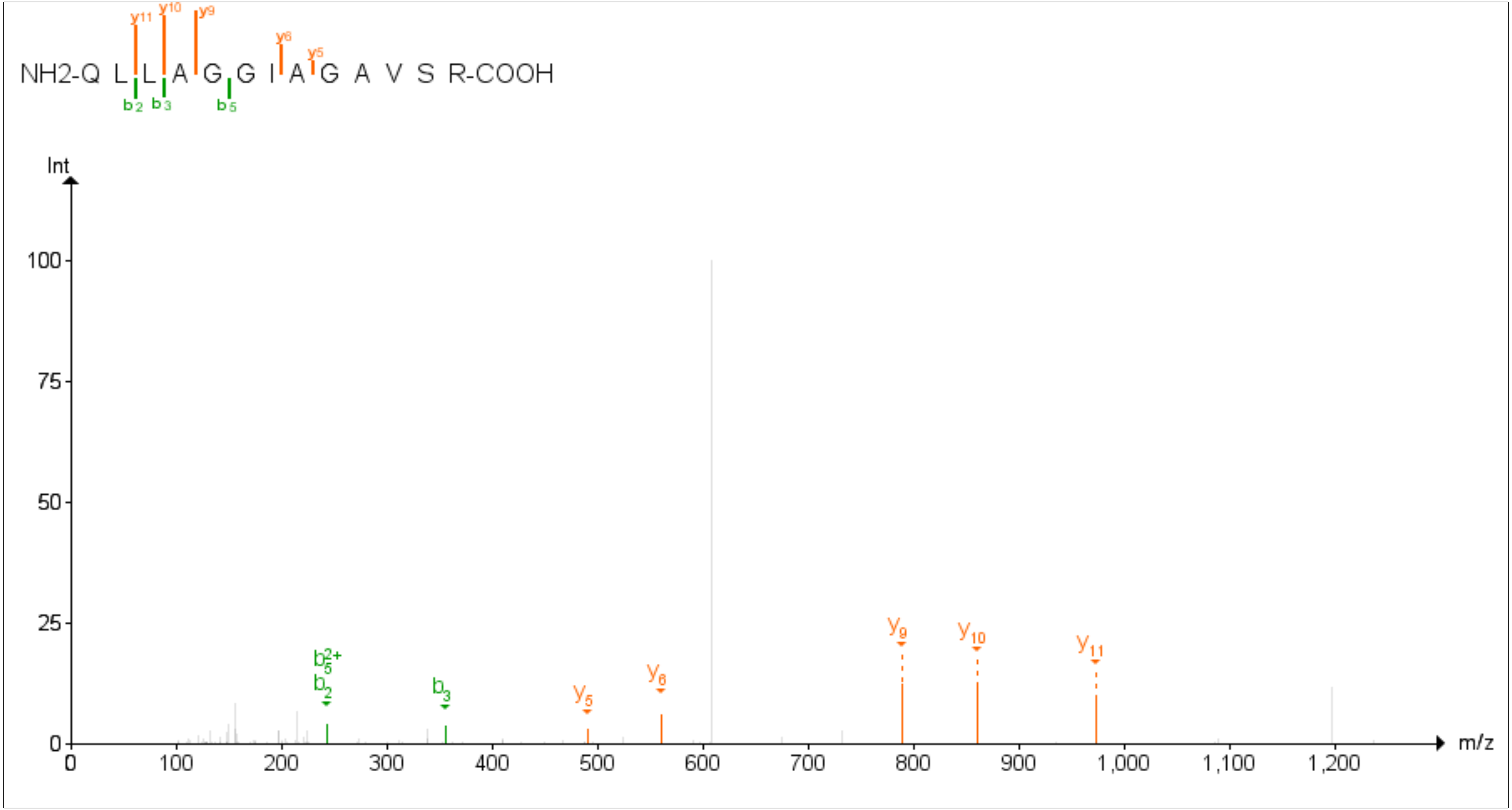

NH2-Q L L A G G I A G A V S R-COOH
y11 y10 y9 y6 y5
b2 b3 b5
Int
100
75
50
25
0
b5 2+
b2
b3
y5
y6
y9
y10
y11
0 100 200 300 400 500 600 700 800 900 1,000 1,100 1,200
m/z

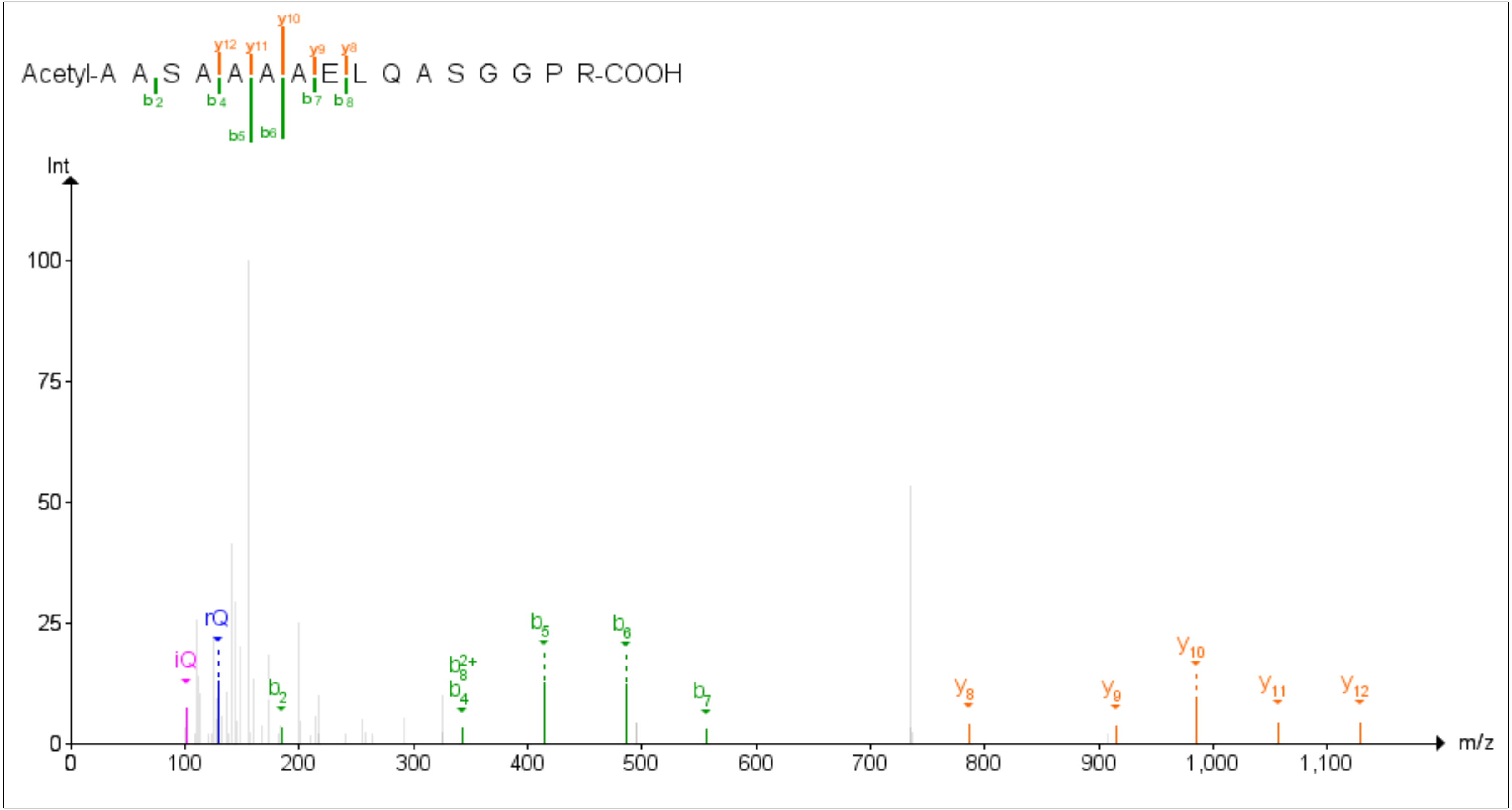

Acetyl-A A S A A A A E L Q A S G G P R-COOH
y12 y11 y10 y9 y8
b2 b4 b5 b6 b7 b8
Int
100
75
50
25
0
iQ
rQ
b2
b8 2+
b4
b5
b6
b7
y8
y9
y10
y11
y12
0 100 200 300 400 500 600 700 800 900 1,000 1,100
m/z

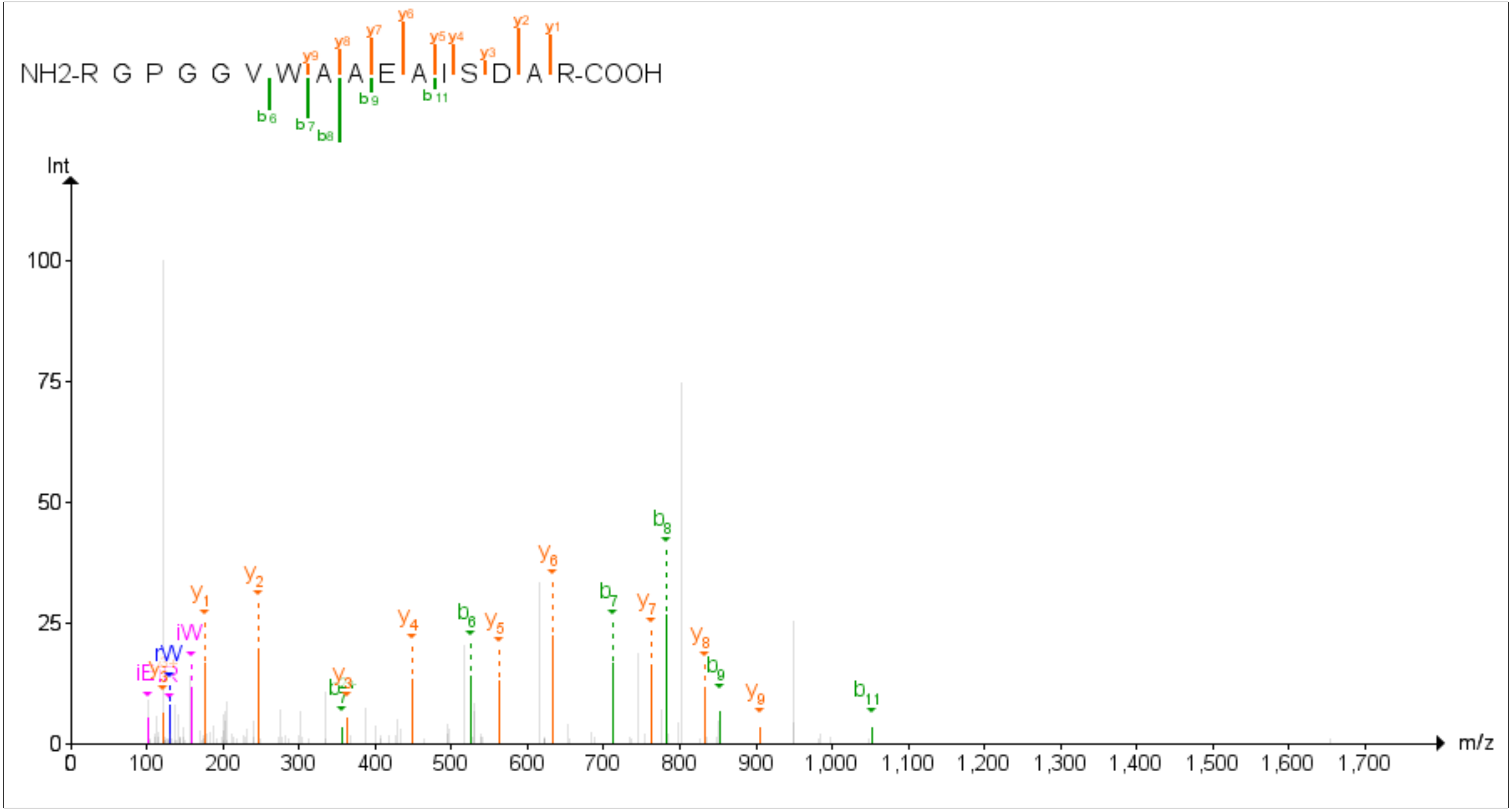

NH2-R G P G G V W A A E A I S D A R-COOH
y9 y8 y7 y6 y5 y4 y3 y2 y1
b6 b7 b8 b9 b11
Int
100
75
50
25
0
m/z
0 100 200 300 400 500 600 700 800 900 1,000 1,100 1,200 1,300 1,400 1,500 1,600 1,700
y1 y2 y3 y4 y5 y6 y7 y8 y9
b6 b7 b8 b9 b11
iW
rW

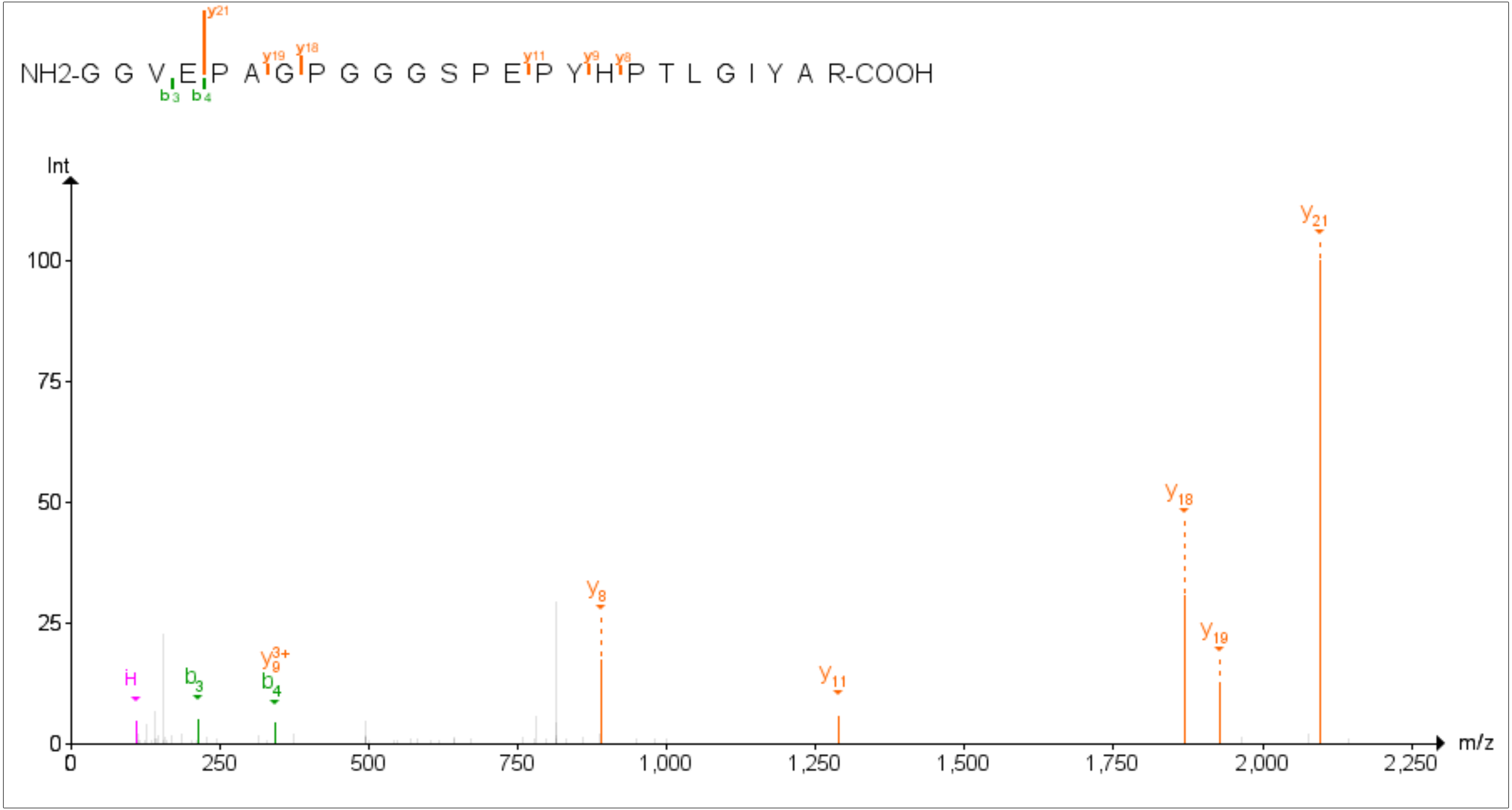
NH2-G G V E P A G P G G G S P E P Y H P T L G I Y A R-COOH
y21
y19
y18
y11
y9
y8
b3
b4
Int
100
75
50
25
0
m/z
0
250
500
750
1,000
1,250
1,500
1,750
2,000
2,250
H
b3
y9 3+
b4
y8
y11
y18
y19
y21

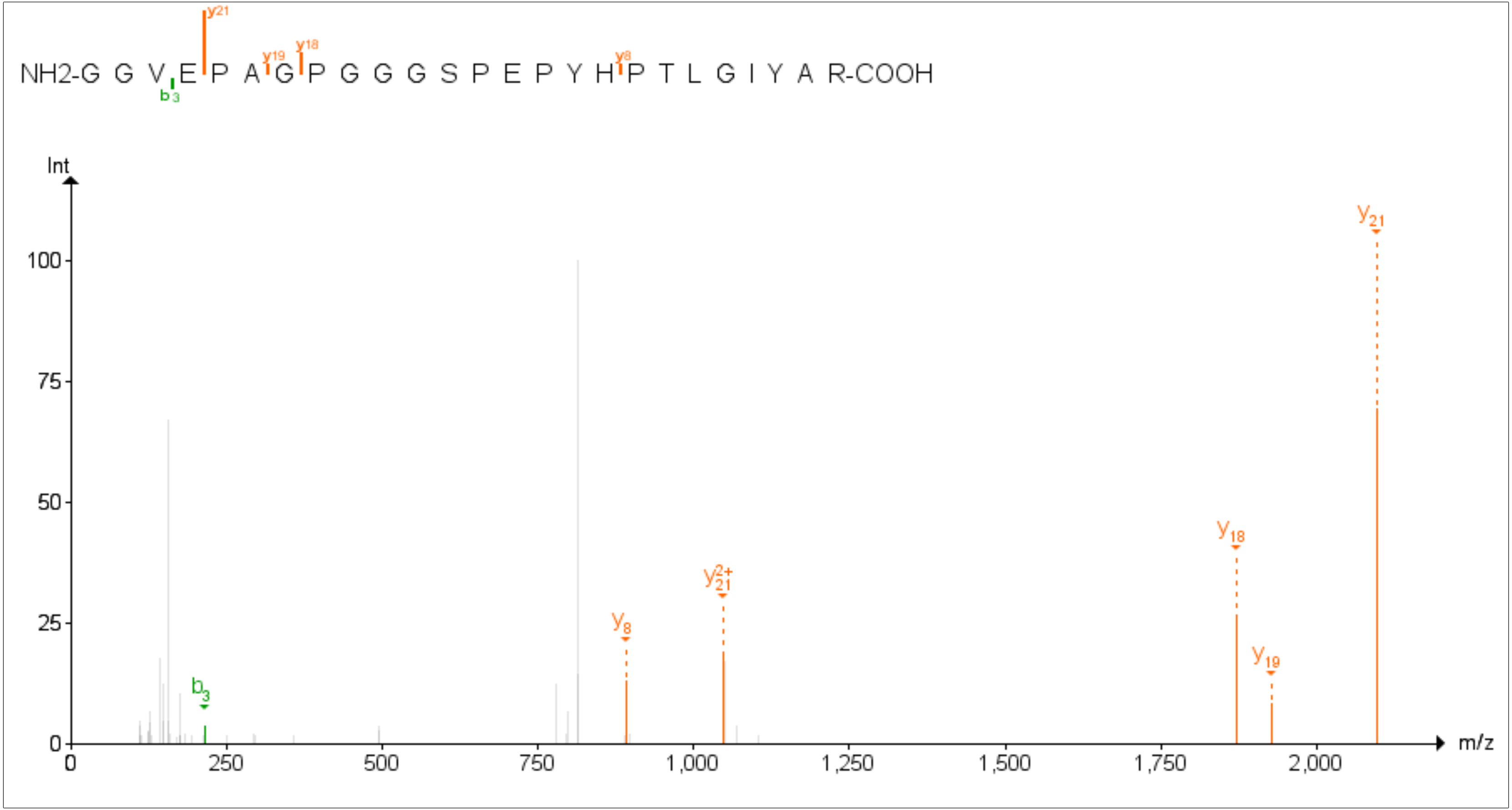

NH2-G G V E P A G P G G G S P E P Y H P T L G I Y A R-COOH
b3
y21
y19
y18
y8
Int
100
75
50
25
0
0
250
500
750
1,000
1,250
1,500
1,750
2,000
m/z
y21
y18
y21 2+
y8
y19
b3

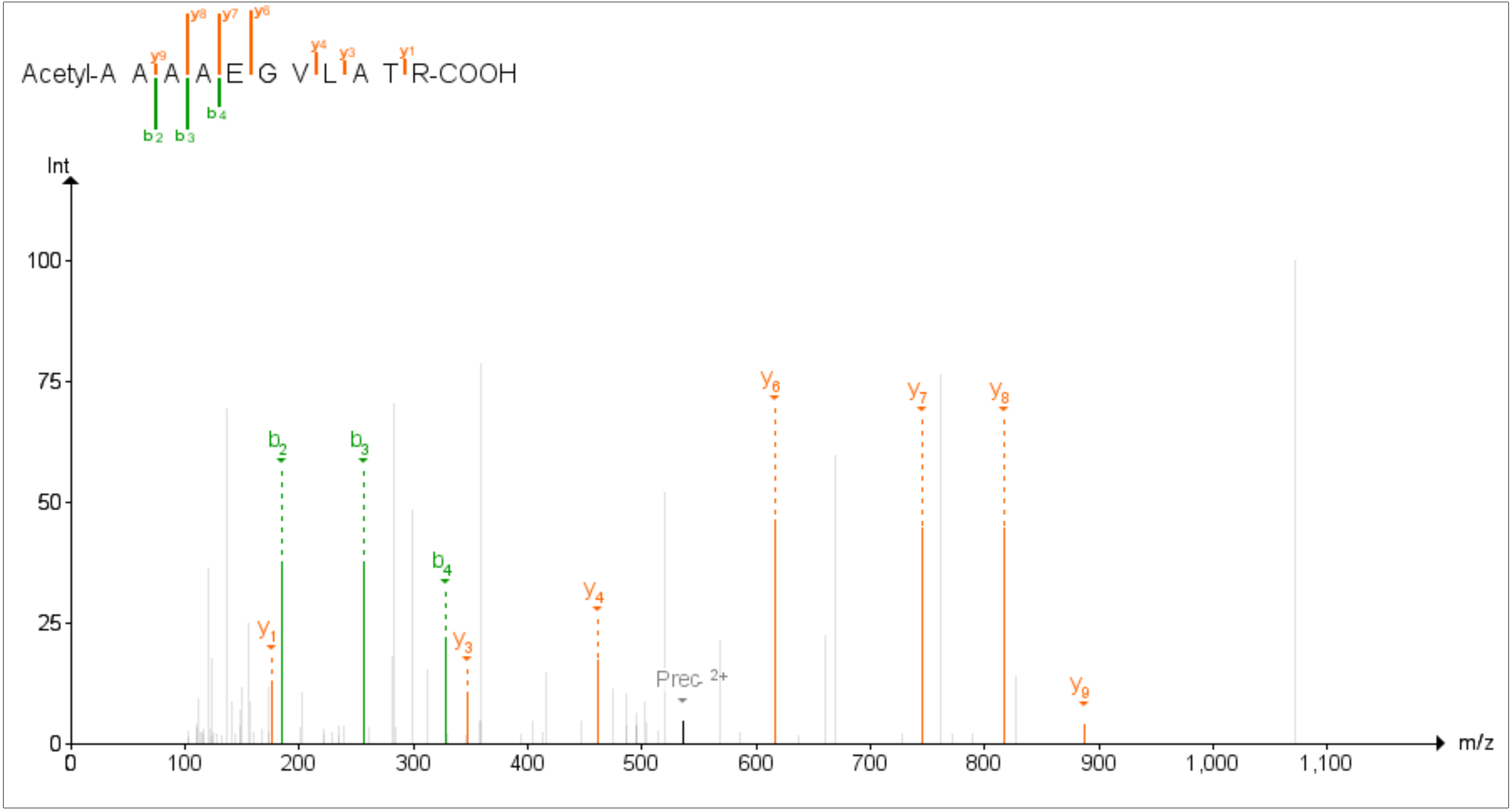

Acetyl-A A A A E G V L A T R-COOH
y9 y8 y7 y6 y4 y3 y1
b2 b3 b4
Int
100
75
50
25
0
b2
b3
b4
y1
y3
y4
Prec 2+
y6
y7
y8
y9
0
100
200
300
400
500
600
700
800
900
1,000
1,100
m/z

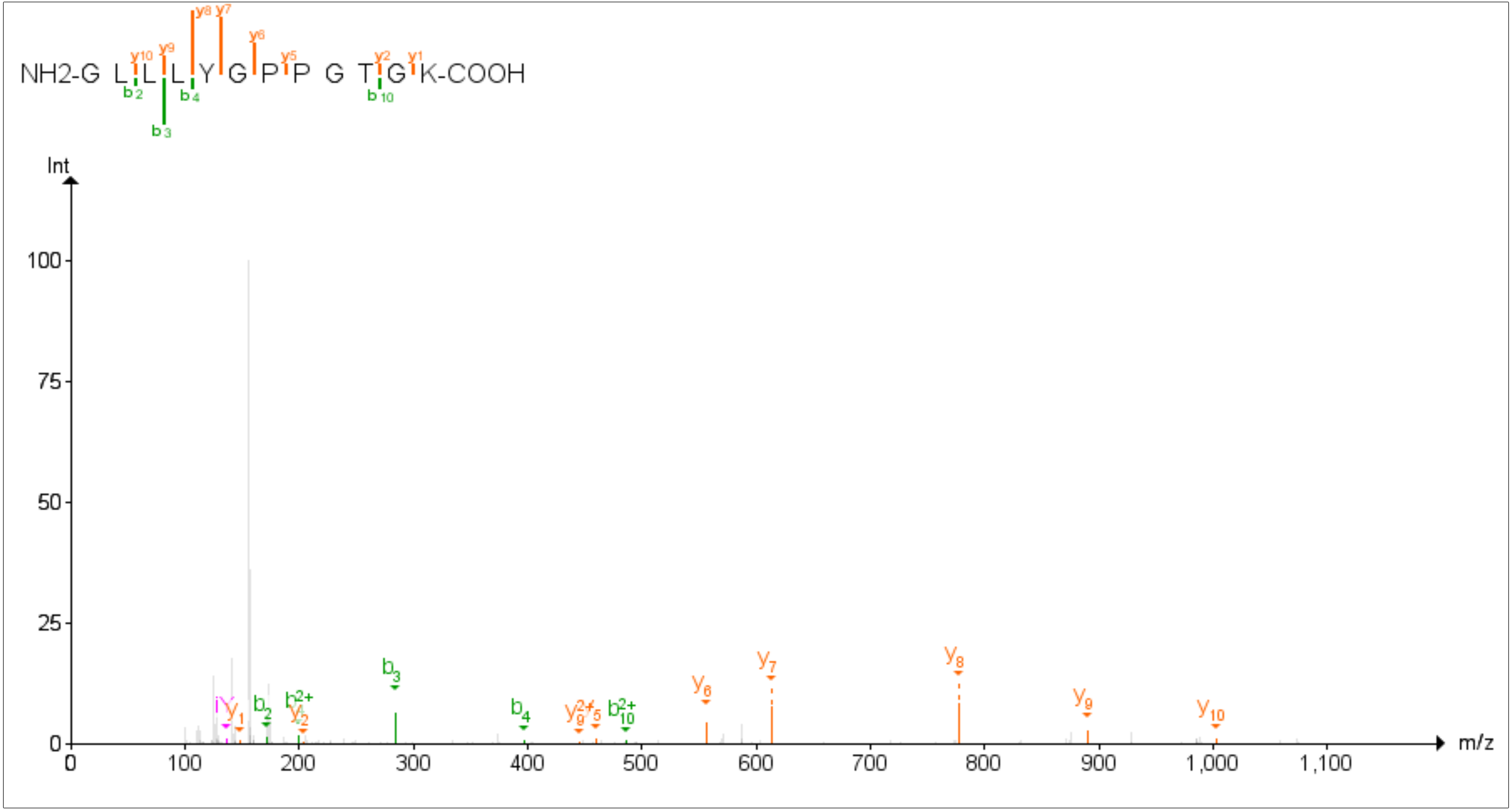
NH2-G L L L Y G P P G T G K-COOH
y10 y9 y8 y7 y6 y5 y2 y1
b2 b3 b4 b10
Int
100
75
50
25
0
m/z
0 100 200 300 400 500 600 700 800 900 1,000 1,100
iY y1 b2 b2+ y2 b3 b4 y92+ y5 b102+ y6 y7 y8 y9 y10

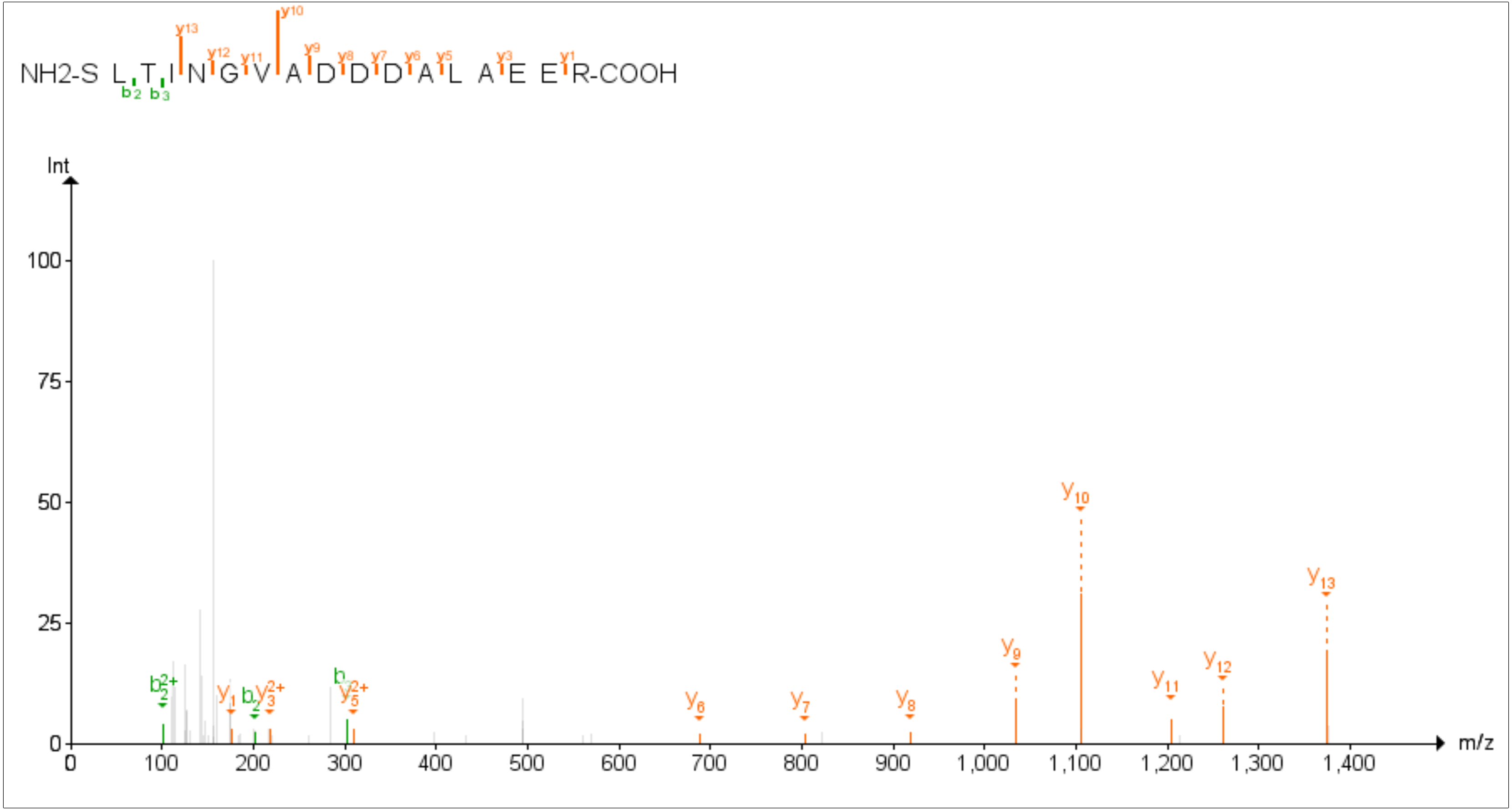
NH2-S L T I N G V A D D D A L A E E R-COOH
Int
m/z
y10
y13
y9
y12
y11
y8
y6
y7
y1
b2
b3
y5
y3
100
75
50
25
0
0 100 200 300 400 500 600 700 800 900 1,000 1,100 1,200 1,300 1,400

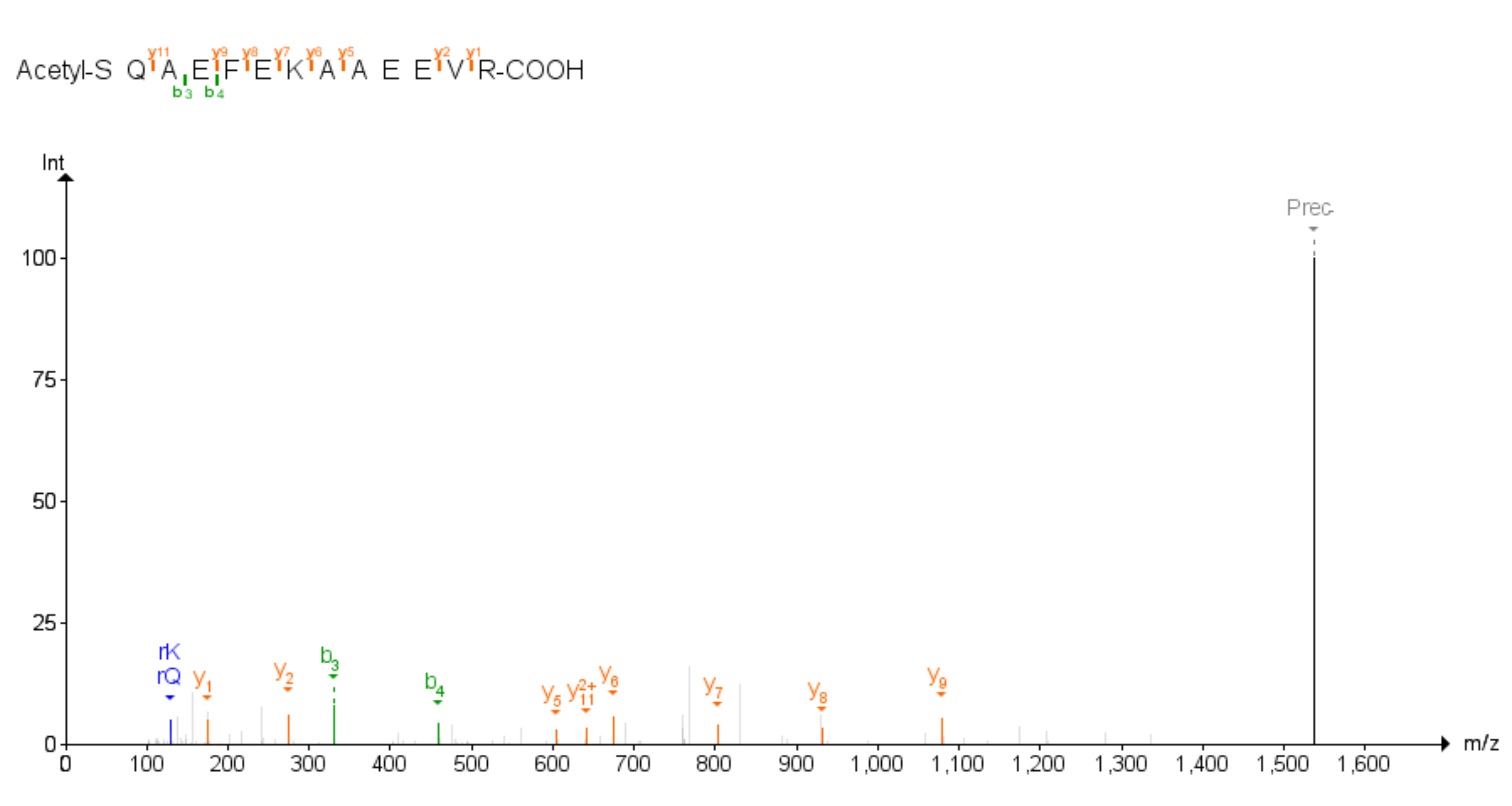

Acetyl-S Q A E F E K A A E E V R-COOH
y11 y9 y8 y7 y6 y5 y2 y1
b3 b4
Int
Prec
rK
rQ
y1
y2
b3
b4
y5
y11 2+
y6
y7
y8
y9
0
25
50
75
100
0 100 200 300 400 500 600 700 800 900 1,000 1,100 1,200 1,300 1,400 1,500 1,600
m/z

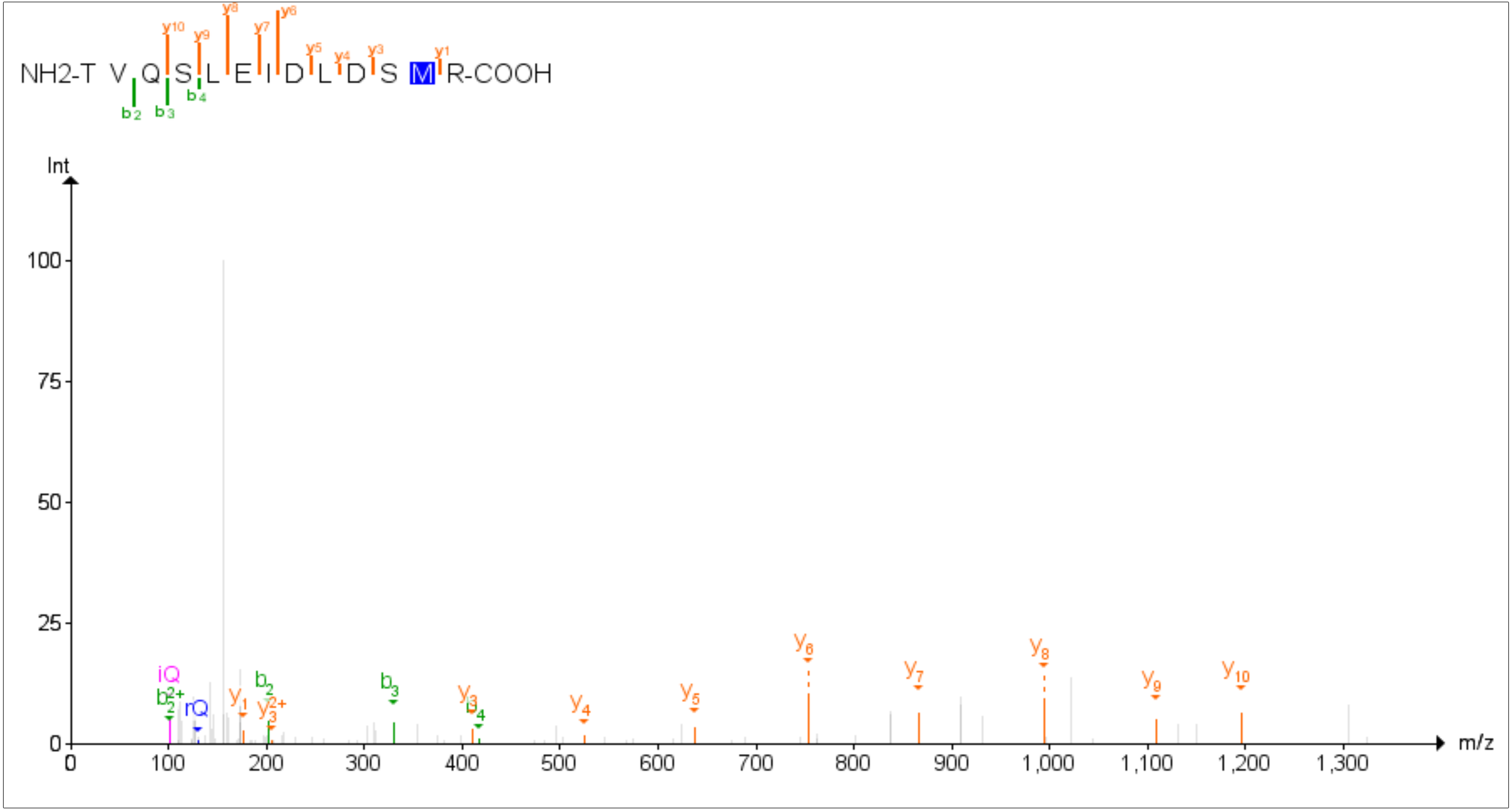
NH2-T V Q S L E I D L D S M R-COOH
y10 y9 y8 y7 y6 y5 y4 y3 y1
b2 b3 b4
Int
100
75
50
25
0
m/z
0 100 200 300 400 500 600 700 800 900 1,000 1,100 1,200 1,300
iQ
rQ
y1
y3
y4
y5
y6
y7
y8
y9
y10
b2
b3
b4

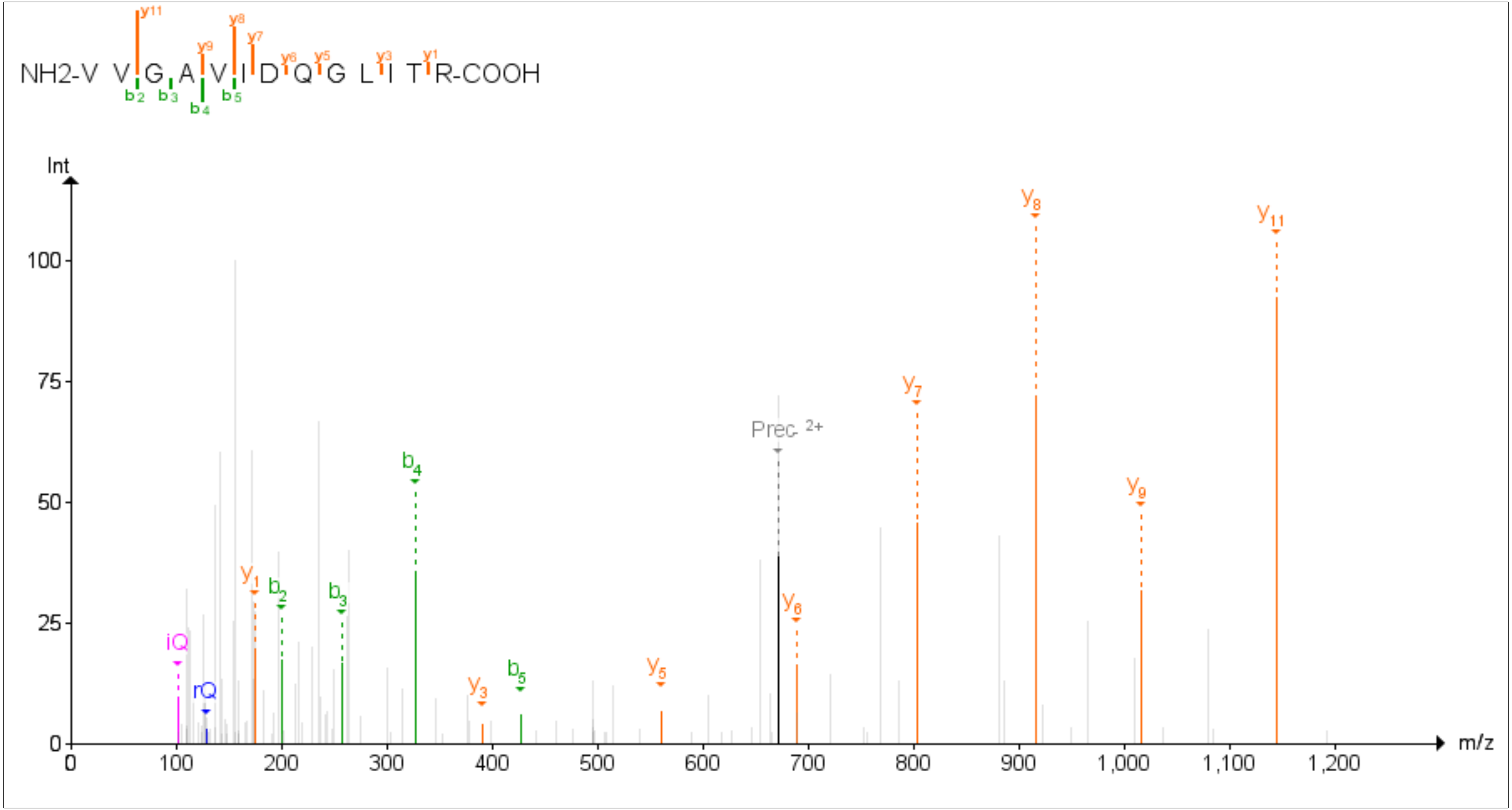

NH2-V V G A V I D Q G L I T R-COOH
y11 y9 y8 y7 y6 y5 y3 y1
b2 b3 b4 b5
Int
m/z
Prec 2+
y1 y3 y5 y6 y7 y8 y9 y11
b2 b3 b4 b5
iQ
rQ
0
25
50
75
100
0
100
200
300
400
500
600
700
800
900
1,000
1,100
1,200

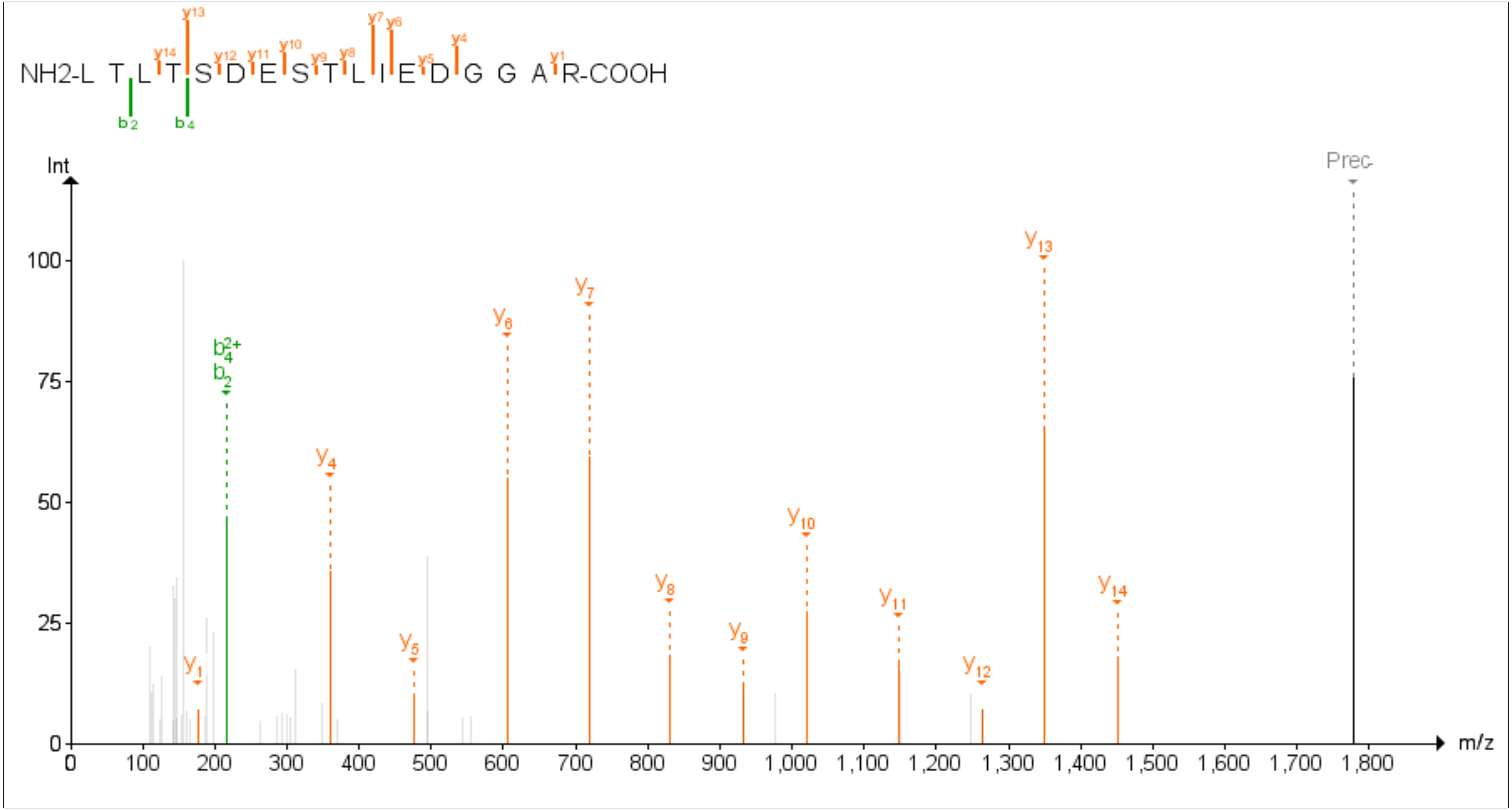
NH2-L T L T S D E S T L I E D G G A R-COOH
y13 y14 y12 y11 y10 y9 y8 y7 y6 y5 y4 y1
b2 b4
Int
Prec
0
25
50
75
100
b_4^{2+}
b_2
y_1
y_4
y_5
y_6
y_7
y_8
y_9
y_{10}
y_{11}
y_{12}
y_{13}
y_{14}
0 100 200 300 400 500 600 700 800 900 1,000 1,100 1,200 1,300 1,400 1,500 1,600 1,700 1,800
m/z

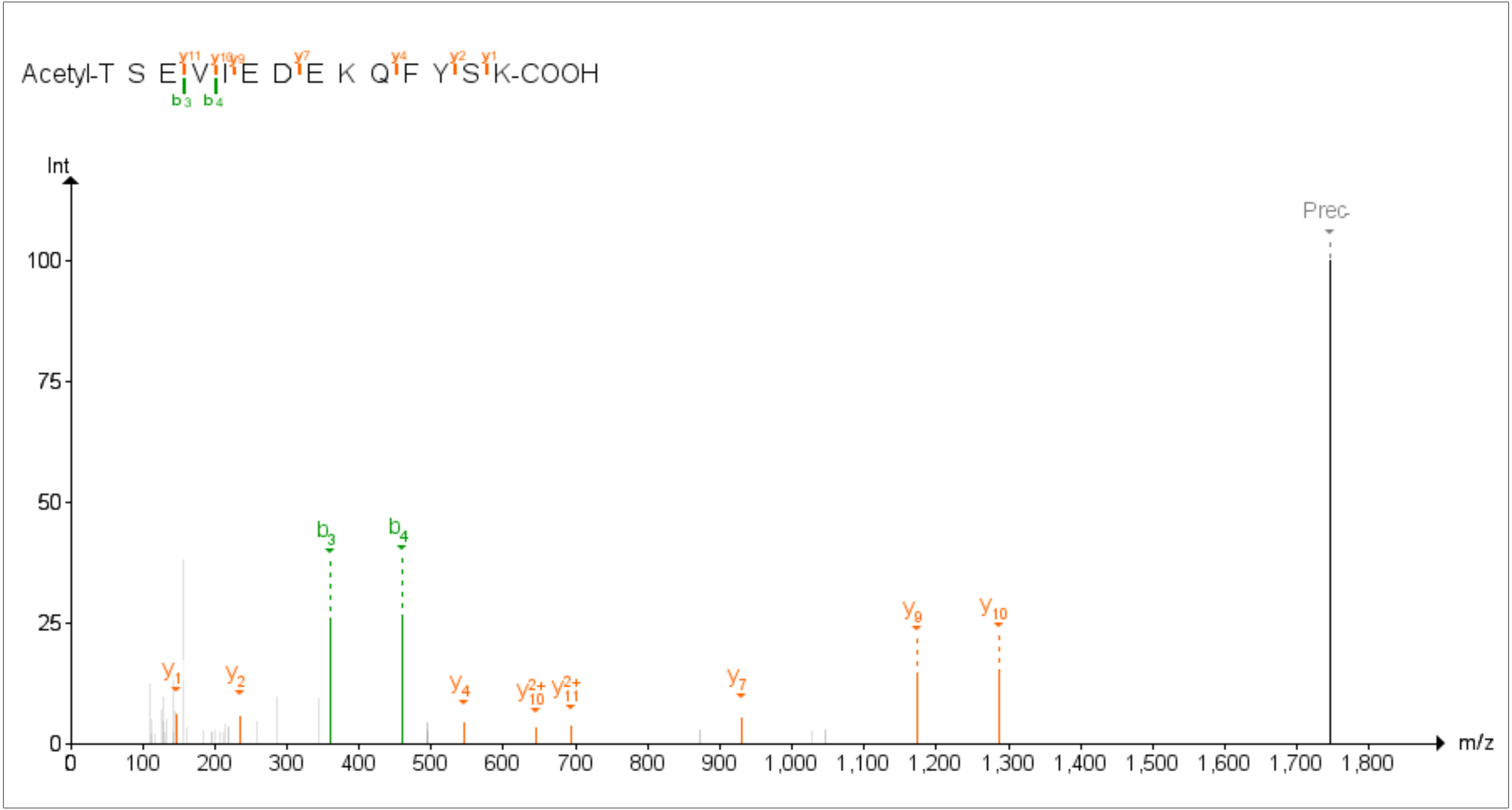

Acetyl-T S E V I E D E K Q F Y S K-COOH
y11 y10 y9 y7 y4 y2 y1
b3 b4
Int
Prec
b3
b4
y1 y2 y4 y10 2+ y11 2+ y7 y9 y10
0 25 50 75 100
0 100 200 300 400 500 600 700 800 900 1,000 1,100 1,200 1,300 1,400 1,500 1,600 1,700 1,800
m/z

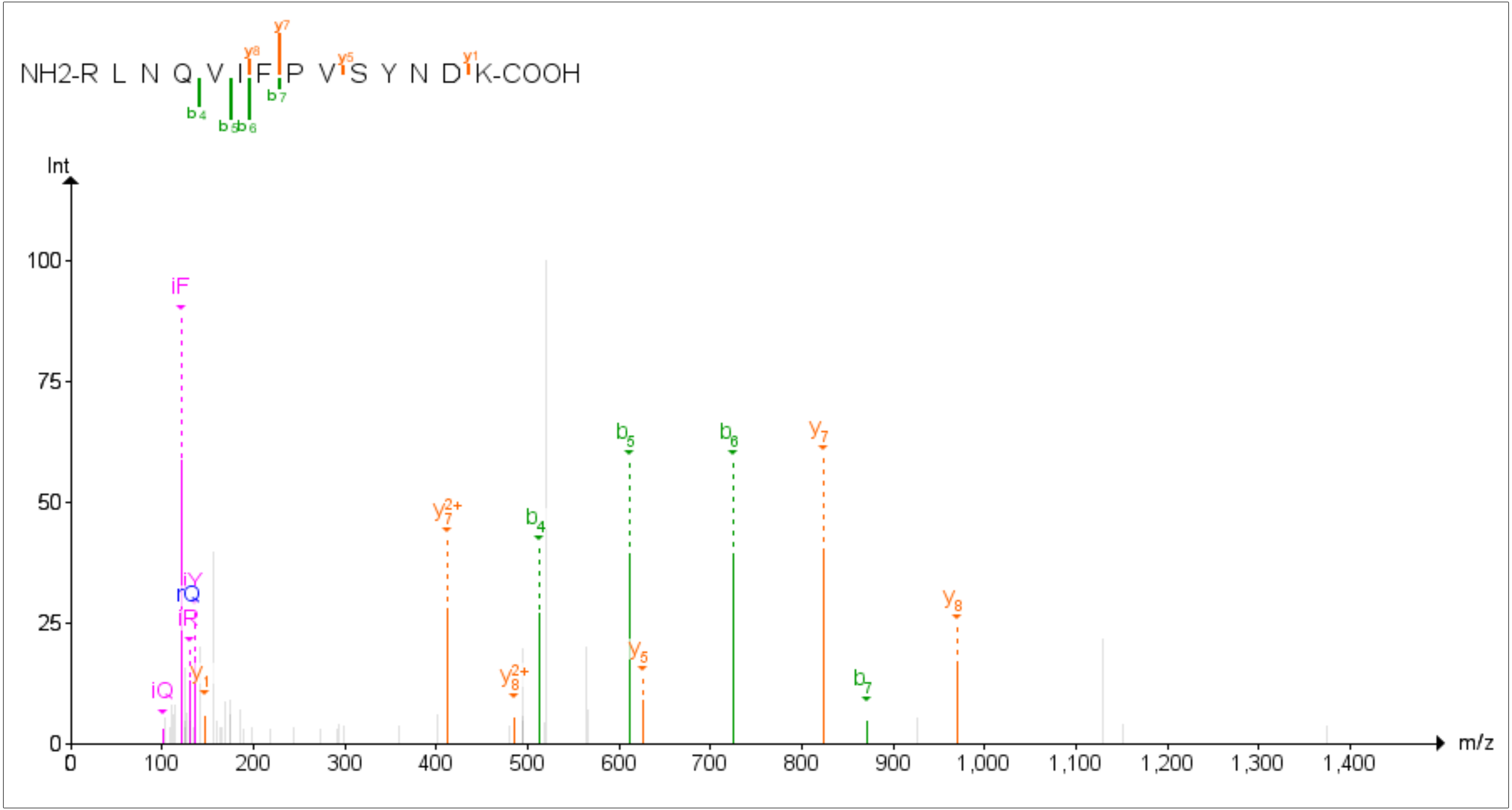

NH2-R L N Q V I F P V S Y N D K-COOH
y7
y8
y5
y1
b4
b5
b6
b7
Int
100
75
50
25
0
iF
iY
rQ
iR
iQ
y1
y7 2+
y8 2+
b4
b5
b6
y7
y5
b7
y8
m/z
0
100
200
300
400
500
600
700
800
900
1,000
1,100
1,200
1,300
1,400

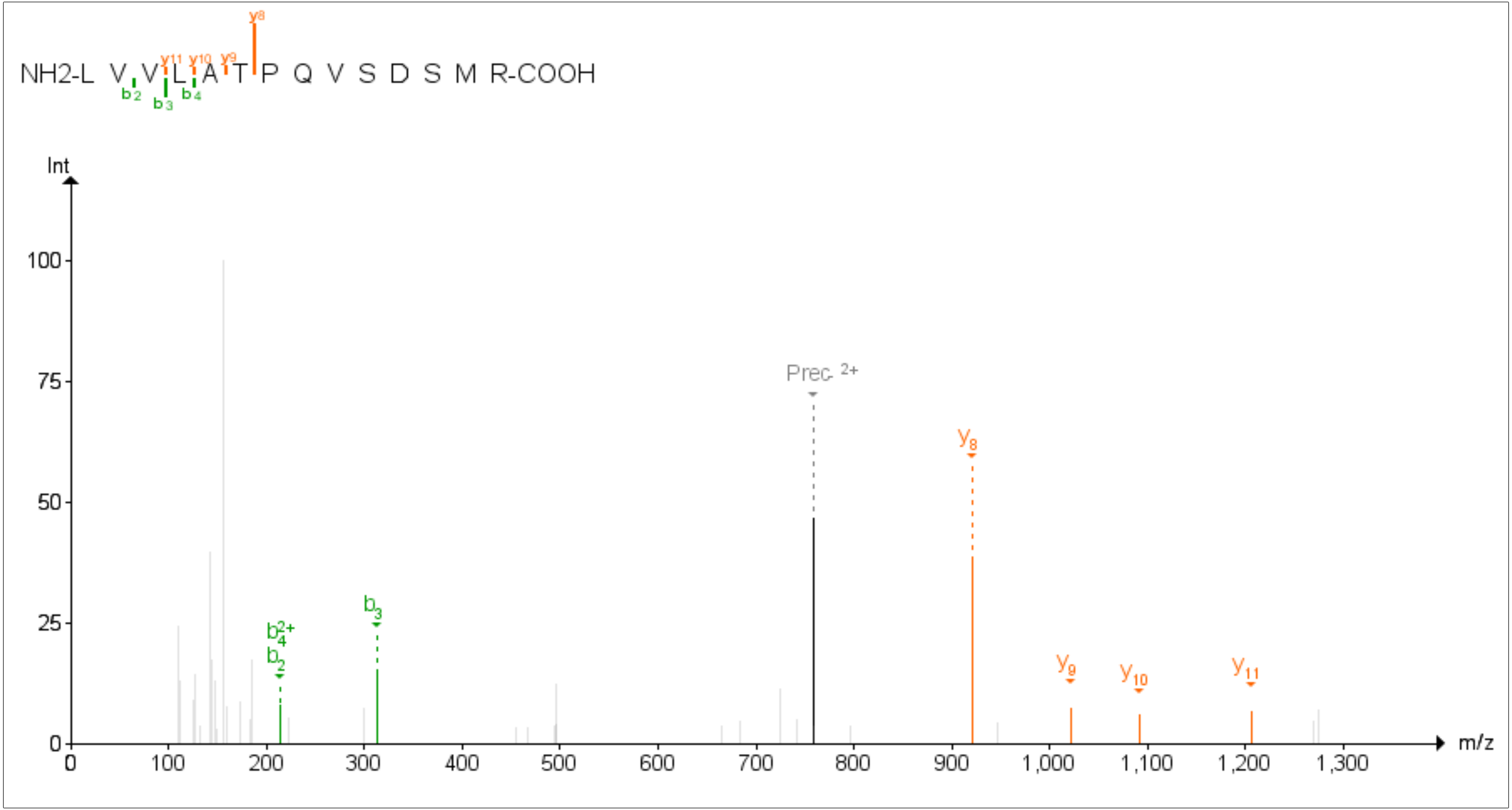

NH2-L V V L A T P Q V S D S M R-COOH
b2
b3
b4
y11
y10
y9
y8
Int
m/z
Prec 2+
y8
y9
y10
y11
b3
b4 2+
b2
0
25
50
75
100
0
100
200
300
400
500
600
700
800
900
1,000
1,100
1,200
1,300

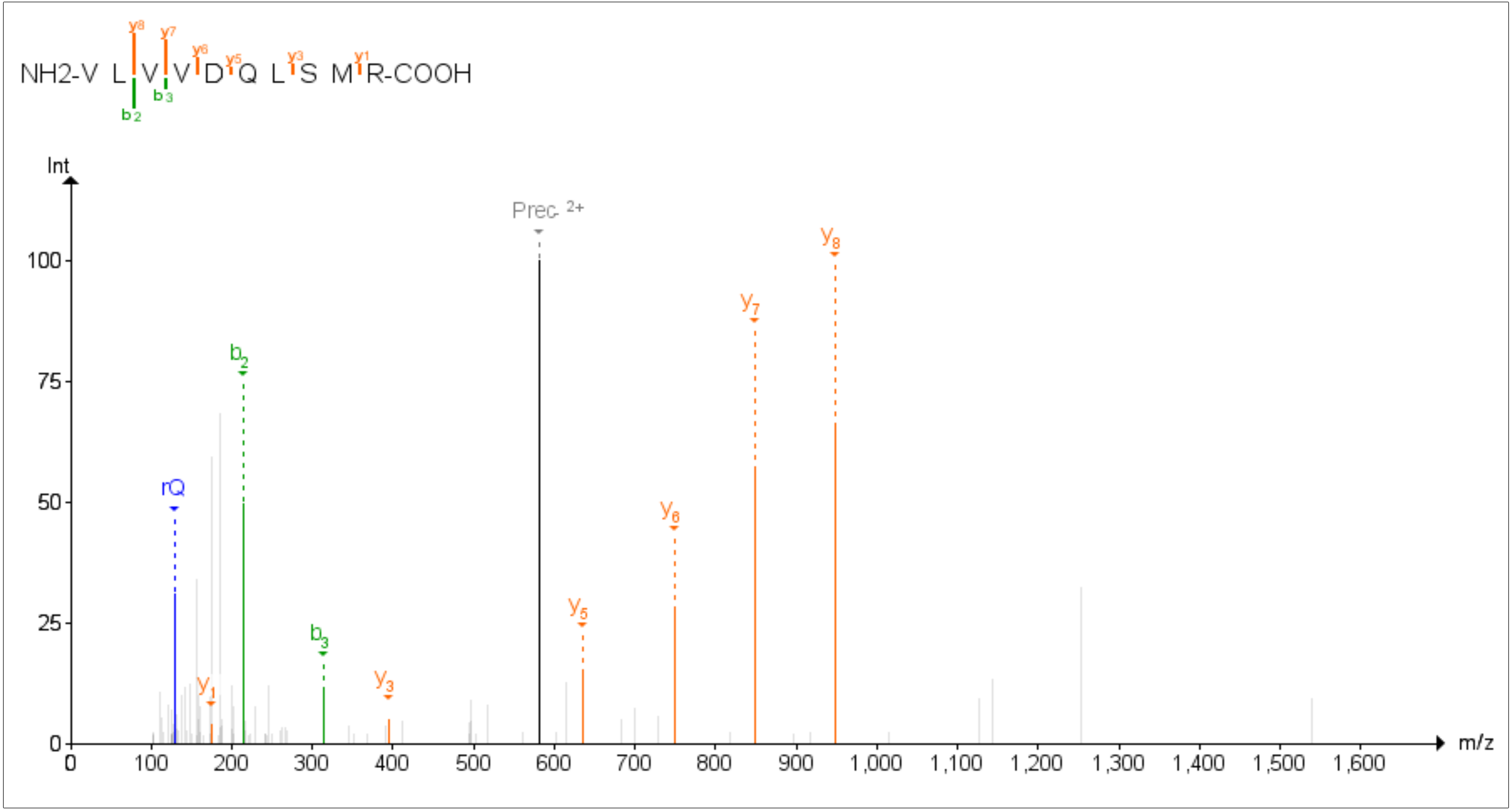
NH2-V L V V D Q L S M R-COOH
y8
y7
y6
y5
y3
y1
b2
b3
Int
Prec 2+
y8
y7
y6
y5
y3
y1
b2
b3
rQ
100
75
50
25
0
0
100
200
300
400
500
600
700
800
900
1,000
1,100
1,200
1,300
1,400
1,500
1,600
m/z

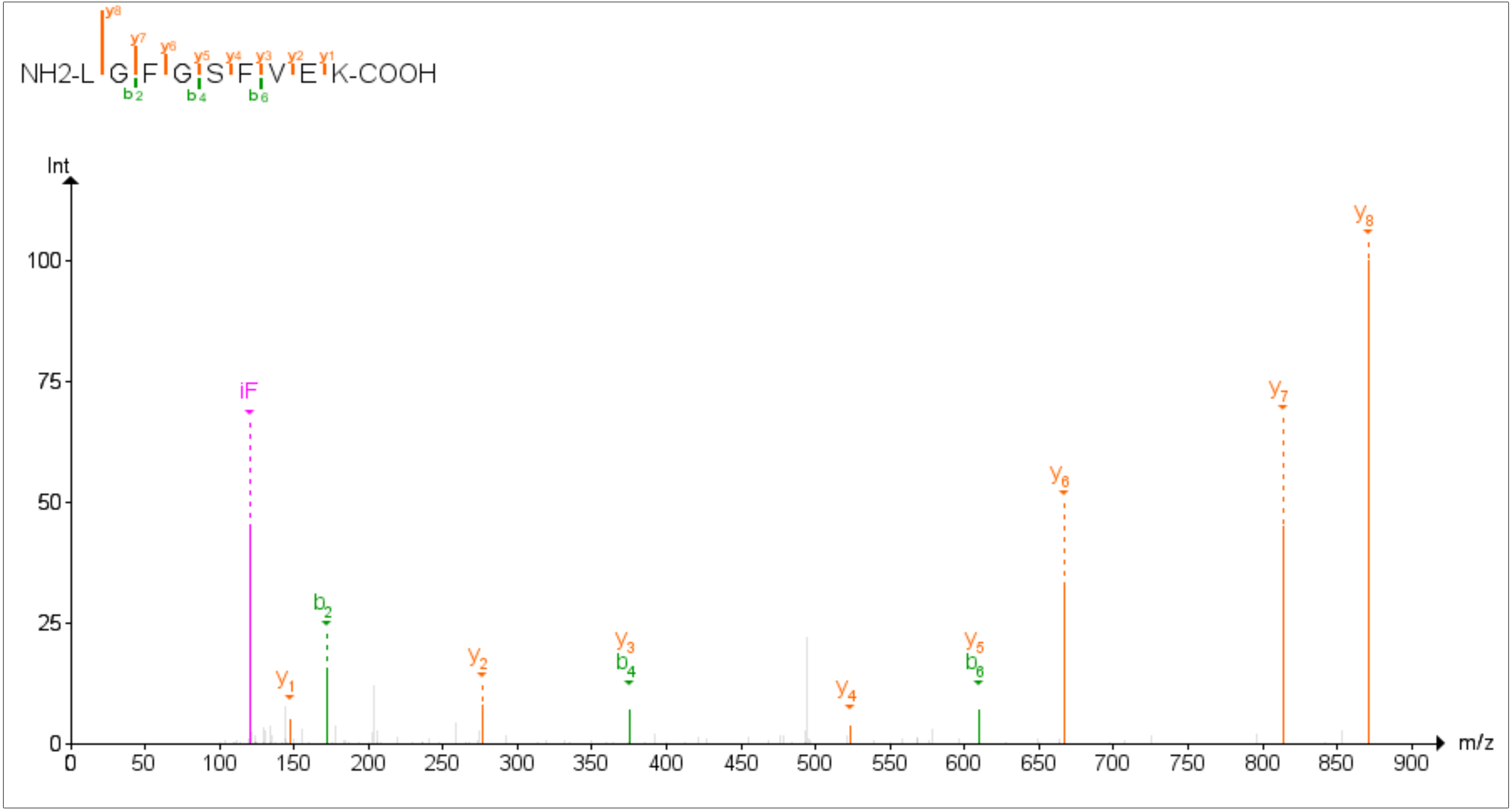

NH2-L G F G S F V E K-COOH
y8
y7
y6
y5
y4
y3
y2
y1
b2
b4
b6
Int
m/z
iF

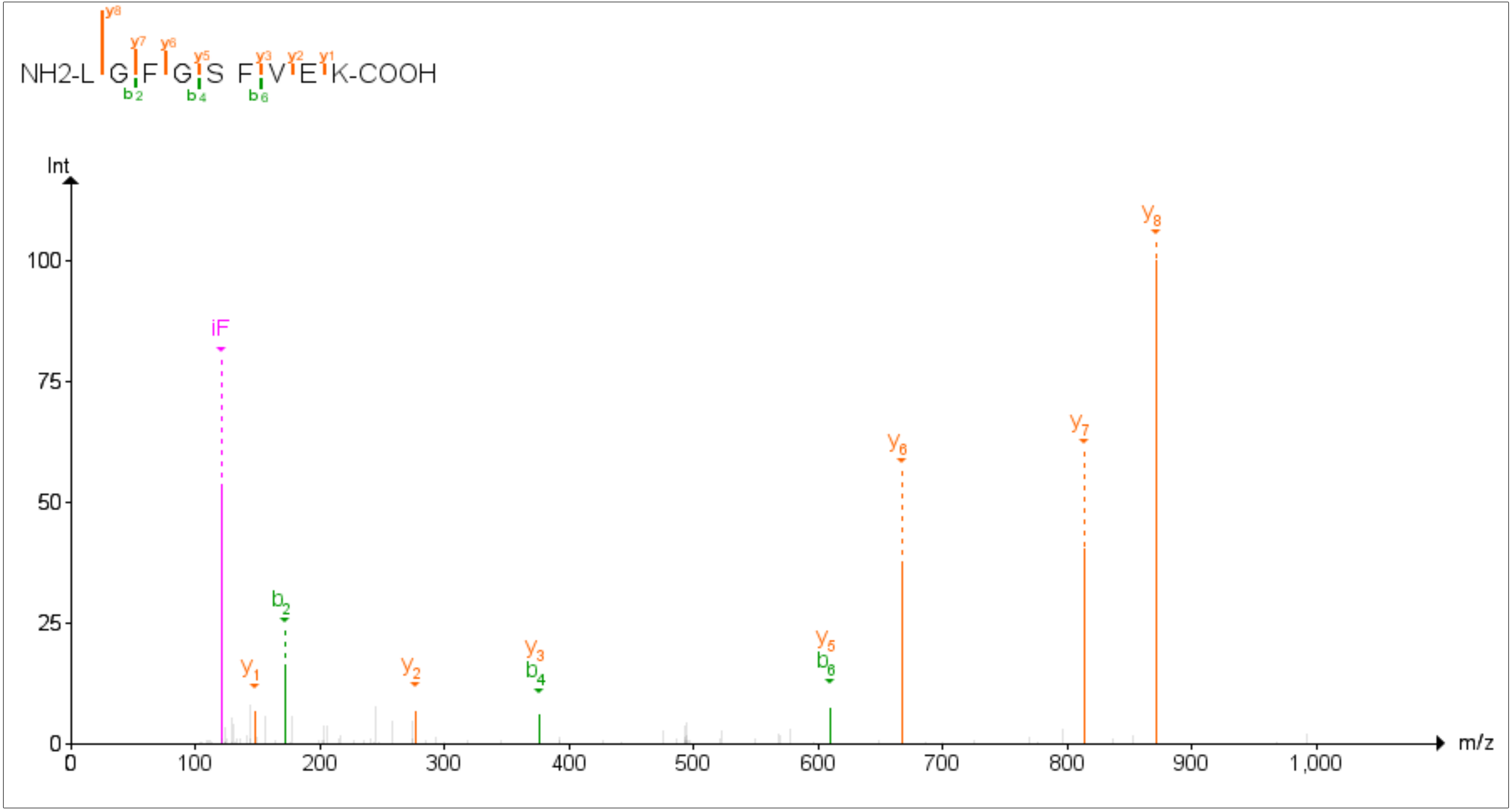

NH2-L G F G S F V E K-COOH
y8
y7
y6
y5
y3
y2
y1
b2
b4
b6
Int
100
75
50
25
0
iF
b2
y1
y2
y3
b4
y5
b6
y6
y7
y8
0
100
200
300
400
500
600
700
800
900
1,000
m/z

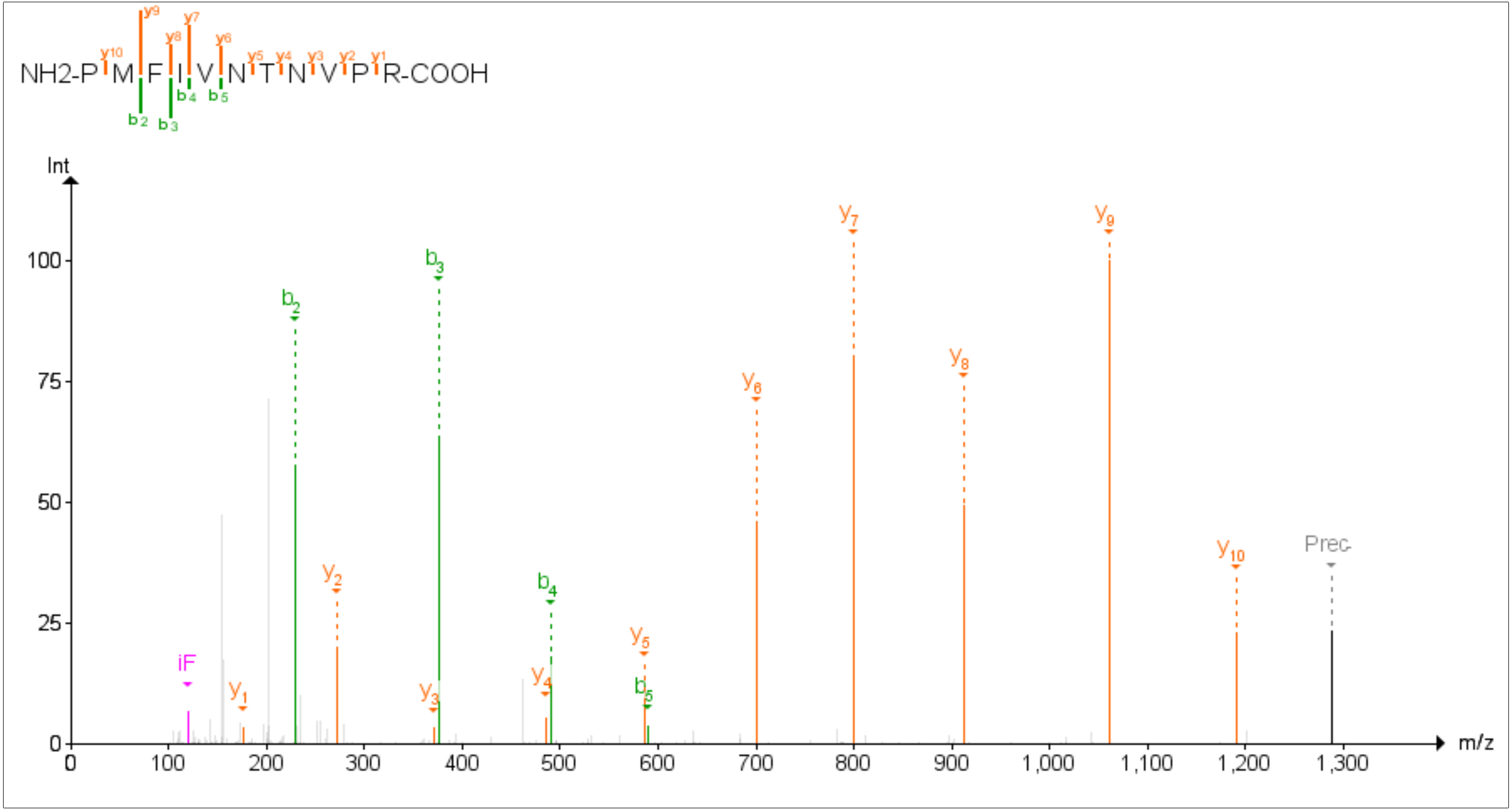

NH2-P M F I V N T N V P R-COOH
y10 y9 y8 y7 y6 y5 y4 y3 y2 y1
b2 b3 b4 b5
Int
100
75
50
25
0
iF
y1
b2
y2
y3
b3
y4
b4
y5
b5
y6
y7
y8
y9
y10
Prec
0 100 200 300 400 500 600 700 800 900 1,000 1,100 1,200 1,300
m/z

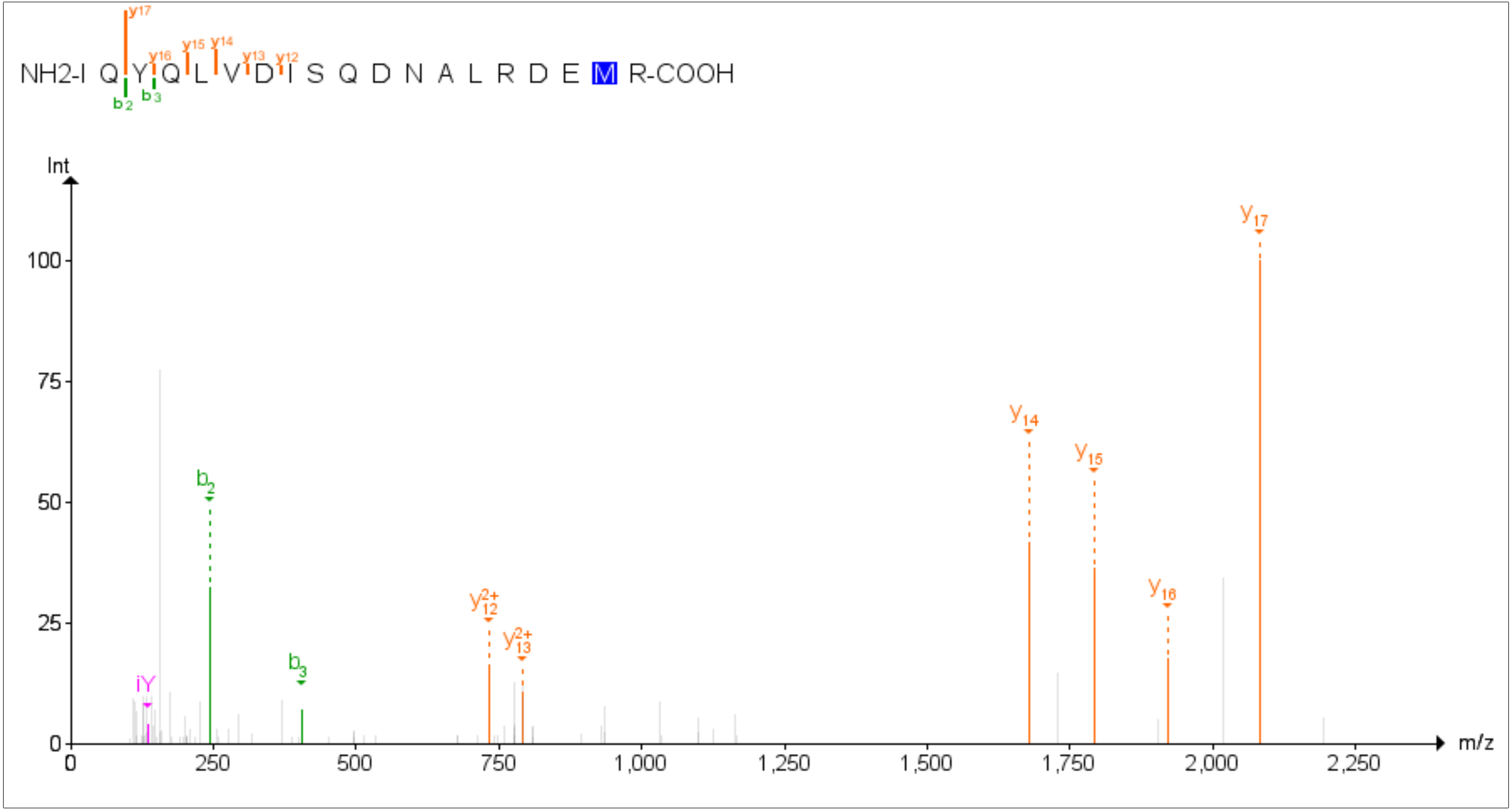

NH2-I Q Y Q L V D I S Q D N A L R D E M R-COOH
y17
y16
y15
y14
y13
y12
b2
b3
Int
100
75
50
25
0
m/z
0
250
500
750
1,000
1,250
1,500
1,750
2,000
2,250
iY
b2
b3
y12 2+
y13 2+
y14
y15
y16
y17

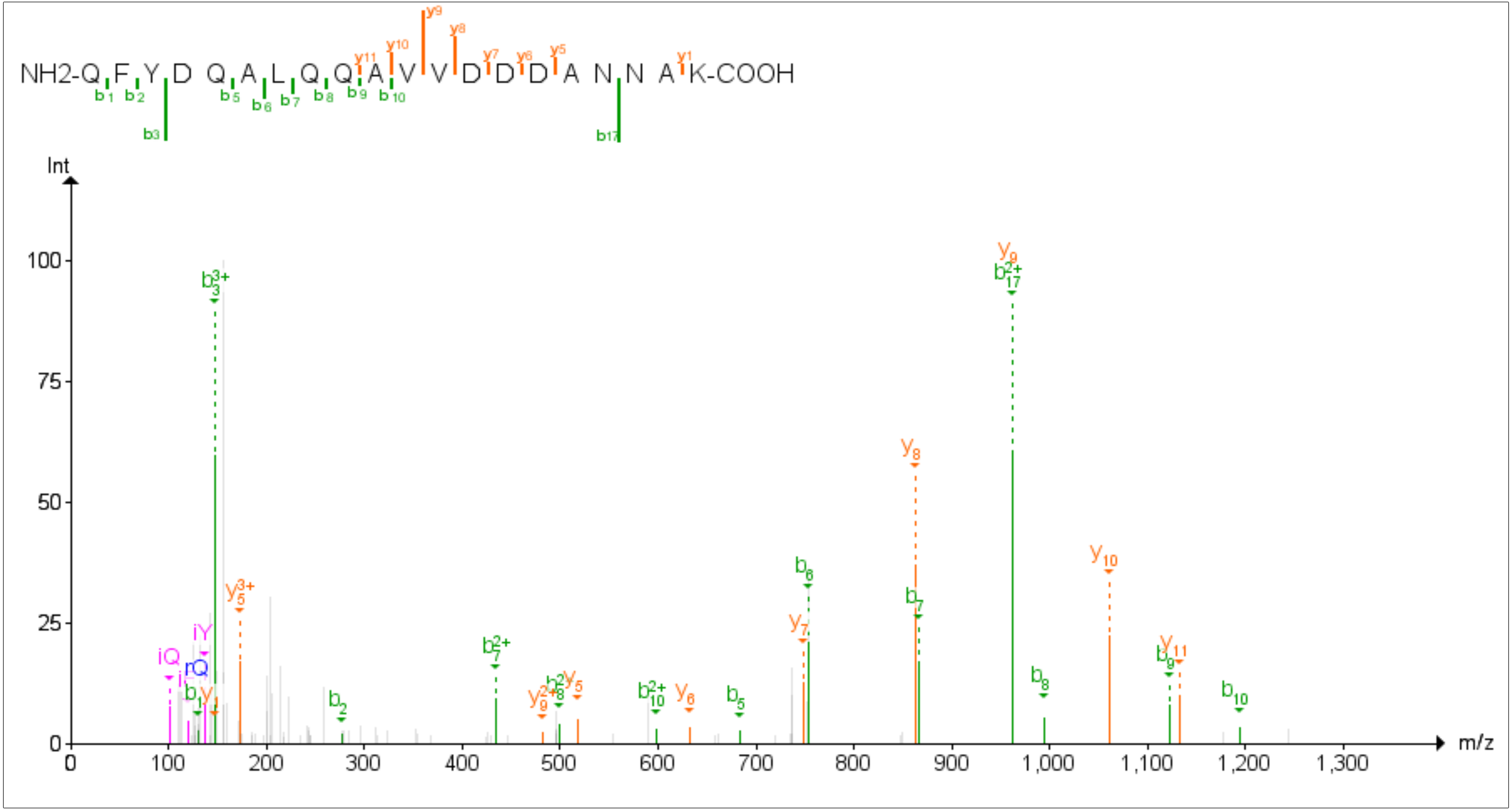

NH2-Q F Y D Q A L Q Q A V V D D D A N N A K-COOH
Int
m/z
0
25
50
75
100
0
100
200
300
400
500
600
700
800
900
1,000
1,100
1,200
1,300

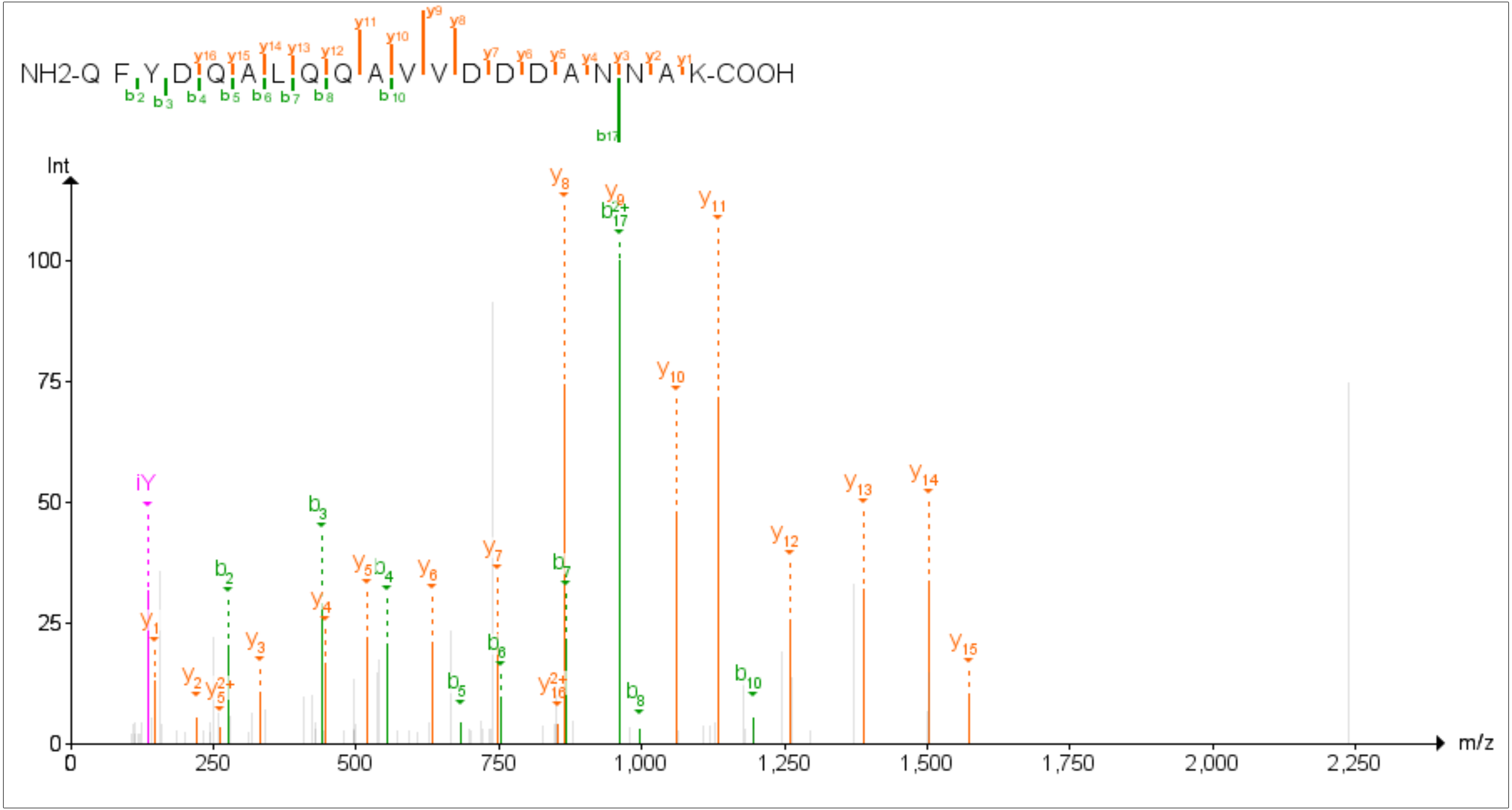

NH2-Q F Y D Q A L Q Q A V V D D D A N N A K-COOH
Int
m/z
iY

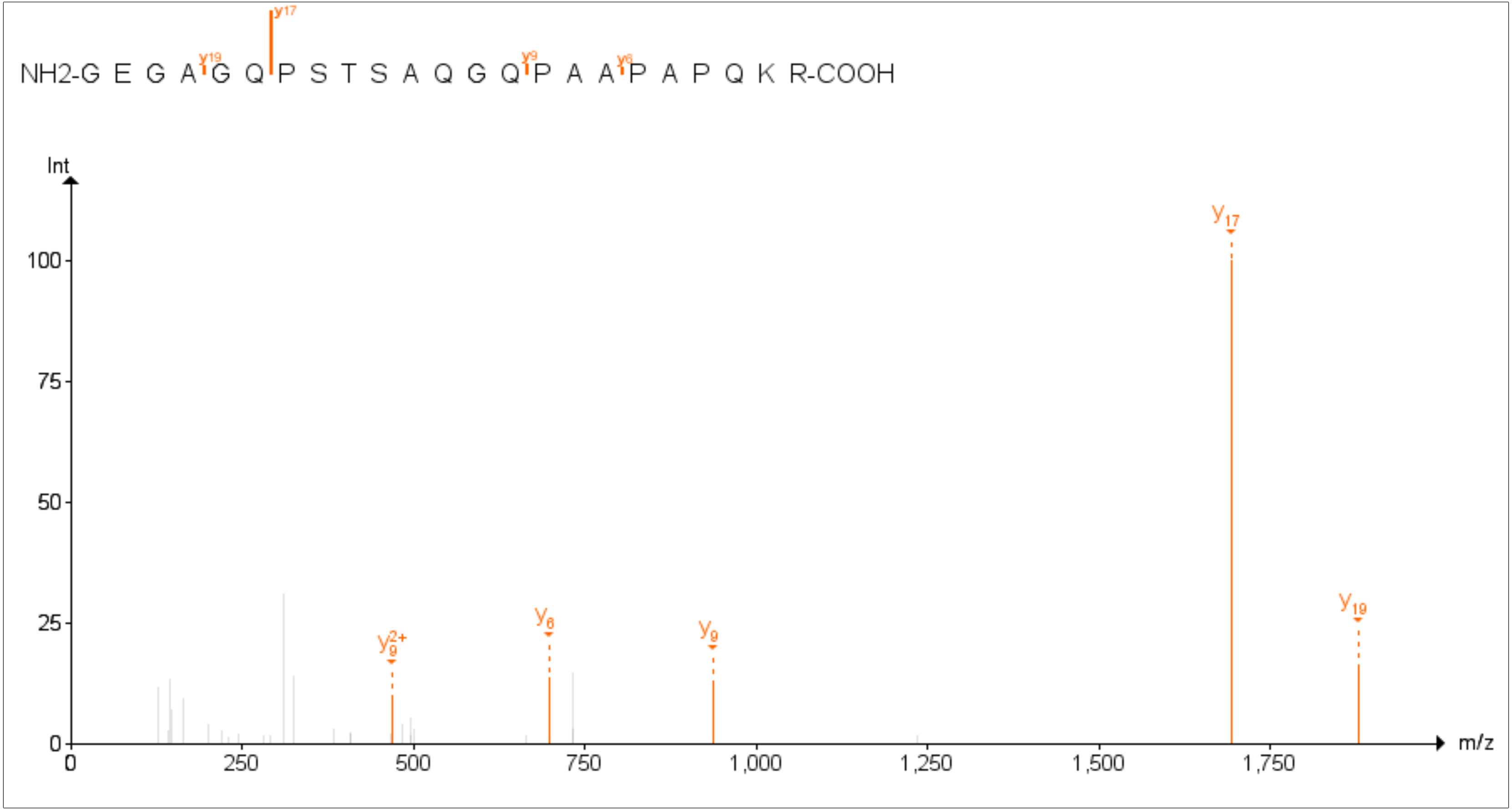

y17
y19
y9
y6
NH2-G E G A G Q P S T S A Q G Q P A A P A P Q K R-COOH
Int
100
75
50
25
0
y17
y19
y6
y9
y9 2+
0
250
500
750
1,000
1,250
1,500
1,750
m/z

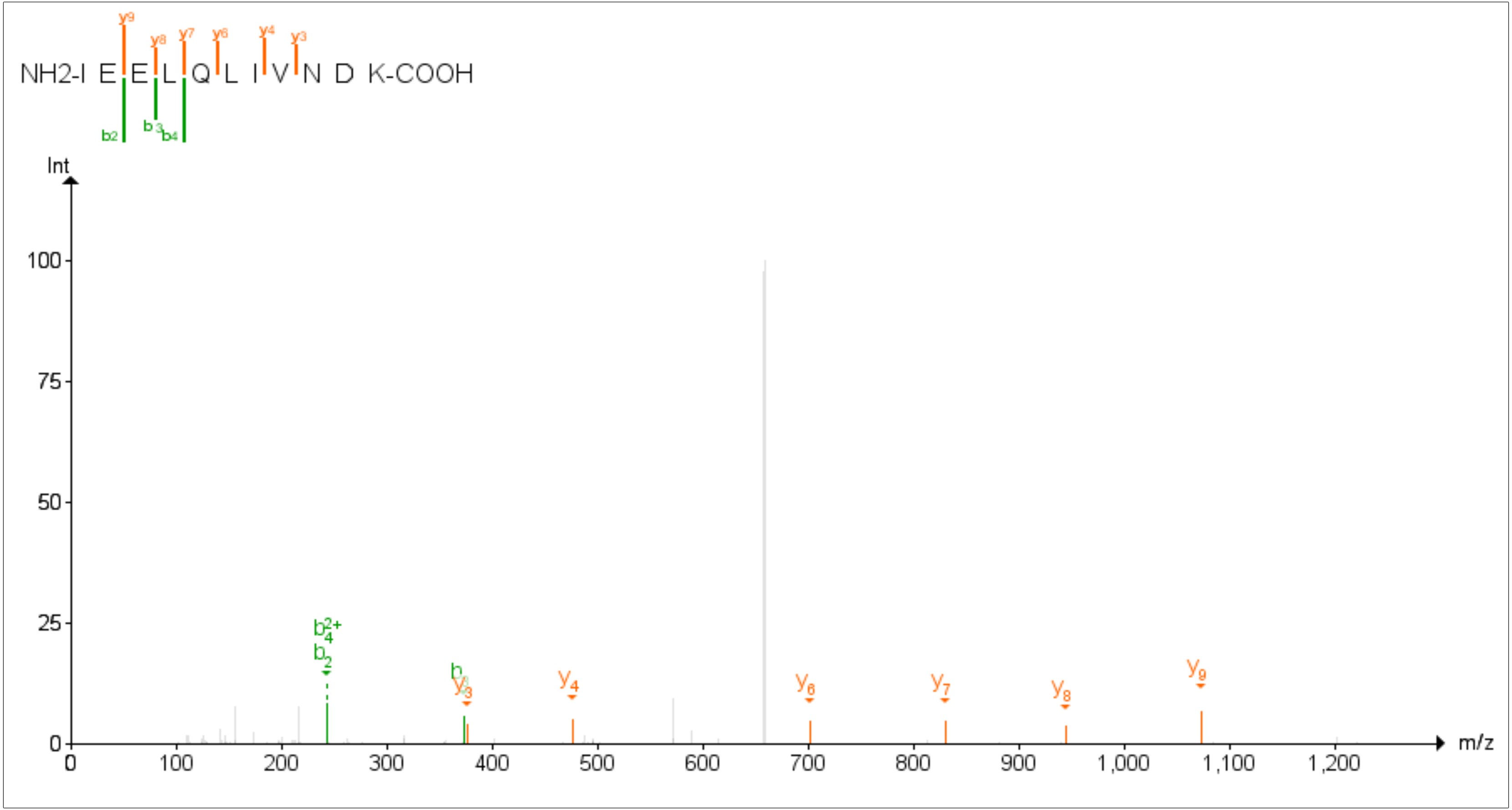

NH2-I E E L Q L I V N D K-COOH
y9
y8
y7
y6
y4
y3
b2
b3
b4
Int
100
75
50
25
0
b4 2+
b2
y3
y4
y6
y7
y8
y9
m/z
0
100
200
300
400
500
600
700
800
900
1,000
1,100
1,200

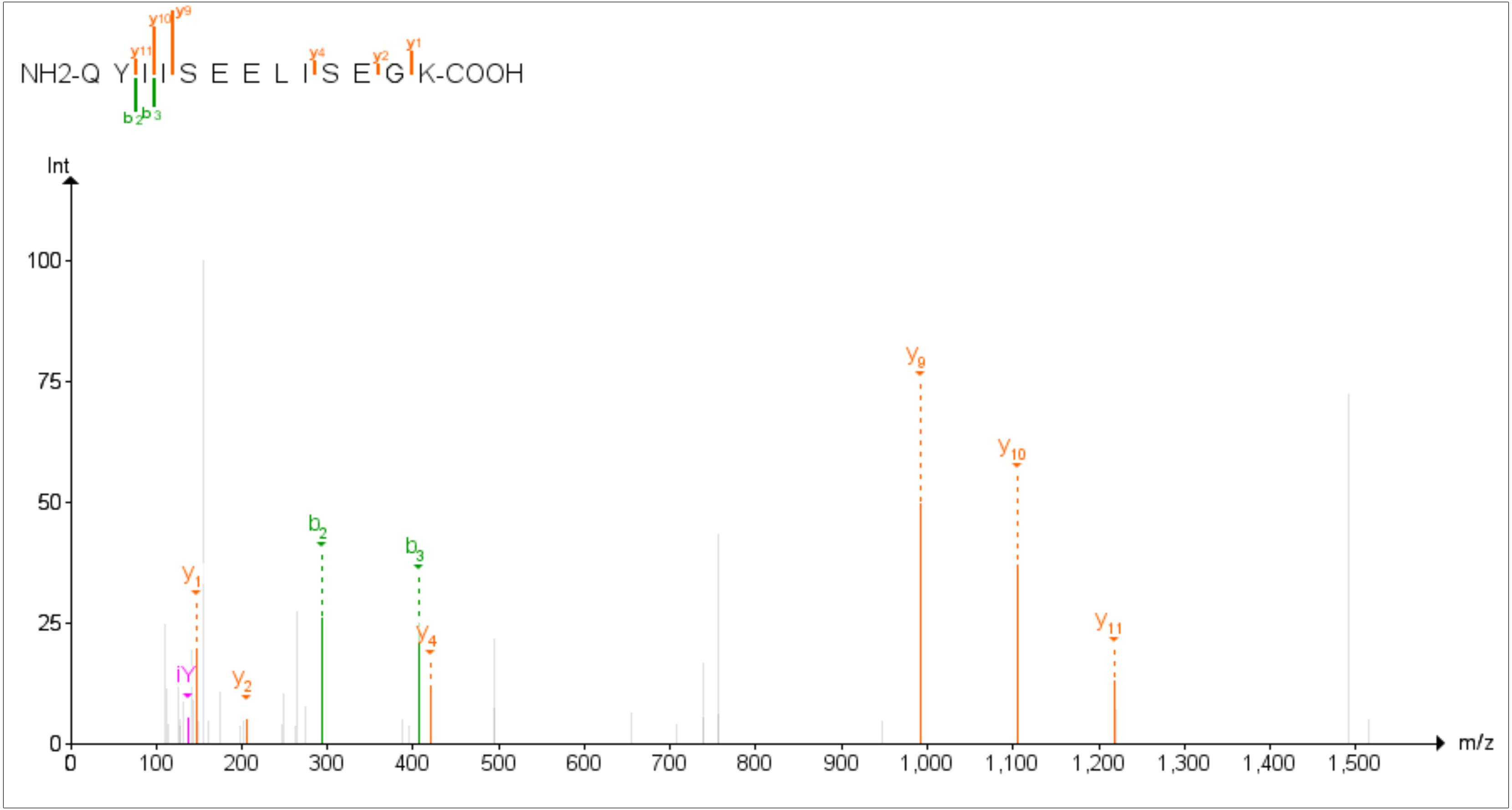

NH2-Q Y I I S E E L I S E G K-COOH
y11 y10 y9 y4 y2 y1
b2 b3
Int
100
75
50
25
0
m/z
0 100 200 300 400 500 600 700 800 900 1,000 1,100 1,200 1,300 1,400 1,500
y1 iY y2 b2 b3 y4 y9 y10 y11

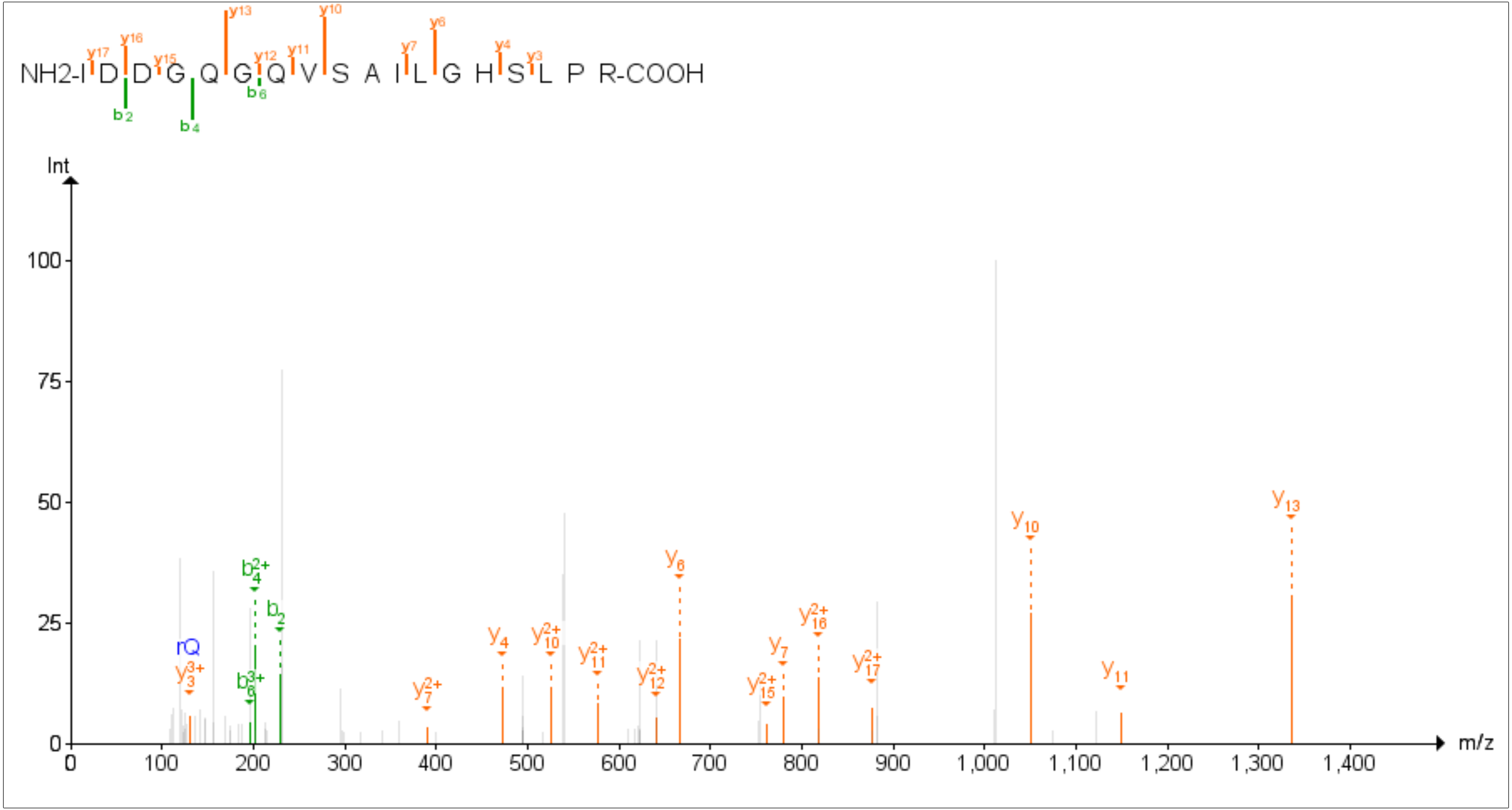

NH2-I D D G Q G Q V S A I L G H S L P R-COOH
y17 y16 y15 y13 y12 y11 y10 y7 y6 y4 y3
b2 b4 b6
Int
m/z
rQ

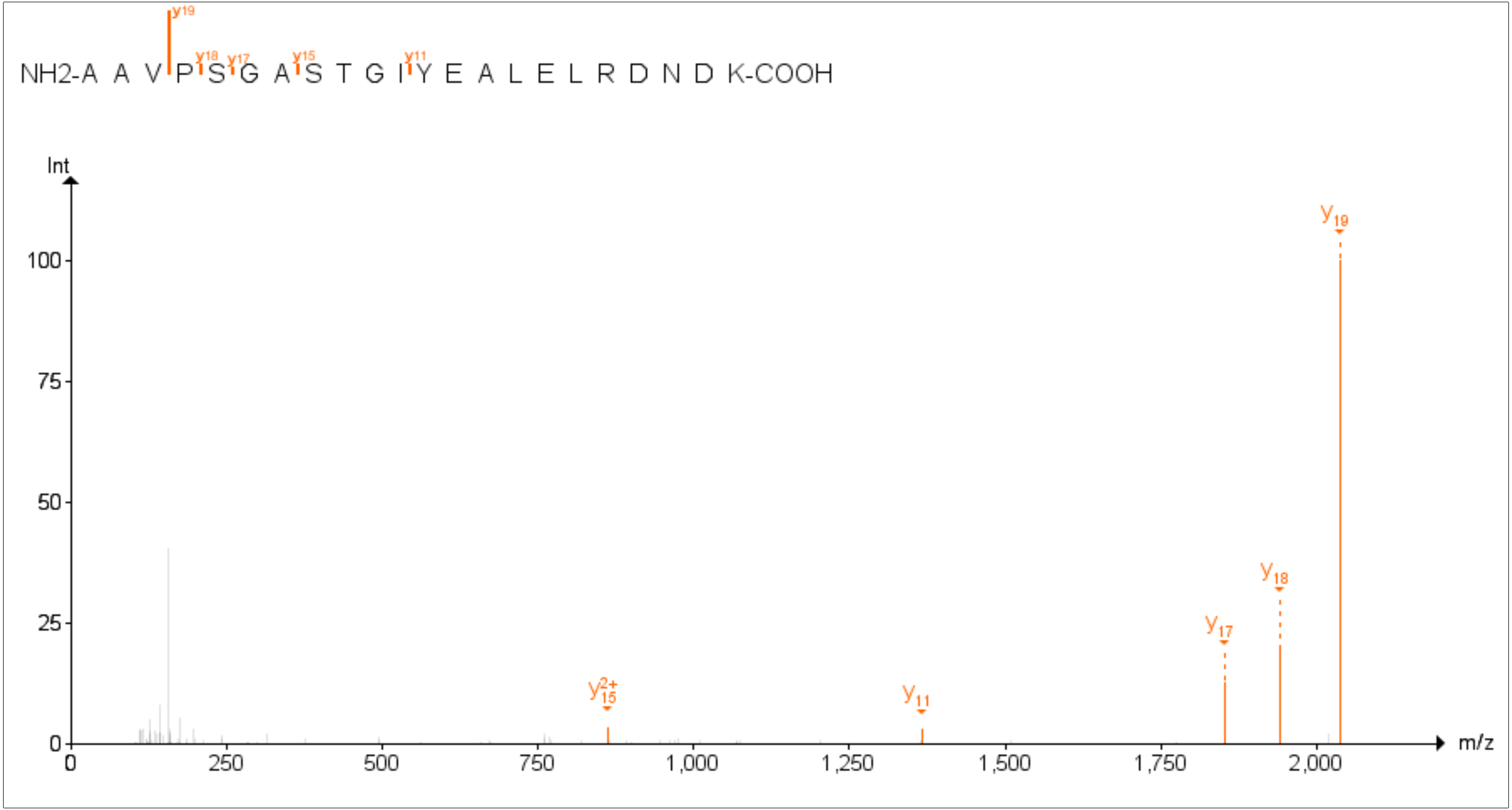

NH2-A A V P S G A S T G I Y E A L E L R D N D K-COOH
y19
y18
y17
y15
y11
Int
100
75
50
25
0
m/z
0
250
500
750
1,000
1,250
1,500
1,750
2,000
y_{15}^{2+}
y_{11}
y_{17}
y_{18}
y_{19}

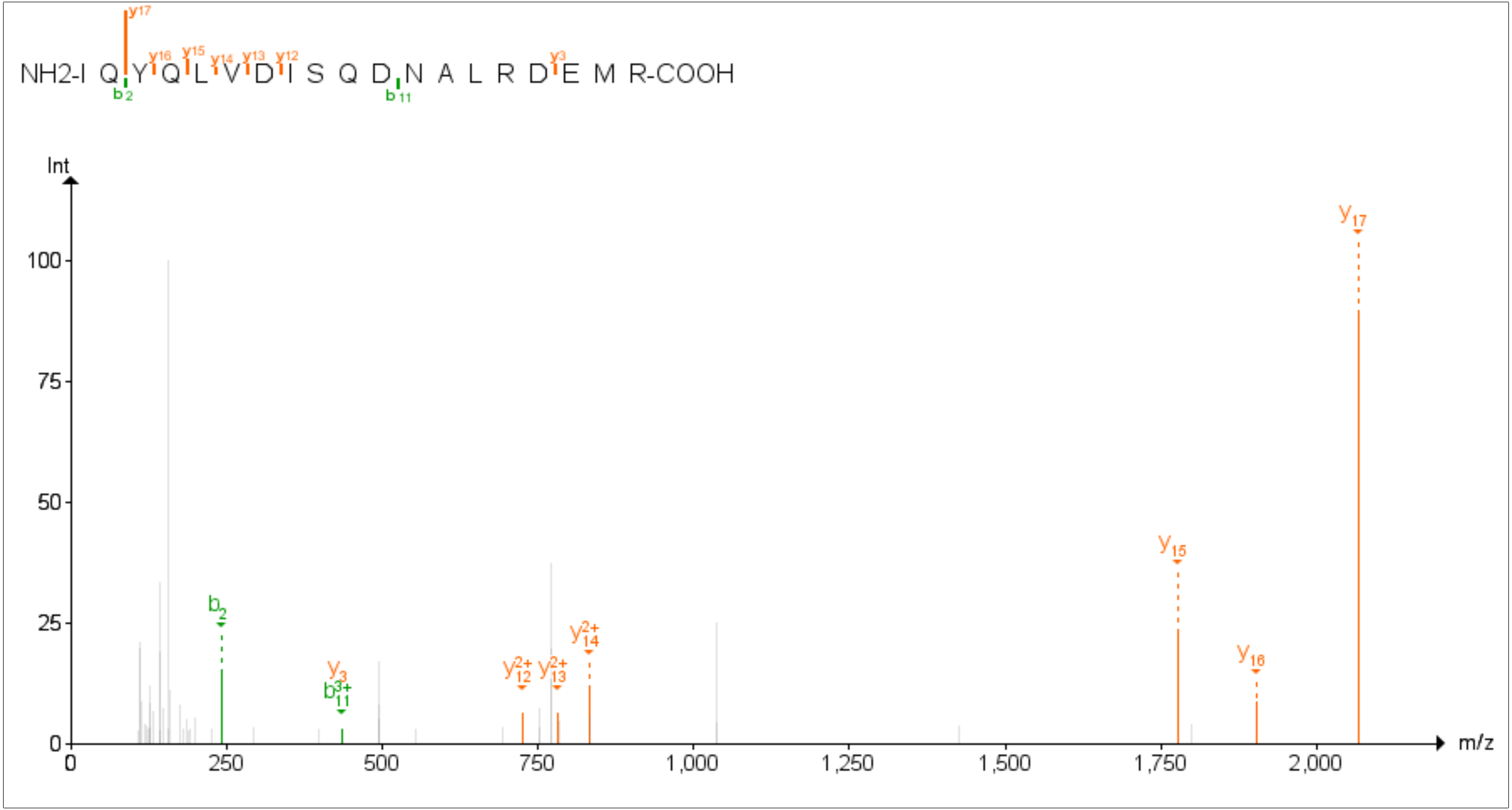

NH2-I Q Y Q L V D I S Q D N A L R D E M R-COOH
y17
y16
y15
y14
y13
y12
y3
b2
b11
Int
100
75
50
25
0
m/z
0
250
500
750
1,000
1,250
1,500
1,750
2,000
b2
y3
b11 3+
y12 2+
y13 2+
y14 2+
y15
y16
y17

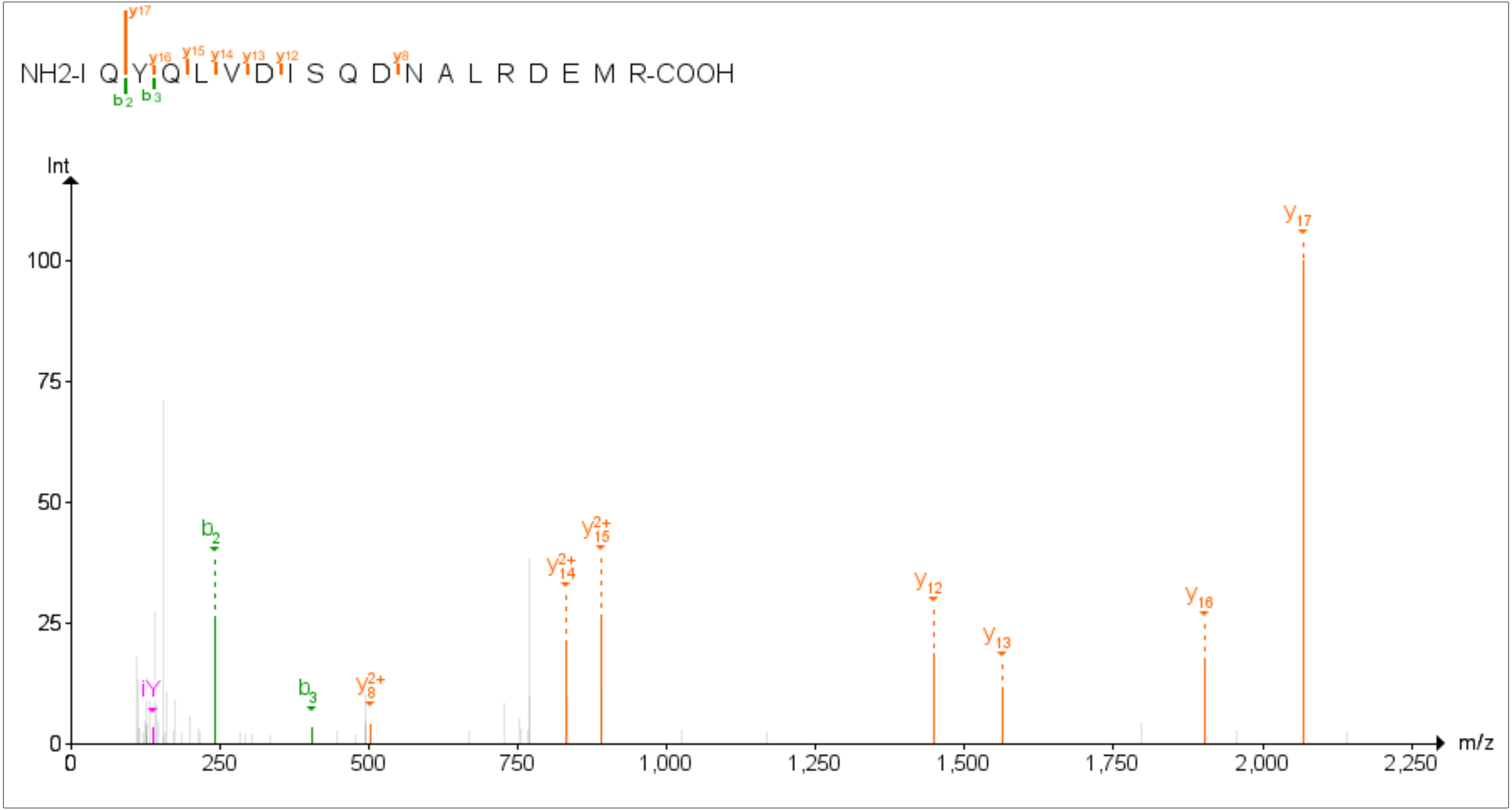
NH2-I Q Y Q L V D I S Q D N A L R D E M R-COOH
Int
m/z
0
25
50
75
100
250
500
750
1,000
1,250
1,500
1,750
2,000
2,250
y17
y16
y15
y14
y13
y12
y8
b2
b3
iY
y8 2+
y14 2+
y15 2+

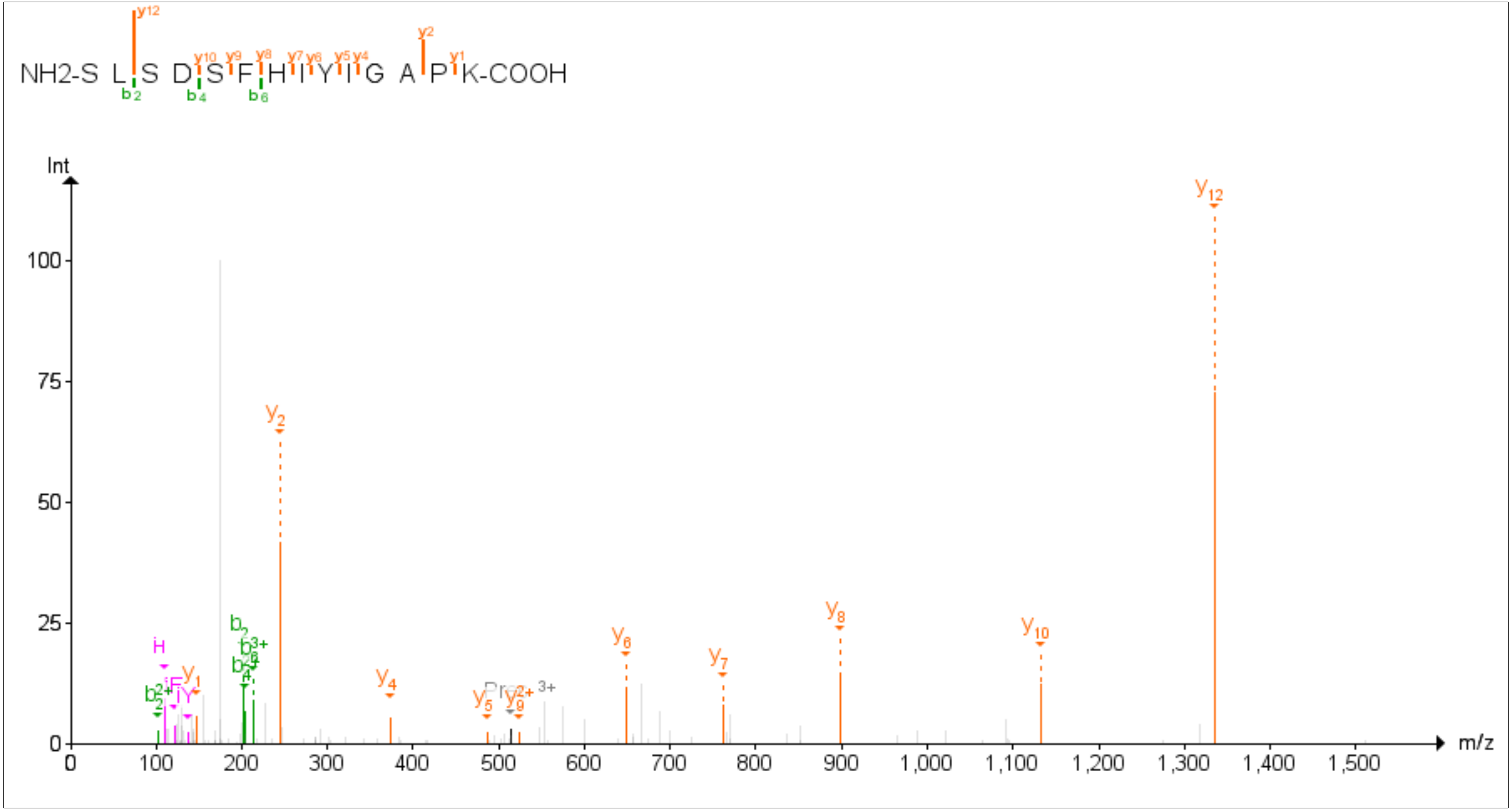
NH2-S L S D S F H I Y I G A P K-COOH
y12 y10 y9 y8 y7 y6 y5 y4 y2 y1
b2 b4 b6
Int
100
75
50
25
0
m/z
0 100 200 300 400 500 600 700 800 900 1,000 1,100 1,200 1,300 1,400 1,500
y_{12}
y_2
y_{10}
y_8
y_6
y_7
y_4
y_5
y_1
H
F
Y
b_2
b_2^{2+}
b_6^{3+}
b_4^{2+}
y_9^{2+}
3+

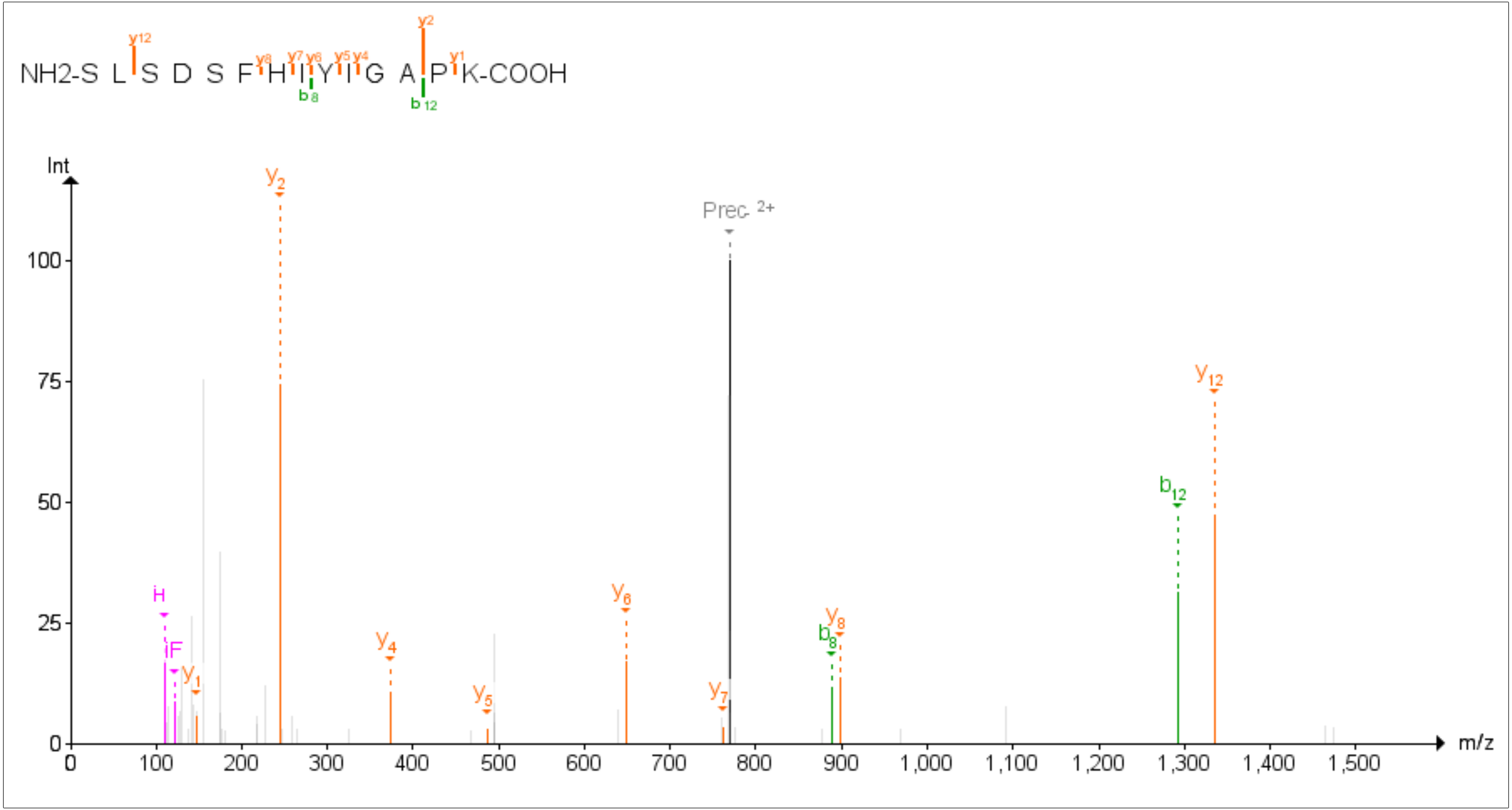
NH2-S L S D S F H I Y I G A P K-COOH
y12 y8 y7 y6 y5 y4 y2 y1
b8 b12
Int
m/z
y2
Prec 2+
y12
b12
y6
y8
b8
y4
y5
y7
y1
iH
iF
100
75
50
25
0
0 100 200 300 400 500 600 700 800 900 1,000 1,100 1,200 1,300 1,400 1,500

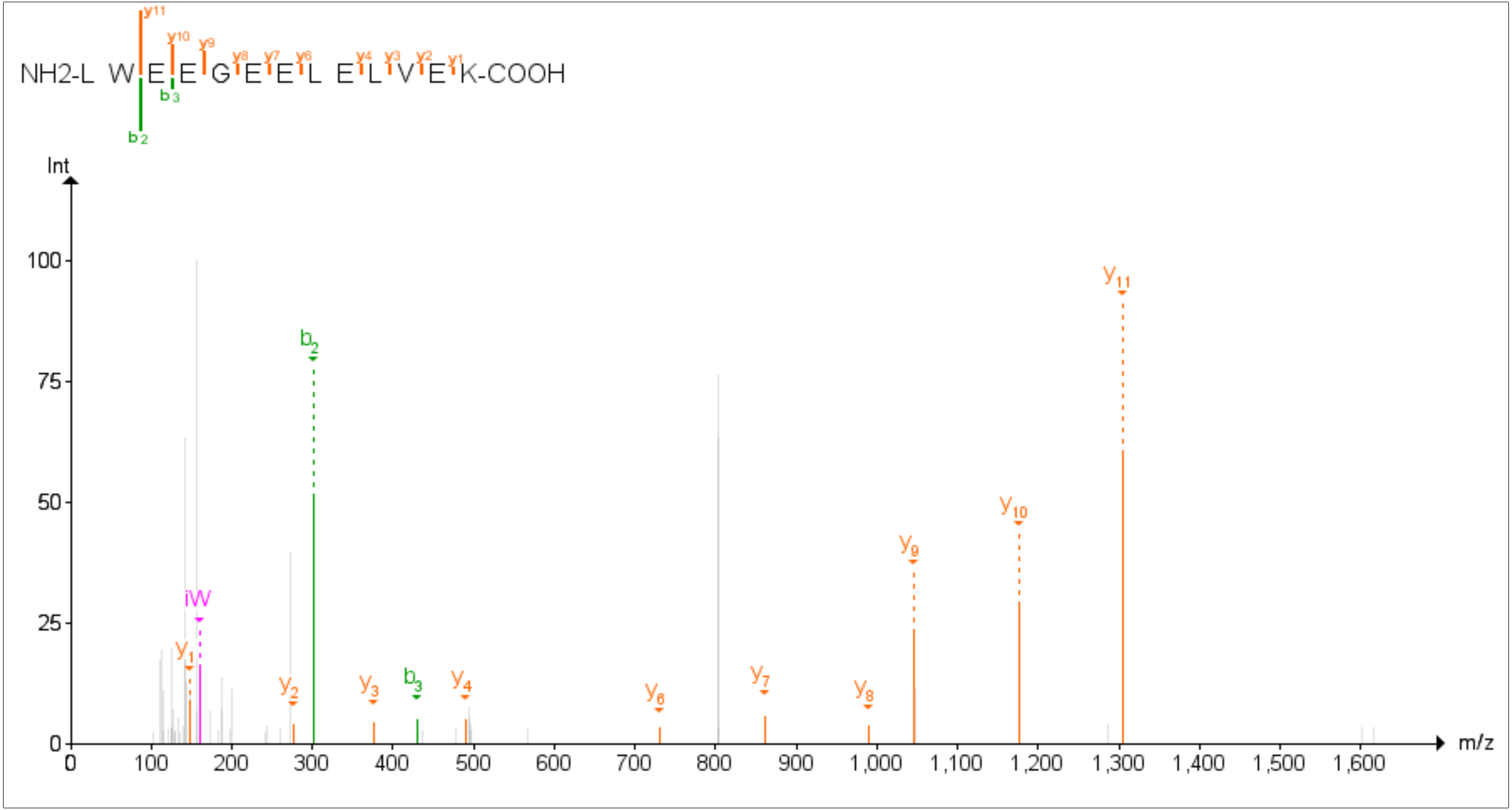

NH2-L W E E G E E L E L V E K-COOH
y11
y10
y9
y8
y7
y6
y4
y3
y2
y1
b2
b3
Int
100
75
50
25
0
iW
m/z
0
100
200
300
400
500
600
700
800
900
1,000
1,100
1,200
1,300
1,400
1,500
1,600

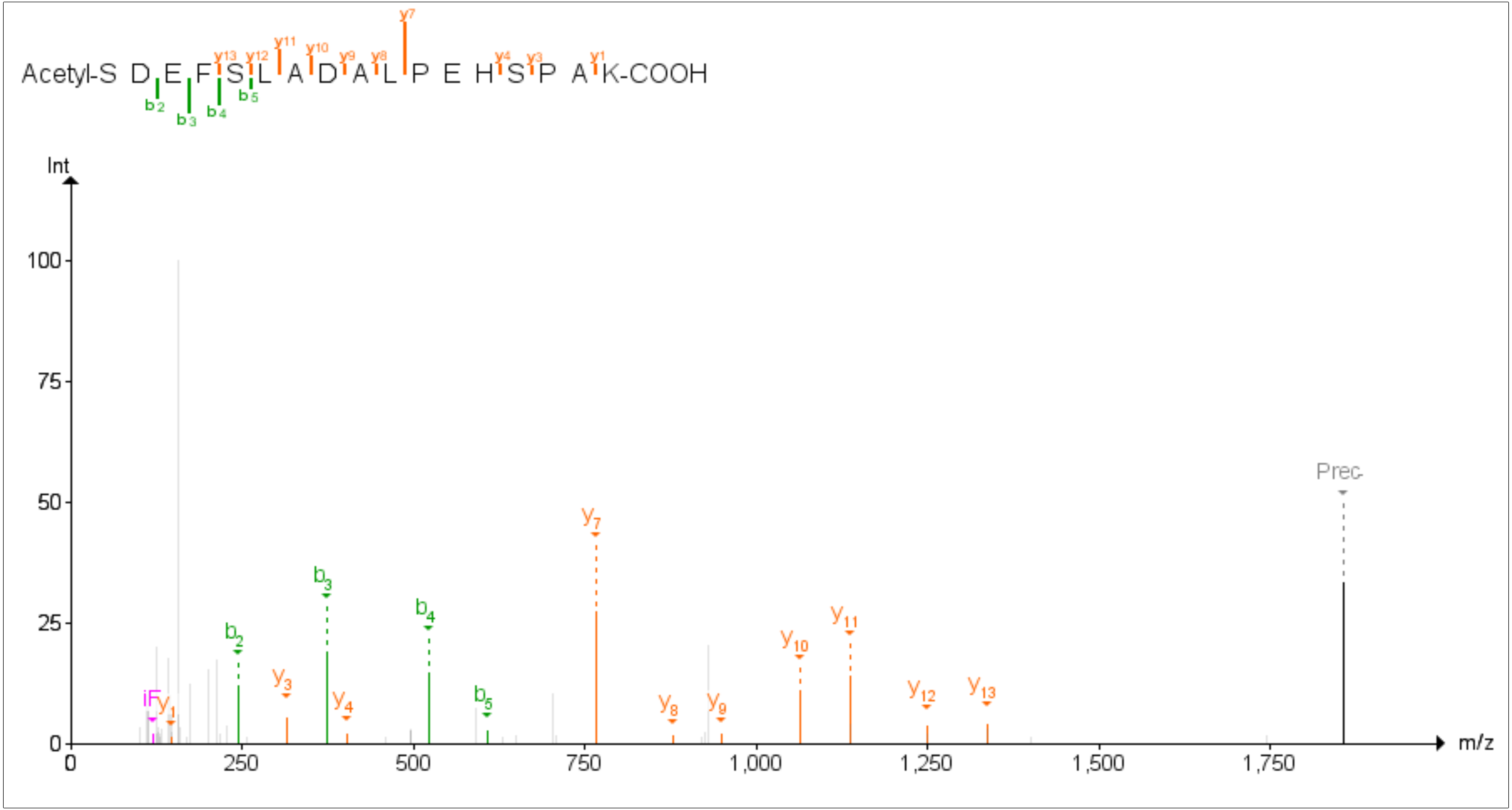

Acetyl-S D E F S L A D A L P E H S P A K-COOH
b2 b3 b4 b5
y13 y12 y11 y10 y9 y8 y7 y4 y3 y1
Int
100
75
50
25
0
m/z
0
250
500
750
1,000
1,250
1,500
1,750
Prec.

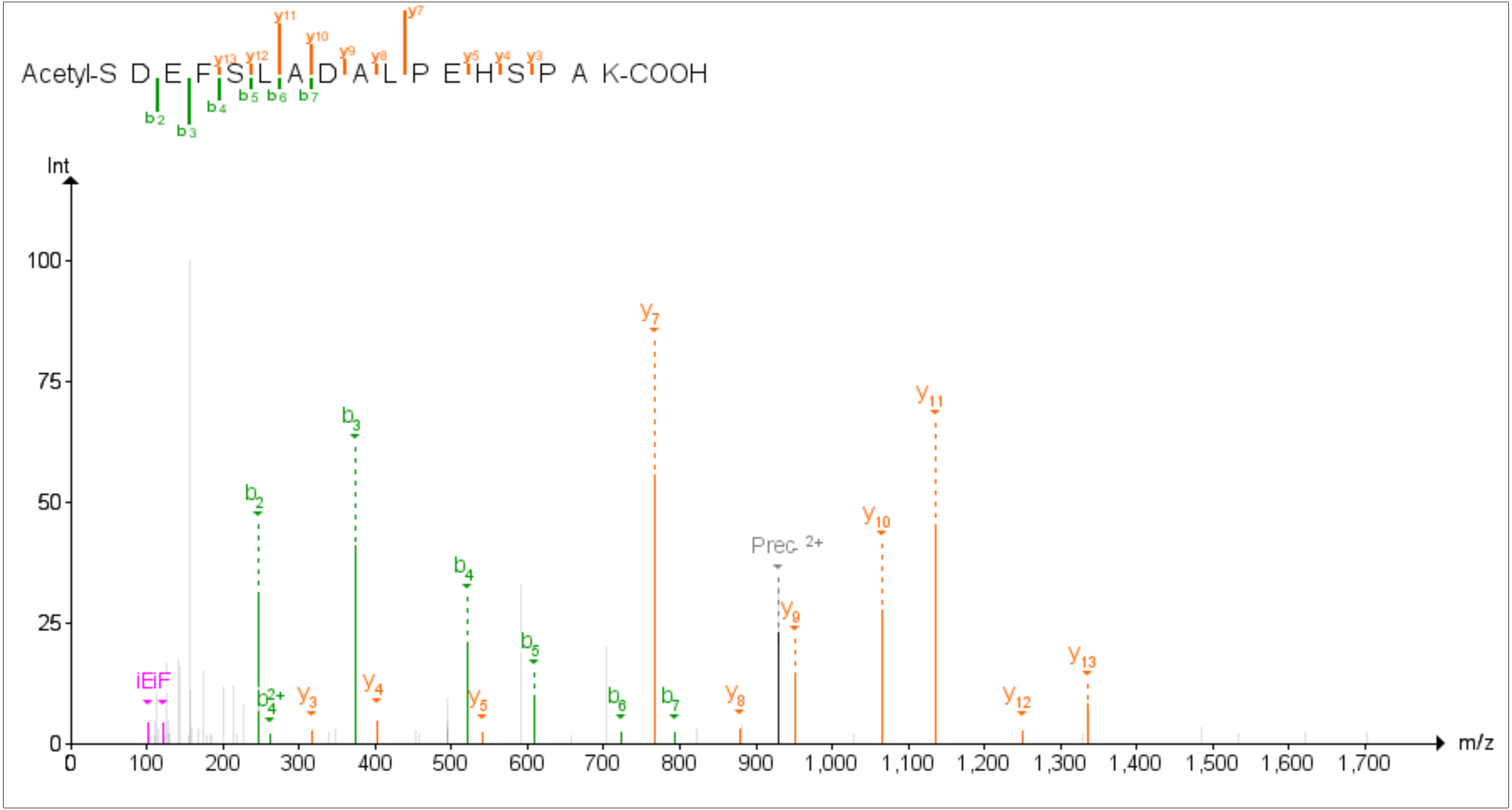

Acetyl-S D E F S L A D A L P E H S P A K-COOH
b2 b3 b4 b5 b6 b7
y13 y12 y11 y10 y9 y8 y7 y5 y4 y3
Int
100
75
50
25
0
iEiF
b2
b3
b4
b4 2+
b5
b6
b7
y3
y4
y5
y7
y8
y9
y10
y11
y12
y13
Prec 2+
0 100 200 300 400 500 600 700 800 900 1,000 1,100 1,200 1,300 1,400 1,500 1,600 1,700
m/z

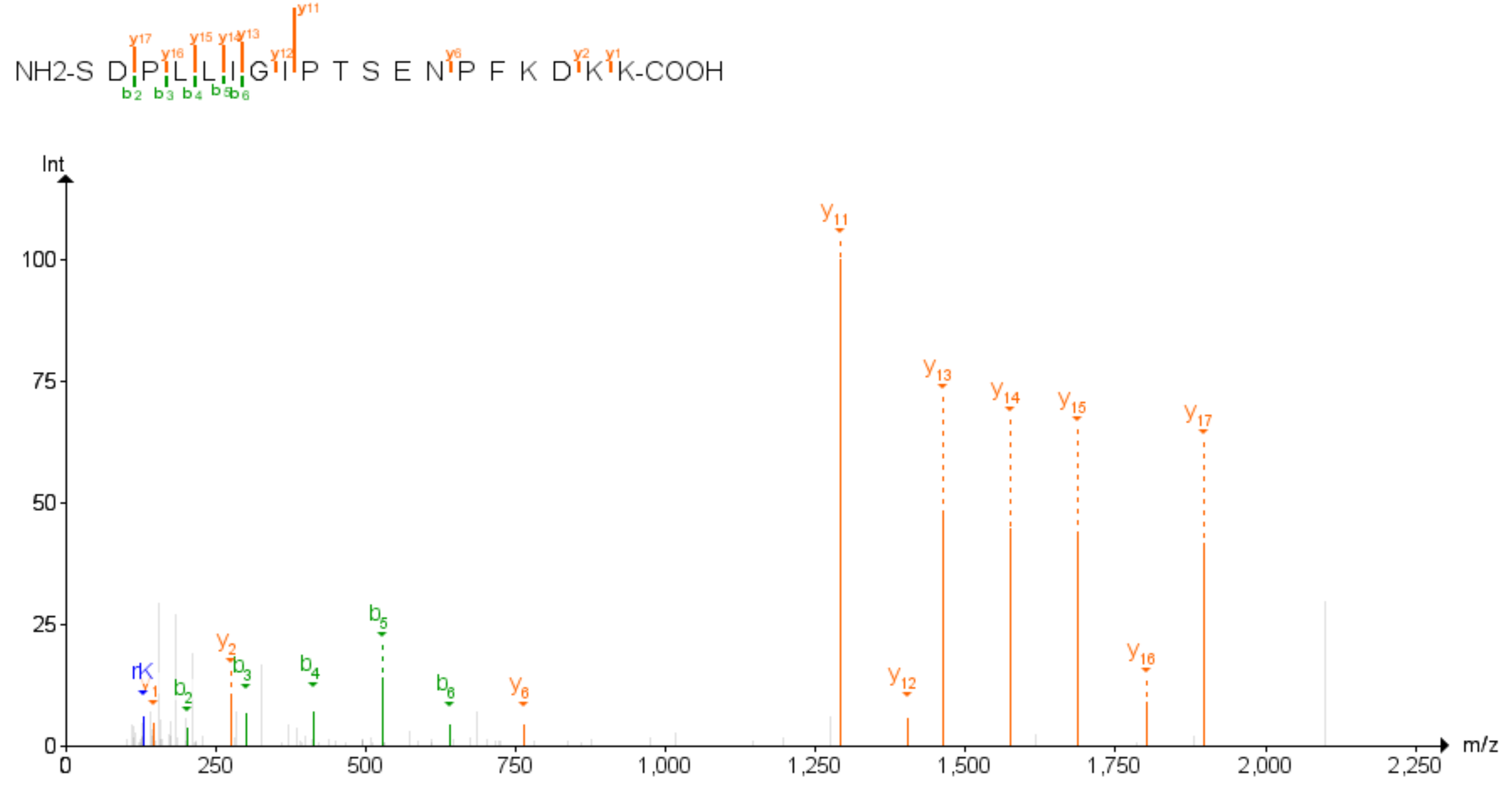

NH2-S D P L L I G I P T S E N P F K D K K-COOH
y17 y16 y15 y14 y13 y12 y11 y6 y2 y1
b2 b3 b4 b5 b6
Int
m/z
0
25
50
75
100
0
250
500
750
1,000
1,250
1,500
1,750
2,000
2,250
rK
y1
b2
y2
b3
b4
b5
b6
y6
y11
y12
y13
y14
y15
y16
y17

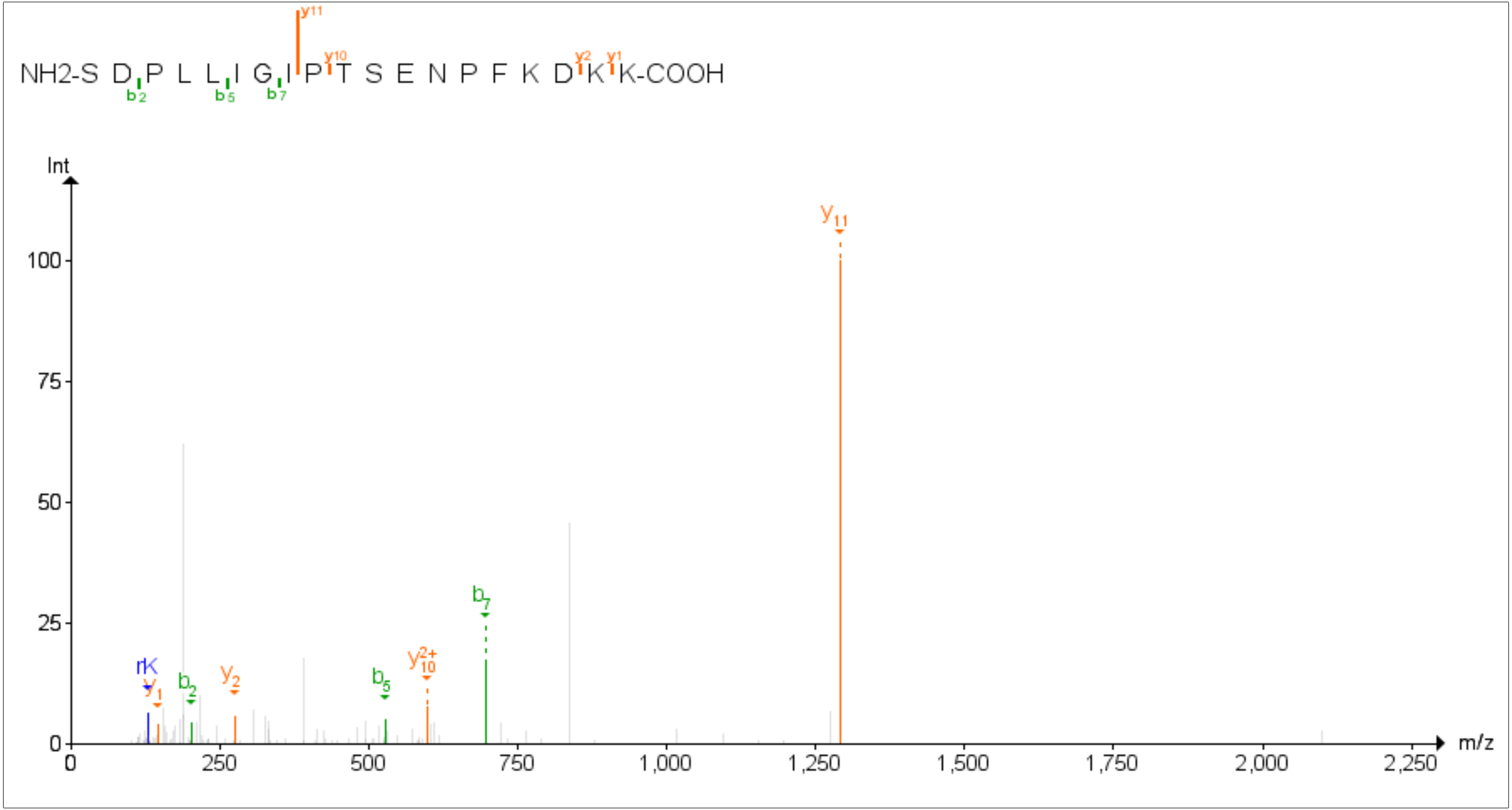
NH2-S D P L L I G I P T S E N P F K D K K-COOH
b2
b5
b7
y11
y10
y2
y1
Int
100
75
50
25
0
rK
y1
b2
y2
b5
y10 2+
b7
y11
0
250
500
750
1,000
1,250
1,500
1,750
2,000
2,250
m/z

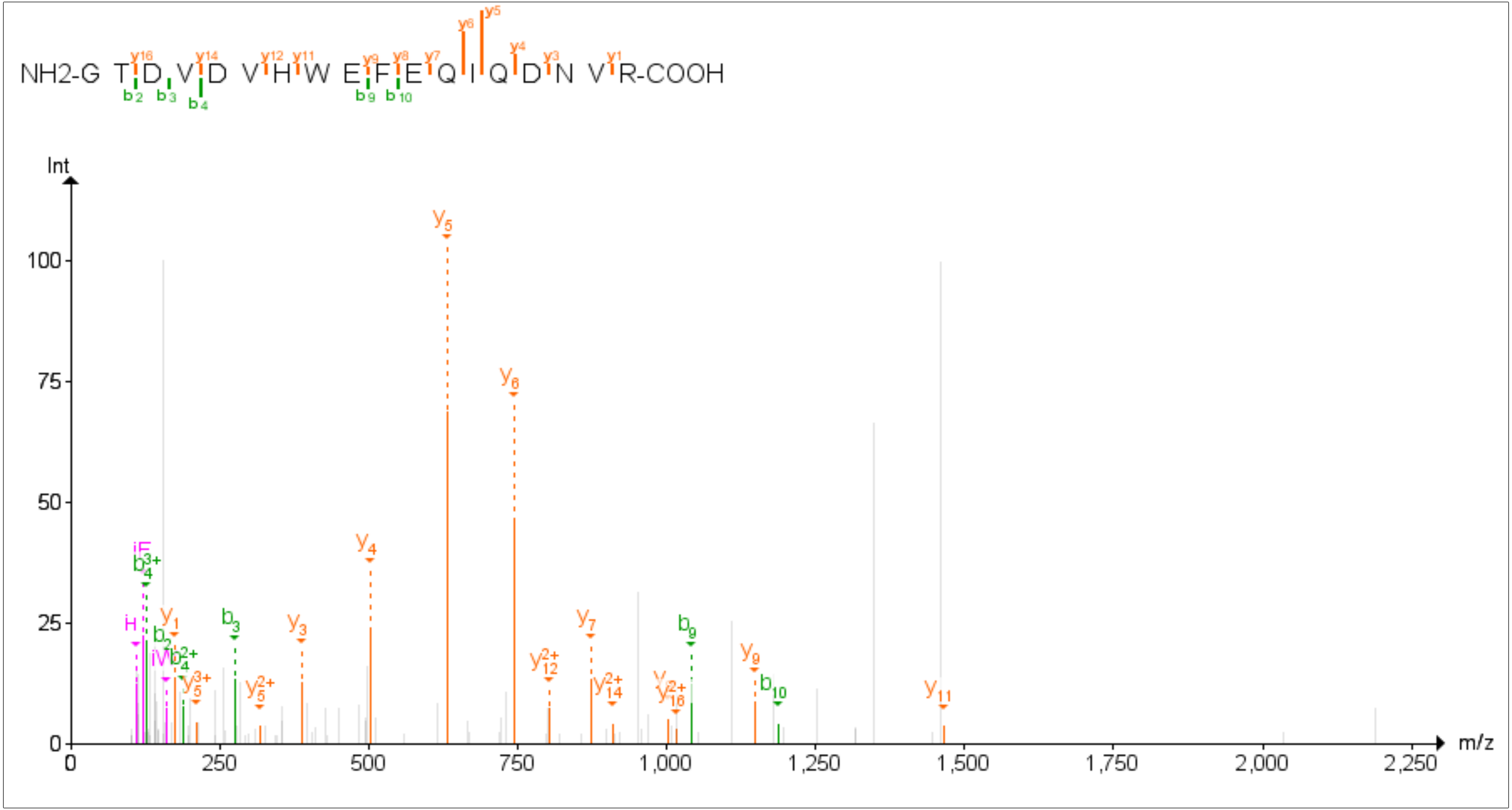
NH2-G T D V D V H W E F E Q I Q D N V R-COOH
Int
m/z
0
25
50
75
100
250
500
750
1,000
1,250
1,500
1,750
2,000
2,250

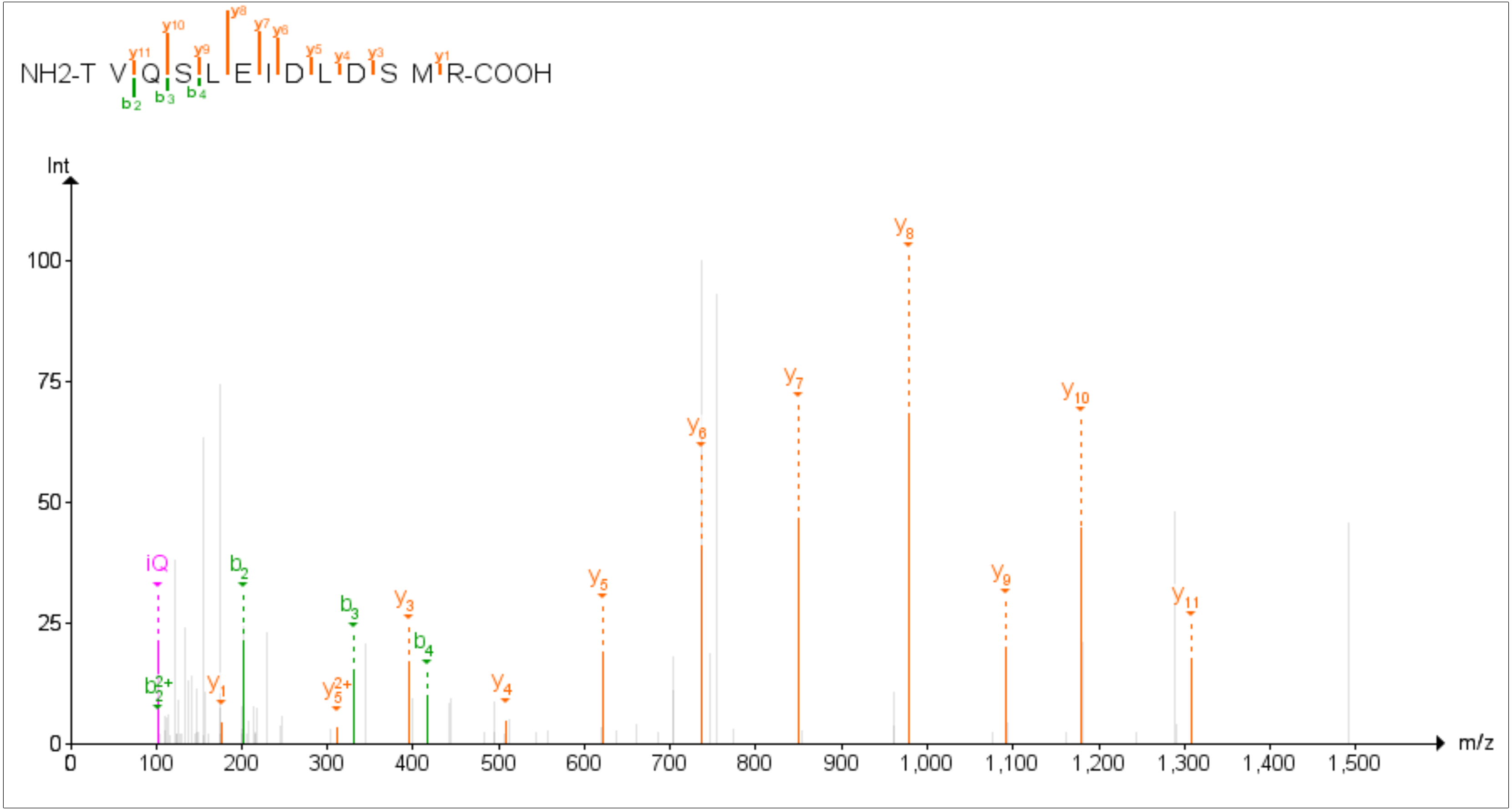

NH2-T V Q S L E I D L D S M R-COOH
y11 y10 y9 y8 y7 y6 y5 y4 y3 y1
b2 b3 b4
Int
m/z
0
25
50
75
100
0 100 200 300 400 500 600 700 800 900 1,000 1,100 1,200 1,300 1,400 1,500
iQ
b_2^{2+}
y_1
b_2
y_5^{2+}
b_3
y_3
b_4
y_4
y_5
y_6
y_7
y_8
y_9
y_{10}
y_{11}

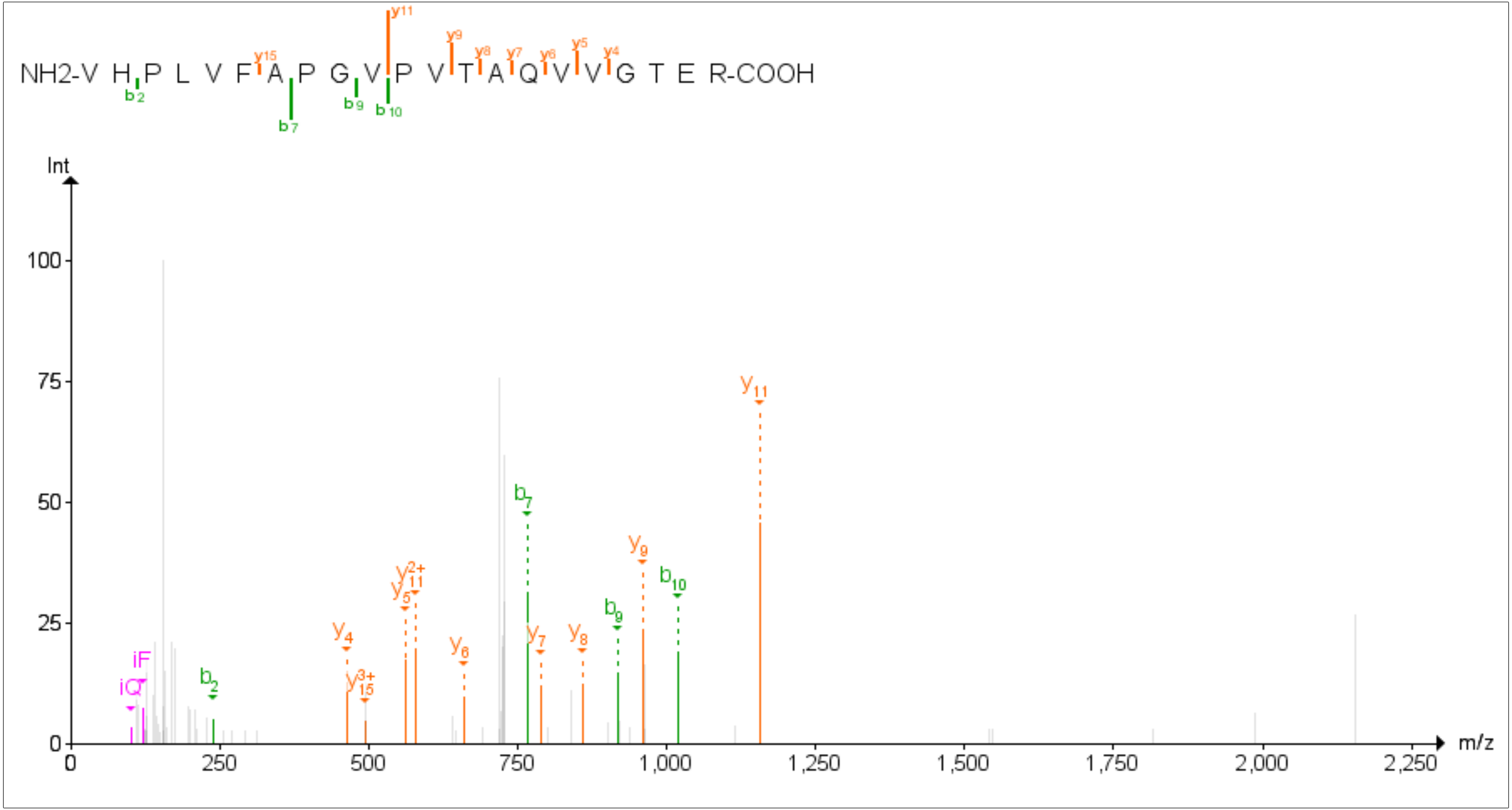

NH2-V H P L V F A P G V P V T A Q V V G T E R-COOH
b2 b7 b9 b10
y15 y11 y9 y8 y7 y6 y5 y4
Int
m/z
0
25
50
75
100
0
250
500
750
1,000
1,250
1,500
1,750
2,000
2,250
iQ
iF
b2
y4
y15 3+
y5
y11 2+
y6
b7
y7
y8
b9
y9
b10
y11

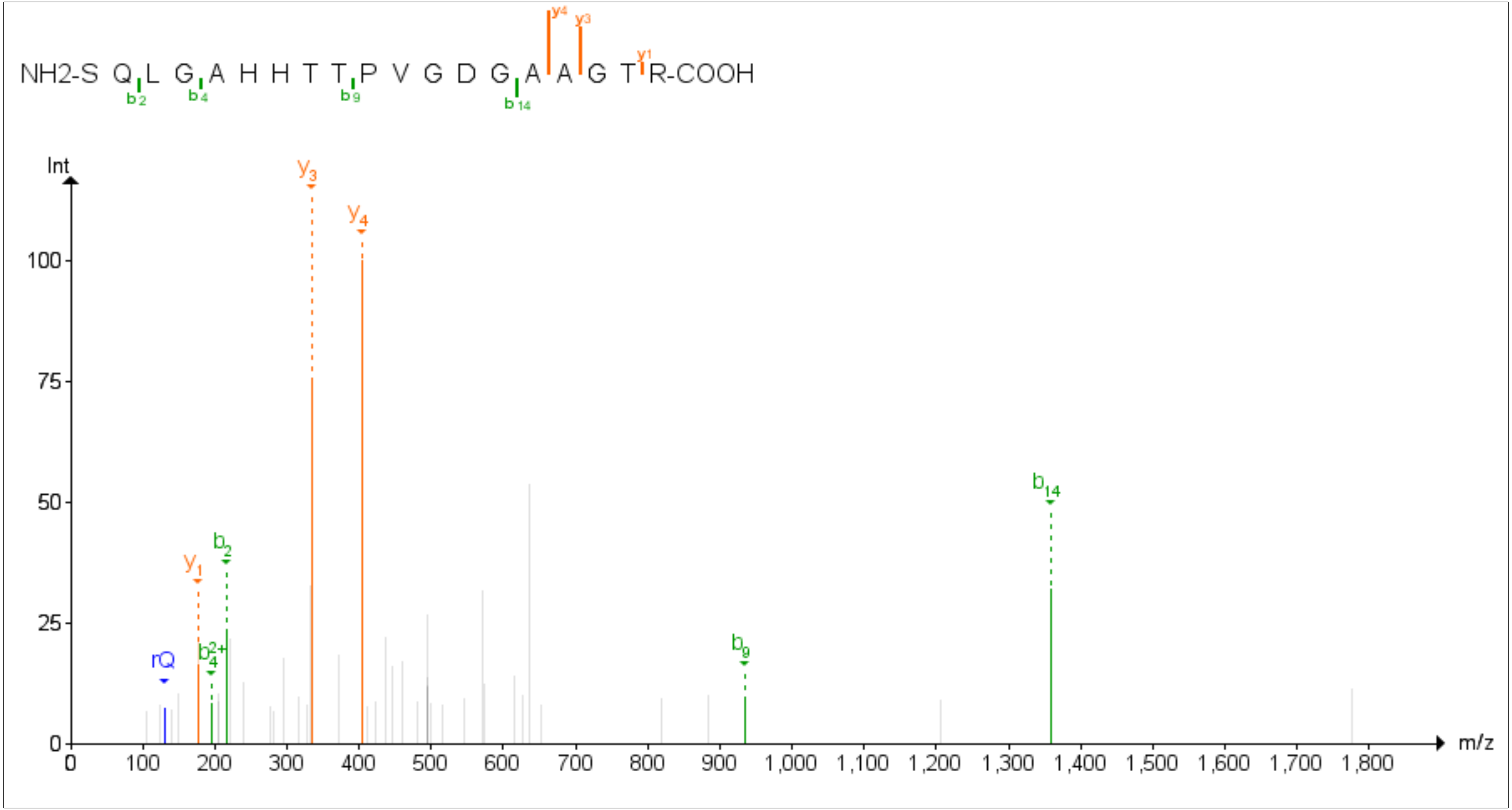

NH2-S Q L G A H H T T P V G D G A A G T R-COOH
b2 b4 b9 b14
y4 y3 y1
Int
m/z
y3
y4
b14
b2
y1
rQ
b4 2+
b9
0 25 50 75 100
0 100 200 300 400 500 600 700 800 900 1,000 1,100 1,200 1,300 1,400 1,500 1,600 1,700 1,800

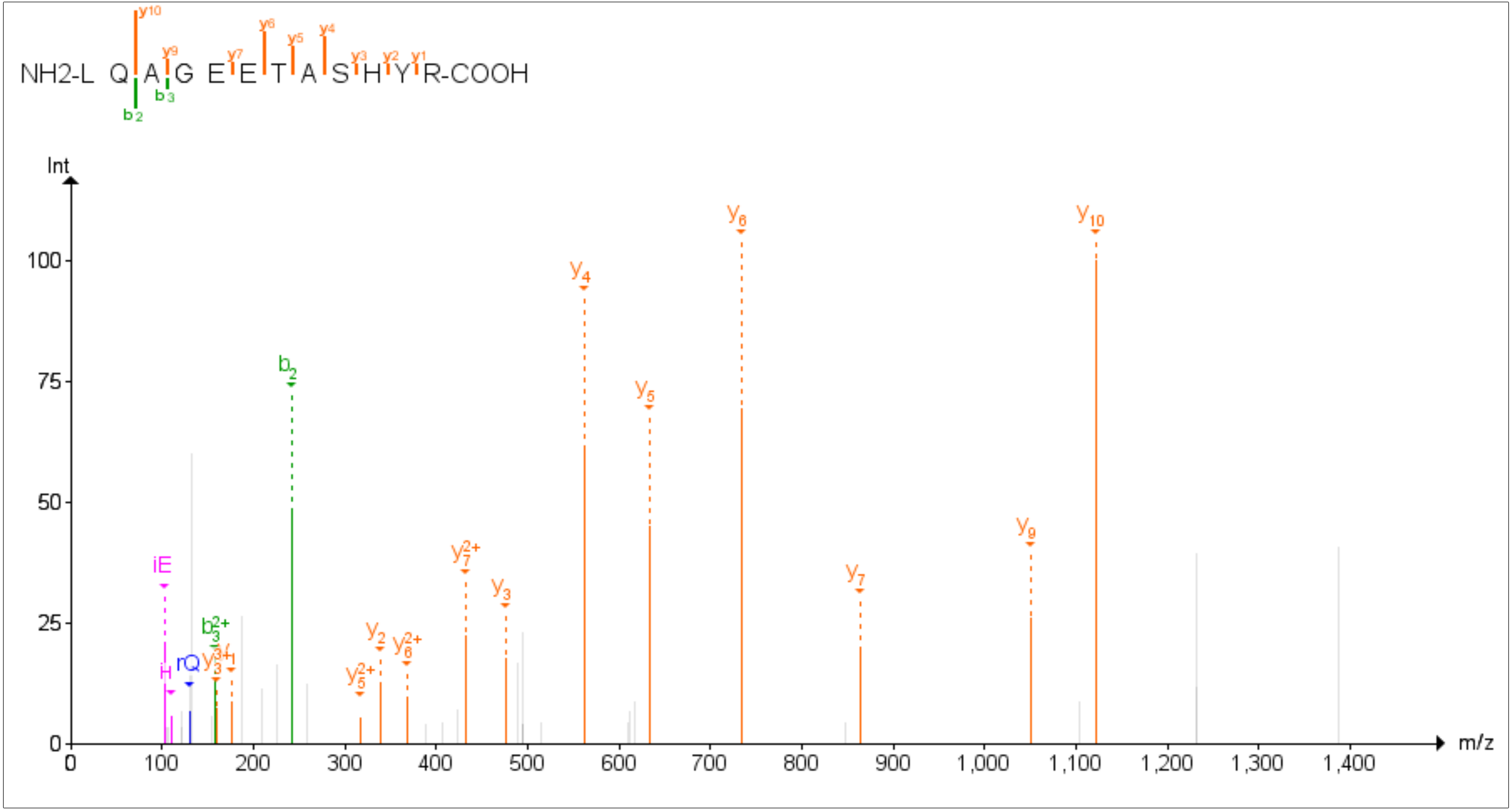

NH2-L Q A G E E T A S H Y R-COOH
y10
y9
y7
y6
y5
y4
y3
y2
y1
b2
b3
Int
100
75
50
25
0
m/z
0
100
200
300
400
500
600
700
800
900
1,000
1,100
1,200
1,300
1,400
y6
y10
y4
y5
b2
y9
y7
y7 2+
y3
iE
b3 2+
y2
y6 2+
rQ
iH
y5 2+

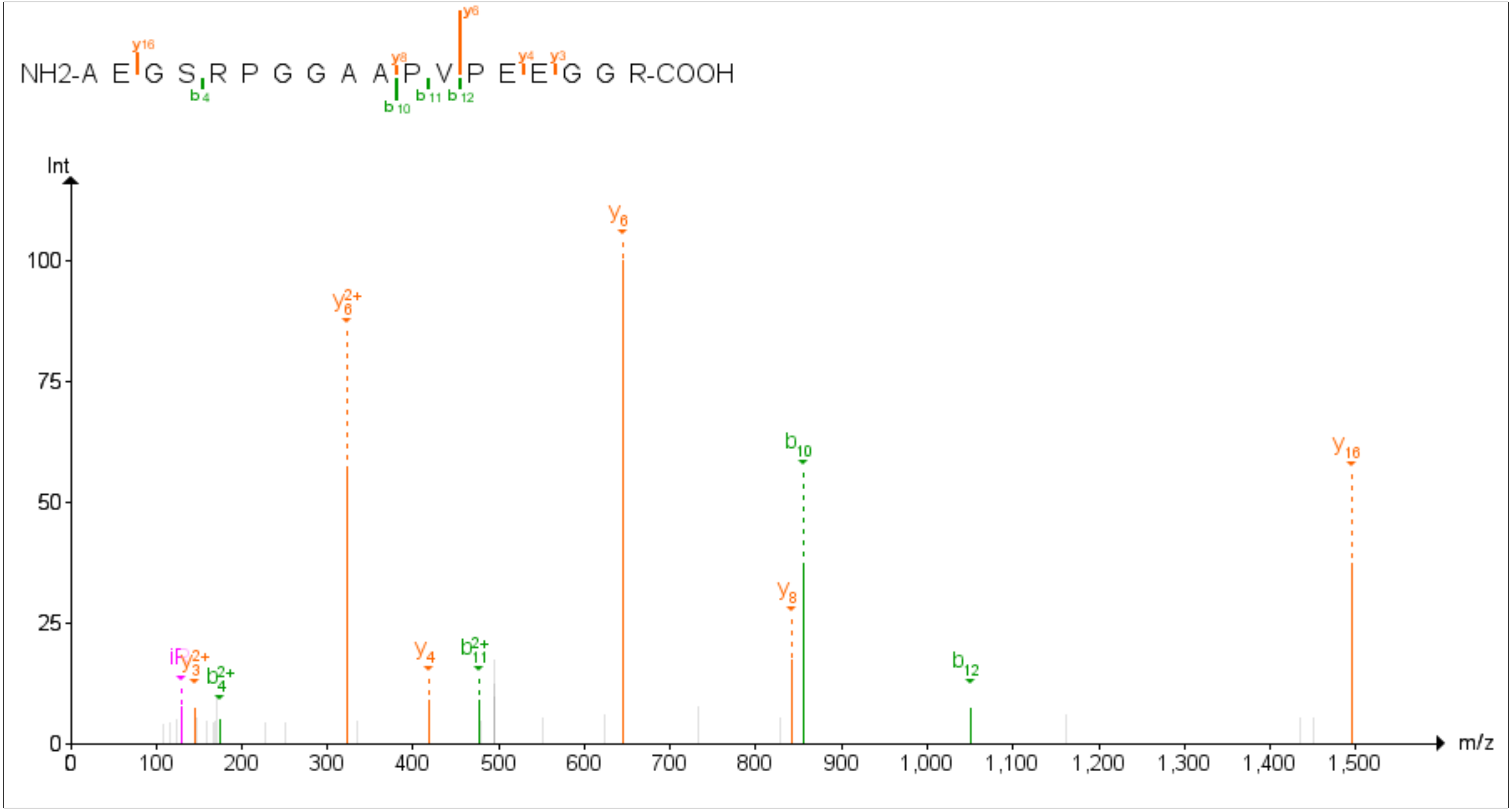

NH2-A E G S R P G G A A P V P E E G G R-COOH
y16
b4
y8
b10
b11
y6
b12
y4
y3
Int
m/z
0
25
50
75
100
100
200
300
400
500
600
700
800
900
1,000
1,100
1,200
1,300
1,400
1,500
y6
y6 2+
b10
y16
y8
y4
b11 2+
b12
y3 2+
b4 2+

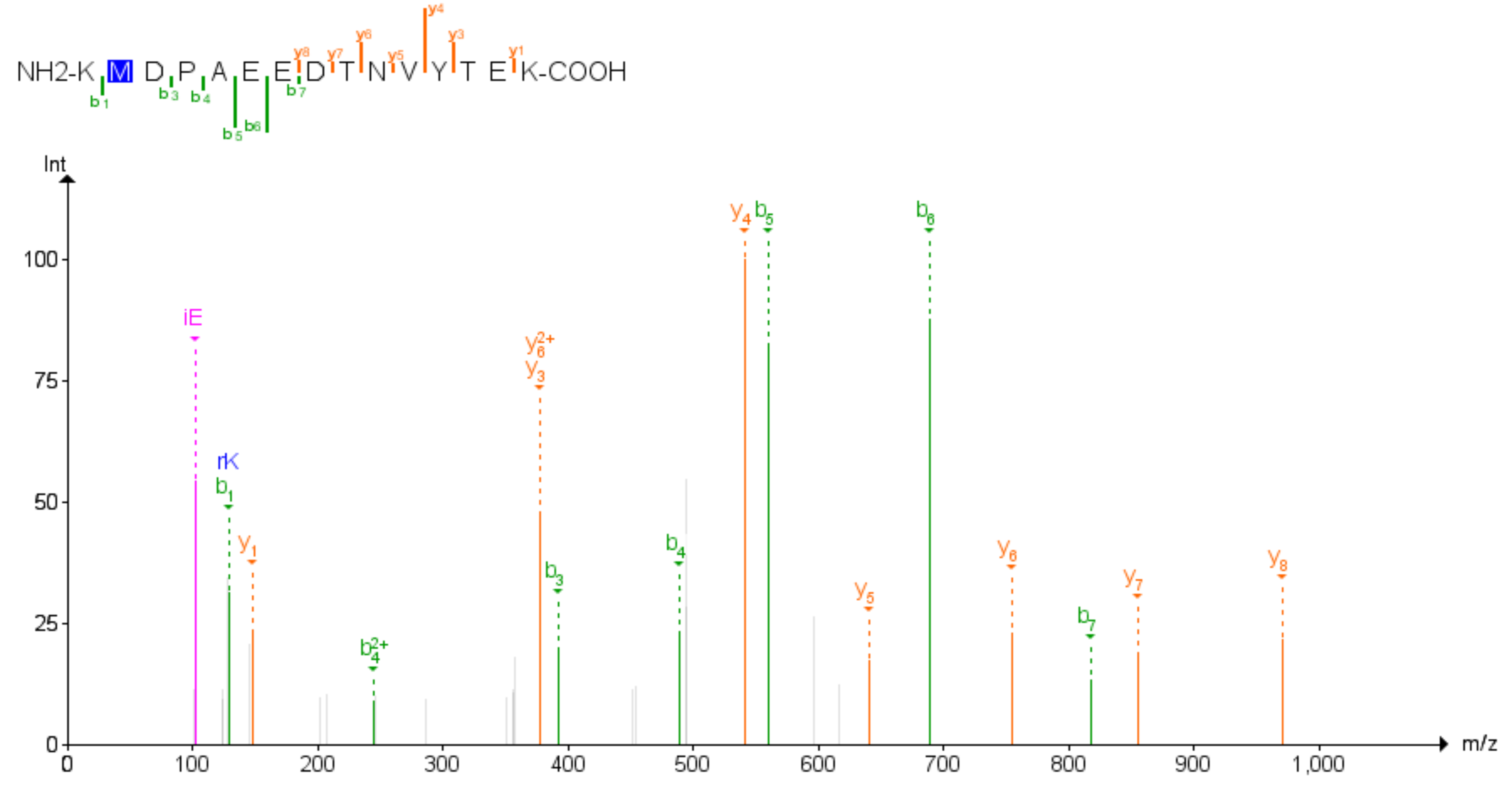

NH2-K M D P A E E D T N V Y T E K-COOH
b1 b3 b4 b5 b6 b7
y8 y7 y6 y5 y4 y3 y1
Int
m/z
iE
rK
b1
y1
b4 2+
y6 2+
y3
b3
b4
y4
b5
y5
b6
y6
b7
y7
y8

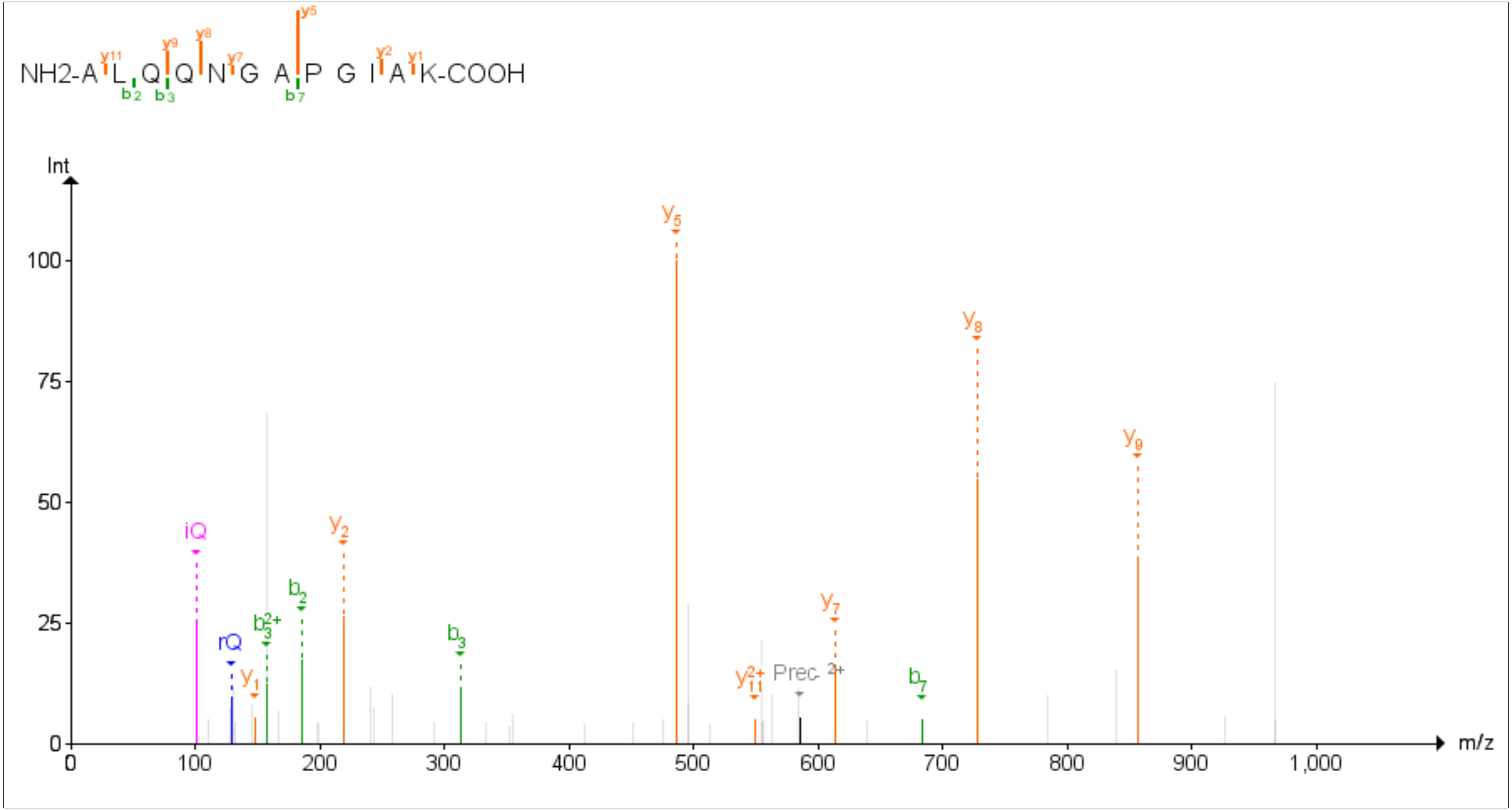

NH2-A L Q Q N G A P G I A K-COOH
y11 y9 y8 y7 y5 y2 y1
b2 b3 b7
Int
100
75
50
25
0
iQ
rQ
y1
b3 2+
b2
y2
b3
y5
y11 2+
Prec 2+
y7
b7
y8
y9
0
100
200
300
400
500
600
700
800
900
1,000
m/z

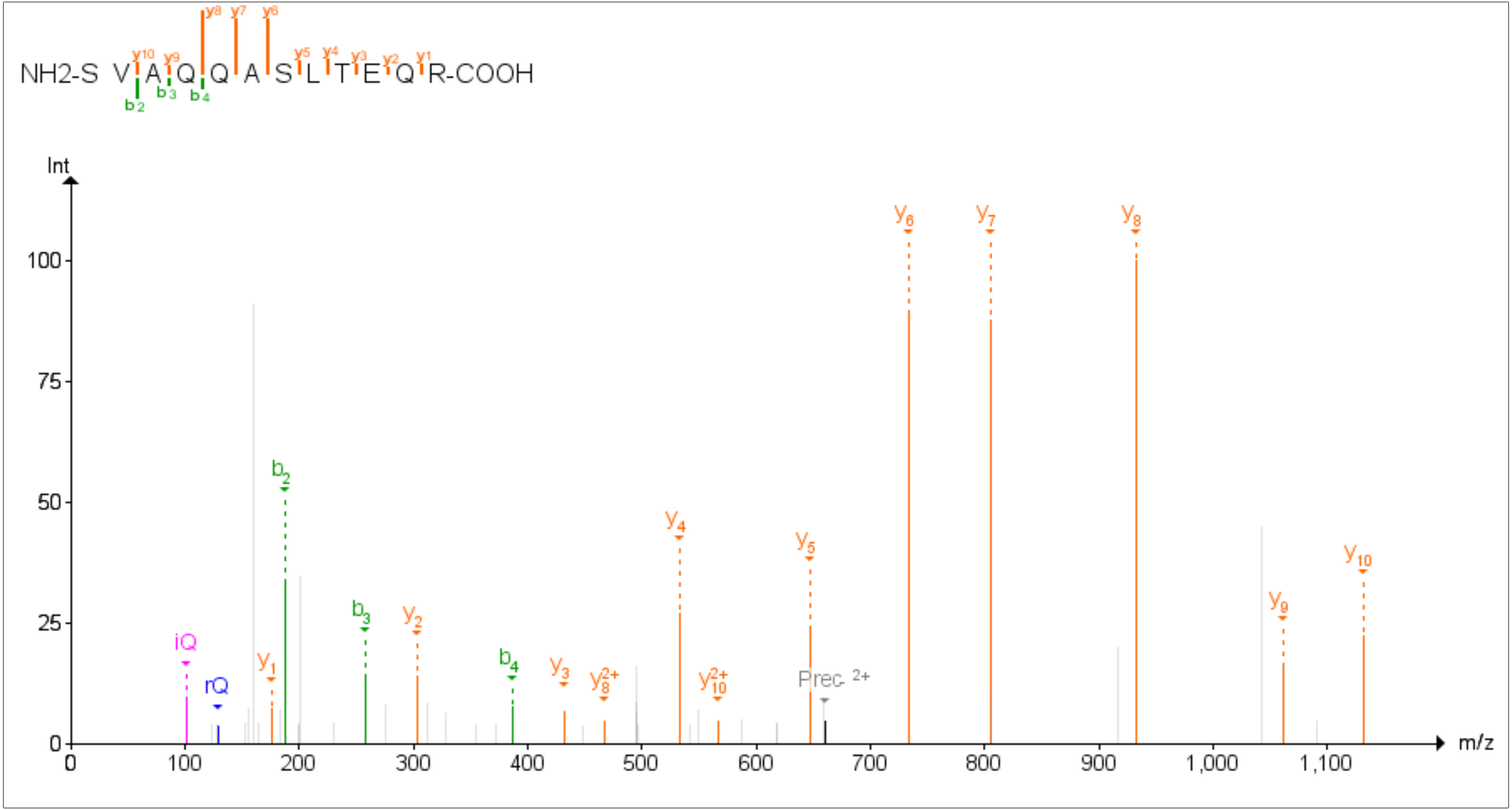

NH2-S V A Q Q A S L T E Q R-COOH
y10 y9 y8 y7 y6 y5 y4 y3 y2 y1
b2 b3 b4
Int
100
75
50
25
0
m/z
0 100 200 300 400 500 600 700 800 900 1,000 1,100
iQ
rQ
y1
b2
b3
y2
b4
y3
y8 2+
y10 2+
y4
y5
Prec 2+
y6
y7
y8
y9
y10

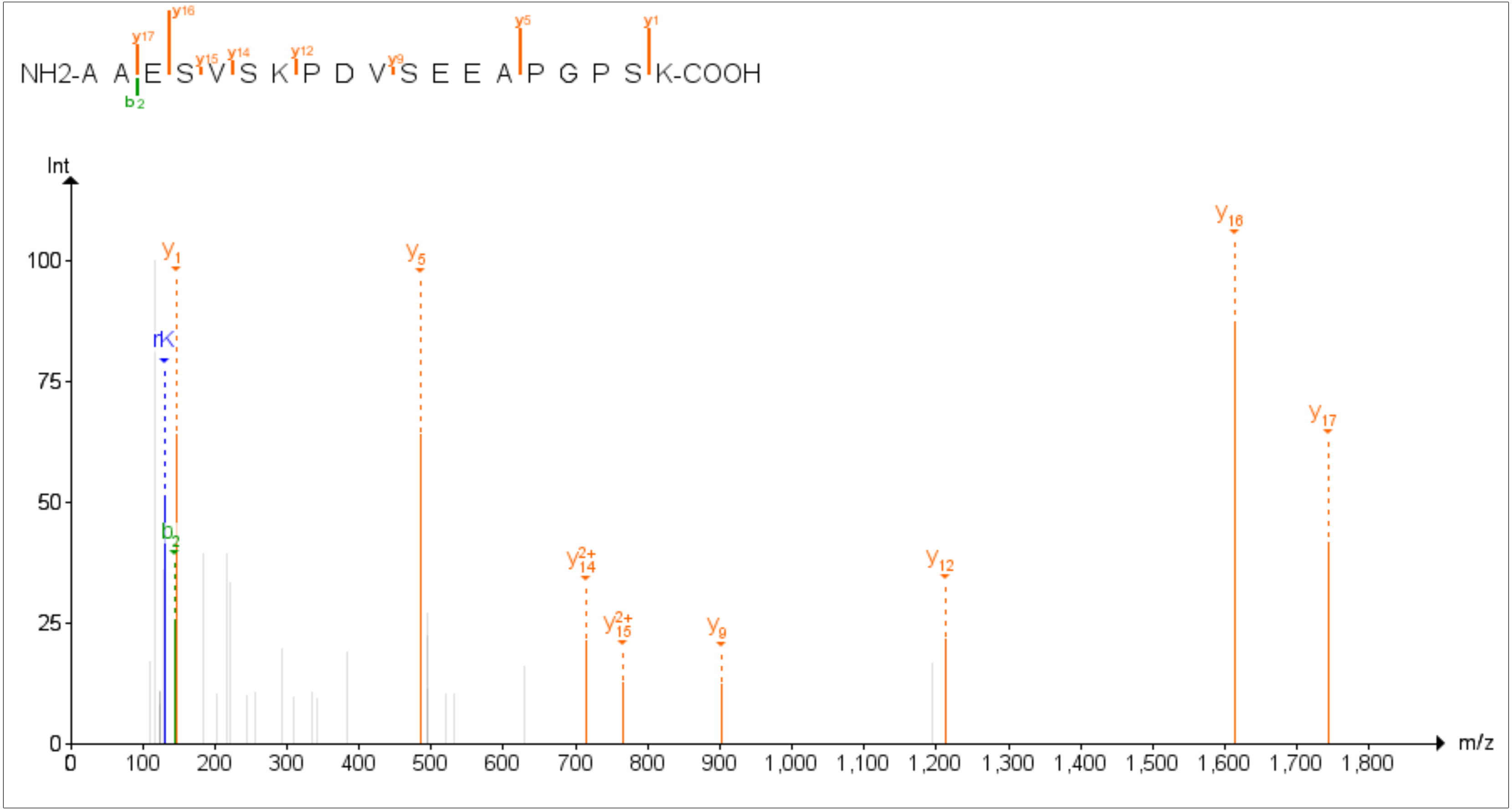
NH2-A A E S V S K P D V S E E A P G P S K-COOH
y17 y16 y15 y14 y12 y9 y5 y1
b2
Int
100
75
50
25
0
y1 rK b2 y5 y14 2+ y15 2+ y9 y12 y16 y17
0 100 200 300 400 500 600 700 800 900 1,000 1,100 1,200 1,300 1,400 1,500 1,600 1,700 1,800
m/z

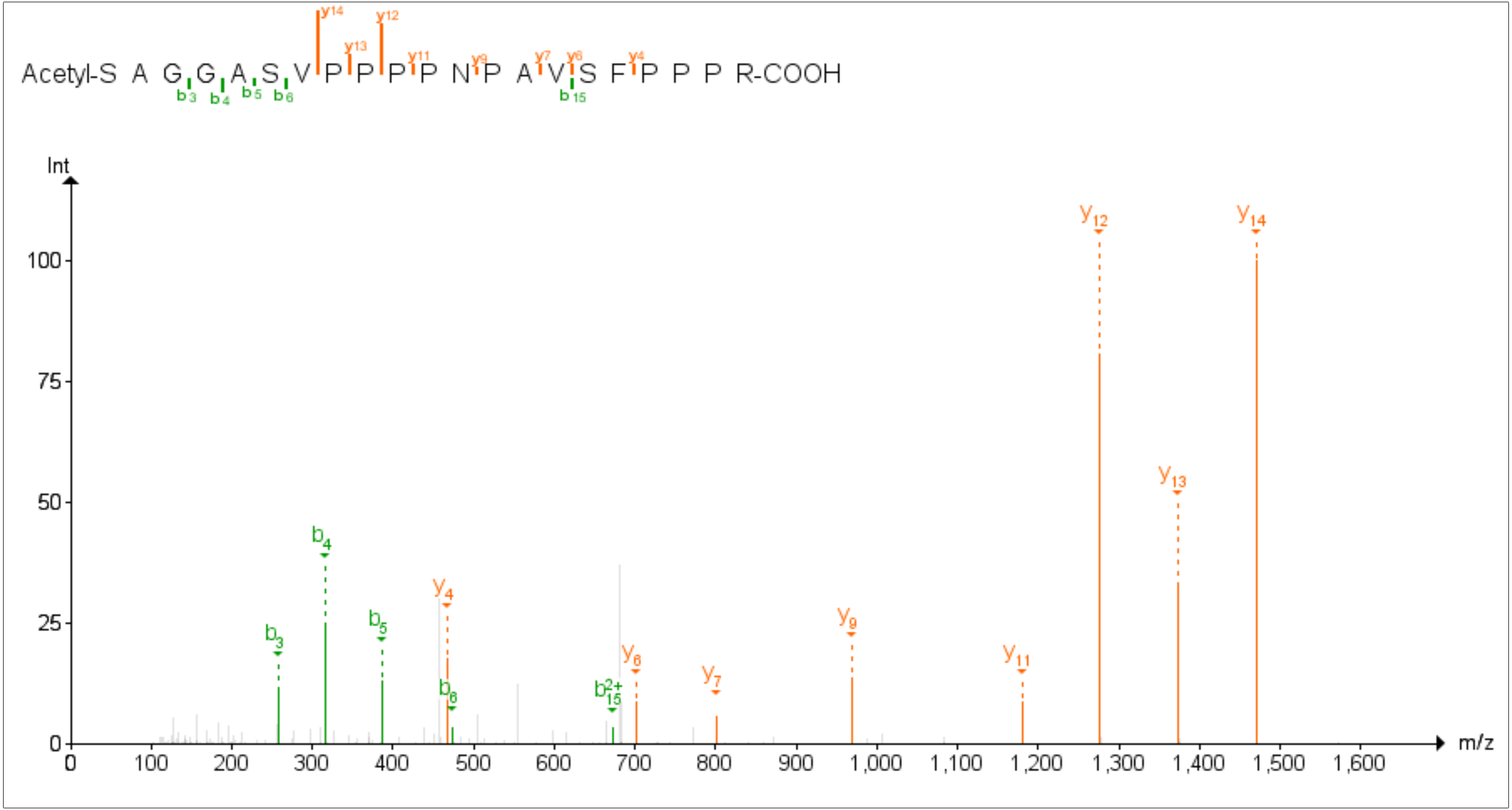
Acetyl-S A G G A S V P P P P N P A V S F P P P R-COOH
Int
m/z
y12
y14
y13
b4
y4
b5
b3
y9
b6
b2+ 15
y6
y7
y11

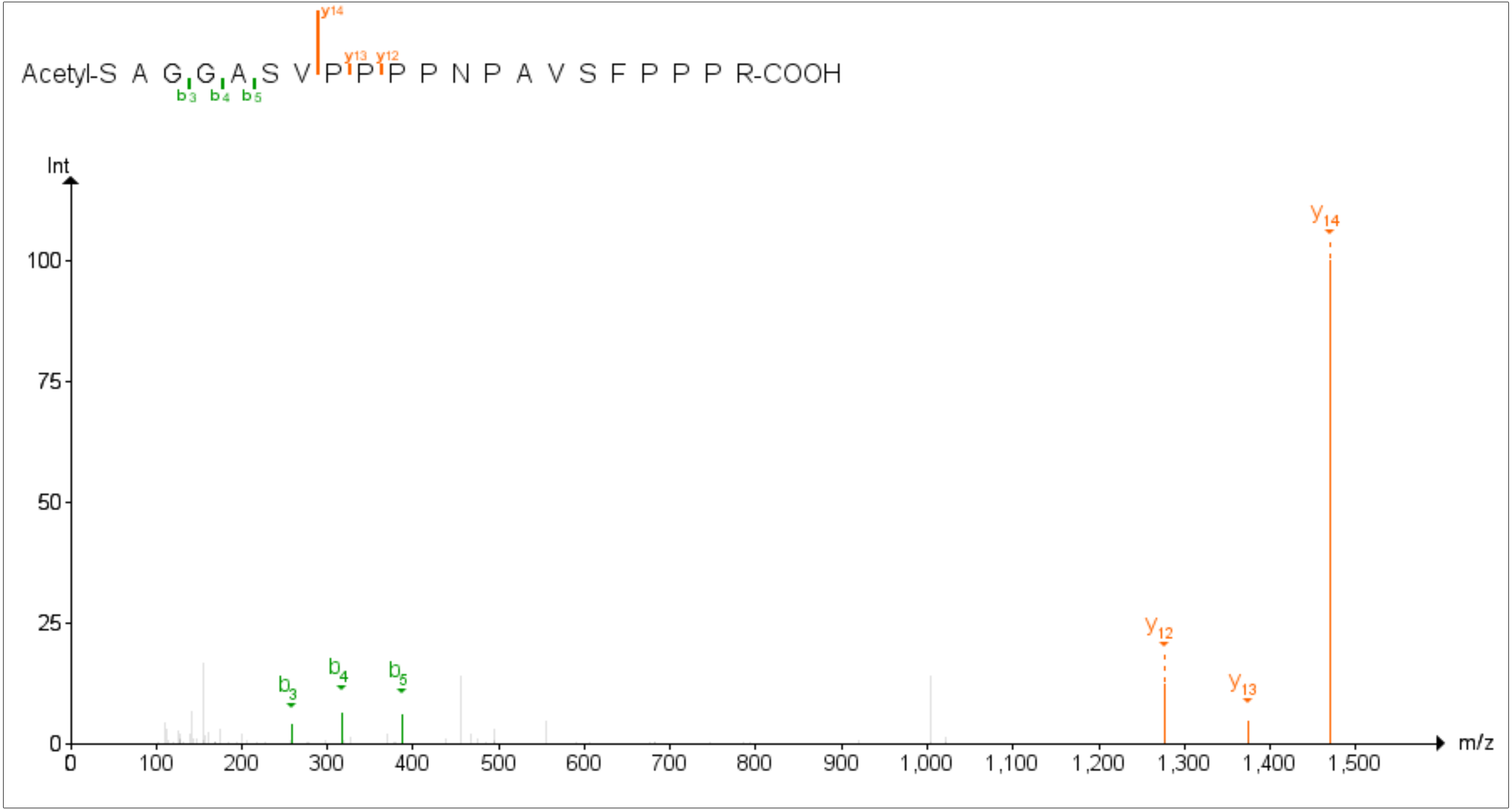

Acetyl-S A G G A S V P P P P N P A V S F P P P R-COOH
b3 b4 b5
y14 y13 y12
Int
100
75
50
25
0
0 100 200 300 400 500 600 700 800 900 1,000 1,100 1,200 1,300 1,400 1,500
m/z
b3 b4 b5
y12 y13 y14

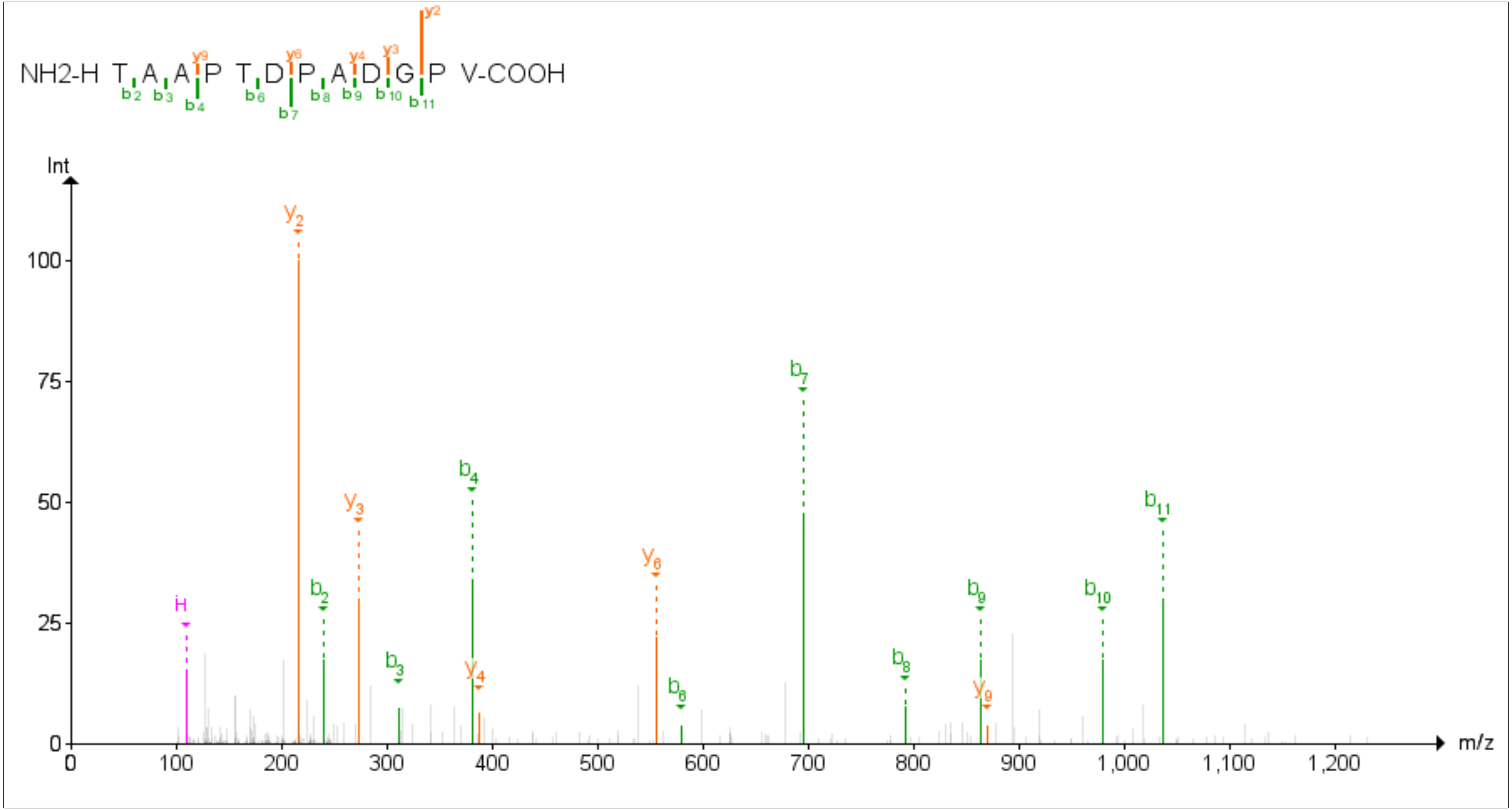
NH2-H T A A P T D P A D G P V-COOH
y9 y6 y4 y3 y2
b2 b3 b4 b6 b7 b8 b9 b10 b11
Int
100
75
50
25
0
m/z
0
100
200
300
400
500
600
700
800
900
1,000
1,100
1,200
H
y2
y3
y4
y6
y9
b2
b3
b4
b6
b7
b8
b9
b10
b11

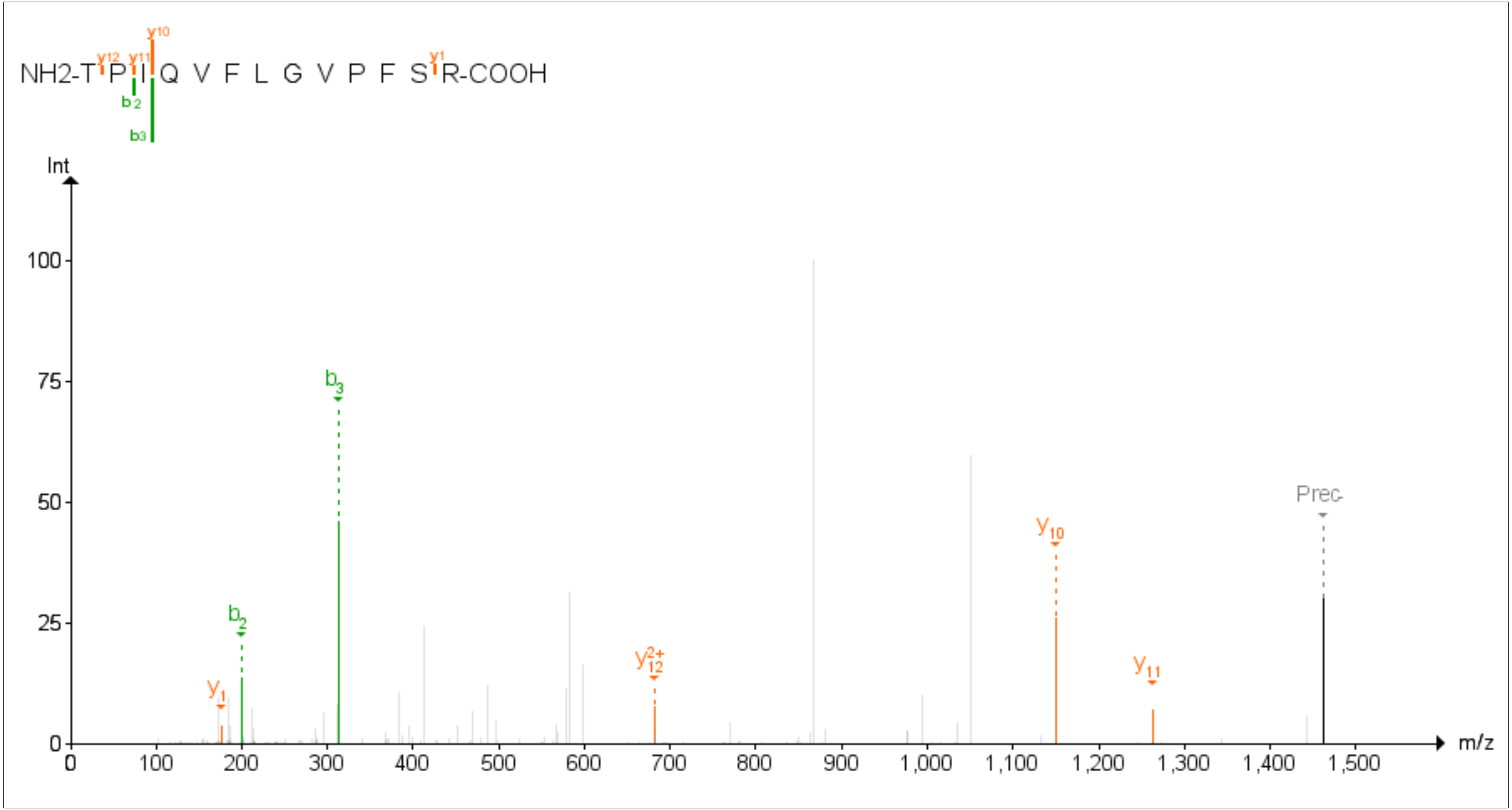

NH2-T P I Q V F L G V P F S R-COOH
y12 y11 y10 y1
b2 b3
Int
100
75
50
25
0
m/z
0 100 200 300 400 500 600 700 800 900 1,000 1,100 1,200 1,300 1,400 1,500
y1
b2
b3
y12 2+
y10
y11
Prec

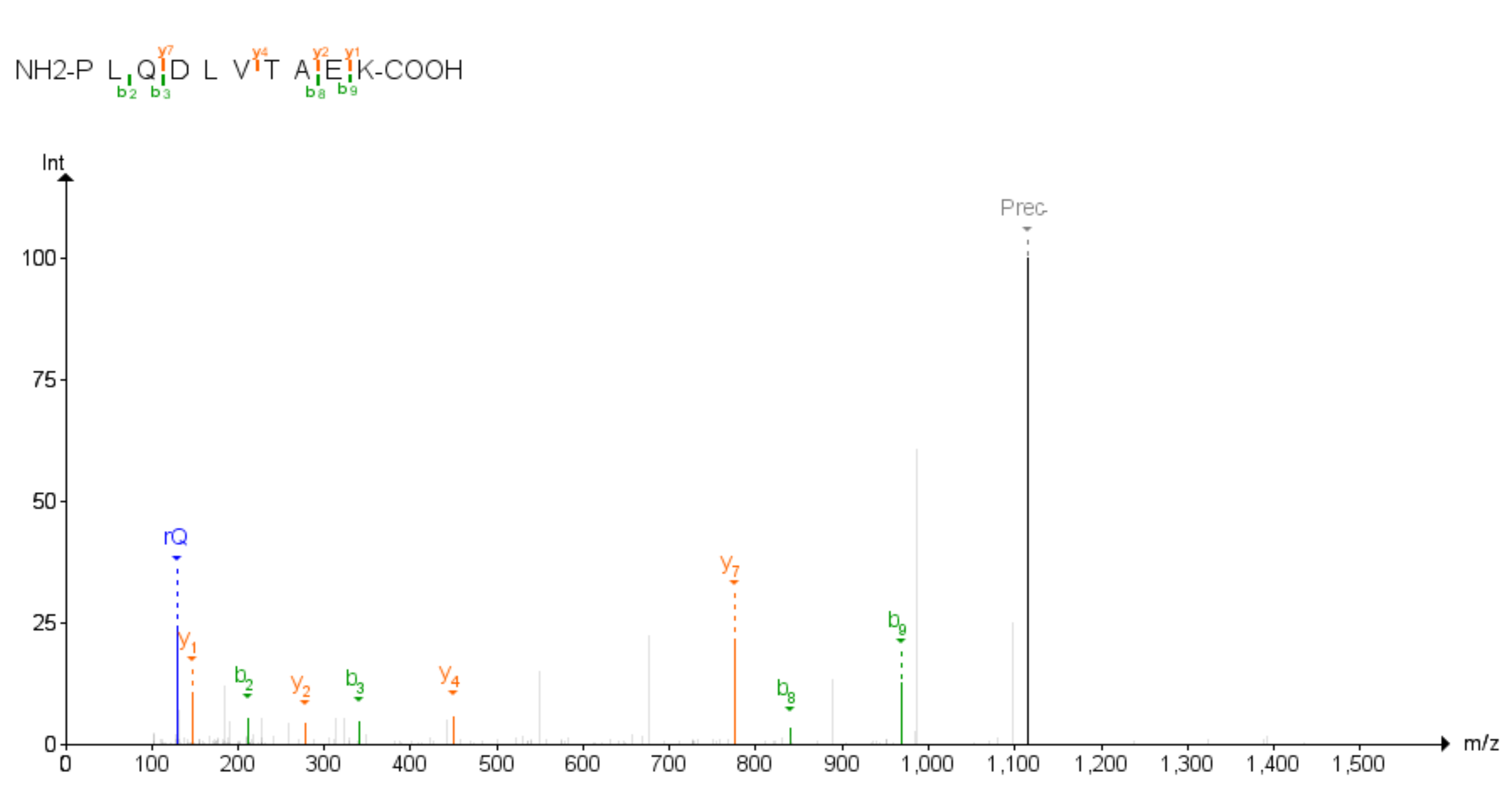

NH2-P L Q D L V T A E K-COOH
y7 y4 y2 y1
b2 b3 b8 b9
Int
Prec
100
75
50
25
0
rQ
y1
b2
y2
b3
y4
y7
b8
b9
0 100 200 300 400 500 600 700 800 900 1,000 1,100 1,200 1,300 1,400 1,500
m/z

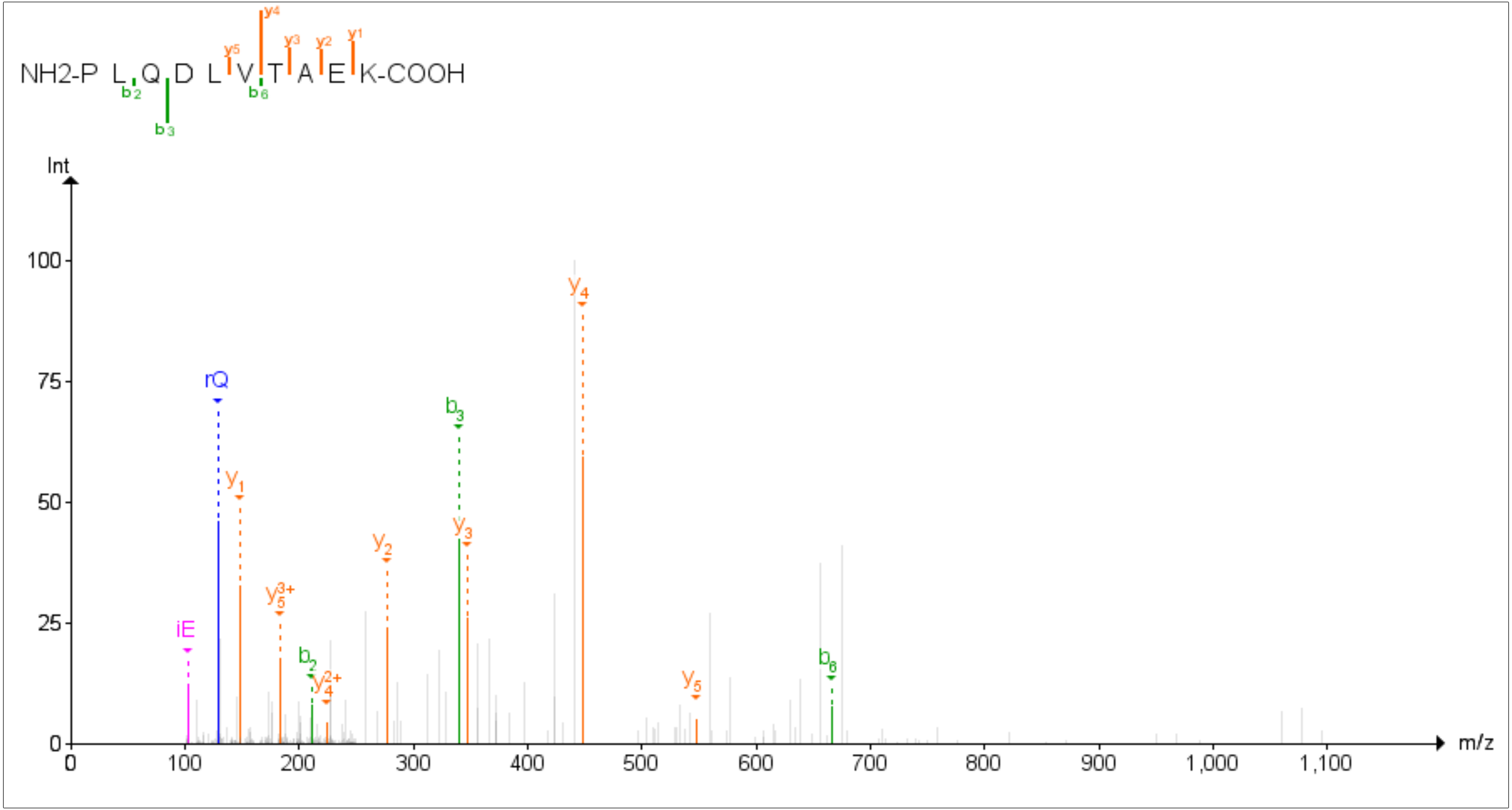

NH2-P L Q D L V T A E K-COOH
y4
y5
y3
y2
y1
b2
b6
b3
Int
100
75
50
25
0
rQ
y1
y5 3+
iE
b2
y4 2+
y2
b3
y3
y4
y5
b6
0
100
200
300
400
500
600
700
800
900
1,000
1,100
m/z

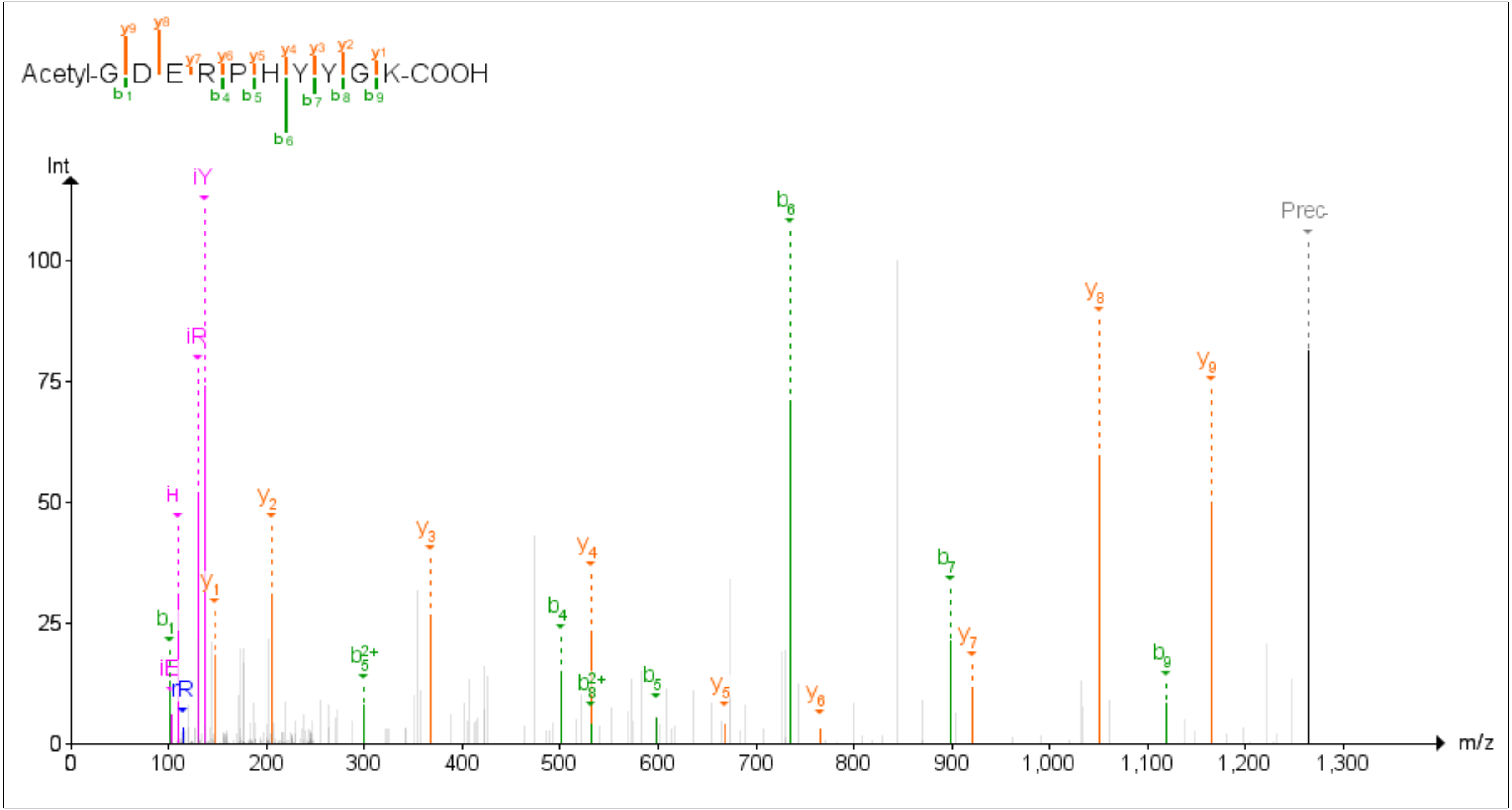

Acetyl-G D E R P H Y Y G K-COOH
y9 y8 y7 y6 y5 y4 y3 y2 y1
b1 b4 b5 b6 b7 b8 b9
Int
m/z
Prec
iY
iR
iH
iE
rR

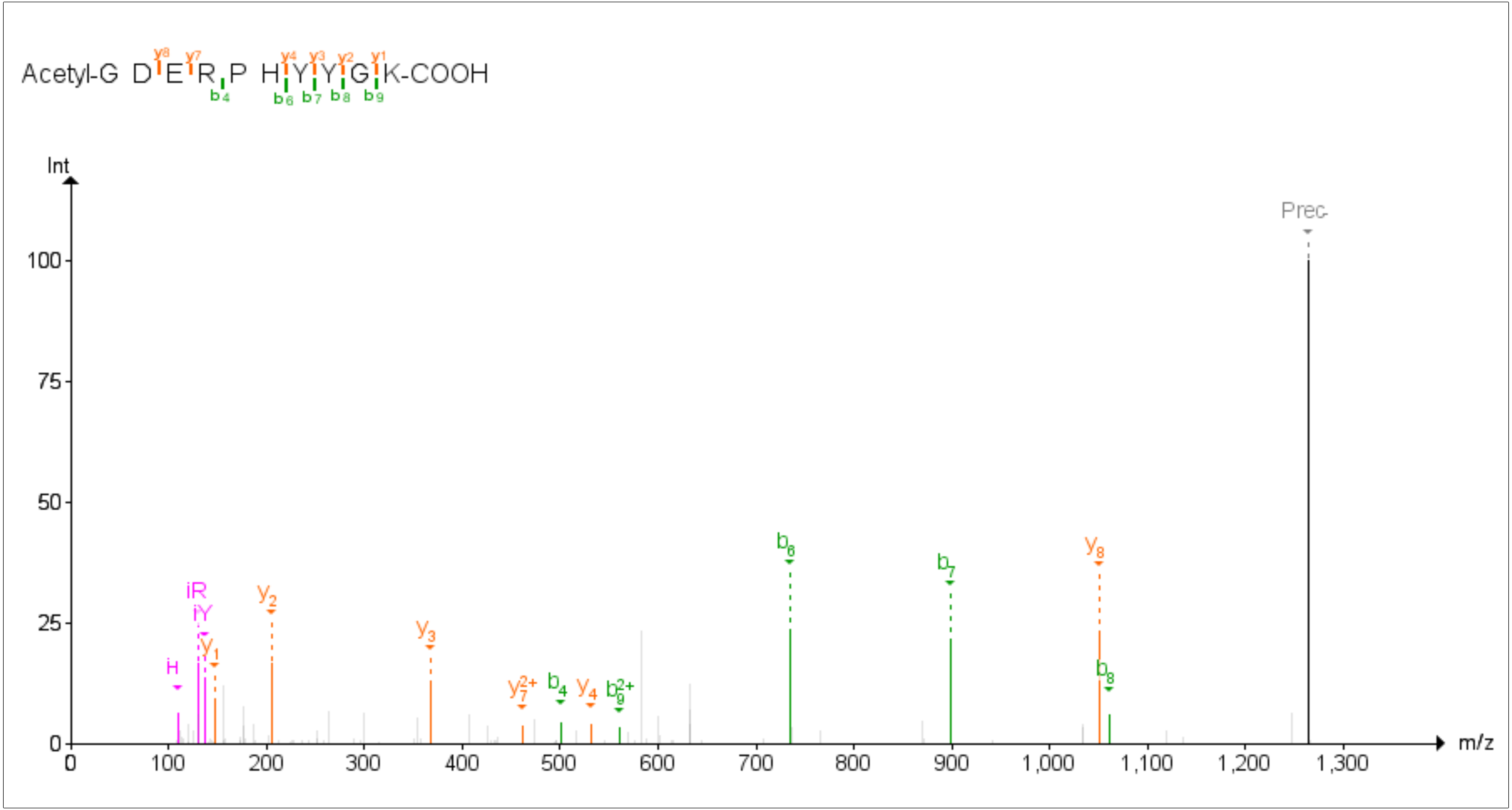

Acetyl-G D E R P H Y Y G K-COOH
y8 y7 y4 y3 y2 y1
b4 b6 b7 b8 b9
Int
100
75
50
25
0
Prec
b6
b7
y8
b8
iR
iY
y1
iH
y2
y3
y7 2+
b4
y4
b9 2+
0 100 200 300 400 500 600 700 800 900 1,000 1,100 1,200 1,300
m/z

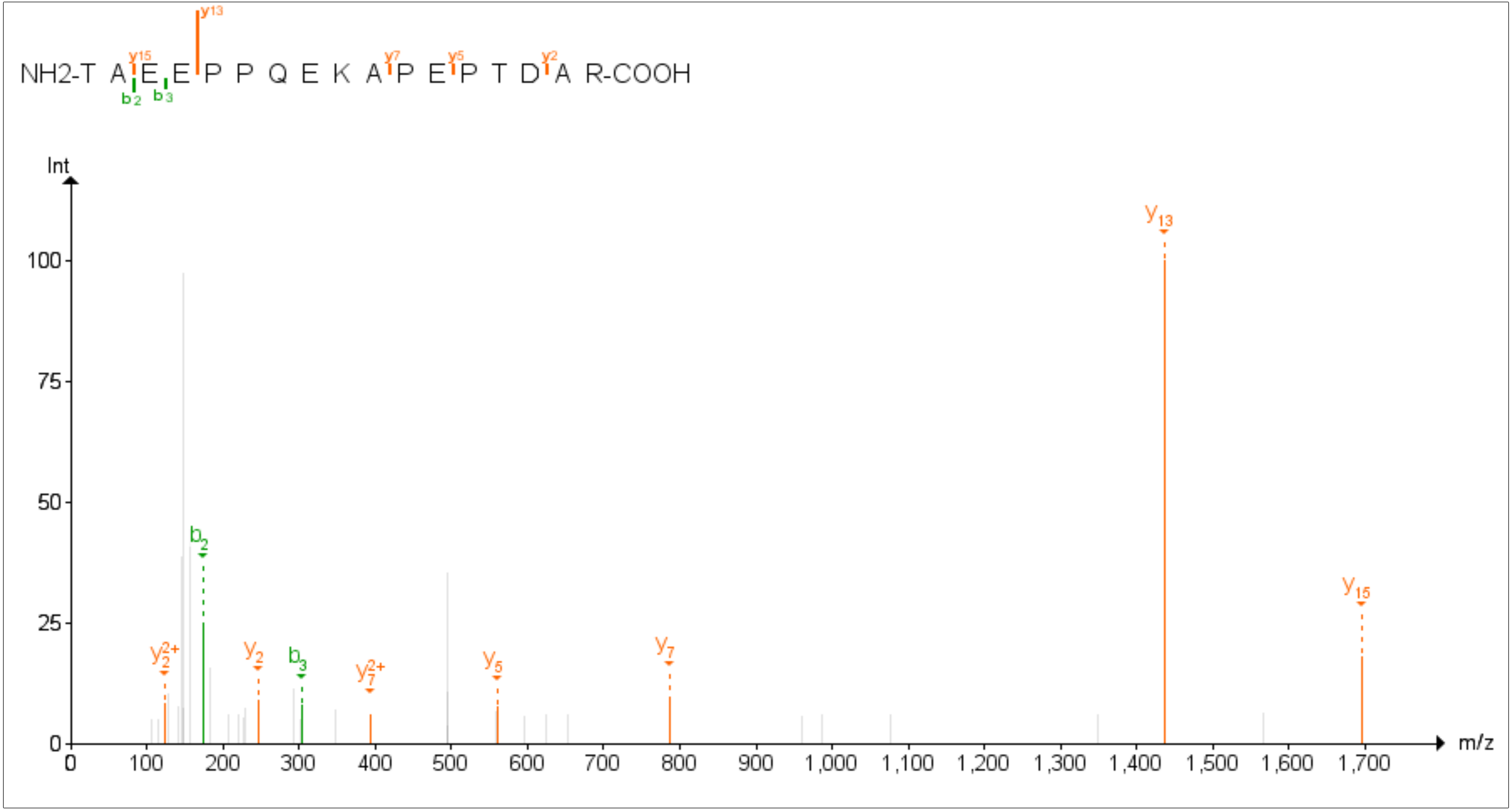

NH2-T A E E P P Q E K A P E P T D A R-COOH
y13
y15
y7
y5
y2
b2
b3
Int
m/z
y_{13}
y_{15}
b_2
y_2^{2+}
y_2
b_3
y_7^{2+}
y_5
y_7
0
25
50
75
100
0
100
200
300
400
500
600
700
800
900
1,000
1,100
1,200
1,300
1,400
1,500
1,600
1,700

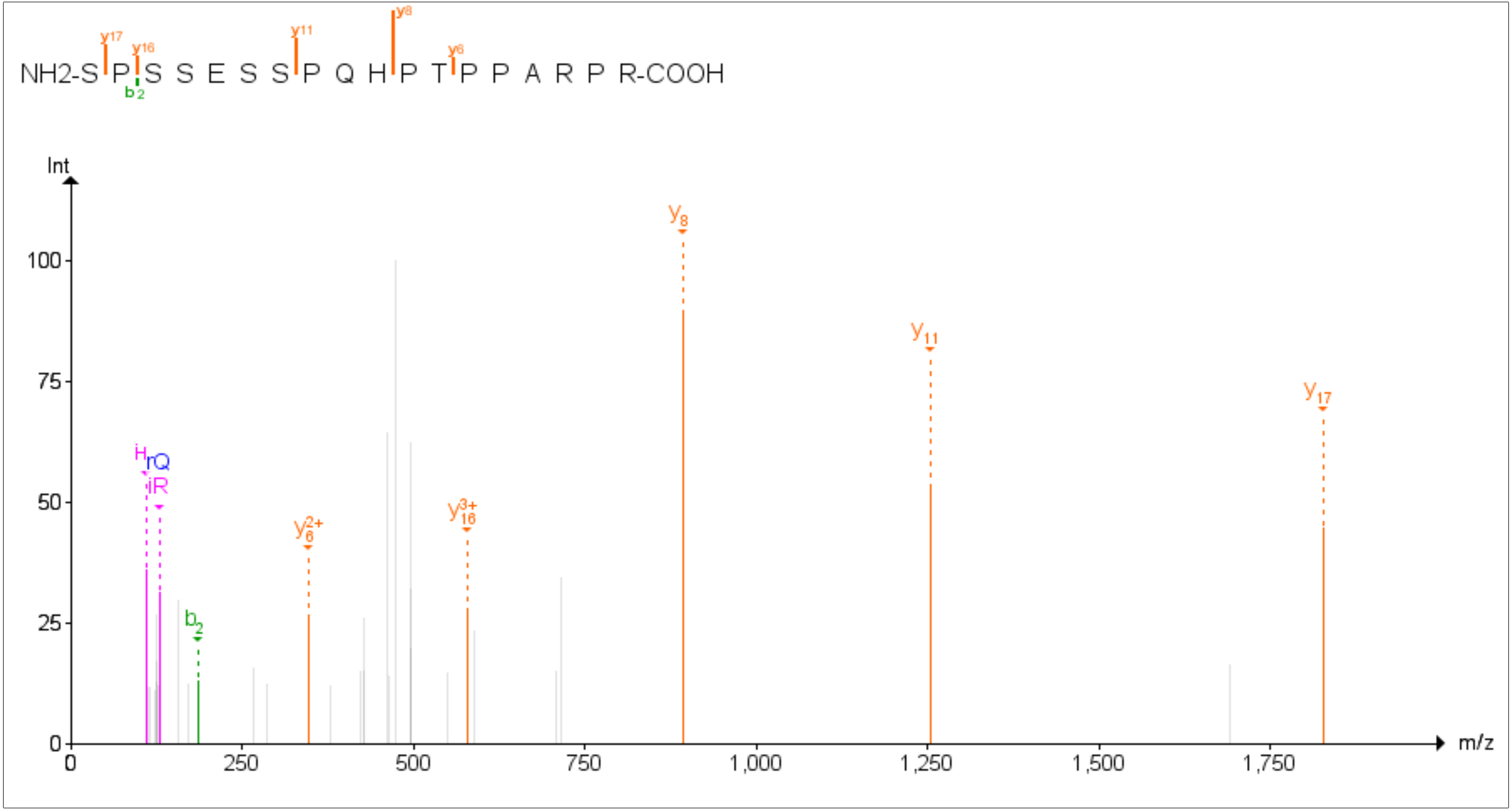

NH2-S P S S E S S P Q H P T P P A R P R-COOH
y17
y16
b2
y11
y8
y6
Int
100
75
50
25
0
y8
y11
y17
H
rQ
iR
b2
y6 2+
y16 3+
0
250
500
750
1,000
1,250
1,500
1,750
m/z

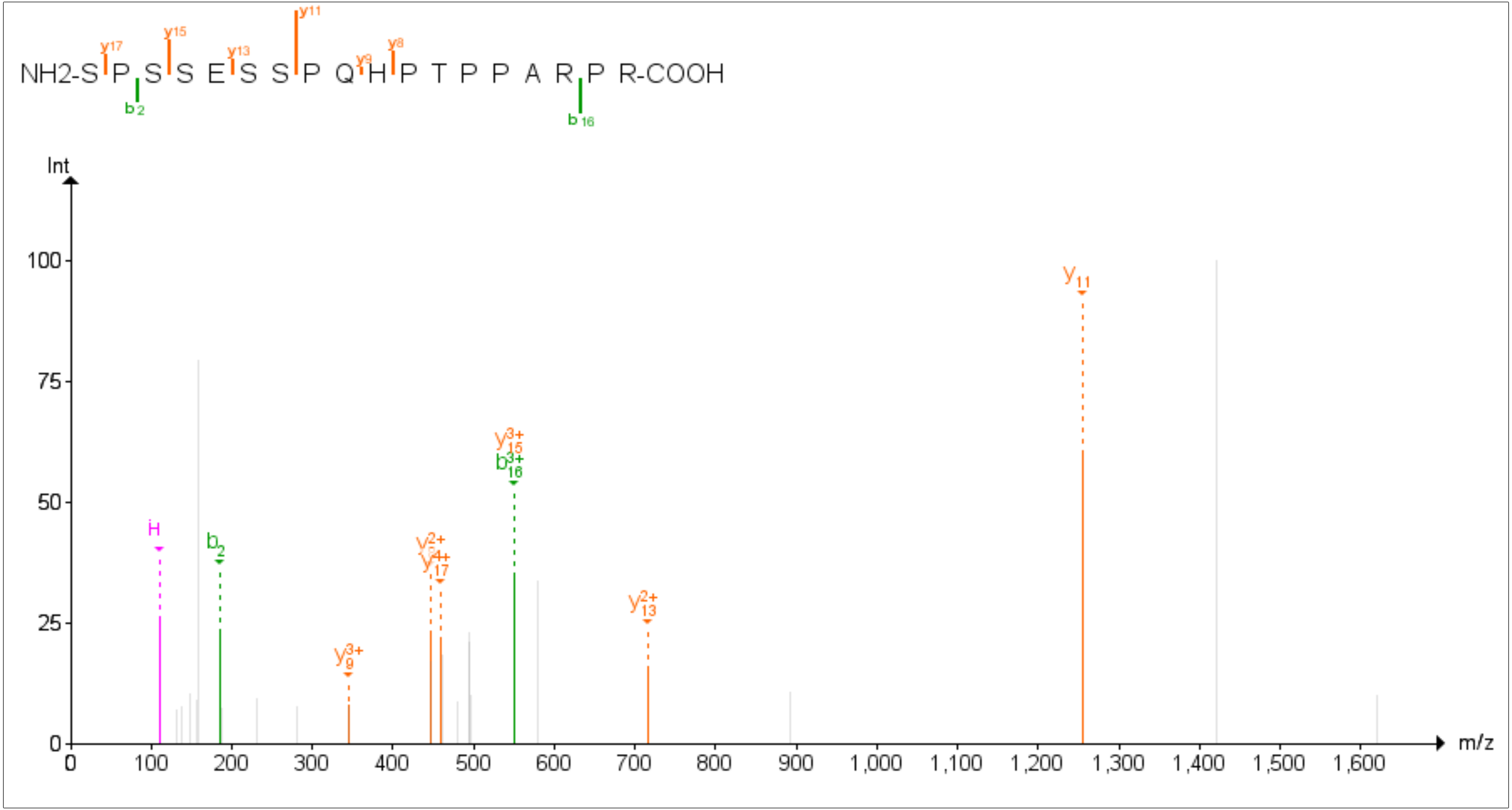

NH2-S P S S E S S P Q H P T P P A R P R-COOH
y17
y15
y13
y11
y9
y8
b 2
b 16
Int
100
75
50
25
0
iH
b2
y9 3+
y17 4+
y15 3+
b16 3+
y13 2+
y11
m/z
0
100
200
300
400
500
600
700
800
900
1,000
1,100
1,200
1,300
1,400
1,500
1,600

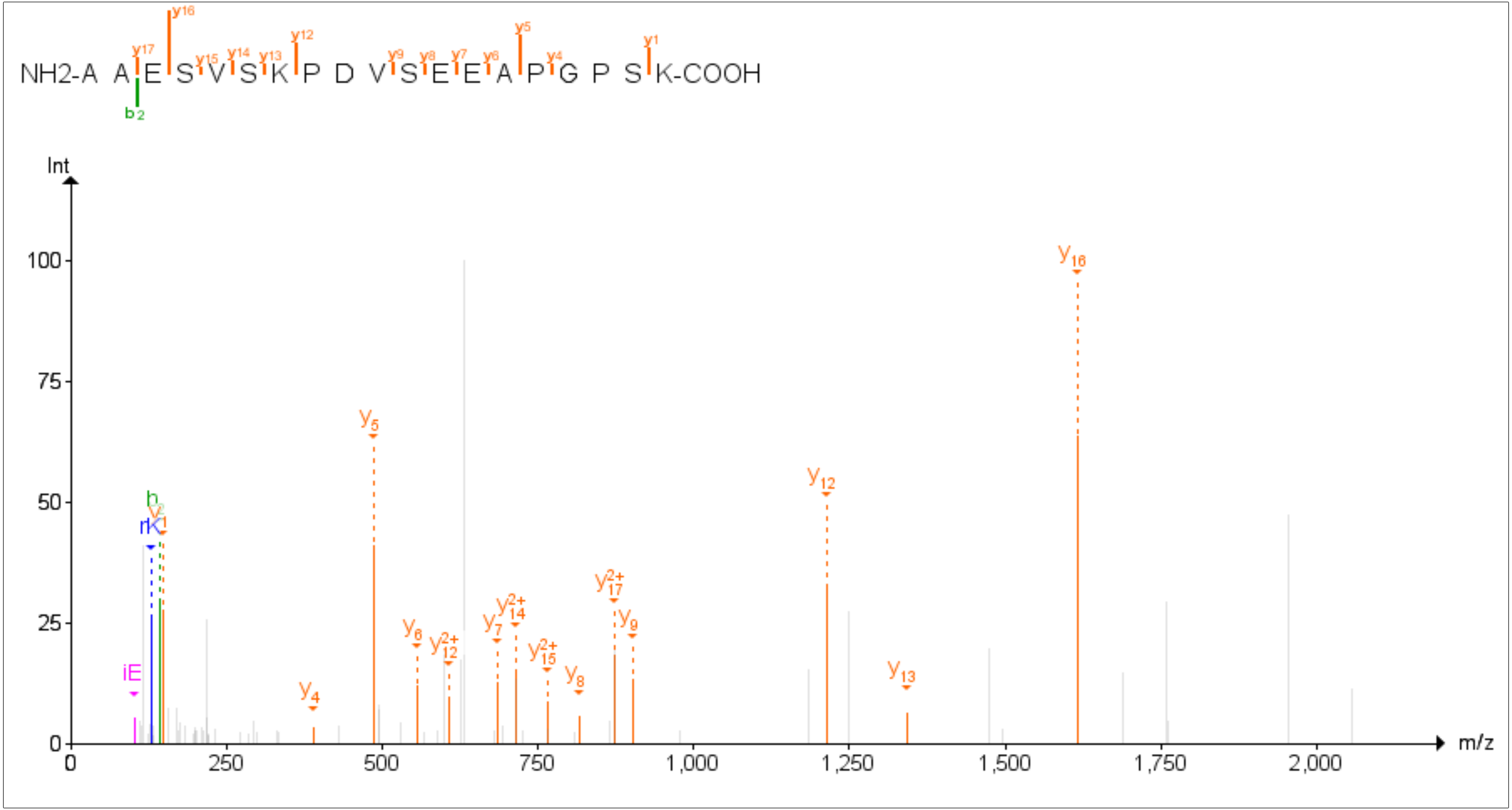

NH2-A A E S V S K P D V S E E A P G P S K-COOH
b2
y17 y16 y15 y14 y13 y12 y9 y8 y7 y6 y5 y4 y1
Int
100
75
50
25
0
0
250
500
750
1,000
1,250
1,500
1,750
2,000
m/z
y16
y5
y12
iE
rK
y4
y6
y7
y8
y9
y13

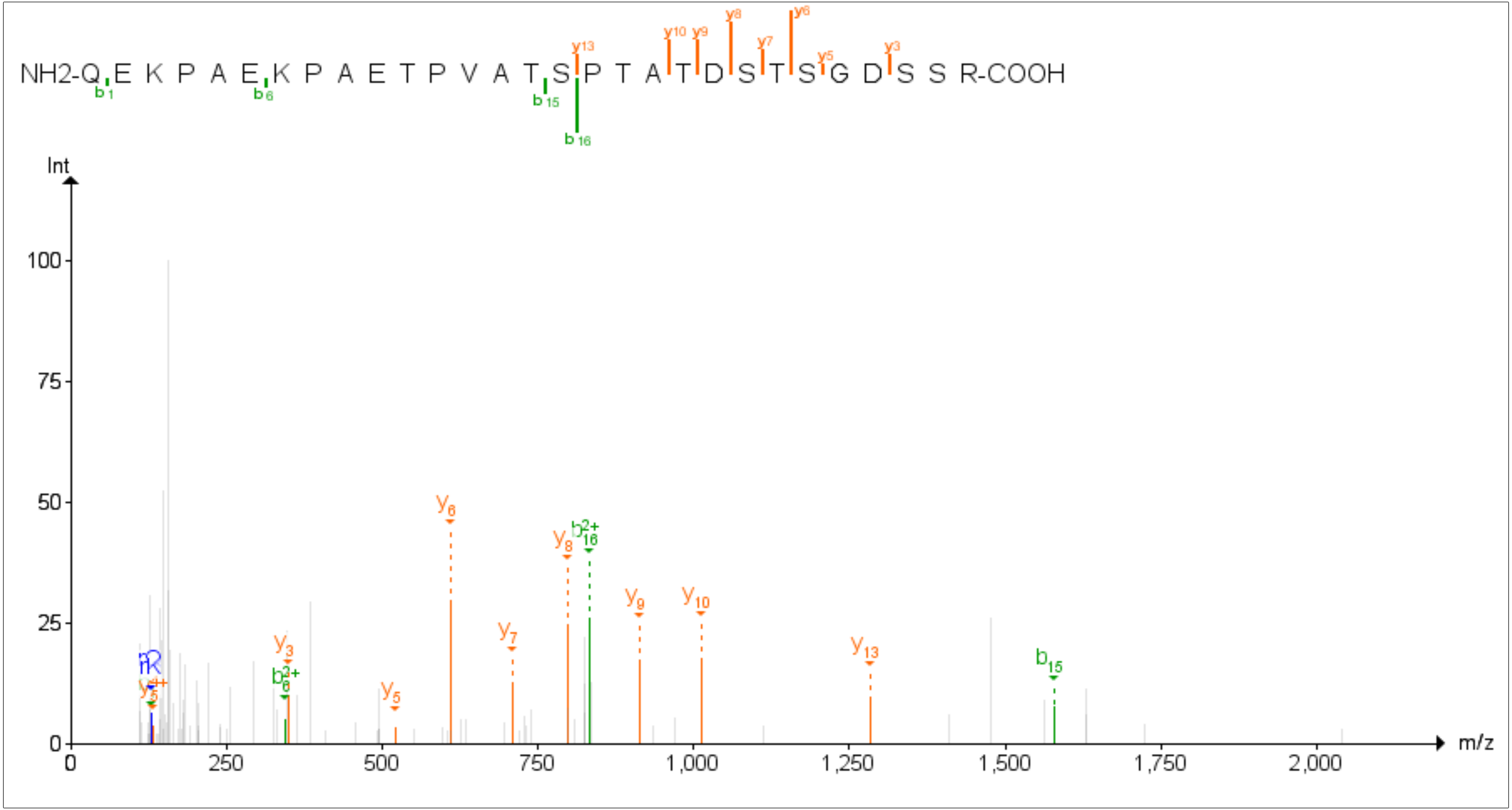

NH2-Q E K P A E K P A E T P V A T S P T A T D S T S G D S S R-COOH
Int
m/z

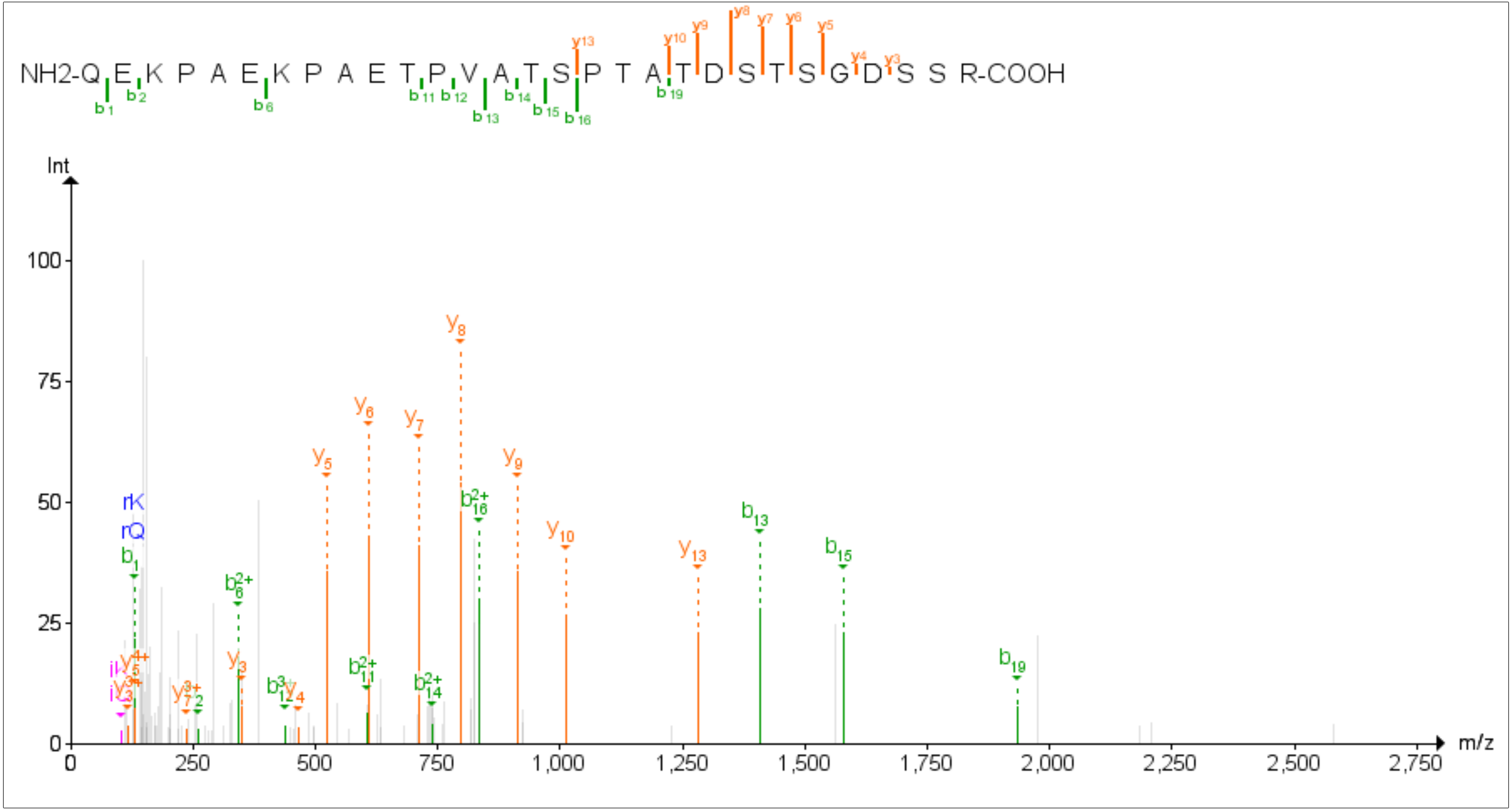

NH2-QEKPAEKPAETPVATSPTATDSTSGDSSR-COOH
Int
m/z
0
25
50
75
100
250
500
750
1,000
1,250
1,500
1,750
2,000
2,250
2,500
2,750
y8
y6
y7
y5
y9
y10
y13
b13
b15
b19
b1
rK
rQ
y3

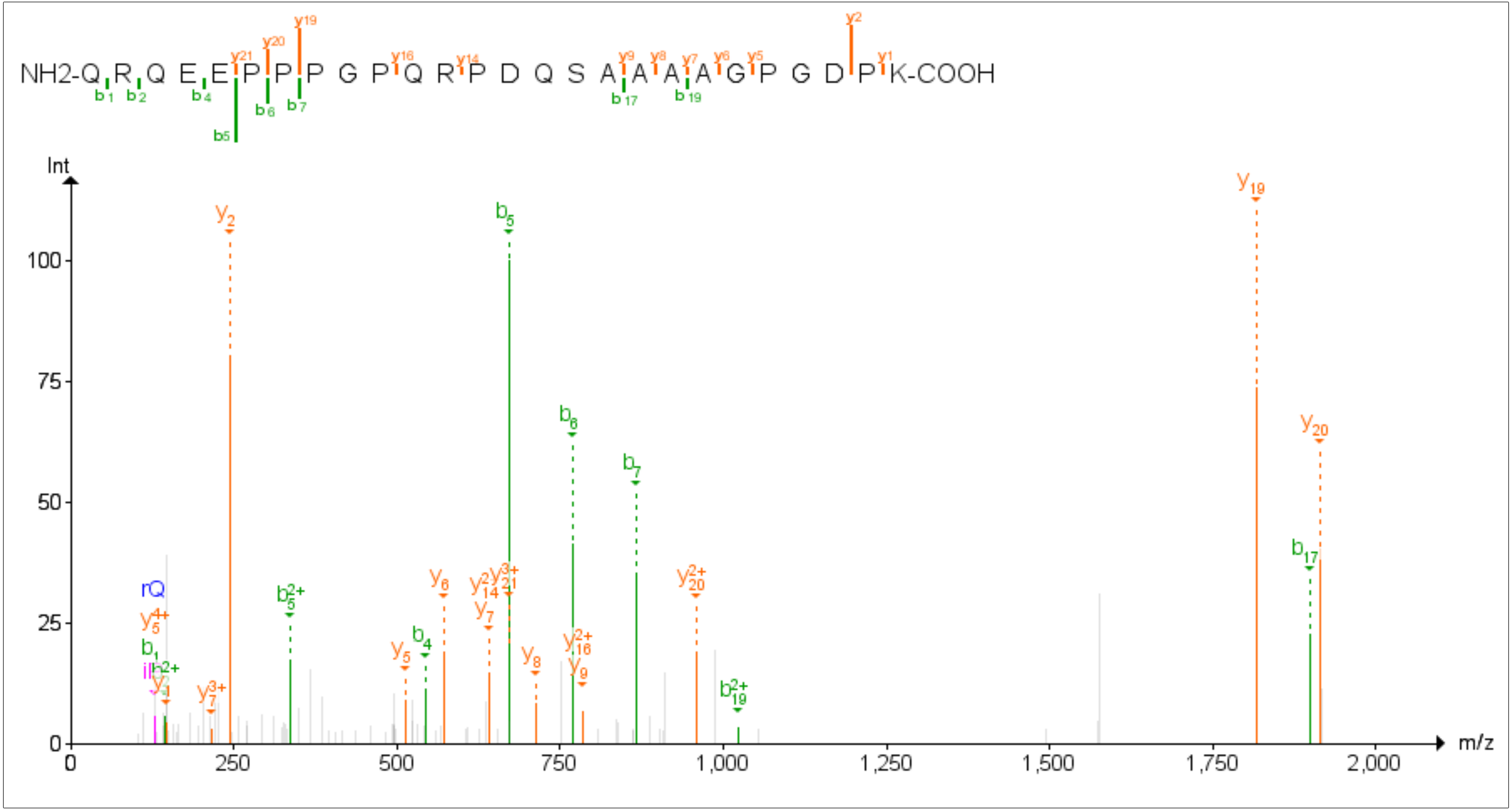

NH2-Q R Q E E P P P G P Q R P D Q S A A A A G P G D P K-COOH
Int
100
75
50
25
0
0
250
500
750
1,000
1,250
1,500
1,750
2,000
m/z

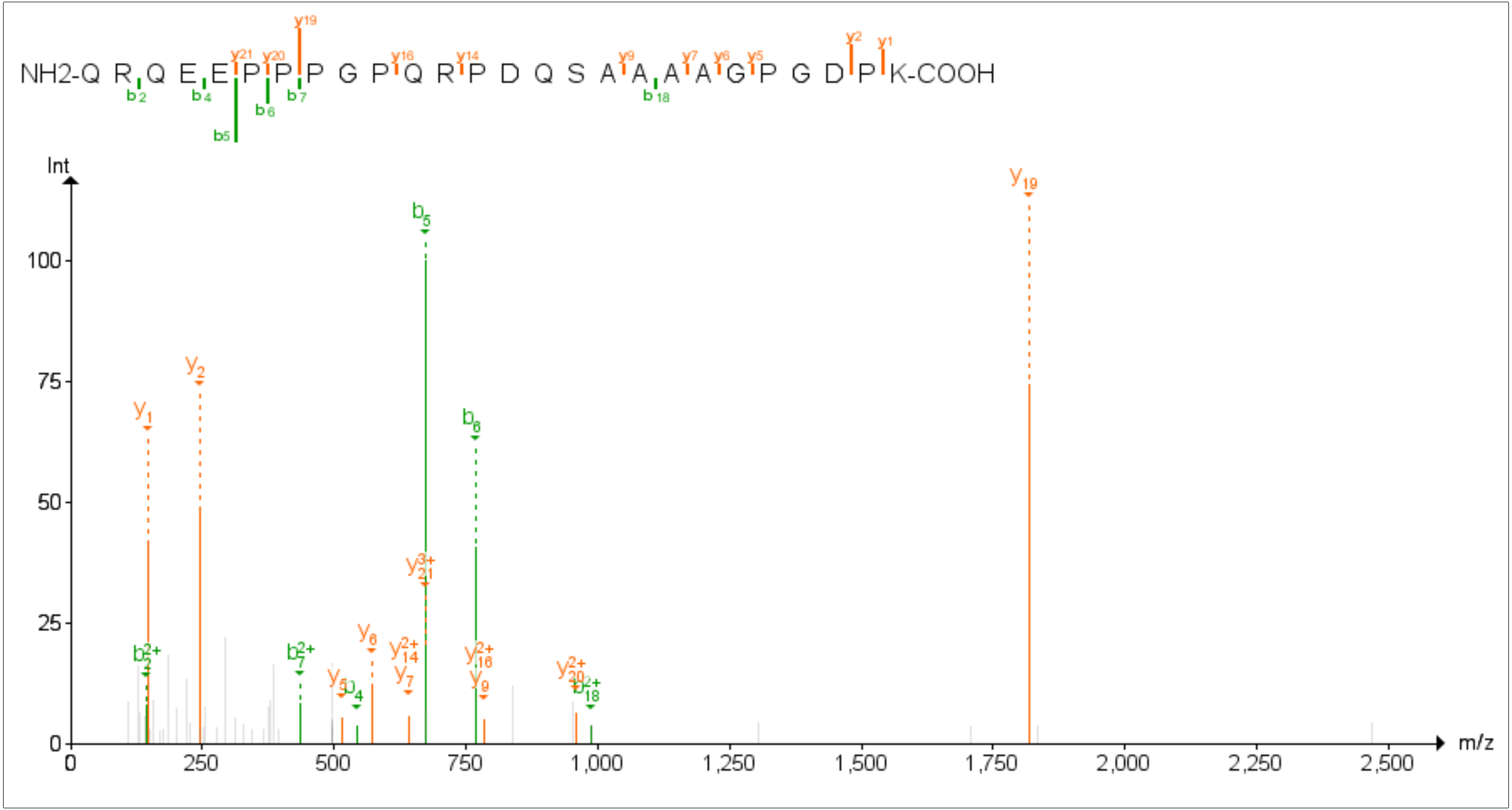

NH2-Q R Q E E P P P G P Q R P D Q S A A A A G P G D P K-COOH
Int
m/z
0
25
50
75
100
250
500
750
1,000
1,250
1,500
1,750
2,000
2,250
2,500

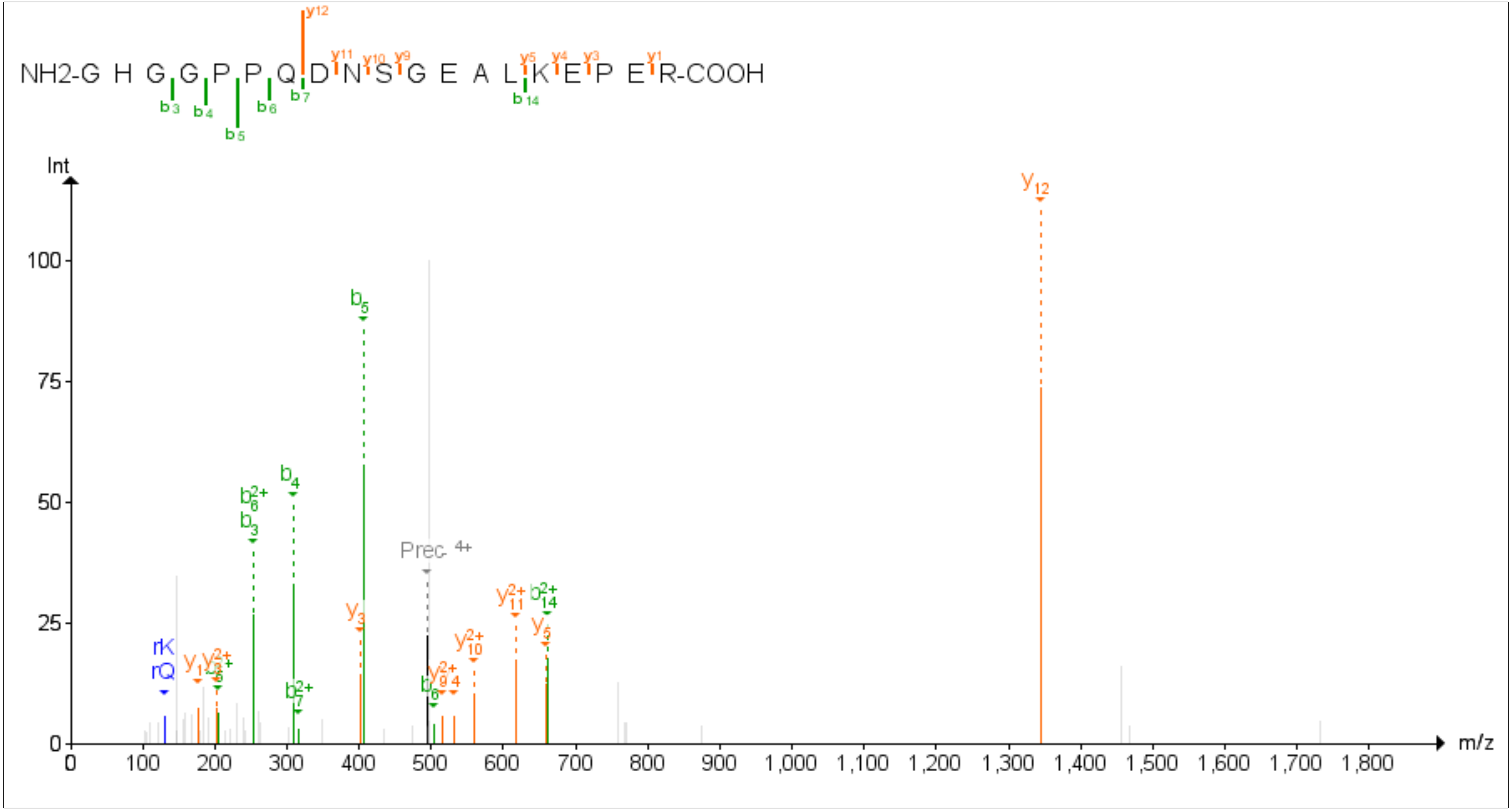

NH2-G H G G P P Q D N S G E A L K E P E R-COOH
b3
b4
b5
b6
b7
b14
y12
y11
y10
y9
y5
y4
y3
y1
Int
m/z
Prec 4+
rK
rQ

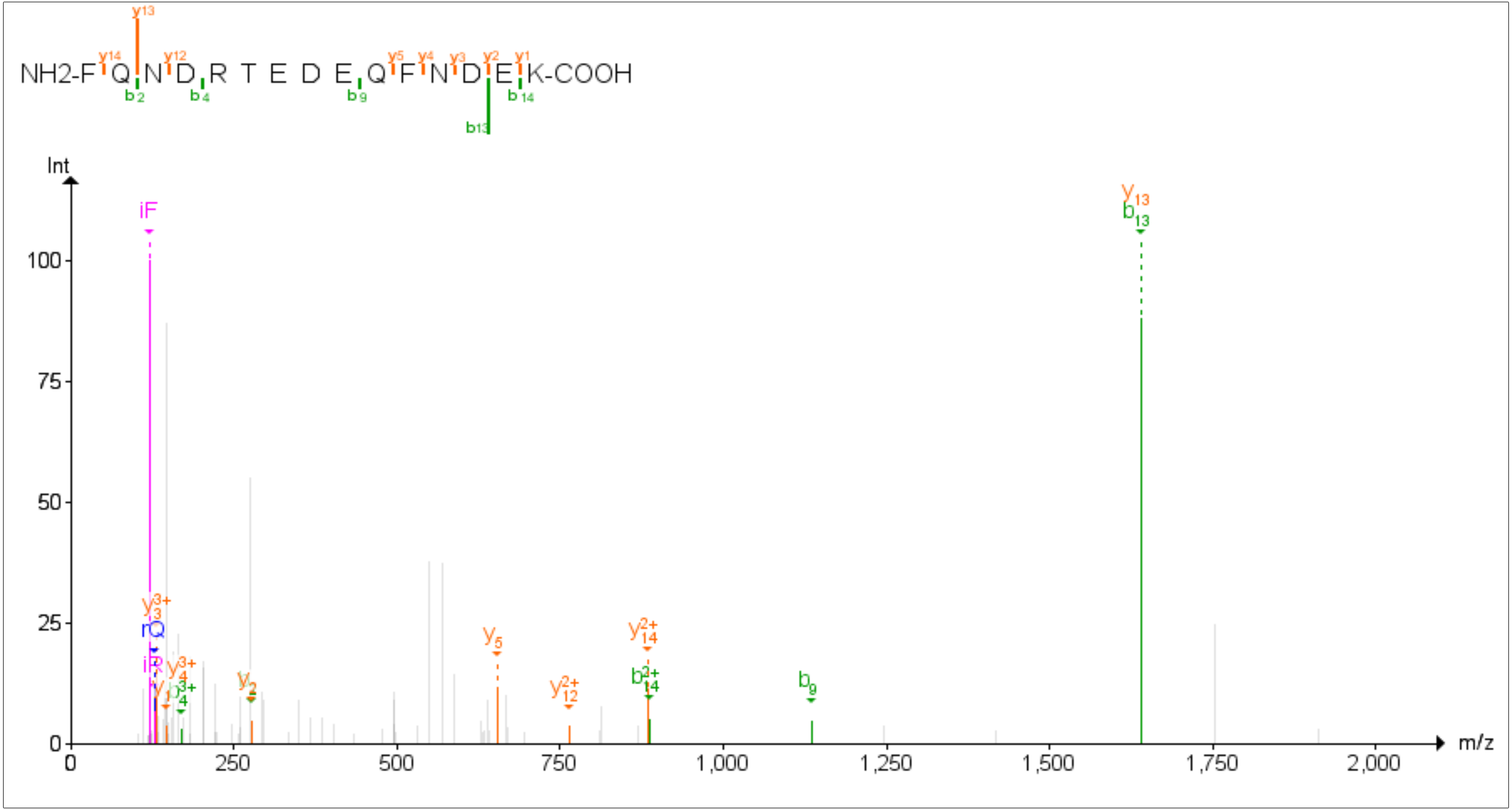

NH2-F Q N D R T E D E Q F N D E K-COOH
y14 y13 y12 y5 y4 y3 y2 y1
b2 b4 b9 b13 b14
Int
100
75
50
25
0
iF
rQ
iR
y_{13} b_{13}
y_3^{3+}
y_4^{3+}
b_4^{3+}
y_1
y_2
y_5
y_{12}^{2+}
y_{14}^{2+}
b_{14}^{2+}
b_9
0
250
500
750
1,000
1,250
1,500
1,750
2,000
m/z

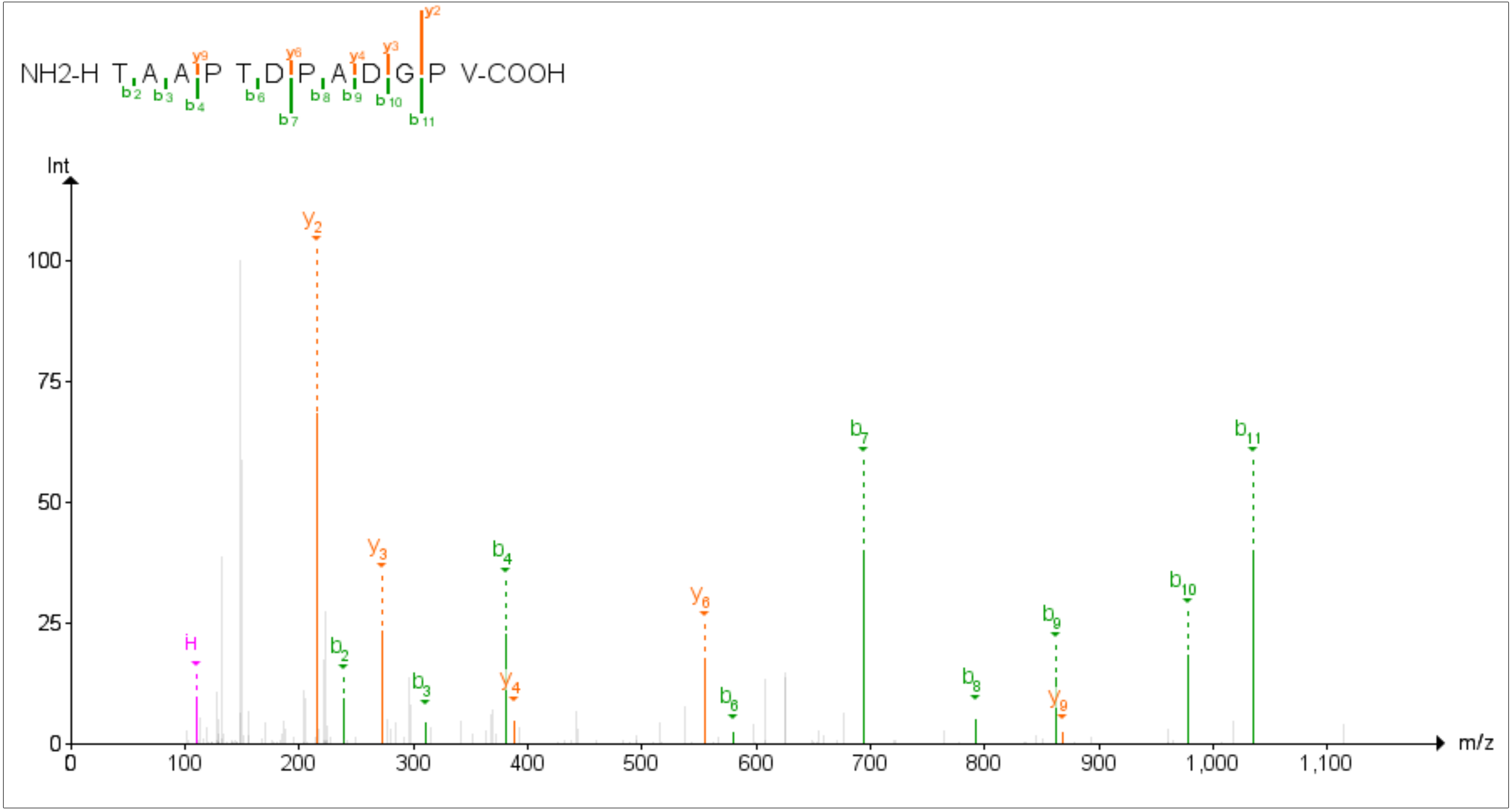

NH2-H T A A P T D P A D G P V-COOH
Int
m/z
y2
y3
b4
y4
y6
b6
b7
b8
b9
y9
b10
b11
b2
b3
H

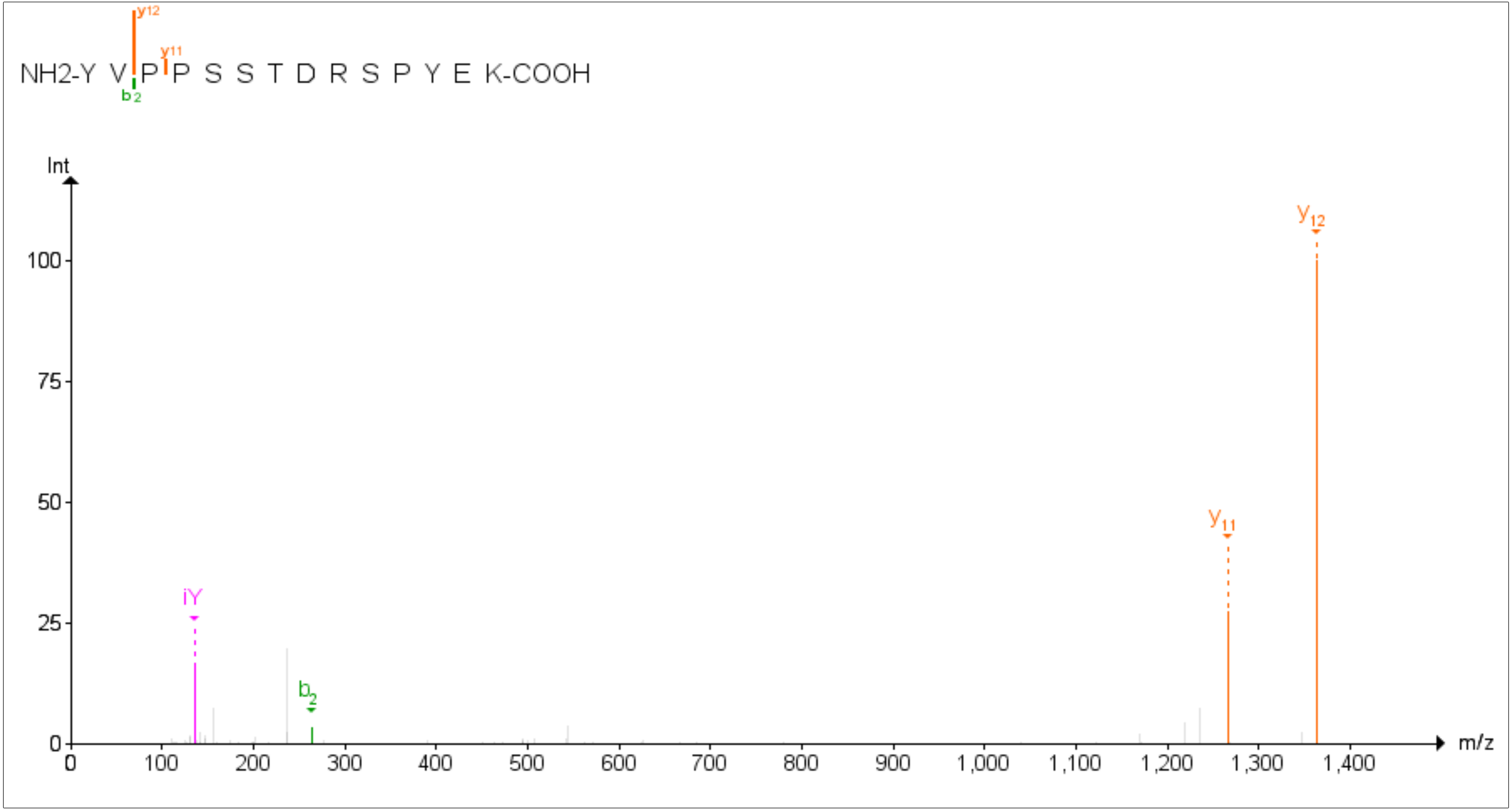

y12
y11
NH2-Y V P P S S T D R S P Y E K-COOH
b2
Int
100
75
50
25
0
iY
b2
y11
y12
0
100
200
300
400
500
600
700
800
900
1,000
1,100
1,200
1,300
1,400
m/z

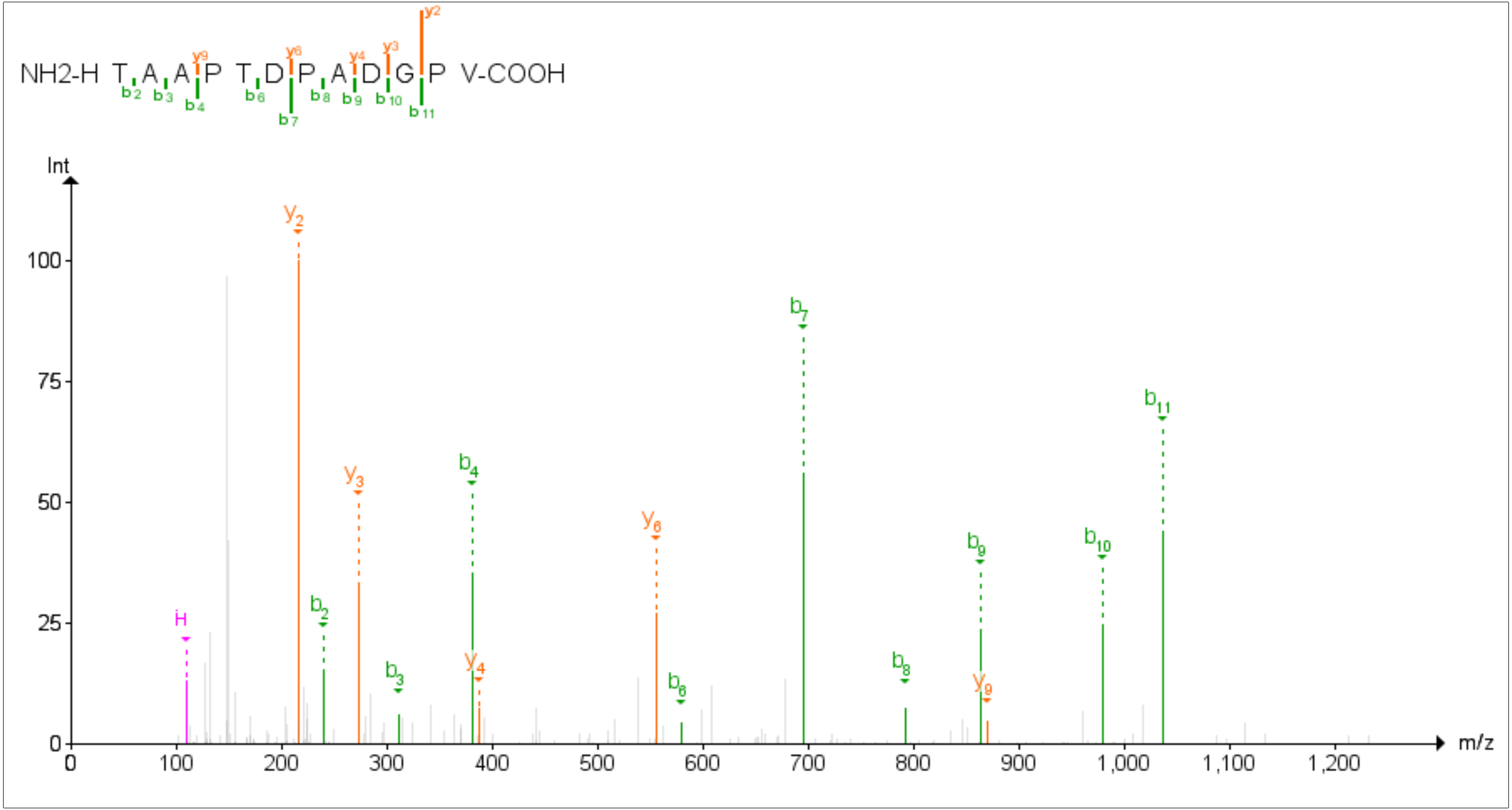

NH2-H T A A P T D P A D G P V-COOH
Int
m/z
y2
y3
y4
y6
y9
b2
b3
b4
b6
b7
b8
b9
b10
b11
H
0
25
50
75
100
0
100
200
300
400
500
600
700
800
900
1,000
1,100
1,200

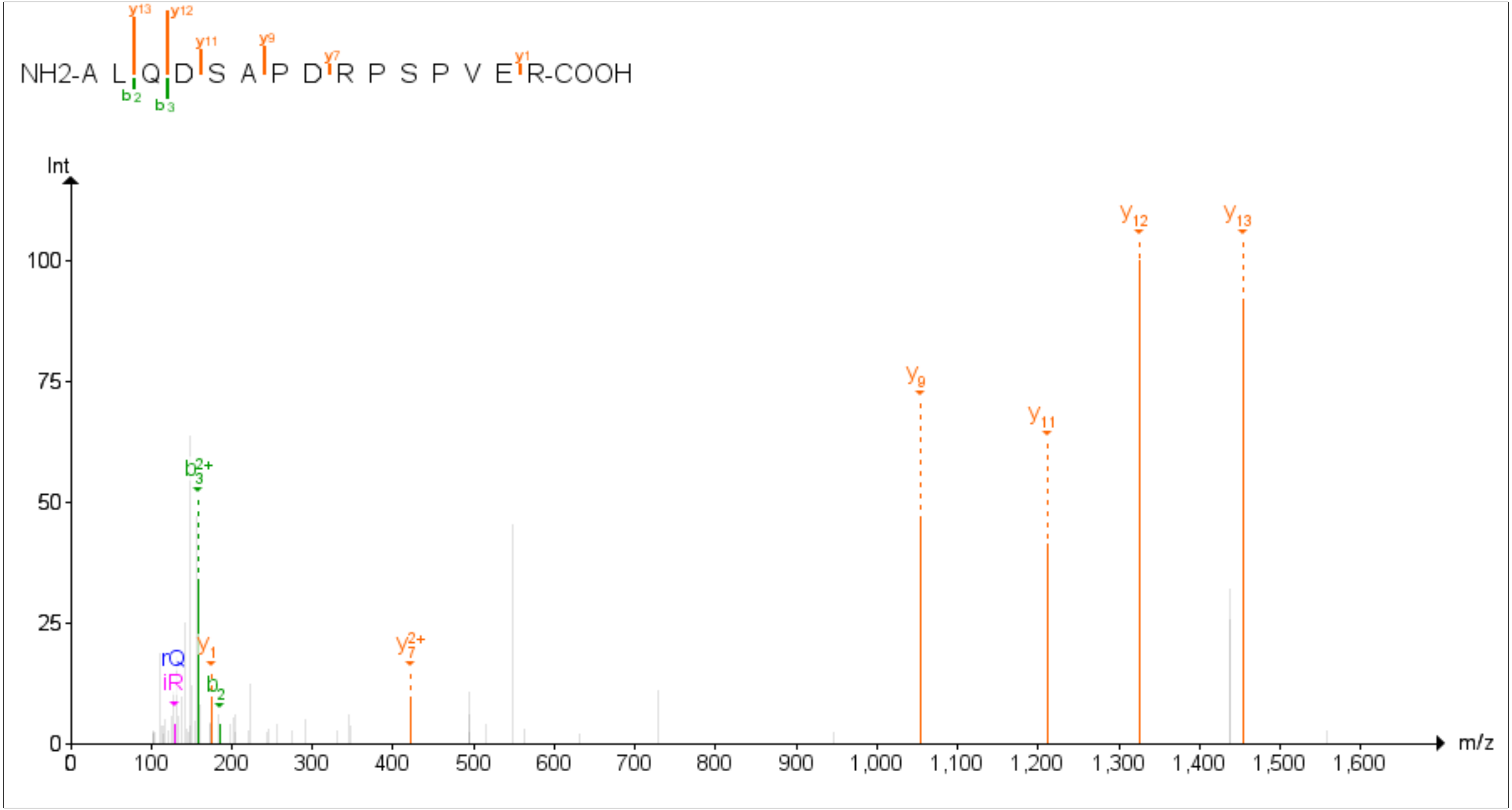

NH2-A L Q D S A P D R P S P V E R-COOH
y13 y12 y11 y9 y7 y1
b2 b3
Int
100
75
50
25
0
m/z
0 100 200 300 400 500 600 700 800 900 1,000 1,100 1,200 1,300 1,400 1,500 1,600
y12 y13 y9 y11
b3 2+
rQ
iR
y1
b2
y7 2+

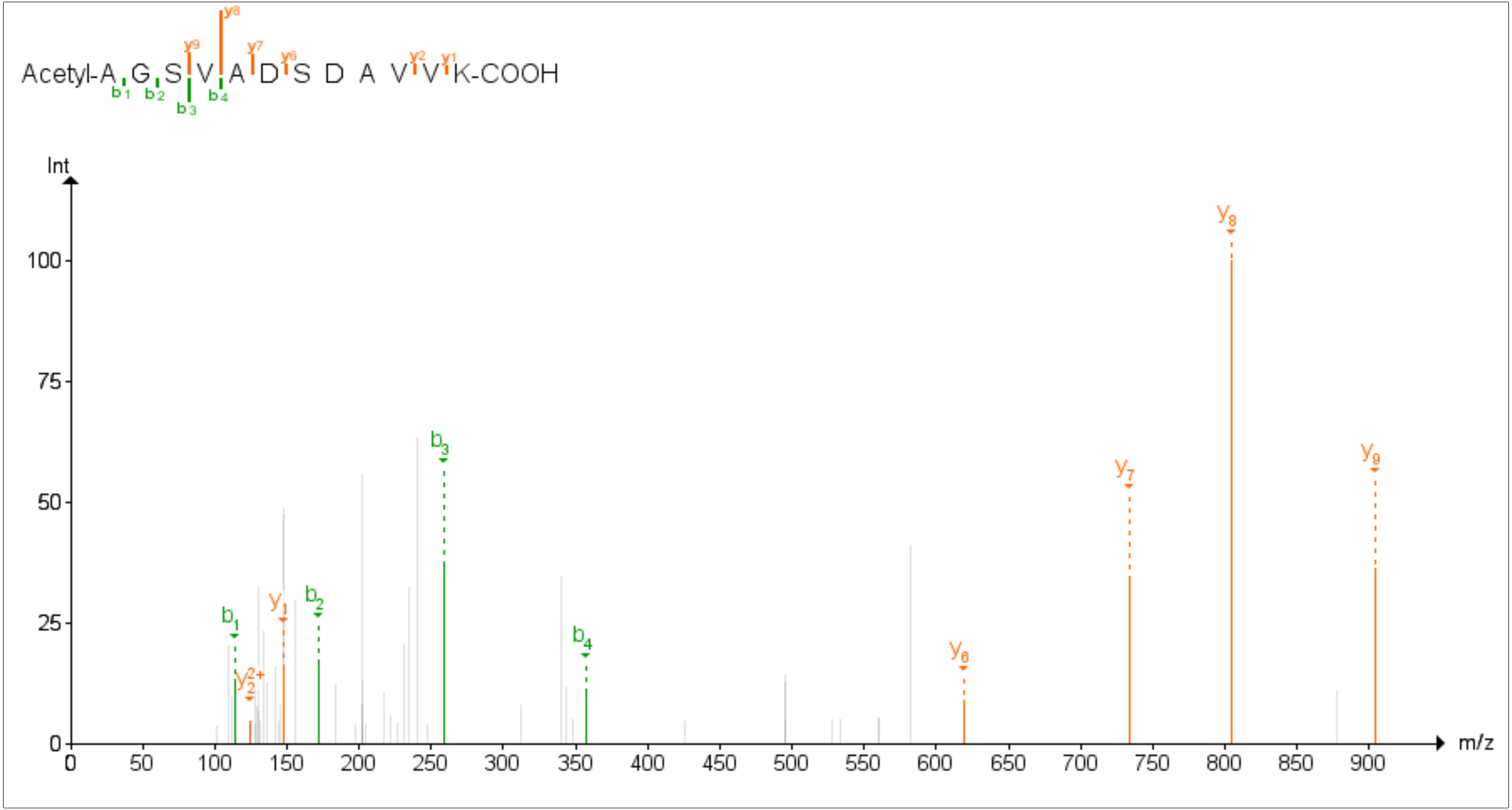

Acetyl-A G S V A D S D A V V K-COOH
y8 y9 y7 y6 y2 y1
b1 b2 b3 b4
Int
100
75
50
25
0
m/z
0 50 100 150 200 250 300 350 400 450 500 550 600 650 700 750 800 850 900
y8 y9 y7 y6 b3 b1 y1 b2 b4 y2 2+

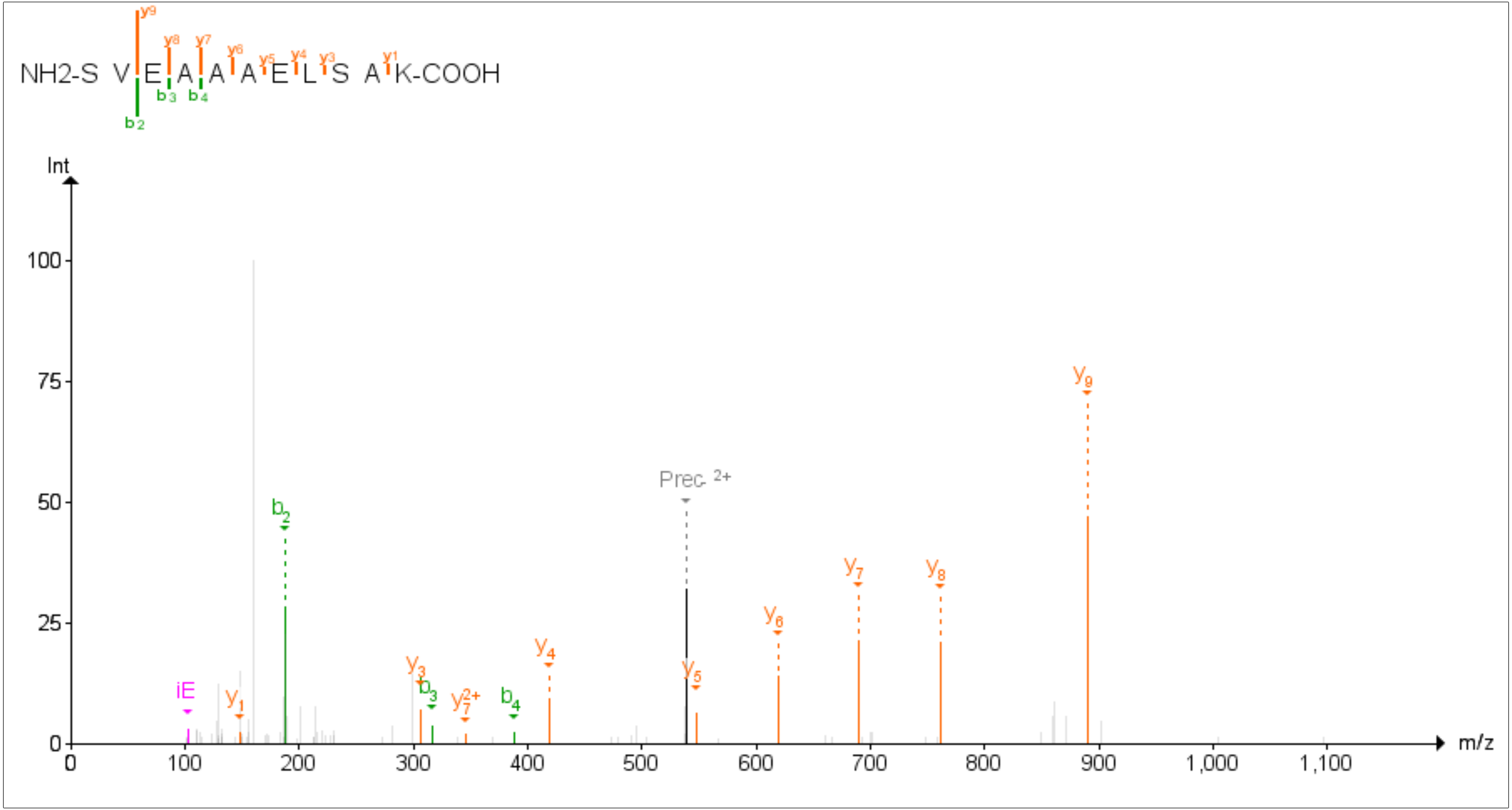

NH2-S V E A A A E L S A K-COOH
y9
y8
y7
y6
y5
y4
y3
y1
b2
b3
b4
Int
100
75
50
25
0
Prec 2+
b2
y9
y7
y8
y6
y4
y5
y3
b3
y7 2+
b4
iE
y1
m/z
0
100
200
300
400
500
600
700
800
900
1,000
1,100

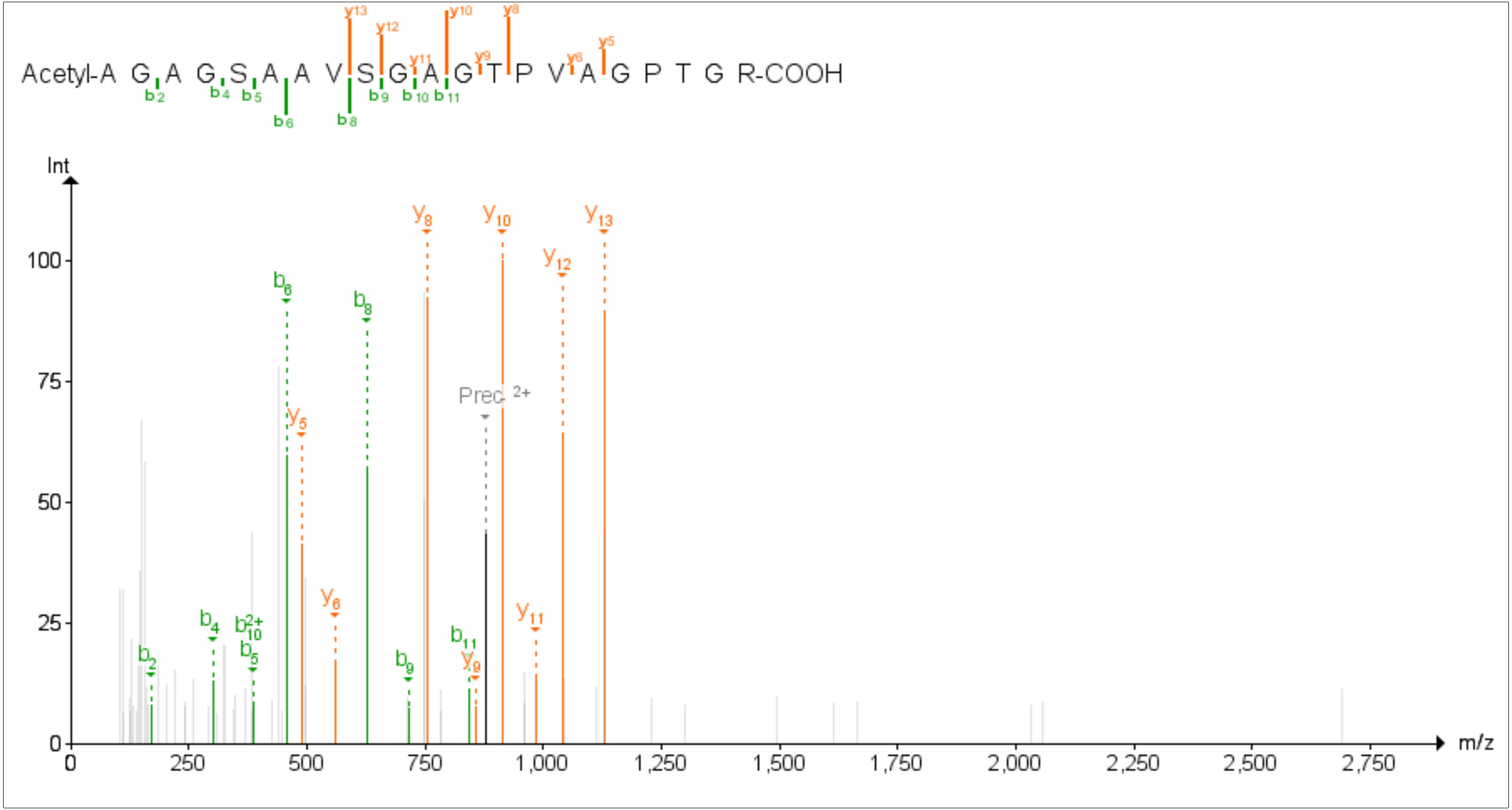

Acetyl-A G A G S A A V S G A G T P V A G P T G R-COOH
b2 b4 b5 b6 b8 b9 b10 b11
y13 y12 y11 y10 y9 y8 y6 y5
Int
m/z
Prec 2+
0
25
50
75
100
250
500
750
1,000
1,250
1,500
1,750
2,000
2,250
2,500
2,750

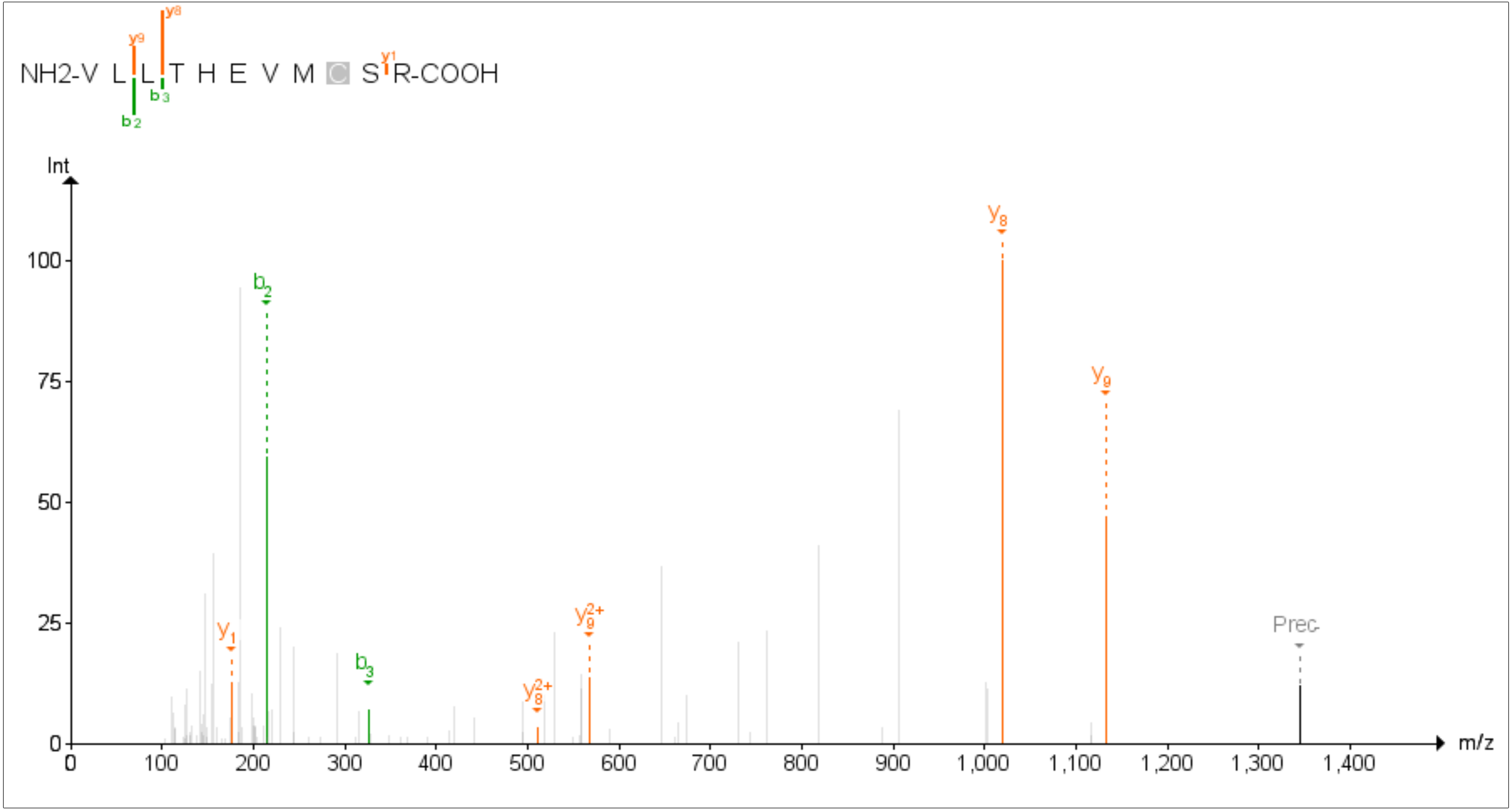

NH2-V L L T H E V M C S R-COOH
y9
y8
y1
b2
b3
Int
m/z
100
75
50
25
0
0
100
200
300
400
500
600
700
800
900
1,000
1,100
1,200
1,300
1,400
y8
y9
b2
y1
b3
y8 2+
y9 2+
Prec

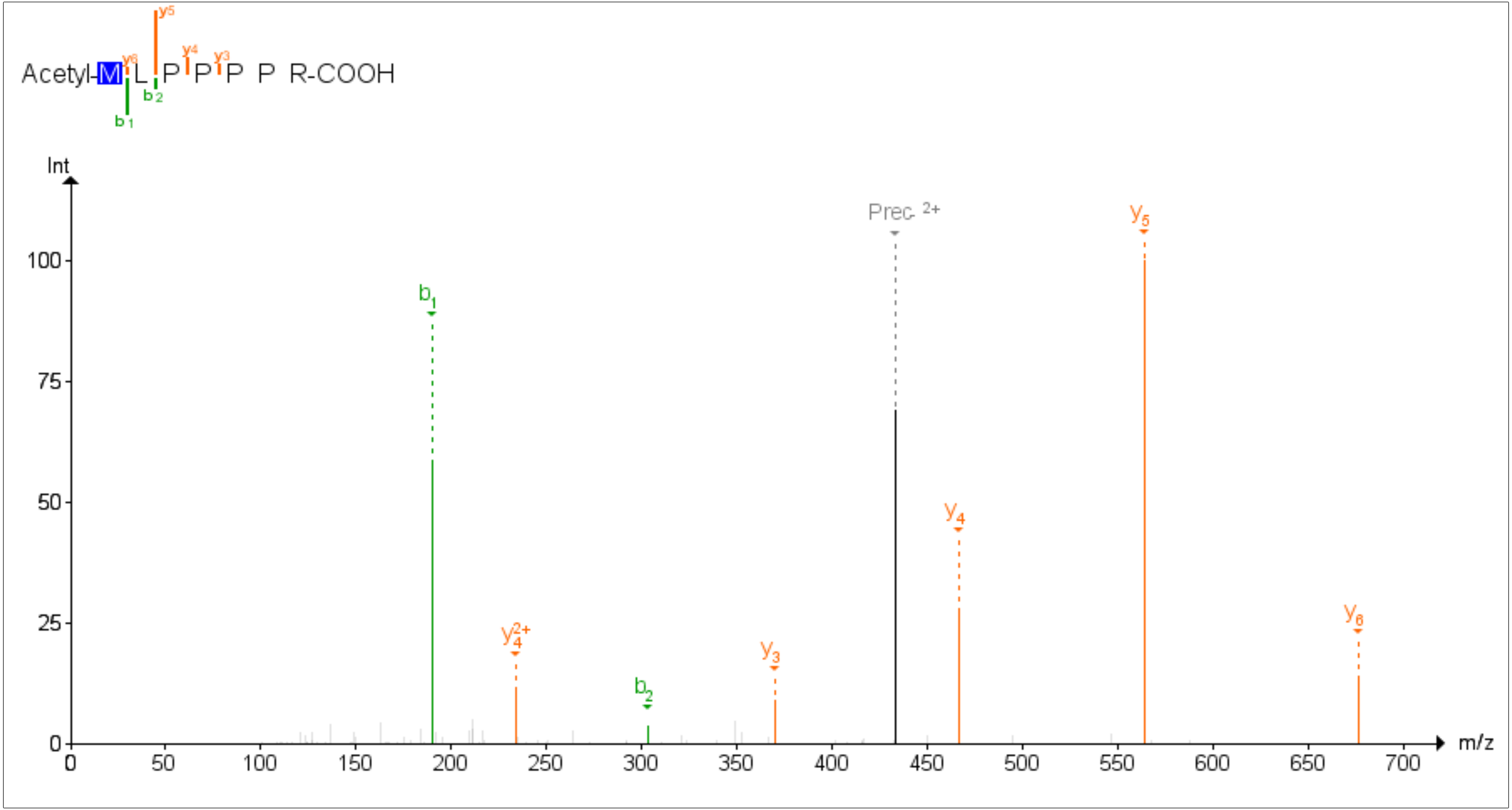

Acetyl-M L P P P P R-COOH
y5
y6
y4
y3
b 1
b 2
Int
m/z
Prec. 2+
y5
b1
y4
y4 2+
b2
y3
y6
0
25
50
75
100
0 50 100 150 200 250 300 350 400 450 500 550 600 650 700

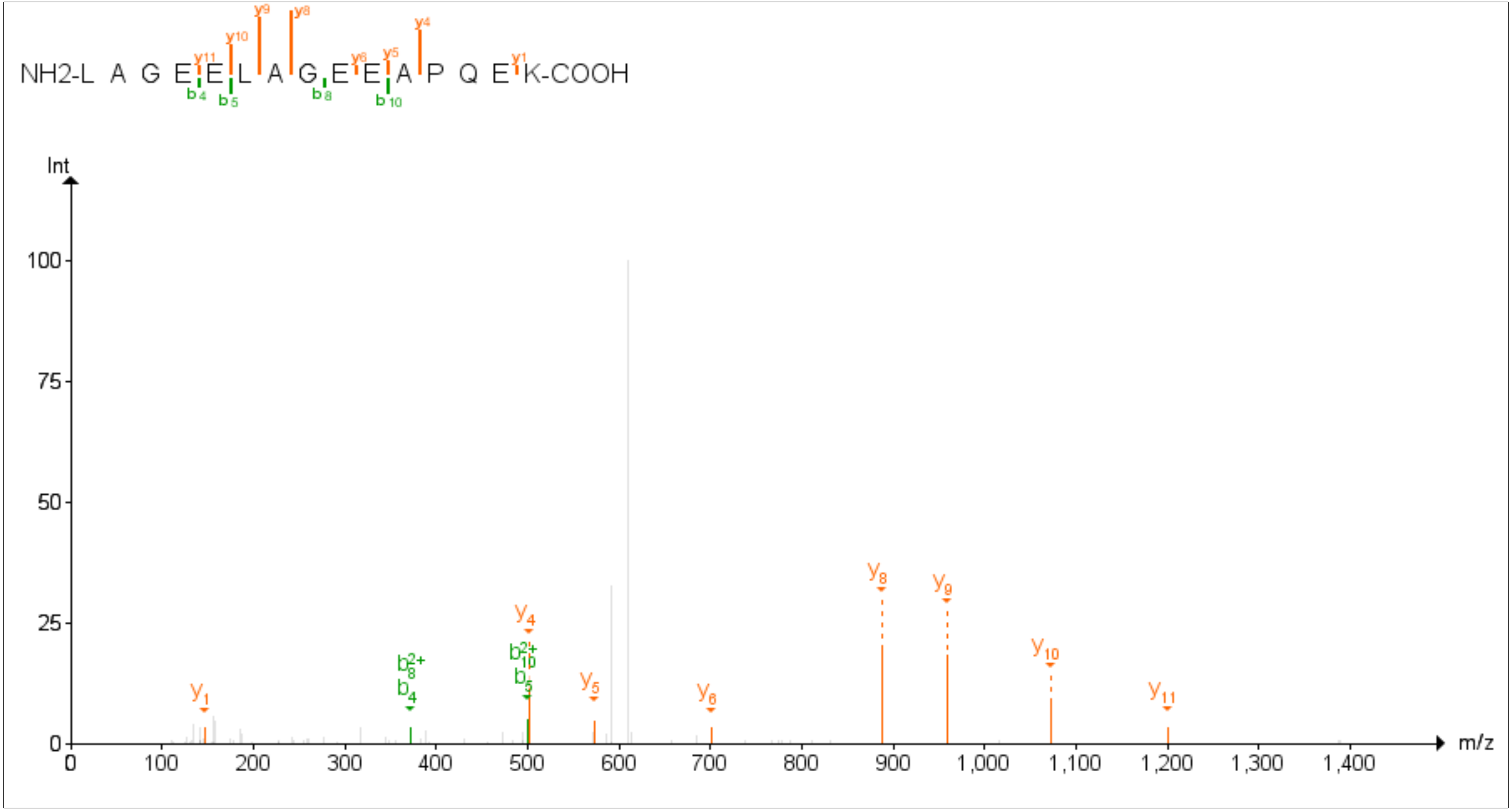

NH2-L A G E E L A G E E A P Q E K-COOH
y11 y10 y9 y8 y6 y5 y4 y1
b4 b5 b8 b10
Int
100
75
50
25
0
m/z
0 100 200 300 400 500 600 700 800 900 1,000 1,100 1,200 1,300 1,400
y1
b8 2+
b4
y4
b10 2+
b5
y5
y6
y8
y9
y10
y11

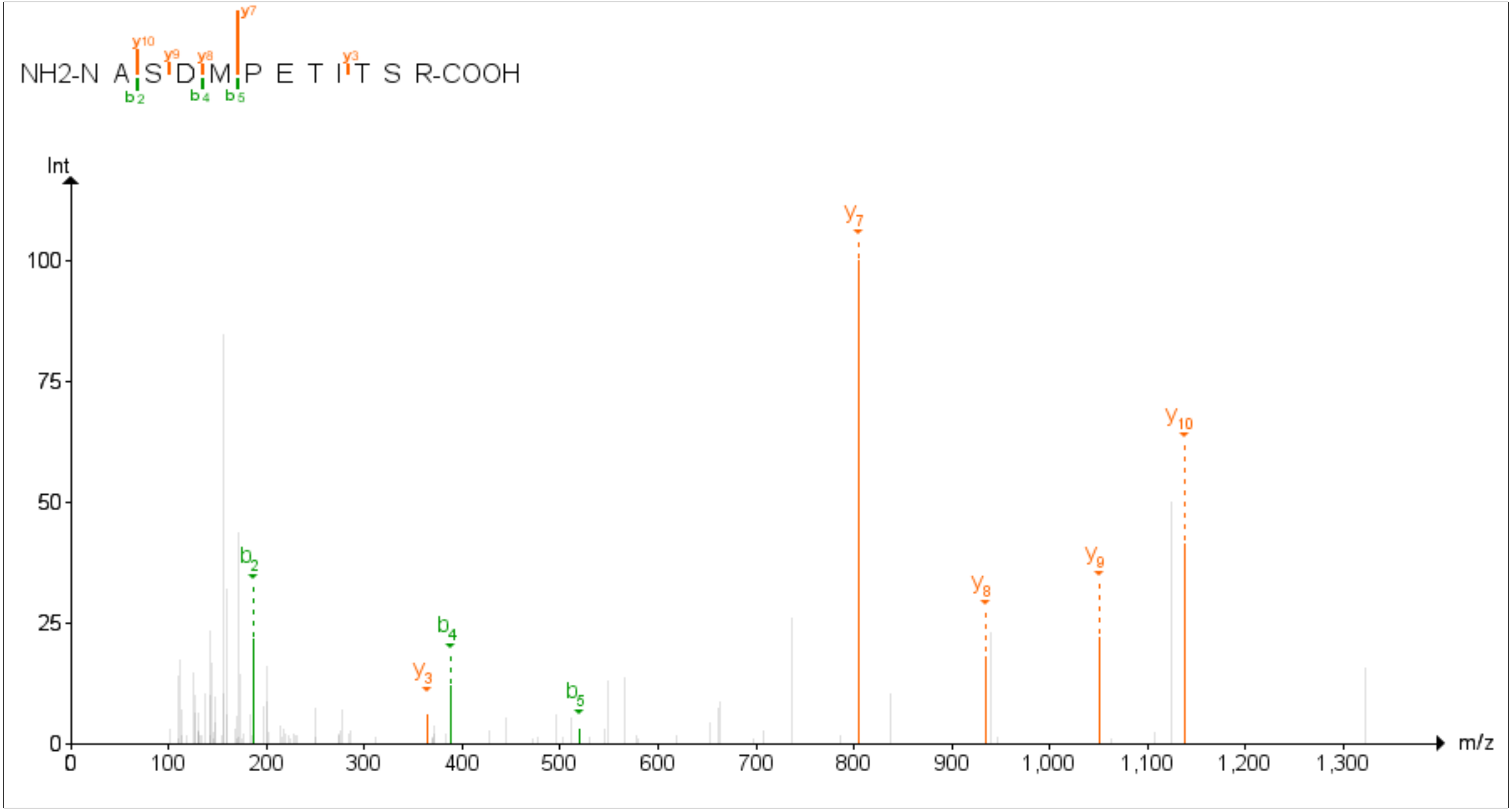

NH2-N A S D M P E T I T S R-COOH
y10 y9 y8 y7 y3
b2 b4 b5
Int
0
25
50
75
100
m/z
0
100
200
300
400
500
600
700
800
900
1,000
1,100
1,200
1,300
y7
y10
y9
y8
y3
b2
b4
b5

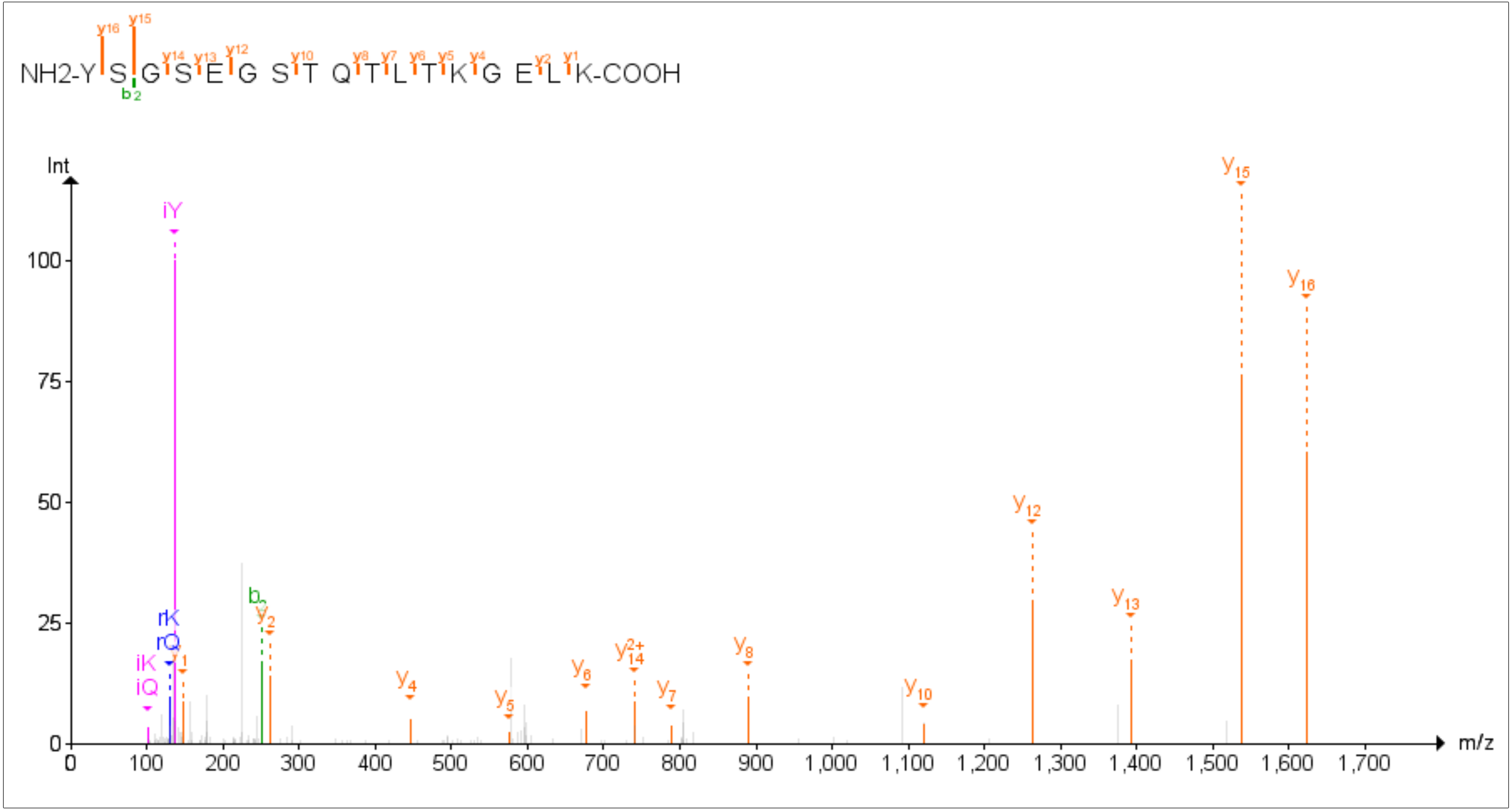

NH2-Y S G S E G S T Q T L T K G E L K-COOH
y16 y15 y14 y13 y12 y10 y8 y7 y6 y5 y4 y2 y1
b2
Int
m/z
iY
rK
rQ
iK
iQ
y1
b2
y2
y4
y5
y6
y14 2+
y7
y8
y10
y12
y13
y15
y16
0
25
50
75
100
0
100
200
300
400
500
600
700
800
900
1,000
1,100
1,200
1,300
1,400
1,500
1,600
1,700

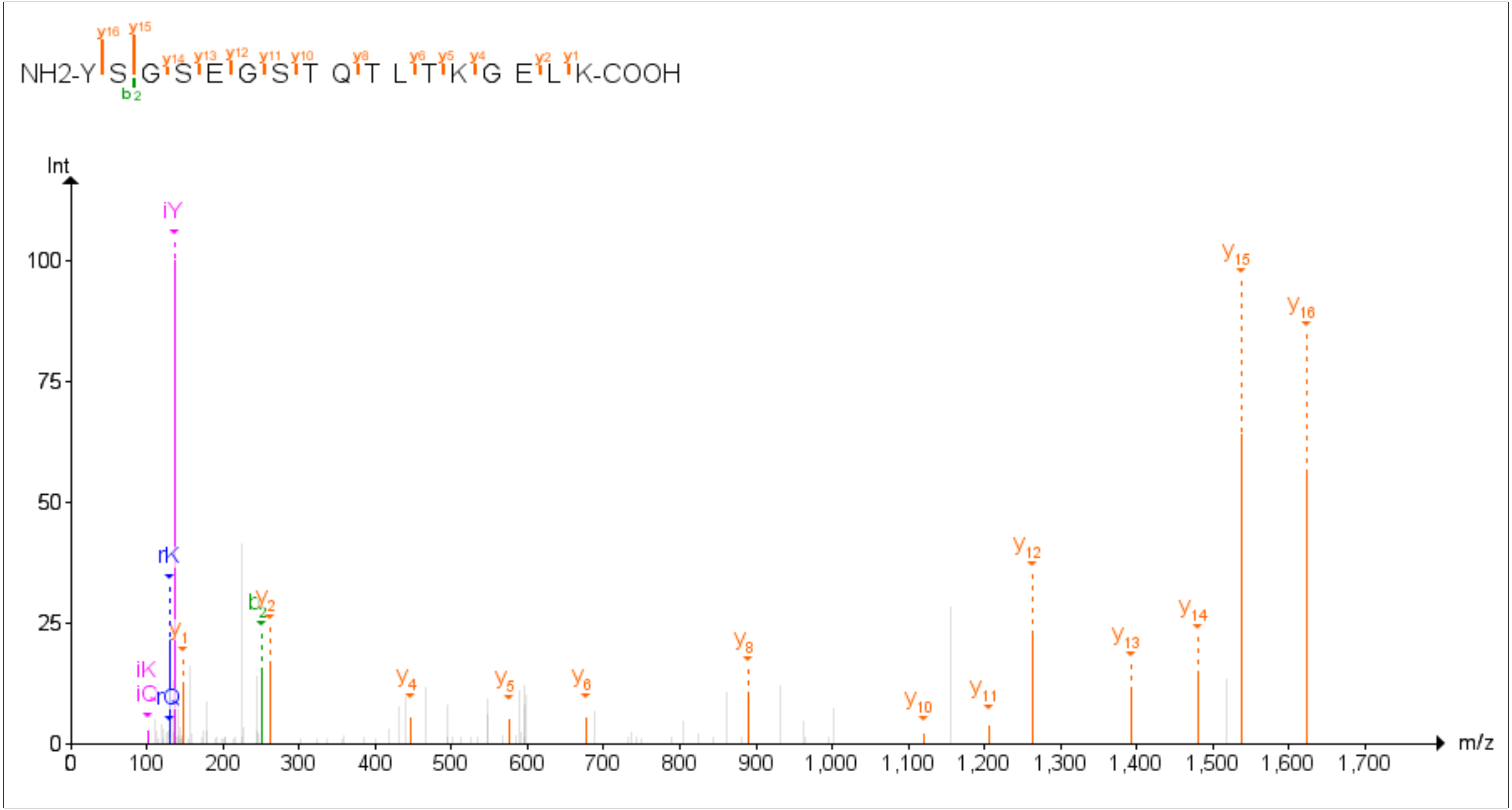
NH2-Y S G S E G S T Q T L T K G E L K-COOH
y16 y15 y14 y13 y12 y11 y10 y8 y6 y5 y4 y2 y1
b2
Int
m/z
iY
rK
iK
iQ
rQ
y1
b2 y2
y4
y5
y6
y8
y10
y11
y12
y13
y14
y15
y16
0
25
50
75
100
0
100
200
300
400
500
600
700
800
900
1,000
1,100
1,200
1,300
1,400
1,500
1,600
1,700

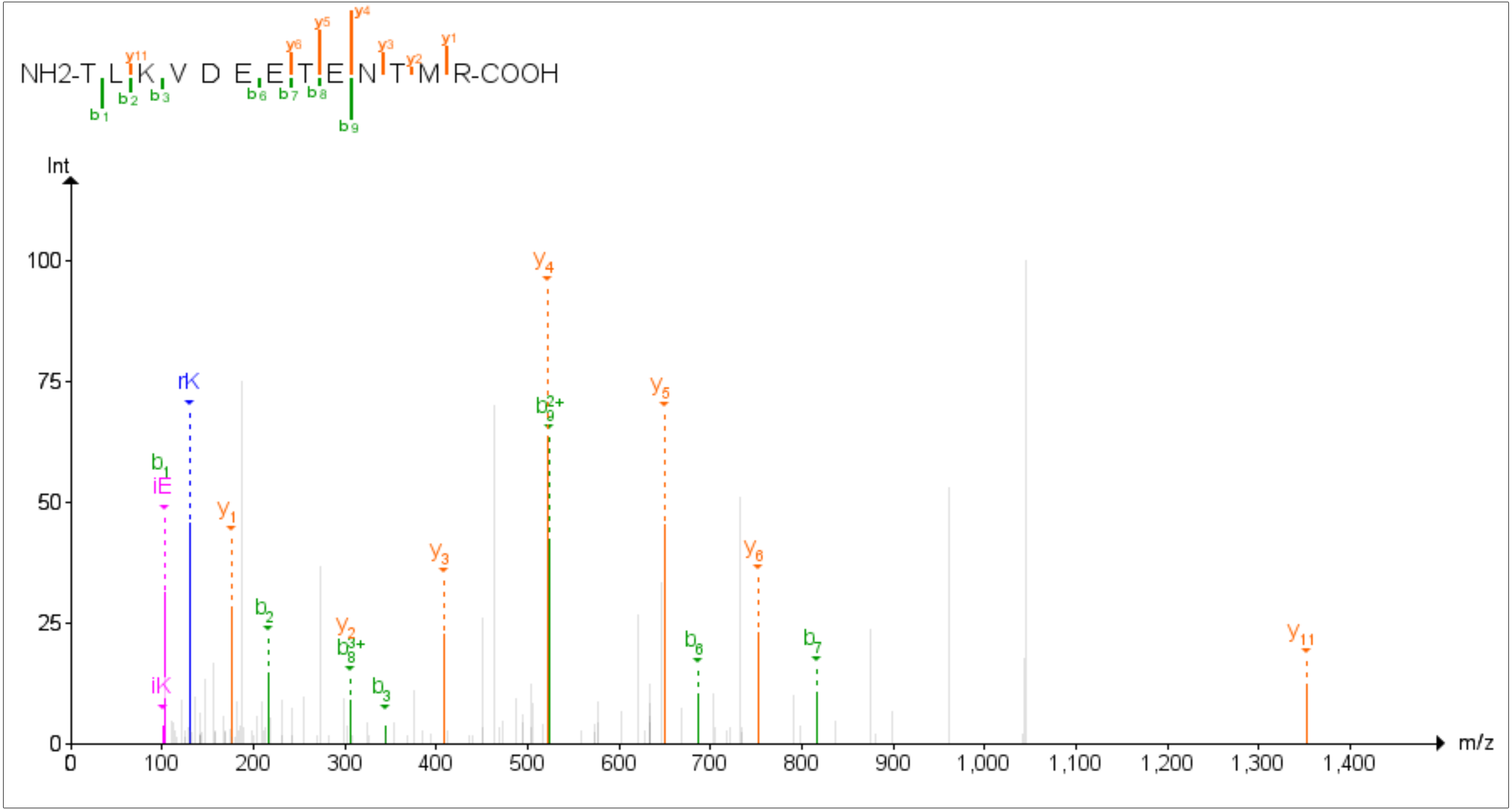

NH2-T L K V D E E T E N T M R-COOH
b1 b2 b3 b6 b7 b8 b9
y11 y6 y5 y4 y3 y2 y1
Int
m/z
0
25
50
75
100
0
100
200
300
400
500
600
700
800
900
1,000
1,100
1,200
1,300
1,400
rK
b1
iE
iK
y1
b2
y2
b8 3+
b3
y3
y4
b9 2+
y5
b6
y6
b7
y11

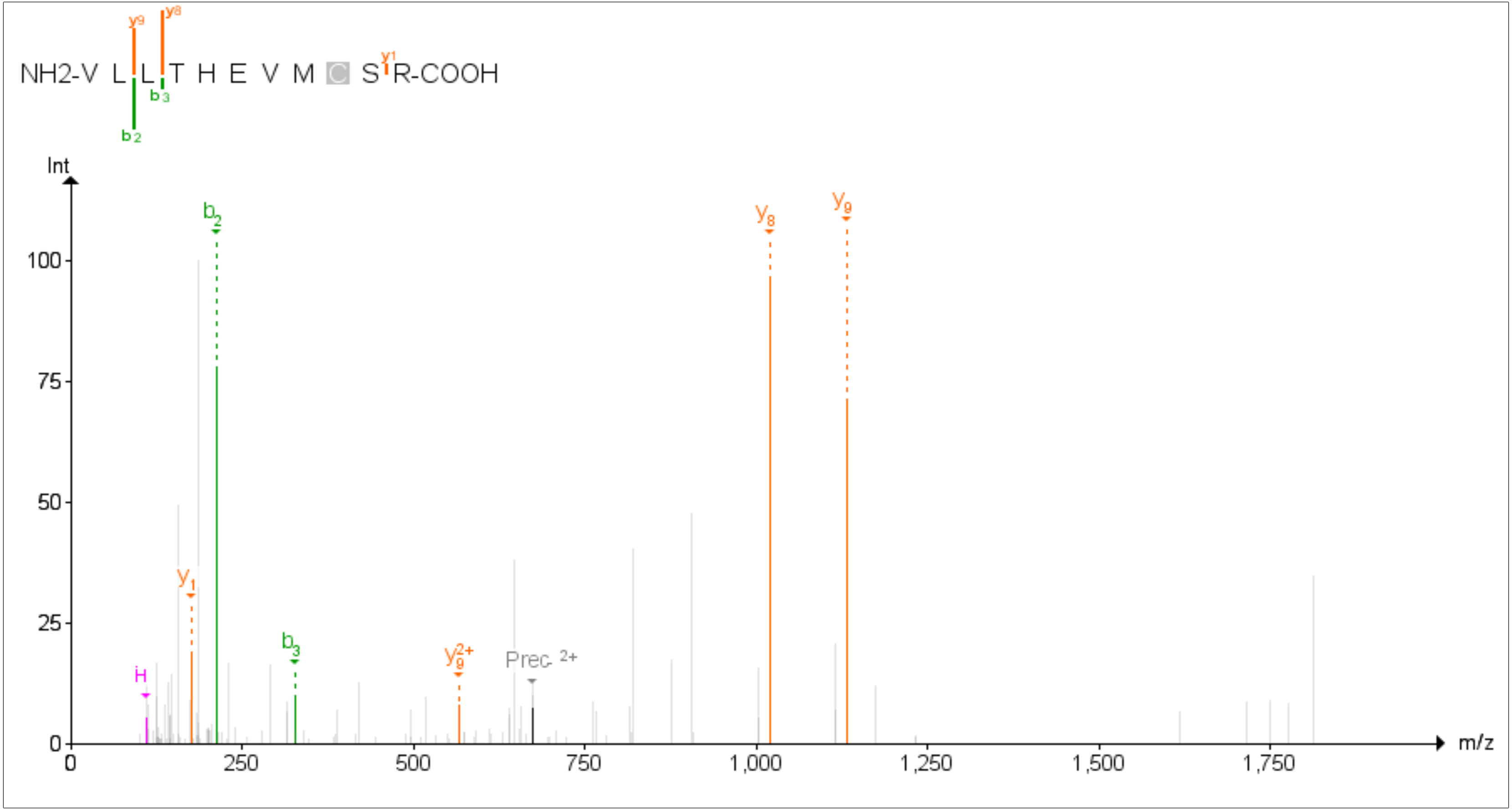

NH2-V L L T H E V M C S R-COOH
y9
y8
y1
b2
b3
Int
100
75
50
25
0
b2
y8
y9
y1
H
b3
y9 2+
Prec- 2+
0
250
500
750
1,000
1,250
1,500
1,750
m/z

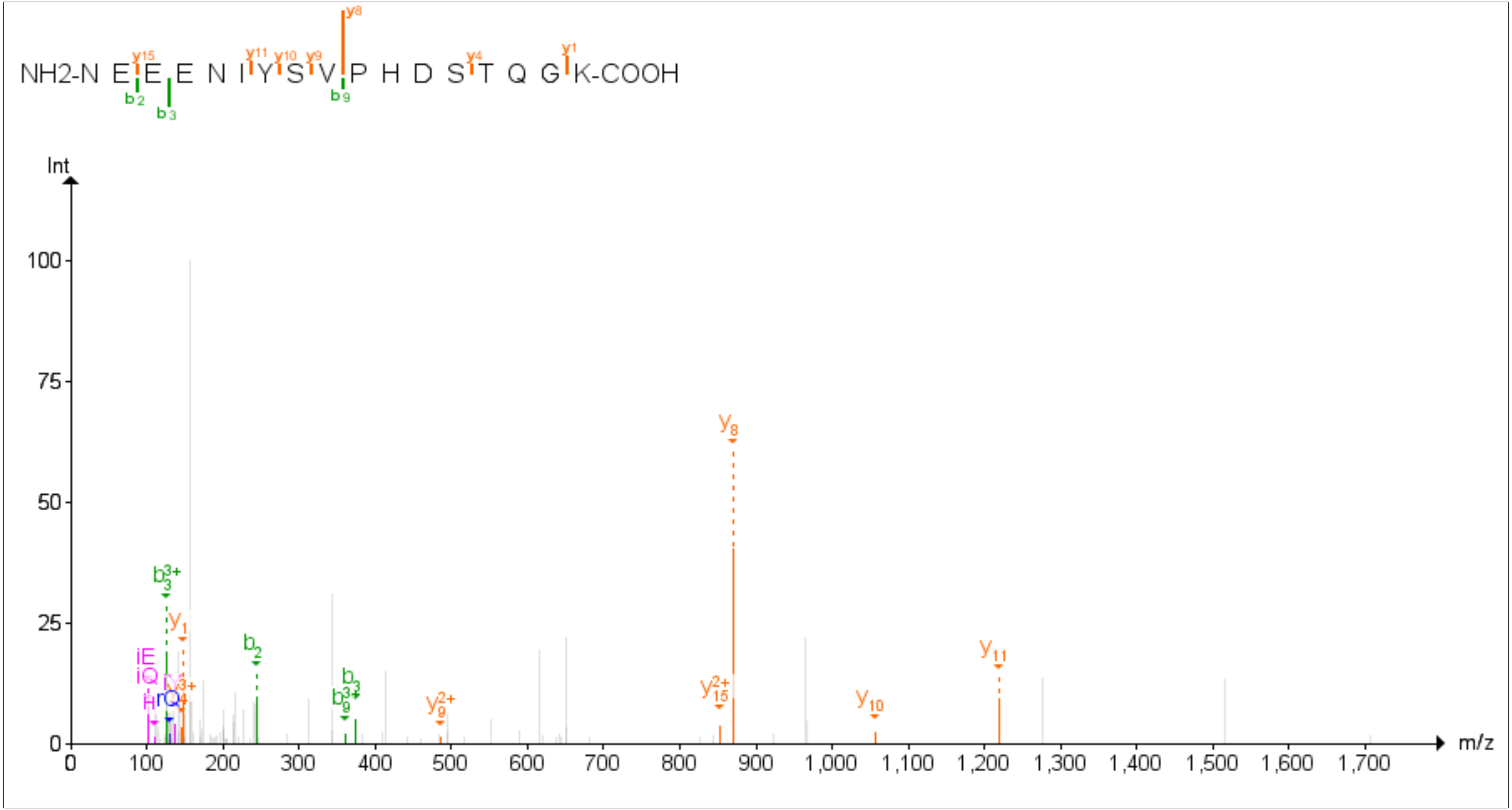

NH2-N E E E N I Y S V P H D S T Q G K-COOH
y15 y11 y10 y9 y8 y4 y1
b2 b3 b9
Int
100
75
50
25
0
m/z
0 100 200 300 400 500 600 700 800 900 1,000 1,100 1,200 1,300 1,400 1,500 1,600 1,700
b_3^{3+}
y_1
iE
iQ
iH
rQ
b_2
b_3
b_9^{3+}
y_9^{2+}
y_8
y_{15}^{2+}
y_{10}
y_{11}

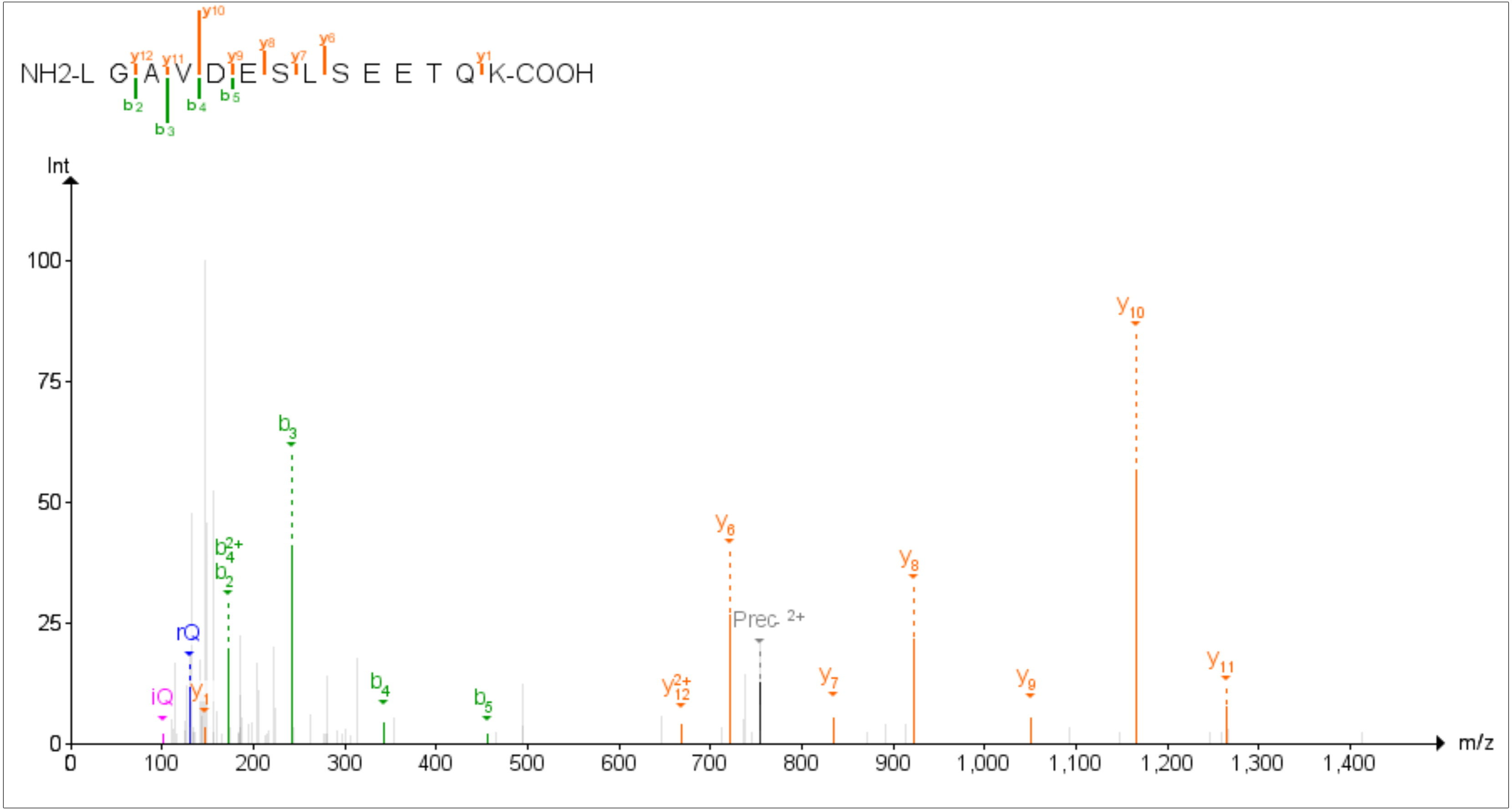

NH2-L G A V D E S L S E E T Q K-COOH
y12 y11 y10 y9 y8 y7 y6 y1
b2 b3 b4 b5
Int
m/z
iQ
rQ
y1
b2
b4 2+
b3
b4
b5
y12 2+
y6
Prec 2+
y7
y8
y9
y10
y11

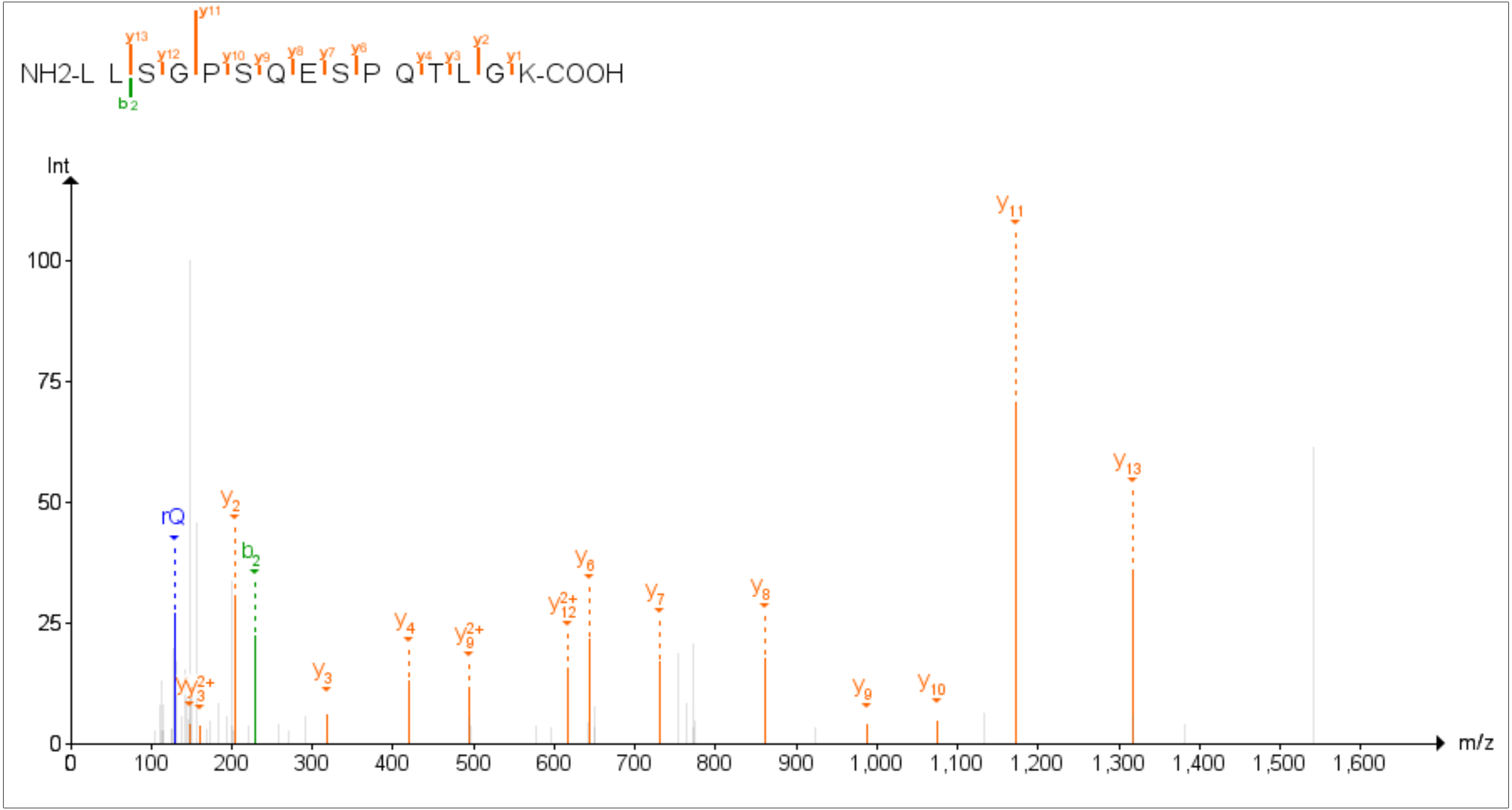

NH2-L L S G P S Q E S P Q T L G K-COOH
y13 y12 y11 y10 y9 y8 y7 y6 y4 y3 y2 y1
b2
Int
m/z
0
25
50
75
100
0
100
200
300
400
500
600
700
800
900
1,000
1,100
1,200
1,300
1,400
1,500
1,600
rQ
y2
b2
y3
y4
y9 2+
y12 2+
y6
y7
y8
y9
y10
y11
y13

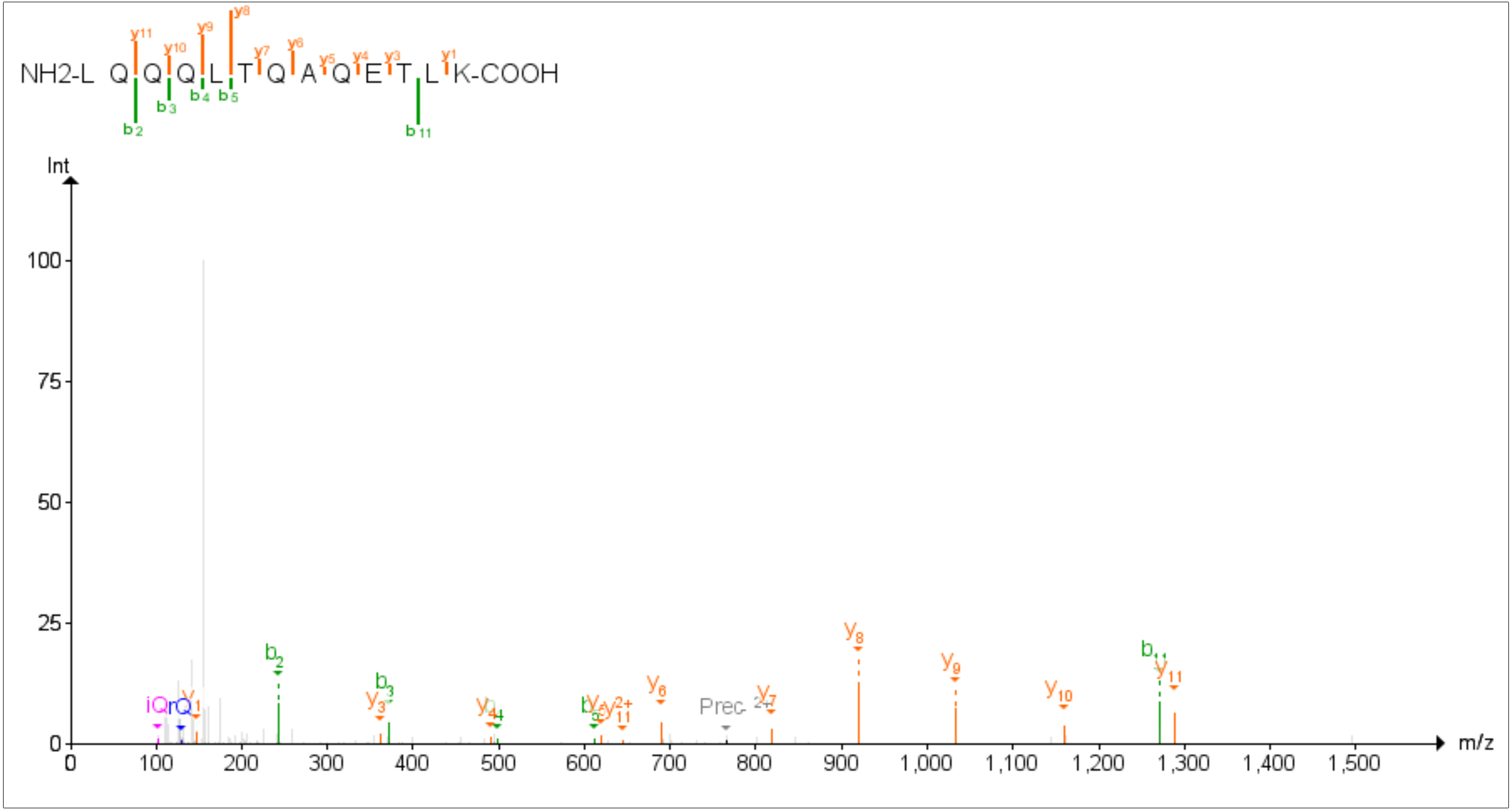
NH2-L Q Q Q L T Q A Q E T L K-COOH
Int
m/z
0
25
50
75
100
0
100
200
300
400
500
600
700
800
900
1,000
1,100
1,200
1,300
1,400
1,500
Prec

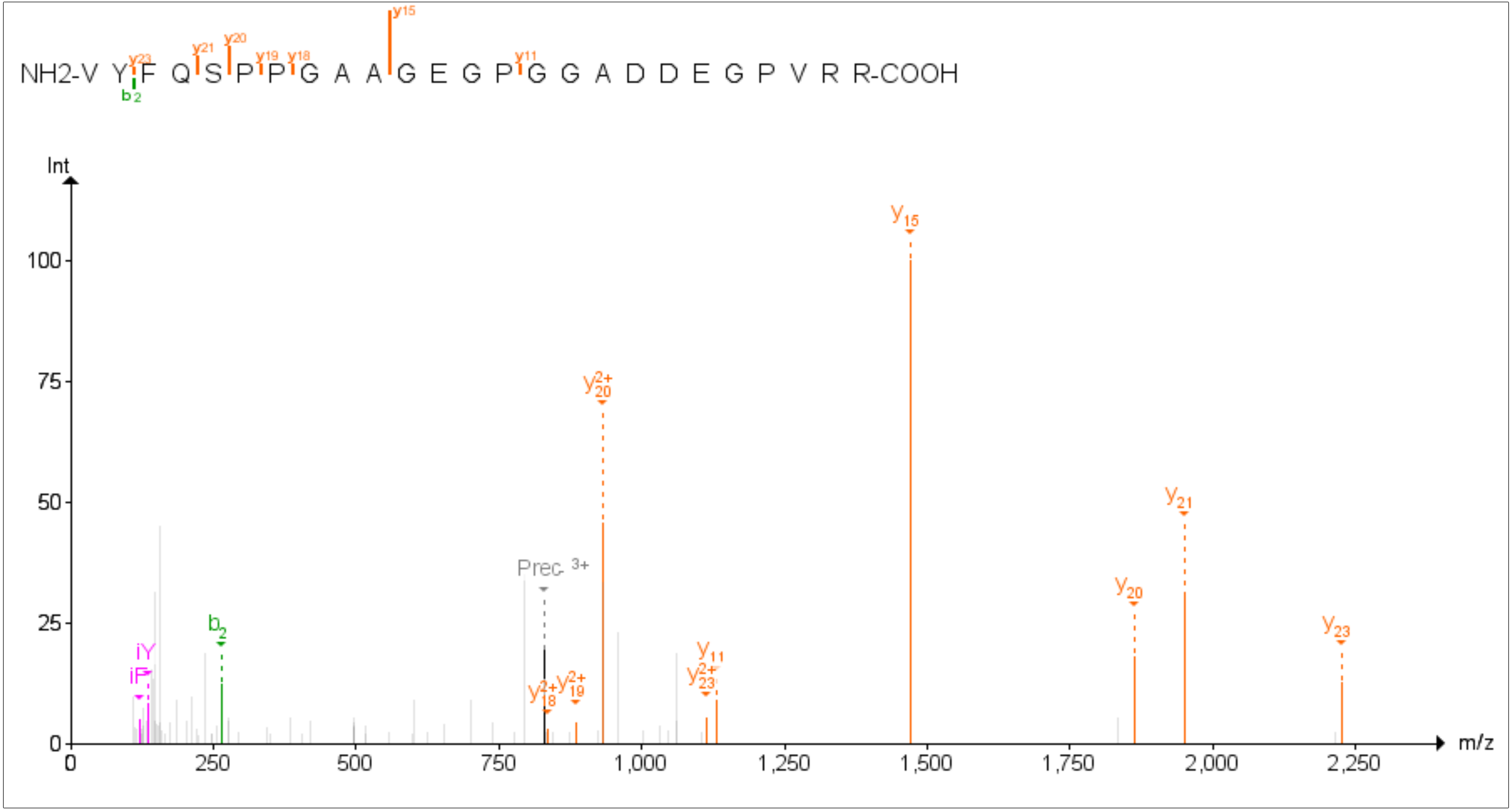

NH2-V Y F Q S P P P G A A G E G P G G A D D E G P V R R-COOH
y23 y21 y20 y19 y18 y15 y11
b2
Int
100
75
50
25
0
m/z
0
250
500
750
1,000
1,250
1,500
1,750
2,000
2,250
iY
iF
b2
Prec. 3+
y18 2+
y19 2+
y20 2+
y23 2+
y11
y15
y20
y21
y23

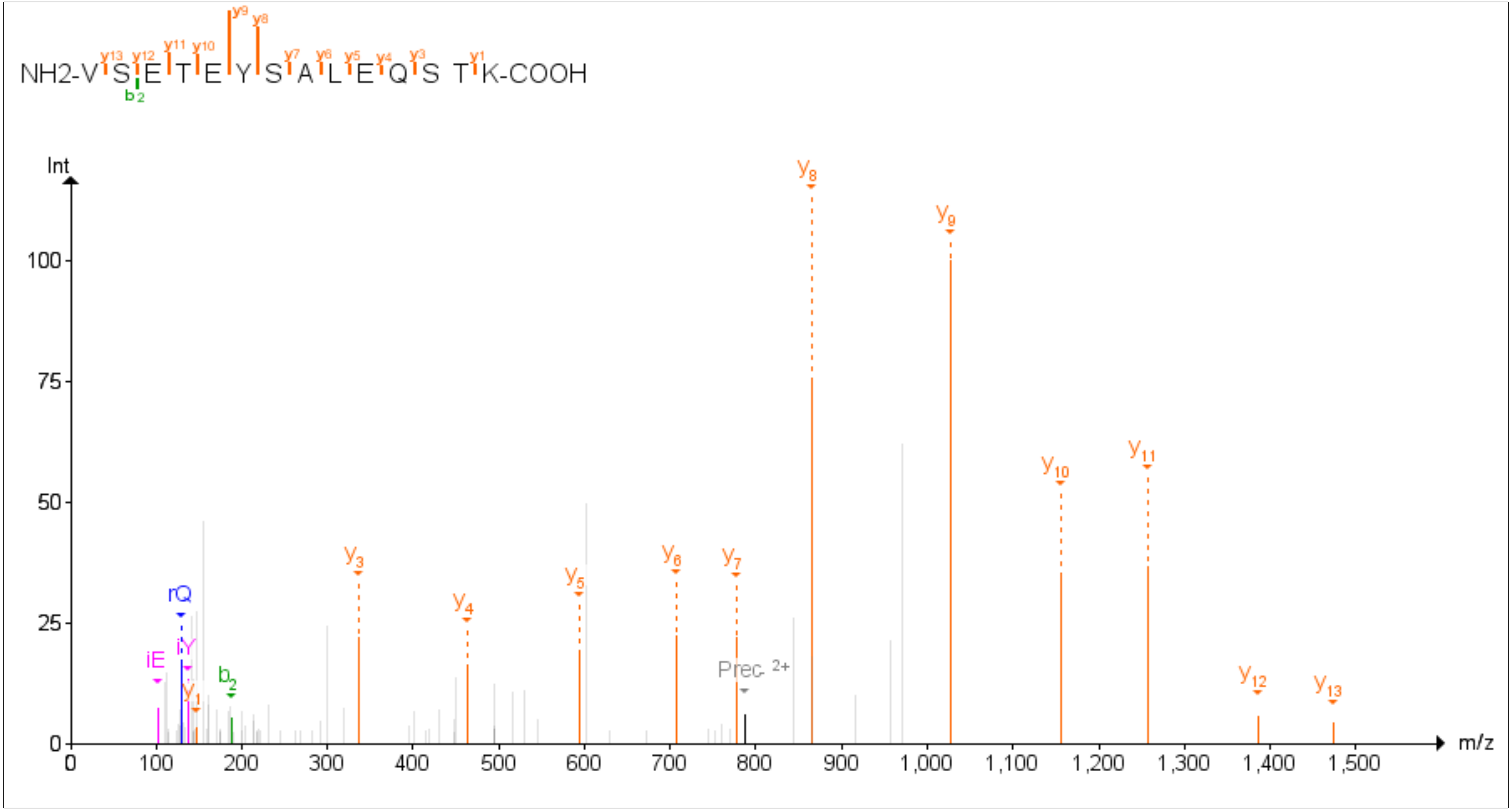

NH2-VSETEYSALEQSTK-COOH
y13 y12 y11 y10 y9 y8 y7 y6 y5 y4 y3 y1
b2
Int
100
75
50
25
0
rQ
iE
iY
y1
b2
y3
y4
y5
y6
y7
Prec. 2+
y8
y9
y10
y11
y12
y13
0 100 200 300 400 500 600 700 800 900 1,000 1,100 1,200 1,300 1,400 1,500
m/z

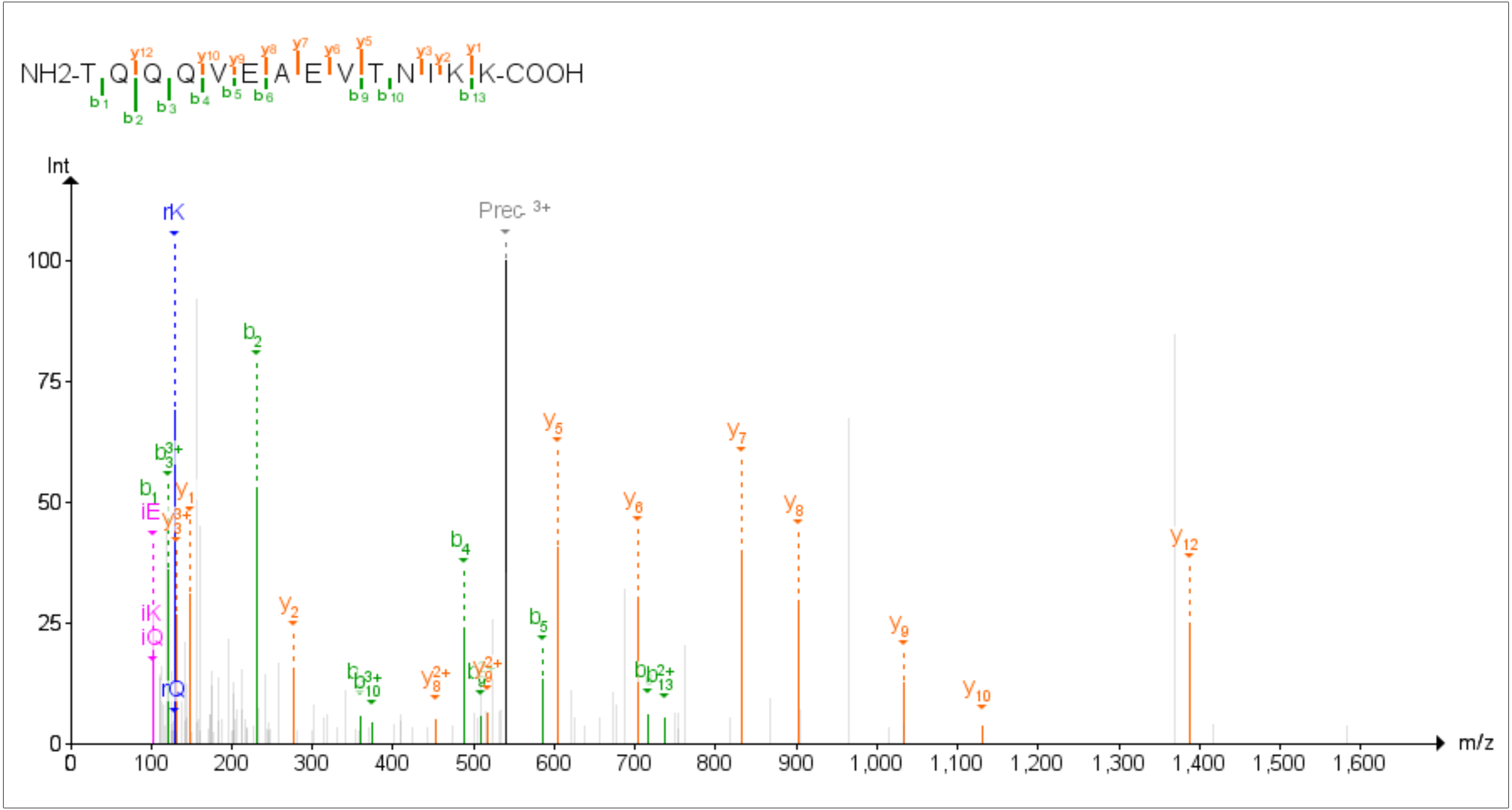
NH2-T Q Q Q V E A E V T N I K K-COOH
Int
m/z
Prec 3+
rK
rQ
iE
iK
iQ
b2
b4
b5
y1
y2
y5
y6
y7
y8
y9
y10
y12

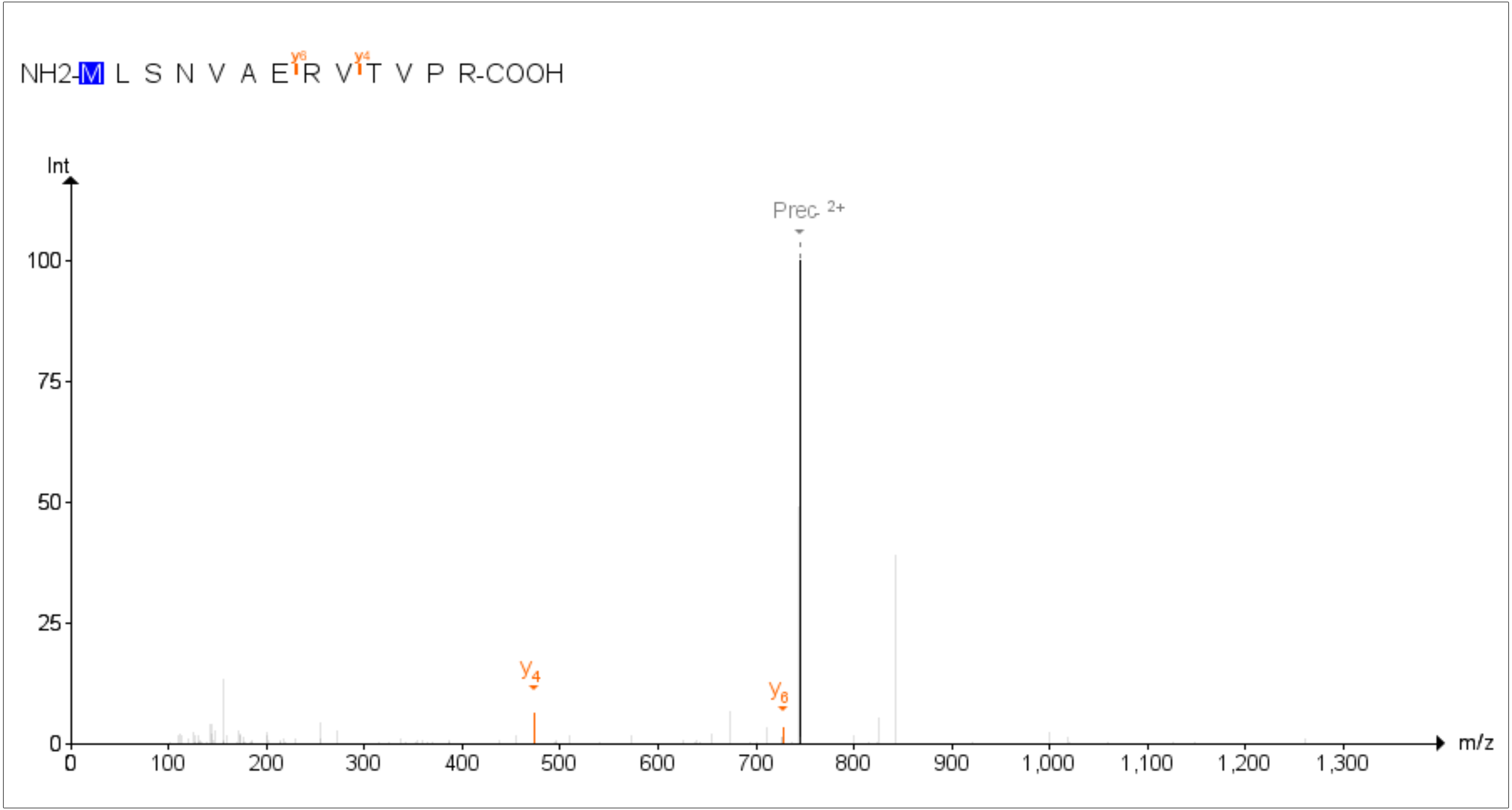

NH2-M L S N V A E R V T V P R-COOH
y6
y4
Int
100
75
50
25
0
Prec. 2+
y4
y6
0
100
200
300
400
500
600
700
800
900
1,000
1,100
1,200
1,300
m/z

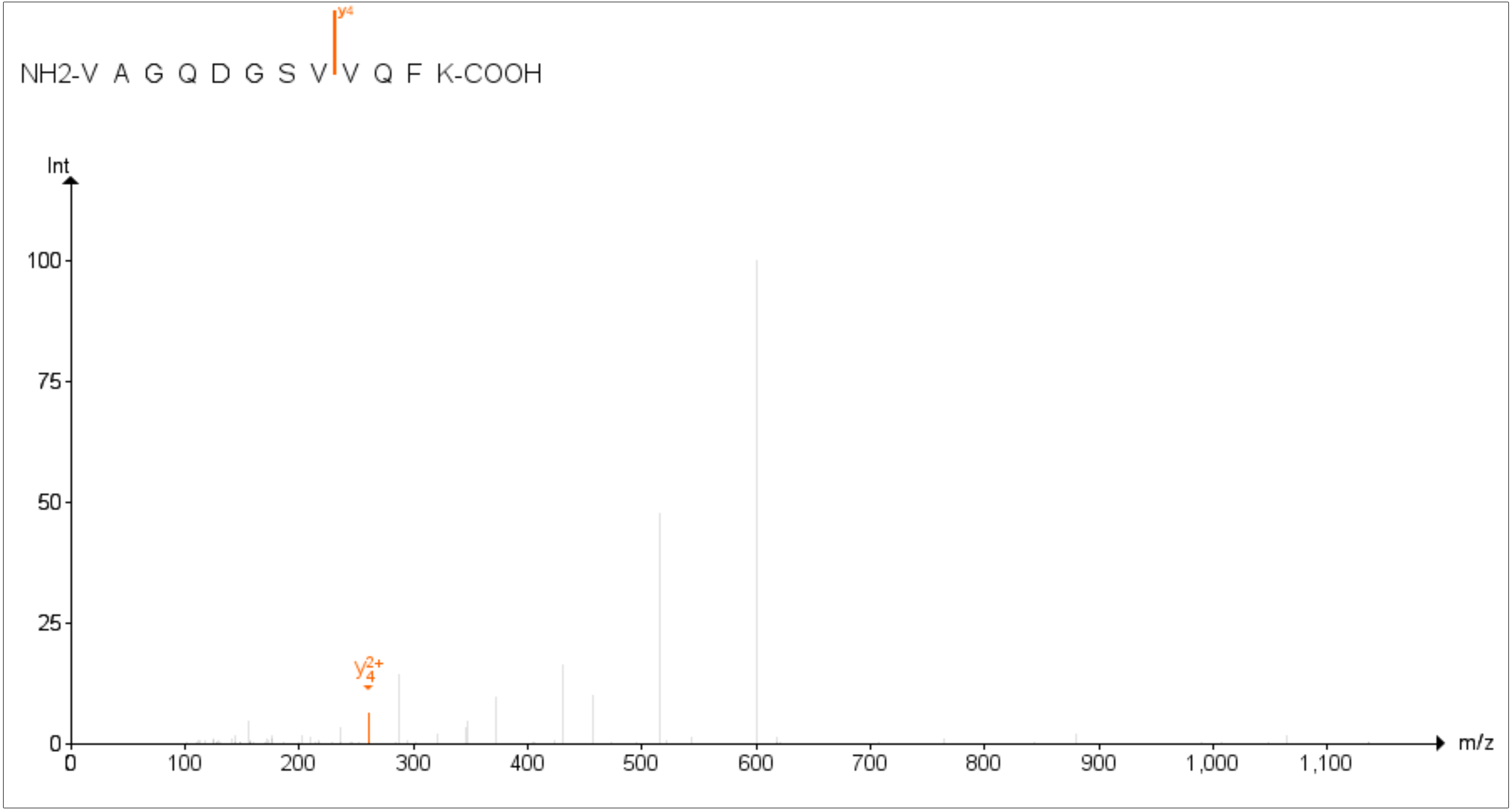

y4
NH2-V A G Q D G S V V Q F K-COOH
Int
100
75
50
25
0
y_4^{2+}
0
100
200
300
400
500
600
700
800
900
1,000
1,100
m/z

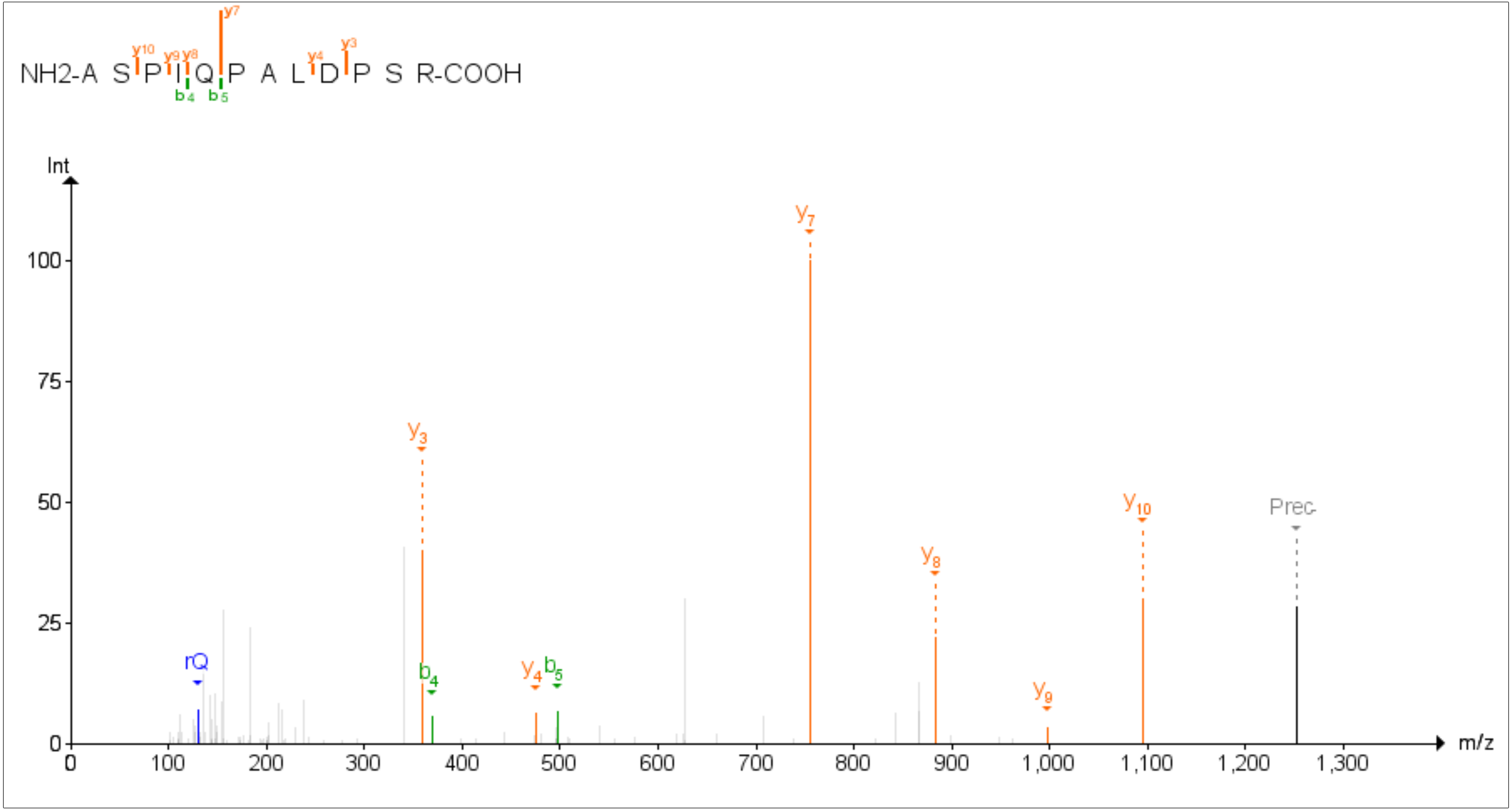

NH2-A S P I Q P A L D P S R-COOH
y10 y9 y8 y7 y4 y3
b4 b5
Int
100
75
50
25
0
y7
y3
y10
y8
y9
y4
b4
b5
rQ
Prec
0 100 200 300 400 500 600 700 800 900 1,000 1,100 1,200 1,300
m/z

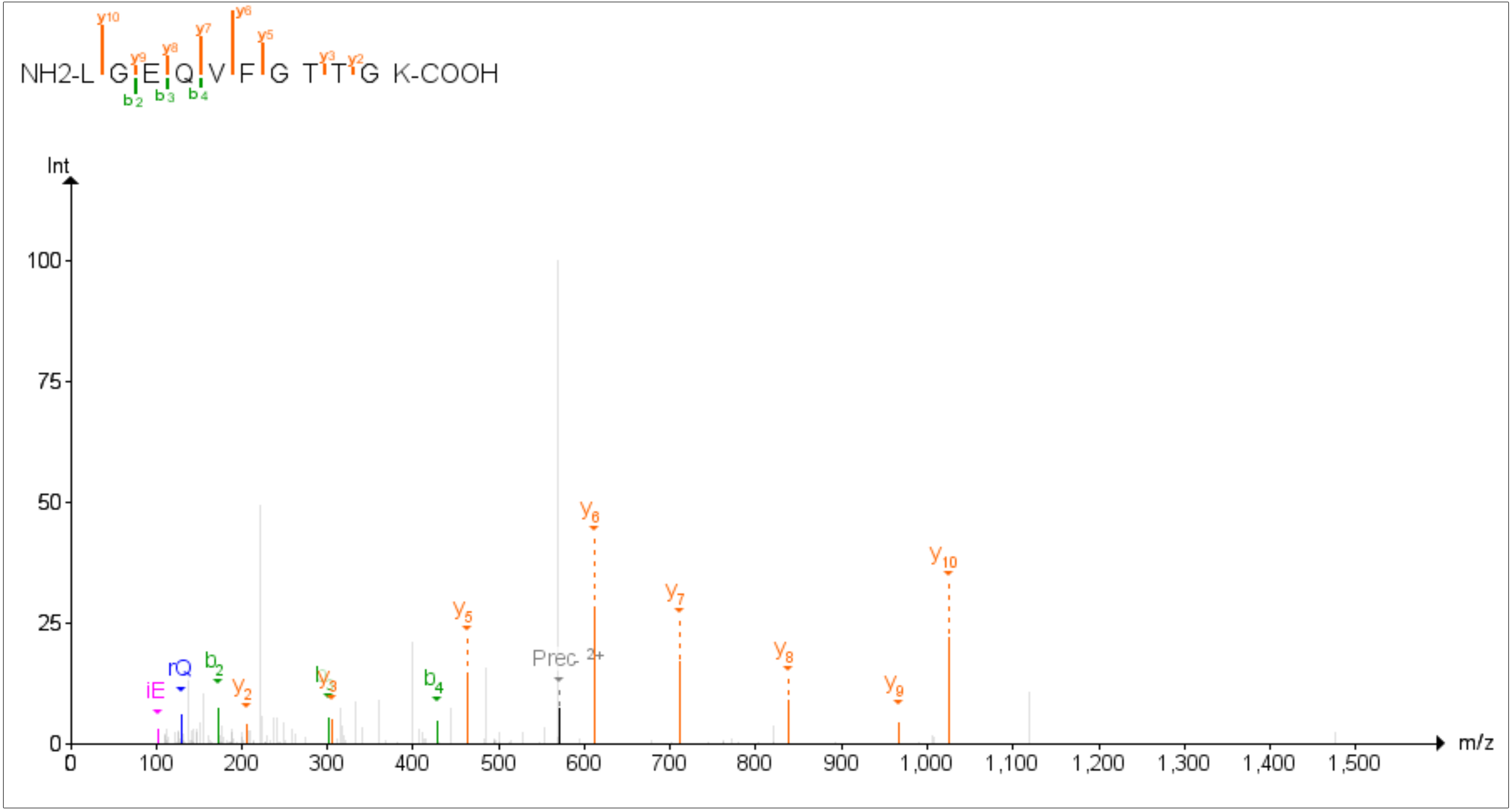

NH2-L G E Q V F G T T G K-COOH
y10 y9 y8 y7 y6 y5 y3 y2
b2 b3 b4
Int
100
75
50
25
0
iE
rQ
b2
y2
y3
b4
y5
Prec 2+
y6
y7
y8
y9
y10
0 100 200 300 400 500 600 700 800 900 1,000 1,100 1,200 1,300 1,400 1,500
m/z

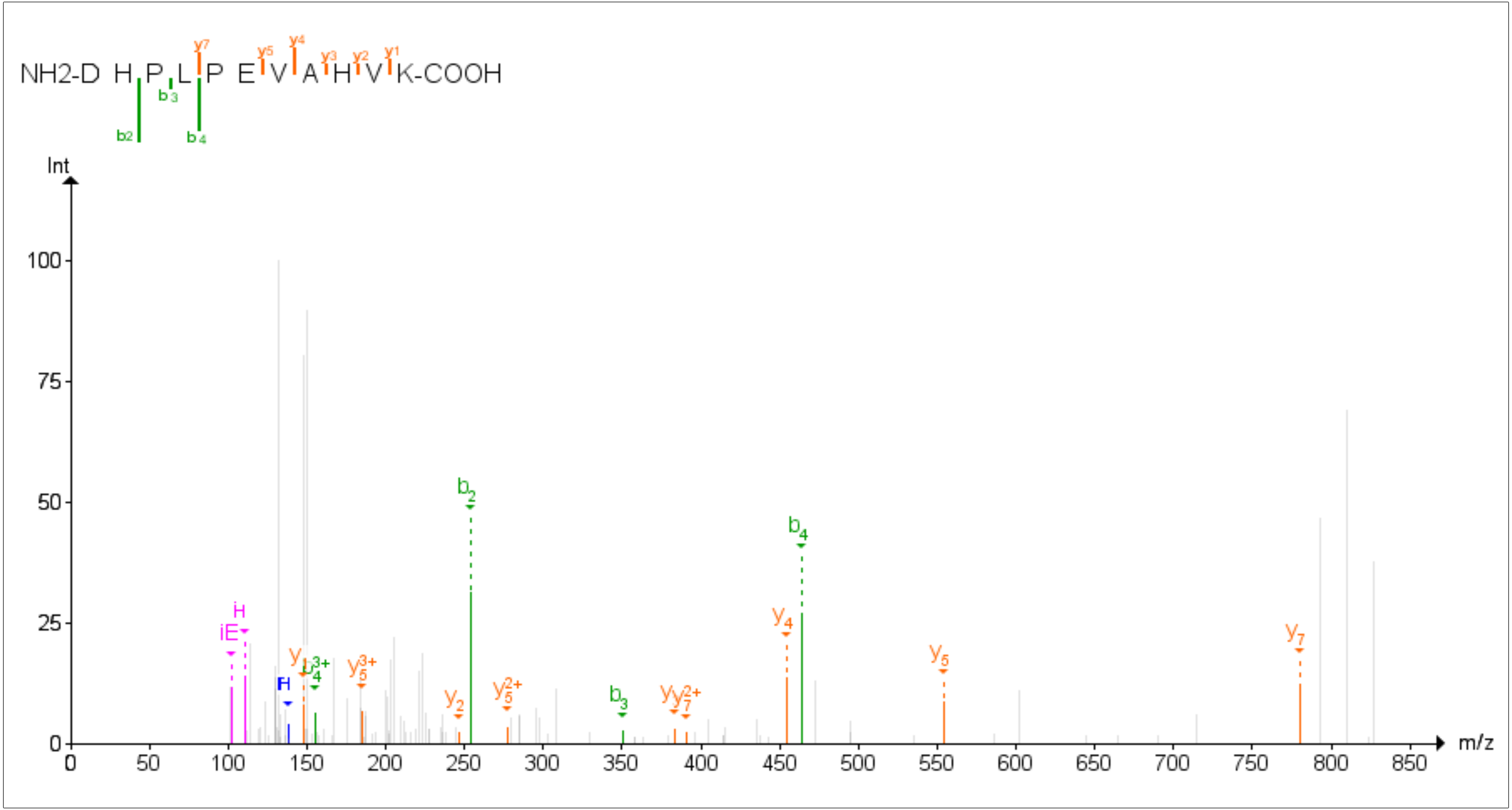

NH2-D H P L P E V A H V K-COOH
y7
y5
y4
y3
y2
y1
b2
b3
b4
Int
100
75
50
25
0
m/z
0
50
100
150
200
250
300
350
400
450
500
550
600
650
700
750
800
850
b2
b4
y4
y5
y7
b3
y2
iE
H

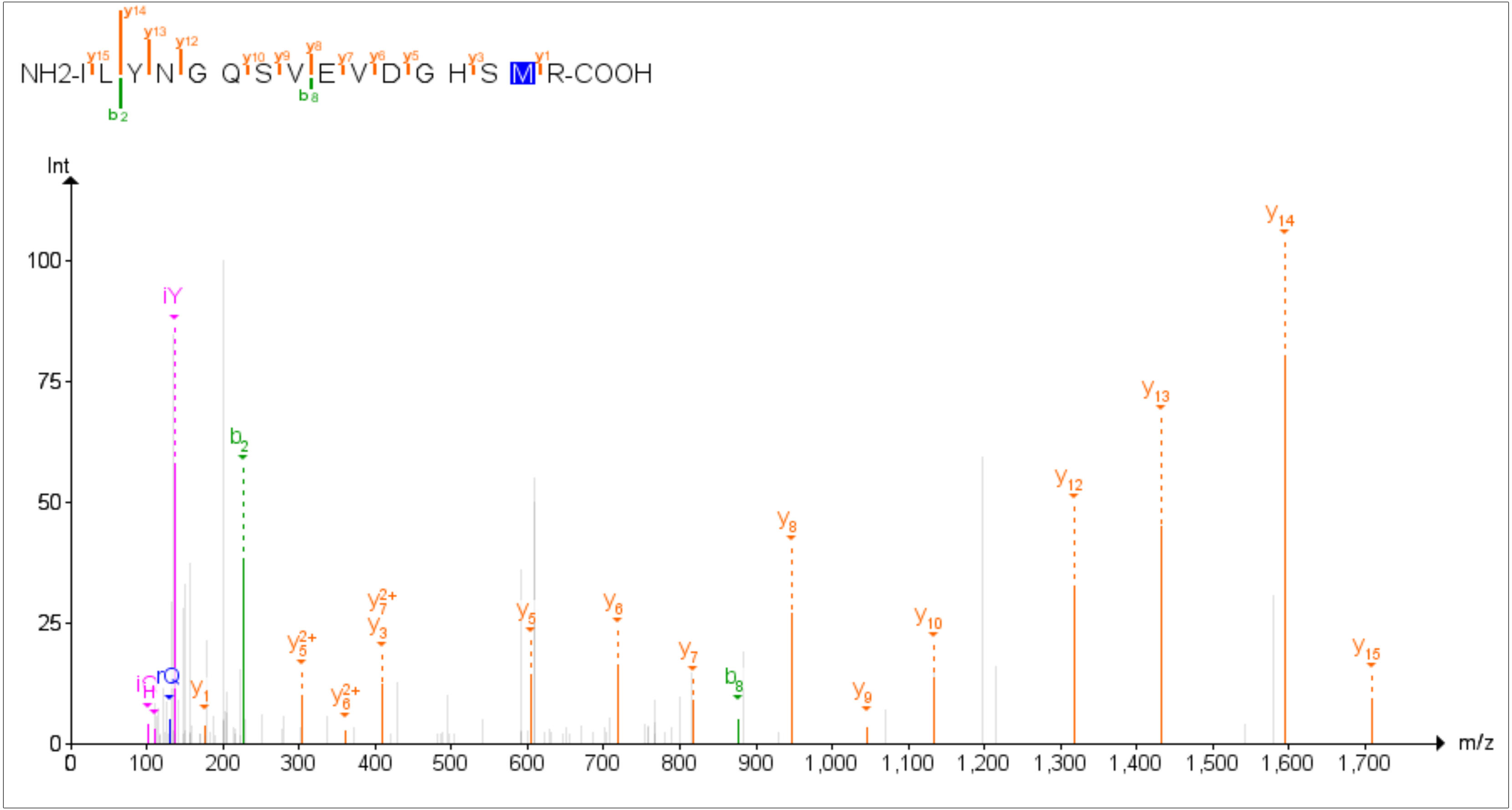
NH2-I L Y N G Q S V E V D G H S M R-COOH
Int
m/z
iY
b2
y14
y13
y12
y8
y6
y7
y5
y3
y1
y9
y10
y15
b8
rQ

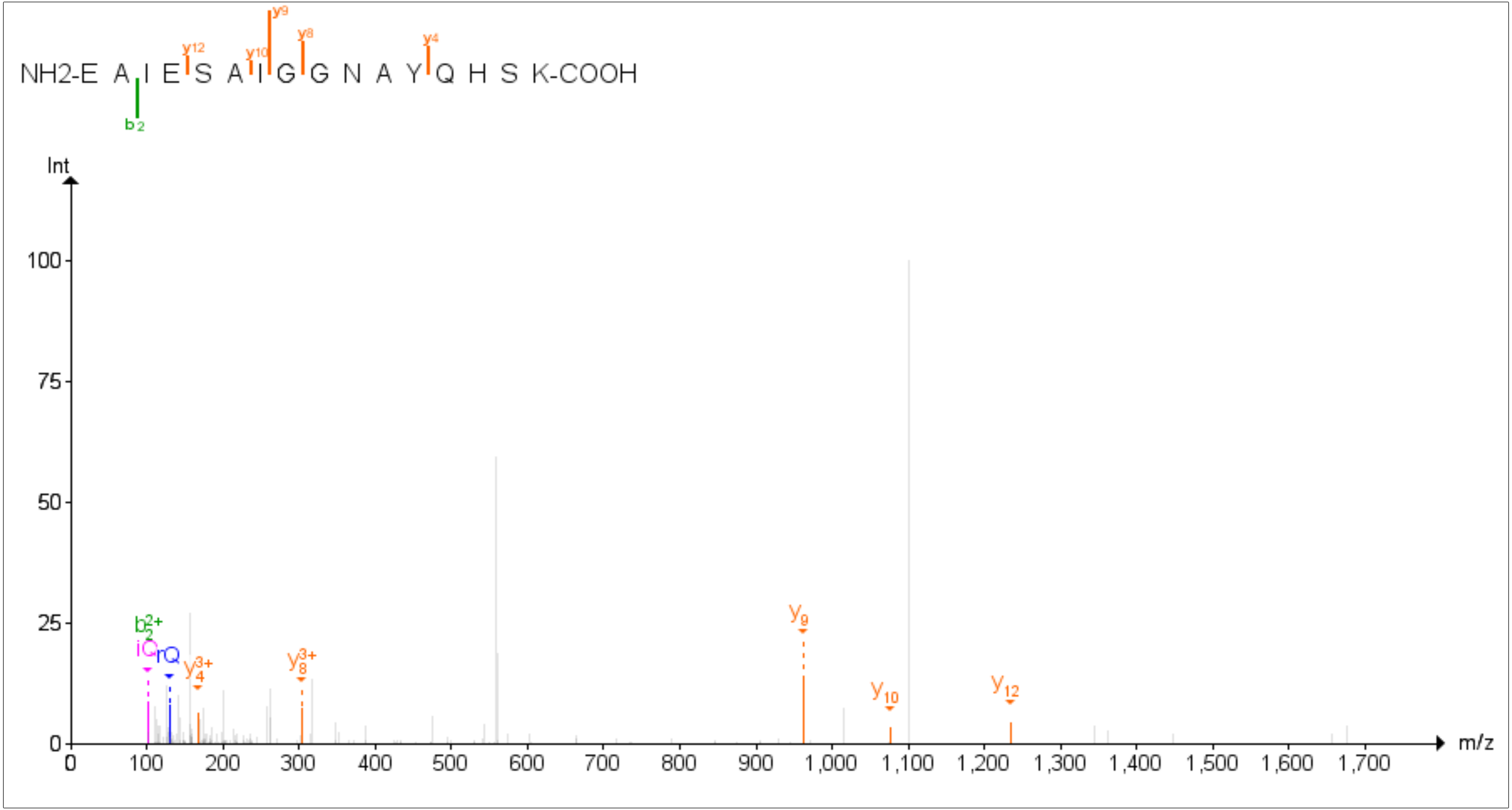

NH2-E A I E S A I G G N A Y Q H S K-COOH
y12
y10
y9
y8
y4
b2
Int
100
75
50
25
0
b2 2+
iQ
rQ
y4 3+
y8 3+
y9
y10
y12
0
100
200
300
400
500
600
700
800
900
1,000
1,100
1,200
1,300
1,400
1,500
1,600
1,700
m/z

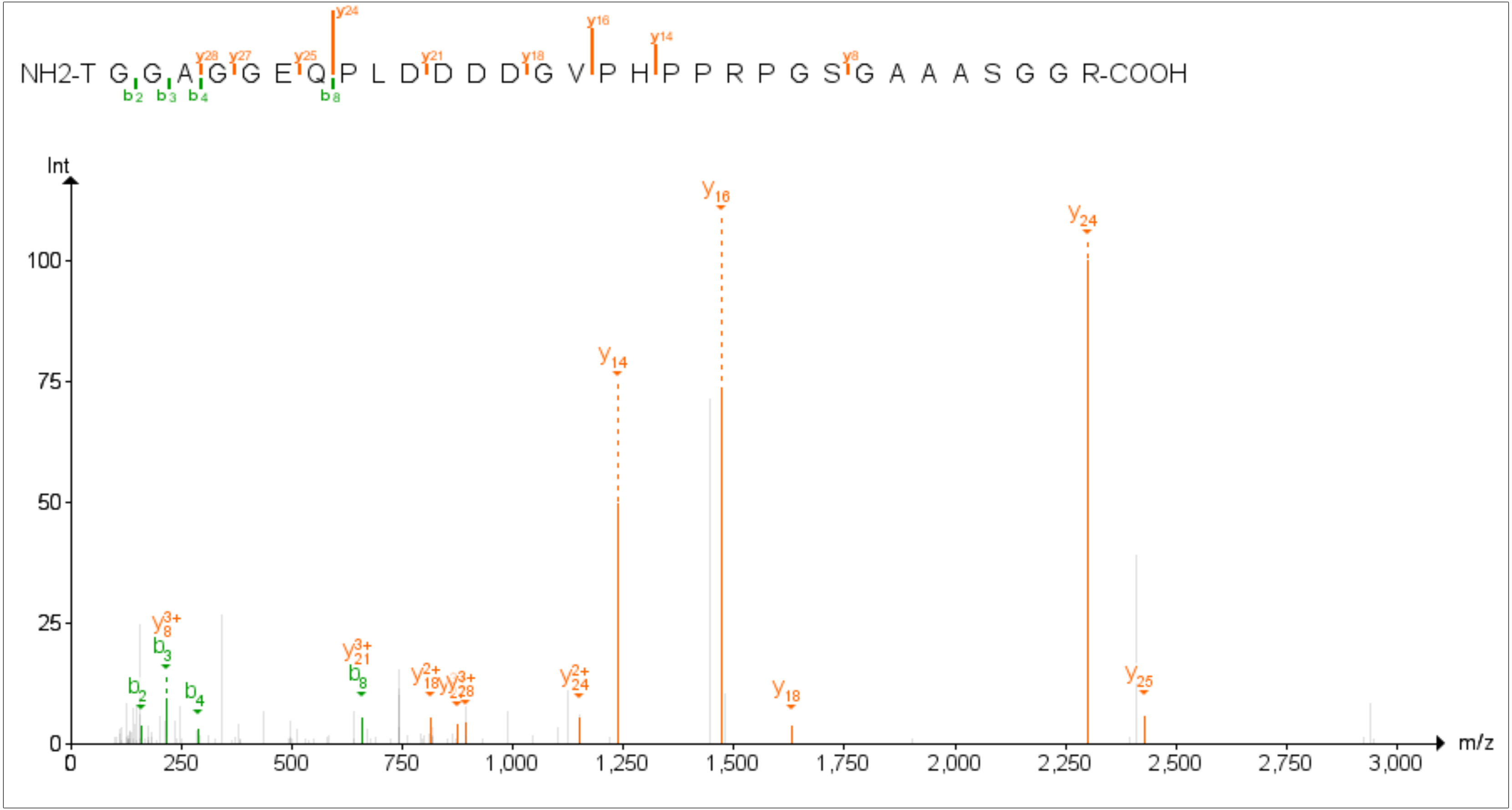

NH2-T G G A G G E Q P L D D D D G V P H P P R P G S G A A A S G G R-COOH
y24
y16
y14
y28
y27
y25
y21
y18
y8
b2
b3
b4
b8
Int
100
75
50
25
0
0
250
500
750
1,000
1,250
1,500
1,750
2,000
2,250
2,500
2,750
3,000
m/z
y16
y24
y14
y8 3+
b3
y21 3+
b8
b2
b4
y18 2+
y28 3+
y24 2+
y18
y25

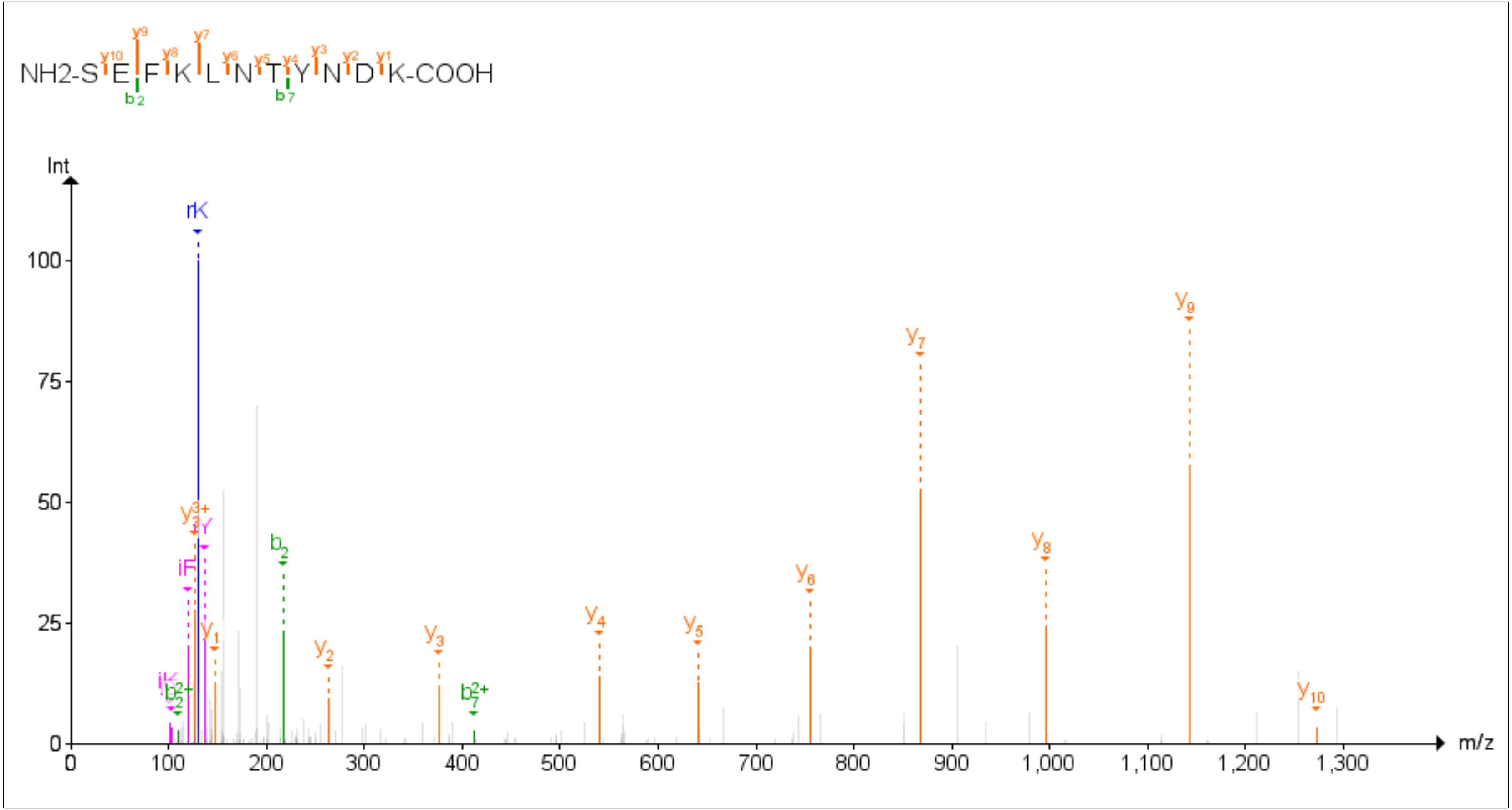
NH2-SEFKLNTYNDK-COOH
Int
m/z
rK
y9
y7
y8
y6
y5
y4
y3
y2
y1
y10
b2
b7
iF
iK
iY
0
25
50
75
100
0
100
200
300
400
500
600
700
800
900
1,000
1,100
1,200
1,300

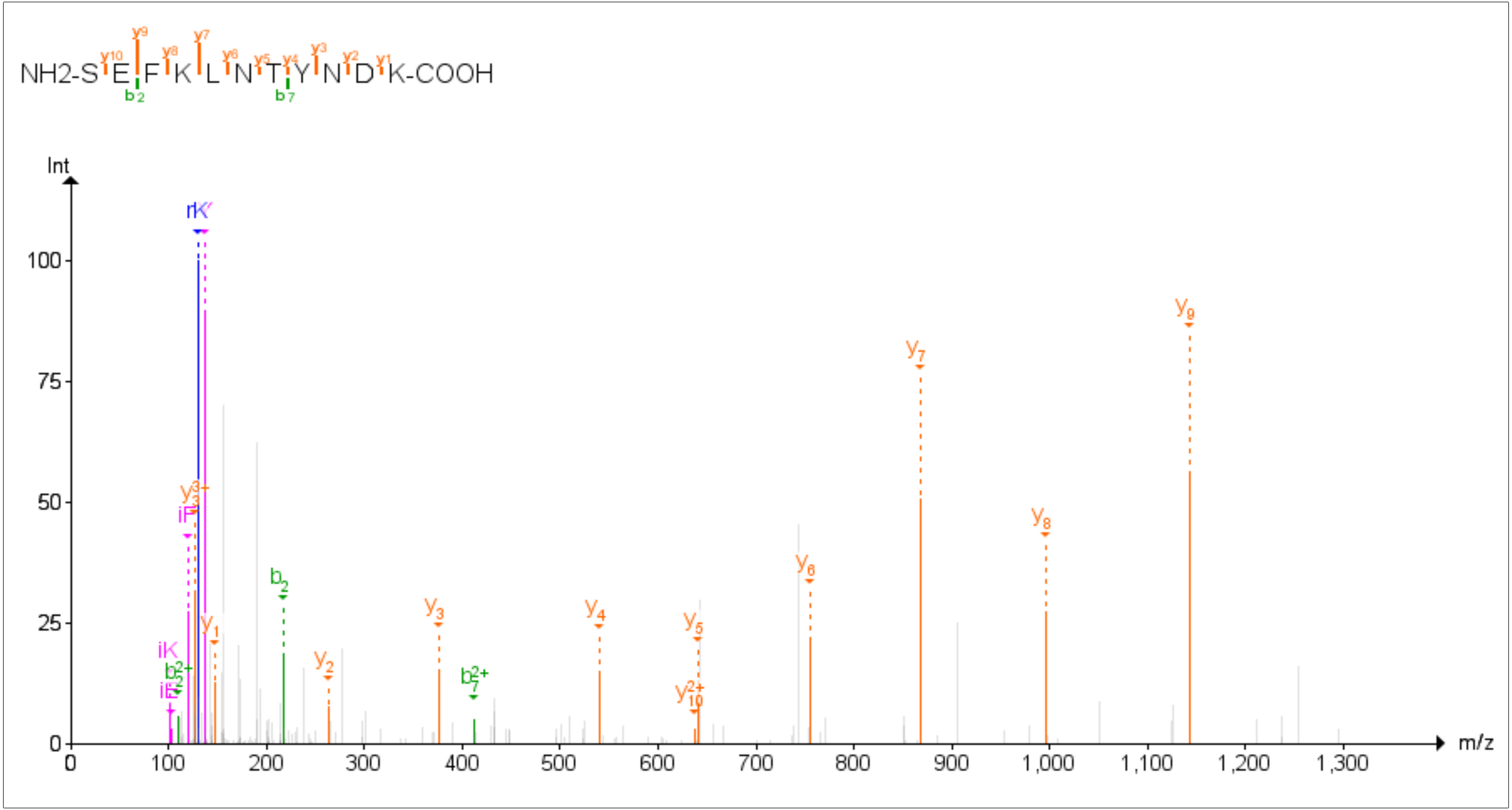

NH2-S E F K L N T Y N D K-COOH
y10 y9 y8 y7 y6 y5 y4 y3 y2 y1
b2 b7
Int
100
75
50
25
0
m/z
0 100 200 300 400 500 600 700 800 900 1,000 1,100 1,200 1,300
rK'
y9
y7
y8
y6
y5
y4
y3
y2
y1
b2
b7 2+
b2 2+
y3 3+
y10 2+
iF
iK
iE

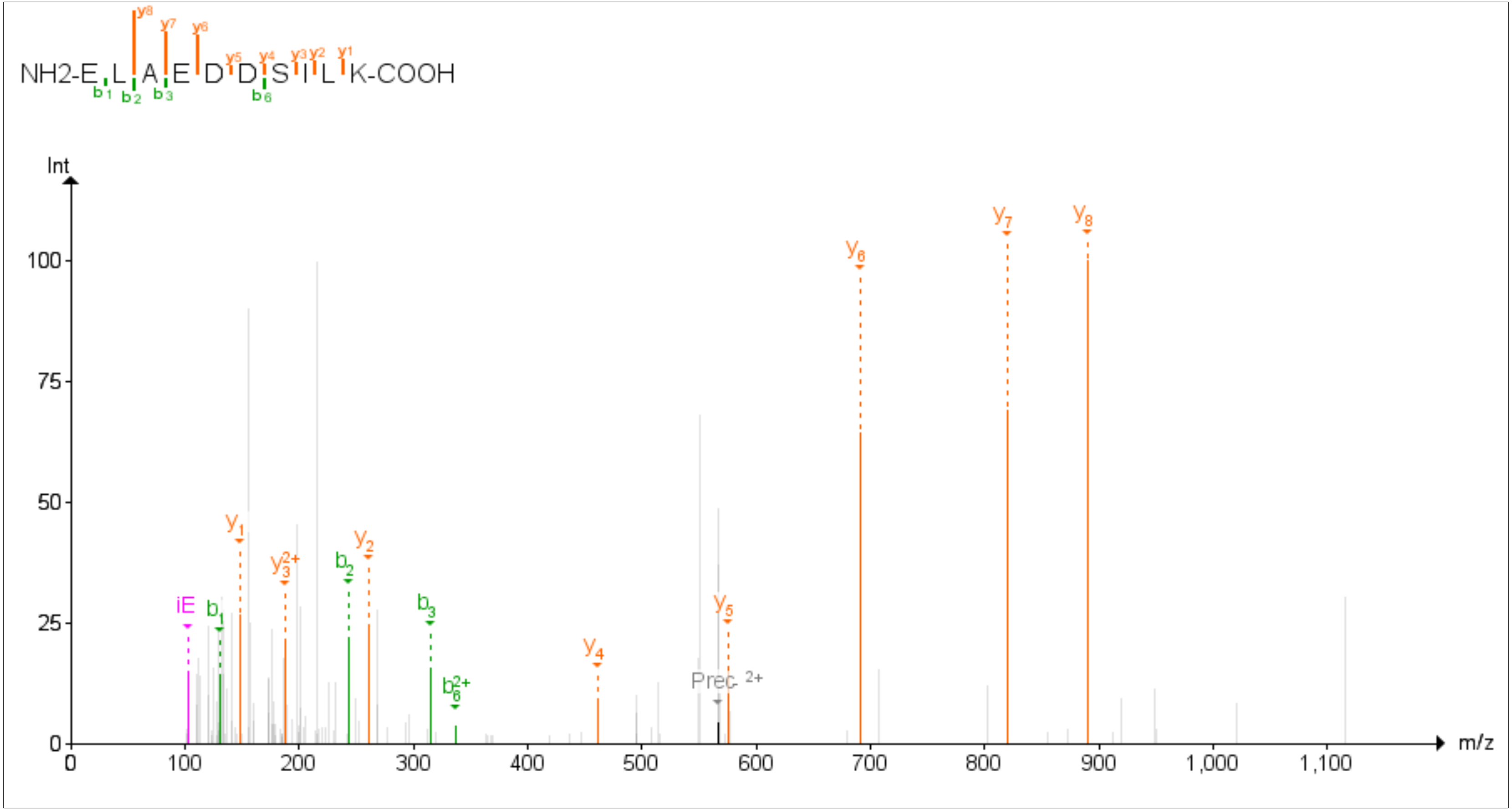

NH2-E L A E D D S I L K-COOH
y8 y7 y6 y5 y4 y3 y2 y1
b1 b2 b3 b6
Int
100
75
50
25
0
m/z
0 100 200 300 400 500 600 700 800 900 1,000 1,100
iE
b1
y1
y3 2+
b2
y2
b3
b6 2+
y4
Prec. 2+
y5
y6
y7
y8

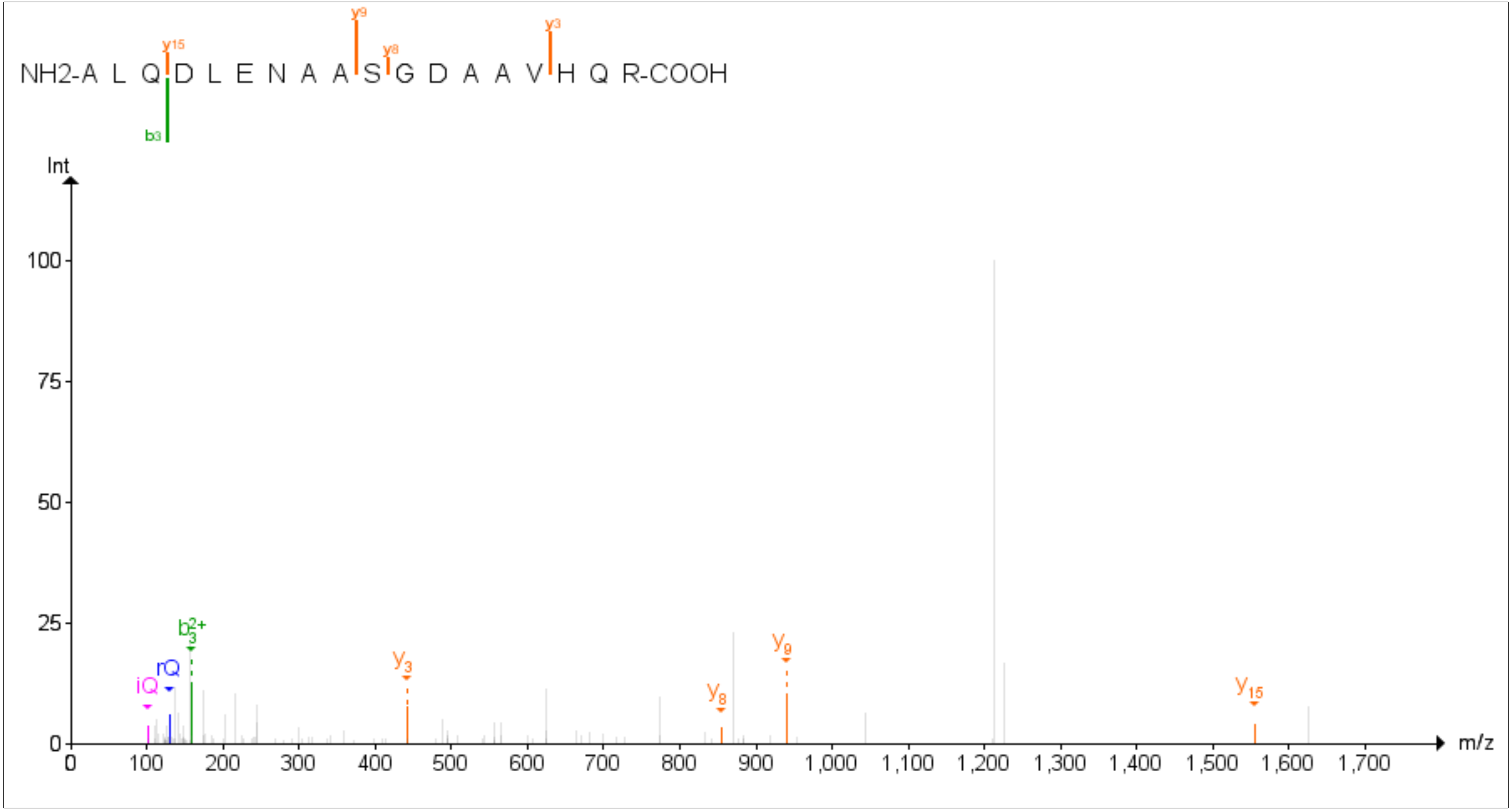
NH2-A L Q D L E N A A S G D A A V H Q R-COOH
y15
y9
y8
y3
b3
Int
100
75
50
25
0
iQ
rQ
b3 2+
y3
y8
y9
y15
0
100
200
300
400
500
600
700
800
900
1,000
1,100
1,200
1,300
1,400
1,500
1,600
1,700
m/z

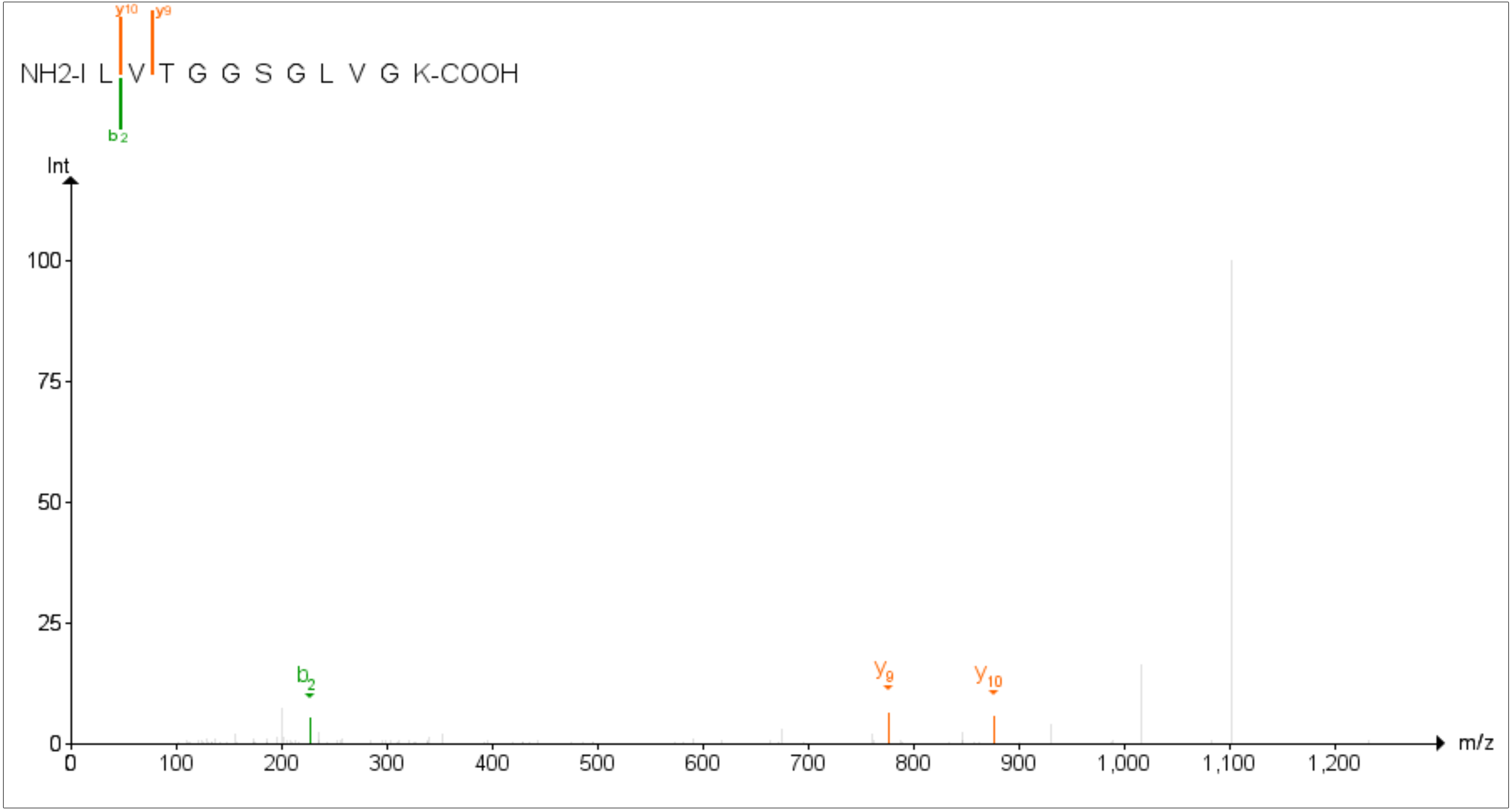

y10
y9
NH2-I L V T G G S G L V G K-COOH
b 2
Int
100
75
50
25
0
b2
y9
y10
0
100
200
300
400
500
600
700
800
900
1,000
1,100
1,200
m/z

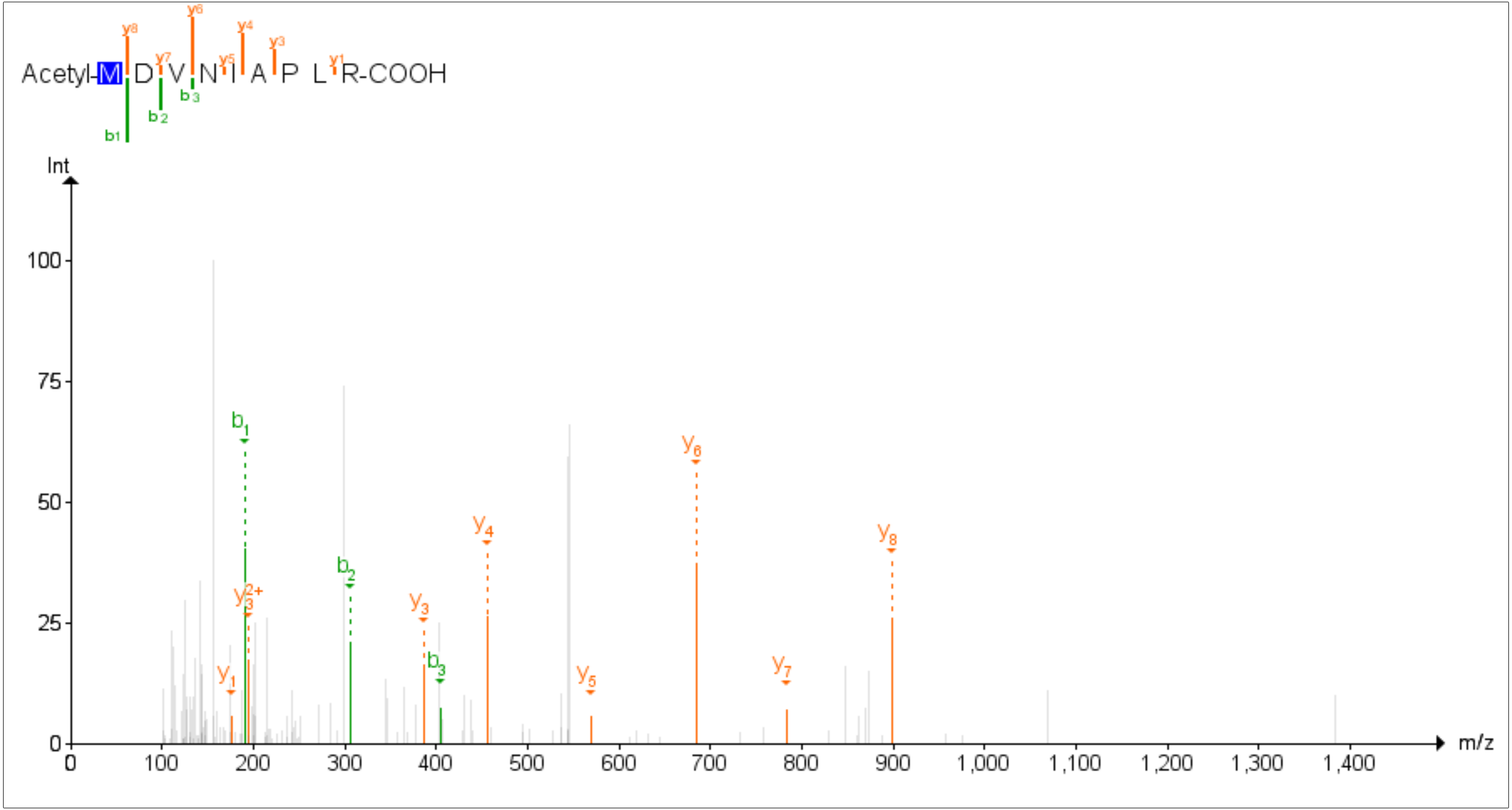

Acetyl-M D V N I A P L R-COOH
y8 y6 y7 y5 y4 y3 y1
b1 b2 b3
Int
100
75
50
25
0
b1
b2
b3
y1
y3 2+
y3
y4
y5
y6
y7
y8
0
100
200
300
400
500
600
700
800
900
1,000
1,100
1,200
1,300
1,400
m/z

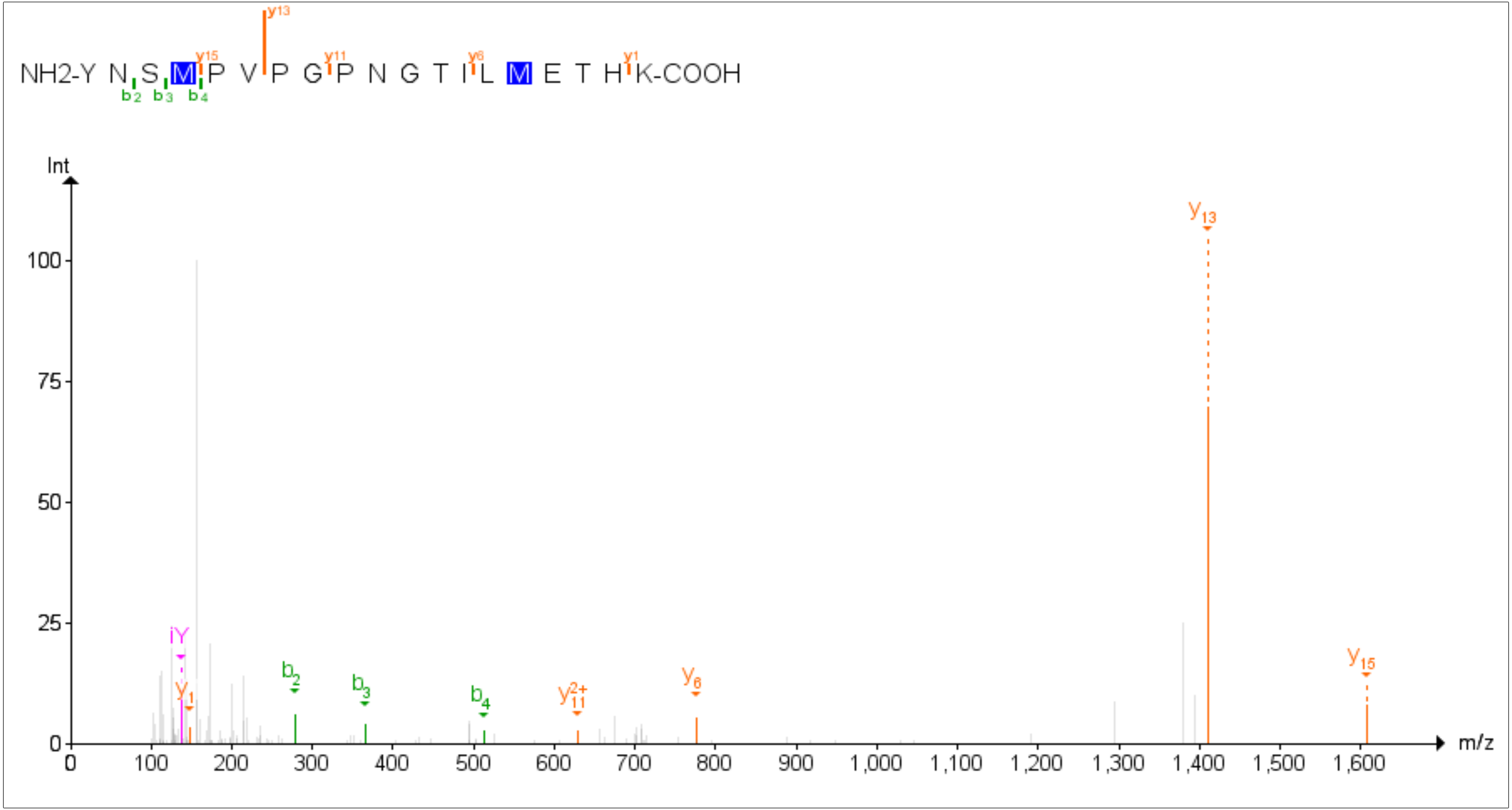

NH2-Y N S M P V P G P N G T I L M E T H K-COOH
b2 b3 b4
y15 y13 y11 y6 y1
Int
100
75
50
25
0
iY
y1
b2
b3
b4
y11 2+
y6
y13
y15
0 100 200 300 400 500 600 700 800 900 1,000 1,100 1,200 1,300 1,400 1,500 1,600
m/z

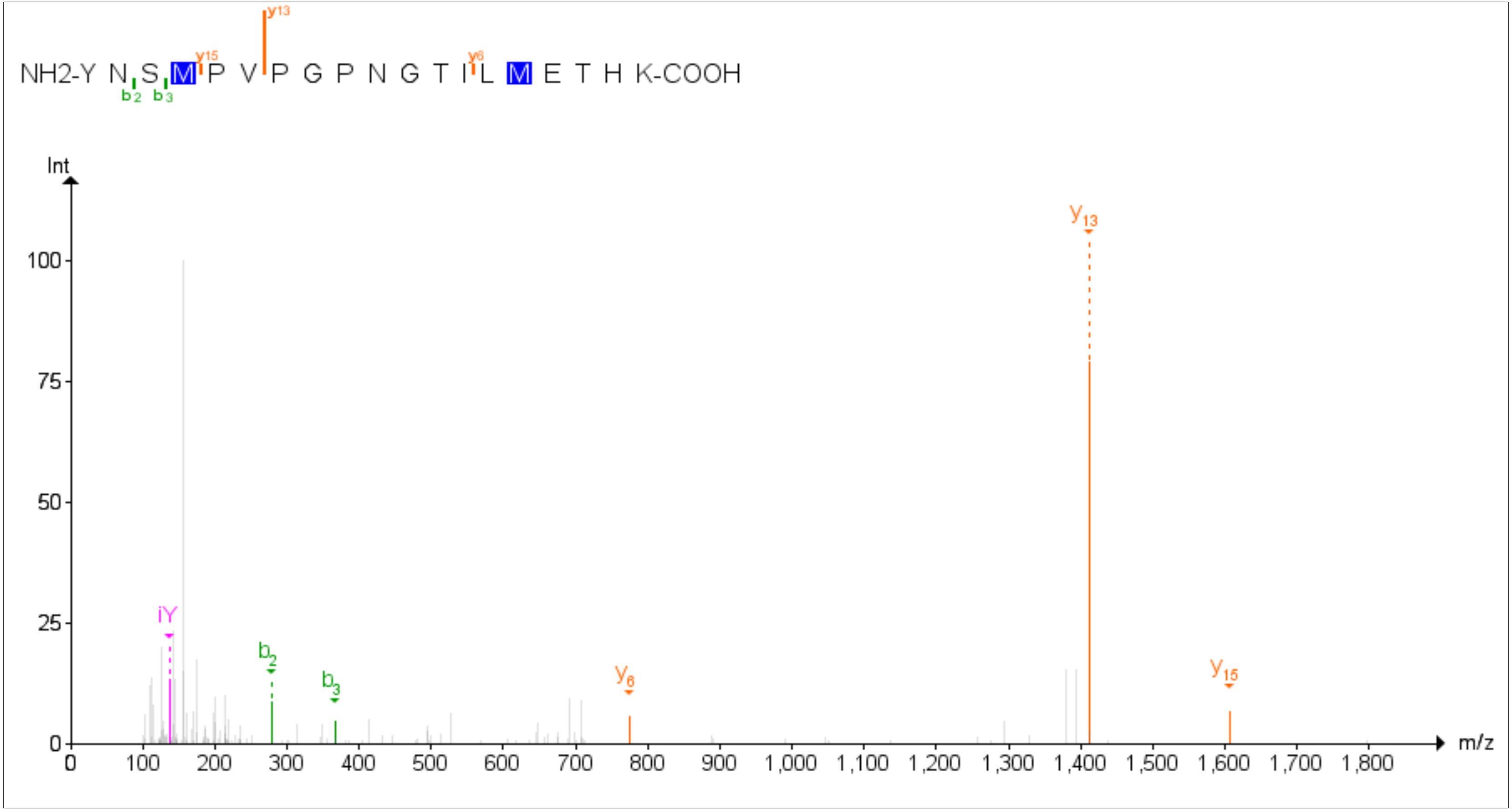

NH2-Y N S M P V P G P N G T I L M E T H K-COOH
b2
b3
y15
y13
y6
Int
m/z
iY
b2
b3
y6
y13
y15
0
25
50
75
100
0
100
200
300
400
500
600
700
800
900
1,000
1,100
1,200
1,300
1,400
1,500
1,600
1,700
1,800

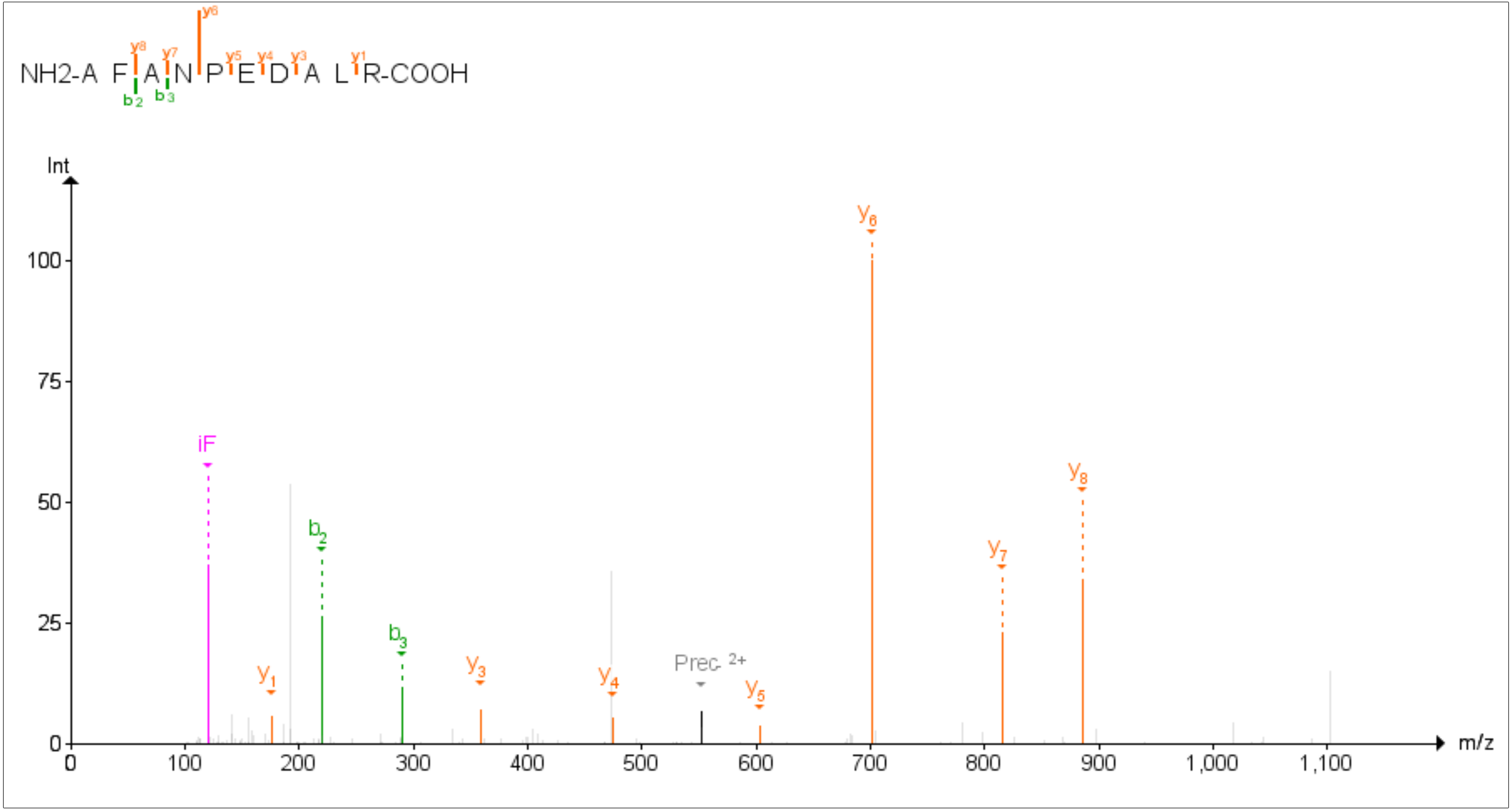

NH2-A F A N P E D A L R-COOH
y8 y7 y6 y5 y4 y3 y1
b2 b3
Int
100
75
50
25
0
0 100 200 300 400 500 600 700 800 900 1,000 1,100
m/z
iF
y1
b2
b3
y3
y4
Prec 2+
y5
y6
y7
y8

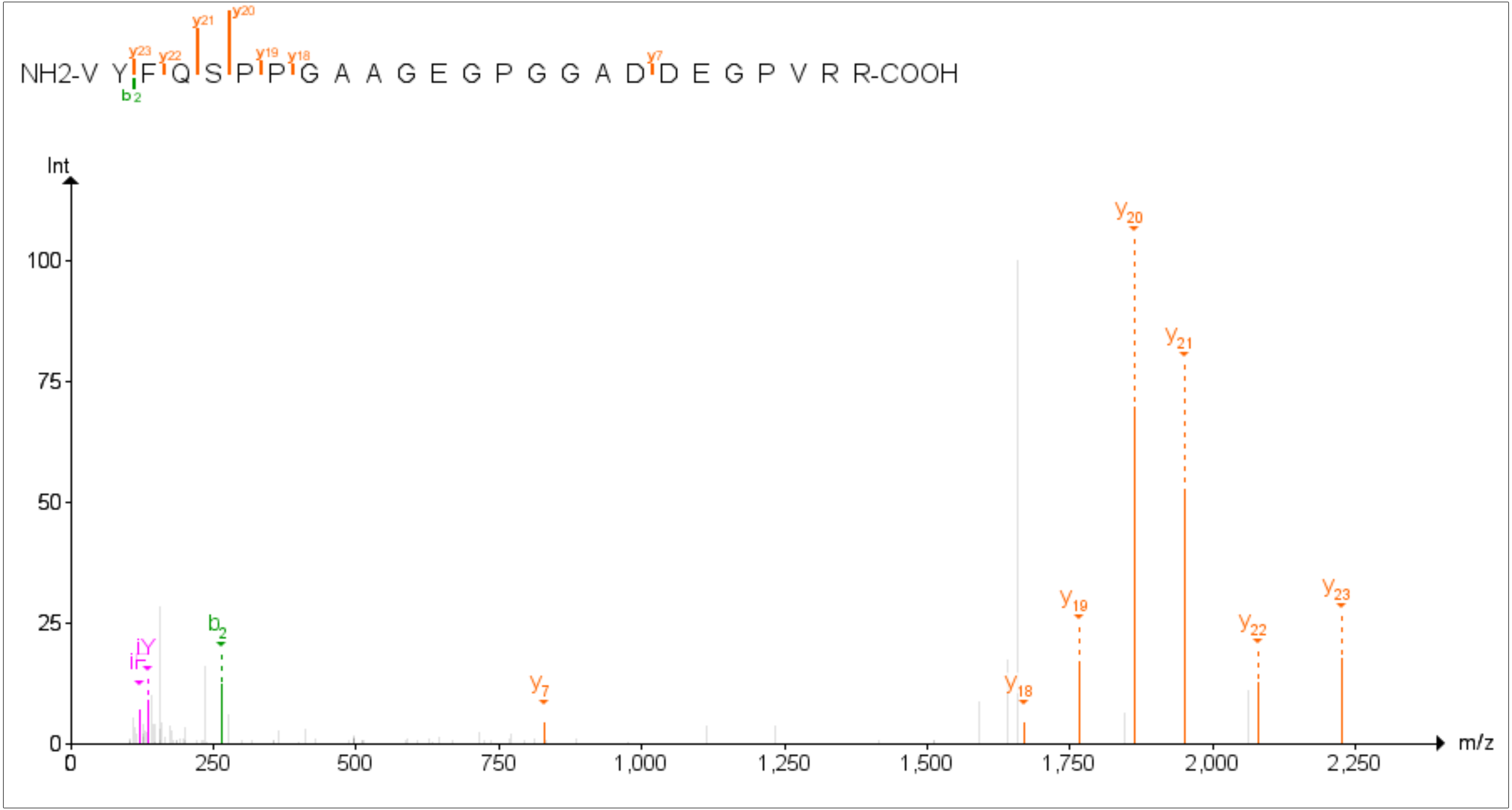

NH2-V Y F Q S P P G A A G E G P G G A D D E G P V R R-COOH
y23 y22 y21 y20 y19 y18 y7
b 2
Int
100
75
50
25
0
iY
iF
b2
y7
y18
y19
y20
y21
y22
y23
0
250
500
750
1,000
1,250
1,500
1,750
2,000
2,250
m/z

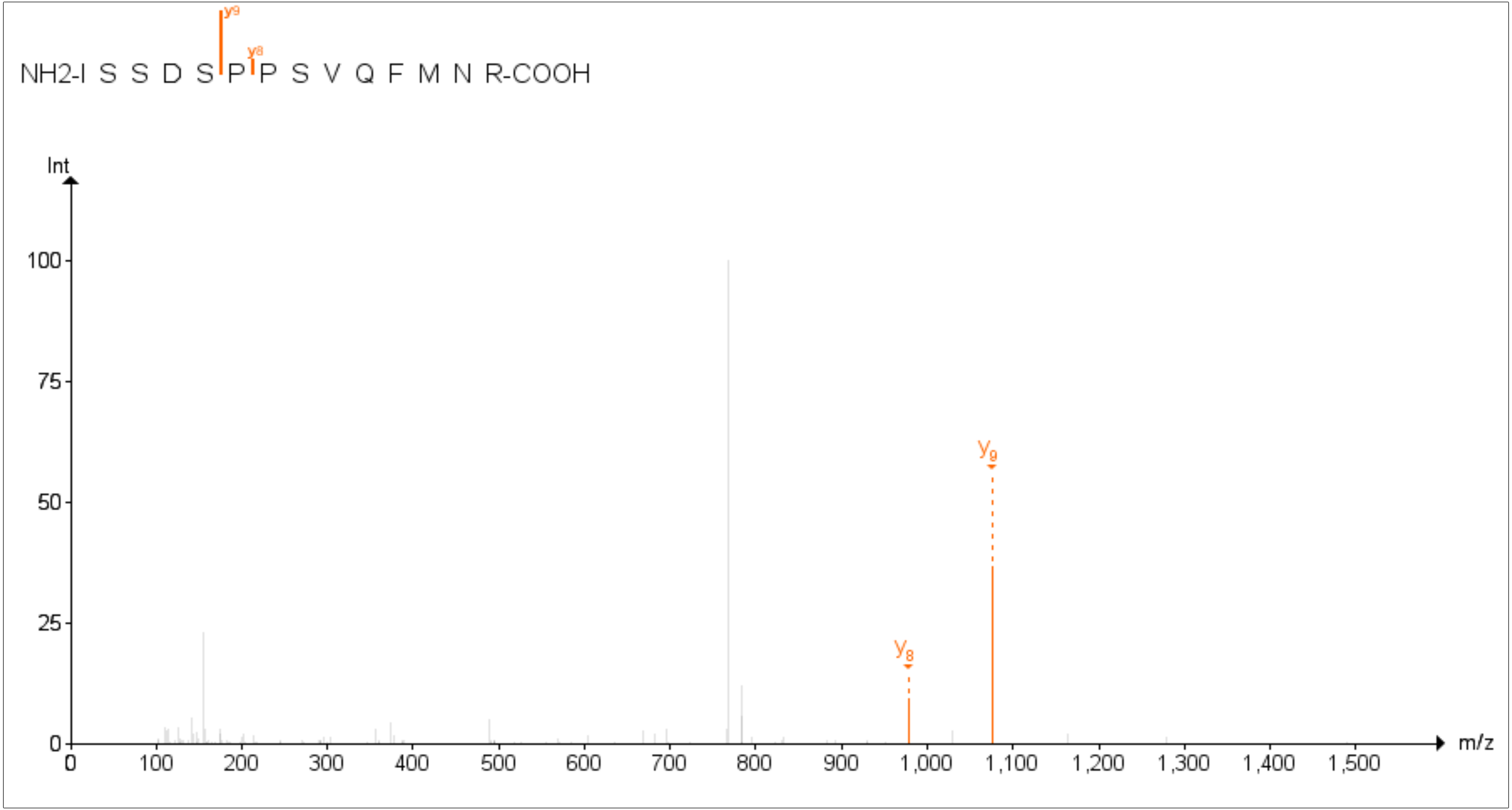

NH2-I S S D S P P S V Q F M N R-COOH
y9
y8
Int
100
75
50
25
0
m/z
0
100
200
300
400
500
600
700
800
900
1,000
1,100
1,200
1,300
1,400
1,500

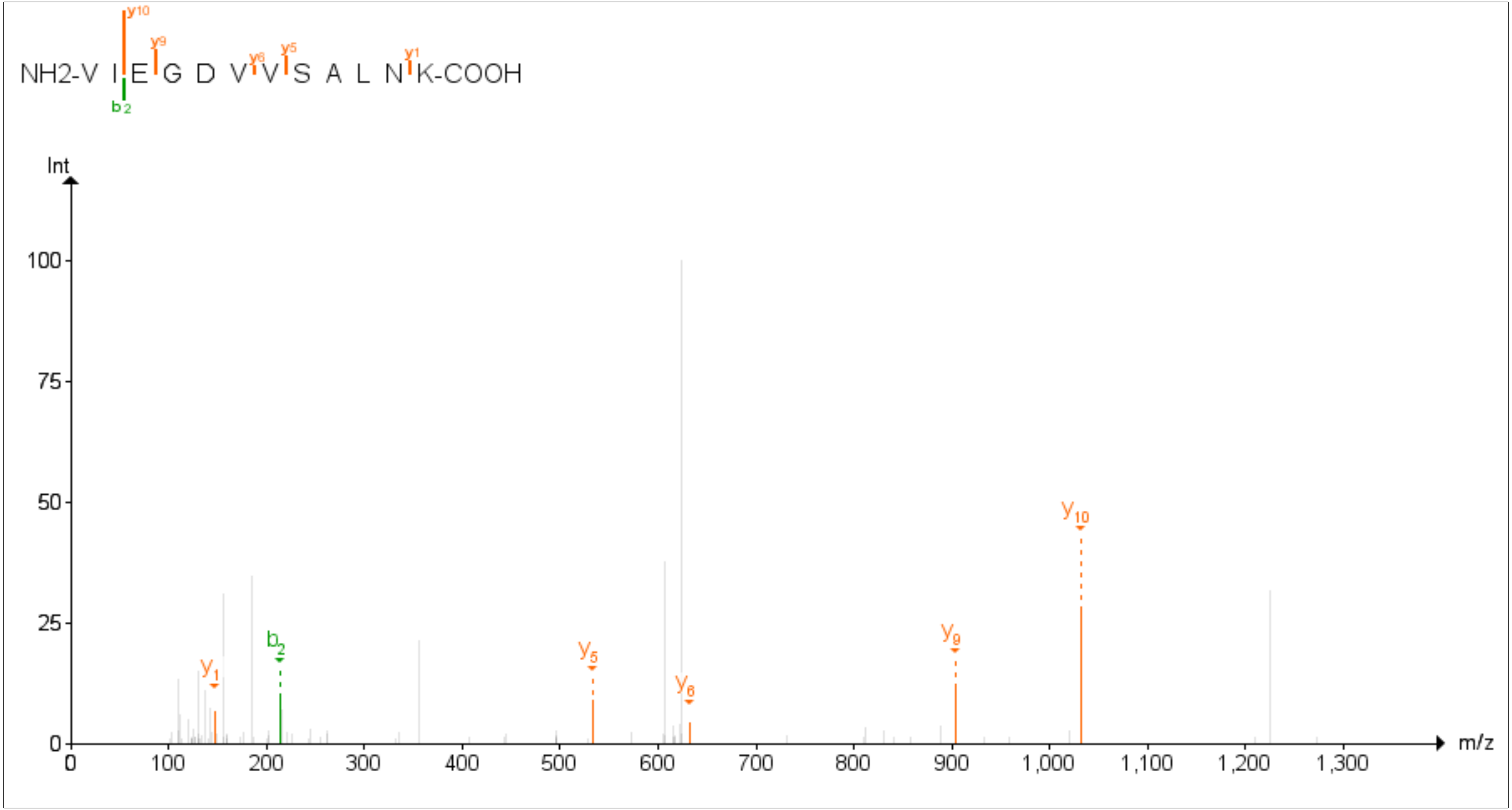

NH2-V I E G D V V S A L N K-COOH
y10
y9
y6
y5
y1
b2
Int
100
75
50
25
0
m/z
0
100
200
300
400
500
600
700
800
900
1,000
1,100
1,200
1,300

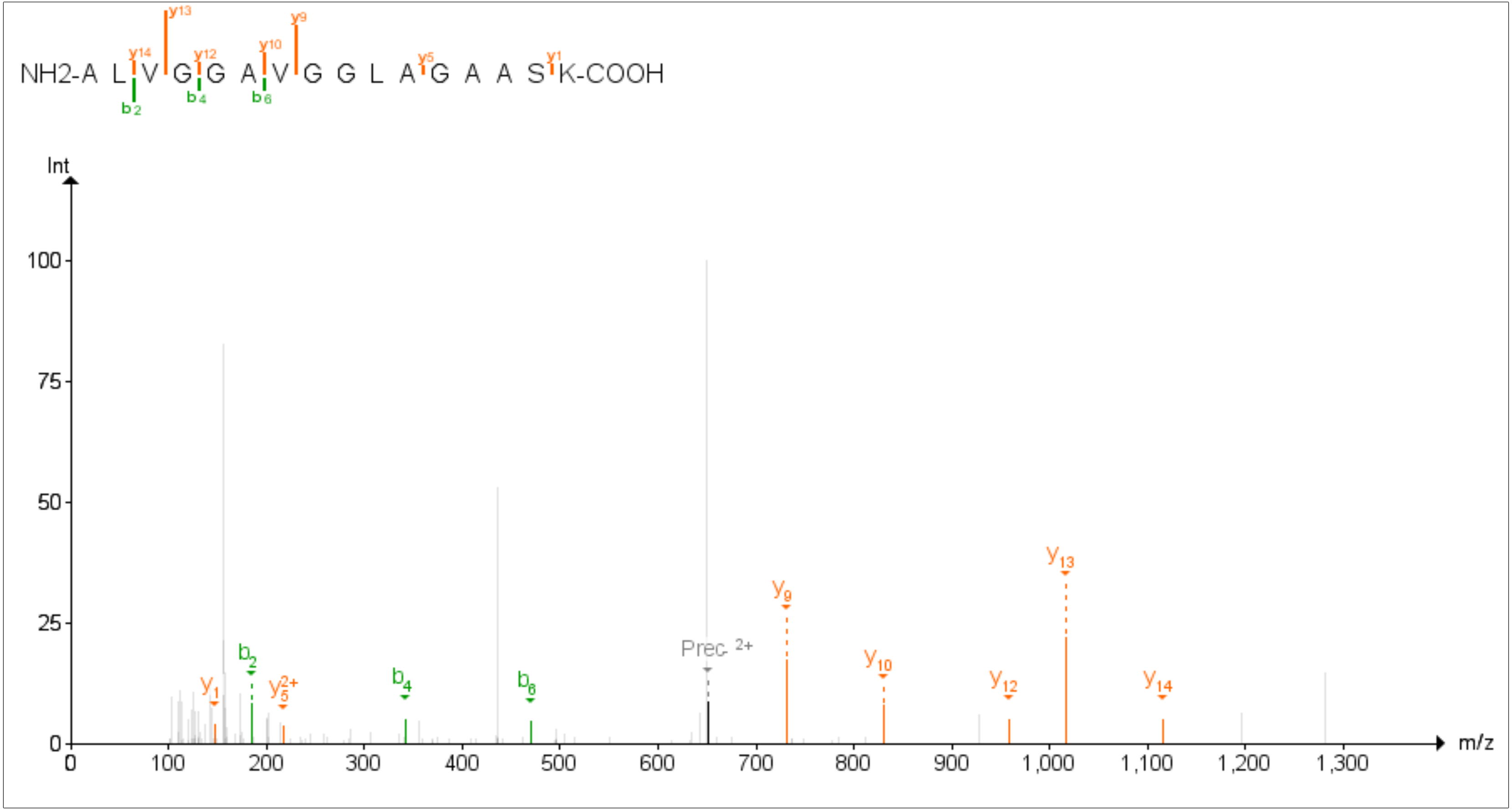
NH2-A L V G G A V G G L A G A A S K-COOH
y13
y9
y14
y12
y10
y5
y1
b2
b4
b6
Int
100
75
50
25
0
Prec. 2+
y9
y13
y10
y12
y14
b2
y1
y5 2+
b4
b6
0
100
200
300
400
500
600
700
800
900
1,000
1,100
1,200
1,300
m/z

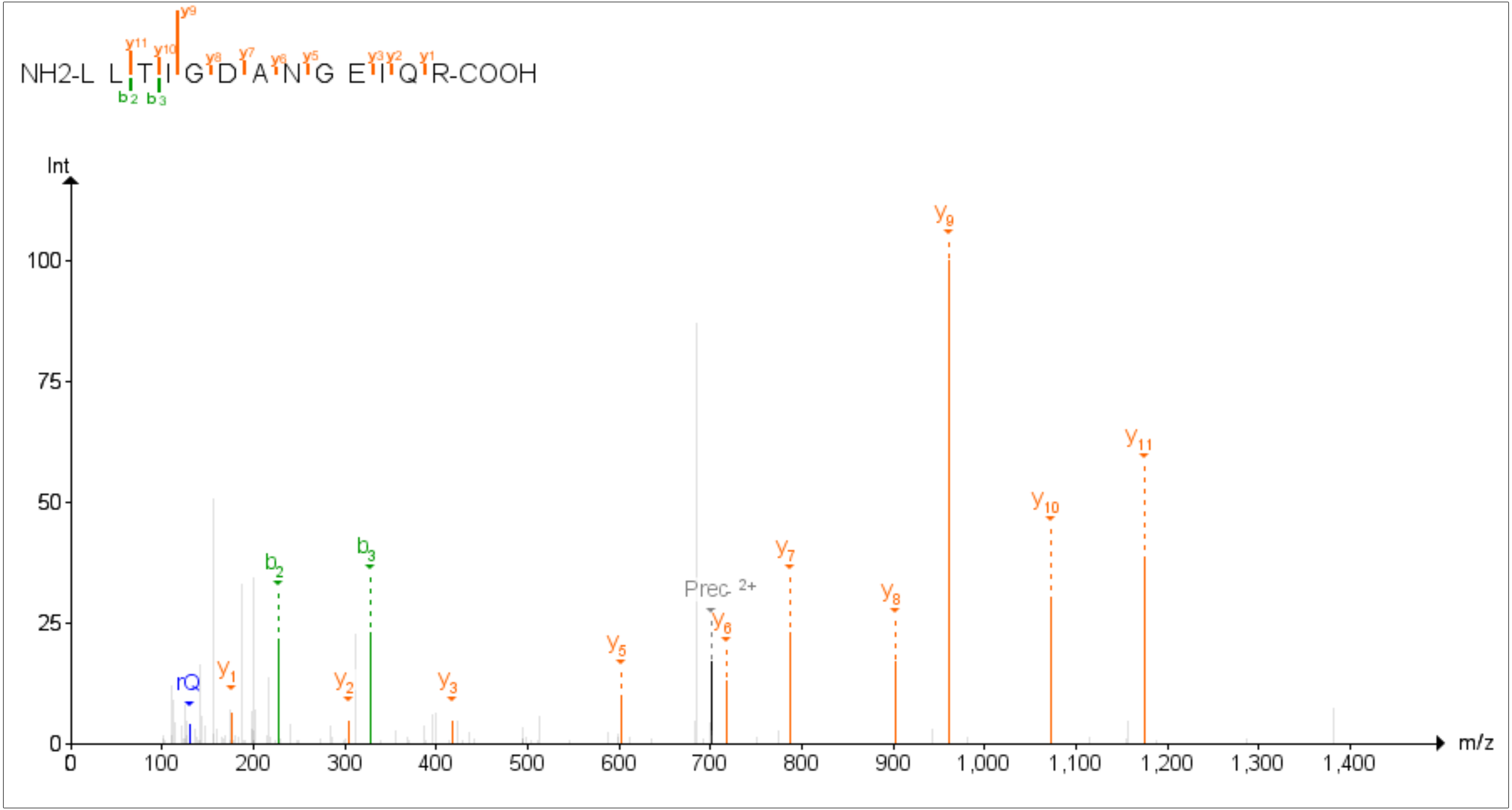

NH2-L L T I G D A N G E I Q R-COOH
y11 y10 y9 y8 y7 y6 y5 y3 y2 y1
b2 b3
Int
m/z
rQ
Prec 2+
0
25
50
75
100
0
100
200
300
400
500
600
700
800
900
1,000
1,100
1,200
1,300
1,400

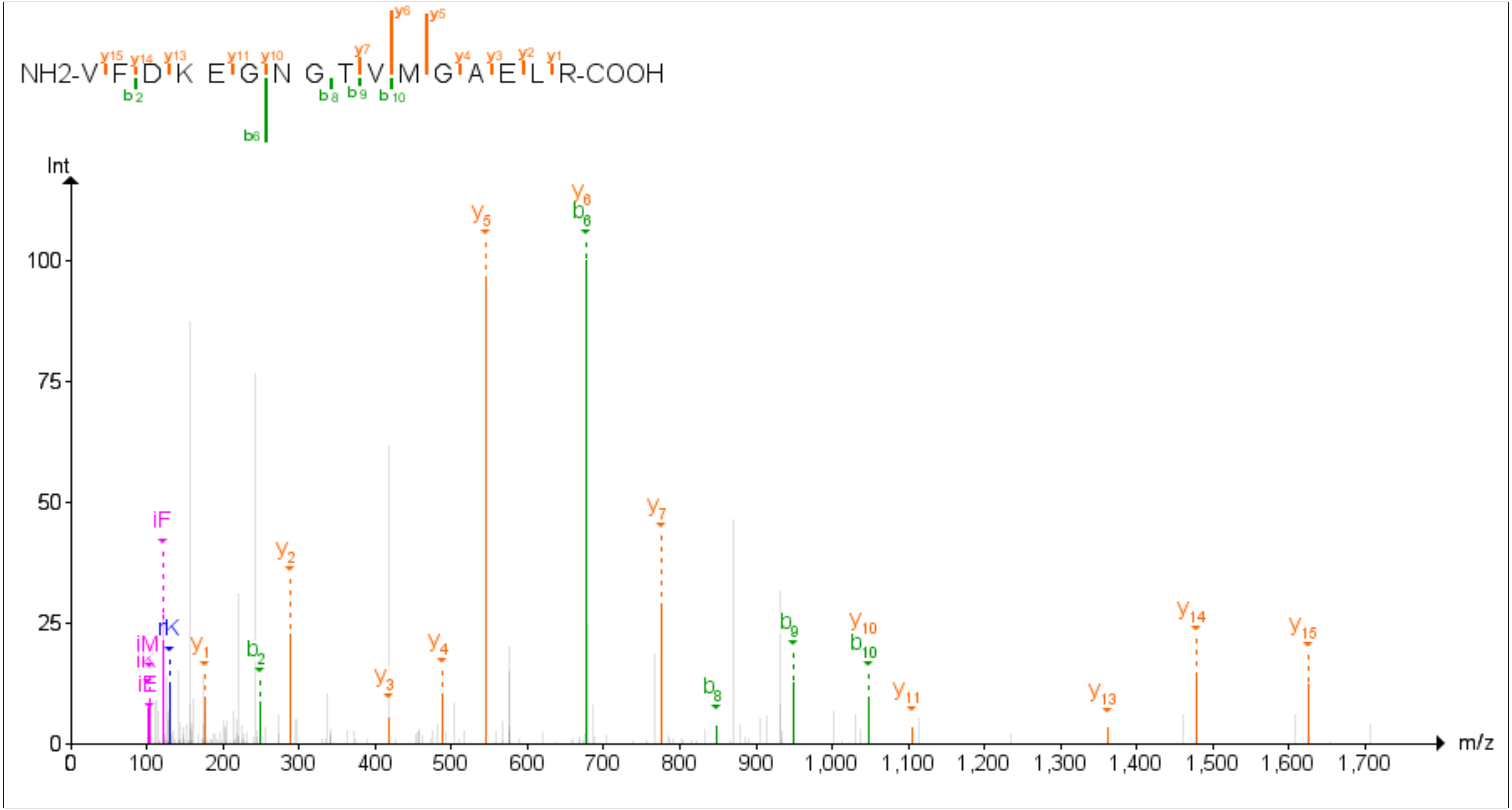

NH2-VFDKEGINGTVMGAELR-COOH
Int
m/z
y15 y14 y13 y11 y10 y7 y6 y5 y4 y3 y2 y1
b2 b6 b8 b9 b10
iF iM iK iE rK

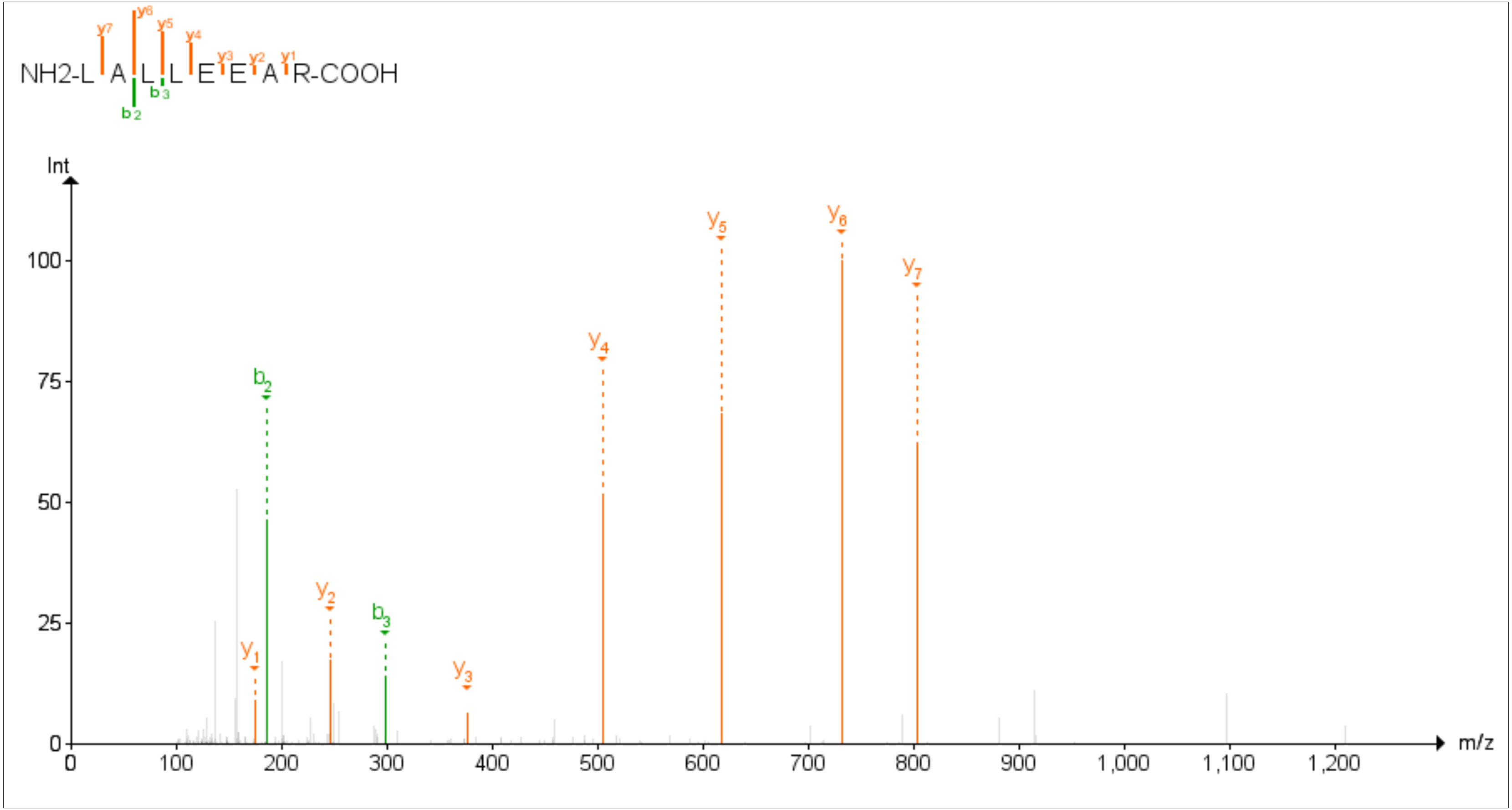
NH2-L A L L E E A R-COOH
y7
y6
y5
y4
y3
y2
y1
b2
b3
Int
100
75
50
25
0
y_1
b_2
y_2
b_3
y_3
y_4
y_5
y_6
y_7
0
100
200
300
400
500
600
700
800
900
1,000
1,100
1,200
m/z

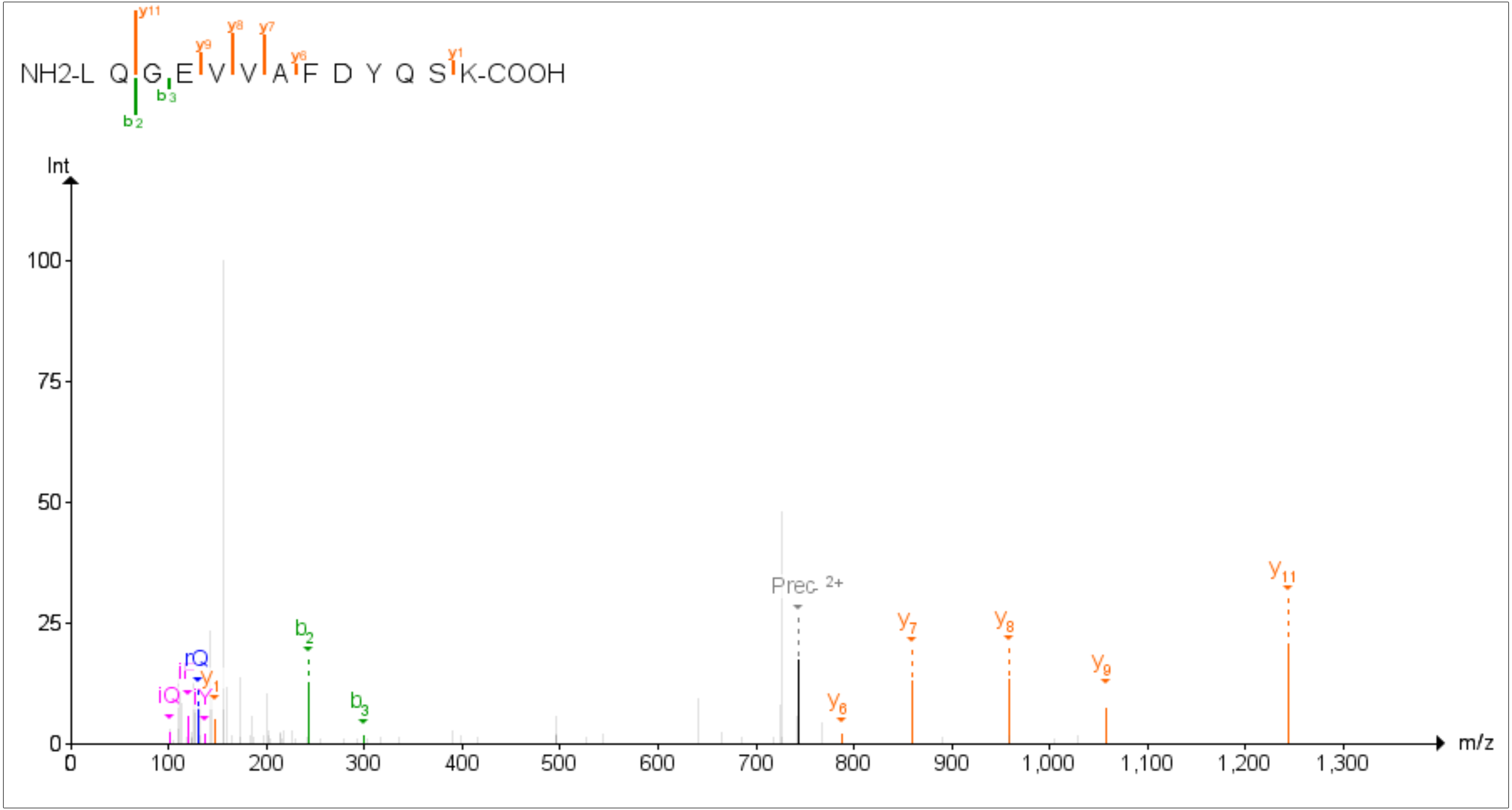

NH2-L Q G E V V A F D Y Q S K-COOH
y11
y9
y8
y7
y6
y1
b2
b3
Int
100
75
50
25
0
Prec 2+
b2
b3
iQ
iF
rQ
iY
y1
y6
y7
y8
y9
y11
0
100
200
300
400
500
600
700
800
900
1,000
1,100
1,200
1,300
m/z

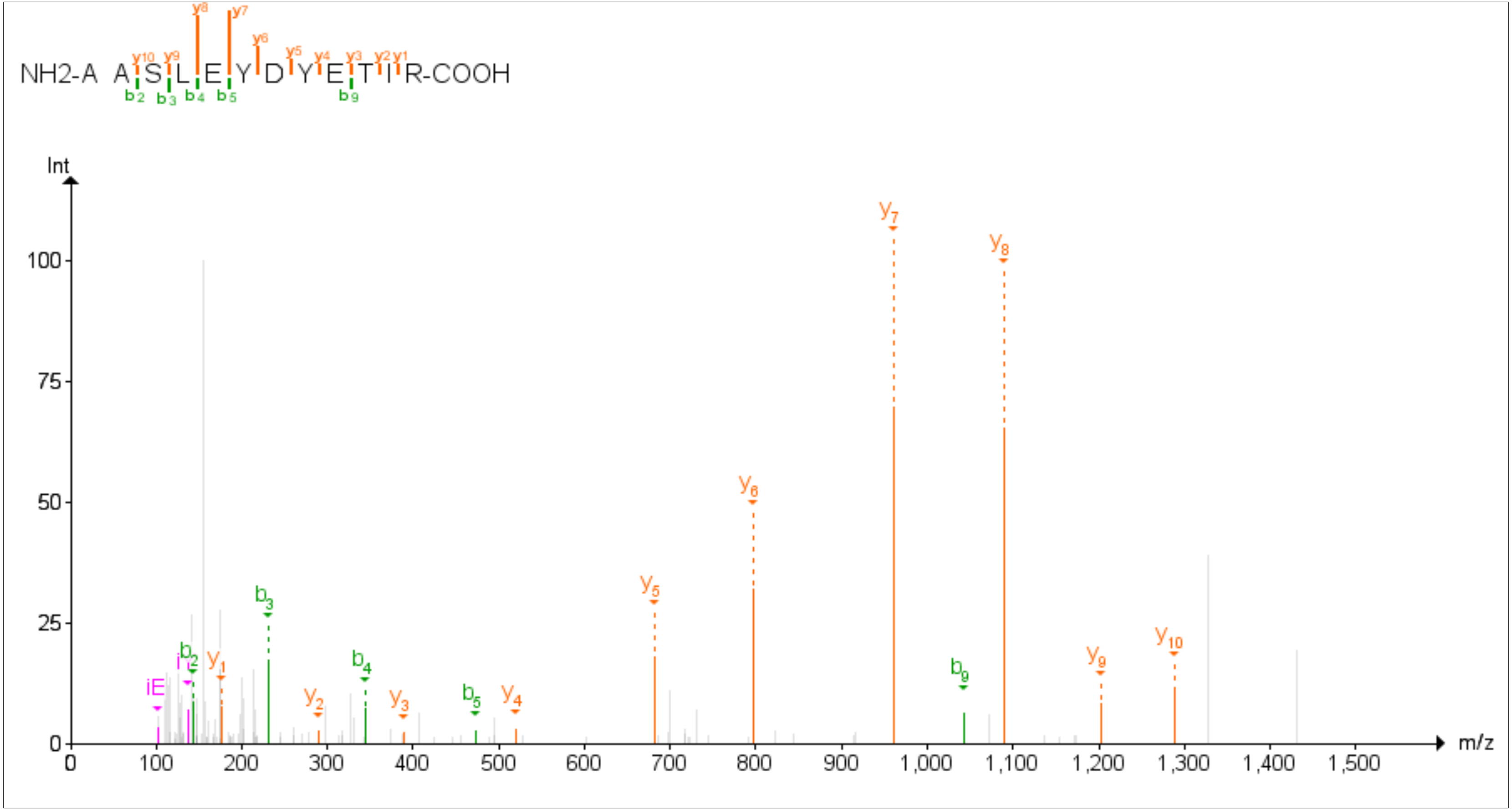
NH2-A A S L E Y D Y E T I R-COOH
y10 y9 y8 y7 y6 y5 y4 y3 y2 y1
b2 b3 b4 b5 b9
Int
100
75
50
25
0
m/z
0 100 200 300 400 500 600 700 800 900 1,000 1,100 1,200 1,300 1,400 1,500
iE
b2
y1
b3
y2
b4
y3
b5
y4
y5
y6
y7
y8
b9
y9
y10

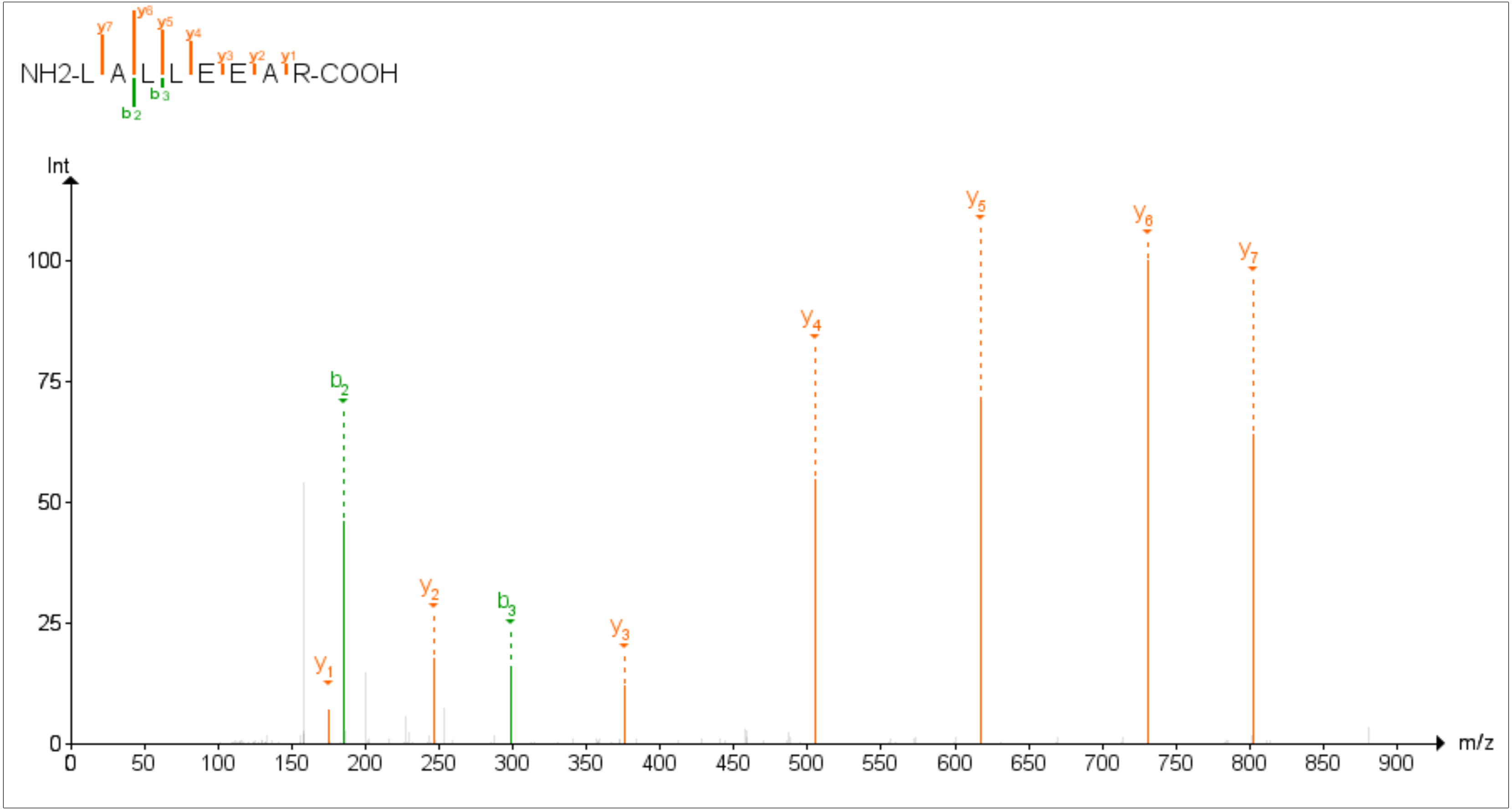

NH2-L A L L E E A R-COOH
y7
y6
y5
y4
y3
y2
y1
b2
b3
Int
100
75
50
25
0
m/z
0
50
100
150
200
250
300
350
400
450
500
550
600
650
700
750
800
850
900
b2
y1
y2
b3
y3
y4
y5
y6
y7

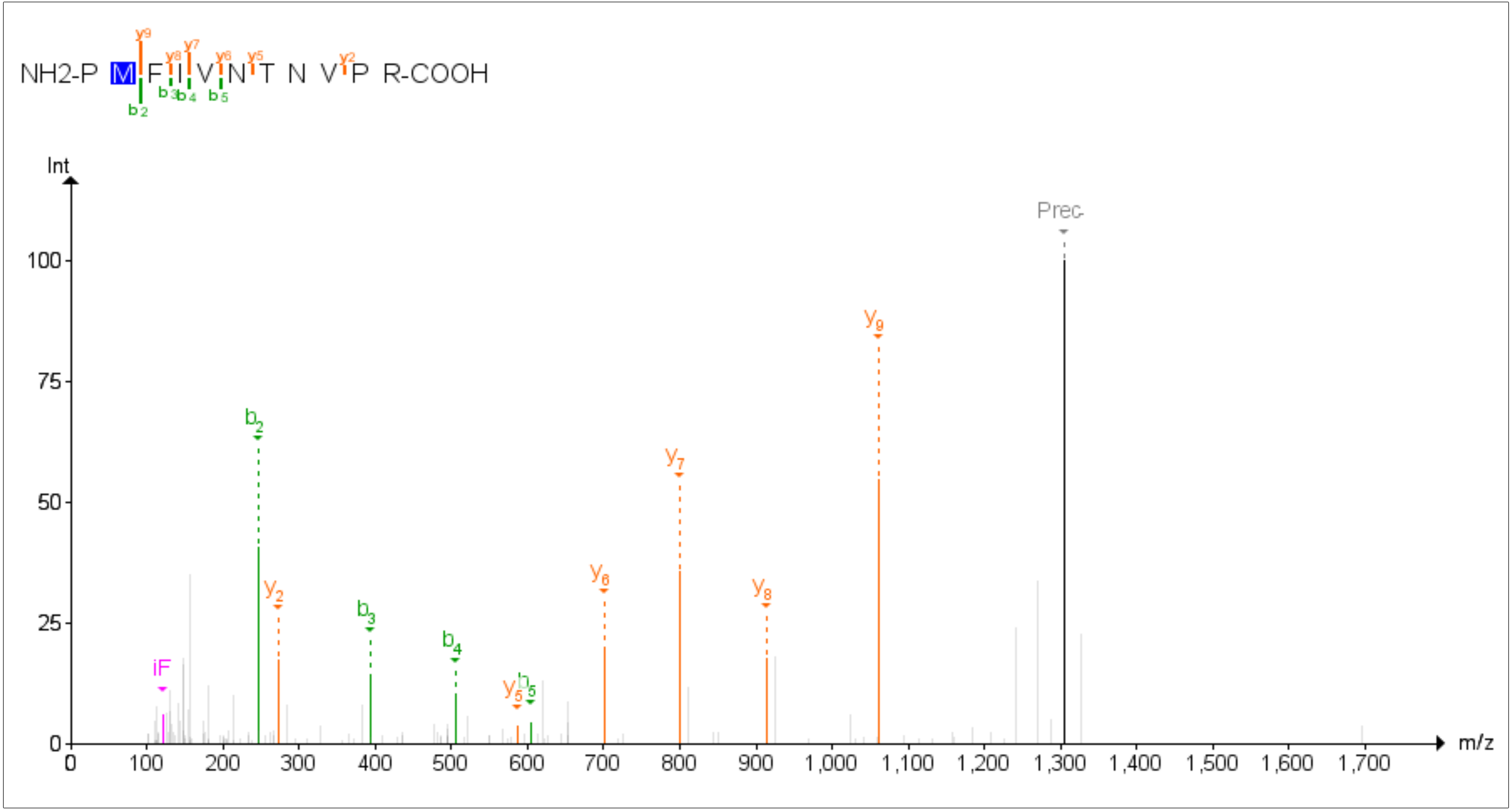

NH2-P M F I I V N T N V P R-COOH
Int
Prec
m/z
y9
y7
b2
y6
y8
y2
b3
b4
y5
b5
iF
0
25
50
75
100
0
100
200
300
400
500
600
700
800
900
1,000
1,100
1,200
1,300
1,400
1,500
1,600
1,700

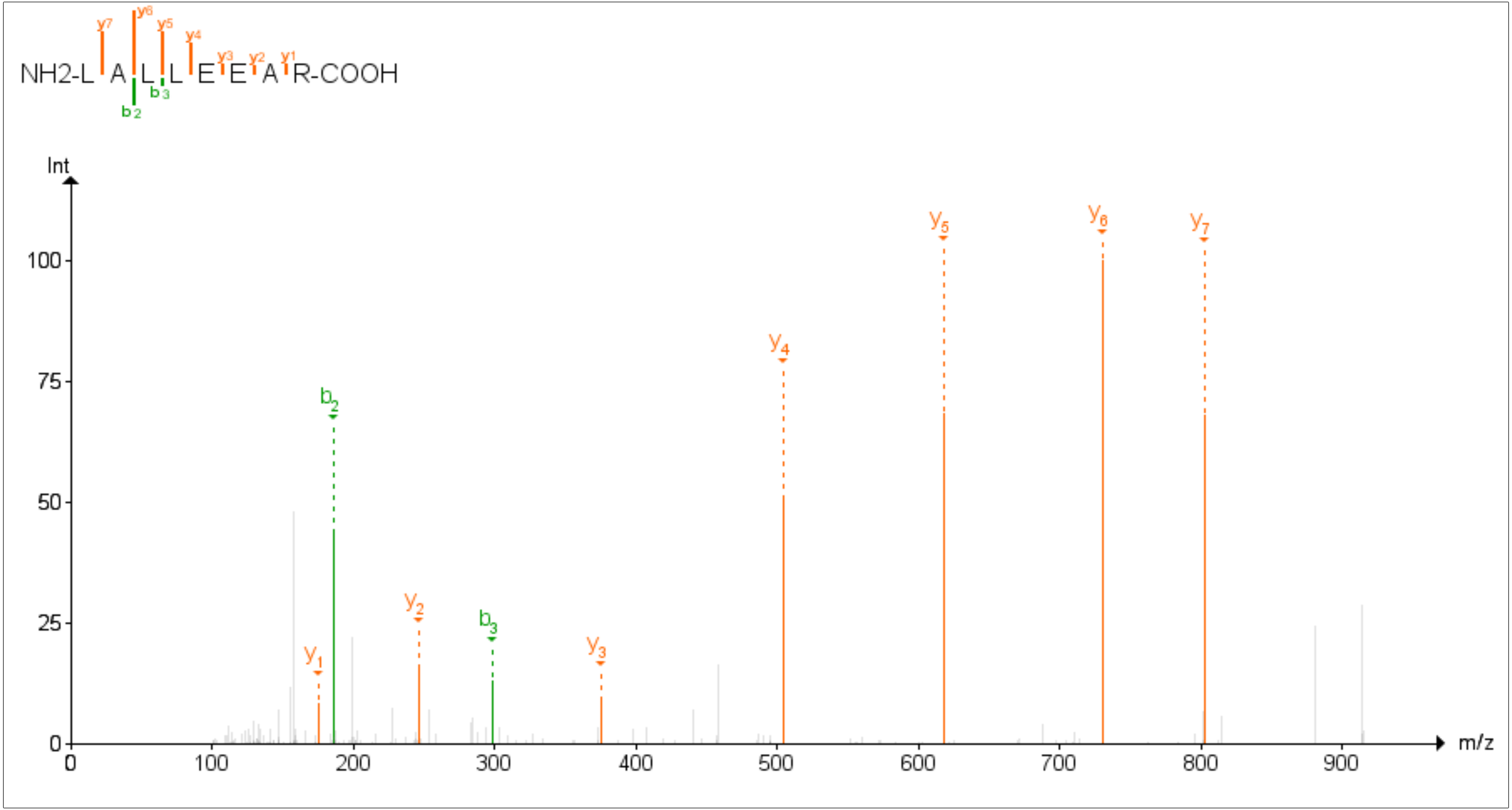

NH2-L A L L E E A R-COOH
y7
y6
y5
y4
y3
y2
y1
b2
b3
Int
100
75
50
25
0
m/z
0
100
200
300
400
500
600
700
800
900
b2
y1
y2
b3
y3
y4
y5
y6
y7

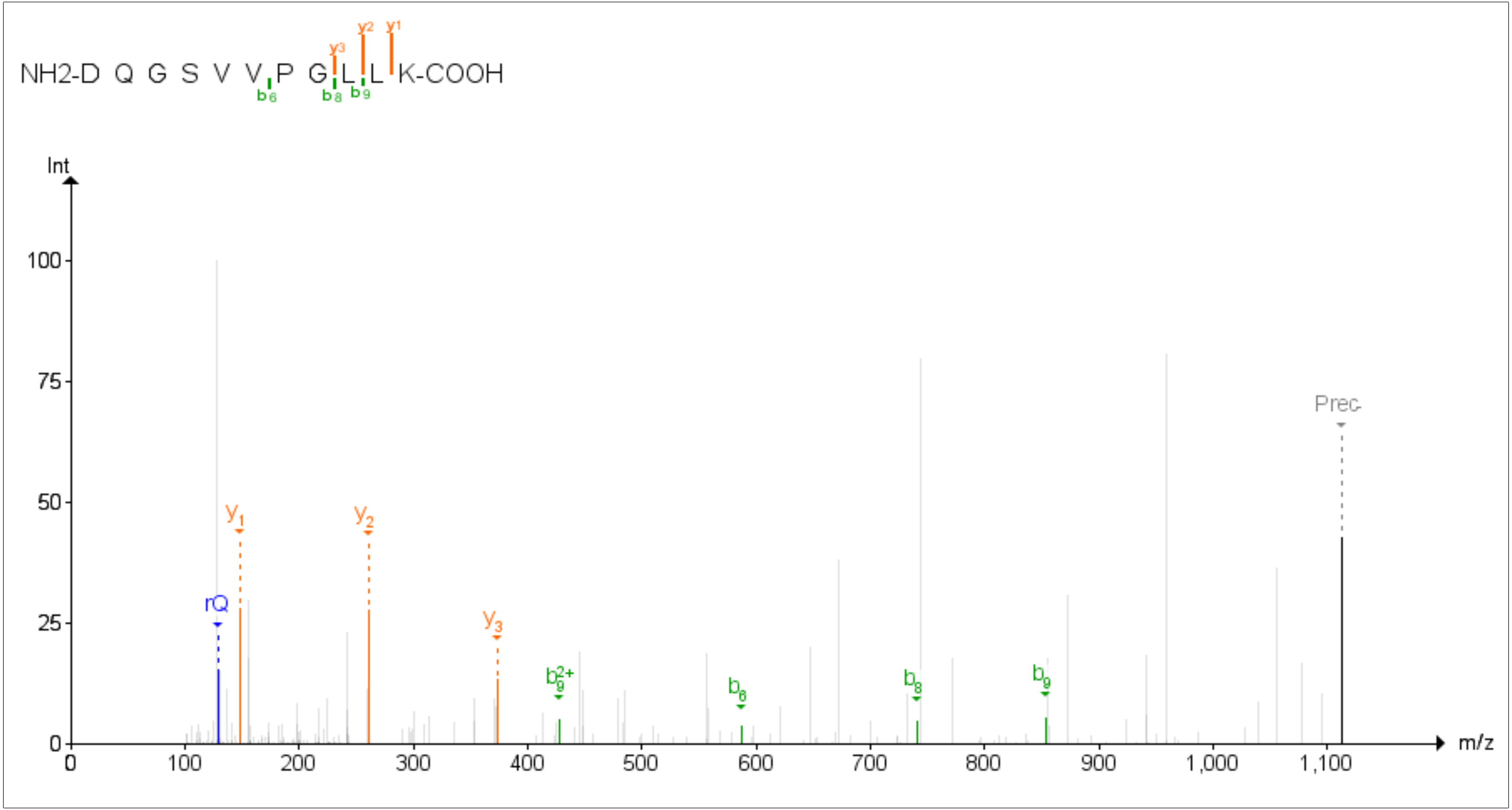

NH2-D Q G S V V P G L L K-COOH
y3 y2 y1
b6 b8 b9
Int
100
75
50
25
0
rQ
y1
y2
y3
b9 2+
b6
b8
b9
Prec
0 100 200 300 400 500 600 700 800 900 1,000 1,100
m/z

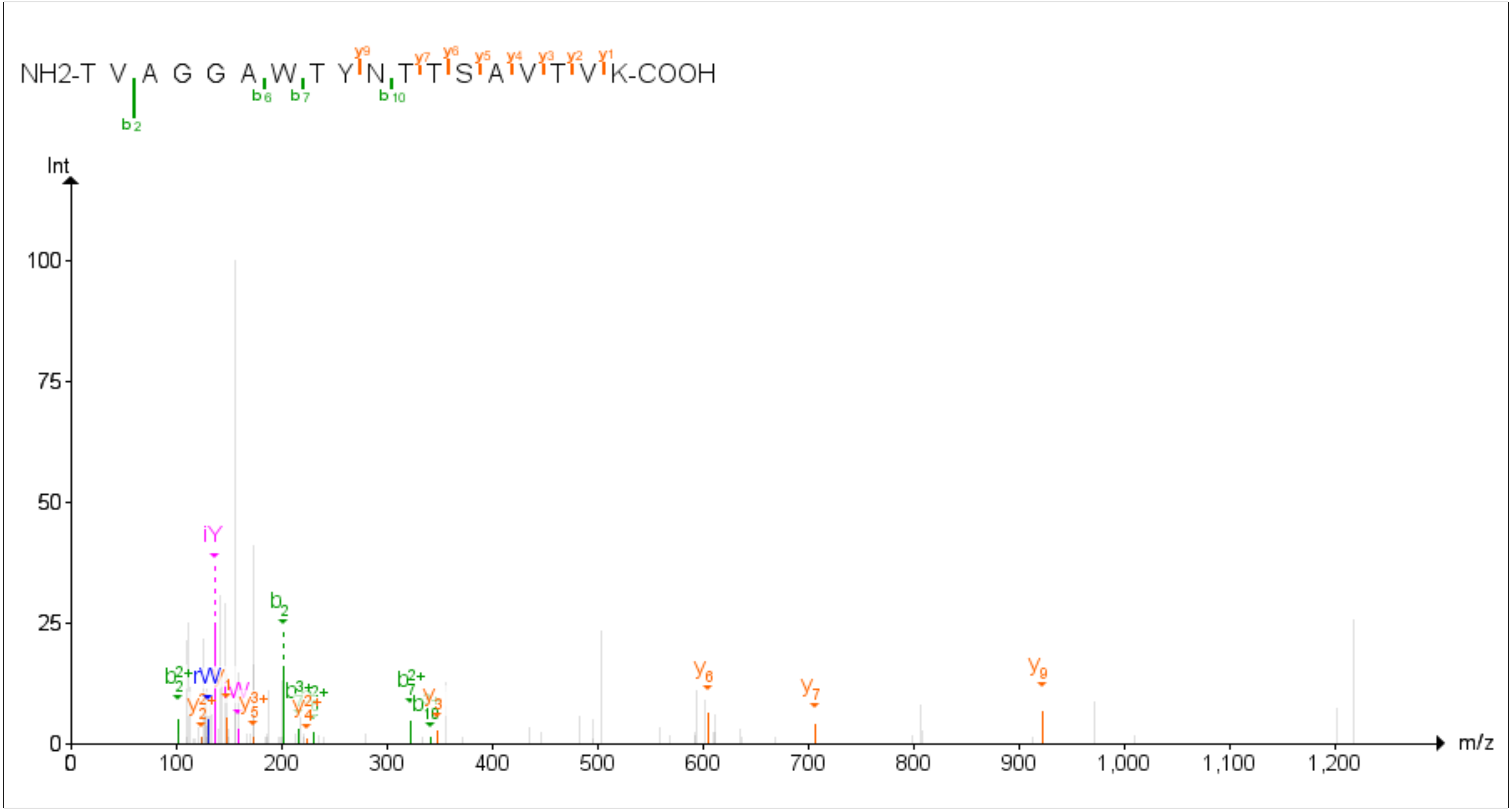

NH2-T V A G G A W T Y N T T S A V T V K-COOH
b2
b6
b7
b10
y9
y7
y6
y5
y4
y3
y2
y1
Int
100
75
50
25
0
iY
b2
y6
y7
y9
m/z
0
100
200
300
400
500
600
700
800
900
1,000
1,100
1,200

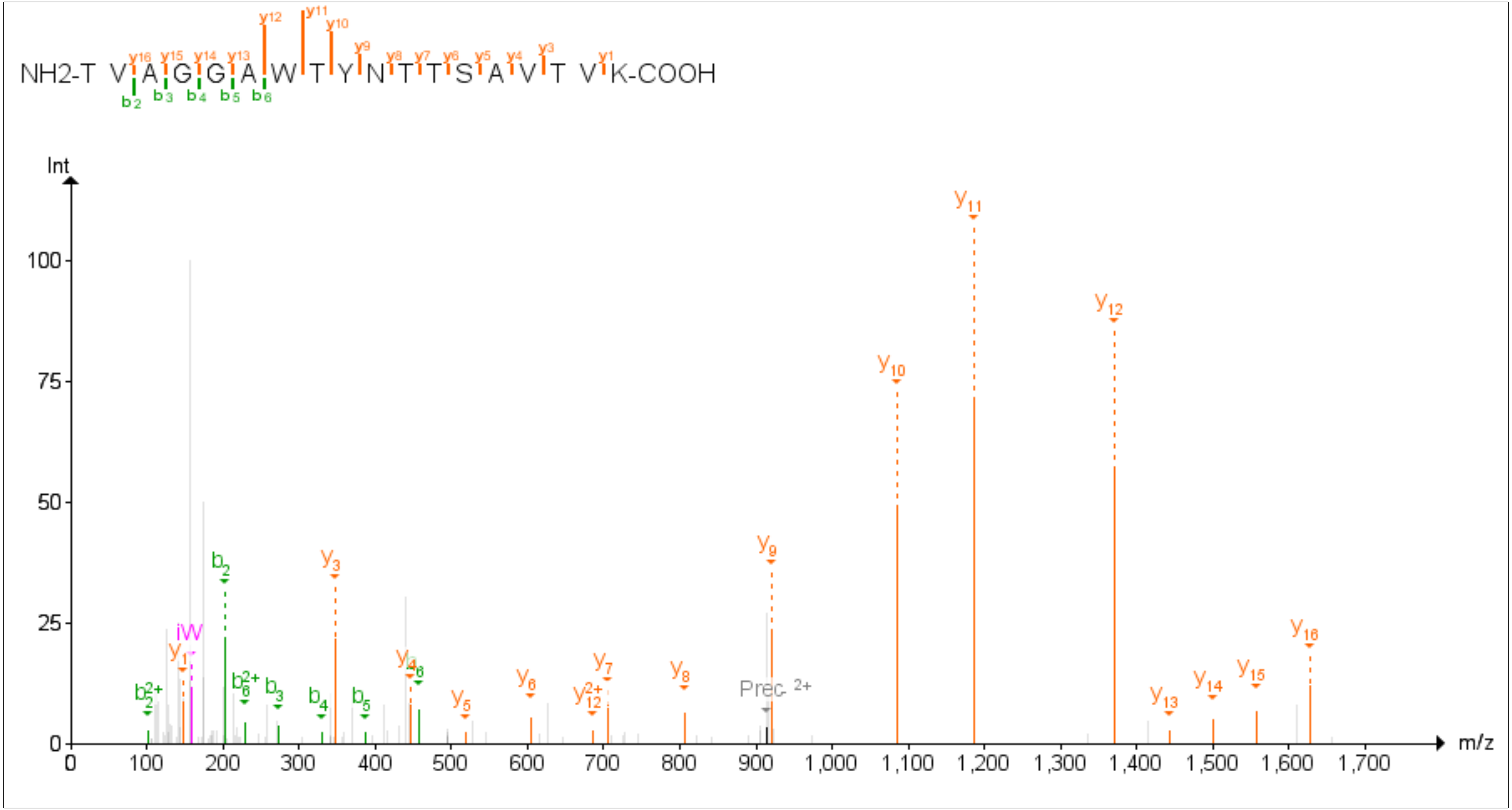

NH2-T V A G G A W T Y N T T S A V T V K-COOH
y16 y15 y14 y13 y12 y11 y10 y9 y8 y7 y6 y5 y4 y3 y1
b2 b3 b4 b5 b6
Int
100
75
50
25
0
m/z
0 100 200 300 400 500 600 700 800 900 1,000 1,100 1,200 1,300 1,400 1,500 1,600 1,700
iW
Prec. 2+

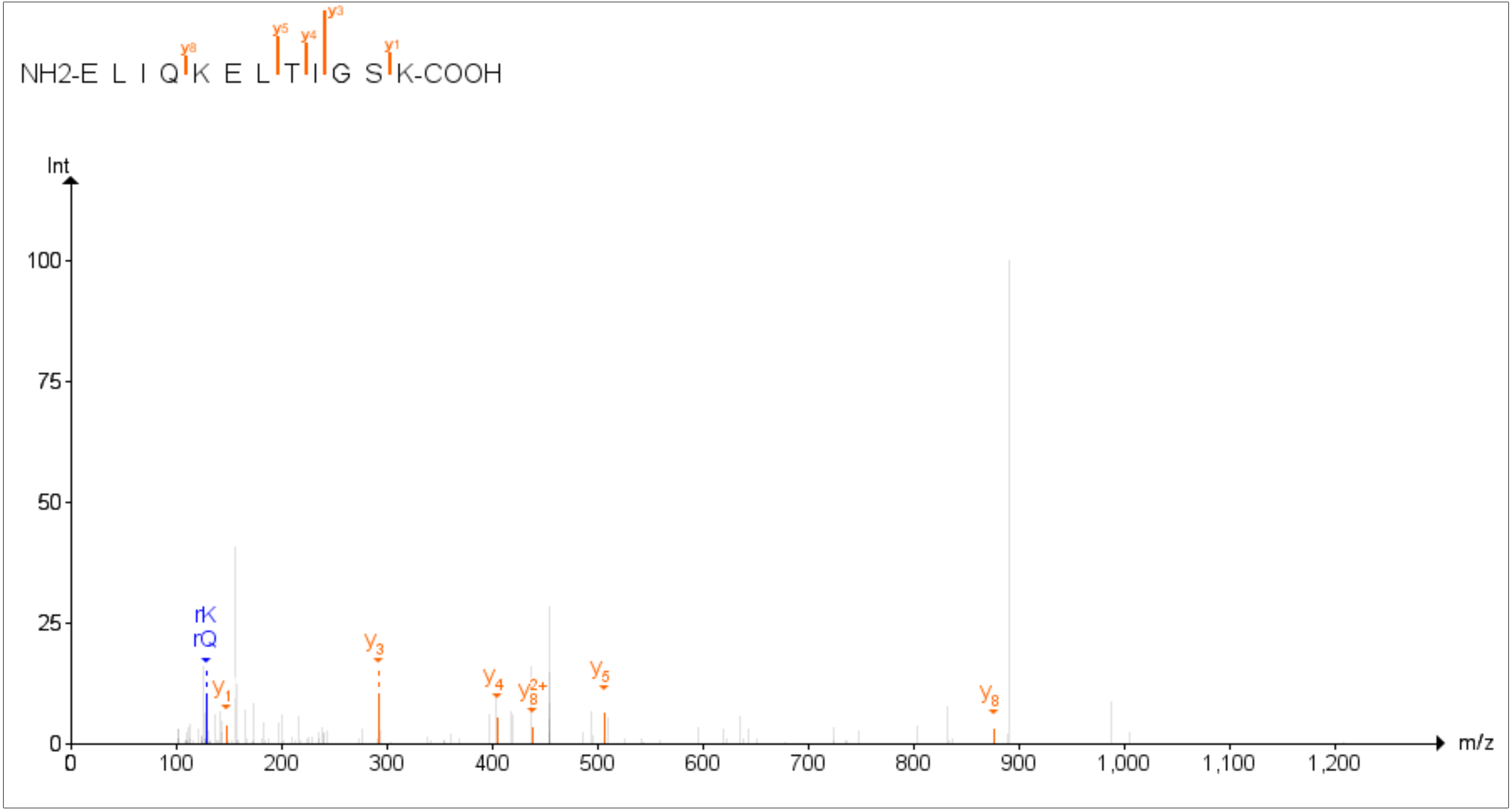

NH2-E L I Q K E L T I G S K-COOH
y8
y5
y4
y3
y1
Int
100
75
50
25
0
rK
rQ
y1
y3
y4
y8 2+
y5
y8
0
100
200
300
400
500
600
700
800
900
1,000
1,100
1,200
m/z

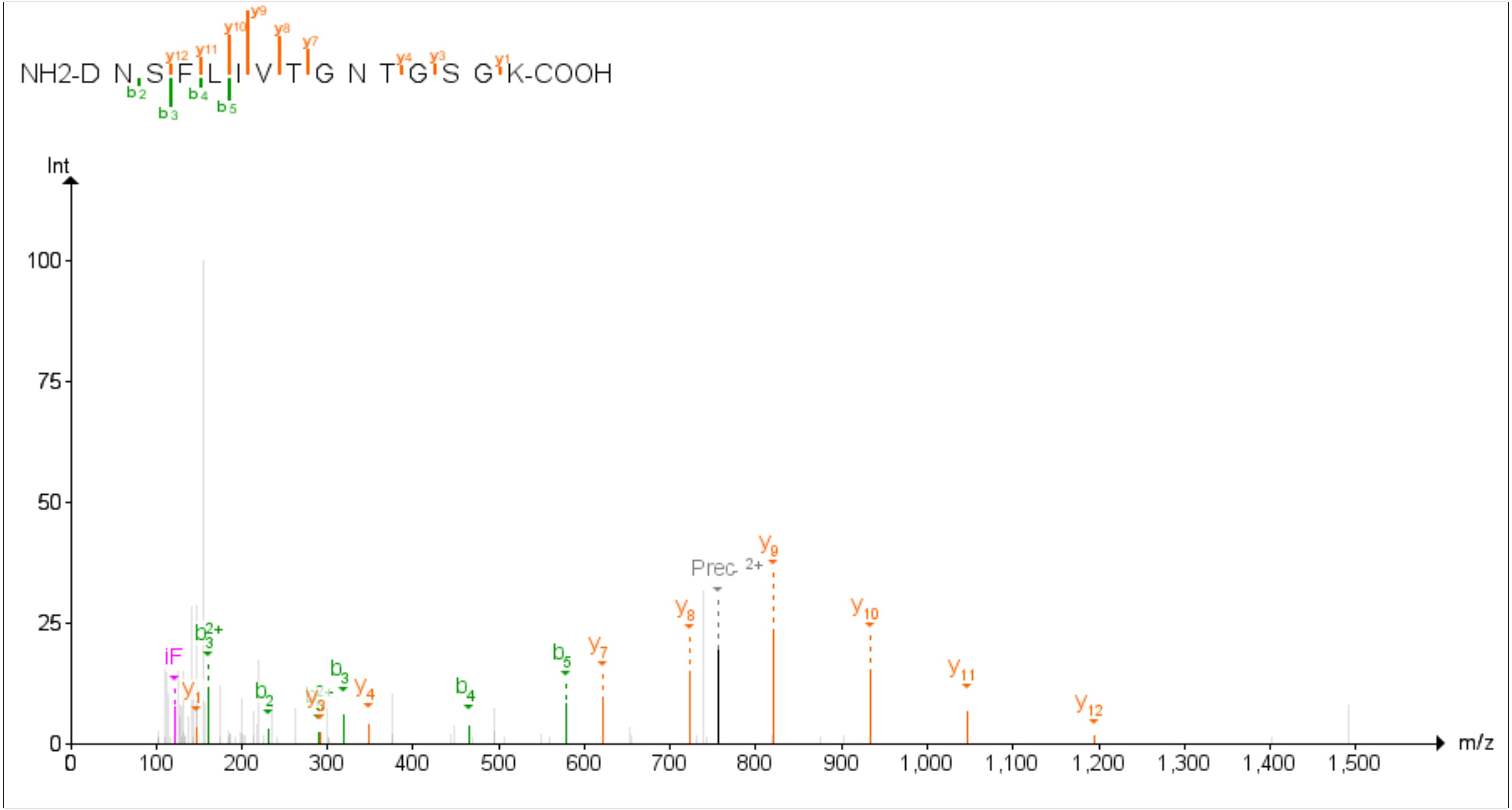
NH2-D N S F L I V T G N T G S G K-COOH
Int
m/z
Prec 2+
iF
0
25
50
75
100
0
100
200
300
400
500
600
700
800
900
1,000
1,100
1,200
1,300
1,400
1,500

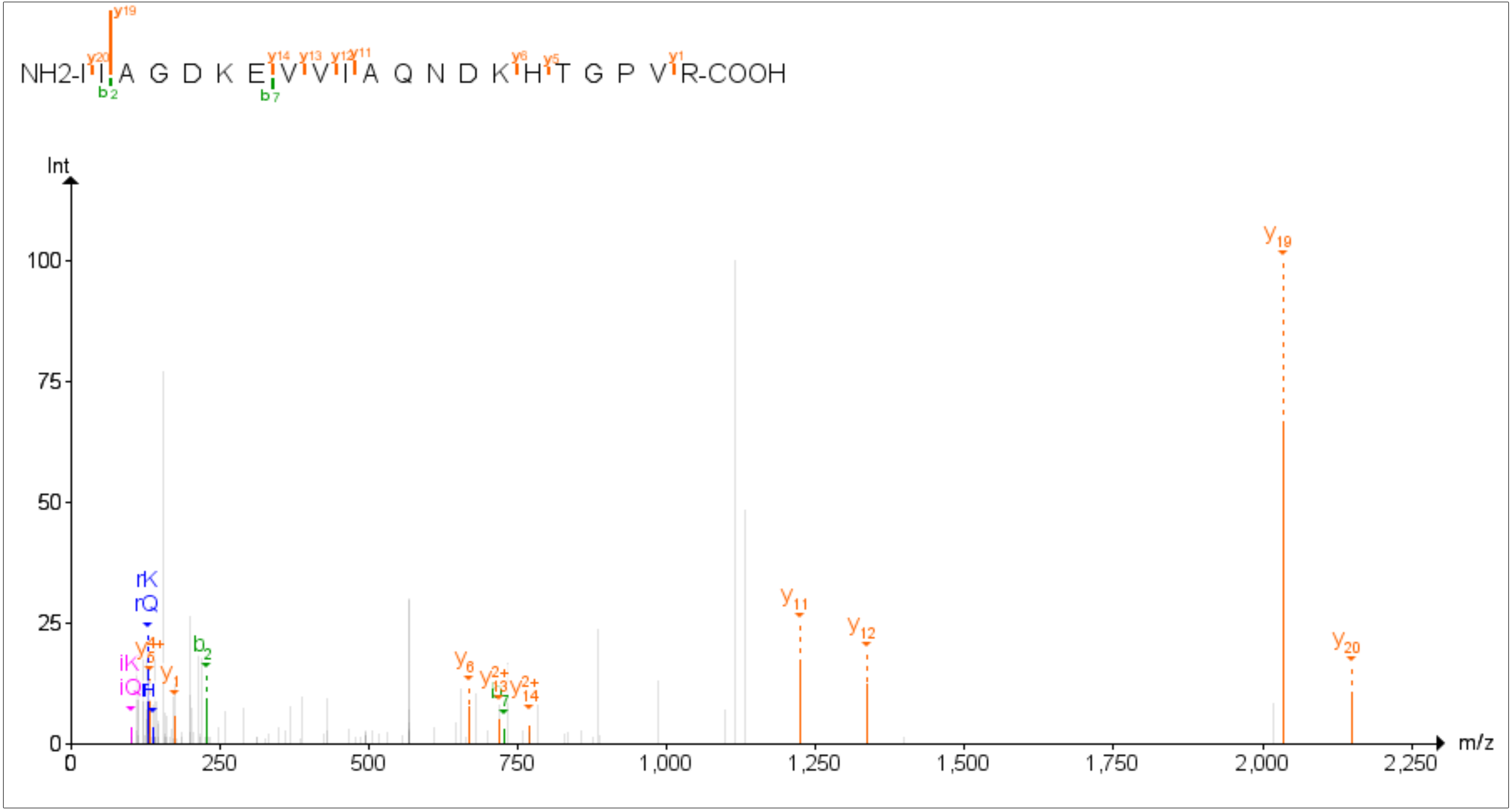

NH2-I I A G D K E V V I A Q N D K H T G P V R-COOH
y19
y20
b2
y14
b7
y13
y12
y11
y6
y5
y1
Int
100
75
50
25
0
rK
rQ
iK
iQ
H
b2
y1
y6
y11
y12
y19
y20
0
250
500
750
1,000
1,250
1,500
1,750
2,000
2,250
m/z

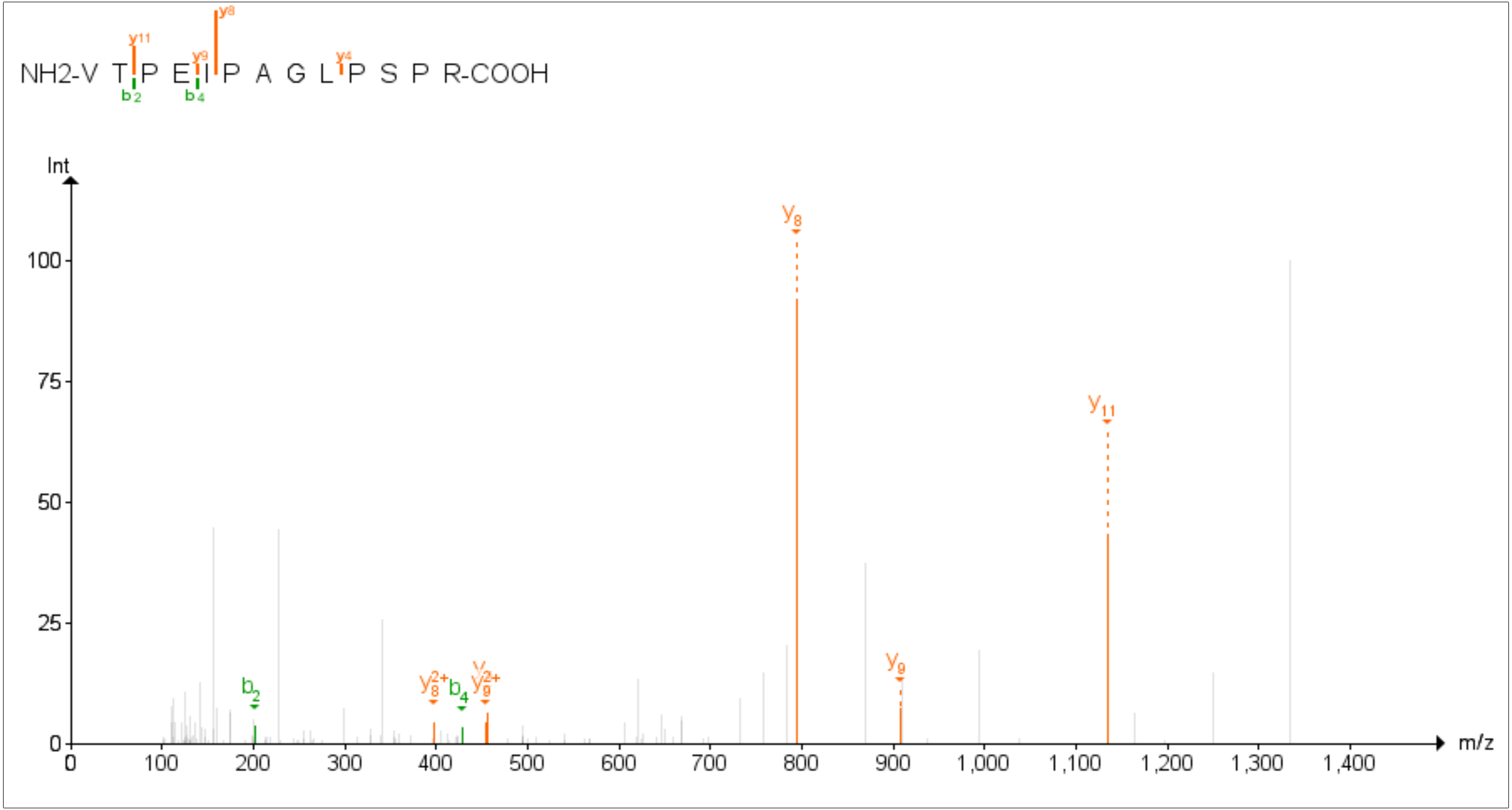

NH2-V T P E I P A G L P S P R-COOH
y11
y9
y8
y4
b2
b4
Int
100
75
50
25
0
m/z
0
100
200
300
400
500
600
700
800
900
1,000
1,100
1,200
1,300
1,400
y8
y11
y9
b2
y8 2+
b4
y9 2+

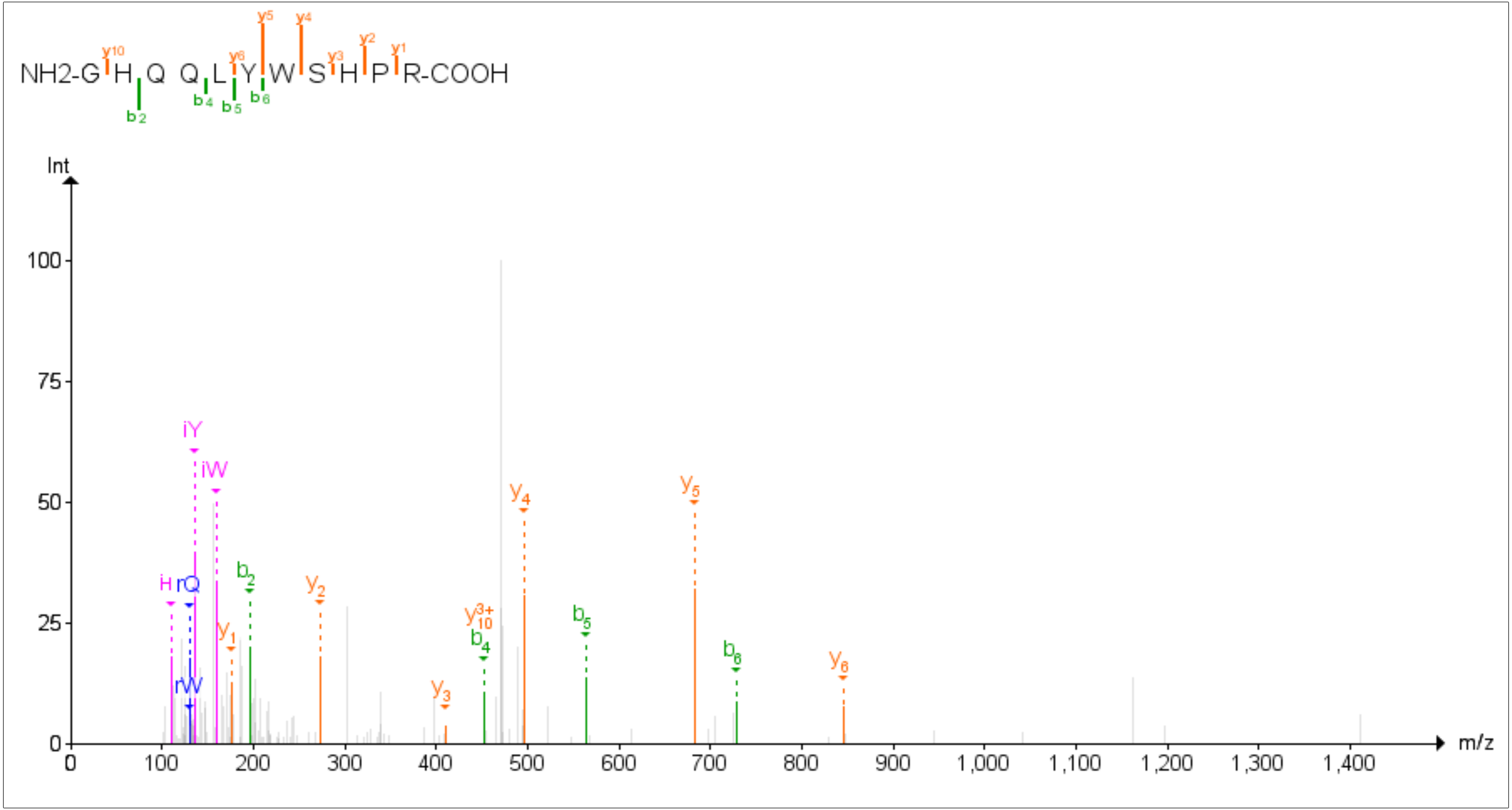

NH2-G H Q Q L Y W S H P R-COOH
y10 y6 y5 y4 y3 y2 y1
b2 b4 b5 b6
Int
m/z
100
75
50
25
0
0 100 200 300 400 500 600 700 800 900 1,000 1,100 1,200 1,300 1,400
iY
iW
iH
rQ
rW
b2
y2
y1
y3
y10 3+
b4
y4
b5
y5
b6
y6

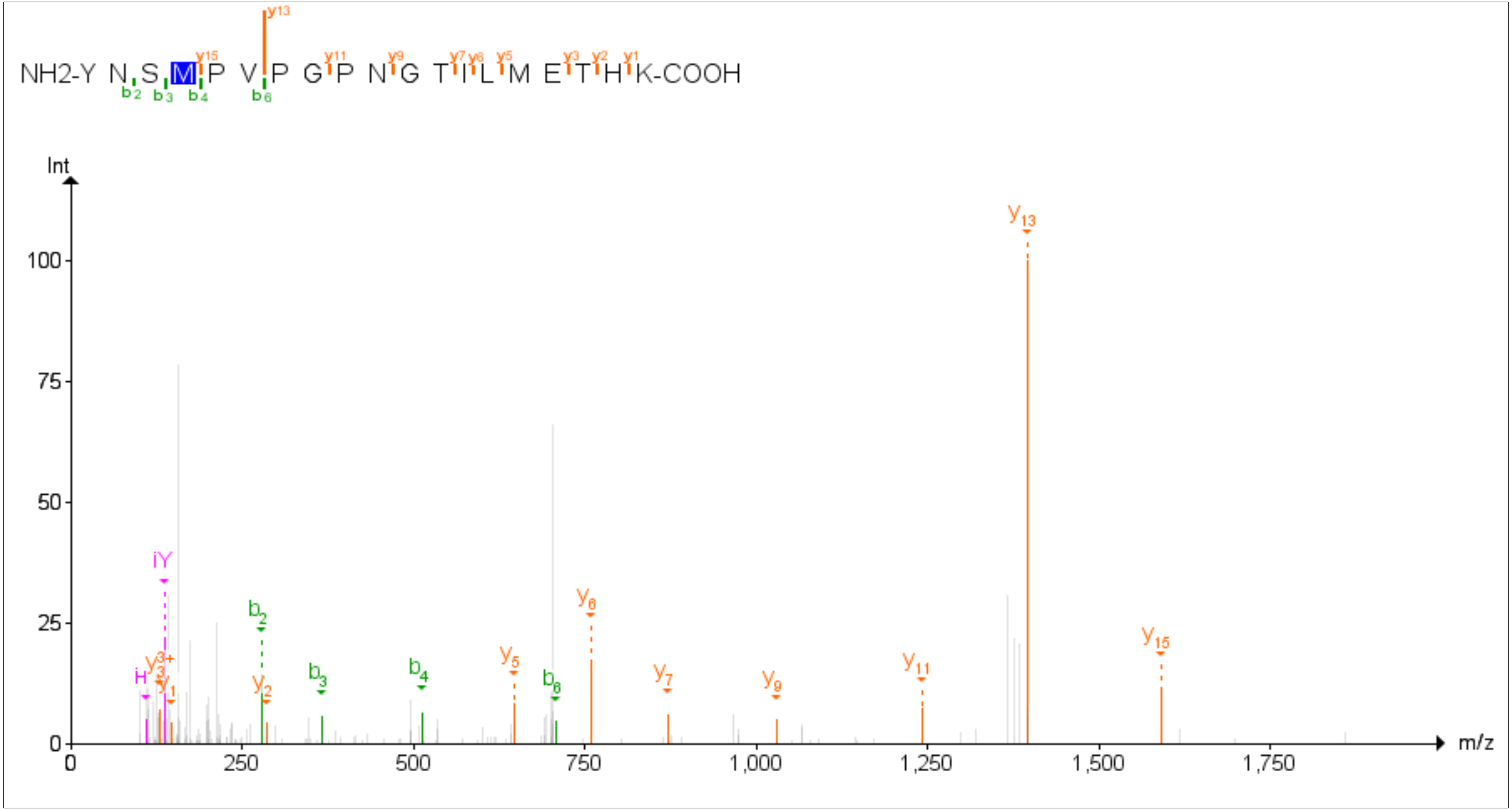

NH2-Y N S M P V P G P N G T I L M E T H K-COOH
b2 b3 b4 b6
y15 y13 y11 y9 y7 y6 y5 y3 y2 y1
Int
m/z
0
25
50
75
100
250
500
750
1,000
1,250
1,500
1,750
iY
iH
y3 3+
y1
y2
b2
b3
b4
y5
b6
y6
y7
y9
y11
y13
y15

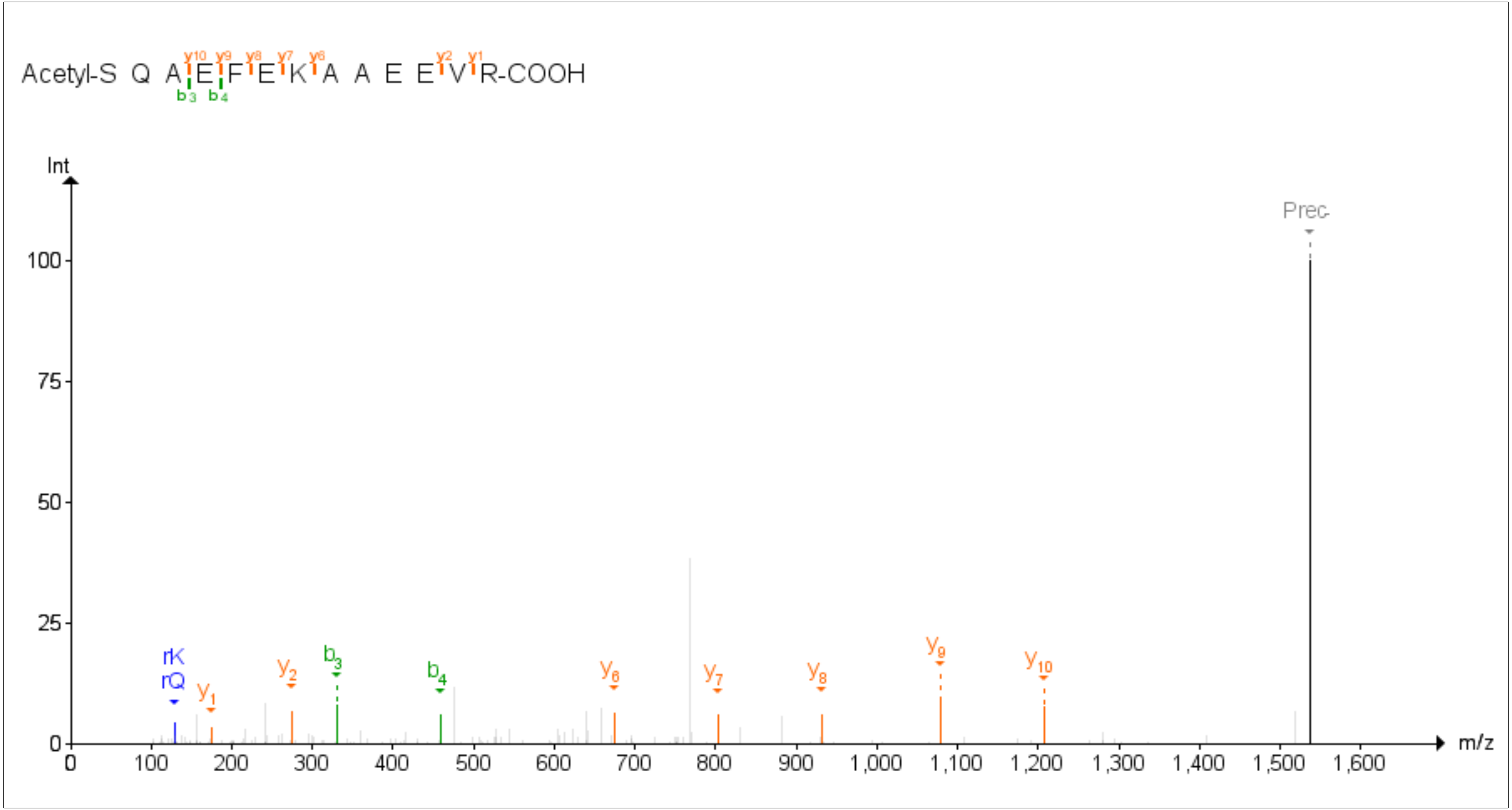

Acetyl-S Q A E F E K A A E E V R-COOH
y10 y9 y8 y7 y6 y2 y1
b3 b4
Int
100
75
50
25
0
Prec
rK
rQ
y1
y2
b3
b4
y6
y7
y8
y9
y10
0 100 200 300 400 500 600 700 800 900 1,000 1,100 1,200 1,300 1,400 1,500 1,600
m/z

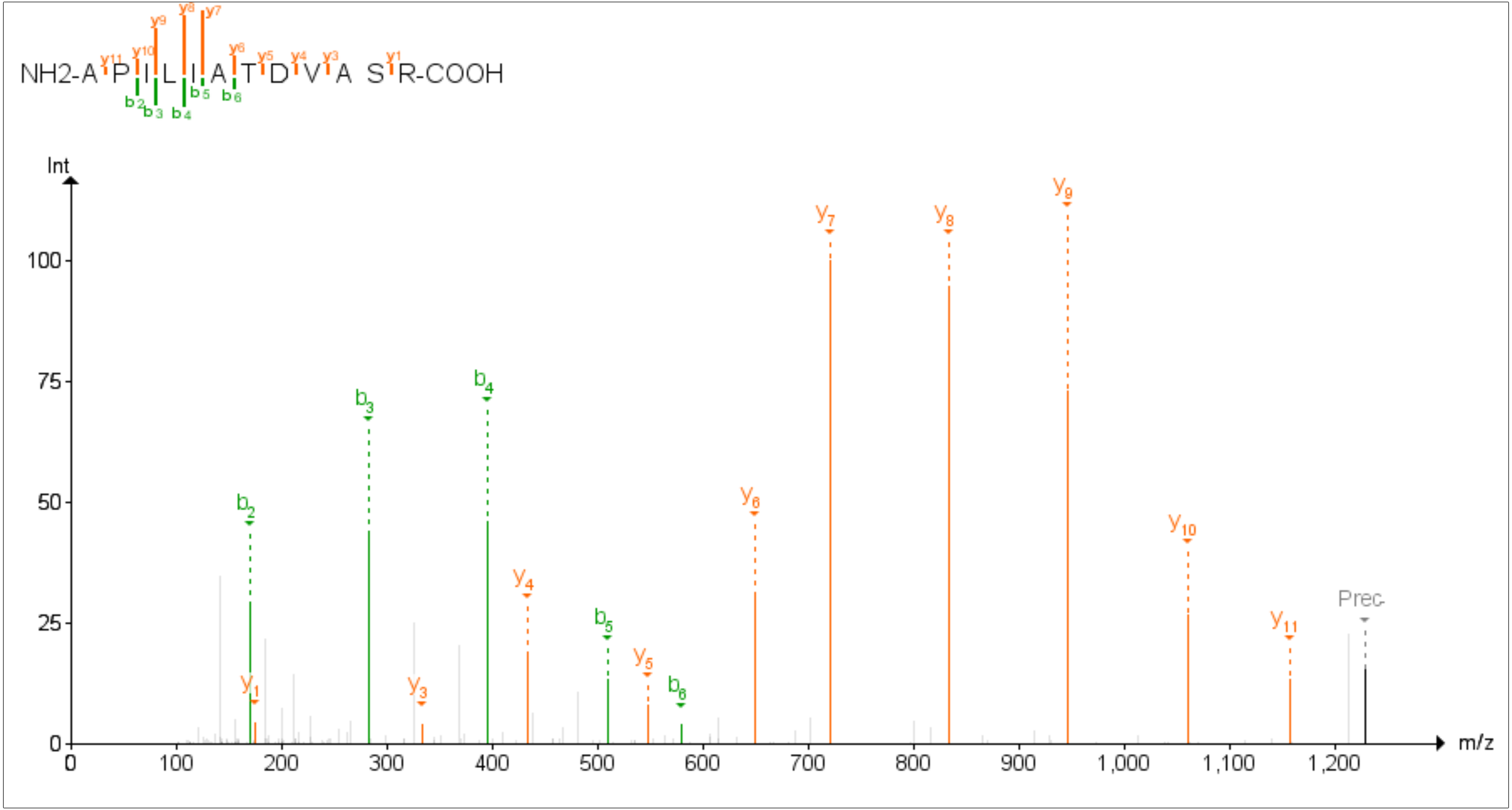

NH2-A P I L I A T D V A S R-COOH
y11 y10 y9 y8 y7 y6 y5 y4 y3 y1
b2 b3 b4 b5 b6
Int
100
75
50
25
0
b2
b3
b4
b5
b6
y1
y3
y4
y5
y6
y7
y8
y9
y10
y11
Prec.
0
100
200
300
400
500
600
700
800
900
1,000
1,100
1,200
m/z

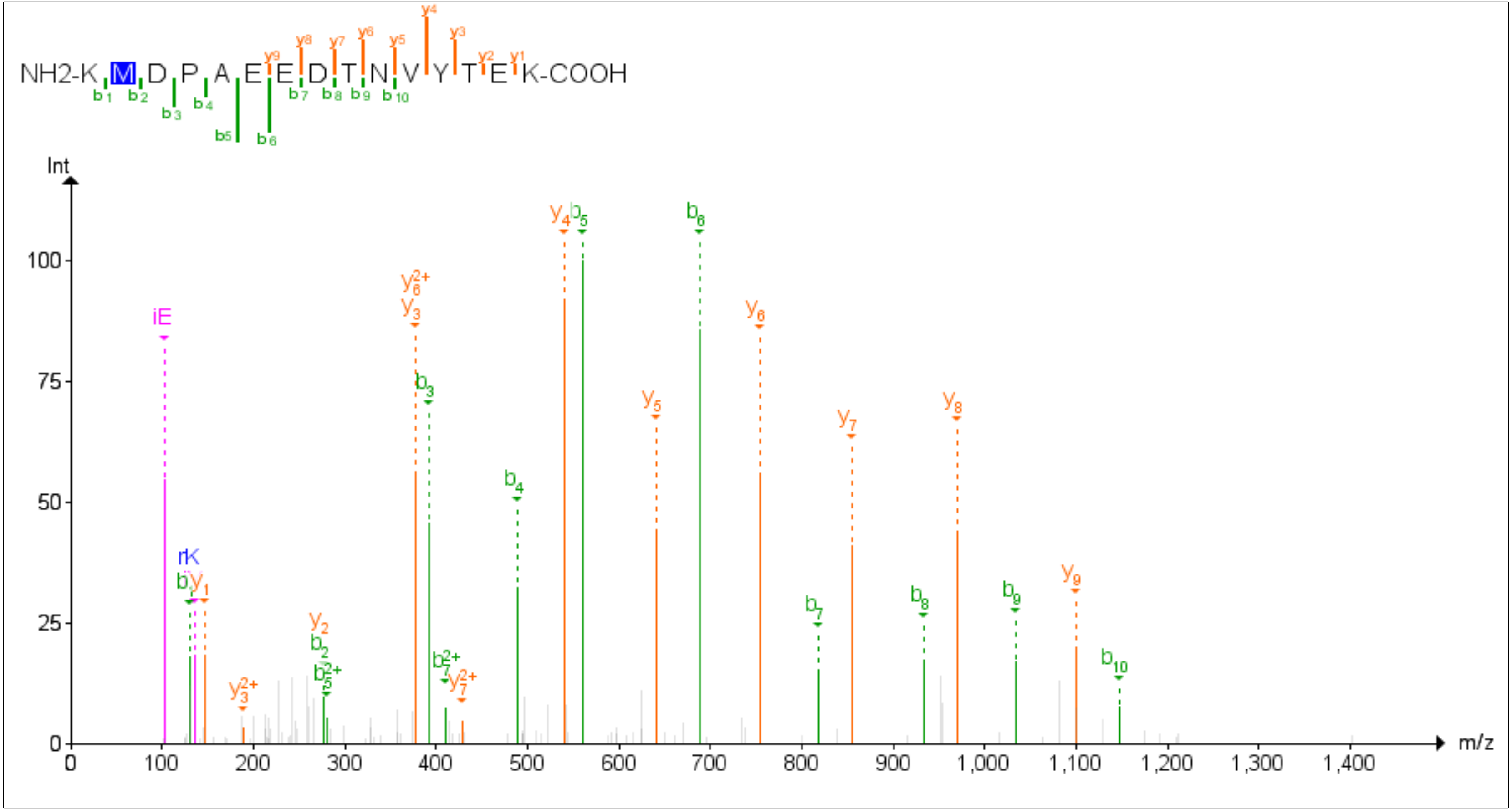

NH2-K M D P A E E D T N V Y T E K-COOH
Int
m/z
iE
rK
y4
b5
b6
y6
y5
y7
y8
y9
b3
b4
b7
b8
b9
b10
y3
y1
y2
b2

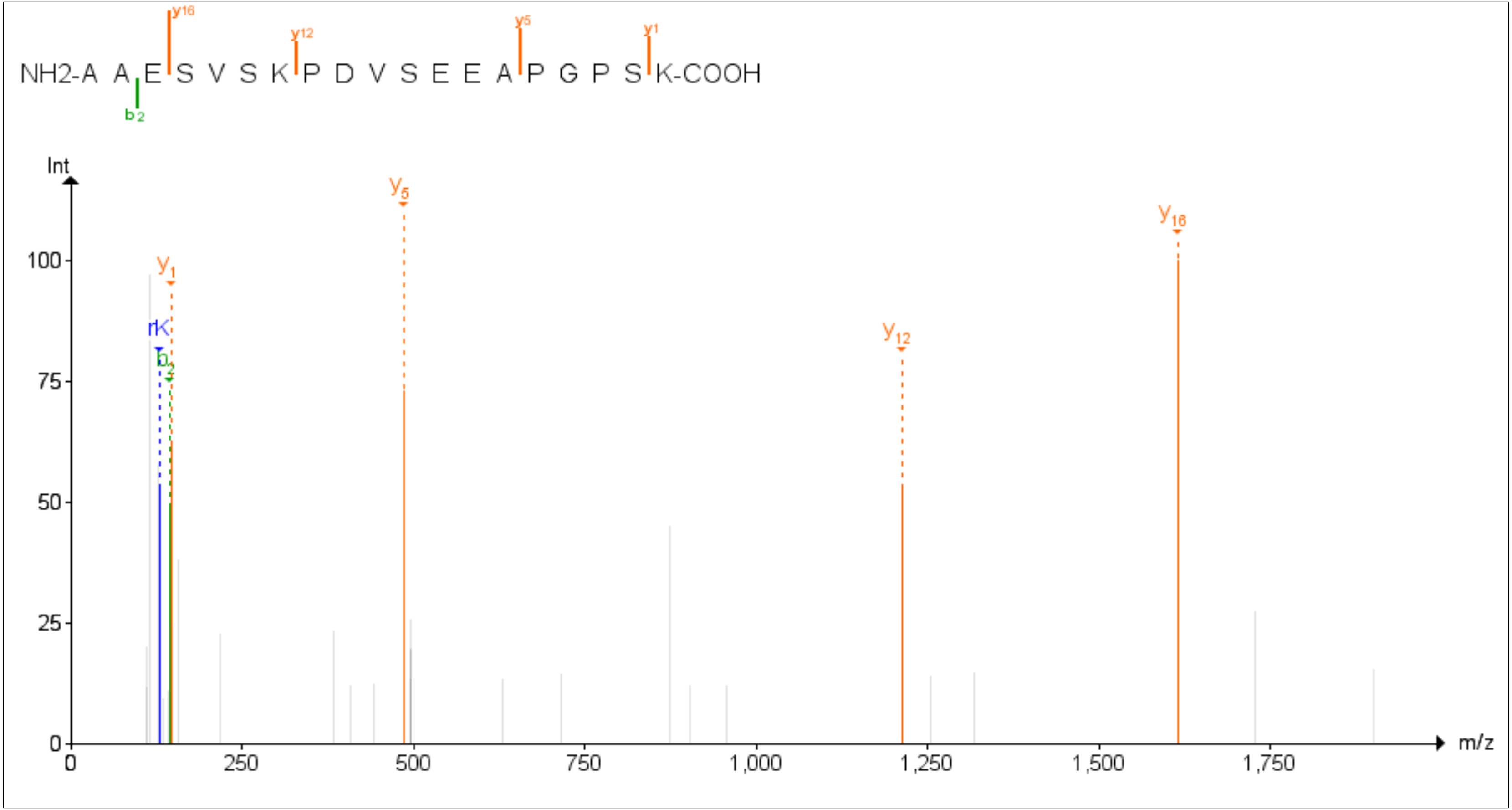

NH2-A A E S V S K P D V S E E A P G P S K-COOH
y16
y12
y5
y1
b2
Int
100
75
50
25
0
y1
rK
b2
y5
y12
y16
0
250
500
750
1,000
1,250
1,500
1,750
m/z

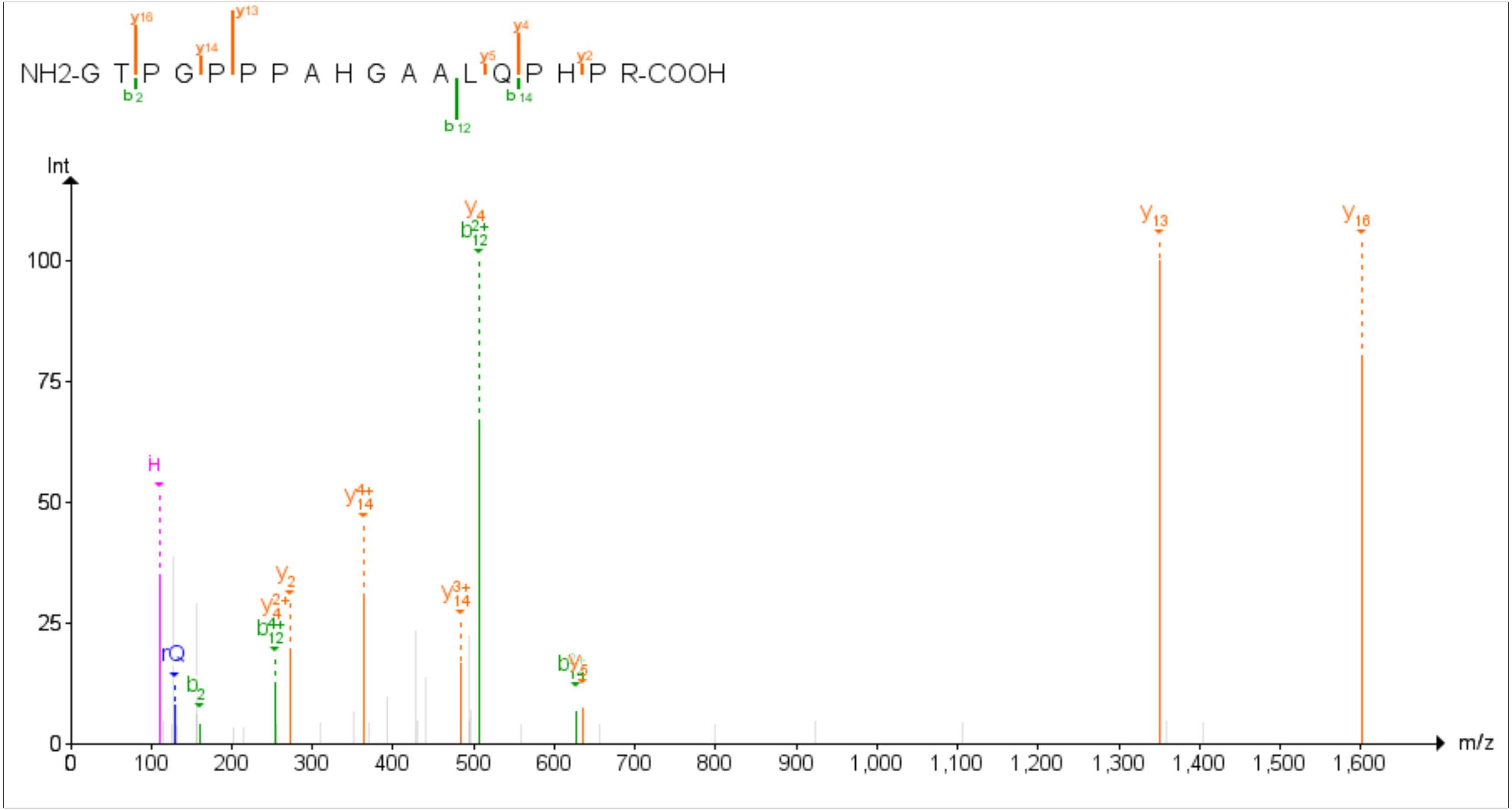

NH2-G T P G P P P A H G A A L Q P H P R-COOH
y16
y14
y13
y5
y4
y2
b2
b12
b14
Int
100
75
50
25
0
y4
b12 2+
y13
y16
H
y14 4+
y2
y4 2+
b12 4+
rQ
b2
y14 3+
b14
y5
0
100
200
300
400
500
600
700
800
900
1,000
1,100
1,200
1,300
1,400
1,500
1,600
m/z

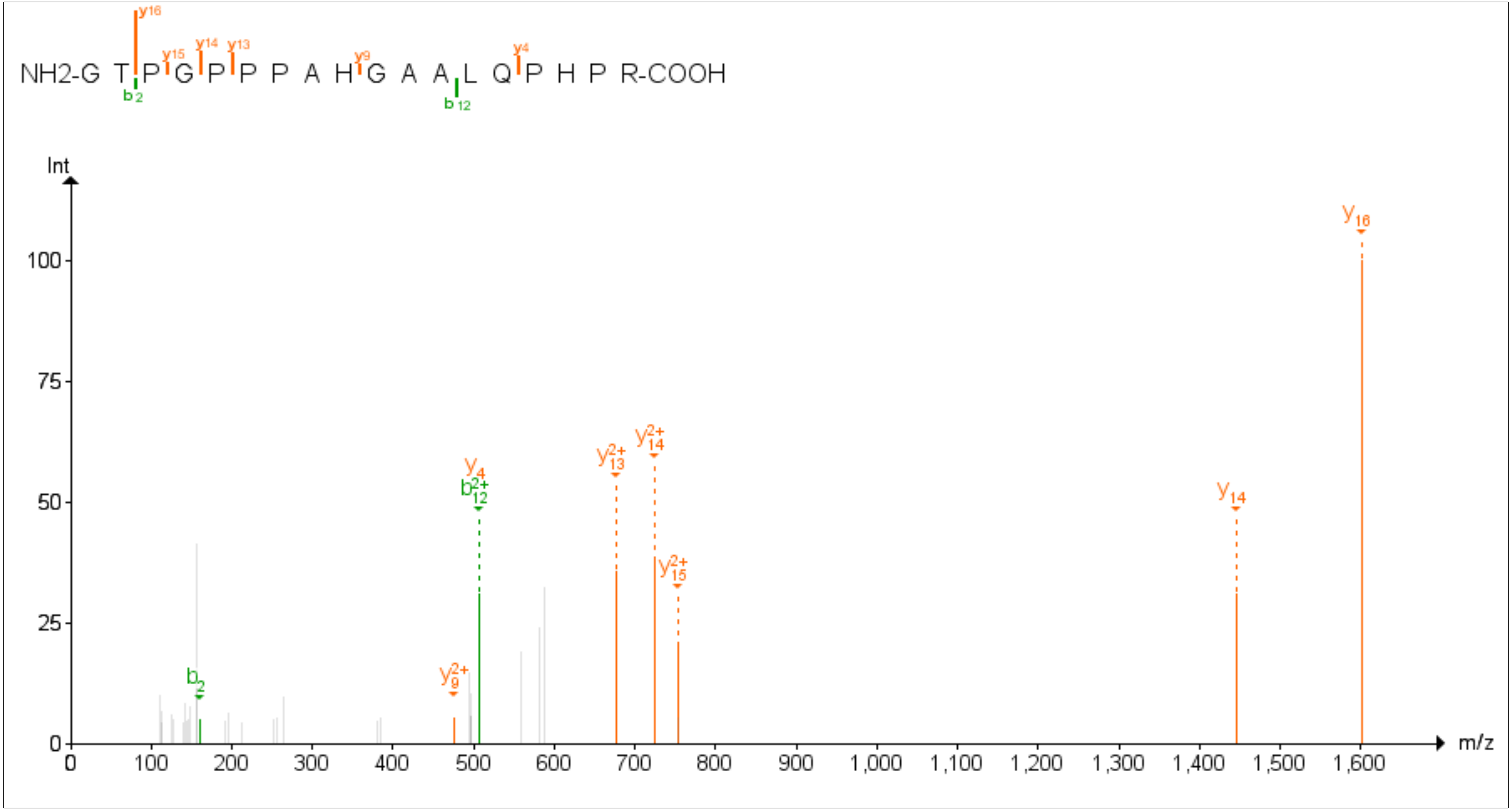

NH2-G T P G P P P A H G A A L Q P H P R-COOH
y16
y15
y14
y13
y9
y4
b 2
b 12
Int
100
75
50
25
0
m/z
0
100
200
300
400
500
600
700
800
900
1,000
1,100
1,200
1,300
1,400
1,500
1,600
y_{16}
y_4
b_{12}^{2+}
y_{13}^{2+}
y_{14}^{2+}
y_{15}^{2+}
y_{14}
b_2
y_9^{2+}

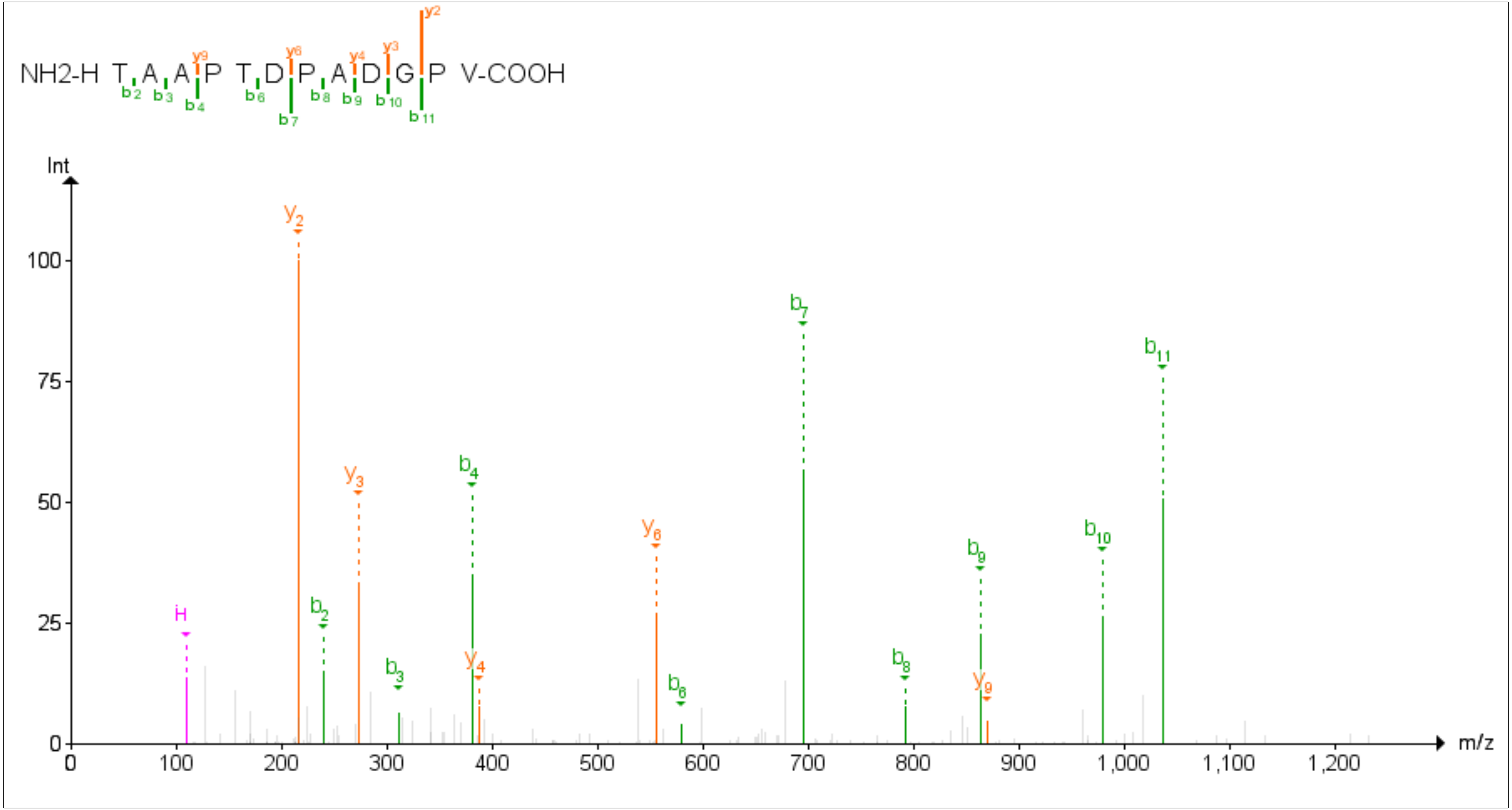

NH2-H T A A P T D P A D G P V-COOH
y9 y6 y4 y3 y2
b2 b3 b4 b6 b7 b8 b9 b10 b11
Int
100
75
50
25
0
m/z
0 100 200 300 400 500 600 700 800 900 1,000 1,100 1,200
H
y2
y3
y4
y6
y9
b2
b3
b4
b6
b7
b8
b9
b10
b11

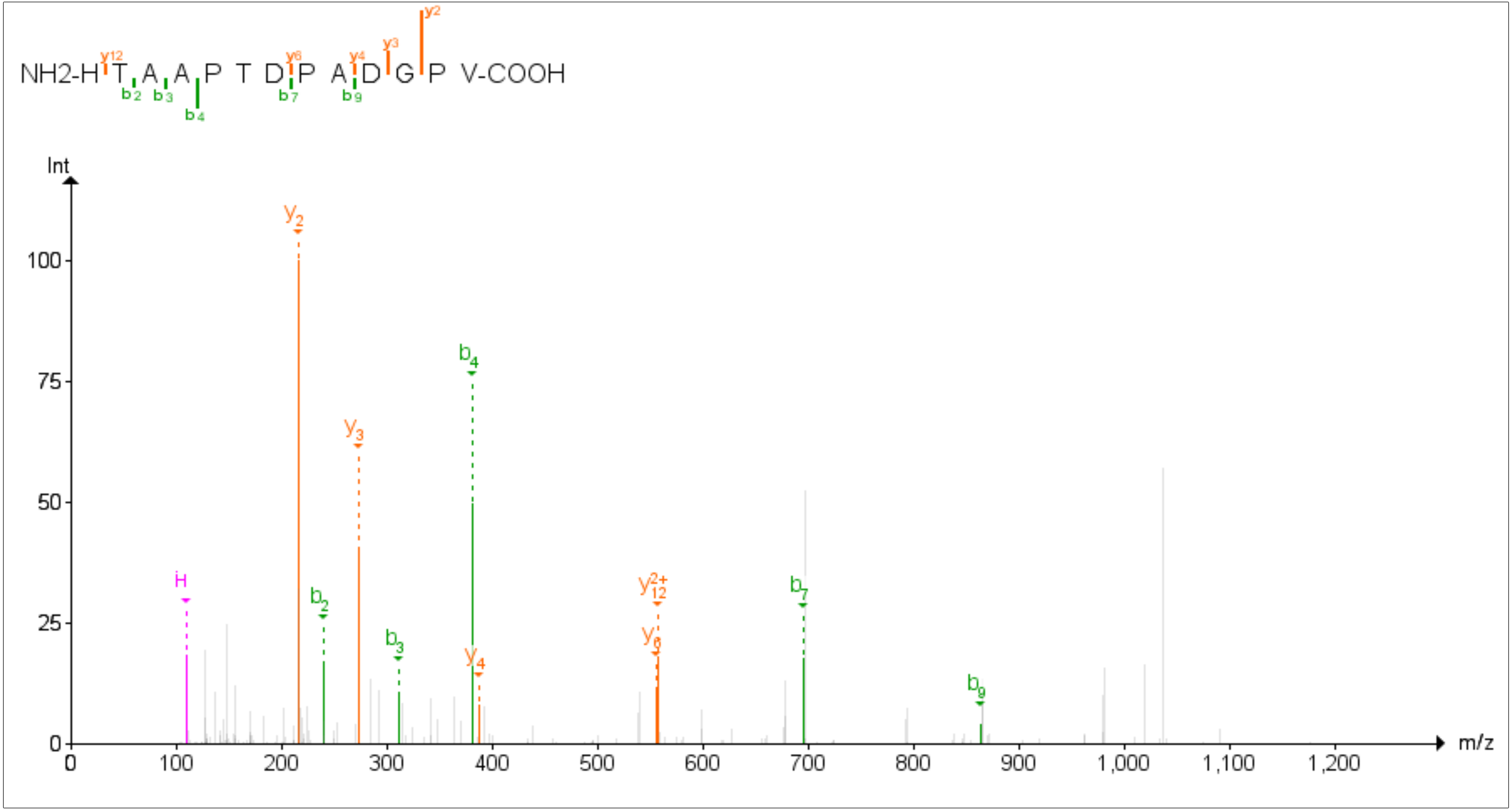

NH2-H T A A P T D P A D G P V-COOH
y12
y6
y4
y3
y2
b2
b3
b4
b7
b9
Int
100
75
50
25
0
y2
y3
b4
H
b2
b3
y4
y12 2+
y6
b7
b9
0
100
200
300
400
500
600
700
800
900
1,000
1,100
1,200
m/z

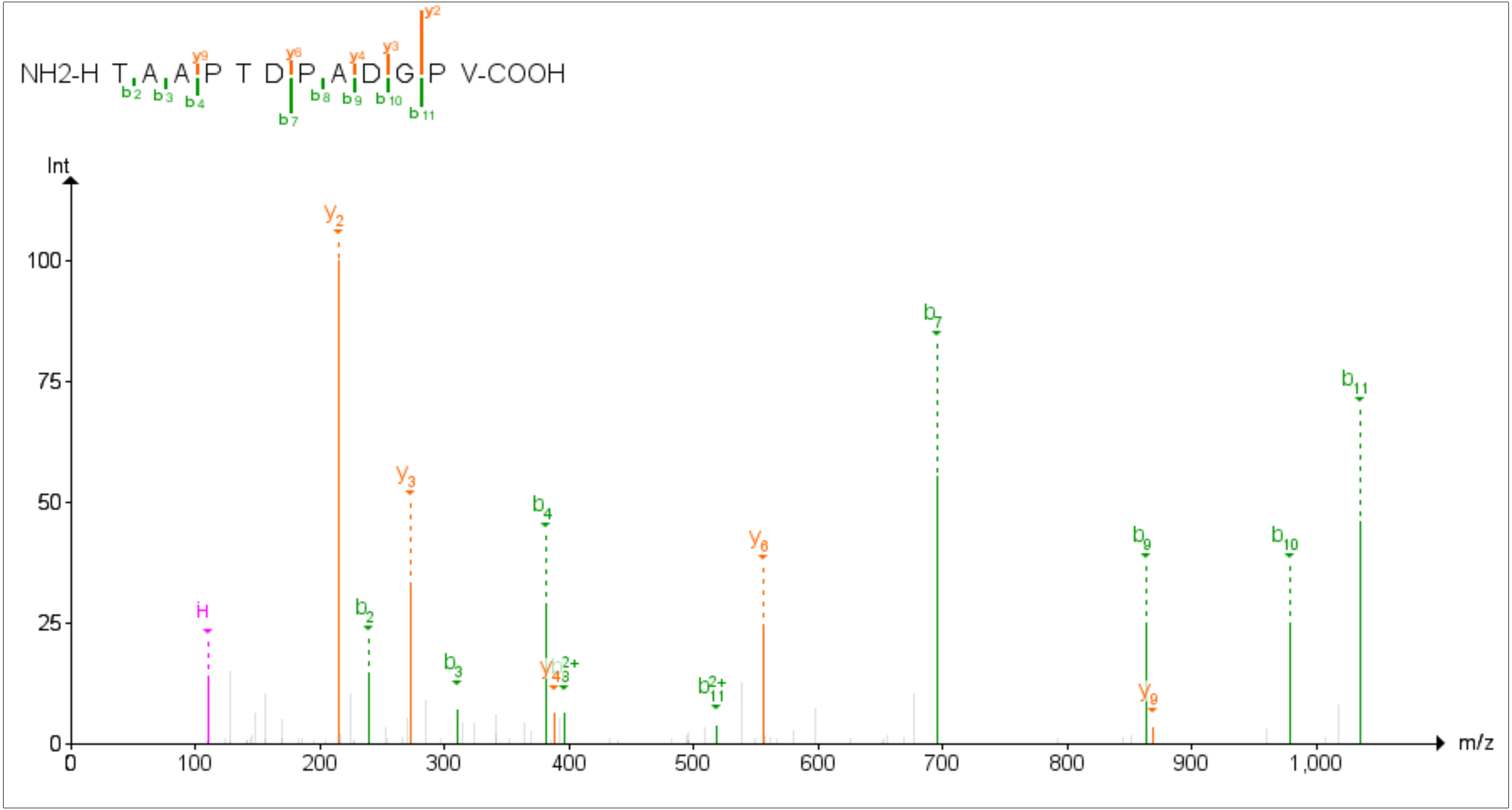

NH2-H T A A P T D P A D G P V-COOH
Int
m/z
y2
y3
b4
y6
b7
b9
b10
b11
b2
b3
y9

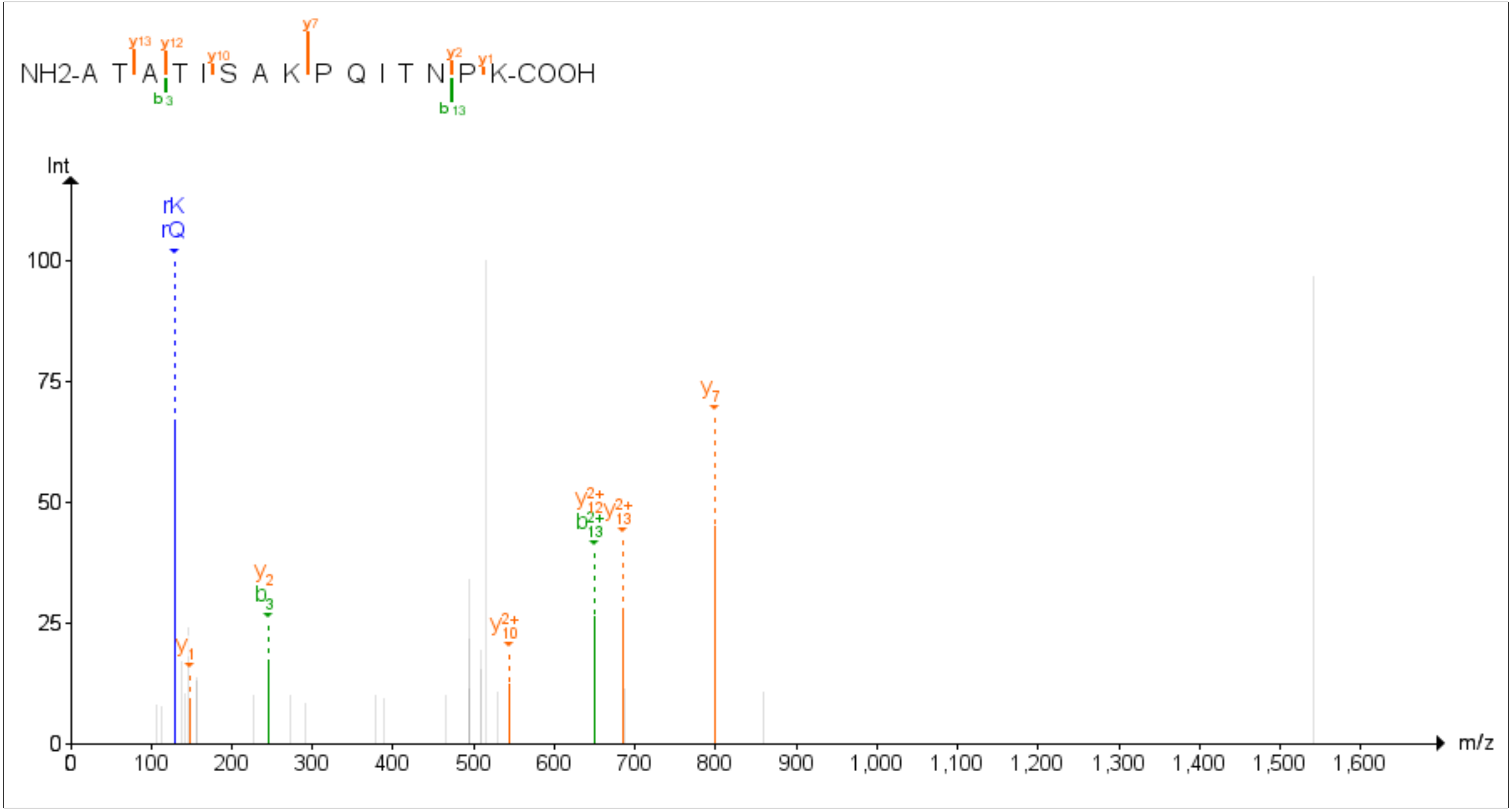

NH2-A T A T I S A K P Q I T N P K-COOH
y13 y12 y10 y7 y2 y1
b3 b13
Int
m/z
rK
rQ
y1
y2
b3
y10 2+
y12 2+
b13 2+
y13 2+
y7
0
25
50
75
100
0
100
200
300
400
500
600
700
800
900
1,000
1,100
1,200
1,300
1,400
1,500
1,600

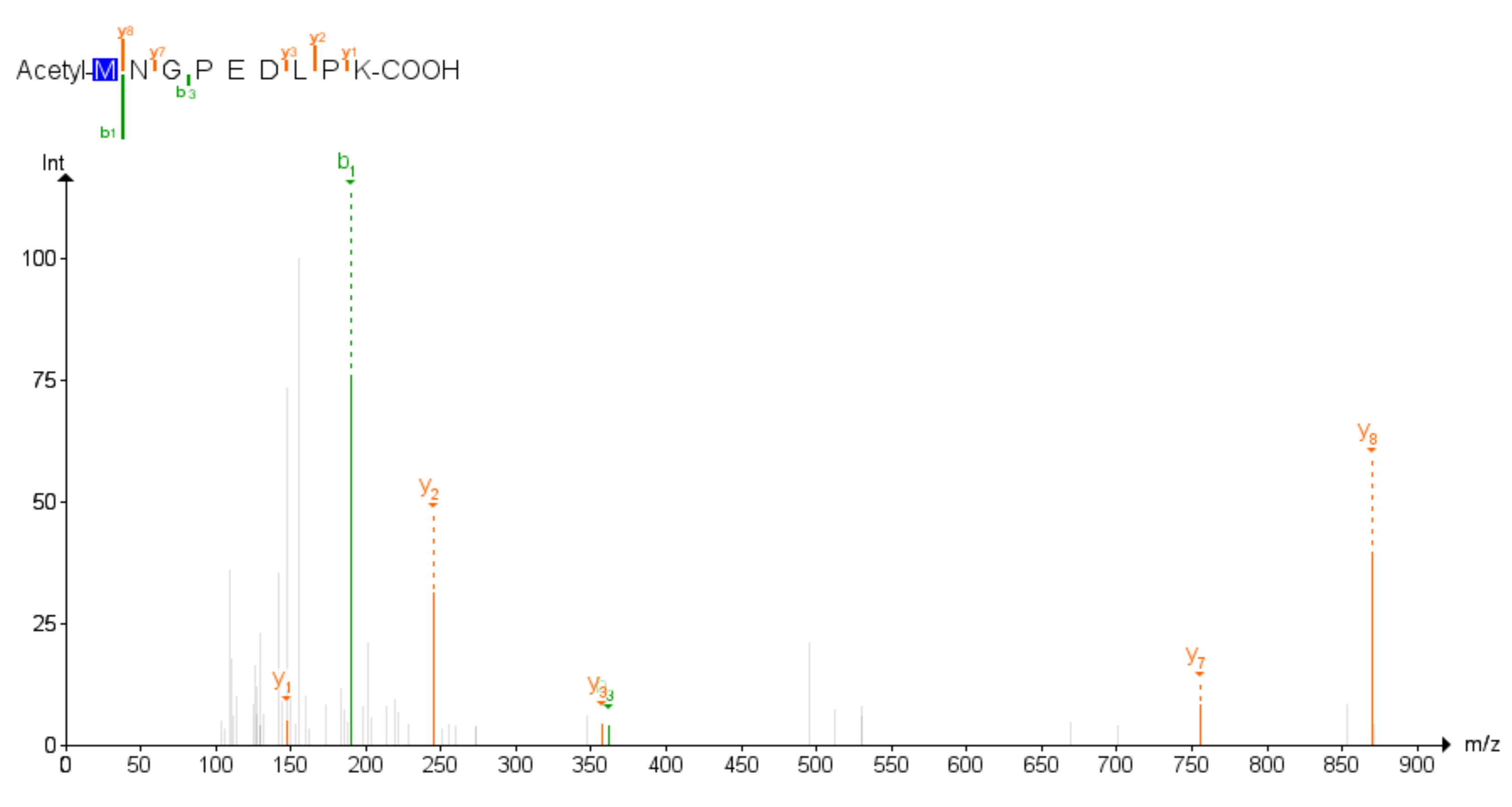

Acetyl-M N G P E D L P K-COOH
y8
y7
y3
y2
y1
b1
b3
Int
100
75
50
25
0
b1
y2
y8
y1
y3
b3
y7
0
50
100
150
200
250
300
350
400
450
500
550
600
650
700
750
800
850
900
m/z

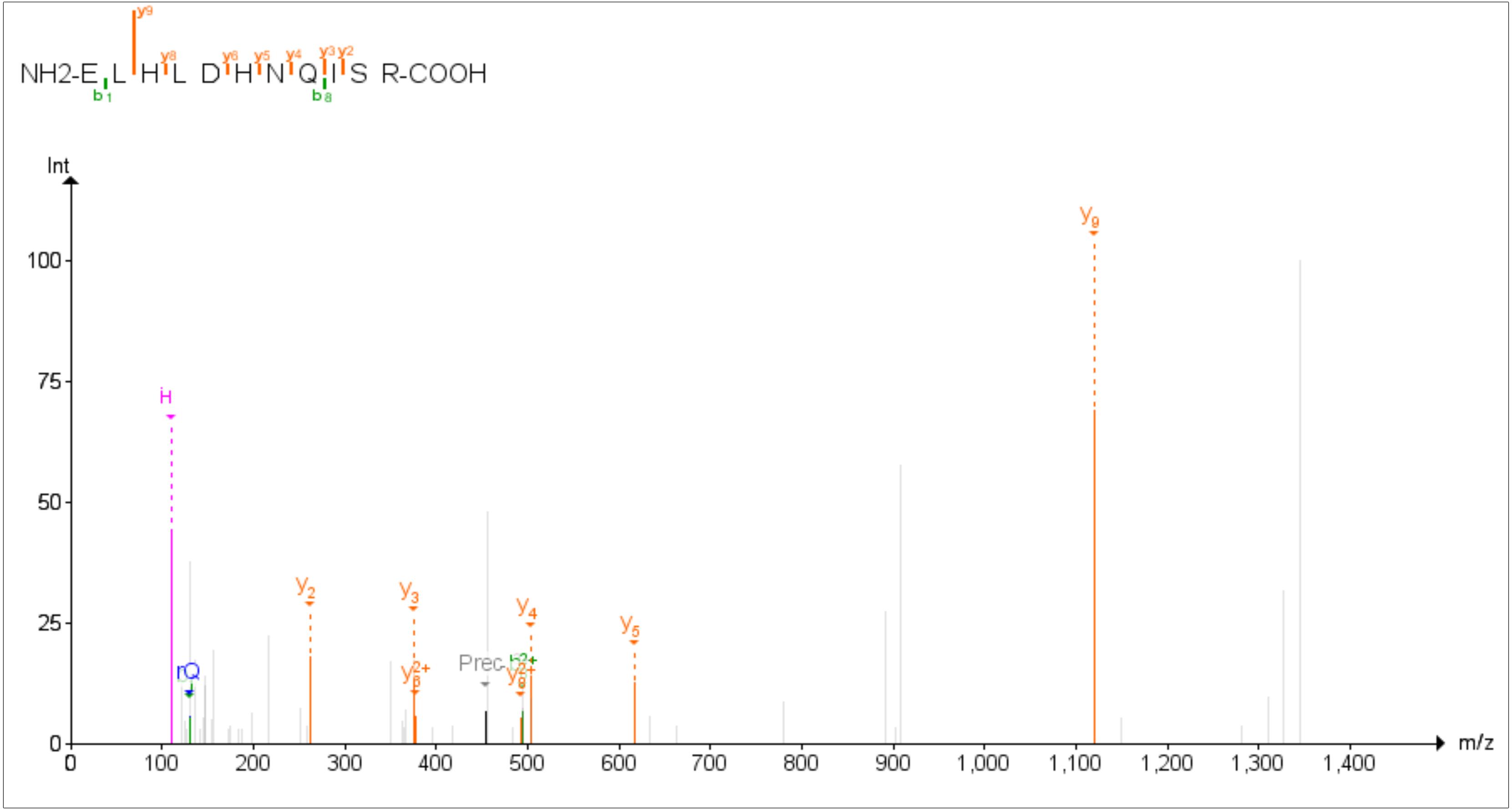
NH2-E L H L D H N Q I S R-COOH
y9
y8
y6
y5
y4
y3
y2
b1
b8
Int
100
75
50
25
0
m/z
0
100
200
300
400
500
600
700
800
900
1,000
1,100
1,200
1,300
1,400
y9
H
y2
y3
y4
y5
rQ
Prec.
y8 2+
y3 2+
b2+

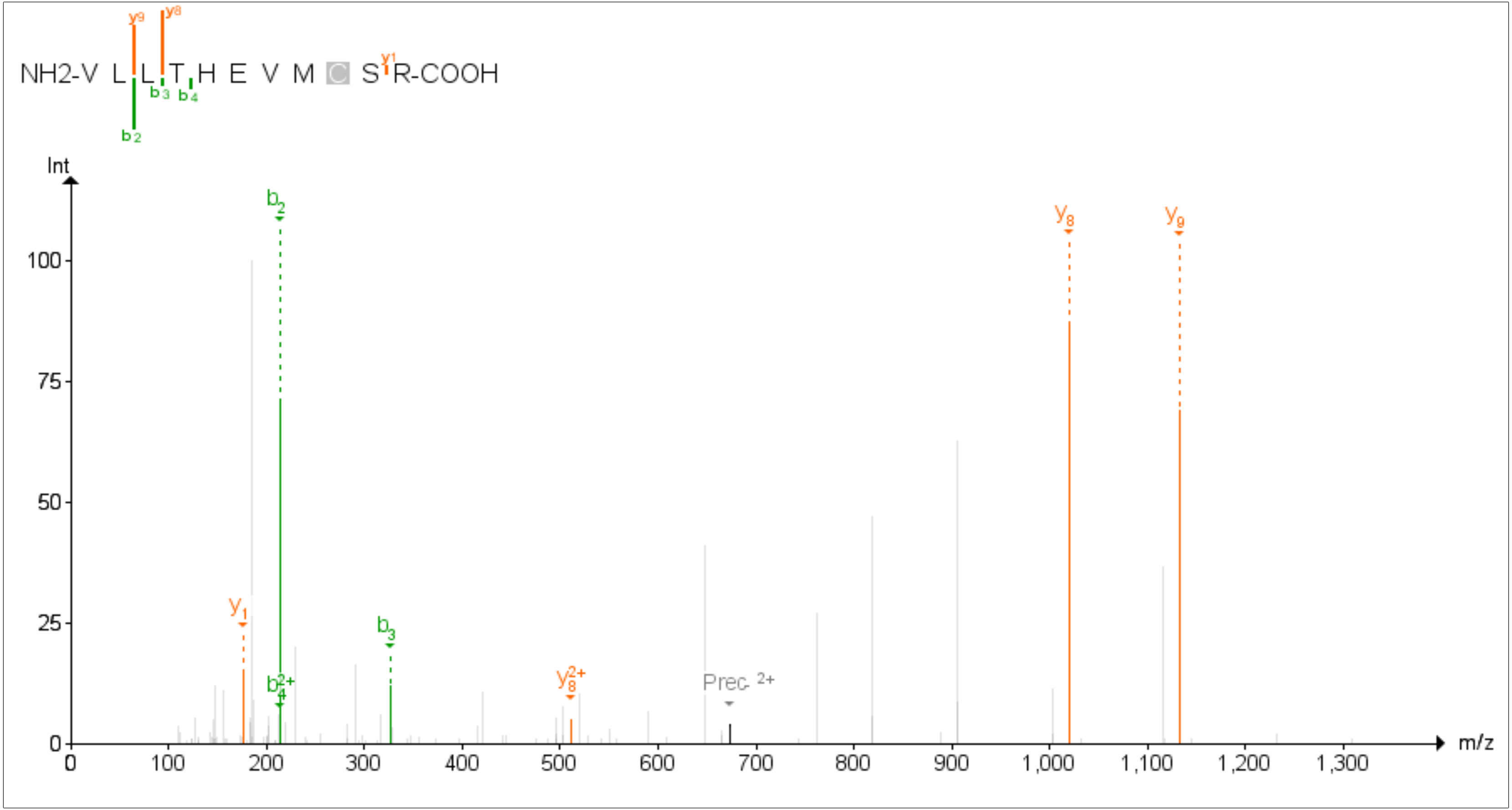

NH2-V L L T H E V M C S R-COOH
y9
y8
y1
b2
b3
b4
Int
100
75
50
25
0
b2
y8
y9
y1
b3
b4 2+
y8 2+
Prec- 2+
0
100
200
300
400
500
600
700
800
900
1,000
1,100
1,200
1,300
m/z

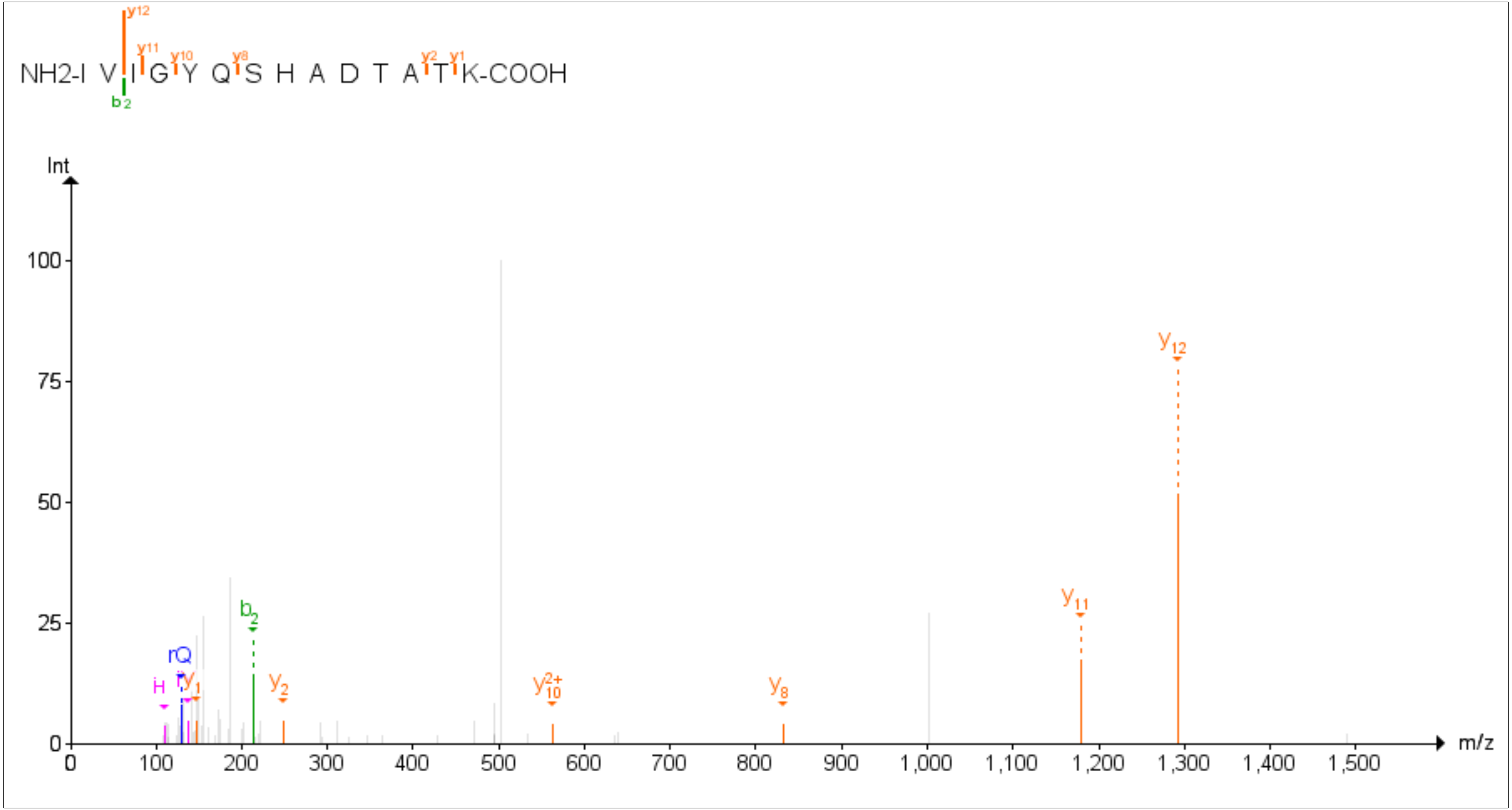

NH2-I V I G Y Q S H A D T A T K-COOH
y12
y11
y10
y8
y2
y1
b2
Int
100
75
50
25
0
m/z
0
100
200
300
400
500
600
700
800
900
1,000
1,100
1,200
1,300
1,400
1,500
y12
y11
b2
rQ
H
y1
y2
y10 2+
y8

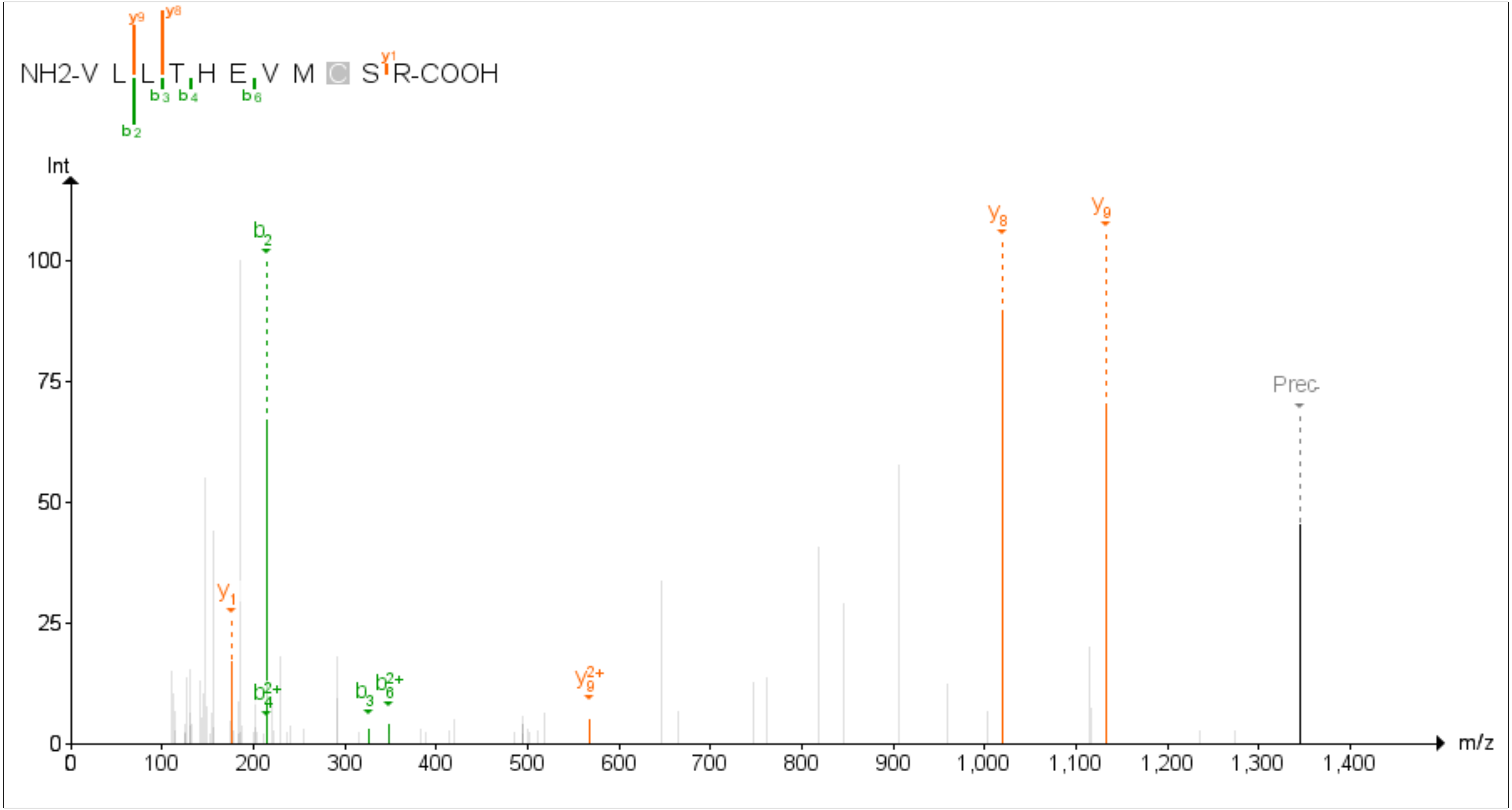
NH2-V L L T H E V M C S R-COOH
y9 y8 y1
b2 b3 b4 b6
Int
m/z
Prec
y8
y9
b2
y1
b4 2+
b3
b6 2+
y9 2+
0
25
50
75
100
0
100
200
300
400
500
600
700
800
900
1,000
1,100
1,200
1,300
1,400

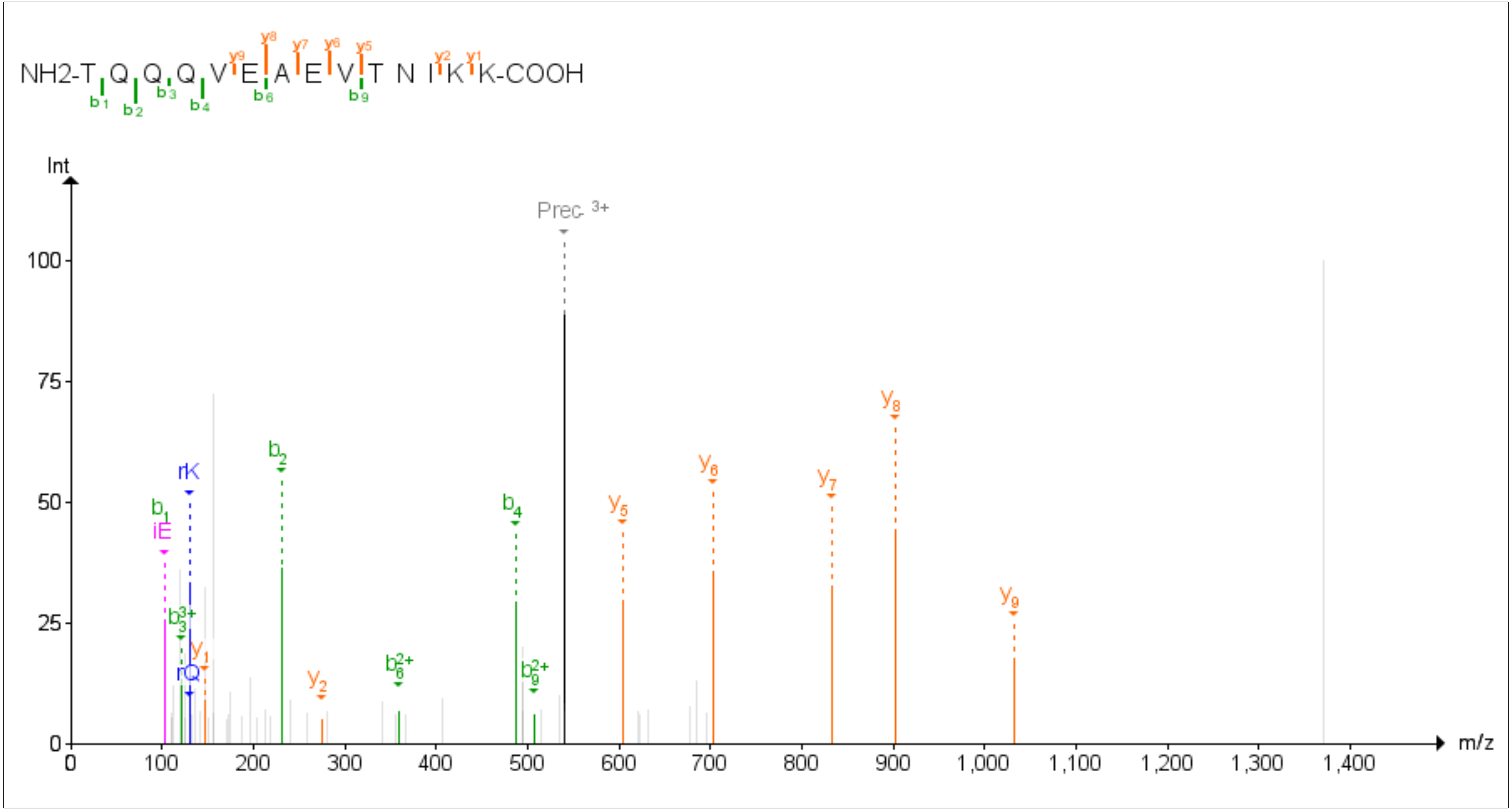

NH2-T Q Q Q V E A E V T N I K K-COOH
b1 b2 b3 b4 b6 b9
y9 y8 y7 y6 y5 y2 y1
Int
Prec 3+
b2
rK
b1
iE
b4
y5
y6
y7
y8
y9
b3 3+
y1
rQ
y2
b6 2+
b9 2+
m/z
0
25
50
75
100
0
100
200
300
400
500
600
700
800
900
1,000
1,100
1,200
1,300
1,400

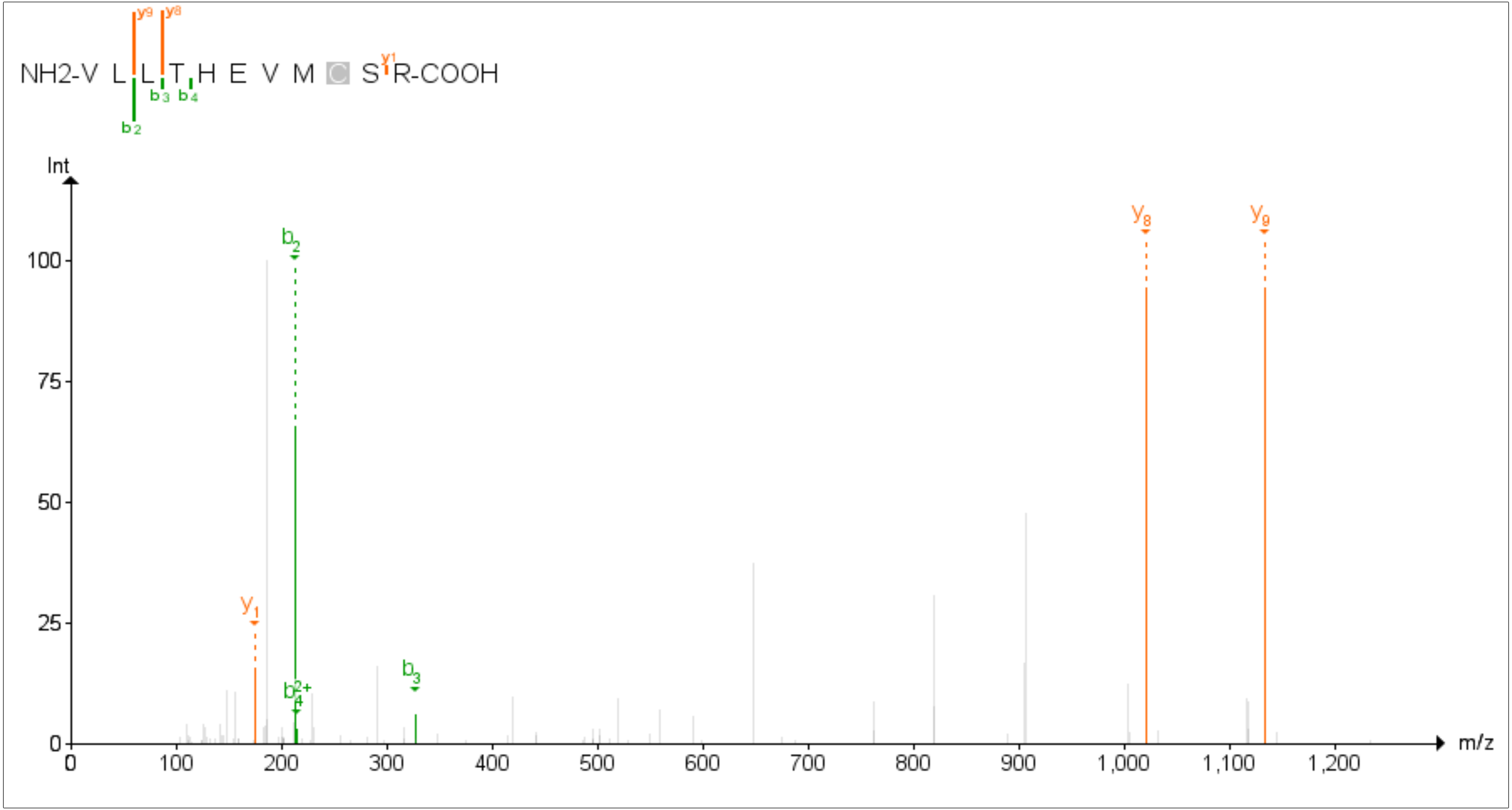

NH2-V L L T H E V M C S R-COOH
y9 y8 y1
b2 b3 b4
Int
100
75
50
25
0
b2
y8
y9
y1
b4 2+
b3
0 100 200 300 400 500 600 700 800 900 1,000 1,100 1,200
m/z

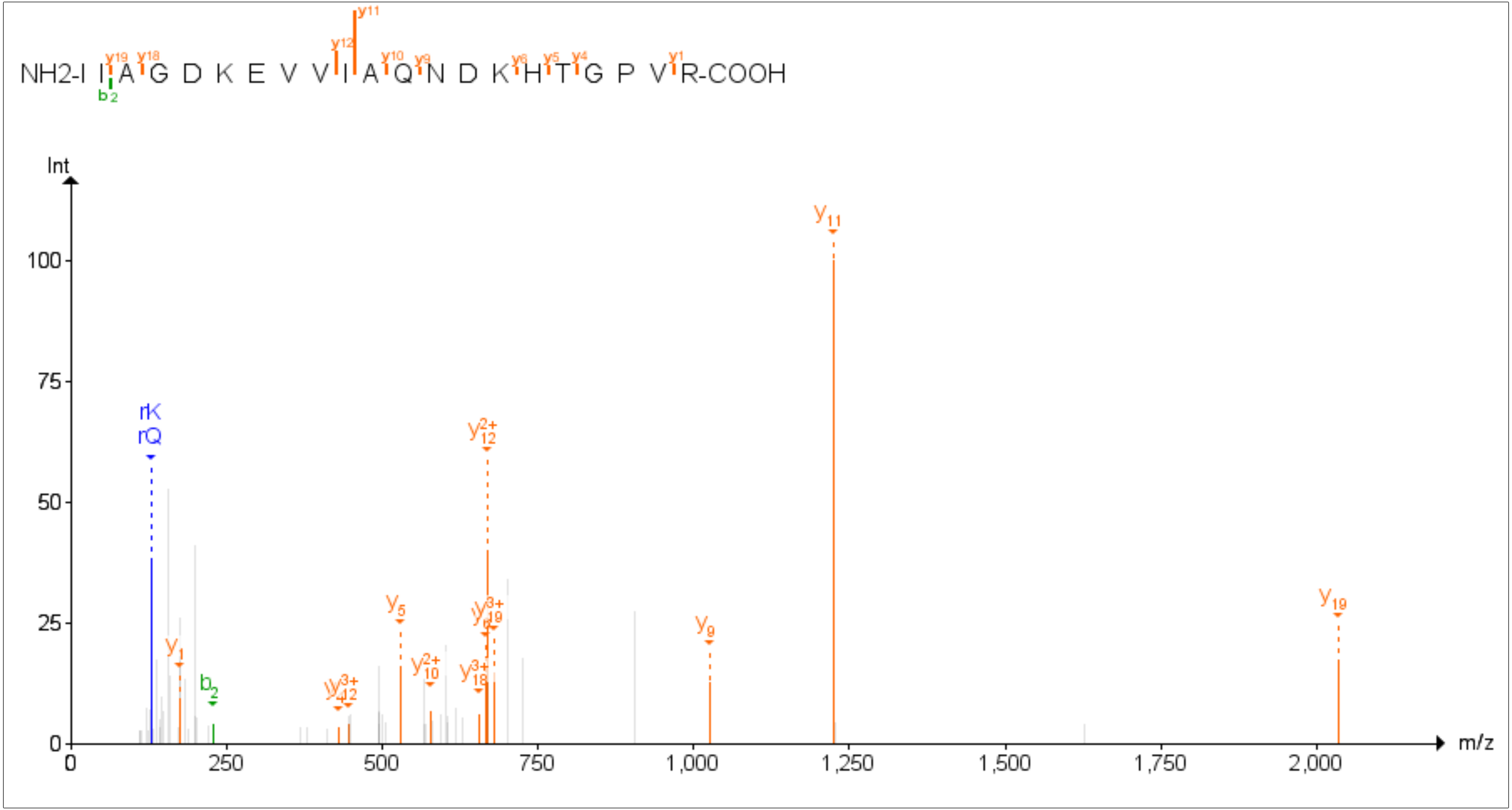

NH2-I I A G D K E V V I A Q N D K H T G P V R-COOH
y19 y18 y12 y11 y10 y9 y6 y5 y4 y1
b2
Int
m/z
rK
rQ

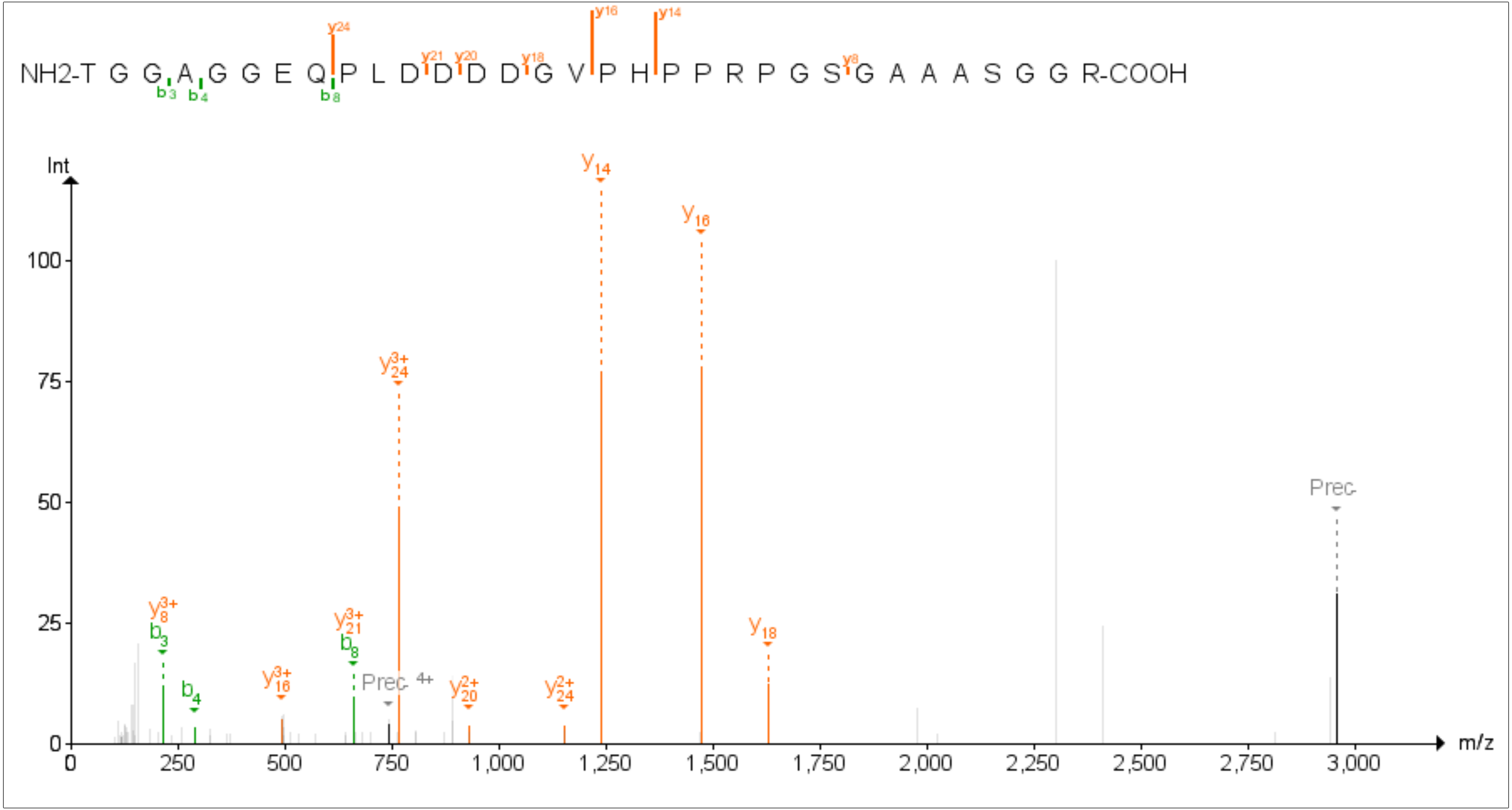

NH2-T G G A G G E Q P L D D D D G V P H P P R P G S G A A A S G G R-COOH
y24
y21
y20
y18
y16
y14
y8
b3
b4
b8
Int
m/z
0
25
50
75
100
250
500
750
1,000
1,250
1,500
1,750
2,000
2,250
2,500
2,750
3,000
y14
y16
y24 3+
y18
Prec
y8 3+
b3
y21 3+
b8
b4
y16 3+
Prec 4+
y20 2+
y24 2+

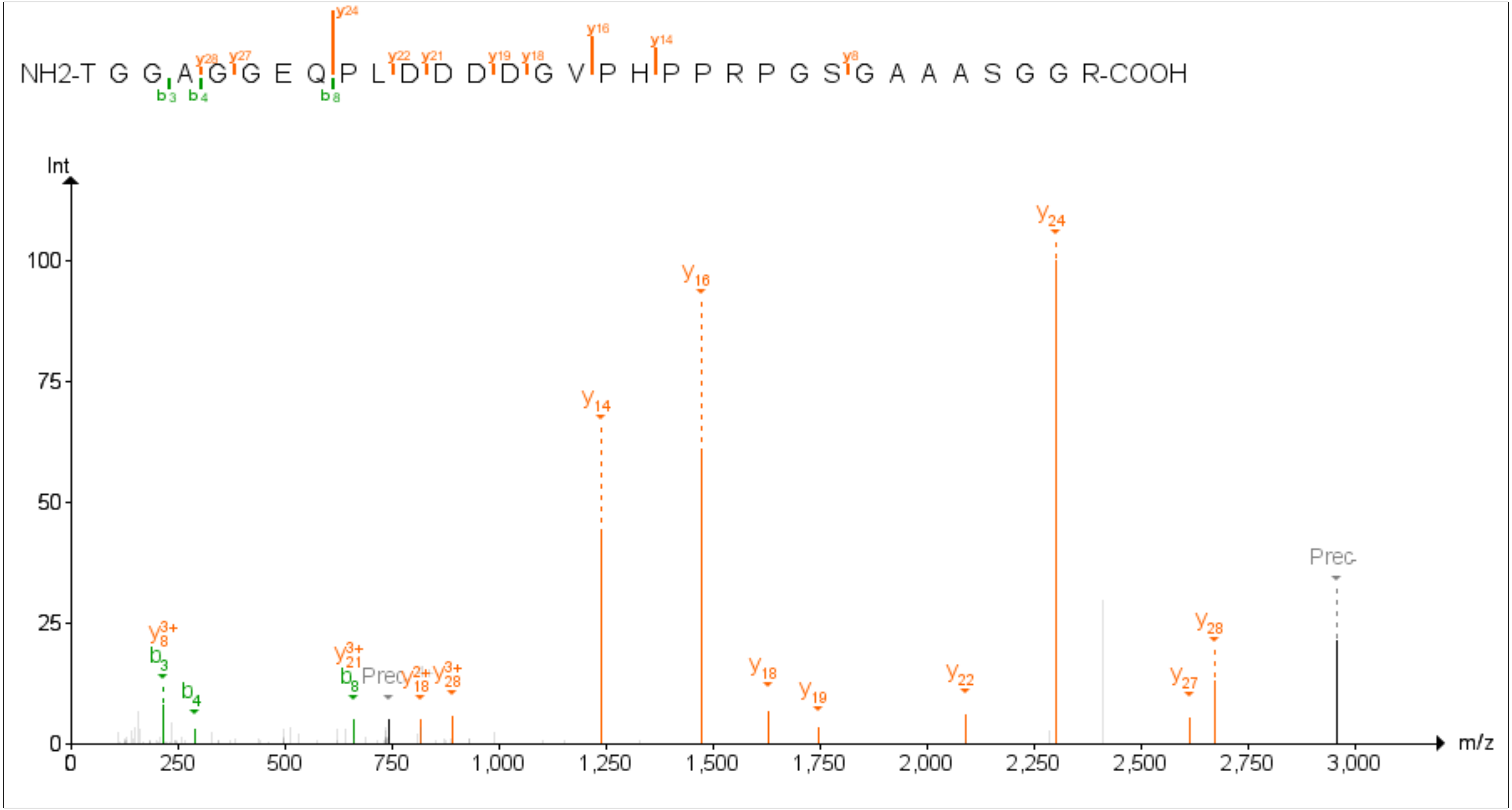

NH2-T G G A G G E Q P L D D D D G V P H P P R P G S G A A A S G G R-COOH
y24
y16
y14
y28
y27
y22
y21
y19
y18
y8
b3
b4
b8
Int
100
75
50
25
0
m/z
0
250
500
750
1,000
1,250
1,500
1,750
2,000
2,250
2,500
2,750
3,000
Prec

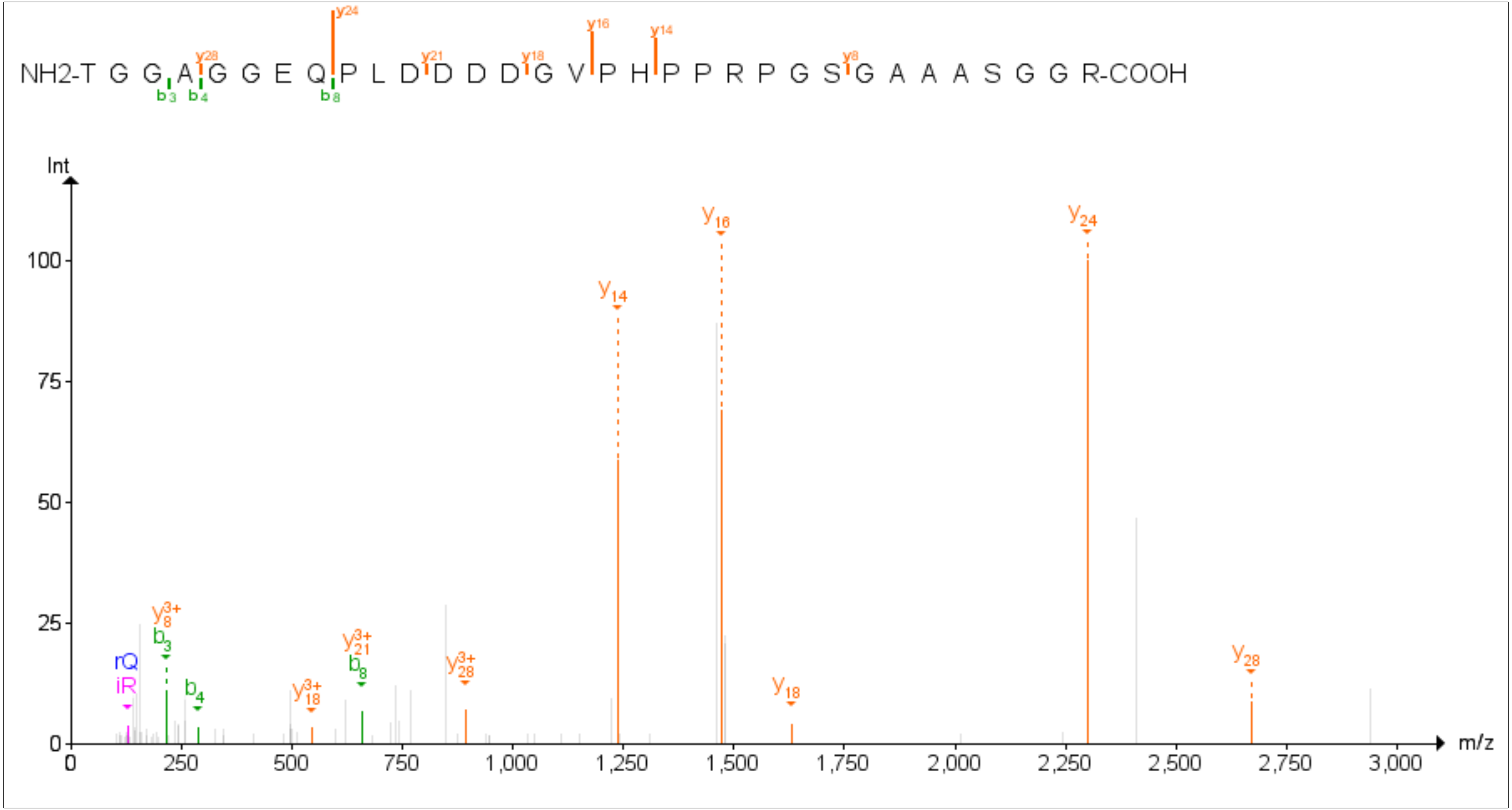

NH2-T G G A G G E Q P L D D D D G V P H P P R P G S G A A A S G G R-COOH
y24
y16
y14
y28
y21
y18
y8
b3
b4
b8
Int
m/z
100
75
50
25
0
250
500
750
1,000
1,250
1,500
1,750
2,000
2,250
2,500
2,750
3,000
y16
y24
y14
y8 3+
b3
rQ
iR
b4
y18 3+
y21 3+
b8
y28 3+
y18
y28

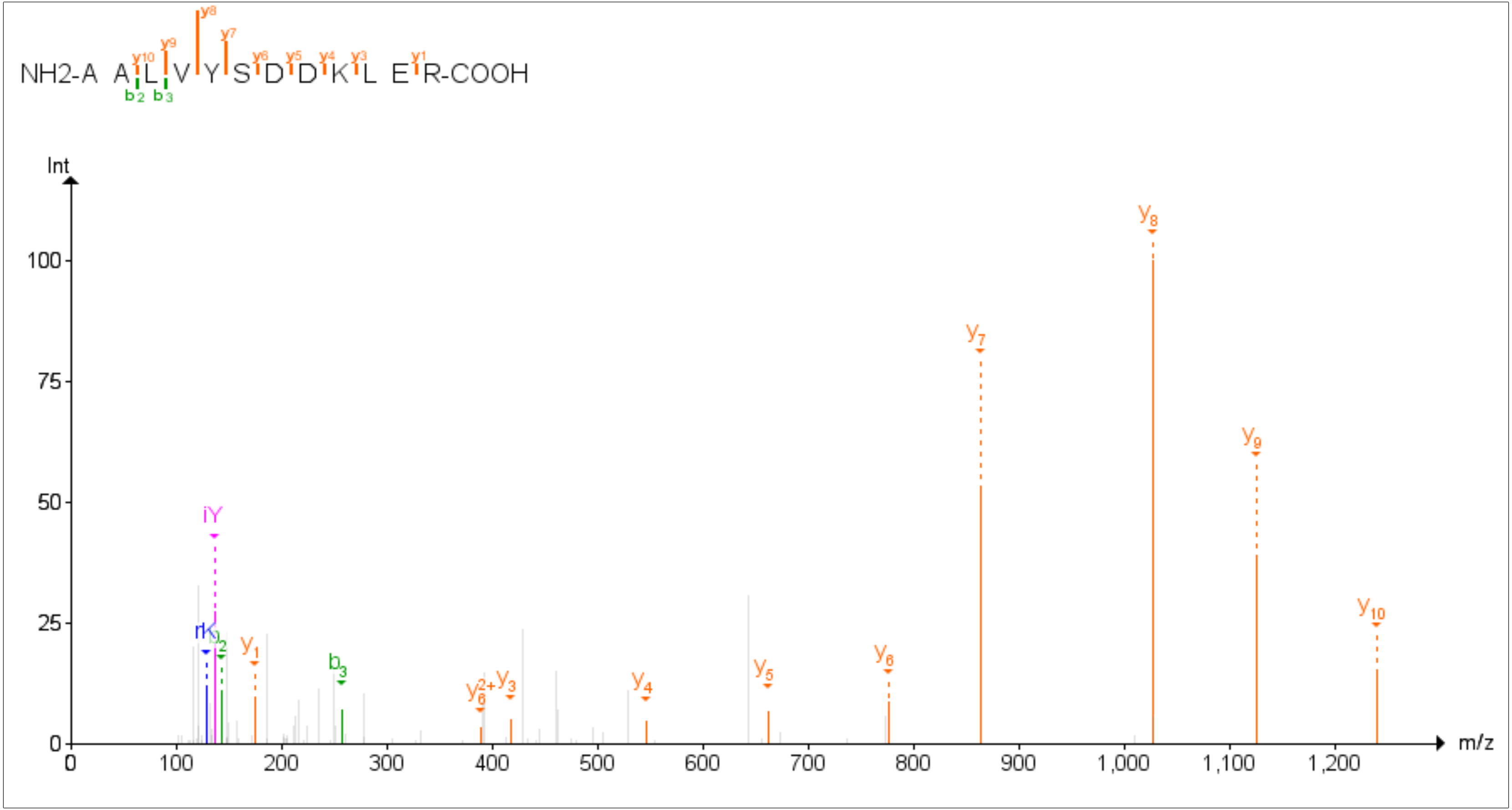

NH2-A A L V Y S D D K L E R-COOH
y10 y9 y8 y7 y6 y5 y4 y3 y1
b2 b3
Int
m/z
iY
rK
b2
y1
b3
y6 2+
y3
y4
y5
y6
y7
y8
y9
y10
0
25
50
75
100
0
100
200
300
400
500
600
700
800
900
1,000
1,100
1,200

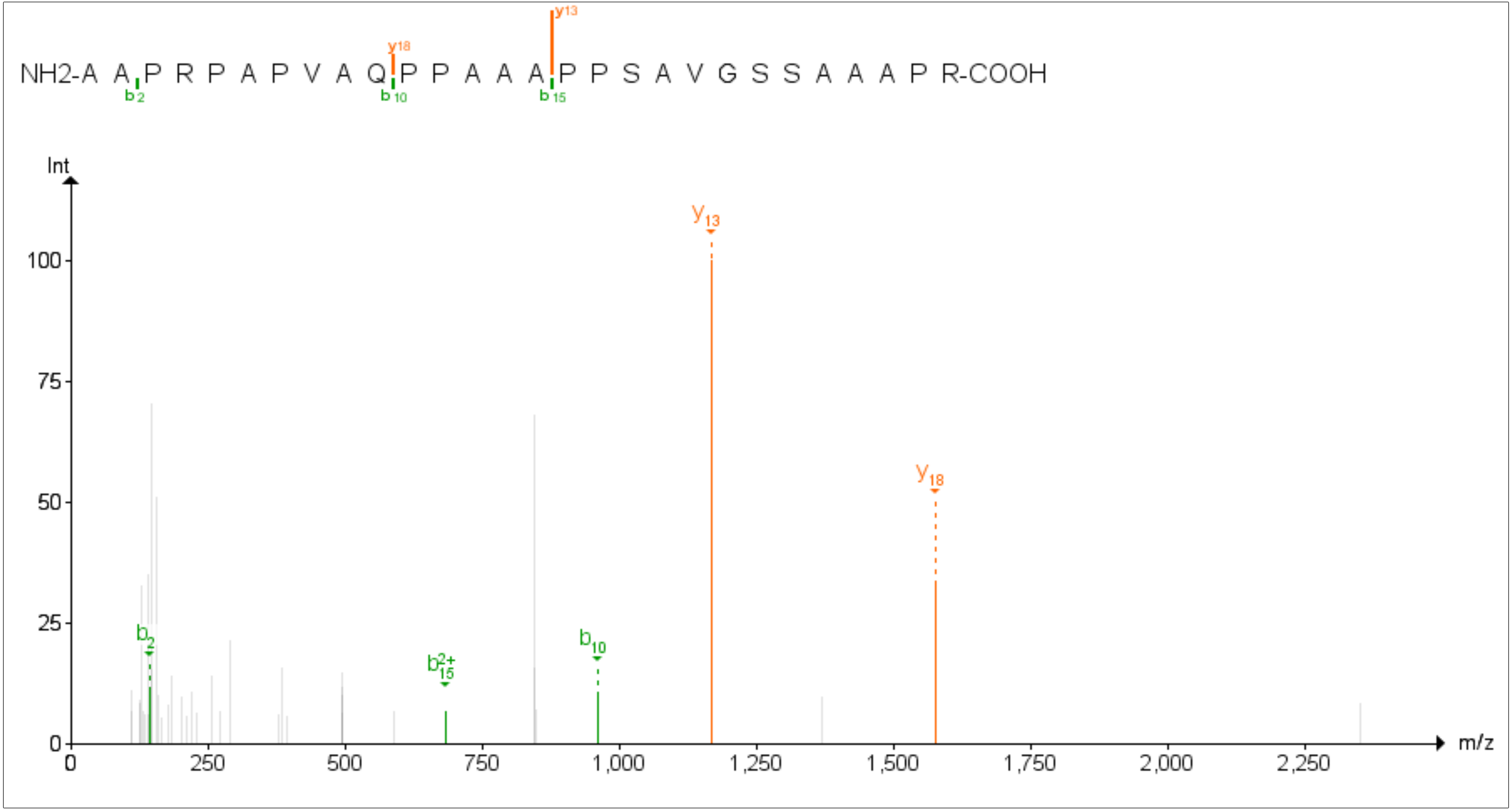

NH2-A A P R P A P V A Q P P A A A P P S A V G S S A A A P R-COOH
y18
y13
b2
b10
b15
Int
100
75
50
25
0
y13
y18
b2
b15 2+
b10
0
250
500
750
1,000
1,250
1,500
1,750
2,000
2,250
m/z

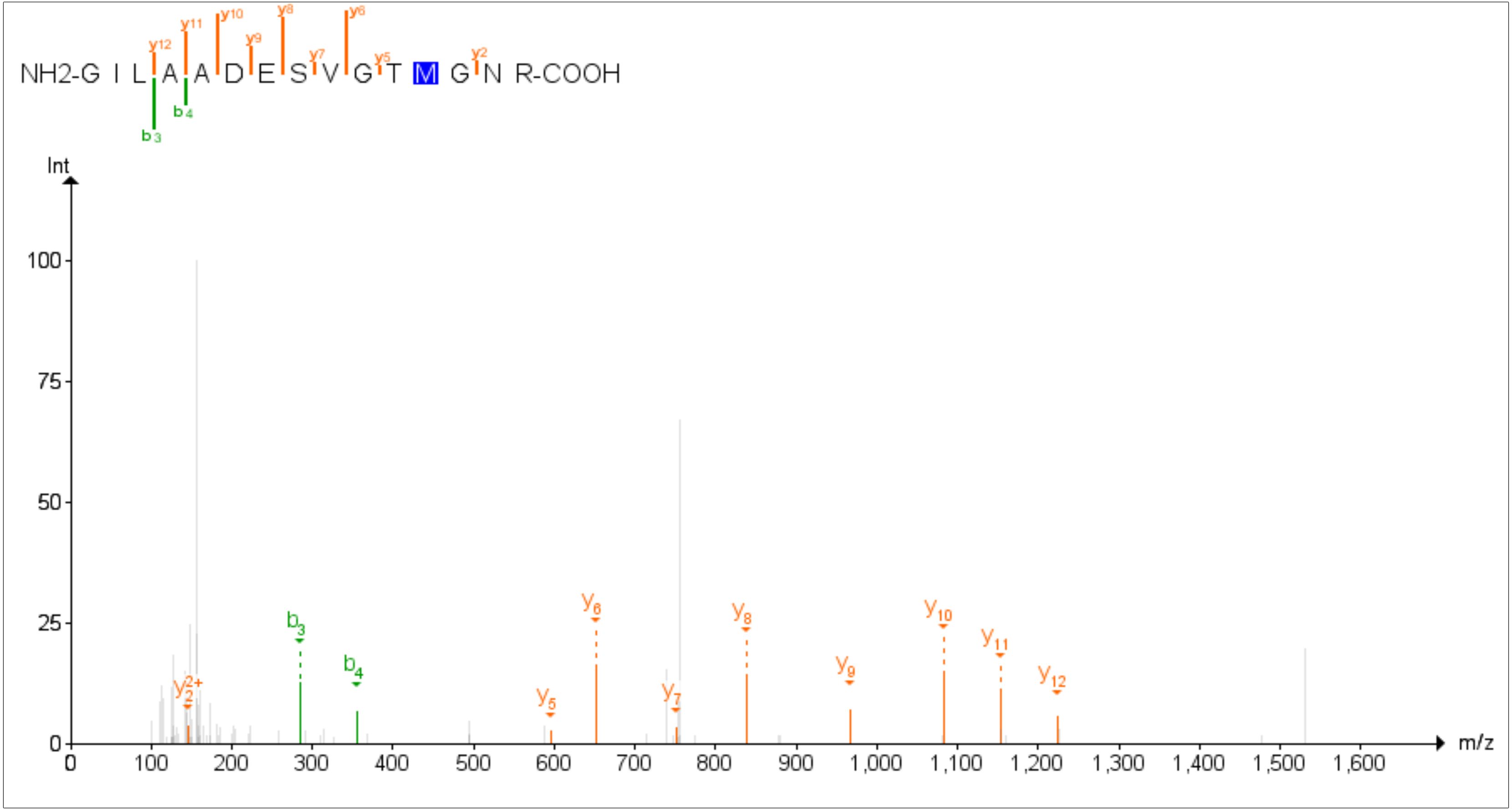

NH2-G I L A A D E S V G T M G N R-COOH
y12
y11
y10
y9
y8
y7
y6
y5
y2
b3
b4
Int
100
75
50
25
0
m/z
0
100
200
300
400
500
600
700
800
900
1,000
1,100
1,200
1,300
1,400
1,500
1,600
y2 2+
b3
b4
y5
y6
y7
y8
y9
y10
y11
y12

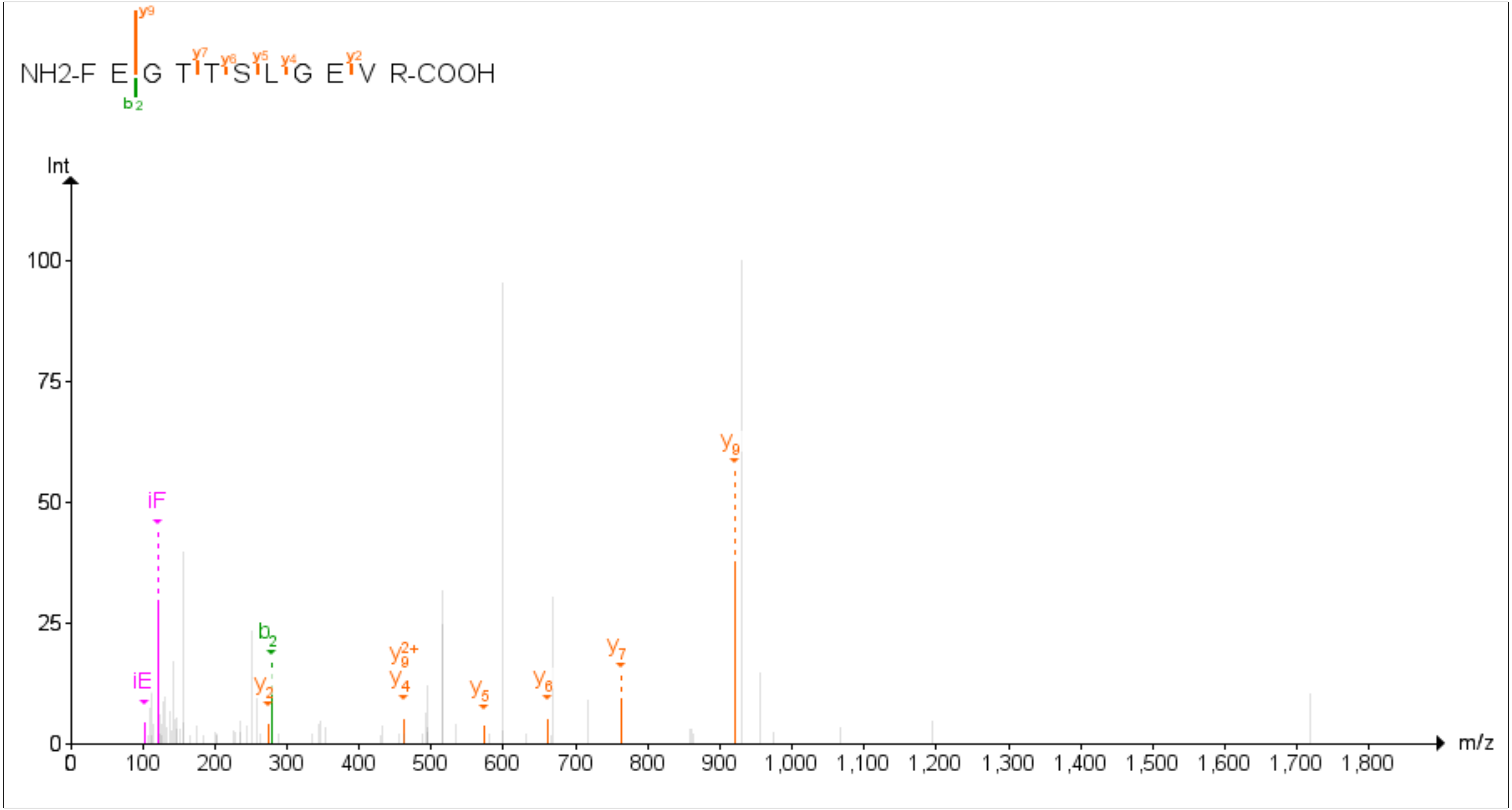

NH2-F E G T T S L G E V R-COOH
y9
y7
y6
y5
y4
y2
b2
Int
100
75
50
25
0
iF
iE
b2
y2
y9 2+
y4
y5
y6
y7
y9
m/z
0
100
200
300
400
500
600
700
800
900
1,000
1,100
1,200
1,300
1,400
1,500
1,600
1,700
1,800

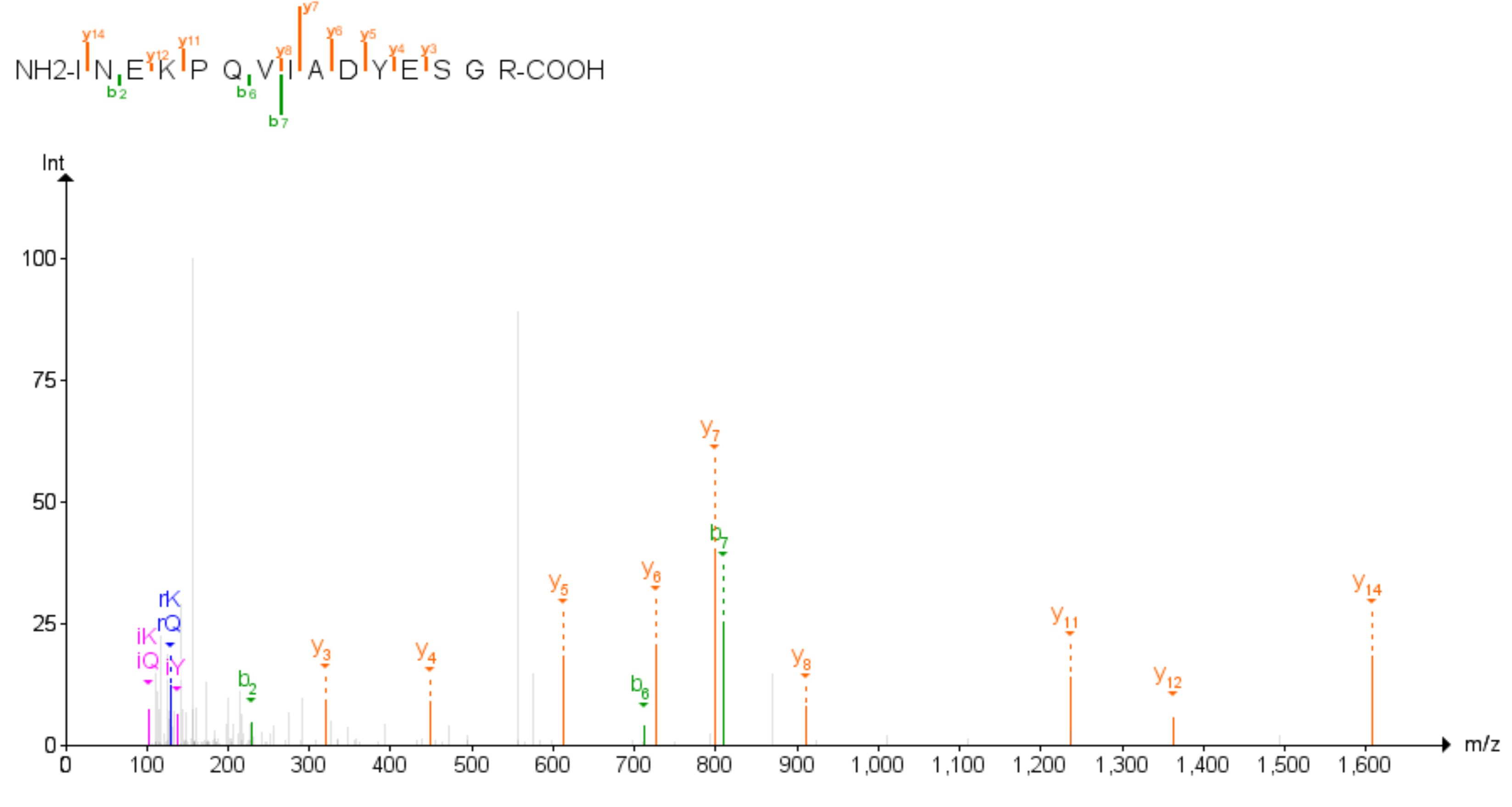

NH2-I N E K P Q V I A D Y E S G R-COOH
y14 y12 y11 y8 y7 y6 y5 y4 y3
b2 b6 b7
Int
100
75
50
25
0
m/z
0
100
200
300
400
500
600
700
800
900
1,000
1,100
1,200
1,300
1,400
1,500
1,600
rK
rQ
iK
iQ
iY
b2
y3
y4
y5
b6
y6
y7
b7
y8
y11
y12
y14

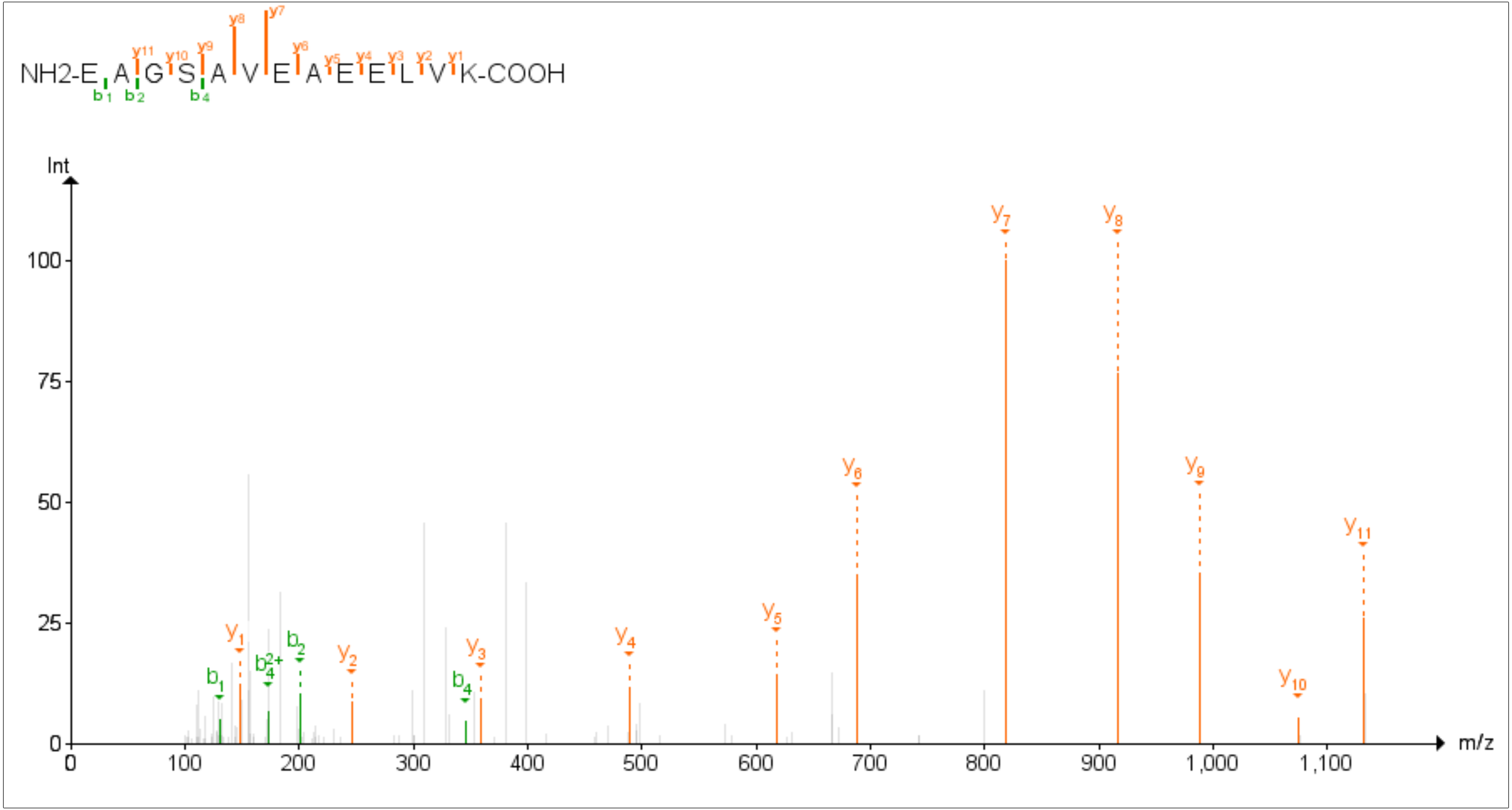

NH2-E A G S A V E A E E L V K-COOH
b1 b2 b4
y11 y10 y9 y8 y7 y6 y5 y4 y3 y2 y1
Int
100
75
50
25
0
m/z
0
100
200
300
400
500
600
700
800
900
1,000
1,100

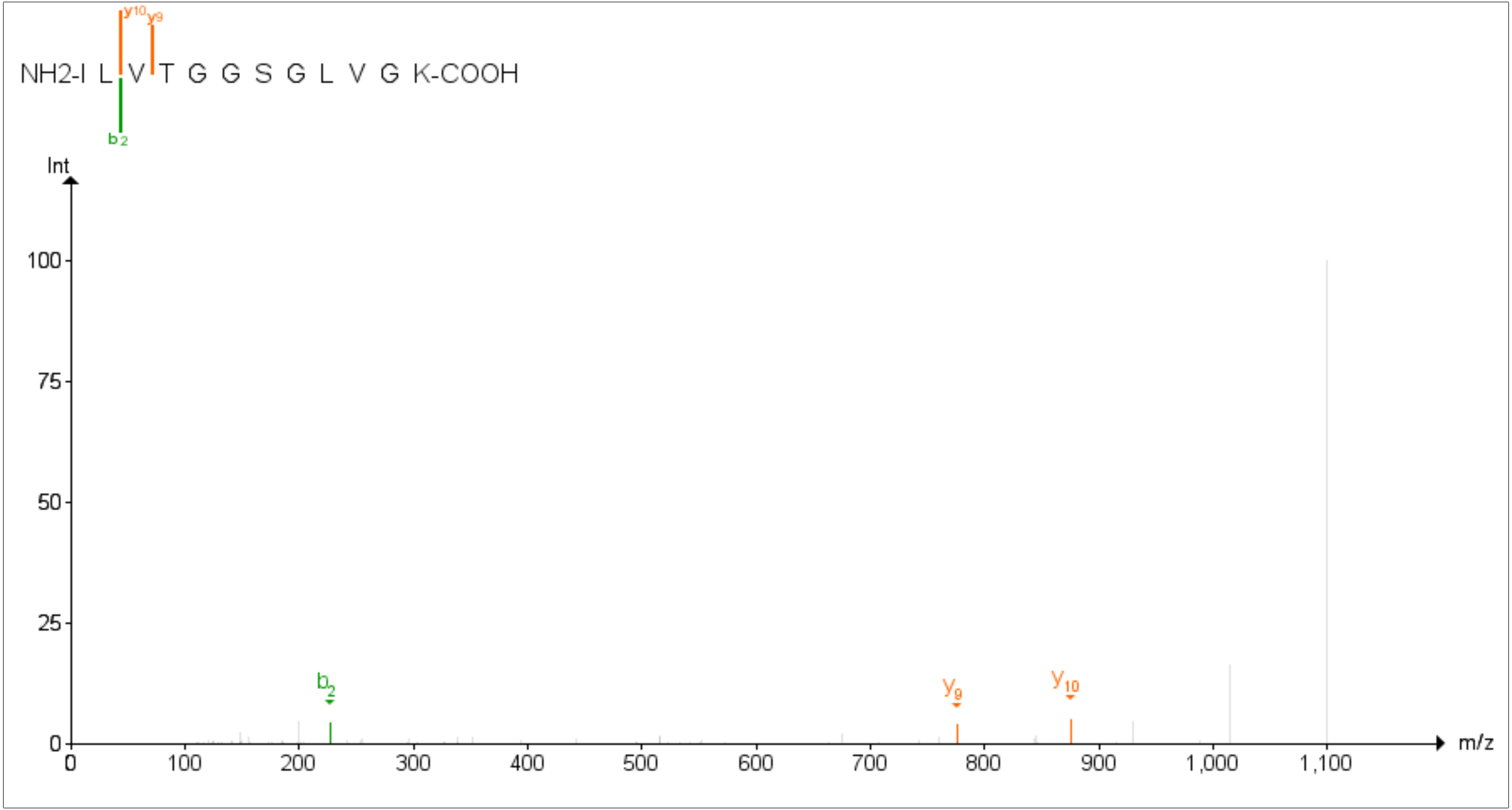

y10
y9
NH2-I L V T G G S G L V G K-COOH
b2
Int
100
75
50
25
0
b2
y9
y10
0
100
200
300
400
500
600
700
800
900
1,000
1,100
m/z

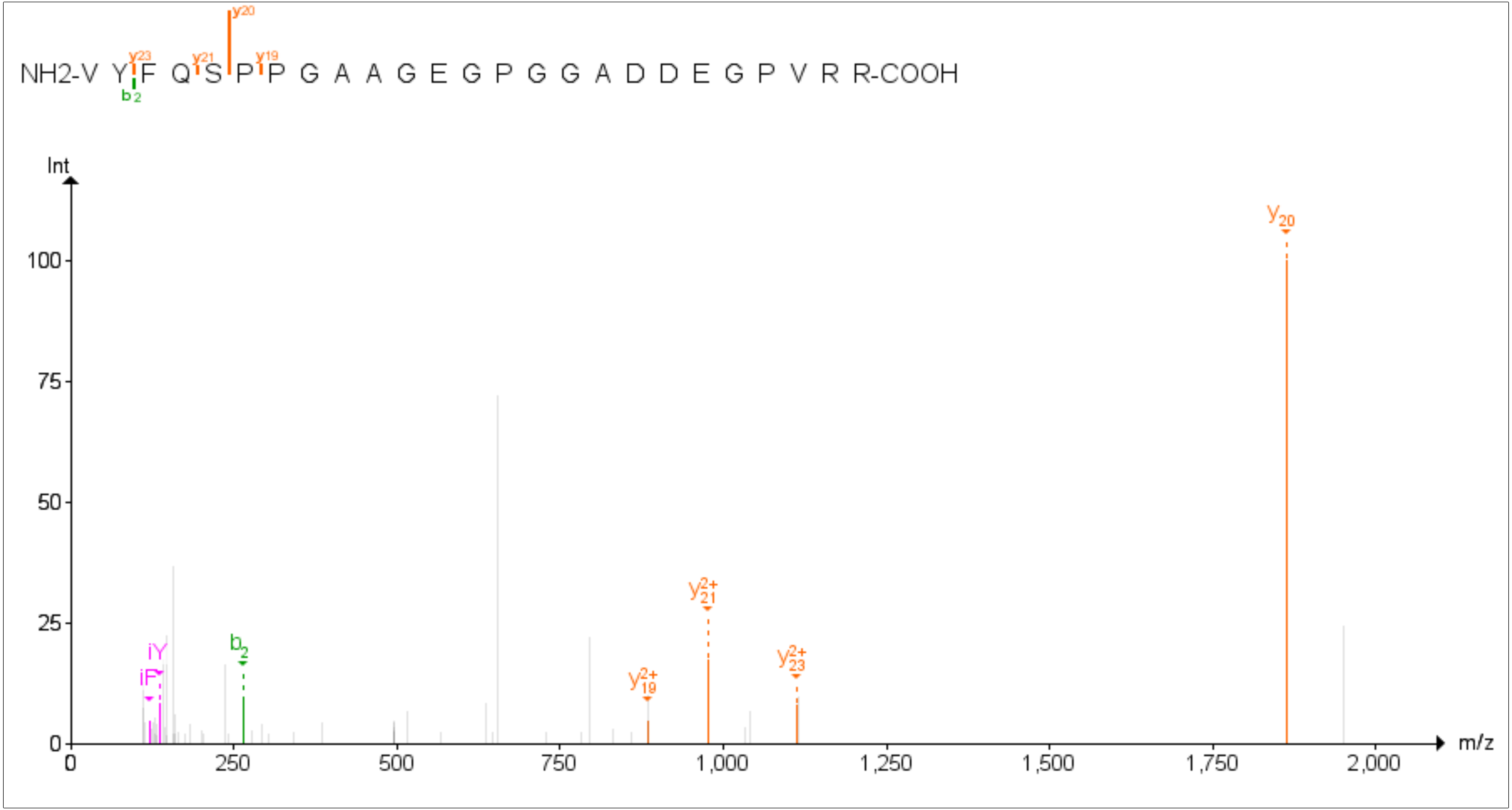

NH2-V Y F Q S P P G A A G E G P G G A D D E G P V R R-COOH
y23
y21
y20
y19
b2
Int
m/z
100
75
50
25
0
250
500
750
1,000
1,250
1,500
1,750
2,000
y20
y21 2+
y23 2+
y19 2+
b2
iY
iF

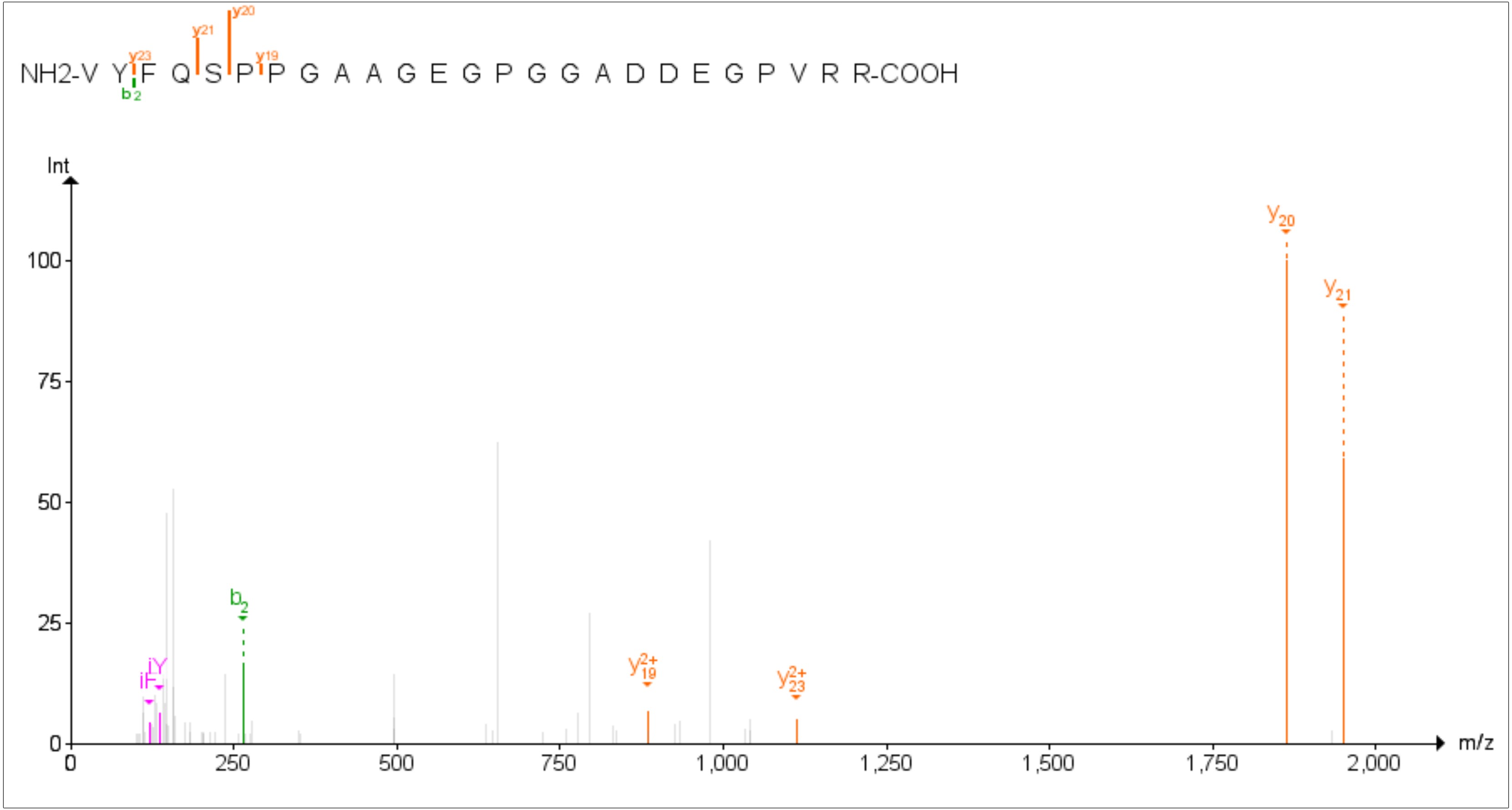

NH2-V Y F Q S P P G A A G E G P G G A D D E G P V R R-COOH
y23
y21
y20
y19
b2
Int
100
75
50
25
0
y20
y21
b2
iY
iF
y19 2+
y23 2+
0
250
500
750
1,000
1,250
1,500
1,750
2,000
m/z

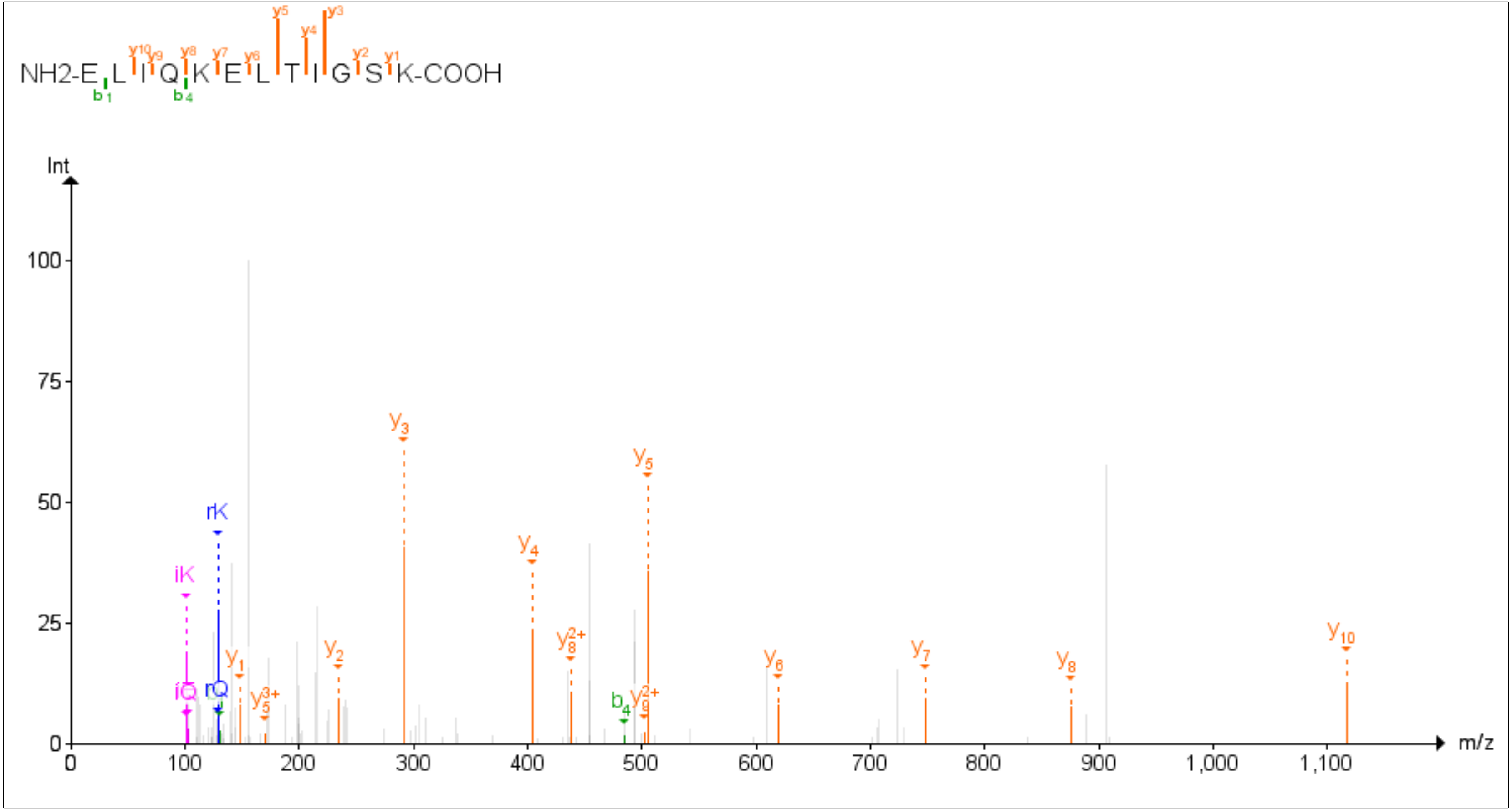
NH2-E L I Q K E L T I G S K-COOH
y10 y9 y8 y7 y6 y5 y4 y3 y2 y1
b1 b4
Int
100
75
50
25
0
m/z
0 100 200 300 400 500 600 700 800 900 1,000 1,100
y3
y5
y4
rK
iK
y1
y2
iQ
rQ
y8 2+
y9 2+
y5 3+
b4
y6
y7
y8
y10

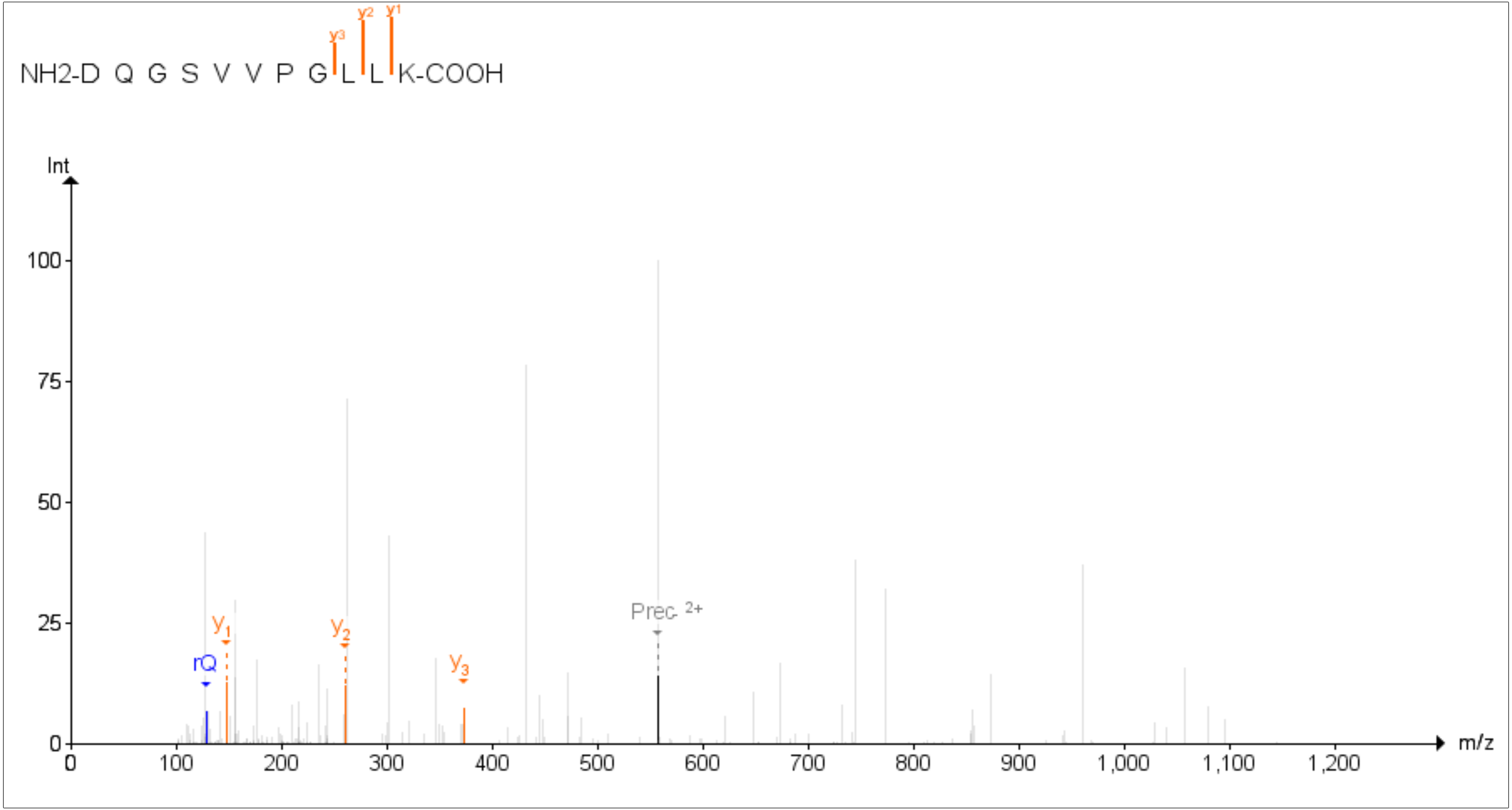

NH2-D Q G S V V P G L L K-COOH
y3
y2
y1
Int
100
75
50
25
0
rQ
y1
y2
y3
Prec 2+
m/z
0
100
200
300
400
500
600
700
800
900
1,000
1,100
1,200

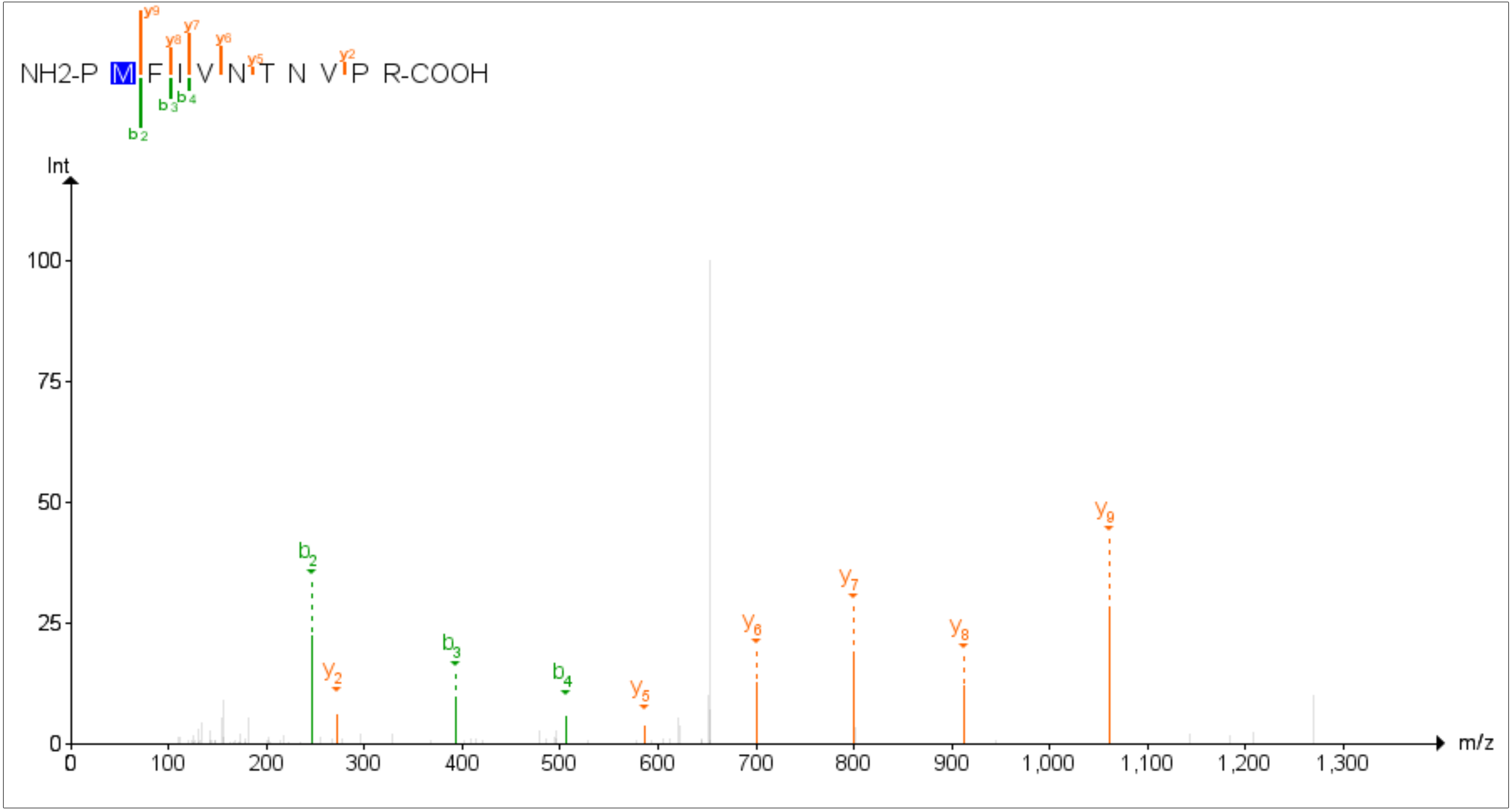

NH2-P M F I V N T N V P R-COOH
y9
y8
y7
y6
y5
y2
b2
b3
b4
Int
100
75
50
25
0
m/z
0
100
200
300
400
500
600
700
800
900
1,000
1,100
1,200
1,300
b2
y2
b3
b4
y5
y6
y7
y8
y9

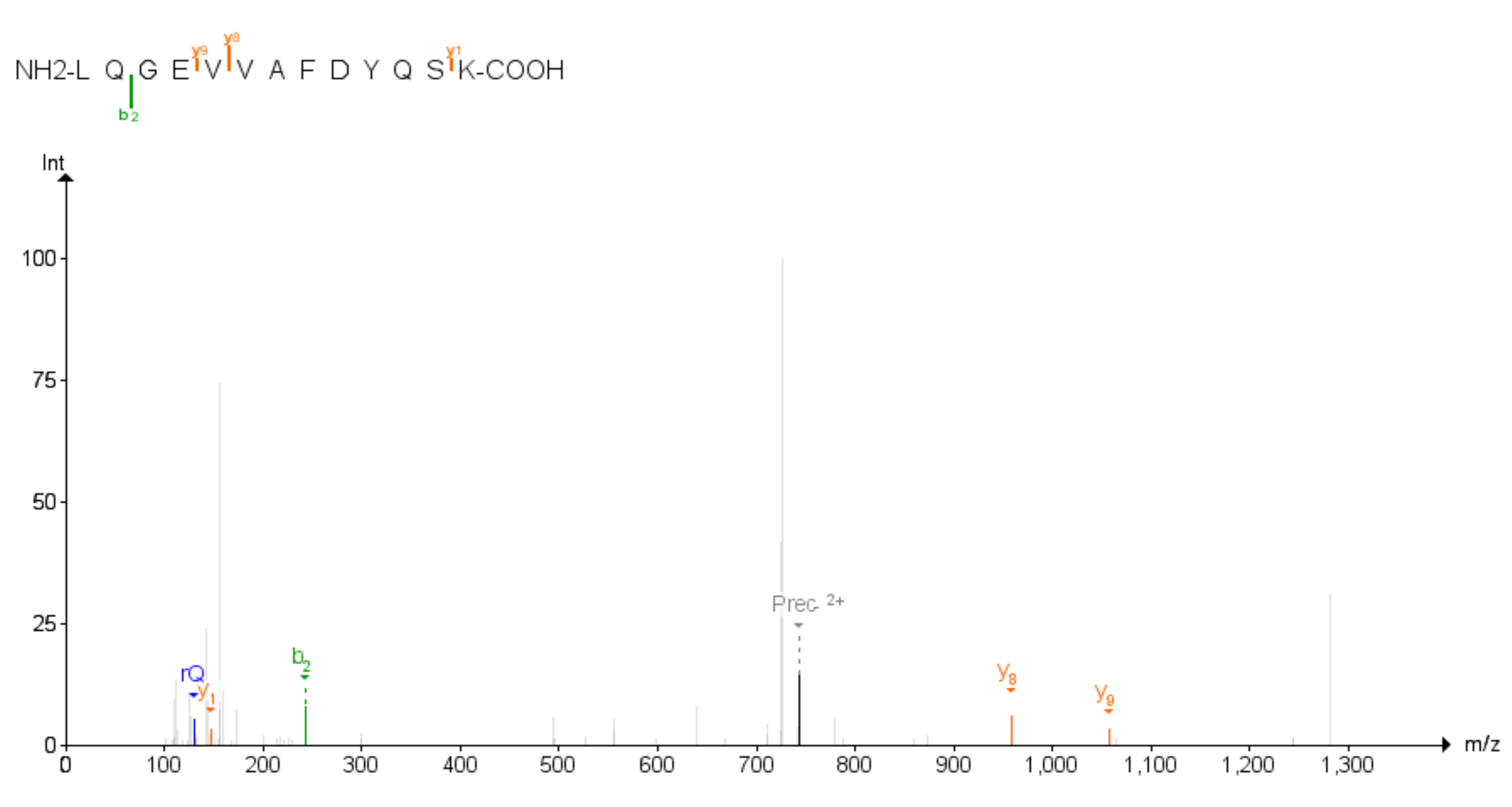
NH2-L Q G E V V A F D Y Q S K-COOH
b2
y9
y8
y1
Int
100
75
50
25
0
Prec- 2+
rQ
y1
b2
y8
y9
0
100
200
300
400
500
600
700
800
900
1,000
1,100
1,200
1,300
m/z

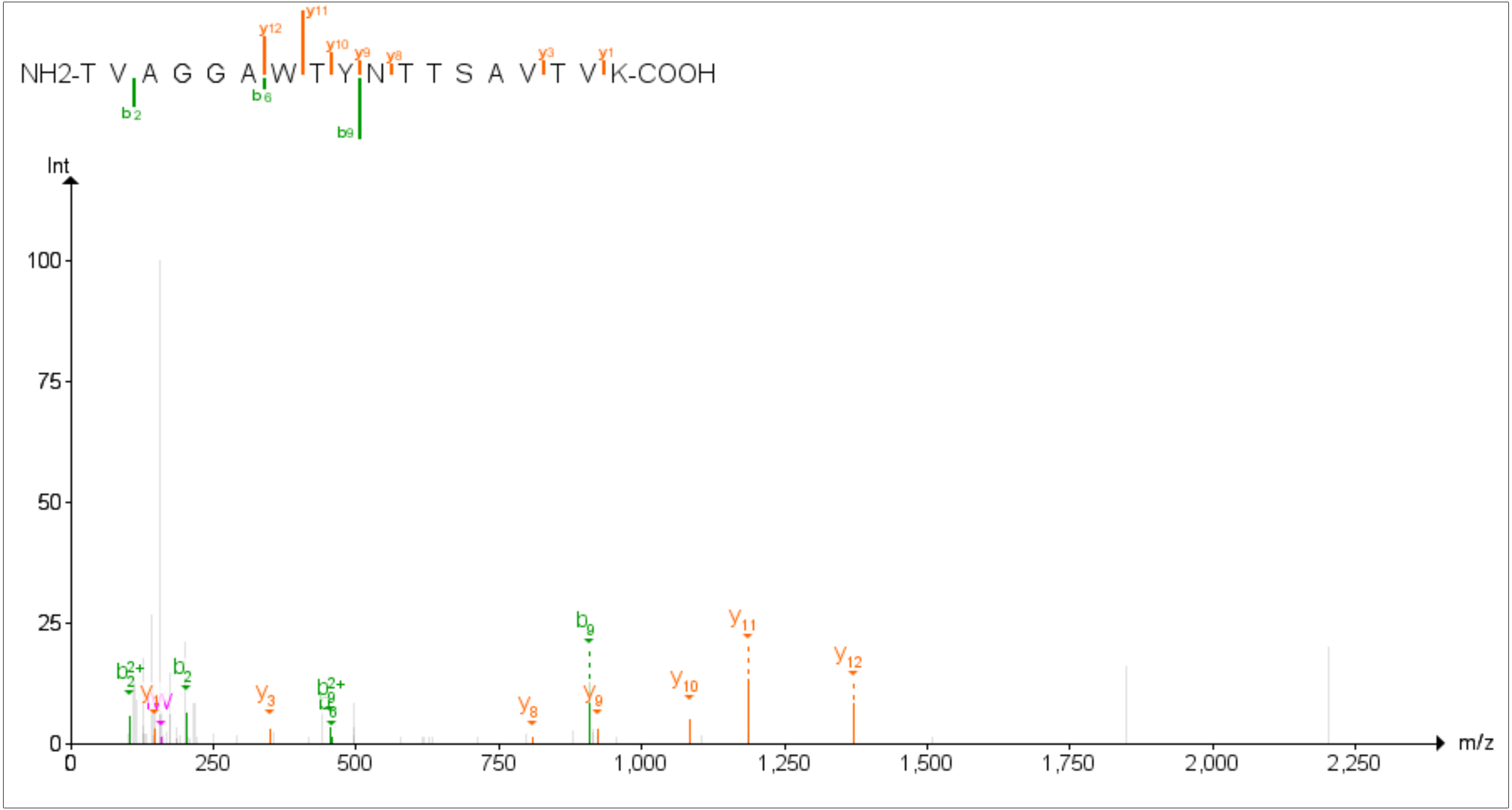

NH2-T V A G G A W T Y N T T S A V T V K-COOH
y12
y11
y10
y9
y8
y3
y1
b2
b6
b9
Int
100
75
50
25
0
m/z
0
250
500
750
1,000
1,250
1,500
1,750
2,000
2,250

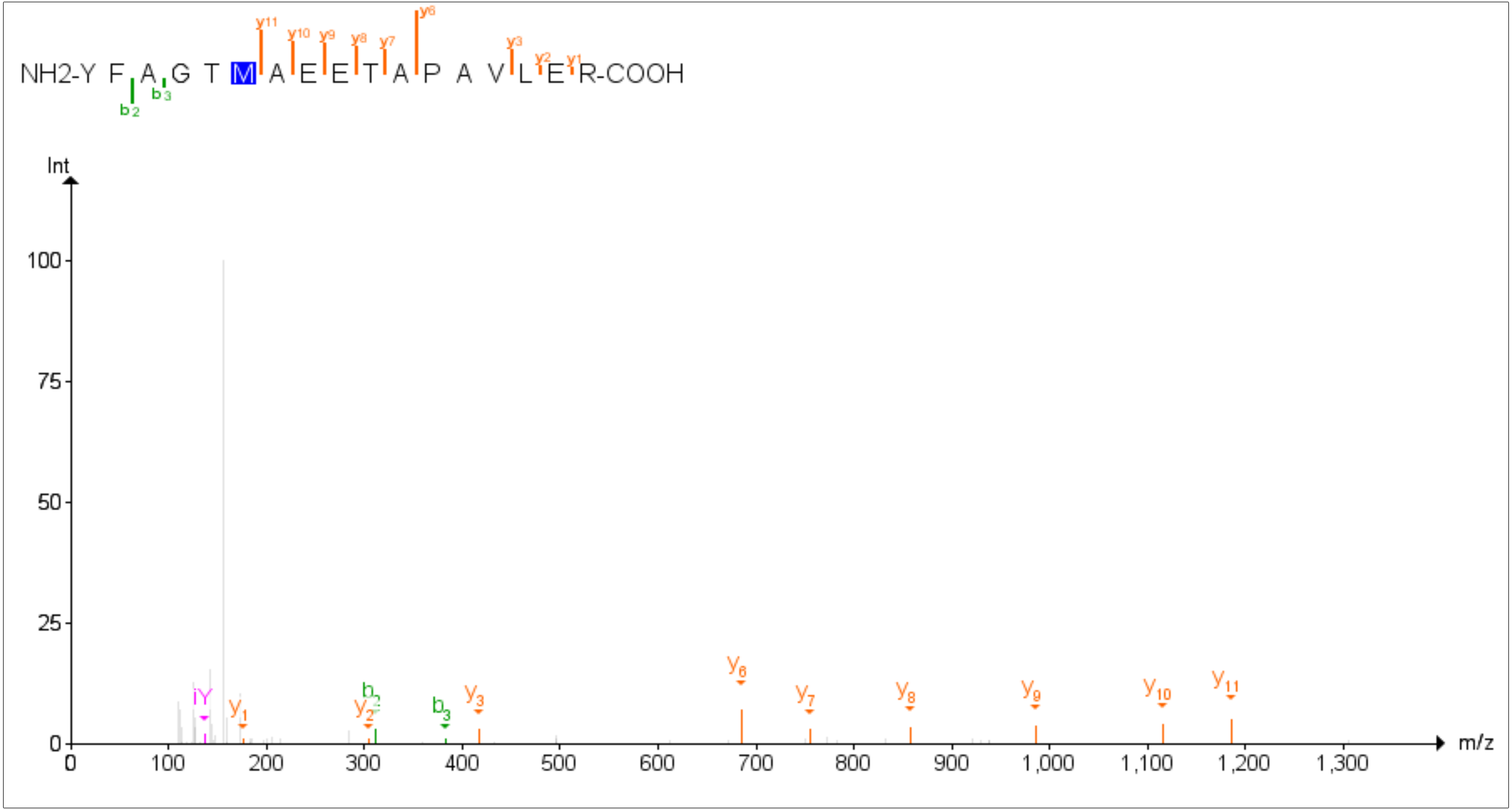

NH2-Y F A G T M A E E T A P A V L E R-COOH
b2
b3
y11
y10
y9
y8
y7
y6
y3
y2
y1
Int
100
75
50
25
0
iY
y1
y2
b2
b3
y3
y6
y7
y8
y9
y10
y11
0
100
200
300
400
500
600
700
800
900
1,000
1,100
1,200
1,300
m/z

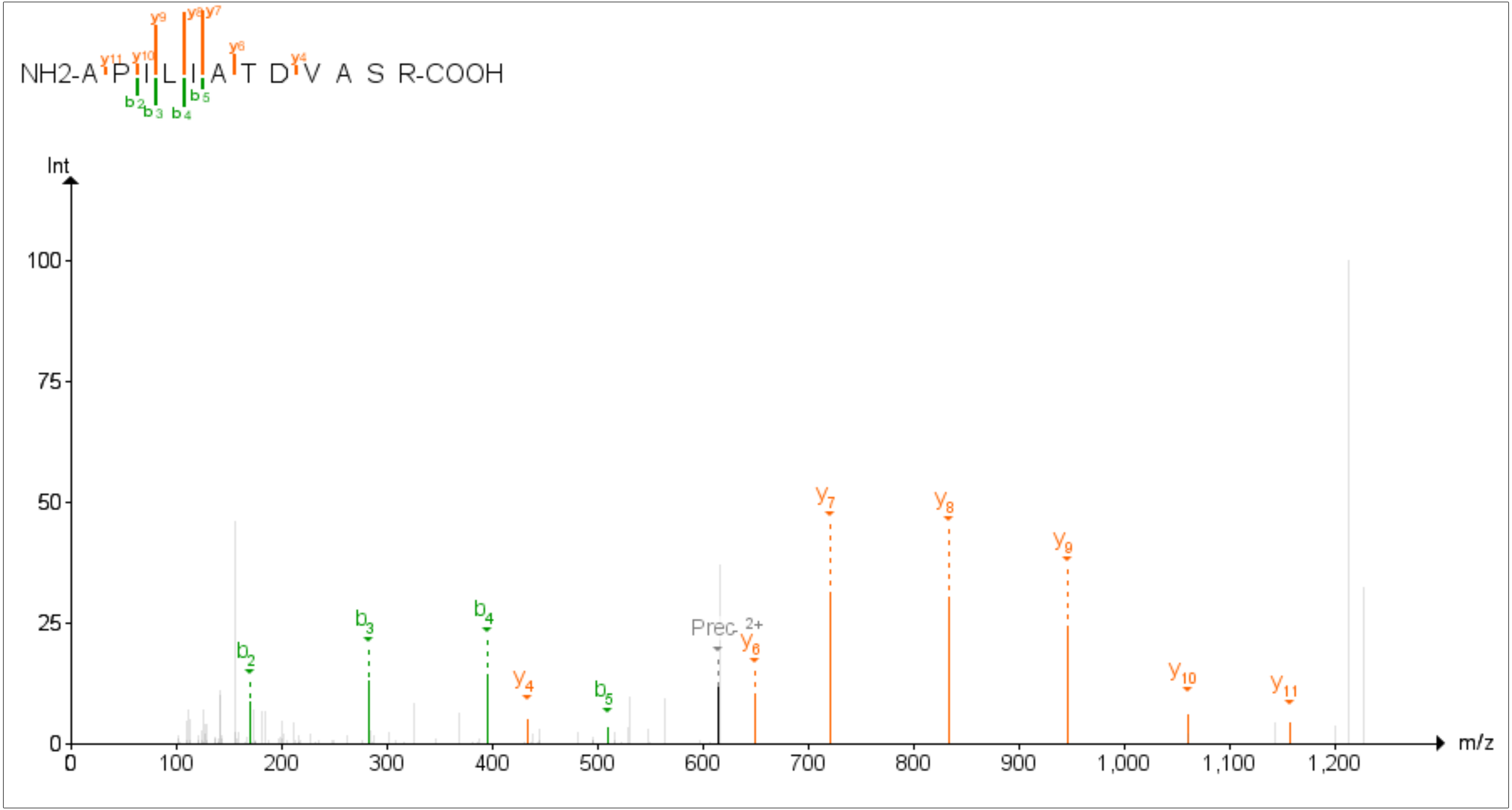

NH2-A P I L I A T D V A S R-COOH
y11 y10 y9 y8 y7 y6 y4
b2 b3 b4 b5
Int
100
75
50
25
0
m/z
0 100 200 300 400 500 600 700 800 900 1,000 1,100 1,200
b2 b3 b4 y4 b5 Prec 2+ y6 y7 y8 y9 y10 y11

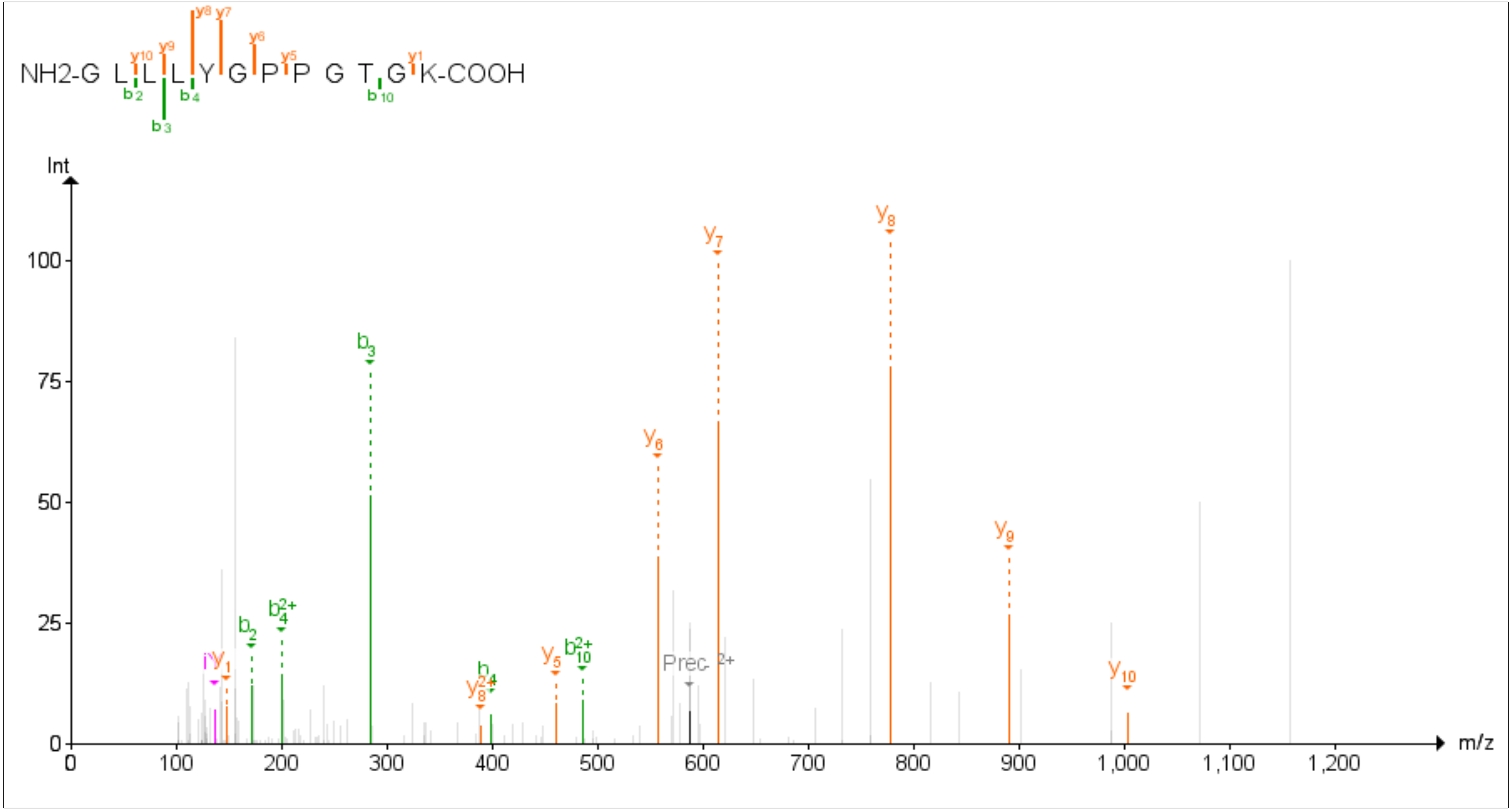

NH2-G L L L Y G P P G T G K-COOH
y10 y9 y8 y7 y6 y5 y1
b2 b3 b4 b10
Int
m/z
0
25
50
75
100
0
100
200
300
400
500
600
700
800
900
1,000
1,100
1,200
b3
y7
y8
y6
y9
y10
b2
b4 2+
y1
y5
b10 2+
b4
y8 2+
Prec. 2+

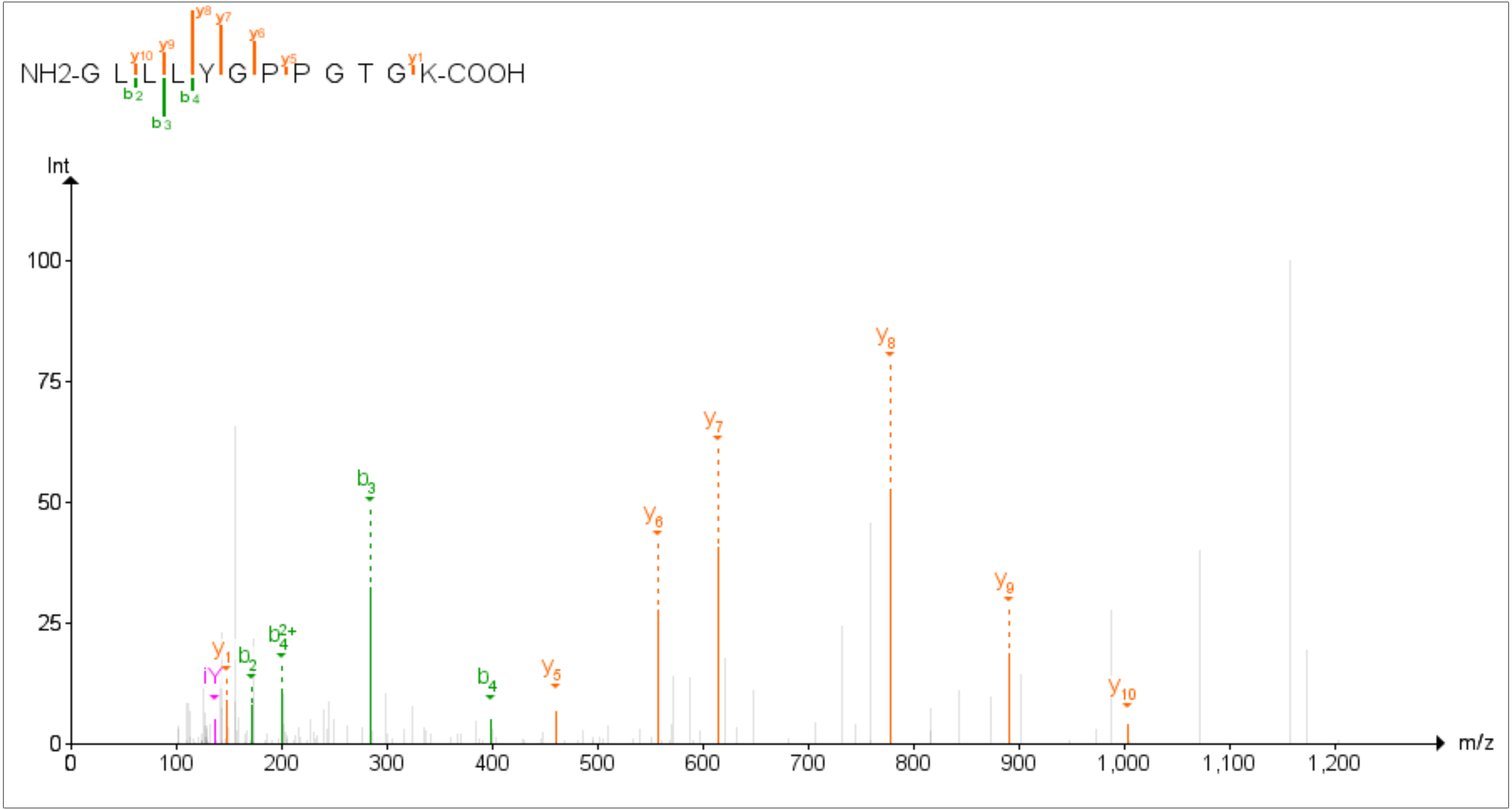

NH2-G L L L Y G P P G T G K-COOH
y10 y9 y8 y7 y6 y5 y1
b2 b3 b4
Int
100
75
50
25
0
b3
y8
y7
y6
y9
y1
iY
b2
b_4^{2+}
b4
y5
y10
0 100 200 300 400 500 600 700 800 900 1,000 1,100 1,200
m/z

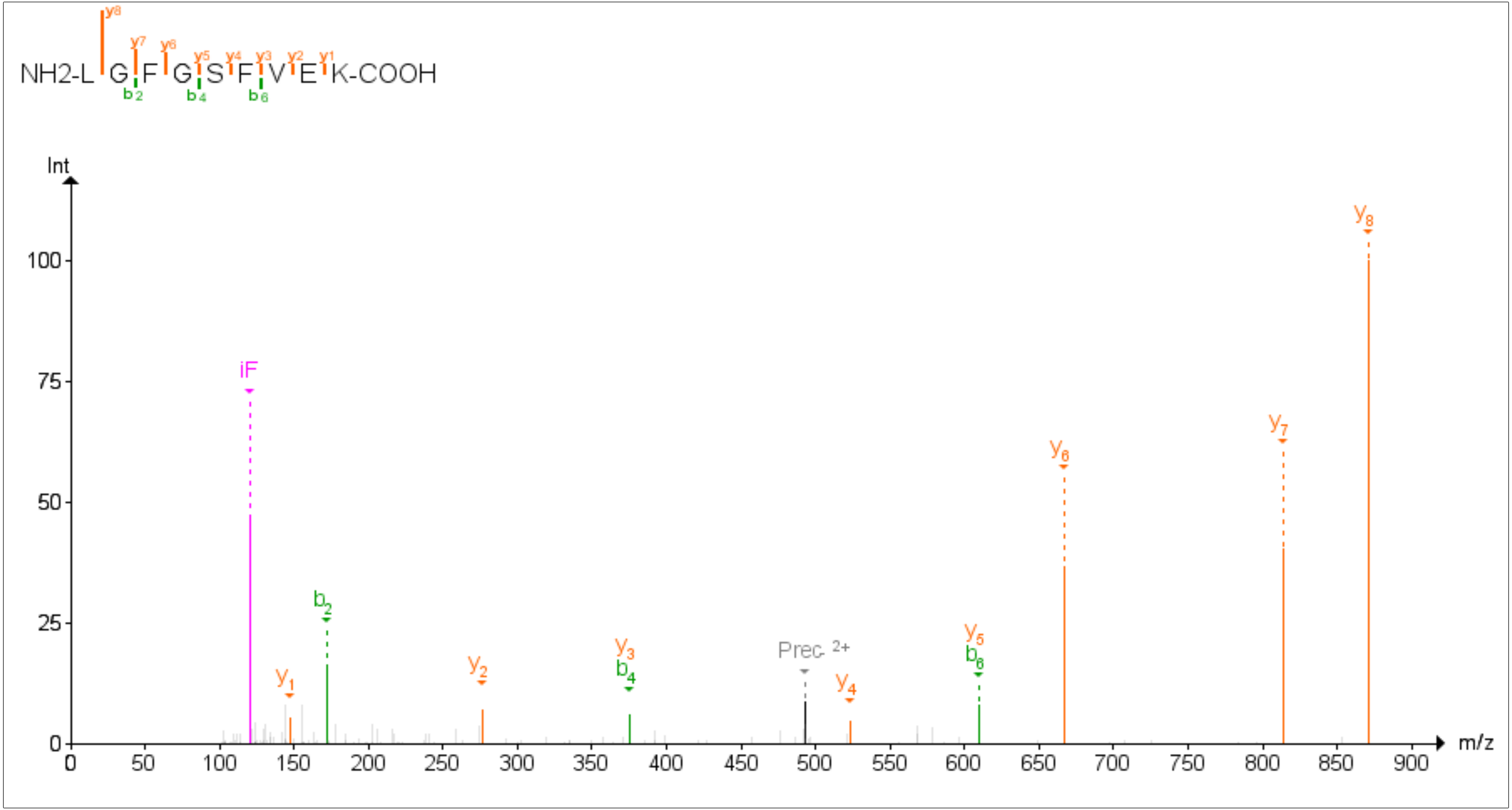

NH2-L G F G S F V E K-COOH
y8
y7
y6
y5
y4
y3
y2
y1
b2
b4
b6
Int
100
75
50
25
0
iF
b2
y1
y2
y3
b4
Prec 2+
y4
y5
b6
y6
y7
y8
0
50
100
150
200
250
300
350
400
450
500
550
600
650
700
750
800
850
900
m/z

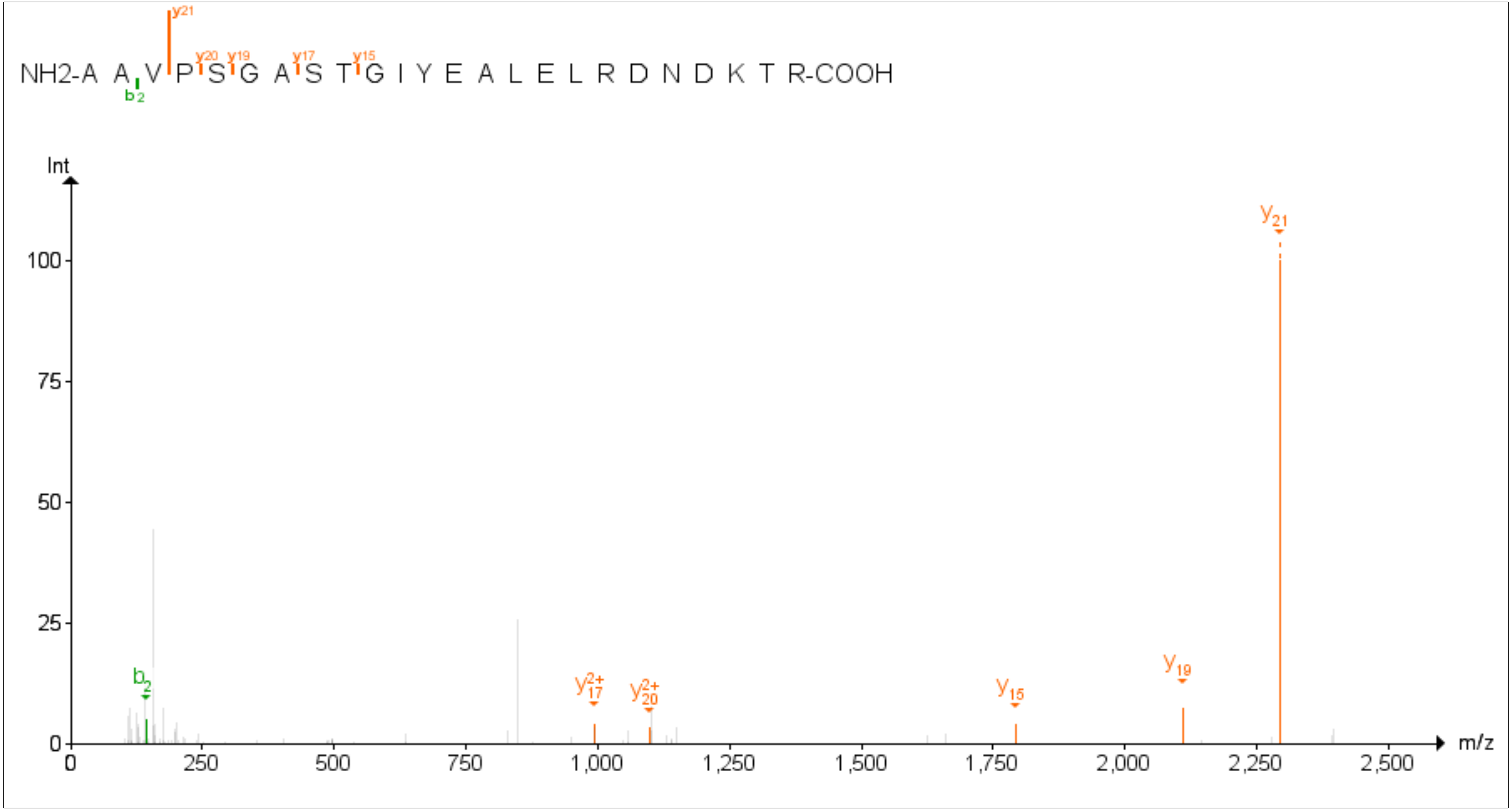

NH2-A A V P S G A S T G I Y E A L E L R D N D K T R-COOH
y21
y20
y19
y17
y15
b2
Int
100
75
50
25
0
250
500
750
1,000
1,250
1,500
1,750
2,000
2,250
2,500
m/z
y21
y19
y15
y17 2+
y20 2+
b2

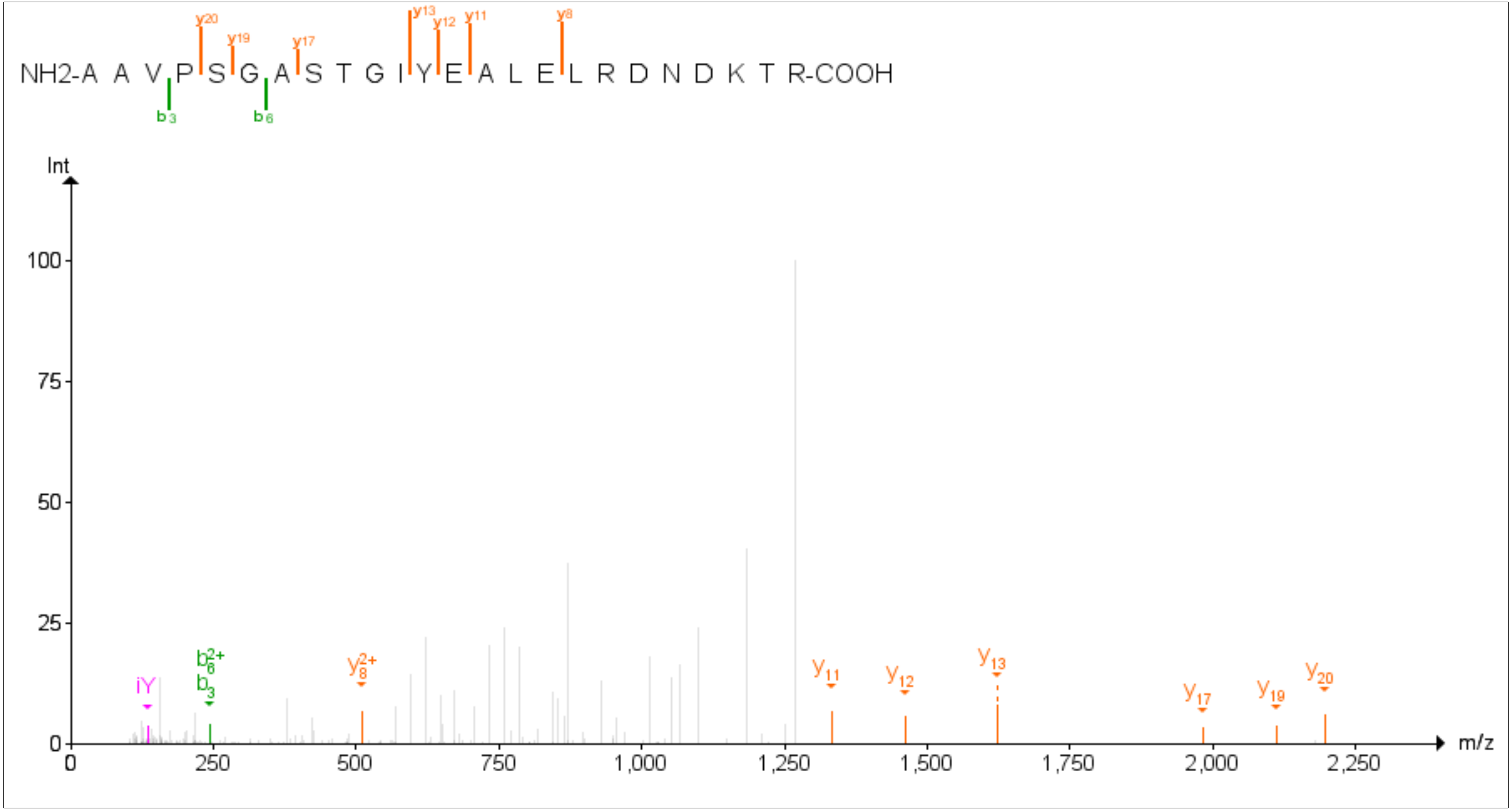
NH2-A A V P S G A S T G I Y E A L E L R D N D K T R-COOH
y20 y19 y17 y13 y12 y11 y8
b3 b6
Int
100
75
50
25
0
iY
b6 2+
b3
y8 2+
y11
y12
y13
y17
y19
y20
0 250 500 750 1,000 1,250 1,500 1,750 2,000 2,250
m/z

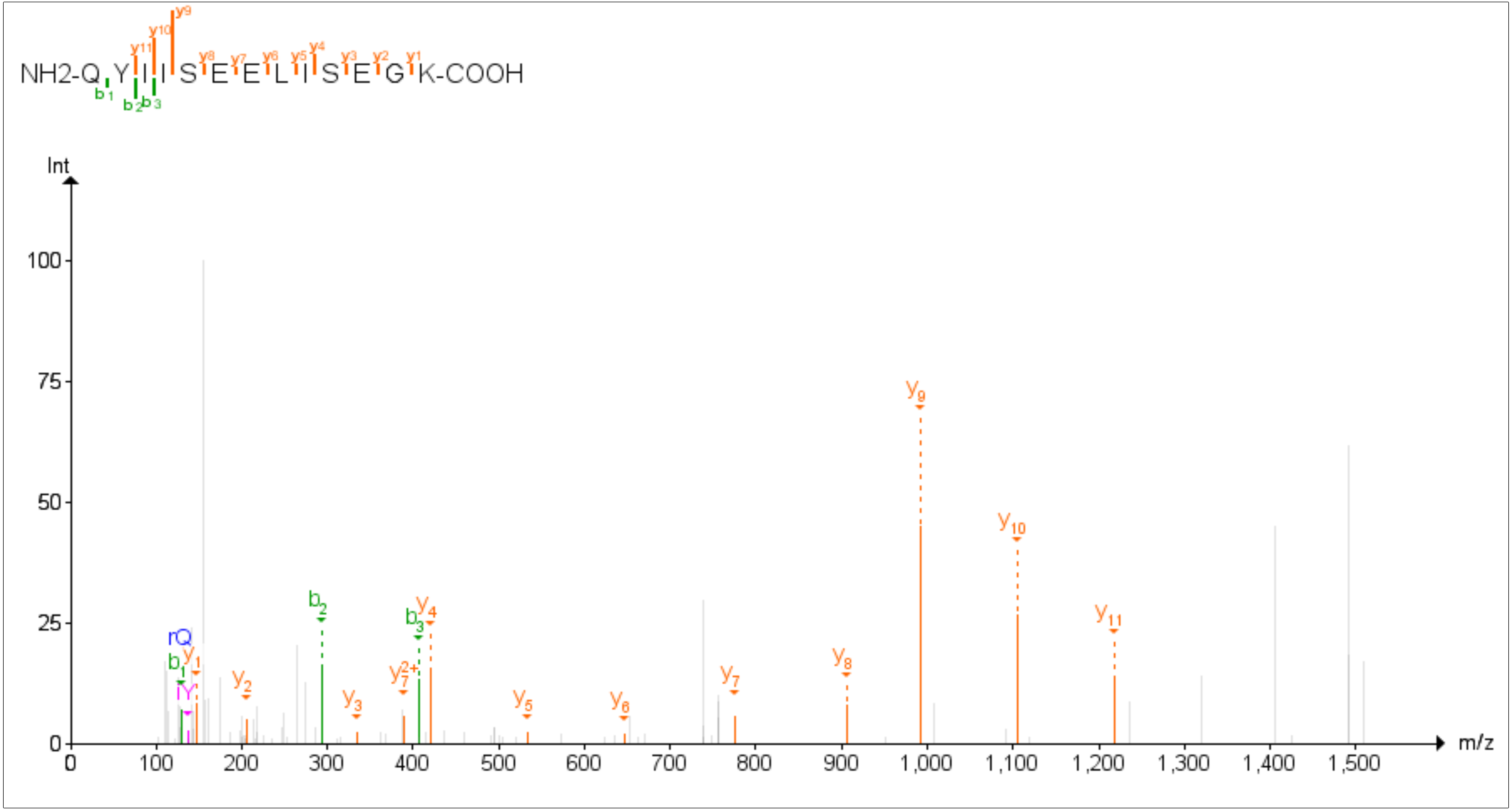
NH2-Q Y I I S E E L I S E G K-COOH
Int
m/z
0
25
50
75
100
100
200
300
400
500
600
700
800
900
1,000
1,100
1,200
1,300
1,400
1,500
rQ
iY
b1
b2
b3
y1
y2
y3
y4
y5
y6
y7
y7 2+
y8
y9
y10
y11

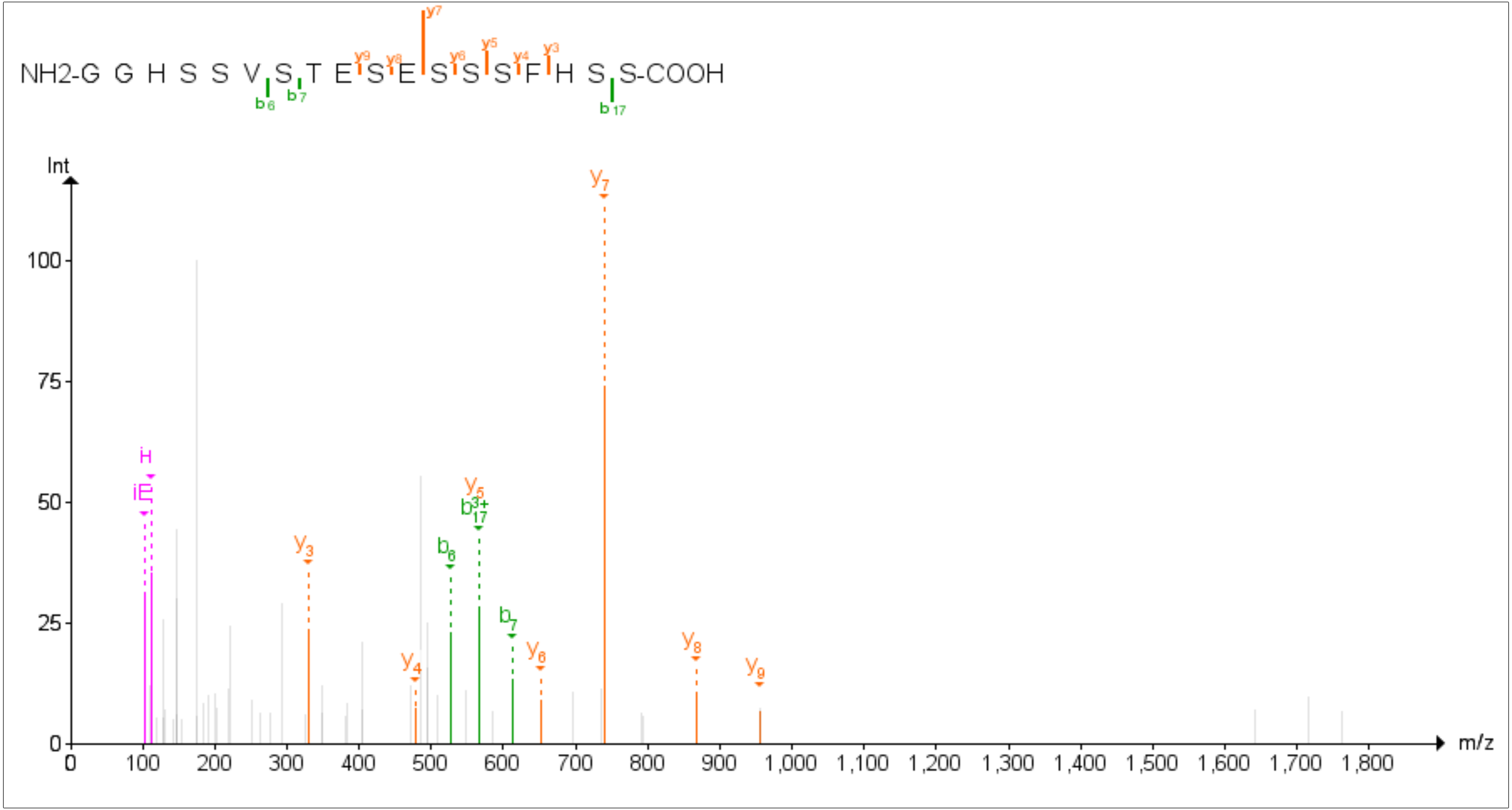

NH2-G G H S S V S T E S E S S S F H S S-COOH
y7 y9 y8 y6 y5 y4 y3
b6 b7 b17
Int
100
75
50
25
0
y7 y3 y4 y5 y6 y8 y9 b6 b7 b17 3+
iH iE
0 100 200 300 400 500 600 700 800 900 1,000 1,100 1,200 1,300 1,400 1,500 1,600 1,700 1,800
m/z

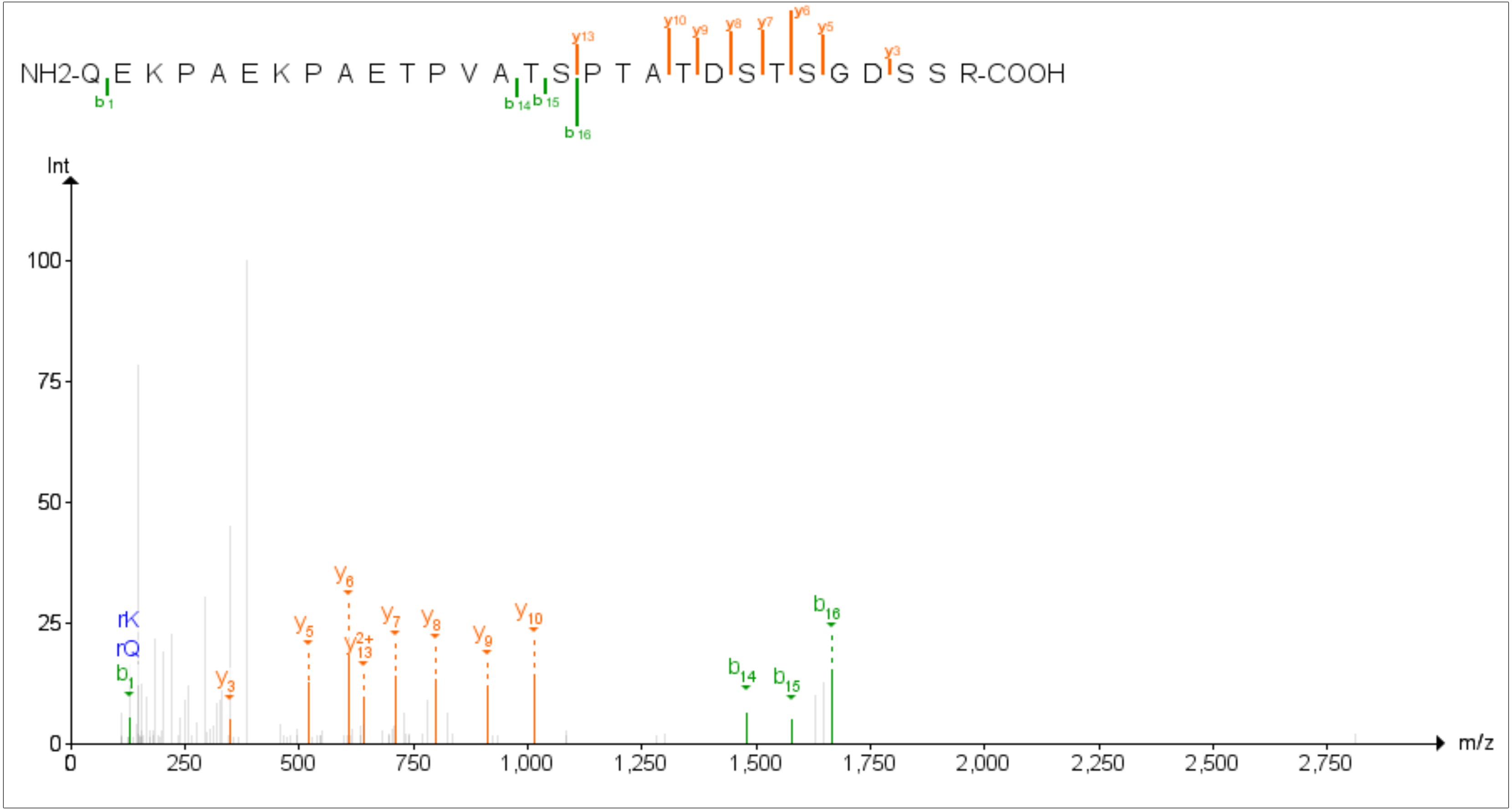

NH2-Q E K P A E K P A E T P V A T S P T A T D S T S G D S S R-COOH
b 1
b 14
b 15
b 16
y13
y10
y9
y8
y7
y6
y5
y3
Int
100
75
50
25
0
rK
rQ
b1
y3
y5
y6
y13 2+
y7
y8
y9
y10
b14
b15
b16
0
250
500
750
1,000
1,250
1,500
1,750
2,000
2,250
2,500
2,750
m/z

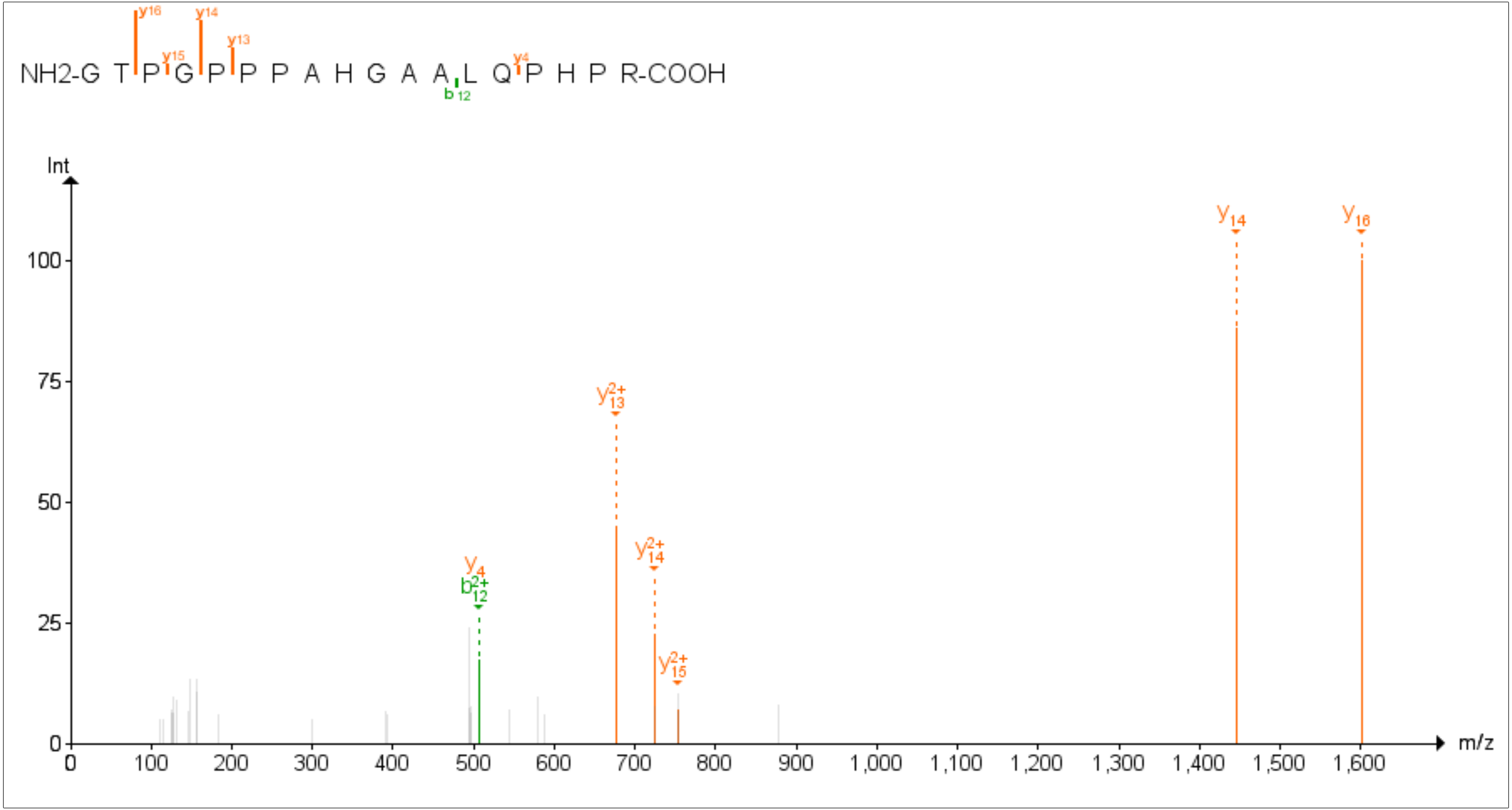

NH2-G T P G P P P A H G A A L Q P H P R-COOH
y16
y15
y14
y13
y4
b12
Int
100
75
50
25
0
y14
y16
y13 2+
y14 2+
y15 2+
y4
b12 2+
0
100
200
300
400
500
600
700
800
900
1,000
1,100
1,200
1,300
1,400
1,500
1,600
m/z

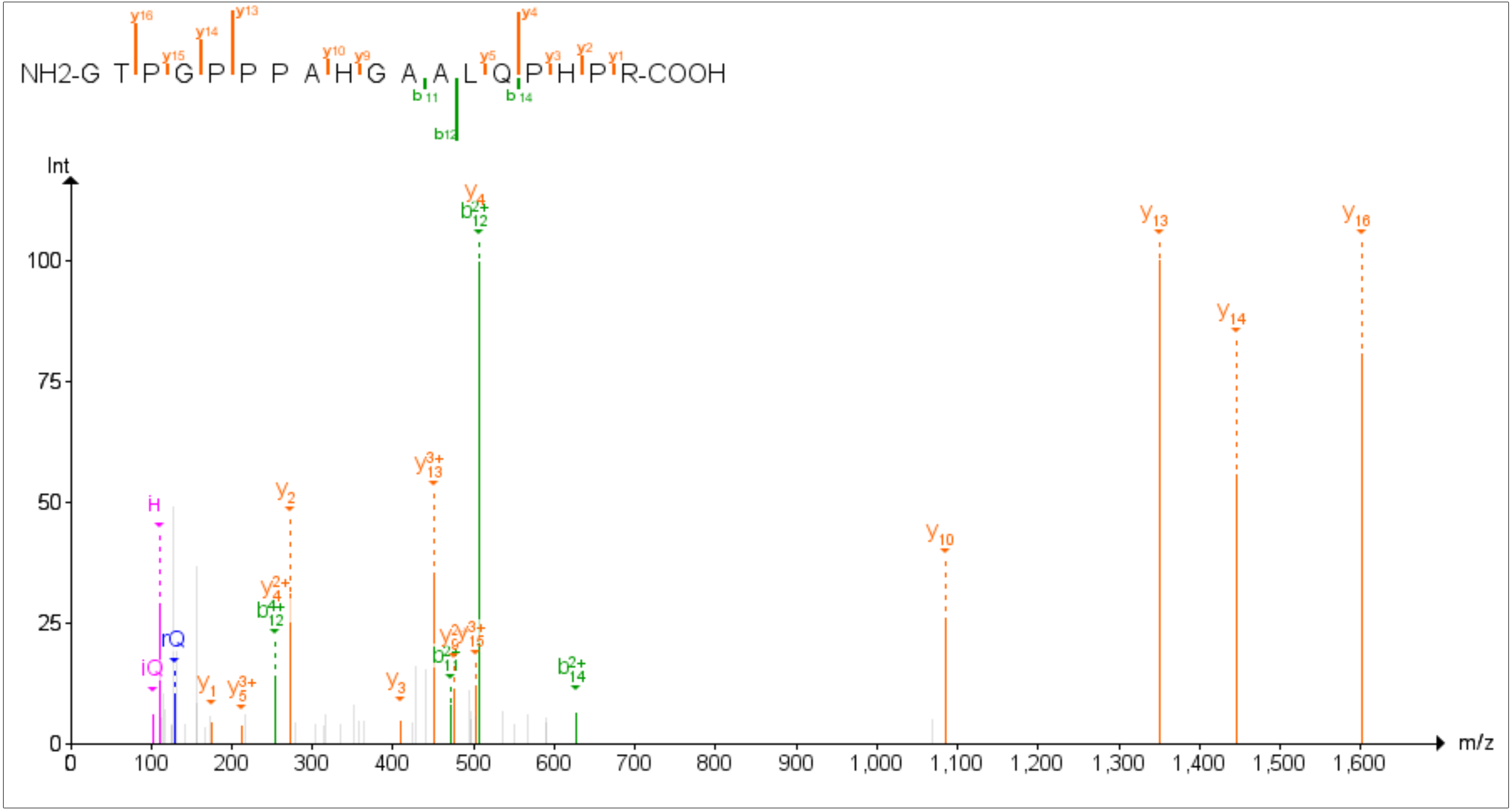

NH2-G T P G P P P A H G A A L Q P H P R-COOH
y16 y15 y14 y13 y10 y9 y5 y4 y3 y2 y1
b11 b12 b14
Int
m/z
0
25
50
75
100
0 100 200 300 400 500 600 700 800 900 1,000 1,100 1,200 1,300 1,400 1,500 1,600
iH
iQ
rQ
y1
y2
y3
y10
y13
y14
y16
y4

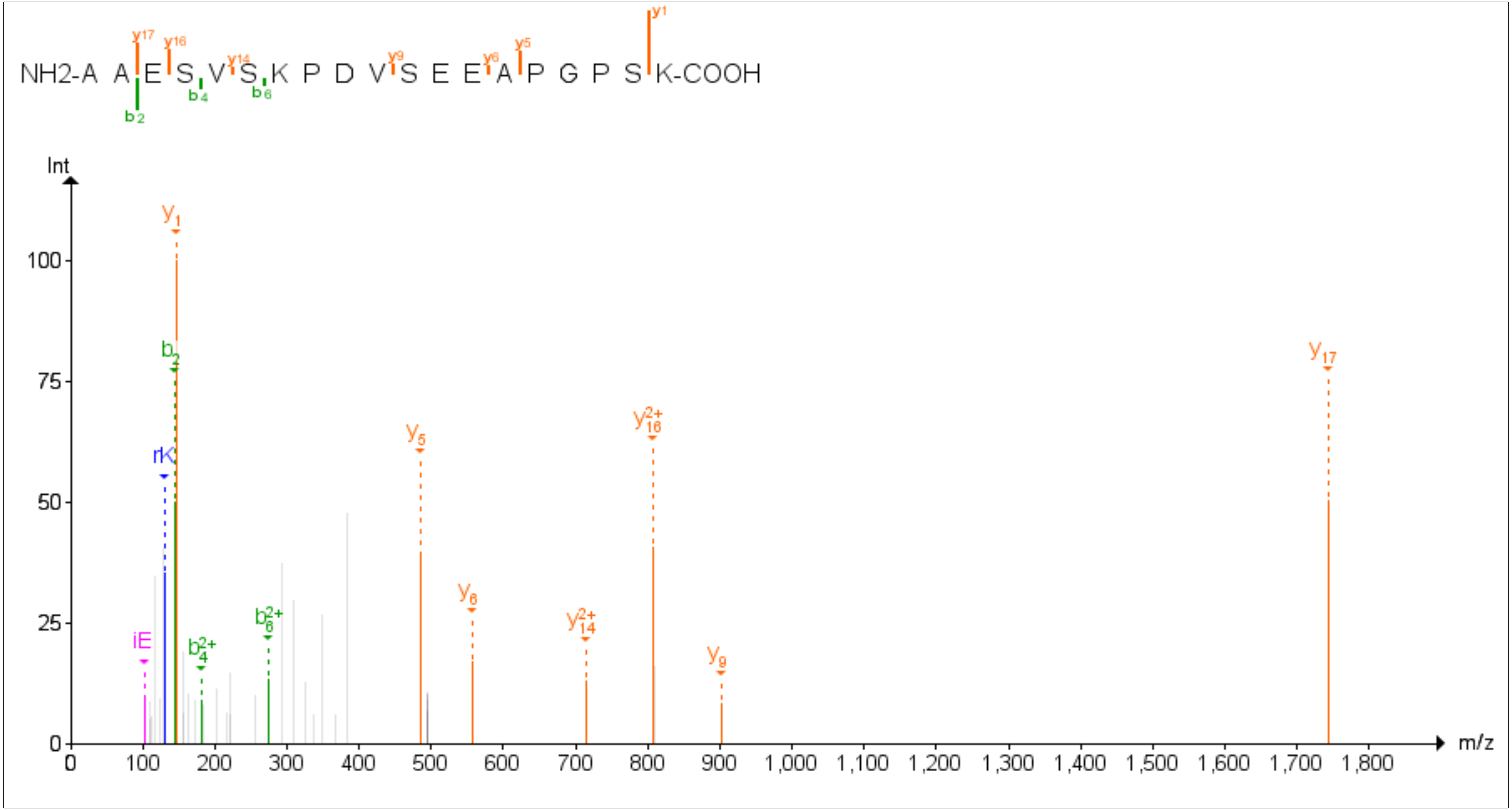
NH2-A A E S V S K P D V S E E A P G P S K-COOH
y17 y16 y14 y9 y6 y5 y1
b2 b4 b6
Int
m/z
y1
b2
rK
iE
b_4^{2+}
b_6^{2+}
y5
y6
y_{14}^{2+}
y_{16}^{2+}
y9
y17
0
25
50
75
100
0
100
200
300
400
500
600
700
800
900
1,000
1,100
1,200
1,300
1,400
1,500
1,600
1,700
1,800

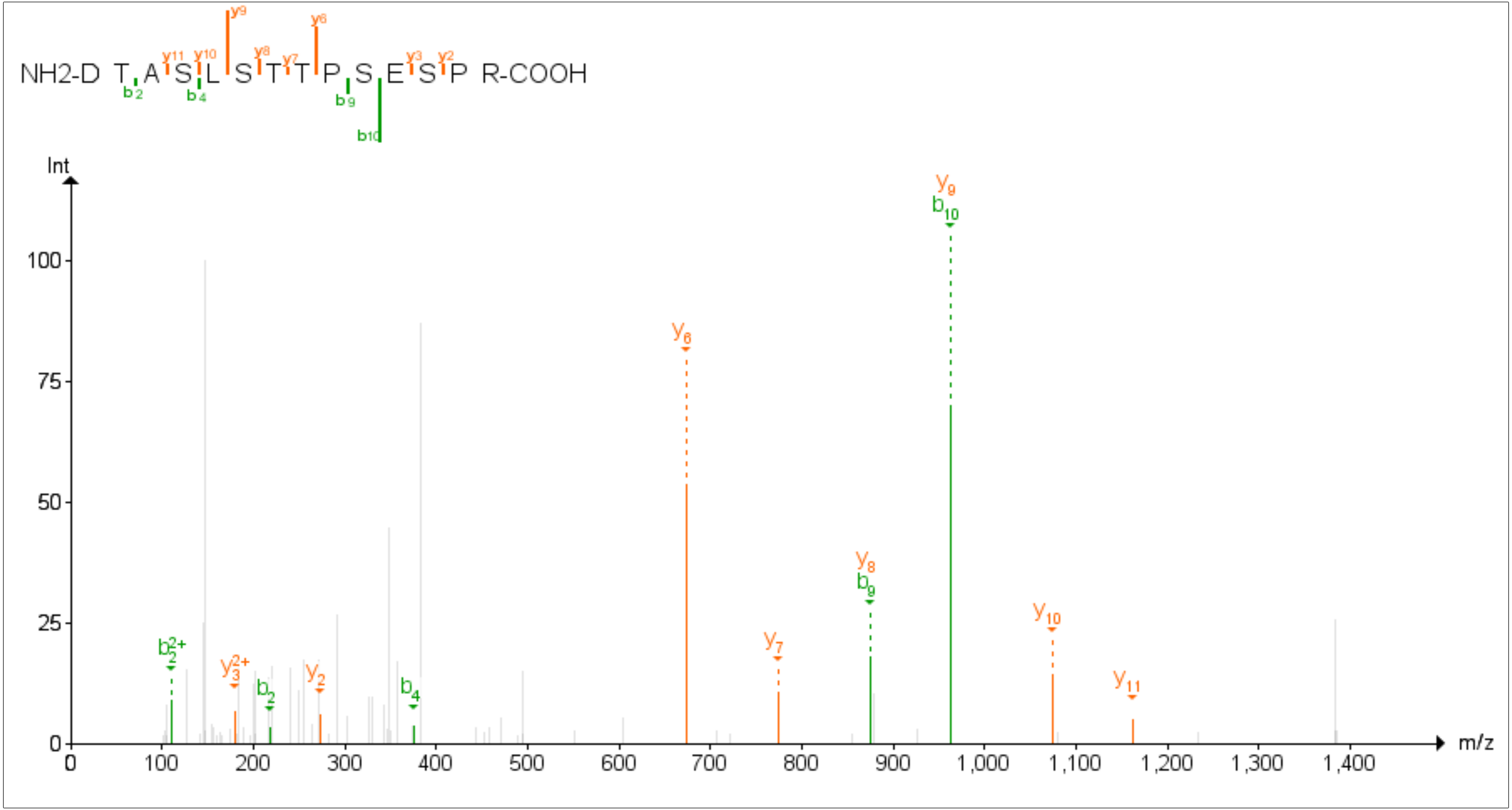

NH2-D T A S L S T T P S E S P R-COOH
y11 y10 y9 y8 y7 y6 y3 y2
b2 b4 b9 b10
Int
100
75
50
25
0
m/z
0 100 200 300 400 500 600 700 800 900 1,000 1,100 1,200 1,300 1,400
b2 2+
y3 2+
b2
y2
b4
y6
y7
y8
b9
y9
b10
y10
y11

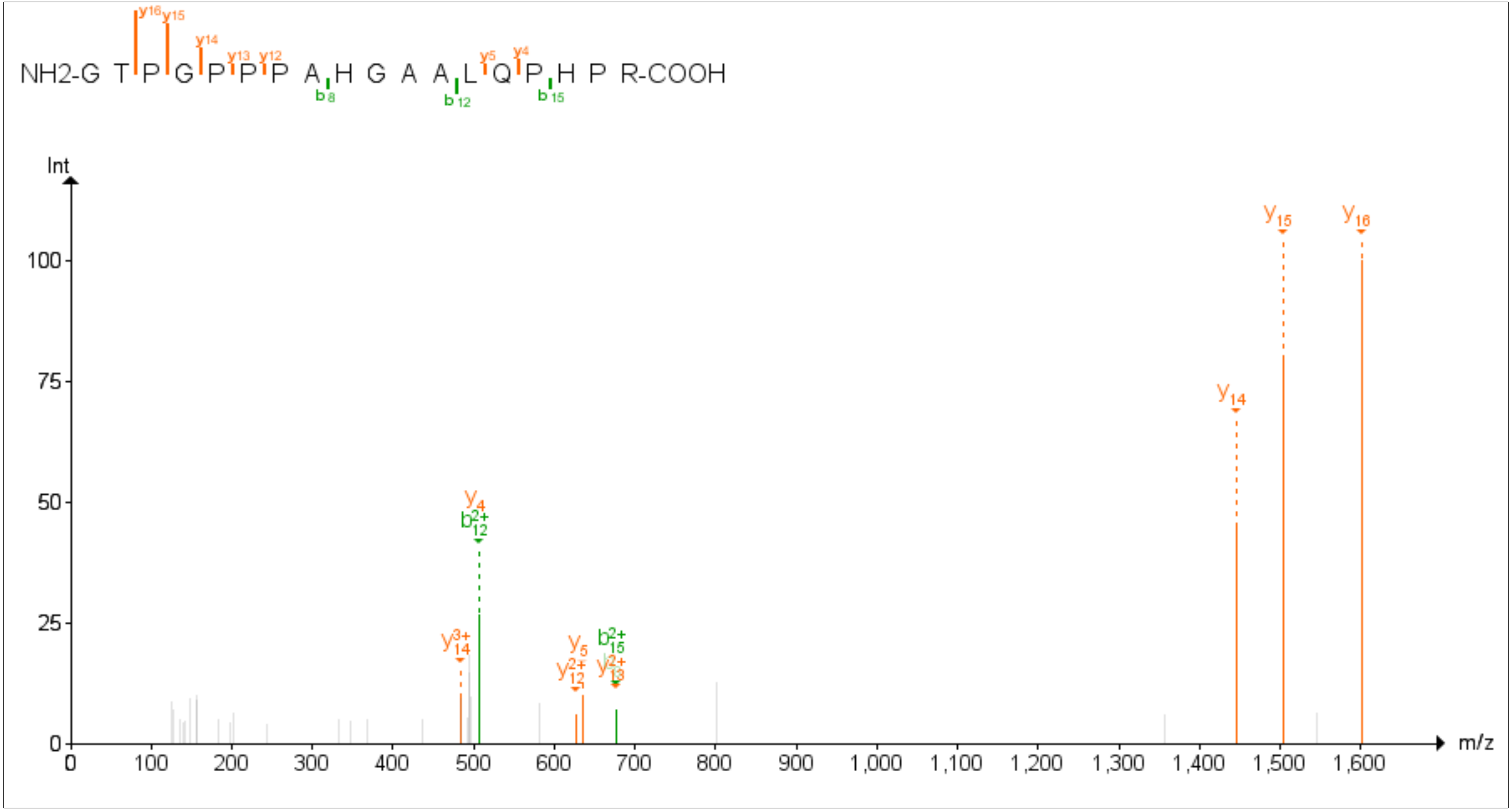

NH2-G T P G P P P A H G A A L Q P H P R-COOH
y16
y15
y14
y13
y12
y5
y4
b8
b12
b15
Int
100
75
50
25
0
m/z
0
100
200
300
400
500
600
700
800
900
1,000
1,100
1,200
1,300
1,400
1,500
1,600
y15
y16
y14
y4
b12 2+
y14 3+
y5
y12 2+
b15 2+
y13 2+

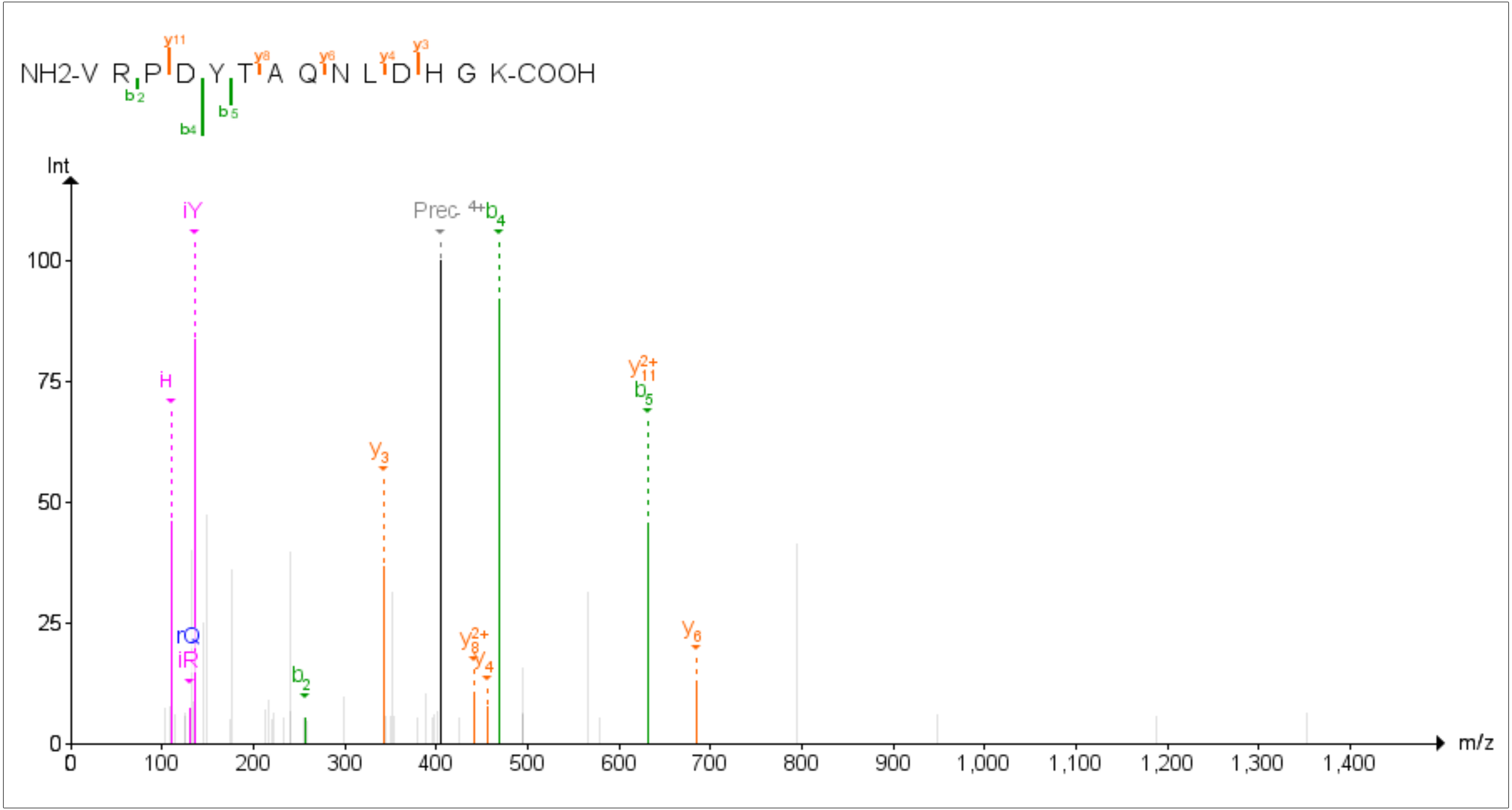

NH2-V R P D Y T A Q N L D H G K-COOH
y11
y8
y6
y4
y3
b2
b4
b5
Int
iY
Prec- 4+
b4
iH
y3
y11 2+
b5
rQ
iR
b2
y8 2+
y4
y6
0
25
50
75
100
0
100
200
300
400
500
600
700
800
900
1,000
1,100
1,200
1,300
1,400
m/z

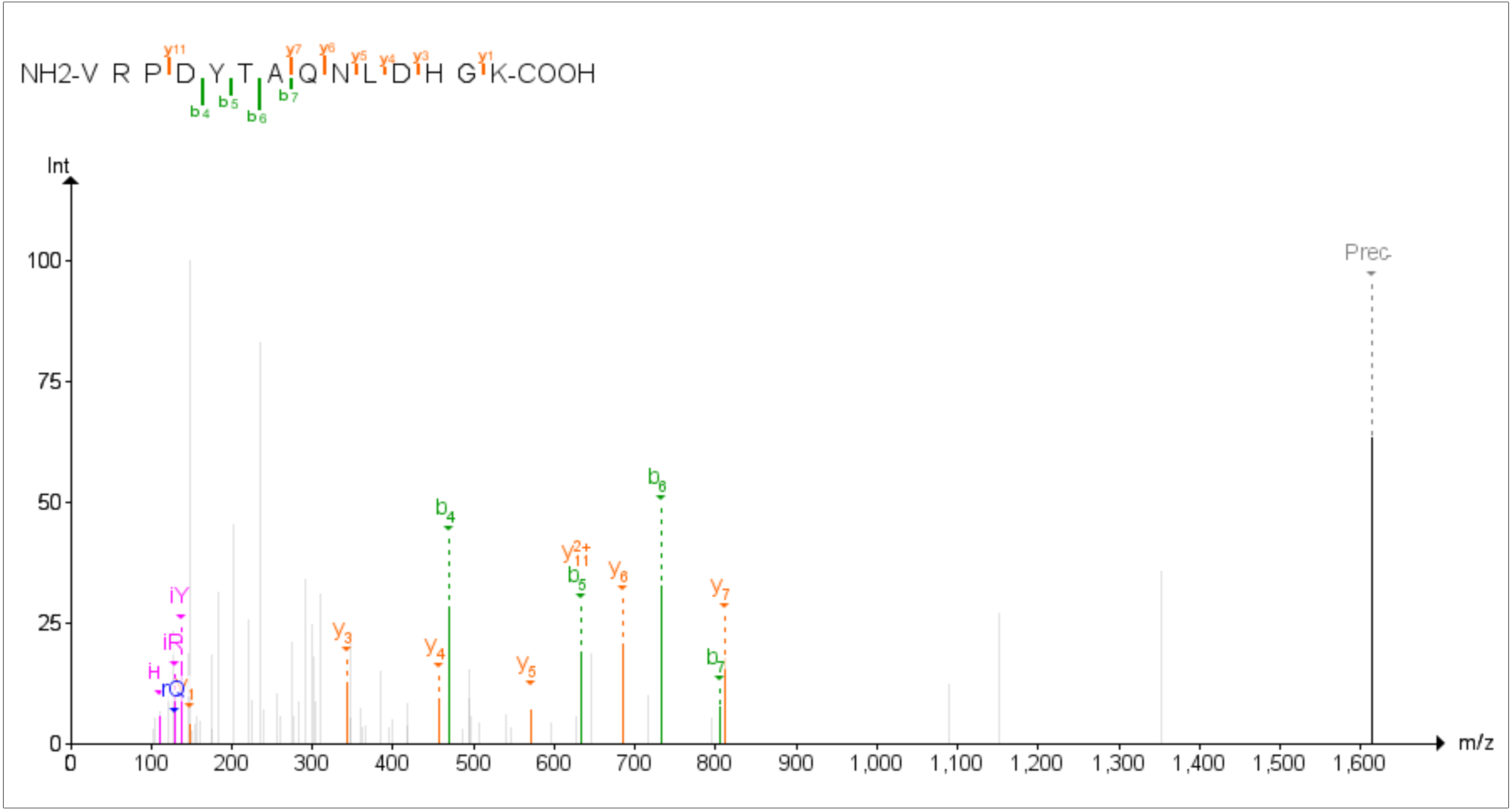

NH2-V R P D Y T A Q N L D H G K-COOH
y11 y7 y6 y5 y4 y3 y1
b4 b5 b6 b7
Int
m/z
Prec.
iY
iR
iH
rQ
y1
y3
y4
y5
b4
b5
y11 2+
y6
b6
y7
b7
0
25
50
75
100
0
100
200
300
400
500
600
700
800
900
1,000
1,100
1,200
1,300
1,400
1,500
1,600

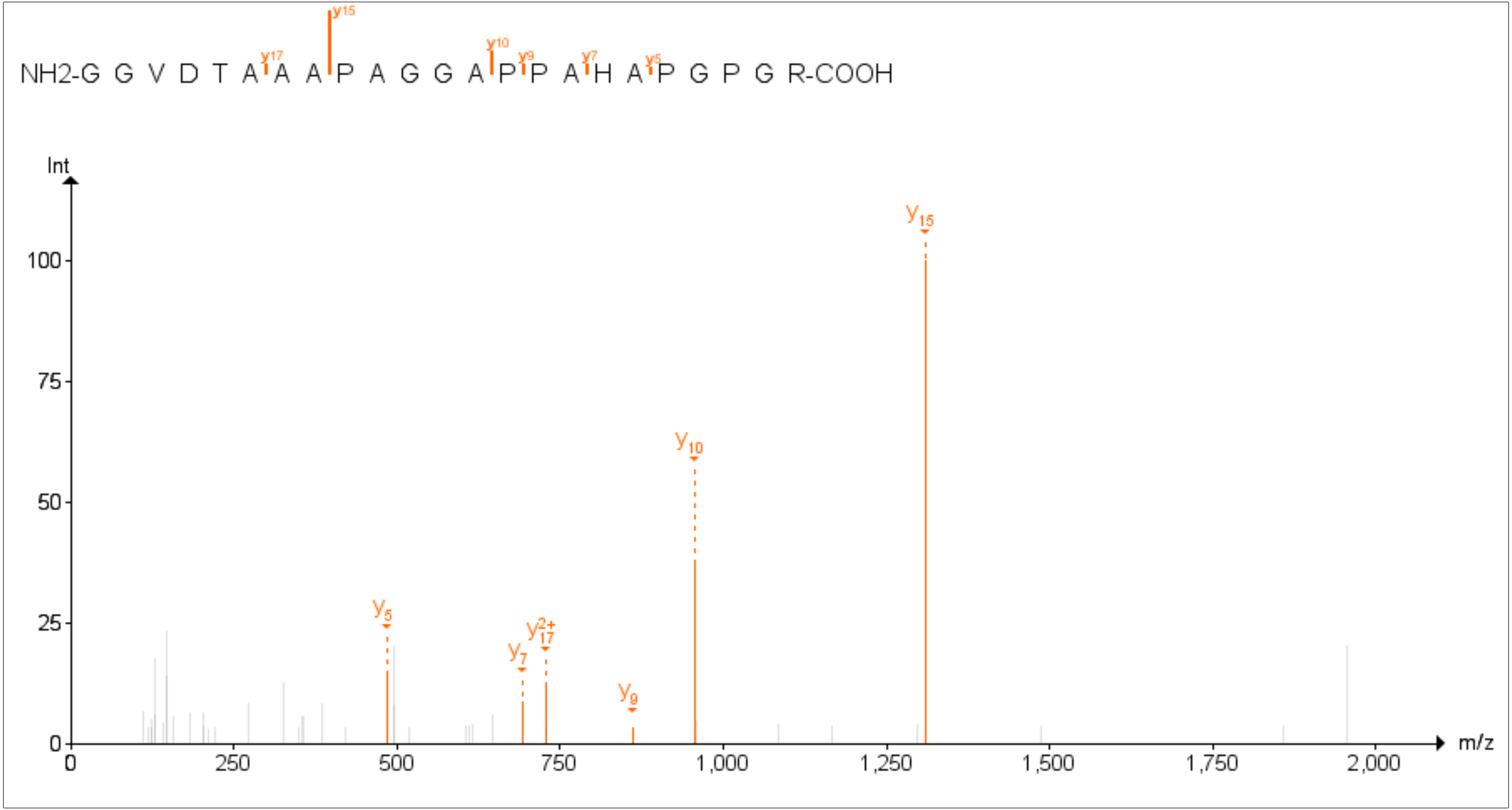

NH2-G G V D T A A A P A G G A P P A H A P G P G R-COOH
y17
y15
y10
y9
y7
y5
Int
100
75
50
25
0
y15
y10
y5
y7
y17 2+
y9
0
250
500
750
1,000
1,250
1,500
1,750
2,000
m/z

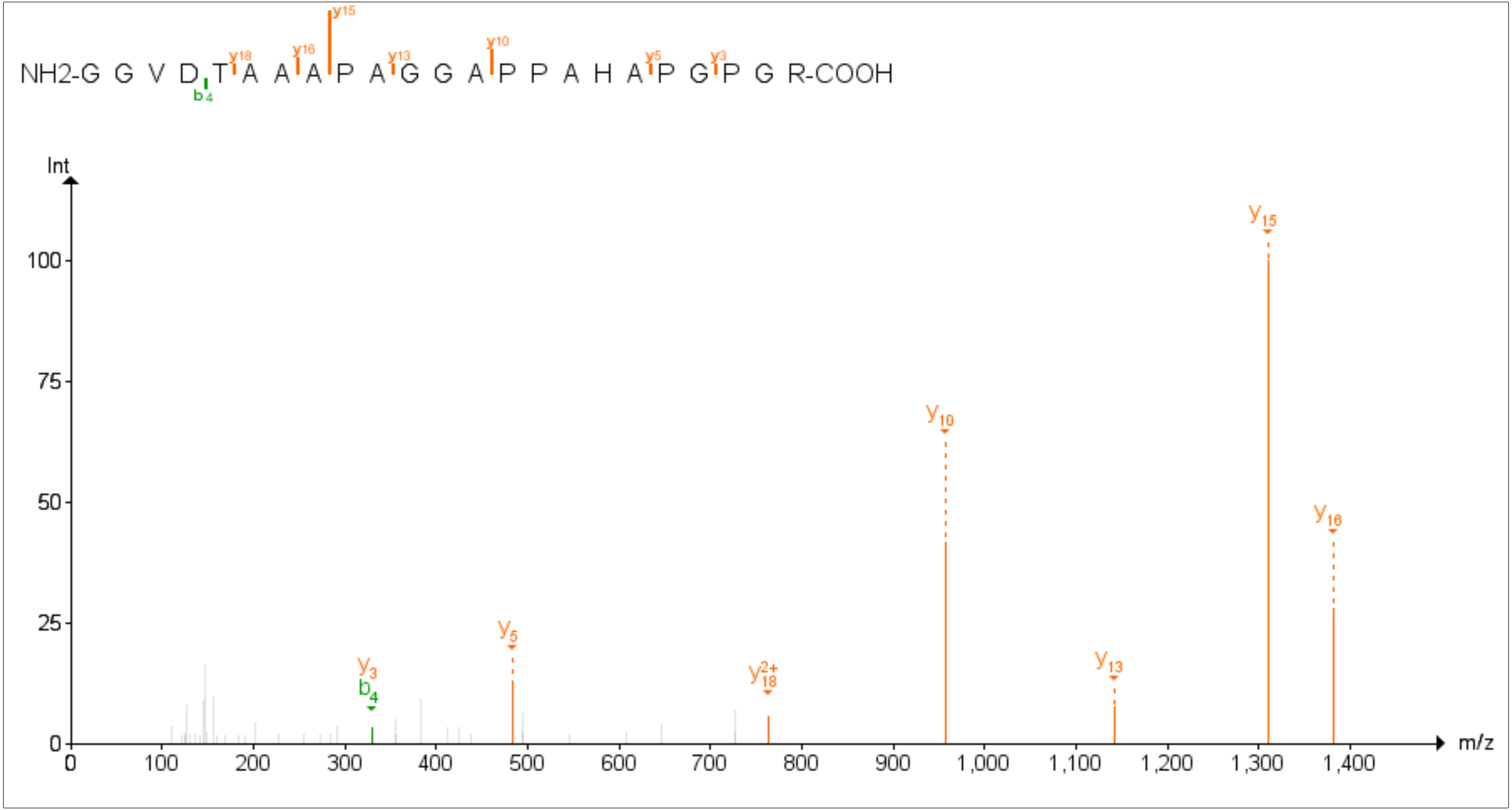
NH2-G G V D T A A A P A G G A P P A H A P G P G R-COOH
b4
y18 y16 y15 y13 y10 y5 y3
Int
100
75
50
25
0
m/z
0 100 200 300 400 500 600 700 800 900 1,000 1,100 1,200 1,300 1,400
y3
b4
y5
y18 2+
y10
y13
y15
y16

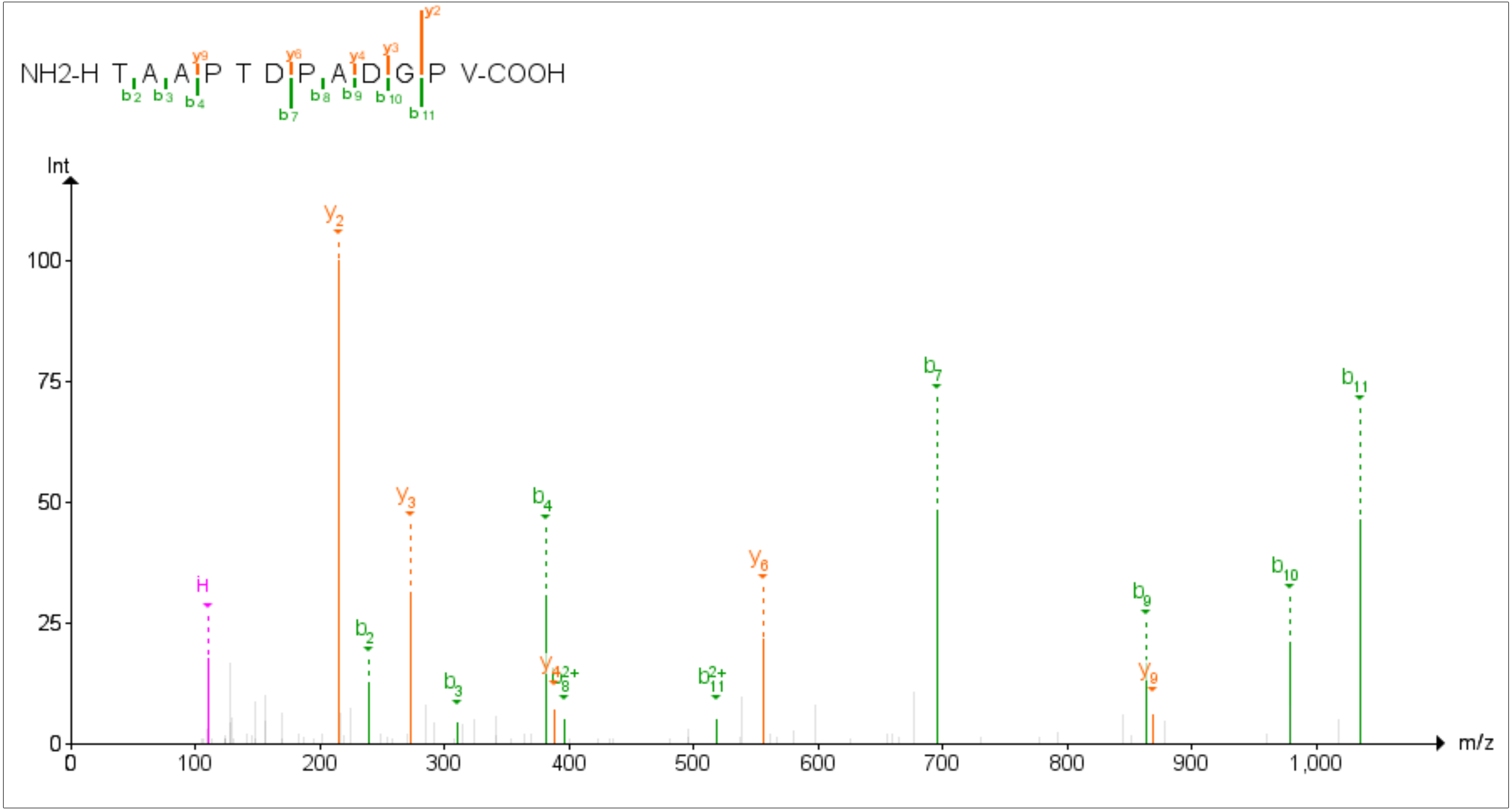
NH2-H T A A P T D P A D G P V-COOH
b2 b3 b4 b7 b8 b9 b10 b11
y9 y6 y4 y3 y2
Int
100
75
50
25
0
0
100
200
300
400
500
600
700
800
900
1,000
m/z
y2
y3
b4
b7
b11
H
b2
b3
y4
b8 2+
b11 2+
y6
b9
y9
b10

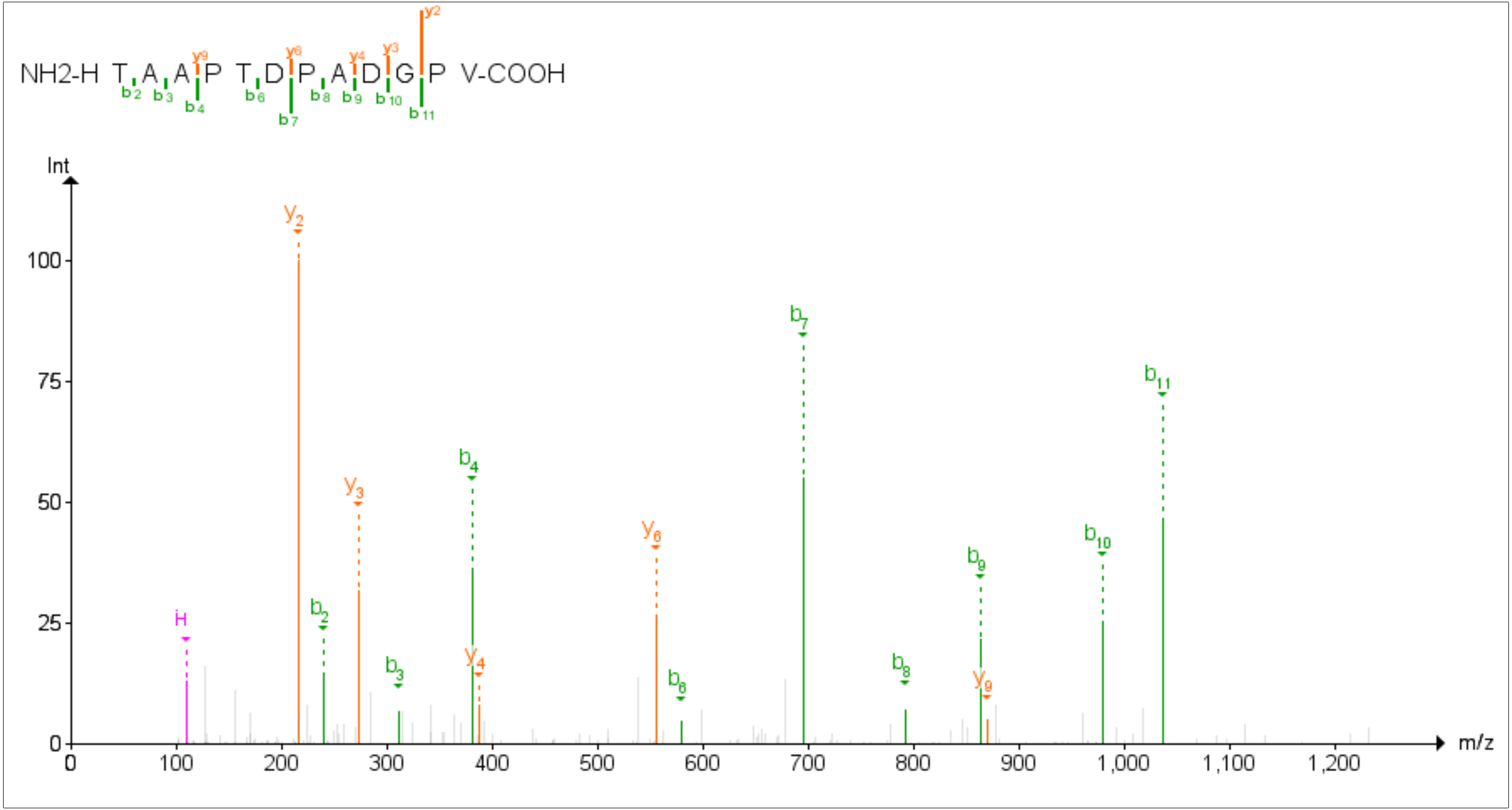

NH2-H T A A P T D P A D G P V-COOH
b2 b3 b4 b6 b7 b8 b9 b10 b11
y9 y6 y4 y3 y2
Int
100
75
50
25
0
m/z
0
100
200
300
400
500
600
700
800
900
1,000
1,100
1,200
H
y2
b2
y3
b3
b4
y4
y6
b6
b7
b8
b9
y9
b10
b11

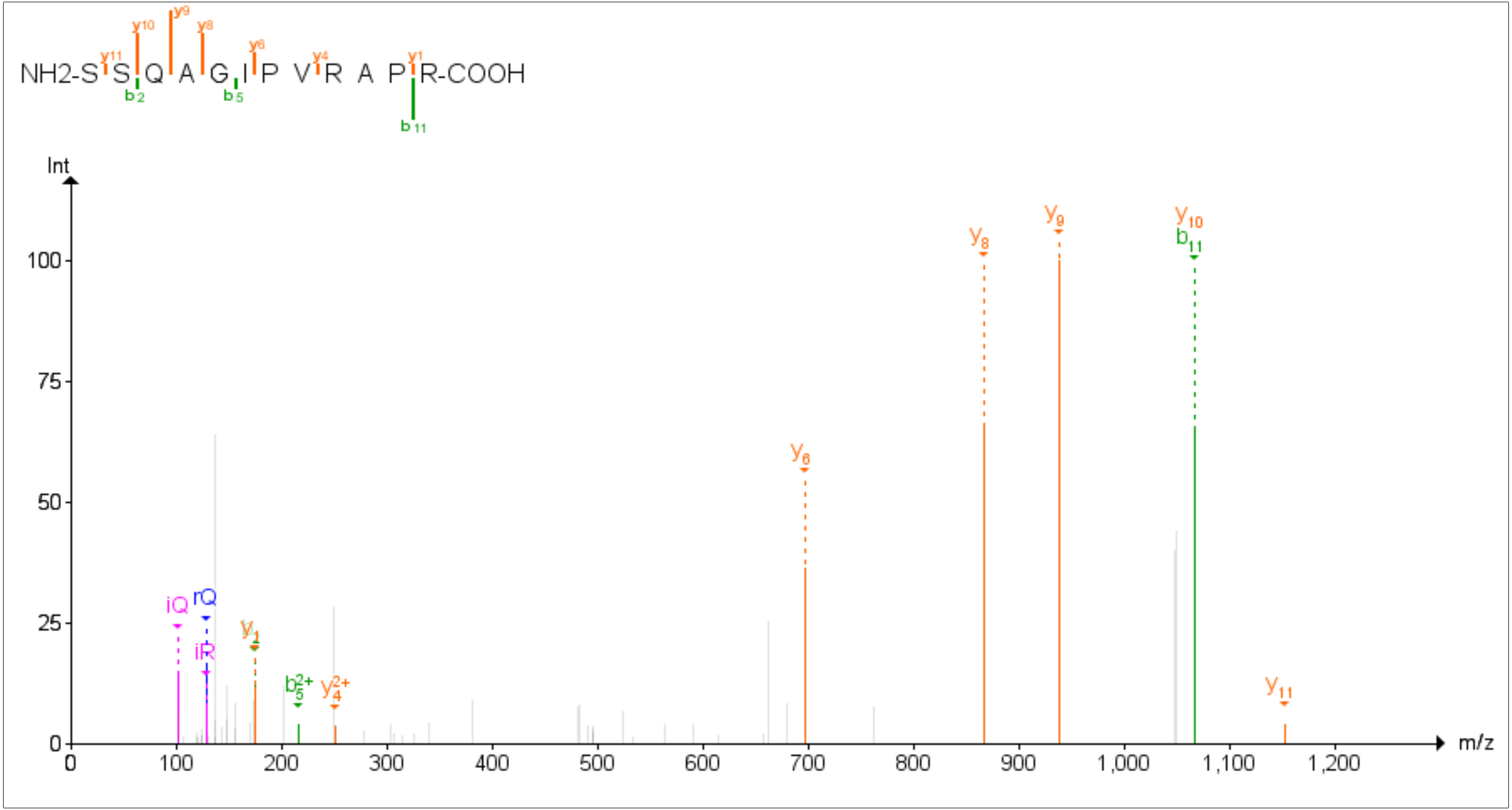

NH2-S S Q A G I P V R A P R-COOH
y11 y10 y9 y8 y6 y4 y1
b2 b5 b11
Int
m/z
0
25
50
75
100
0 100 200 300 400 500 600 700 800 900 1,000 1,100 1,200
iQ
rQ
iR
y1
b5 2+
y4 2+
y6
y8
y9
y10
b11
y11

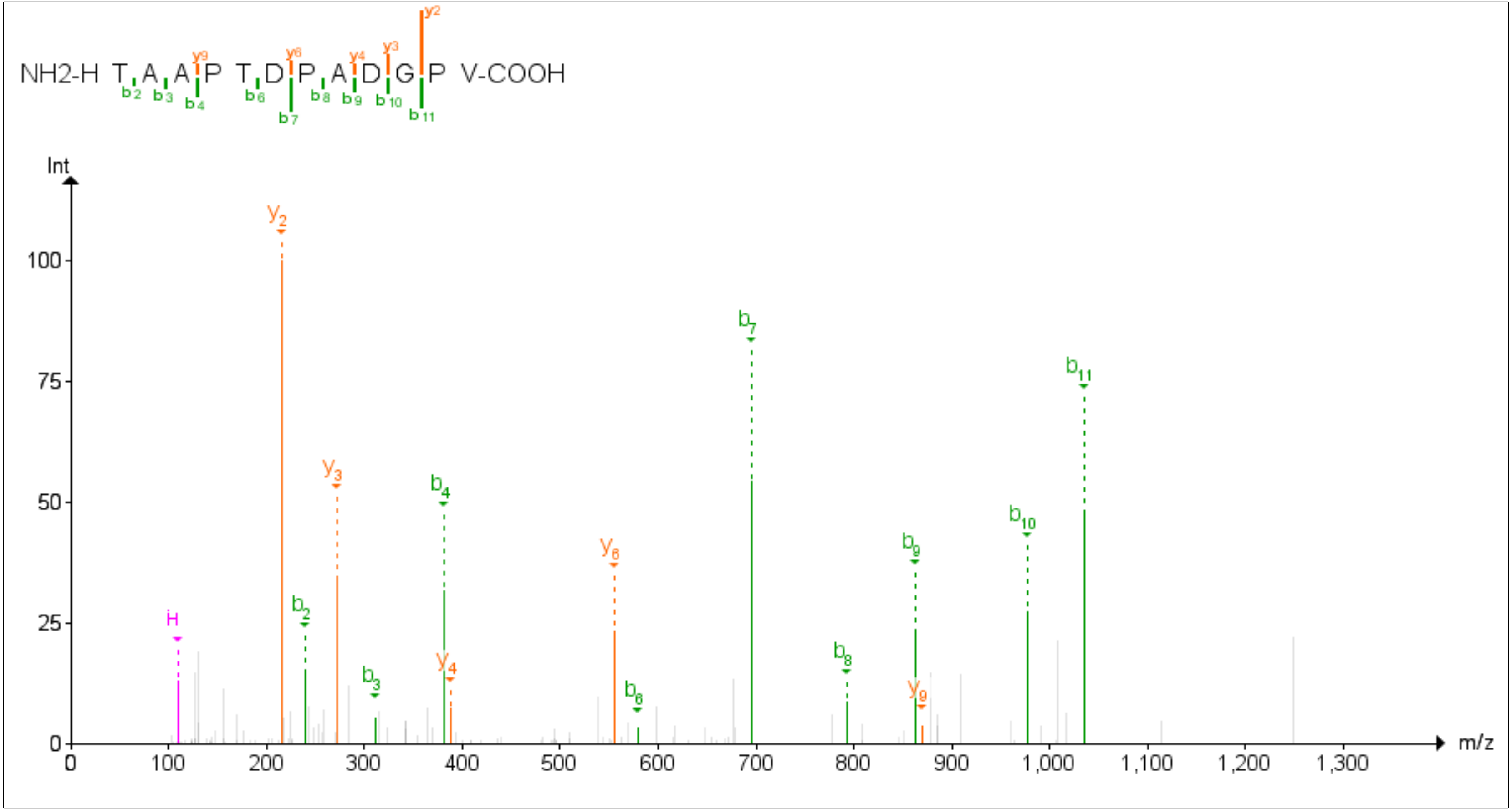

NH2-H T A A P T D P A D G P V-COOH
Int
m/z
y2
y3
y4
y6
y9
b2
b3
b4
b6
b7
b8
b9
b10
b11
H

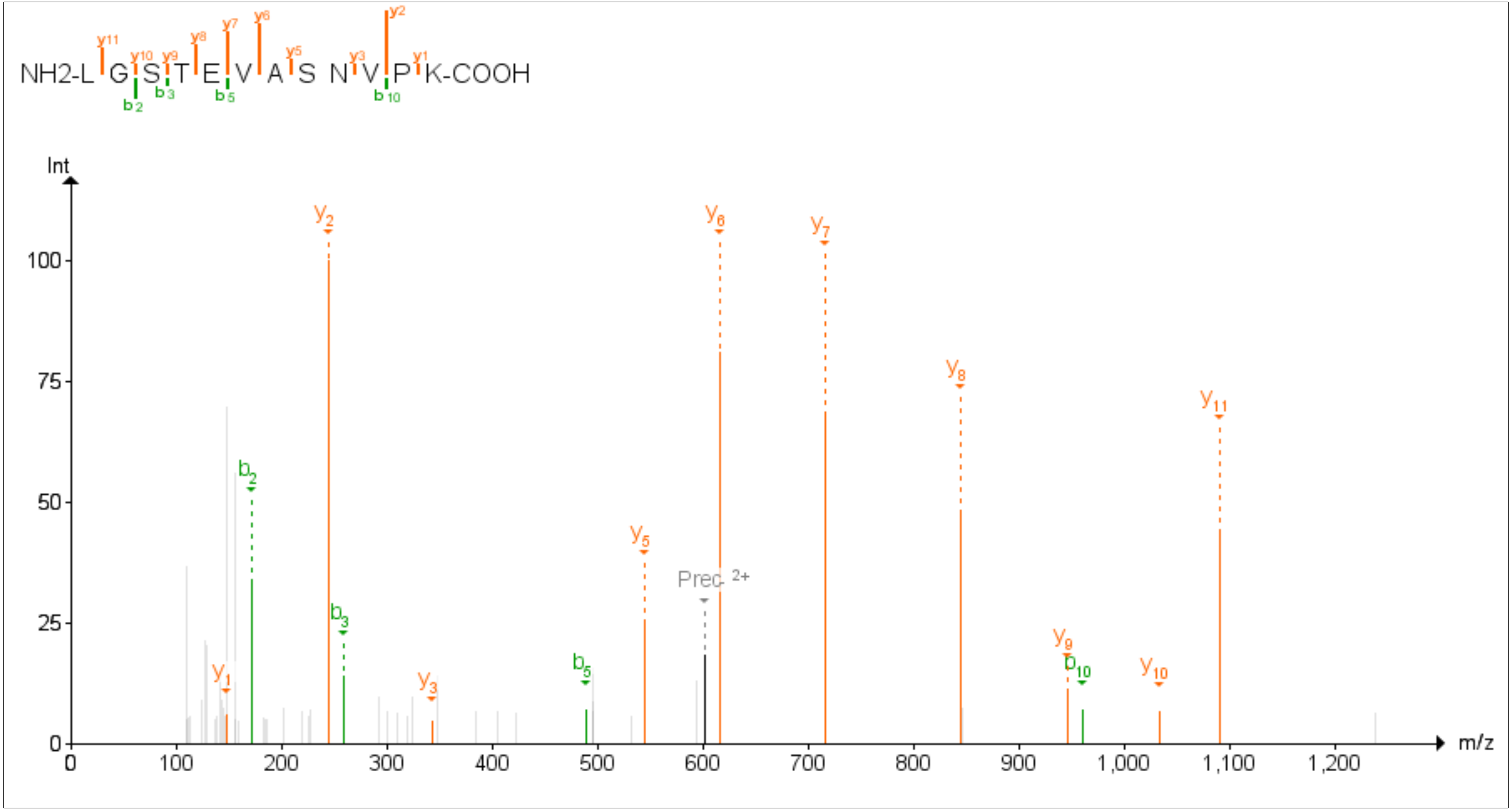
NH2-L G S T E V A S N V P K-COOH
y11 y10 y9 y8 y7 y6 y5 y3 y2 y1
b2 b3 b5 b10
Int
100
75
50
25
0
m/z
0
100
200
300
400
500
600
700
800
900
1,000
1,100
1,200
y2
y6
y7
y8
y11
b2
y5
Prec 2+
b3
y1
y3
b5
y9
b10
y10

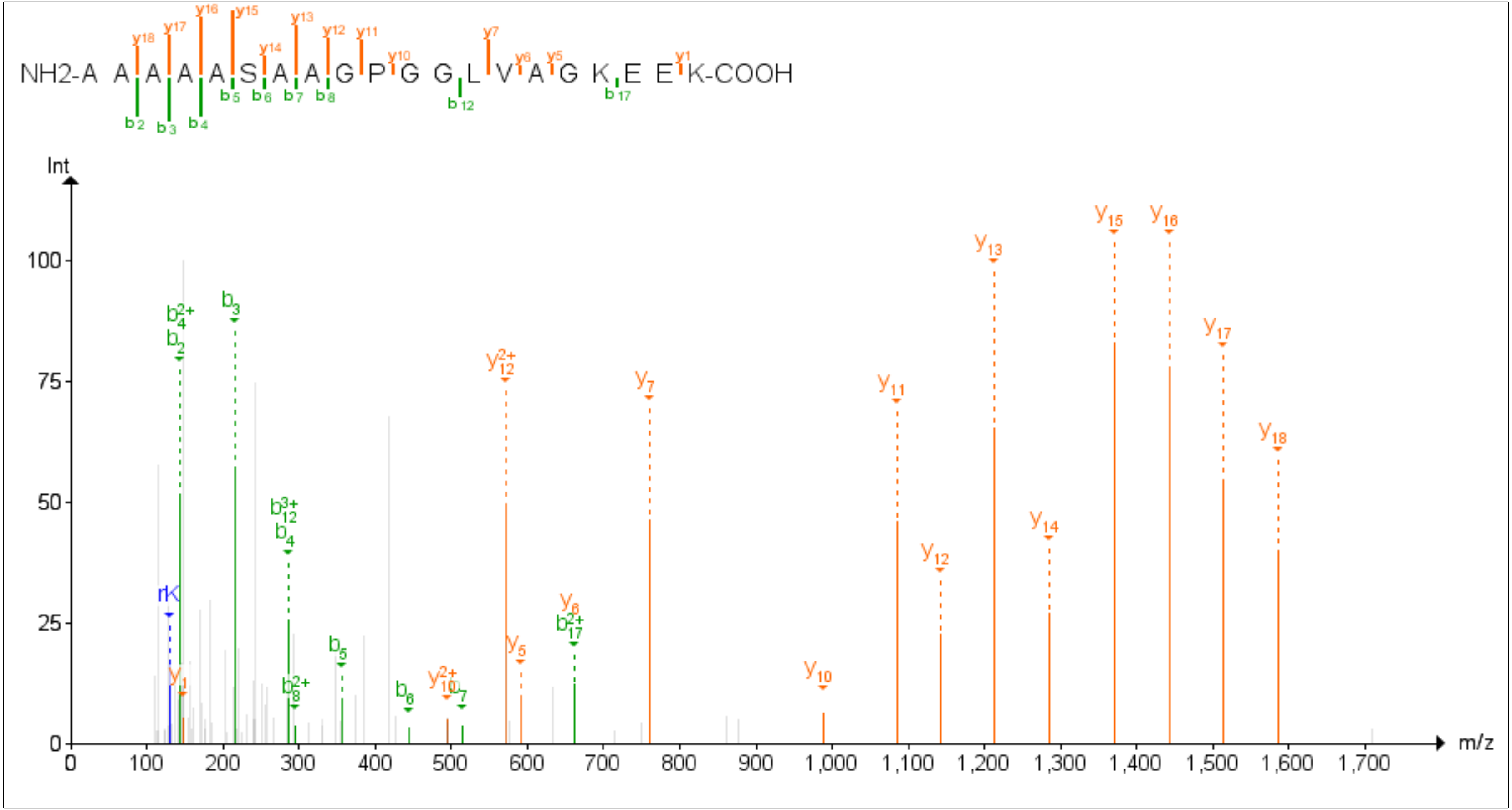

NH2-A A A A A S A A G P G G L V A G K E E K-COOH
y18 y17 y16 y15 y14 y13 y12 y11 y10 y7 y6 y5 y1
b2 b3 b4 b5 b6 b7 b8 b12 b17
Int
100
75
50
25
0
m/z
0 100 200 300 400 500 600 700 800 900 1,000 1,100 1,200 1,300 1,400 1,500 1,600 1,700
rK
y1
b4 2+
b2
b3
b12 3+
b4
b8 2+
b5
b6
y10 2+
b7
y12 2+
y5
y6
b17 2+
y7
y10
y11
y12
y13
y14
y15
y16
y17
y18

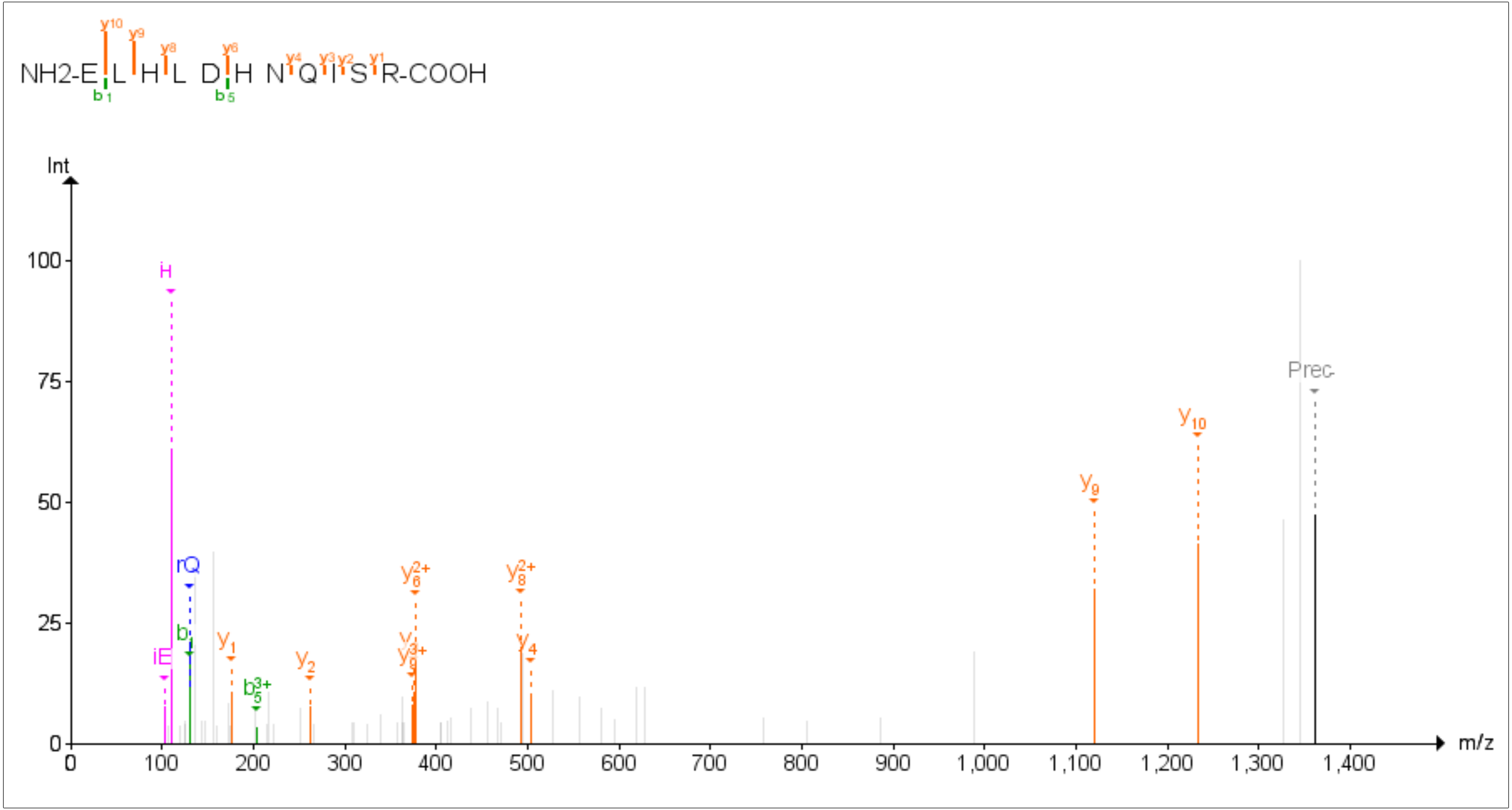

NH2-E L H L D H N Q I S R-COOH
y10 y9 y8 y6 y4 y3 y2 y1
b1 b5
Int
m/z
iH
iE
rQ
b
y1
y2
b5 3+
y6 2+
y9 3+
y8 2+
y4
y9
y10
Prec

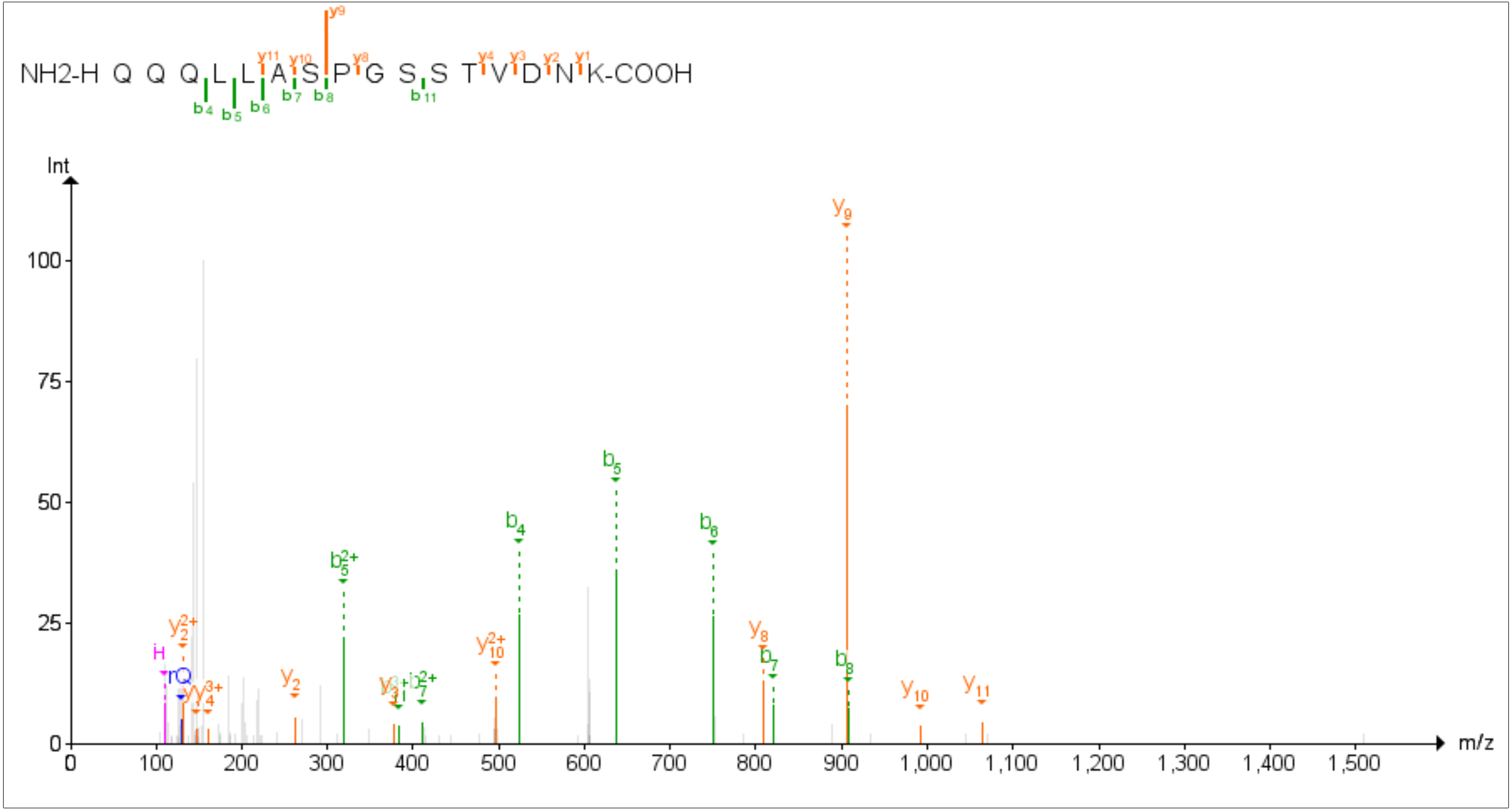

NH2-H Q Q Q L L A S P G S S T V D N K-COOH
y9
y11
y10
y8
y4
y3
y2
y1
b4
b5
b6
b7
b8
b11
Int
100
75
50
25
0
m/z
0
100
200
300
400
500
600
700
800
900
1,000
1,100
1,200
1,300
1,400
1,500
y9
b5
b4
b6
b5 2+
y2 2+
y8
b7
b8
y10 2+
H
rQ
y2
y10
y11

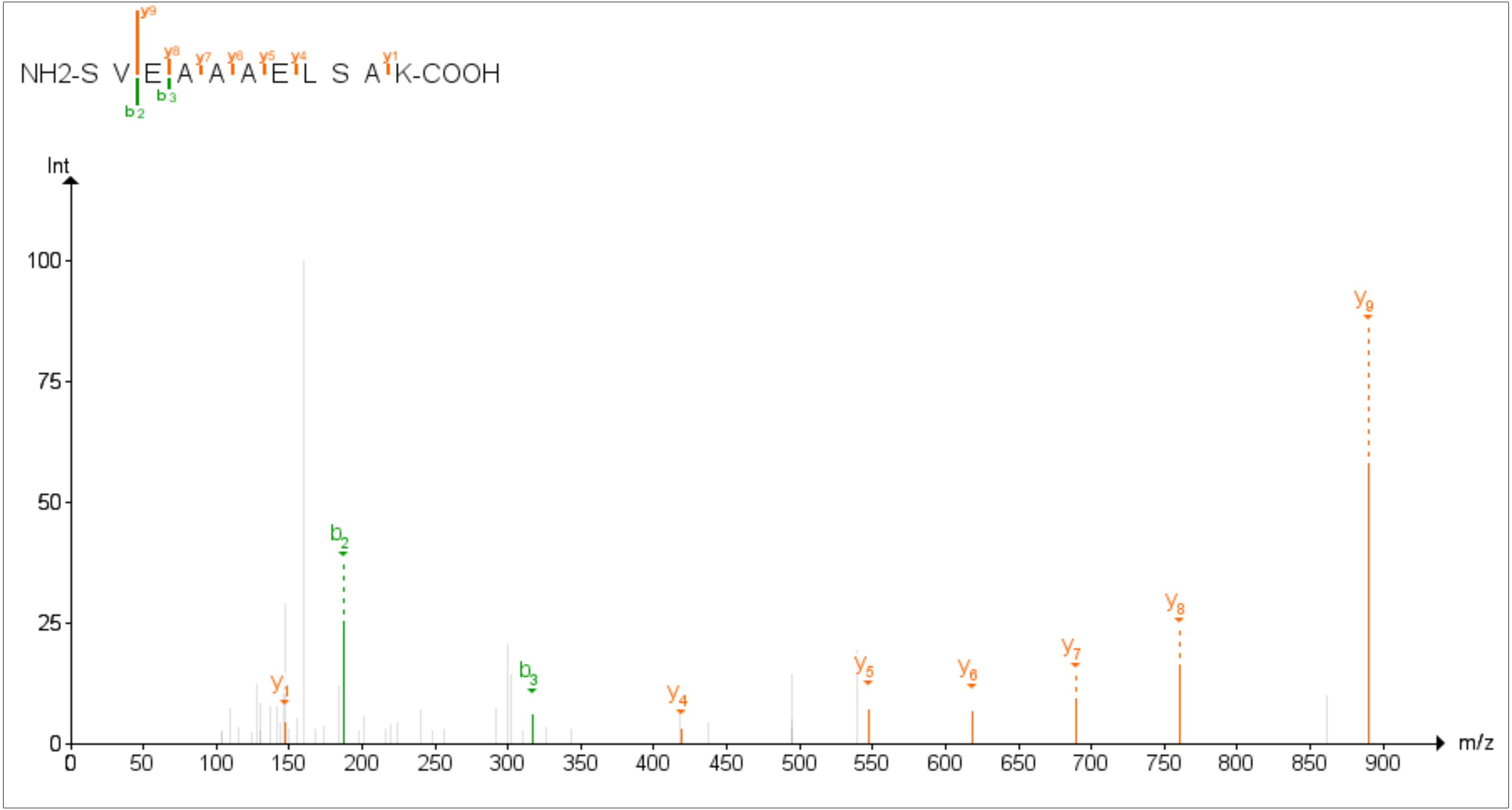
NH2-S V E A A A E L S A K-COOH
y9 y8 y7 y6 y5 y4 y1
b2 b3
Int
m/z
0 25 50 75 100
0 50 100 150 200 250 300 350 400 450 500 550 600 650 700 750 800 850 900
y1 b2 b3 y4 y5 y6 y7 y8 y9

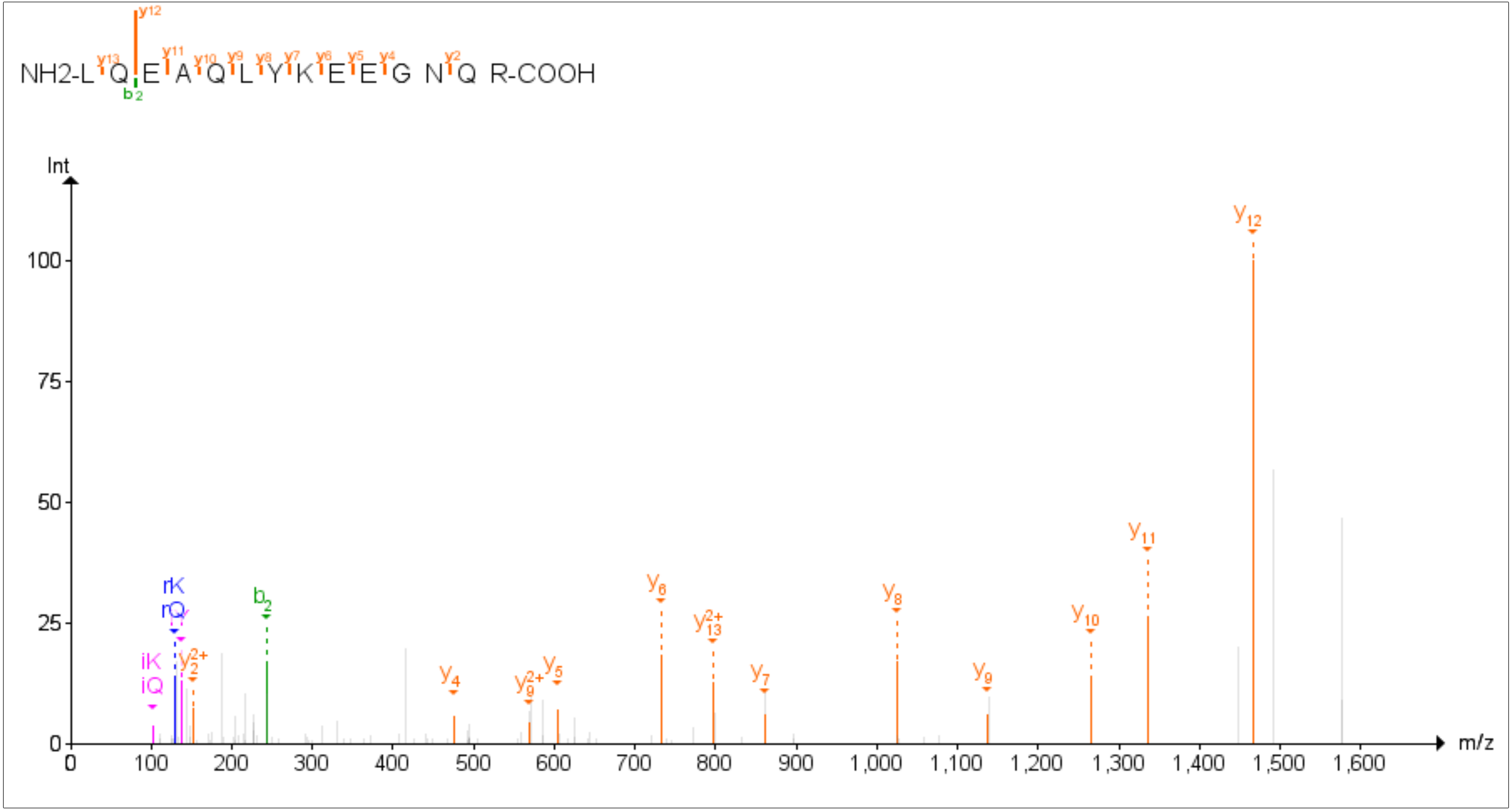

NH2-L Q E A Q L Y K E E G N Q R-COOH
y12
y13
y11
y10
y9
y8
y7
y6
y5
y4
y2
b2
Int
m/z
rK
rQ
iK
iQ
b2
y4
y5
y6
y7
y8
y9
y10
y11
y12

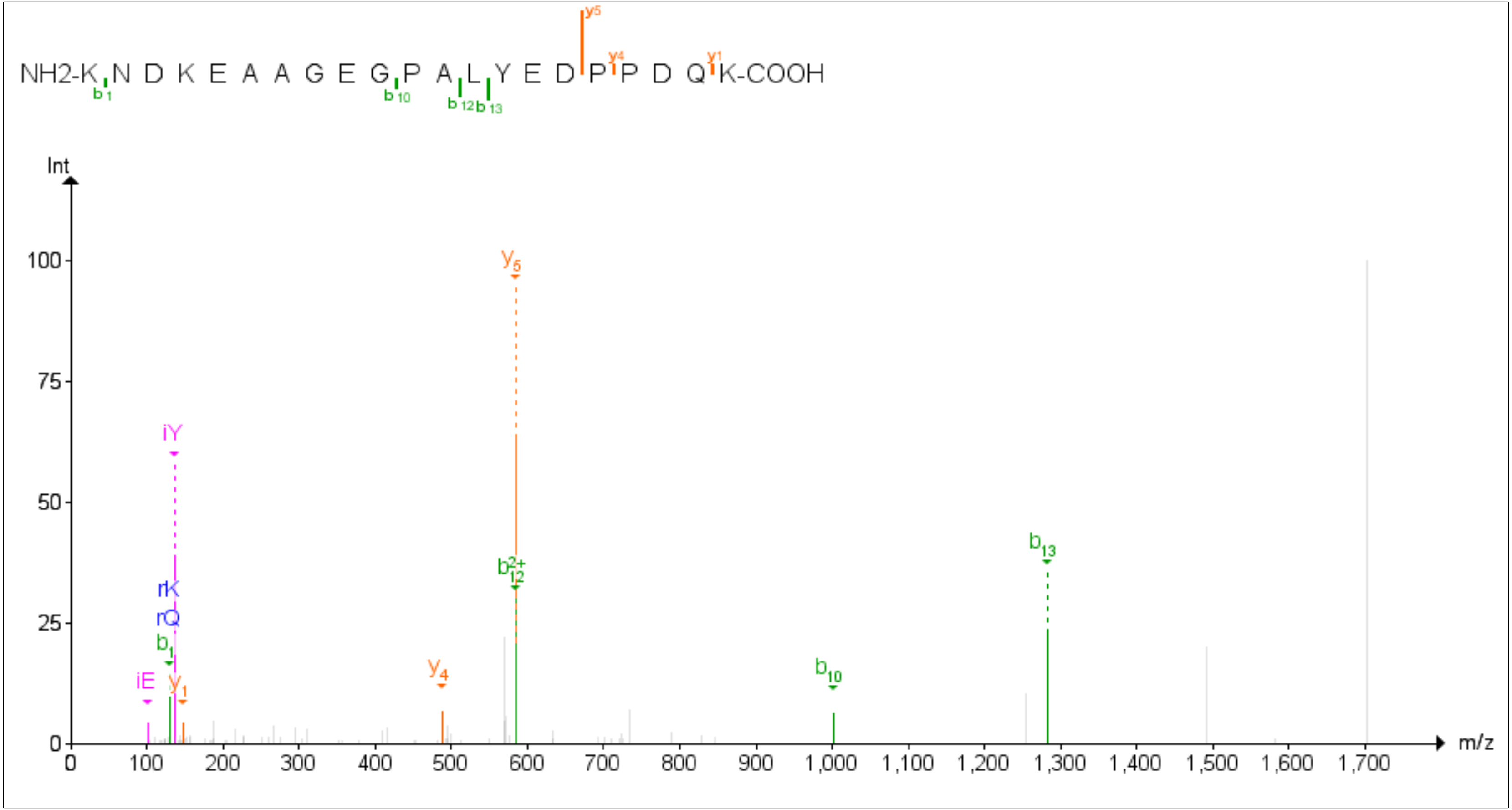
NH2-K N D K E A A G E G P A L Y E D P P D Q K-COOH
y5
y4
y1
b1
b10
b12
b13
Int
100
75
50
25
0
y5
iY
b12 2+
b13
rK
rQ
b1
iE
y1
y4
b10
0
100
200
300
400
500
600
700
800
900
1,000
1,100
1,200
1,300
1,400
1,500
1,600
1,700
m/z

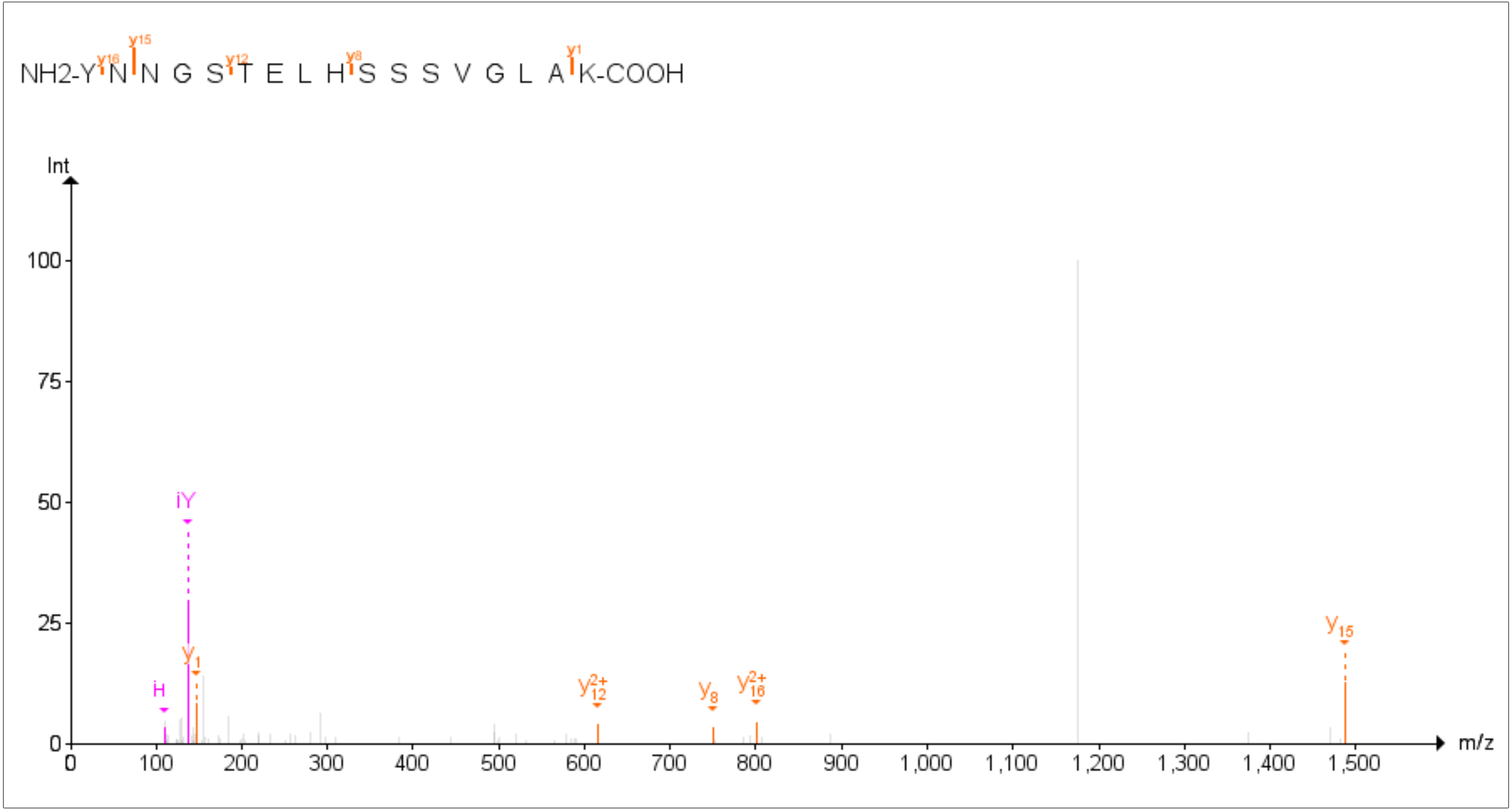

NH2-Y N N G S T E L H S S S V G L A K-COOH
y16
y15
y12
y8
y1
Int
100
75
50
25
0
iY
H
y1
y12 2+
y8
y16 2+
y15
0
100
200
300
400
500
600
700
800
900
1,000
1,100
1,200
1,300
1,400
1,500
m/z

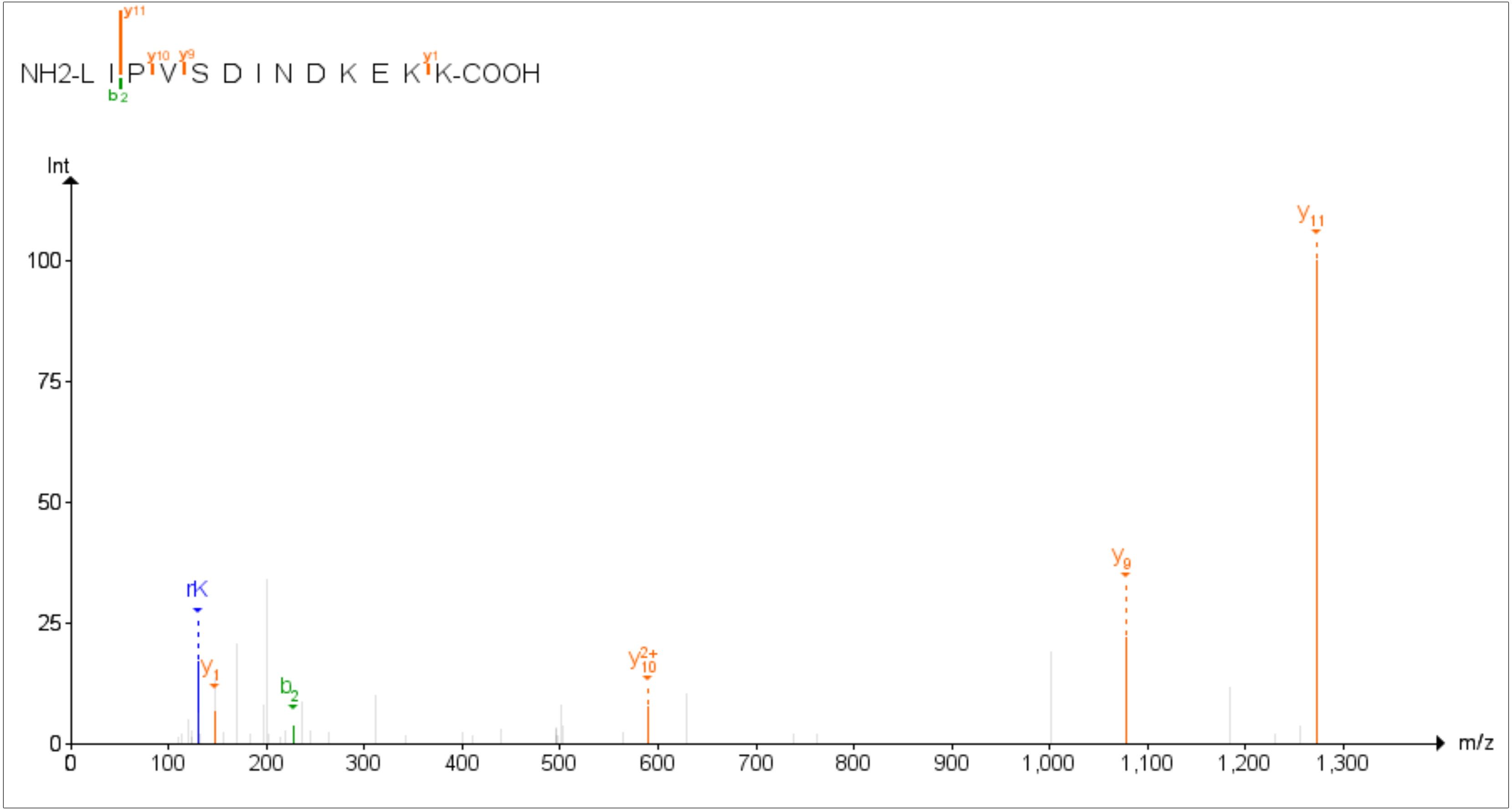

NH2-L I P V S D I N D K E K K-COOH
y11
y10
y9
y1
b2
Int
100
75
50
25
0
m/z
0
100
200
300
400
500
600
700
800
900
1,000
1,100
1,200
1,300
rK
y1
b2
y10 2+
y9
y11

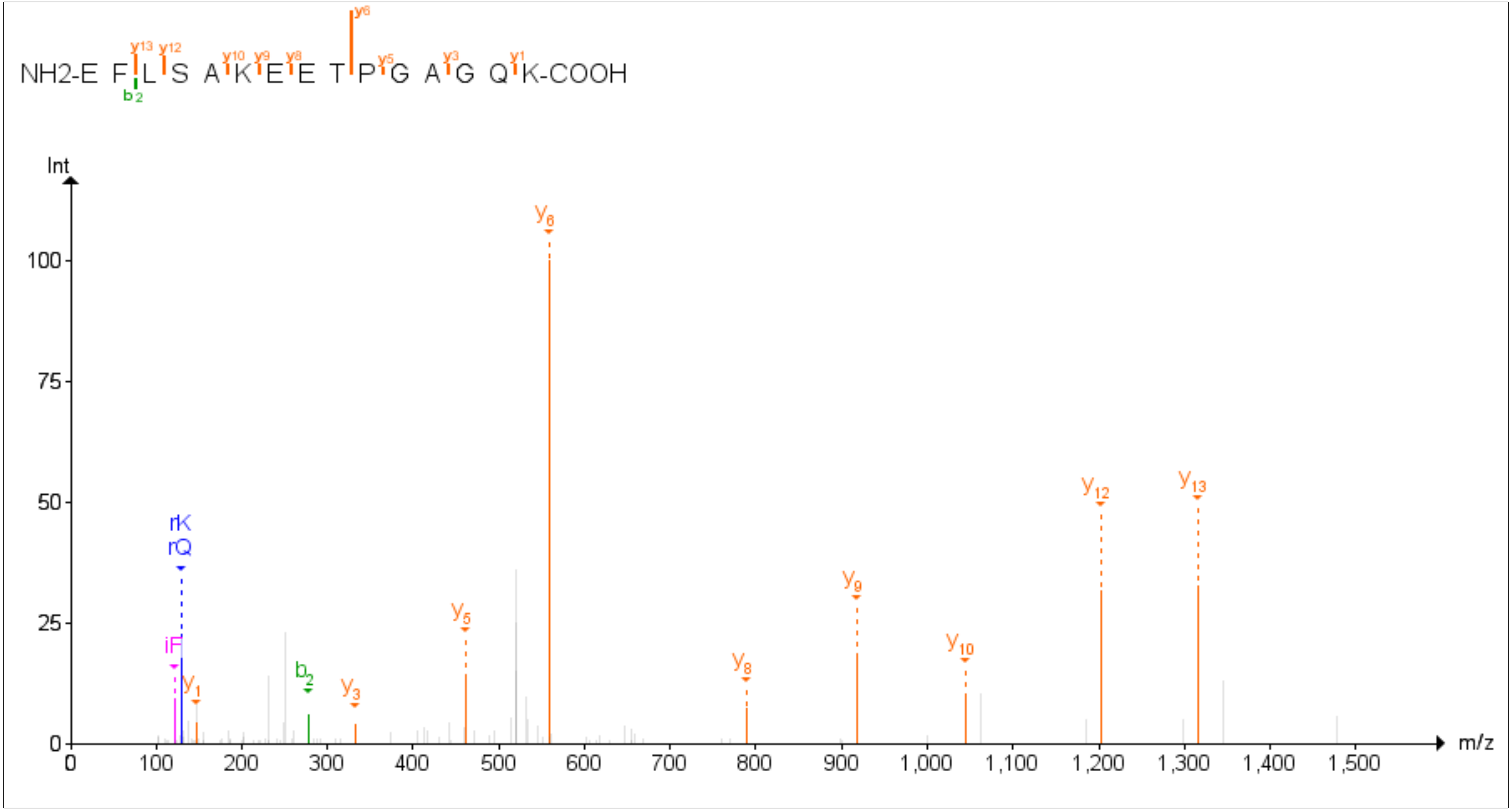

NH2-E F L S A K E E T P G A G Q K-COOH
y13 y12 y10 y9 y8 y6 y5 y3 y1
b2
Int
100
75
50
25
0
y6
y12
y13
rK
rQ
y9
y5
iF
y10
y8
b2
y1
y3
0 100 200 300 400 500 600 700 800 900 1,000 1,100 1,200 1,300 1,400 1,500
m/z

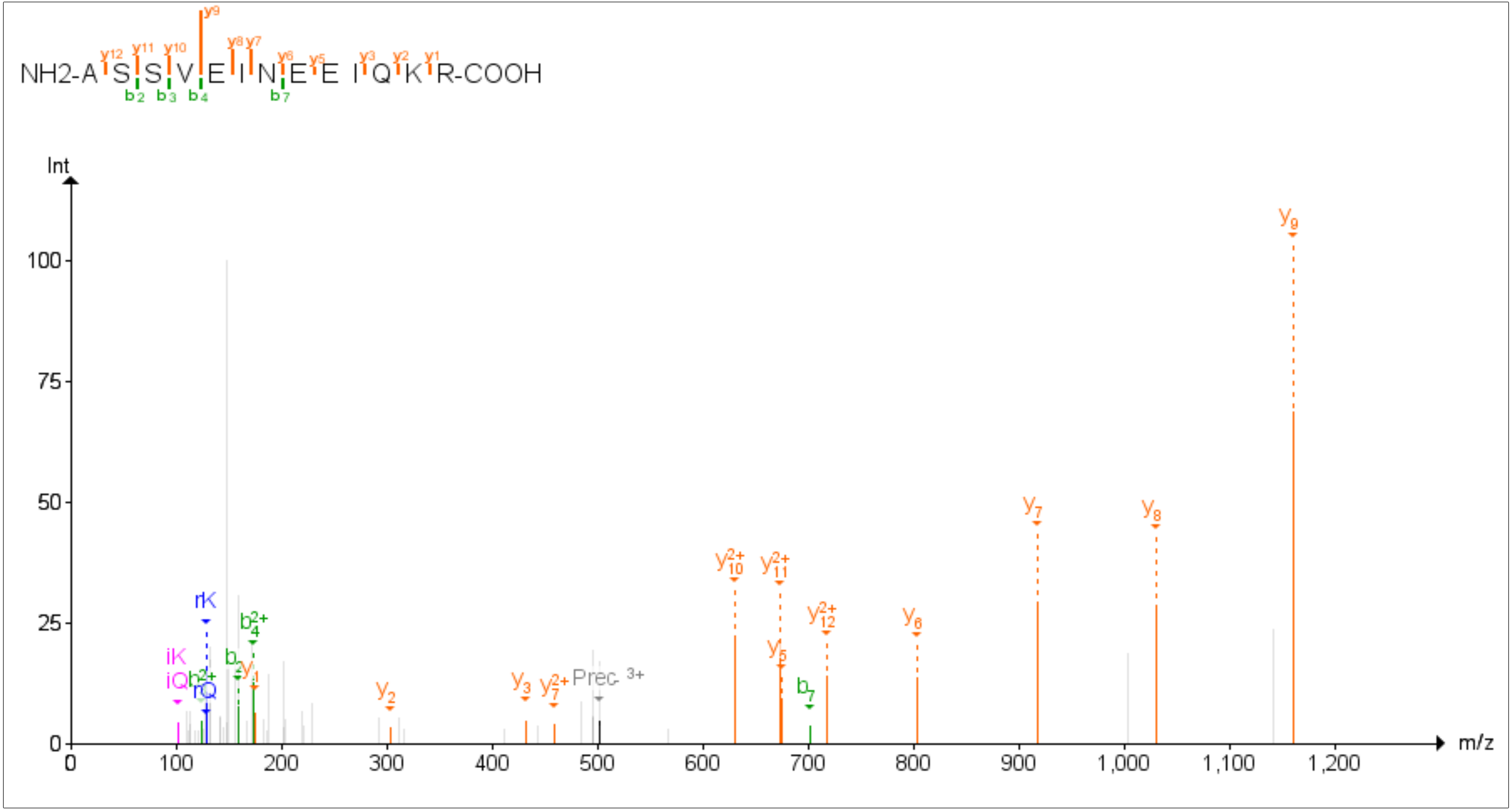

NH2-A S S V E I N E E I Q K R-COOH
y12 y11 y10 y9 y8 y7 y6 y5 y3 y2 y1
b2 b3 b4 b7
Int
100
75
50
25
0
m/z
0 100 200 300 400 500 600 700 800 900 1,000 1,100 1,200
y9
y7
y8
y10 2+
y11 2+
y12 2+
y6
y5
b7
rK
b4 2+
iK
b2
y1
iQ
b2+
rQ
y2
y3
y7 2+
Prec 3+

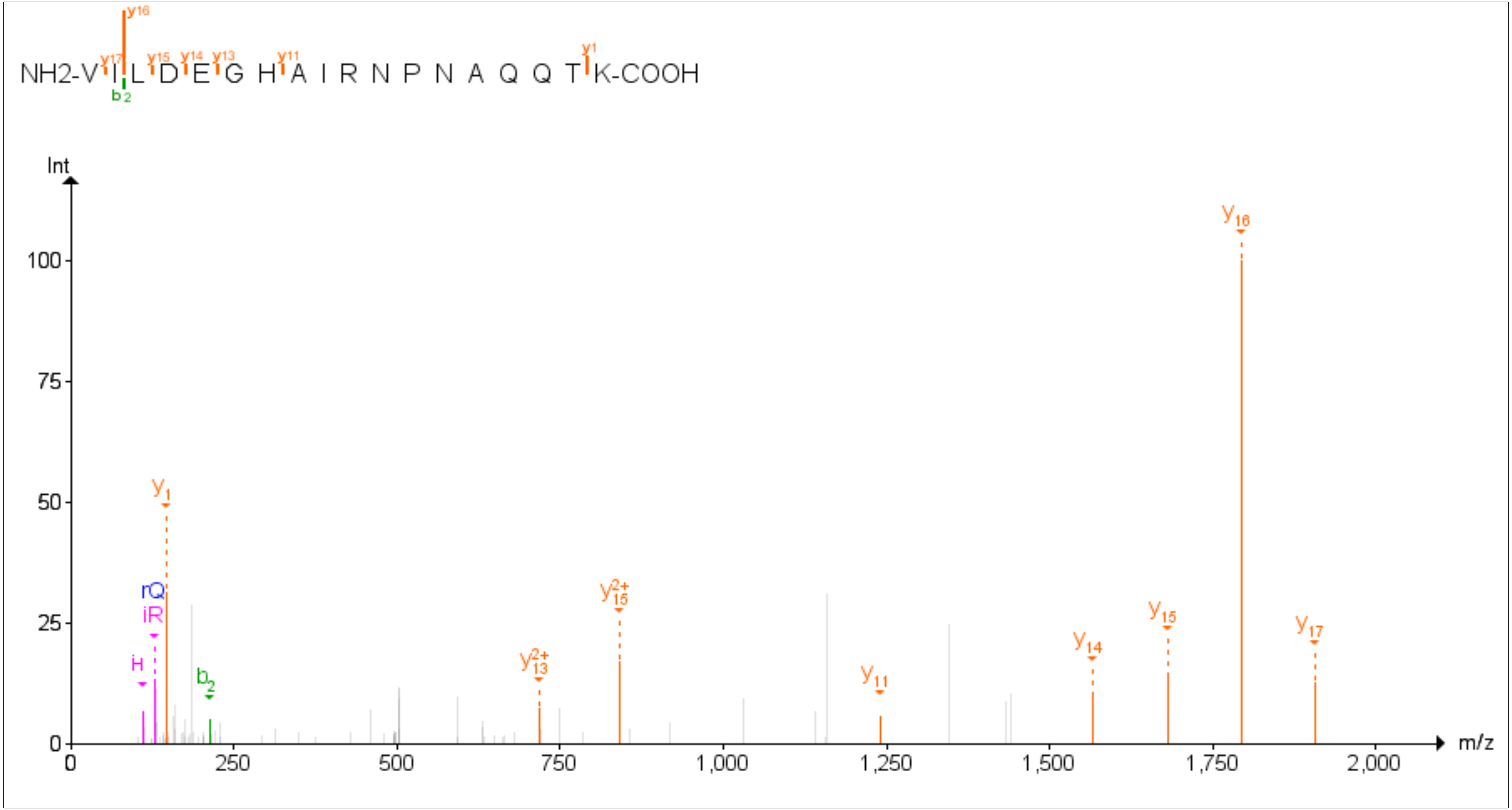

NH2-V I L D E G H A I R N P N A Q Q T K-COOH
y16
y17
y15
y14
y13
y11
y1
b2
Int
100
75
50
25
0
y1
rQ
iR
iH
b2
y_{13}^{2+}
y_{15}^{2+}
y11
y14
y15
y16
y17
0
250
500
750
1,000
1,250
1,500
1,750
2,000
m/z

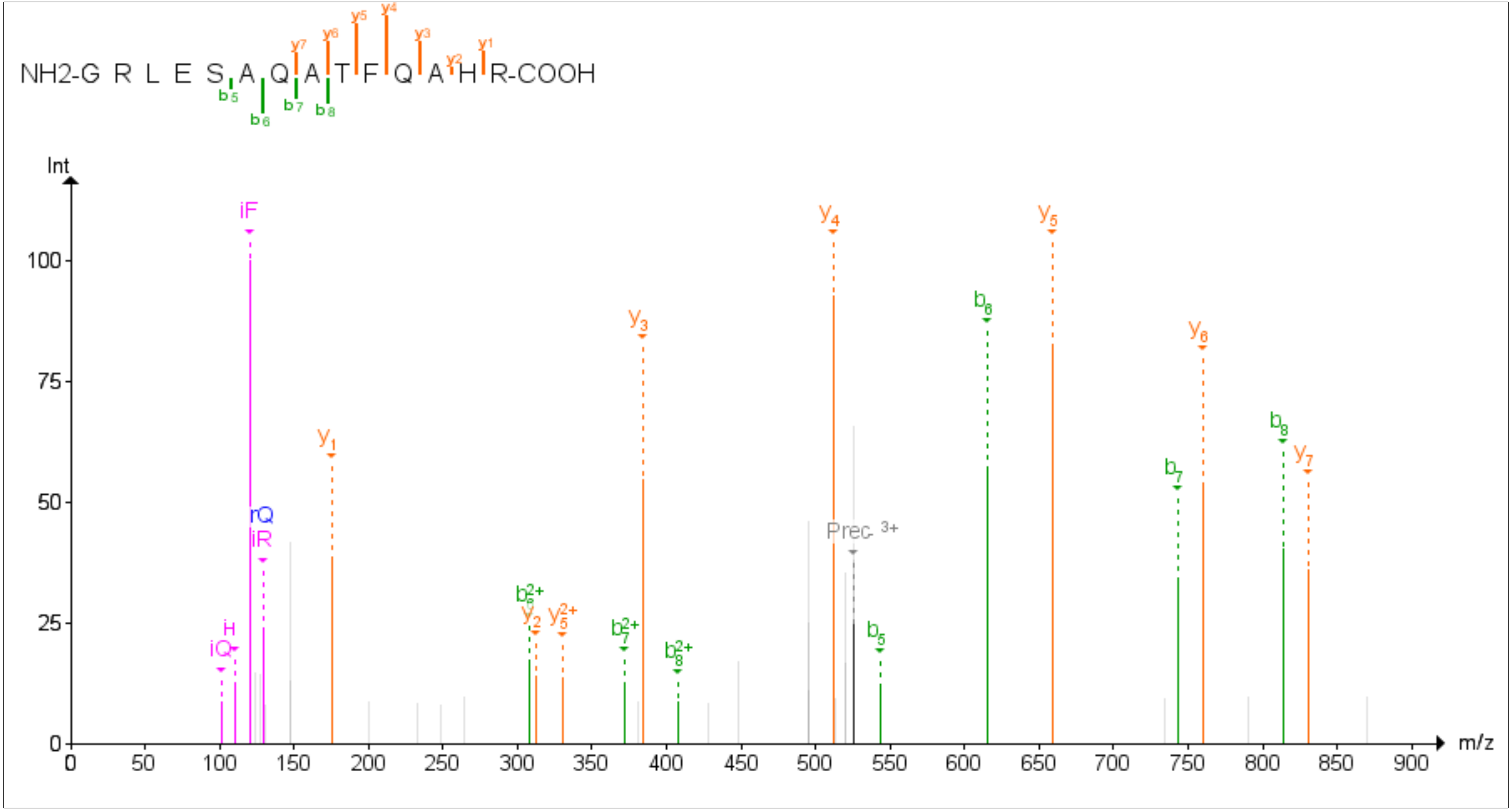
NH2-G R L E S A Q A T F Q A H R-COOH
Int
m/z
iF
rQ
iR
iH
iQ
y1
y2
y3
y4
y5
y6
y7
b5
b6
b7
b8
Prec. 3+
0
25
50
75
100
0 50 100 150 200 250 300 350 400 450 500 550 600 650 700 750 800 850 900

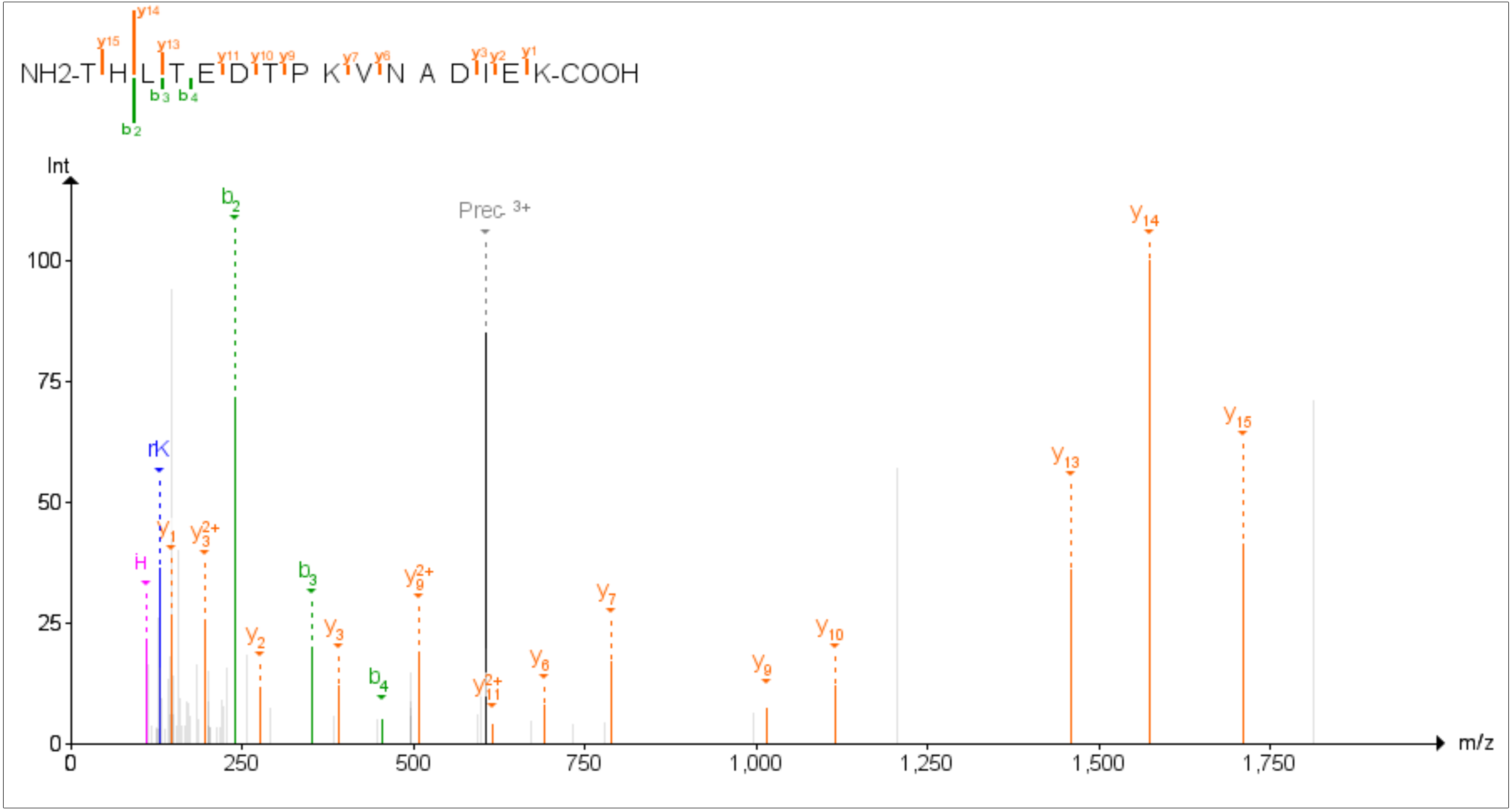

NH2-T H L T E D T P K V N A D I E K-COOH
Int
m/z
Prec. 3+

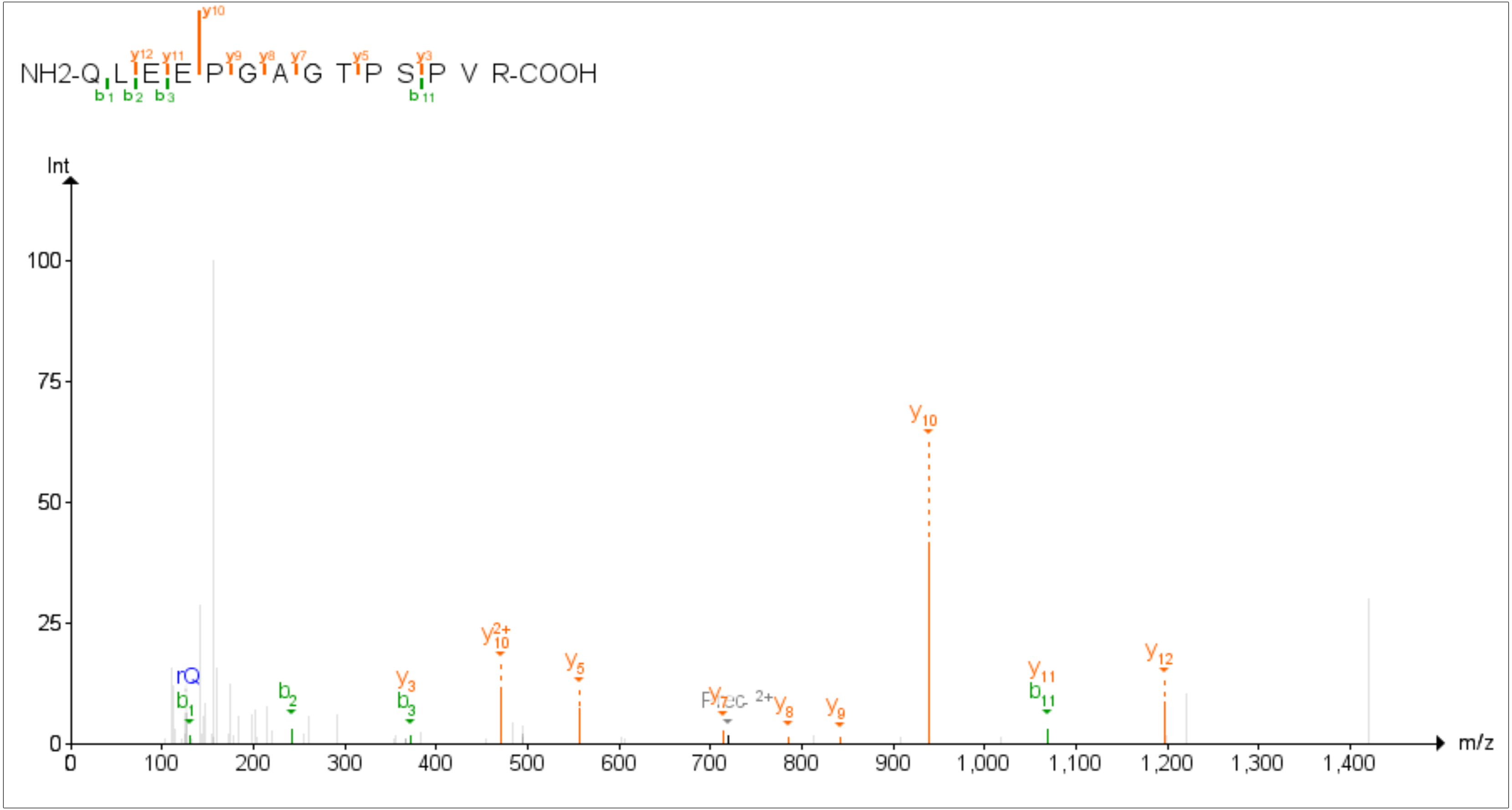

NH2-Q L E E P G A G T P S P V R-COOH
y10
y12
y11
y9
y8
y7
y5
y3
b1
b2
b3
b11
Int
100
75
50
25
0
m/z
0
100
200
300
400
500
600
700
800
900
1,000
1,100
1,200
1,300
1,400
rQ
b1
b2
y3
b3
y10 2+
y5
y7
Prec- 2+
y8
y9
y10
y11
b11
y12

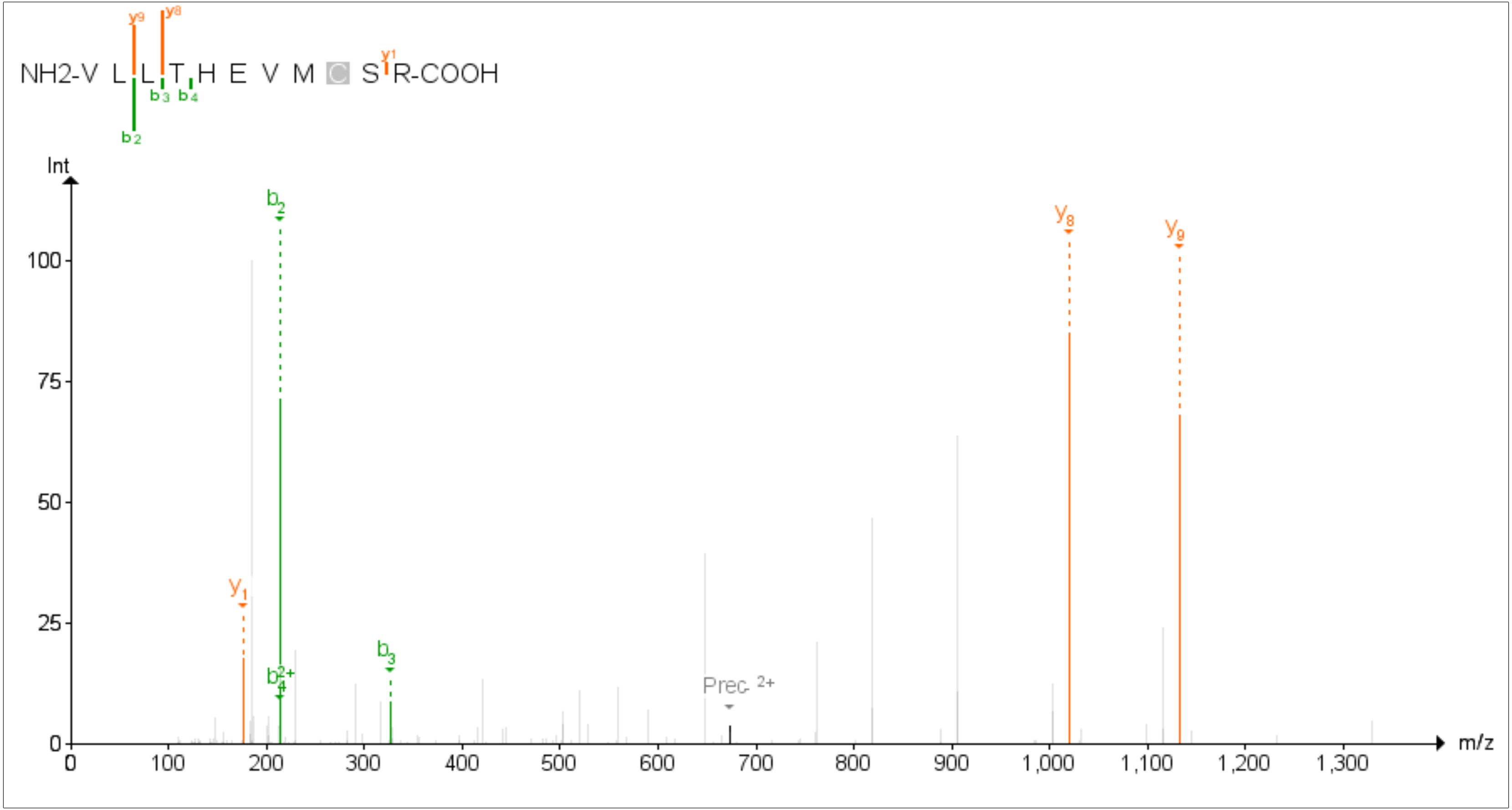

NH2-V L L T H E V M C S R-COOH
y9
y8
y1
b2
b3
b4
Int
100
75
50
25
0
b2
y8
y9
y1
b4 2+
b3
Prec- 2+
0
100
200
300
400
500
600
700
800
900
1,000
1,100
1,200
1,300
m/z

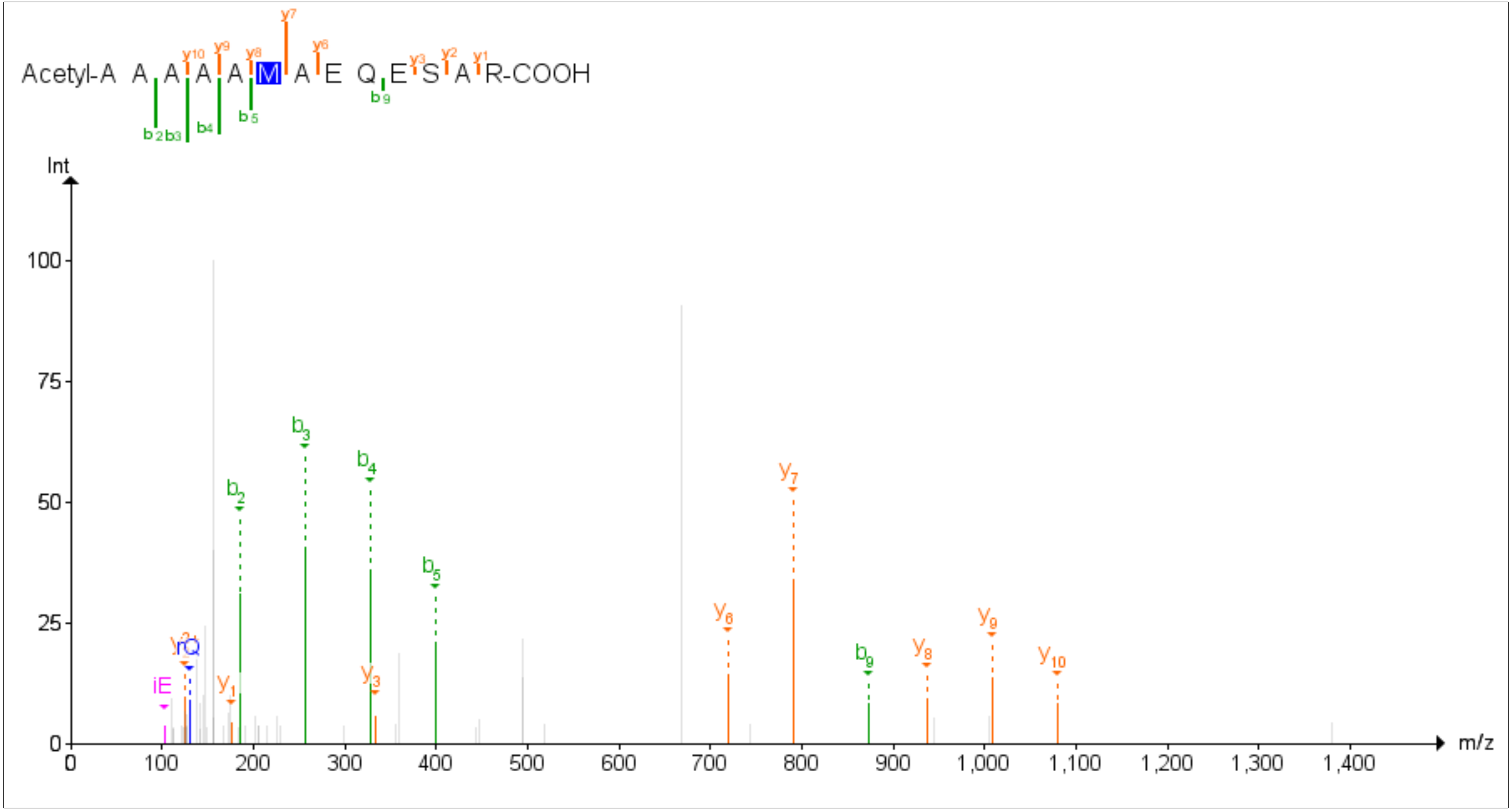

Acetyl-A A A A A M A E Q E S A R-COOH
y10 y9 y8 y7 y6 y3 y2 y1
b2 b3 b4 b5 b9
Int
100
75
50
25
0
0 100 200 300 400 500 600 700 800 900 1,000 1,100 1,200 1,300 1,400
m/z
b3
b4
b2
b5
y7
y6
y9
b9
y8
y10
rQ
iE
y1
y3

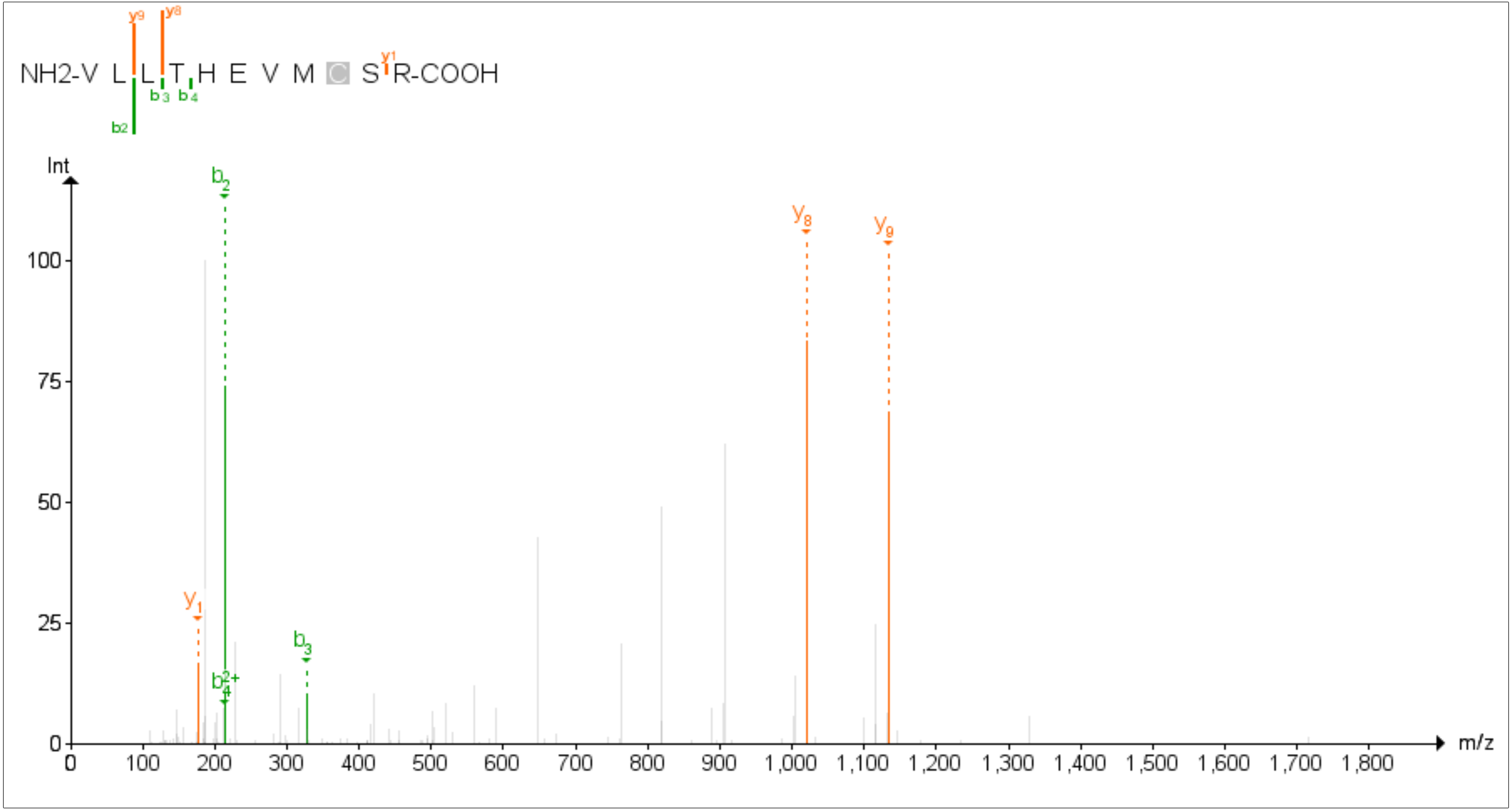

NH2-V L L T H E V M C S R-COOH
y9
y8
y1
b2
b3
b4
Int
b2
y8
y9
y1
b3
b4 2+
100
75
50
25
0
0
100
200
300
400
500
600
700
800
900
1,000
1,100
1,200
1,300
1,400
1,500
1,600
1,700
1,800
m/z

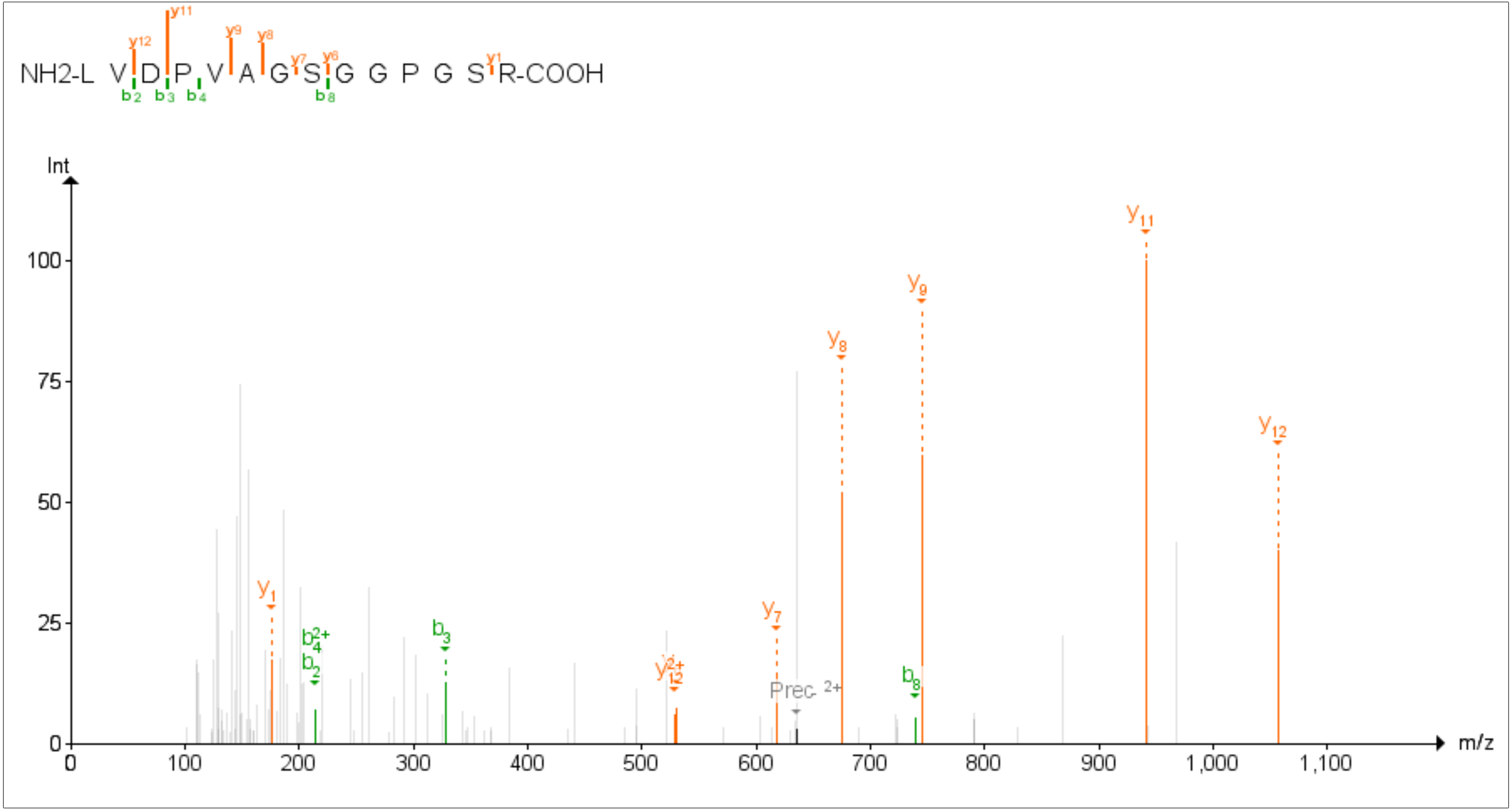
NH2-L V D P V A G S G G P G S R-COOH
y12 y11 y9 y8 y7 y6 y1
b2 b3 b4 b8
Int
100
75
50
25
0
y11
y9
y8
y12
y1
b4 2+
b2
b3
y12 2+
y7
Prec 2+
b8
0 100 200 300 400 500 600 700 800 900 1,000 1,100
m/z

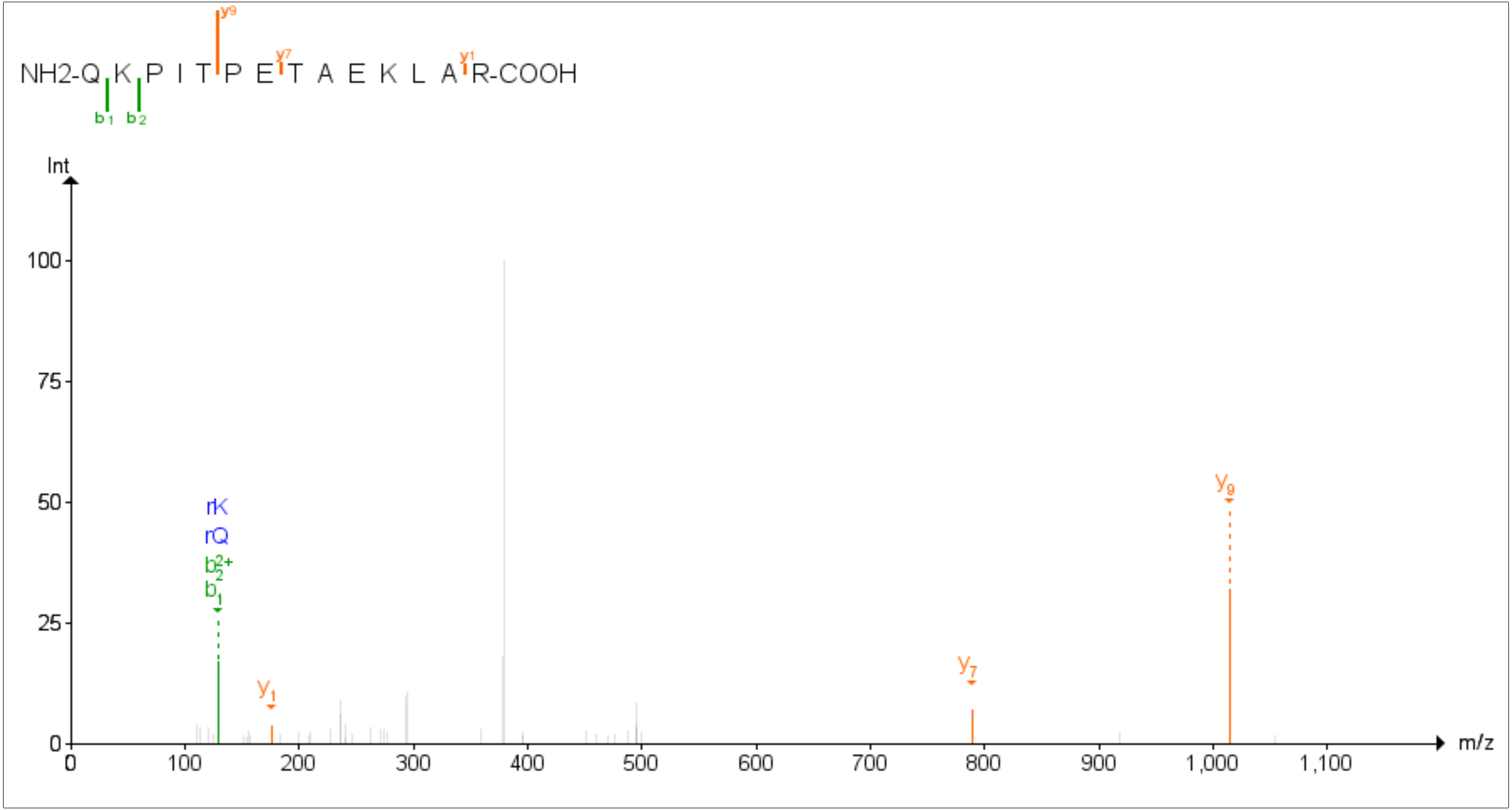

NH2-Q K P I T P E T A E K L A R-COOH
y9
y7
y1
b1
b2
Int
100
75
50
25
0
rK
rQ
b_2^{2+}
b_1
y_1
y_7
y_9
0
100
200
300
400
500
600
700
800
900
1,000
1,100
m/z

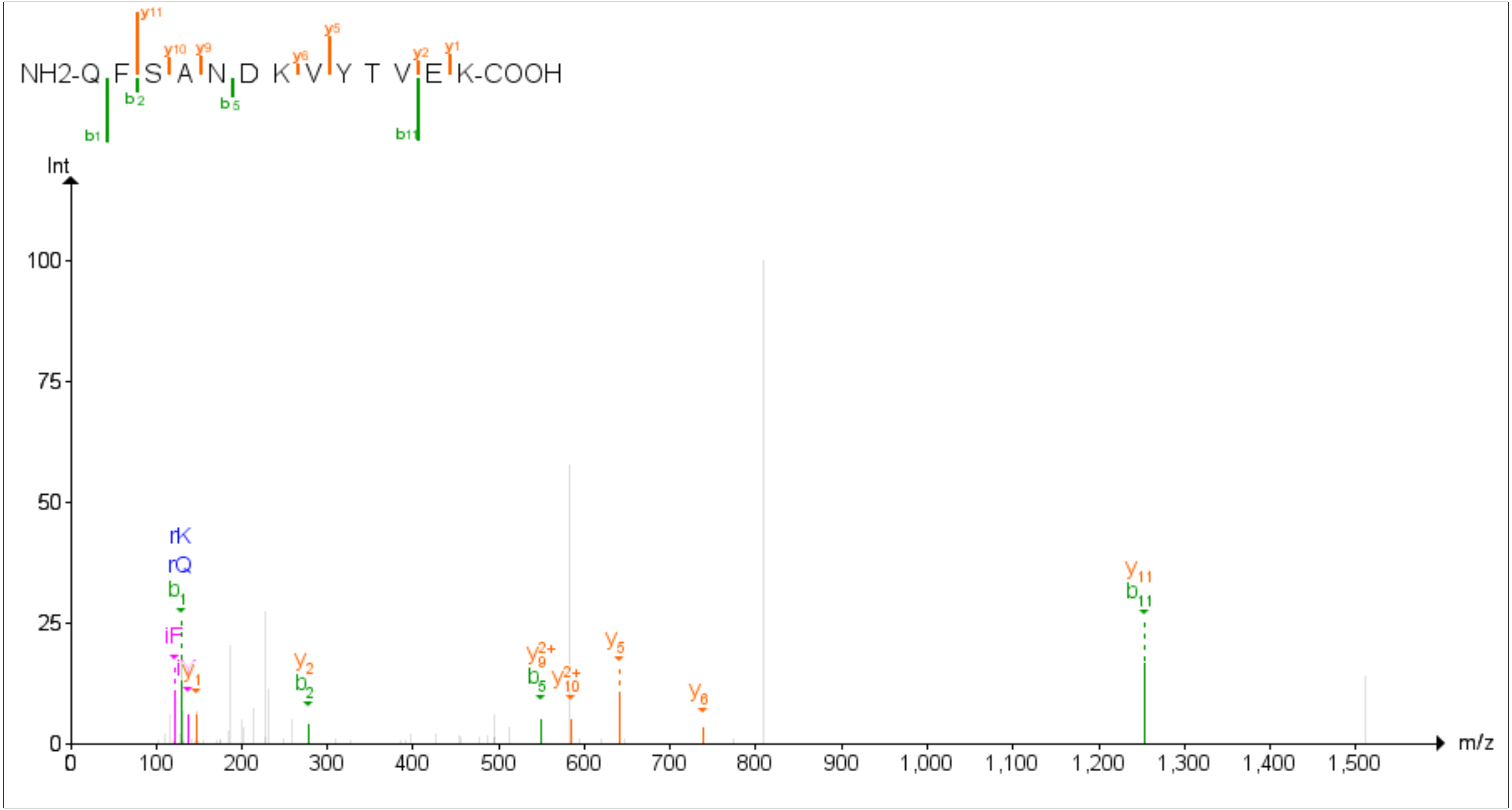

NH2-Q F S A N D K V Y T V E K-COOH
y11
y10
y9
y6
y5
y2
y1
b1
b2
b5
b11
Int
100
75
50
25
0
rK
rQ
b1
iF
y1
y2
b2
y9 2+
b5
y10 2+
y5
y6
y11
b11
m/z
0
100
200
300
400
500
600
700
800
900
1,000
1,100
1,200
1,300
1,400
1,500

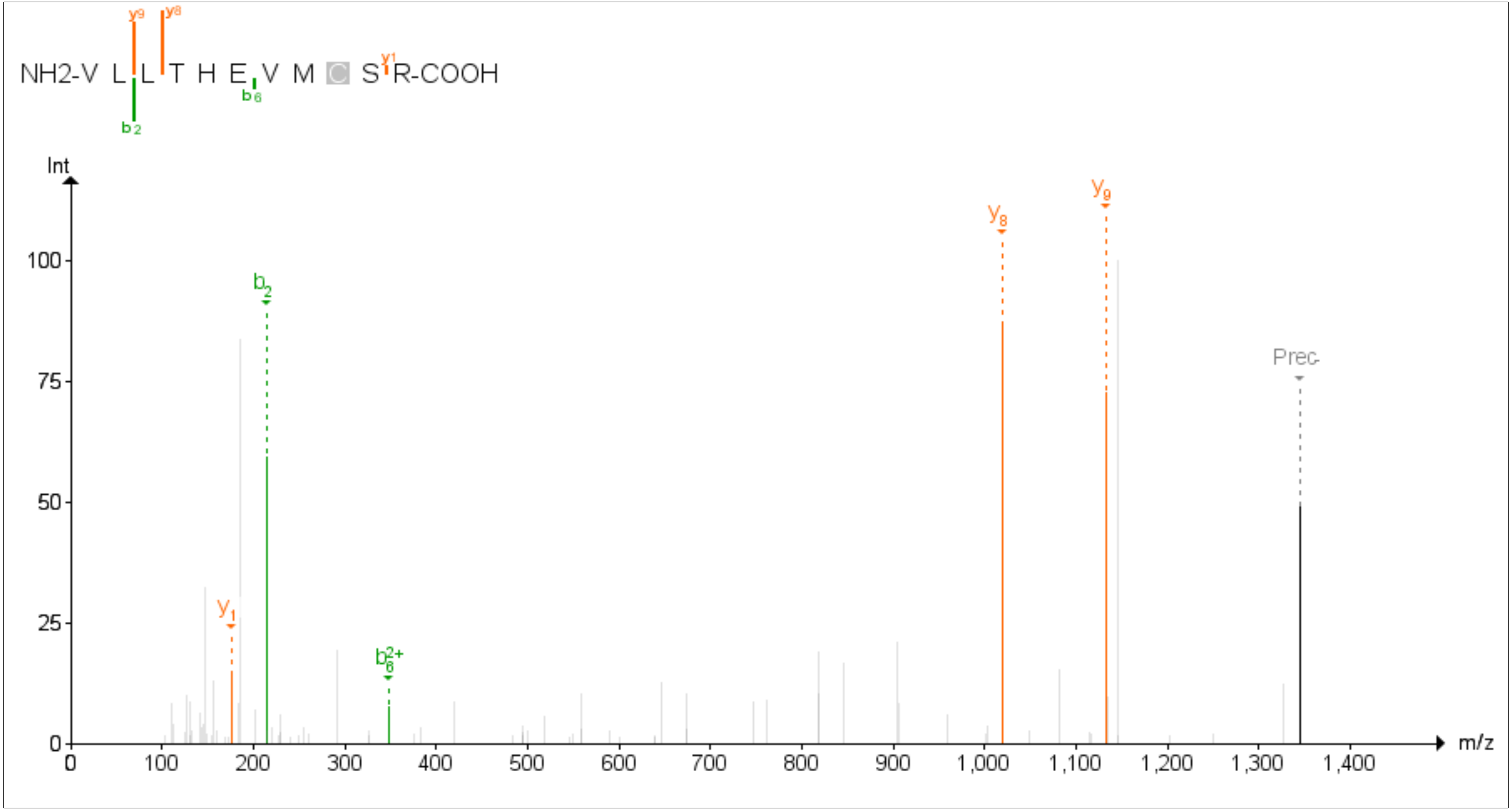

NH2-V L L T H E V M C S R-COOH
y9
y8
y1
b2
b6
Int
100
75
50
25
0
b2
y1
b6 2+
y8
y9
Prec
0
100
200
300
400
500
600
700
800
900
1,000
1,100
1,200
1,300
1,400
m/z

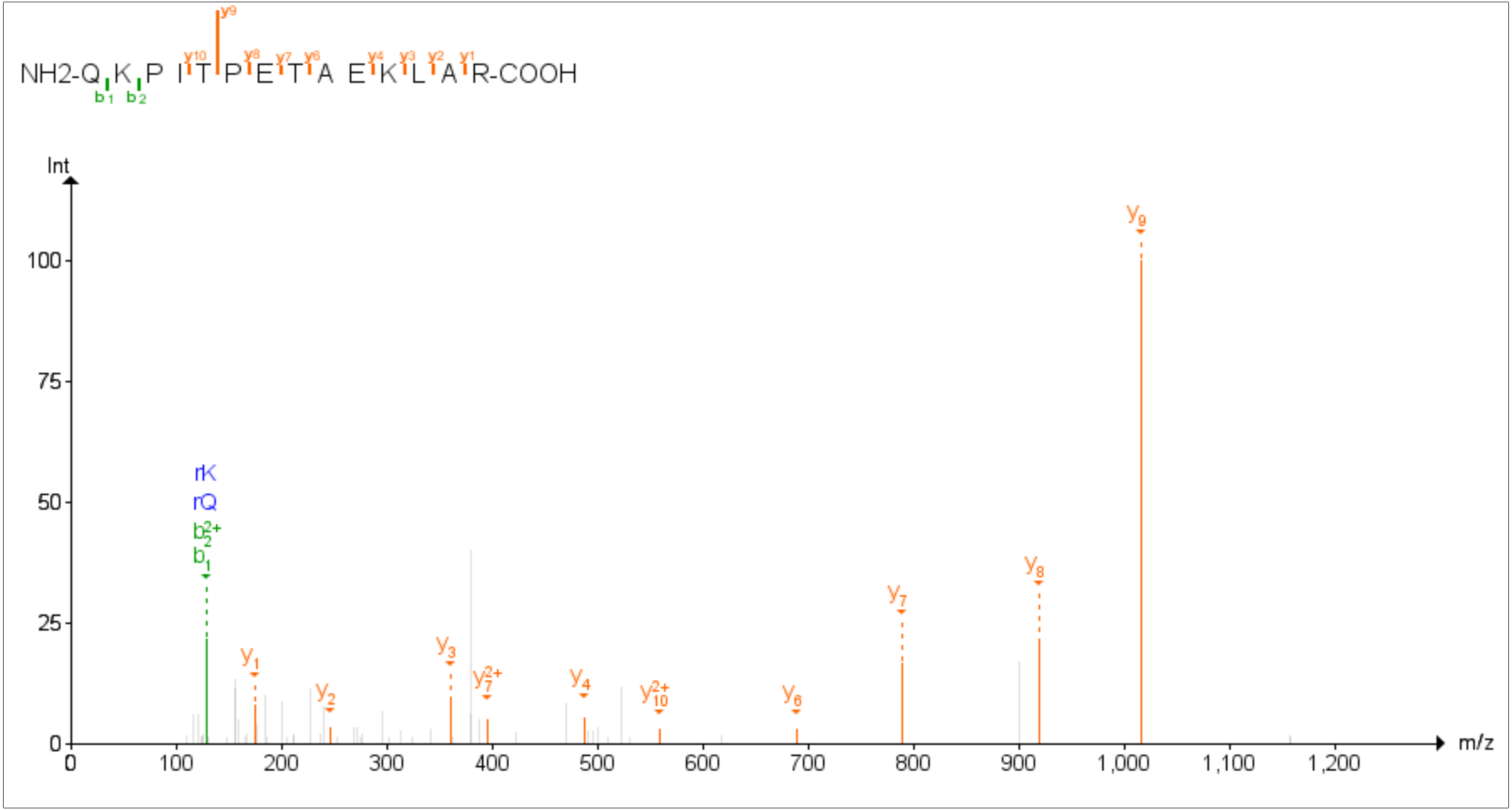

NH2-Q K P I T P E T A E K L A R-COOH
b1 b2
y10 y9 y8 y7 y6 y4 y3 y2 y1
Int
m/z
rK
rQ
b2 2+
b1
y1 y2 y3 y7 2+ y4 y10 2+ y6 y7 y8 y9
0 25 50 75 100
0 100 200 300 400 500 600 700 800 900 1,000 1,100 1,200

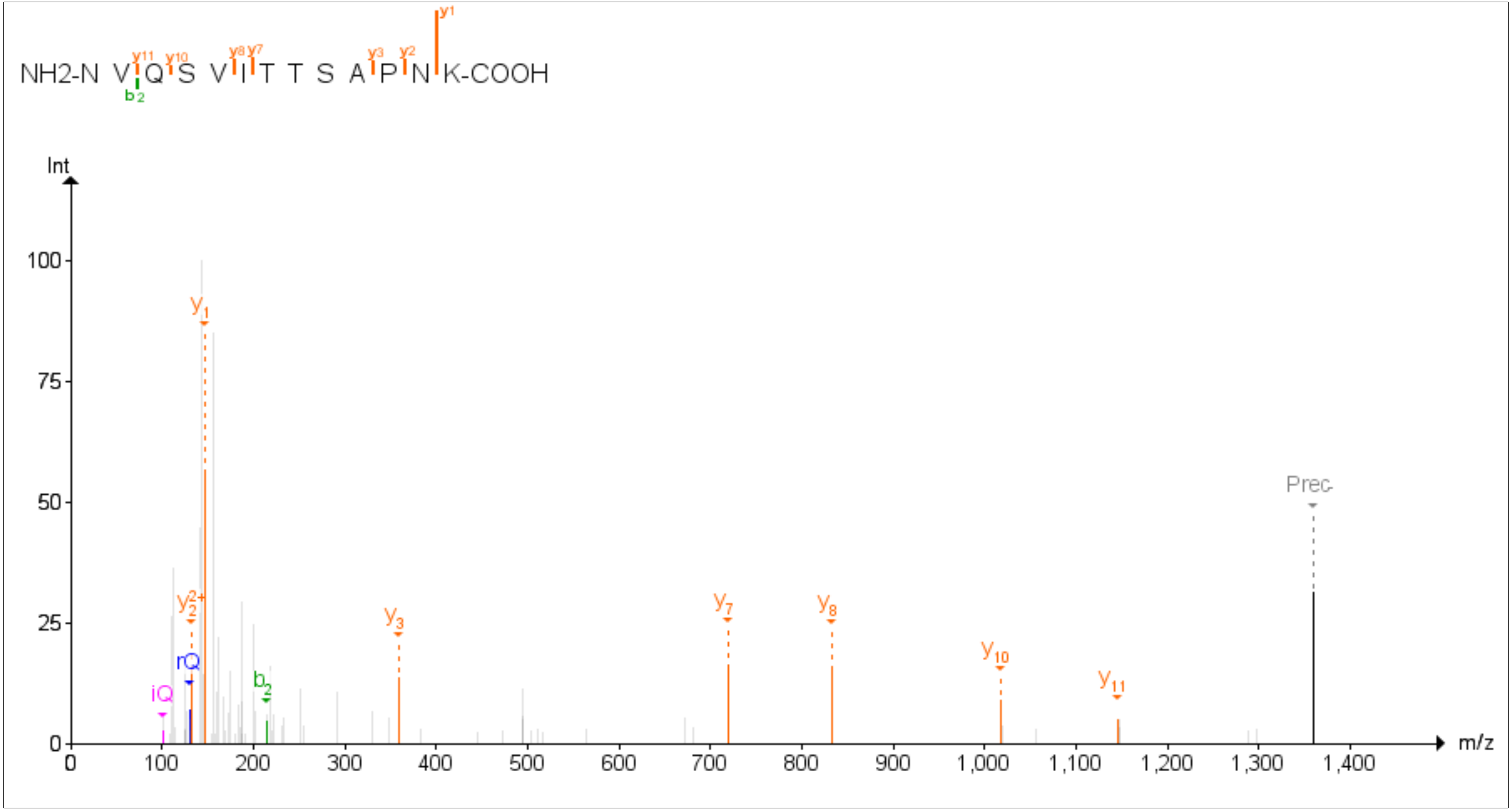

NH2-N V Q S V I T T S A P N K-COOH
y11 y10 y8 y7 y3 y2 y1
b2
Int
100
75
50
25
0
y1
y2 2+
rQ
iQ
b2
y3
y7
y8
y10
y11
Prec
0
100
200
300
400
500
600
700
800
900
1,000
1,100
1,200
1,300
1,400
m/z

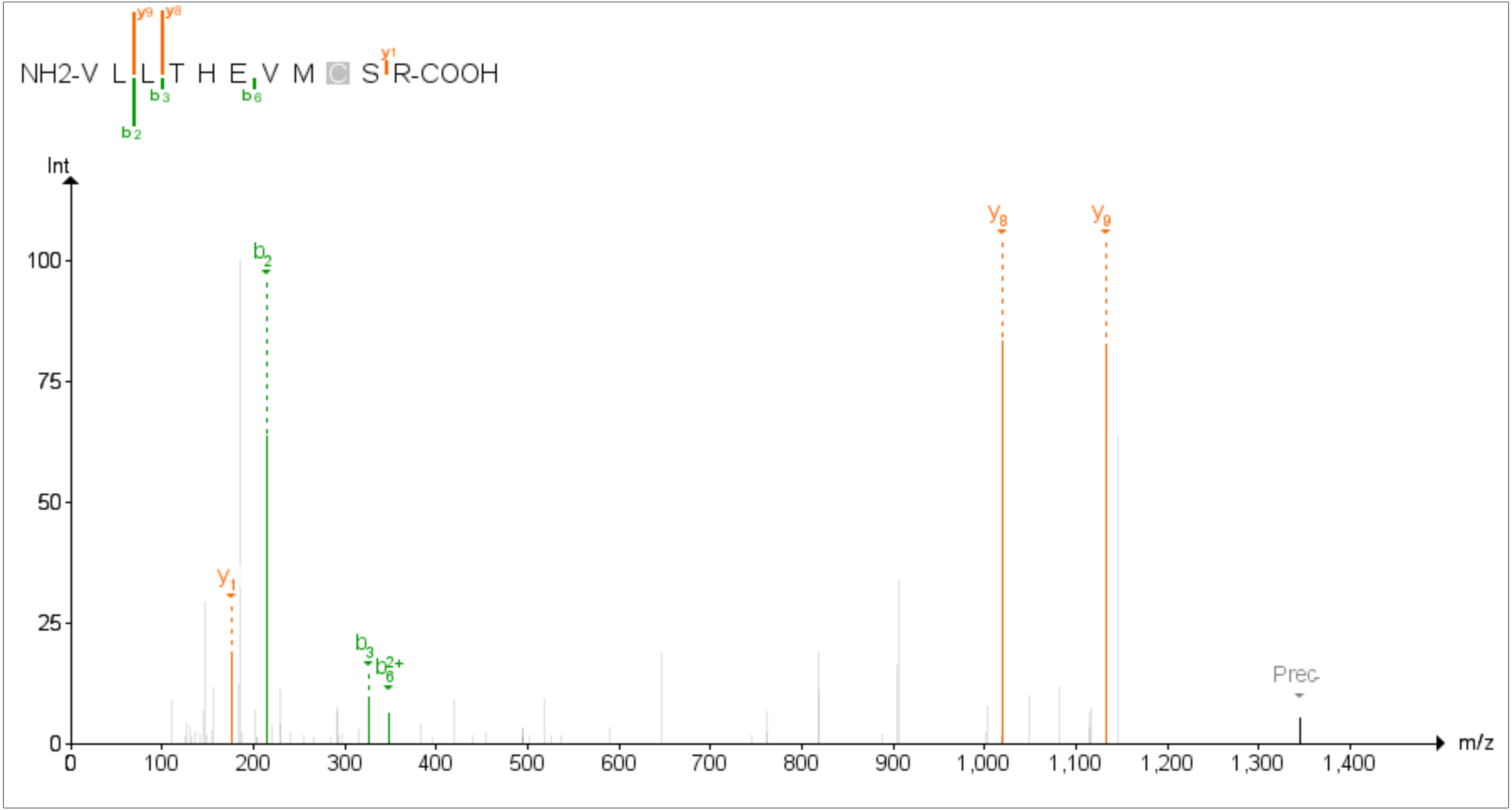

NH2-V L L T H E V M C S R-COOH
y9
y8
y1
b2
b3
b6
Int
100
75
50
25
0
y8
y9
b2
y1
b3
b6 2+
Prec.
0
100
200
300
400
500
600
700
800
900
1,000
1,100
1,200
1,300
1,400
m/z

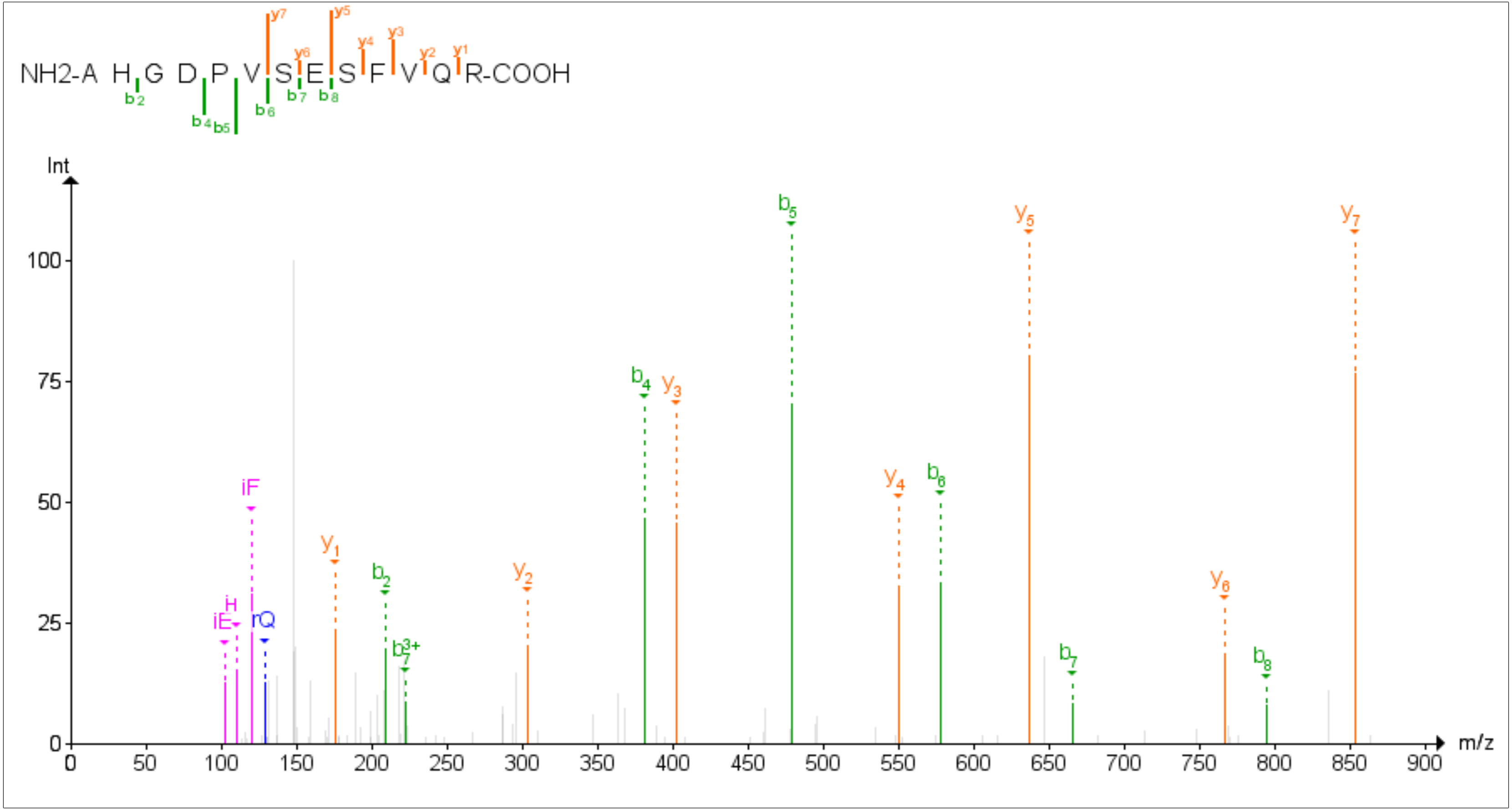
NH2-A H G D P V S E S F V Q R-COOH
y7
y5
y6
y4
y3
y2
y1
b2
b4
b5
b6
b7
b8
Int
m/z
100
75
50
25
0
0
50
100
150
200
250
300
350
400
450
500
550
600
650
700
750
800
850
900
iF
iH
iE
rQ
y1
b2
b7 3+
y2
b4
y3
b5
y4
b6
y5
b7
y6
b8
y7

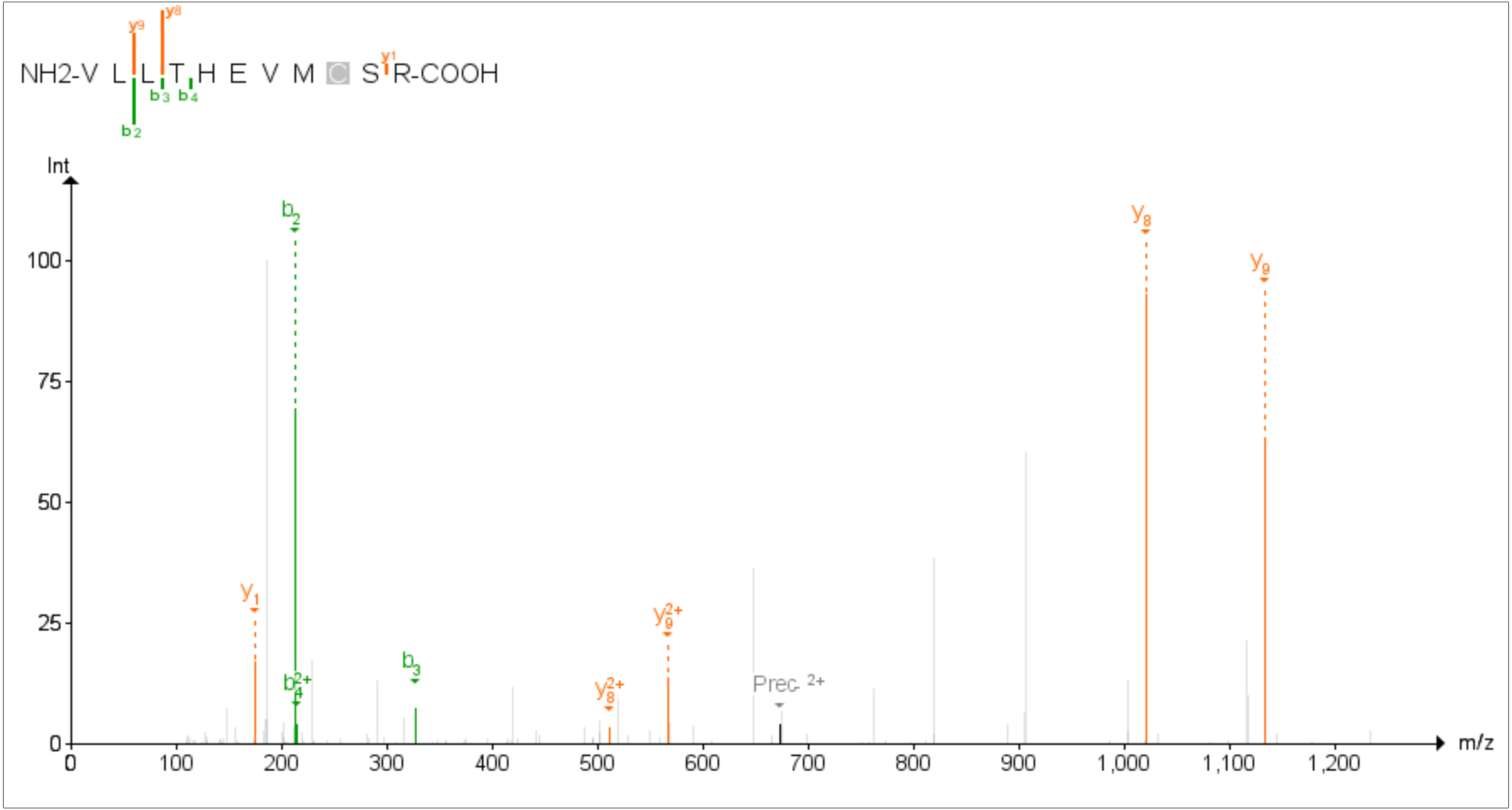

NH2-V L L T H E V M C S R-COOH
y9
y8
y1
b2
b3
b4
Int
100
75
50
25
0
b2
y8
y9
y1
b4 2+
b3
y8 2+
y9 2+
Prec- 2+
0
100
200
300
400
500
600
700
800
900
1,000
1,100
1,200
m/z

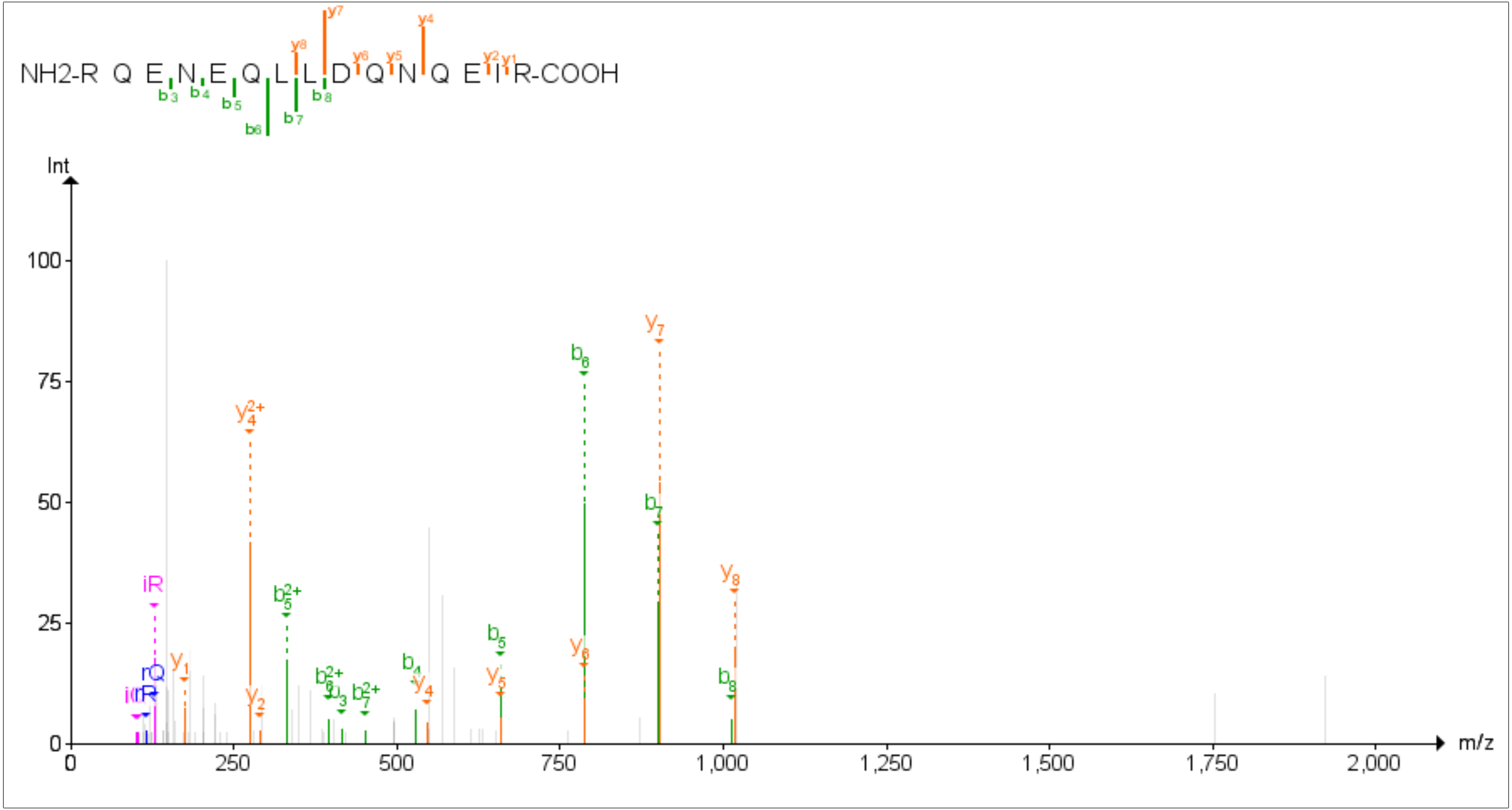
NH2-R Q E N E Q L L D Q N Q E I R-COOH
Int
m/z
100
75
50
25
0
250
500
750
1,000
1,250
1,500
1,750
2,000

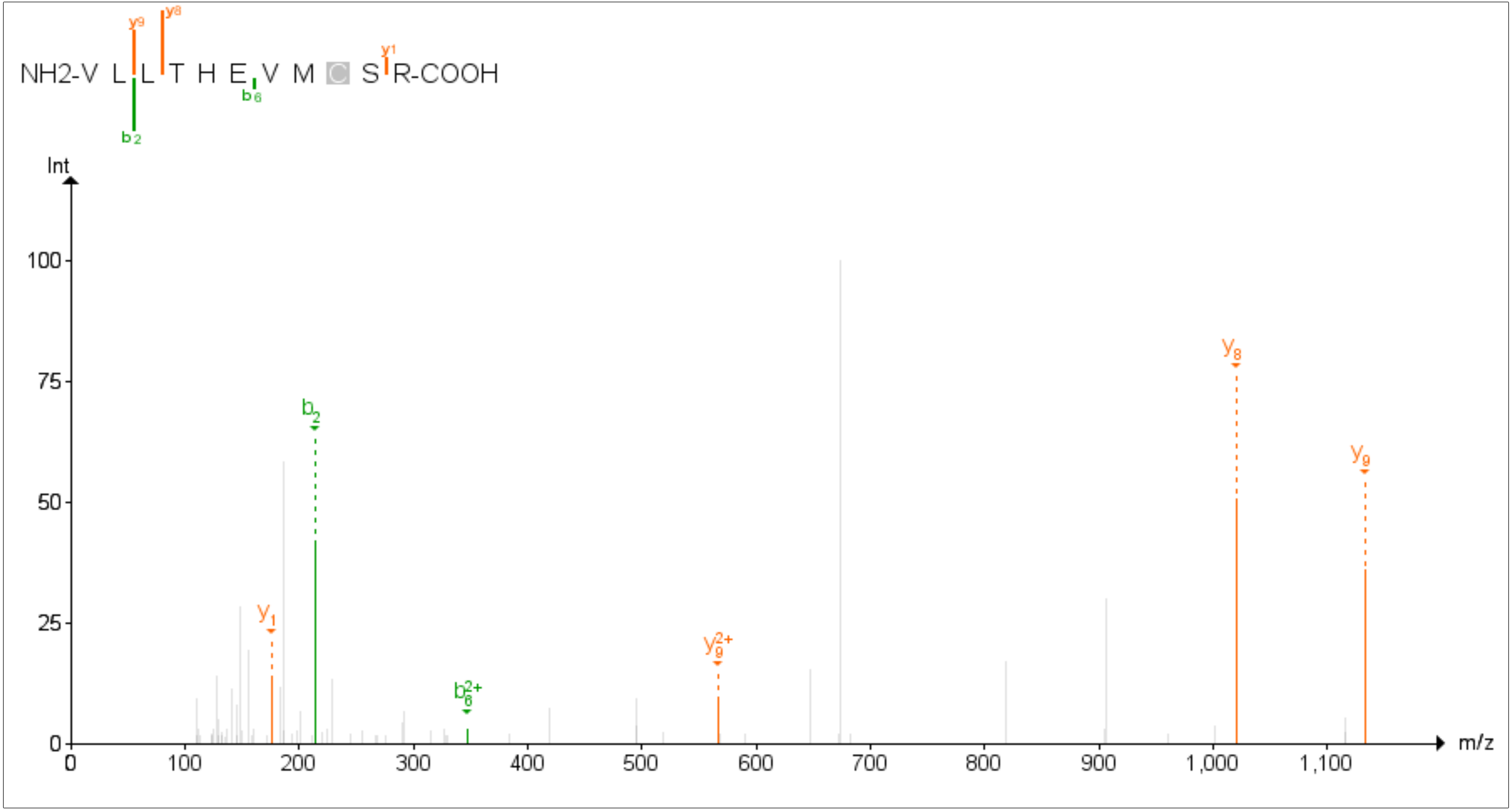

NH2-V L L T H E V M C S R-COOH
y9
y8
y1
b2
b6
Int
100
75
50
25
0
b2
y8
y9
y1
y9 2+
b6 2+
0
100
200
300
400
500
600
700
800
900
1,000
1,100
m/z

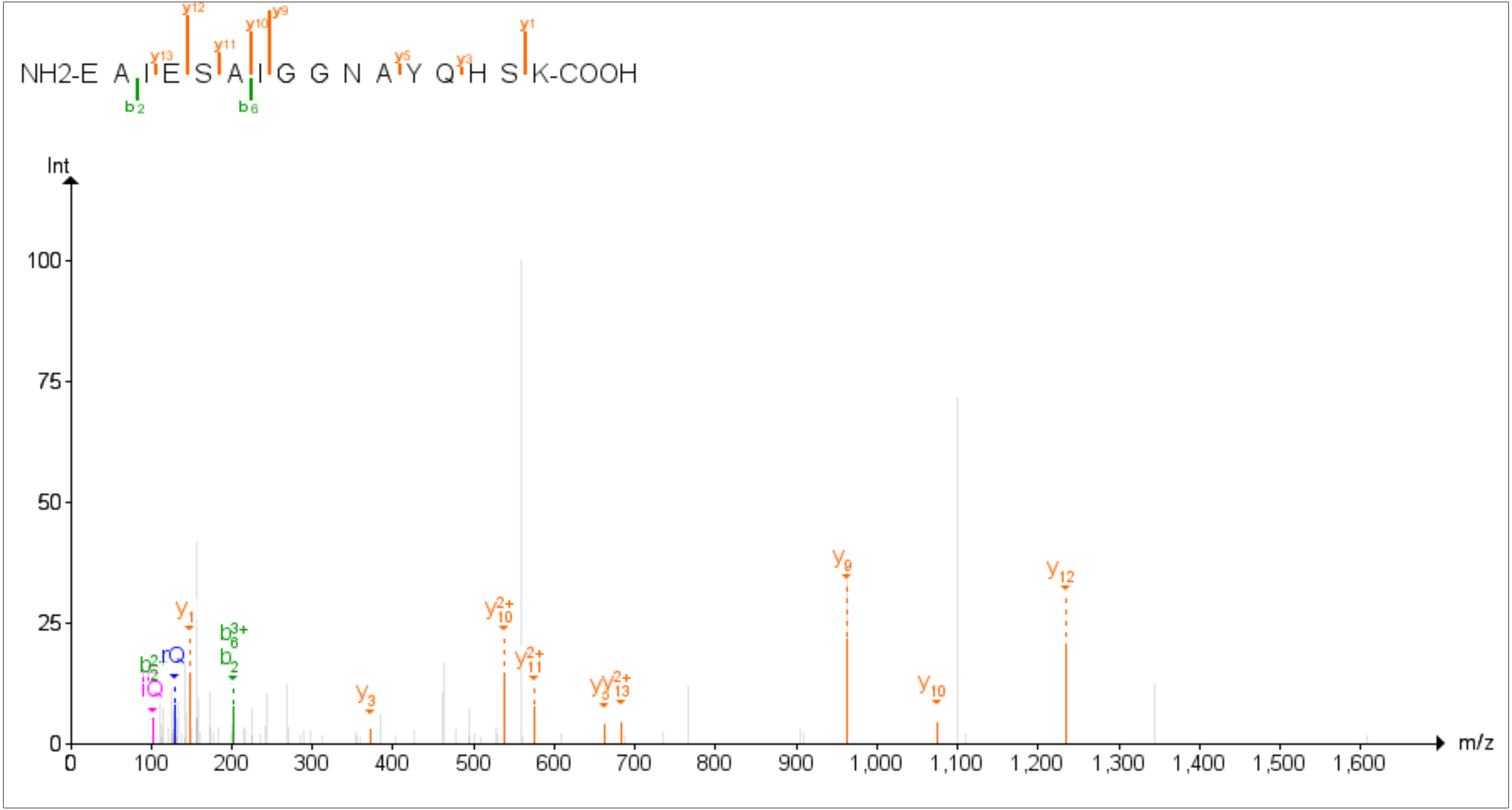

NH2-E A I E S A I G G N A Y Q H S K-COOH
y12
y13
y11
y10
y9
y5
y3
y1
b2
b6
Int
100
75
50
25
0
m/z
0
100
200
300
400
500
600
700
800
900
1,000
1,100
1,200
1,300
1,400
1,500
1,600

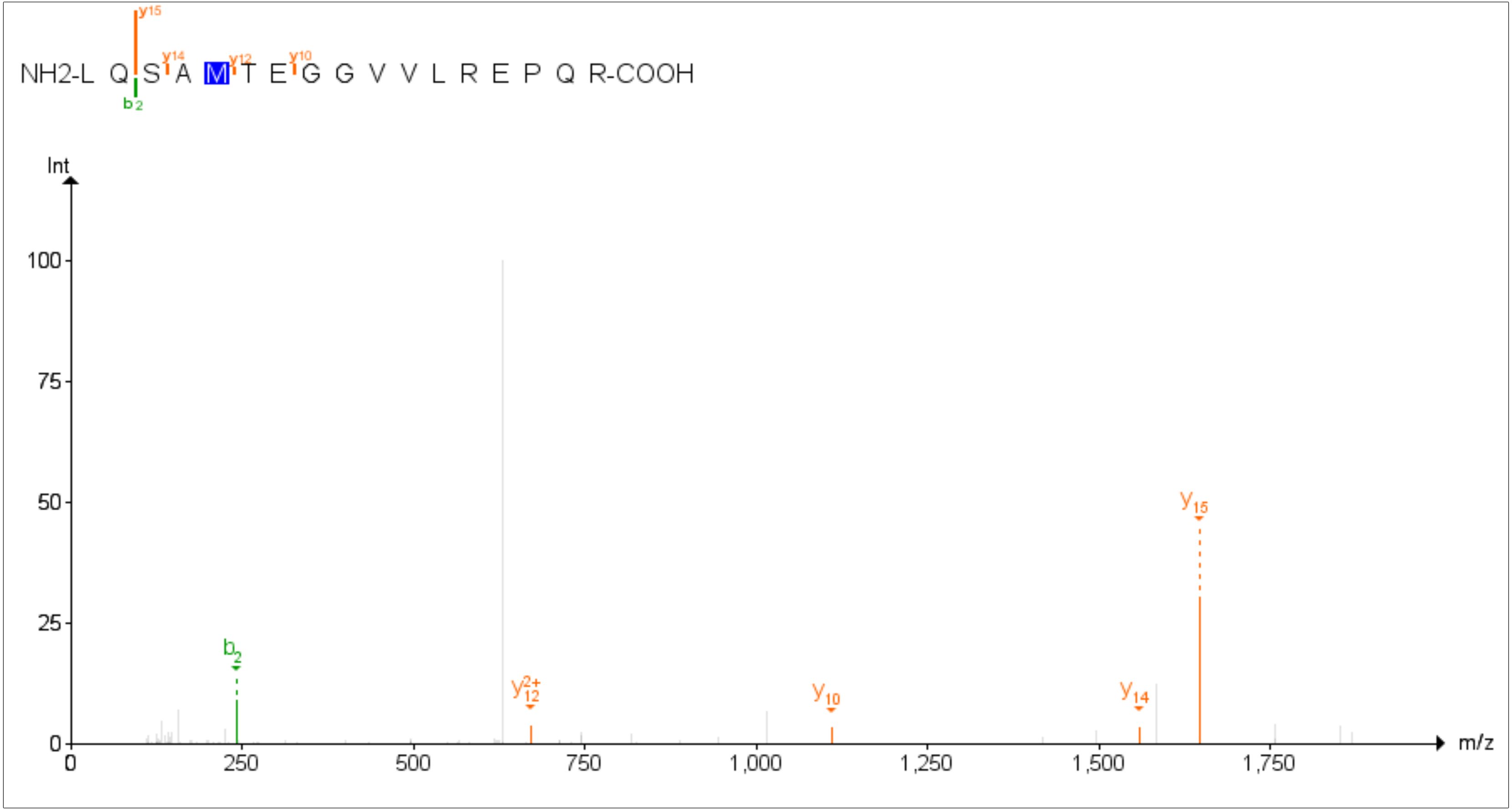

NH2-L Q S A M T E G G V V L R E P Q R-COOH
y15
y14
y12
y10
b2
Int
100
75
50
25
0
m/z
0
250
500
750
1,000
1,250
1,500
1,750
b2
y2+12
y10
y14
y15

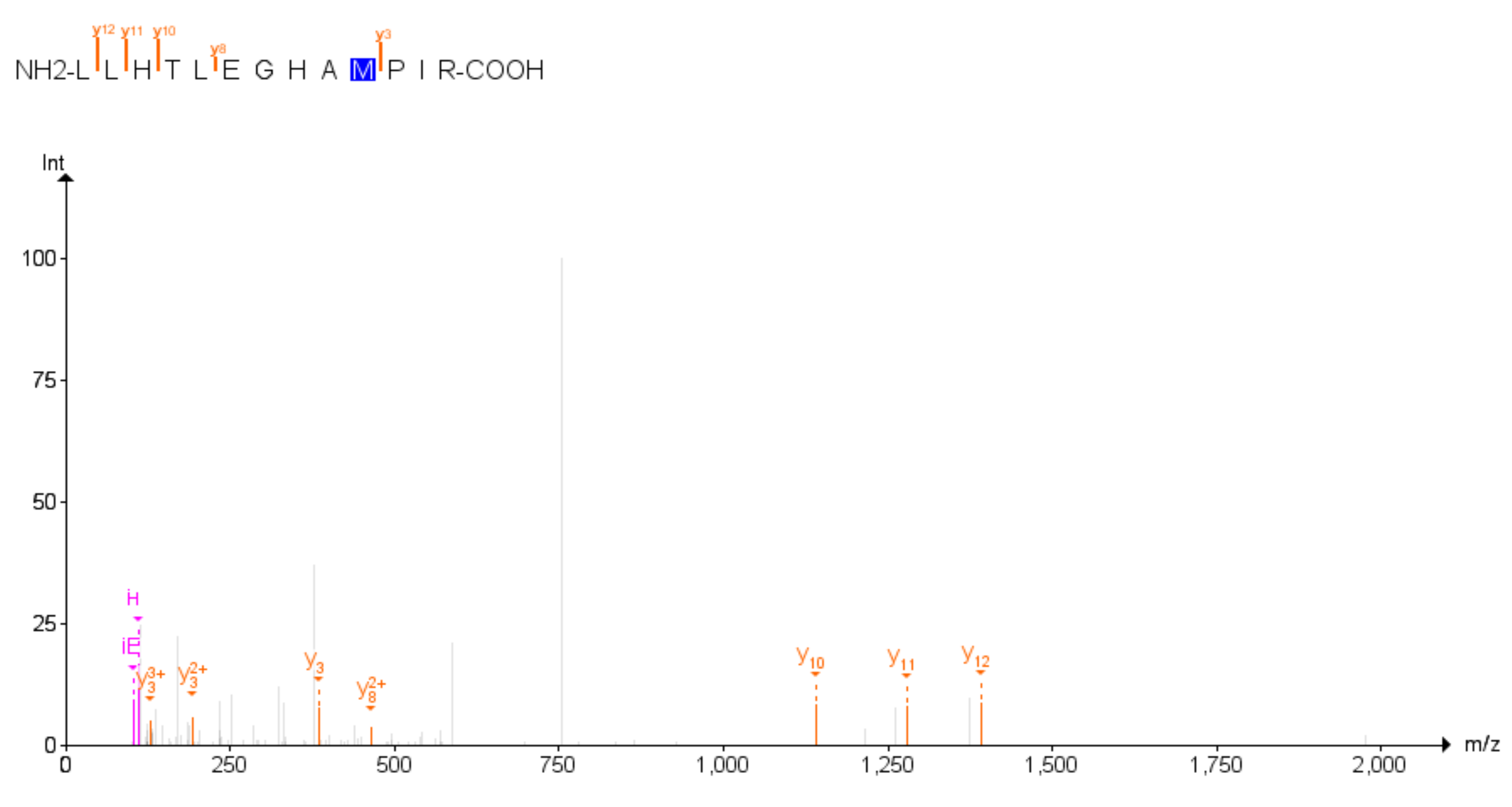

NH2-L L H T L E G H A M P I R-COOH
y12 y11 y10 y8 y3
Int
100
75
50
25
0
m/z
0
250
500
750
1,000
1,250
1,500
1,750
2,000
H
iE
y3 3+
y3 2+
y3
y8 2+
y10
y11
y12

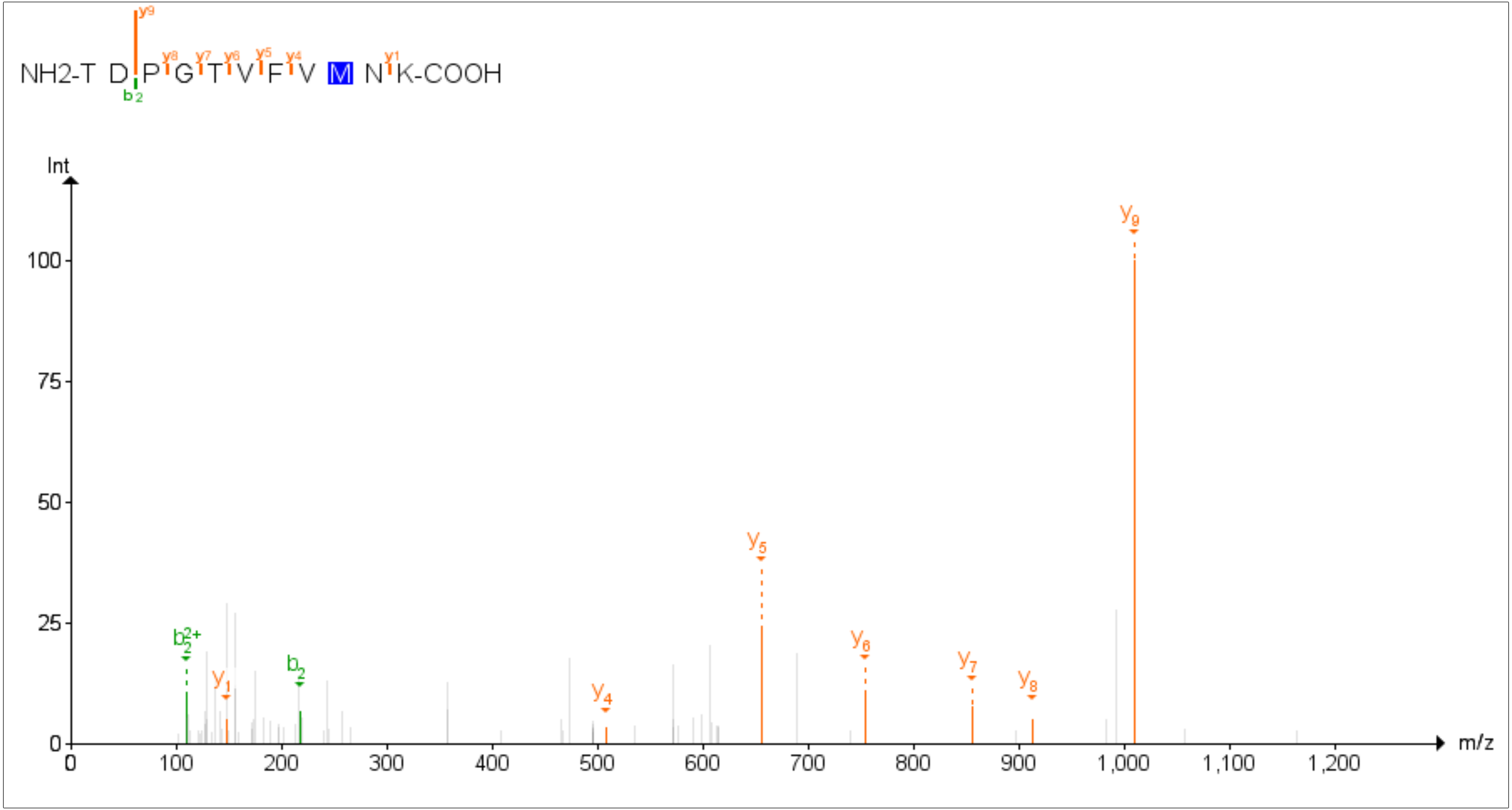

NH2-T D P G T V F V M N K-COOH
y9 y8 y7 y6 y5 y4 y1
b2
Int
100
75
50
25
0
y9
y5
y6
y7
y8
y4
y1
b2
b2 2+
0
100
200
300
400
500
600
700
800
900
1,000
1,100
1,200
m/z

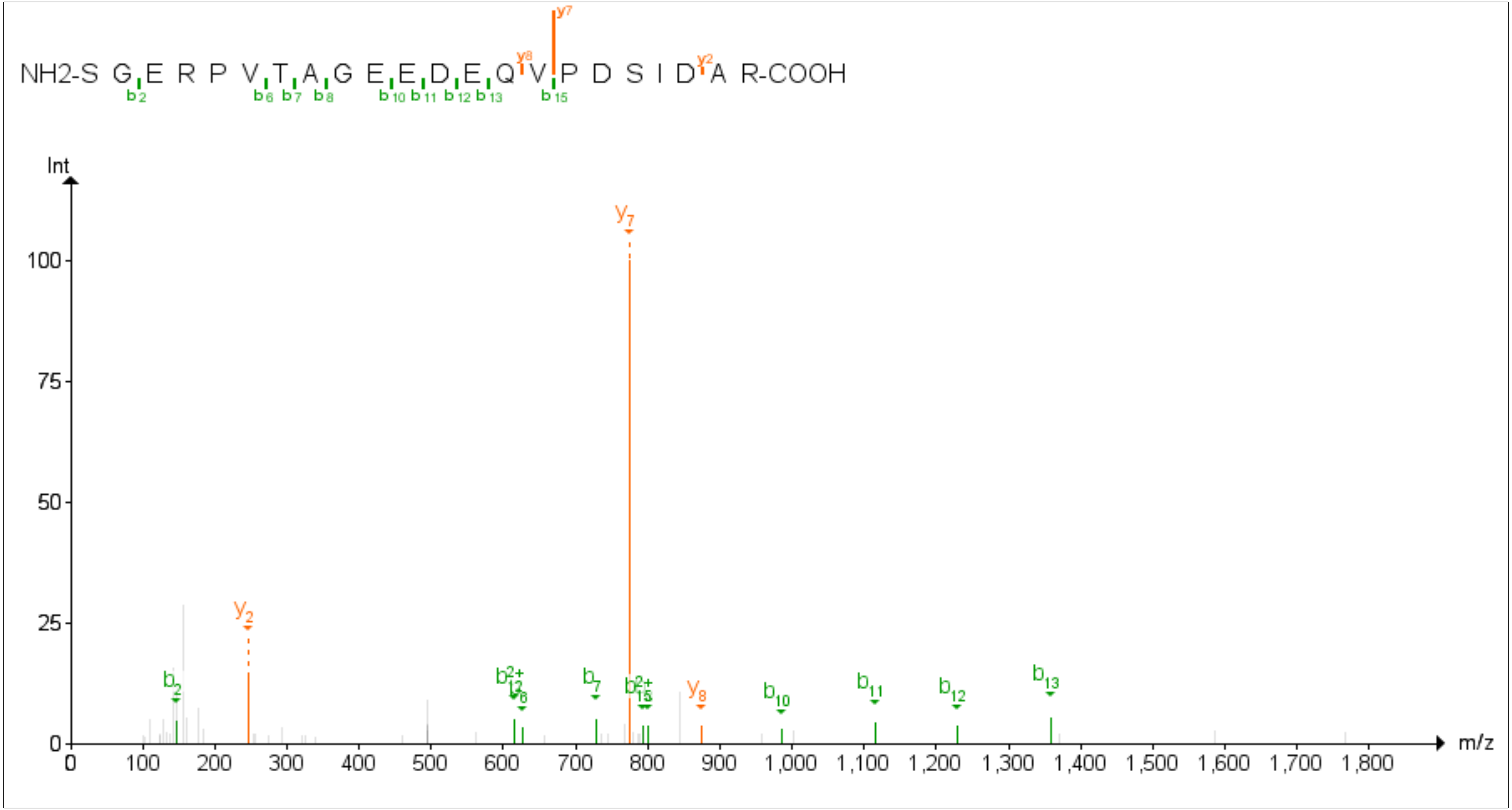

NH2-S G E R P V T A G E E D E Q V P D S I D A R-COOH
y7 y8 y2
b2 b6 b7 b8 b10 b11 b12 b13 b15
Int
100
75
50
25
0
y7
y2
b2
b12 2+
y6
b7
b15 2+
y8
b10
b11
b12
b13
0 100 200 300 400 500 600 700 800 900 1,000 1,100 1,200 1,300 1,400 1,500 1,600 1,700 1,800
m/z

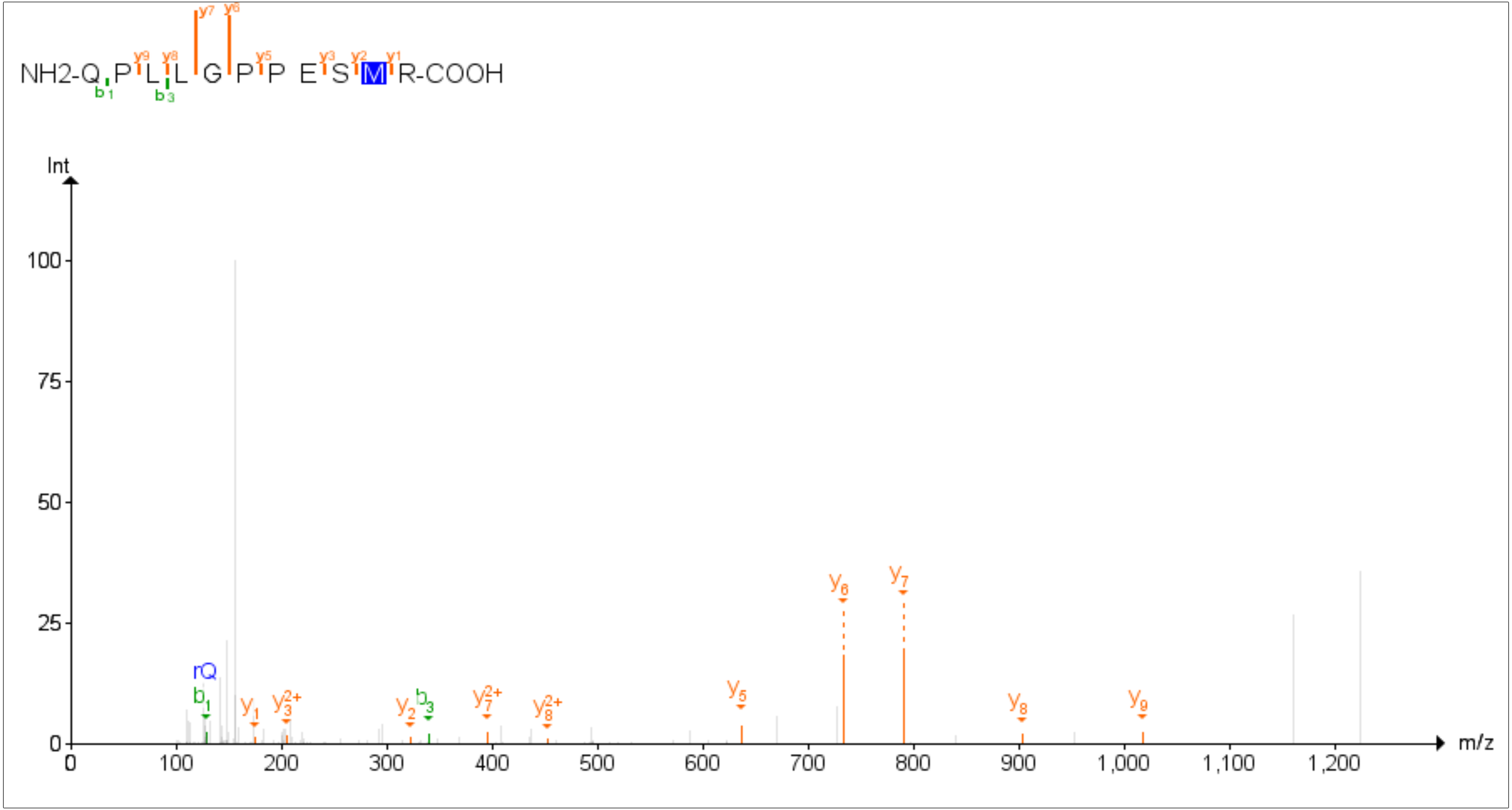

NH2-Q P L L G P P E S M R-COOH
y9 y8 y7 y6 y5 y3 y2 y1
b1 b3
Int
100
75
50
25
0
rQ
b1
y1
y2
b3
y5
y6
y7
y8
y9
0 100 200 300 400 500 600 700 800 900 1,000 1,100 1,200
m/z

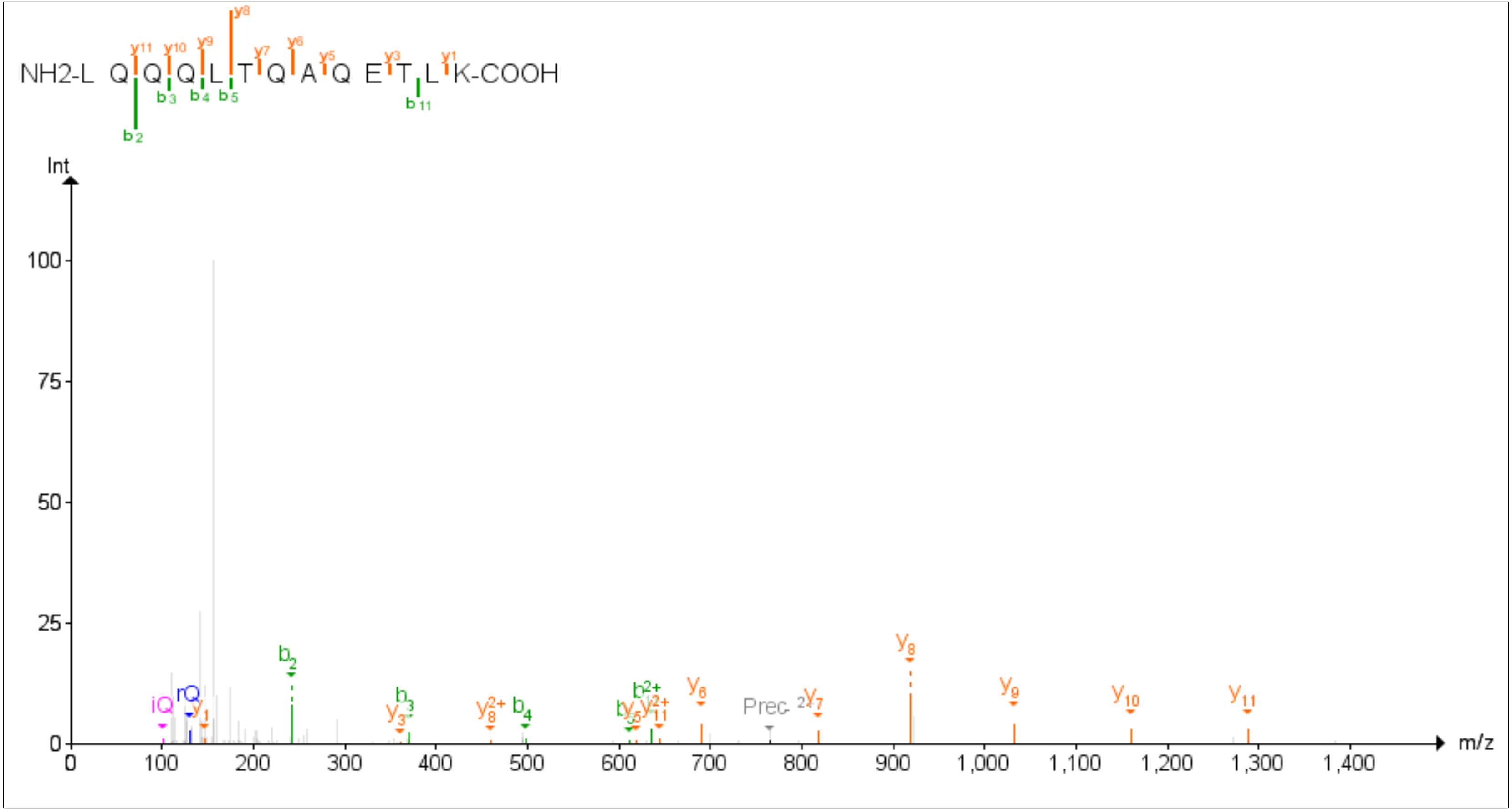

NH2-L Q Q Q L T Q A Q E T L K-COOH
Int
100
75
50
25
0
m/z
0
100
200
300
400
500
600
700
800
900
1,000
1,100
1,200
1,300
1,400
iQ
rQ
Prec

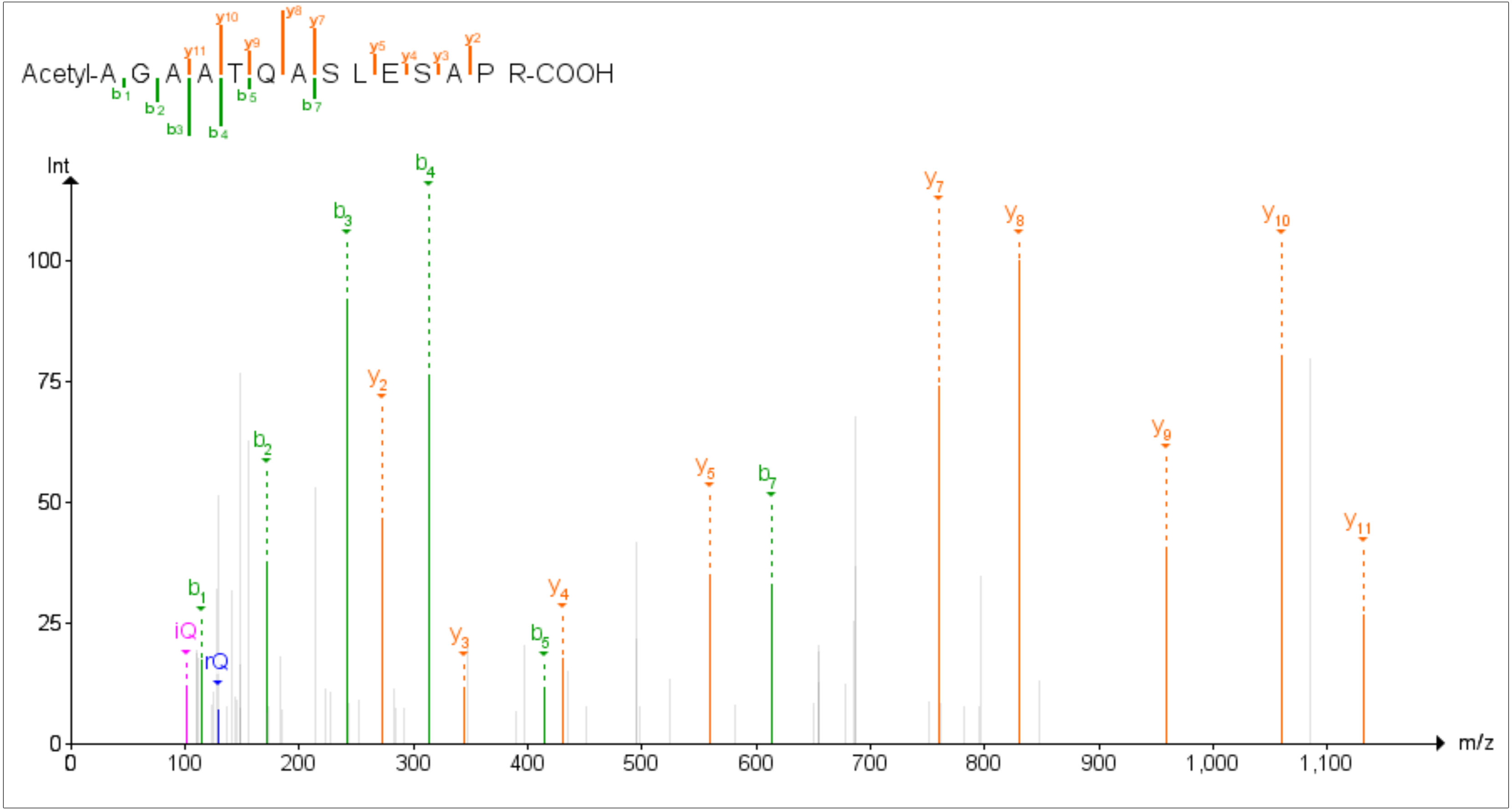

Acetyl-A G A A T Q A S L E S A P R-COOH
b1 b2 b3 b4 b5 b7
y11 y10 y9 y8 y7 y5 y4 y3 y2
Int
m/z
0
25
50
75
100
100
200
300
400
500
600
700
800
900
1,000
1,100
iQ
rQ

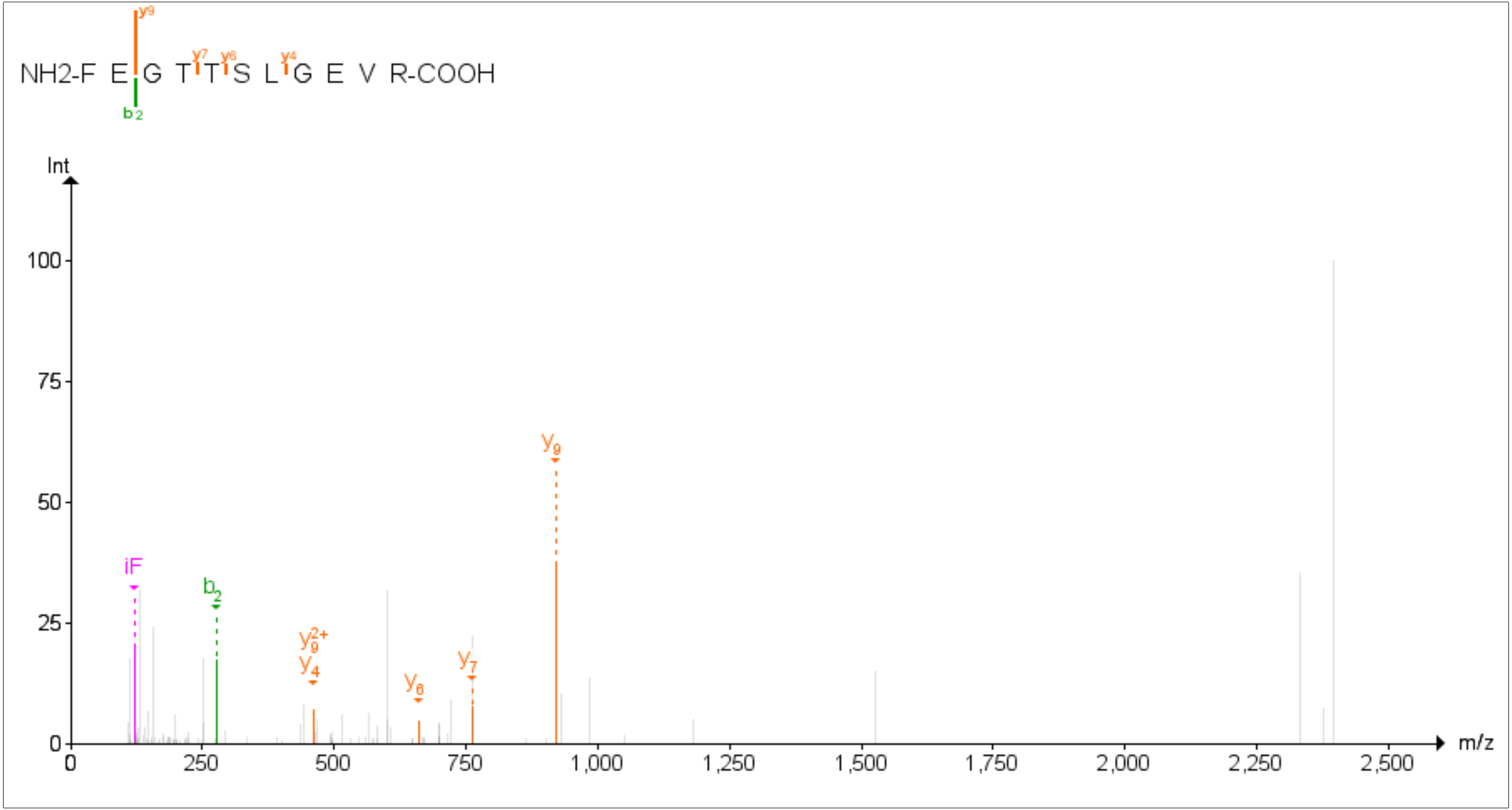

NH2-F E G T T S L G E V R-COOH
y9
y7
y6
y4
b2
Int
100
75
50
25
0
iF
b2
y9
y9 2+
y4
y6
y7
0
250
500
750
1,000
1,250
1,500
1,750
2,000
2,250
2,500
m/z

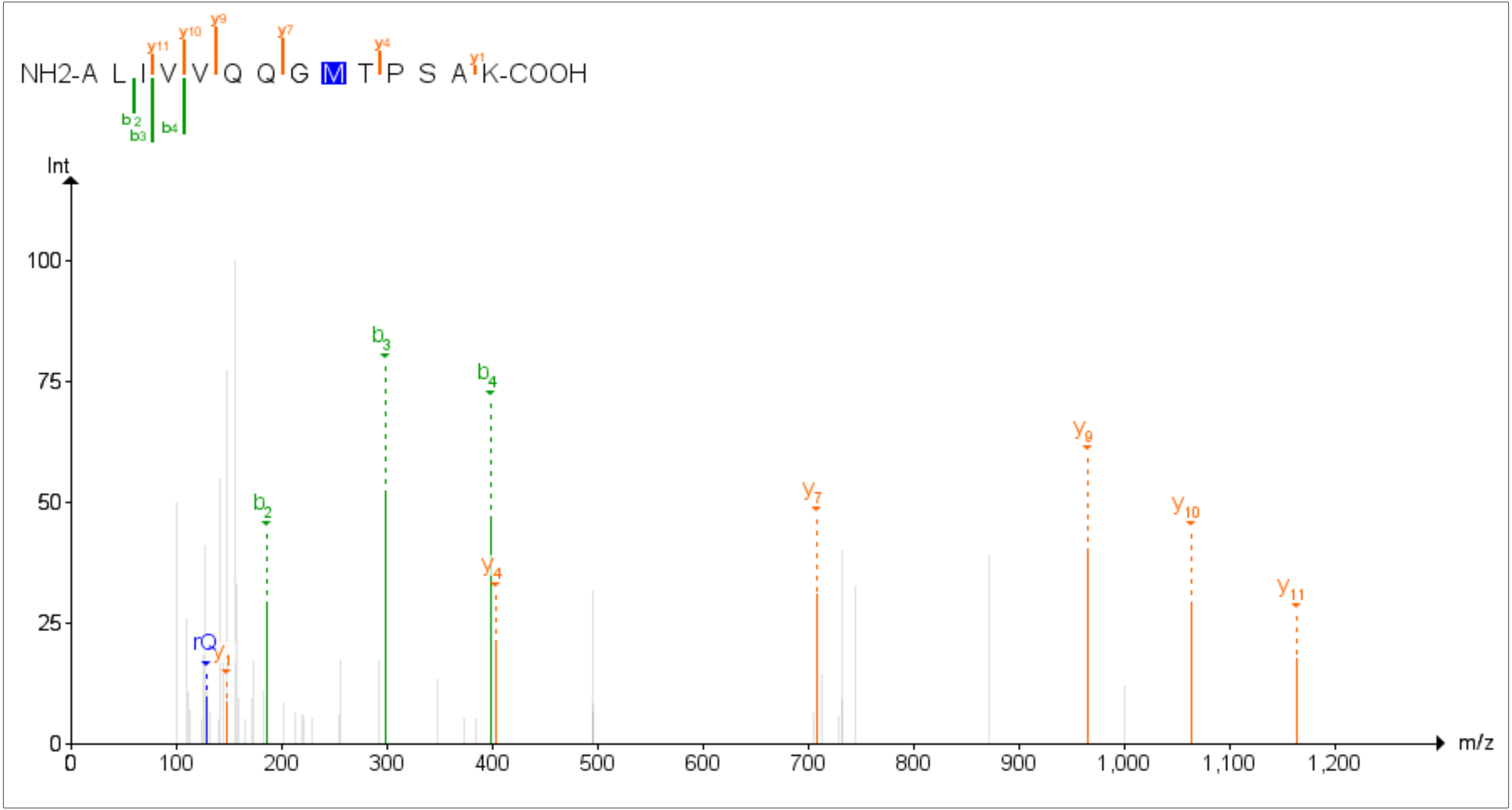
NH2-A L I V V Q Q G M T P S A K-COOH
y11 y10 y9 y7 y4 y1
b2 b3 b4
Int
100
75
50
25
0
b3
b4
b2
y4
y7
y9
y10
y11
rQ
y1
0 100 200 300 400 500 600 700 800 900 1,000 1,100 1,200
m/z

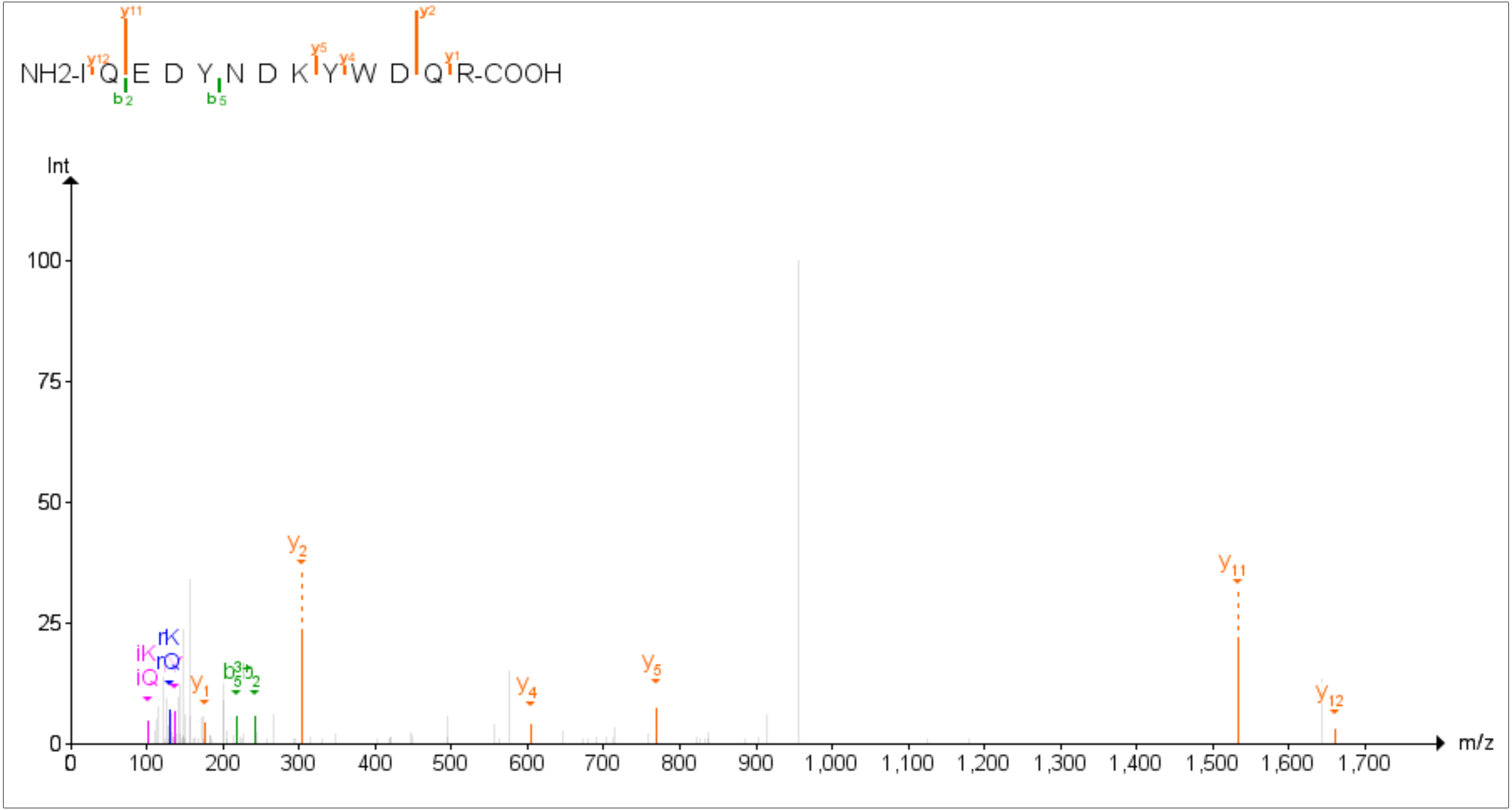

NH2-I Q E D Y N D K Y W D Q R-COOH
y11
y12
y2
y5
y4
y1
b2
b5
Int
100
75
50
25
0
m/z
0
100
200
300
400
500
600
700
800
900
1,000
1,100
1,200
1,300
1,400
1,500
1,600
1,700

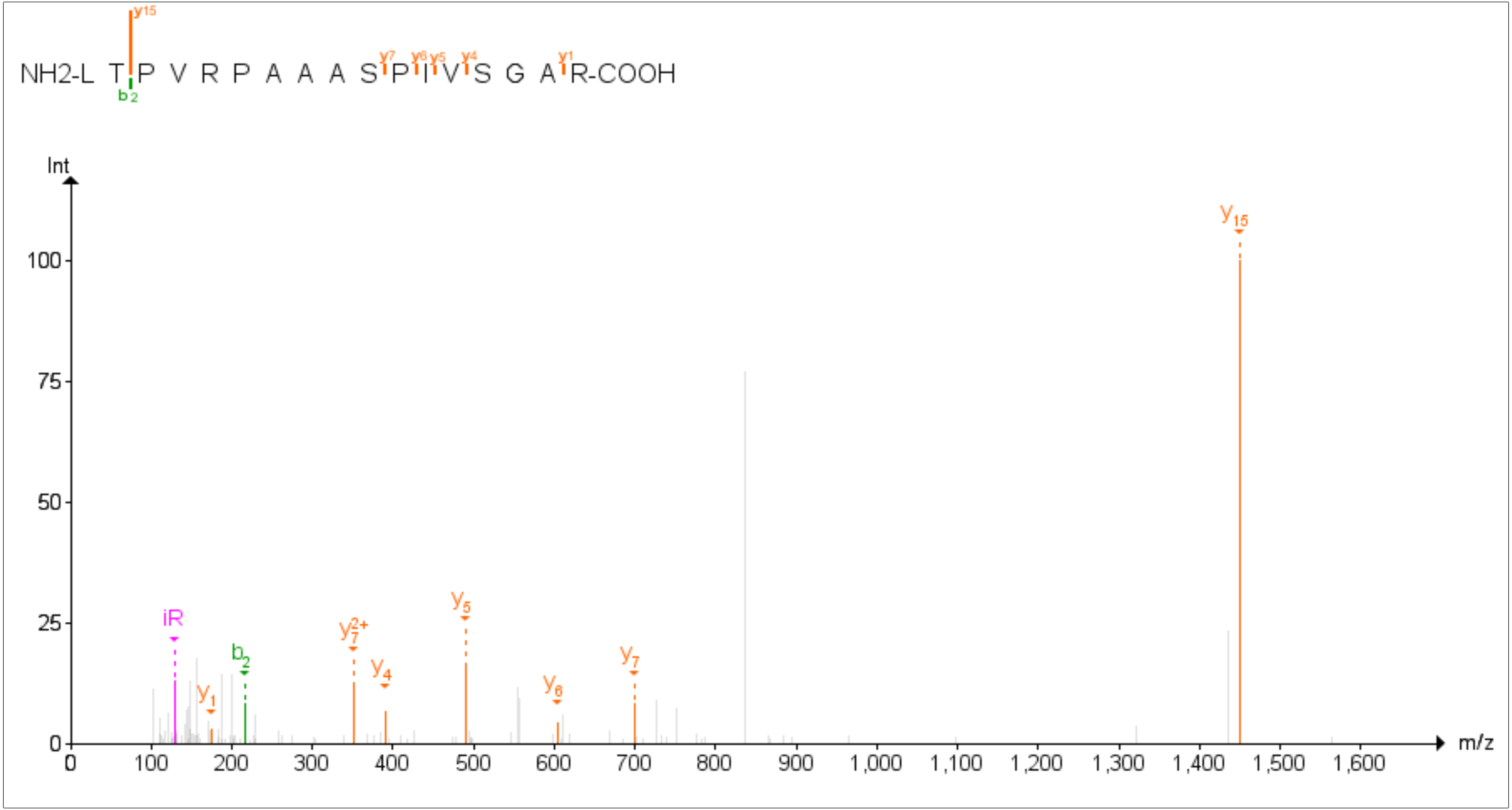
NH2-L T P V R P A A A S P I V S G A R-COOH
y15
b2
y7
y6
y5
y4
y1
Int
100
75
50
25
0
iR
y1
b2
y7 2+
y4
y5
y6
y7
y15
0
100
200
300
400
500
600
700
800
900
1,000
1,100
1,200
1,300
1,400
1,500
1,600
m/z

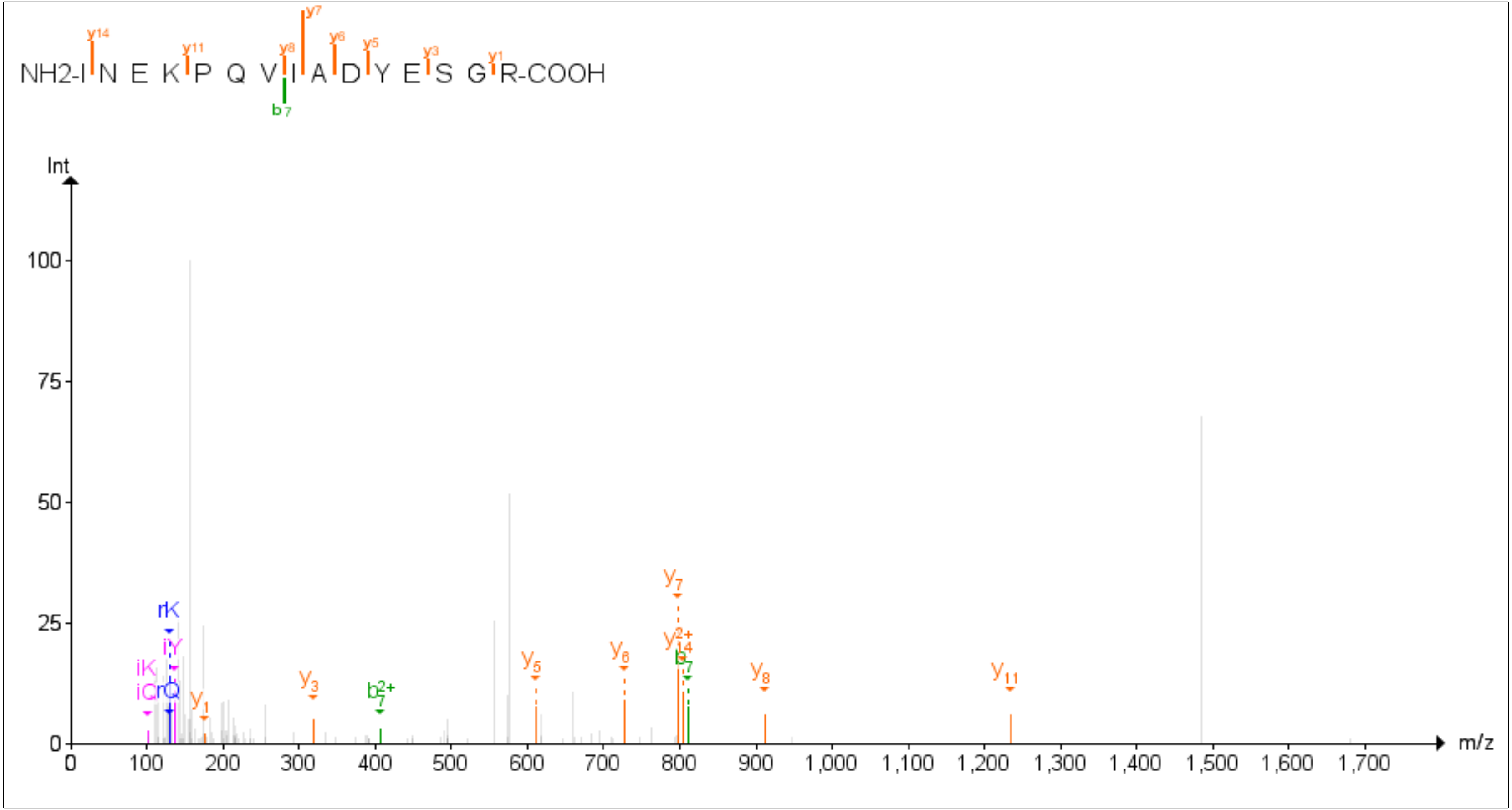

NH2-I N E K P Q V I A D Y E S G R-COOH
y14
y11
y8
y7
y6
y5
y3
y1
b7
Int
m/z
100
75
50
25
0
rK
iY
iK
iQ
rQ
y1
y3
y5
y6
y7
y8
y11

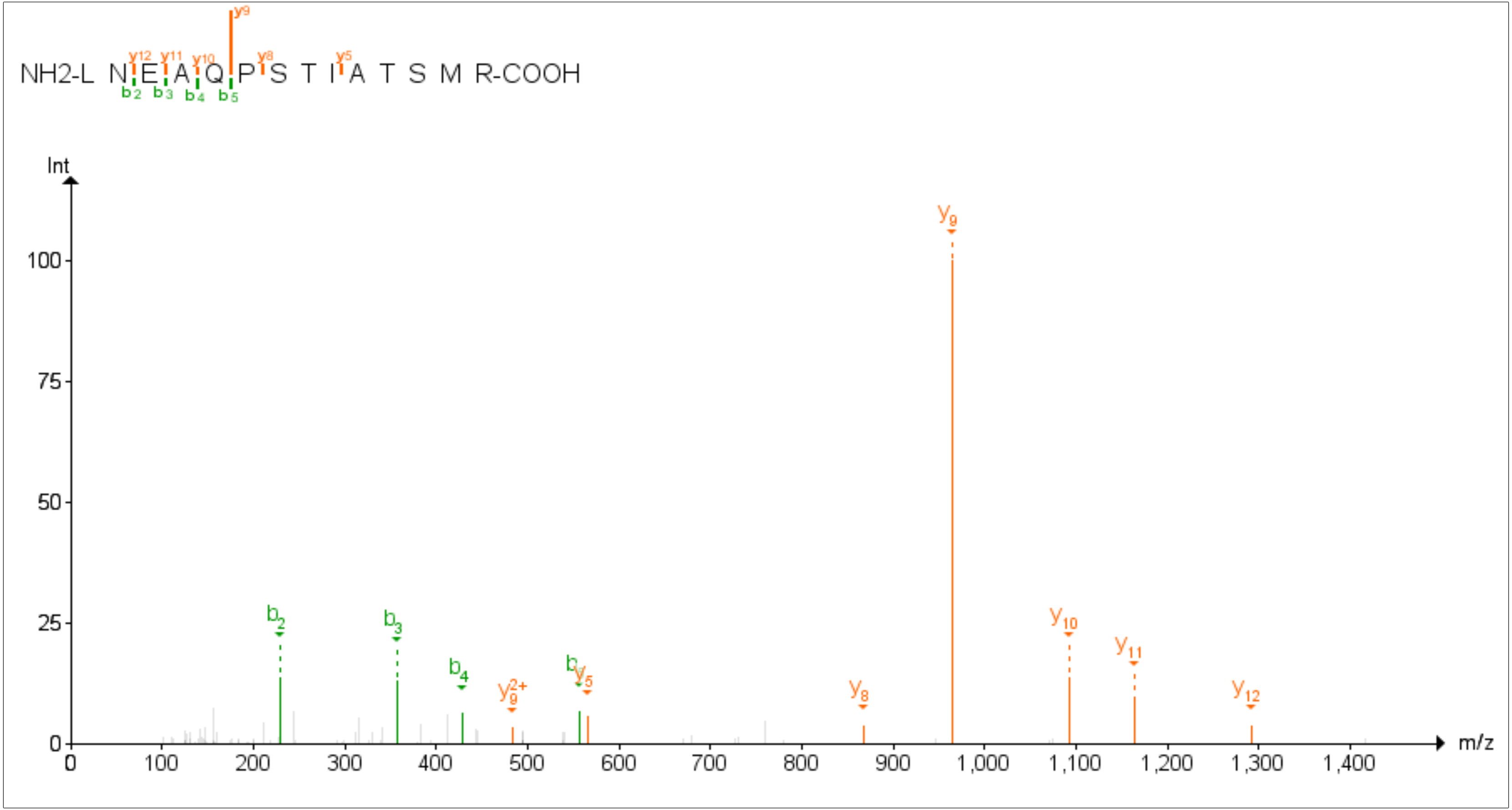

NH2-L N E A Q P S T I A T S M R-COOH
y12 y11 y10 y9 y8 y5
b2 b3 b4 b5
Int
100
75
50
25
0
y9
b2
b3
b4
y9 2+
b5
y5
y8
y10
y11
y12
0
100
200
300
400
500
600
700
800
900
1,000
1,100
1,200
1,300
1,400
m/z

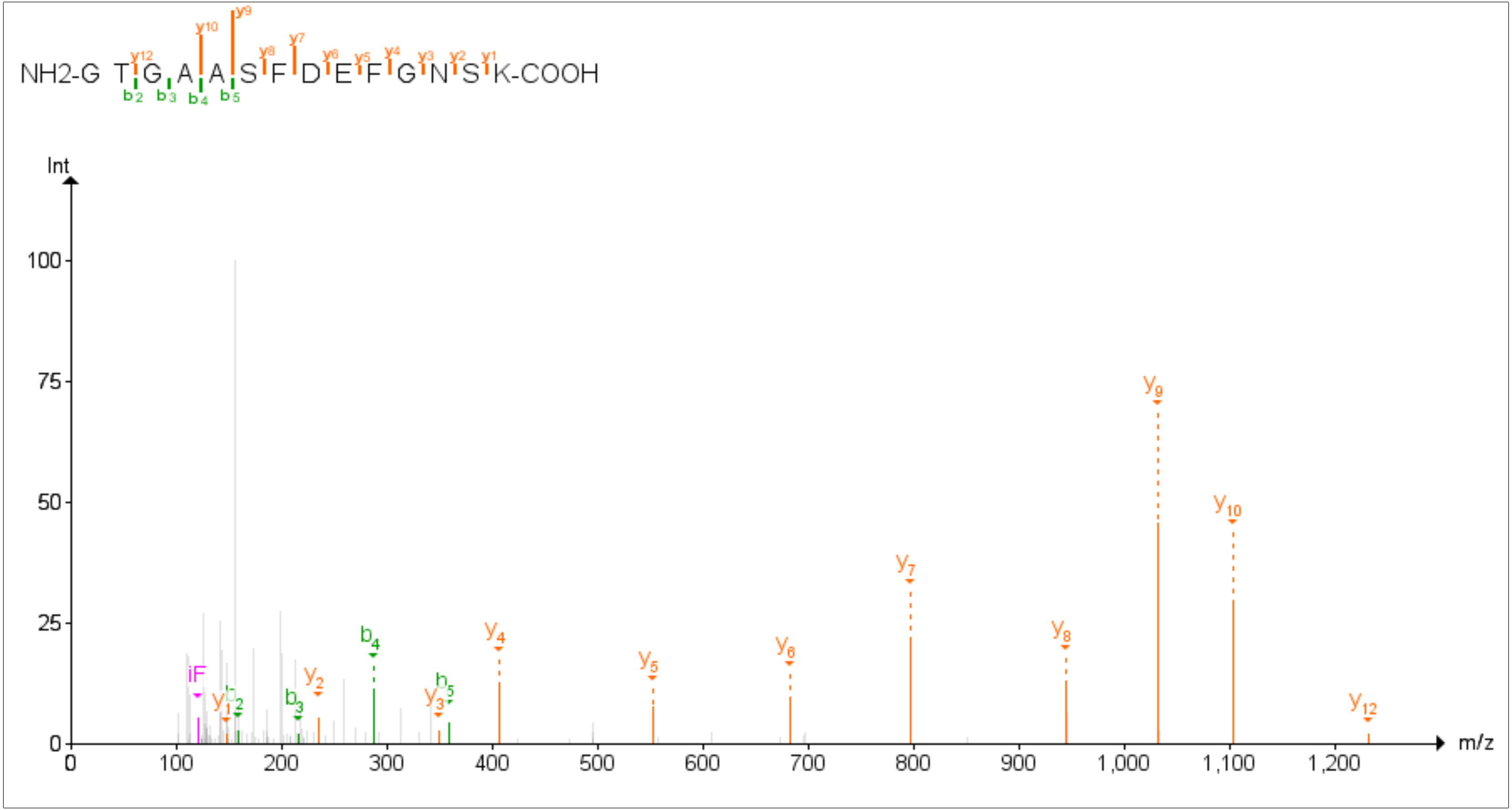

NH2-G T G A A S F D E F G N S K-COOH
y12 y10 y9 y8 y7 y6 y5 y4 y3 y2 y1
b2 b3 b4 b5
Int
100
75
50
25
0
m/z
0
100
200
300
400
500
600
700
800
900
1,000
1,100
1,200
iF
y1
b2
y2
b3
b4
y3
b5
y4
y5
y6
y7
y8
y9
y10
y12

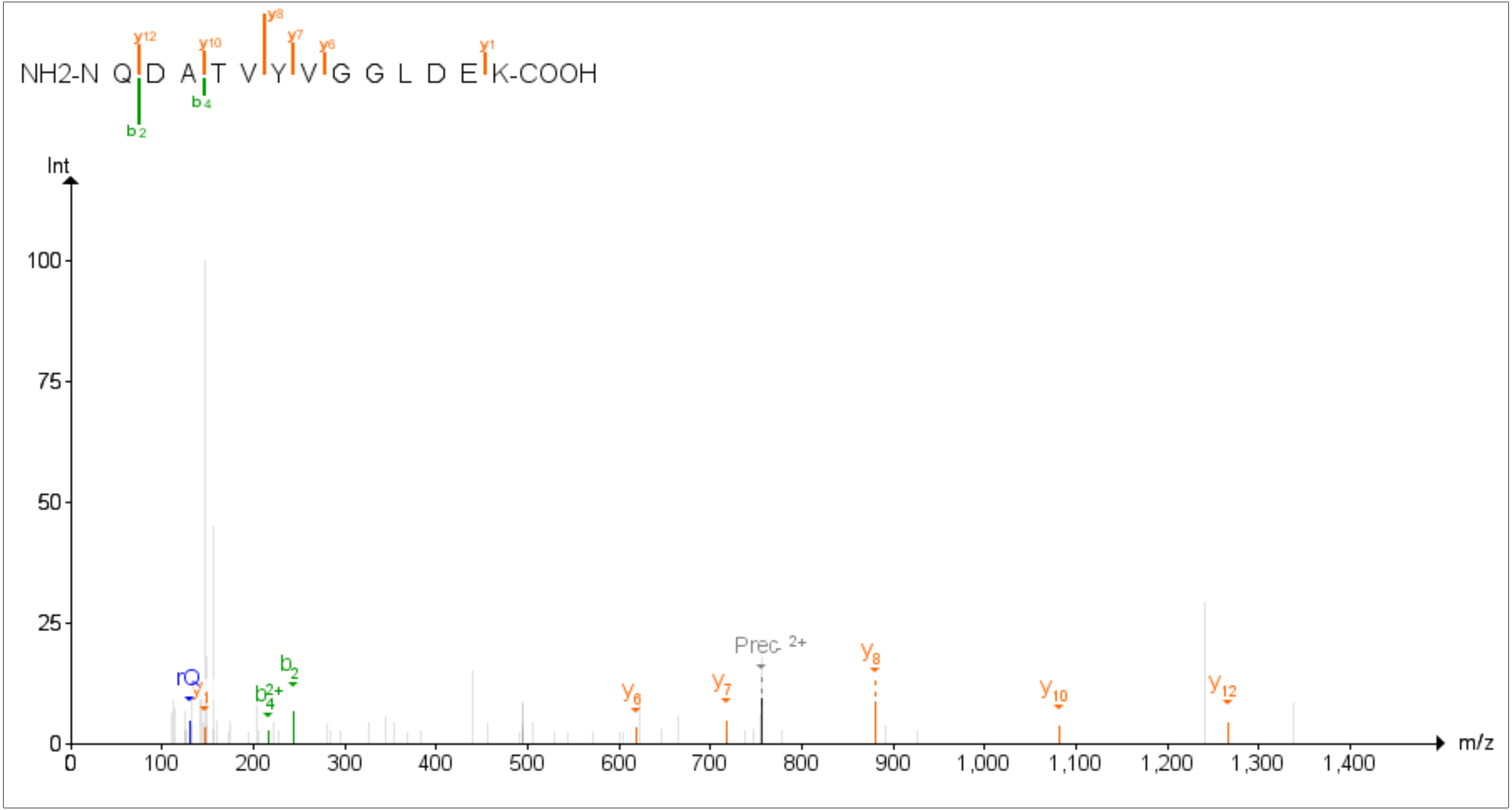

NH2-N Q D A T V Y V G G L D E K-COOH
y12
y10
y8
y7
y6
y1
b2
b4
Int
100
75
50
25
0
rQ
y1
b4 2+
b2
Prec 2+
y6
y7
y8
y10
y12
0
100
200
300
400
500
600
700
800
900
1,000
1,100
1,200
1,300
1,400
m/z

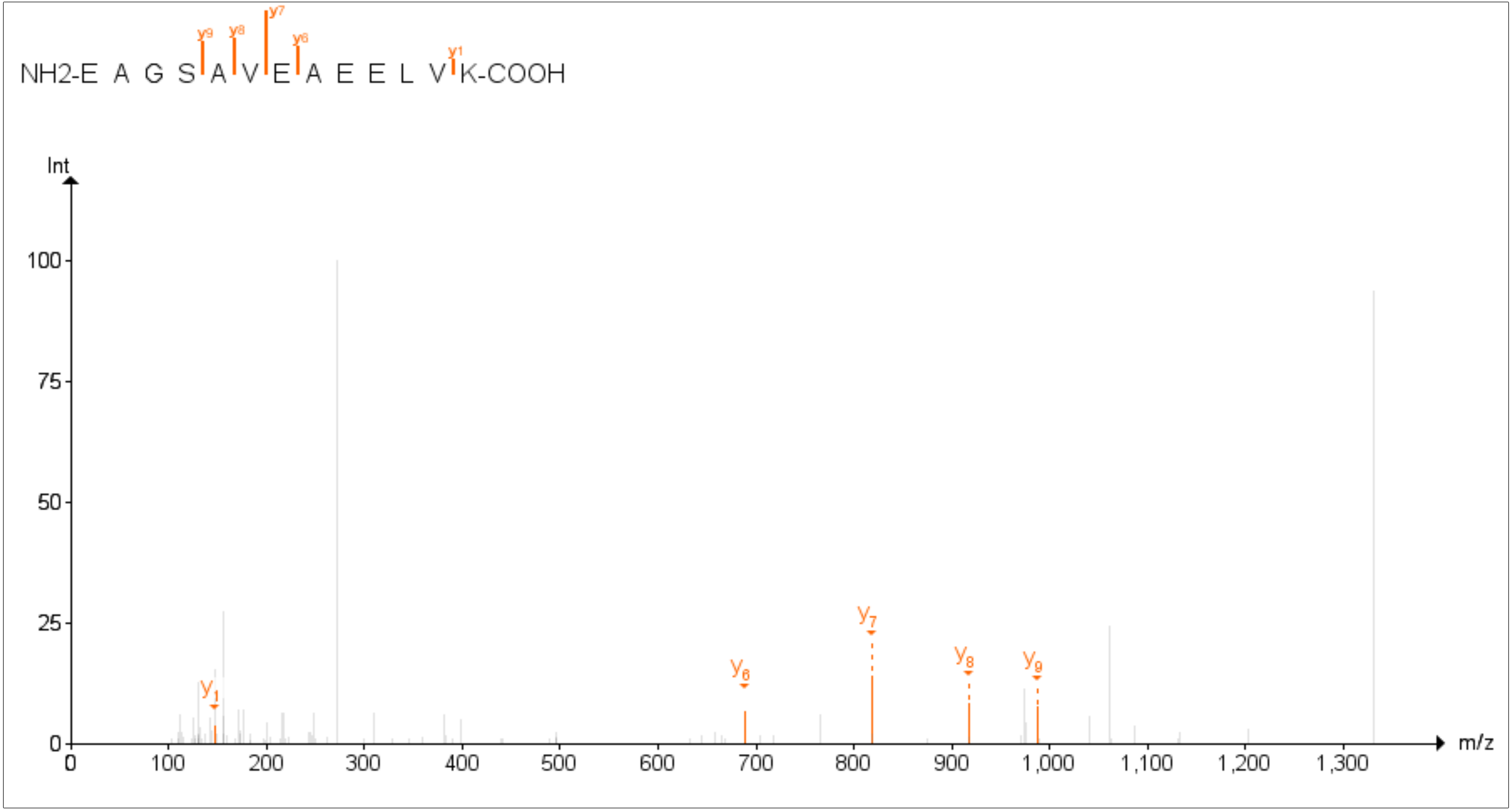

NH2-E A G S A V E A E E L V K-COOH
y9
y8
y7
y6
y1
Int
100
75
50
25
0
0
100
200
300
400
500
600
700
800
900
1,000
1,100
1,200
1,300
m/z
y1
y6
y7
y8
y9

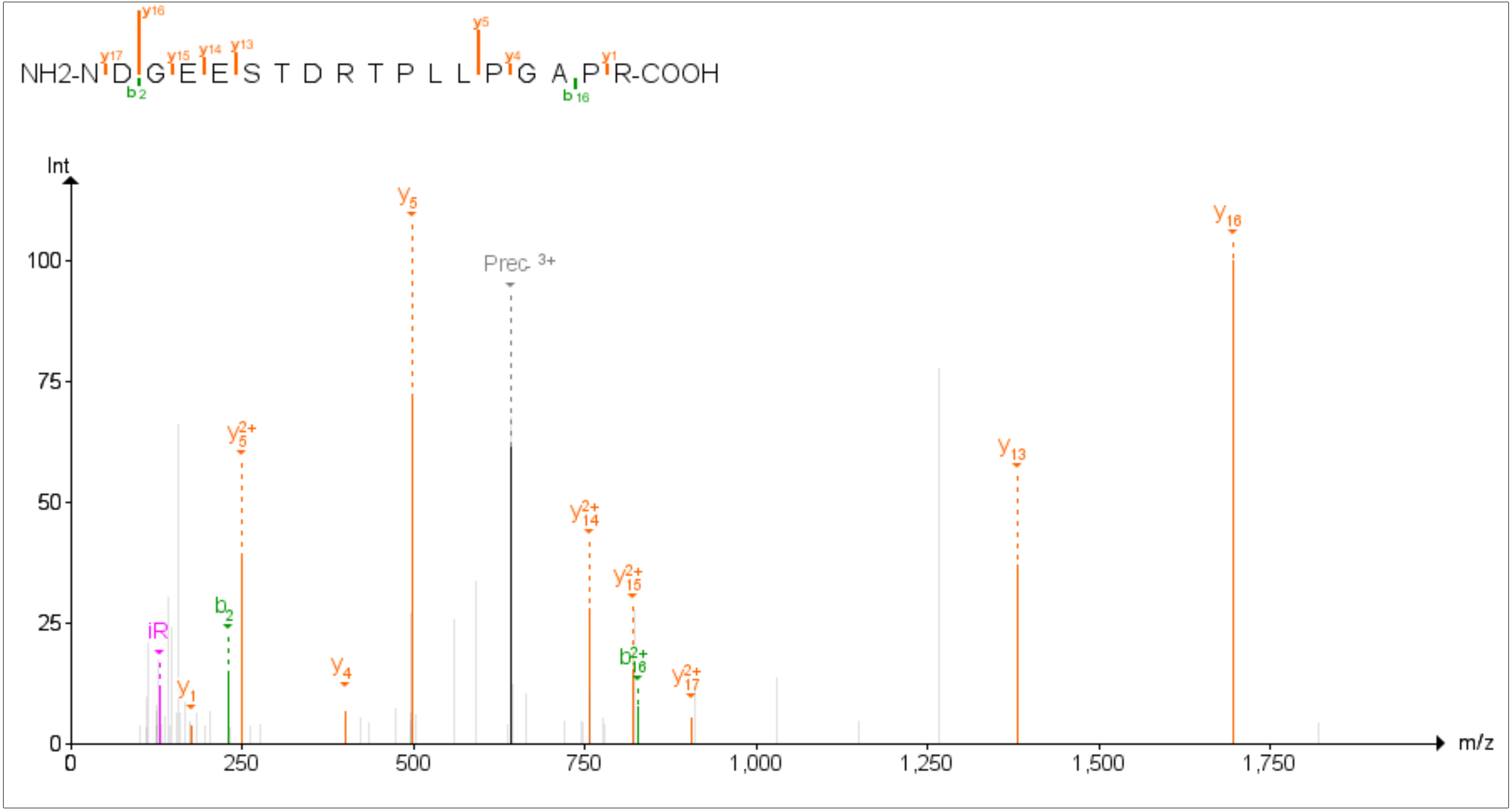

NH2-N D G E E S T D R T P L L P G A P R-COOH
y16
y17
y15
y14
y13
y5
y4
y1
b 2
b 16
Int
100
75
50
25
0
y5
Prec 3+
y16
y5 2+
y13
y14 2+
y15 2+
b2
iR
y1
y4
b16 2+
y17 2+
0
250
500
750
1,000
1,250
1,500
1,750
m/z

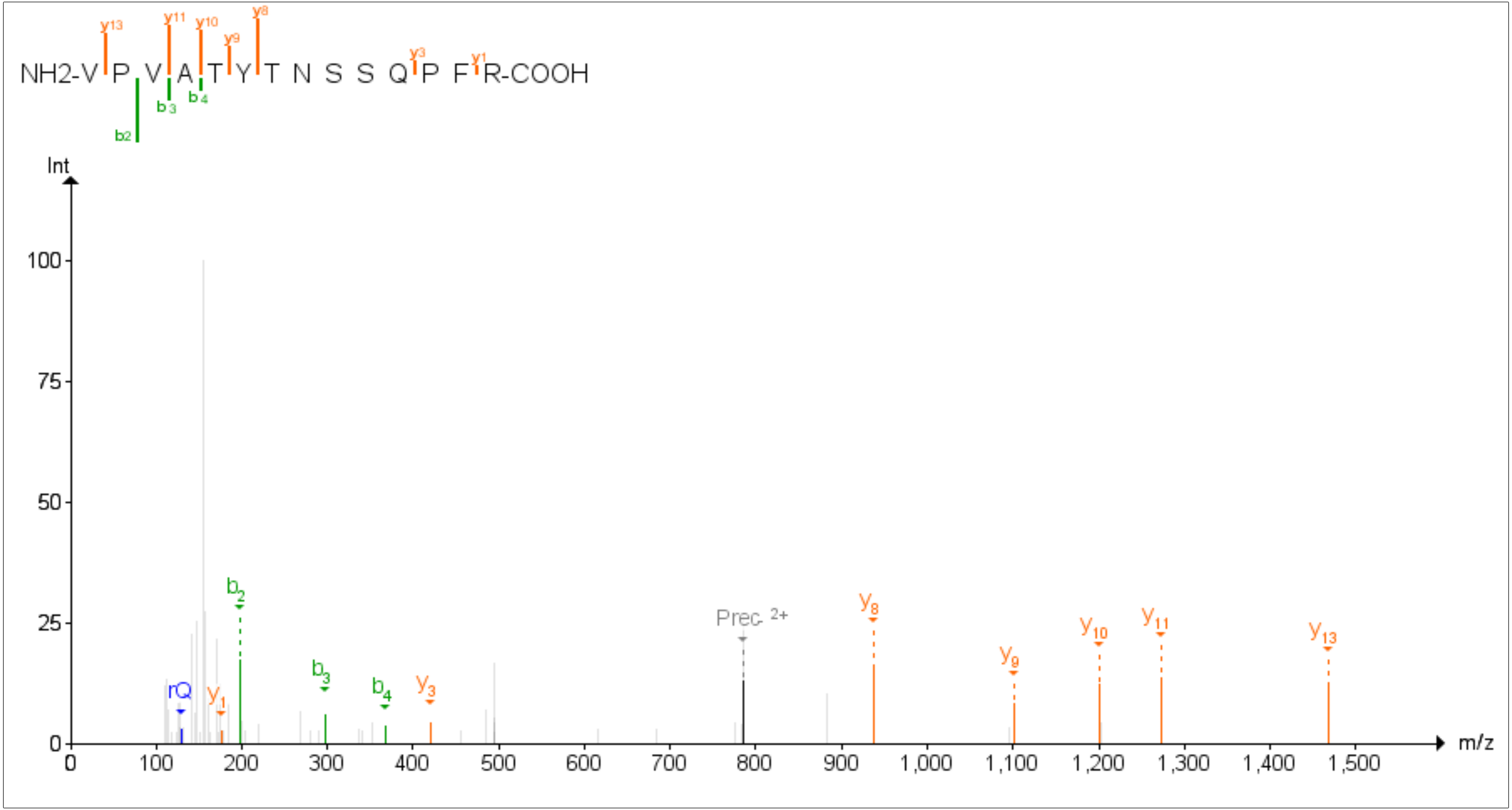
NH2-V P V A T Y T N S S Q P F R-COOH
y13 y11 y10 y9 y8 y3 y1
b2 b3 b4
Int
100
75
50
25
0
rQ
y1
b2
b3
b4
y3
Prec 2+
y8
y9
y10
y11
y13
0 100 200 300 400 500 600 700 800 900 1,000 1,100 1,200 1,300 1,400 1,500
m/z

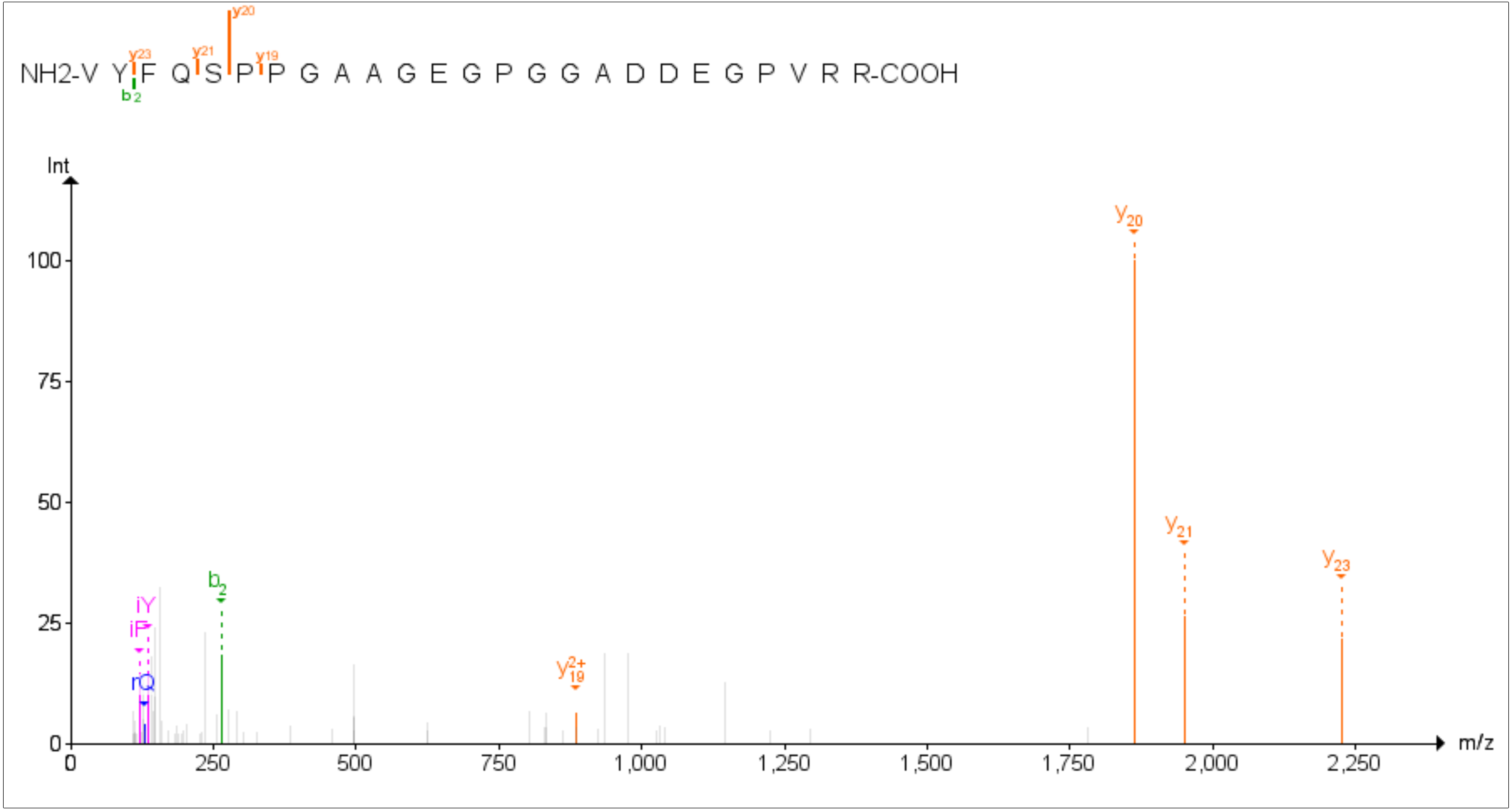
NH2-V Y F Q S P P G A A G E G P G G A D D E G P V R R-COOH
y23
y21
y20
y19
b2
Int
100
75
50
25
0
iY
iF
rQ
b2
y19 2+
y20
y21
y23
0
250
500
750
1,000
1,250
1,500
1,750
2,000
2,250
m/z

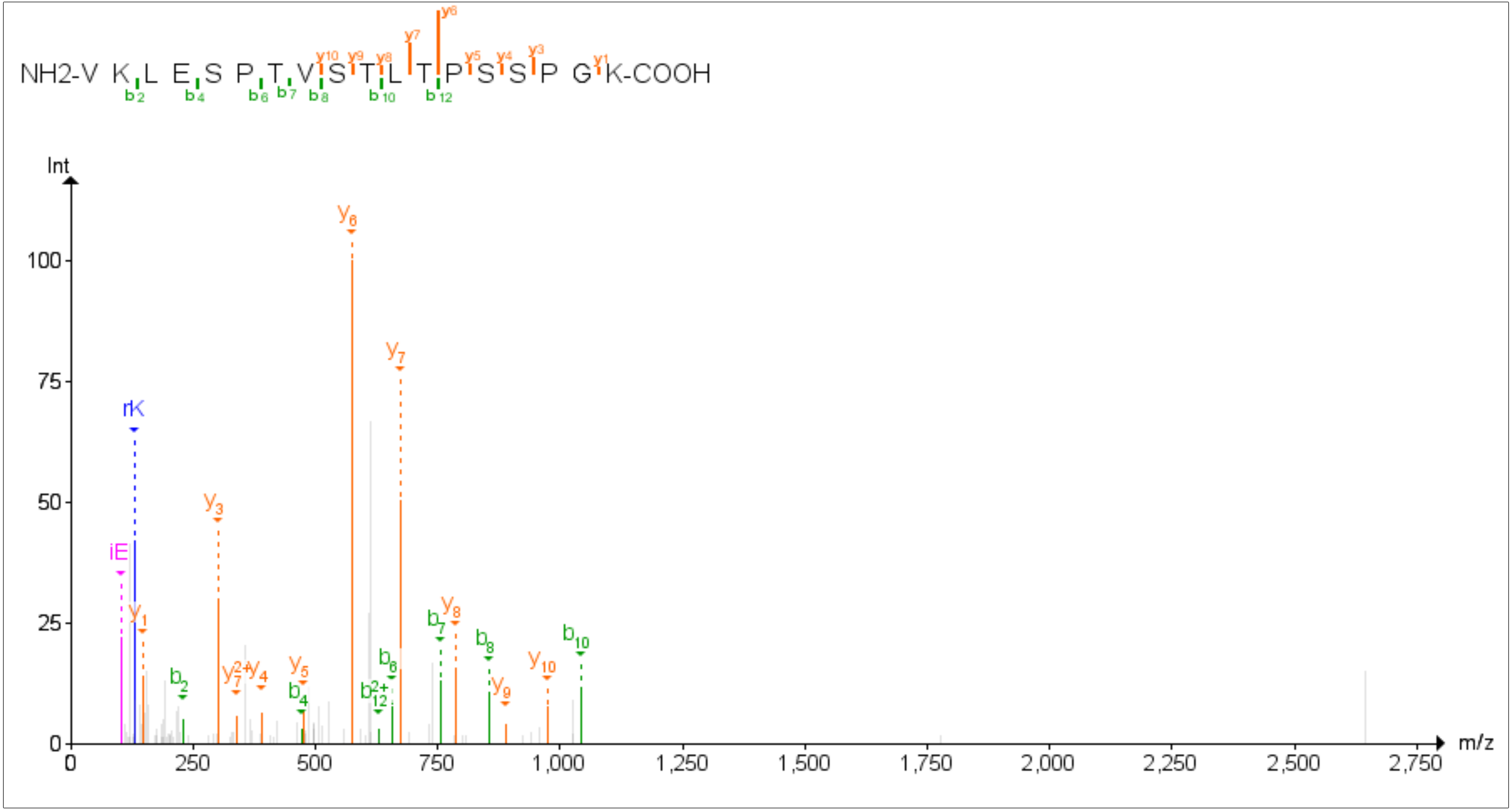

NH2-V K L E S P T V S T L T P S S P G K-COOH
Int
m/z

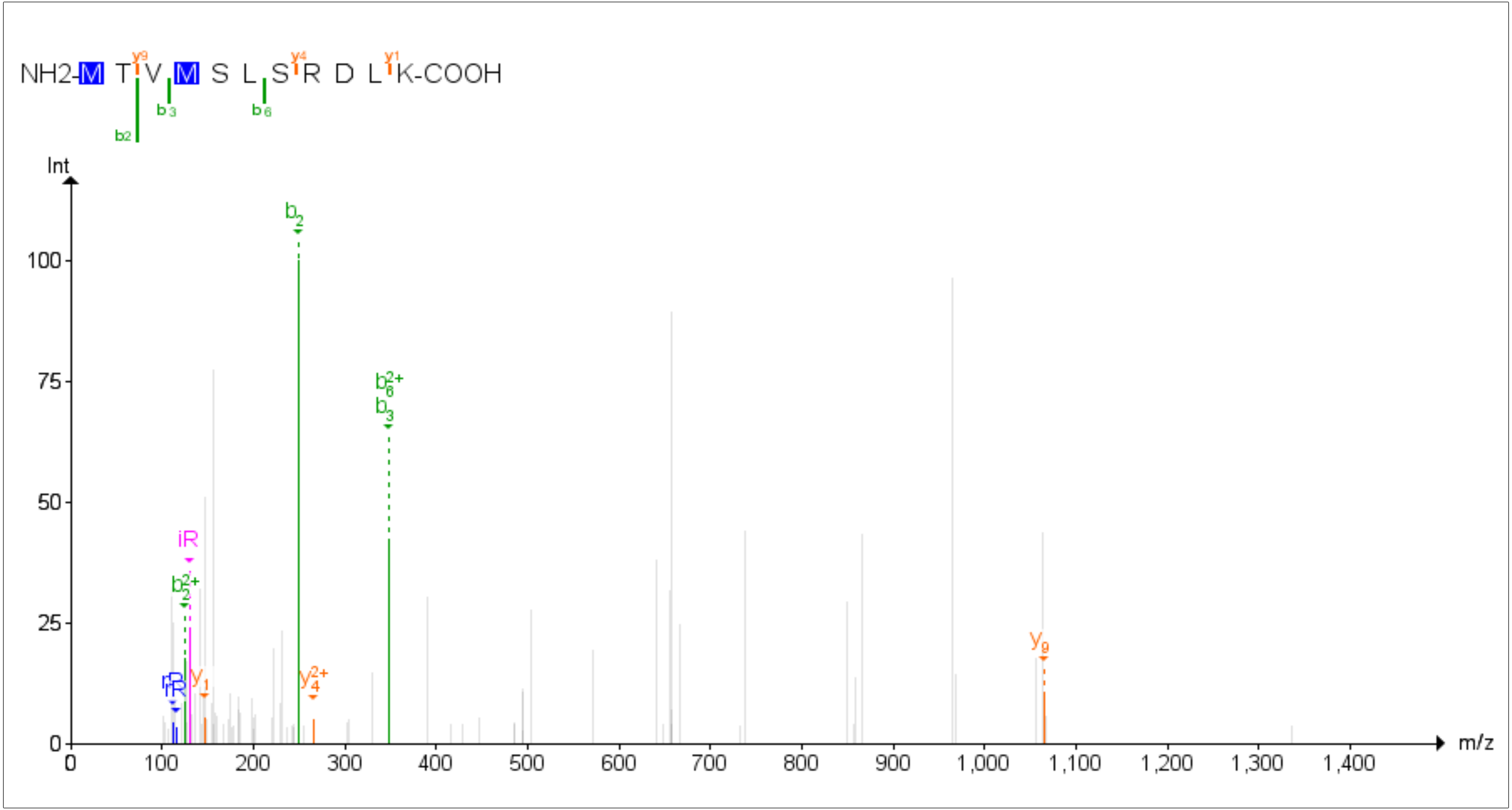

NH2-M T V M S L S R D L K-COOH
y9
y4
y1
b2
b3
b6
Int
m/z
b2
b6 2+
b3
iR
b2 2+
iR
iR
y1
y4 2+
y9
0
25
50
75
100
0
100
200
300
400
500
600
700
800
900
1,000
1,100
1,200
1,300
1,400

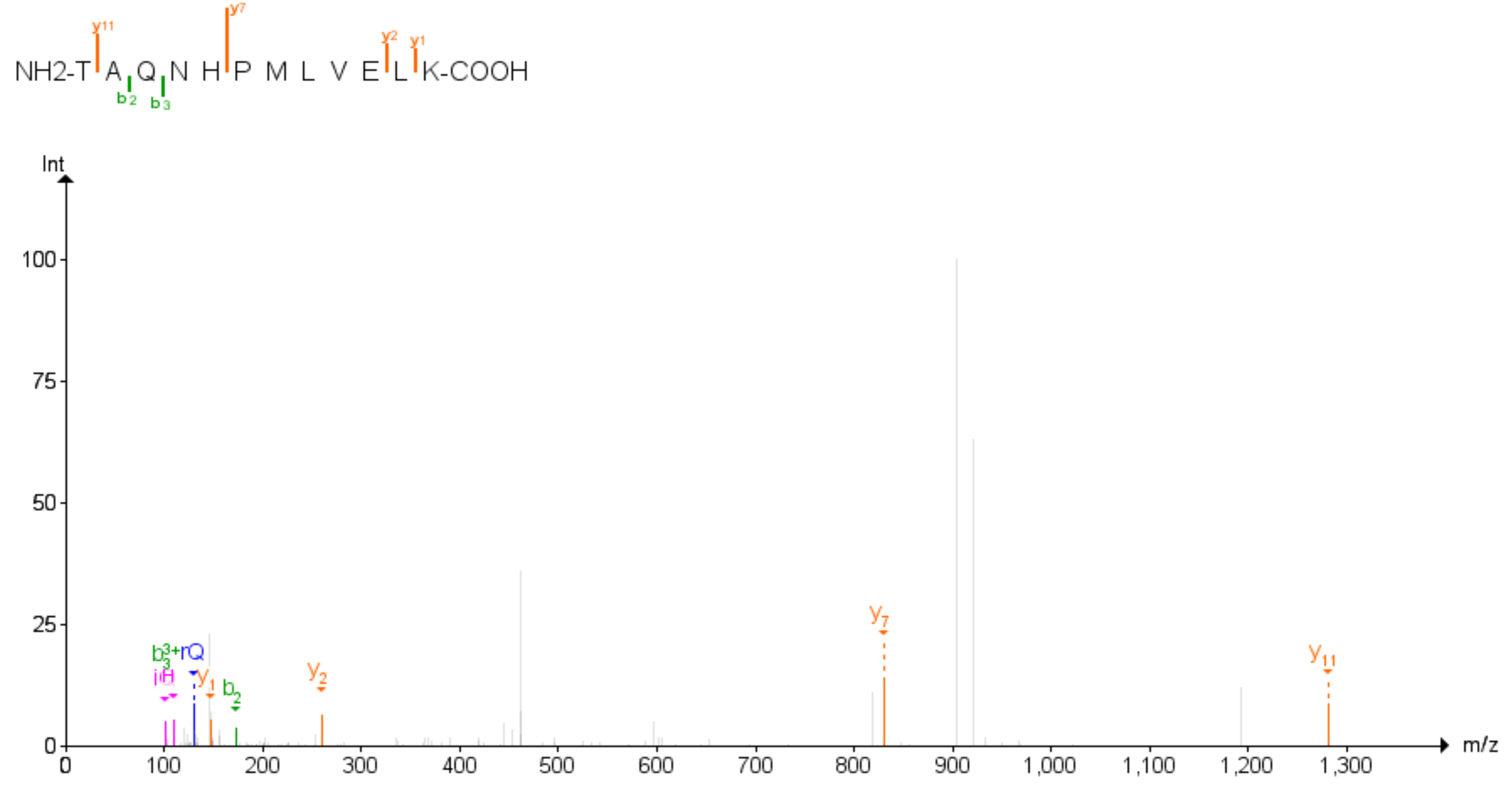

NH2-T A Q N H P M L V E L K-COOH
y11
y7
y2
y1
b2
b3
Int
100
75
50
25
0
b3+rQ
iH
y1
b2
y2
y7
y11
m/z
0
100
200
300
400
500
600
700
800
900
1,000
1,100
1,200
1,300

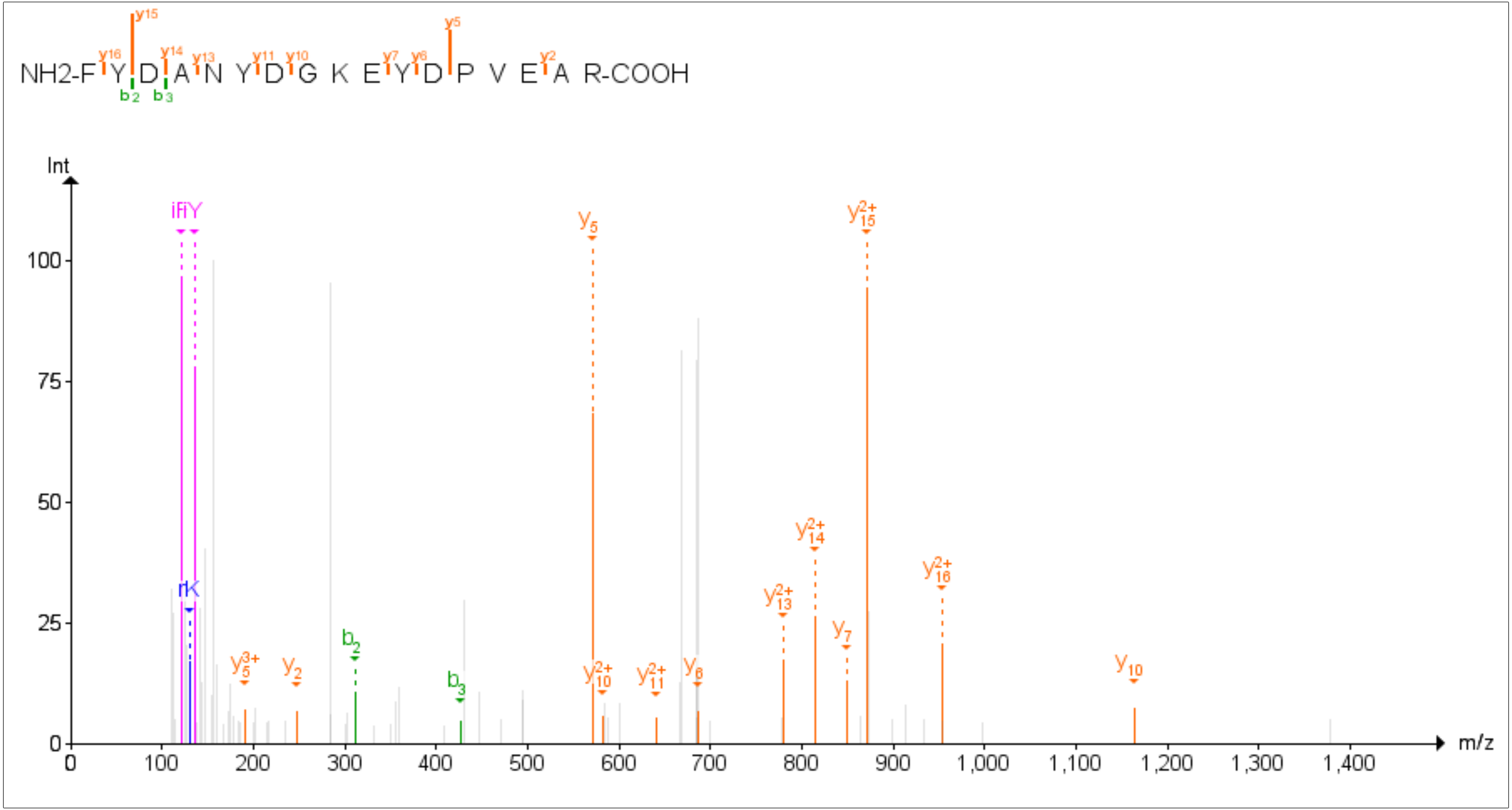

NH2-F Y D A N Y D G K E Y D P V E A R-COOH
y16 y15 y14 y13 y11 y10 y7 y6 y5 y2
b2 b3
Int
m/z
iFiY
rK
y_5
y_{15}^{2+}
y_{14}^{2+}
y_{13}^{2+}
y_{16}^{2+}
y_7
y_{5}^{3+}
y_2
b_2
b_3
y_{10}^{2+}
y_{11}^{2+}
y_6
y_{10}
0
25
50
75
100
0
100
200
300
400
500
600
700
800
900
1,000
1,100
1,200
1,300
1,400

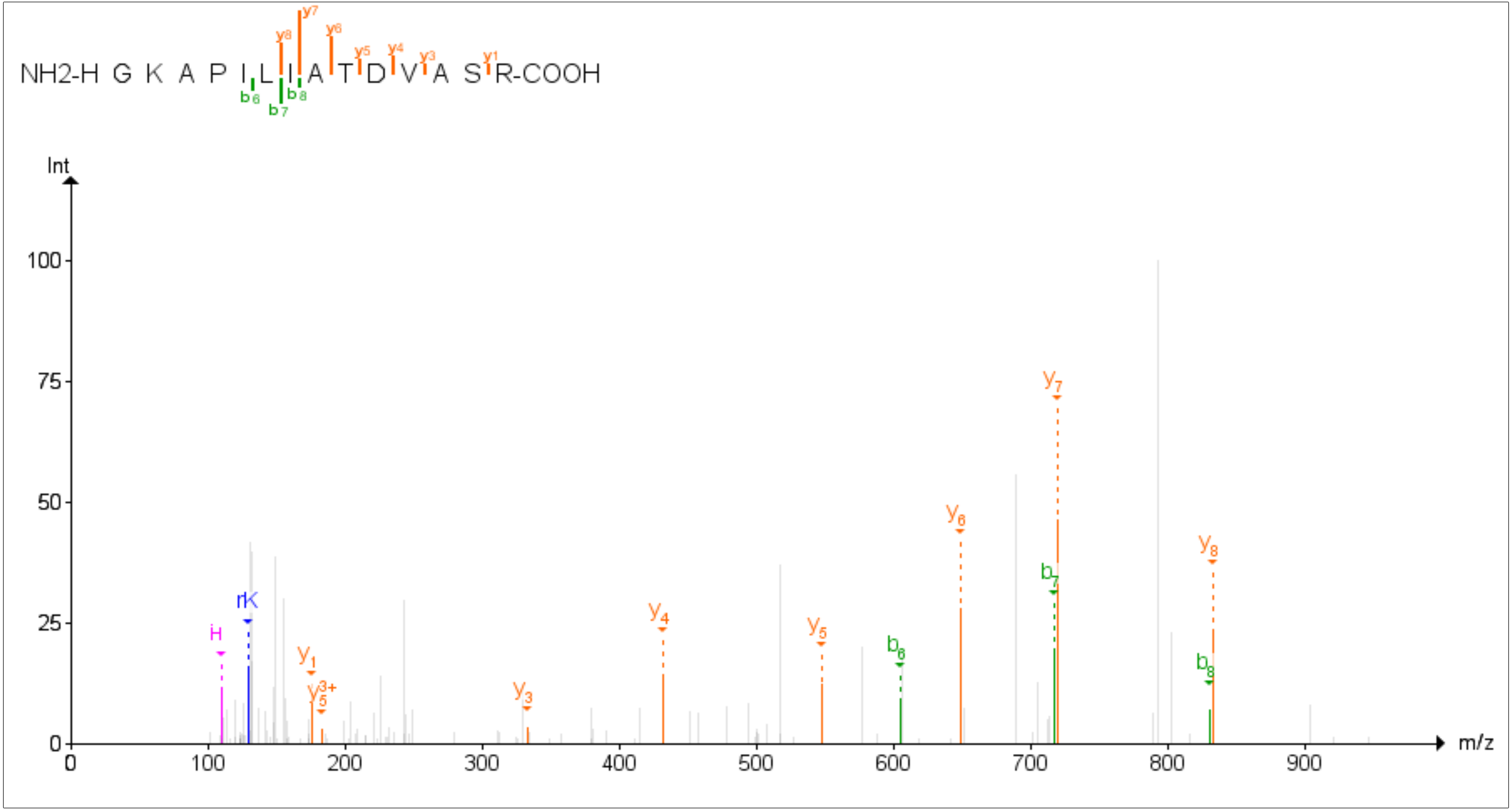
NH2-H G K A P I L I A T D V A S R-COOH
y8 y7 y6 y5 y4 y3 y1
b6 b7 b8
Int
100
75
50
25
0
m/z
0 100 200 300 400 500 600 700 800 900
iH
rK
y1
y5 3+
y3
y4
y5
b6
y6
b7
y7
b8
y8

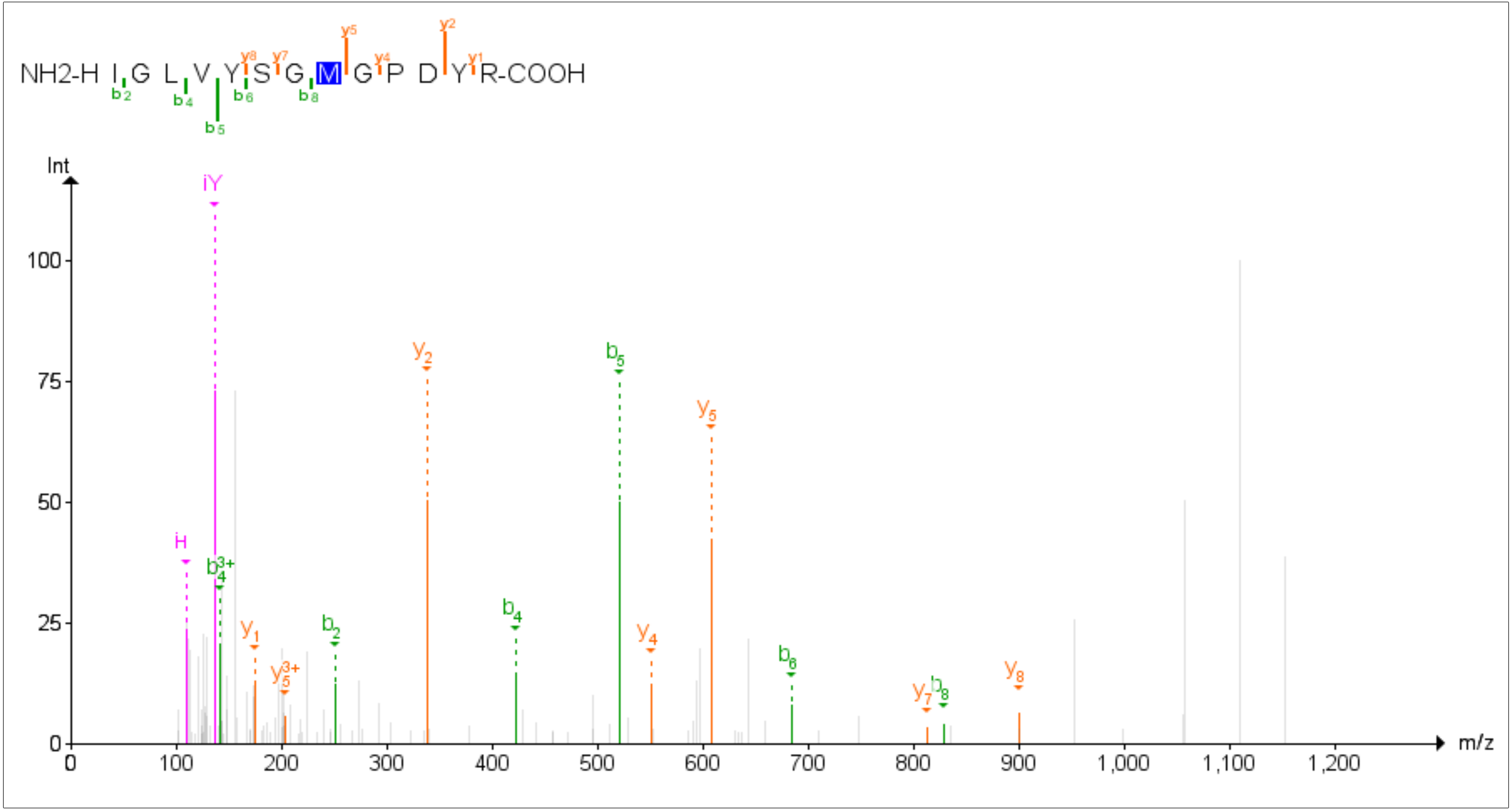

NH2-H I G L V Y S G M G P D Y R-COOH
b2
b4
b5
b6
b8
y8
y7
y5
y4
y2
y1
Int
iY
iH
y2
b5
y5
b4
y4
b6
b2
y1
y5 3+
b4 3+
y7
b8
y8
m/z
0
25
50
75
100
0
100
200
300
400
500
600
700
800
900
1,000
1,100
1,200

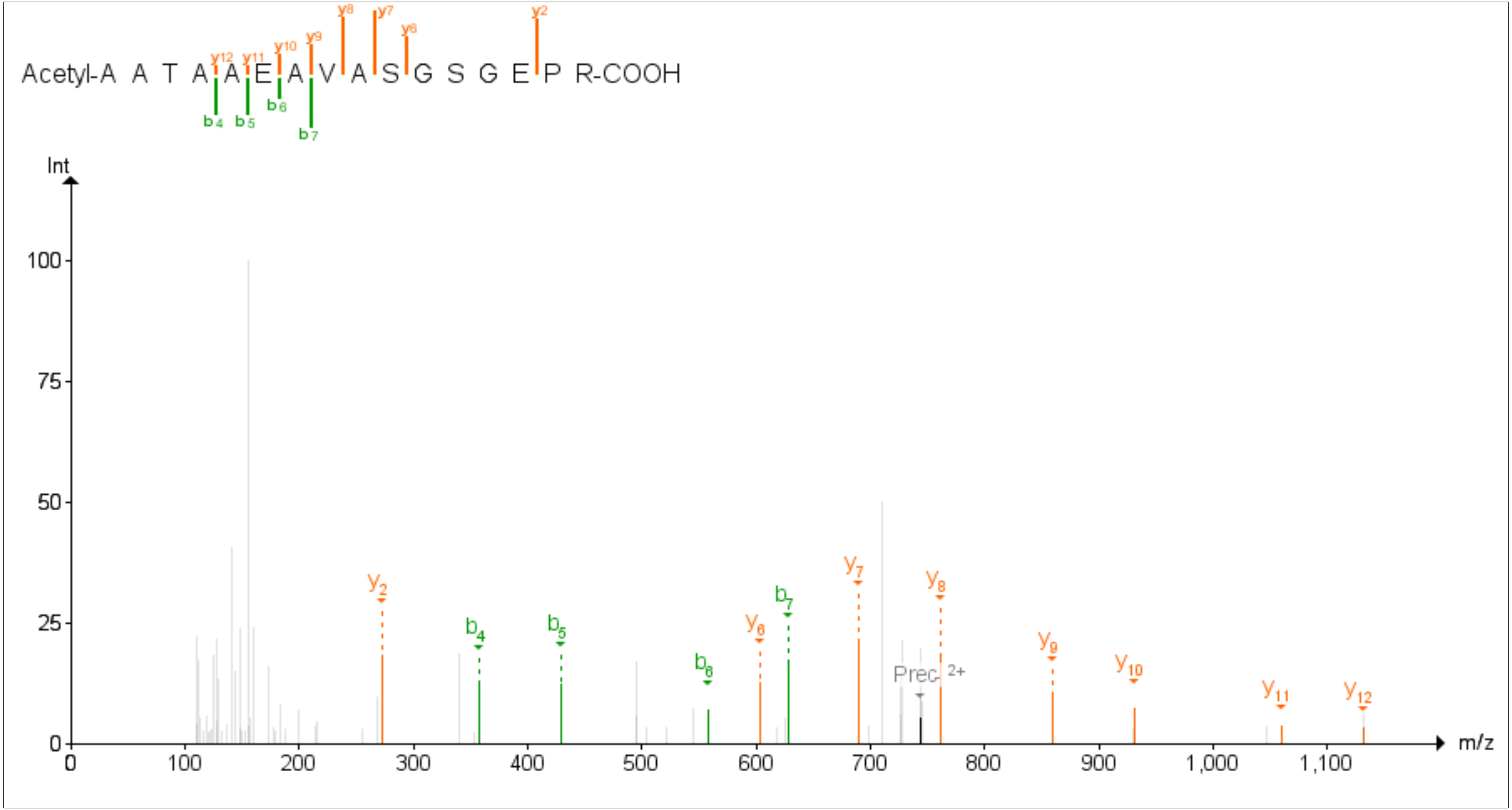

Acetyl-A A T A A E A V A S G S G E P R-COOH
y12 y11 y10 y9 y8 y7 y6 y2
b4 b5 b6 b7
Int
100
75
50
25
0
y2
b4
b5
b6
y6
b7
y7
y8
Prec 2+
y9
y10
y11
y12
0 100 200 300 400 500 600 700 800 900 1,000 1,100
m/z

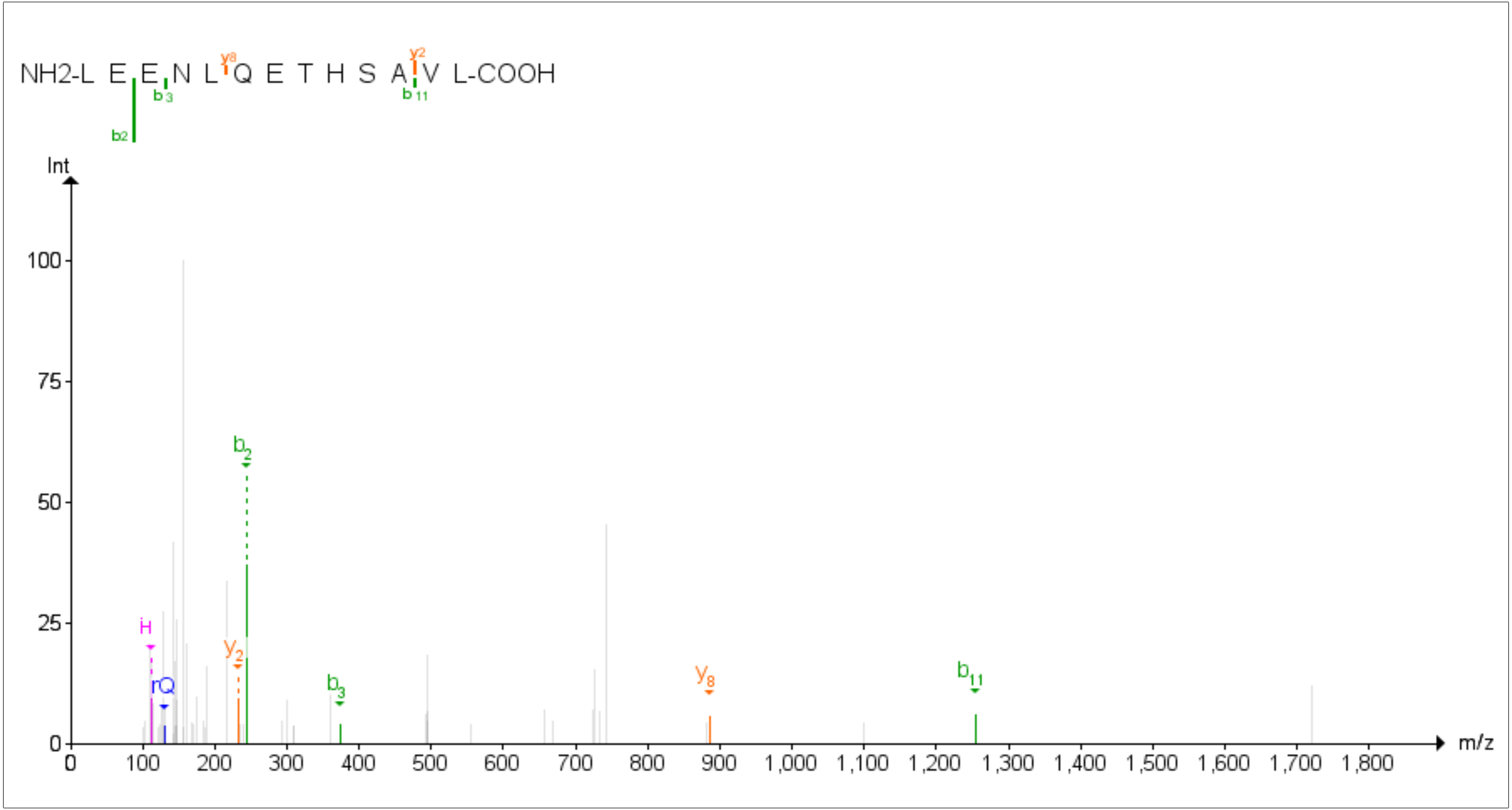

NH2-L E E N L Q E T H S A V L-COOH
b2
b3
y8
y2
b11
Int
100
75
50
25
0
b2
H
y2
rQ
b3
y8
b11
0
100
200
300
400
500
600
700
800
900
1,000
1,100
1,200
1,300
1,400
1,500
1,600
1,700
1,800
m/z

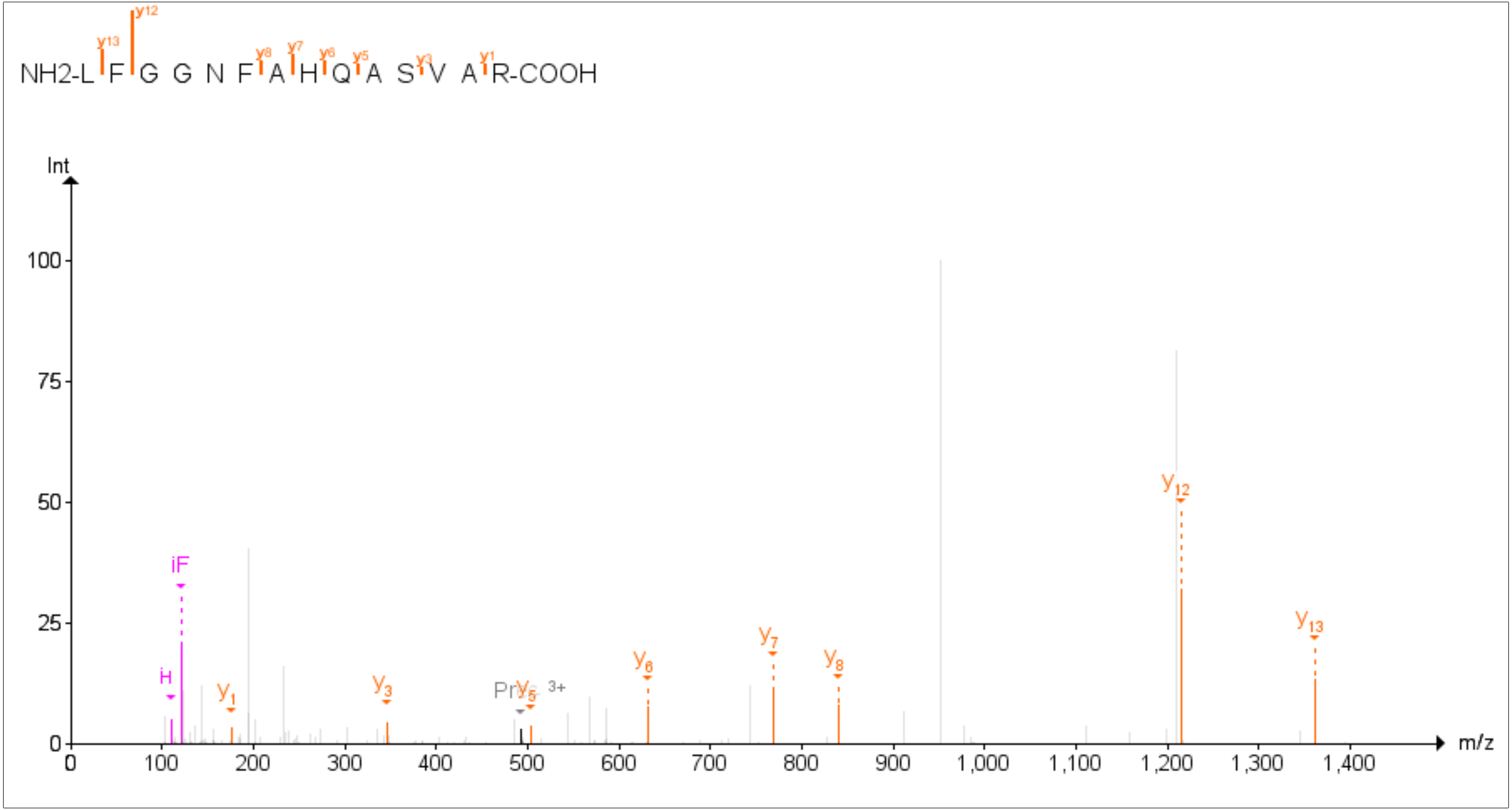

NH2-L F G G N F A H Q A S V A R-COOH
y13 y12 y8 y7 y6 y5 y3 y1
Int
m/z
iF
H
y1
y3
Prec 3+
y5
y6
y7
y8
y12
y13
0
25
50
75
100
0
100
200
300
400
500
600
700
800
900
1,000
1,100
1,200
1,300
1,400

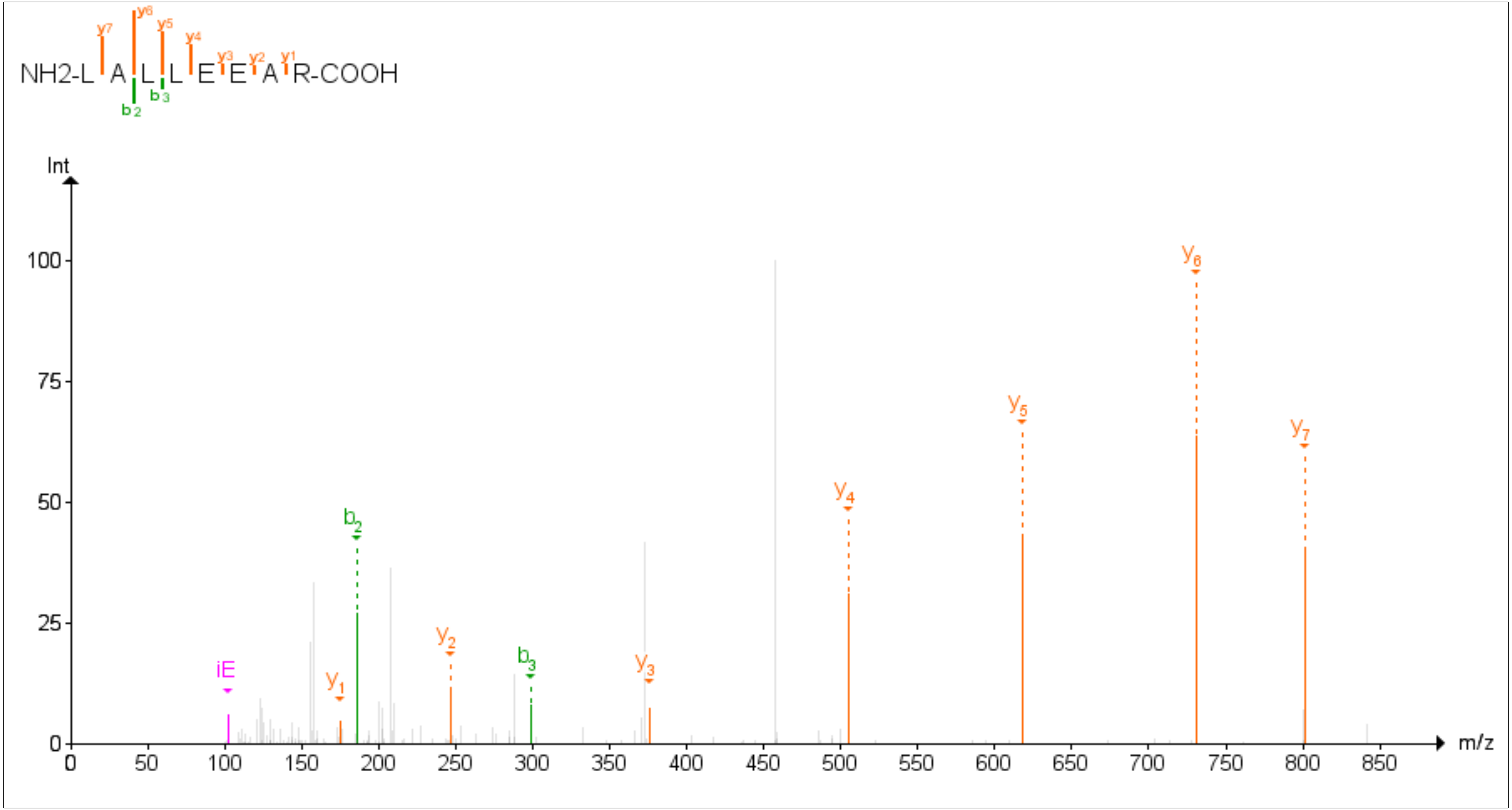
NH2-L A L L E E A R-COOH
y7
y6
y5
y4
y3
y2
y1
b2
b3
Int
m/z
iE
y1
b2
y2
b3
y3
y4
y5
y6
y7
0
25
50
75
100
0
50
100
150
200
250
300
350
400
450
500
550
600
650
700
750
800
850

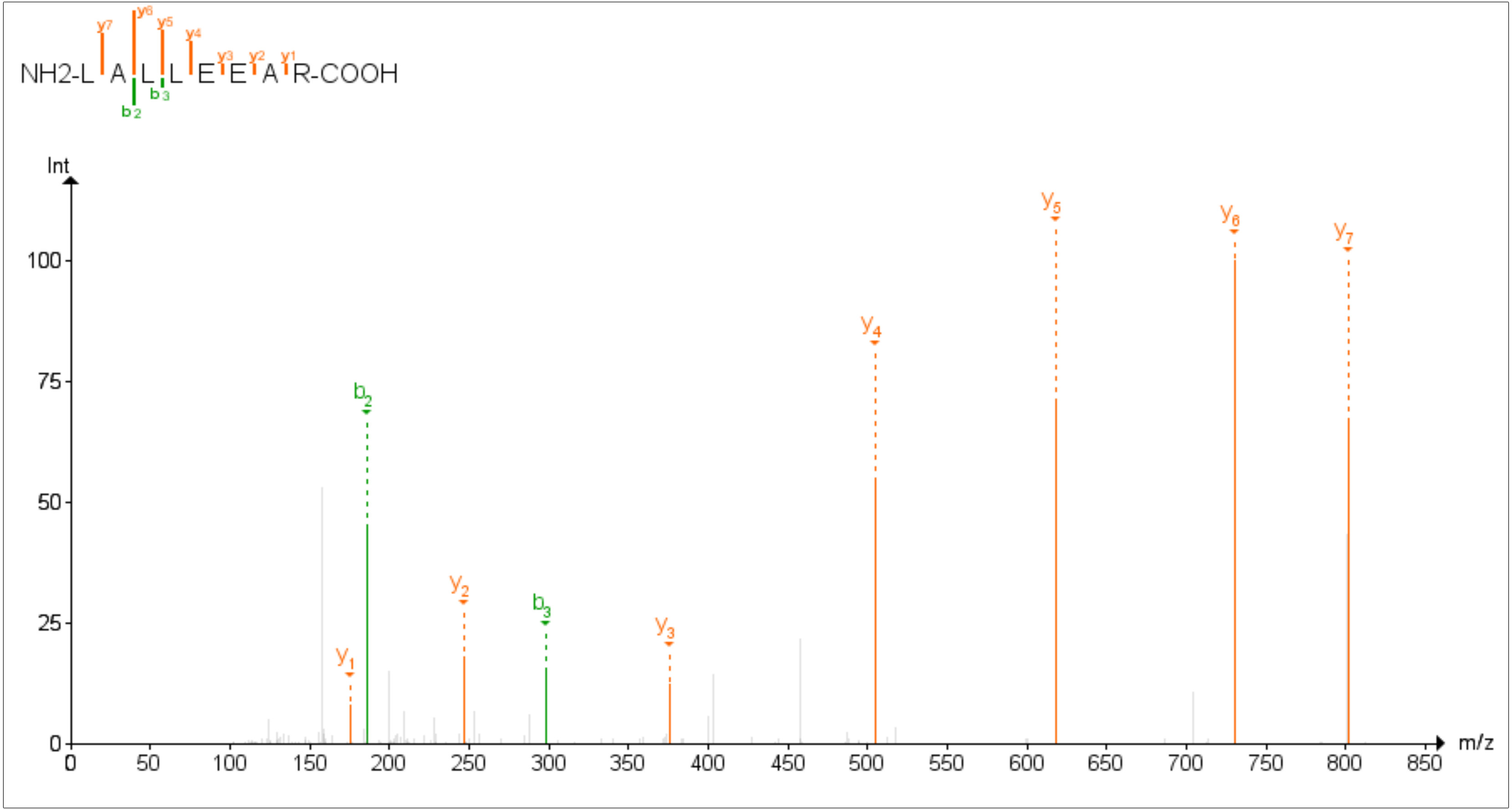

NH2-L A L L E E A R-COOH
y7 y6 y5 y4 y3 y2 y1
b2 b3
Int
100
75
50
25
0
y5 y6 y7 y4 b2 y2 b3 y3 y1
0 50 100 150 200 250 300 350 400 450 500 550 600 650 700 750 800 850
m/z

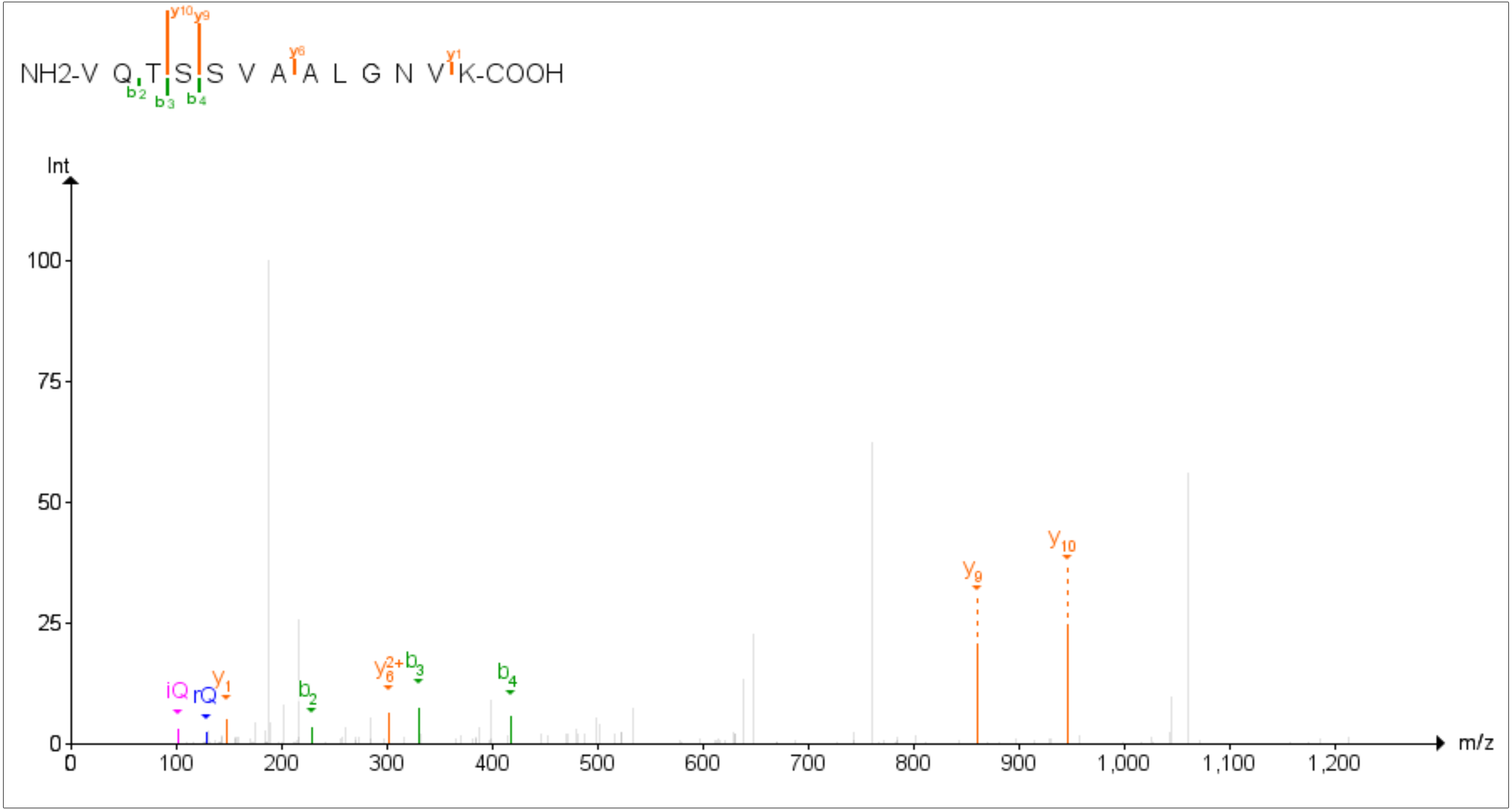
NH2-V Q T S S V A A L G N V K-COOH
y10 y9 y6 y1
b2 b3 b4
Int
100
75
50
25
0
m/z
0 100 200 300 400 500 600 700 800 900 1,000 1,100 1,200
iQ
rQ
y1
b2
y6 2+
b3
b4
y9
y10

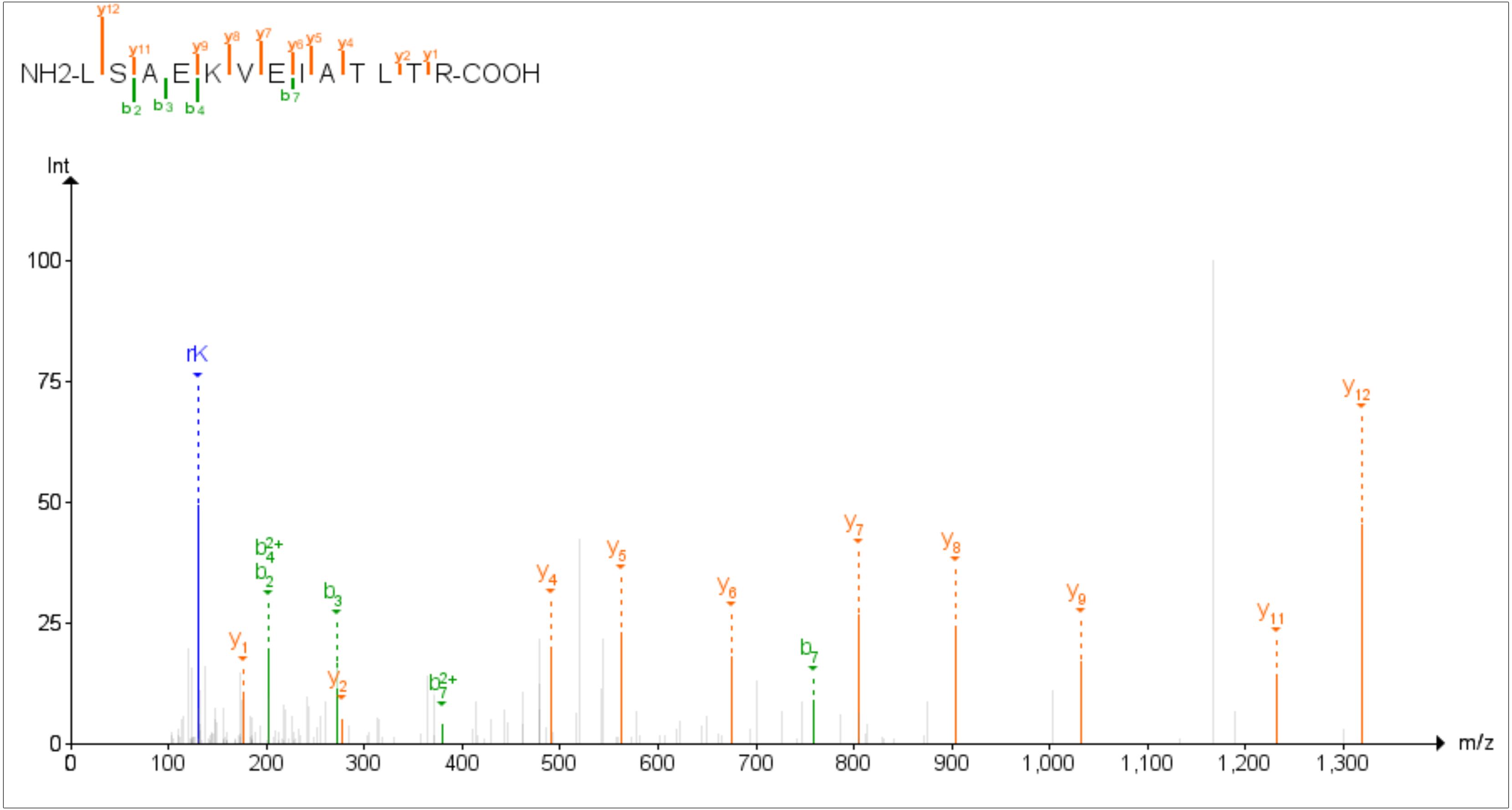

NH2-L S A E K V E I A T L T R-COOH
y12 y11 y9 y8 y7 y6 y5 y4 y2 y1
b2 b3 b4 b7
Int
m/z
100
75
50
25
0
0 100 200 300 400 500 600 700 800 900 1,000 1,100 1,200 1,300
rK
y1 y2 y4 y5 y6 y7 y8 y9 y11 y12
b4 2+ b2 b3 b7 2+ b7

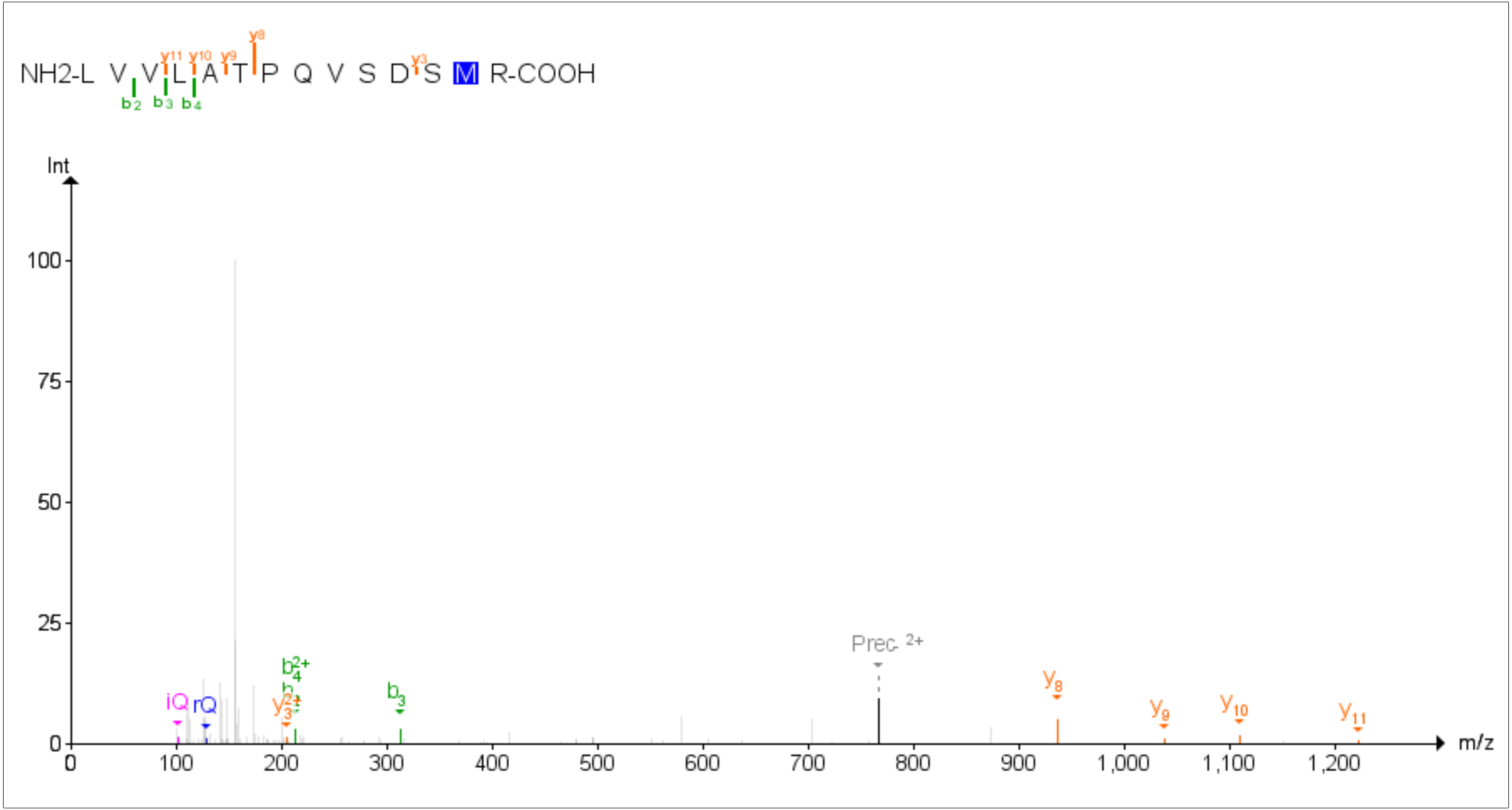
NH2-L V V L A T P Q V S D S M R-COOH
b2
b3
b4
y11
y10
y9
y8
y3
Int
100
75
50
25
0
iQ
rQ
y3 2+
b4 2+
b3
Prec 2+
y8
y9
y10
y11
m/z
0
100
200
300
400
500
600
700
800
900
1,000
1,100
1,200

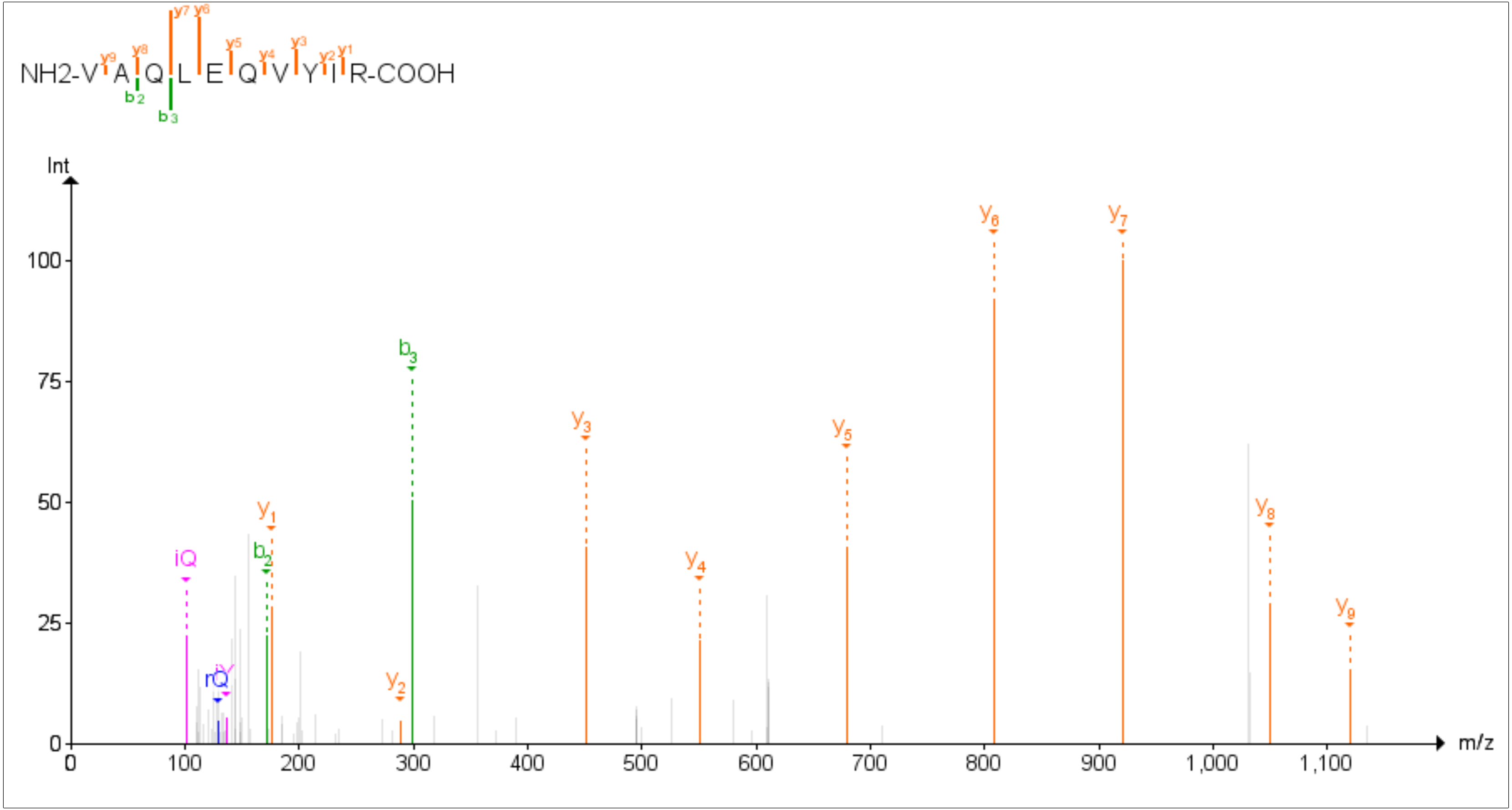
NH2-V A Q L E Q V Y I R-COOH
y9 y8 y7 y6 y5 y4 y3 y2 y1
b2 b3
Int
100
75
50
25
0
m/z
0
100
200
300
400
500
600
700
800
900
1,000
1,100
y6
y7
b3
y3
y5
y1
y8
iQ
b2
y4
y9
rQ
y2

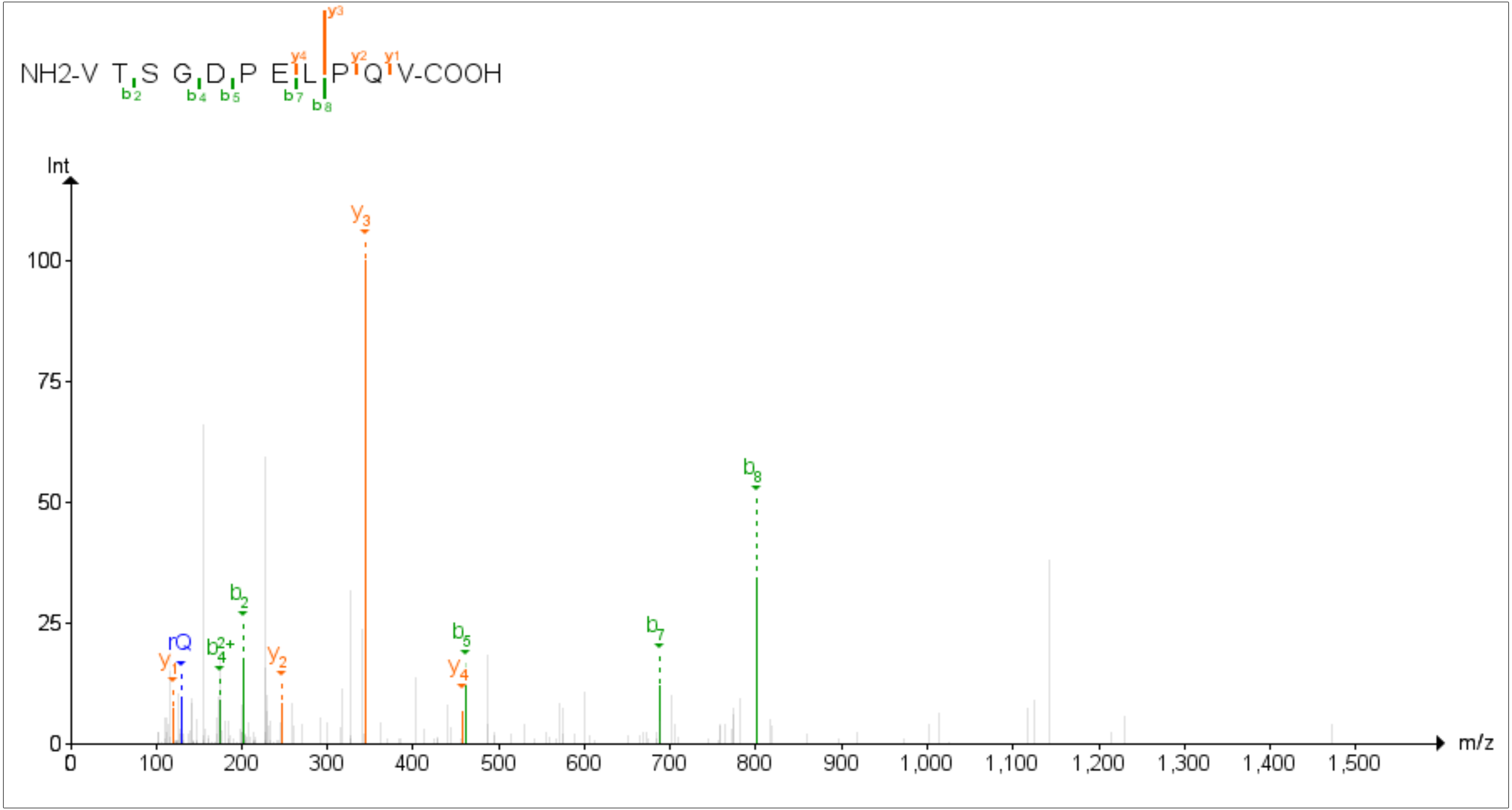

NH2-V T S G D P E L P Q V-COOH
b2
b4
b5
b7
b8
y4
y3
y2
y1
Int
100
75
50
25
0
m/z
0
100
200
300
400
500
600
700
800
900
1,000
1,100
1,200
1,300
1,400
1,500

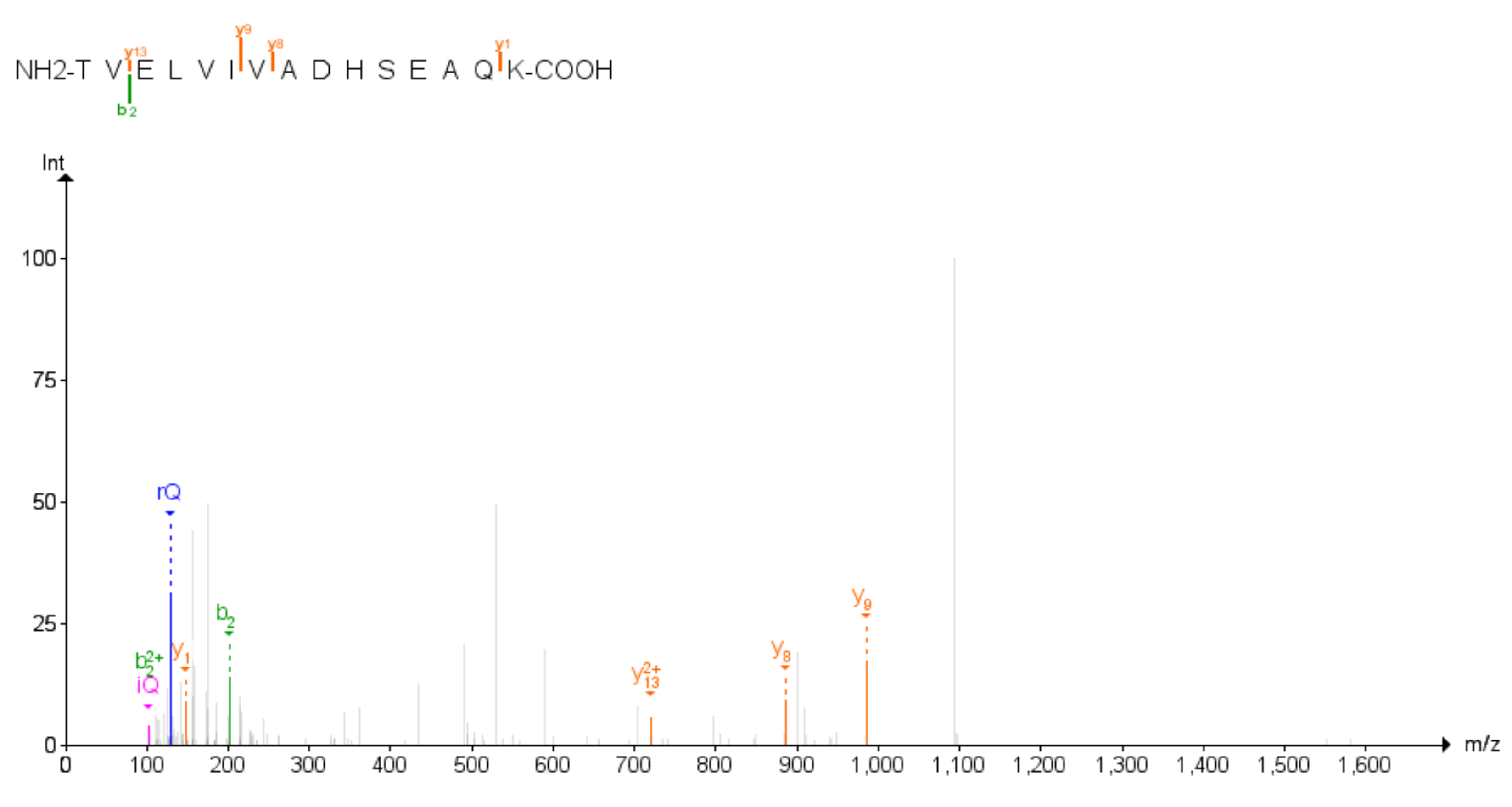

NH2-T V E L V I V A D H S E A Q K-COOH
y13
y9
y8
y1
b2
Int
100
75
50
25
0
rQ
b2
y1
iQ
y9
y8
y13 2+
b2 2+
0
100
200
300
400
500
600
700
800
900
1,000
1,100
1,200
1,300
1,400
1,500
1,600
m/z

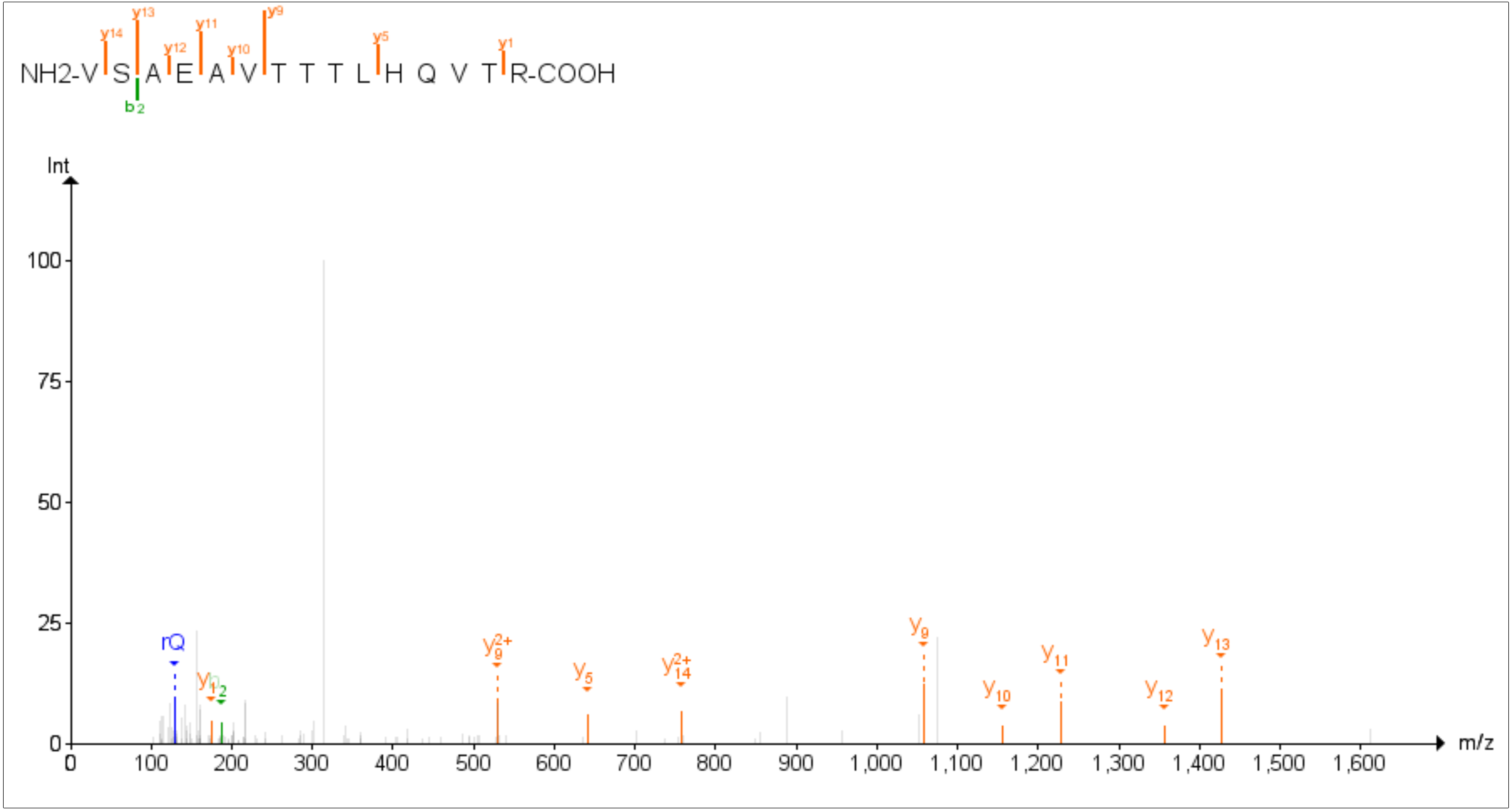

NH2-V S A E A V T T T L H Q V T R-COOH
y14
y13
y12
y11
y10
y9
y5
y1
b2
Int
100
75
50
25
0
rQ
y9 2+
y5
y14 2+
y9
y10
y11
y12
y13
0
100
200
300
400
500
600
700
800
900
1,000
1,100
1,200
1,300
1,400
1,500
1,600
m/z

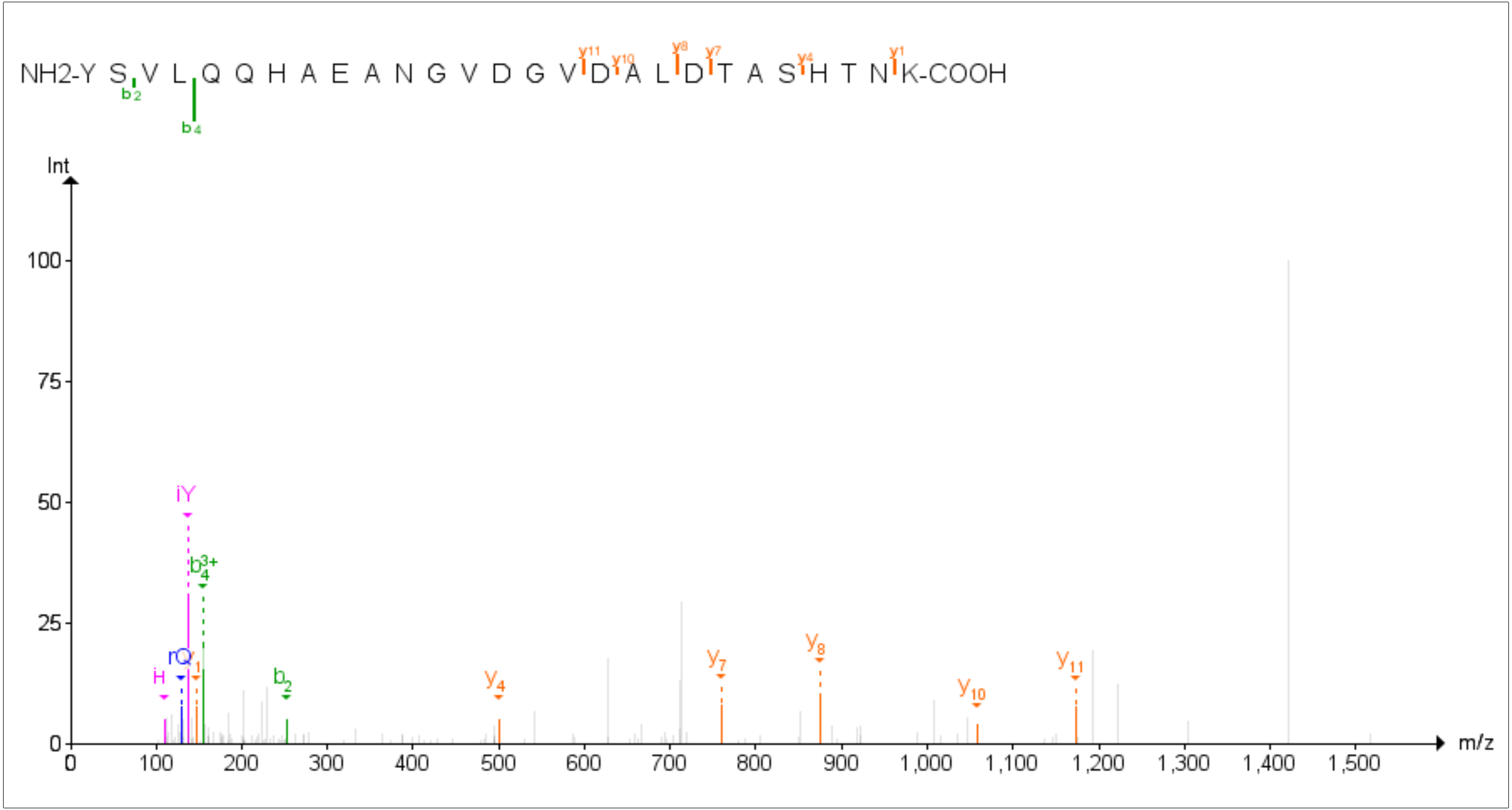

NH2-Y S V L Q Q H A E A N G V D G V D A L D T A S H T N K-COOH
b2
b4
y11
y10
y8
y7
y4
y1
Int
100
75
50
25
0
iY
b_4^{3+}
rQ
y_1
iH
b_2
y_4
y_7
y_8
y_{10}
y_{11}
0
100
200
300
400
500
600
700
800
900
1,000
1,100
1,200
1,300
1,400
1,500
m/z

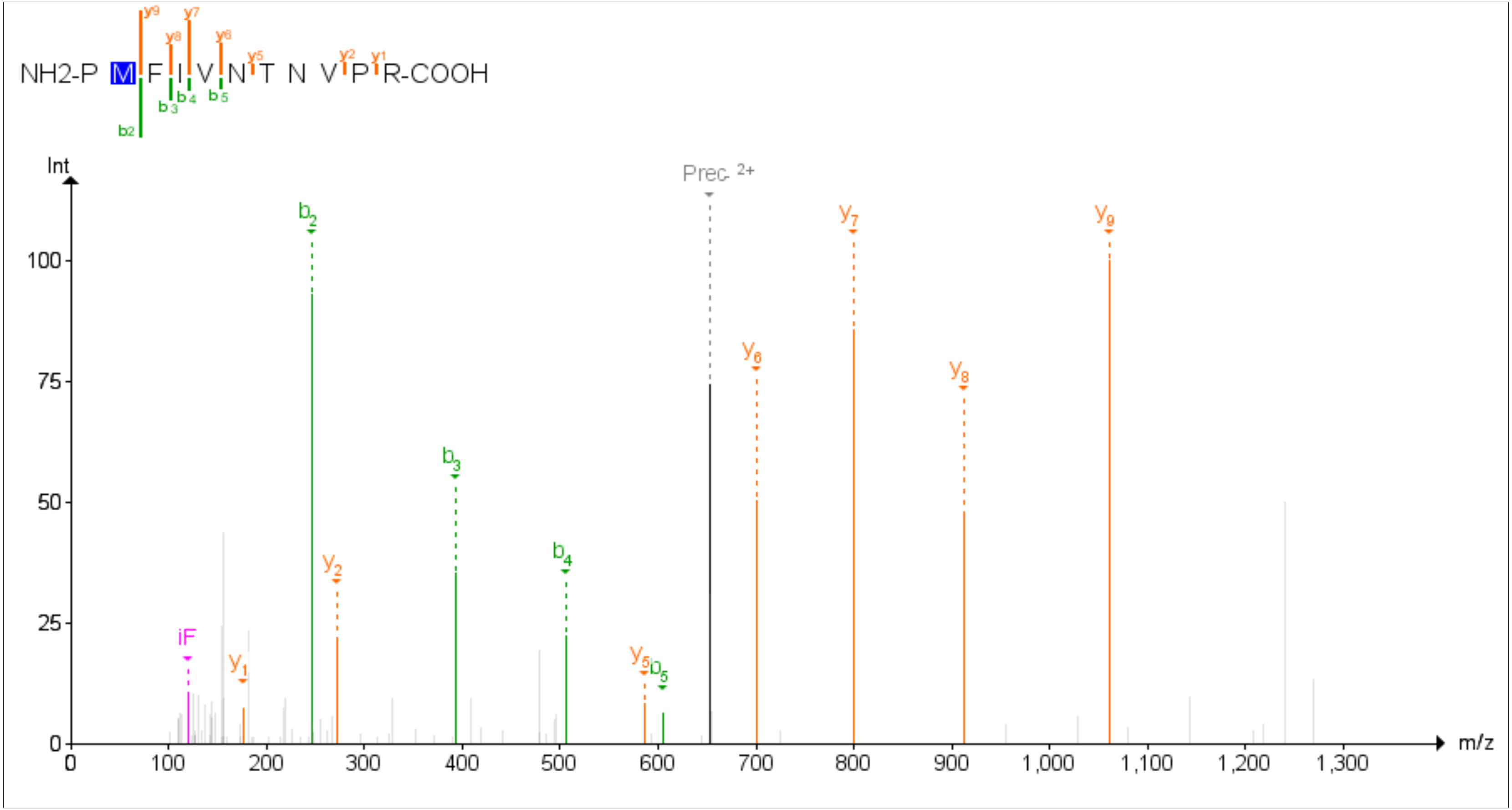

NH2-P M F I V N T N V P R-COOH
y9 y8 y7 y6 y5 y2 y1
b2 b3 b4 b5
Int
Prec 2+
b2
y7
y9
y6
y8
b3
b4
y2
iF
y1
y5
b5
100
75
50
25
0
0
100
200
300
400
500
600
700
800
900
1,000
1,100
1,200
1,300
m/z

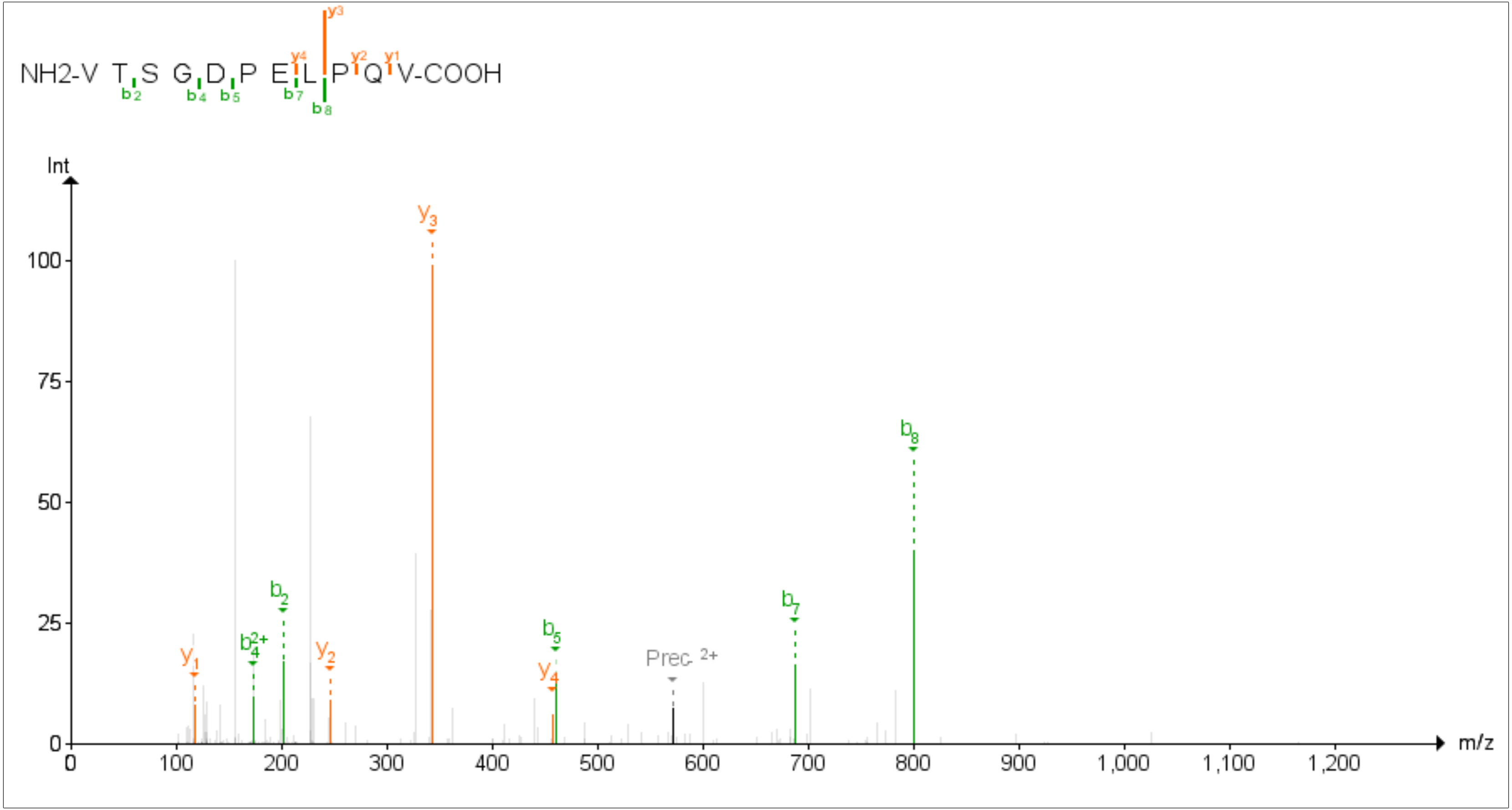

NH2-V T S G D P E L P Q V-COOH
b2 b4 b5 b7 b8
y4 y3 y2 y1
Int
m/z
y3
b8
b2
b7
b5
y1
b4 2+
y2
y4
Prec 2+
0
25
50
75
100
100
200
300
400
500
600
700
800
900
1,000
1,100
1,200

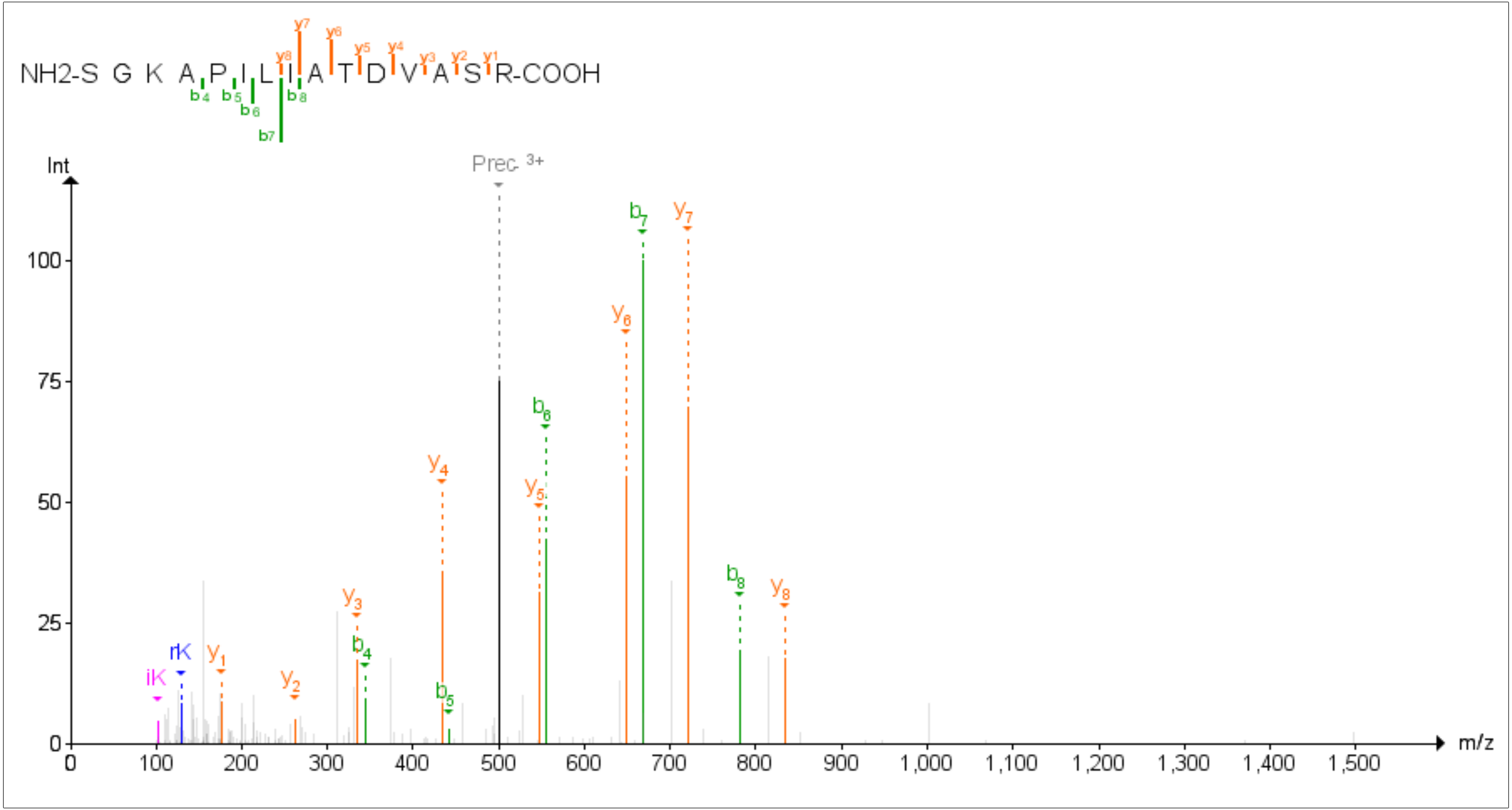

NH2-S G K A P I L I A T D V A S R-COOH
y8 y7 y6 y5 y4 y3 y2 y1
b4 b5 b6 b7 b8
Int
Prec 3+
100
75
50
25
0
iK
rK
y1
y2
y3
b4
b5
y4
y5
b6
y6
b7
y7
b8
y8
0
100
200
300
400
500
600
700
800
900
1,000
1,100
1,200
1,300
1,400
1,500
m/z

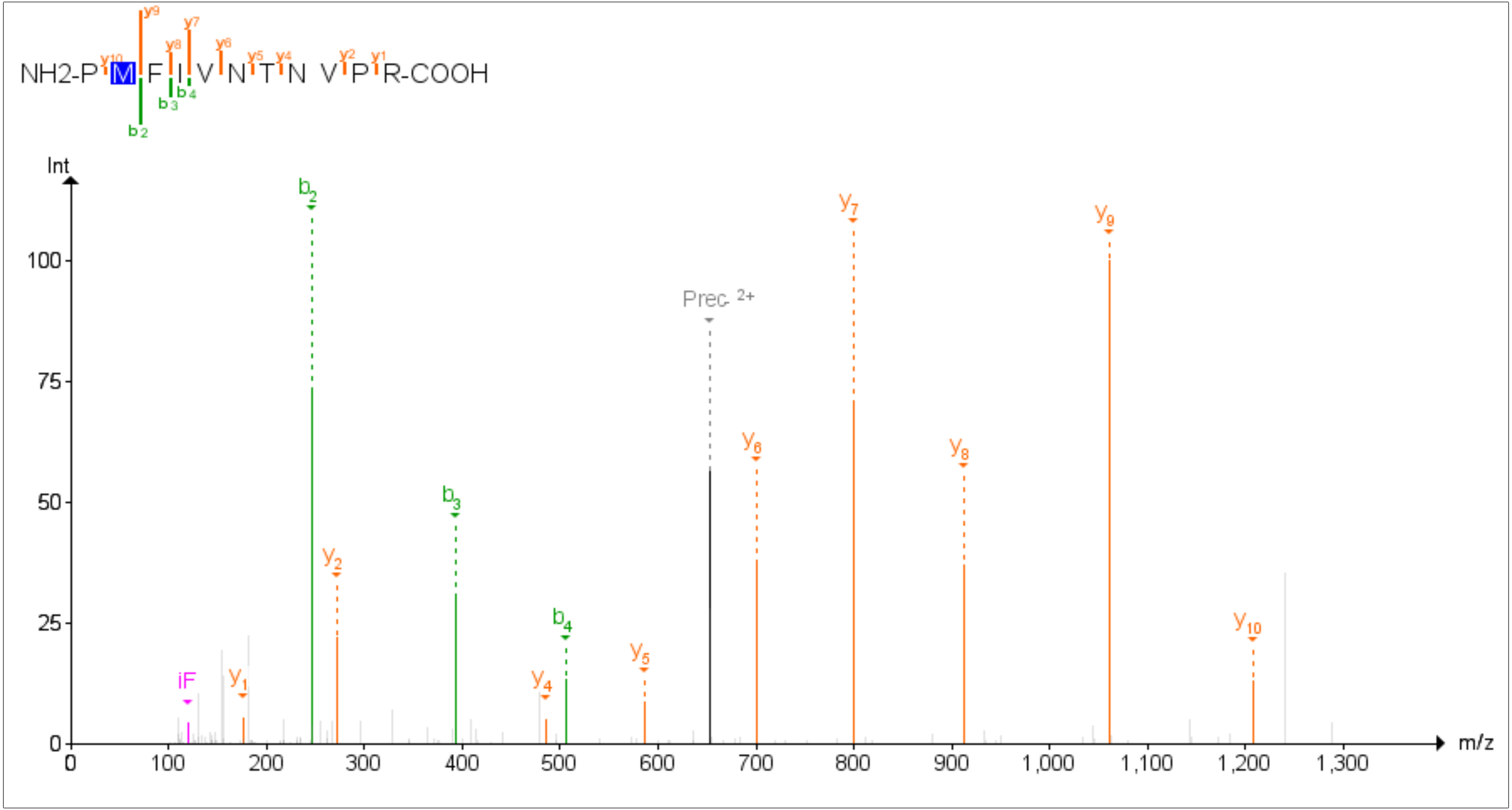
NH2-P M F I V N T N V P R-COOH
y10 y9 y8 y7 y6 y5 y4 y2 y1
b2 b3 b4
Int
m/z
Prec. 2+
iF

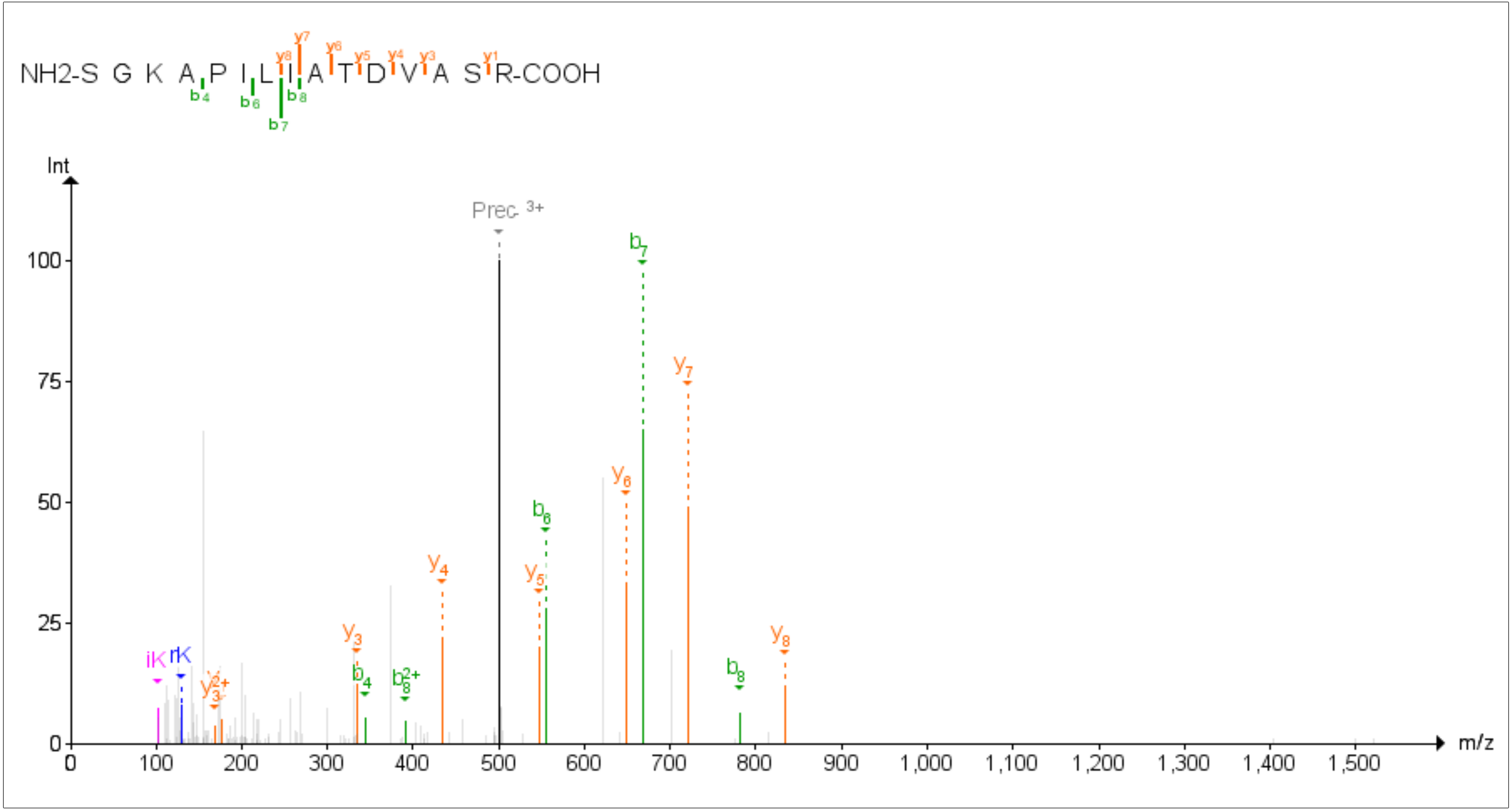
NH2-S G K A P I L I A T D V A S R-COOH
Int
Prec 3+
m/z
iK
rK
0
25
50
75
100
200
300
400
500
600
700
800
900
1,000
1,100
1,200
1,300
1,400
1,500

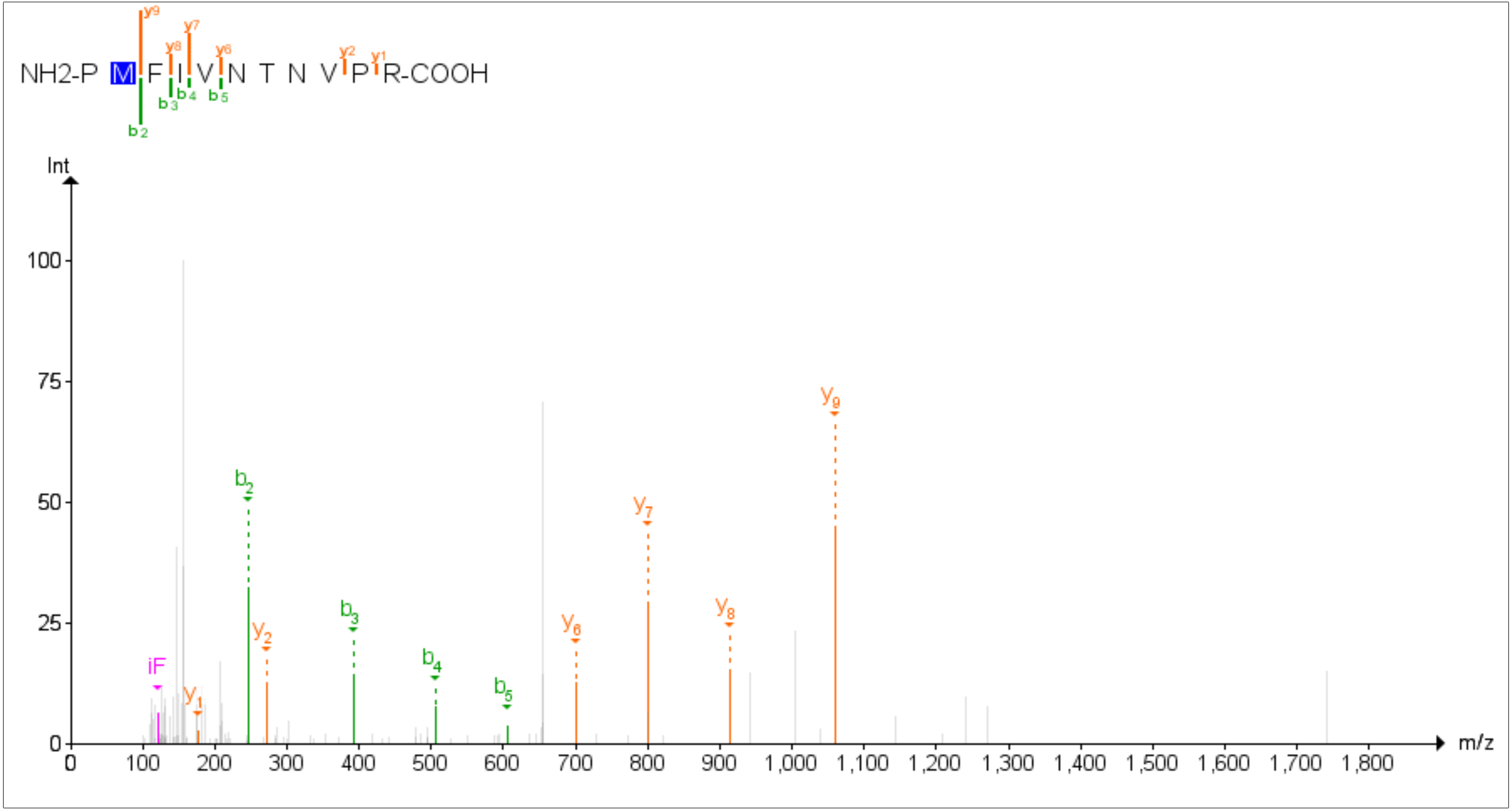

NH2-P M F I V N T N V P R-COOH
y9
y8
y7
y6
y2
y1
b 2
b 3
b 4
b 5
Int
100
75
50
25
0
b2
b3
b4
b5
y1
y2
y6
y7
y8
y9
iF
m/z
0
100
200
300
400
500
600
700
800
900
1,000
1,100
1,200
1,300
1,400
1,500
1,600
1,700
1,800

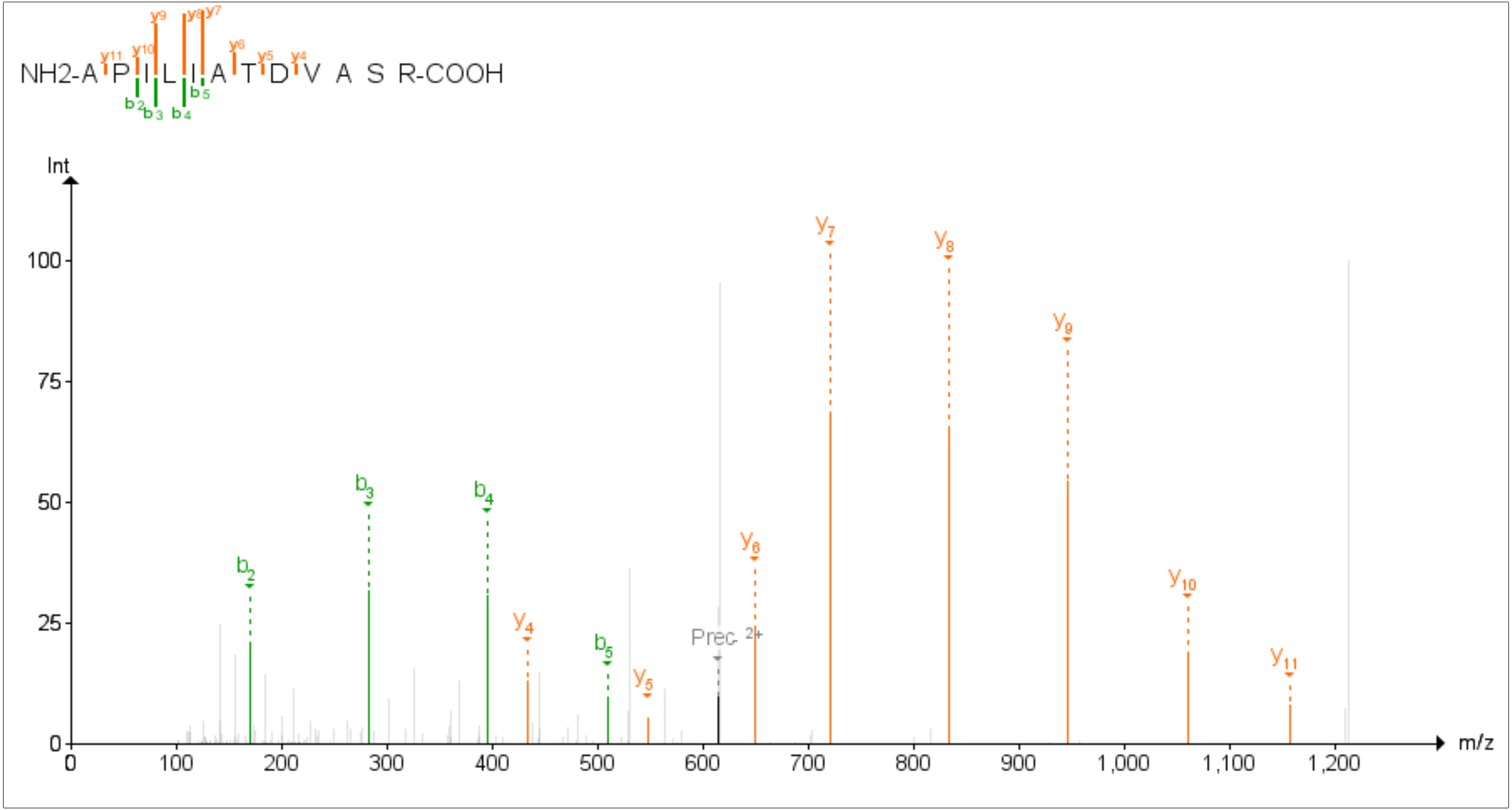

NH2-A P I L I A T D V A S R-COOH
y11 y10 y9 y8 y7 y6 y5 y4
b2 b3 b4 b5
Int
100
75
50
25
0
b2
b3
b4
y4
b5
y5
Prec 2+
y6
y7
y8
y9
y10
y11
0
100
200
300
400
500
600
700
800
900
1,000
1,100
1,200
m/z

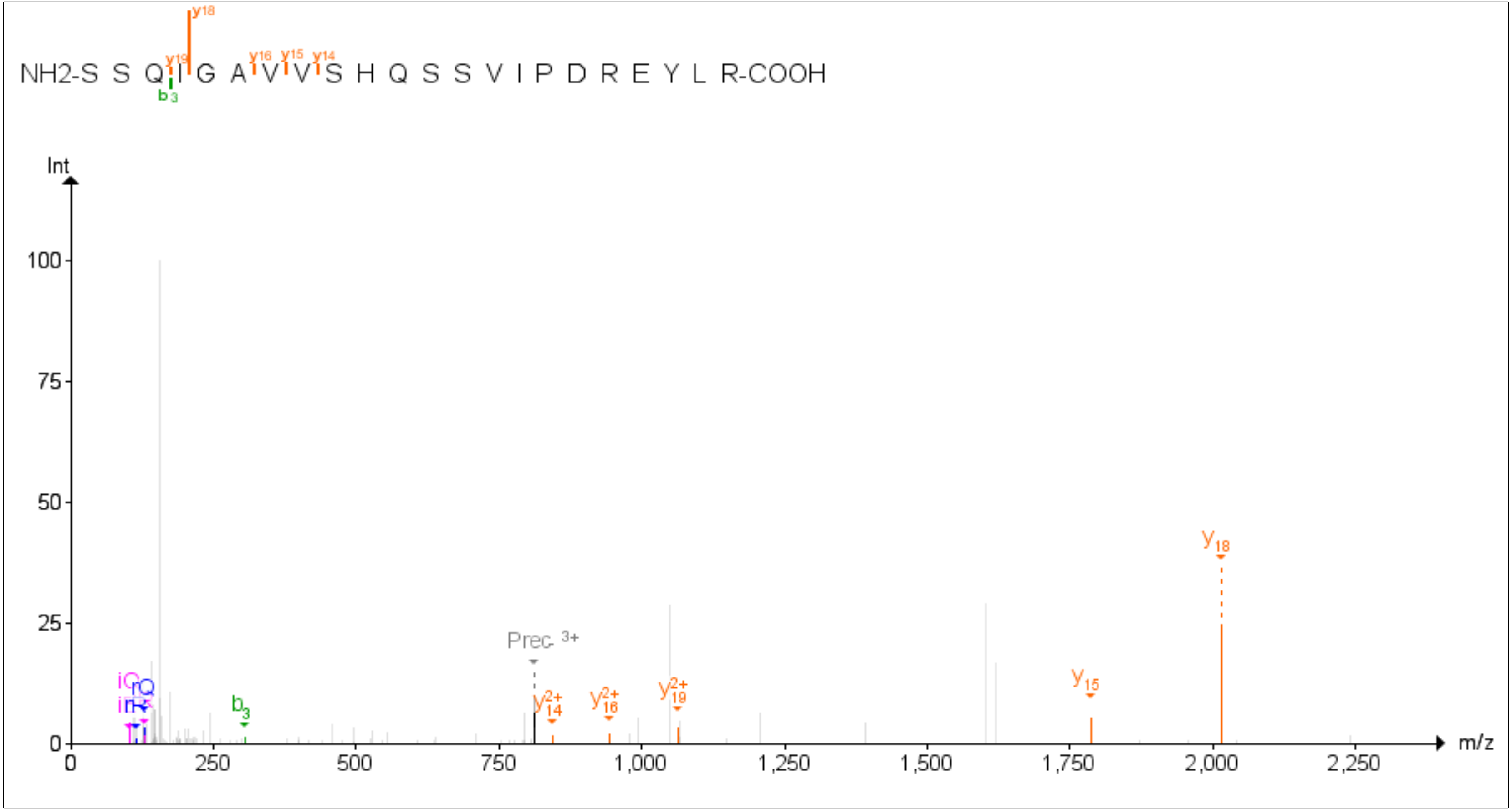

NH2-S S Q I G A V V S H Q S S V I P D R E Y L R-COOH
y18
y19
y16
y15
y14
b3
Int
100
75
50
25
0
Prec 3+
y14 2+
y16 2+
y19 2+
y15
y18
b3
iQ
rQ
0
250
500
750
1,000
1,250
1,500
1,750
2,000
2,250
m/z

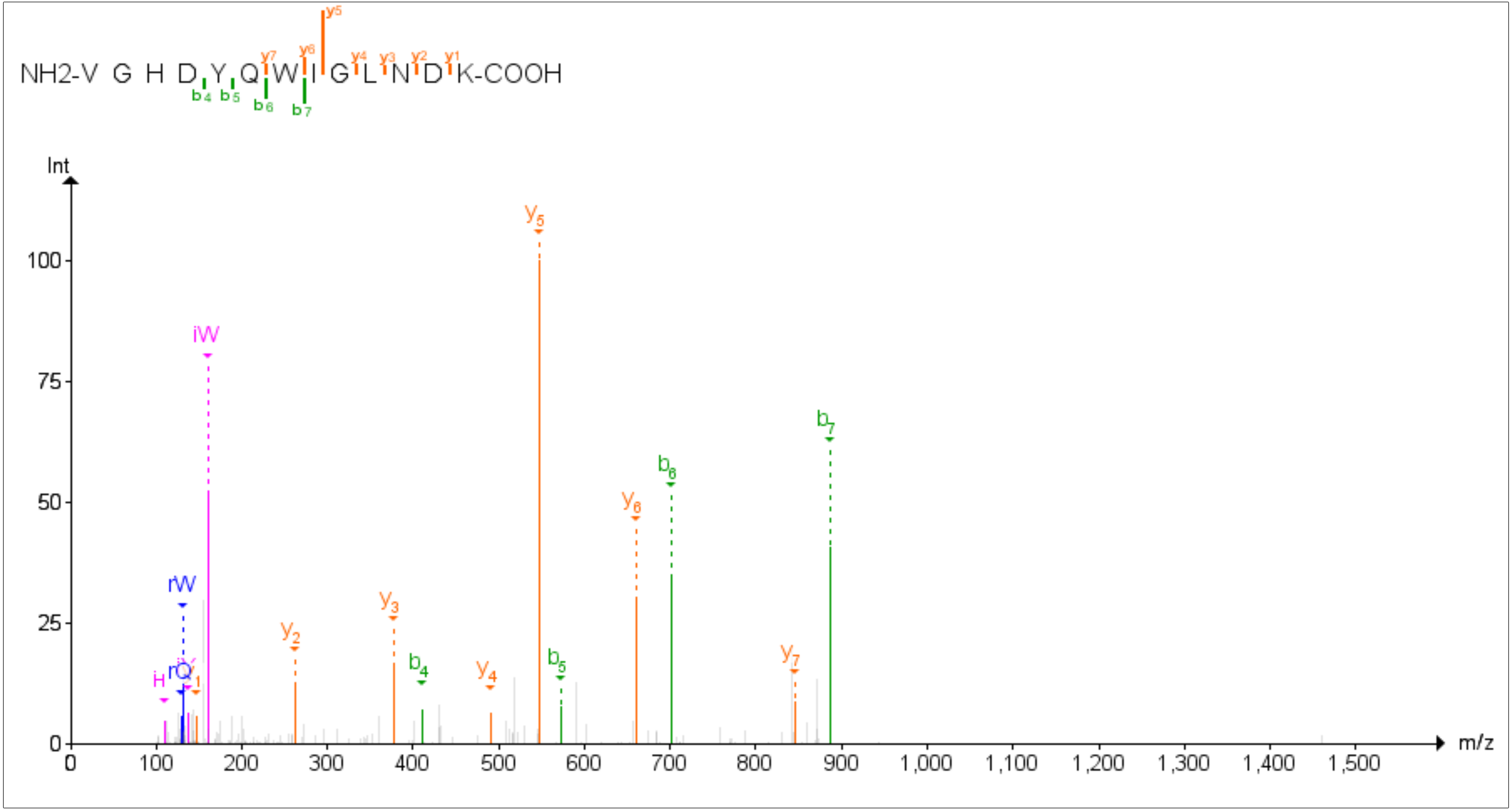

NH2-V G H D Y Q W I G L N D K-COOH
y5 y7 y6 y4 y3 y2 y1
b4 b5 b6 b7
Int
100
75
50
25
0
m/z
0 100 200 300 400 500 600 700 800 900 1,000 1,100 1,200 1,300 1,400 1,500
iW
rW
rQ
iH
y5
b7
b6
y6
y3
y2
b4
y4
b5
y7

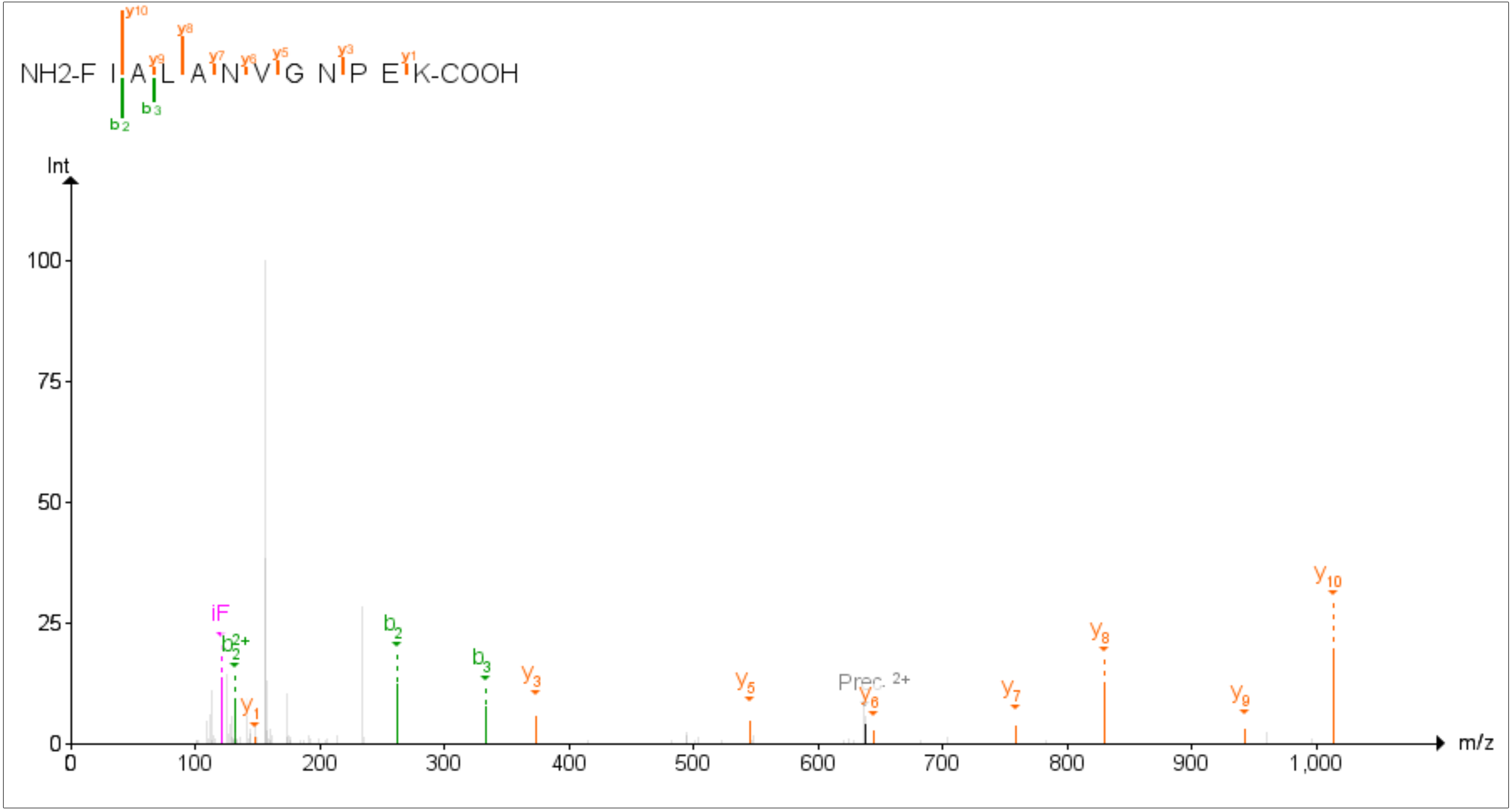

NH2-F I A L A N V G N P E K-COOH
y10
y9
y8
y7
y6
y5
y3
y1
b2
b3
Int
100
75
50
25
0
iF
b2 2+
y1
b2
b3
y3
y5
Prec. 2+
y6
y7
y8
y9
y10
0
100
200
300
400
500
600
700
800
900
1,000
m/z

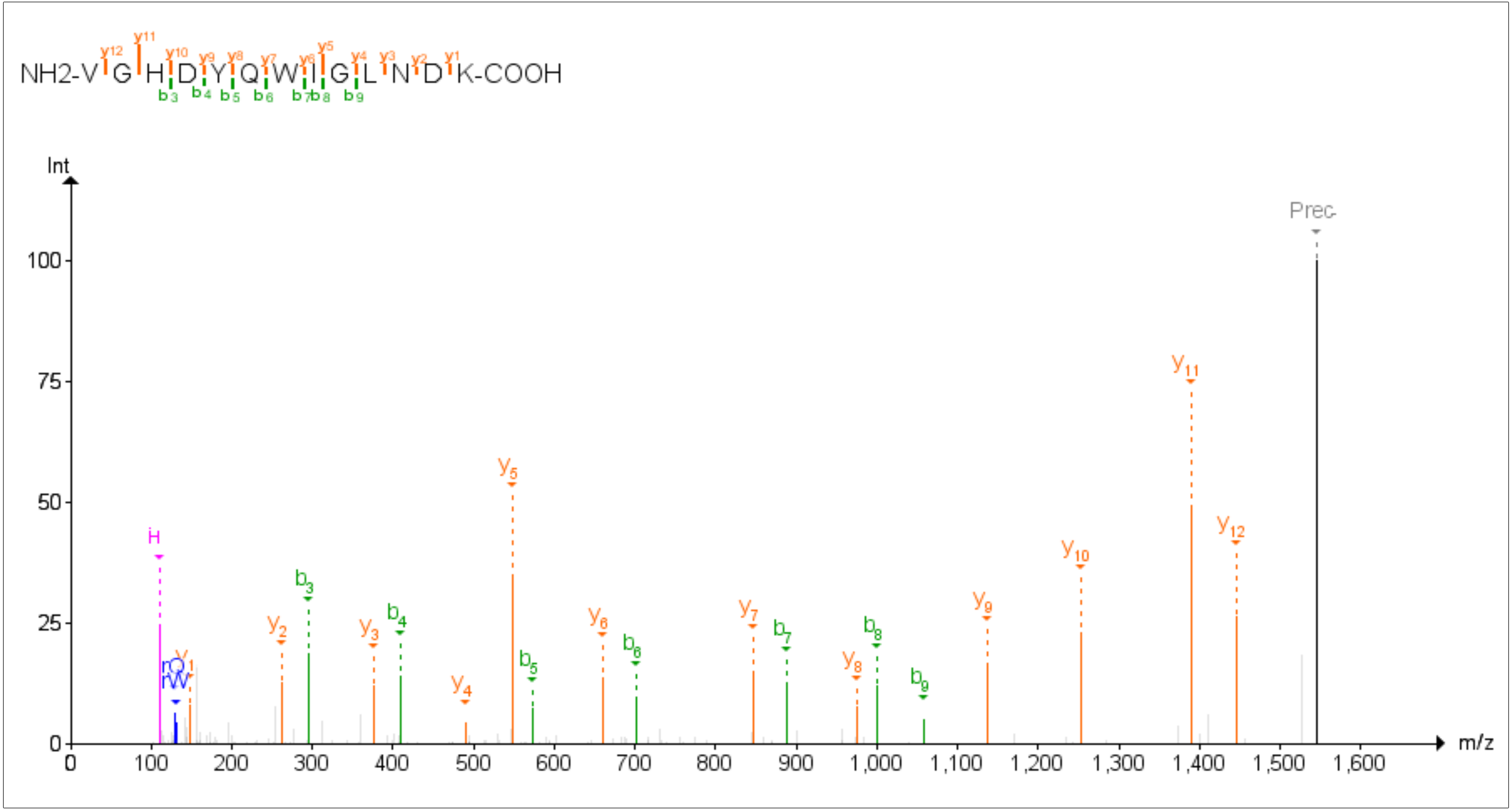

NH2-V G H D Y Q W I I G L N D K-COOH
y12 y11 y10 y9 y8 y7 y6 y5 y4 y3 y2 y1
b3 b4 b5 b6 b7 b8 b9
Int
Prec
100
75
50
25
0
i H
rQ
rW
y1
y2
b3
y3
b4
y4
y5
b5
y6
b6
y7
b7
y8
b8
b9
y9
y10
y11
y12
0
100
200
300
400
500
600
700
800
900
1,000
1,100
1,200
1,300
1,400
1,500
1,600
m/z

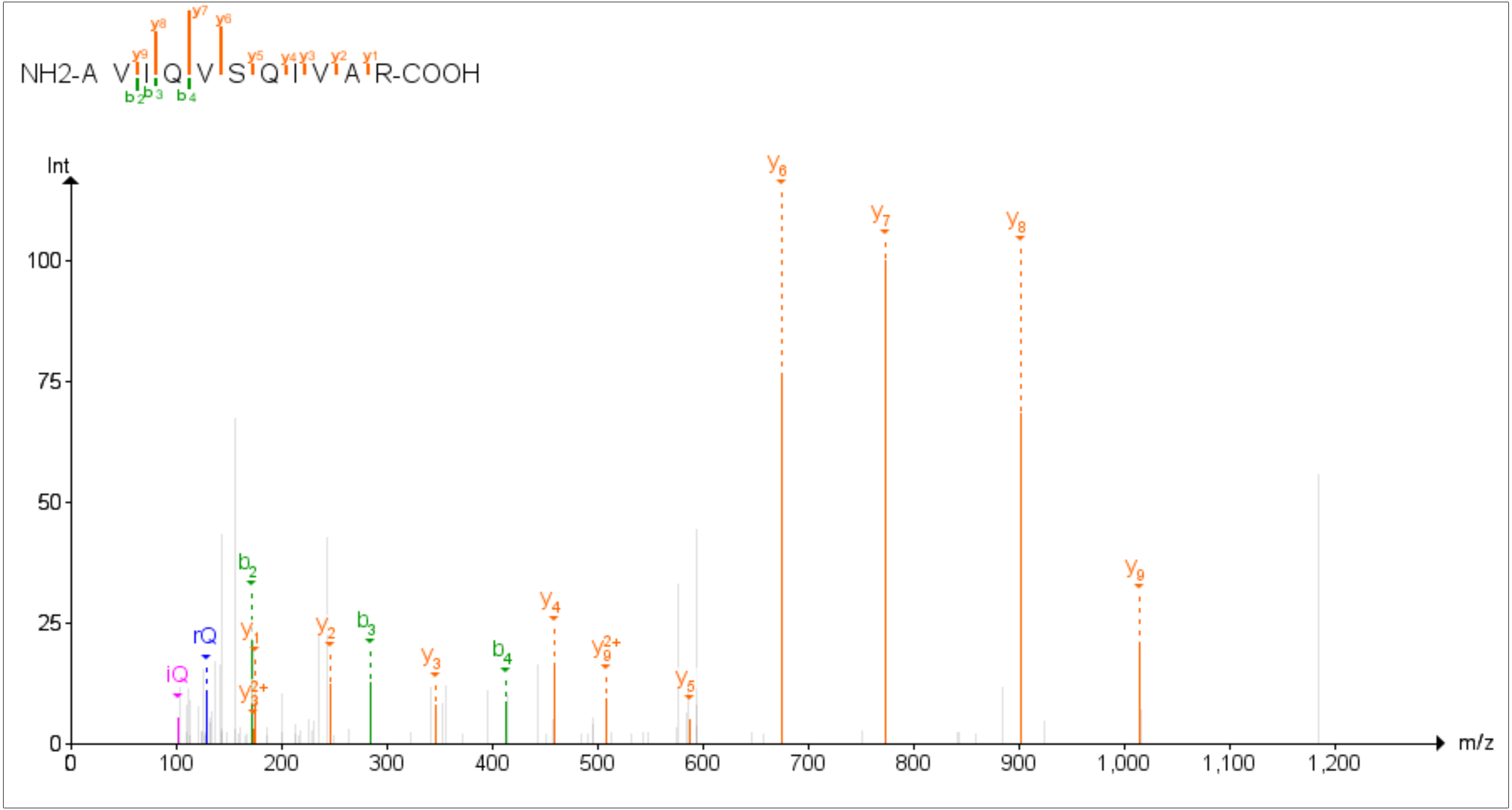
NH2-A V I Q V S Q I V A R-COOH
y9 y8 y7 y6 y5 y4 y3 y2 y1
b2 b3 b4
Int
100
75
50
25
0
m/z
0 100 200 300 400 500 600 700 800 900 1,000 1,100 1,200
y6
y7
y8
y9
b2
rQ
iQ
y1
y2
b3
y3
b4
y4
y9 2+
y5
y3 2+

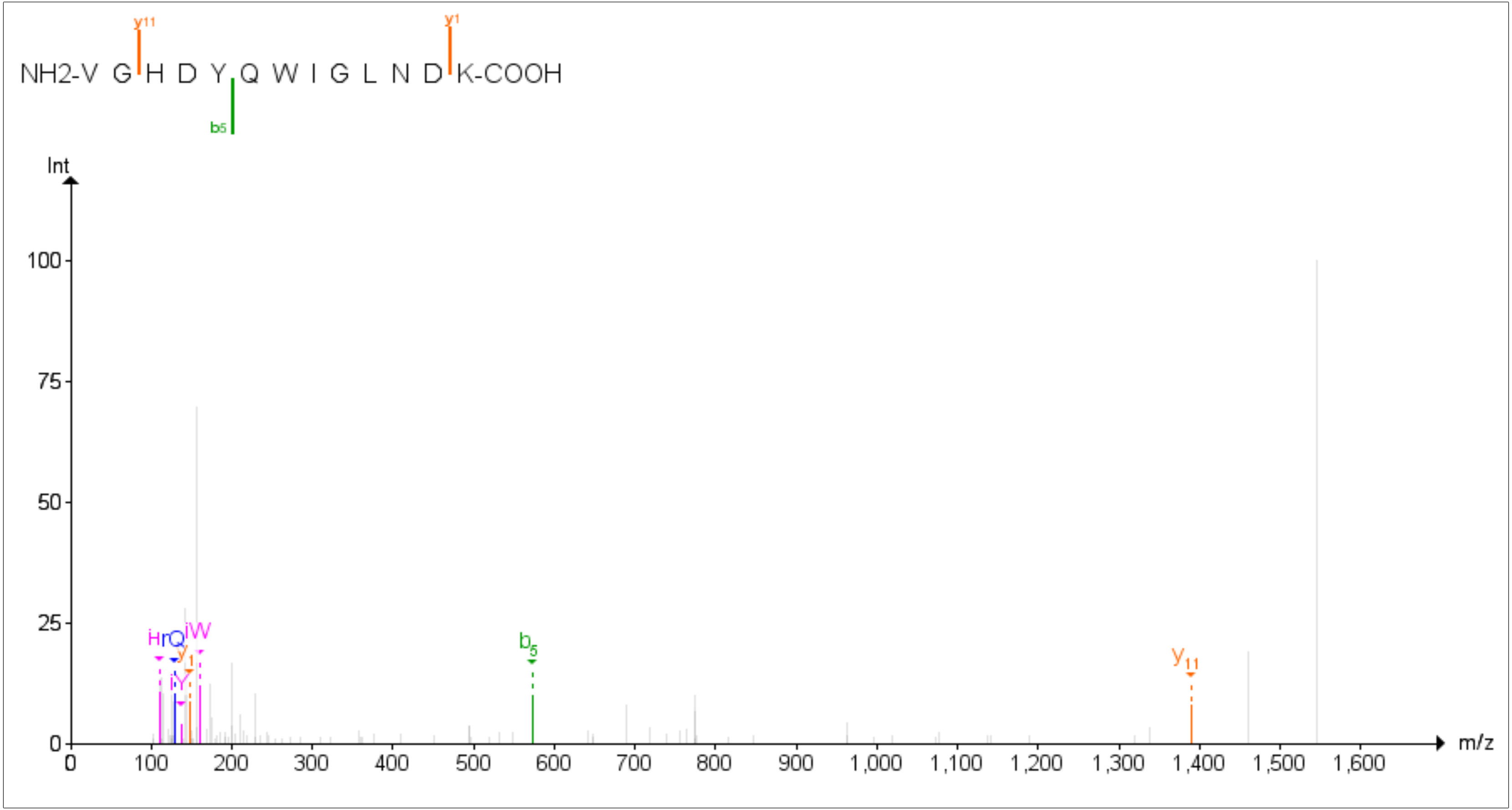

NH2-V G H D Y Q W I G L N D K-COOH
y11
y1
b5
Int
m/z
iH
rQ
iW
y1
iY
b5
y11
0
25
50
75
100
0
100
200
300
400
500
600
700
800
900
1,000
1,100
1,200
1,300
1,400
1,500
1,600

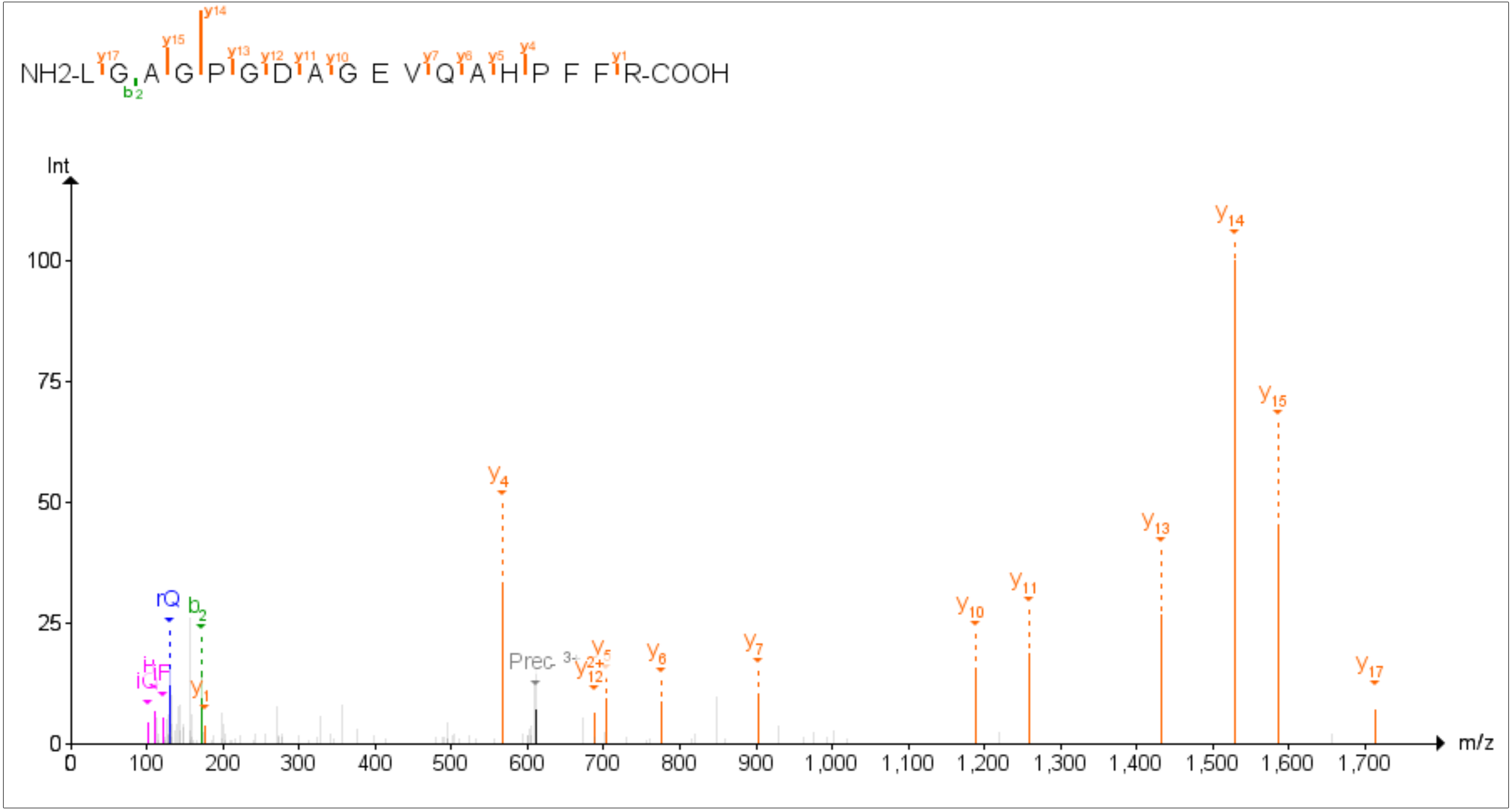

NH2-L G A G P G D A G E V Q A H P F F R-COOH
y17 y15 y14 y13 y12 y11 y10 y7 y6 y5 y4 y1
b2
Int
m/z
100
75
50
25
0
0 100 200 300 400 500 600 700 800 900 1,000 1,100 1,200 1,300 1,400 1,500 1,600 1,700
rQ
b2
iQ
iF
y1
y4
Prec- 3+
y5
y12 2+
y6
y7
y10
y11
y13
y14
y15
y17

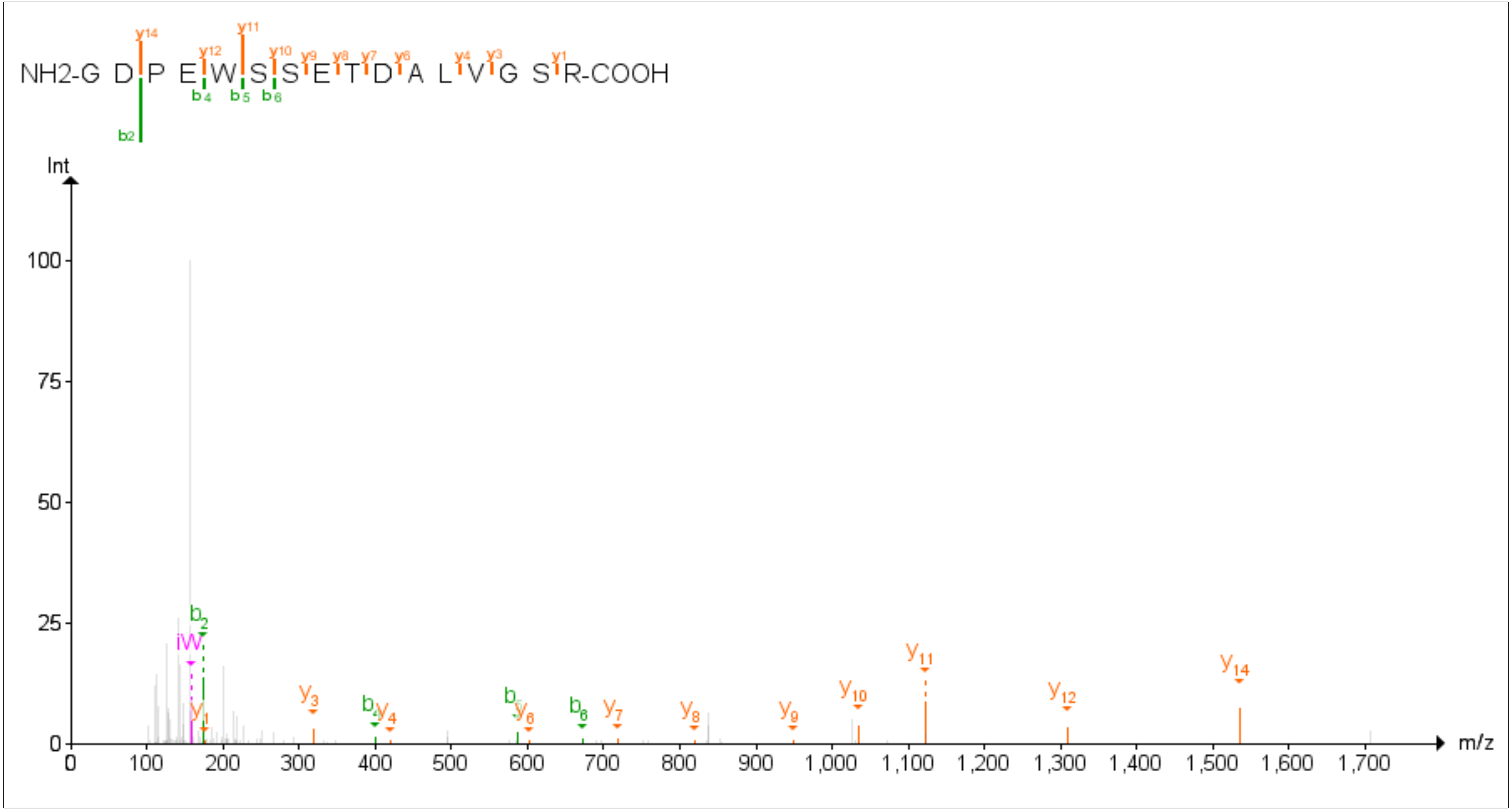
NH2-G D P E W S S E T D A L V G S R-COOH
y14
y12
y11
y10
y9
y8
y7
y6
y4
y3
y1
b2
b4
b5
b6
Int
100
75
50
25
0
iW
m/z
0
100
200
300
400
500
600
700
800
900
1,000
1,100
1,200
1,300
1,400
1,500
1,600
1,700

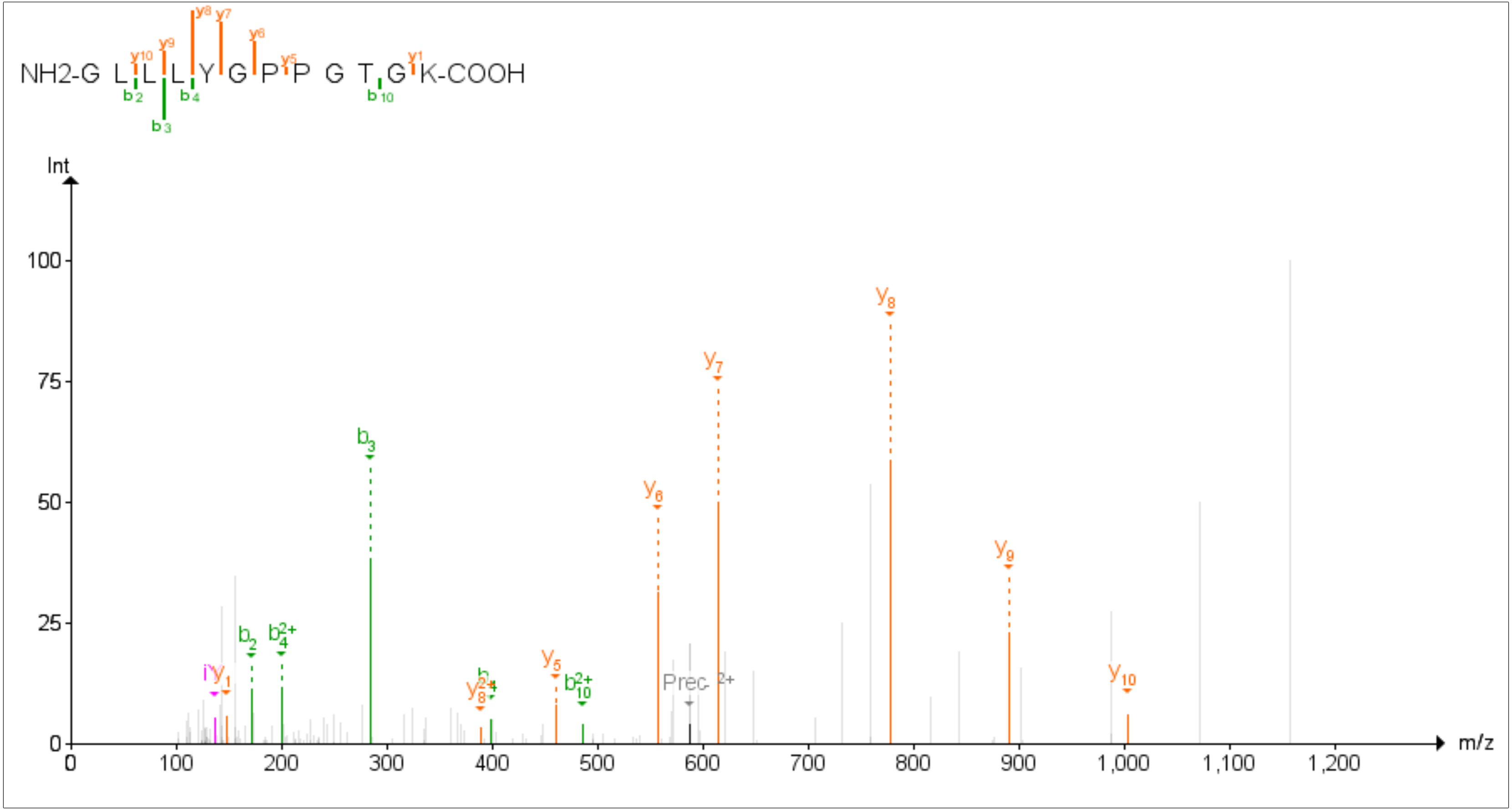

NH2-G L L L Y G P P G T G K-COOH
y10 y9 y8 y7 y6 y5 y1
b2 b3 b4 b10
Int
100
75
50
25
0
m/z
0 100 200 300 400 500 600 700 800 900 1,000 1,100 1,200
b3
y8
y7
y6
y9
y10
y5
b2
y1
Prec 2+

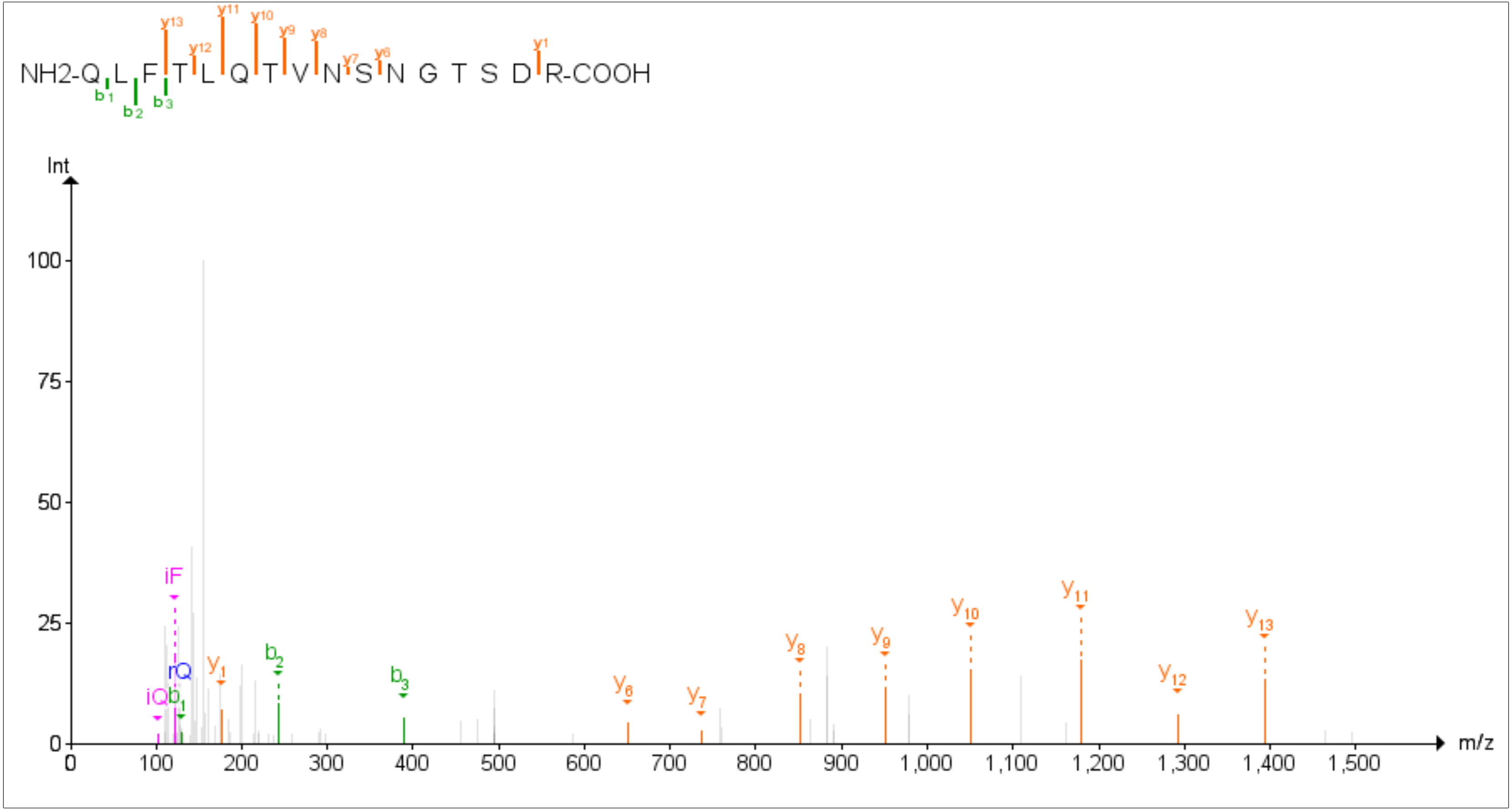

NH2-Q L F T L Q T V N S N G T S D R-COOH
y13 y12 y11 y10 y9 y8 y7 y6 y1
b1 b2 b3
Int
100
75
50
25
0
iF
rQ
iQ
b1
y1
b2
b3
y6
y7
y8
y9
y10
y11
y12
y13
0 100 200 300 400 500 600 700 800 900 1,000 1,100 1,200 1,300 1,400 1,500
m/z

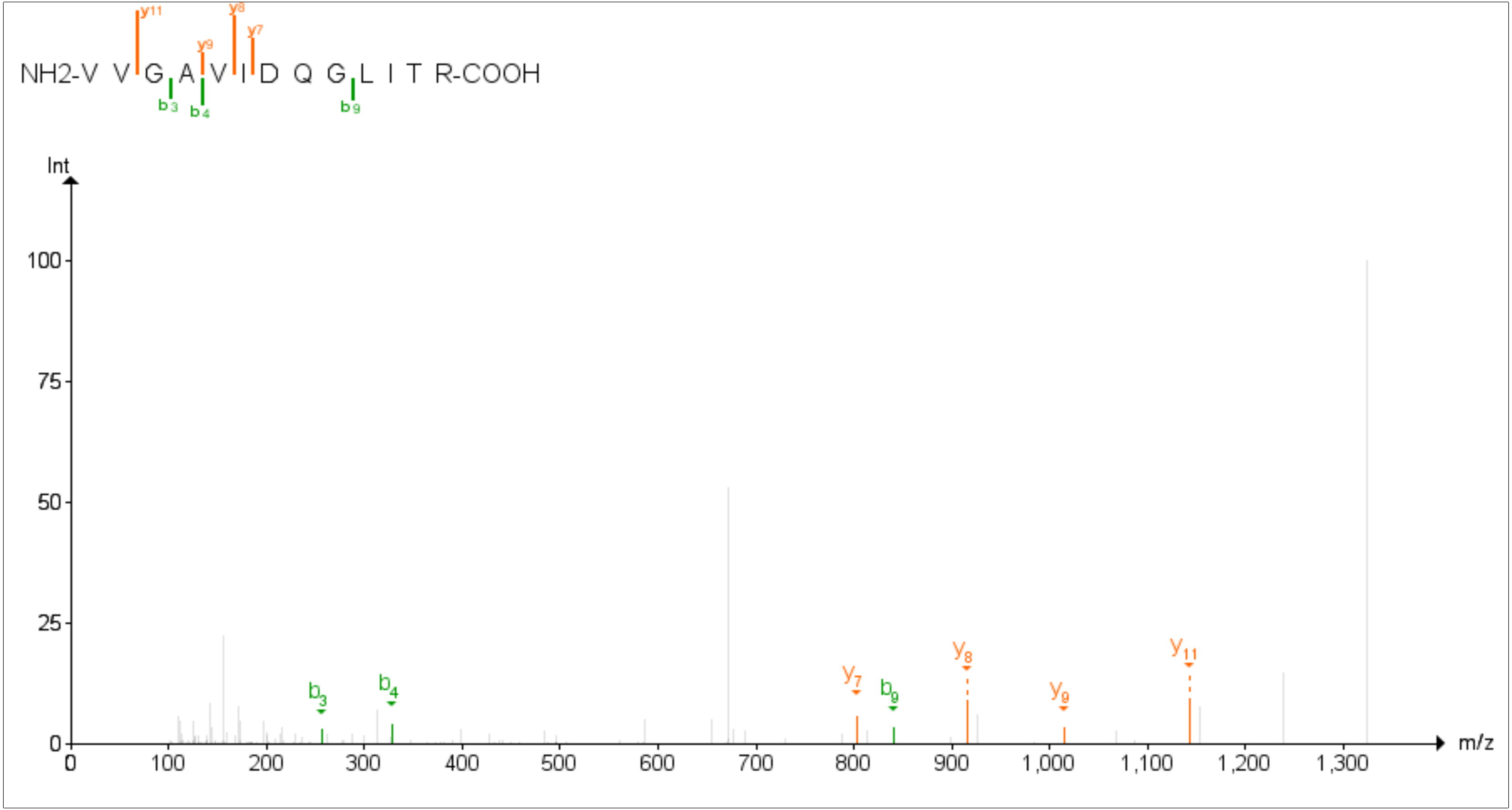

NH2-V V G A V I D Q G L I T R-COOH
y11
y9
y8
y7
b3
b4
b9
Int
100
75
50
25
0
m/z
0
100
200
300
400
500
600
700
800
900
1,000
1,100
1,200
1,300
b3
b4
y7
b9
y8
y9
y11

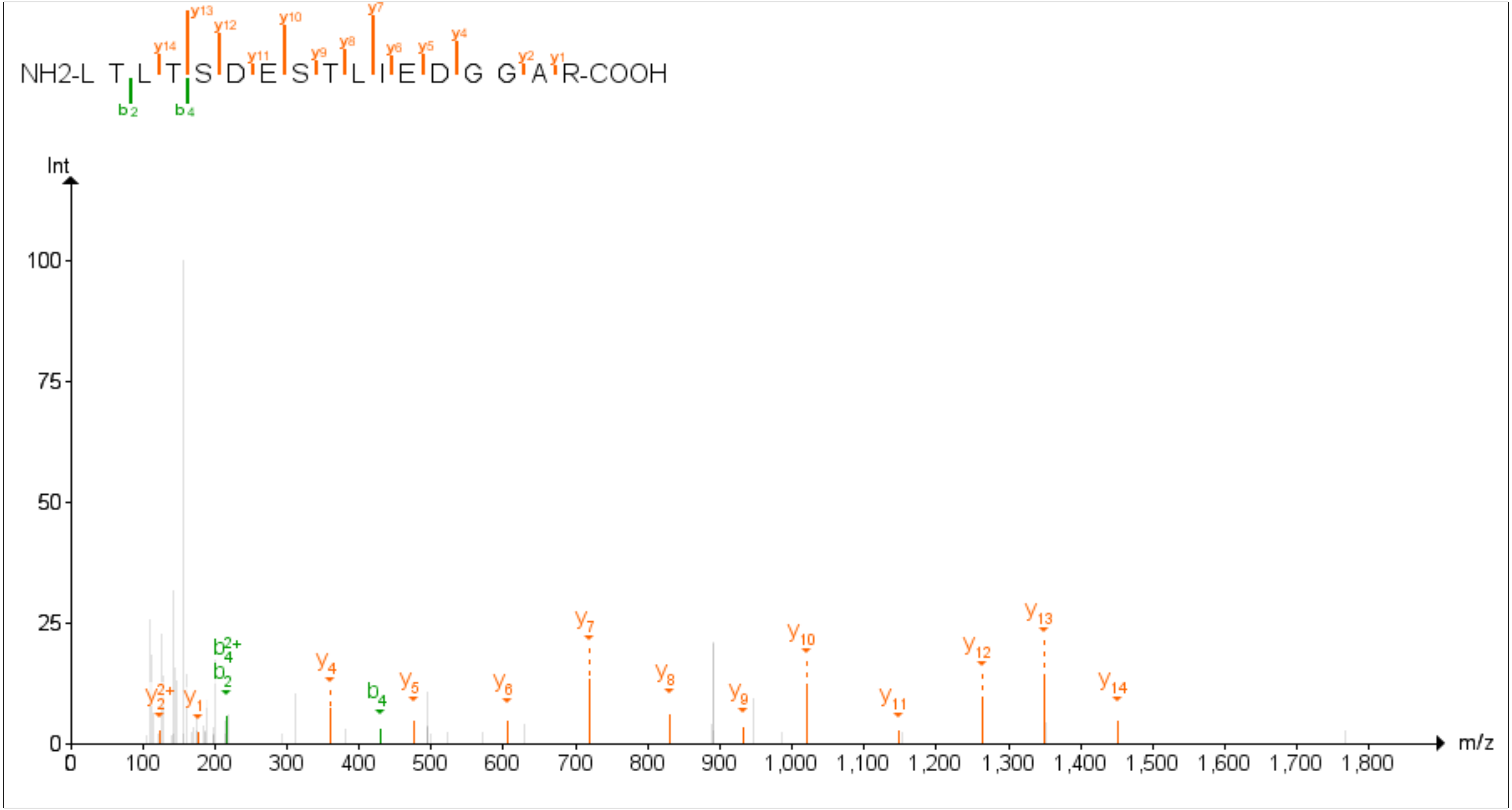

NH2-L T L T S D E S T L I E D G G A R-COOH
y13 y12 y14 y11 y10 y9 y8 y7 y6 y5 y4 y2 y1
b2 b4
Int
100
75
50
25
0
0 100 200 300 400 500 600 700 800 900 1,000 1,100 1,200 1,300 1,400 1,500 1,600 1,700 1,800
m/z

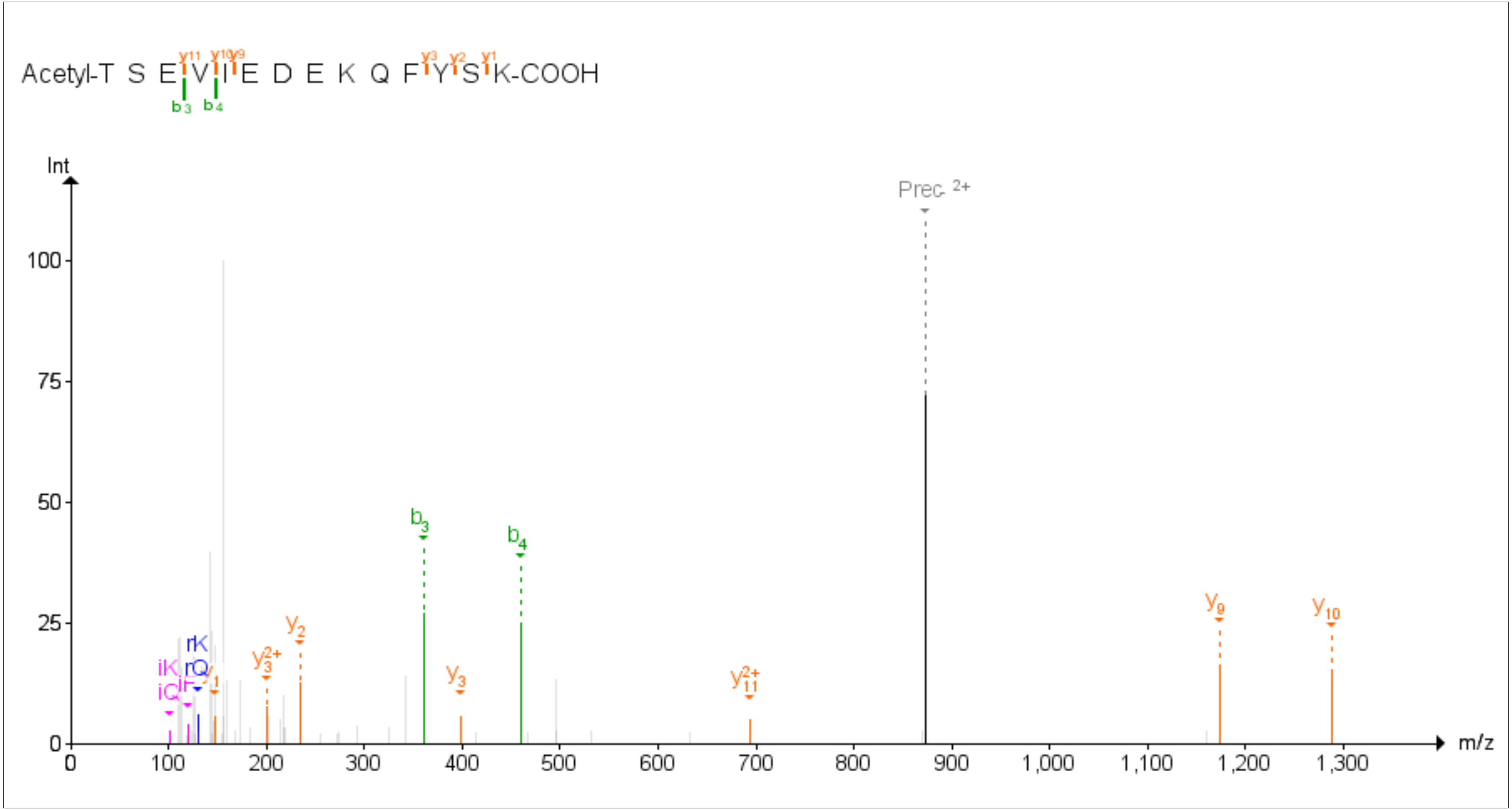

Acetyl-T S E V I E D E K Q F Y S K-COOH
y11 y10 y9 y3 y2 y1
b3 b4
Int
Prec 2+
b3
b4
y2
y3
y9
y10
rK
rQ
iK
iQ
iF
m/z
0
25
50
75
100
0
100
200
300
400
500
600
700
800
900
1,000
1,100
1,200
1,300

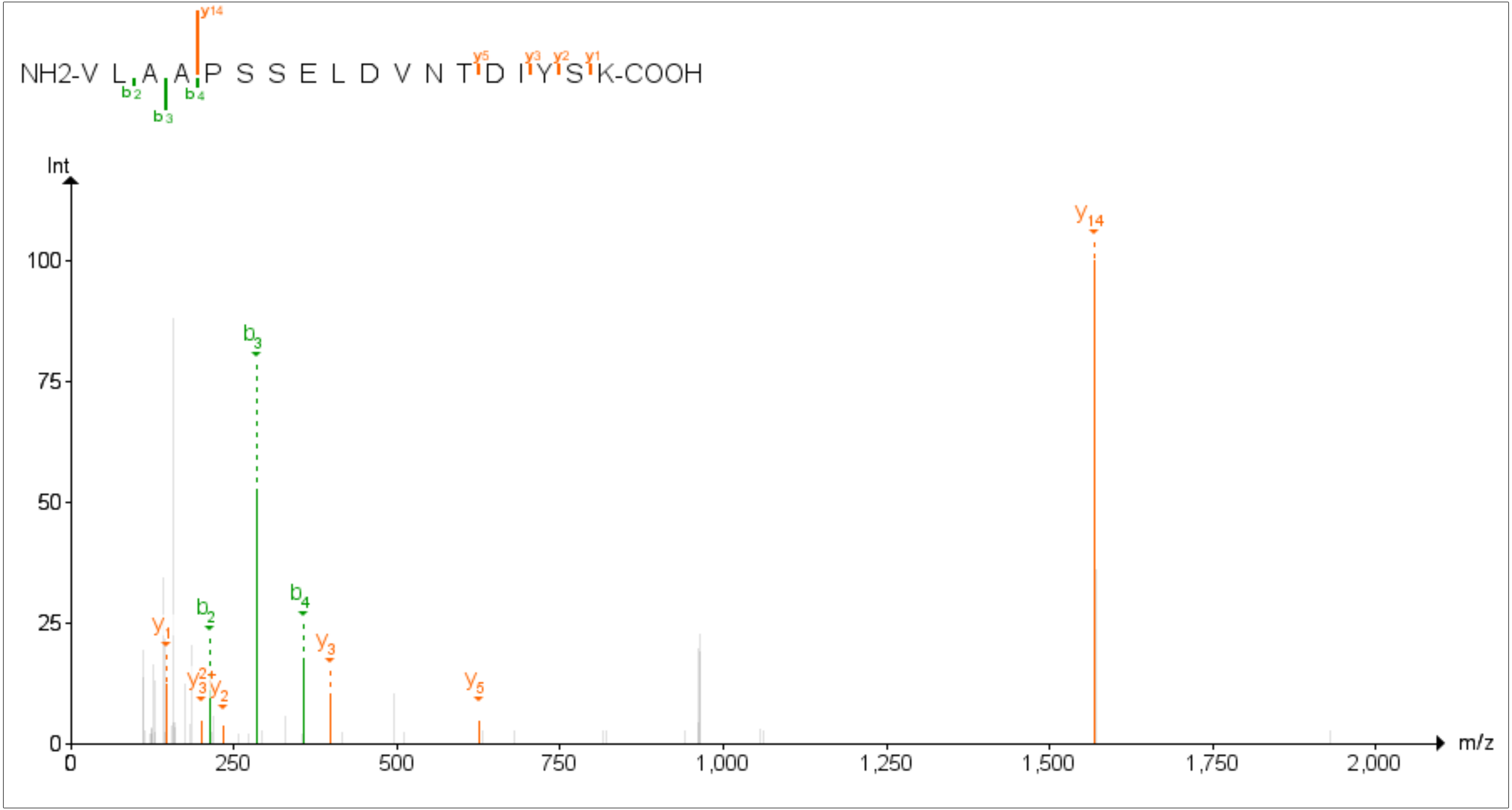

NH2-V L A A P S S E L D V N T D I Y S K-COOH
y14
y5
y3
y2
y1
b2
b3
b4
Int
100
75
50
25
0
y14
b3
b4
b2
y1
y3
y3 2+
y2
y5
0
250
500
750
1,000
1,250
1,500
1,750
2,000
m/z

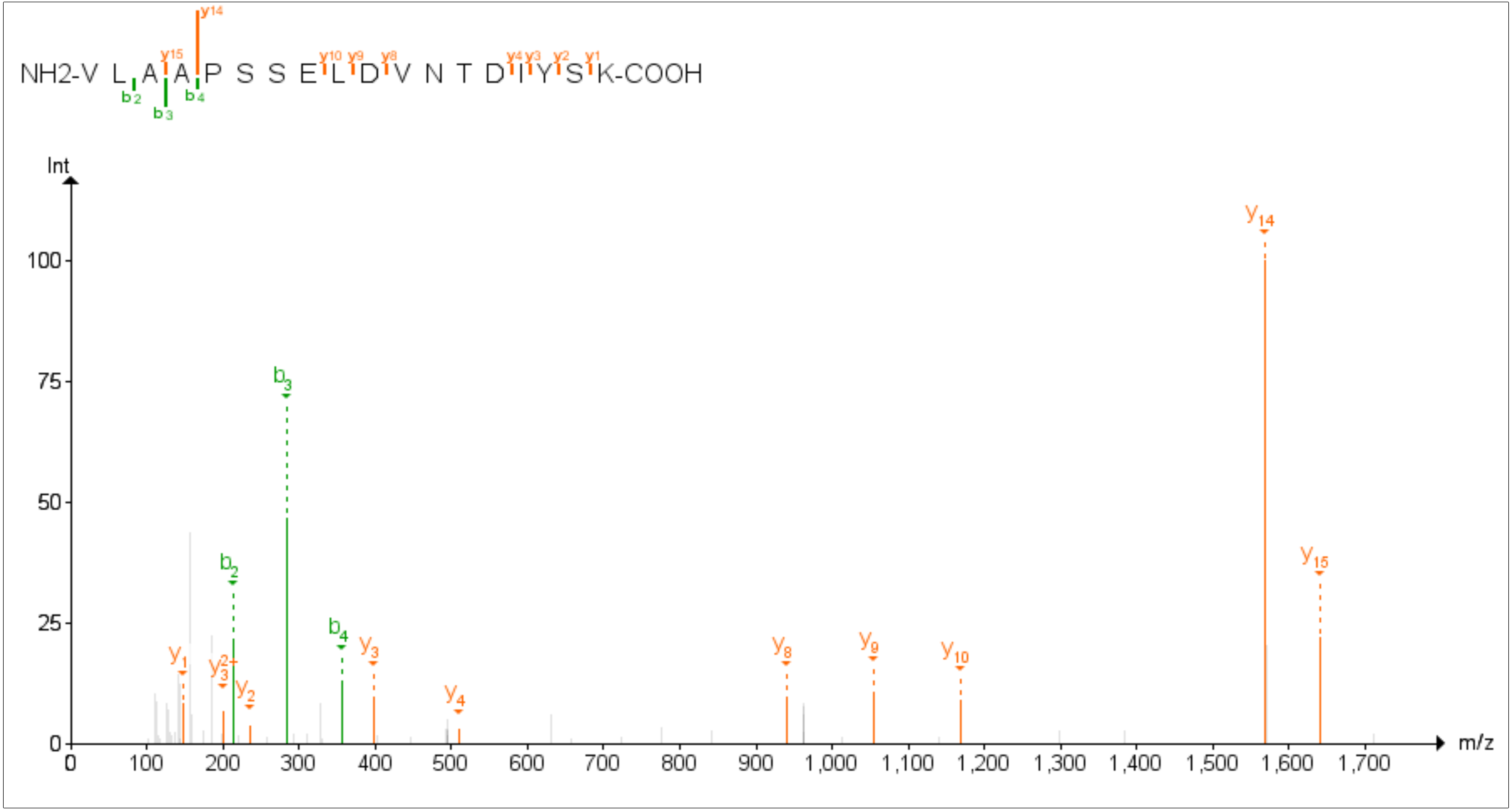

NH2-V L A A P S S E L D V N T D I Y S K-COOH
y14 y15 y10 y9 y8 y4 y3 y2 y1
b2 b3 b4
Int
m/z
y14
y15
b3
b2
b4
y1
y3 2+
y2
y3
y4
y8
y9
y10
0 25 50 75 100
0 100 200 300 400 500 600 700 800 900 1,000 1,100 1,200 1,300 1,400 1,500 1,600 1,700

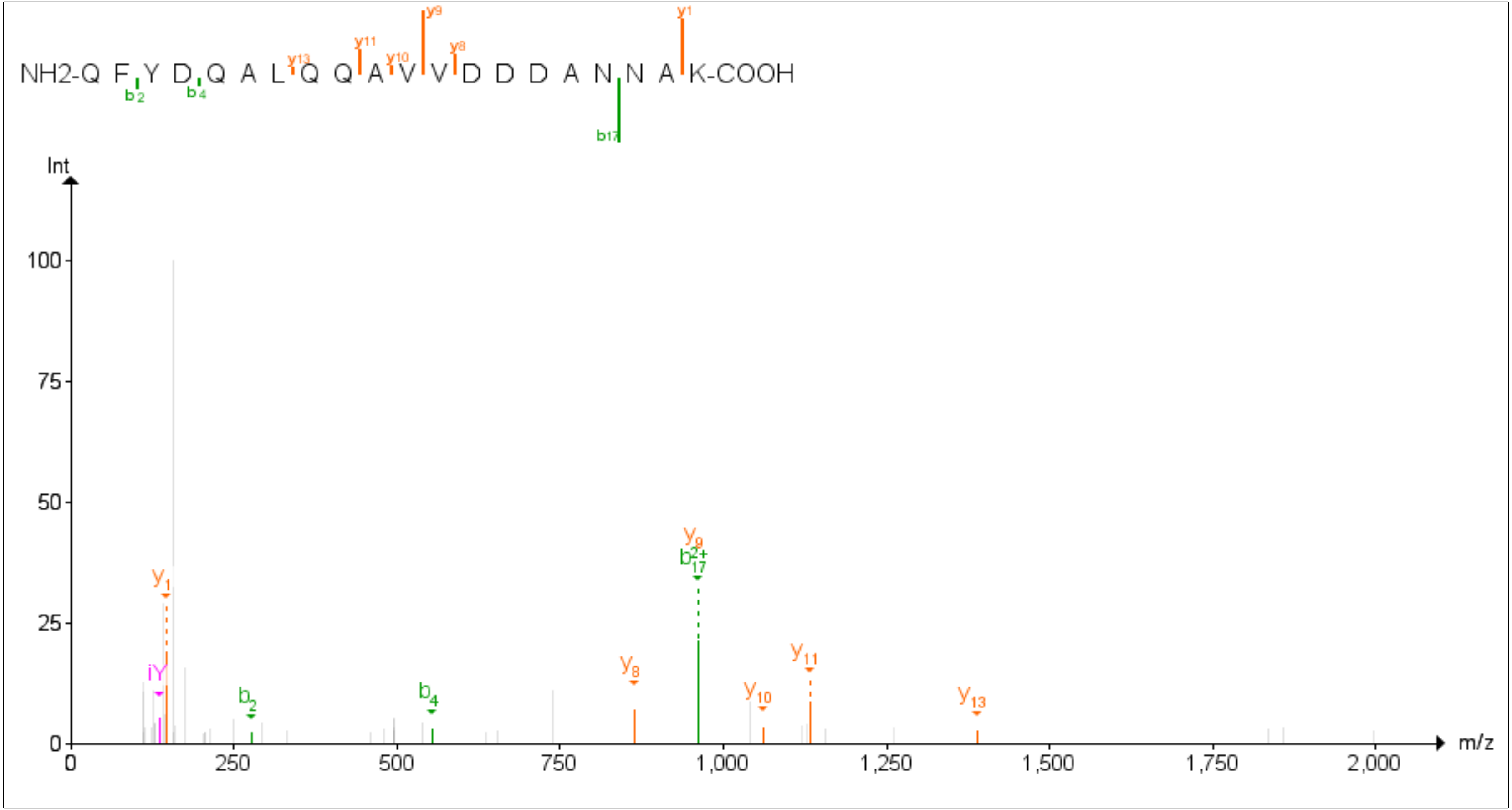

NH2-Q F Y D Q A L Q Q A V V D D D A N N A K-COOH
b2
b4
b17
y13
y11
y10
y9
y8
y1
Int
100
75
50
25
0
y1
iY
b2
b4
y8
y9
b17 2+
y10
y11
y13
0
250
500
750
1,000
1,250
1,500
1,750
2,000
m/z

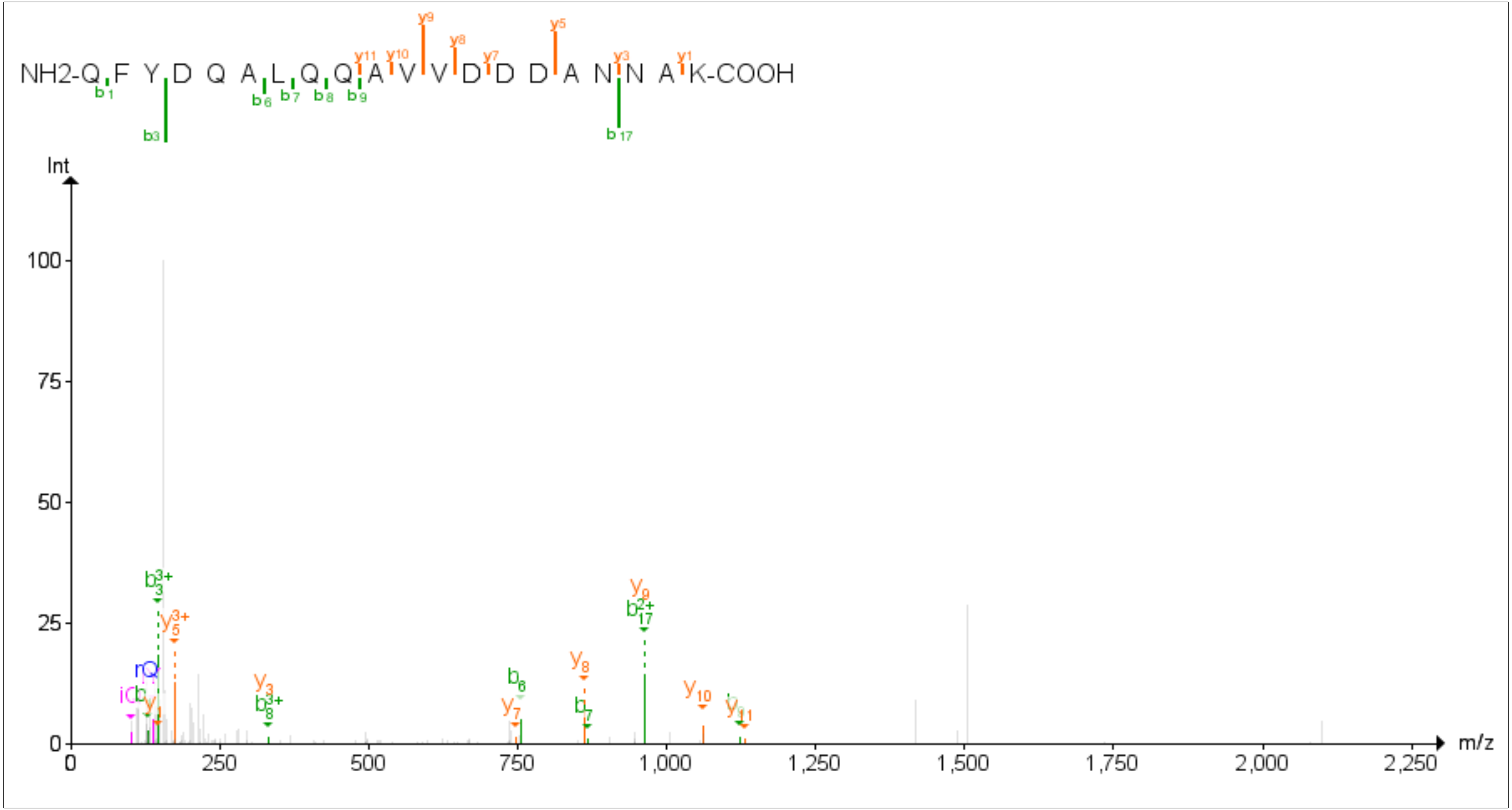

NH2-Q F Y D Q A L Q Q A V V D D D A N N A K-COOH
Int
m/z

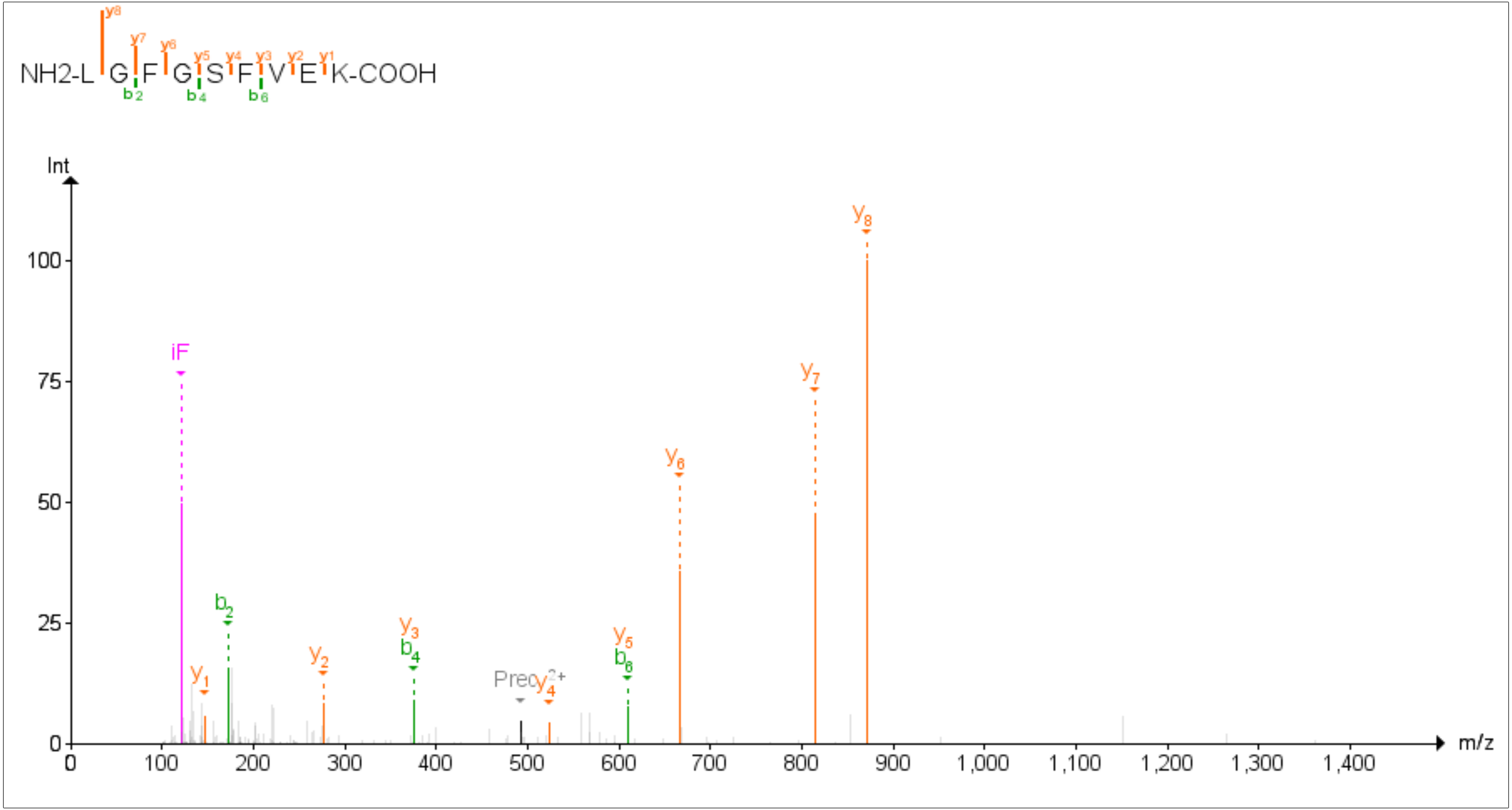

NH2-L G F G S F V E K-COOH
y8 y7 y6 y5 y4 y3 y2 y1
b2 b4 b6
Int
100
75
50
25
0
iF
b2
y1
y2
y3
b4
Prec
y4 2+
y5
b6
y6
y7
y8
0 100 200 300 400 500 600 700 800 900 1,000 1,100 1,200 1,300 1,400
m/z

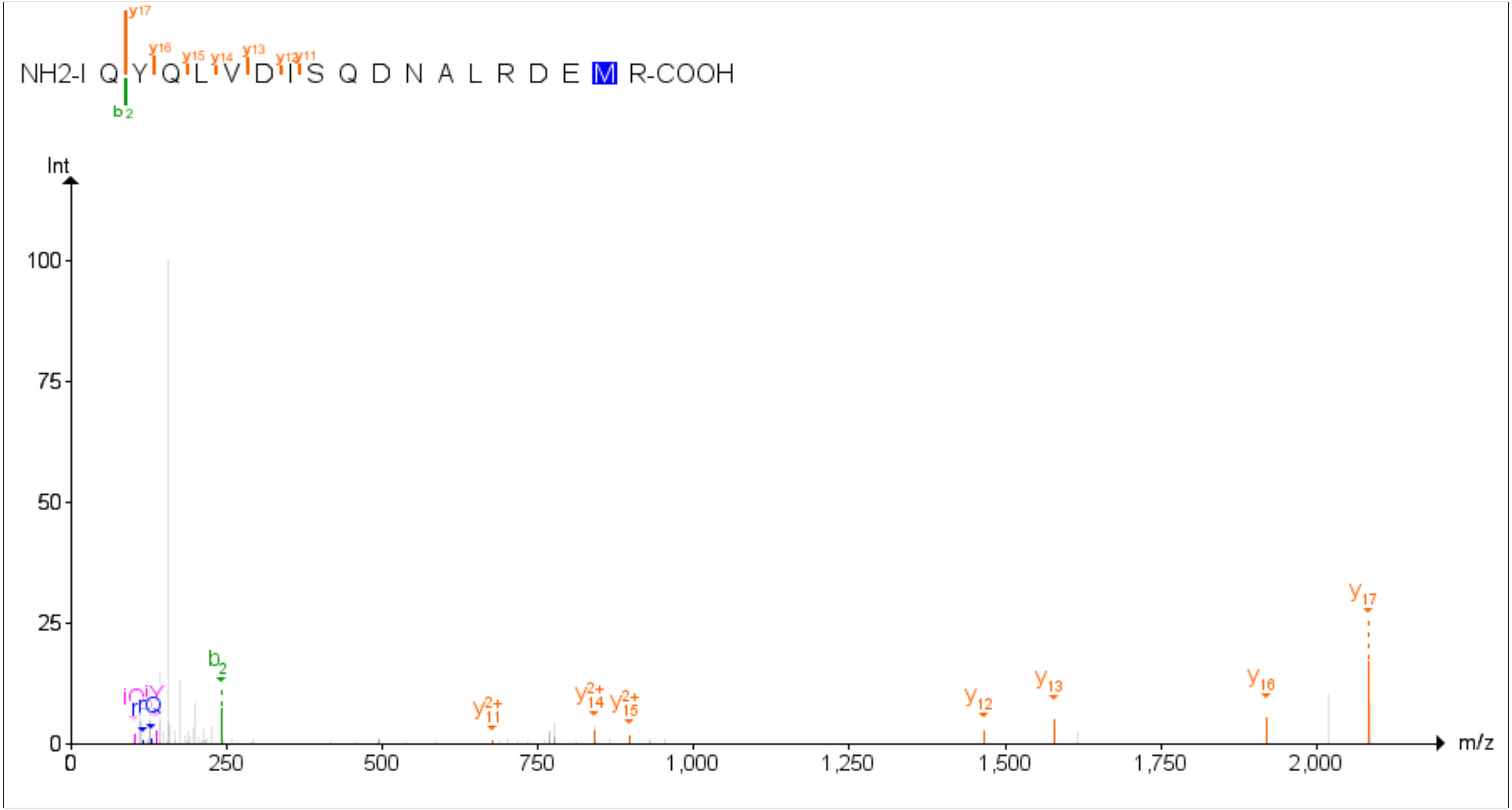

NH2-I Q Y Q L V D I S Q D N A L R D E M R-COOH
y17
y16
y15
y14
y13
y12
y11
b2
Int
100
75
50
25
0
m/z
0
250
500
750
1,000
1,250
1,500
1,750
2,000
b2
y11 2+
y14 2+
y15 2+
y12
y13
y16
y17

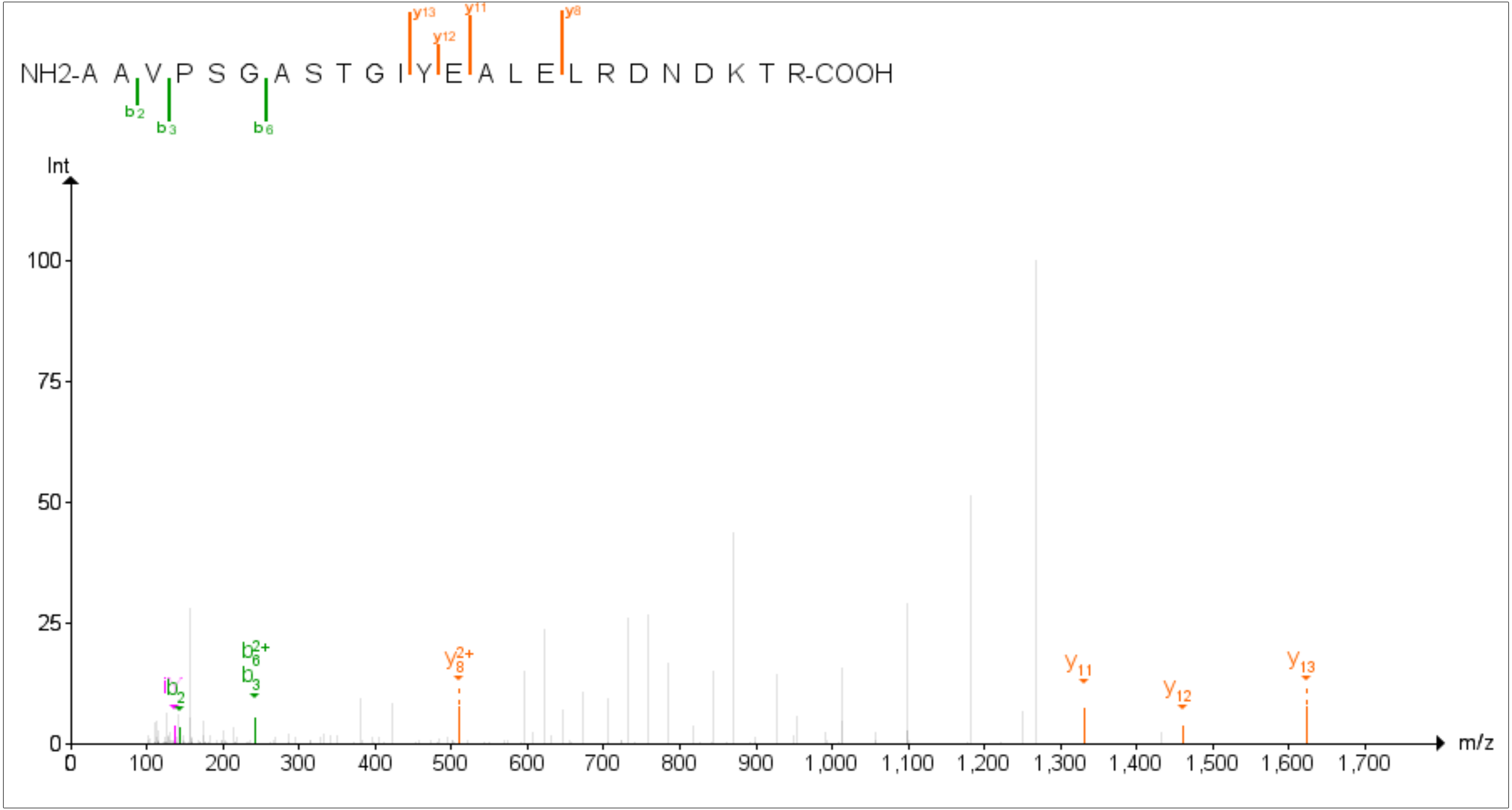
NH2-A A V P S G A S T G I Y E A L E L R D N D K T R-COOH
y13
y12
y11
y8
b2
b3
b6
Int
100
75
50
25
0
m/z
0
100
200
300
400
500
600
700
800
900
1,000
1,100
1,200
1,300
1,400
1,500
1,600
1,700
ib2
b6 2+
b3
y8 2+
y11
y12
y13

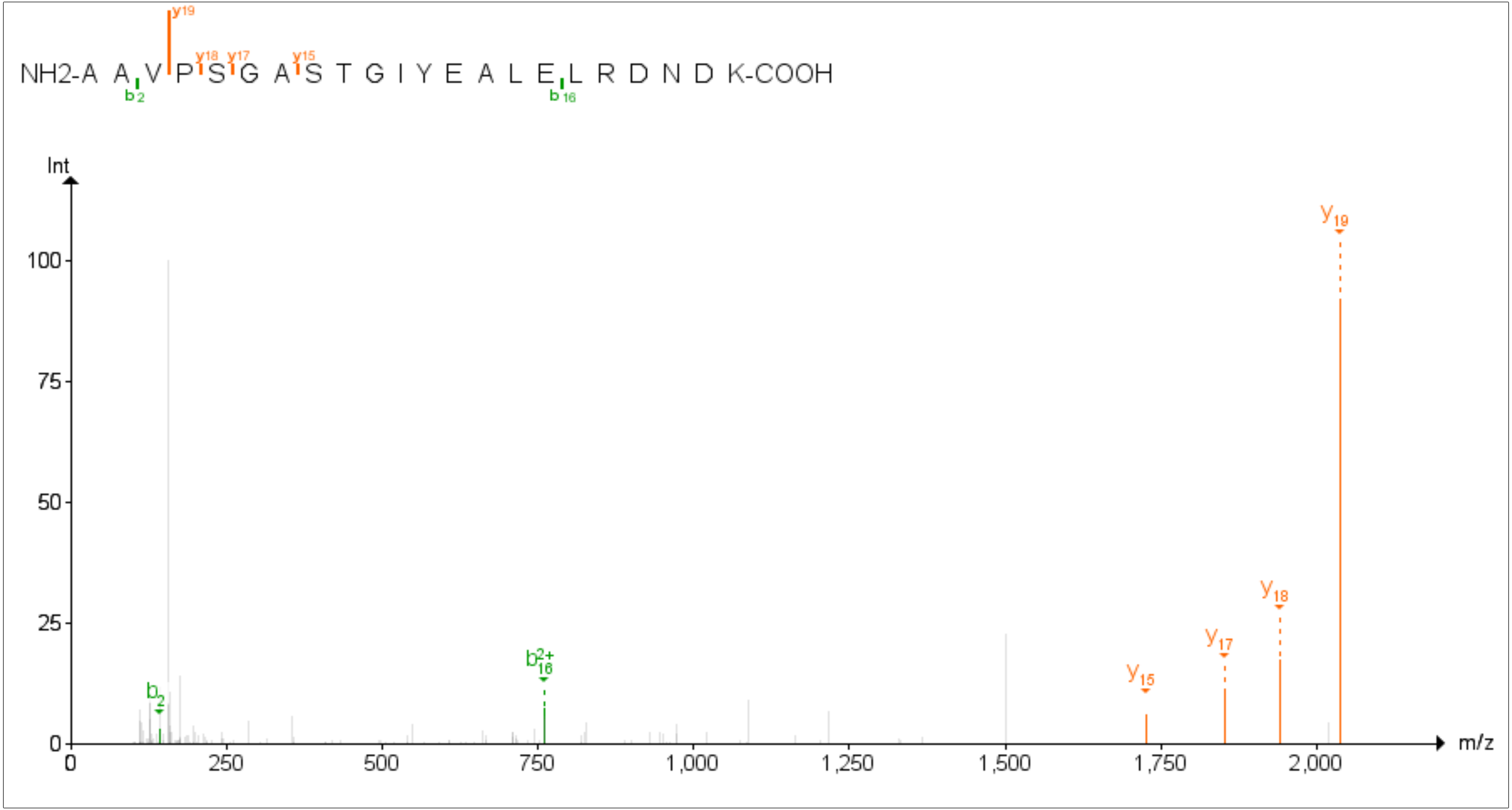
NH2-A A V P S G A S T G I Y E A L E L R D N D K-COOH
y19
y18
y17
y15
b2
b16
Int
100
75
50
25
0
m/z
0
250
500
750
1,000
1,250
1,500
1,750
2,000
y19
y18
y17
y15
b2+16
b2

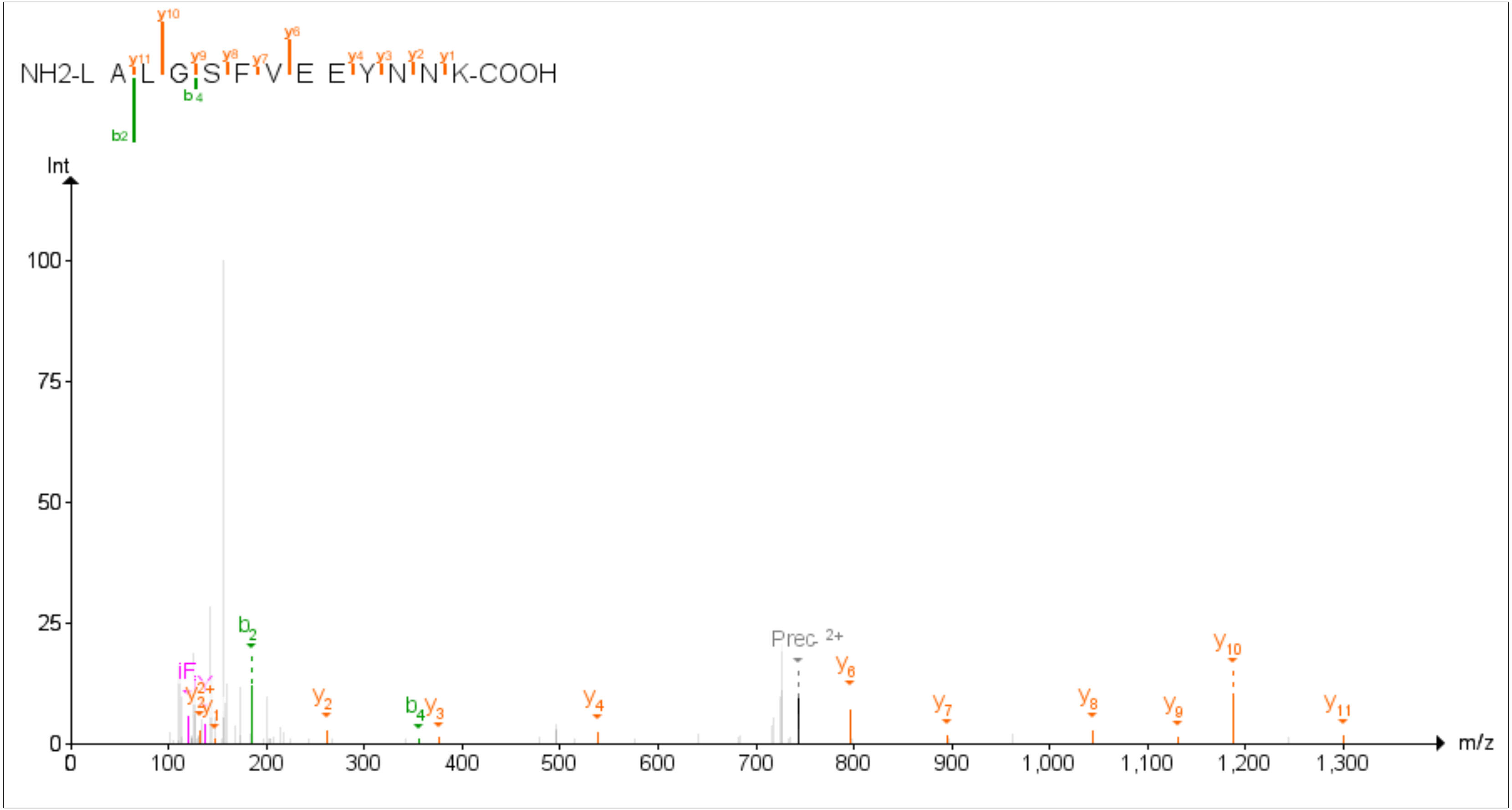

NH2-L A L G S F V E E Y N N K-COOH
y10
y11
y9
y8
y7
y6
y4
y3
y2
y1
b2
b4
Int
100
75
50
25
0
m/z
0
100
200
300
400
500
600
700
800
900
1,000
1,100
1,200
1,300
b2
Prec 2+
y10
y6
y7
y8
y9
y11
y4
y2
b4
y3
iF
y1

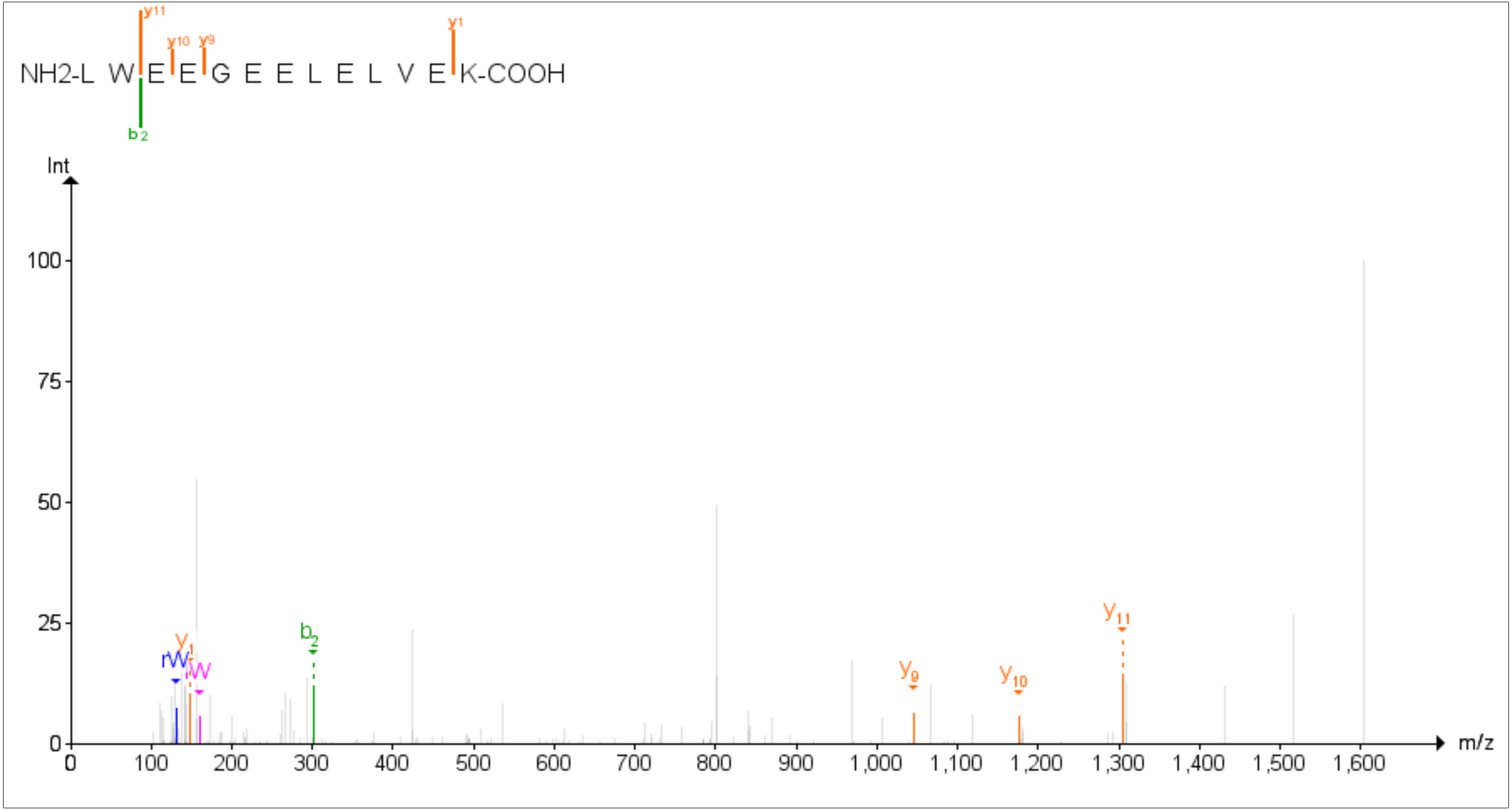

NH2-L W E E G E E L E L V E K-COOH
y11
y10
y9
y1
b2
Int
100
75
50
25
0
rW
y1
iW
b2
y9
y10
y11
0
100
200
300
400
500
600
700
800
900
1,000
1,100
1,200
1,300
1,400
1,500
1,600
m/z

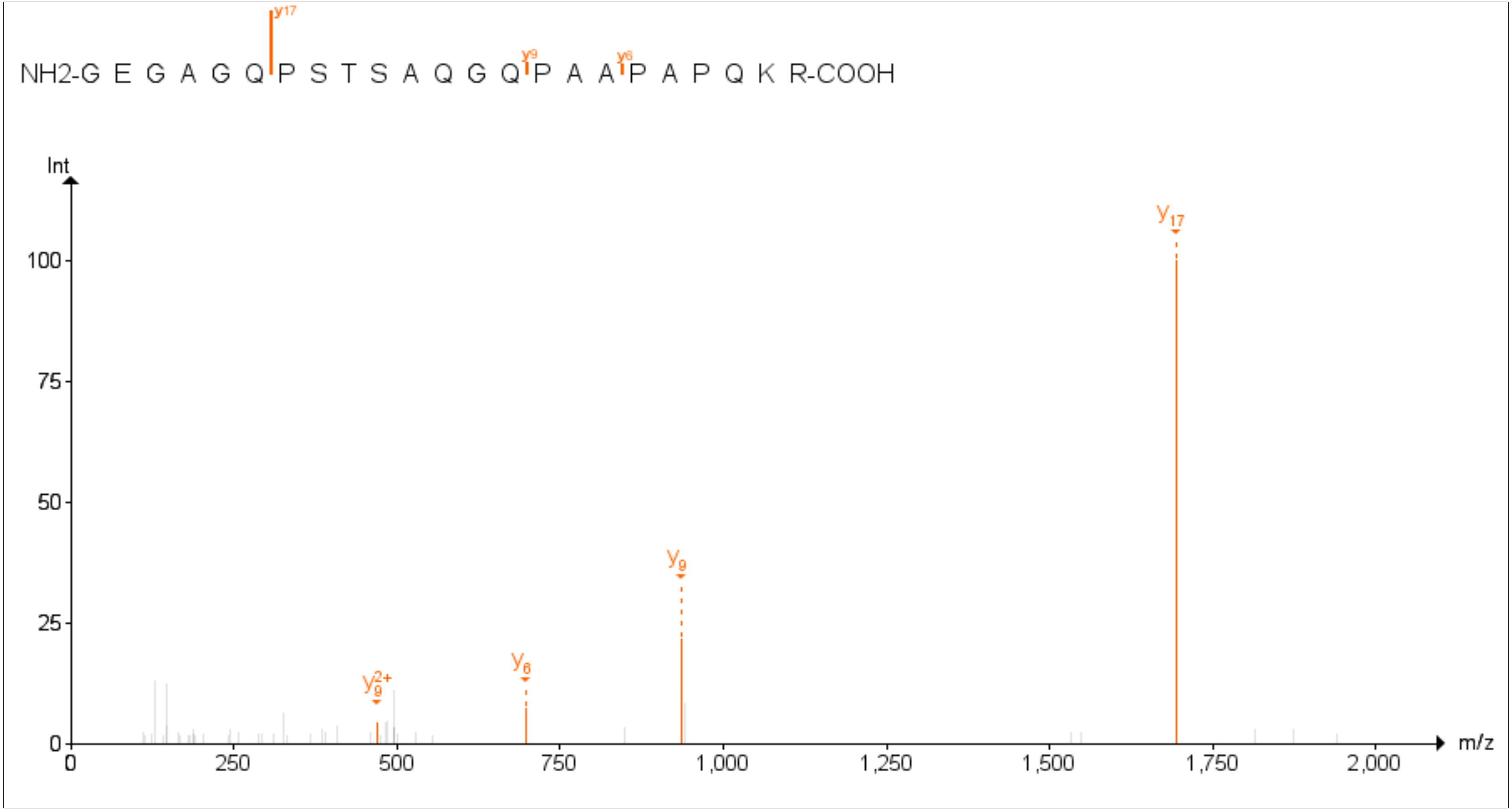

NH2-G E G A G Q P S T S A Q G Q P A A P A P Q K R-COOH
y17
y9
y6
Int
100
75
50
25
0
y_{17}
y_9
y_6
y_9^{2+}
0
250
500
750
1,000
1,250
1,500
1,750
2,000
m/z

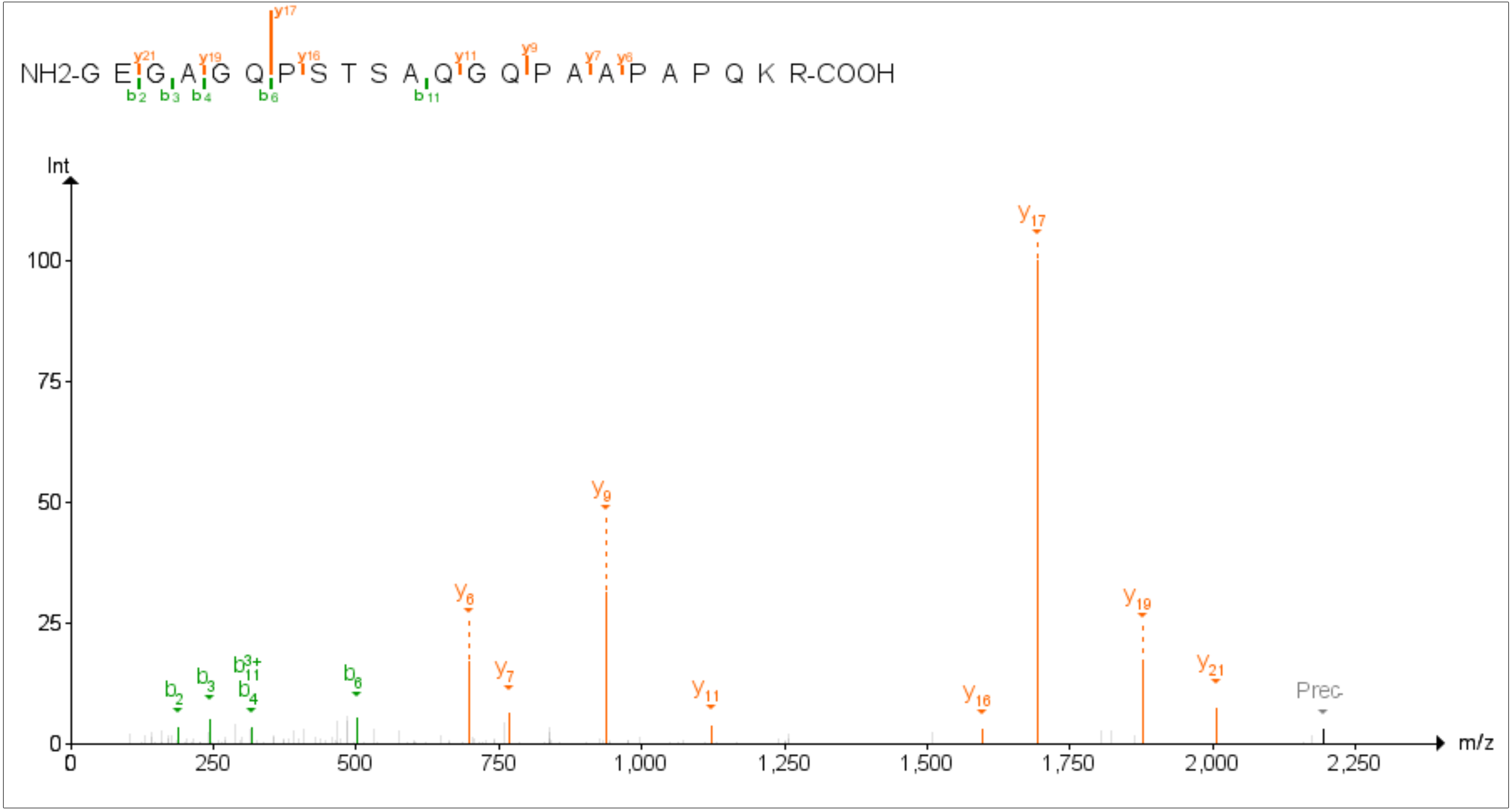

NH2-G E G A G Q P S T S A Q G Q P A A P A P Q K R-COOH
y21 y19 y17 y16 y11 y9 y7 y6
b2 b3 b4 b6 b11
Int
100
75
50
25
0
y17
y9
y6
y7
y11
y16
y19
y21
b2
b3
b11 3+
b4
b6
Prec
0 250 500 750 1,000 1,250 1,500 1,750 2,000 2,250
m/z

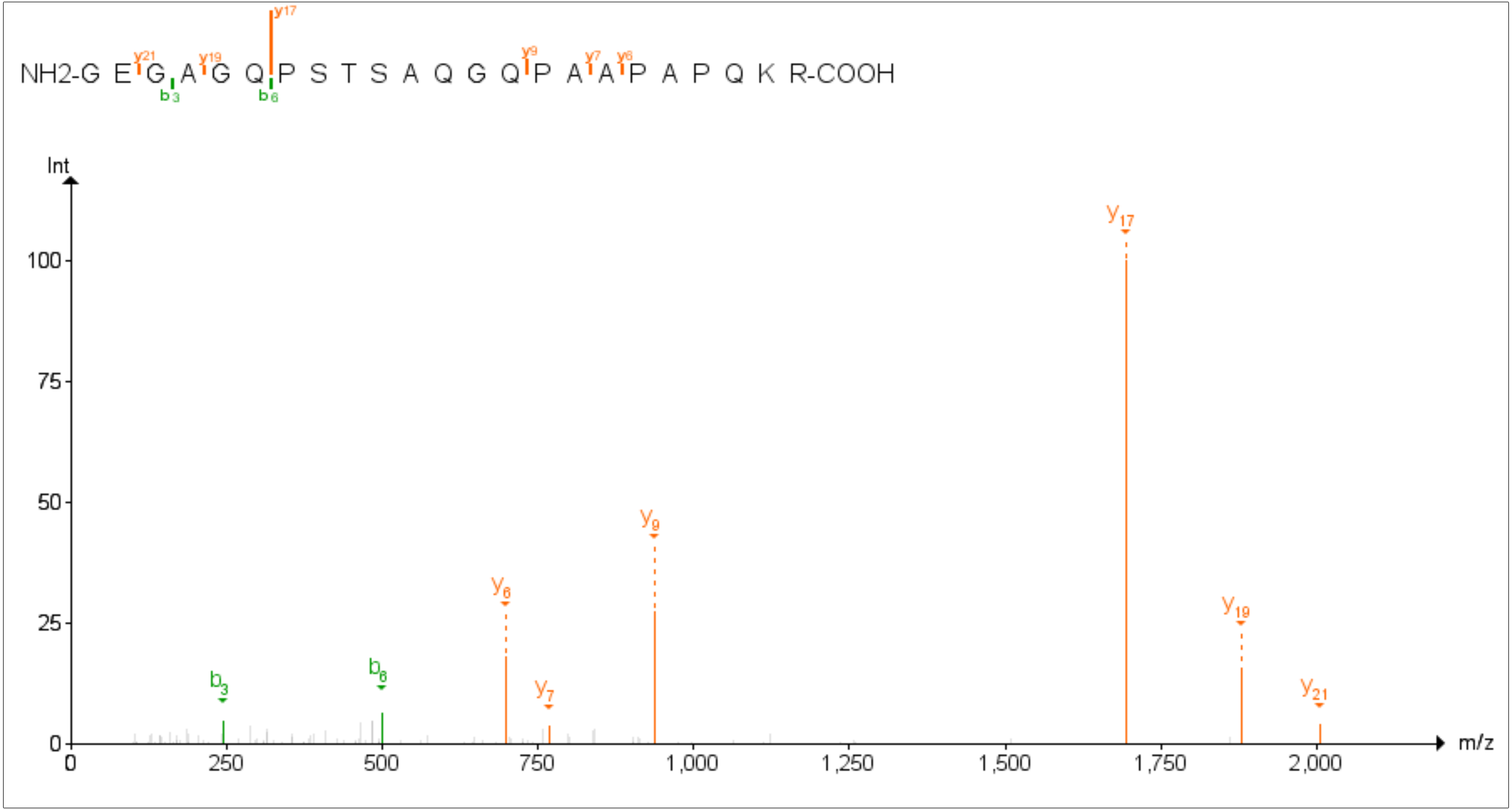
NH2-G E G A G Q P S T S A Q G G Q P A A P A P Q K R-COOH
y21 y19 y17 y9 y7 y6
b3 b6
Int
100
75
50
25
0
y17
y9
y6
y7
y19
y21
b3
b6
0 250 500 750 1,000 1,250 1,500 1,750 2,000
m/z

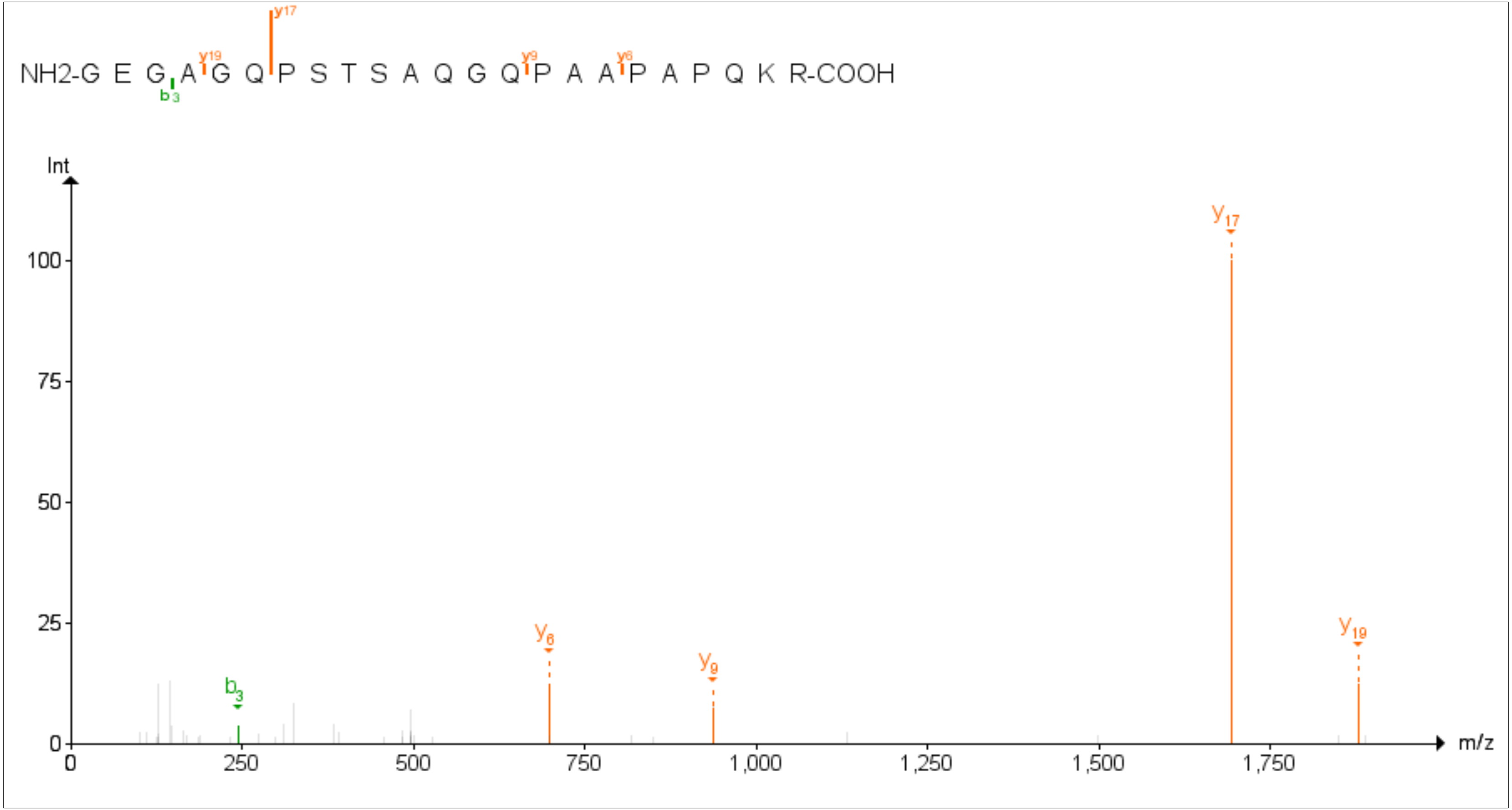

NH2-G E G A G Q P S T S A Q G Q P A A P A P Q K R-COOH
y17
y19
y9
y6
b3
Int
100
75
50
25
0
y17
y6
y9
b3
y19
0
250
500
750
1,000
1,250
1,500
1,750
m/z

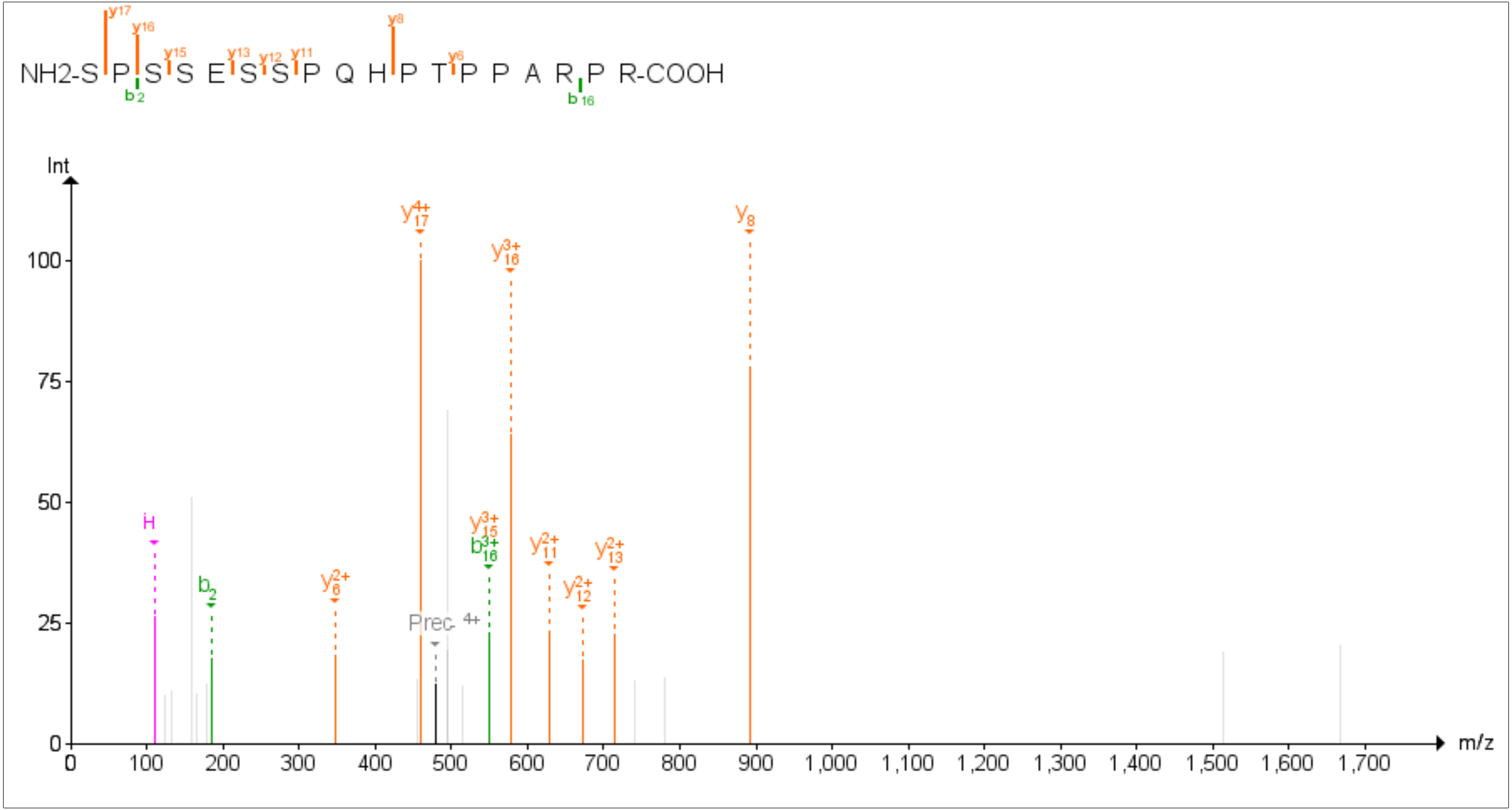

NH2-S P S S E S S P Q H P T P P A R P R-COOH
y17 y16 y15 y13 y12 y11 y8 y6
b2 b16
Int
m/z
y17 4+
y8
y16 3+
y15 3+
b16 3+
y11 2+
y13 2+
y12 2+
y6 2+
b2
Prec 4+
0
25
50
75
100
0 100 200 300 400 500 600 700 800 900 1,000 1,100 1,200 1,300 1,400 1,500 1,600 1,700

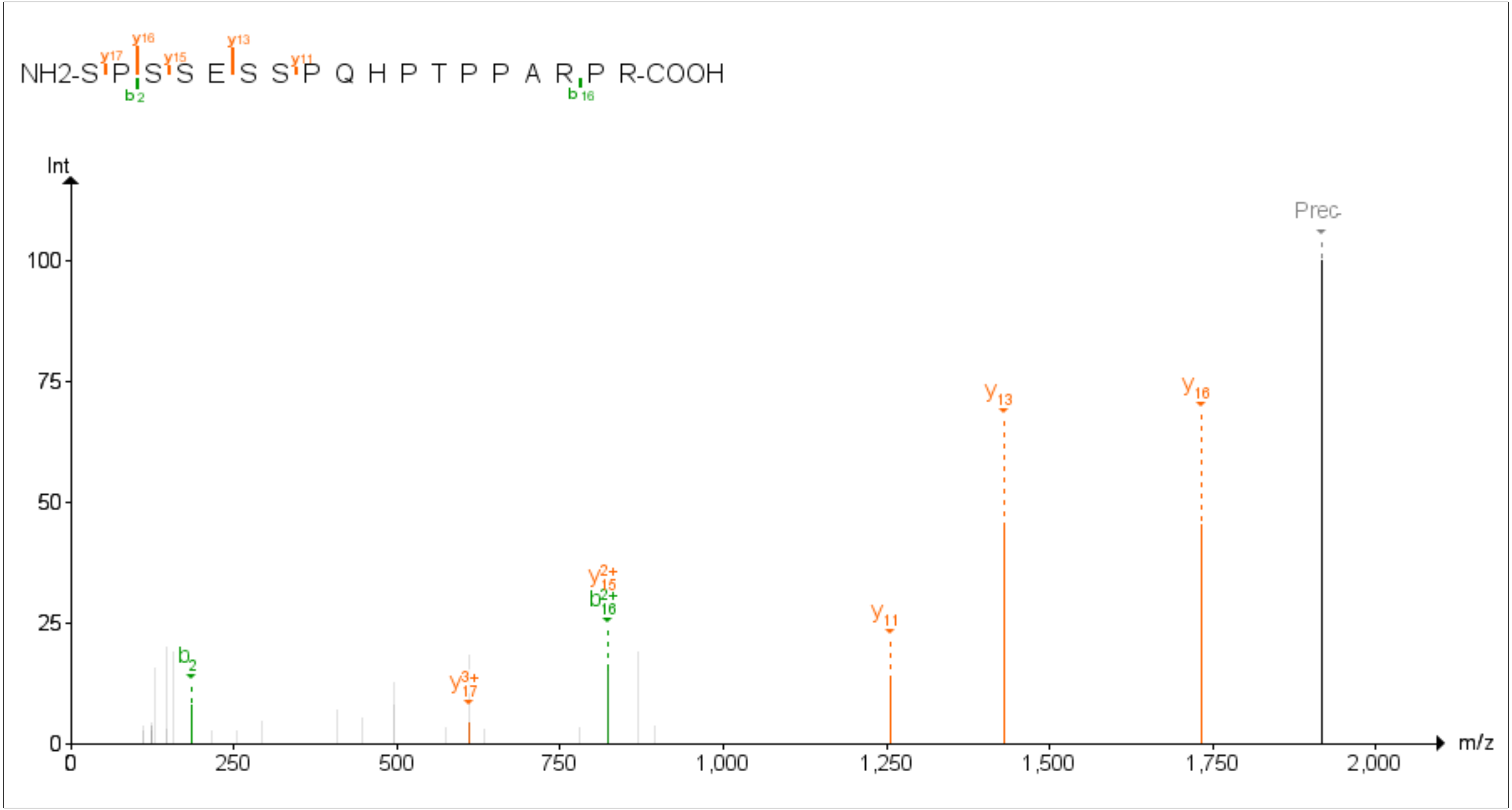

NH2-S P S S E S S P Q H P T P P A R P R-COOH
y17
y16
y15
y13
y11
b2
b16
Int
100
75
50
25
0
Prec
y13
y16
y11
y15 2+
b16 2+
y17 3+
b2
0
250
500
750
1,000
1,250
1,500
1,750
2,000
m/z

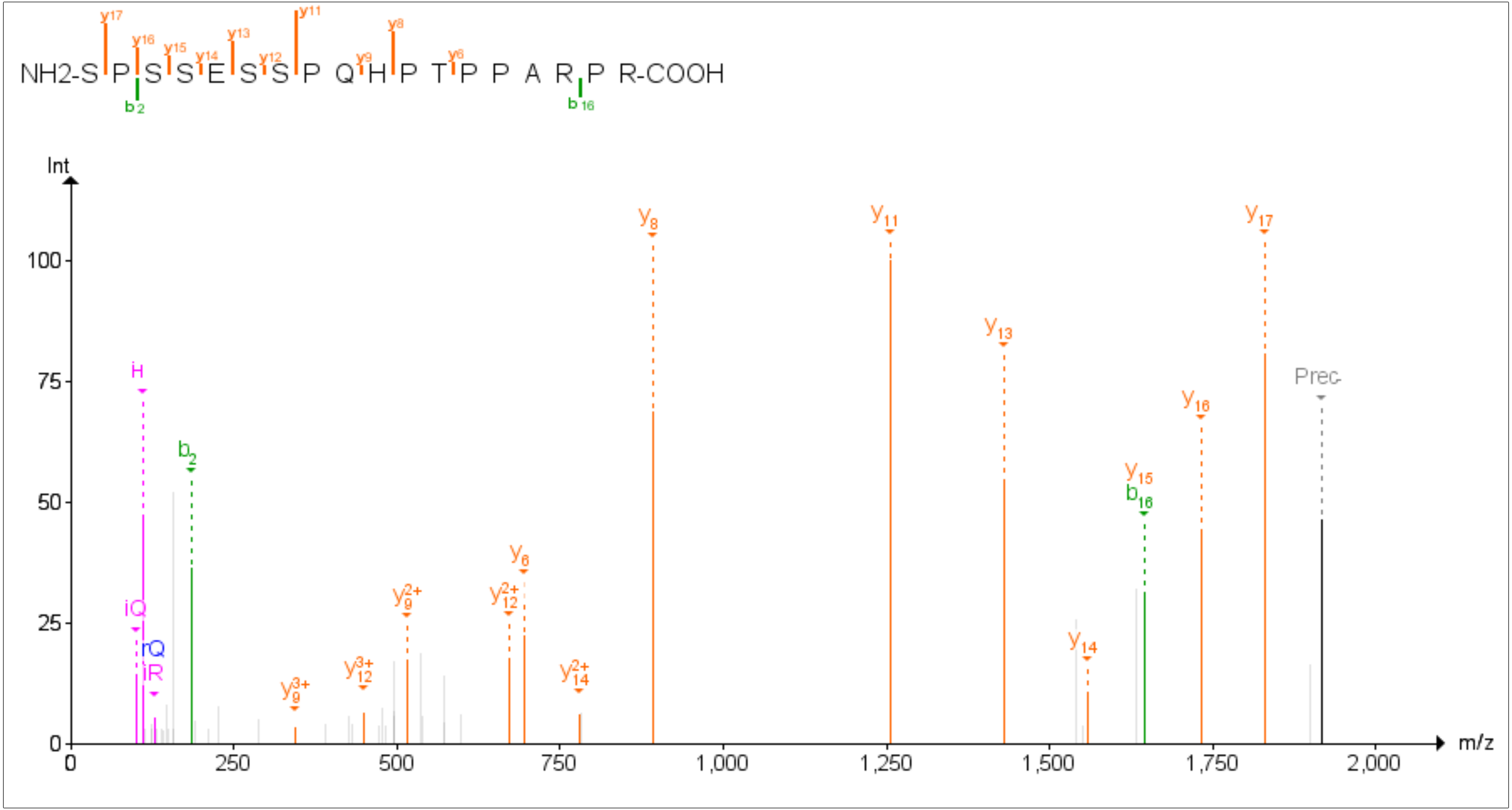

NH2-S P S S E S S P Q H P T P P A R P R-COOH
y17 y16 y15 y14 y13 y12 y11 y9 y8 y6
b2 b16
Int
100
75
50
25
0
iH
b2
iQ
rQ
iR
y8
y11
y13
y17
Prec
y16
y15
b16
y14
y6
y9 2+
y12 2+
y14 2+
y9 3+
y12 3+
0
250
500
750
1,000
1,250
1,500
1,750
2,000
m/z

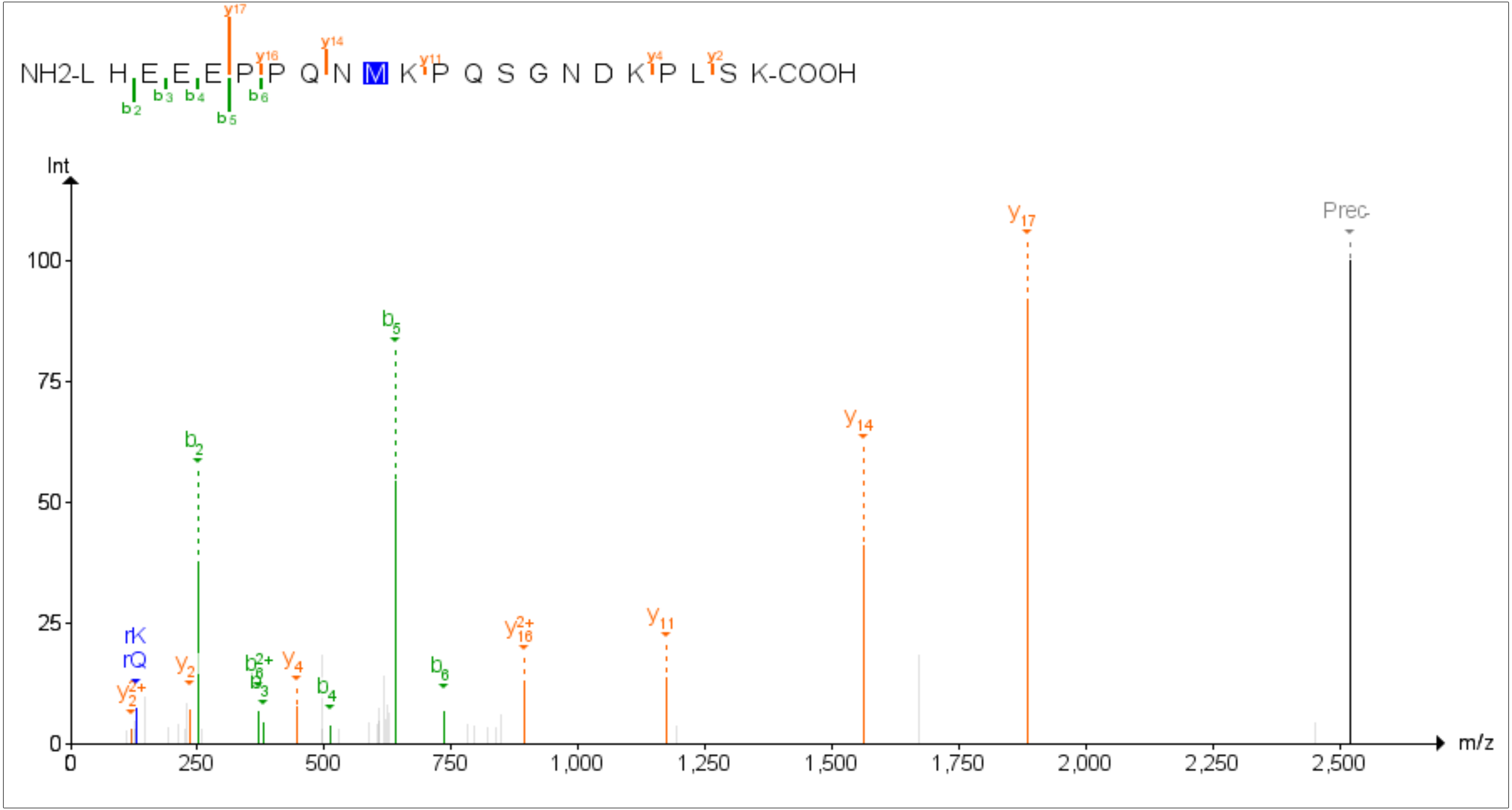

NH2-L H E E E P P Q N M K P Q S G N D K P L S K-COOH
y17 y16 y14 y11 y4 y2
b2 b3 b4 b5 b6
Int
m/z
Prec
y17
y14
b5
b2
y11
y16 2+
b6
b4
y4
b3
y2
y2 2+
rK
rQ
0
25
50
75
100
250
500
750
1,000
1,250
1,500
1,750
2,000
2,250
2,500

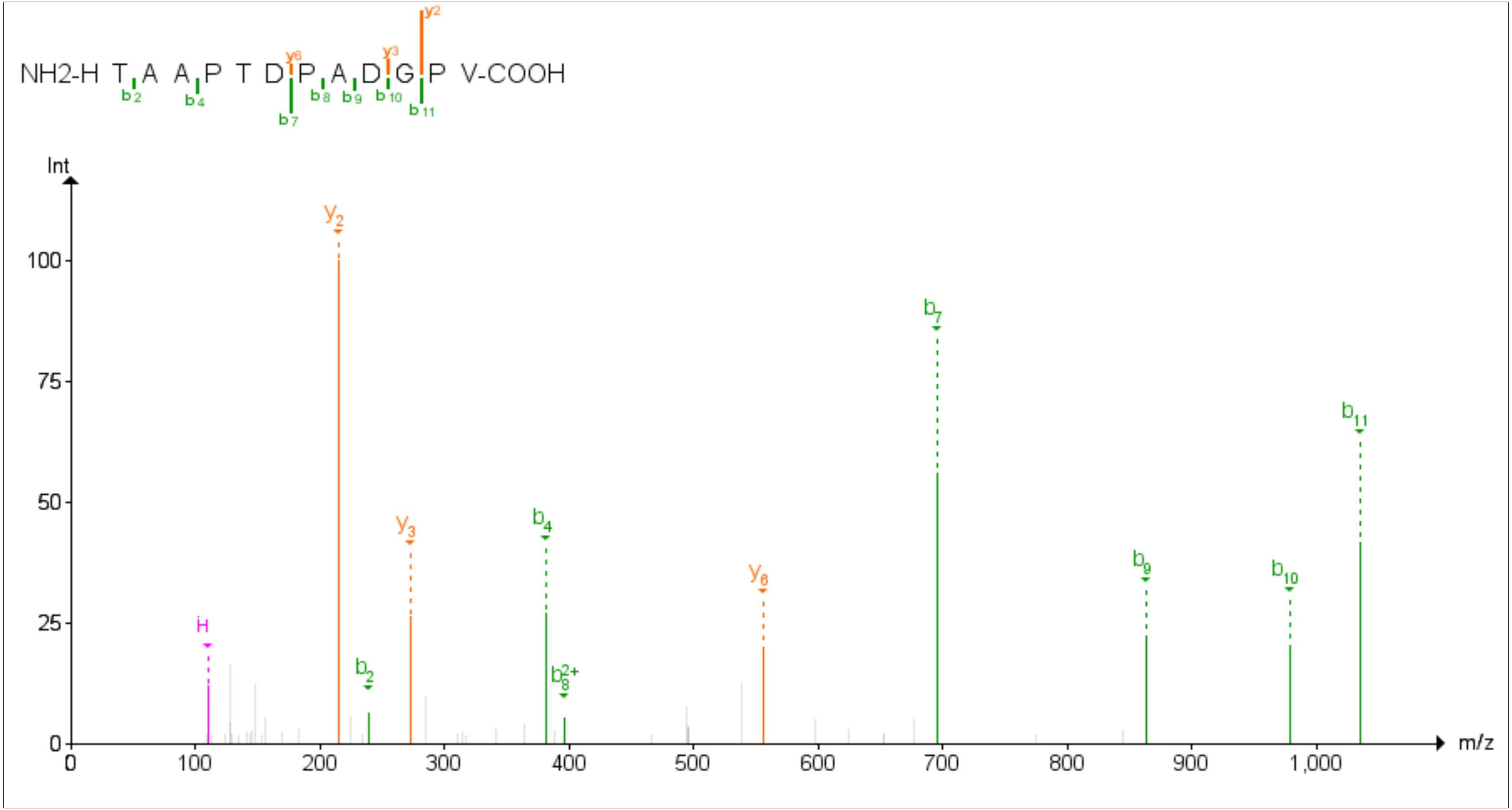
NH2-H T A A P T D P A D G P V-COOH
b2
b4
b7
b8
b9
b10
b11
y6
y3
y2
Int
100
75
50
25
0
H
y2
y3
y6
b2
b4
b8 2+
b7
b9
b10
b11
0
100
200
300
400
500
600
700
800
900
1,000
m/z

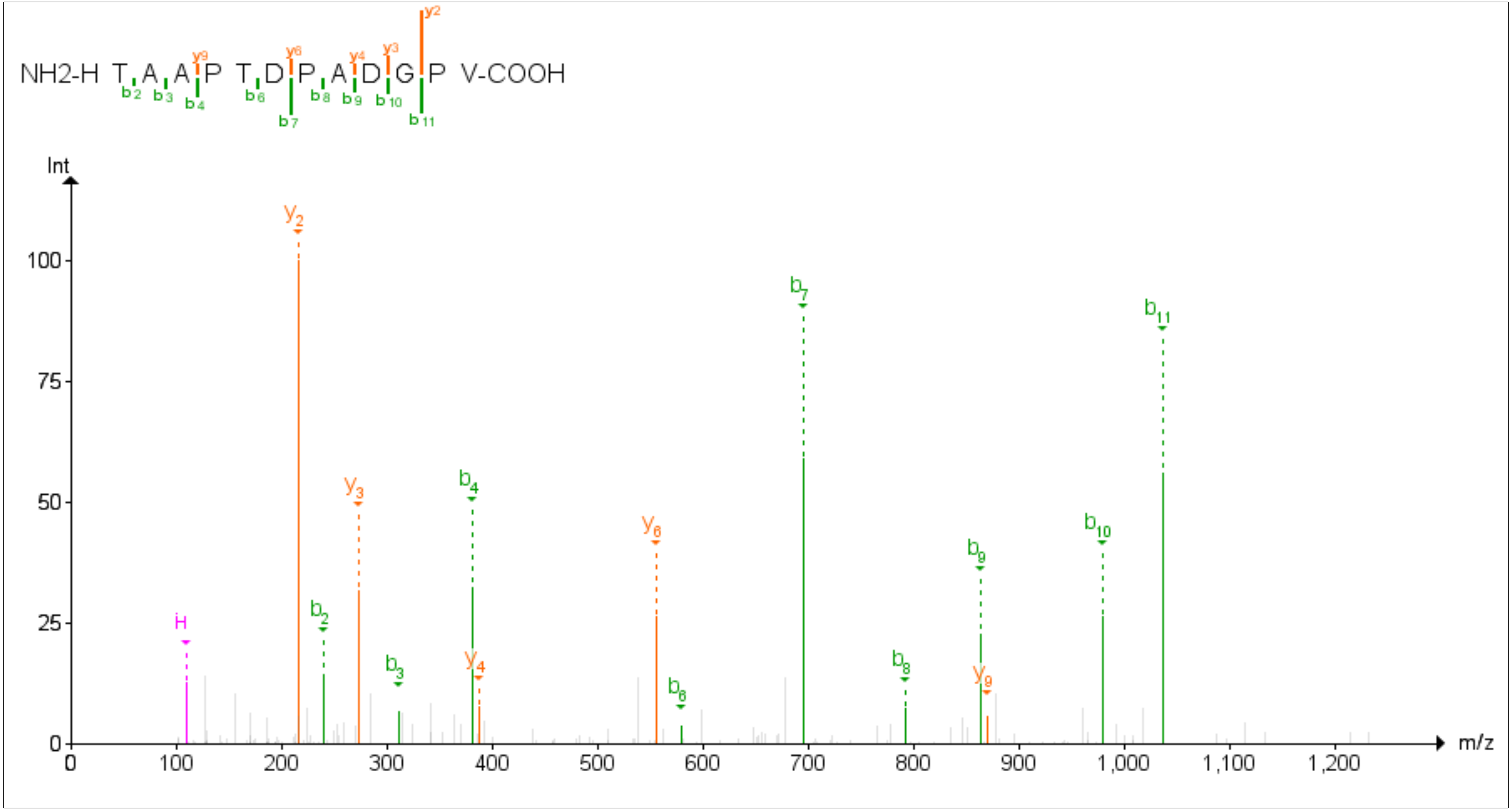

NH2-H T A A P T D P A D G P V-COOH
y9 y6 y4 y3 y2
b2 b3 b4 b6 b7 b8 b9 b10 b11
Int
100
75
50
25
0
m/z
0
100
200
300
400
500
600
700
800
900
1,000
1,100
1,200
y2
y3
y4
y6
y9
b2
b3
b4
b6
b7
b8
b9
b10
b11
H

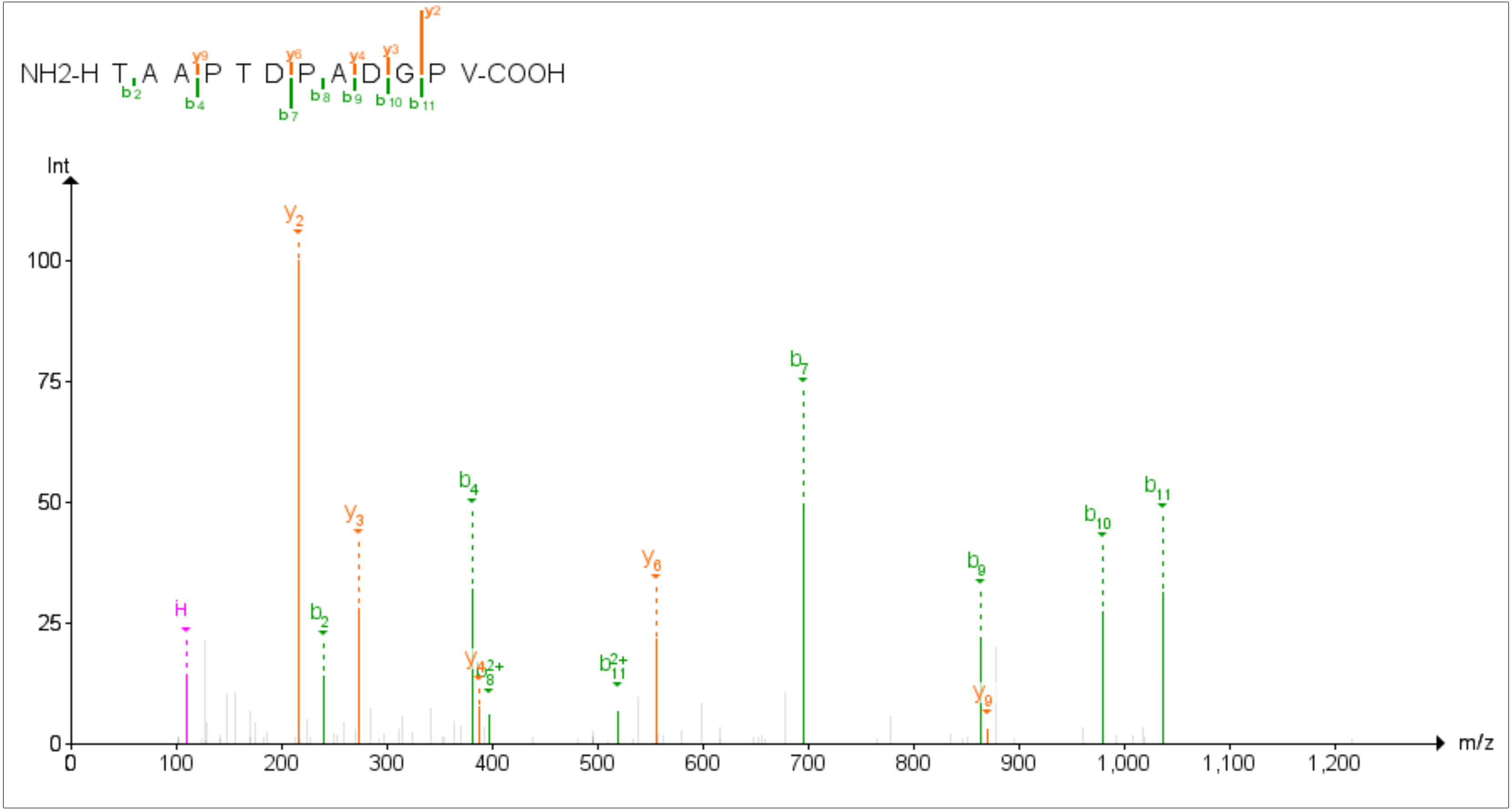
NH2-H T A A P T D P A D G P V-COOH
y9 y6 y4 y3 y2
b2 b4 b7 b8 b9 b10 b11
Int
m/z
y2
y3
b2
b4
y4
b7
b10
b11
b9
y6
y9
H

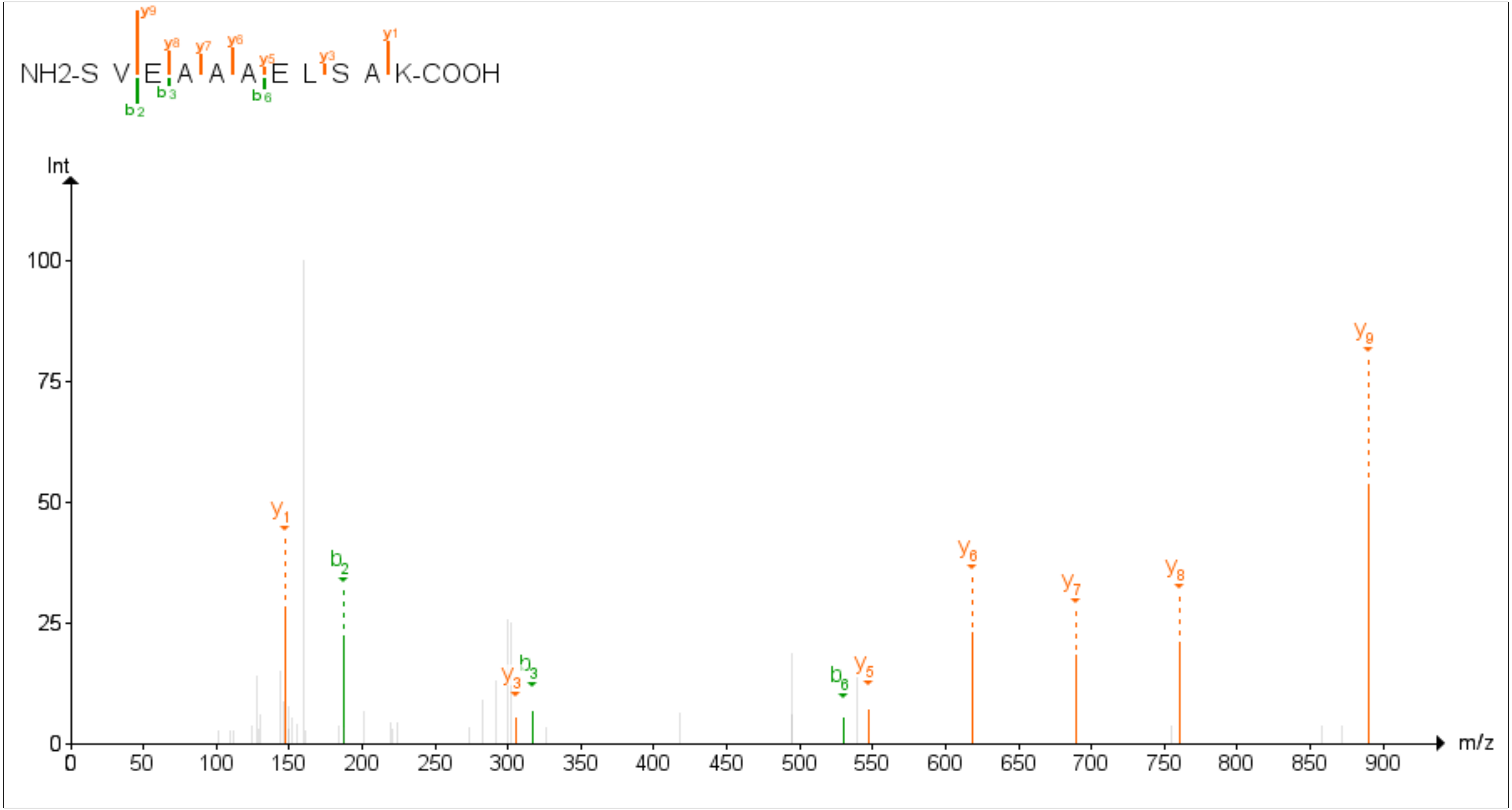

NH2-S V E A A A E L S A K-COOH
y9
y8
y7
y6
y5
y3
y1
b2
b3
b6
Int
100
75
50
25
0
m/z
0
50
100
150
200
250
300
350
400
450
500
550
600
650
700
750
800
850
900
y1
b2
y3
b3
b6
y5
y6
y7
y8
y9

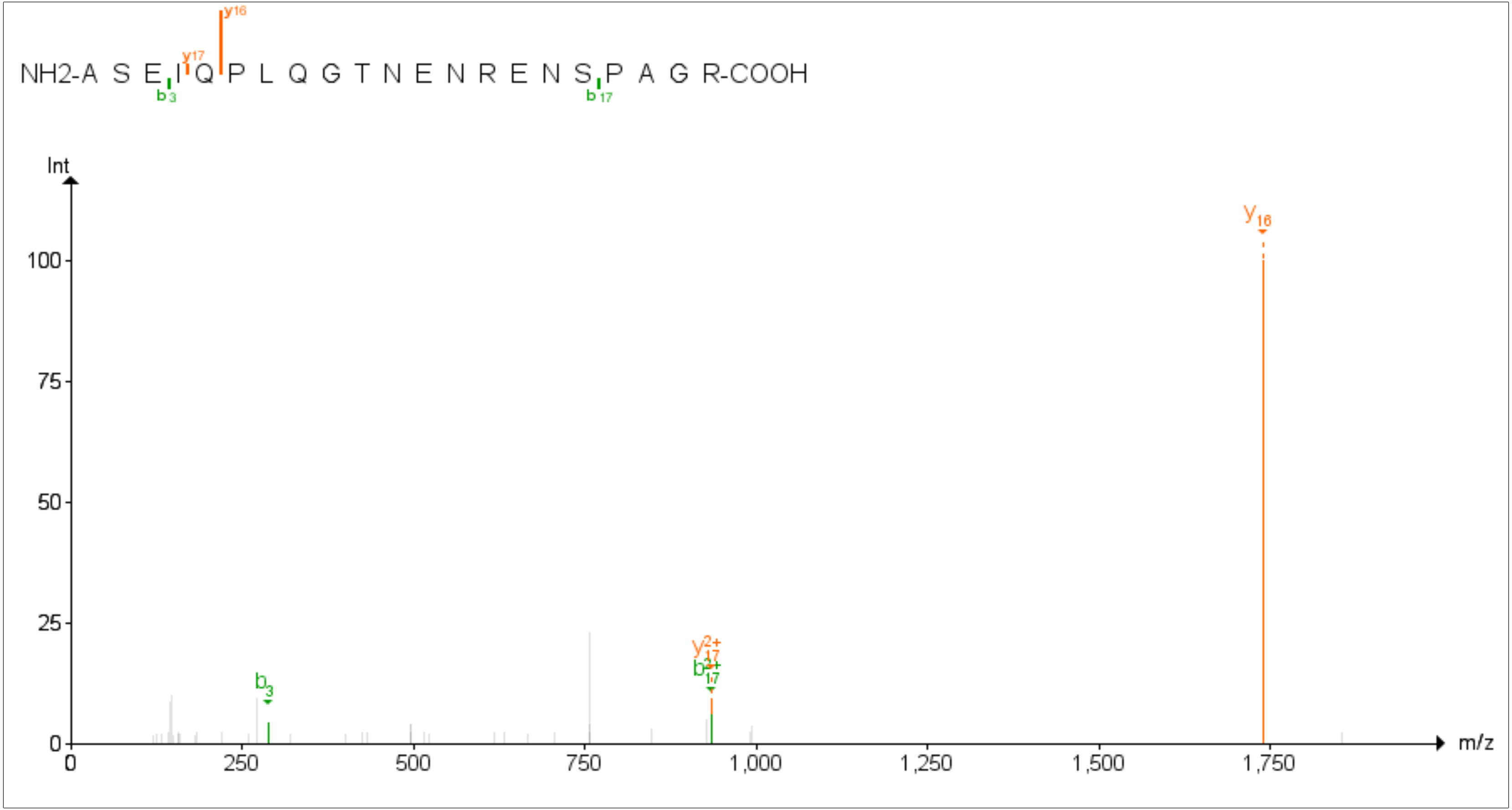

NH2-A S E I Q P L Q G T N E N R E N S P A G R-COOH
y16
y17
b3
b17
Int
100
75
50
25
0
m/z
0
250
500
750
1,000
1,250
1,500
1,750
y16
y17 2+
b17 2+
b3

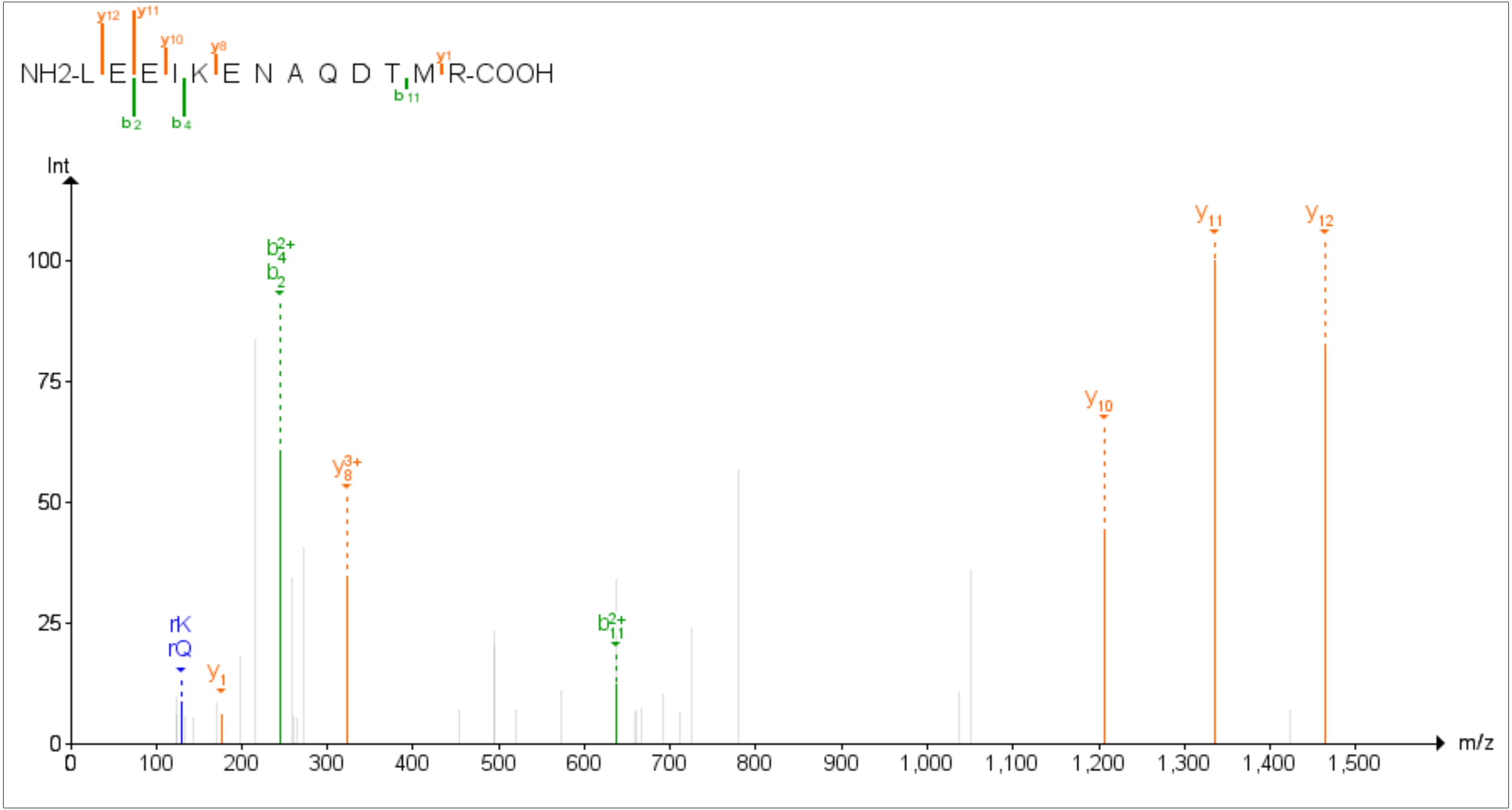

NH2-L E E I K E N A Q D T M R-COOH
y12
y11
y10
y8
y1
b2
b4
b11
Int
m/z
b_4^{2+}
b_2
y_8^{3+}
y_{10}
y_{11}
y_{12}
b_{11}^{2+}
rK
rQ
y_1

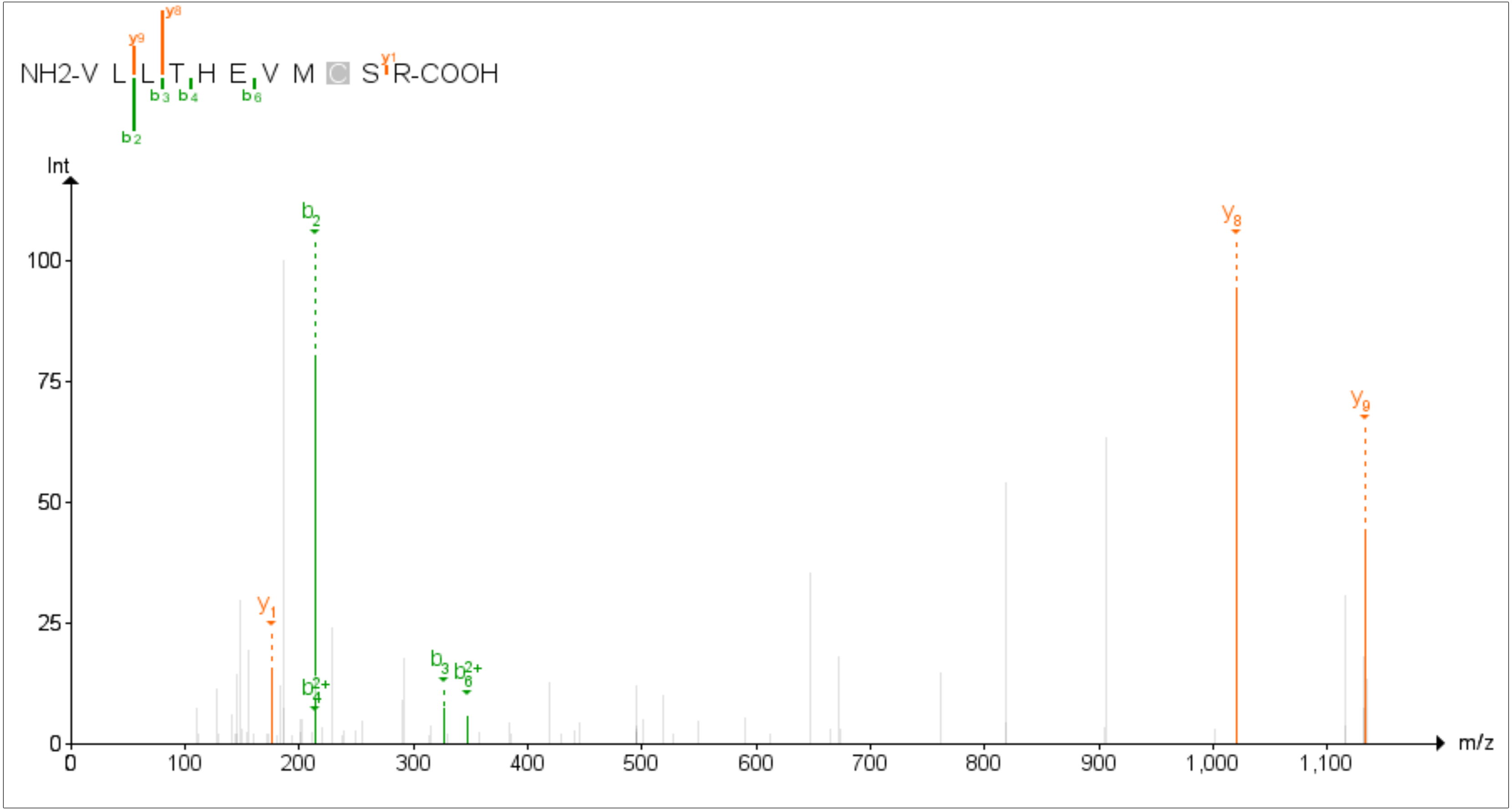

NH2-V L L T H E V M C S R-COOH
y9
y8
y1
b2
b3
b4
b6
Int
100
75
50
25
0
b2
y8
y9
y1
b4 2+
b3
b6 2+
0
100
200
300
400
500
600
700
800
900
1,000
1,100
m/z

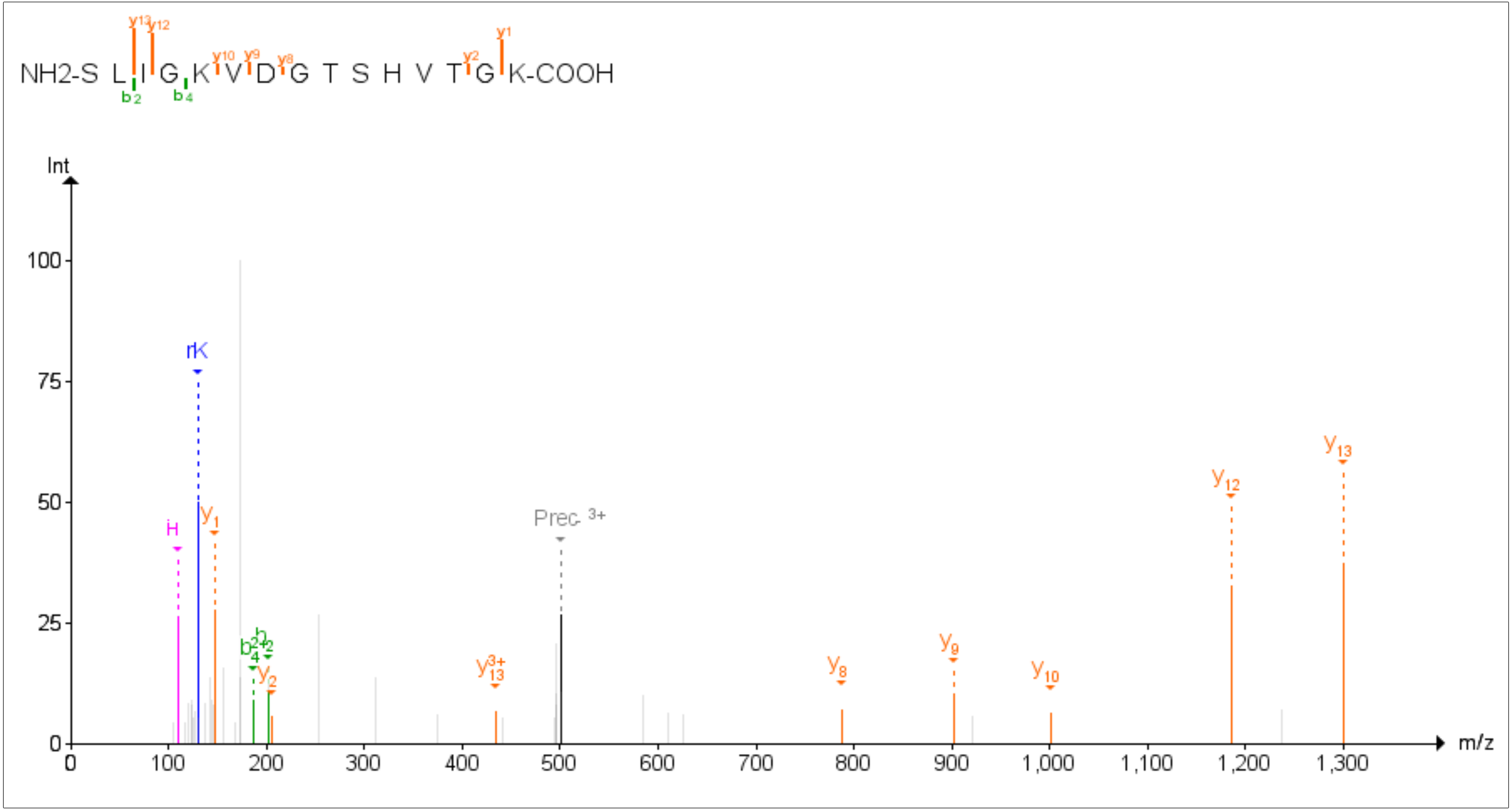

NH2-S L I G K V D G T S H V T G K-COOH
y13 y12 y10 y9 y8 y2 y1
b2 b4
Int
100
75
50
25
0
rK
iH
y1
Prec 3+
b4 2+
b2
y2
y13 3+
y8
y9
y10
y12
y13
0
100
200
300
400
500
600
700
800
900
1,000
1,100
1,200
1,300
m/z

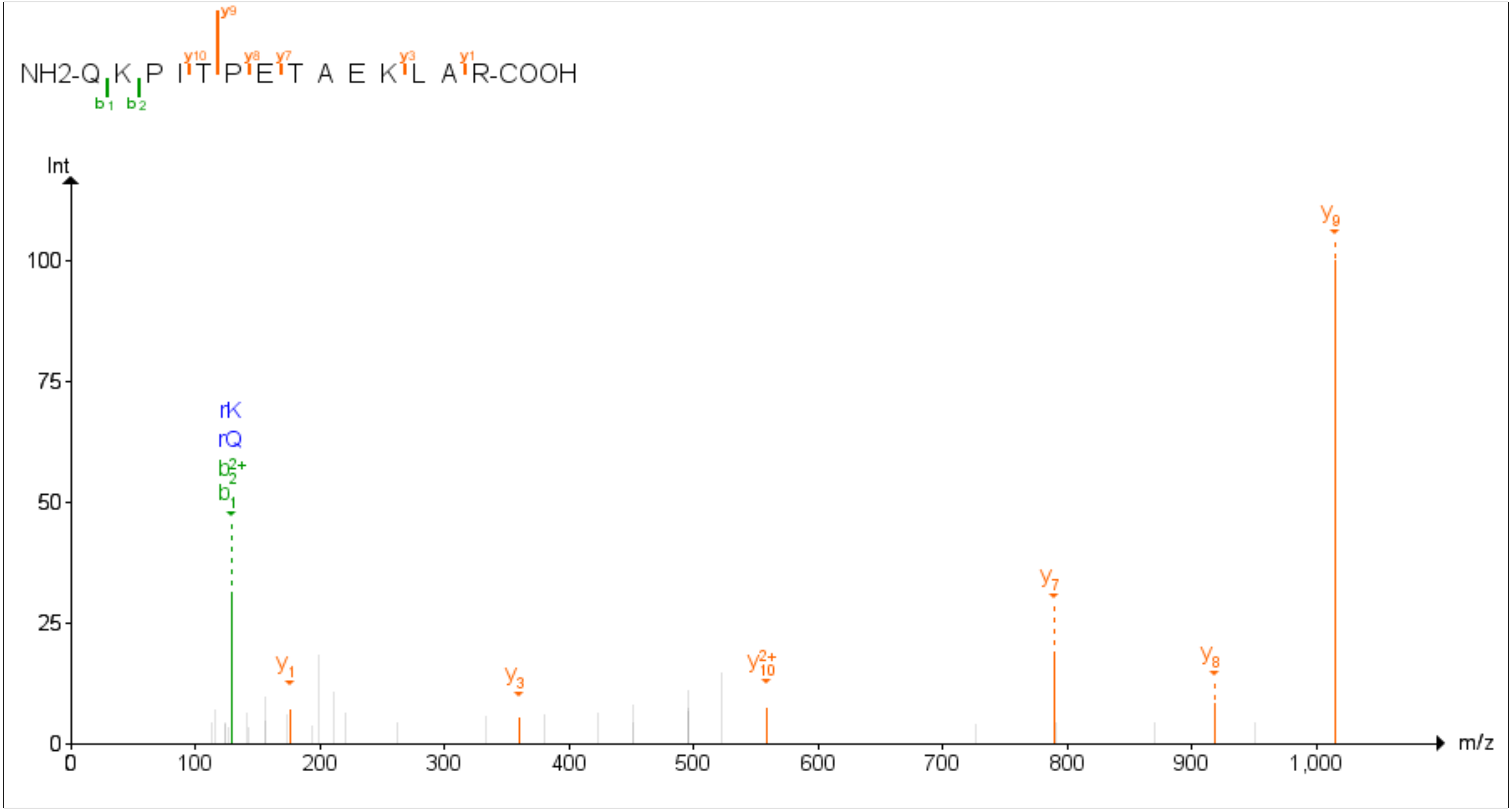

NH2-Q K P I T P E T A E K L A R-COOH
b1 b2
y10 y9 y8 y7 y3 y1
Int
m/z
rK
rQ
b_2^{2+}
b_1
y_1
y_3
y_{10}^{2+}
y_7
y_8
y_9
0
25
50
75
100
0
100
200
300
400
500
600
700
800
900
1,000

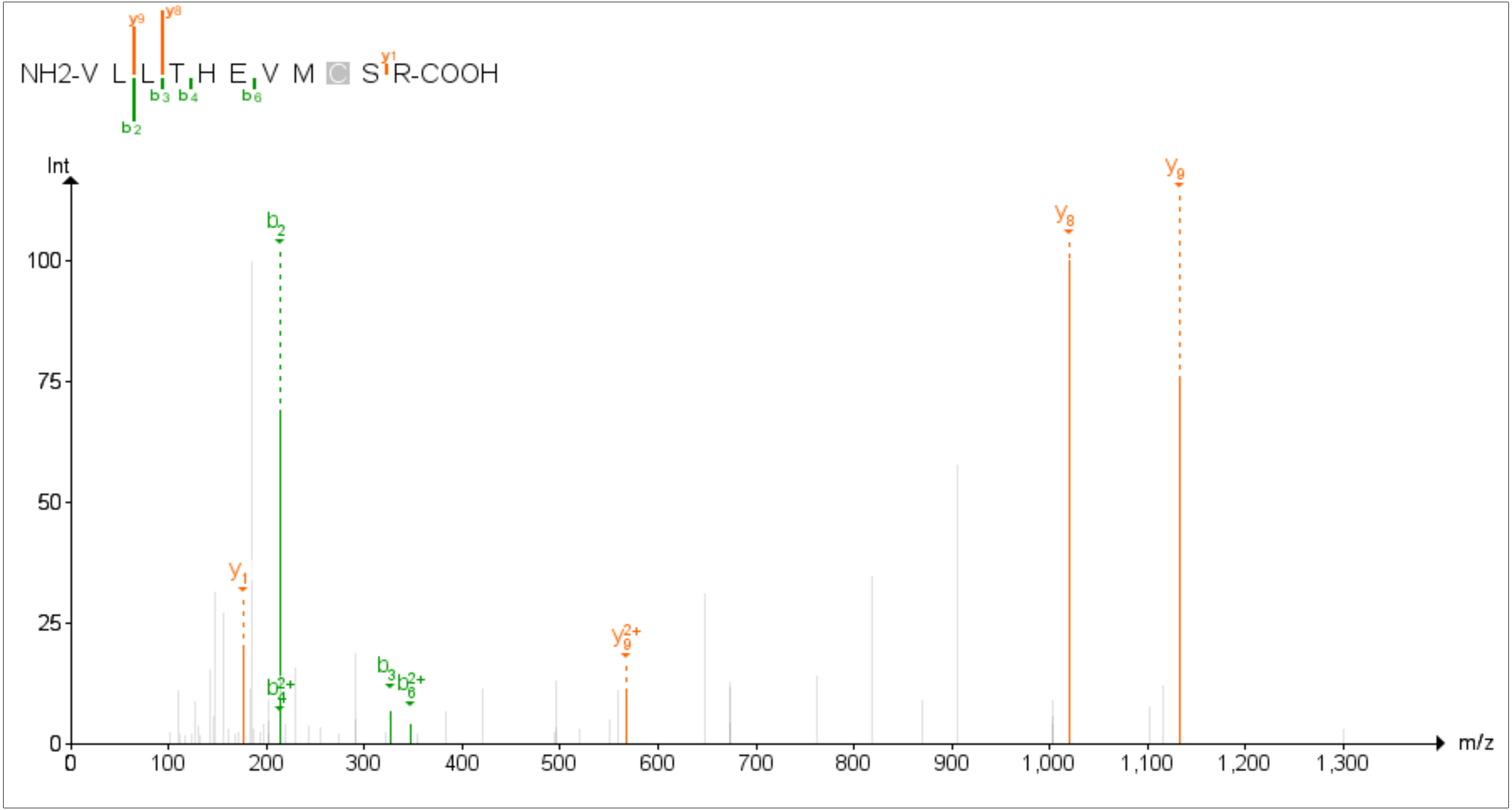
NH2-V L L T H E V M C S R-COOH
y9
y8
y1
b2
b3
b4
b6
Int
m/z
100
75
50
25
0
0
100
200
300
400
500
600
700
800
900
1,000
1,100
1,200
1,300
b_2
y_8
y_9
y_1
b_4^{2+}
b_3
b_6^{2+}
y_9^{2+}

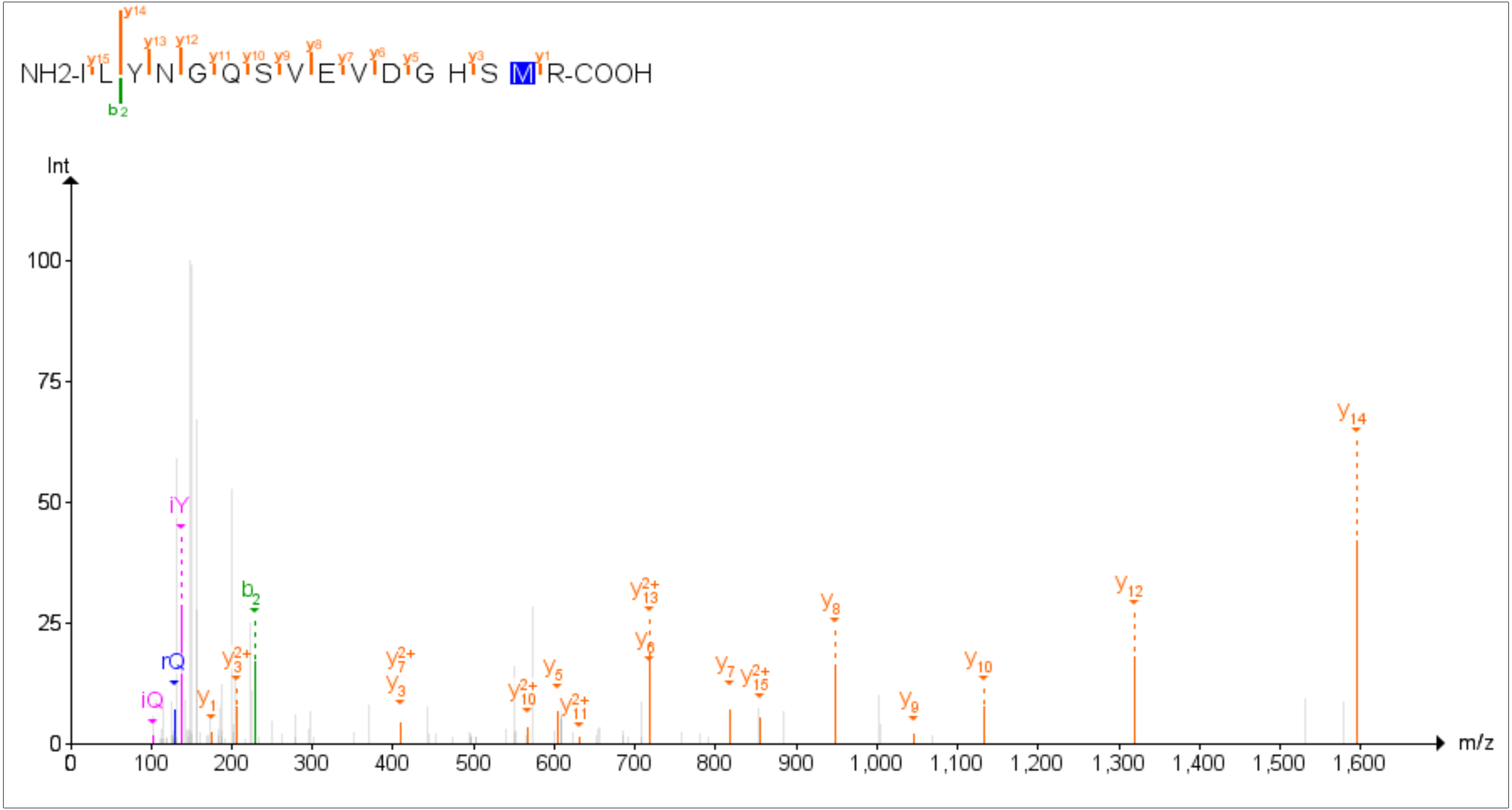

NH2-I L Y N G Q S V E V D G H S M R-COOH
y15 y14 y13 y12 y11 y10 y9 y8 y7 y6 y5 y3 y1
b2
Int
100
75
50
25
0
m/z
0 100 200 300 400 500 600 700 800 900 1,000 1,100 1,200 1,300 1,400 1,500 1,600
iQ
rQ
iY
y1
y3 2+
b2
y7 2+
y3
y10 2+
y5
y11 2+
y13 2+
y6
y7
y15 2+
y8
y9
y10
y12
y14

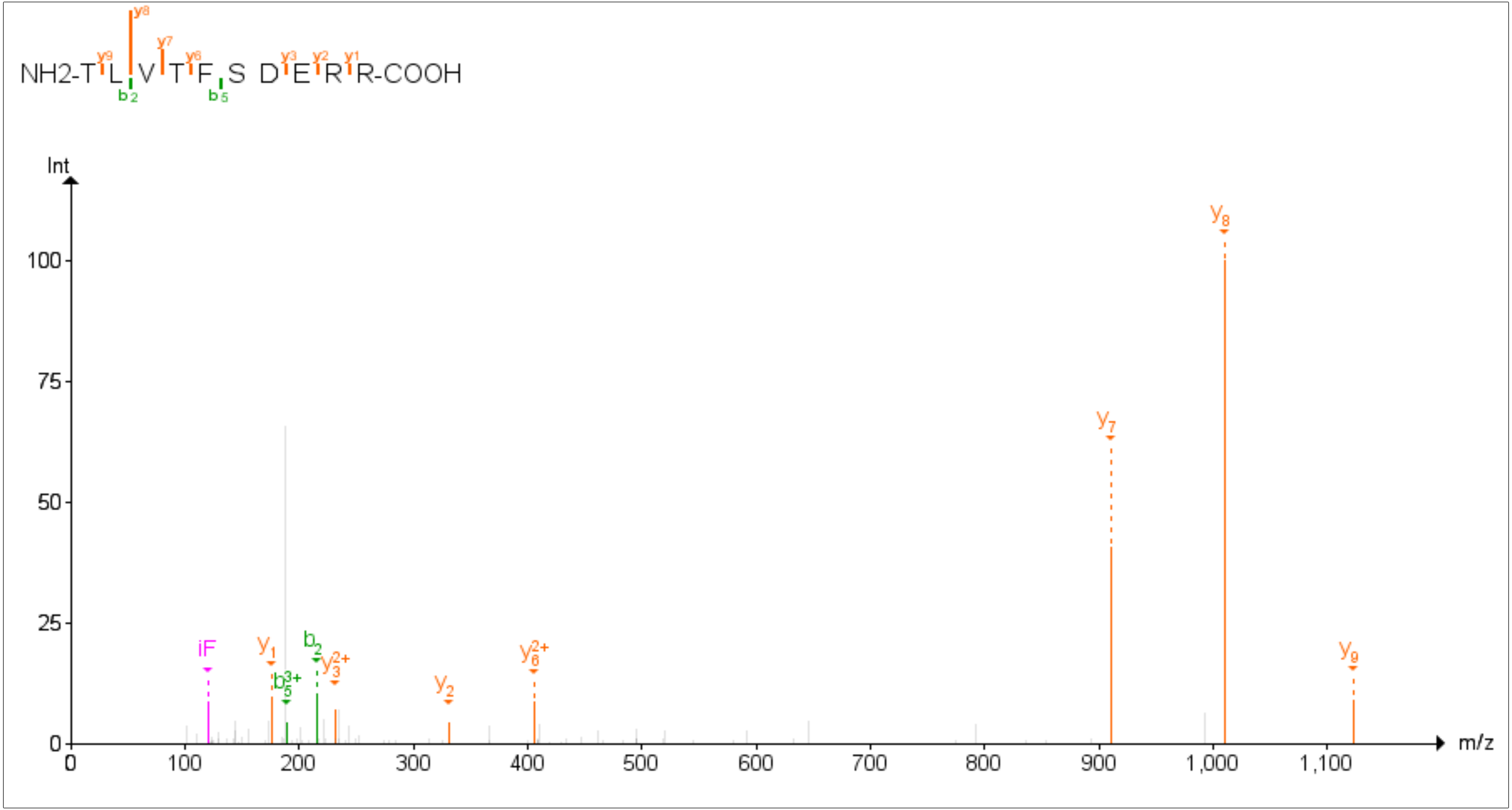

NH2-T L V T F S D E R R-COOH
y9 y8 y7 y6 y3 y2 y1
b2 b5
Int
100
75
50
25
0
iF
y1
b5 3+
b2
y3 2+
y2
y6 2+
y7
y8
y9
0 100 200 300 400 500 600 700 800 900 1,000 1,100
m/z

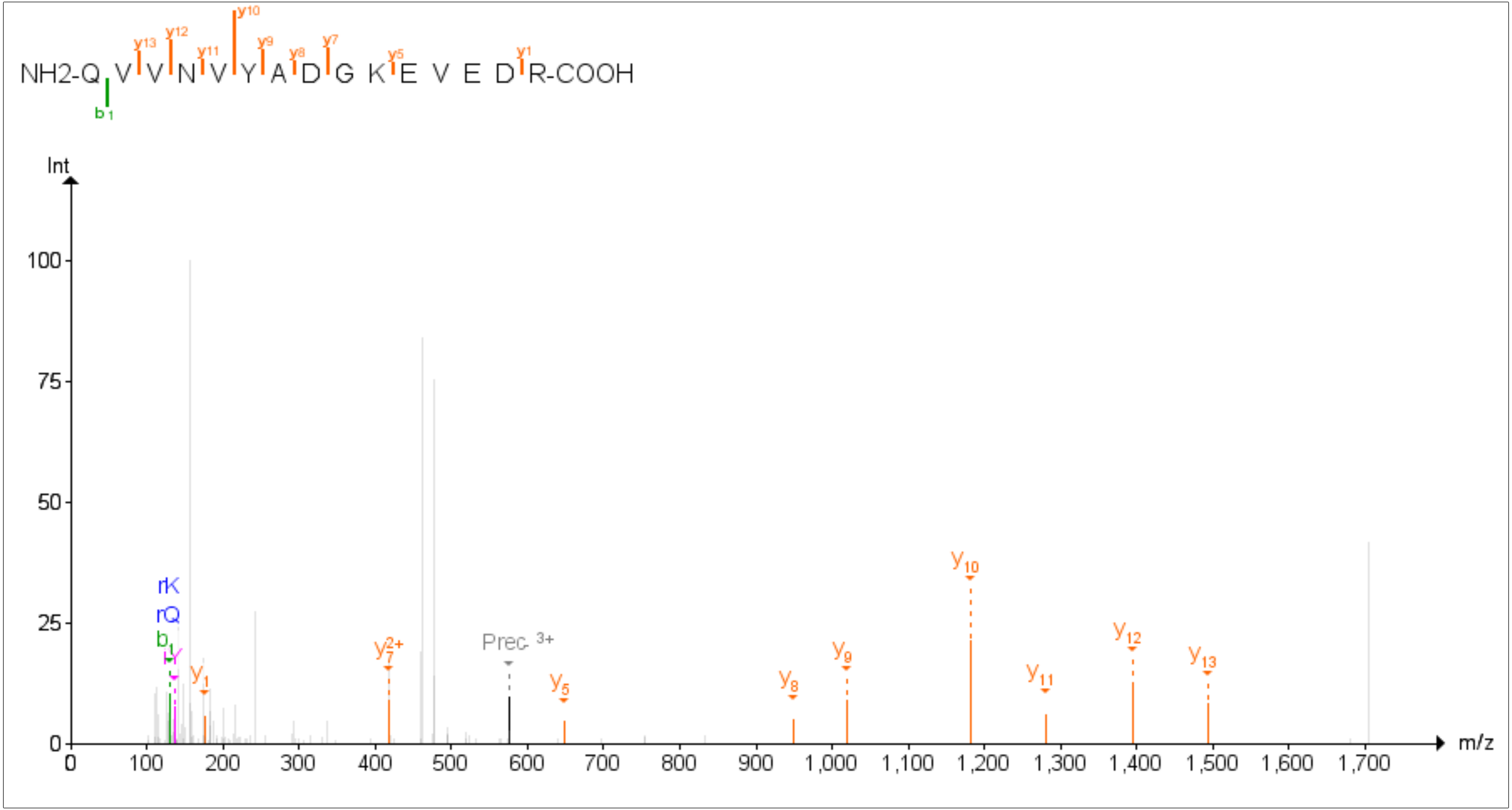

NH2-Q V V N V Y A D G K E V E D R-COOH
b1
y13 y12 y11 y10 y9 y8 y7 y5 y1
Int
m/z
rK
rQ
b1
rY
y1
y7 2+
Prec 3+
y5
y8
y9
y10
y11
y12
y13

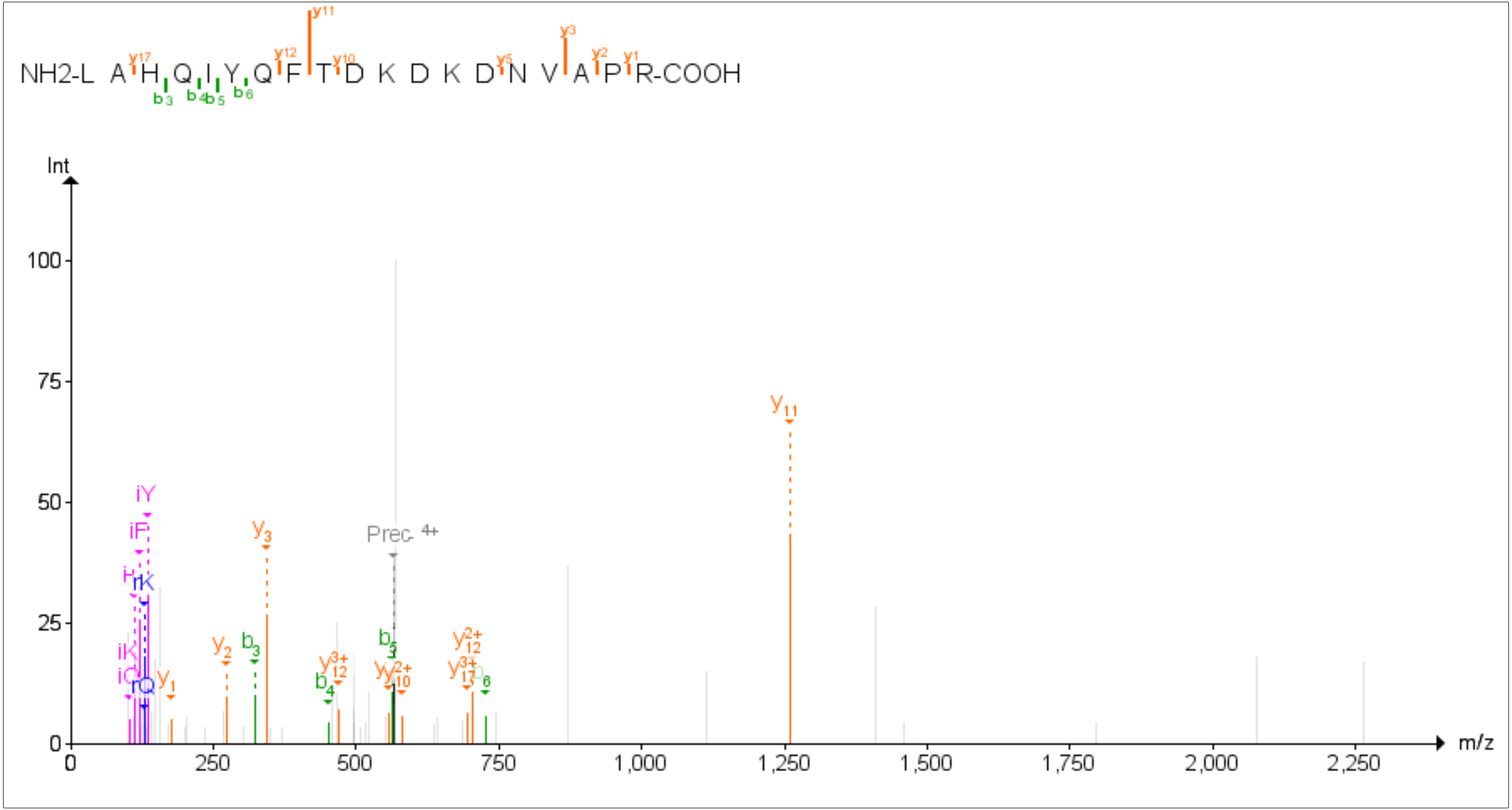

NH2-L A H Q I Y Q F T D K D K D N V A P R-COOH
y17 y12 y11 y10 y5 y3 y2 y1
b3 b4 b5 b6
Int
100
75
50
25
0
m/z
0
250
500
750
1,000
1,250
1,500
1,750
2,000
2,250
iY
iF
iK
iQ
rK
rQ
y1
y2
b3
y3
b4
Prec 4+
b5
y11

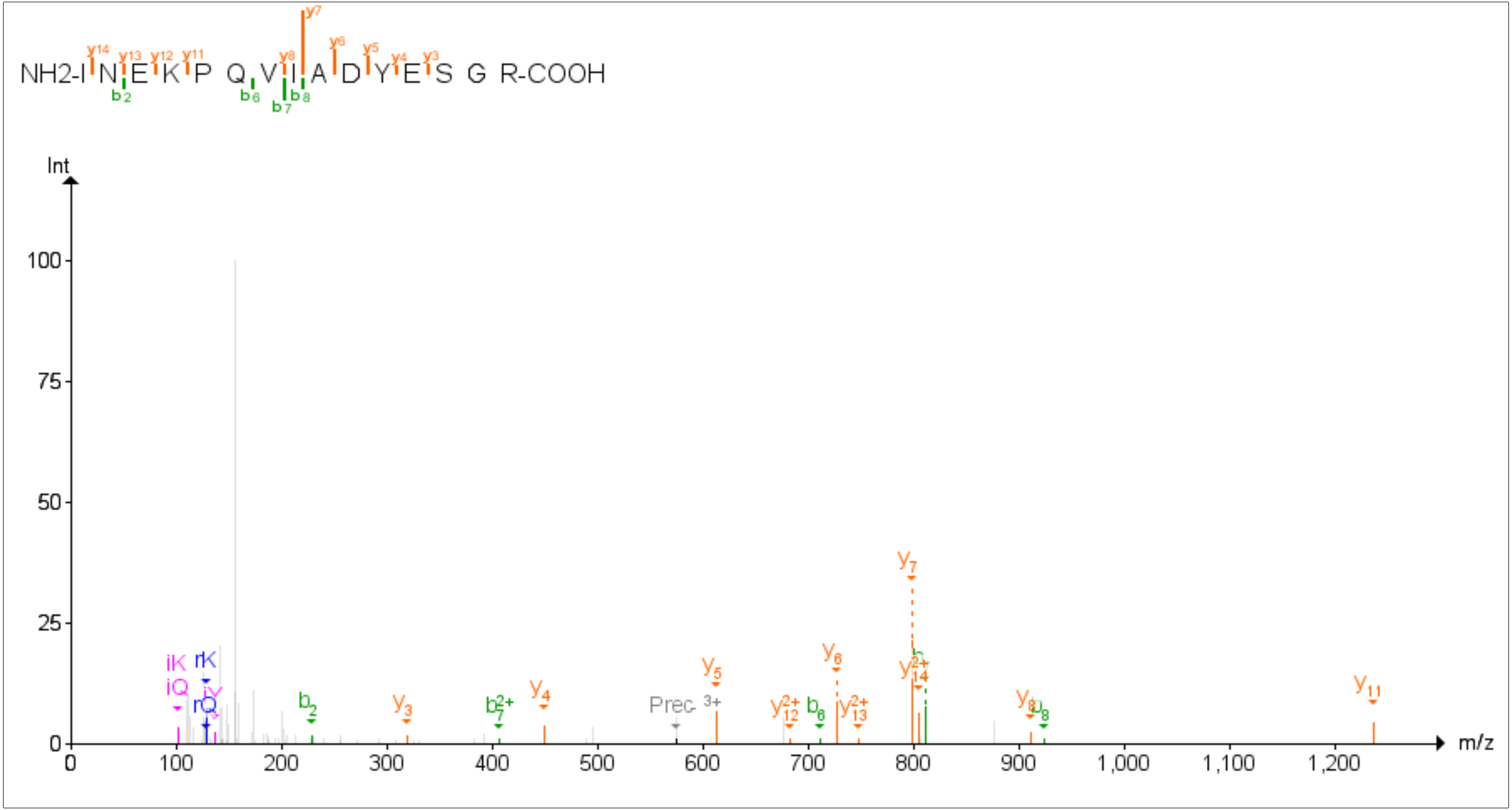

NH2-I N E K P Q V I I A D Y E S G R-COOH
y14 y13 y12 y11 y8 y7 y6 y5 y4 y3
b2 b6 b7 b8
Int
100
75
50
25
0
iK rK
iQ rQ
b2
y3
b7 2+
y4
Prec 3+
y5
y12 2+
b6
y6
y13 2+
y7
y14 2+
y8
b8
y11
0 100 200 300 400 500 600 700 800 900 1,000 1,100 1,200
m/z

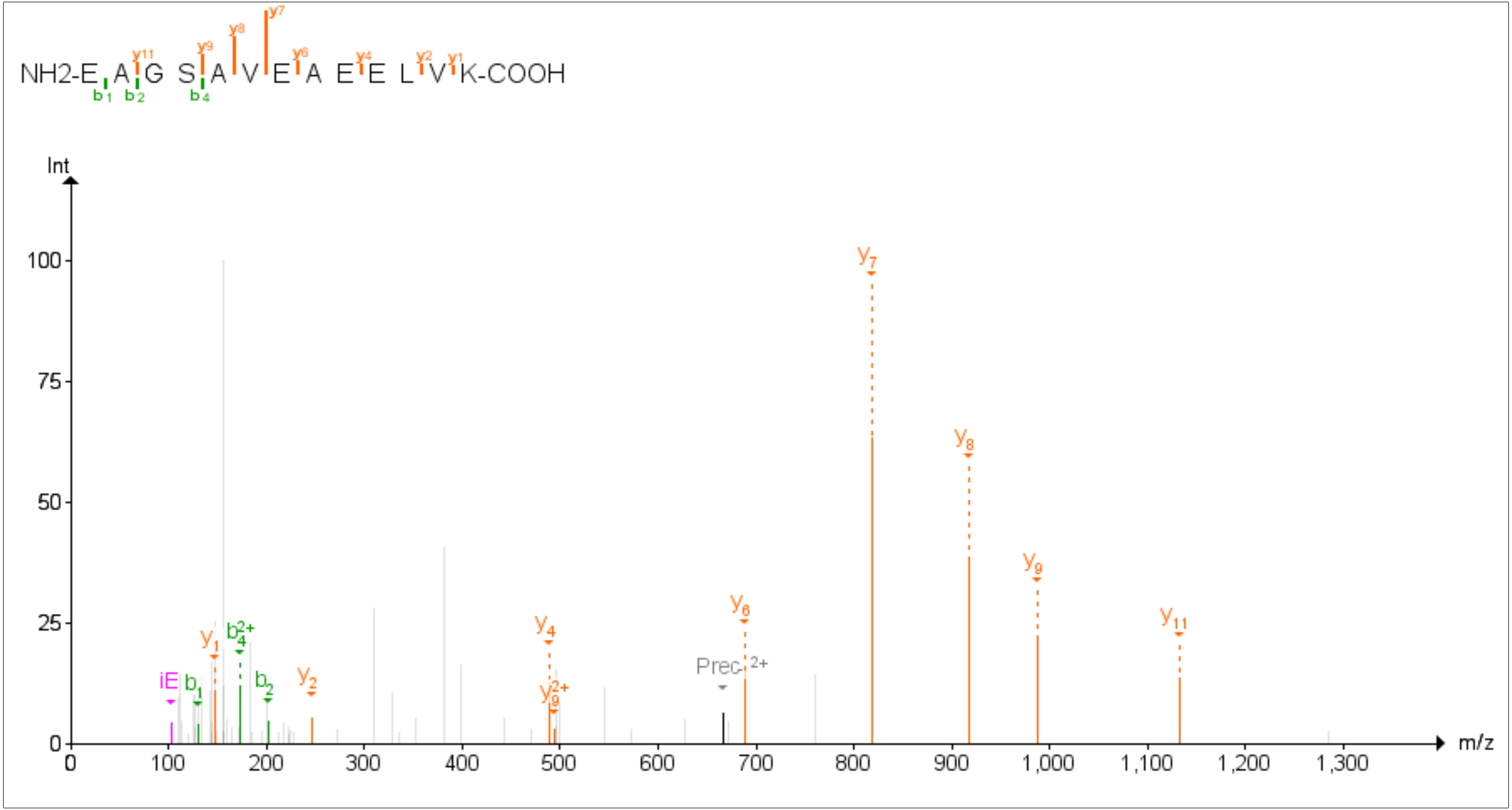

NH2-E A G S A V E A E E L V K-COOH
Int
m/z
y7
y8
y9
y11
y6
y4
y2
y1
iE
b1
b2
b4 2+
y9 2+
Prec 2+
0
25
50
75
100
0
100
200
300
400
500
600
700
800
900
1,000
1,100
1,200
1,300

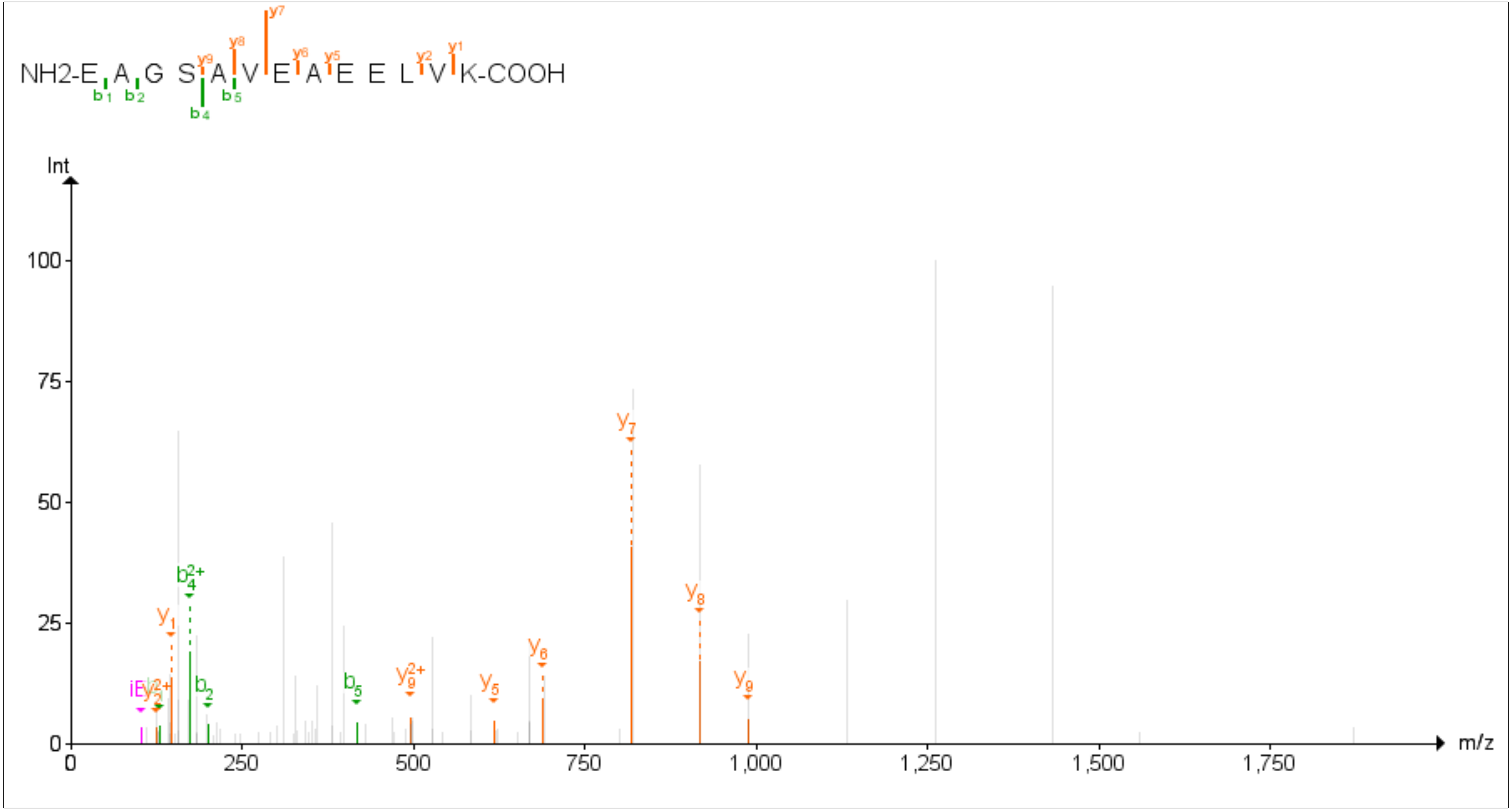
NH2-E A G S A V E A E E L V K-COOH
b1 b2 b4 b5
y9 y8 y7 y6 y5 y2 y1
Int
m/z
0
25
50
75
100
0
250
500
750
1,000
1,250
1,500
1,750
y7
y8
b4 2+
y1
b2
b5
y9 2+
y5
y6
y9
iE
y2 2+
y2

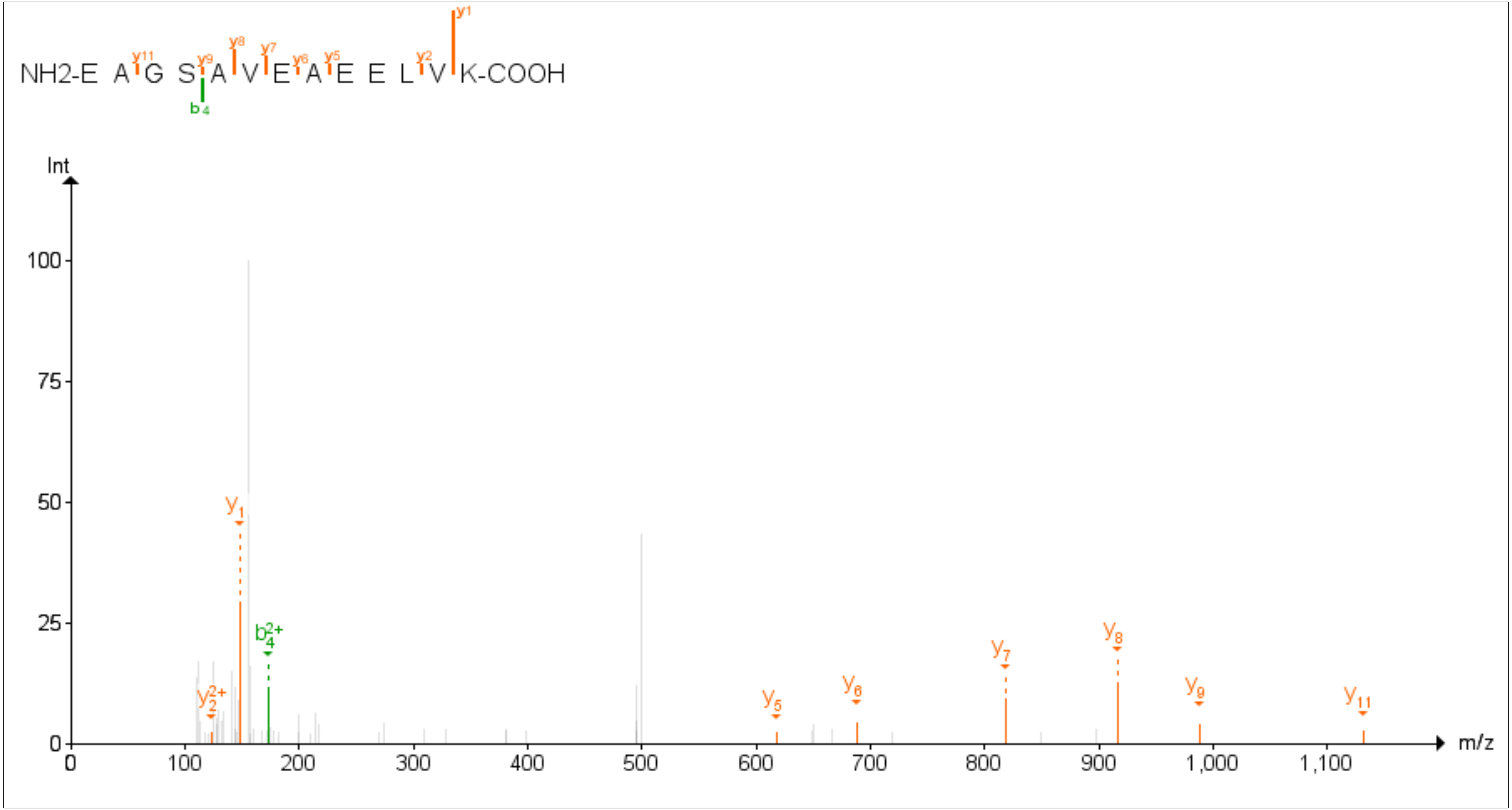

NH2-E A G S A V E A E E L V K-COOH
y11 y9 y8 y7 y6 y5 y2 y1
b4
Int
100
75
50
25
0
y1
b4 2+
y2 2+
y5
y6
y7
y8
y9
y11
0
100
200
300
400
500
600
700
800
900
1,000
1,100
m/z

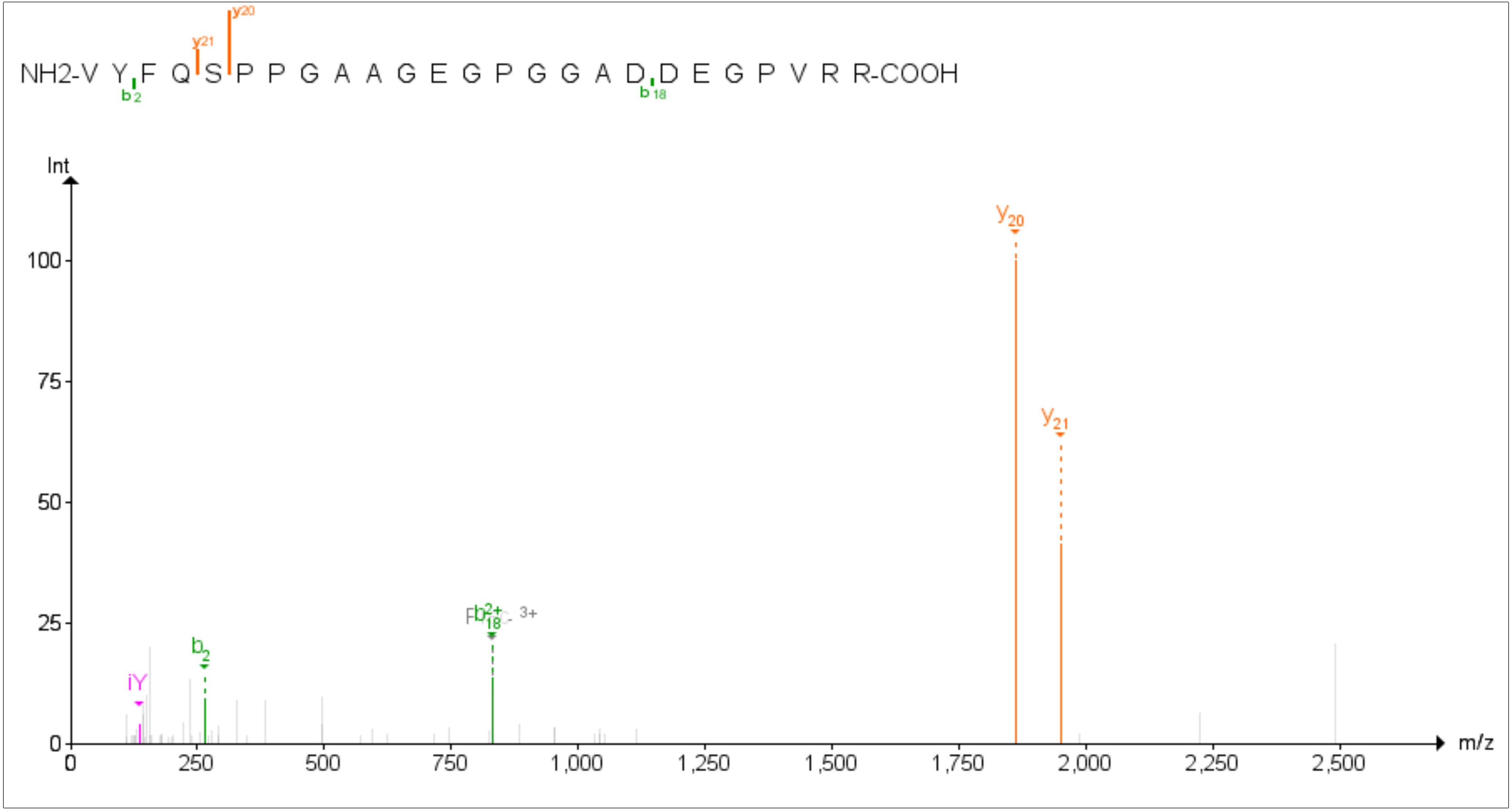

NH2-V Y F Q S P P G A A G E G P G G A D D E G P V R R-COOH
b2
y21
y20
b18
Int
100
75
50
25
0
m/z
0
250
500
750
1,000
1,250
1,500
1,750
2,000
2,250
2,500
y20
y21
iY
b2
b18 2+
3+

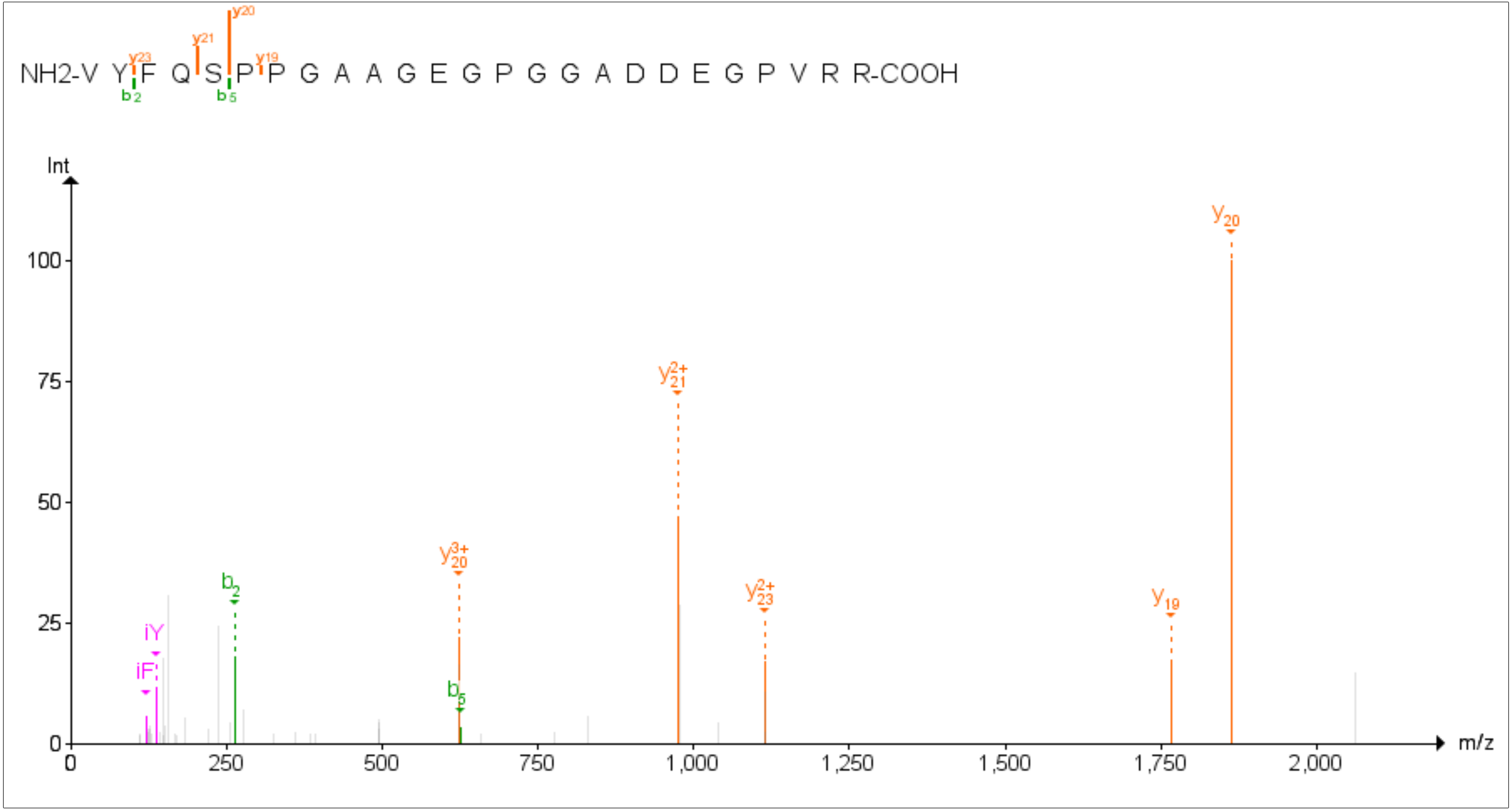

NH2-V Y F Q S P P G A A G E G P G G A D D E G P V R R-COOH
y23
y21
y20
y19
b2
b5
Int
100
75
50
25
0
iY
iF
b2
b5
y20 3+
y21 2+
y23 2+
y19
y20
0
250
500
750
1,000
1,250
1,500
1,750
2,000
m/z

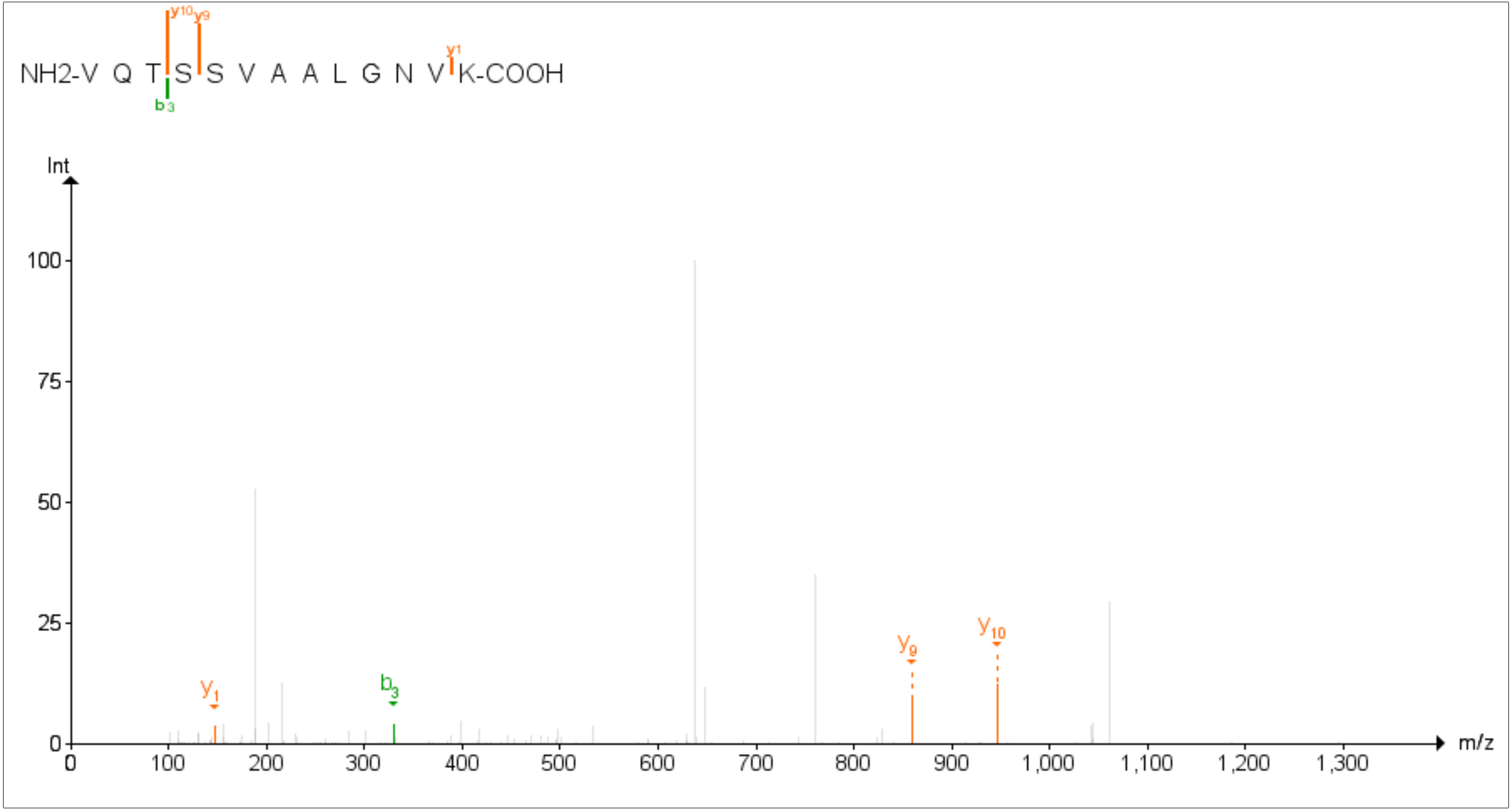

y10 y9
y1
NH2-V Q T S S V A A L G N V K-COOH
b3
Int
100
75
50
25
0
y1
b3
y9
y10
0
100
200
300
400
500
600
700
800
900
1,000
1,100
1,200
1,300
m/z

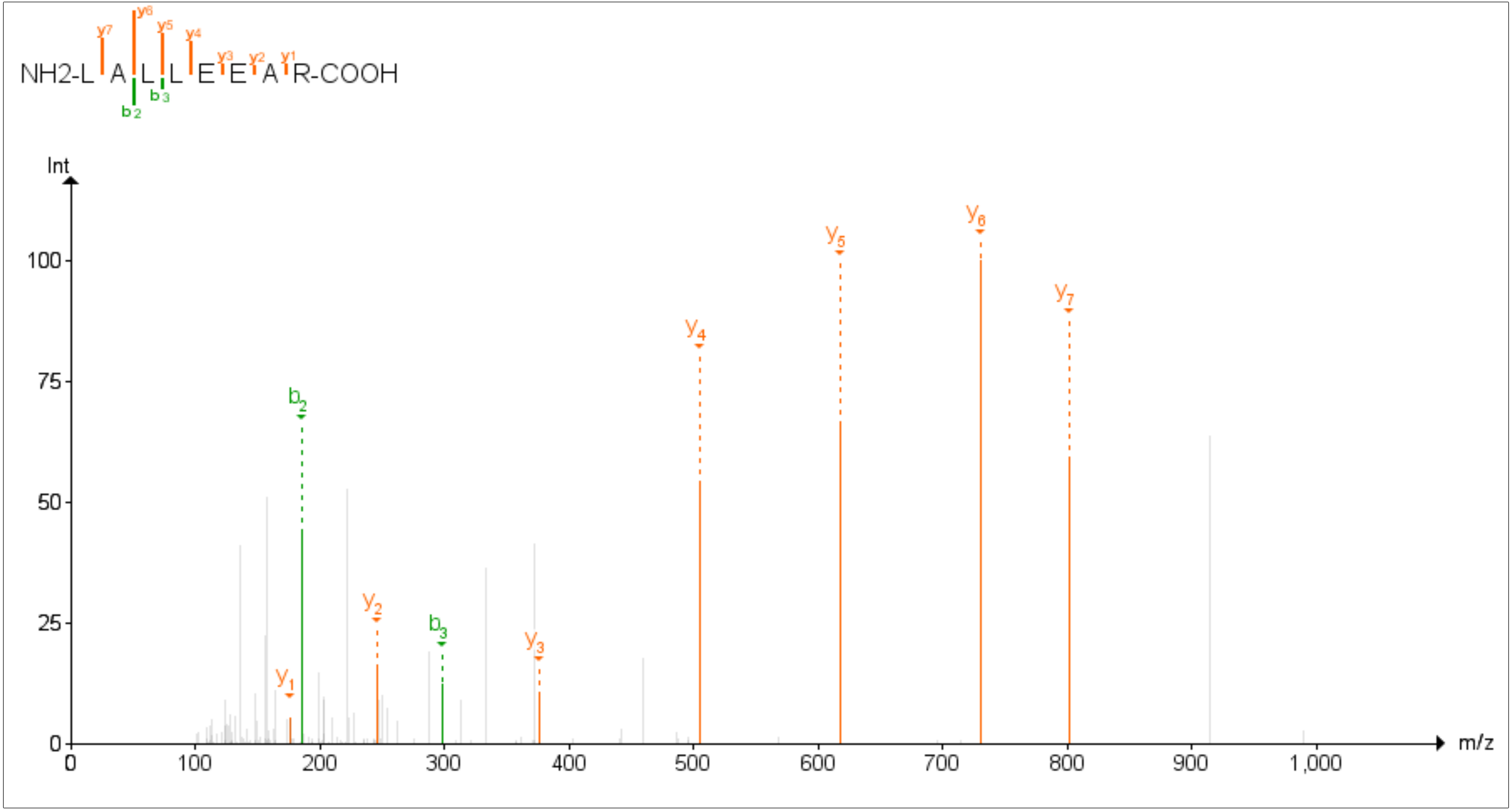

NH2-L A L L E E A R-COOH
y7
y6
y5
y4
y3
y2
y1
b2
b3
Int
100
75
50
25
0
m/z
0
100
200
300
400
500
600
700
800
900
1,000
b2
y1
y2
b3
y3
y4
y5
y6
y7

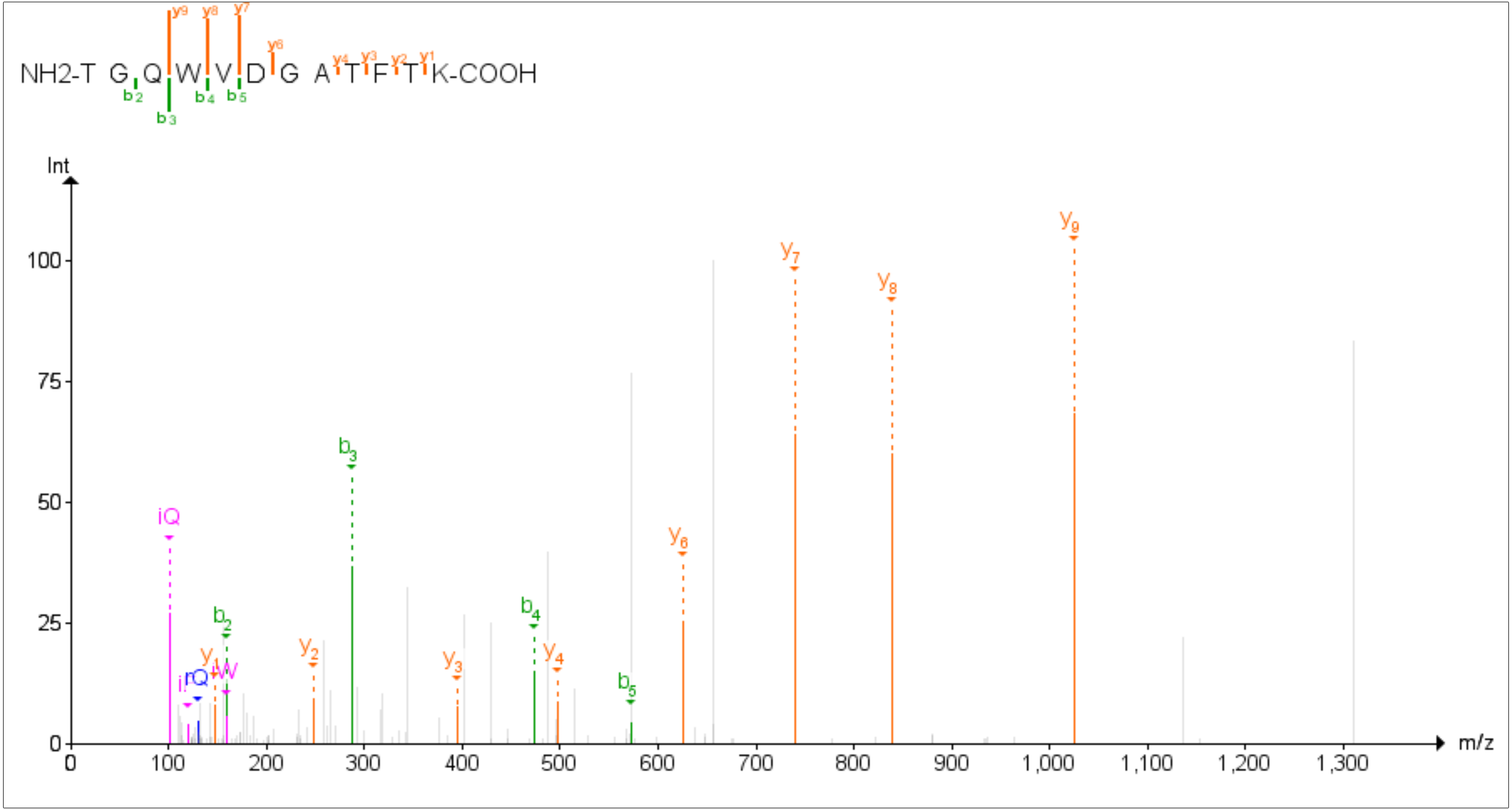

NH2-T G Q W V D G A T F T K-COOH
y9
y8
y7
y6
y4
y3
y2
y1
b2
b3
b4
b5
Int
m/z
iQ
rQ
W

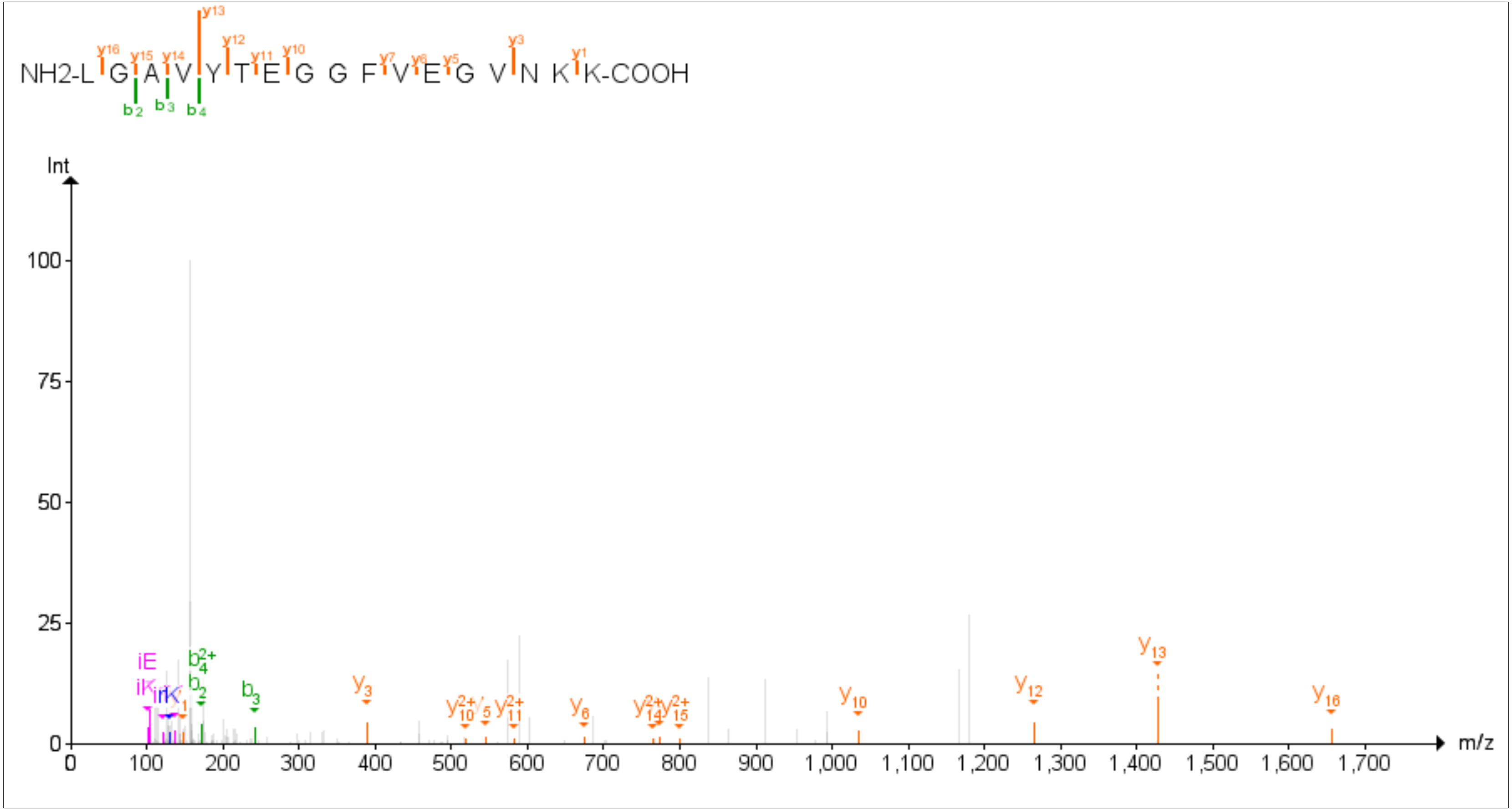
NH2-L G A V Y T E G G F V E G V N K K-COOH
Int
m/z

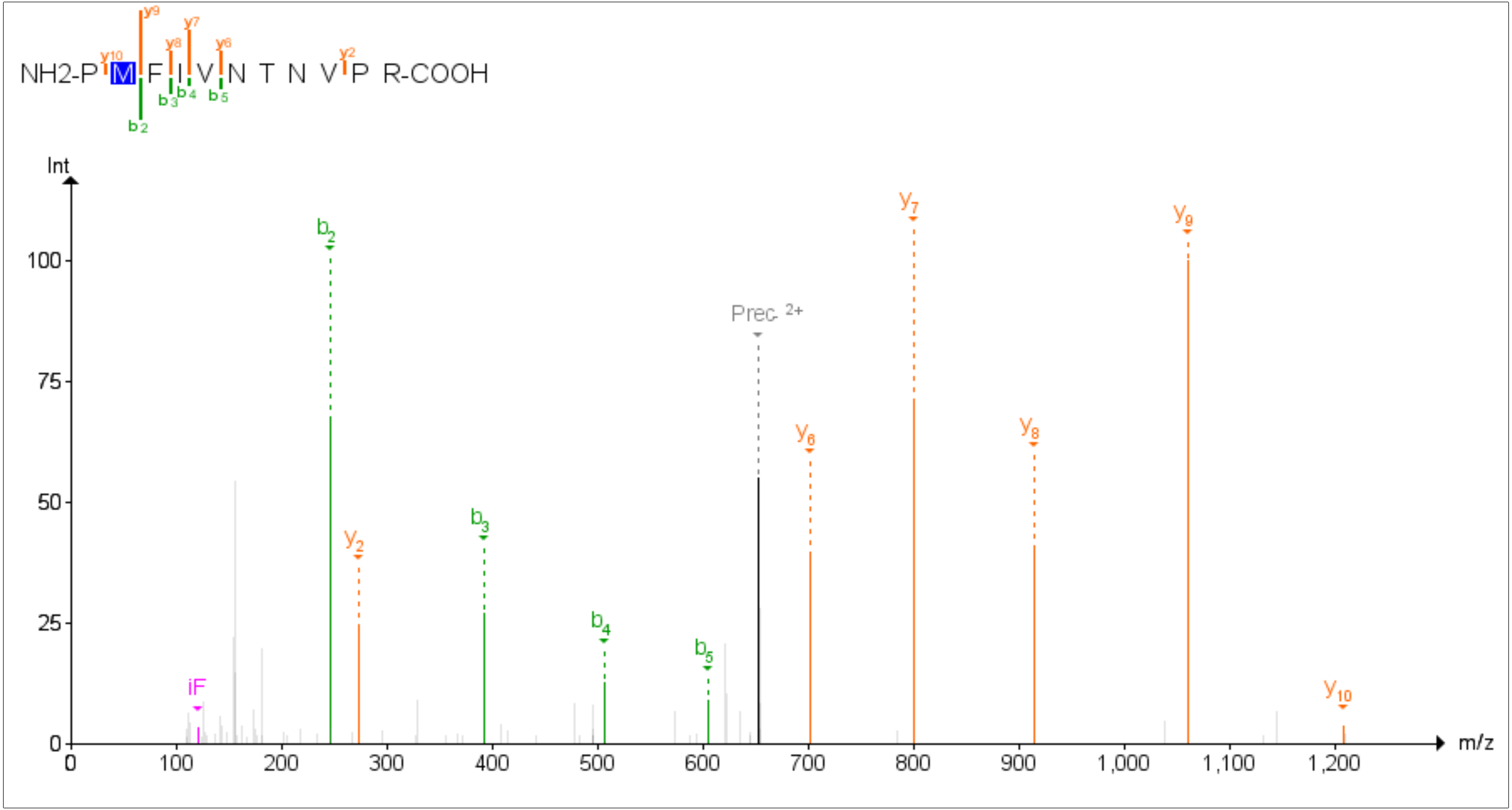

NH2-P M F I V N T N V P R-COOH
y10
y9
y8
y7
y6
y2
b2
b3
b4
b5
Int
100
75
50
25
0
m/z
0
100
200
300
400
500
600
700
800
900
1,000
1,100
1,200
iF
Prec 2+

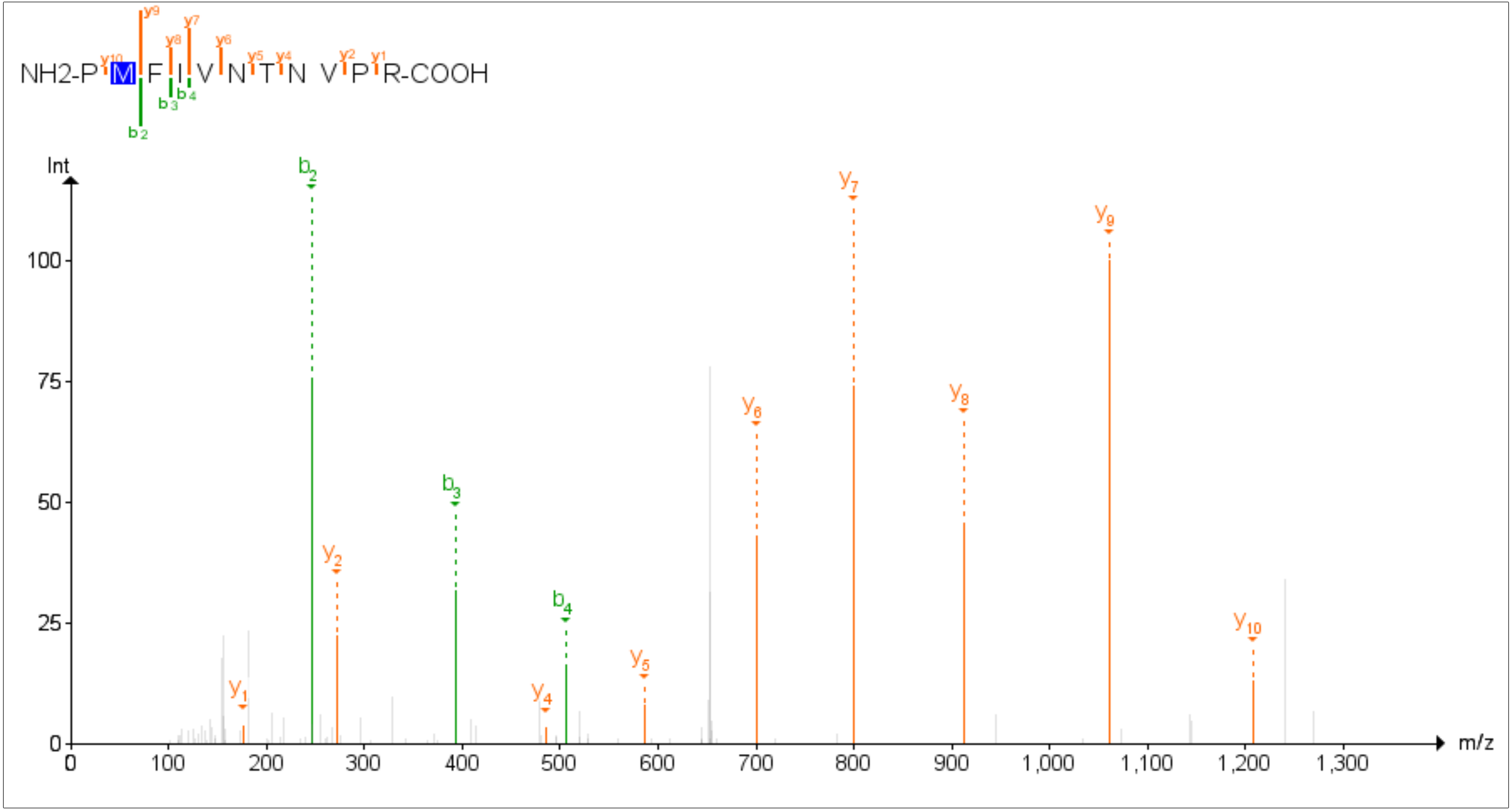

NH2-P M F I V N T N V P R-COOH
y10 y9 y8 y7 y6 y5 y4 y2 y1
b2 b3 b4
Int
100
75
50
25
0
b2
y7
y9
y6
y8
b3
y2
b4
y10
y5
y1
y4
0 100 200 300 400 500 600 700 800 900 1,000 1,100 1,200 1,300
m/z

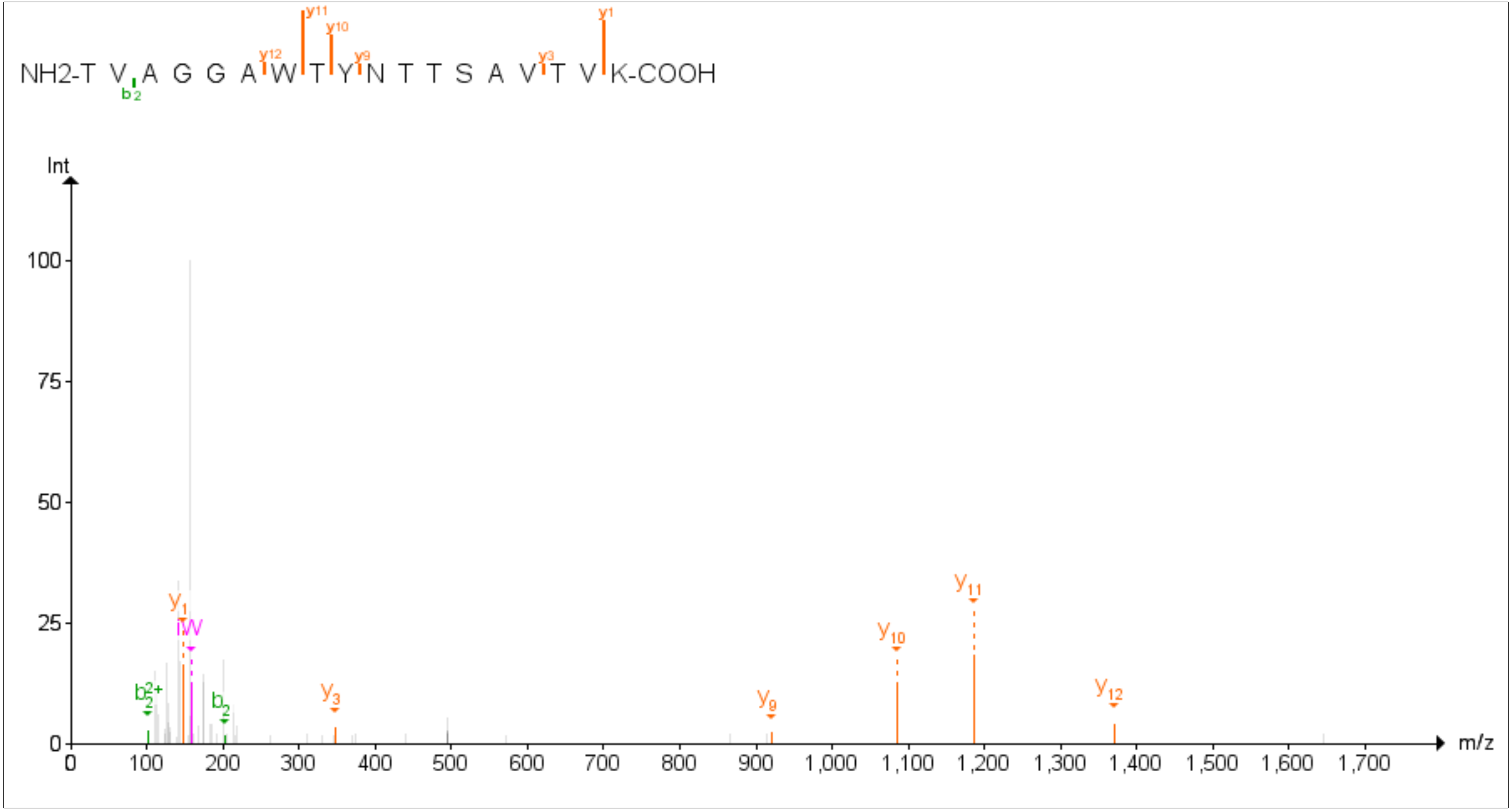

NH2-T V A G G A W T Y N T T S A V T V K-COOH
b2
y12
y11
y10
y9
y3
y1
Int
100
75
50
25
0
m/z
0
100
200
300
400
500
600
700
800
900
1,000
1,100
1,200
1,300
1,400
1,500
1,600
1,700
y1
iW
b2 2+
b2
y3
y9
y10
y11
y12

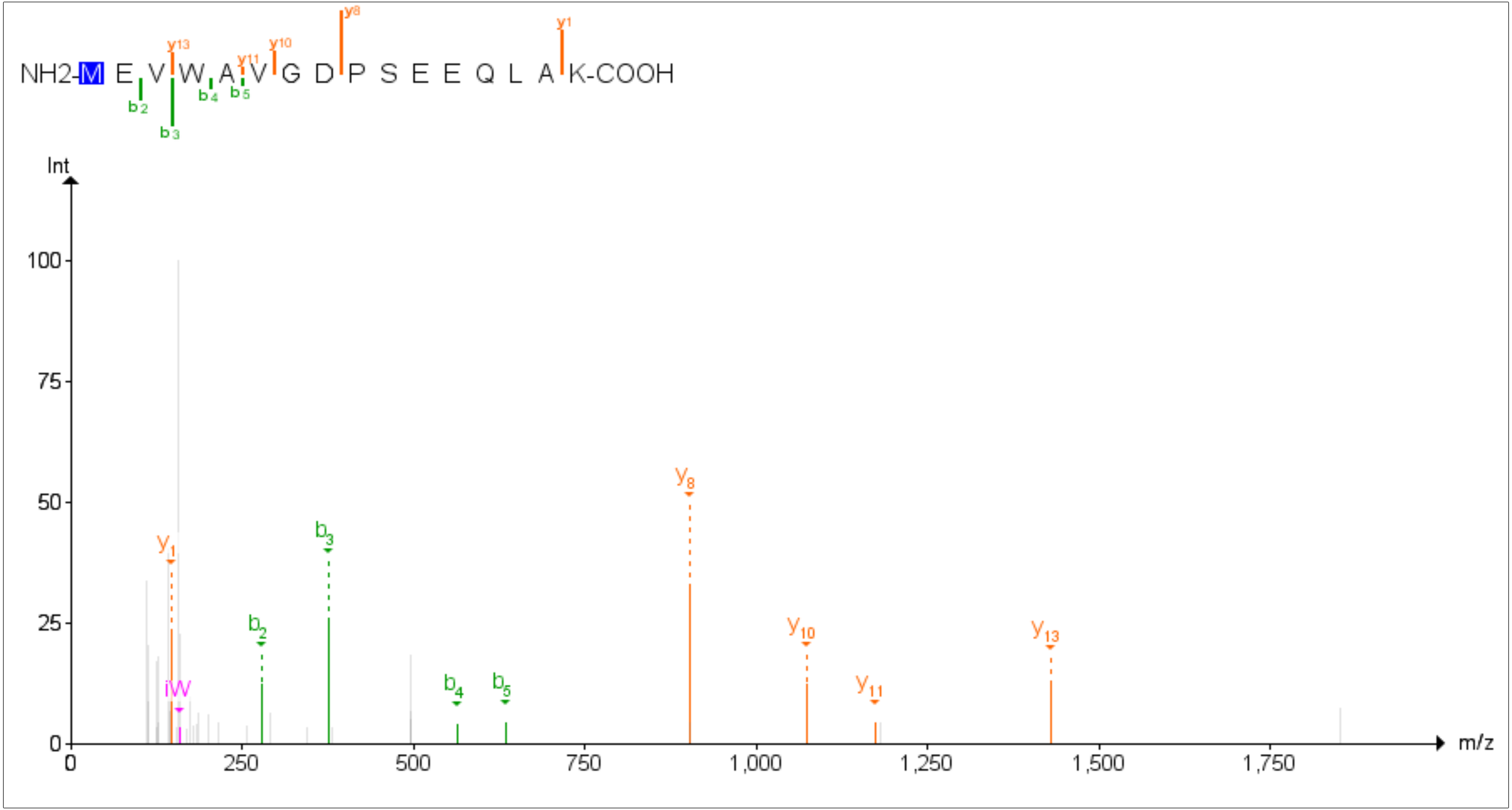

NH2-M E V W A V G D P S E E Q L A K-COOH
y13
y11
y10
y8
y1
b2
b3
b4
b5
Int
100
75
50
25
0
y1
iW
b2
b3
b4
b5
y8
y10
y11
y13
0
250
500
750
1,000
1,250
1,500
1,750
m/z

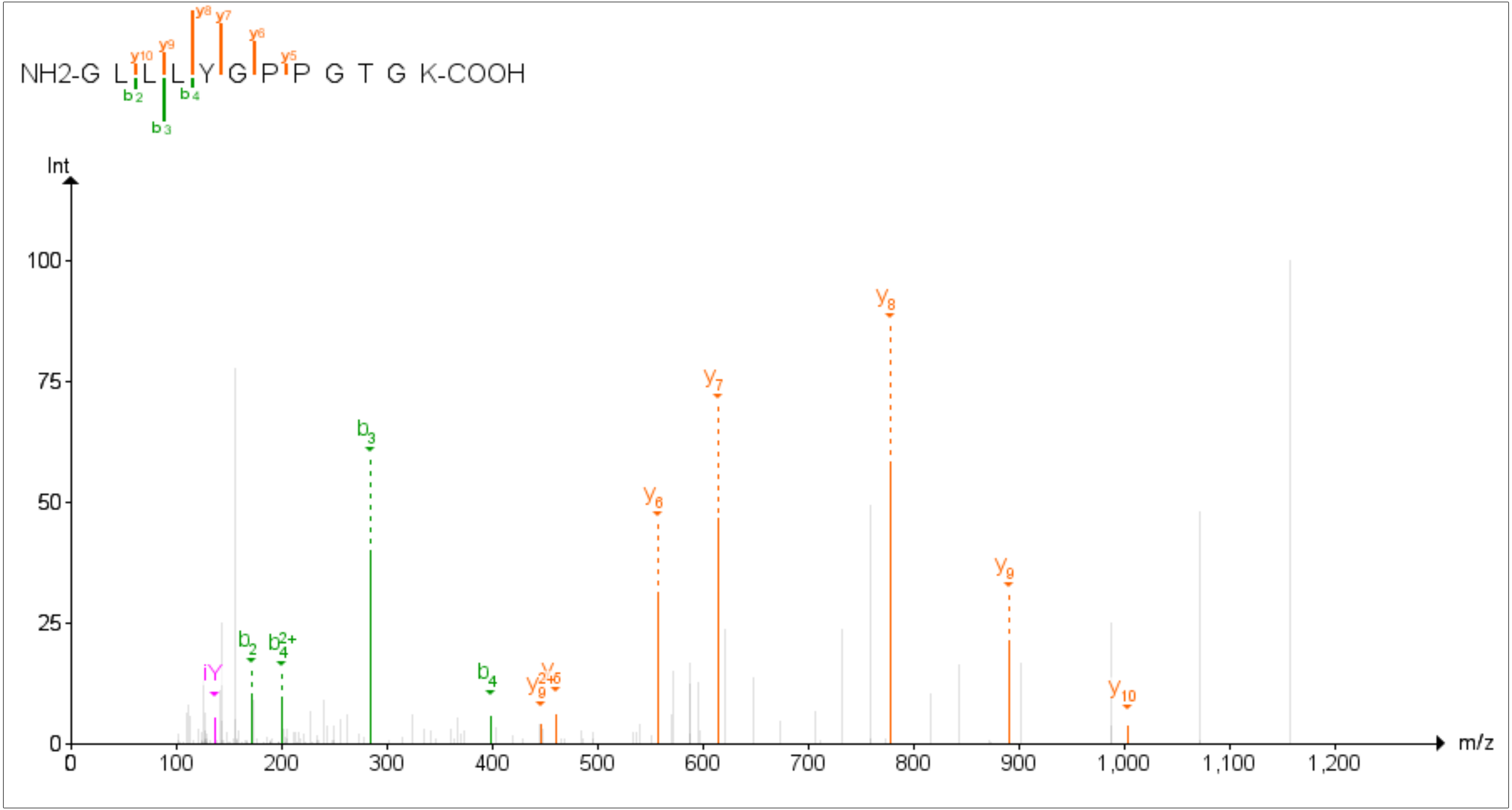

NH2-G L L L Y G P P G T G K-COOH
y10 y9 y8 y7 y6 y5
b2 b3 b4
Int
100
75
50
25
0
m/z
0 100 200 300 400 500 600 700 800 900 1,000 1,100 1,200
iY
b2
b4 2+
b3
b4
y9 2+
y6
y7
y8
y9
y10

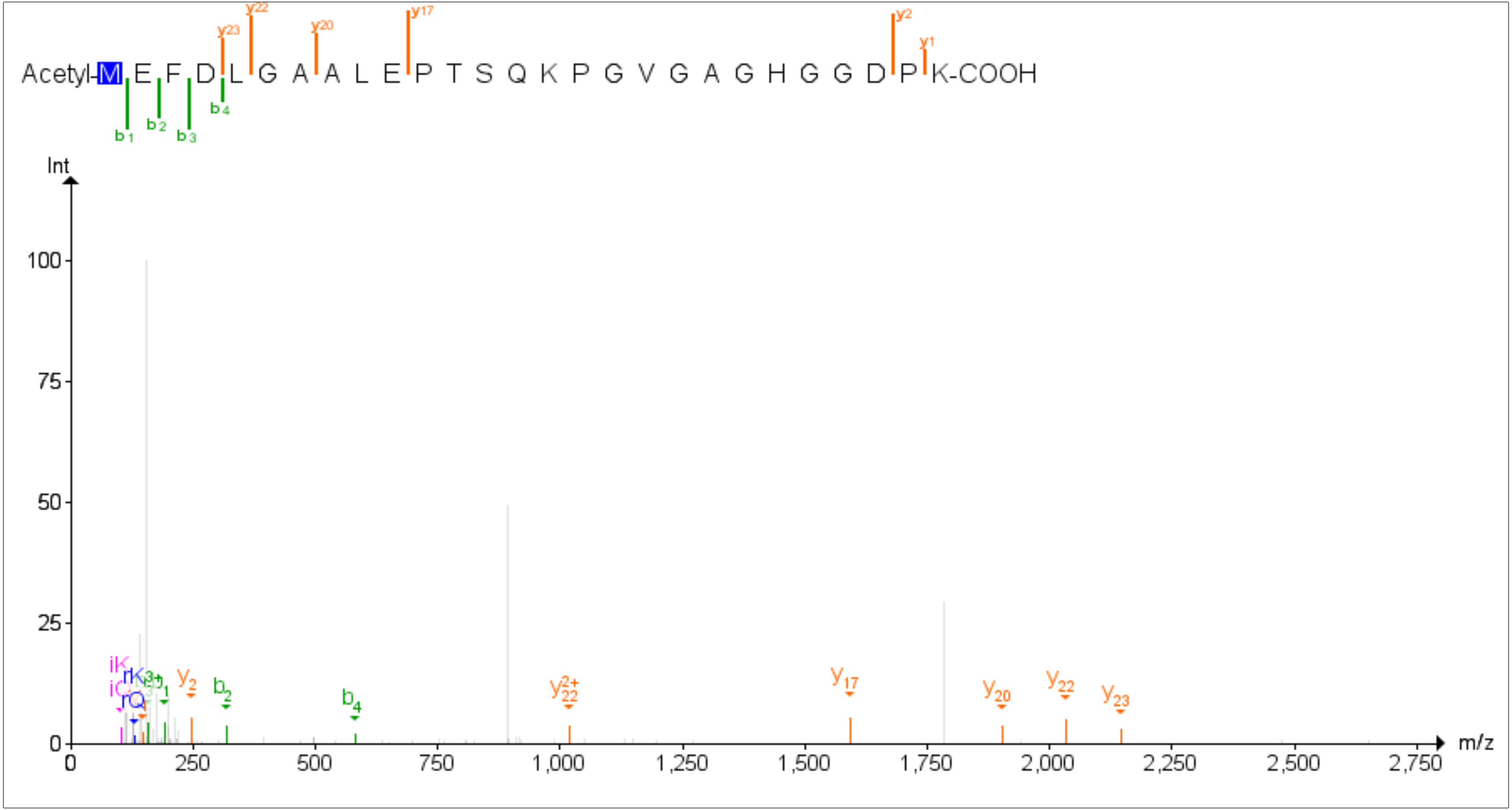

Acetyl-M E F D L G A A L E P T S Q K P G V G A G H G G D P K-COOH
y23
y22
y20
y17
y2
y1
b1
b2
b3
b4
Int
100
75
50
25
0
y2
b2
b4
y22 2+
y17
y20
y22
y23
0
250
500
750
1,000
1,250
1,500
1,750
2,000
2,250
2,500
2,750
m/z

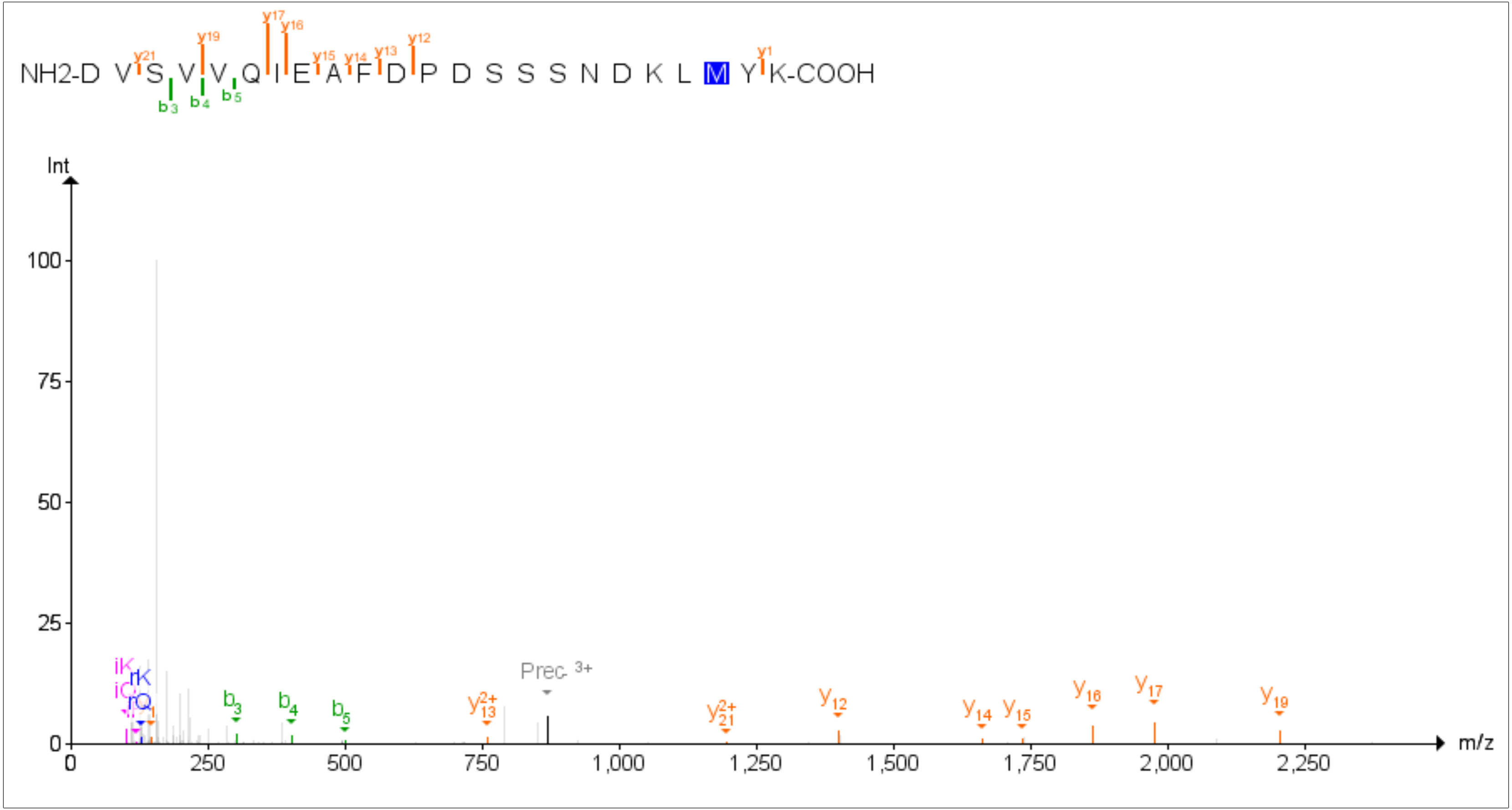

NH2-D V S V V Q I E A F D P D S S S N D K L M Y K-COOH
y21 y19 y17 y16 y15 y14 y13 y12 y1
b3 b4 b5
Int
100
75
50
25
0
Prec. 3+
b3 b4 b5
y13 2+ y21 2+ y12 y14 y15 y16 y17 y19
iK rK iQ rQ
0 250 500 750 1,000 1,250 1,500 1,750 2,000 2,250
m/z

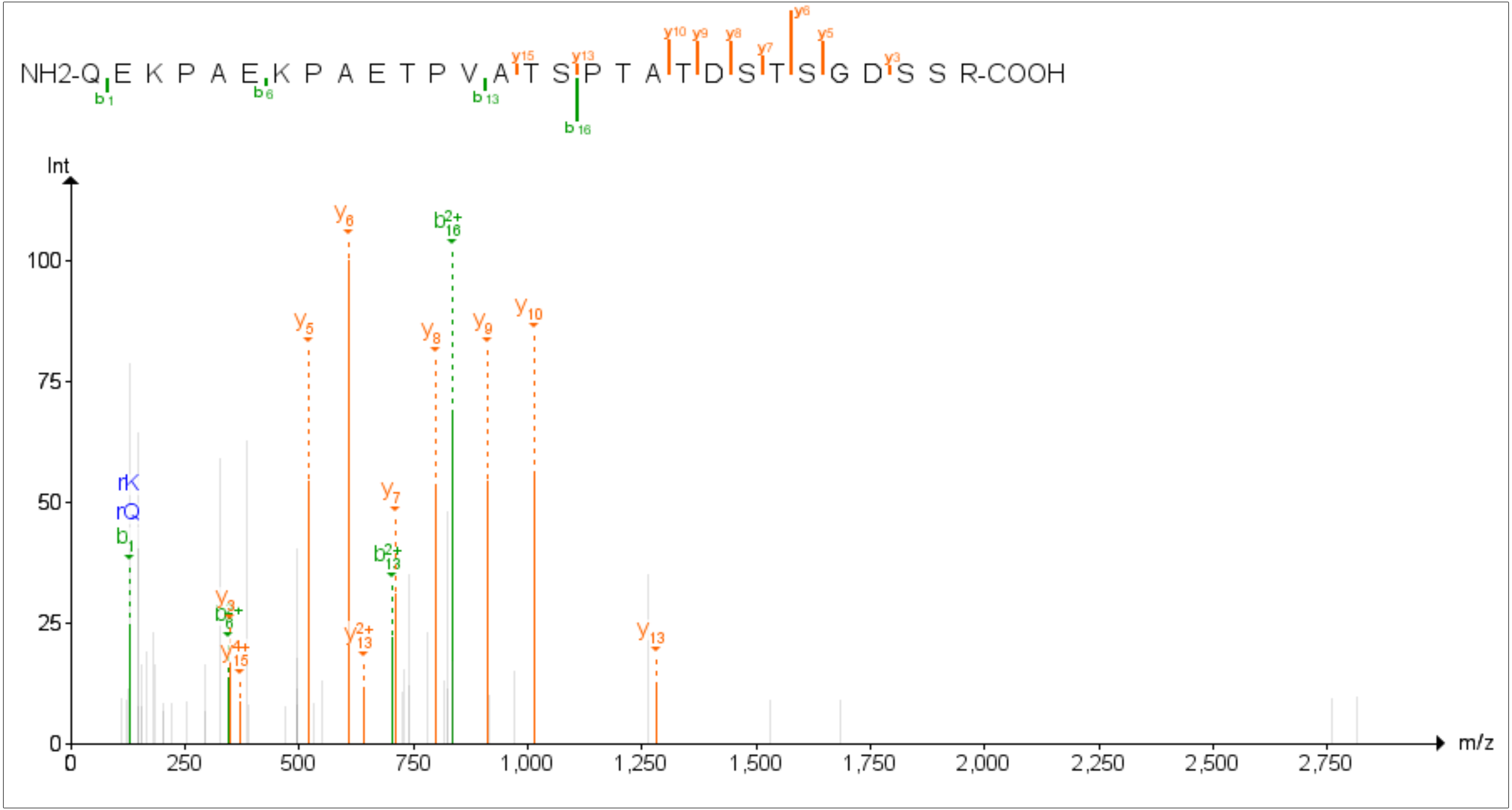

NH2-Q E K P A E K P A E T P V A T S P T A T D S T S G D S S R-COOH
b1
b6
b13
b16
y15
y13
y10
y9
y8
y7
y6
y5
y3
Int
m/z
rK
rQ

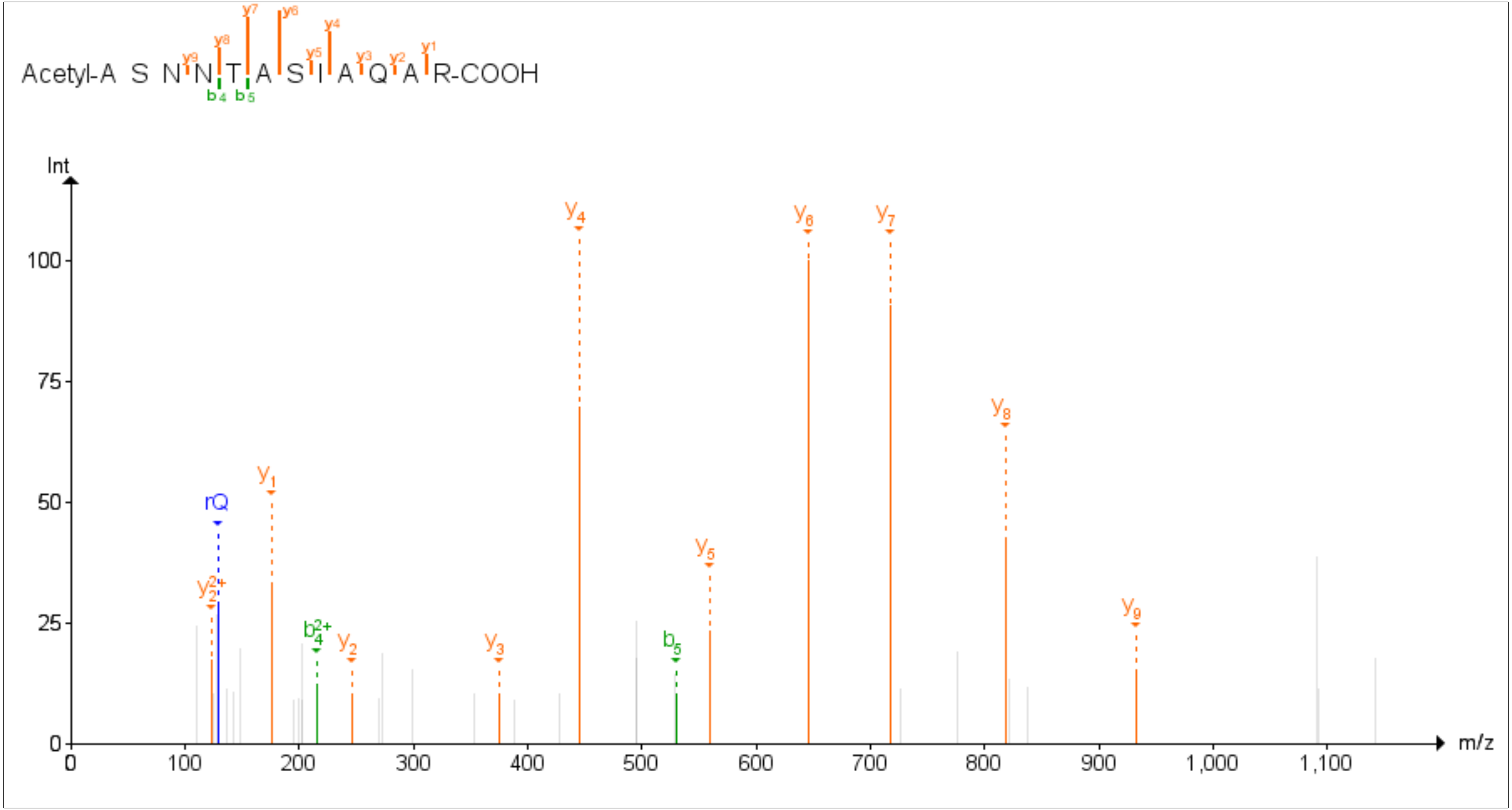

Acetyl-A S N N T A S I A Q A R-COOH
y9 y8 y7 y6 y5 y4 y3 y2 y1
b4 b5
Int
m/z
rQ

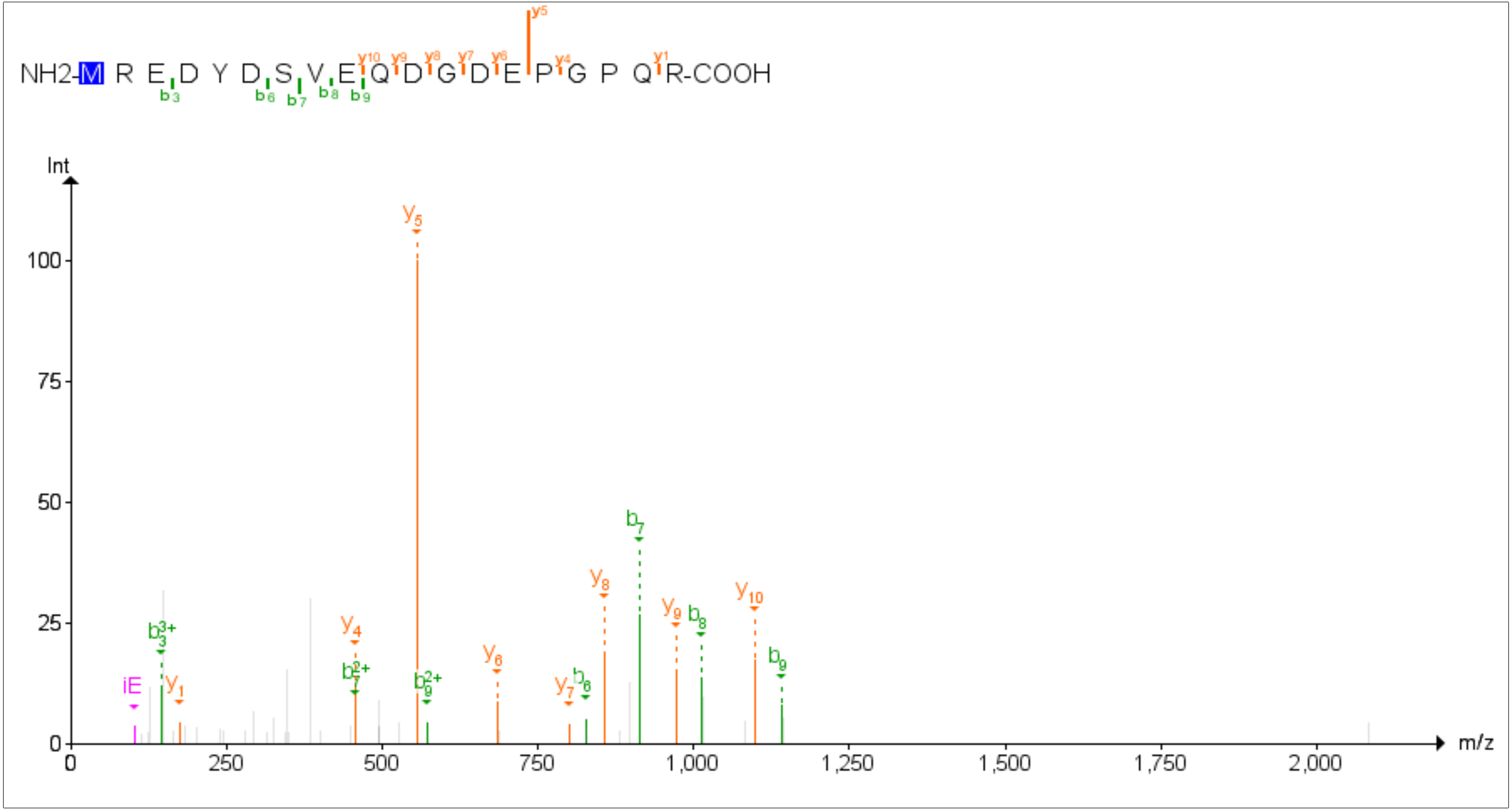

NH2-M R E D Y D S V E Q D G D E P G P Q R-COOH
y5
y10 y9 y8 y7 y6 y4 y1
b3 b6 b7 b8 b9
Int
100
75
50
25
0
m/z
0
250
500
750
1,000
1,250
1,500
1,750
2,000

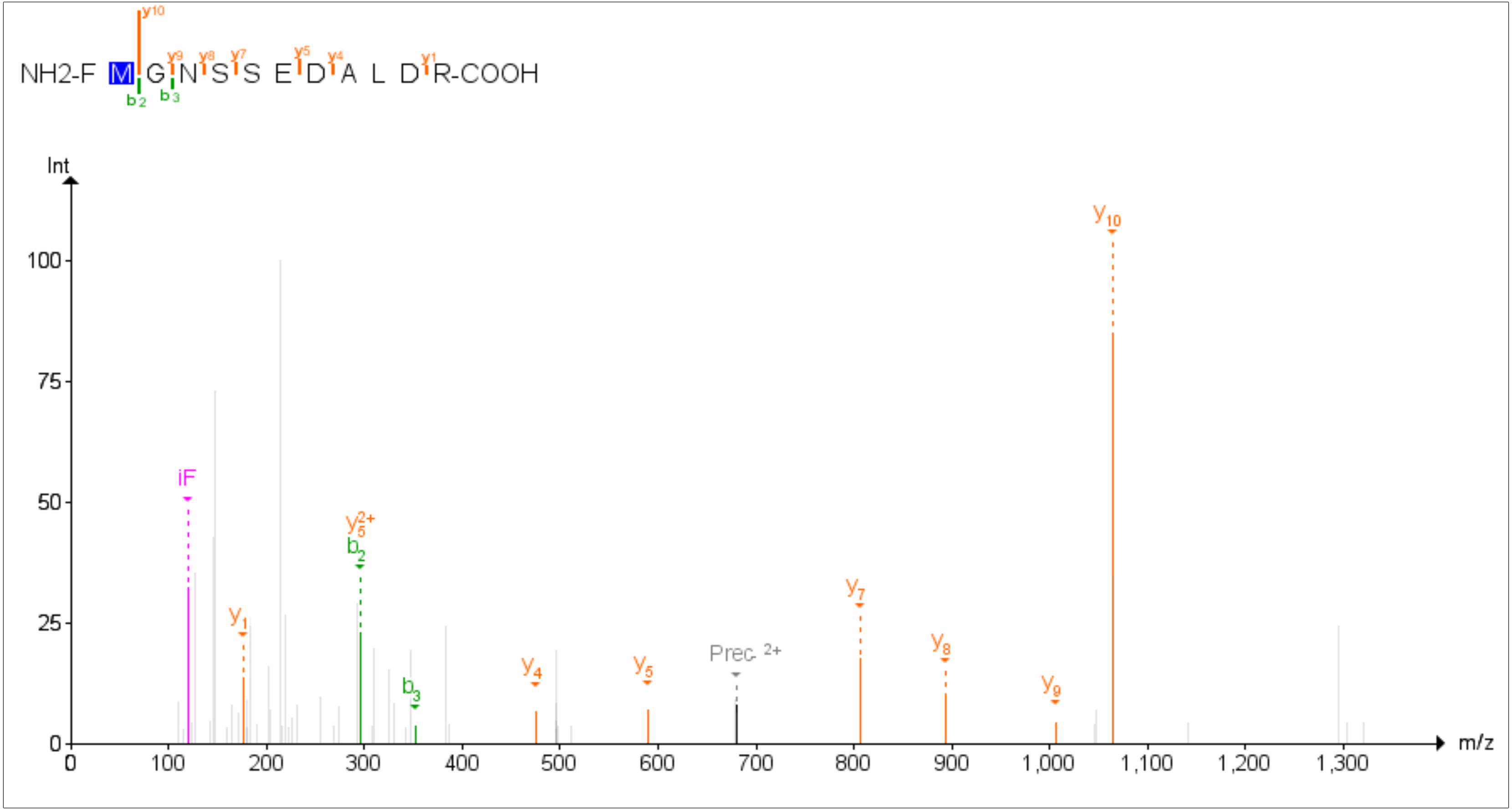

NH2-F M G N S S E D A L D R-COOH
y10
y9
y8
y7
y5
y4
y1
b2
b3
Int
100
75
50
25
0
iF
y1
y5 2+
b2
b3
y4
y5
Prec 2+
y7
y8
y9
y10
0
100
200
300
400
500
600
700
800
900
1,000
1,100
1,200
1,300
m/z

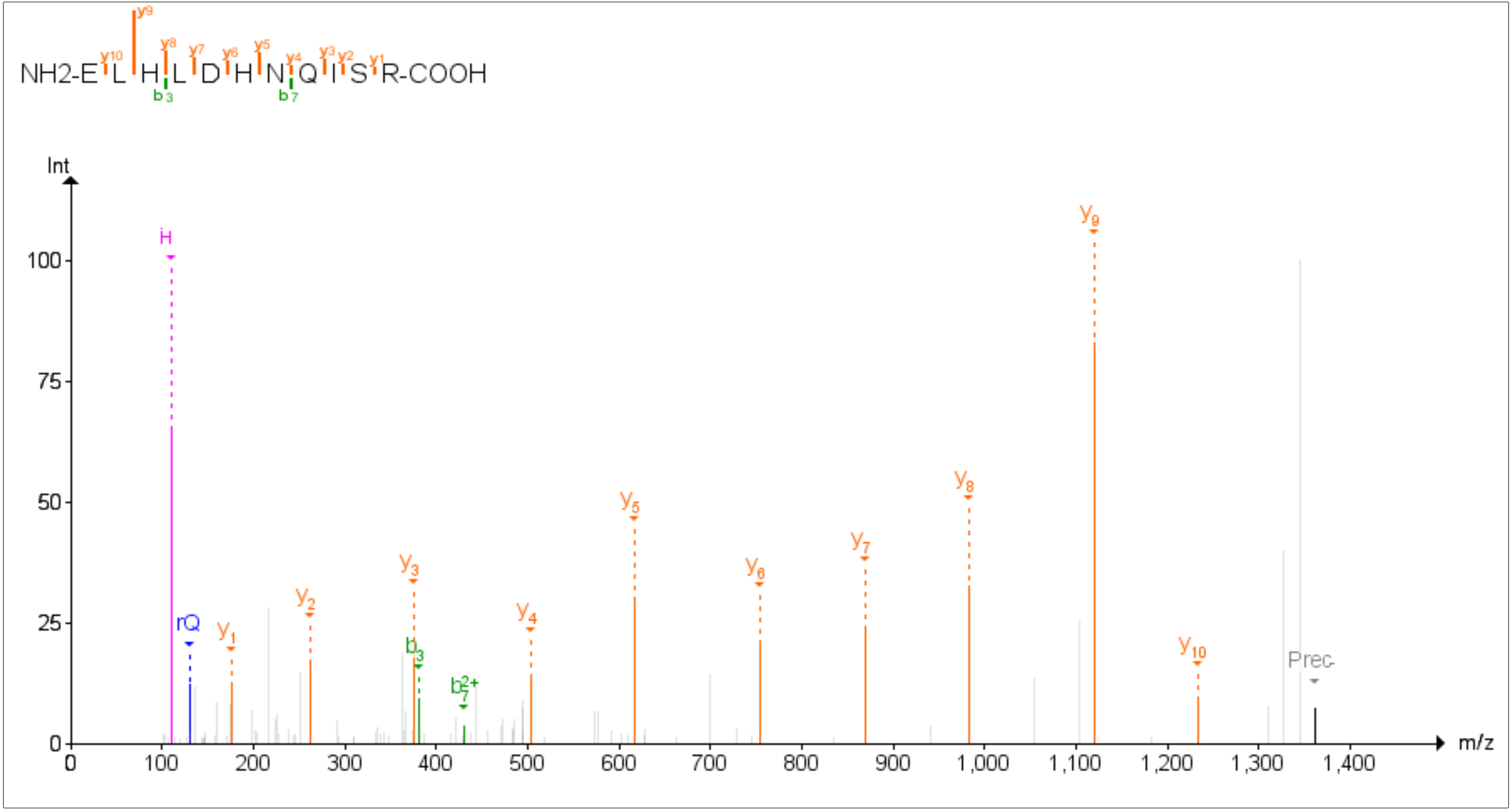

NH2-E L H L D H N Q I S R-COOH
y10 y9 y8 y7 y6 y5 y4 y3 y2 y1
b3 b7
Int
m/z
iH
rQ
y1 y2 y3 b3 b7 2+ y4 y5 y6 y7 y8 y9 y10
Prec
0 25 50 75 100
0 100 200 300 400 500 600 700 800 900 1,000 1,100 1,200 1,300 1,400

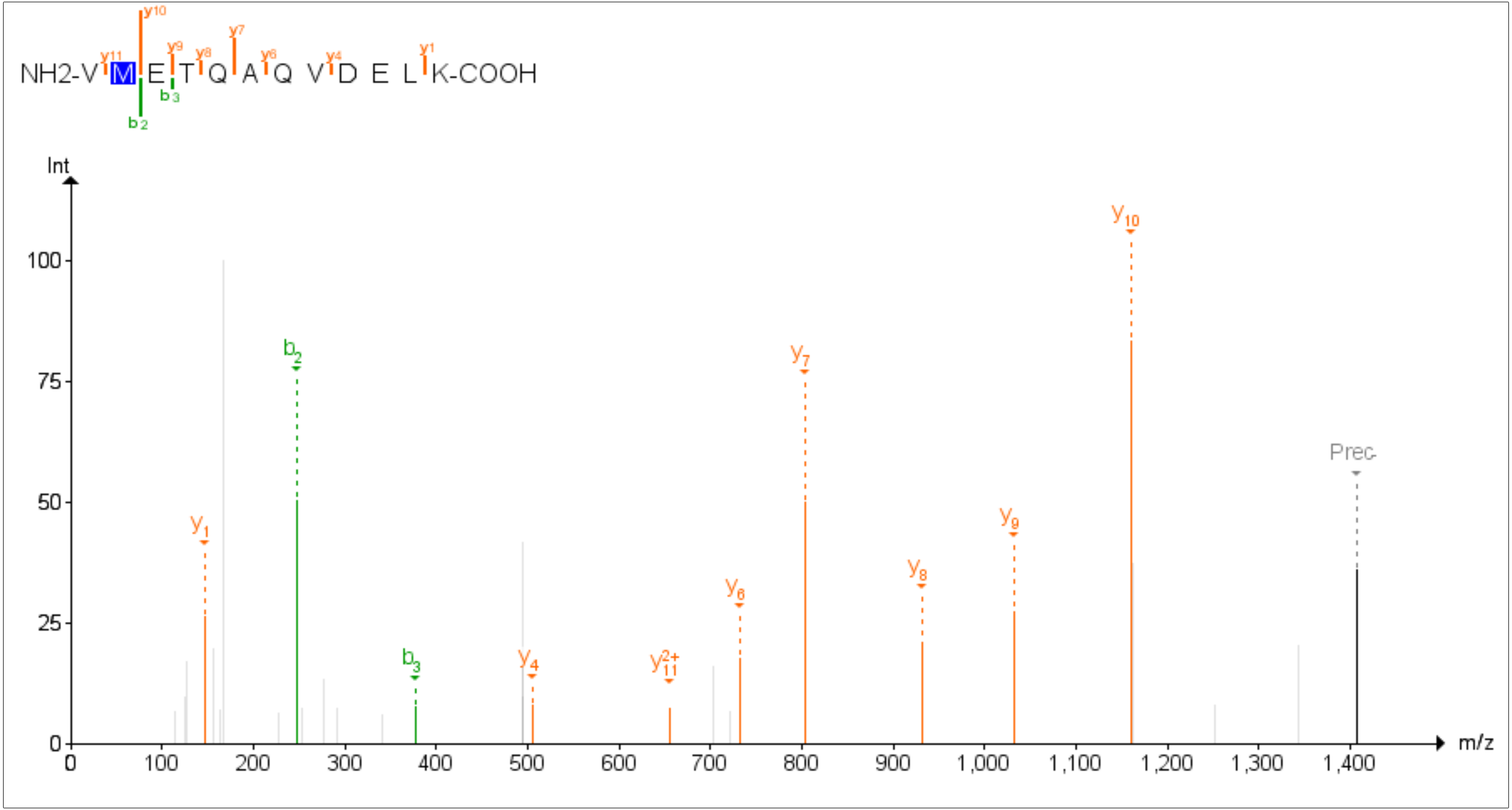

NH2-V M E T Q A Q V D E L K-COOH
y11 y10 y9 y8 y7 y6 y4 y1
b2 b3
Int
m/z
0
25
50
75
100
0 100 200 300 400 500 600 700 800 900 1,000 1,100 1,200 1,300 1,400
y1
b2
b3
y4
y11 2+
y6
y7
y8
y9
y10
Prec

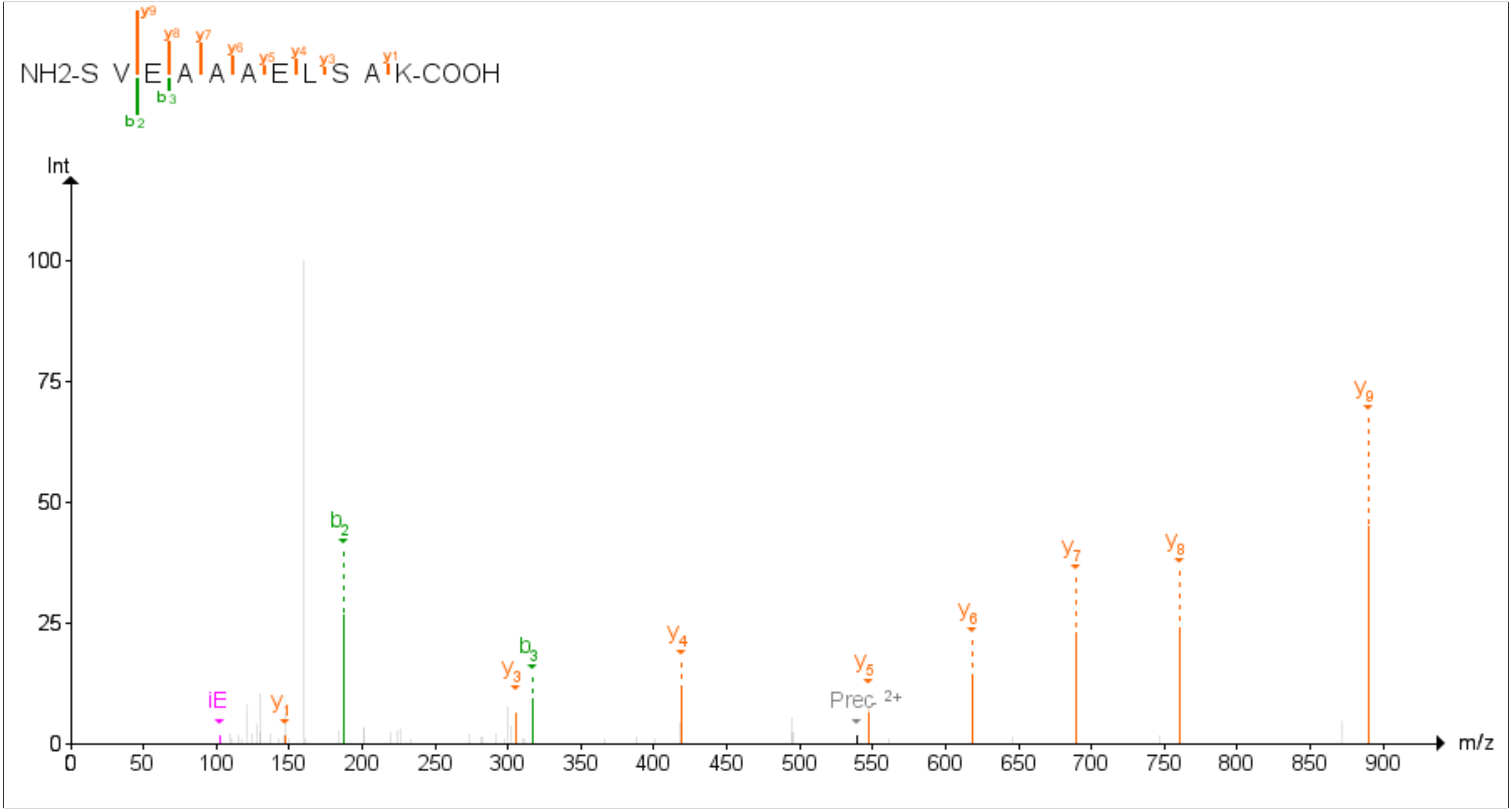

NH2-S V E A A A E L S A K-COOH
y9
y8
y7
y6
y5
y4
y3
y1
b2
b3
Int
100
75
50
25
0
m/z
0
50
100
150
200
250
300
350
400
450
500
550
600
650
700
750
800
850
900
iE
y1
b2
y3
b3
y4
Prec 2+
y5
y6
y7
y8
y9

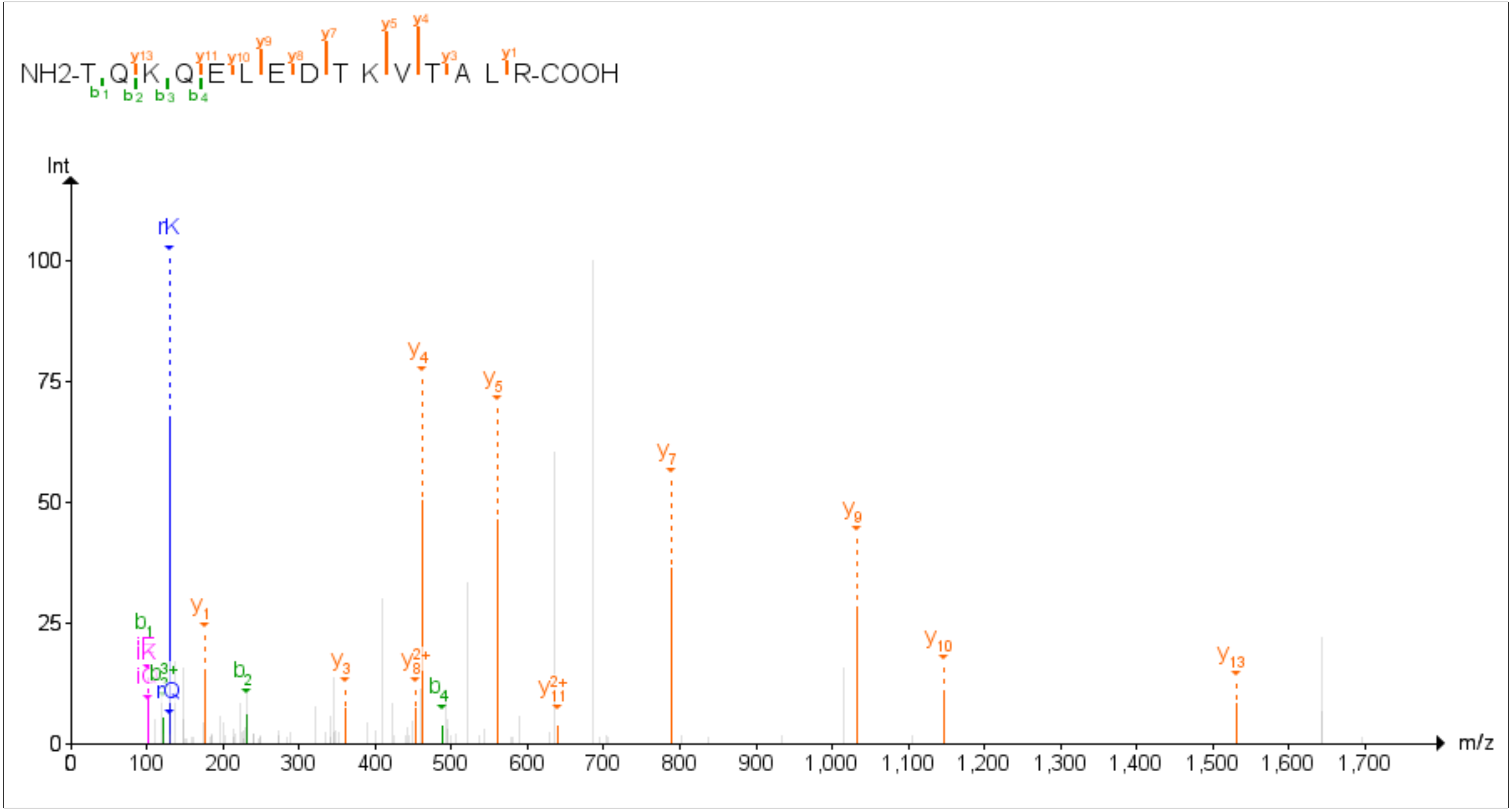

NH2-T Q K Q E L E D T K V T A L R-COOH
Int
m/z
rK
rQ
y4
y5
y7
y9
y10
y13
y1
y3
b1
b2
b4
0
25
50
75
100
0
100
200
300
400
500
600
700
800
900
1,000
1,100
1,200
1,300
1,400
1,500
1,600
1,700

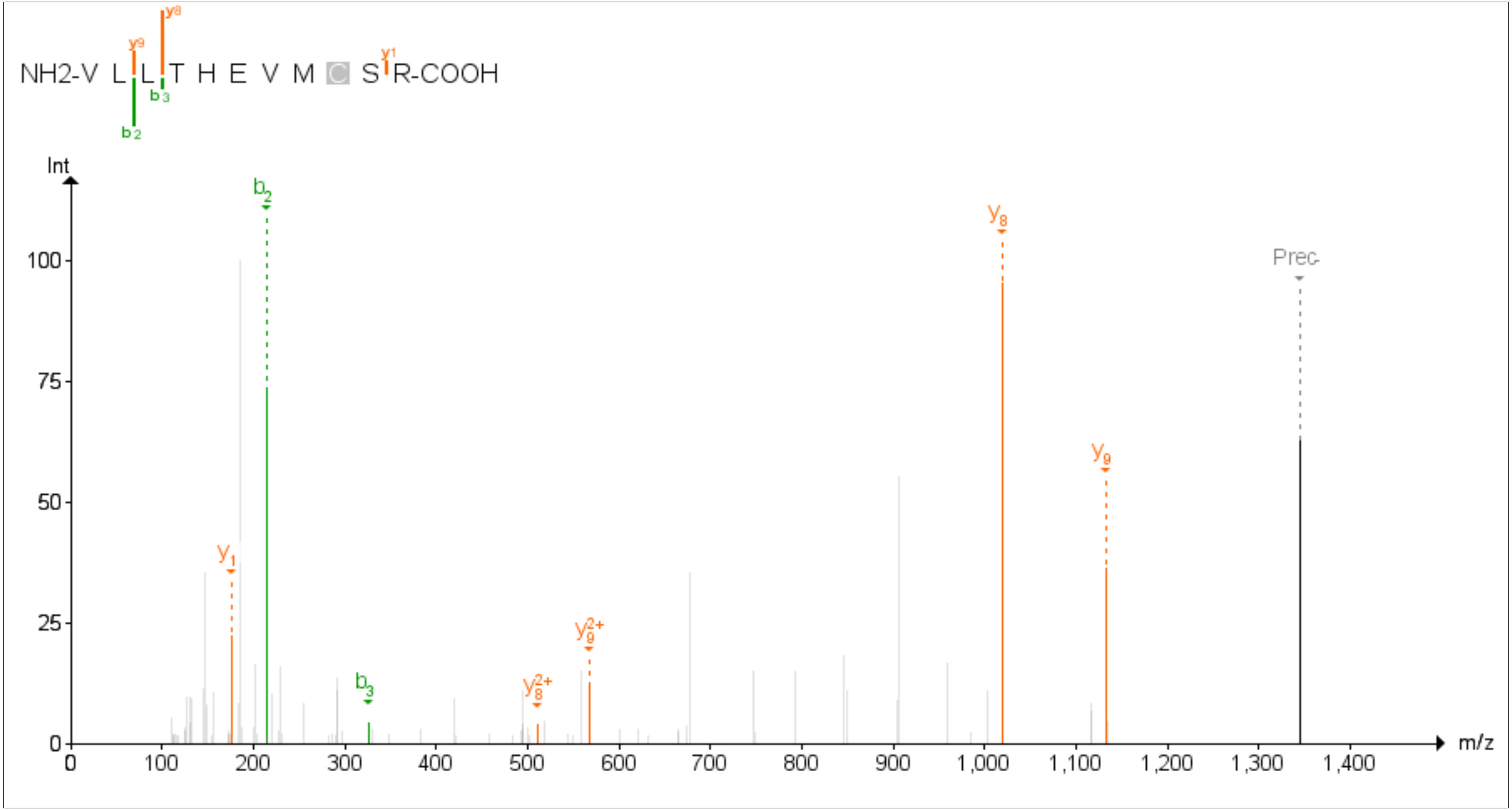

NH2-V L L T H E V M C S R-COOH
y9
y8
y1
b2
b3
Int
m/z
b2
y8
Prec.
y9
y1
y9 2+
y8 2+
b3
100
75
50
25
0
0
100
200
300
400
500
600
700
800
900
1,000
1,100
1,200
1,300
1,400

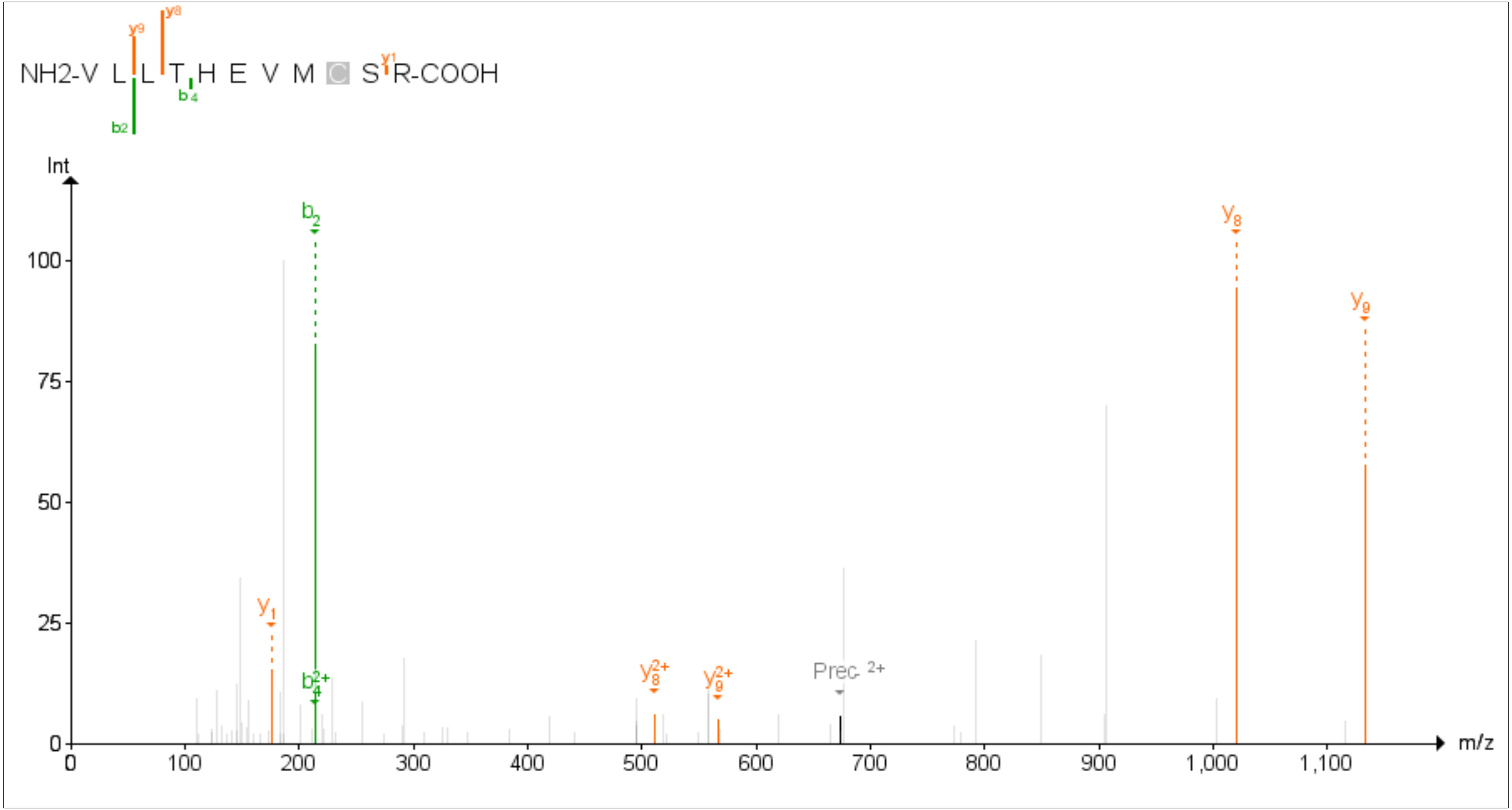

NH2-V L L T H E V M C S R-COOH
y9
y8
y1
b2
b4
Int
100
75
50
25
0
b2
y8
y9
y1
b4 2+
y8 2+
y9 2+
Prec 2+
0
100
200
300
400
500
600
700
800
900
1,000
1,100
m/z

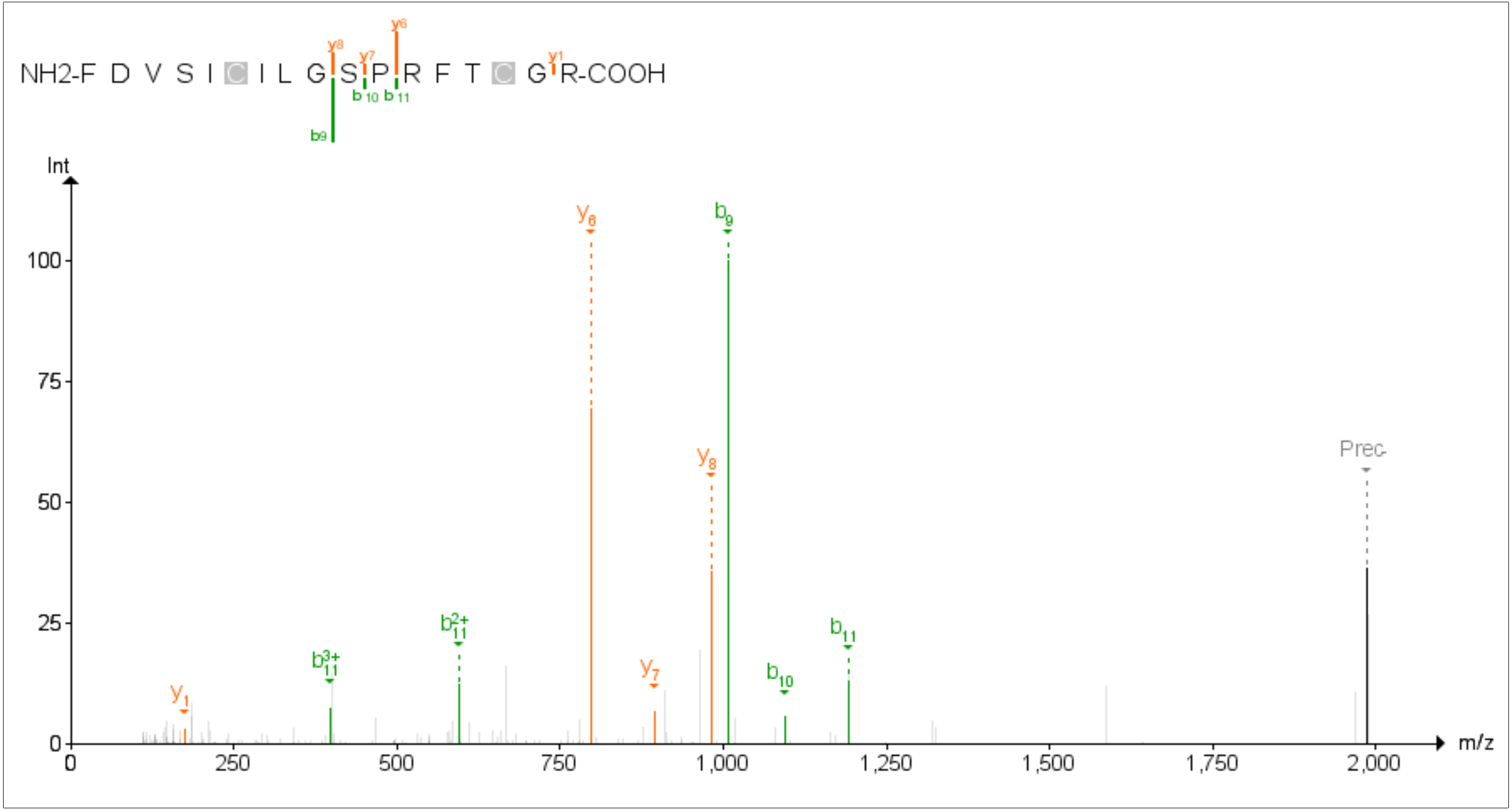
NH2-F D V S I C I L G S P R F T C G R-COOH
y8 y7 y6 y1
b9 b10 b11
Int
100
75
50
25
0
y6
b9
y8
Prec.
b11^2+
b11^3+
y1
y7
b10
b11
0
250
500
750
1,000
1,250
1,500
1,750
2,000
m/z

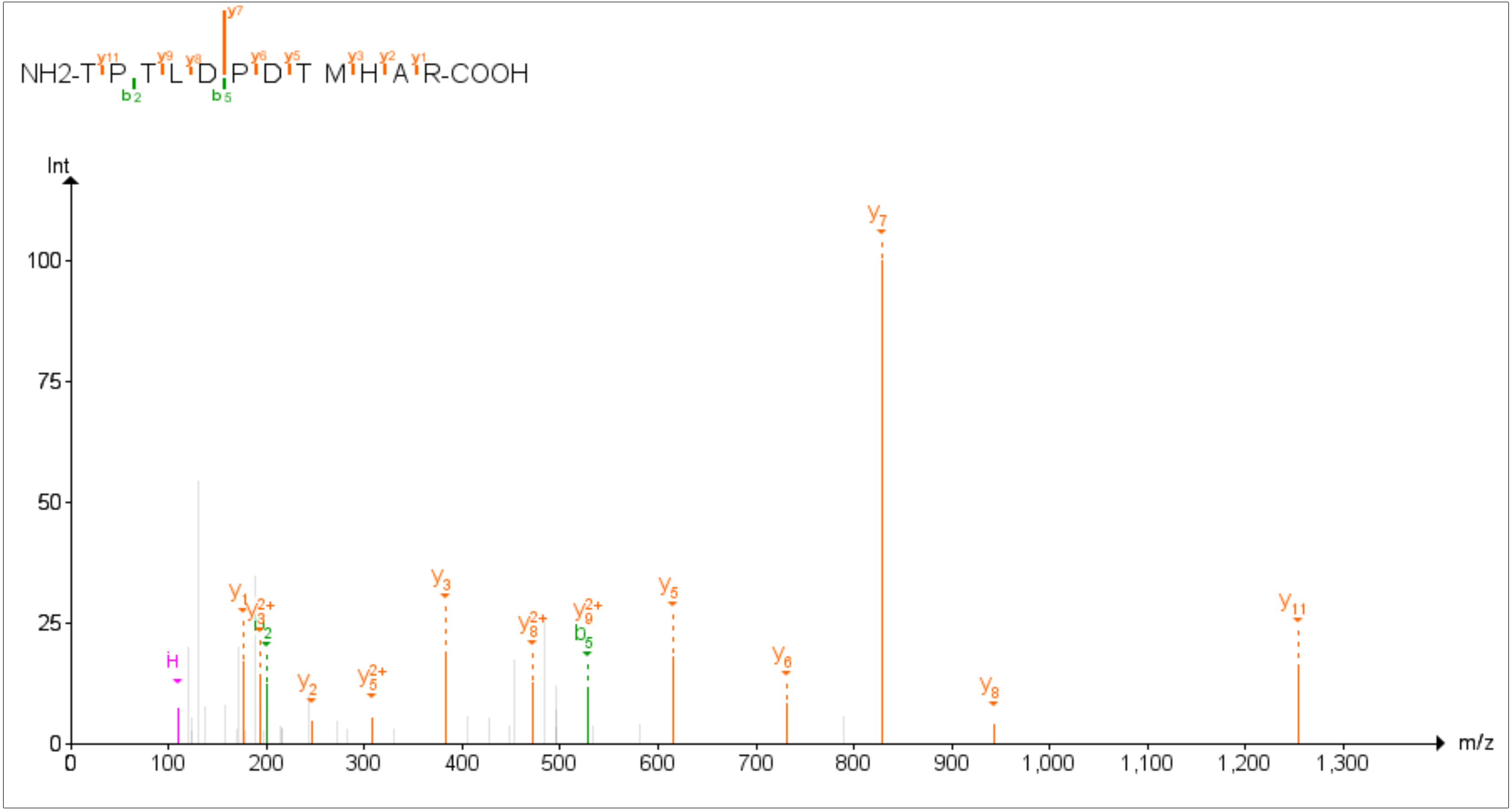
NH2-T P T L D P D T M H A R-COOH
y11 y9 y8 y7 y6 y5 y3 y2 y1
b2 b5
Int
m/z
y7
y1
y3 2+
b2
y2
y5 2+
y3
y8 2+
y9 2+
b5
y5
y6
y8
y11
H
0
25
50
75
100
0
100
200
300
400
500
600
700
800
900
1,000
1,100
1,200
1,300

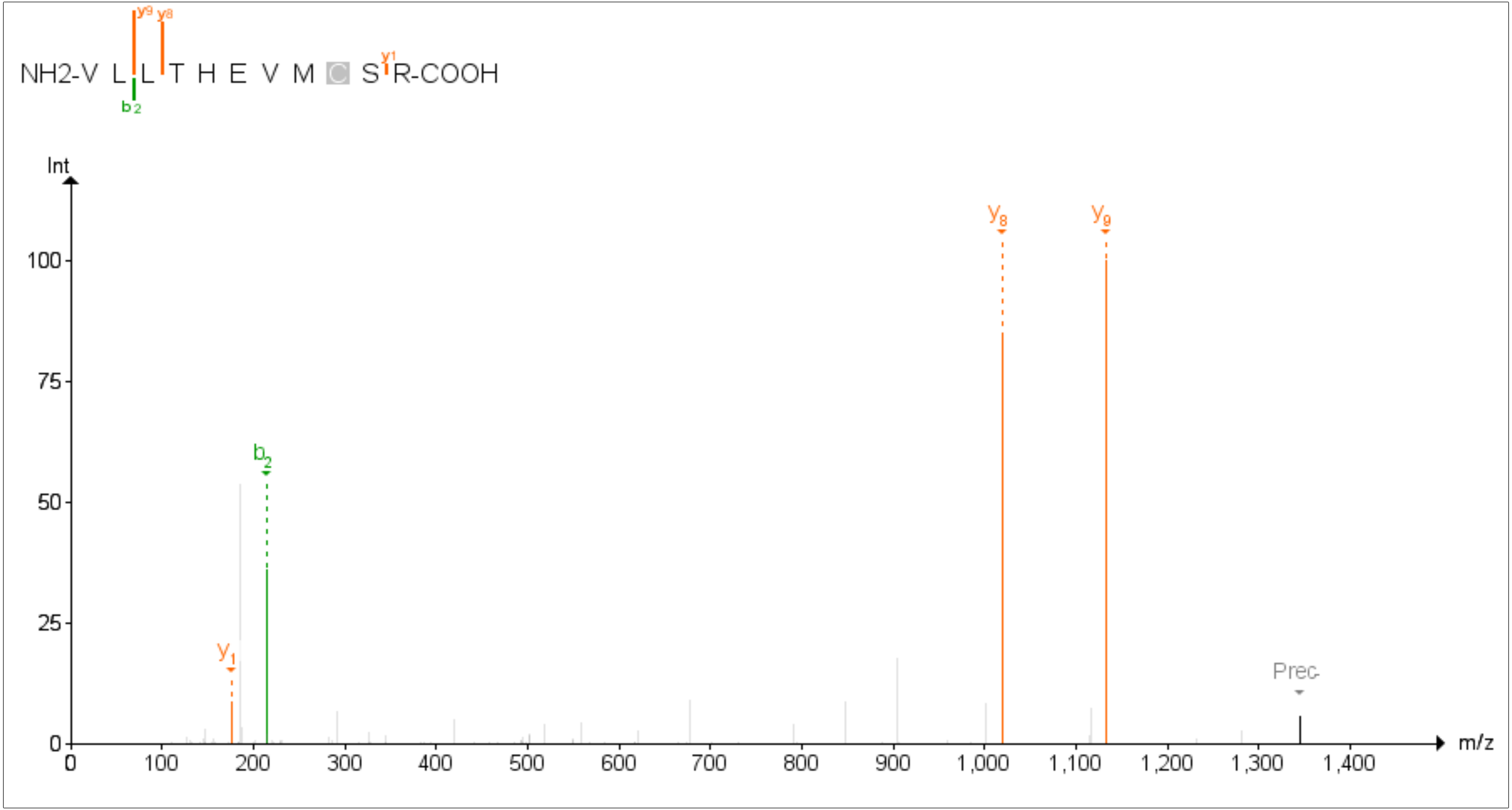

NH2-V L L T H E V M C S R-COOH
y9 y8 y1 b2
Int
100
75
50
25
0
y8
y9
b2
y1
Prec.
0 100 200 300 400 500 600 700 800 900 1,000 1,100 1,200 1,300 1,400
m/z

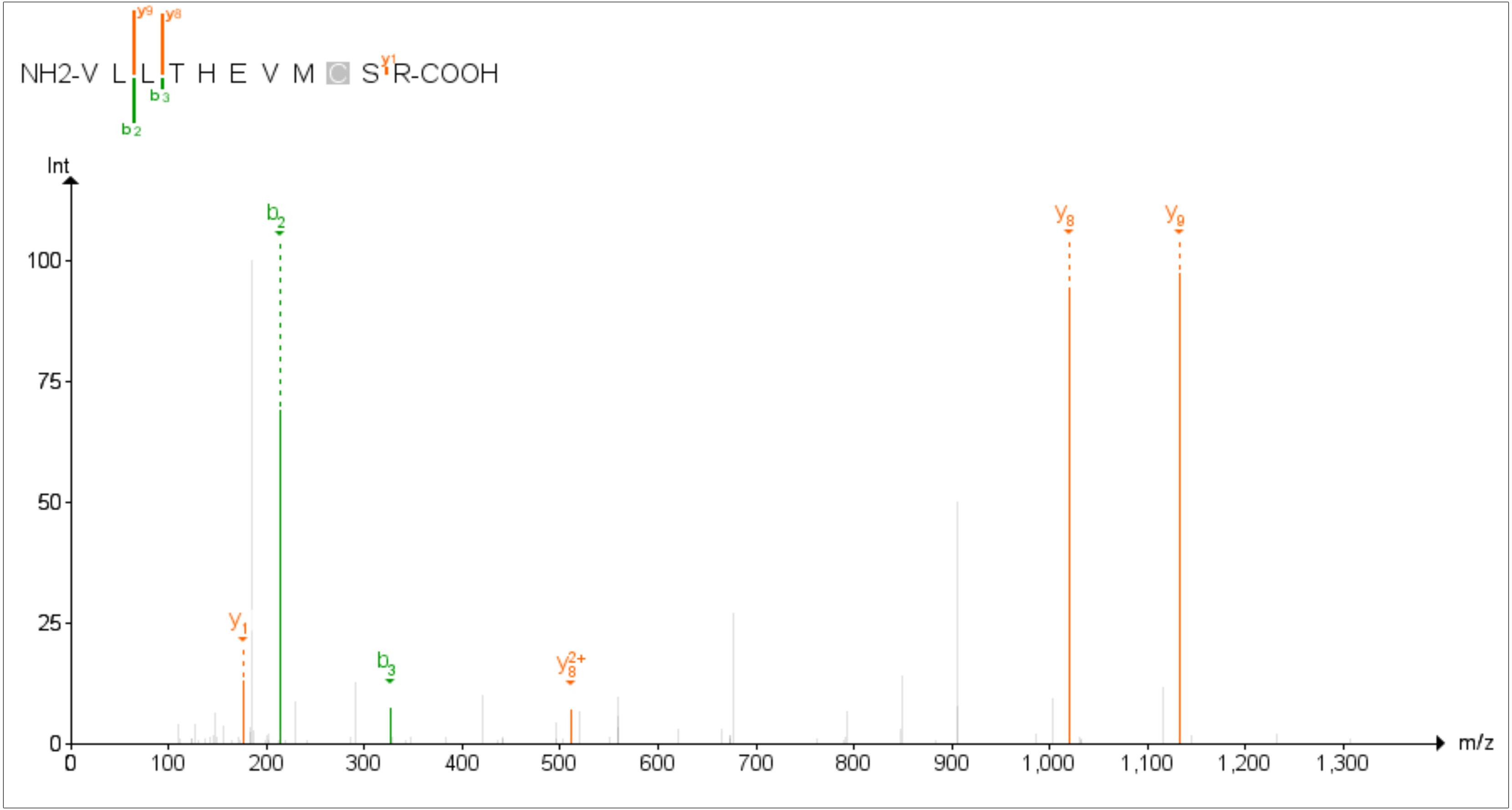

NH2-V L L T H E V M C S R-COOH
y9
y8
y1
b2
b3
Int
100
75
50
25
0
b2
y8
y9
y1
b3
y8 2+
0
100
200
300
400
500
600
700
800
900
1,000
1,100
1,200
1,300
m/z

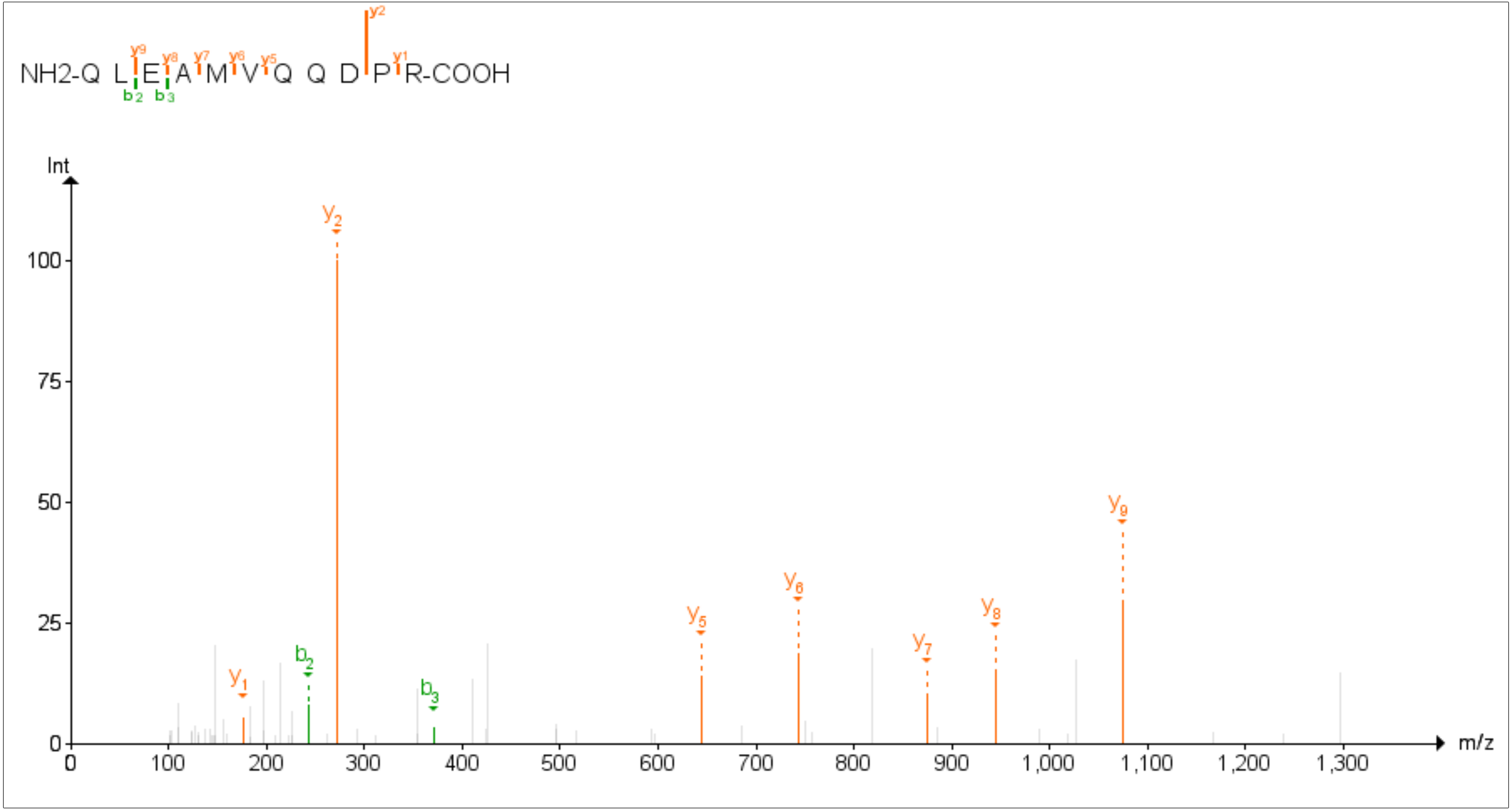

NH2-Q L E A M V Q Q D P R-COOH
y9 y8 y7 y6 y5 y2 y1
b2 b3
Int
100
75
50
25
0
m/z
0 100 200 300 400 500 600 700 800 900 1,000 1,100 1,200 1,300
y2
y9
y6
y5
y8
y7
b2
y1
b3

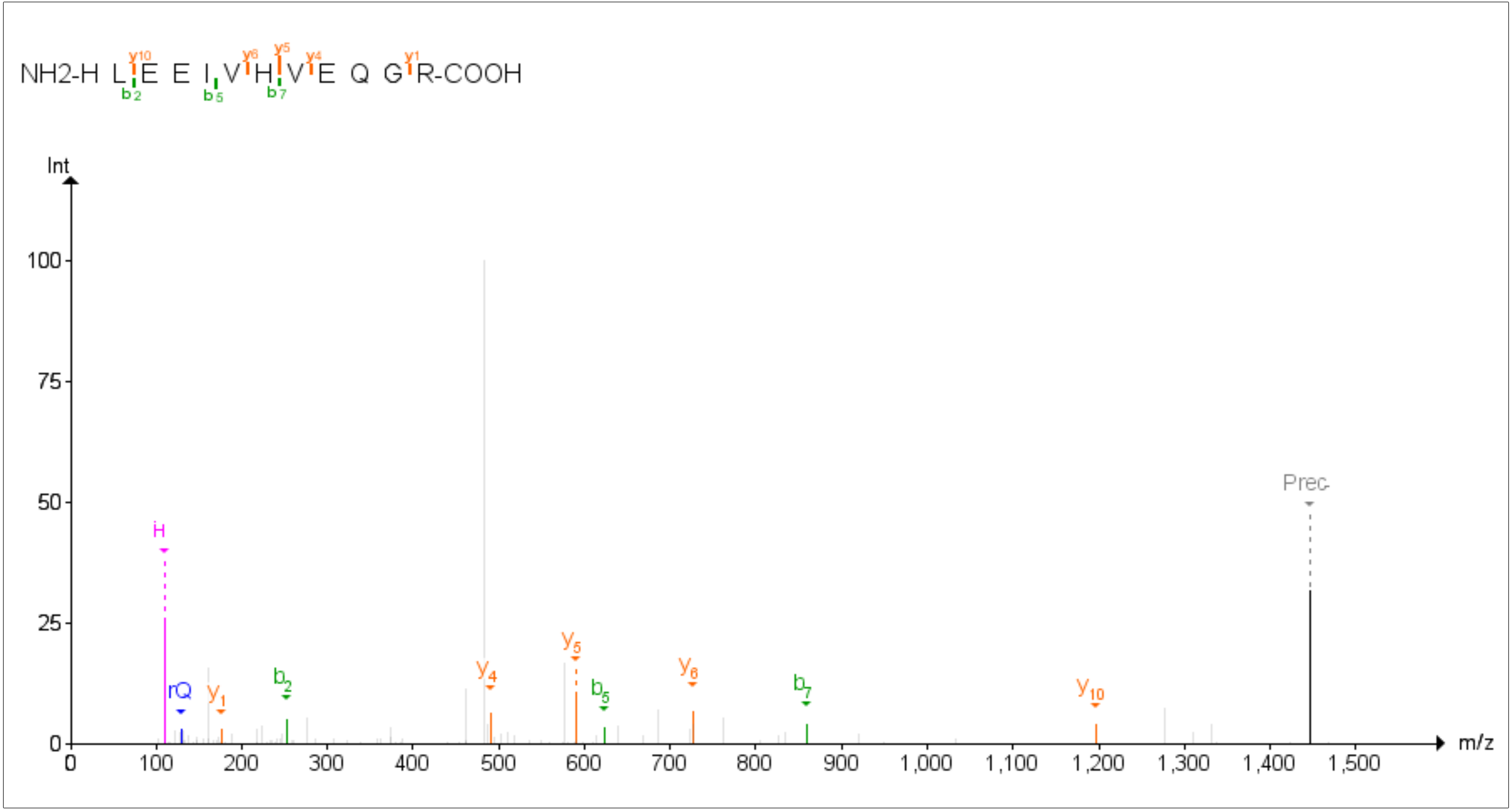

NH2-H L E E I V H V E Q G R-COOH
y10
y6
y5
y4
y1
b2
b5
b7
Int
100
75
50
25
0
H
rQ
y1
b2
y4
y5
b5
y6
b7
y10
Prec
0
100
200
300
400
500
600
700
800
900
1,000
1,100
1,200
1,300
1,400
1,500
m/z

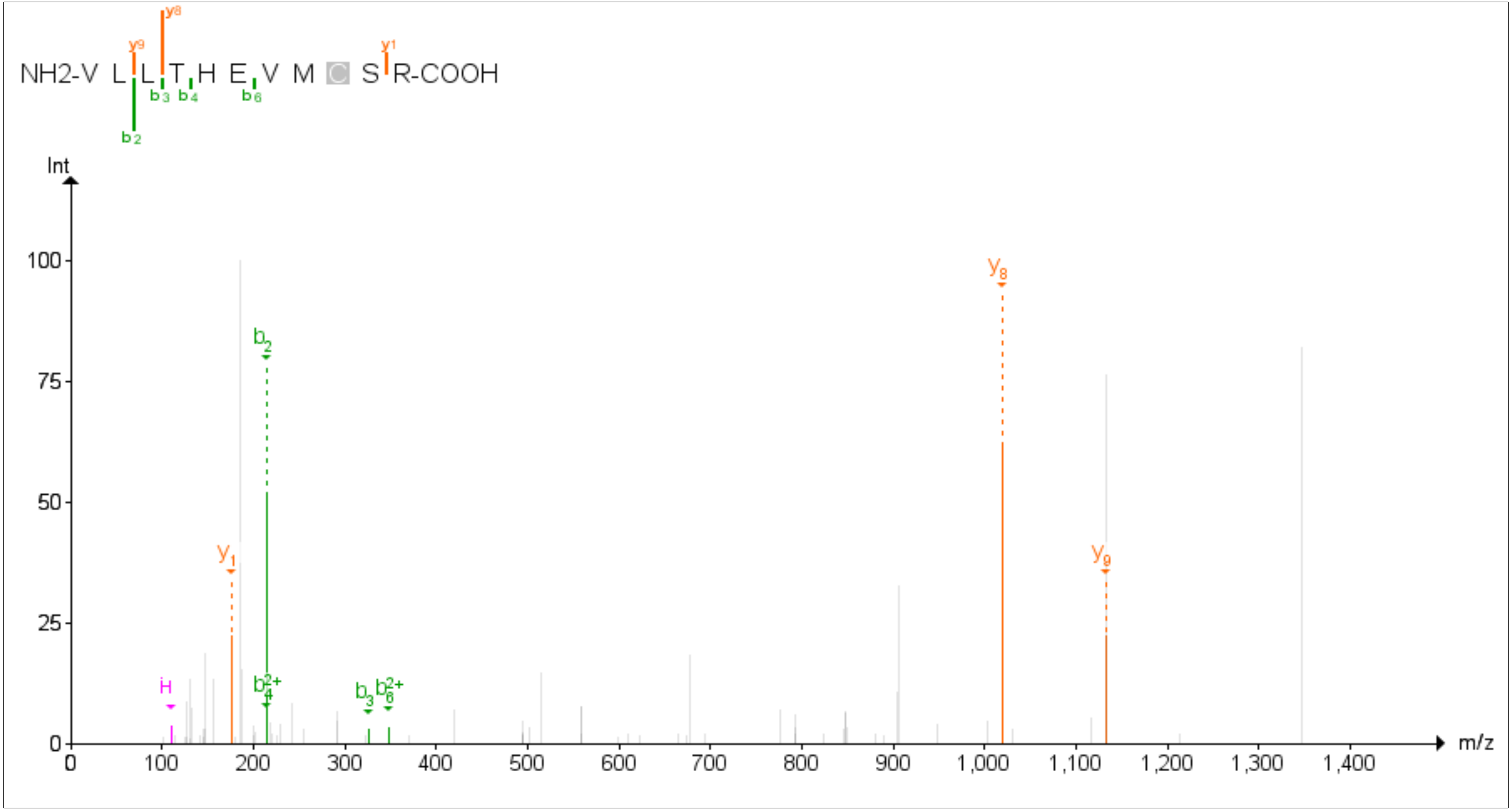

NH2-V L L T H E V M C S R-COOH
y9 y8 y1
b2 b3 b4 b6
Int
100
75
50
25
0
y8
b2
y1
y9
H
b4 2+
b3
b6 2+
0 100 200 300 400 500 600 700 800 900 1,000 1,100 1,200 1,300 1,400
m/z

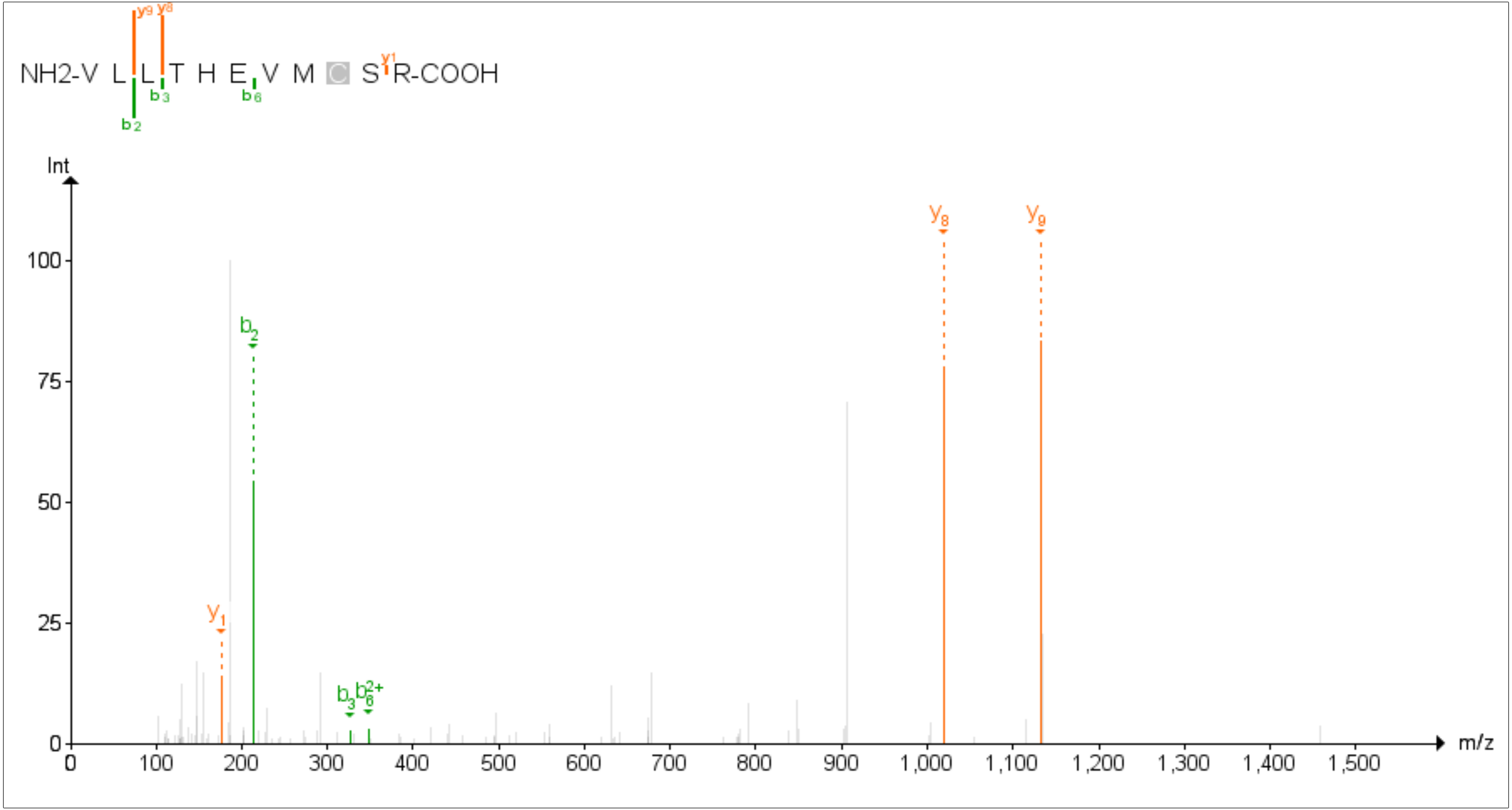
NH2-V L L T H E V M C S R-COOH
y9 y8 y1
b2 b3 b6
Int
100
75
50
25
0
y1
b2
b3 b6 2+
y8
y9
0 100 200 300 400 500 600 700 800 900 1,000 1,100 1,200 1,300 1,400 1,500
m/z

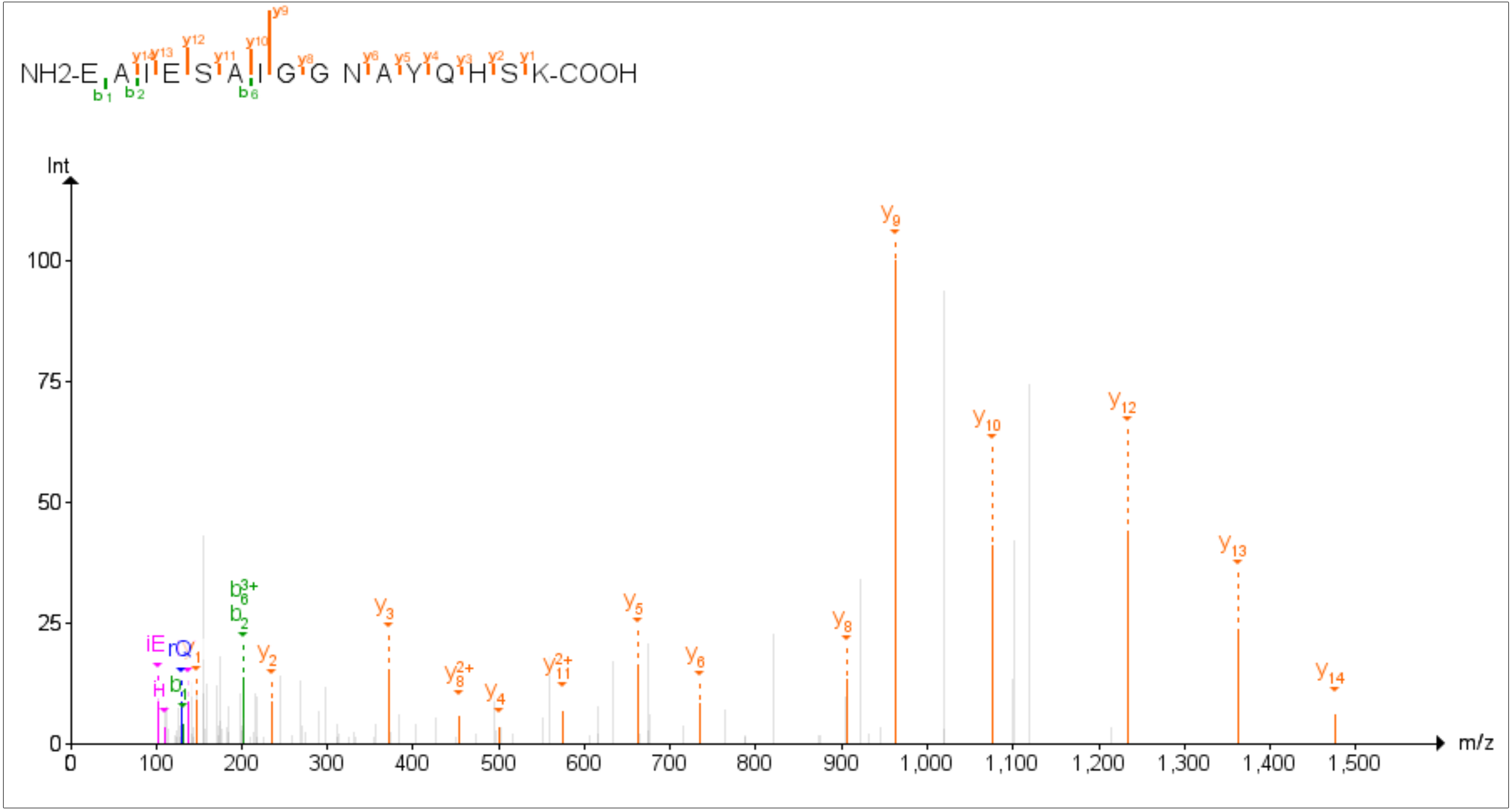

NH2-E A I E S A I G G N A Y Q H S K-COOH
b1 b2 b6
y14 y13 y12 y11 y10 y9 y8 y6 y5 y4 y3 y2 y1
Int
100
75
50
25
0
m/z
0 100 200 300 400 500 600 700 800 900 1,000 1,100 1,200 1,300 1,400 1,500
y9
y10
y12
y13
y14
y8
y6
y5
y4
y3
y2
y1
y8 2+
y11 2+
b6 3+
b2
b1
iE
rQ
H

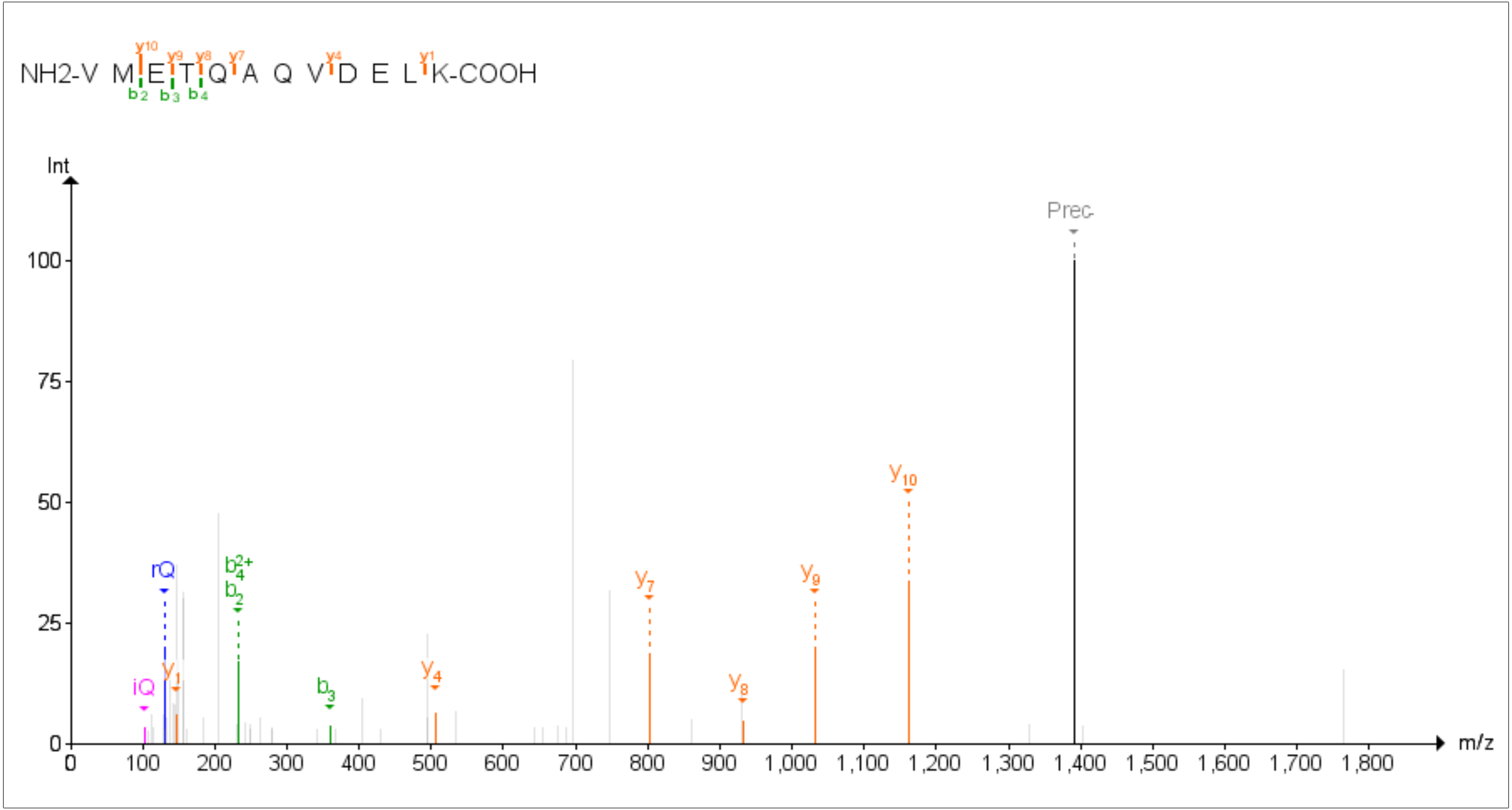

NH2-V M E T Q A Q V D E L K-COOH
y10 y9 y8 y7 y4 y1
b2 b3 b4
Int
m/z
Prec.
y10
y9
y7
y8
y4
y1
b3
b4 2+
b2
rQ
iQ
0
25
50
75
100
0 100 200 300 400 500 600 700 800 900 1,000 1,100 1,200 1,300 1,400 1,500 1,600 1,700 1,800

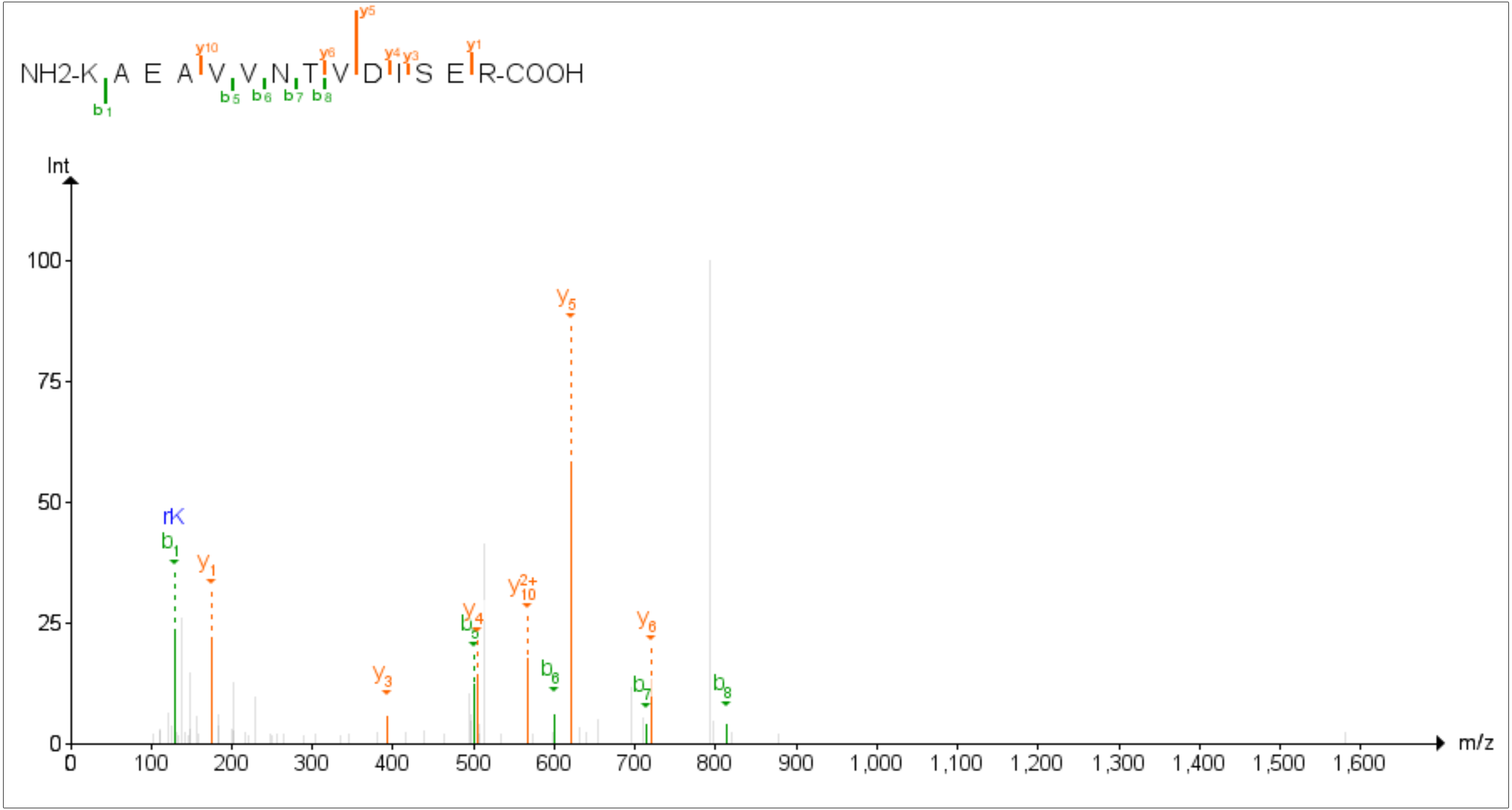

NH2-K A E A V V N T V D I S E R-COOH
b1 b5 b6 b7 b8
y10 y6 y5 y4 y3 y1
Int
100
75
50
25
0
rK
b1
y1
y3
b5
y4
y10 2+
y5
b6
b7
y6
b8
0 100 200 300 400 500 600 700 800 900 1,000 1,100 1,200 1,300 1,400 1,500 1,600
m/z

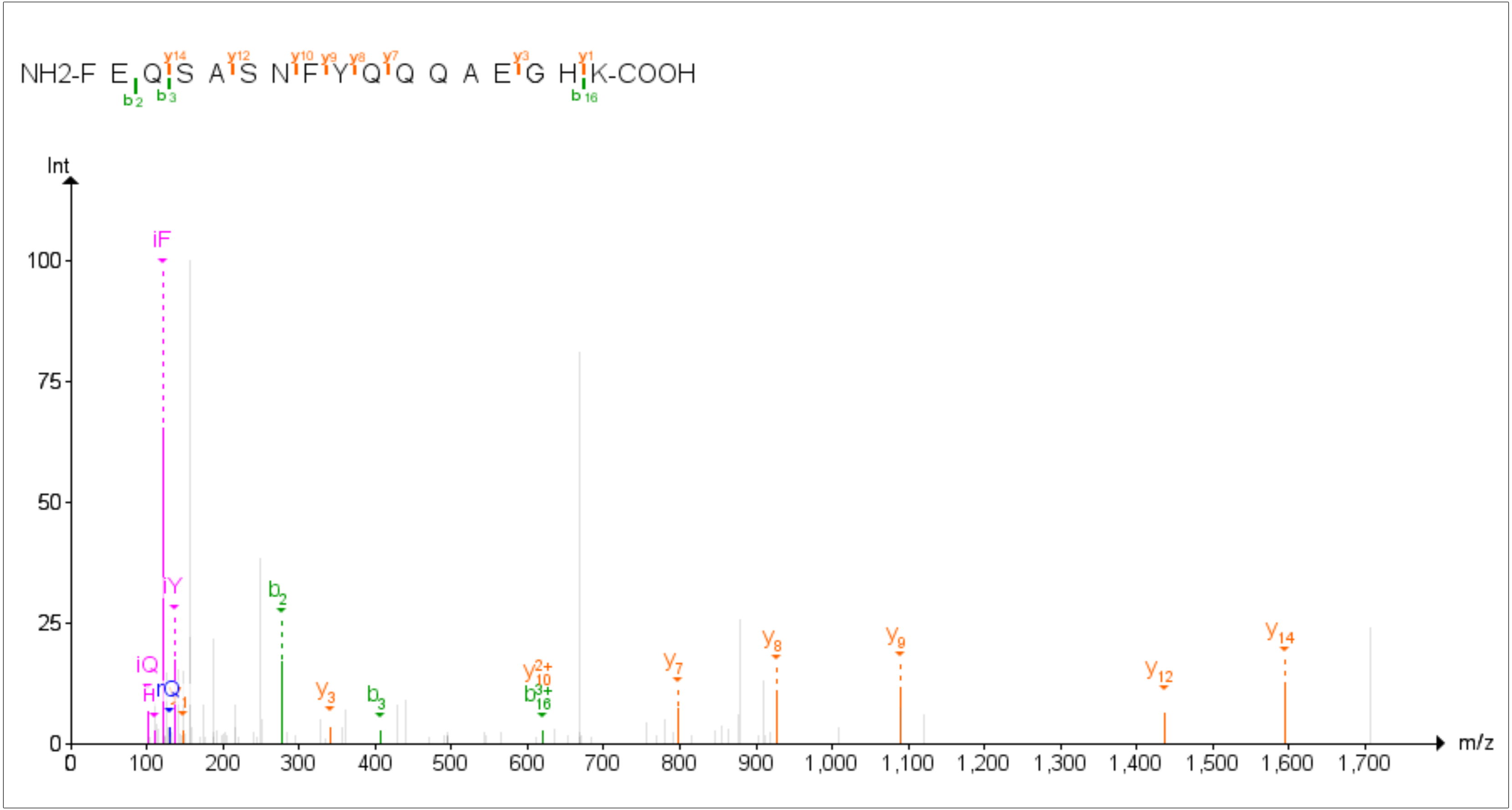

NH2-F E Q S A S N F Y Q Q Q A E G H K-COOH
y14
y12
y10
y9
y8
y7
y3
y1
b2
b3
b16
Int
iF
iY
iQ
iH
rQ
y1
b2
y3
b3
y_{10}^{2+}
b_{16}^{3+}
y7
y8
y9
y12
y14
m/z
0
25
50
75
100
0
100
200
300
400
500
600
700
800
900
1,000
1,100
1,200
1,300
1,400
1,500
1,600
1,700

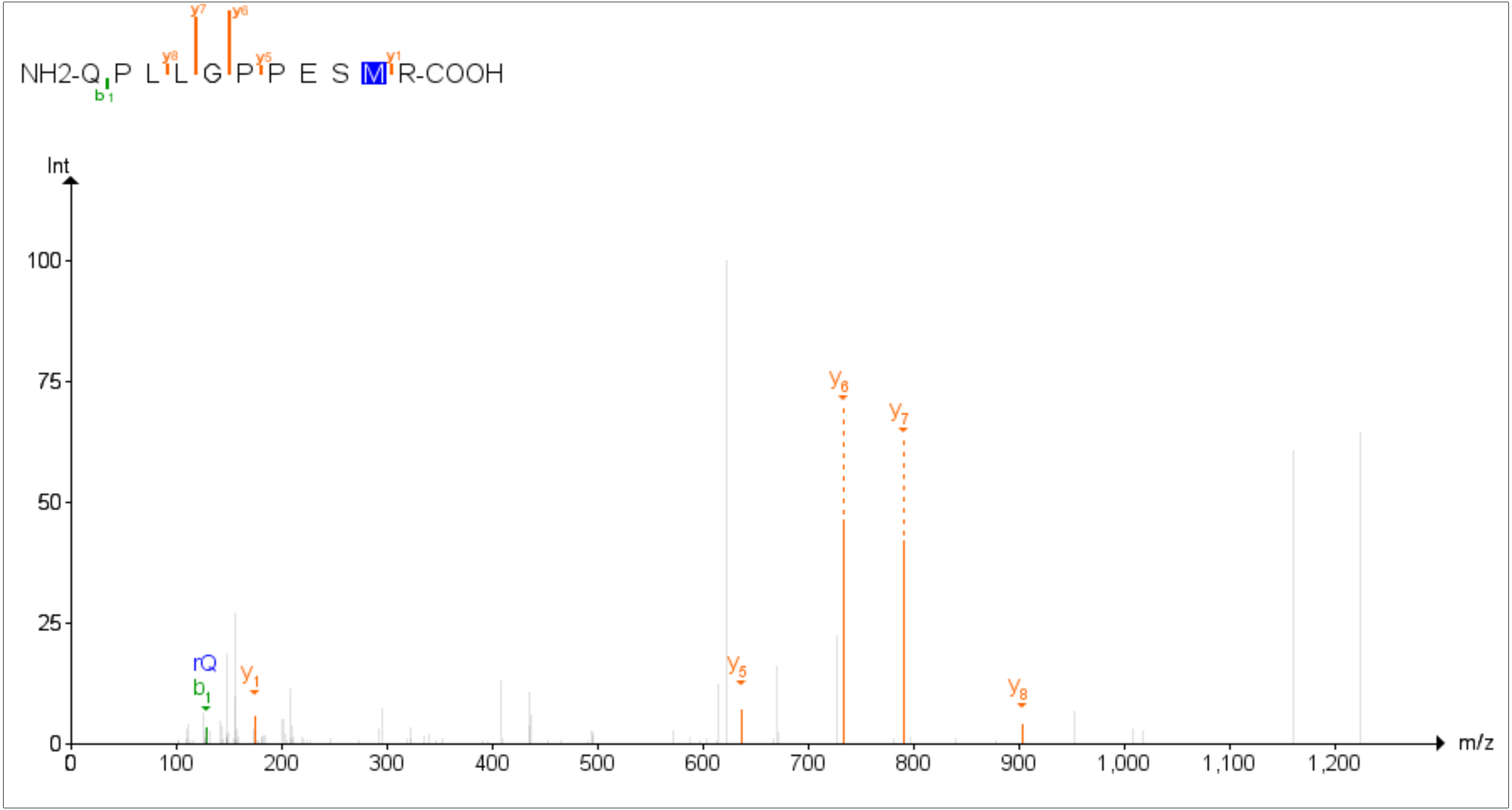

NH2-Q P L L G P P E S M R-COOH
b1
y8 y7 y6 y5 y1
Int
100
75
50
25
0
rQ
b1
y1
y5
y6
y7
y8
0 100 200 300 400 500 600 700 800 900 1,000 1,100 1,200
m/z

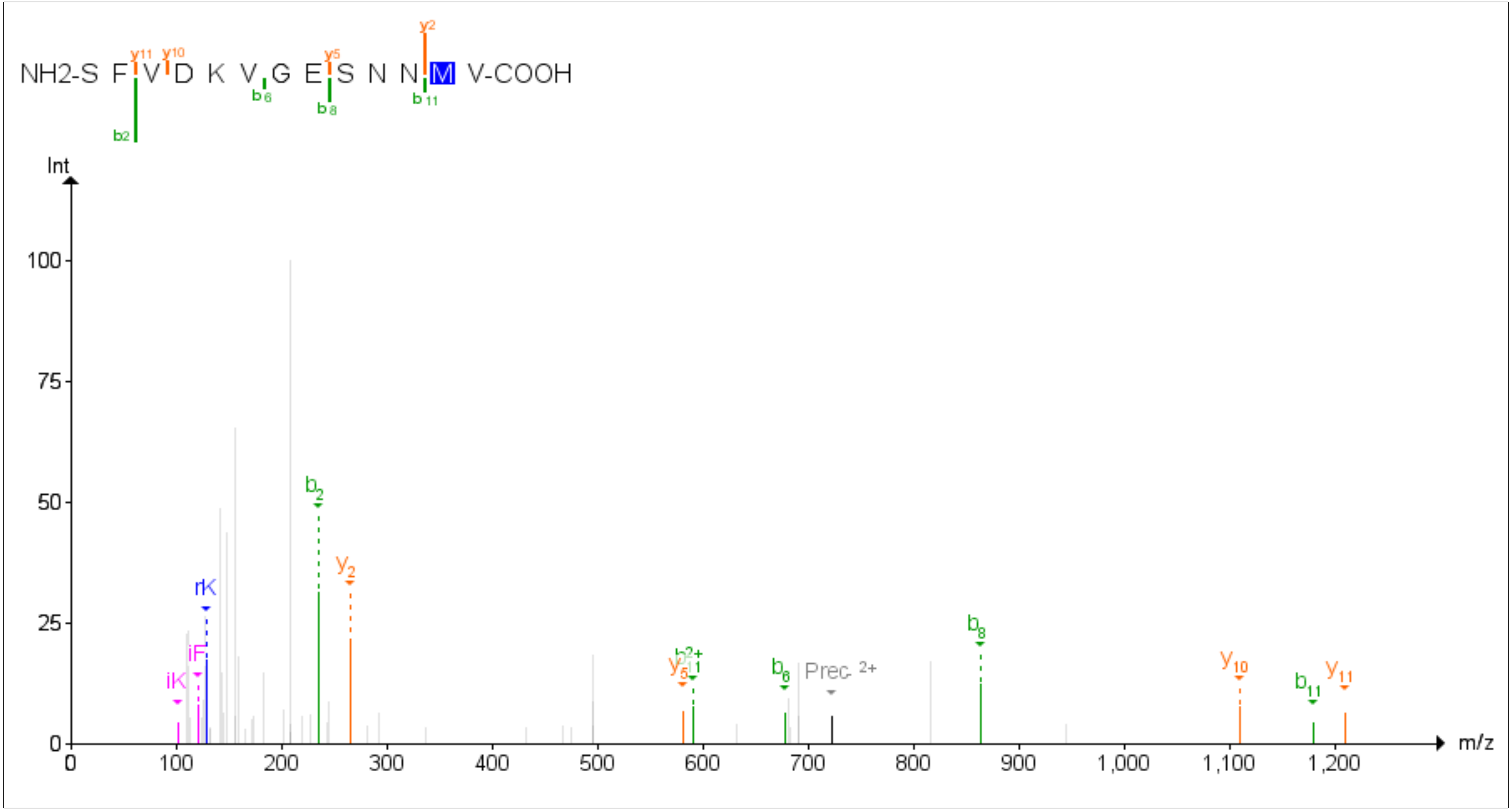

NH2-S F V D K V G E S N N M V-COOH
y11 y10 y5 y2
b2 b6 b8 b11
Int
100
75
50
25
0
b2
y2
rK
iF
iK
y5
b11 2+
b6
Prec 2+
b8
y10
b11
y11
0
100
200
300
400
500
600
700
800
900
1,000
1,100
1,200
m/z

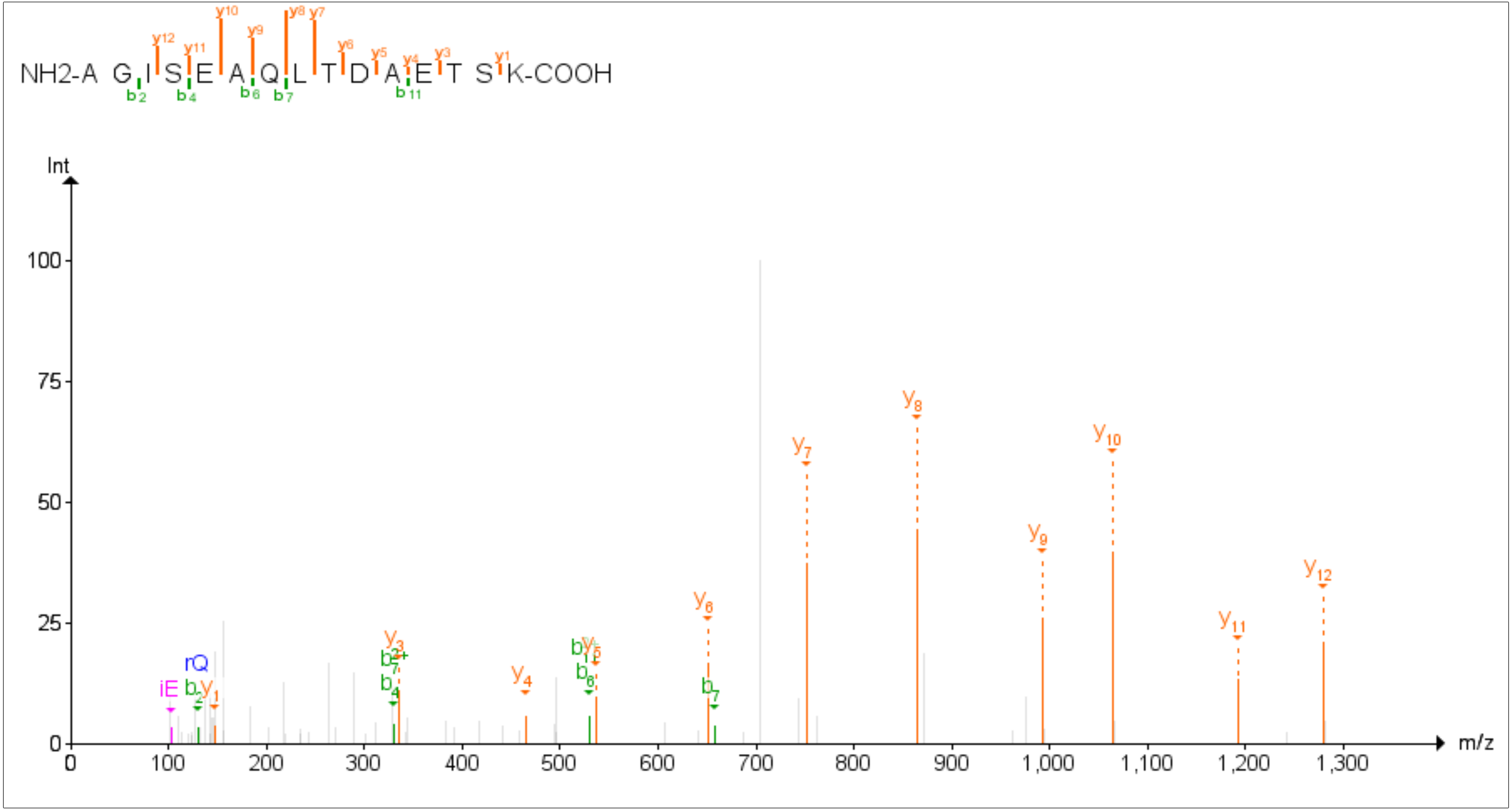

NH2-A G I S E A Q L T D A E T S K-COOH
Int
m/z

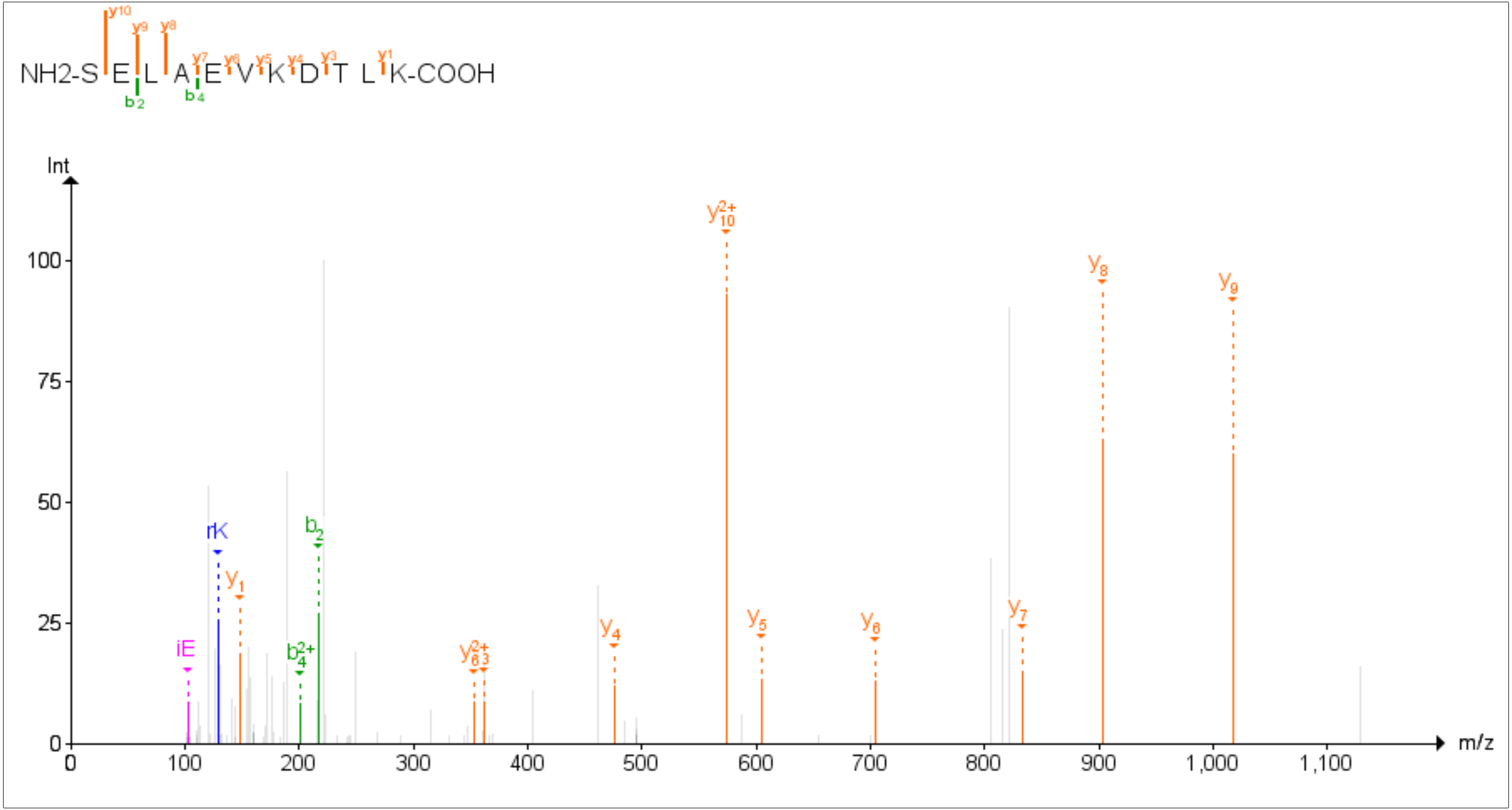
NH2-S E L A E V K D T L K-COOH
y10 y9 y8 y7 y6 y5 y4 y3 y1
b2 b4
Int
m/z
0
25
50
75
100
0 100 200 300 400 500 600 700 800 900 1,000 1,100
iE
rK
y1
b4 2+
b2
y6 2+ y3
y4
y10 2+
y5
y6
y7
y8
y9

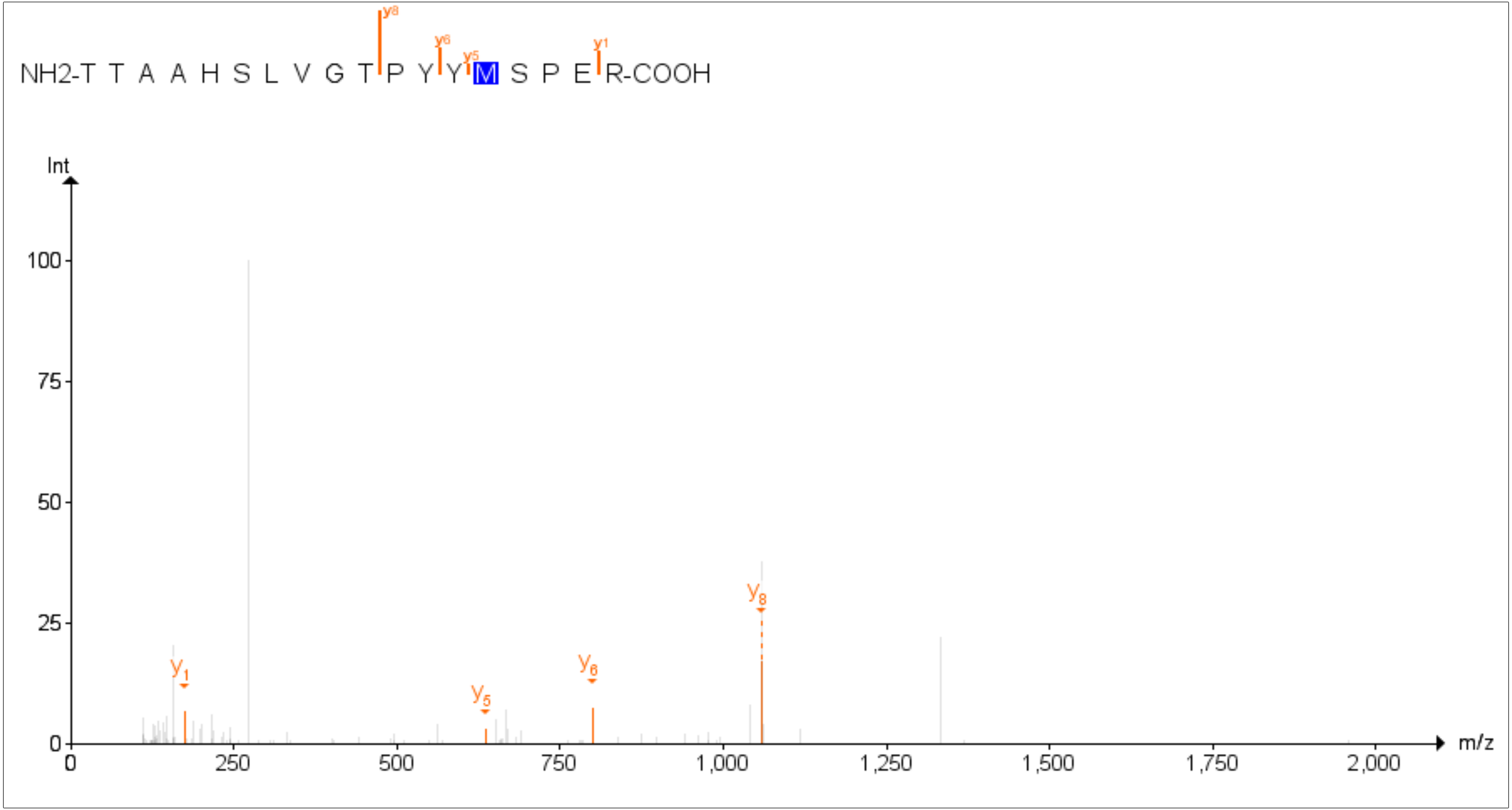

NH2-T T A A H S L V G T P Y Y M S P E R-COOH
y8
y6
y5
y1
Int
100
75
50
25
0
y1
y5
y6
y8
0
250
500
750
1,000
1,250
1,500
1,750
2,000
m/z

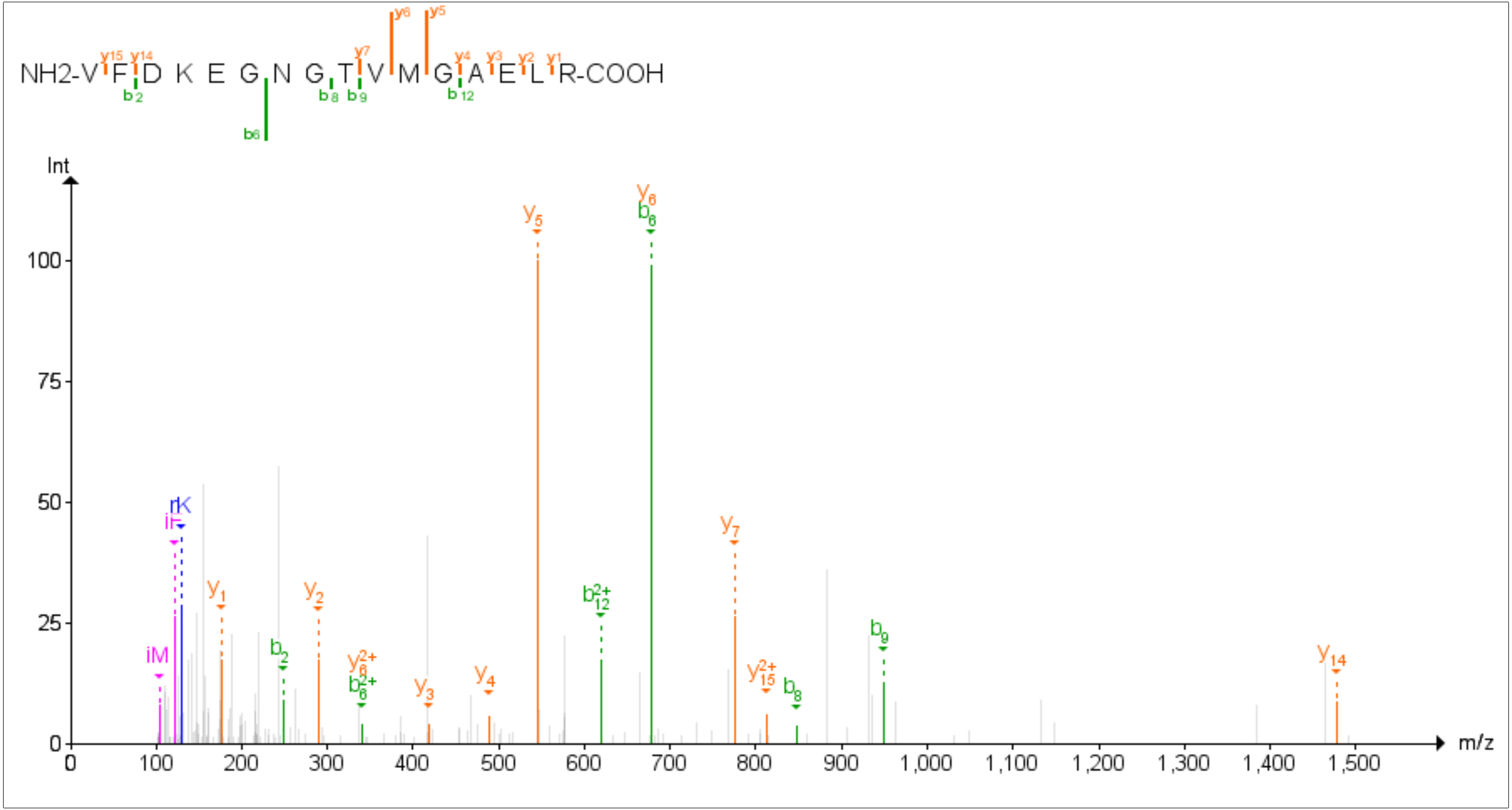
NH2-VFDKEGNGTVMGAELR-COOH
Int
m/z
0
25
50
75
100
200
300
400
500
600
700
800
900
1,000
1,100
1,200
1,300
1,400
1,500
y5
y6
b6
y7
iM
iF
rK
y1
y2
b2
y3
y4
b9
b8
y14

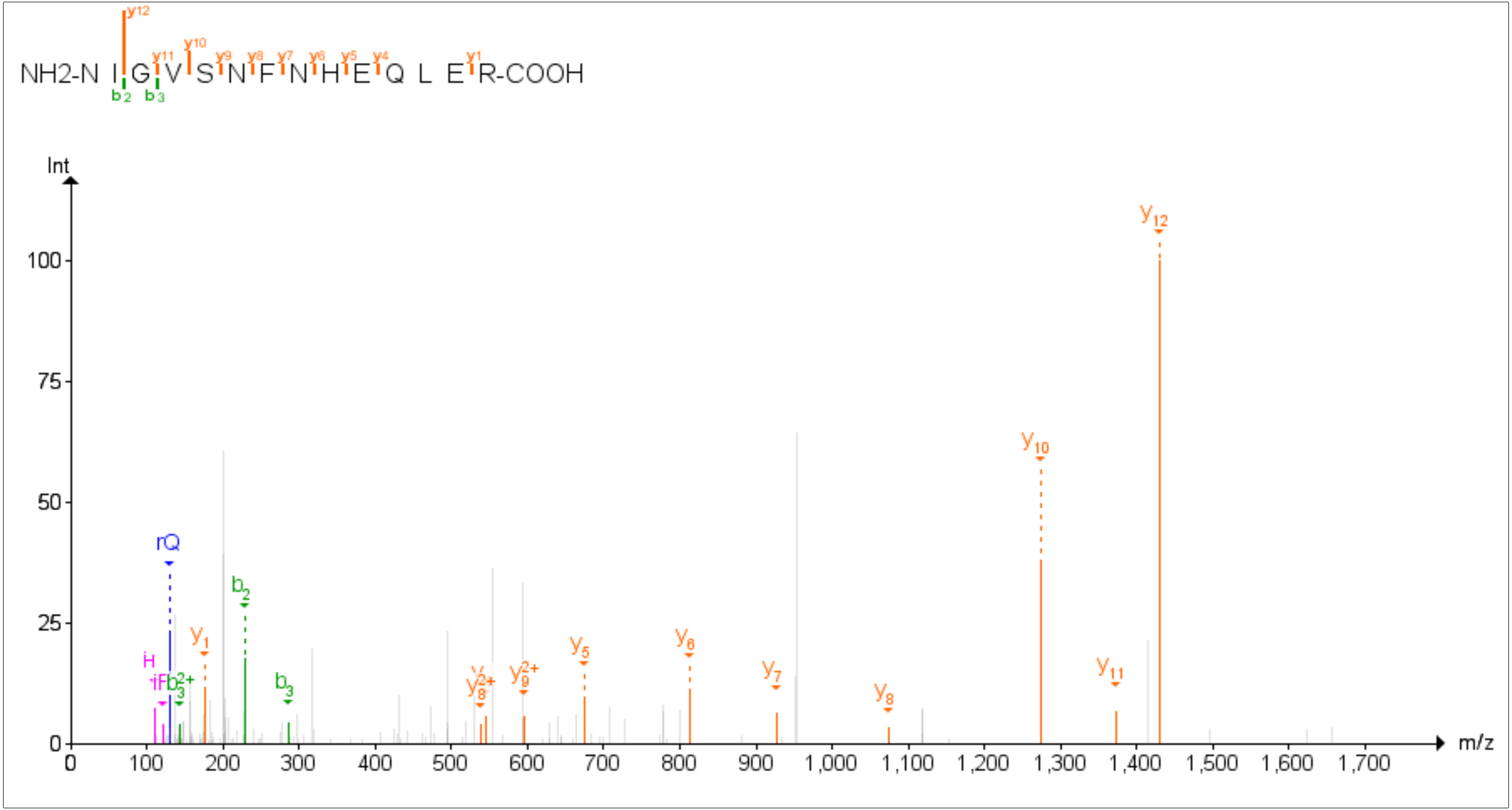

NH2-N I G V S N F N H E Q L E R-COOH
y12
y11
y10
y9
y8
y7
y6
y5
y4
y1
b2
b3
Int
100
75
50
25
0
m/z
0
100
200
300
400
500
600
700
800
900
1,000
1,100
1,200
1,300
1,400
1,500
1,600
1,700
rQ
iH
iF
b_3^{2+}
b_2
y_1
b_3
y_8^{2+}
y_9^{2+}
y_5
y_6
y_7
y_8
y_{10}
y_{11}
y_{12}

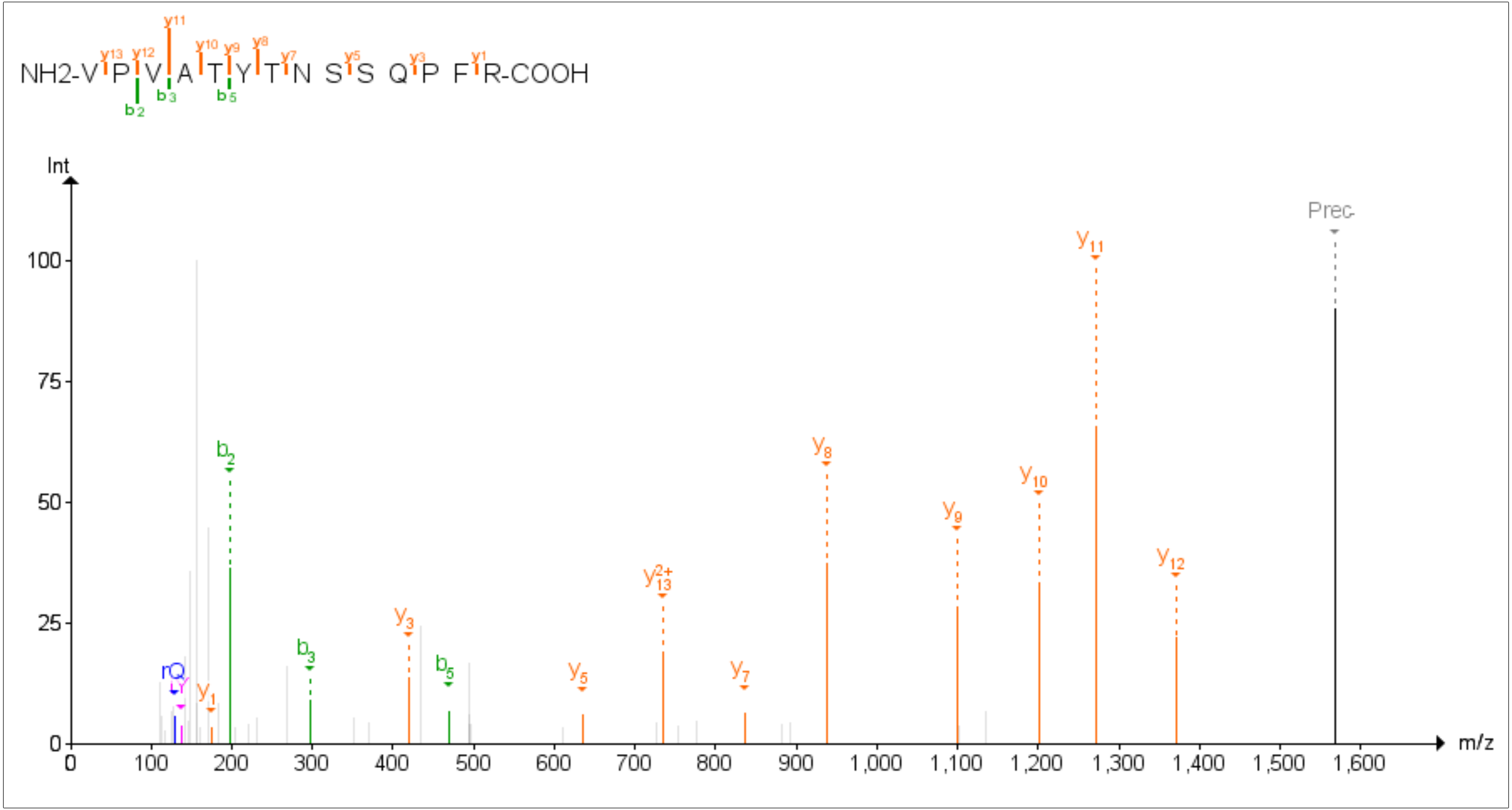

NH2-V P V A T Y T N S S Q P F R-COOH
y13 y12 y11 y10 y9 y8 y7 y5 y3 y1
b2 b3 b5
Int
100
75
50
25
0
m/z
0 100 200 300 400 500 600 700 800 900 1,000 1,100 1,200 1,300 1,400 1,500 1,600
Prec
y11
b2
y8
y10
y9
y12
y13 2+
y3
b3
b5
rQ
y5
y7
y1

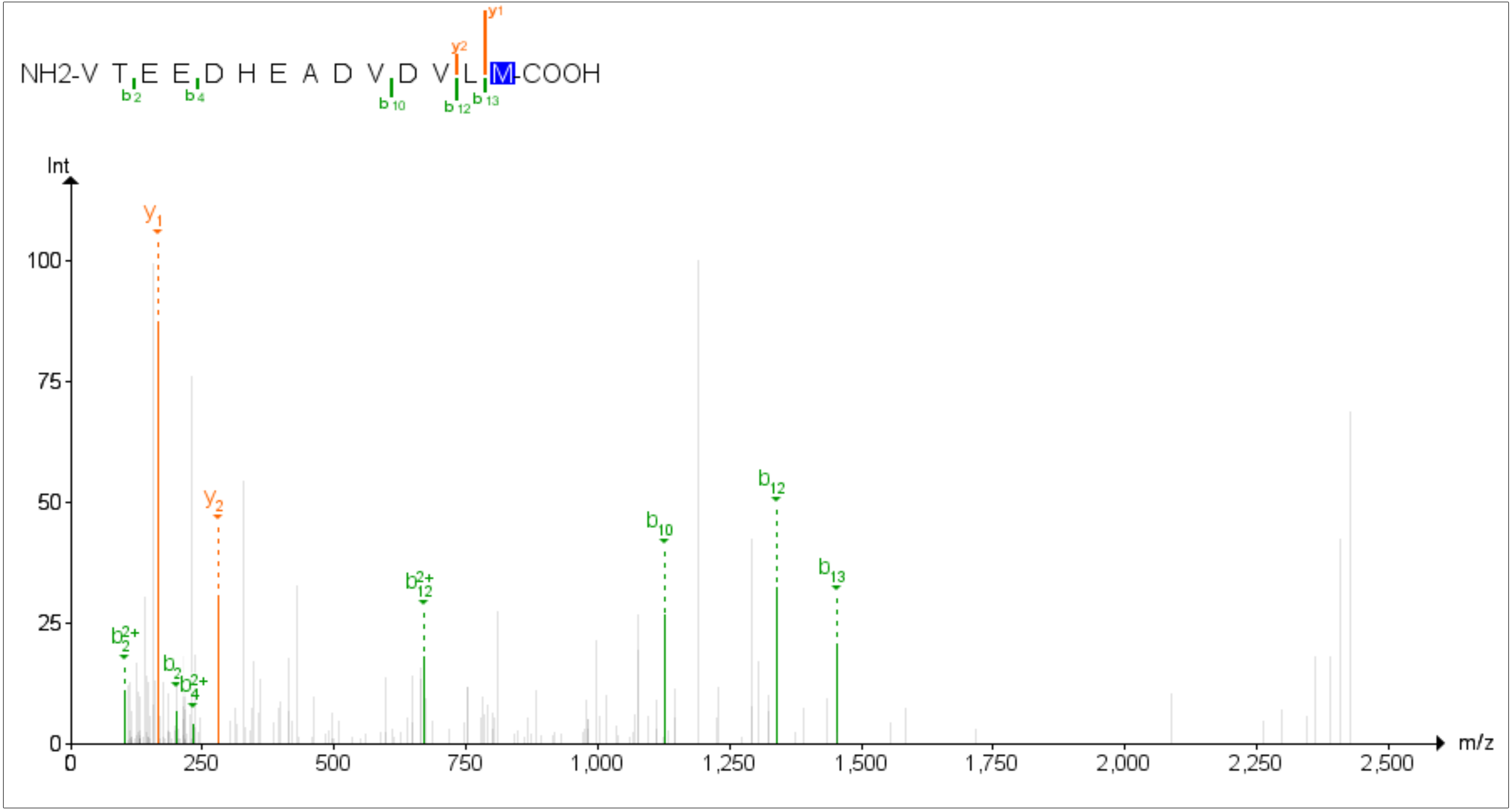

NH2-V T E E D H E A D V D V L M-COOH
y1
y2
b 2
b 4
b 10
b 12
b 13
Int
100
75
50
25
0
y1
y2
b2 2+
b2
b4 2+
b12 2+
b10
b12
b13
0
250
500
750
1,000
1,250
1,500
1,750
2,000
2,250
2,500
m/z

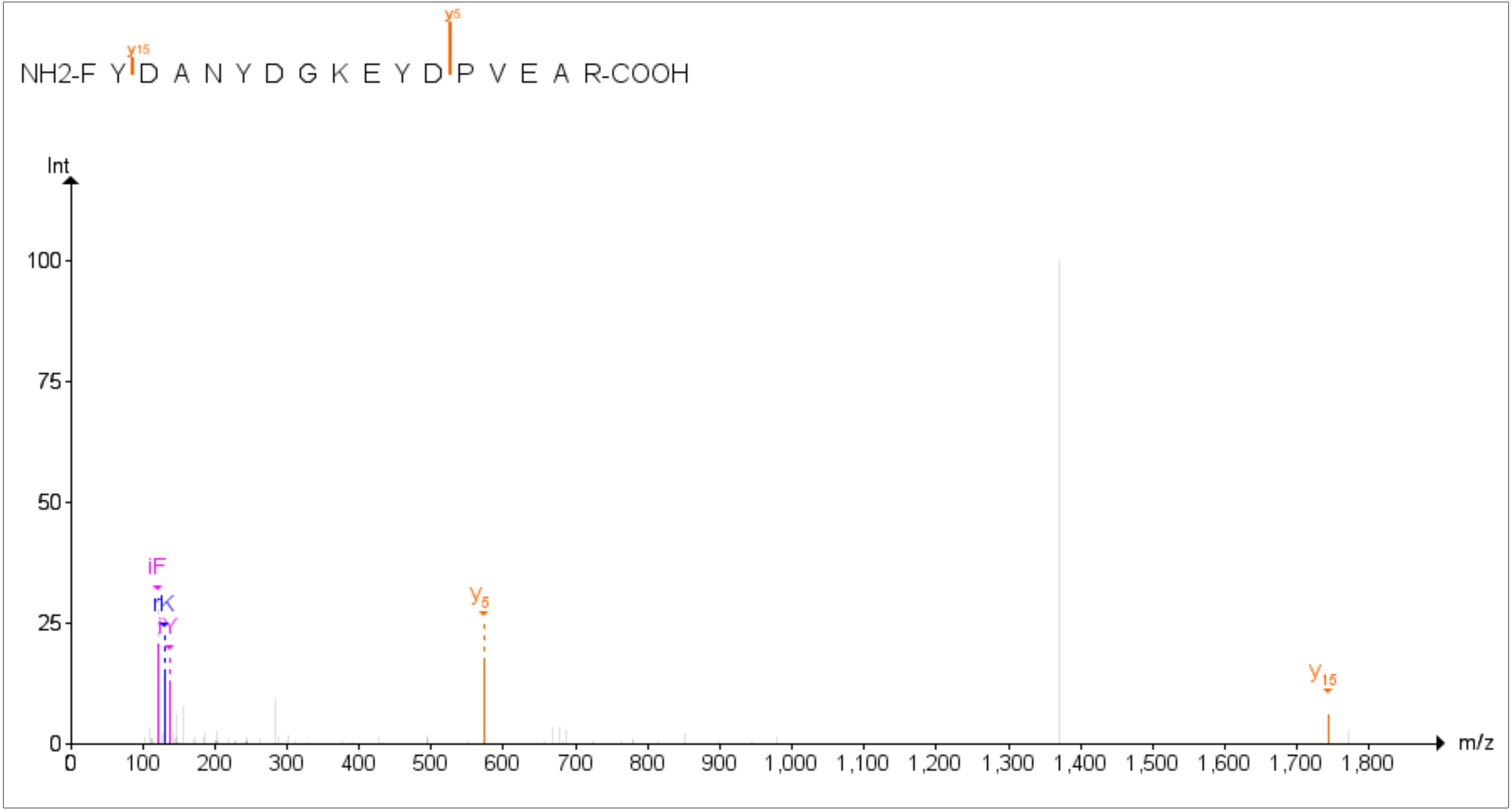
NH2-F Y D A N Y D G K E Y D P V E A R-COOH
y15
y5
Int
100
75
50
25
0
iF
rK
iY
y5
y15
m/z
0
100
200
300
400
500
600
700
800
900
1,000
1,100
1,200
1,300
1,400
1,500
1,600
1,700
1,800

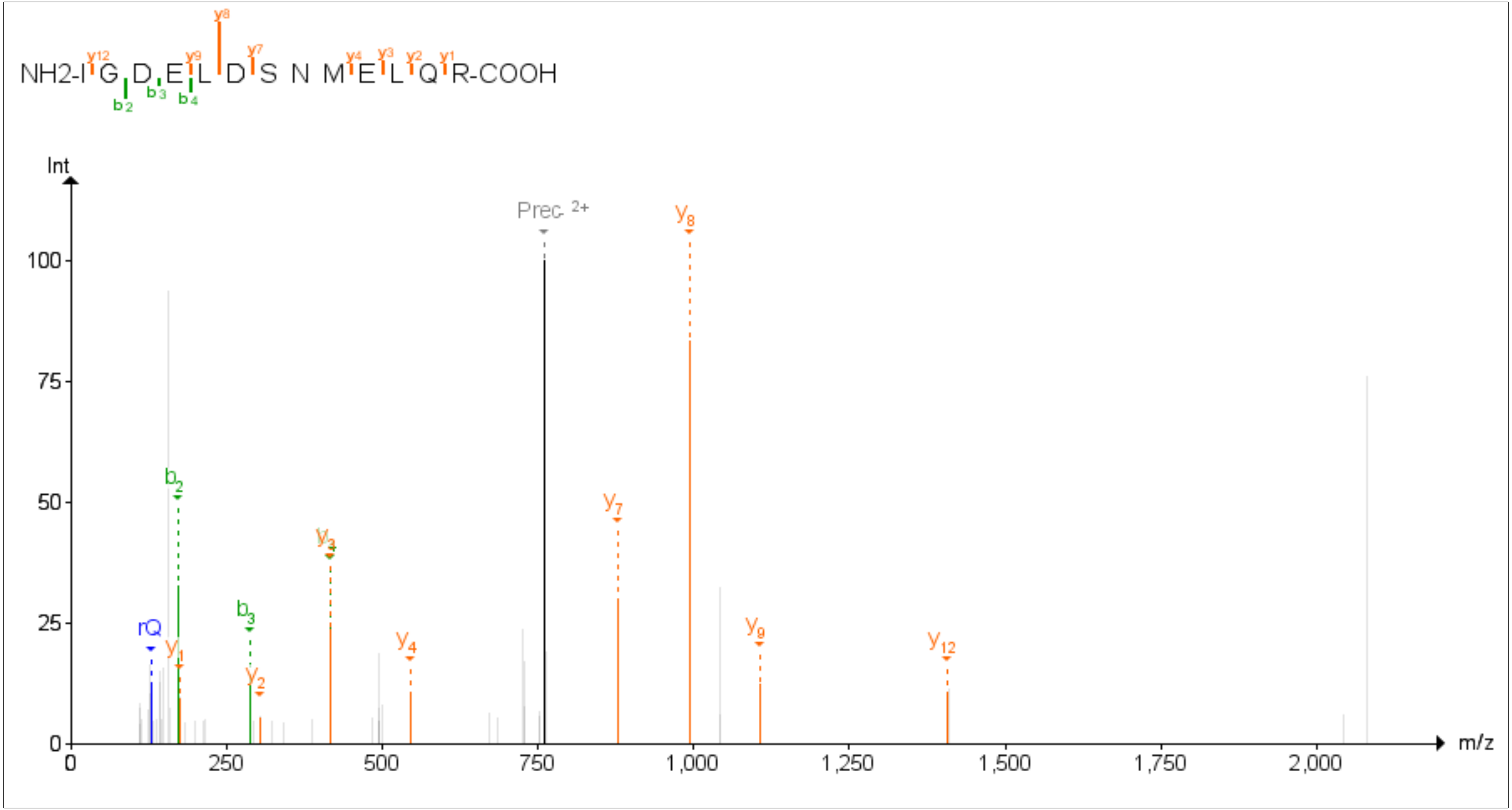

NH2-IGDELDSNMELQR-COOH
y12 y9 y8 y7 y4 y3 y2 y1
b2 b3 b4
Int
Prec 2+
y8
y7
y3
b2
b3
rQ
y1
y2
y4
y9
y12
100
75
50
25
0
250
500
750
1,000
1,250
1,500
1,750
2,000
m/z

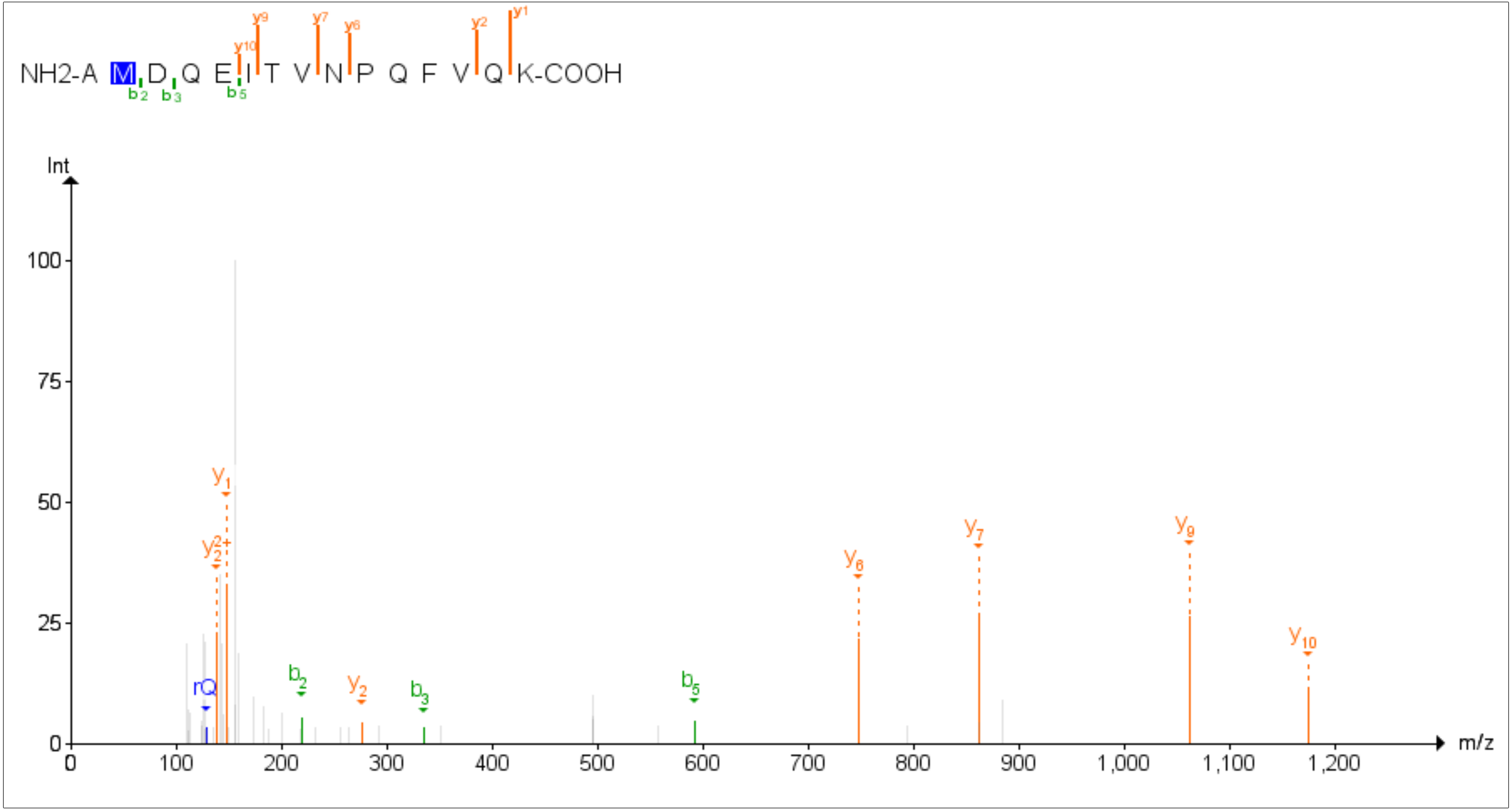

NH2-A M D Q E I T V N P Q F V Q K-COOH
y9 y7 y6 y2 y1 y10
b2 b3 b5
Int
100
75
50
25
0
m/z
0
100
200
300
400
500
600
700
800
900
1,000
1,100
1,200
y1
y2 2+
rQ
b2
y2
b3
b5
y6
y7
y9
y10

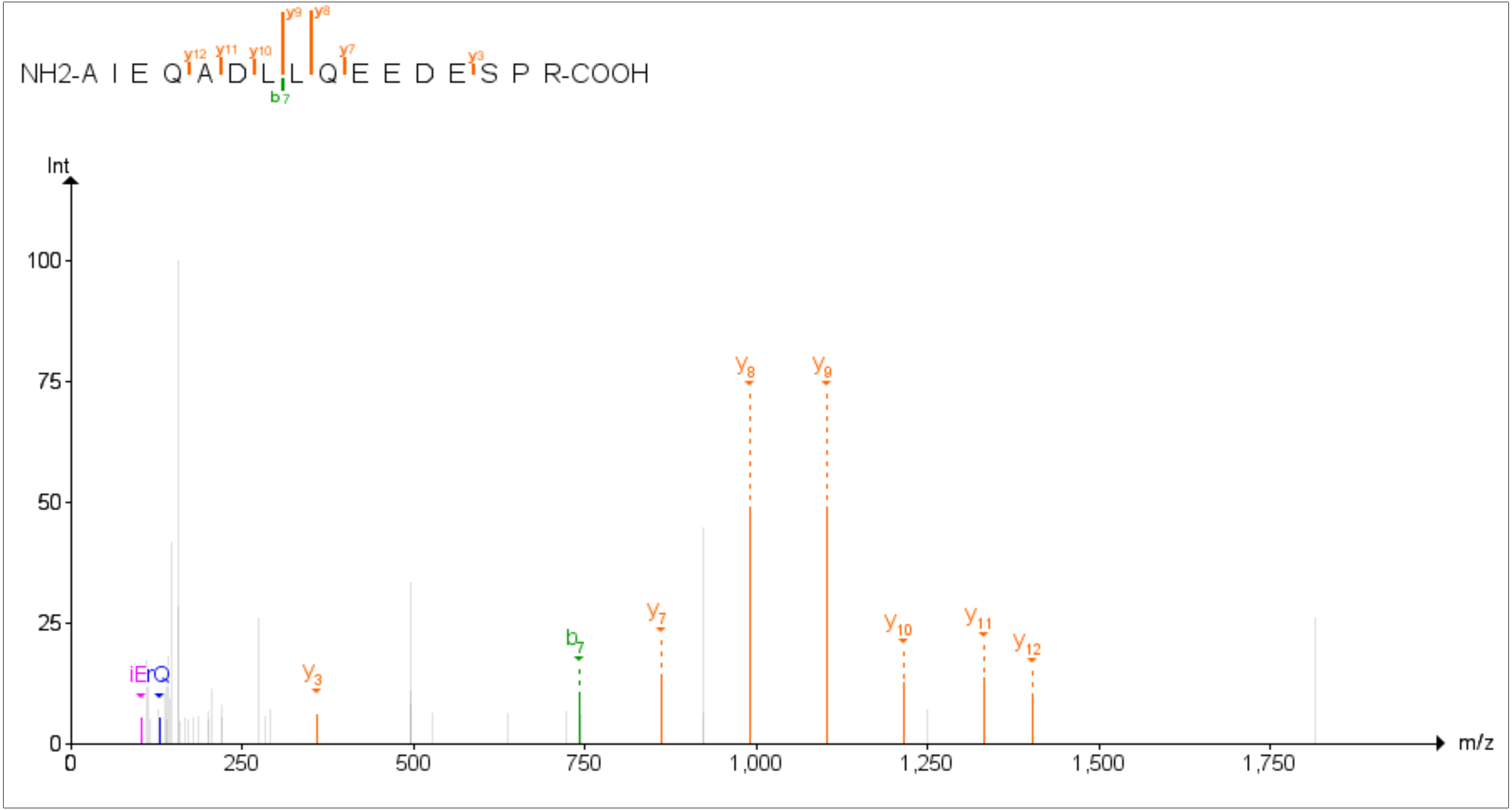
NH2-A I E Q A D L L Q E E D E S P R-COOH
y12 y11 y10 y9 y8 y7 y3
b7
Int
100
75
50
25
0
iE rQ
y3 b7 y7 y8 y9 y10 y11 y12
0 250 500 750 1,000 1,250 1,500 1,750
m/z

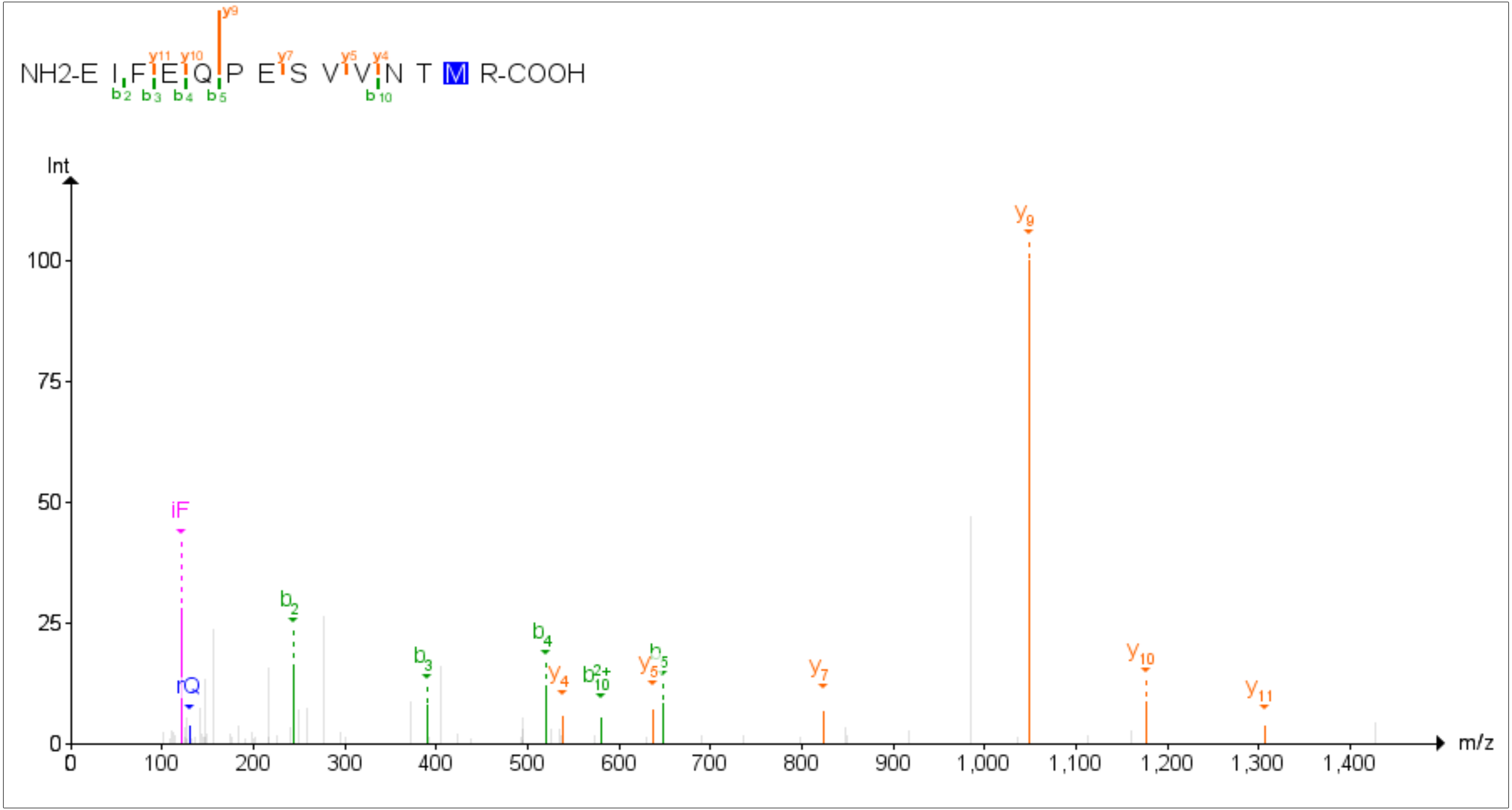

NH2-E I F E Q P E S V V N T M R-COOH
y9
y11 y10 y7 y5 y4
b2 b3 b4 b5 b10
Int
100
75
50
25
0
m/z
0 100 200 300 400 500 600 700 800 900 1,000 1,100 1,200 1,300 1,400
iF
rQ
b2
b3
b4
y4
b10 2+
y5
b5
y7
y9
y10
y11

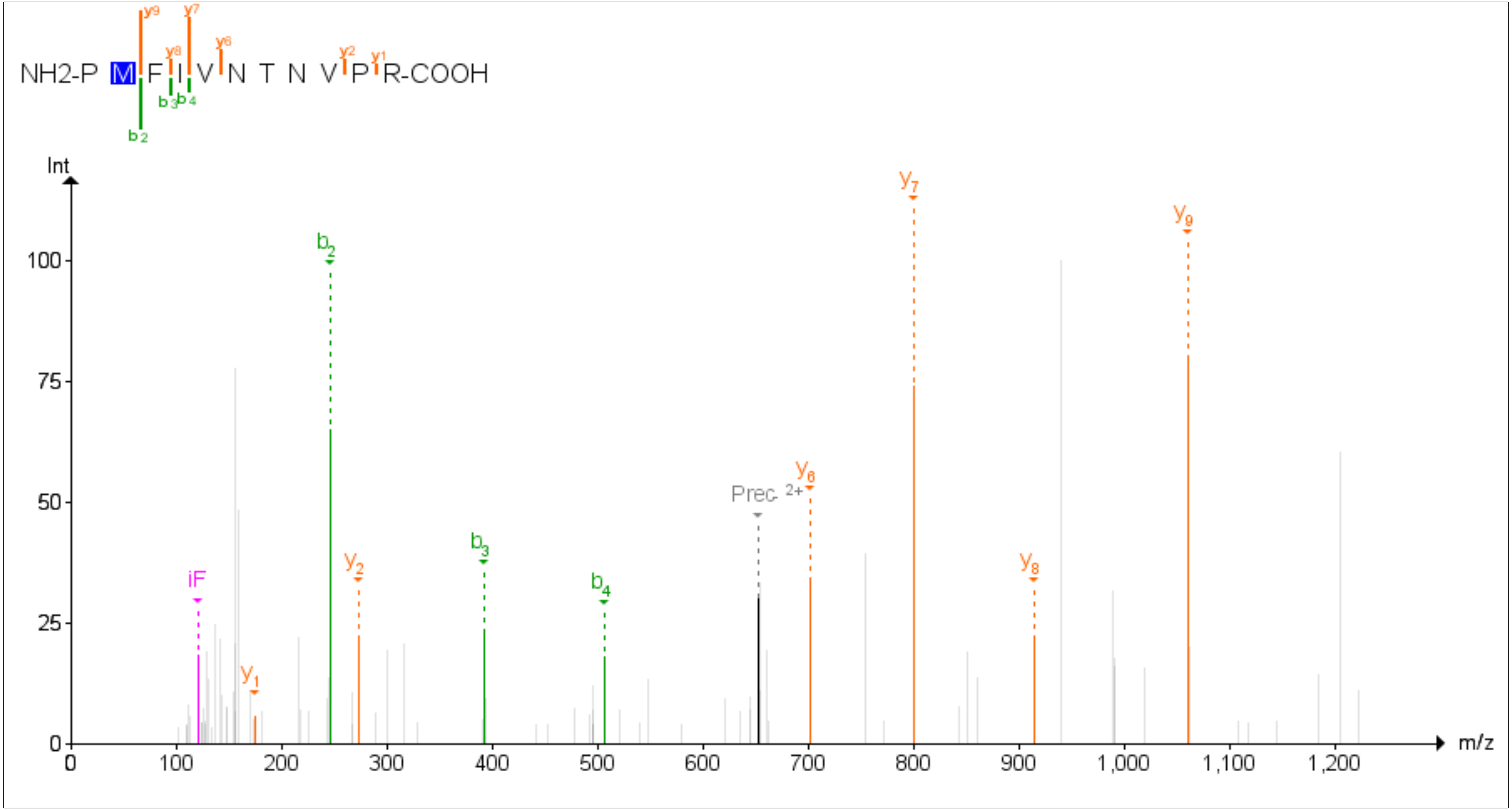
NH2-P M F I V N T N V P R-COOH
y9
y7
y8
y6
y2
y1
b2
b3
b4
Int
100
75
50
25
0
m/z
0
100
200
300
400
500
600
700
800
900
1,000
1,100
1,200
iF
y1
b2
y2
b3
b4
Prec 2+
y6
y7
y8
y9

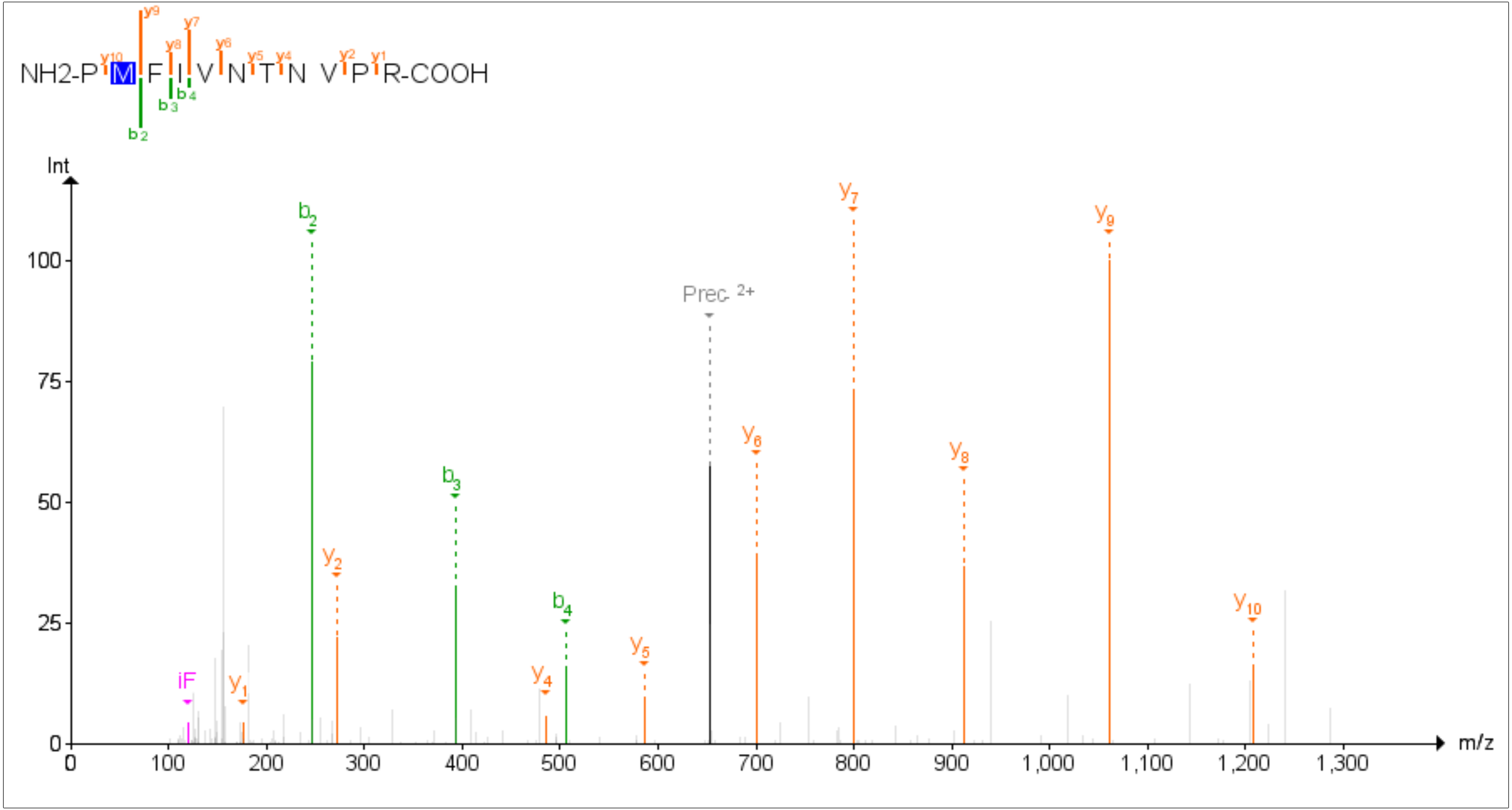

NH2-P M F I V N T N V P R-COOH
y10 y9 y8 y7 y6 y5 y4 y2 y1
b2 b3 b4
Int
100
75
50
25
0
m/z
0 100 200 300 400 500 600 700 800 900 1,000 1,100 1,200 1,300
iF
y1
b2
y2
b3
y4
b4
y5
Prec 2+
y6
y7
y8
y9
y10

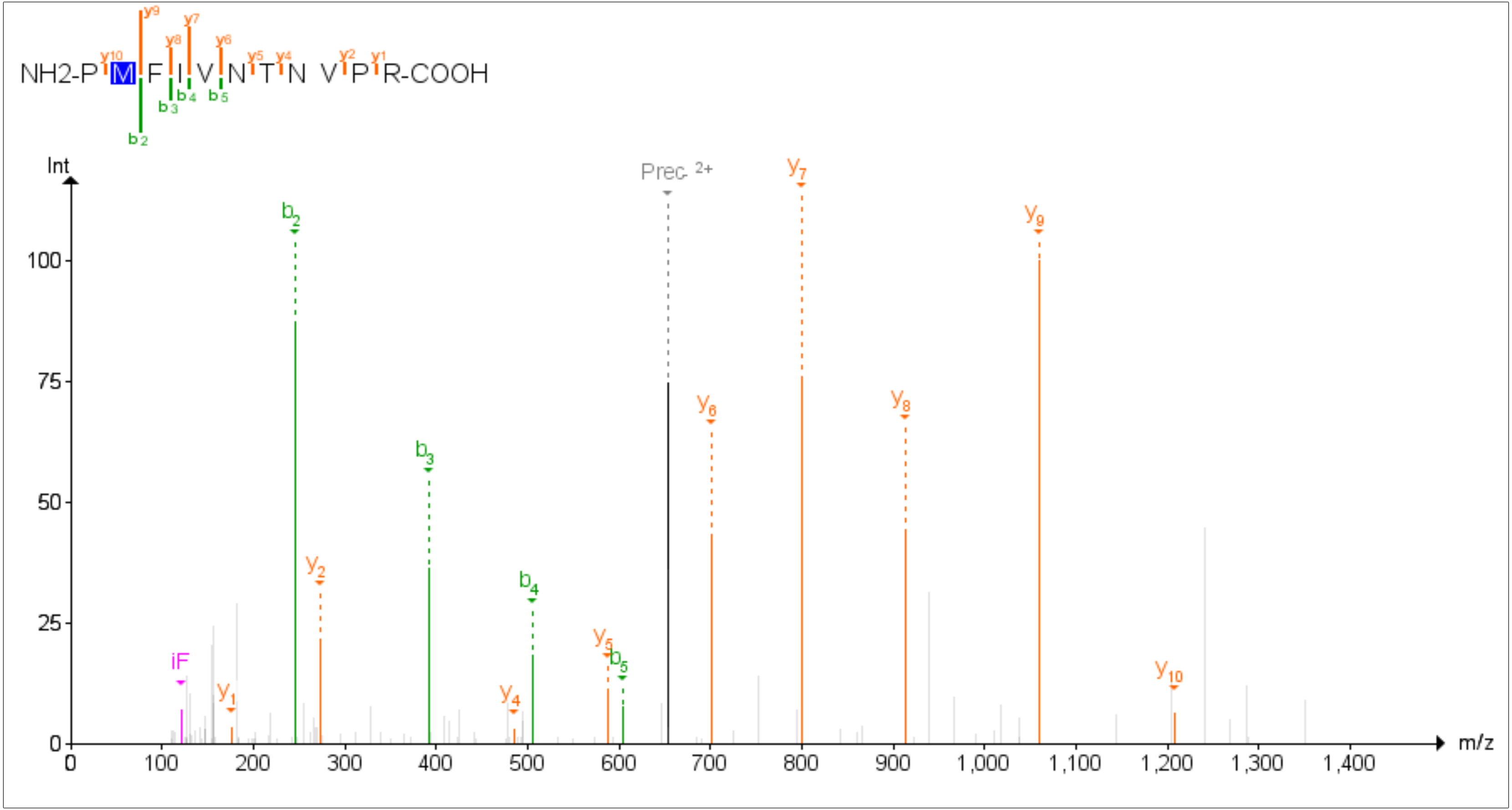
NH2-PMFIVNTNVPR-COOH
y10 y9 y8 y7 y6 y5 y4 y2 y1
b2 b3 b4 b5
Int
m/z
Prec 2+
b2 b3 b4 b5
y1 y2 y4 y5 y6 y7 y8 y9 y10
iF
0 25 50 75 100
0 100 200 300 400 500 600 700 800 900 1,000 1,100 1,200 1,300 1,400

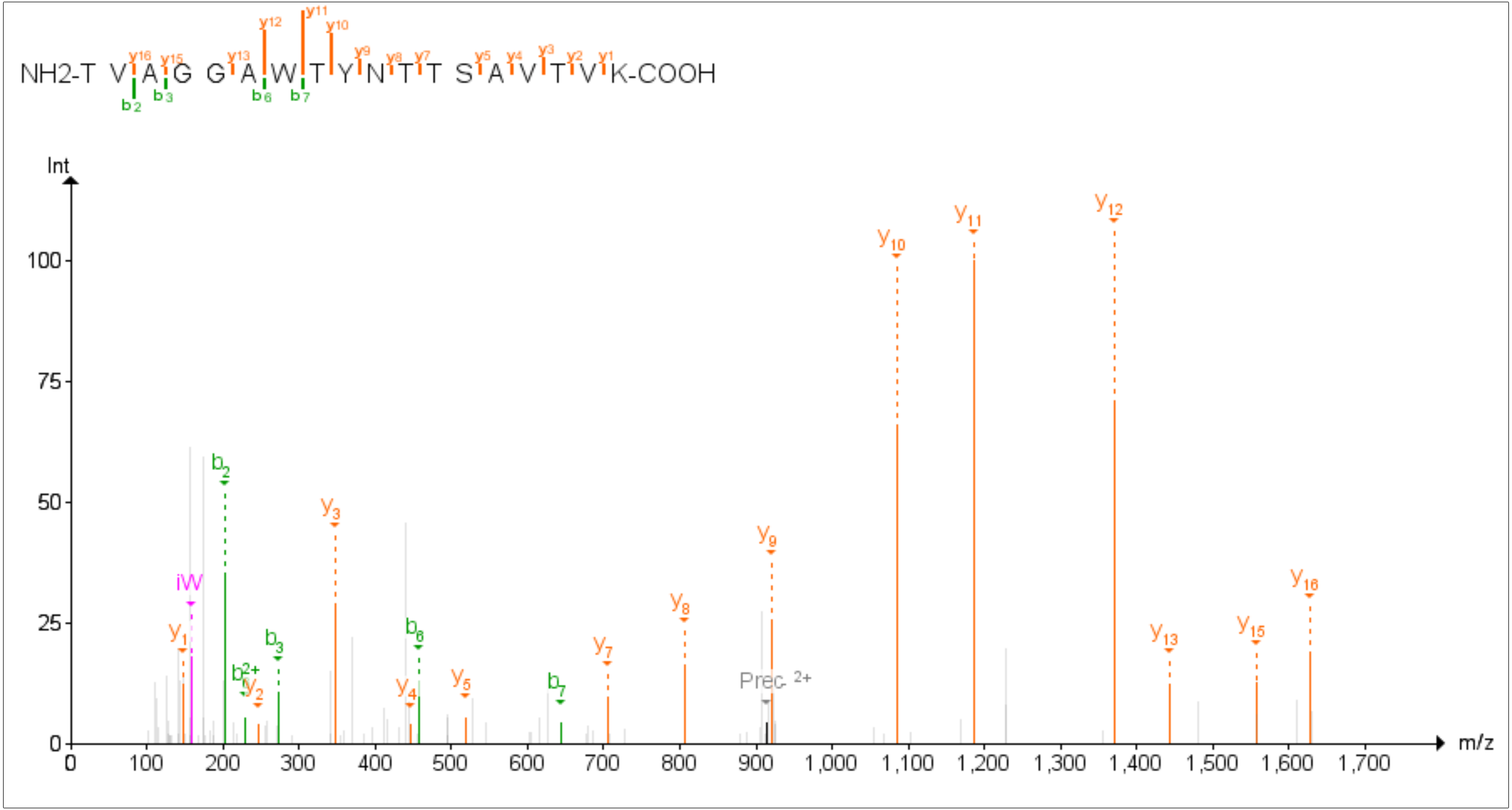
NH2-T V A G G A W T Y N T T S A V T V K-COOH
Int
m/z
Prec. 2+
iW

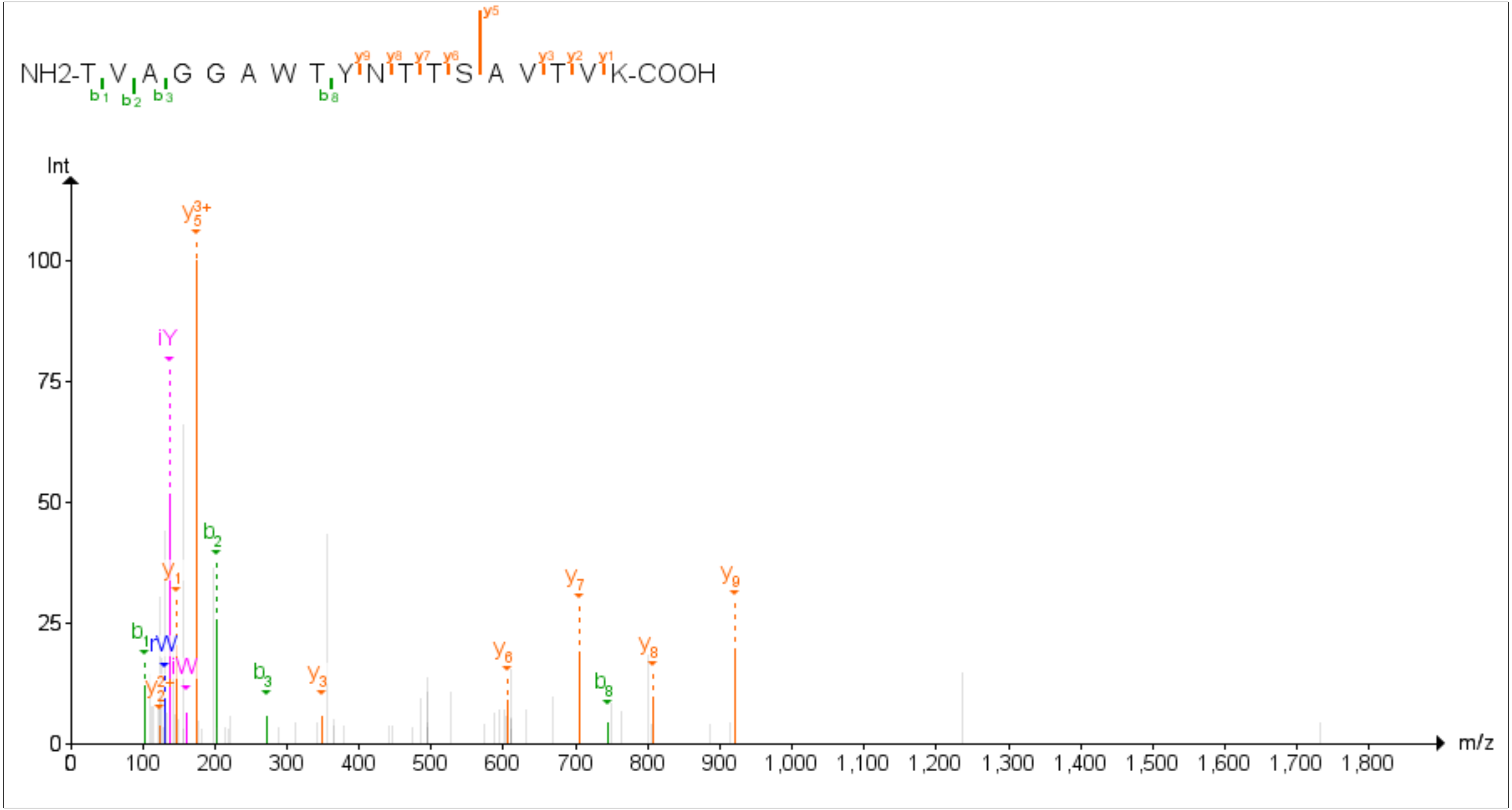

NH2-T V A G G A W T Y N T T S A V T V K-COOH
b1 b2 b3 b8
y9 y8 y7 y6 y5 y3 y2 y1
Int
0
25
50
75
100
m/z
0 100 200 300 400 500 600 700 800 900 1,000 1,100 1,200 1,300 1,400 1,500 1,600 1,700 1,800
y5 3+
iY
b2
y1
b1
rW
iW
y2 2+
b3
y3
y6
y7
b8
y8
y9

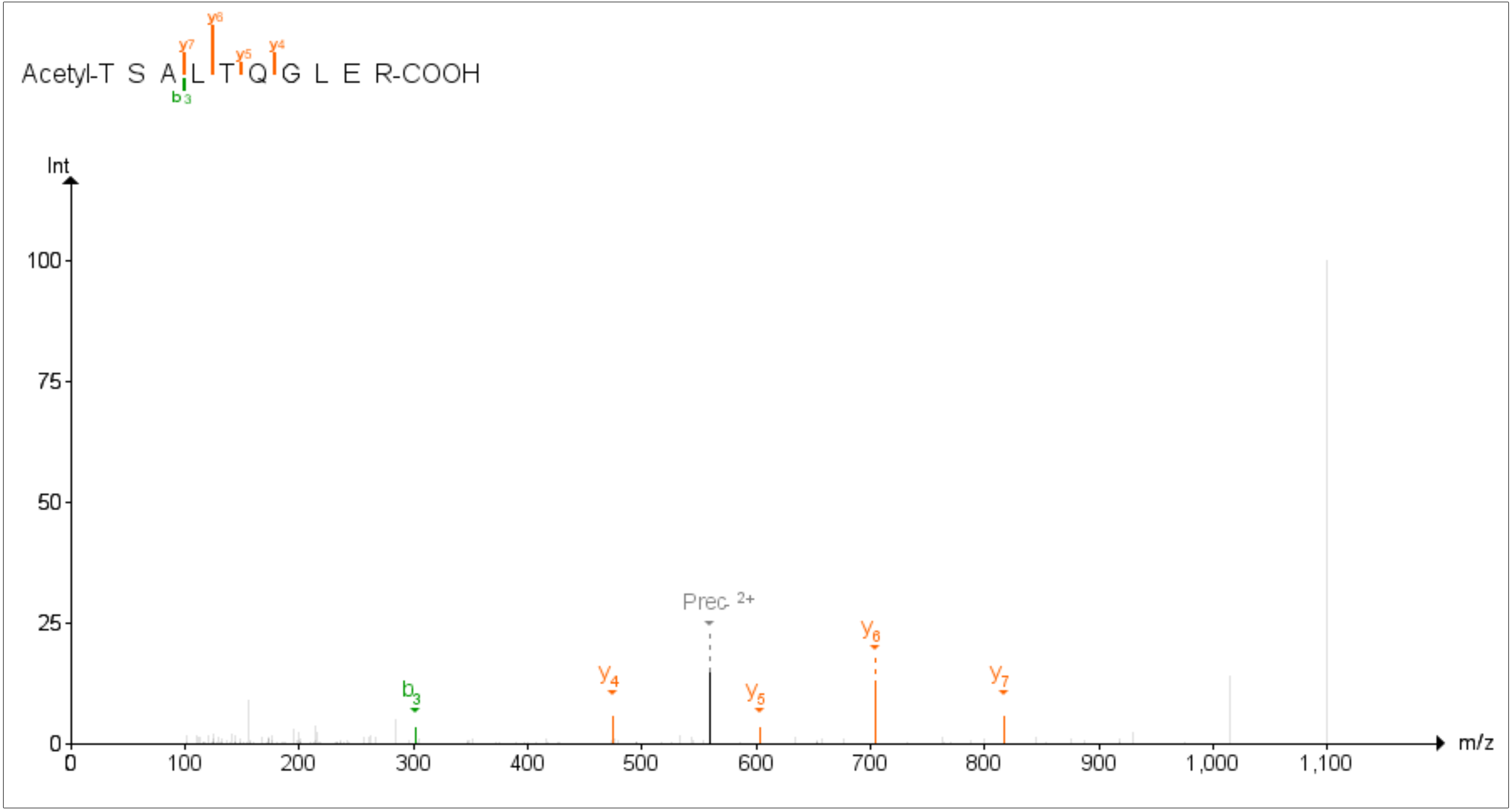

Acetyl-T S A L T Q G L E R-COOH
y7
y6
y5
y4
b3
Int
100
75
50
25
0
Prec 2+
b3
y4
y5
y6
y7
0
100
200
300
400
500
600
700
800
900
1,000
1,100
m/z

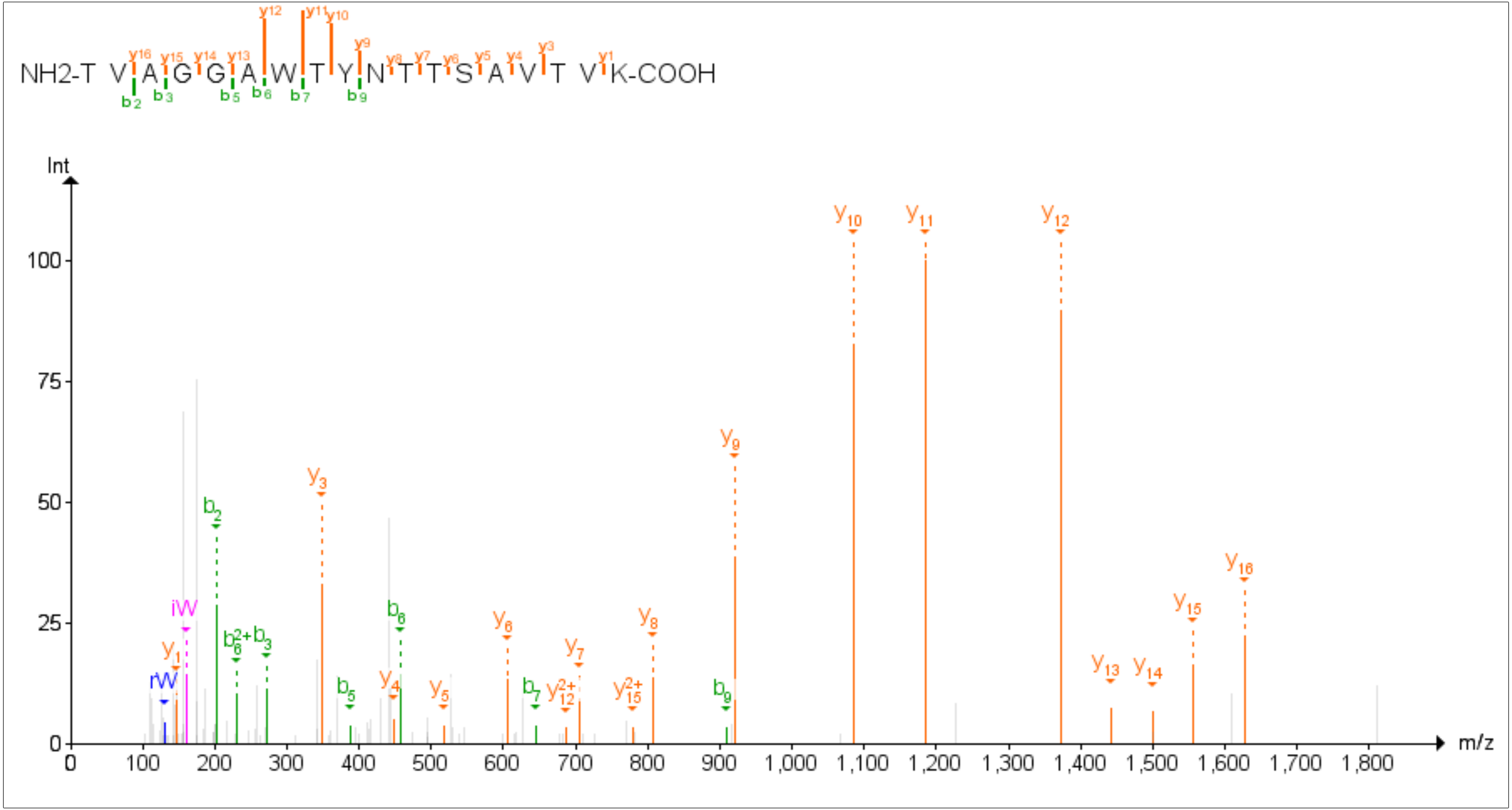

NH2-T V A G G A W T Y N T T S A V T V K-COOH
y16 y15 y14 y13 y12 y11 y10 y9 y8 y7 y6 y5 y4 y3 y1
b2 b3 b5 b6 b7 b9
Int
m/z
rW
iW
y1
b2
b6 2+
b3
y3
b5
y4
b6
y5
y6
b7
y12 2+
y7
y15 2+
y8
b9
y9
y10
y11
y12
y13
y14
y15
y16
0
25
50
75
100
0 100 200 300 400 500 600 700 800 900 1,000 1,100 1,200 1,300 1,400 1,500 1,600 1,700 1,800

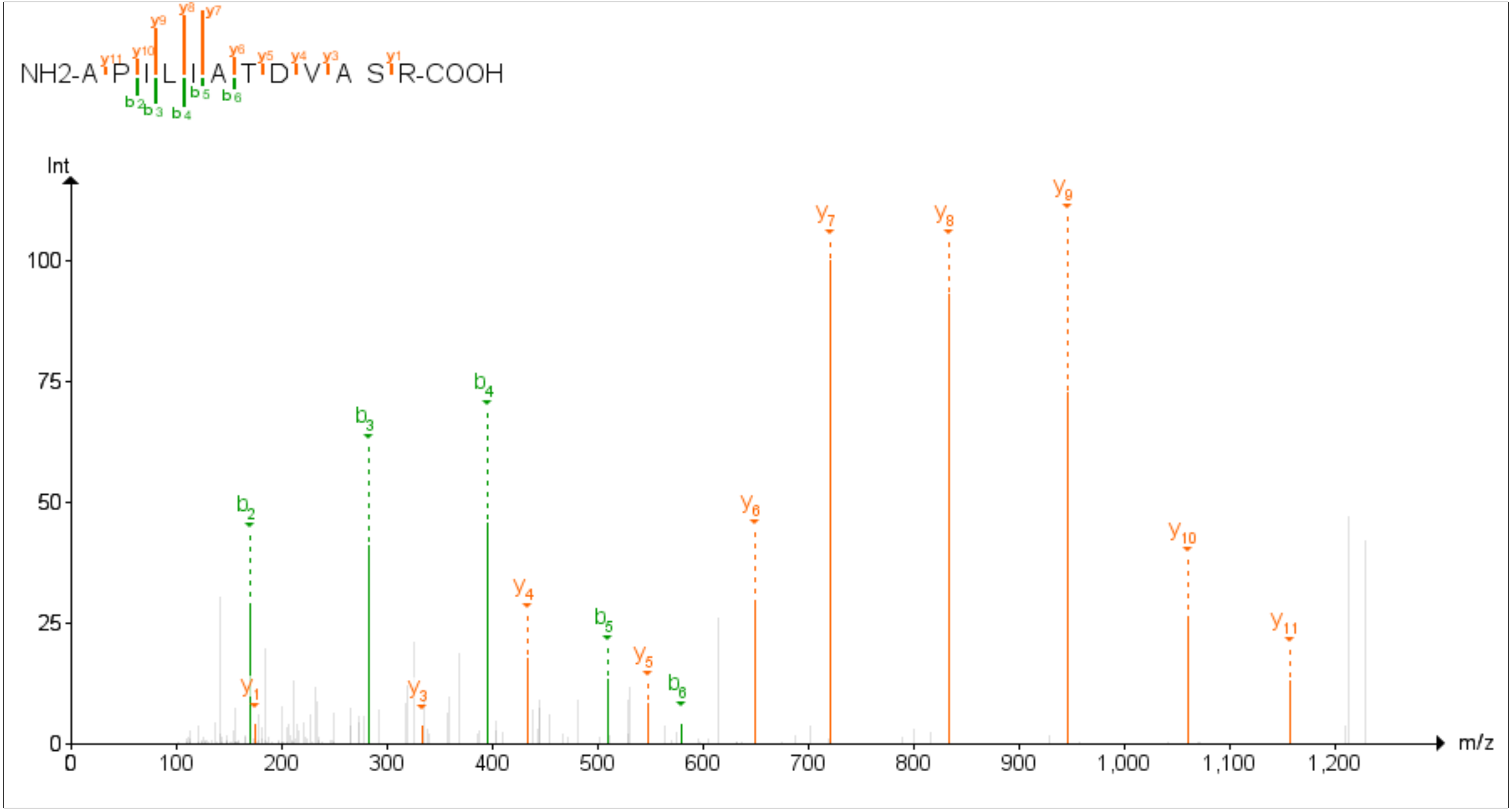
NH2-A P I L I I A T D V A S R-COOH
y11 y10 y9 y8 y7 y6 y5 y4 y3 y1
b2 b3 b4 b5 b6
Int
100
75
50
25
0
m/z
0
100
200
300
400
500
600
700
800
900
1,000
1,100
1,200
b2 b3 b4 b5 b6
y1 y3 y4 y5 y6 y7 y8 y9 y10 y11

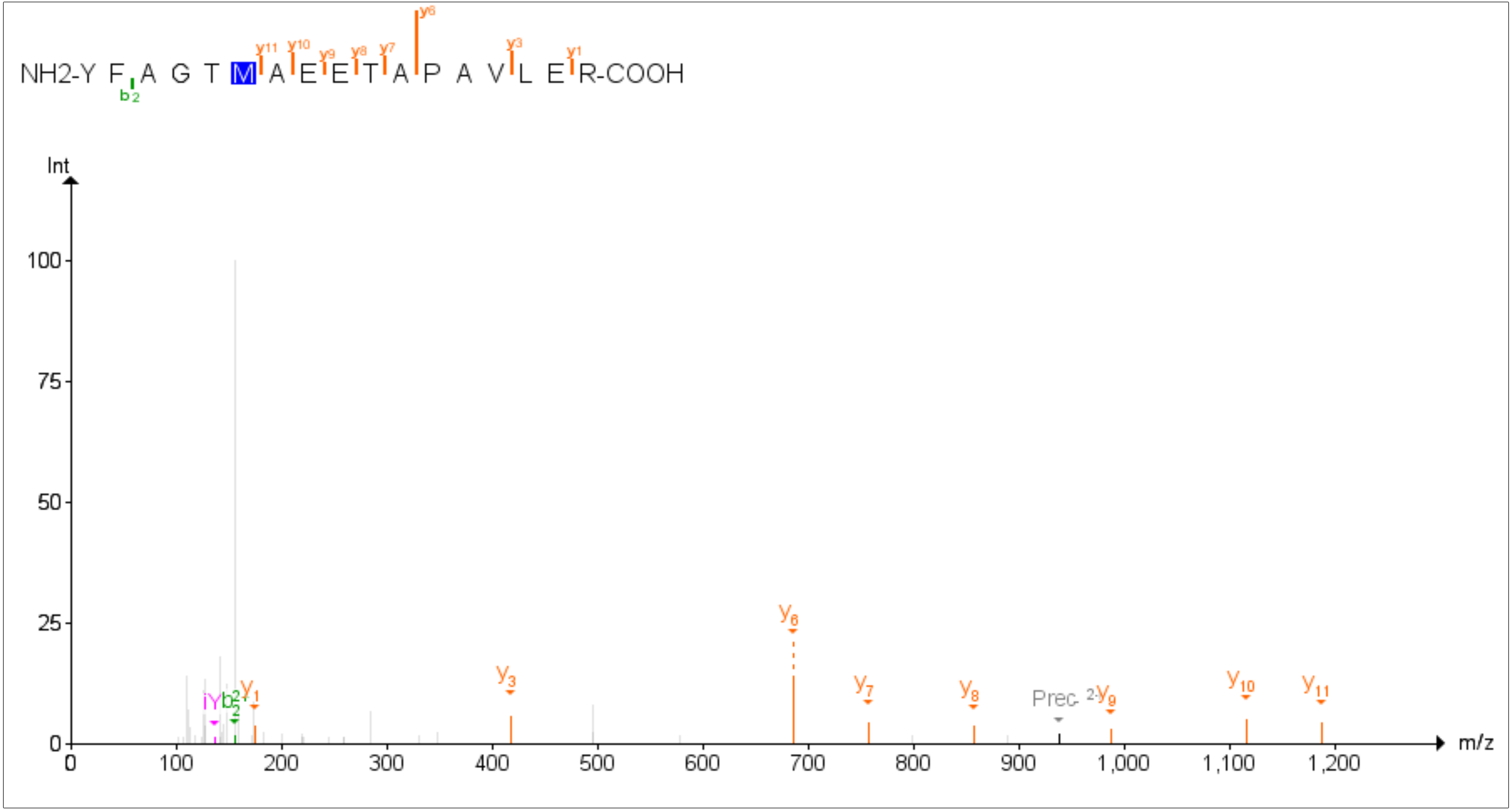

NH2-Y F A G T M A E E T A P A V L E R-COOH
b2
y11 y10 y9 y8 y7 y6 y3 y1
Int
100
75
50
25
0
iY
b2
y1
y3
y6
y7
y8
Prec- 2-
y9
y10
y11
0 100 200 300 400 500 600 700 800 900 1,000 1,100 1,200
m/z

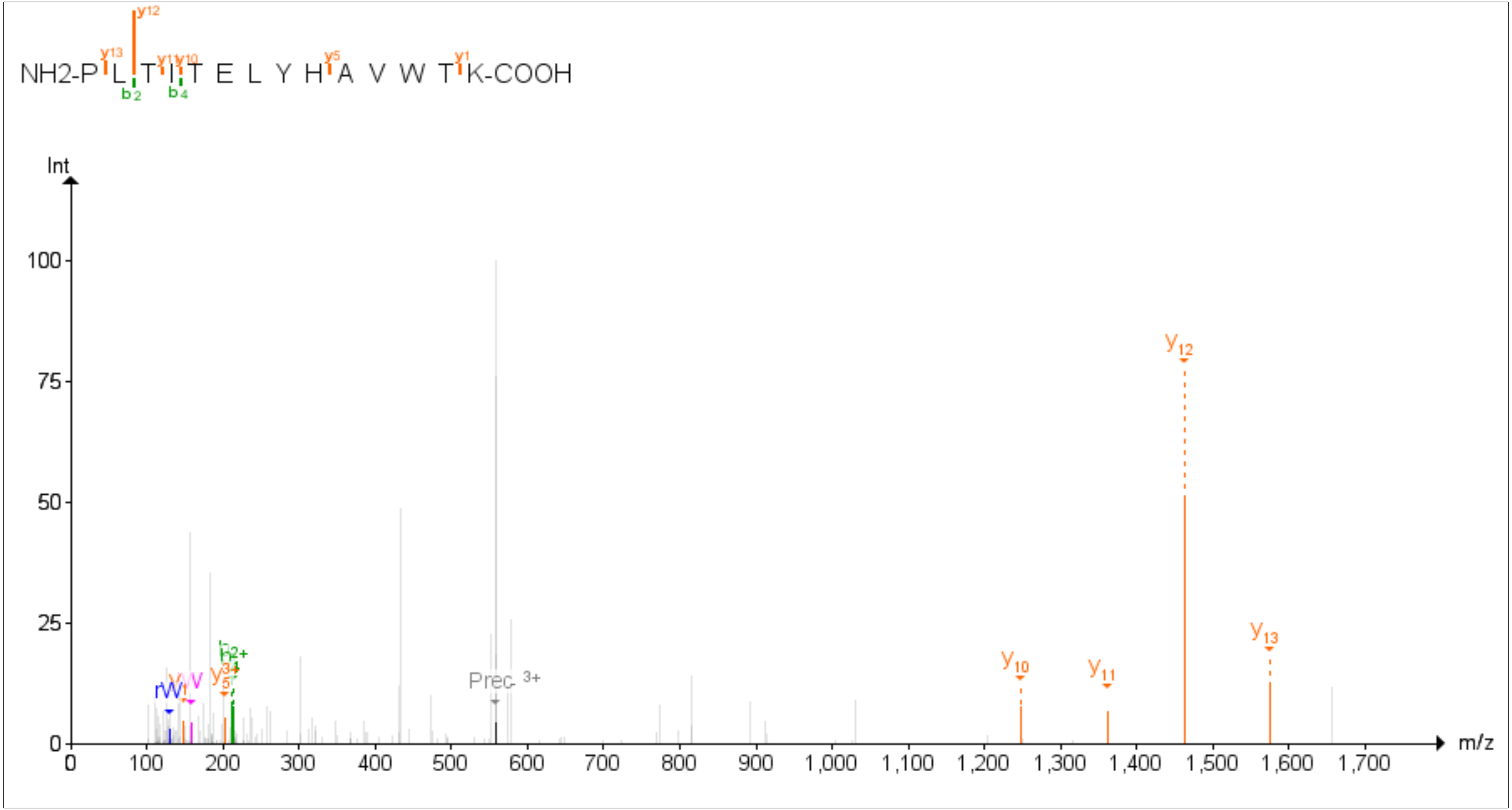

NH2-P L T I T E L Y H A V W T K-COOH
y13 y12 y11 y10 y5 y1
b2 b4
Int
m/z
y12
y13
y10
y11
Prec 3+

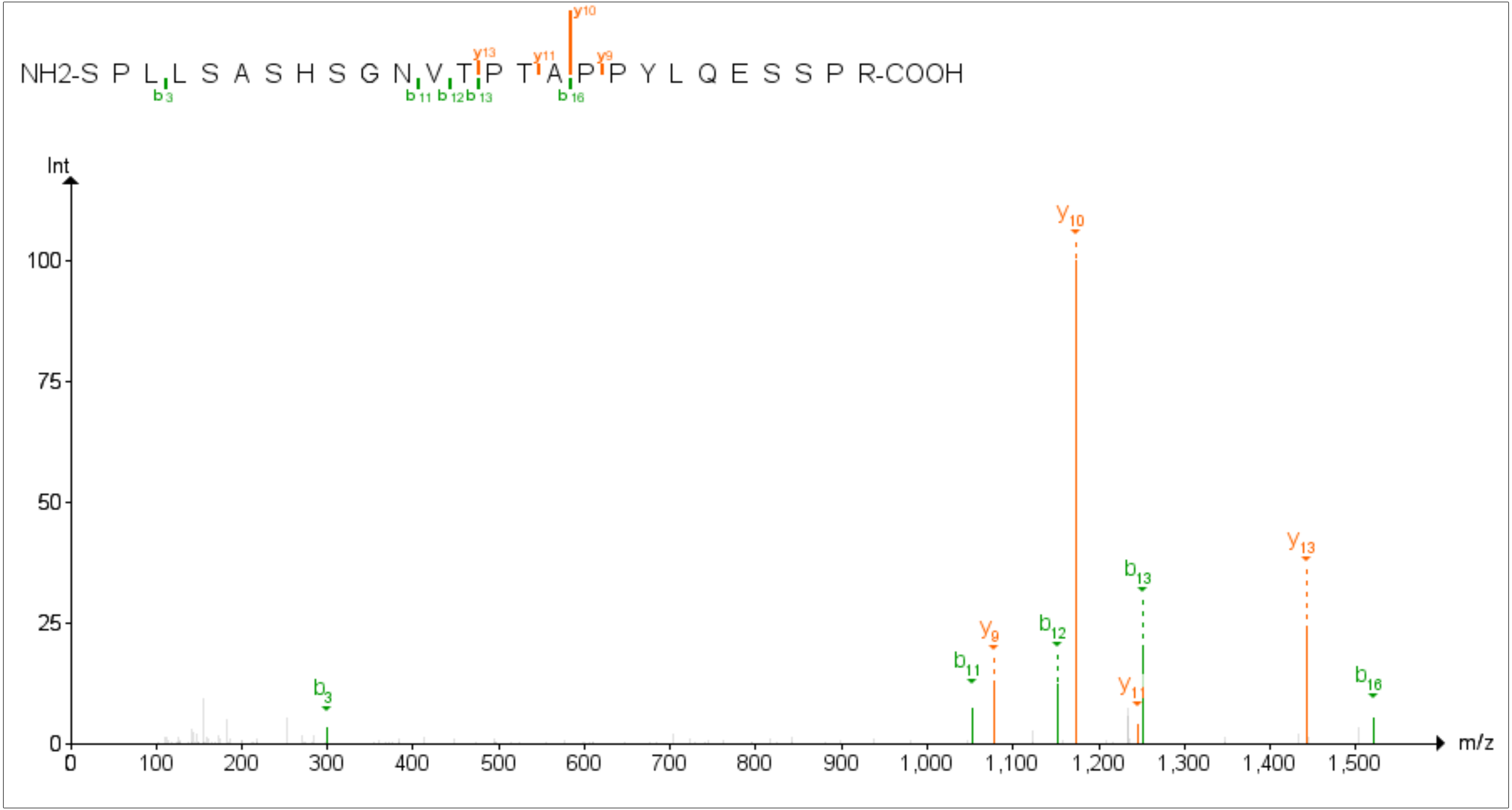

NH2-S P L L S A S H S G N V T P T A P P Y L Q E S S P R-COOH
y10
y13
y11
y9
b3
b11
b12
b13
b16
Int
100
75
50
25
0
m/z
0
100
200
300
400
500
600
700
800
900
1,000
1,100
1,200
1,300
1,400
1,500

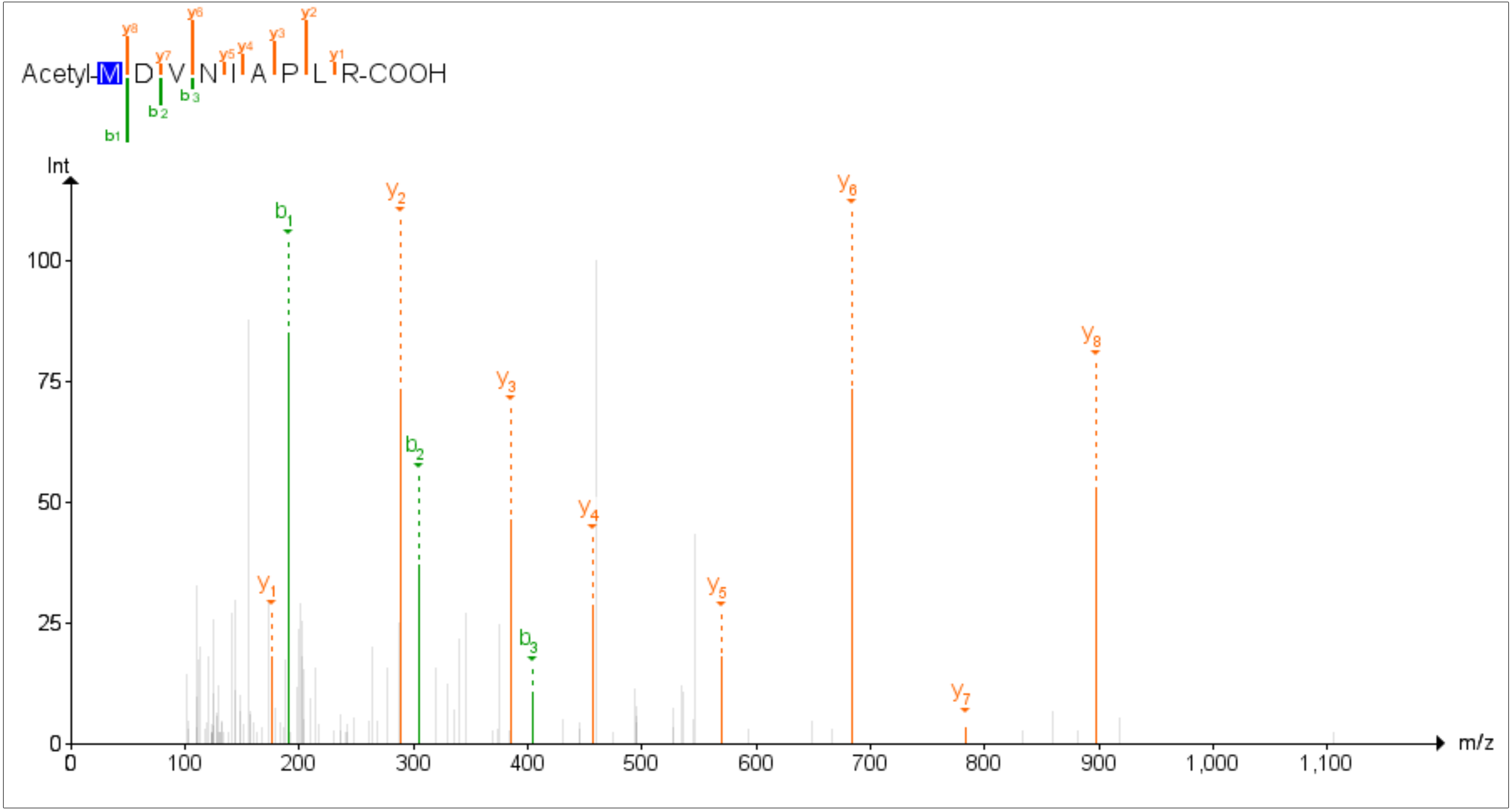

Acetyl-M D V N I A P L R-COOH
y8 y7 y6 y5 y4 y3 y2 y1
b1 b2 b3
Int
m/z
0
25
50
75
100
0
100
200
300
400
500
600
700
800
900
1,000
1,100
b1
y2
y6
y8
y3
b2
y4
y1
y5
b3
y7

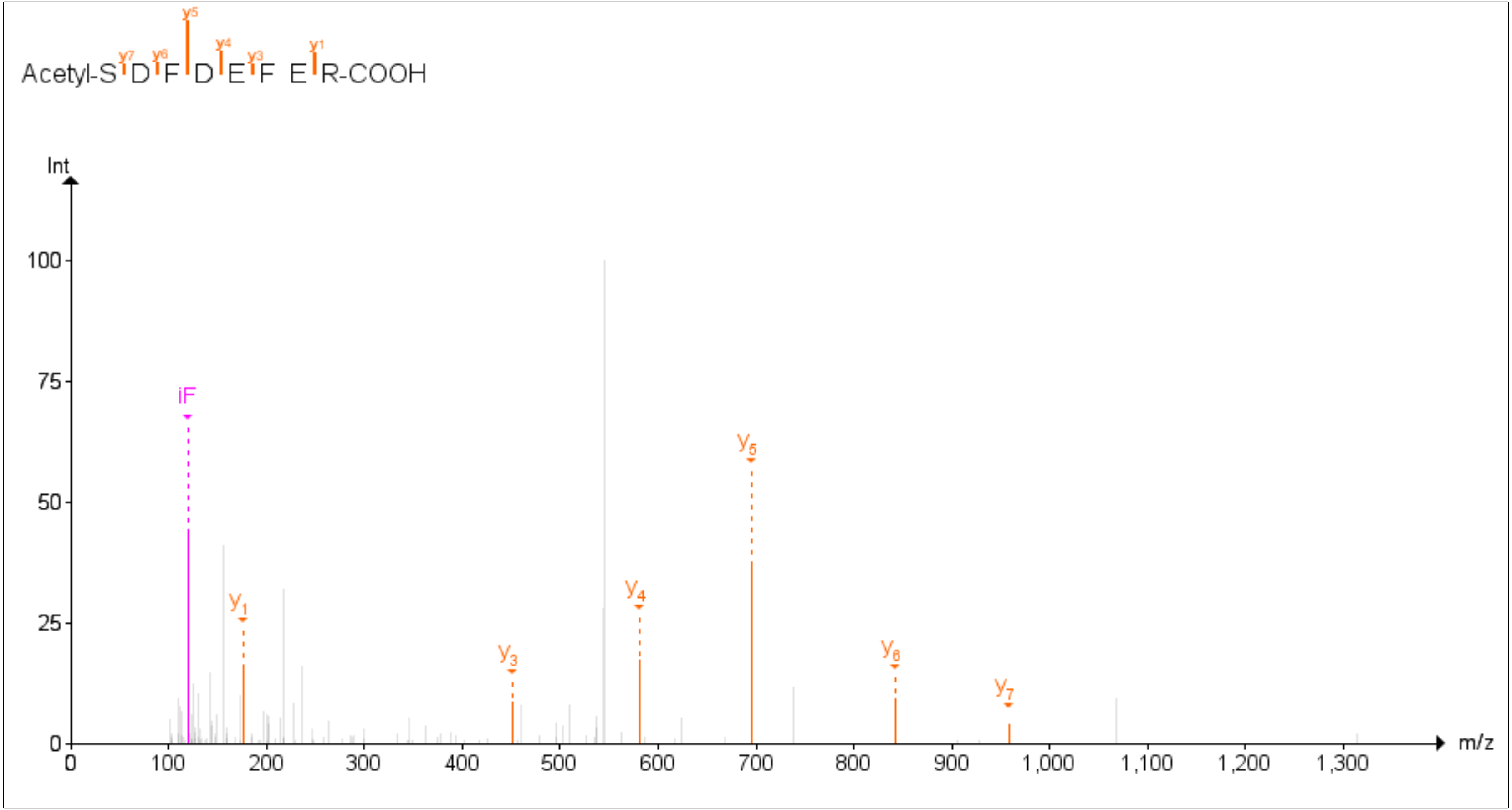

Acetyl-S D F D E F E R-COOH
y7 y6 y5 y4 y3 y1
Int
100
75
50
25
0
iF
y1
y3
y4
y5
y6
y7
0 100 200 300 400 500 600 700 800 900 1,000 1,100 1,200 1,300
m/z

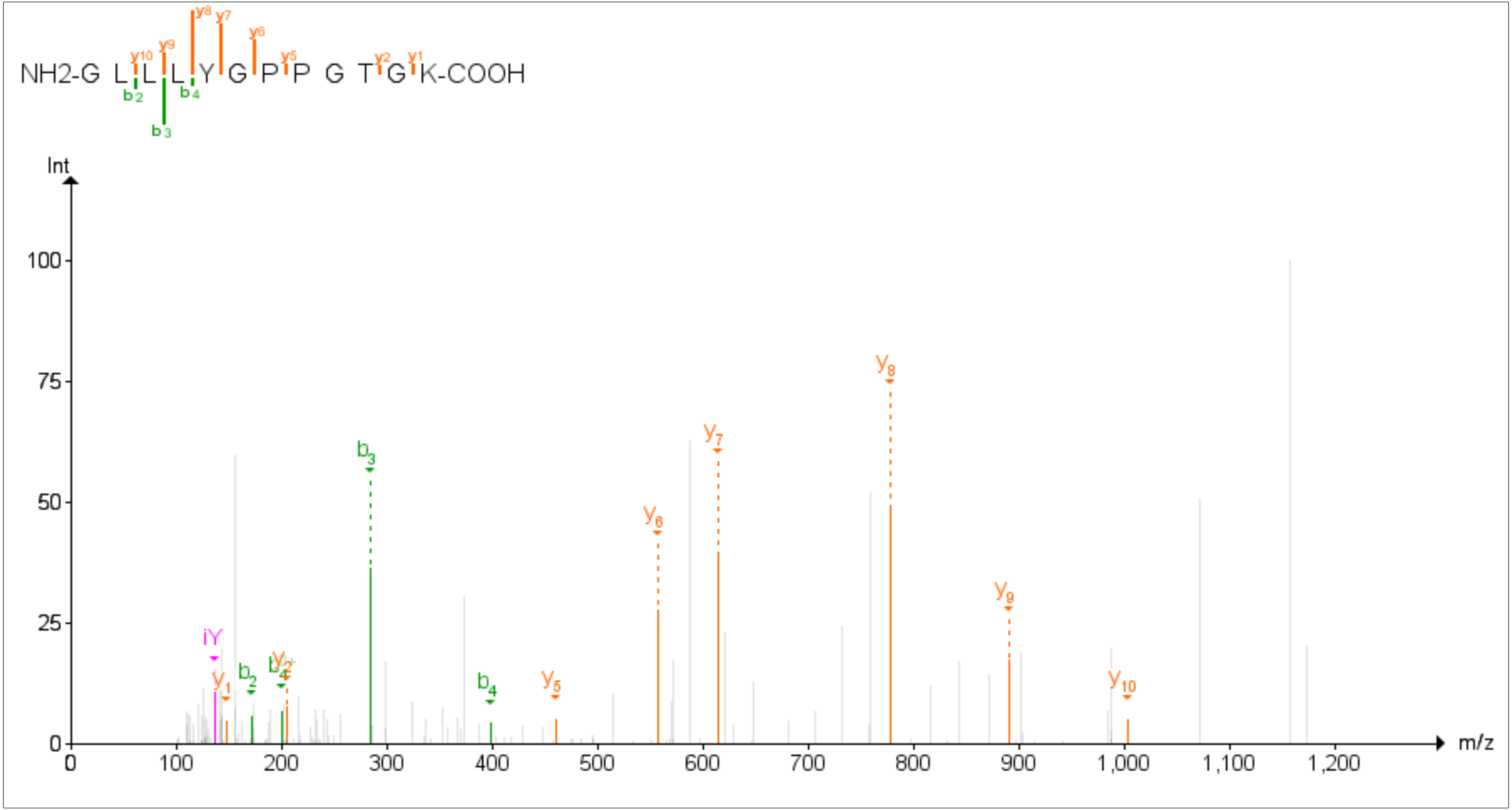

NH2-G L L L Y G P P G T G K-COOH
y10 y9 y8 y7 y6 y5 y2 y1
b2 b3 b4
Int
100
75
50
25
0
0 100 200 300 400 500 600 700 800 900 1,000 1,100 1,200
m/z
iY
y1
b2
b4
y2
b3
b4
y5
y6
y7
y8
y9
y10

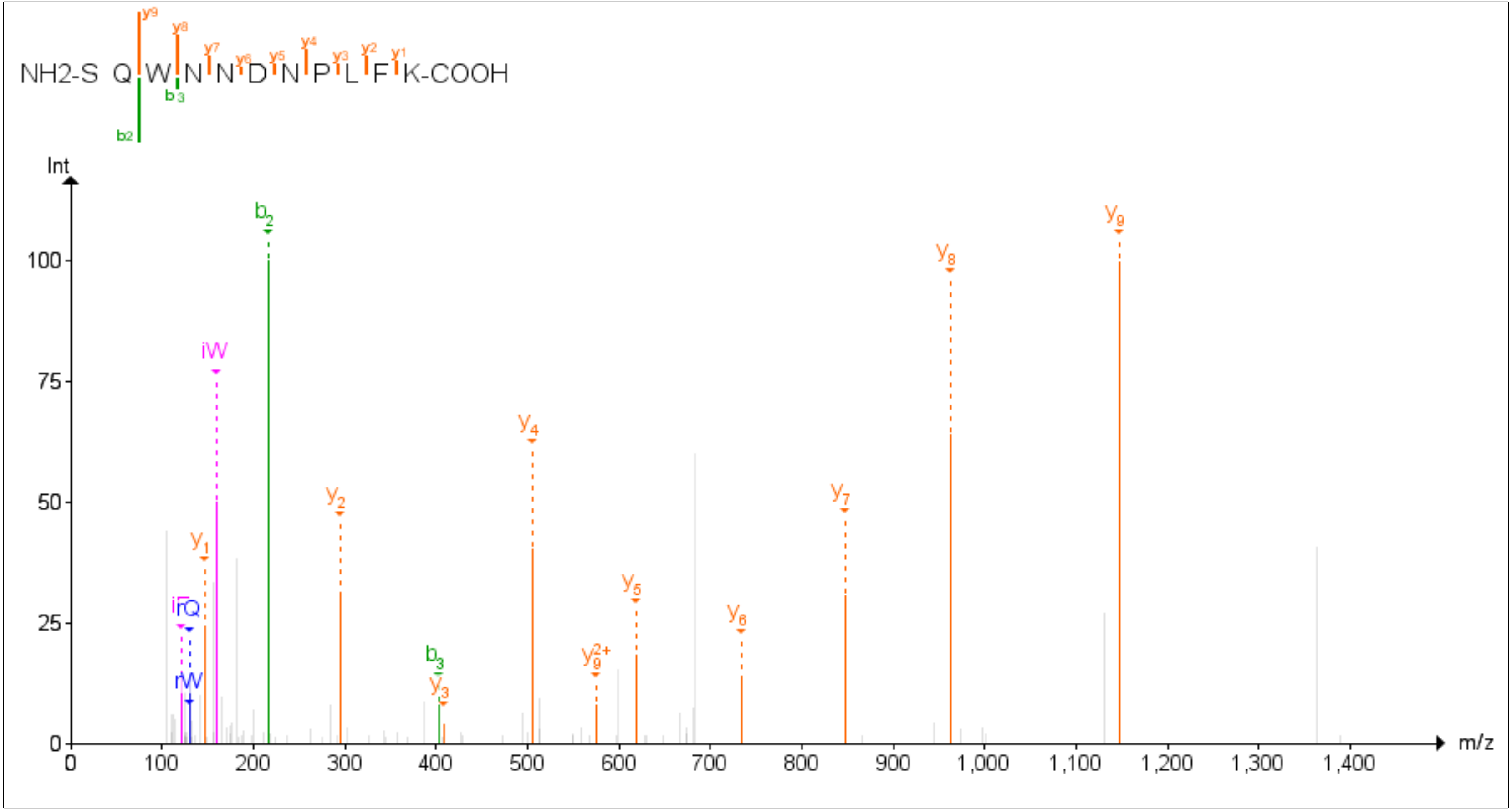

NH2-S Q W N N D N P L F K-COOH
y9 y8 y7 y6 y5 y4 y3 y2 y1
b2 b3
Int
100
75
50
25
0
b2
y9
y8
iW
y4
y2
y7
y1
y5
y6
iF
rQ
b3
y3
y9 2+
rW
m/z
0
100
200
300
400
500
600
700
800
900
1,000
1,100
1,200
1,300
1,400

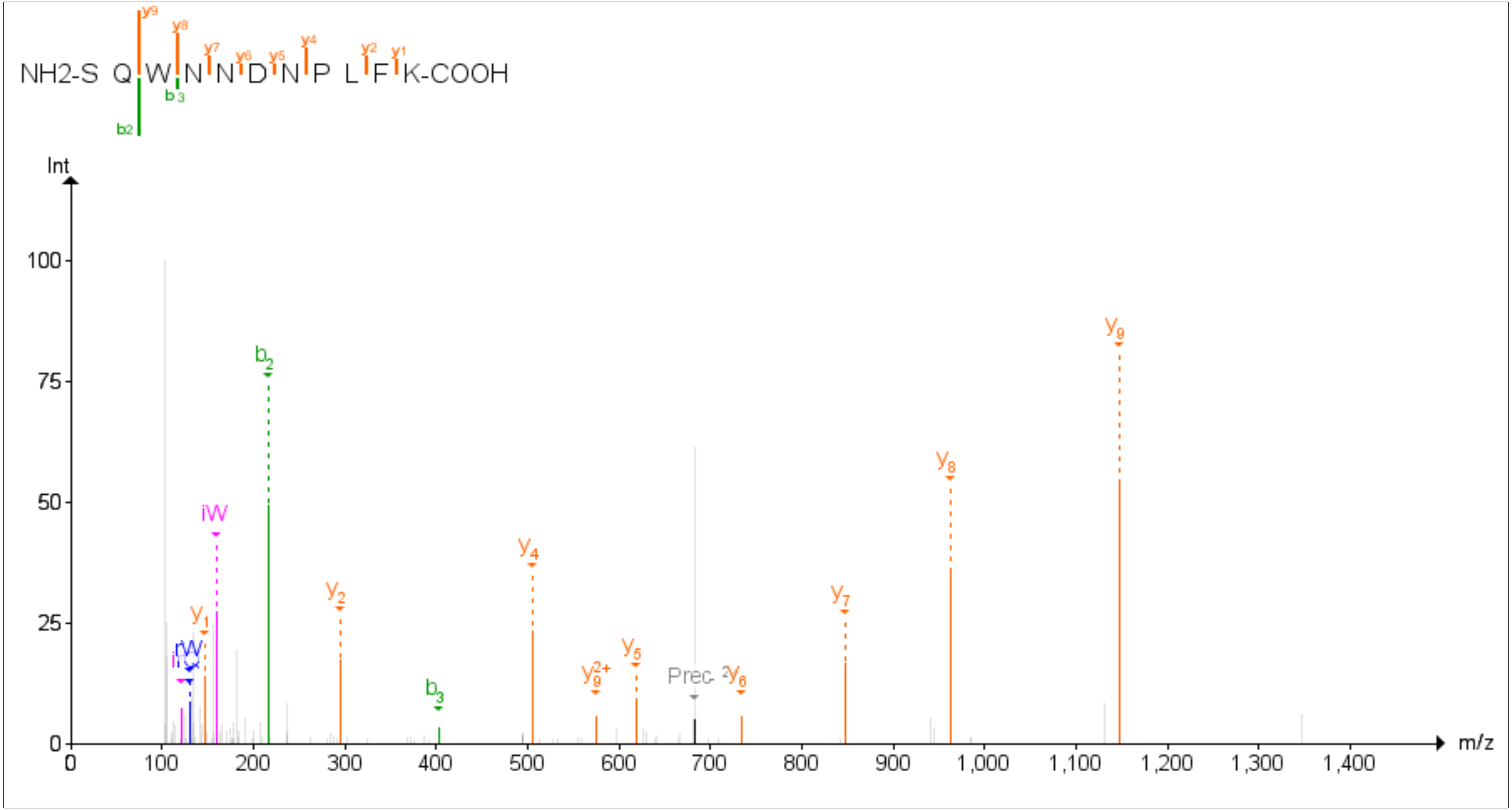

NH2-S Q W N N D N P L F K-COOH
y9
y8
y7
y6
y5
y4
y2
y1
b2
b3
Int
100
75
50
25
0
b2
y9
y8
iW
y4
y2
y1
y7
y5
y_9^{2+}
Prec- 2
y6
b3
m/z
0
100
200
300
400
500
600
700
800
900
1,000
1,100
1,200
1,300
1,400

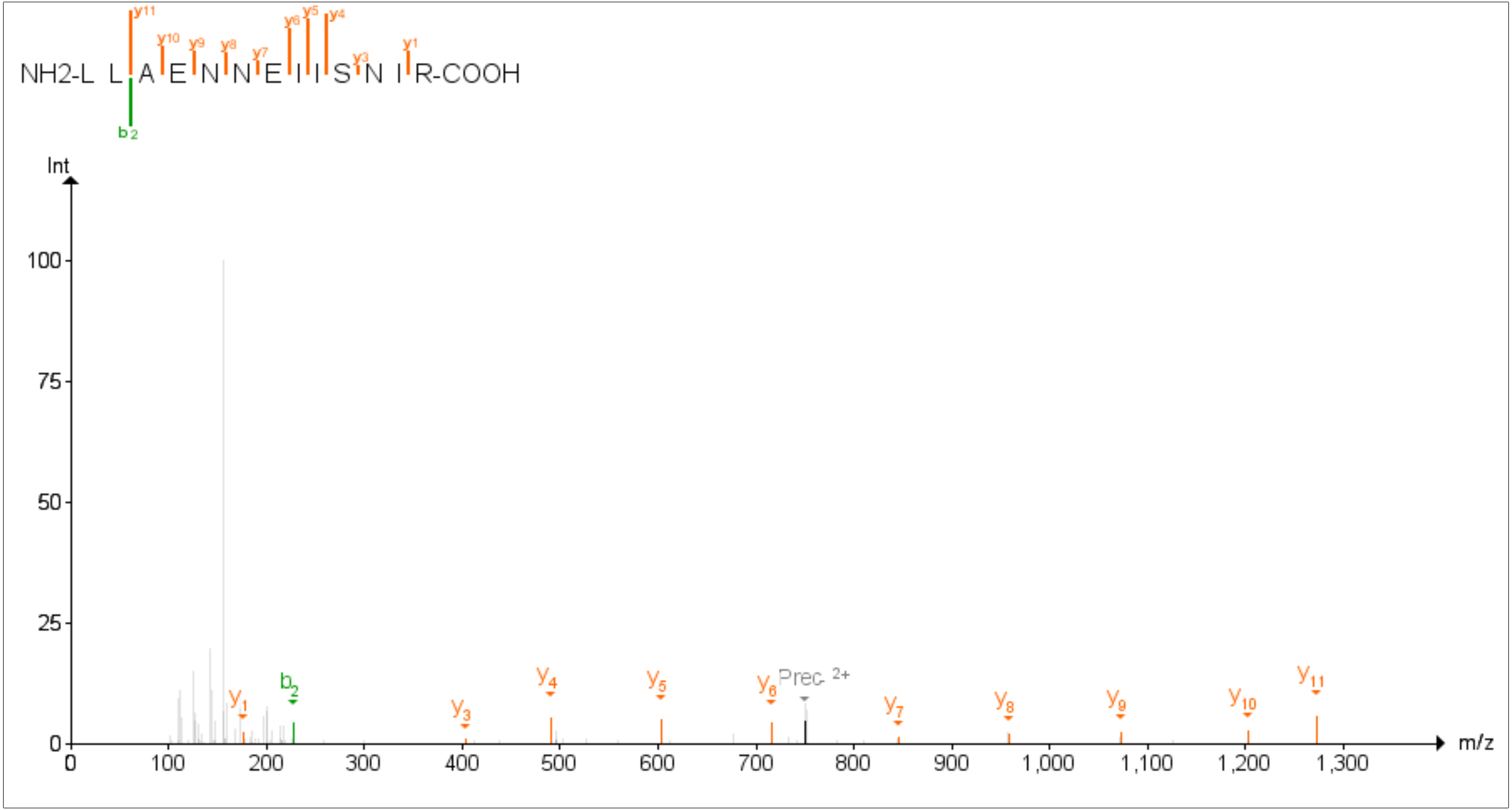

NH2-L L A E N N E I I S N I R-COOH
y11 y10 y9 y8 y7 y6 y5 y4 y3 y1
b2
Int
100
75
50
25
0
y1 b2 y3 y4 y5 y6 Prec 2+ y7 y8 y9 y10 y11
0 100 200 300 400 500 600 700 800 900 1,000 1,100 1,200 1,300
m/z

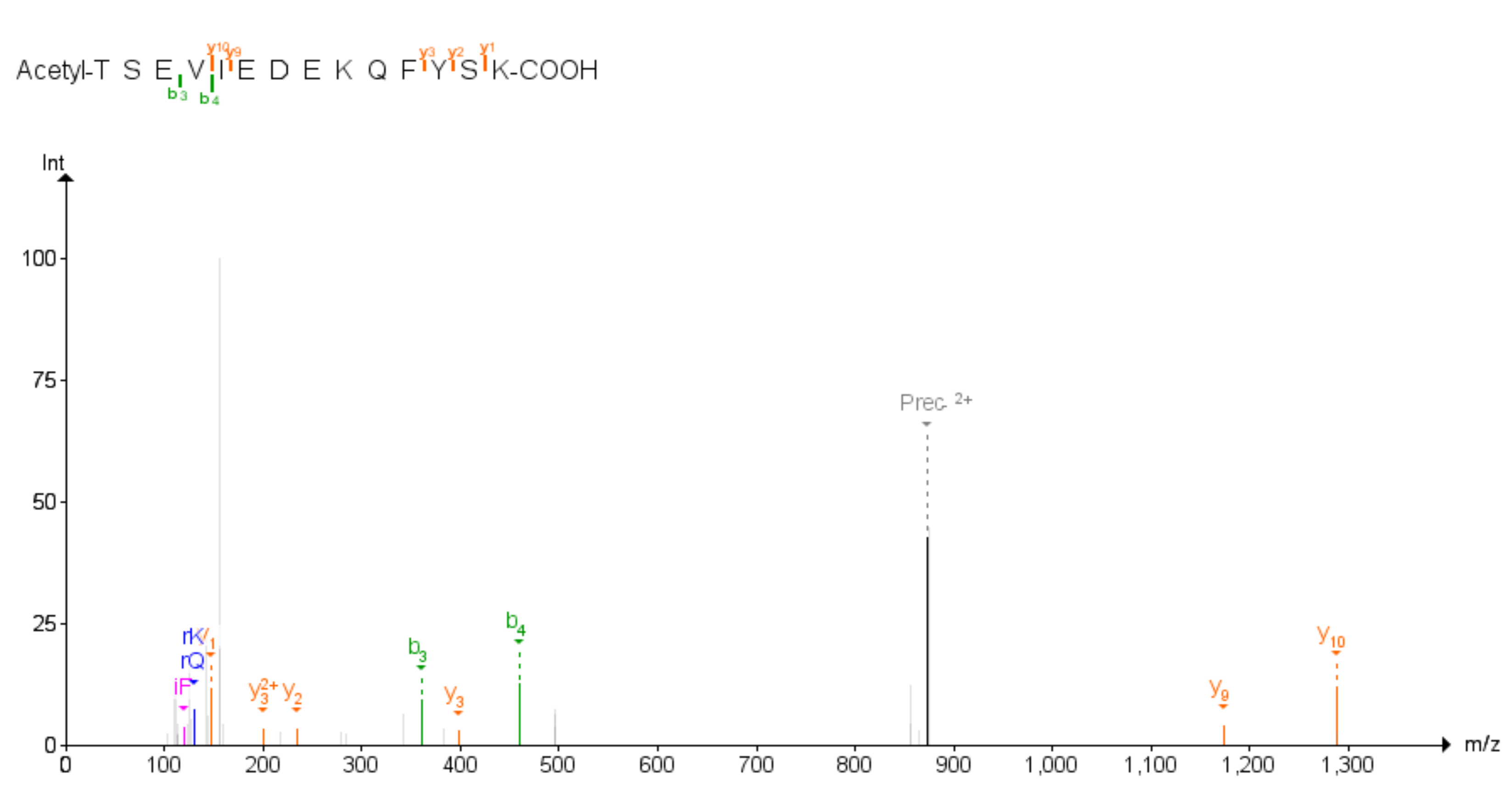

Acetyl-T S E V I E D E K Q F Y S K-COOH
y10 y9 y3 y2 y1
b3 b4
Int
100
75
50
25
0
Prec 2+
rK
y1
rQ
iF
y3 2+
y2
b3
y3
b4
y9
y10
0
100
200
300
400
500
600
700
800
900
1,000
1,100
1,200
1,300
m/z

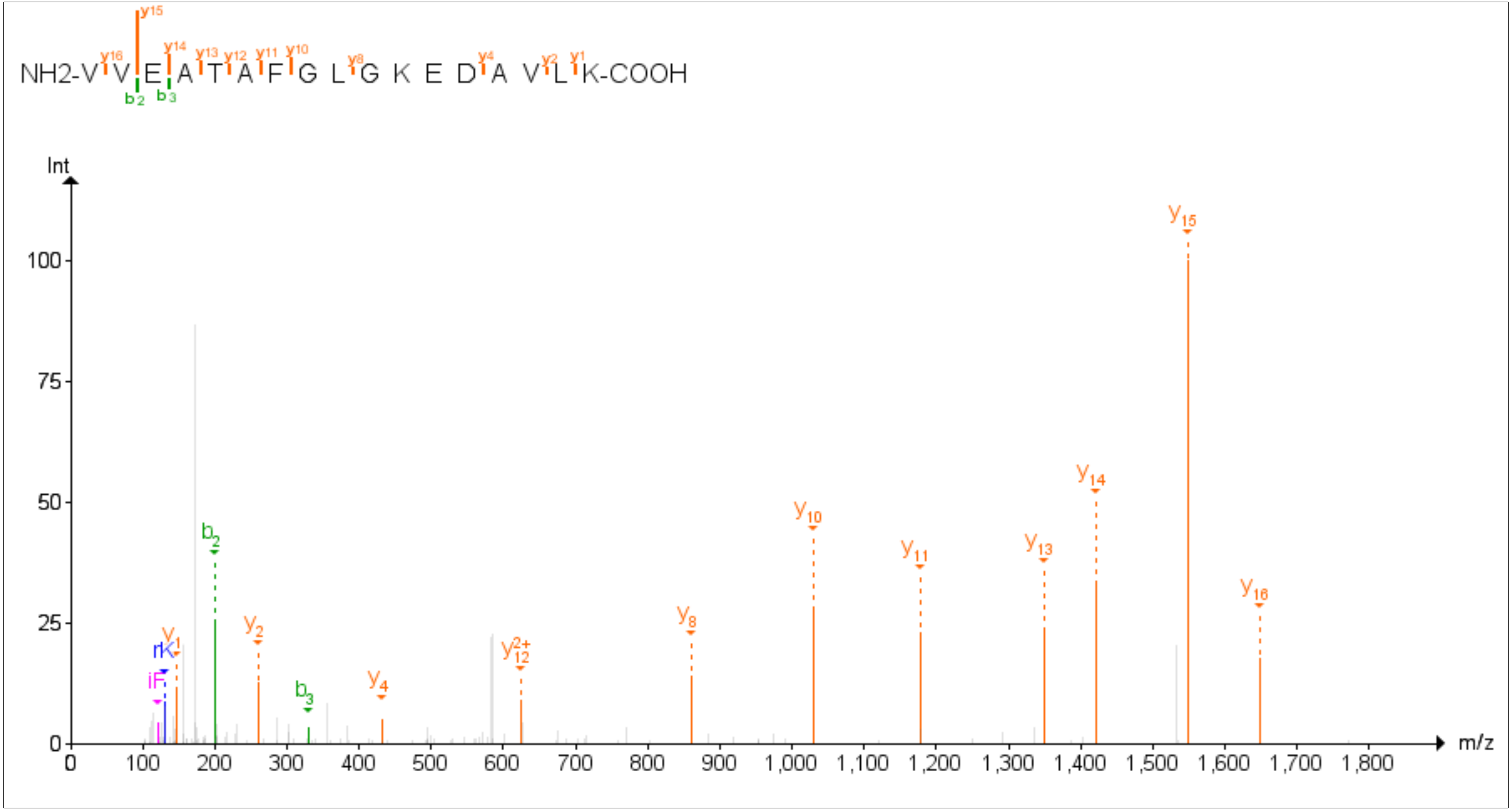

NH2-V V E A T A F G L G K E D A V L K-COOH
y16 y15 y14 y13 y12 y11 y10 y8 y4 y2 y1
b2 b3
Int
m/z
0
25
50
75
100
0 100 200 300 400 500 600 700 800 900 1,000 1,100 1,200 1,300 1,400 1,500 1,600 1,700 1,800
y15
y14
y10
y11
y13
y16
b2
y8
y2
y1
rK
iF
b3
y4
y12 2+

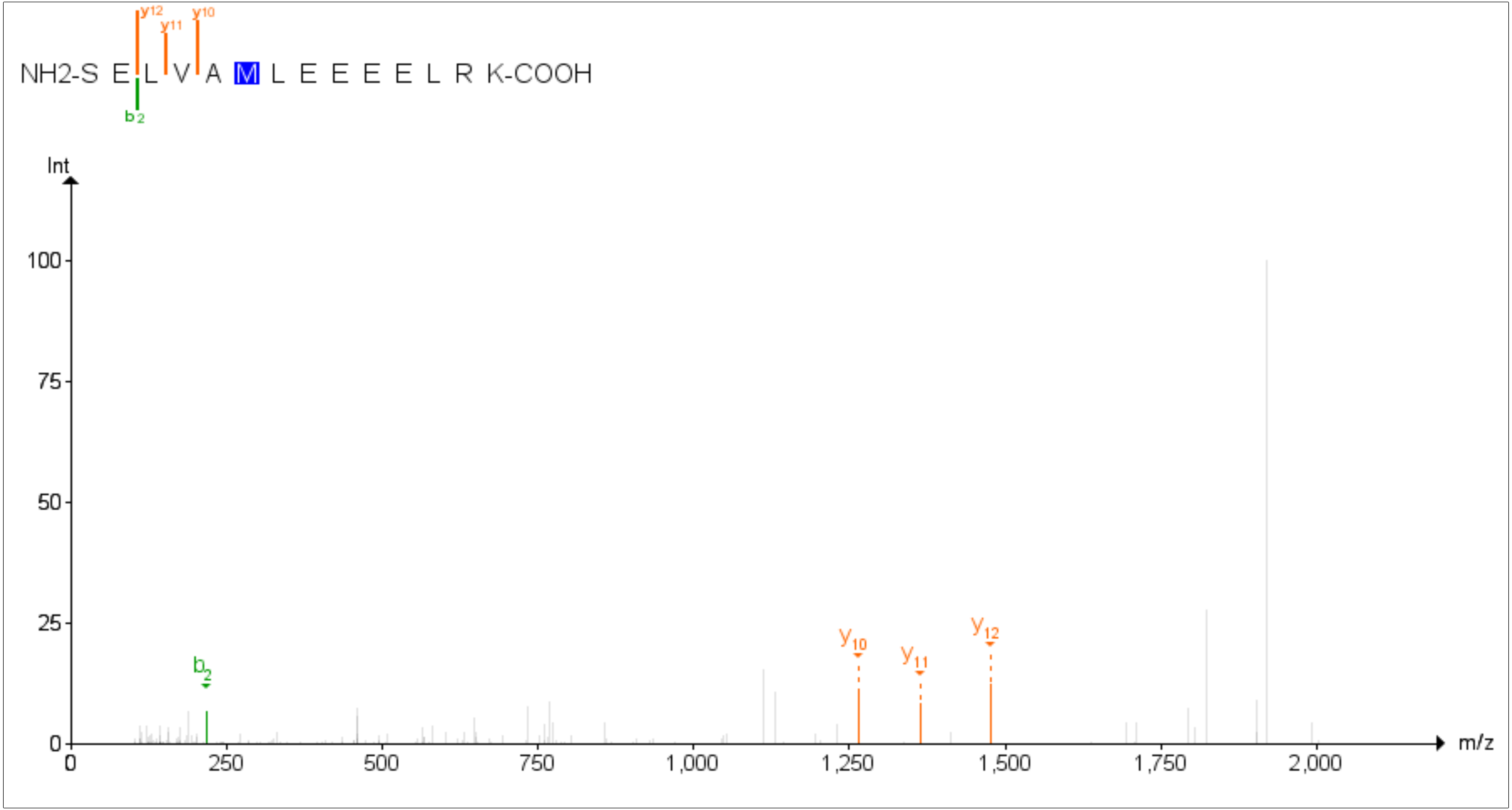

NH2-S E L V A M L E E E E L R K-COOH
y12
y11
y10
b2
Int
100
75
50
25
0
250
500
750
1,000
1,250
1,500
1,750
2,000
m/z

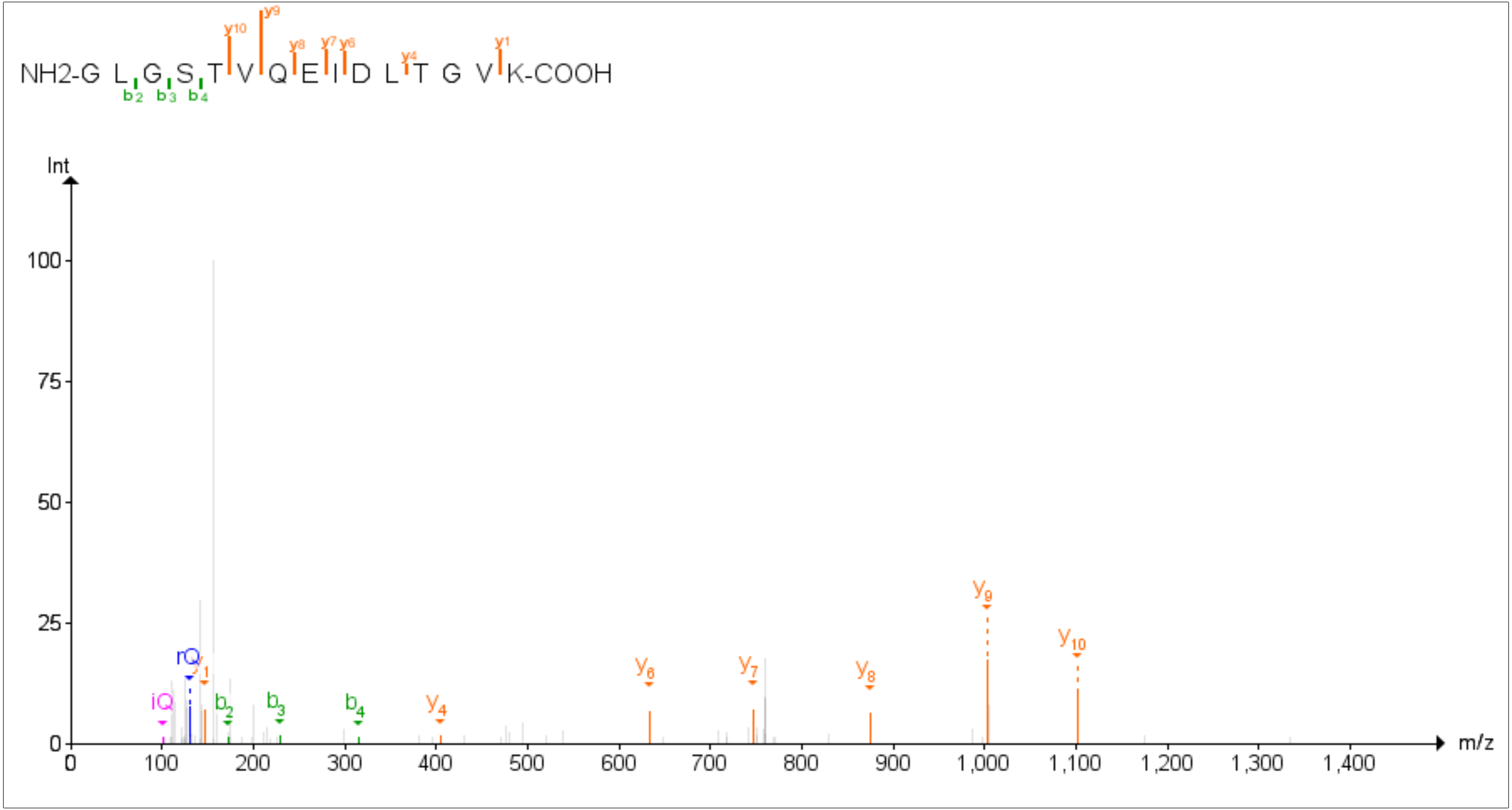

NH2-G L G S T V Q E I D L T G V K-COOH
y10 y9 y8 y7 y6 y4 y1
b2 b3 b4
Int
100
75
50
25
0
iQ
rQ
y1
b2
b3
b4
y4
y6
y7
y8
y9
y10
0 100 200 300 400 500 600 700 800 900 1,000 1,100 1,200 1,300 1,400
m/z

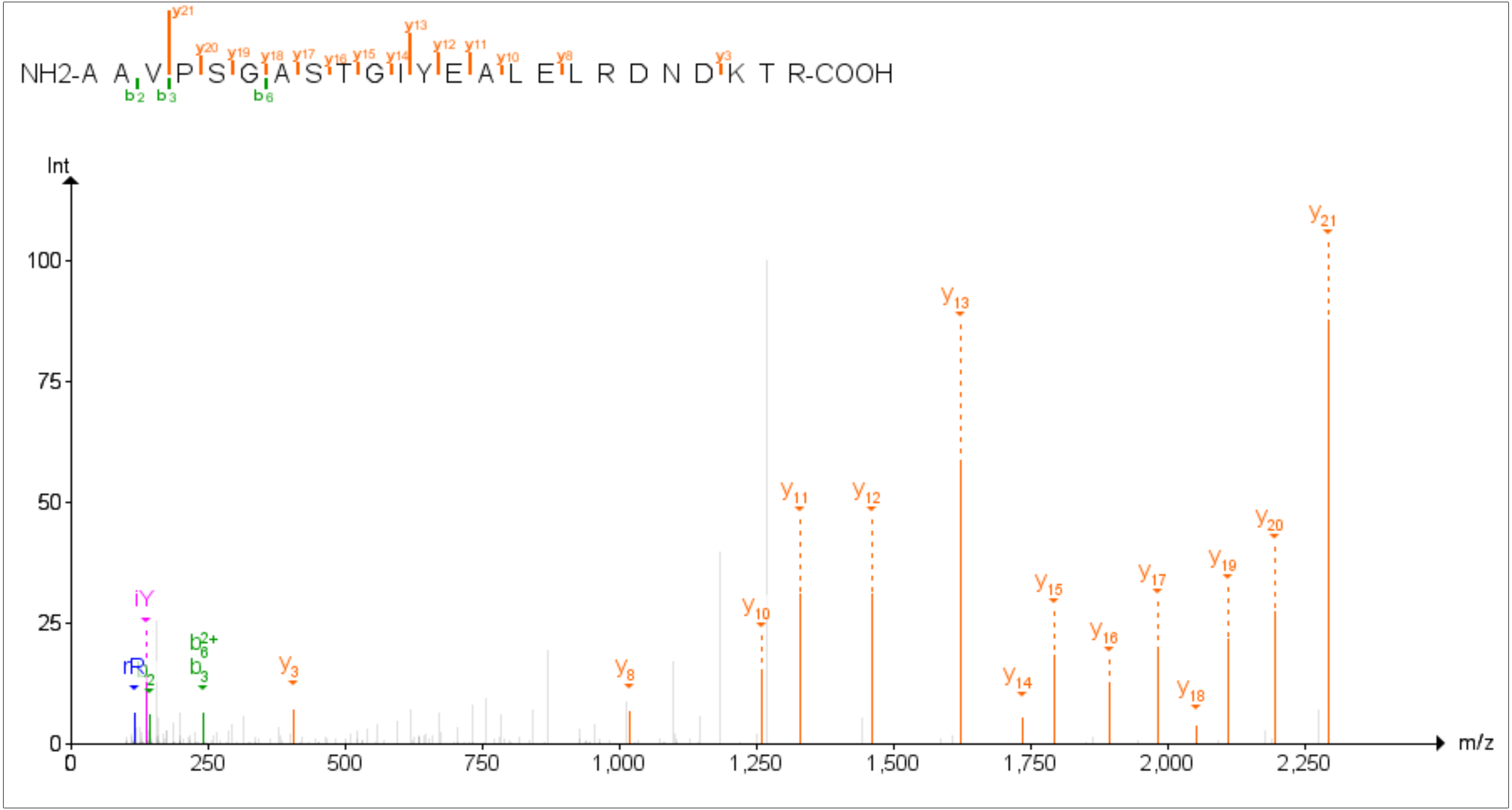

NH2-A A V P S G A S T G I Y E A L E L R D N D K T R-COOH
y21
y20
y19
y18
y17
y16
y15
y14
y13
y12
y11
y10
y8
y3
b2
b3
b6
Int
100
75
50
25
0
m/z
250
500
750
1,000
1,250
1,500
1,750
2,000
2,250
iY
rR
b2
b6 2+
b3

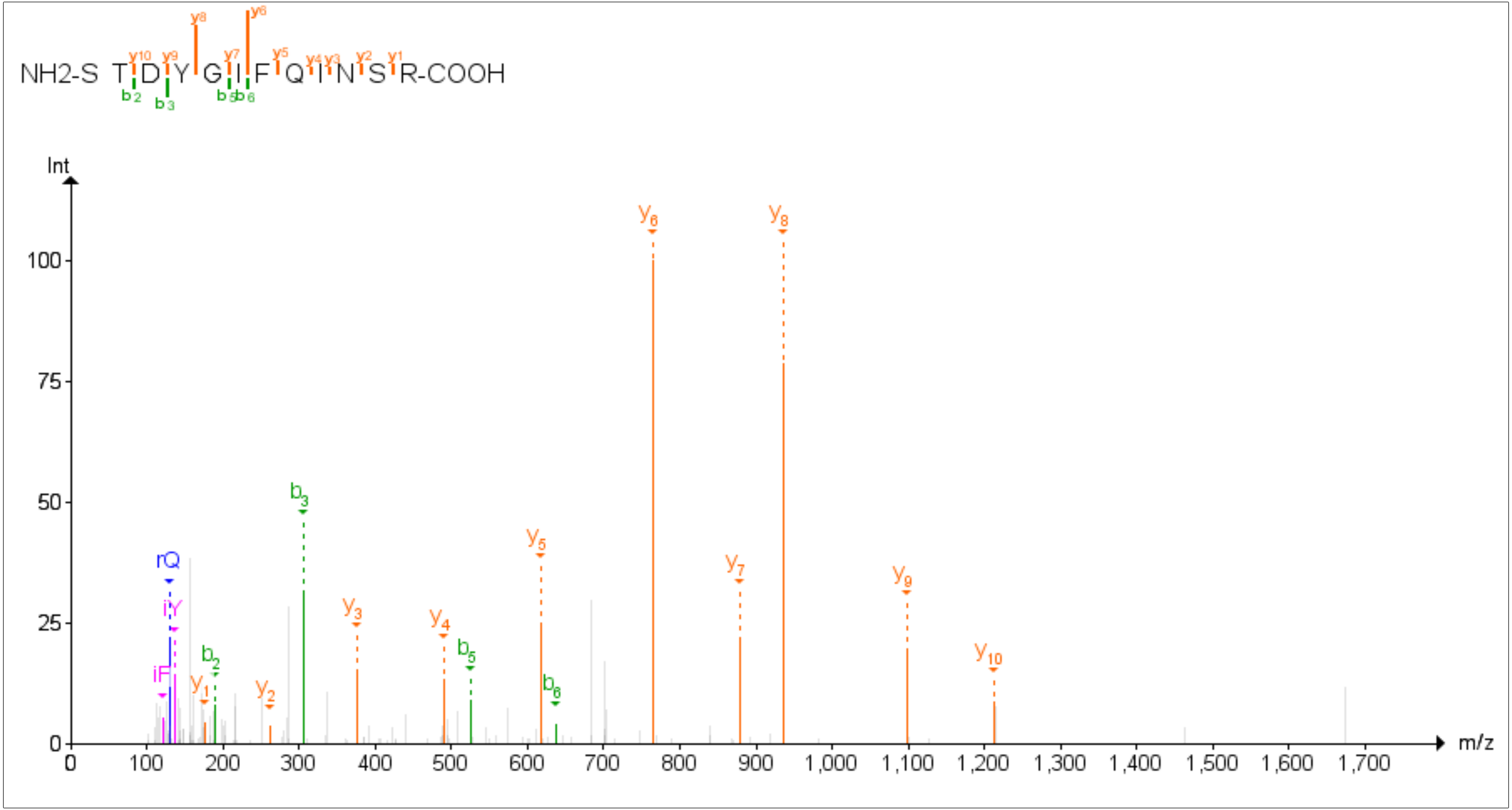

NH2-S T D Y G I I F Q I N S R-COOH
y10 y9 y8 y7 y6 y5 y4 y3 y2 y1
b2 b3 b5 b6
Int
m/z
0
25
50
75
100
0 100 200 300 400 500 600 700 800 900 1,000 1,100 1,200 1,300 1,400 1,500 1,600 1,700
rQ
iY
iF
y1
b2
y2
b3
y3
y4
b5
y5
b6
y6
y7
y8
y9
y10

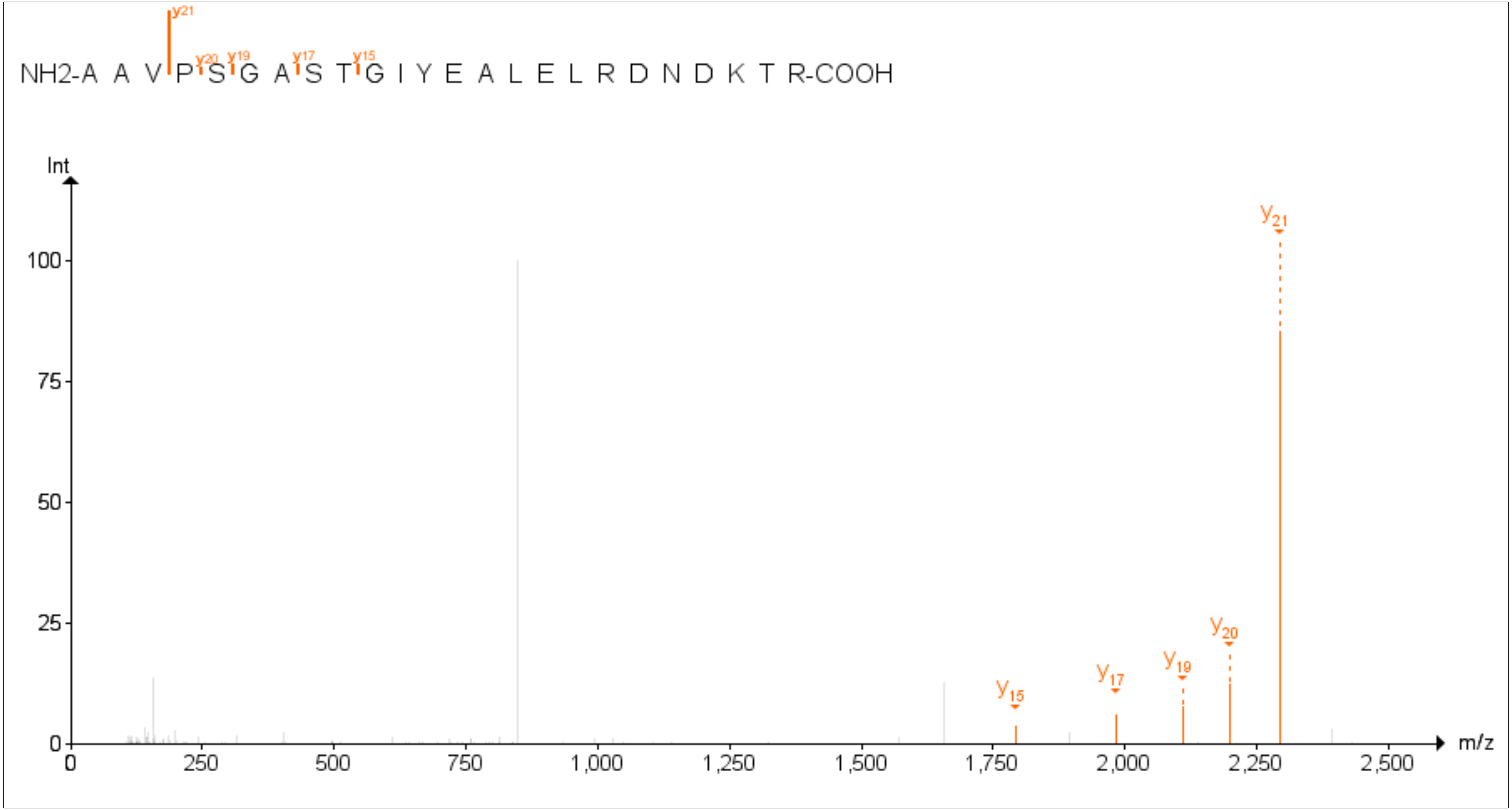

NH2-A A V P S G A S T G I Y E A L E L R D N D K T R-COOH
y21
y20
y19
y17
y15
Int
100
75
50
25
0
m/z
250
500
750
1,000
1,250
1,500
1,750
2,000
2,250
2,500

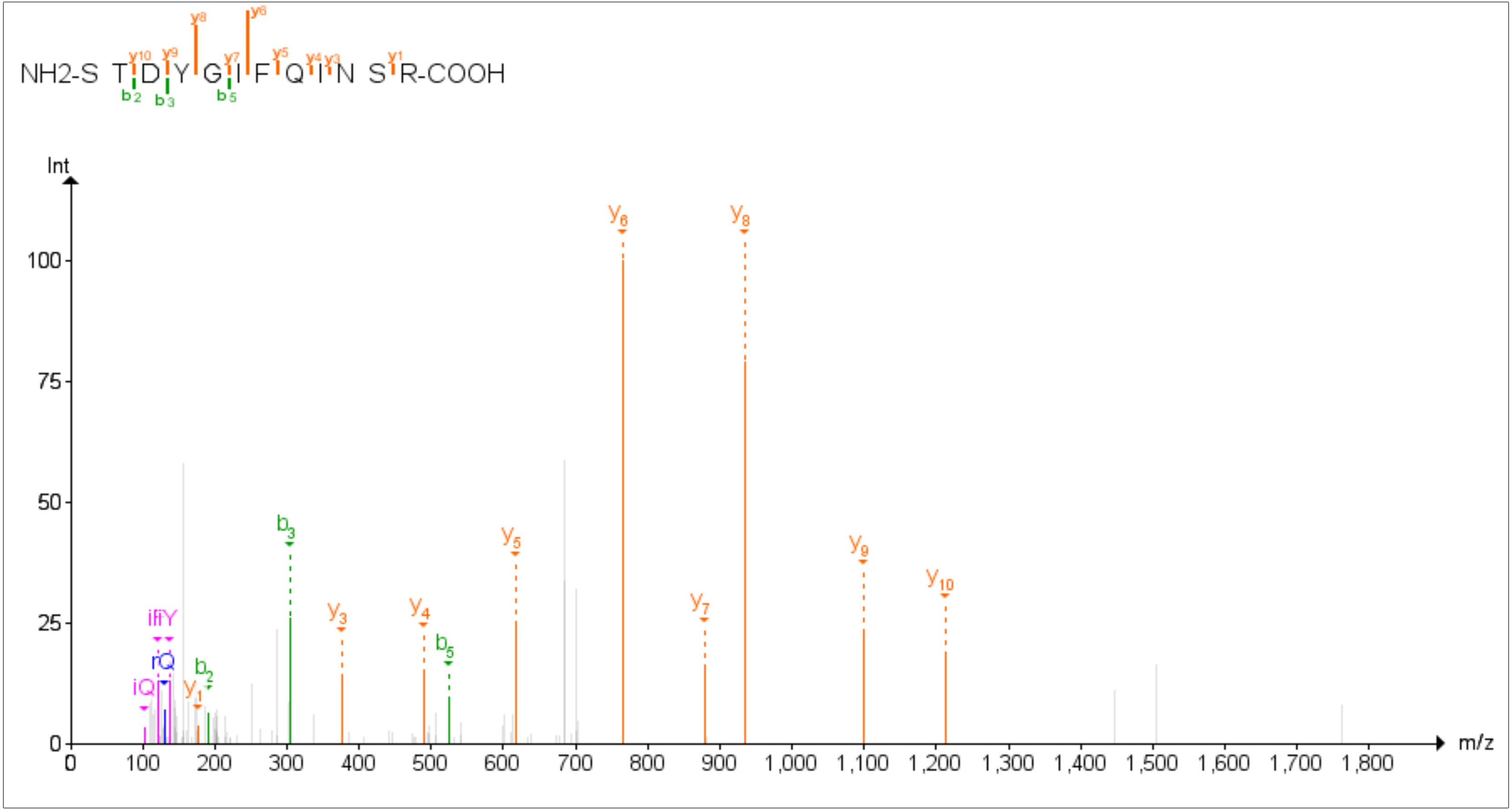
NH2-S T D Y G I F Q I N S R-COOH
y10 y9 y8 y7 y6 y5 y4 y3 y1
b2 b3 b5
Int
m/z
0
25
50
75
100
0 100 200 300 400 500 600 700 800 900 1,000 1,100 1,200 1,300 1,400 1,500 1,600 1,700 1,800
y6 y8 y5 y9 y10 y3 y4 y7 y1
b3 b2 b5
ifiY
rQ
iQ

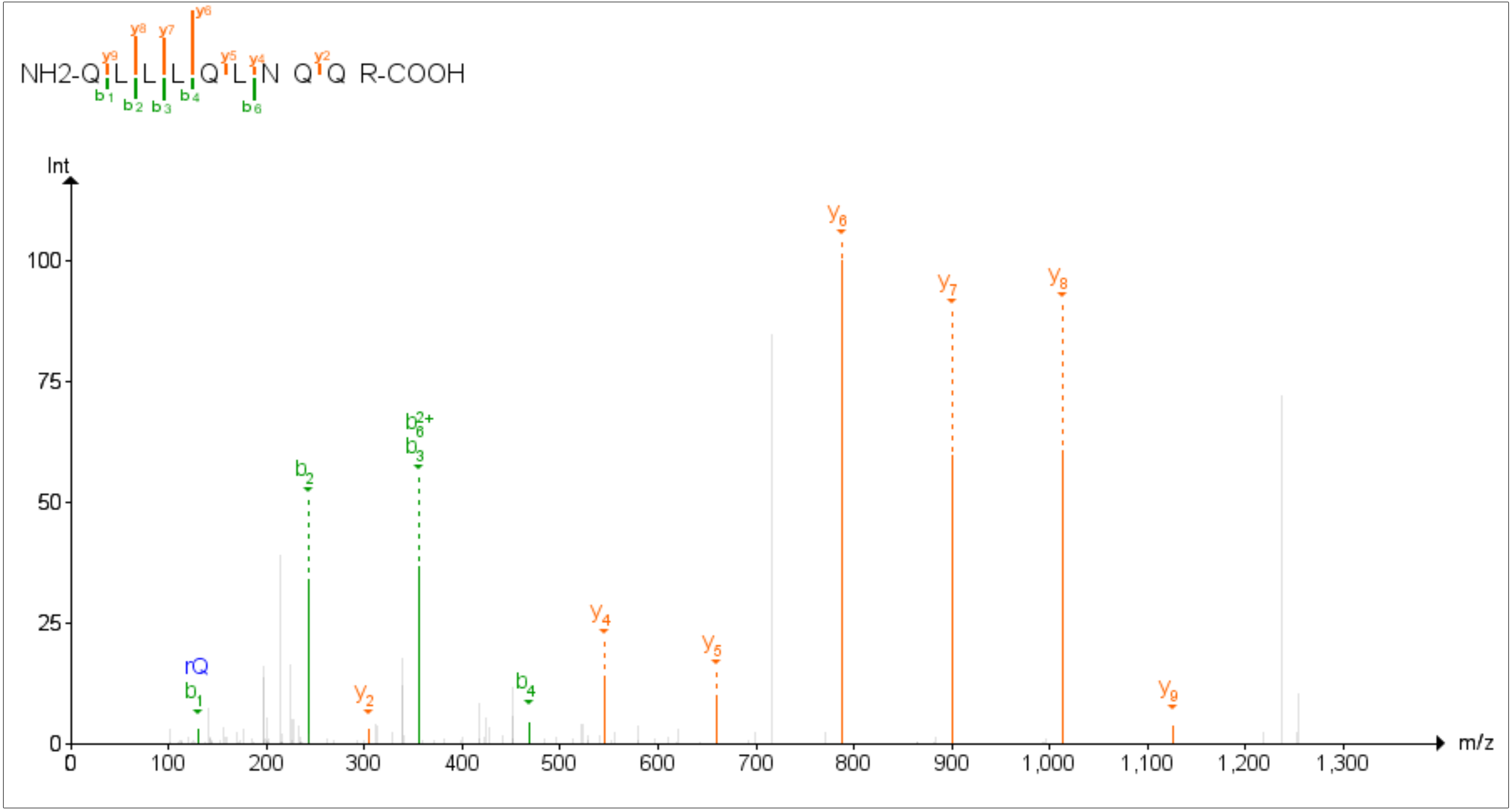

NH2-Q L L L Q L N Q Q R-COOH
y9
y8
y7
y6
y5
y4
y2
b1
b2
b3
b4
b6
Int
100
75
50
25
0
m/z
0
100
200
300
400
500
600
700
800
900
1,000
1,100
1,200
1,300
rQ
b1
b2
b6 2+
b3
y2
b4
y4
y5
y6
y7
y8
y9

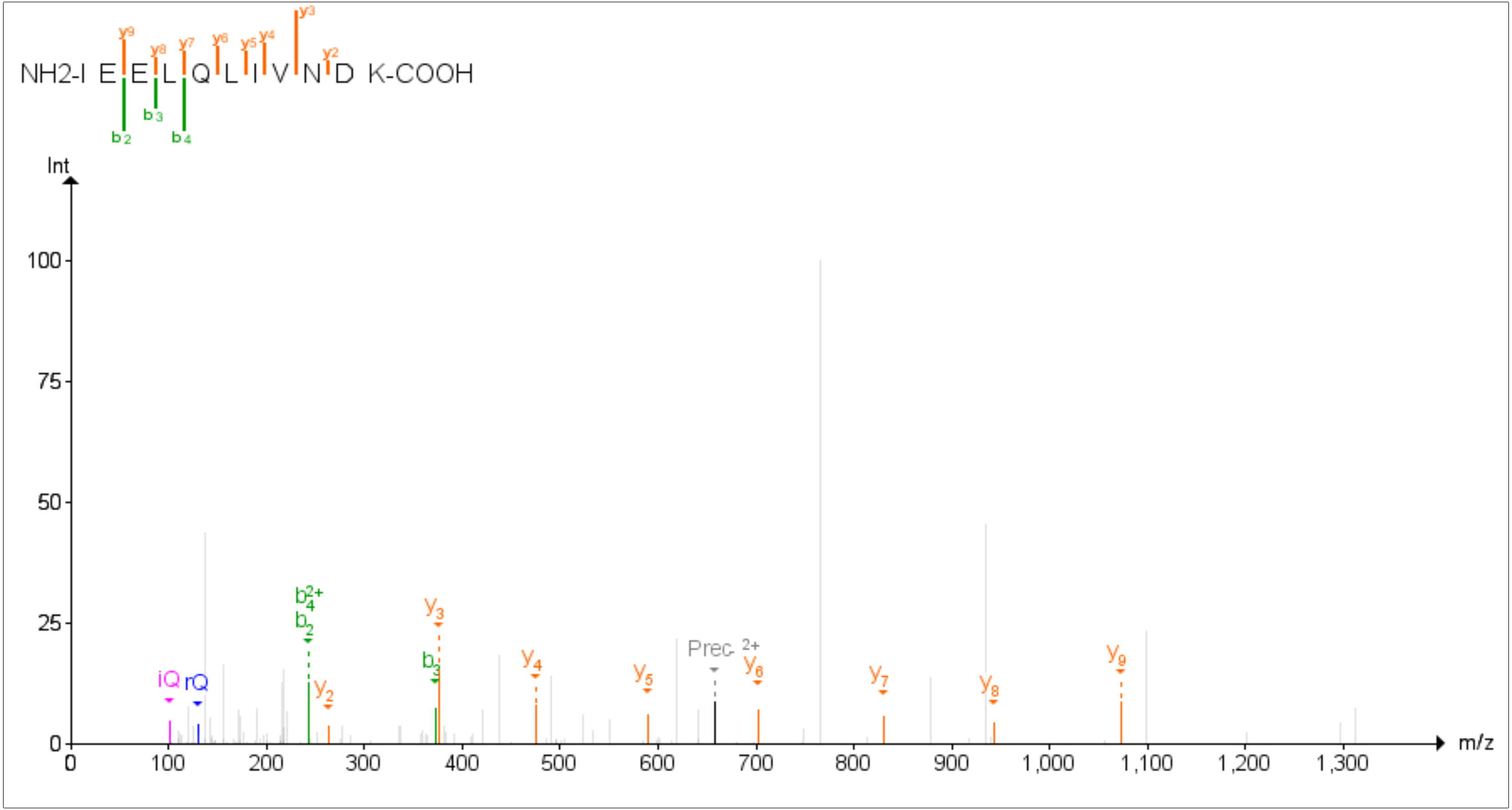

NH2-I E E L Q L I V N D K-COOH
y9 y8 y7 y6 y5 y4 y3 y2
b2 b3 b4
Int
100
75
50
25
0
m/z
0 100 200 300 400 500 600 700 800 900 1,000 1,100 1,200 1,300
iQ
rQ
b4 2+
b2
y2
b3
y3
y4
y5
Prec 2+
y6
y7
y8
y9

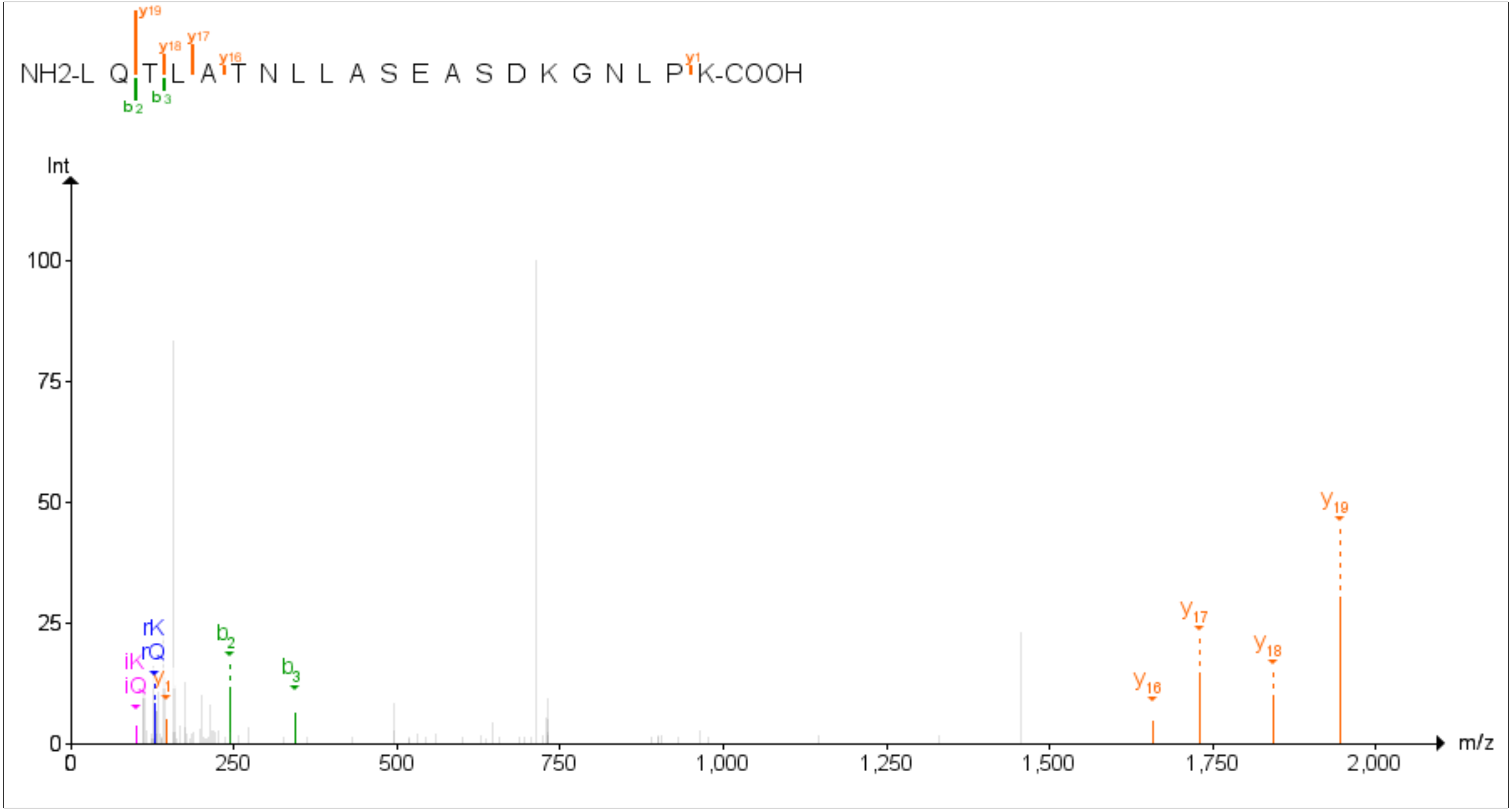

NH2-L Q T L A T N L L A S E A S D K G N L P K-COOH
y19
y18
y17
y16
y1
b 2
b 3
Int
100
75
50
25
0
m/z
0
250
500
750
1,000
1,250
1,500
1,750
2,000
rK
rQ
iK
iQ
y1
b2
b3
y16
y17
y18
y19

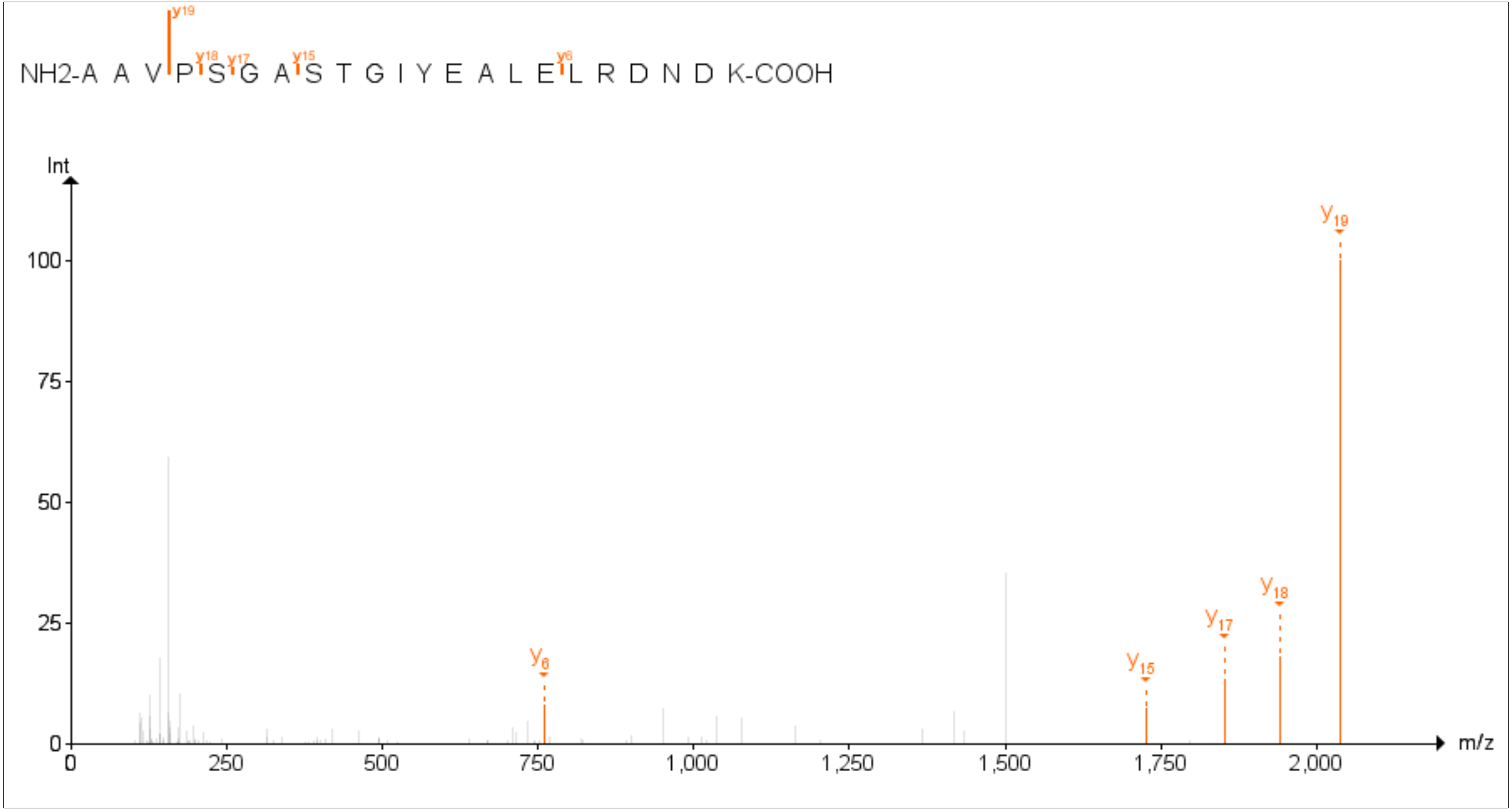

NH2-A A V P S G A S T G I Y E A L E L R D N D K-COOH
y19
y18
y17
y15
y6
Int
100
75
50
25
0
250
500
750
1,000
1,250
1,500
1,750
2,000
m/z

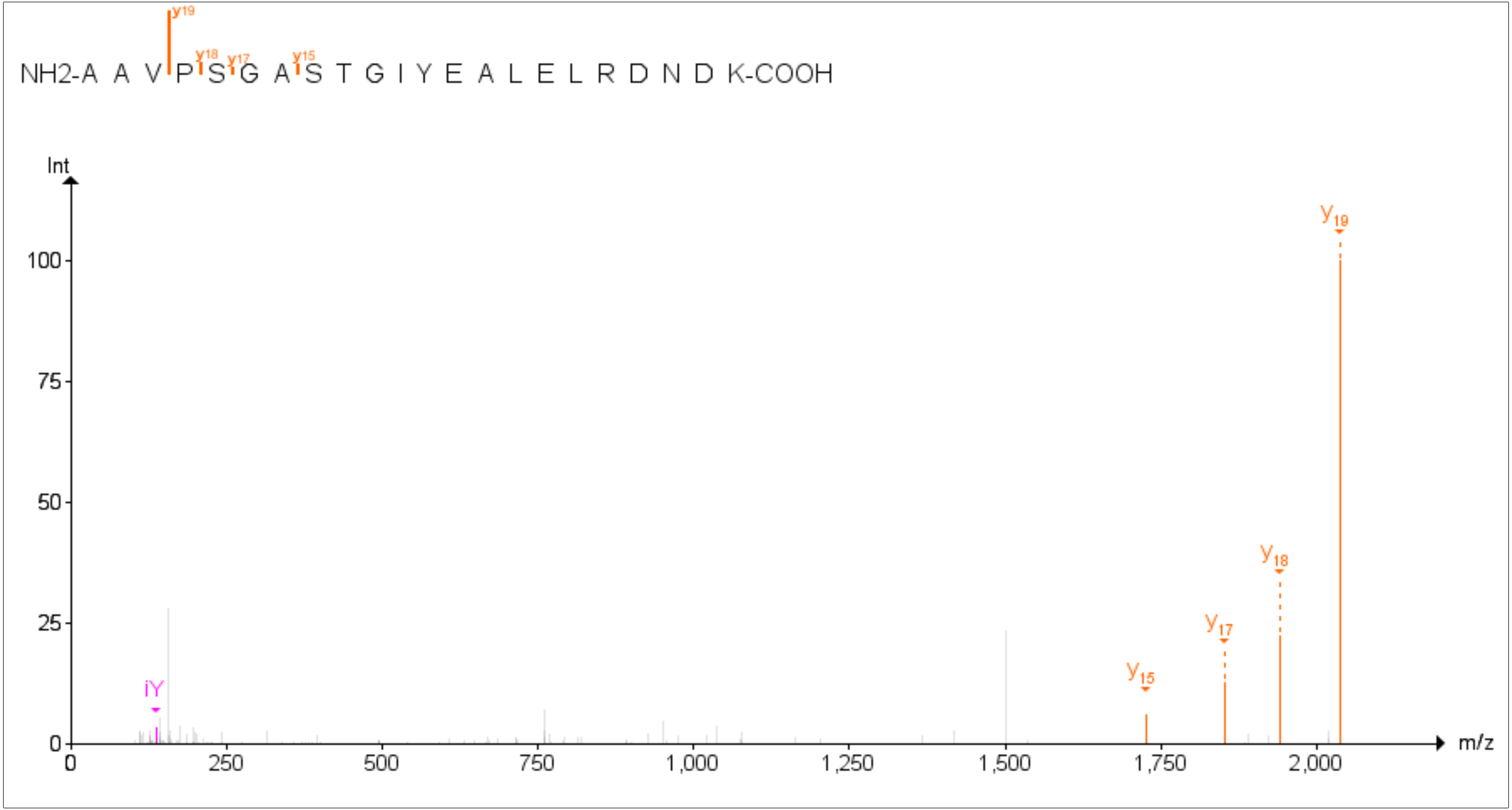

NH2-A A V P S G A S T G I Y E A L E L R D N D K-COOH
y19
y18
y17
y15
Int
100
75
50
25
0
iY
y19
y18
y17
y15
0
250
500
750
1,000
1,250
1,500
1,750
2,000
m/z

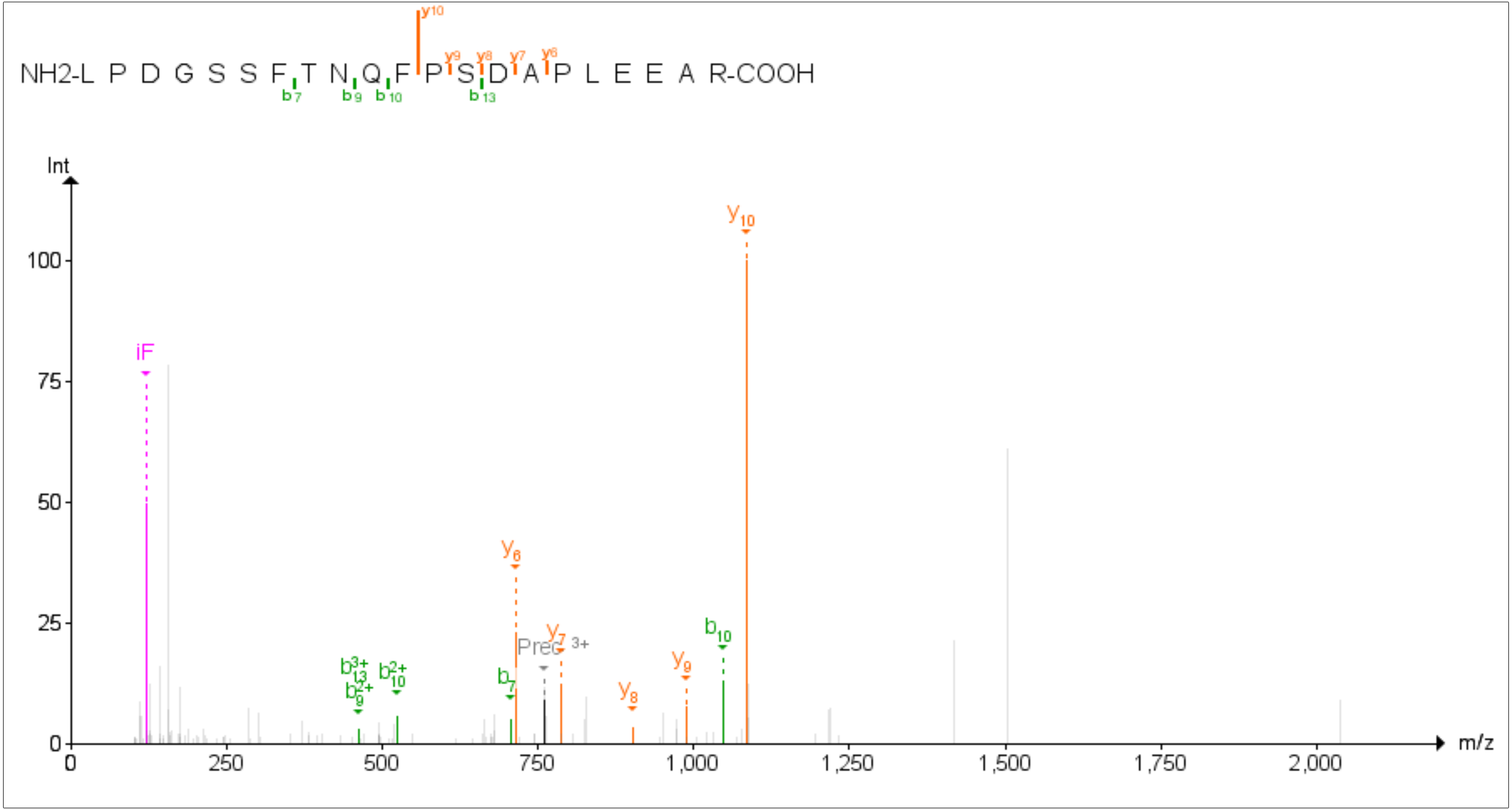

NH2-L P D G S S F T N Q F P S D A P L E E A R-COOH
y10
y9
y8
y7
y6
b7
b9
b10
b13
Int
iF
y_{10}
y_6
y_7
Prec 3+
y_8
y_9
b_{10}
b_{13}^{3+}
b_9^{2+}
b_{10}^{2+}
b_7
m/z
0
25
50
75
100
250
500
750
1,000
1,250
1,500
1,750
2,000

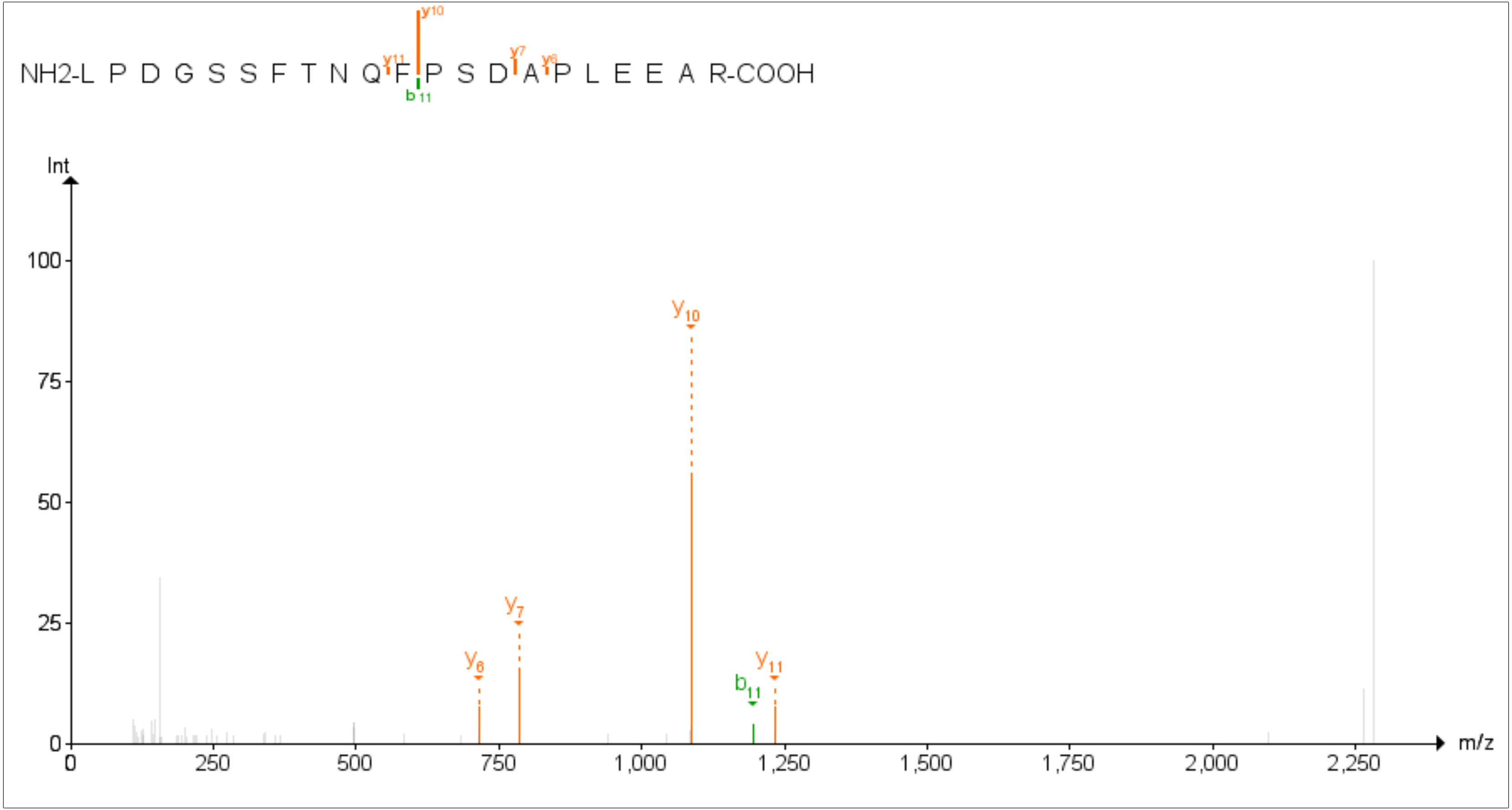

NH2-L P D G S S F T N Q F P S D A P L E E A R-COOH
y10
y11
y7
y6
b 11
Int
100
75
50
25
0
y_{10}
y_7
y_6
b_{11}
y_{11}
0
250
500
750
1,000
1,250
1,500
1,750
2,000
2,250
m/z

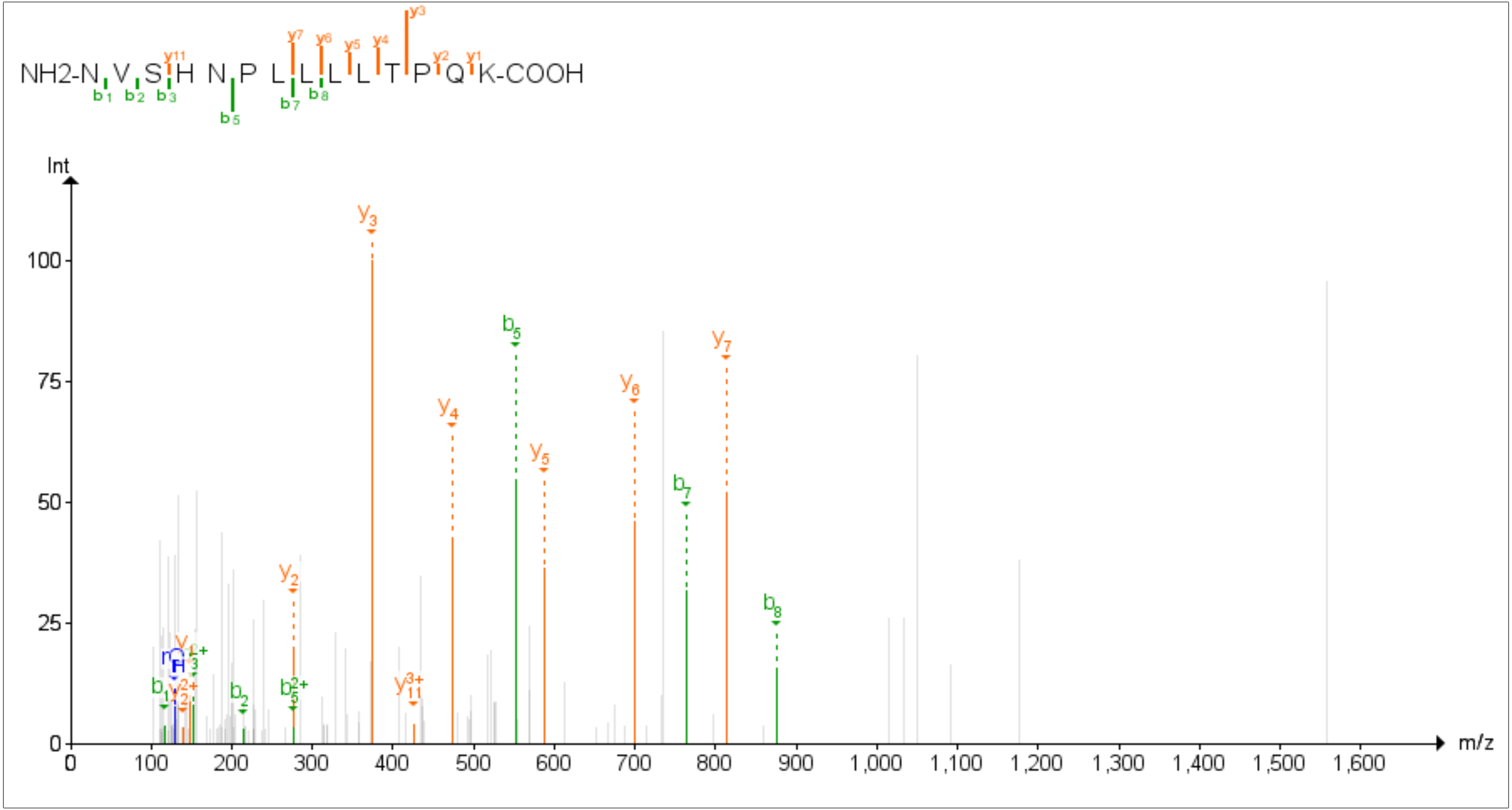

NH2-N V S H N P L L L L T P Q K-COOH
b1 b2 b3 b5 b7 b8
y11 y7 y6 y5 y4 y3 y2 y1
Int
m/z

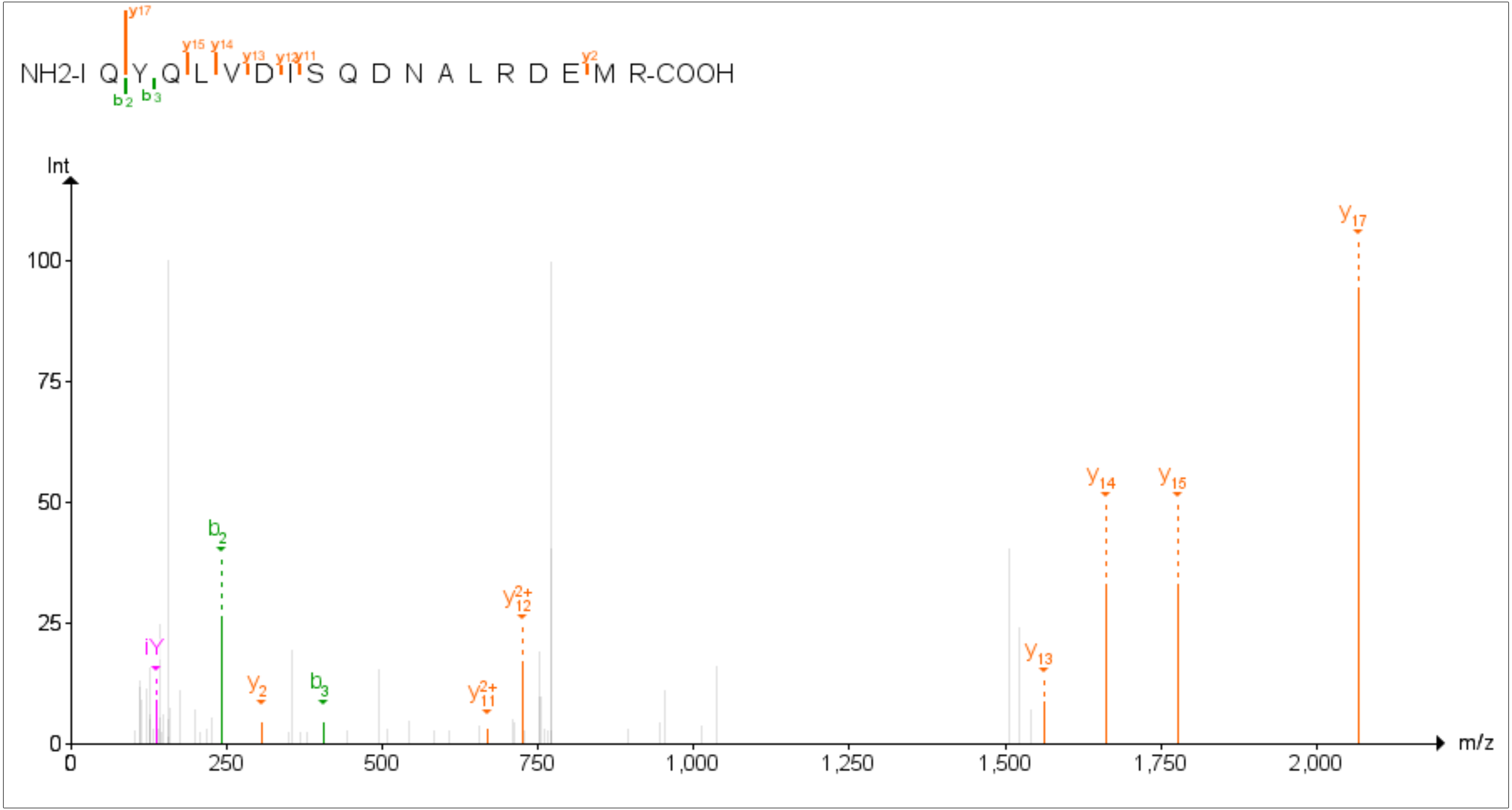

NH2-I Q Y Q L V D I S Q D N A L R D E M R-COOH
y17
y15
y14
y13
y12
y11
y2
b2
b3
Int
100
75
50
25
0
m/z
0
250
500
750
1,000
1,250
1,500
1,750
2,000
iY
y17
y14
y15
y13
y12 2+
y11 2+

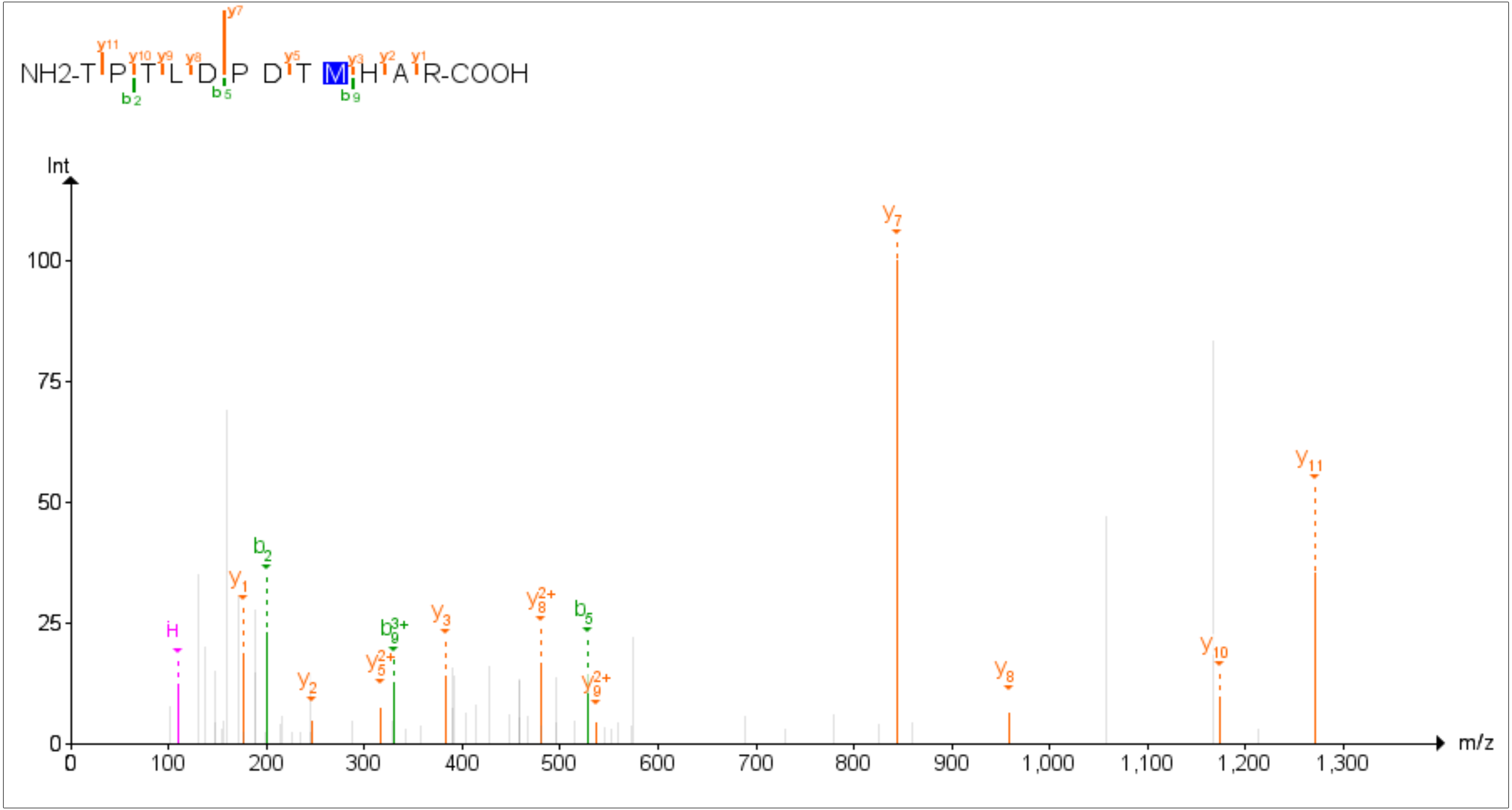
NH2-T P T L D P D T M H A R-COOH
y11 y10 y9 y8 y7 y5 y3 y2 y1
b2 b5 b9
Int
100
75
50
25
0
y7
y11
b2
y1
H
y2
y5 2+
b9 3+
y3
y8 2+
b5
y9 2+
y8
y10
0 100 200 300 400 500 600 700 800 900 1,000 1,100 1,200 1,300
m/z

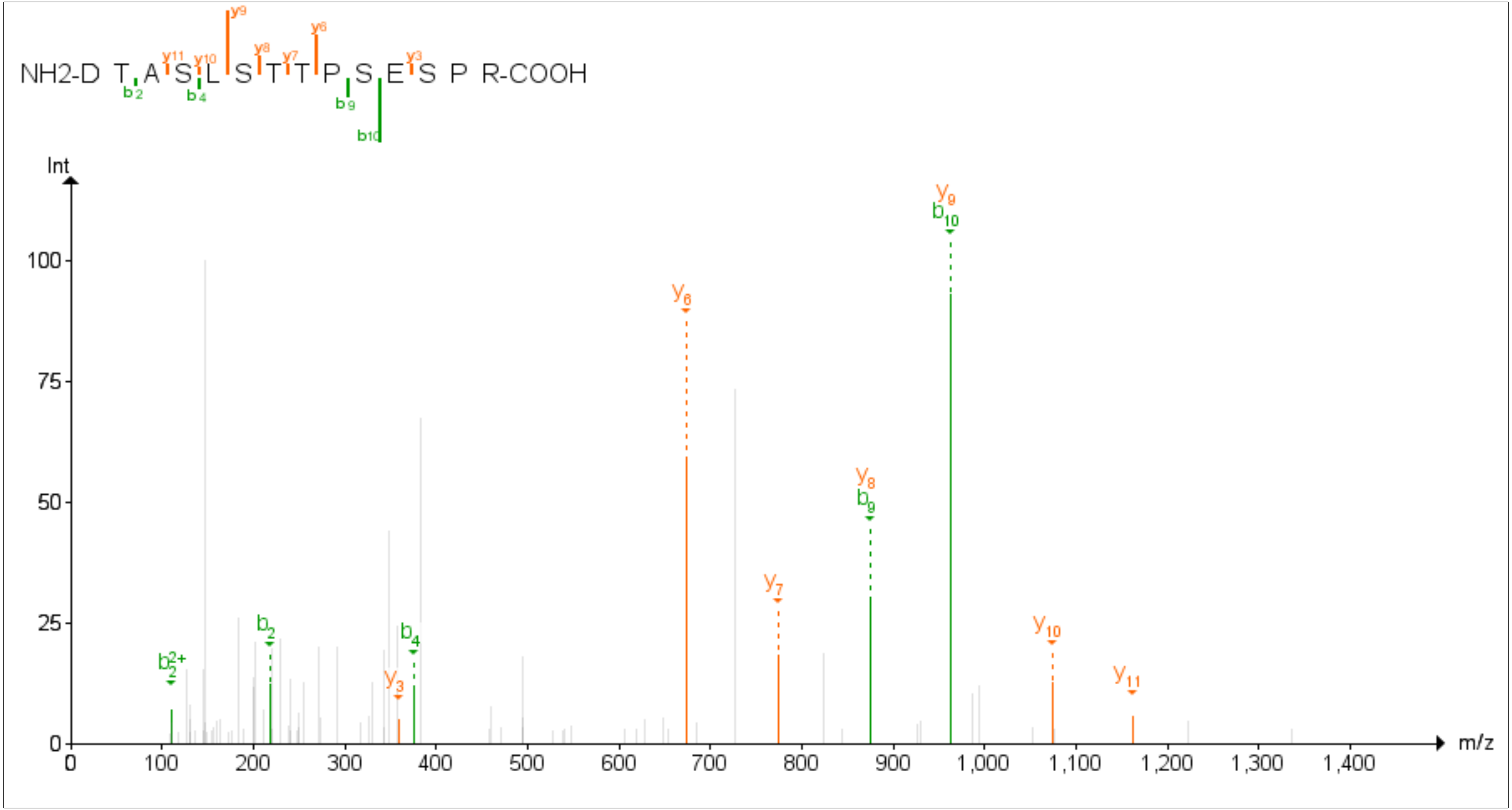

NH2-D T A S L S T T P S E S P R-COOH
y9 y6 y11 y10 y8 y7 y3
b2 b4 b9 b10
Int
100
75
50
25
0
y_9 b_{10}
y_6
y_8 b_9
y_7
y_{10}
y_{11}
b_2
b_2^{2+}
b_4
y_3
0 100 200 300 400 500 600 700 800 900 1,000 1,100 1,200 1,300 1,400
m/z

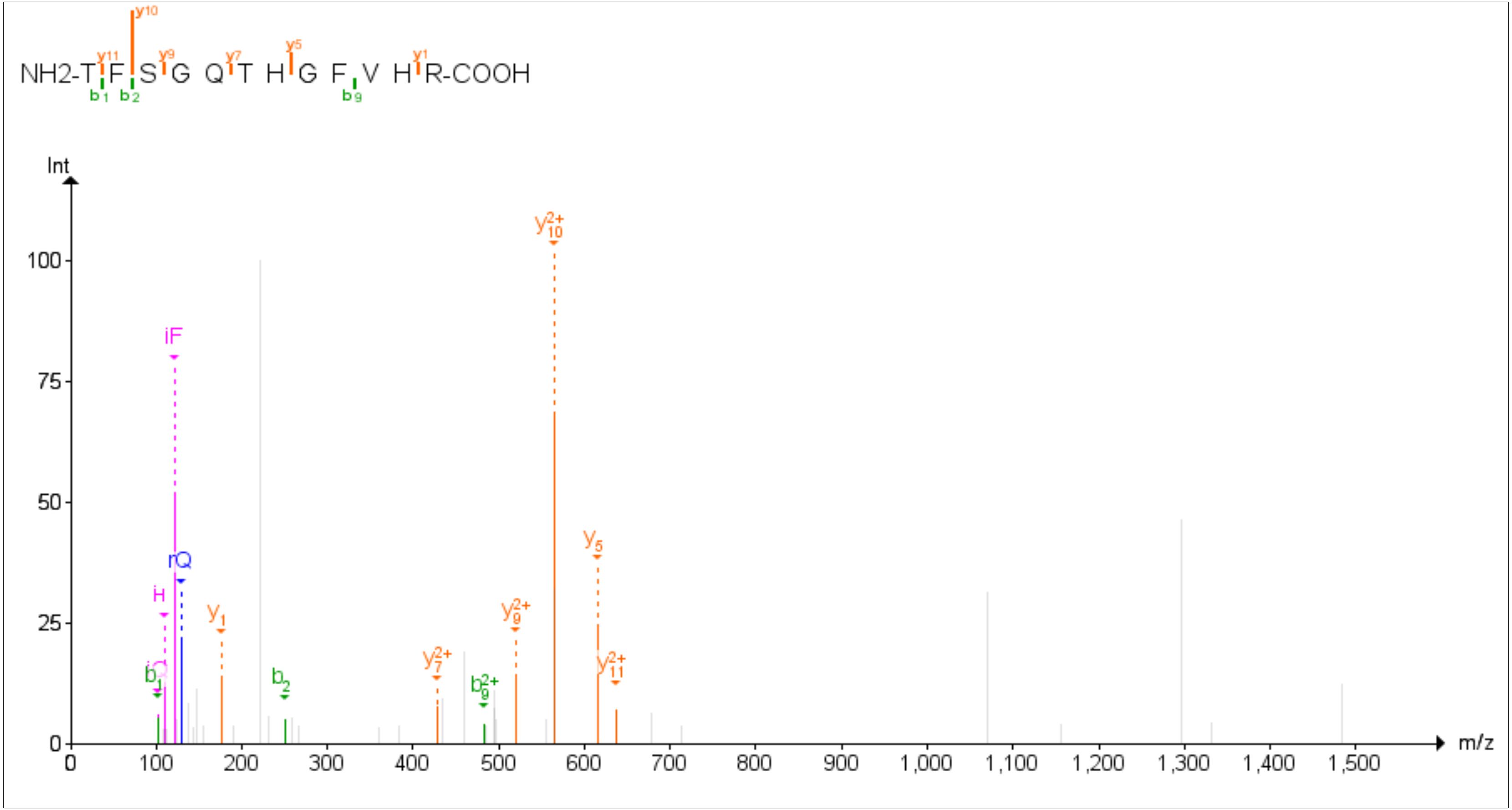

NH2-T I F S G Q T H G F V H R-COOH
y10
y11
y9
y7
y5
y1
b1
b2
b9
Int
100
75
50
25
0
iF
rQ
iH
iQ
b1
y1
b2
y7 2+
b9 2+
y9 2+
y10 2+
y5
y11 2+
m/z
0
100
200
300
400
500
600
700
800
900
1,000
1,100
1,200
1,300
1,400
1,500

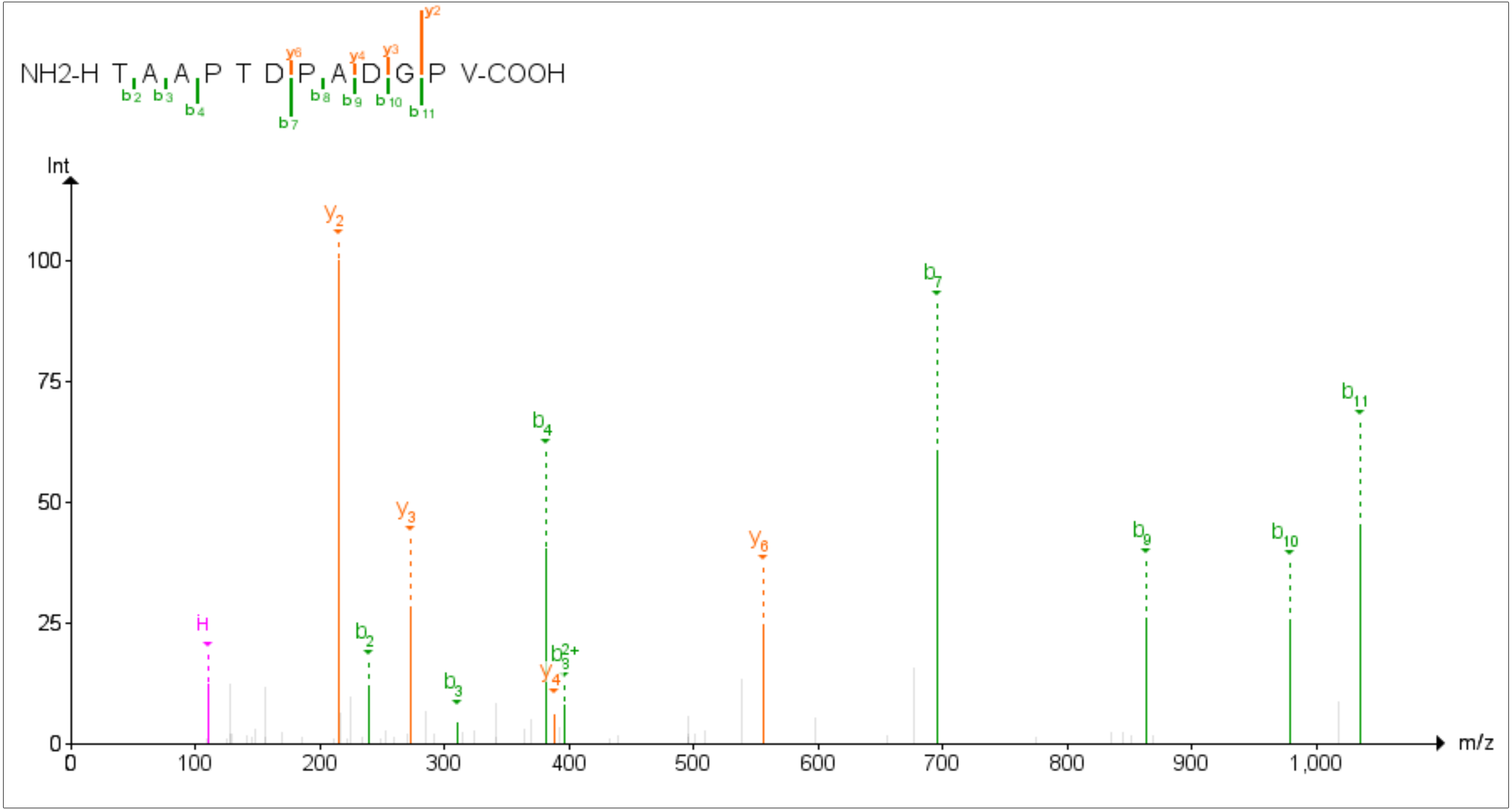

NH2-H T A A P T D P A D G P V-COOH
b2 b3 b4 b7 b8 b9 b10 b11
y6 y4 y3 y2
Int
100
75
50
25
0
m/z
0
100
200
300
400
500
600
700
800
900
1,000
y2
b7
b11
b4
y3
y6
b9
b10
H
b2
b3
y4
b8 2+

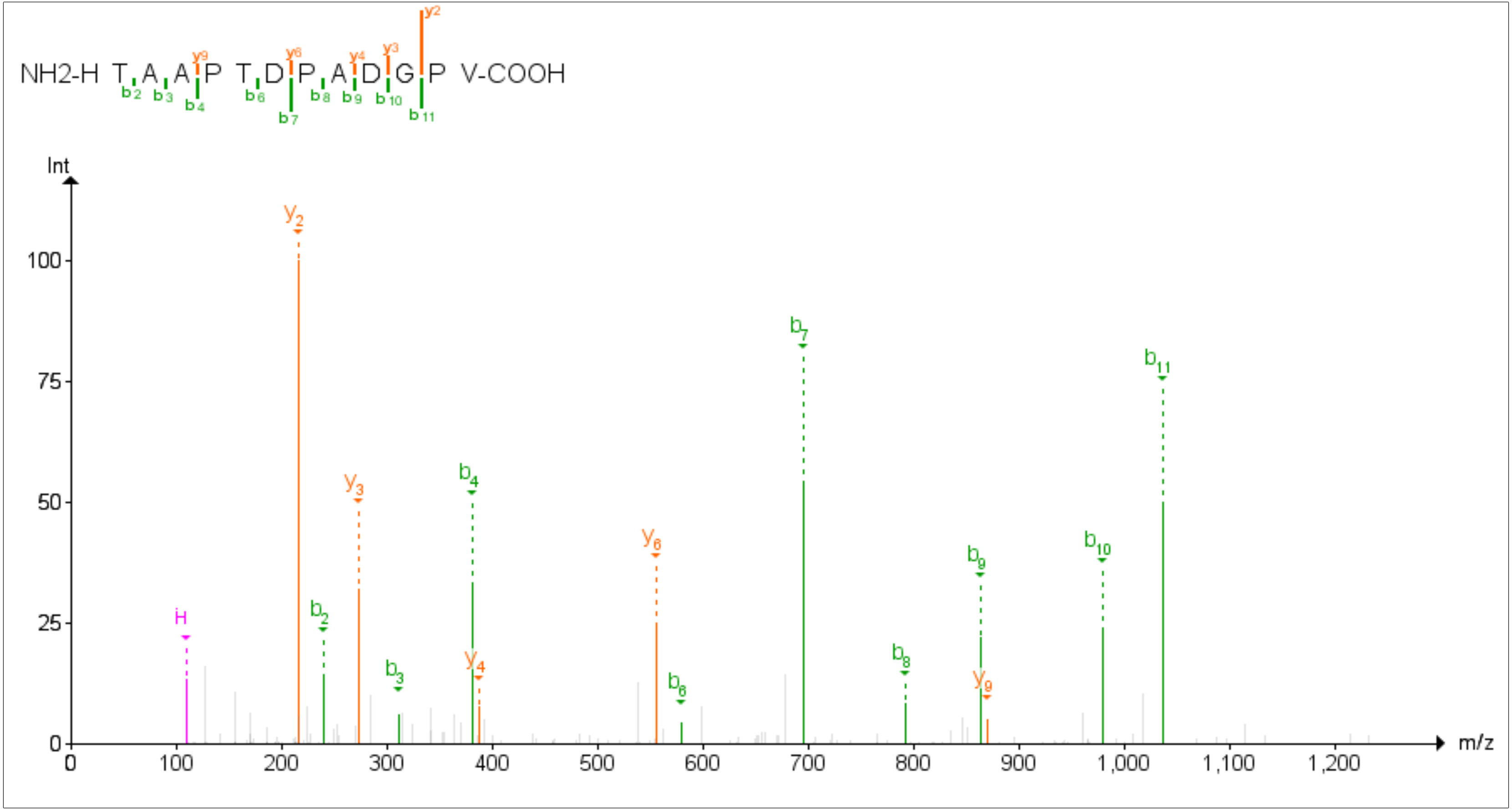

NH2-H T A A P T D P A D G P V-COOH
b2 b3 b4 b6 b7 b8 b9 b10 b11
y9 y6 y4 y3 y2
Int
m/z
0
25
50
75
100
0
100
200
300
400
500
600
700
800
900
1,000
1,100
1,200
H
y2
b2
y3
b3
b4
y4
y6
b6
b7
b8
b9
y9
b10
b11

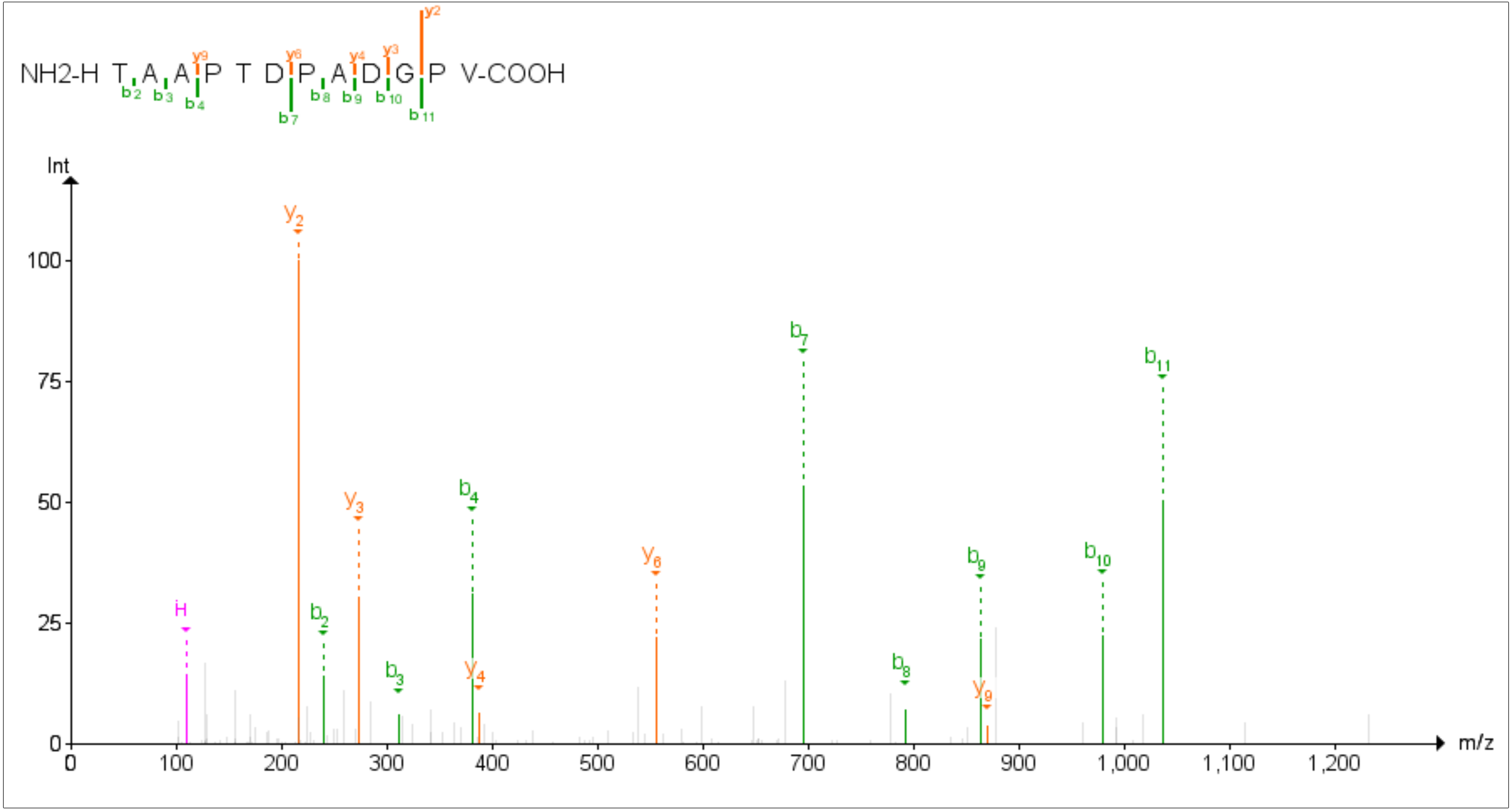

NH2-H T A A P T D P A D G P V-COOH
y9 y6 y4 y3 y2
b2 b3 b4 b7 b8 b9 b10 b11
Int
100
75
50
25
0
y2
y3
y4
y6
y9
b2
b3
b4
b7
b8
b9
b10
b11
H
0 100 200 300 400 500 600 700 800 900 1,000 1,100 1,200
m/z

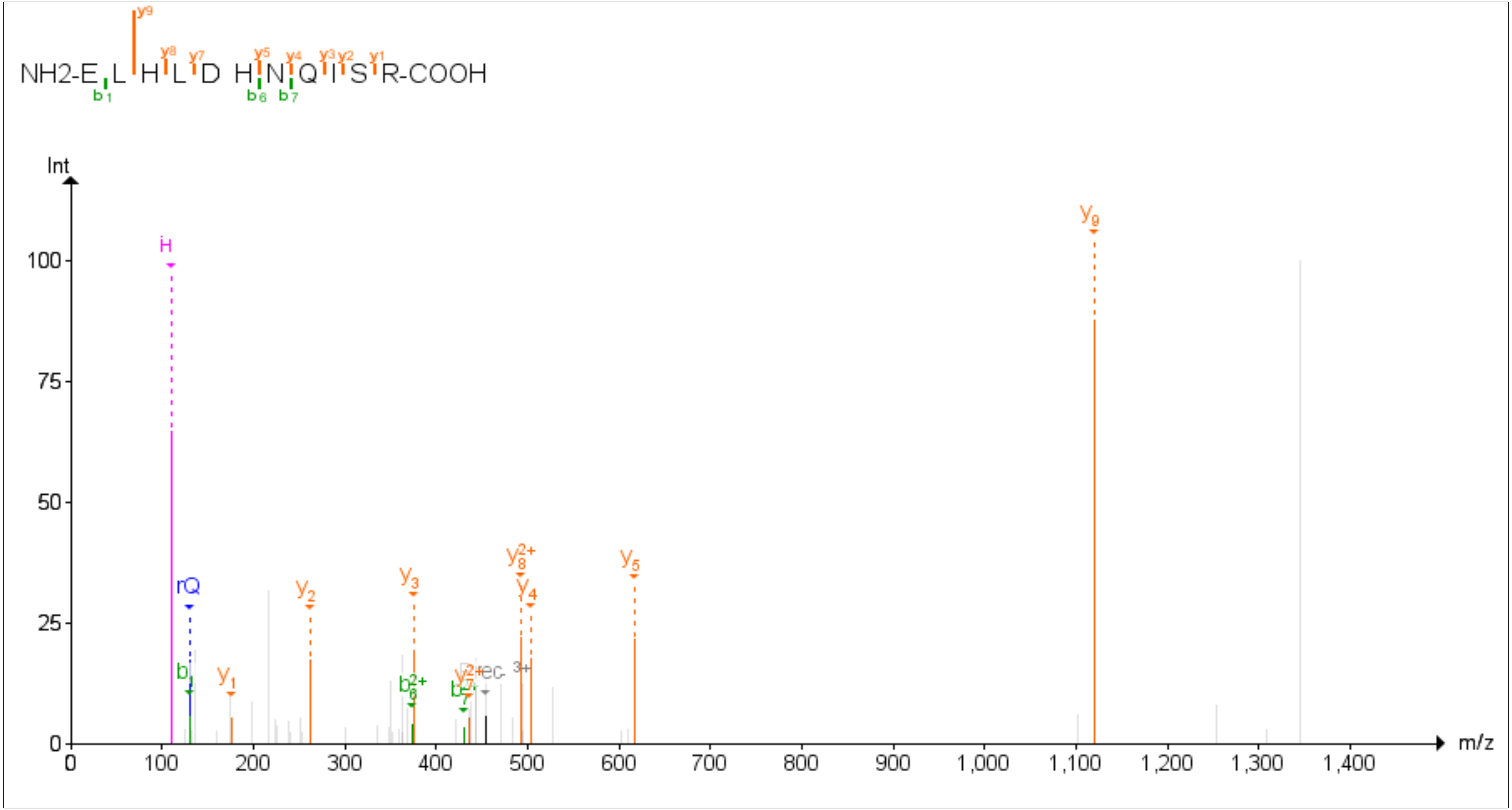
NH2-E L H L D H N Q I S R-COOH
y9 y8 y7 y5 y4 y3 y2 y1
b1 b6 b7
Int
m/z
iH
rQ
y9
y8 2+
y5
y4
y3
y2
y1
b6 2+
Prec 3+

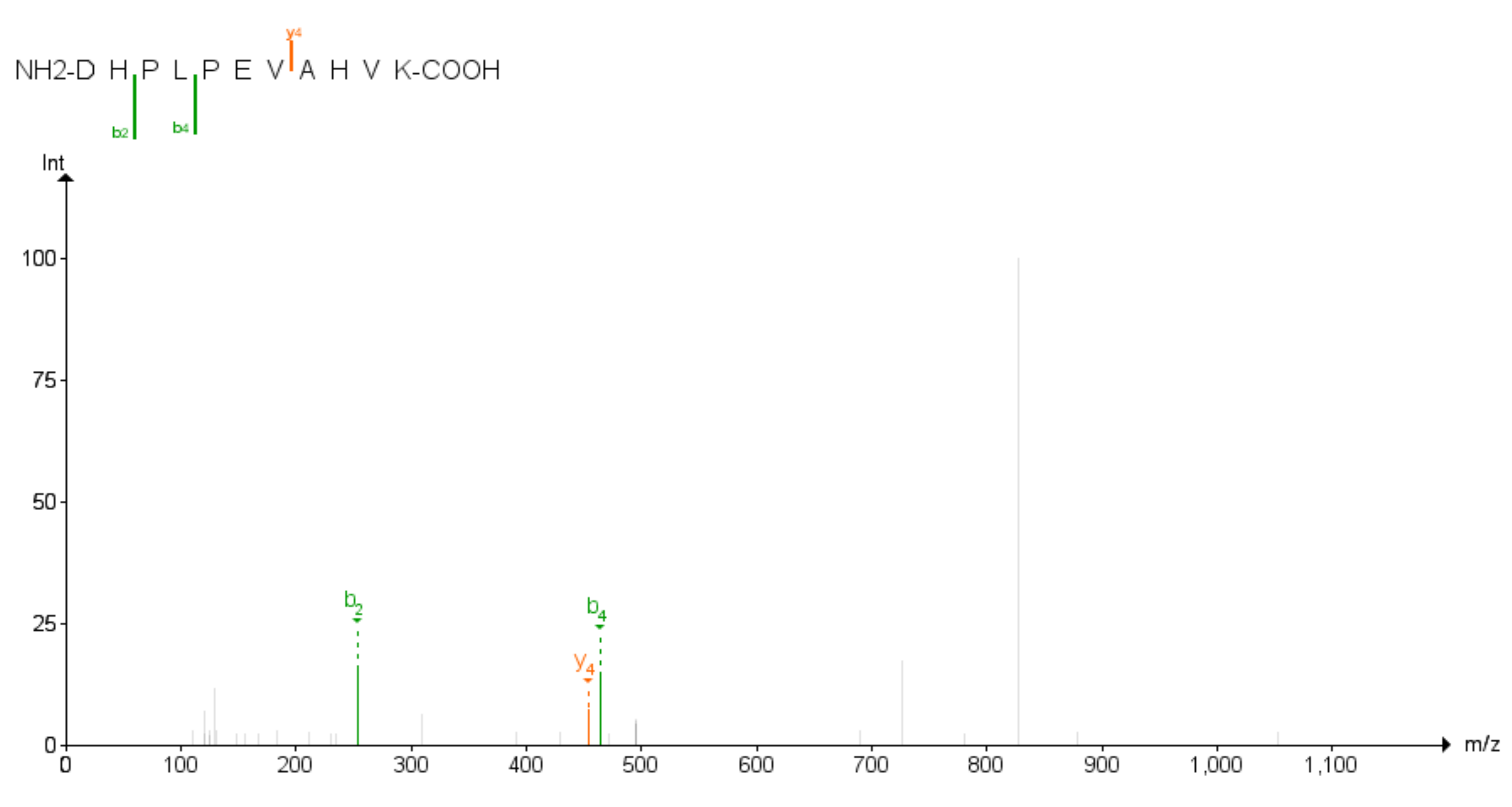

NH2-D H P L P E V A H V K-COOH
y4
b2
b4
Int
100
75
50
25
0
b2
b4
y4
0
100
200
300
400
500
600
700
800
900
1,000
1,100
m/z

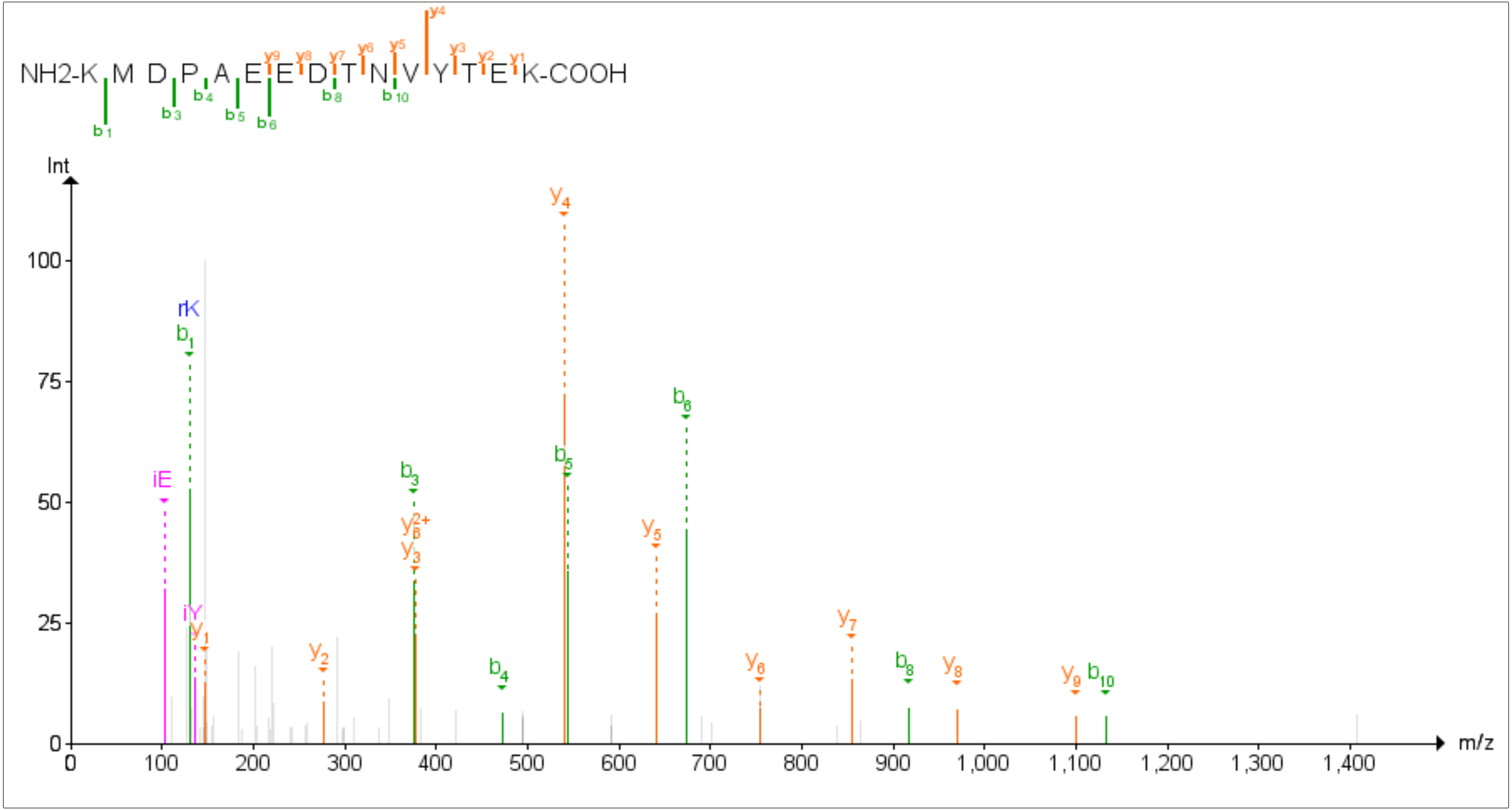

NH2-K M D P A E E D T N V Y T E K-COOH
b1 b3 b4 b5 b6 b8 b10
y9 y8 y7 y6 y5 y4 y3 y2 y1
Int
m/z
0
25
50
75
100
0
100
200
300
400
500
600
700
800
900
1,000
1,100
1,200
1,300
1,400
rK
b1
iE
iY
y1
y2
b3
y3
b4
y4
b5
y5
b6
y6
y7
b8
y8
y9
b10

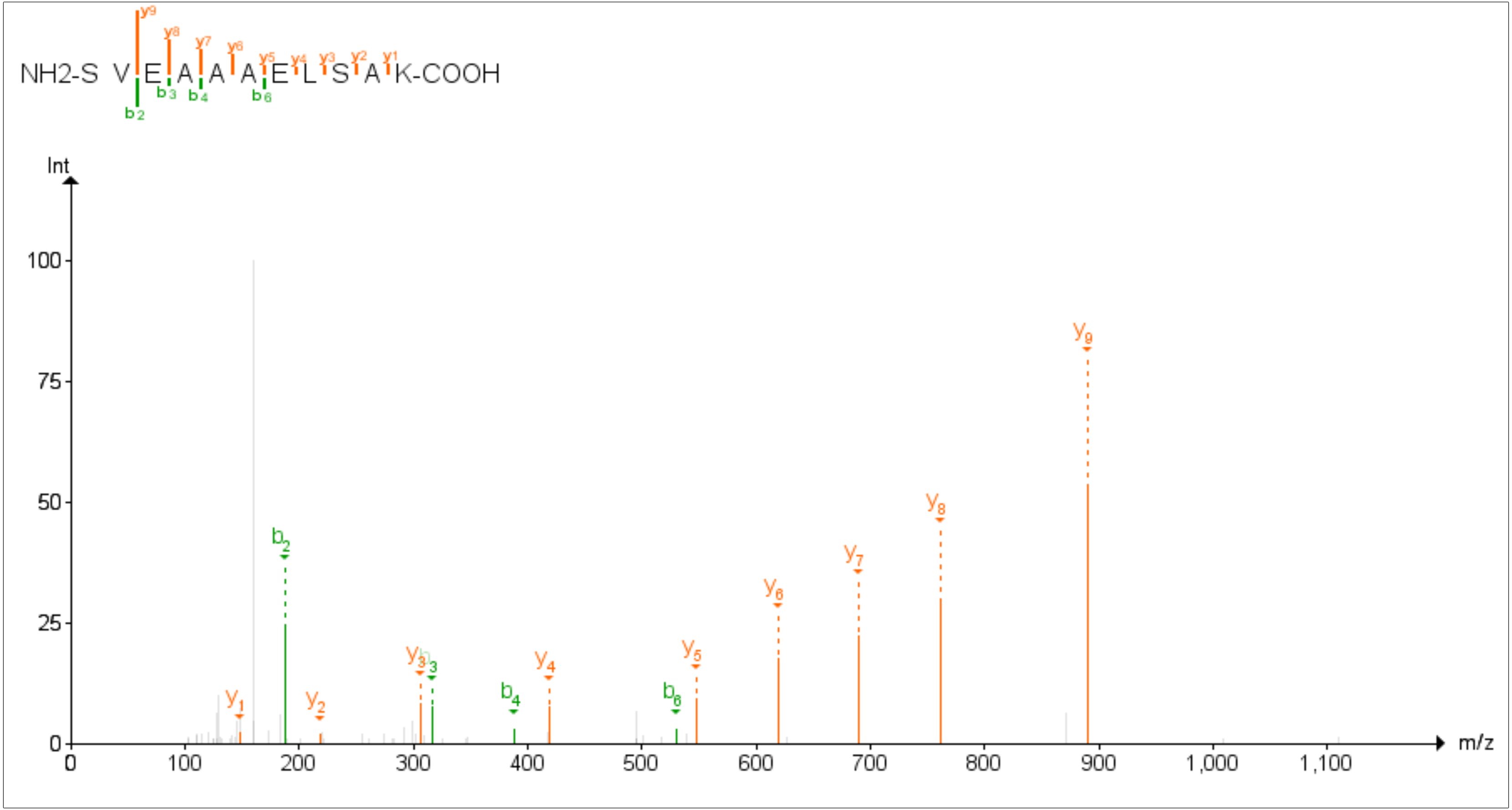

NH2-S V E A A A E L S A K-COOH
y9 y8 y7 y6 y5 y4 y3 y2 y1
b2 b3 b4 b6
Int
100
75
50
25
0
0 100 200 300 400 500 600 700 800 900 1,000 1,100
m/z
y1 y2 y3 y4 y5 y6 y7 y8 y9
b2 b3 b4 b6

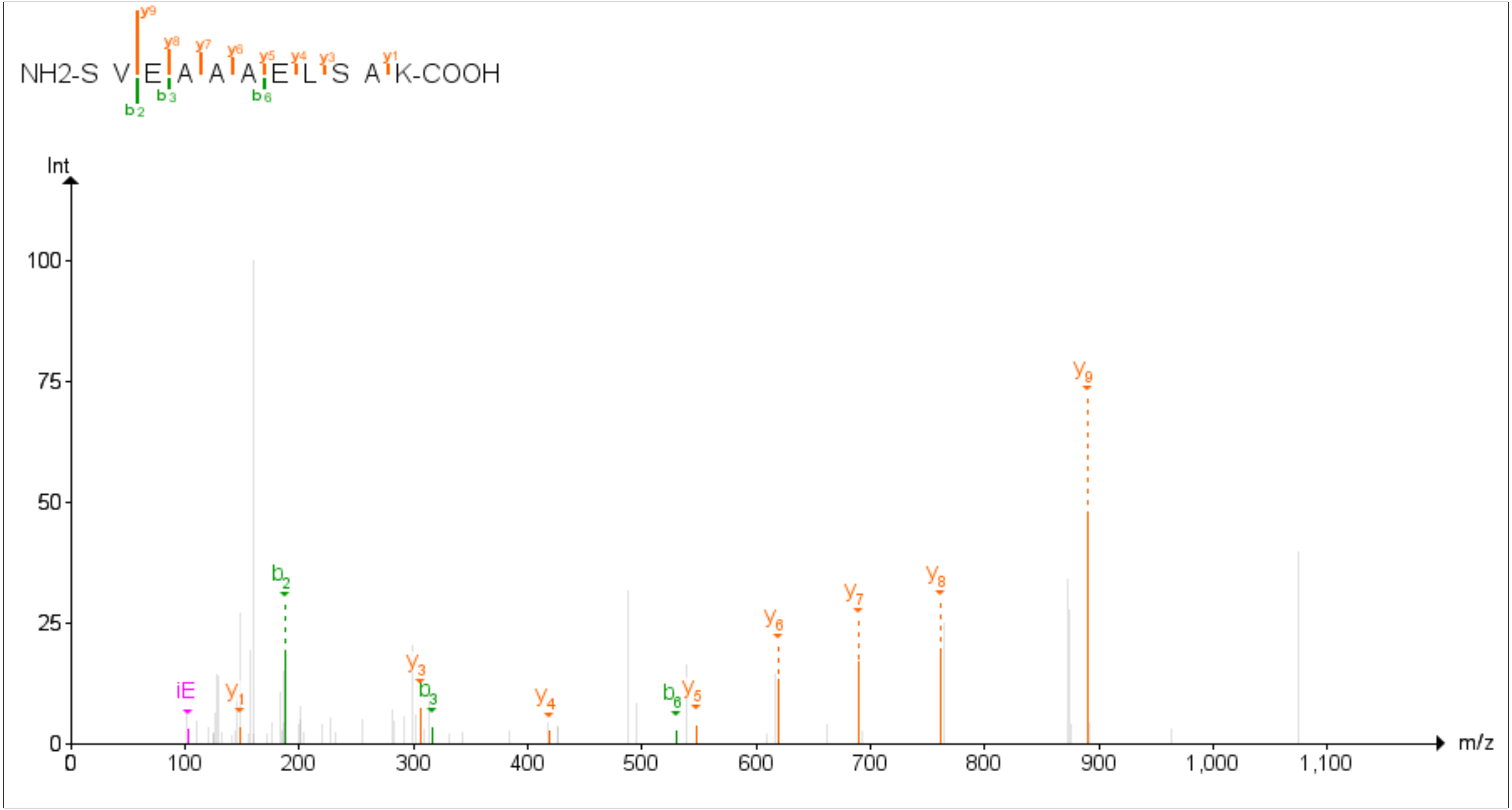

NH2-S V E A A A E L S A K-COOH
y9
y8
y7
y6
y5
y4
y3
y1
b2
b3
b6
Int
100
75
50
25
0
m/z
0
100
200
300
400
500
600
700
800
900
1,000
1,100
iE
y1
b2
y3
b3
y4
b6
y5
y6
y7
y8
y9

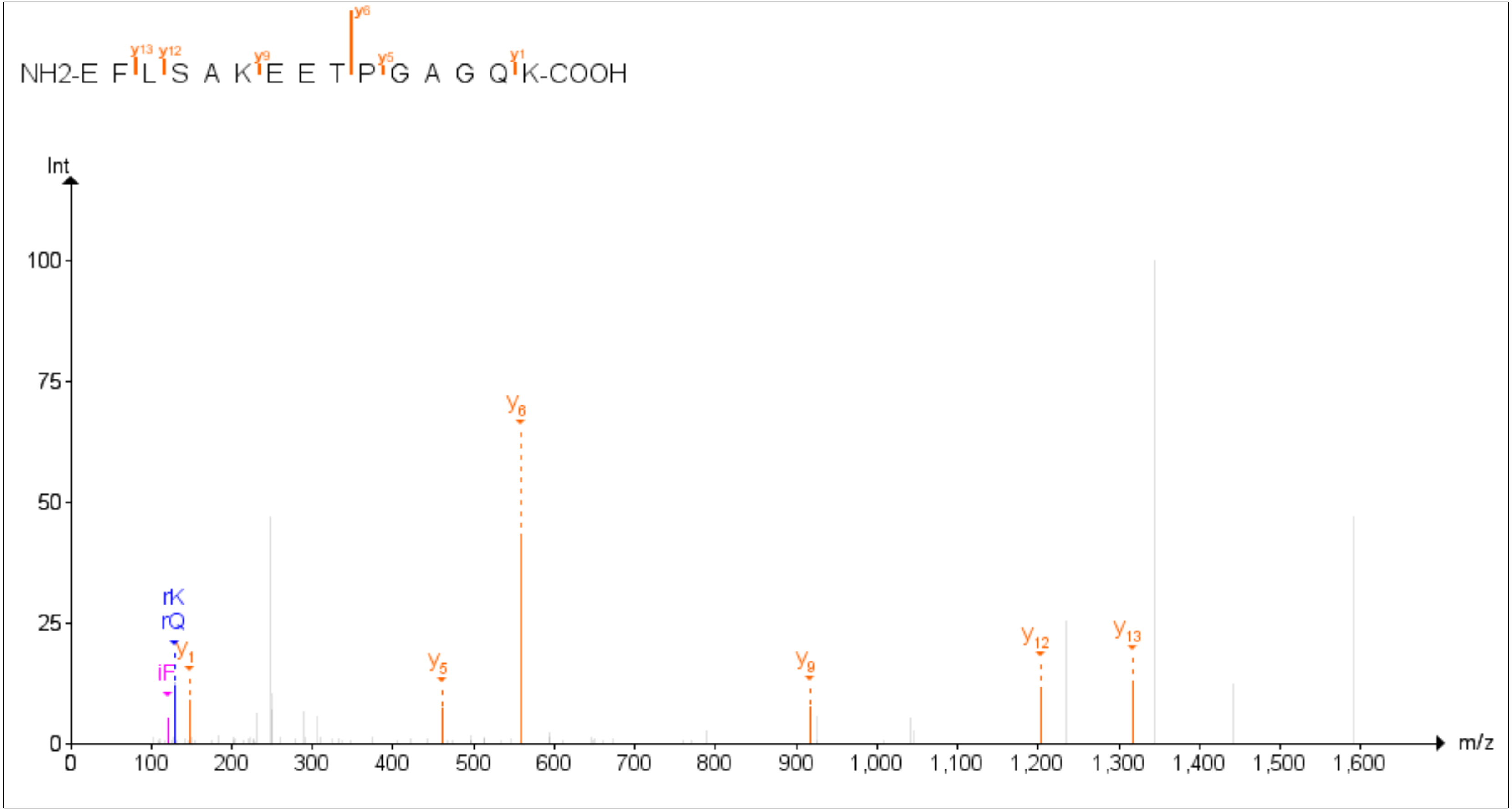

NH2-E F L S A K E E T P G A G Q K-COOH
y13
y12
y9
y6
y5
y1
Int
100
75
50
25
0
rK
rQ
iF
y1
y5
y6
y9
y12
y13
0
100
200
300
400
500
600
700
800
900
1,000
1,100
1,200
1,300
1,400
1,500
1,600
m/z

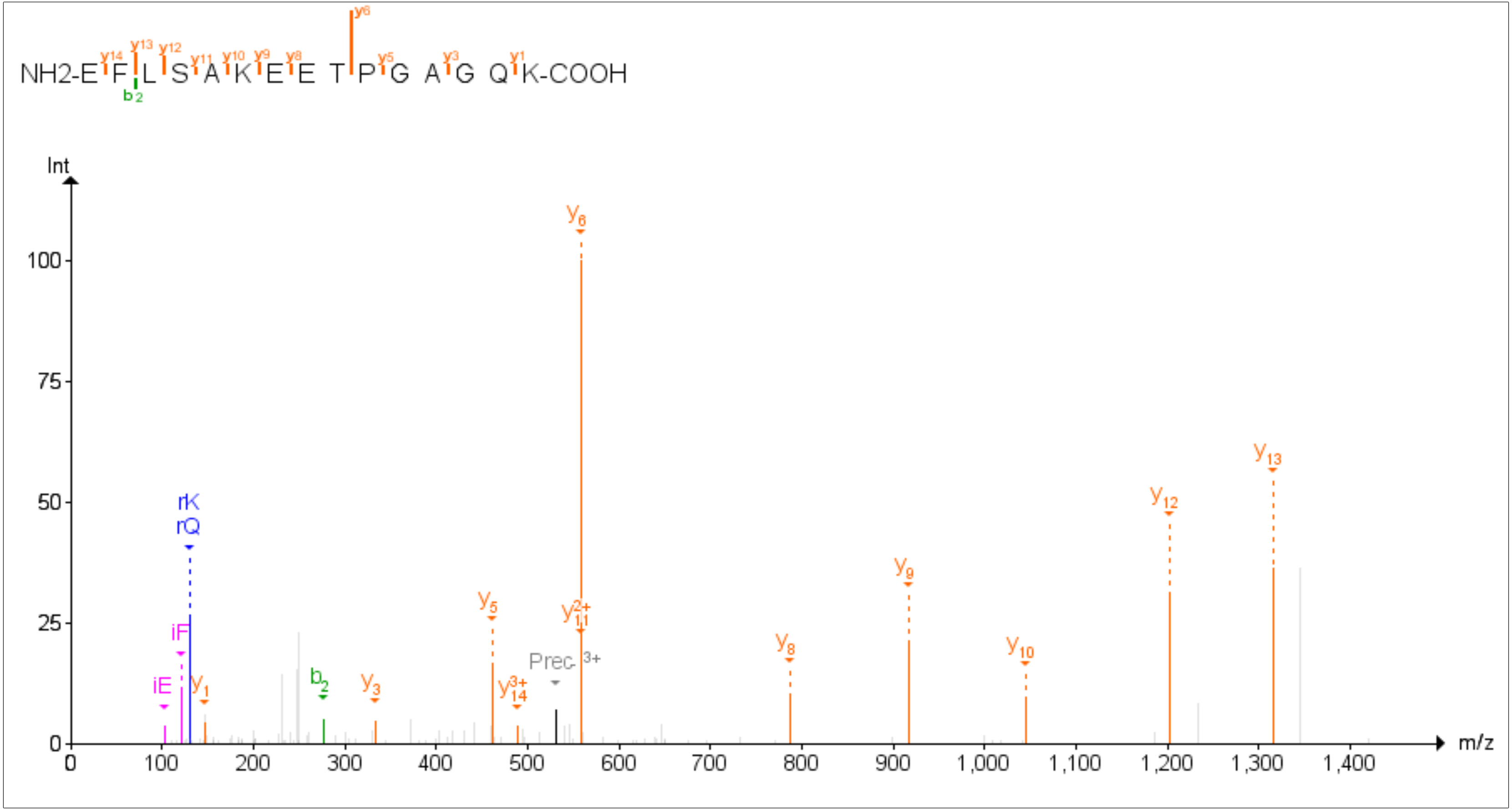

NH2-E F L S A K E E T P G A G Q K-COOH
y14 y13 y12 y11 y10 y9 y8 y6 y5 y3 y1
b2
Int
m/z
100
75
50
25
0
y6
rK
rQ
iF
iE
y1
b2
y3
y5
y_{14}^{3+}
Prec 3+
y_{11}^{2+}
y8
y9
y10
y12
y13
0
100
200
300
400
500
600
700
800
900
1,000
1,100
1,200
1,300
1,400

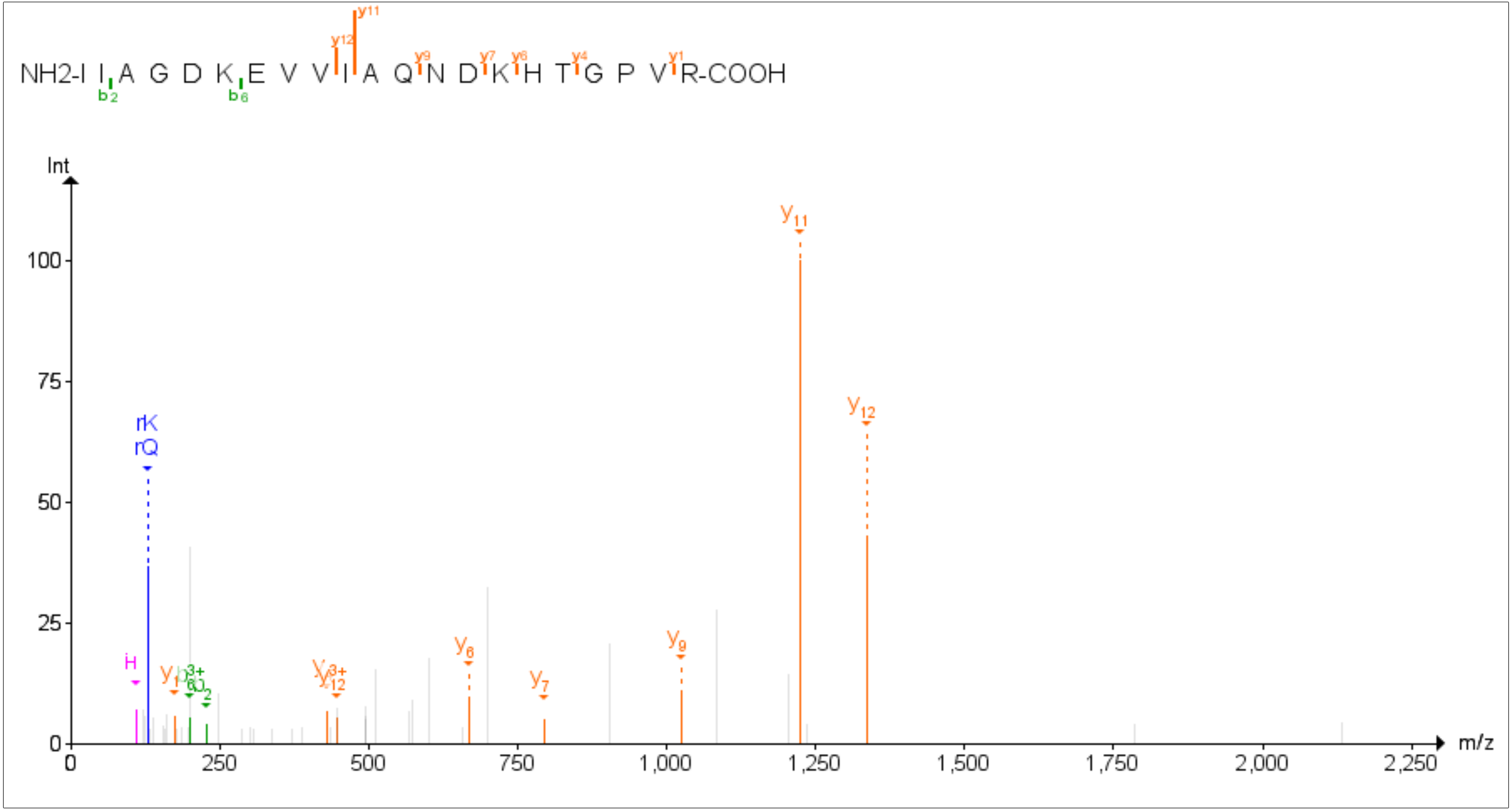

NH2-I I A G D K E V V I A Q N D K H T G P V R-COOH
b2
b6
y12
y11
y9
y7
y6
y4
y1
Int
m/z
rK
rQ
y11
y12
y6
y9
y7
iH
0
25
50
75
100
0
250
500
750
1,000
1,250
1,500
1,750
2,000
2,250

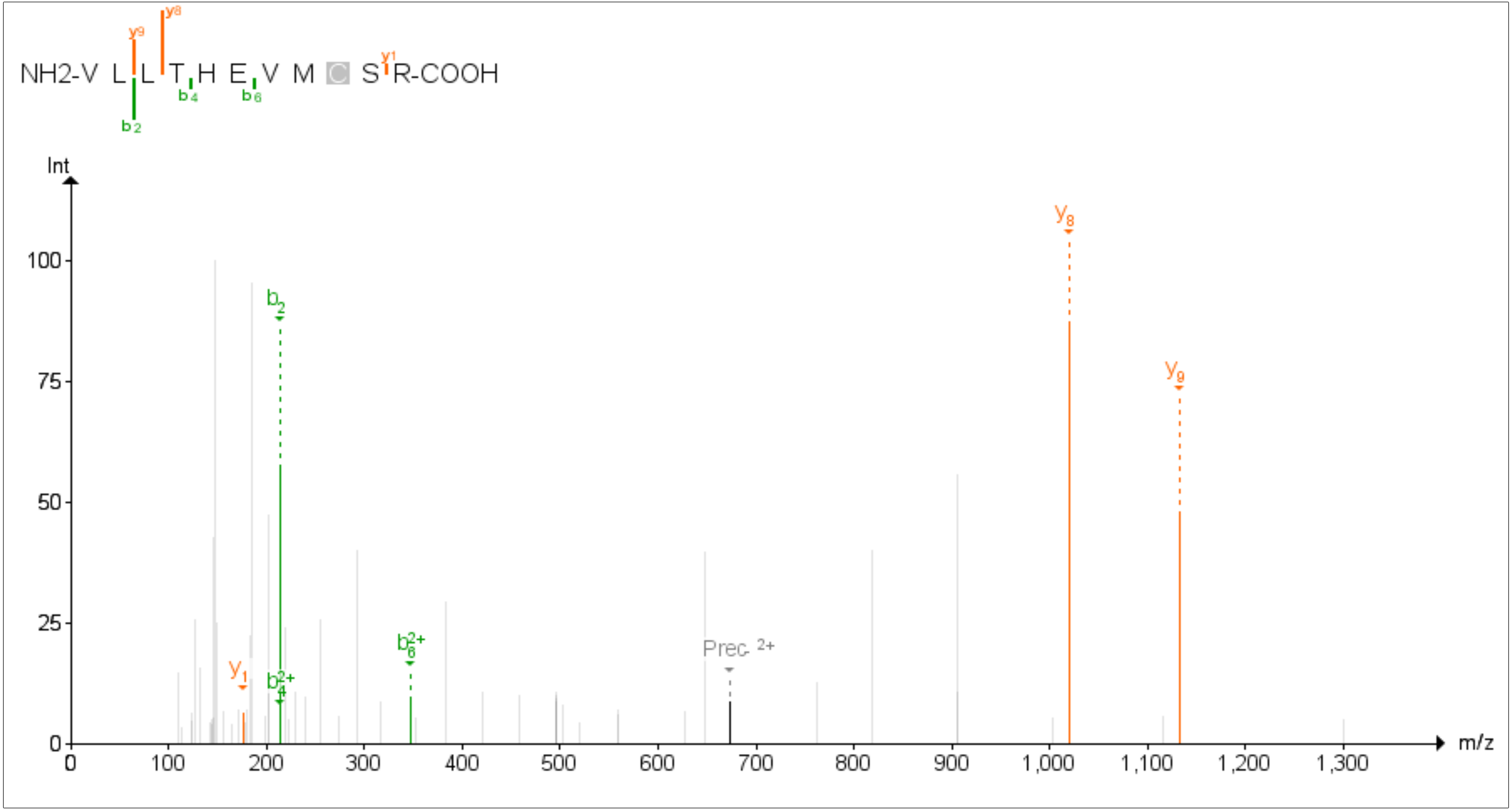

NH2-V L L T H E V M C S R-COOH
y9
y8
y1
b2
b4
b6
Int
100
75
50
25
0
y8
y9
b2
y1
b4 2+
b6 2+
Prec 2+
0
100
200
300
400
500
600
700
800
900
1,000
1,100
1,200
1,300
m/z

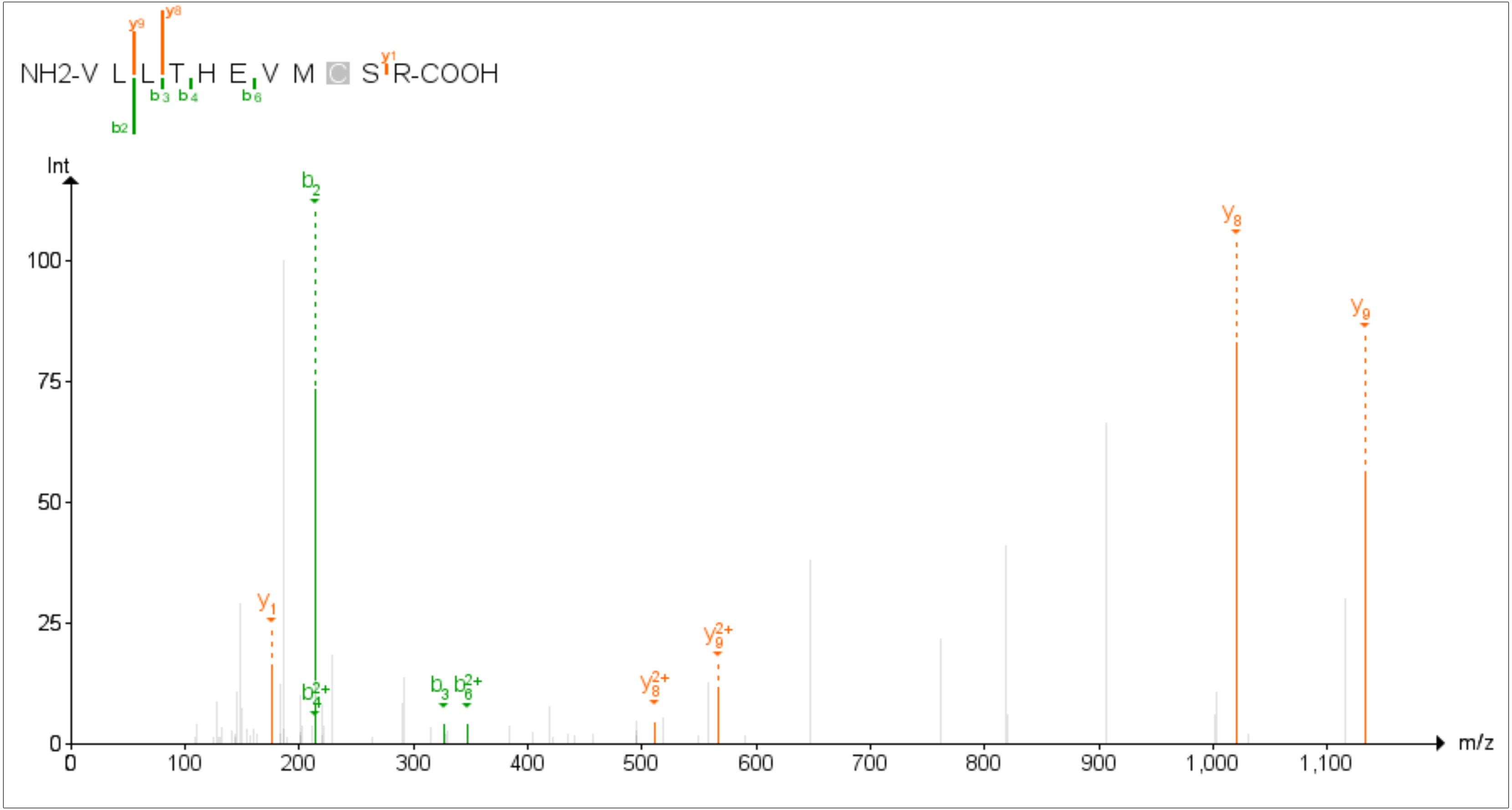

NH2-V L L T H E V M C S R-COOH
y9
y8
y1
b2
b3
b4
b6
Int
100
75
50
25
0
b2
y8
y9
y1
b4 2+
b3
b6 2+
y8 2+
y9 2+
0
100
200
300
400
500
600
700
800
900
1,000
1,100
m/z

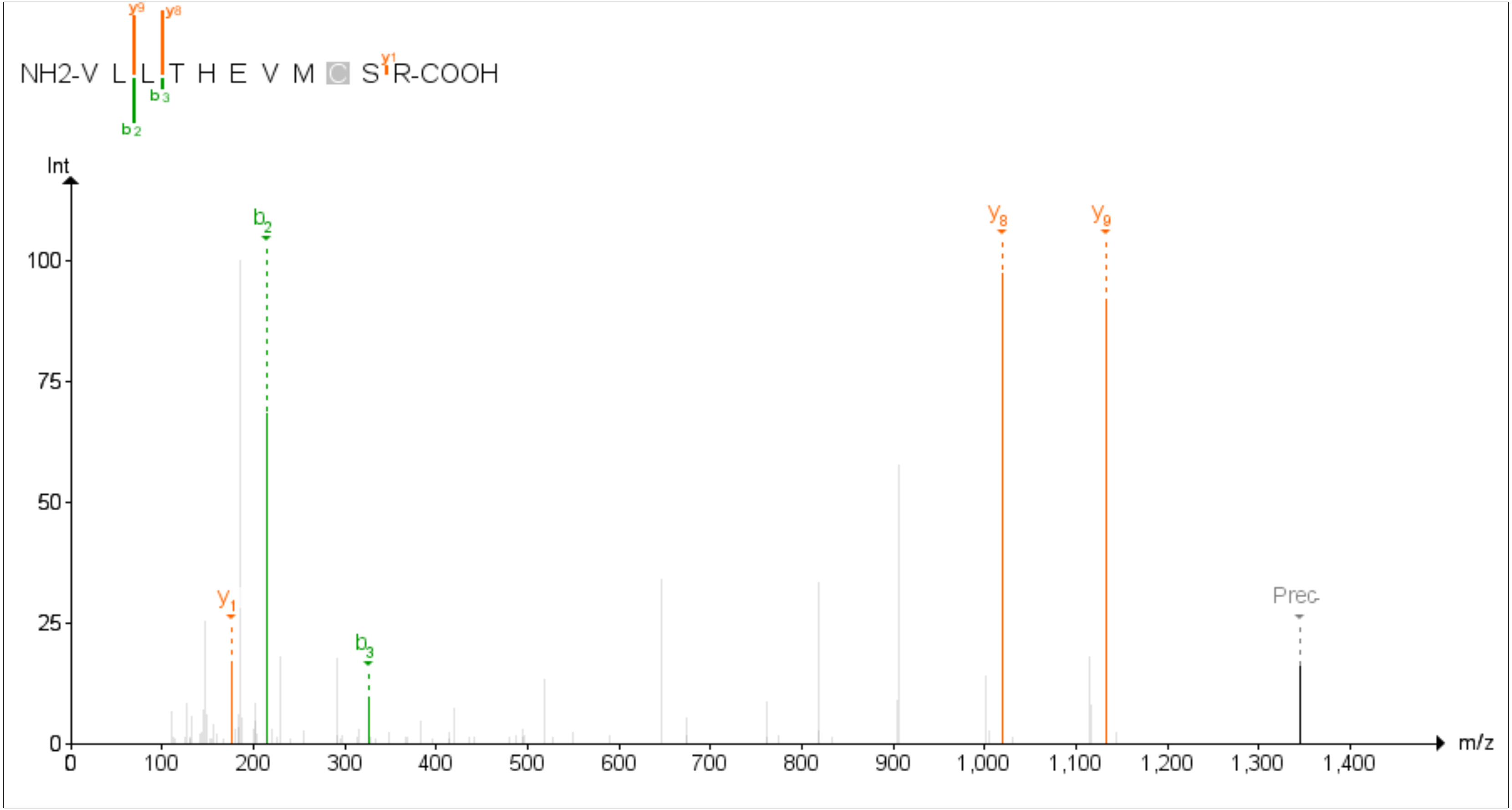

NH2-V L L T H E V M C S R-COOH
y9
y8
b2
b3
y1
Int
100
75
50
25
0
b2
y8
y9
y1
b3
Prec
0
100
200
300
400
500
600
700
800
900
1,000
1,100
1,200
1,300
1,400
m/z

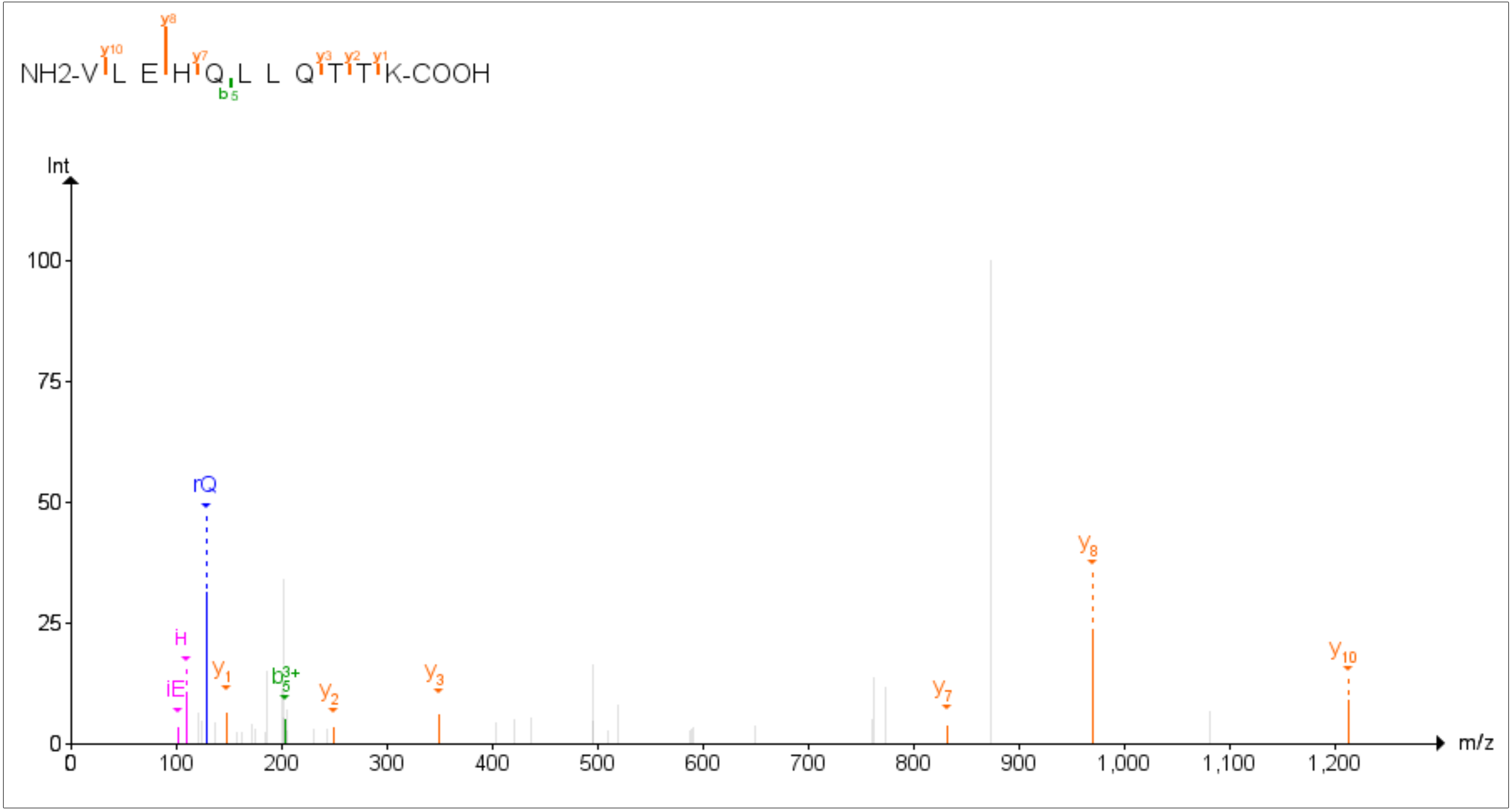

NH2-V L E H Q L L Q T T K-COOH
y10
y8
y7
y3
y2
y1
b5
Int
100
75
50
25
0
rQ
H
iE
y1
b5 3+
y2
y3
y7
y8
y10
0
100
200
300
400
500
600
700
800
900
1,000
1,100
1,200
m/z

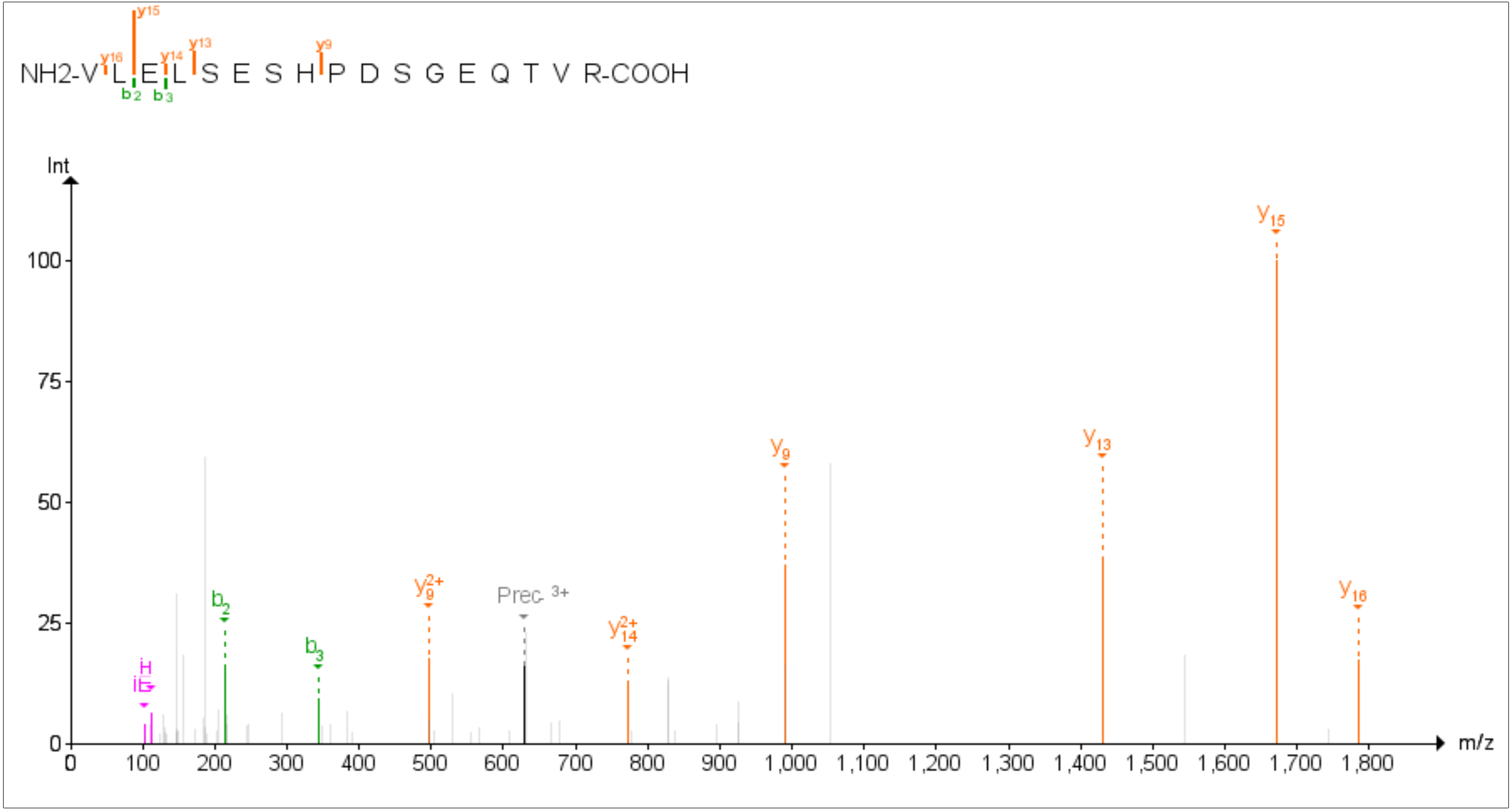
NH2-V L E L S E S H P D S G E Q T V R-COOH
y16
y15
y14
y13
y9
b2
b3
Int
m/z
Prec. 3+
y15
y13
y9
y16
b2
b3
y_9^{2+}
y_{14}^{2+}
iH
iE
0
25
50
75
100
0
100
200
300
400
500
600
700
800
900
1,000
1,100
1,200
1,300
1,400
1,500
1,600
1,700
1,800

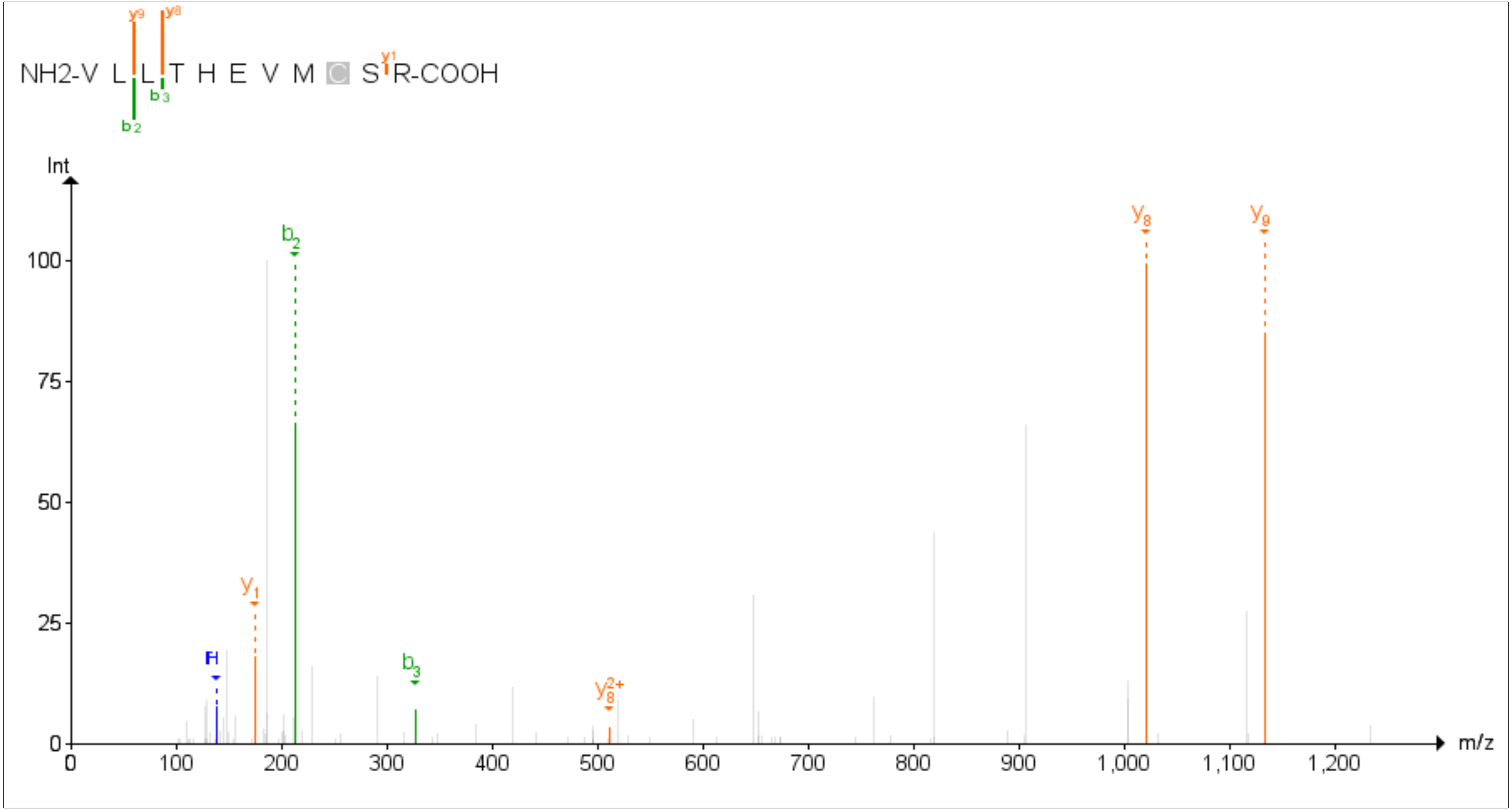

NH2-V L L T H E V M C S R-COOH
y9
y8
y1
b2
b3
Int
100
75
50
25
0
b2
y8
y9
y1
H
b3
y8 2+
0
100
200
300
400
500
600
700
800
900
1,000
1,100
1,200
m/z

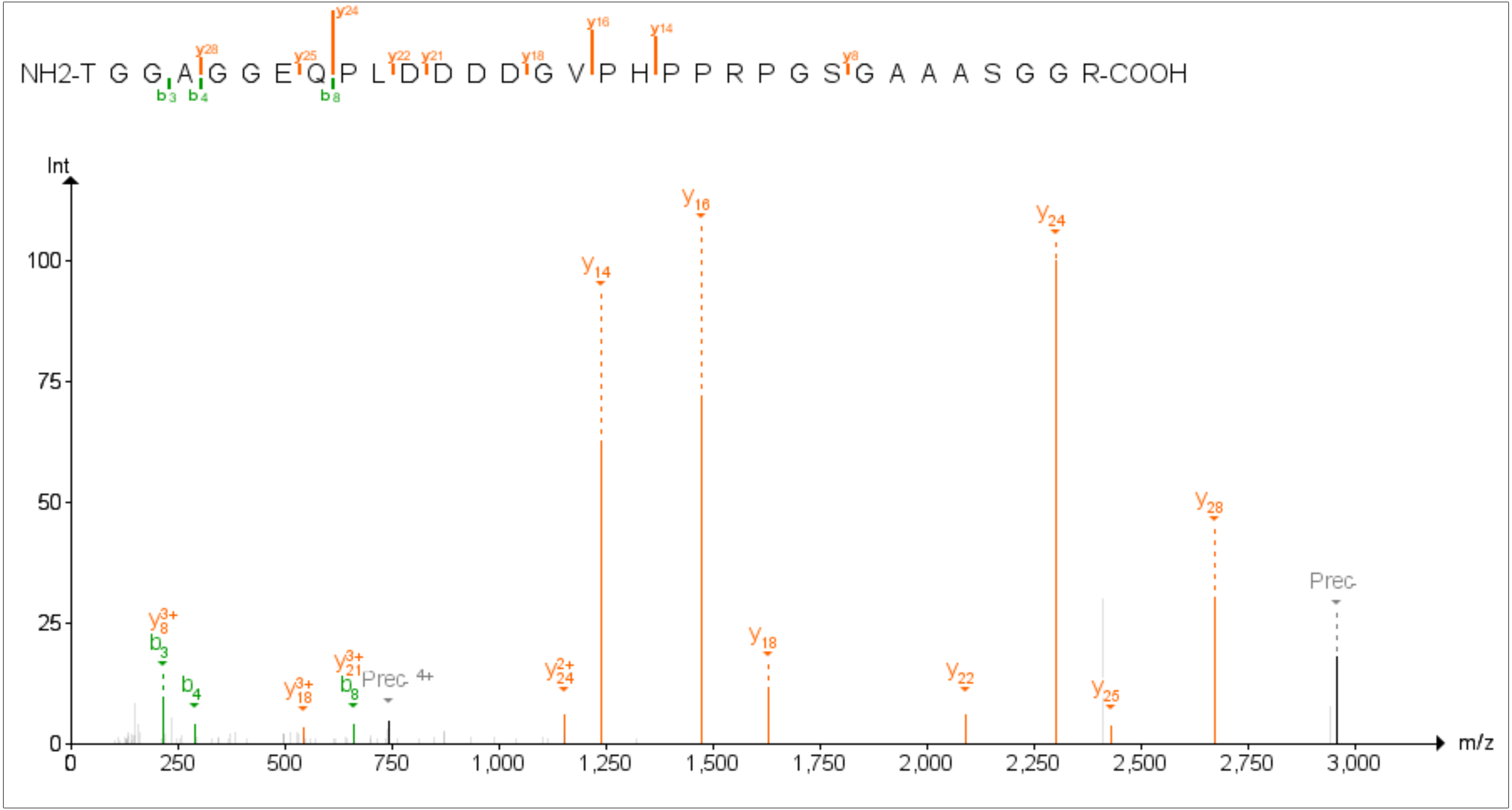

NH2-T G G A G G E Q P L D D D D D G V P H P P R P G S G A A A S G G R-COOH
y24 y16 y14 y28 y25 y22 y21 y18 y8
b3 b4 b8
Int
100
75
50
25
0
m/z
0 250 500 750 1,000 1,250 1,500 1,750 2,000 2,250 2,500 2,750 3,000
y16
y24
y14
y28
Prec
y8 3+
b3
b4
y18 3+
y21 3+
b8
Prec 4+
y24 2+
y18
y22
y25

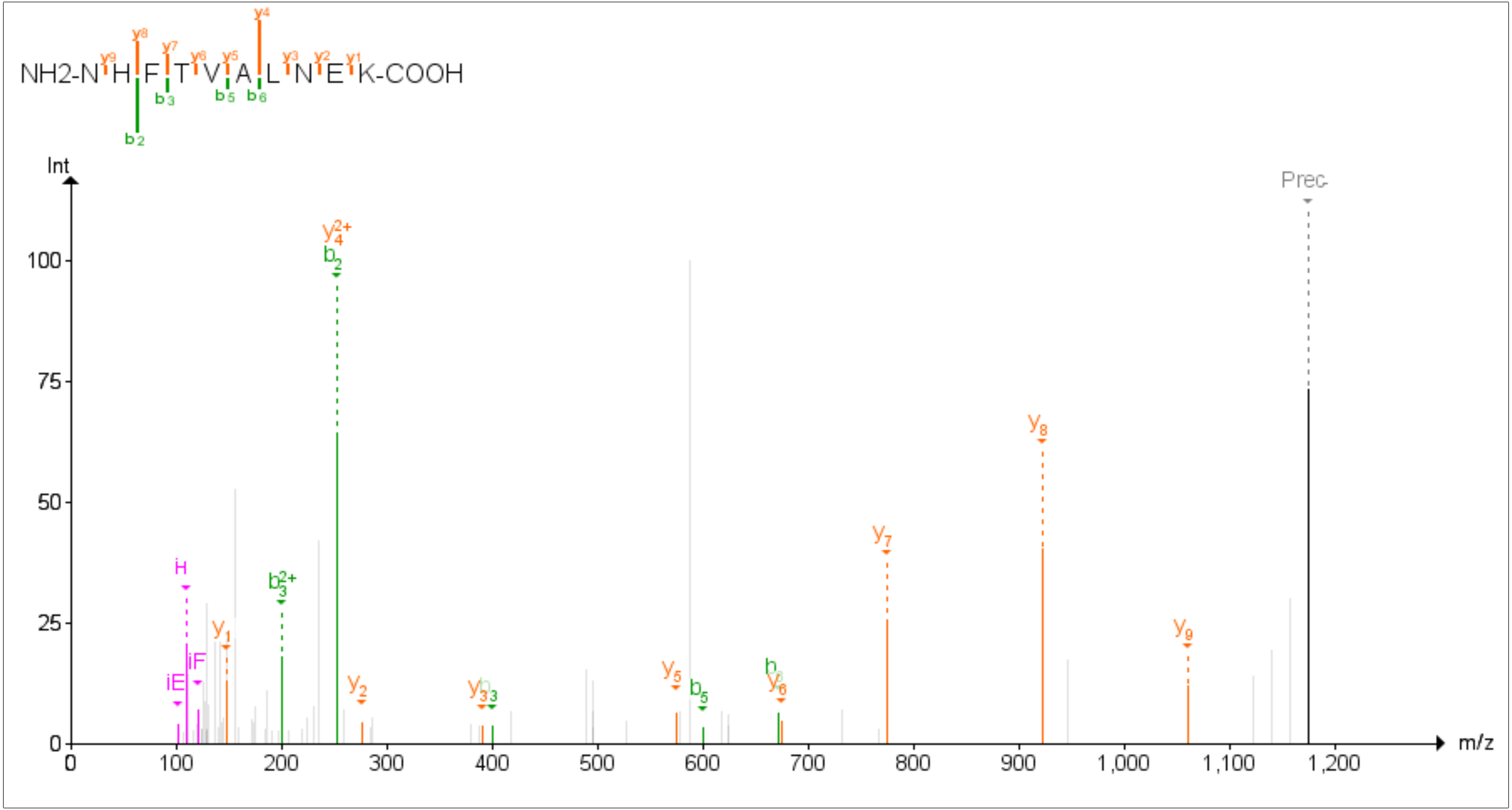
NH2-N H F T V A L N E K-COOH
y9 y8 y7 y6 y5 y4 y3 y2 y1
b2 b3 b5 b6
Int
m/z
Prec
y4 2+
b2
b3 2+
y1
y2
y3 b3
y5
b5
b6 y6
y7
y8
y9
iH
iF
iE
0
25
50
75
100
0
100
200
300
400
500
600
700
800
900
1,000
1,100
1,200

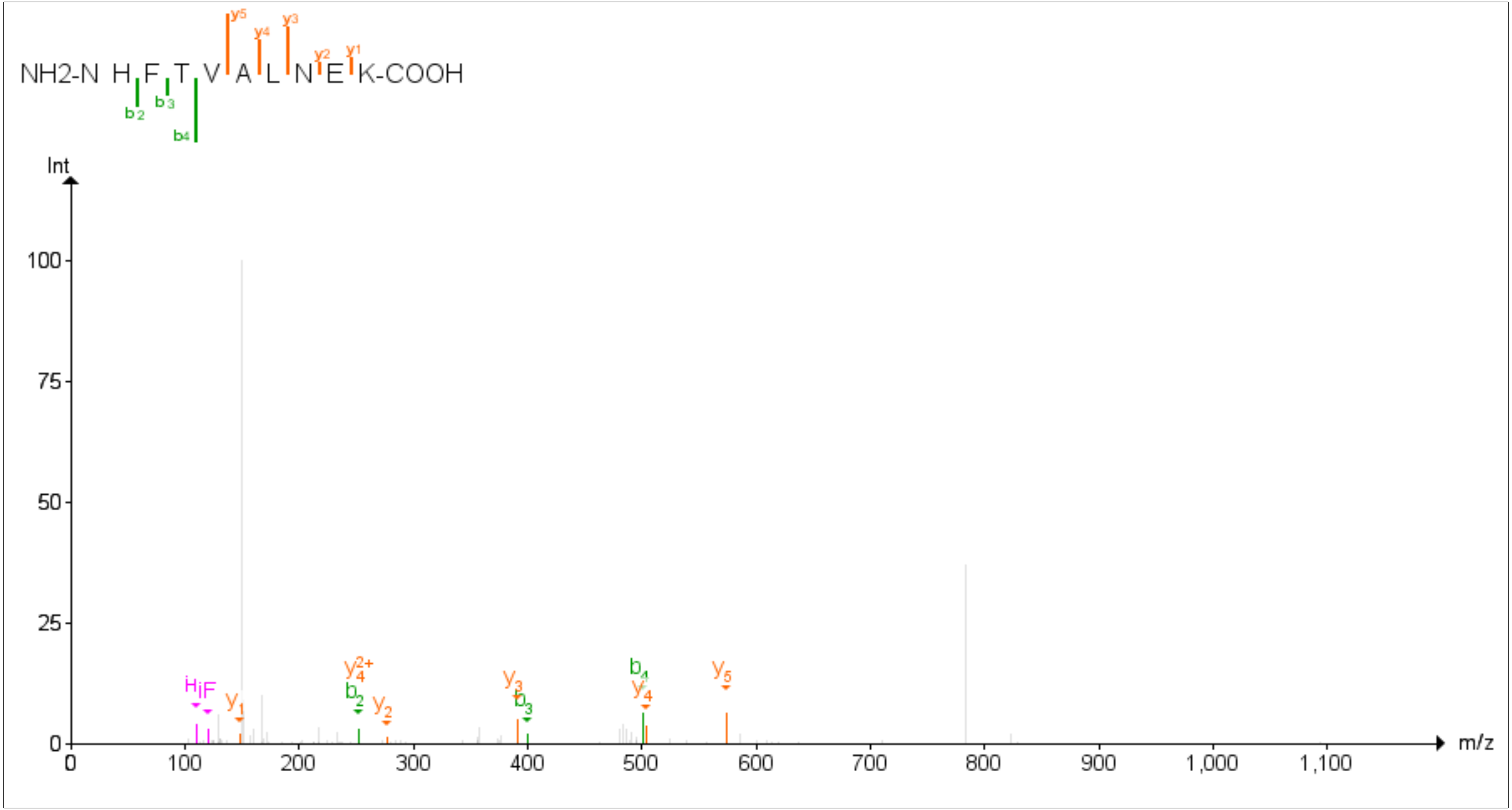

NH2-N H F T V A L N E K-COOH
y5 y4 y3 y2 y1
b2 b3 b4
Int
100
75
50
25
0
m/z
0
100
200
300
400
500
600
700
800
900
1,000
1,100

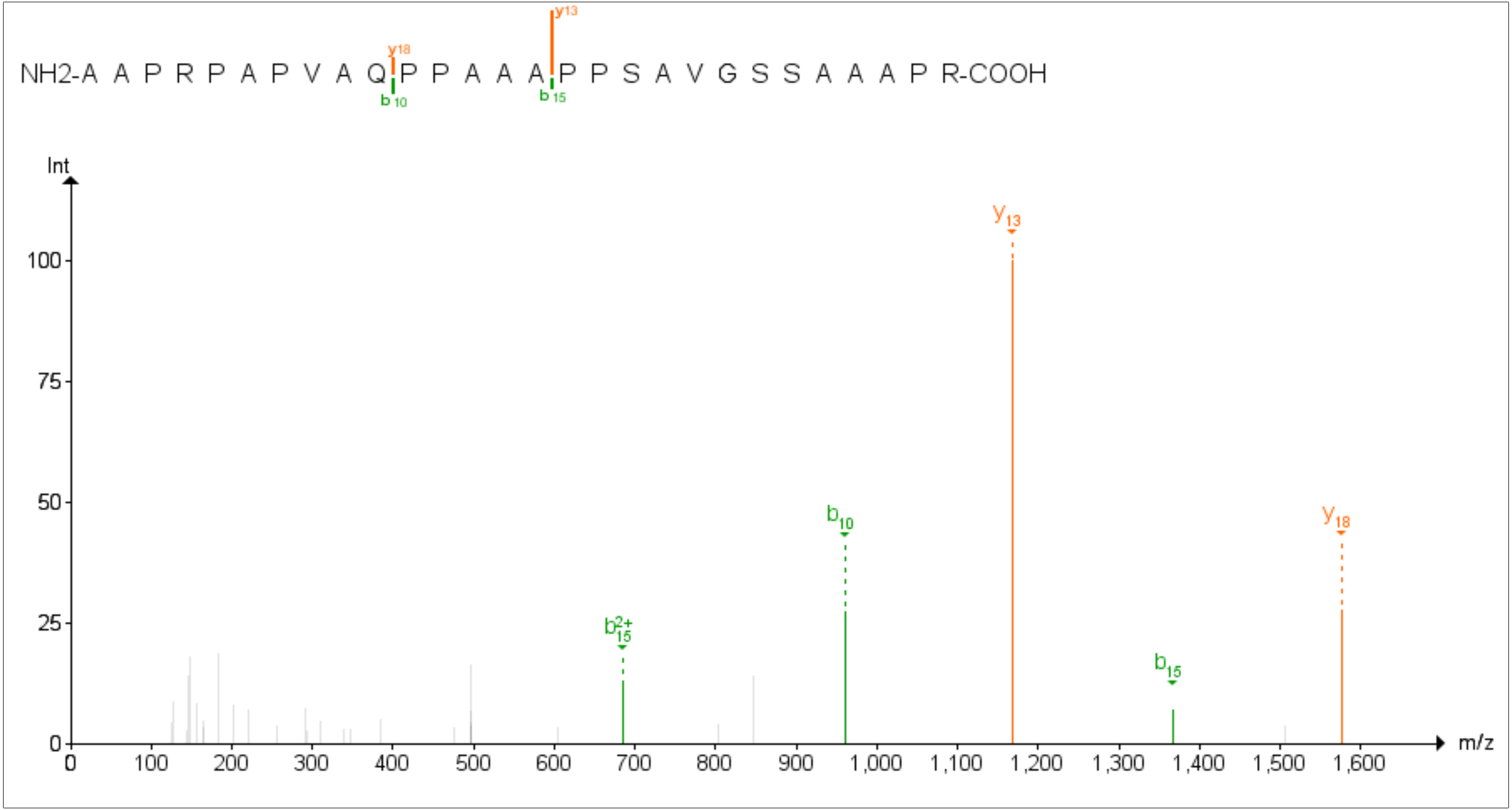

NH2-A A P R P A P V A Q P P A A A P P S A V G S S A A A P R-COOH
y18
y13
b 10
b 15
Int
100
75
50
25
0
m/z
0
100
200
300
400
500
600
700
800
900
1,000
1,100
1,200
1,300
1,400
1,500
1,600
y_{13}
b_{10}
y_{18}
b_{15}^{2+}
b_{15}

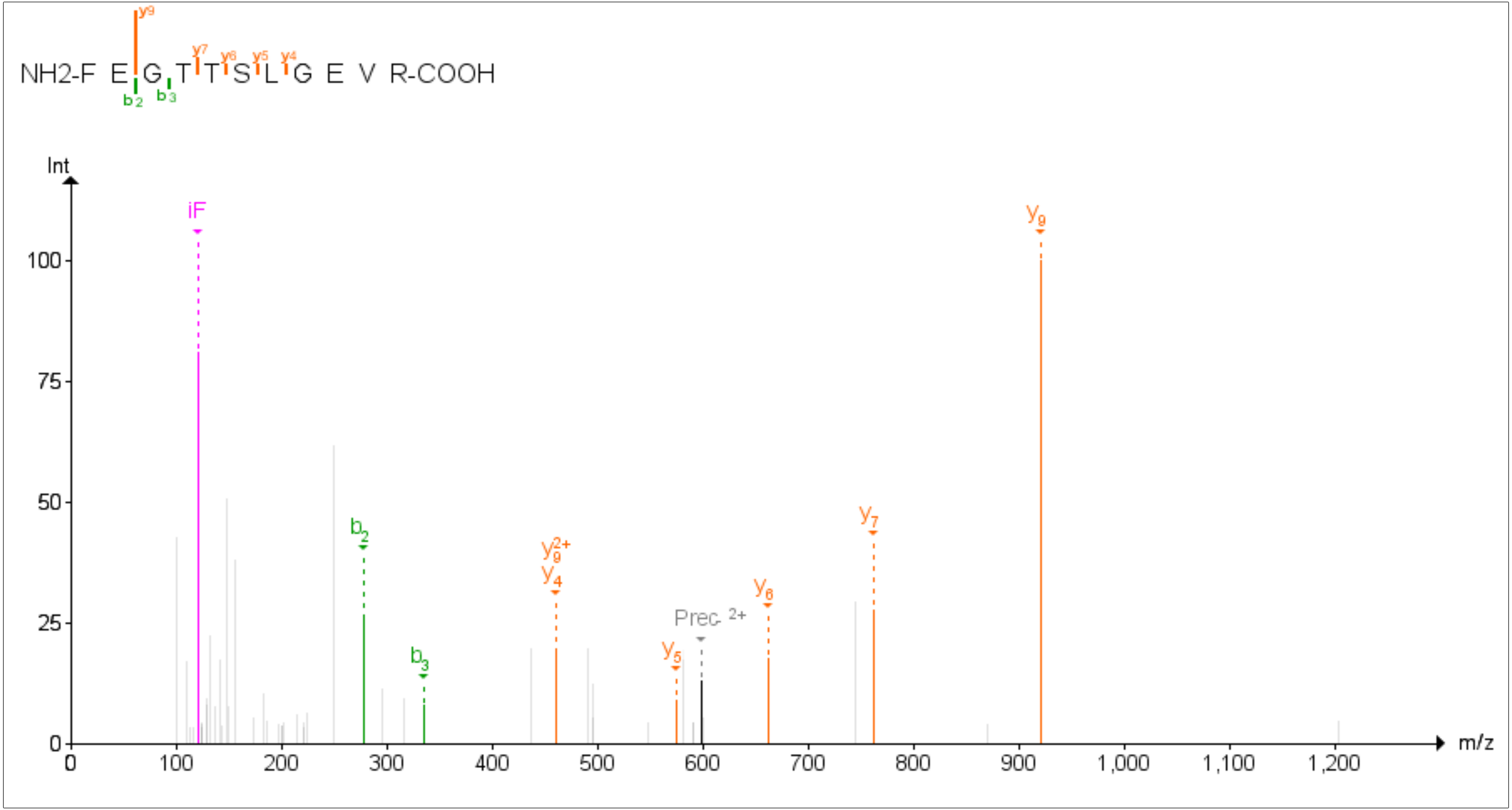
NH2-F E G T T S L G E V R-COOH
y9
y7
y6
y5
y4
b2
b3
Int
iF
y9
b2
b3
y9 2+
y4
y5
Prec 2+
y6
y7
m/z
0
25
50
75
100
0
100
200
300
400
500
600
700
800
900
1,000
1,100
1,200

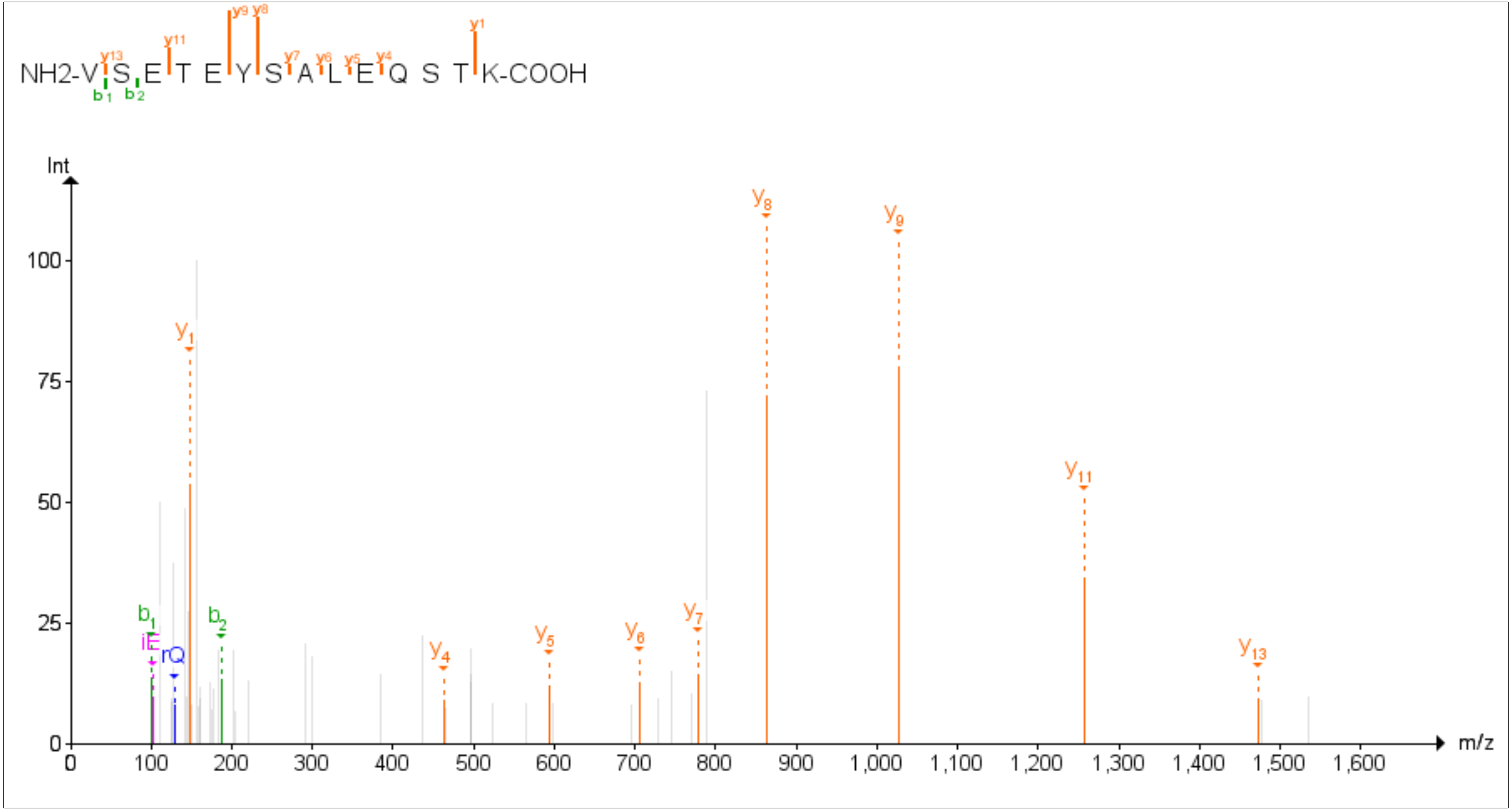
NH2-V S E T E Y S A L E Q S T K-COOH
y13 y11 y9 y8 y7 y6 y5 y4 y1
b1 b2
Int
m/z
0
25
50
75
100
0
100
200
300
400
500
600
700
800
900
1,000
1,100
1,200
1,300
1,400
1,500
1,600
y1
b1
iE
rQ
b2
y4
y5
y6
y7
y8
y9
y11
y13

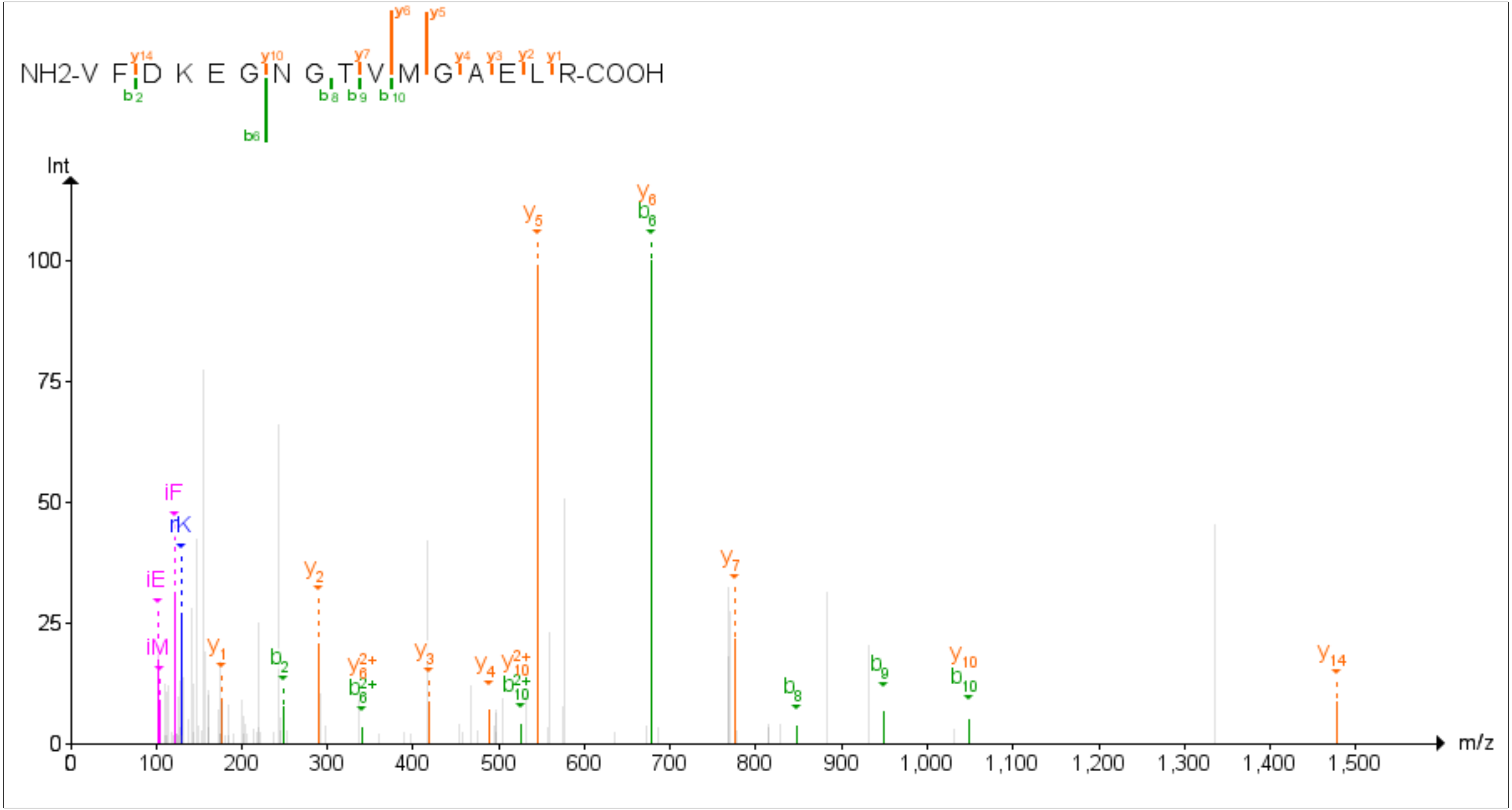

NH2-V F D K E G N G T V M G A E L R-COOH
y14 y10 y7 y6 y5 y4 y3 y2 y1
b2 b6 b8 b9 b10
Int
m/z
iF
rK
iE
iM
y1
b2
y2
y6 2+
b6 2+
y3
y4
y10 2+
b10 2+
y5
y6
b6
y7
b8
b9
y10
b10
y14
0
25
50
75
100
0
100
200
300
400
500
600
700
800
900
1,000
1,100
1,200
1,300
1,400
1,500

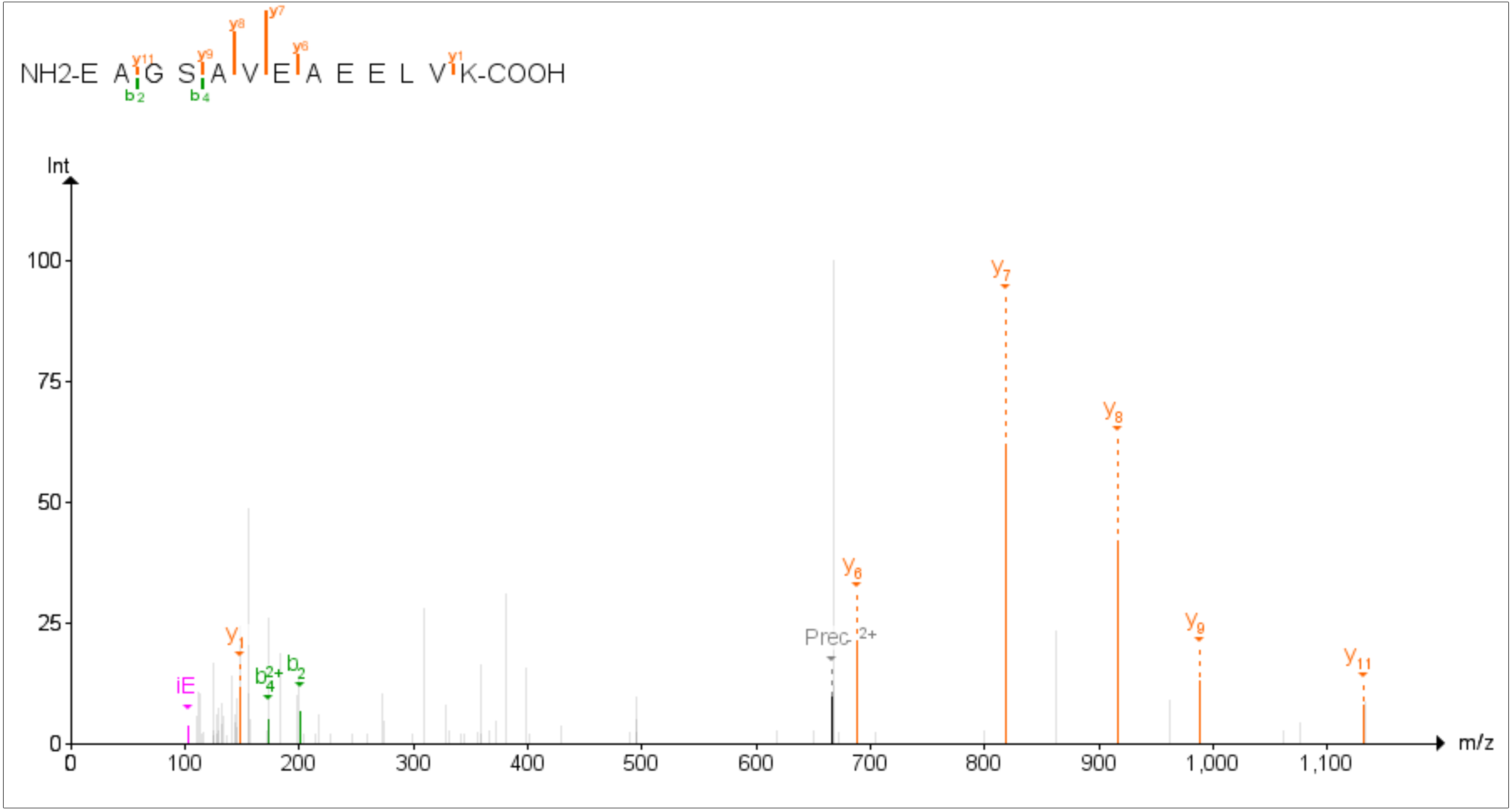

NH2-E A G S A V E A E E L V K-COOH
y11 y9 y8 y7 y6 y1
b2 b4
Int
100
75
50
25
0
m/z
0 100 200 300 400 500 600 700 800 900 1,000 1,100
y7
y8
y6
Prec 2+
y9
y11
y1
iE
b4 2+
b2

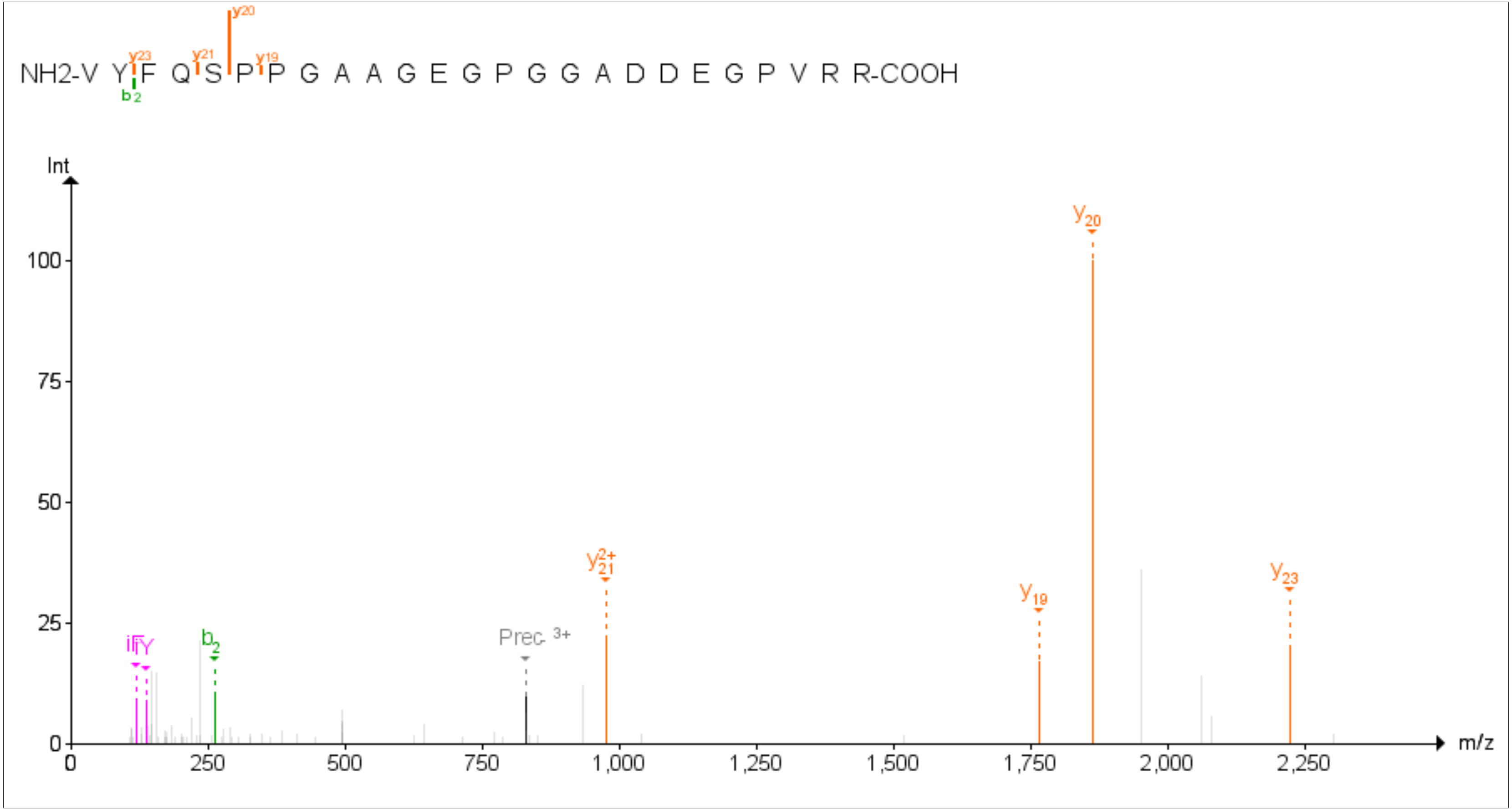

NH2-V Y F Q S P P G A A G E G P G G A D D E G P V R R-COOH
y20
y23
y21
y19
b2
Int
100
75
50
25
0
0
250
500
750
1,000
1,250
1,500
1,750
2,000
2,250
m/z
y20
y21 2+
y19
y23
b2
Prec 3+
iF
iY

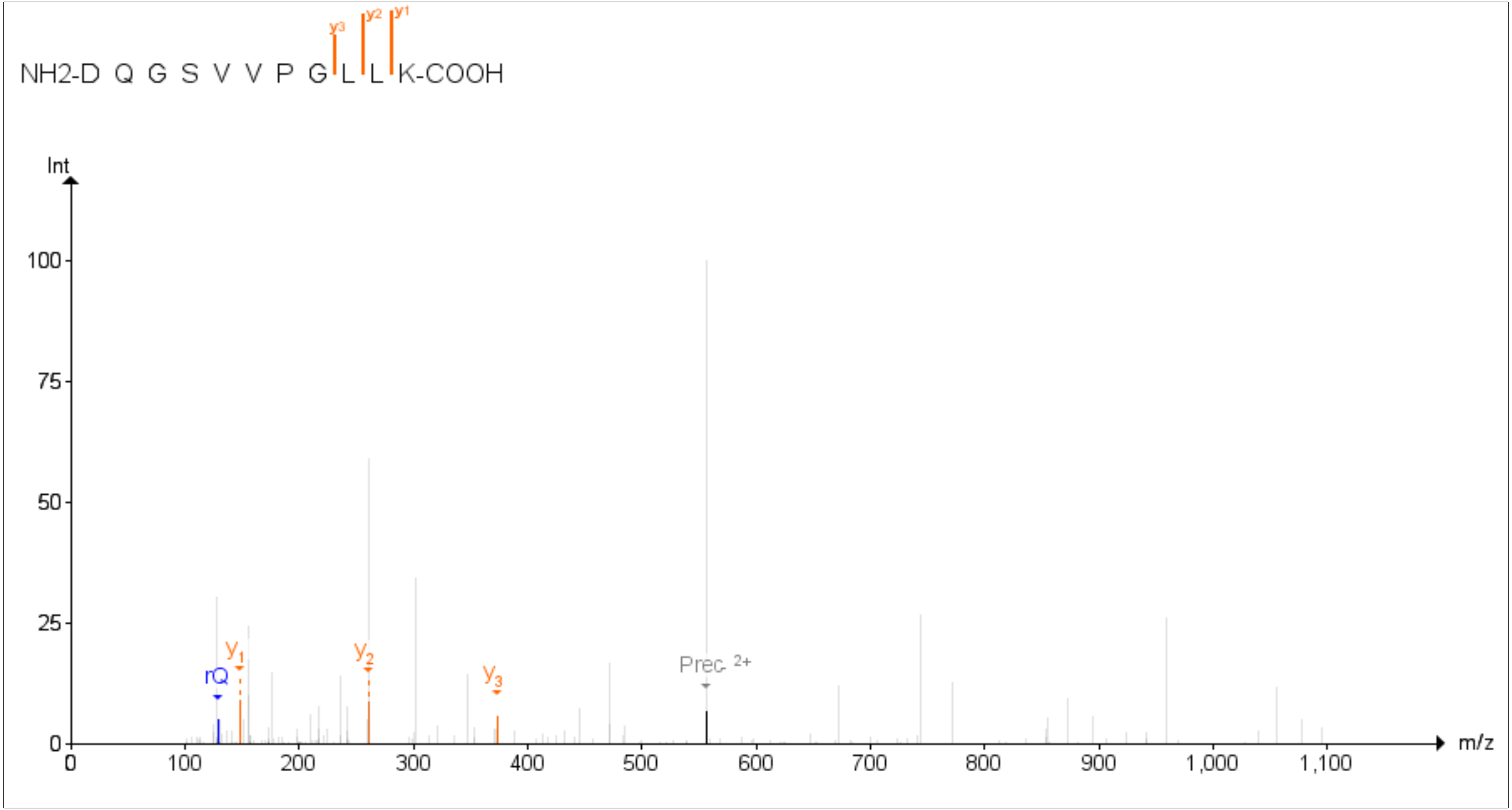

NH2-D Q G S V V P G L L K-COOH
y3
y2
y1
Int
100
75
50
25
0
rQ
y1
y2
y3
Prec 2+
0
100
200
300
400
500
600
700
800
900
1,000
1,100
m/z

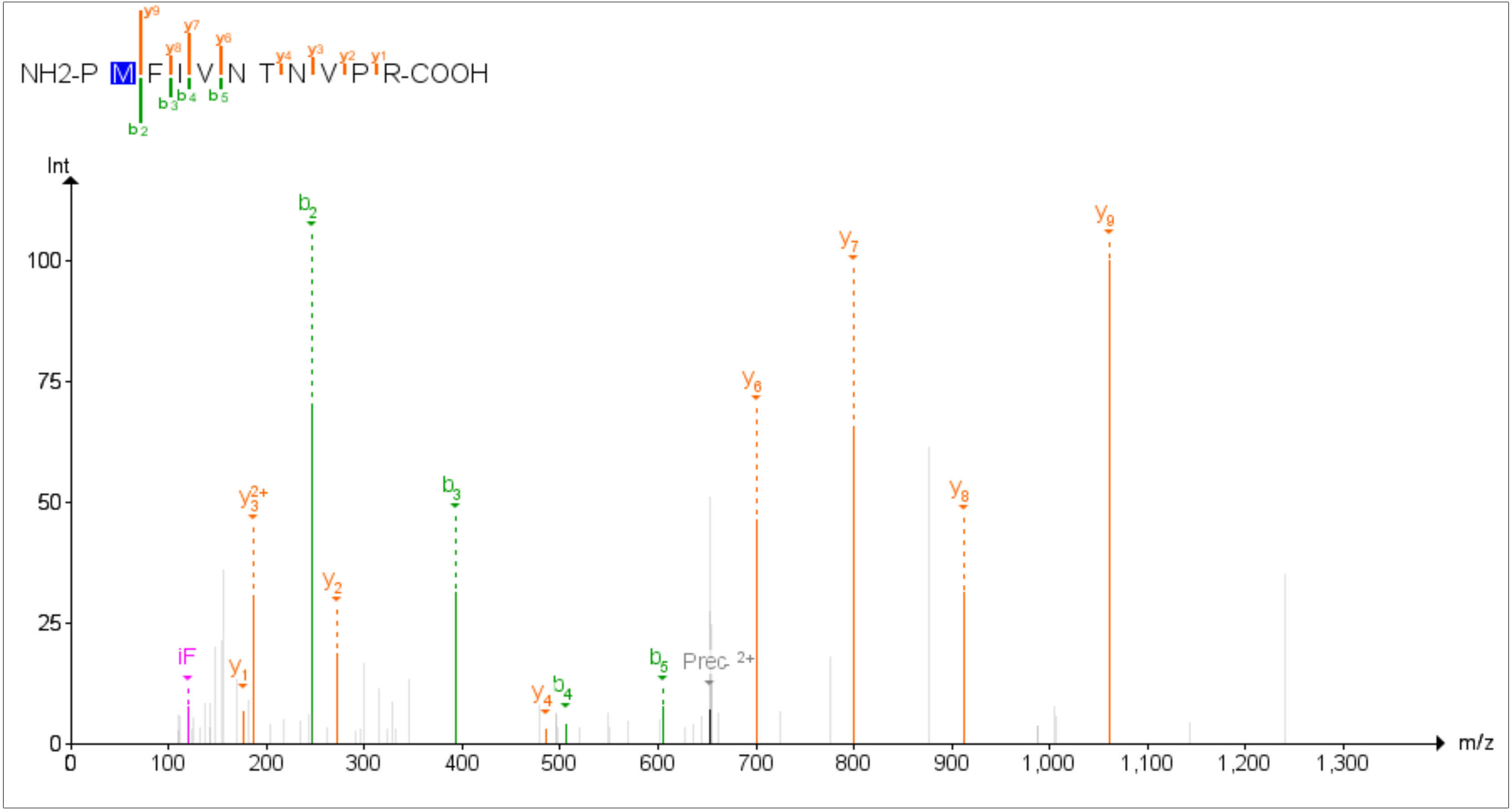

NH2-P M F I V N T N V P R-COOH
y9 y8 y7 y6 y4 y3 y2 y1
b2 b3 b4 b5
Int
100
75
50
25
0
m/z
0 100 200 300 400 500 600 700 800 900 1,000 1,100 1,200 1,300
iF
y1
y3 2+
b2
y2
b3
y4
b4
b5
Prec 2+
y6
y7
y8
y9

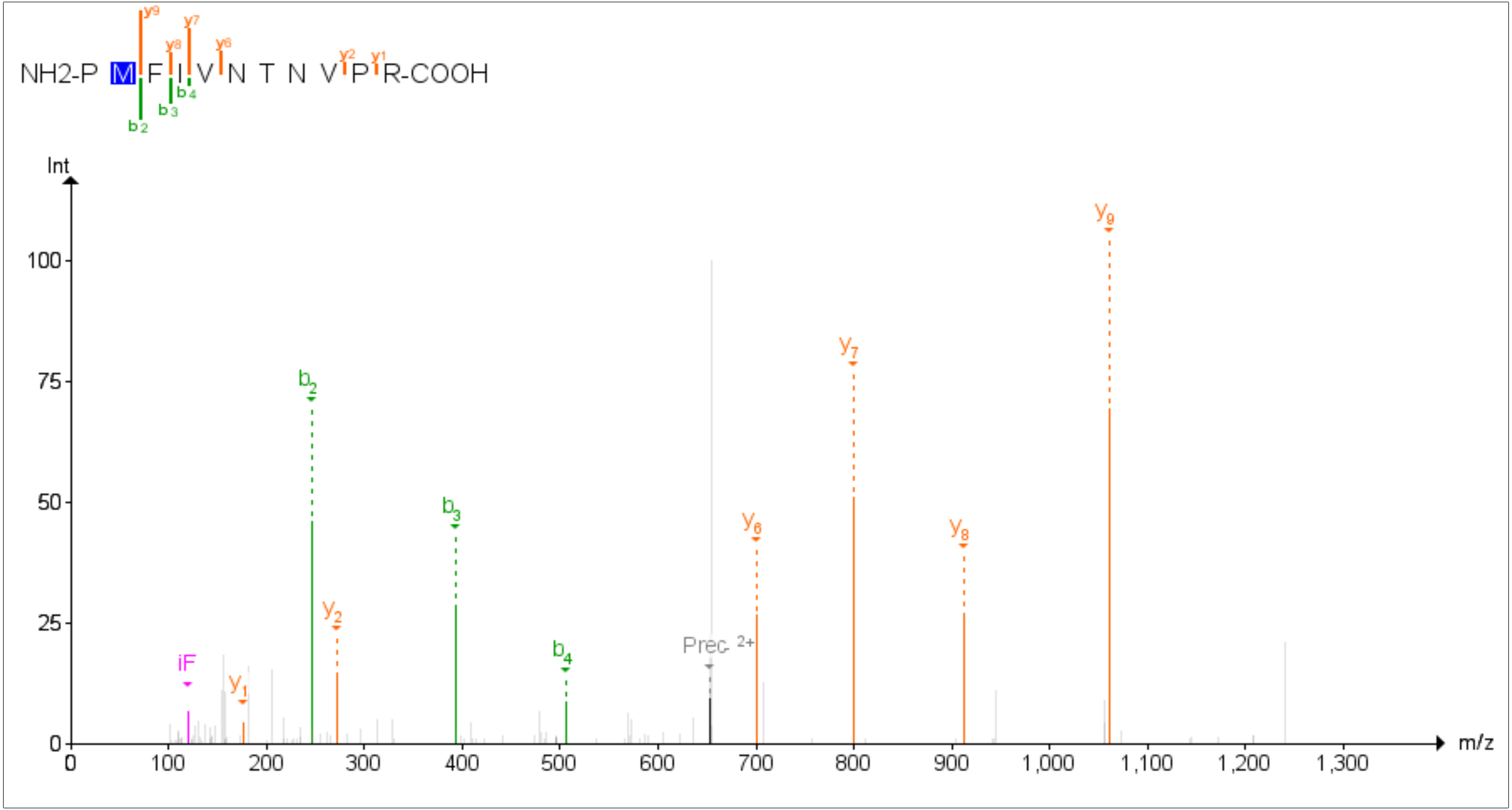

NH2-P M F I V N T N V P R-COOH
y9
y8
y7
y6
y2
y1
b2
b3
b4
Int
100
75
50
25
0
b2
b3
b4
y1
y2
y6
y7
y8
y9
iF
Prec 2+
0
100
200
300
400
500
600
700
800
900
1,000
1,100
1,200
1,300
m/z

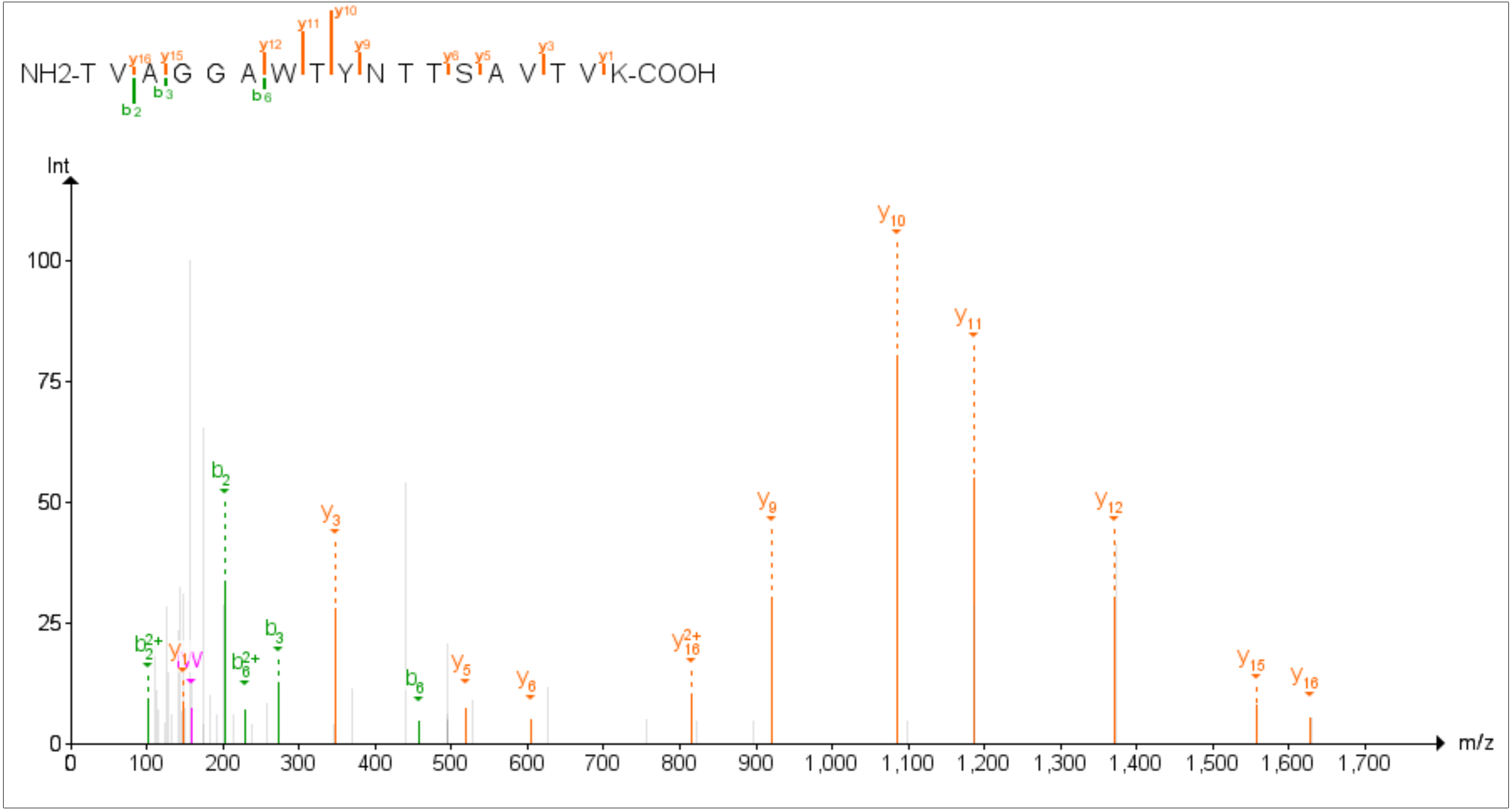

NH2-T V A G G A W T Y N T T S A V T V K-COOH
y16 y15 y12 y11 y10 y9 y6 y5 y3 y1
b2 b3 b6
Int
100
75
50
25
0
m/z
0 100 200 300 400 500 600 700 800 900 1,000 1,100 1,200 1,300 1,400 1,500 1,600 1,700

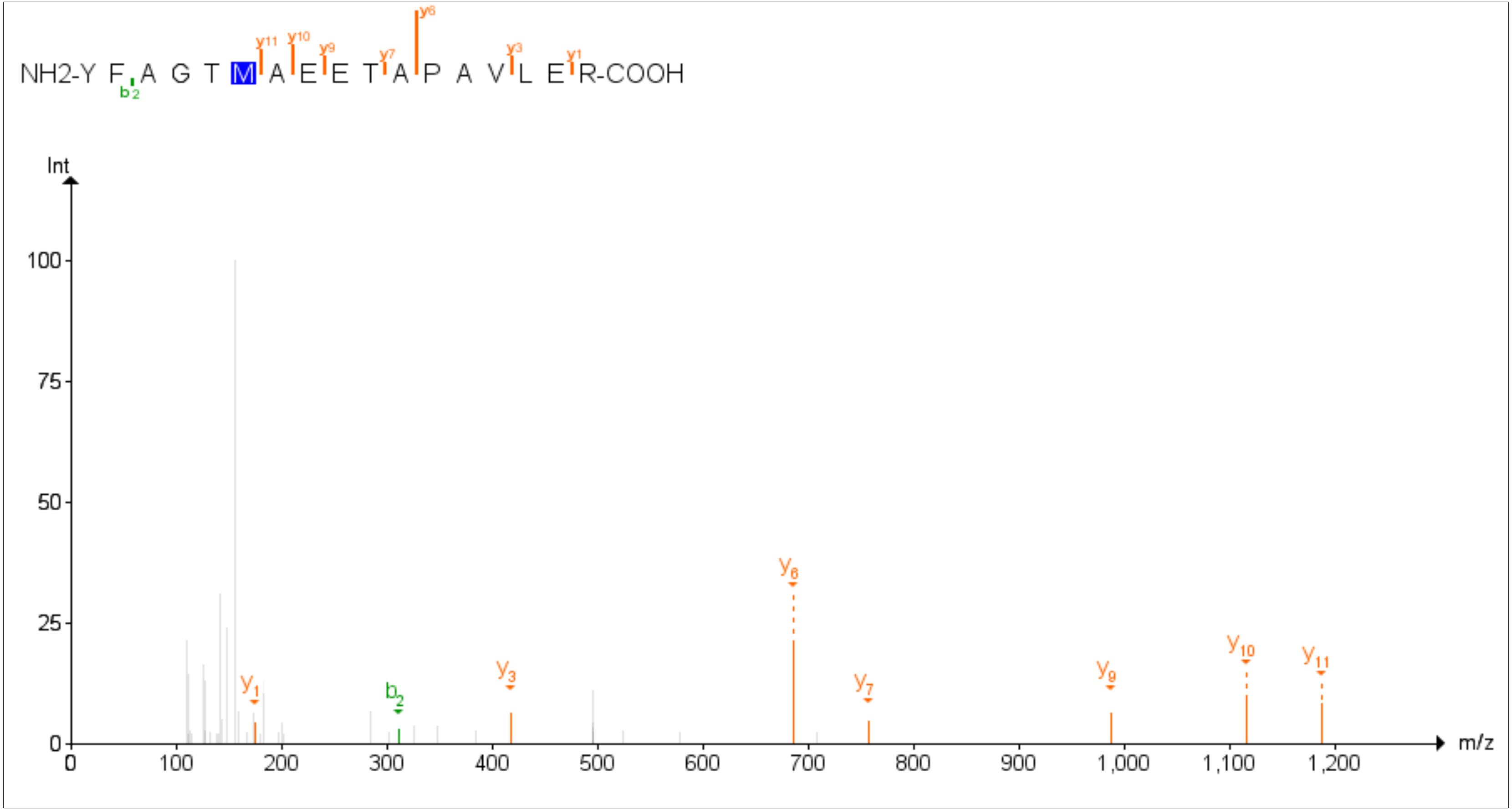

NH2-Y F A G T M A E E T A P A V L E R-COOH
b2
y11 y10 y9 y7 y6 y3 y1
Int
100
75
50
25
0
m/z
0 100 200 300 400 500 600 700 800 900 1,000 1,100 1,200

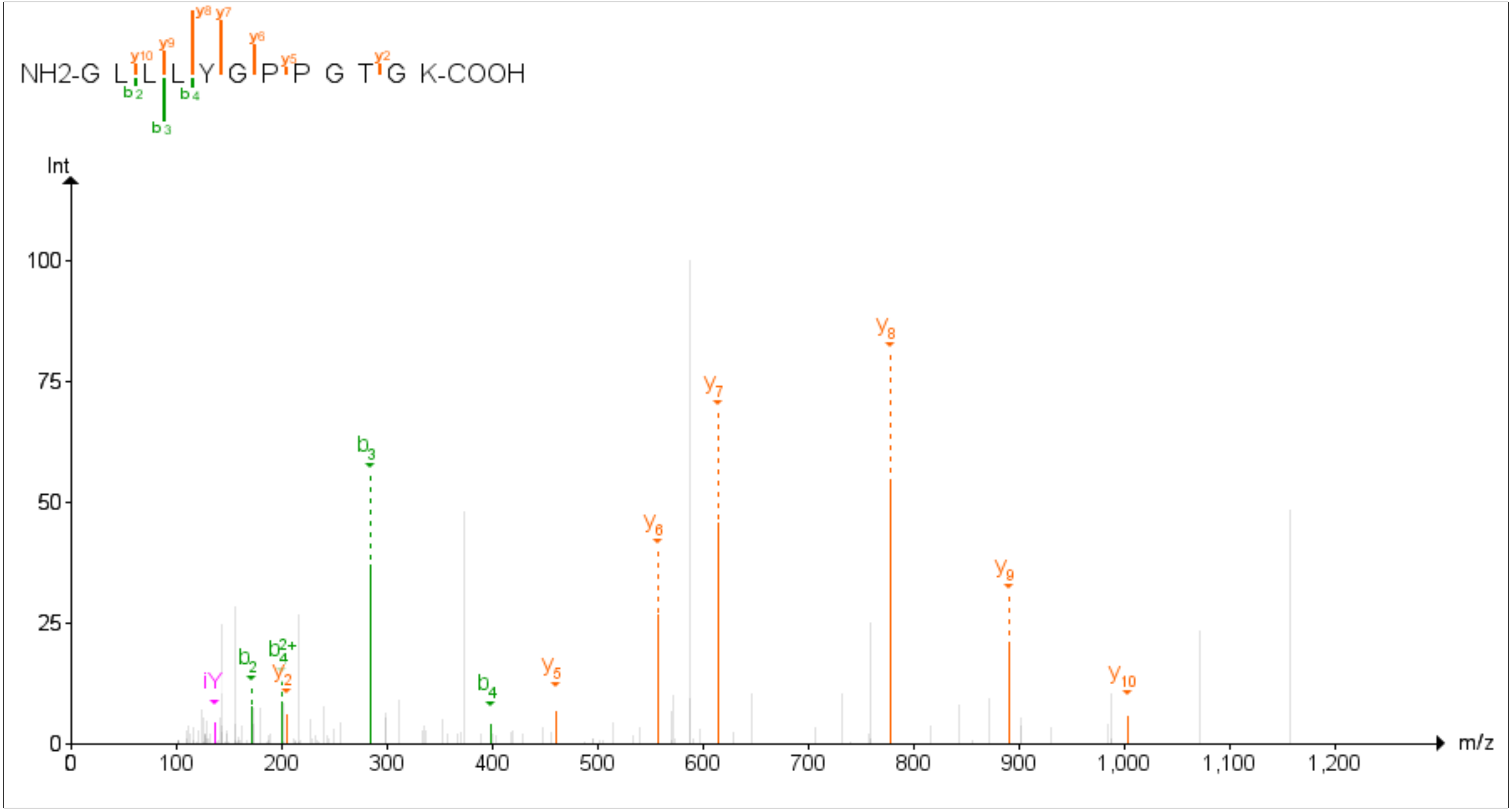

NH2-G L L L Y G P P G T G K-COOH
y10 y9 y8 y7 y6 y5 y2
b2 b3 b4
Int
100
75
50
25
0
m/z
0
100
200
300
400
500
600
700
800
900
1,000
1,100
1,200
iY
b2
b4 2+
y2
b3
b4
y5
y6
y7
y8
y9
y10

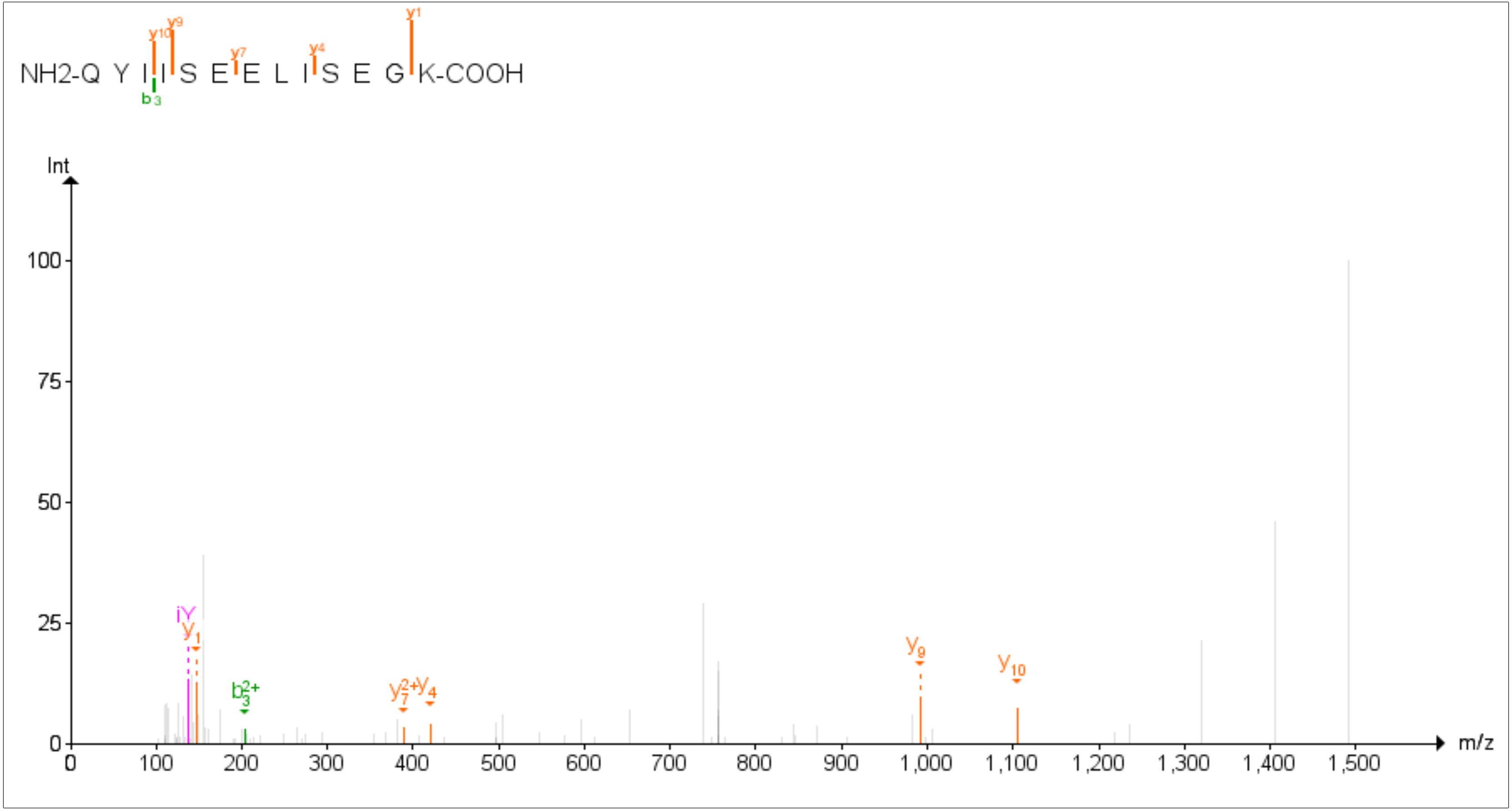

NH2-Q Y I I S E E L I S E G K-COOH
y10
y9
y7
y4
y1
b3
Int
100
75
50
25
0
iY
y1
b3 2+
y7 2+
y4
y9
y10
0
100
200
300
400
500
600
700
800
900
1,000
1,100
1,200
1,300
1,400
1,500
m/z

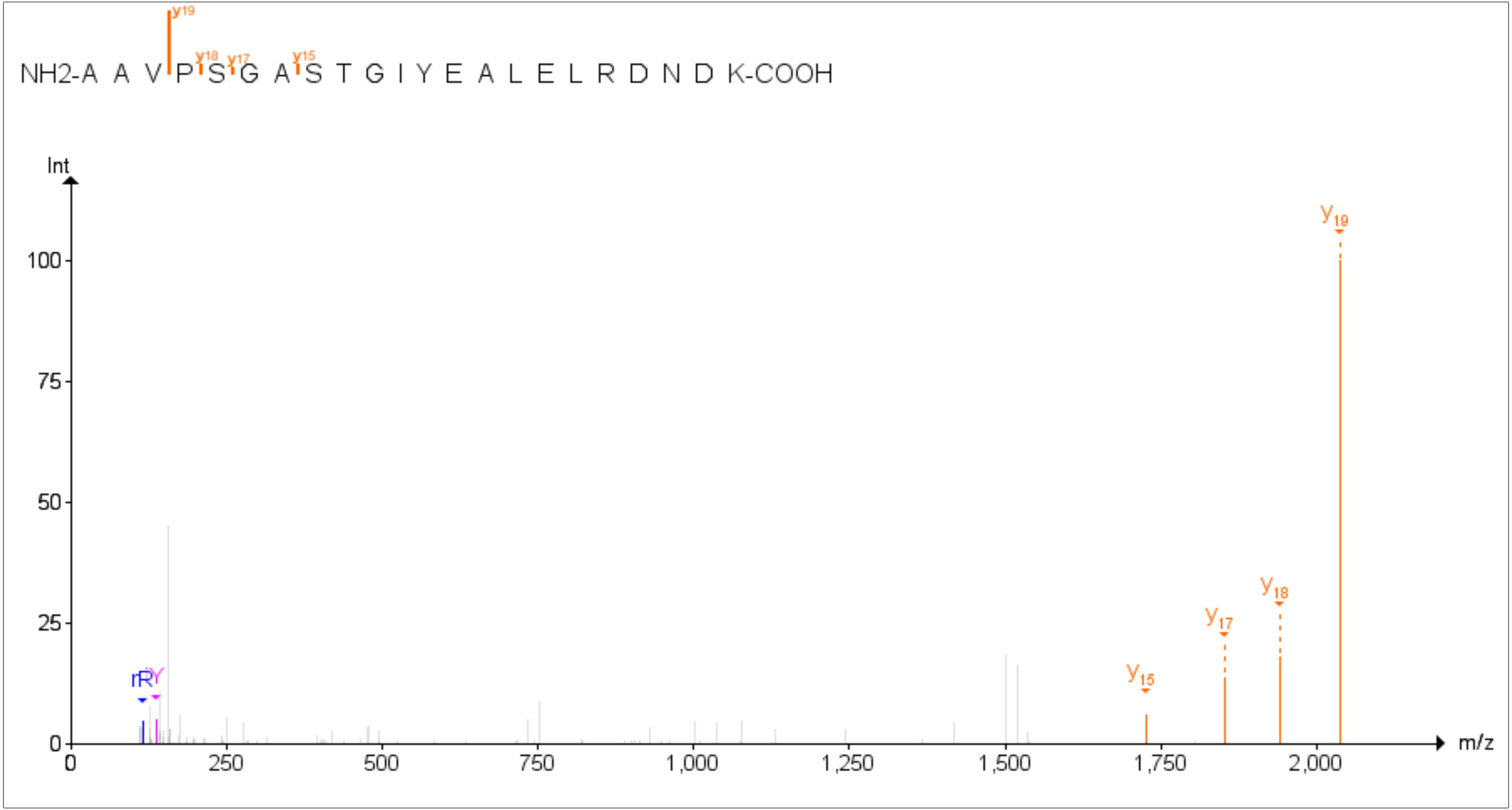

NH2-A A V P S G A S T G I Y E A L E L R D N D K-COOH
y19
y18
y17
y15
Int
100
75
50
25
0
0
250
500
750
1,000
1,250
1,500
1,750
2,000
m/z
rR
Y

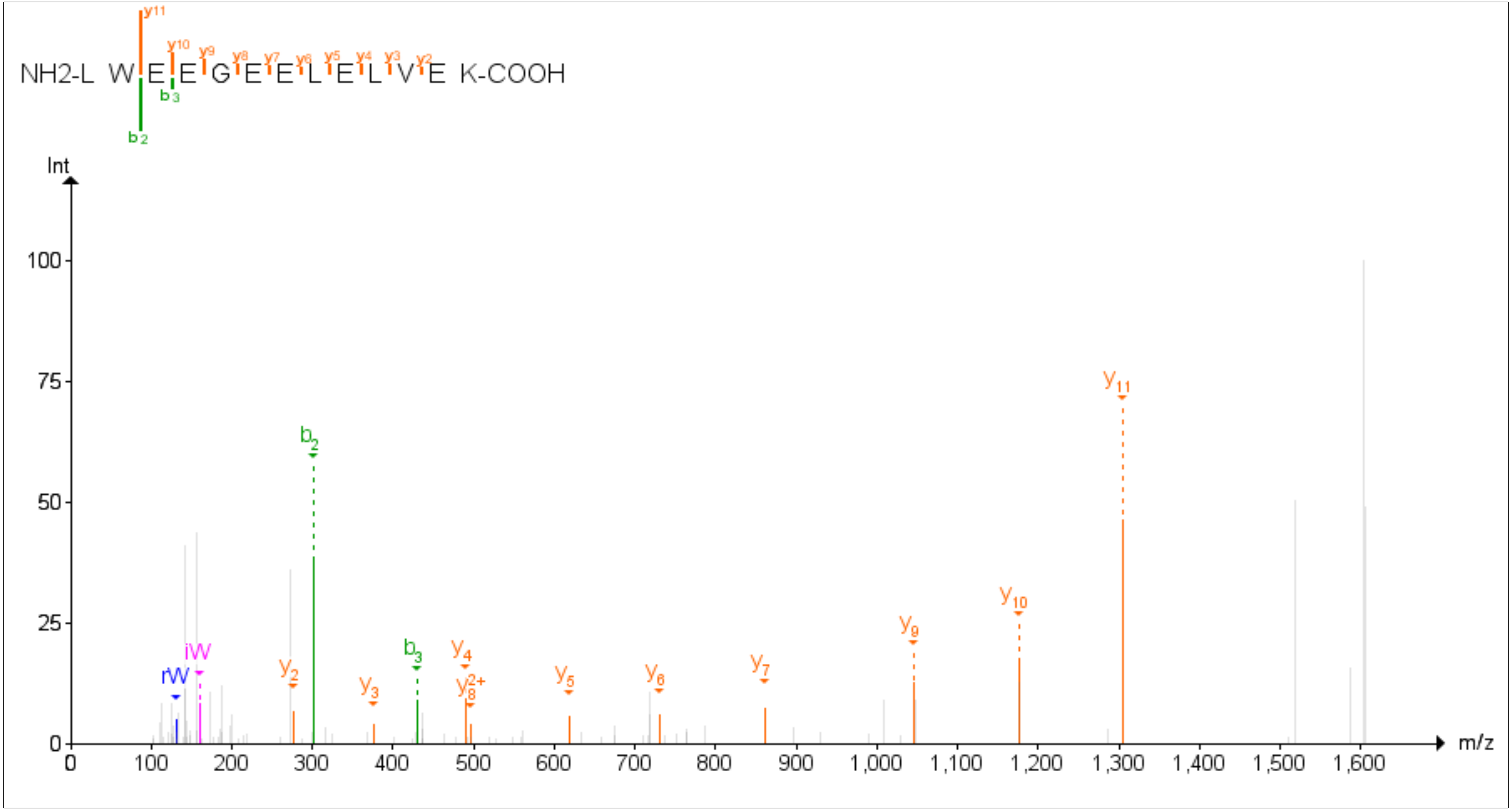
NH2-L W E E G E E L E L V E K-COOH
y11
y10
y9
y8
y7
y6
y5
y4
y3
y2
b2
b3
Int
100
75
50
25
0
rW
iW
y2
b2
y3
b3
y4
y8 2+
y5
y6
y7
y9
y10
y11
0
100
200
300
400
500
600
700
800
900
1,000
1,100
1,200
1,300
1,400
1,500
1,600
m/z

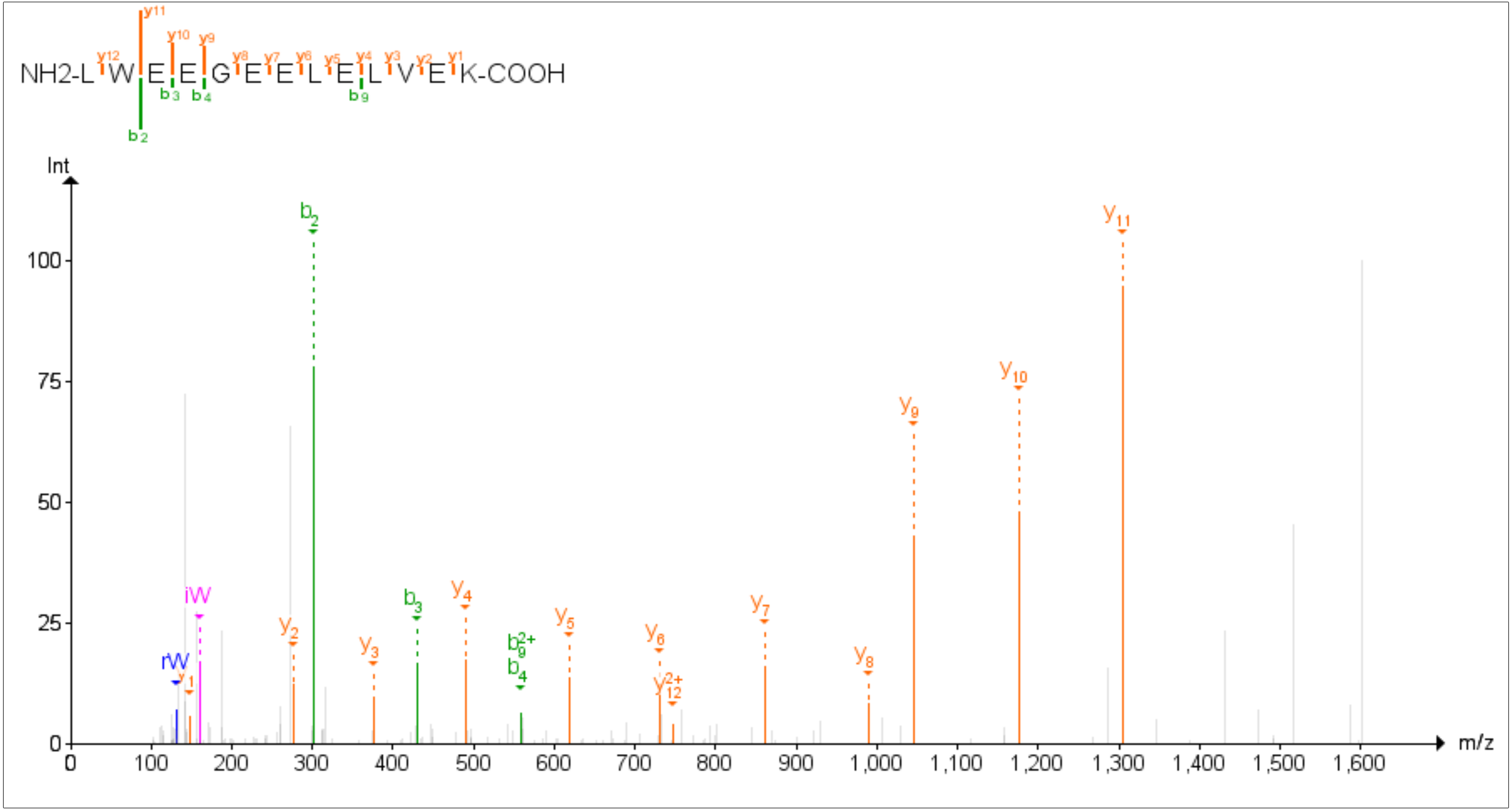

NH2-L W E E G E E L E L V E K-COOH
y12 y11 y10 y9 y8 y7 y6 y5 y4 y3 y2 y1
b2 b3 b4 b9
Int
100
75
50
25
0
m/z
0 100 200 300 400 500 600 700 800 900 1,000 1,100 1,200 1,300 1,400 1,500 1,600
b2
y11
y10
y9
iW
rW
y1
y2
y3
b3
y4
b9 2+
b4
y5
y6
y12 2+
y7
y8

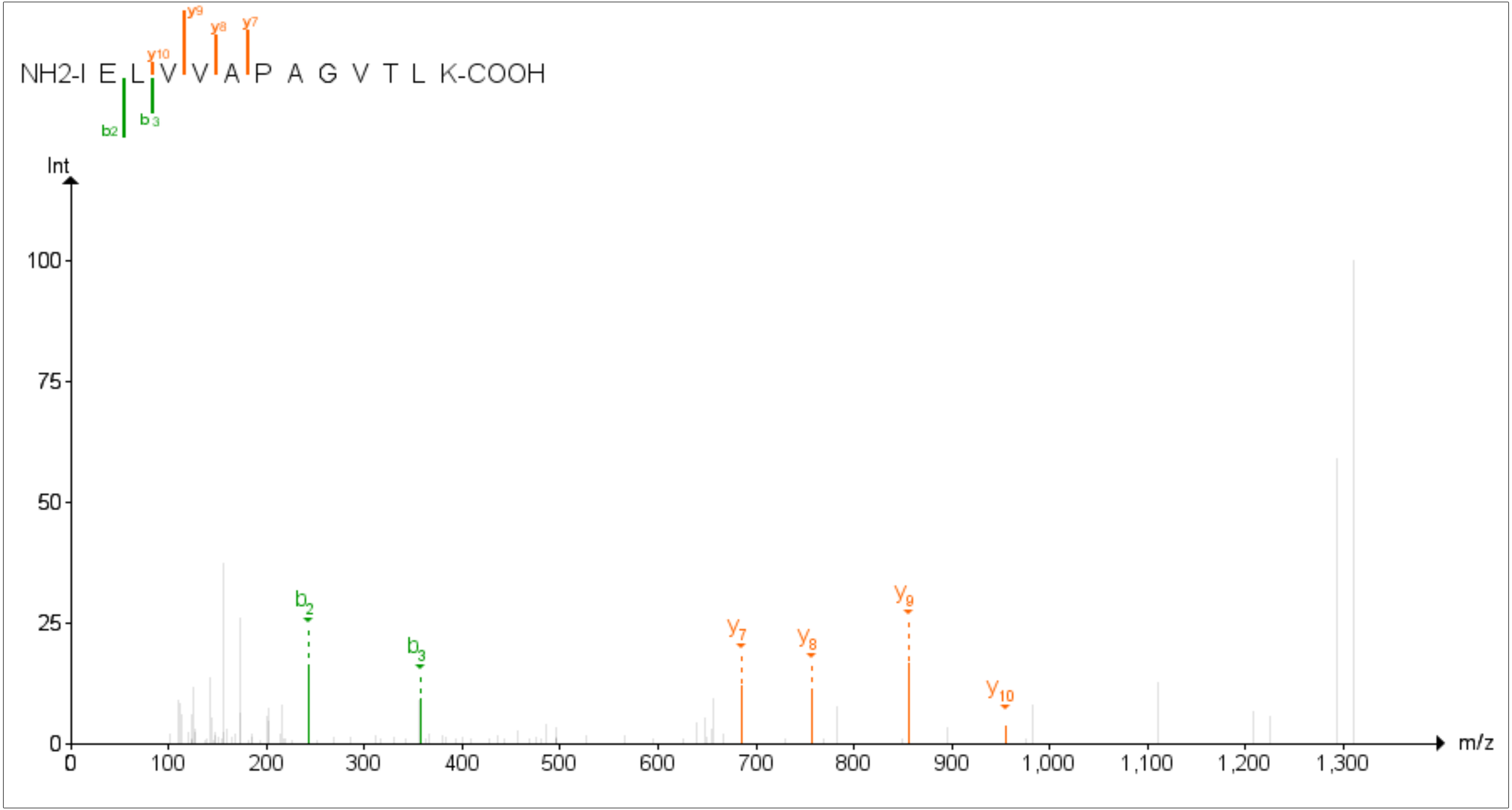

NH2-I E L V V A P A G V T L K-COOH
y9
y8
y7
y10
b2
b3
Int
100
75
50
25
0
b2
b3
y7
y8
y9
y10
0
100
200
300
400
500
600
700
800
900
1,000
1,100
1,200
1,300
m/z

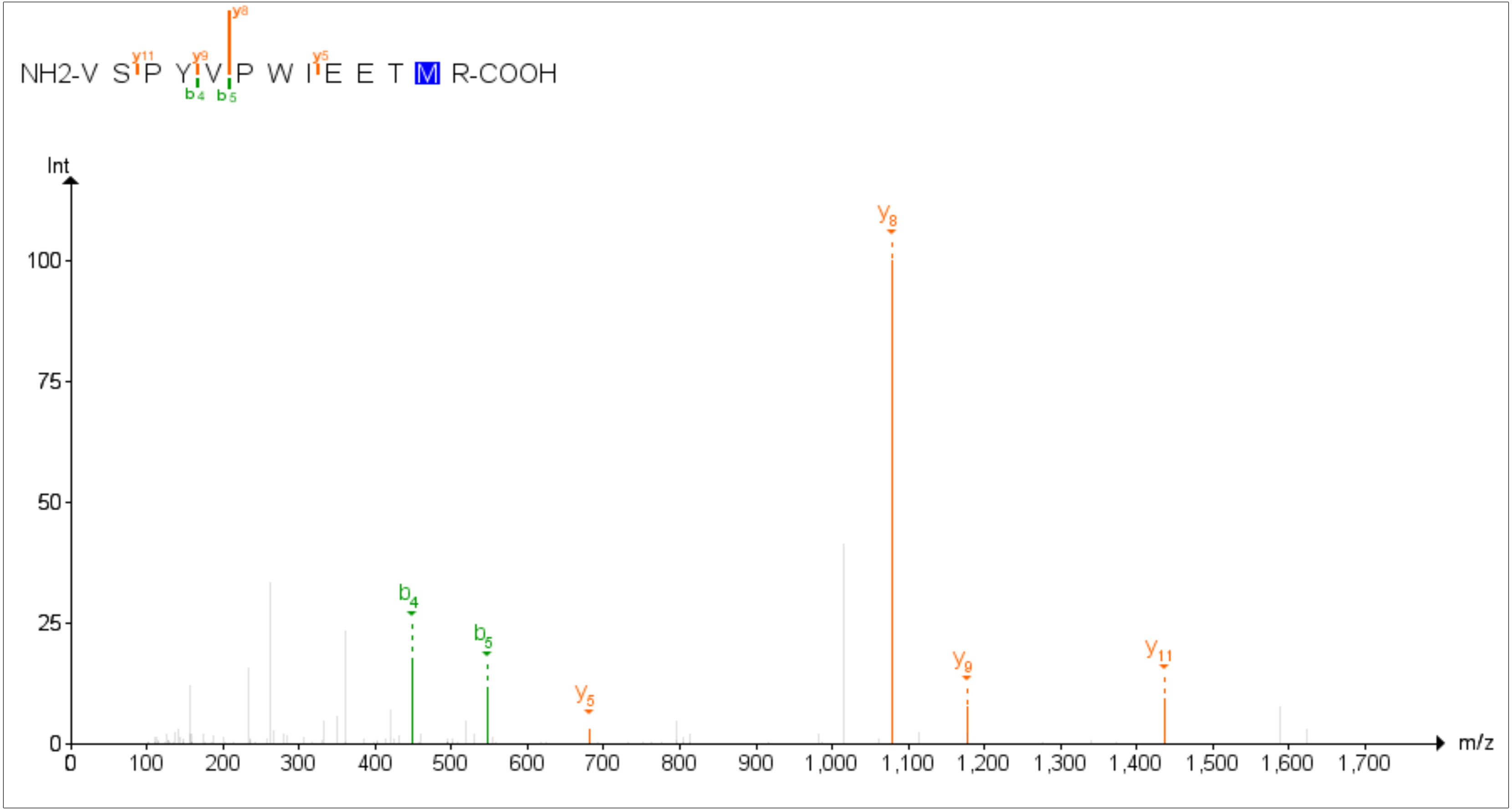

NH2-V S P Y V P W I E E T M R-COOH
y11
y9
y8
y5
b4
b5
Int
100
75
50
25
0
b4
b5
y5
y8
y9
y11
0
100
200
300
400
500
600
700
800
900
1,000
1,100
1,200
1,300
1,400
1,500
1,600
1,700
m/z

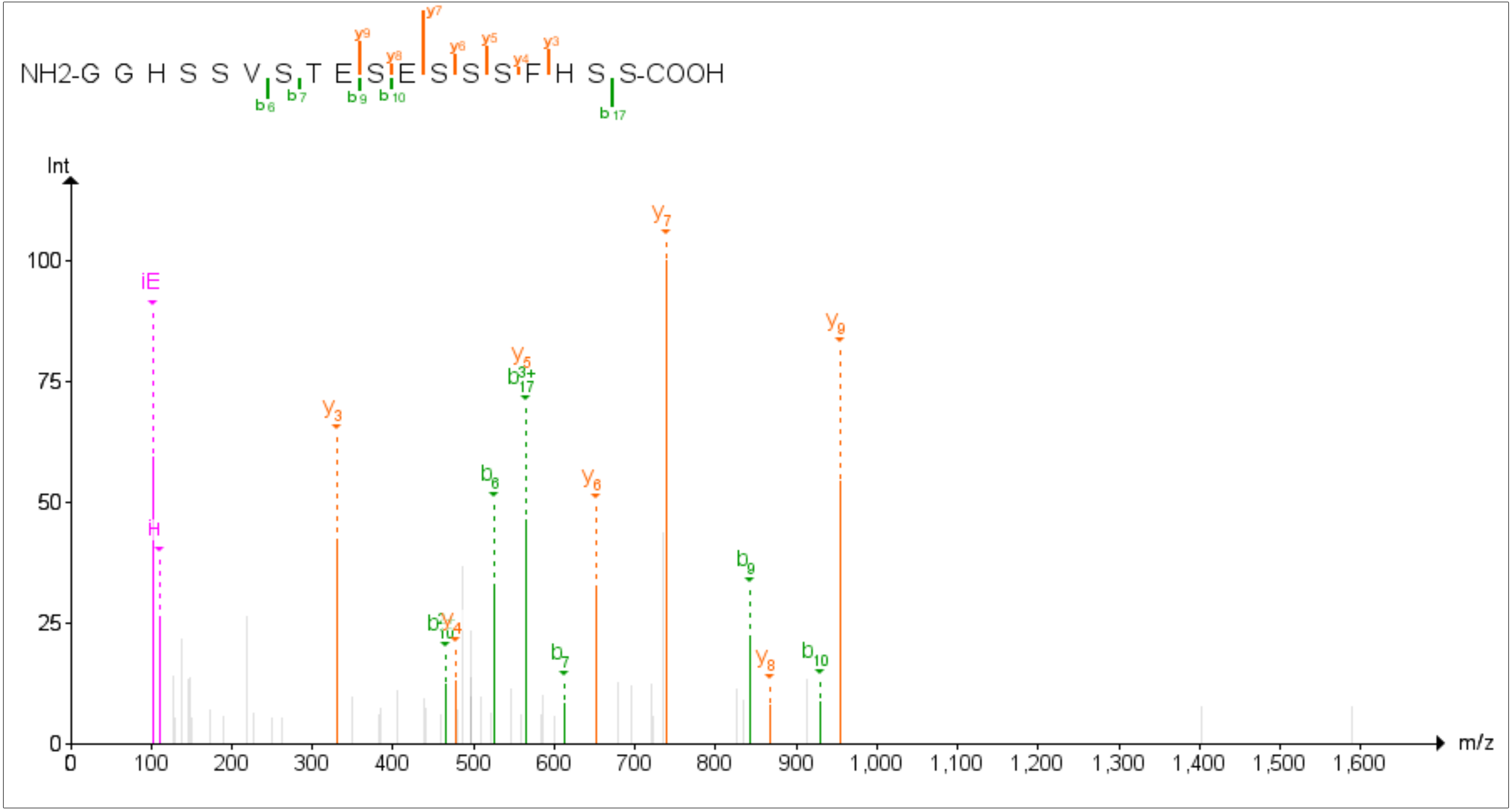
NH2-G G H S S V S T E S E S S S F H S S-COOH
y9 y8 y7 y6 y5 y4 y3
b6 b7 b9 b10 b17
Int
m/z
0
25
50
75
100
0
100
200
300
400
500
600
700
800
900
1,000
1,100
1,200
1,300
1,400
1,500
1,600
iE
iH
y3
y5
b3+ 17
b6
y6
y7
y9
b9
y8
b10
b7
b10
y4

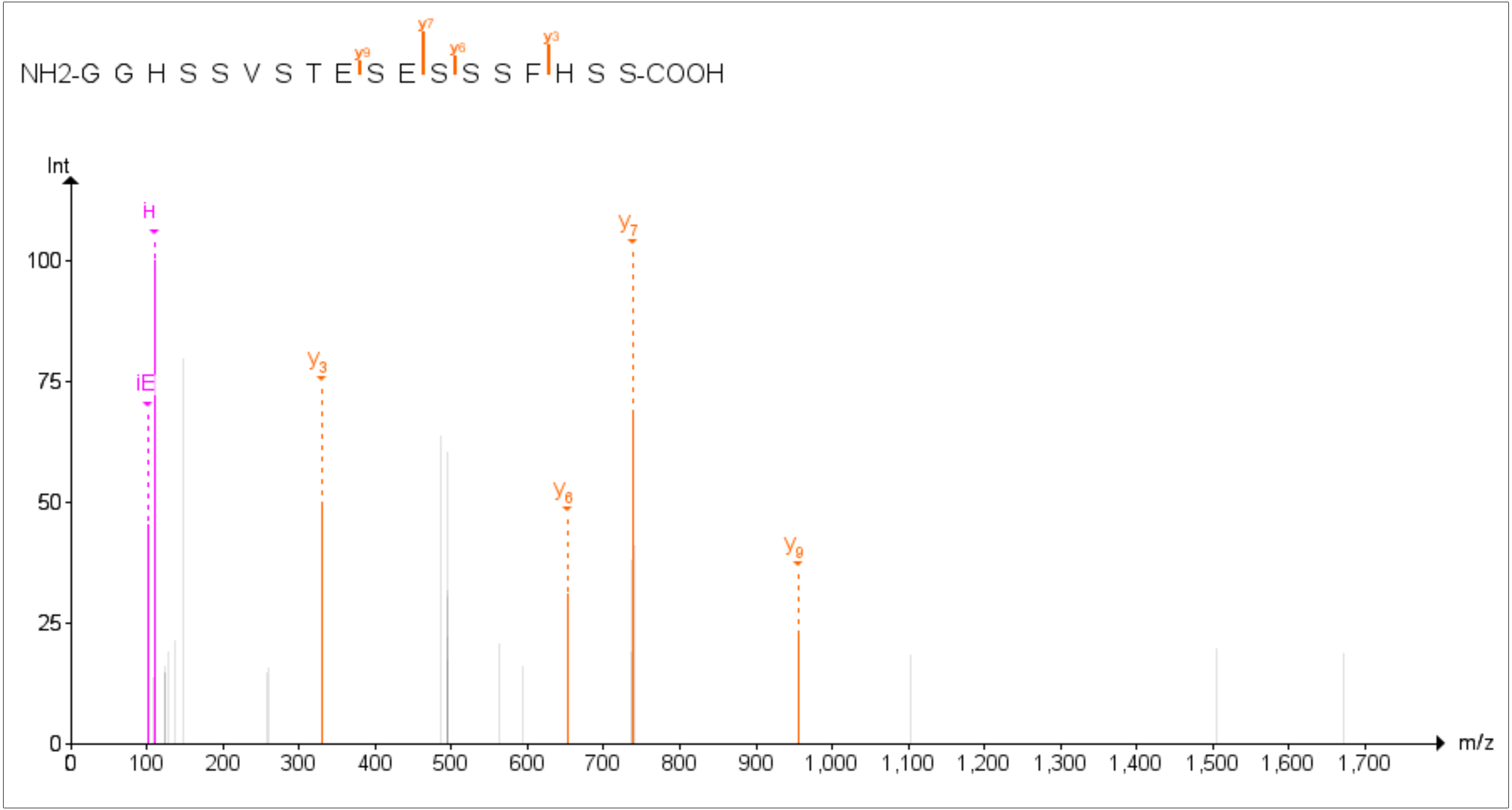

NH2-G G H S S V S T E S E S S S F H S S-COOH
y9
y7
y6
y3
Int
100
75
50
25
0
iH
iE
y3
y7
y6
y9
0
100
200
300
400
500
600
700
800
900
1,000
1,100
1,200
1,300
1,400
1,500
1,600
1,700
m/z

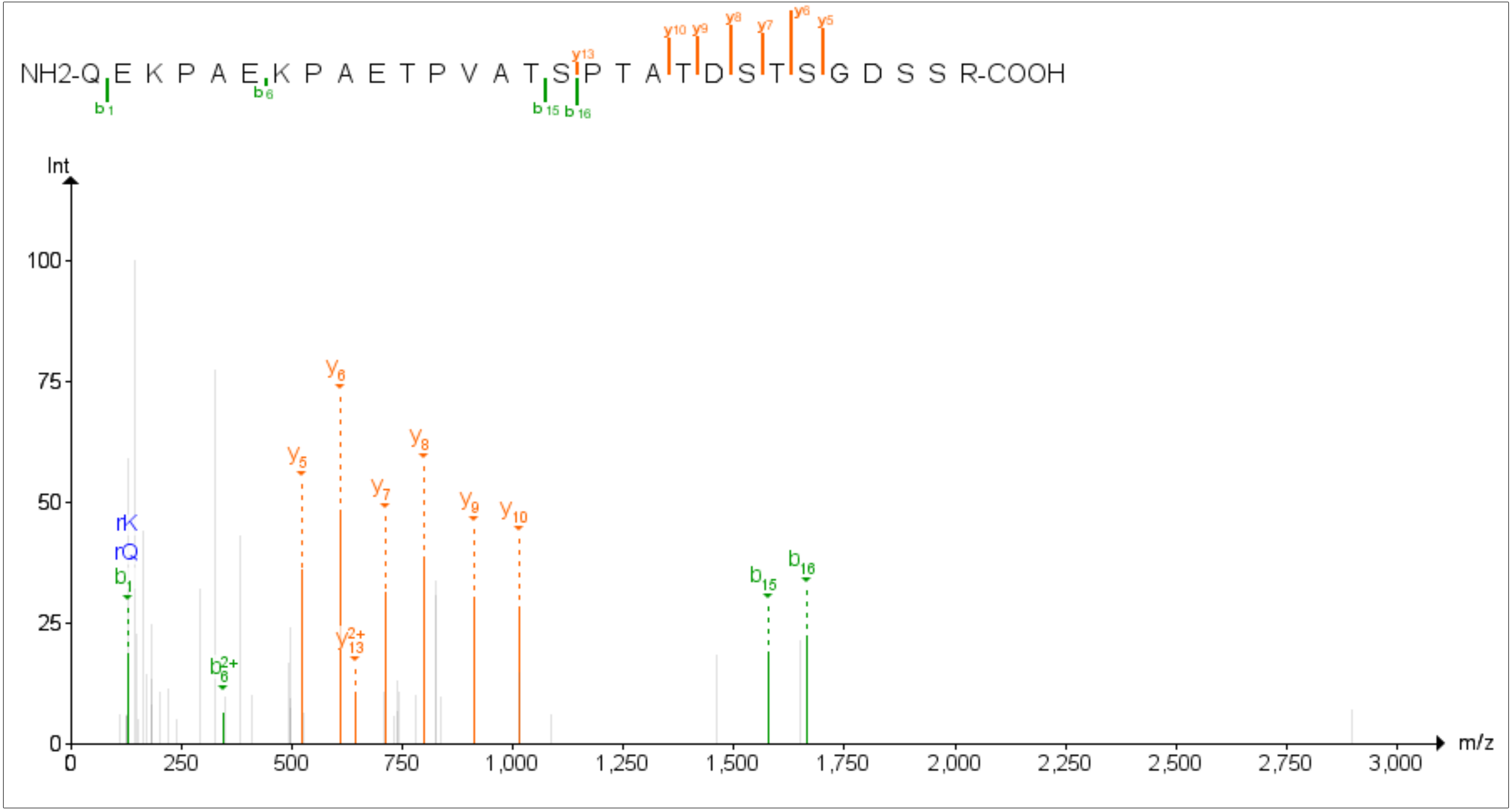

NH2-Q E K P A E K P A E T P V A T S P T A T D S T S G D S S R-COOH
b1 b6 b15 b16
y13 y10 y9 y8 y7 y6 y5
Int
100
75
50
25
0
rK
rQ
b1
b6 2+
y5
y6
y13 2+
y7
y8
y9
y10
b15
b16
0
250
500
750
1,000
1,250
1,500
1,750
2,000
2,250
2,500
2,750
3,000
m/z

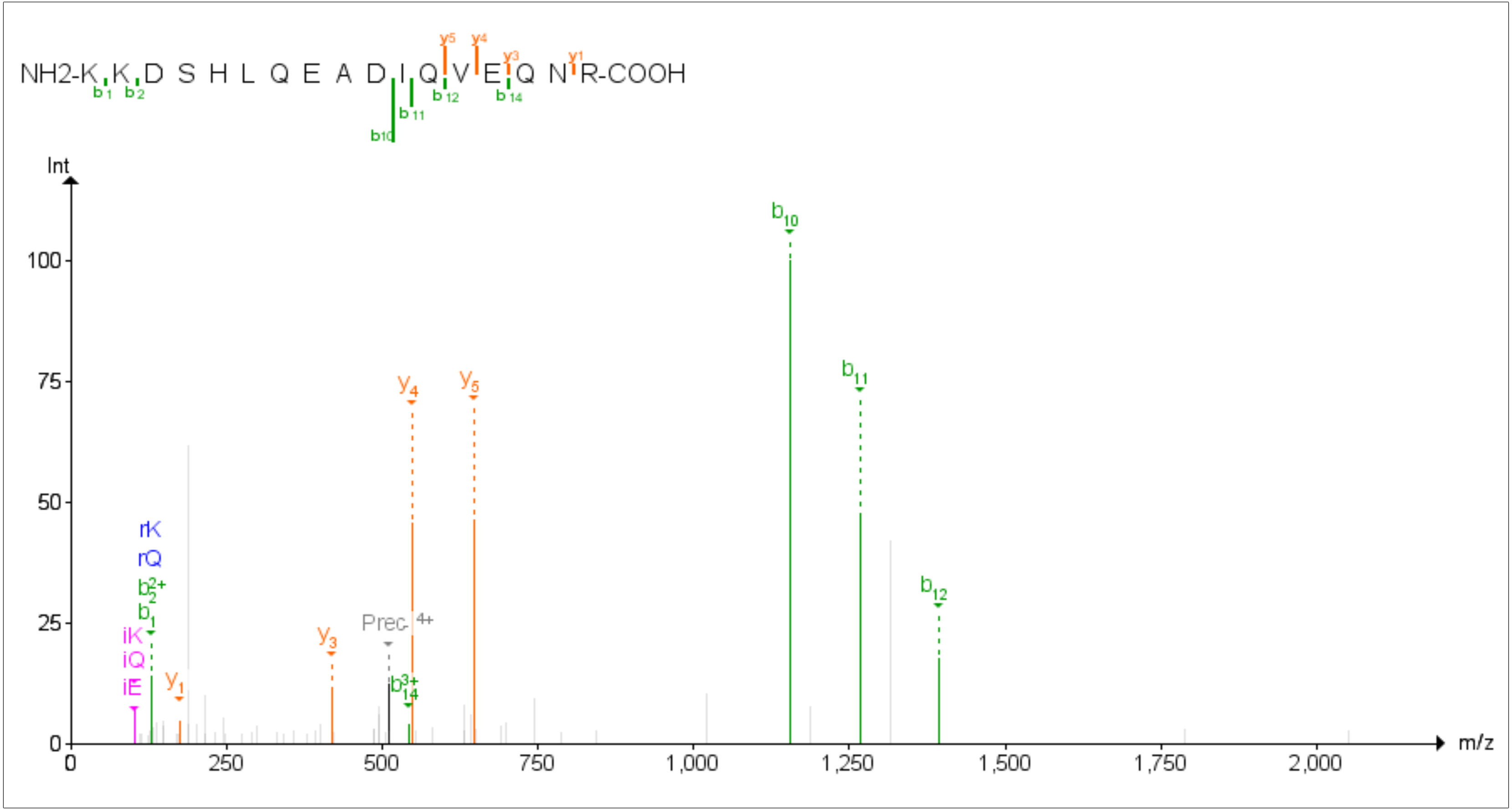

NH2-K K D S H L Q E A D I Q V E Q N R-COOH
b1 b2 b10 b11 b12 b14
y5 y4 y3 y1
Int
m/z
0 25 50 75 100
0 250 500 750 1,000 1,250 1,500 1,750 2,000
b10
b11
b12
y4
y5
y3
y1
Prec 4+
b14 3+
rK
rQ
b2 2+
b1
iK
iQ
iE

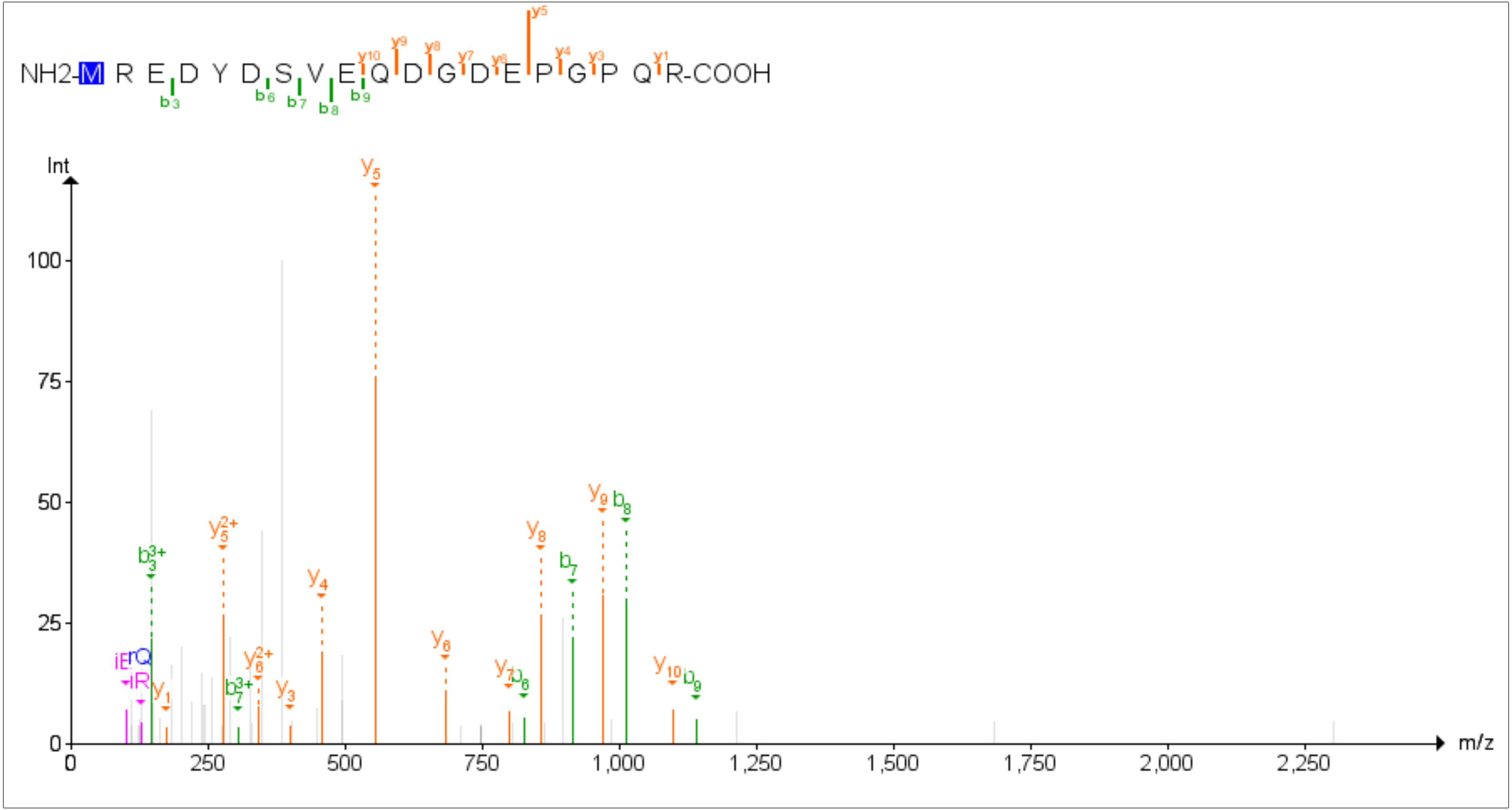

NH2-M R E D Y D S V E Q D G D E P G P Q R-COOH
b3 b6 b7 b8 b9
y10 y9 y8 y7 y6 y5 y4 y3 y1
Int
100
75
50
25
0
m/z
0
250
500
750
1,000
1,250
1,500
1,750
2,000
2,250
y5
y5 2+
b3 3+
y4
y9
b8
y8
b7
y6
y6 2+
y7
b6
y10
b9
y3
y1
b7 3+
iE
rQ
iR

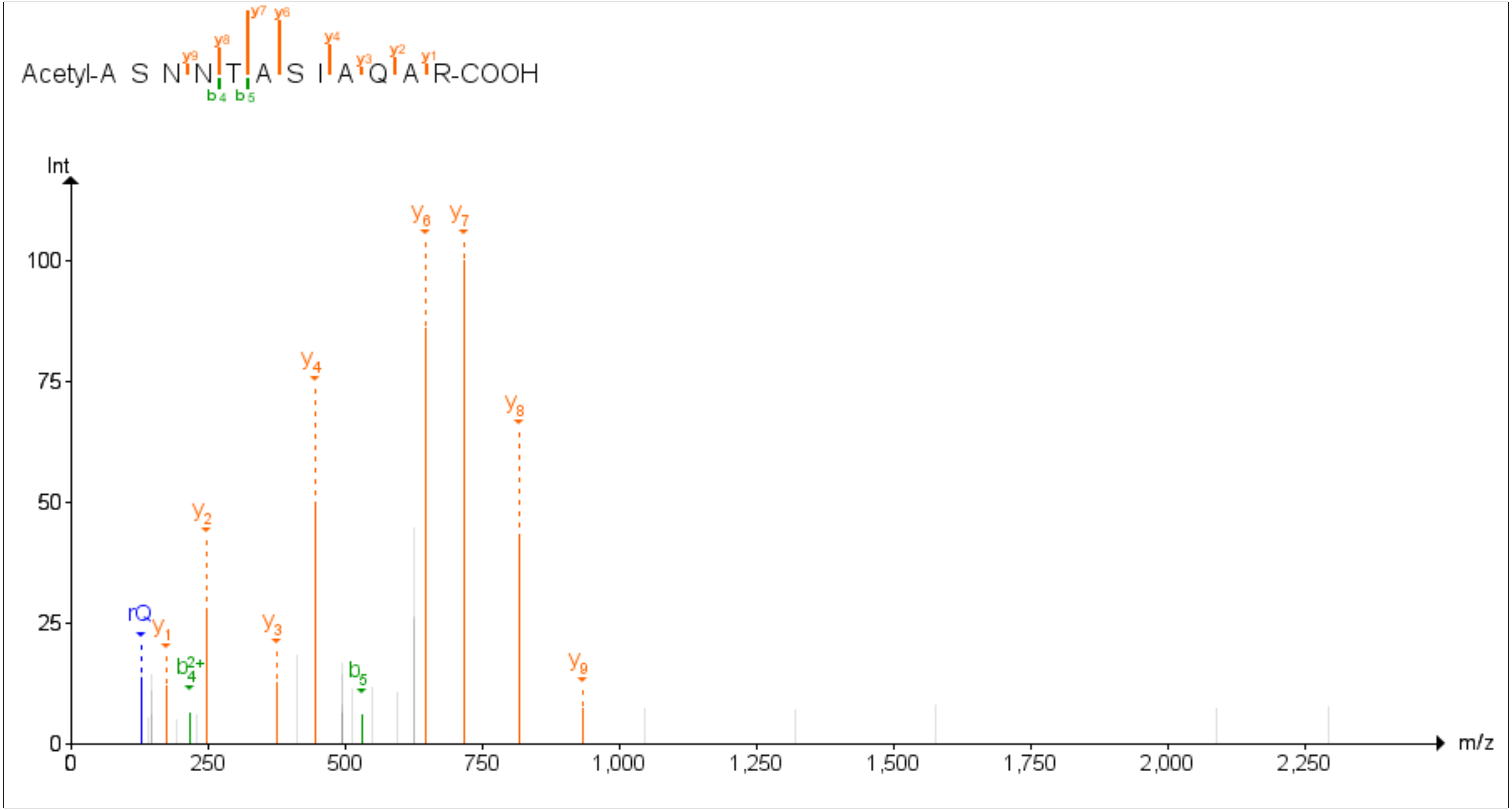
Acetyl-A S N N T A S I A Q A R-COOH
y9 y8 y7 y6 y4 y3 y2 y1
b4 b5
Int
100
75
50
25
0
rQ
y1
y2
b4 2+
y3
y4
b5
y6
y7
y8
y9
0
250
500
750
1,000
1,250
1,500
1,750
2,000
2,250
m/z

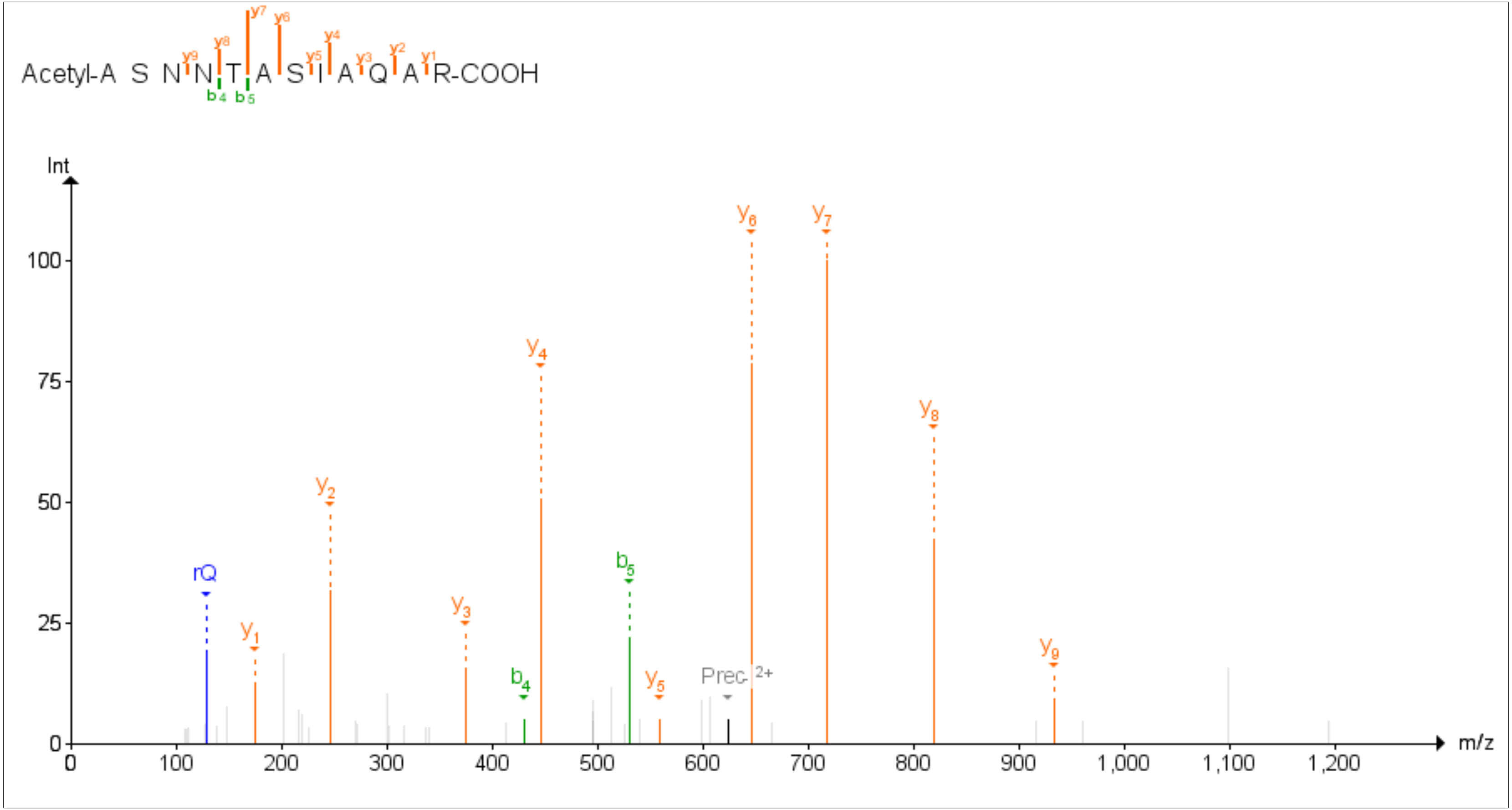

Acetyl-A S N N T A S I A Q A R-COOH
y9 y8 y7 y6 y5 y4 y3 y2 y1
b4 b5
Int
m/z
rQ
y1
y2
y3
b4
y4
b5
y5
Prec. 2+
y6
y7
y8
y9
0
25
50
75
100
0
100
200
300
400
500
600
700
800
900
1,000
1,100
1,200

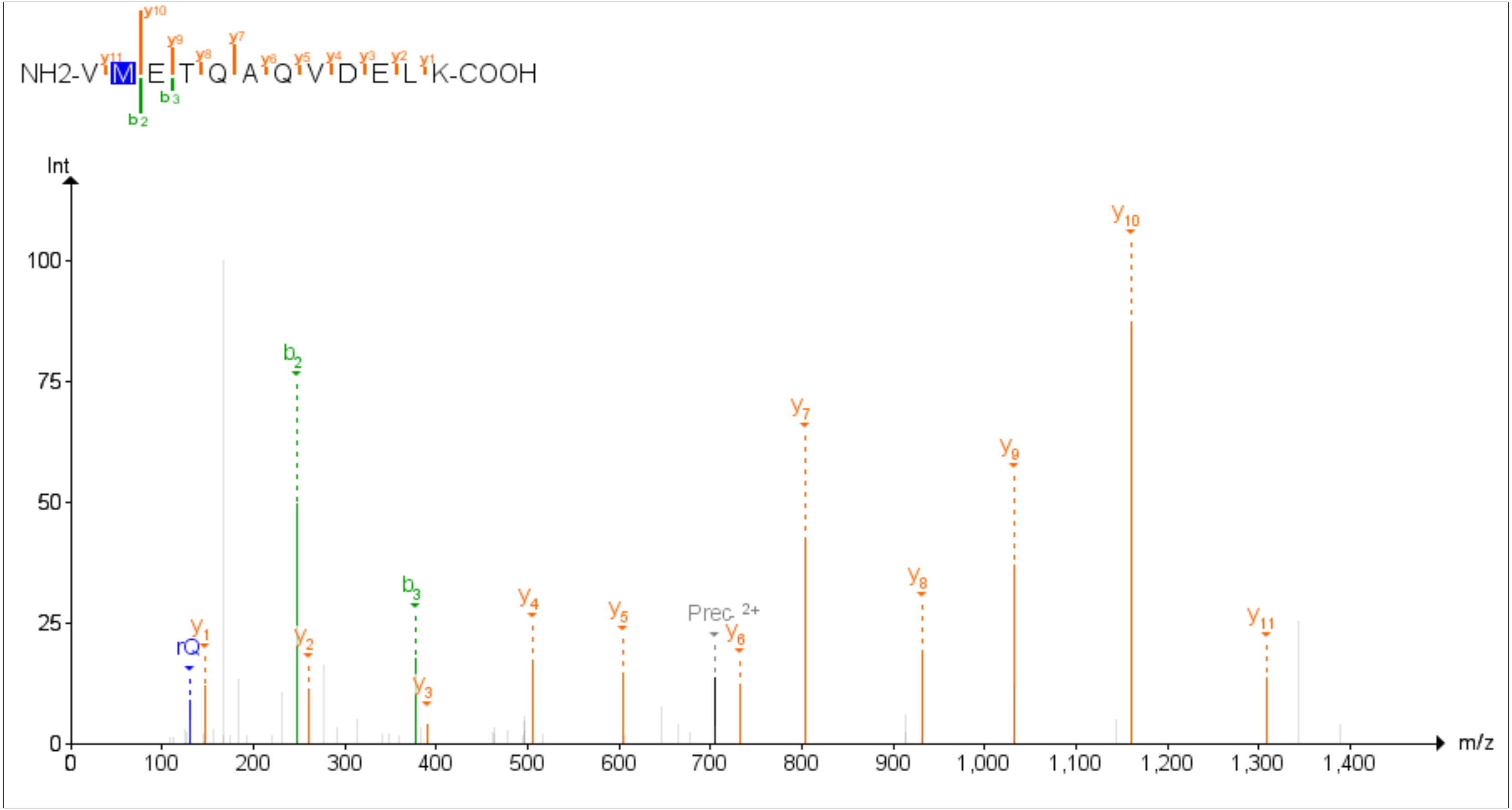

NH2-V M E T Q A Q V D E L K-COOH
y11 y10 y9 y8 y7 y6 y5 y4 y3 y2 y1
b2 b3
Int
100
75
50
25
0
rQ
y1
b2
y2
b3
y3
y4
y5
Prec 2+
y6
y7
y8
y9
y10
y11
0 100 200 300 400 500 600 700 800 900 1,000 1,100 1,200 1,300 1,400
m/z

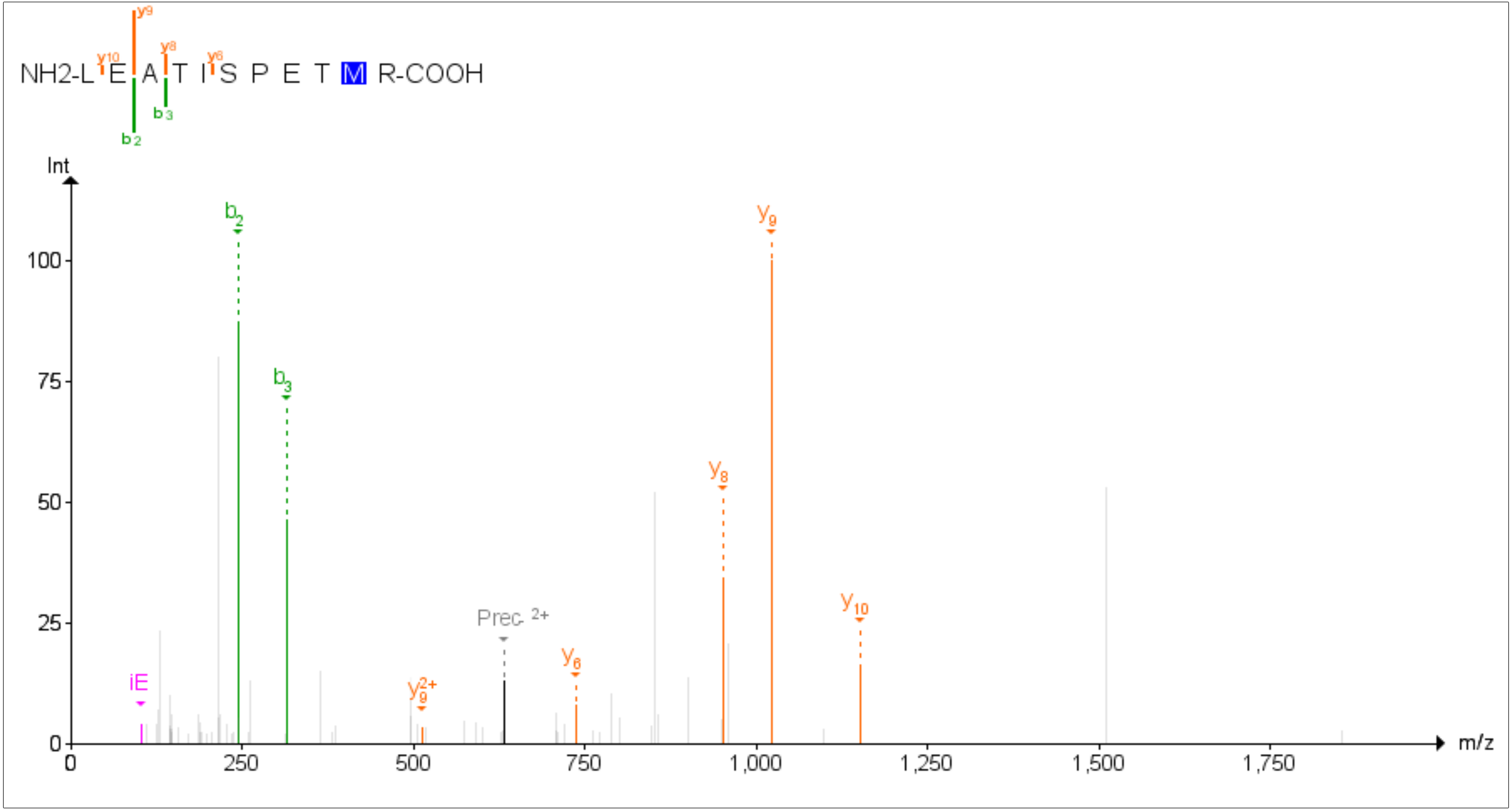

NH2-L E A T I S P E T M R-COOH
y10
y9
y8
y6
b2
b3
Int
100
75
50
25
0
b2
b3
y9
y8
y10
Prec 2+
y6
y9 2+
iE
250
500
750
1,000
1,250
1,500
1,750
m/z

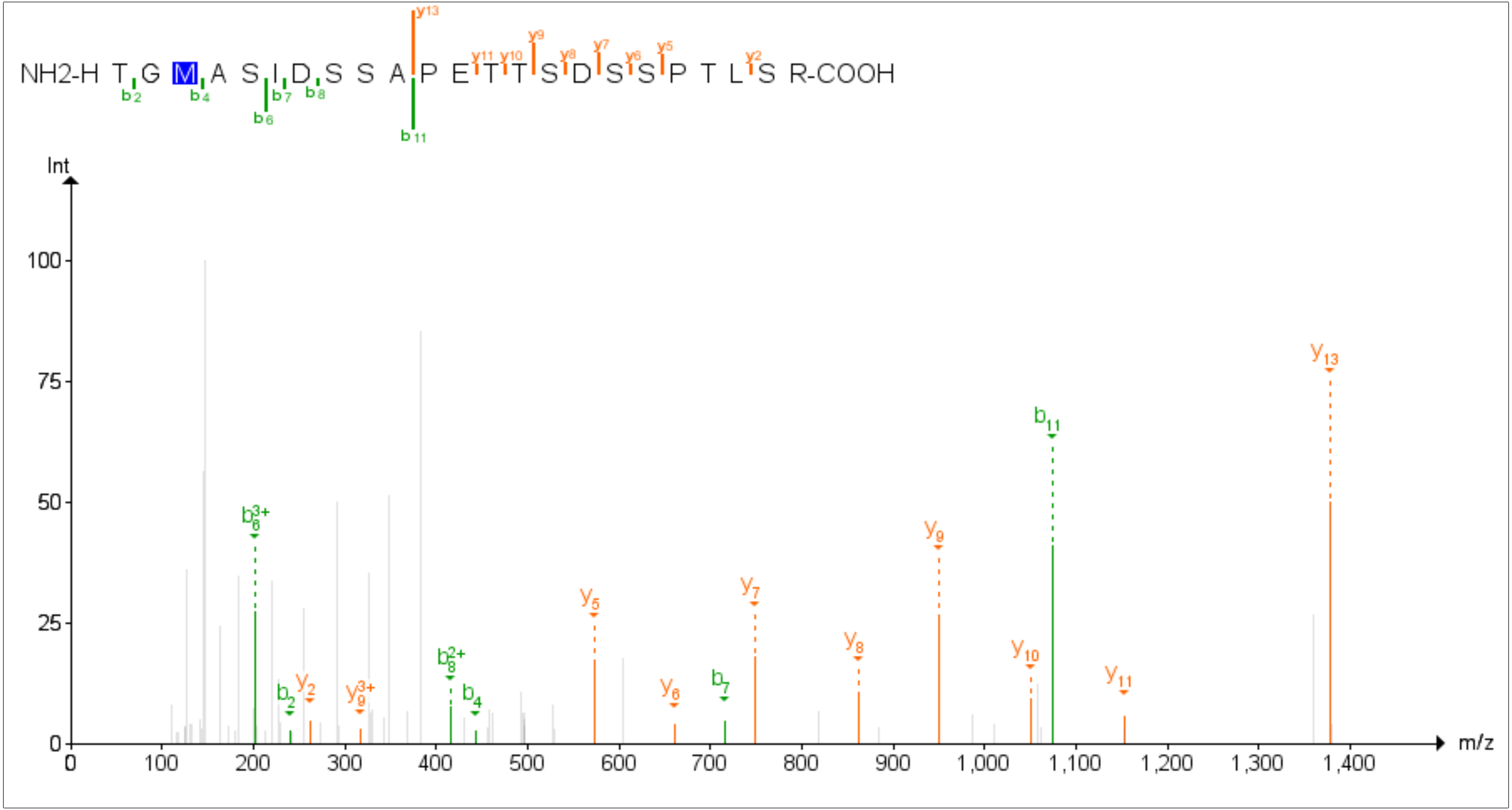

NH2-H T G M A S I D S S A P E T T S D S S P T L S R-COOH
b2 b4 b6 b7 b8 b11
y13 y11 y10 y9 y8 y7 y6 y5 y2
Int
m/z

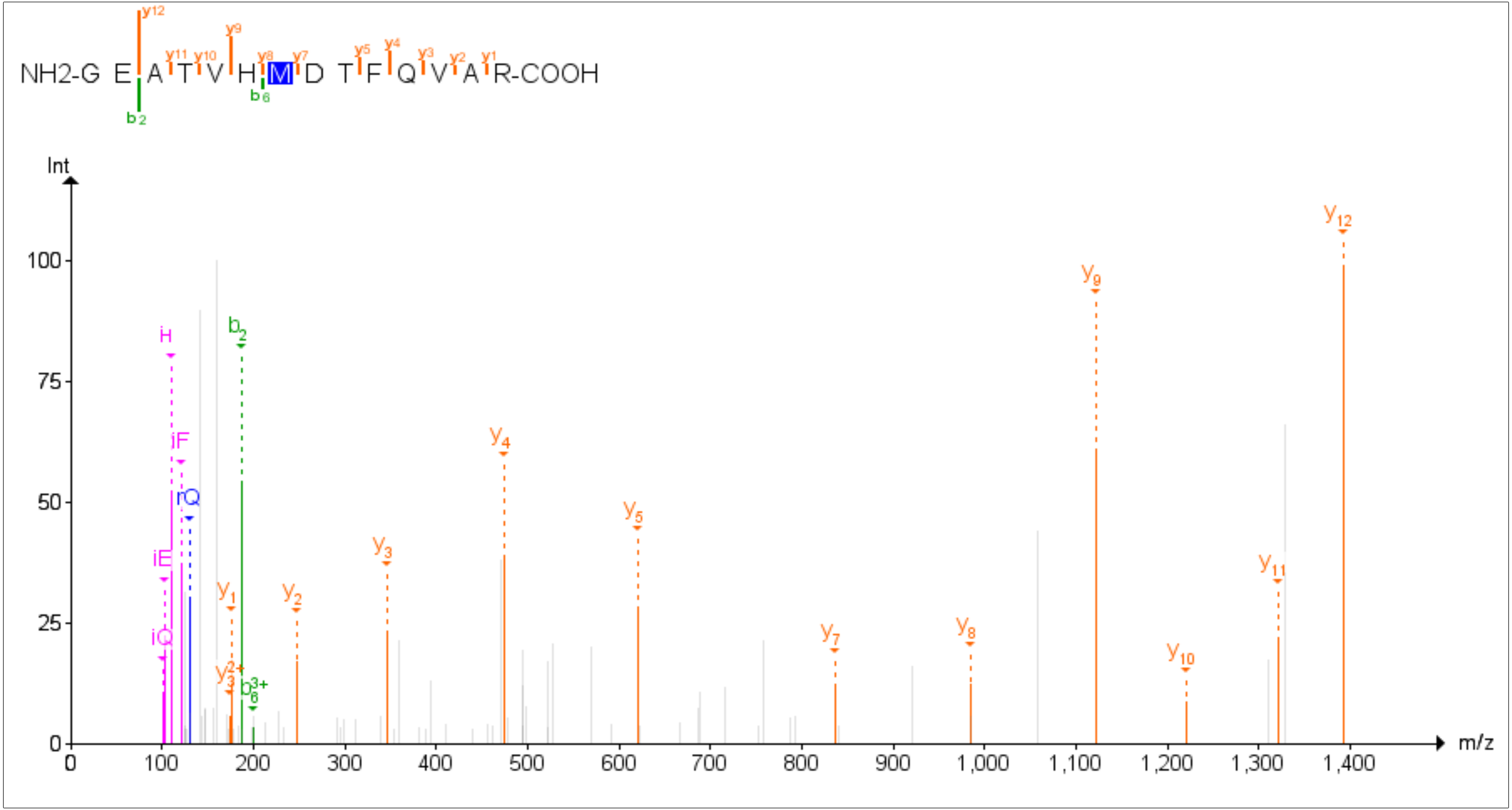
NH2-G E A T V H M D T F Q V A R-COOH
y12 y11 y10 y9 y8 y7 y5 y4 y3 y2 y1
b2 b6
Int
100
75
50
25
0
m/z
0 100 200 300 400 500 600 700 800 900 1,000 1,100 1,200 1,300 1,400
iH iF rQ iE iQ b2 y1 y2 y3 y4 y5 y7 y8 y9 y10 y11 y12

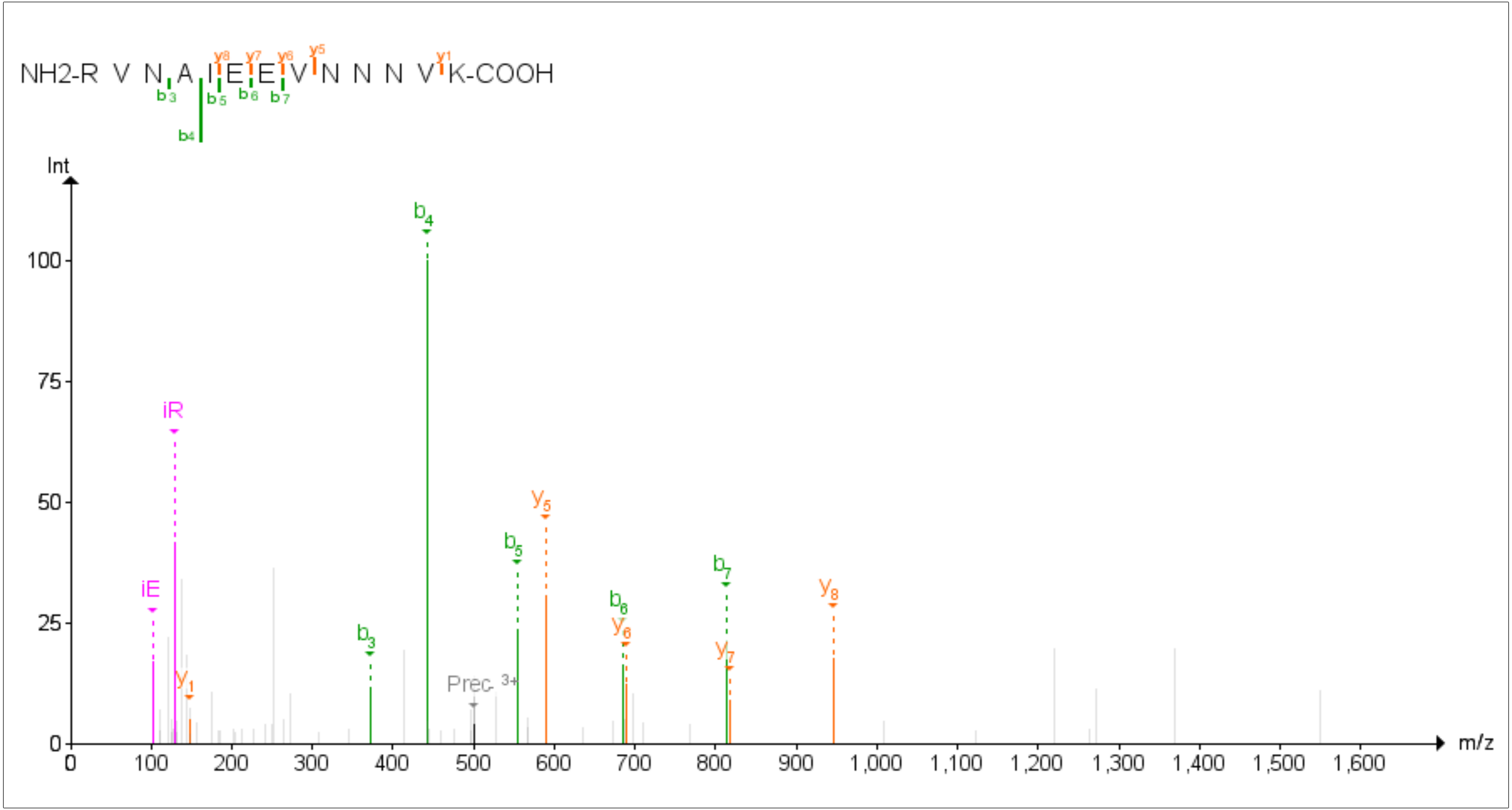

NH2-R V N A I E E V N N N V K-COOH
y8 y7 y6 y5 y1
b3 b4 b5 b6 b7
Int
100
75
50
25
0
b4
iR
y5
b5
iE
b7
b6
y6
y8
y7
b3
y1
Prec 3+
0 100 200 300 400 500 600 700 800 900 1,000 1,100 1,200 1,300 1,400 1,500 1,600
m/z

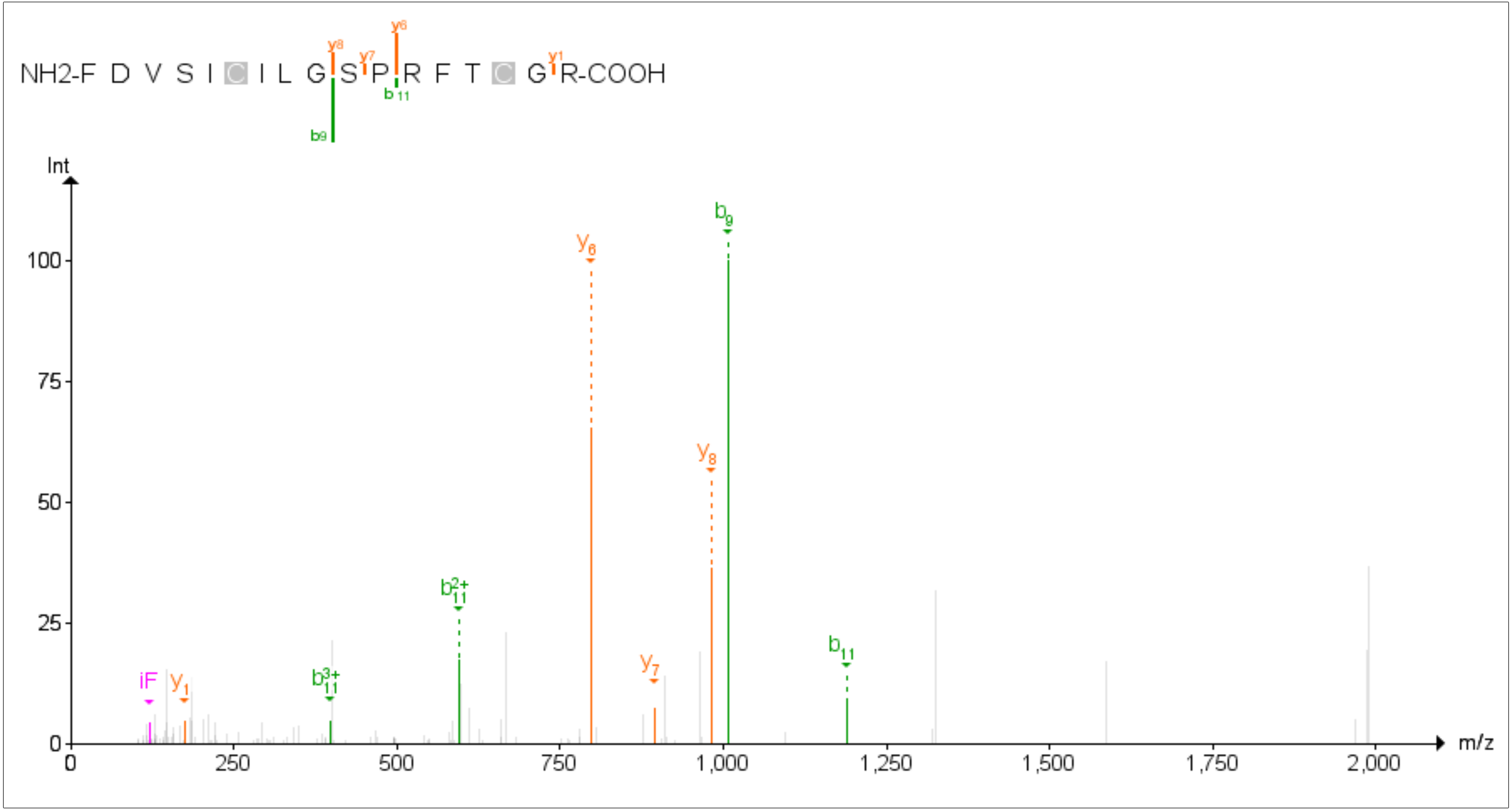

NH2-F D V S I C I L G S P R F T C G R-COOH
y8
y7
y6
y1
b9
b 11
Int
100
75
50
25
0
iF
y_1
b_{11}^{3+}
b_{11}^{2+}
y_6
y_7
y_8
b_9
b_{11}
0
250
500
750
1,000
1,250
1,500
1,750
2,000
m/z

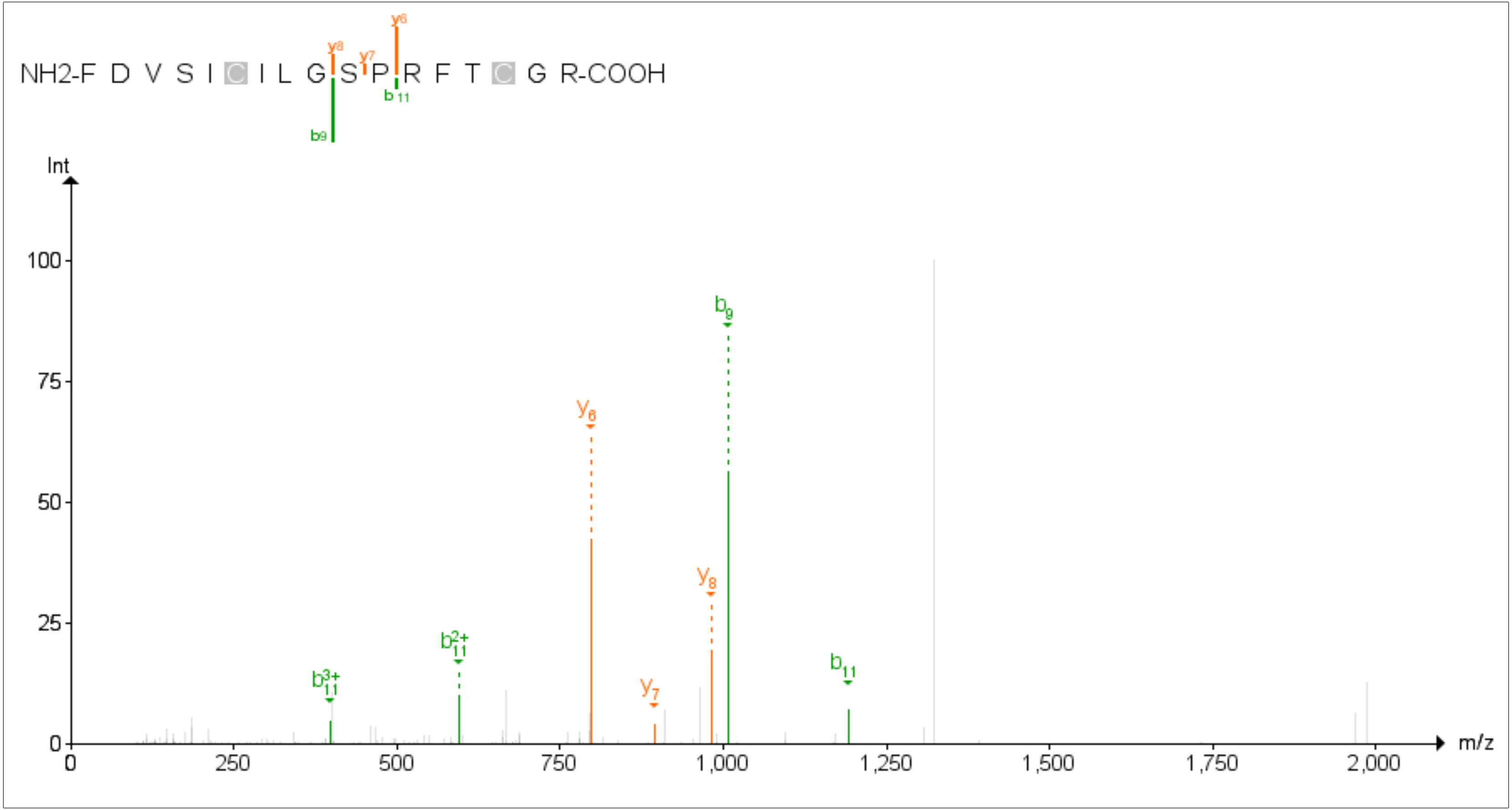

NH2-F D V S I C I L G S P R F T C G R-COOH
y8
y7
y6
b9
b 11
Int
100
75
50
25
0
b9
y6
y8
y7
b11
b11 2+
b11 3+
0
250
500
750
1,000
1,250
1,500
1,750
2,000
m/z

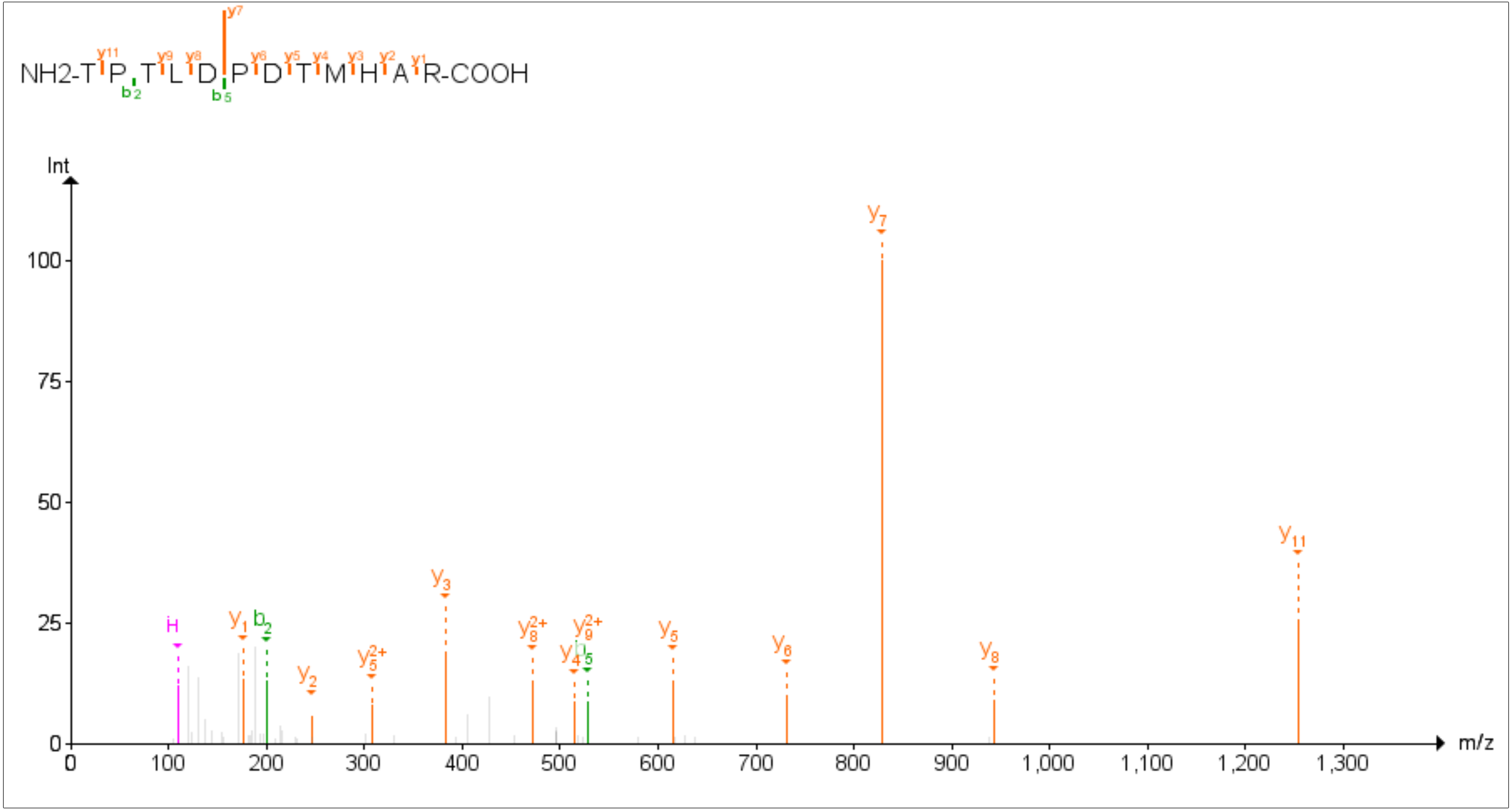

NH2-TPTLDPDTMHAR-COOH
y11 y9 y8 y7 y6 y5 y4 y3 y2 y1
b2 b5
Int
m/z
y7
y11
y3
y1
b2
H
y2
y5 2+
y8 2+
y9 2+
y4
b5
y5
y6
y8
0 25 50 75 100
0 100 200 300 400 500 600 700 800 900 1,000 1,100 1,200 1,300

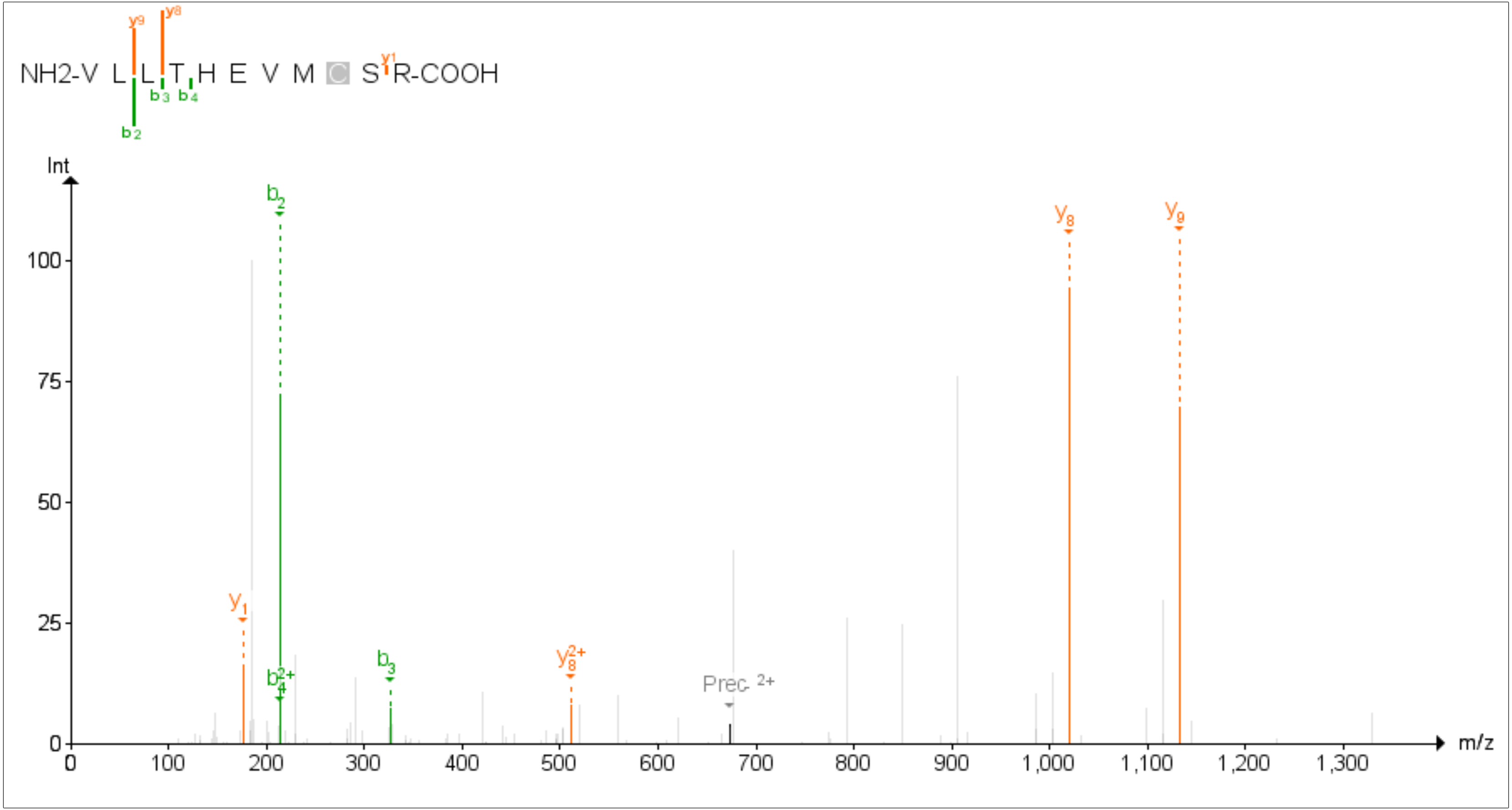
NH2-V L L T H E V M C S R-COOH
y9
y8
y1
b2
b3
b4
Int
100
75
50
25
0
b2
y8
y9
y1
b4 2+
b3
y8 2+
Prec- 2+
0
100
200
300
400
500
600
700
800
900
1,000
1,100
1,200
1,300
m/z

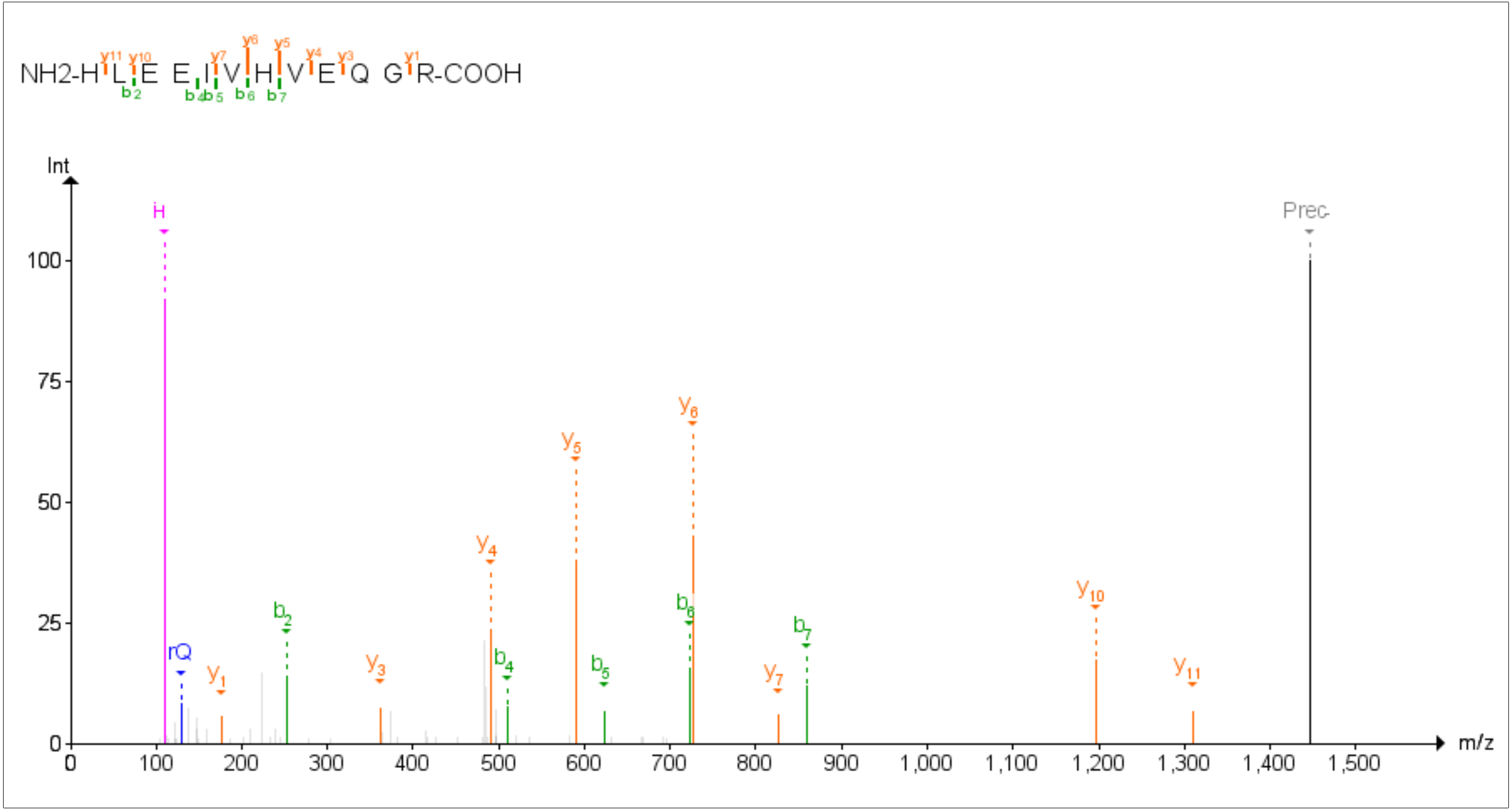
NH2-H L E E I V H V E Q G R-COOH
y11 y10 y7 y6 y5 y4 y3 y1
b2 b4 b5 b6 b7
Int
100
75
50
25
0
H
rQ
y1
b2
y3
y4
b4
y5
b5
y6
b6
y7
b7
y10
y11
Prec
m/z
0
100
200
300
400
500
600
700
800
900
1,000
1,100
1,200
1,300
1,400
1,500

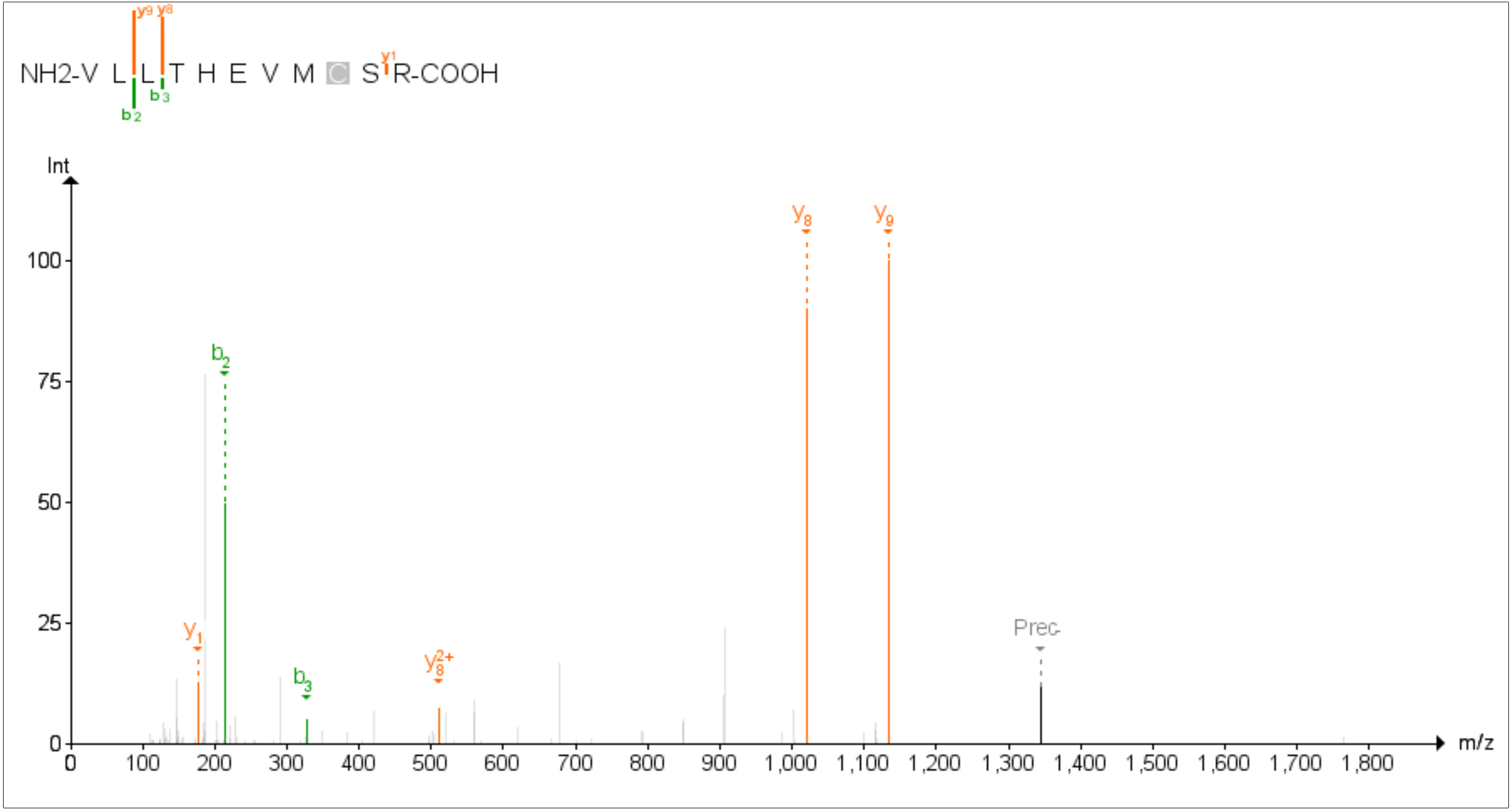

NH2-V L L T H E V M C S R-COOH
y9 y8 y1
b2 b3
Int
100
75
50
25
0
y8
y9
b2
y1
b3
y_8^{2+}
Prec
m/z
0 100 200 300 400 500 600 700 800 900 1,000 1,100 1,200 1,300 1,400 1,500 1,600 1,700 1,800

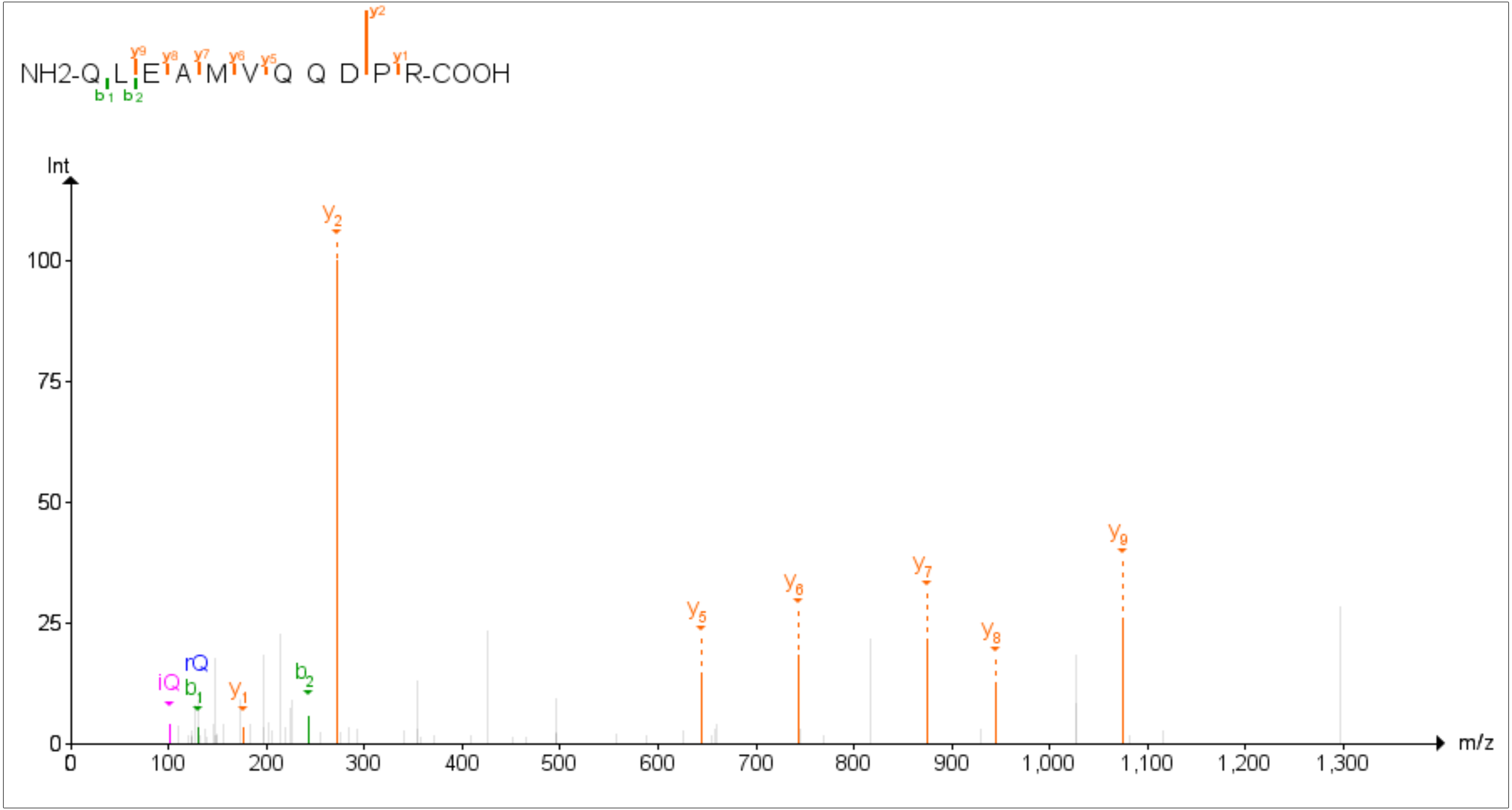

NH2-Q L E A M V Q Q D P R-COOH
y9 y8 y7 y6 y5 y2 y1
b1 b2
Int
100
75
50
25
0
y2
y9
y7
y6
y5
y8
rQ
iQ
b1
y1
b2
0 100 200 300 400 500 600 700 800 900 1,000 1,100 1,200 1,300
m/z

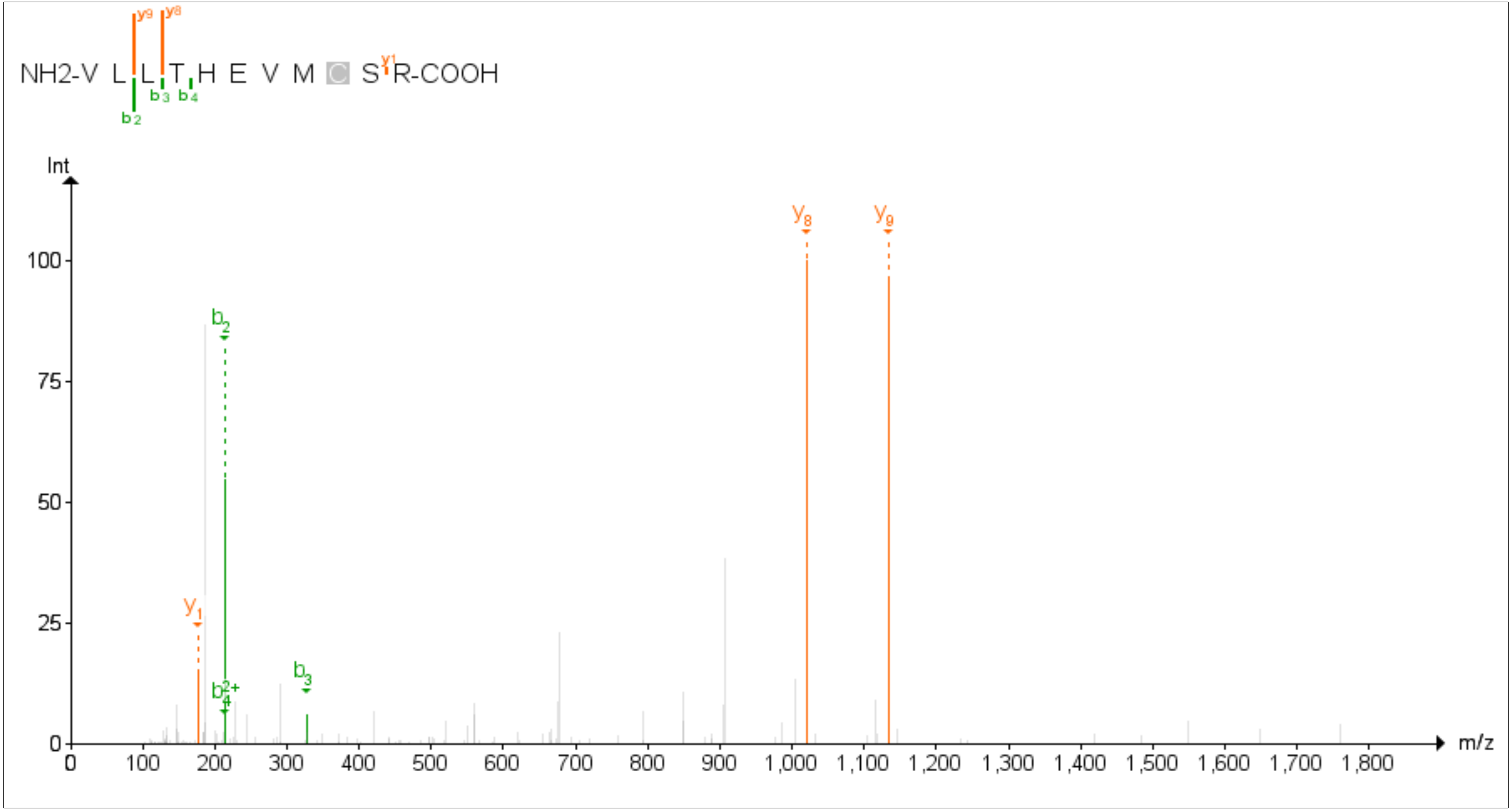

NH2-V L L T H E V M C S R-COOH
y9
y8
y1
b2
b3
b4
Int
m/z
y8
y9
b2
y1
b4 2+
b3
0
25
50
75
100
0
100
200
300
400
500
600
700
800
900
1,000
1,100
1,200
1,300
1,400
1,500
1,600
1,700
1,800

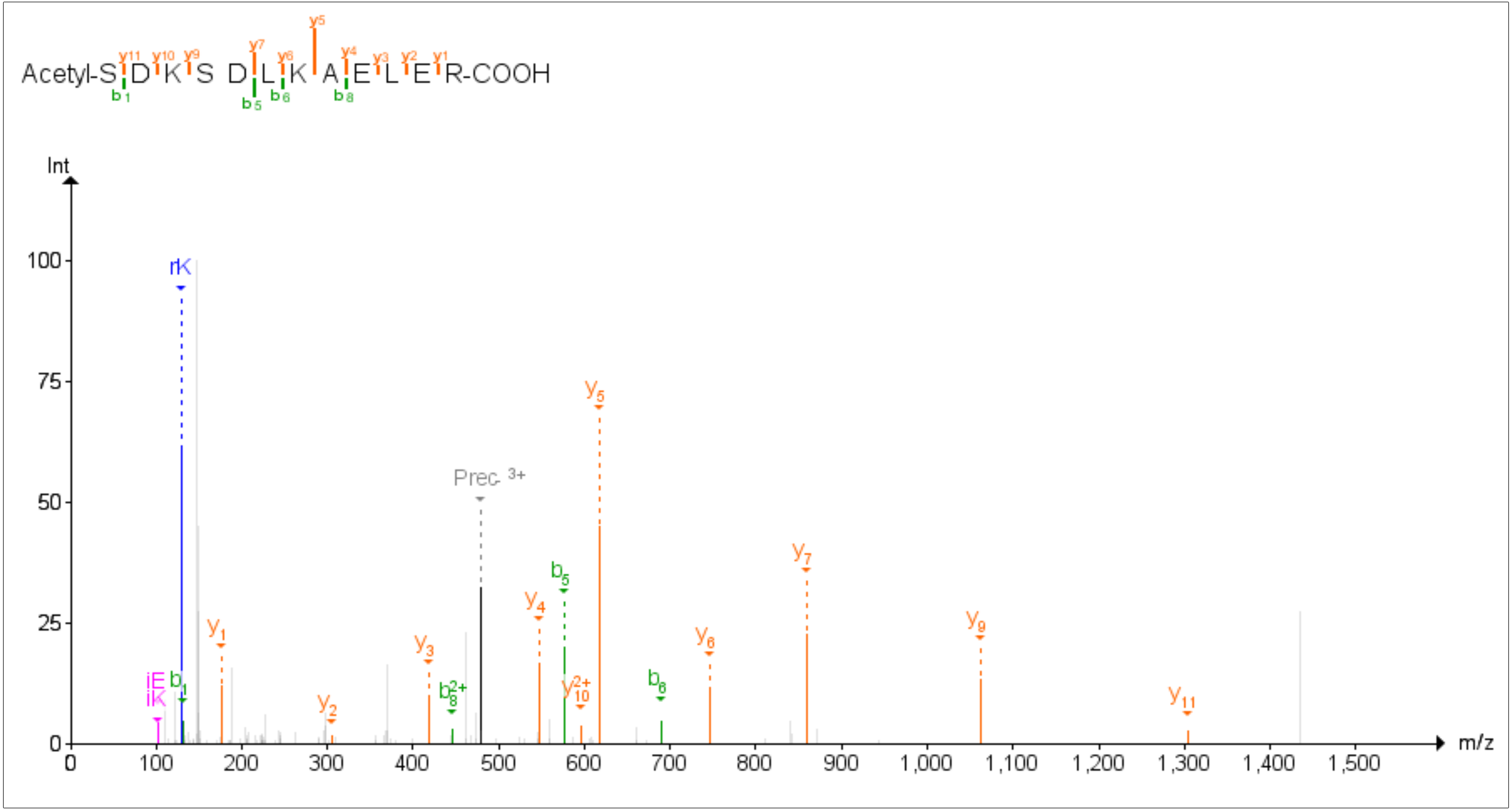

Acetyl-S D K S D L K A E L E R-COOH
y11 y10 y9 y7 y6 y5 y4 y3 y2 y1
b1 b5 b6 b8
Int
100
75
50
25
0
rK
Prec 3+
y5
y7
b5
y4
y9
y1
y3
y6
iE
iK
b1
b8 2+
y10 2+
b6
y2
y11
0
100
200
300
400
500
600
700
800
900
1,000
1,100
1,200
1,300
1,400
1,500
m/z

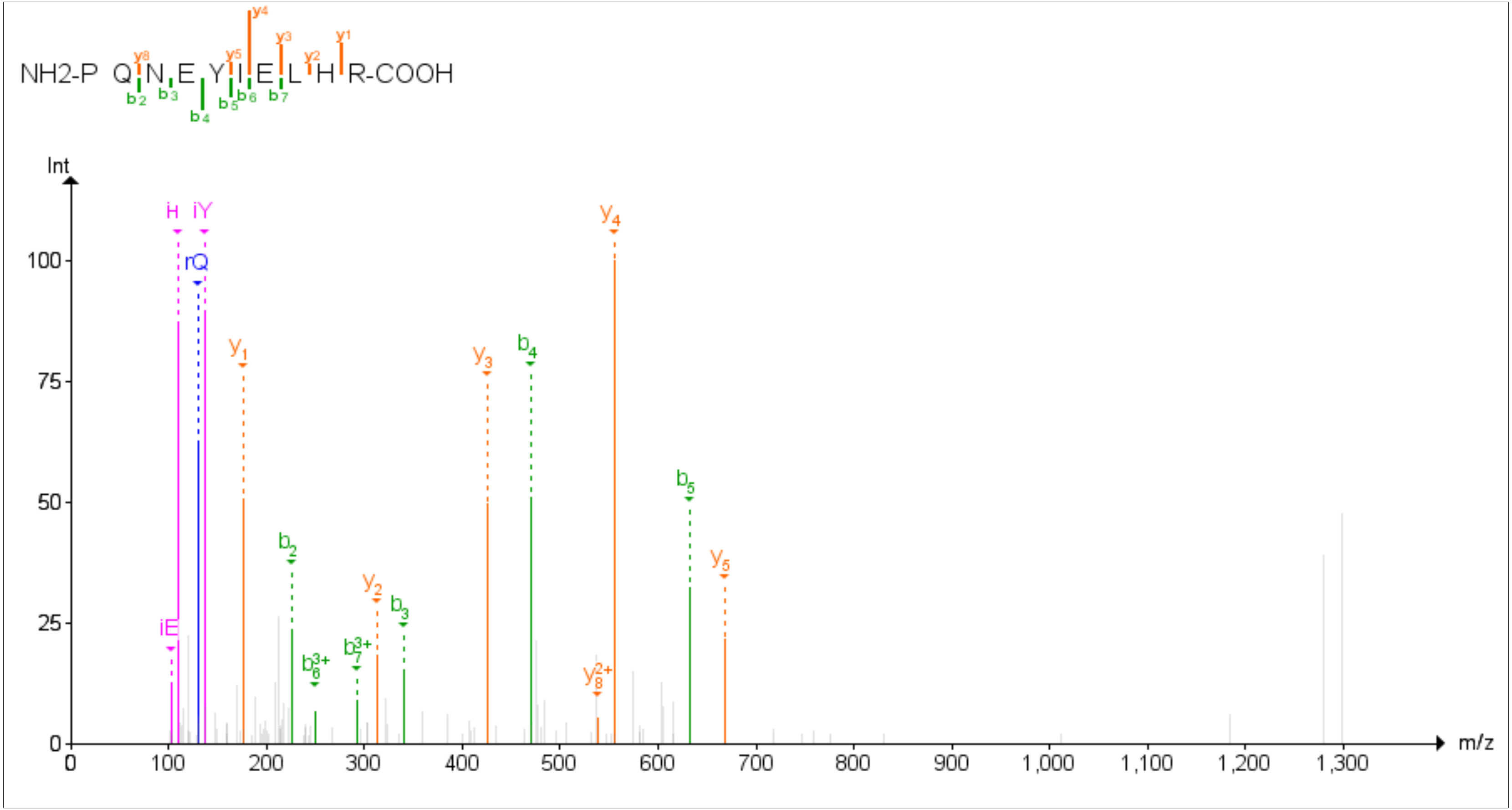
NH2-P Q N E Y I E L H R-COOH
y8 y5 y4 y3 y2 y1
b2 b3 b4 b5 b6 b7
Int
m/z
iH iY rQ iE
y1 y2 y3 y4 y5 y8 2+
b2 b3 b4 b5 b6 3+ b7 3+

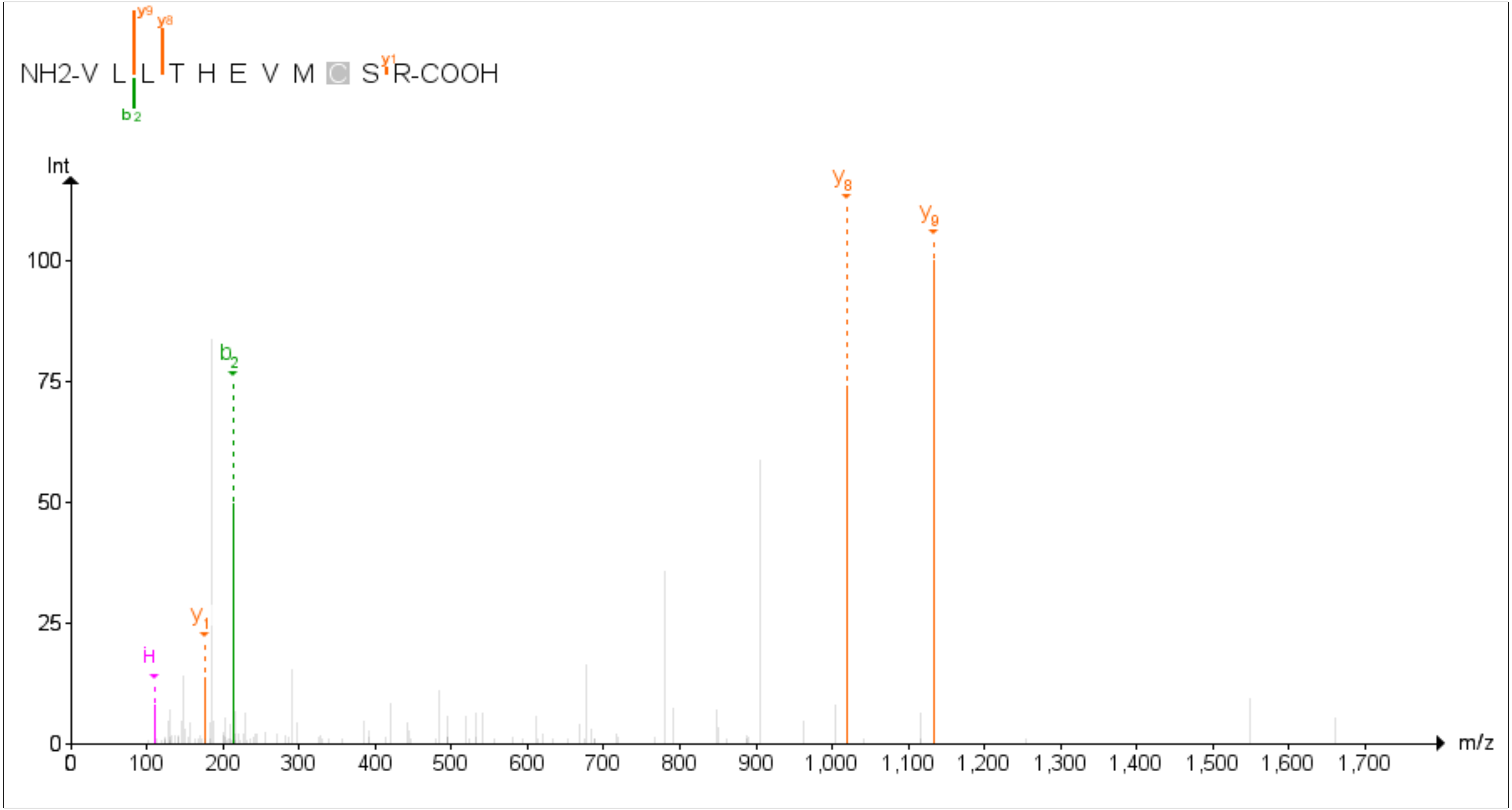

NH2-V L L T H E V M C S R-COOH
y9
y8
y1
b2
Int
m/z
y8
y9
b2
y1
H

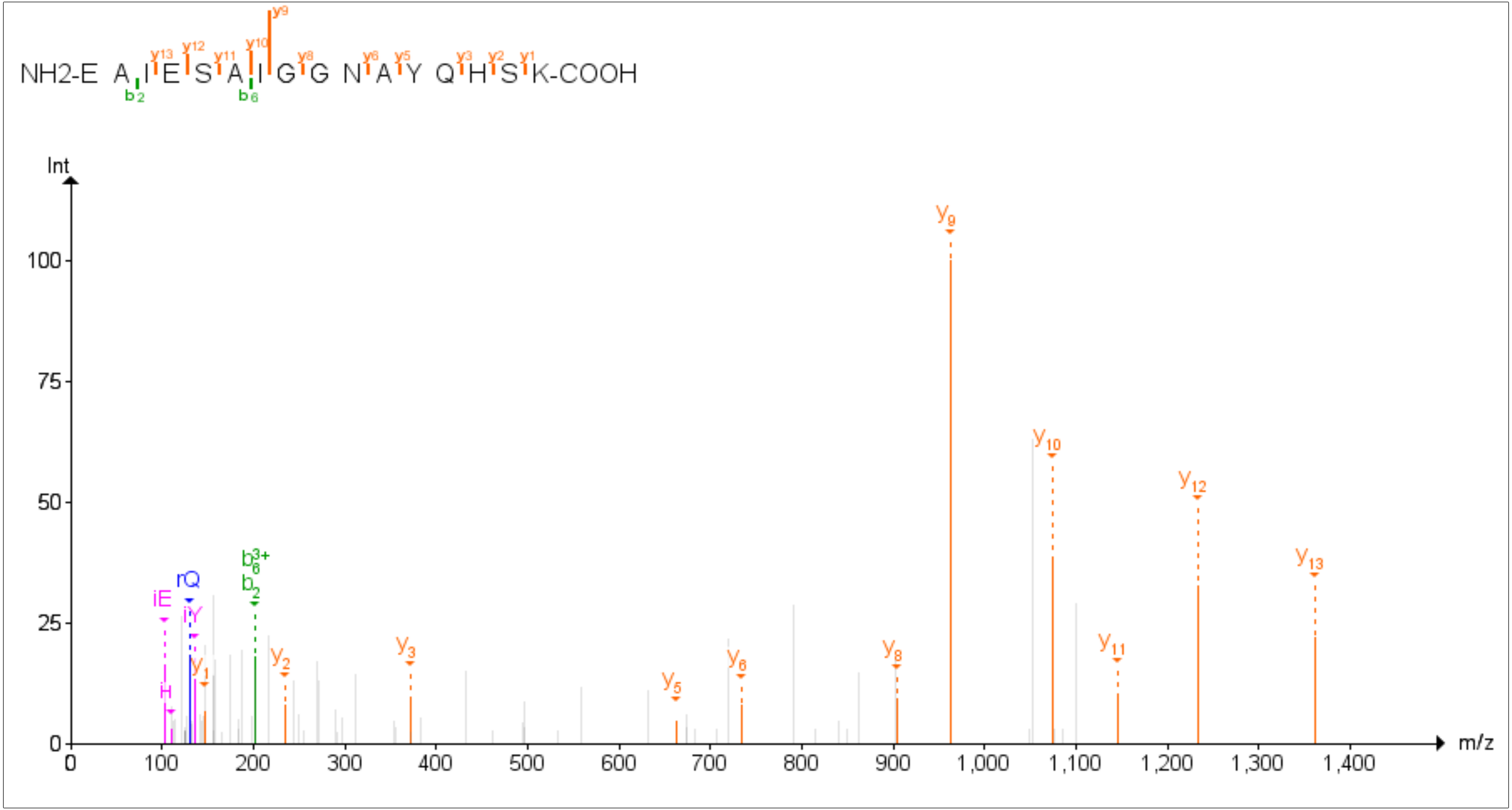

NH2-E A I E S A I G G N A Y Q H S K-COOH
y13 y12 y11 y10 y9 y8 y6 y5 y3 y2 y1
b2 b6
Int
100
75
50
25
0
m/z
0 100 200 300 400 500 600 700 800 900 1,000 1,100 1,200 1,300 1,400
y9 y10 y12 y13 y11 y8 y6 y5 y3 y2 y1
iE rQ iY iH
b6 3+
b2

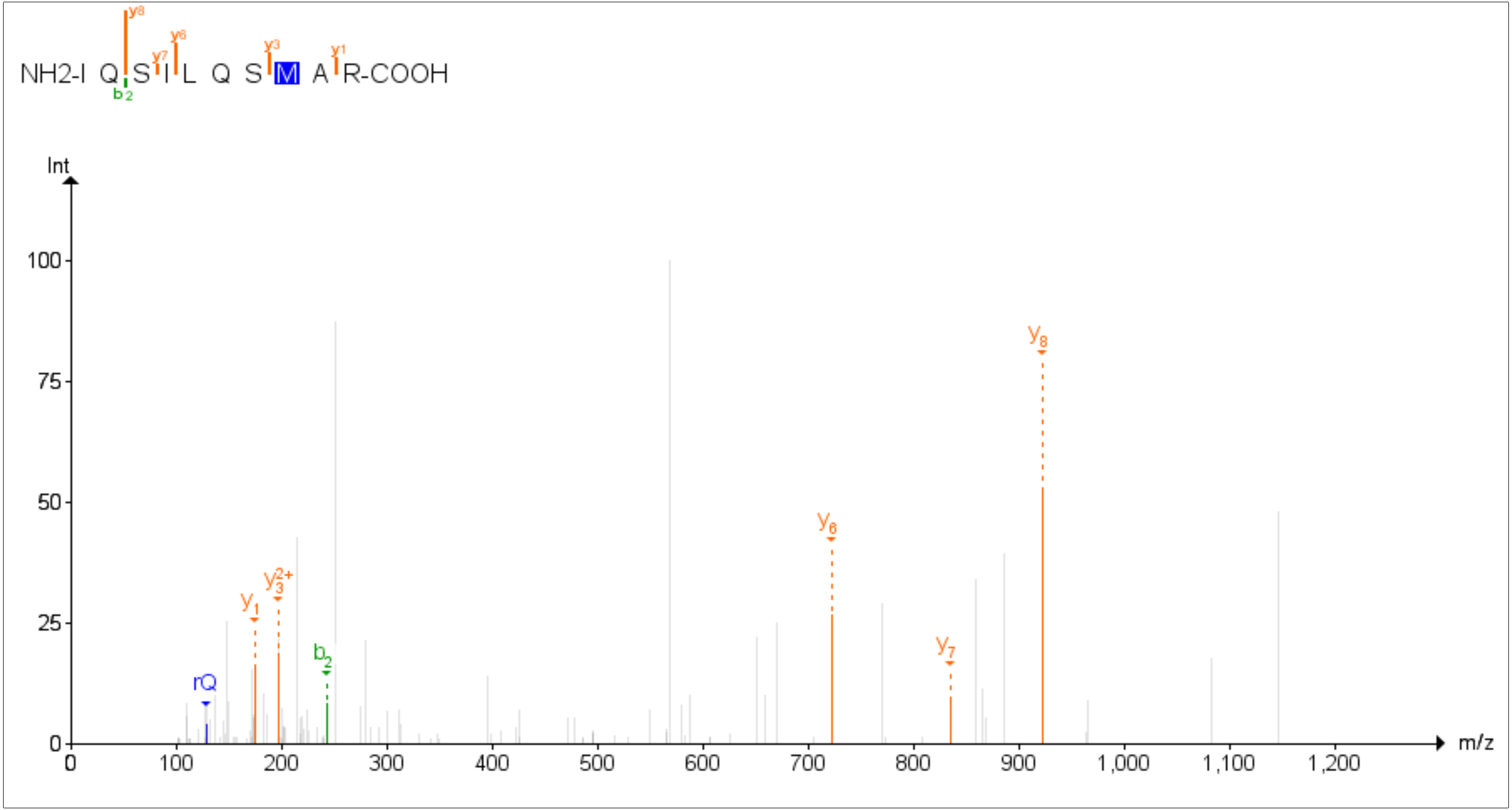
NH2-I Q S I L Q S M A R-COOH
y8
y7
y6
y3
y1
b2
Int
100
75
50
25
0
rQ
y1
y3 2+
b2
y6
y7
y8
0
100
200
300
400
500
600
700
800
900
1,000
1,100
1,200
m/z

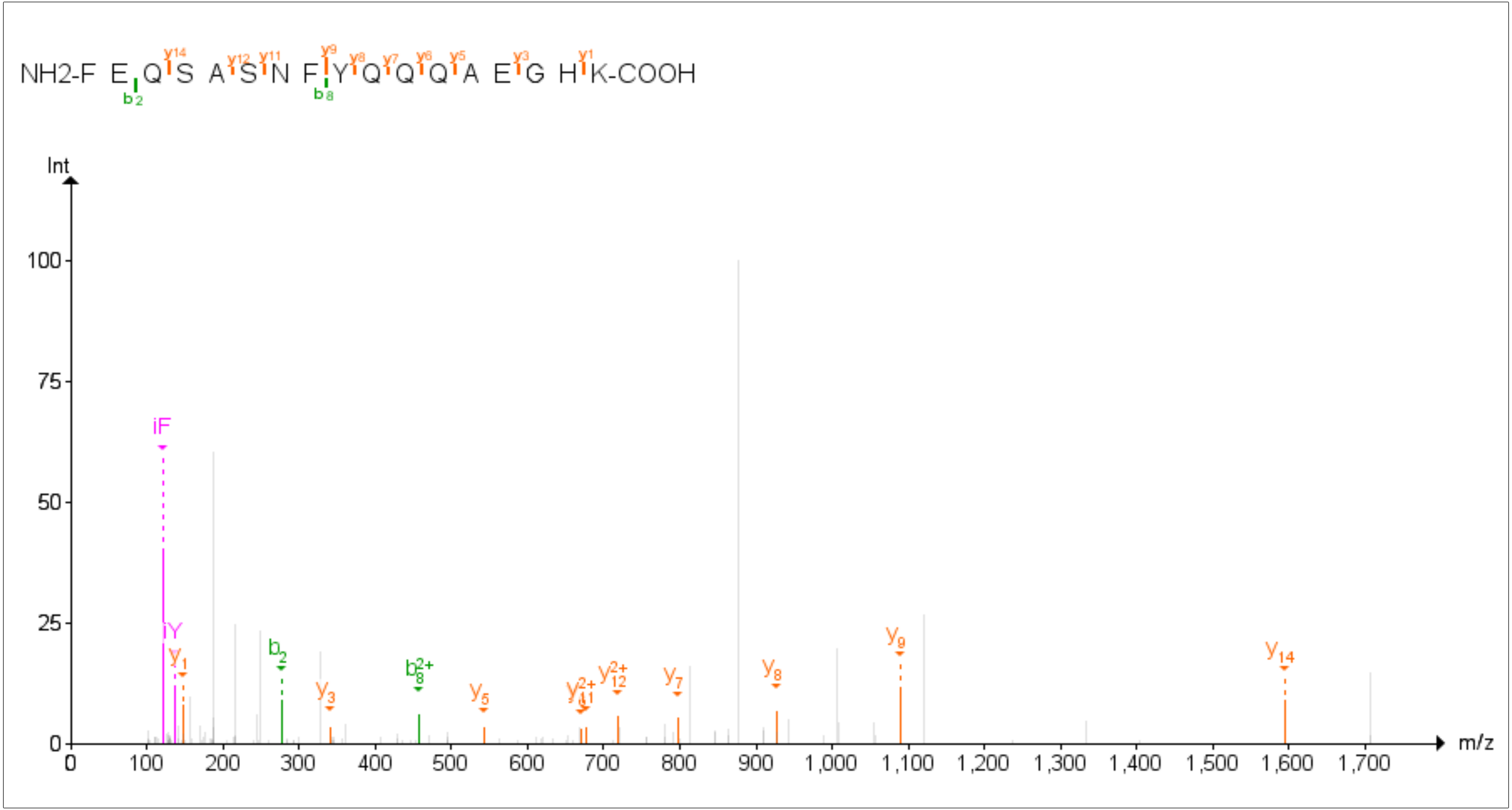

NH2-F E Q S A S N F Y Q Q Q A E G H K-COOH
y14 y12 y11 y9 y8 y7 y6 y5 y3 y1
b2 b8
Int
100
75
50
25
0
iF
iY
y1
b2
y3
b8 2+
y5
y11 2+
y12 2+
y7
y8
y9
y14
0 100 200 300 400 500 600 700 800 900 1,000 1,100 1,200 1,300 1,400 1,500 1,600 1,700
m/z

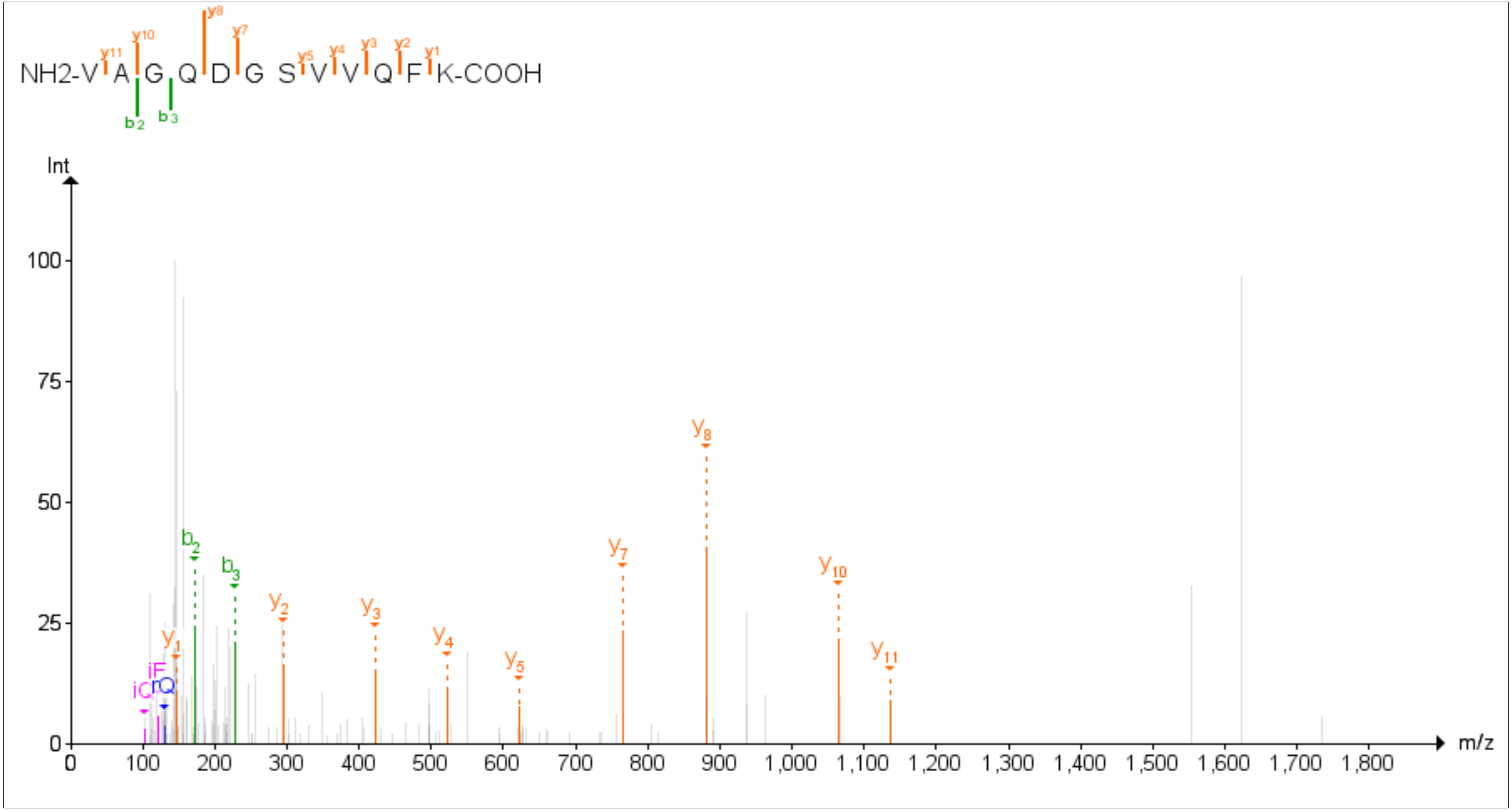

NH2-V A G Q D G S V V Q F K-COOH
y11 y10 y8 y7 y5 y4 y3 y2 y1
b2 b3
Int
100
75
50
25
0
m/z
0 100 200 300 400 500 600 700 800 900 1,000 1,100 1,200 1,300 1,400 1,500 1,600 1,700 1,800
y1 y2 y3 y4 y5 y7 y8 y10 y11
b2 b3
iC iF rQ

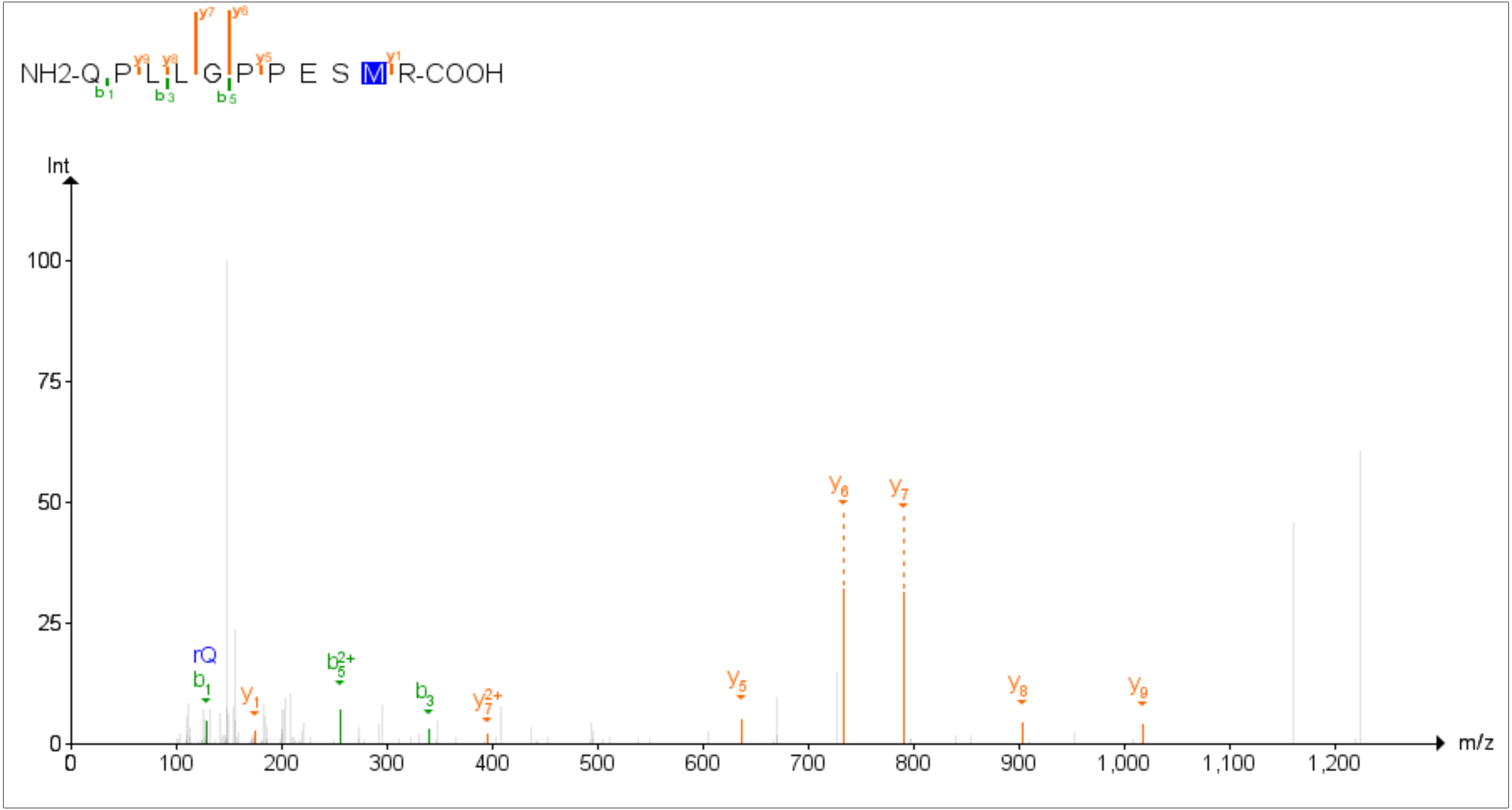

NH2-Q P L L G P P E S M R-COOH
y9 y8 y7 y6 y5 y1
b1 b3 b5
Int
100
75
50
25
0
rQ
b1
y1
b5 2+
b3
y7 2+
y5
y6
y7
y8
y9
0 100 200 300 400 500 600 700 800 900 1,000 1,100 1,200
m/z

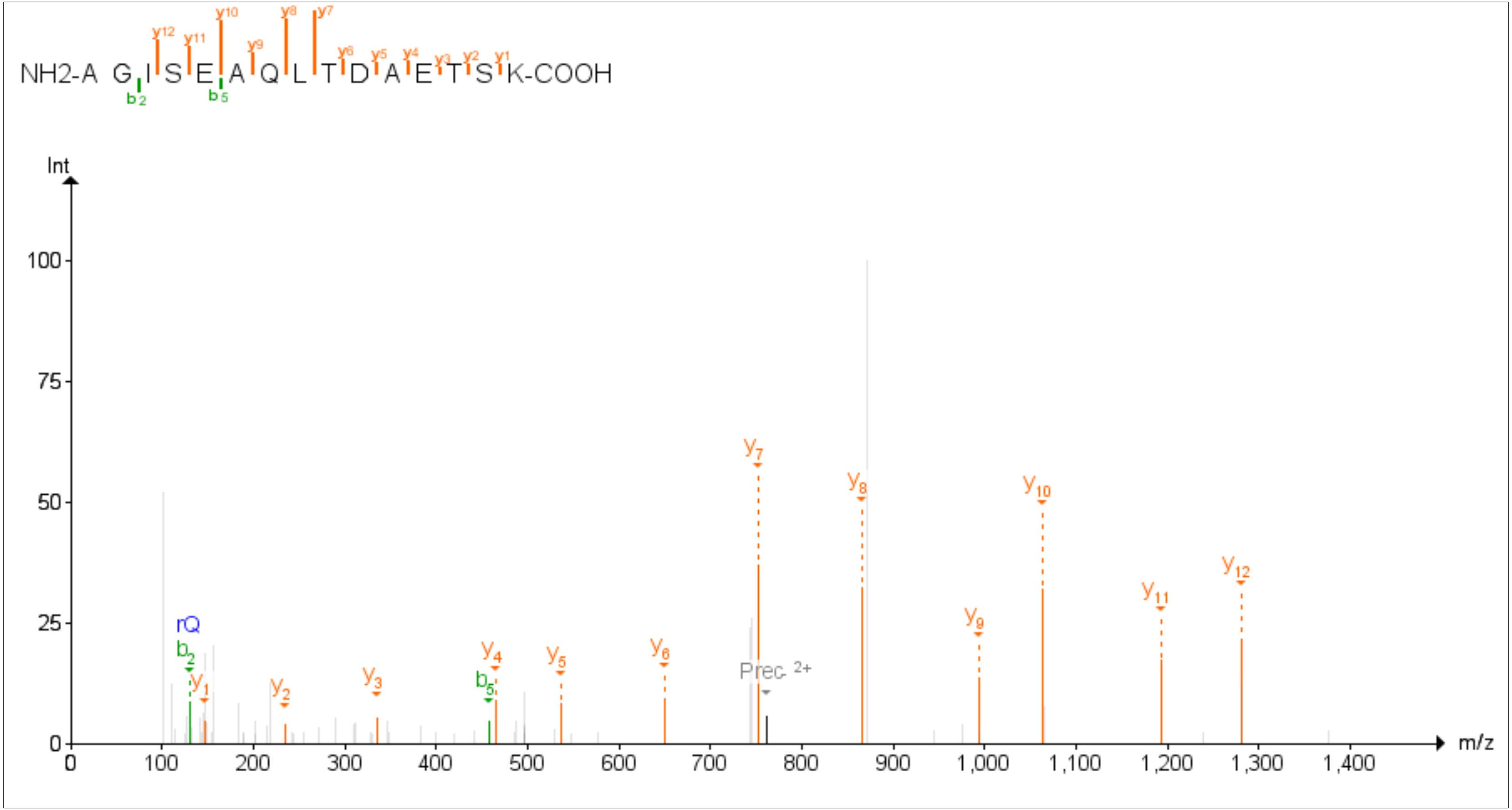

NH2-A G I S E A Q L T D A E T S K-COOH
y12 y11 y10 y9 y8 y7 y6 y5 y4 y3 y2 y1
b2 b5
Int
100
75
50
25
0
rQ
b2
y1
y2
y3
y4
b5
y5
y6
Prec. 2+
y7
y8
y9
y10
y11
y12
0 100 200 300 400 500 600 700 800 900 1,000 1,100 1,200 1,300 1,400
m/z

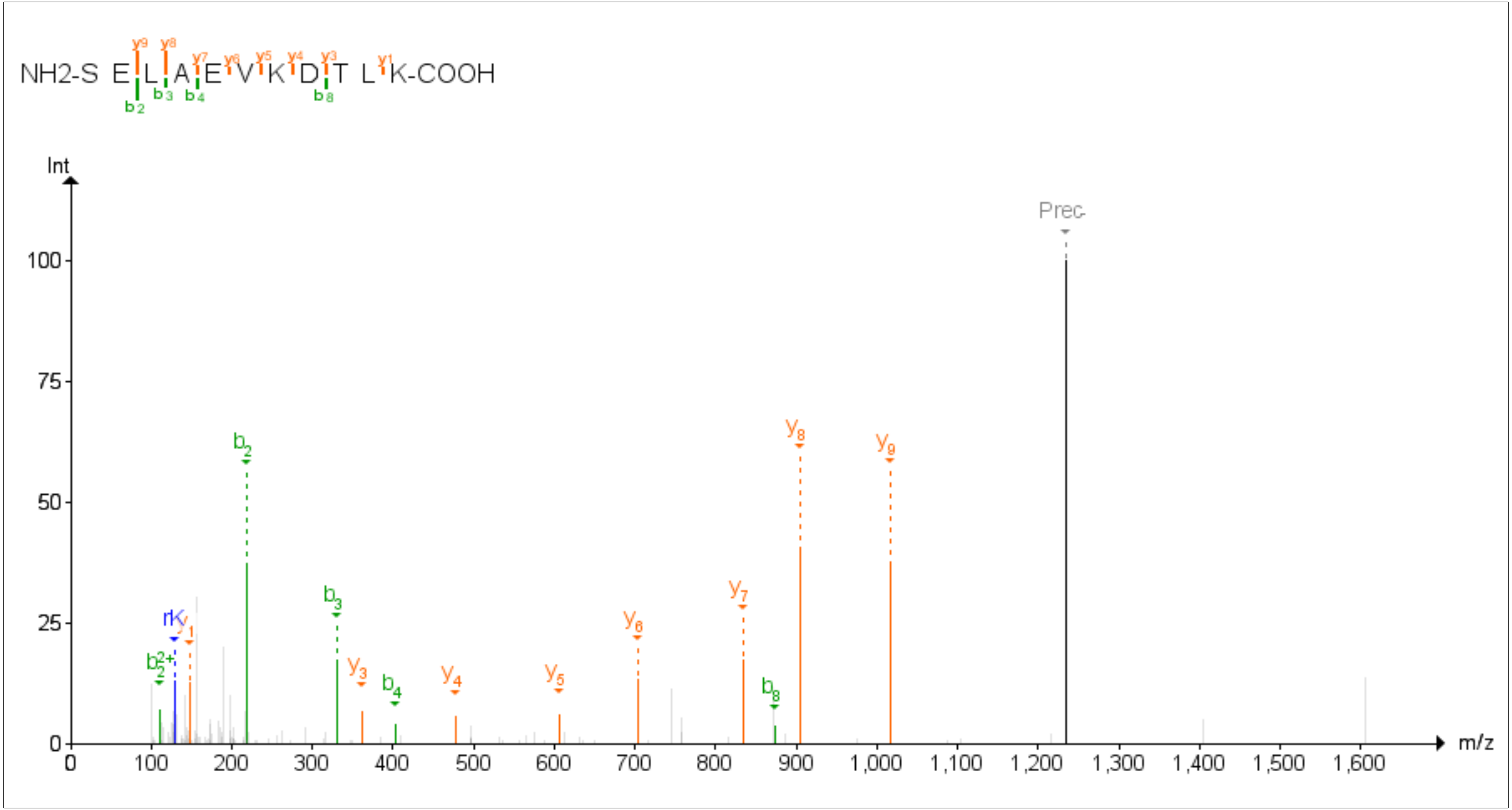

NH2-S E L A E V K D T L K-COOH
y9 y8 y7 y6 y5 y4 y3 y1
b2 b3 b4 b8
Int
m/z
Prec
b2
b3
b4
b8
y1
y3
y4
y5
y6
y7
y8
y9
rK
b2 2+
0
25
50
75
100
0 100 200 300 400 500 600 700 800 900 1,000 1,100 1,200 1,300 1,400 1,500 1,600

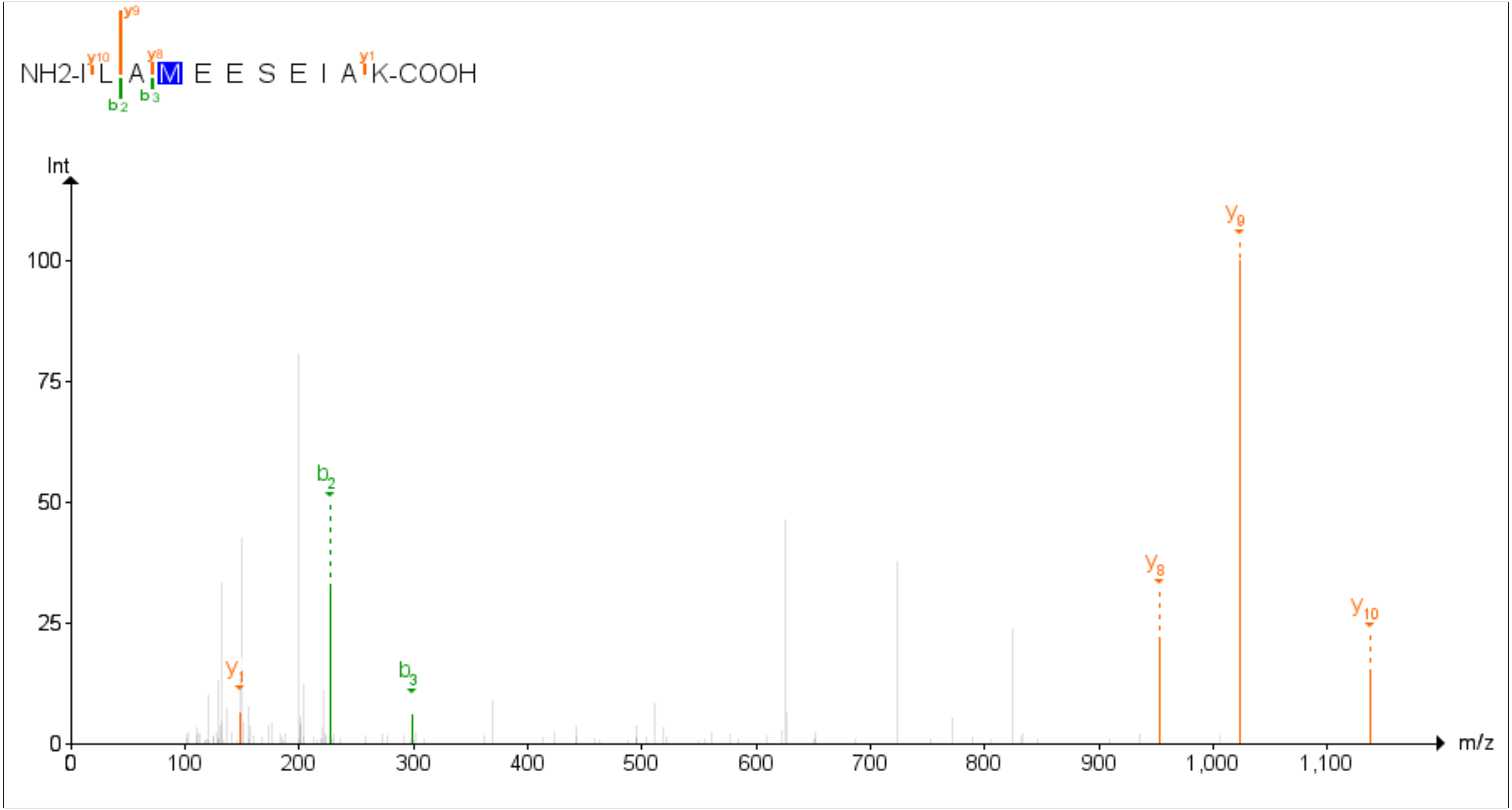

NH2-I L A M E E S E I A K-COOH
y10
y9
y8
y1
b2
b3
Int
100
75
50
25
0
m/z
0
100
200
300
400
500
600
700
800
900
1,000
1,100
y9
b2
y8
y10
y1
b3

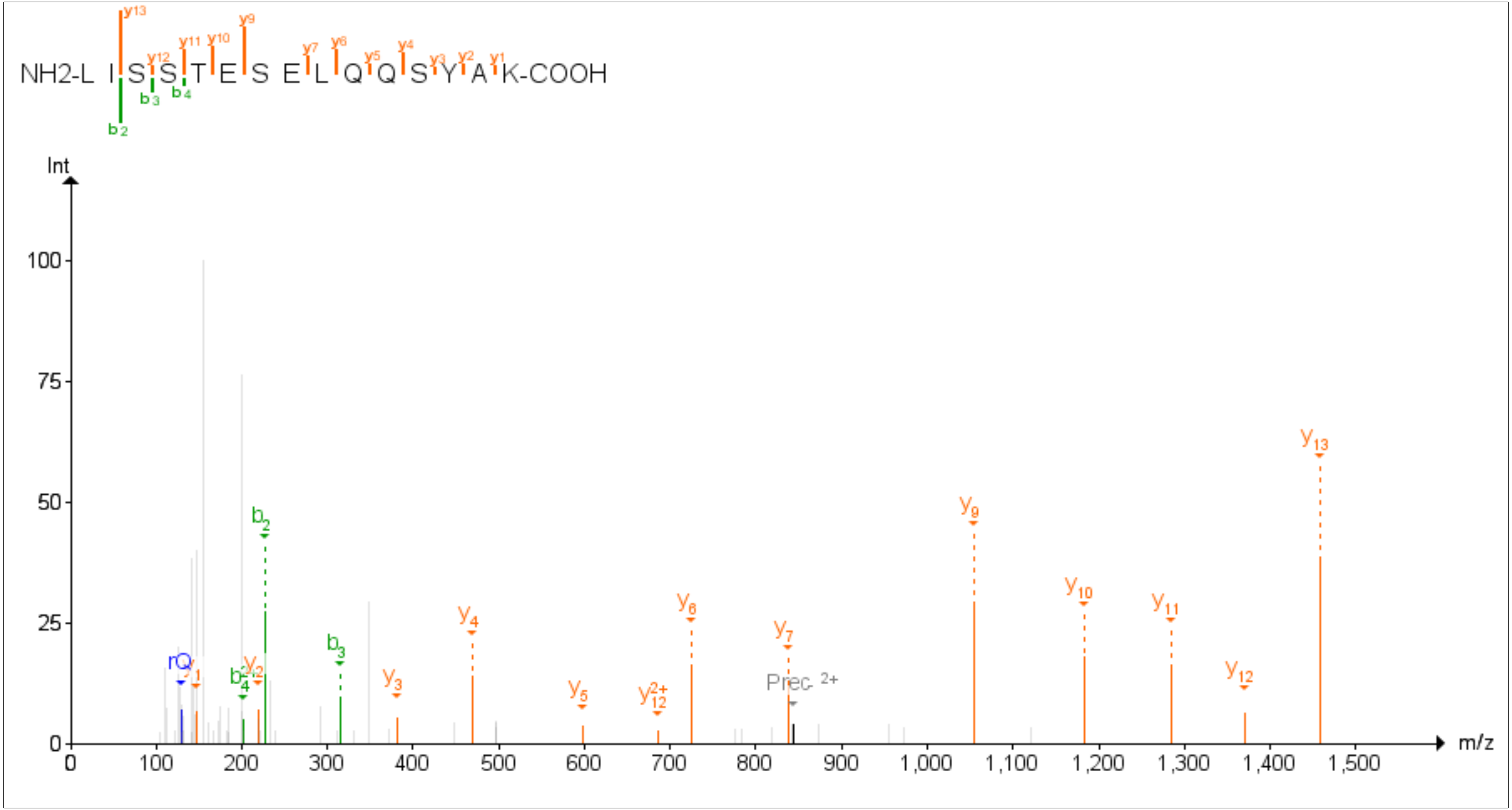

NH2-L I S S T E S E L Q Q S Y A K-COOH
y13 y12 y11 y10 y9 y7 y6 y5 y4 y3 y2 y1
b2 b3 b4
Int
100
75
50
25
0
m/z
0 100 200 300 400 500 600 700 800 900 1,000 1,100 1,200 1,300 1,400 1,500
rQ
y1
b4
y2
b2
b3
y3
y4
y5
y12 2+
y6
y7
Prec. 2+
y9
y10
y11
y12
y13

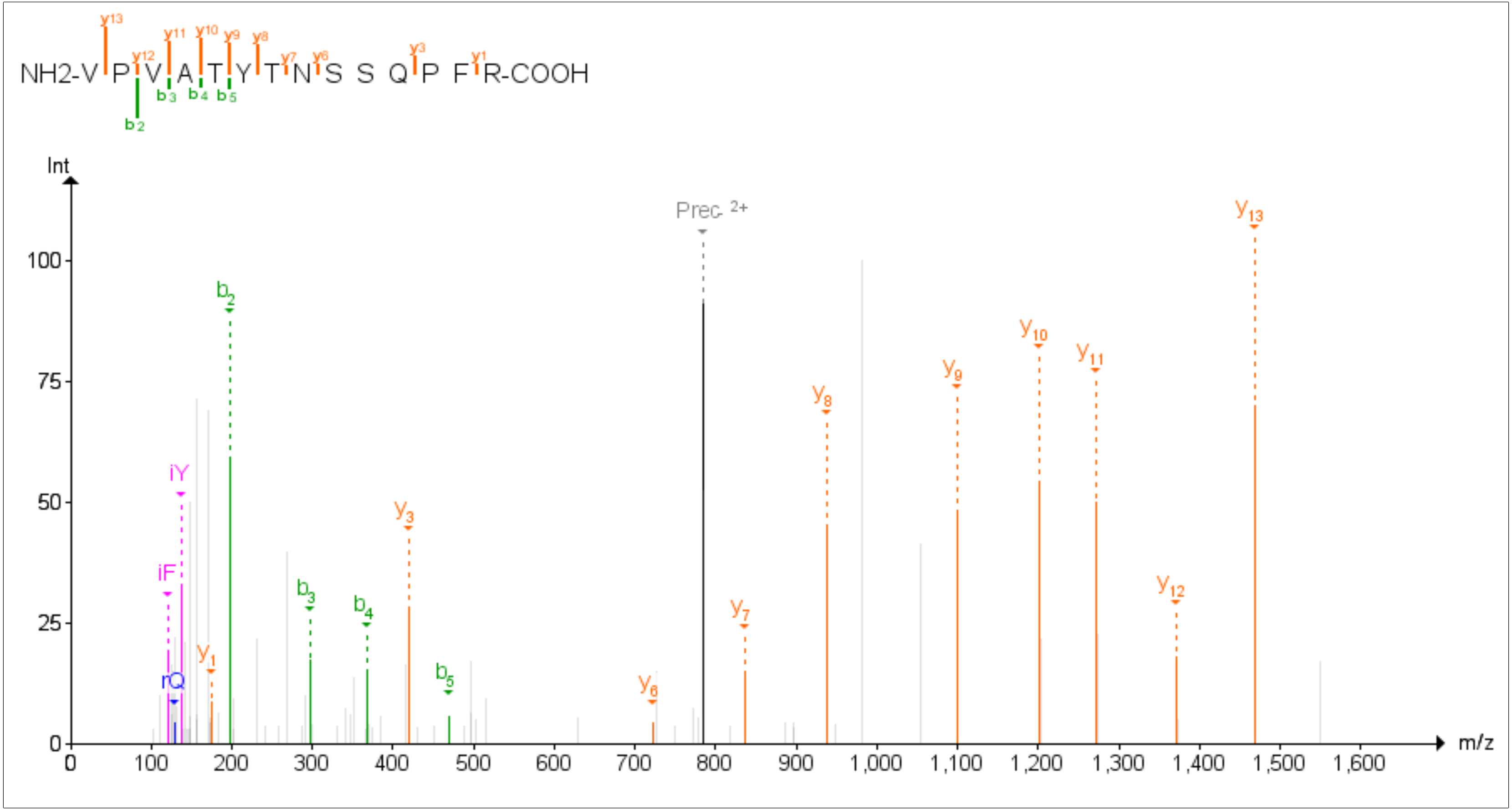

NH2-V P V A T Y T N S S Q P F R-COOH
y13 y12 y11 y10 y9 y8 y7 y6 y3 y1
b2 b3 b4 b5
Int
m/z
Prec 2+
iY
iF
rQ

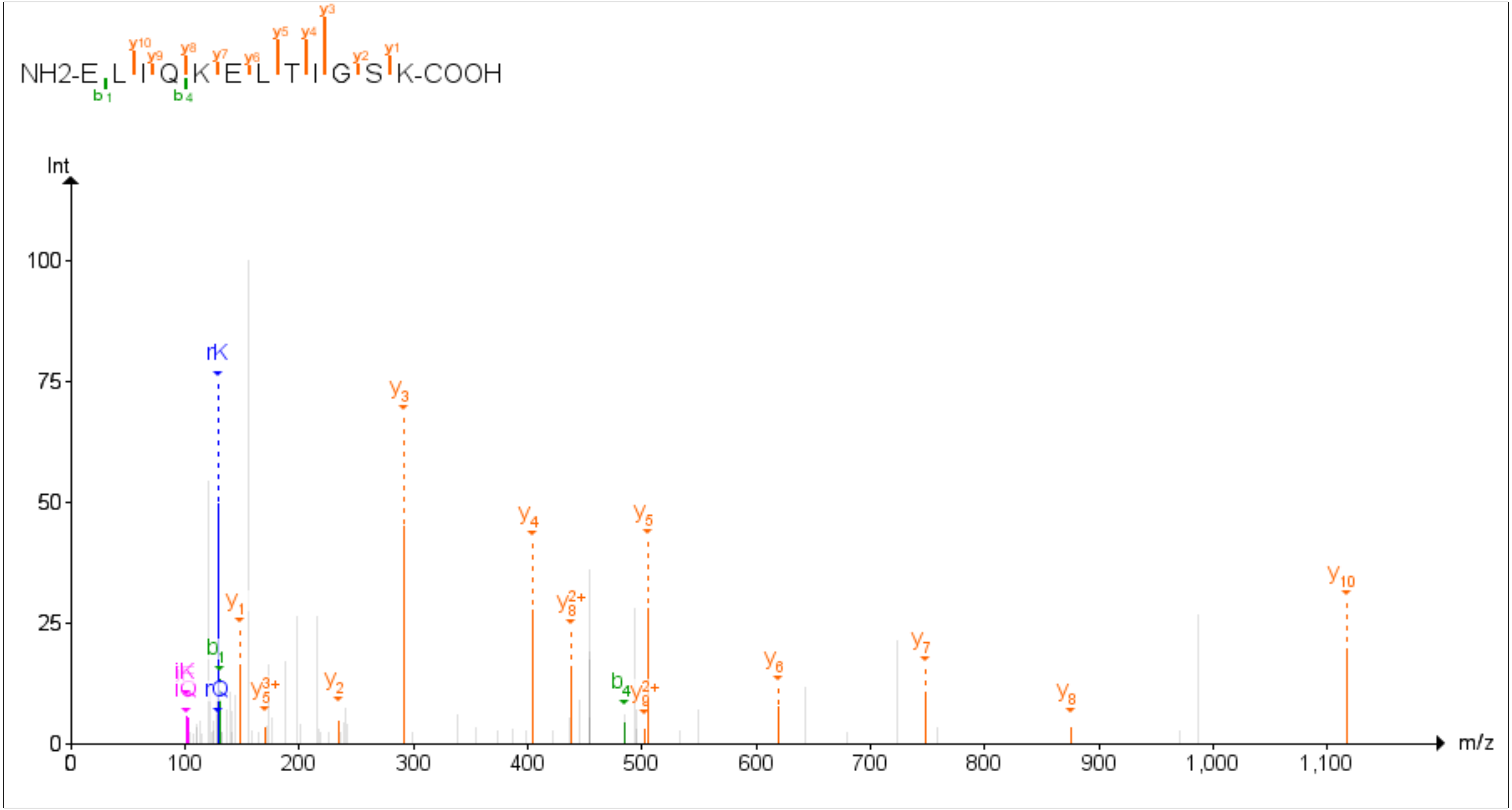

NH2-E L I Q K E L T I G S K-COOH
y10 y9 y8 y7 y6 y5 y4 y3 y2 y1
b1 b4
Int
100
75
50
25
0
rK
y3
y4
y5
y1
y8 2+
b1
iK
iQ
rQ
y5 3+
y2
b4
y9 2+
y6
y7
y8
y10
0
100
200
300
400
500
600
700
800
900
1,000
1,100
m/z

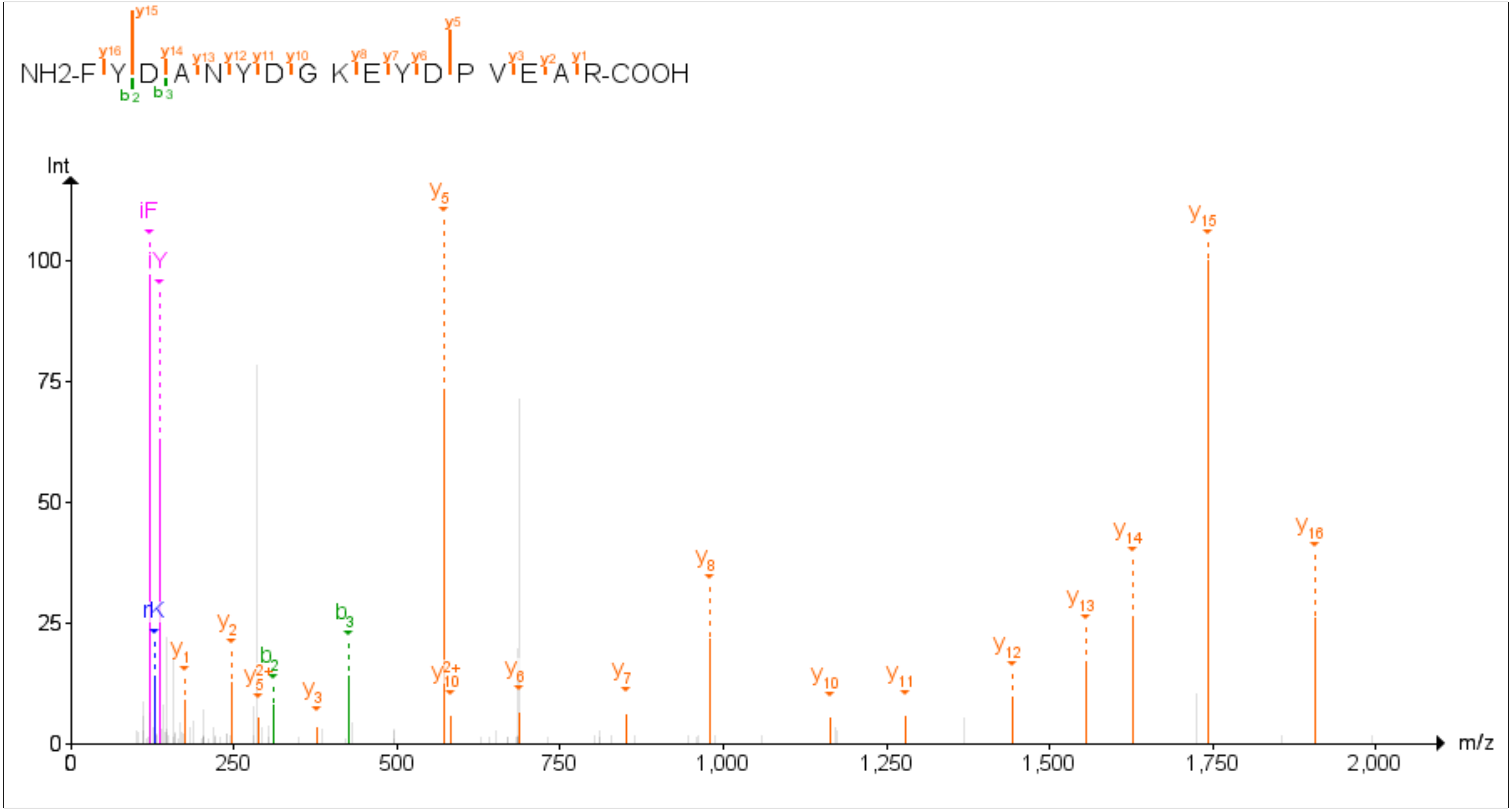
NH2-FYDANYDG KEYDP VEAR-COOH
Int
m/z
iF
iY
iK
y5
y15
y16
y14
y13
y12
y11
y10
y8
y7
y6
y3
y2
y1
b2
b3
0
25
50
75
100
250
500
750
1,000
1,250
1,500
1,750
2,000

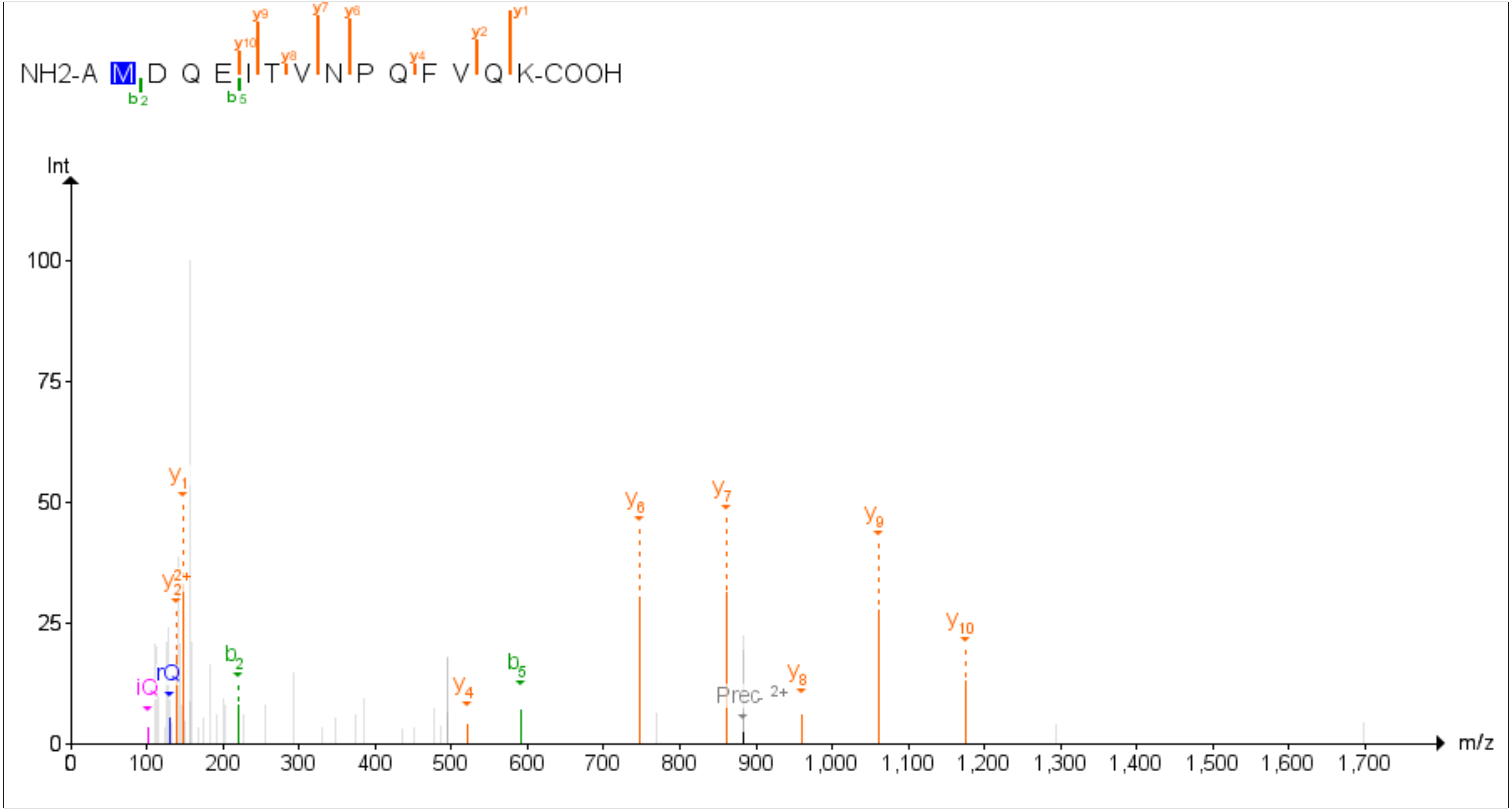
NH2-A M D Q E I T V N P Q F V Q K-COOH
b2
b5
y10
y9
y8
y7
y6
y4
y2
y1
Int
100
75
50
25
0
m/z
0
100
200
300
400
500
600
700
800
900
1,000
1,100
1,200
1,300
1,400
1,500
1,600
1,700
y1
y2 2+
iQ
rQ
b2
y4
b5
y6
y7
Prec 2+
y8
y9
y10

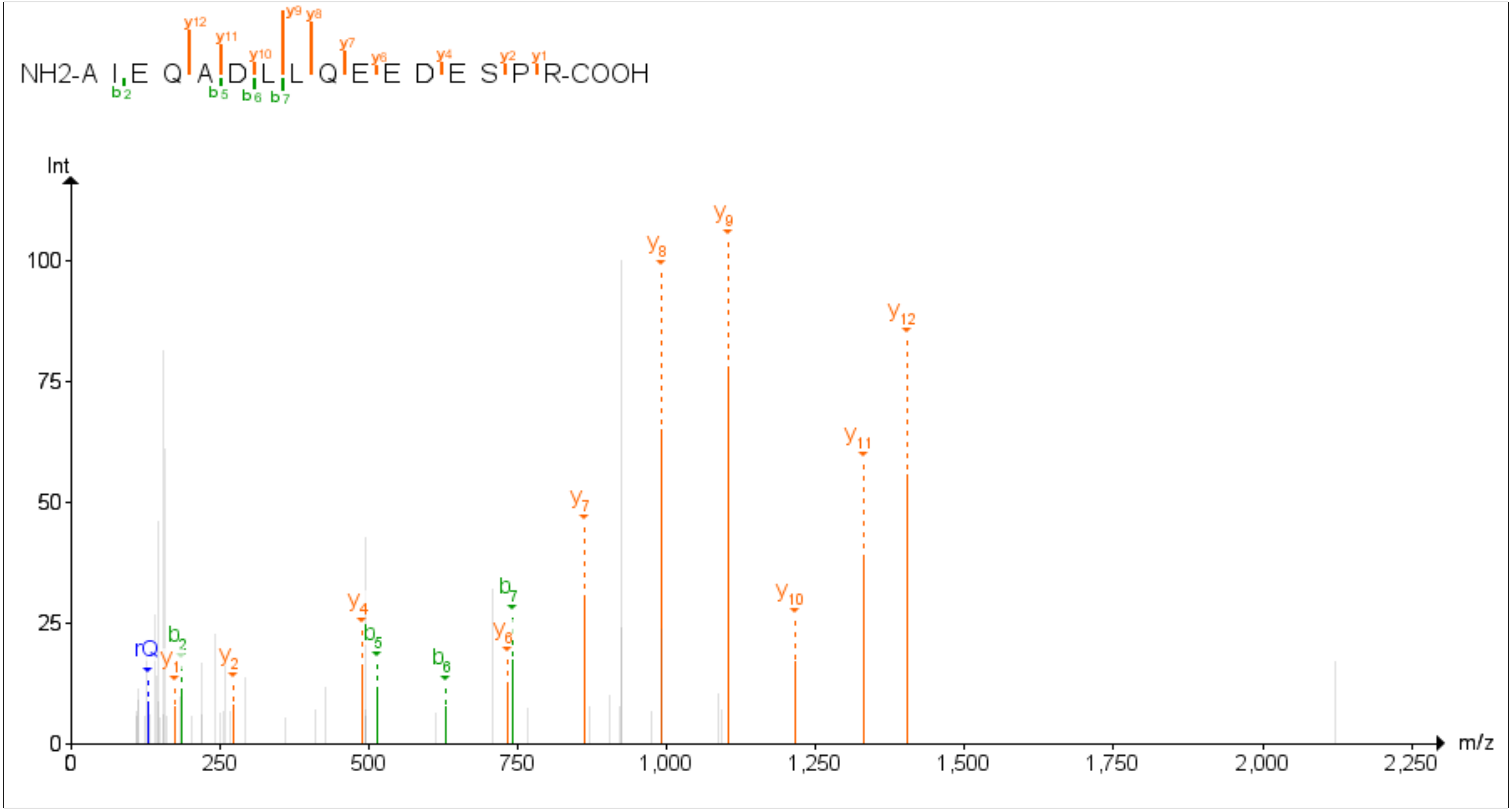

NH2-A I E Q A D L L Q E E D E S P R-COOH
y12 y11 y10 y9 y8 y7 y6 y4 y2 y1
b2 b5 b6 b7
Int
100
75
50
25
0
rQ
y1 b2 y2 y4 b5 b6 y6 b7 y7 y8 y9 y10 y11 y12
0 250 500 750 1,000 1,250 1,500 1,750 2,000 2,250
m/z

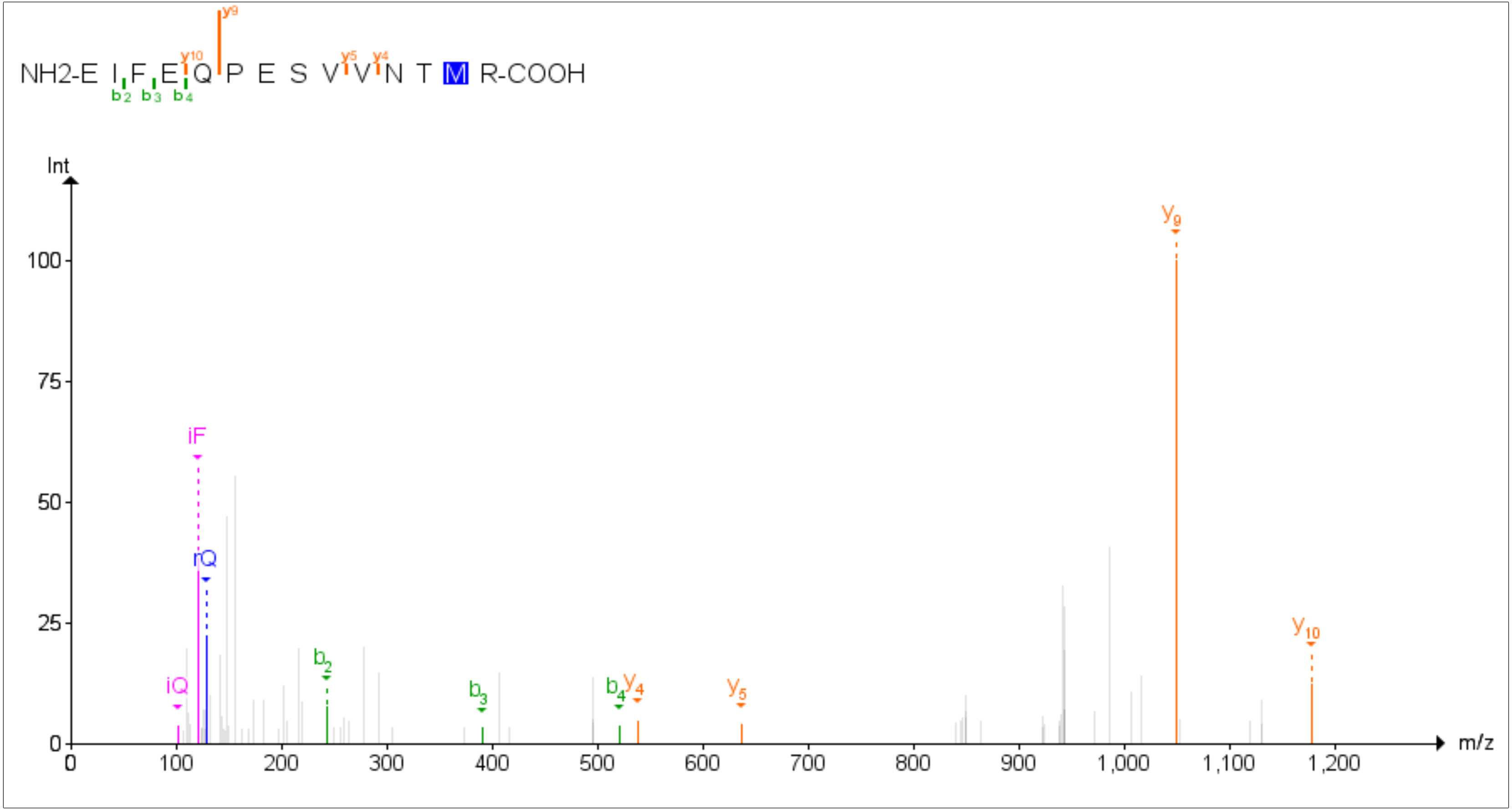

NH2-E I F E Q P E S V V N T M R-COOH
b2 b3 b4
y10 y9 y5 y4
Int
100
75
50
25
0
iF
rQ
iQ
b2
b3
b4
y4
y5
y9
y10
0 100 200 300 400 500 600 700 800 900 1,000 1,100 1,200
m/z

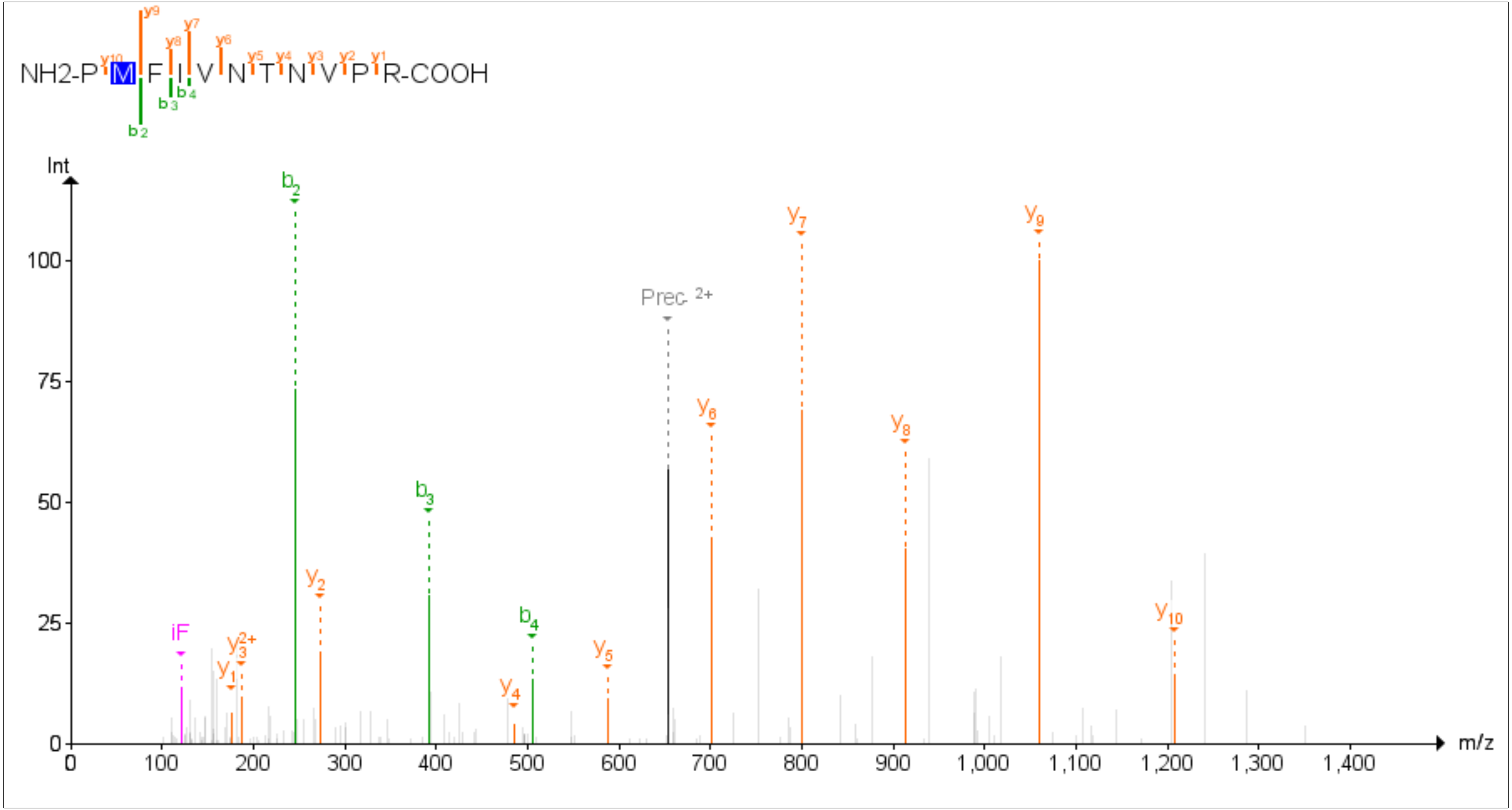

NH2-P M F I V N T N V P R-COOH
y10 y9 y8 y7 y6 y5 y4 y3 y2 y1
b2 b3 b4
Int
100
75
50
25
0
b2
b3
b4
y7
y9
Prec 2+
y6
y8
y2
y10
iF
y1
y3 2+
y4
y5
0 100 200 300 400 500 600 700 800 900 1,000 1,100 1,200 1,300 1,400
m/z

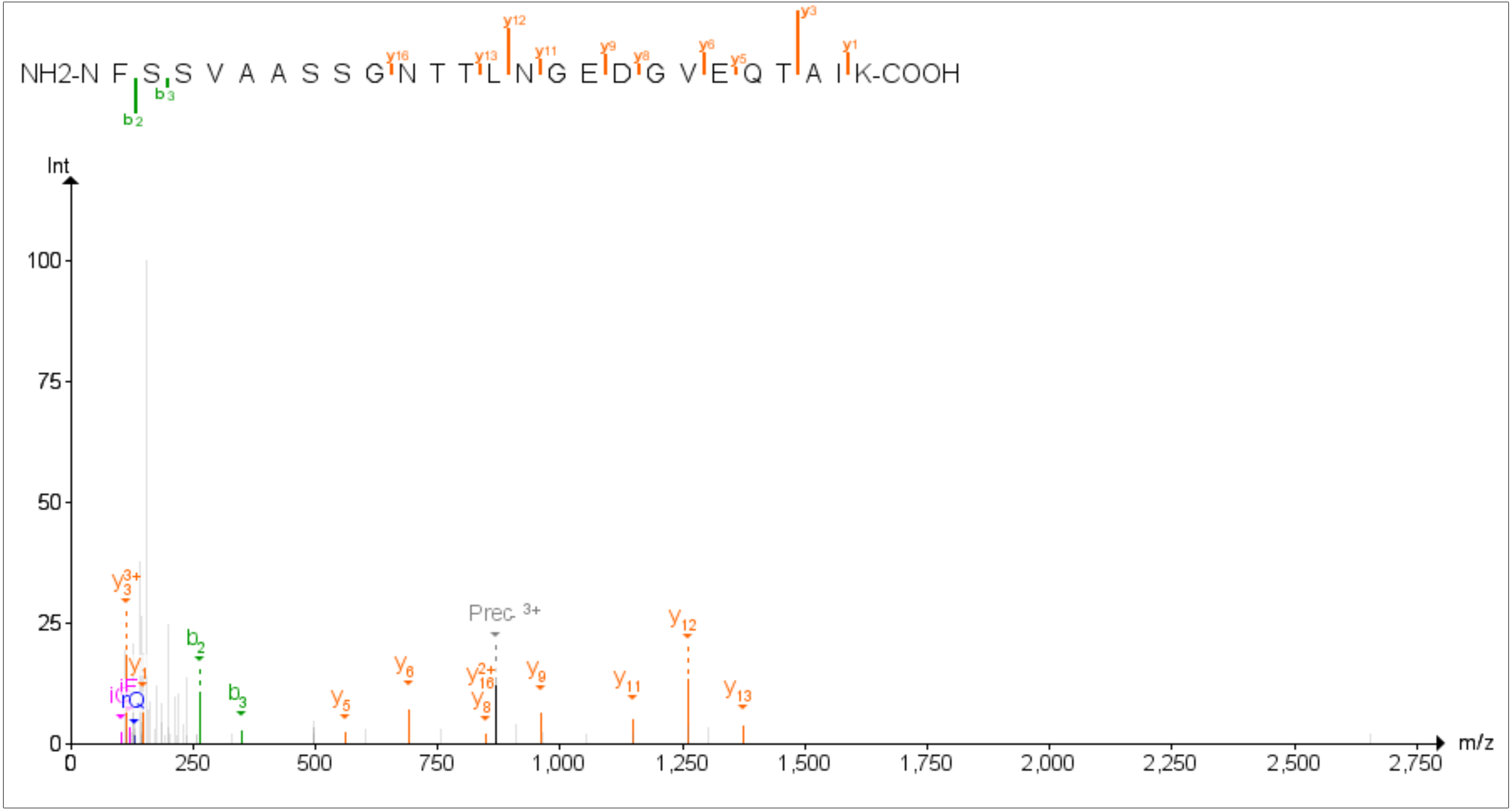

NH2-N F S S V A A S S G N T T L N G E D G V E Q T A I K-COOH
Int
m/z
Prec 3+

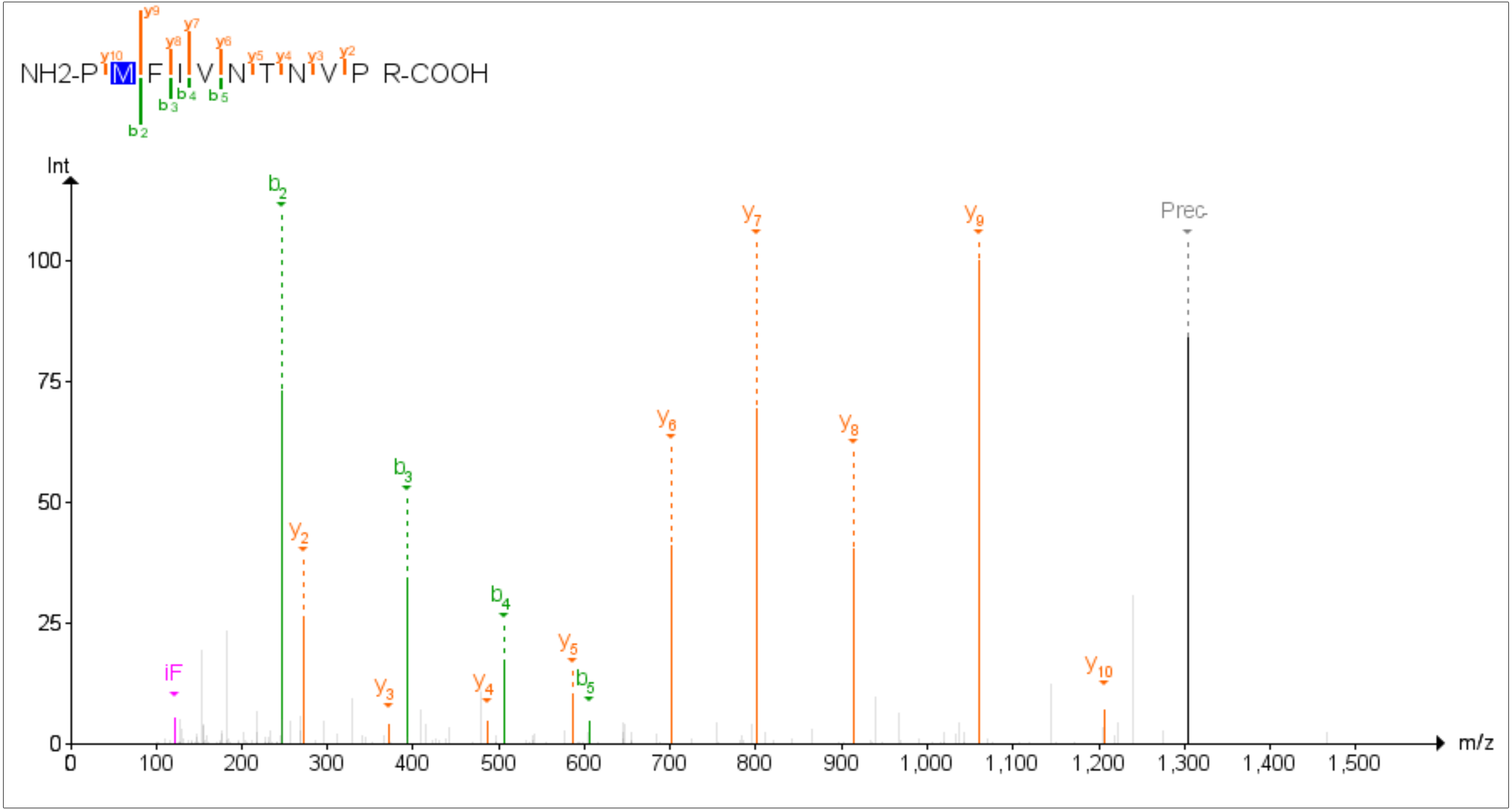

NH2-P M F I V N T N V P R-COOH
y10 y9 y8 y7 y6 y5 y4 y3 y2
b2 b3 b4 b5
Int
100
75
50
25
0
b2
y7
y9
Prec
y6
y8
b3
y2
b4
y5
b5
iF
y3
y4
y10
0 100 200 300 400 500 600 700 800 900 1,000 1,100 1,200 1,300 1,400 1,500
m/z

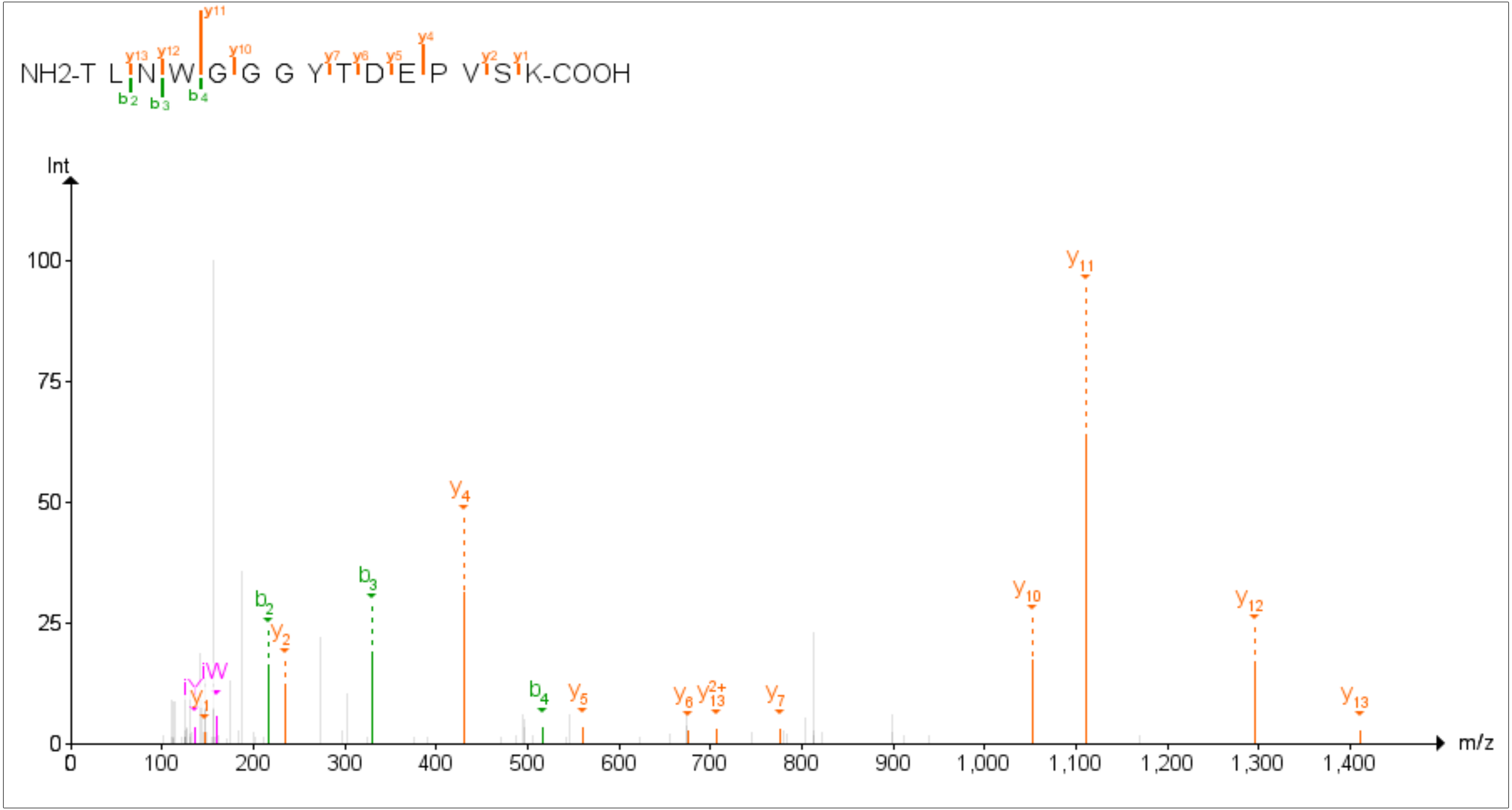

NH2-T L N W G G G Y T D E P V S K-COOH
y13 y12 y11 y10 y7 y6 y5 y4 y2 y1
b2 b3 b4
Int
100
75
50
25
0
m/z
0 100 200 300 400 500 600 700 800 900 1,000 1,100 1,200 1,300 1,400
y11
y4
b3
b2
y2
iY
iW
y1
b4
y5
y6
y13 2+
y7
y10
y12
y13

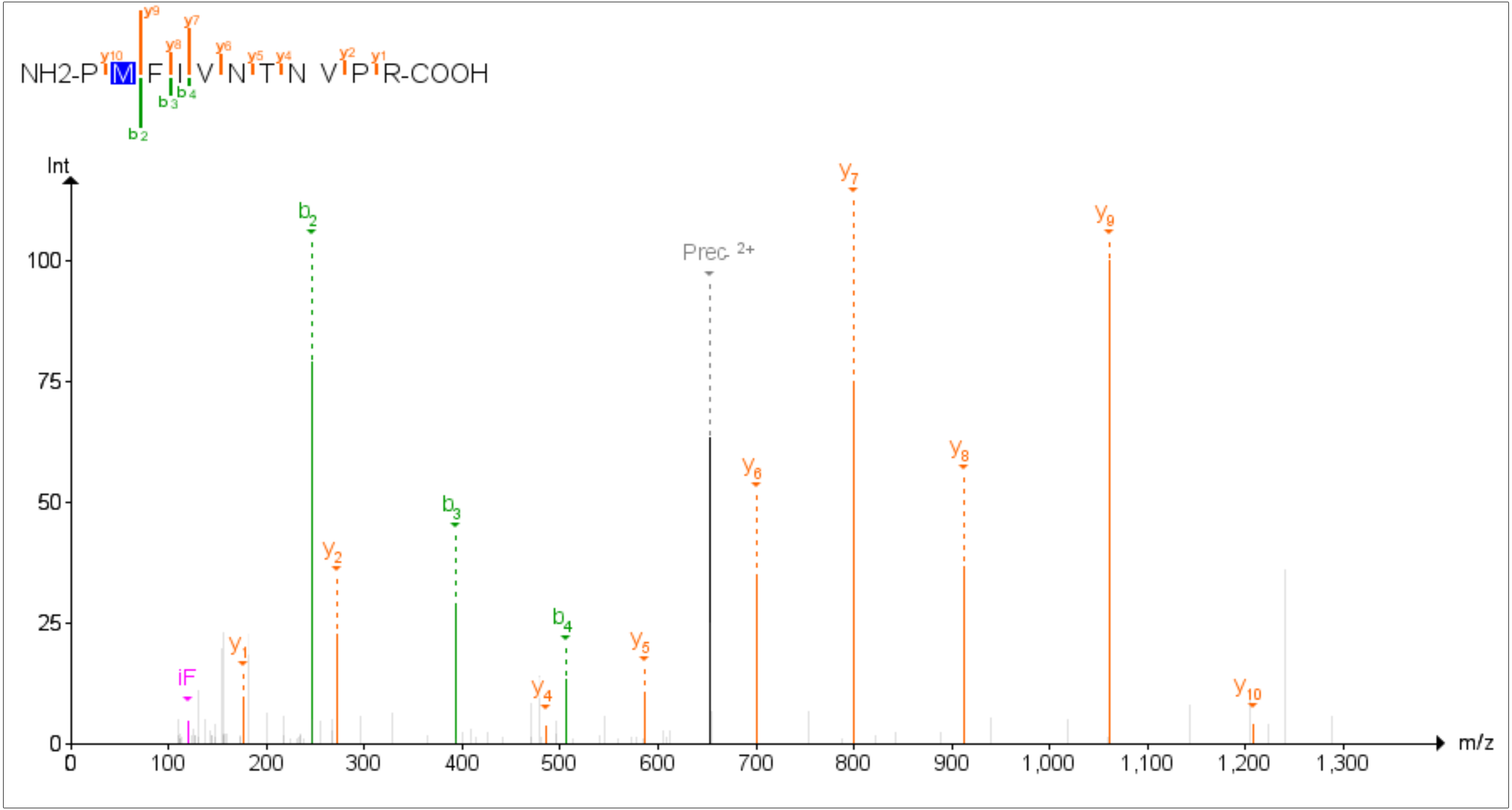
NH2-P M F I V N T N V P R-COOH
y10
y9
y8
y7
y6
y5
y4
y2
y1
b2
b3
b4
Int
100
75
50
25
0
m/z
0
100
200
300
400
500
600
700
800
900
1,000
1,100
1,200
1,300
iF
y1
b2
y2
b3
y4
b4
y5
Prec 2+
y6
y7
y8
y9
y10

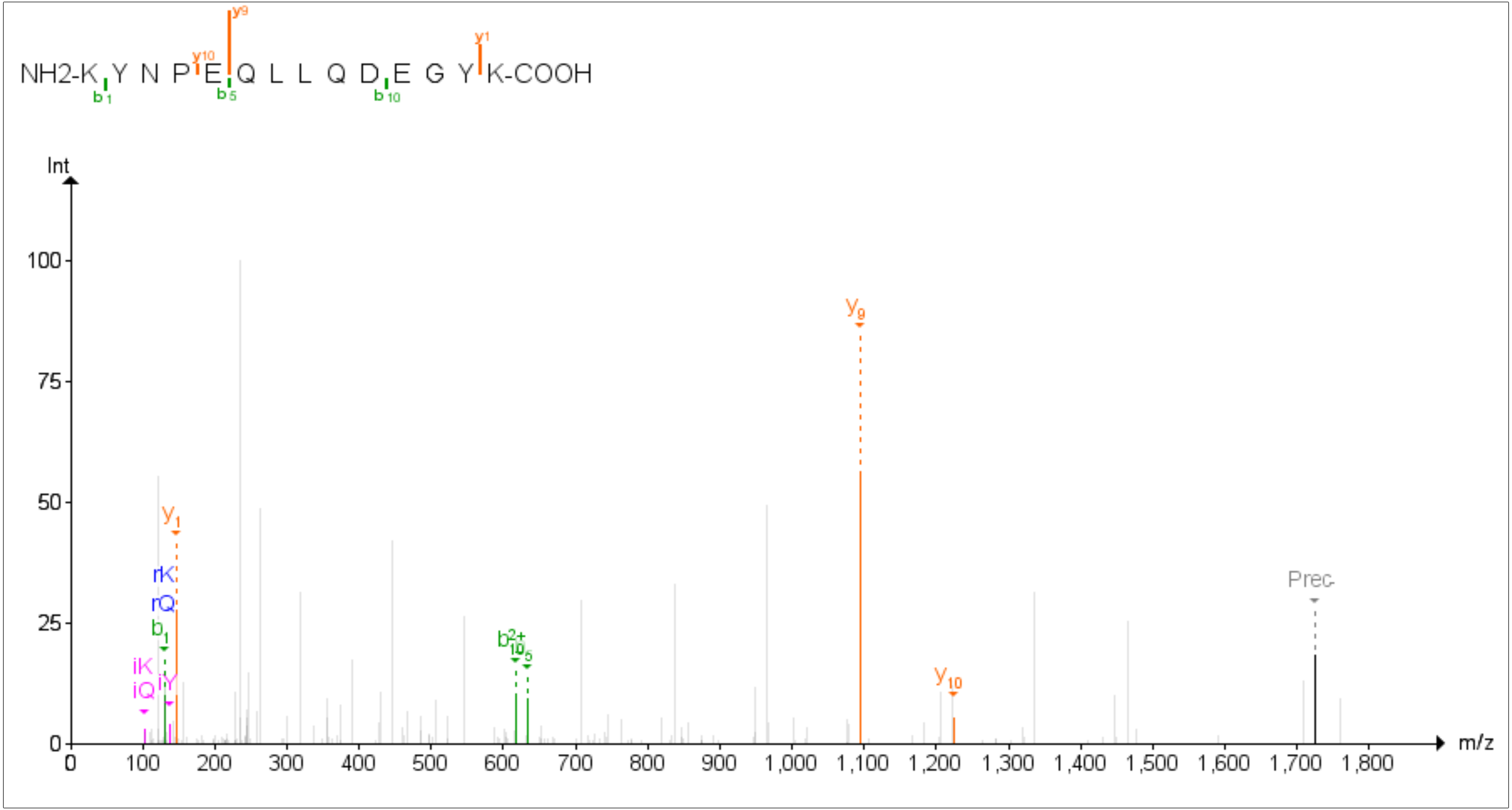

NH2-K Y N P E Q L L Q D E G Y K-COOH
b1 b5 b10
y10 y9 y1
Int
m/z
Prec
y9
y1
y10
rK
rQ
b1
iK
iQ
iY
b10 2+
b5

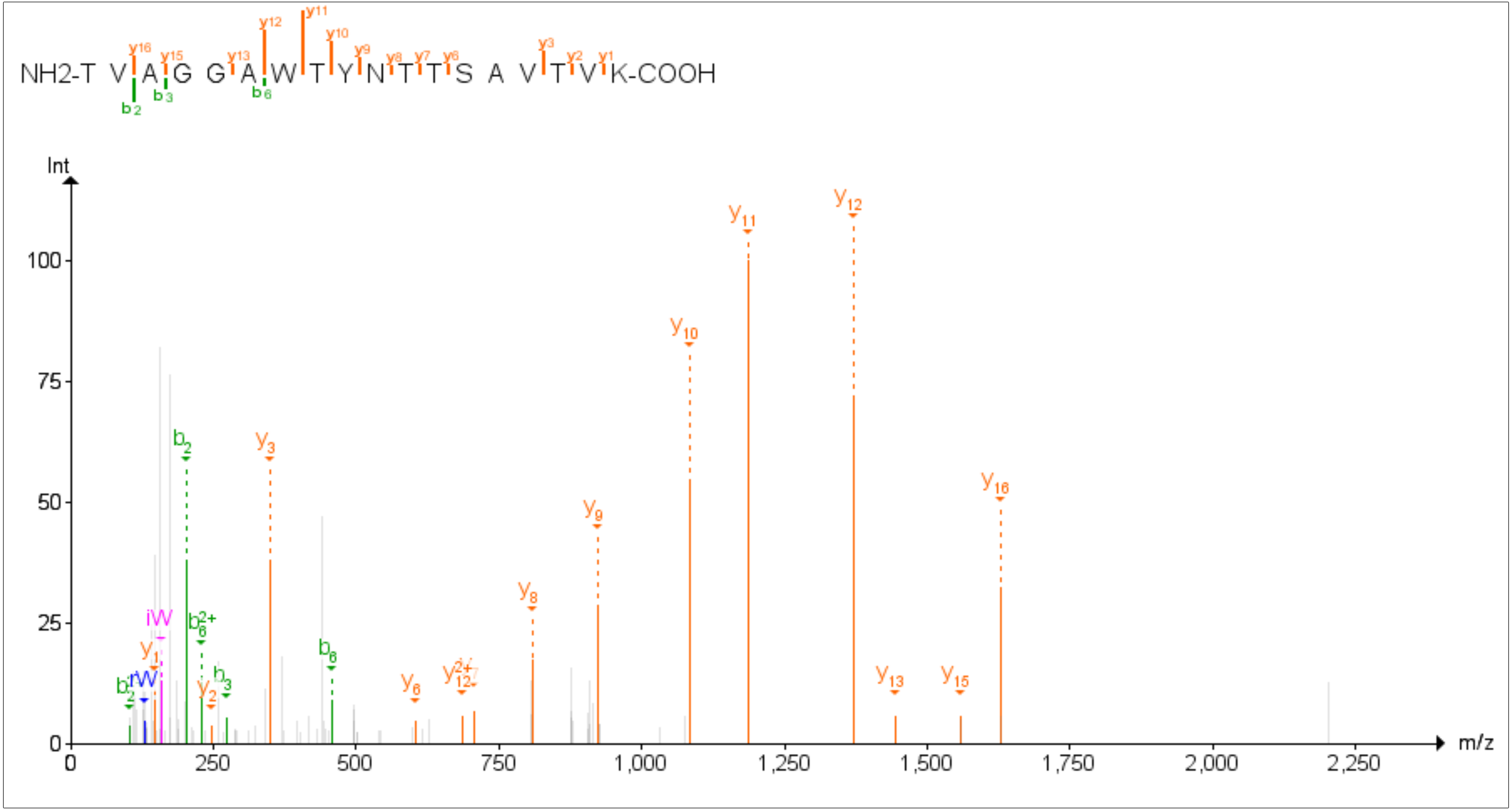
NH2-T V A G G A W T Y N T T S A V T V K-COOH
y16 y15 y13 y12 y11 y10 y9 y8 y7 y6 y3 y2 y1
b2 b3 b6
Int
100
75
50
25
0
m/z
0
250
500
750
1,000
1,250
1,500
1,750
2,000
2,250
y11
y12
y10
y16
y9
y8
y3
b2
iW
b6 2+
y1
rW
b2
b3
y2
b6
y6
y12 2+
y13
y15

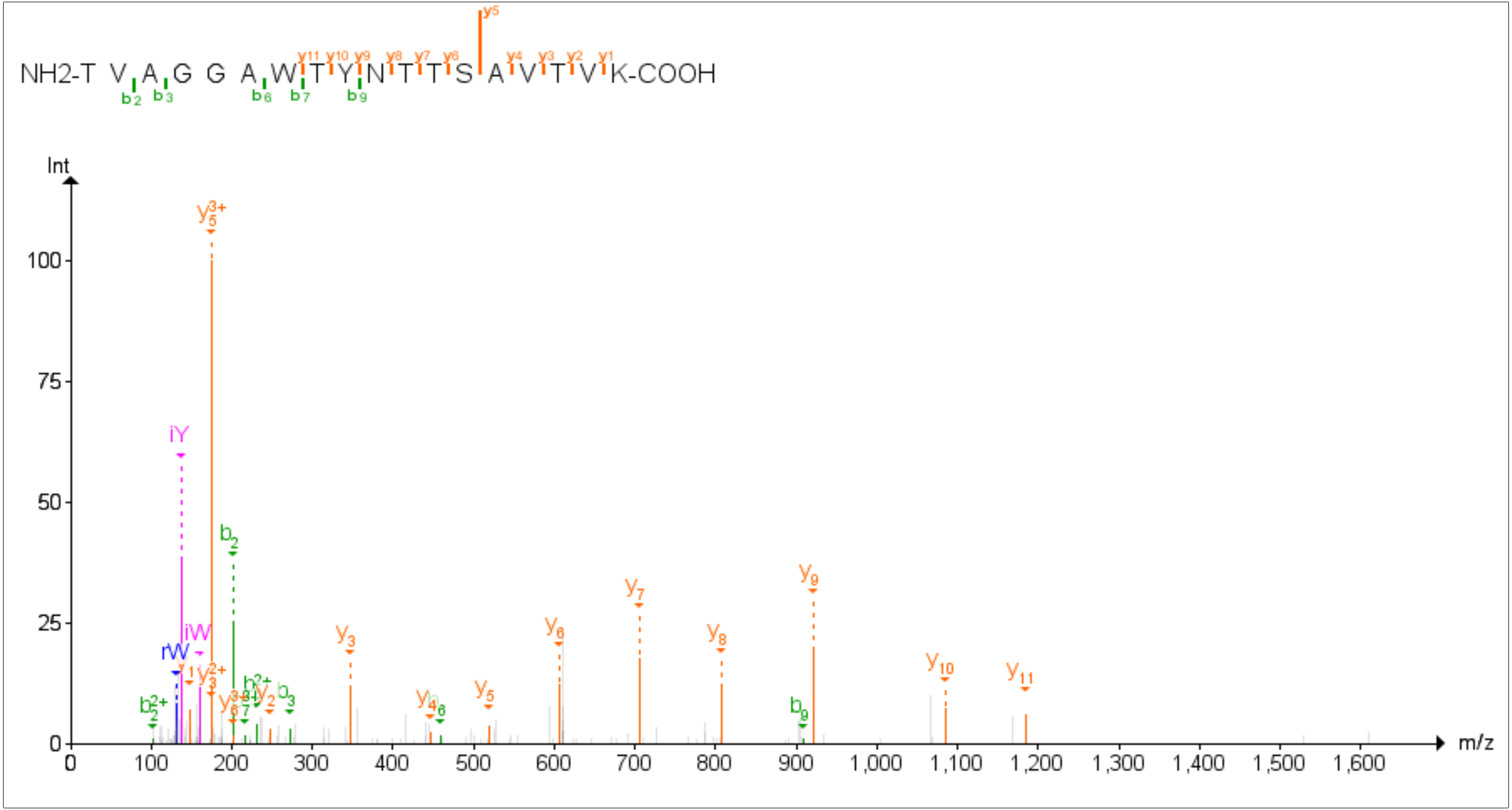

NH2-T V A G G A W T Y N T T S A V T V K-COOH
y11 y10 y9 y8 y7 y6 y5 y4 y3 y2 y1
b2 b3 b6 b7 b9
Int
m/z
iY
rW
iW
0
25
50
75
100
100
200
300
400
500
600
700
800
900
1,000
1,100
1,200
1,300
1,400
1,500
1,600

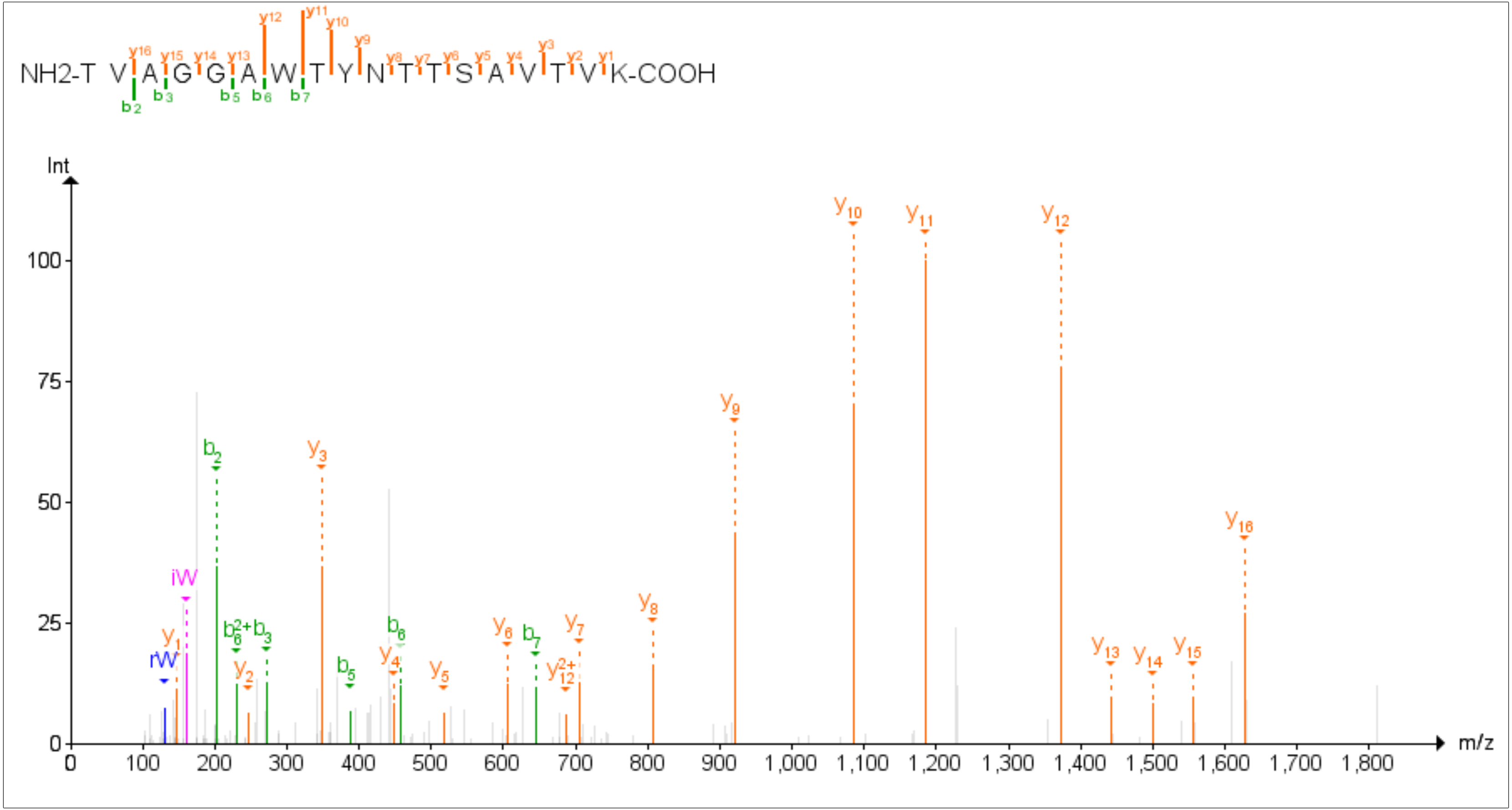
NH2-T V A G G A W T Y N T T S A V T V K-COOH
Int
m/z
y10
y11
y12
y9
y3
b2
y16
iW
y1
rW
y2
b3
b5
y4
b6
y5
y6
b7
y7
y8
y13
y14
y15

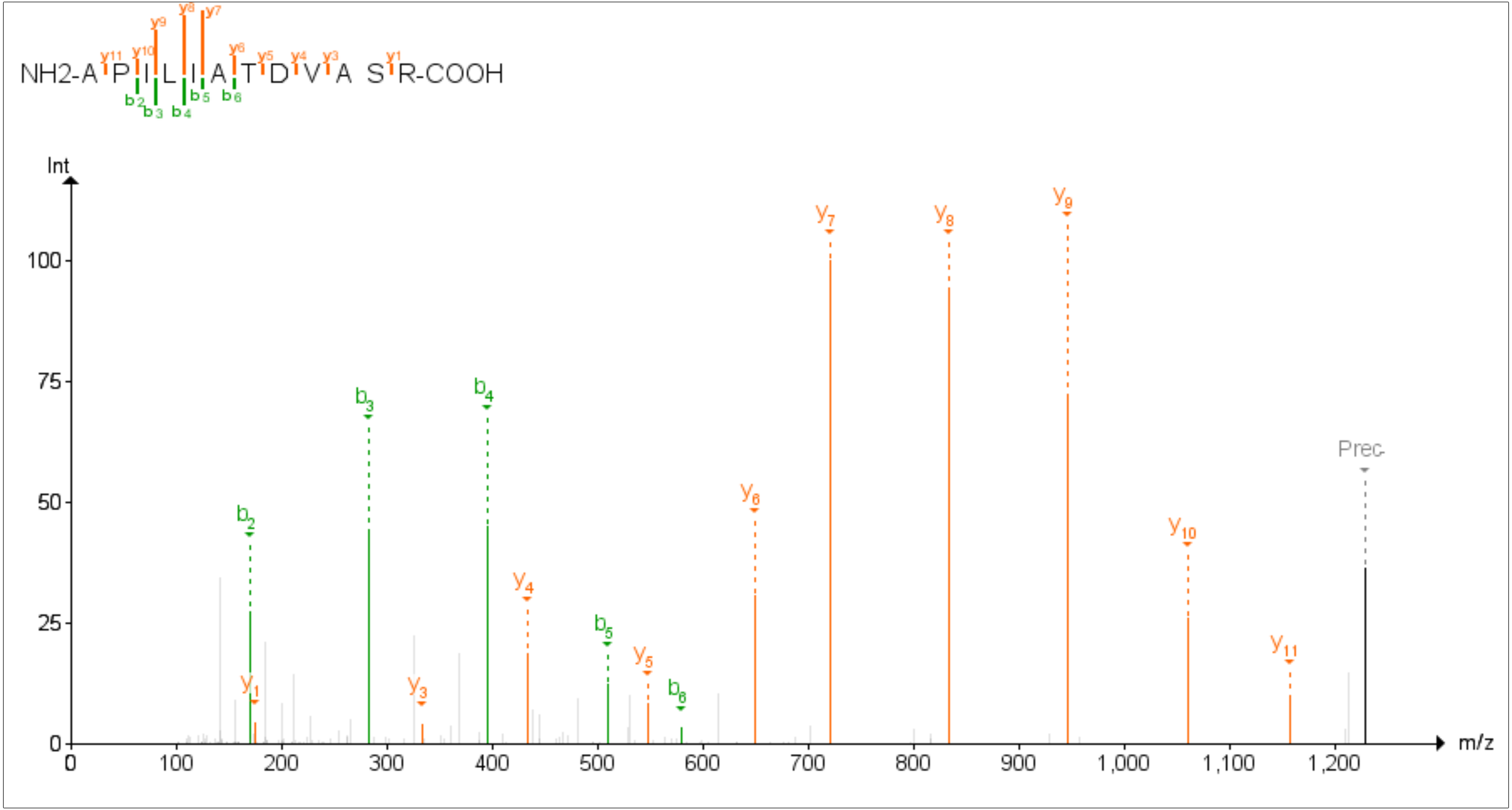

NH2-A P I L I A T D V A S R-COOH
Int
100
75
50
25
0
m/z
0
100
200
300
400
500
600
700
800
900
1,000
1,100
1,200
b2
b3
b4
b5
b6
y1
y3
y4
y5
y6
y7
y8
y9
y10
y11
Prec.

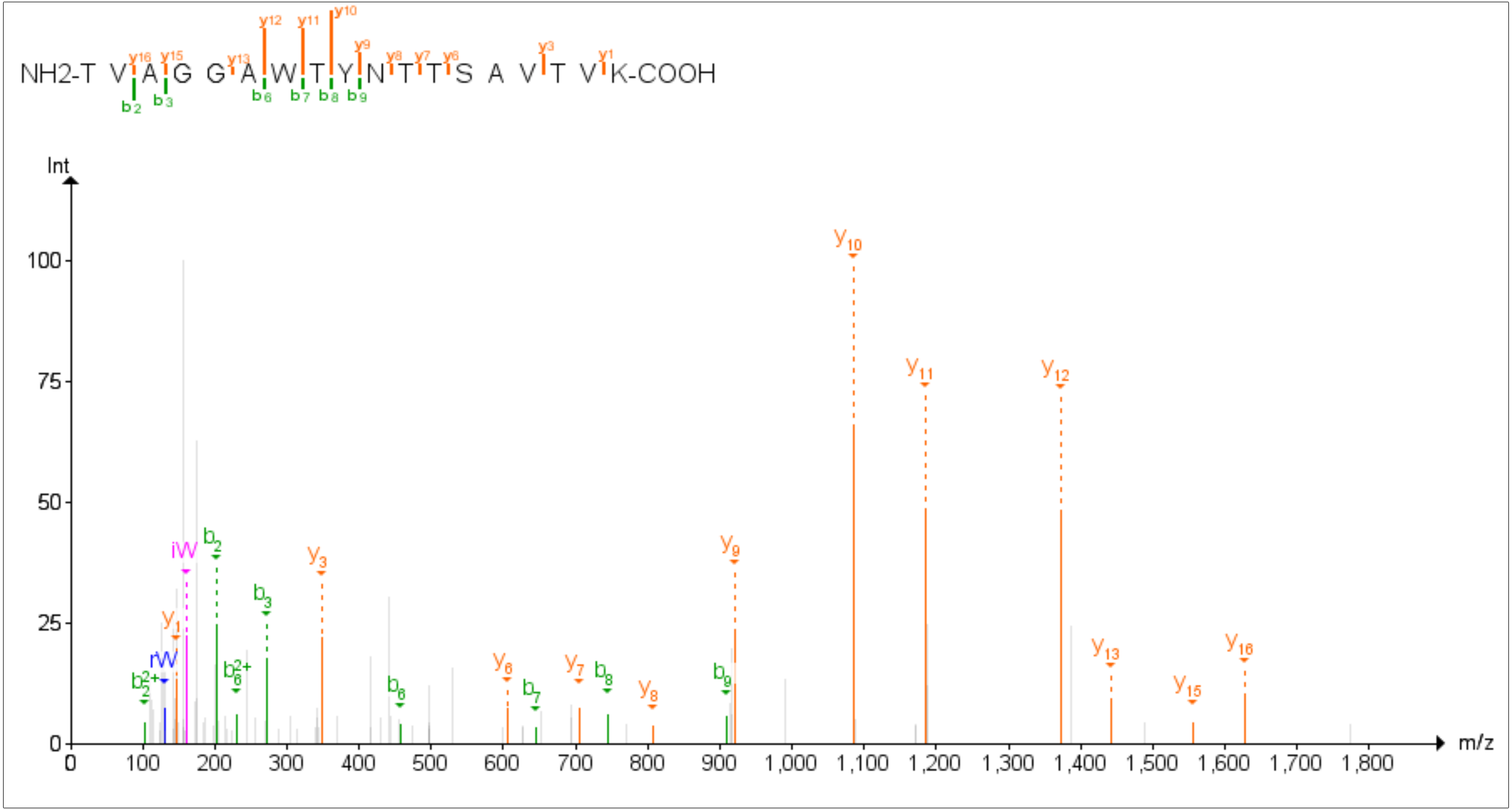
NH2-T V A G G A W T Y N T T S A V T V K-COOH
y16 y15 y13 y12 y11 y10 y9 y8 y7 y6 y3 y1
b2 b3 b6 b7 b8 b9
Int
m/z
0
25
50
75
100
100
200
300
400
500
600
700
800
900
1,000
1,100
1,200
1,300
1,400
1,500
1,600
1,700
1,800
y10
y11
y12
y13
y15
y16
y9
y8
y7
y6
y3
y1
b2
b3
b6
b7
b8
b9
b_2^{2+}
b_6^{2+}
iW
rW

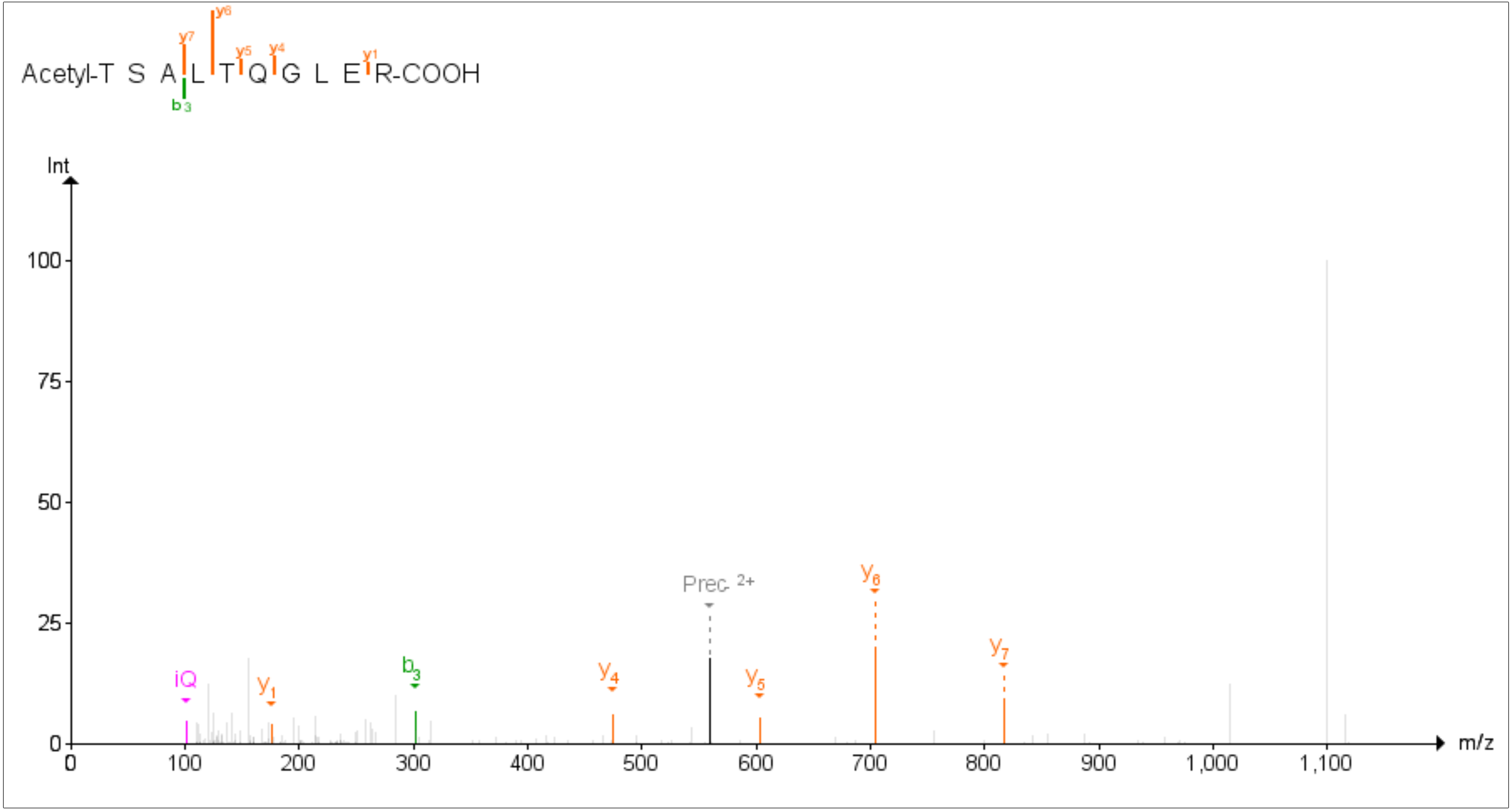

Acetyl-T S A L T Q G L E R-COOH
y7
y6
y5
y4
y1
b3
Int
100
75
50
25
0
iQ
y1
b3
y4
Prec 2+
y5
y6
y7
0
100
200
300
400
500
600
700
800
900
1,000
1,100
m/z

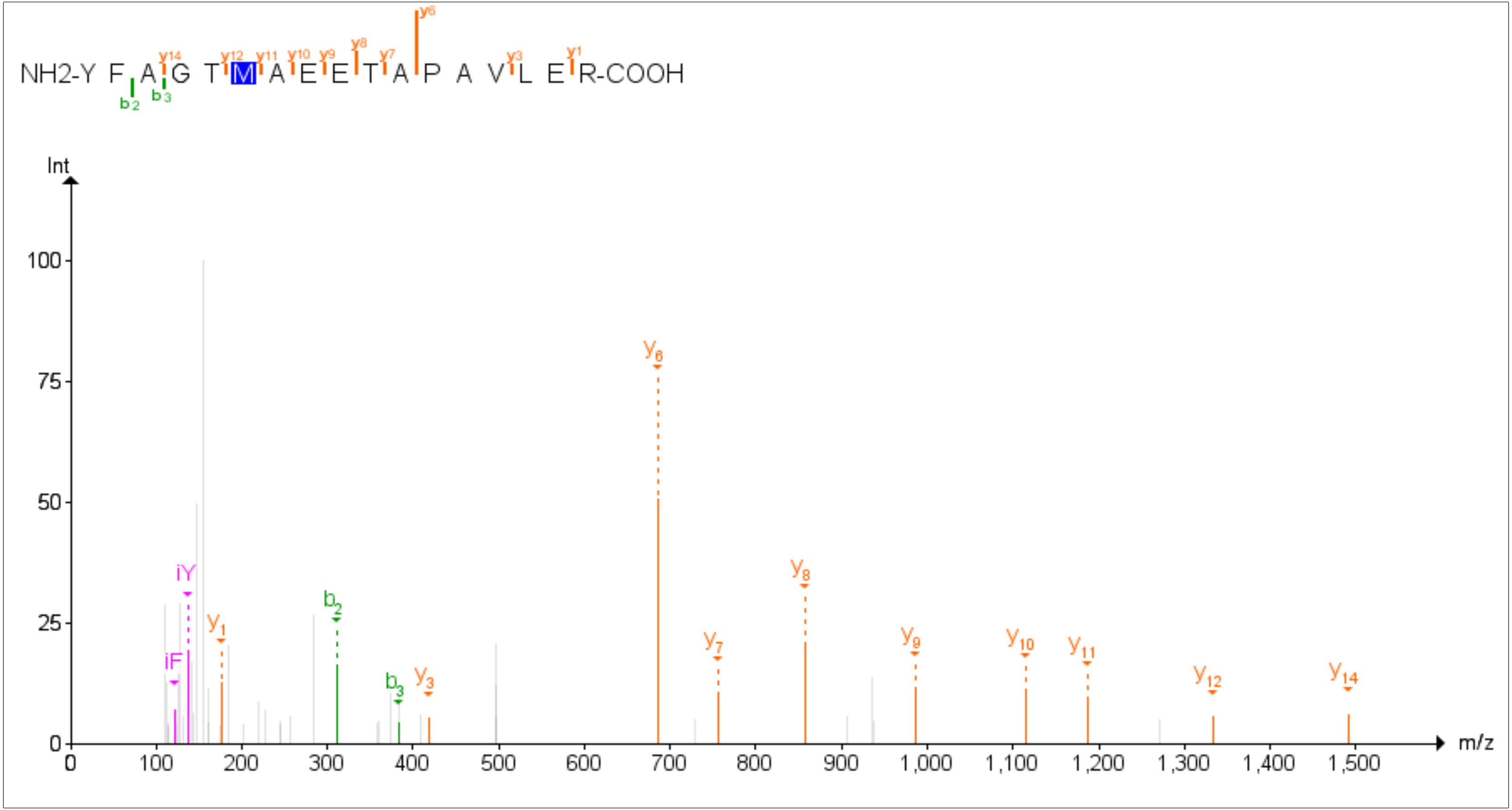

NH2-Y F A G T M A E E T A P A V L E R-COOH
y14 y12 y11 y10 y9 y8 y7 y6 y3 y1
b2 b3
Int
100
75
50
25
0
iY
iF
y1
b2
b3
y3
y6
y7
y8
y9
y10
y11
y12
y14
0 100 200 300 400 500 600 700 800 900 1,000 1,100 1,200 1,300 1,400 1,500
m/z

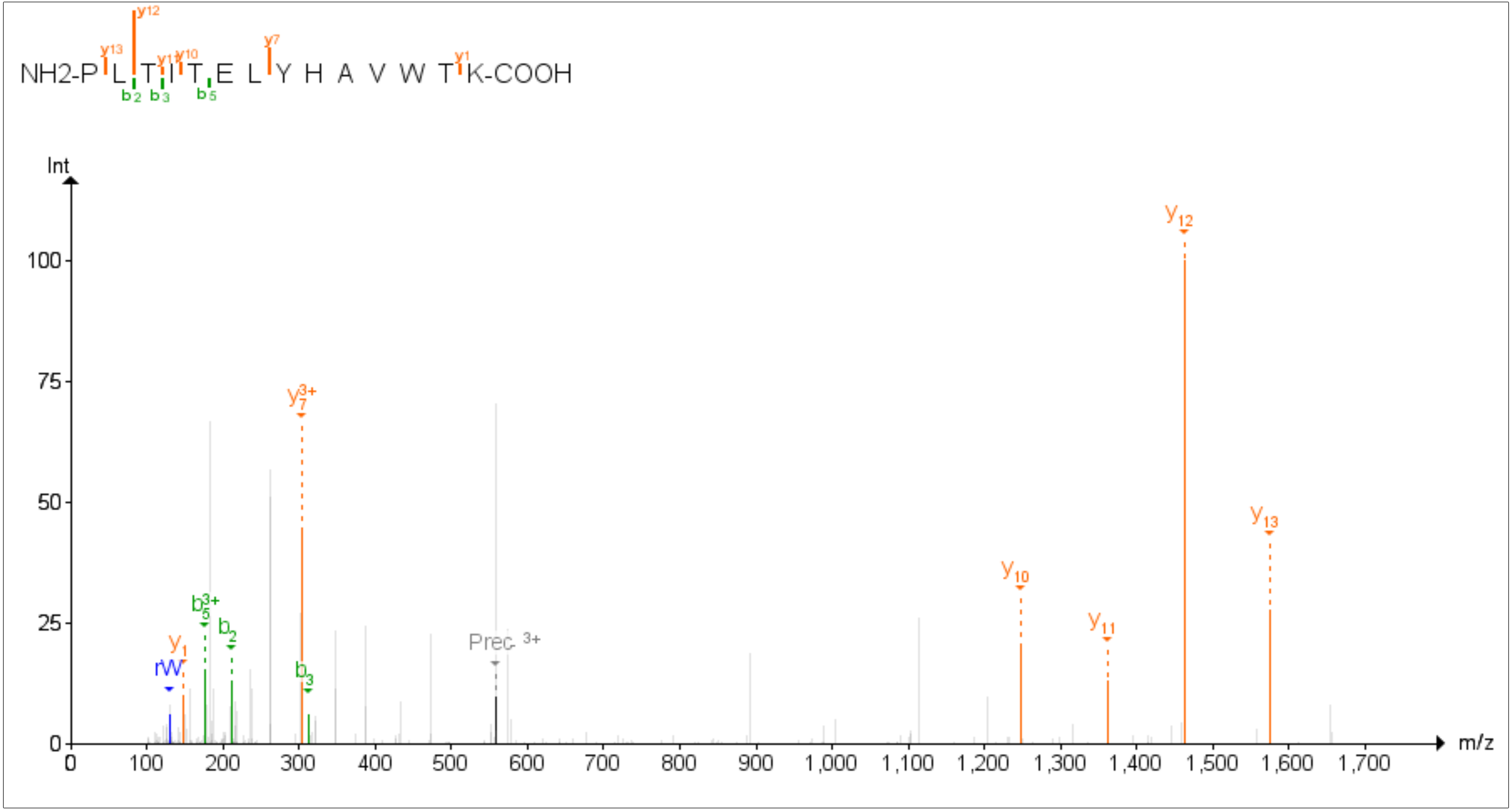

NH2-P L T I T E L Y H A V W T K-COOH
y12
y13
y11
y10
y7
y1
b2
b3
b5
Int
100
75
50
25
0
y12
y13
y10
y11
y7 3+
b5 3+
b2
b3
y1
rW
Prec 3+
0
100
200
300
400
500
600
700
800
900
1,000
1,100
1,200
1,300
1,400
1,500
1,600
1,700
m/z

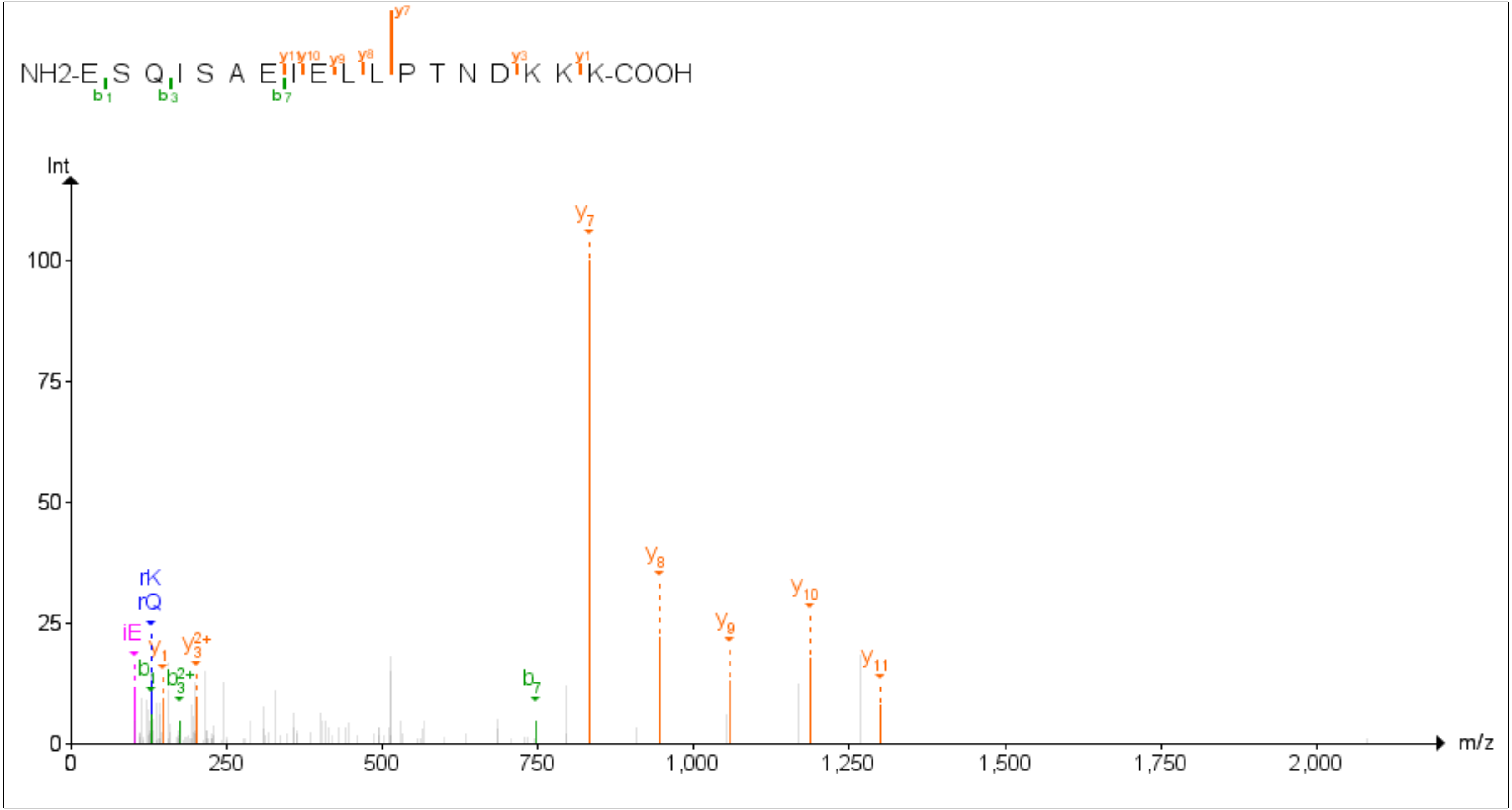

NH2-E S Q I S A E I I E L L P T N D K K K-COOH
b1
b3
b7
y11
y10
y9
y8
y7
y3
y1
Int
m/z
y7
y8
y9
y10
y11
b7
rK
rQ
iE
y1
b1
y3 2+
b3 2+
0
25
50
75
100
250
500
750
1,000
1,250
1,500
1,750
2,000

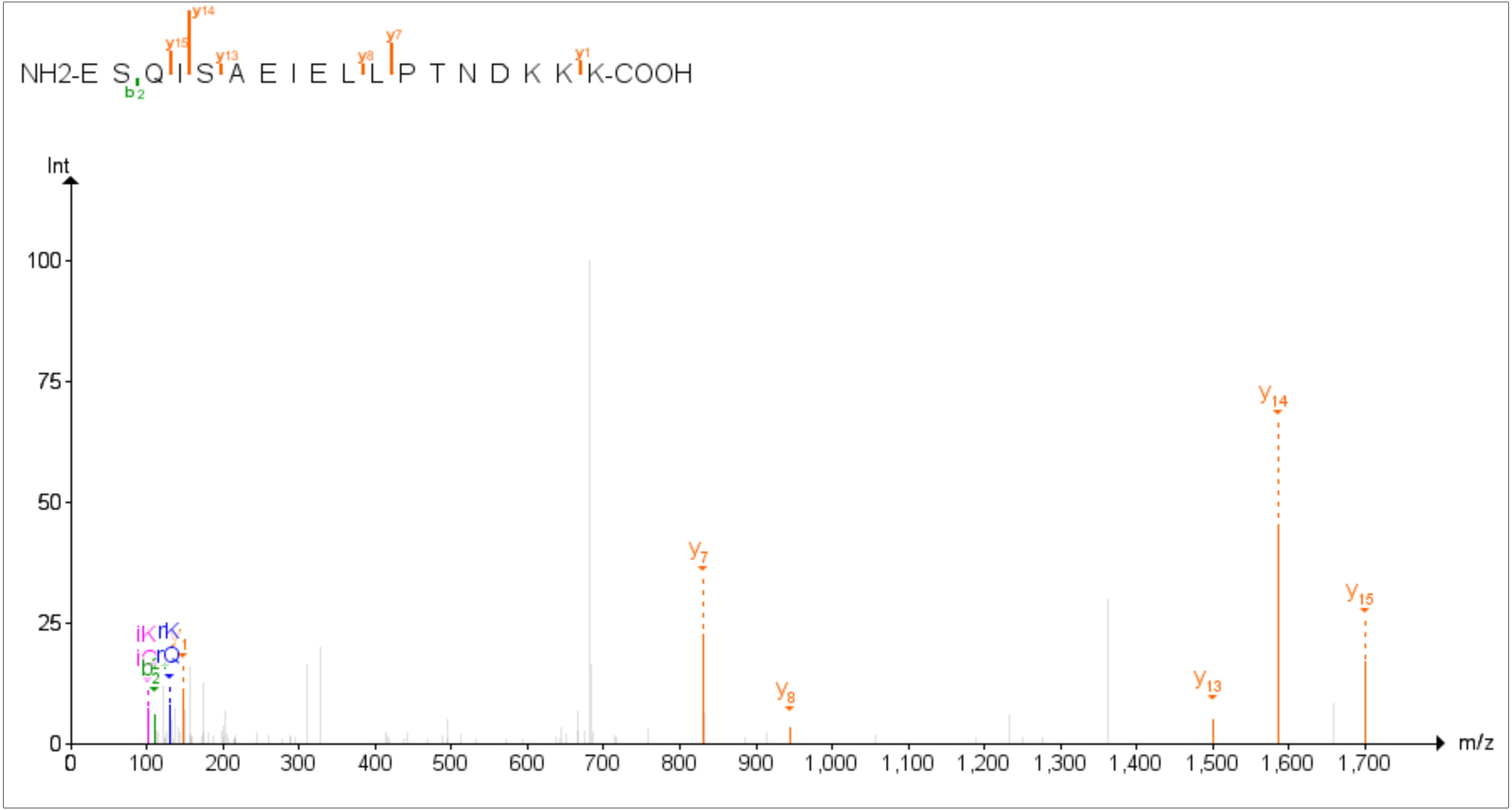

NH2-E S Q I S A E I E L L P T N D K K K-COOH
y14
y15
y13
y8
y7
y1
b2
Int
100
75
50
25
0
y7
y8
y13
y14
y15
iK
rK
rQ
m/z
0
100
200
300
400
500
600
700
800
900
1,000
1,100
1,200
1,300
1,400
1,500
1,600
1,700

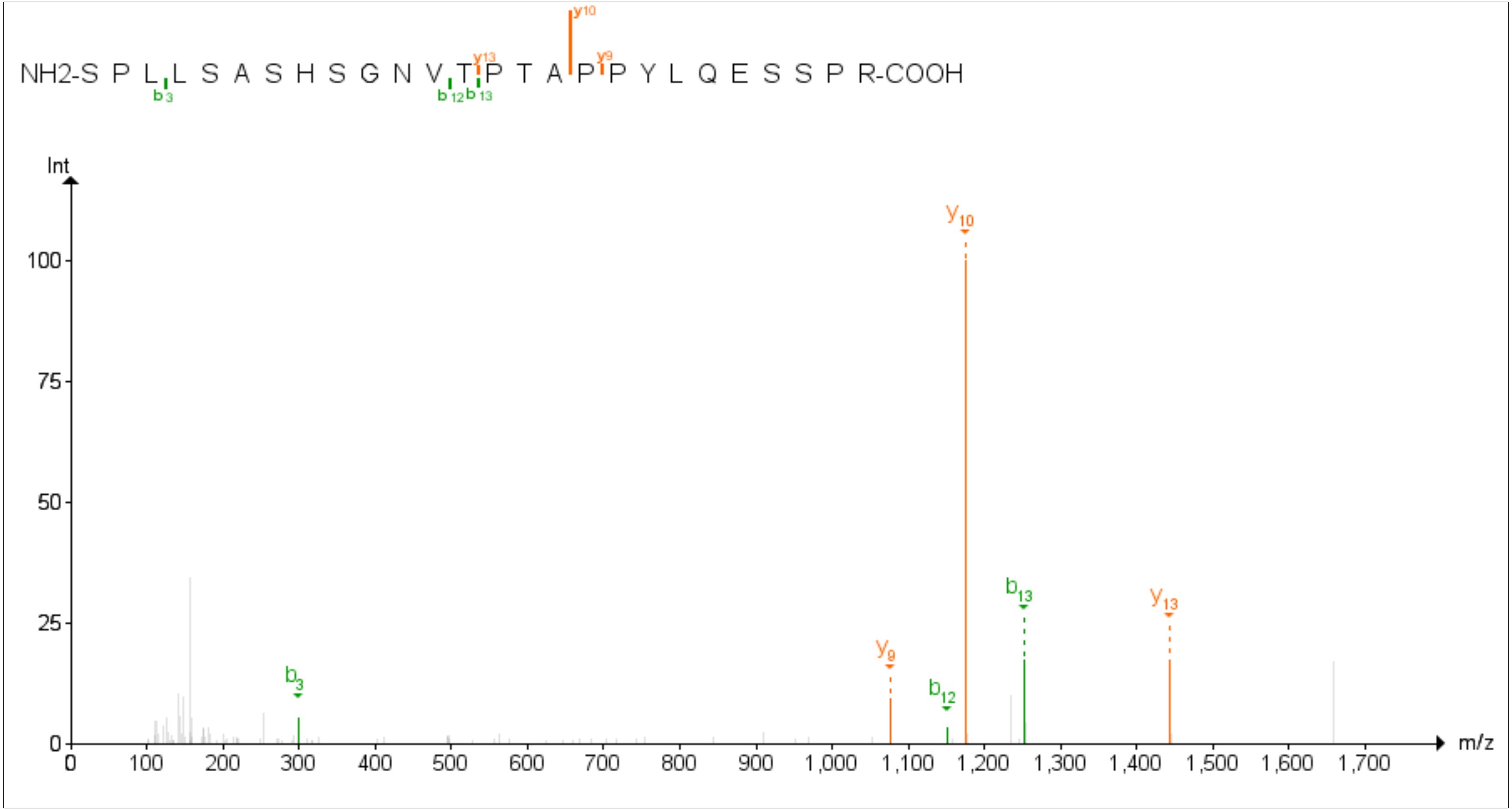

NH2-S P L L S A S H S G N V T P T A P P Y L Q E S S P R-COOH
b3
b12
b13
y13
y10
y9
Int
100
75
50
25
0
m/z
0
100
200
300
400
500
600
700
800
900
1,000
1,100
1,200
1,300
1,400
1,500
1,600
1,700

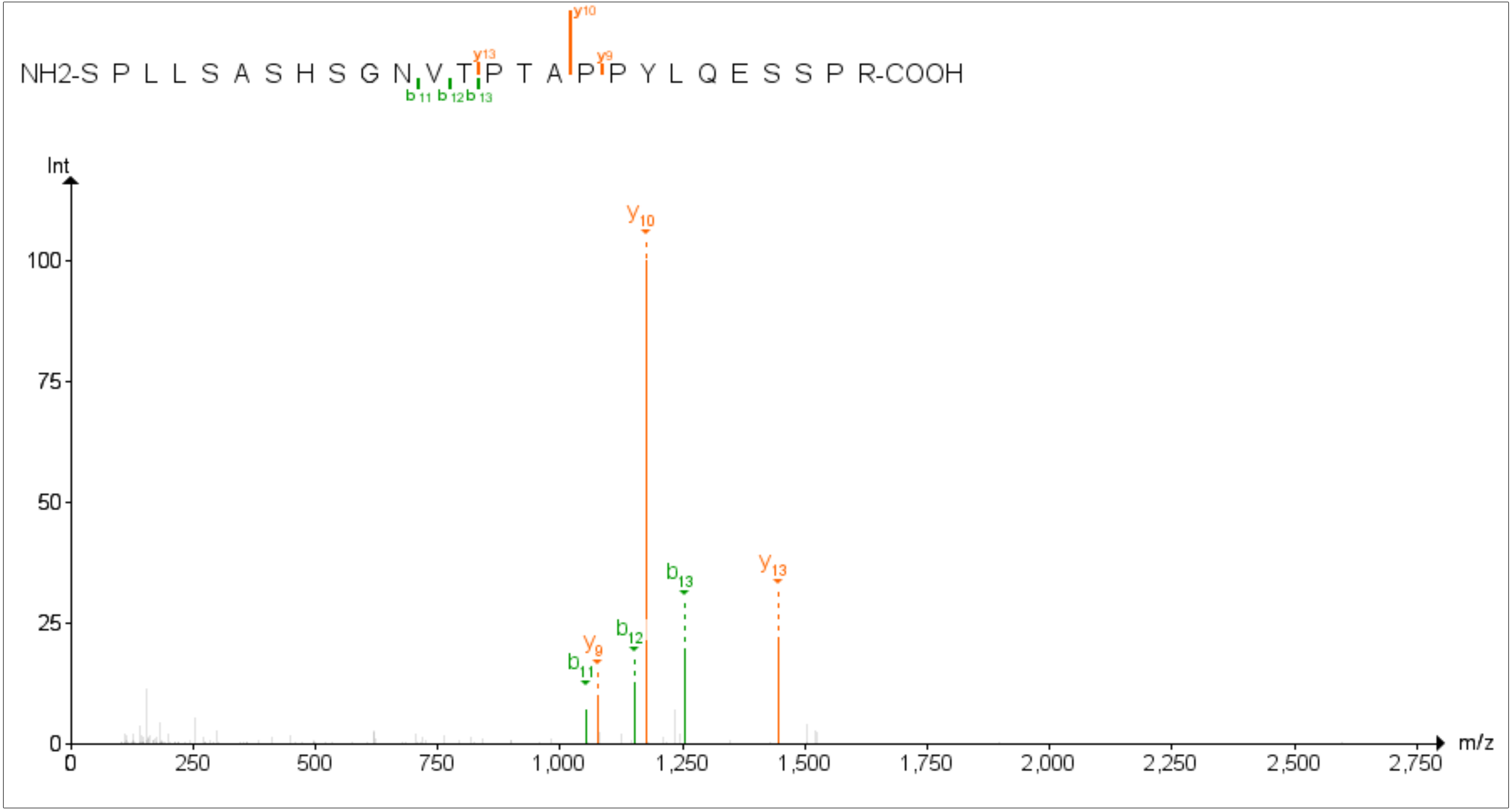
NH2-S P L L S A S H S G N V T P T A P P Y L Q E S S P R-COOH
y10
y13
y9
b11
b12
b13
Int
100
75
50
25
0
0
250
500
750
1,000
1,250
1,500
1,750
2,000
2,250
2,500
2,750
m/z
y10
b13
y13
b12
y9
b11

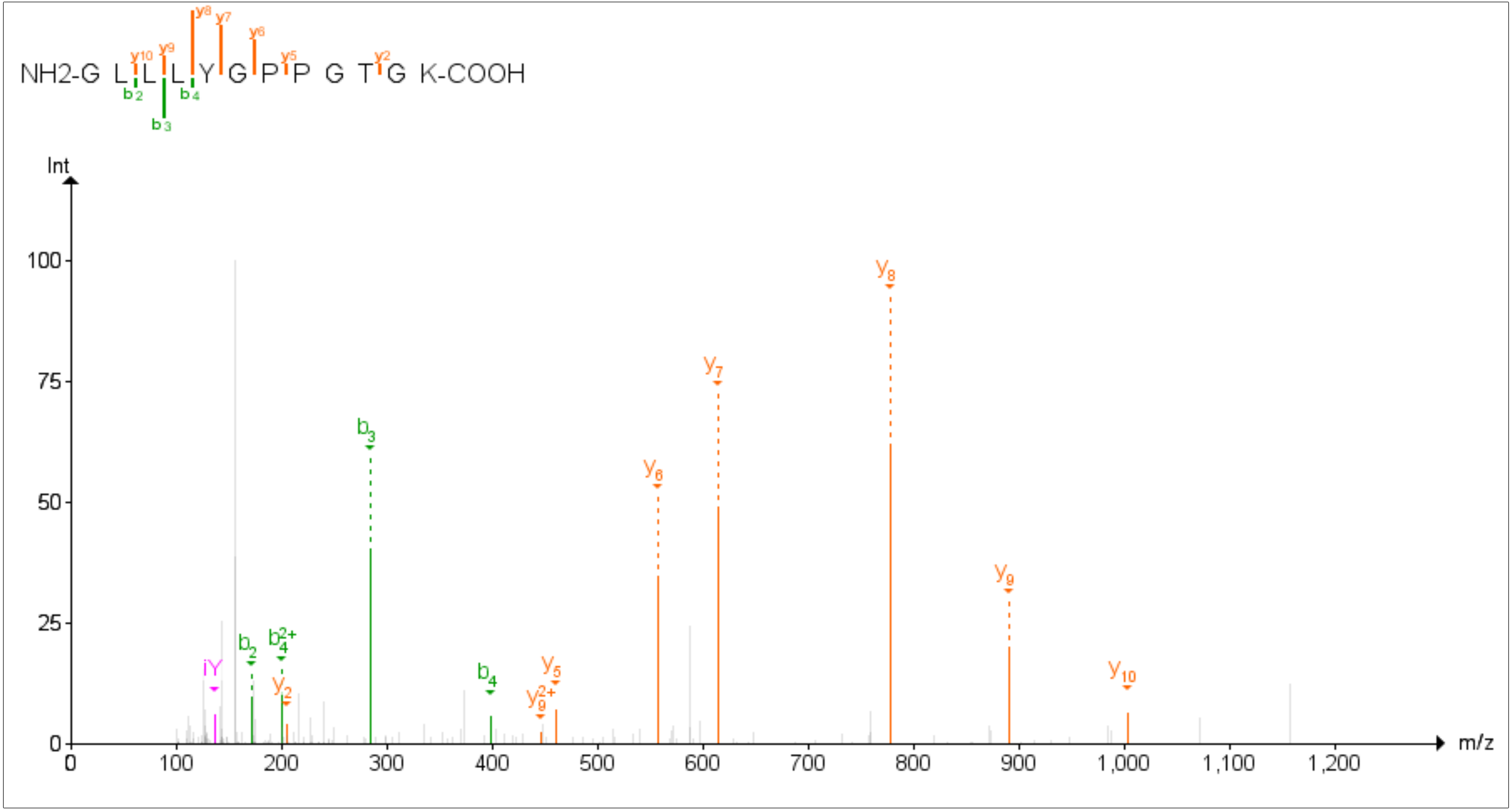

NH2-G L L L Y G P P G T G K-COOH
y10 y9 y8 y7 y6 y5 y2
b2 b3 b4
Int
100
75
50
25
0
m/z
0 100 200 300 400 500 600 700 800 900 1,000 1,100 1,200
iY
b2
b4 2+
y2
b3
b4
y9 2+
y5
y6
y7
y8
y9
y10

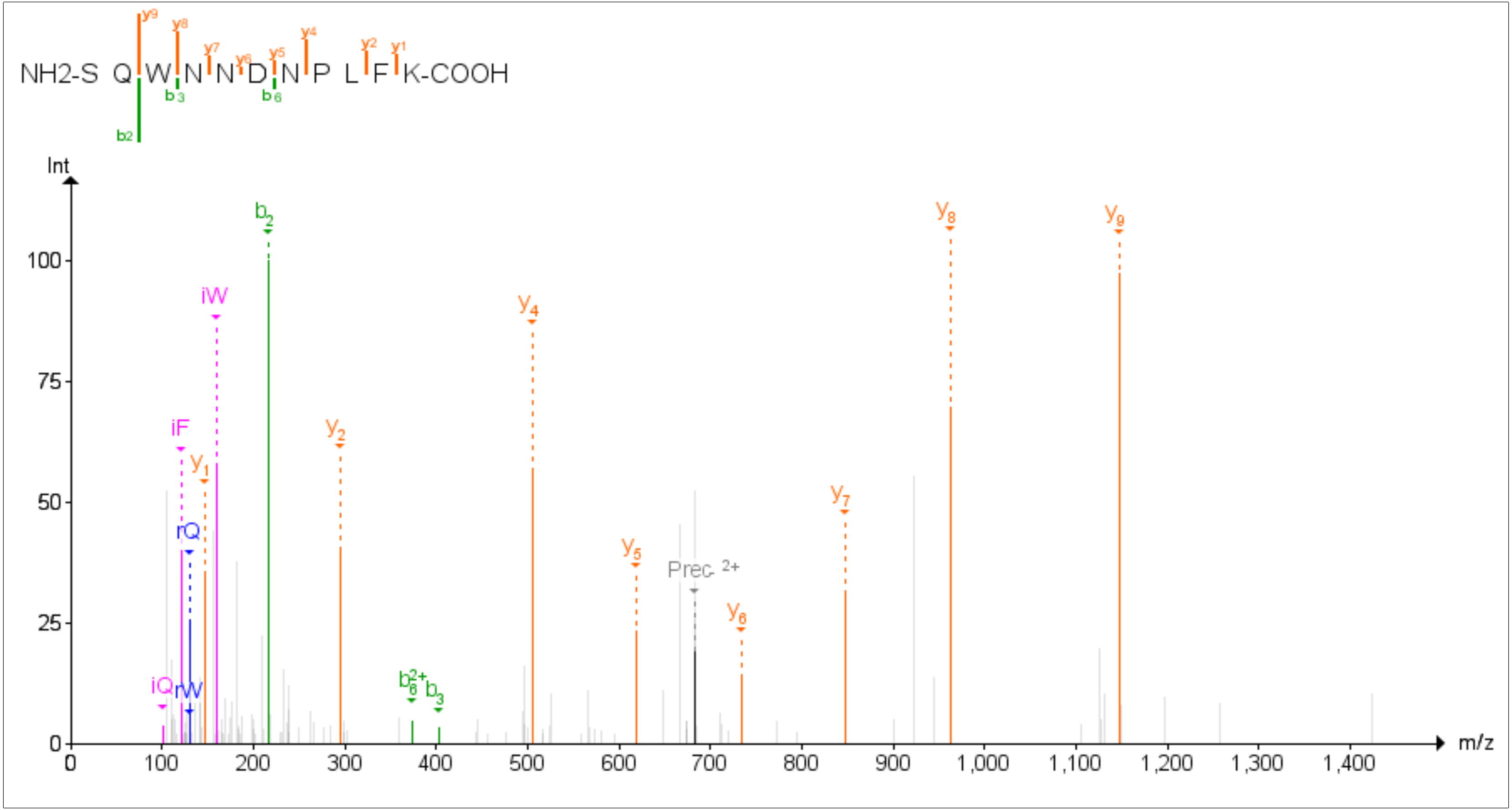

NH2-S Q W N N D N P L F K-COOH
y9
y8
y7
y6
y5
y4
y2
y1
b2
b3
b6
Int
m/z
b2
y8
y9
iW
y4
iF
y2
y1
rQ
y7
y5
Prec- 2+
y6
iQ
rW
b6 2+
b3
0
25
50
75
100
0
100
200
300
400
500
600
700
800
900
1,000
1,100
1,200
1,300
1,400

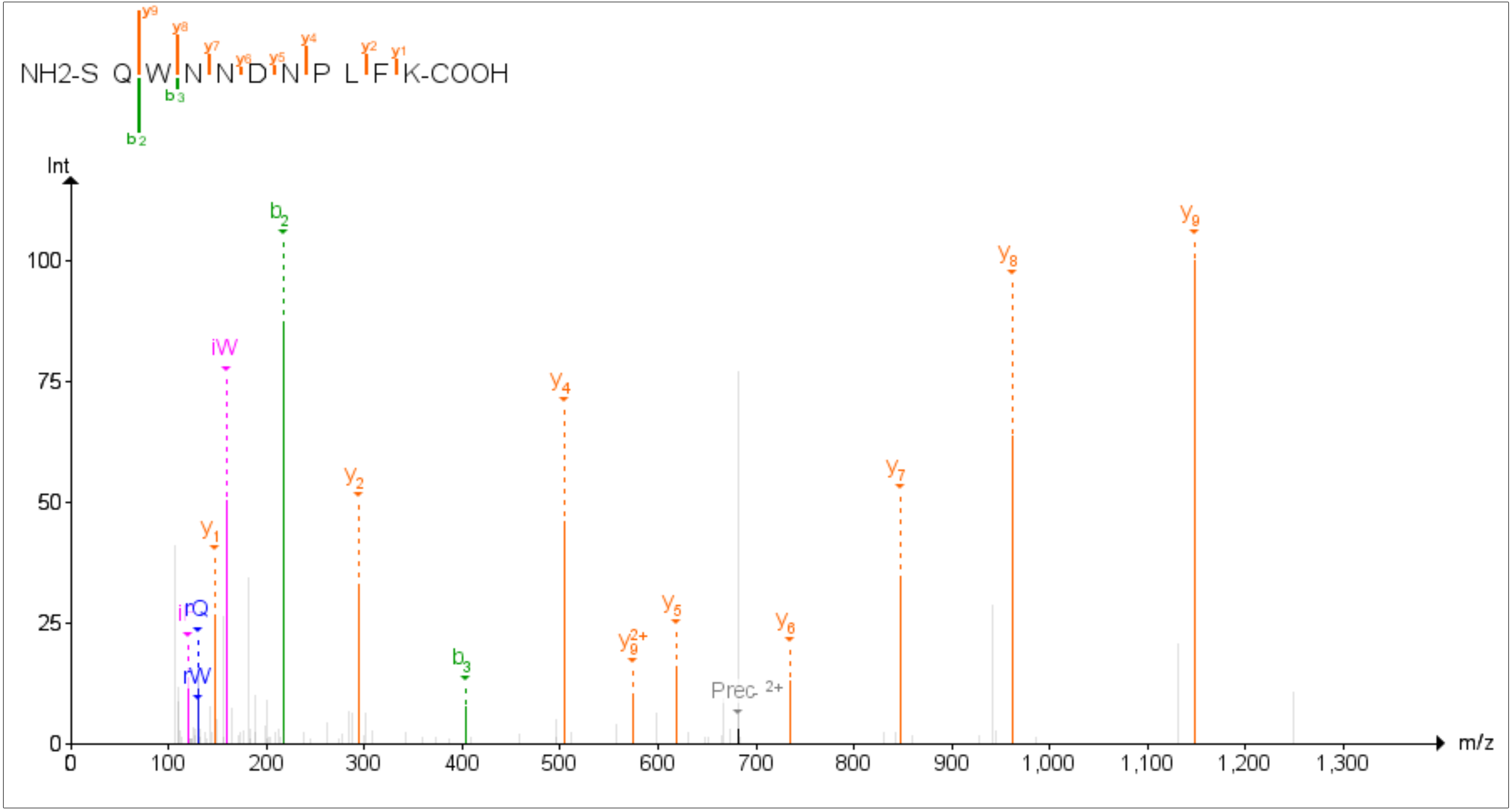
NH2-S Q W N N D N P L F K-COOH
y9
y8
y7
y6
y5
y4
y2
y1
b3
b2
Int
m/z
iW
iQ
rQ
rW
y1
b2
y2
y4
b3
y9 2+
y5
Prec- 2+
y6
y7
y8
y9
0
25
50
75
100
0
100
200
300
400
500
600
700
800
900
1,000
1,100
1,200
1,300

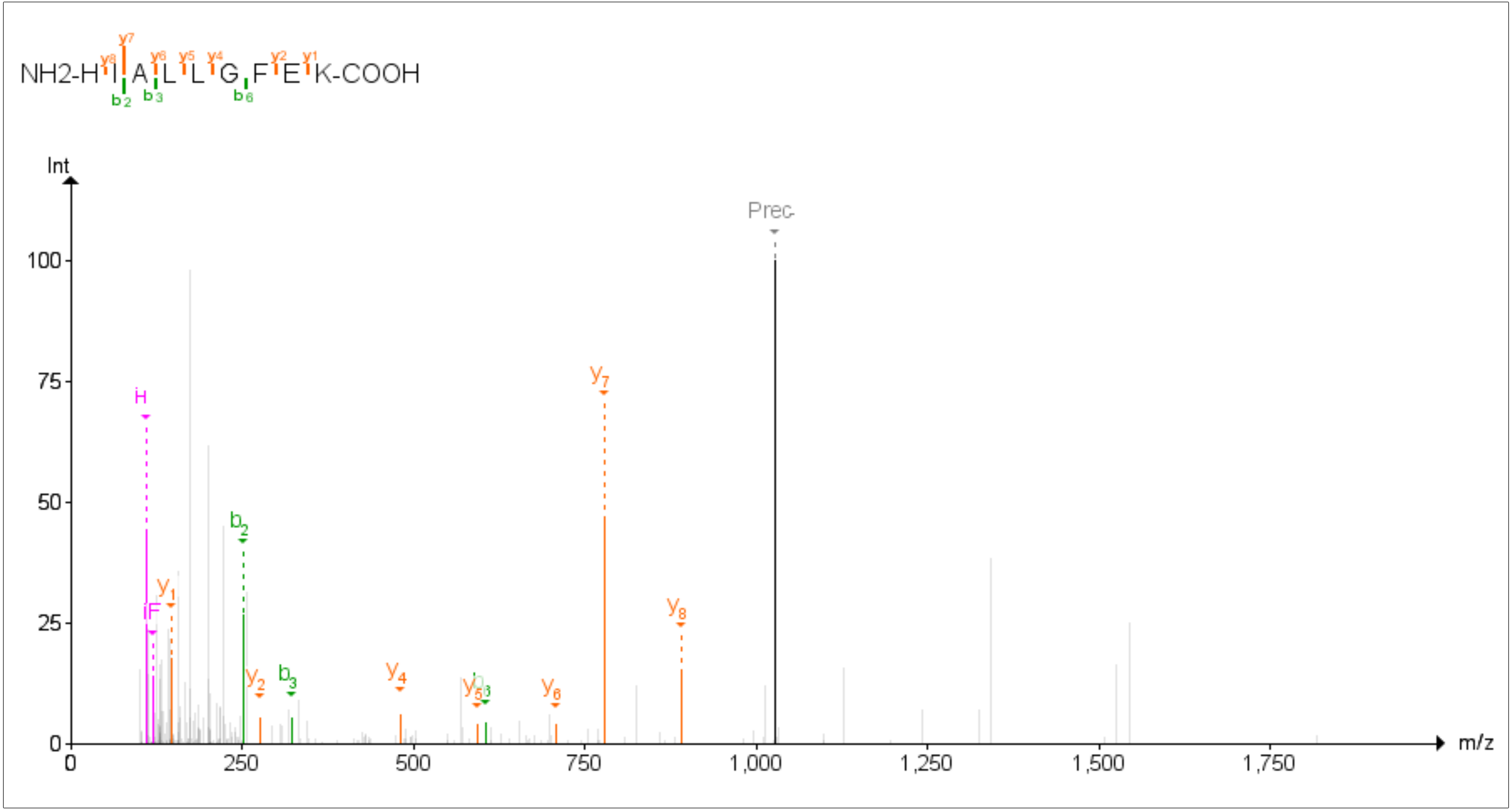

NH2-HIALLGFEK-COOH
y8 y7 y6 y5 y4 y2 y1
b2 b3 b6
Int
Prec
100
75
50
25
0
iH
iF
y1
b2
y2
b3
y4
y5
b6
y6
y7
y8
0
250
500
750
1,000
1,250
1,500
1,750
m/z

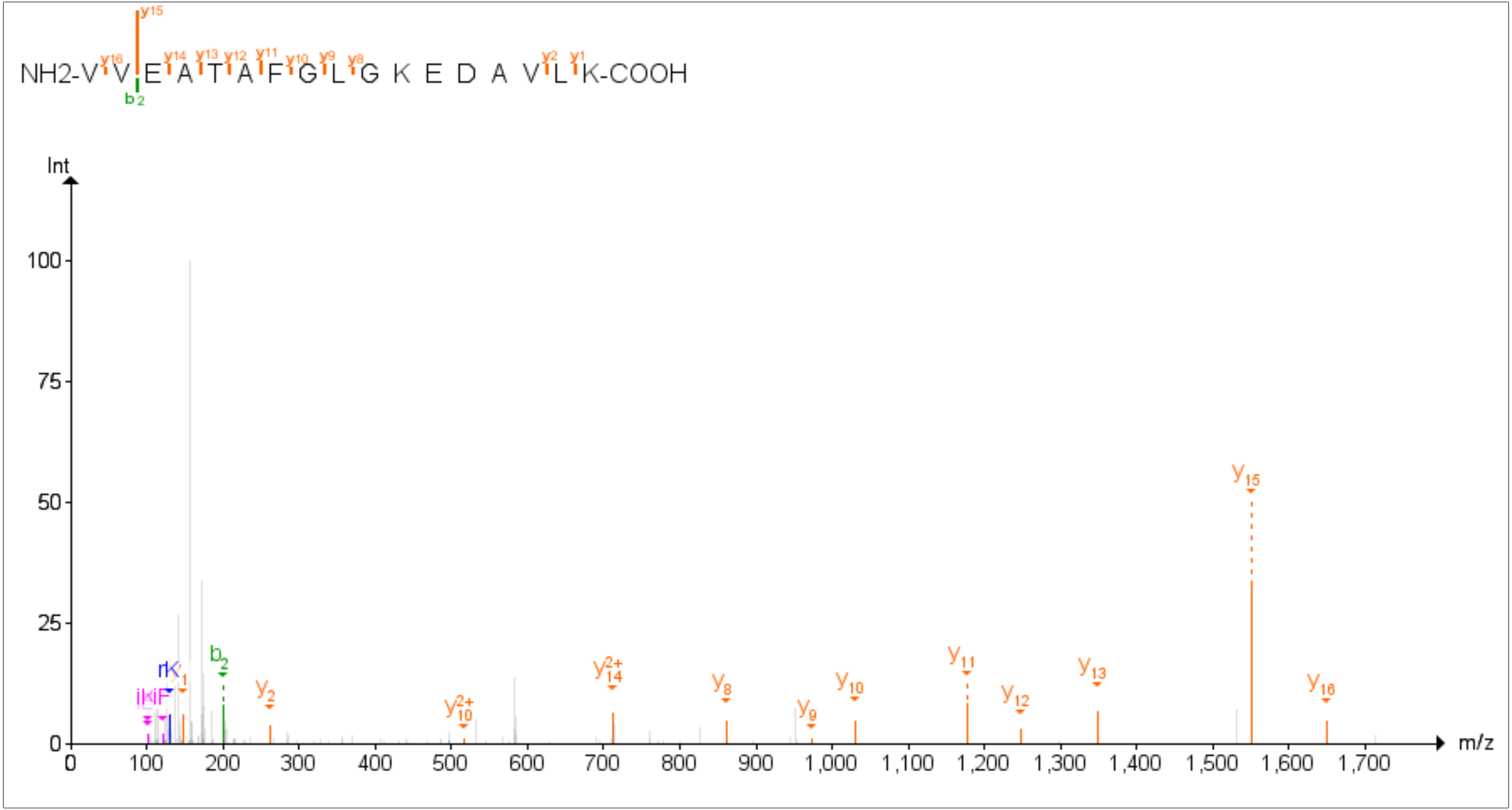
NH2-V V E A T A F G L G K E D A V L K-COOH
y16 y15 y14 y13 y12 y11 y10 y9 y8 y2 y1
b2
Int
m/z
y15
y16
y13
y12
y11
y10
y9
y8
y14 2+
y10 2+
y2
y1
b2
rK
iK iF

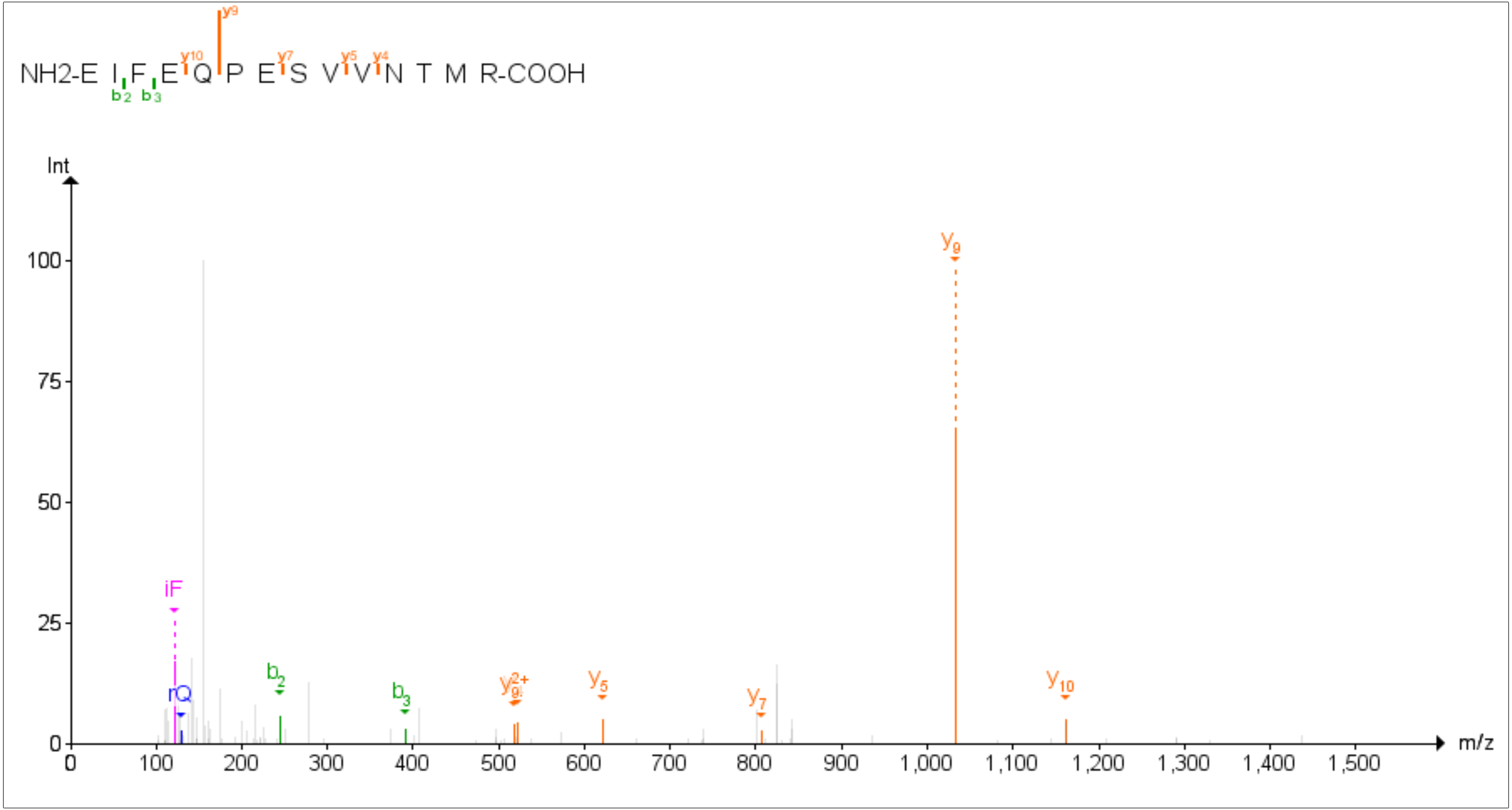

NH2-E I F E Q P E S V V N T M R-COOH
b2
b3
y10
y9
y7
y5
y4
Int
100
75
50
25
0
m/z
0
100
200
300
400
500
600
700
800
900
1,000
1,100
1,200
1,300
1,400
1,500
y9
iF
rQ
b2
b3
y9 2+
y5
y7
y10

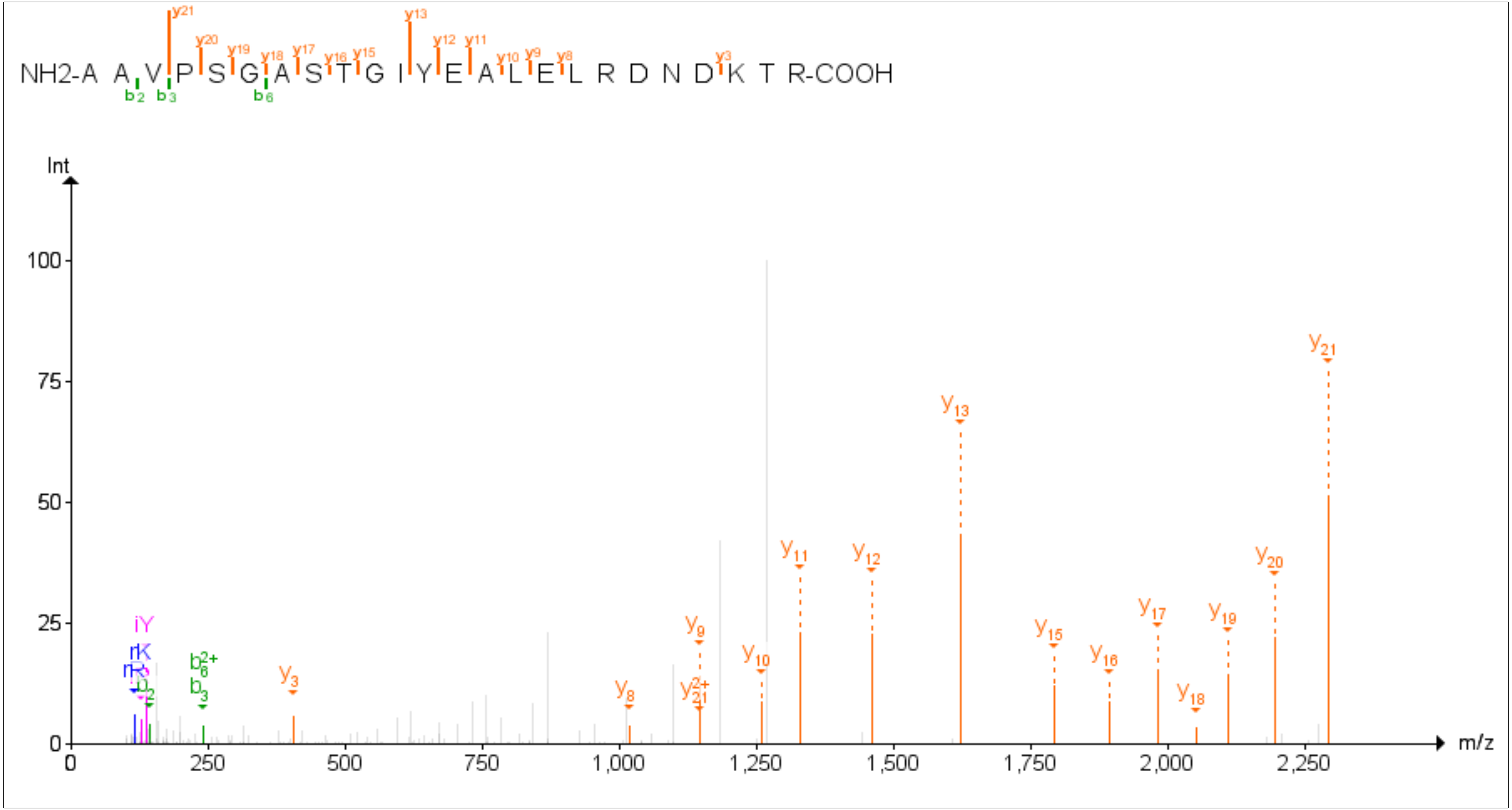

NH2-A A V P S G A S T G I Y E A L E L R D N D K T R-COOH
y21 y20 y19 y18 y17 y16 y15 y13 y12 y11 y10 y9 y8 y3
b2 b3 b6
Int
100
75
50
25
0
iY
rK
rR
b2
b6 2+
b3
y3
y8
y9
y21 2+
y10
y11
y12
y13
y15
y16
y17
y18
y19
y20
y21
0
250
500
750
1,000
1,250
1,500
1,750
2,000
2,250
m/z

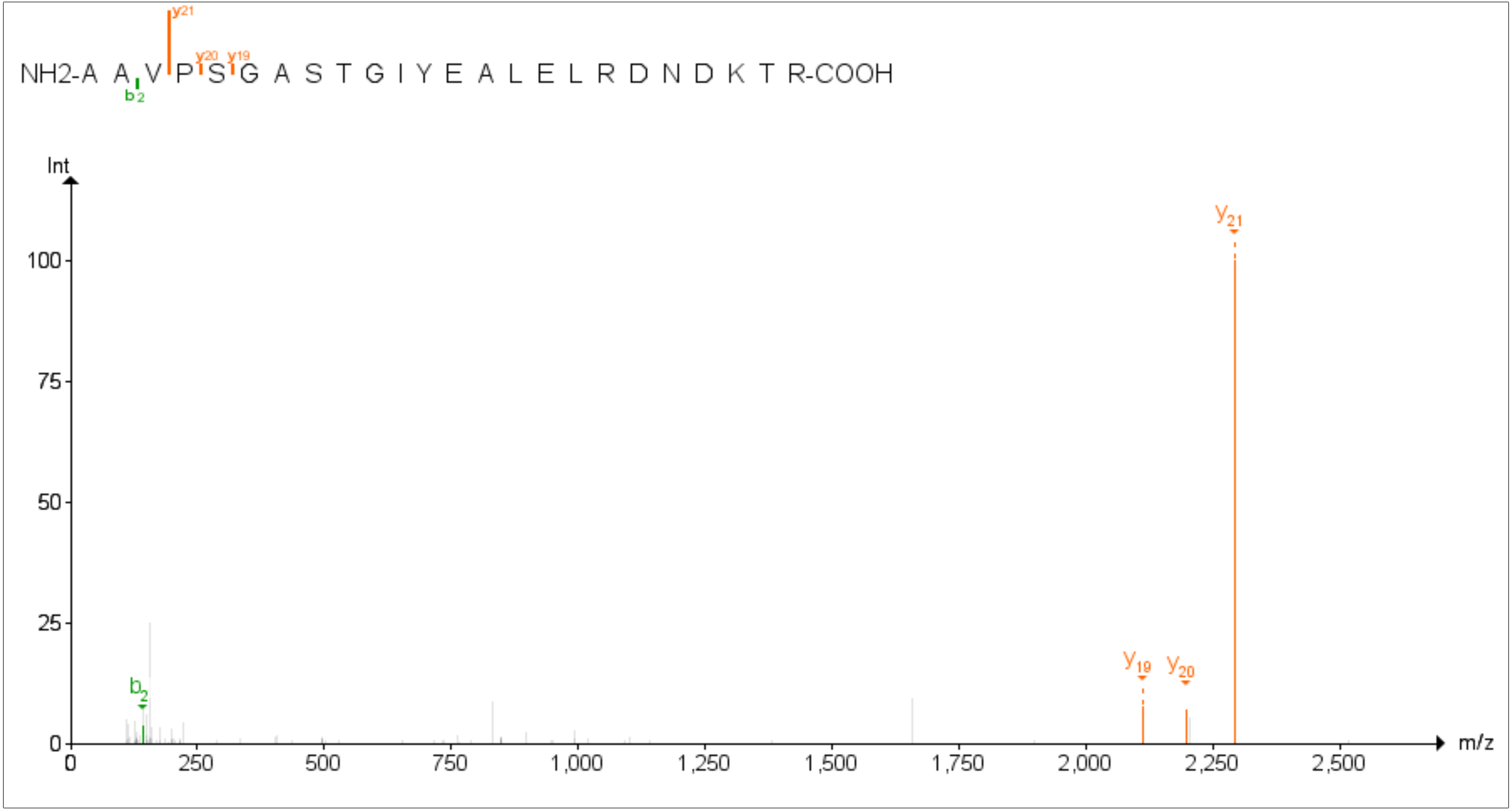

NH2-A A V P S G A S T G I Y E A L E L R D N D K T R-COOH
y21
y20
y19
b2
Int
100
75
50
25
0
0
250
500
750
1,000
1,250
1,500
1,750
2,000
2,250
2,500
m/z

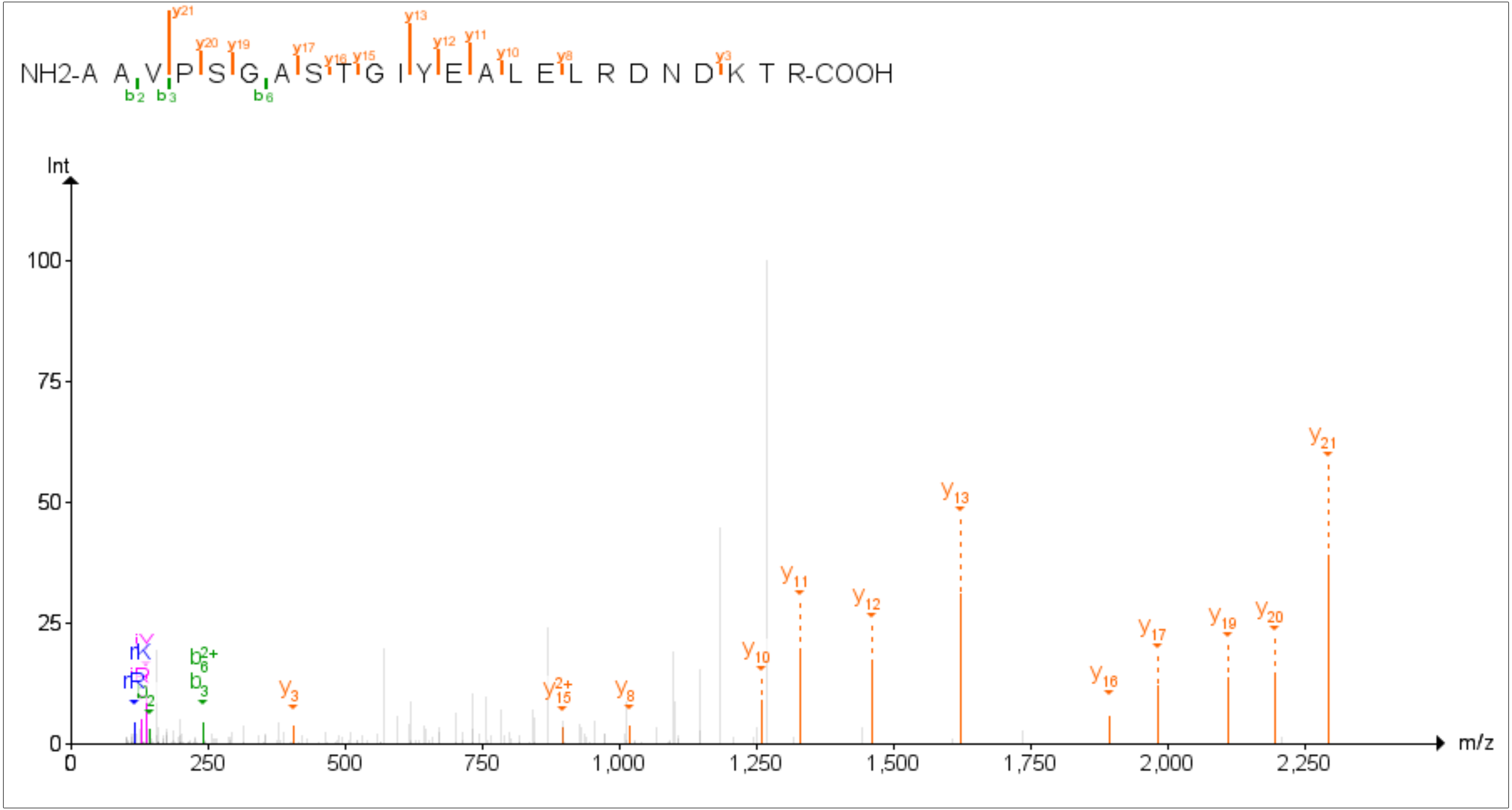

NH2-A A V P S G A S T G I Y E A L E L R D N D K T R-COOH
y21 y20 y19 y17 y16 y15 y13 y12 y11 y10 y8 y3
b2 b3 b6
Int
100
75
50
25
0
0 250 500 750 1,000 1,250 1,500 1,750 2,000 2,250
m/z
y21
y13
y11
y12
y10
y16
y17
y19
y20
y3
y8
b3
rK
rR

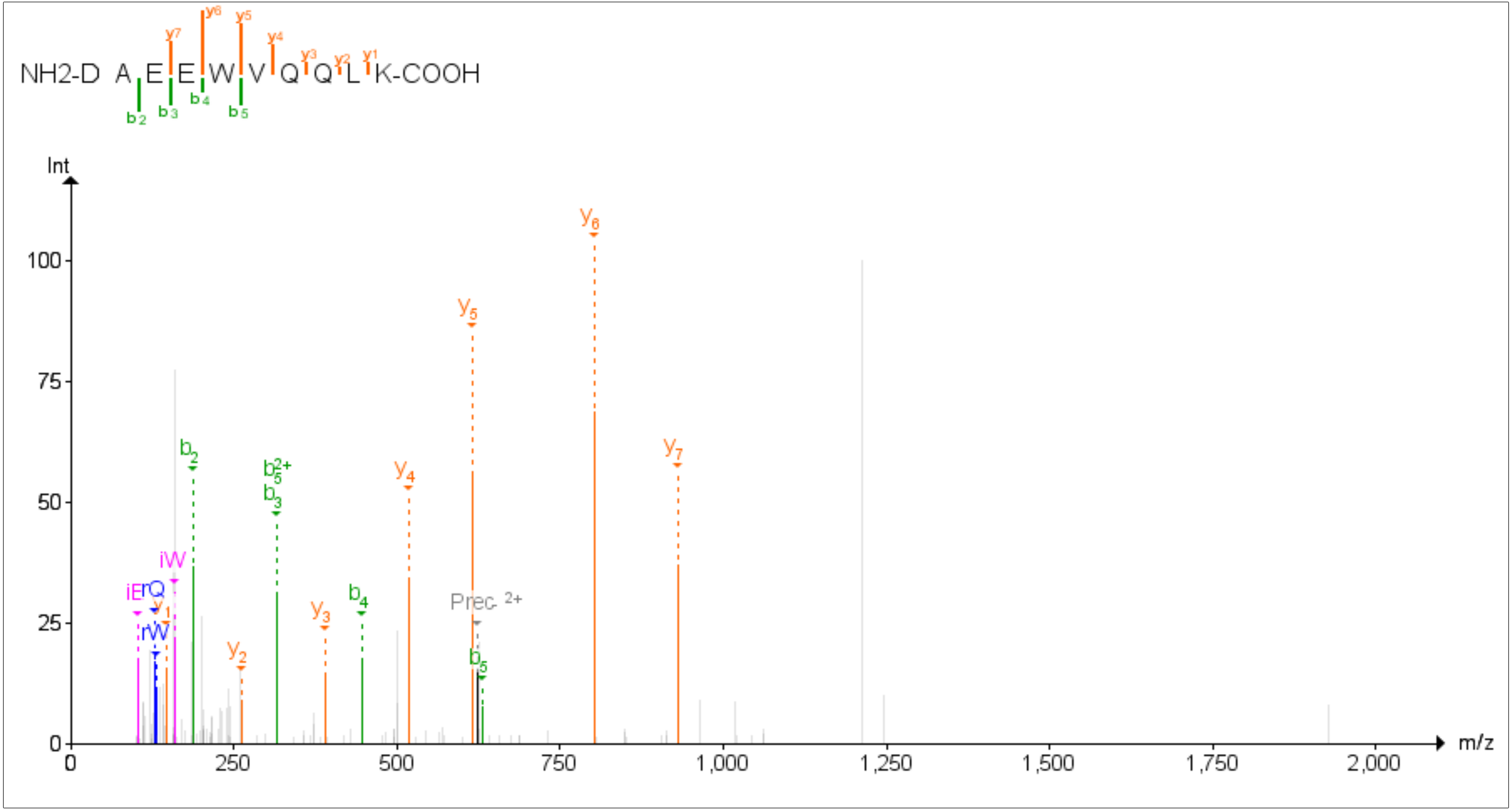

NH2-D A E E W V Q Q L K-COOH
y7 y6 y5 y4 y3 y2 y1
b2 b3 b4 b5
Int
100
75
50
25
0
y6
y5
y7
y4
b2
b5 2+
b3
iW
iE
rQ
y1
rW
y2
y3
b4
Prec 2+
b5
0
250
500
750
1,000
1,250
1,500
1,750
2,000
m/z

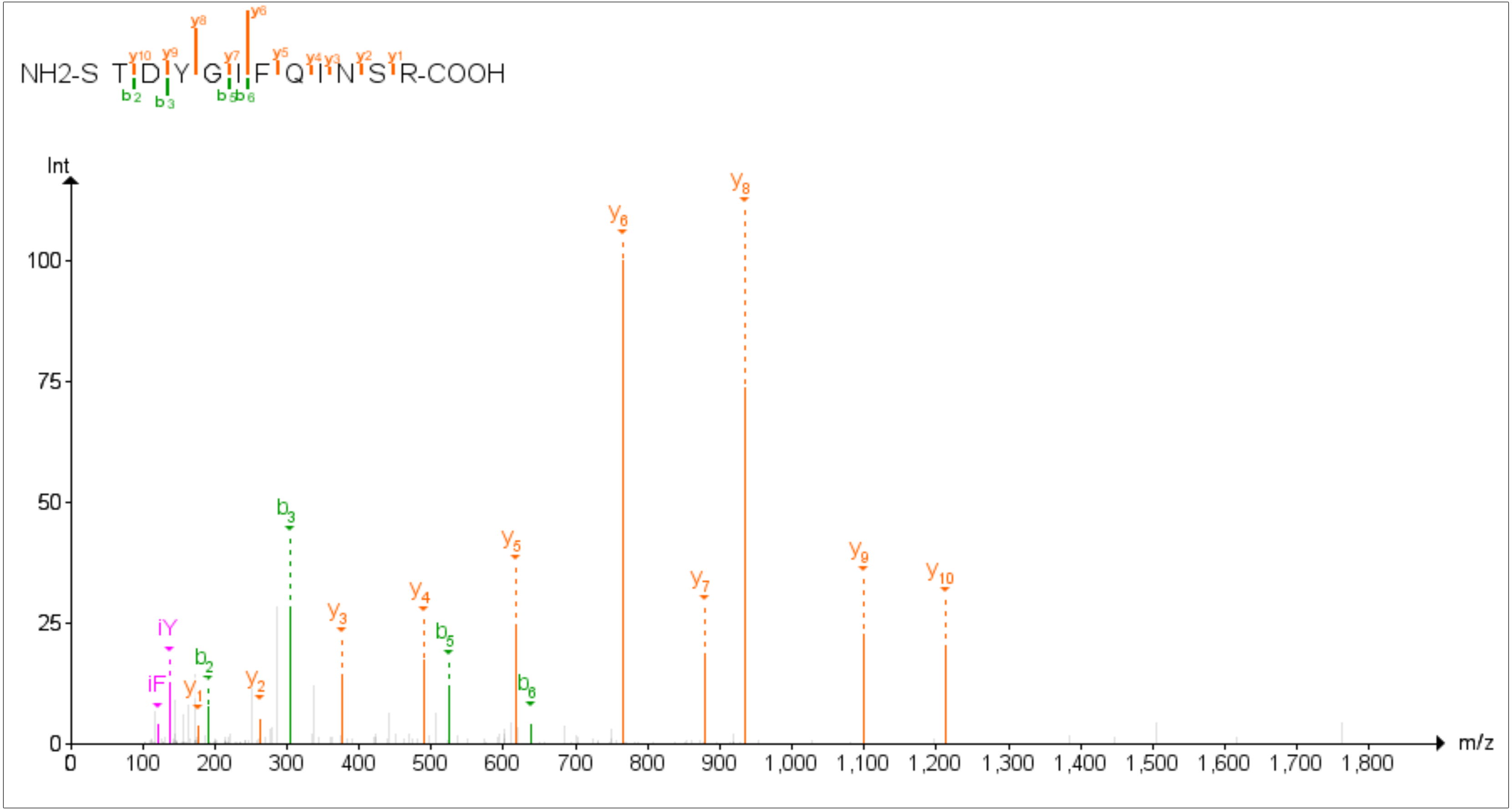
NH2-S T D Y G I I F Q I N S R-COOH
y10 y9 y8 y7 y6 y5 y4 y3 y2 y1
b2 b3 b5 b6
Int
100
75
50
25
0
iY
iF
y1
b2
y2
b3
y3
y4
b5
y5
b6
y6
y7
y8
y9
y10
0 100 200 300 400 500 600 700 800 900 1,000 1,100 1,200 1,300 1,400 1,500 1,600 1,700 1,800
m/z

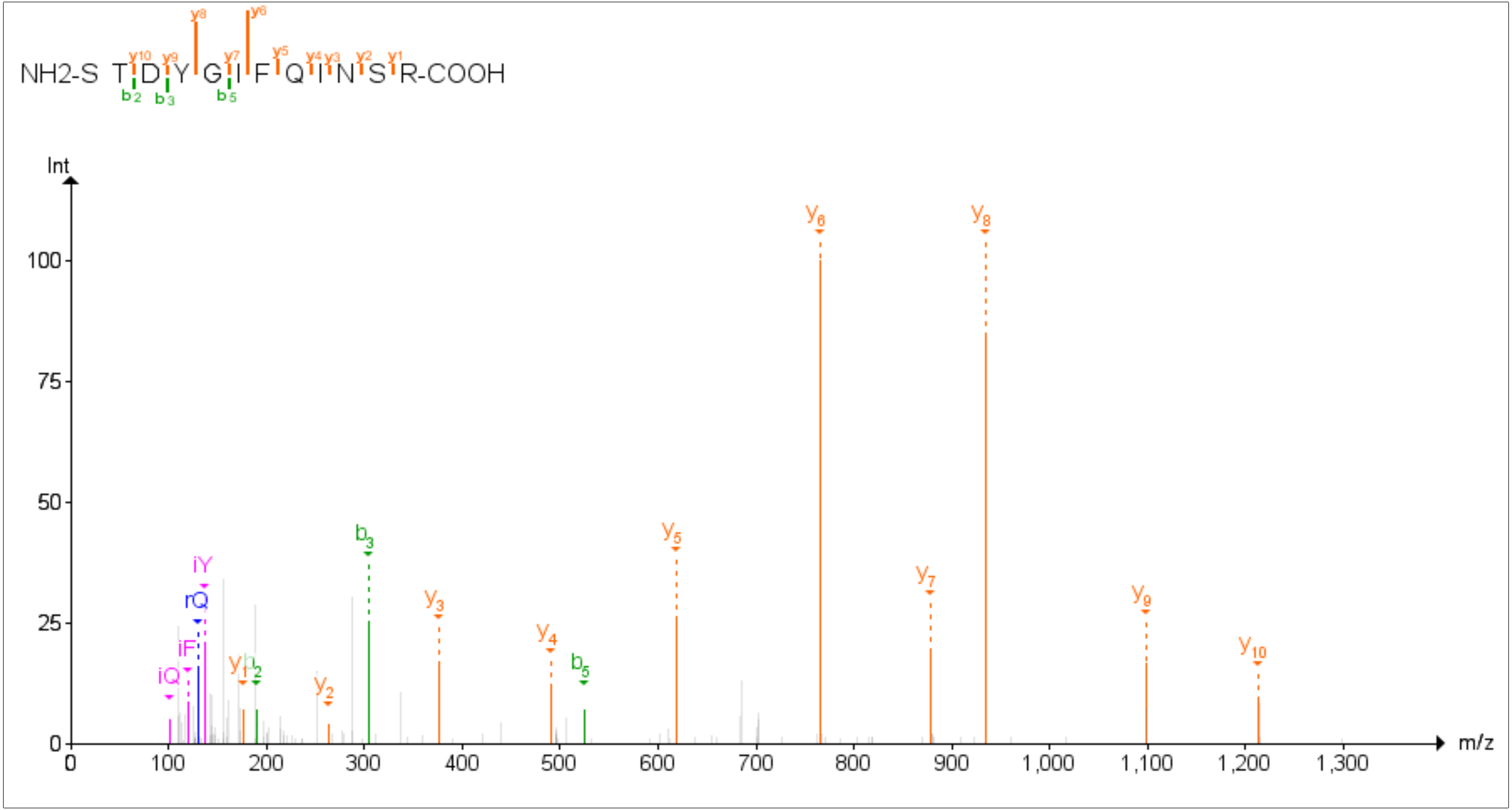

NH2-S T D Y G I F Q I N S R-COOH
y10 y9 y8 y7 y6 y5 y4 y3 y2 y1
b2 b3 b5
Int
100
75
50
25
0
m/z
0 100 200 300 400 500 600 700 800 900 1,000 1,100 1,200 1,300
iQ iF rQ iY y1 b2 y2 b3 y3 y4 b5 y5 y6 y7 y8 y9 y10

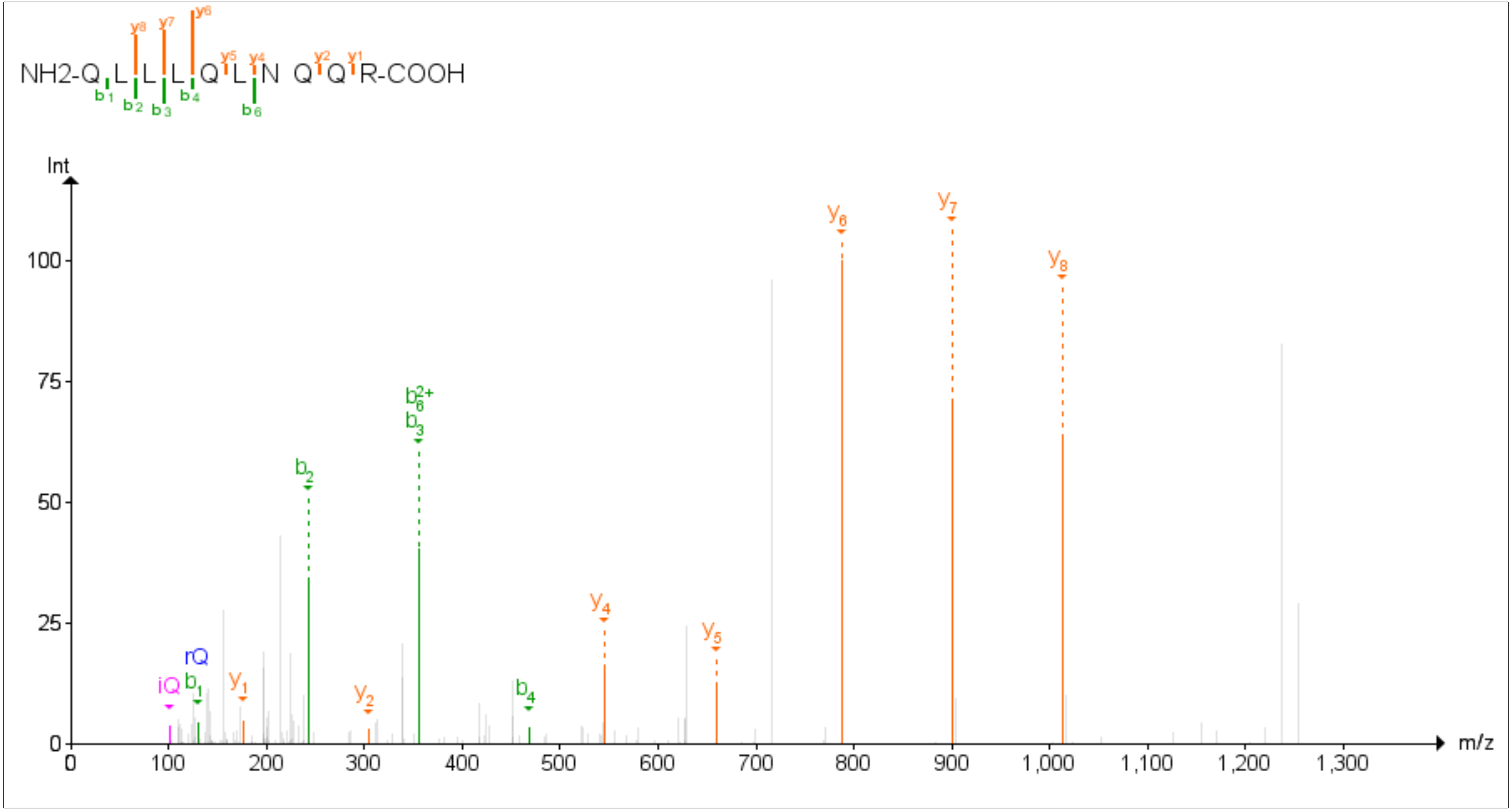
NH2-Q L L L Q L N Q Q R-COOH
y8 y7 y6 y5 y4 y2 y1
b1 b2 b3 b4 b6
Int
100
75
50
25
0
y6
y7
y8
b_6^{2+}
b_3
b_2
y_4
y_5
rQ
iQ
b_1
y_1
y_2
b_4
0 100 200 300 400 500 600 700 800 900 1,000 1,100 1,200 1,300
m/z

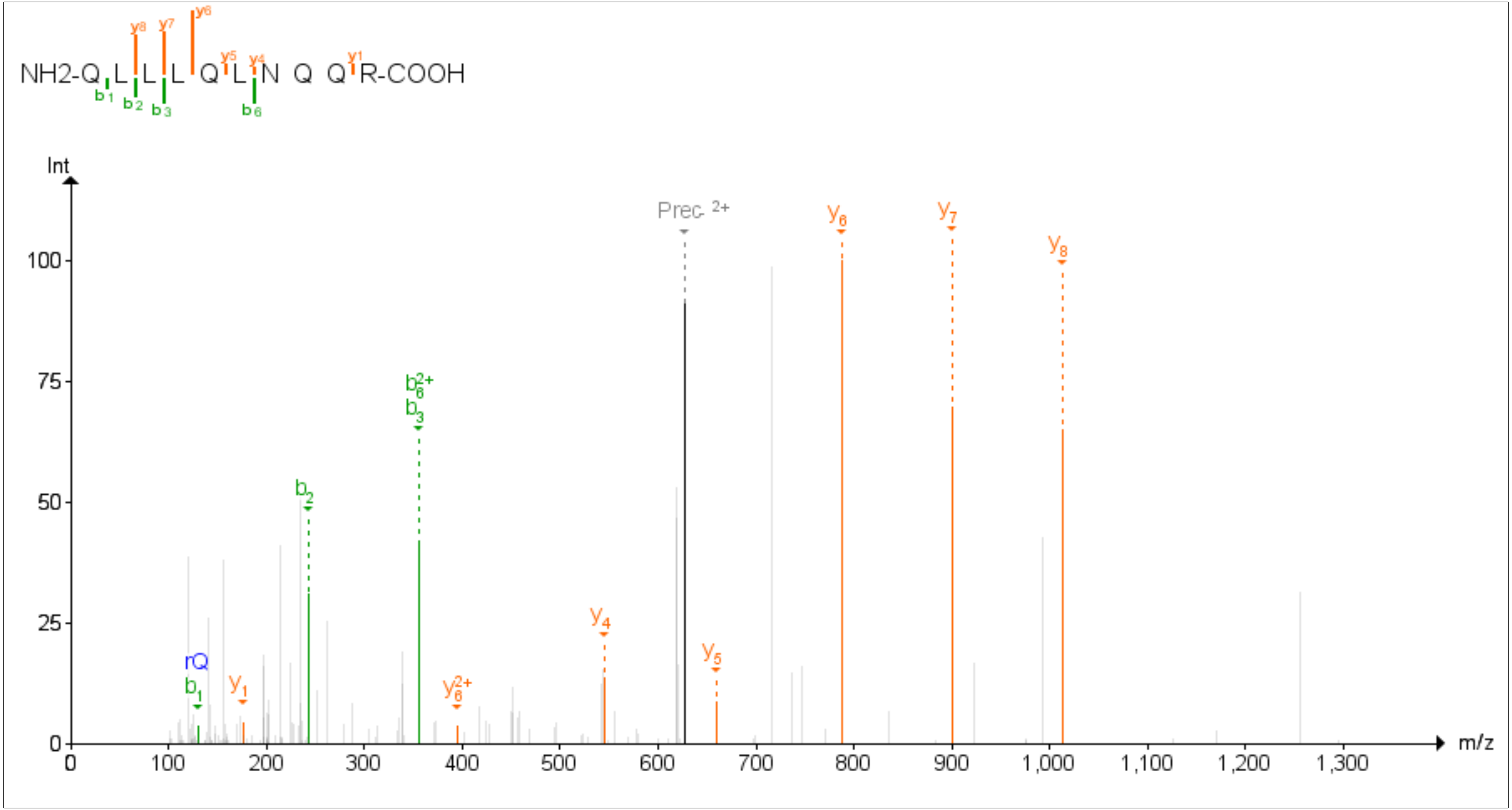

NH2-Q L L L Q L N Q Q R-COOH
b1 b2 b3 b6
y8 y7 y6 y5 y4 y1
Int
100
75
50
25
0
Prec 2+
y6
y7
y8
b6 2+
b3
b2
rQ
b1
y1
y6 2+
y4
y5
0 100 200 300 400 500 600 700 800 900 1,000 1,100 1,200 1,300
m/z

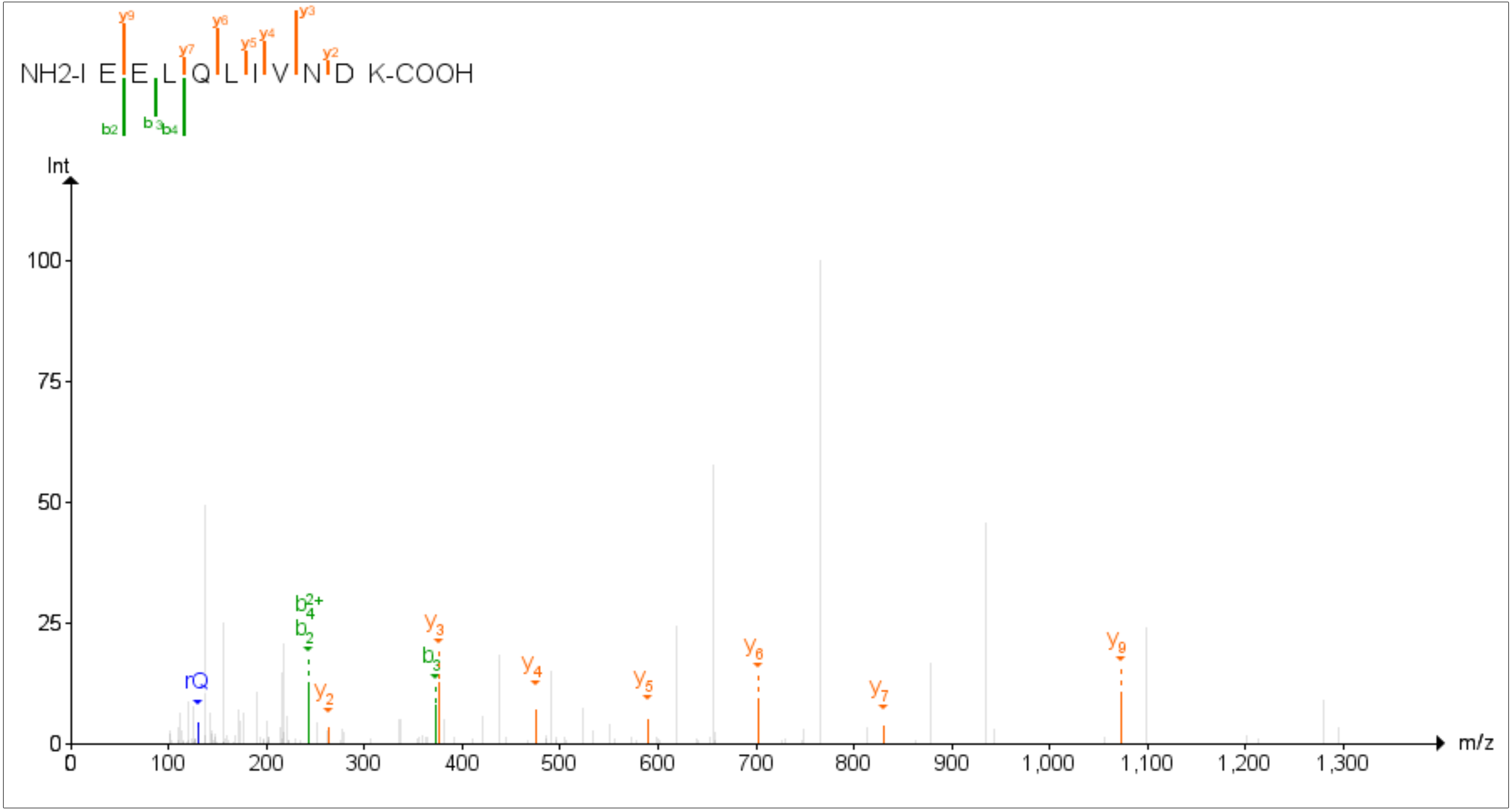

NH2-I E E L Q L I V N D K-COOH
y9
y8
y7
y5
y4
y3
y2
b2
b3
b4
Int
100
75
50
25
0
rQ
b_4^{2+}
b_2
y_2
y_3
b_3
y_4
y_5
y_6
y_7
y_9
0
100
200
300
400
500
600
700
800
900
1,000
1,100
1,200
1,300
m/z

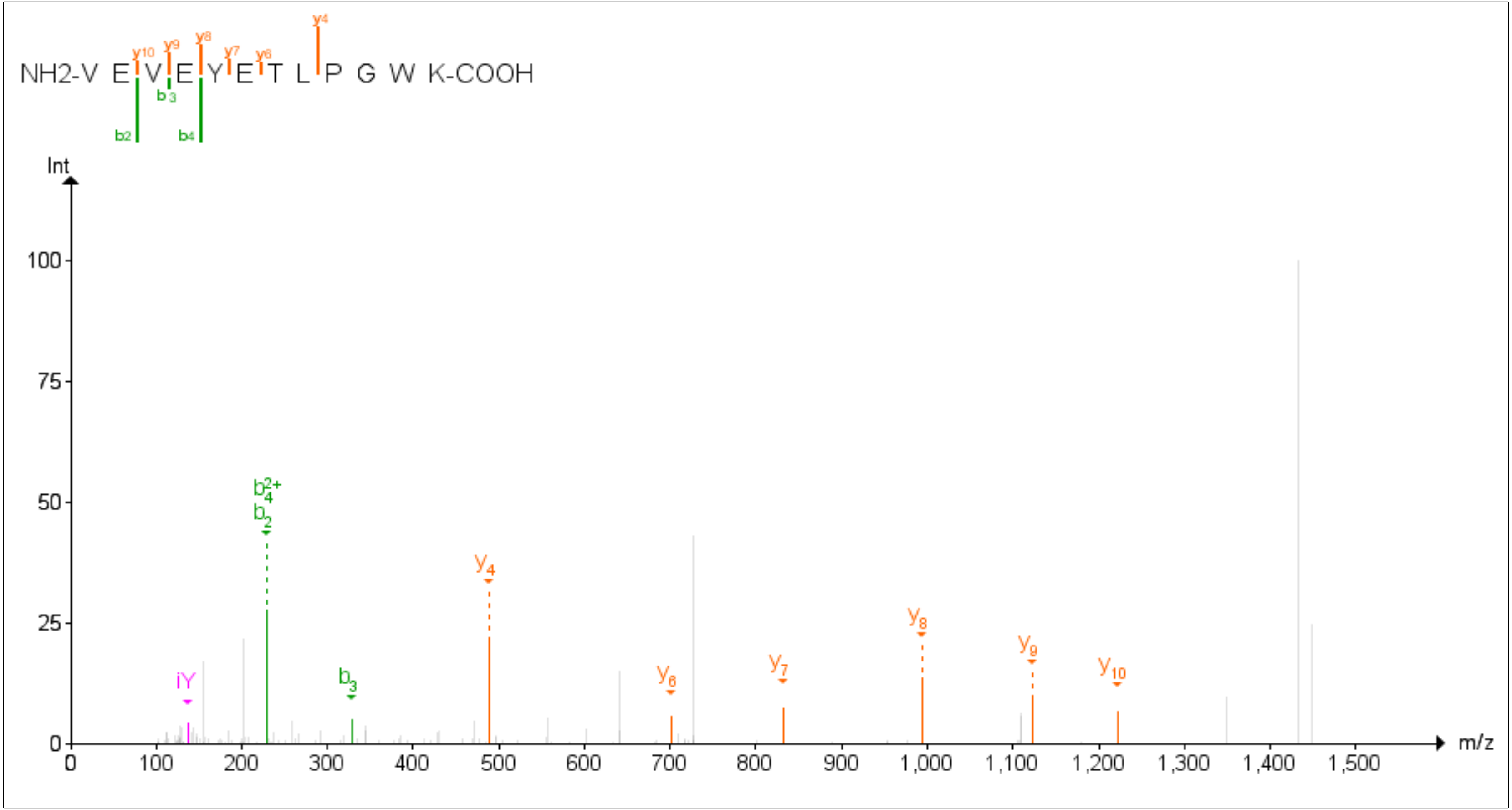
NH2-V E V E Y E T L P G W K-COOH
y10 y9 y8 y7 y6 y4
b2 b3 b4
Int
m/z
100
75
50
25
0
0 100 200 300 400 500 600 700 800 900 1,000 1,100 1,200 1,300 1,400 1,500
iY
b_4^{2+}
b_2
b_3
y_4
y_6
y_7
y_8
y_9
y_{10}

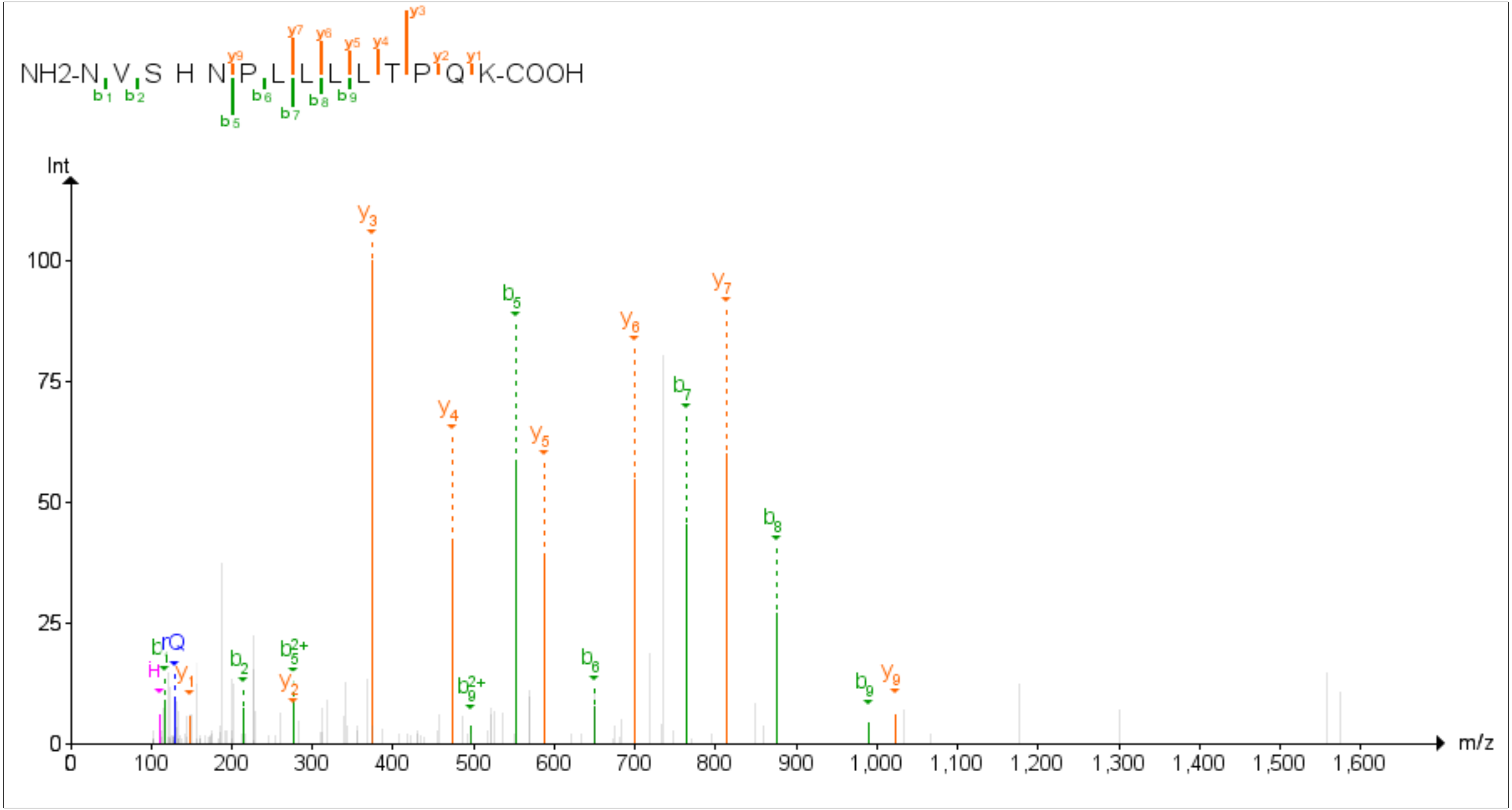

NH2-N V S H N P L L L L T P Q K-COOH
Int
100
75
50
25
0
m/z
0
100
200
300
400
500
600
700
800
900
1,000
1,100
1,200
1,300
1,400
1,500
1,600
y3
b5
y6
y7
y4
b7
y5
b8
b2
b5 2+
y2
y1
b9 2+
b6
b9
y9

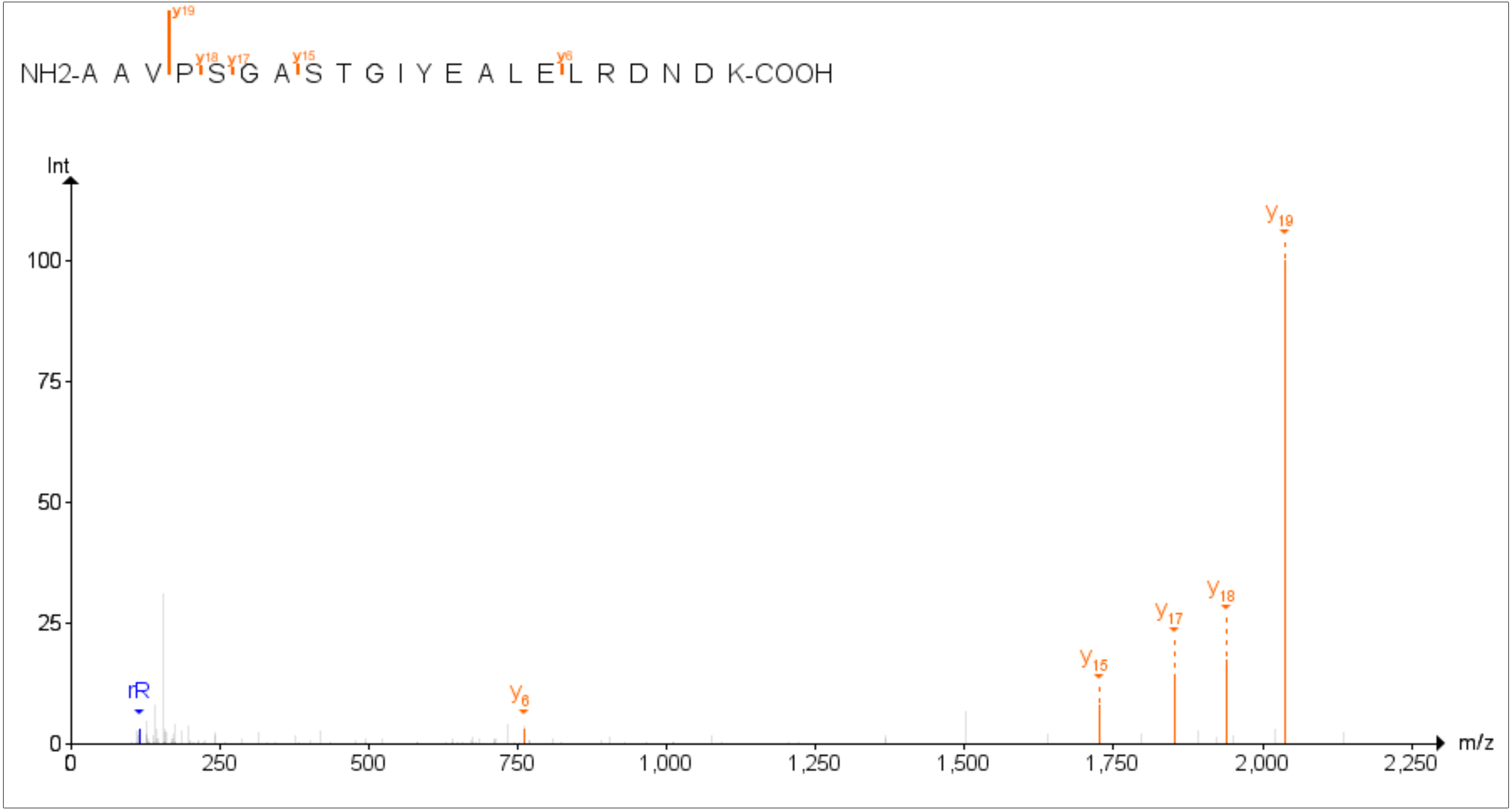

NH2-A A V P S G A S T G I Y E A L E L R D N D K-COOH
y19
y18
y17
y15
y6
Int
100
75
50
25
0
rR
0
250
500
750
1,000
1,250
1,500
1,750
2,000
2,250
m/z

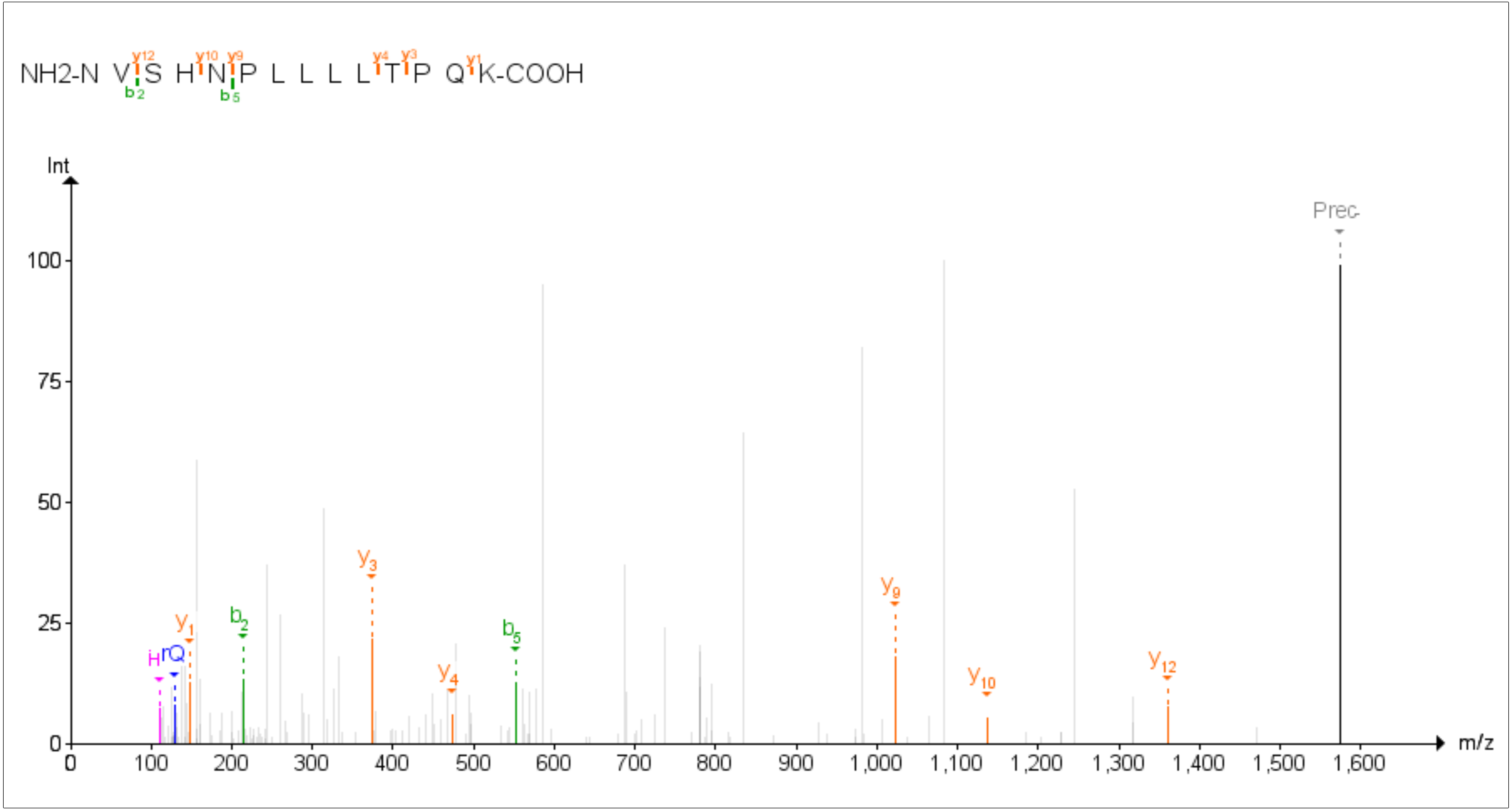
NH2-N V S H N P L L L L T P Q K-COOH
y12 y10 y9 y4 y3 y1
b2 b5
Int
m/z
Prec.
0
25
50
75
100
0 100 200 300 400 500 600 700 800 900 1,000 1,100 1,200 1,300 1,400 1,500 1,600
H
rQ
y1
b2
y3
y4
b5
y9
y10
y12

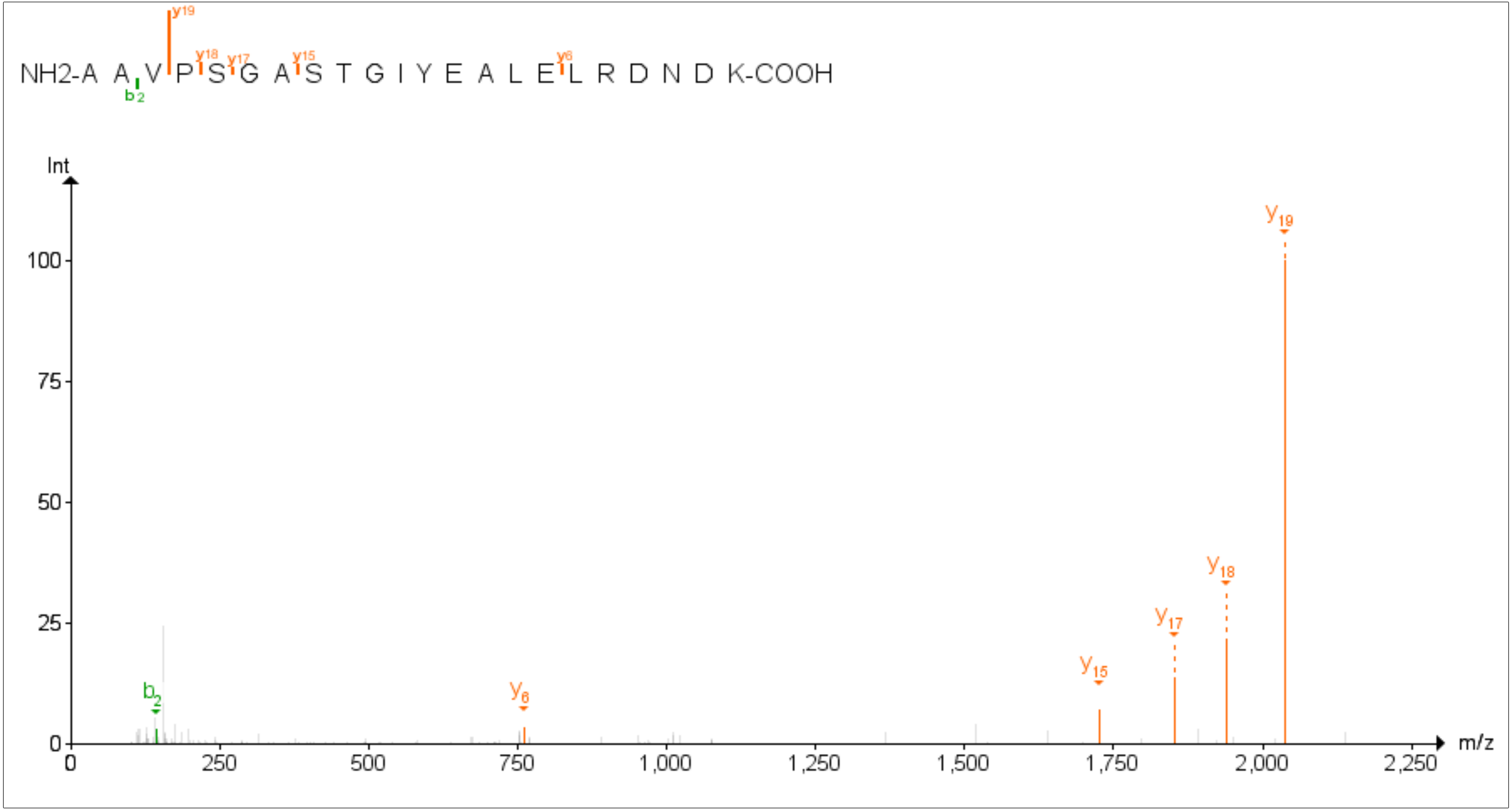

NH2-A A V P S G A S T G I Y E A L E L R D N D K-COOH
y19
y18
y17
y15
y6
b2
Int
100
75
50
25
0
250
500
750
1,000
1,250
1,500
1,750
2,000
2,250
m/z

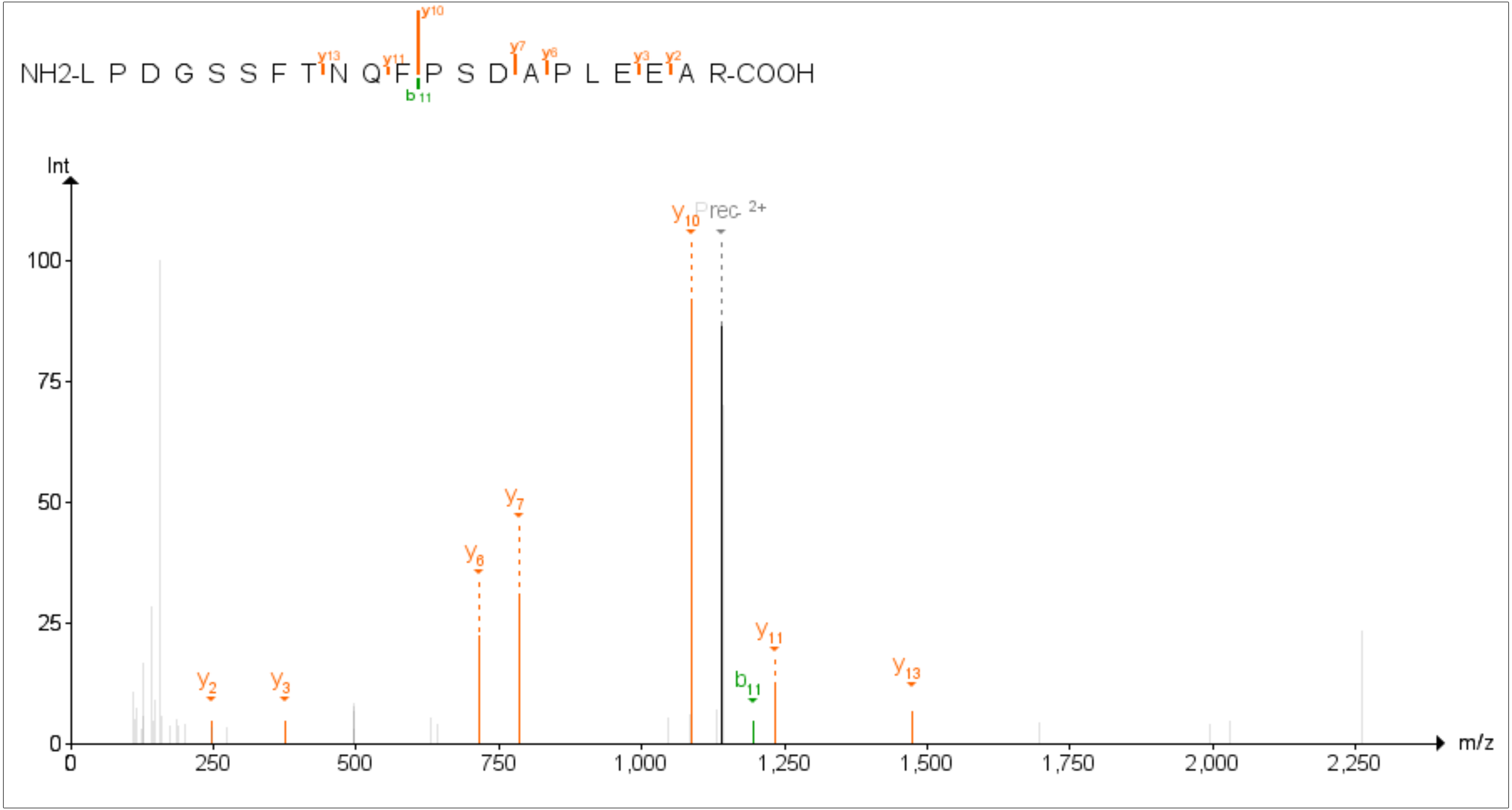

NH2-L P D G S S F T N Q F P S D A P L E E A R-COOH
y13
y11
y10
y7
y6
y3
y2
b 11
Int
100
75
50
25
0
y10
Prec 2+
y7
y6
y2
y3
y11
b11
y13
0
250
500
750
1,000
1,250
1,500
1,750
2,000
2,250
m/z

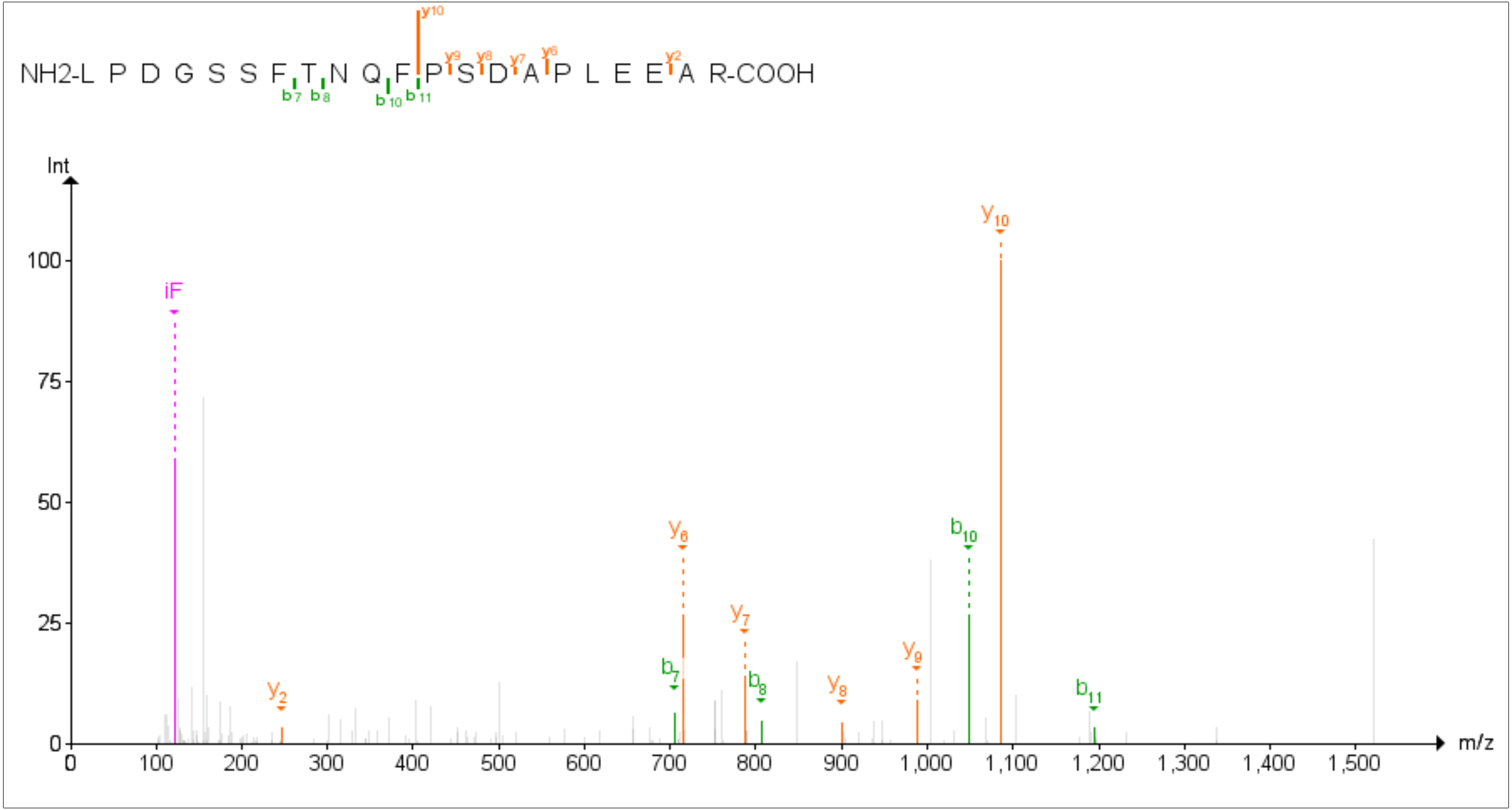

NH2-L P D G S S F T N Q F P S D A P L E E A R-COOH
y10 y9 y8 y7 y6 y2
b7 b8 b10 b11
Int
m/z
0
25
50
75
100
0 100 200 300 400 500 600 700 800 900 1,000 1,100 1,200 1,300 1,400 1,500
iF
y2
b7
y6
y7
b8
y8
y9
b10
y10
b11

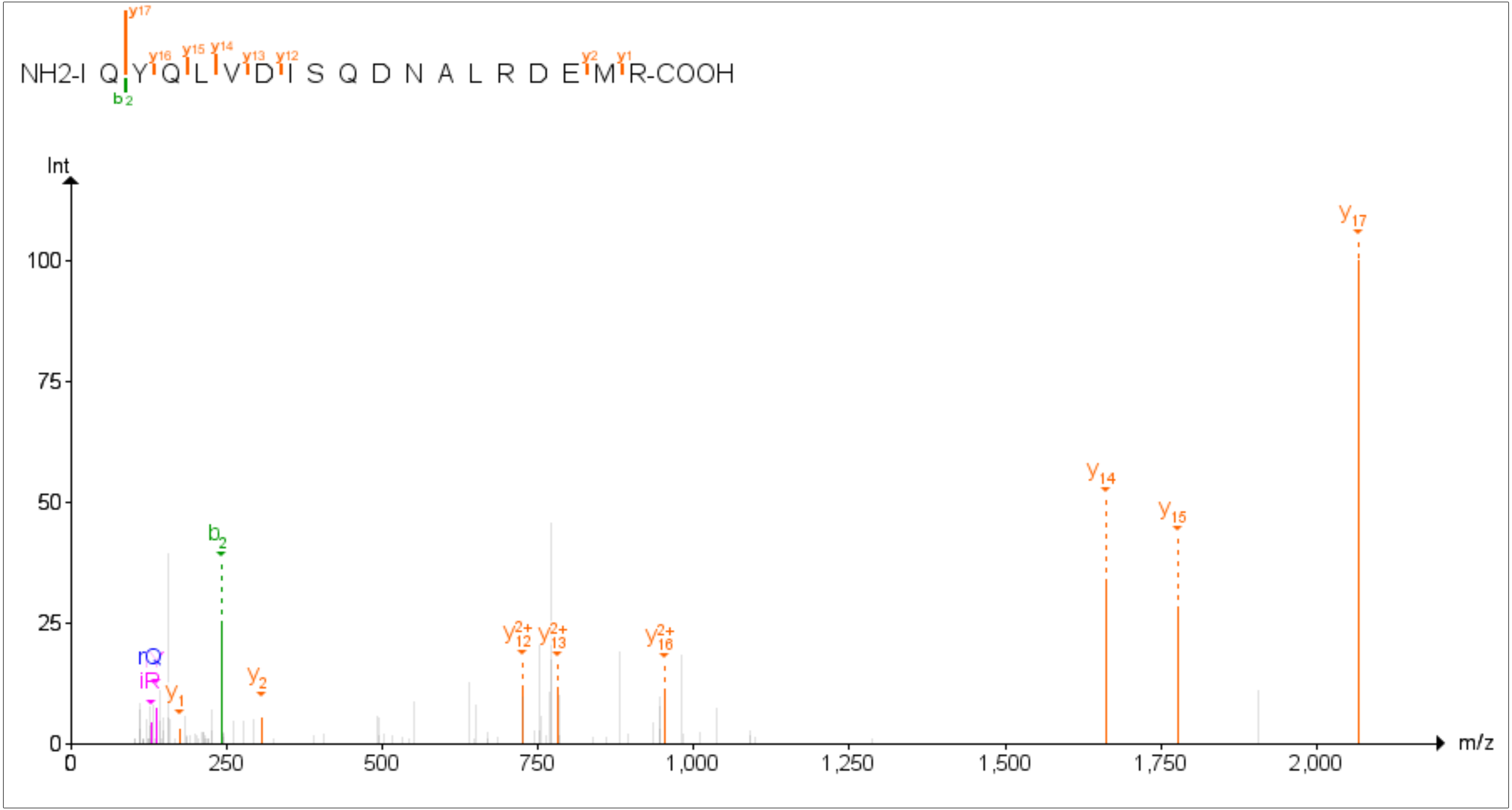
NH2-I Q Y Q L V D I S Q D N A L R D E M R-COOH
y17 y16 y15 y14 y13 y12 y2 y1
b2
Int
m/z
0
25
50
75
100
0
250
500
750
1,000
1,250
1,500
1,750
2,000
y17
y14
y15
b2
y2+12
y2+13
y2+16
rQ
iR
y1
y2

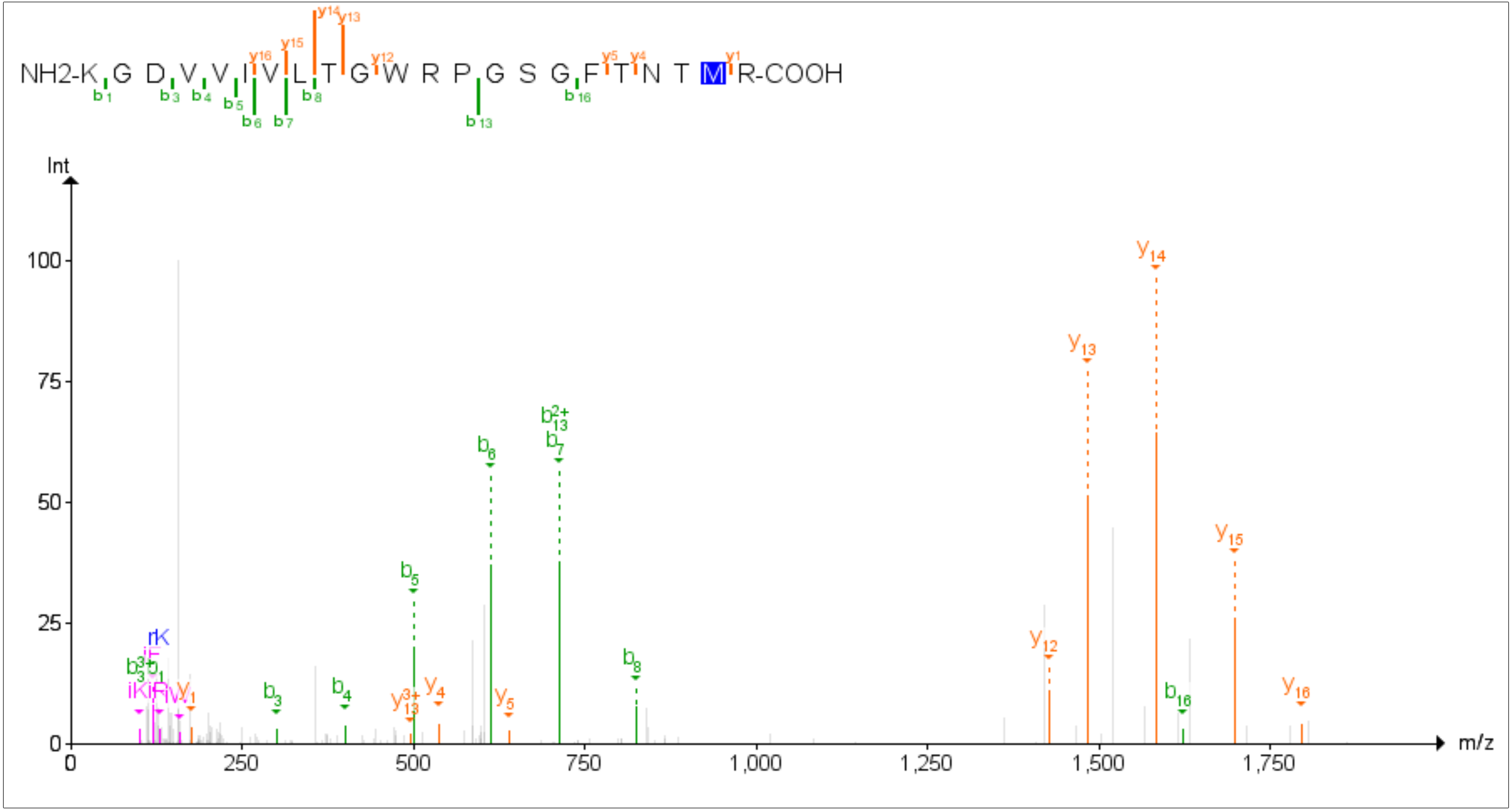

NH2-K G D V V I V L T G W R P G S G F T N T M R-COOH
Int
100
75
50
25
0
250
500
750
1,000
1,250
1,500
1,750
m/z

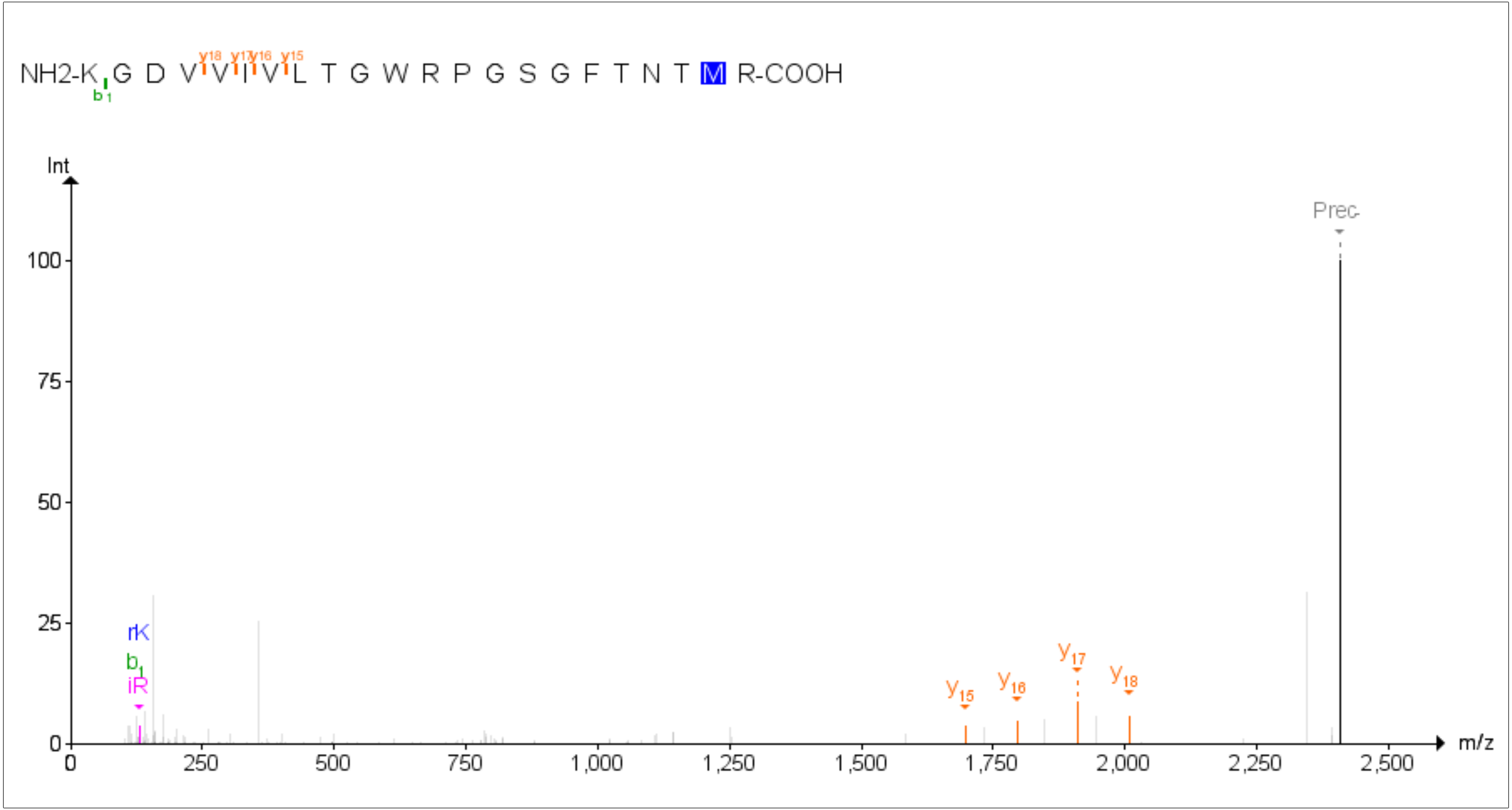

NH2-K G D V V I V L T G W R P G S G F T N T M R-COOH
b1
y18 y17 y16 y15
Int
100
75
50
25
0
Prec
rK
b1
iR
y15
y16
y17
y18
0
250
500
750
1,000
1,250
1,500
1,750
2,000
2,250
2,500
m/z

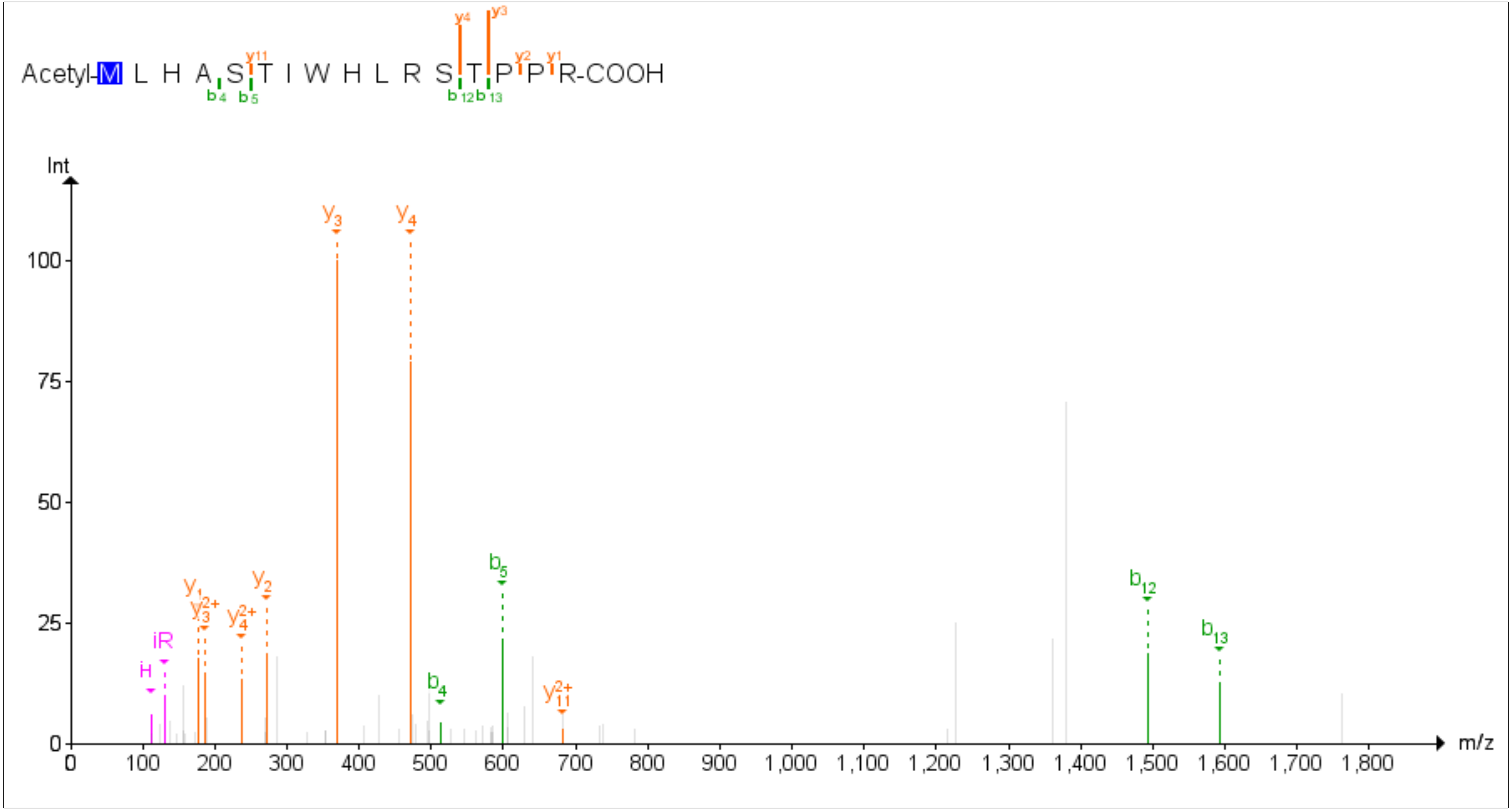

Acetyl-M L H A S T I W H L R S T P P R-COOH
y4
y3
y11
y2
y1
b4
b5
b12
b13
Int
100
75
50
25
0
y3
y4
b5
b12
b13
y1
y2
y3 2+
y4 2+
iR
H
b4
y11 2+
0
100
200
300
400
500
600
700
800
900
1,000
1,100
1,200
1,300
1,400
1,500
1,600
1,700
1,800
m/z

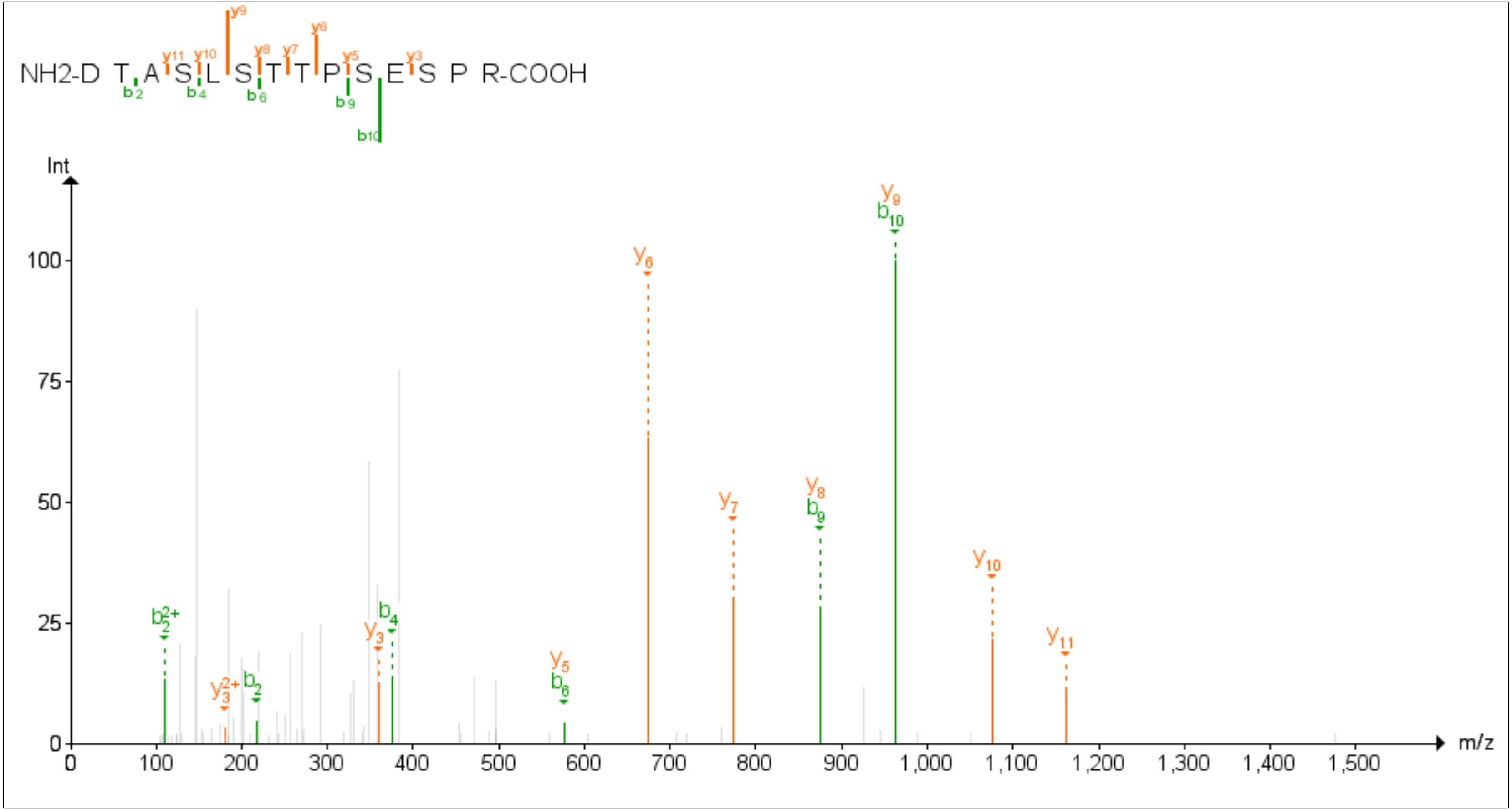

NH2-D T A S L S T T P S E S P R-COOH
y11 y10 y9 y8 y7 y6 y5 y3
b2 b4 b6 b9 b10
Int
100
75
50
25
0
m/z
0 100 200 300 400 500 600 700 800 900 1,000 1,100 1,200 1,300 1,400 1,500

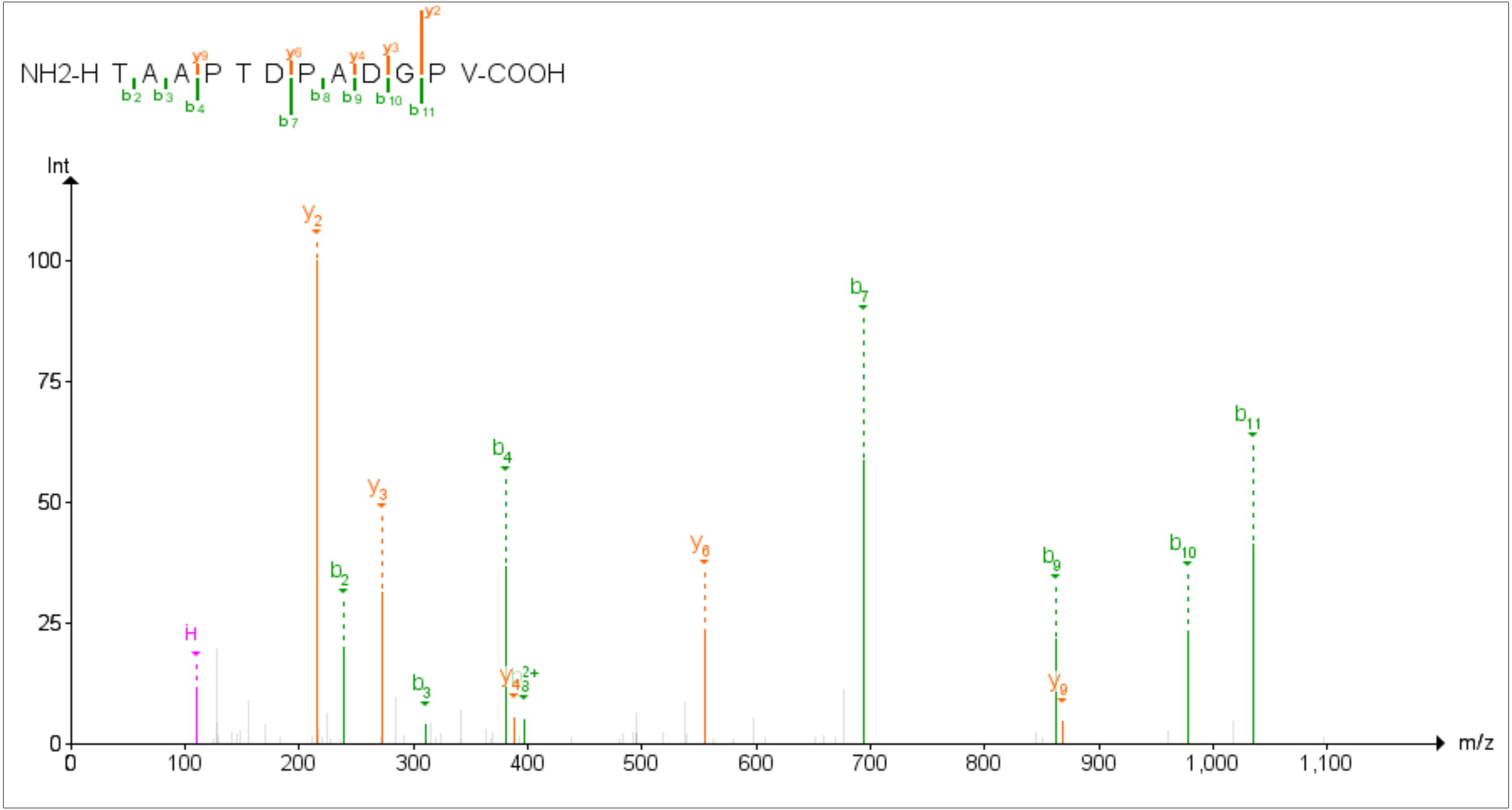

NH2-H T A A P T D P A D G P V-COOH
Int
m/z

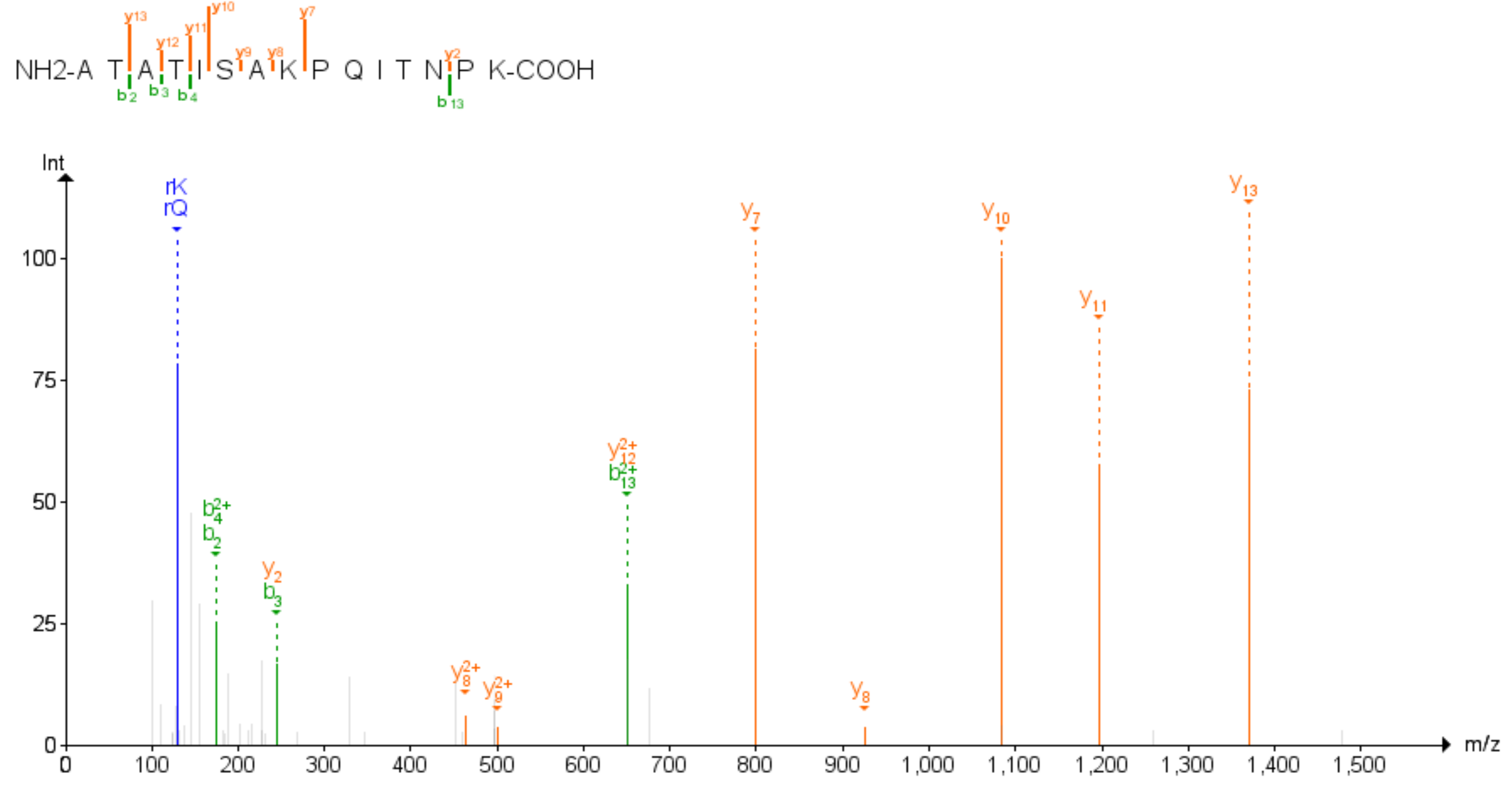

NH2-A T A T I S A K P Q I T N P K-COOH
y13
y12
y11
y10
y9
y8
y7
y2
b2
b3
b4
b13
Int
rK
rQ
y7
y10
y13
y11
y12 2+
b13 2+
b4 2+
b2
y2
b3
y8 2+
y9 2+
y8
0
25
50
75
100
0
100
200
300
400
500
600
700
800
900
1,000
1,100
1,200
1,300
1,400
1,500
m/z

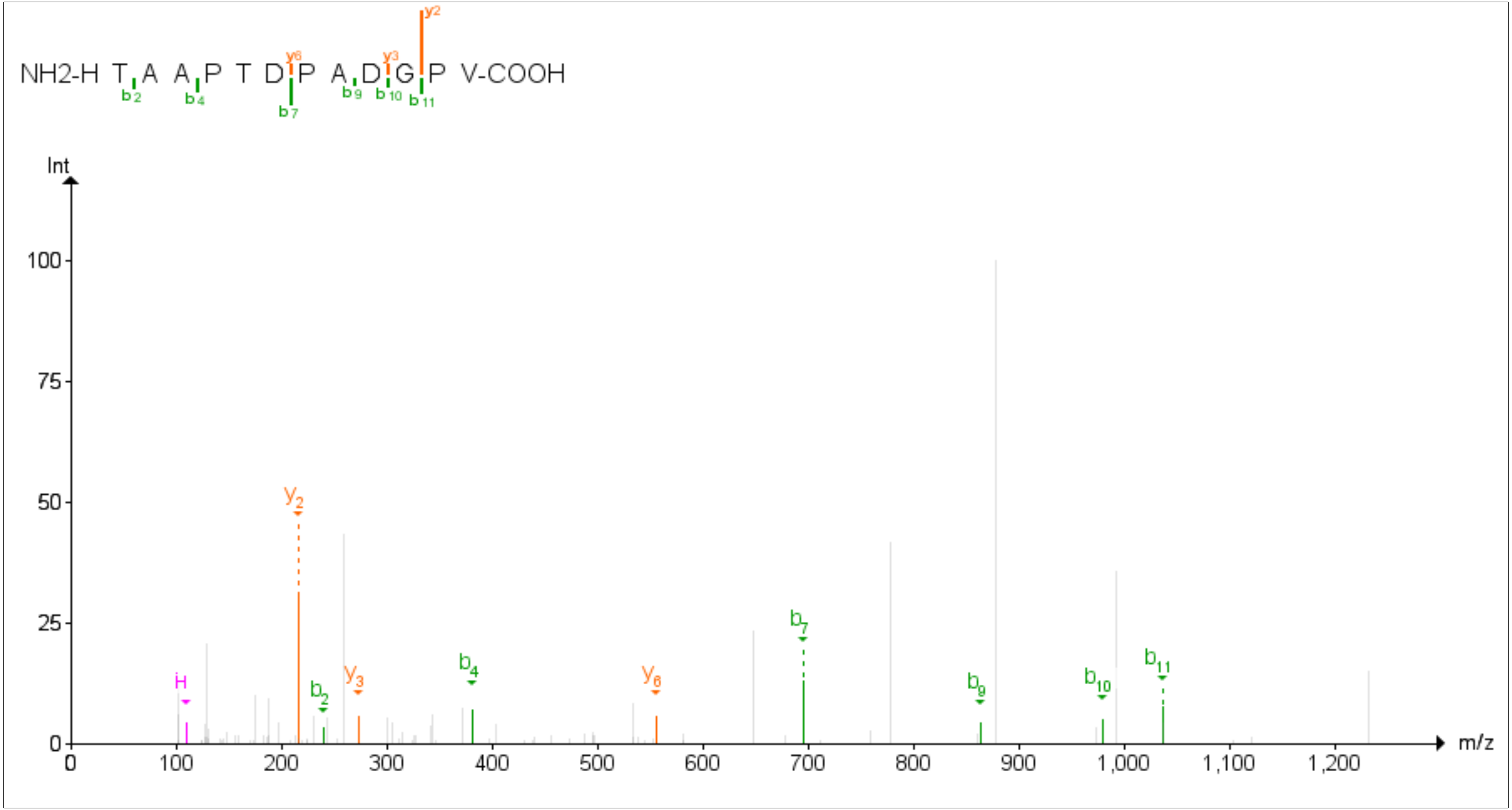

NH2-H T A A P T D P A D G P V-COOH
b2
b4
b7
b9
b10
b11
y6
y3
y2
Int
100
75
50
25
0
H
y2
b2
y3
b4
y6
b7
b9
b10
b11
0
100
200
300
400
500
600
700
800
900
1,000
1,100
1,200
m/z

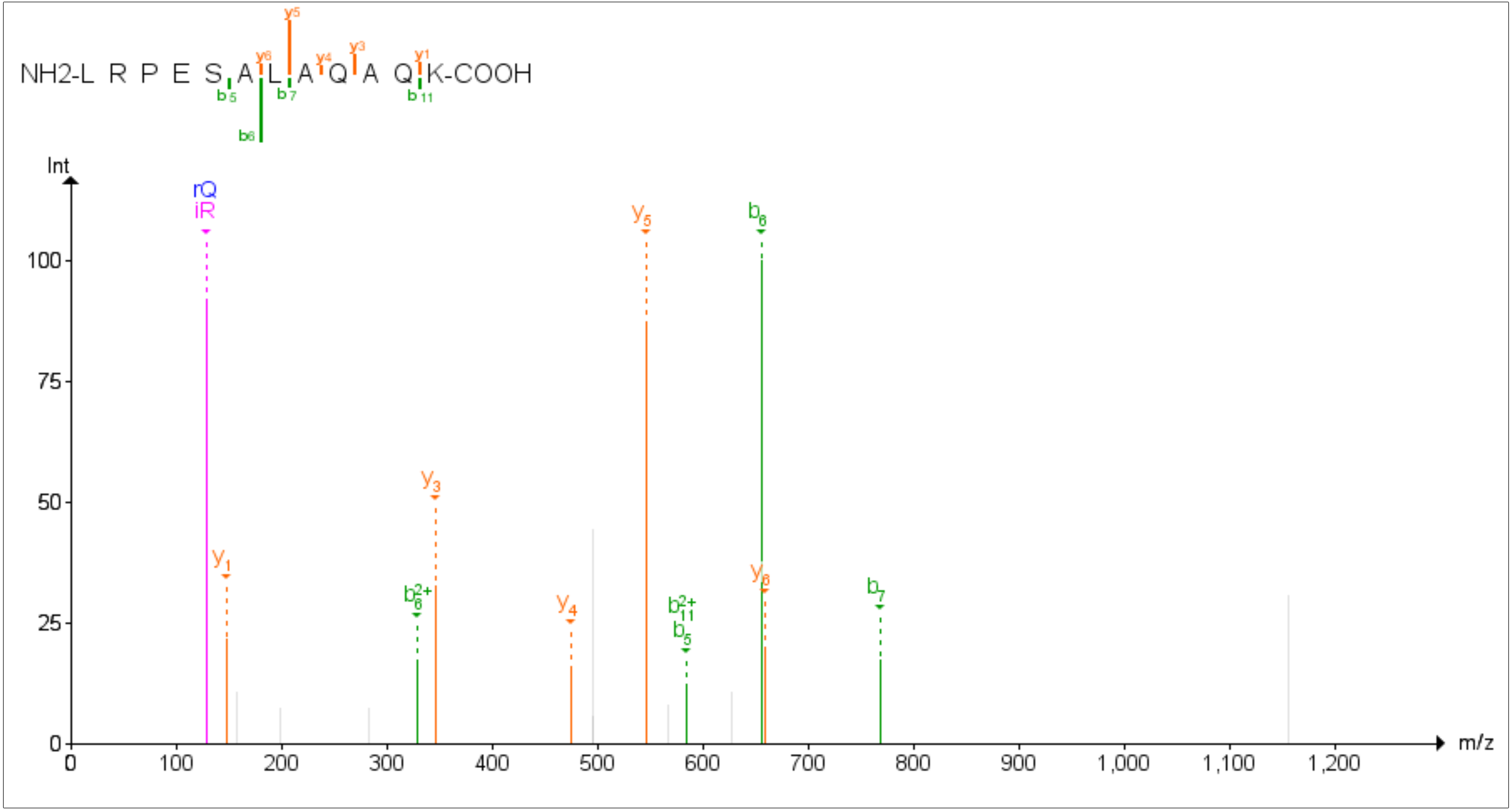

NH2-L R P E S A L A Q A Q K-COOH
b5 b6 b7 b11
y6 y5 y4 y3 y1
Int
rQ
iR
y5
b6
y3
y1
b6 2+
y4
b11 2+
b5
y6
b7
100
75
50
25
0
0 100 200 300 400 500 600 700 800 900 1,000 1,100 1,200
m/z

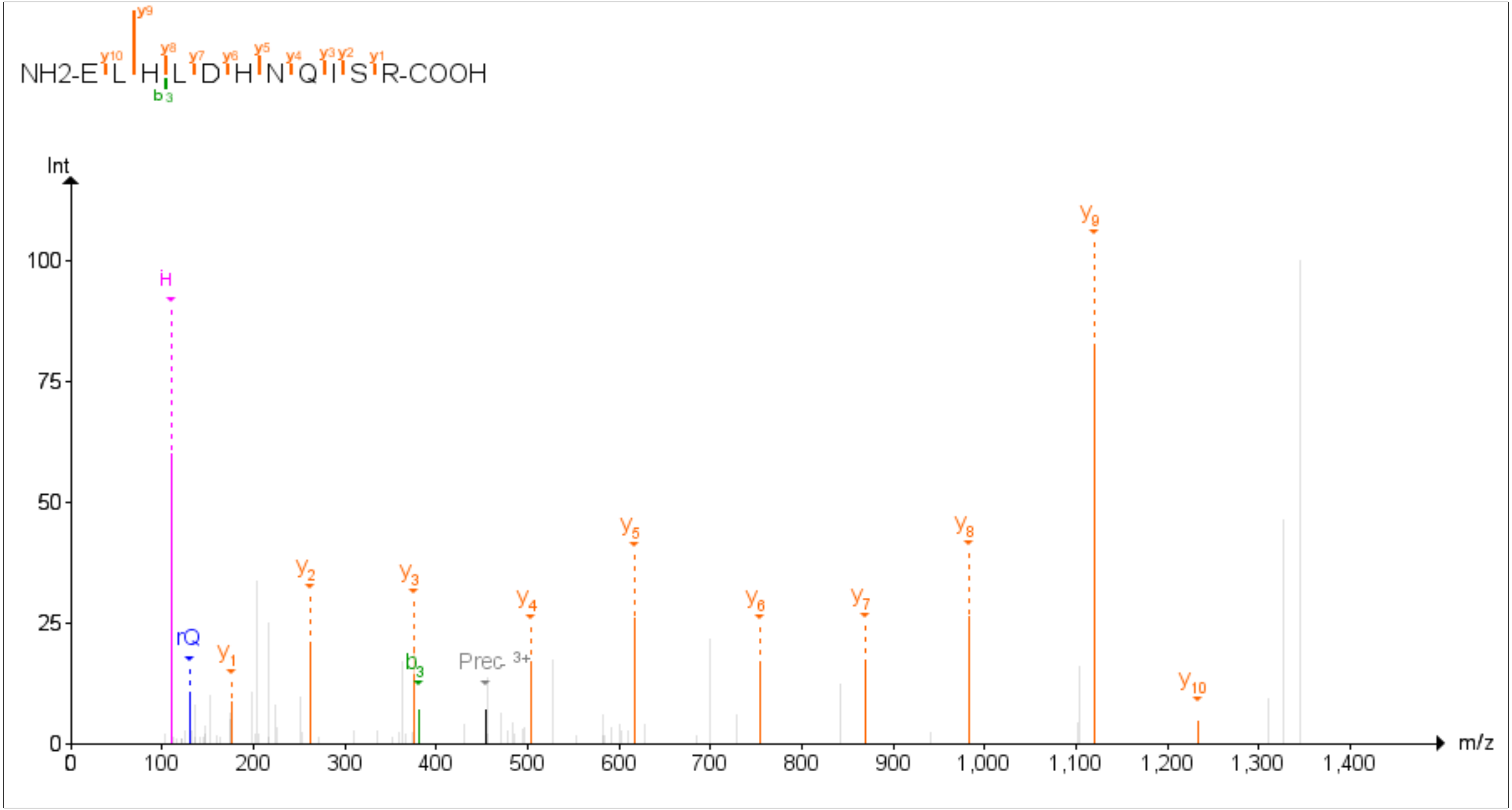

NH2-E L H L D H N Q I S R-COOH
y10 y9 y8 y7 y6 y5 y4 y3 y2 y1
b3
Int
m/z
H
rQ
y1
y2
y3
b3
Prec 3+
y4
y5
y6
y7
y8
y9
y10
0
25
50
75
100
0
100
200
300
400
500
600
700
800
900
1,000
1,100
1,200
1,300
1,400

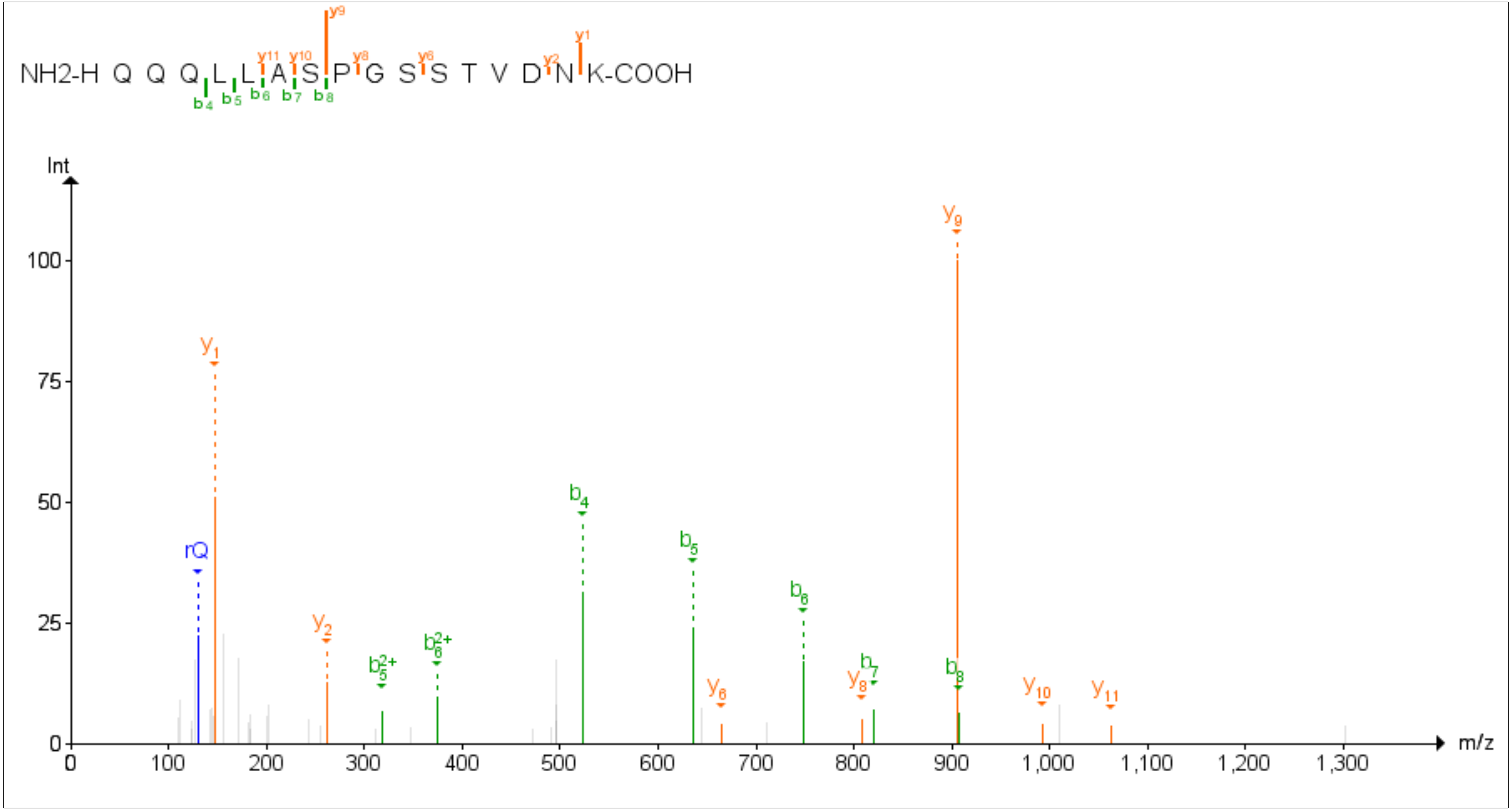

NH2-H Q Q Q L L A S P G S S T V D N K-COOH
y9
y1
y11 y10 y8 y6 y2
b4 b5 b6 b7 b8
Int
m/z
y9
y1
rQ
y2
b4
b5
b6
b5 2+
b6 2+
y6
y8
b7
b8
y10
y11
0
25
50
75
100
0
100
200
300
400
500
600
700
800
900
1,000
1,100
1,200
1,300

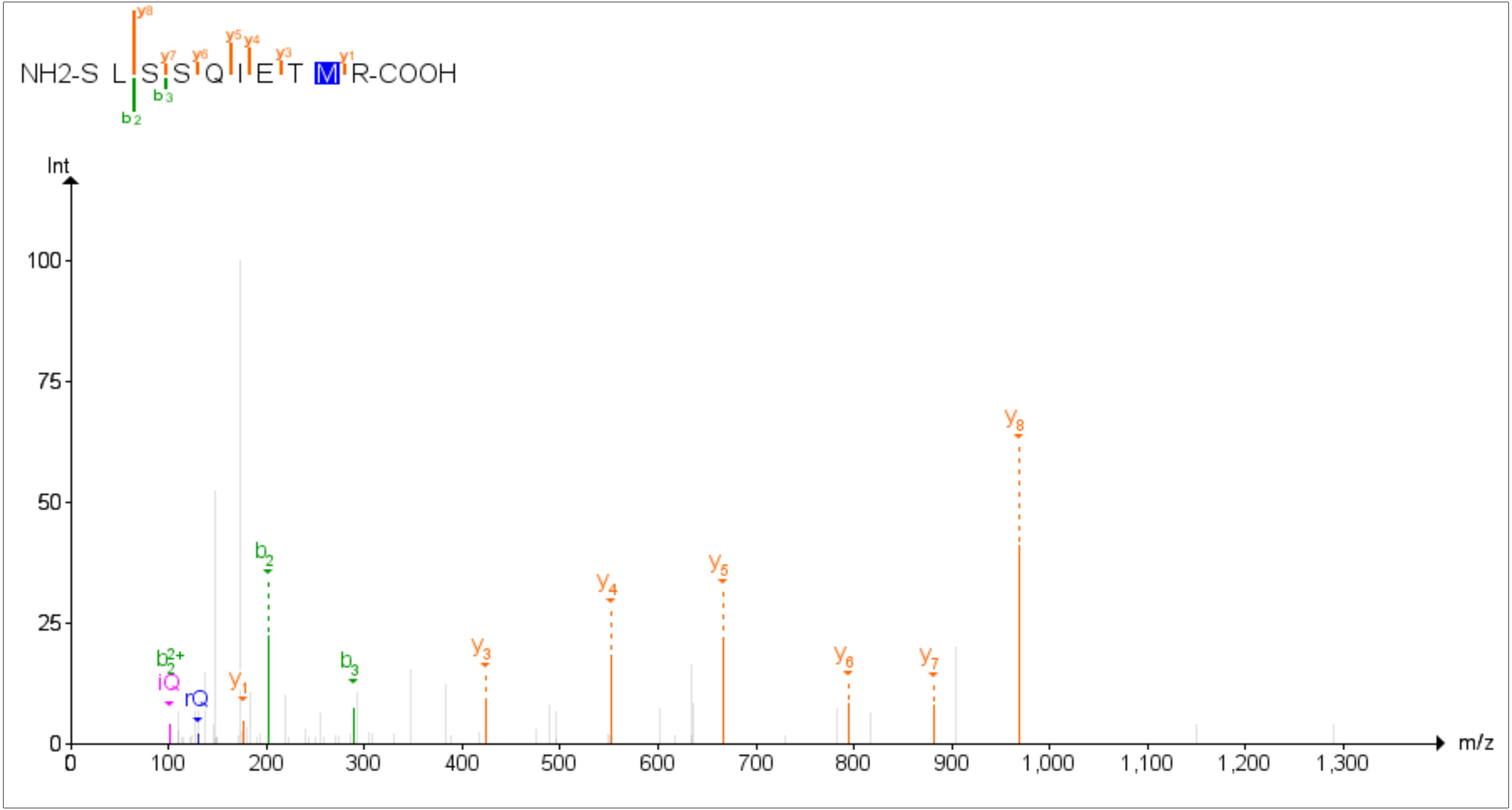

NH2-S L S S Q I E T M R-COOH
y8
y7
y6
y5
y4
y3
y1
b2
b3
Int
100
75
50
25
0
m/z
0
100
200
300
400
500
600
700
800
900
1,000
1,100
1,200
1,300
b2
b2 2+
iQ
rQ
y1
b3
y3
y4
y5
y6
y7
y8

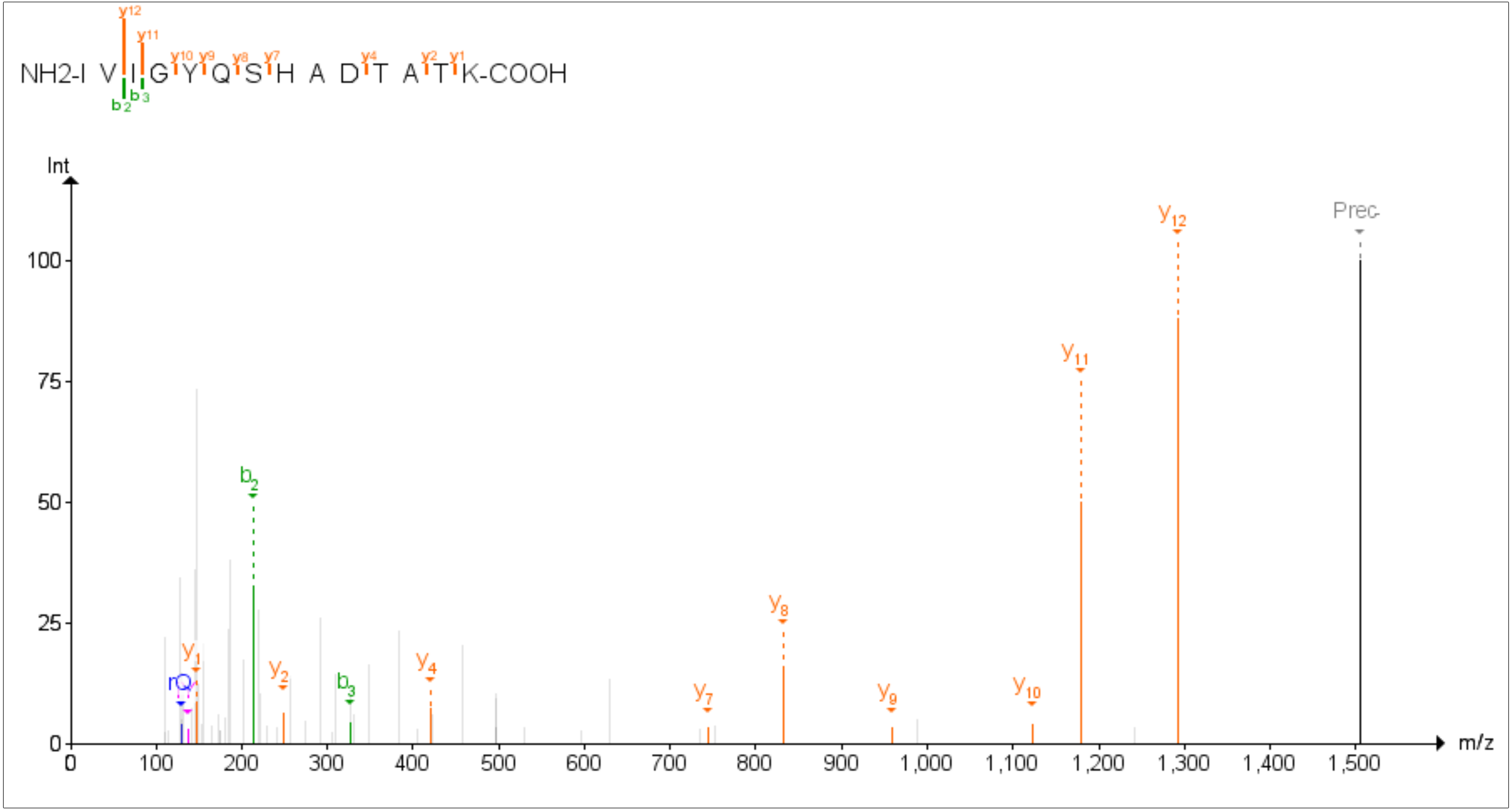

NH2-I V I G Y Q S H A D T A T K-COOH
y12
y11
y10
y9
y8
y7
y4
y2
y1
b2
b3
Int
100
75
50
25
0
Prec.
y12
y11
b2
y8
y1
rQ
y2
b3
y4
y7
y9
y10
0
100
200
300
400
500
600
700
800
900
1,000
1,100
1,200
1,300
1,400
1,500
m/z

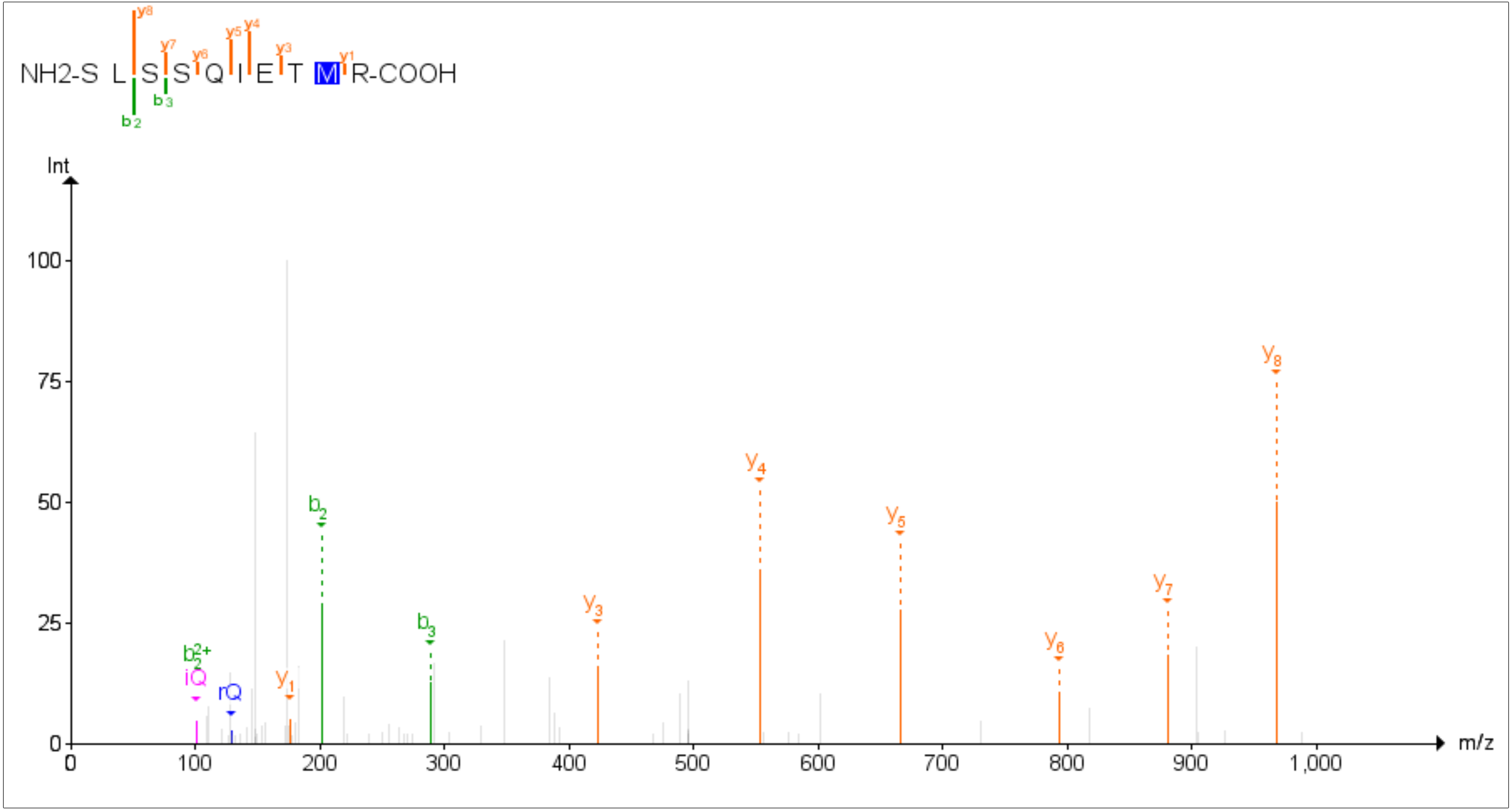
NH2-S L S S Q I E T M R-COOH
y8
y7
y6
y5
y4
y3
y1
b2
b3
Int
100
75
50
25
0
m/z
0
100
200
300
400
500
600
700
800
900
1,000
iQ
rQ
y1
b2
b3
y3
y4
y5
y6
y7
y8

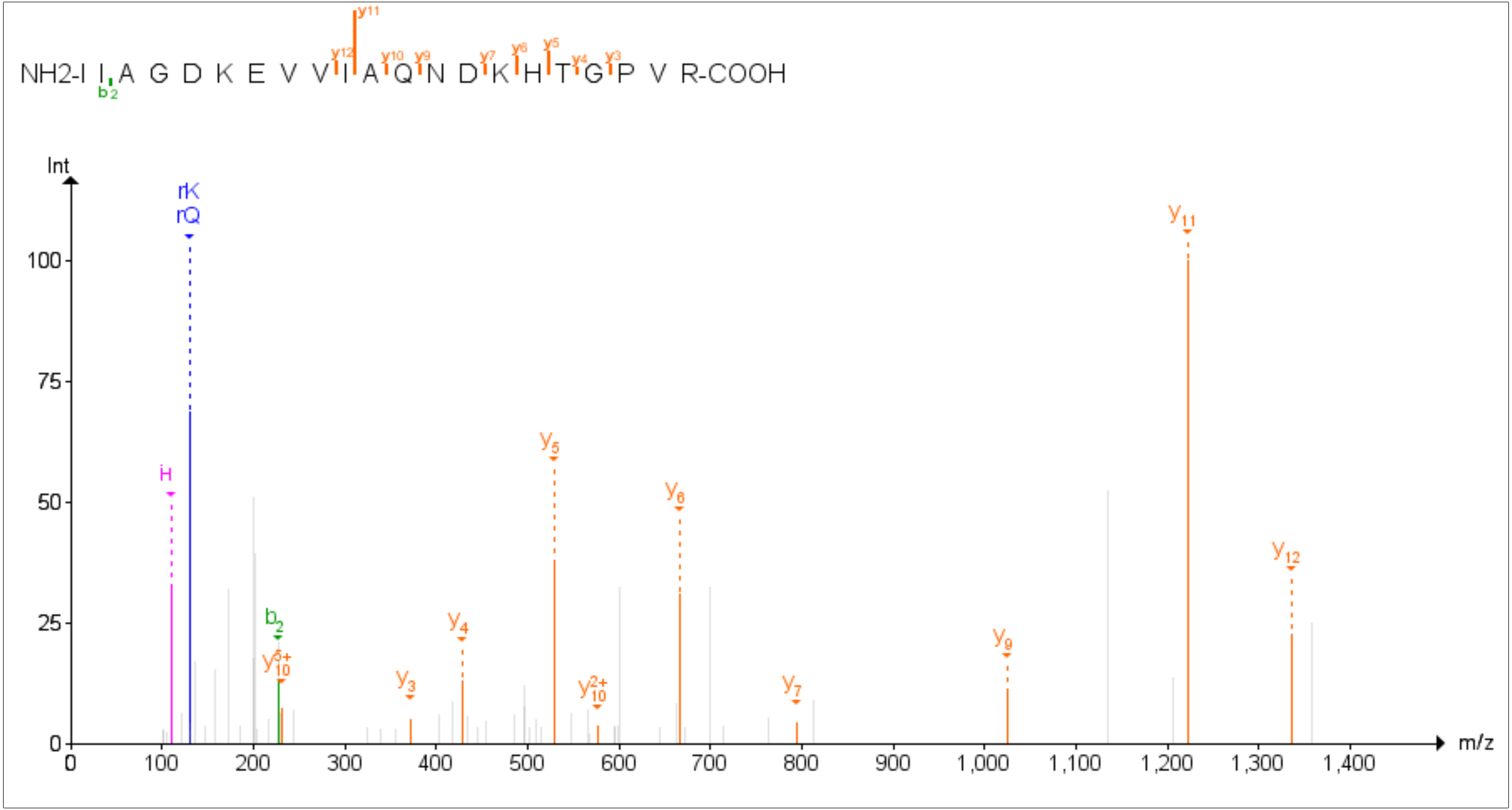

NH2-I I A G D K E V V I A Q N D K H T G P V R-COOH
b2
y12
y11
y10
y9
y7
y6
y5
y4
y3
Int
100
75
50
25
0
rK
rQ
iH
b2
y$^{5+}_{10}$
y3
y4
y5
y$^{2+}_{10}$
y6
y7
y9
y11
y12
0
100
200
300
400
500
600
700
800
900
1,000
1,100
1,200
1,300
1,400
m/z

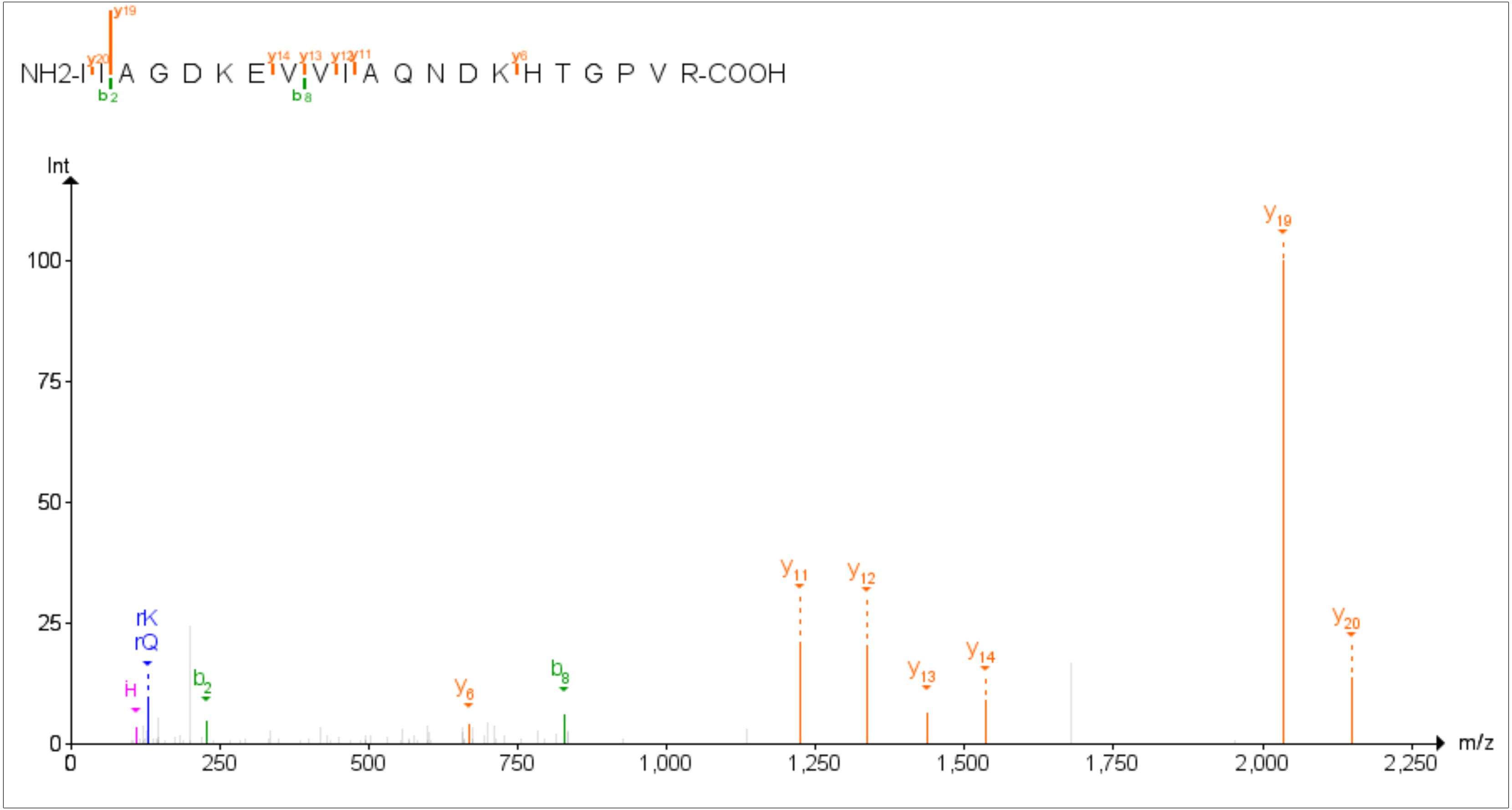

NH2-I I A G D K E V V V I A Q N D K H T G P V R-COOH
y19
y20
b 2
y14
y13
y12
y11
b 8
y6
Int
100
75
50
25
0
rK
rQ
iH
b2
y6
b8
y11
y12
y13
y14
y19
y20
0
250
500
750
1,000
1,250
1,500
1,750
2,000
2,250
m/z

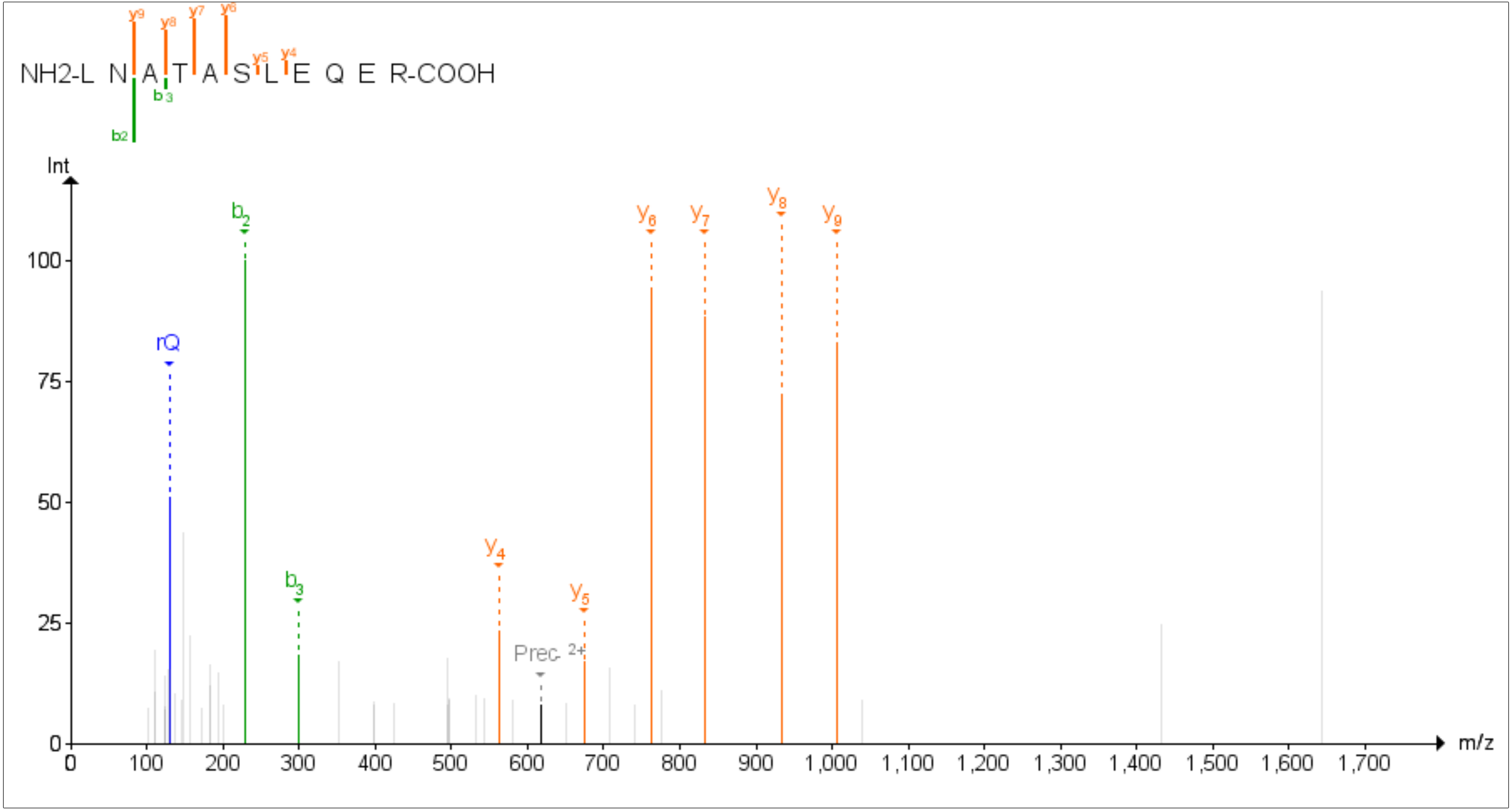

NH2-L N A T A S L E Q E R-COOH
y9
y8
y7
y6
y5
y4
b3
b2
Int
m/z
b2
b3
rQ
y4
y5
y6
y7
y8
y9
Prec- 2+
0
25
50
75
100
0
100
200
300
400
500
600
700
800
900
1,000
1,100
1,200
1,300
1,400
1,500
1,600
1,700

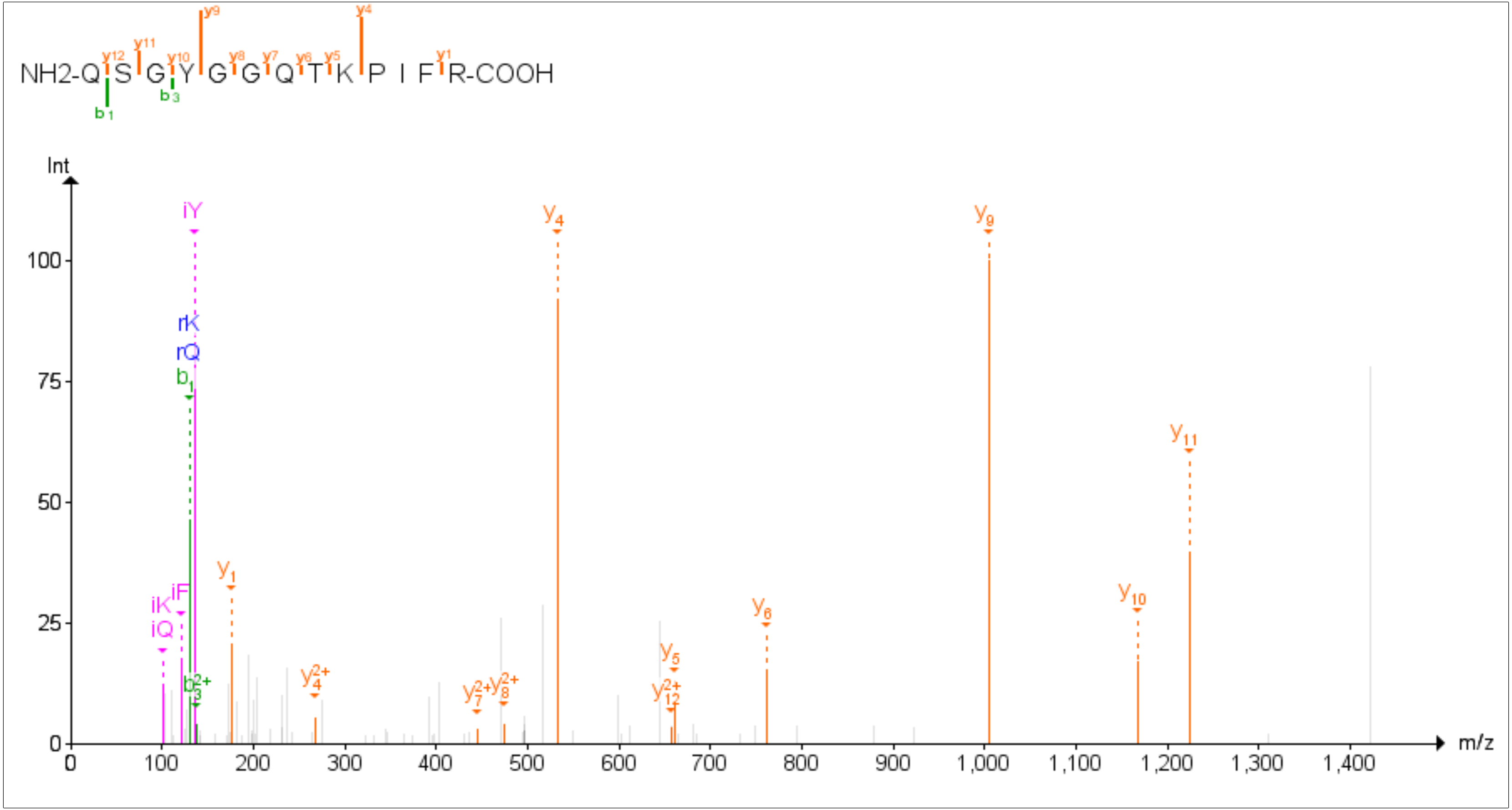

NH2-Q S G Y G G Q T K P I F R-COOH
y12 y11 y10 y9 y8 y7 y6 y5 y4 y1
b1 b3
Int
m/z
iY
rK
rQ
b1
y4
y9
y11
y10
y1
iK
iF
iQ
y6
y5
y4 2+
b3 2+
y7 2+
y8 2+
y12 2+
0
25
50
75
100
0
100
200
300
400
500
600
700
800
900
1,000
1,100
1,200
1,300
1,400

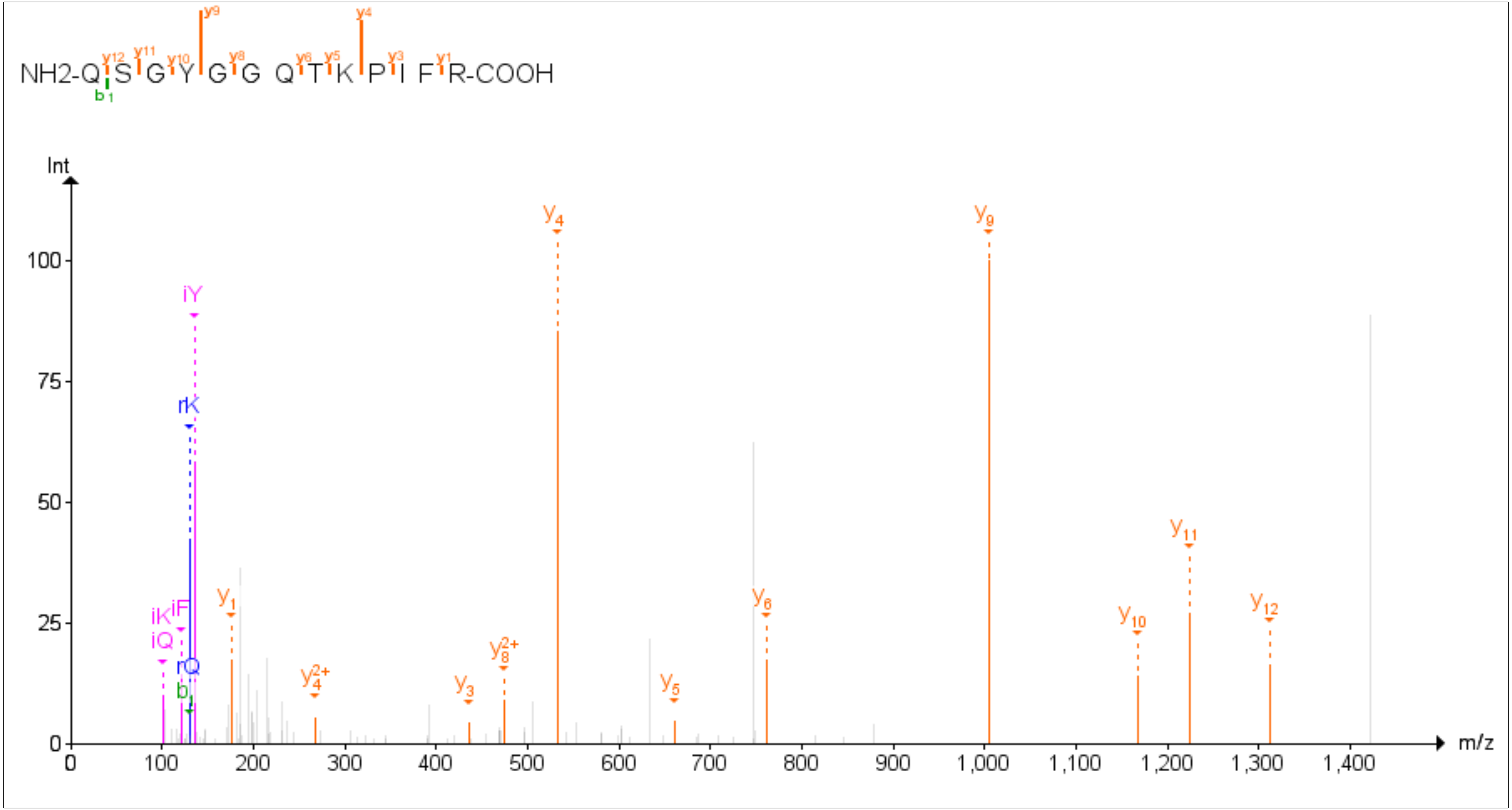

NH2-Q S G Y G G Q T K P I F R-COOH
b1
y12 y11 y10 y9 y8 y6 y5 y4 y3 y1
Int
m/z
0
25
50
75
100
0
100
200
300
400
500
600
700
800
900
1,000
1,100
1,200
1,300
1,400
iY
rK
iK
iF
iQ
rQ
b1
y1
y4 2+
y3
y8 2+
y4
y5
y6
y9
y10
y11
y12

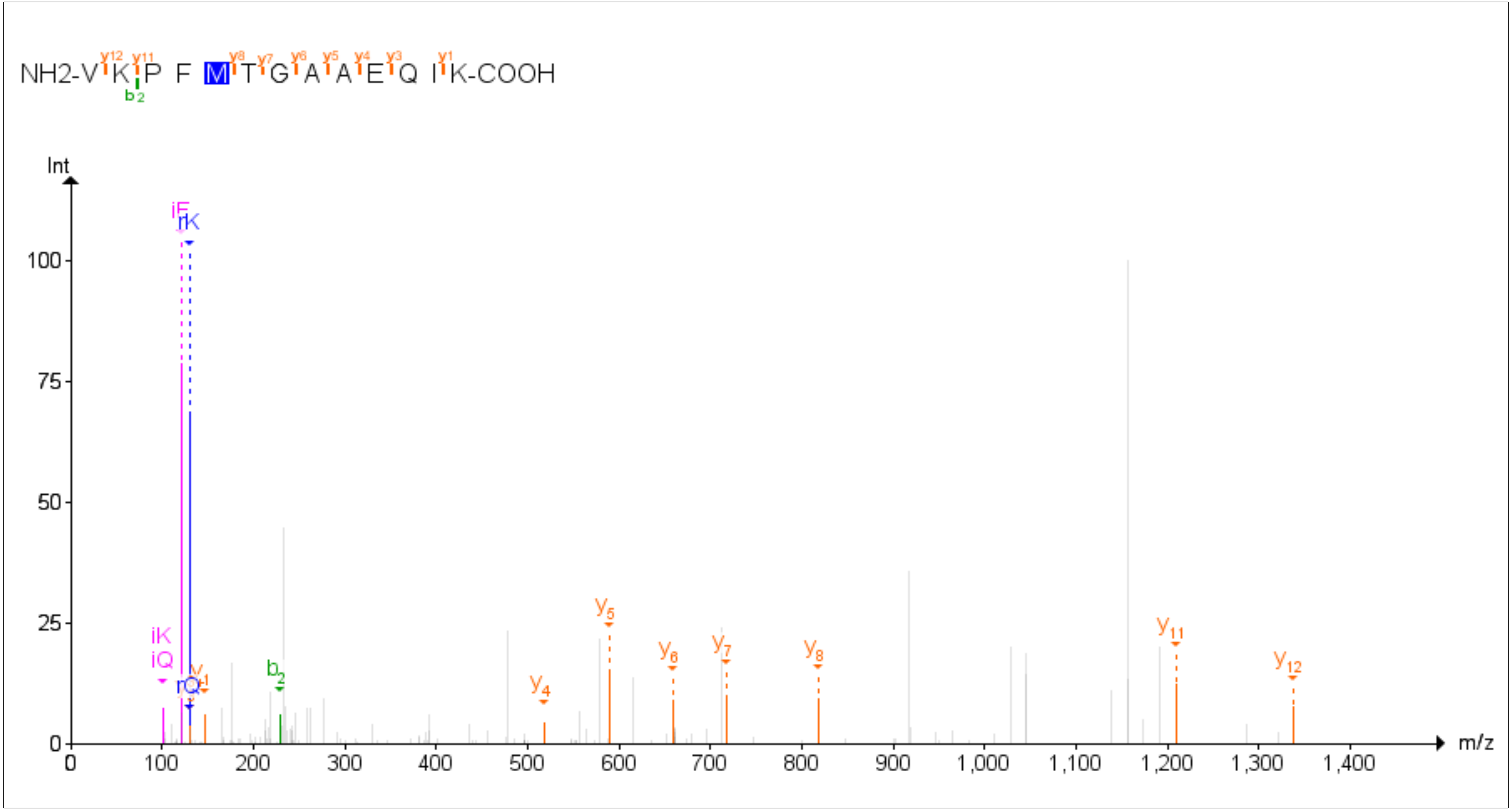

NH2-V K P F M T G A A E Q I K-COOH
y12 y11 y8 y7 y6 y5 y4 y3 y1
b2
Int
100
75
50
25
0
iF
rK
iK
iQ
rQ
y1
b2
y4
y5
y6
y7
y8
y11
y12
0
100
200
300
400
500
600
700
800
900
1,000
1,100
1,200
1,300
1,400
m/z

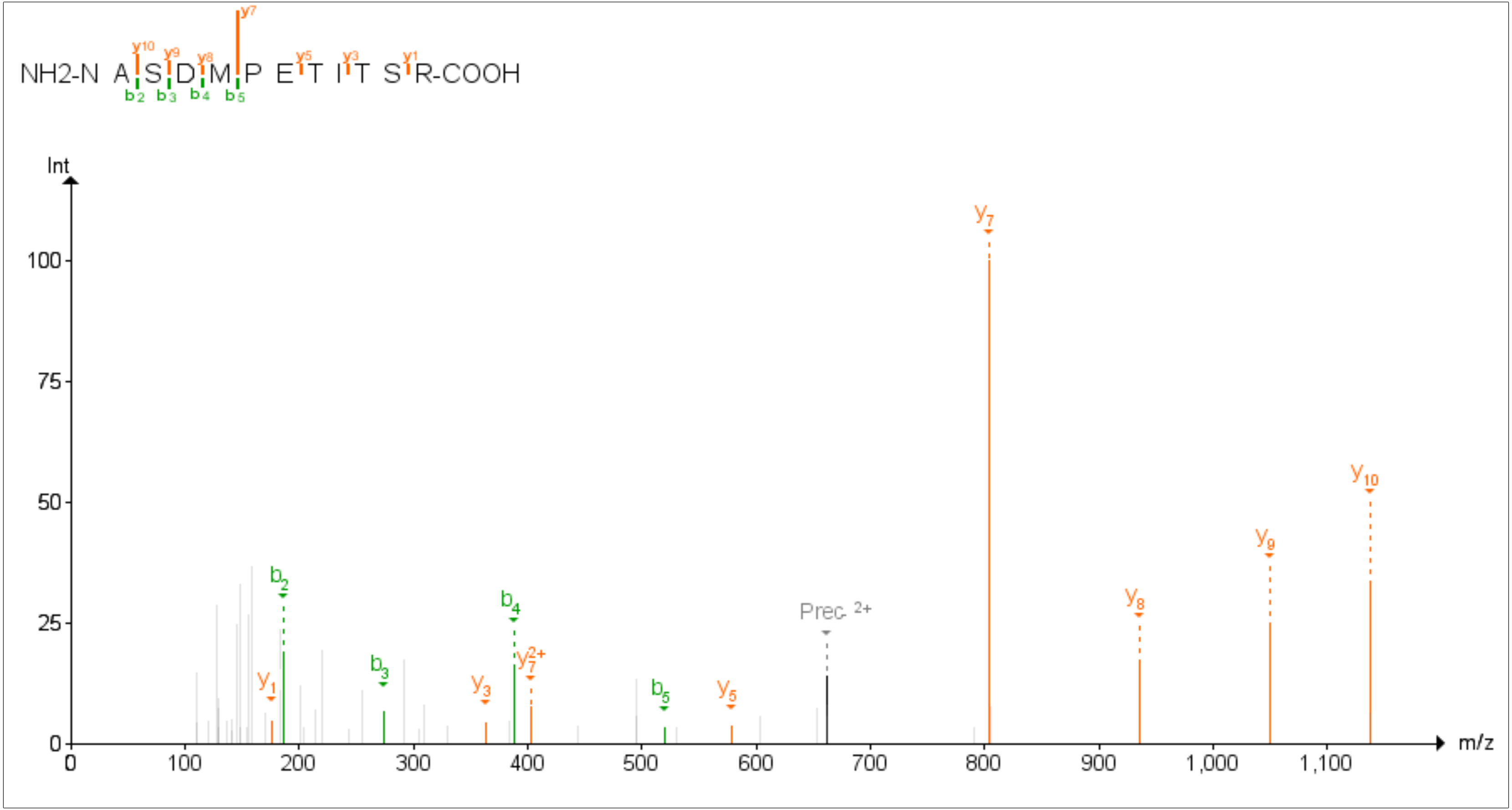

NH2-N A S D M P E T I T S R-COOH
y10 y9 y8 y7 y5 y3 y1
b2 b3 b4 b5
Int
100
75
50
25
0
y7
y10
y9
y8
b2
b4
Prec 2+
y_7^{2+}
b3
y1
y3
b5
y5
0 100 200 300 400 500 600 700 800 900 1,000 1,100
m/z

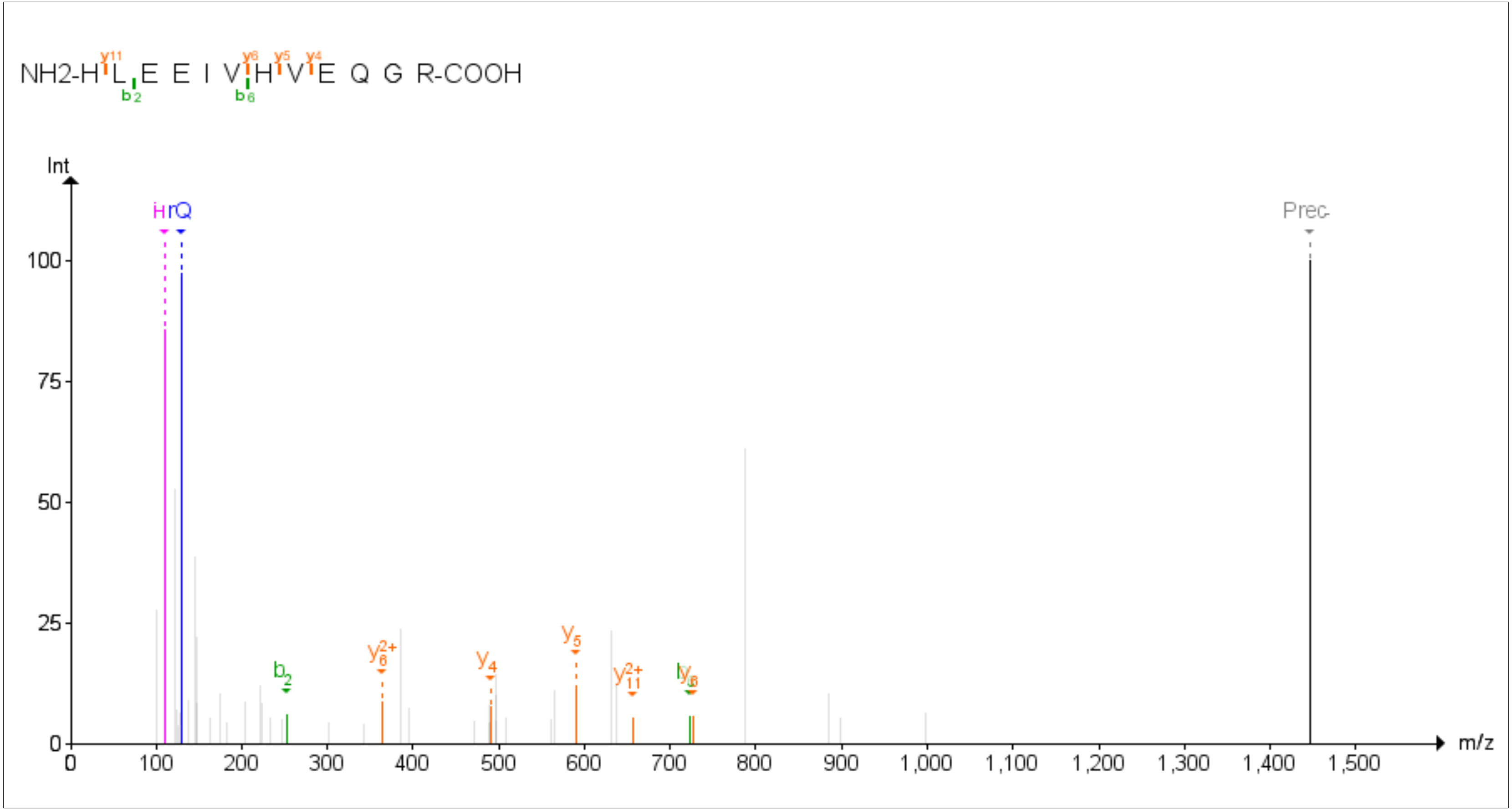

NH2-H L E E I V H V E Q G R-COOH
y11 y6 y5 y4
b2 b6
Int
iH
rQ
Prec
100
75
50
25
0
b2
y6 2+
y4
y5
y11 2+
y6
m/z
0
100
200
300
400
500
600
700
800
900
1,000
1,100
1,200
1,300
1,400
1,500

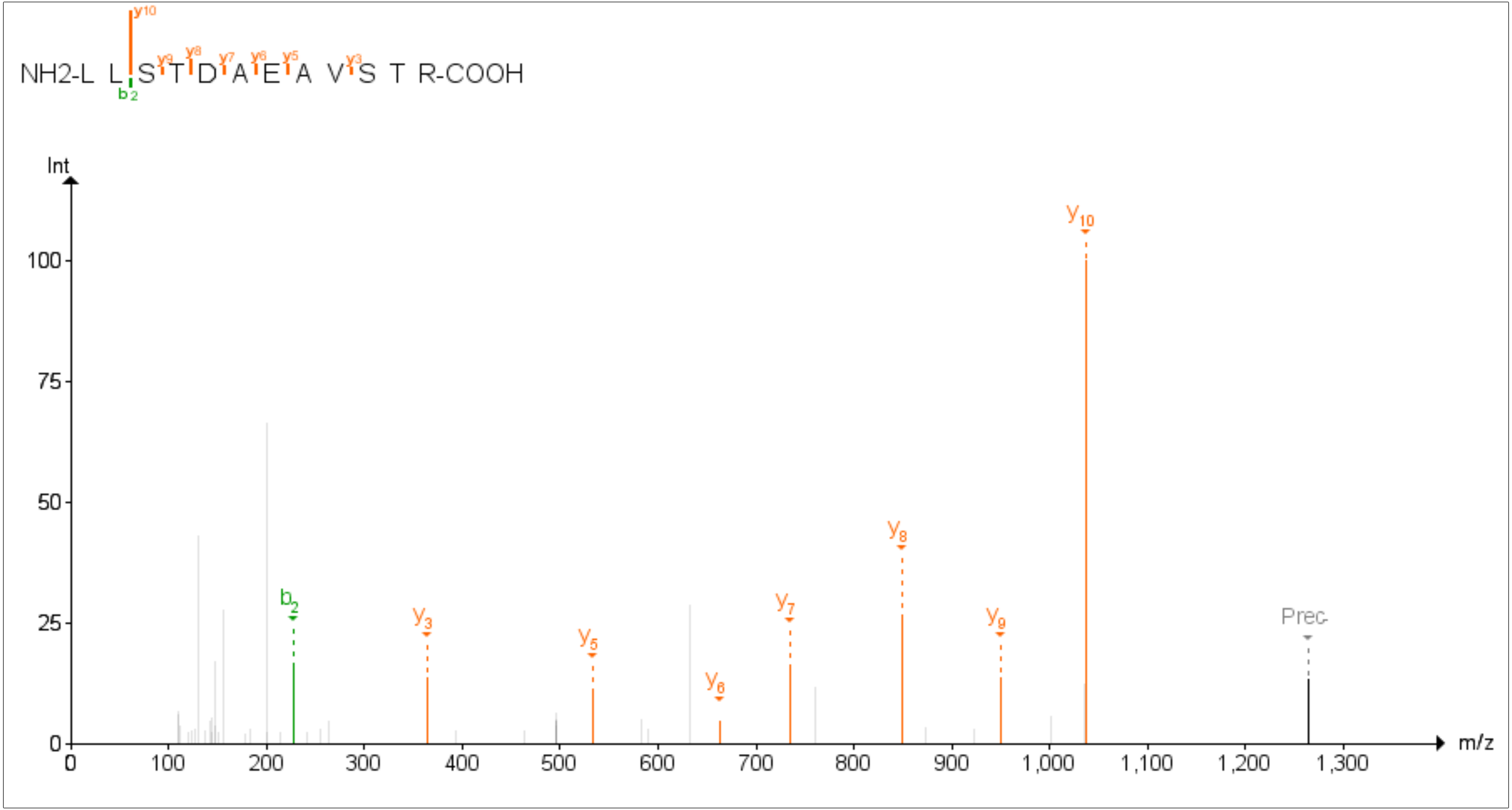
NH2-L L S T D A E A V S T R-COOH
y10 y9 y8 y7 y6 y5 y3
b2
Int
100
75
50
25
0
b2
y3
y5
y6
y7
y8
y9
y10
Prec.
0 100 200 300 400 500 600 700 800 900 1,000 1,100 1,200 1,300
m/z

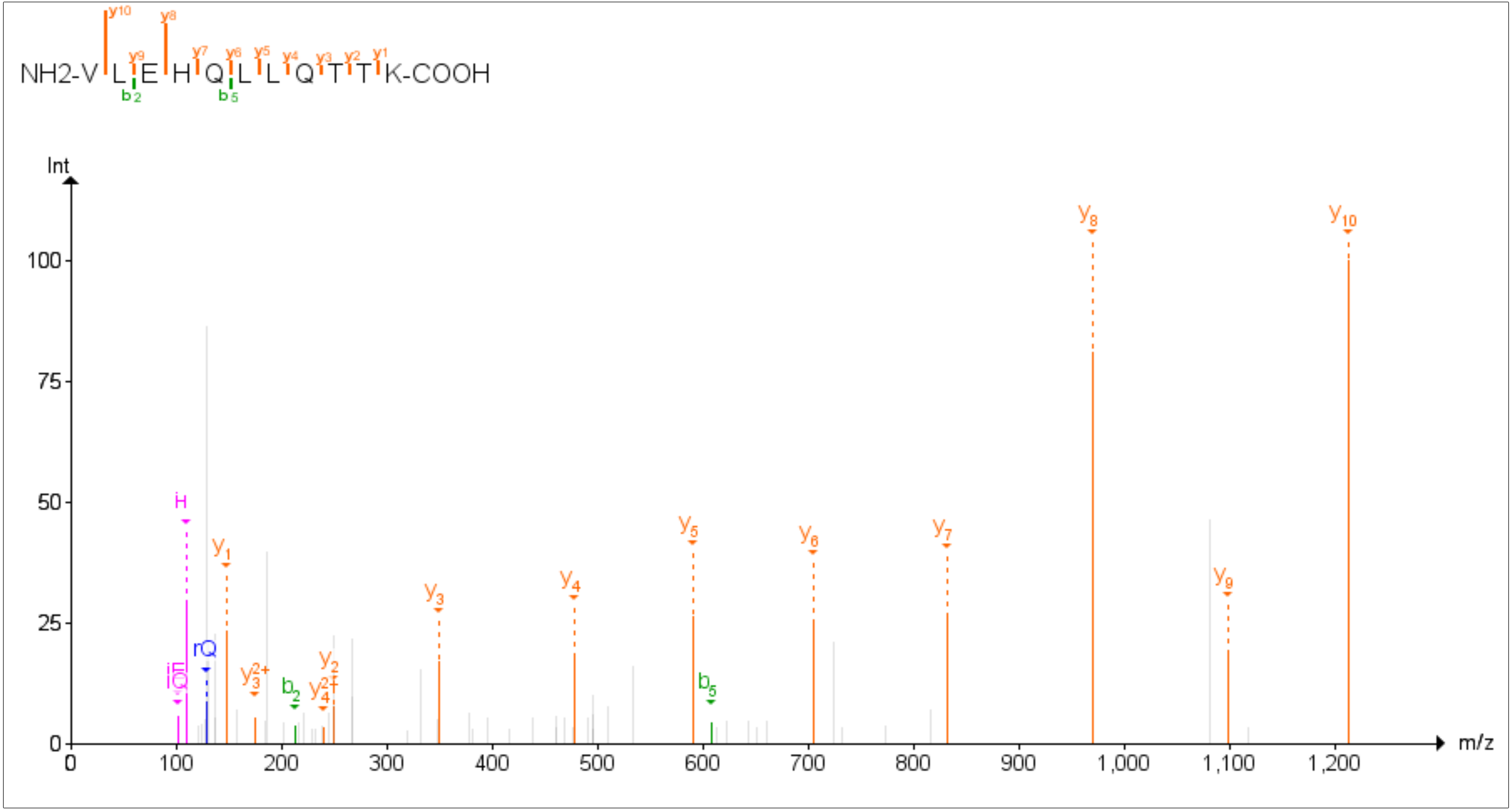

NH2-V L E H Q L L Q T T K-COOH
y10 y9 y8 y7 y6 y5 y4 y3 y2 y1
b2 b5
Int
m/z
0
25
50
75
100
100
200
300
400
500
600
700
800
900
1,000
1,100
1,200
iH
iE
iQ
rQ
y1
y2
y3
y4
y5
y6
y7
y8
y9
y10
b2
b5

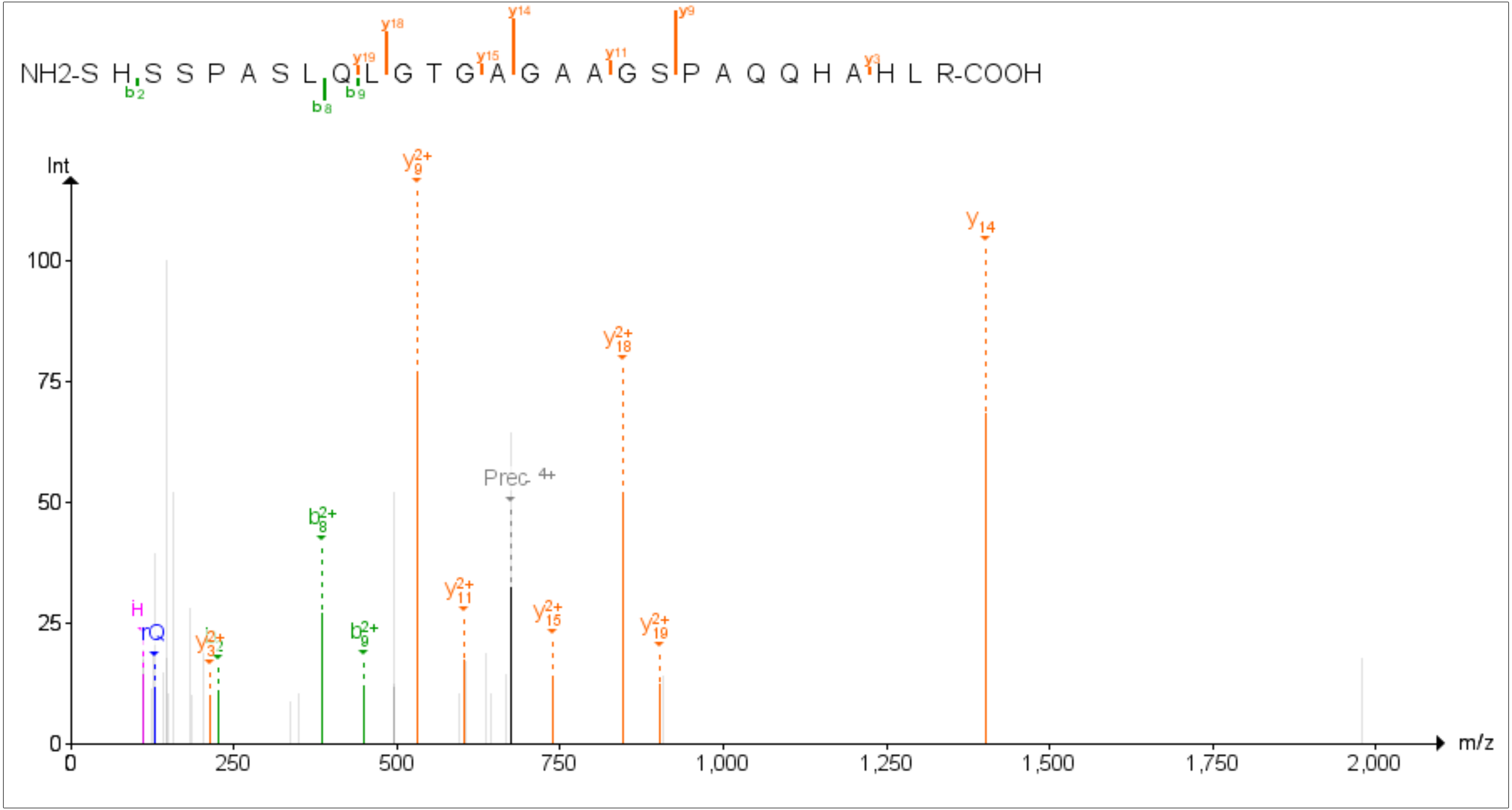

NH2-S H S S P A S L Q L G T G A G A A G S P A Q Q H A H L R-COOH
b2
b8
b9
y19
y18
y15
y14
y11
y9
y3
Int
100
75
50
25
0
m/z
0
250
500
750
1,000
1,250
1,500
1,750
2,000
Prec 4+
rQ
H

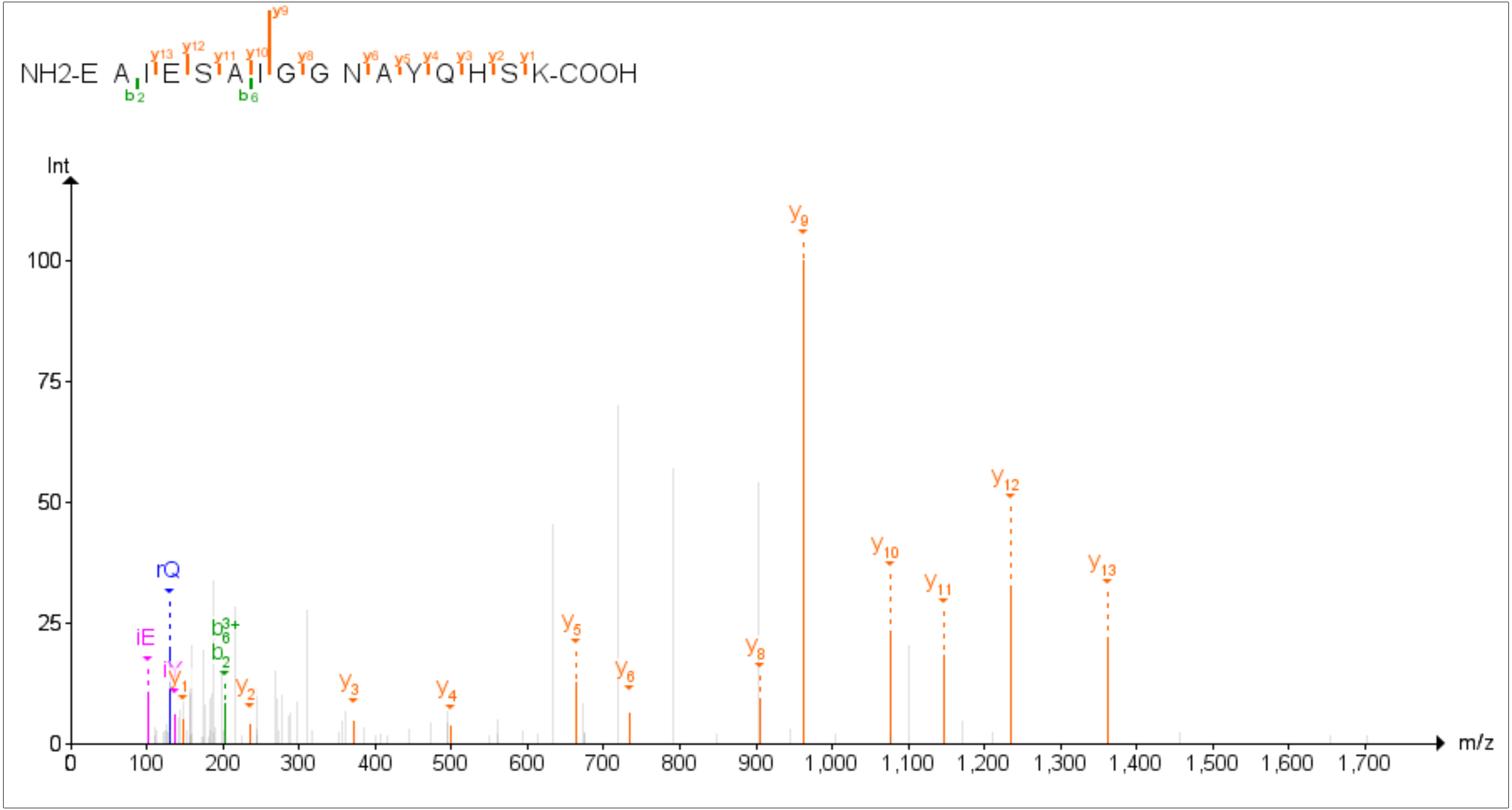

NH2-E A I E S A I G G N A Y Q H S K-COOH
y13
y12
y11
y10
y9
y8
y6
y5
y4
y3
y2
y1
b2
b6
Int
100
75
50
25
0
y9
y12
y10
y11
y13
y5
y6
y8
rQ
iE
iY
y1
b_6^{3+}
b_2
y2
y3
y4
m/z
0
100
200
300
400
500
600
700
800
900
1,000
1,100
1,200
1,300
1,400
1,500
1,600
1,700

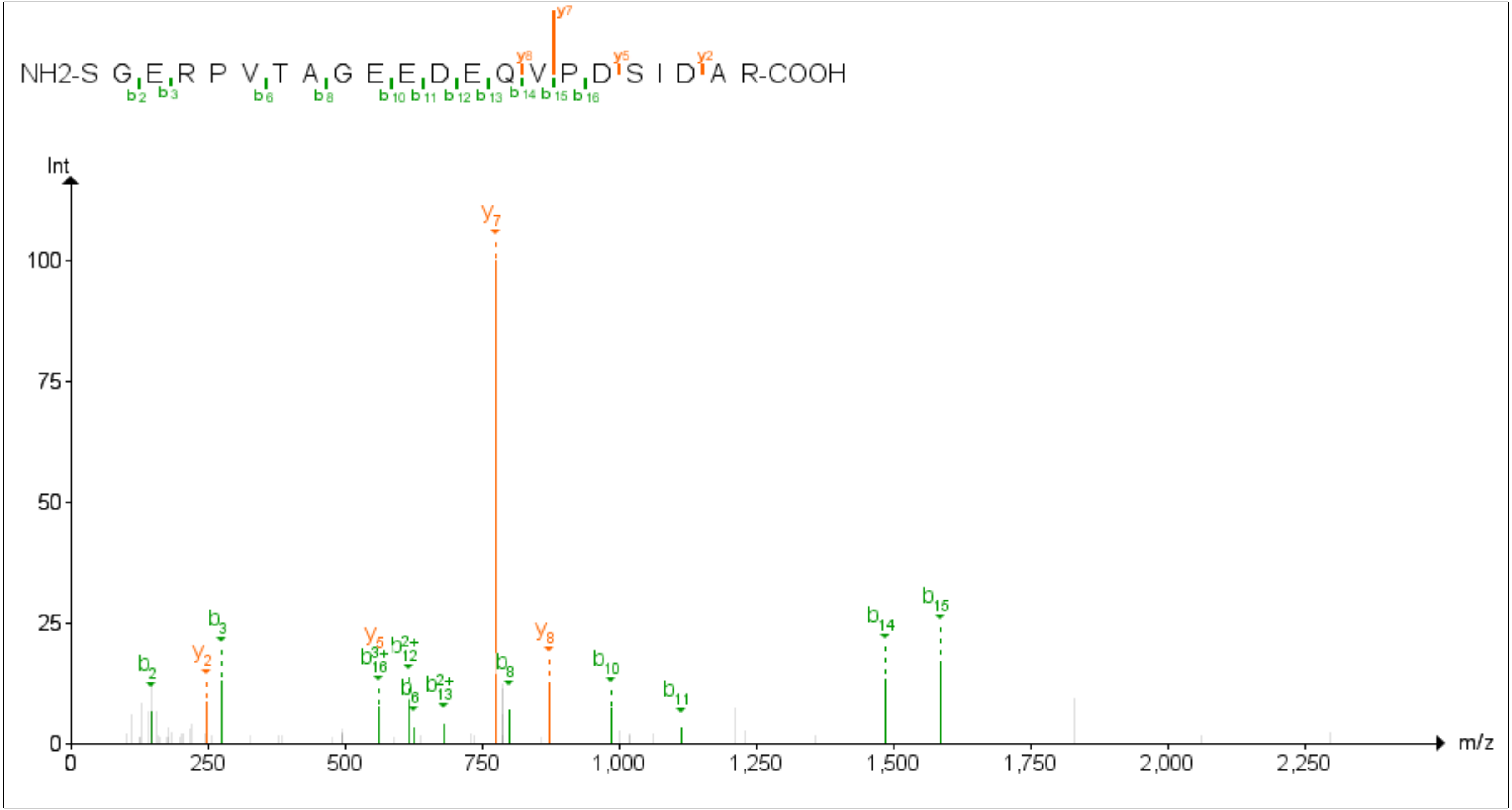

NH2-S G E R P V T A G E E D E Q V P D S I D A R-COOH
b2 b3 b6 b8 b10 b11 b12 b13 b14 b15 b16
y8 y7 y5 y2
Int
100
75
50
25
0
y7
y2
b2
b3
y5
b3+16
b2+12
b6
b2+13
b8
y8
b10
b11
b14
b15
0
250
500
750
1,000
1,250
1,500
1,750
2,000
2,250
m/z

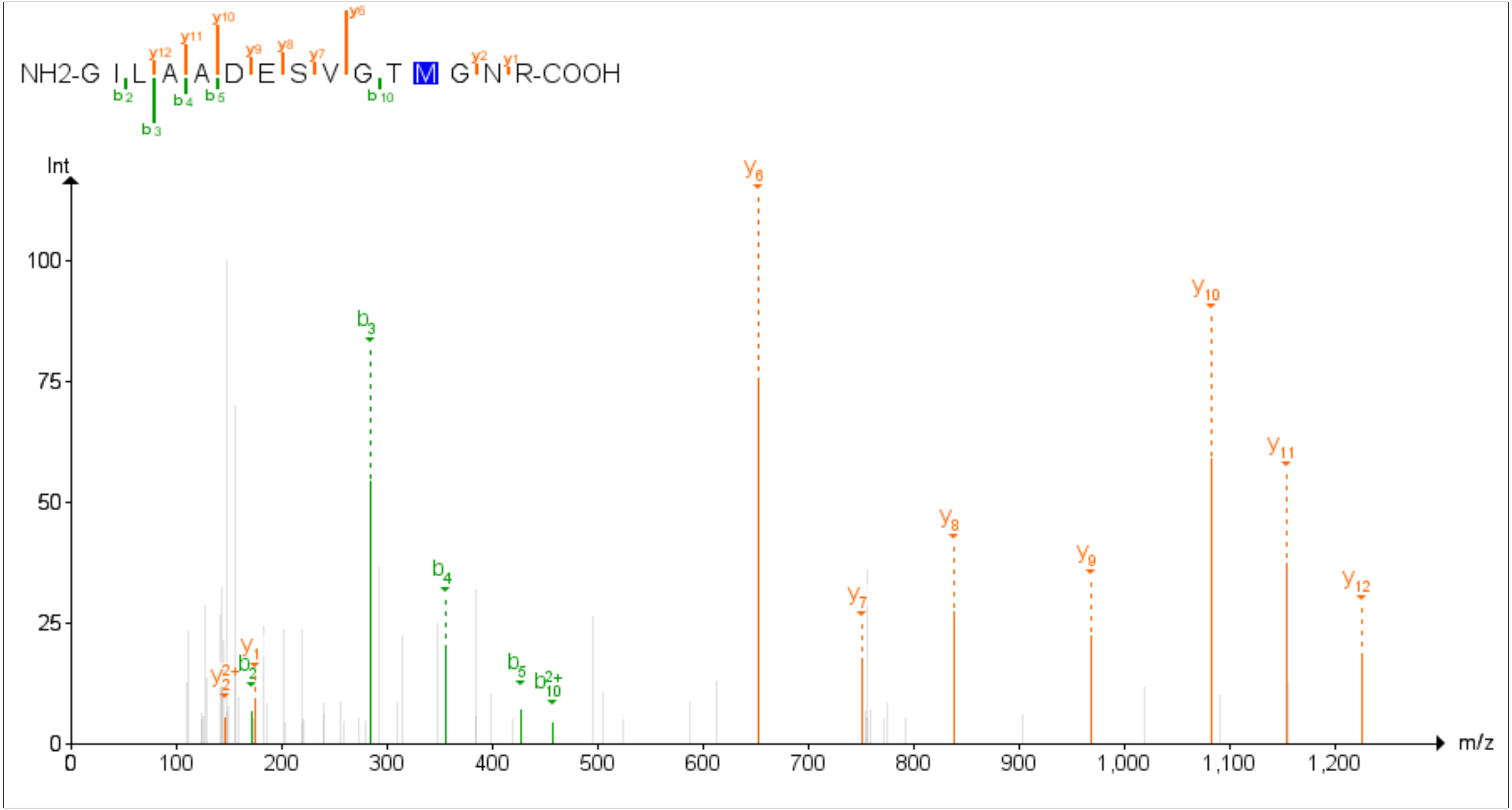

NH2-G I L A A D E S V G T M G N R-COOH
Int
m/z
0
25
50
75
100
0
100
200
300
400
500
600
700
800
900
1,000
1,100
1,200

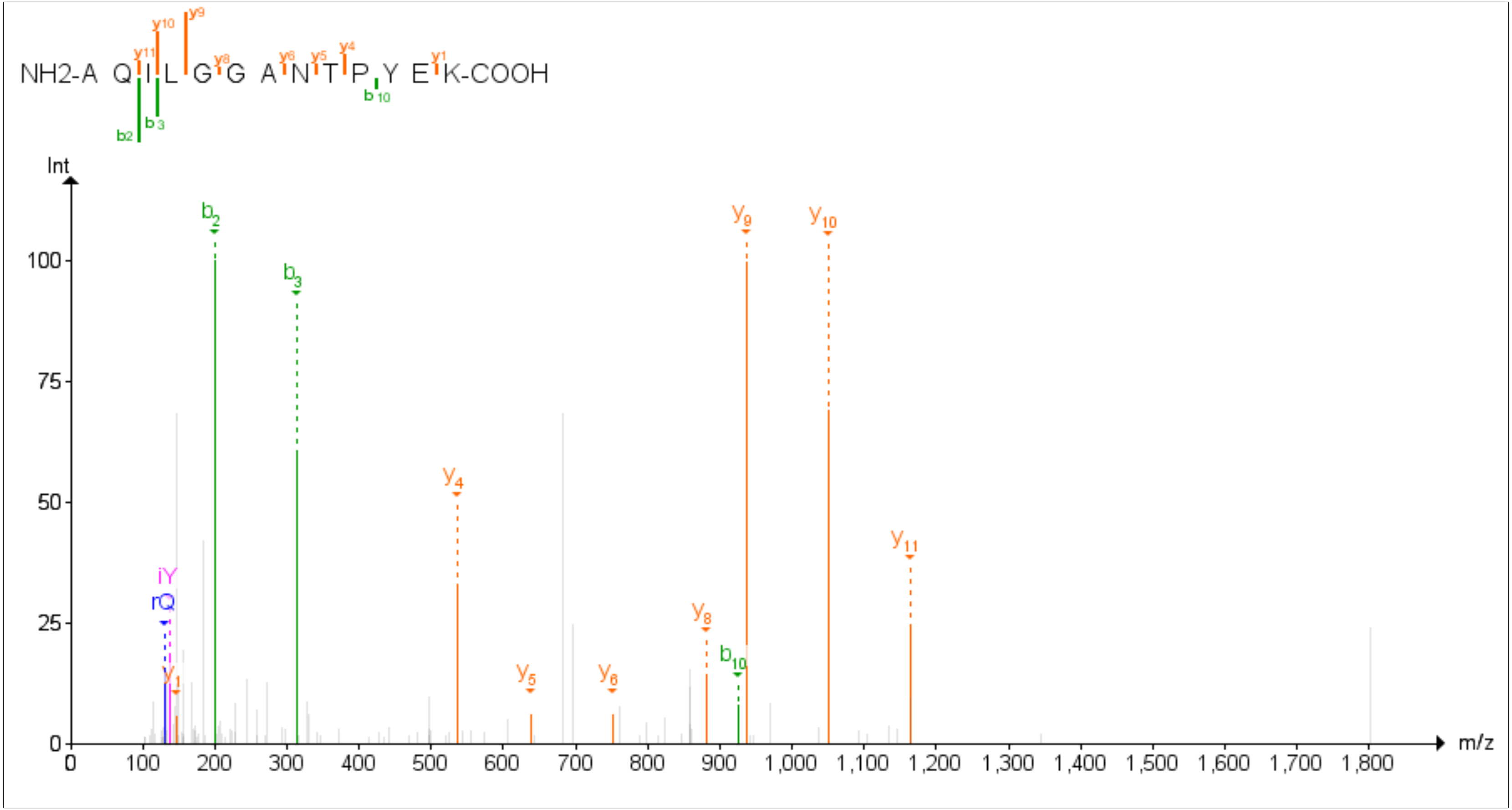

NH2-A Q I L G G A N T P Y E K-COOH
y11 y10 y9 y8 y6 y5 y4 y1
b2 b3 b10
Int
100
75
50
25
0
b2
b3
y9
y10
y4
y11
iY
rQ
y1
y5
y6
y8
b10
m/z
0 100 200 300 400 500 600 700 800 900 1,000 1,100 1,200 1,300 1,400 1,500 1,600 1,700 1,800

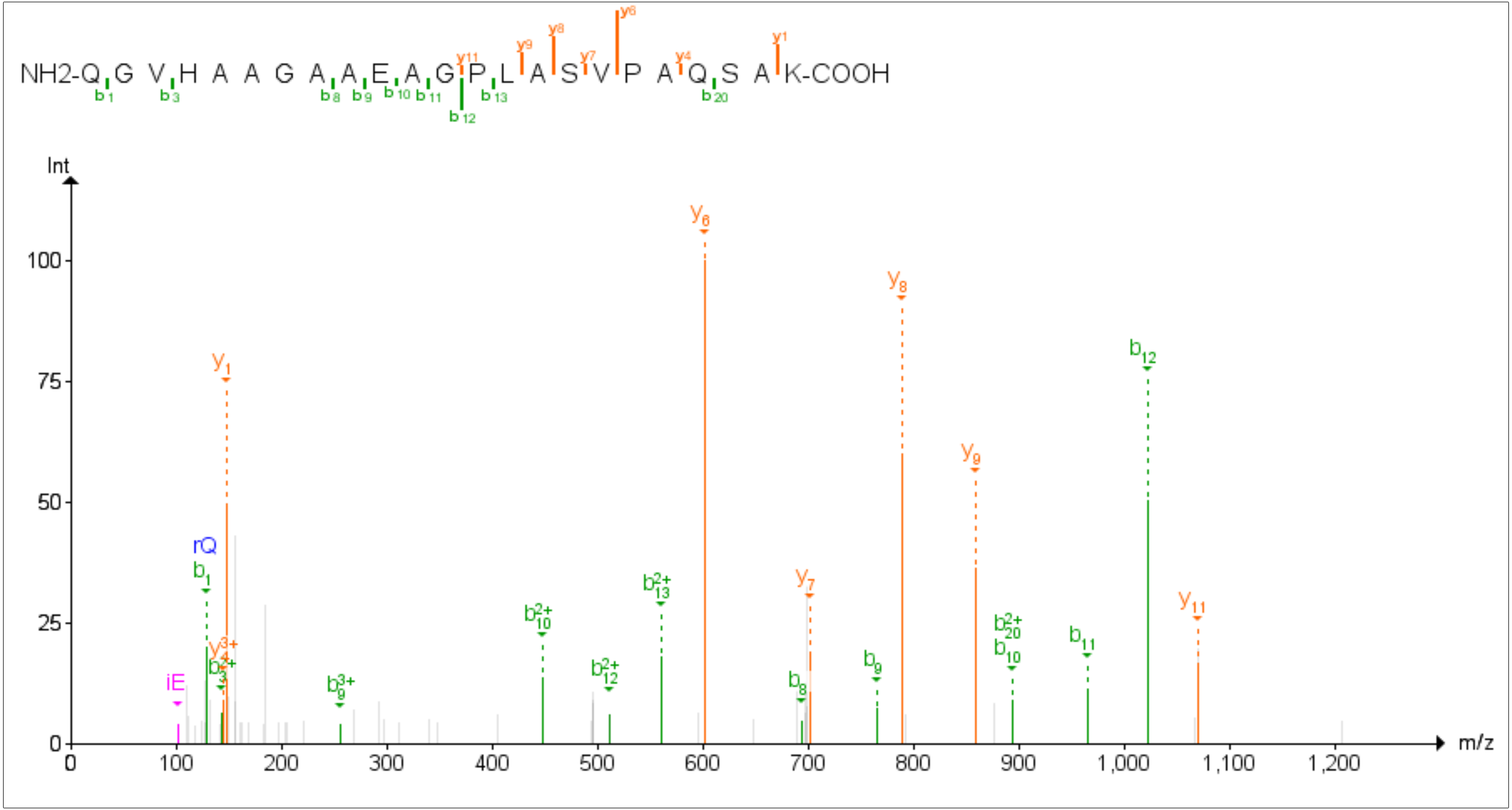

NH2-Q G V H A A G A A E A G P L A S V P A Q S A K-COOH
b1 b3 b8 b9 b10 b11 b12 b13 b20
y11 y9 y8 y7 y6 y4 y1
Int
100
75
50
25
0
y6
y8
b12
y1
y9
rQ
b1
b13 2+
y7
y11
b10 2+
b20 2+
b10
b11
y4 3+
b3 2+
iE
b9 3+
b12 2+
b8
b9
0 100 200 300 400 500 600 700 800 900 1,000 1,100 1,200
m/z

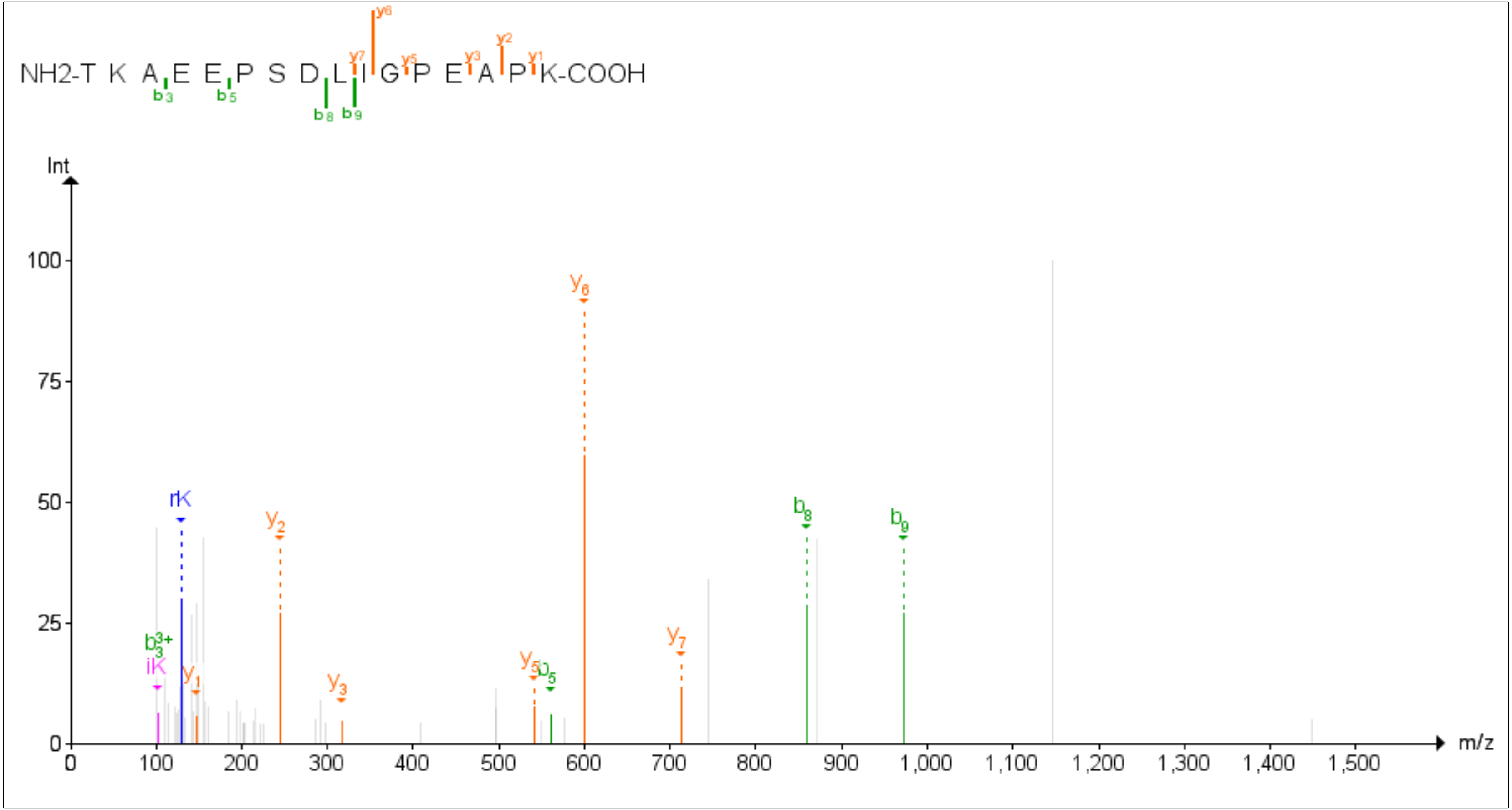

NH2-T K A E E P S D L I G P E A P K-COOH
b3 b5 b8 b9
y7 y6 y5 y3 y2 y1
Int
100
75
50
25
0
m/z
0 100 200 300 400 500 600 700 800 900 1,000 1,100 1,200 1,300 1,400 1,500
rK
iK
y6
y2
y7
y5
y3
y1
b8
b9
b5

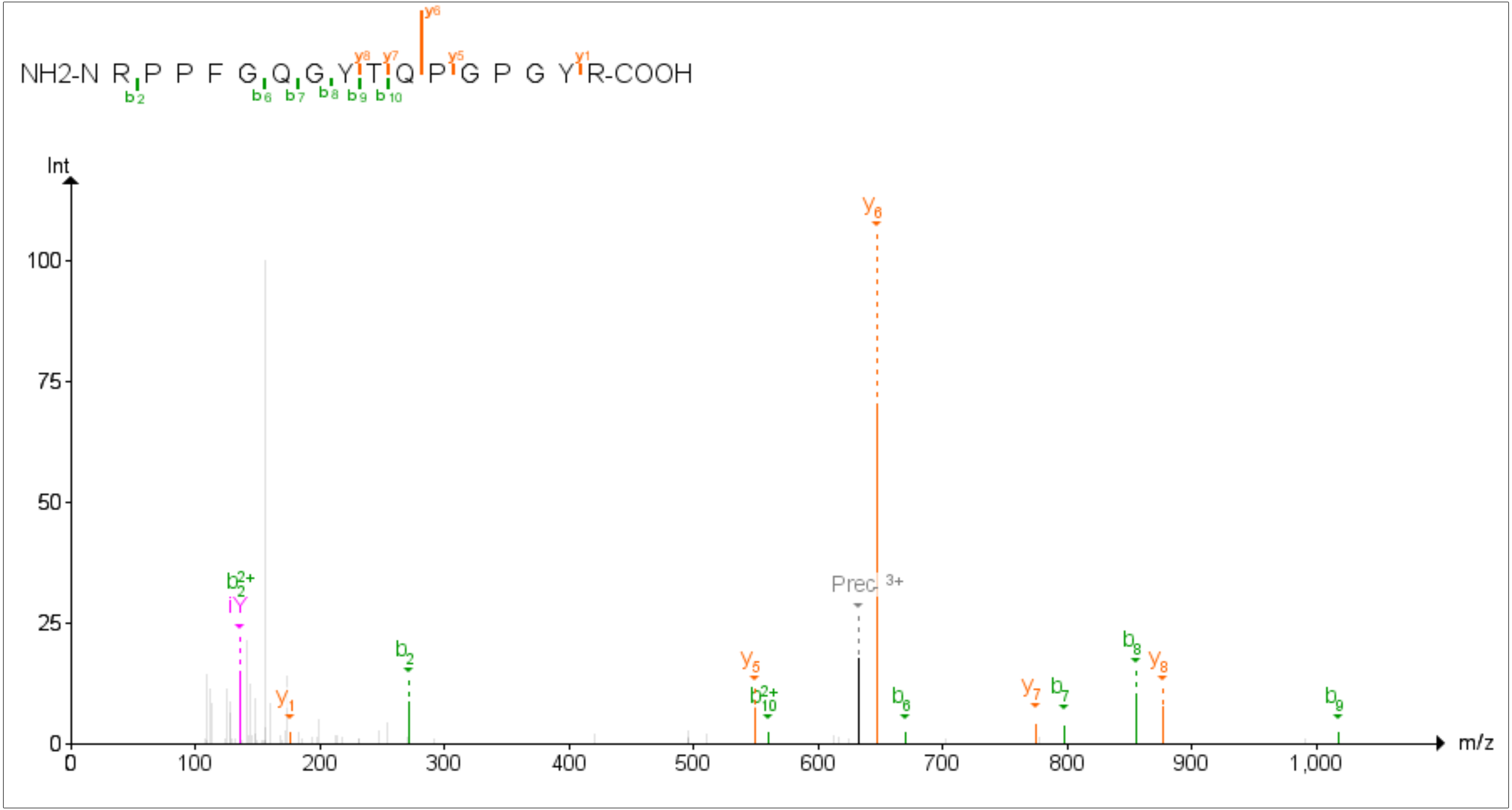

NH2-N R P P F G Q G Y T Q P G P G Y R-COOH
y6
y8
y7
y5
y1
b2
b6
b7
b8
b9
b10
Int
100
75
50
25
0
m/z
0
100
200
300
400
500
600
700
800
900
1,000
iY
y1
b2
y5
Prec 3+
y6
b6
y7
b7
b8
y8
b9

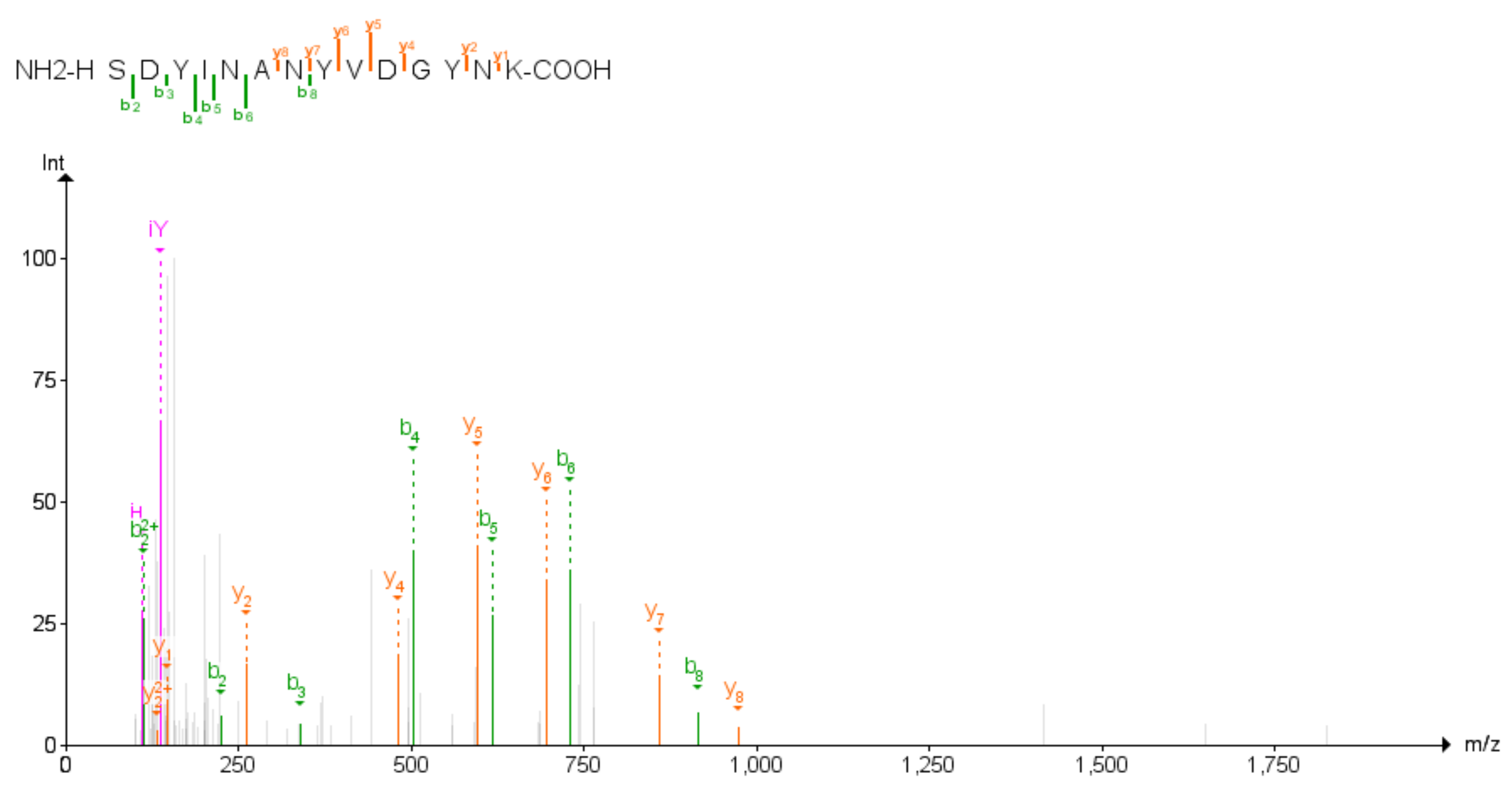

NH2-H S D Y I N A N Y V D G Y N K-COOH
y8 y7 y6 y5 y4 y2 y1
b2 b3 b4 b5 b6 b8
Int
m/z
iY
iH
b2 2+
y1
y2 2+
b2
y2
b3
y4
b4
y5
b5
y6
b6
y7
b8
y8
0
25
50
75
100
250
500
750
1,000
1,250
1,500
1,750

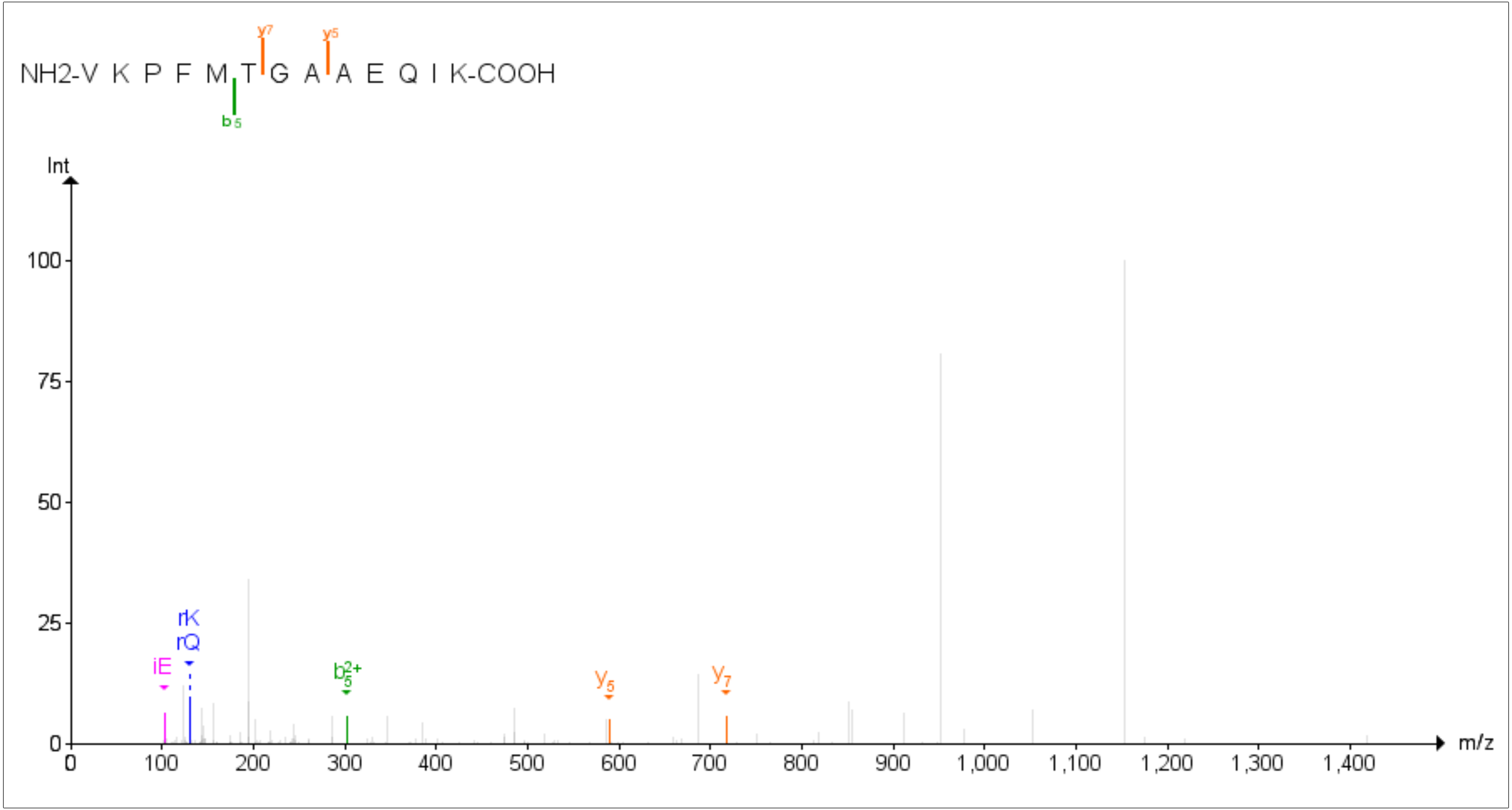

NH2-V K P F M T G A A E Q I K-COOH
y7
y5
b5
Int
100
75
50
25
0
rK
rQ
iE
b5 2+
y5
y7
0
100
200
300
400
500
600
700
800
900
1,000
1,100
1,200
1,300
1,400
m/z

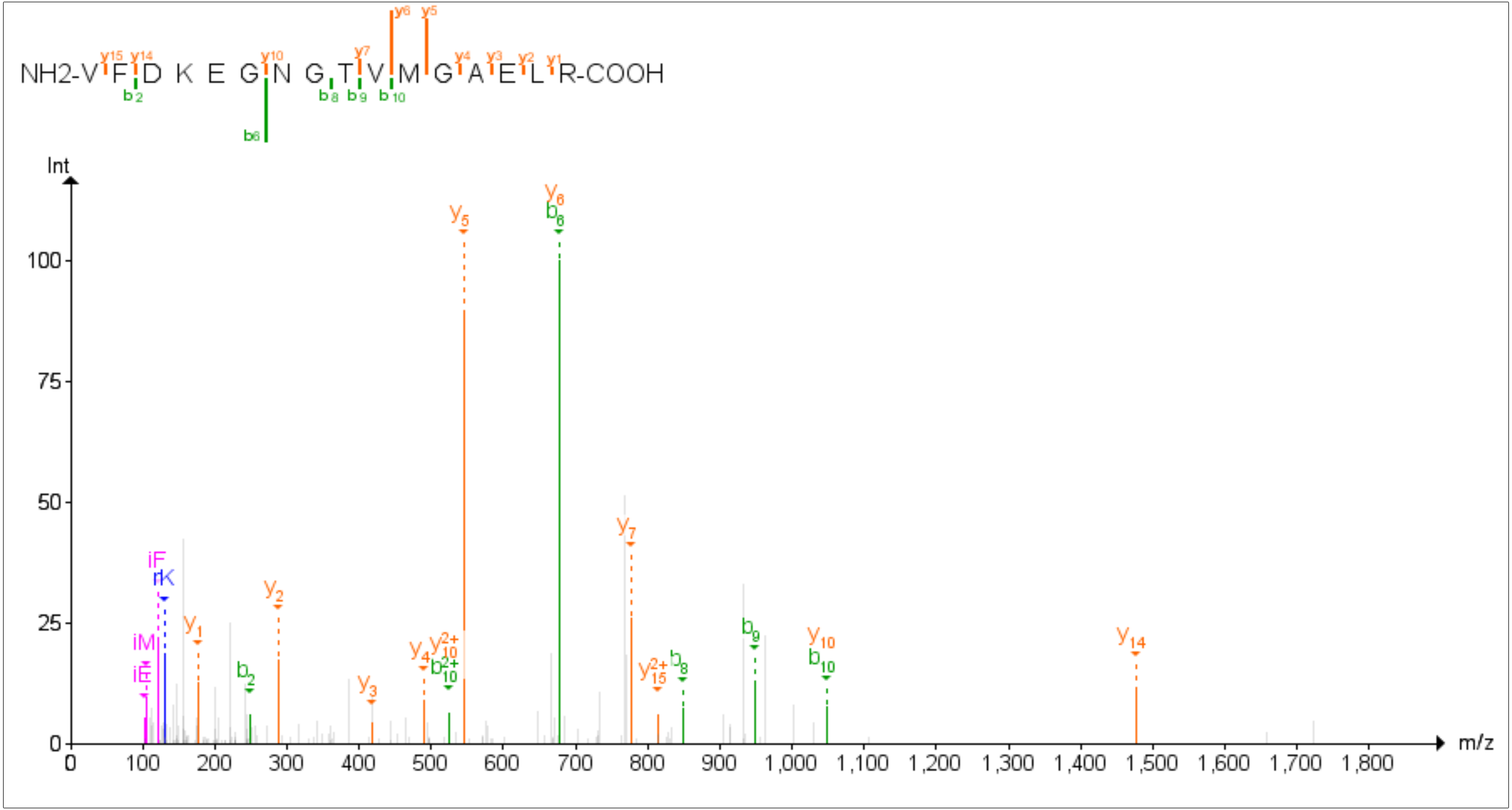
NH2-V F D K E G N G T V M G A E L R-COOH
Int
m/z
0
25
50
75
100
0
100
200
300
400
500
600
700
800
900
1,000
1,100
1,200
1,300
1,400
1,500
1,600
1,700
1,800

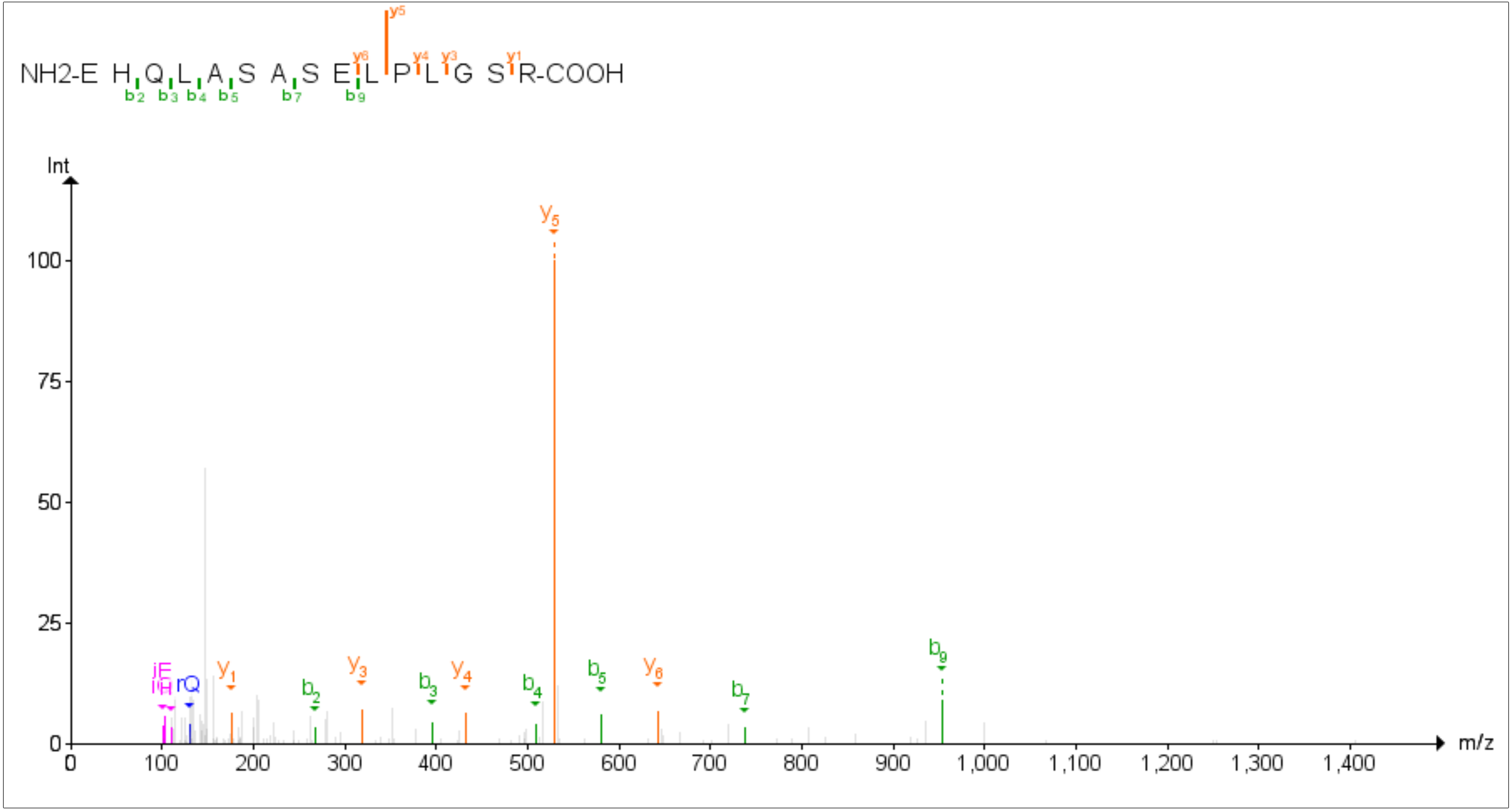
NH2-E H Q L A S A S E L P L G S R-COOH
b2 b3 b4 b5 b7 b9
y6 y5 y4 y3 y1
Int
100
75
50
25
0
y5
iE
iH
rQ
y1
b2
y3
b3
y4
b4
b5
y6
b7
b9
0 100 200 300 400 500 600 700 800 900 1,000 1,100 1,200 1,300 1,400
m/z

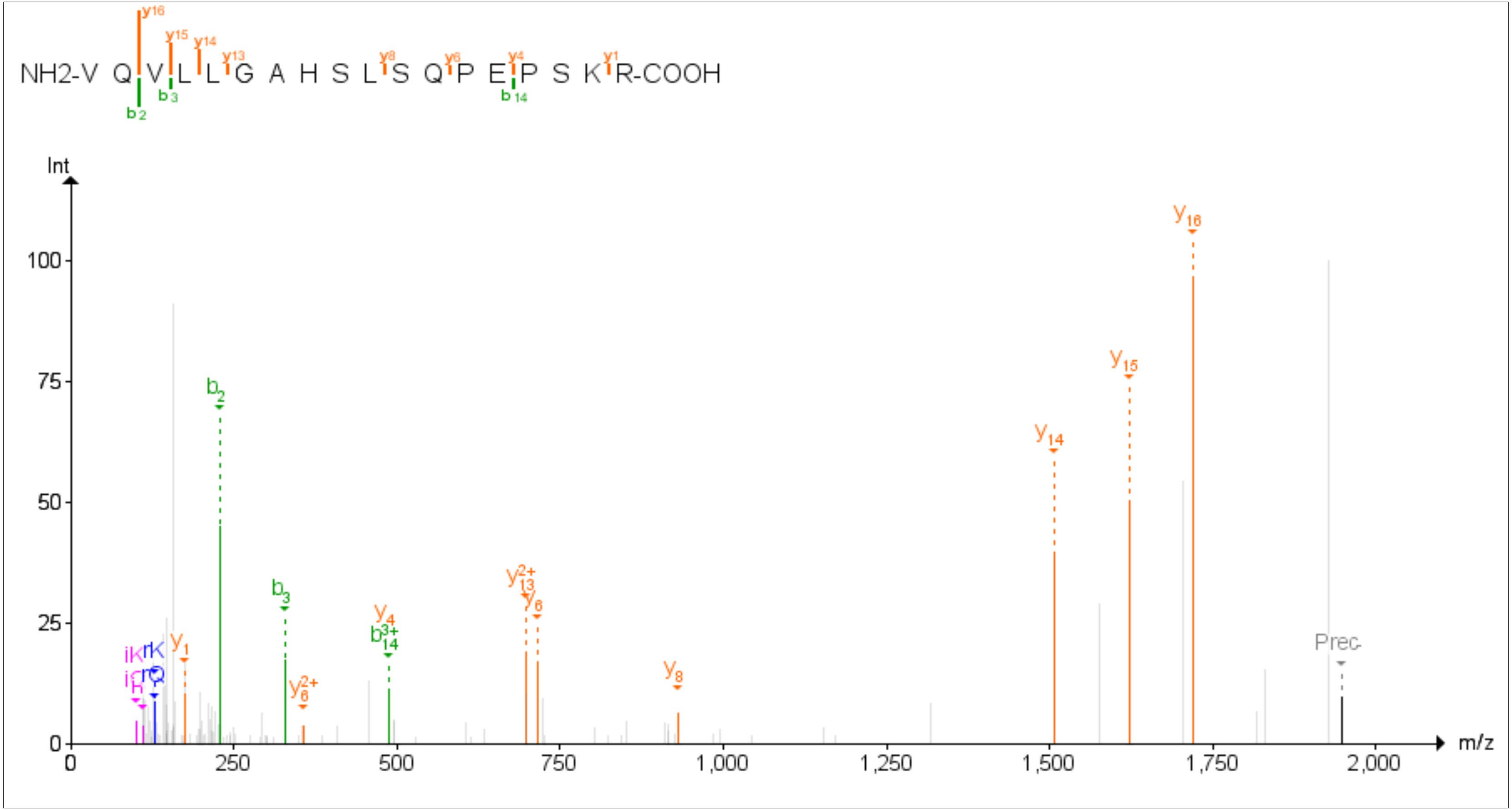
NH2-V Q V L L G A H S L S Q P E P S K R-COOH
y16
y15
y14
y13
y8
y6
y4
y1
b2
b3
b14
Int
100
75
50
25
0
m/z
0
250
500
750
1,000
1,250
1,500
1,750
2,000
Prec

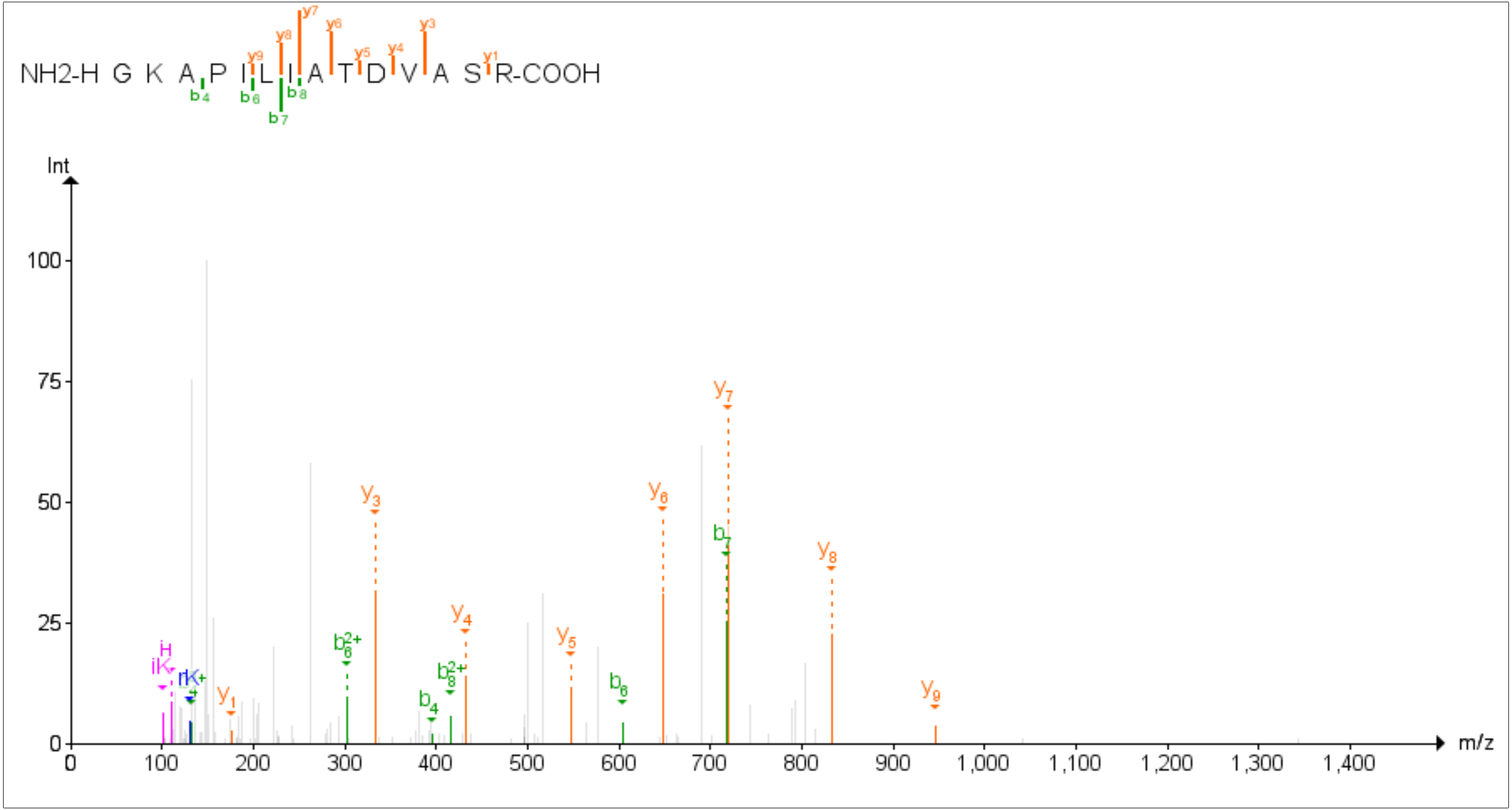

NH2-H G K A P I L I A T D V A S R-COOH
Int
m/z

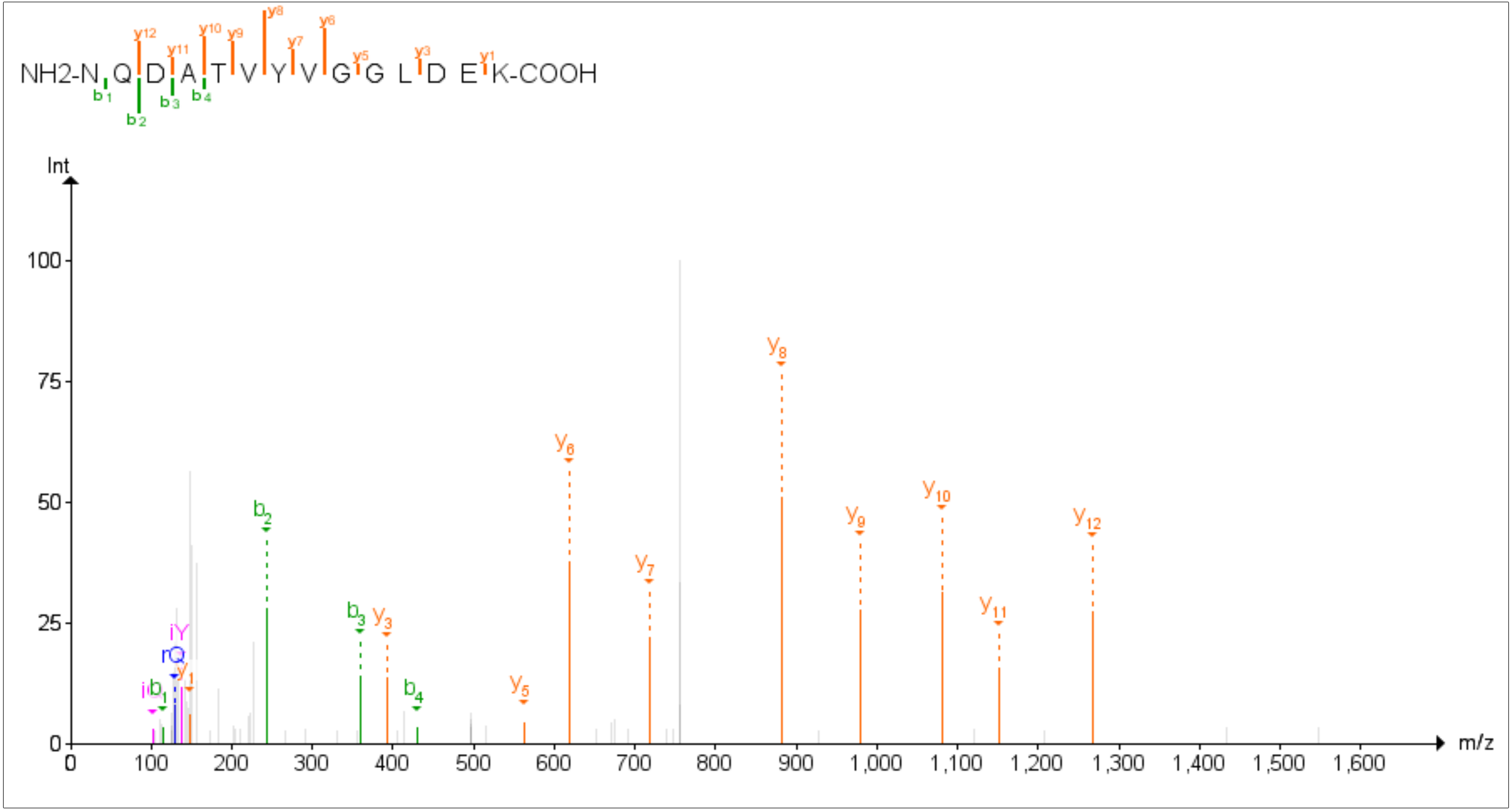

NH2-N Q D A T V Y V G G L D E K-COOH
Int
m/z

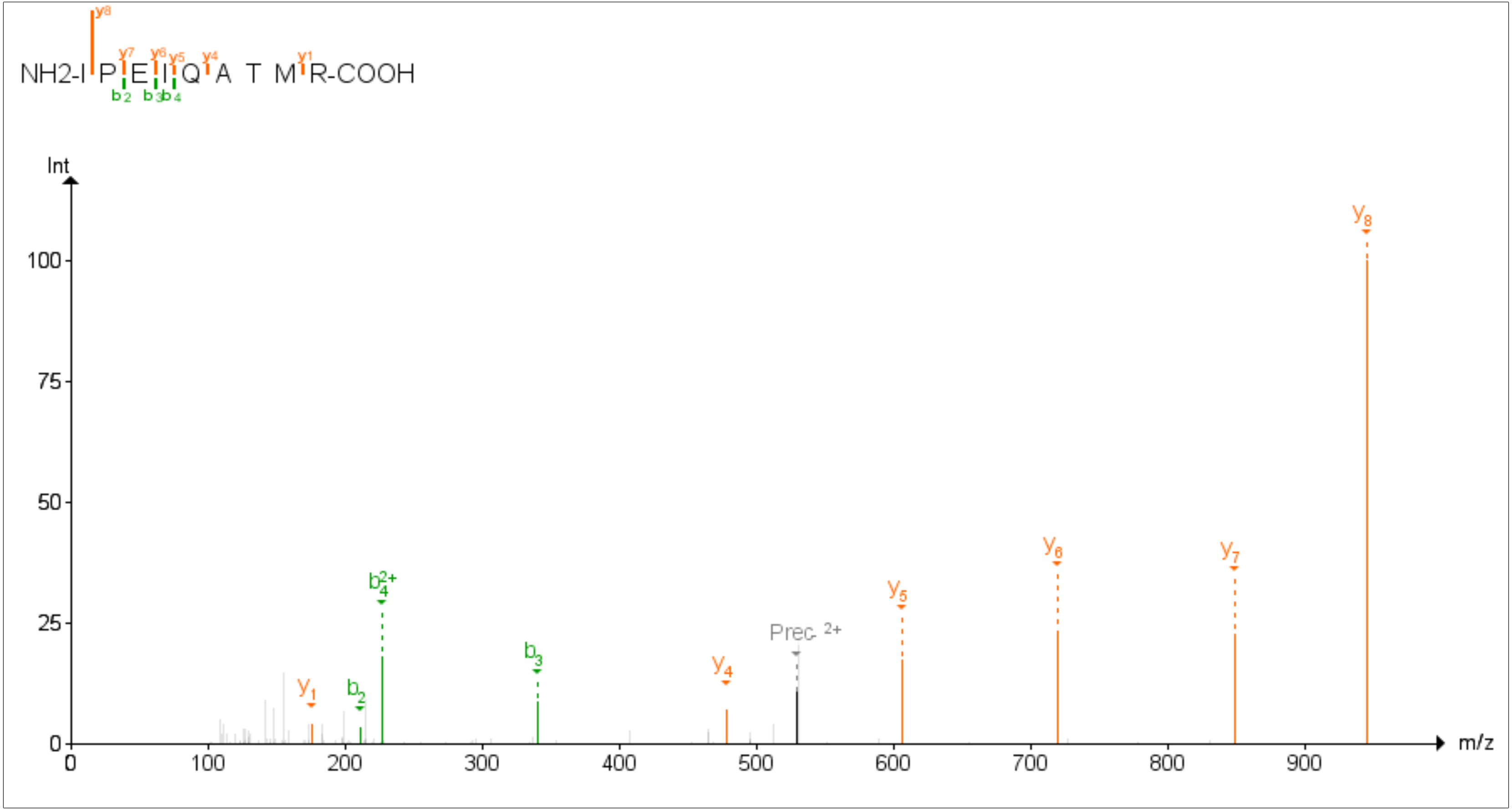

NH2-I P E I Q A T M R-COOH
y8
y7
y6
y5
y4
y1
b2
b3
b4
Int
100
75
50
25
0
m/z
0
100
200
300
400
500
600
700
800
900
y8
y6
y7
y5
b4 2+
Prec 2+
b3
y4
y1
b2

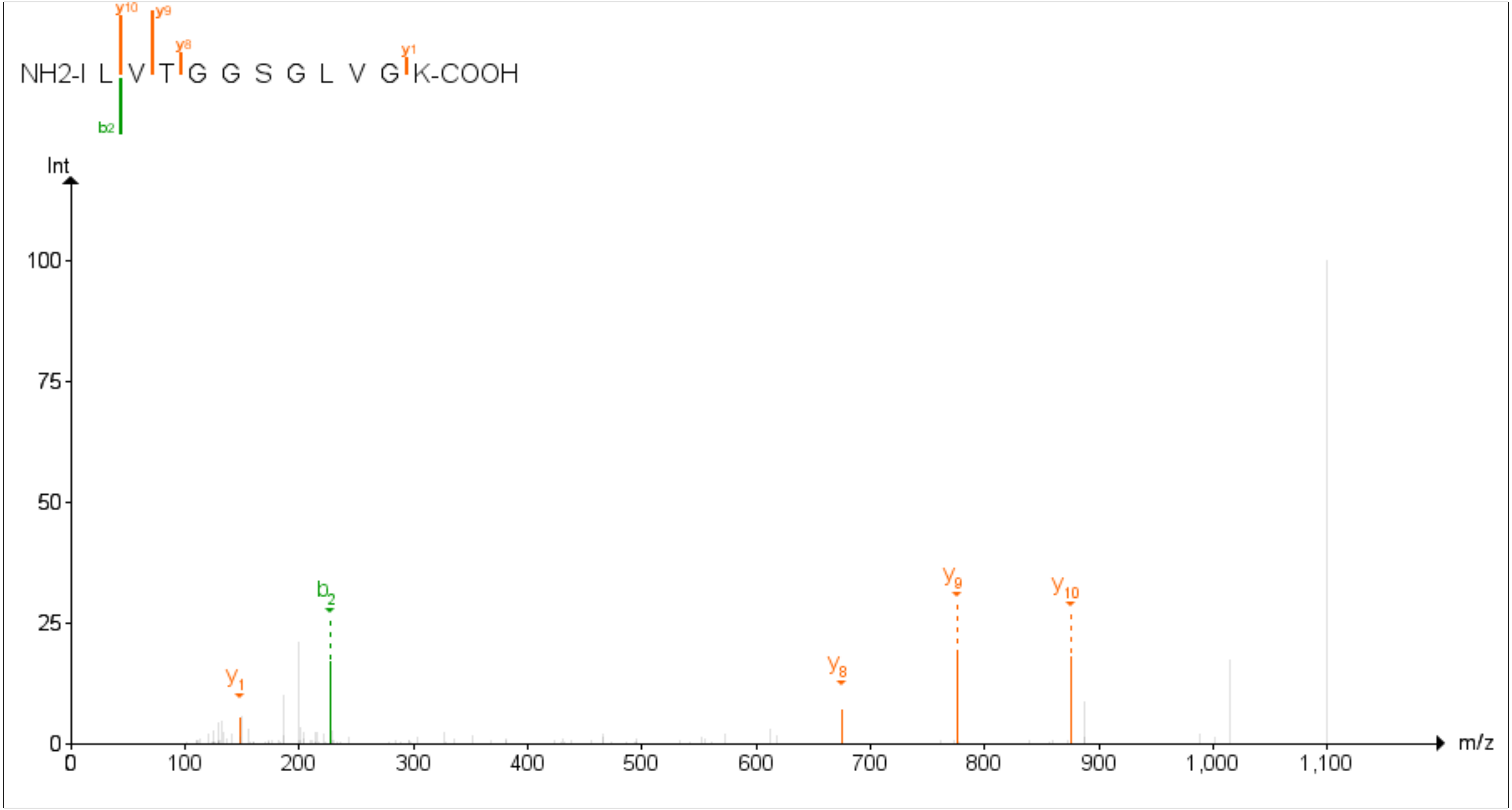

y10
y9
y8
y1
NH2-I L V T G G S G L V G K-COOH
b2
Int
100
75
50
25
0
b2
y1
y8
y9
y10
0
100
200
300
400
500
600
700
800
900
1,000
1,100
m/z

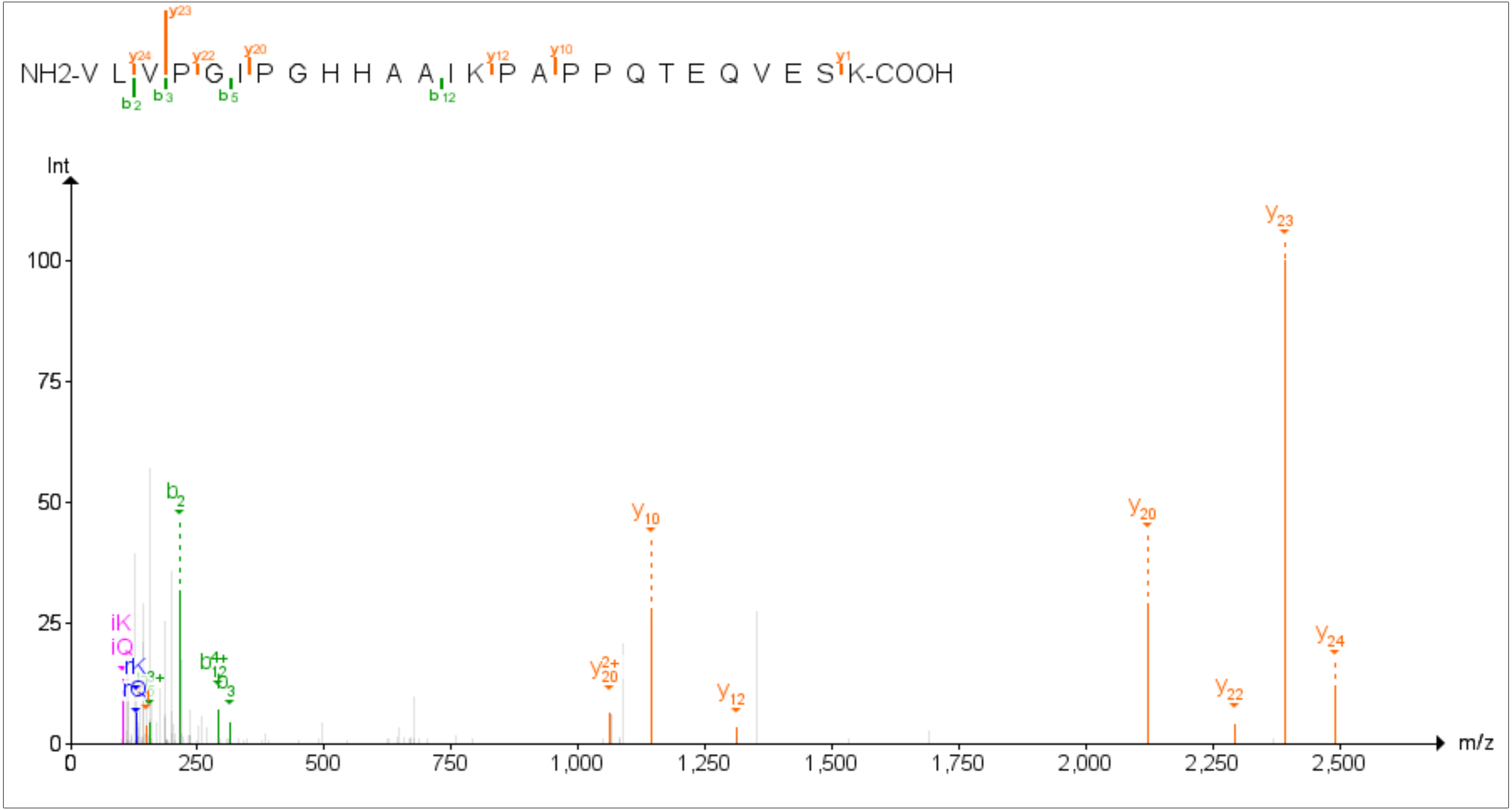

NH2-V L V P G I P G H H A A I K P A P P Q T E Q V E S K-COOH
y24 y23 y22 y20 y12 y10 y1
b2 b3 b5 b12
Int
100
75
50
25
0
m/z
0
250
500
750
1,000
1,250
1,500
1,750
2,000
2,250
2,500
b2
iK
iQ
rK
rQ
y10
y20
y23
y24
y22
y12
y20 2+
b12 4+
b3

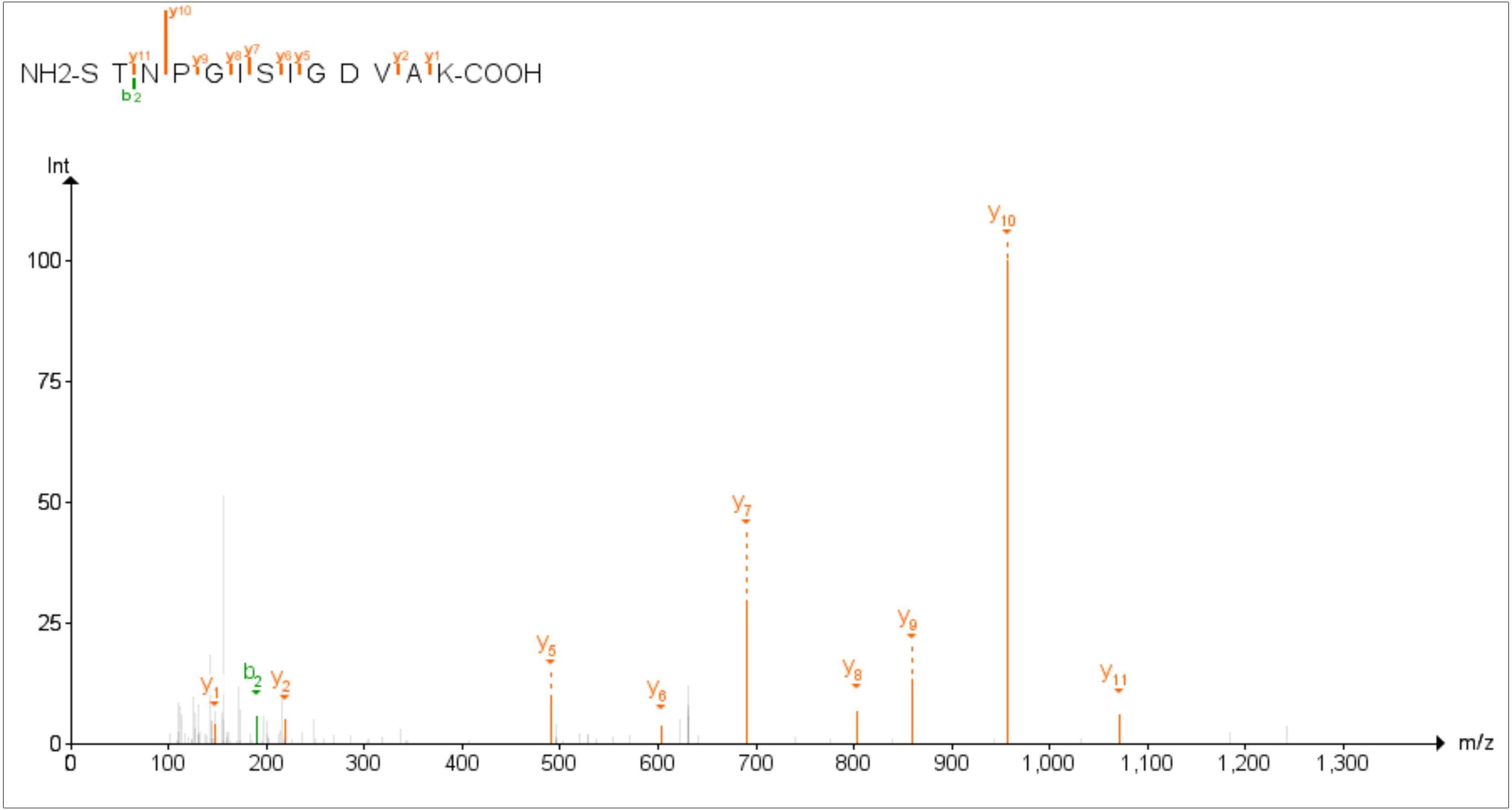
NH2-S T N P G I S I G D V A K-COOH
Int
m/z
y10
y7
y9
y5
y8
y11
y6
y1
b2
y2
100
75
50
25
0
100
200
300
400
500
600
700
800
900
1,000
1,100
1,200
1,300

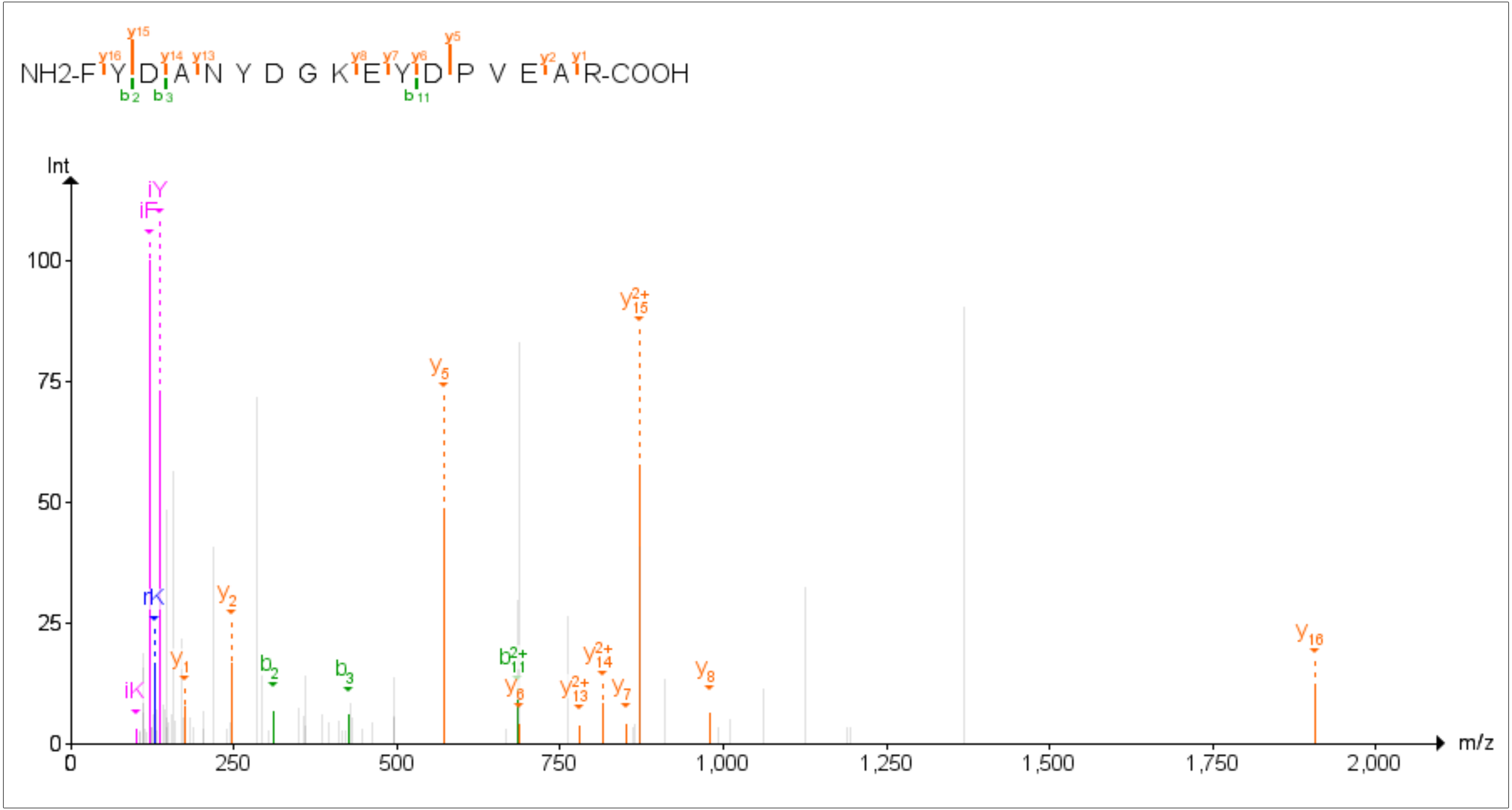
NH2-F Y D A N Y D G K E Y D P V E A R-COOH
y16 y15 y14 y13 y8 y7 y6 y5 y2 y1
b2 b3 b11
Int
m/z
0
25
50
75
100
250
500
750
1,000
1,250
1,500
1,750
2,000
iY
iF
rK
iK
y2
y1
b2
b3
y5
y15 2+
b11 2+
y6
y13 2+
y14 2+
y7
y8
y16

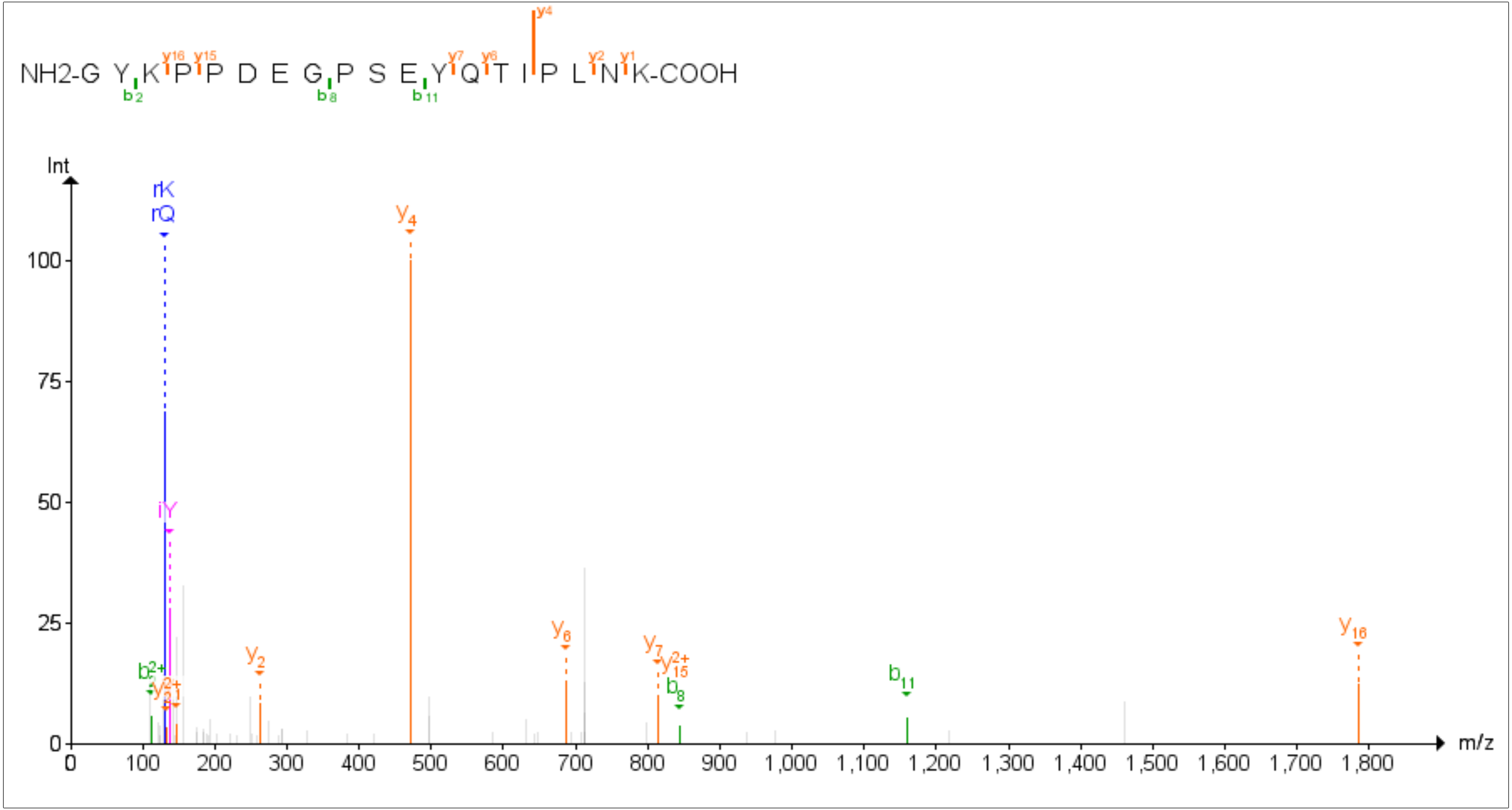
NH2-G Y K P P D E G P S E Y Q T I P L N K-COOH
b2
b8
b11
y16
y15
y7
y6
y4
y2
y1
Int
rK
rQ
y4
iY
y6
y7
y2+ 15
b8
b11
y16
y2
b2+ 2
0
25
50
75
100
0
100
200
300
400
500
600
700
800
900
1,000
1,100
1,200
1,300
1,400
1,500
1,600
1,700
1,800
m/z

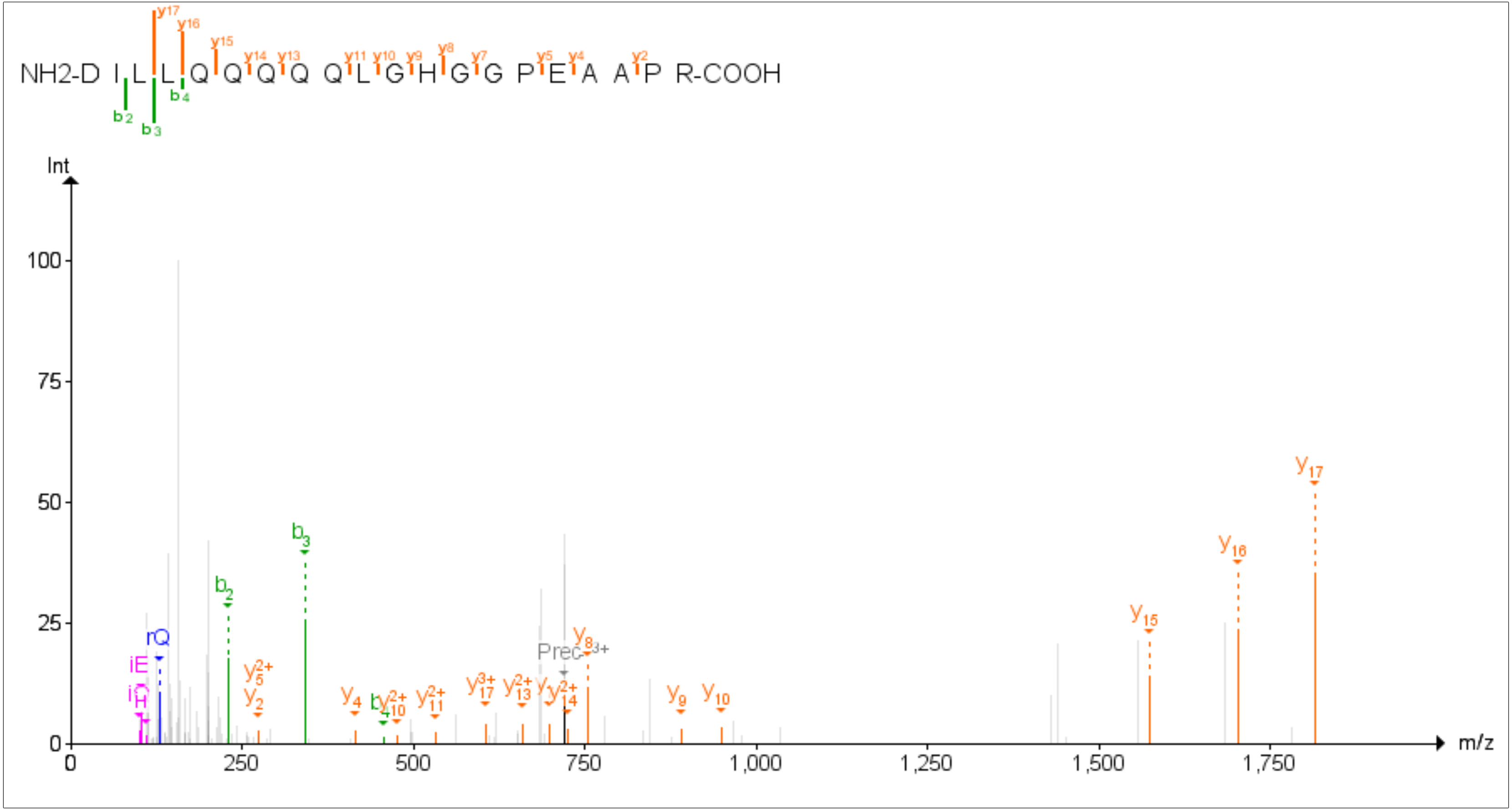

NH2-D I L L Q Q Q Q Q L G H G G P E A A P R-COOH
y17 y16 y15 y14 y13 y11 y10 y9 y8 y7 y5 y4 y2
b2 b3 b4
Int
100
75
50
25
0
m/z
0
250
500
750
1,000
1,250
1,500
1,750
rQ
iE
iH
b2
b3
y17
y16
y15
Prec

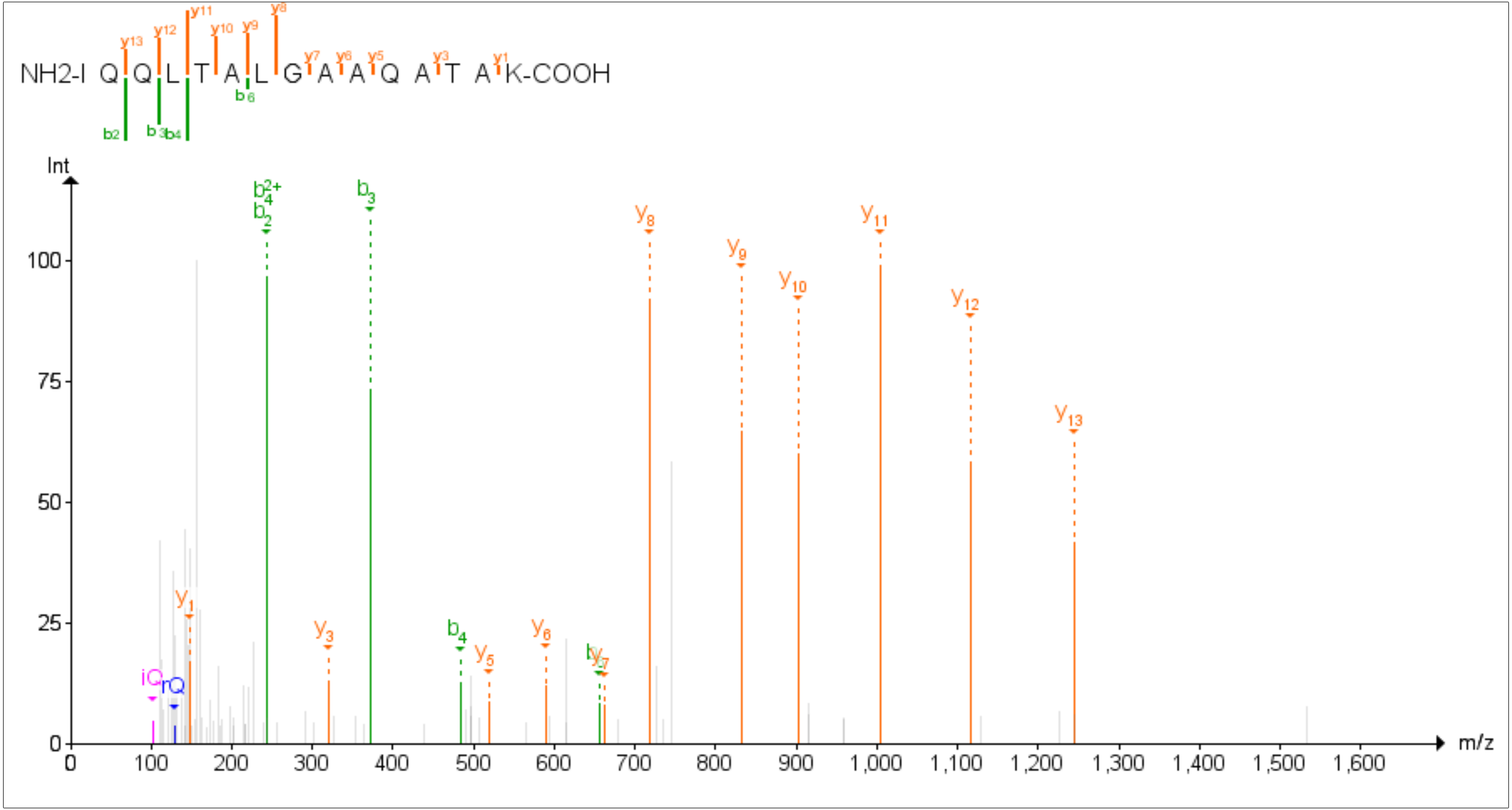

NH2-I Q Q L T A L G A A Q A T A K-COOH
y13 y12 y11 y10 y9 y8 y7 y6 y5 y3 y1
b2 b3 b4 b6
Int
100
75
50
25
0
b_4^{2+}
b_2
b_3
y_8
y_9
y_{10}
y_{11}
y_{12}
y_{13}
y_1
y_3
b_4
y_5
y_6
y_7
iQ
rQ
0 100 200 300 400 500 600 700 800 900 1,000 1,100 1,200 1,300 1,400 1,500 1,600
m/z

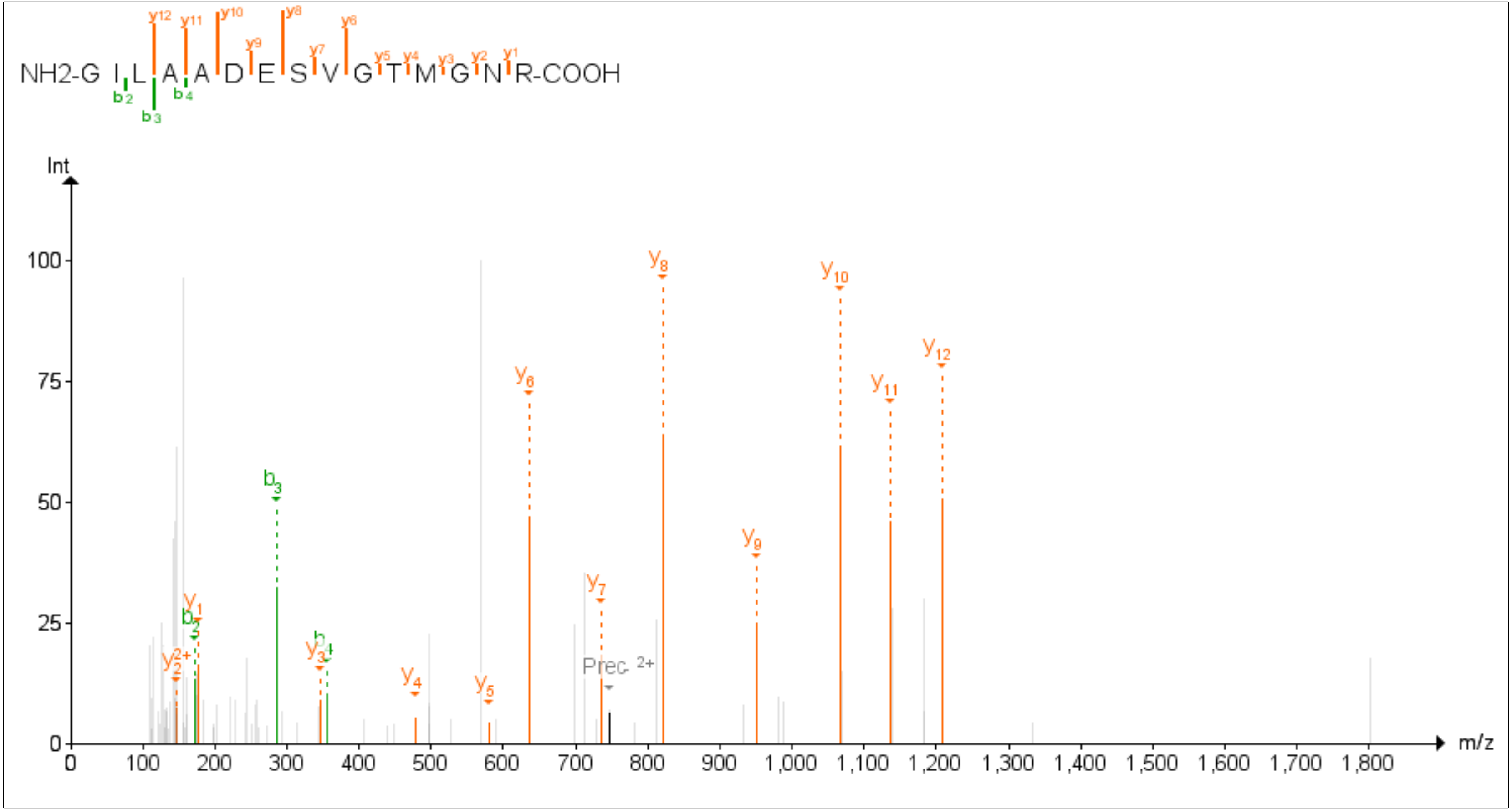

NH2-G I L A A D E S V G T M G N R-COOH
y12 y11 y10 y9 y8 y7 y6 y5 y4 y3 y2 y1
b2 b3 b4
Int
100
75
50
25
0
m/z
0 100 200 300 400 500 600 700 800 900 1,000 1,100 1,200 1,300 1,400 1,500 1,600 1,700 1,800
y8
y10
y12
y11
y6
b3
y9
y7
y1
b2
y2 2+
y3
b4
y4
y5
Prec- 2+

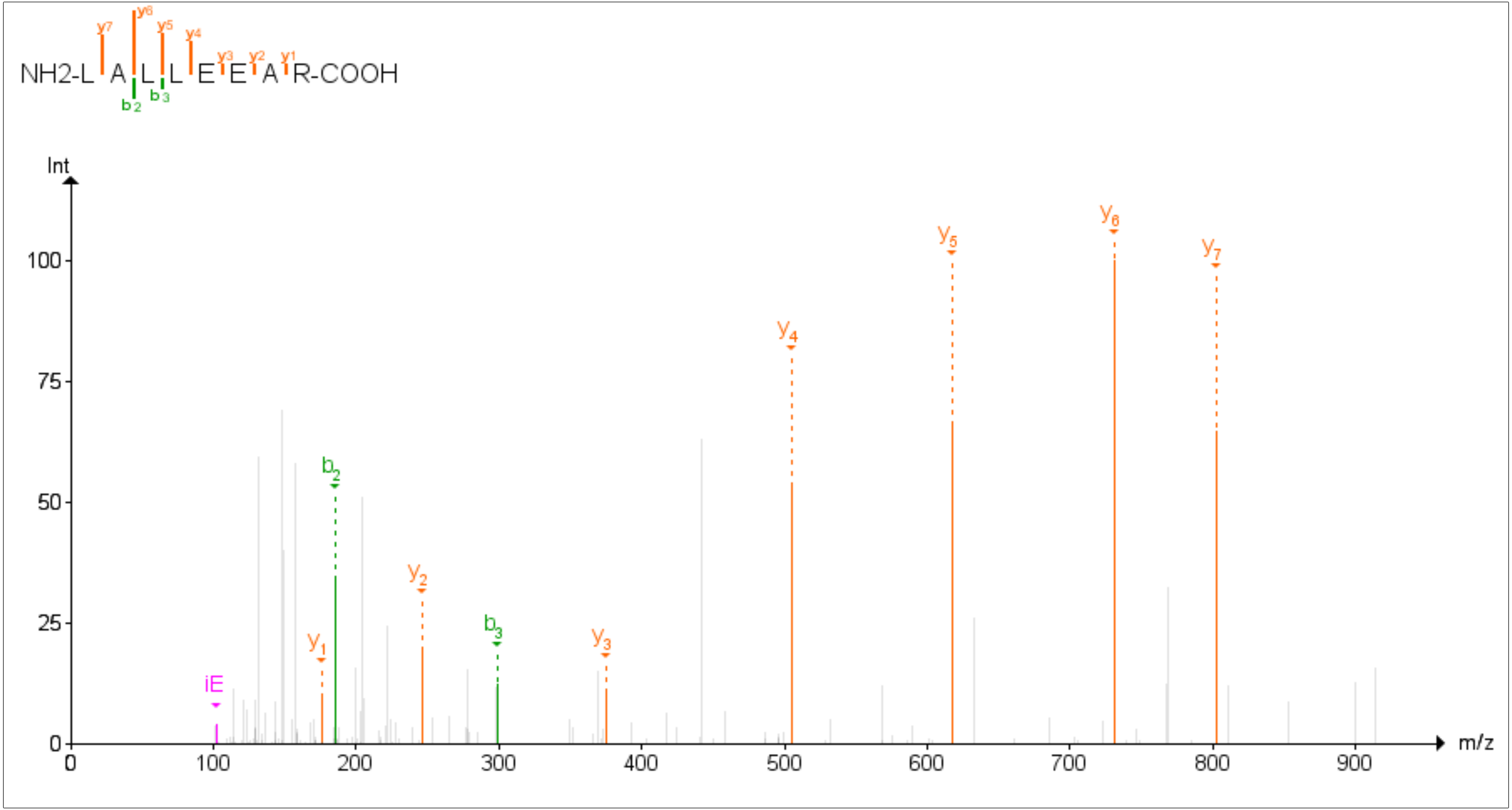

NH2-L A L L E E A R-COOH
y7 y6 y5 y4 y3 y2 y1
b2 b3
Int
100
75
50
25
0
m/z
0 100 200 300 400 500 600 700 800 900
iE
y1
b2
y2
b3
y3
y4
y5
y6
y7

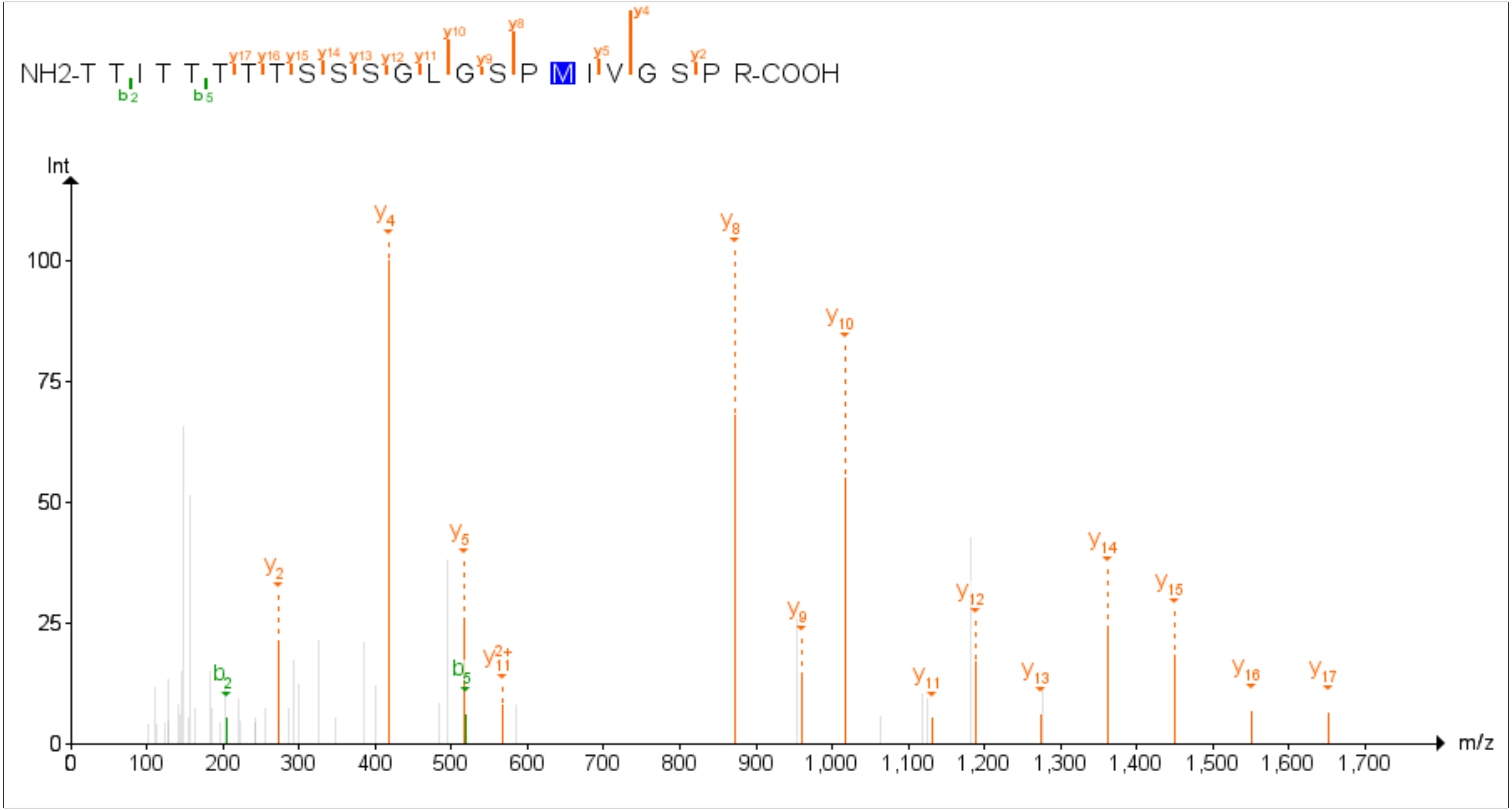

NH2-T T I T T T T T S S S G L G S P M I V G S P R-COOH
b2 b5
y17 y16 y15 y14 y13 y12 y11 y10 y9 y8 y5 y4 y2
Int
m/z
0 25 50 75 100
0 100 200 300 400 500 600 700 800 900 1,000 1,100 1,200 1,300 1,400 1,500 1,600 1,700
y_4 y_8 y_{10} y_5 y_2 y_9 y_{12} y_{14} y_{15} b_2 b_5 y_{11}^{2+} y_{11} y_{13} y_{16} y_{17}

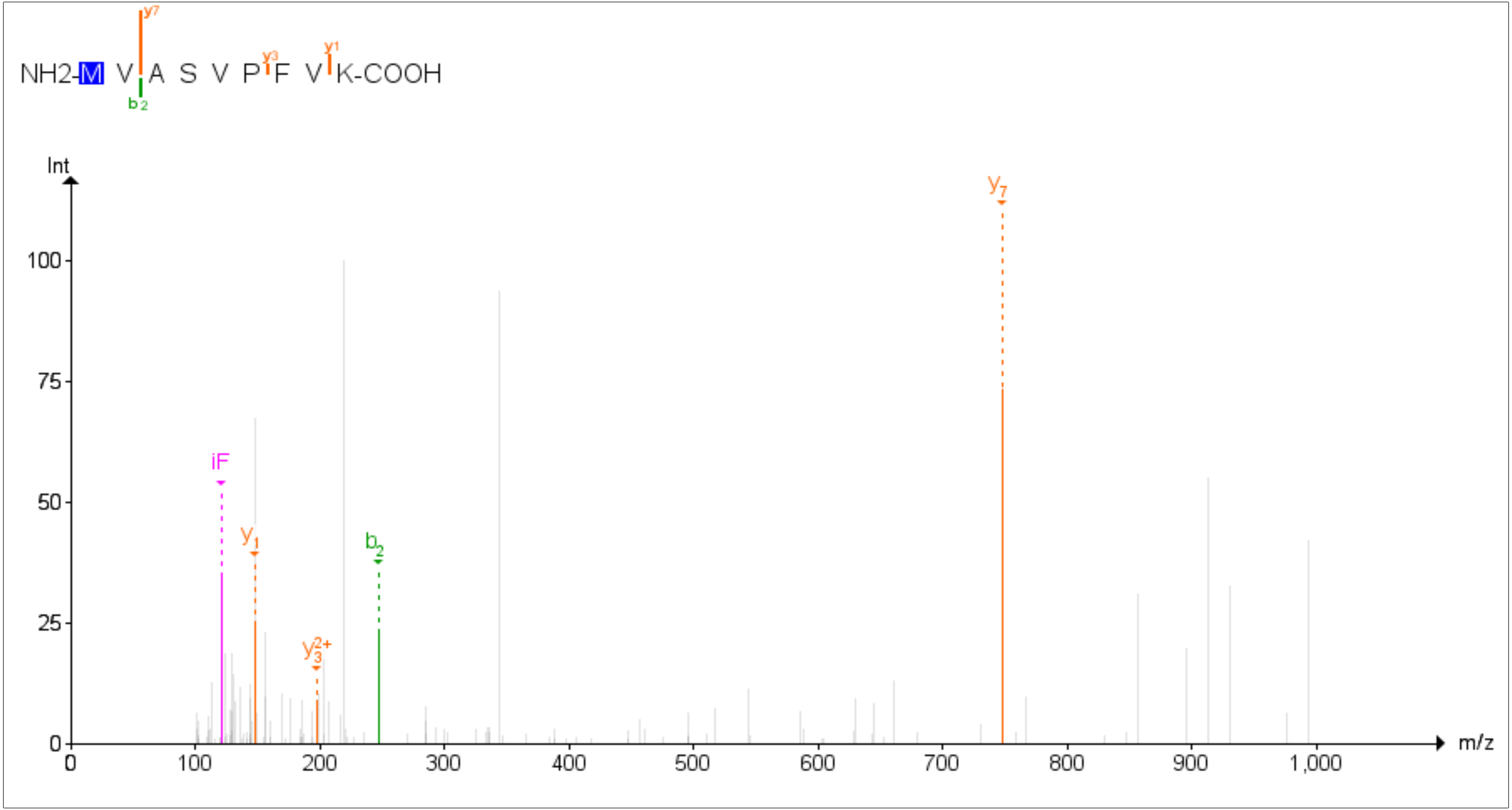

NH2-M V A S V P F V K-COOH
y7
b2
y3
y1
Int
100
75
50
25
0
y7
iF
y1
b2
y3 2+
0
100
200
300
400
500
600
700
800
900
1,000
m/z

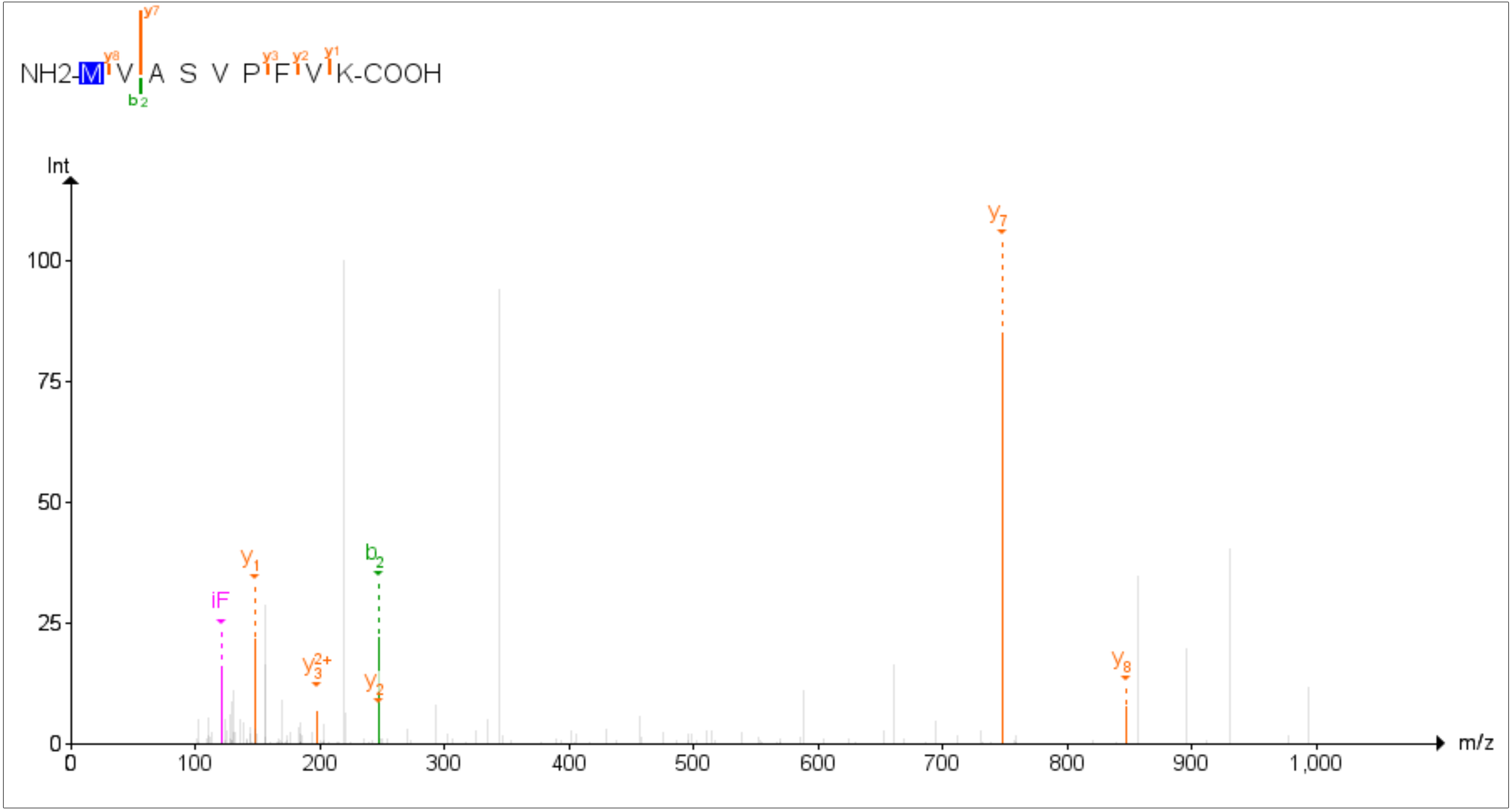

NH2-M V A S V P F V K-COOH
y7
y8
y3
y2
y1
b2
Int
100
75
50
25
0
y7
y1
b2
iF
y3 2+
y2
y8
0
100
200
300
400
500
600
700
800
900
1,000
m/z

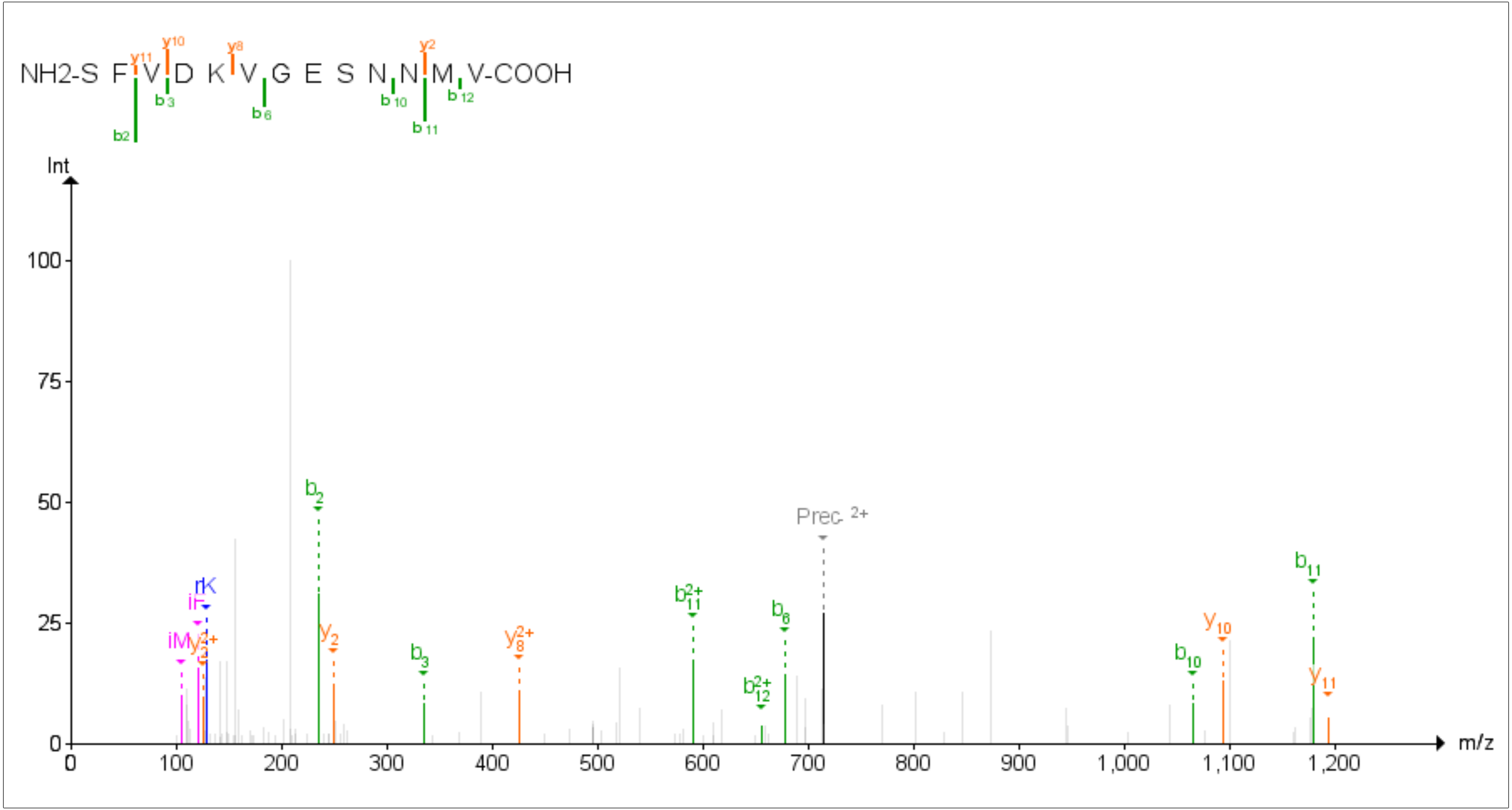

NH2-S F V D K V G E S N N M V-COOH
y11
y10
y8
y2
b2
b3
b6
b10
b11
b12
Int
100
75
50
25
0
m/z
0
100
200
300
400
500
600
700
800
900
1,000
1,100
1,200
b2
Prec- 2+
b11
rK
iF
iM
y2
y2 2+
b3
y8 2+
b11 2+
b6
b12 2+
y10
b10
y11

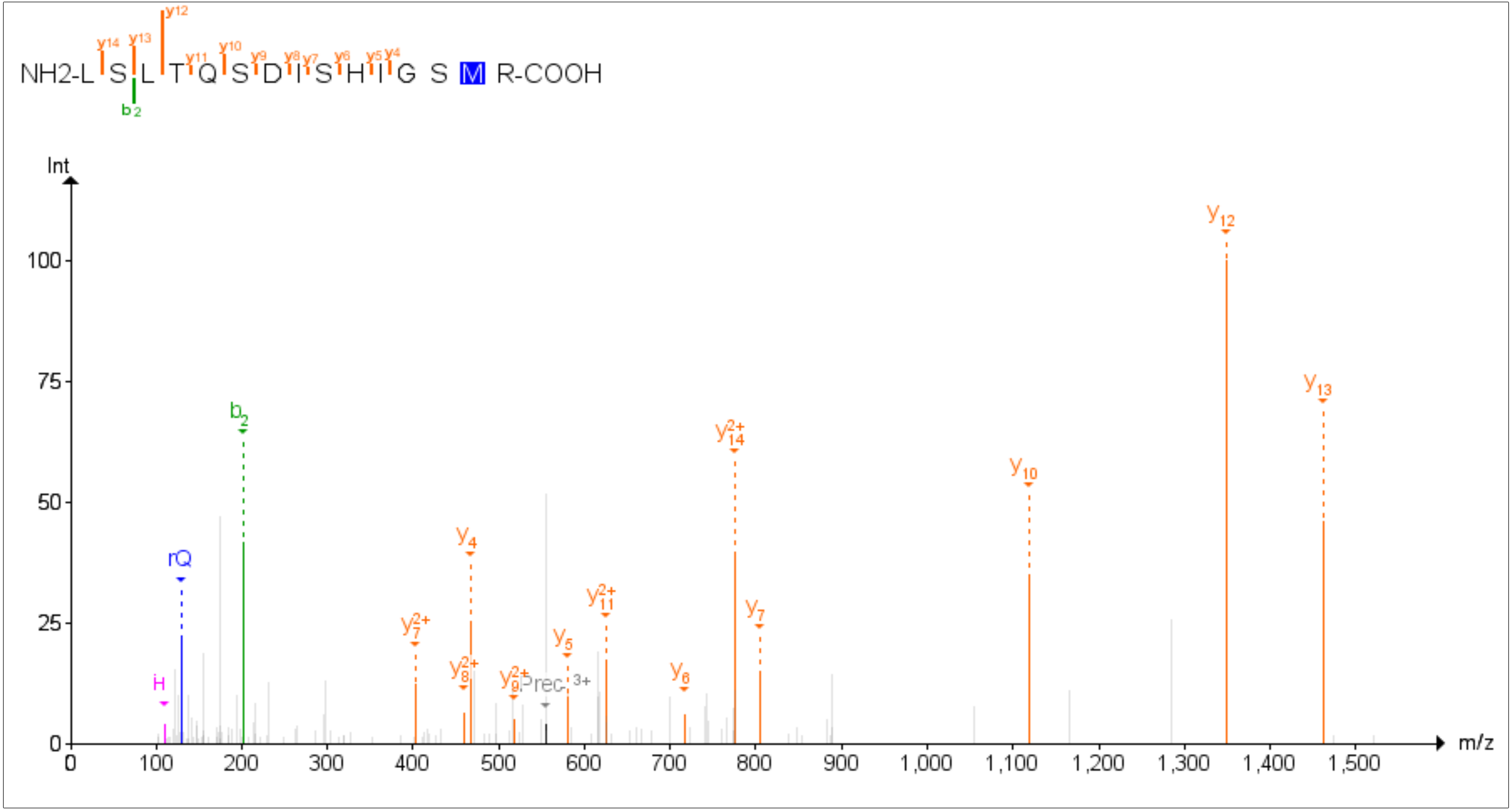

NH2-L S L T Q S D I S H I G S M R-COOH
y14 y13 y12 y11 y10 y9 y8 y7 y6 y5 y4
b2
Int
m/z
rQ
H
b2
y4
y5
y6
y7
y10
y12
y13
Prec 3+

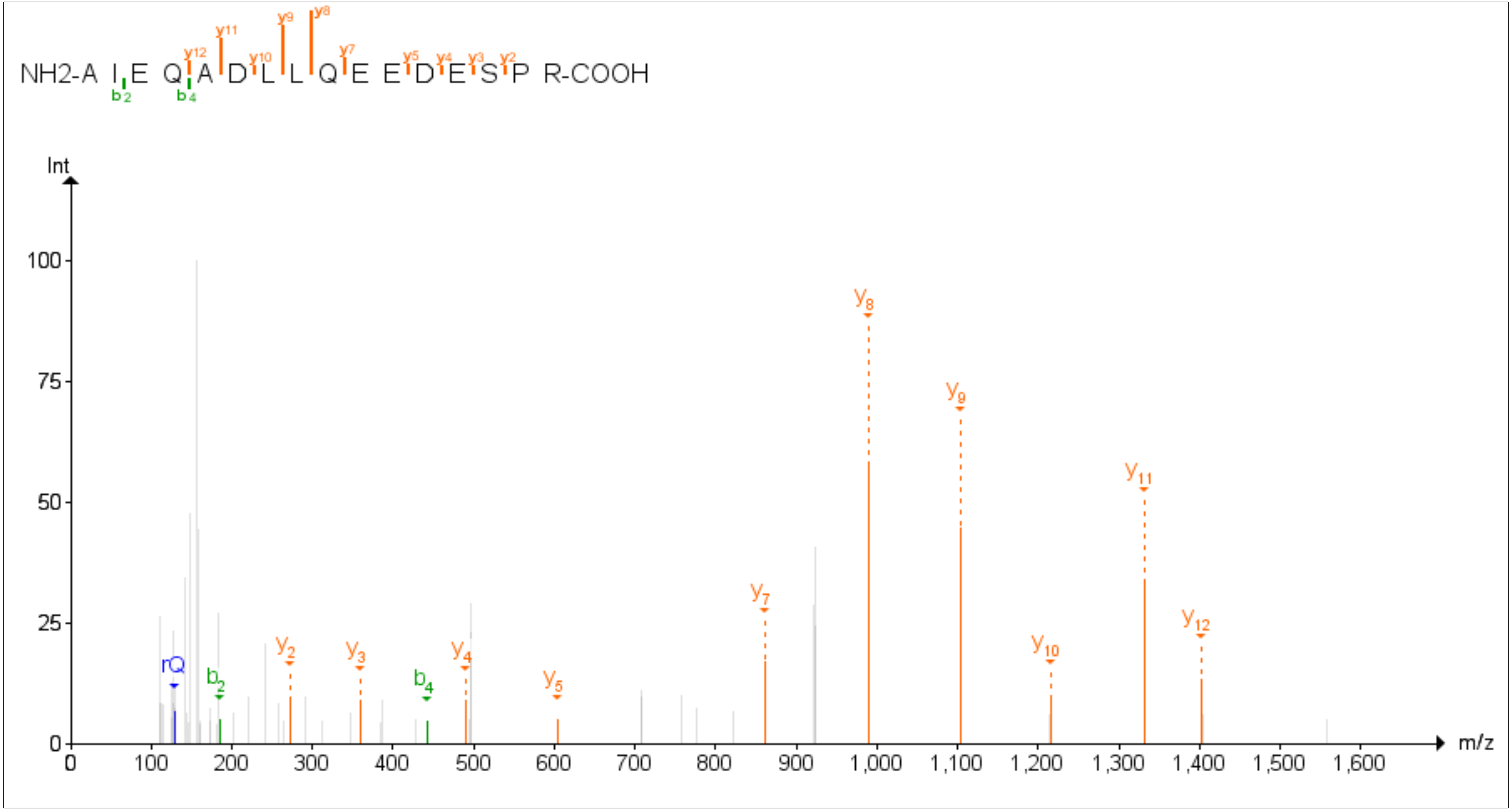

NH2-A I E Q A D L L Q E E D E S P R-COOH
b2
b4
y12
y11
y10
y9
y8
y7
y5
y4
y3
y2
Int
100
75
50
25
0
rQ
b2
y2
y3
b4
y4
y5
y7
y8
y9
y10
y11
y12
0
100
200
300
400
500
600
700
800
900
1,000
1,100
1,200
1,300
1,400
1,500
1,600
m/z

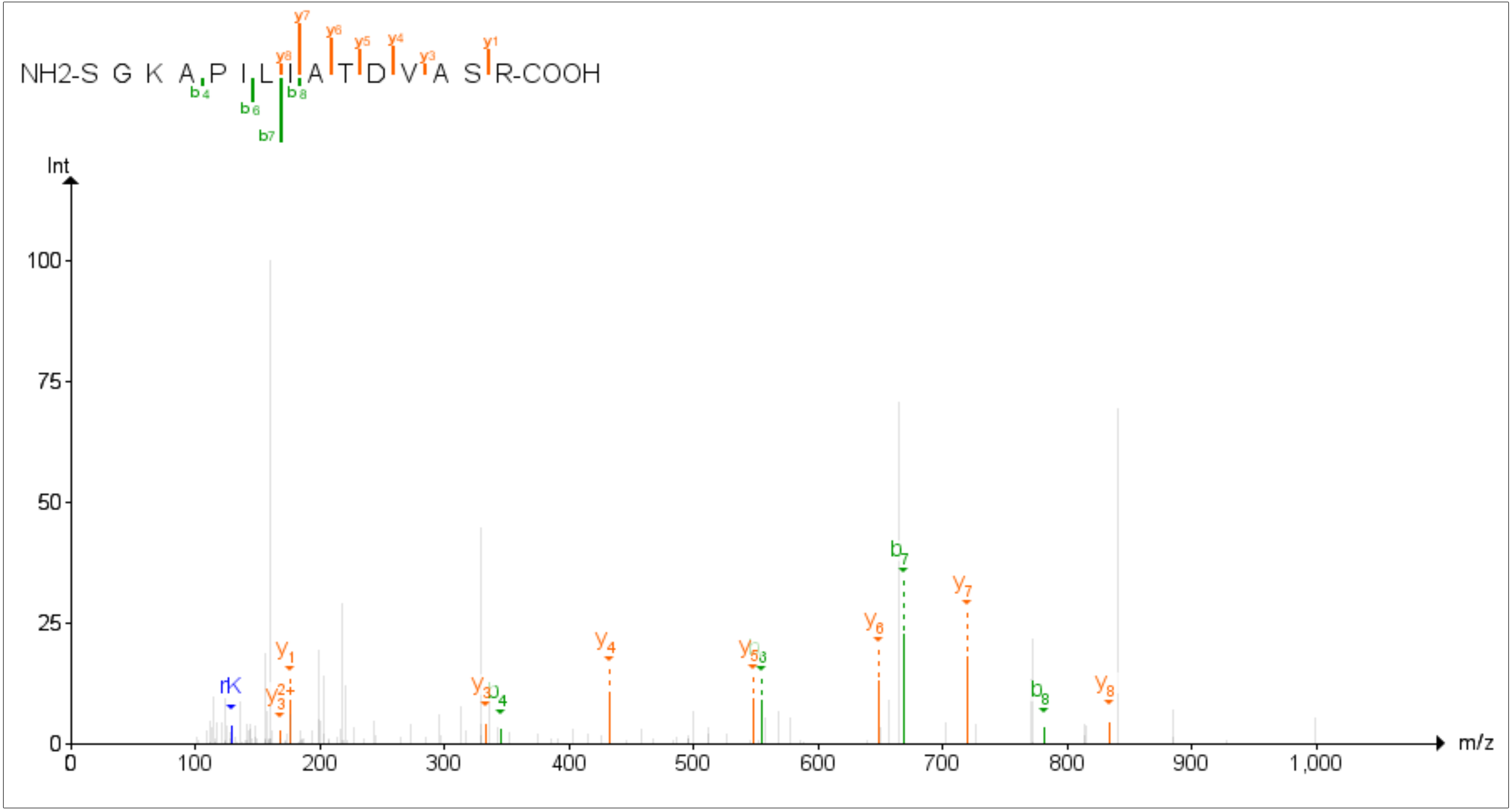

NH2-S G K A P I L I A T D V A S R-COOH
b4
b6
b7
b8
y8
y7
y6
y5
y4
y3
y1
Int
100
75
50
25
0
m/z
0
100
200
300
400
500
600
700
800
900
1,000

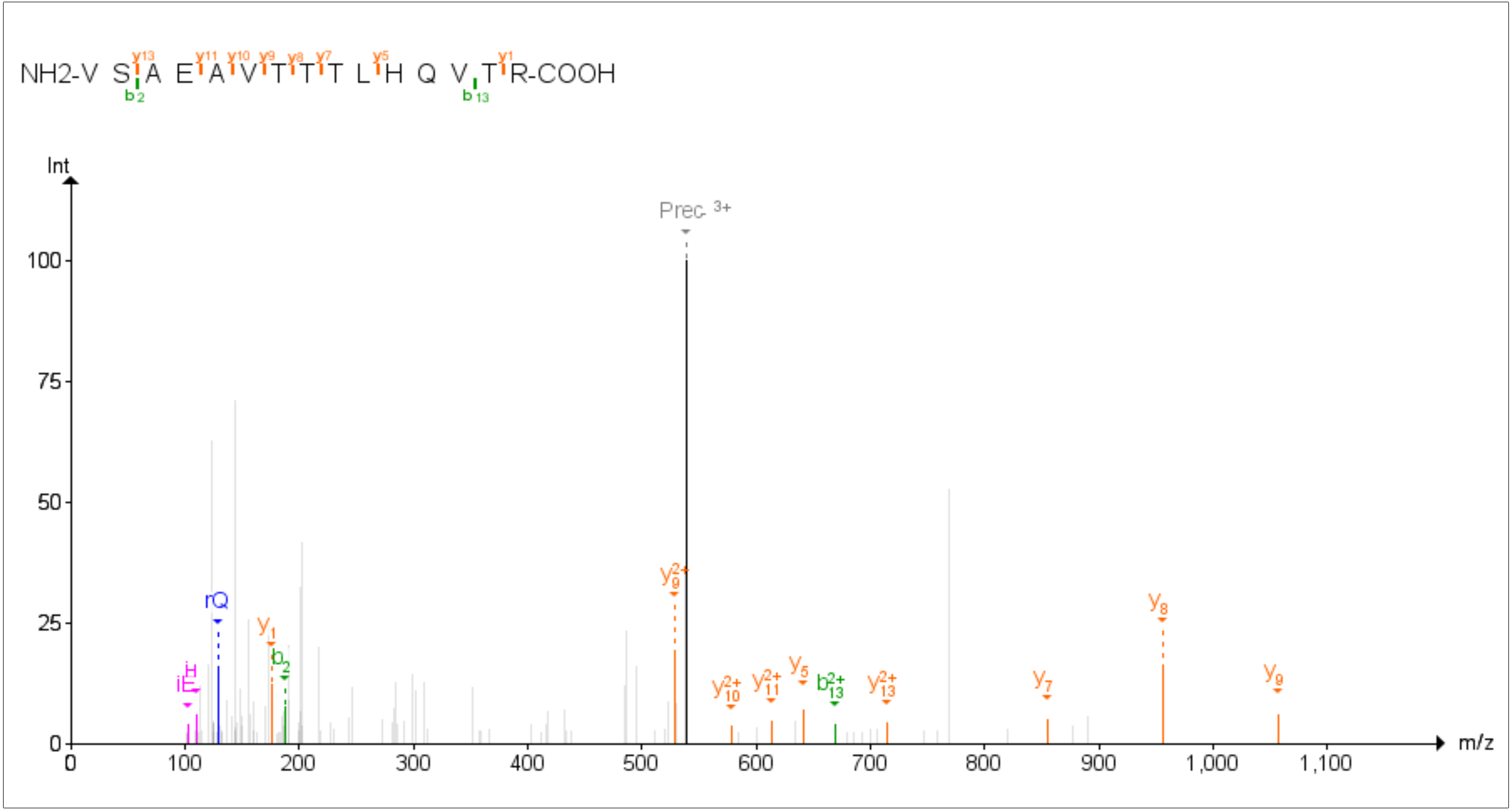

NH2-V S A E A V T T T L H Q V T R-COOH
y13 y11 y10 y9 y8 y7 y5 y1
b2 b13
Int
100
75
50
25
0
Prec- 3+
rQ
iH
iE
y1
b2
y9 2+
y10 2+
y11 2+
y5
b13 2+
y13 2+
y7
y8
y9
0
100
200
300
400
500
600
700
800
900
1,000
1,100
m/z

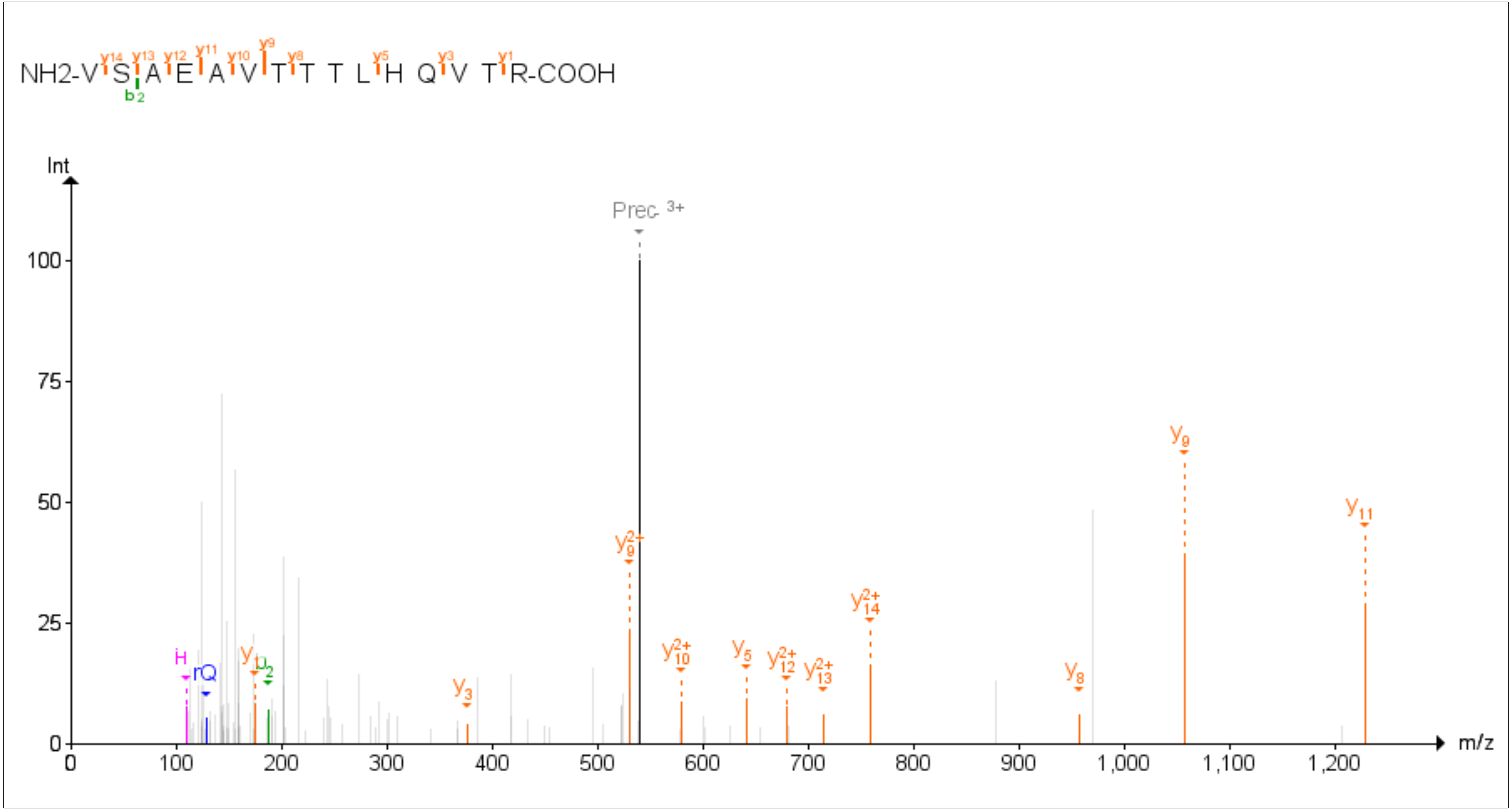

NH2-VSAEAVTTTLHQVTR-COOH
y14 y13 y12 y11 y10 y9 y8 y5 y3 y1
b2
Int
100
75
50
25
0
Prec 3+
y9
y11
y9 2+
y14 2+
y10 2+
y5
y12 2+
y13 2+
y8
y3
H
rQ
b2
0
100
200
300
400
500
600
700
800
900
1,000
1,100
1,200
m/z

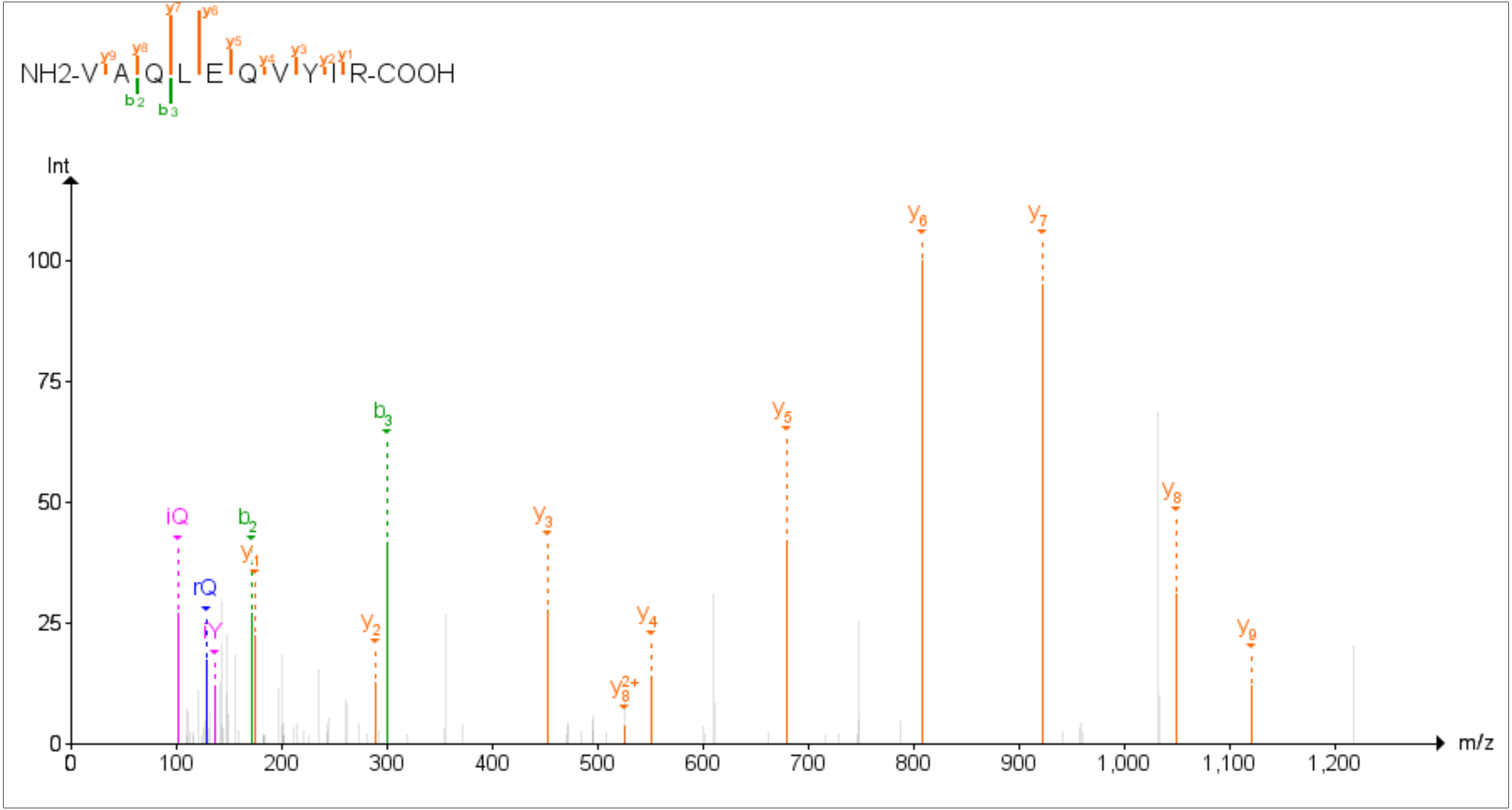

NH2-V A Q L E Q V Y I R-COOH
y9 y8 y7 y6 y5 y4 y3 y2 y1
b2 b3
Int
m/z
0
25
50
75
100
0
100
200
300
400
500
600
700
800
900
1,000
1,100
1,200
iQ
rQ
iY
b2
y1
y2
b3
y3
y8 2+
y4
y5
y6
y7
y8
y9

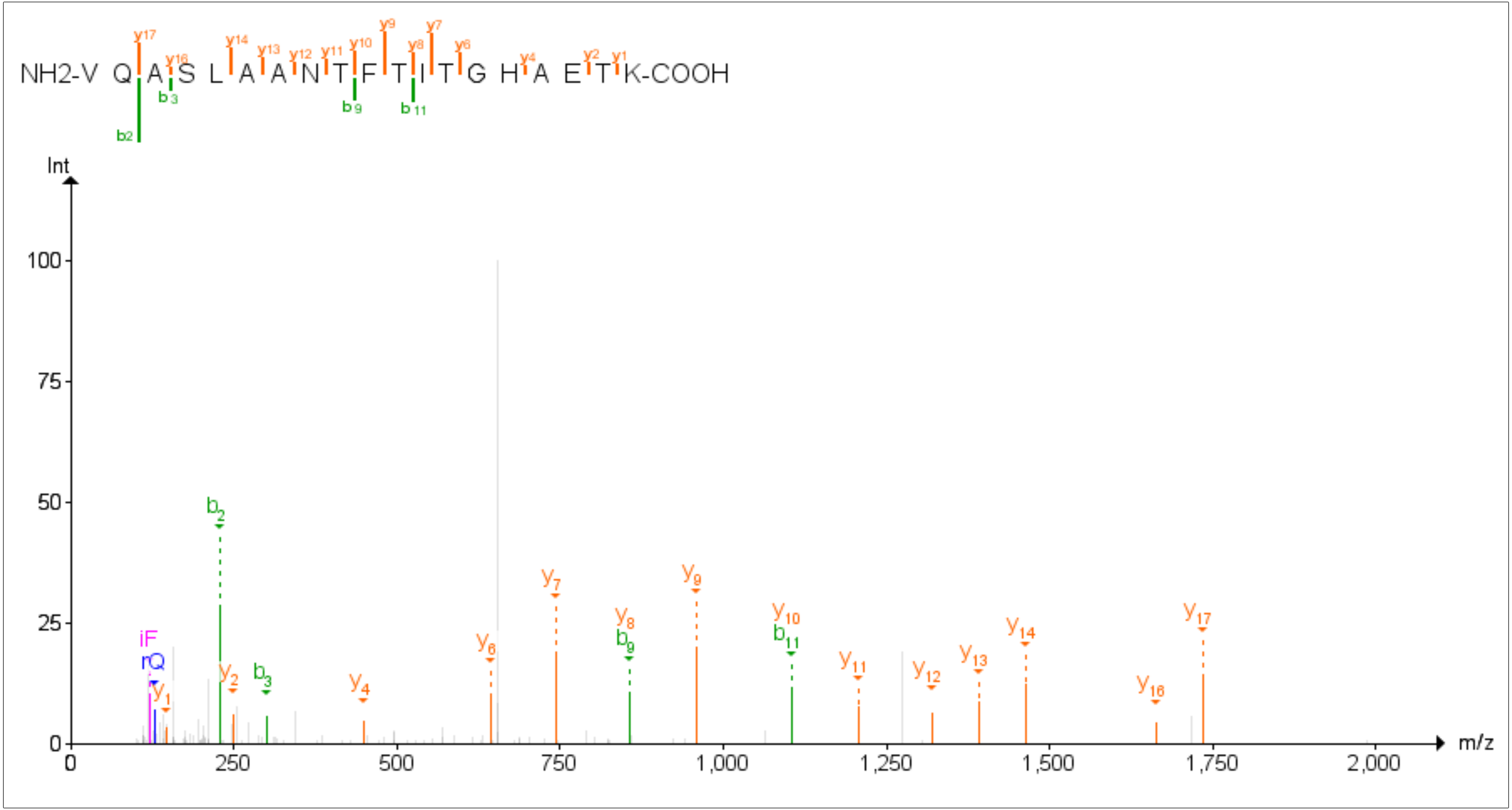

NH2-V Q A S L A A N T F T I T G H A E T K-COOH
y17 y16 y14 y13 y12 y11 y10 y9 y8 y7 y6 y4 y2 y1
b2 b3 b9 b11
Int
100
75
50
25
0
m/z
0
250
500
750
1,000
1,250
1,500
1,750
2,000
iF
rQ
y1 y2 b2 b3 y4 y6 y7 y8 b9 y9 y10 b11 y11 y12 y13 y14 y16 y17

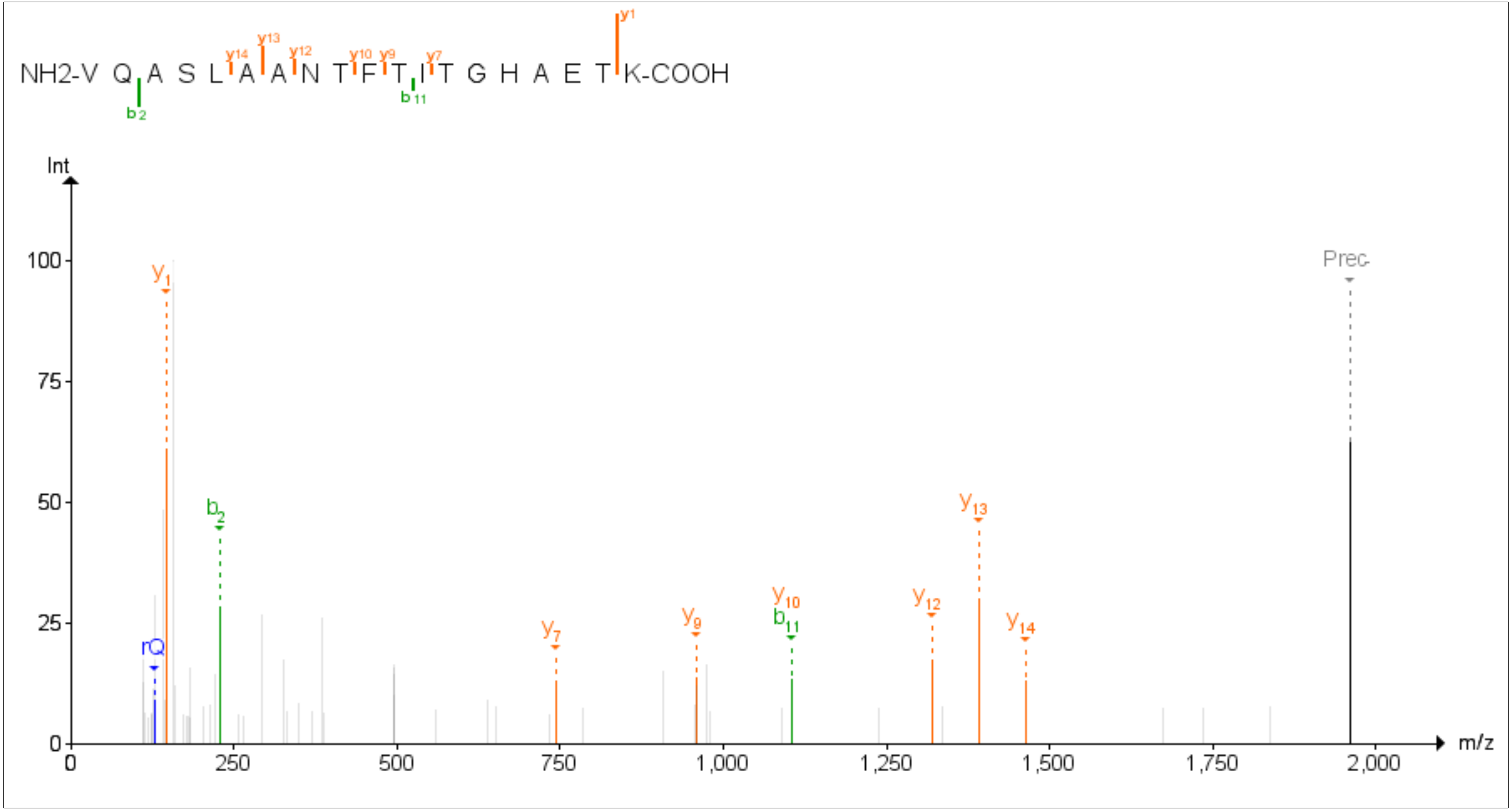

NH2-V Q A S L A A N T F T I T G H A E T K-COOH
y14 y13 y12 y10 y9 y7 y1
b2 b11
Int
100
75
50
25
0
y1
b2
rQ
y7
y9
y10
b11
y12
y13
y14
Prec
0
250
500
750
1,000
1,250
1,500
1,750
2,000
m/z

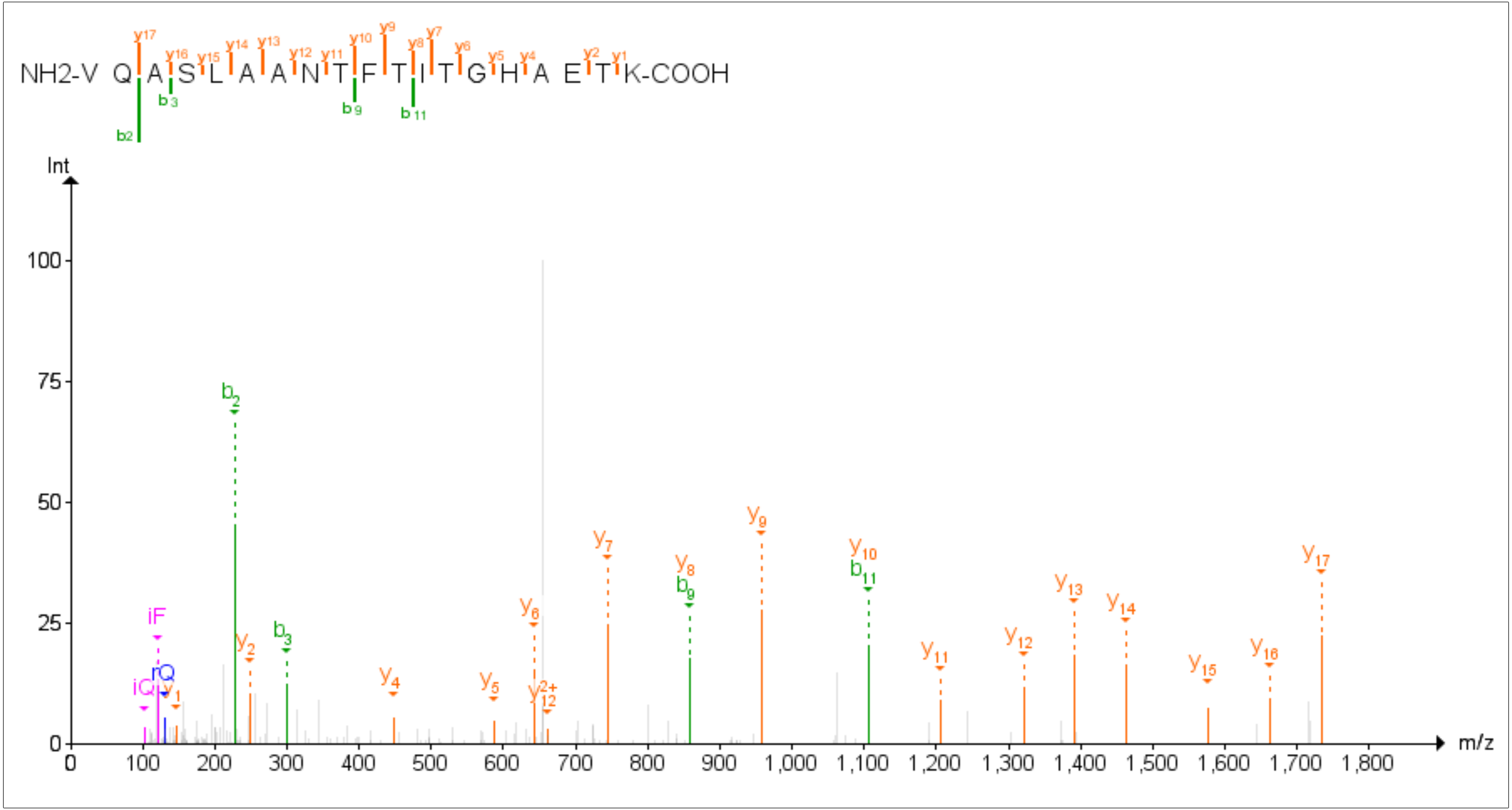

NH2-V Q A S L A A N T F T I T G H A E T K-COOH
y17 y16 y15 y14 y13 y12 y11 y10 y9 y8 y7 y6 y5 y4 y2 y1
b2 b3 b9 b11
Int
100
75
50
25
0
m/z
0 100 200 300 400 500 600 700 800 900 1,000 1,100 1,200 1,300 1,400 1,500 1,600 1,700 1,800
iF
iQ
rQ
y1
y2
b2
b3
y4
y5
y6
y12 2+
y7
y8
b9
y9
y10
b11
y11
y12
y13
y14
y15
y16
y17

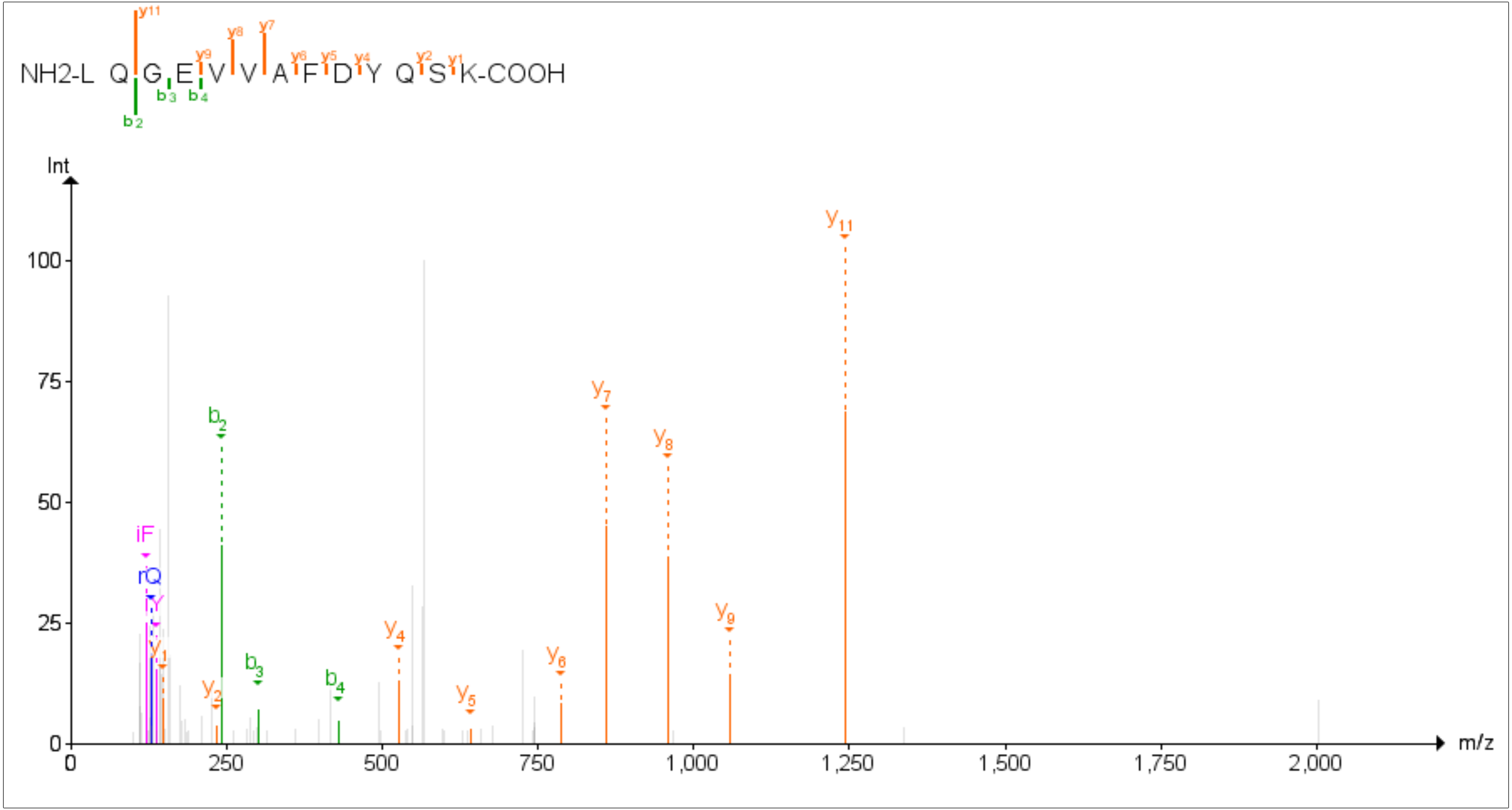

NH2-L Q G E V V A F D Y Q S K-COOH
y11
y9
y8
y7
y6
y5
y4
y2
y1
b2
b3
b4
Int
100
75
50
25
0
iF
rQ
iY
y1
y2
b2
b3
b4
y4
y5
y6
y7
y8
y9
y11
0
250
500
750
1,000
1,250
1,500
1,750
2,000
m/z

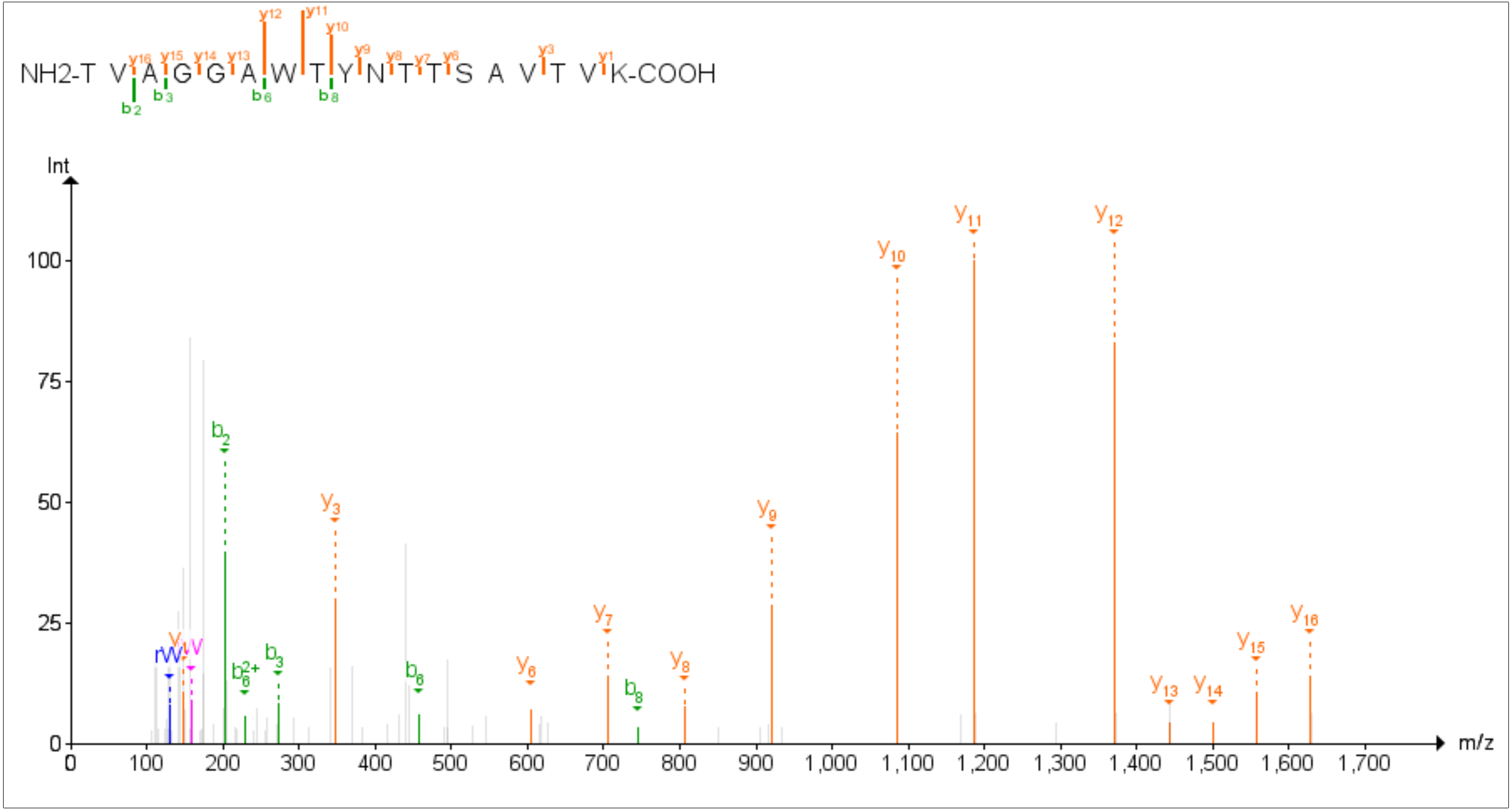

NH2-T V A G G A W T Y N T T S A V T V K-COOH
Int
m/z
0
25
50
75
100
100
200
300
400
500
600
700
800
900
1,000
1,100
1,200
1,300
1,400
1,500
1,600
1,700

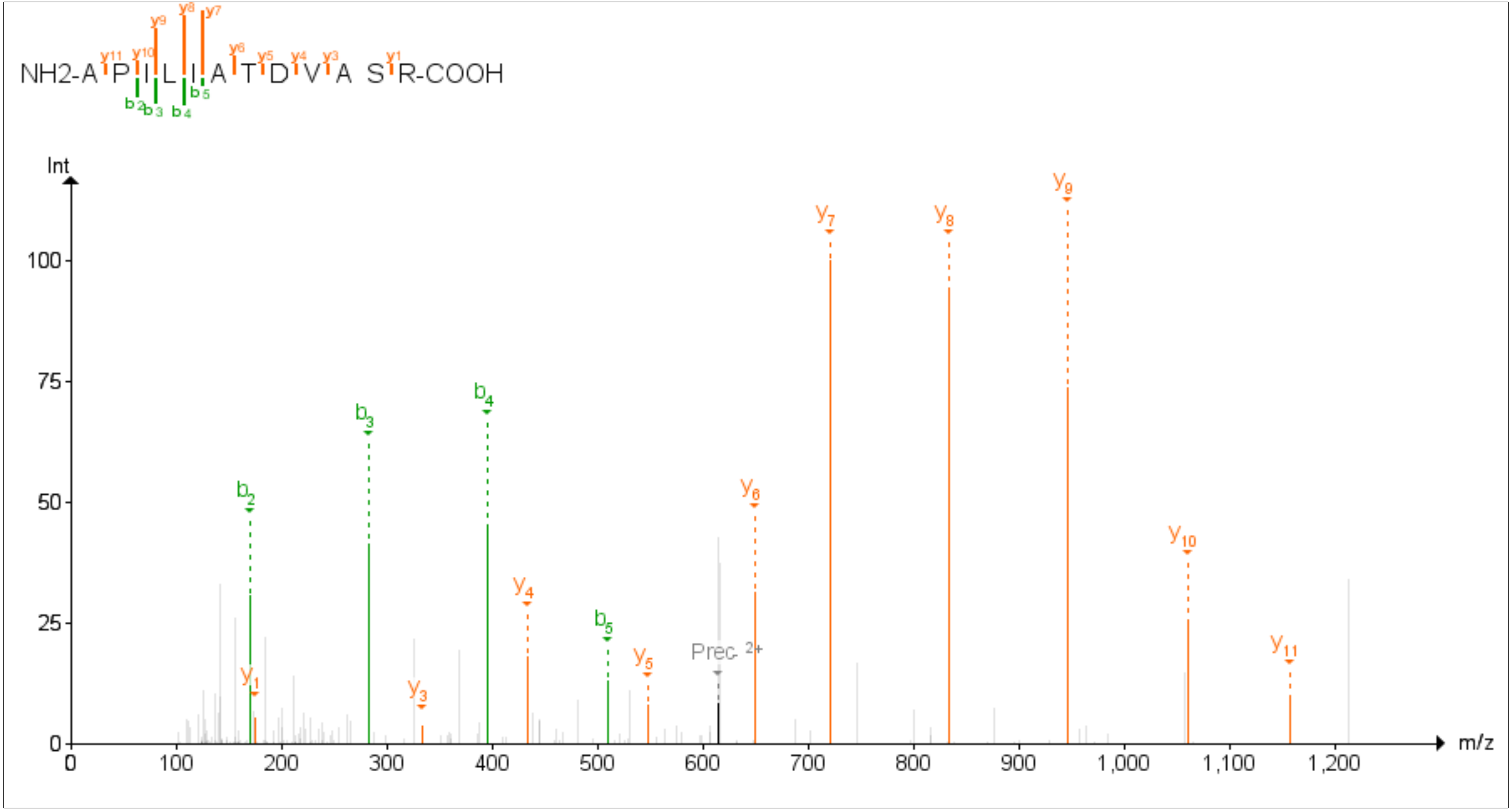

NH2-A P I L I A T D V A S R-COOH
y11 y10 y9 y8 y7 y6 y5 y4 y3 y1
b2 b3 b4 b5
Int
100
75
50
25
0
b2
b3
b4
b5
y1
y3
y4
y5
y6
y7
y8
y9
y10
y11
Prec 2+
0 100 200 300 400 500 600 700 800 900 1,000 1,100 1,200
m/z

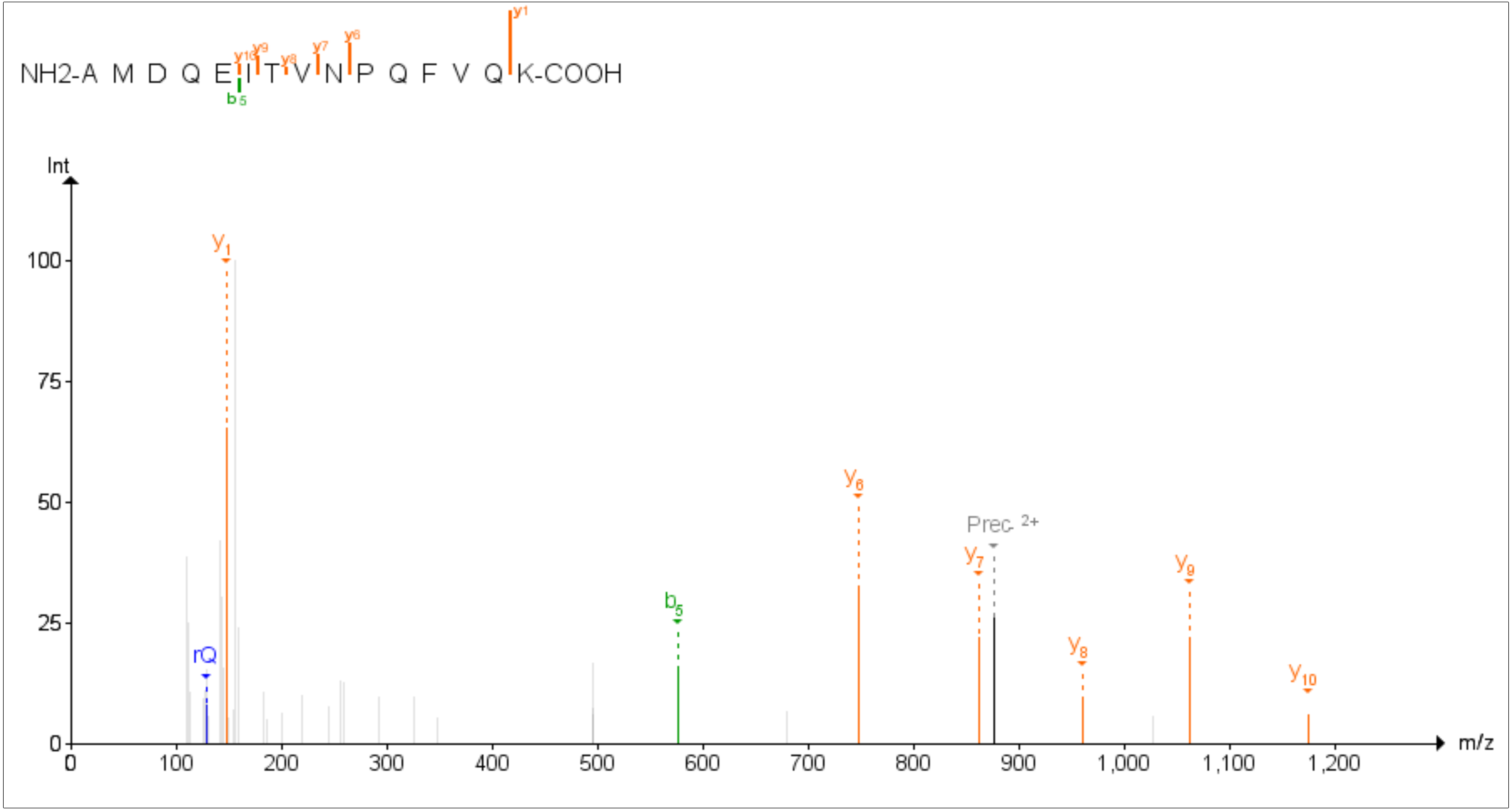

NH2-A M D Q E I I T V N P Q F V Q K-COOH
y1
y6
y7
y8
y9
y10
b5
Int
100
75
50
25
0
y1
y6
y7
y8
y9
y10
b5
rQ
Prec 2+
m/z
0
100
200
300
400
500
600
700
800
900
1,000
1,100
1,200

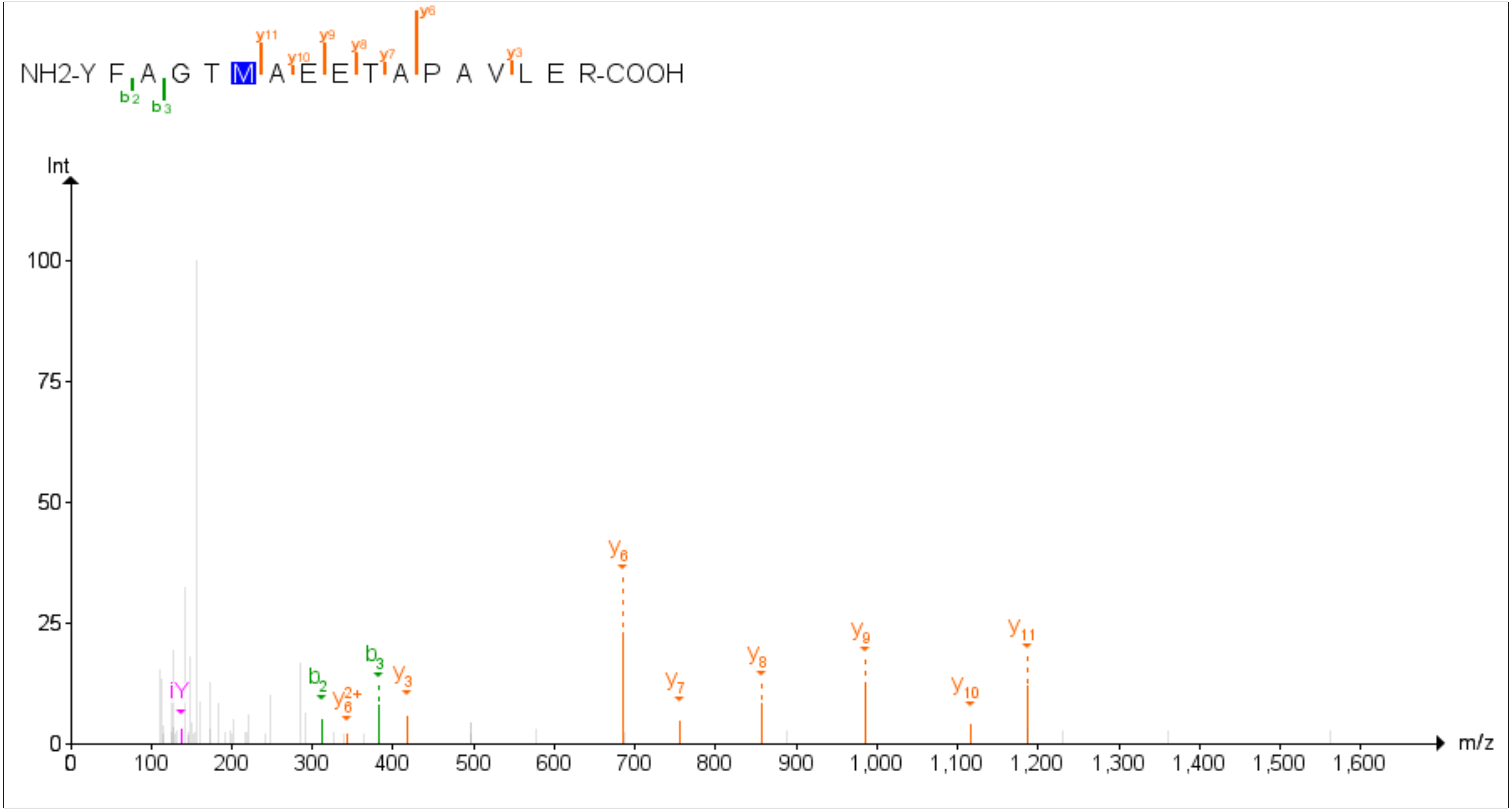

NH2-Y F A G T M A E E T A P A V L E R-COOH
b2 b3
y11 y10 y9 y8 y7 y6 y3
Int
100
75
50
25
0
m/z
0 100 200 300 400 500 600 700 800 900 1,000 1,100 1,200 1,300 1,400 1,500 1,600
iY
b2
b3
y6 2+
y3
y6
y7
y8
y9
y10
y11

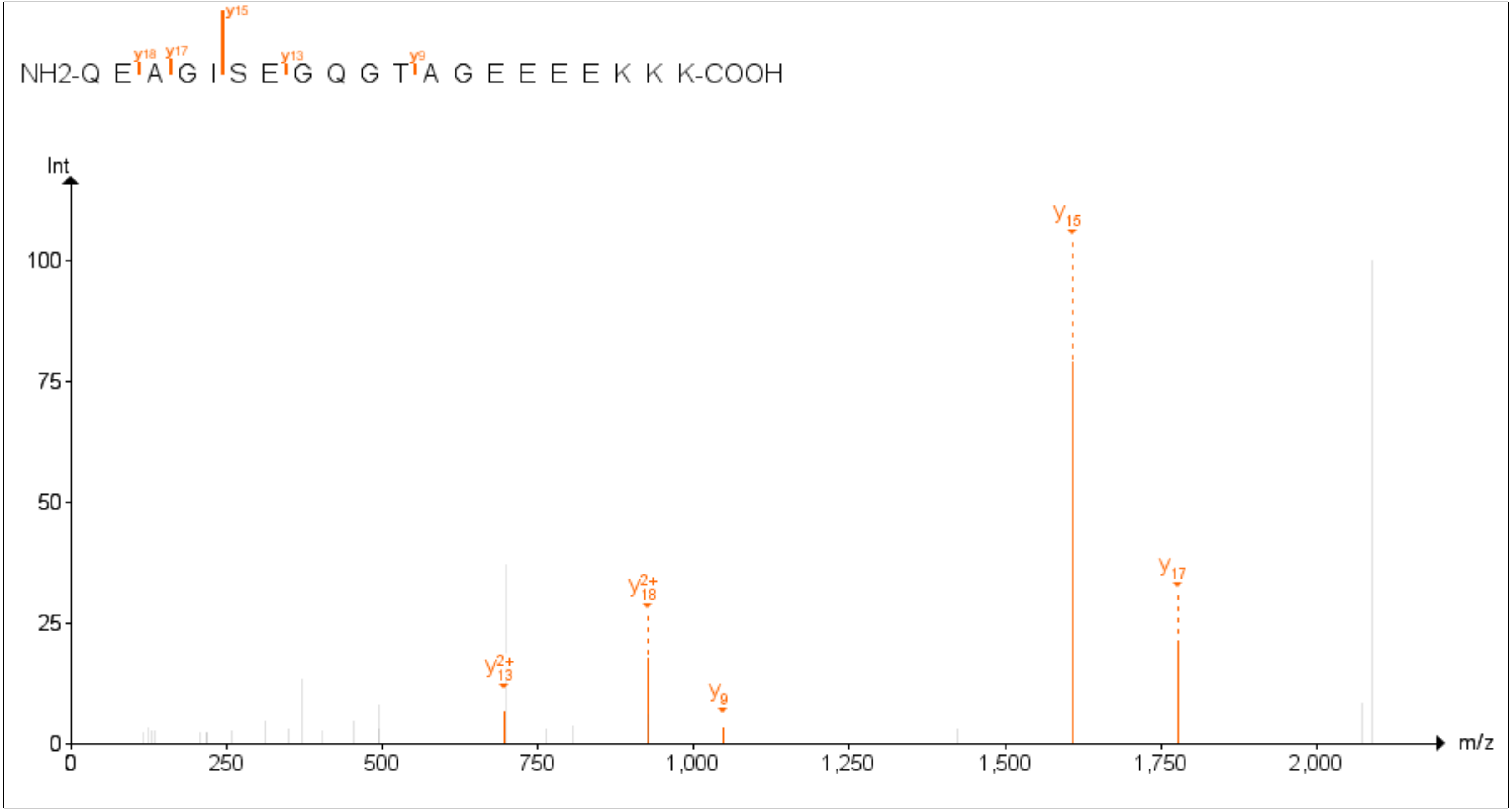

NH2-Q E A G I S E G Q G T A G E E E E K K K-COOH
y18 y17 y15 y13 y9
Int
100
75
50
25
0
0 250 500 750 1,000 1,250 1,500 1,750 2,000
m/z
y15
y17
y18 2+
y13 2+
y9

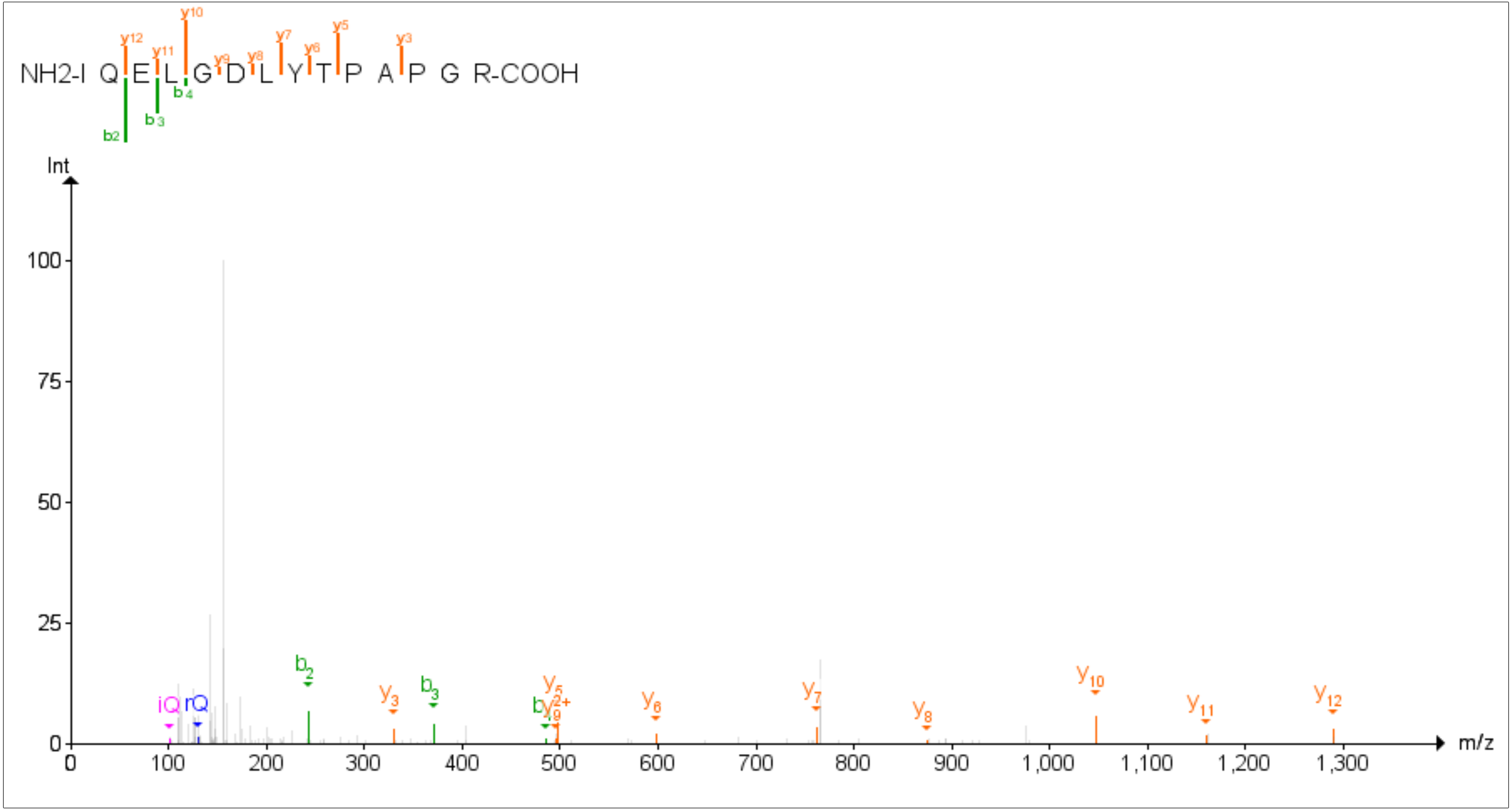

NH2-I Q E L G D L Y T P A P G R-COOH
y12
y11
y10
y9
y8
y7
y6
y5
y3
b2
b3
b4
Int
100
75
50
25
0
iQ
rQ
b2
y3
b3
y5
b9
y9 2+
y6
y7
y8
y10
y11
y12
m/z
0
100
200
300
400
500
600
700
800
900
1,000
1,100
1,200
1,300

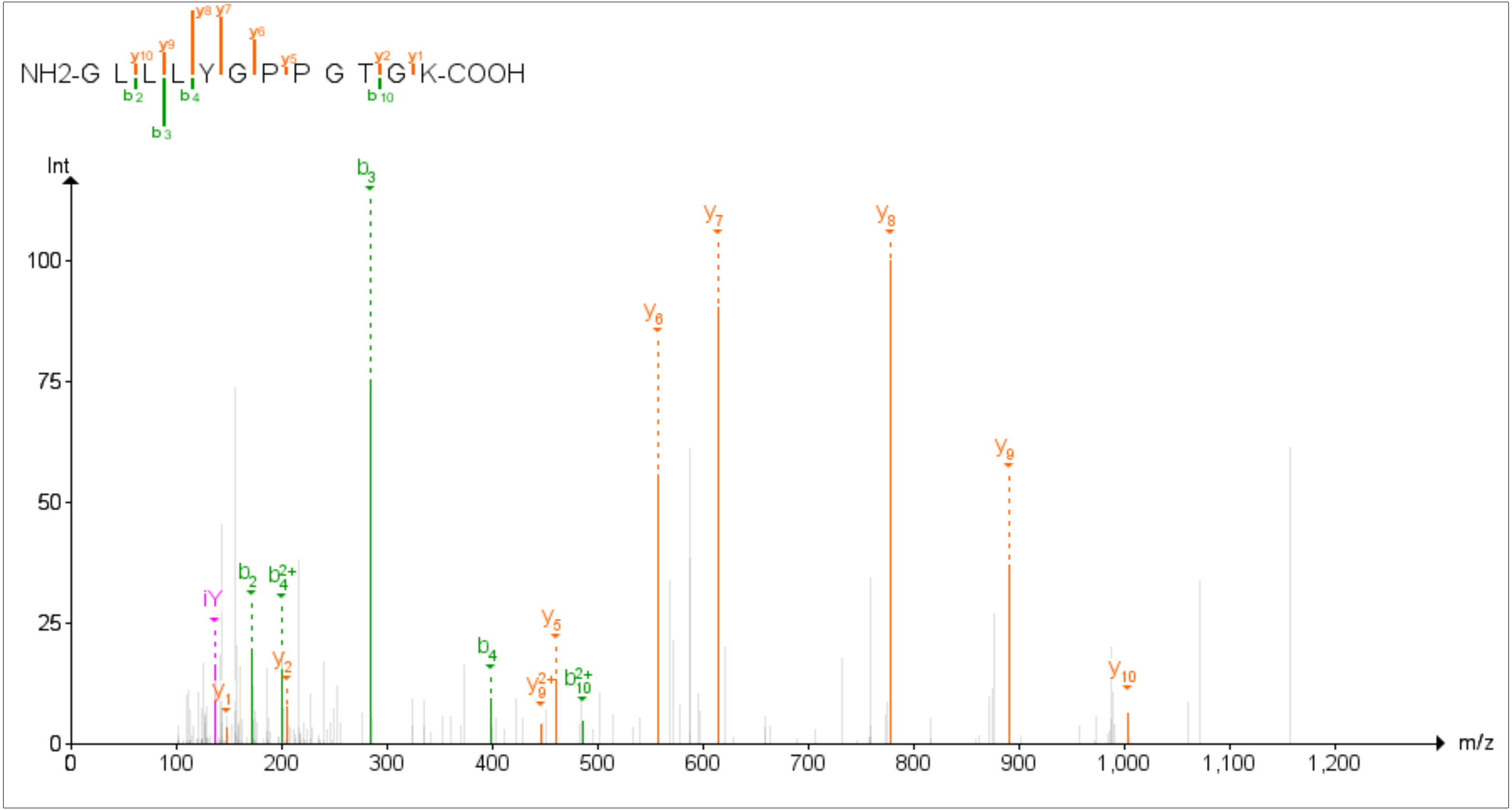

NH2-G L L L Y G P P G T G K-COOH
y10 y9 y8 y7 y6 y5 y2 y1
b2 b3 b4 b10
Int
m/z
b3
y7
y8
y6
y9
iY
b2
b_4^{2+}
y2
y1
b4
y5
y_9^{2+}
b_{10}^{2+}
y10
0
25
50
75
100
0
100
200
300
400
500
600
700
800
900
1,000
1,100
1,200

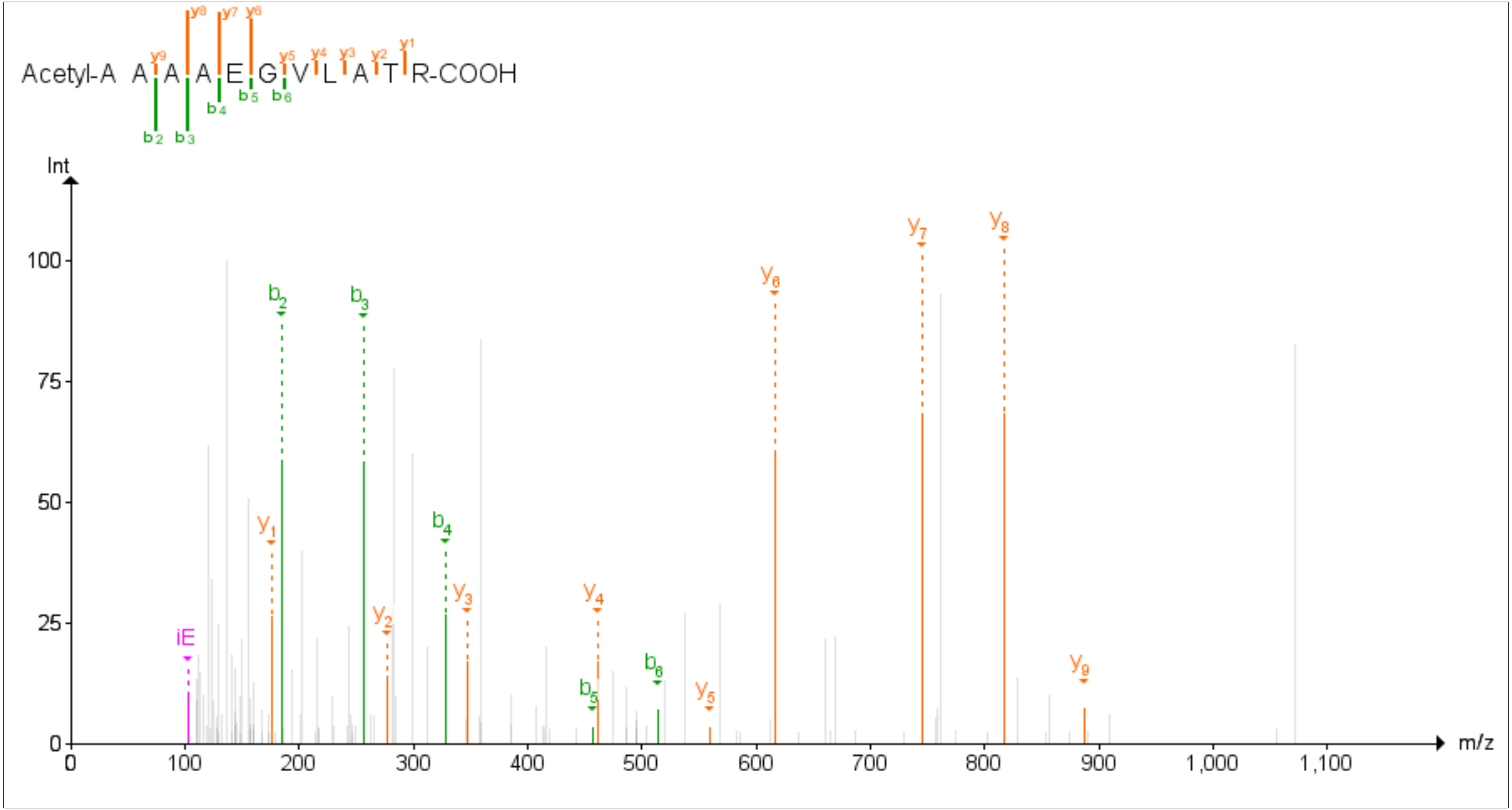

Acetyl-A A A A E G V L A T R-COOH
y9 y8 y7 y6 y5 y4 y3 y2 y1
b2 b3 b4 b5 b6
Int
100
75
50
25
0
iE
b_2
b_3
b_4
b_5
b_6
y_1
y_2
y_3
y_4
y_5
y_6
y_7
y_8
y_9
0
100
200
300
400
500
600
700
800
900
1,000
1,100
m/z

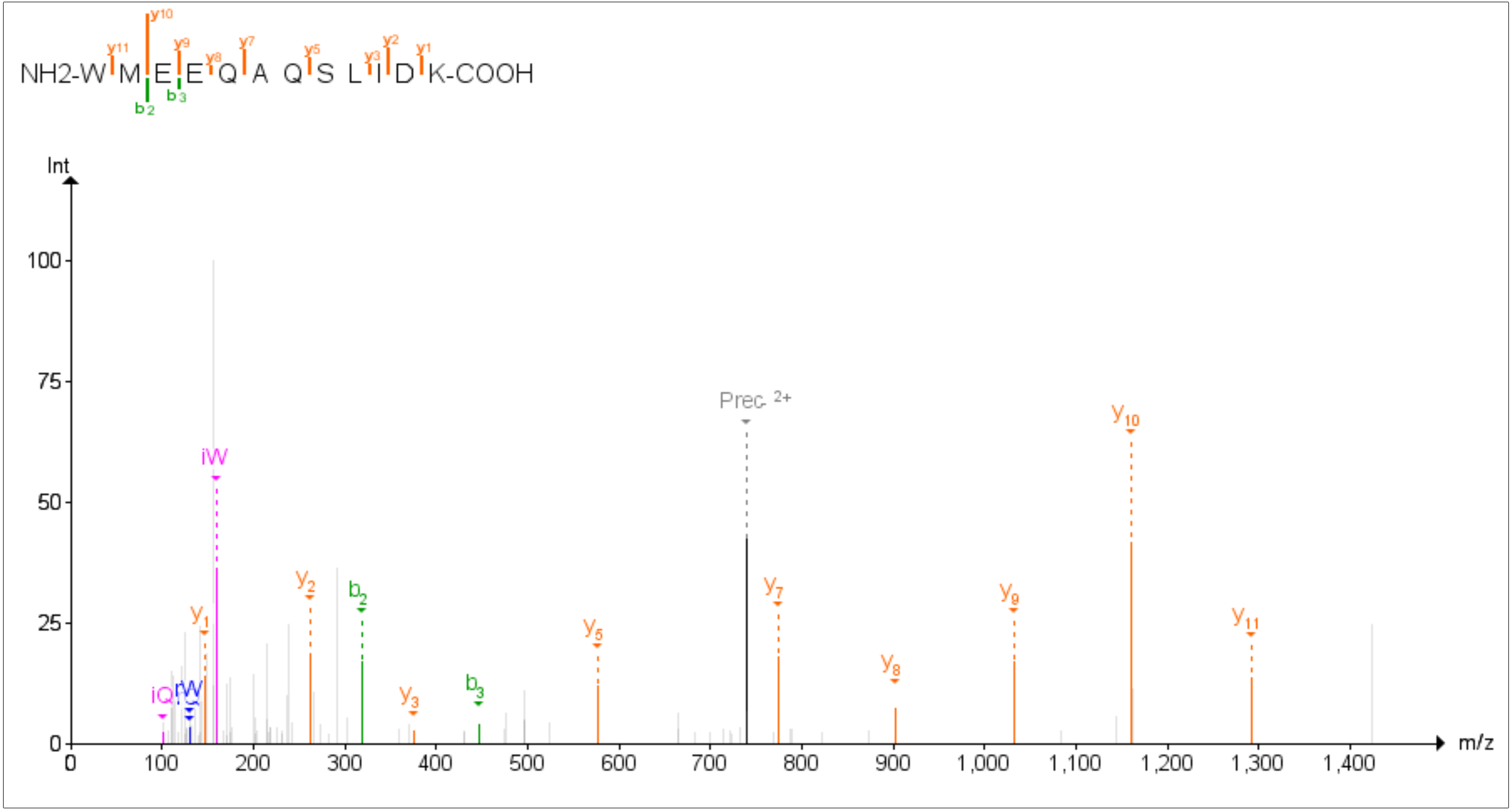

NH2-W M E E Q A Q S L I D K-COOH
y11 y10 y9 y8 y7 y5 y3 y2 y1
b2 b3
Int
100
75
50
25
0
iW
iQ
rW
y1
y2
b2
y3
b3
y5
Prec 2+
y7
y8
y9
y10
y11
0 100 200 300 400 500 600 700 800 900 1,000 1,100 1,200 1,300 1,400
m/z

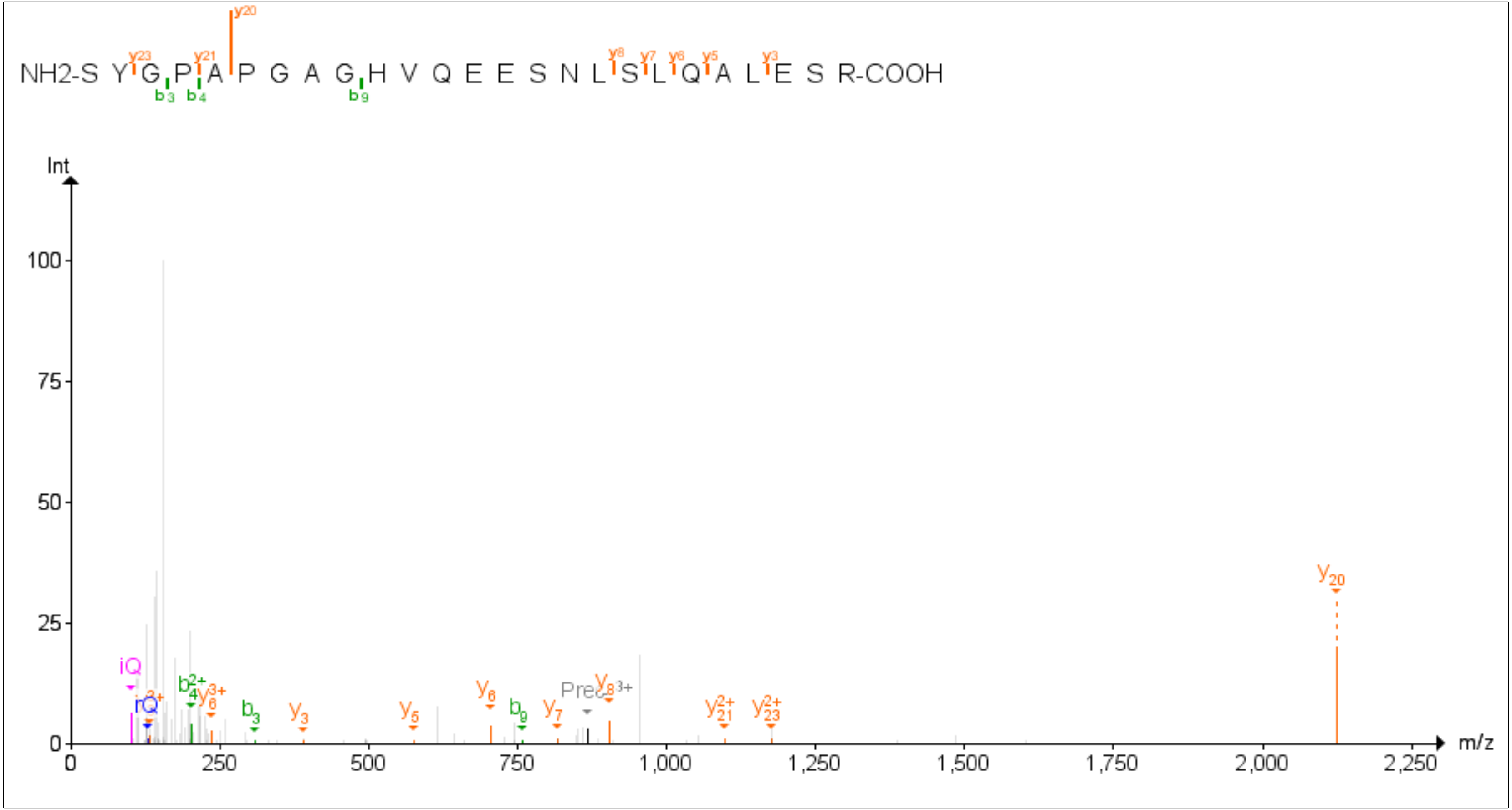

NH2-S Y G P A P G A G H V Q E E S N L S L Q A L E S R-COOH
y23
y21
y20
y8
y7
y6
y5
y3
b3
b4
b9
Int
100
75
50
25
0
iQ
rQ
b4 2+
y6 3+
b3
y3
y5
y6
b9
y7
Prec 3+
y8
y21 2+
y23 2+
y20
m/z
0
250
500
750
1,000
1,250
1,500
1,750
2,000
2,250

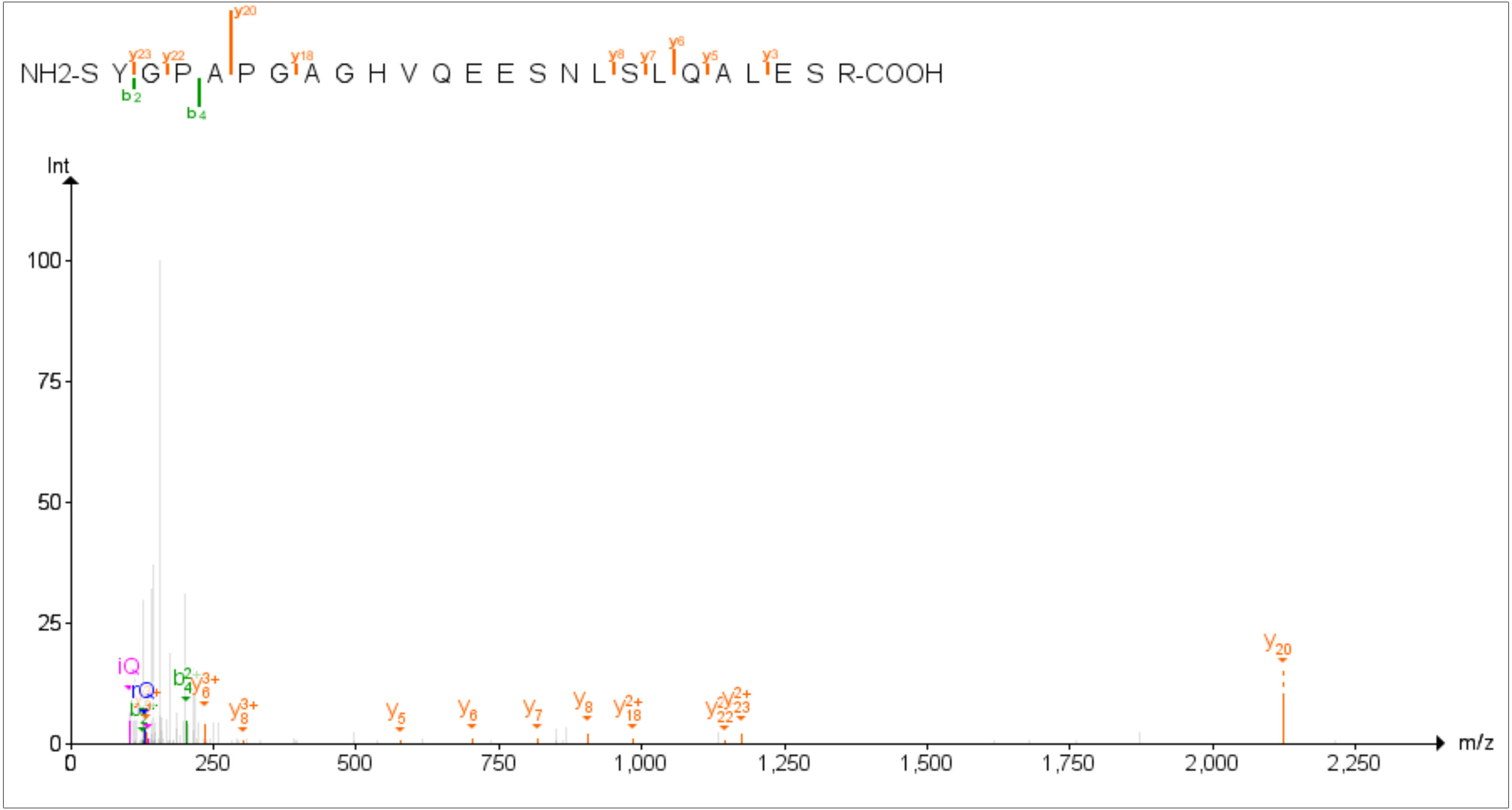

NH2-S Y G P A P G A G H V Q E E S N L S L Q A L E S R-COOH
y23
y22
y20
y18
y8
y7
y6
y5
y3
b2
b4
Int
100
75
50
25
0
m/z
0
250
500
750
1,000
1,250
1,500
1,750
2,000
2,250
iQ
rQ
y20
y5
y6
y7
y8

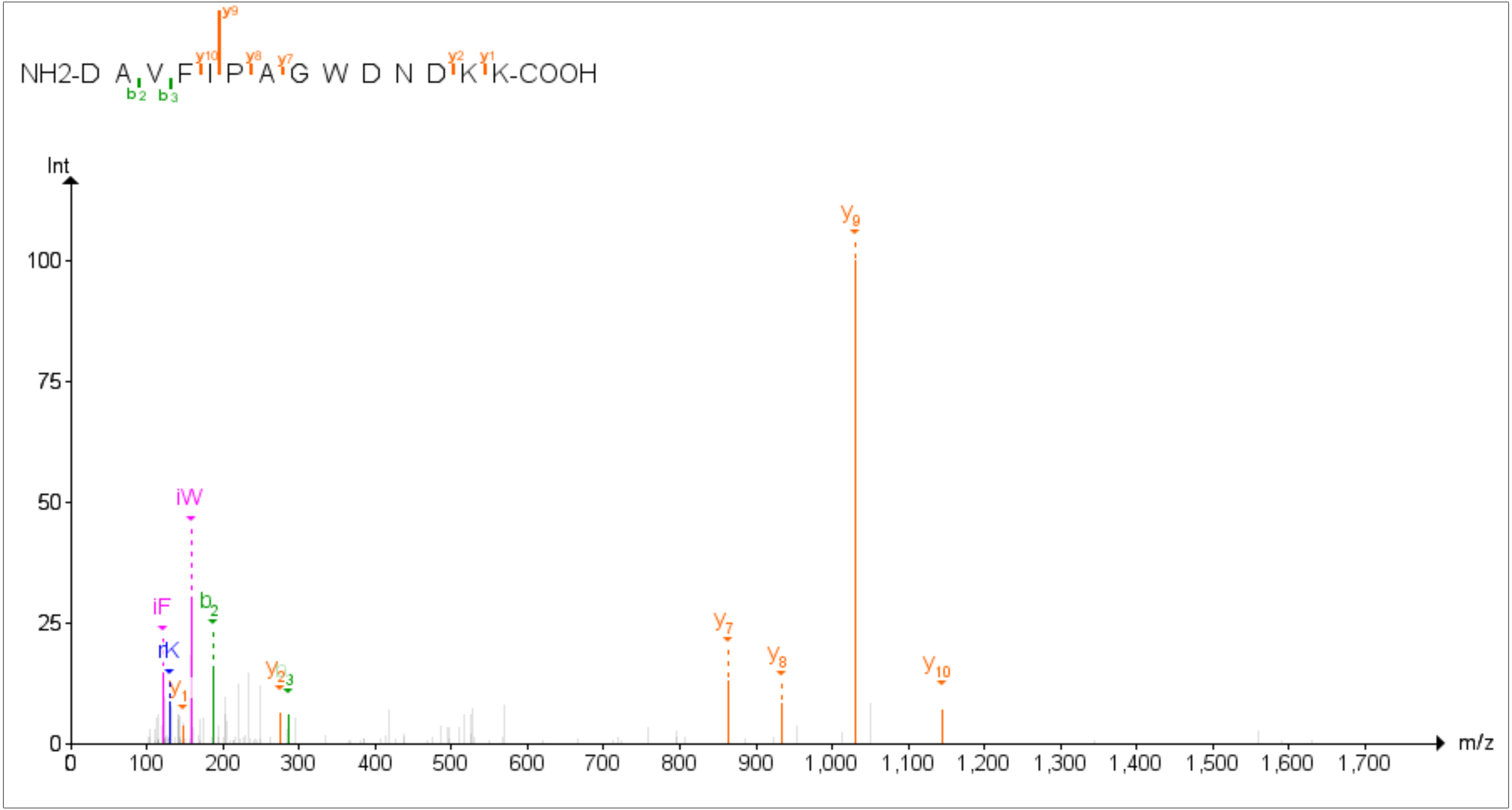

NH2-D A V F I P A G W D N D K K-COOH
b2
b3
y10
y9
y8
y7
y2
y1
Int
100
75
50
25
0
iW
iF
iK
b2
y1
y2
b3
y7
y8
y9
y10
m/z
0
100
200
300
400
500
600
700
800
900
1,000
1,100
1,200
1,300
1,400
1,500
1,600
1,700

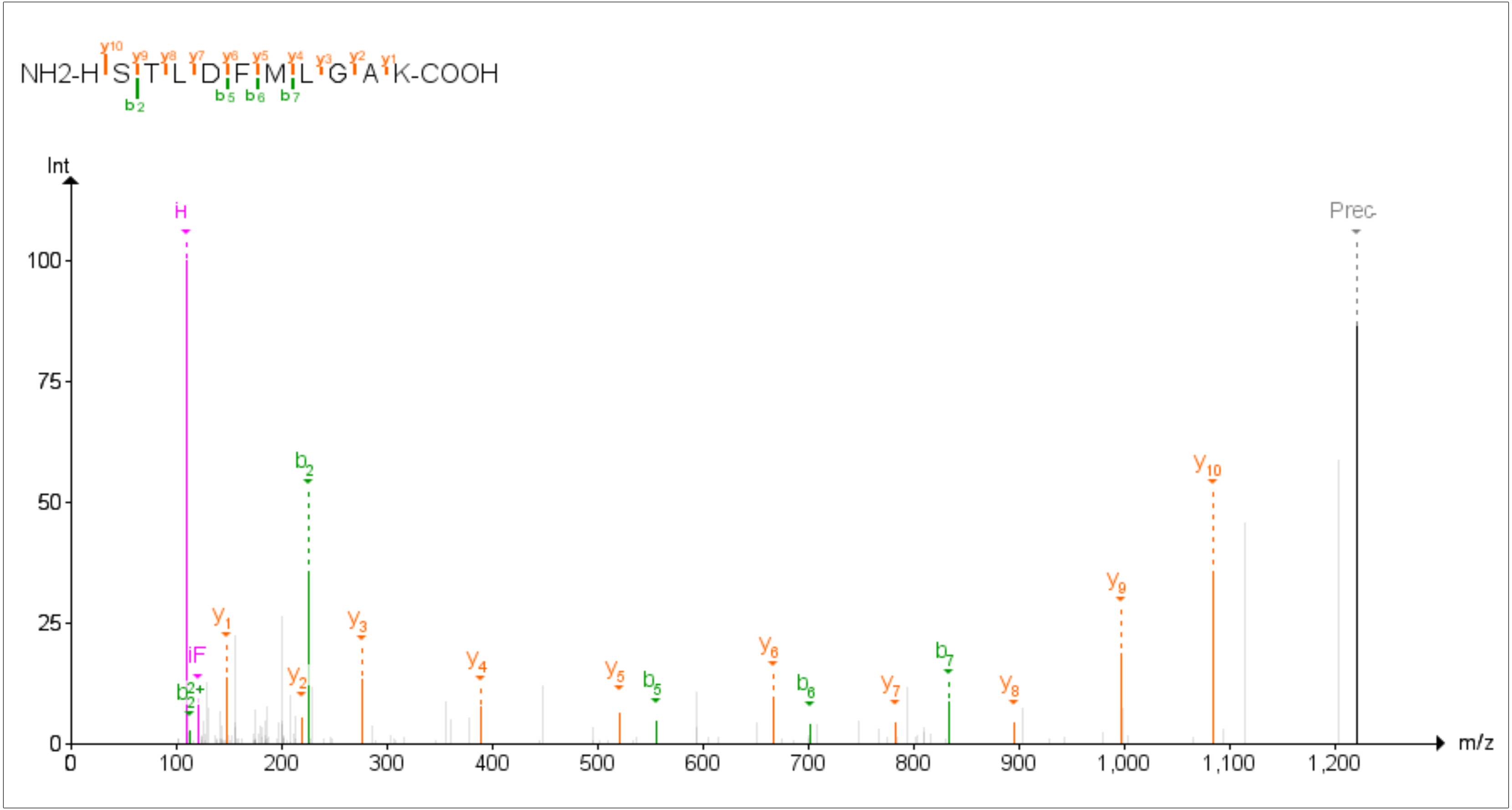

NH2-H S T L D F M L G A K-COOH
y10 y9 y8 y7 y6 y5 y4 y3 y2 y1
b2 b5 b6 b7
Int
m/z
0
25
50
75
100
0 100 200 300 400 500 600 700 800 900 1,000 1,100 1,200
iH
iF
b2 2+
y1
y2
b2
y3
y4
y5
b5
y6
b6
y7
b7
y8
y9
y10
Prec

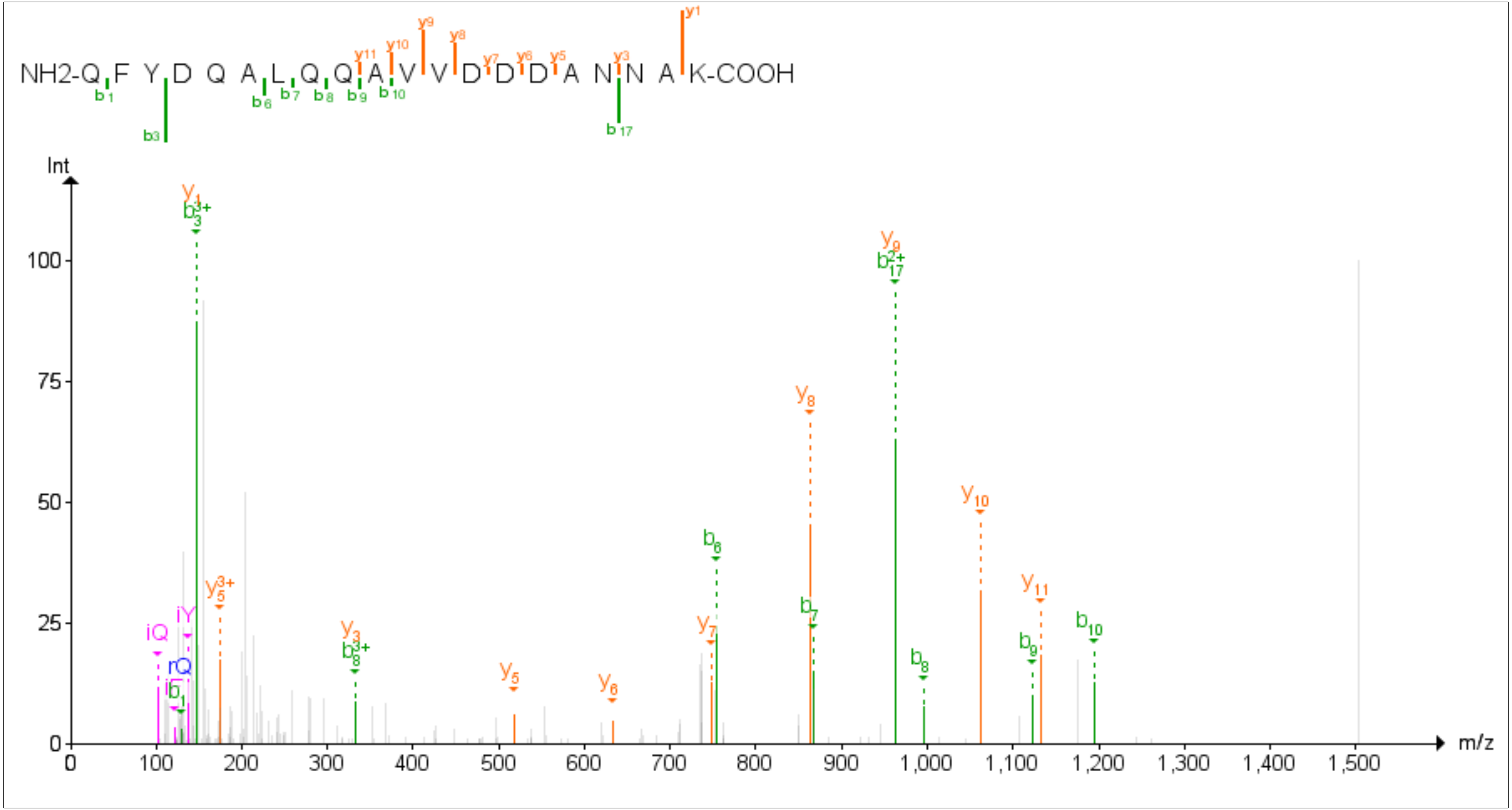

NH2-Q F Y D Q A L Q Q A V V D D D A N N A K-COOH
b1 b3 b6 b7 b8 b9 b10 b17
y11 y10 y9 y8 y7 y6 y5 y3 y1
Int
m/z
0 25 50 75 100
0 100 200 300 400 500 600 700 800 900 1,000 1,100 1,200 1,300 1,400 1,500

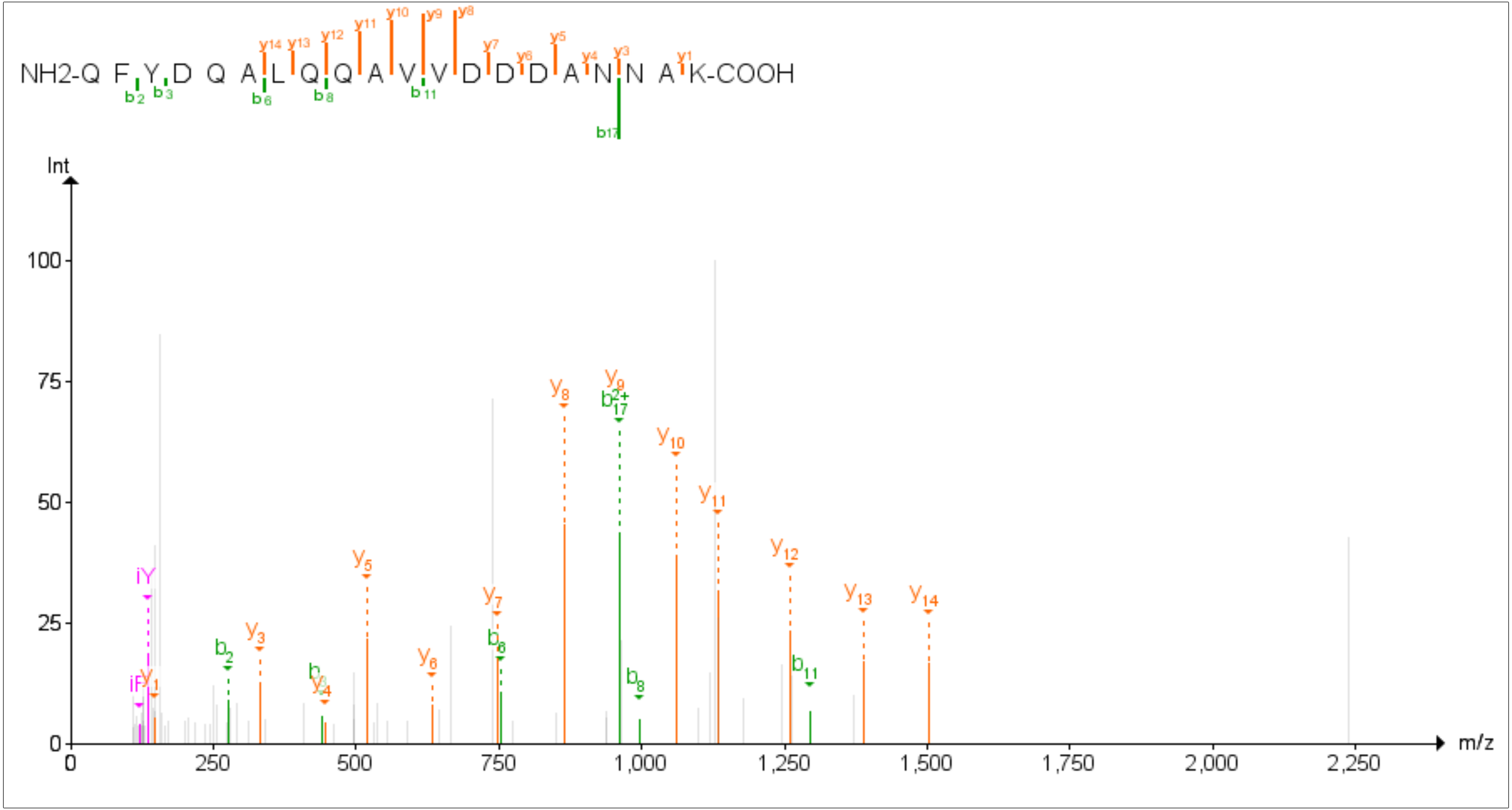

NH2-Q F Y D Q A L Q Q A V V D D D A N N A K-COOH
Int
100
75
50
25
0
0
250
500
750
1,000
1,250
1,500
1,750
2,000
2,250
m/z

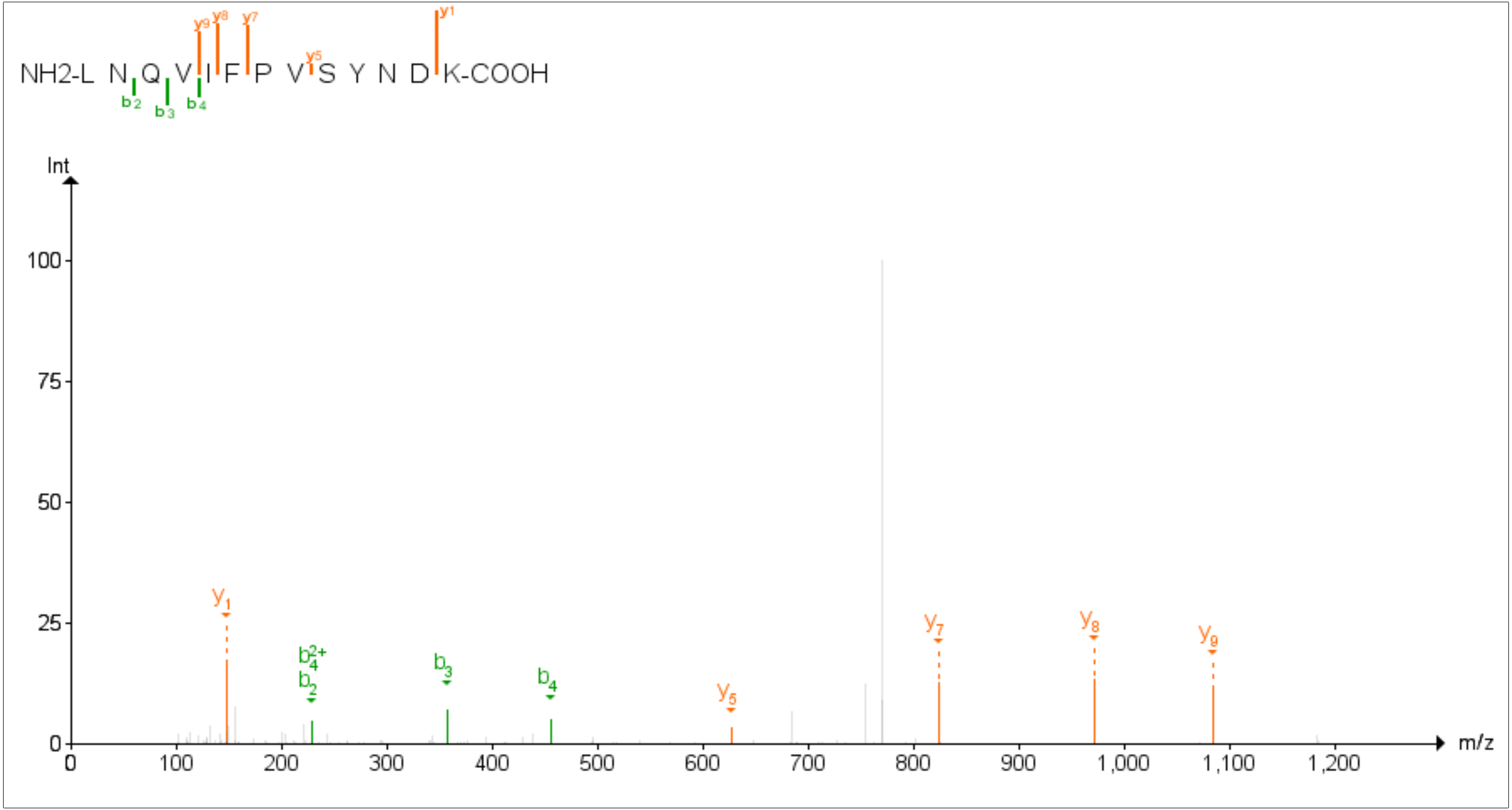
NH2-L N Q V I F P V S Y N D K-COOH
y9 y8 y7 y5 y1
b2 b3 b4
Int
100
75
50
25
0
y1
b4 2+
b2
b3
b4
y5
y7
y8
y9
0 100 200 300 400 500 600 700 800 900 1,000 1,100 1,200
m/z

NH2-I E E L Q L I V N D K-COOH

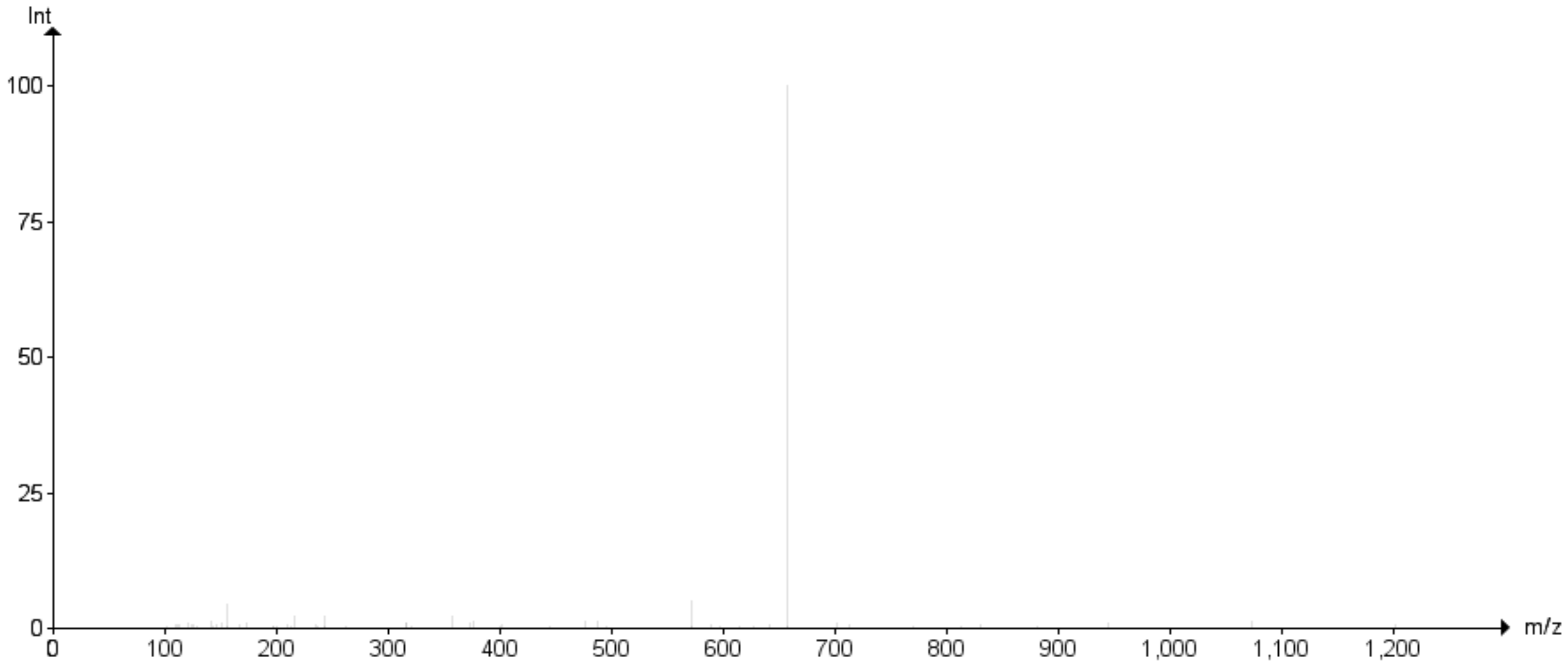

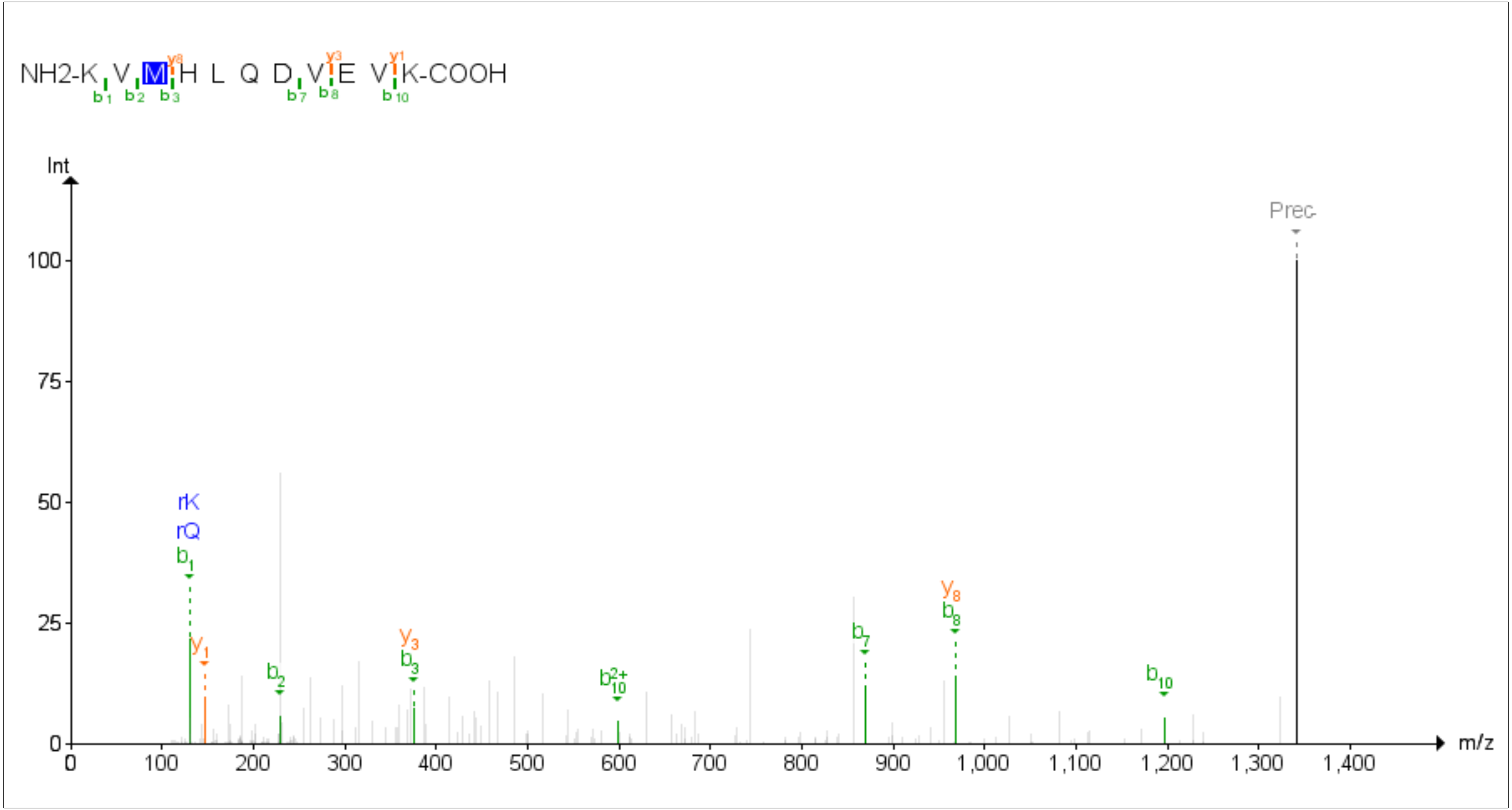

NH2-K V M H L Q D V E V K-COOH
b1 b2 b3 b7 b8 b10
y8 y3 y1
Int
100
75
50
25
0
Prec.
rK
rQ
b1
y1
b2
y3
b3
b10 2+
b7
y8
b8
b10
0 100 200 300 400 500 600 700 800 900 1,000 1,100 1,200 1,300 1,400
m/z

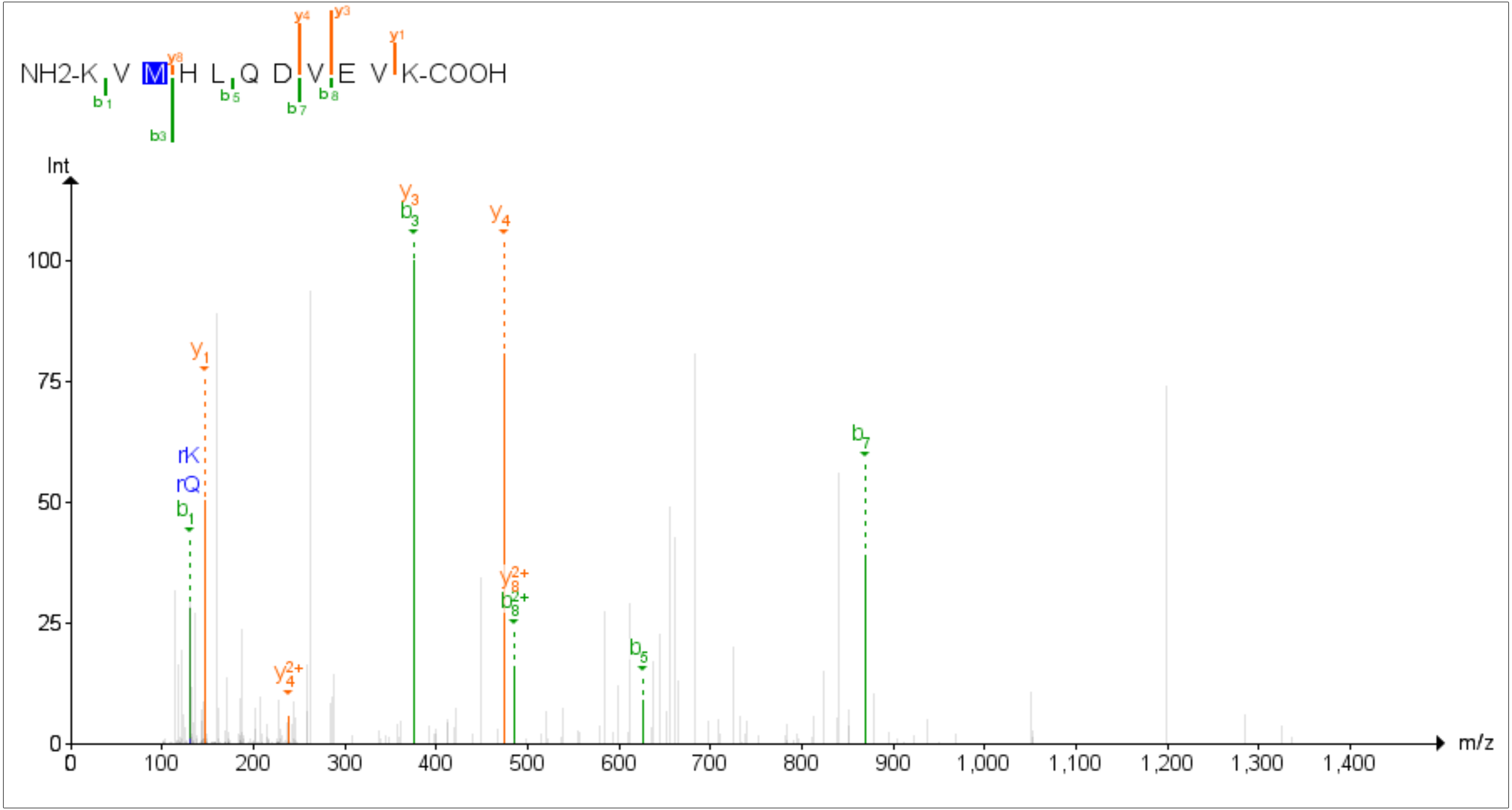
NH2-K V M H L Q D V E V K-COOH
b1
b3
b5
b7
b8
y8
y4
y3
y1
Int
100
75
50
25
0
m/z
0
100
200
300
400
500
600
700
800
900
1,000
1,100
1,200
1,300
1,400
y3
b3
y4
y1
rK
rQ
b1
b7
y8 2+
b8 2+
b5
y4 2+

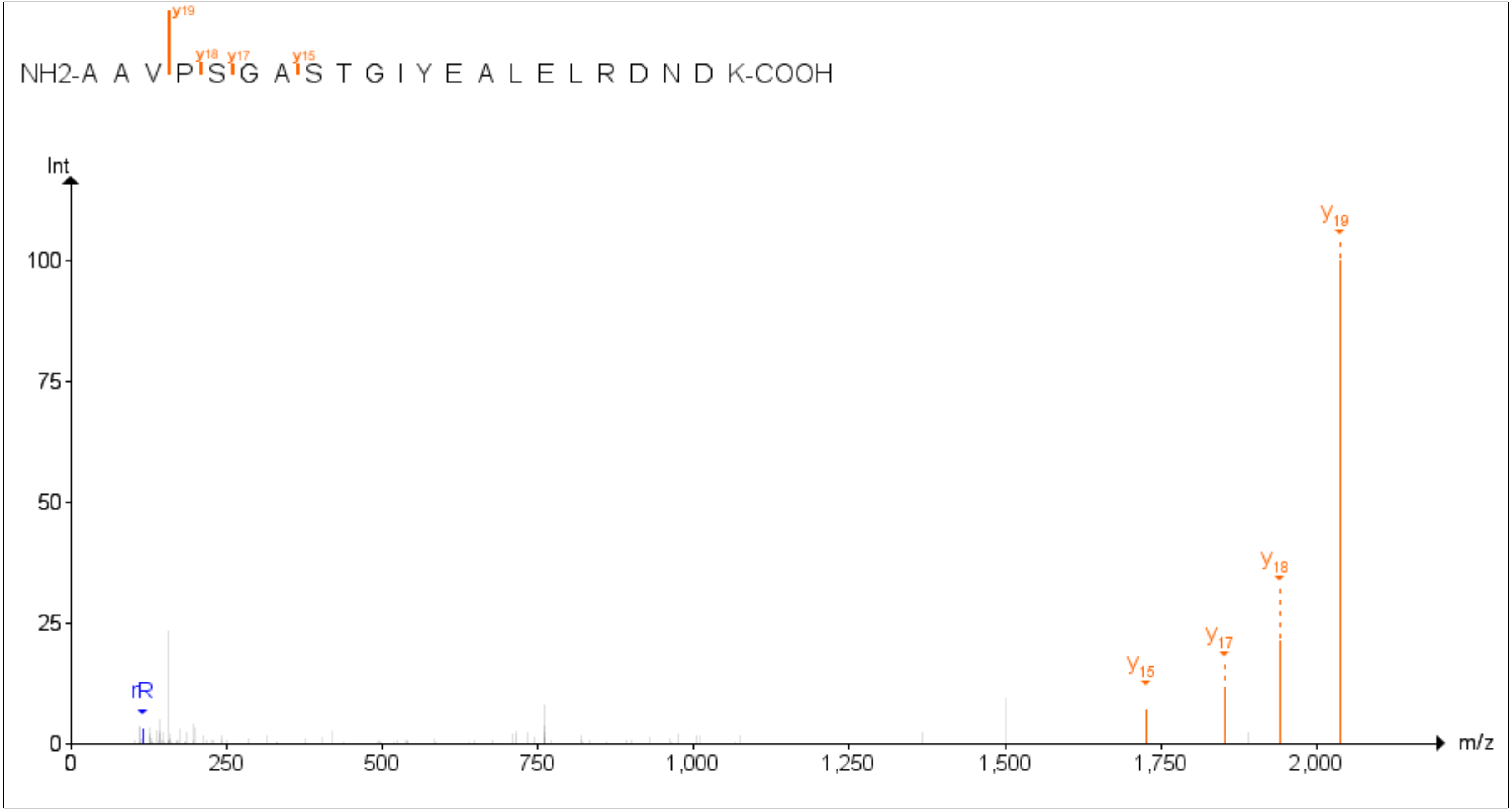

NH2-A A V P S G A S T G I Y E A L E L R D N D K-COOH
y19
y18
y17
y15
Int
100
75
50
25
0
rR
m/z
0
250
500
750
1,000
1,250
1,500
1,750
2,000

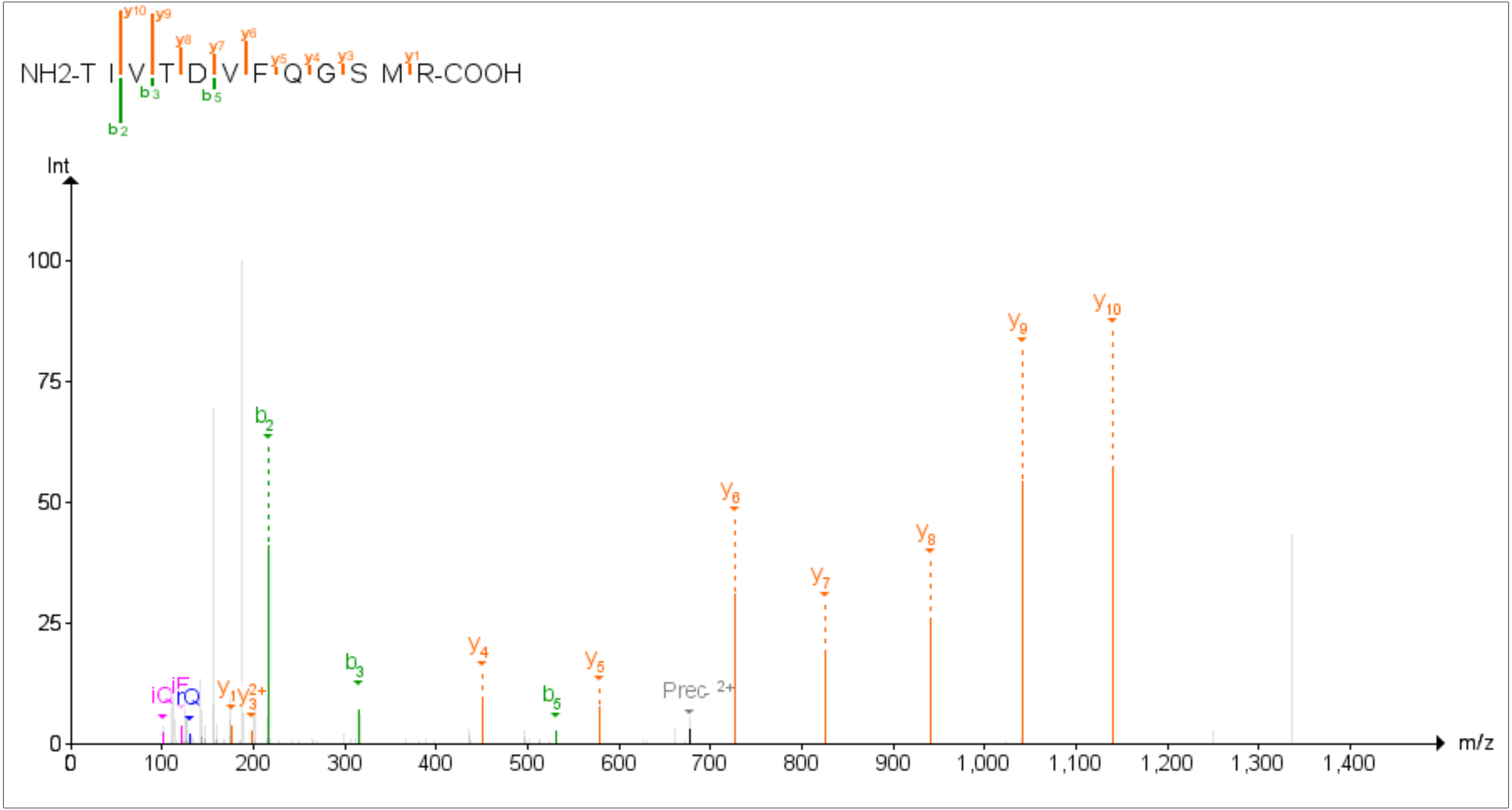

NH2-T I V T D V F Q G S M R-COOH
y10 y9 y8 y7 y6 y5 y4 y3 y1
b2 b3 b5
Int
100
75
50
25
0
b2
b3
b5
y1
y3 2+
y4
y5
y6
y7
y8
y9
y10
iQ
iF
rQ
Prec 2+
0 100 200 300 400 500 600 700 800 900 1,000 1,100 1,200 1,300 1,400
m/z

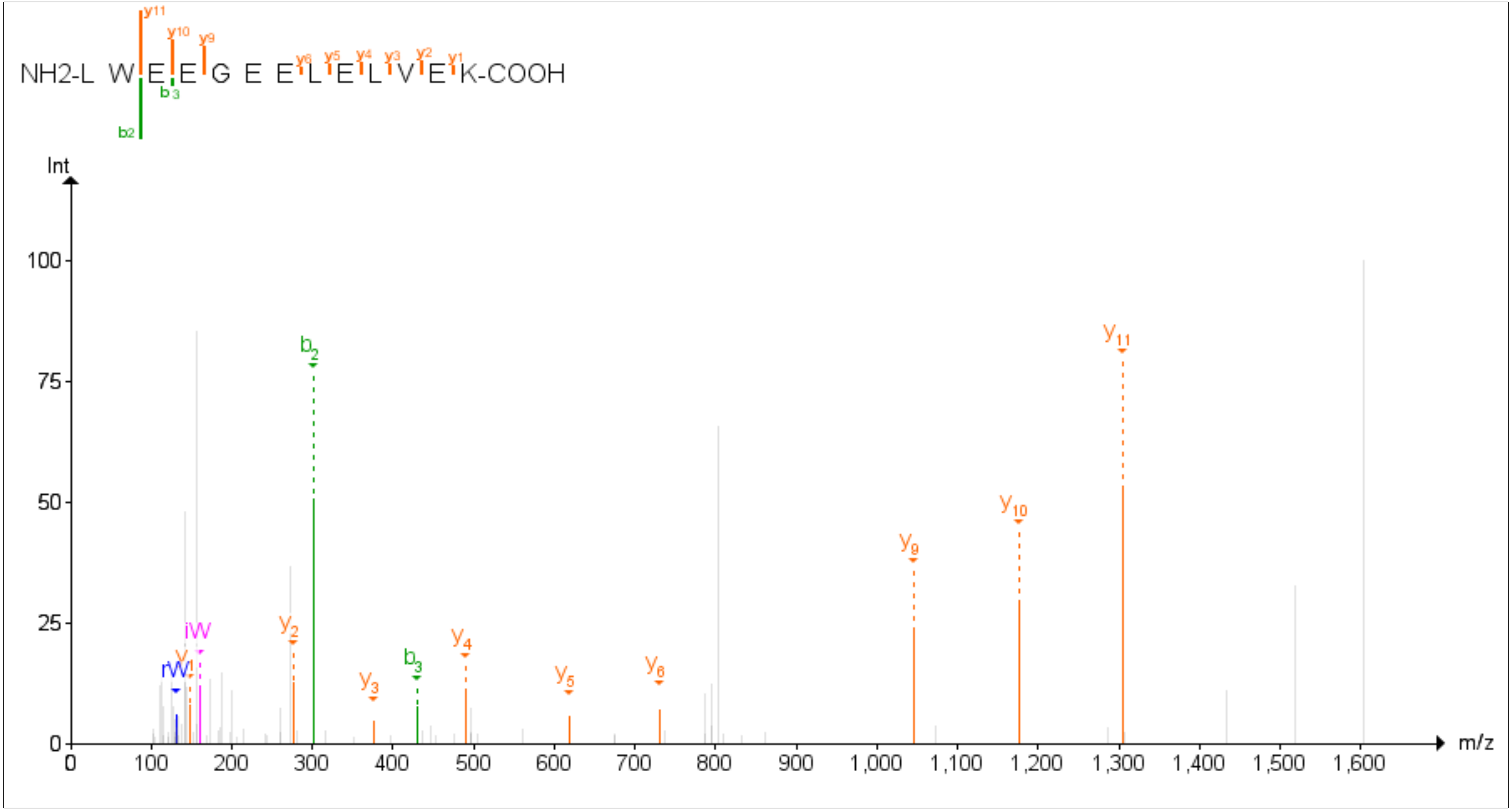

NH2-L W E E G E E L E L V E K-COOH
y11
y10
y9
y6
y5
y4
y3
y2
y1
b2
b3
Int
100
75
50
25
0
m/z
0
100
200
300
400
500
600
700
800
900
1,000
1,100
1,200
1,300
1,400
1,500
1,600
rW
iW
y1
y2
b2
y3
b3
y4
y5
y6
y9
y10
y11

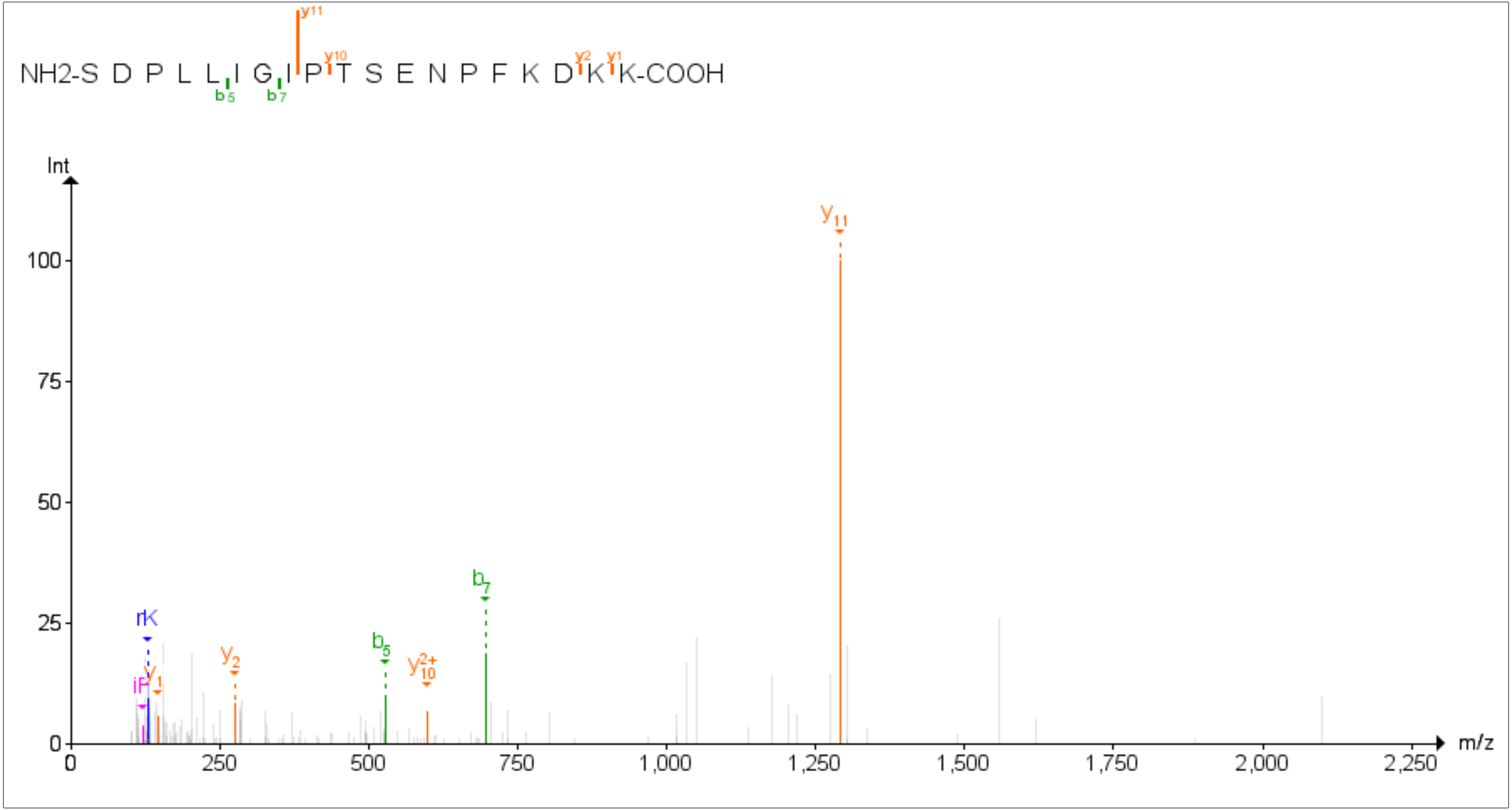

NH2-S D P L L I G I P T S E N P F K D K K-COOH
b5
b7
y11
y10
y2
y1
Int
100
75
50
25
0
rK
iF
y1
y2
b5
y10 2+
b7
y11
0
250
500
750
1,000
1,250
1,500
1,750
2,000
2,250
m/z

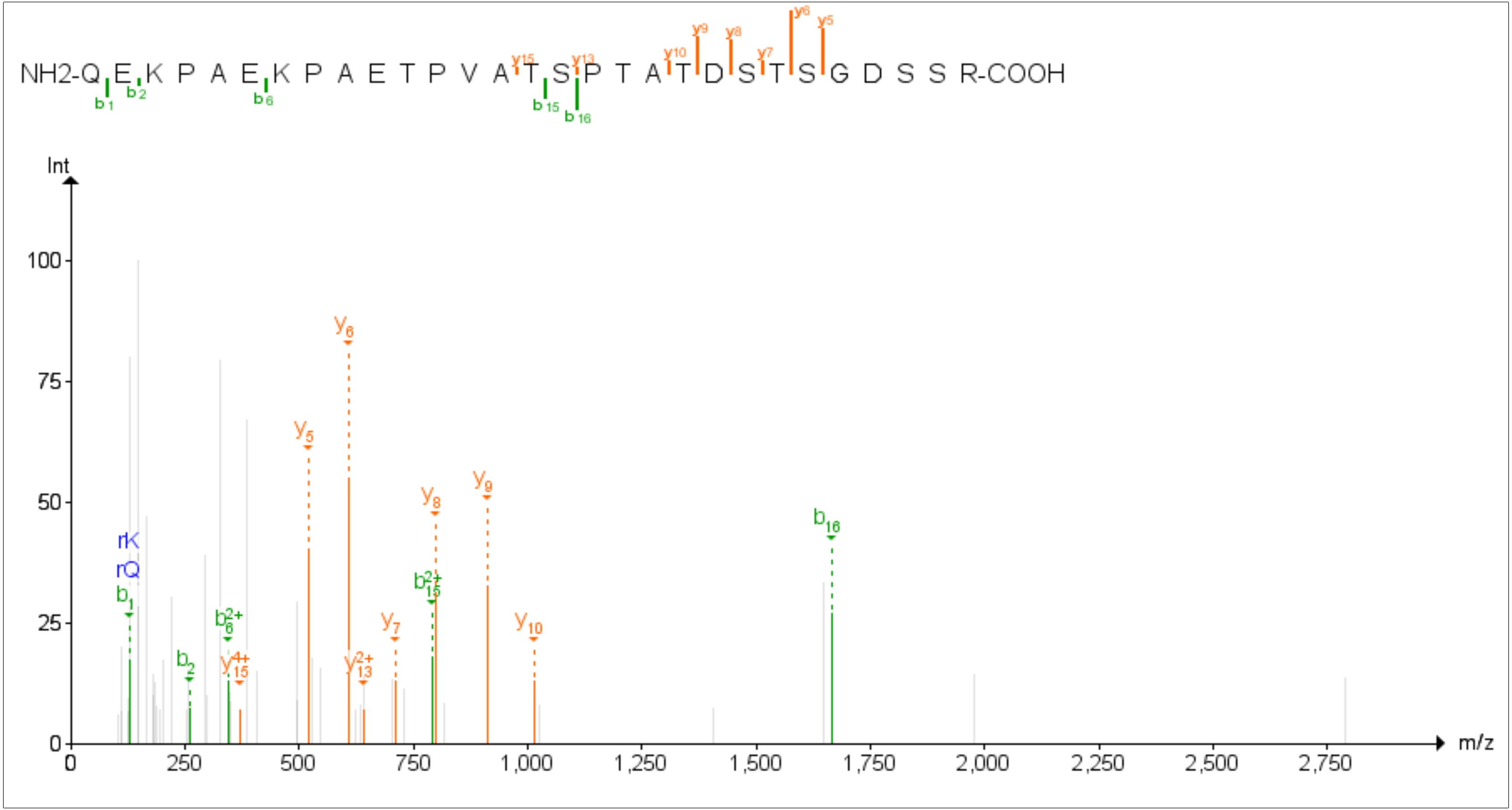

NH2-Q E K P A E K P A E T P V A T S P T A T D S T S G D S S R-COOH
b1 b2 b6 b15 b16
y15 y13 y10 y9 y8 y7 y6 y5
Int
100
75
50
25
0
rK
rQ
b1
b2
b6 2+
y15 4+
y5
y6
y13 2+
y7
b15 2+
y8
y9
y10
b16
0
250
500
750
1,000
1,250
1,500
1,750
2,000
2,250
2,500
2,750
m/z

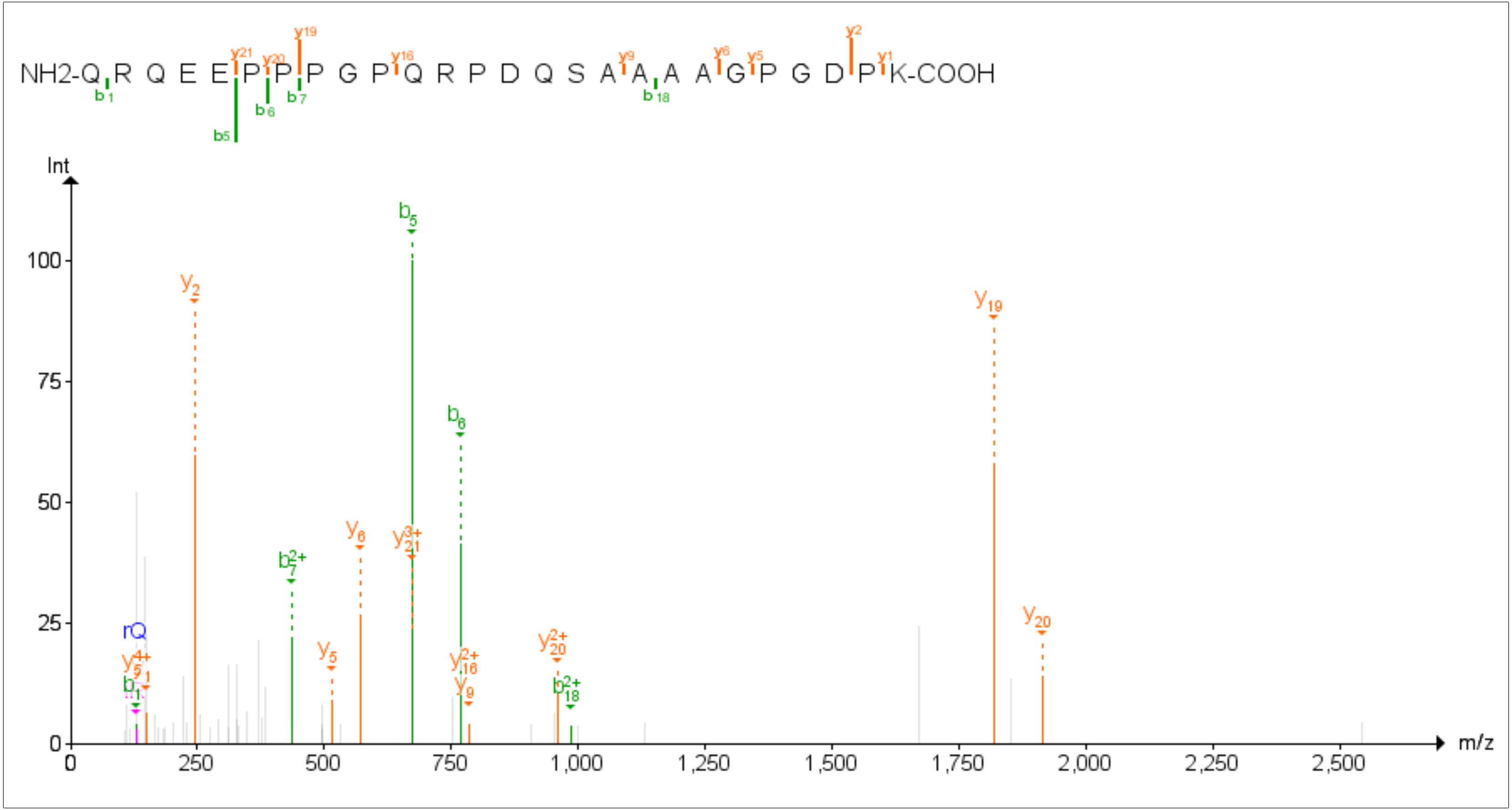
NH2-Q R Q E E P P P G P Q R P D Q S A A A A G P G D P K-COOH
Int
100
75
50
25
0
m/z
0
250
500
750
1,000
1,250
1,500
1,750
2,000
2,250
2,500
rQ

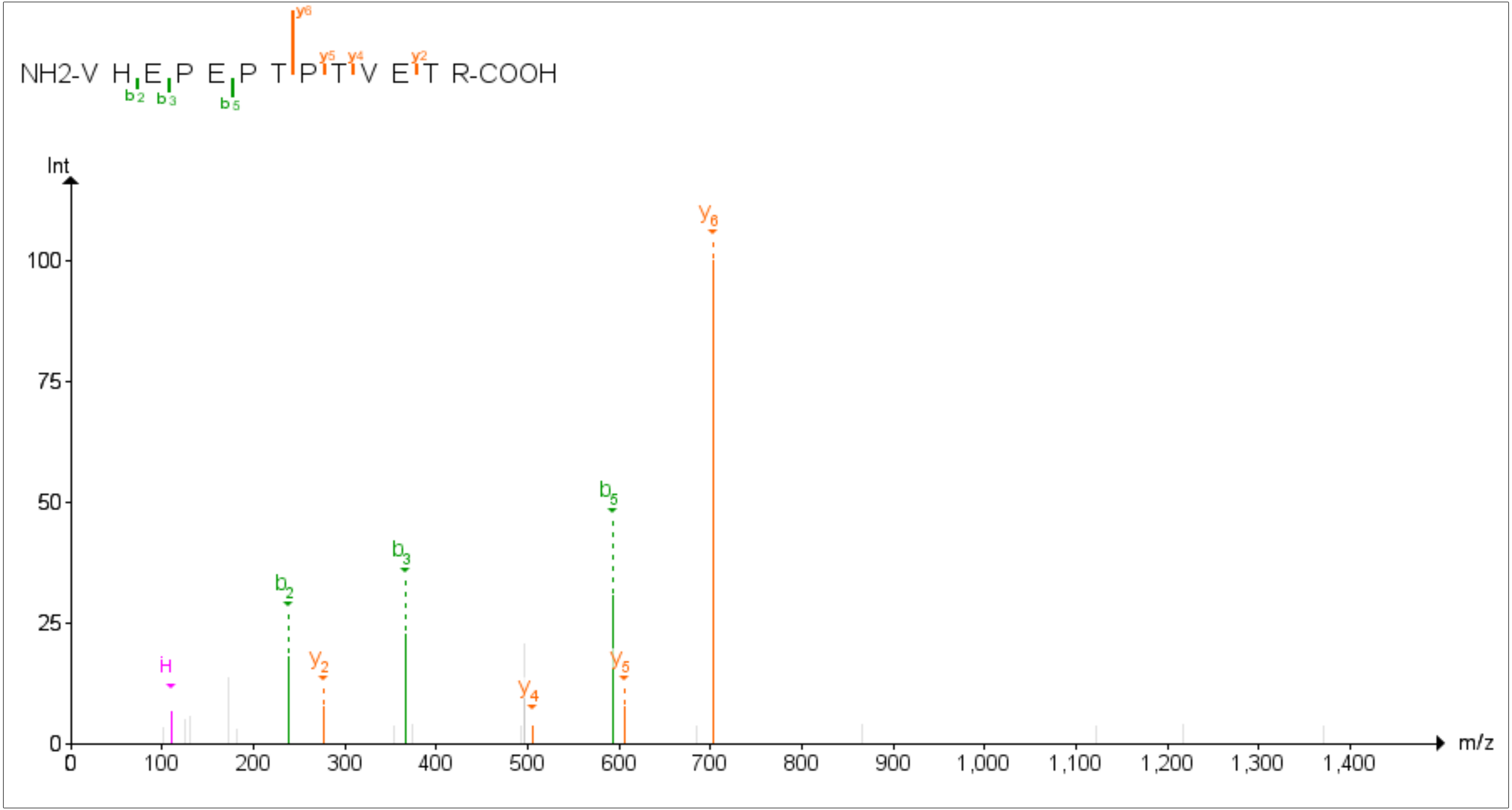

NH2-V H E P E P T P T V E T R-COOH
y6 y5 y4 y2
b2 b3 b5
Int
m/z
0 25 50 75 100
0 100 200 300 400 500 600 700 800 900 1,000 1,100 1,200 1,300 1,400
H
b2 y2 b3 y4 b5 y5 y6

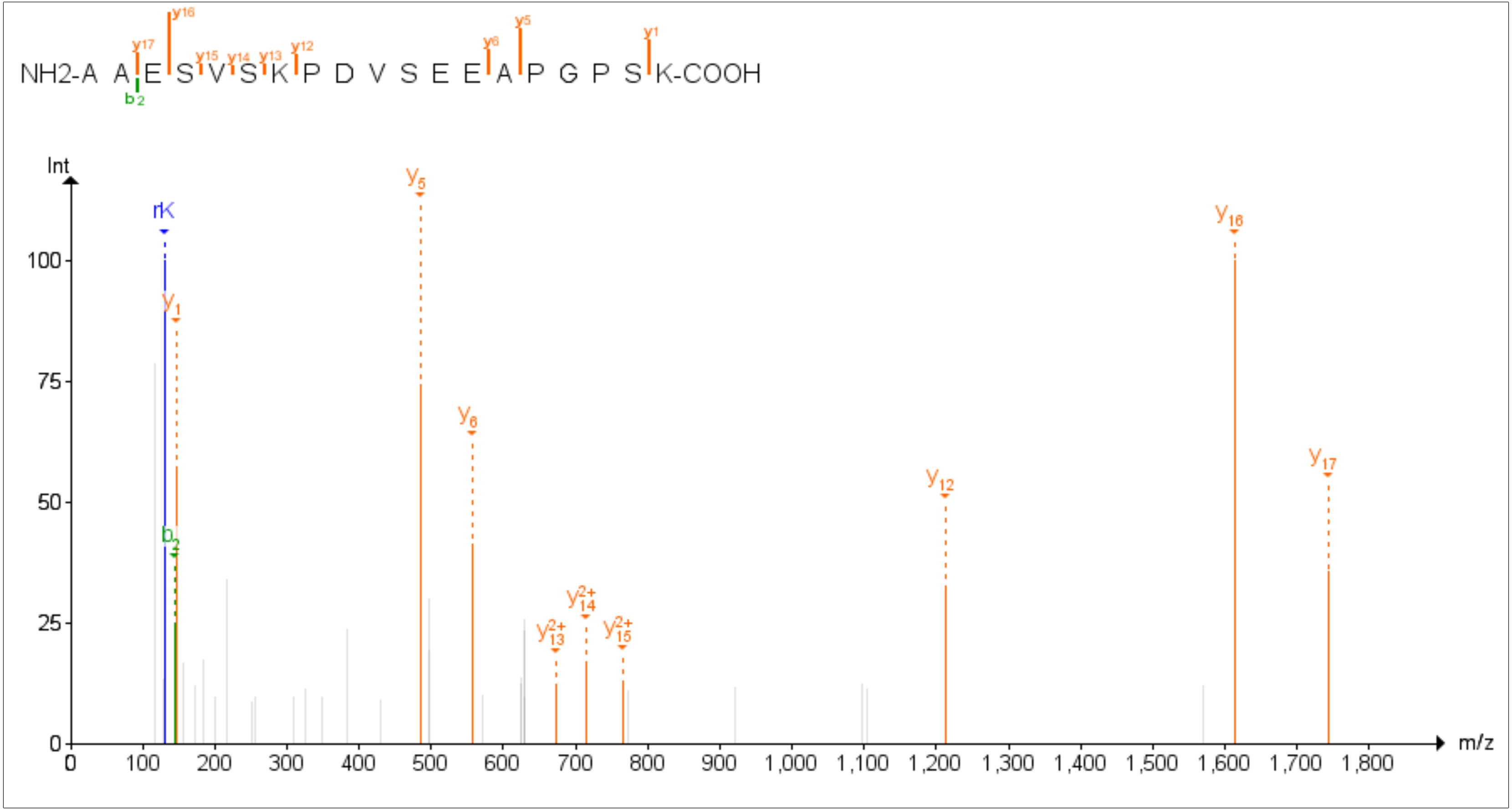

NH2-A A E S V S K P D V S E E A P G P S K-COOH
y17 y16 y15 y14 y13 y12 y6 y5 y1
b2
Int
m/z
rK
y5
y16
y1
y6
y12
y17
b2
y2+ 14
y2+ 13
y2+ 15
0
25
50
75
100
0
100
200
300
400
500
600
700
800
900
1,000
1,100
1,200
1,300
1,400
1,500
1,600
1,700
1,800

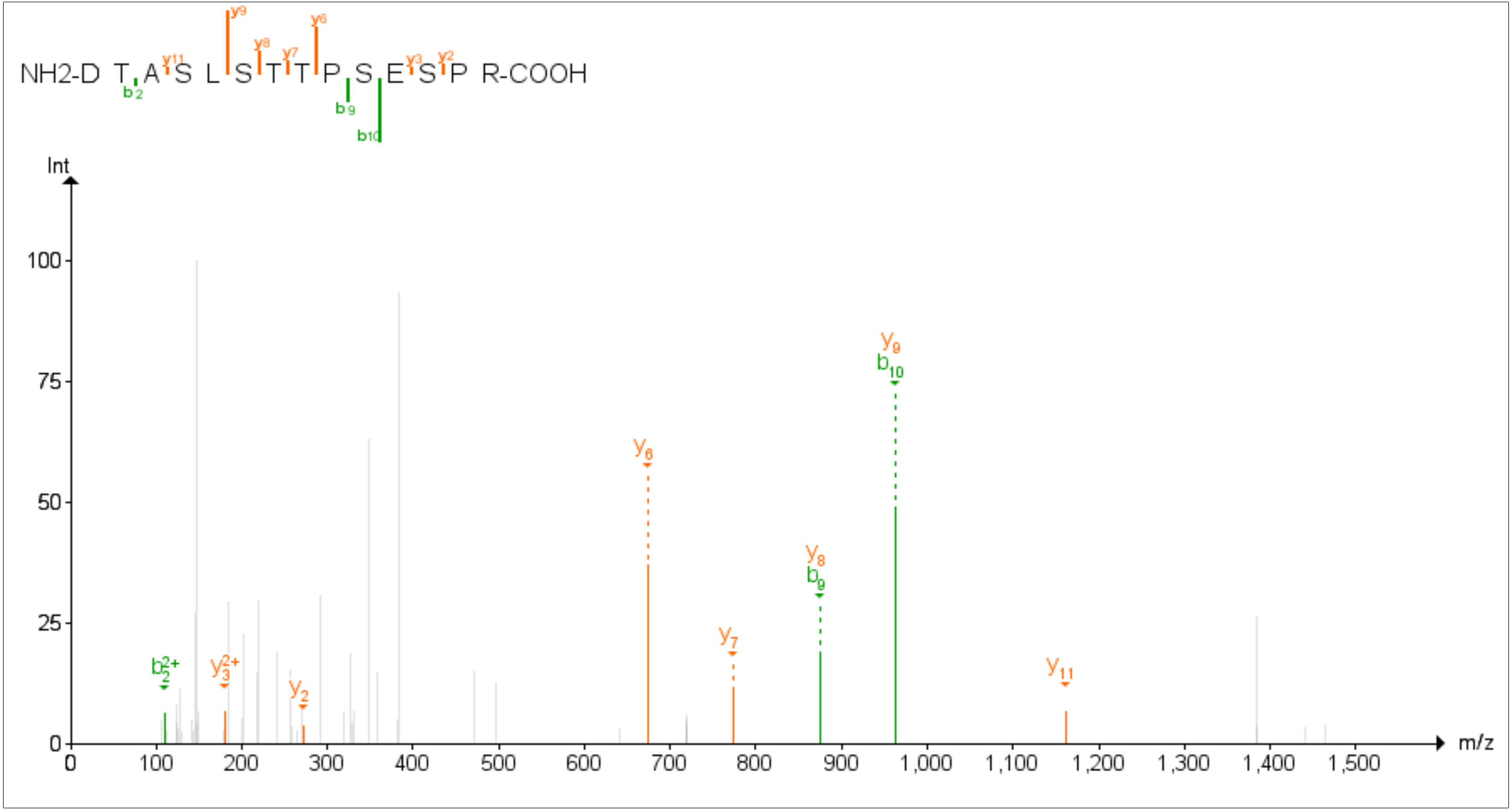

NH2-D T A S L S T T P S E S P R-COOH
b2
y11
y9
y8
y7
y6
y3
y2
b9
b10
Int
100
75
50
25
0
y9
b10
y6
y8
b9
y7
y11
b2 2+
y3 2+
y2
0
100
200
300
400
500
600
700
800
900
1,000
1,100
1,200
1,300
1,400
1,500
m/z

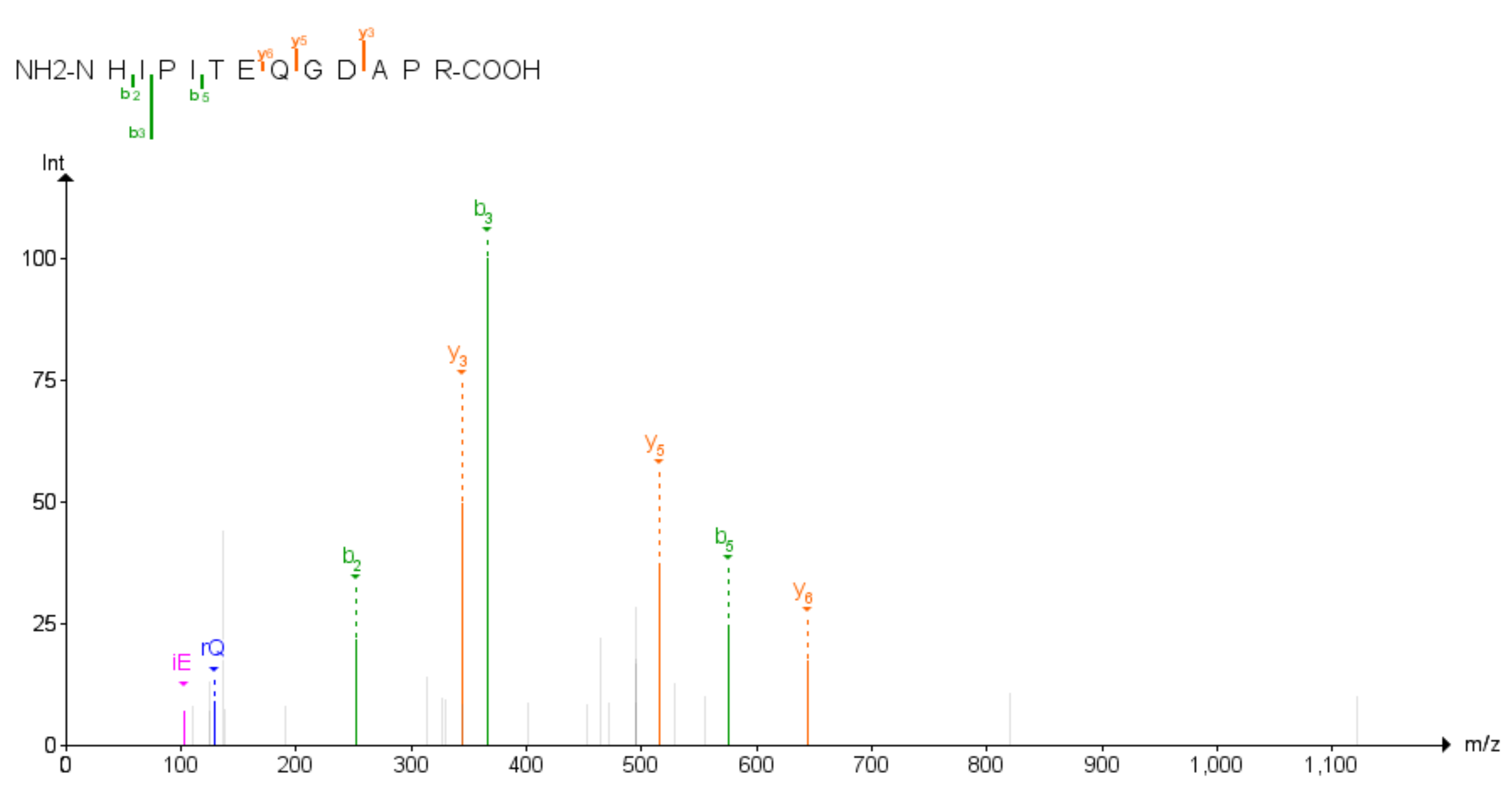

NH2-N H I P I T E Q G D A P R-COOH
b2
b3
b5
y6
y5
y3
Int
100
75
50
25
0
b3
y3
y5
b5
b2
y6
iE
rQ
m/z
0
100
200
300
400
500
600
700
800
900
1,000
1,100

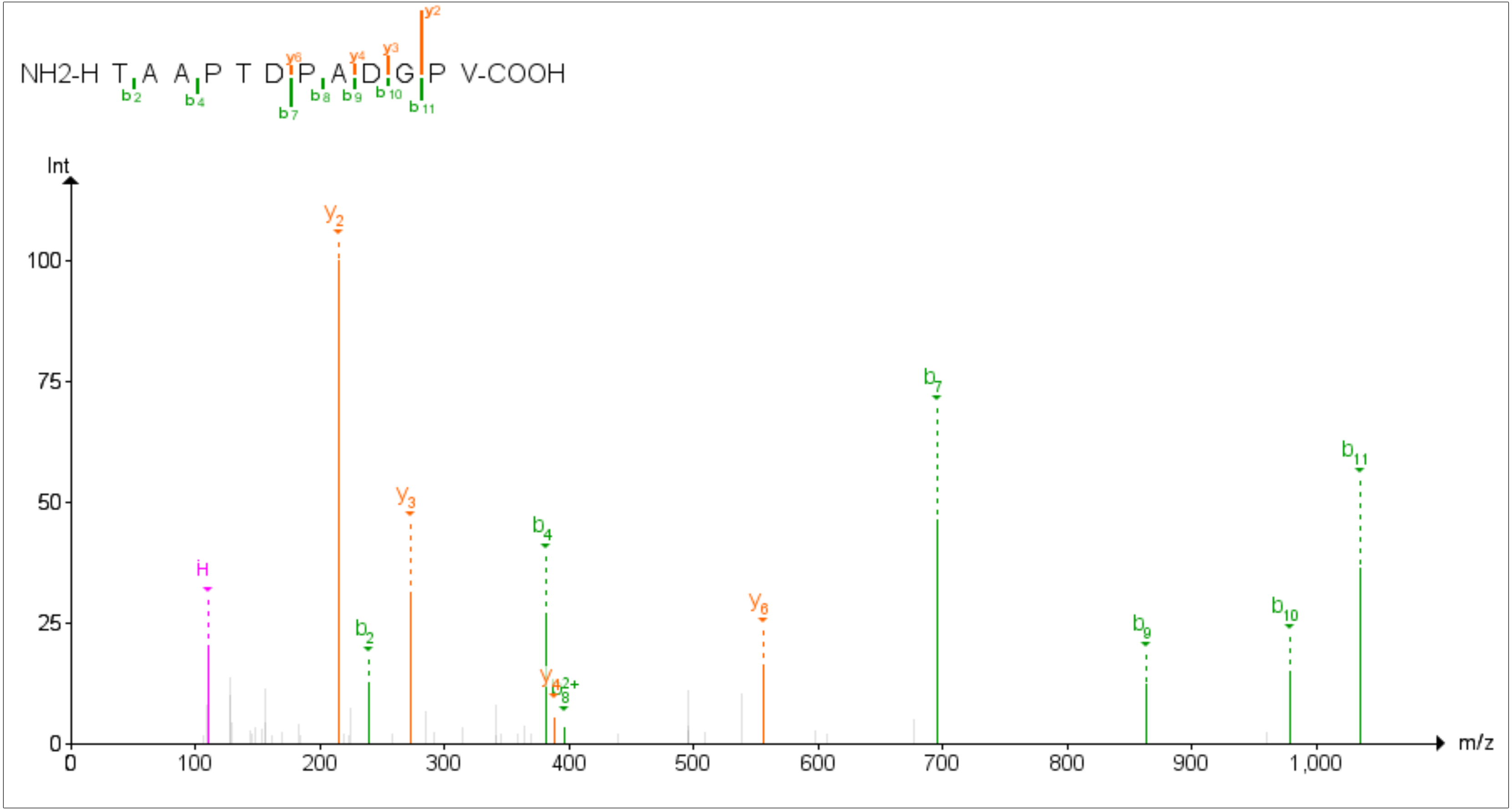

NH2-H T A A P T D P A D G P V-COOH
Int
m/z
y2
y3
y4
y6
b2
b4
b7
b8
b9
b10
b11
H
100
75
50
25
0
0
100
200
300
400
500
600
700
800
900
1,000

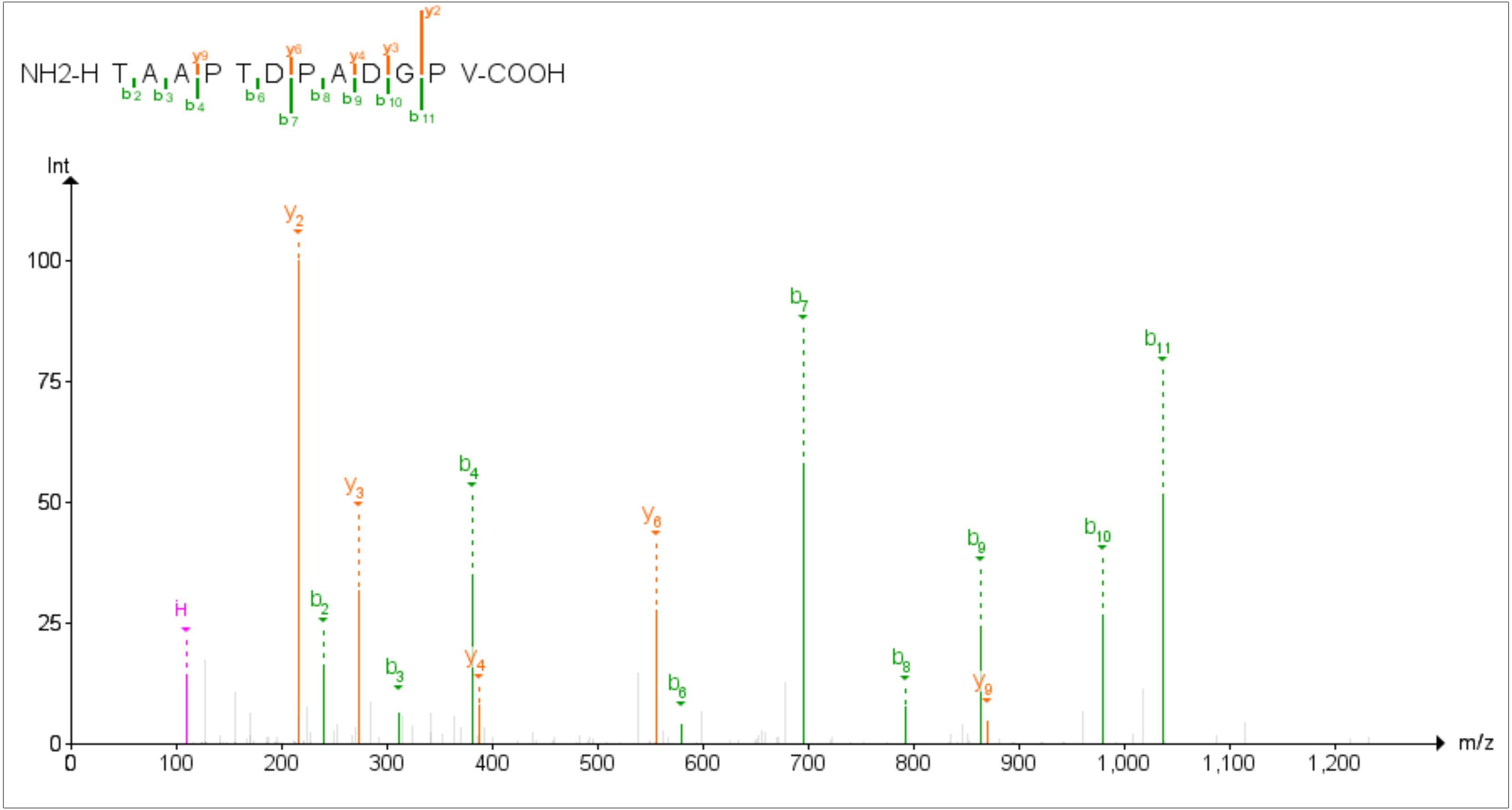

NH2-H T A A P T D P A D G P V-COOH
y2 y3 y4 y6 y9
b2 b3 b4 b6 b7 b8 b9 b10 b11
Int
100
75
50
25
0
H
m/z
0
100
200
300
400
500
600
700
800
900
1,000
1,100
1,200

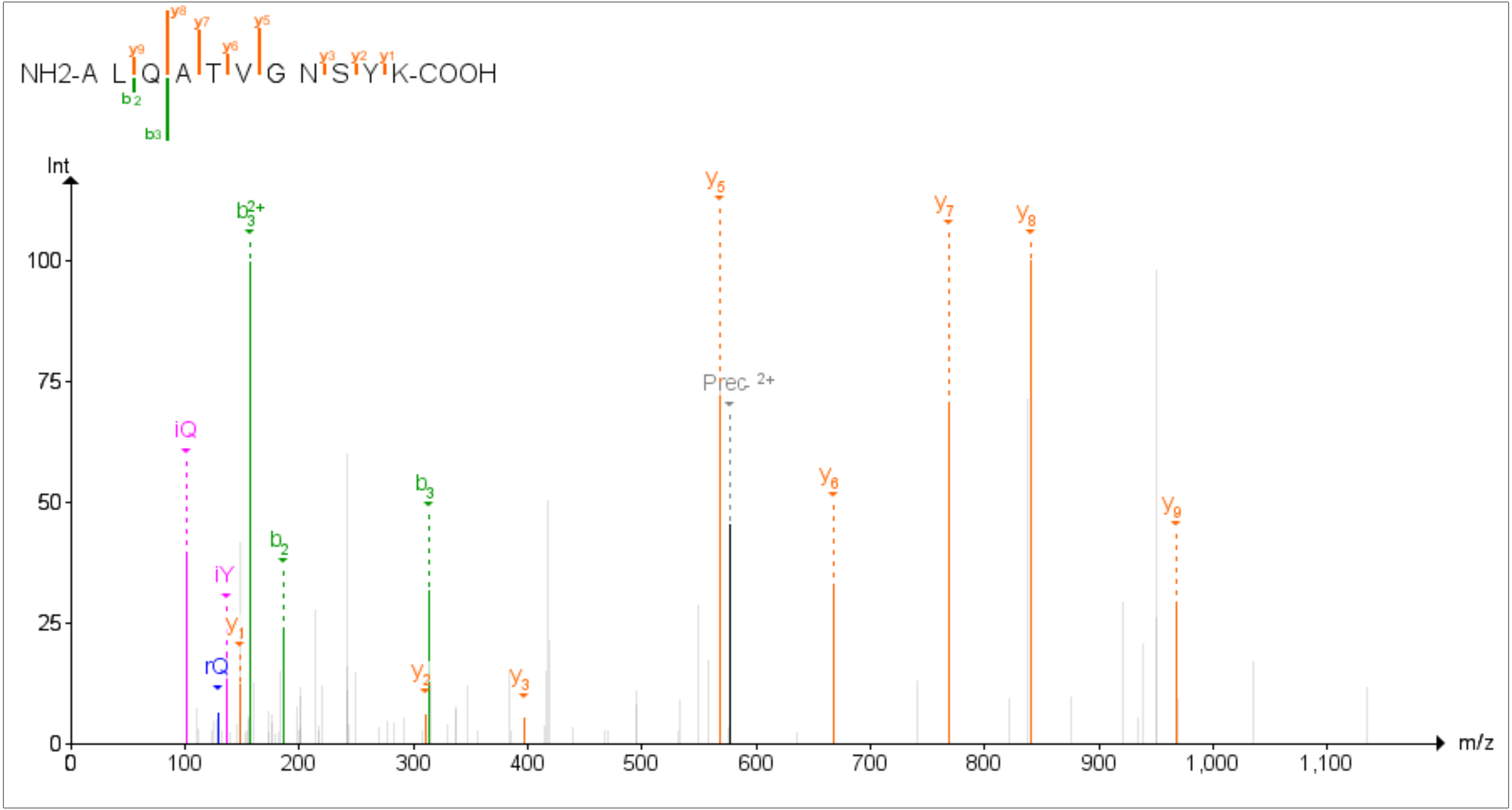

NH2-A L Q A T V G N S Y K-COOH
y9 y8 y7 y6 y5 y3 y2 y1
b2 b3
Int
m/z
b_3^{2+}
y_5
y_7
y_8
Prec. 2+
iQ
iY
rQ
y_1
b_2
b_3
y_2
y_3
y_6
y_9
0
25
50
75
100
0
100
200
300
400
500
600
700
800
900
1,000
1,100

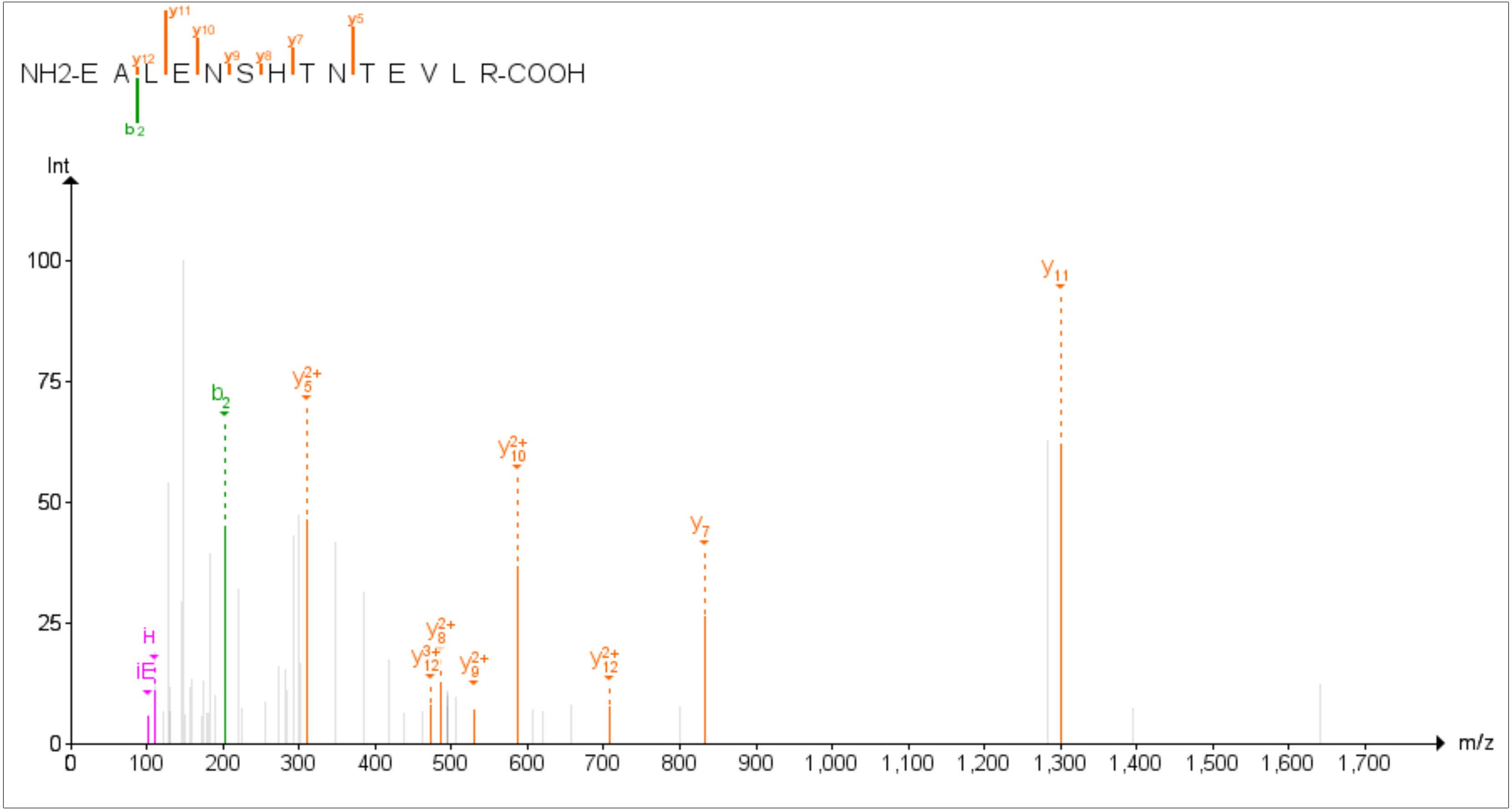

NH2-E A L E N S H T N T E V L R-COOH
y11
y10
y12
y9
y8
y7
y5
b2
Int
100
75
50
25
0
m/z
0
100
200
300
400
500
600
700
800
900
1,000
1,100
1,200
1,300
1,400
1,500
1,600
1,700
y11
b2
y5 2+
y10 2+
y7
y8 2+
y12 3+
y9 2+
y12 2+
iH
iE

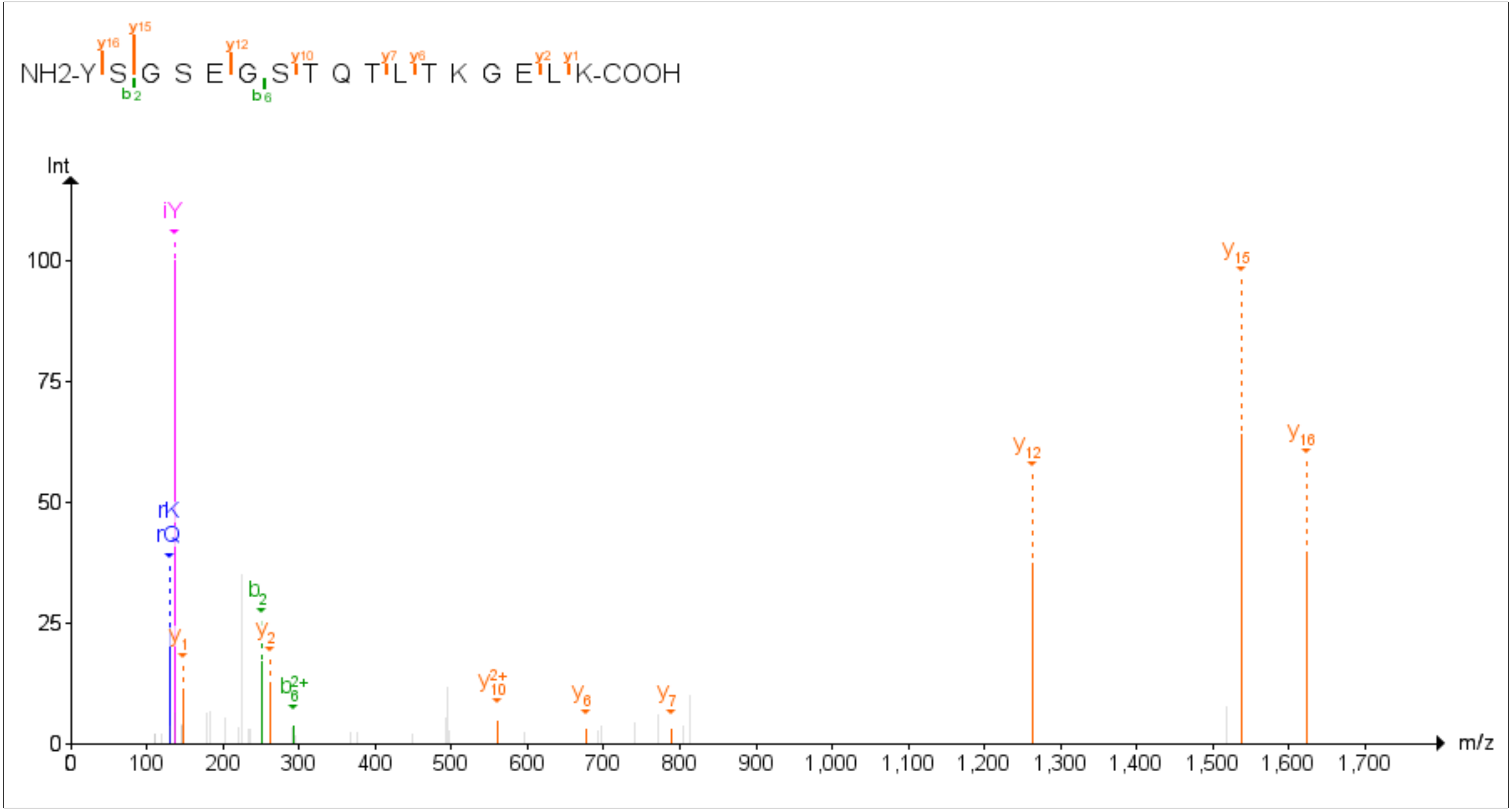

NH2-Y S G S E G S T Q T L T K G E L K-COOH
y16 y15 y12 y10 y7 y6 y2 y1
b2 b6
Int
iY
rK
rQ
y1
b2
y2
b6 2+
y10 2+
y6
y7
y12
y15
y16
0
25
50
75
100
0 100 200 300 400 500 600 700 800 900 1,000 1,100 1,200 1,300 1,400 1,500 1,600 1,700
m/z

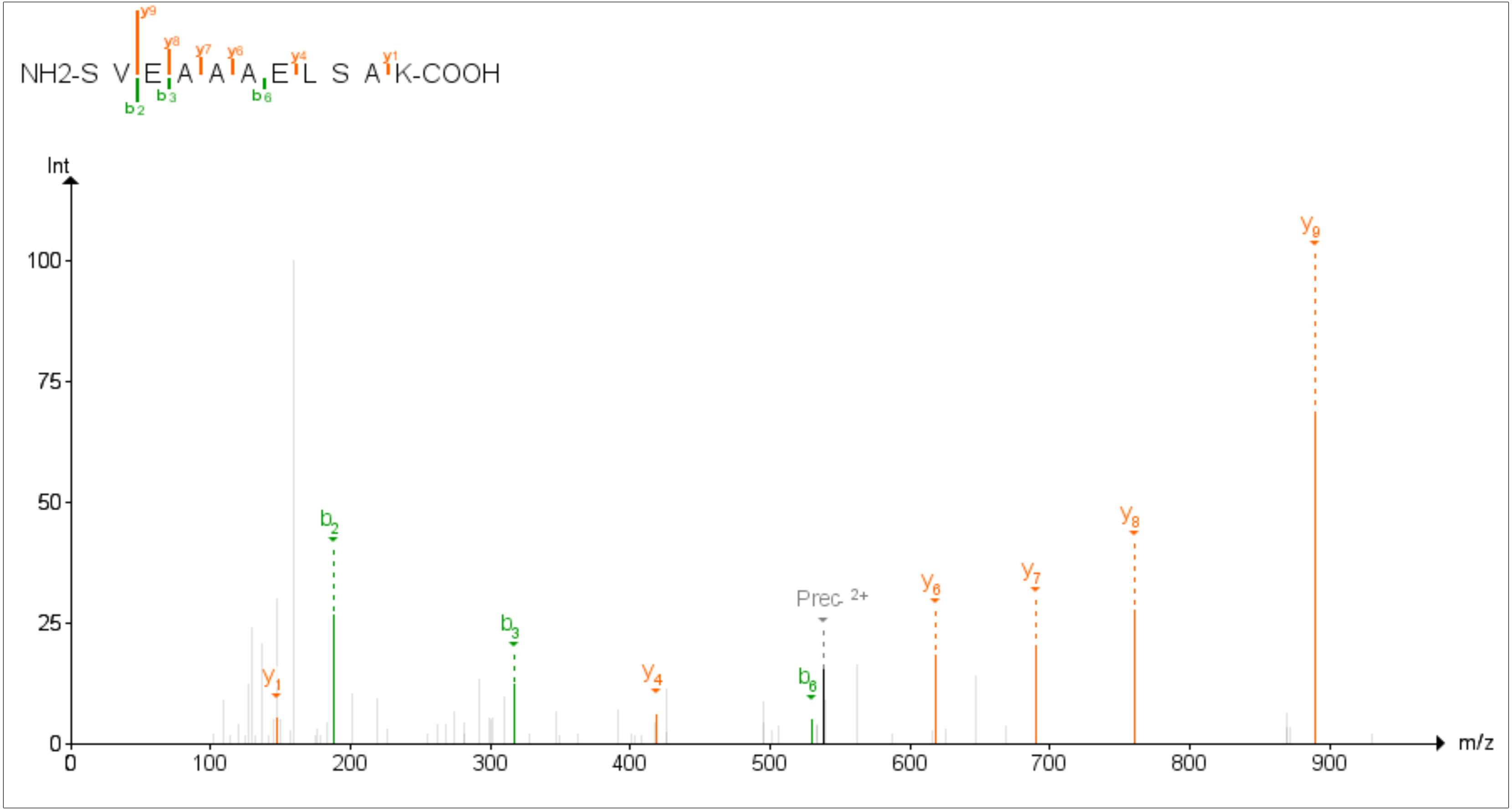

NH2-S V E A A A E L S A K-COOH
y9
y8
y7
y6
y4
y1
b2
b3
b6
Int
100
75
50
25
0
m/z
0
100
200
300
400
500
600
700
800
900
y1
b2
b3
y4
b6
Prec. 2+
y6
y7
y8
y9

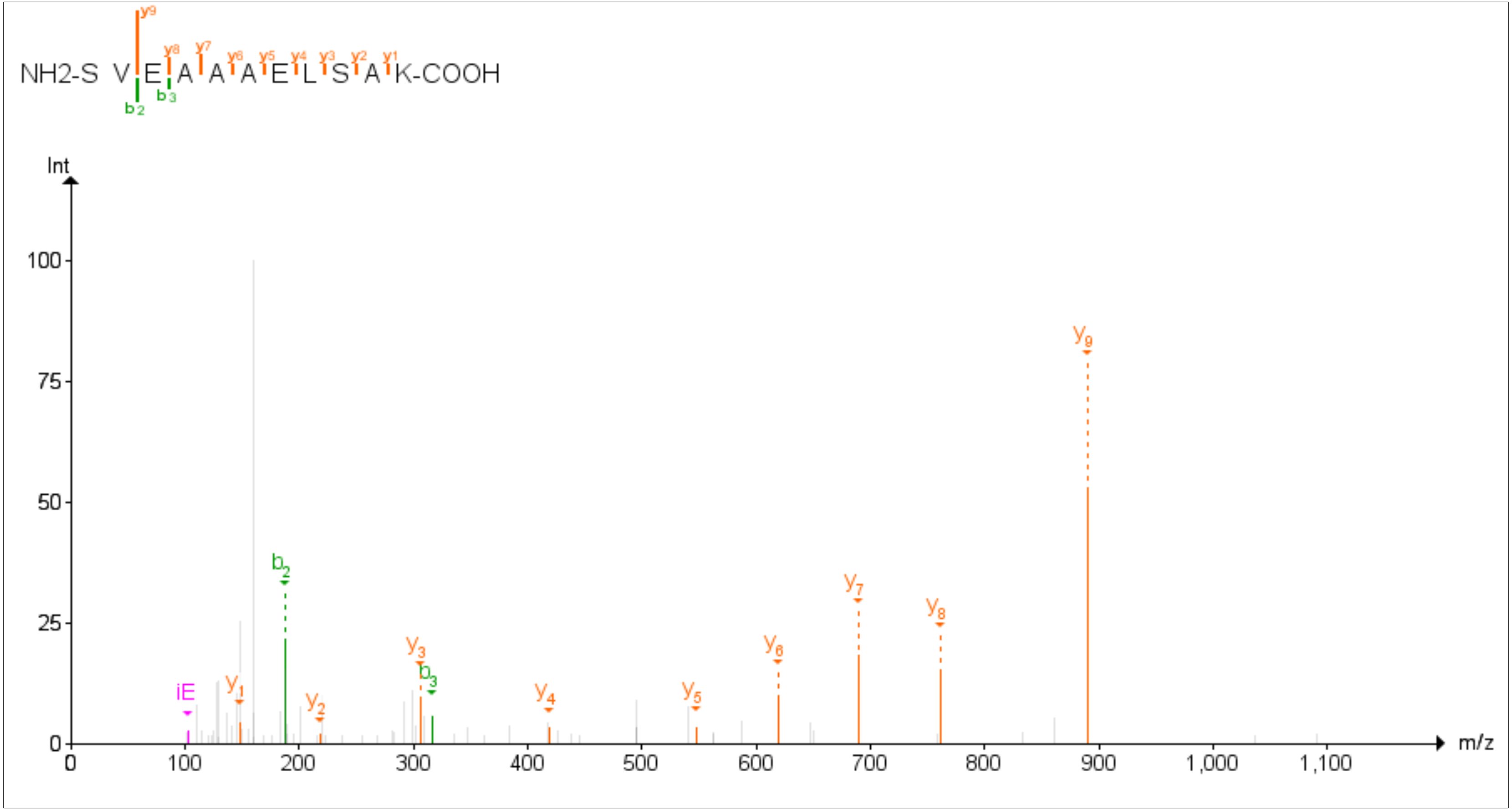
NH2-S V E A A A E L S A K-COOH
y9 y8 y7 y6 y5 y4 y3 y2 y1
b2 b3
Int
100
75
50
25
0
iE
y1
b2
y2
y3
b3
y4
y5
y6
y7
y8
y9
0 100 200 300 400 500 600 700 800 900 1,000 1,100
m/z

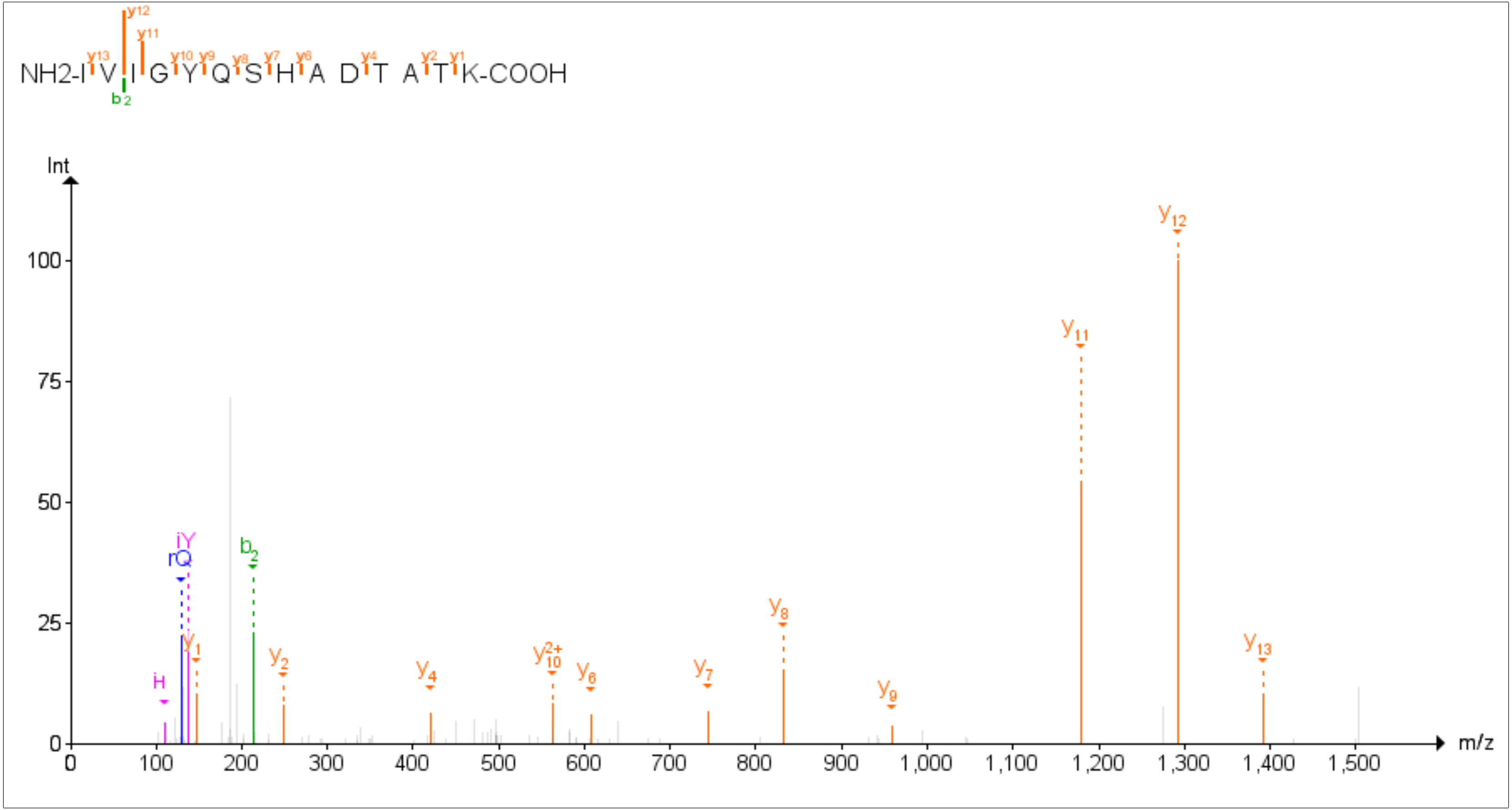

NH2-IVIGYQSHADTATK-COOH
Int
m/z
y12
y11
y13
y1
y2
y4
y6
y7
y8
y9
y10
b2
iY
rQ
iH
0
25
50
75
100
100
200
300
400
500
600
700
800
900
1,000
1,100
1,200
1,300
1,400
1,500

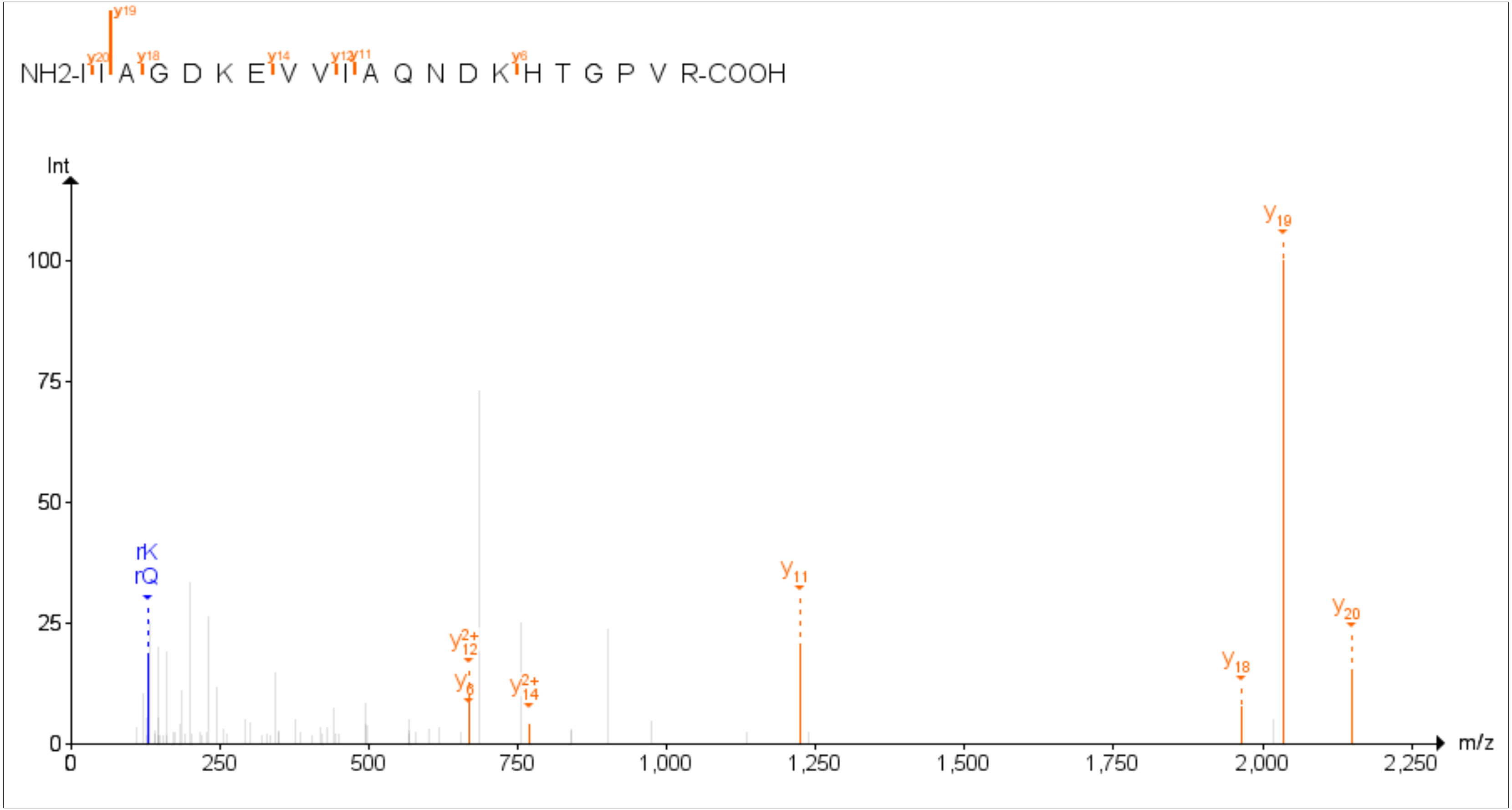

NH2-I I A G D K E V V I A Q N D K H T G P V R-COOH
y20
y19
y18
y14
y12
y11
y6
Int
100
75
50
25
0
rK
rQ
y2+ 12
y6
y2+ 14
y11
y18
y19
y20
0
250
500
750
1,000
1,250
1,500
1,750
2,000
2,250
m/z

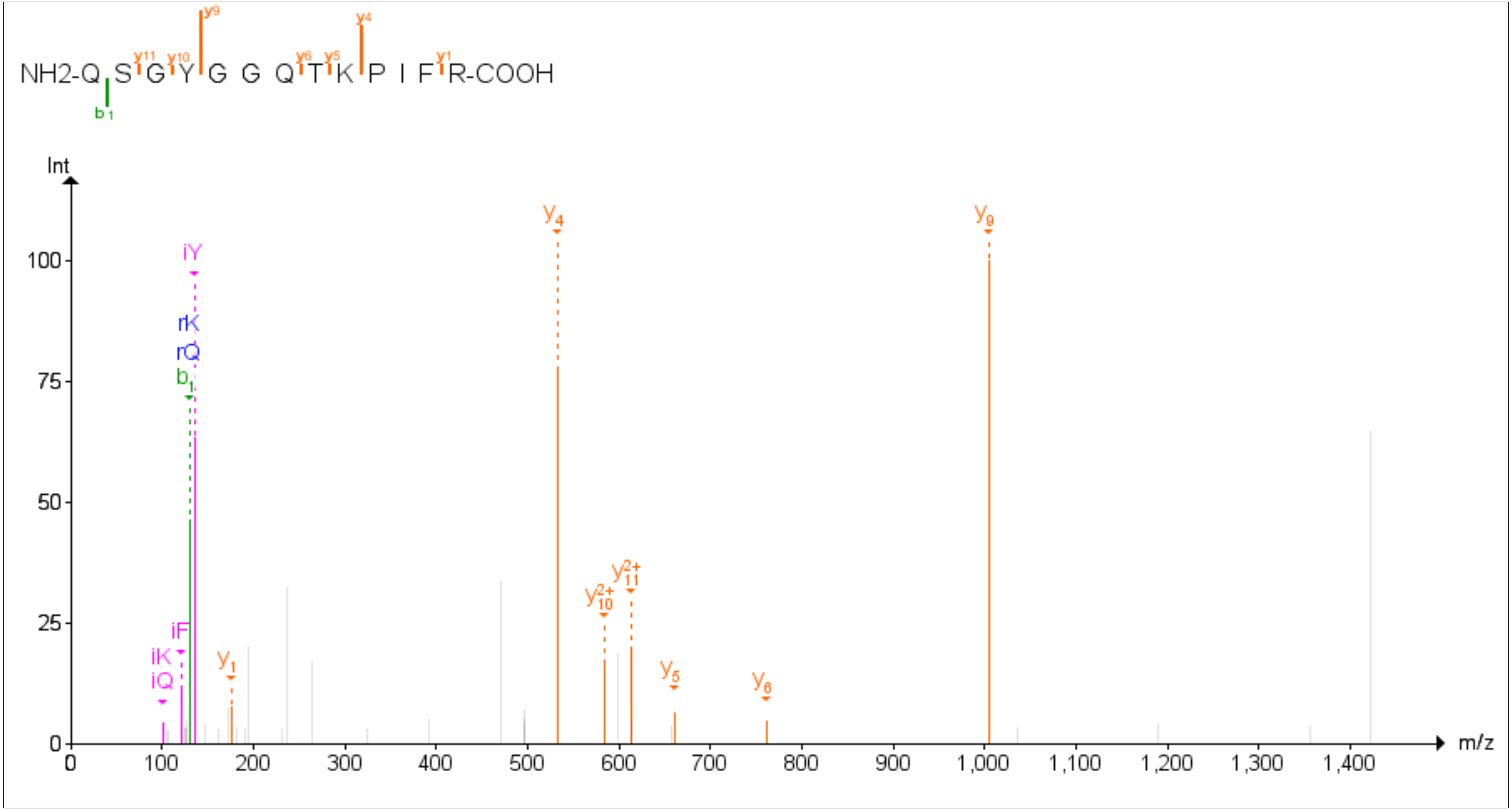
NH2-Q S G Y G G Q T K P I F R-COOH
b1
y11
y10
y9
y6
y5
y4
y1
Int
100
75
50
25
0
iY
rK
rQ
b1
iF
iK
iQ
y1
y4
y9
y10 2+
y11 2+
y5
y6
0
100
200
300
400
500
600
700
800
900
1,000
1,100
1,200
1,300
1,400
m/z

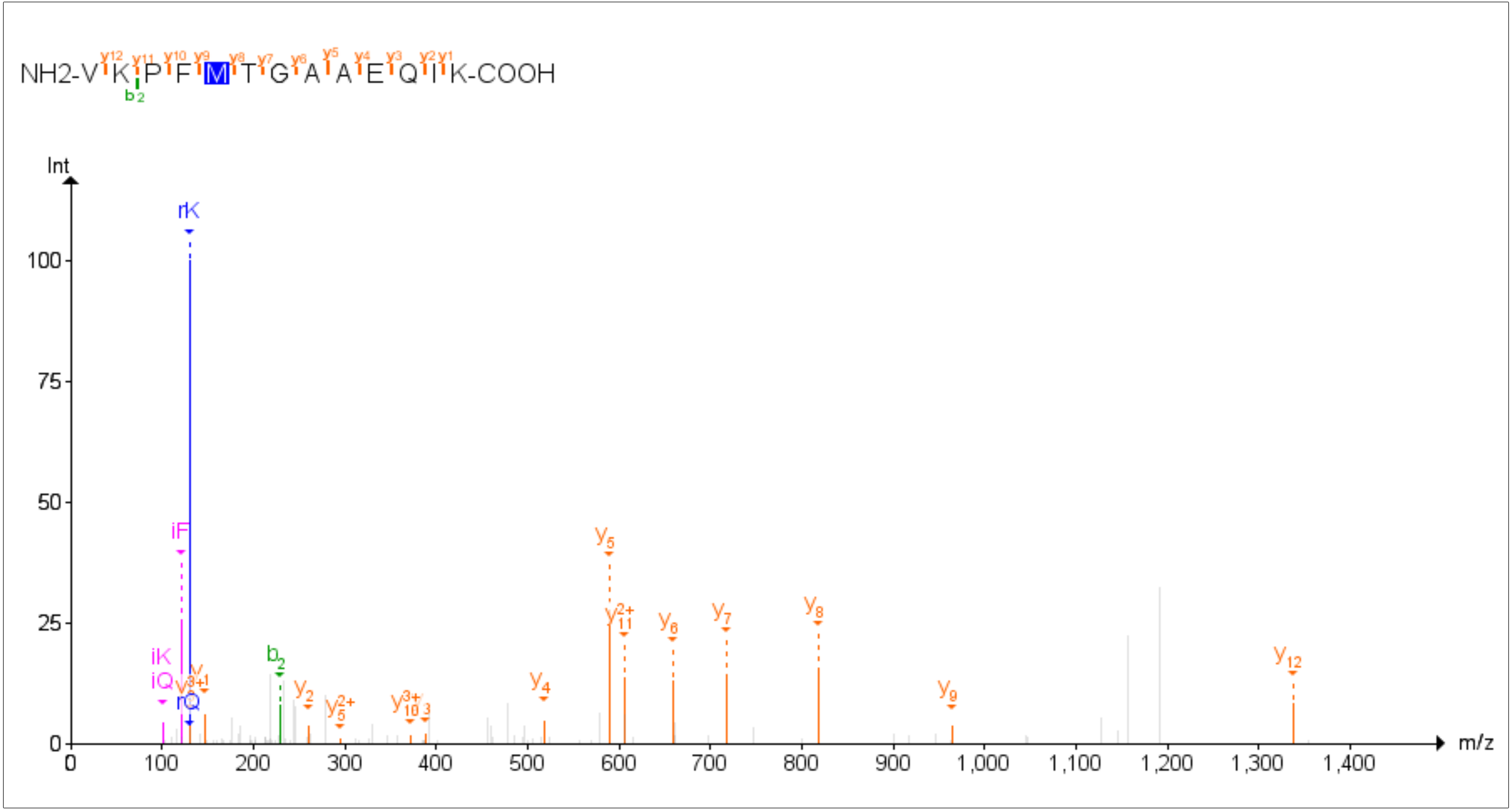

NH2-VKPF M TGAAEQIK-COOH
y12 y11 y10 y9 y8 y7 y6 y5 y4 y3 y2 y1
b2
Int
100
75
50
25
0
rK
iF
iK
iQ
rQ
b2
y2
y4
y5
y6
y7
y8
y9
y12
m/z
0
100
200
300
400
500
600
700
800
900
1,000
1,100
1,200
1,300
1,400

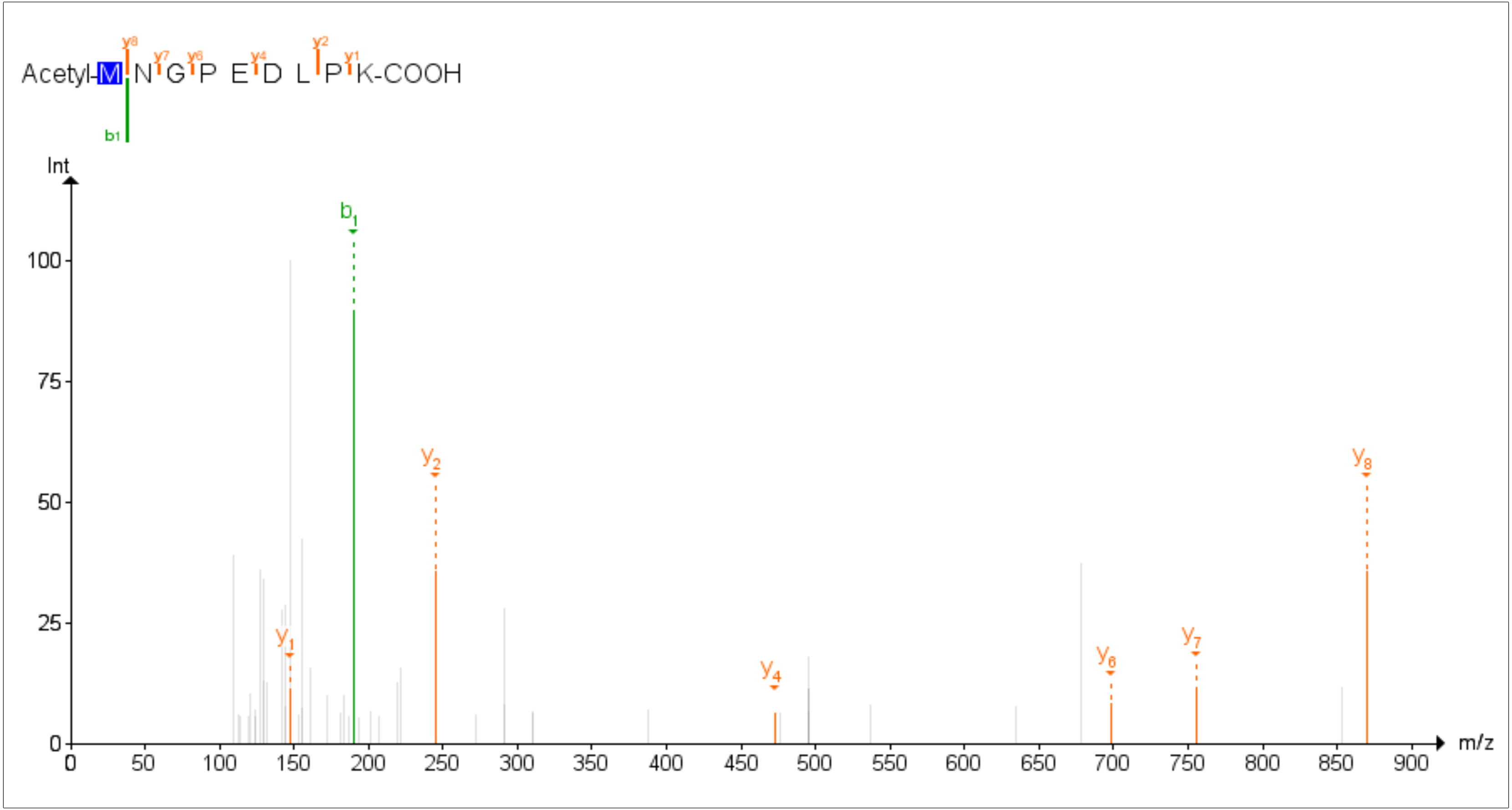

Acetyl-M N G P E D L P K-COOH
y8 y7 y6 y4 y2 y1
b1
Int
100
75
50
25
0
b1
y2
y8
y1
y4
y6
y7
0 50 100 150 200 250 300 350 400 450 500 550 600 650 700 750 800 850 900
m/z

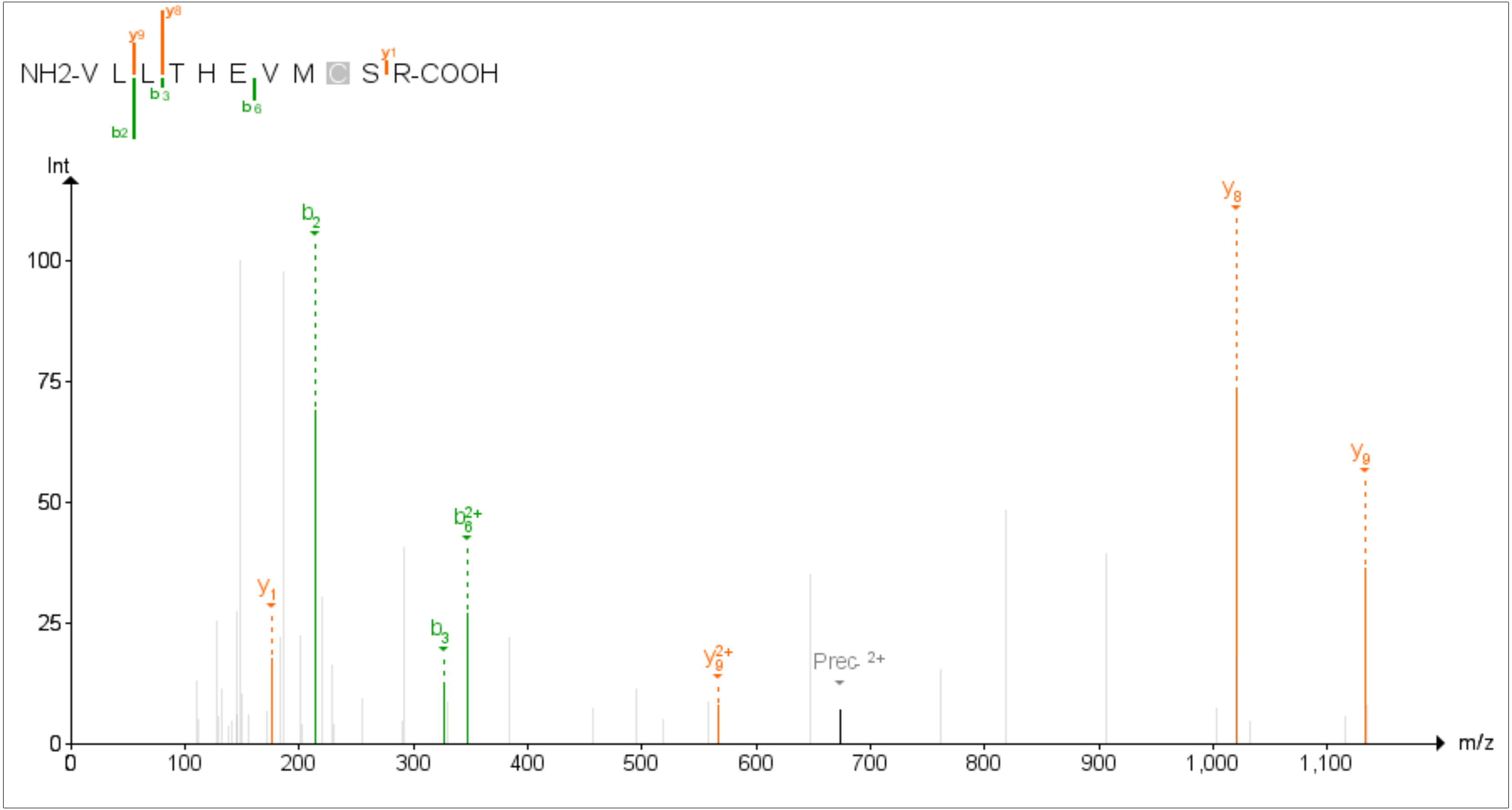
NH2-V L L T H E V M C S R-COOH
y9
y8
y1
b2
b3
b6
Int
100
75
50
25
0
b2
y8
y9
y1
b3
b6 2+
y9 2+
Prec. 2+
0
100
200
300
400
500
600
700
800
900
1,000
1,100
m/z

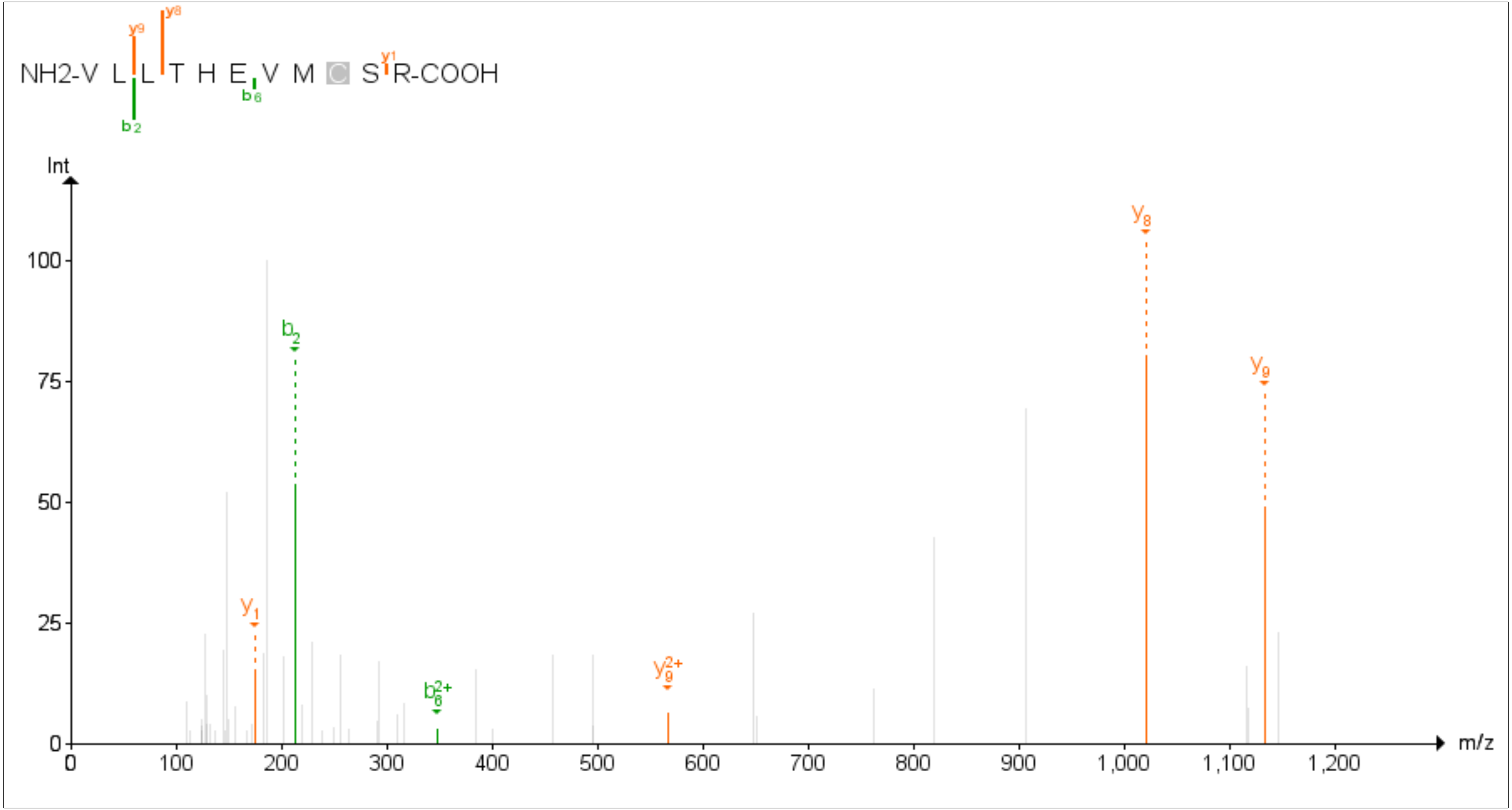

NH2-V L L T H E V M C S R-COOH
y9
y8
y1
b2
b6
Int
m/z
y8
y9
b2
y1
b6 2+
y9 2+

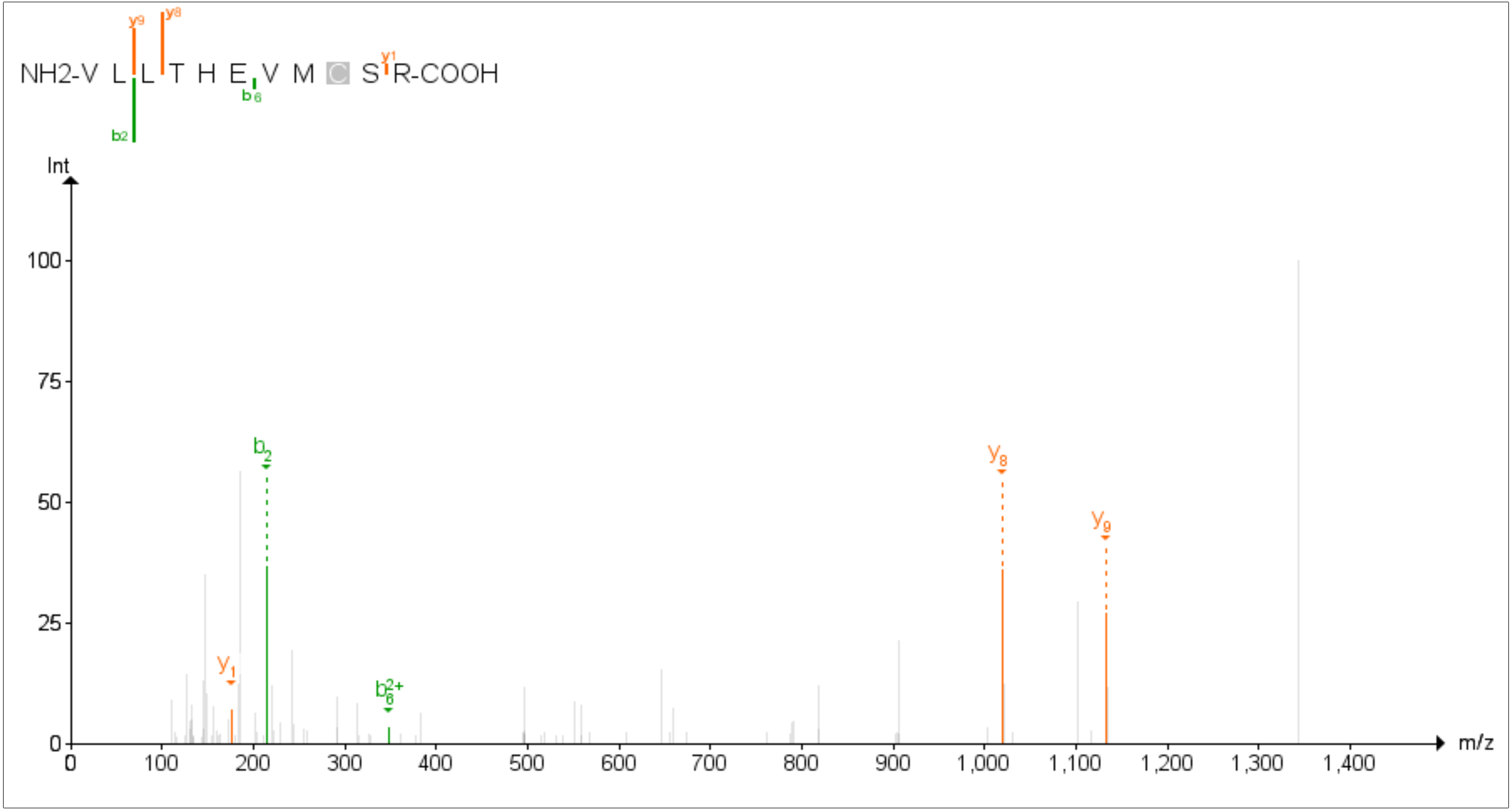

NH2-V L L T H E V M C S R-COOH
y9
y8
y1
b2
b6
Int
100
75
50
25
0
b2
y8
y9
y1
b6 2+
0
100
200
300
400
500
600
700
800
900
1,000
1,100
1,200
1,300
1,400
m/z

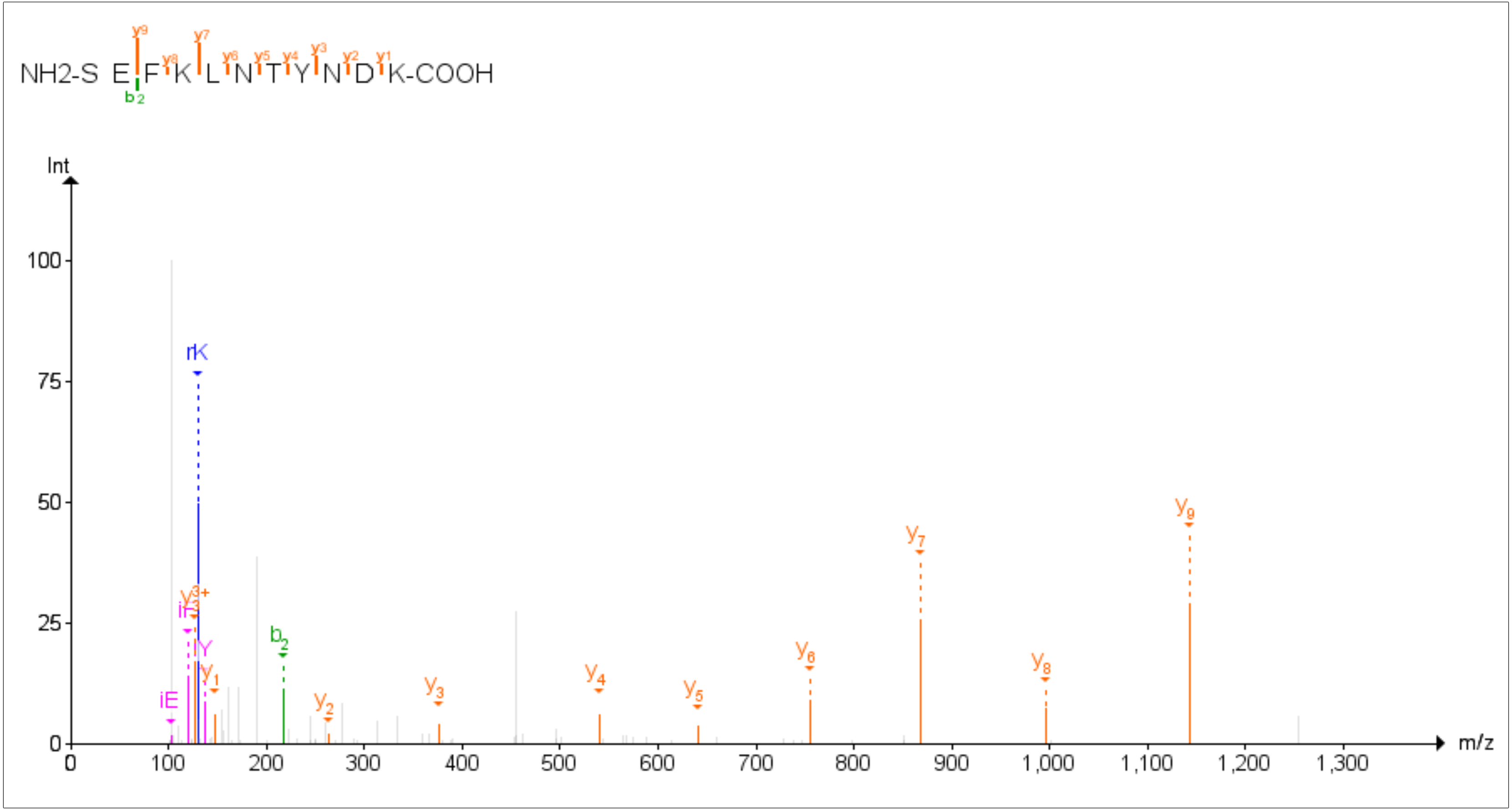
NH2-S E F K L N T Y N D K-COOH
Int
m/z
y9
y8
y7
y6
y5
y4
y3
y2
y1
b2
rK
y3 3+
iF
iY
iE
0
25
50
75
100
0
100
200
300
400
500
600
700
800
900
1,000
1,100
1,200
1,300

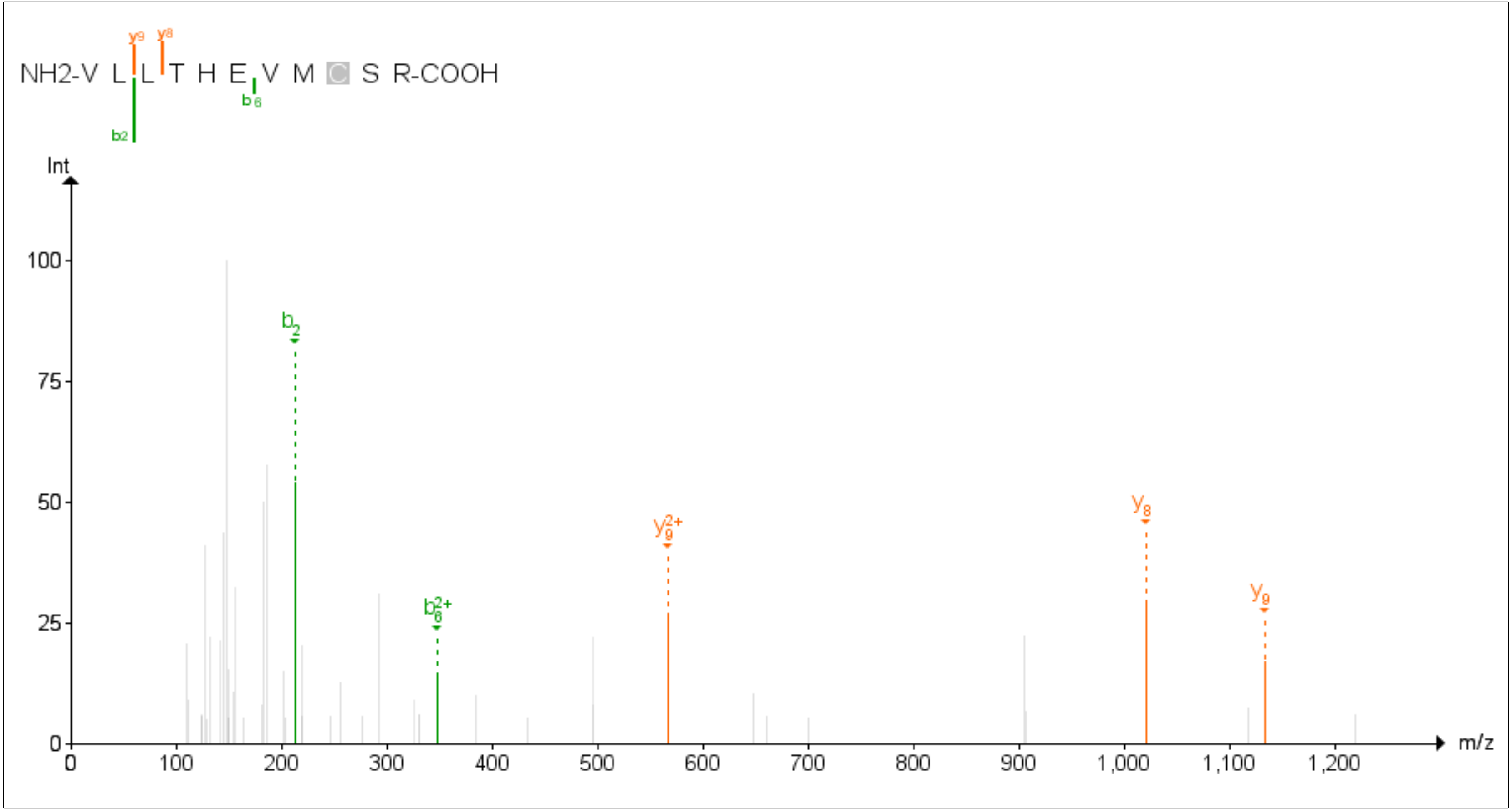
NH2-V L L T H E V M C S R-COOH
y9
y8
b2
b6
Int
100
75
50
25
0
b2
b6 2+
y9 2+
y8
y9
0
100
200
300
400
500
600
700
800
900
1,000
1,100
1,200
m/z

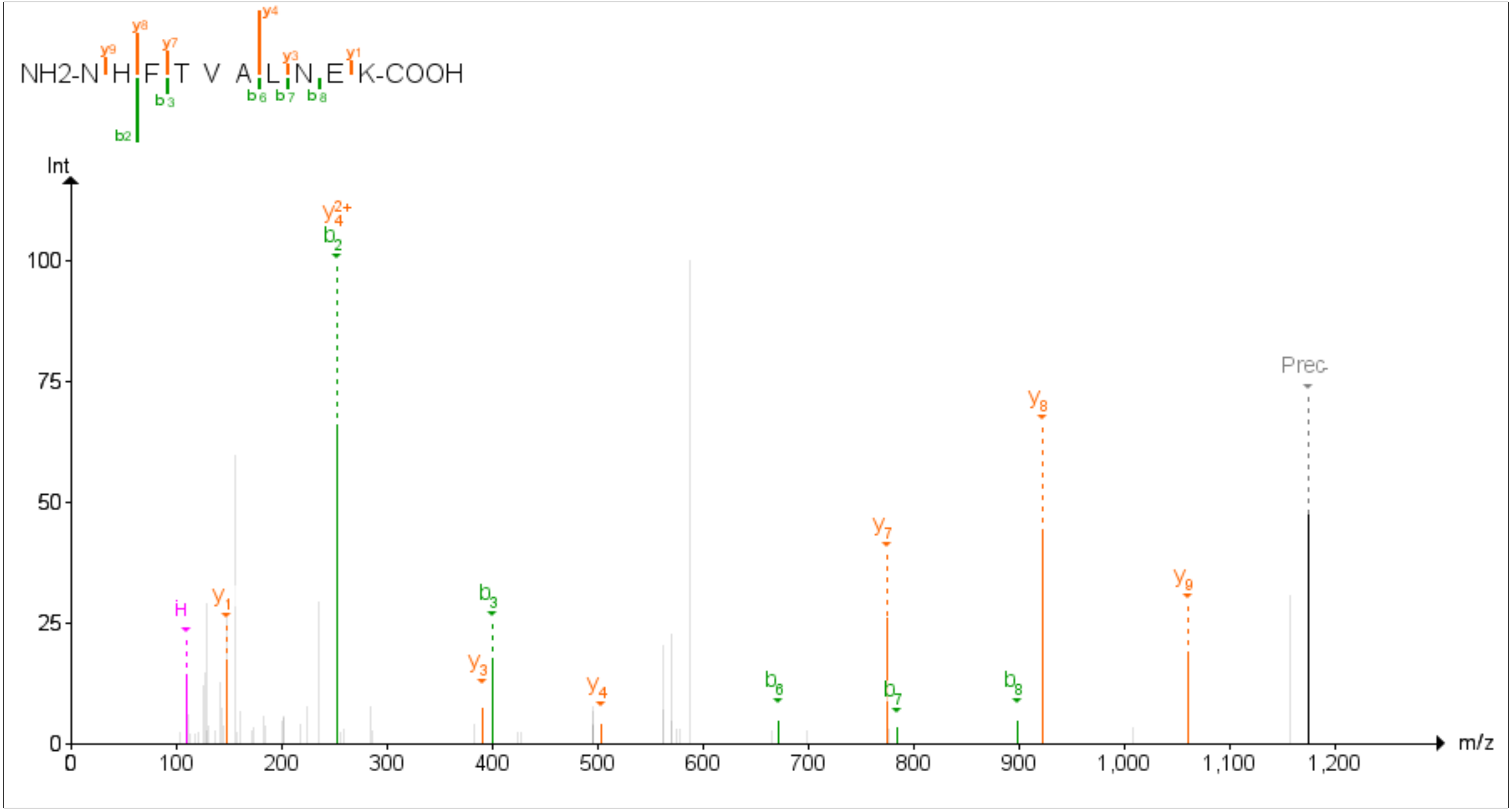

NH2-N H F T V A L N E K-COOH
y9 y8 y7 y4 y3 y1
b2 b3 b6 b7 b8
Int
m/z
0
25
50
75
100
0 100 200 300 400 500 600 700 800 900 1,000 1,100 1,200
y_4^{2+}
b_2
Prec
y_8
y_7
y_9
y_1
b_3
y_3
y_4
b_6
b_7
b_8

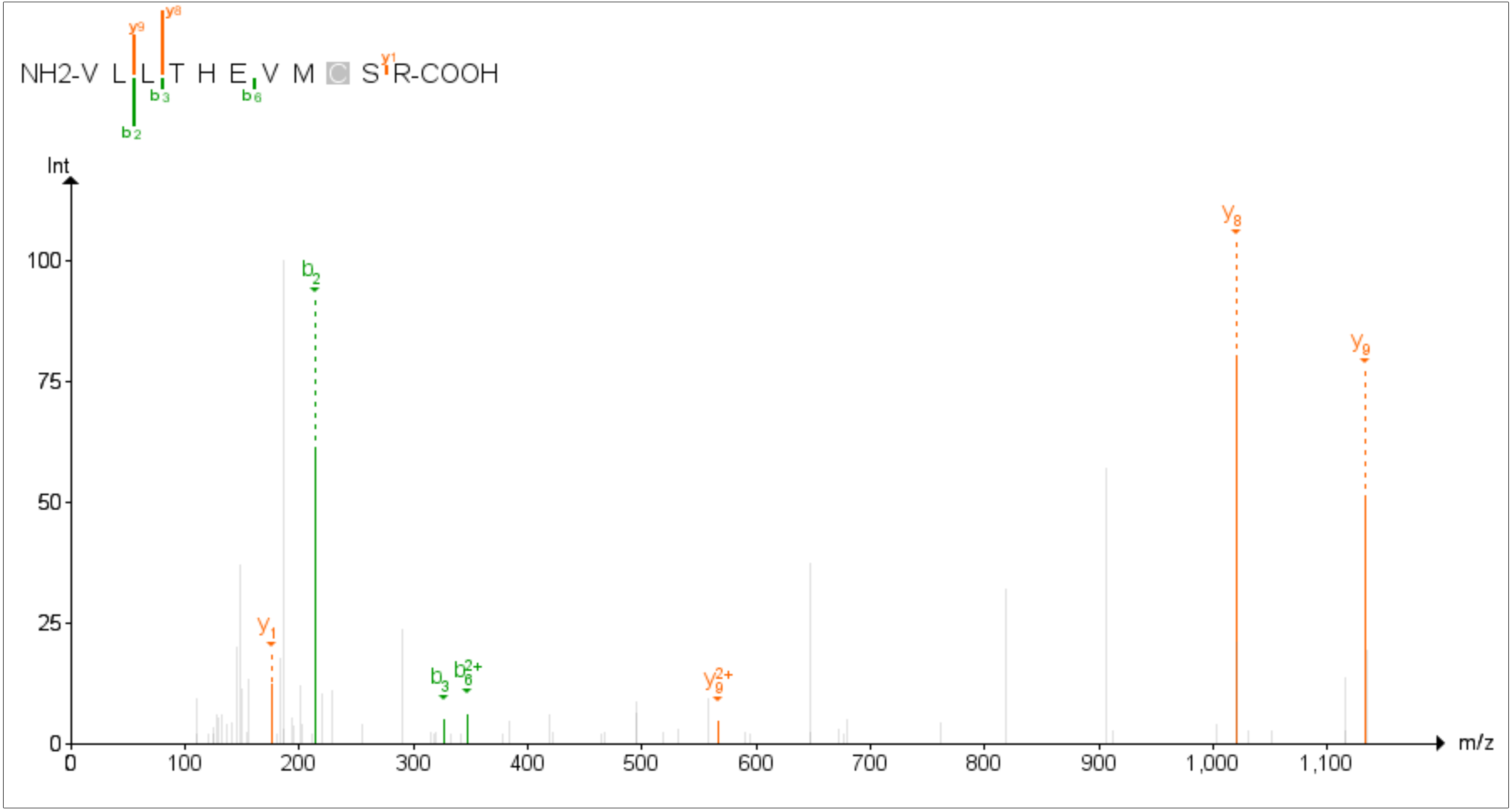

NH2-V L L T H E V M C S R-COOH
y9
y8
y1
b2
b3
b6
Int
100
75
50
25
0
b2
y8
y9
y1
b3
b6 2+
y9 2+
0
100
200
300
400
500
600
700
800
900
1,000
1,100
m/z

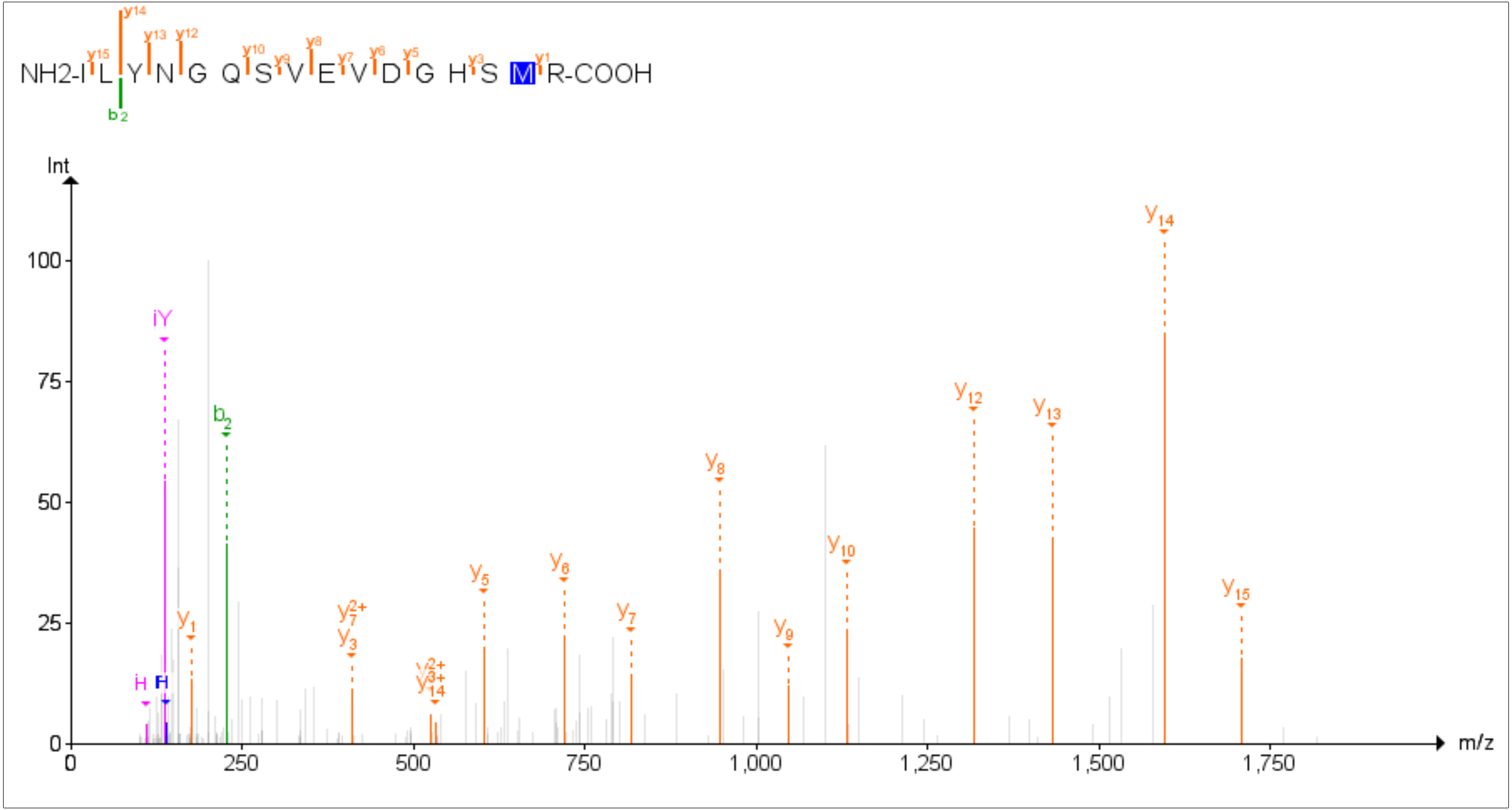

NH2-I L Y N G Q S V E V D G H S M R-COOH
y15 y14 y13 y12 y10 y9 y8 y7 y6 y5 y3 y1
b2
Int
100
75
50
25
0
m/z
0
250
500
750
1,000
1,250
1,500
1,750
iY
b2
y1
y3
y5
y6
y7
y8
y9
y10
y12
y13
y14
y15

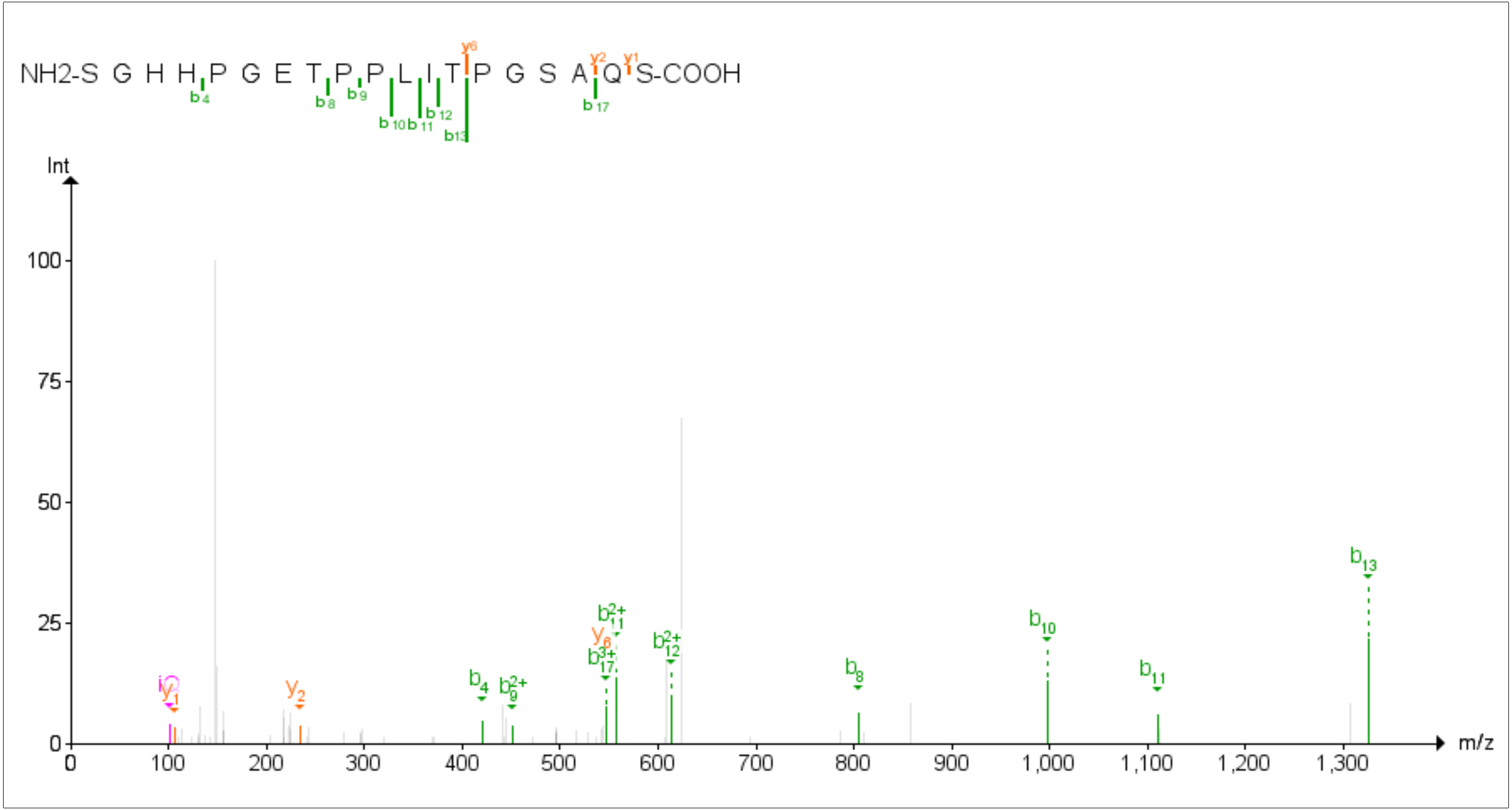

NH2-S G H H P G E T P P L I T P G S A Q S-COOH
y6
y2
y1
b 4
b 8
b 9
b 10
b 11
b 12
b 13
b 17
Int
100
75
50
25
0
m/z
0
100
200
300
400
500
600
700
800
900
1,000
1,100
1,200
1,300
b13
b10
b11
b8
b2+ 11
b2+ 12
y6
b3+ 17
b4
b2+ 9
y2
y1

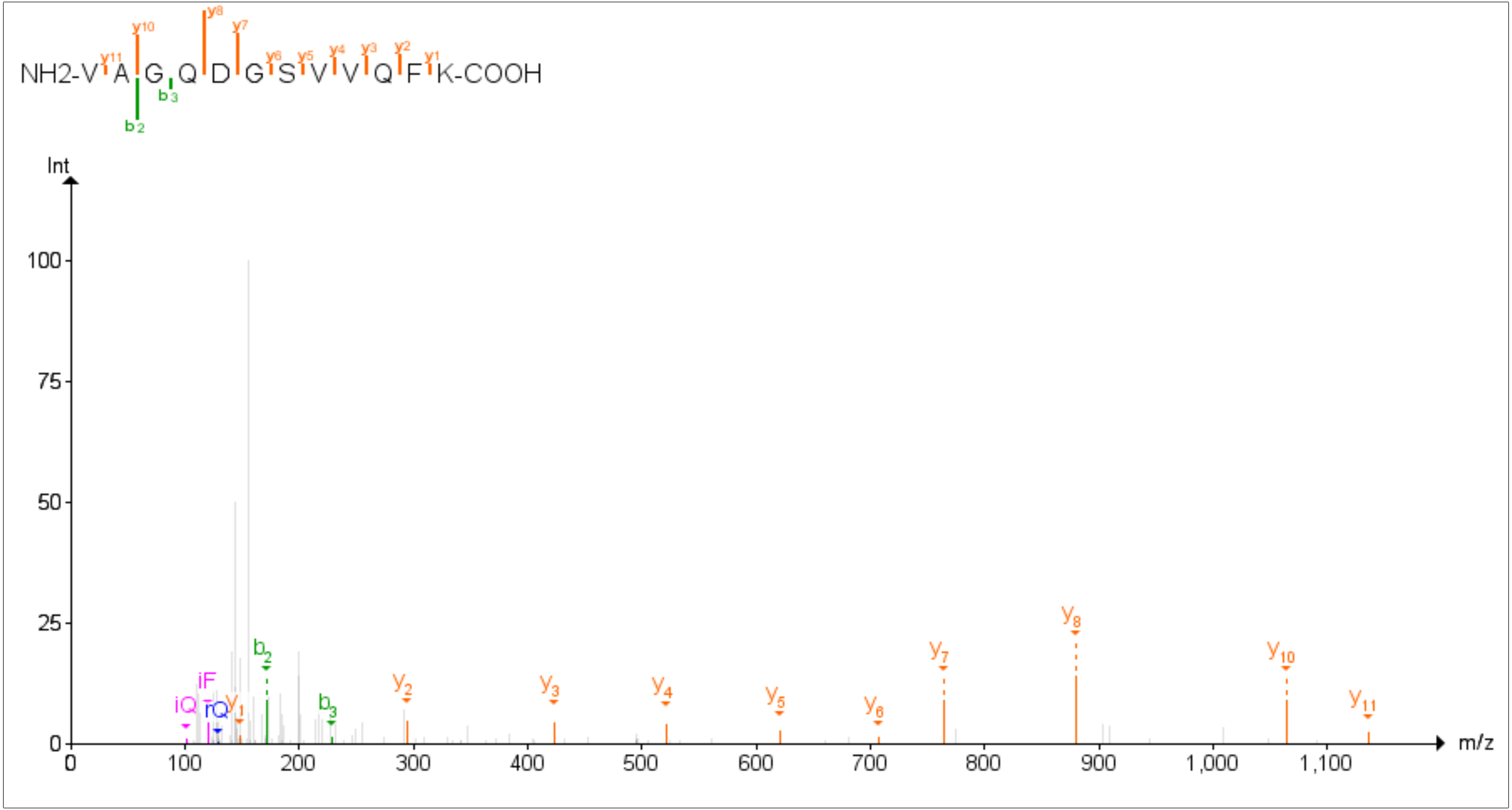
NH2-VAGQDGSVVQFK-COOH
y11 y10 y8 y7 y6 y5 y4 y3 y2 y1
b2 b3
Int
100
75
50
25
0
m/z
0 100 200 300 400 500 600 700 800 900 1,000 1,100
iQ iF rQ y1 b2 b3 y2 y3 y4 y5 y6 y7 y8 y10 y11

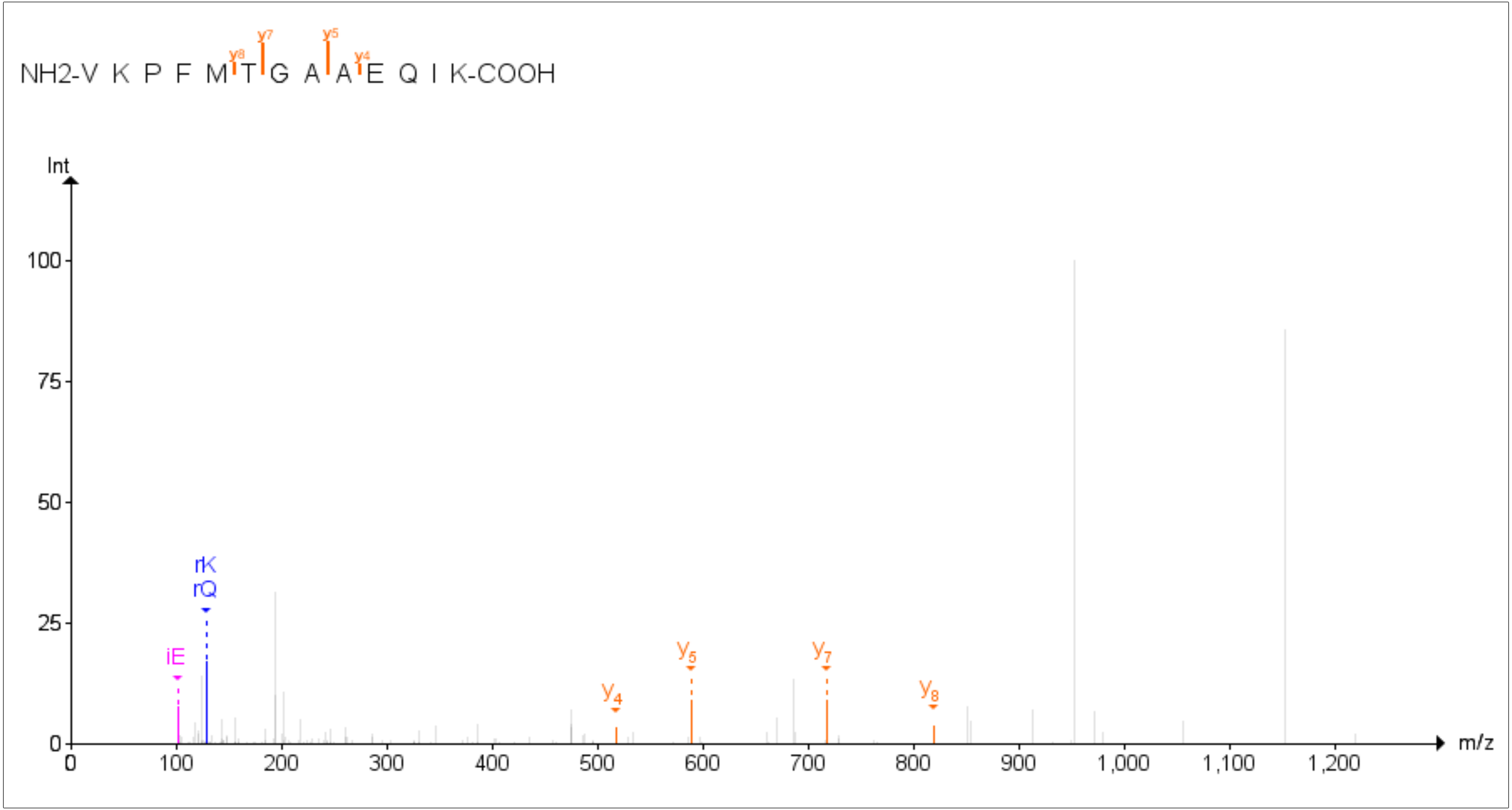

NH2-V K P F M T G A A E Q I K-COOH
y8
y7
y5
y4
Int
100
75
50
25
0
rK
rQ
iE
y4
y5
y7
y8
0
100
200
300
400
500
600
700
800
900
1,000
1,100
1,200
m/z

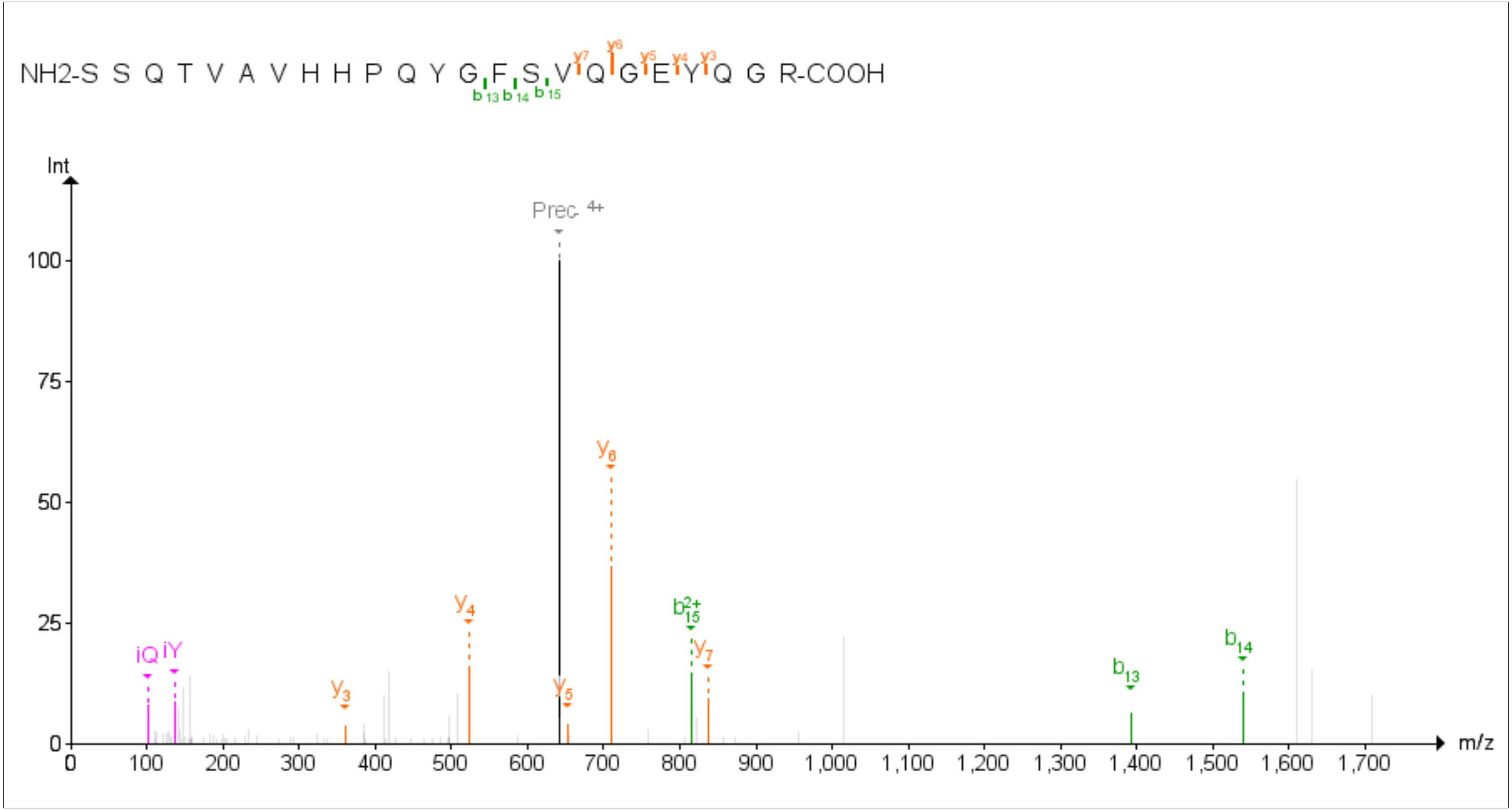

NH2-S S Q T V A V H H P Q Y G F S V Q G E Y Q G R-COOH
y7 y6 y5 y4 y3
b13 b14 b15
Int
Prec 4+
100
75
50
25
0
iQ iY y3 y4 y5 y6 b15 2+ y7 b13 b14
0 100 200 300 400 500 600 700 800 900 1,000 1,100 1,200 1,300 1,400 1,500 1,600 1,700
m/z

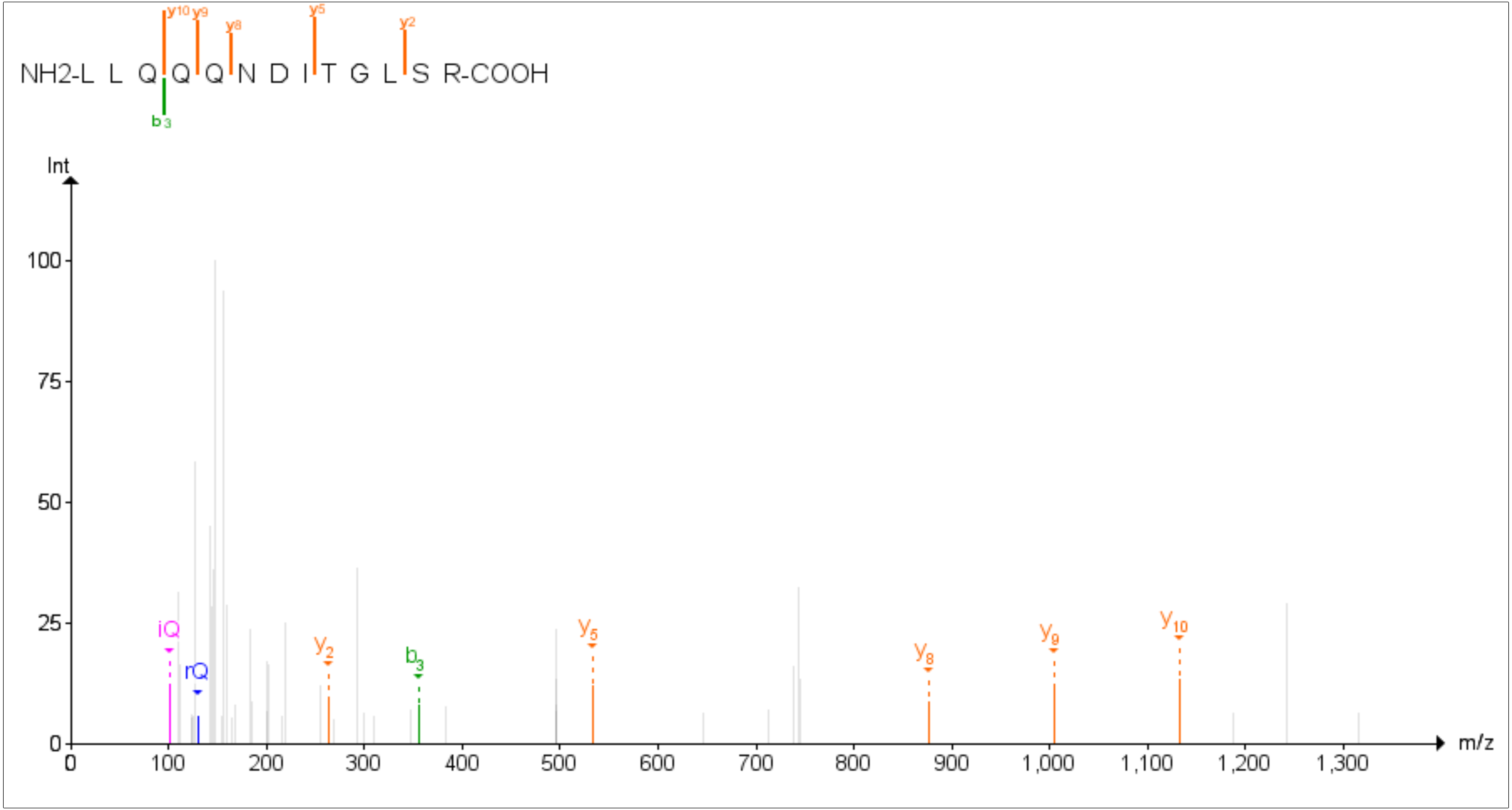

NH2-L L Q Q Q N D I T G L S R-COOH
y10 y9 y8 y5 y2
b3
Int
100
75
50
25
0
iQ
rQ
y2
b3
y5
y8
y9
y10
0 100 200 300 400 500 600 700 800 900 1,000 1,100 1,200 1,300
m/z

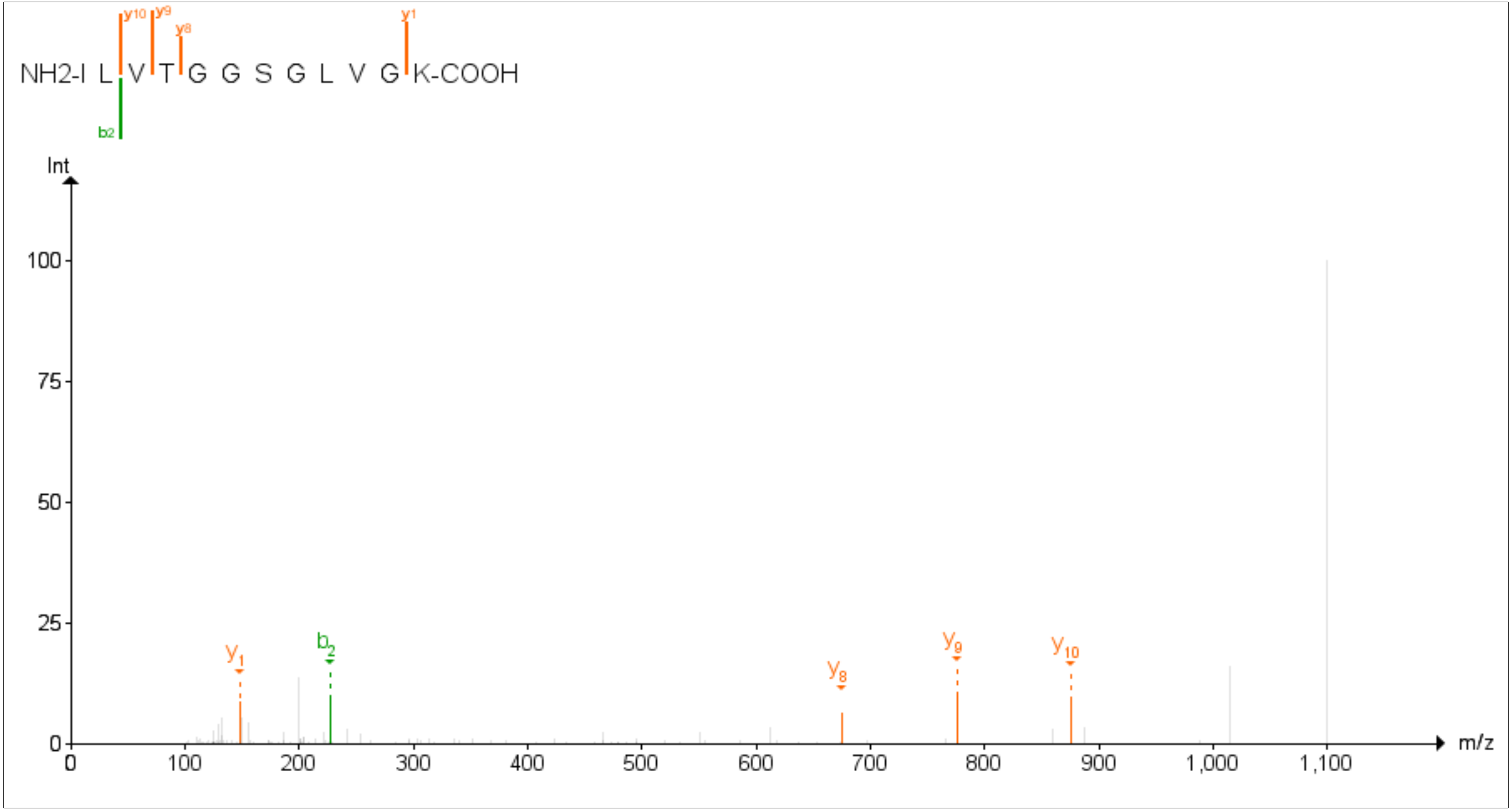

NH2-I L V T G G S G L V G K-COOH
y10
y9
y8
y1
b2
Int
100
75
50
25
0
0
100
200
300
400
500
600
700
800
900
1,000
1,100
m/z
y_1
b_2
y_8
y_9
y_{10}

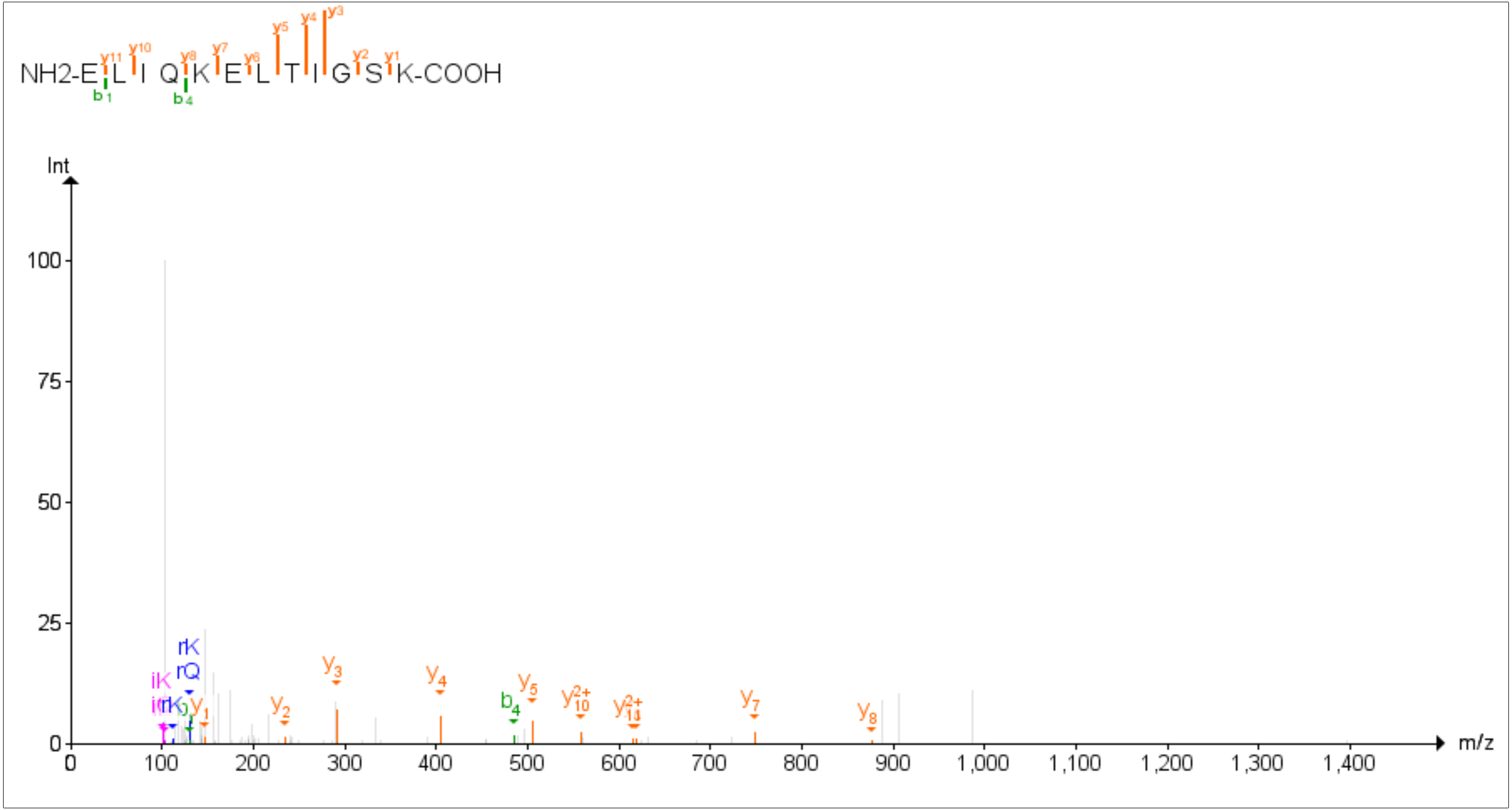

NH2-E L I Q K E L T I G S K-COOH
Int
m/z

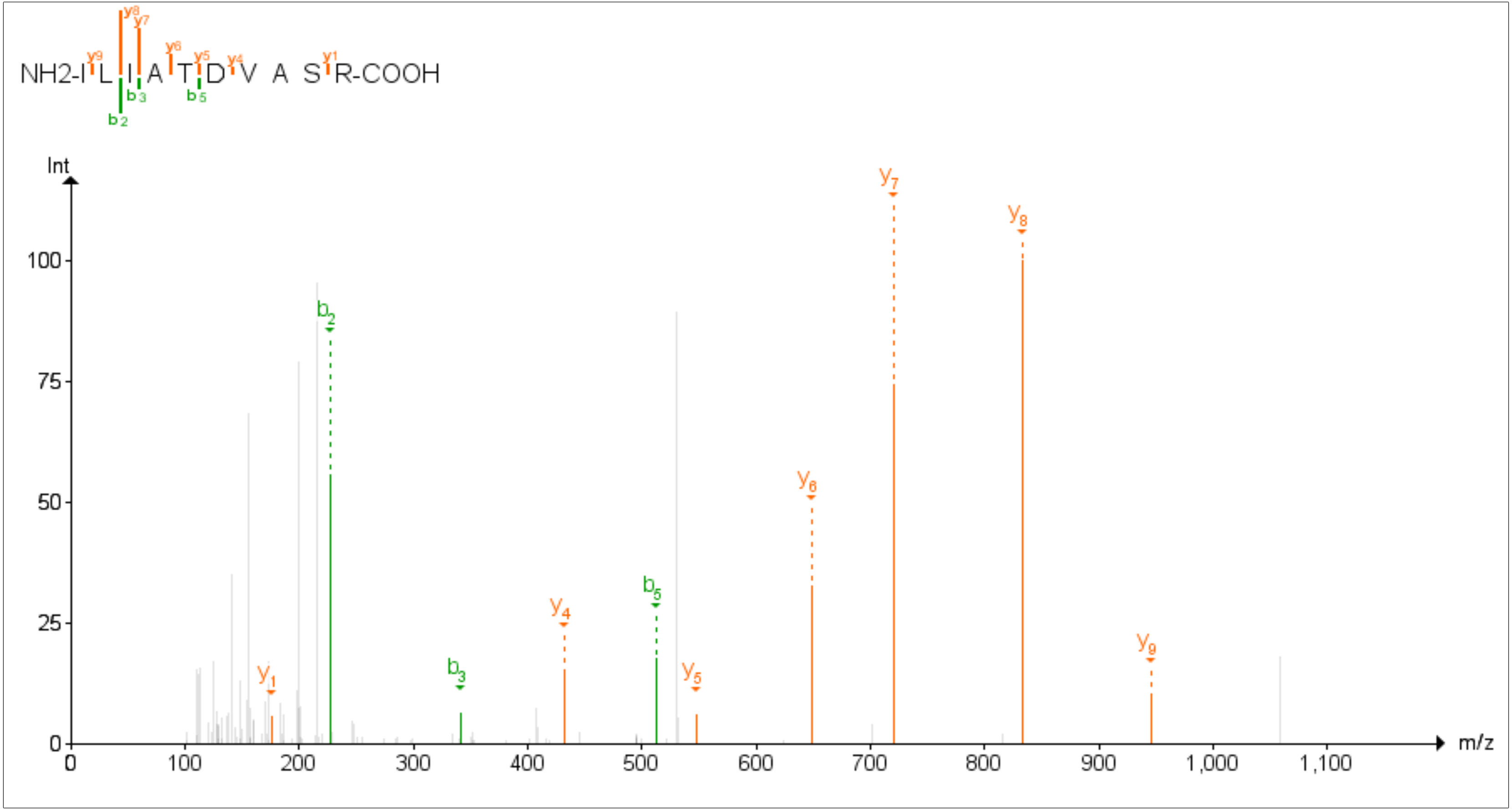

NH2-I L I A T D V A S R-COOH
y9 y8 y7 y6 y5 y4 y1
b2 b3 b5
Int
0
25
50
75
100
m/z
0 100 200 300 400 500 600 700 800 900 1,000 1,100
y_7
y_8
b_2
y_6
b_5
y_4
y_9
b_3
y_5
y_1

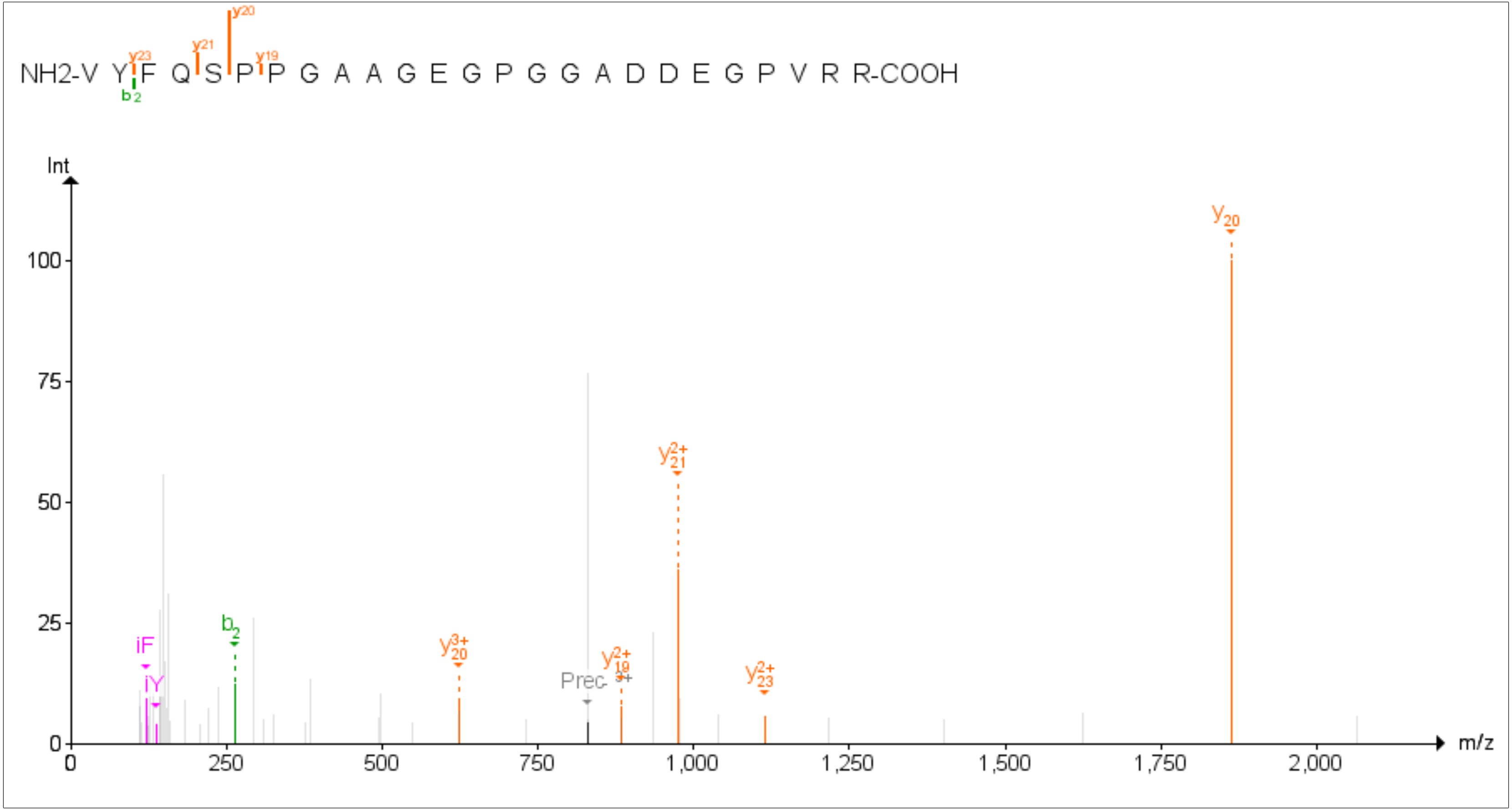

NH2-V Y F Q S P P G A A G E G P G G A D D E G P V R R-COOH
y23
y21
y20
y19
b2
Int
100
75
50
25
0
y20
y21 2+
iF
iY
b2
y20 3+
Prec 3+
y19 2+
y23 2+
0
250
500
750
1,000
1,250
1,500
1,750
2,000
m/z

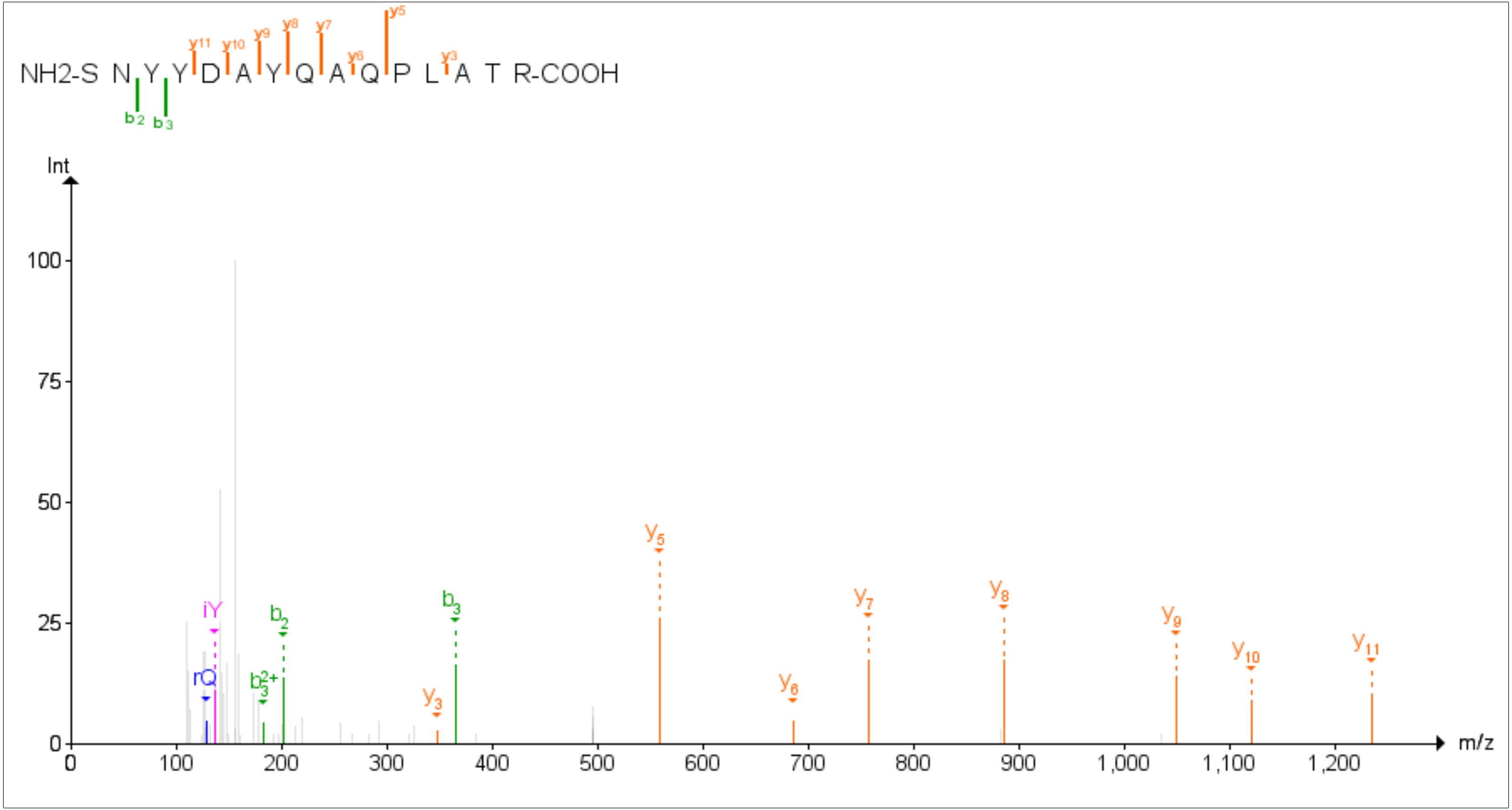
NH2-S N Y Y D A Y Q A Q P L A T R-COOH
y11 y10 y9 y8 y7 y6 y5 y3
b2 b3
Int
100
75
50
25
0
m/z
0 100 200 300 400 500 600 700 800 900 1,000 1,100 1,200
iY
rQ
b2
b3
b3 2+
y3
y5
y6
y7
y8
y9
y10
y11

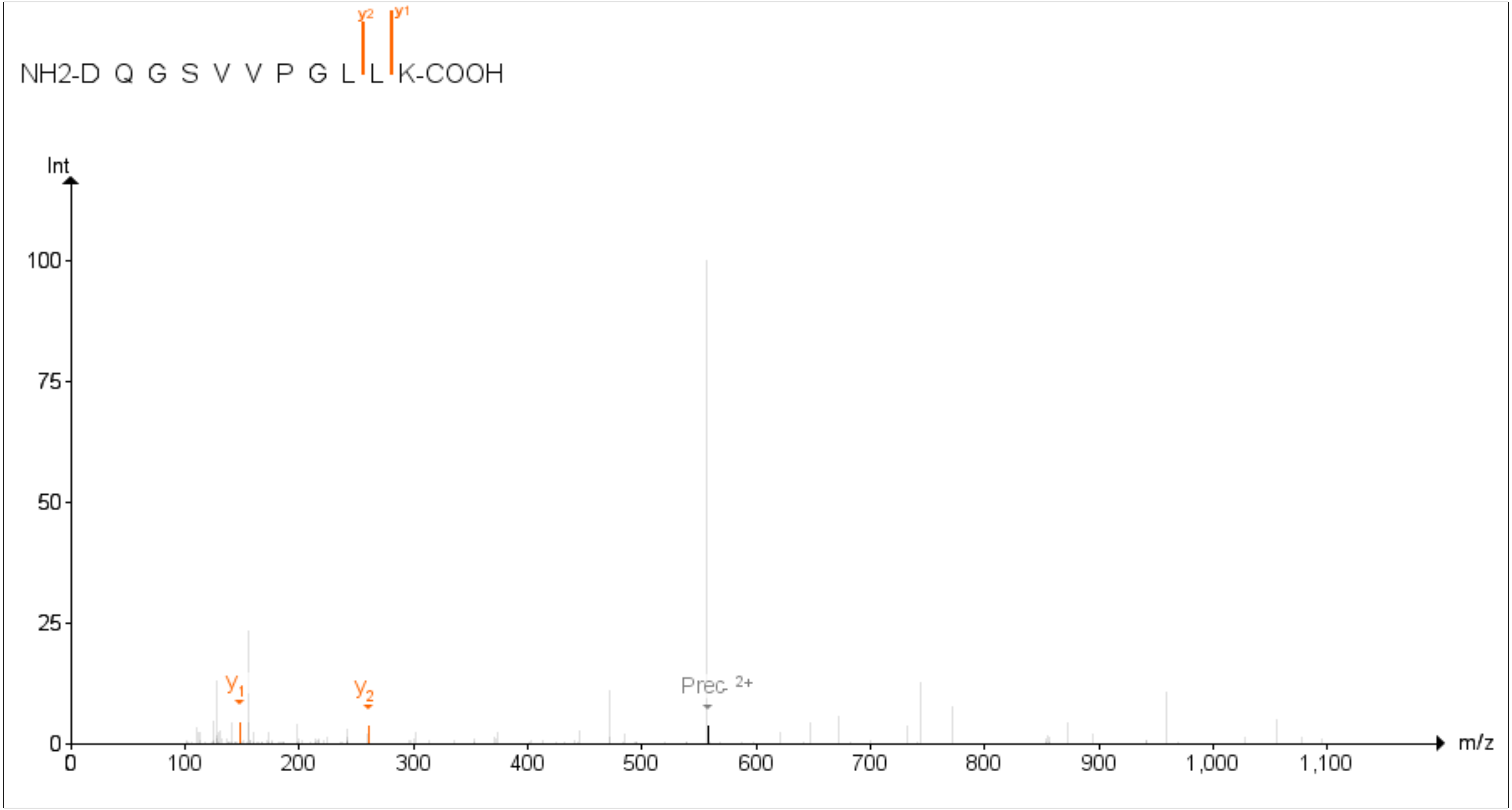

NH2-D Q G S V V P G L L K-COOH
y2
y1
Int
100
75
50
25
0
y1
y2
Prec. 2+
0
100
200
300
400
500
600
700
800
900
1,000
1,100
m/z

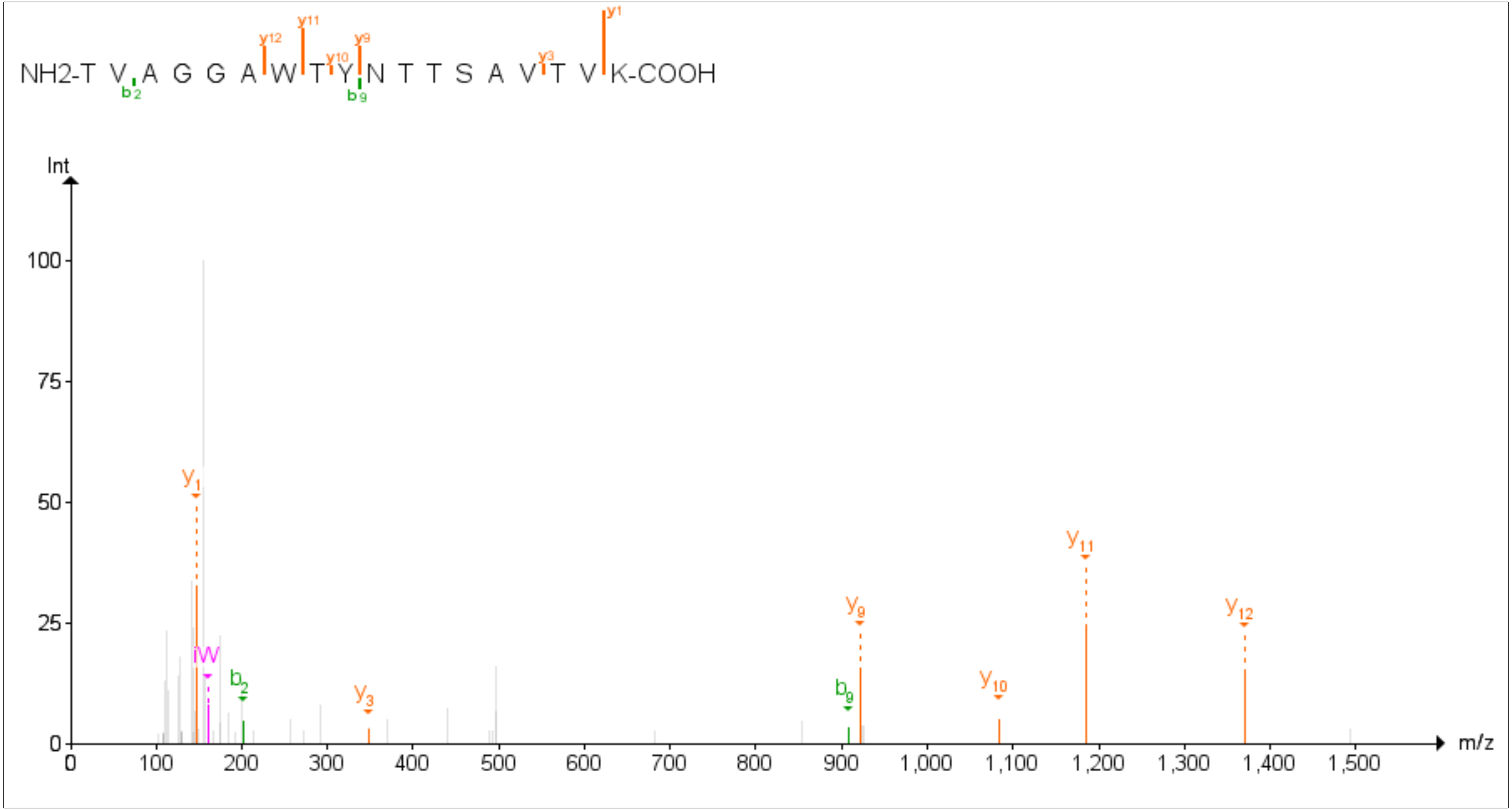

NH2-T V A G G A W T Y N T T S A V T V K-COOH
y12
y11
y10
y9
y3
y1
b2
b9
Int
100
75
50
25
0
y1
iW
b2
y3
b9
y9
y10
y11
y12
0
100
200
300
400
500
600
700
800
900
1,000
1,100
1,200
1,300
1,400
1,500
m/z

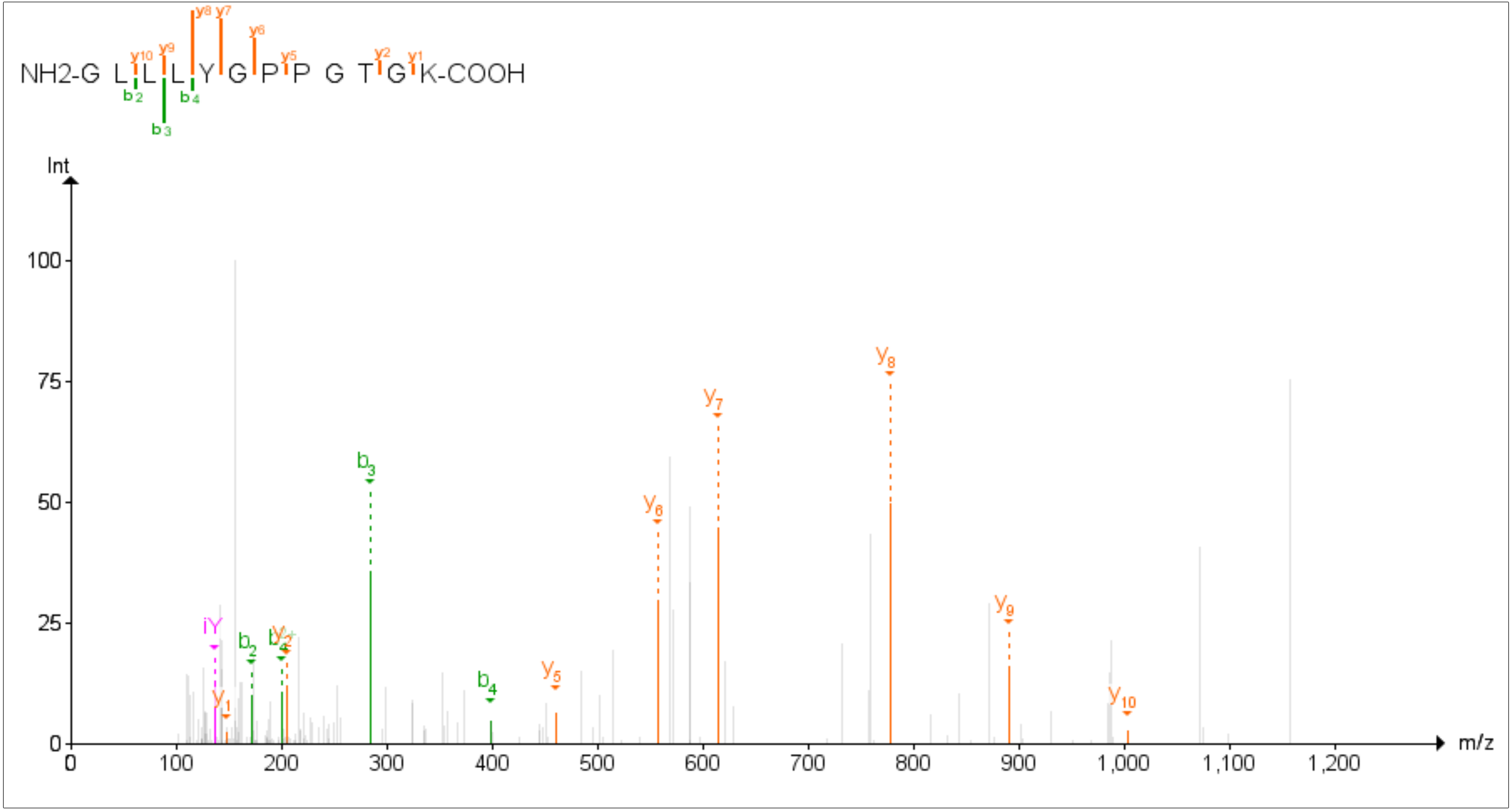
NH2-G L L L Y G P P G T G K-COOH
y10 y9 y8 y7 y6 y5 y2 y1
b2 b3 b4
Int
100
75
50
25
0
m/z
0
100
200
300
400
500
600
700
800
900
1,000
1,100
1,200
iY
y1
b2
b4
b3
y5
y6
y7
y8
y9
y10

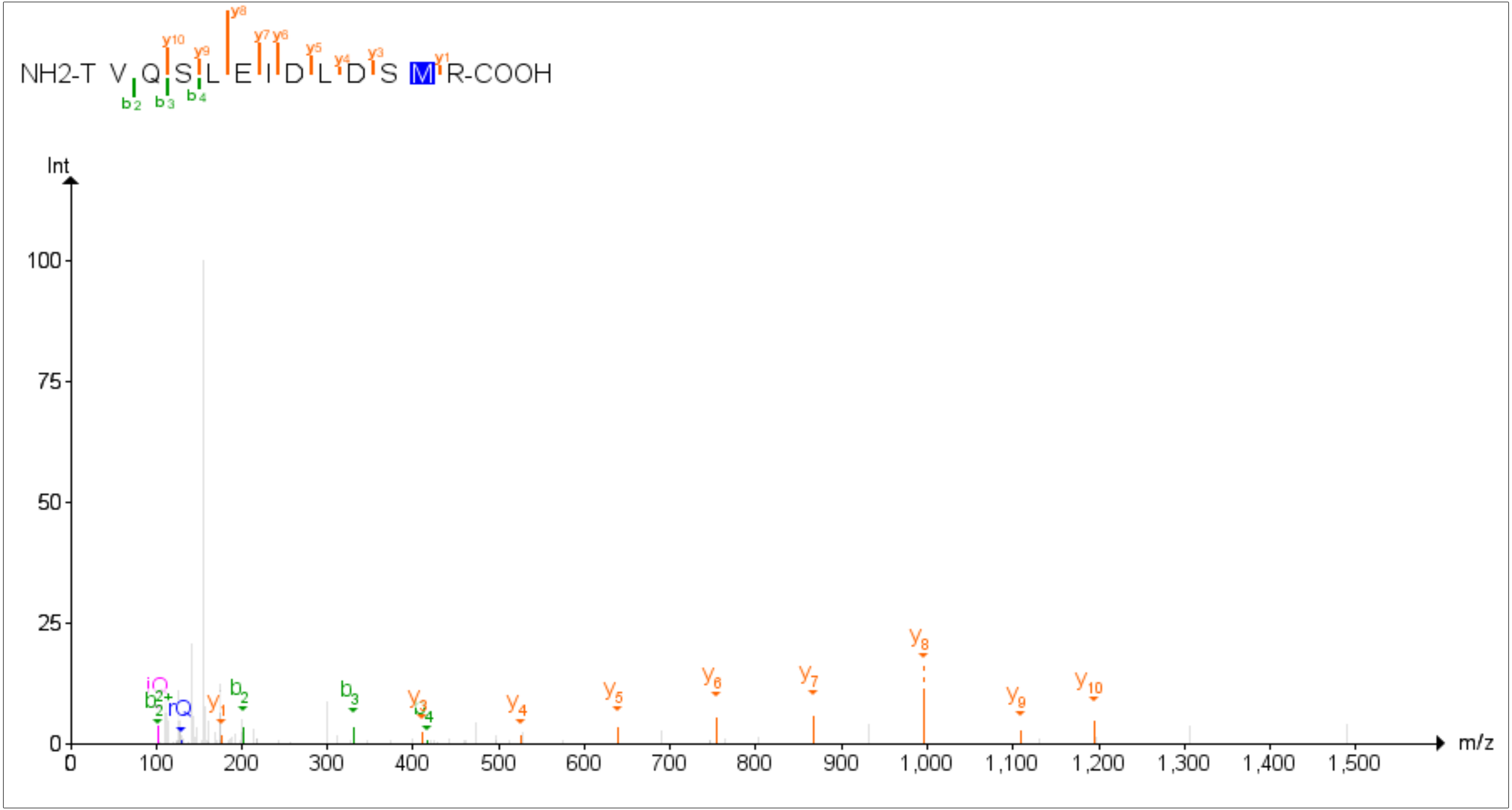

NH2-T V Q S L E I D L D S M R-COOH
Int
m/z

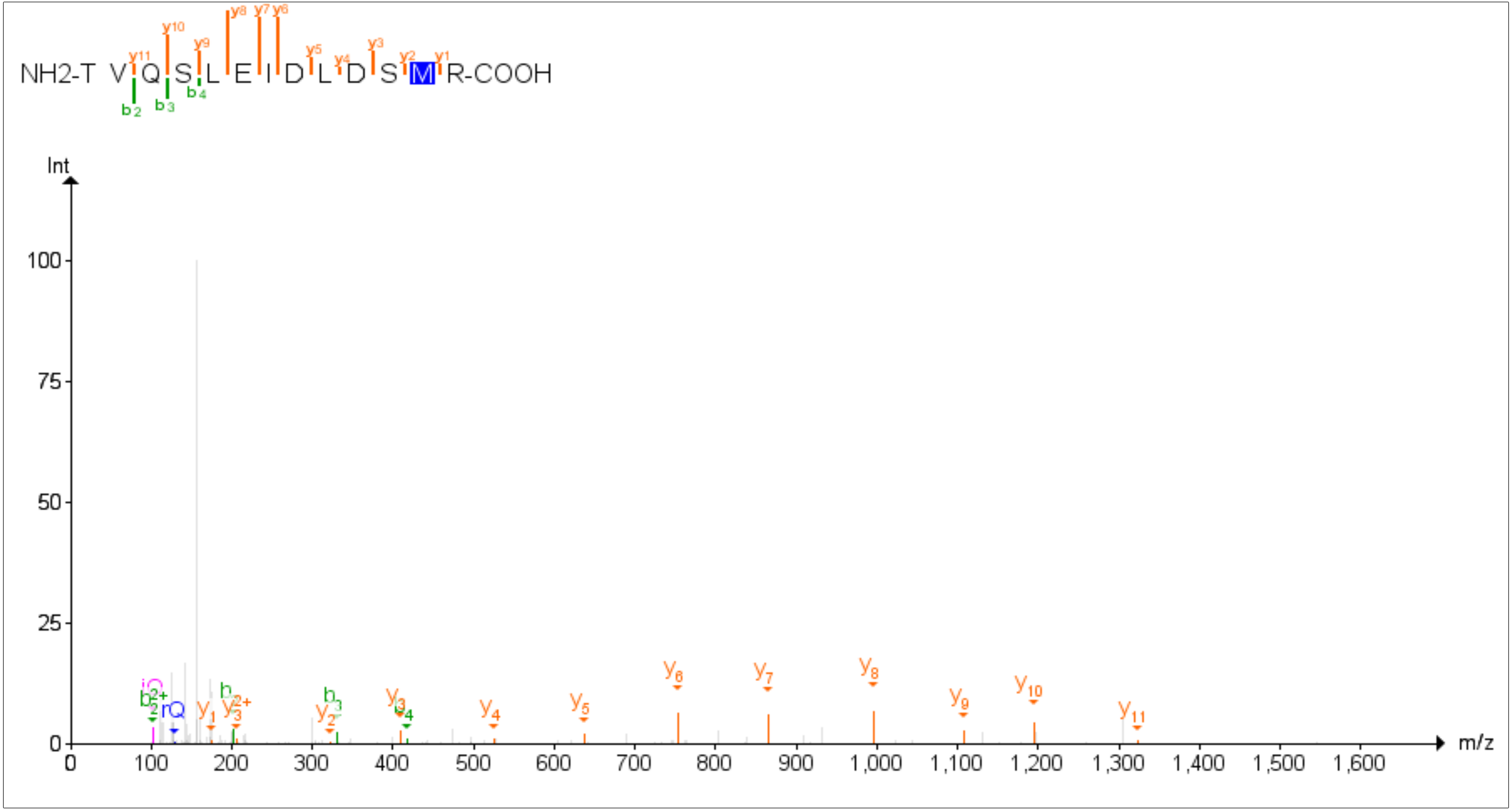

NH2-T V Q S L E I D L D S M R-COOH
y11 y10 y9 y8 y7 y6 y5 y4 y3 y2 y1
b2 b3 b4
Int
100
75
50
25
0
m/z
0 100 200 300 400 500 600 700 800 900 1,000 1,100 1,200 1,300 1,400 1,500 1,600
y1 y2 y3 y4 y5 y6 y7 y8 y9 y10 y11
b3 b4
rQ

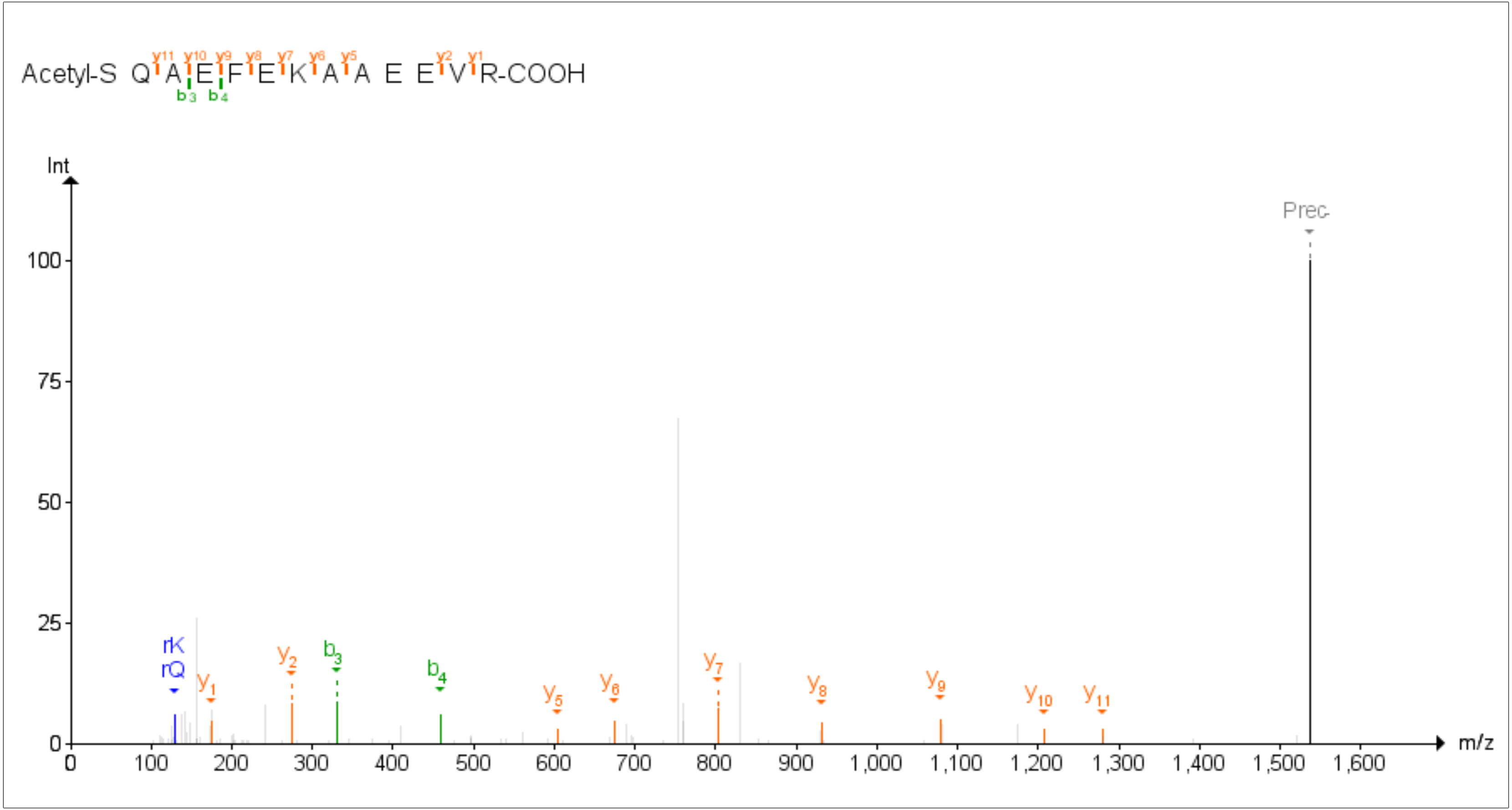

Acetyl-S Q A E F E K A A E E V R-COOH
y11 y10 y9 y8 y7 y6 y5 y2 y1
b3 b4
Int
Prec
rK
rQ
y1
y2
b3
b4
y5
y6
y7
y8
y9
y10
y11
0
25
50
75
100
0 100 200 300 400 500 600 700 800 900 1,000 1,100 1,200 1,300 1,400 1,500 1,600
m/z

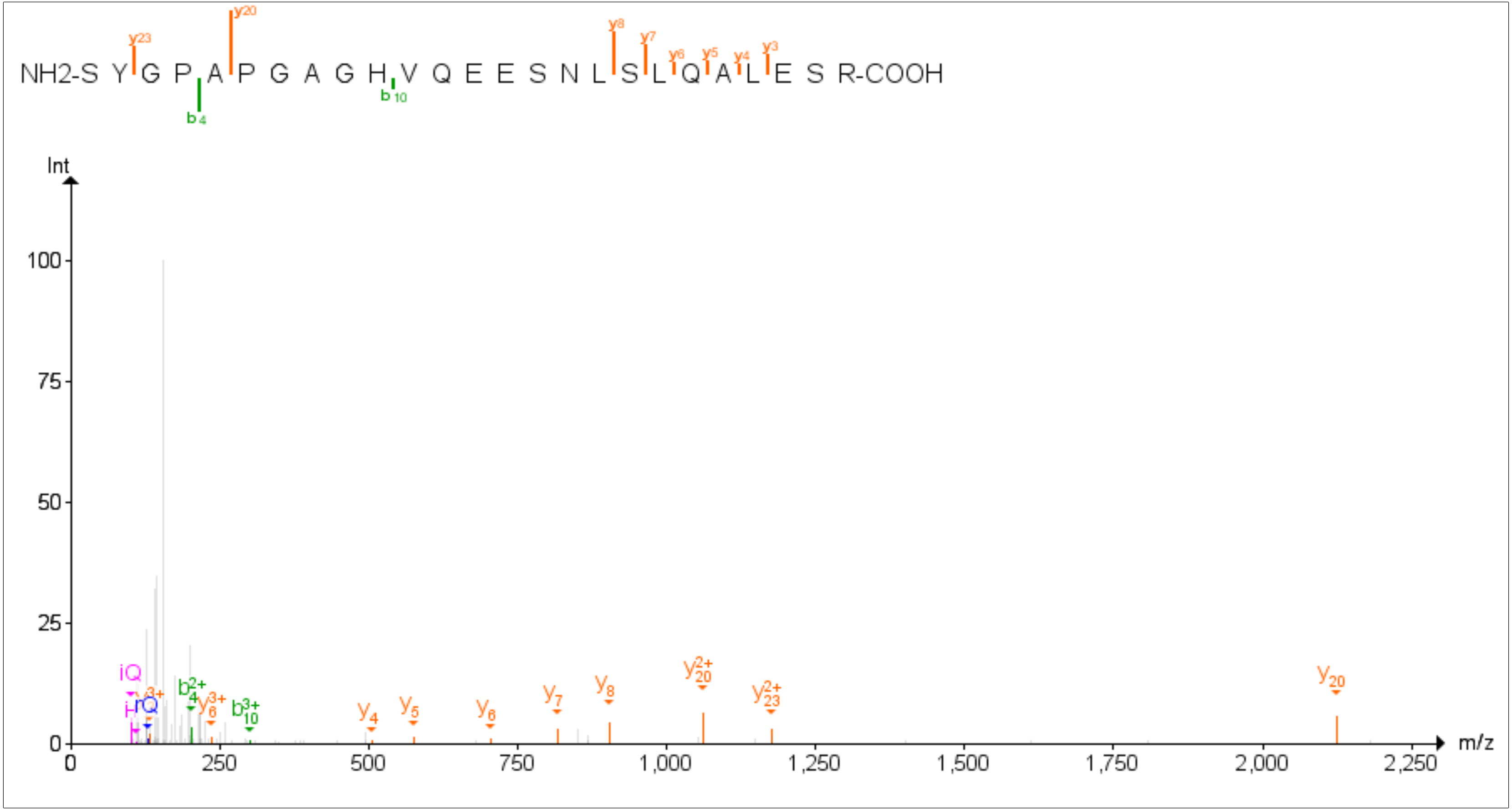

NH2-S Y G P A P G A G H V Q E E S N L S L Q A L E S R-COOH
y23
y20
y8
y7
y6
y5
y4
y3
b4
b10
Int
100
75
50
25
0
iQ
rQ
b4 2+
y6 3+
b10 3+
y4
y5
y6
y7
y8
y20 2+
y23 2+
y20
0
250
500
750
1,000
1,250
1,500
1,750
2,000
2,250
m/z

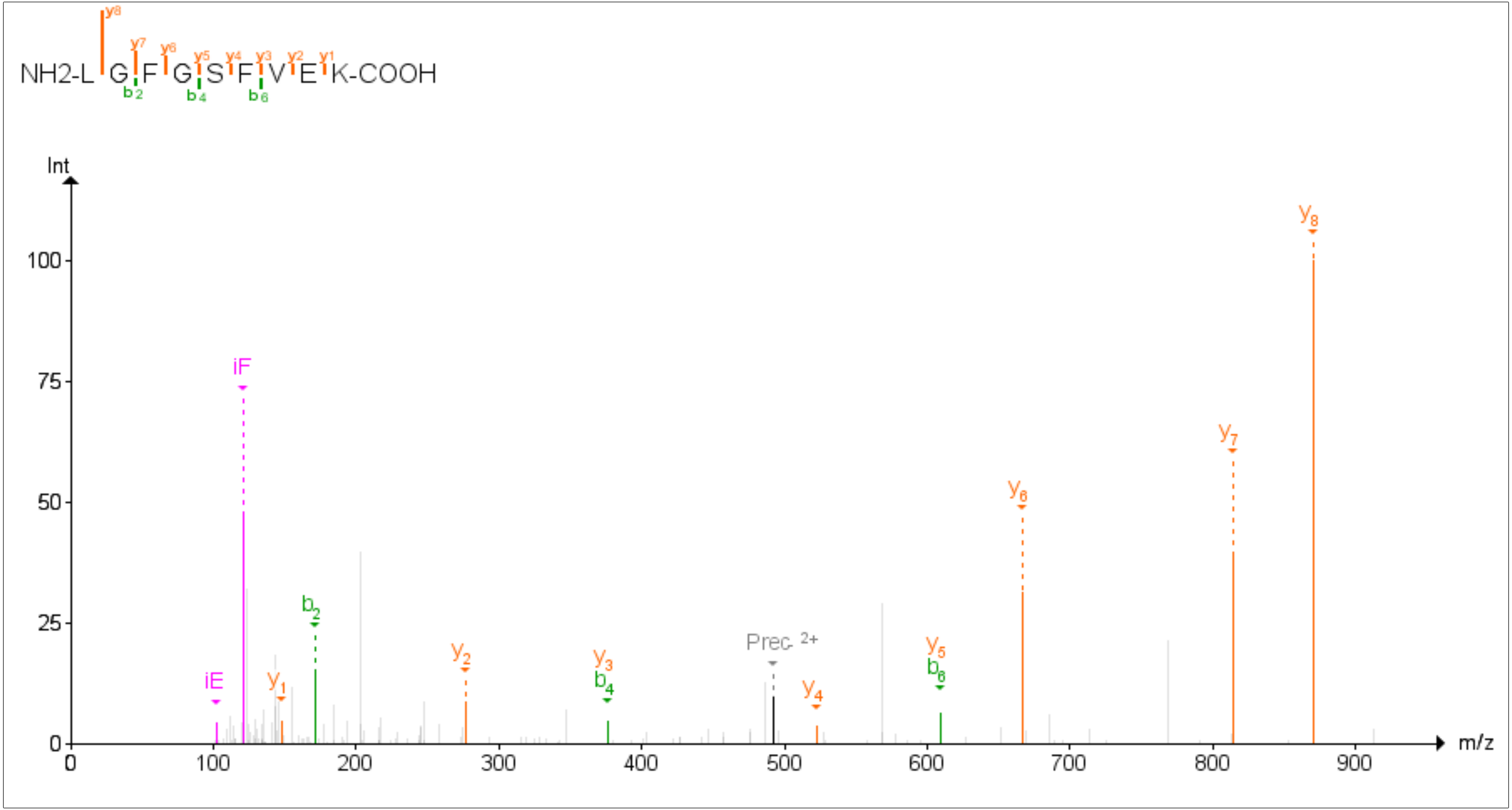

NH2-L G F G S F V E K-COOH
y8
y7
y6
y5
y4
y3
y2
y1
b2
b4
b6
Int
100
75
50
25
0
iF
iE
y1
b2
y2
y3
b4
Prec. 2+
y4
y5
b6
y6
y7
y8
0
100
200
300
400
500
600
700
800
900
m/z

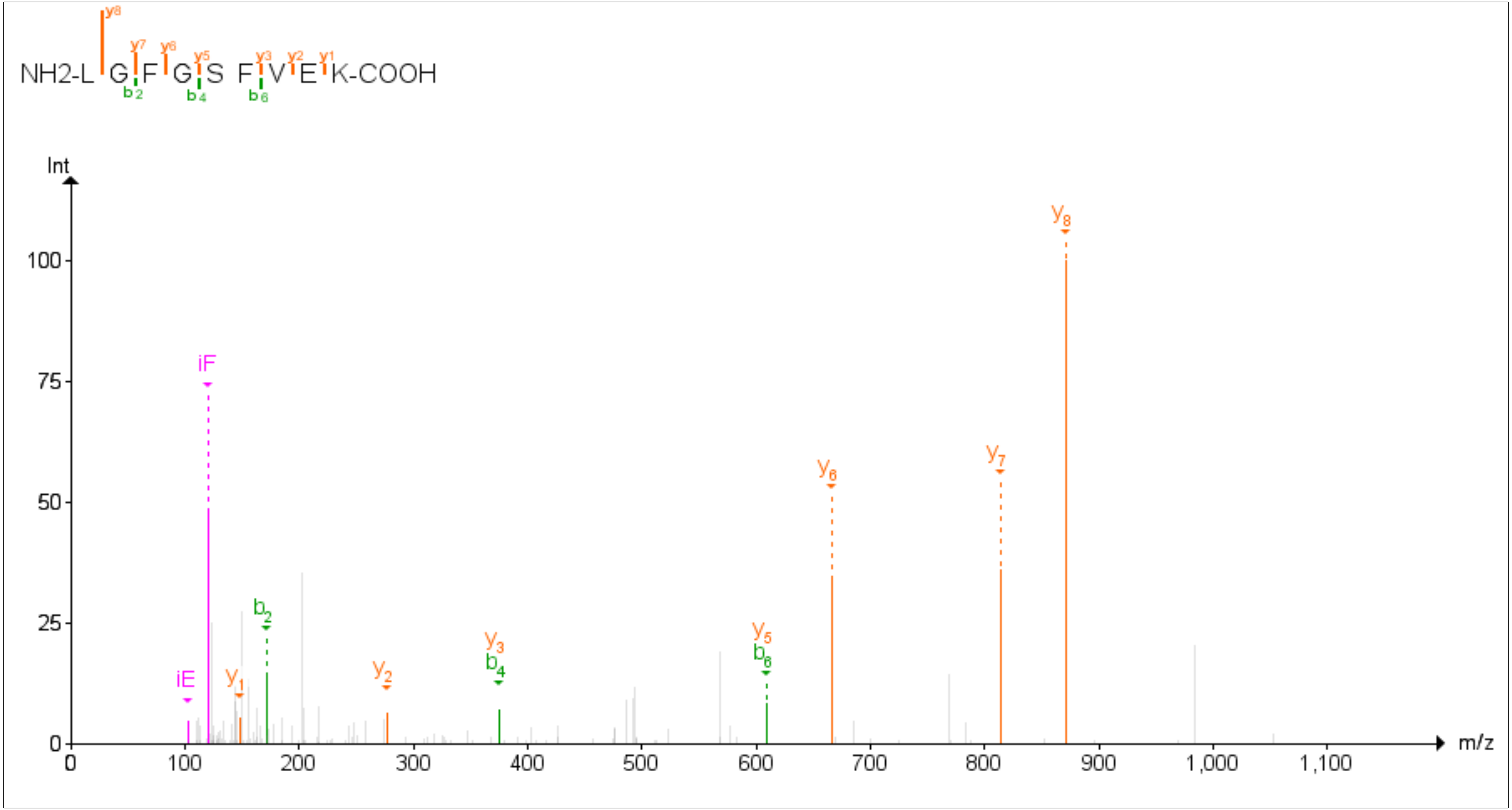

NH2-L G F G S F V E K-COOH
y8
y7
y6
y5
y3
y2
y1
b2
b4
b6
Int
100
75
50
25
0
iF
iE
y1
b2
y2
y3
b4
y5
b6
y6
y7
y8
0
100
200
300
400
500
600
700
800
900
1,000
1,100
m/z

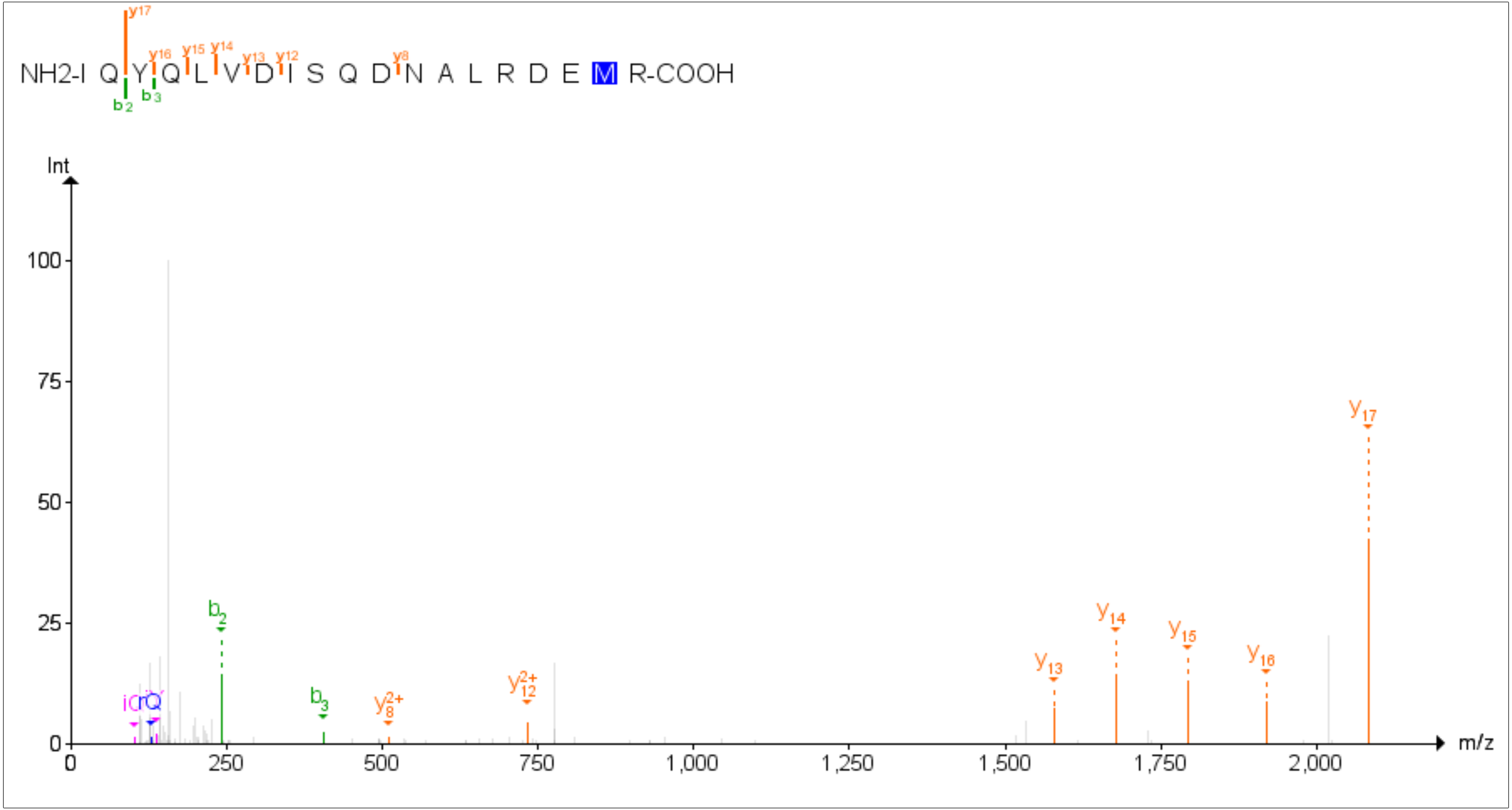

NH2-I Q Y Q L V D I S Q D N A L R D E M R-COOH
y17 y16 y15 y14 y13 y12 y8
b2 b3
Int
100
75
50
25
0
m/z
0 250 500 750 1,000 1,250 1,500 1,750 2,000
b2
b3
y_8^{2+}
y_{12}^{2+}
y13
y14
y15
y16
y17

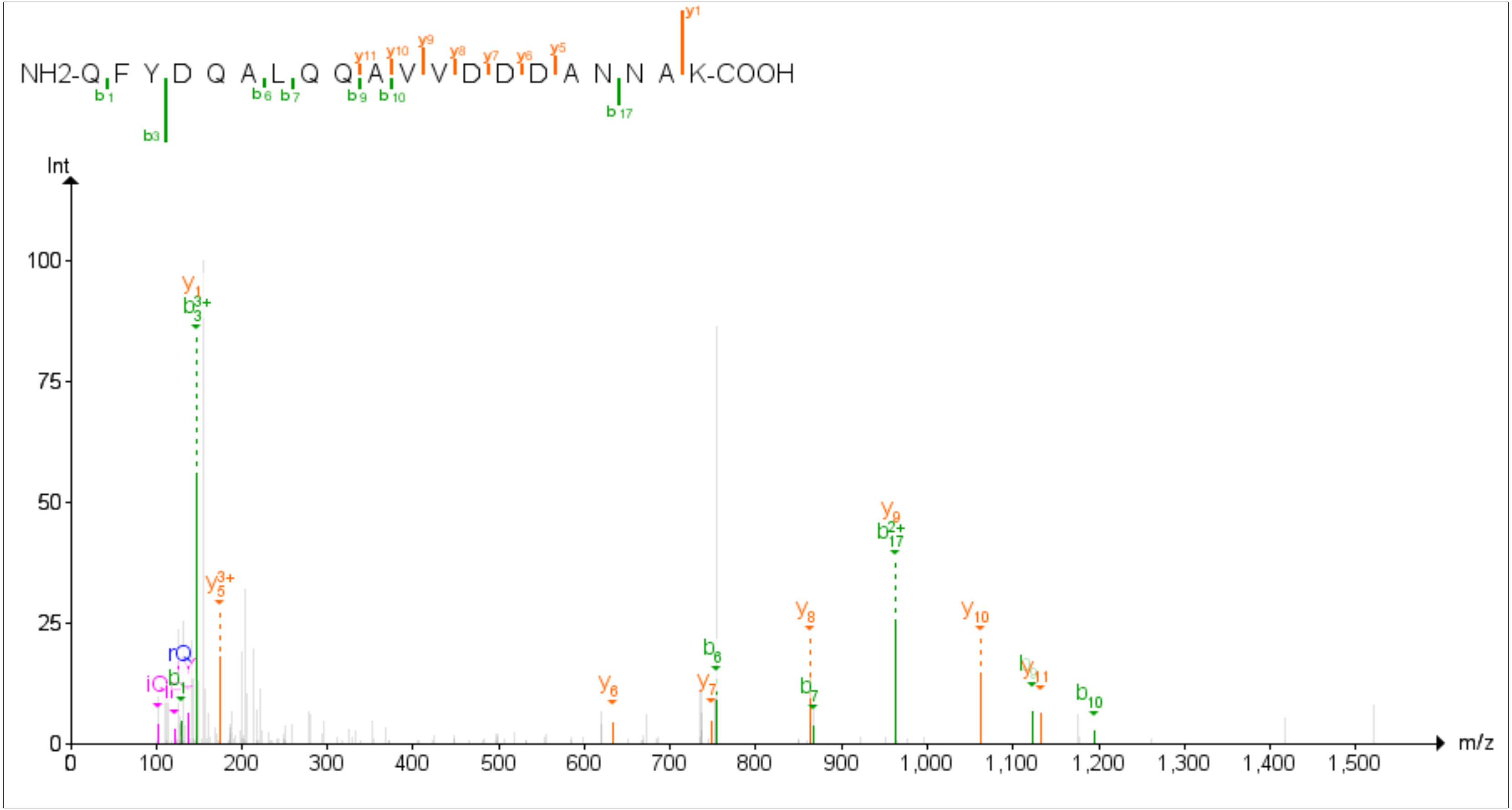

NH2-Q F Y D Q A L Q Q A V V D D D A N N A K-COOH
Int
m/z
0
25
50
75
100
0
100
200
300
400
500
600
700
800
900
1,000
1,100
1,200
1,300
1,400
1,500

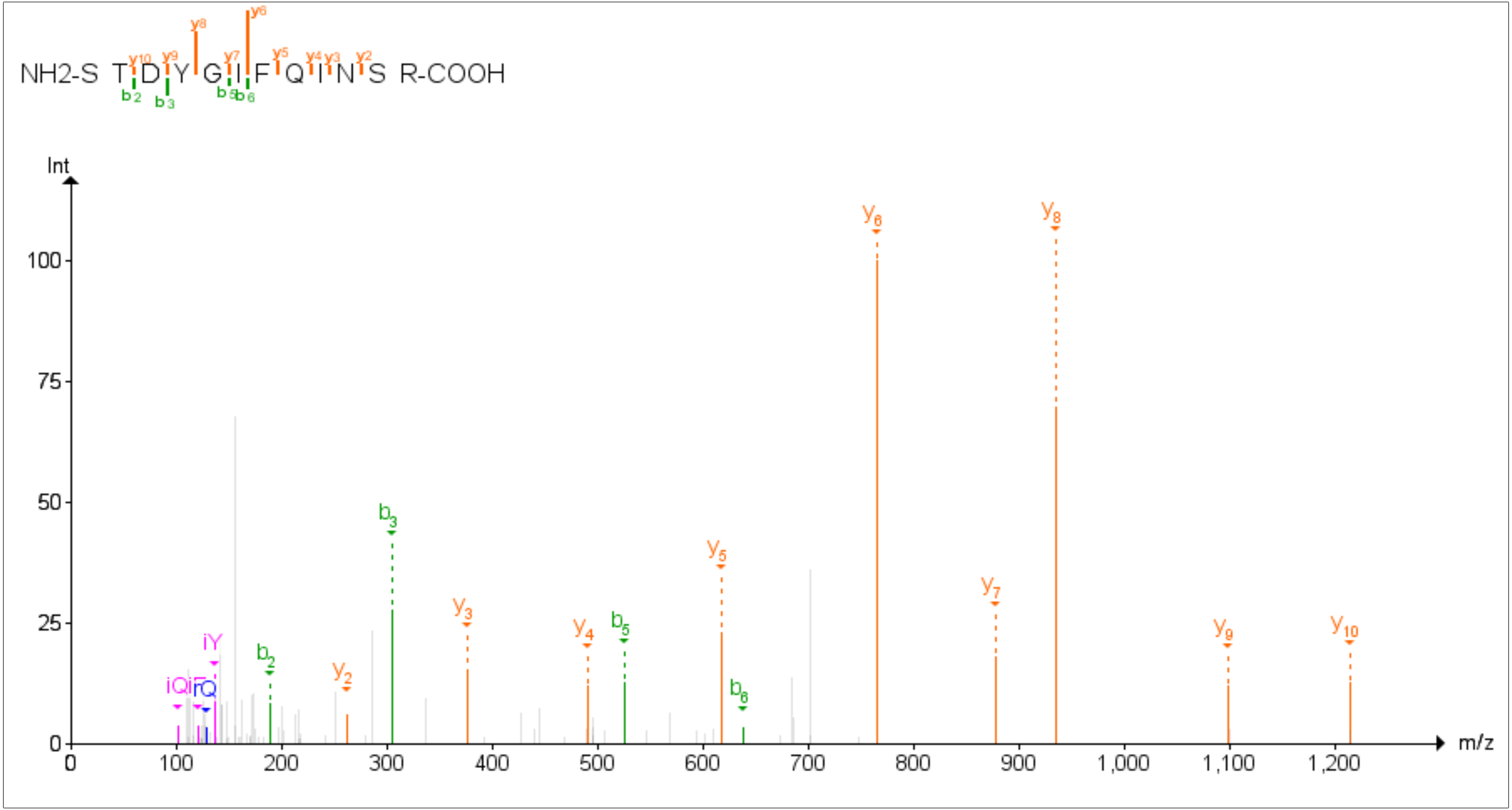
NH2-S T D Y G I I F Q I N S R-COOH
y10 y9 y8 y7 y6 y5 y4 y3 y2
b2 b3 b5 b6
Int
100
75
50
25
0
m/z
0 100 200 300 400 500 600 700 800 900 1,000 1,100 1,200
y6 y8 b3 y5 y7 y3 y4 b5 y9 y10 iY b2 y2 b6 iQ iF rQ

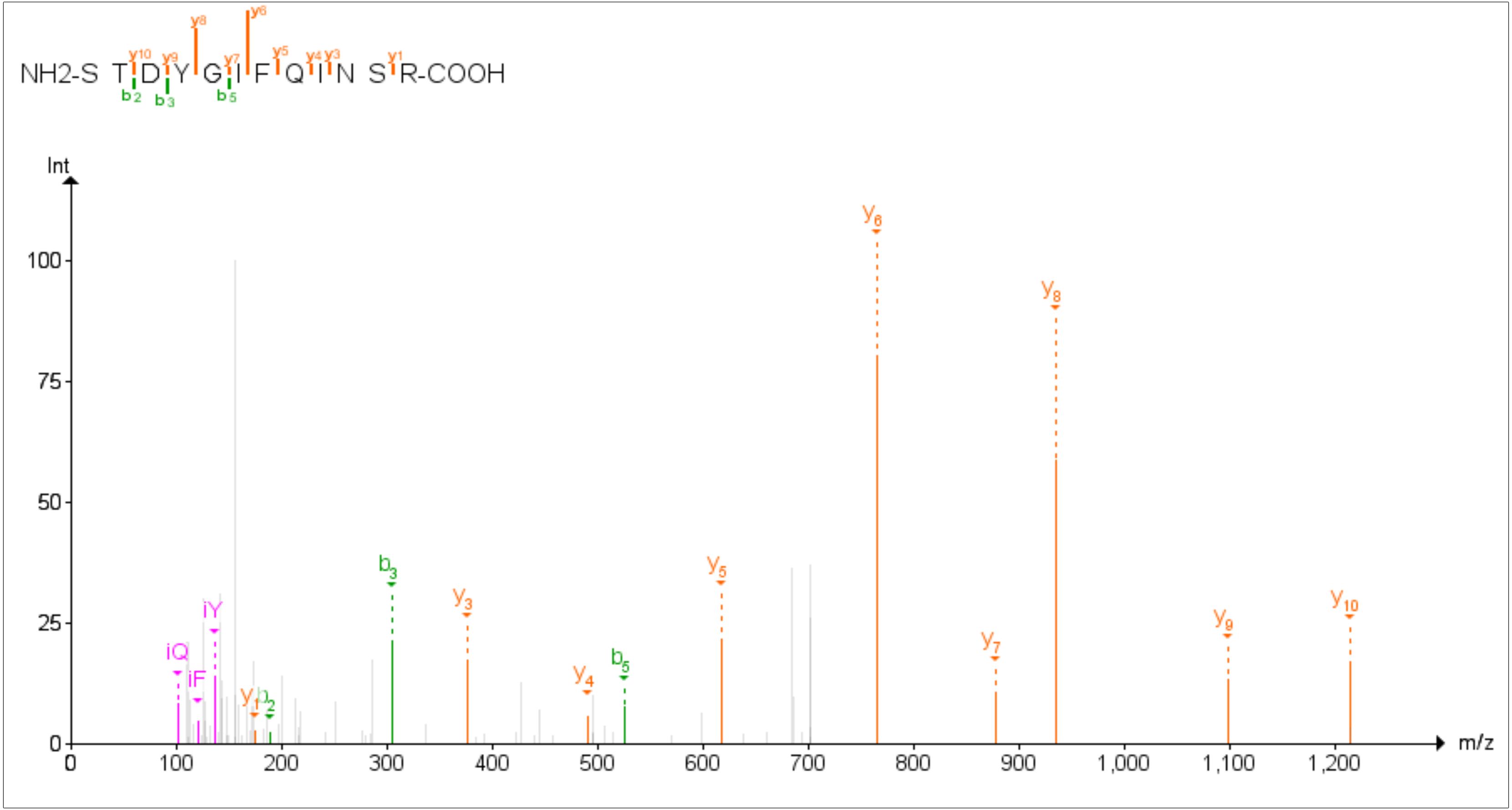
NH2-S T D Y G I F Q I N S R-COOH
y10 y9 y8 y7 y6 y5 y4 y3 y1
b2 b3 b5
Int
100
75
50
25
0
m/z
0 100 200 300 400 500 600 700 800 900 1,000 1,100 1,200
y6
y8
b3
y5
y3
y10
y9
iY
iQ
iF
y1
b2
y4
b5
y7

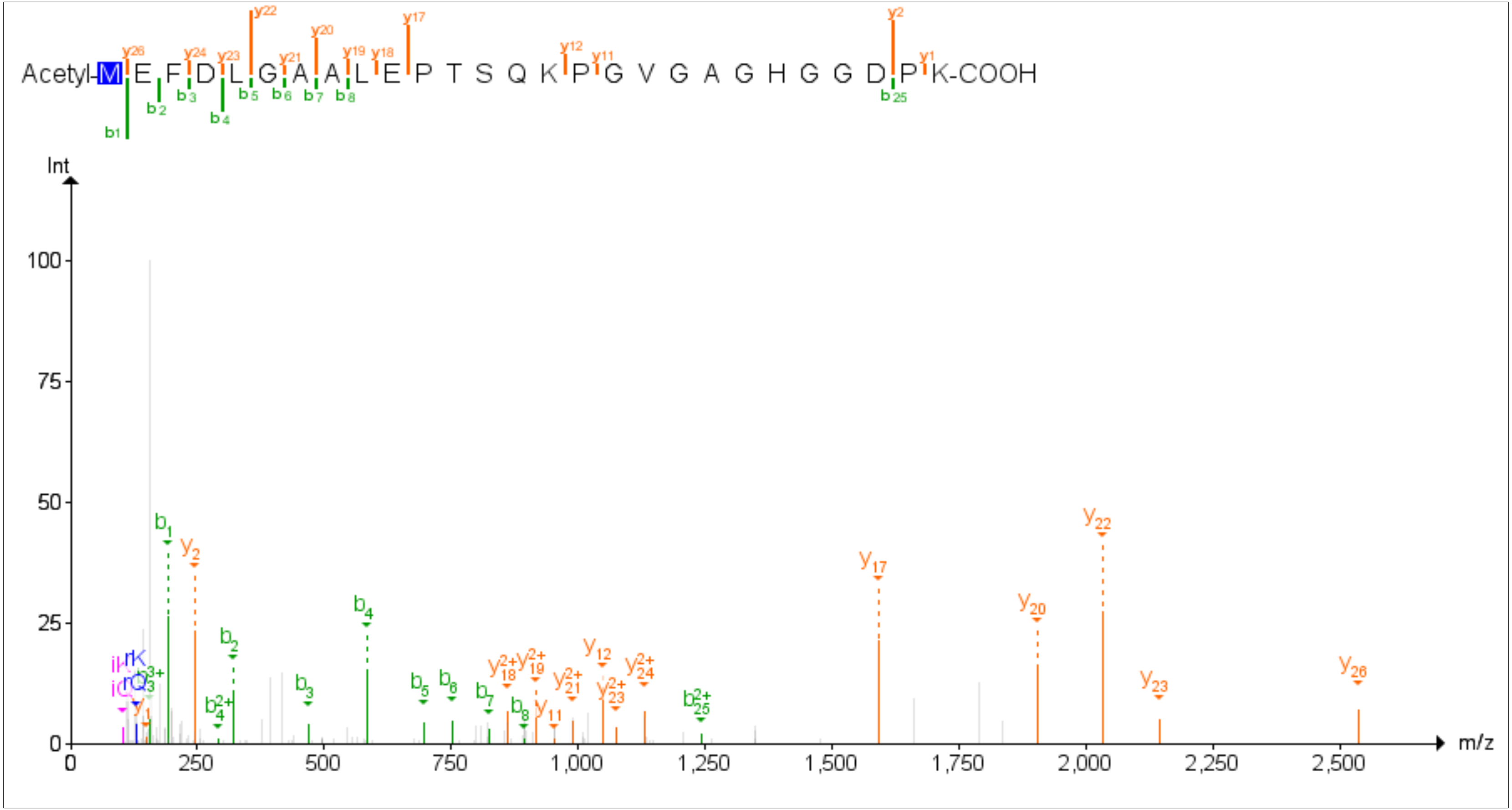
Acetyl-M E F D L G A A L E P T S Q K P G V G A G H G G D P K-COOH
Int
m/z
0
25
50
75
100
250
500
750
1,000
1,250
1,500
1,750
2,000
2,250
2,500

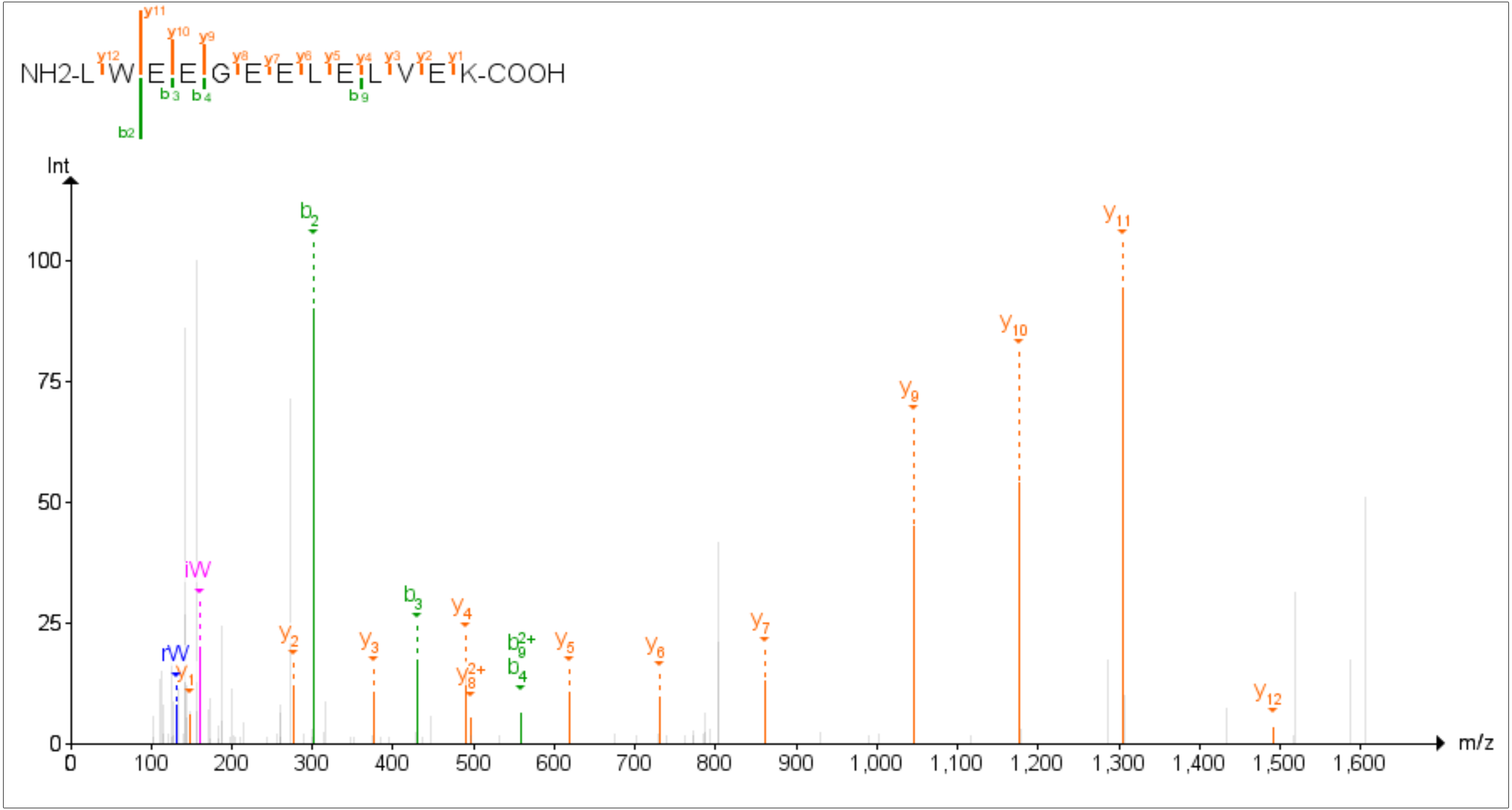

NH2-L W E E G E E L E L V E K-COOH
y12 y11 y10 y9 y8 y7 y6 y5 y4 y3 y2 y1
b2 b3 b4 b9
Int
m/z
0 25 50 75 100
0 100 200 300 400 500 600 700 800 900 1,000 1,100 1,200 1,300 1,400 1,500 1,600
b2
y11
y10
y9
iW
rW
y1
y2
y3
b3
y4
y8 2+
b9 2+
b4
y5
y6
y7
y12

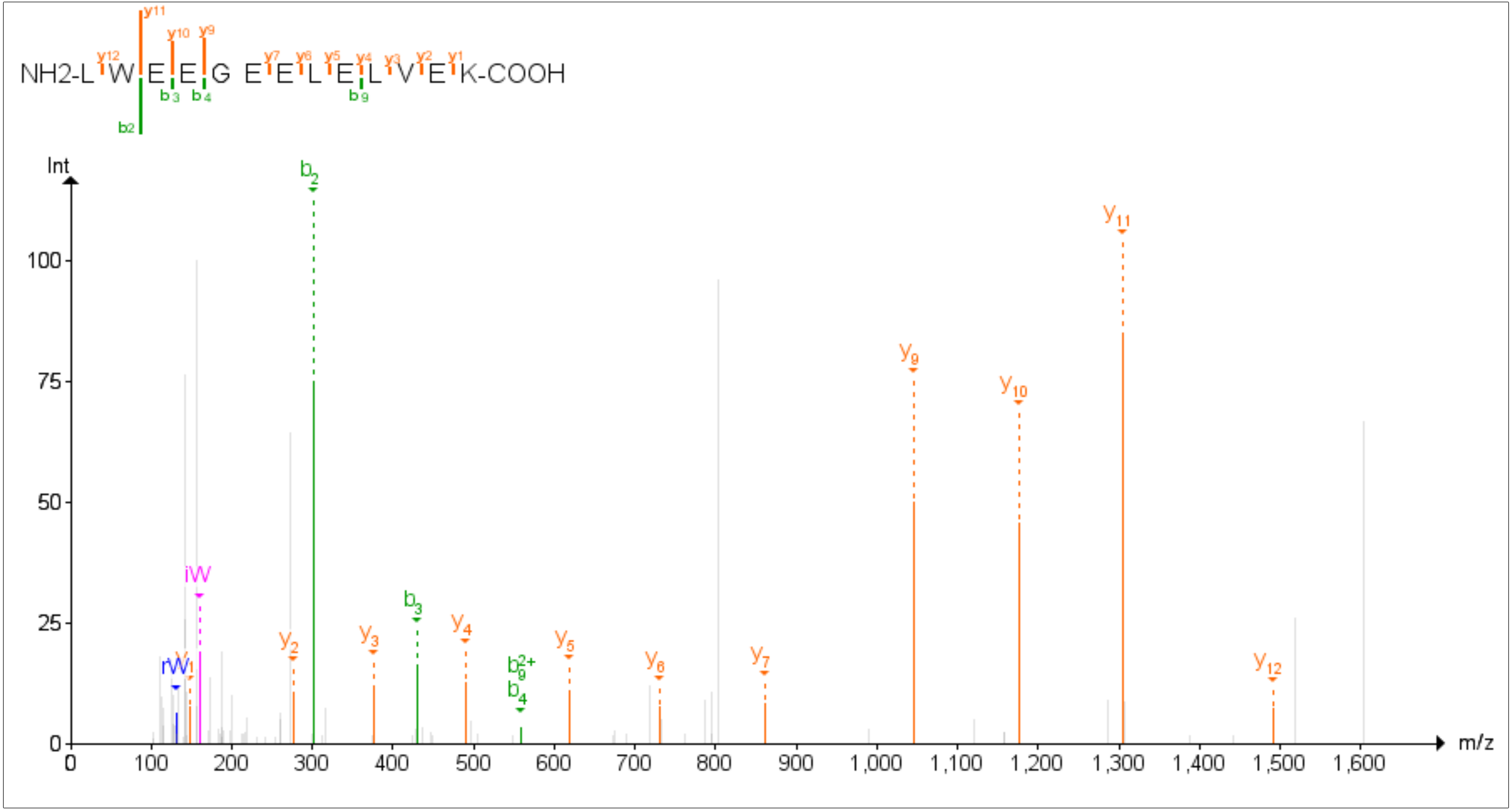

NH2-L W E E G E E L E L V E K-COOH
y12 y11 y10 y9 y7 y6 y5 y4 y3 y2 y1
b2 b3 b4 b9
Int
m/z
b2
y11
y9
y10
iW
rW
y1
y2
y3
b3
y4
b9 2+
b4
y5
y6
y7
y12
0
25
50
75
100
0
100
200
300
400
500
600
700
800
900
1,000
1,100
1,200
1,300
1,400
1,500
1,600

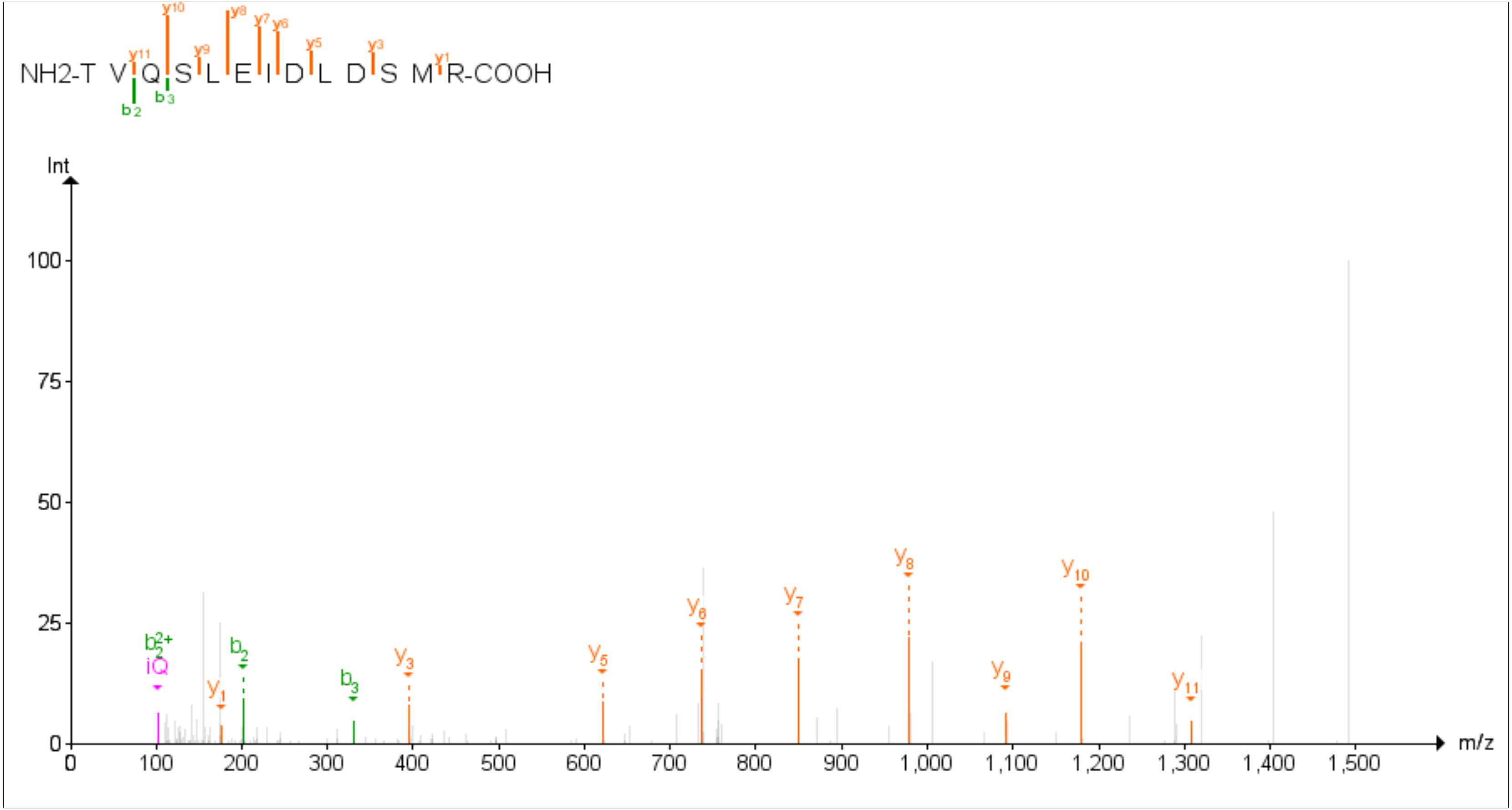

NH2-T V Q S L E I D L D S M R-COOH
y11 y10 y9 y8 y7 y6 y5 y3 y1
b2 b3
Int
100
75
50
25
0
m/z
0 100 200 300 400 500 600 700 800 900 1,000 1,100 1,200 1,300 1,400 1,500
b_2^{2+}
iQ
y_1
b_2
b_3
y_3
y_5
y_6
y_7
y_8
y_9
y_{10}
y_{11}

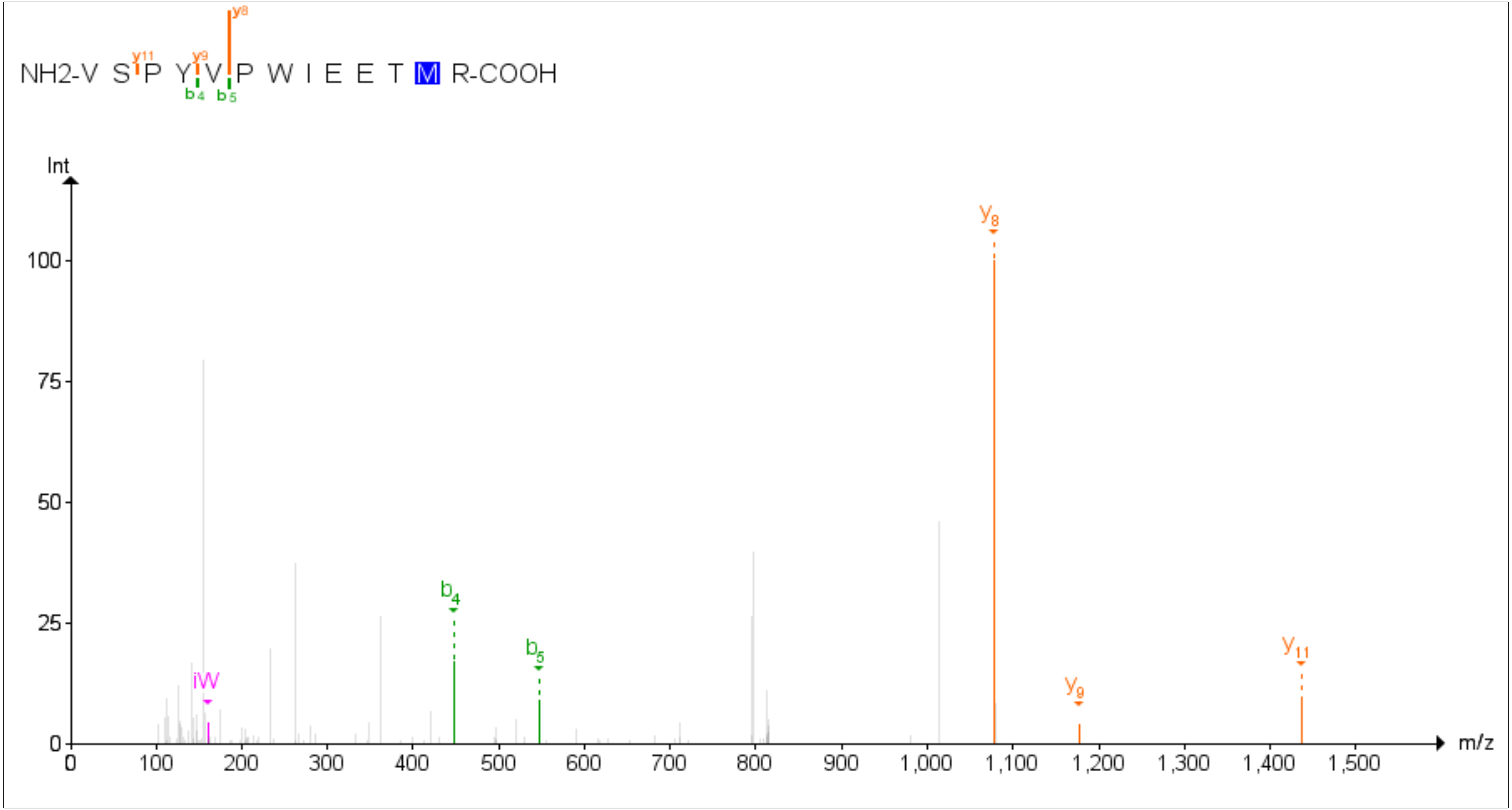

NH2-V S P Y V P W I E E T M R-COOH
y11
y9
y8
b4
b5
Int
100
75
50
25
0
iW
b4
b5
y8
y9
y11
0
100
200
300
400
500
600
700
800
900
1,000
1,100
1,200
1,300
1,400
1,500
m/z

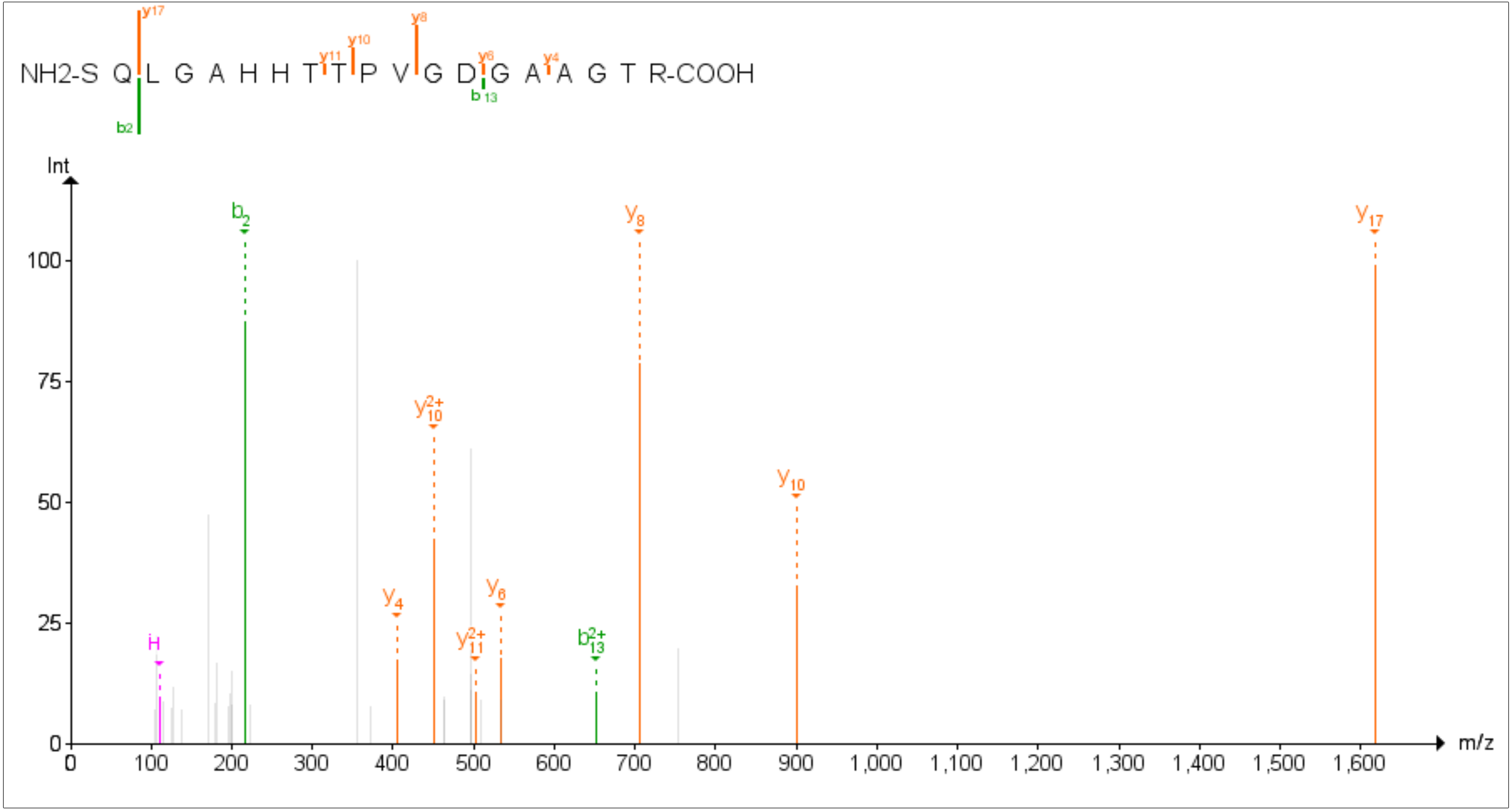

NH2-S Q L G A H H T T P V G D G A A G T R-COOH
y17
y11
y10
y8
y6
y4
b2
b 13
Int
m/z
b2
y8
y17
y10 2+
y10
y4
y6
y11 2+
b13 2+
H
0
25
50
75
100
0
100
200
300
400
500
600
700
800
900
1,000
1,100
1,200
1,300
1,400
1,500
1,600

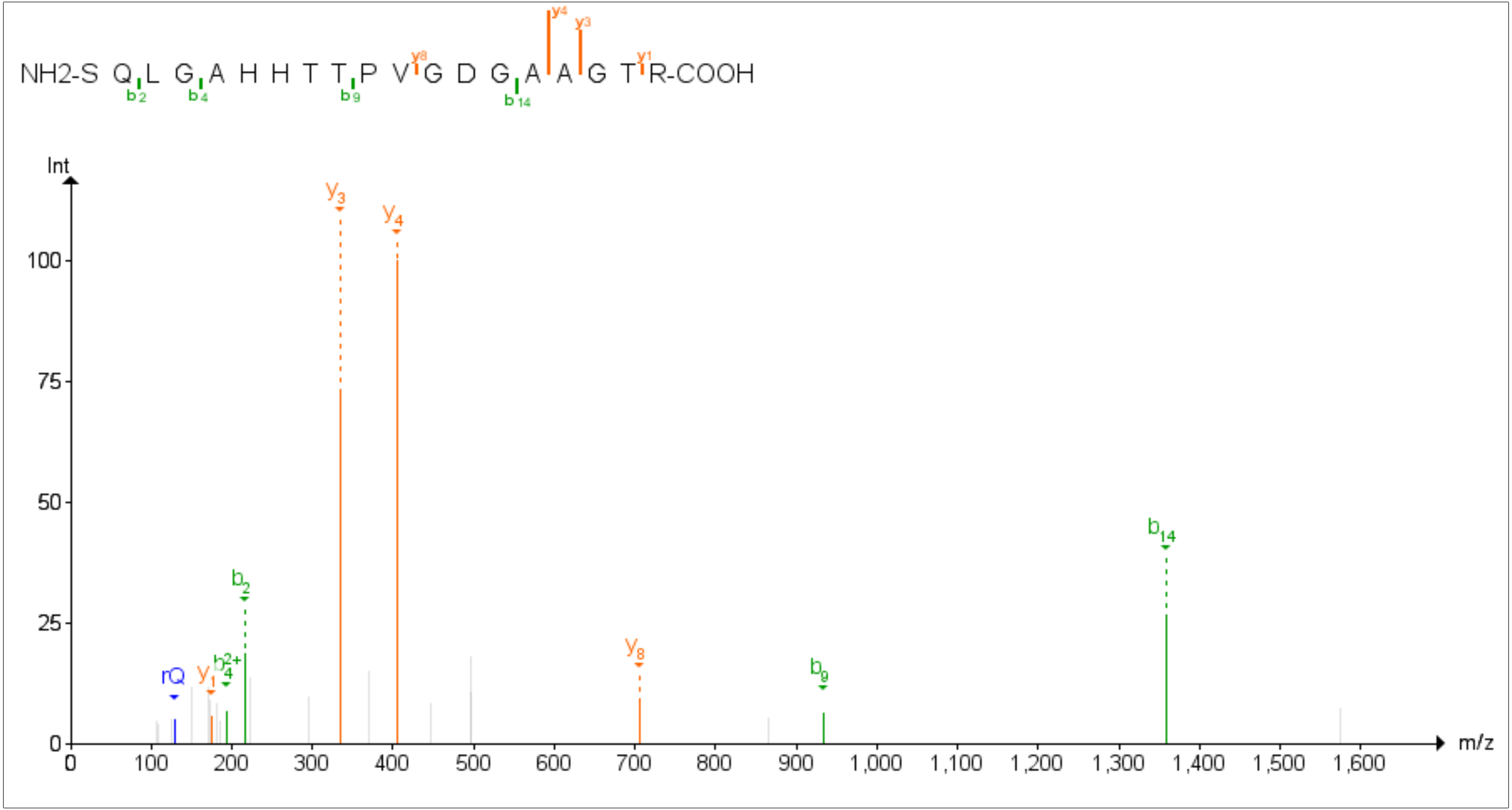

NH2-S Q L G A H H T T P V G D G A A G T R-COOH
b2
b4
b9
b14
y8
y4
y3
y1
Int
100
75
50
25
0
y3
y4
b14
b2
rQ
y1
b4 2+
y8
b9
0
100
200
300
400
500
600
700
800
900
1,000
1,100
1,200
1,300
1,400
1,500
1,600
m/z

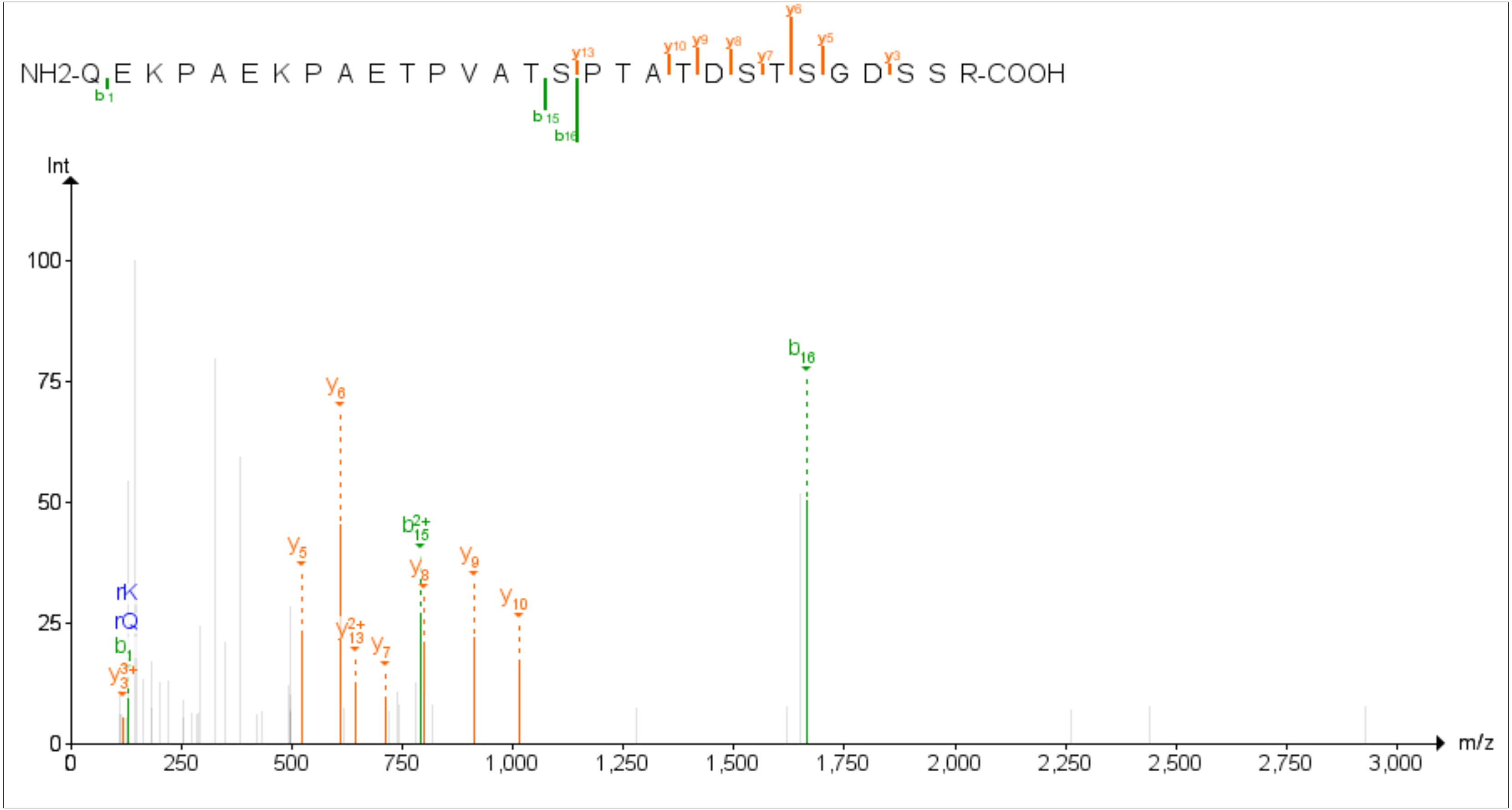

NH2-Q E K P A E K P A E T P V A T S P T A T D S T S G D S S R-COOH
b1
b15
b16
y13
y10
y9
y8
y7
y6
y5
y3
Int
100
75
50
25
0
m/z
0
250
500
750
1,000
1,250
1,500
1,750
2,000
2,250
2,500
2,750
3,000
rK
rQ
b1
y3 3+
y5
y6
y13 2+
y7
b15 2+
y8
y9
y10
b16

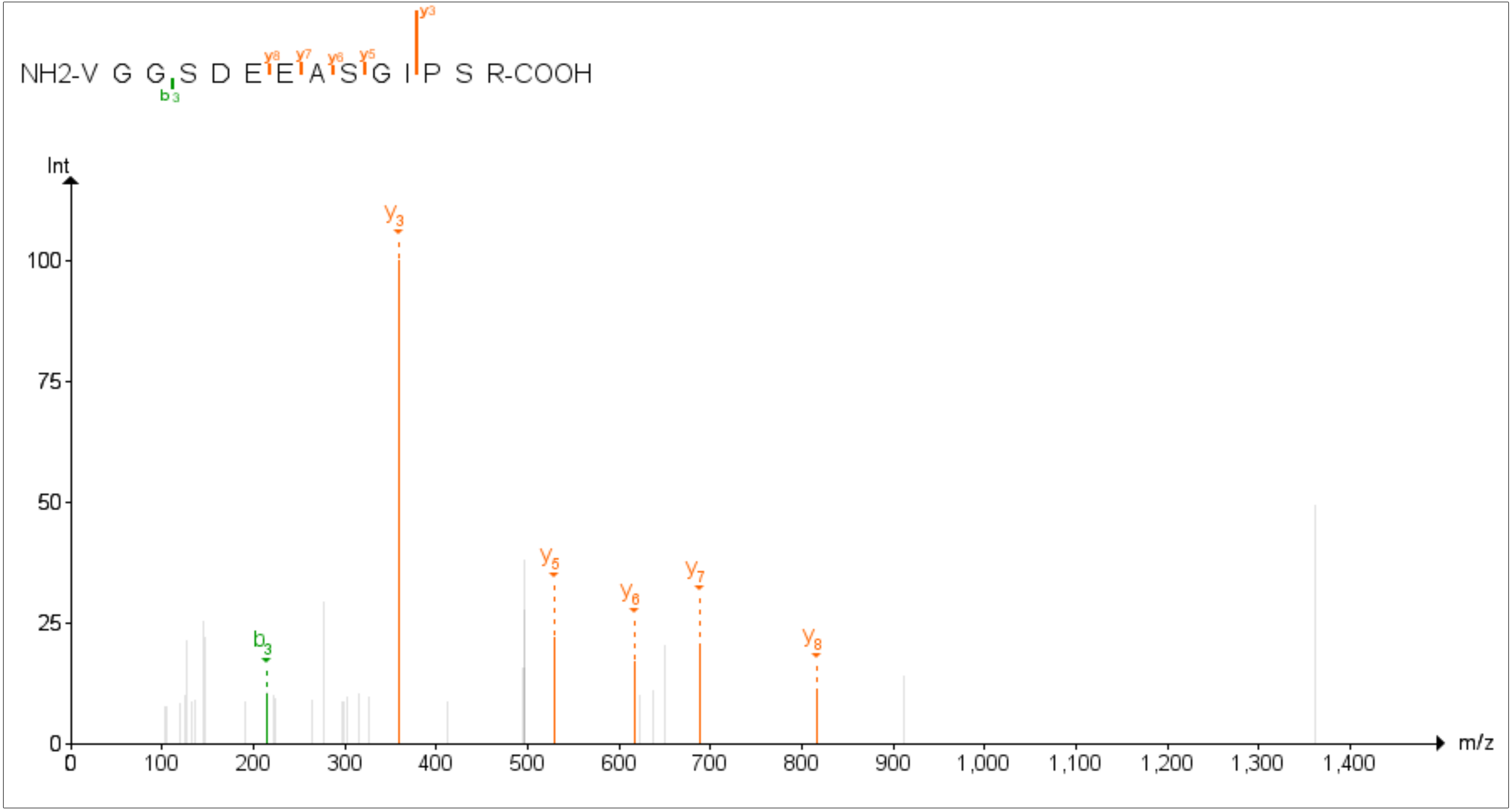

NH2-V G G S D E E A S G I P S R-COOH
b3
y8 y7 y6 y5 y3
Int
100
75
50
25
0
y3
y5
y6
y7
y8
b3
0 100 200 300 400 500 600 700 800 900 1,000 1,100 1,200 1,300 1,400
m/z

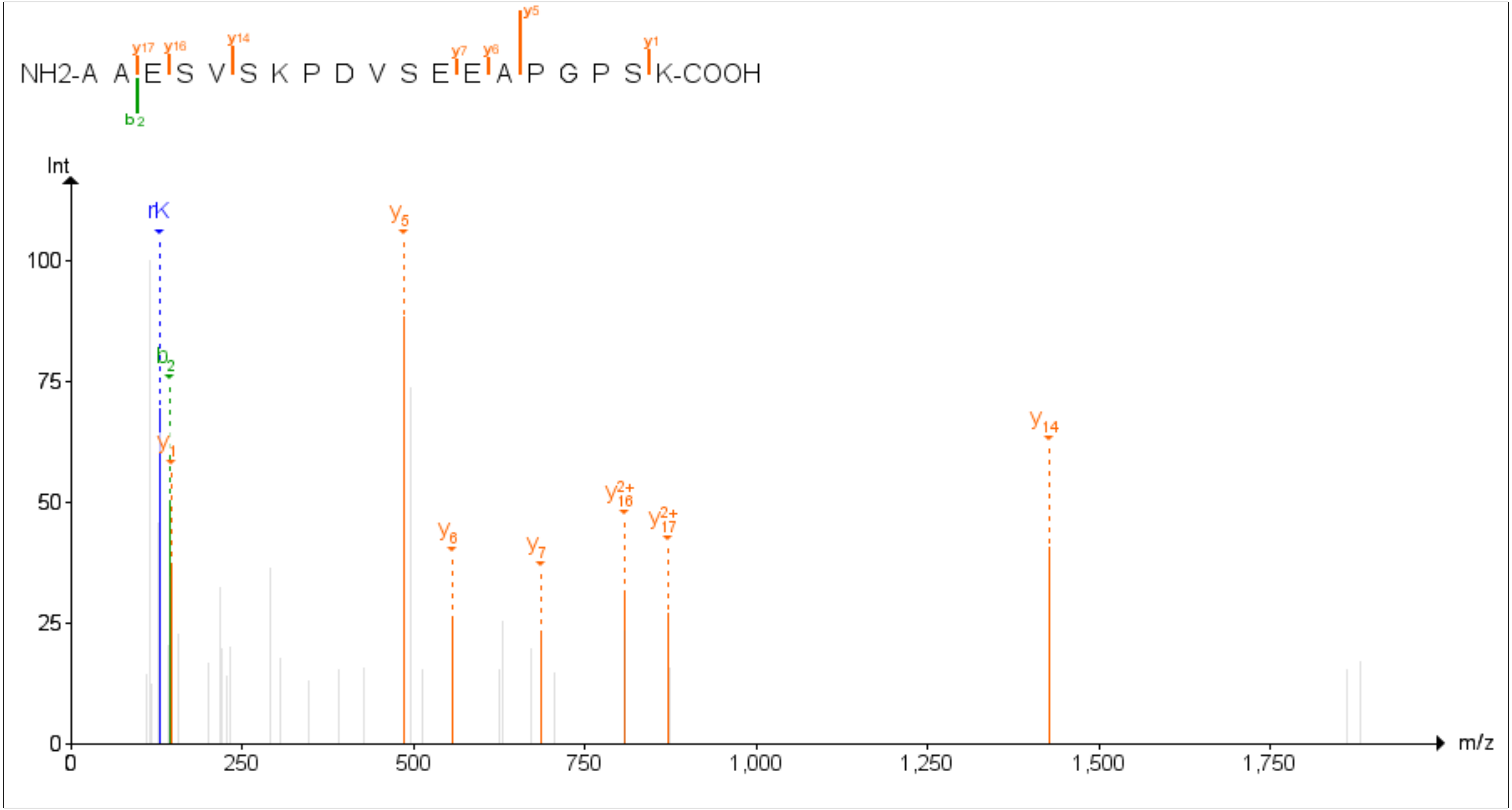

NH2-A A E S V S K P D V S E E A P G P S K-COOH
y17
y16
y14
y7
y6
y5
y1
b2
Int
rK
y5
b2
y1
y6
y7
y16 2+
y17 2+
y14
100
75
50
25
0
250
500
750
1,000
1,250
1,500
1,750
m/z

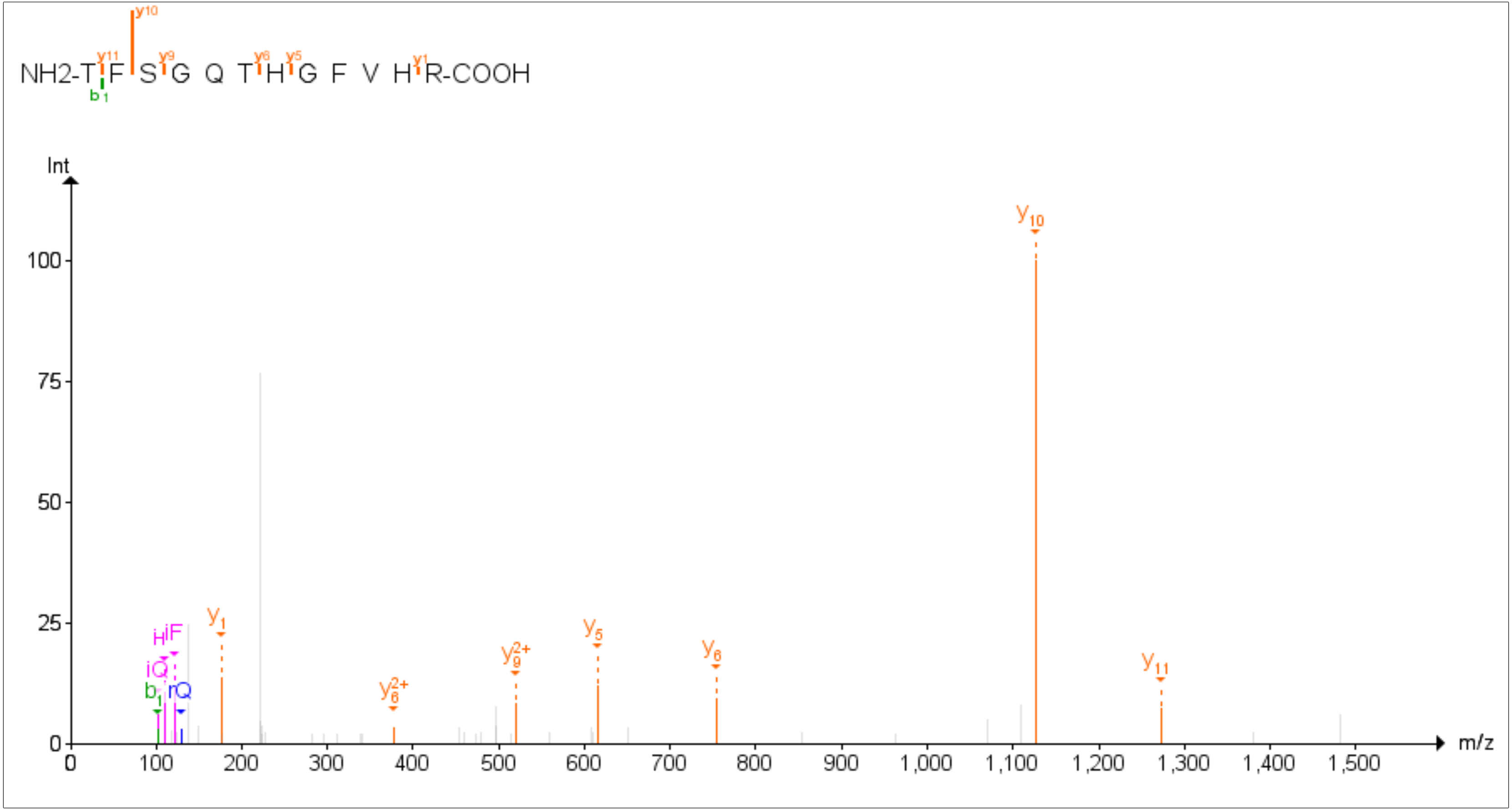

NH2-T I F S G Q T H G F V H R-COOH
y10
y11
y9
y6
y5
y1
b1
Int
100
75
50
25
0
m/z
0
100
200
300
400
500
600
700
800
900
1,000
1,100
1,200
1,300
1,400
1,500
y10
y11
y6
y5
y9 2+
y6 2+
y1
iH
iF
iQ
b1
rQ

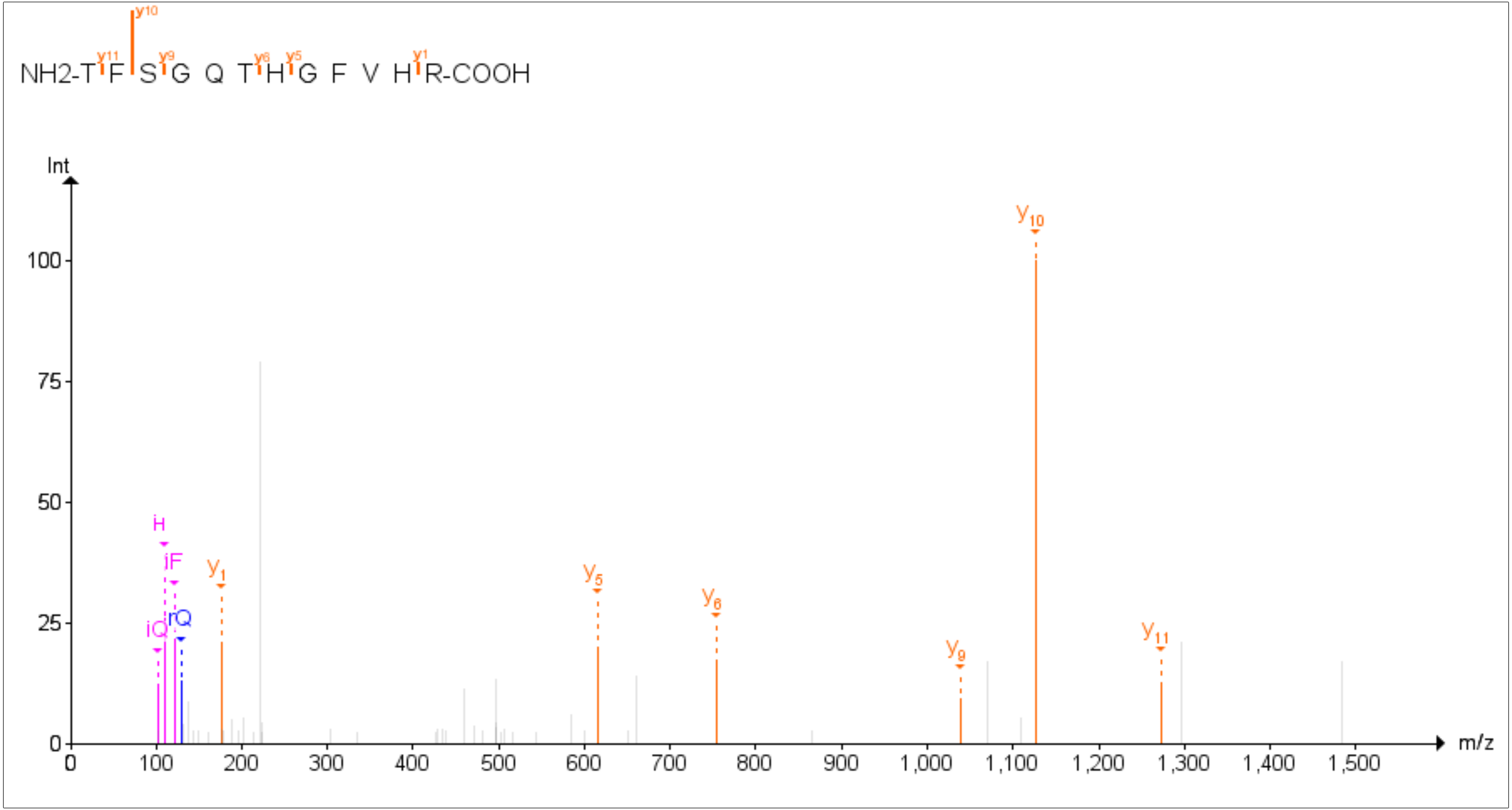

NH2-T F S G Q T H G F V H R-COOH
y10
y11
y9
y6
y5
y1
Int
100
75
50
25
0
iH
iF
iQ
rQ
y1
y5
y6
y9
y10
y11
m/z
0
100
200
300
400
500
600
700
800
900
1,000
1,100
1,200
1,300
1,400
1,500

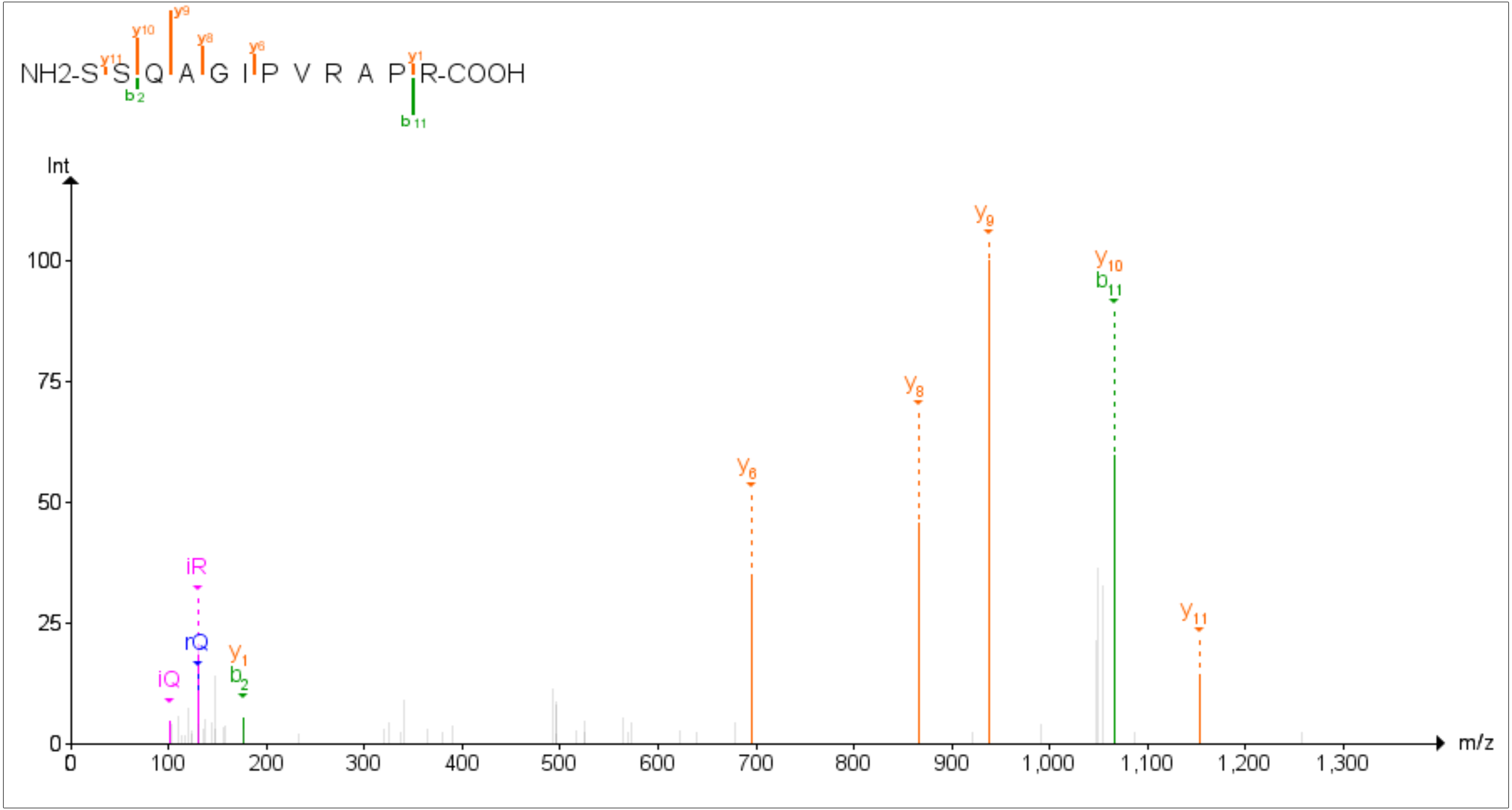

NH2-S S Q A G I P V R A P R-COOH
y11
y10
y9
y8
y6
y1
b2
b11
Int
100
75
50
25
0
m/z
0
100
200
300
400
500
600
700
800
900
1,000
1,100
1,200
1,300
iR
rQ
iQ
y1
b2
y6
y8
y9
y10
b11
y11

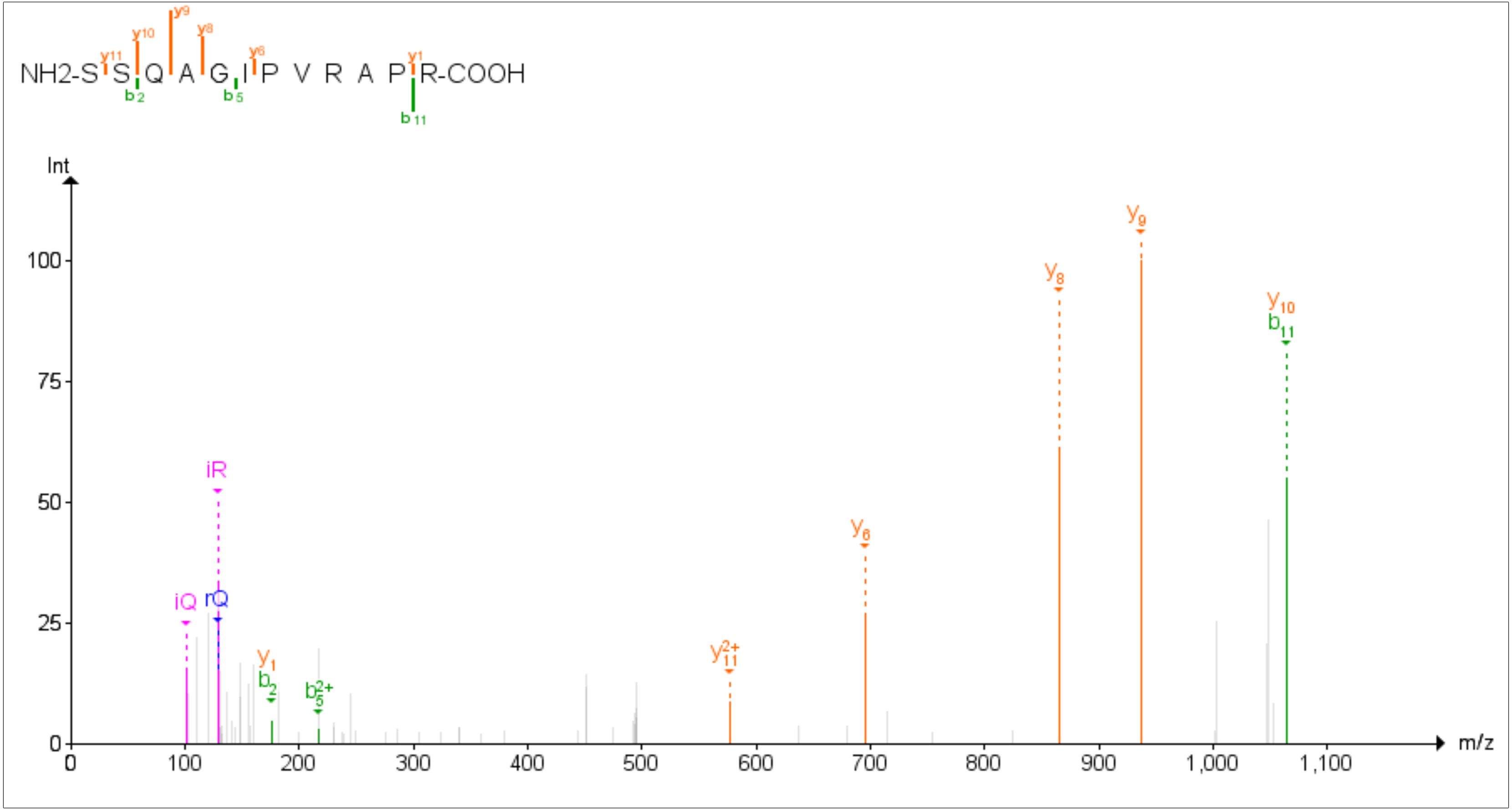

NH2-S S Q A G I P V R A P R-COOH
y11 y10 y9 y8 y6 y1
b2 b5 b11
Int
m/z
y9
y8
y10
b11
iR
iQ
rQ
y1
b2
b5 2+
y11 2+
y6
0
25
50
75
100
0
100
200
300
400
500
600
700
800
900
1,000
1,100

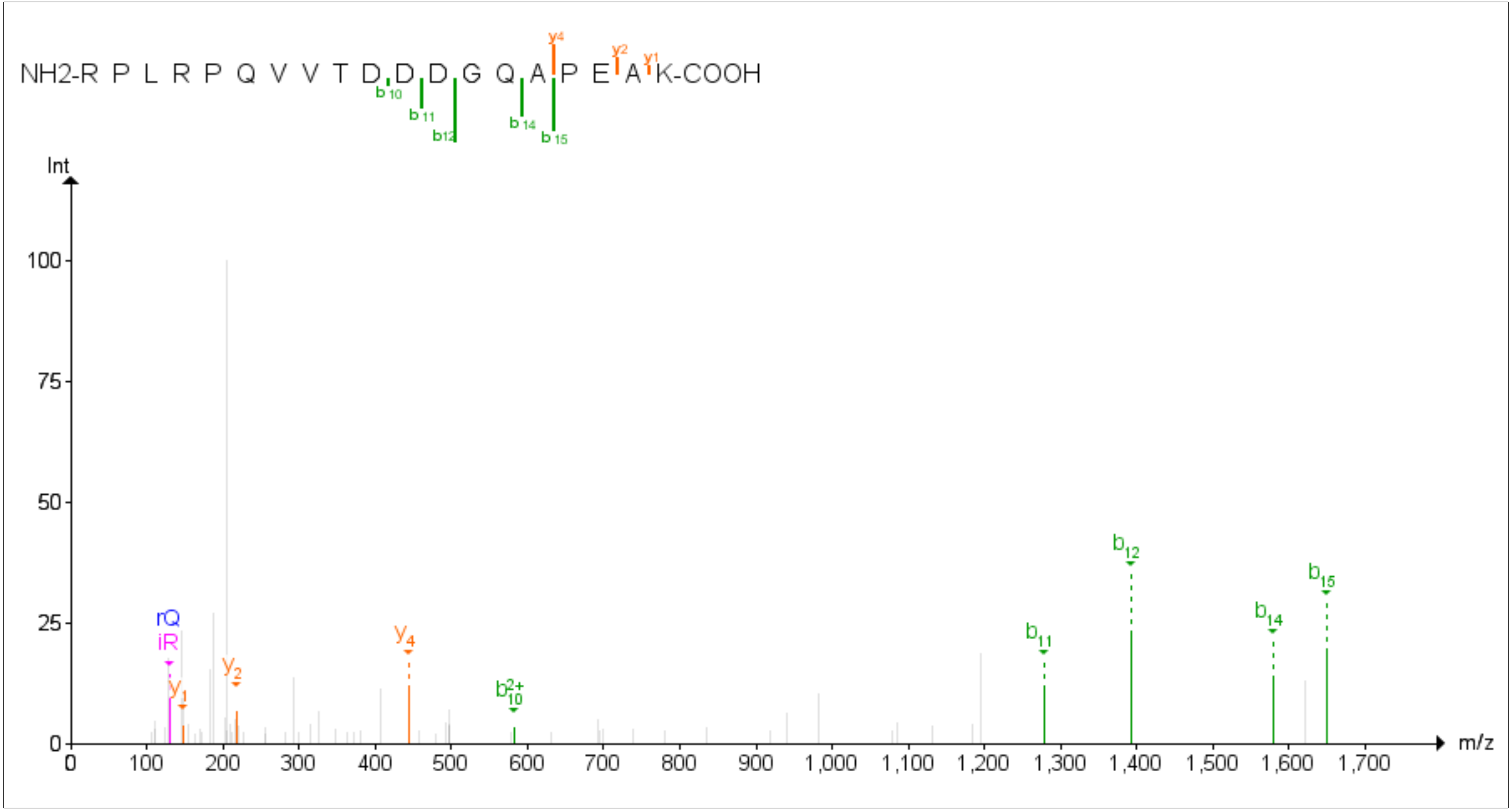

NH2-R P L R P Q V V T D D D G Q A P E A K-COOH
y4
y2
y1
b 10
b 11
b12
b 14
b 15
Int
100
75
50
25
0
rQ
iR
y1
y2
y4
b10 2+
b11
b12
b14
b15
0
100
200
300
400
500
600
700
800
900
1,000
1,100
1,200
1,300
1,400
1,500
1,600
1,700
m/z

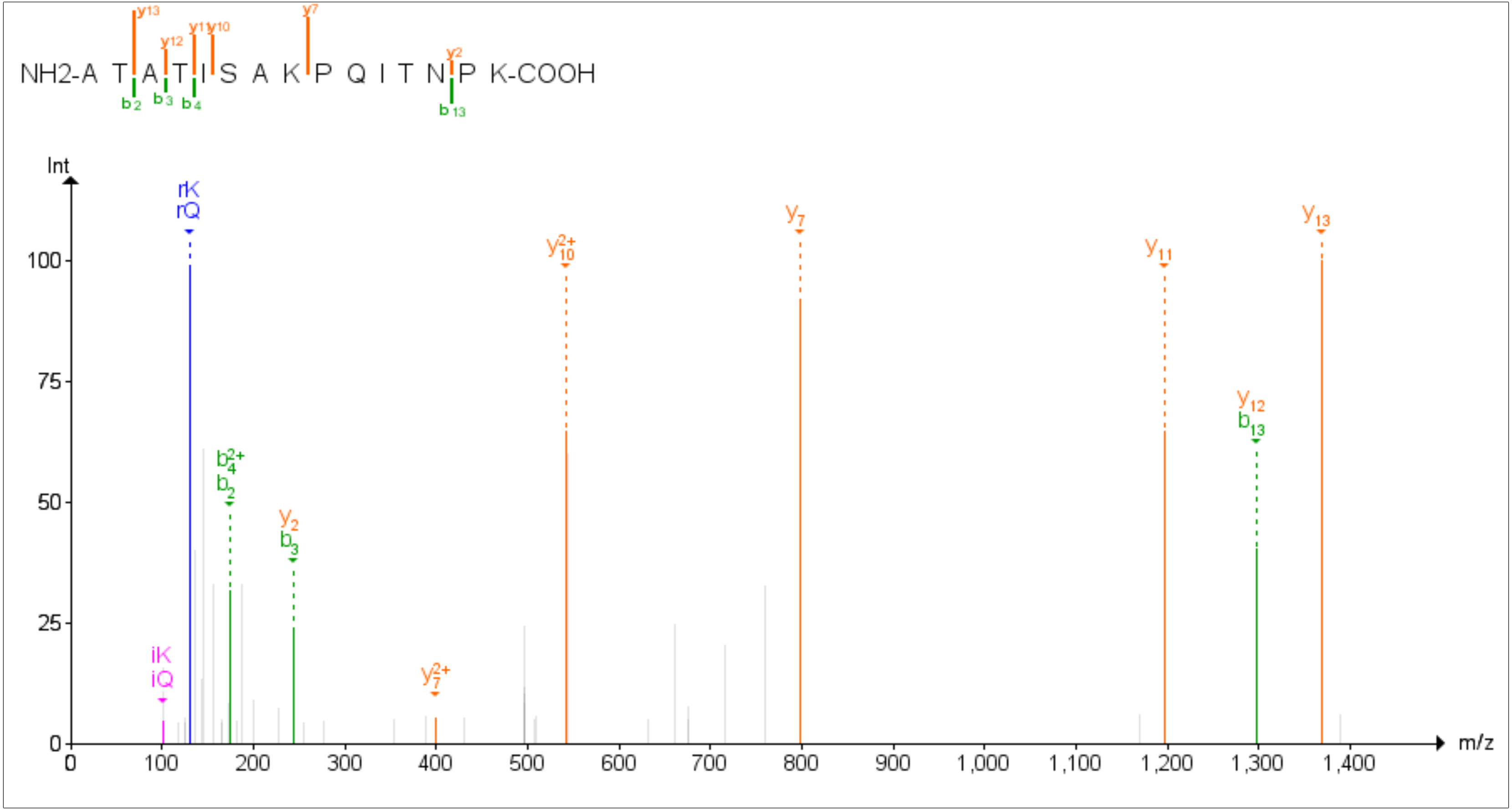

NH2-A T A T I S A K P Q I T N P K-COOH
y13
y12
y11
y10
y7
y2
b2
b3
b4
b13
Int
m/z
rK
rQ
iK
iQ
y_{10}^{2+}
y_7
y_{11}
y_{13}
y_{12}
b_{13}
b_4^{2+}
b_2
y_2
b_3
y_7^{2+}
0
25
50
75
100
0
100
200
300
400
500
600
700
800
900
1,000
1,100
1,200
1,300
1,400

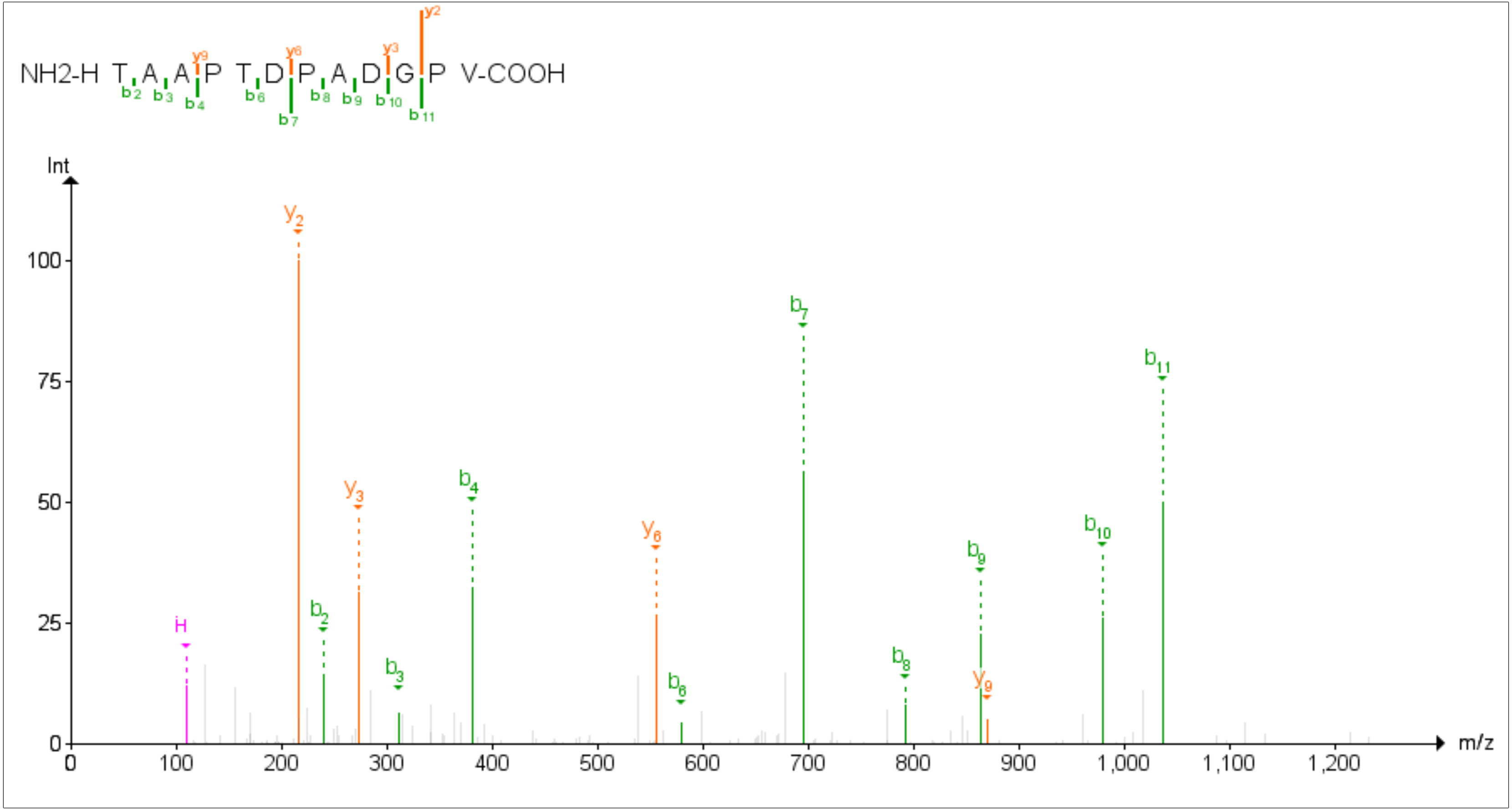

NH2-H T A A P T D P A D G P V-COOH
b2 b3 b4 b6 b7 b8 b9 b10 b11
y9 y6 y3 y2
Int
100
75
50
25
0
m/z
0 100 200 300 400 500 600 700 800 900 1,000 1,100 1,200
H
y2 b2 y3 b3 b4 y6 b6 b7 b8 b9 y9 b10 b11

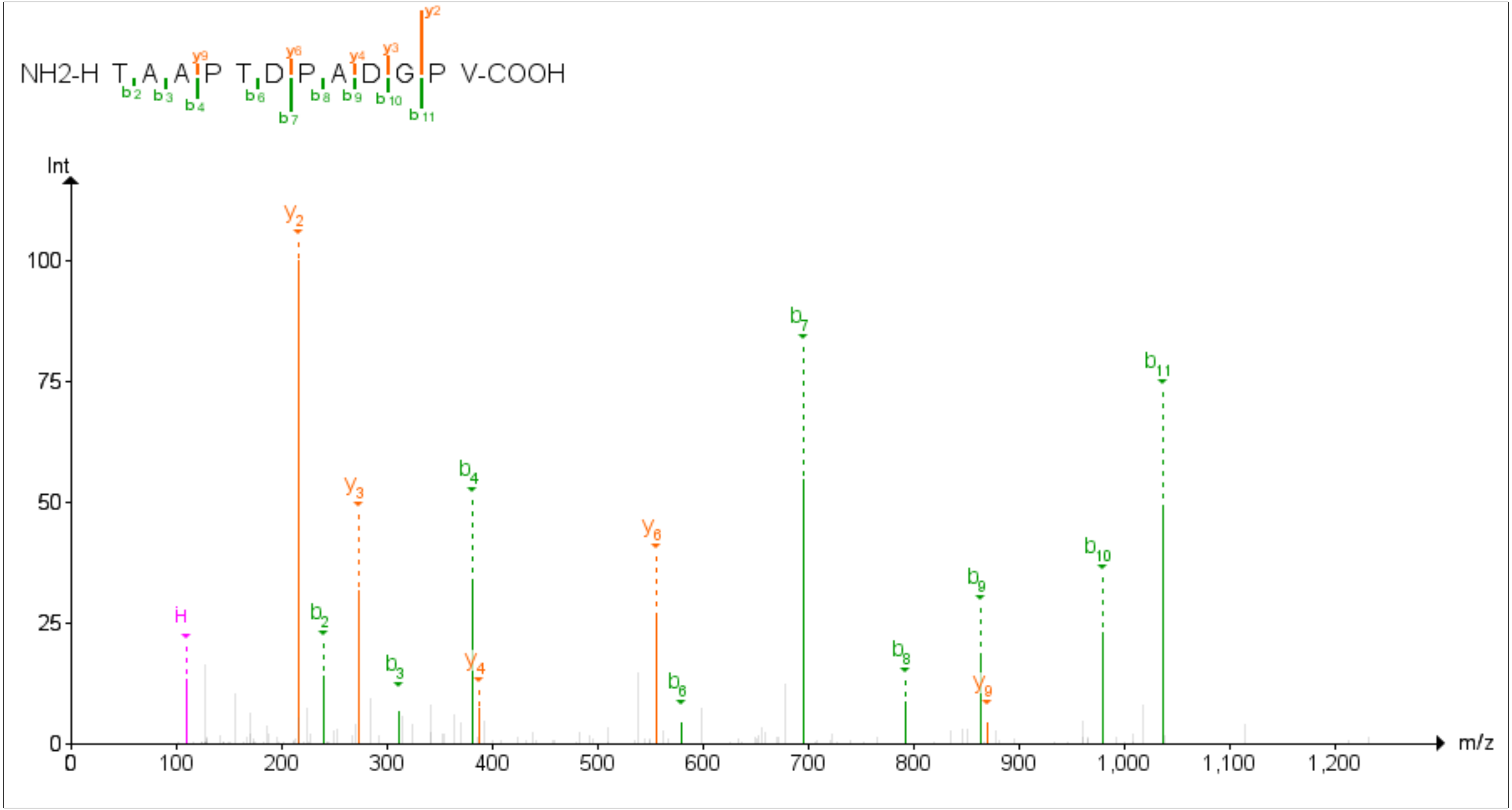

NH2-H T A A P T D P A D G P V-COOH
b2 b3 b4 b6 b7 b8 b9 b10 b11
y9 y6 y4 y3 y2
Int
100
75
50
25
0
m/z
0
100
200
300
400
500
600
700
800
900
1,000
1,100
1,200
H
y2
y3
y4
y6
y9
b2
b3
b4
b6
b7
b8
b9
b10
b11

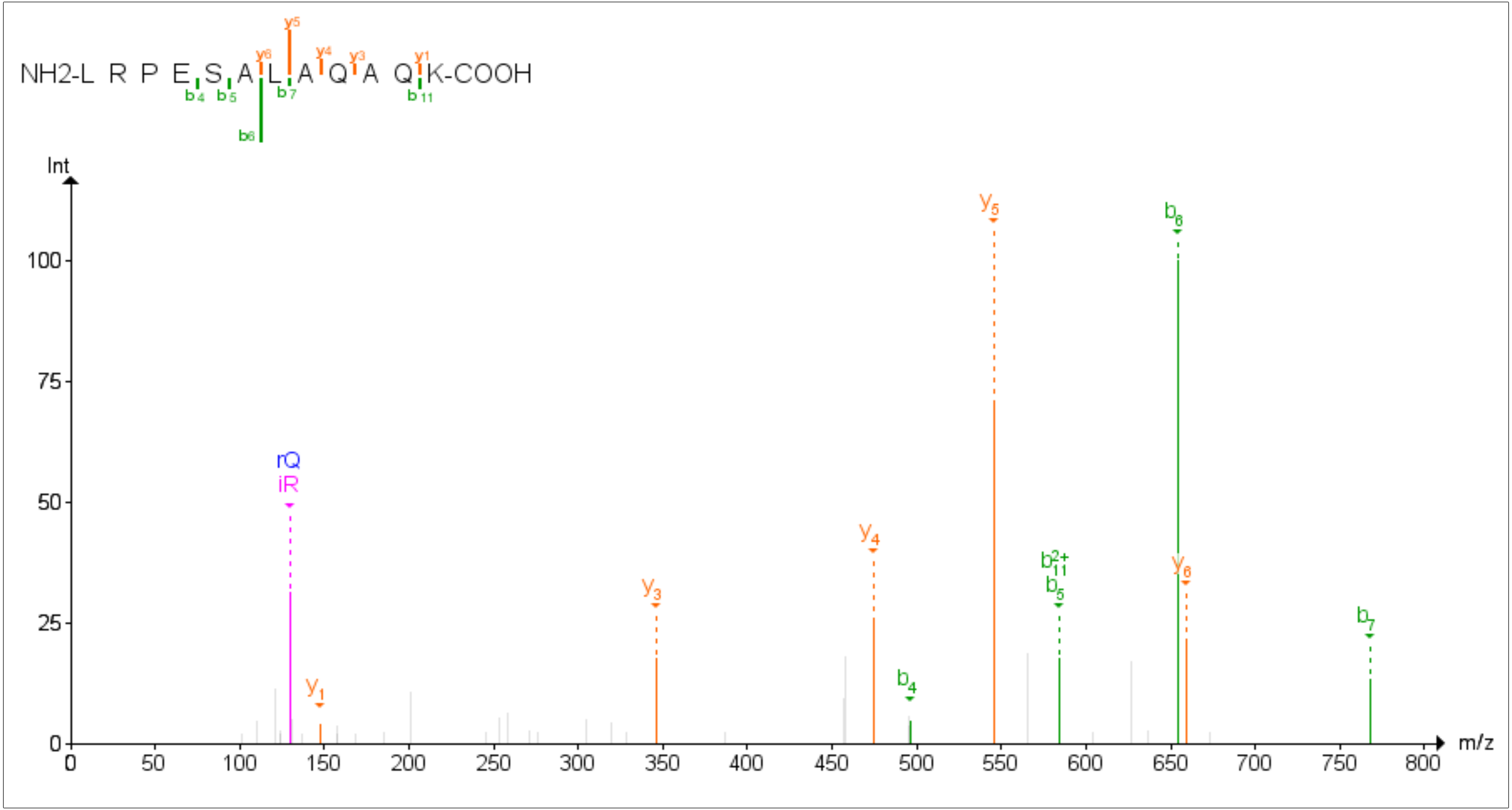

NH2-L R P E S A L A Q A Q K-COOH
b4 b5 b6 b7 b11
y6 y5 y4 y3 y1
Int
100
75
50
25
0
m/z
0 50 100 150 200 250 300 350 400 450 500 550 600 650 700 750 800
rQ
iR
y1
y3
y4
b4
y5
b11 2+
b5
b6
y6
b7

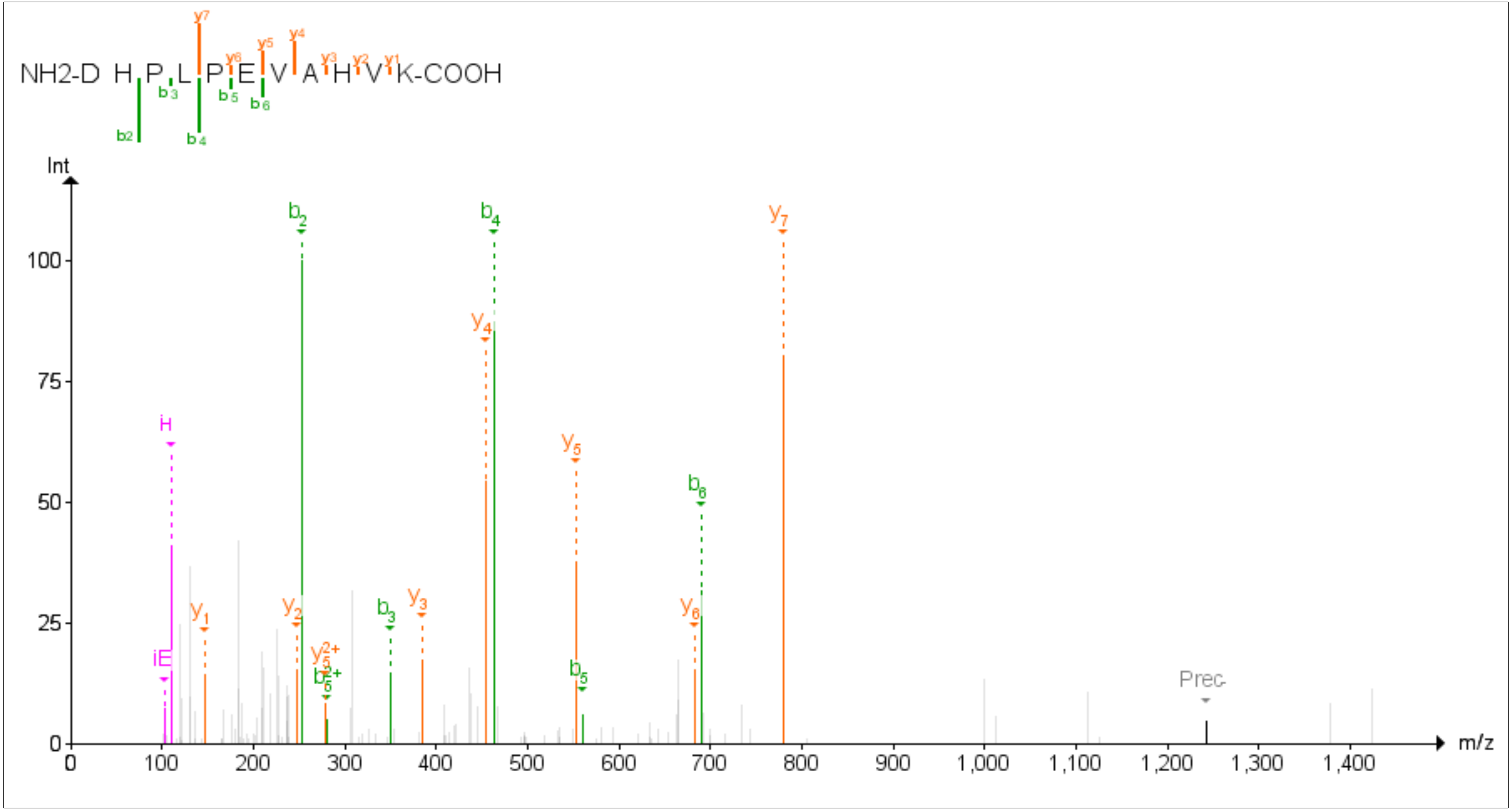
NH2-D H P L P E V A H V K-COOH
b2 b3 b4 b5 b6
y7 y6 y5 y4 y3 y2 y1
Int
100
75
50
25
0
b2
b4
y7
y4
iH
y5
b6
y1
y2
y3
b3
y6
iE
y5 2+
b5 2+
b5
Prec
0 100 200 300 400 500 600 700 800 900 1,000 1,100 1,200 1,300 1,400
m/z

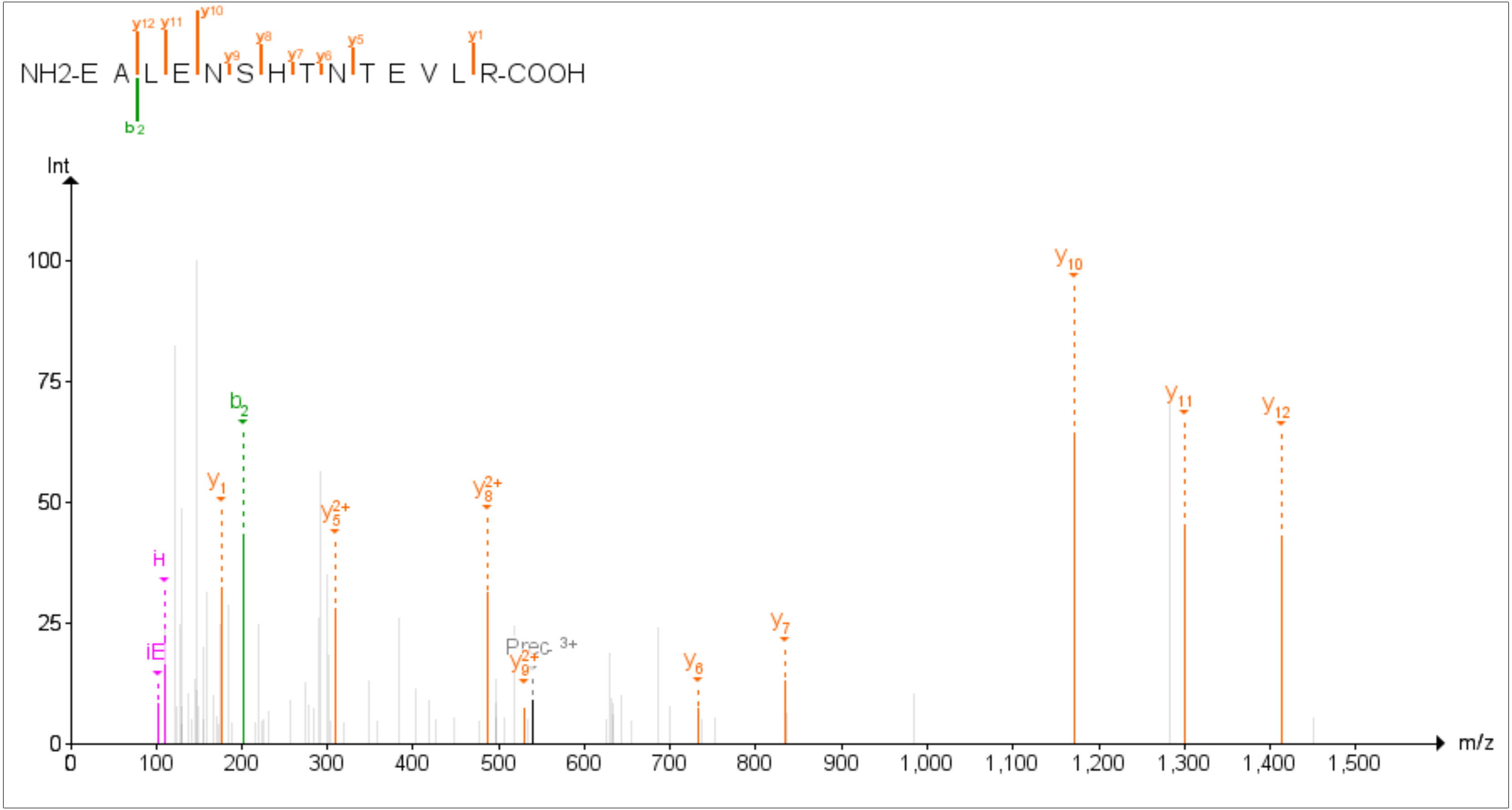

NH2-E A L E N S H T N T E V L R-COOH
y12 y11 y10 y9 y8 y7 y6 y5 y1
b2
Int
100
75
50
25
0
y10
y11
y12
b2
y1
y5 2+
y8 2+
iH
iE
Prec- 3+
y9 2+
y6
y7
0 100 200 300 400 500 600 700 800 900 1,000 1,100 1,200 1,300 1,400 1,500
m/z

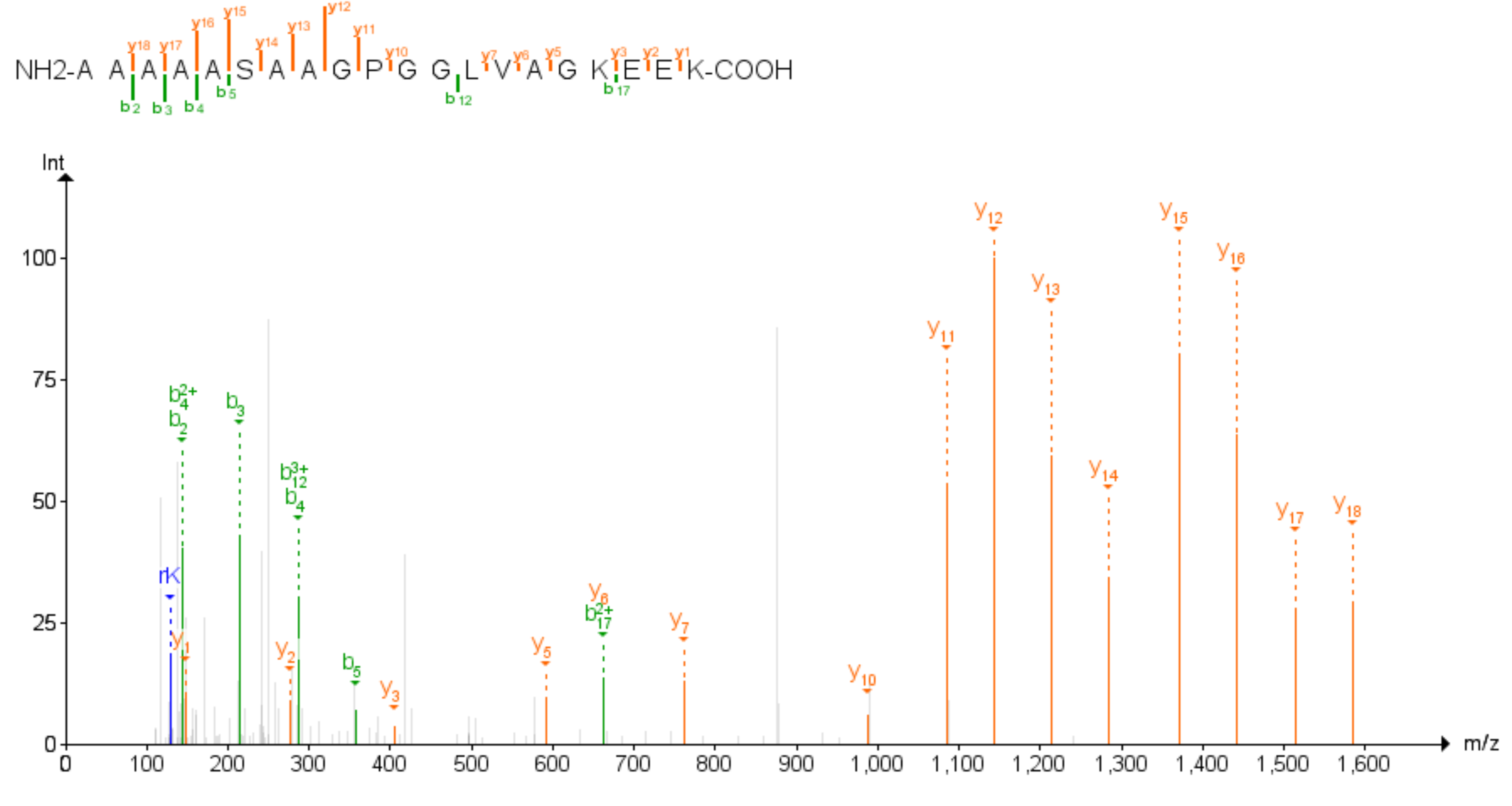

NH2-A A A A A S A A G P G G L V A G K E E K-COOH
Int
m/z
0
25
50
75
100
0
100
200
300
400
500
600
700
800
900
1,000
1,100
1,200
1,300
1,400
1,500
1,600

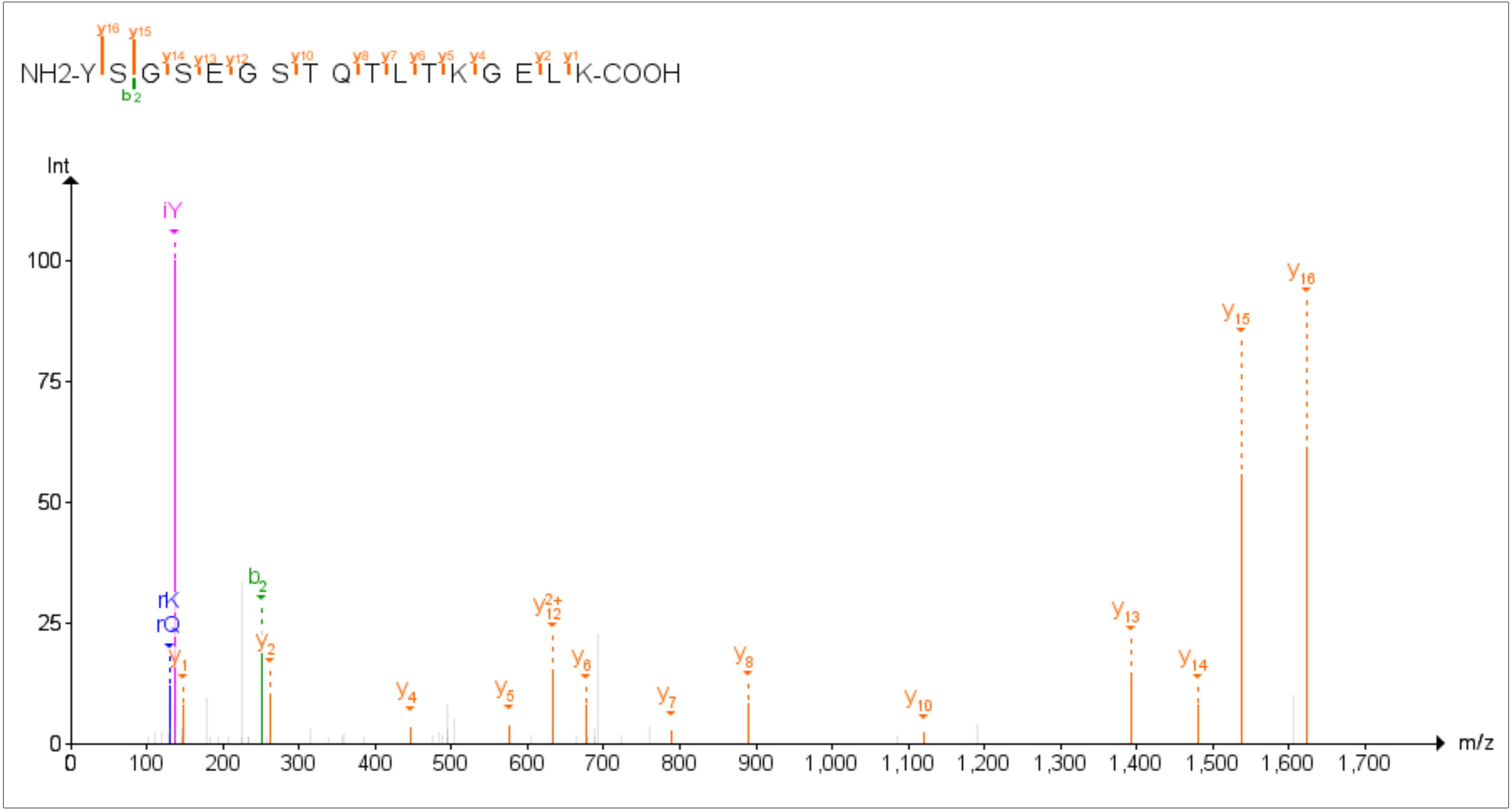

NH2-Y S G S E G S T Q T L T K G E L K-COOH
y16 y15 y14 y13 y12 y10 y8 y7 y6 y5 y4 y2 y1
b2
Int
m/z
iY
rK
rQ
y1
b2
y2
y4
y5
y12 2+
y6
y7
y8
y10
y13
y14
y15
y16
0
25
50
75
100
0
100
200
300
400
500
600
700
800
900
1,000
1,100
1,200
1,300
1,400
1,500
1,600
1,700

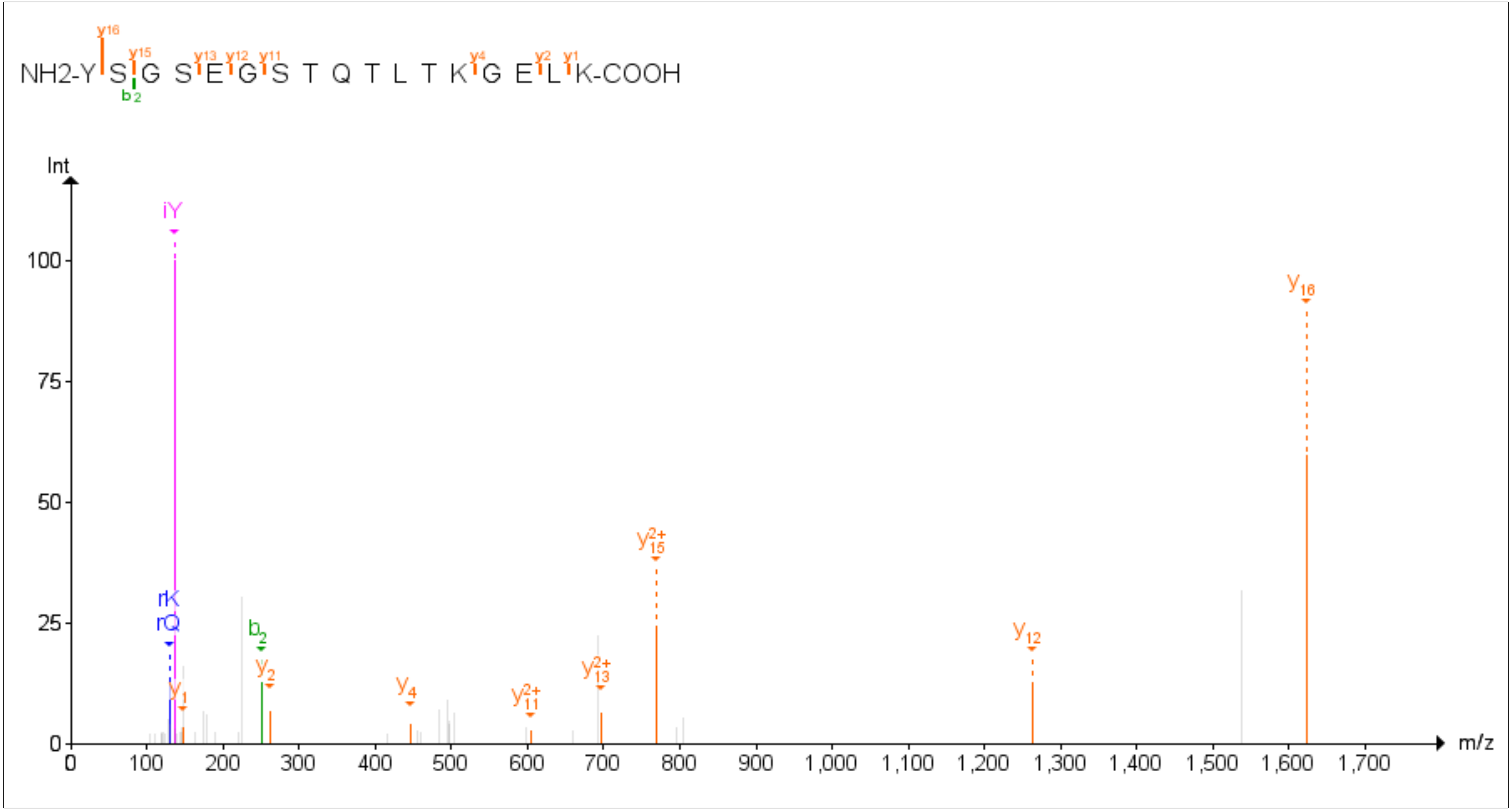
NH2-Y S G S E G S T Q T L T K G E L K-COOH
y16
y15
y13
y12
y11
y4
y2
y1
b2
Int
iY
100
75
50
25
0
rK
rQ
b2
y1
y2
y4
y11 2+
y13 2+
y15 2+
y12
y16
0
100
200
300
400
500
600
700
800
900
1,000
1,100
1,200
1,300
1,400
1,500
1,600
1,700
m/z

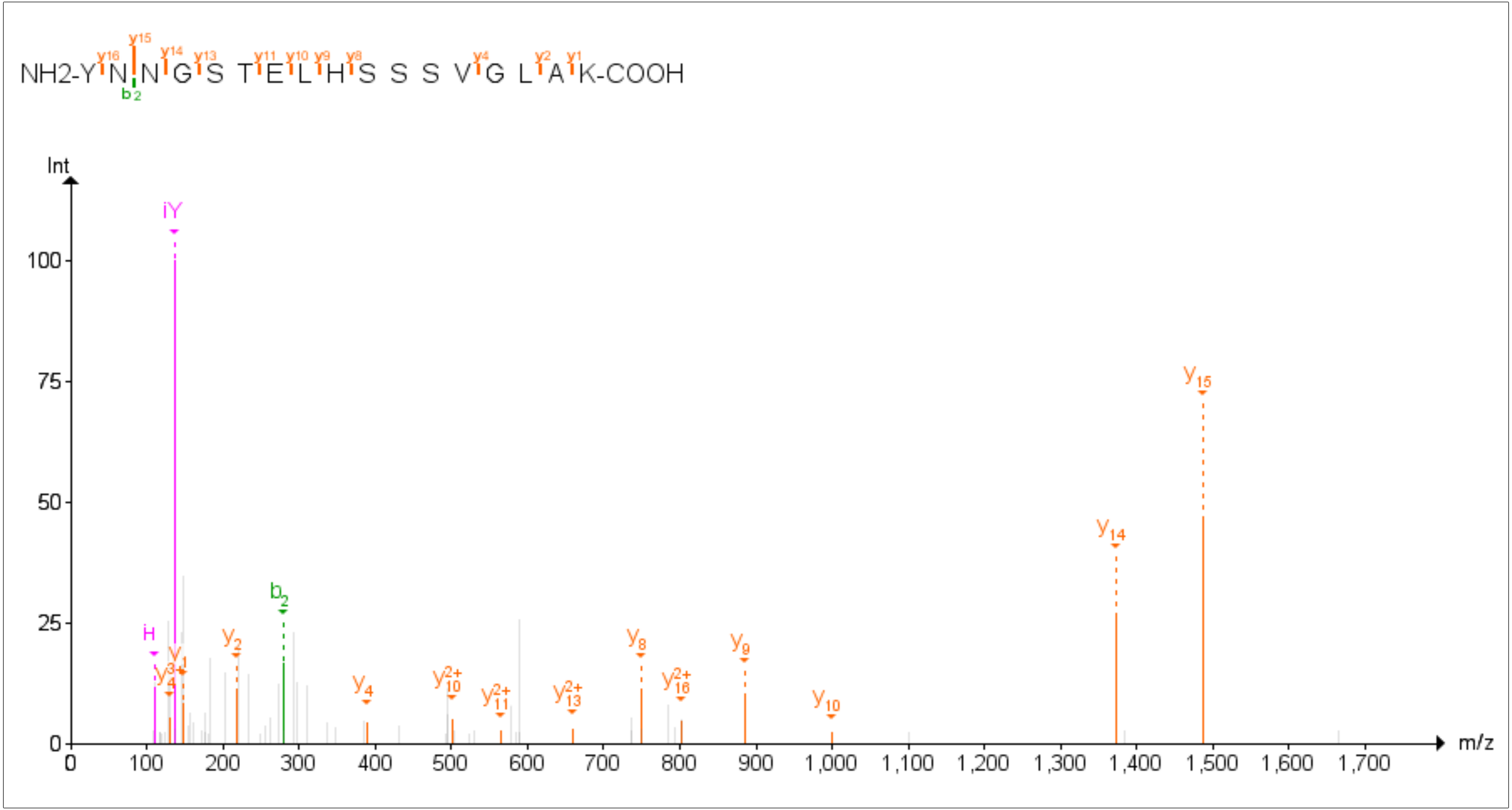
NH2-Y N N G S T E L H S S S V G L A K-COOH
y16 y15 y14 y13 y11 y10 y9 y8 y4 y2 y1
b2
Int
iY
H
y2
b2
y4
y8
y9
y10
y14
y15
m/z
0
25
50
75
100
0 100 200 300 400 500 600 700 800 900 1,000 1,100 1,200 1,300 1,400 1,500 1,600 1,700

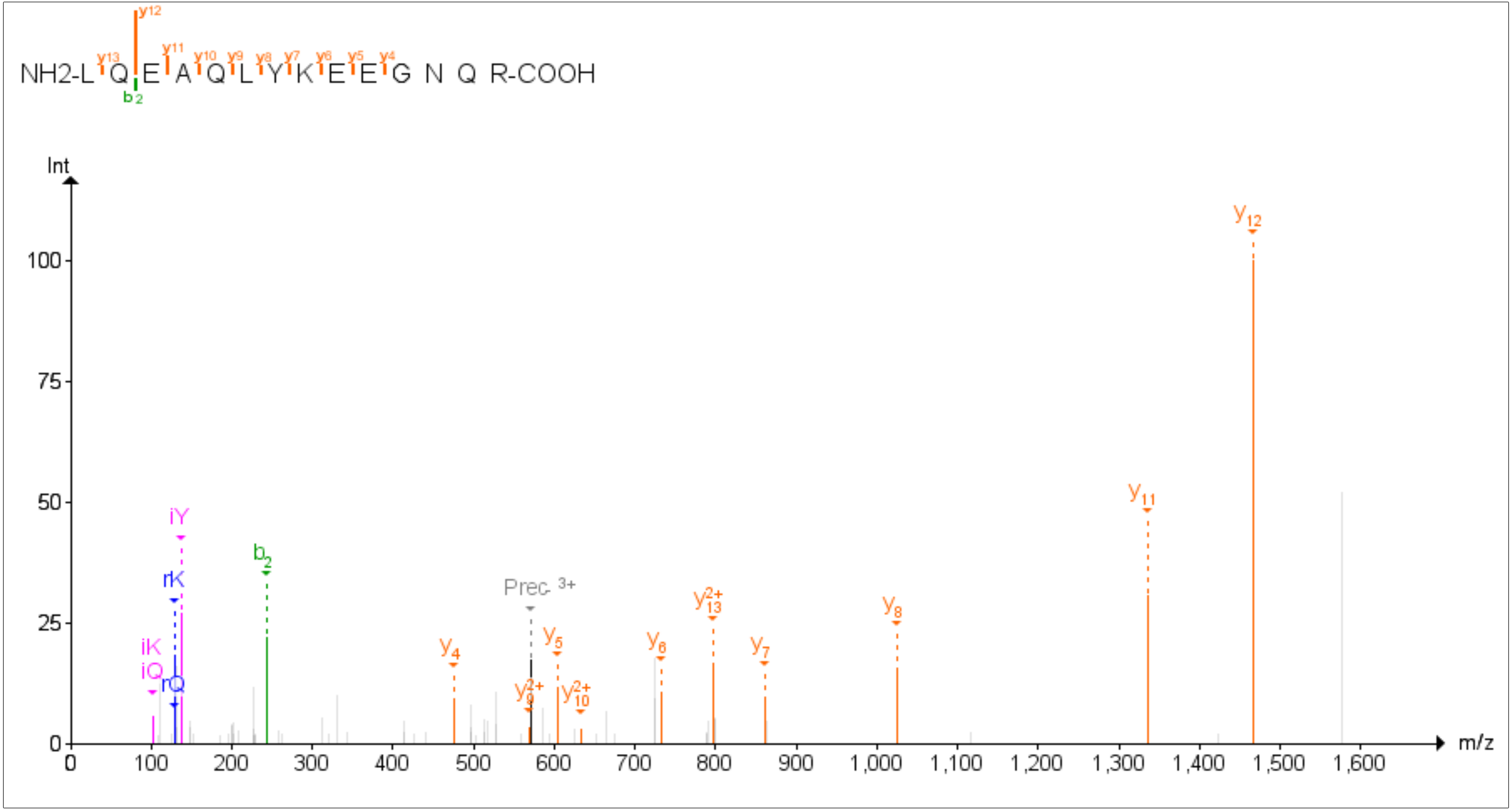

NH2-L Q E A Q L Y K E E G N Q R-COOH
y13 y12 y11 y10 y9 y8 y7 y6 y5 y4
b2
Int
m/z
iY
b2
rK
iK
iQ
rQ
Prec 3+
y4
y5
y6
y7
y8
y11
y12
y9 2+
y10 2+
y13 2+
0
25
50
75
100
0
100
200
300
400
500
600
700
800
900
1,000
1,100
1,200
1,300
1,400
1,500
1,600

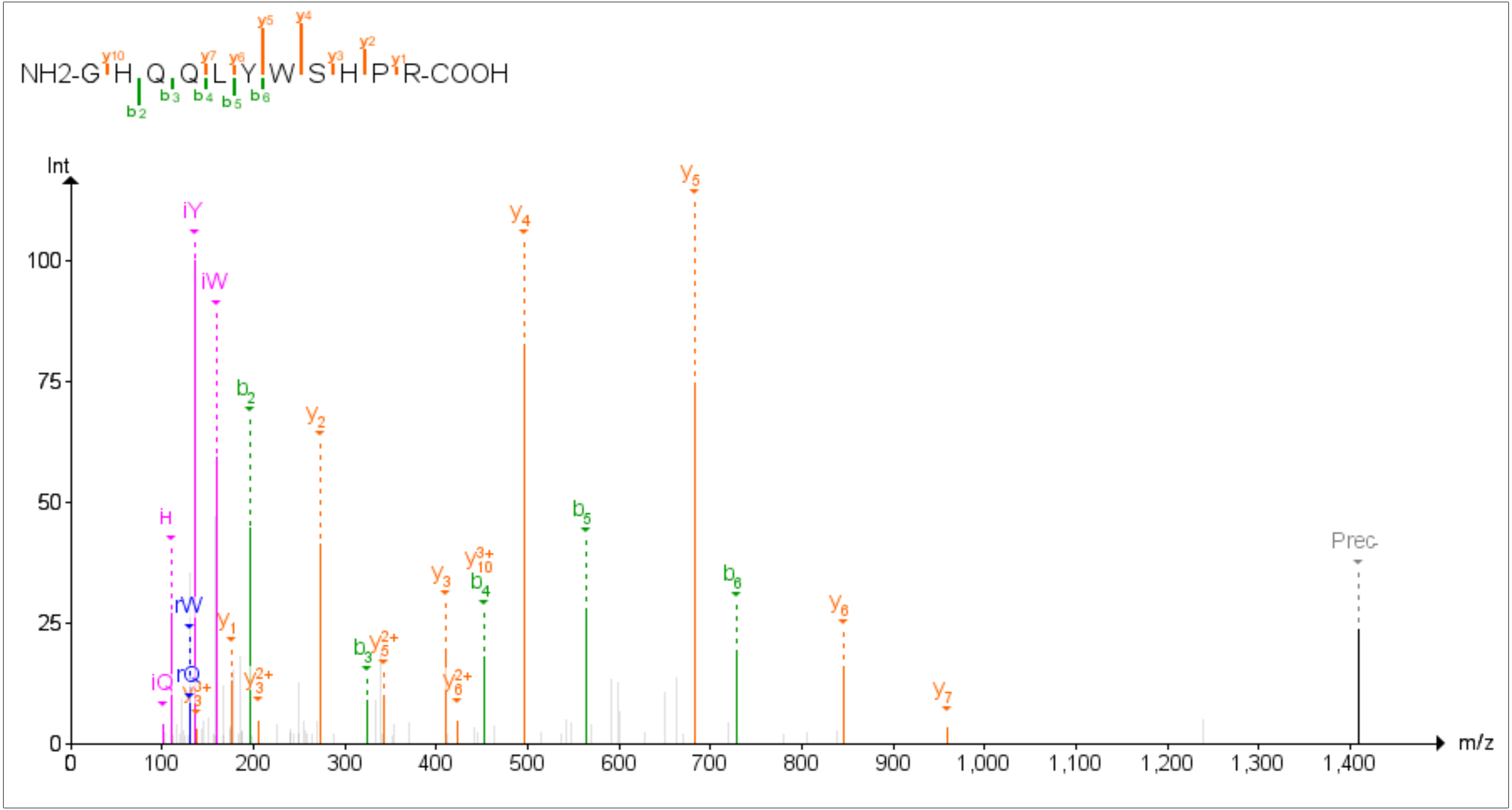

NH2-G H Q Q L Y W S H P R-COOH
y10 y7 y6 y5 y4 y3 y2 y1
b2 b3 b4 b5 b6
Int
100
75
50
25
0
iY
iW
b2
y2
y4
y5
iH
rW
y1
iQ
rQ
y3
b3
y3
b4
b5
b6
y6
y7
Prec
m/z
0
100
200
300
400
500
600
700
800
900
1,000
1,100
1,200
1,300
1,400

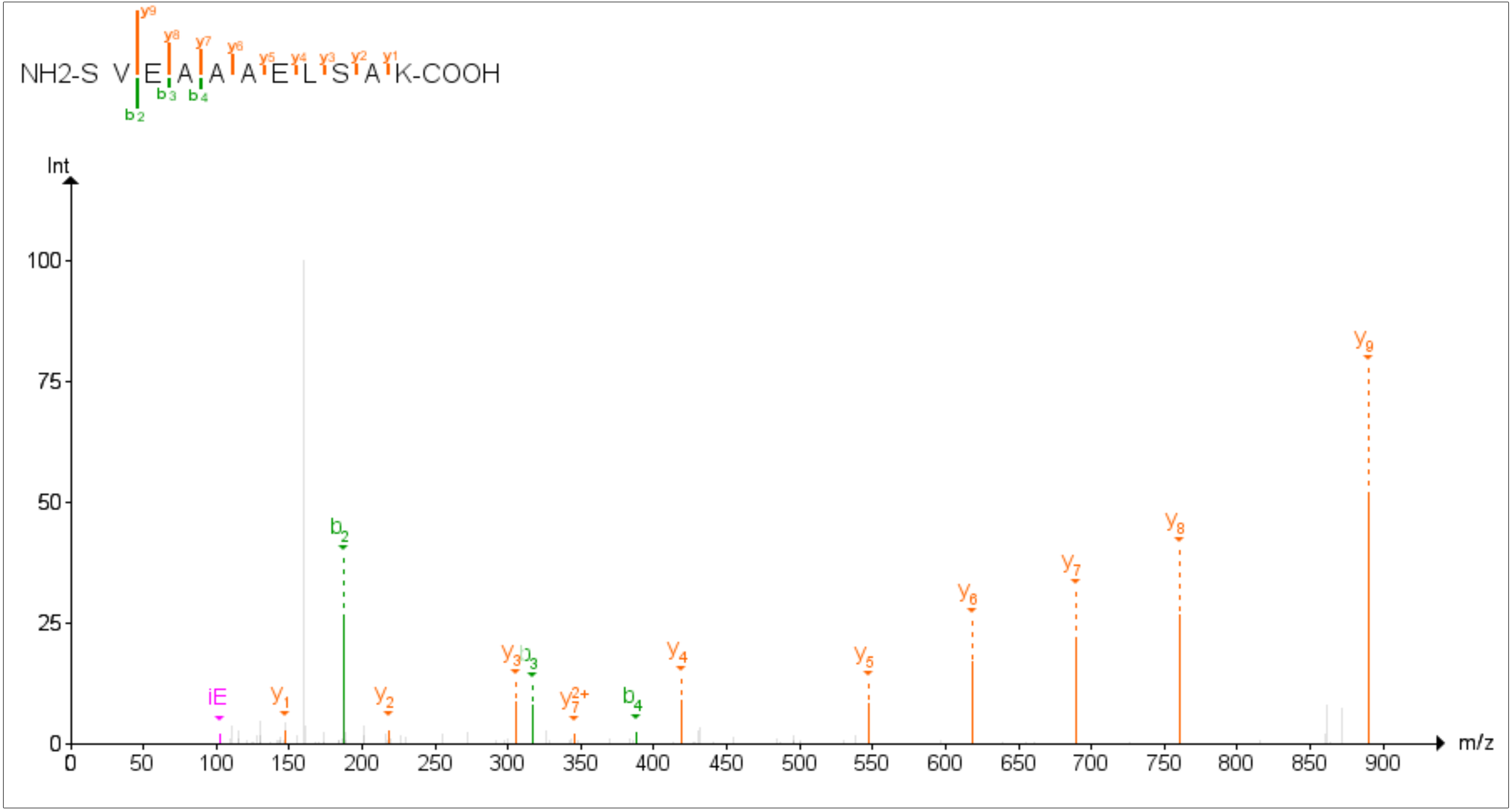

NH2-S V E A A A E L S A K-COOH
y9 y8 y7 y6 y5 y4 y3 y2 y1
b2 b3 b4
Int
m/z
0 25 50 75 100
0 50 100 150 200 250 300 350 400 450 500 550 600 650 700 750 800 850 900
iE
y1 y2 y3 b3 b2 y7 2+ b4 y4 y5 y6 y7 y8 y9

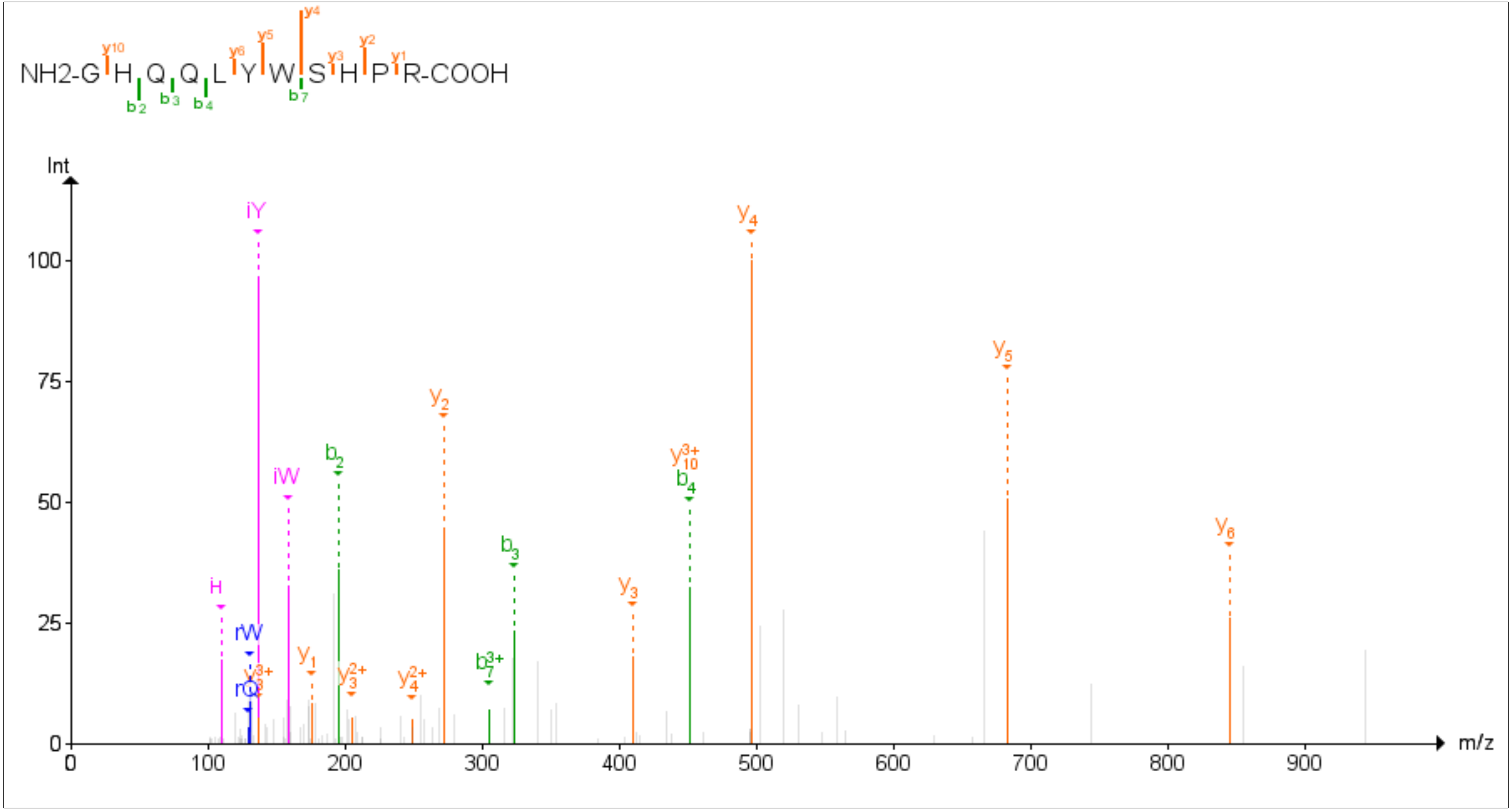

NH2-GHQQLYWSHPR-COOH
y10
y6
y5
y4
y3
y2
y1
b2
b3
b4
b7
Int
100
75
50
25
0
iY
y4
y5
y2
b2
iW
y10 3+
b4
b3
y3
y6
iH
rW
y1
y3 2+
y4 2+
b7 3+
rQ
m/z
0
100
200
300
400
500
600
700
800
900

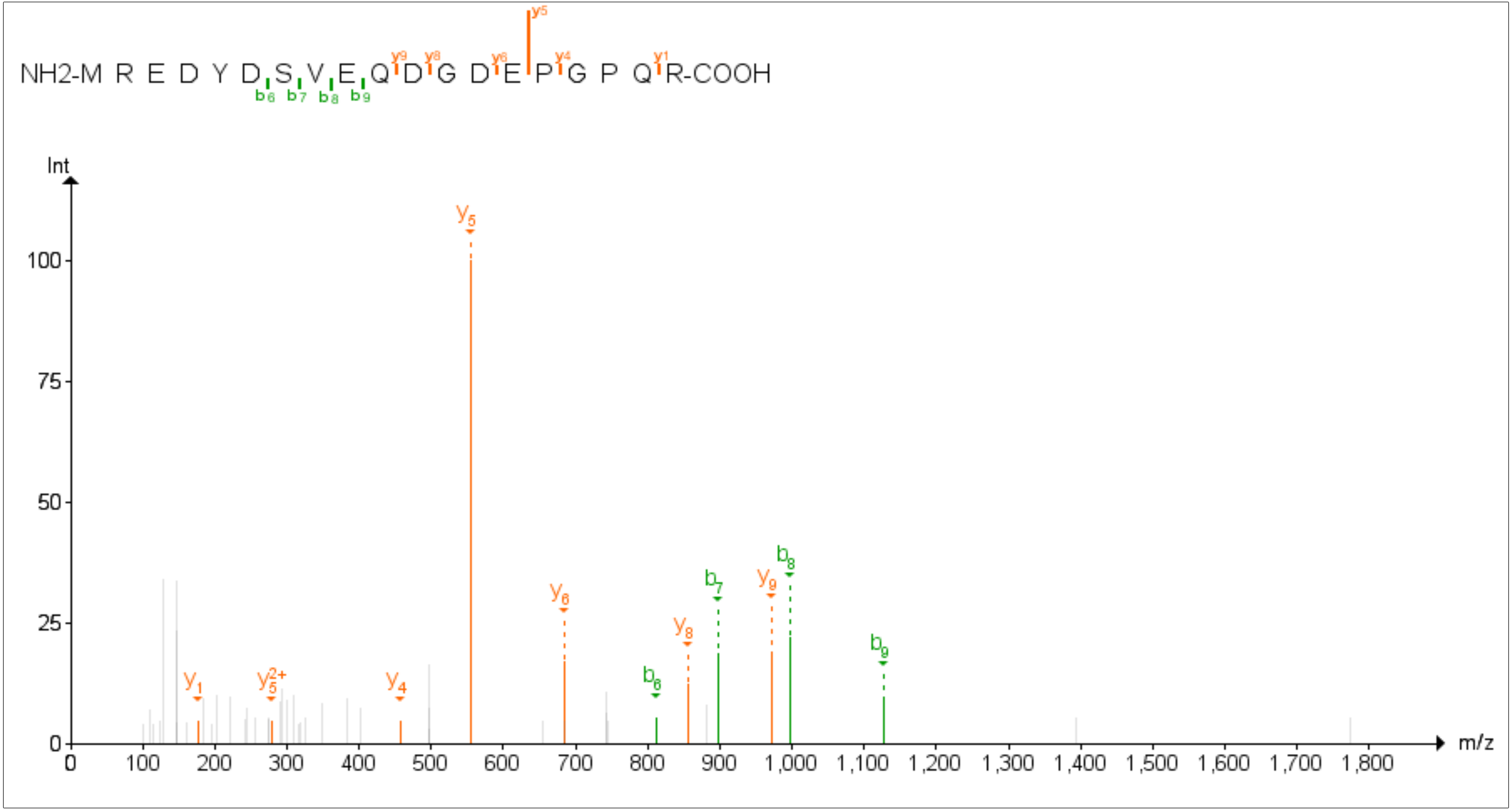
NH2-M R E D Y D S V E Q D G D E P G P Q R-COOH
y9 y8 y6 y5 y4 y1
b6 b7 b8 b9
Int
100
75
50
25
0
y5
y6
b8
b7
y9
y8
b9
b6
y1
y5 2+
y4
0 100 200 300 400 500 600 700 800 900 1,000 1,100 1,200 1,300 1,400 1,500 1,600 1,700 1,800
m/z

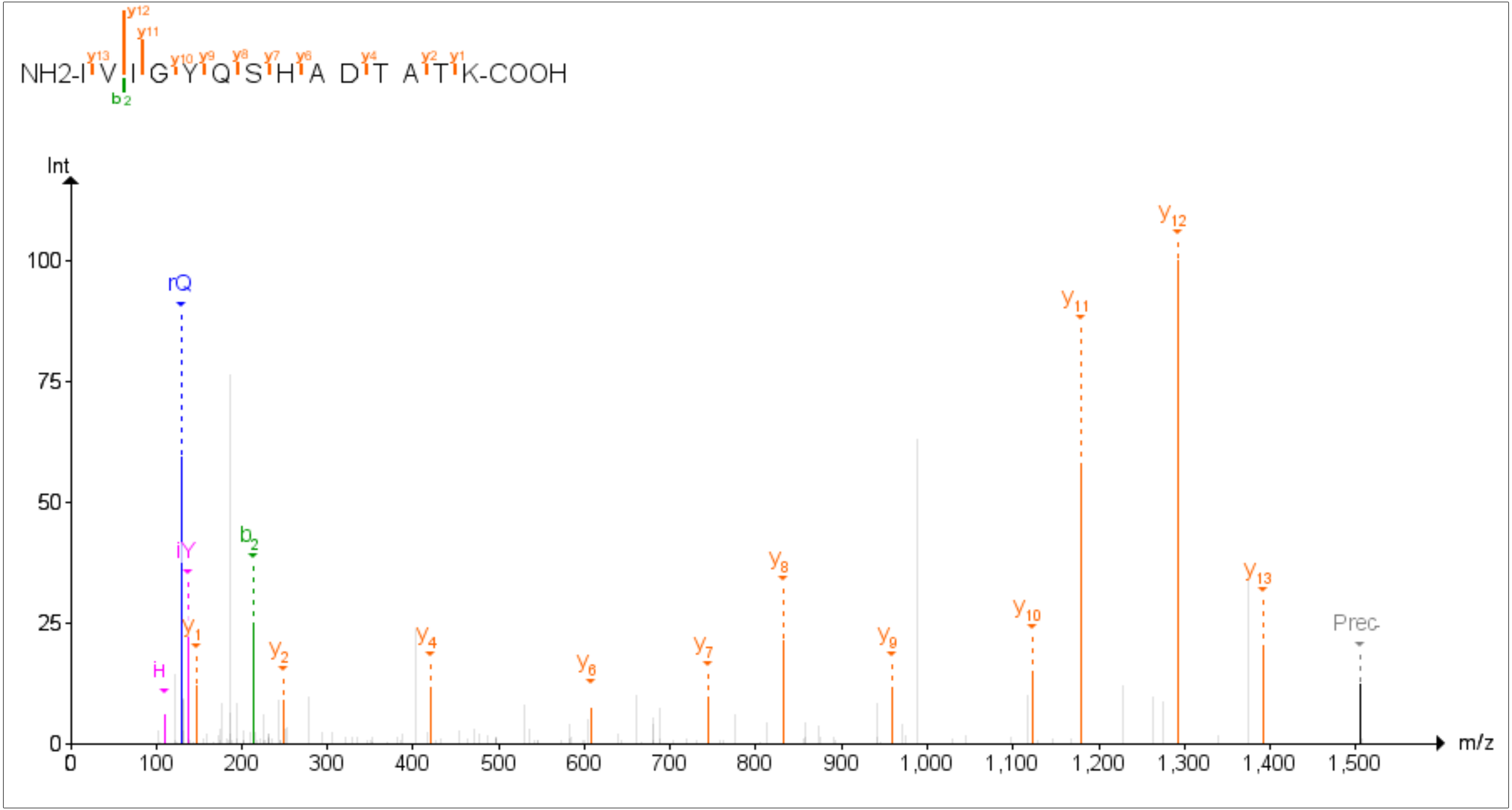

NH2-I V I G Y Q S H A D T A T K-COOH
y13 y12 y11 y10 y9 y8 y7 y6 y4 y2 y1
b2
Int
100
75
50
25
0
rQ
iY
iH
b2
y1
y2
y4
y6
y7
y8
y9
y10
y11
y12
y13
Prec.
0
100
200
300
400
500
600
700
800
900
1,000
1,100
1,200
1,300
1,400
1,500
m/z

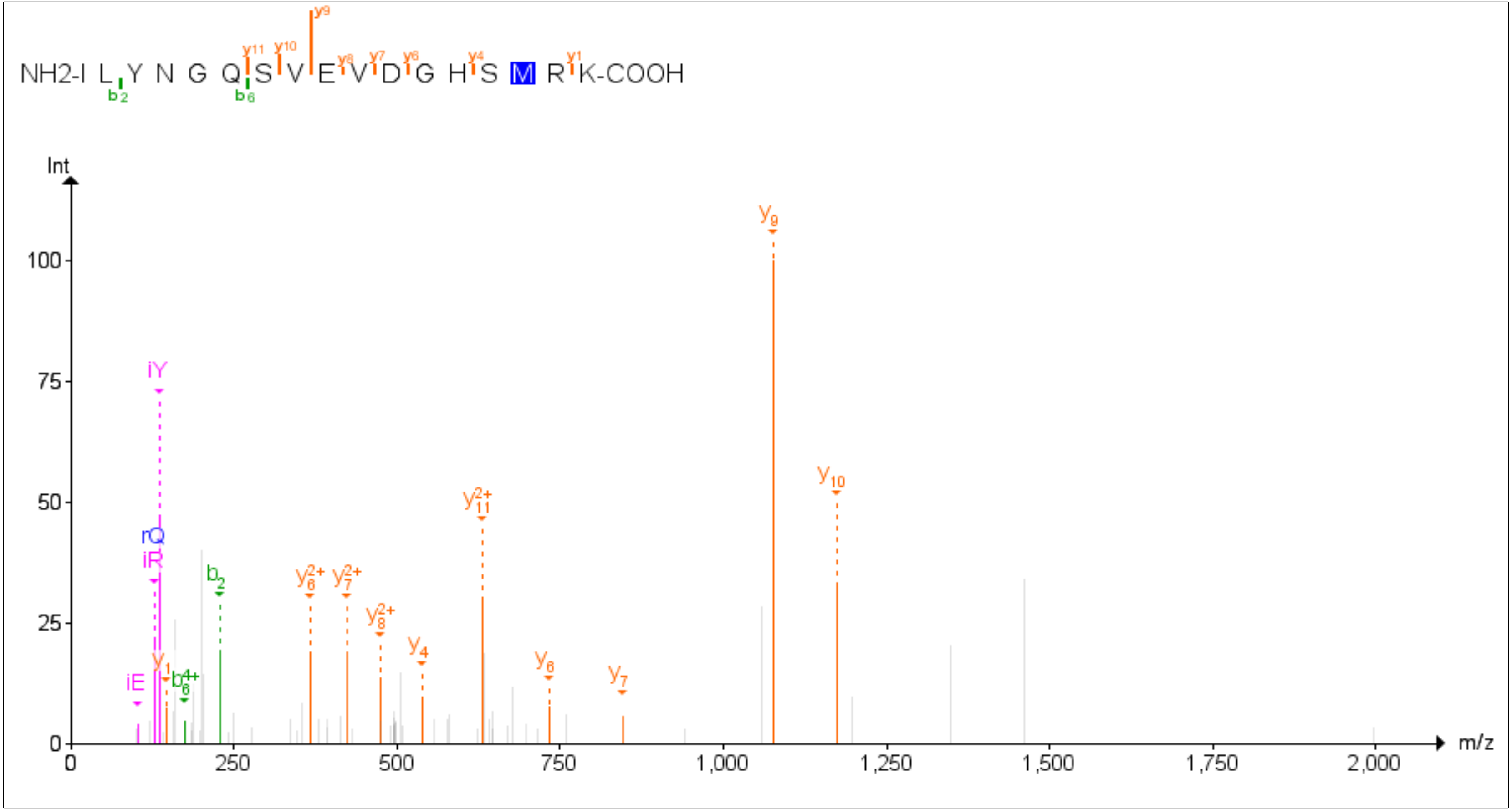

NH2-I L Y N G Q S V E V D G H S M R K-COOH
b2
b6
y11
y10
y9
y8
y7
y6
y4
y1
Int
m/z
100
75
50
25
0
250
500
750
1,000
1,250
1,500
1,750
2,000
y9
y10
y11 2+
iY
rQ
iR
b2
y6 2+
y7 2+
y8 2+
y4
y6
y7
y1
iE
b6 4+

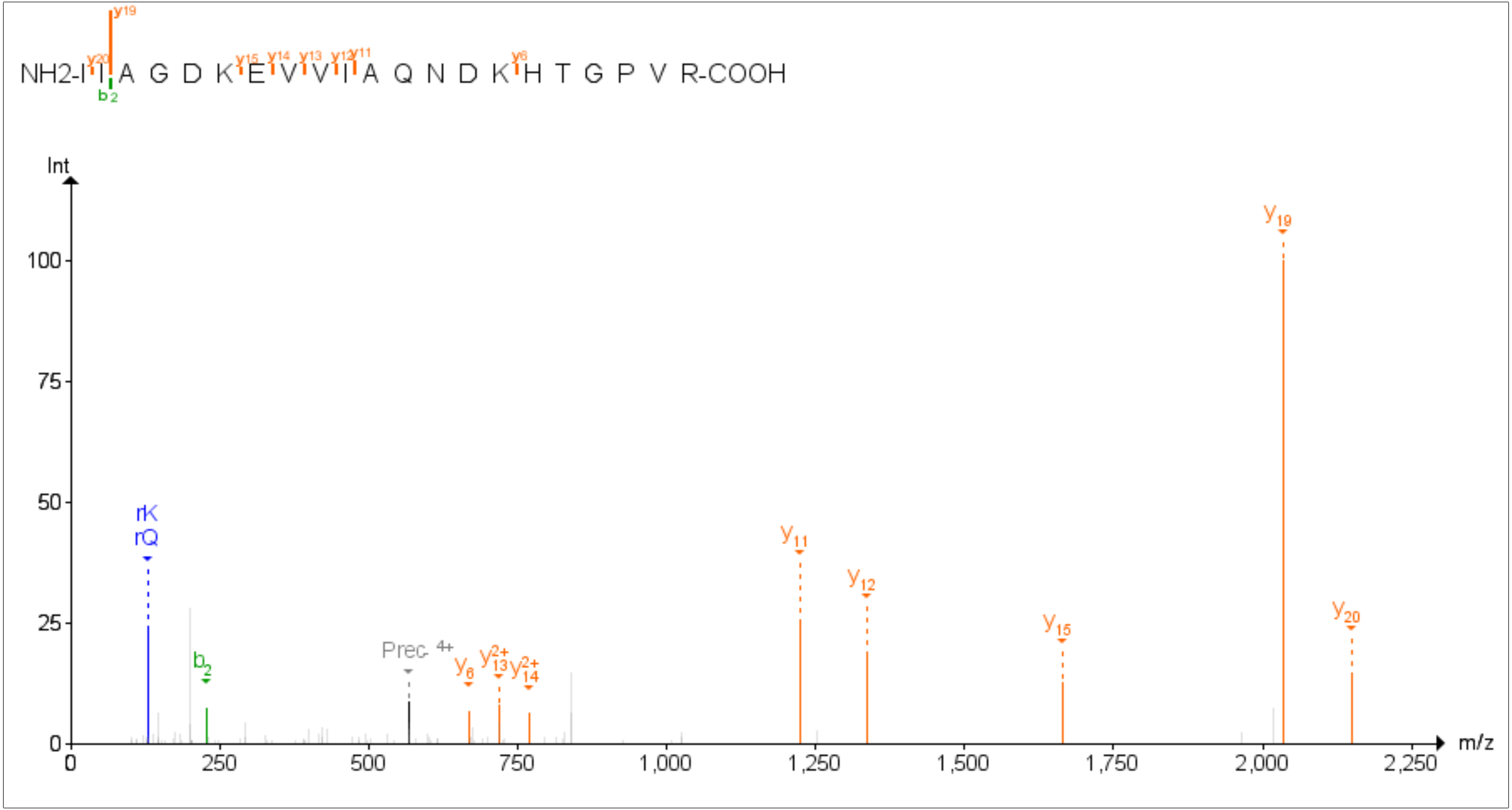
NH2-I I A G D K E V V I A Q N D K H T G P V R-COOH
y19
y20
y15
y14
y13
y12
y11
y6
b2
Int
100
75
50
25
0
rK
rQ
b2
Prec 4+
y6
y13 2+
y14 2+
y11
y12
y15
y19
y20
0
250
500
750
1,000
1,250
1,500
1,750
2,000
2,250
m/z

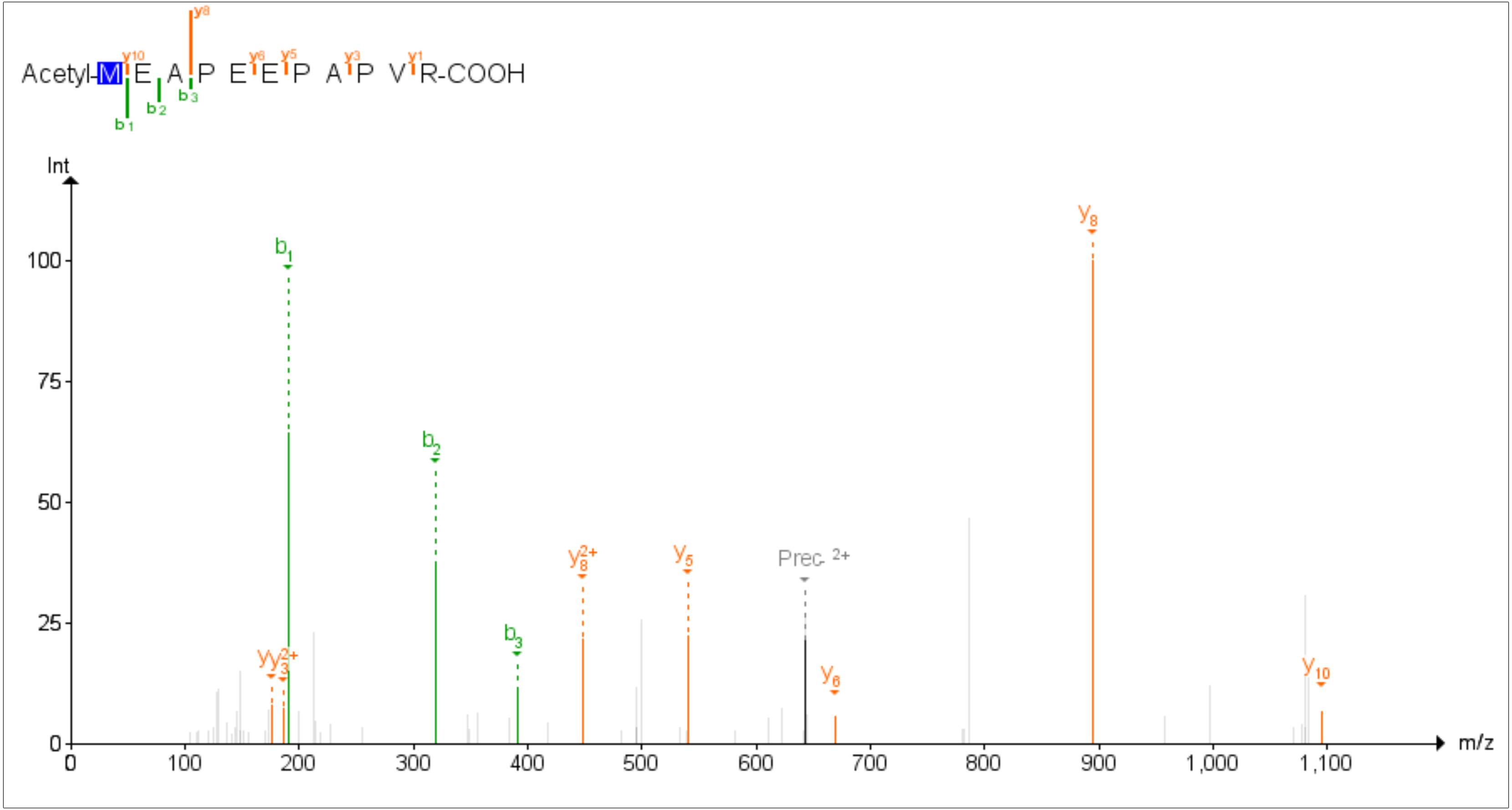

Acetyl-M E A P E E P A P V R-COOH
y10
y8
y6
y5
y3
y1
b1
b2
b3
Int
100
75
50
25
0
b_1
b_2
b_3
y_8
y_8^{2+}
y_5
Prec. 2+
y_6
y_{10}
y
y_3^{2+}
m/z
0
100
200
300
400
500
600
700
800
900
1,000
1,100

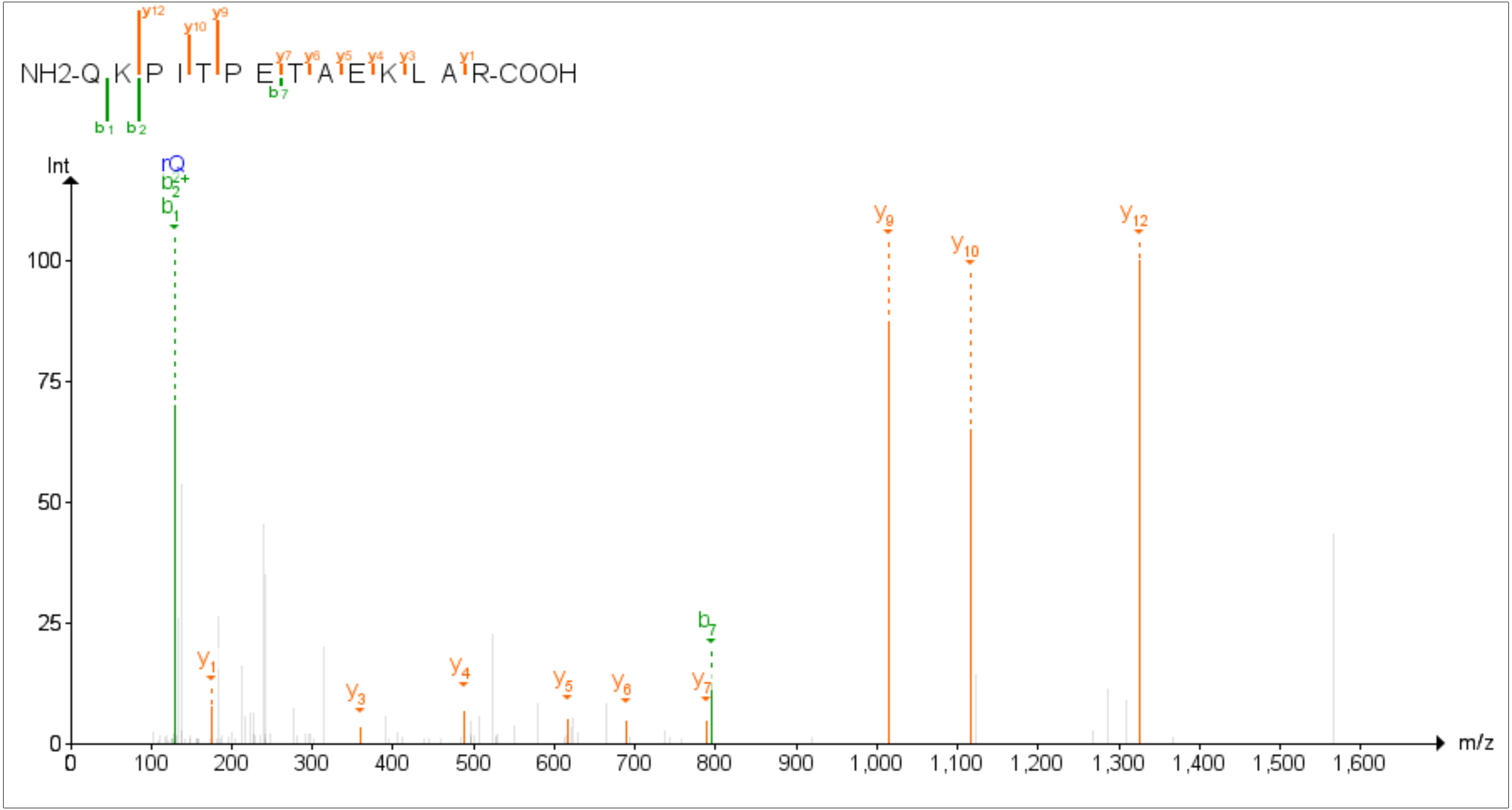
NH2-Q K P I T P E T A E K L A R-COOH
y12
y10
y9
y7
y6
y5
y4
y3
y1
b1
b2
b7
Int
rQ
b2 2+
b1
y9
y10
y12
100
75
50
25
0
y1
y3
y4
y5
y6
y7
b7
0
100
200
300
400
500
600
700
800
900
1,000
1,100
1,200
1,300
1,400
1,500
1,600
m/z

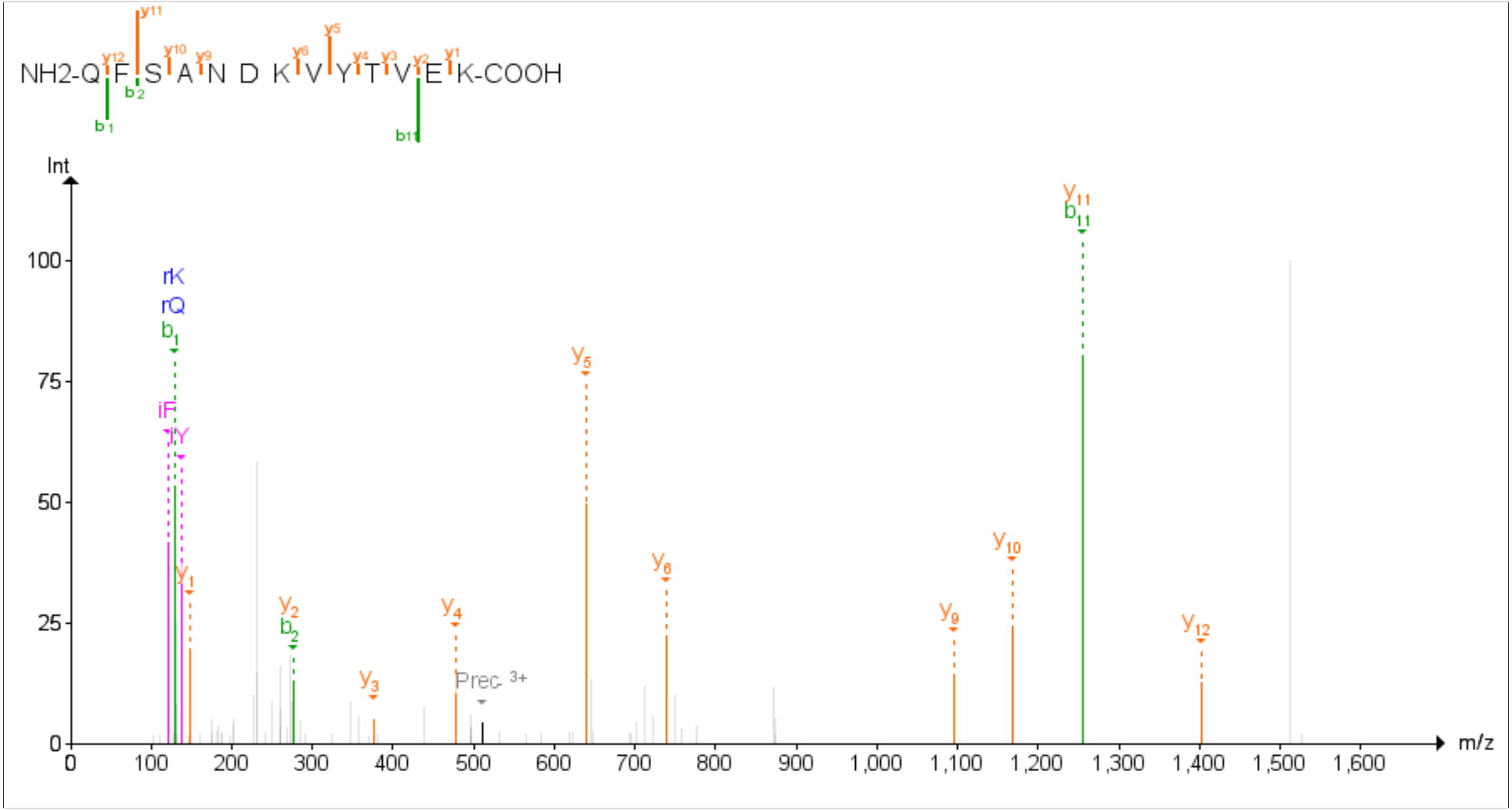

NH2-Q F S A N D K V Y T V E K-COOH
y11
y12
y10
y9
y6
y5
y4
y3
y2
y1
b1
b2
b11
Int
rK
rQ
b1
iF
iY
y1
y2
b2
y3
y4
Prec. 3+
y5
y6
y9
y10
y11
b11
y12
100
75
50
25
0
0
100
200
300
400
500
600
700
800
900
1,000
1,100
1,200
1,300
1,400
1,500
1,600
m/z

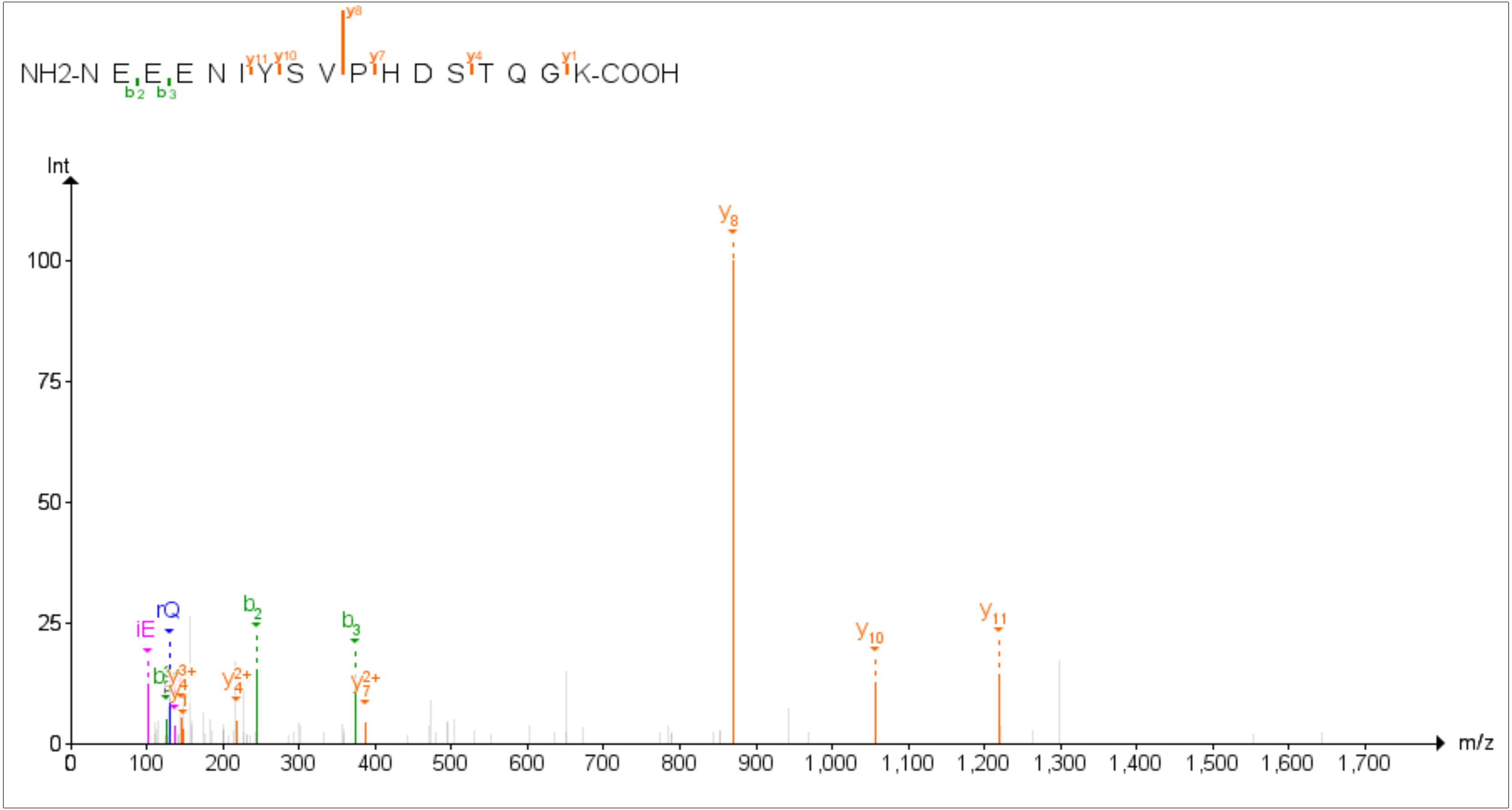

NH2-N E E E N I Y S V P H D S T Q G K-COOH
b2 b3
y11 y10 y8 y7 y4 y1
Int
100
75
50
25
0
m/z
0 100 200 300 400 500 600 700 800 900 1,000 1,100 1,200 1,300 1,400 1,500 1,600 1,700
y8
y10
y11
b2
b3
iE
rQ

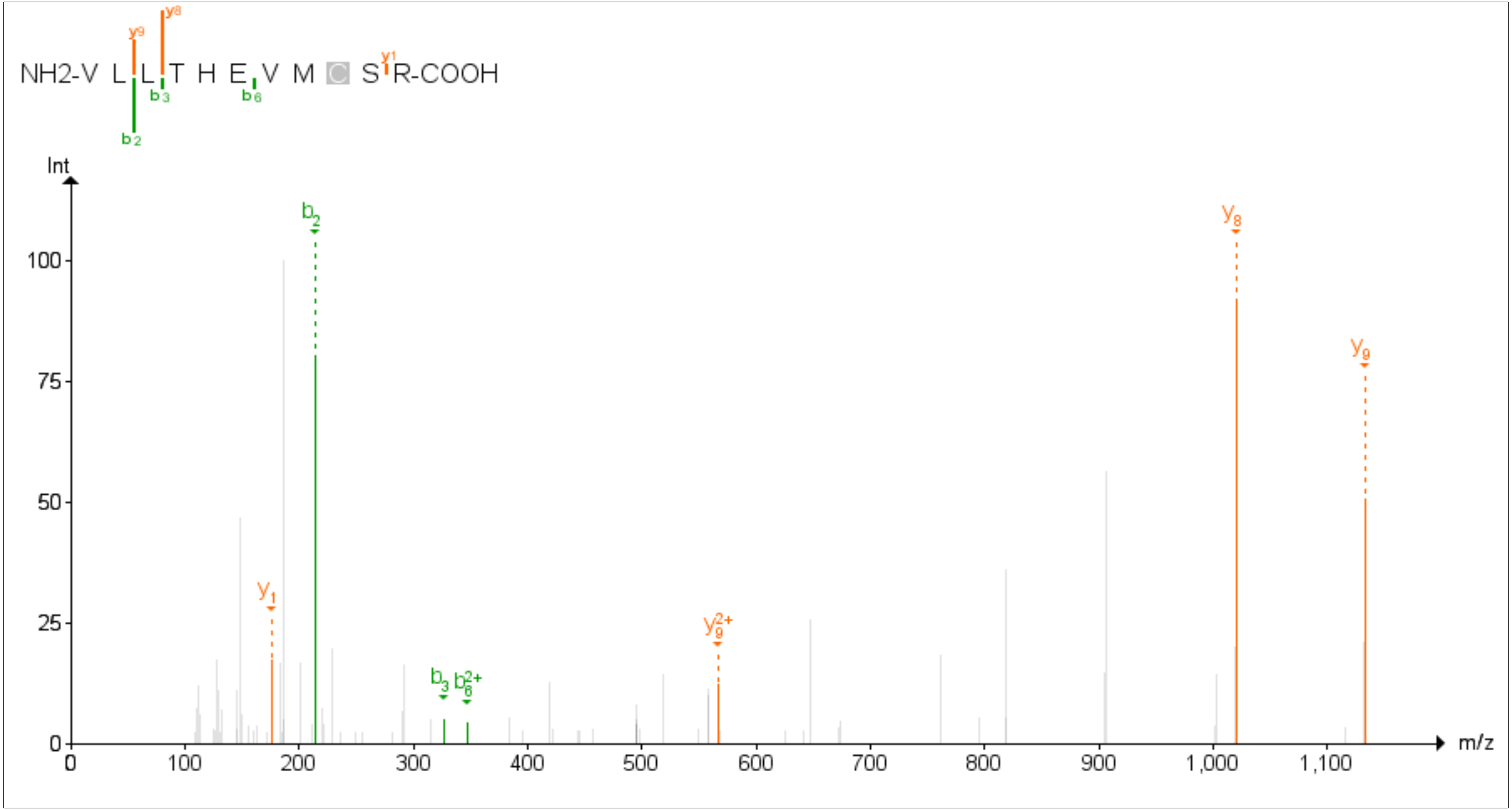

NH2-V L L T H E V M C S R-COOH
y9
y8
y1
b2
b3
b6
Int
100
75
50
25
0
b2
y8
y9
y1
y9 2+
b3
b6 2+
0
100
200
300
400
500
600
700
800
900
1,000
1,100
m/z

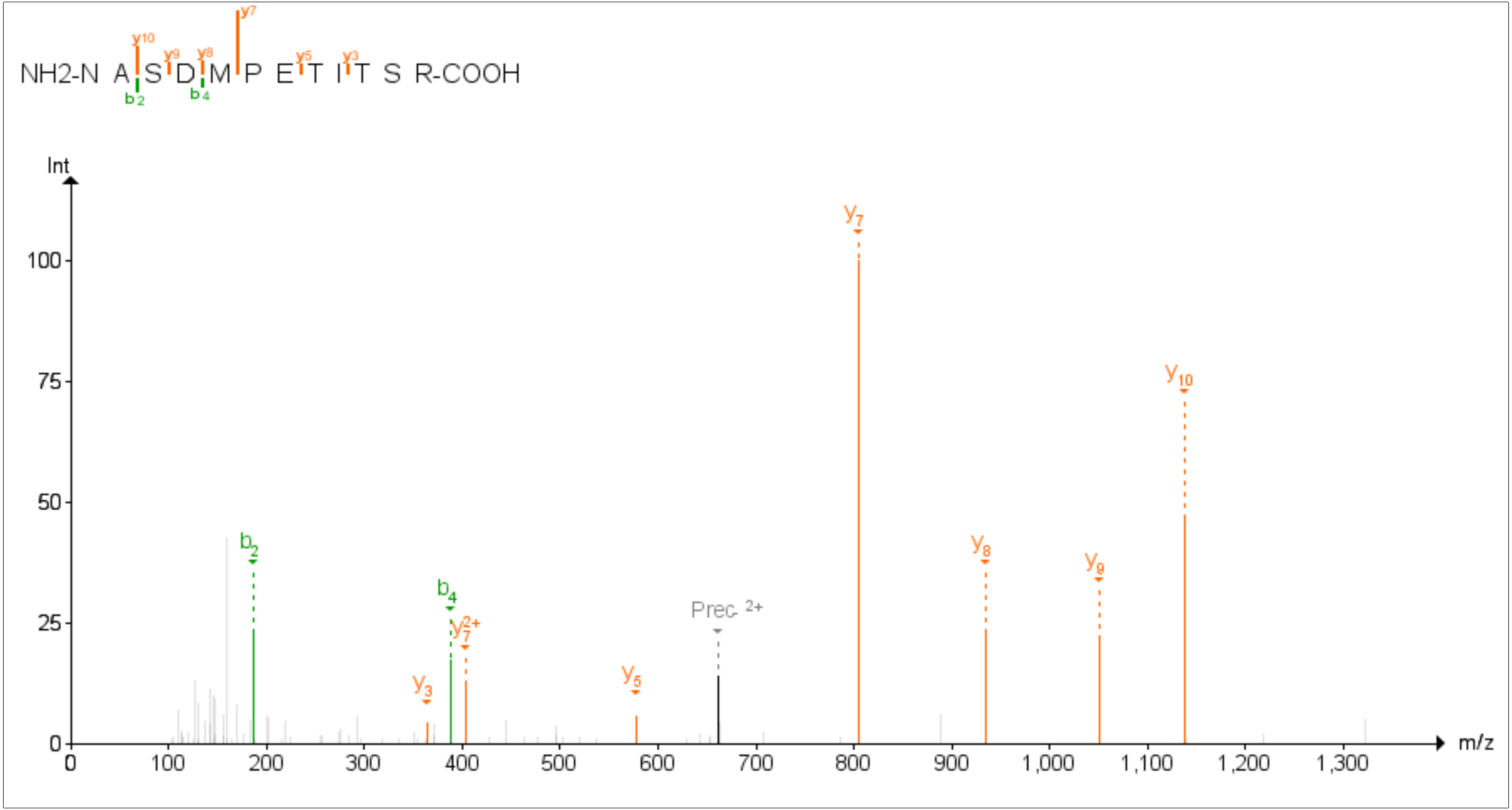

NH2-N A S D M P E T I T S R-COOH
y10 y9 y8 y7 y5 y3
b2 b4
Int
100
75
50
25
0
b2
b4
y3
y7 2+
y5
Prec 2+
y7
y8
y9
y10
0 100 200 300 400 500 600 700 800 900 1,000 1,100 1,200 1,300
m/z

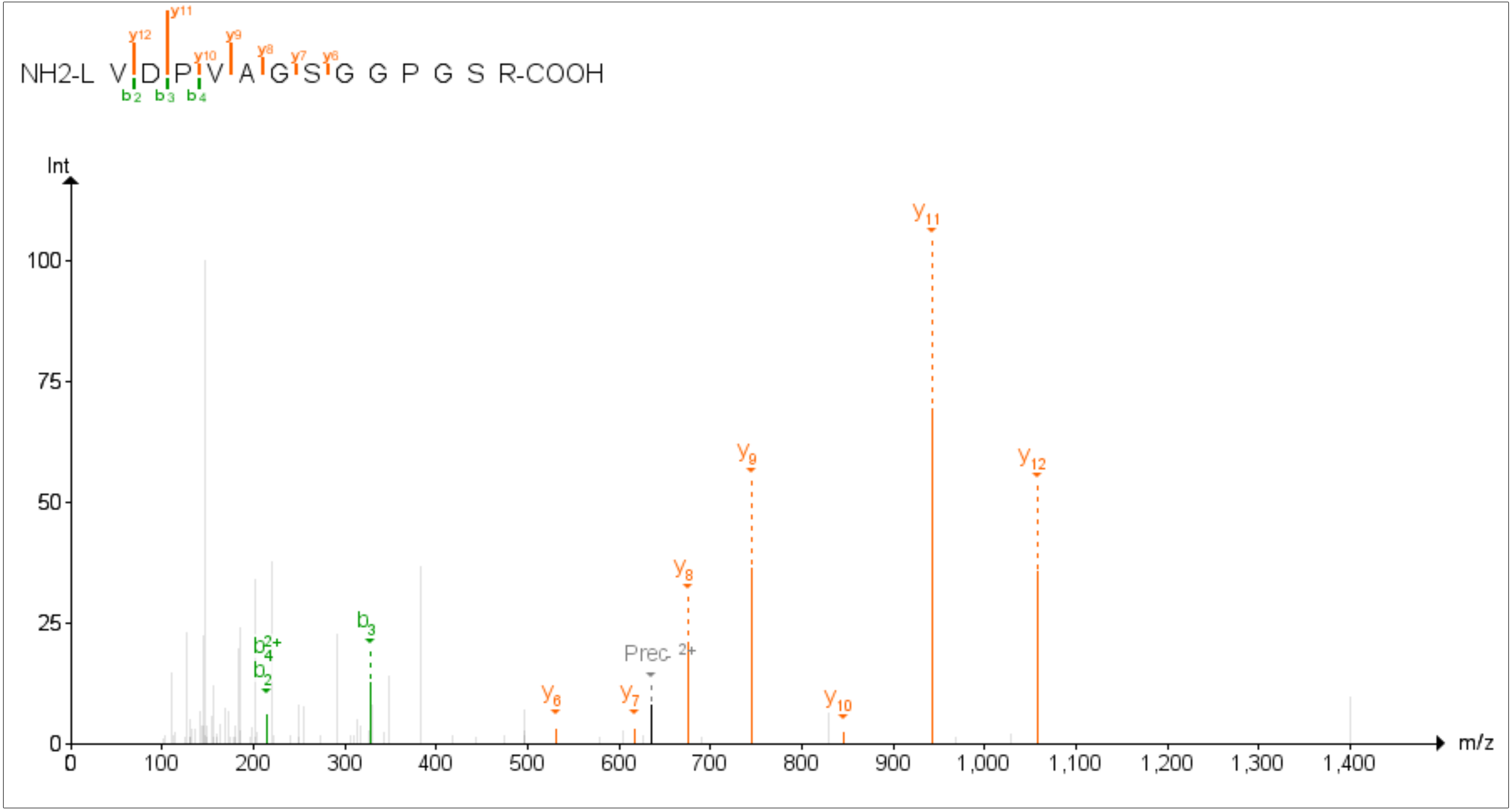

NH2-L V D P V A G S G G P G S R-COOH
y12 y11 y10 y9 y8 y7 y6
b2 b3 b4
Int
m/z
0 25 50 75 100
0 100 200 300 400 500 600 700 800 900 1,000 1,100 1,200 1,300 1,400
y11
y9
y12
y8
b3
b4 2+
b2
Prec 2+
y6
y7
y10

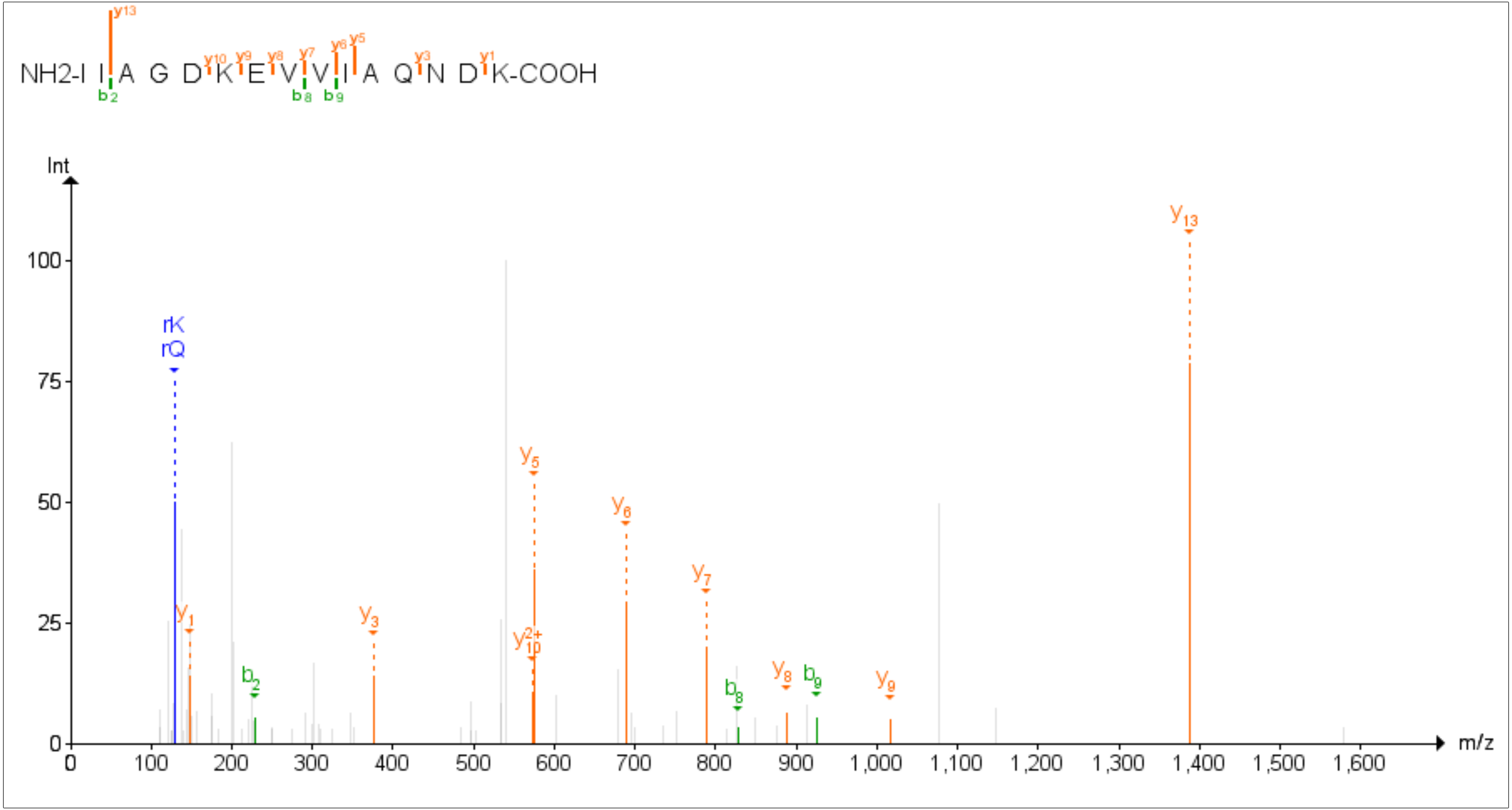

NH2-I I A G D K E V V V I A Q N D K-COOH
y13 y10 y9 y8 y7 y6 y5 y3 y1
b2 b8 b9
Int
m/z
rK
rQ
y_1 y_3 y_5 y_{10}^{2+} y_6 y_7 b_2 b_8 y_8 b_9 y_9 y_{13}

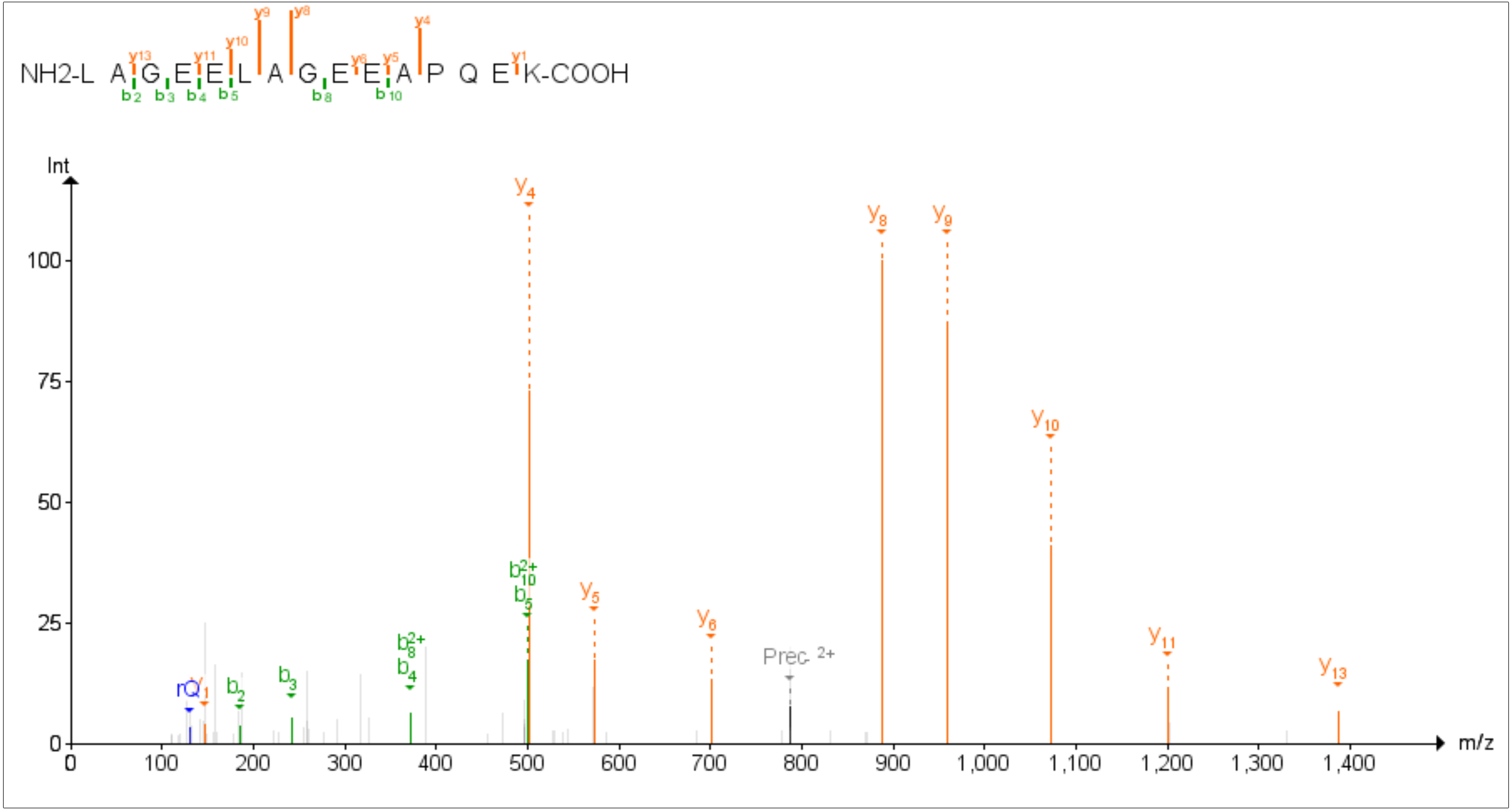

NH2-L A G E E L A G E E A P Q E K-COOH
y13 y11 y10 y9 y8 y6 y5 y4 y1
b2 b3 b4 b5 b8 b10
Int
100
75
50
25
0
y4
y8
y9
y10
y5
y6
y11
y13
Prec- 2+
b10 2+
b5
b8 2+
b4
b3
b2
rQ
y1
m/z
0
100
200
300
400
500
600
700
800
900
1,000
1,100
1,200
1,300
1,400

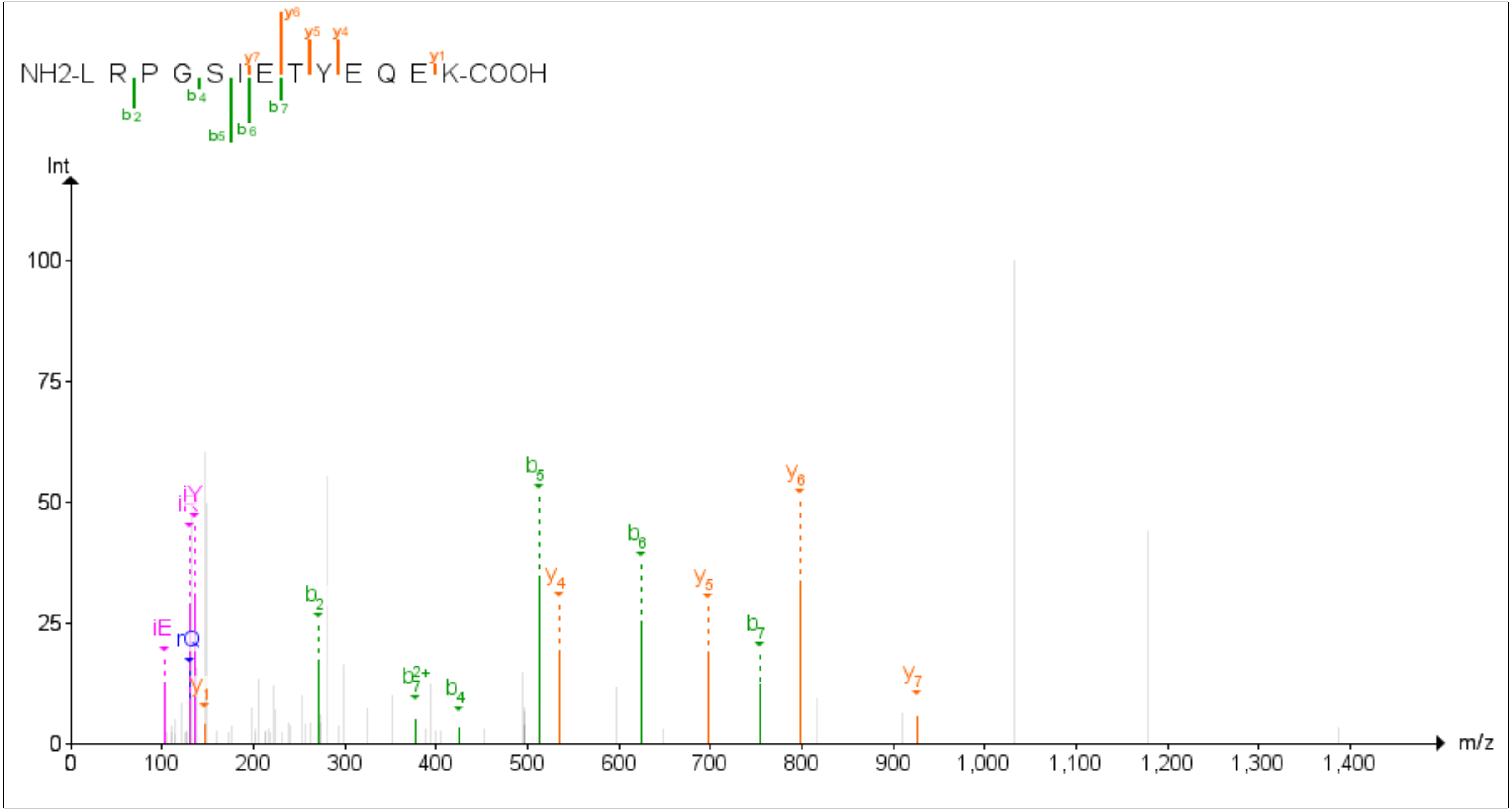
NH2-L R P G S I E T Y E Q E K-COOH
b2
b4
b5
b6
b7
y7
y6
y5
y4
y1
Int
100
75
50
25
0
iY
iE
rQ
y1
b2
b7 2+
b4
b5
y4
b6
y5
b7
y6
y7
0
100
200
300
400
500
600
700
800
900
1,000
1,100
1,200
1,300
1,400
m/z

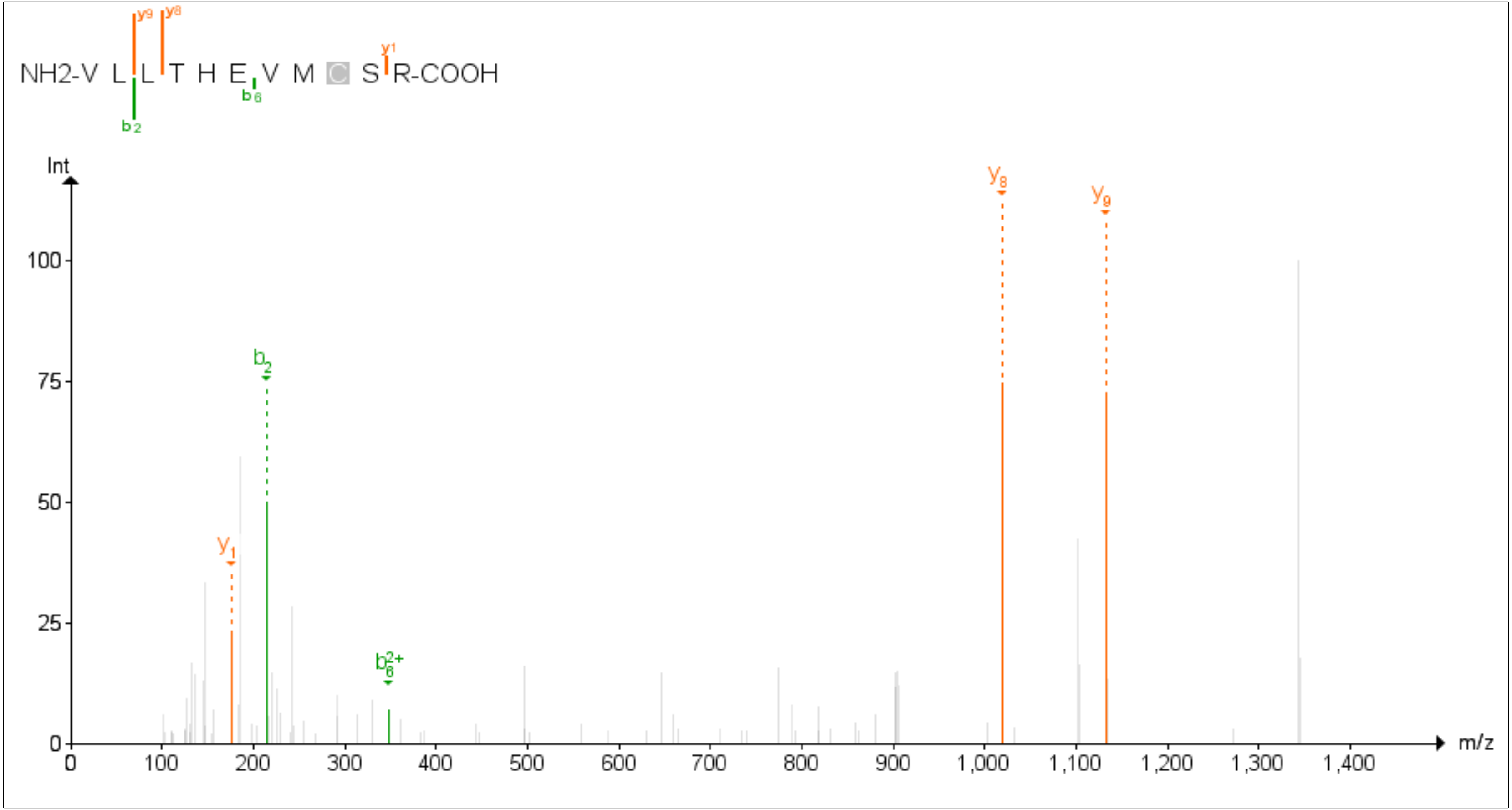

NH2-V L L T H E V M C S R-COOH
y9
y8
y1
b2
b6
Int
100
75
50
25
0
y8
y9
b2
y1
b6 2+
0
100
200
300
400
500
600
700
800
900
1,000
1,100
1,200
1,300
1,400
m/z

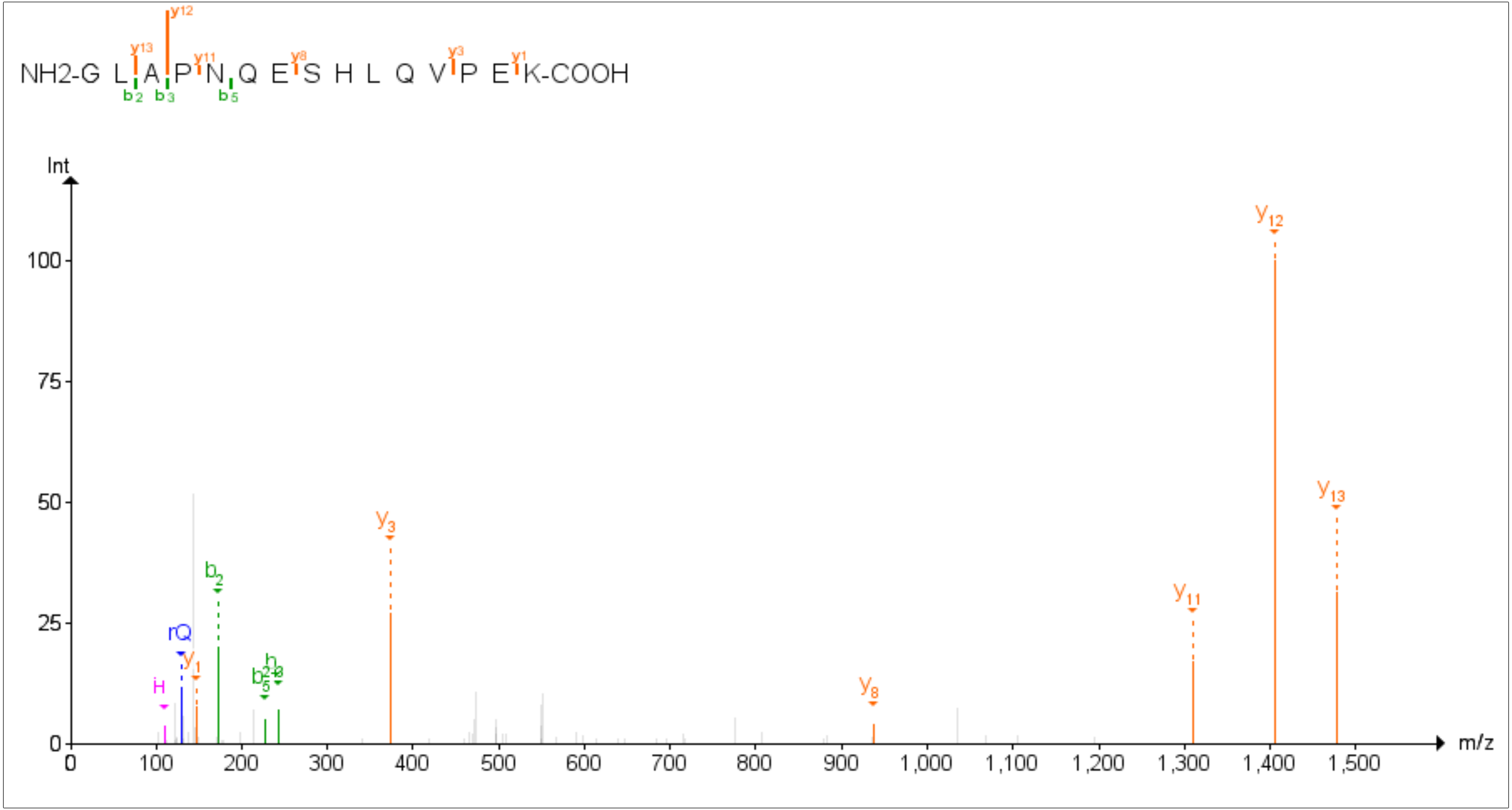

NH2-G L A P N Q E S H L Q V P E K-COOH
y12
y13
y11
y8
y3
y1
b2
b3
b5
Int
100
75
50
25
0
m/z
0
100
200
300
400
500
600
700
800
900
1,000
1,100
1,200
1,300
1,400
1,500
y12
y13
y3
b2
rQ
y1
H
y11
y8

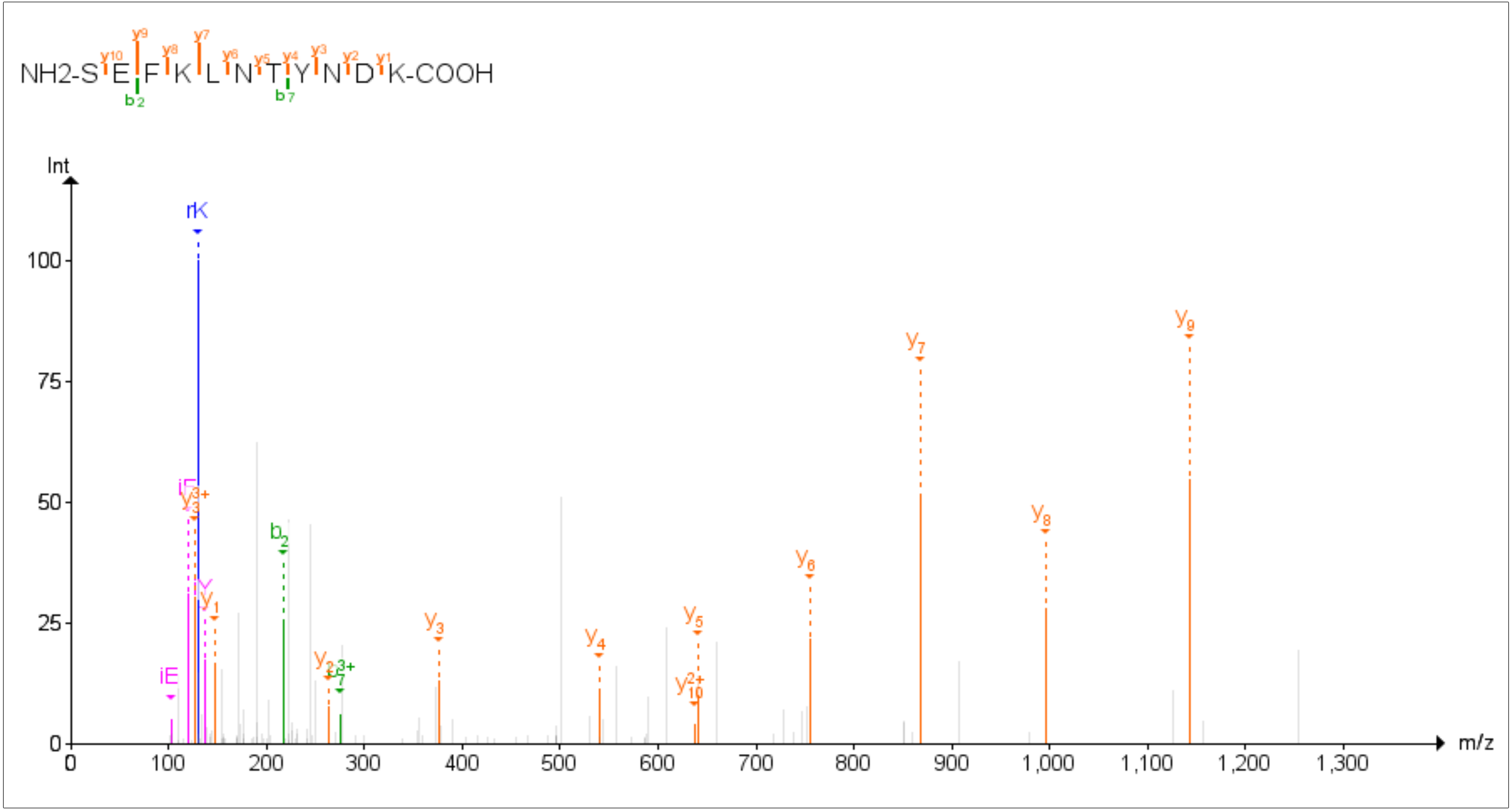

NH2-SEFKLNTYNDK-COOH
y10
y9
y8
y7
y6
y5
y4
y3
y2
y1
b2
b7
Int
rK
iE
iY
y1
b2
y3
y4
y5
y6
y7
y8
y9
m/z
0
25
50
75
100
0
100
200
300
400
500
600
700
800
900
1,000
1,100
1,200
1,300

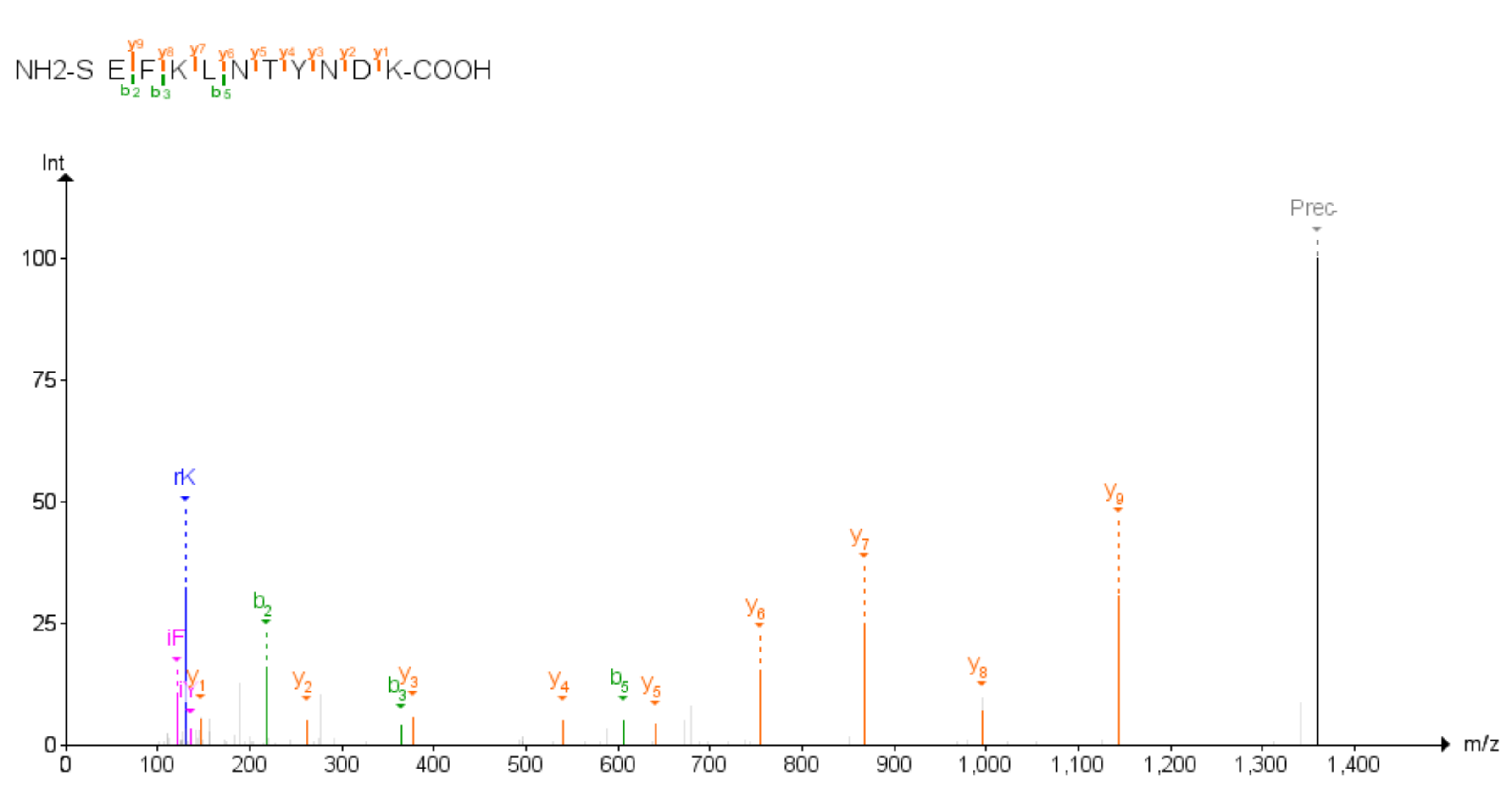

NH2-S E F K L N T Y N D K-COOH
y9 y8 y7 y6 y5 y4 y3 y2 y1
b2 b3 b5
Int
m/z
Prec.
rK
iF
iY
y1 y2 y3 y4 y5 y6 y7 y8 y9
b2 b3 b5
0
25
50
75
100
0 100 200 300 400 500 600 700 800 900 1,000 1,100 1,200 1,300 1,400

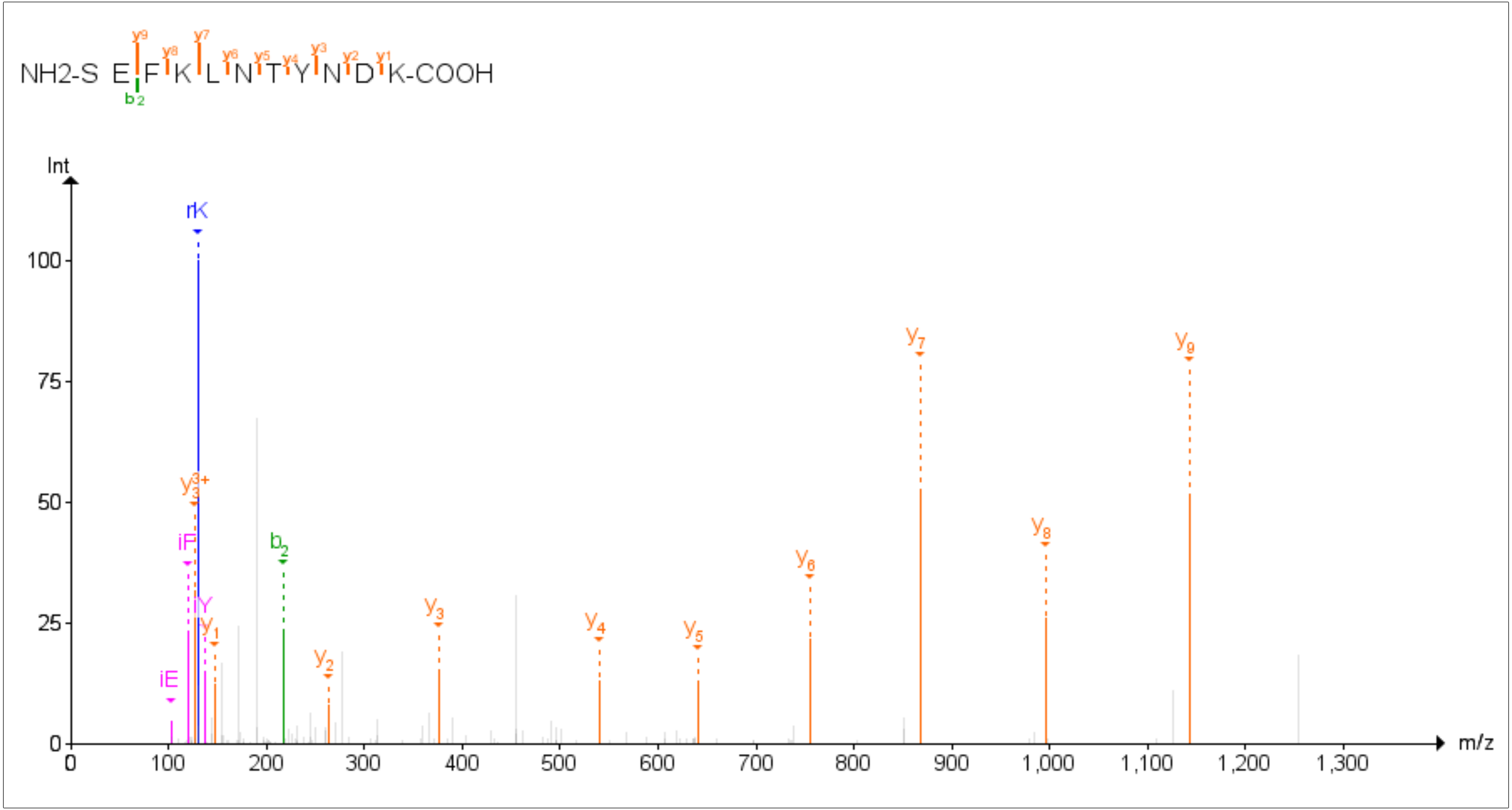

NH2-S E F K L N T Y N D K-COOH
y9 y8 y7 y6 y5 y4 y3 y2 y1
b2
Int
m/z
rK
y7
y9
y8
y6
y5
y4
y3
y2
y1
iY
iF
iE
b2
y3 3+
0
25
50
75
100
0
100
200
300
400
500
600
700
800
900
1,000
1,100
1,200
1,300

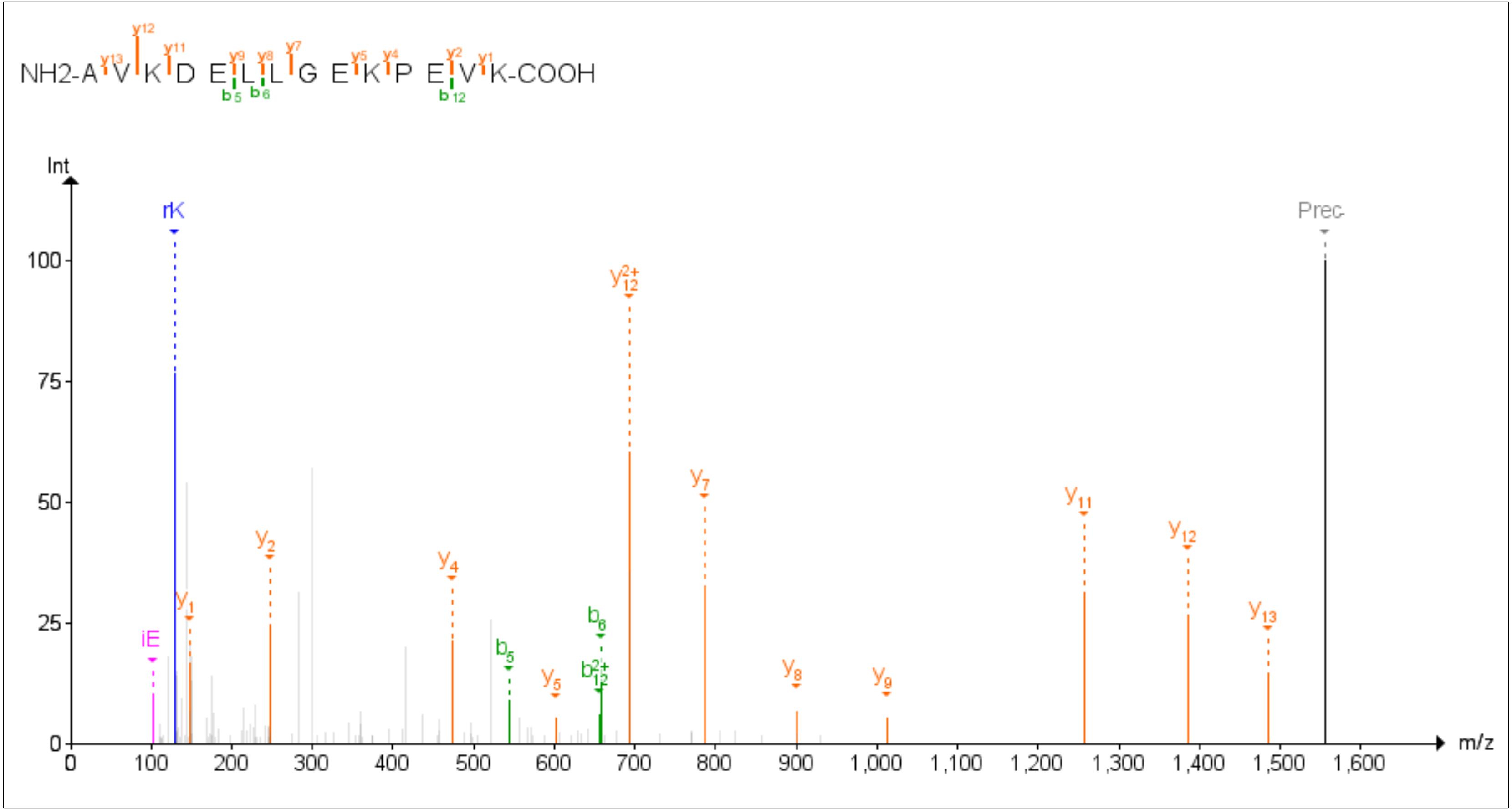
NH2-A V K D E L L G E K P E V K-COOH
y13 y12 y11 y9 y8 y7 y5 y4 y2 y1
b5 b6 b12
Int
m/z
rK
Prec
iE
y1
y2
y4
b5
y5
b6
b12 2+
y12 2+
y7
y8
y9
y11
y12
y13
0
25
50
75
100
0
100
200
300
400
500
600
700
800
900
1,000
1,100
1,200
1,300
1,400
1,500
1,600

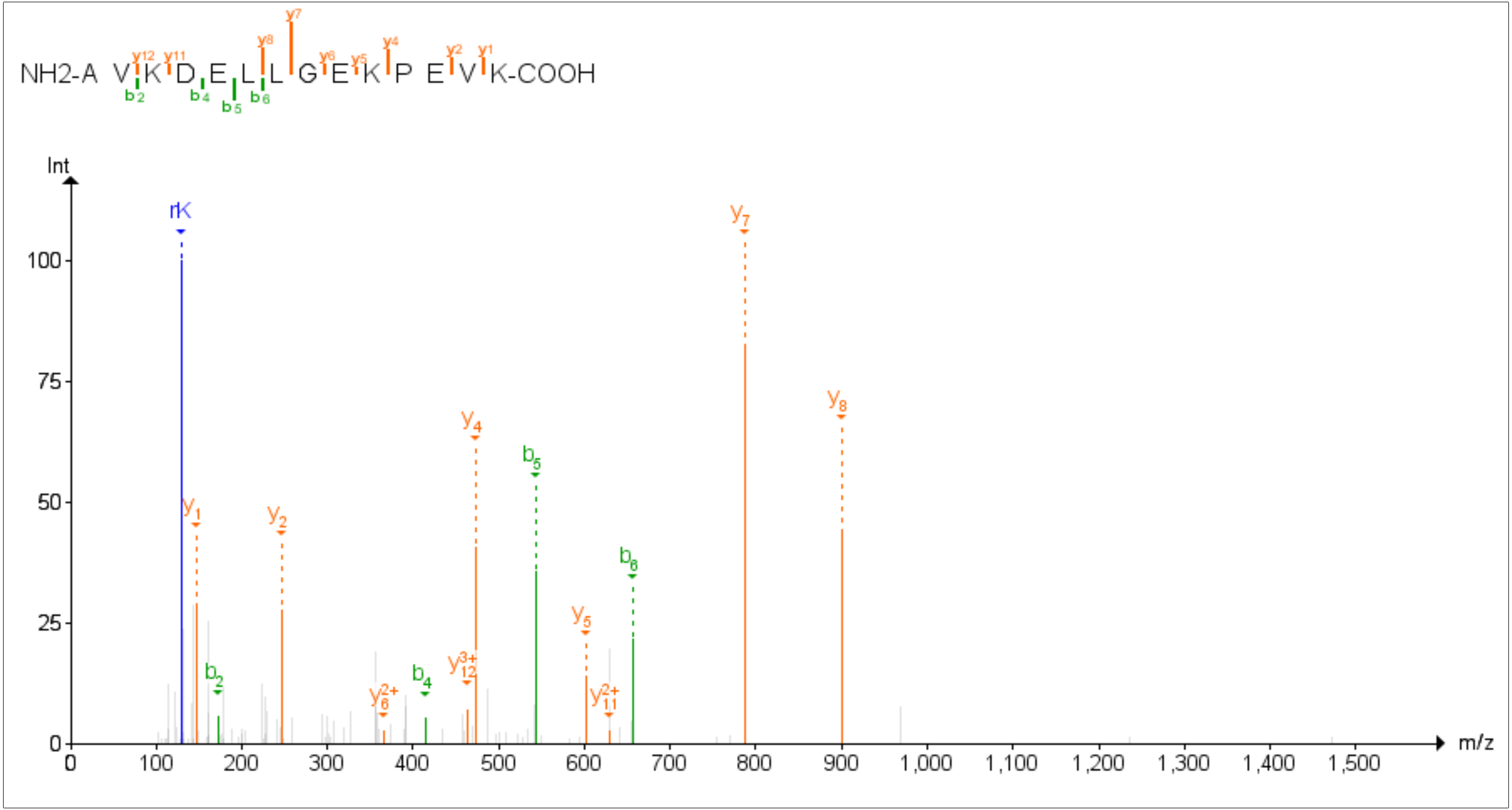

NH2-A V K D E L L G E K P E V K-COOH
y12 y11 y8 y7 y6 y5 y4 y2 y1
b2 b4 b5 b6
Int
rK
y7
y8
y4
b5
y1
y2
b6
y5
b2
b4
y6 2+
y12 3+
y11 2+
0
25
50
75
100
0 100 200 300 400 500 600 700 800 900 1,000 1,100 1,200 1,300 1,400 1,500
m/z

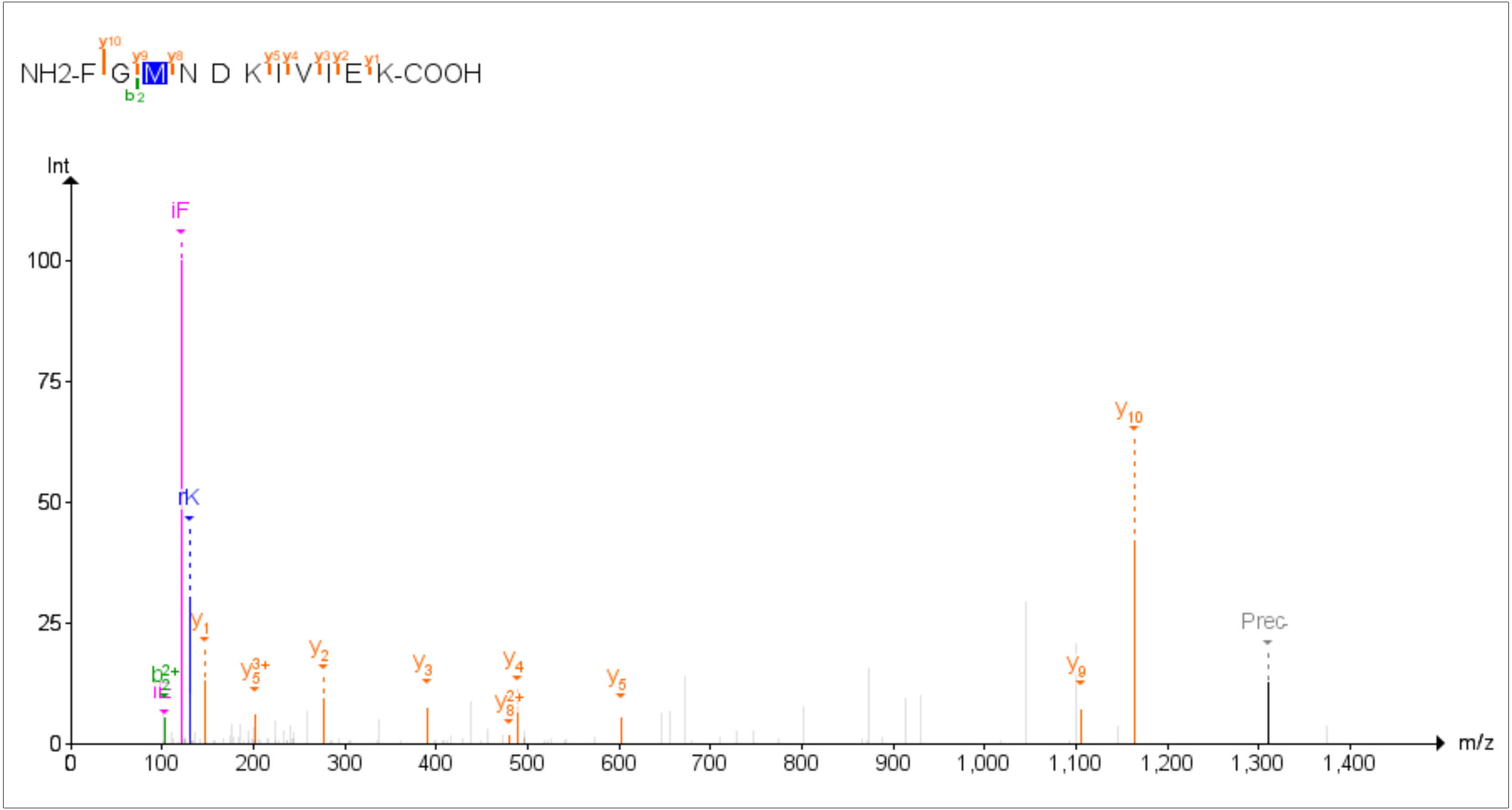

NH2-F G M N D K I V I E K-COOH
y10 y9 y8 y5 y4 y3 y2 y1
b2
Int
iF
rK
y10
Prec
y1
y2
y3
y4
y5
y9
b2 2+
y5 3+
y8 2+
iE
m/z
0 25 50 75 100
0 100 200 300 400 500 600 700 800 900 1,000 1,100 1,200 1,300 1,400

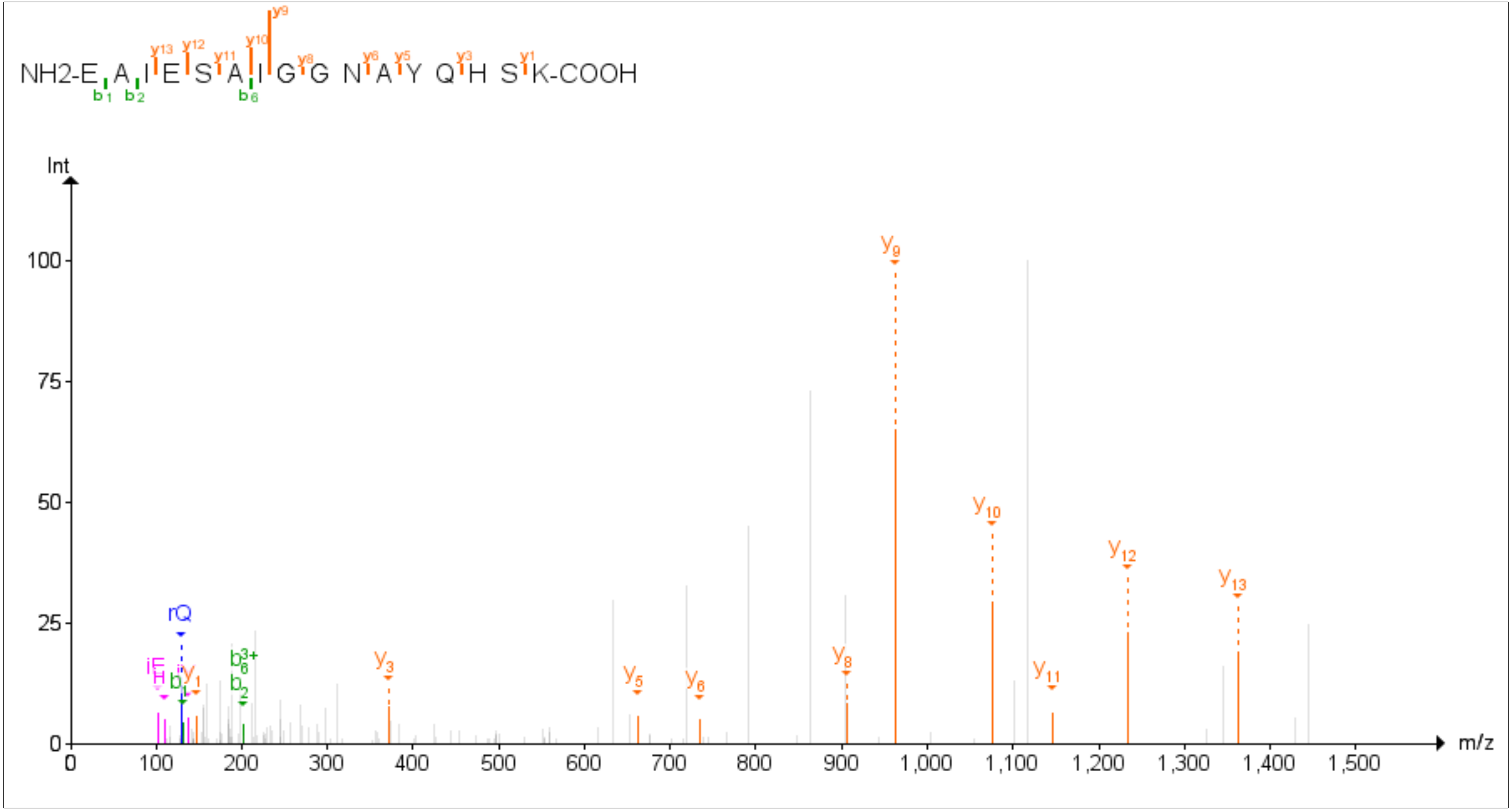

NH2-E A I E S A I G G N A Y Q H S K-COOH
Int
m/z
y9
y10
y12
y13
y8
y11
y3
y5
y6
y1
rQ
b1
b2
b6

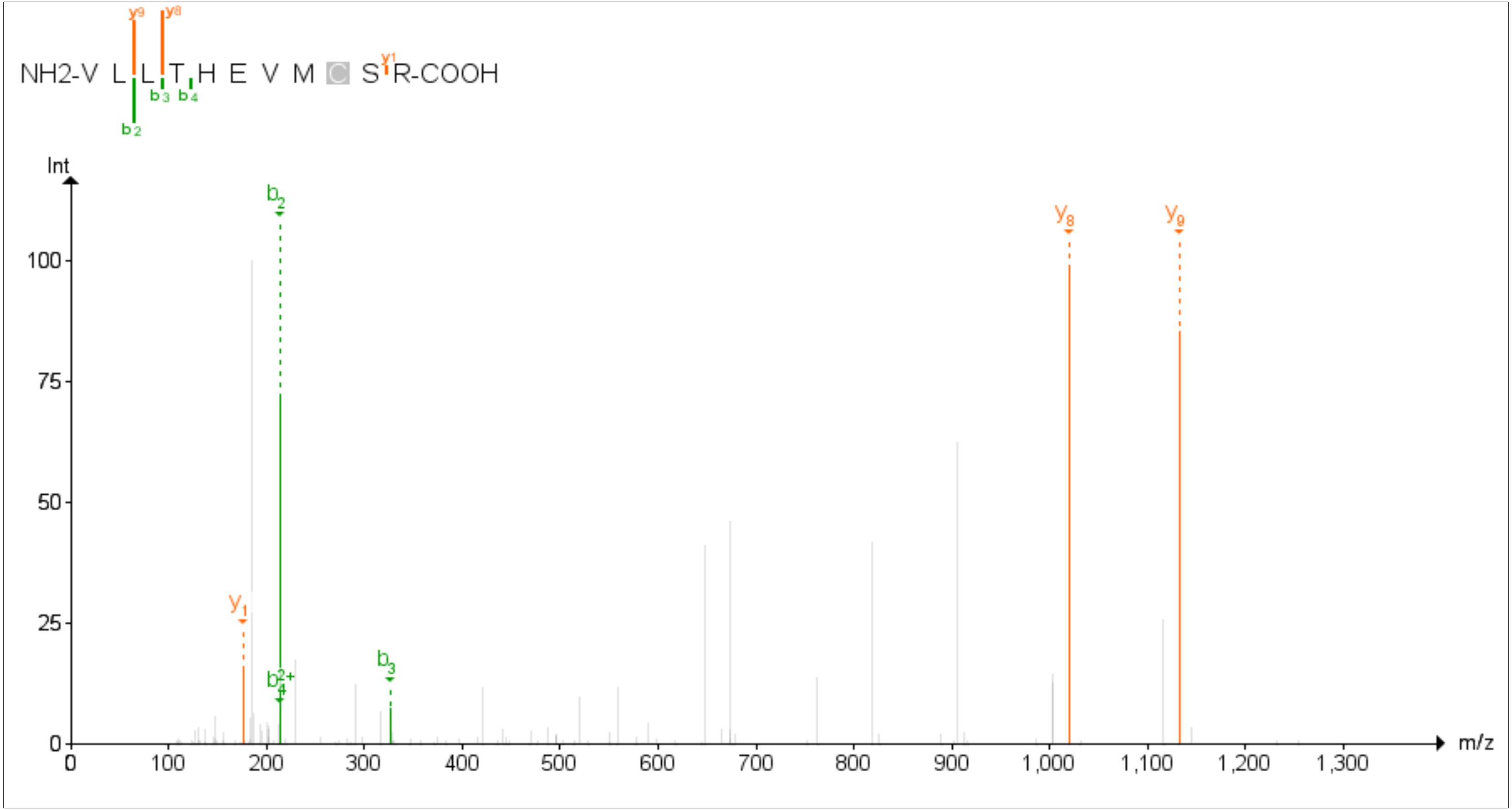

NH2-V L L T H E V M C S R-COOH
y9
y8
y1
b2
b3
b4
Int
m/z
y1
b2
b2+4
b3
y8
y9
0
25
50
75
100
0
100
200
300
400
500
600
700
800
900
1,000
1,100
1,200
1,300

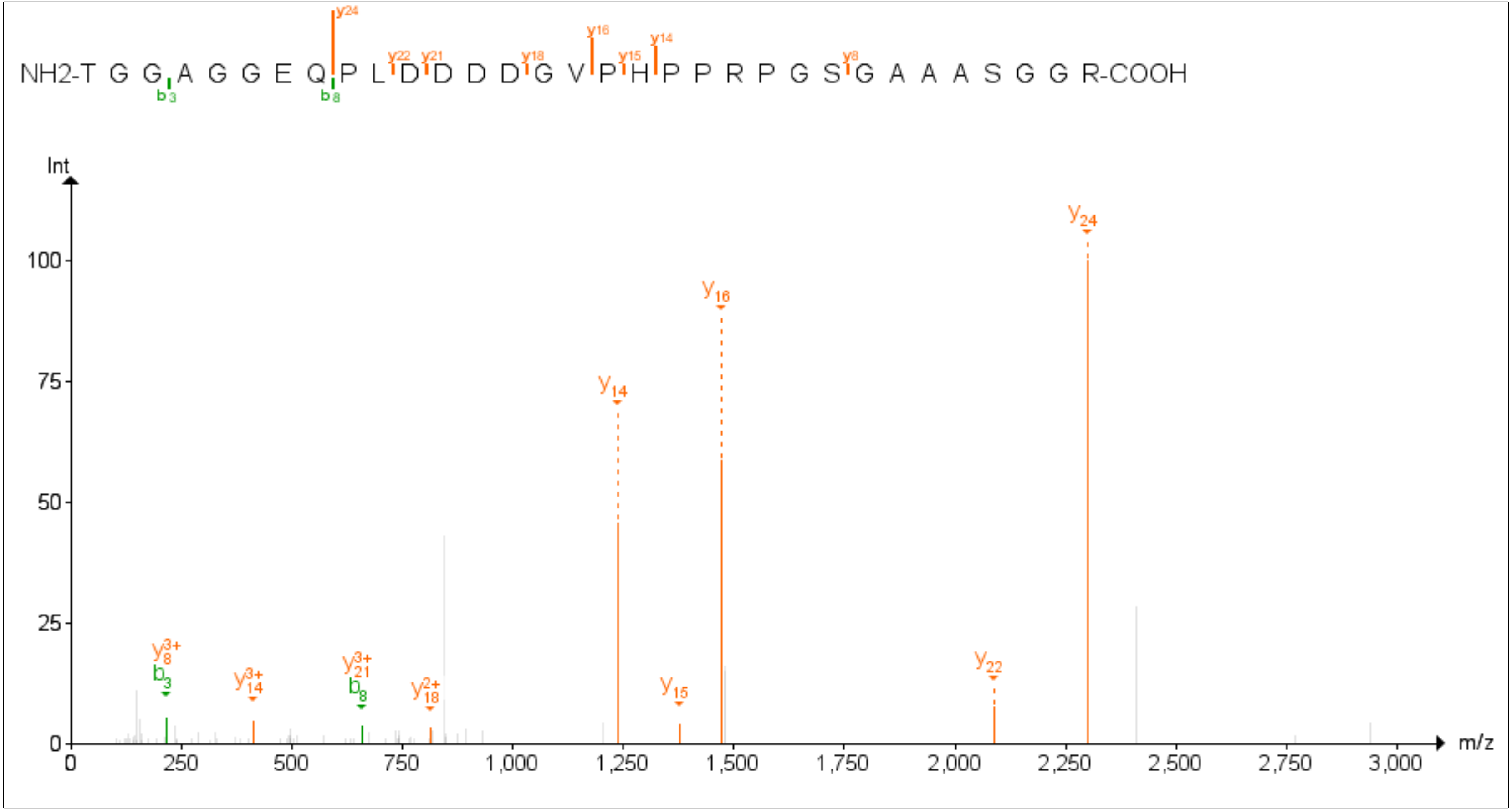
NH2-T G G A G G E Q P L D D D D G V P H P P R P G S G A A A S G G R-COOH
b3
b8
y24
y22
y21
y18
y16
y15
y14
y8
Int
100
75
50
25
0
y24
y16
y14
y22
y15
y8 3+
b3
y14 3+
y21 3+
b8
y18 2+
0
250
500
750
1,000
1,250
1,500
1,750
2,000
2,250
2,500
2,750
3,000
m/z

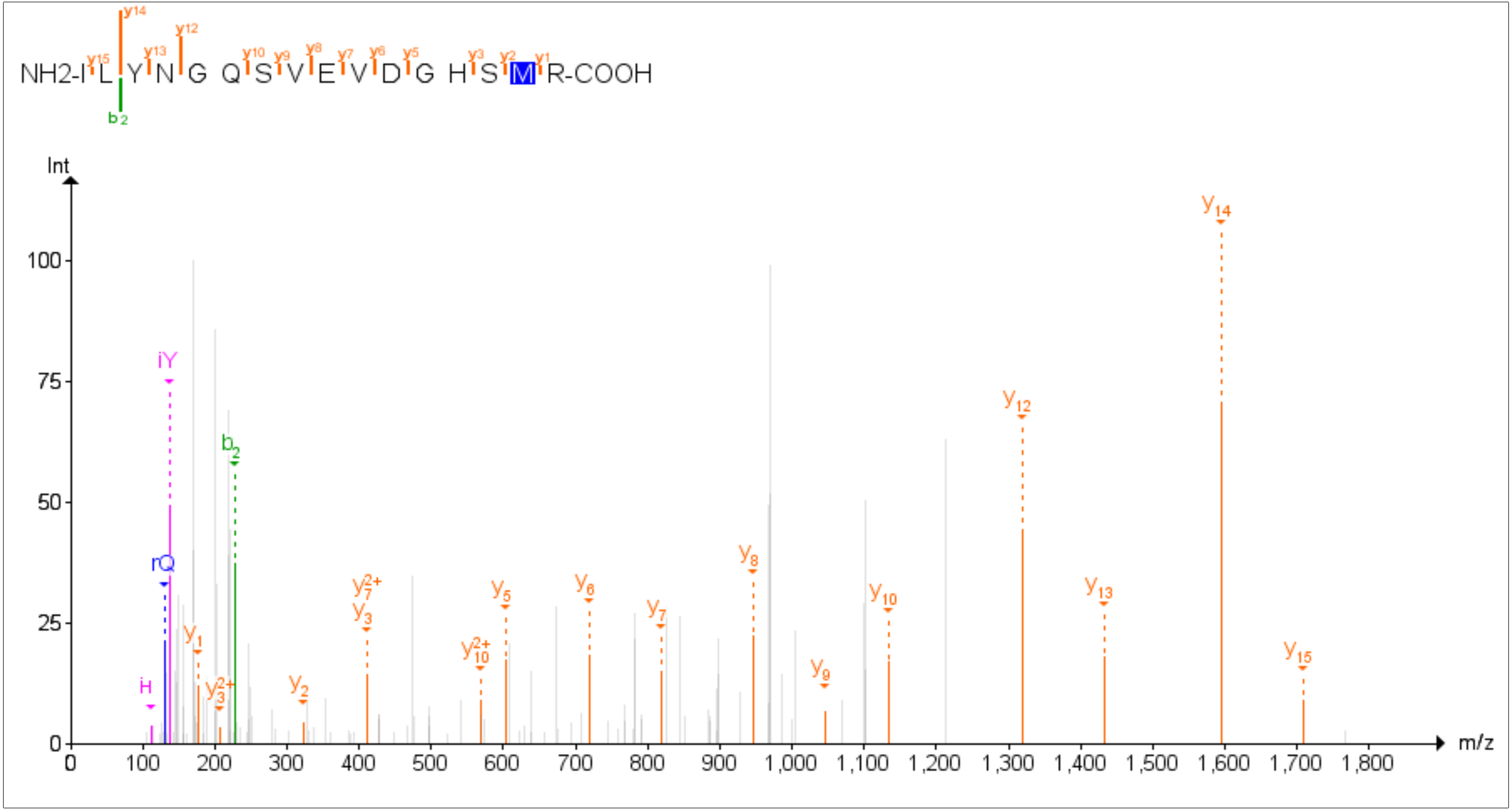

NH2-I L Y N G Q S V E V D G H S M R-COOH
y15 y14 y13 y12 y10 y9 y8 y7 y6 y5 y3 y2 y1
b2
Int
m/z
0
25
50
75
100
0
100
200
300
400
500
600
700
800
900
1,000
1,100
1,200
1,300
1,400
1,500
1,600
1,700
1,800
iY
rQ
iH
b2
y1
y2
y3
y5
y6
y7
y8
y9
y10
y12
y13
y14
y15
y3 2+
y7 2+
y10 2+

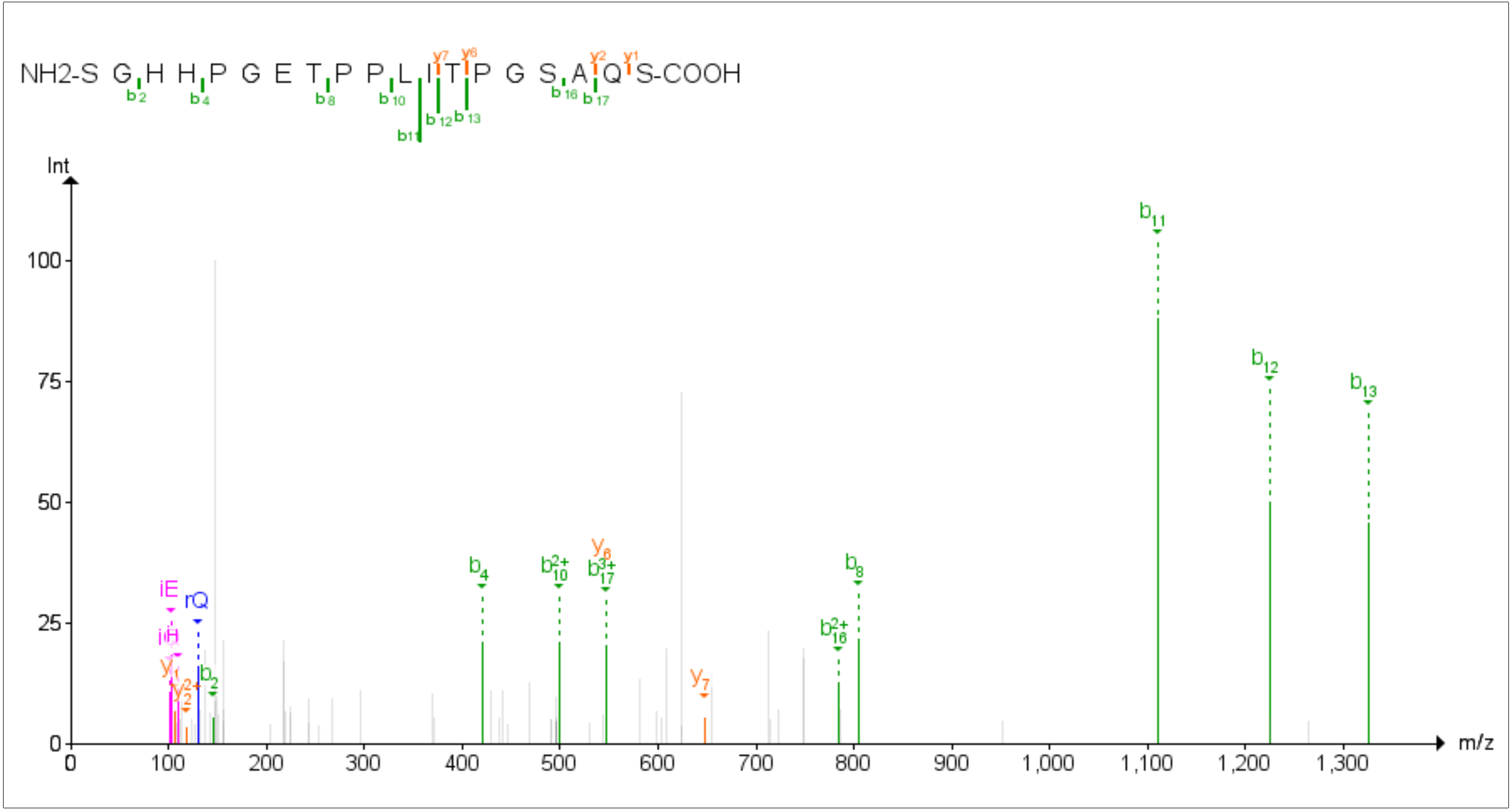

NH2-S G H H P G E T P P L I T P G S A Q S-COOH
b2 b4 b8 b10 b11 b12 b13 b16 b17
y7 y6 y2 y1
Int
100
75
50
25
0
m/z
0 100 200 300 400 500 600 700 800 900 1,000 1,100 1,200 1,300
b11
b12
b13
b4
b10 2+
y6
b17 3+
b8
b16 2+
y7
iE
rQ
iH
y1
y2 2+
b2

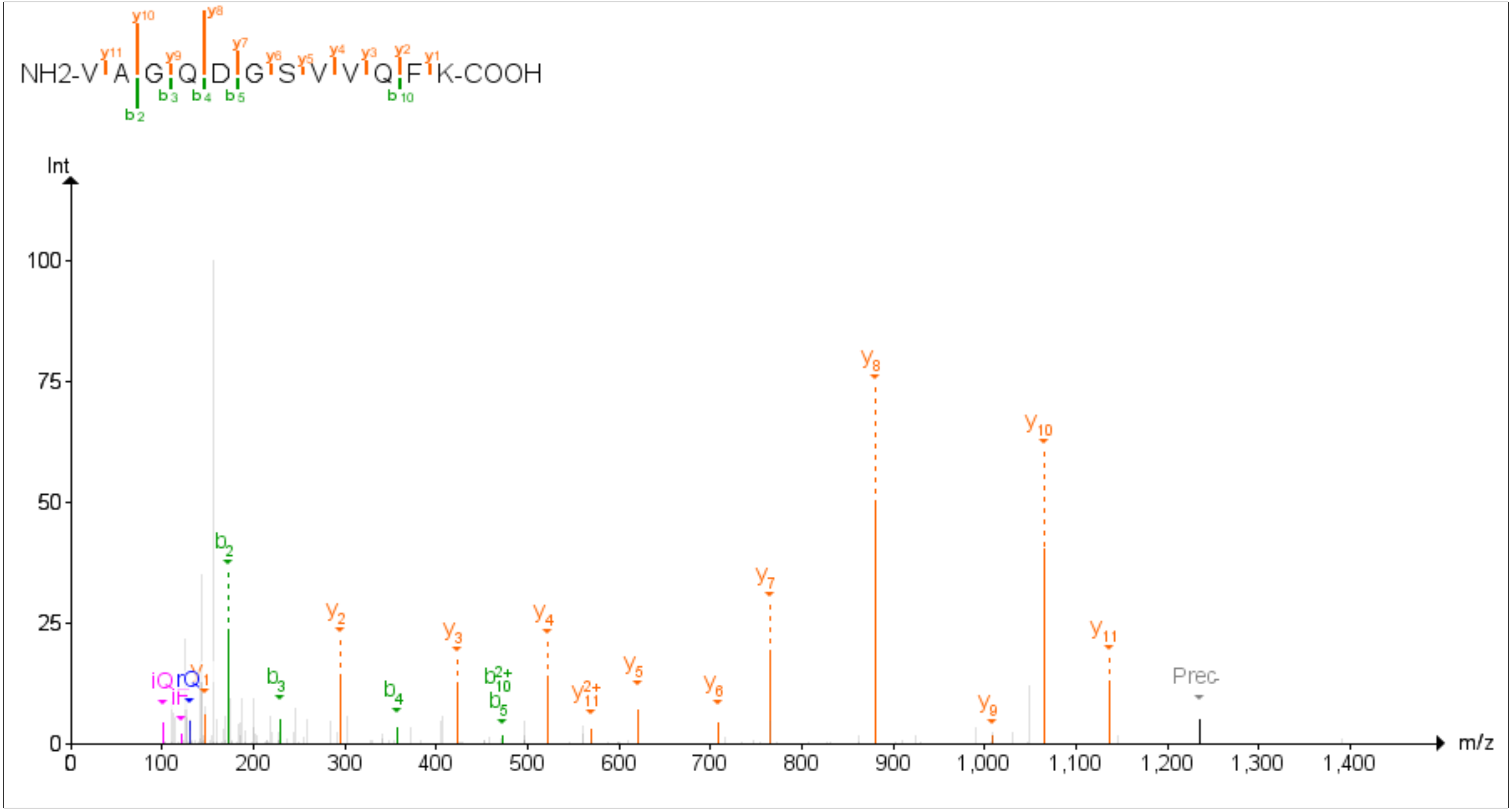

NH2-VAGQDGSVVQFK-COOH
y11 y10 y9 y8 y7 y6 y5 y4 y3 y2 y1
b2 b3 b4 b5 b10
Int
100
75
50
25
0
m/z
0
100
200
300
400
500
600
700
800
900
1,000
1,100
1,200
1,300
1,400
y8
y10
b2
y7
y2
y4
y3
y11
y1
iQ
rQ
iF
b3
b4
b10 2+
b5
y11 2+
y5
y6
y9
Prec

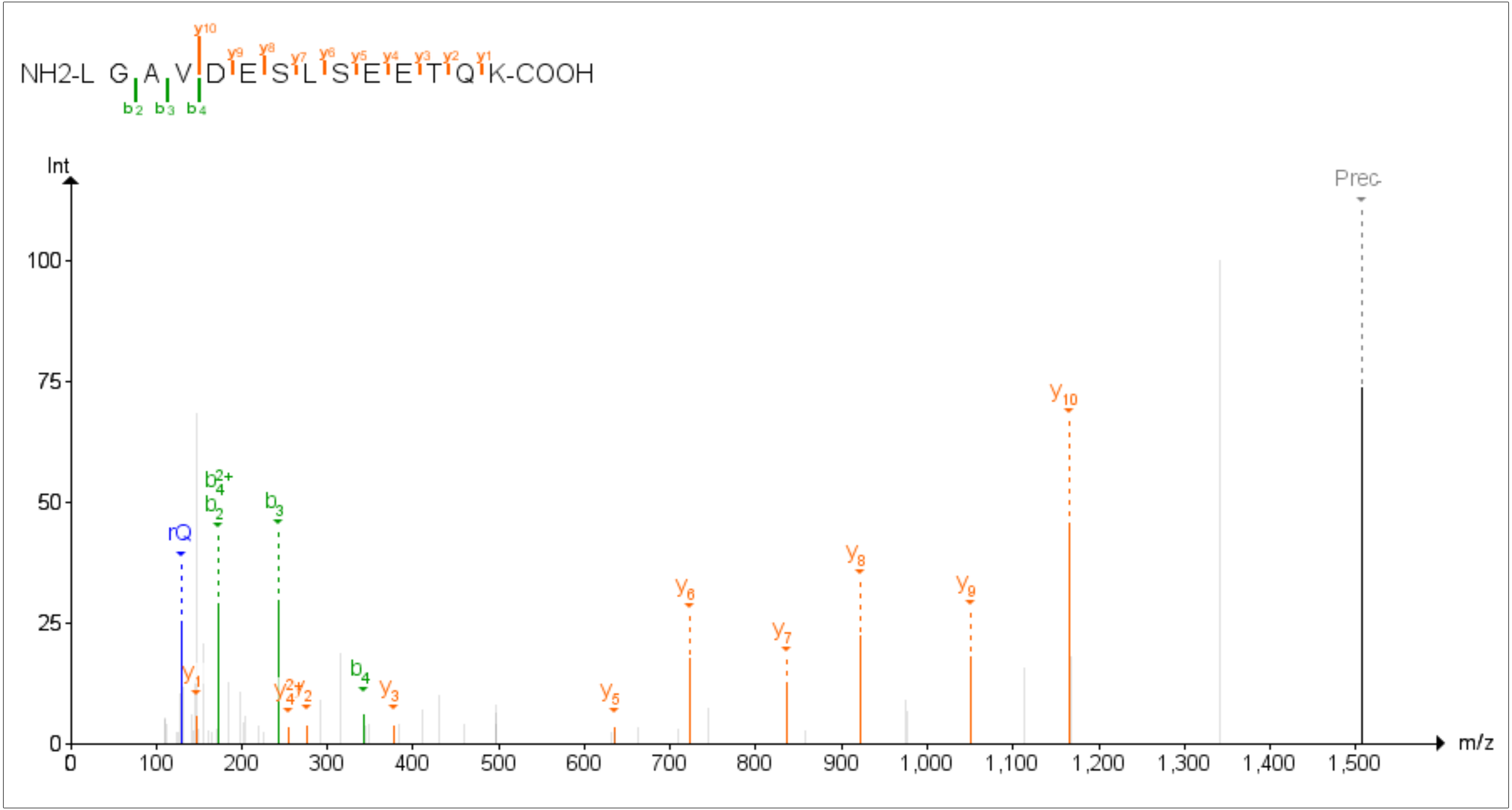

NH2-L G A V D E S L S E E T Q K-COOH
y10 y9 y8 y7 y6 y5 y4 y3 y2 y1
b2 b3 b4
Int
100
75
50
25
0
Prec
rQ
b_4^{2+}
b_2
b_3
b_4
y_1
y_4^{2+}
y_2
y_3
y_5
y_6
y_7
y_8
y_9
y_{10}
0 100 200 300 400 500 600 700 800 900 1,000 1,100 1,200 1,300 1,400 1,500
m/z

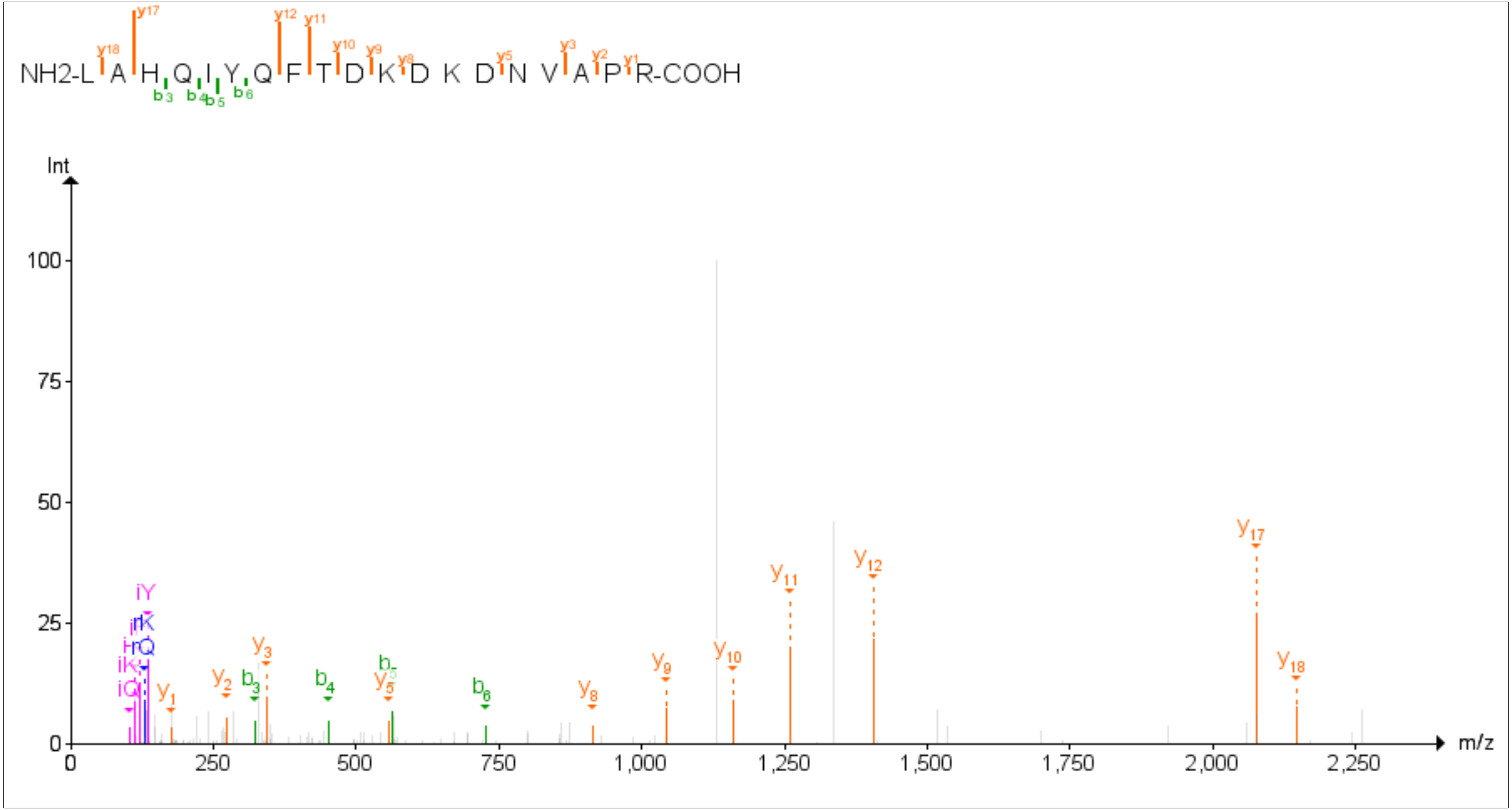

NH2-L A H Q I Y Q F T D K D K D N V A P R-COOH
y18 y17 y12 y11 y10 y9 y8 y5 y3 y2 y1
b3 b4 b5 b6
Int
100
75
50
25
0
iY
iK
iQ
y1 y2 y3 y5 y8 y9 y10 y11 y12 y17 y18
b3 b4 b5 b6
0
250
500
750
1,000
1,250
1,500
1,750
2,000
2,250
m/z

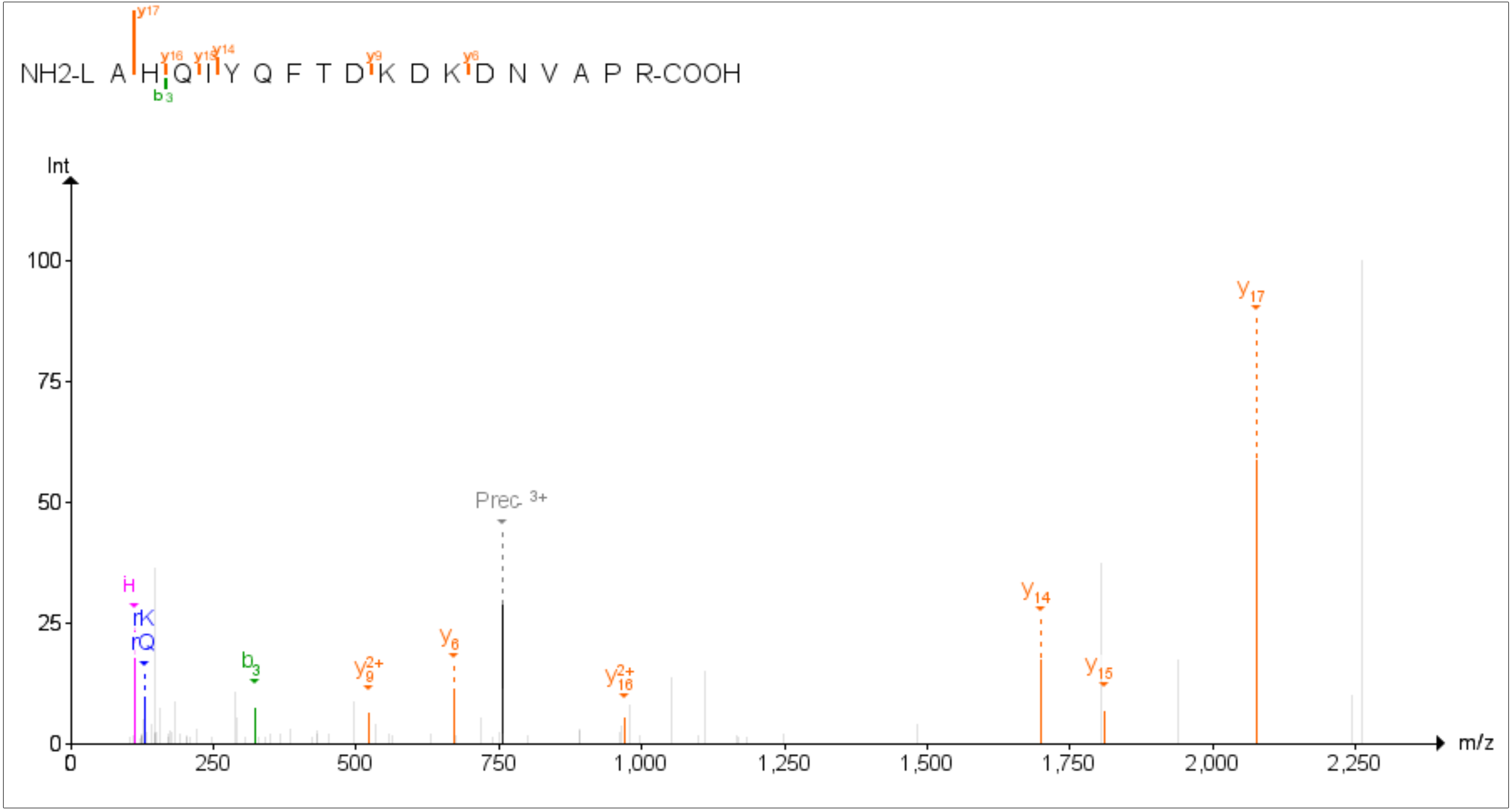

NH2-L A H Q I Y Q F T D K D K D N V A P R-COOH
y17
y16
y15
y14
y9
y6
b3
Int
100
75
50
25
0
Prec 3+
y17
y14
y15
y6
y_9^{2+}
y_{16}^{2+}
b3
iH
rK
rQ
0
250
500
750
1,000
1,250
1,500
1,750
2,000
2,250
m/z

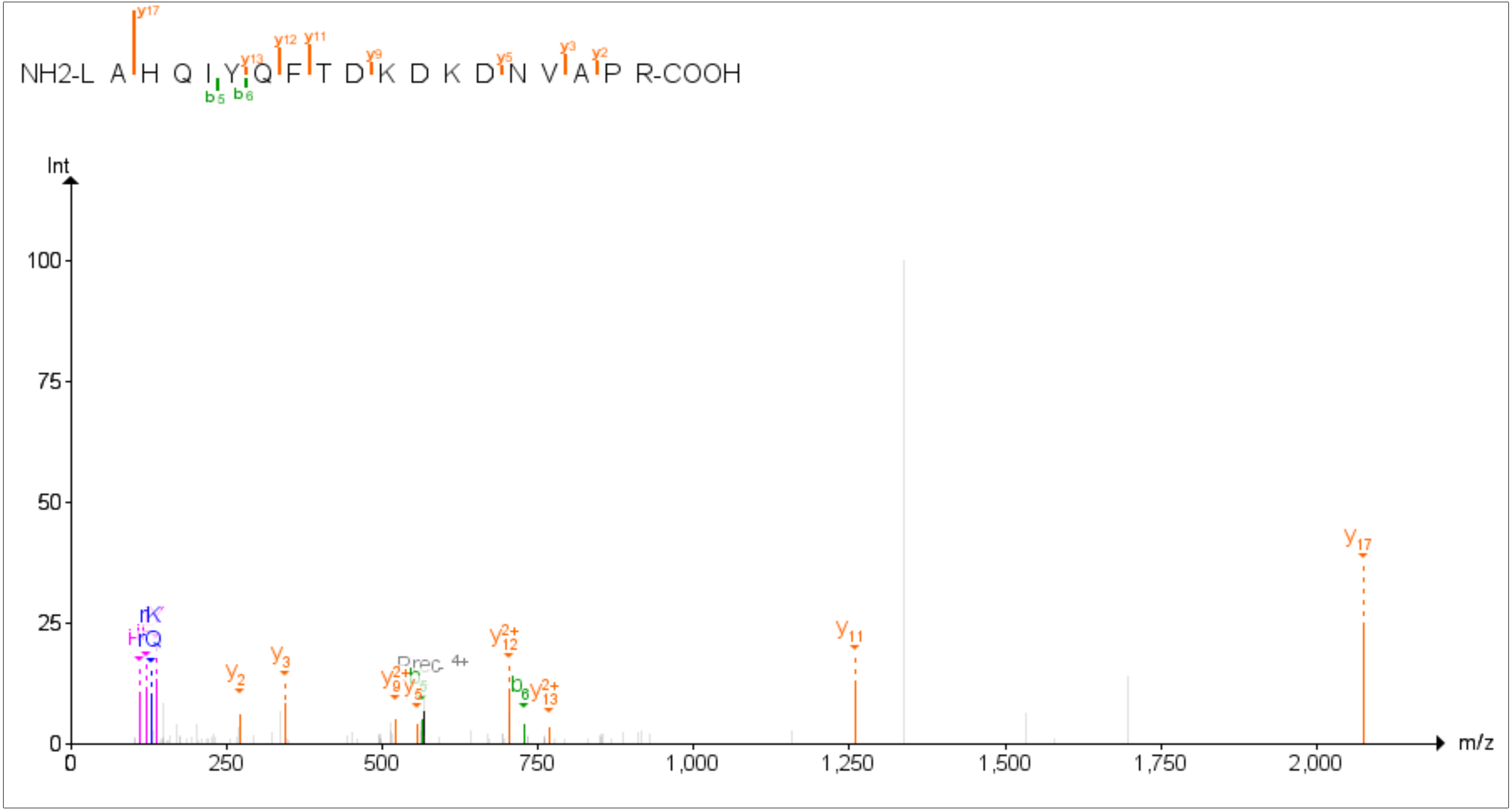
NH2-L A H Q I Y Q F T D K D K D N V A P R-COOH
y17
y13
y12
y11
y9
y5
y3
y2
b5
b6
Int
100
75
50
25
0
m/z
0
250
500
750
1,000
1,250
1,500
1,750
2,000
y17
y11
y2+12
y2+13
y2+9
y5
b5
b6
y2
y3
Prec 4+
rK
rQ

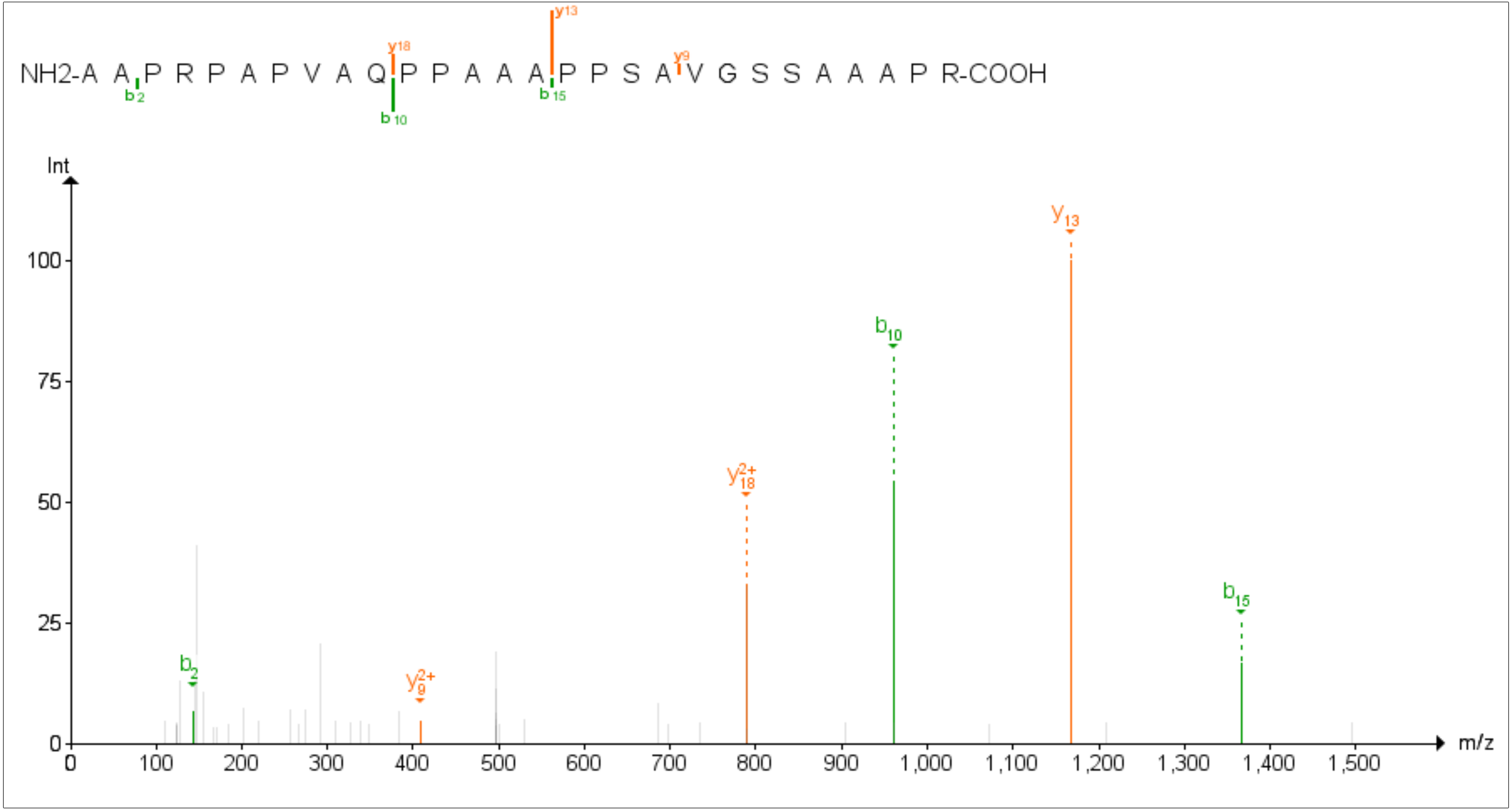

NH2-A A P R P A P V A Q P P A A A P P S A V G S S A A A P R-COOH
y18
y13
y9
b2
b10
b15
Int
m/z
100
75
50
25
0
0
100
200
300
400
500
600
700
800
900
1,000
1,100
1,200
1,300
1,400
1,500
y_{13}
b_{10}
y_{18}^{2+}
b_{15}
b_2
y_9^{2+}

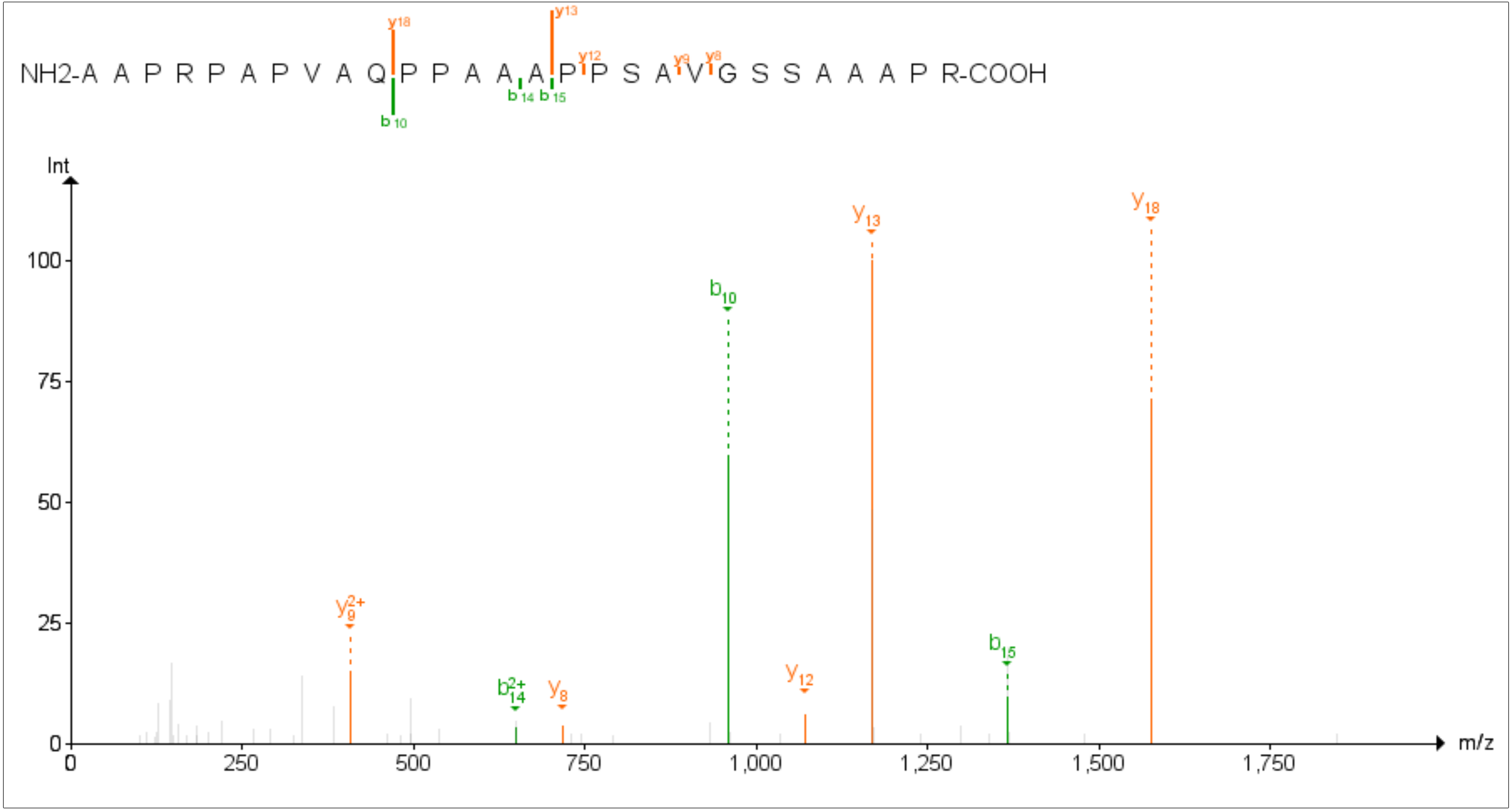
NH2-A A P R P A P V A Q P P A A A P P S A V G S S A A A P R-COOH
y18
y13
y12
y9
y8
b 10
b 14
b 15
Int
m/z
0
25
50
75
100
0
250
500
750
1,000
1,250
1,500
1,750
y9 2+
b14 2+
y8
b10
y12
y13
b15
y18

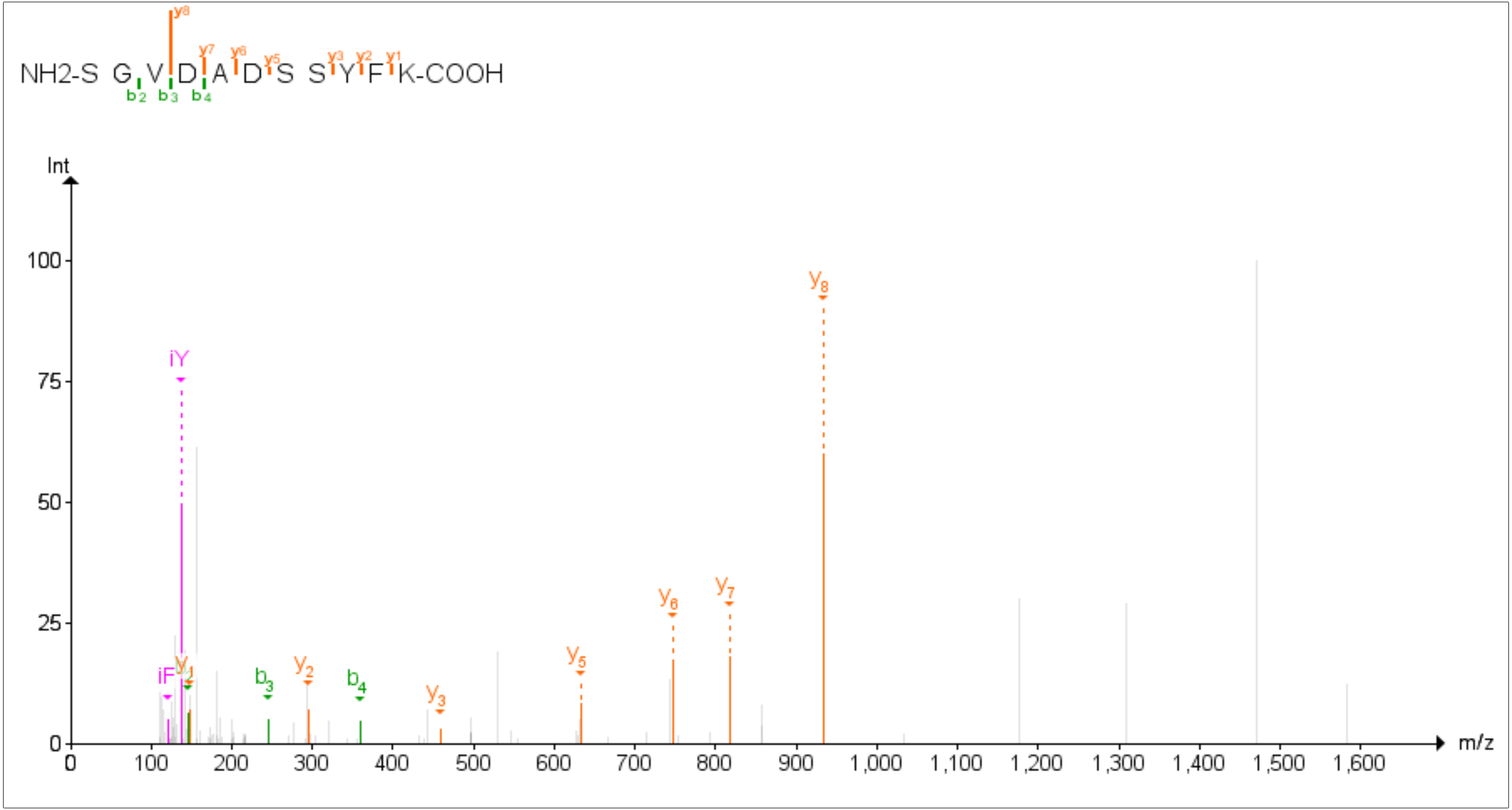

NH2-S G V D A D S S Y F K-COOH
b2 b3 b4
y8 y7 y6 y5 y3 y2 y1
Int
iY
iF
y1
b3
y2
b4
y3
y5
y6
y7
y8
m/z

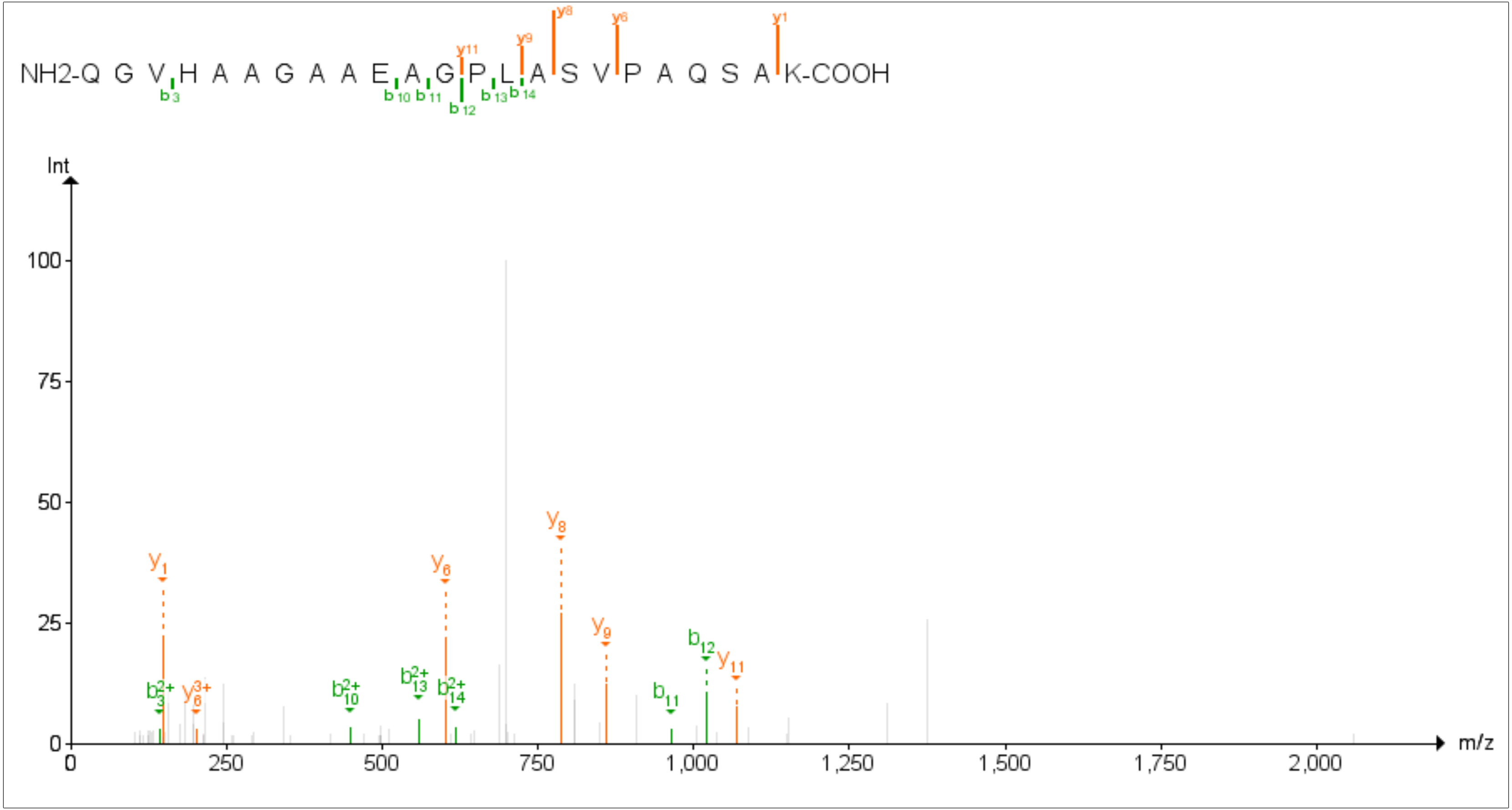
NH2-Q G V H A A G A A E A G P L A S V P A Q S A K-COOH
b3 b10 b11 b12 b13 b14
y11 y9 y8 y6 y1
Int
100
75
50
25
0
y1
b3 2+
y6 3+
b10 2+
b13 2+
y6
b14 2+
y8
y9
b11
b12
y11
0
250
500
750
1,000
1,250
1,500
1,750
2,000
m/z

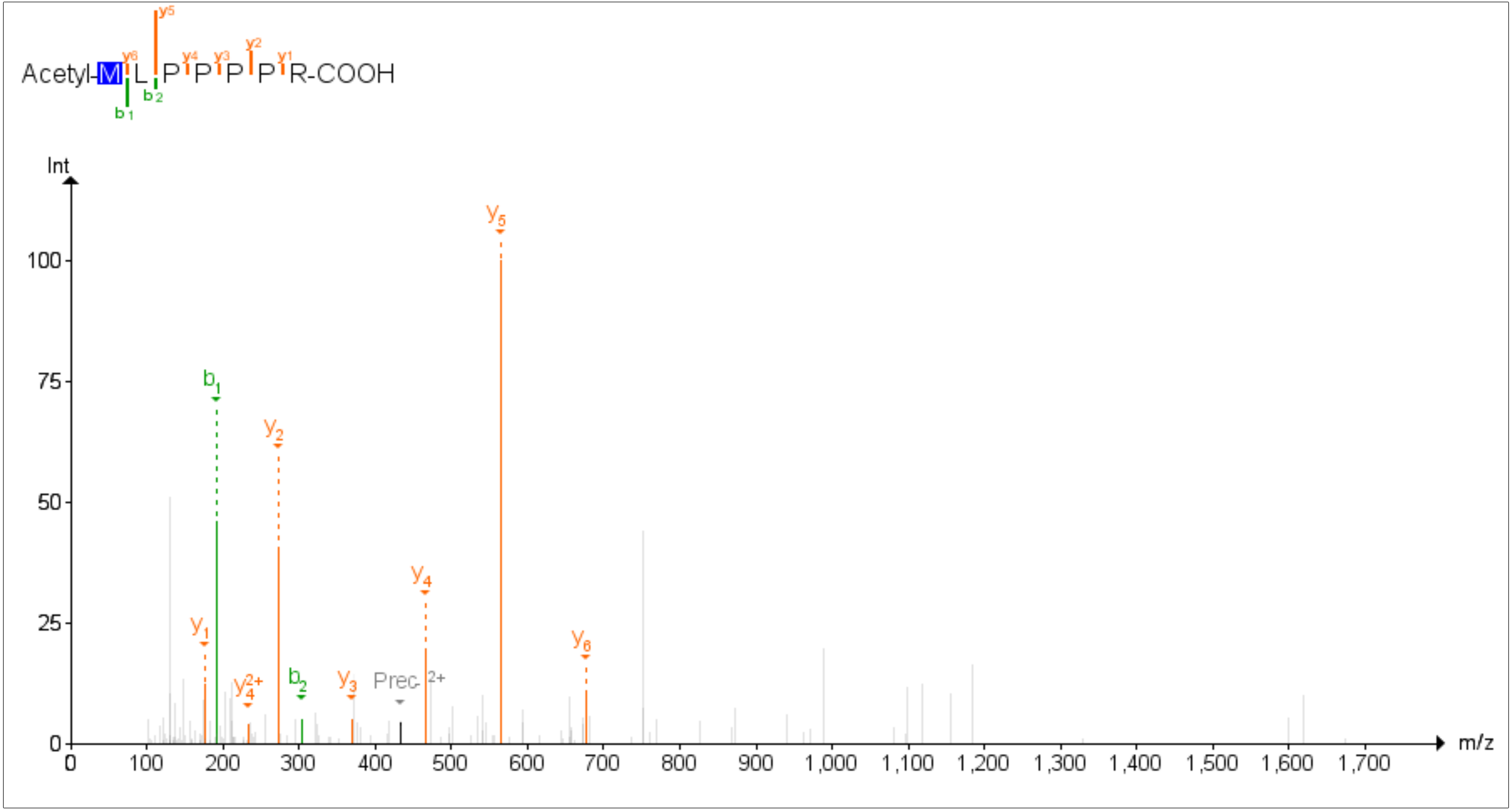

Acetyl-M L P P P P R-COOH
y6 y5 y4 y3 y2 y1
b 1 b 2
Int
100
75
50
25
0
y5
b1
y2
y4
y1
y6
y4 2+
b2
y3
Prec 2+
0 100 200 300 400 500 600 700 800 900 1,000 1,100 1,200 1,300 1,400 1,500 1,600 1,700
m/z

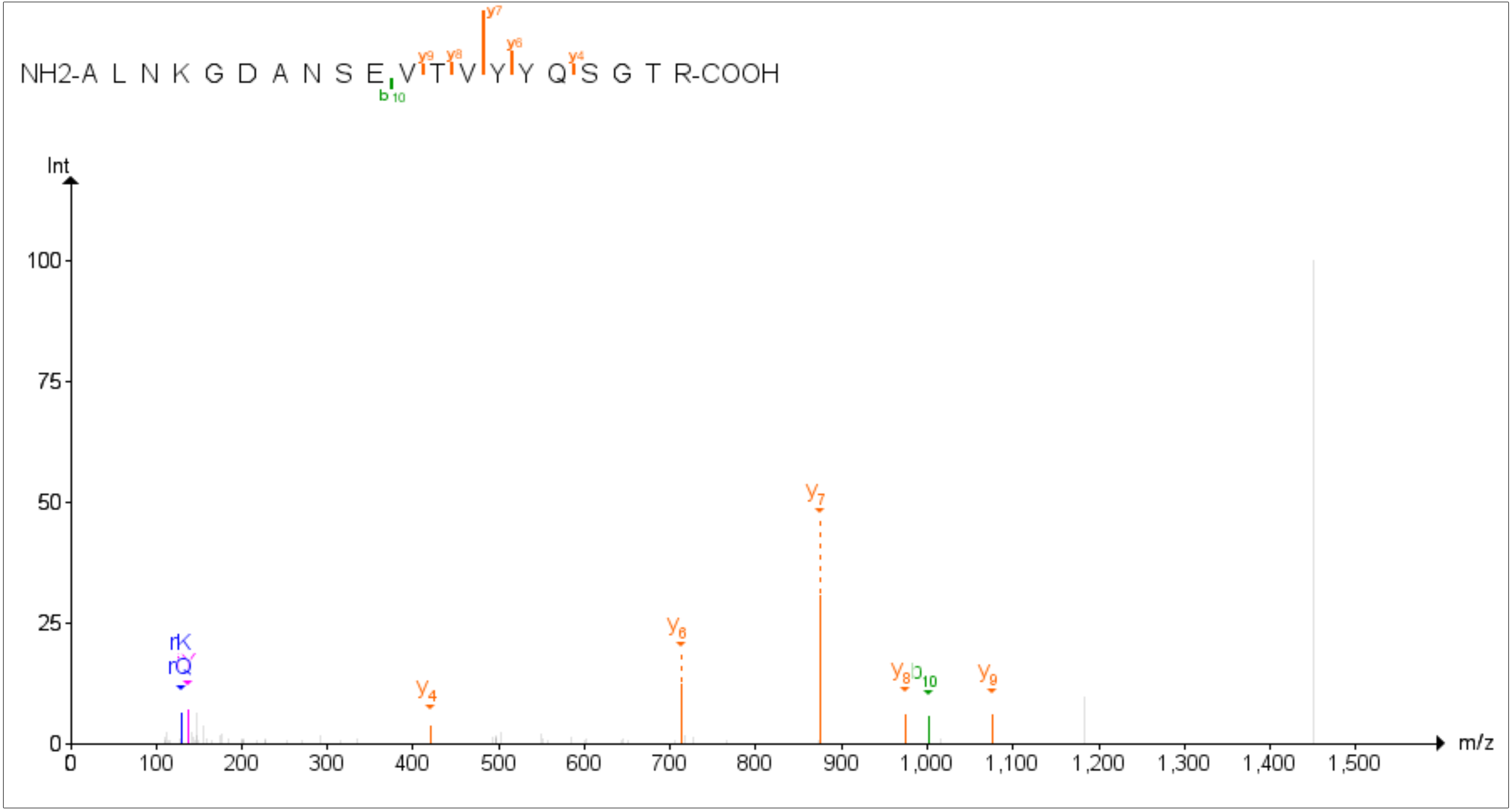

NH2-A L N K G D A N S E V T V Y Y Q S G T R-COOH
b10
y9
y8
y7
y6
y4
Int
100
75
50
25
0
rK
rQ
y4
y6
y7
y8
b10
y9
0
100
200
300
400
500
600
700
800
900
1,000
1,100
1,200
1,300
1,400
1,500
m/z

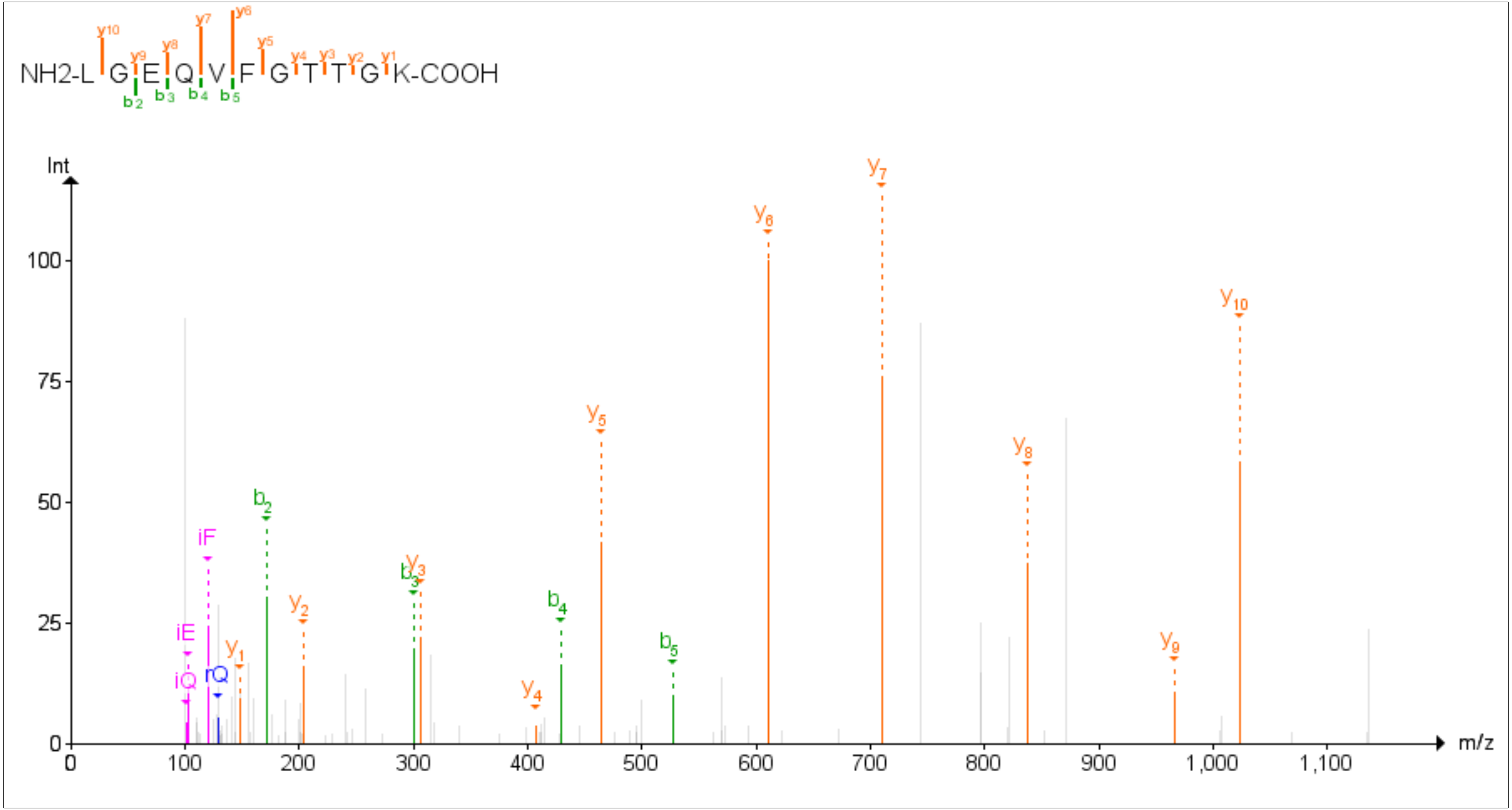

NH2-L G E Q V F G T T G K-COOH
y10 y9 y8 y7 y6 y5 y4 y3 y2 y1
b2 b3 b4 b5
Int
m/z
0
25
50
75
100
0
100
200
300
400
500
600
700
800
900
1,000
1,100
iQ
iE
iF
rQ
y1
b2
y2
b3
y3
y4
b4
y5
b5
y6
y7
y8
y9
y10

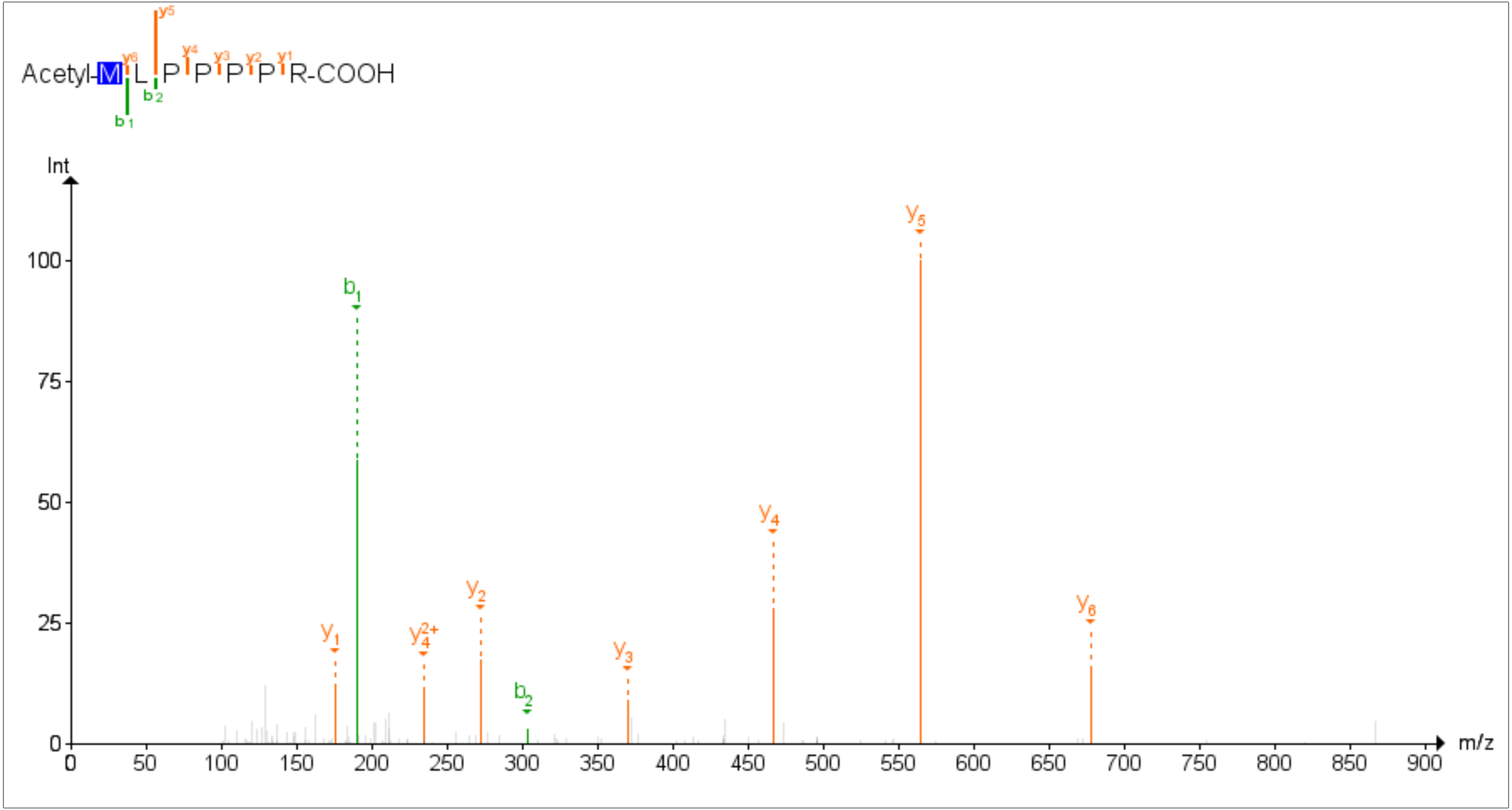

Acetyl-M L P P P P R-COOH
y6 y5 y4 y3 y2 y1
b1 b2
Int
100
75
50
25
0
m/z
0 50 100 150 200 250 300 350 400 450 500 550 600 650 700 750 800 850 900
y5
b1
y4
y2
y6
y1
y4 2+
y3
b2

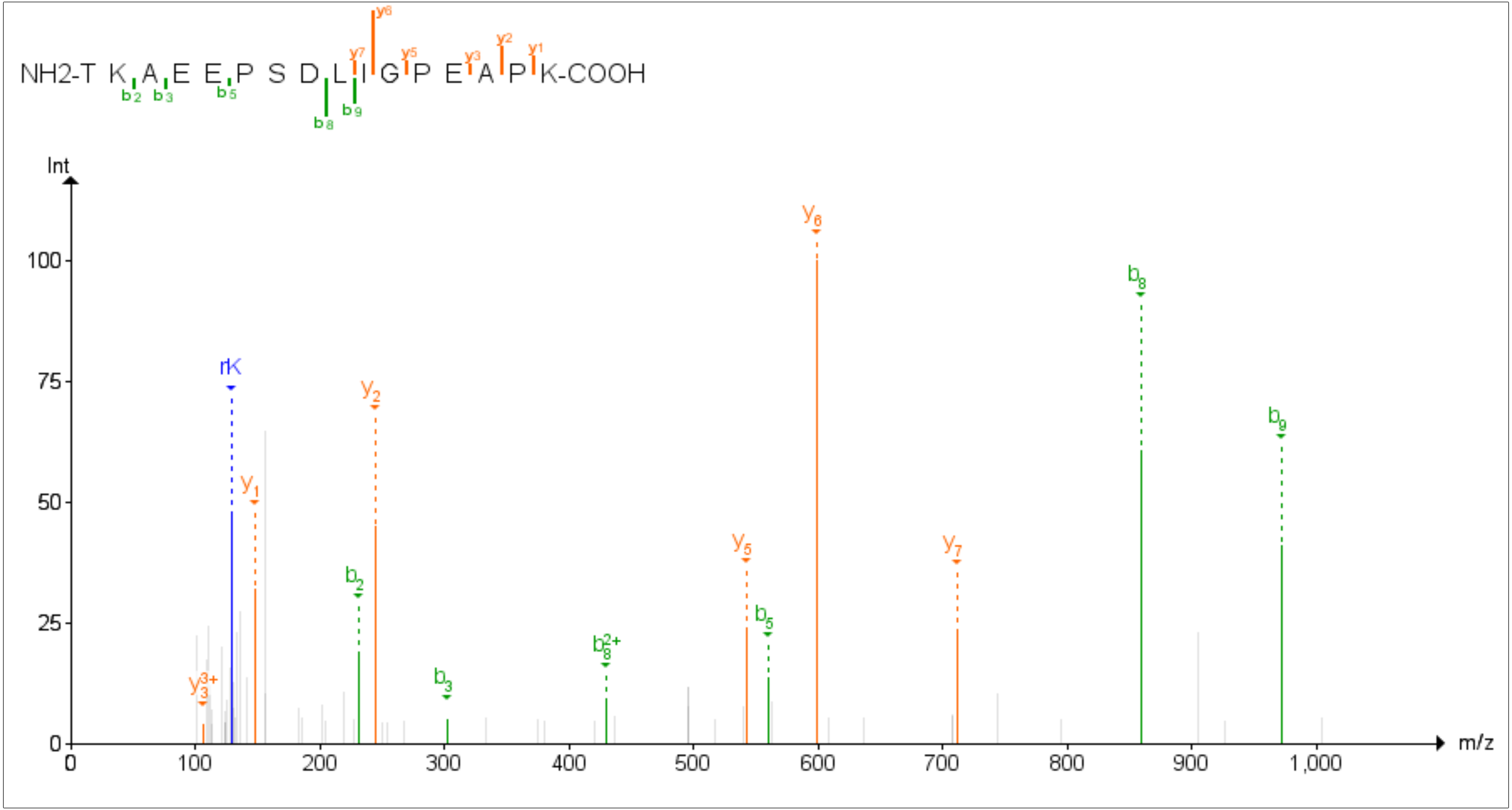

NH2-T K A E E P S D L I G P E A P K-COOH
b2 b3 b5 b8 b9
y7 y6 y5 y3 y2 y1
Int
100
75
50
25
0
rK
y1
y2
y6
y5
y7
b2
b3
b8 2+
b5
b8
b9
y3 3+
0 100 200 300 400 500 600 700 800 900 1,000
m/z

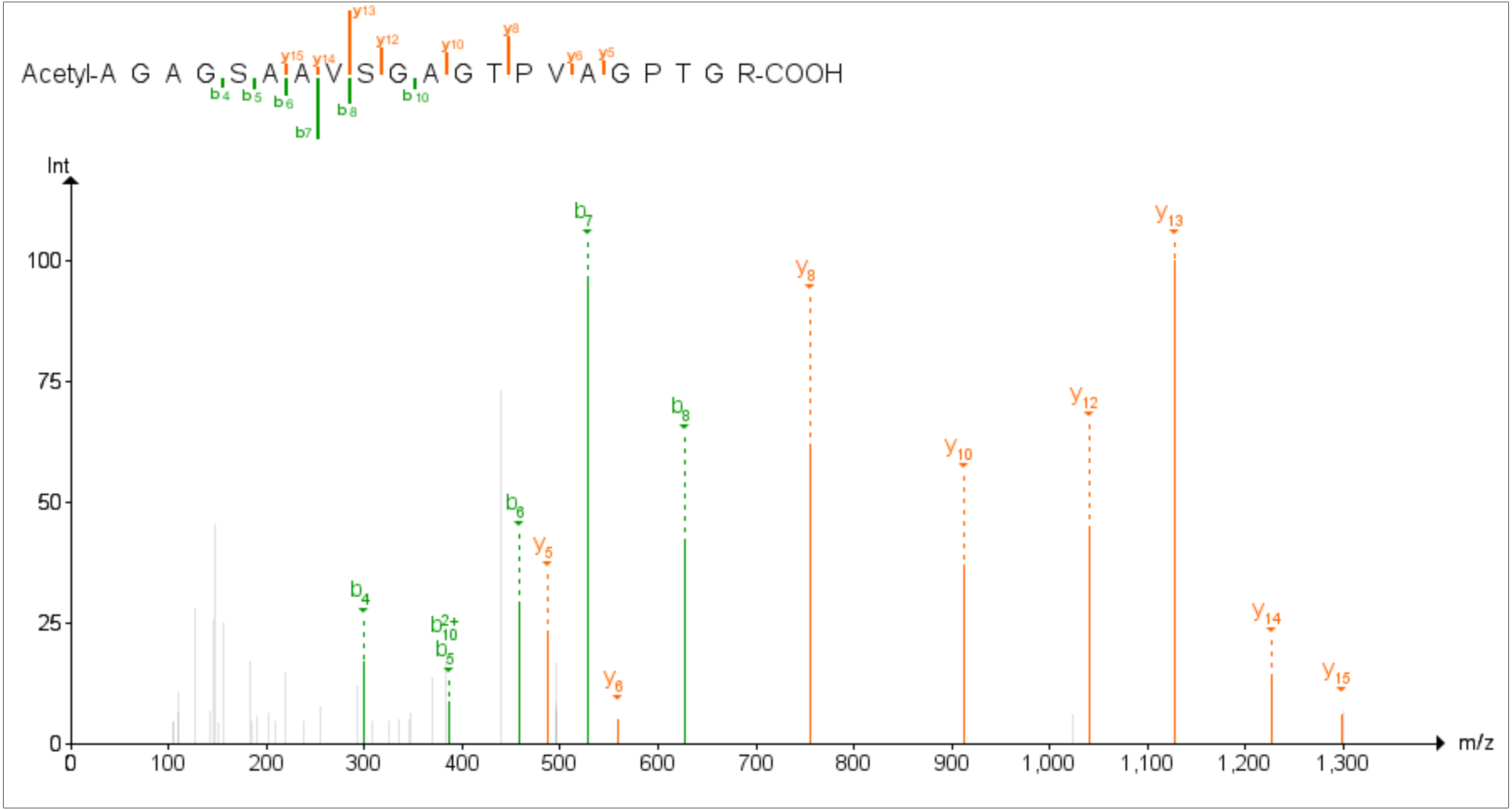

Acetyl-A G A G S A A V S G A G T P V A G P T G R-COOH
Int
m/z
b4
b5
b6
b7
b8
b10
y5
y6
y8
y10
y12
y13
y14
y15
b10 2+
0
25
50
75
100
0
100
200
300
400
500
600
700
800
900
1,000
1,100
1,200
1,300

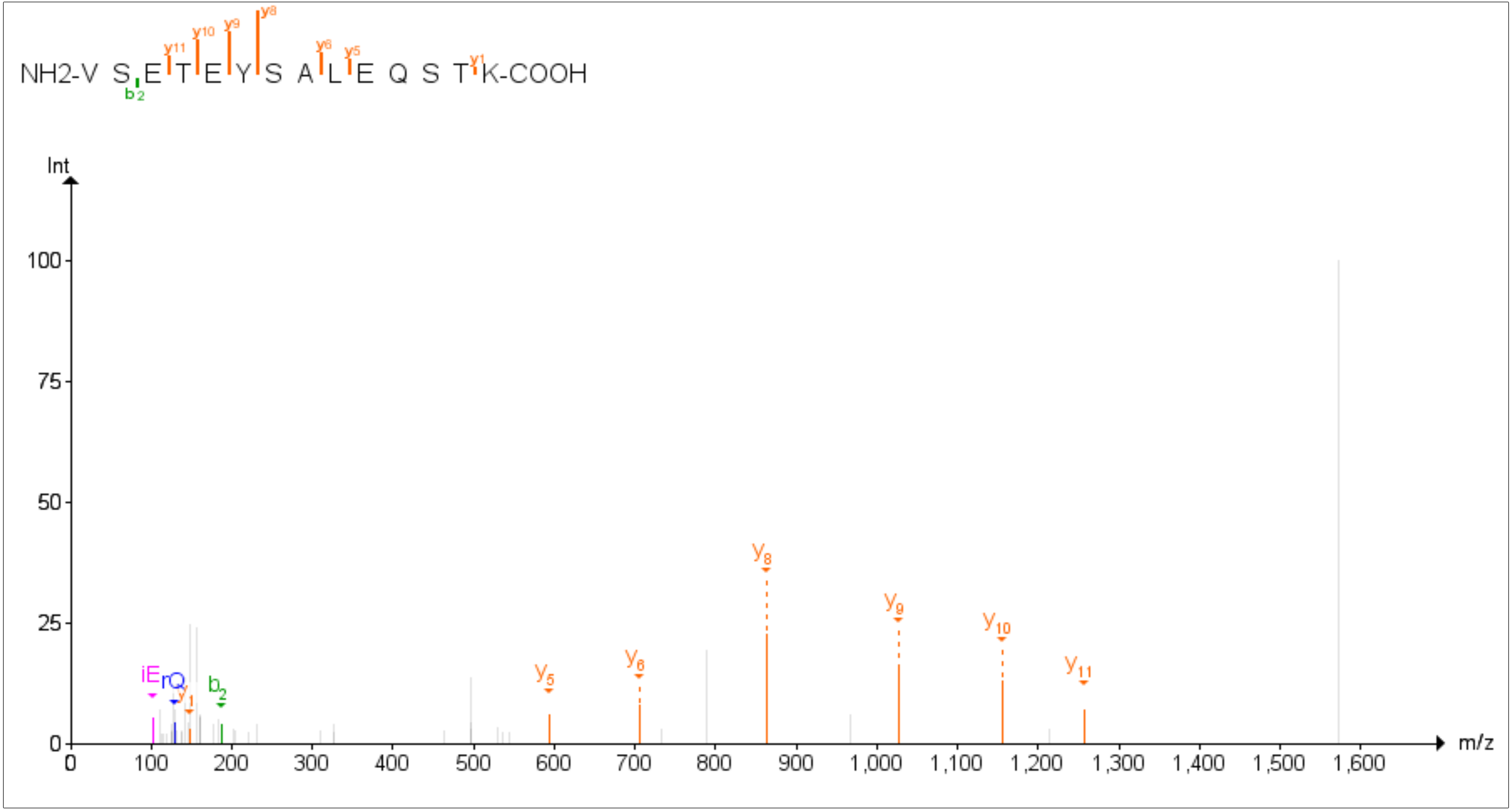

NH2-V S E T E Y S A L E Q S T K-COOH
y11
y10
y9
y8
y6
y5
y1
b2
Int
100
75
50
25
0
m/z
0
100
200
300
400
500
600
700
800
900
1,000
1,100
1,200
1,300
1,400
1,500
1,600
iE
rQ
y1
b2
y5
y6
y8
y9
y10
y11

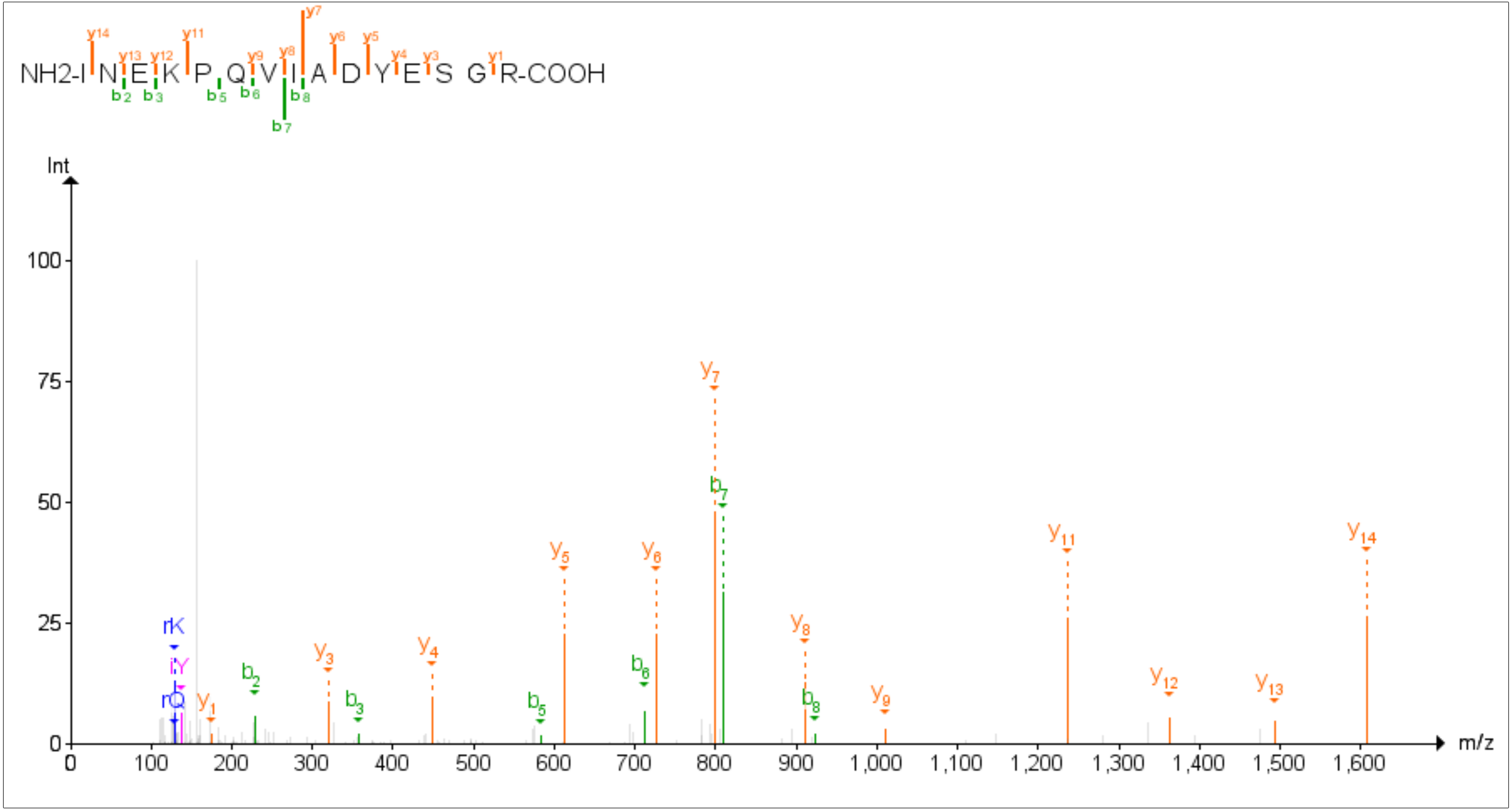

NH2-I N E K P Q V I A D Y E S G R-COOH
Int
m/z
y1 y3 y4 y5 y6 y7 y8 y9 y11 y12 y13 y14
b2 b3 b5 b6 b7 b8
rK
iY
rQ

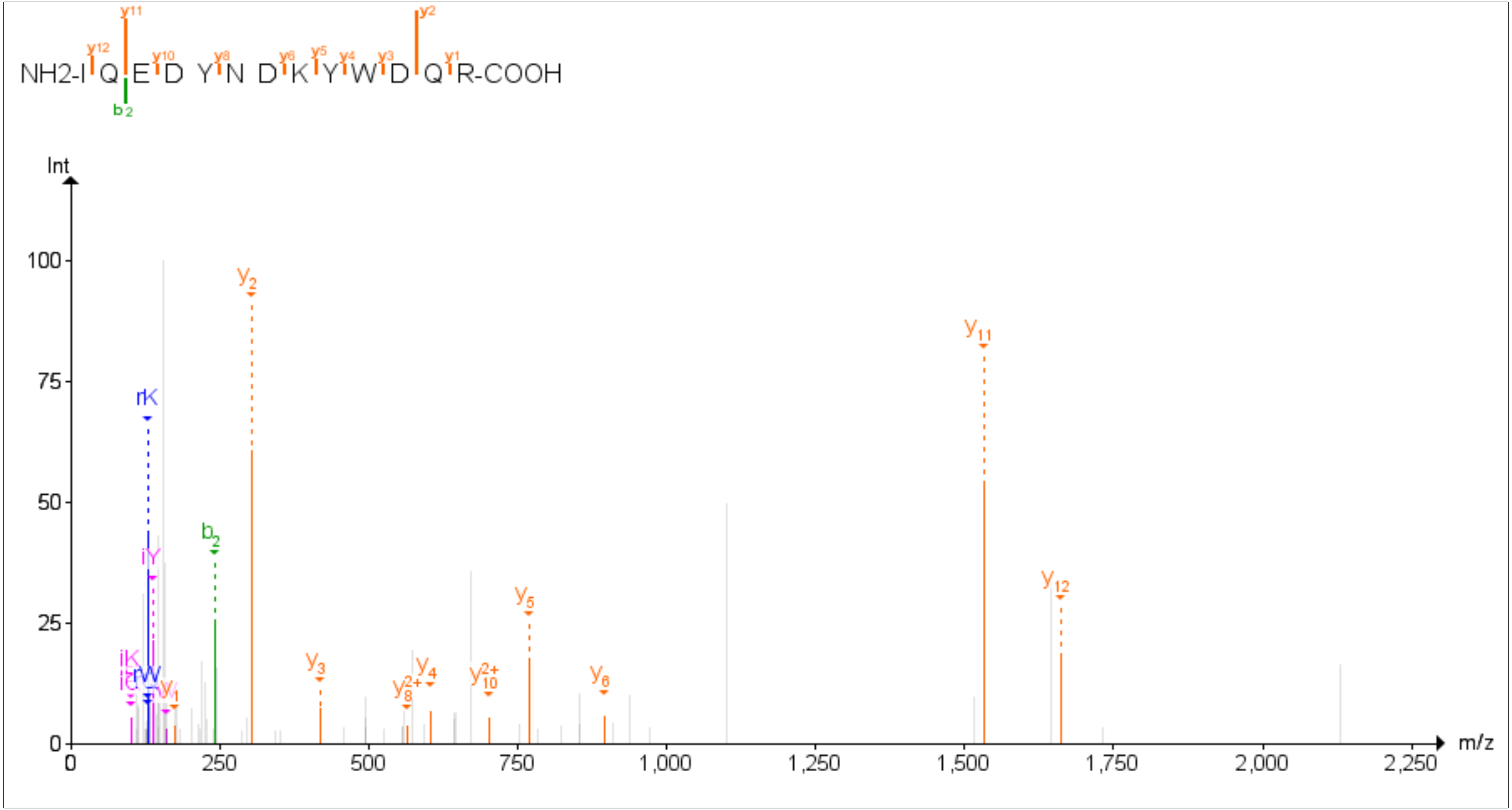

NH2-I Q E D Y N D K Y W D Q R-COOH
y11
y12
y10
y8
y6
y5
y4
y3
y2
y1
b2
Int
100
75
50
25
0
rK
iY
b2
y2
iK
rW
y1
y3
y8 2+
y4
y10 2+
y5
y6
y11
y12
0
250
500
750
1,000
1,250
1,500
1,750
2,000
2,250
m/z

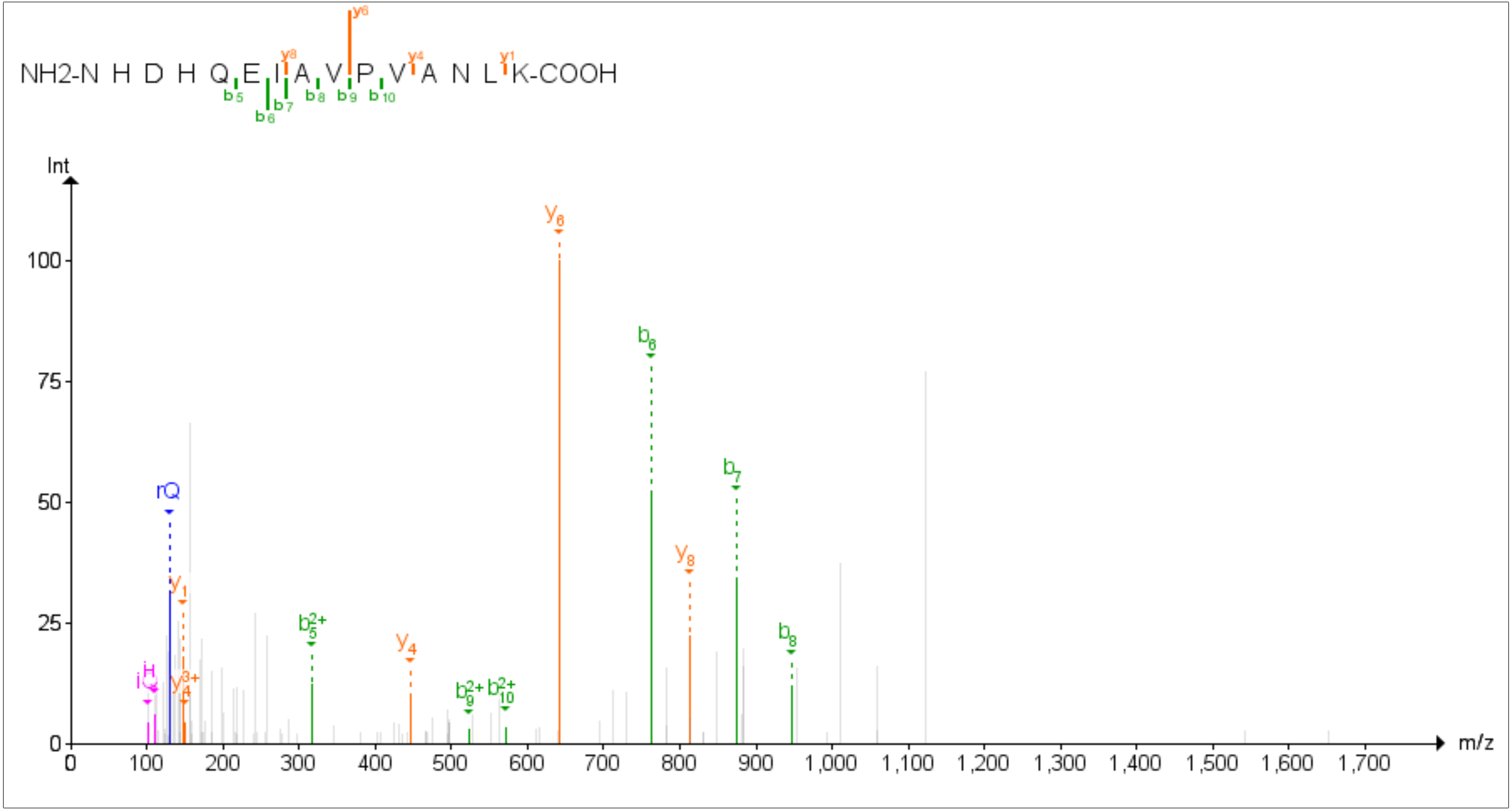

NH2-N H D H Q E I A V P V A N L K-COOH
Int
m/z
rQ
iQ
iH
y1
y3+ 4
b2+ 5
y4
b2+ 9
b2+ 10
y6
b6
b7
y8
b8

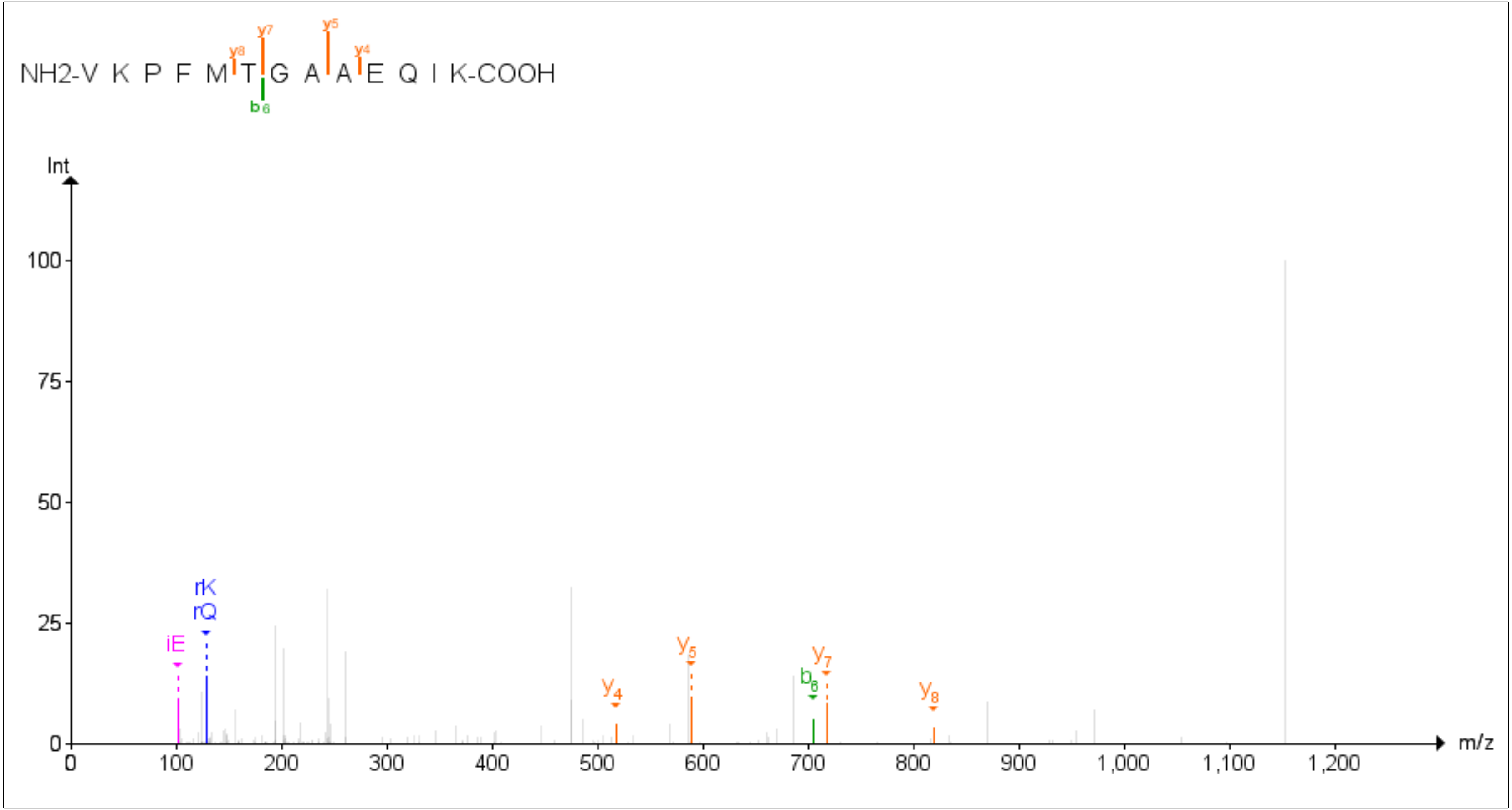
NH2-V K P F M T G A A E Q I K-COOH
y8
y7
y5
y4
b6
Int
100
75
50
25
0
rK
rQ
iE
y4
y5
b6
y7
y8
0
100
200
300
400
500
600
700
800
900
1,000
1,100
1,200
m/z

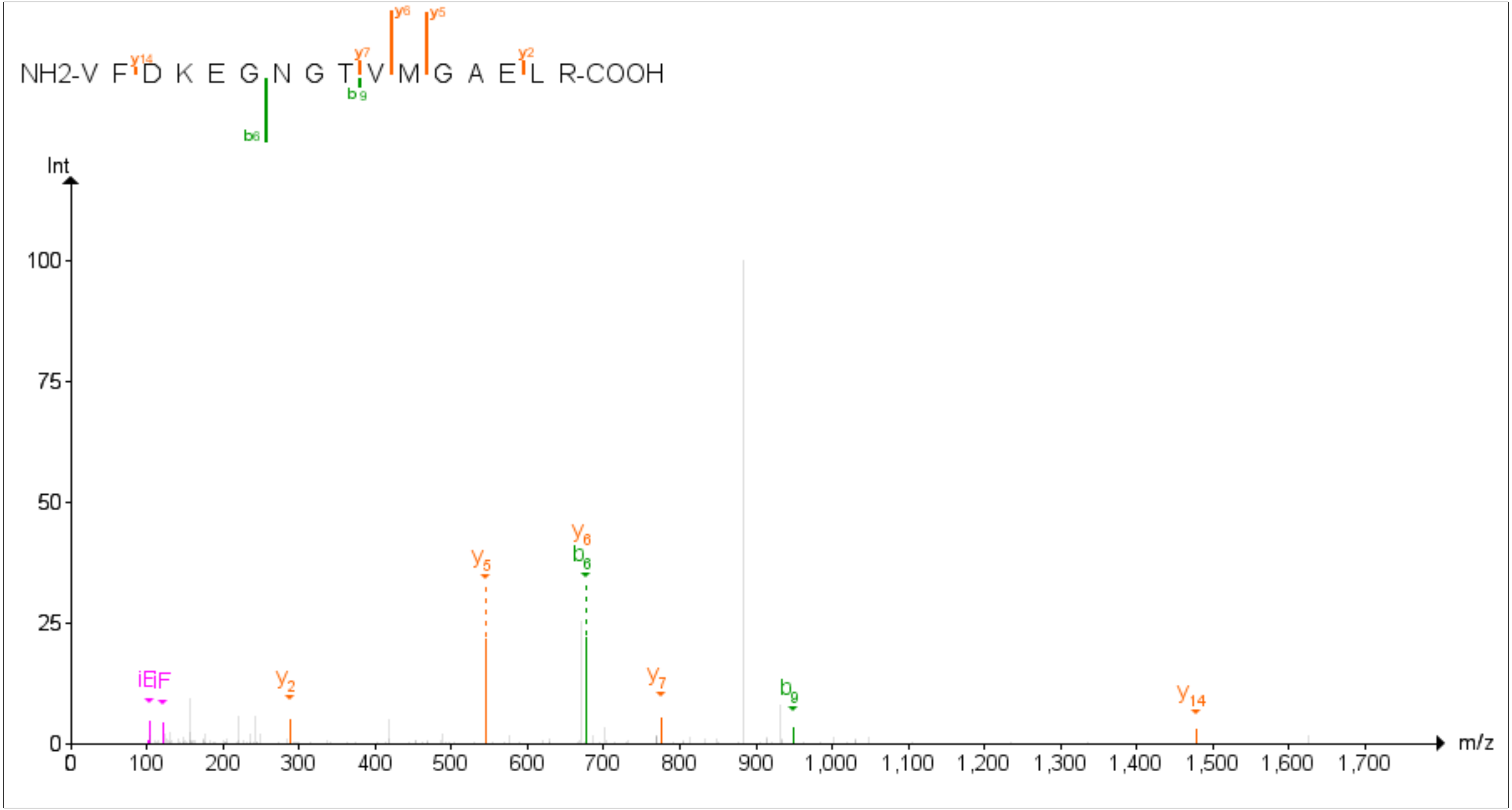

NH2-V F D K E G N G T V M G A E L R-COOH
y14 y7 y6 y5 y2
b6 b9
Int
100
75
50
25
0
iE iF y2 y5 y6 b6 y7 b9 y14
0 100 200 300 400 500 600 700 800 900 1,000 1,100 1,200 1,300 1,400 1,500 1,600 1,700
m/z

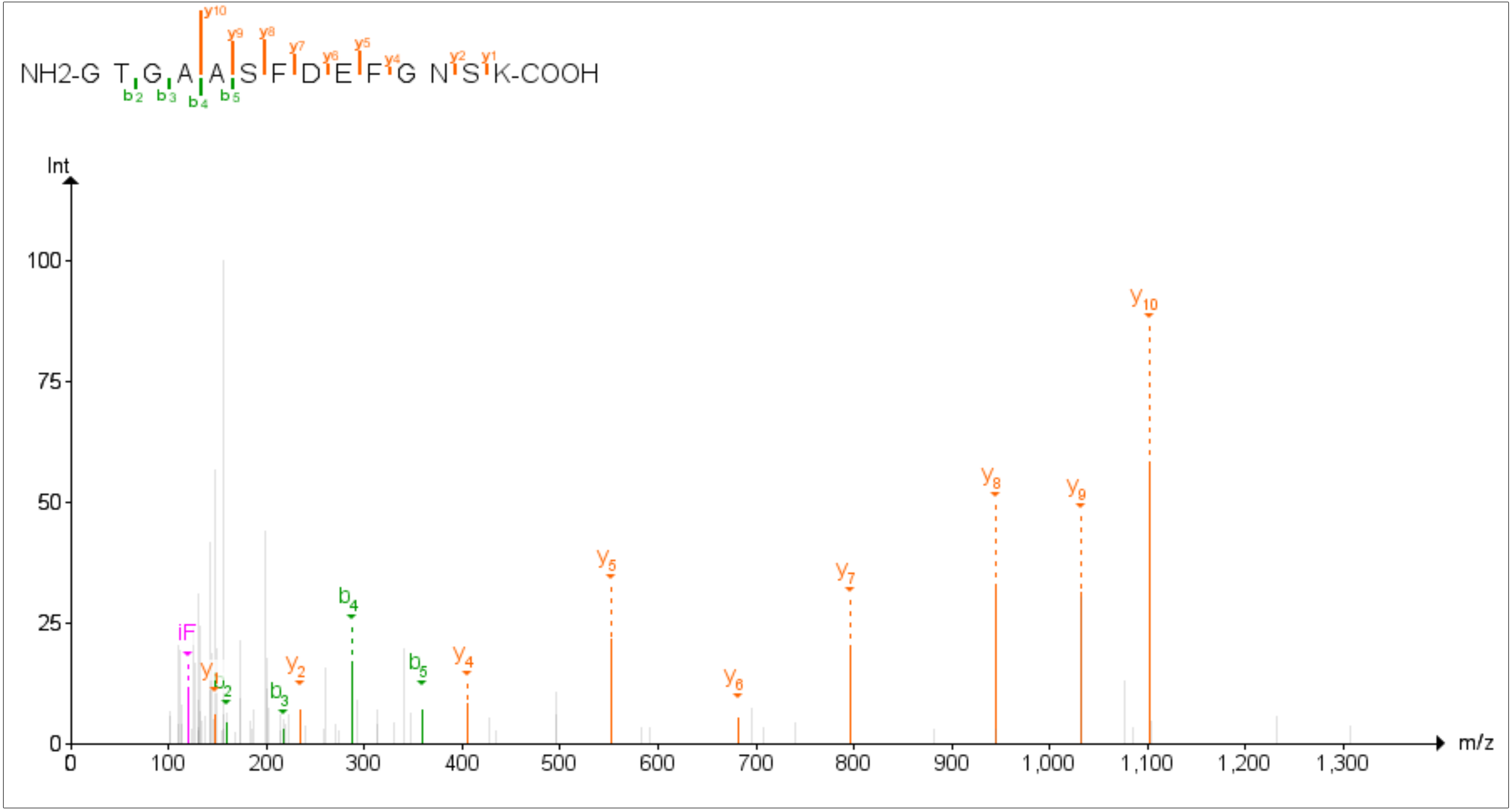

NH2-G T G A A S F D E F G N S K-COOH
b2 b3 b4 b5
y10 y9 y8 y7 y6 y5 y4 y2 y1
Int
100
75
50
25
0
m/z
0
100
200
300
400
500
600
700
800
900
1,000
1,100
1,200
1,300
iF
y1
b2
b3
y2
b4
b5
y4
y5
y6
y7
y8
y9
y10

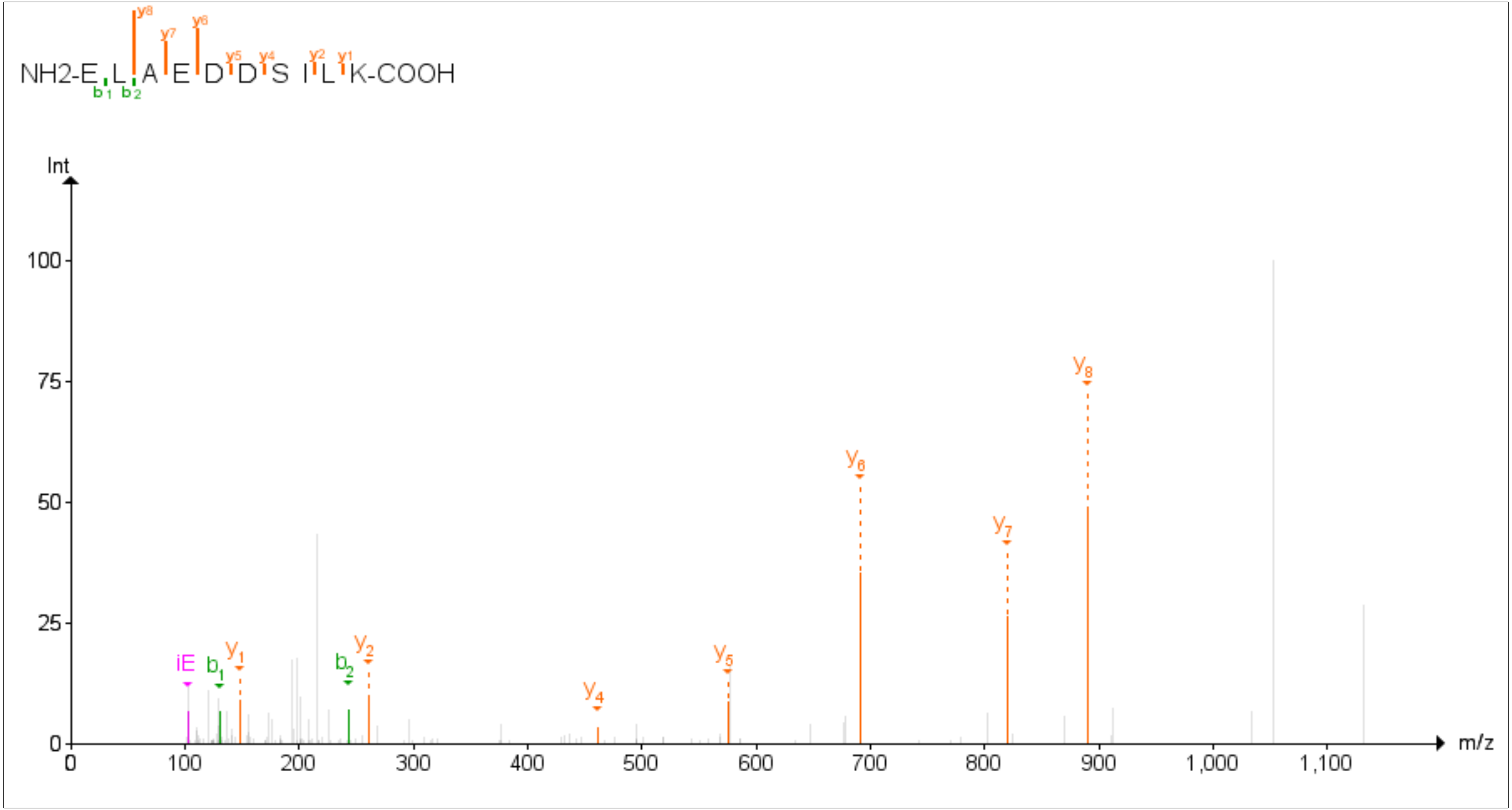

NH2-E L A E D D S I L K-COOH
y8
y7
y6
y5
y4
y2
y1
b1
b2
Int
100
75
50
25
0
iE
b1
y1
b2
y2
y4
y5
y6
y7
y8
0
100
200
300
400
500
600
700
800
900
1,000
1,100
m/z

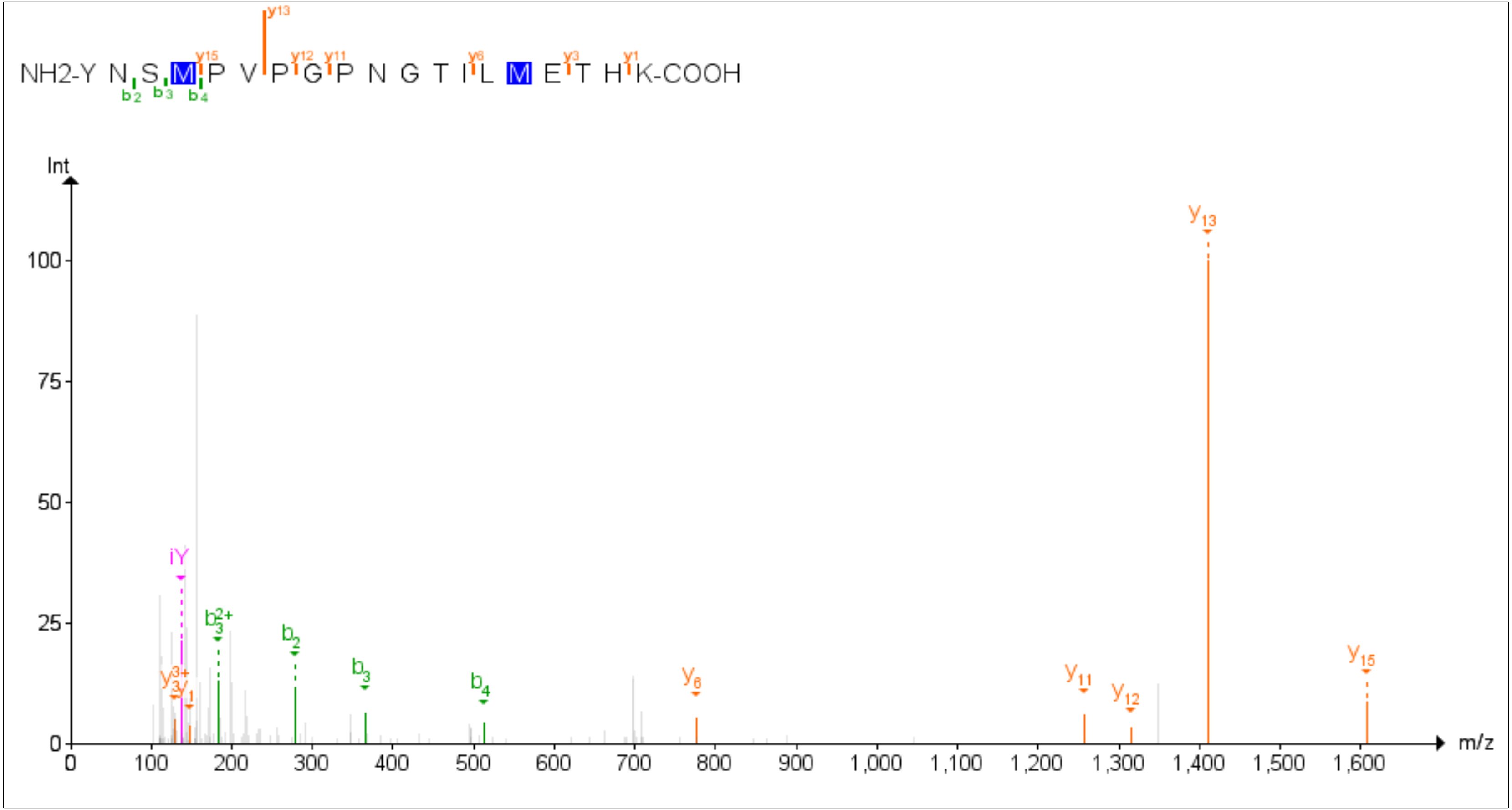

NH2-Y N S M P V P G P N G T I L M E T H K-COOH
y13 y15 y12 y11 y6 y3 y1
b2 b3 b4
Int
100
75
50
25
0
iY
b_3^{2+}
b_2
b_3
b_4
y_3^{3+}
y_1
y_6
y_{11}
y_{12}
y_{13}
y_{15}
0 100 200 300 400 500 600 700 800 900 1,000 1,100 1,200 1,300 1,400 1,500 1,600
m/z

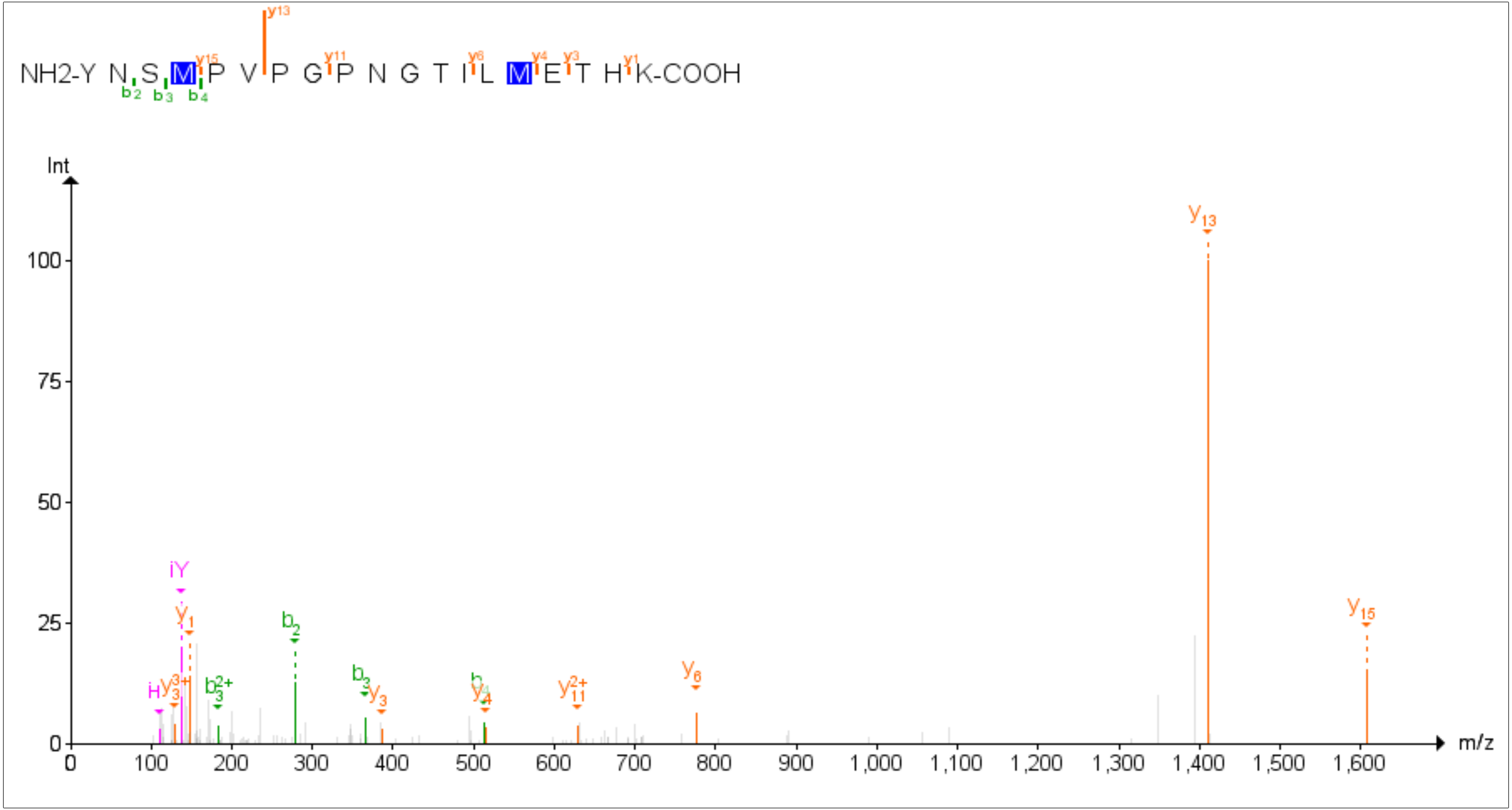

NH2-Y N S M P V P G P N G T I L M E T H K-COOH
b2
b3
b4
y15
y13
y11
y6
y4
y3
y1
Int
100
75
50
25
0
iY
y1
b2
iH
y3 3+
b3 2+
b3
y3
b4
y4
y11 2+
y6
y13
y15
0
100
200
300
400
500
600
700
800
900
1,000
1,100
1,200
1,300
1,400
1,500
1,600
m/z

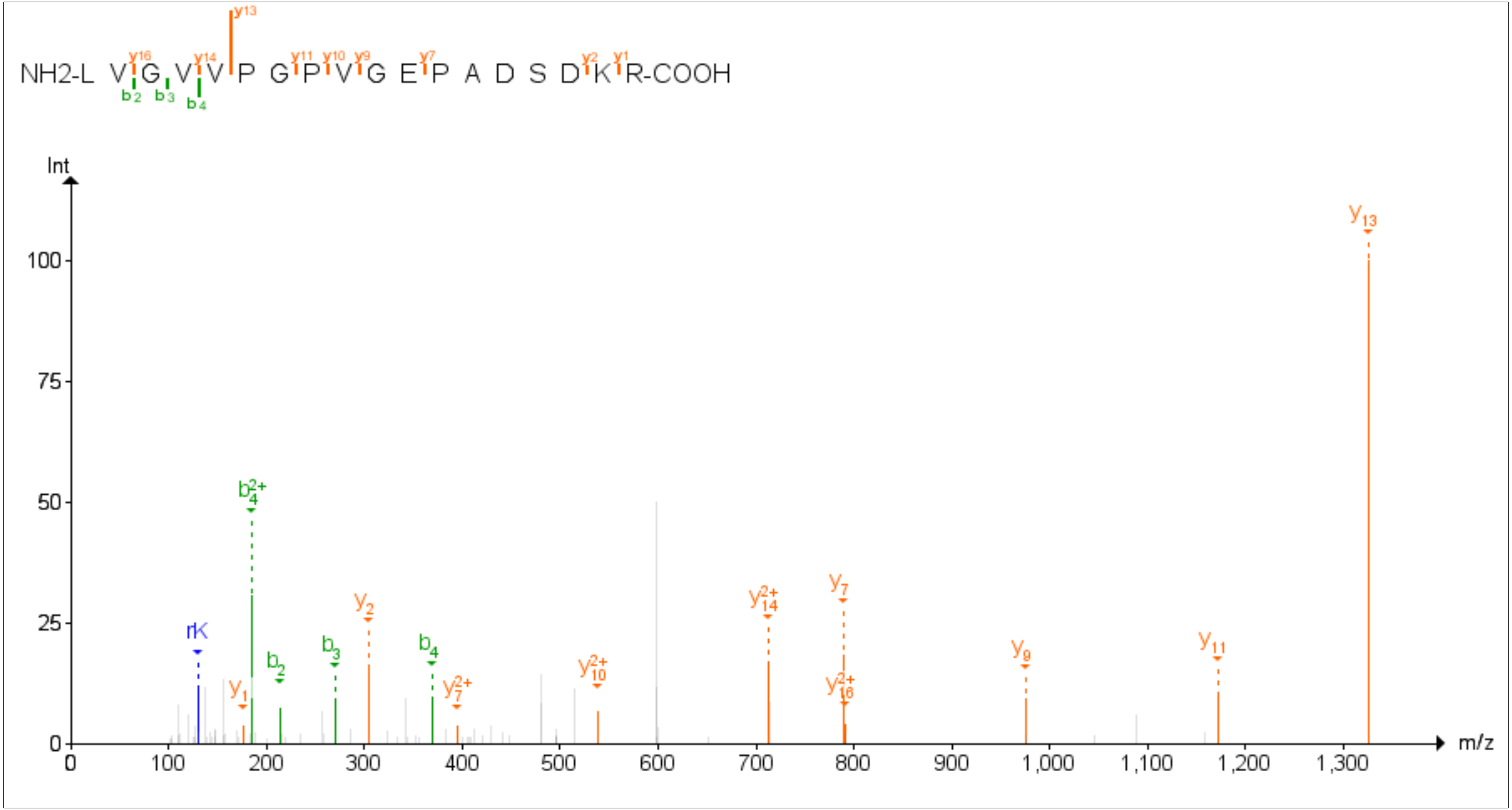

NH2-L V G V V P G P V G E P A D S D K R-COOH
y16 y14 y13 y11 y10 y9 y7 y2 y1
b2 b3 b4
Int
m/z
y13
b4 2+
y2
y7
y14 2+
rK
b2
b3
b4
y1
y7 2+
y10 2+
y16 2+
y9
y11

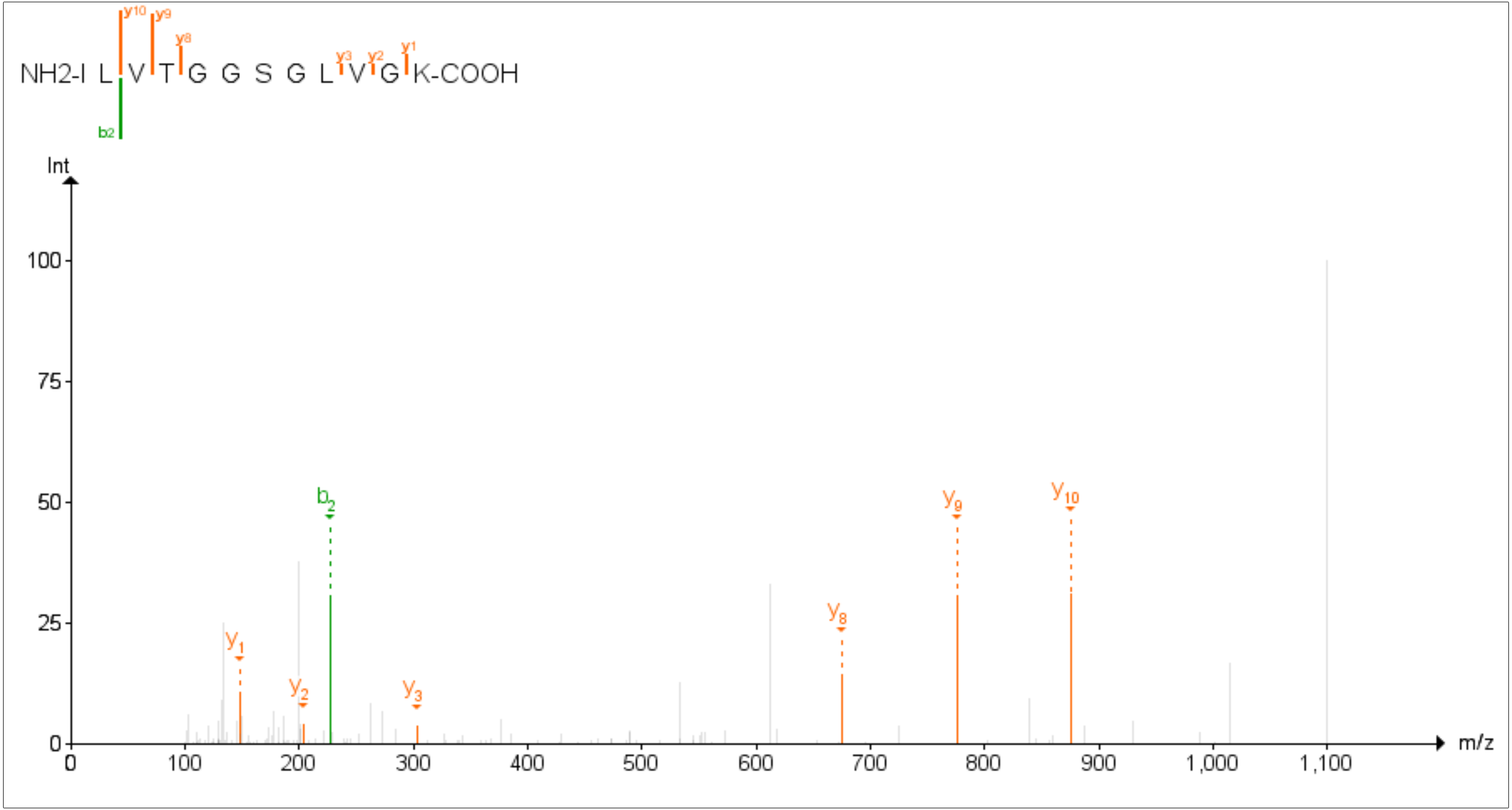

NH2-I L V T G G S G L V G K-COOH
y10
y9
y8
y3
y2
y1
b2
Int
100
75
50
25
0
b2
y9
y10
y8
y1
y2
y3
0
100
200
300
400
500
600
700
800
900
1,000
1,100
m/z

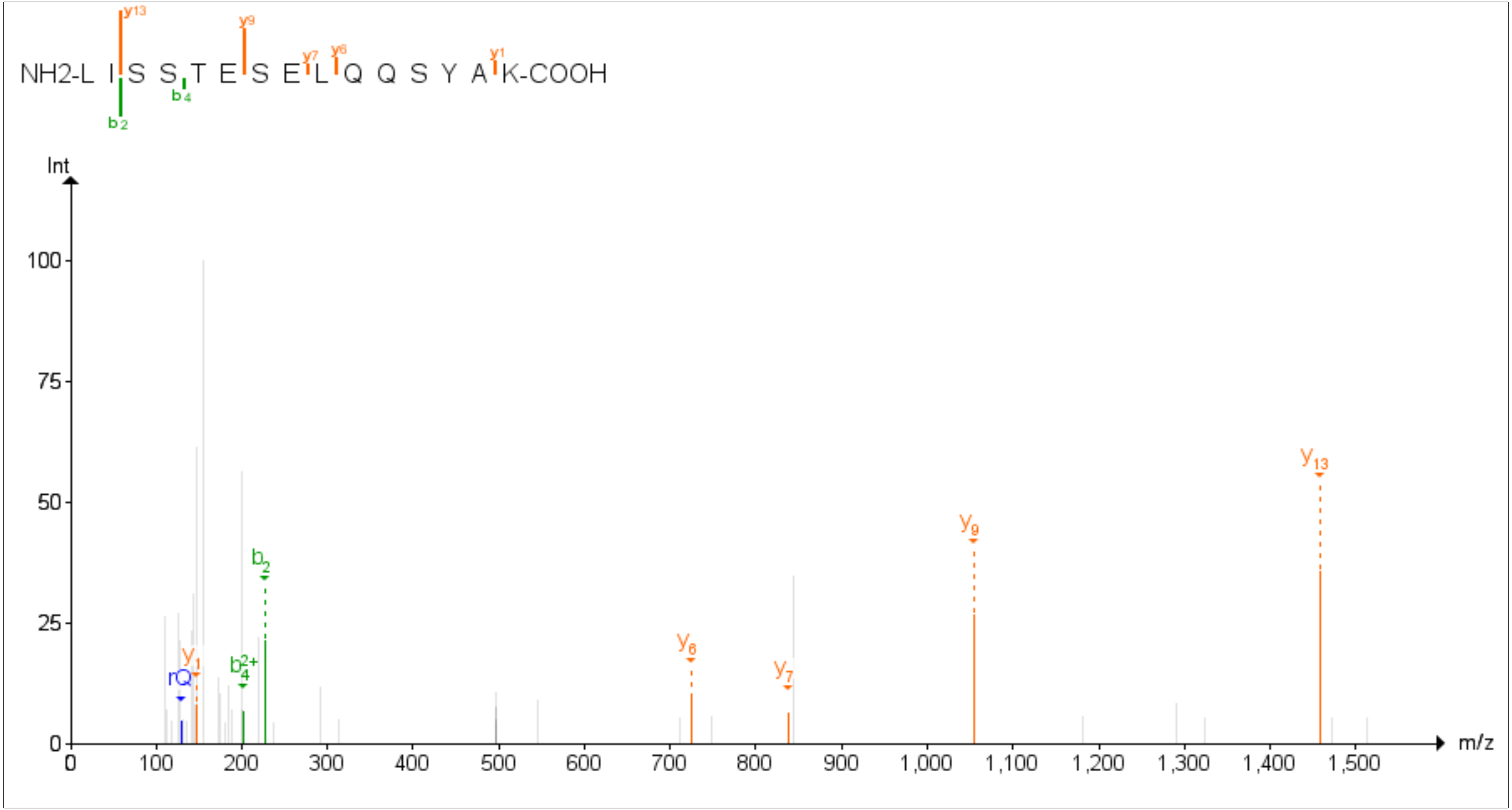

NH2-L I S S T E S E L Q Q S Y A K-COOH
y13
y9
y7
y6
y1
b2
b4
Int
100
75
50
25
0
m/z
0
100
200
300
400
500
600
700
800
900
1,000
1,100
1,200
1,300
1,400
1,500
rQ
y1
b4 2+
b2
y6
y7
y9
y13

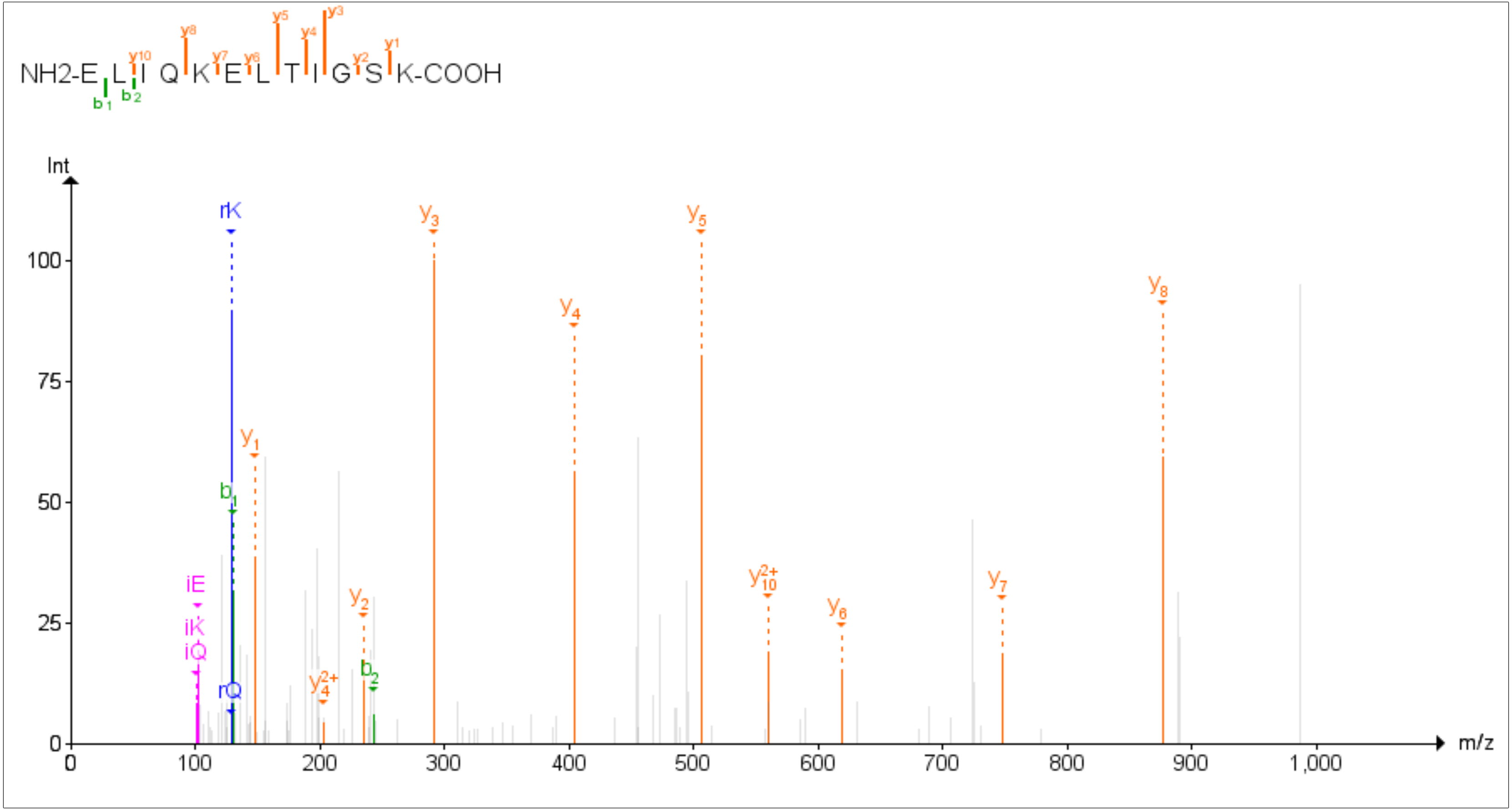

NH2-E L I Q K E L T I G S K-COOH
y10 y8 y7 y6 y5 y4 y3 y2 y1
b 1 b 2
Int
rK
y3
y5
y4
y8
y1
b1
iE
iK
iQ
rQ
y2
y4 2+
b2
y10 2+
y6
y7
0
25
50
75
100
0
100
200
300
400
500
600
700
800
900
1,000
m/z

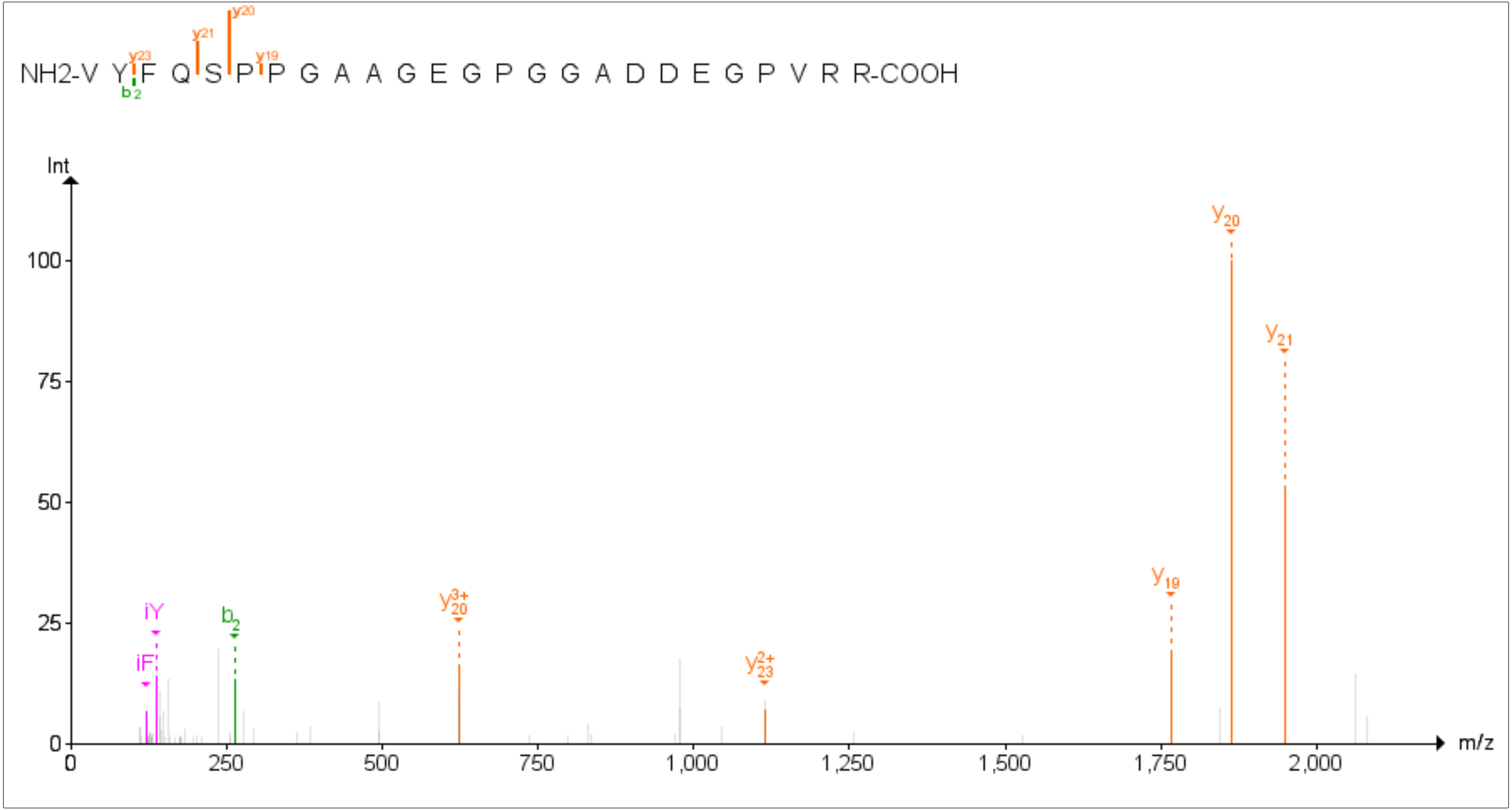

NH2-V Y F Q S P P G A A G E G P G G A D D E G P V R R-COOH
y23
y21
y20
y19
b2
Int
100
75
50
25
0
iY
iF
b2
y20 3+
y23 2+
y19
y20
y21
0
250
500
750
1,000
1,250
1,500
1,750
2,000
m/z

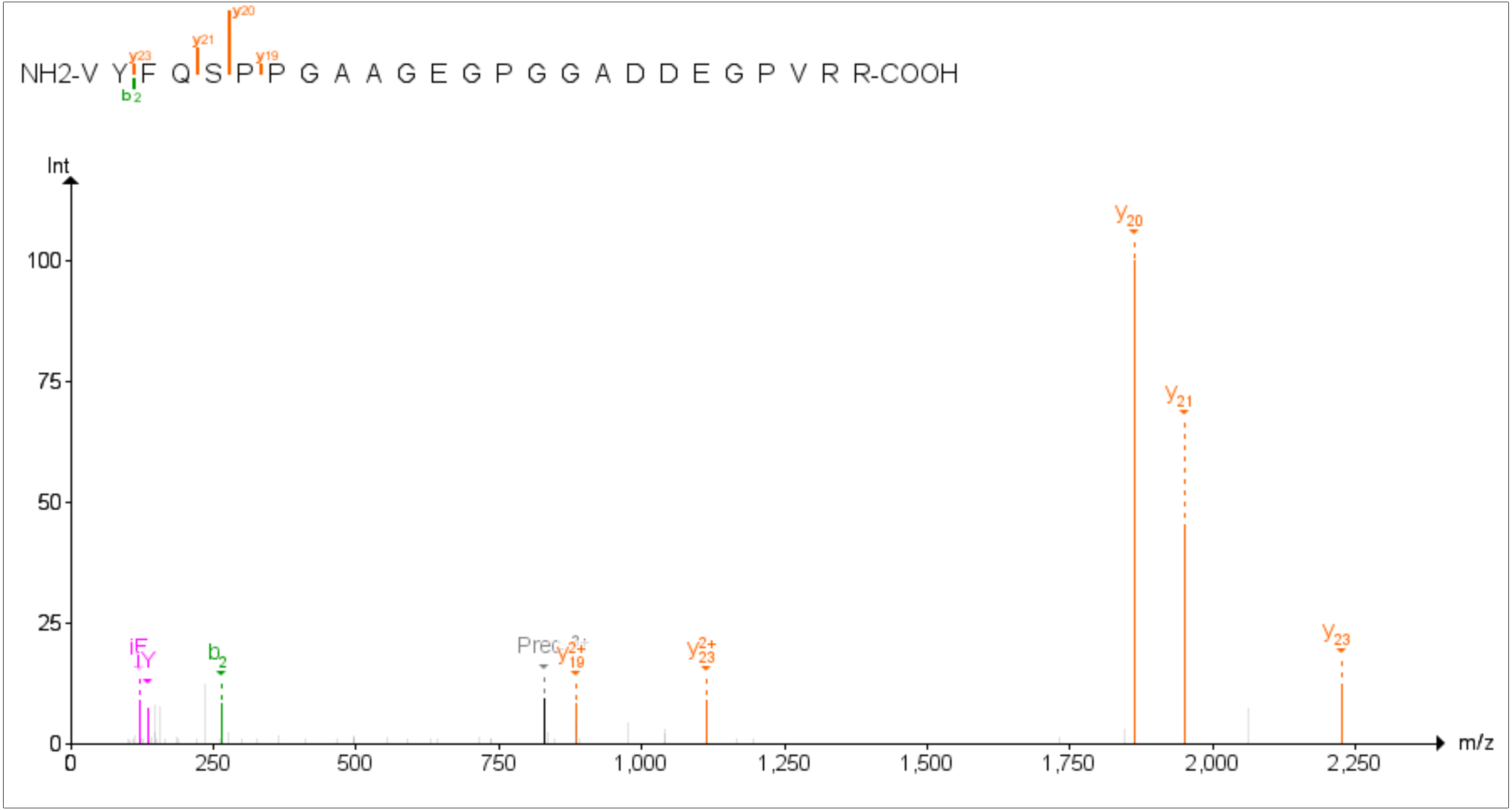

NH2-V Y F Q S P P G A A G E G P G G A D D E G P V R R-COOH
y23
y21
y20
y19
b2
Int
100
75
50
25
0
y20
y21
y23
iF
iY
b2
Prec
y19 2+
y23 2+
0
250
500
750
1,000
1,250
1,500
1,750
2,000
2,250
m/z

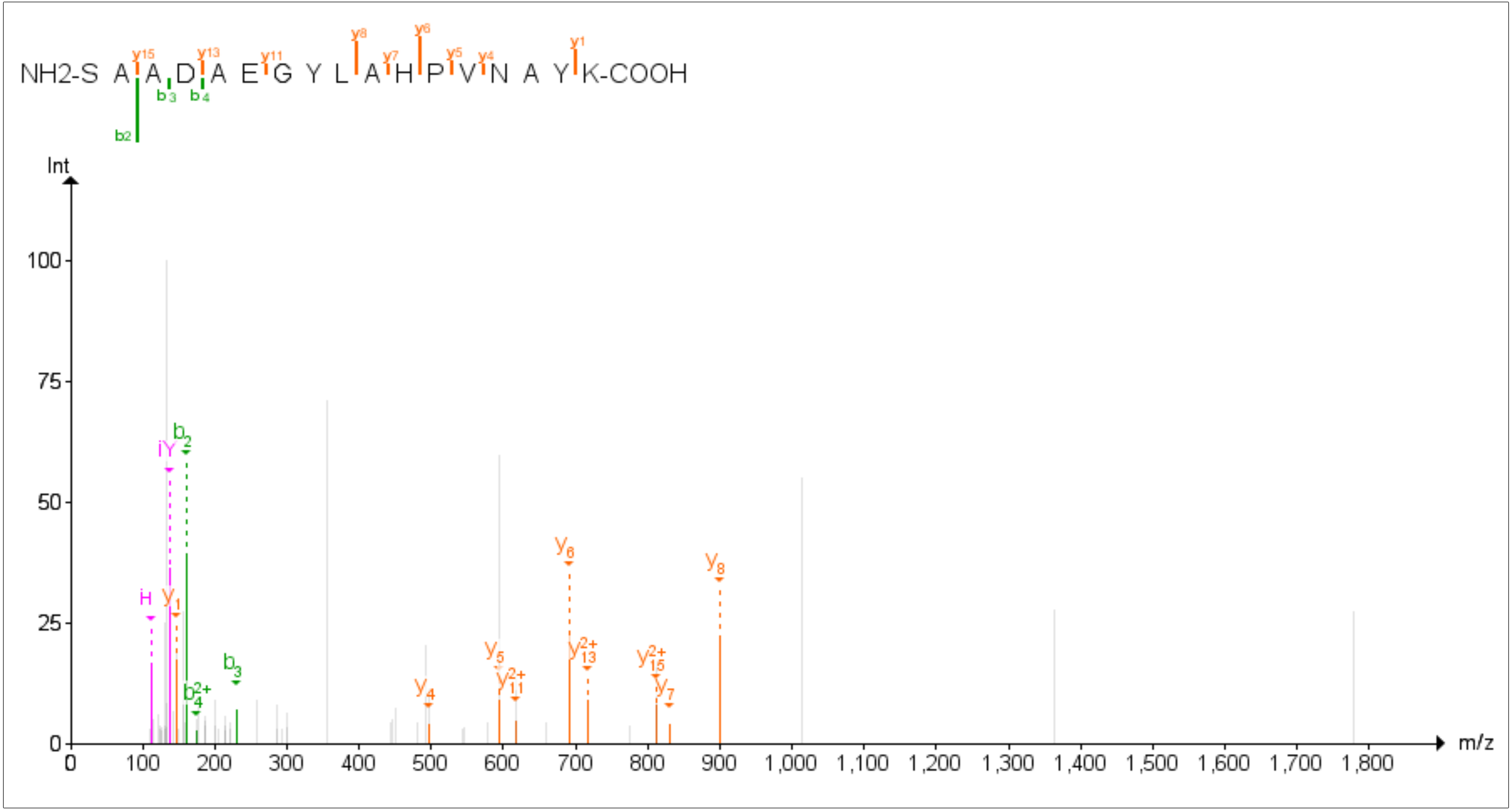

NH2-S A A D A E G Y L A H P V N A Y K-COOH
y15 y13 y11 y8 y7 y6 y5 y4 y1
b2 b3 b4
Int
100
75
50
25
0
iY
iH
b2
y1
b3
b4 2+
y4
y5
y11 2+
y6
y13 2+
y15 2+
y7
y8
0 100 200 300 400 500 600 700 800 900 1,000 1,100 1,200 1,300 1,400 1,500 1,600 1,700 1,800
m/z

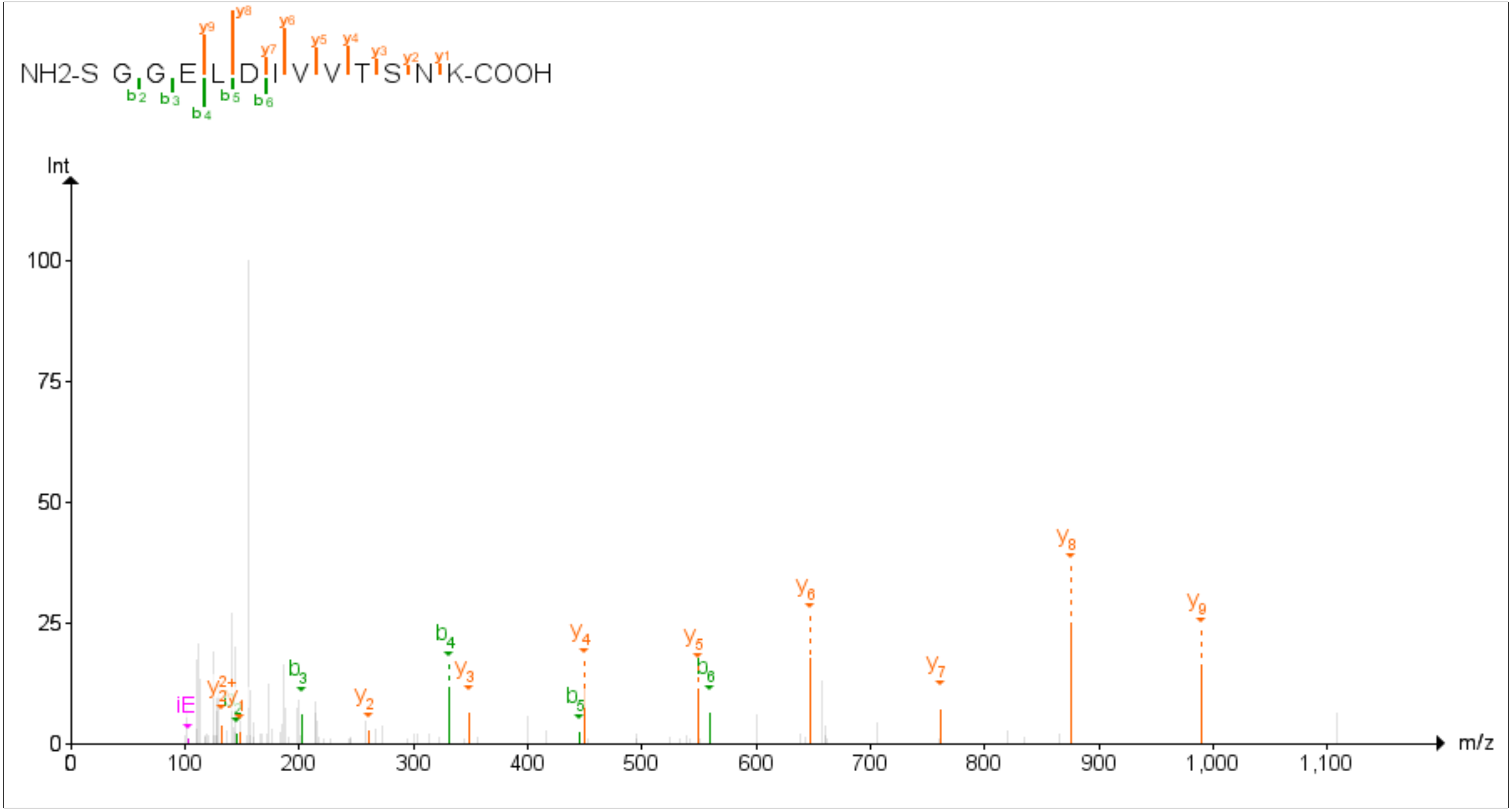

NH2-S G G E L D I V V T S N K-COOH
y9 y8 y7 y6 y5 y4 y3 y2 y1
b2 b3 b4 b5 b6
Int
100
75
50
25
0
m/z
0 100 200 300 400 500 600 700 800 900 1,000 1,100
iE
b3
b4
b5
b6
y2
y3
y4
y5
y6
y7
y8
y9

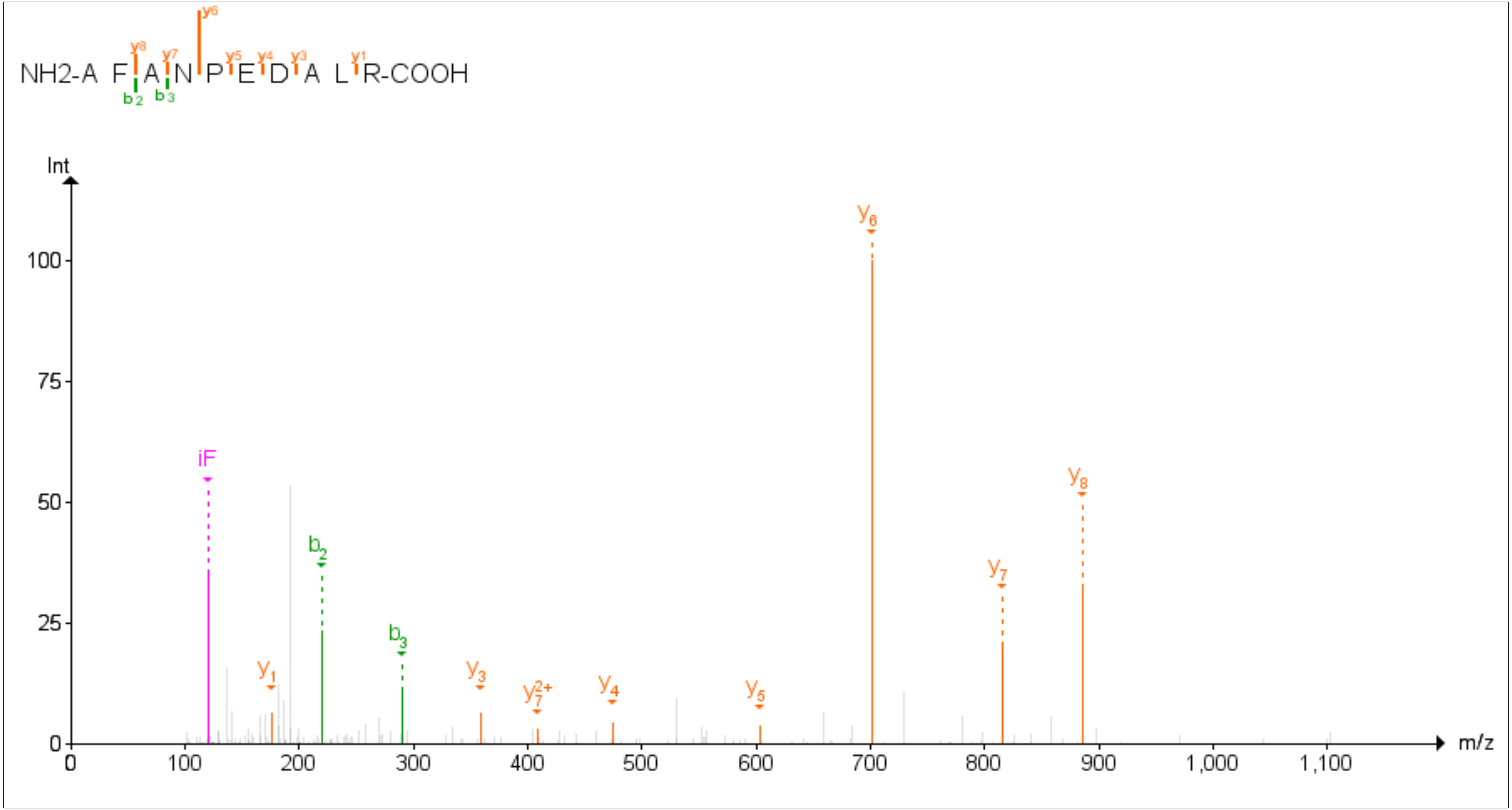
NH2-A F A N P E D A L R-COOH
y8 y7 y6 y5 y4 y3 y1
b2 b3
Int
100
75
50
25
0
iF
b2
b3
y1
y3
y7 2+
y4
y5
y6
y7
y8
0 100 200 300 400 500 600 700 800 900 1,000 1,100
m/z

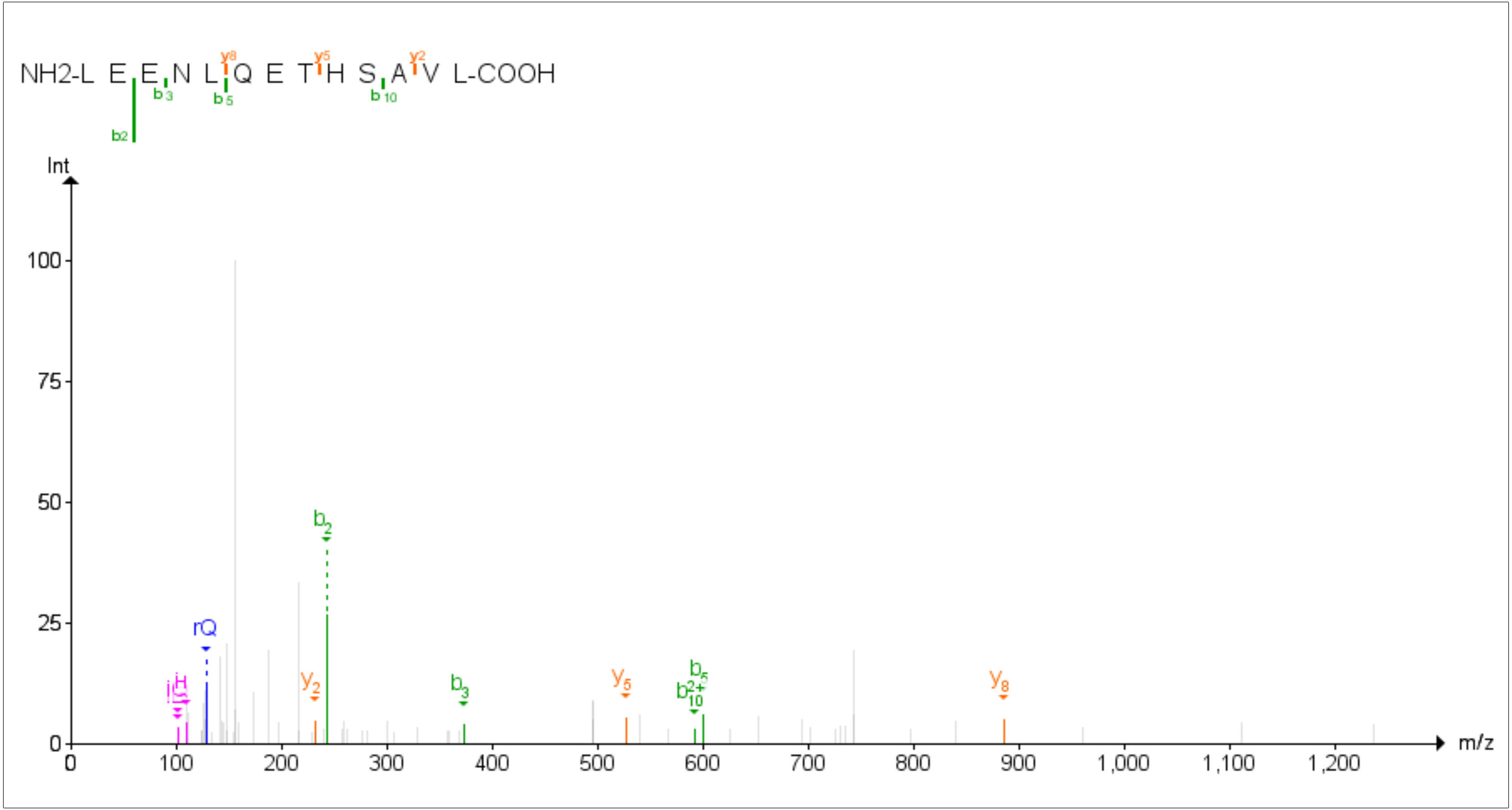

NH2-L E E N L Q E T H S A V L-COOH
b2
b3
b5
b10
y8
y5
y2
Int
100
75
50
25
0
b2
rQ
y2
b3
y5
b5
b10 2+
y8
0
100
200
300
400
500
600
700
800
900
1,000
1,100
1,200
m/z

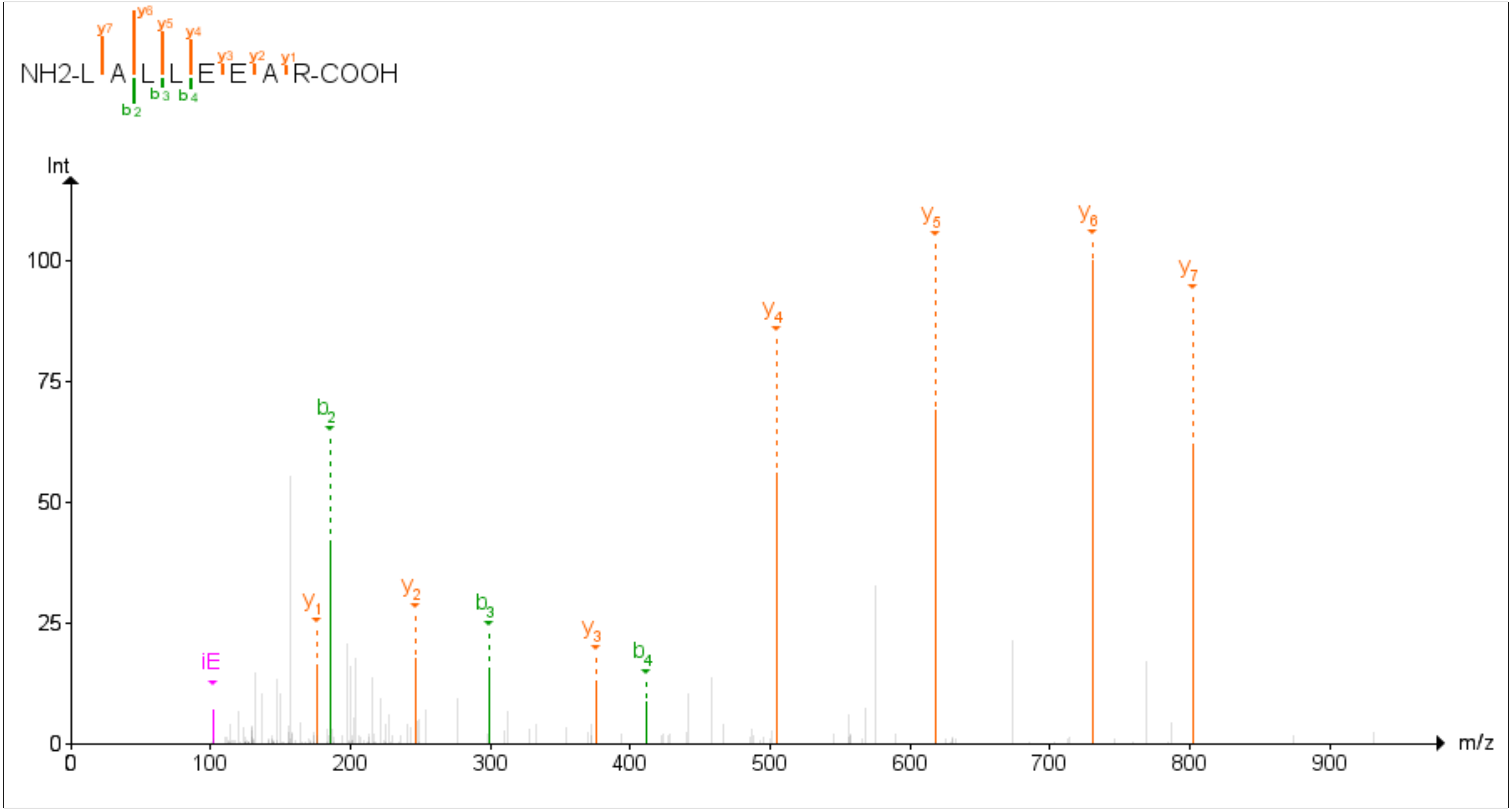

NH2-L A L L E E A R-COOH
y7 y6 y5 y4 y3 y2 y1
b2 b3 b4
Int
m/z
iE
y1 y2 y3 y4 y5 y6 y7
b2 b3 b4
0 25 50 75 100
0 100 200 300 400 500 600 700 800 900

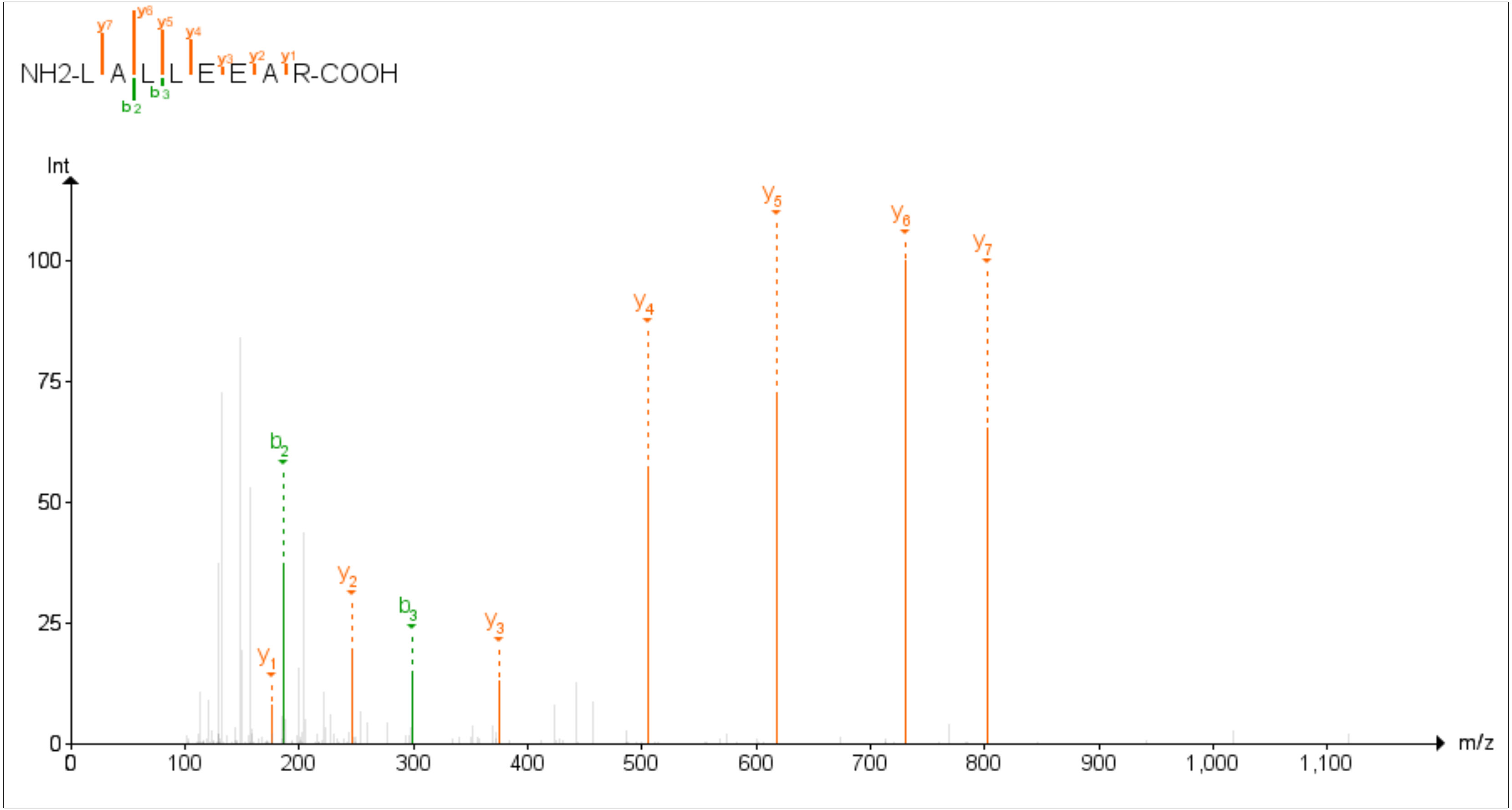

NH2-L A L L E E A R-COOH
y7 y6 y5 y4 y3 y2 y1
b2 b3
Int
100
75
50
25
0
y5
y6
y7
y4
b2
y2
b3
y3
y1
0 100 200 300 400 500 600 700 800 900 1,000 1,100
m/z

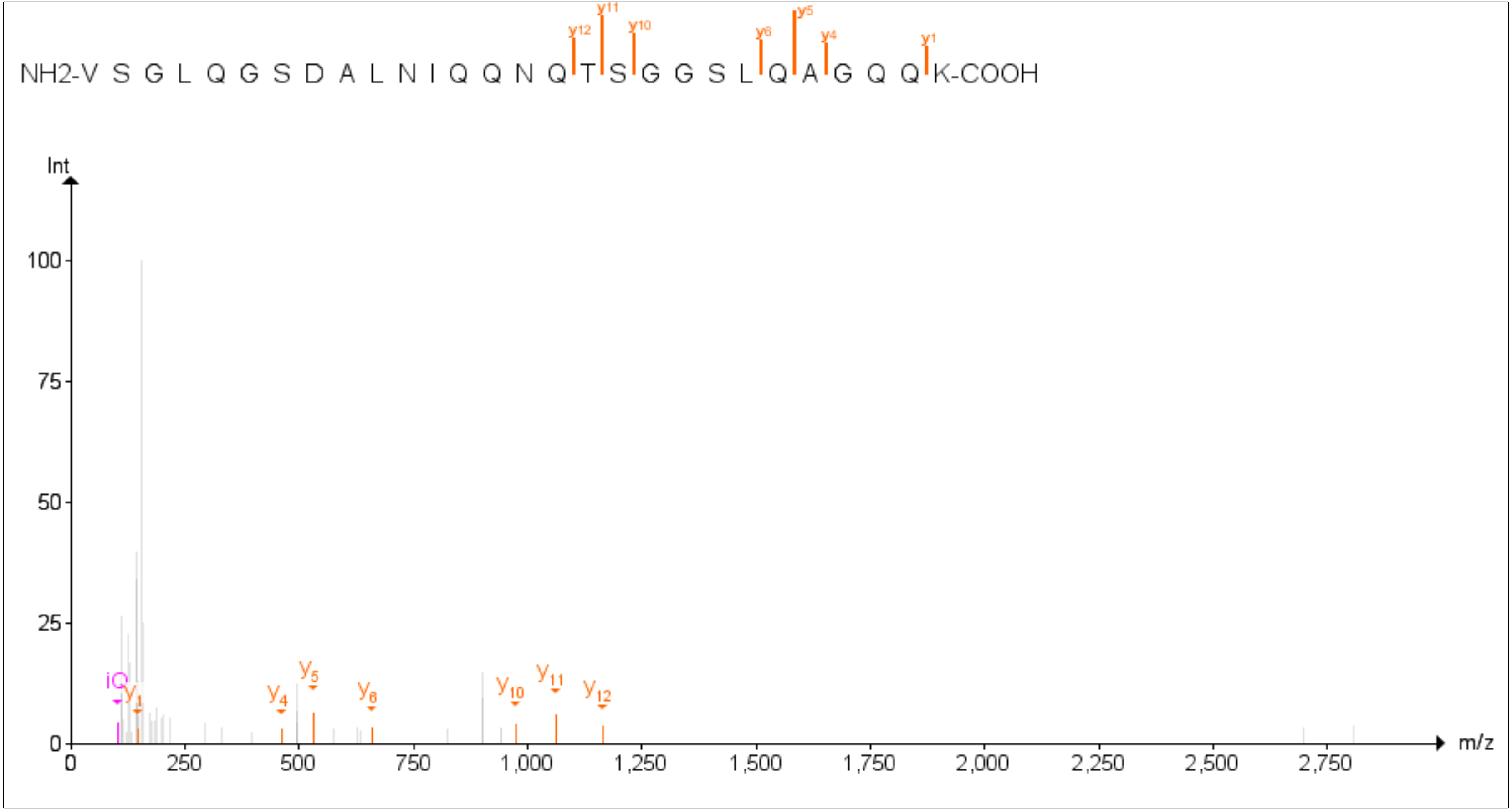

NH2-V S G L Q G S D A L N I Q Q N Q T S G G S L Q A G Q Q K-COOH
y12
y11
y10
y6
y5
y4
y1
Int
100
75
50
25
0
iQ
y1
y4
y5
y6
y10
y11
y12
0
250
500
750
1,000
1,250
1,500
1,750
2,000
2,250
2,500
2,750
m/z

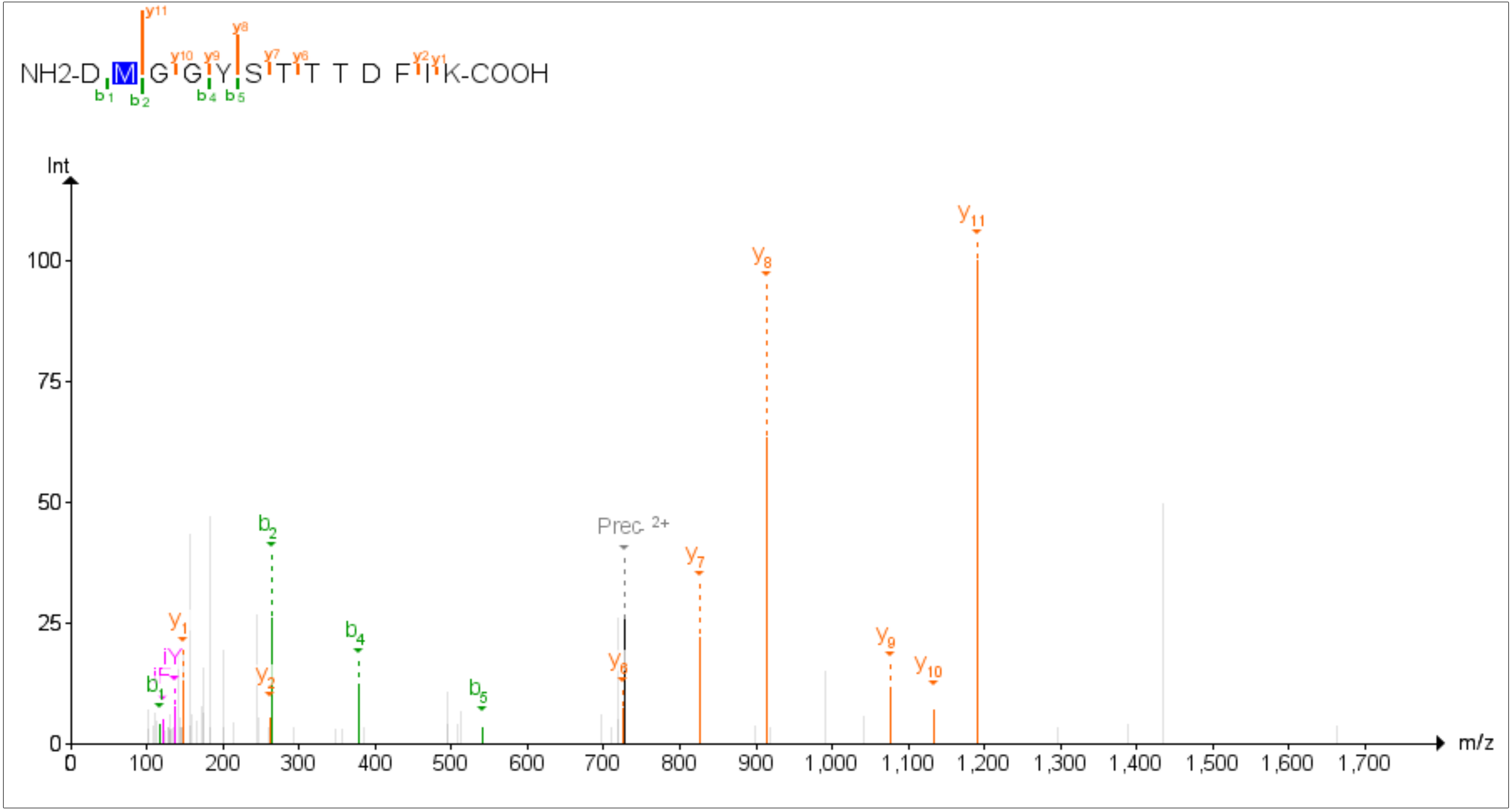

NH2-D M G G Y S T T T D F I K-COOH
y11 y10 y9 y8 y7 y6 y2 y1
b1 b2 b4 b5
Int
m/z
Prec 2+
0
25
50
75
100
0
100
200
300
400
500
600
700
800
900
1,000
1,100
1,200
1,300
1,400
1,500
1,600
1,700

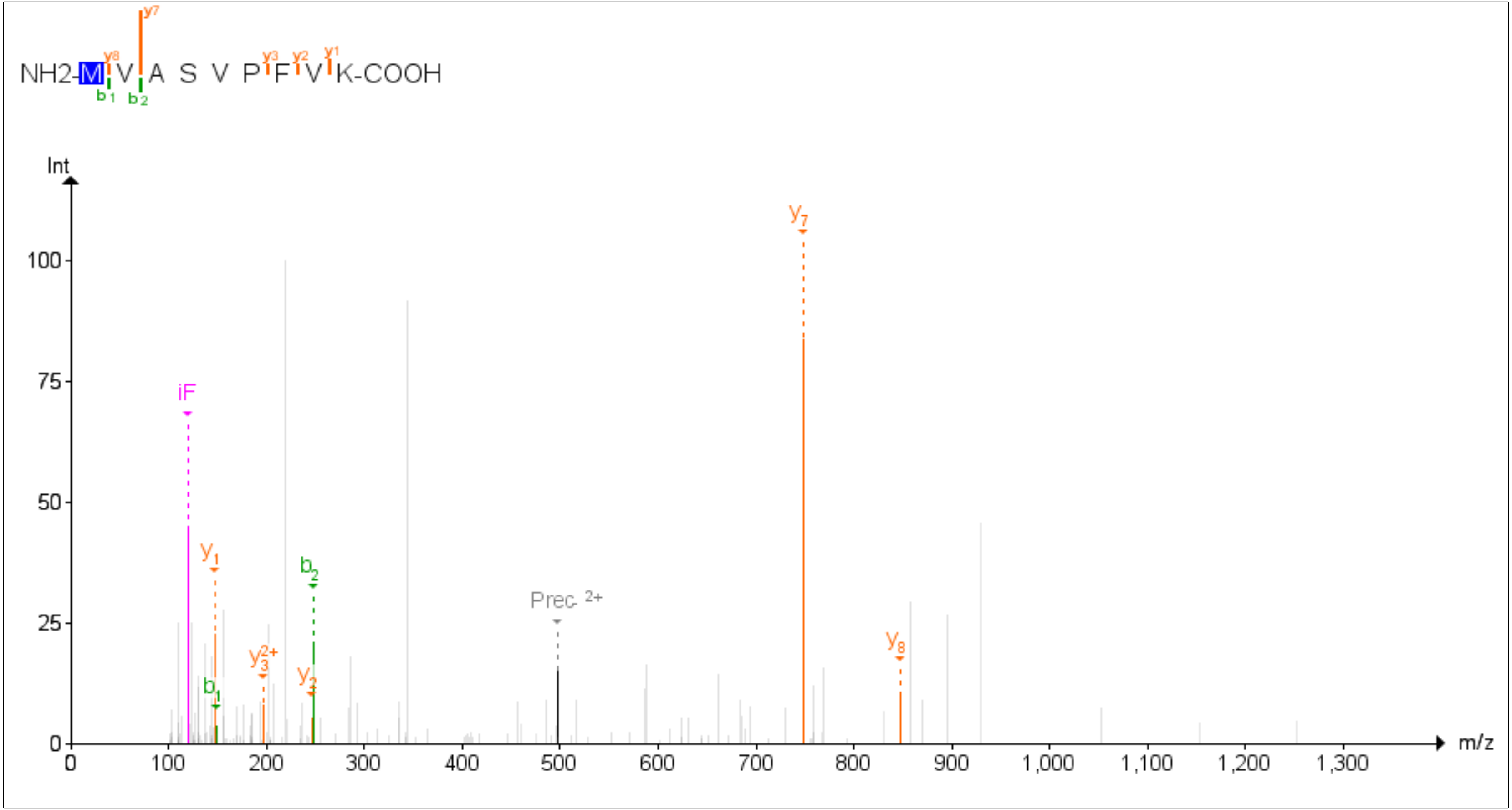

NH2-M V A S V P F V K-COOH
y7
y8
y3
y2
y1
b1
b2
Int
100
75
50
25
0
iF
y1
b2
y3 2+
y2
b1
Prec 2+
y7
y8
0
100
200
300
400
500
600
700
800
900
1,000
1,100
1,200
1,300
m/z

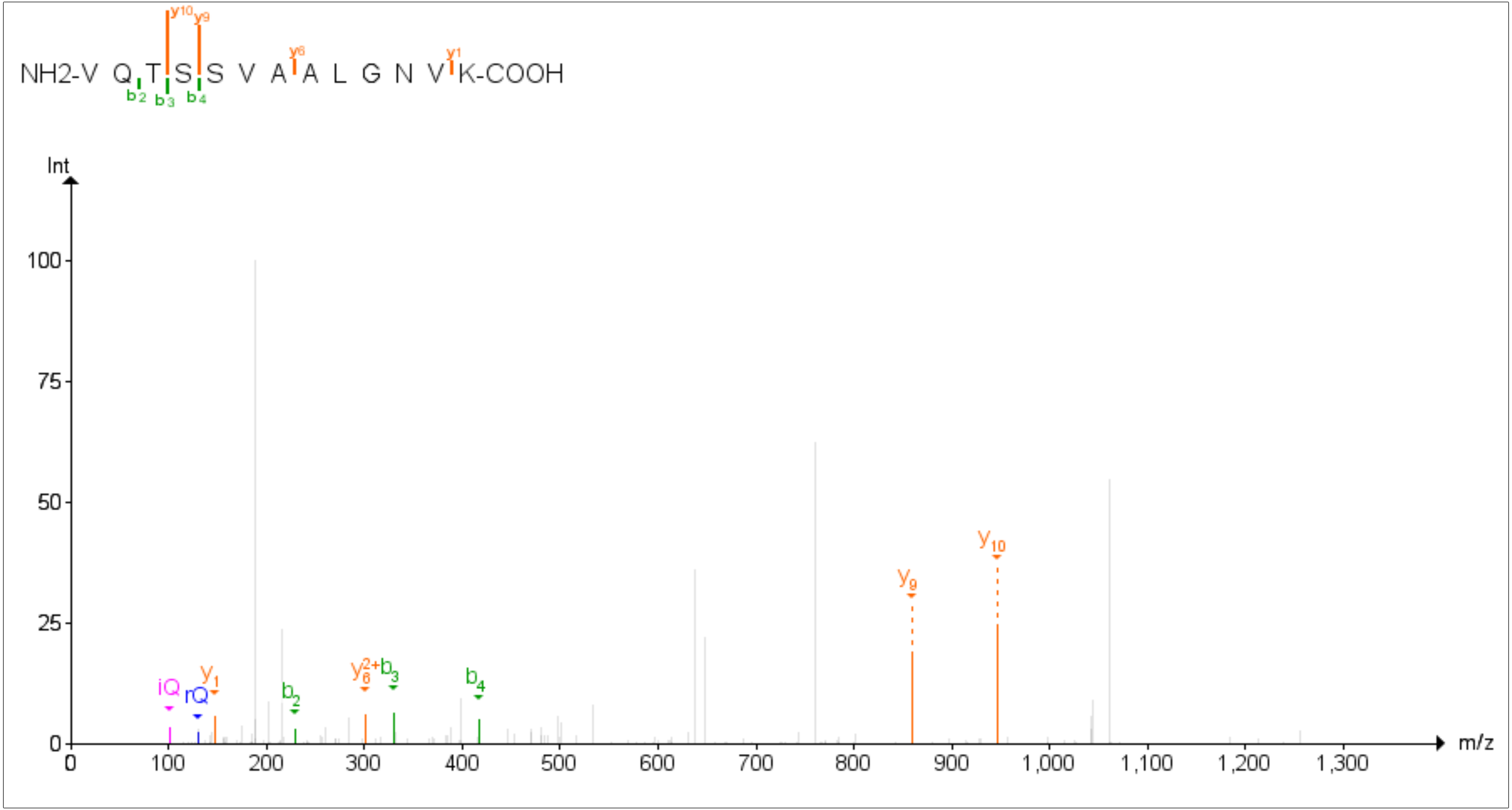
NH2-V Q T S S V A A L G N V K-COOH
y10 y9 y6 y1
b2 b3 b4
Int
100
75
50
25
0
iQ
rQ
y1
b2
y6 2+b3
b4
y9
y10
0 100 200 300 400 500 600 700 800 900 1,000 1,100 1,200 1,300
m/z

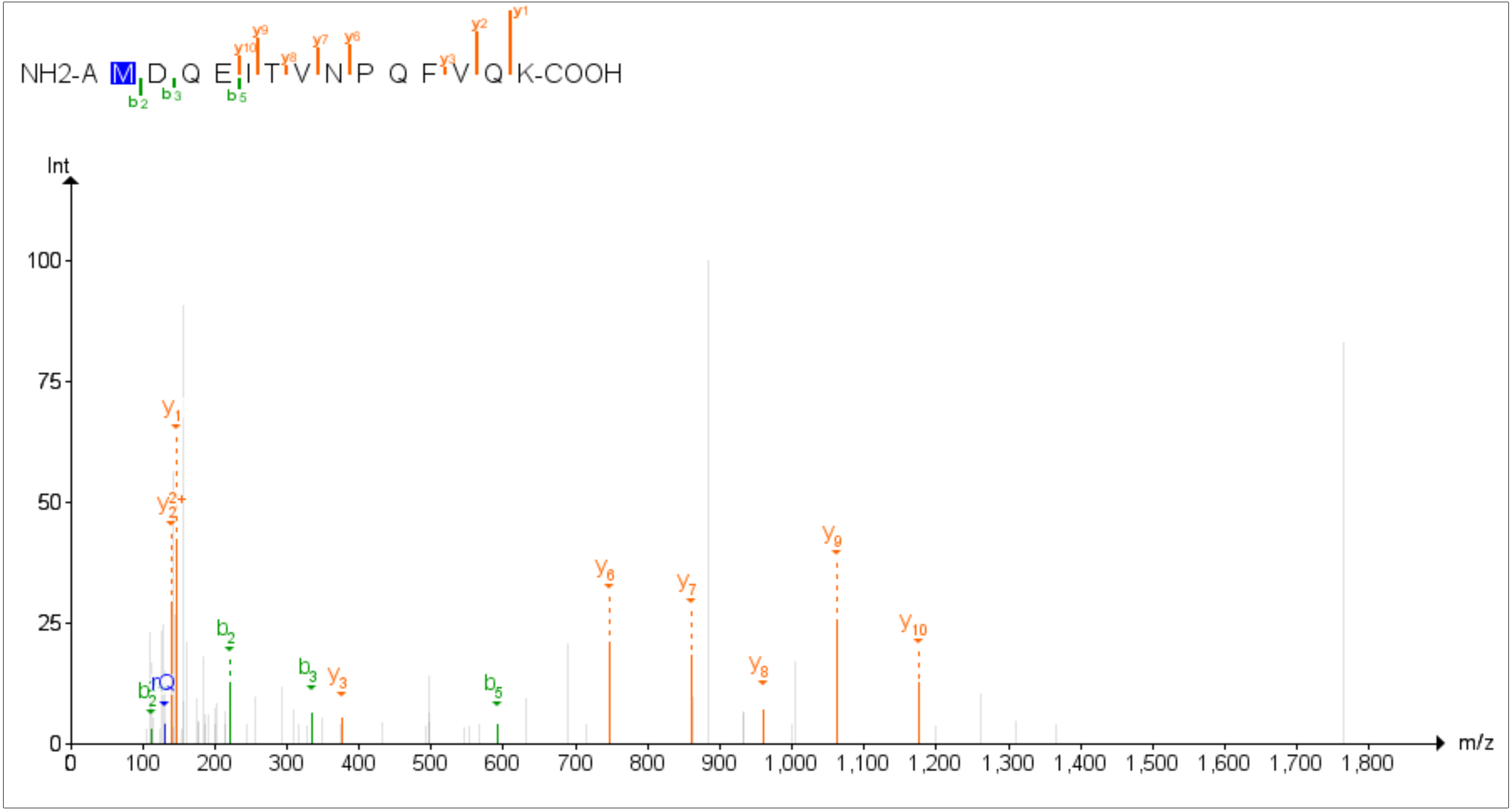
NH2-A M D Q E I T V N P Q F V Q K-COOH
b2
b3
b5
y10
y9
y8
y7
y6
y3
y2
y1
Int
100
75
50
25
0
y1
y2 2+
y6
y7
y9
y10
b2
b3
y3
b5
y8
b2
rQ
0
100
200
300
400
500
600
700
800
900
1,000
1,100
1,200
1,300
1,400
1,500
1,600
1,700
1,800
m/z

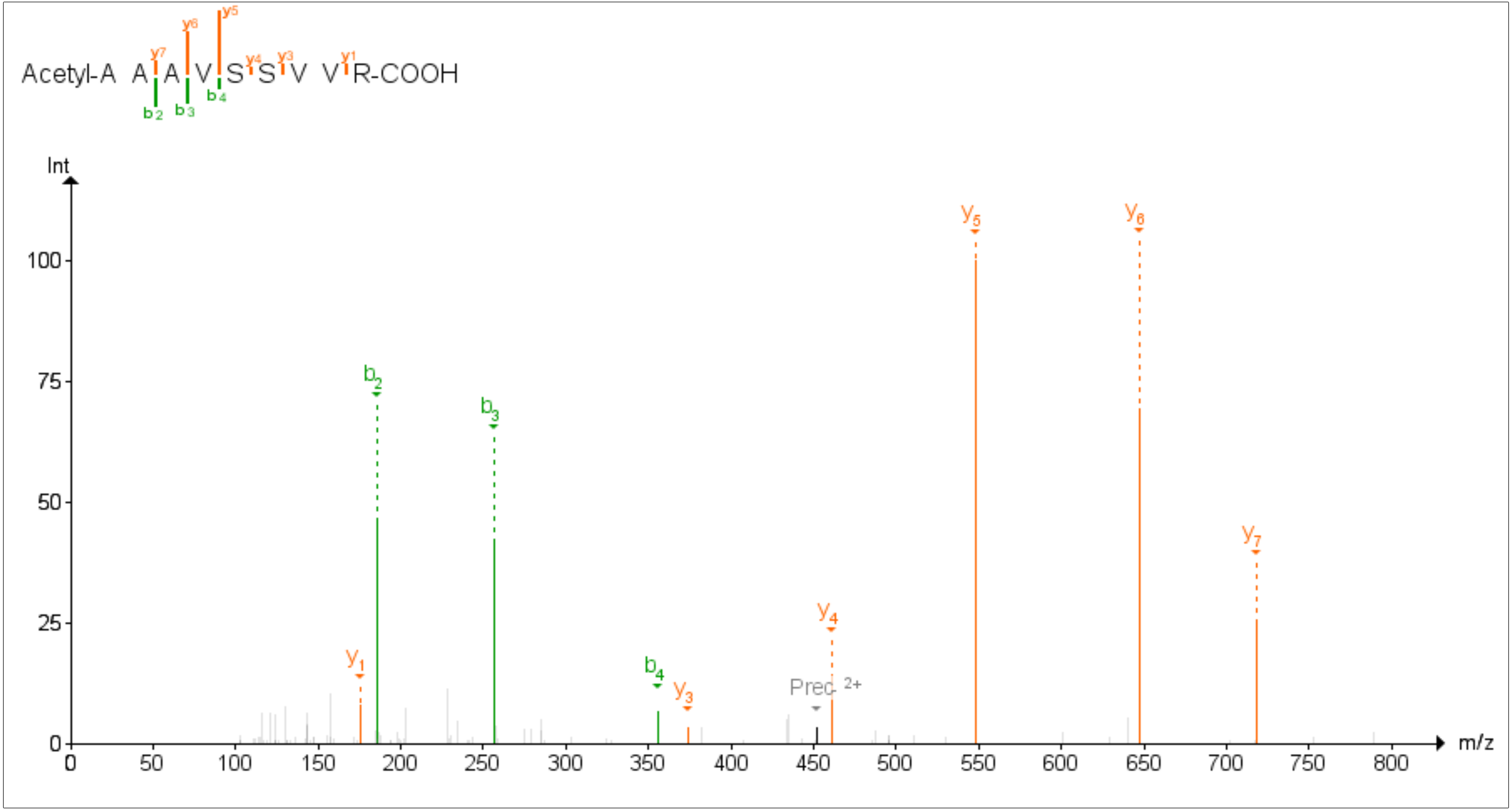

Acetyl-A A A V S S V V R-COOH
y7 y6 y5 y4 y3 y1
b2 b3 b4
Int
100
75
50
25
0
y5
y6
b2
b3
y7
y4
y1
b4
y3
Prec 2+
0 50 100 150 200 250 300 350 400 450 500 550 600 650 700 750 800
m/z

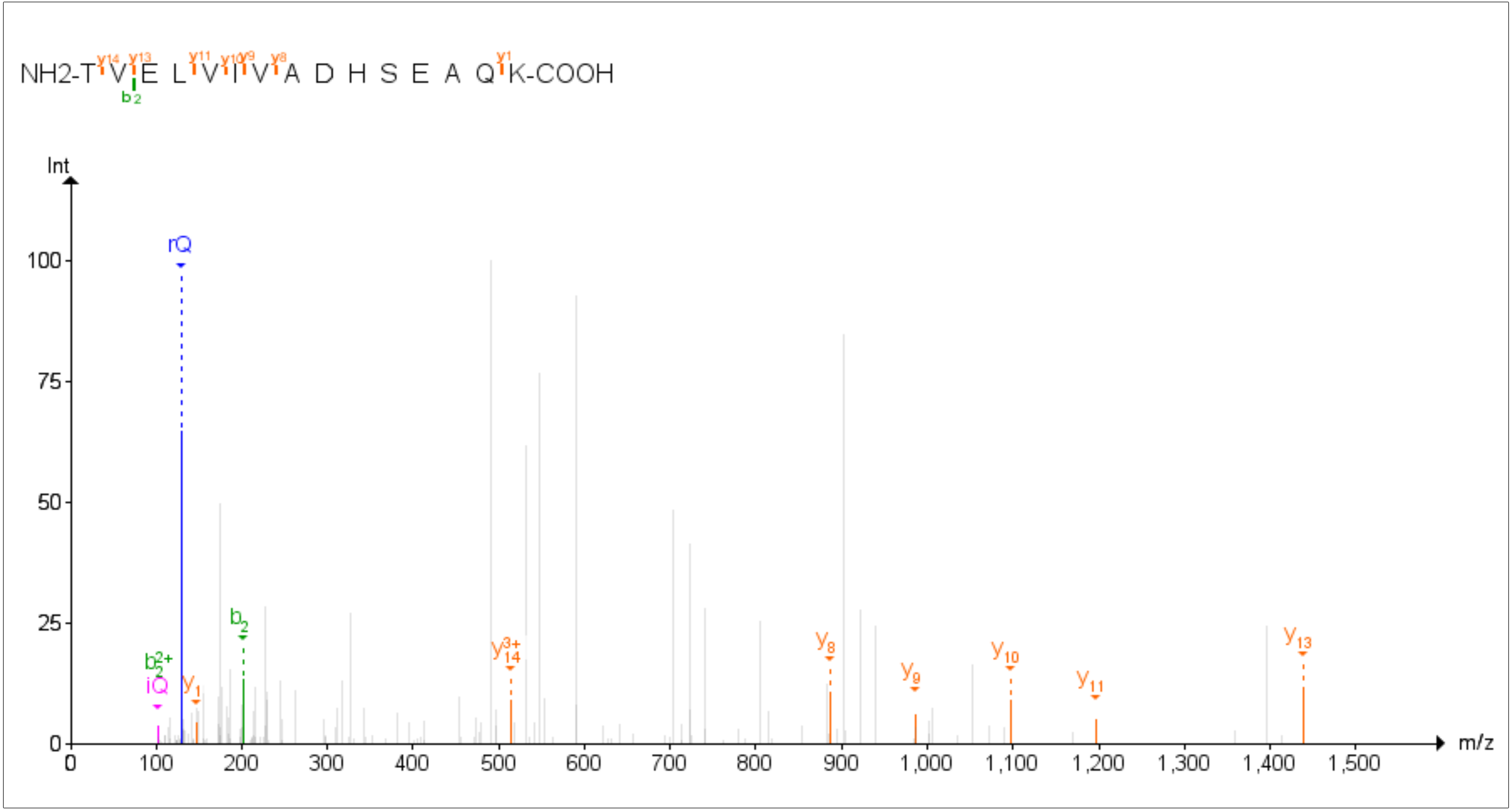

NH2-T V E L V I V A D H S E A Q K-COOH
y14
y13
y11
y10
y9
y8
y1
b2
Int
100
75
50
25
0
rQ
b2
iQ
y1
y14 3+
y8
y9
y10
y11
y13
0
100
200
300
400
500
600
700
800
900
1,000
1,100
1,200
1,300
1,400
1,500
m/z

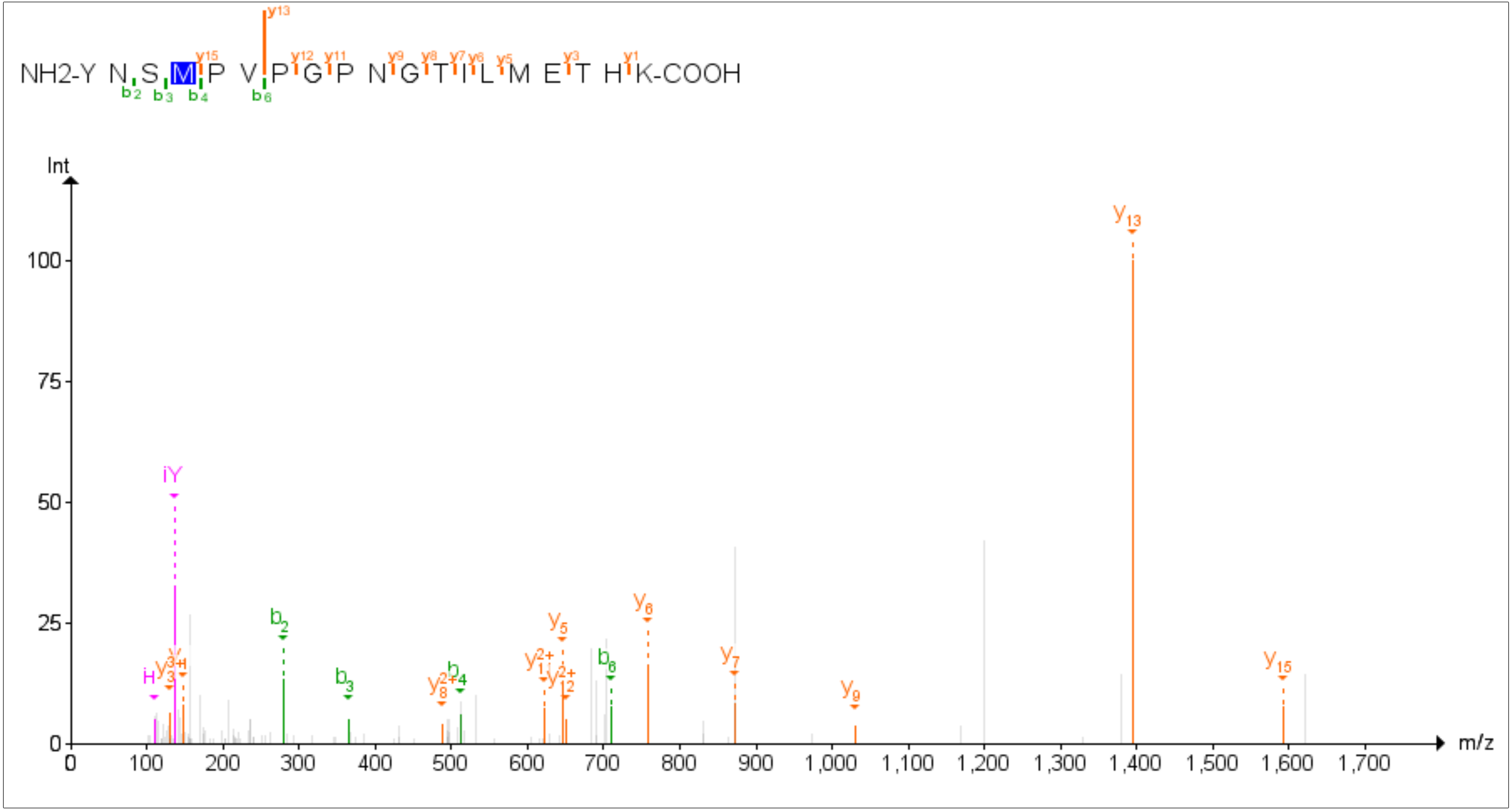

NH2-Y N S M P V P G P N G T I L M E T H K-COOH
b2 b3 b4 b6
y15 y13 y12 y11 y9 y8 y7 y6 y5 y3 y1
Int
m/z
iY
iH
b2
b3
b4
b6
y5
y6
y7
y9
y13
y15

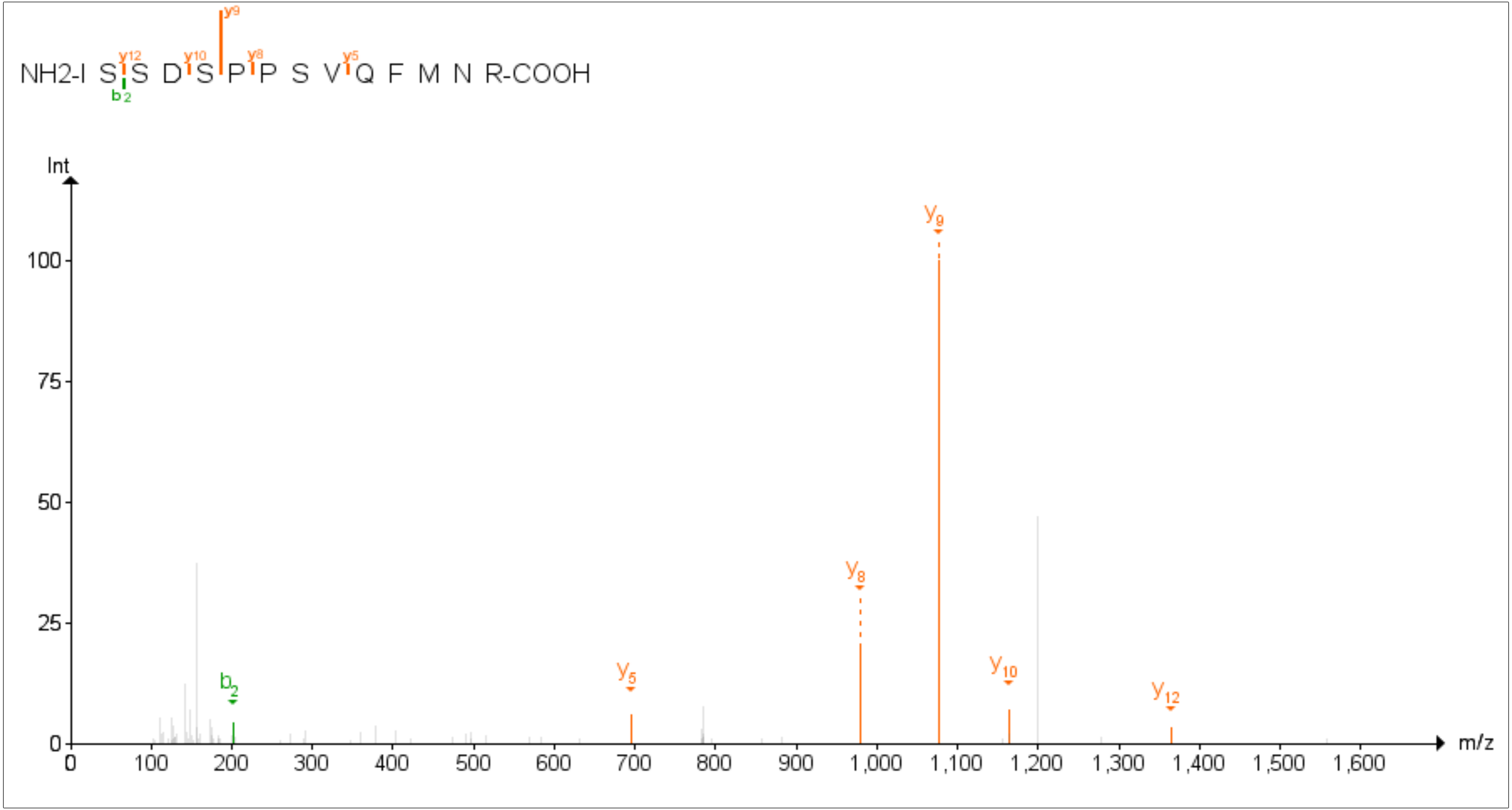

NH2-I S S D S P P S V Q F M N R-COOH
y12
y10
y9
y8
y5
b2
Int
100
75
50
25
0
m/z
0
100
200
300
400
500
600
700
800
900
1,000
1,100
1,200
1,300
1,400
1,500
1,600
y9
y8
y5
y10
y12
b2

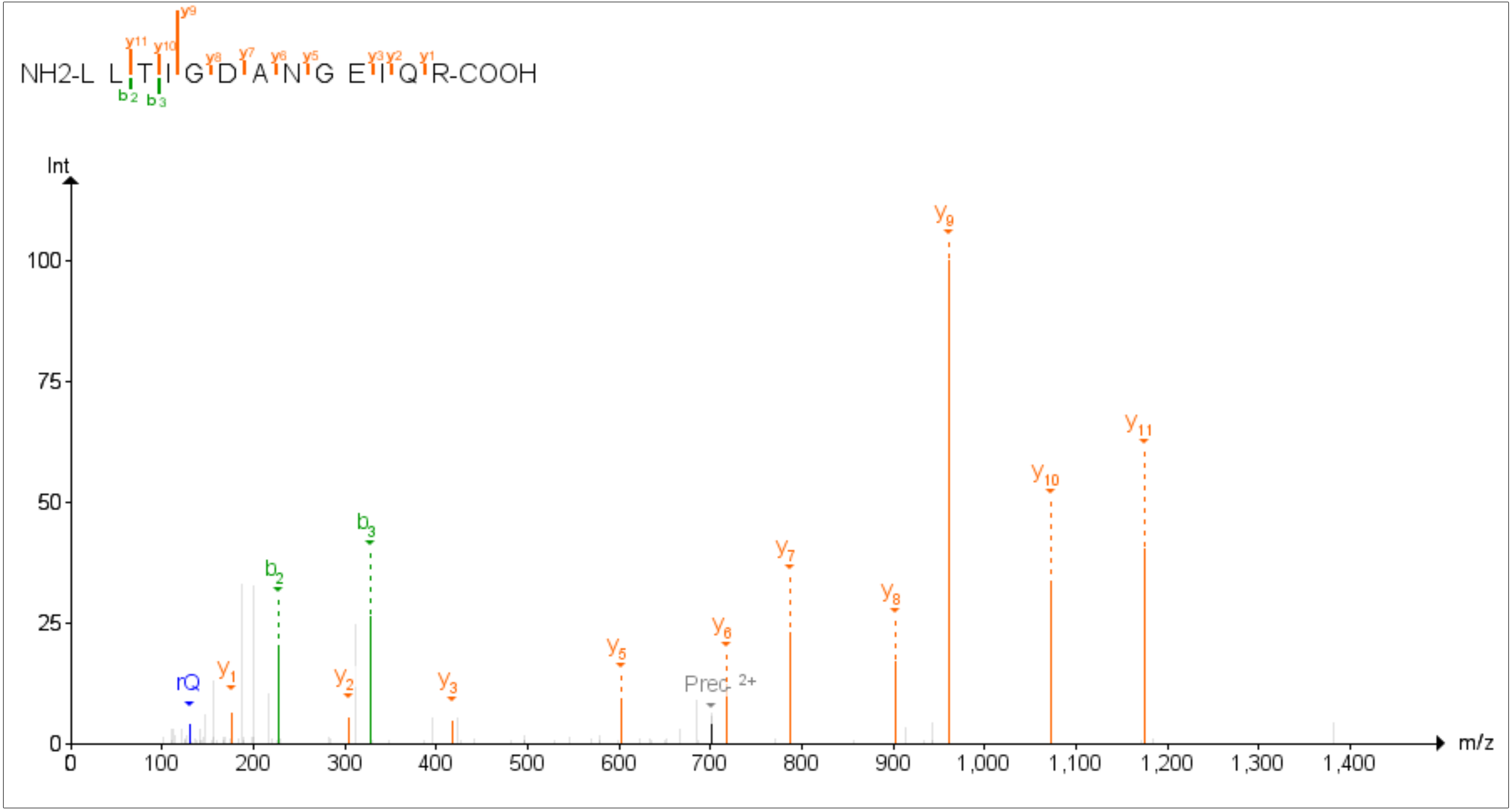

NH2-L L T I G D A N G E I Q R-COOH
y9
y11
y10
y8
y7
y6
y5
y3
y2
y1
b2
b3
Int
100
75
50
25
0
rQ
y1
b2
y2
b3
y3
y5
Prec 2+
y6
y7
y8
y9
y10
y11
0
100
200
300
400
500
600
700
800
900
1,000
1,100
1,200
1,300
1,400
m/z

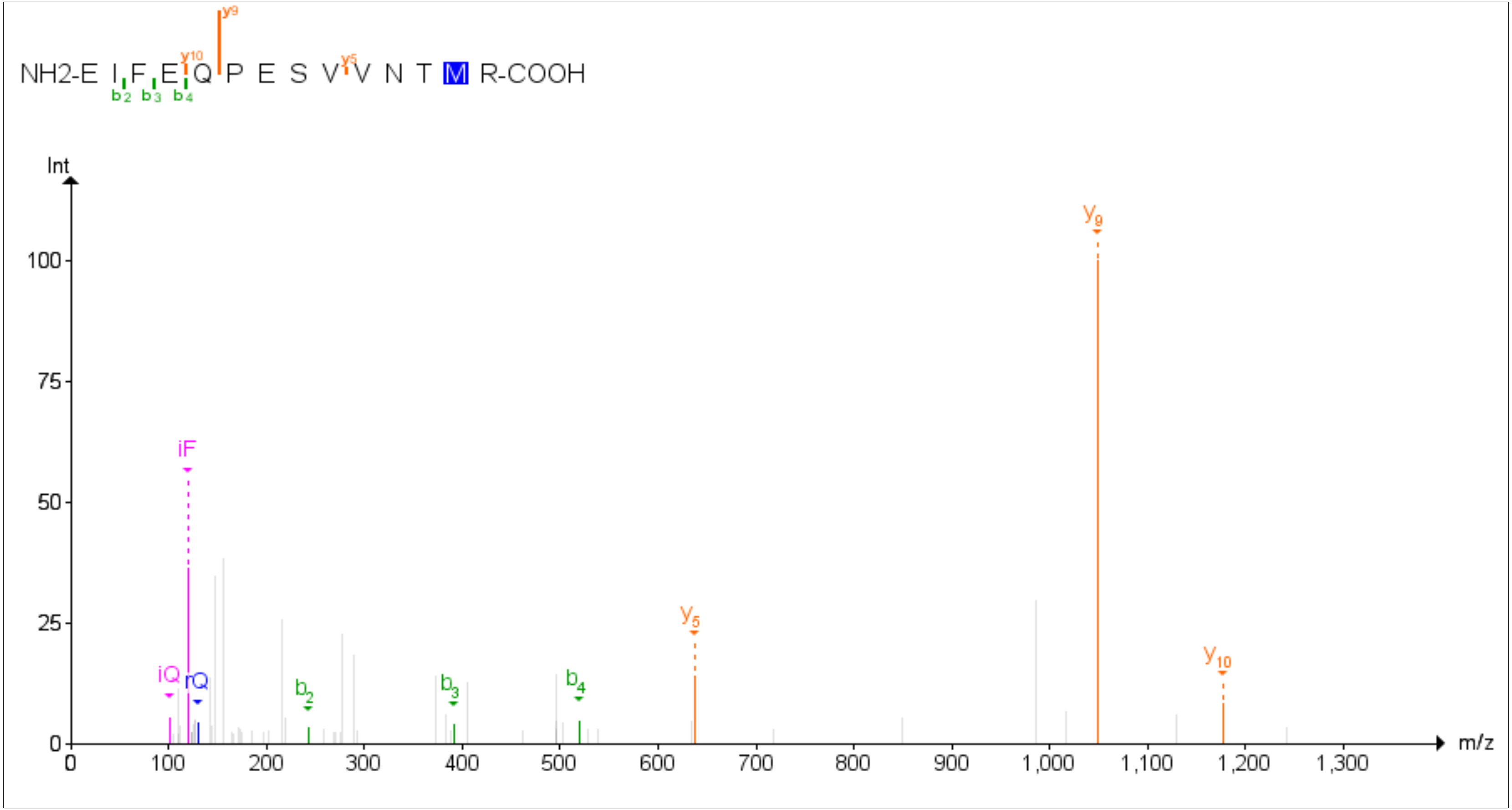

NH2-E I F E Q P E S V V N T M R-COOH
y9
y10
y5
b2
b3
b4
Int
100
75
50
25
0
iF
iQ
rQ
b2
b3
b4
y5
y9
y10
0
100
200
300
400
500
600
700
800
900
1,000
1,100
1,200
1,300
m/z

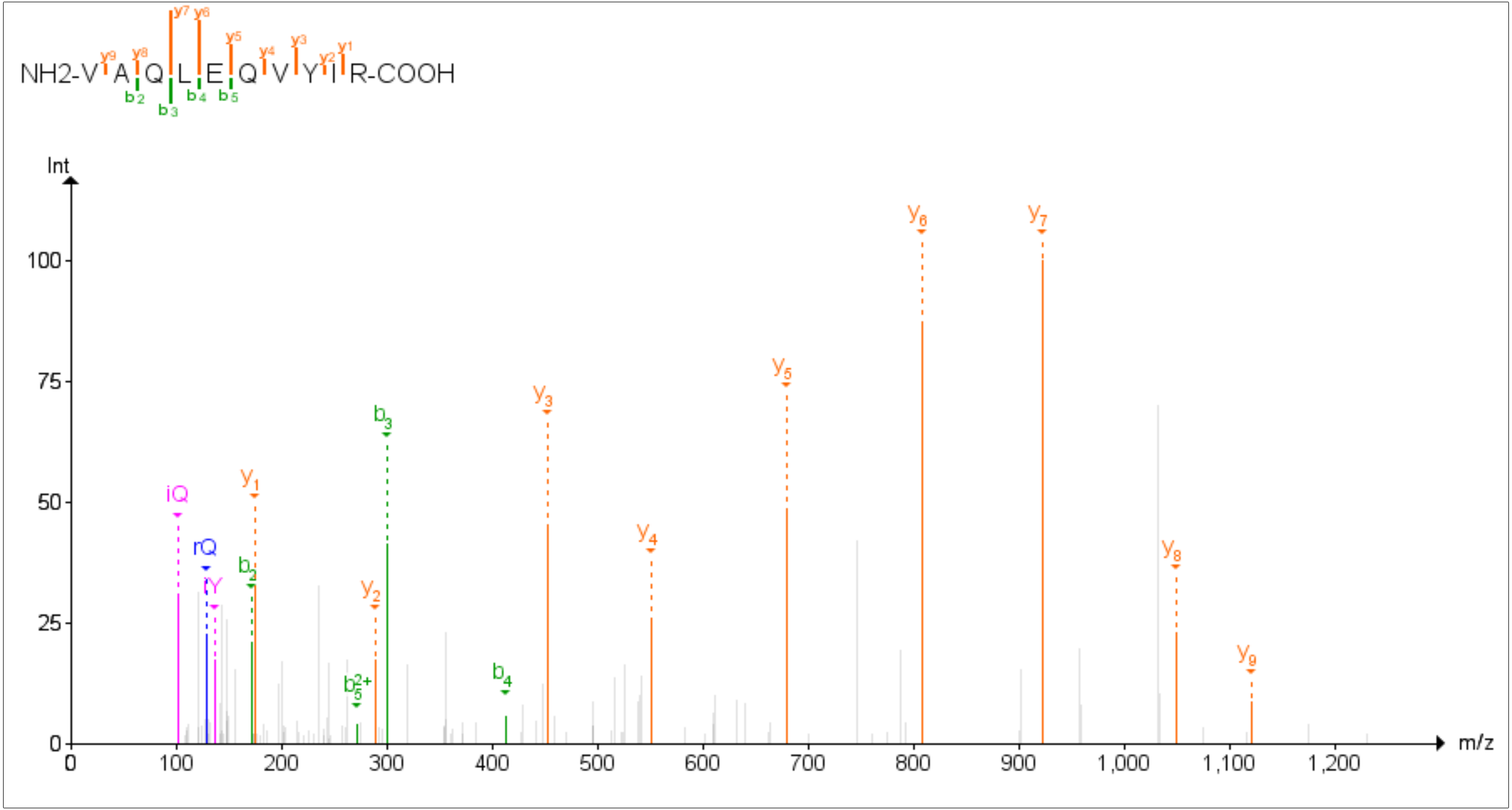

NH2-V A Q L E Q V Y I R-COOH
y9 y8 y7 y6 y5 y4 y3 y2 y1
b2 b3 b4 b5
Int
m/z
iQ
rQ
iY
y1
b2
b3
y2
b5 2+
b4
y3
y4
y5
y6
y7
y8
y9
0
25
50
75
100
0
100
200
300
400
500
600
700
800
900
1,000
1,100
1,200

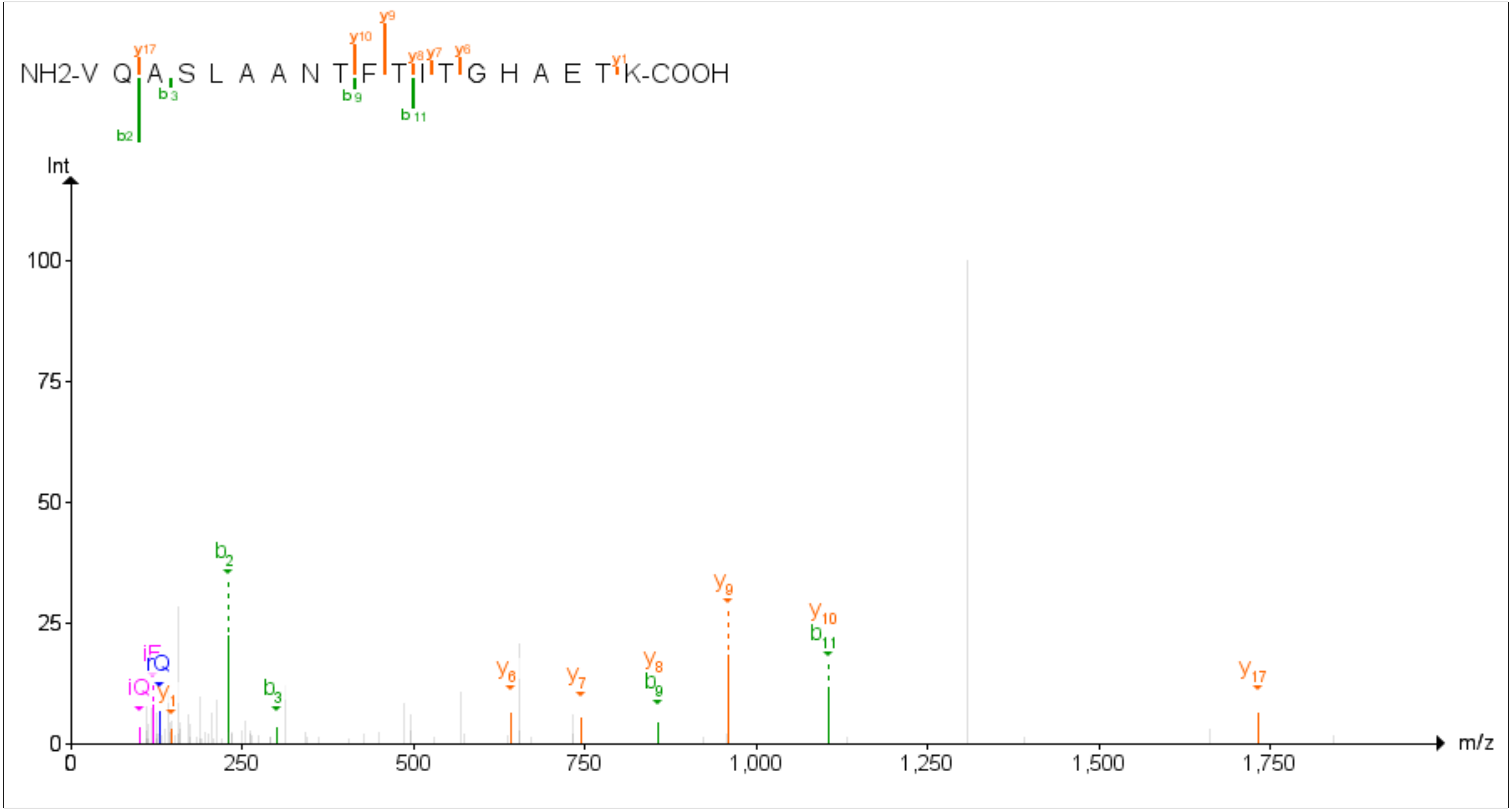

NH2-V Q A S L A A N T F T I T G H A E T K-COOH
y17 y10 y9 y8 y7 y6 y1
b2 b3 b9 b11
Int
100
75
50
25
0
0 250 500 750 1,000 1,250 1,500 1,750
m/z
b2 b3 y6 y7 y8 b9 y9 y10 b11 y17 iF rQ iQ y1

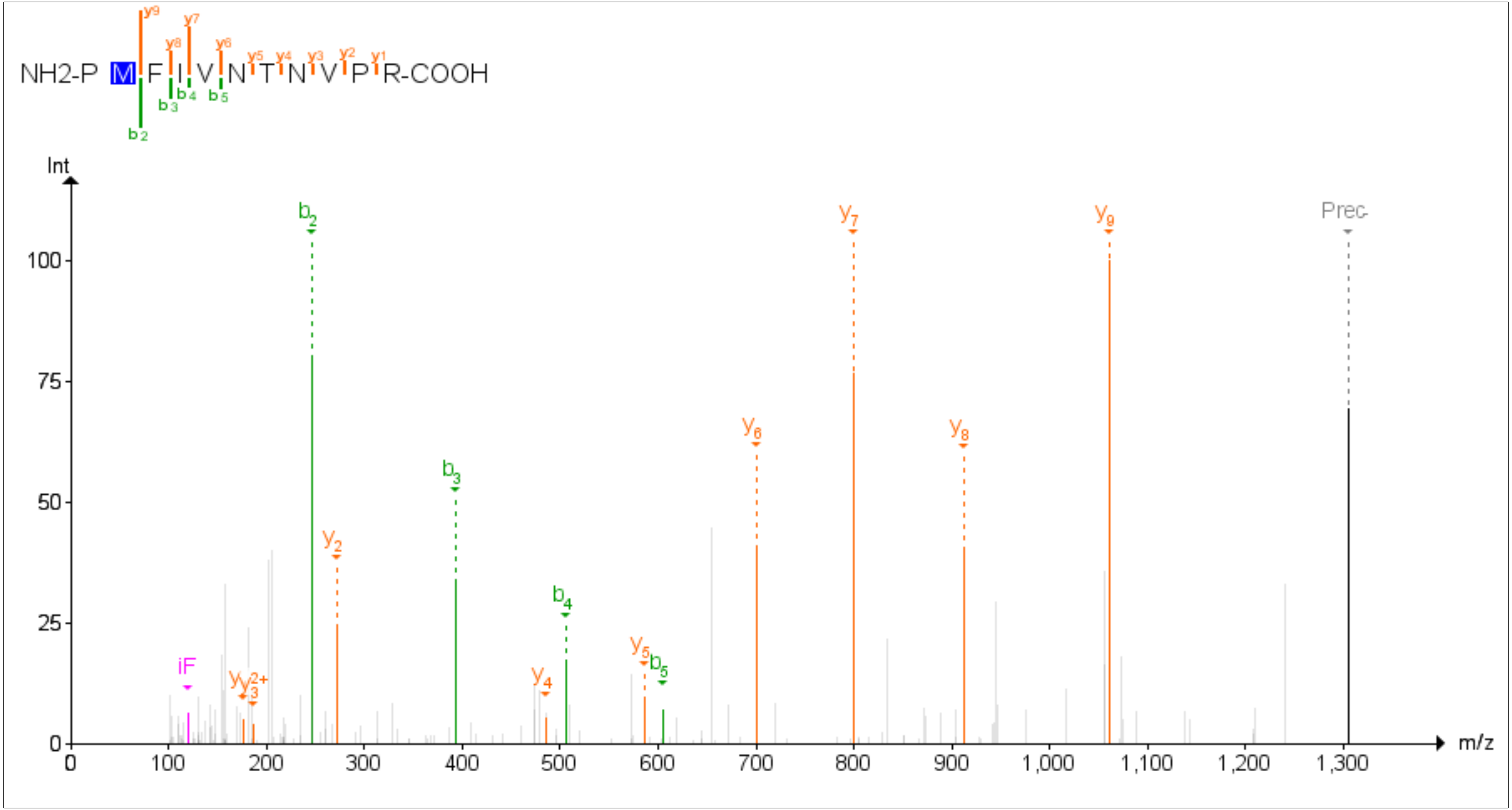

NH2-P M F I V N T N V P R-COOH
y9 y8 y7 y6 y5 y4 y3 y2 y1
b2 b3 b4 b5
Int
m/z
Prec.
b2
y7
y9
y6
y8
b3
y2
b4
y5
b5
y4
iF
y3
y2+
0
25
50
75
100
0
100
200
300
400
500
600
700
800
900
1,000
1,100
1,200
1,300

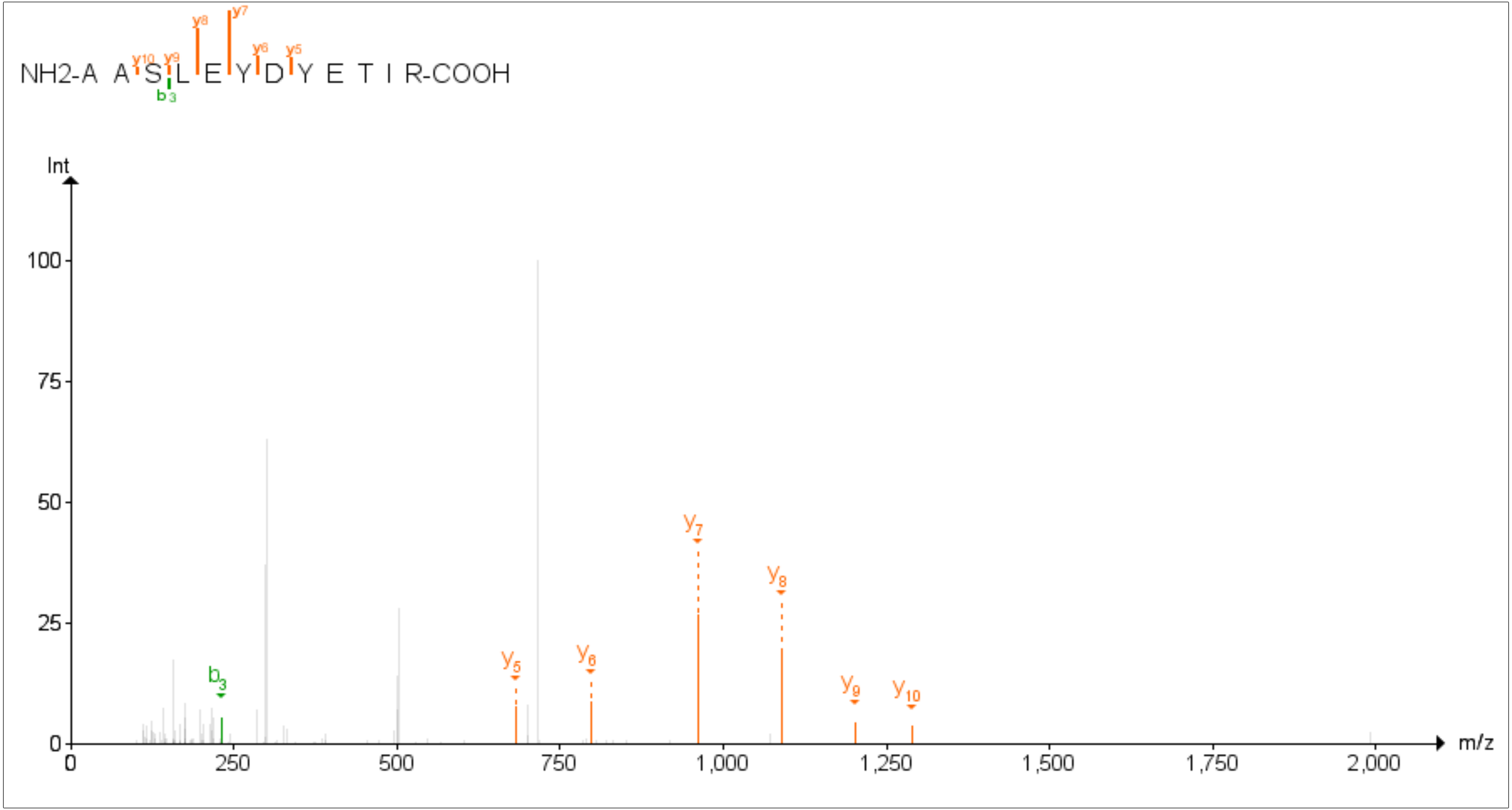

NH2-A A S L E Y D Y E T I R-COOH
y10 y9 y8 y7 y6 y5
b3
Int
100
75
50
25
0
250
500
750
1,000
1,250
1,500
1,750
2,000
m/z
y5 y6 y7 y8 y9 y10
b3

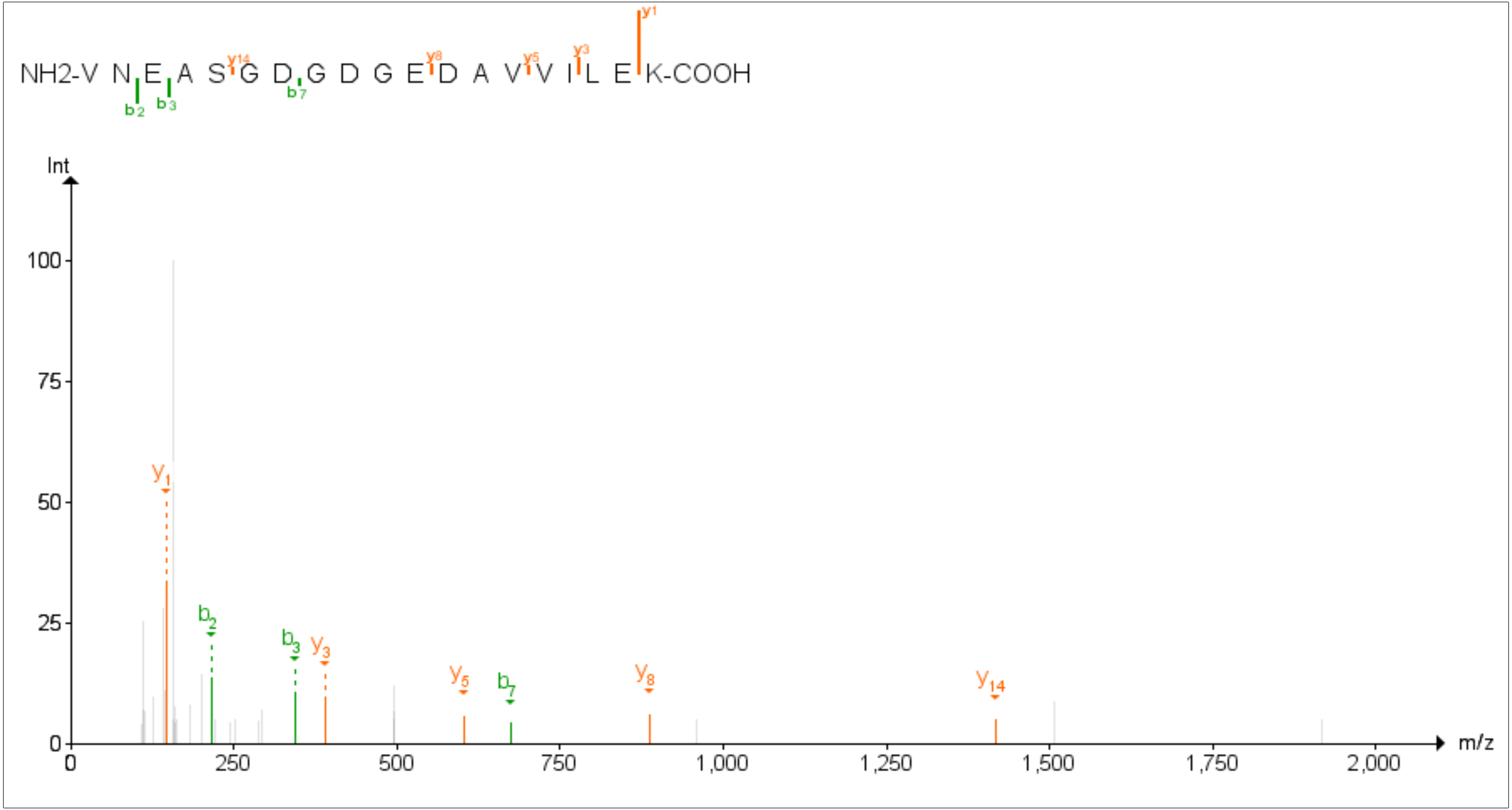

NH2-V N E A S G D G D G E D A V V I L E K-COOH
y1 y3 y5 y8 y14
b2 b3 b7
Int
100
75
50
25
0
y1
b2
b3
y3
y5
b7
y8
y14
0
250
500
750
1,000
1,250
1,500
1,750
2,000
m/z

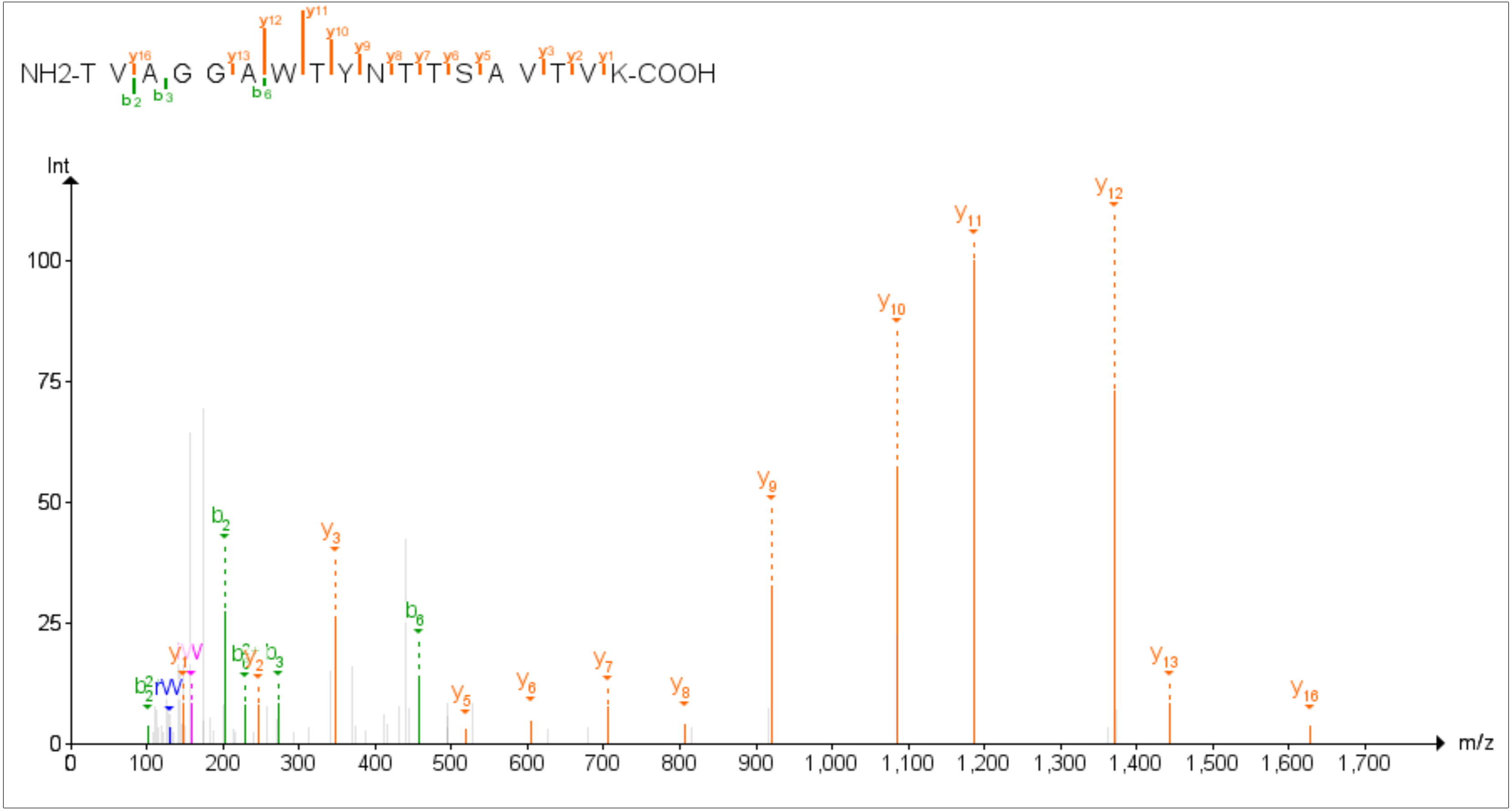
NH2-T V A G G A W T Y N T T S A V T V K-COOH
y16 y13 y12 y11 y10 y9 y8 y7 y6 y5 y3 y2 y1
b2 b3 b6
Int
100
75
50
25
0
m/z
0 100 200 300 400 500 600 700 800 900 1,000 1,100 1,200 1,300 1,400 1,500 1,600 1,700
y12 y11 y10 y9 y3 b2 b6 y1 y2 b3 y5 y6 y7 y8 y13 y16

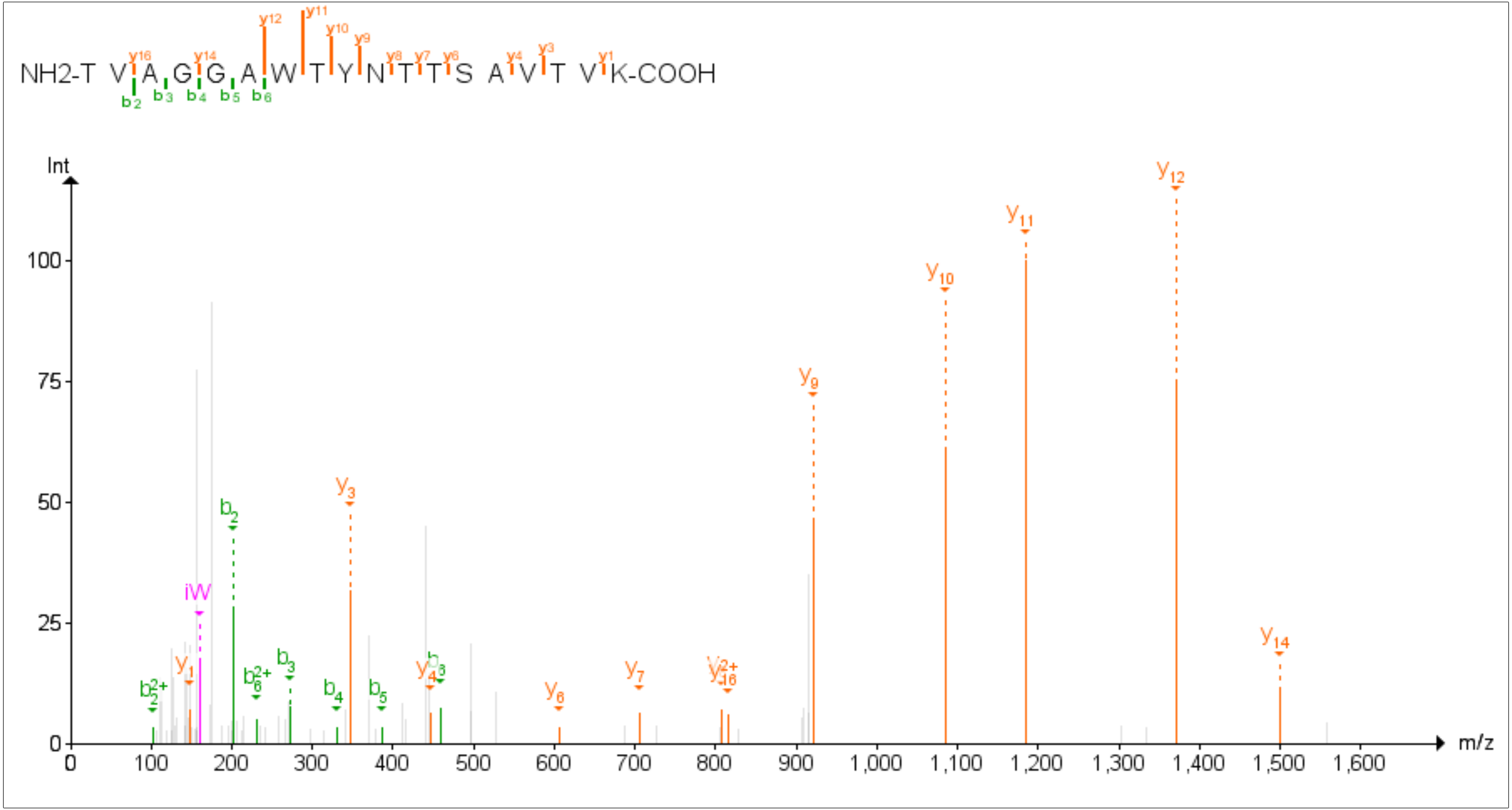

NH2-T V A G G A W T Y N T T S A V T V K-COOH
y16 y14 y12 y11 y10 y9 y8 y7 y6 y4 y3 y1
b2 b3 b4 b5 b6
Int
m/z
iW

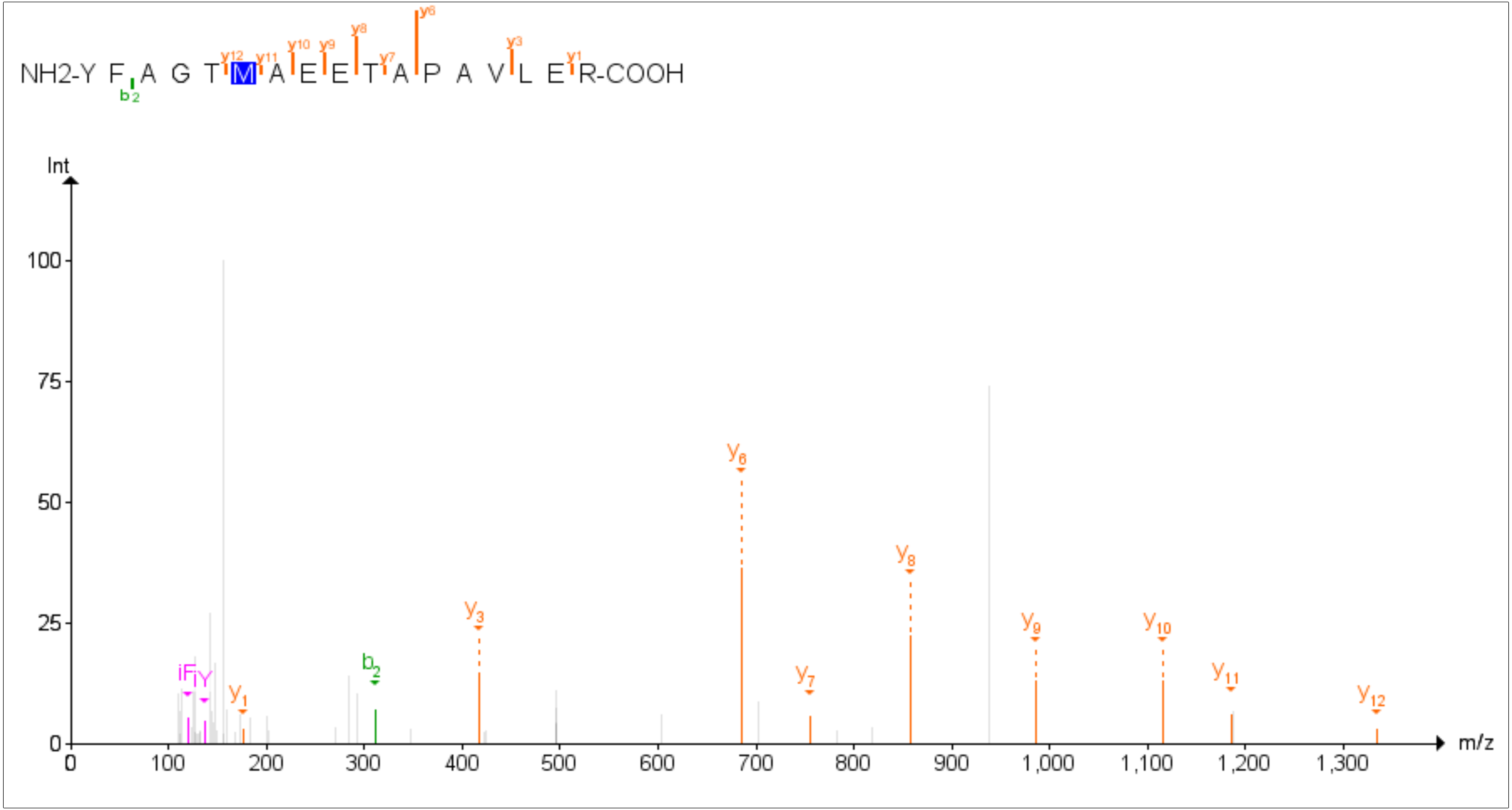
NH2-Y F A G T M A E E T A P A V L E R-COOH
b2
y12 y11 y10 y9 y8 y7 y6 y3 y1
Int
100
75
50
25
0
iF iY y1 b2 y3 y6 y7 y8 y9 y10 y11 y12
0 100 200 300 400 500 600 700 800 900 1,000 1,100 1,200 1,300
m/z

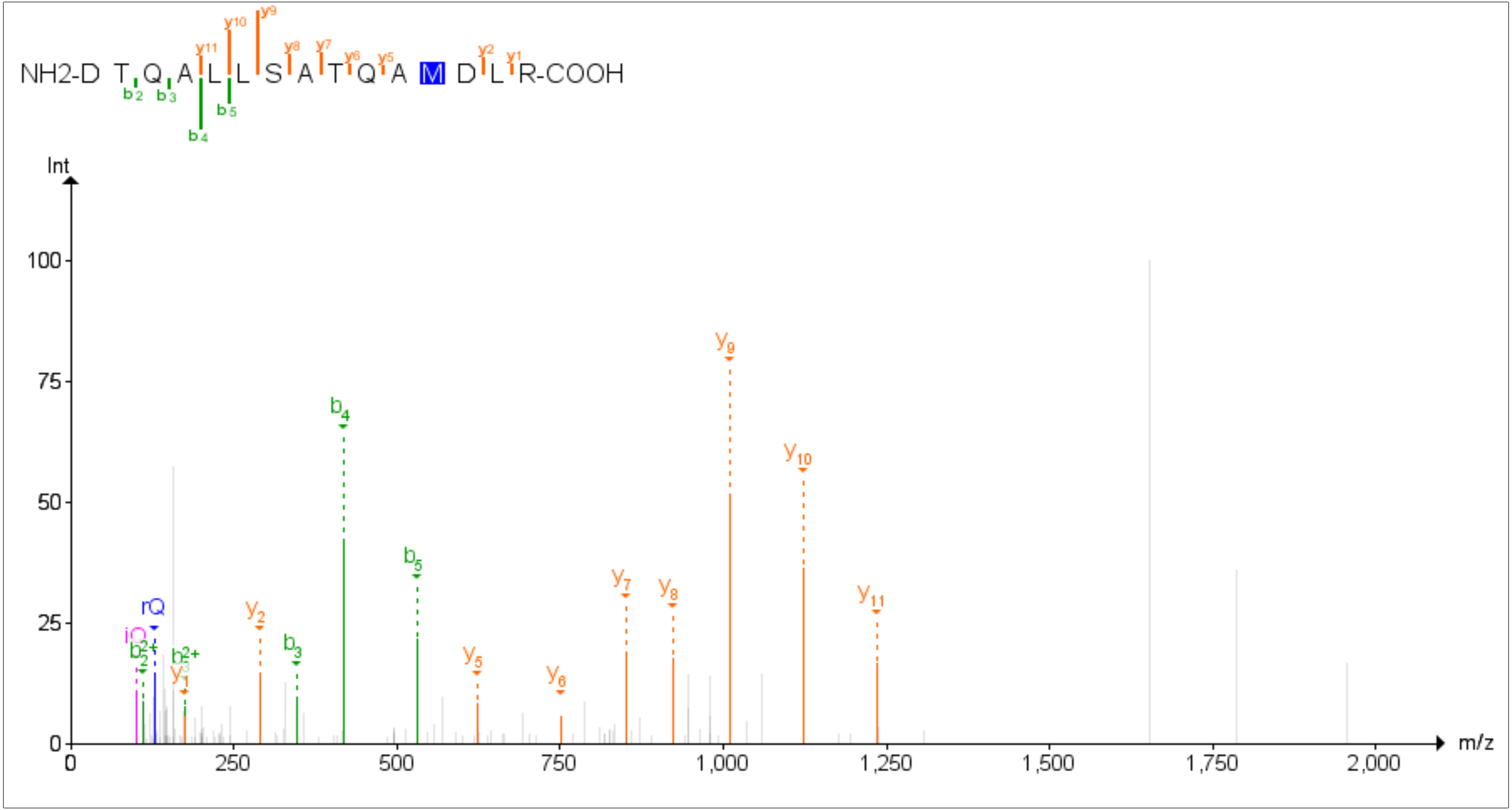

NH2-D T Q A L L S A T Q A M D L R-COOH
Int
m/z
0
25
50
75
100
250
500
750
1,000
1,250
1,500
1,750
2,000
b4
b5
b3
y9
y10
y11
y7
y8
y5
y6
y2
y1
rQ

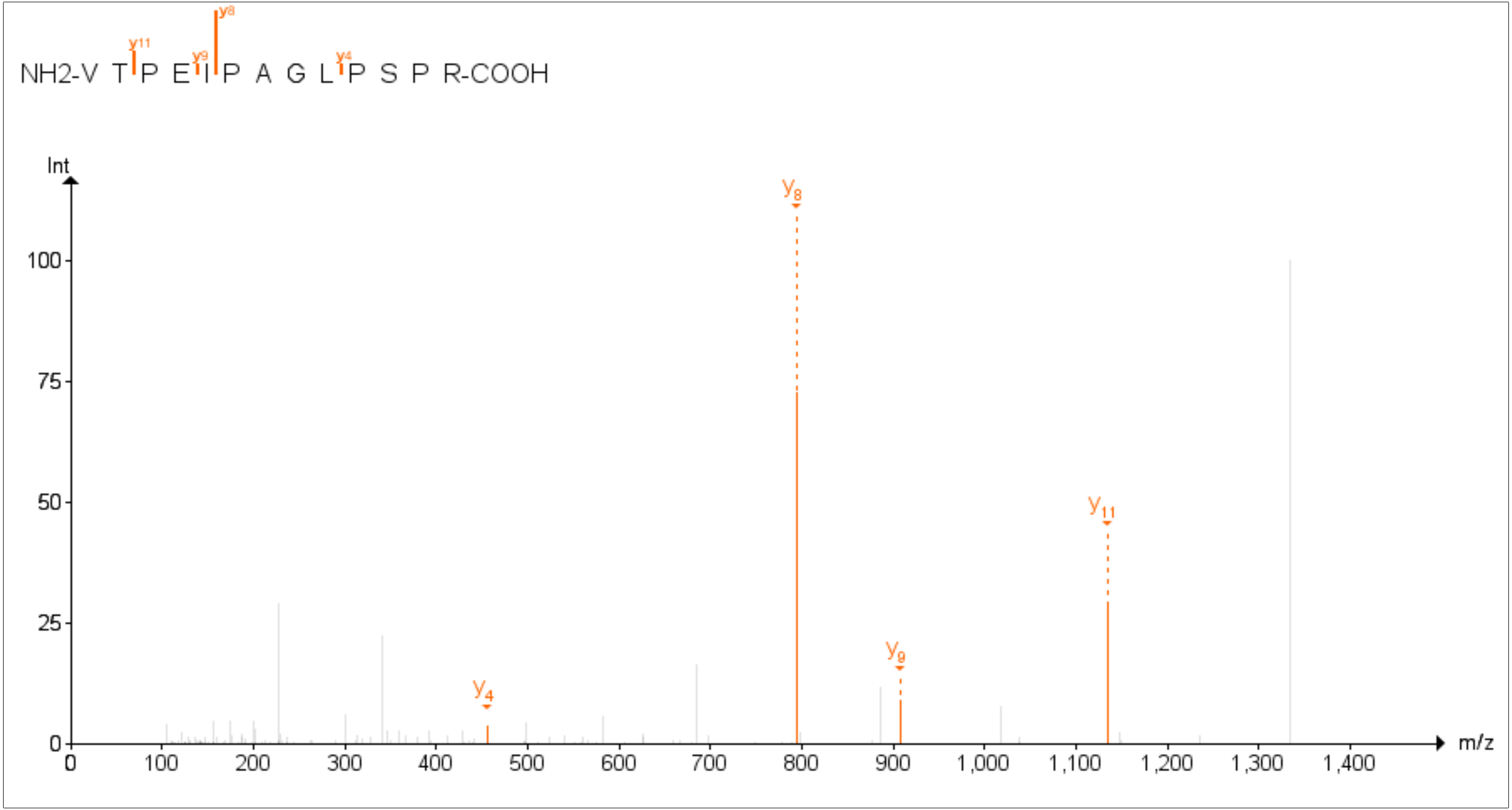

NH2-V T P E I P A G L P S P R-COOH
y11
y9
y8
y4
Int
100
75
50
25
0
y8
y9
y11
y4
0
100
200
300
400
500
600
700
800
900
1,000
1,100
1,200
1,300
1,400
m/z

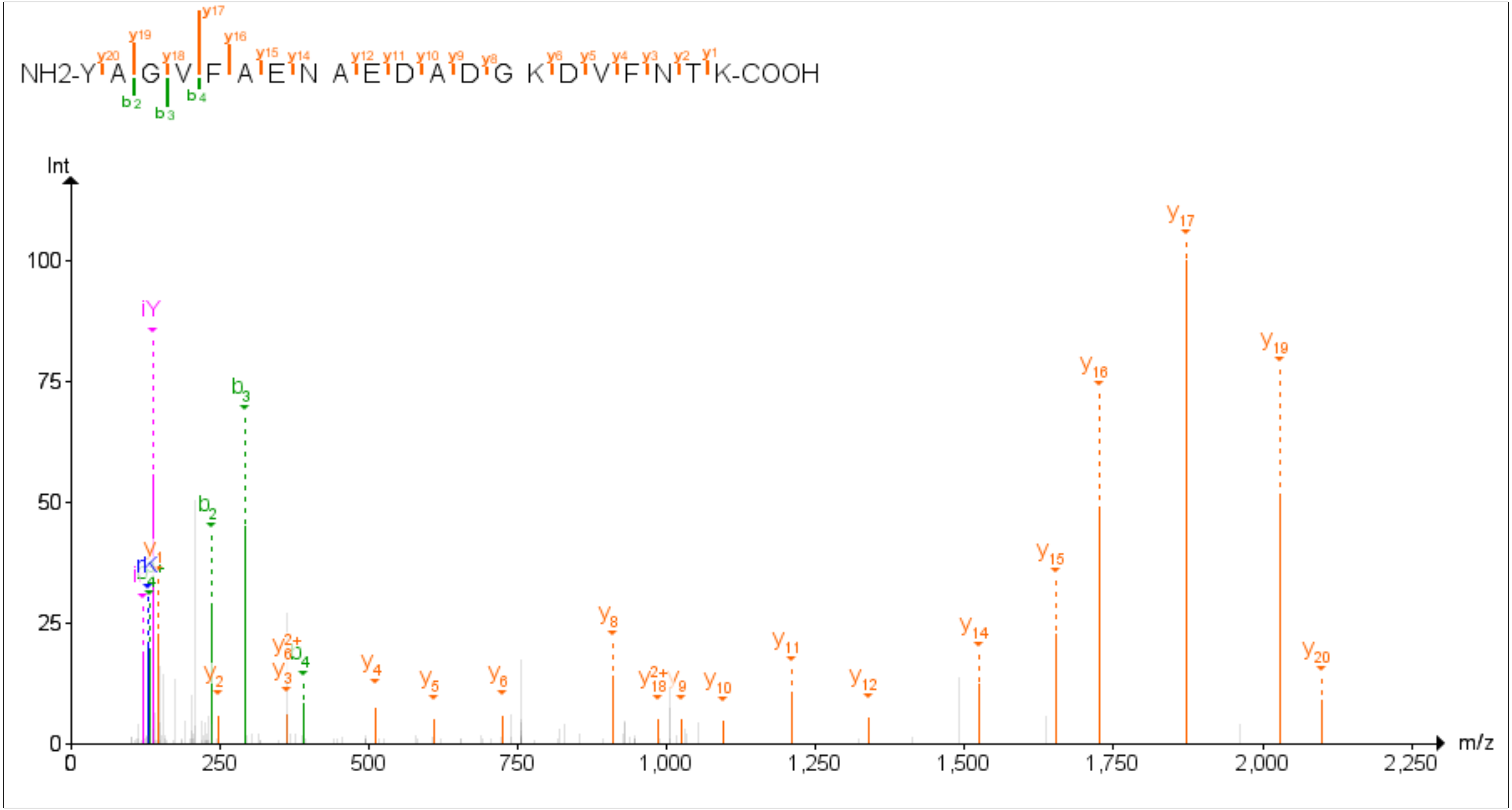

NH2-Y A G V F A E N A E D A D G K D V F N T K-COOH
Int
m/z
0
25
50
75
100
0
250
500
750
1,000
1,250
1,500
1,750
2,000
2,250
iY
b_3
b_2
b_4
y_2
y_3
y_4
y_5
y_6
y_8
y_9
y_{10}
y_{11}
y_{12}
y_{14}
y_{15}
y_{16}
y_{17}
y_{19}
y_{20}
y_{18}^{2+}

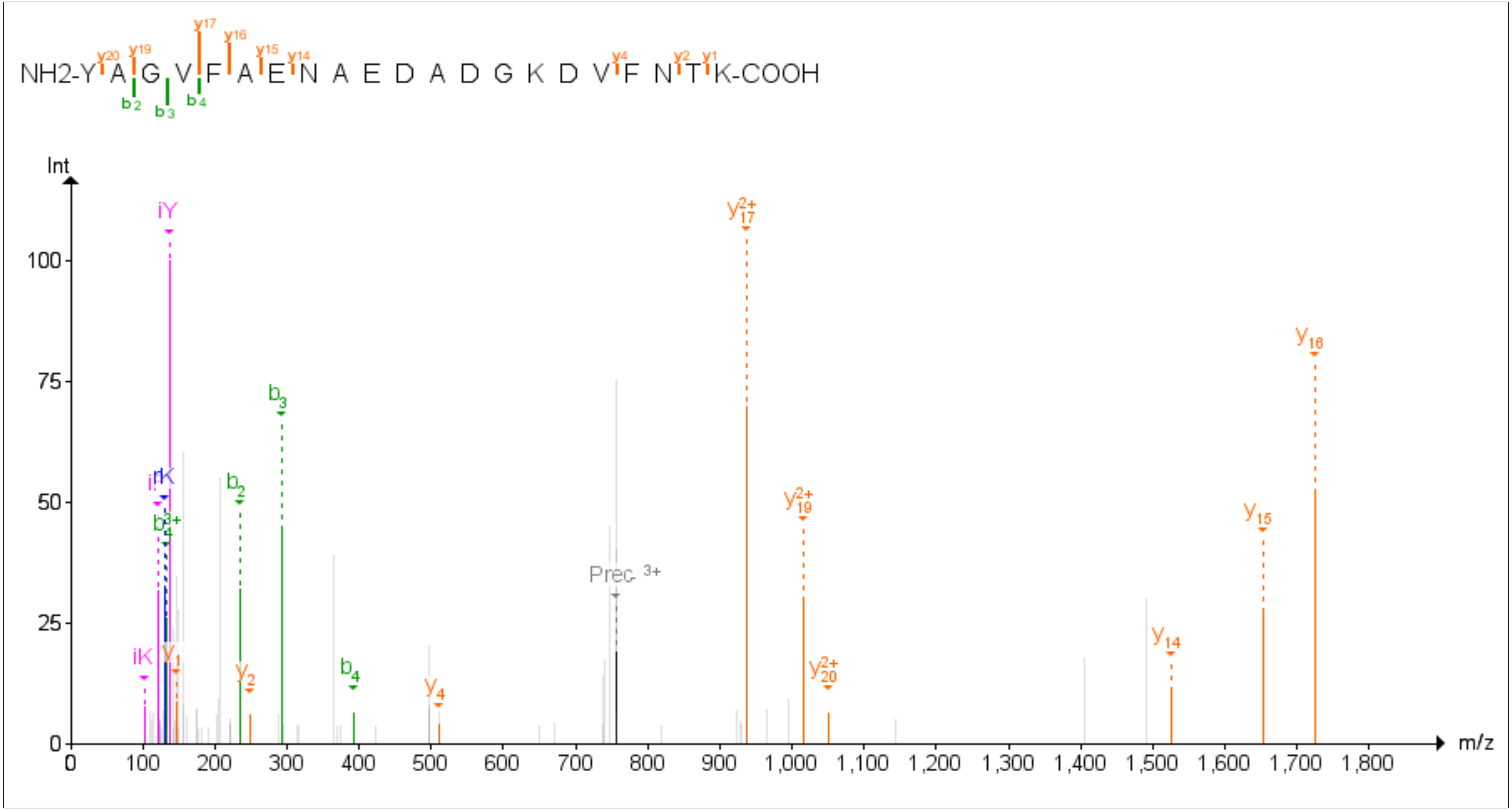

NH2-Y A G V F A E N A E D A D G K D V F N T K-COOH
y20 y19 y17 y16 y15 y14 y4 y2 y1
b2 b3 b4
Int
100
75
50
25
0
iY
iK
rK
b4 3+
iK
y1
b2
b3
y2
b4
y4
Prec 3+
y17 2+
y19 2+
y20 2+
y14
y15
y16
0 100 200 300 400 500 600 700 800 900 1,000 1,100 1,200 1,300 1,400 1,500 1,600 1,700 1,800
m/z

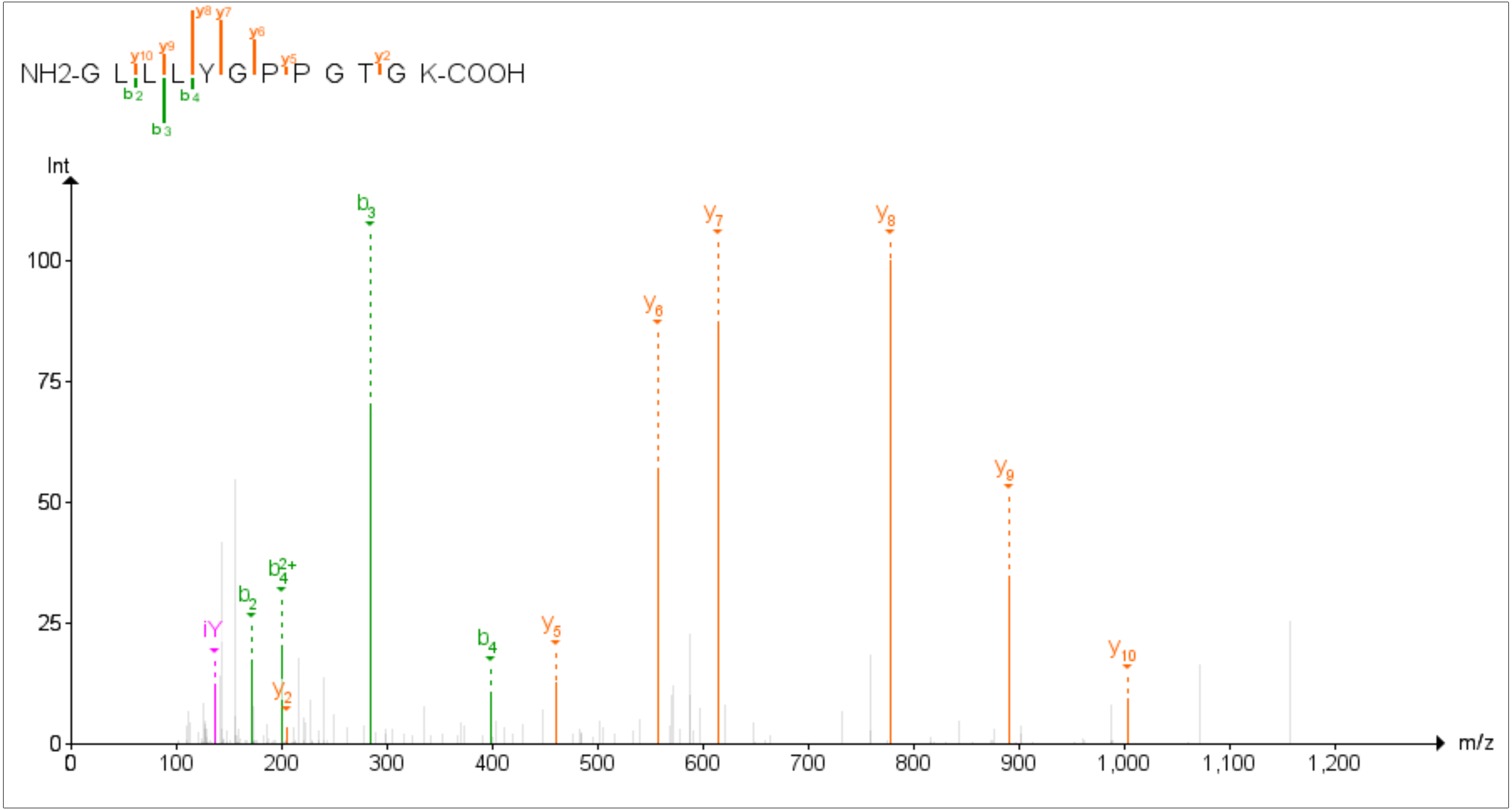

NH2-G L L L Y G P P G T G K-COOH
y10 y9 y8 y7 y6 y5 y2
b2 b3 b4
Int
m/z
b3
y7
y8
y6
y9
b4 2+
b2
iY
y2
b4
y5
y10
0
25
50
75
100
0
100
200
300
400
500
600
700
800
900
1,000
1,100
1,200

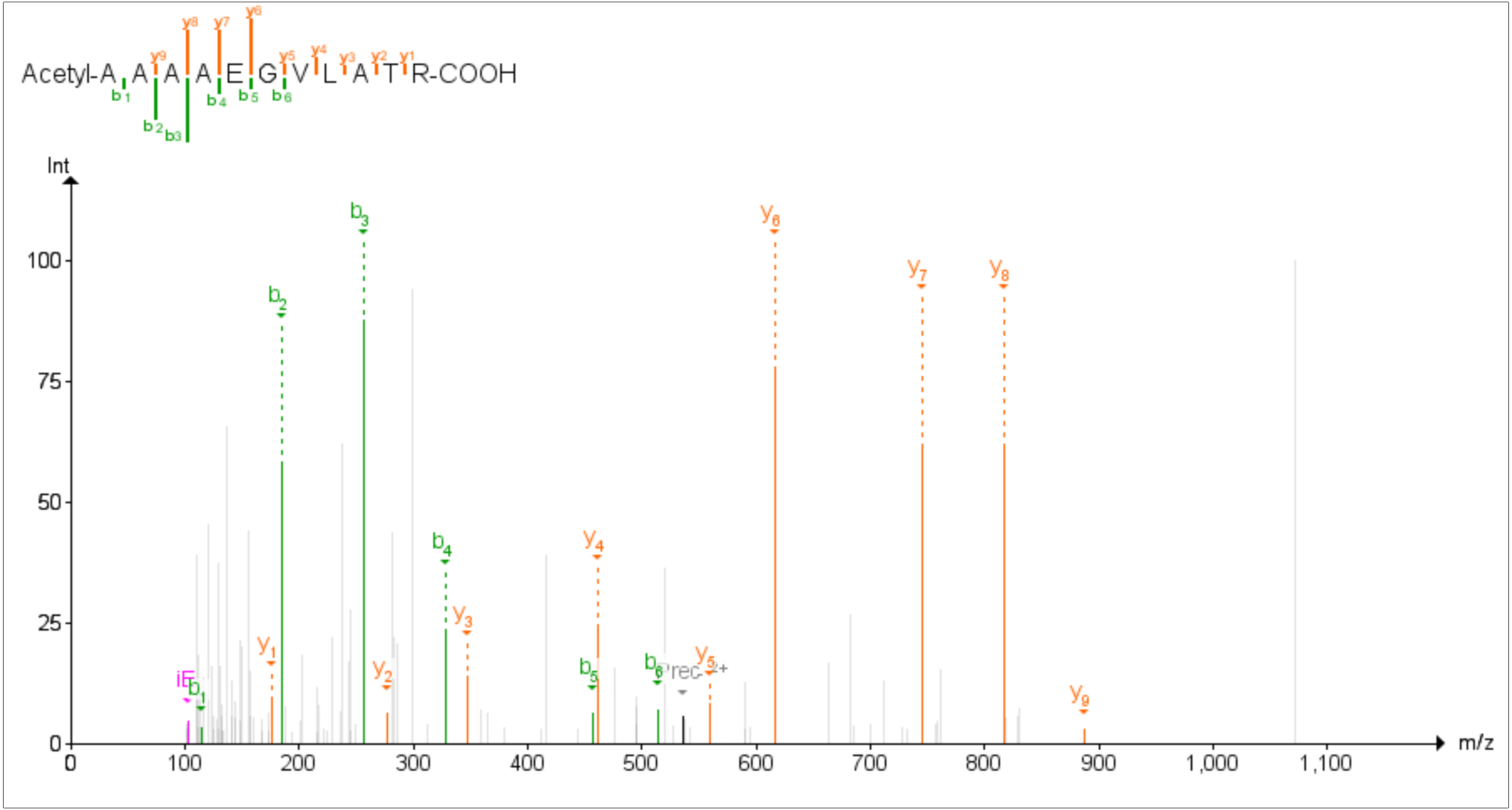

Acetyl-A A A A E G V L A T R-COOH
y9 y8 y7 y6 y5 y4 y3 y2 y1
b1 b2 b3 b4 b5 b6
Int
100
75
50
25
0
b3
b2
y6
y7
y8
b4
y4
y3
y1
y2
iE
b1
b5
b6
Prec 2+
y5
y9
0 100 200 300 400 500 600 700 800 900 1,000 1,100
m/z

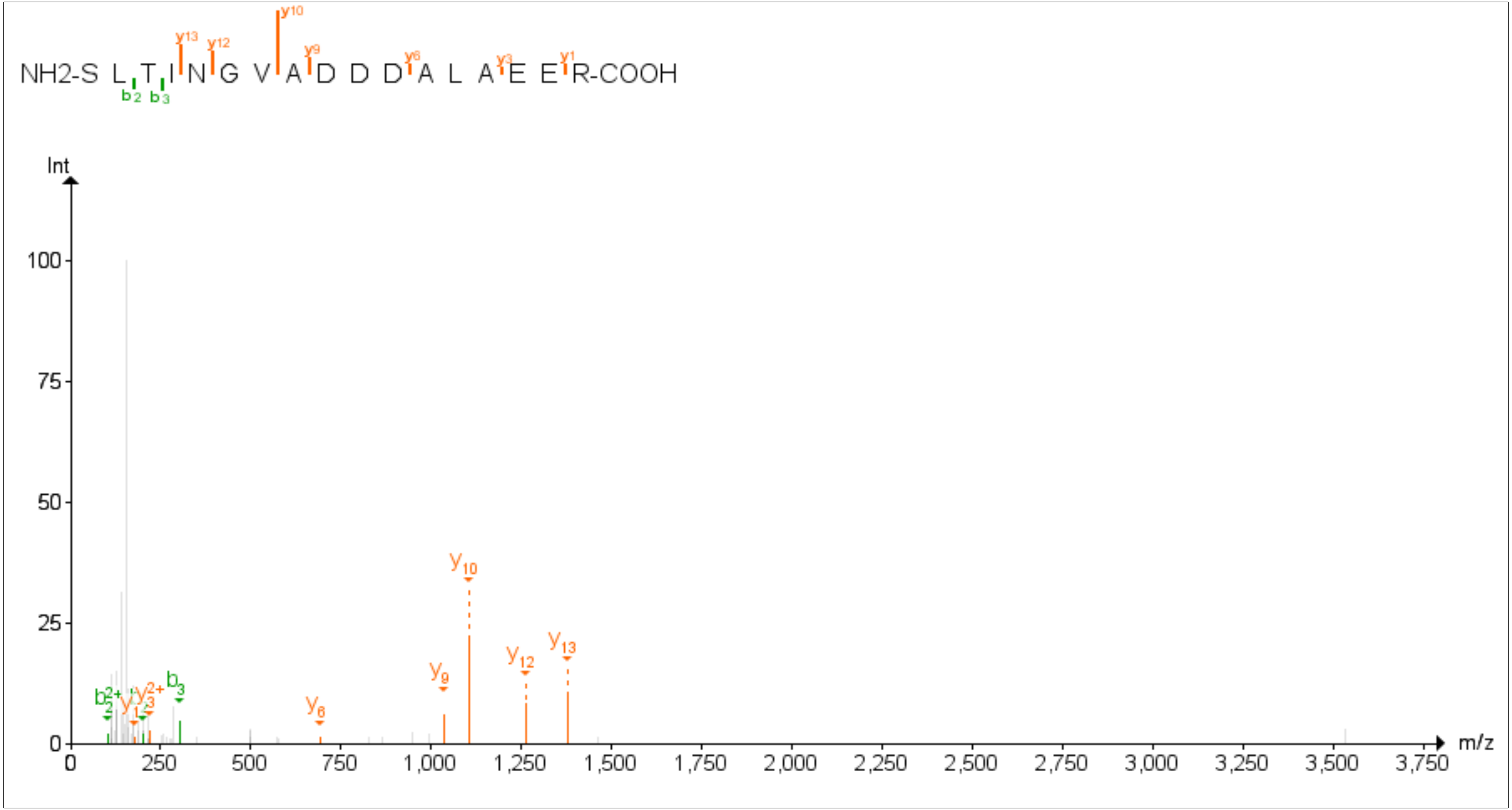

NH2-S L T I N G V A D D D A L A E E R-COOH
y13
y12
y10
y9
y6
y3
y1
b2
b3
Int
100
75
50
25
0
b_2^{2+}
y_1
y_3^{2+}
b_3
y_6
y_9
y_{10}
y_{12}
y_{13}
0
250
500
750
1,000
1,250
1,500
1,750
2,000
2,250
2,500
2,750
3,000
3,250
3,500
3,750
m/z

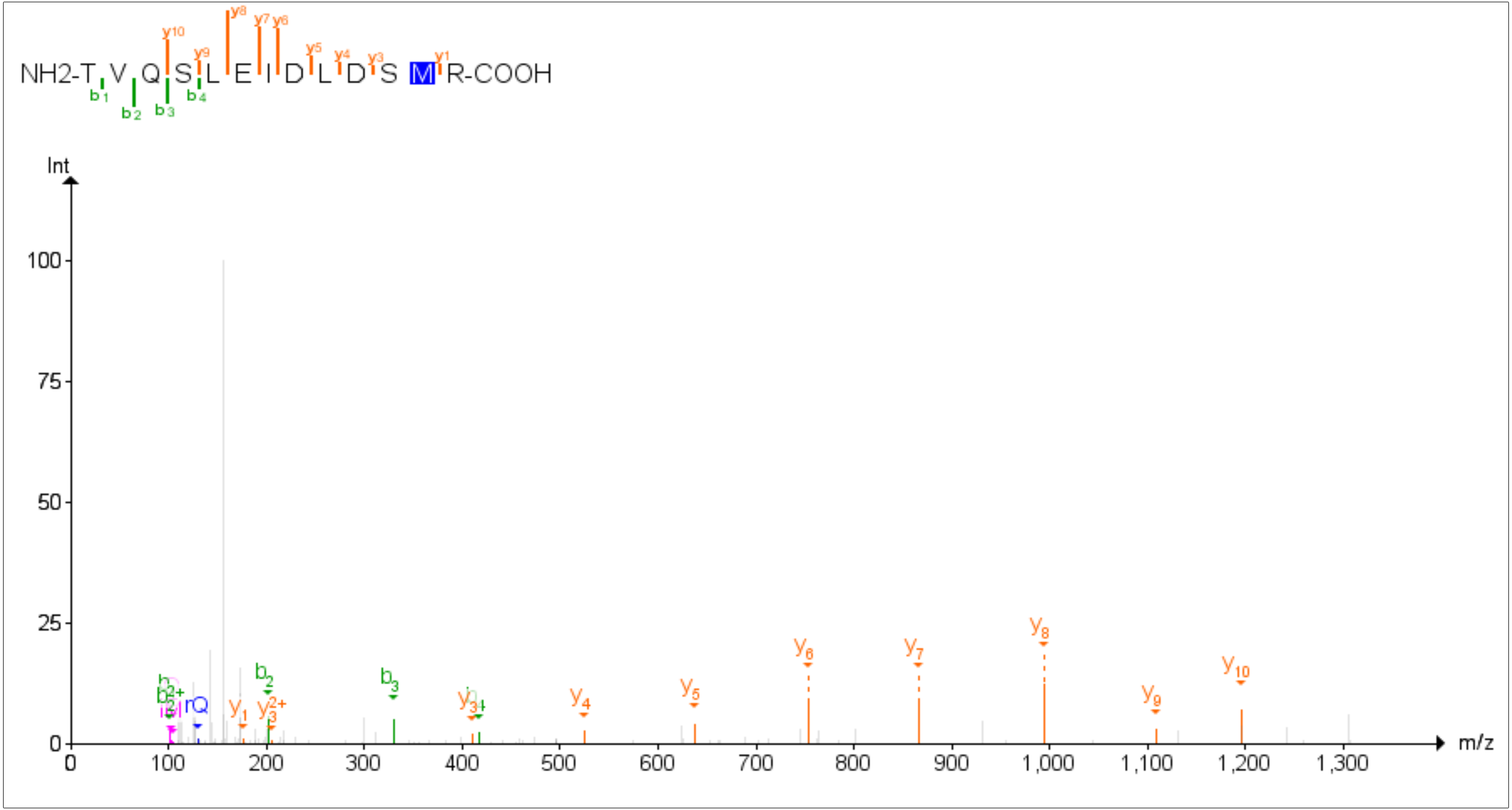
NH2-T V Q S L E I D L D S M R-COOH
Int
100
75
50
25
0
m/z
0
100
200
300
400
500
600
700
800
900
1,000
1,100
1,200
1,300
y6
y7
y8
y9
y10
y4
y5
b3
b2
rQ

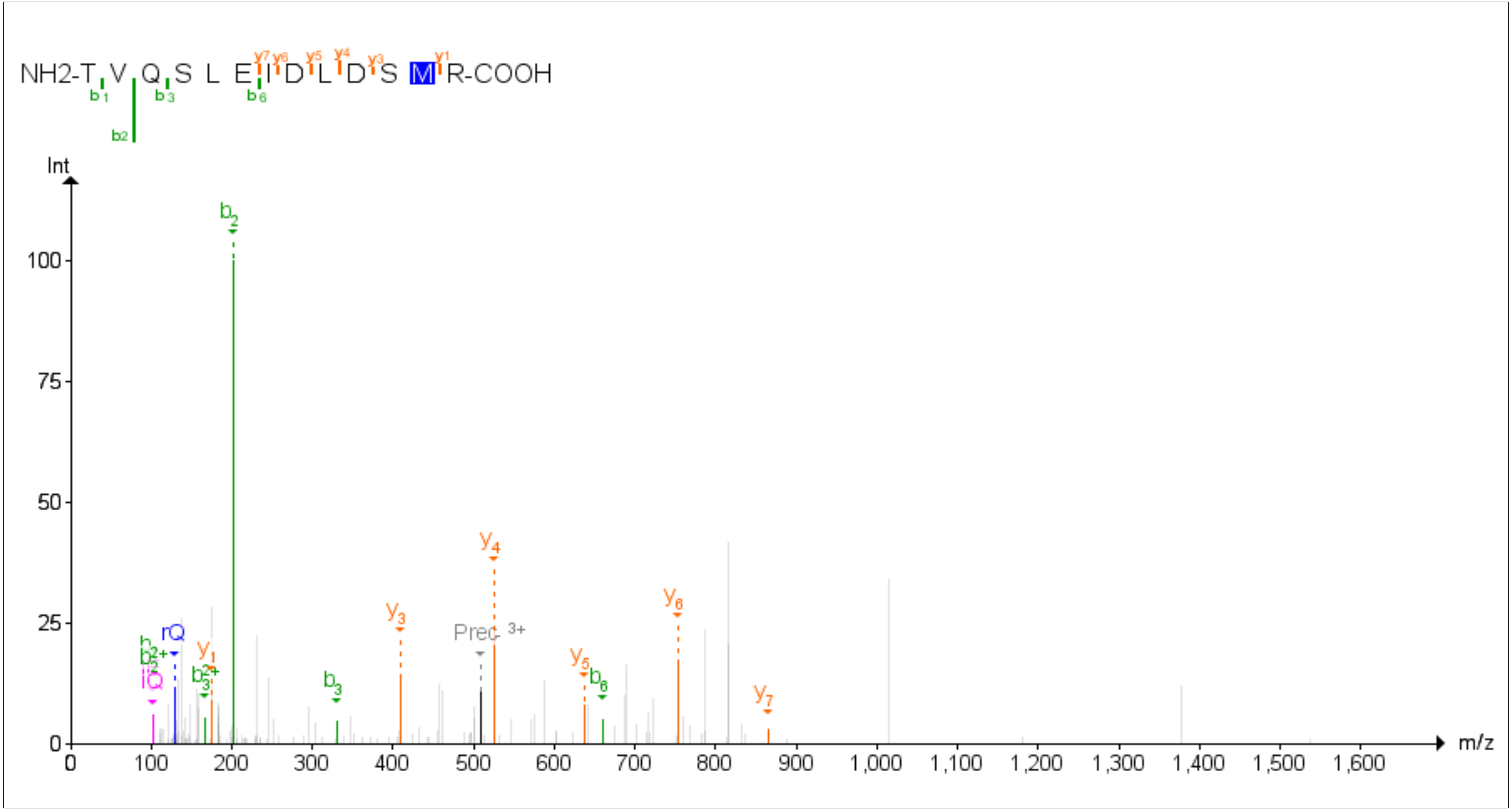

NH2-T V Q S L E I D L D S M R-COOH
b1 b2 b3 b6
y7 y6 y5 y4 y3 y1
Int
100
75
50
25
0
b2
y4
y6
y3
Prec 3+
y5
b6
y7
b3
y1
rQ
iQ
m/z
0 100 200 300 400 500 600 700 800 900 1,000 1,100 1,200 1,300 1,400 1,500 1,600

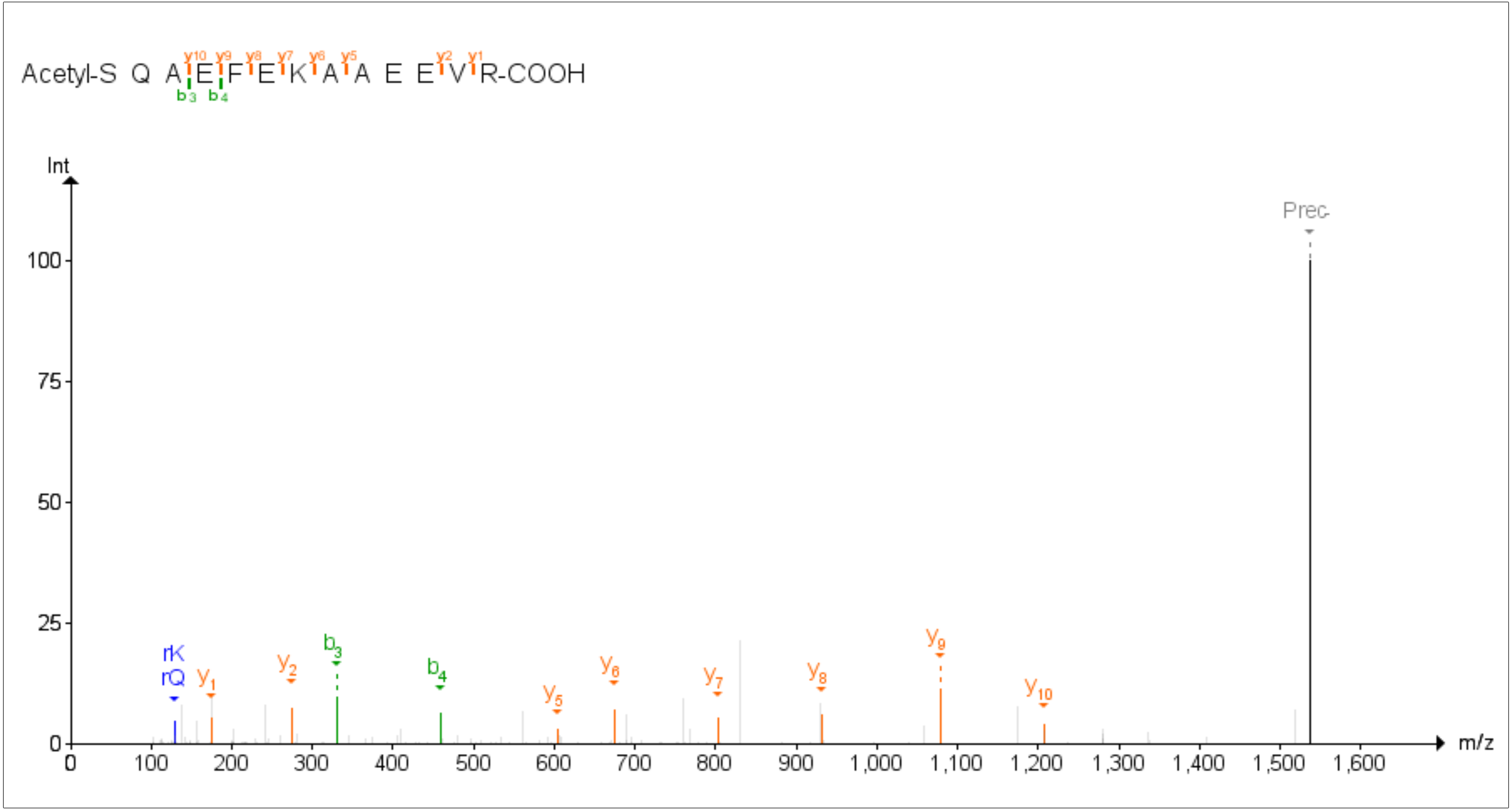

Acetyl-S Q A E F E K A A E E V R-COOH
y10 y9 y8 y7 y6 y5 y2 y1
b3 b4
Int
100
75
50
25
0
Prec
rK
rQ
y1
y2
b3
b4
y5
y6
y7
y8
y9
y10
0
100
200
300
400
500
600
700
800
900
1,000
1,100
1,200
1,300
1,400
1,500
1,600
m/z

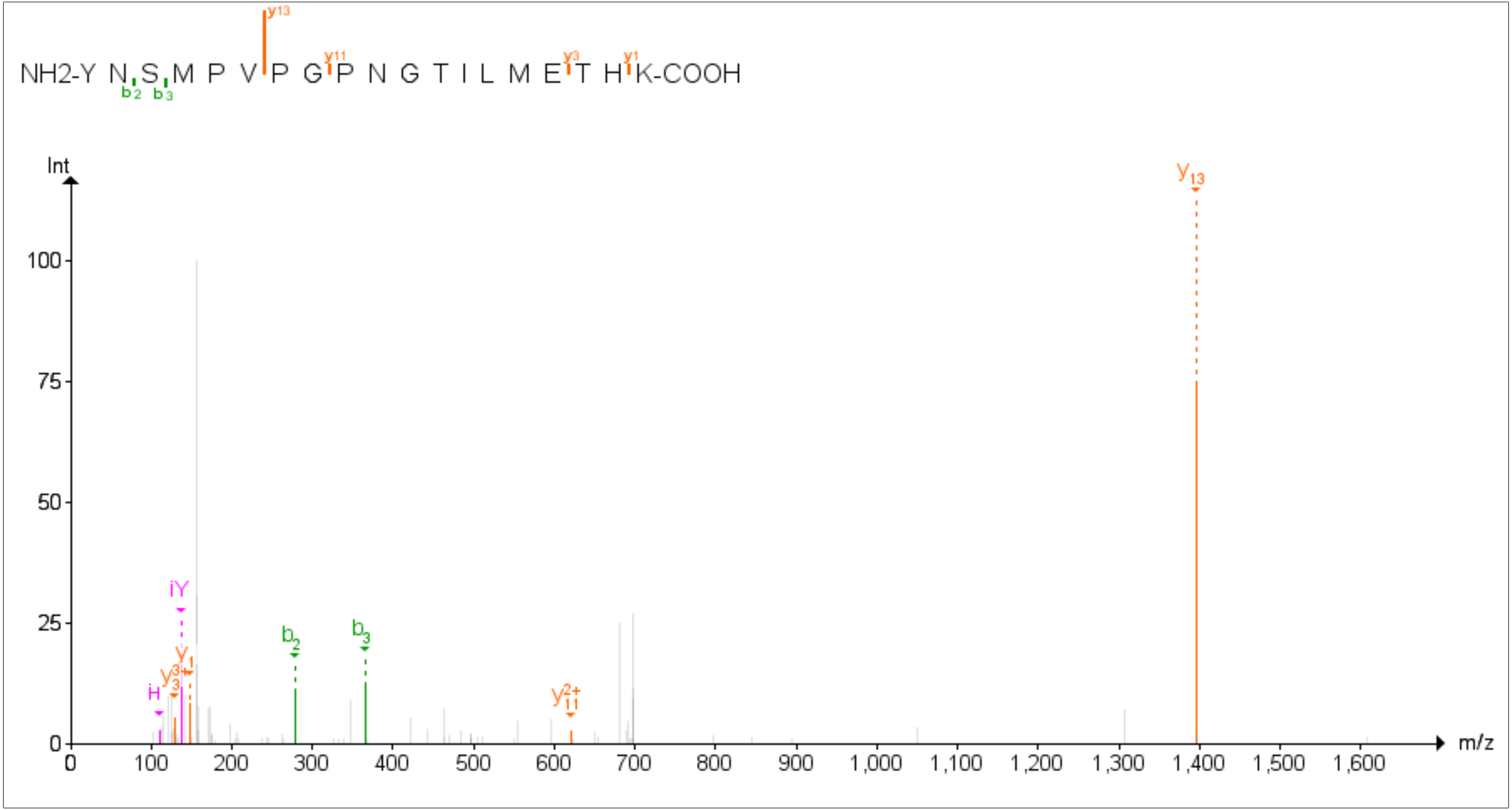

NH2-Y N S M P V P G P N G T I L M E T H K-COOH
y13
y11
y3
y1
b2
b3
Int
m/z
y13
iY
iH
y3 3+
y1
b2
b3
y11 2+
0
25
50
75
100
0
100
200
300
400
500
600
700
800
900
1,000
1,100
1,200
1,300
1,400
1,500
1,600

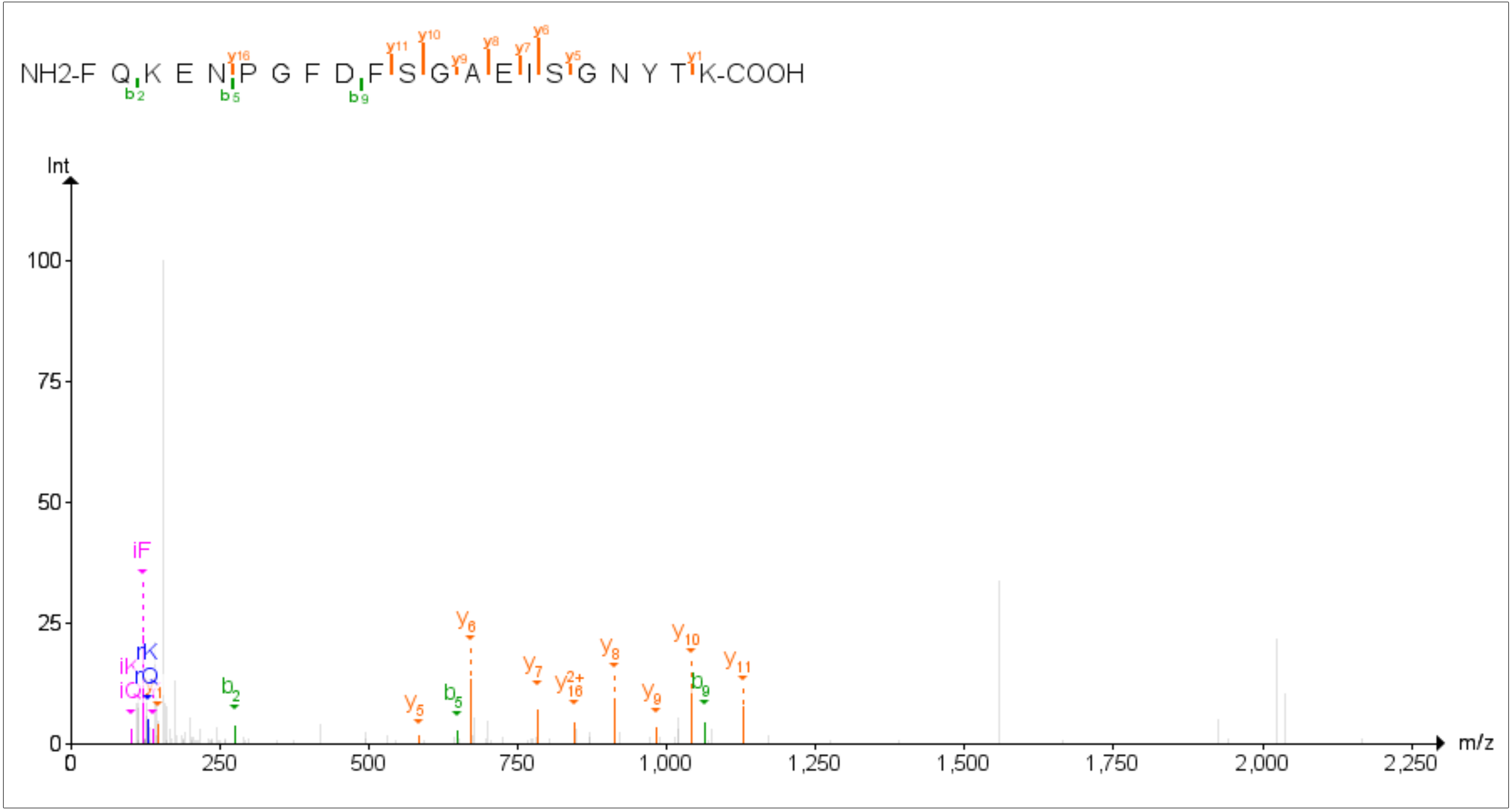

NH2-F Q K E N P G F D F S G A E I S G N Y T K-COOH
b2
b5
b9
y16
y11
y10
y9
y8
y7
y6
y5
y1
Int
100
75
50
25
0
iF
rK
iK
rQ
iQ
y1
b2
y5
b5
y6
y7
y16 2+
y8
y9
y10
b9
y11
0
250
500
750
1,000
1,250
1,500
1,750
2,000
2,250
m/z

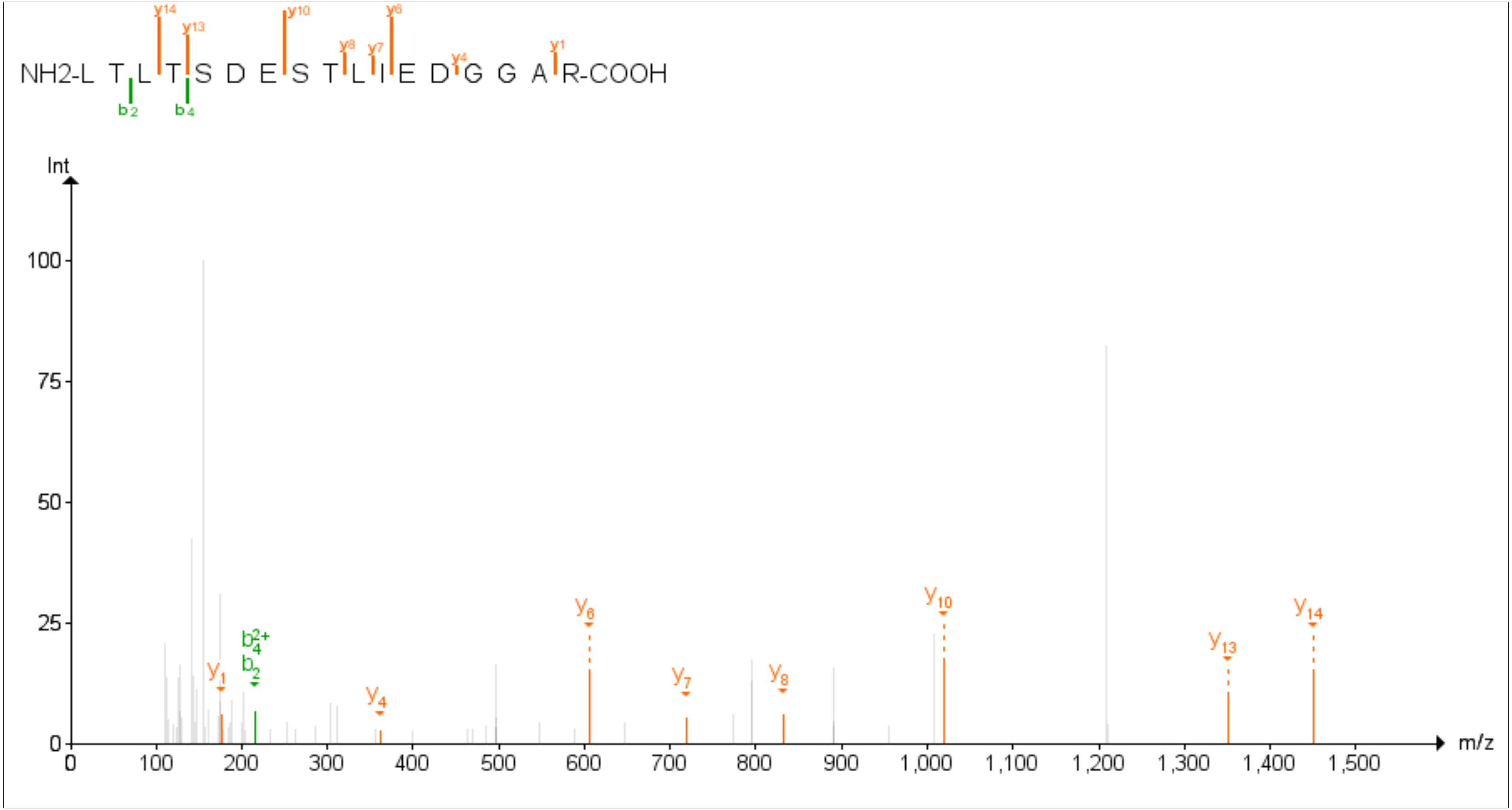

NH2-L T L T S D E S T L I E D G G A R-COOH
y14 y13 y10 y8 y7 y6 y4 y1
b2 b4
Int
100
75
50
25
0
m/z
0 100 200 300 400 500 600 700 800 900 1,000 1,100 1,200 1,300 1,400 1,500
b$_4^{2+}$
b$_2$
y$_1$
y$_4$
y$_6$
y$_7$
y$_8$
y$_{10}$
y$_{13}$
y$_{14}$

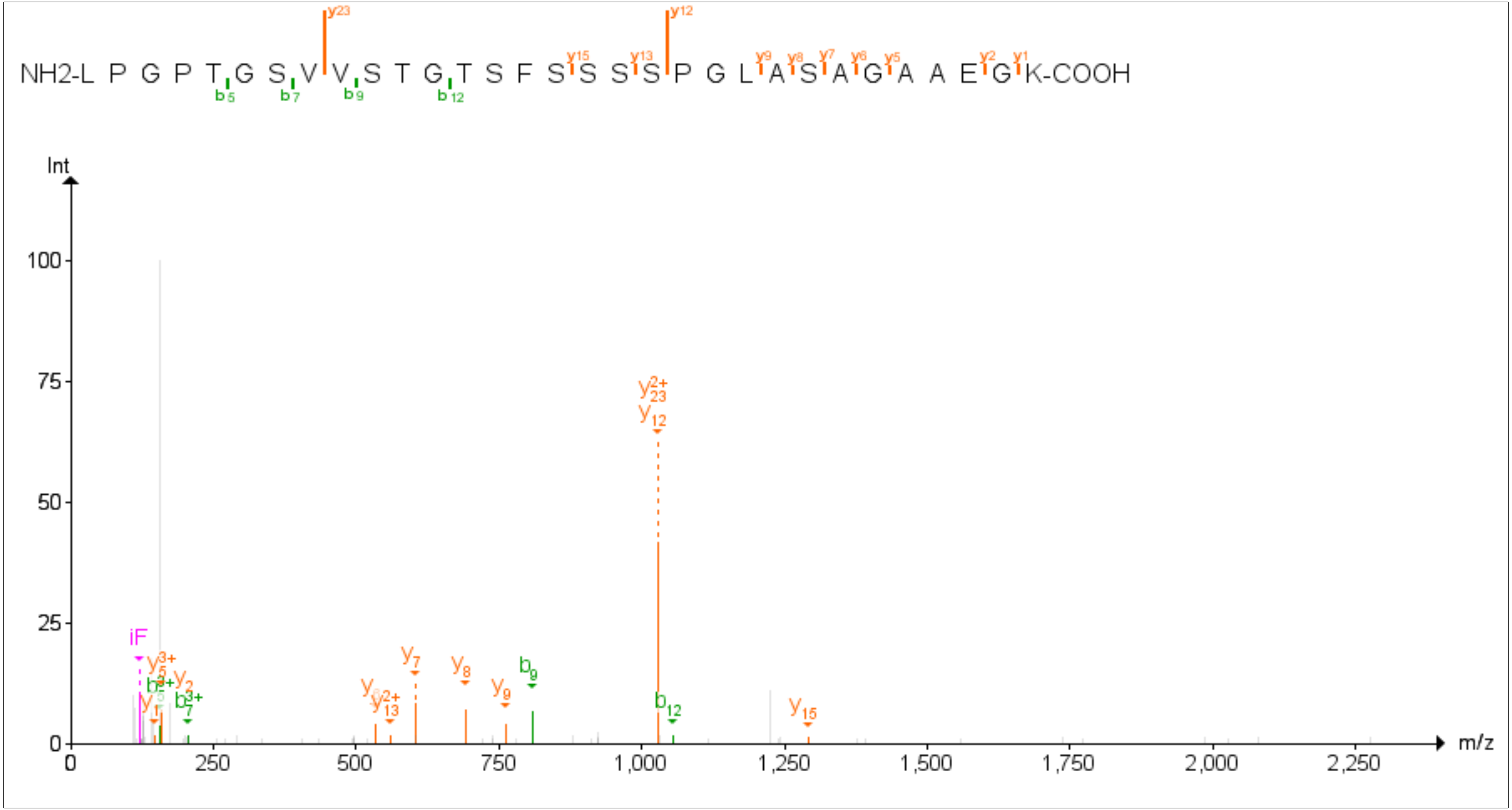
NH2-L P G P T G S V V S T G T S F S S S S P G L A S A G A A E G K-COOH
y23
y12
y15
y13
y9
y8
y7
y6
y5
y2
y1
b5
b7
b9
b12
Int
100
75
50
25
0
m/z
0
250
500
750
1,000
1,250
1,500
1,750
2,000
2,250
iF
y7
y8
y9
b9
b12
y15

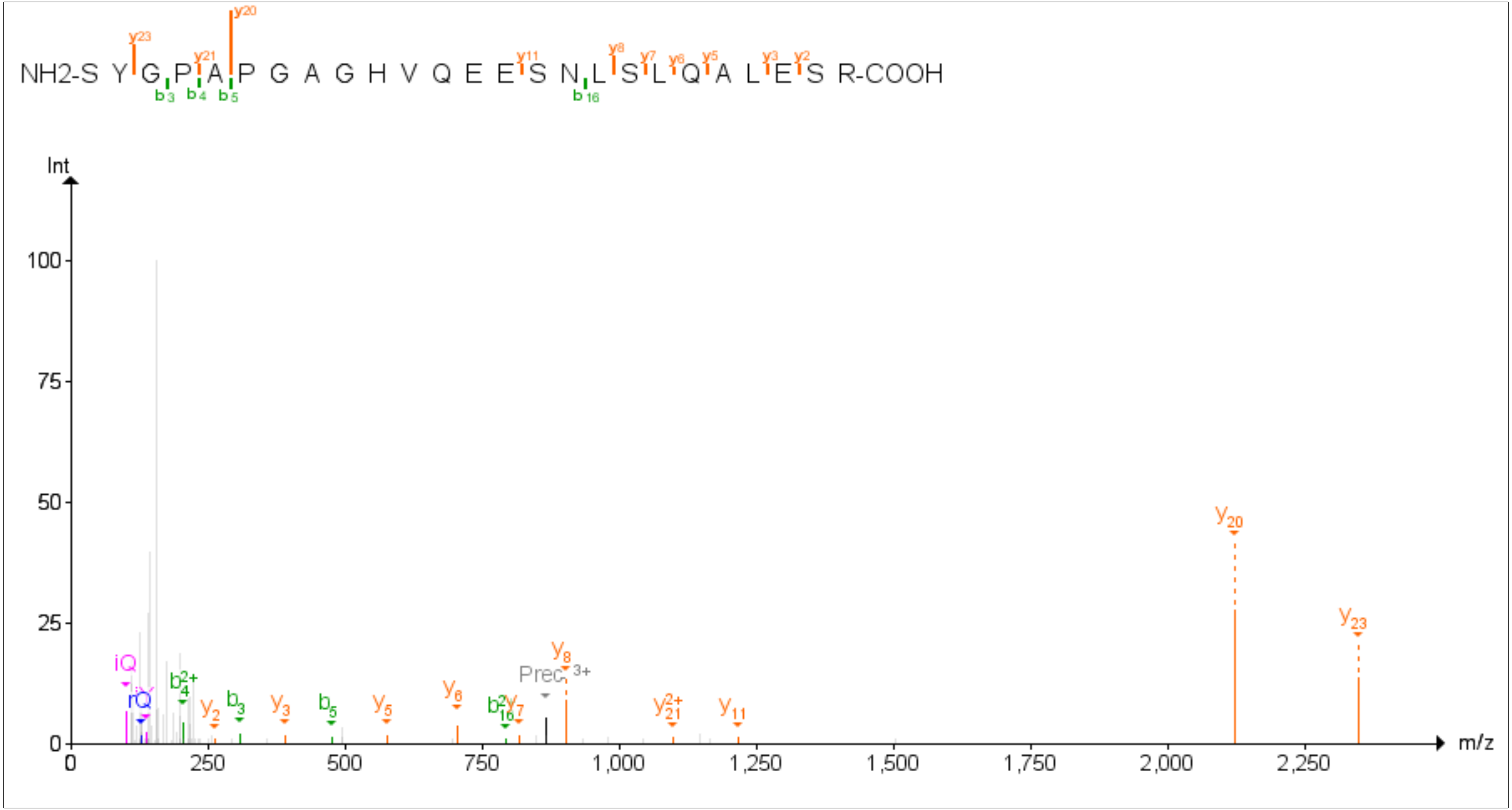

NH2-S Y G P A P G A G H V Q E E S N L S L Q A L E S R-COOH
y23 y21 y20 y11 y8 y7 y6 y5 y3 y2
b3 b4 b5 b16
Int
100
75
50
25
0
m/z
0 250 500 750 1,000 1,250 1,500 1,750 2,000 2,250
iQ
rQ
b4 2+
y2
b3
y3
b5
y5
y6
b16 2+
y7
Prec 3+
y8
y21 2+
y11
y20
y23

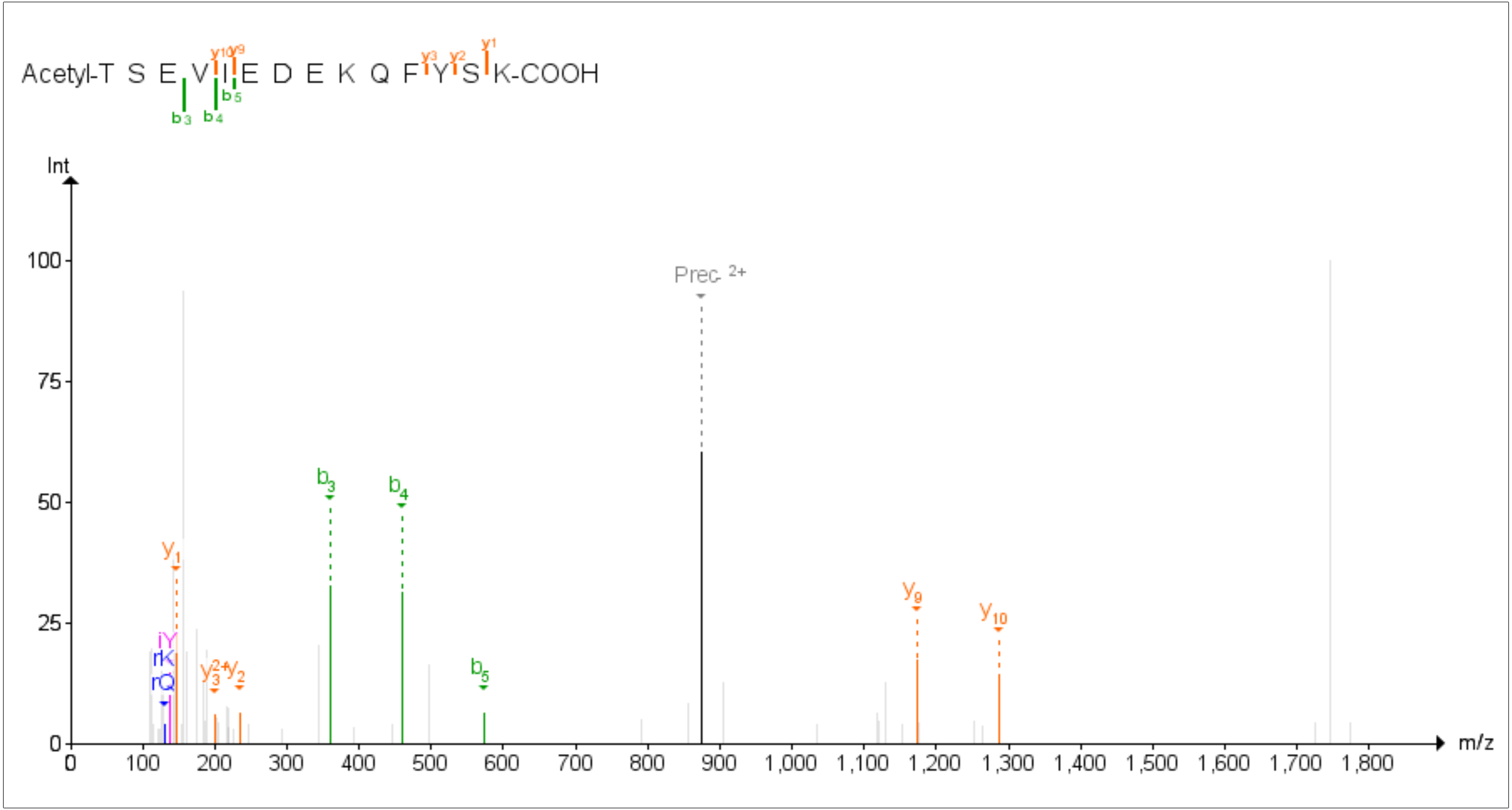

Acetyl-T S E V I E D E K Q F Y S K-COOH
y10 y9 y3 y2 y1
b3 b4 b5
Int
m/z
Prec. 2+
b3
b4
b5
y1
y9
y10
iY
rK
rQ
y3 2+
y2

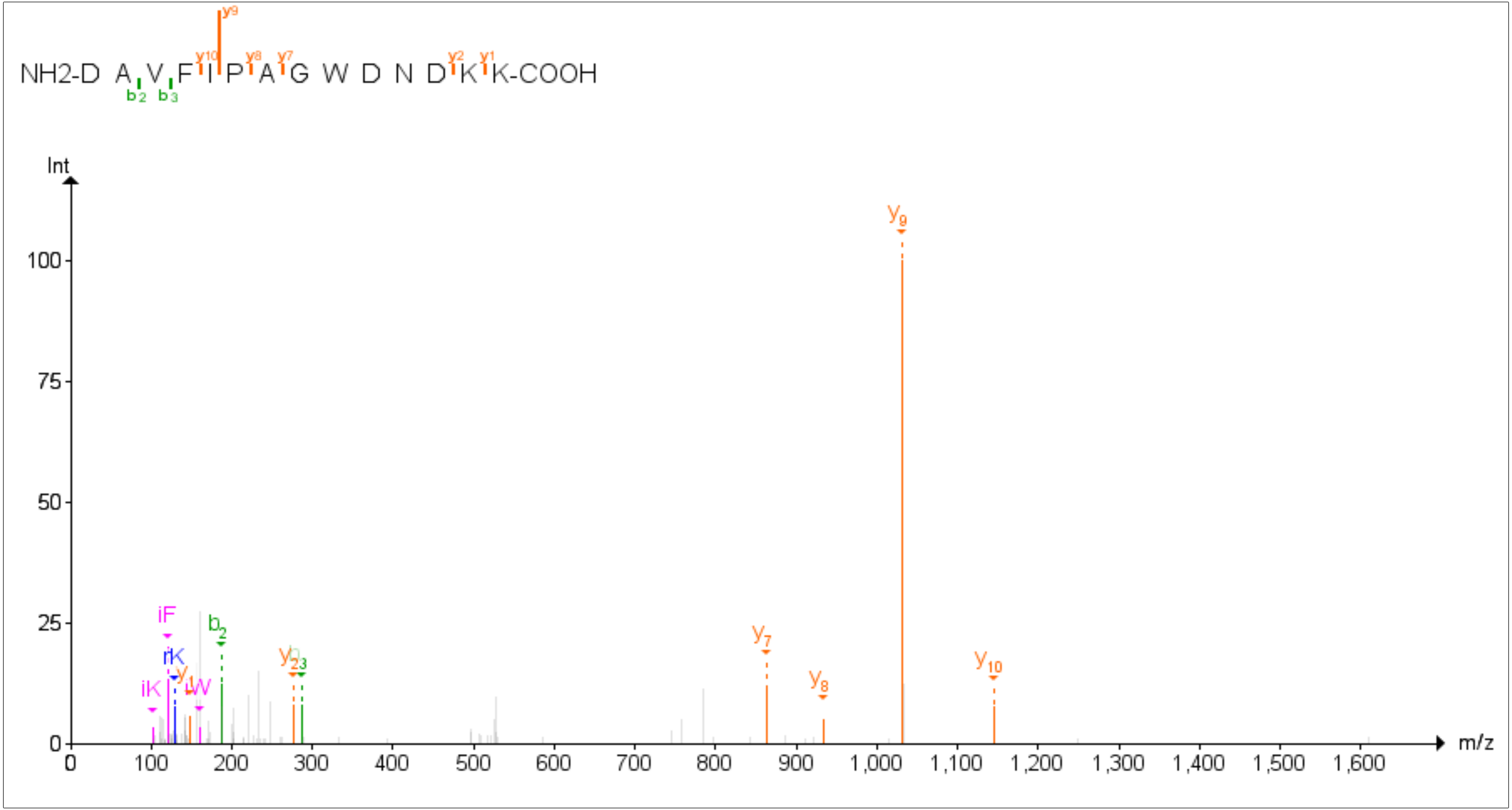

NH2-D A V F I P A G W D N D K K-COOH
y9
y10
y8
y7
y2
y1
b2
b3
Int
100
75
50
25
0
y9
iF
b2
rK
iK
y1
iW
y2
b3
y7
y8
y10
0
100
200
300
400
500
600
700
800
900
1,000
1,100
1,200
1,300
1,400
1,500
1,600
m/z

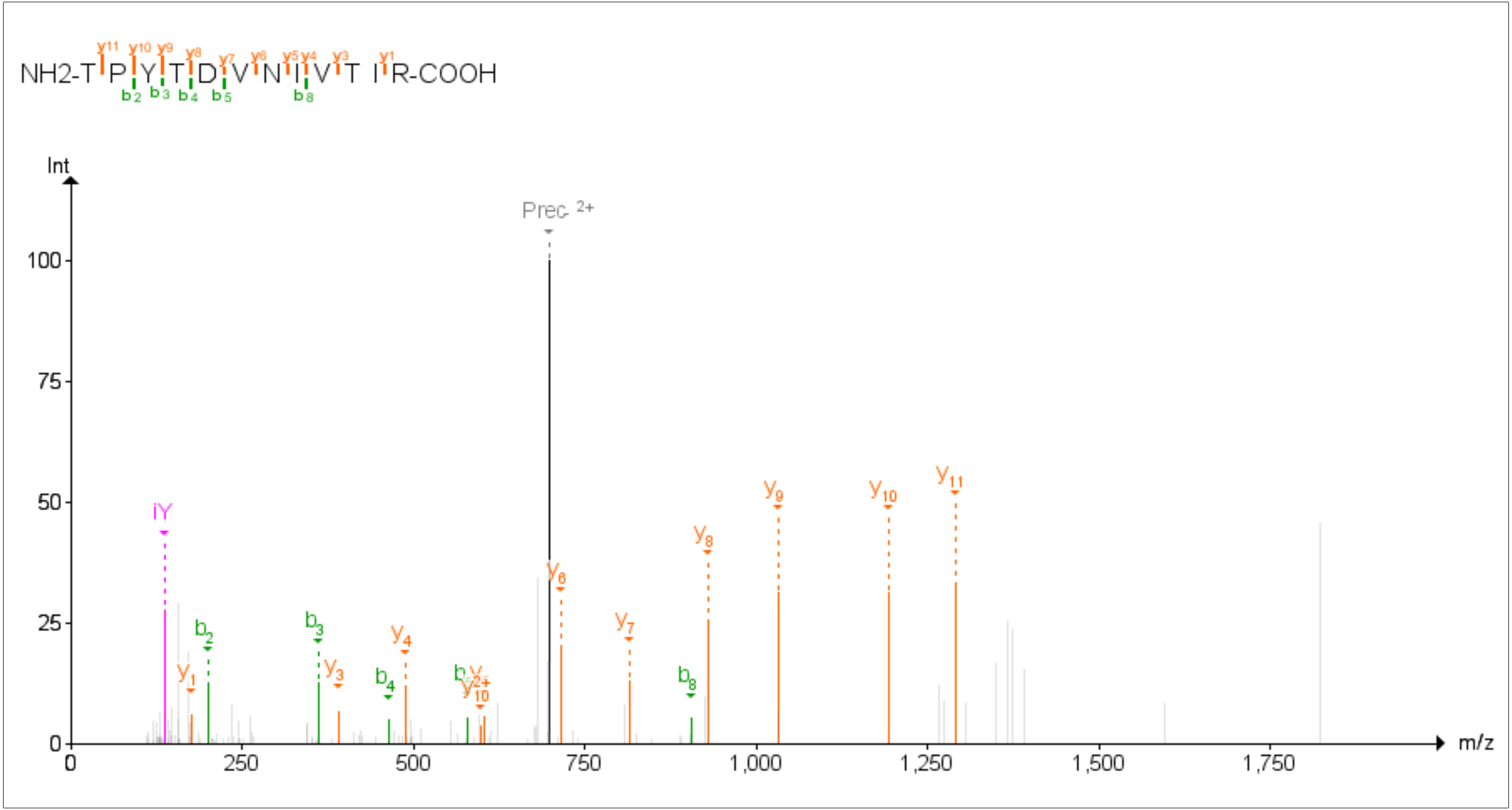

NH2-T P Y T D V N I V T I R-COOH
y11 y10 y9 y8 y7 y6 y5 y4 y3 y1
b2 b3 b4 b5 b8
Int
Prec 2+
iY
y1
b2
b3
y3
y4
b4
y6
y7
y8
b8
y9
y10
y11
m/z
0
25
50
75
100
0
250
500
750
1,000
1,250
1,500
1,750

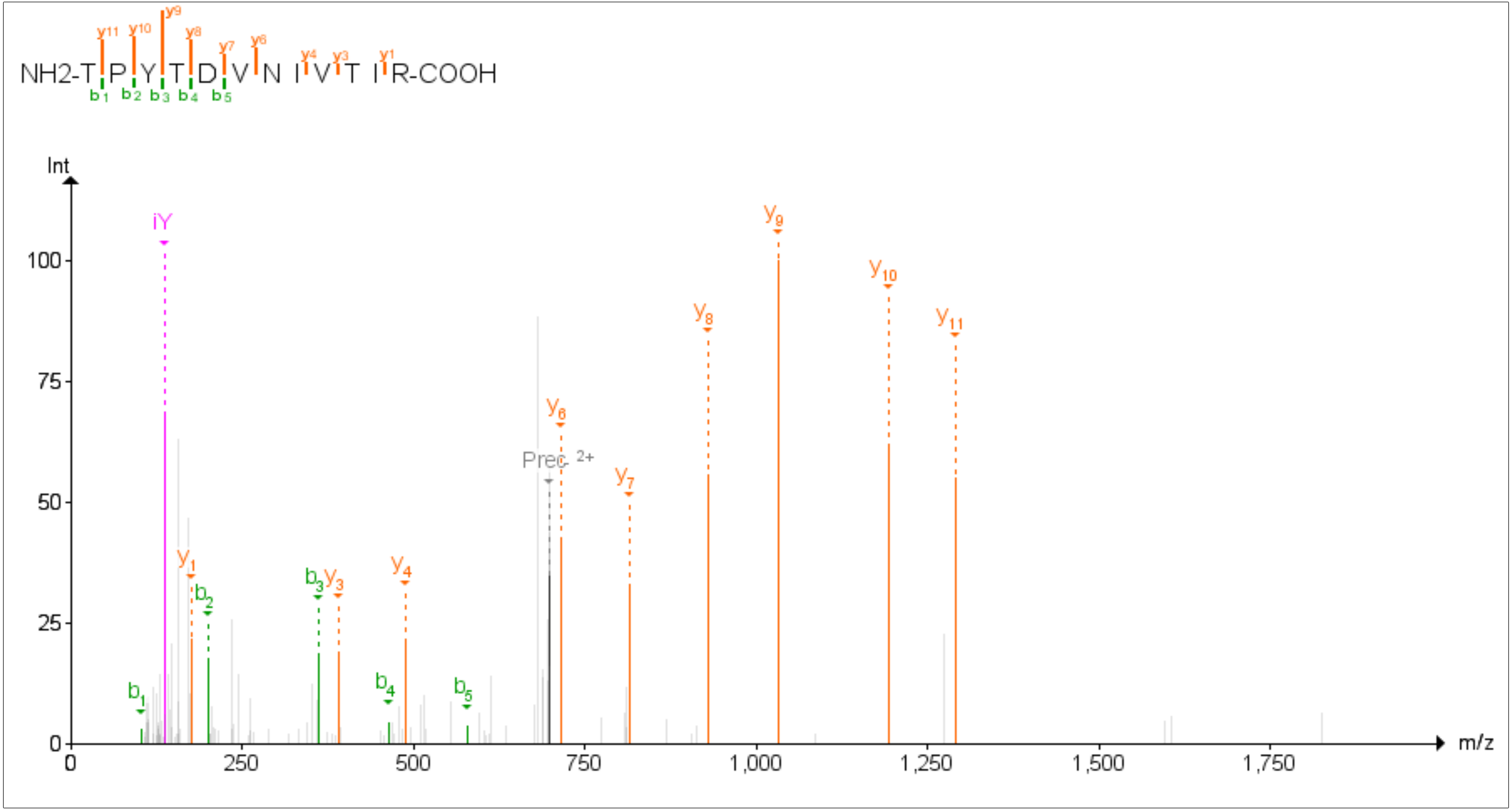

NH2-T P Y T D V N I V T I R-COOH
y11 y10 y9 y8 y7 y6 y4 y3 y1
b1 b2 b3 b4 b5
Int
m/z
iY
Prec 2+
y9
y10
y8
y11
y6
y7
y1
b2
b3
y3
y4
b1
b4
b5
0
25
50
75
100
0
250
500
750
1,000
1,250
1,500
1,750

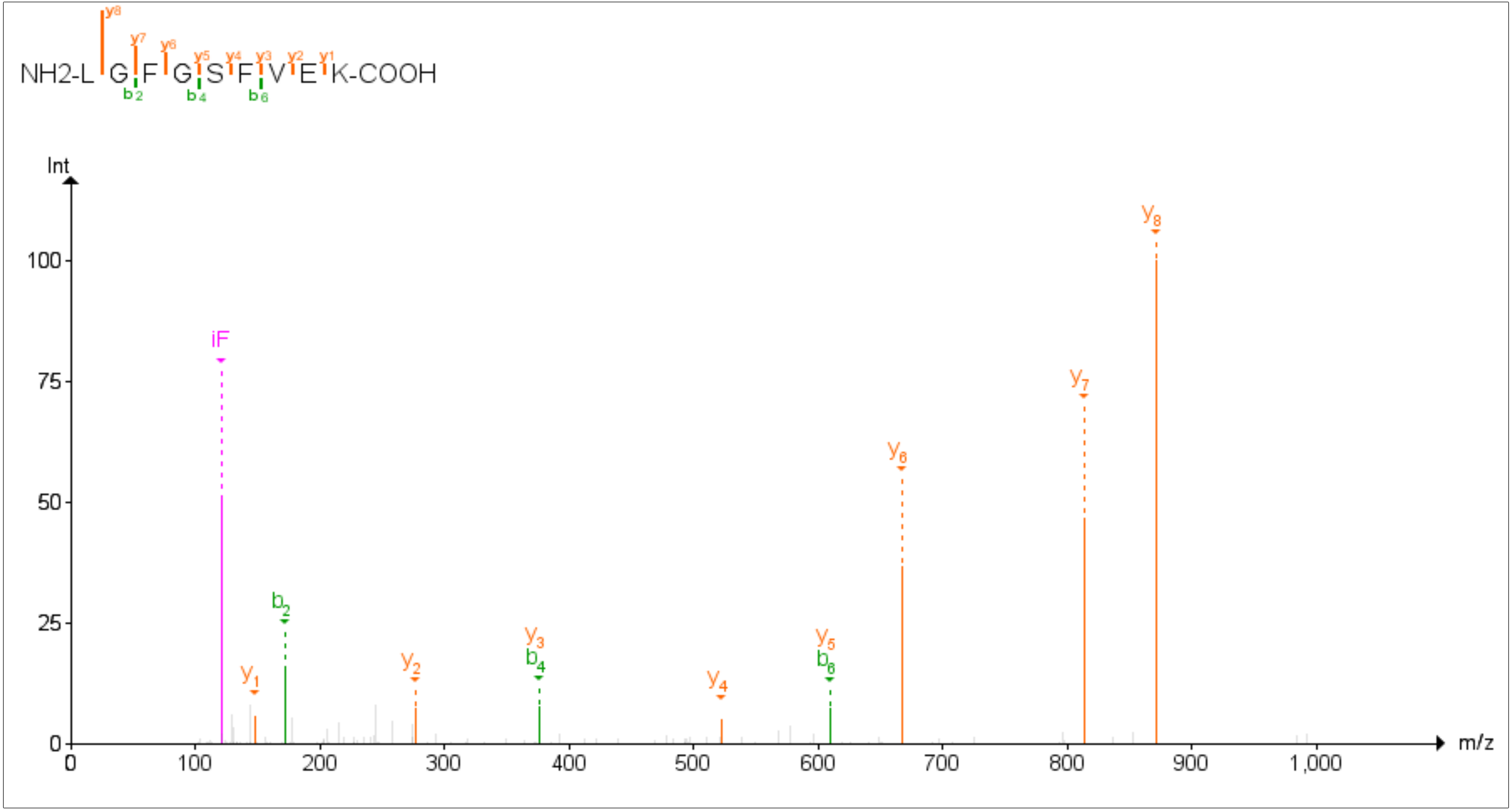
y8
y7
y6
y5
y4
y3
y2
y1
NH2-L G F G S F V E K-COOH
b2
b4
b6
Int
100
75
50
25
0
iF
y8
y7
y6
b2
y1
y2
y3
b4
y4
y5
b6
0
100
200
300
400
500
600
700
800
900
1,000
m/z

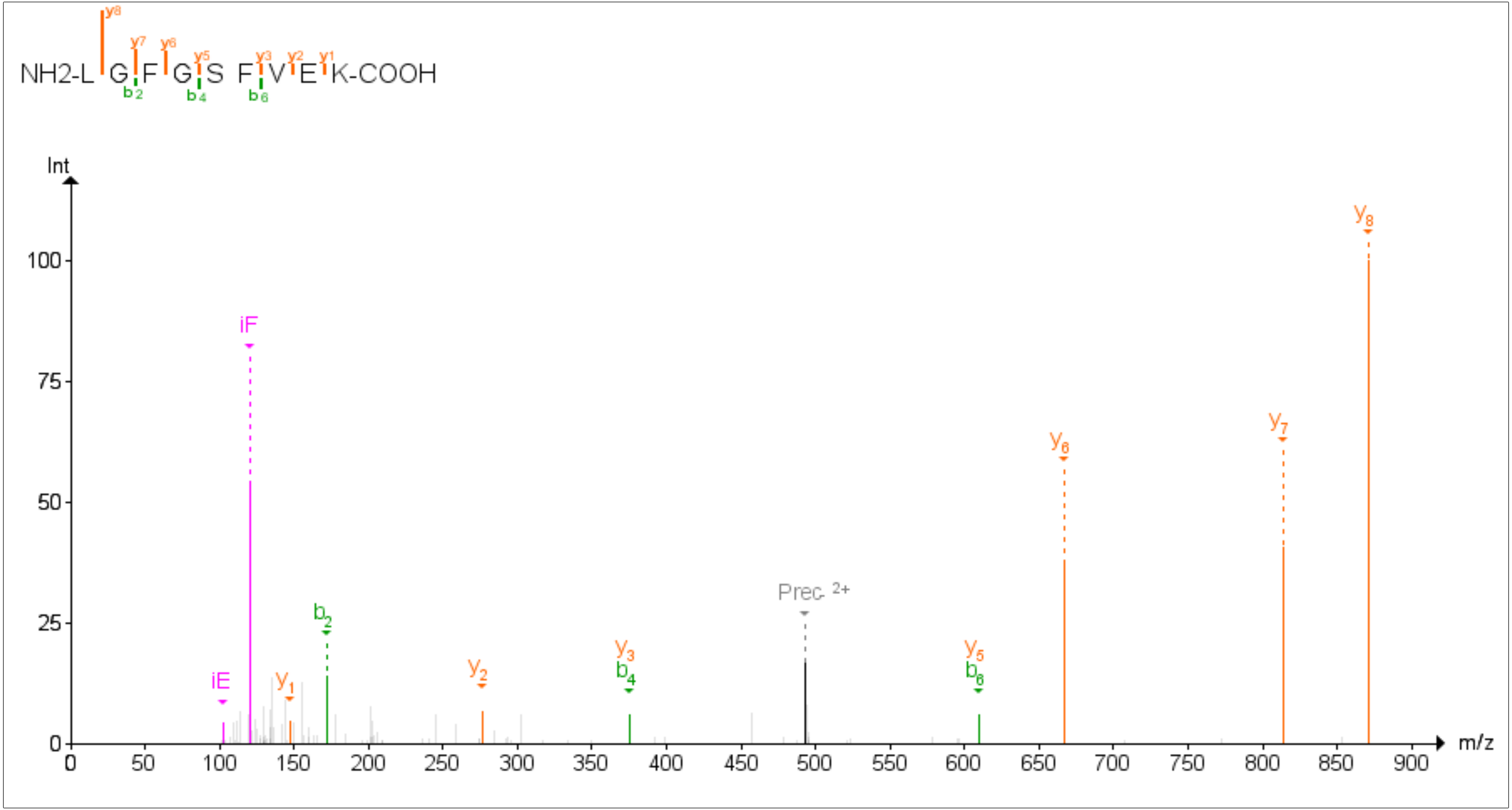

NH2-L G F G S F V E K-COOH
y8
y7
y6
y5
y3
y2
y1
b2
b4
b6
Int
m/z
iF
iE
y1
b2
y2
y3
b4
Prec 2+
y5
b6
y6
y7
y8
0
25
50
75
100
0
50
100
150
200
250
300
350
400
450
500
550
600
650
700
750
800
850
900

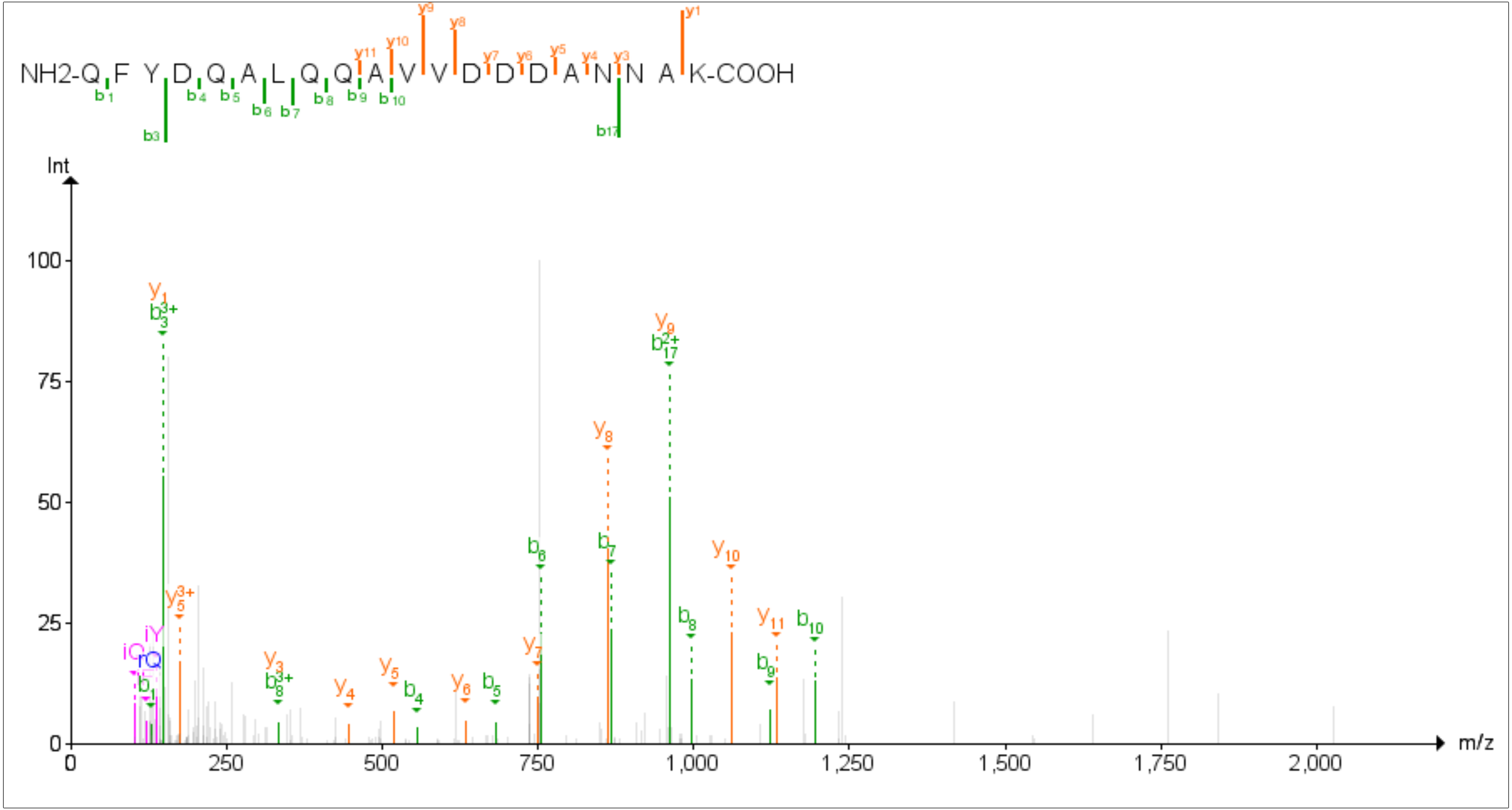

NH2-Q F Y D Q A L Q Q A V V D D D A N N A K-COOH
Int
m/z
0
25
50
75
100
250
500
750
1,000
1,250
1,500
1,750
2,000

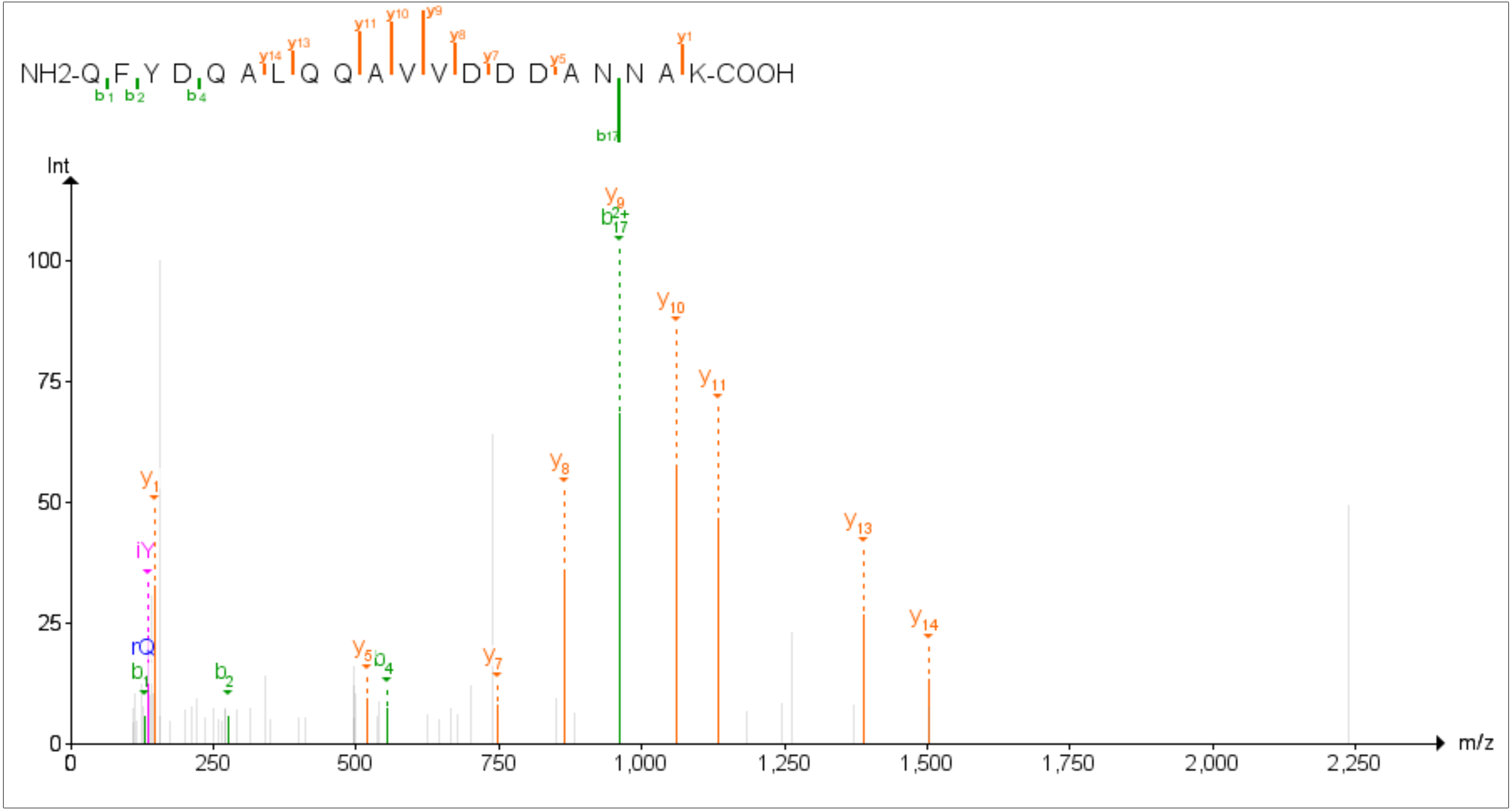

NH2-Q F Y D Q A L Q Q A V V D D D A N N A K-COOH
b1 b2 b4 b17
y14 y13 y11 y10 y9 y8 y7 y5 y1
Int
m/z
0 25 50 75 100
0 250 500 750 1,000 1,250 1,500 1,750 2,000 2,250
y1 iY rQ b1 b2 y5 b4 y7 y8 y9 b17 2+ y10 y11 y13 y14

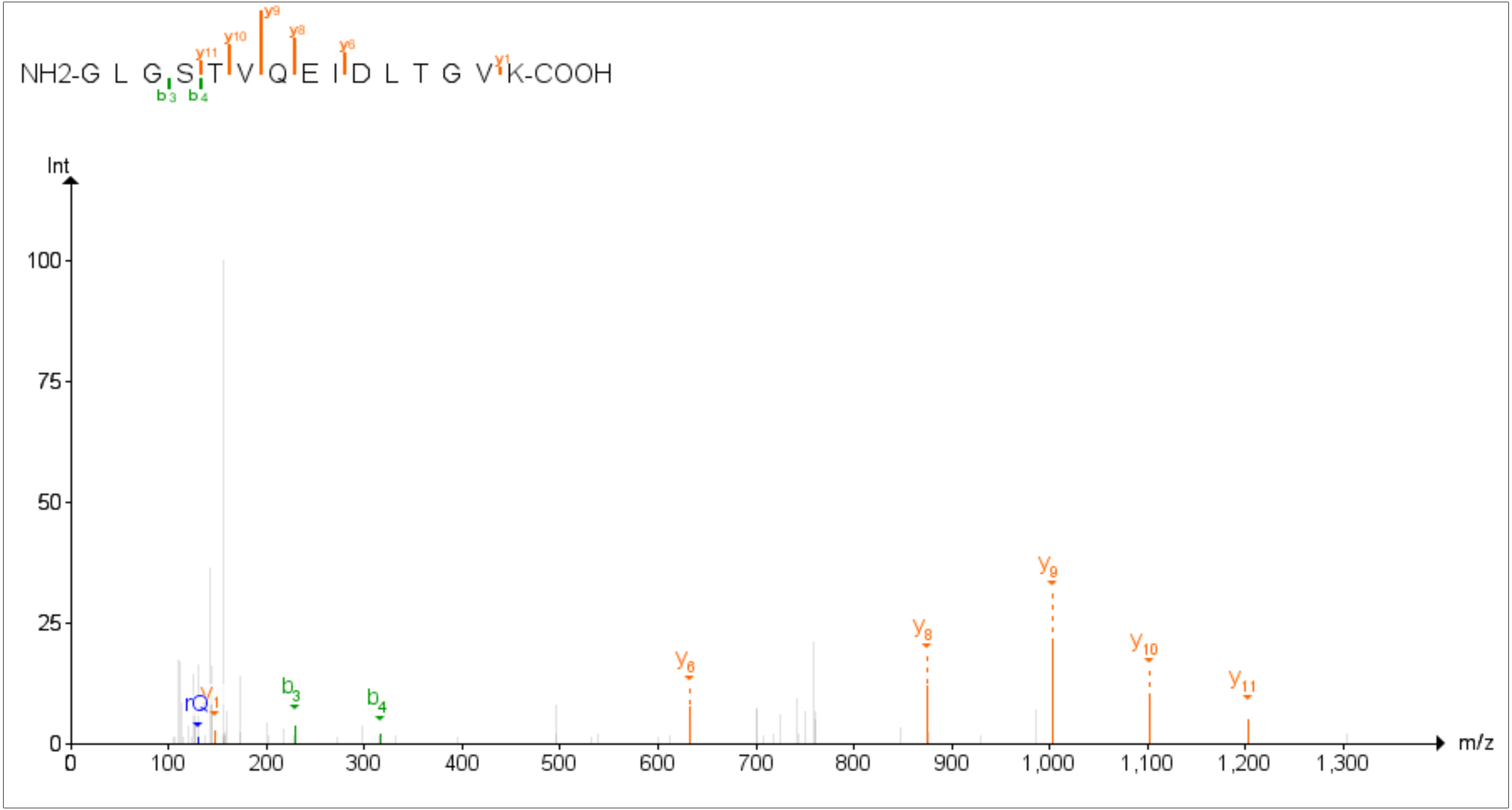
NH2-G L G S T V Q E I D L T G V K-COOH
y9
y10
y8
y11
y6
y1
b3
b4
Int
100
75
50
25
0
rQ
y1
b3
b4
y6
y8
y9
y10
y11
0
100
200
300
400
500
600
700
800
900
1,000
1,100
1,200
1,300
m/z

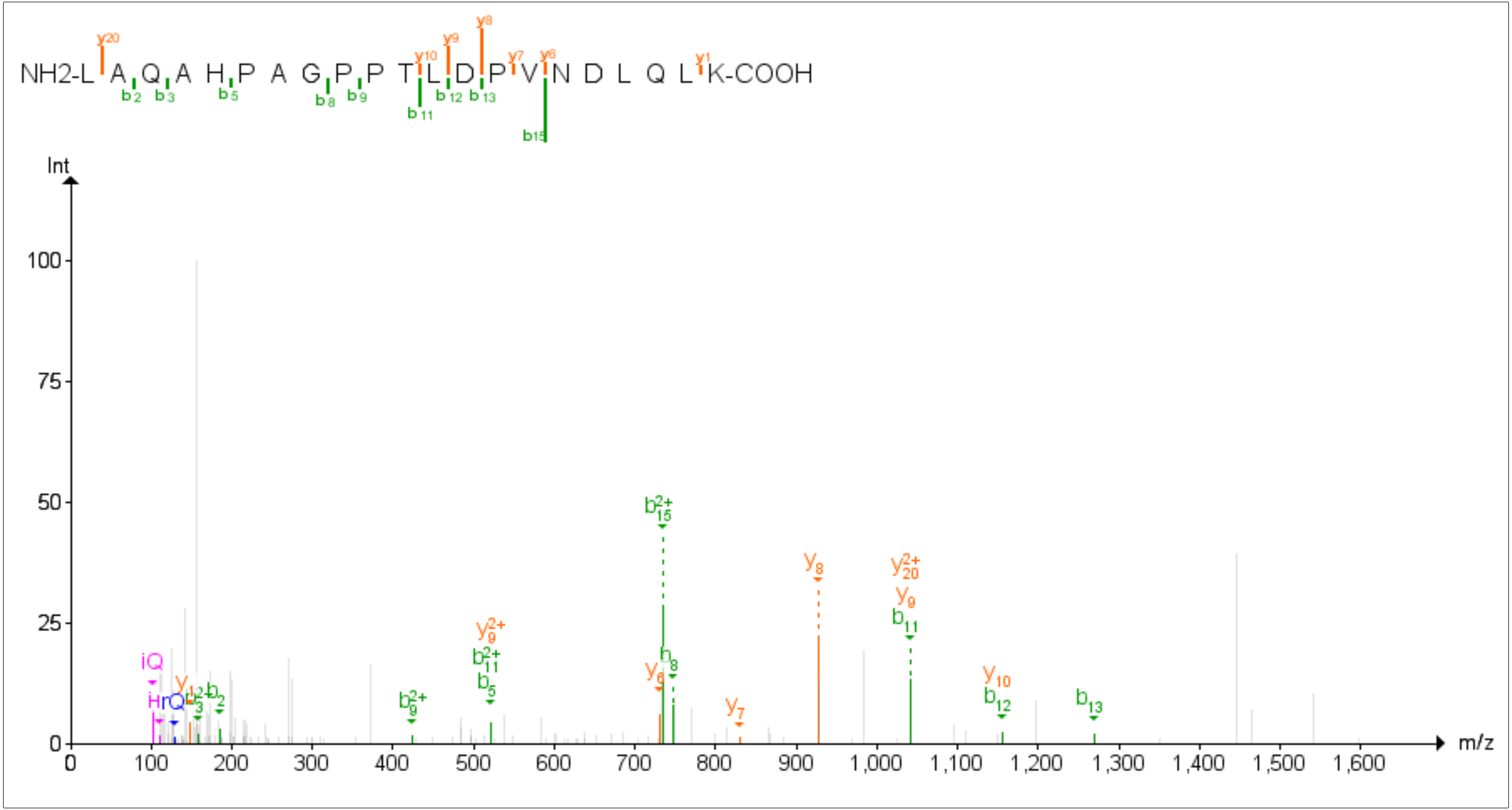
NH2-L A Q A H P A G P P T L D P V N D L Q L K-COOH
Int
m/z
y8
y20
y9
b11
b15
y10
b12
b13
y7
y6
b8
b5
b11
y9
b9
iQ
rQ
b2
b3
0
25
50
75
100
0
100
200
300
400
500
600
700
800
900
1,000
1,100
1,200
1,300
1,400
1,500
1,600

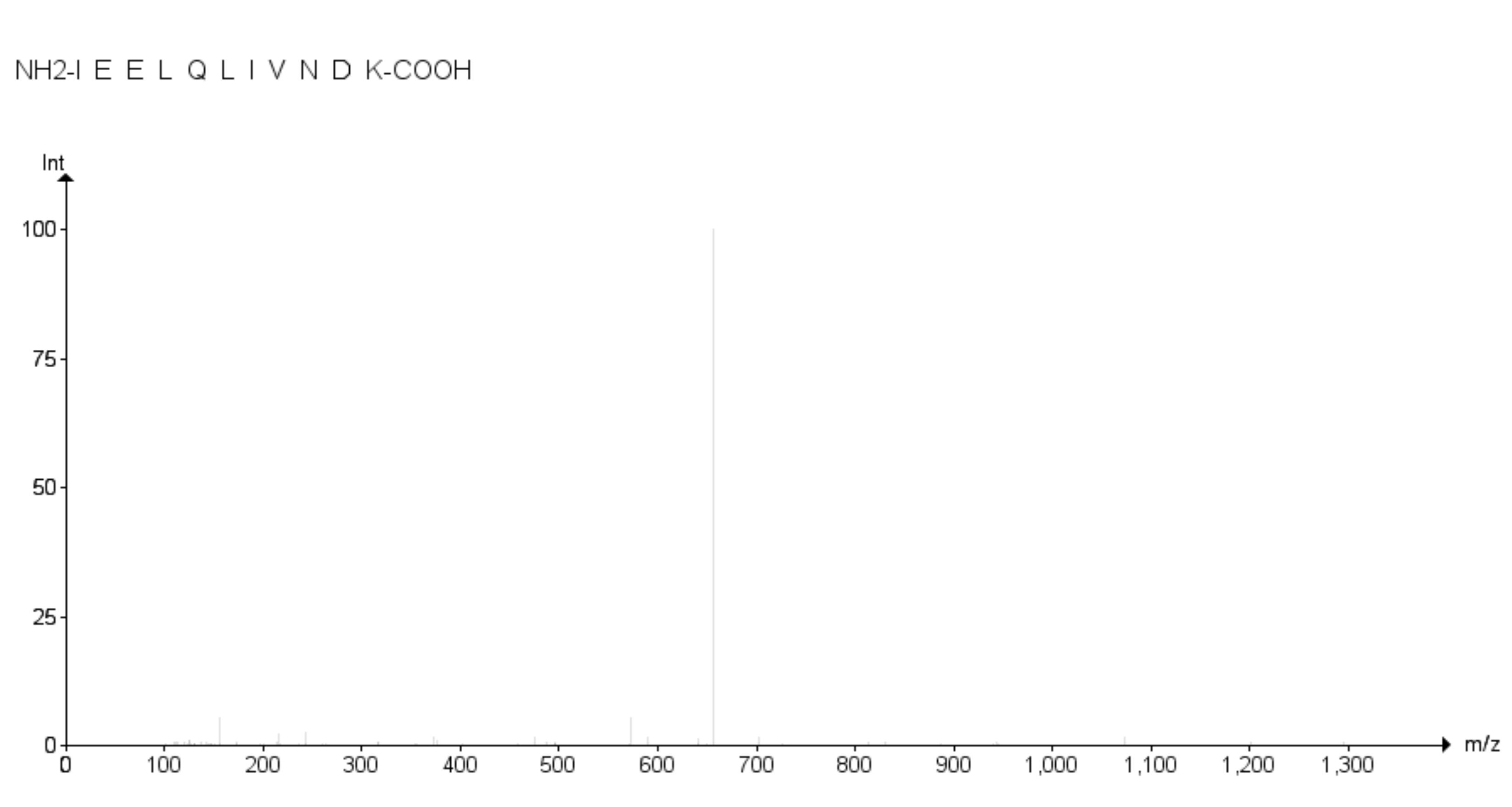
NH2-I E E L Q L I V N D K-COOH
Int
100
75
50
25
0
m/z
0
100
200
300
400
500
600
700
800
900
1,000
1,100
1,200
1,300

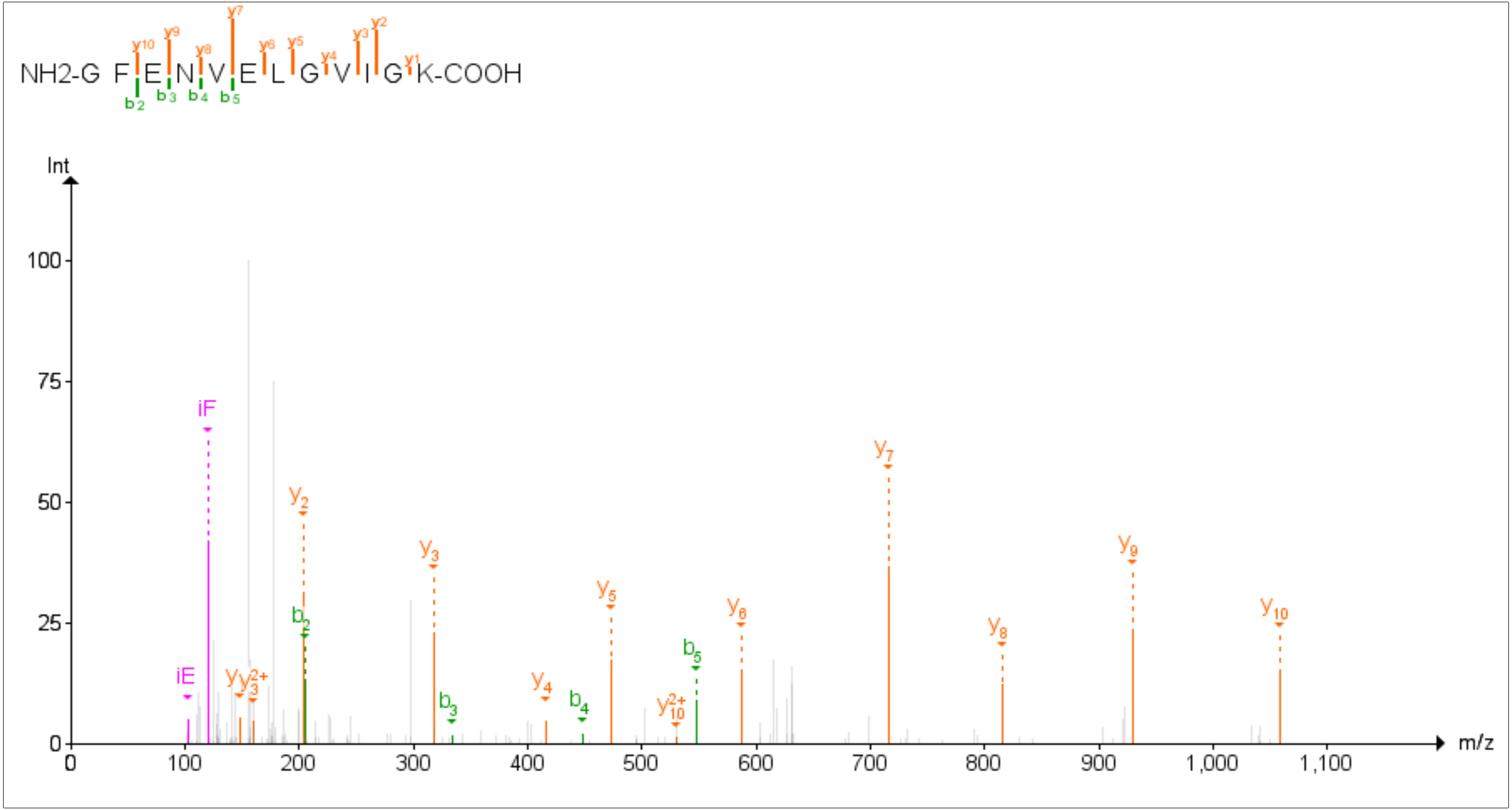

NH2-G F E N V E L G V I G K-COOH
y10 y9 y8 y7 y6 y5 y4 y3 y2 y1
b2 b3 b4 b5
Int
100
75
50
25
0
iF
iE
y2
y3
y4
y5
y6
y7
y8
y9
y10
y_3^{2+}
y_{10}^{2+}
b2
b3
b4
b5
0 100 200 300 400 500 600 700 800 900 1,000 1,100
m/z

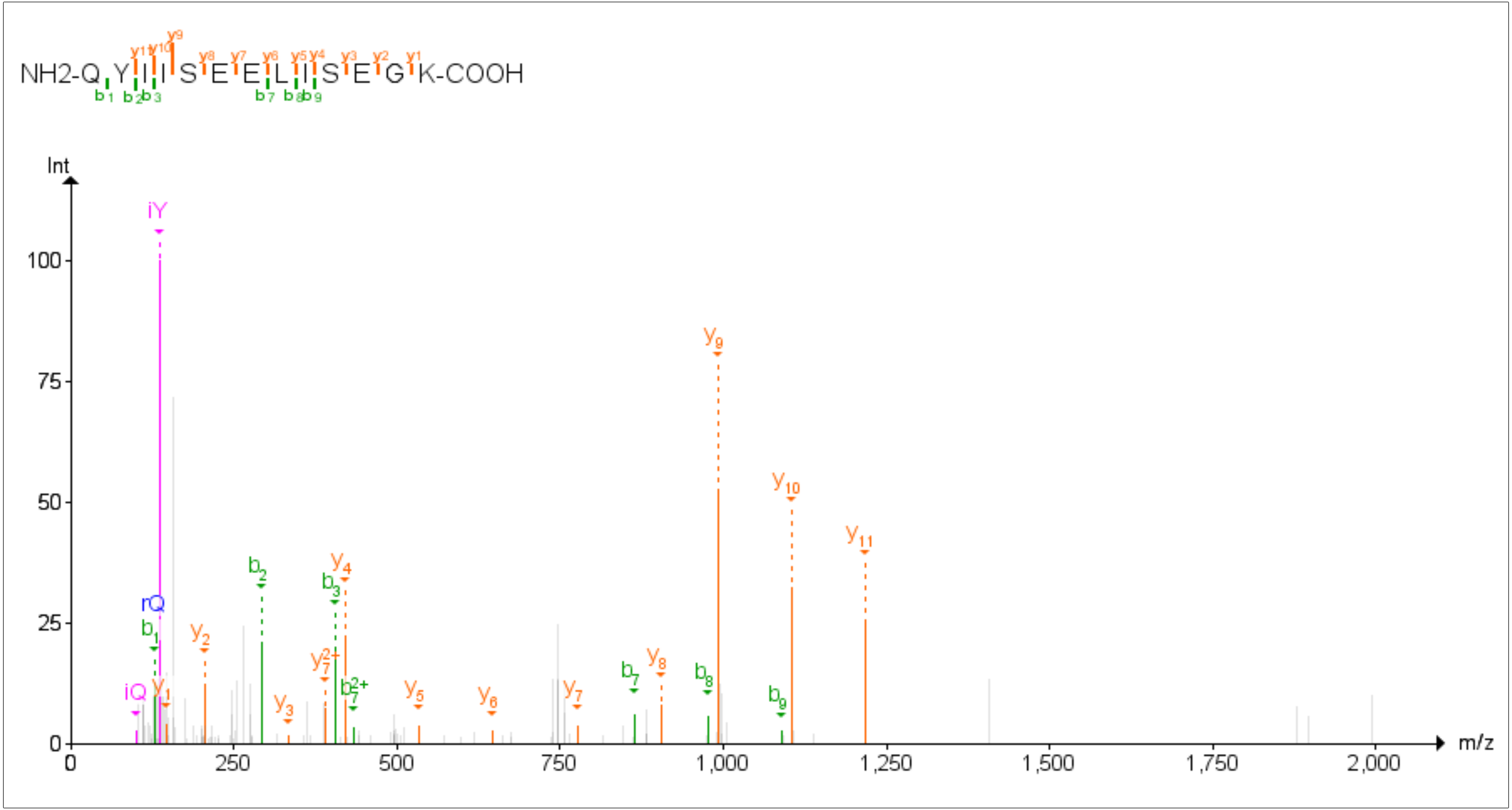

NH2-Q Y I I S E E L I I S E G K-COOH
y11 y10 y9 y8 y7 y6 y5 y4 y3 y2 y1
b1 b2 b3 b7 b8 b9
Int
iY
y9
y10
y11
b2
y4
b3
rQ
b1
y2
y7 2+
b7 2+
iQ
y1
y3
y5
y6
y7
b7
y8
b8
b9
100
75
50
25
0
250
500
750
1,000
1,250
1,500
1,750
2,000
m/z

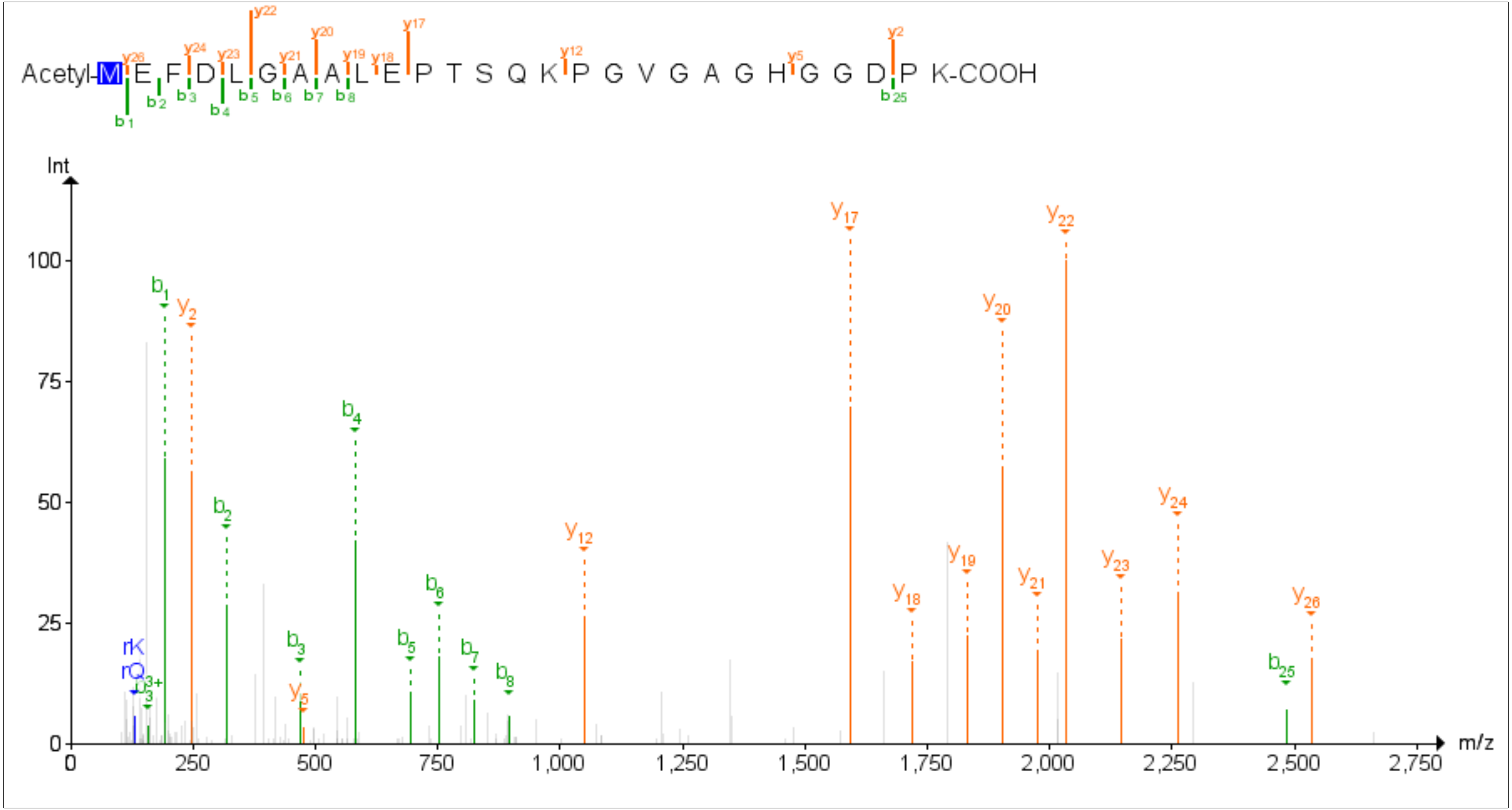

Acetyl-M E F D L G A A L E P T S Q K P G V G A G H G G D P K-COOH
y26 y24 y23 y22 y21 y20 y19 y18 y17 y12 y5 y2
b1 b2 b3 b4 b5 b6 b7 b8 b25
Int
m/z
0
25
50
75
100
250
500
750
1,000
1,250
1,500
1,750
2,000
2,250
2,500
2,750
rK
rQ
b3 3+

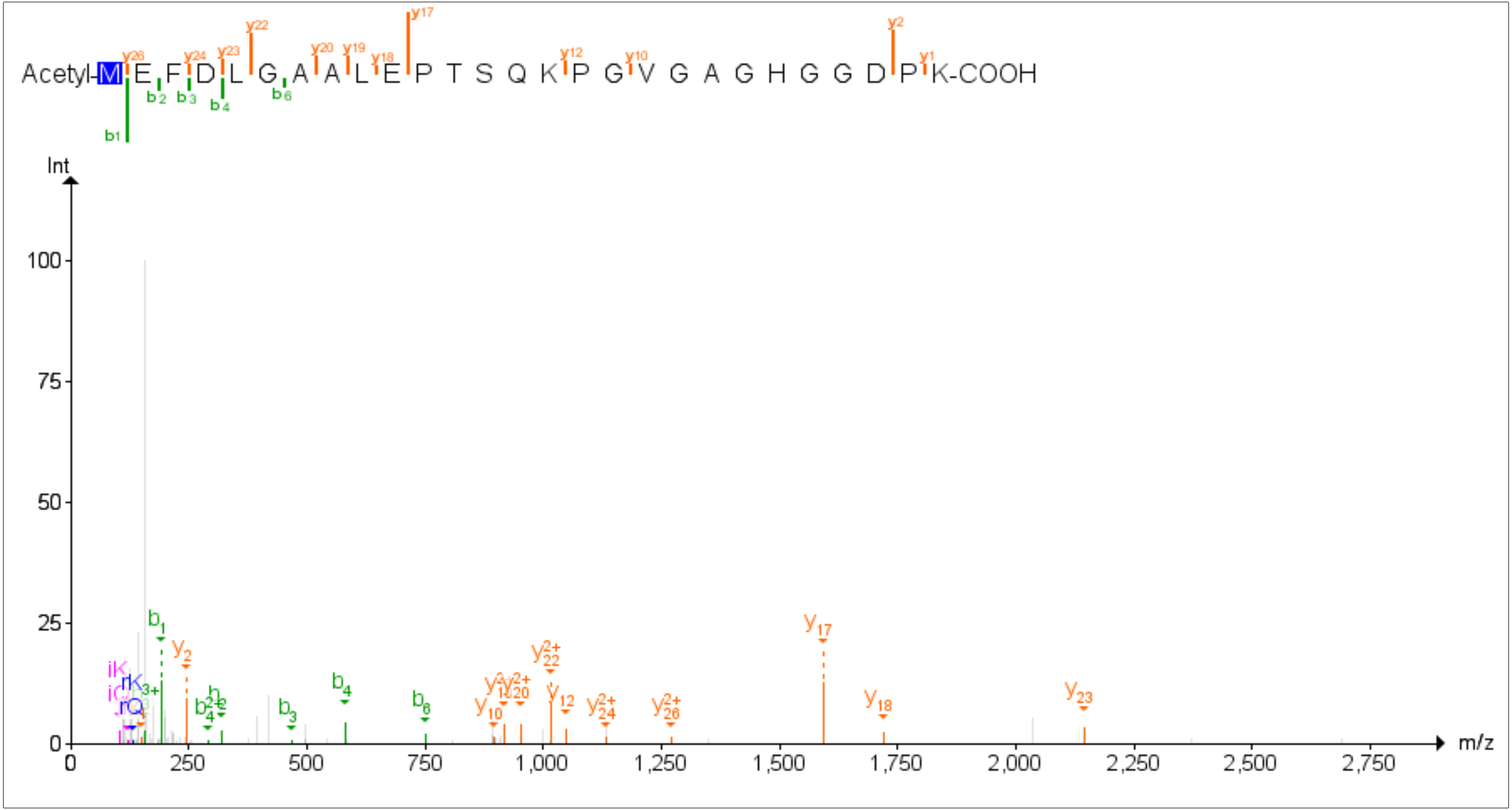

Acetyl-M E F D L G A A L E P T S Q K P G V G A G H G G D P K-COOH
y26 y24 y23 y22 y20 y19 y18 y17 y12 y10 y2 y1
b1 b2 b3 b4 b6
Int
100
75
50
25
0
b1
y2
b4
b6
b3
y17
y18
y23
y12
y10
m/z
0 250 500 750 1,000 1,250 1,500 1,750 2,000 2,250 2,500 2,750

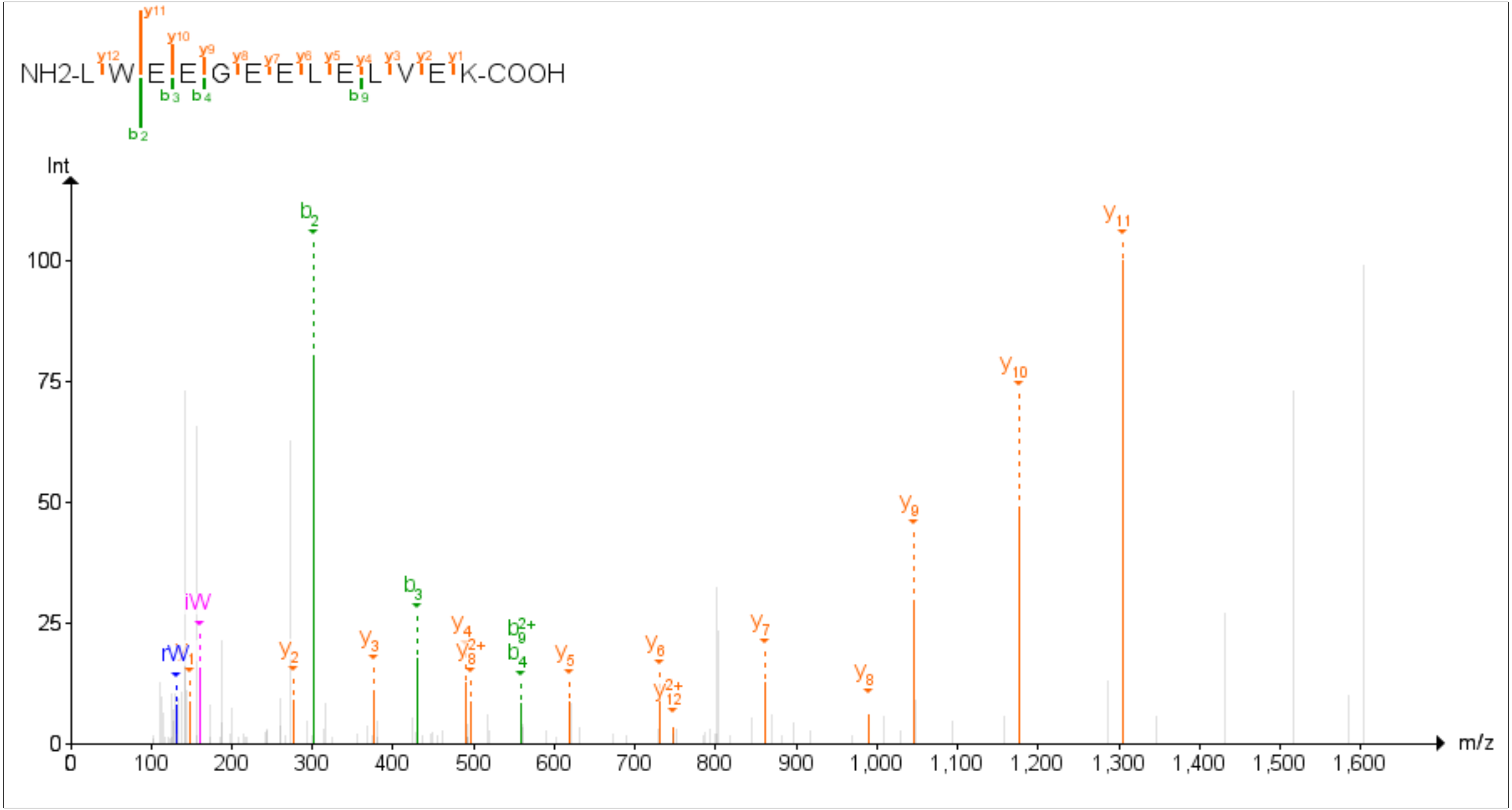

NH2-L W E E G E E L E L V E K-COOH
y12 y11 y10 y9 y8 y7 y6 y5 y4 y3 y2 y1
b2 b3 b4 b9
Int
100
75
50
25
0
m/z
0 100 200 300 400 500 600 700 800 900 1,000 1,100 1,200 1,300 1,400 1,500 1,600
b2
y11
y10
y9
b3
iW
rW
y1
y2
y3
y4
y8 2+
b9 2+
b4
y5
y6
y12 2+
y7
y8

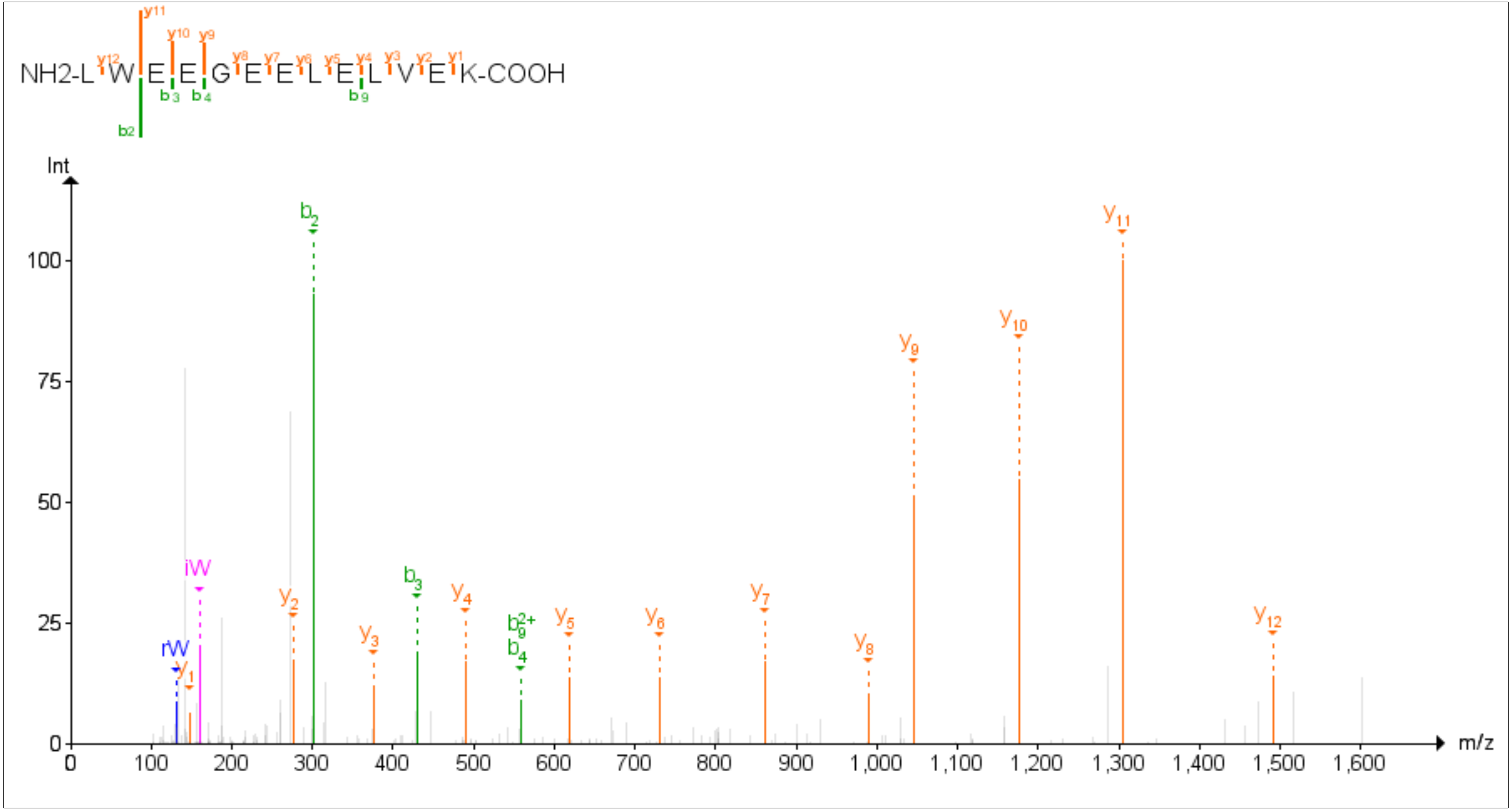

NH2-L W E E G E E L E L V E K-COOH
y12
y11
y10
y9
y8
y7
y6
y5
y4
y3
y2
y1
b2
b3
b4
b9
Int
100
75
50
25
0
b2
y11
y10
y9
iW
rW
y1
y2
y3
b3
y4
b9 2+
b4
y5
y6
y7
y8
y12
0
100
200
300
400
500
600
700
800
900
1,000
1,100
1,200
1,300
1,400
1,500
1,600
m/z

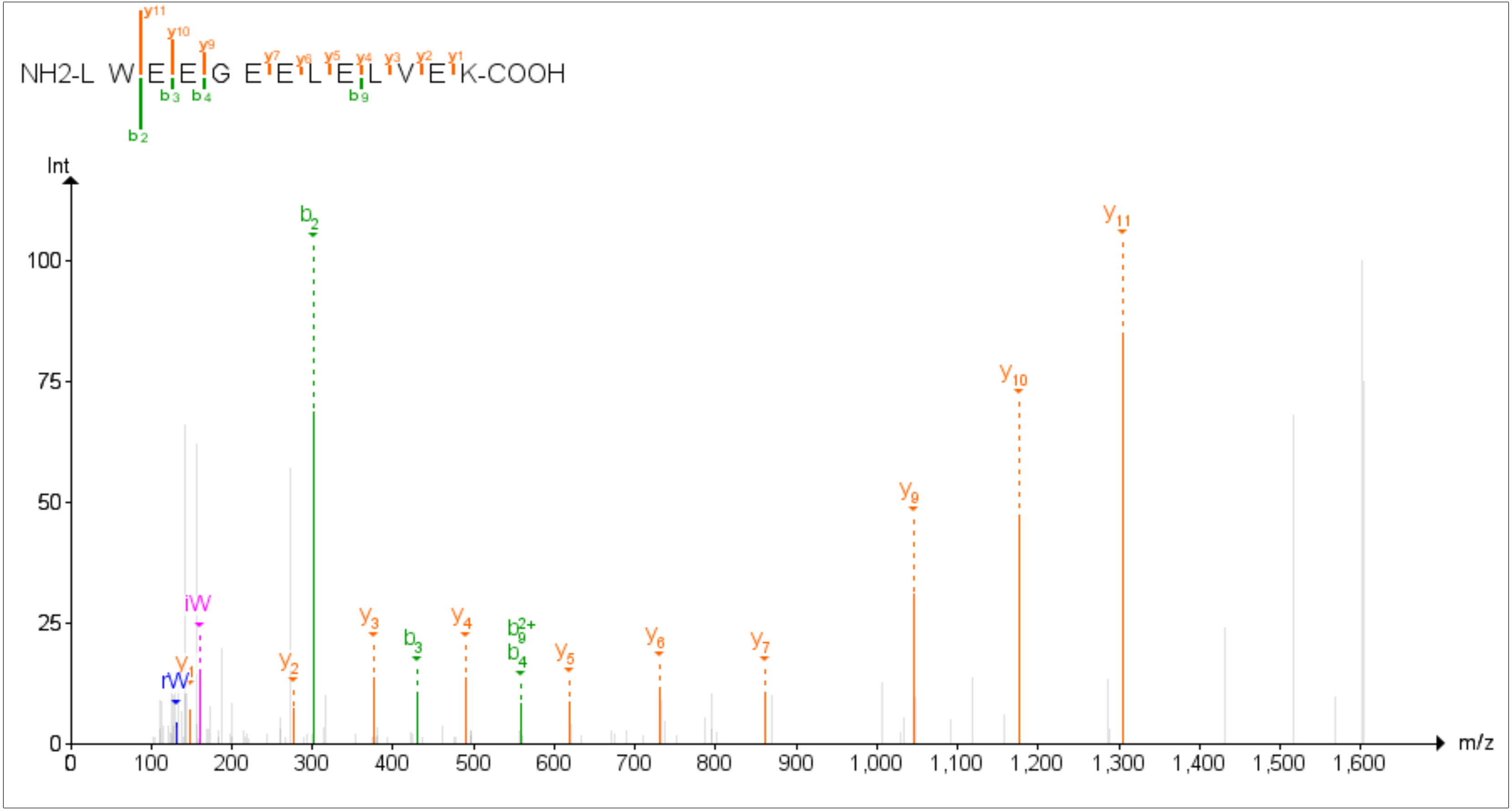

NH2-L W E E G E E L E L V E K-COOH
y11
y10
y9
y7
y6
y5
y4
y3
y2
y1
b2
b3
b4
b9
Int
100
75
50
25
0
m/z
0
100
200
300
400
500
600
700
800
900
1,000
1,100
1,200
1,300
1,400
1,500
1,600
iW
rW

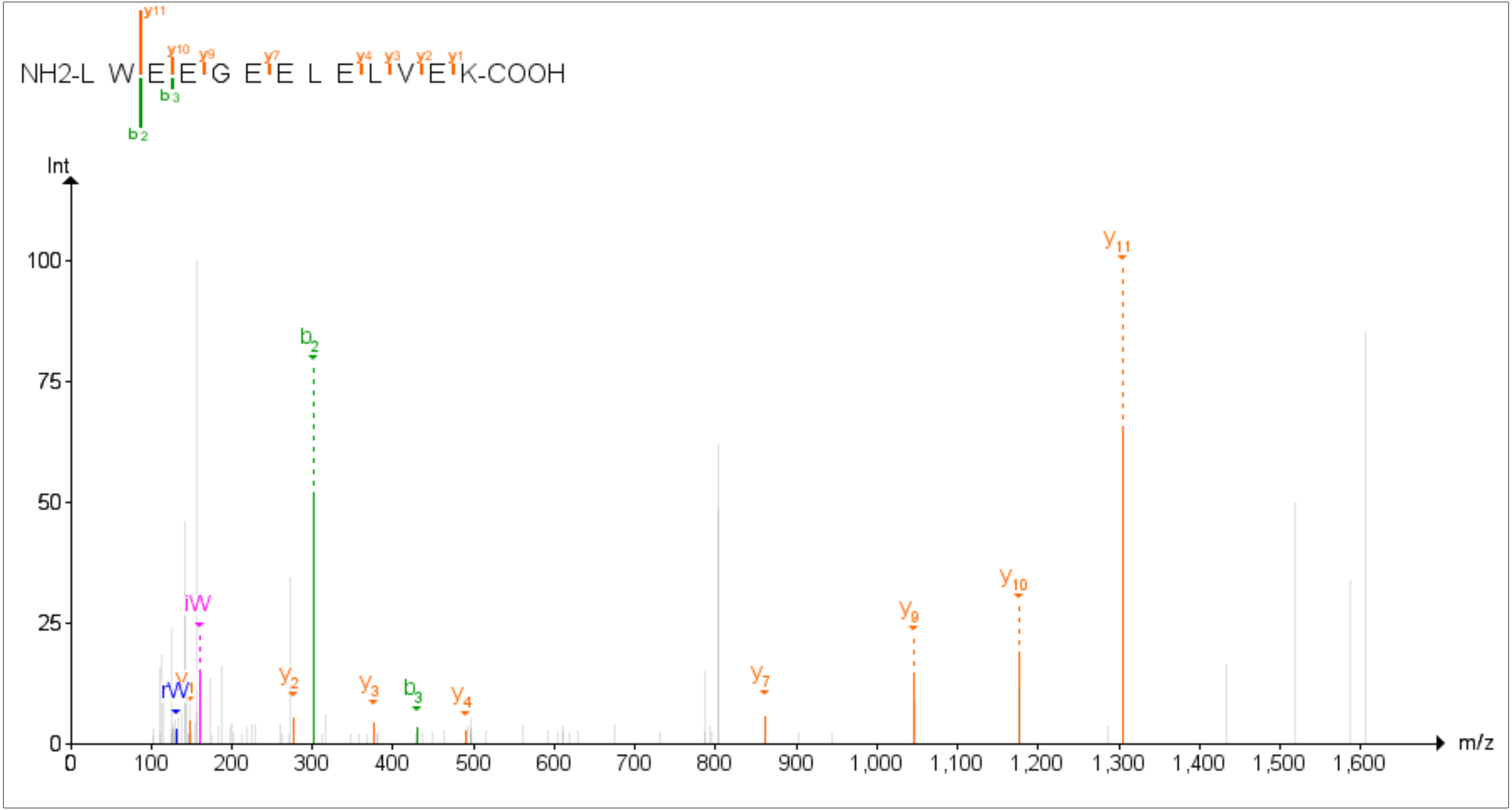
NH2-L W E E G E E L E L V E K-COOH
y11 y10 y9 y7 y4 y3 y2 y1
b2 b3
Int
100
75
50
25
0
y11
b2
iW
y10
y9
rW
y1
y2
y3
b3
y4
y7
0 100 200 300 400 500 600 700 800 900 1,000 1,100 1,200 1,300 1,400 1,500 1,600
m/z

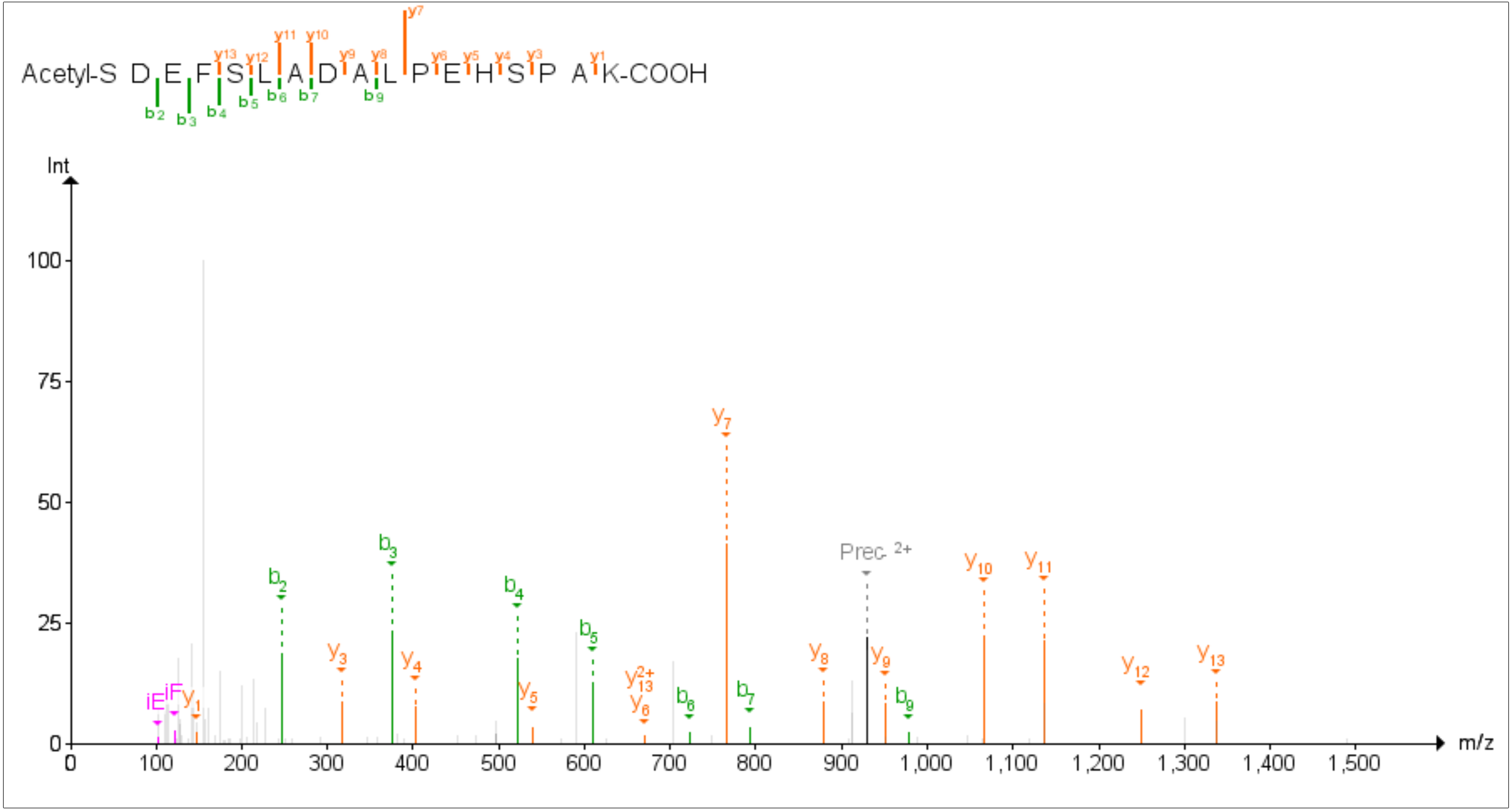

Acetyl-S D E F S L A D A L P E H S P A K-COOH
y13 y12 y11 y10 y9 y8 y7 y6 y5 y4 y3 y1
b2 b3 b4 b5 b6 b7 b9
Int
100
75
50
25
0
Prec. 2+
m/z
0 100 200 300 400 500 600 700 800 900 1,000 1,100 1,200 1,300 1,400 1,500

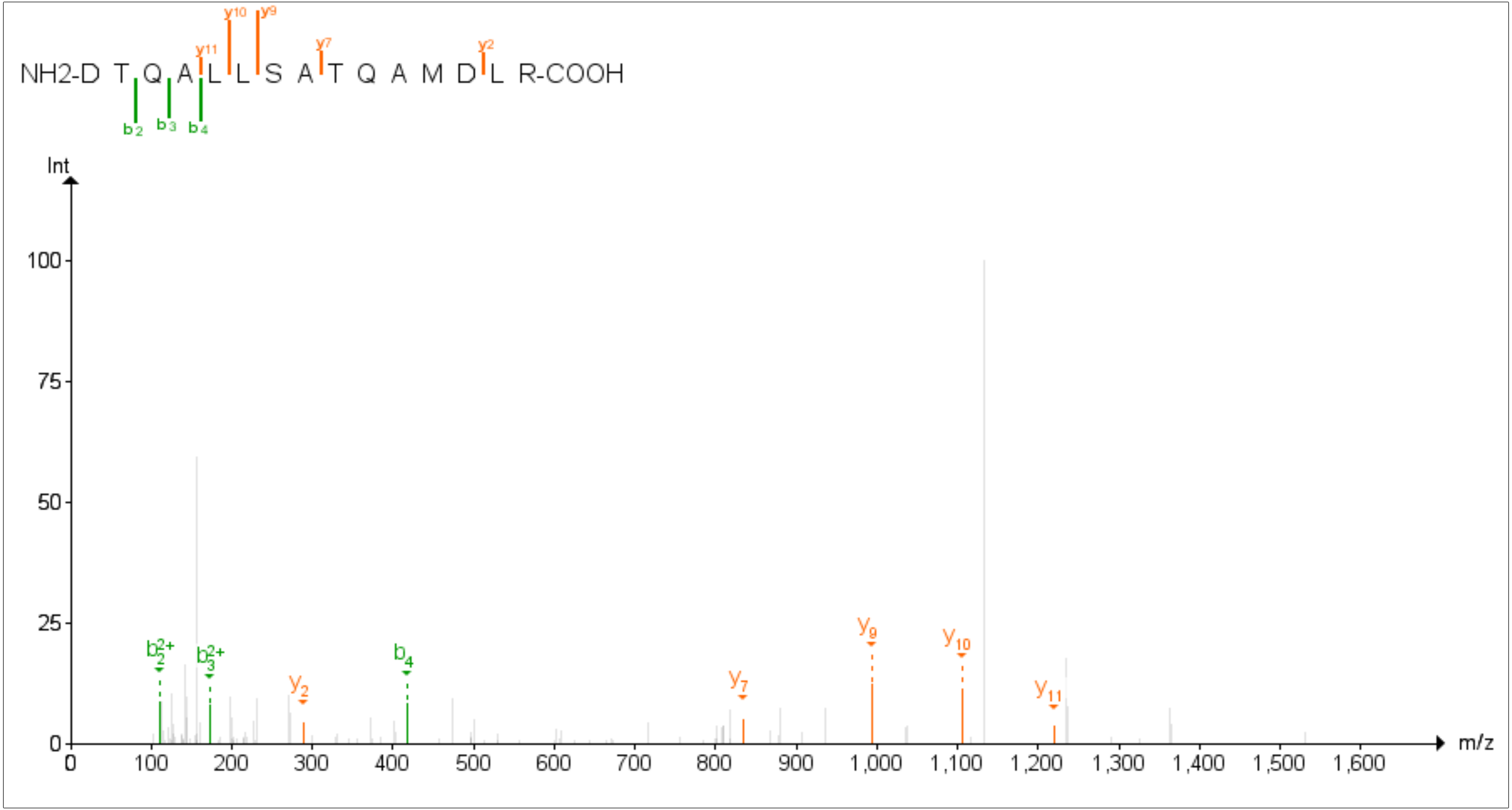

NH2-D T Q A L L S A T Q A M D L R-COOH
y10 y9 y11 y7 y2
b2 b3 b4
Int
100
75
50
25
0
b2 2+
b3 2+
y2
b4
y7
y9
y10
y11
0 100 200 300 400 500 600 700 800 900 1,000 1,100 1,200 1,300 1,400 1,500 1,600
m/z

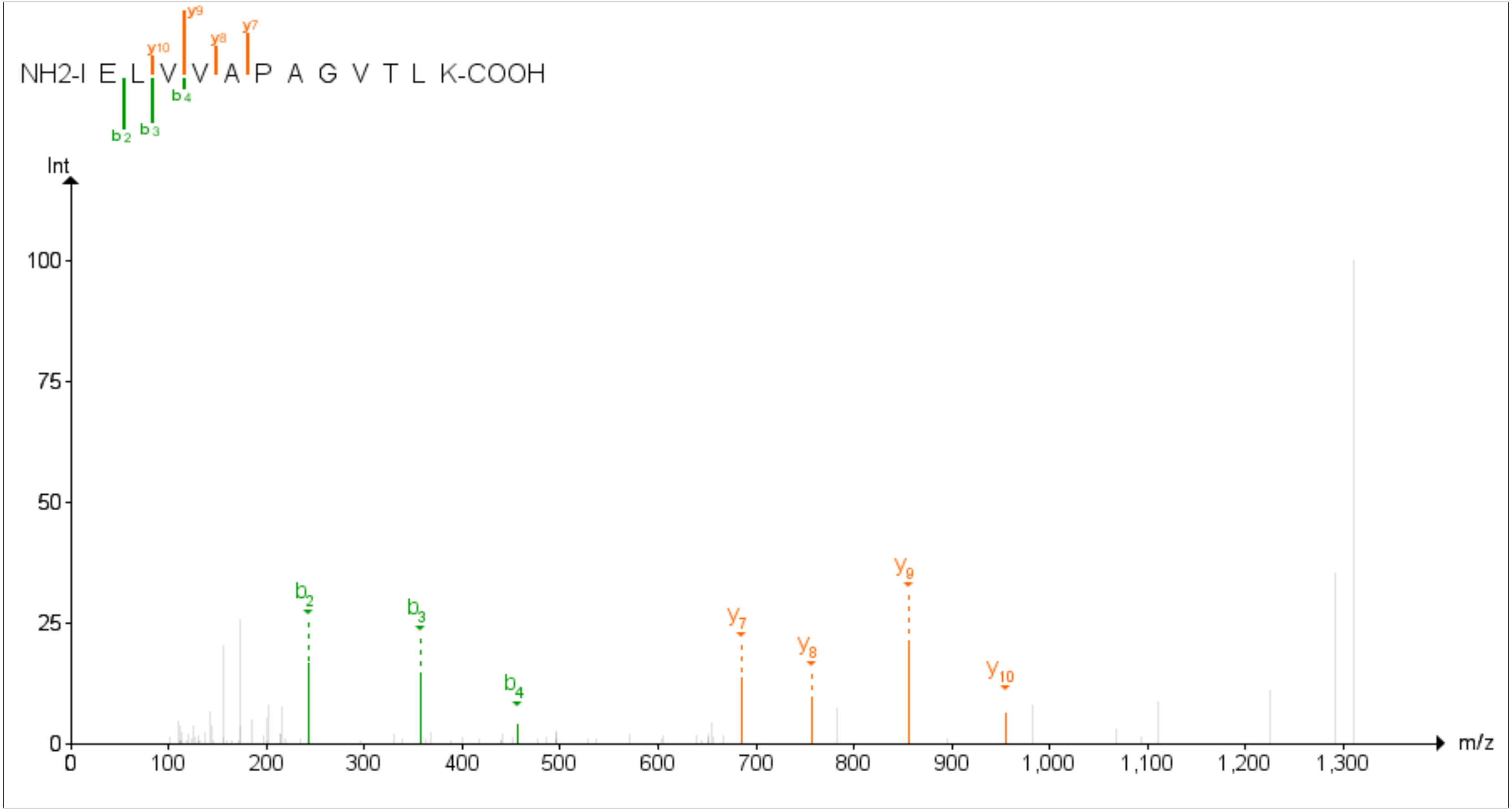

NH2-I E L V V A P A G V T L K-COOH
y10 y9 y8 y7
b2 b3 b4
Int
100
75
50
25
0
0 100 200 300 400 500 600 700 800 900 1,000 1,100 1,200 1,300
m/z
b2 b3 b4 y7 y8 y9 y10

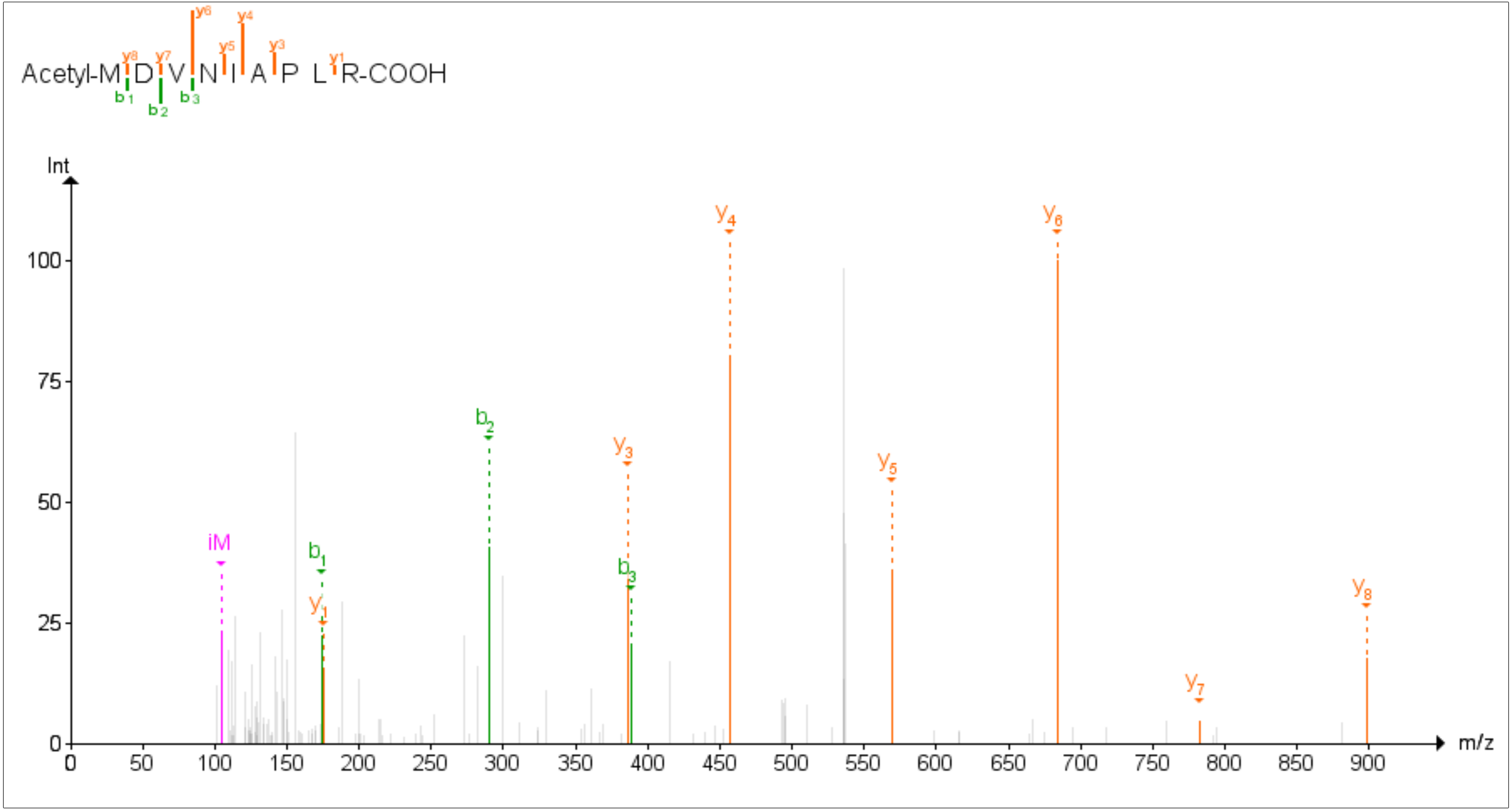

Acetyl-M D V N I A P L R-COOH
y8 y7 y6 y5 y4 y3 y1
b 1 b 2 b 3
Int
100
75
50
25
0
iM
b1
y1
b2
y3
b3
y4
y5
y6
y7
y8
0 50 100 150 200 250 300 350 400 450 500 550 600 650 700 750 800 850 900
m/z

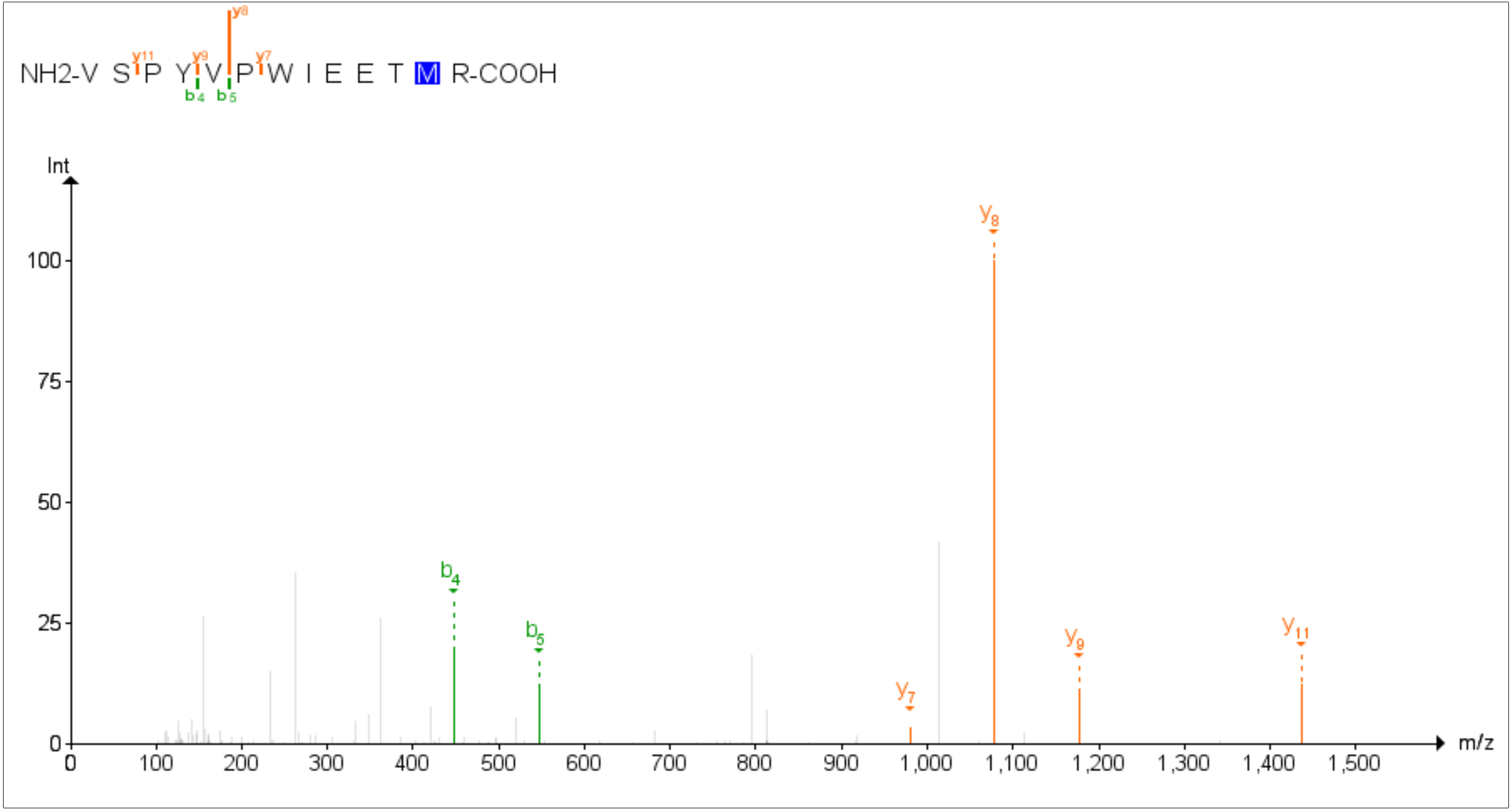
NH2-V S P Y V P W I E E T M R-COOH
y11 y9 y8 y7
b4 b5
Int
100
75
50
25
0
b4
b5
y7
y8
y9
y11
0 100 200 300 400 500 600 700 800 900 1,000 1,100 1,200 1,300 1,400 1,500
m/z

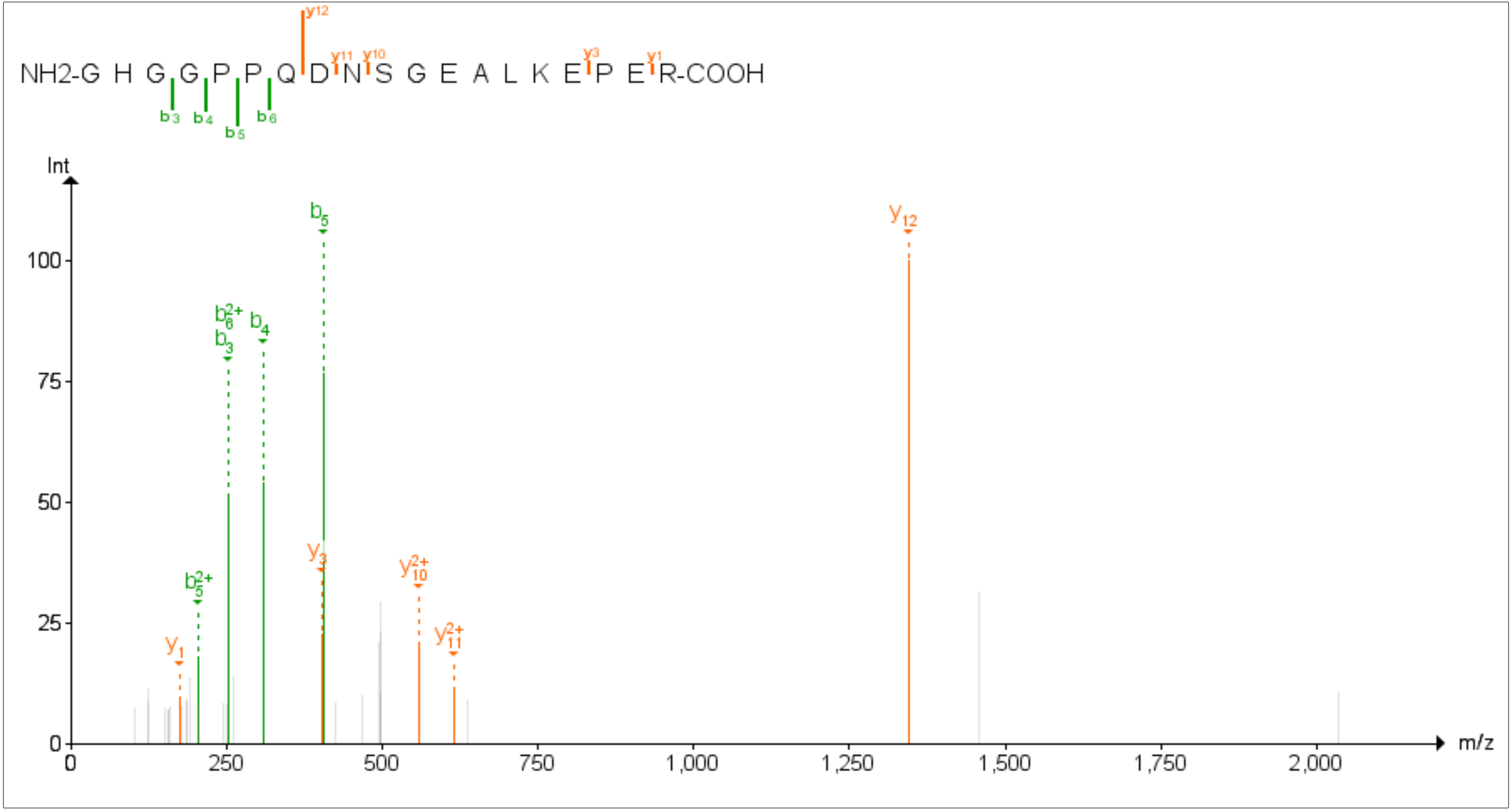

NH2-G H G G P P Q D N S G E A L K E P E R-COOH
y12
y11
y10
y3
y1
b3
b4
b5
b6
Int
100
75
50
25
0
b5
y12
b6 2+
b4
b3
b5 2+
y1
y3
y10 2+
y11 2+
0
250
500
750
1,000
1,250
1,500
1,750
2,000
m/z

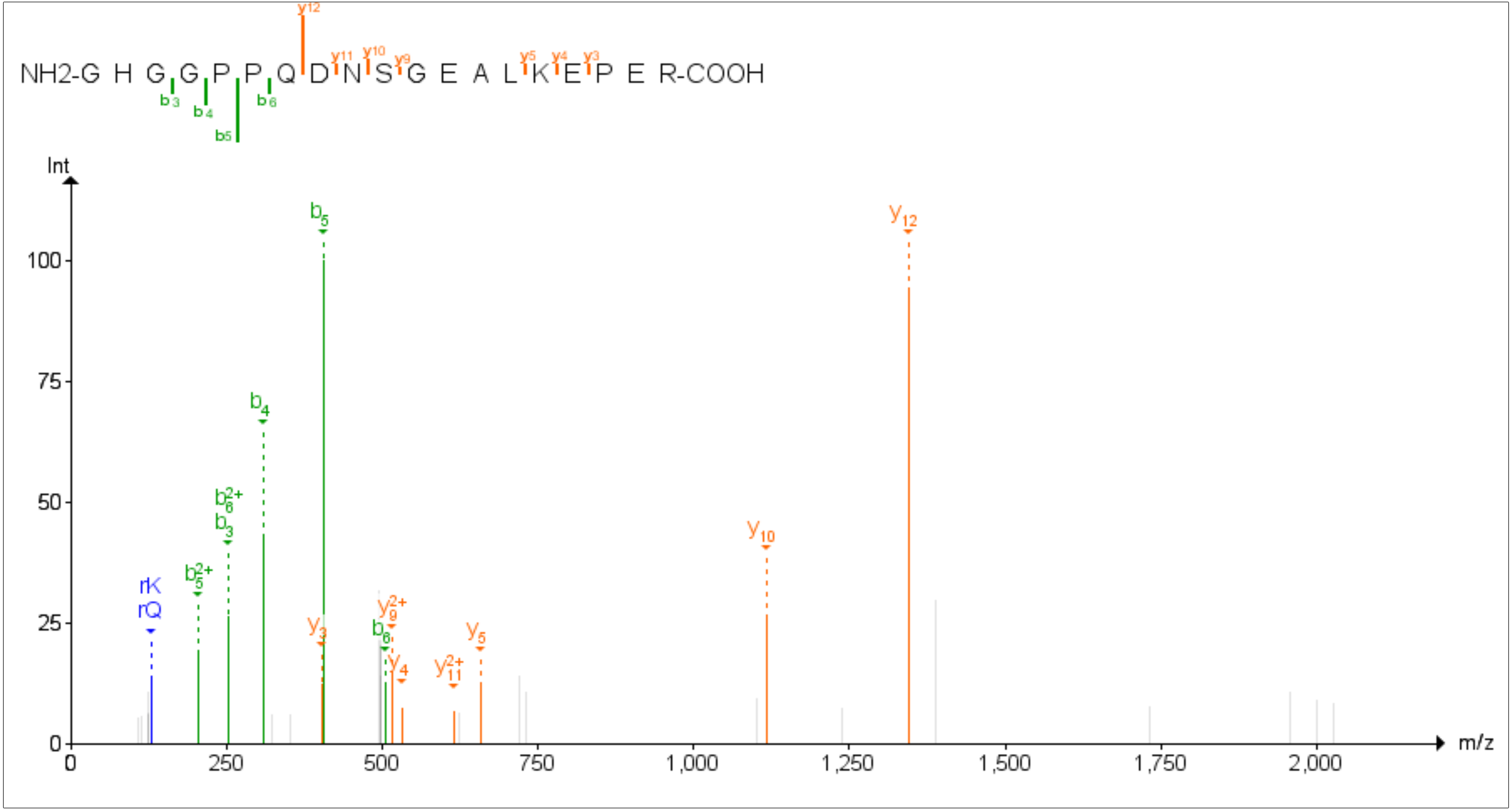

NH2-G H G G P P Q D N S G E A L K E P E R-COOH
b3 b4 b5 b6
y12 y11 y10 y9 y5 y4 y3
Int
m/z
0
25
50
75
100
250
500
750
1,000
1,250
1,500
1,750
2,000
rK
rQ
b5
b4
b3
b6
y3
y4
y5
y10
y12

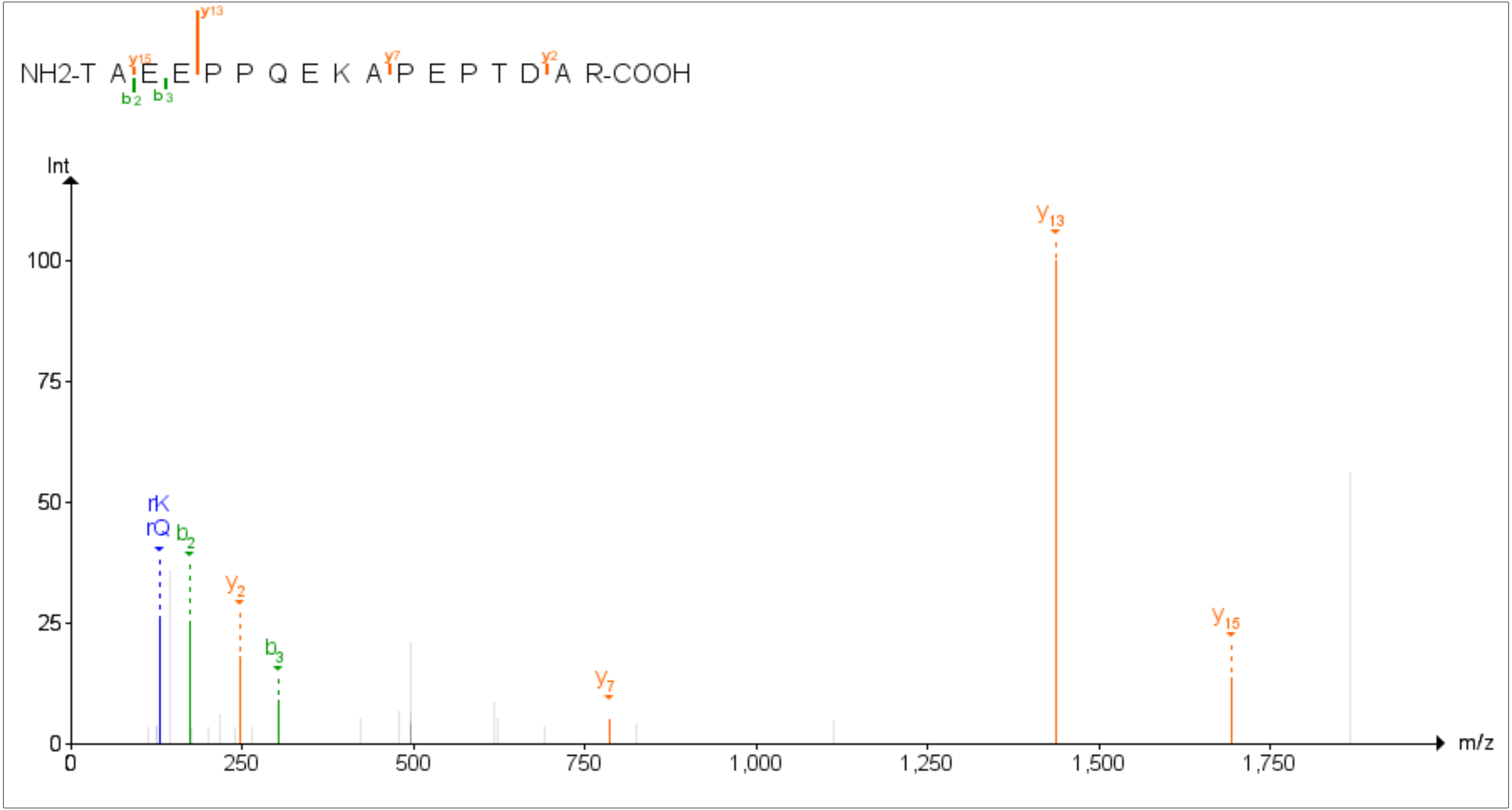

NH2-T A E E P P Q E K A P E P T D A R-COOH
y13
y15
y7
y2
b2
b3
Int
100
75
50
25
0
rK
rQ
b2
y2
b3
y7
y13
y15
0
250
500
750
1,000
1,250
1,500
1,750
m/z

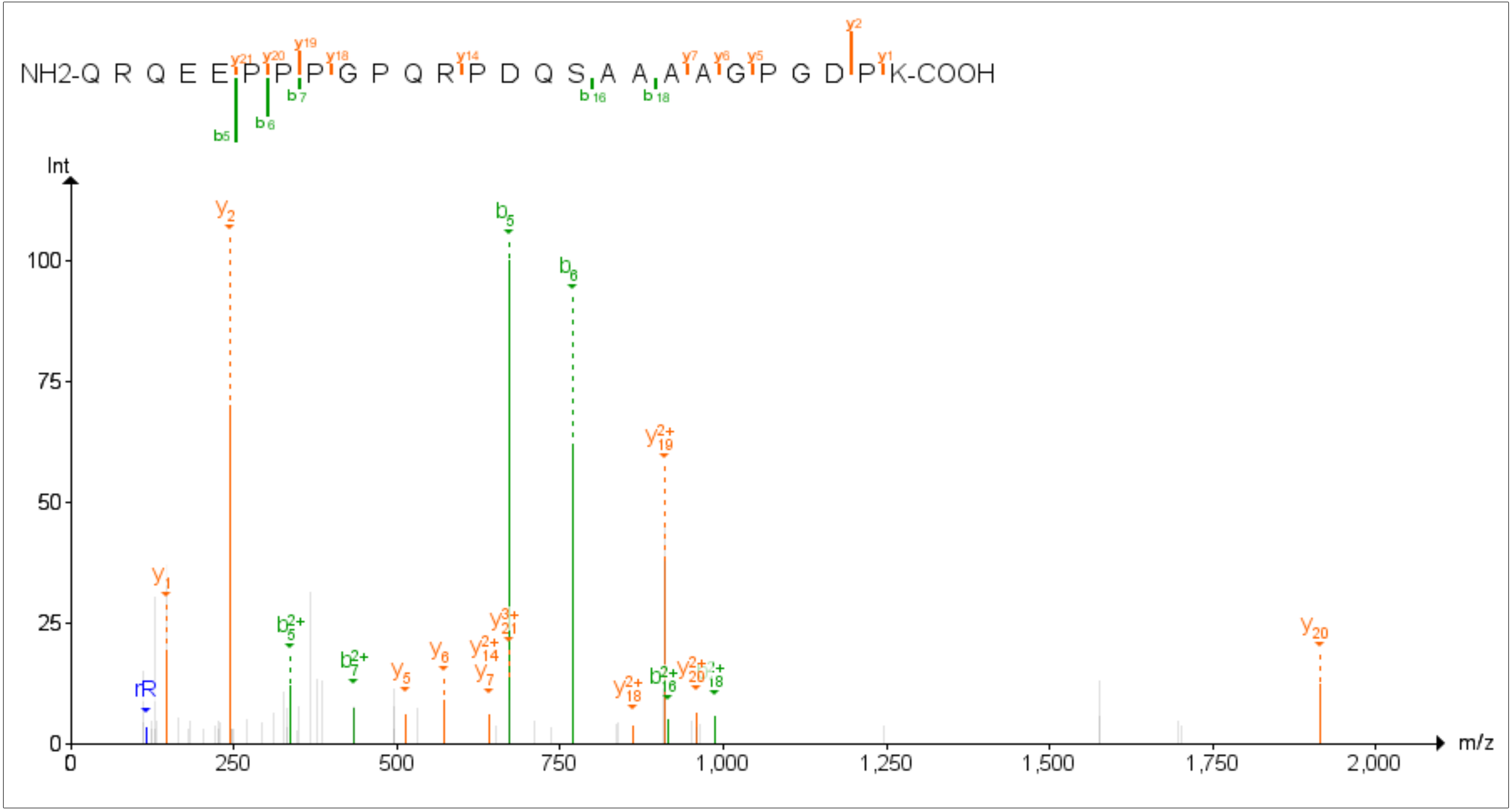

NH2-Q R Q E E P P P G P Q R P D Q S A A A A G P G D P K-COOH
y21 y20 y19 y18 y14 y7 y6 y5 y2 y1
b5 b6 b7 b16 b18
Int
100
75
50
25
0
m/z
0
250
500
750
1,000
1,250
1,500
1,750
2,000
y2
b5
b6
y1
rR
y19 2+
b5 2+
b7 2+
y5
y6
y14 2+
y7
y21 3+
y18 2+
b16 2+
y20 2+
b18 2+
y20

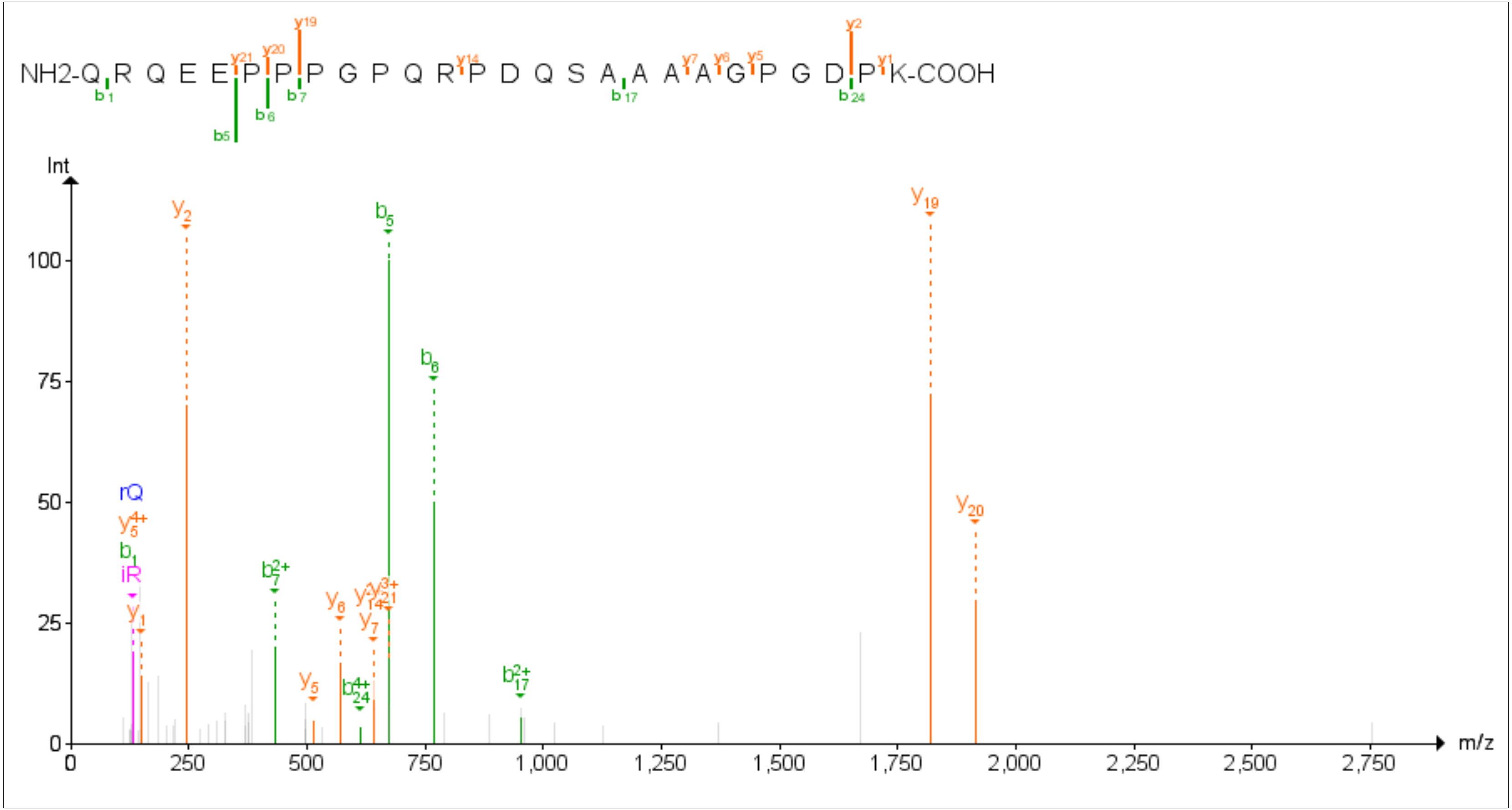

NH2-Q R Q E E P P P G P Q R P D Q S A A A A G P G D P K-COOH
Int
m/z
y2
b5
y19
b6
y20
rQ
y5 4+
b1
iR
b7 2+
y6
y14 4+
y21 3+
y7
y1
y5
b24 4+
b17 2+
0
25
50
75
100
250
500
750
1,000
1,250
1,500
1,750
2,000
2,250
2,500
2,750

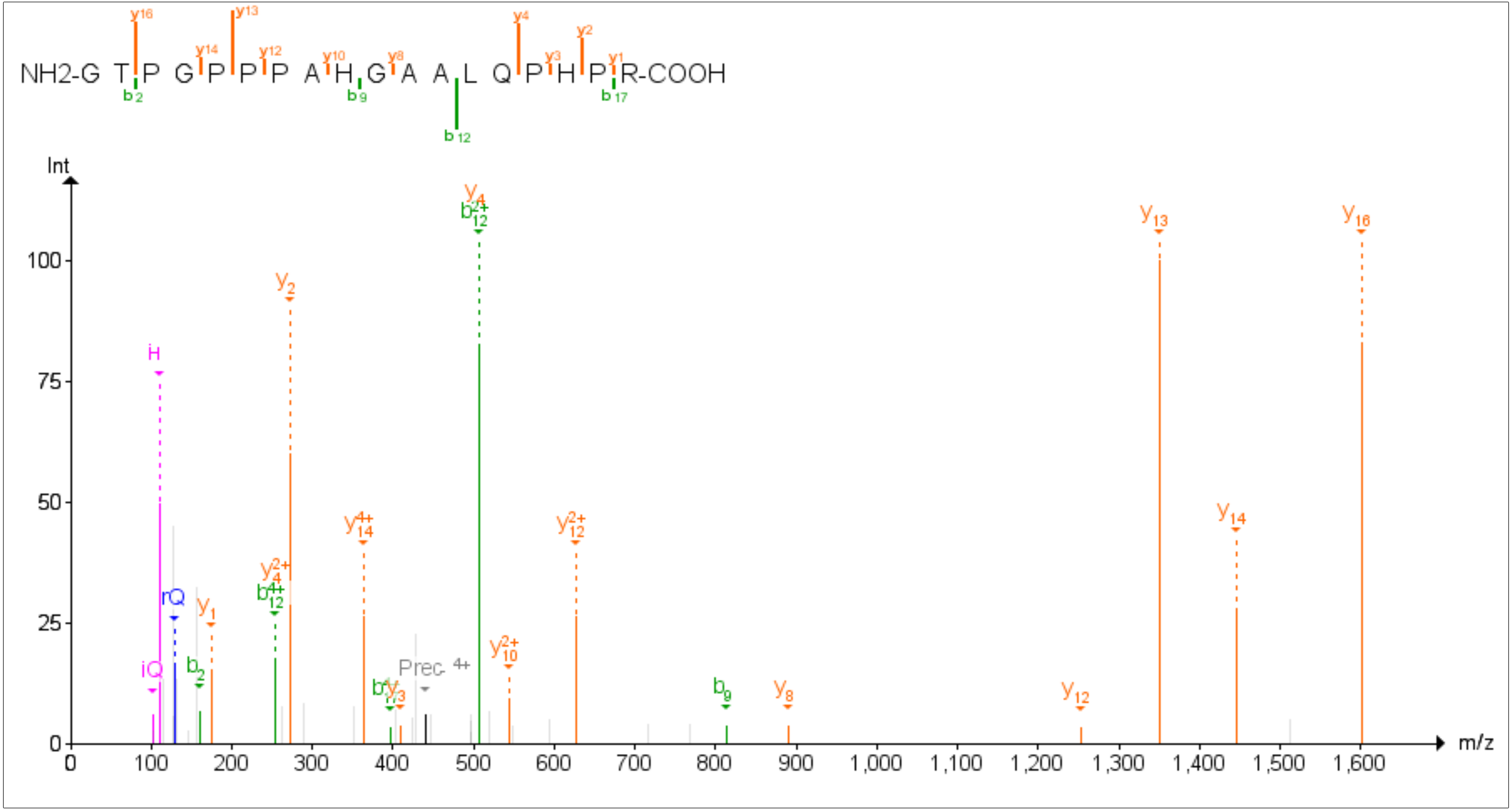
NH2-G T P G P P P A H G A A L Q P H P R-COOH
y16 y14 y13 y12 y10 y8 y4 y3 y2 y1
b2 b9 b12 b17
Int
m/z
0
25
50
75
100
0 100 200 300 400 500 600 700 800 900 1,000 1,100 1,200 1,300 1,400 1,500 1,600
iH
iQ
rQ
y1
b2
y2
b4+12
y2+4
y4+14
y3
Prec- 4+
y4
b2+12
y2+10
y2+12
b9
y8
y12
y13
y14
y16

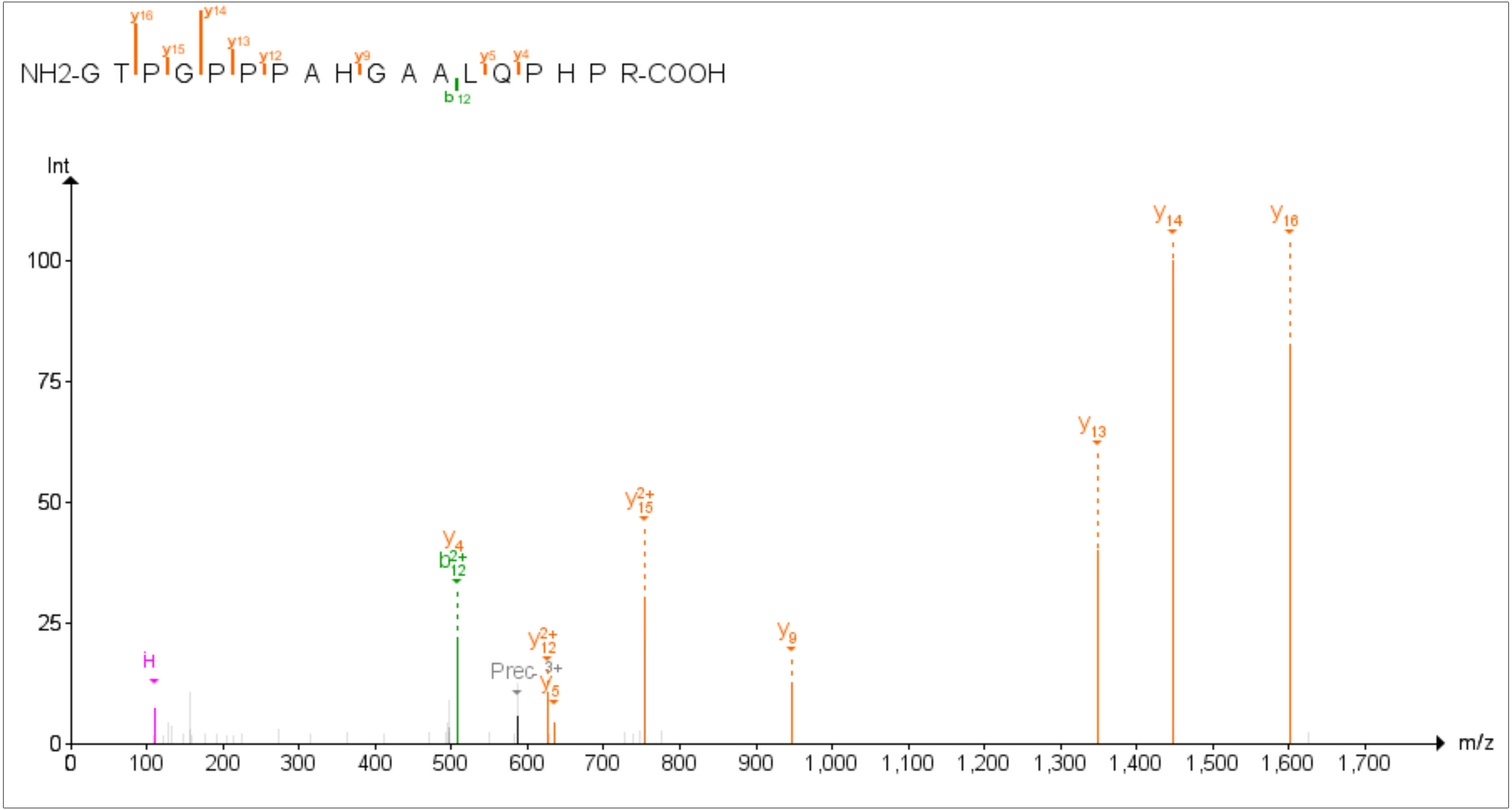

NH2-G T P G P P P A H G A A L Q P H P R-COOH
y16
y15
y14
y13
y12
y9
y5
y4
b 12
Int
100
75
50
25
0
y14
y16
y13
y15 2+
y4
b12 2+
y12 2+
Prec- 3+
y5
y9
H
m/z
0
100
200
300
400
500
600
700
800
900
1,000
1,100
1,200
1,300
1,400
1,500
1,600
1,700

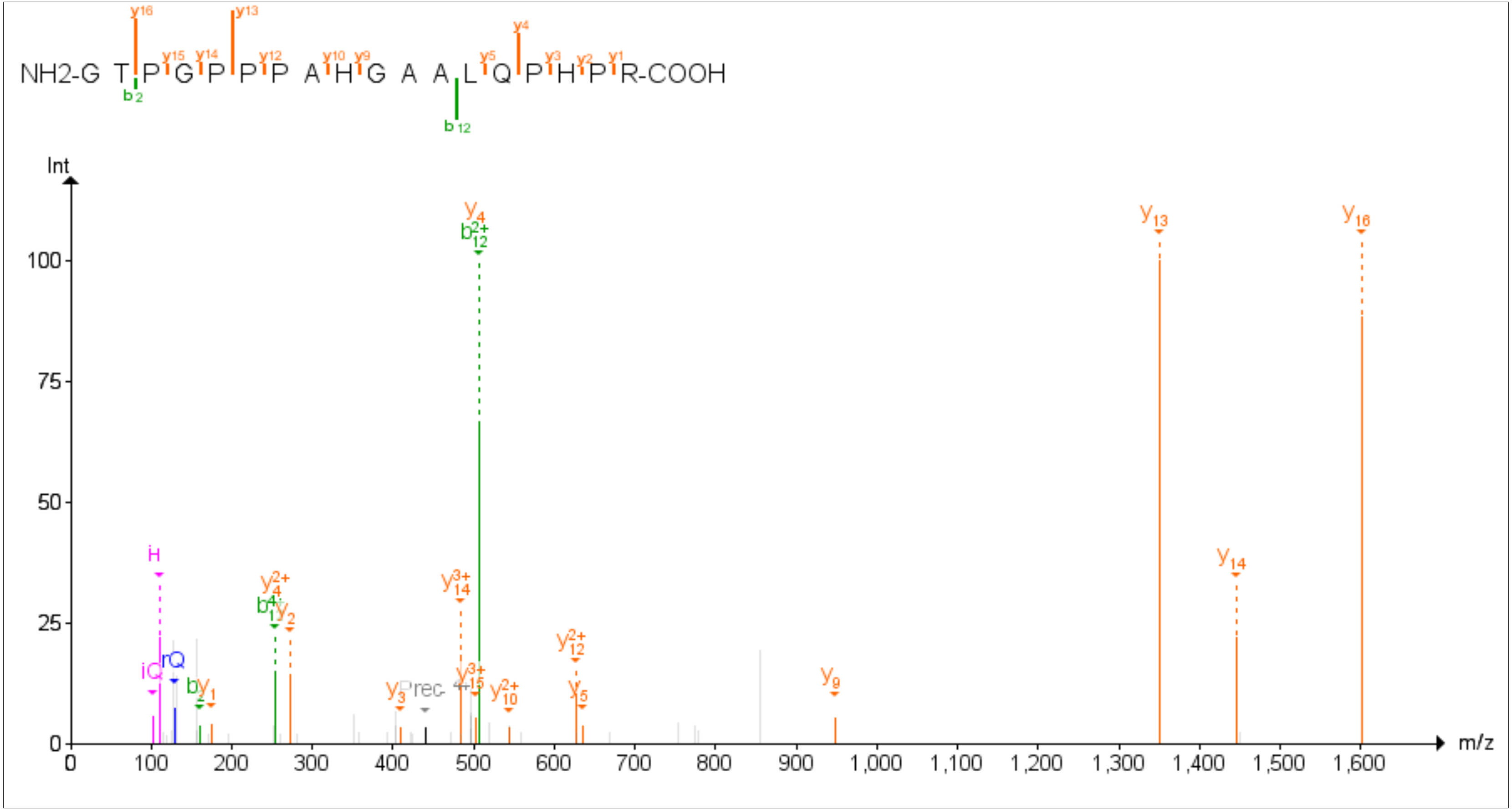

NH2-G T P G P P P A H G A A L Q P H P R-COOH
y16
y15
y14
y13
y12
y10
y9
y5
y4
y3
y2
y1
b2
b12
Int
m/z
0
25
50
75
100
0
100
200
300
400
500
600
700
800
900
1,000
1,100
1,200
1,300
1,400
1,500
1,600

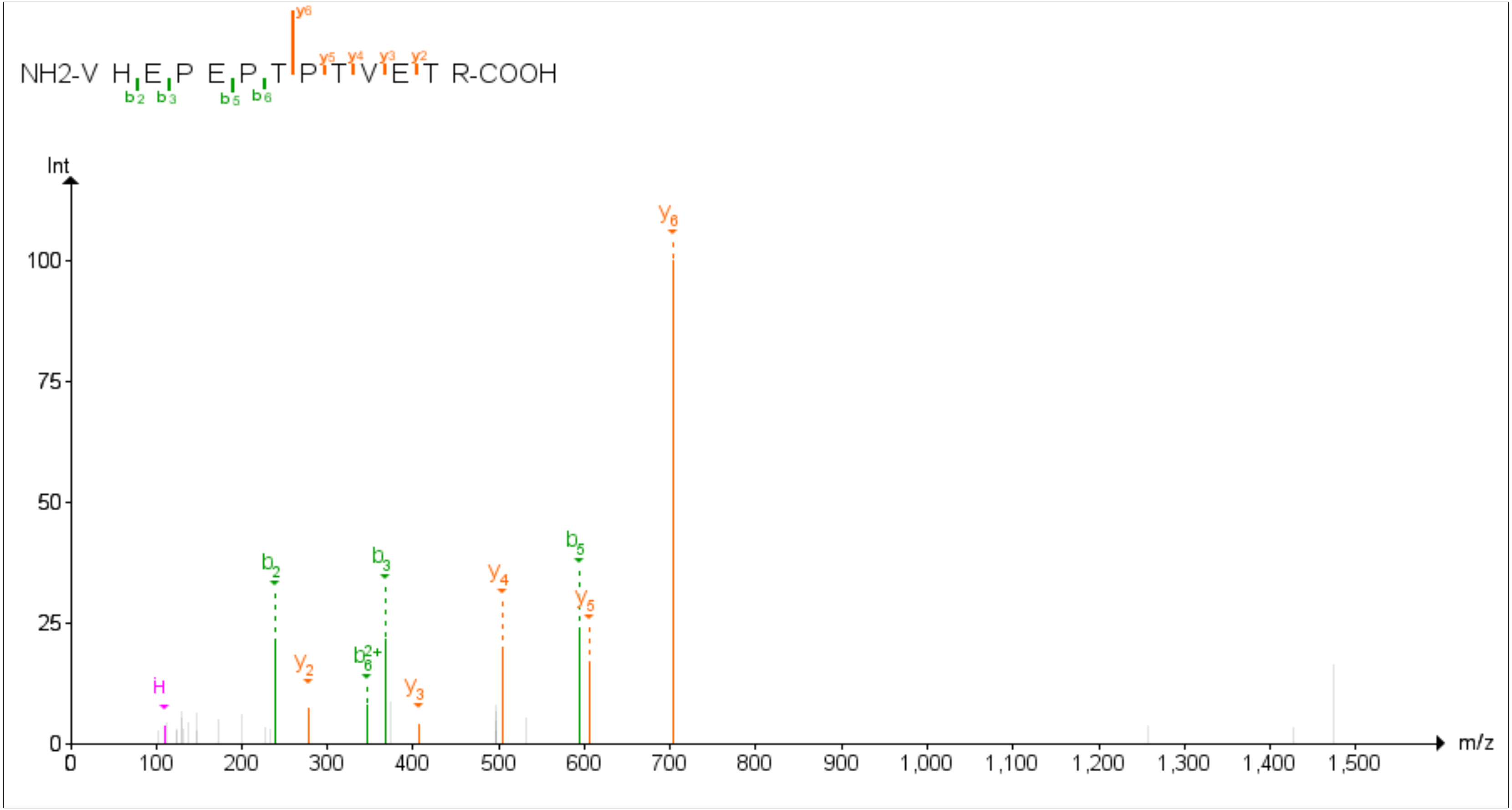
NH2-V H E P E P T P T V E T R-COOH
b2 b3 b5 b6
y6 y5 y4 y3 y2
Int
100
75
50
25
0
m/z
0 100 200 300 400 500 600 700 800 900 1,000 1,100 1,200 1,300 1,400 1,500

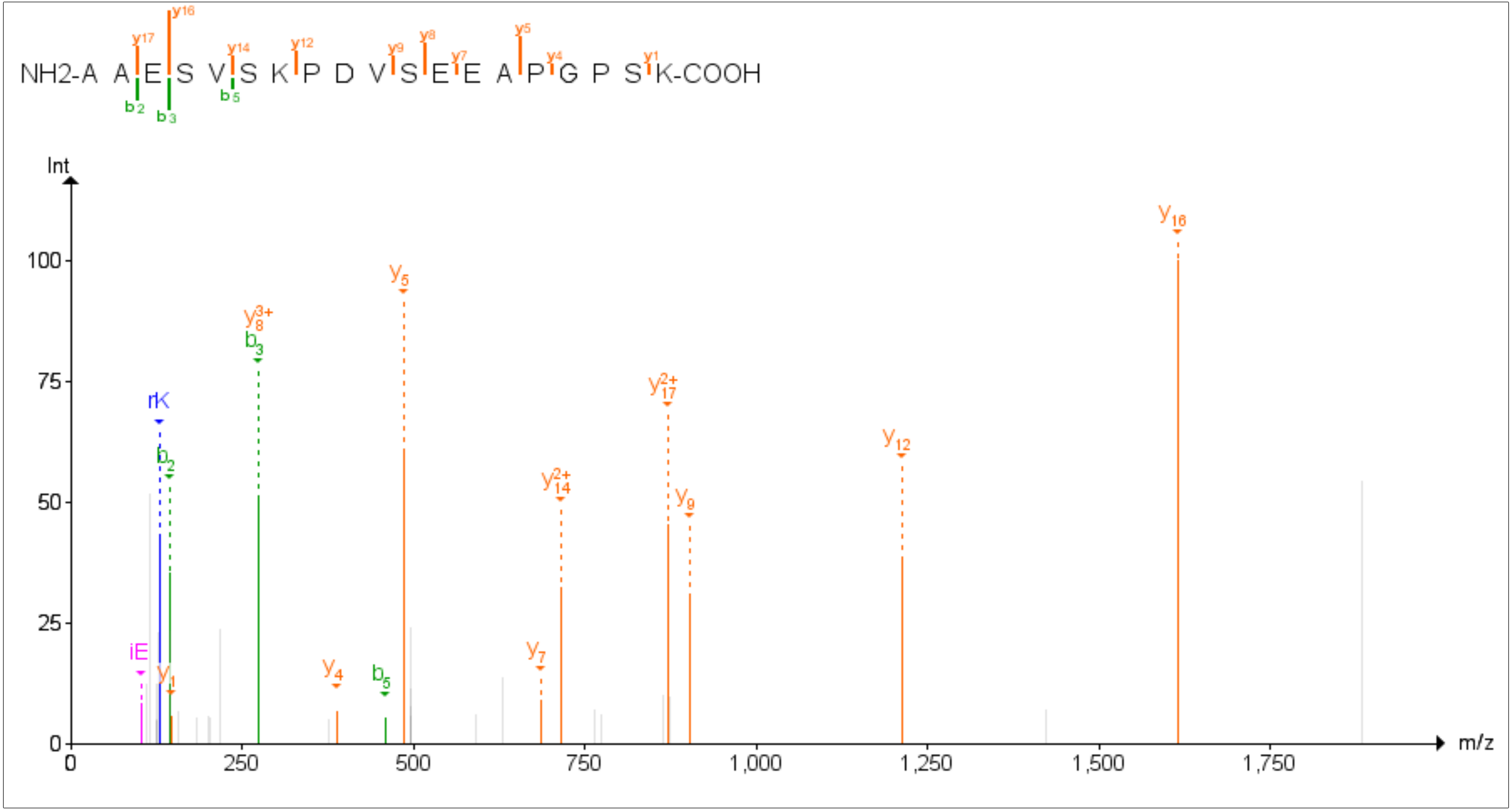

NH2-A A E S V S K P D V S E E A P G P S K-COOH
y17
y16
y14
y12
y9
y8
y7
y5
y4
y1
b 2
b 3
b 5
Int
m/z
iE
rK
b_2
y_1
y_8^{3+}
b_3
y_4
b_5
y_5
y_7
y_{14}^{2+}
y_{17}^{2+}
y_9
y_{12}
y_{16}
0
25
50
75
100
250
500
750
1,000
1,250
1,500
1,750

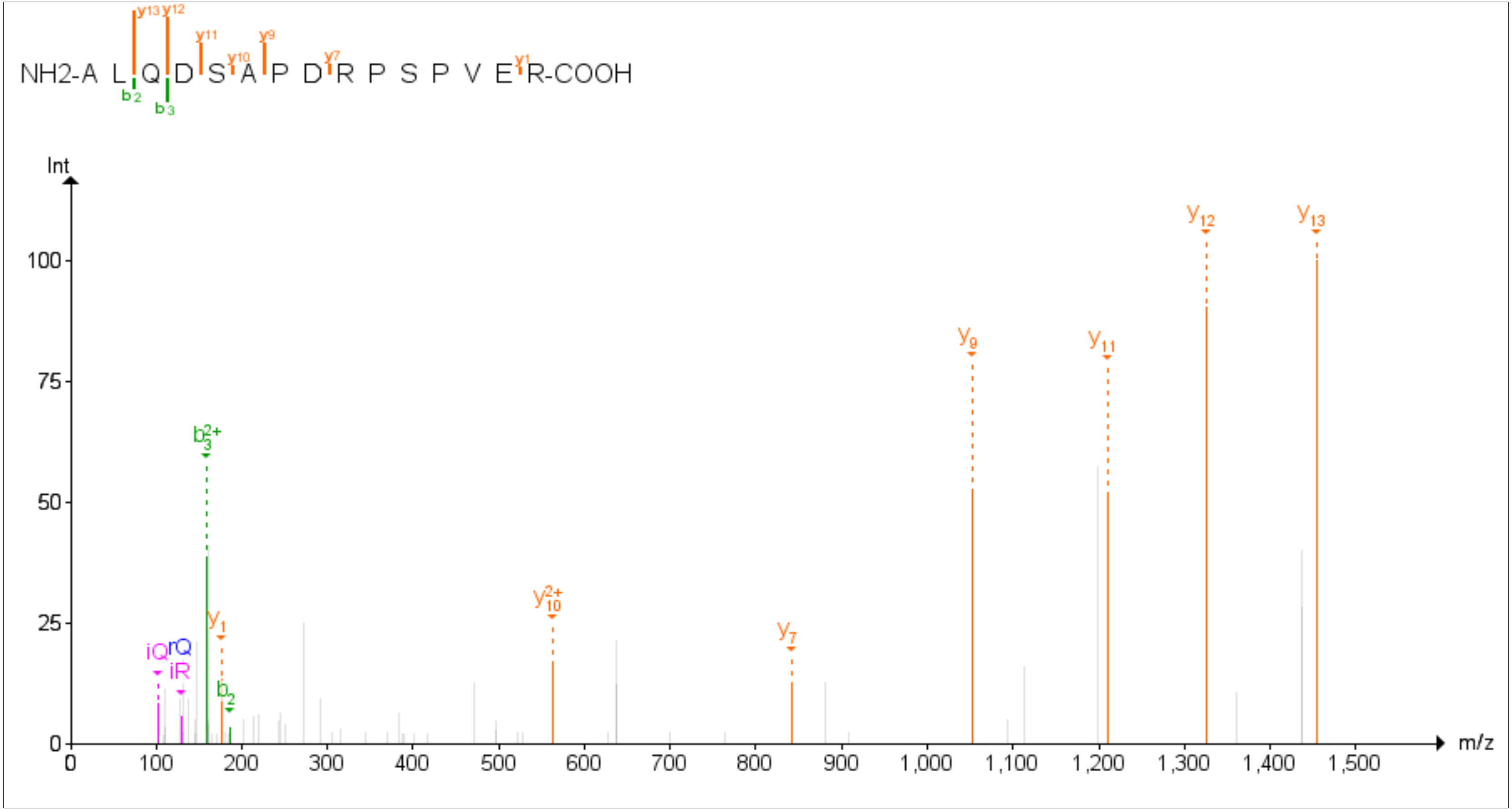

NH2-A L Q D S A P D R P S P V E R-COOH
y13 y12 y11 y10 y9 y7 y1
b2 b3
Int
m/z
iQ
rQ
iR

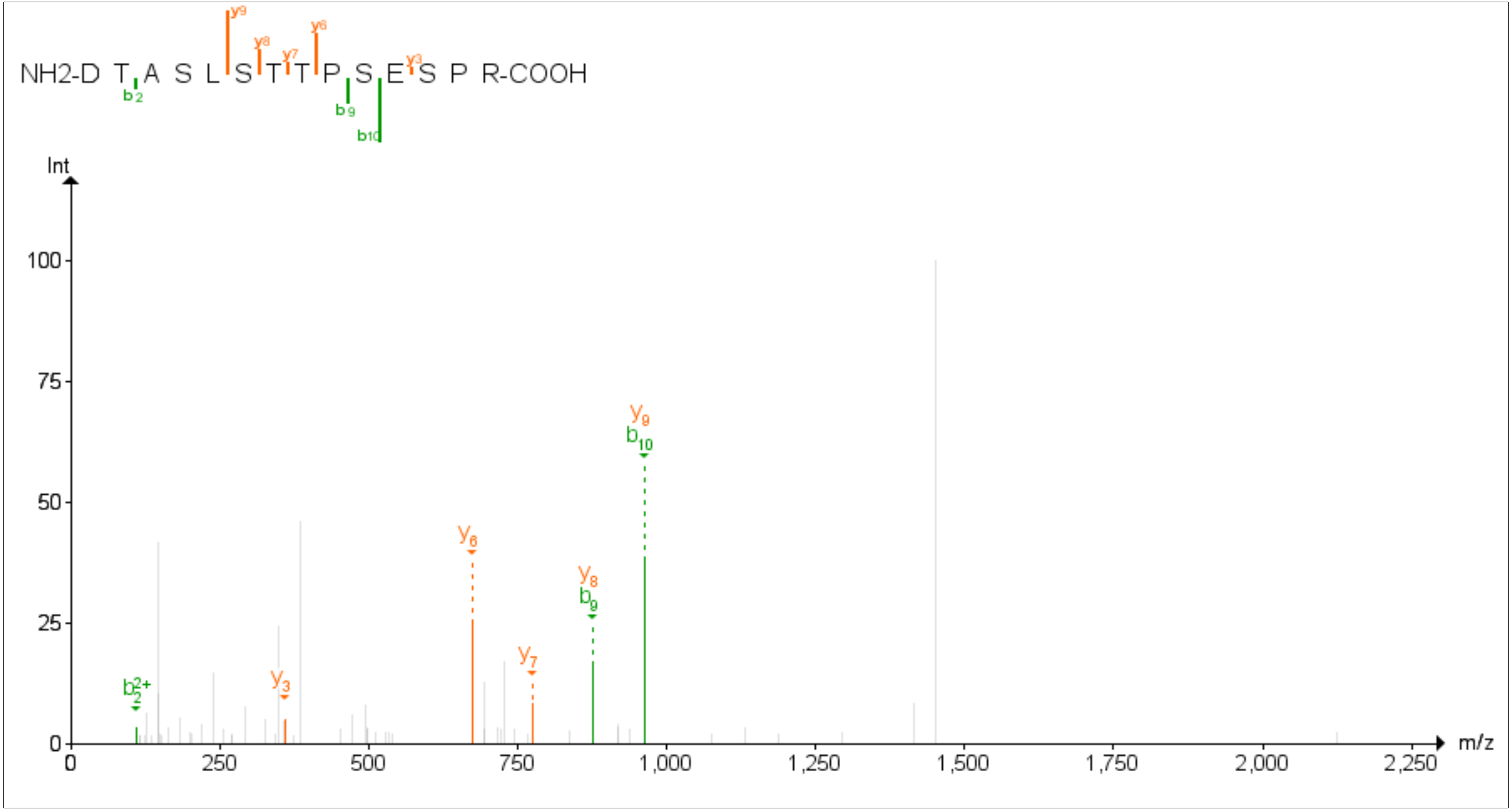

NH2-D T A S L S T T P S E S P R-COOH
y9
y8
y7
y6
y3
b2
b9
b10
Int
100
75
50
25
0
m/z
0
250
500
750
1,000
1,250
1,500
1,750
2,000
2,250
b_2^{2+}
y_3
y_6
y_7
y_8
b_9
y_9
b_{10}

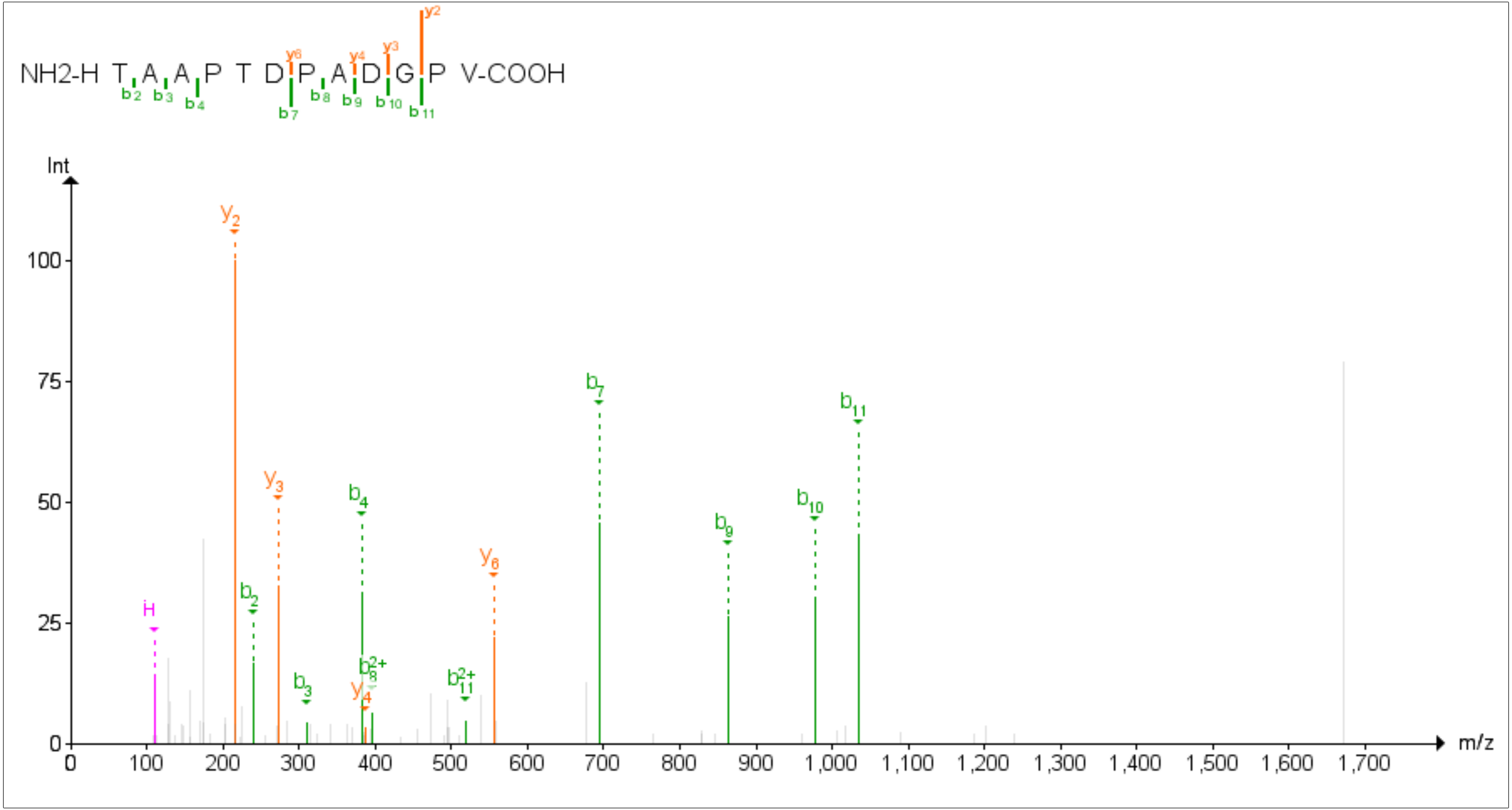

NH2-H T A A P T D P A D G P V-COOH
Int
m/z

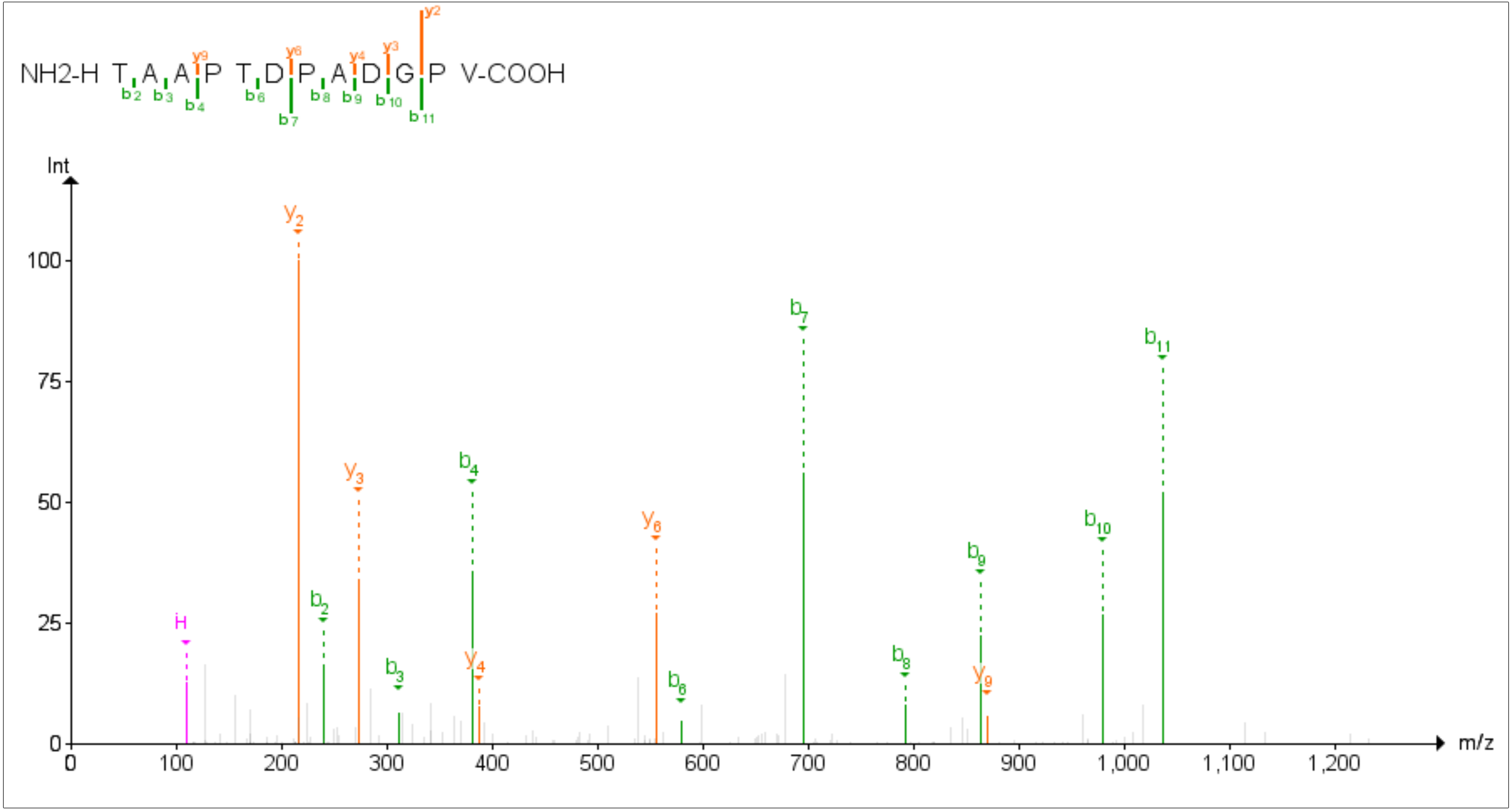

NH2-H T A A P T D P A D G P V-COOH
y9 y6 y4 y3 y2
b2 b3 b4 b6 b7 b8 b9 b10 b11
Int
m/z
0
25
50
75
100
0
100
200
300
400
500
600
700
800
900
1,000
1,100
1,200
H
y2
y3
y4
y6
y9
b2
b3
b4
b6
b7
b8
b9
b10
b11

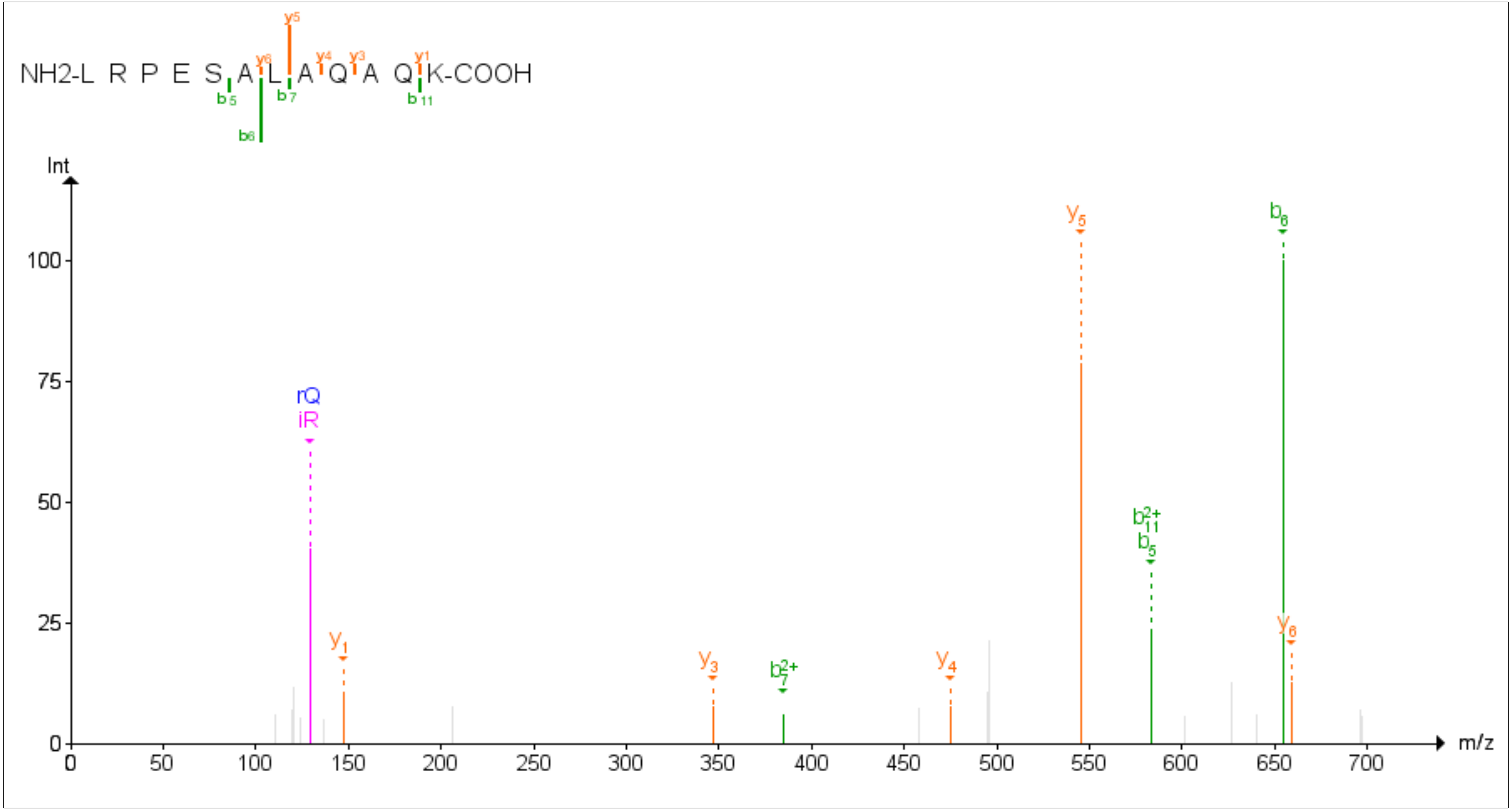
NH2-L R P E S A L A Q A Q K-COOH
y5
y6
y4
y3
y1
b5
b6
b7
b11
Int
m/z
0
25
50
75
100
0
50
100
150
200
250
300
350
400
450
500
550
600
650
700
rQ
iR
y1
y3
b7 2+
y4
y5
b11 2+
b5
b6
y6

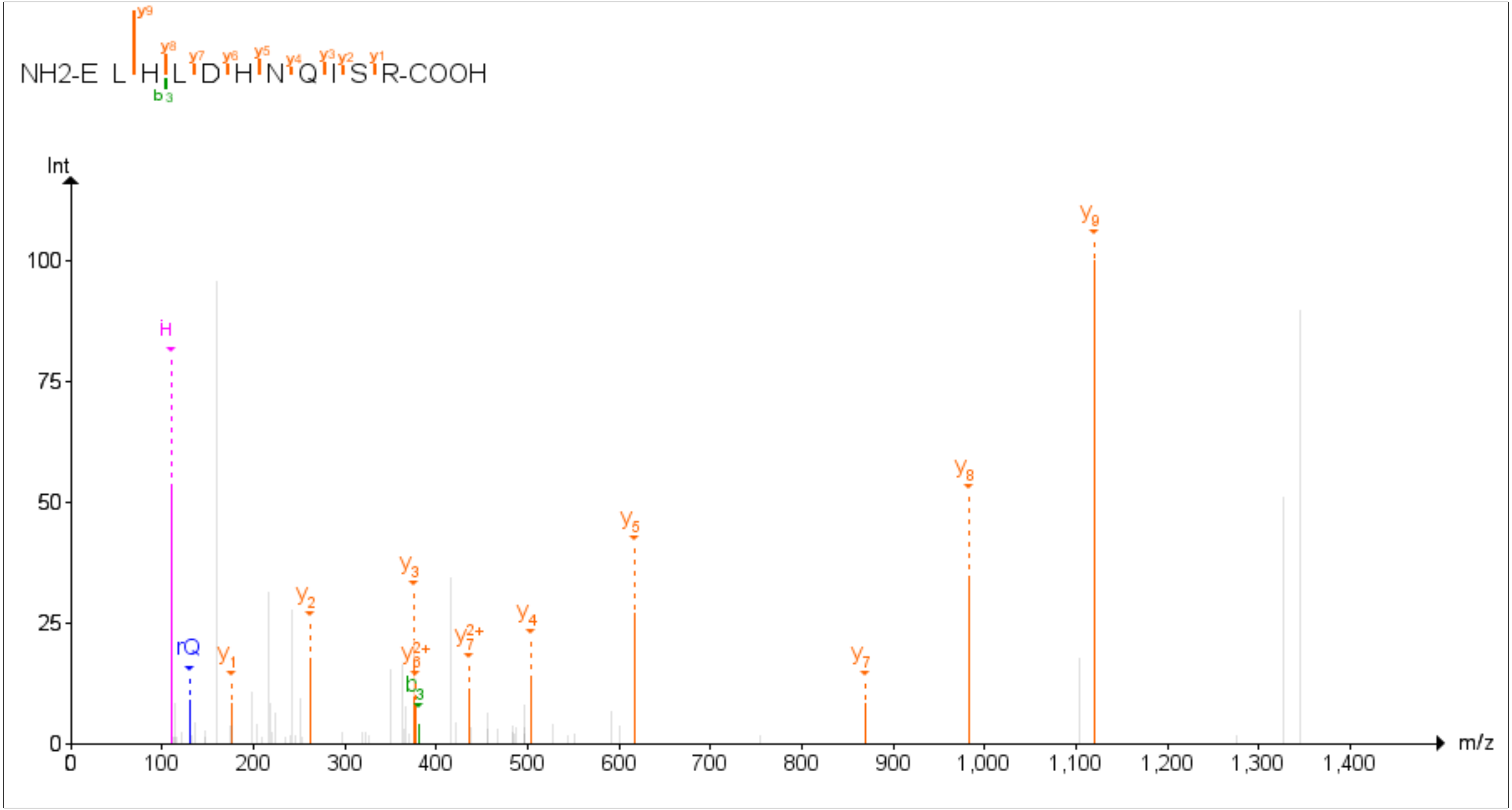
NH2-E L H L D H N Q I S R-COOH
y9 y8 y7 y6 y5 y4 y3 y2 y1
b3
Int
100
75
50
25
0
m/z
0 100 200 300 400 500 600 700 800 900 1,000 1,100 1,200 1,300 1,400
H
rQ
y1 y2 y3 y4 y5 y7 y8 y9
b3

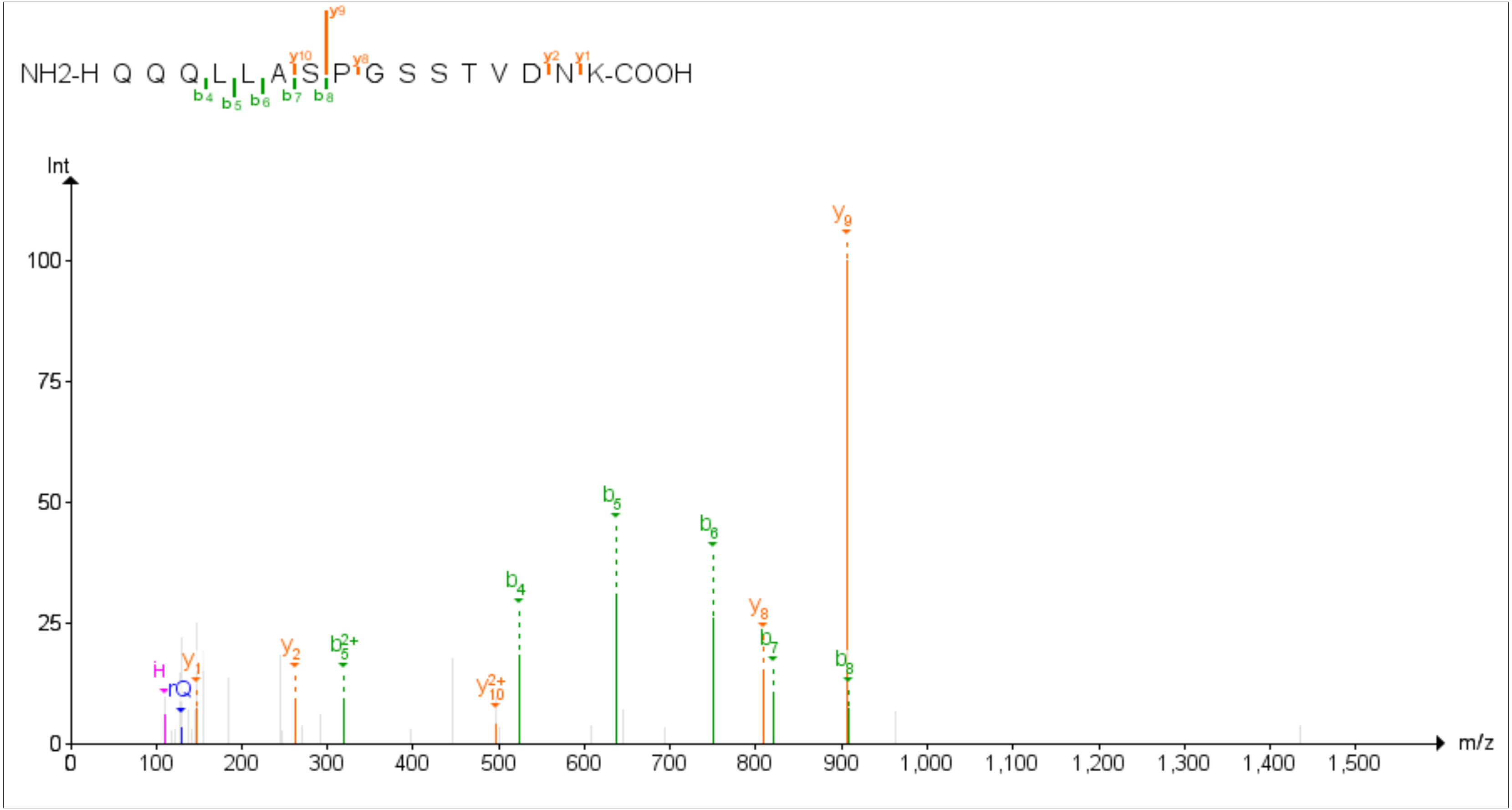

NH2-H Q Q Q L L A S P G S S T V D N K-COOH
y9
y10
y8
y2
y1
b4
b5
b6
b7
b8
Int
100
75
50
25
0
m/z
0
100
200
300
400
500
600
700
800
900
1,000
1,100
1,200
1,300
1,400
1,500
H
rQ
y1
y2
b5 2+
y10 2+
b4
b5
b6
y8
b7
b8
y9

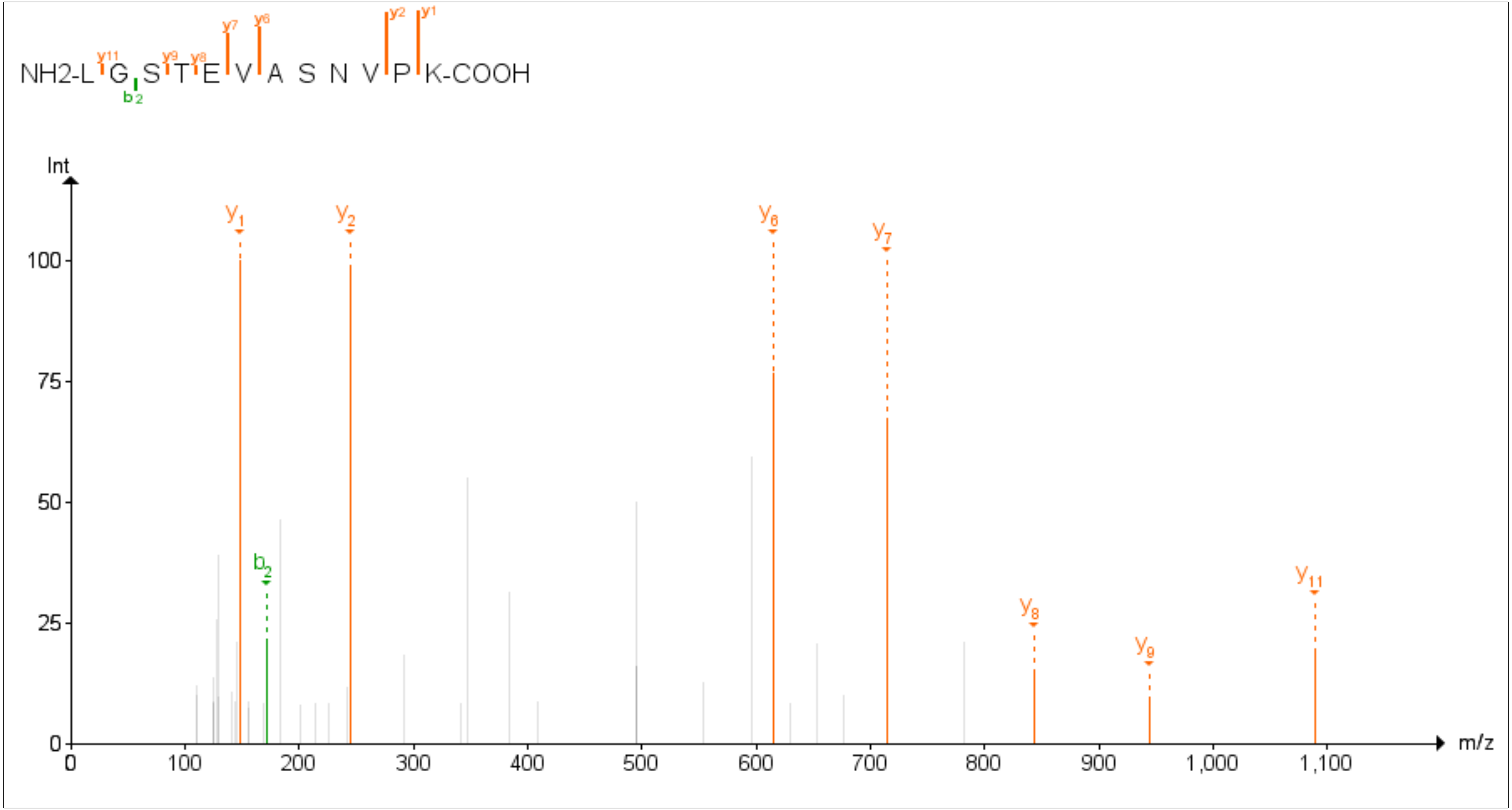

NH2-L G S T E V A S N V P K-COOH
y11 y9 y8 y7 y6 y2 y1
b2
Int
100
75
50
25
0
y1 y2 y6 y7 b2 y8 y9 y11
0 100 200 300 400 500 600 700 800 900 1,000 1,100
m/z

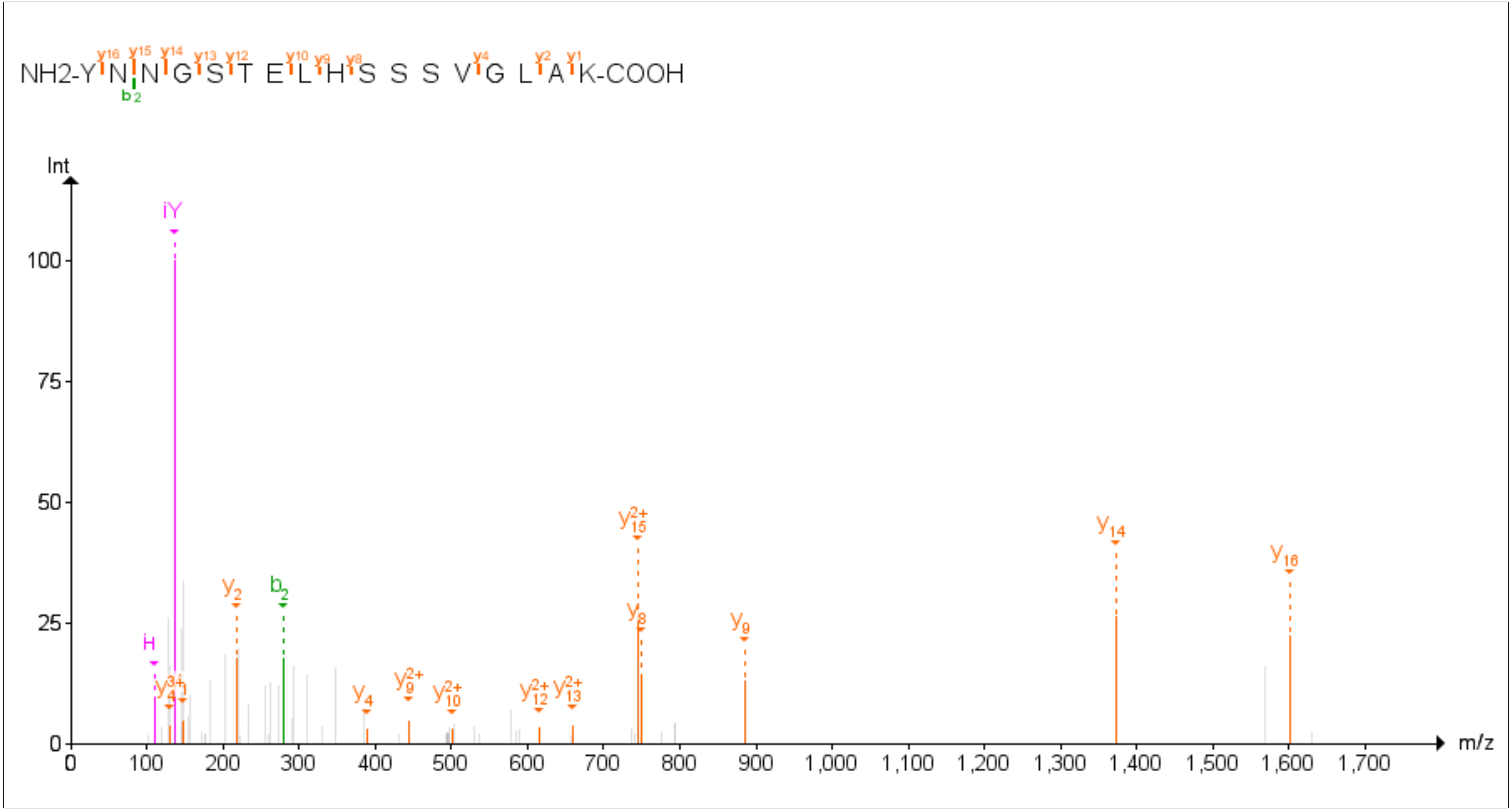

NH2-YNNGSTELHSSSVGLAK-COOH
y16 y15 y14 y13 y12 y10 y9 y8 y4 y2 y1
b2
Int
iY
iH
y2
b2
y4
y15 2+
y14
y16
y8
y9
y9 2+
y10 2+
y12 2+
y13 2+
y4 3+
y1
m/z
0
25
50
75
100
0
100
200
300
400
500
600
700
800
900
1,000
1,100
1,200
1,300
1,400
1,500
1,600
1,700

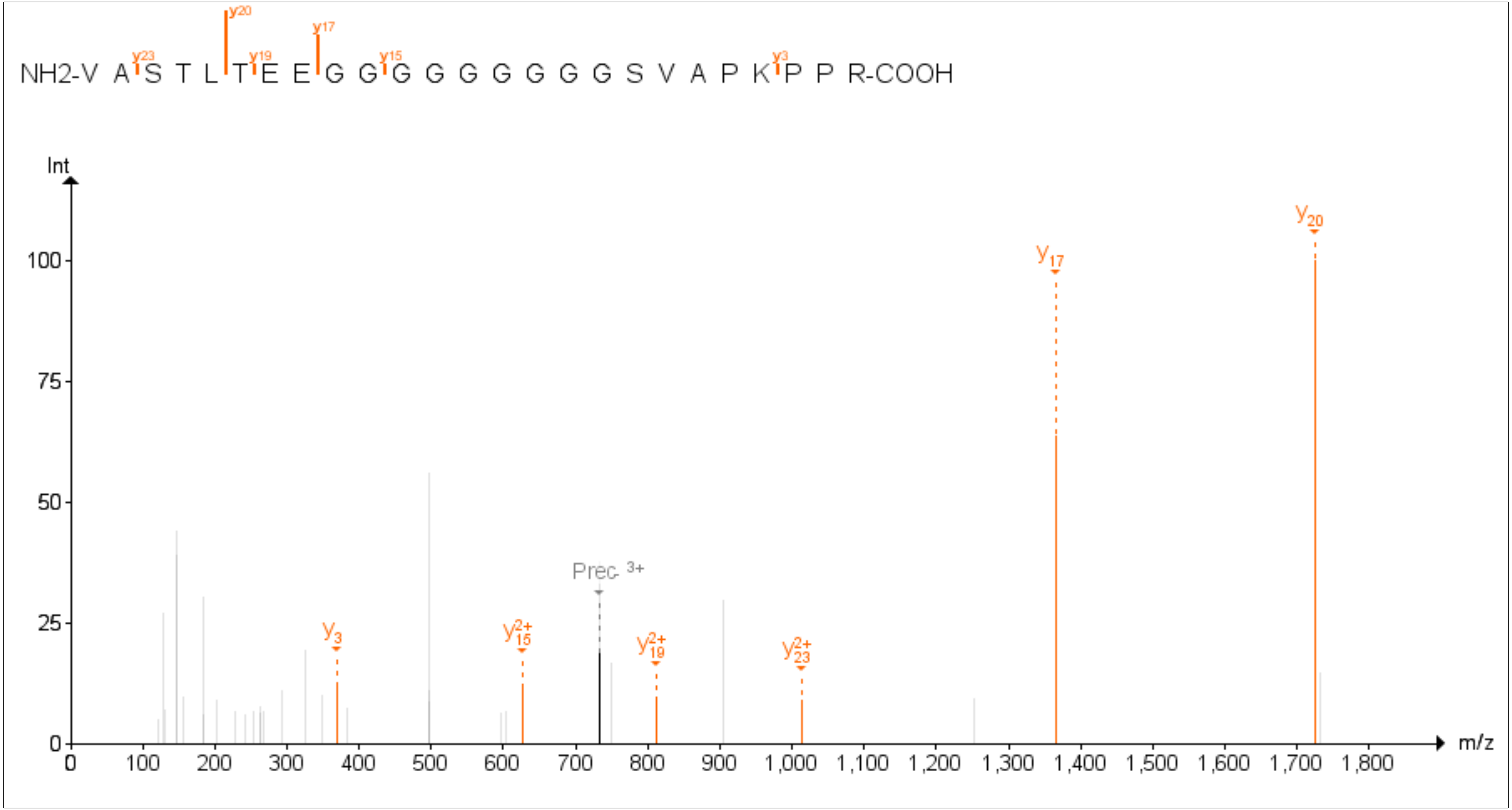

NH2-V A S T L T E E G G G G G G G G G S V A P K P P R-COOH
y23
y20
y19
y17
y15
y3
Int
m/z
Prec 3+
y3
y15 2+
y19 2+
y23 2+
y17
y20
0
25
50
75
100
0
100
200
300
400
500
600
700
800
900
1,000
1,100
1,200
1,300
1,400
1,500
1,600
1,700
1,800

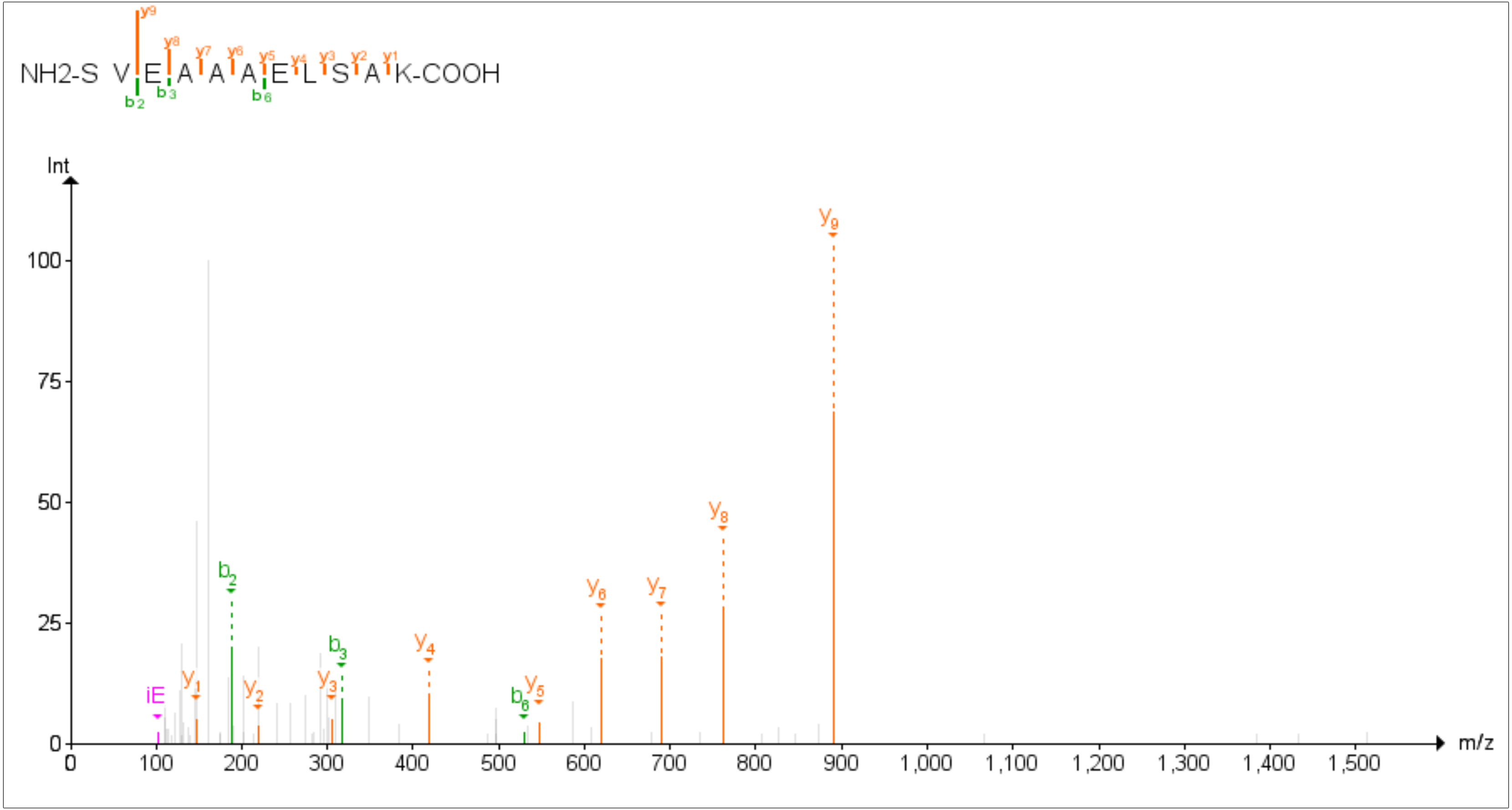

NH2-S V E A A A E L S A K-COOH
y9
y8
y7
y6
y5
y4
y3
y2
y1
b2
b3
b6
Int
100
75
50
25
0
m/z
0
100
200
300
400
500
600
700
800
900
1,000
1,100
1,200
1,300
1,400
1,500
iE
y1
b2
y2
y3
b3
y4
b6
y5
y6
y7
y8
y9

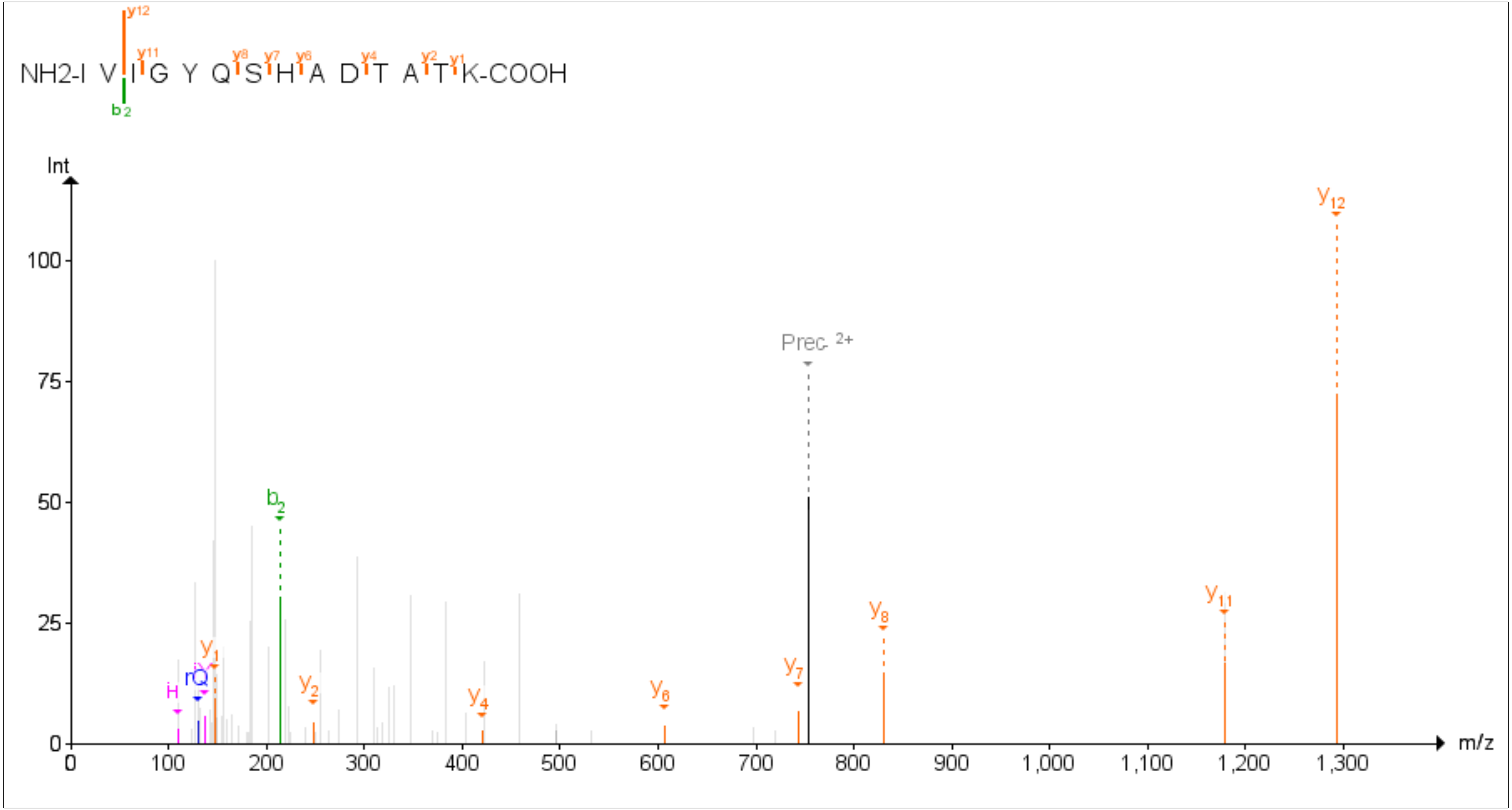

NH2-I V I G Y Q S H A D T A T K-COOH
y12
y11
y8
y7
y6
y4
y2
y1
b2
Int
m/z
Prec 2+

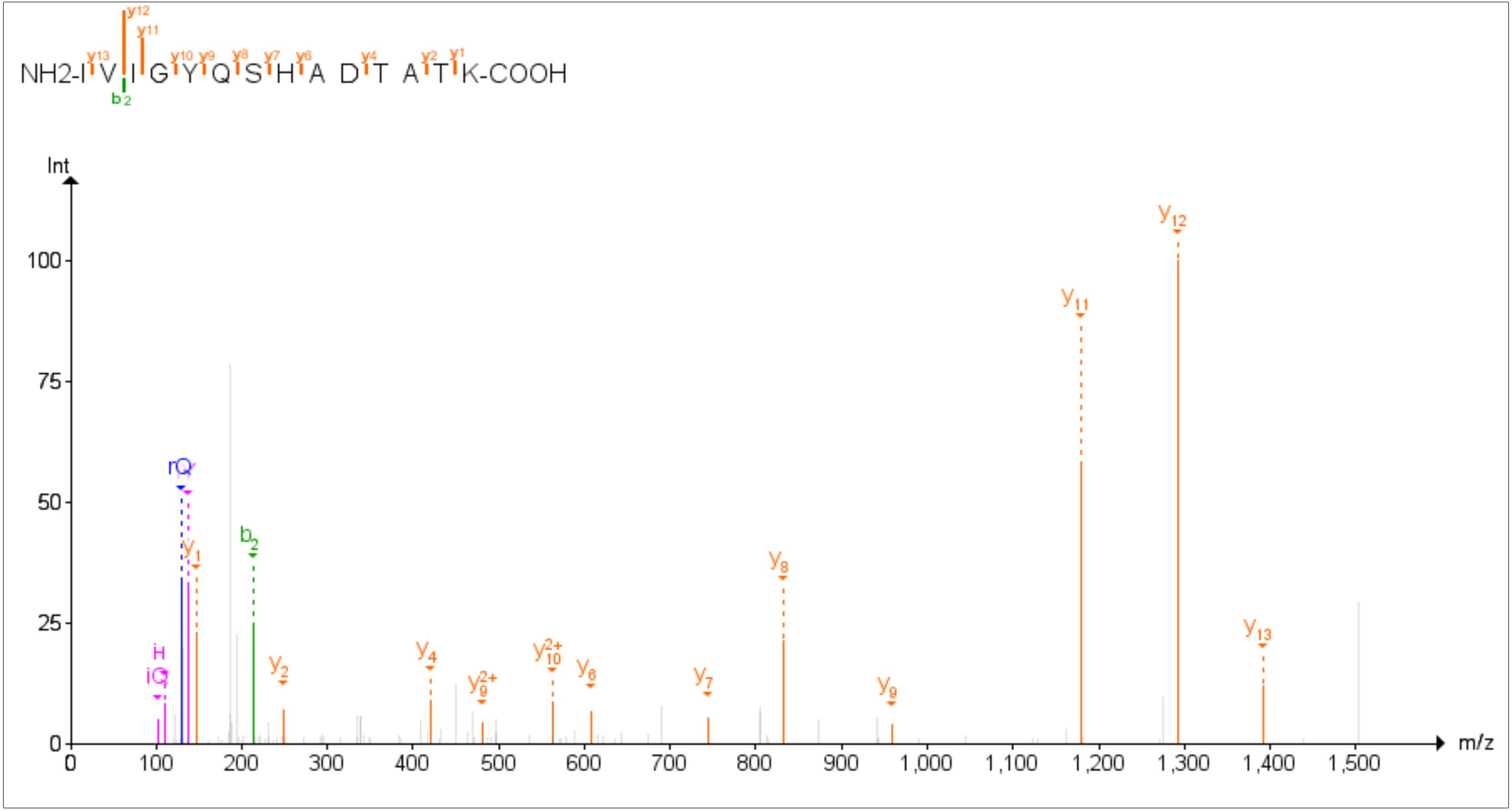

NH2-I V I G Y Q S H A D T A T K-COOH
y13 y12 y11 y10 y9 y8 y7 y6 y4 y2 y1
b2
Int
m/z
rQ
b2
y1
iH
iQ
y2
y4
y9 2+
y10 2+
y6
y7
y8
y9
y11
y12
y13

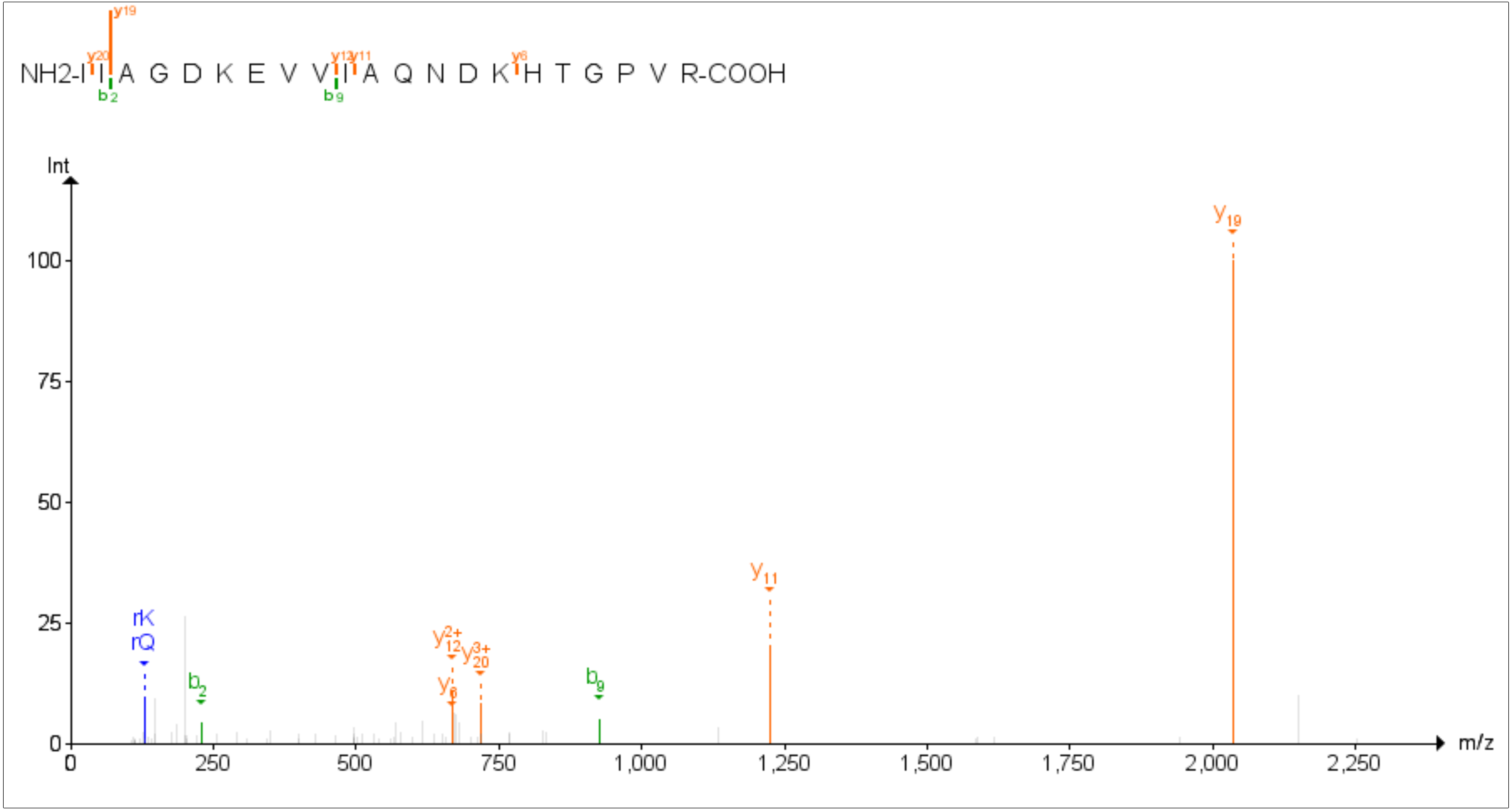

NH2-I I A G D K E V V I A Q N D K H T G P V R-COOH
y19
y20
y12
y11
y6
b2
b9
Int
100
75
50
25
0
m/z
0
250
500
750
1,000
1,250
1,500
1,750
2,000
2,250
y19
y11
rK
rQ
b2
y12 2+
y20 3+
y6
b9

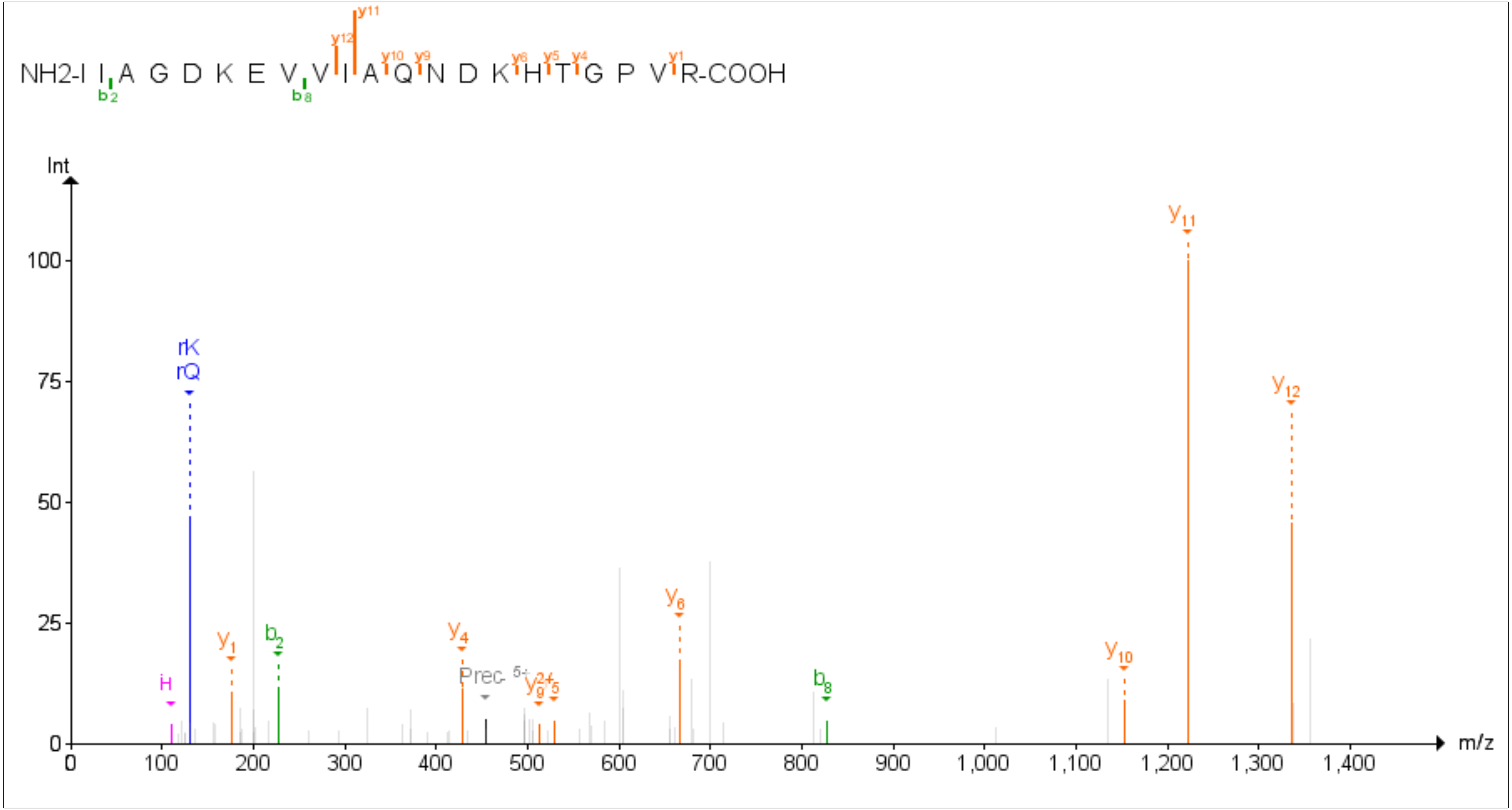

NH2-I I A G D K E V V I A Q N D K H T G P V R-COOH
b2 b8 y12 y11 y10 y9 y6 y5 y4 y1
Int
m/z
rK
rQ
H
y1
b2
y4
Prec 5+
y9 2+
y5
y6
b8
y10
y11
y12

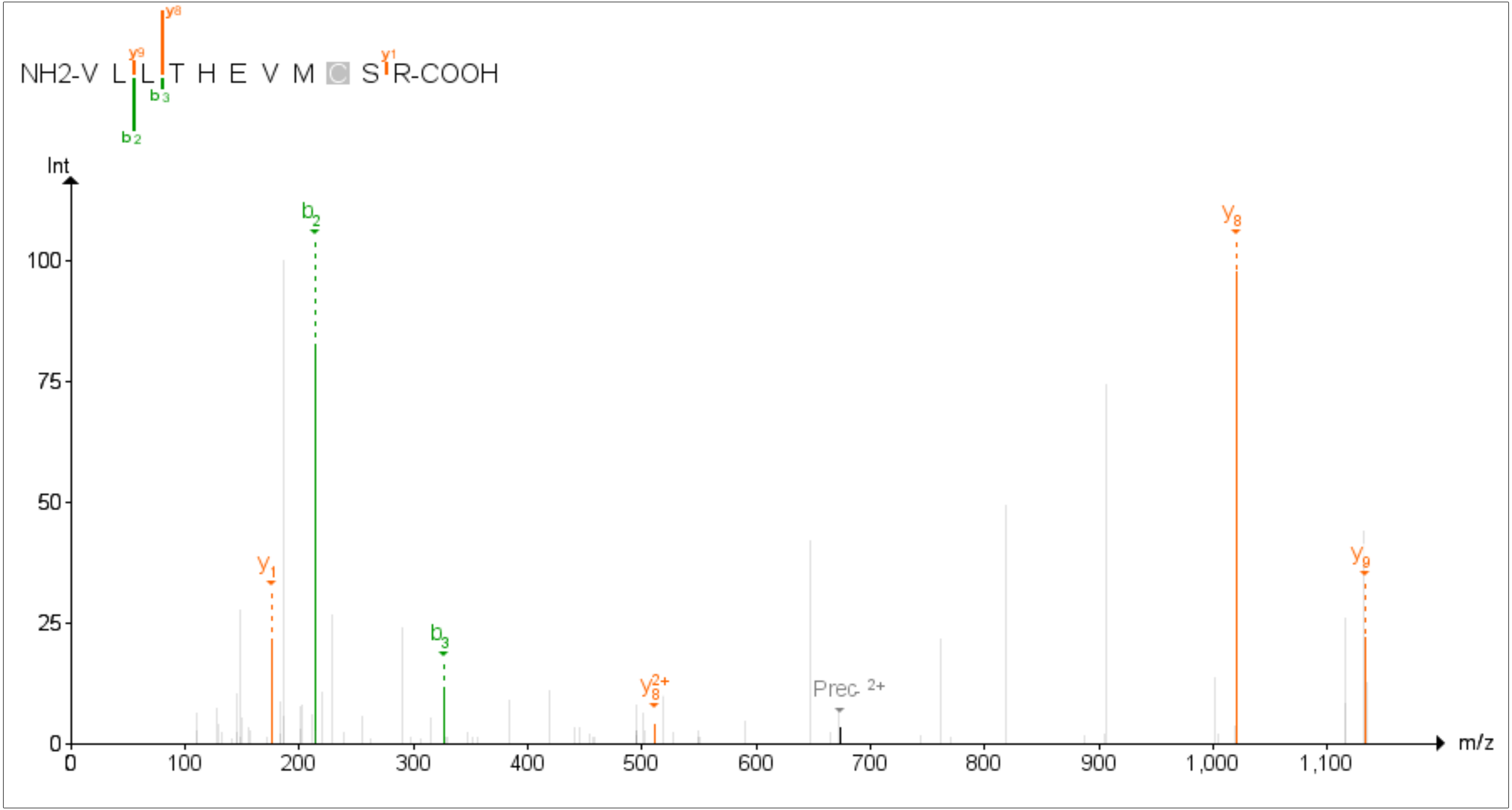

NH2-V L L T H E V M C S R-COOH
y8
y9
y1
b3
b2
Int
b2
y8
100
75
50
25
0
y1
b3
y9
y8 2+
Prec. 2+
0
100
200
300
400
500
600
700
800
900
1,000
1,100
m/z

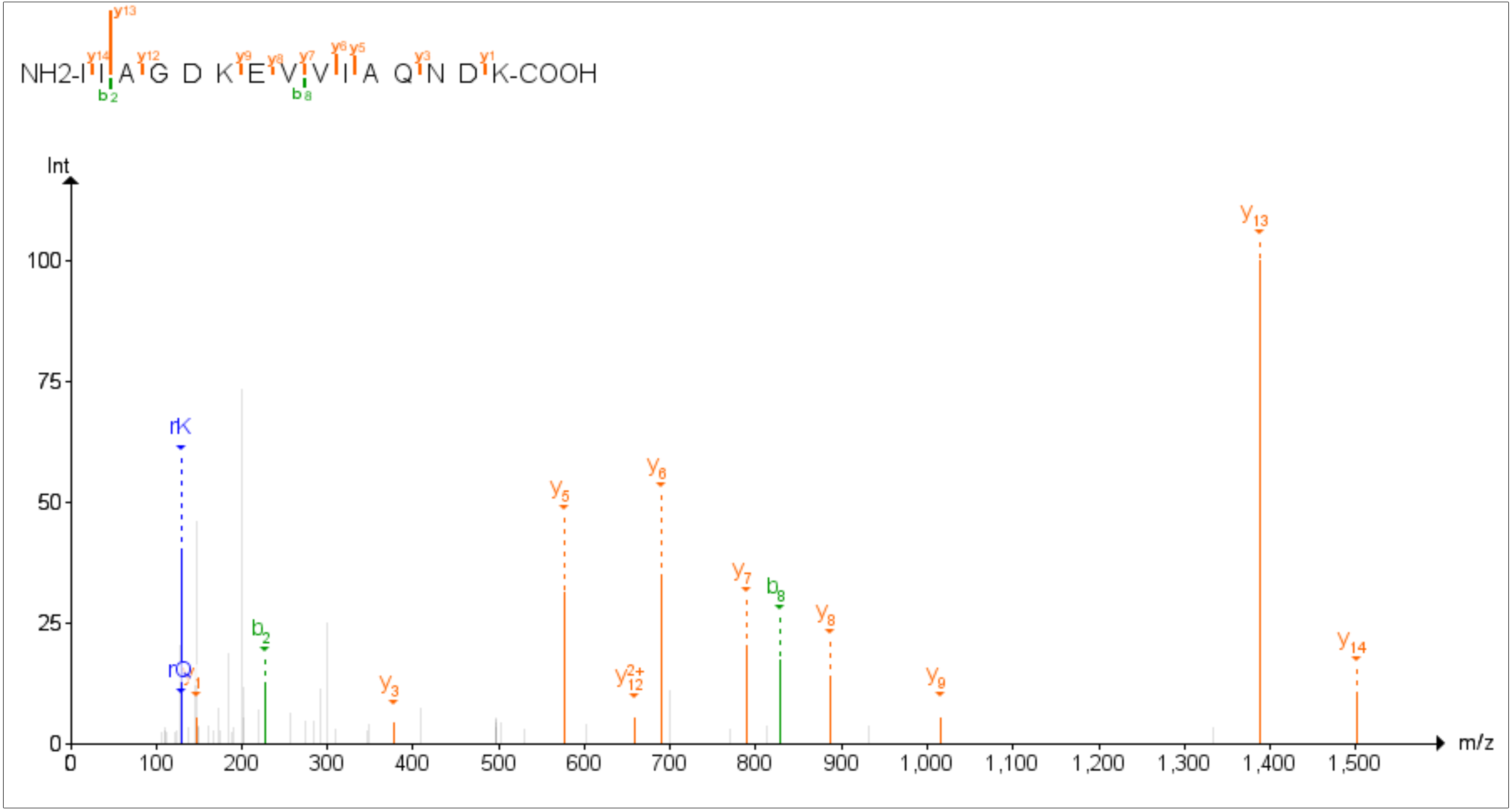
NH2-I I A G D K E V V I A Q N D K-COOH
Int
m/z
rK
rQ
y1
b2
y3
y5
y6
y7
b8
y8
y9
y12 2+
y13
y14
y14
y13
y12
y9
y8
y7
y6
y5
y3
y1
b2
b8

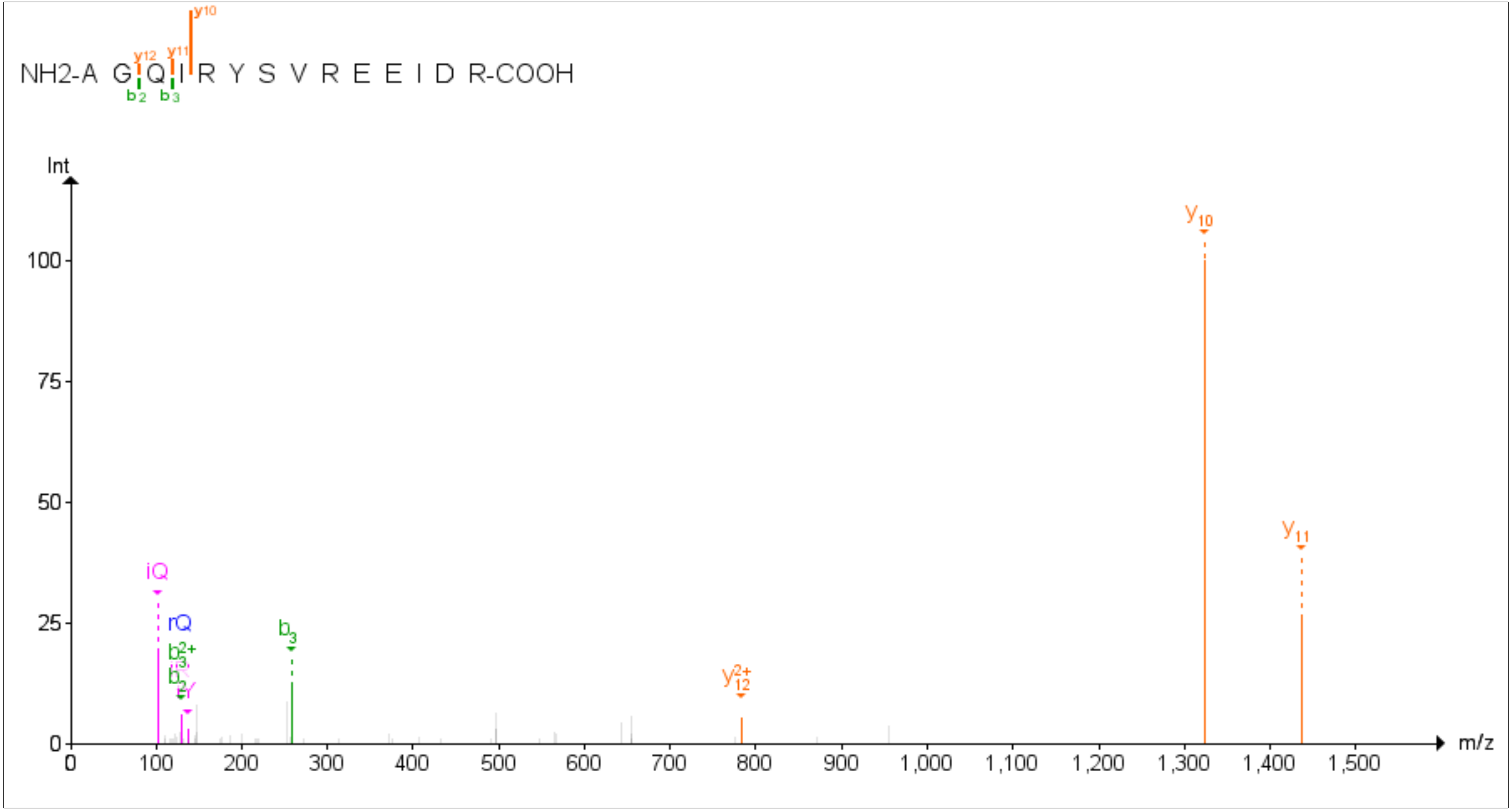

NH2-A G Q I R Y S V R E E I D R-COOH
y10
y12
y11
b2
b3
Int
100
75
50
25
0
y10
y11
iQ
rQ
b3
b3 2+
b2
y12 2+
0
100
200
300
400
500
600
700
800
900
1,000
1,100
1,200
1,300
1,400
1,500
m/z

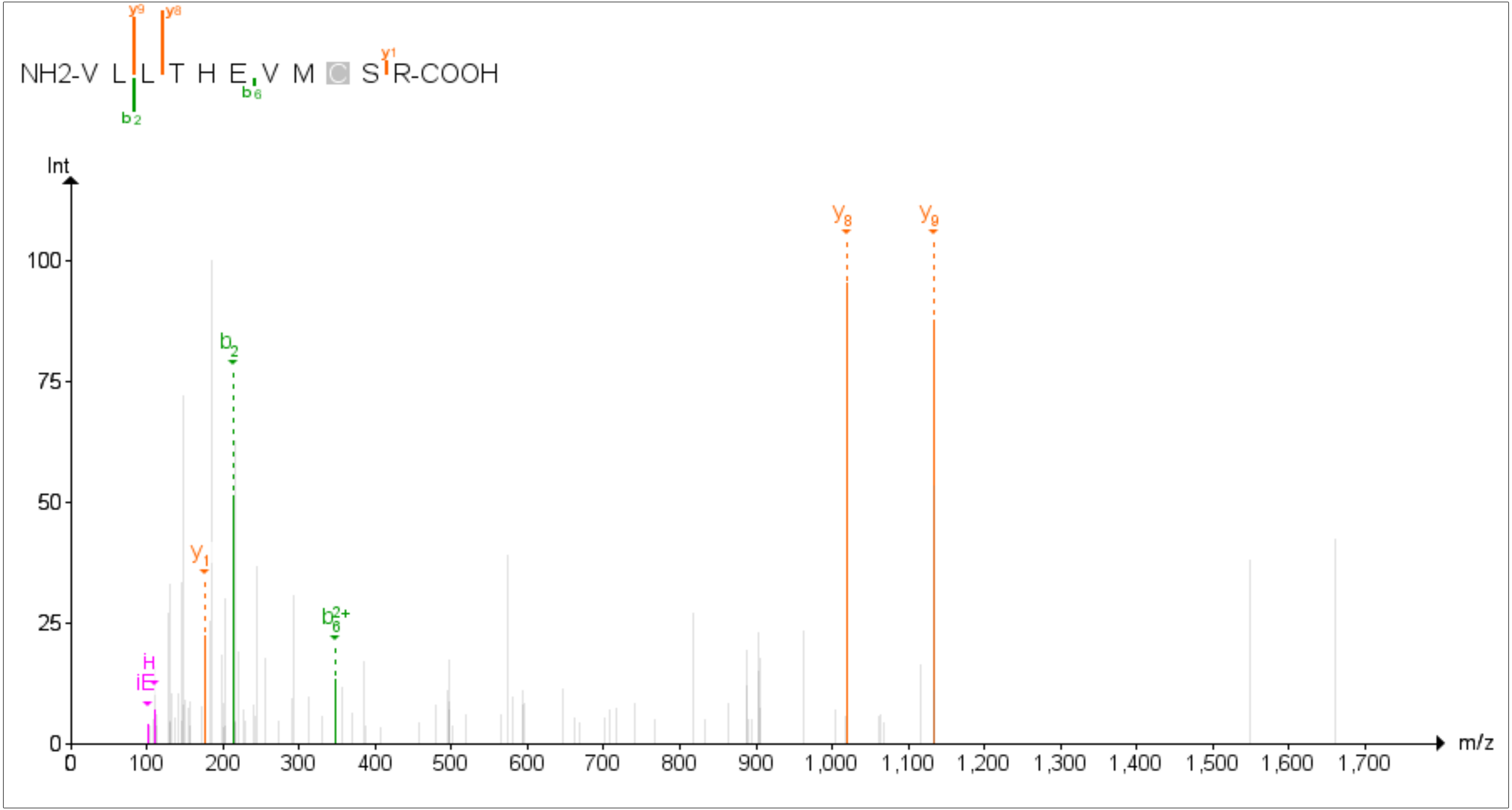

NH2-V L L T H E V M C S R-COOH
y9
y8
y1
b2
b6
Int
100
75
50
25
0
y8
y9
b2
y1
b6 2+
iH
iE
0
100
200
300
400
500
600
700
800
900
1,000
1,100
1,200
1,300
1,400
1,500
1,600
1,700
m/z

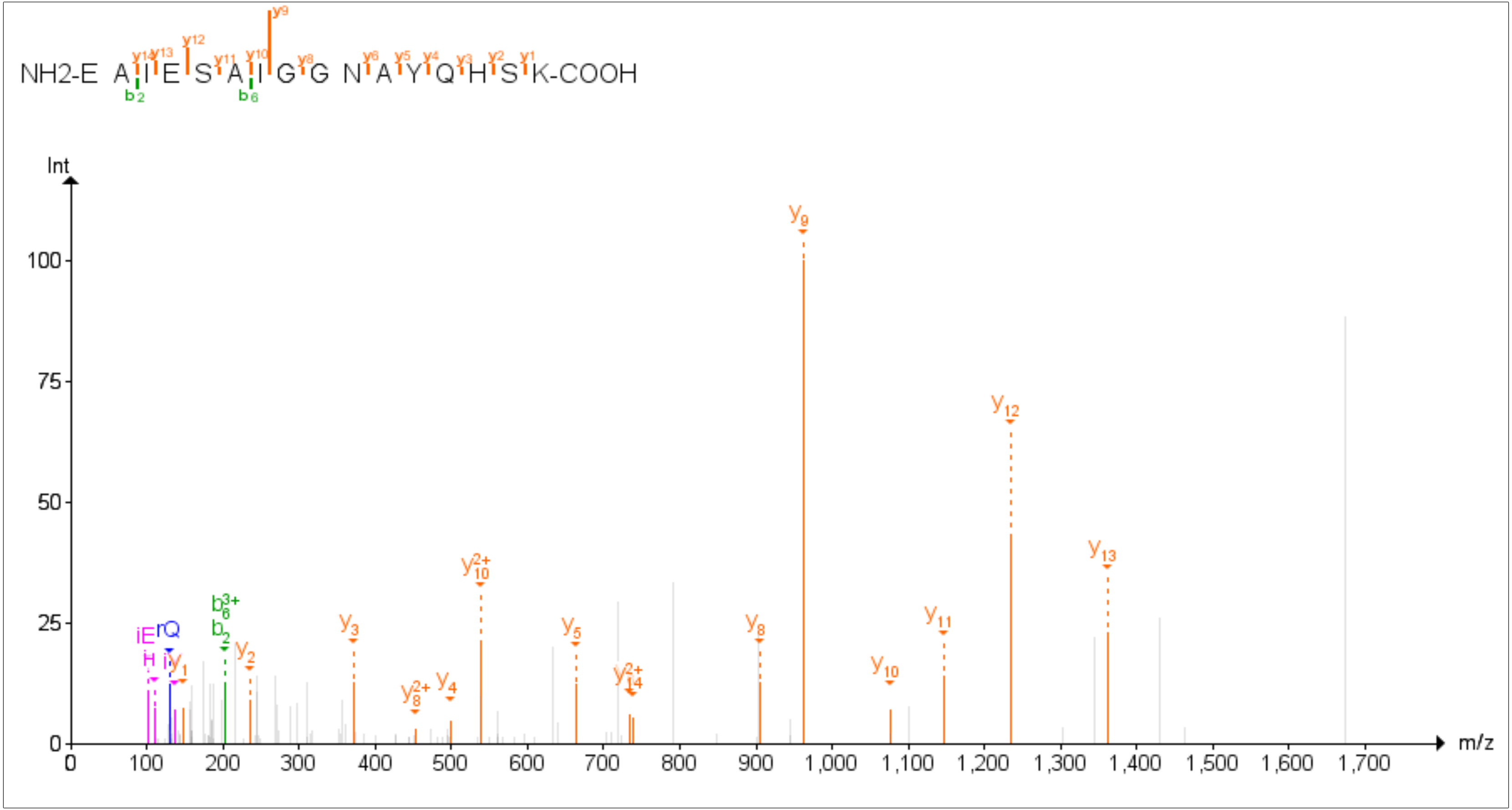

NH2-E A I E S A I G G N A Y Q H S K-COOH
Int
m/z
y9
y12
y13
y11
y10
y8
y5
y3
y2
y1
y4
b2
b6
iE
rQ
iH
iY
y10 2+
y8 2+
y14 2+
b6 3+

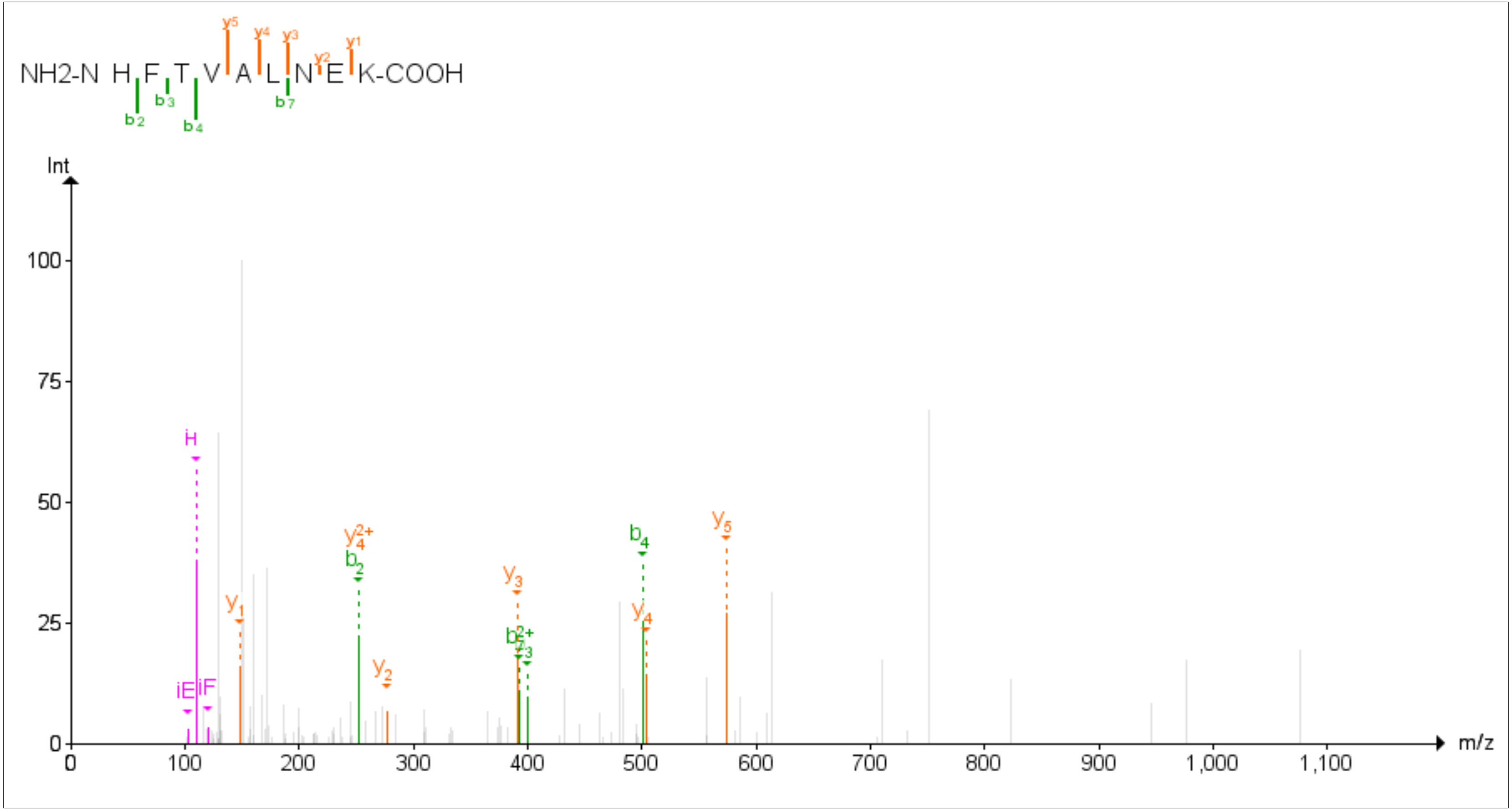

NH2-N H F T V A L N E K-COOH
y5 y4 y3 y2 y1
b2 b3 b4 b7
Int
100
75
50
25
0
m/z
0 100 200 300 400 500 600 700 800 900 1,000 1,100
iH
iE iF
y1
y4 2+
b2
y2
y3
b7 2+
y3
b4
y4
y5

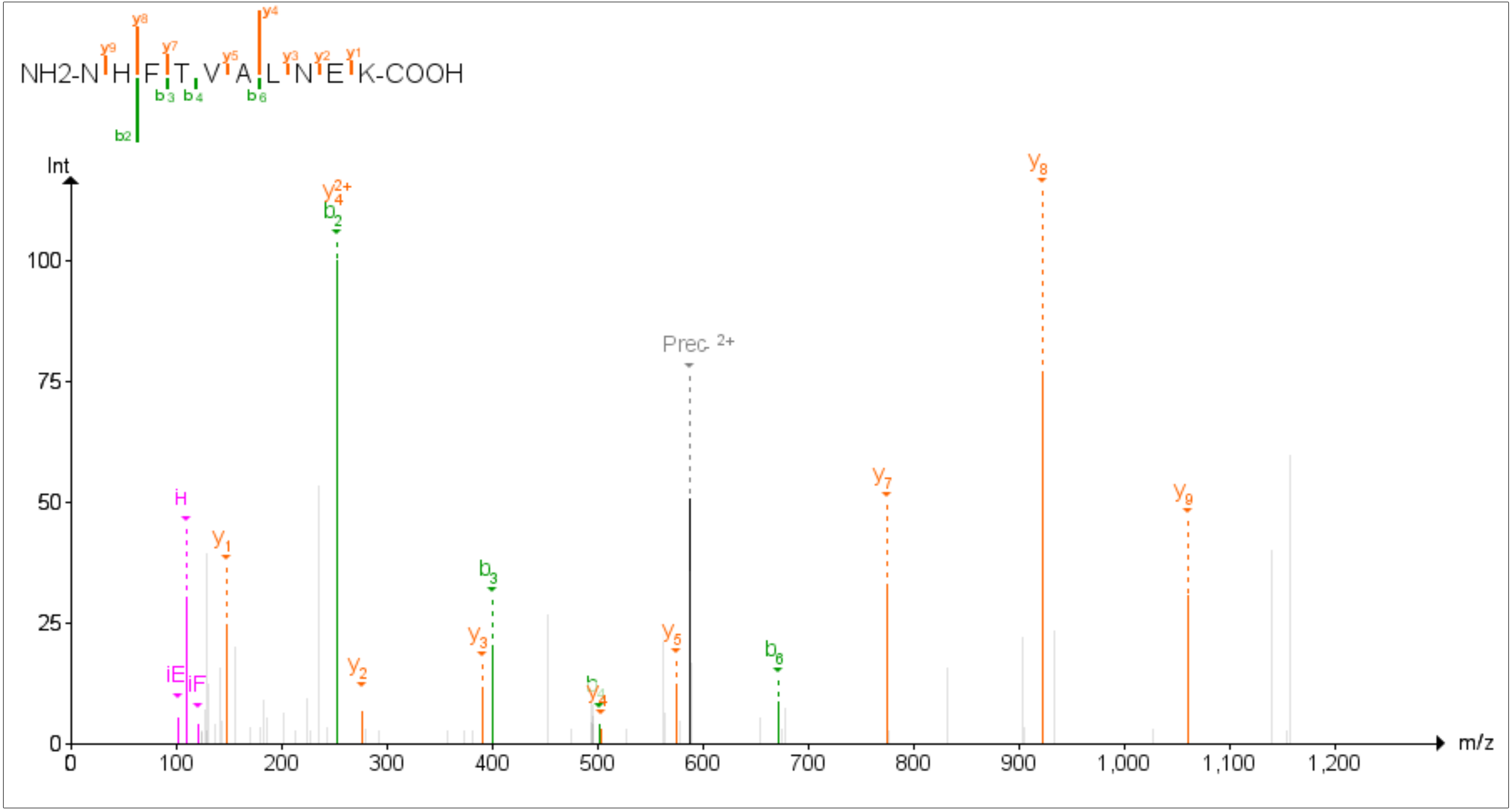

NH2-N H F T V A L N E K-COOH
y9 y8 y7 y5 y4 y3 y2 y1
b2 b3 b4 b6
Int
m/z
Prec. 2+
y8
y7
y9
y1
y2
y3
y5
b2
b3
b6
iH
iE
iF
0 25 50 75 100
0 100 200 300 400 500 600 700 800 900 1,000 1,100 1,200

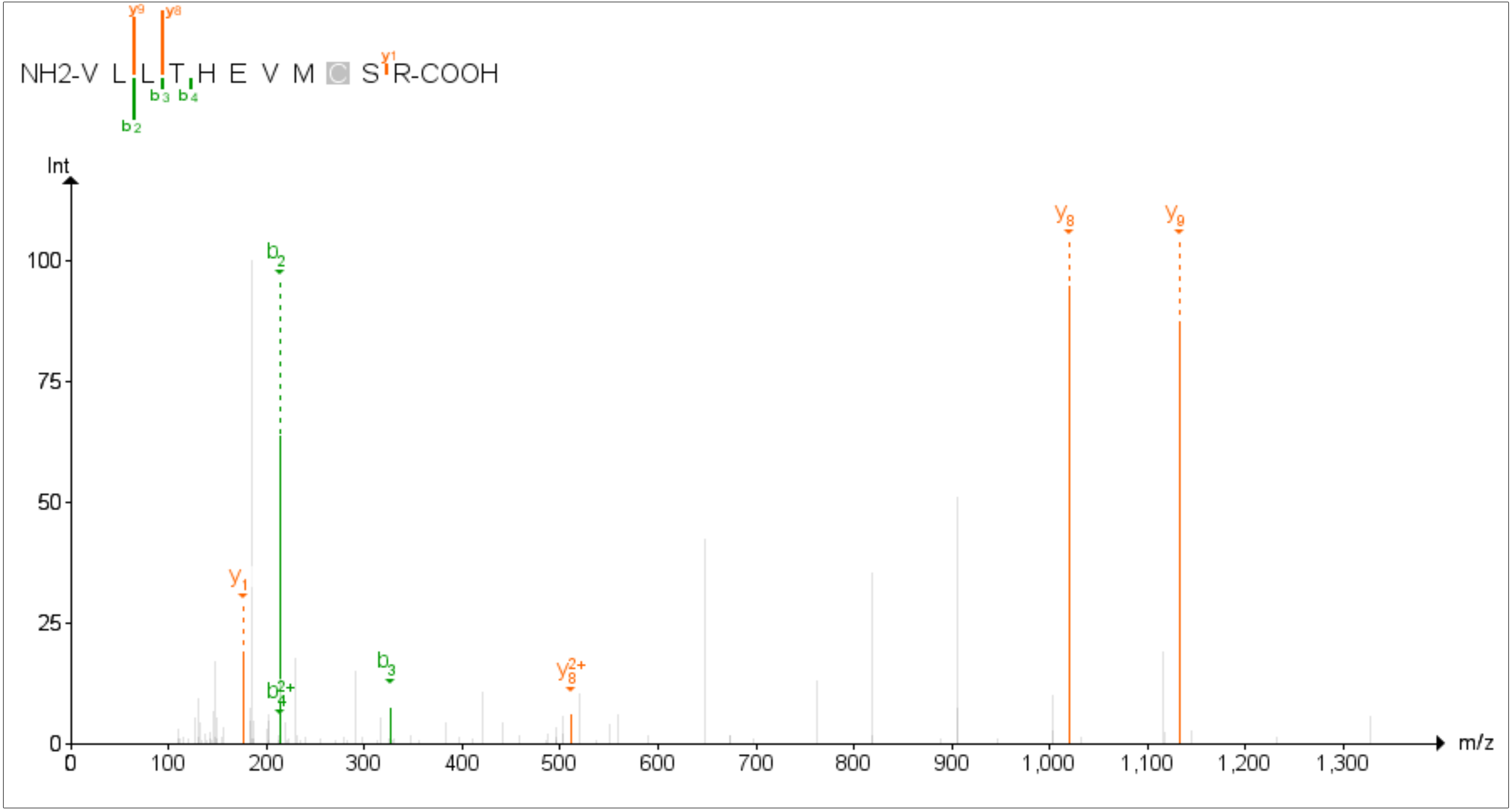

NH2-V L L T H E V M C S R-COOH
y9
y8
y1
b2
b3
b4
Int
100
75
50
25
0
y8
y9
b2
y1
b3
b_4^{2+}
y_8^{2+}
m/z
0
100
200
300
400
500
600
700
800
900
1,000
1,100
1,200
1,300

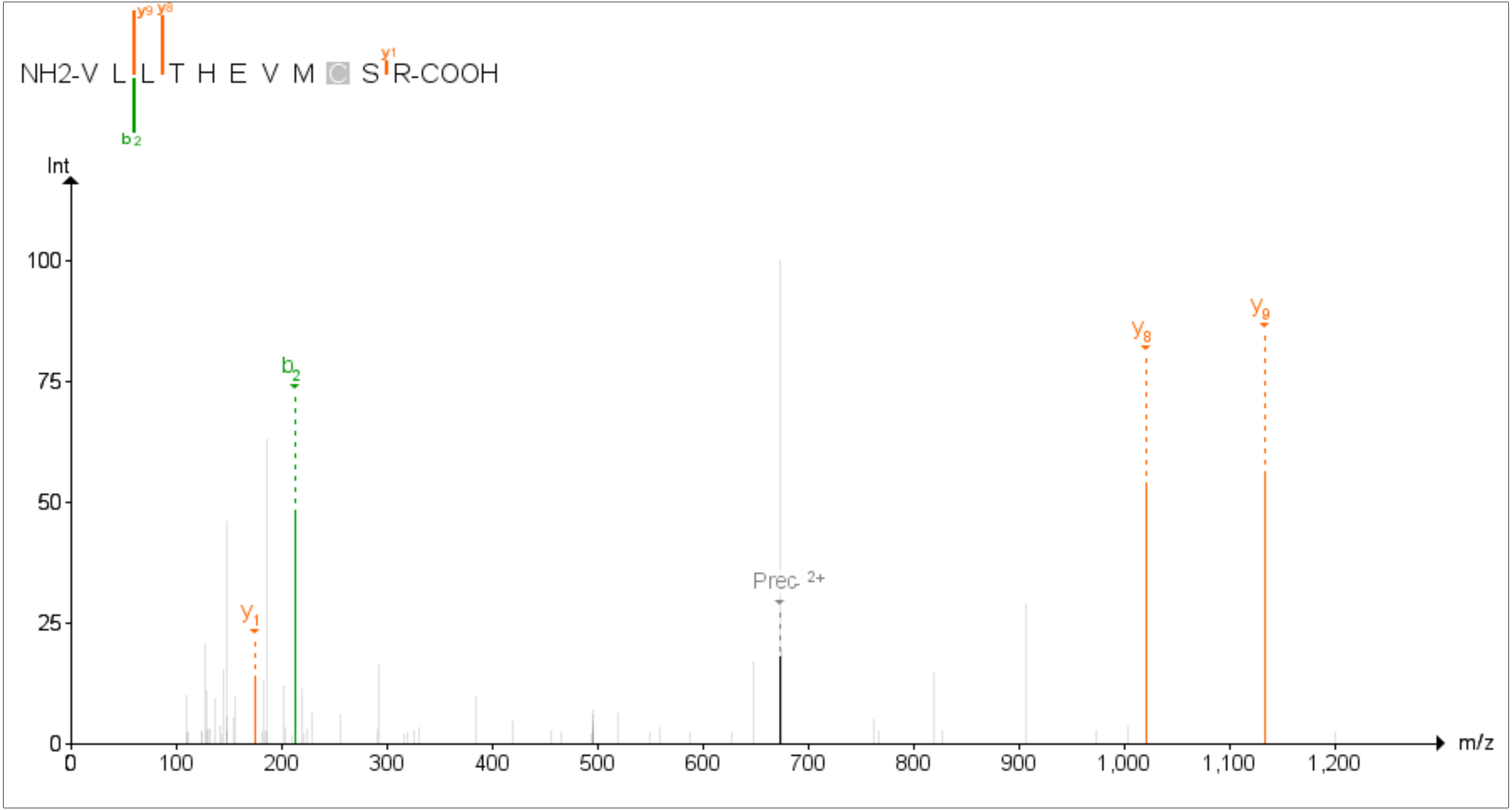

NH2-V L L T H E V M C S R-COOH
y9 y8 y1 b2
Int
100
75
50
25
0
b2
y1
Prec 2+
y8
y9
0 100 200 300 400 500 600 700 800 900 1,000 1,100 1,200
m/z

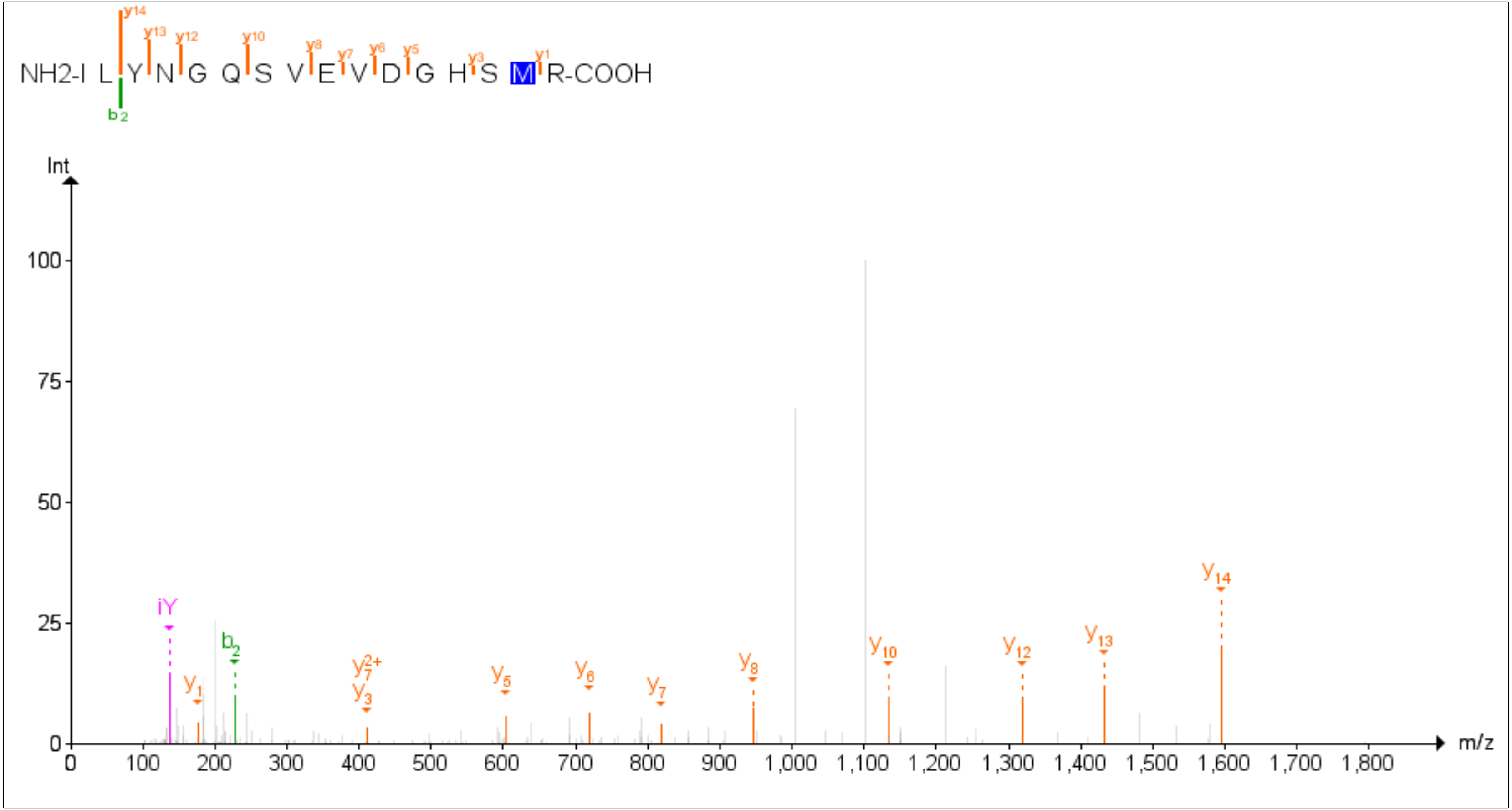

NH2-I L Y N G Q S V E V D G H S M R-COOH
y14 y13 y12 y10 y8 y7 y6 y5 y3 y1
b2
Int
100
75
50
25
0
iY
b2
y1
y7 2+
y3
y5
y6
y7
y8
y10
y12
y13
y14
0 100 200 300 400 500 600 700 800 900 1,000 1,100 1,200 1,300 1,400 1,500 1,600 1,700 1,800
m/z

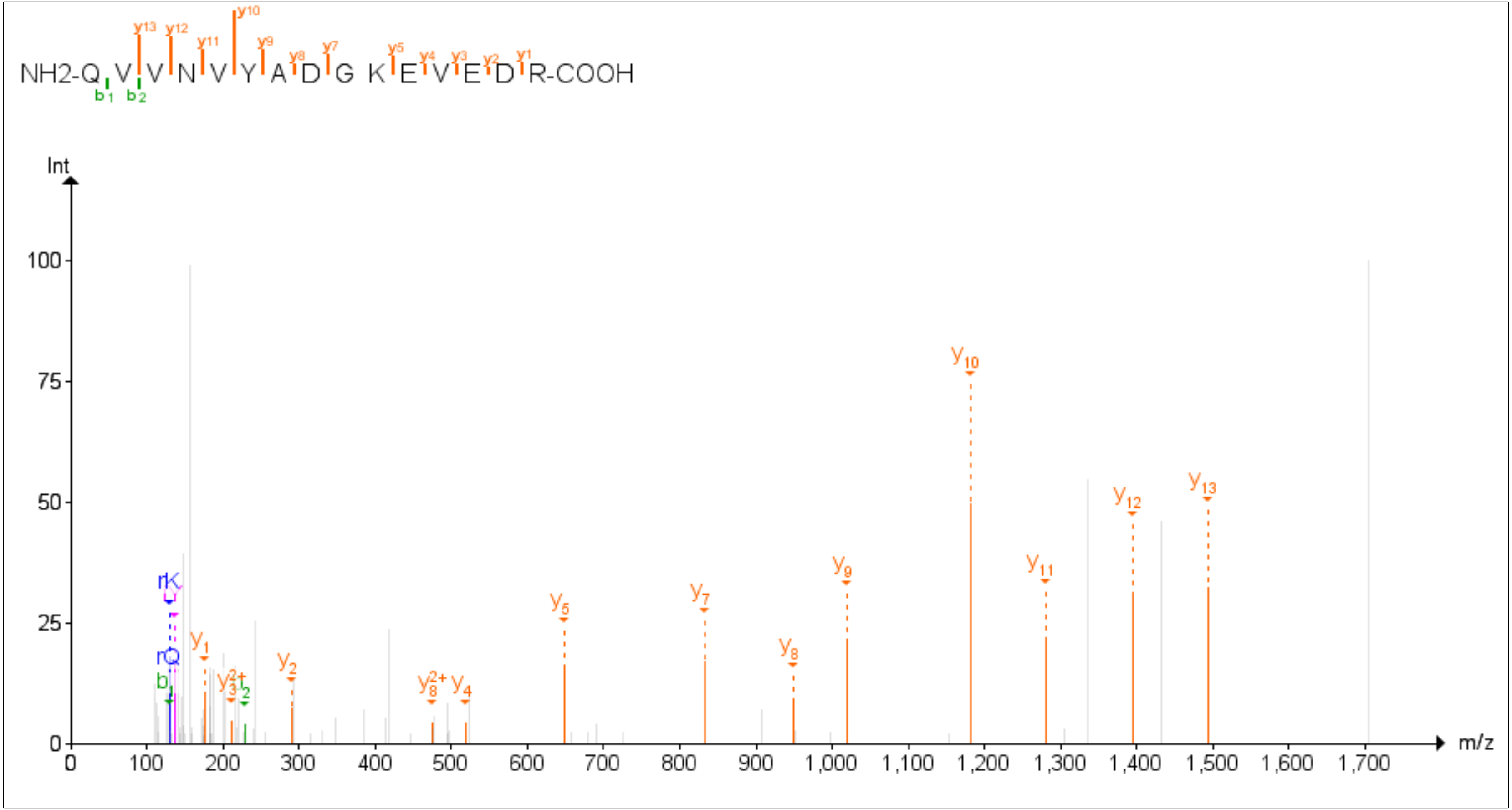

NH2-QVVNVYADGKEVEDR-COOH
y13 y12 y11 y10 y9 y8 y7 y5 y4 y3 y2 y1
b1 b2
Int
100
75
50
25
0
m/z
0 100 200 300 400 500 600 700 800 900 1,000 1,100 1,200 1,300 1,400 1,500 1,600 1,700
rK
rQ
b1
y1
y2
y8 2+
y4
y5
y7
y8
y9
y10
y11
y12
y13

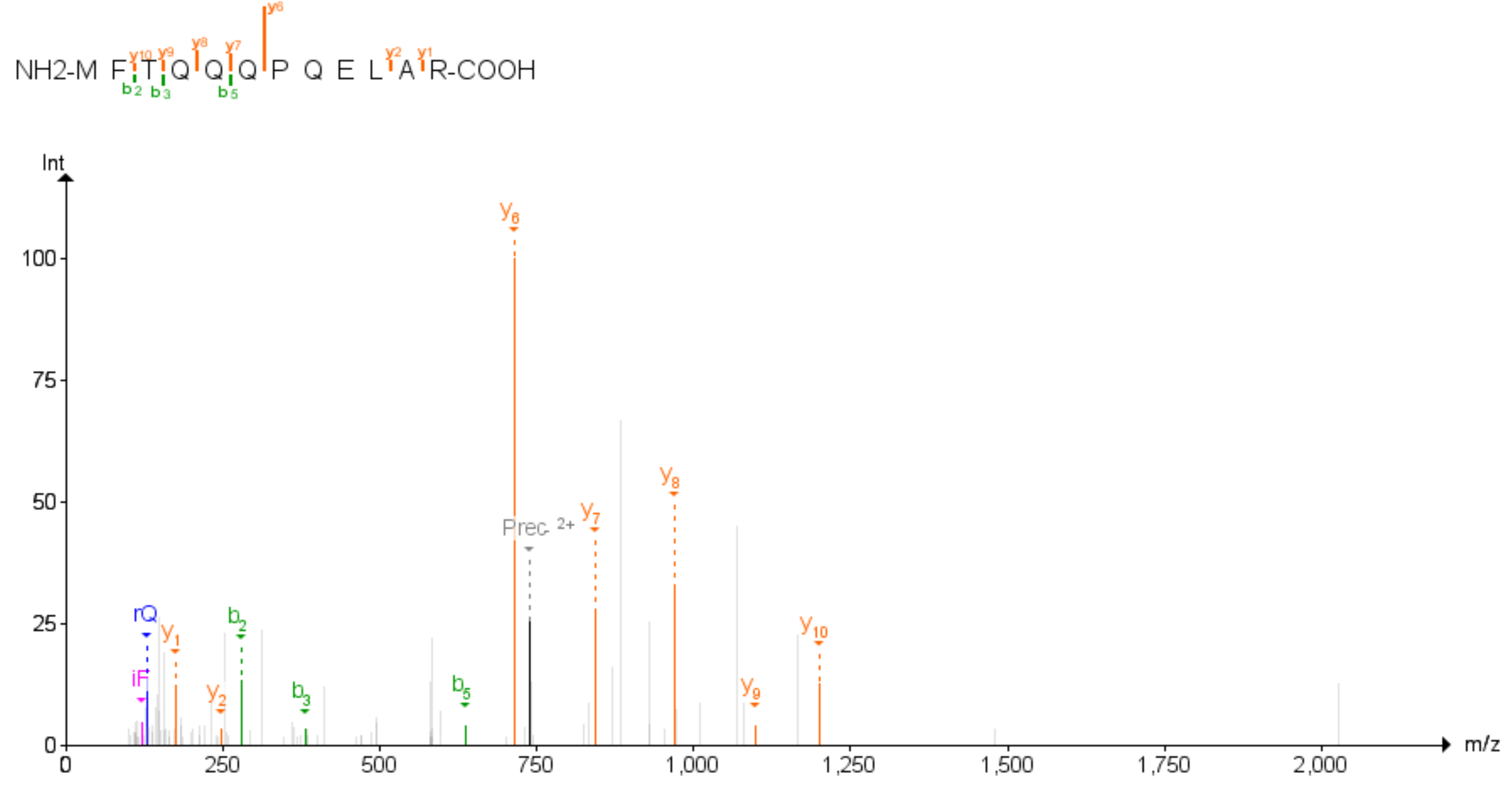

NH2-M F T Q Q Q P Q E L A R-COOH
y10 y9 y8 y7 y6 y2 y1
b2 b3 b5
Int
100
75
50
25
0
y6
Prec- 2+
y7
y8
y10
y9
rQ
y1
iF
y2
b2
b3
b5
0
250
500
750
1,000
1,250
1,500
1,750
2,000
m/z

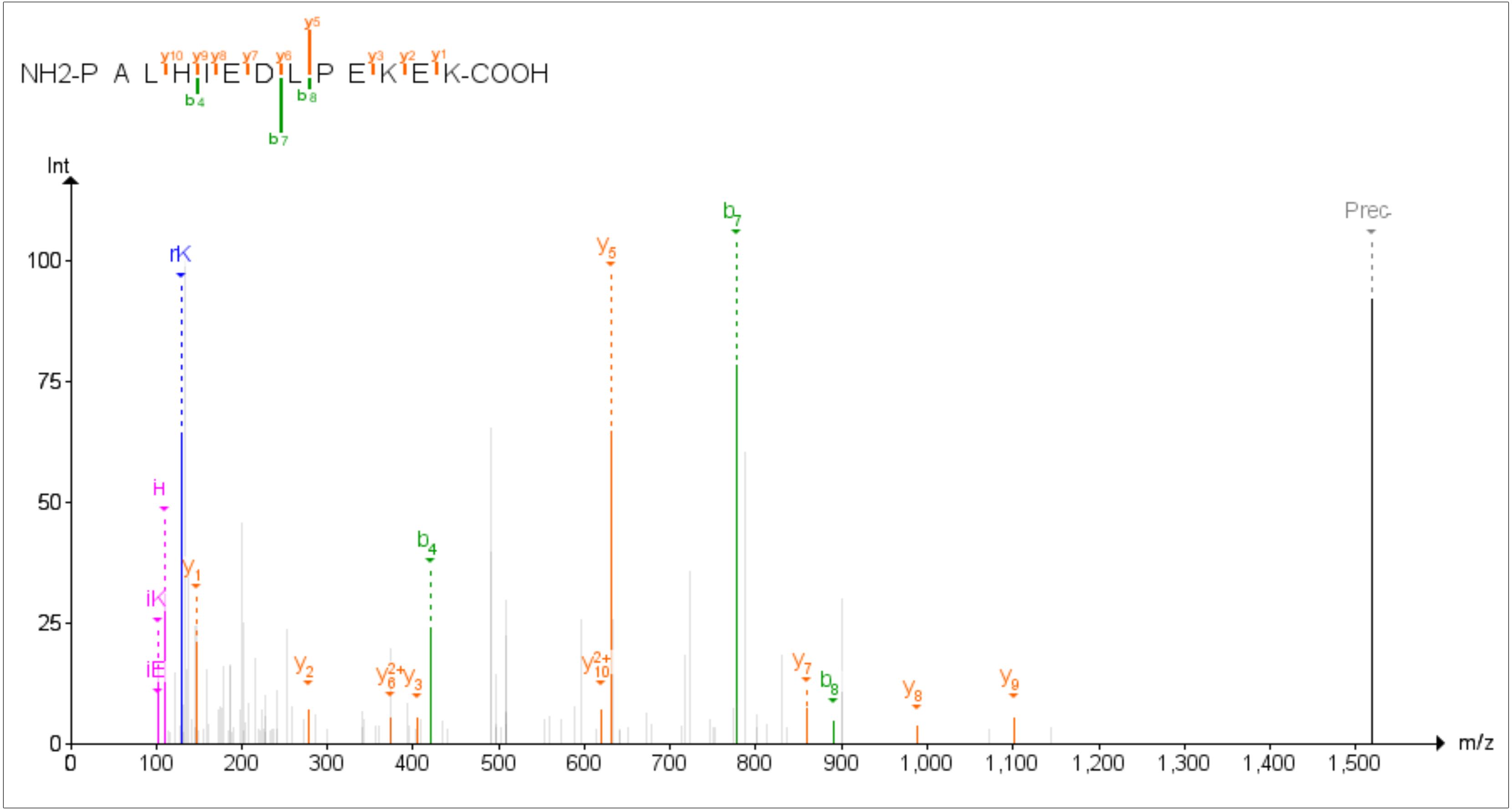

NH2-P A L H I E D L P E K E K-COOH
y10 y9 y8 y7 y6 y5 y3 y2 y1
b4 b7 b8
Int
100
75
50
25
0
rK
iH
iK
iE
y1
y2
y6 2+
y3
b4
y5
y10 2+
b7
y7
b8
y8
y9
Prec
0 100 200 300 400 500 600 700 800 900 1,000 1,100 1,200 1,300 1,400 1,500
m/z

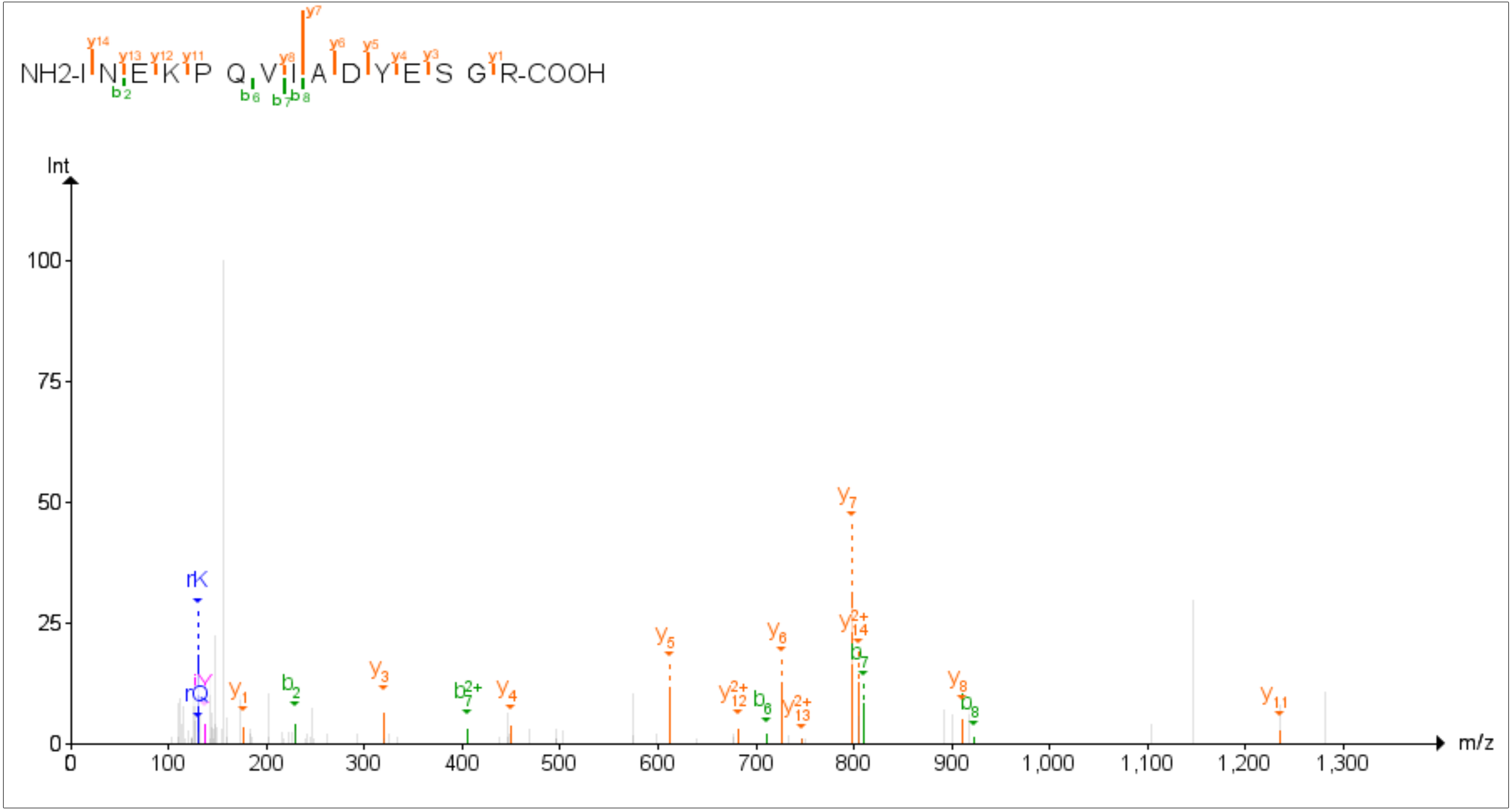

NH2-I N E K P Q V I I A D Y E S G R-COOH
y14 y13 y12 y11 y8 y7 y6 y5 y4 y3 y1
b2 b6 b7 b8
Int
100
75
50
25
0
rK
iY
rQ
y1
b2
y3
b7 2+
y4
y5
y12 2+
b6
y6
y13 2+
y7
y14 2+
b7
y8
b8
y11
0 100 200 300 400 500 600 700 800 900 1,000 1,100 1,200 1,300
m/z

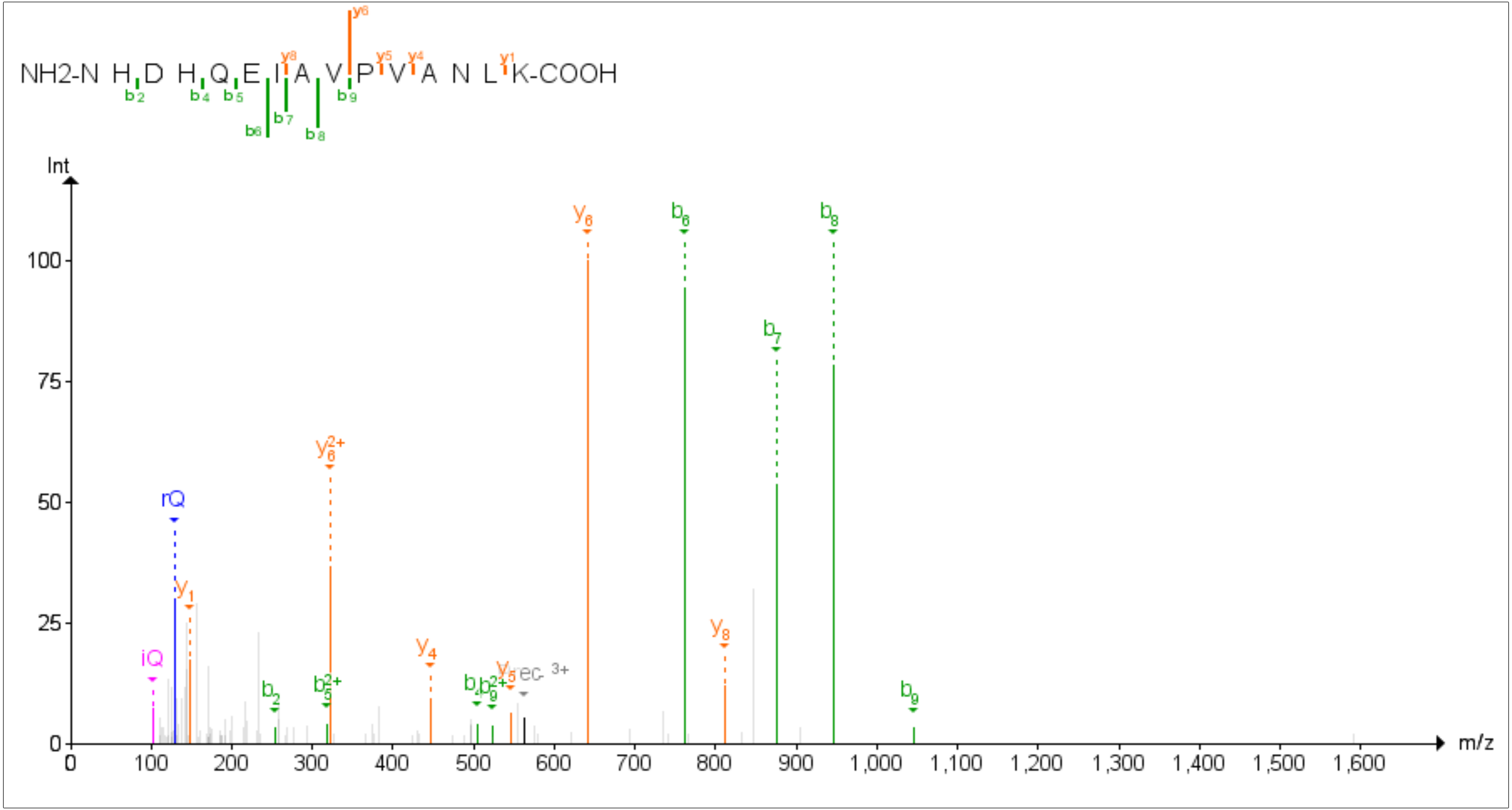

NH2-N H D H Q E I A V P V A N L K-COOH
Int
m/z
y6
b6
b8
b7
y6 2+
rQ
y1
iQ
y4
y8
b2
b5 2+
b4
b9 2+
y5
prec- 3+
b9

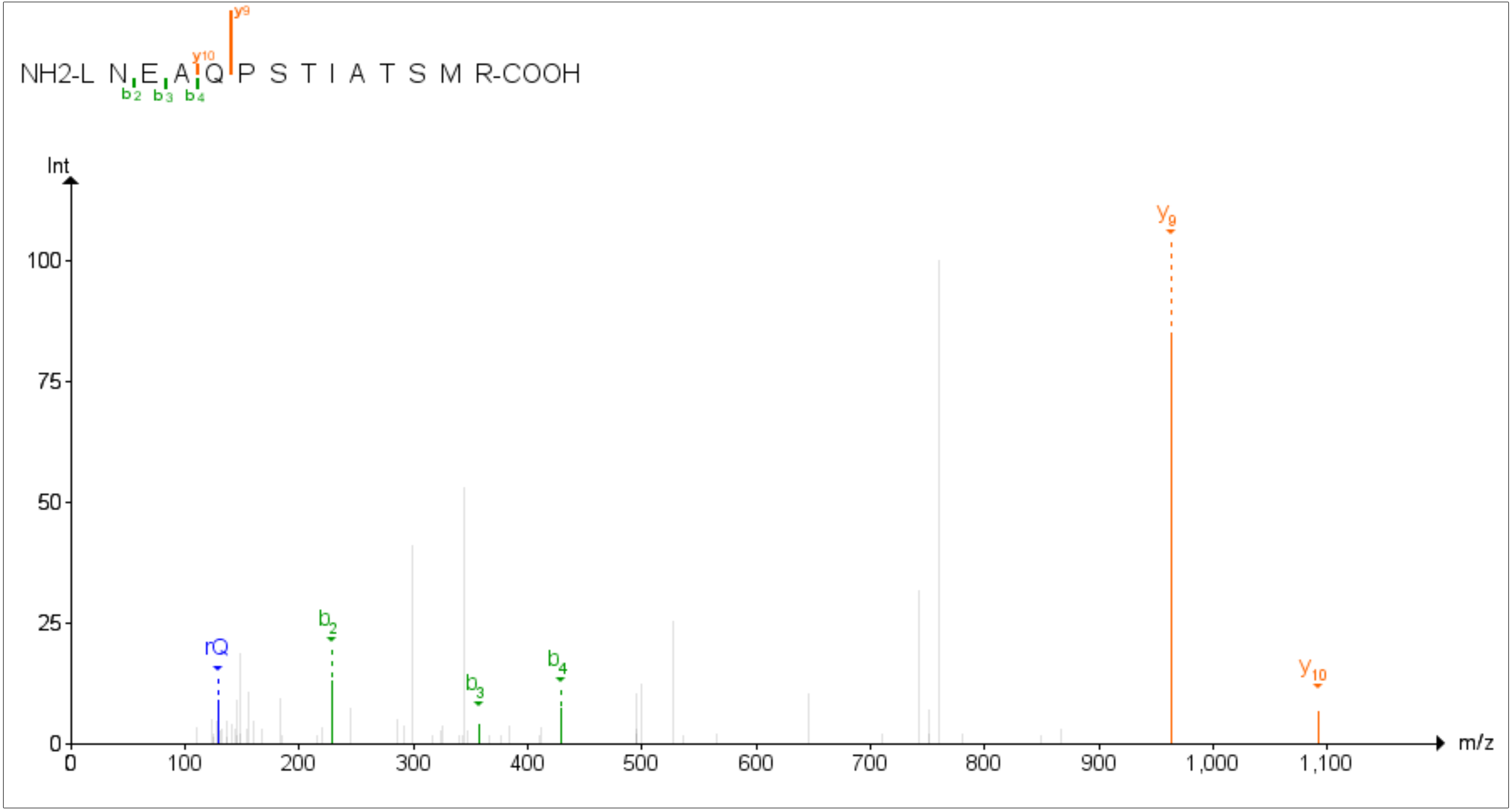

NH2-L N E A Q P S T I A T S M R-COOH
y9
y10
b2
b3
b4
Int
rQ
m/z
0
25
50
75
100
0
100
200
300
400
500
600
700
800
900
1,000
1,100

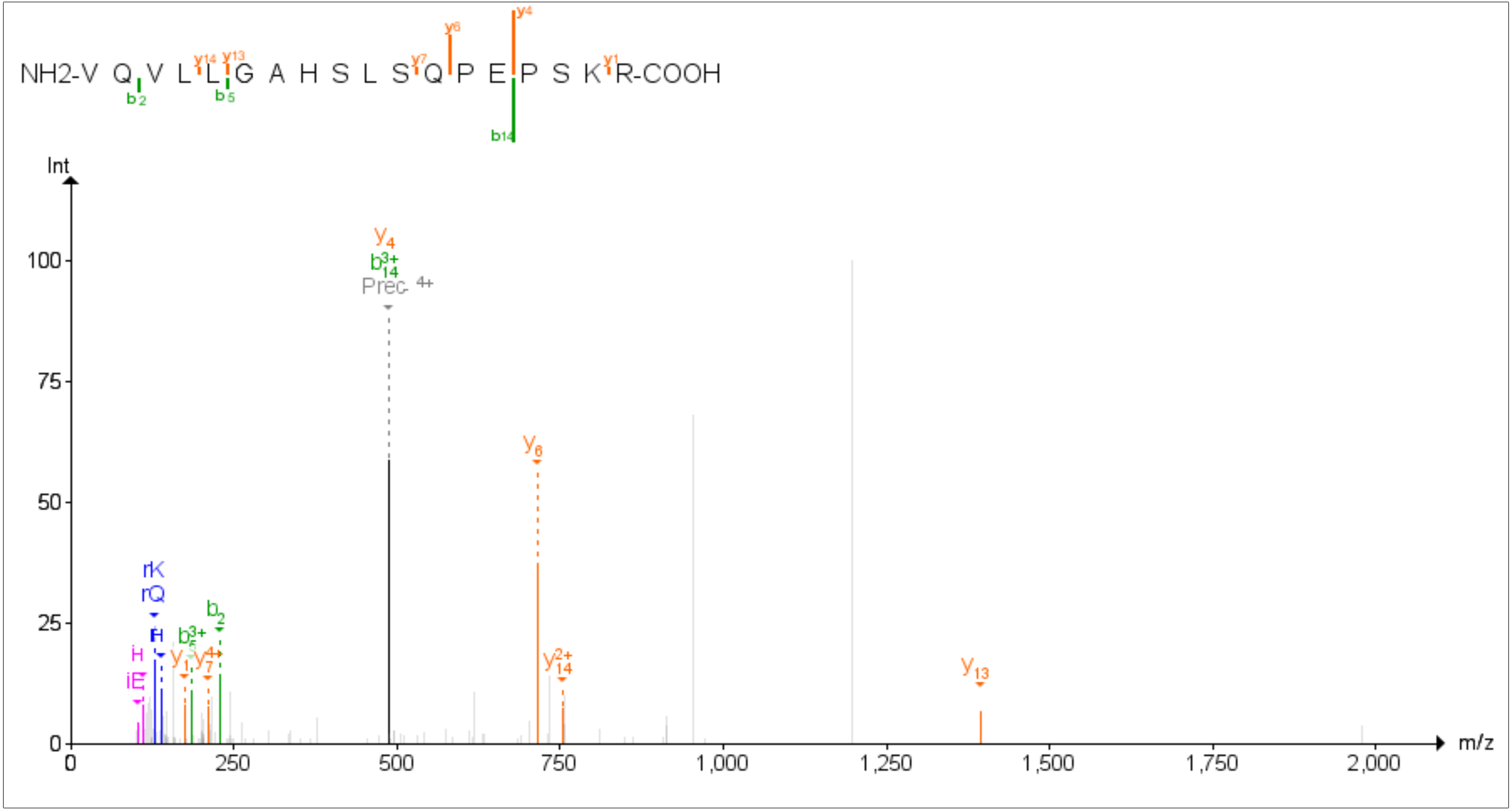

NH2-V Q V L L G A H S L S Q P E P S K R-COOH
y14 y13 y7 y6 y4 y1
b 2 b 5 b14
Int
m/z
0
25
50
75
100
250
500
750
1,000
1,250
1,500
1,750
2,000
y4
b3+14
Prec- 4+
y6
y2+14
y13
rK
rQ
b2
b3+5
y1
y4+7
iH
iE

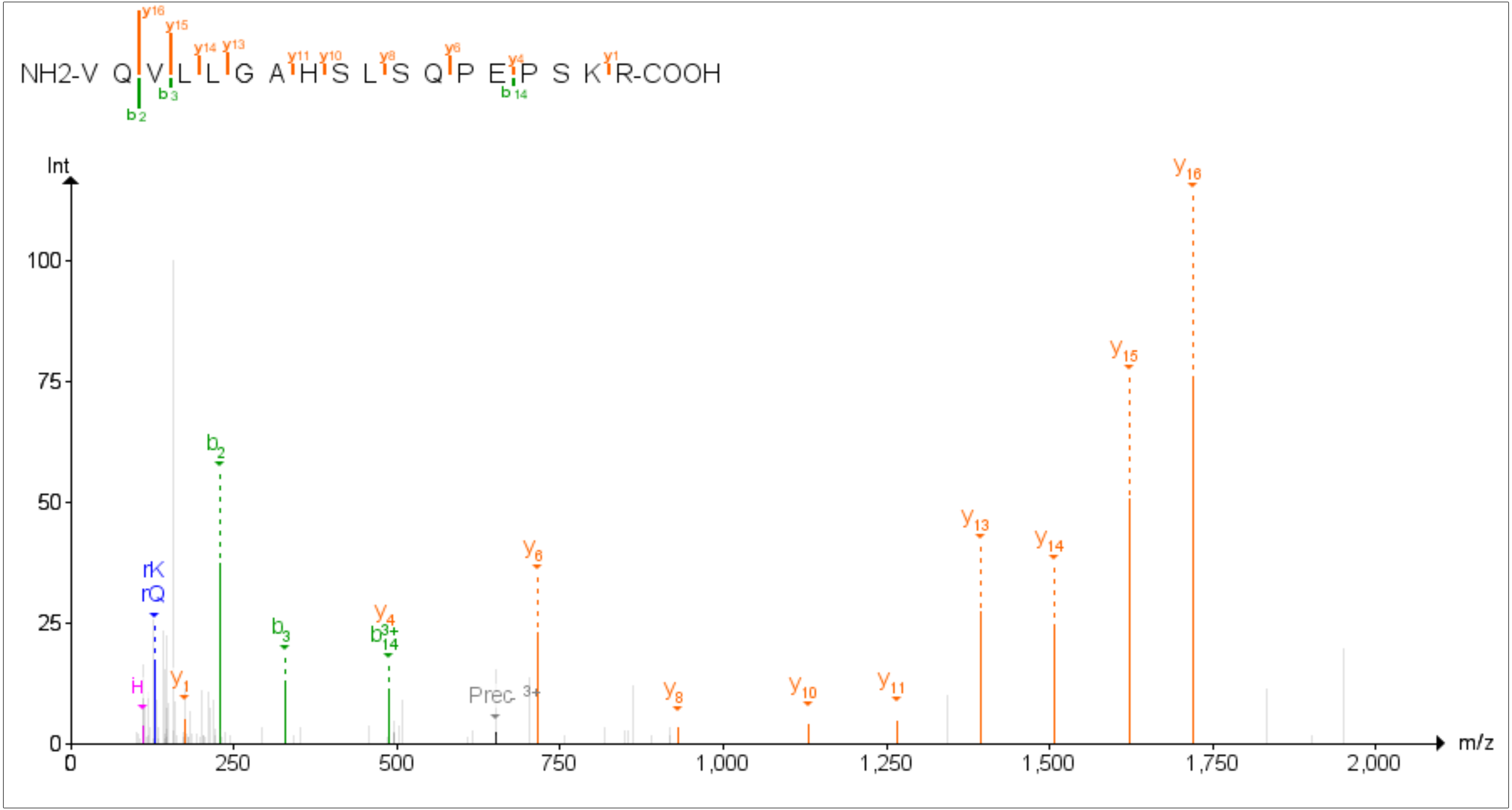

NH2-V Q V L L G A H S L S Q P E P S K R-COOH
y16
y15
y14
y13
y11
y10
y8
y6
y4
y1
b2
b3
b14
Int
100
75
50
25
0
m/z
0
250
500
750
1,000
1,250
1,500
1,750
2,000
y16
y15
y13
y14
y6
b2
rK
rQ
b3
y4
b14 3+
H
y1
Prec 3+
y8
y10
y11

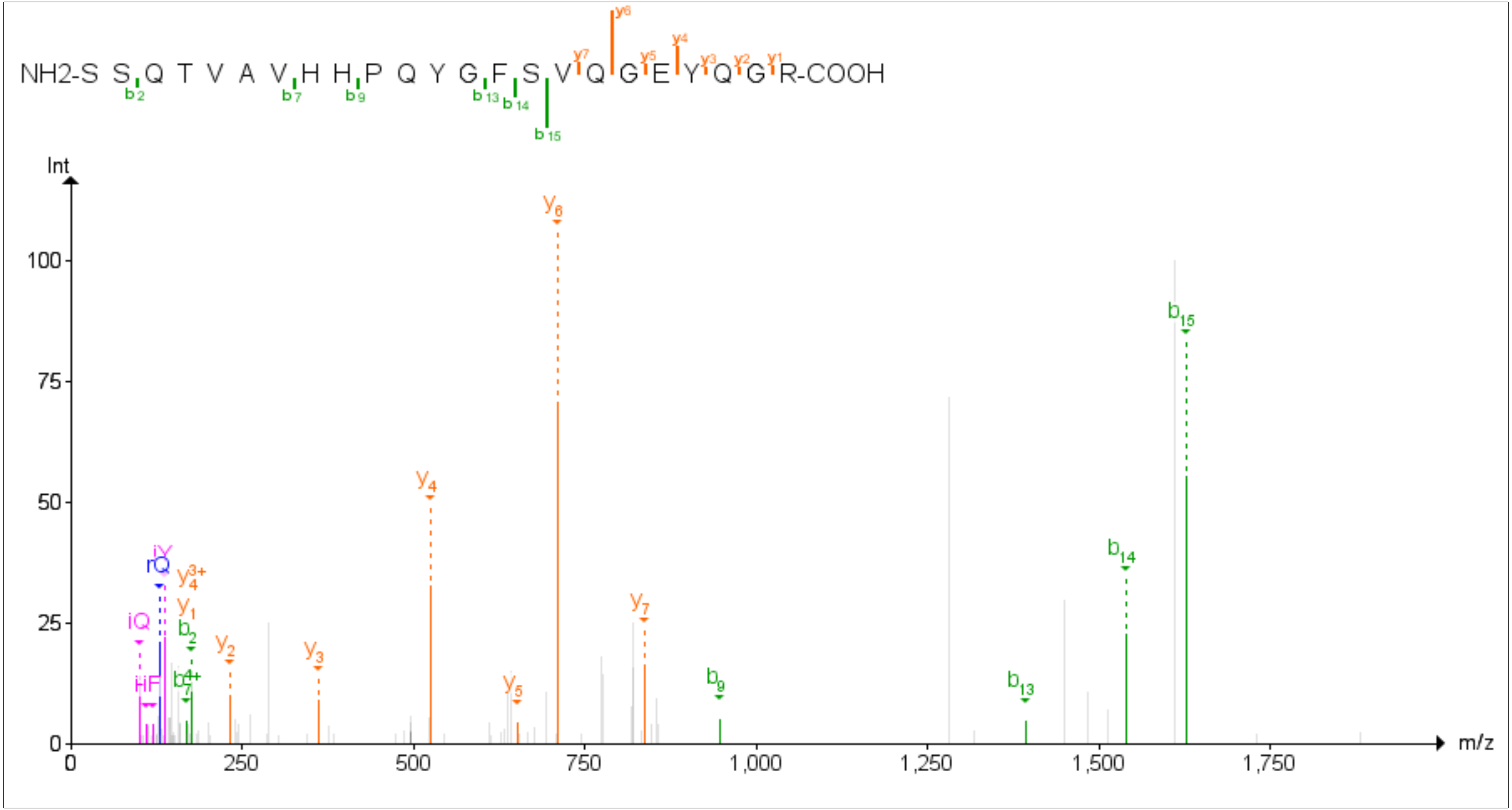

NH2-S S Q T V A V H H P Q Y G F S V Q G E Y Q G R-COOH
Int
m/z

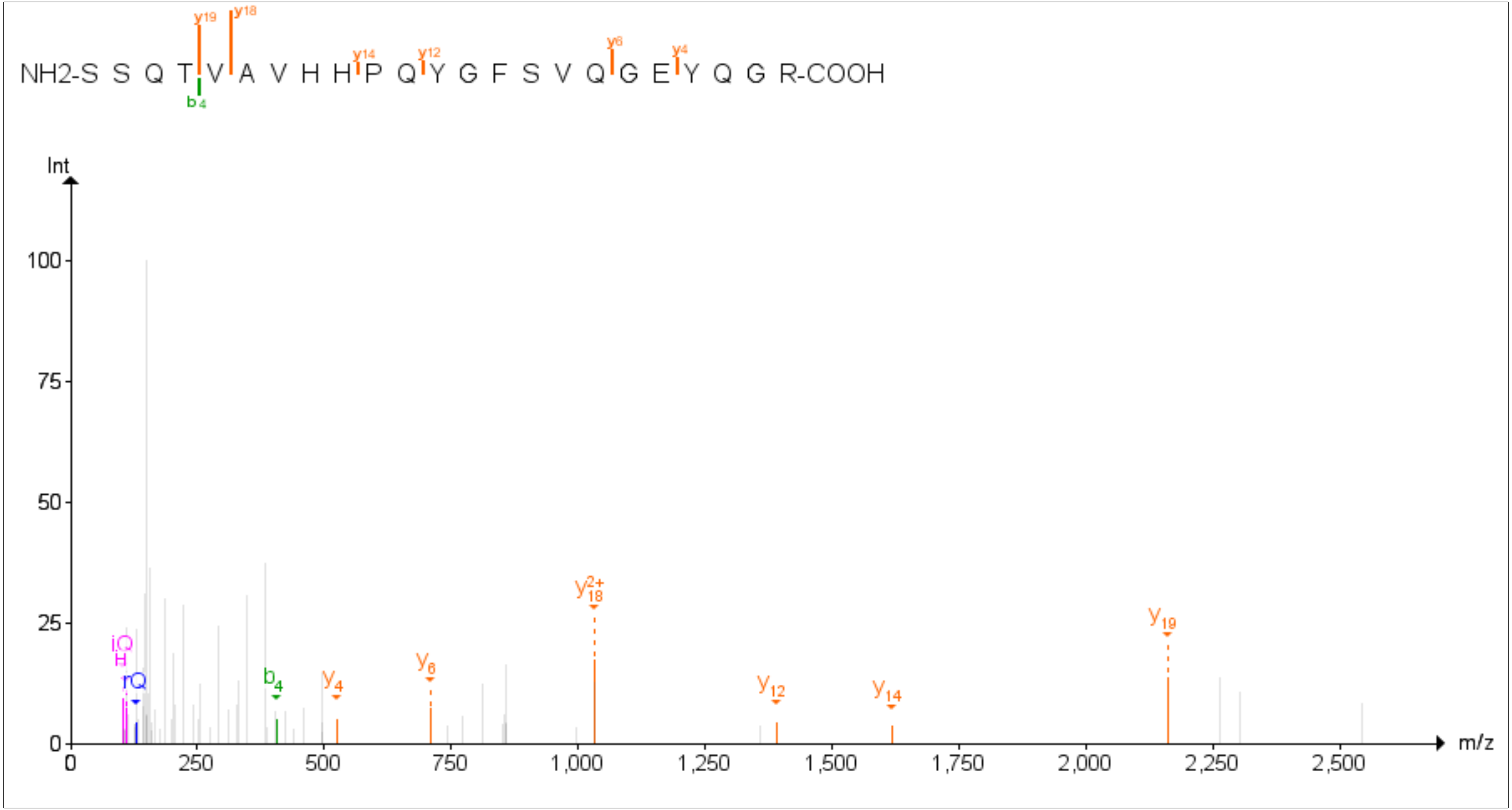

NH2-S S Q T V A V H H P Q Y G F S V Q G E Y Q G R-COOH
y19
y18
y14
y12
y6
y4
b4
Int
100
75
50
25
0
iQ
H
rQ
b4
y4
y6
y18 2+
y12
y14
y19
m/z
0
250
500
750
1,000
1,250
1,500
1,750
2,000
2,250
2,500

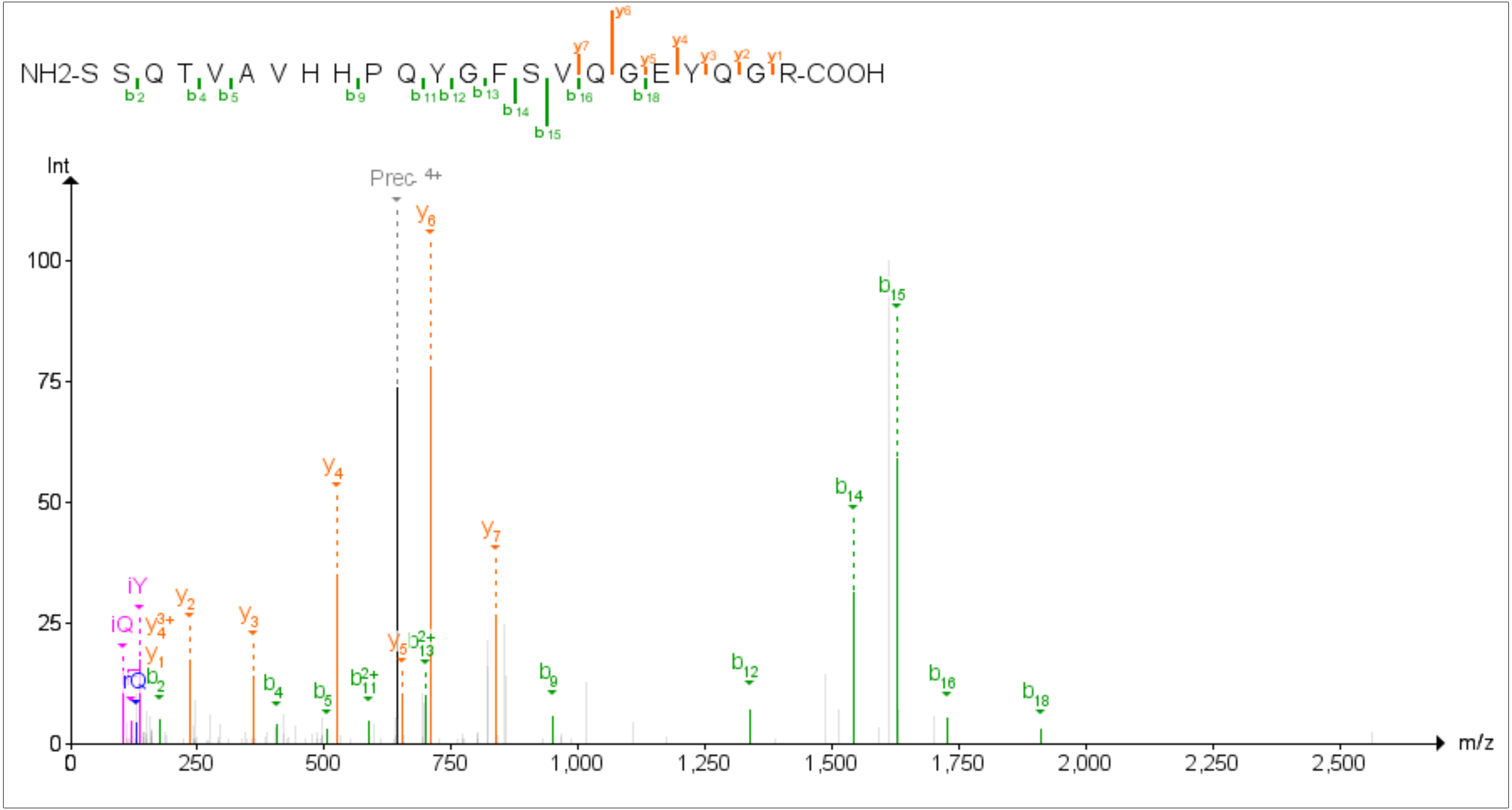
NH2-S S Q T V A V H H P Q Y G F S V Q G E Y Q G R-COOH
Int
Prec 4+
m/z

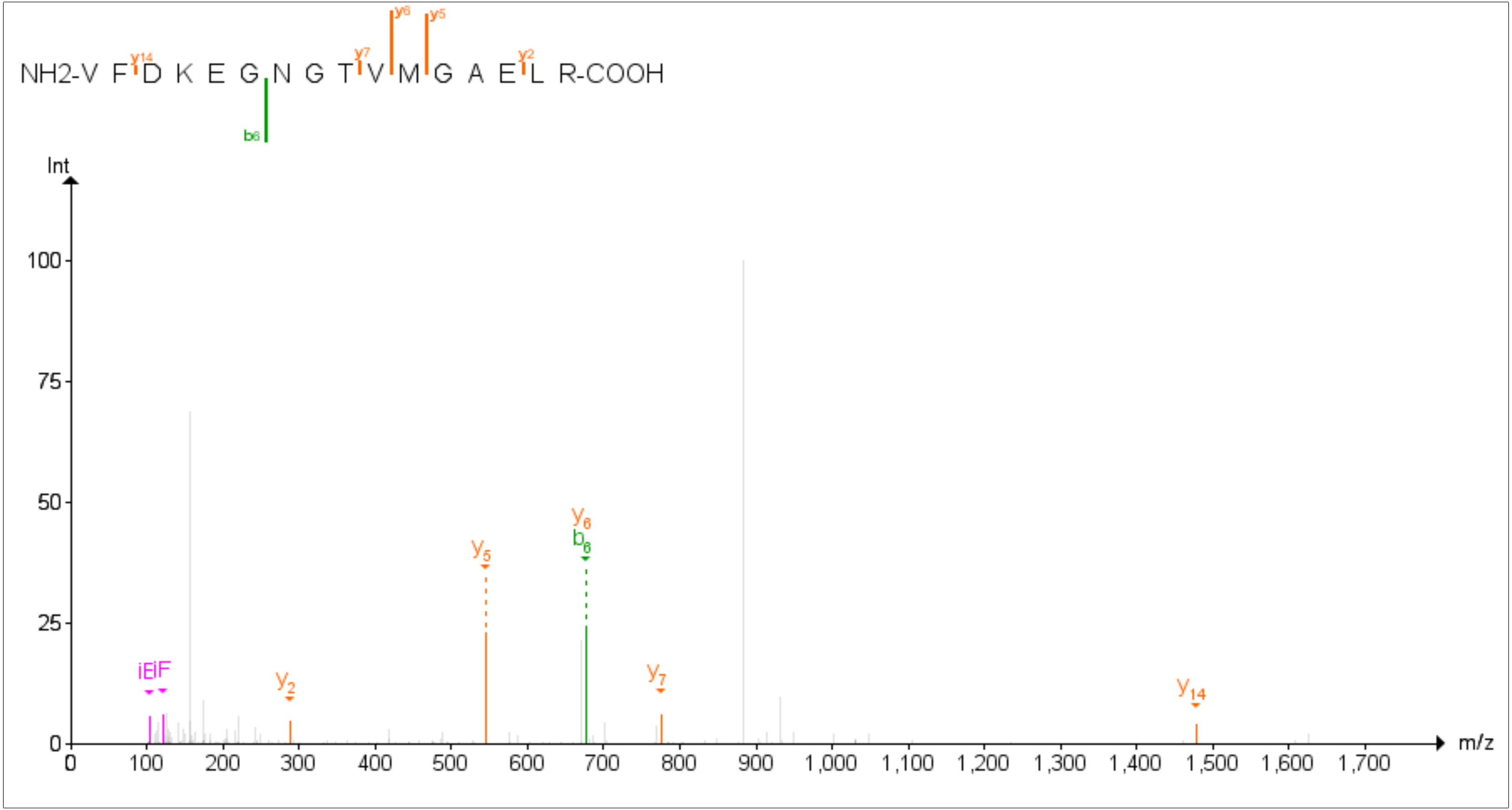

NH2-V F D K E G N G T V M G A E L R-COOH
y14
y7
y6
y5
y2
b6
Int
100
75
50
25
0
iEiF
y2
y5
y6
b6
y7
y14
0
100
200
300
400
500
600
700
800
900
1,000
1,100
1,200
1,300
1,400
1,500
1,600
1,700
m/z

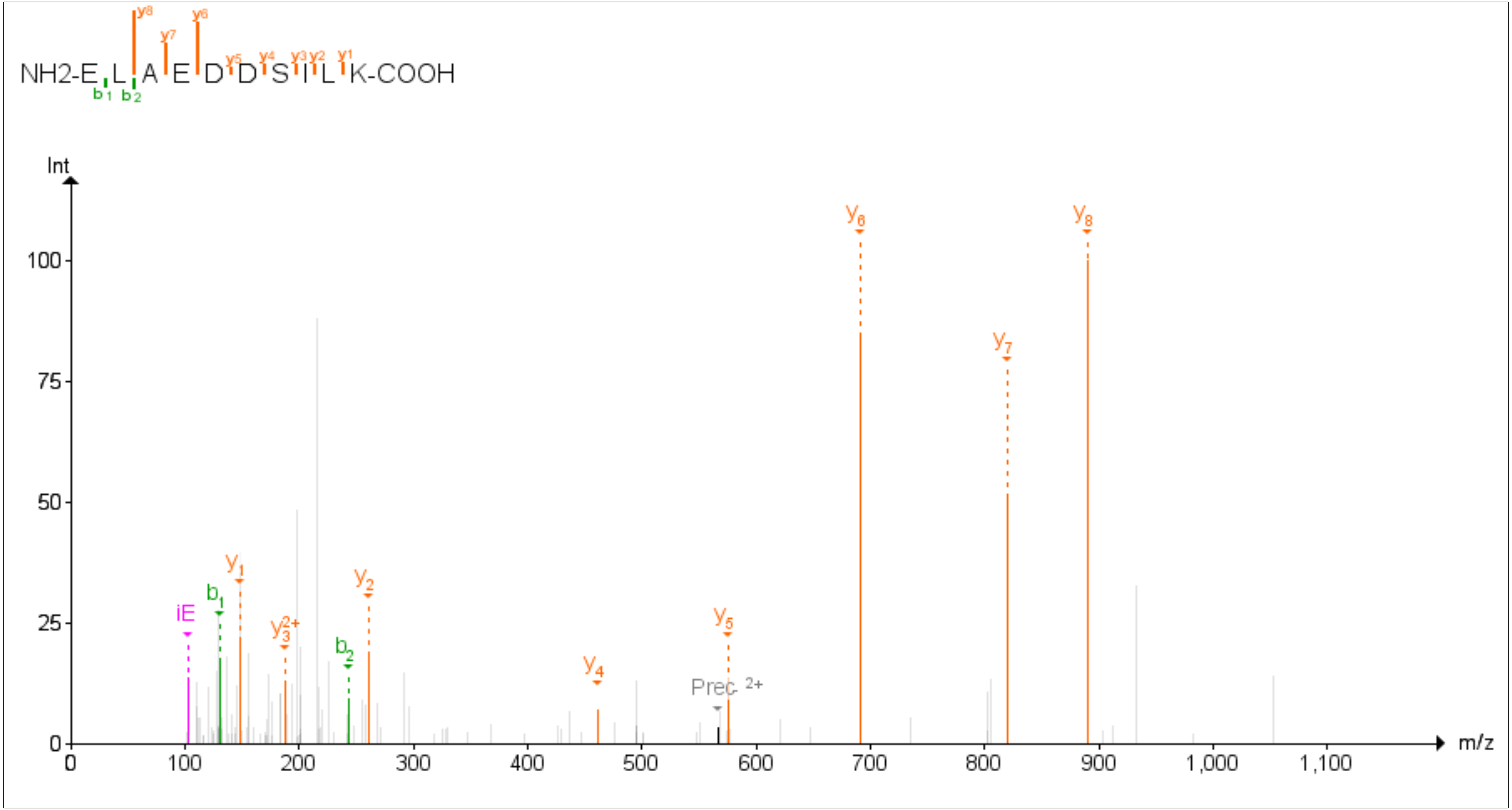

NH2-E L A E D D S I L K-COOH
y8 y7 y6 y5 y4 y3 y2 y1
b1 b2
Int
m/z
y6
y8
y7
y1
b1
iE
y2
y3 2+
b2
y5
y4
Prec. 2+
100
75
50
25
0
0
100
200
300
400
500
600
700
800
900
1,000
1,100

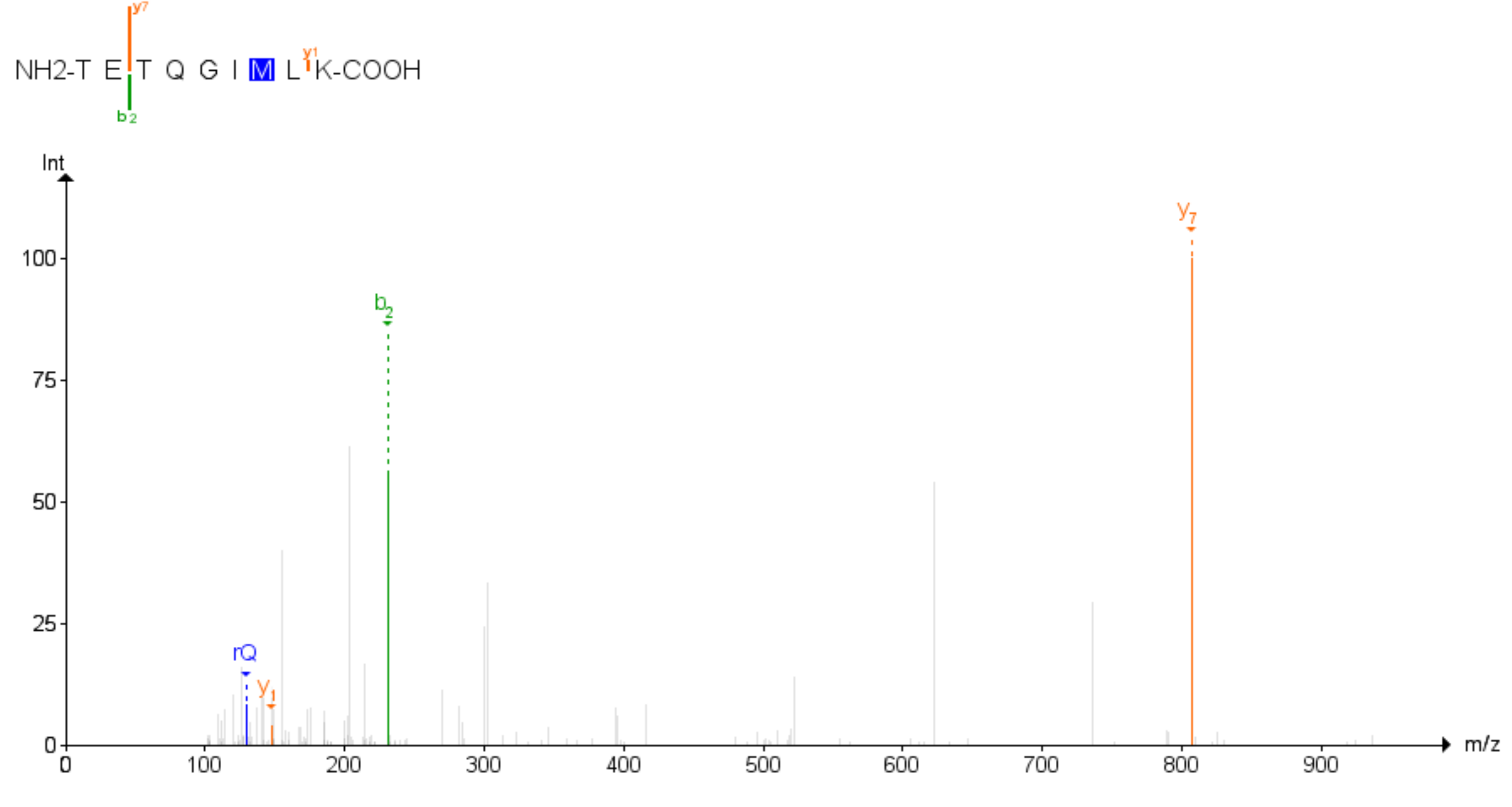

NH2-T E T Q G I M L K-COOH
y7
y1
b2
Int
100
75
50
25
0
y7
b2
rQ
y1
0
100
200
300
400
500
600
700
800
900
m/z

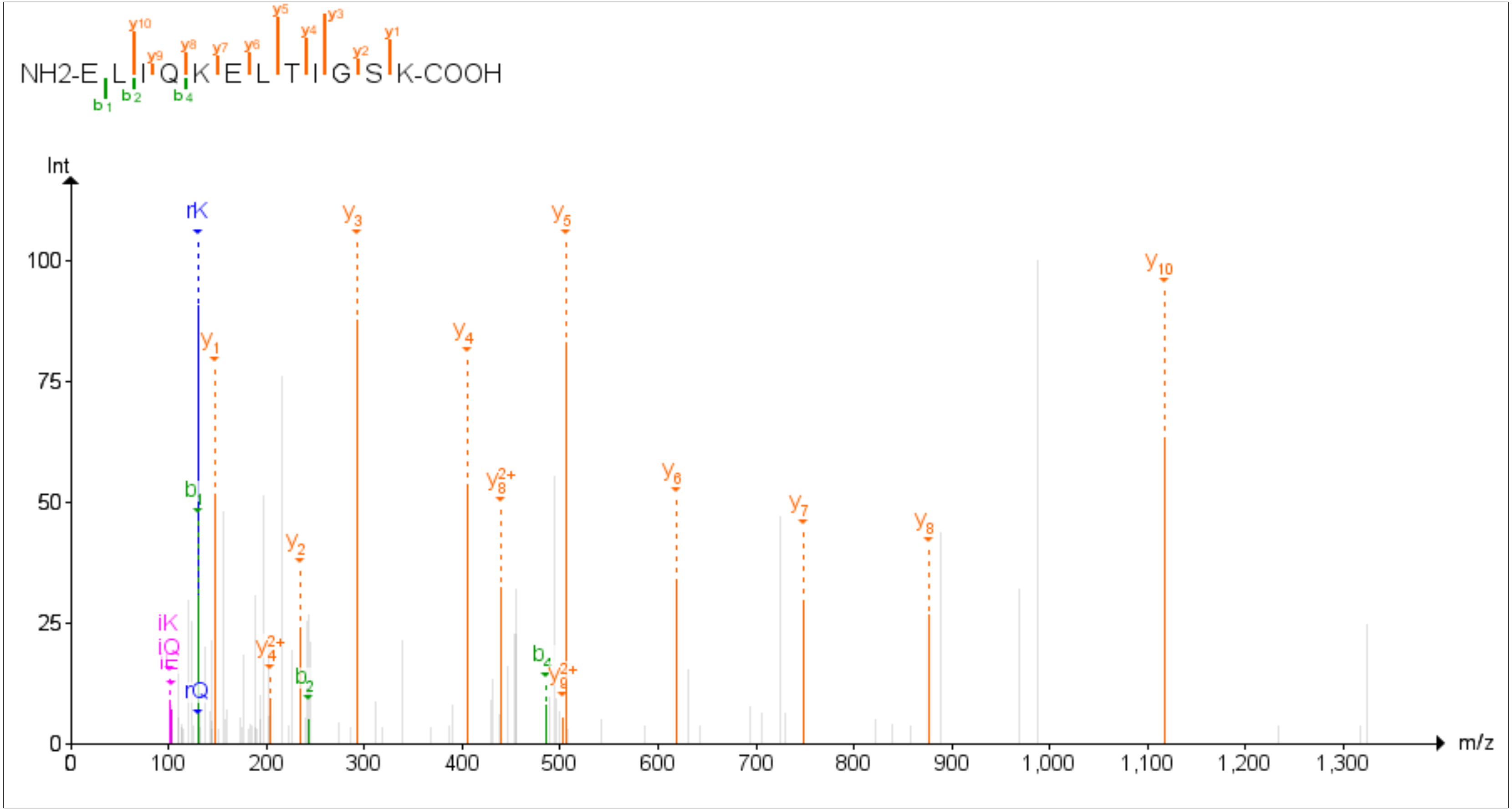

NH2-E L I Q K E L T I G S K-COOH
y10 y9 y8 y7 y6 y5 y4 y3 y2 y1
b1 b2 b4
Int
m/z
0
25
50
75
100
200
300
400
500
600
700
800
900
1,000
1,100
1,200
1,300
rK
y3
y5
y10
y1
y4
b1
y8 2+
y6
y7
y8
y2
iK
iQ
iE
y4 2+
b2
rQ
b4
y9 2+

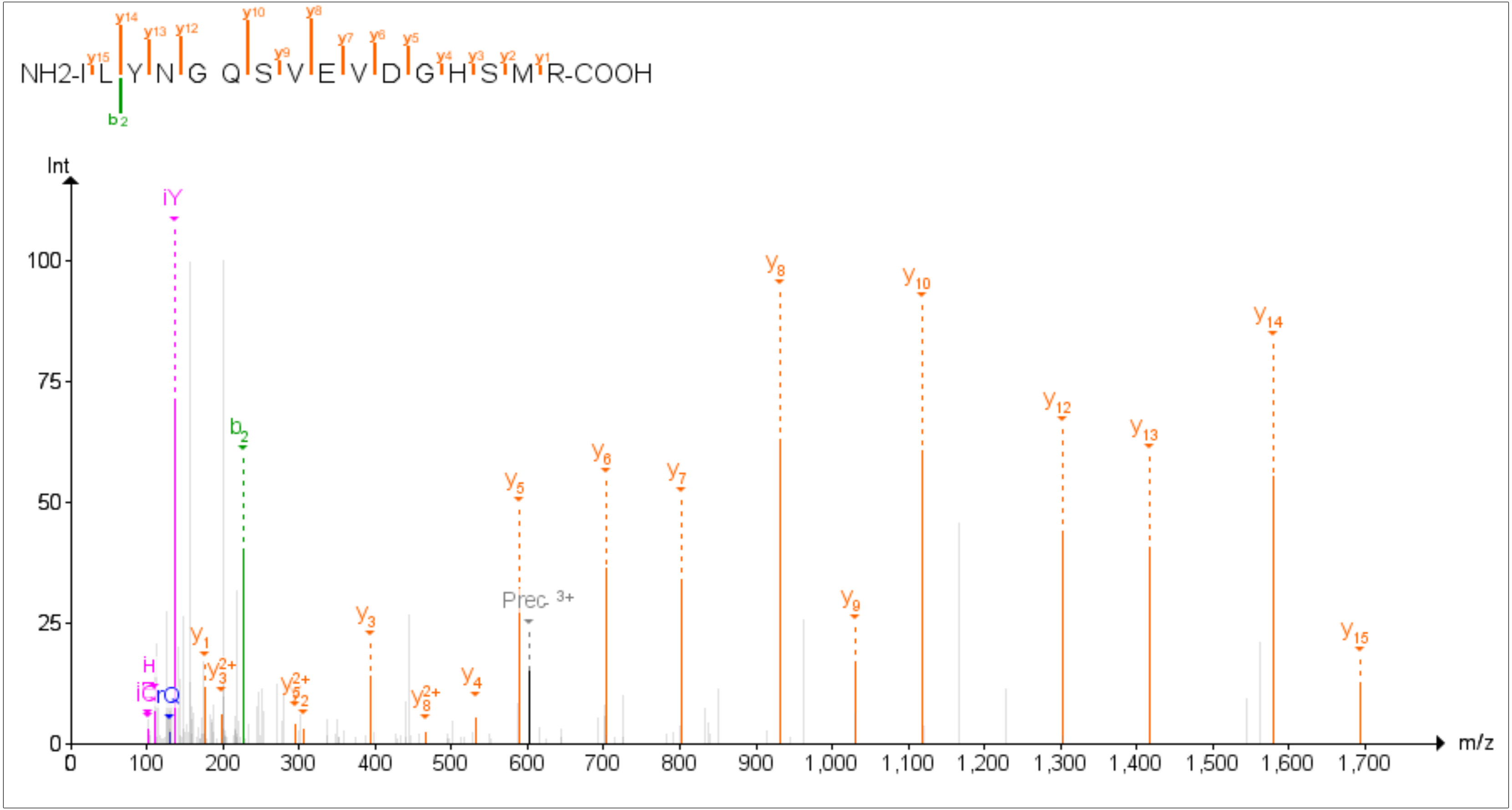
NH2-I L Y N G Q S V E V D G H S M R-COOH
y15
y14
y13
y12
y10
y9
y8
y7
y6
y5
y4
y3
y2
y1
b2
Int
iY
b2
y8
y10
y14
y12
y13
y6
y7
y5
Prec 3+
y9
y15
y3
y1
H
iC
rQ
y4
0
25
50
75
100
m/z
0
100
200
300
400
500
600
700
800
900
1,000
1,100
1,200
1,300
1,400
1,500
1,600
1,700

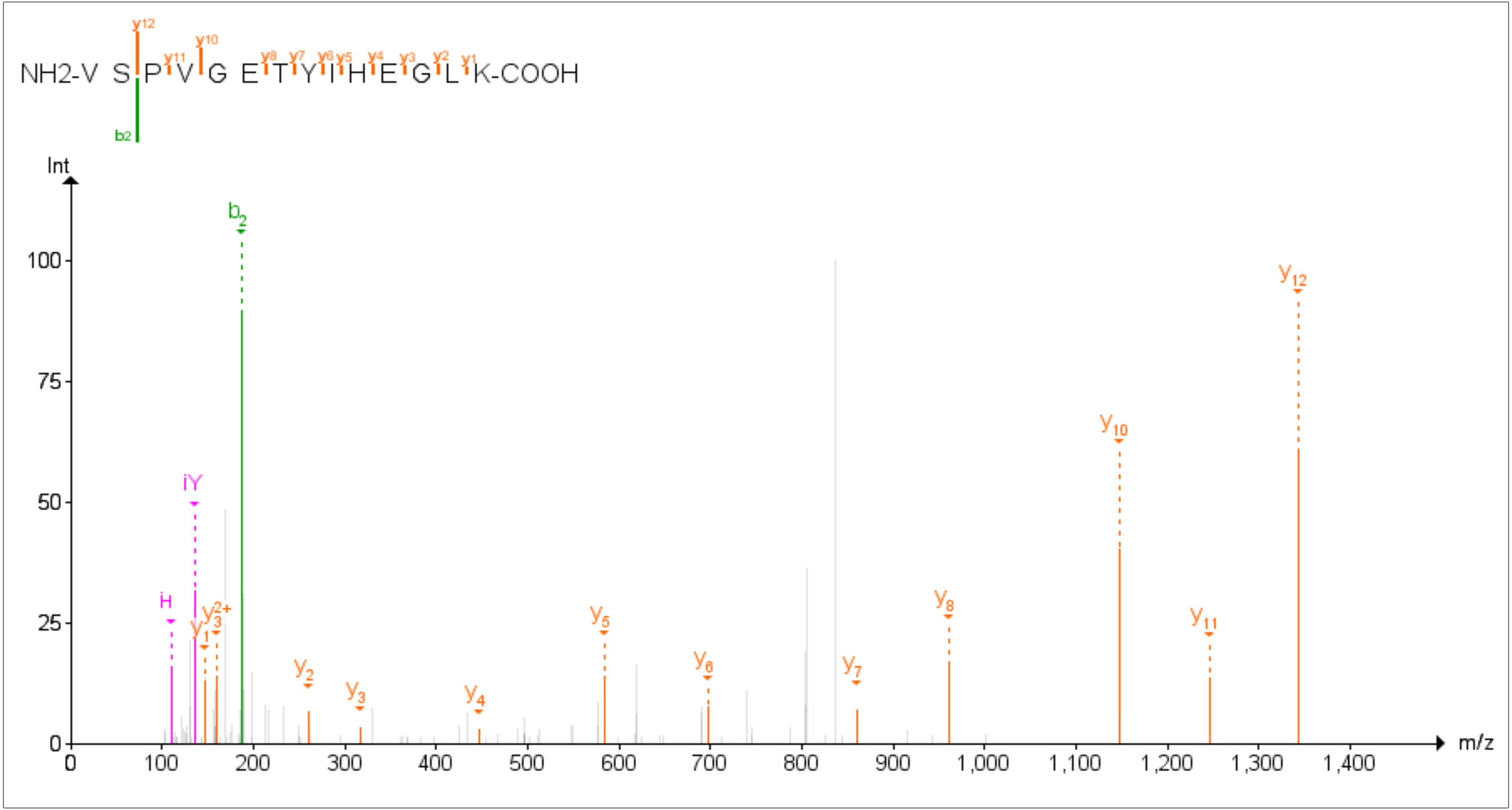

NH2-V S P V G E T Y I H E G L K-COOH
y12
y11
y10
y8
y7
y6
y5
y4
y3
y2
y1
b2
Int
m/z
0
25
50
75
100
100
200
300
400
500
600
700
800
900
1,000
1,100
1,200
1,300
1,400
iH
iY
y1
y3 2+
y2
y3
y4
y5
y6
y7
y8
y10
y11
y12

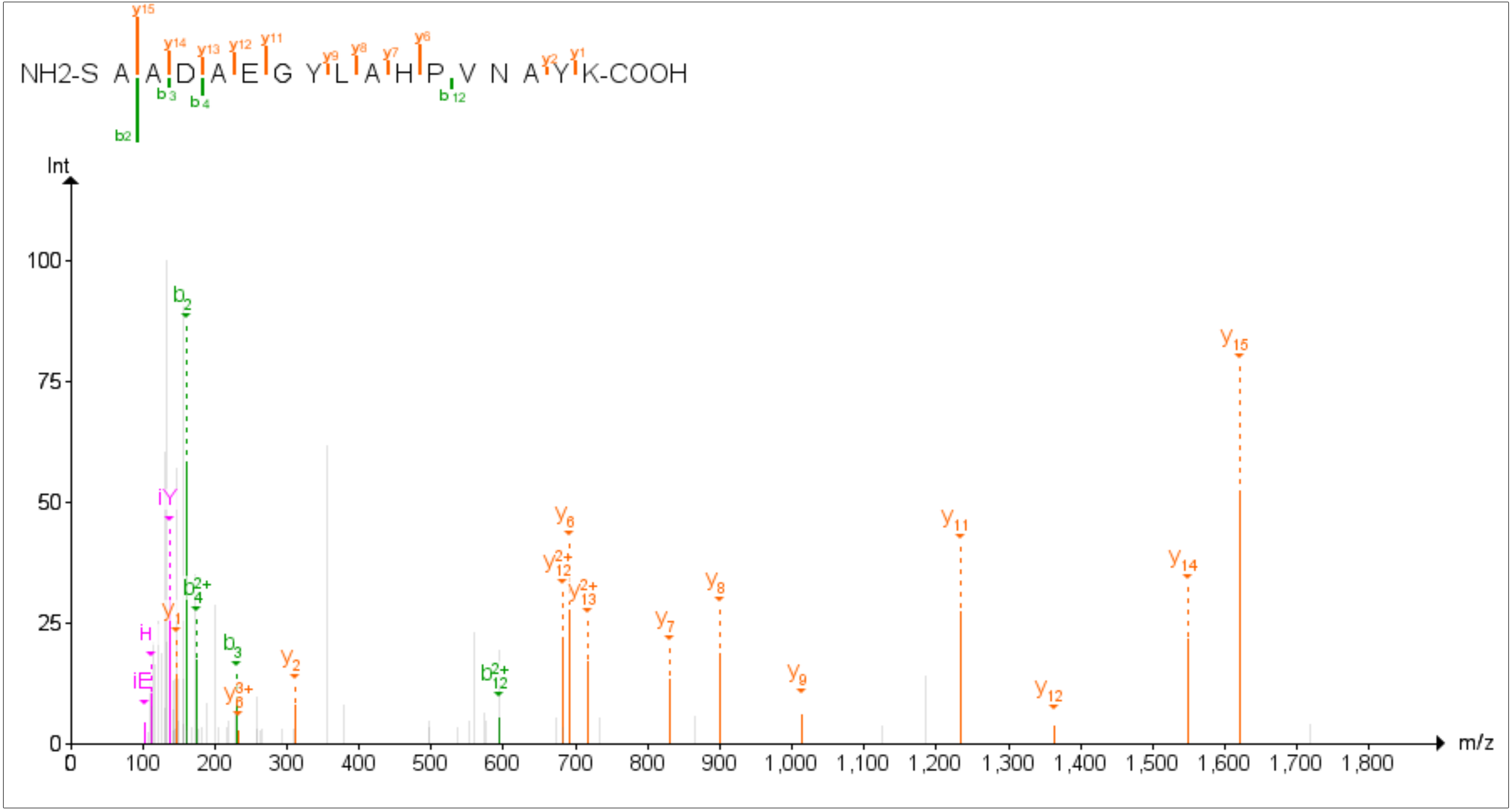

NH2-S A A D A E G Y L A H P V N A Y K-COOH
y15
y14
y13
y12
y11
y9
y8
y7
y6
y2
y1
b2
b3
b4
b12
Int
100
75
50
25
0
m/z
0
100
200
300
400
500
600
700
800
900
1,000
1,100
1,200
1,300
1,400
1,500
1,600
1,700
1,800
iY
iH
iE
y1
y2
y3+6
b2
b2+4
b3
b2+12
y6
y2+12
y2+13
y7
y8
y9
y11
y12
y14
y15

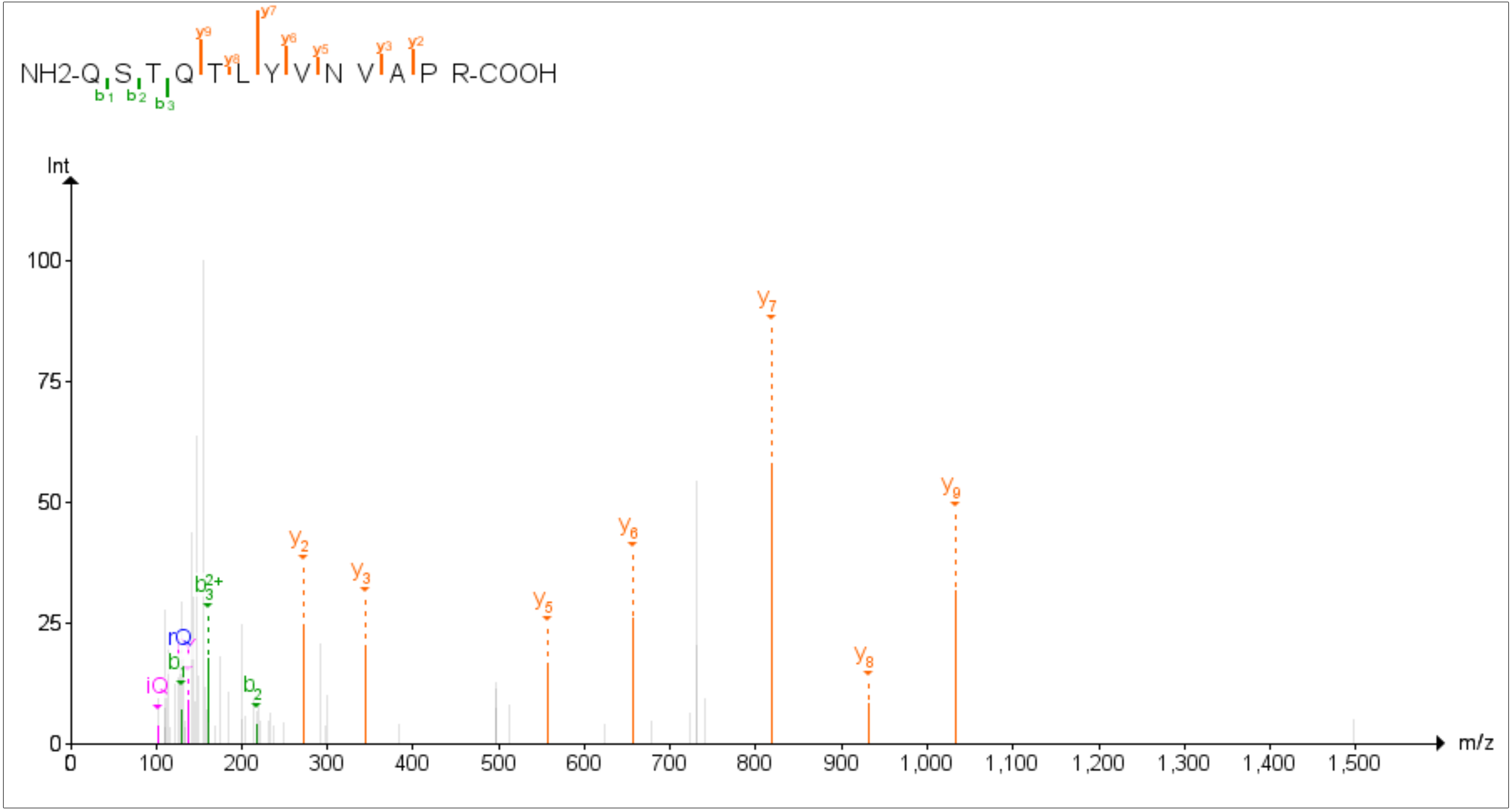
NH2-Q S T Q T L Y V N V A P R-COOH
b1 b2 b3
y9 y8 y7 y6 y5 y3 y2
Int
100
75
50
25
0
y7
y9
y6
y2
y3
b3 2+
y5
rQ
b1
iQ
b2
y8
0 100 200 300 400 500 600 700 800 900 1,000 1,100 1,200 1,300 1,400 1,500
m/z

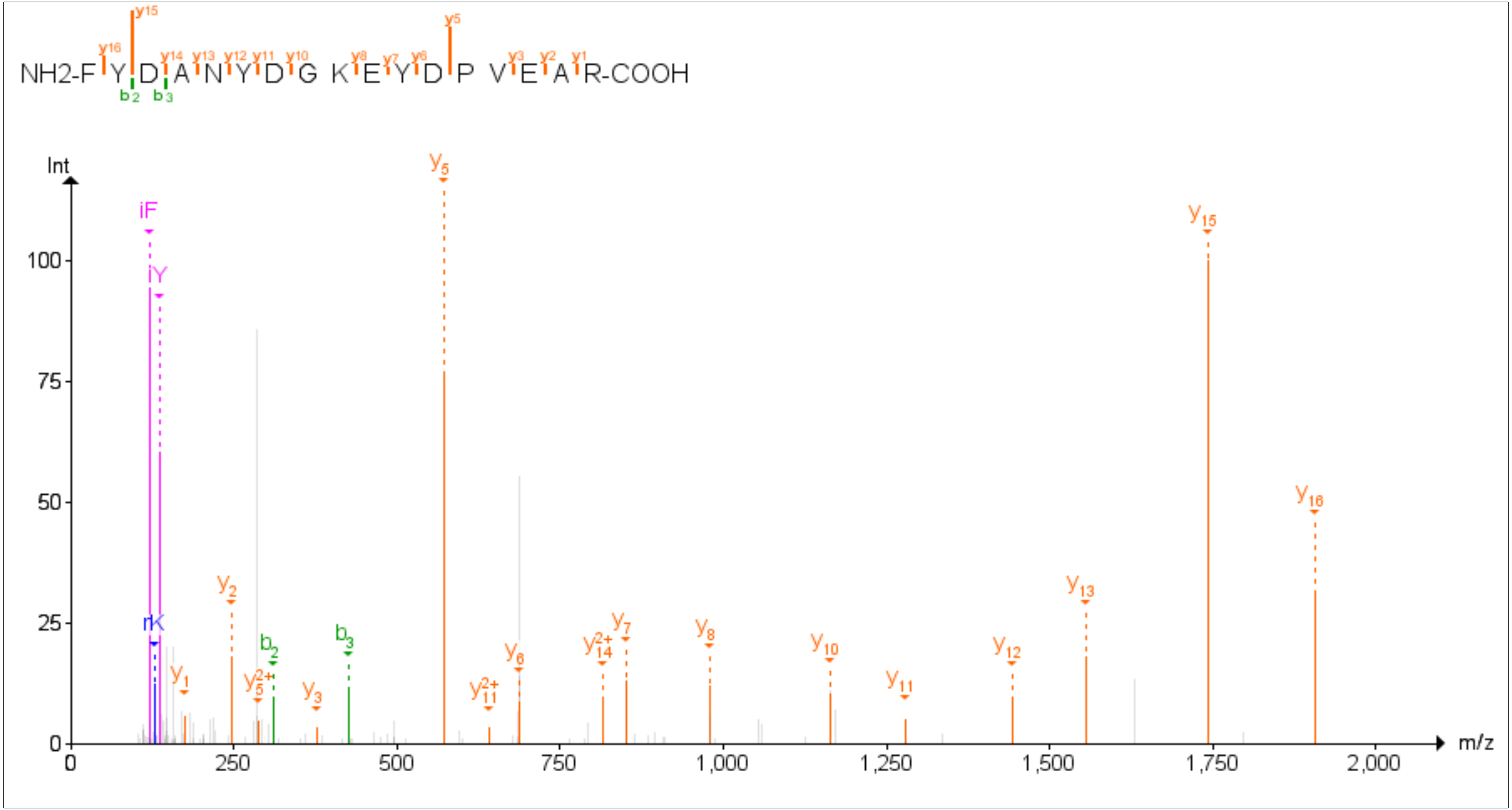

NH2-F Y D A N Y D G K E Y D P V E A R-COOH
y16 y15 y14 y13 y12 y11 y10 y8 y7 y6 y5 y3 y2 y1
b2 b3
Int
m/z
iF
iY
rK
y1
y2
b2
b3
y3
y5
y6
y7
y8
y10
y11
y12
y13
y15
y16
0
25
50
75
100
0
250
500
750
1,000
1,250
1,500
1,750
2,000

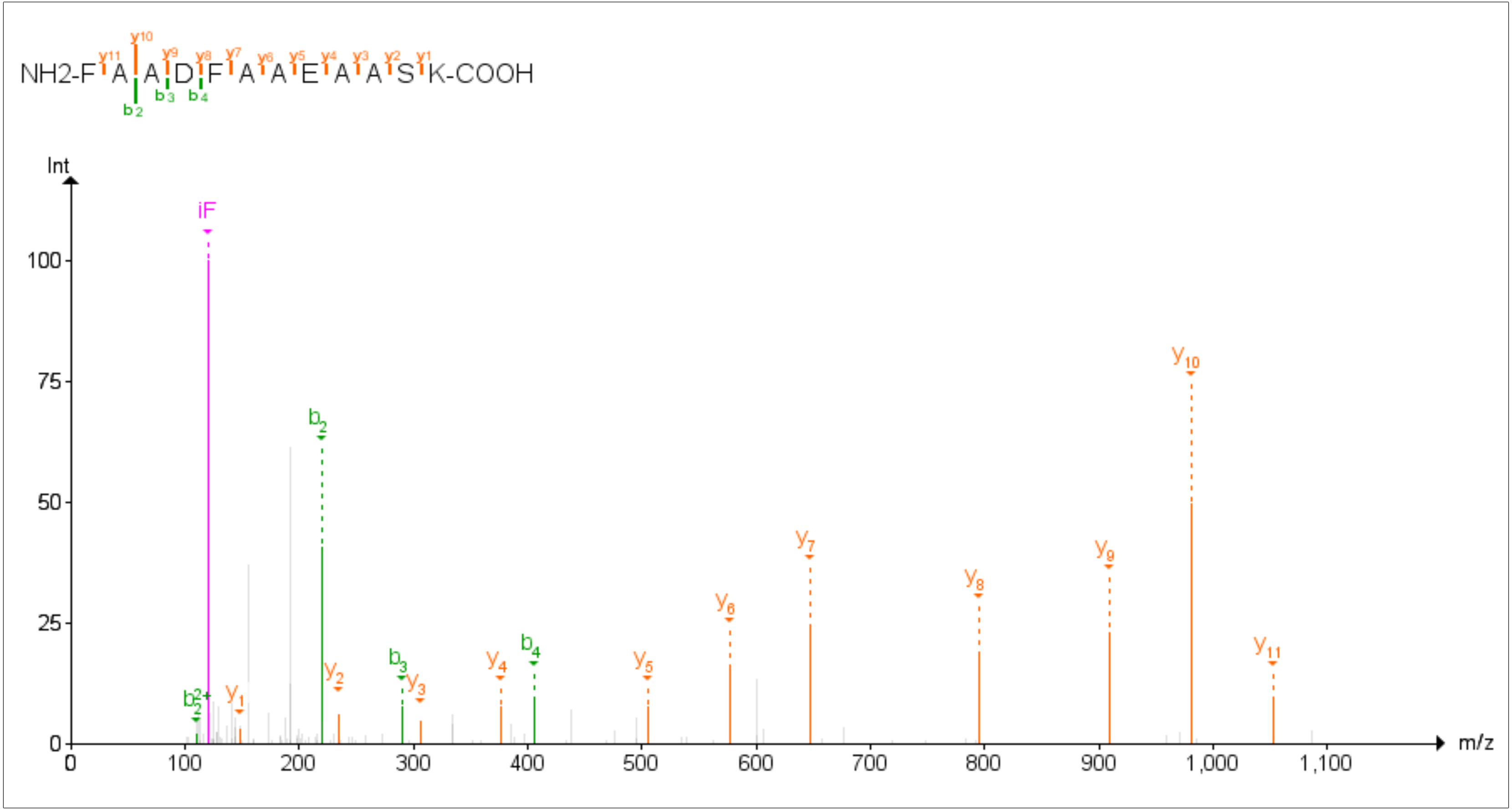
NH2-F A A D F A A E A A S K-COOH
y11 y10 y9 y8 y7 y6 y5 y4 y3 y2 y1
b2 b3 b4
Int
iF
b2
y10
y7
y9
y8
y6
y11
b4
y4
y5
b3
y2
y3
y1
b2 2+
100
75
50
25
0
0 100 200 300 400 500 600 700 800 900 1,000 1,100
m/z

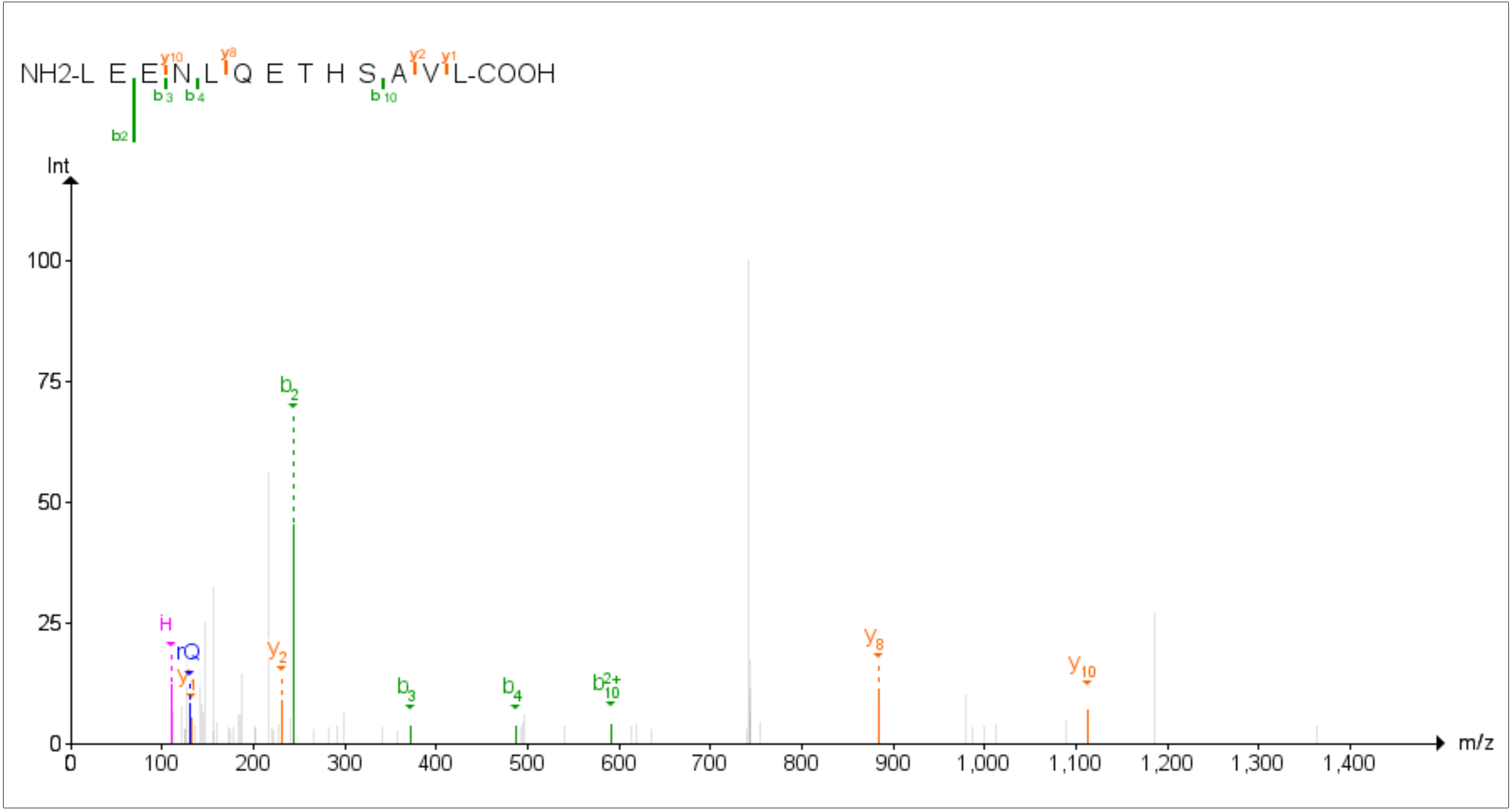

NH2-L E E N L Q E T H S A V L-COOH
y10
y8
y2
y1
b2
b3
b4
b10
Int
100
75
50
25
0
m/z
0
100
200
300
400
500
600
700
800
900
1,000
1,100
1,200
1,300
1,400
iH
rQ
y1
y2
b2
b3
b4
b10 2+
y8
y10

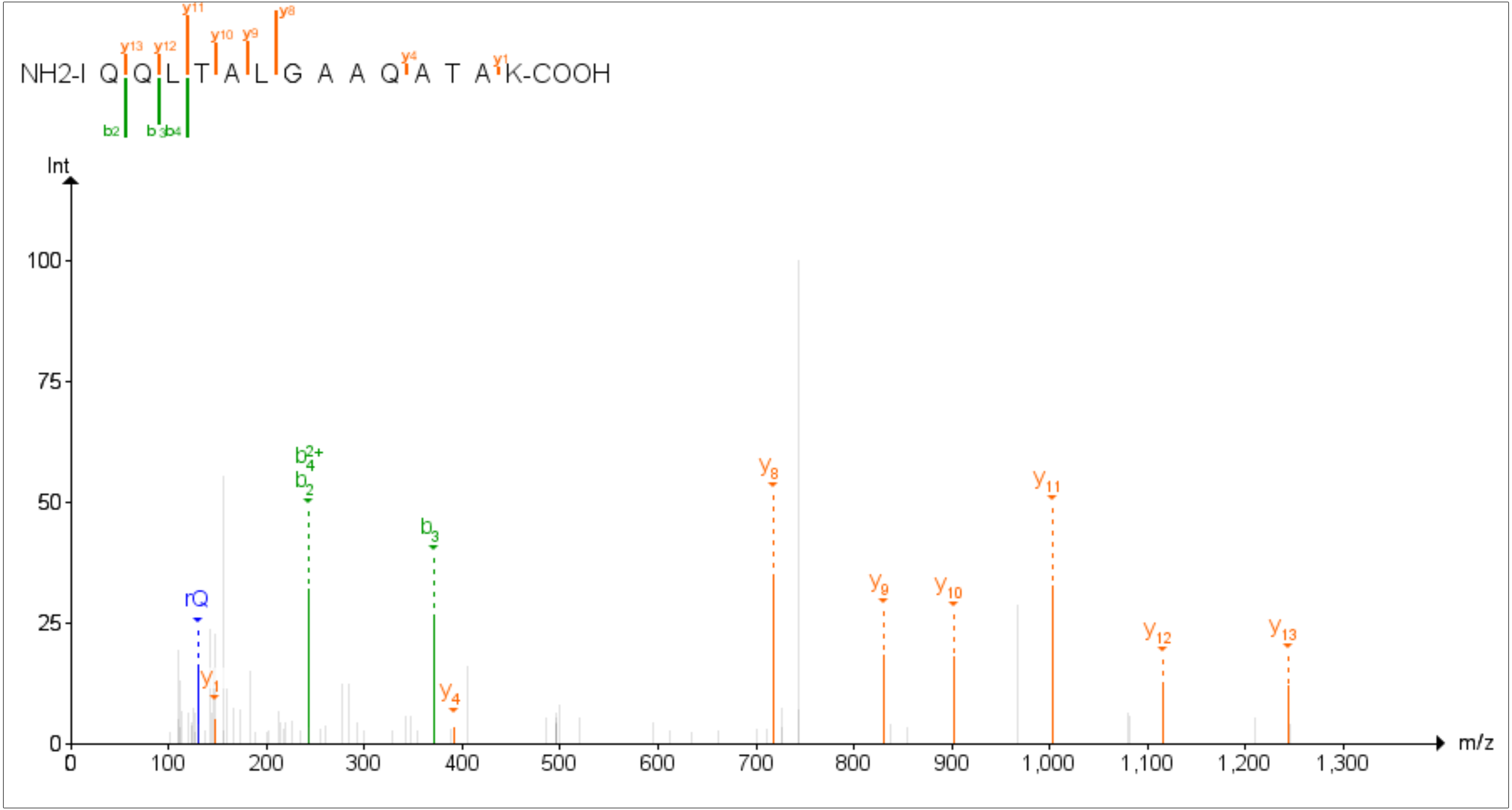
NH2-I Q Q L T A L G A A Q A T A K-COOH
y13 y12 y11 y10 y9 y8 y4 y1
b2 b3 b4
Int
100
75
50
25
0
m/z
0 100 200 300 400 500 600 700 800 900 1,000 1,100 1,200 1,300
rQ
y1
b4 2+
b2
b3
y4
y8
y9
y10
y11
y12
y13

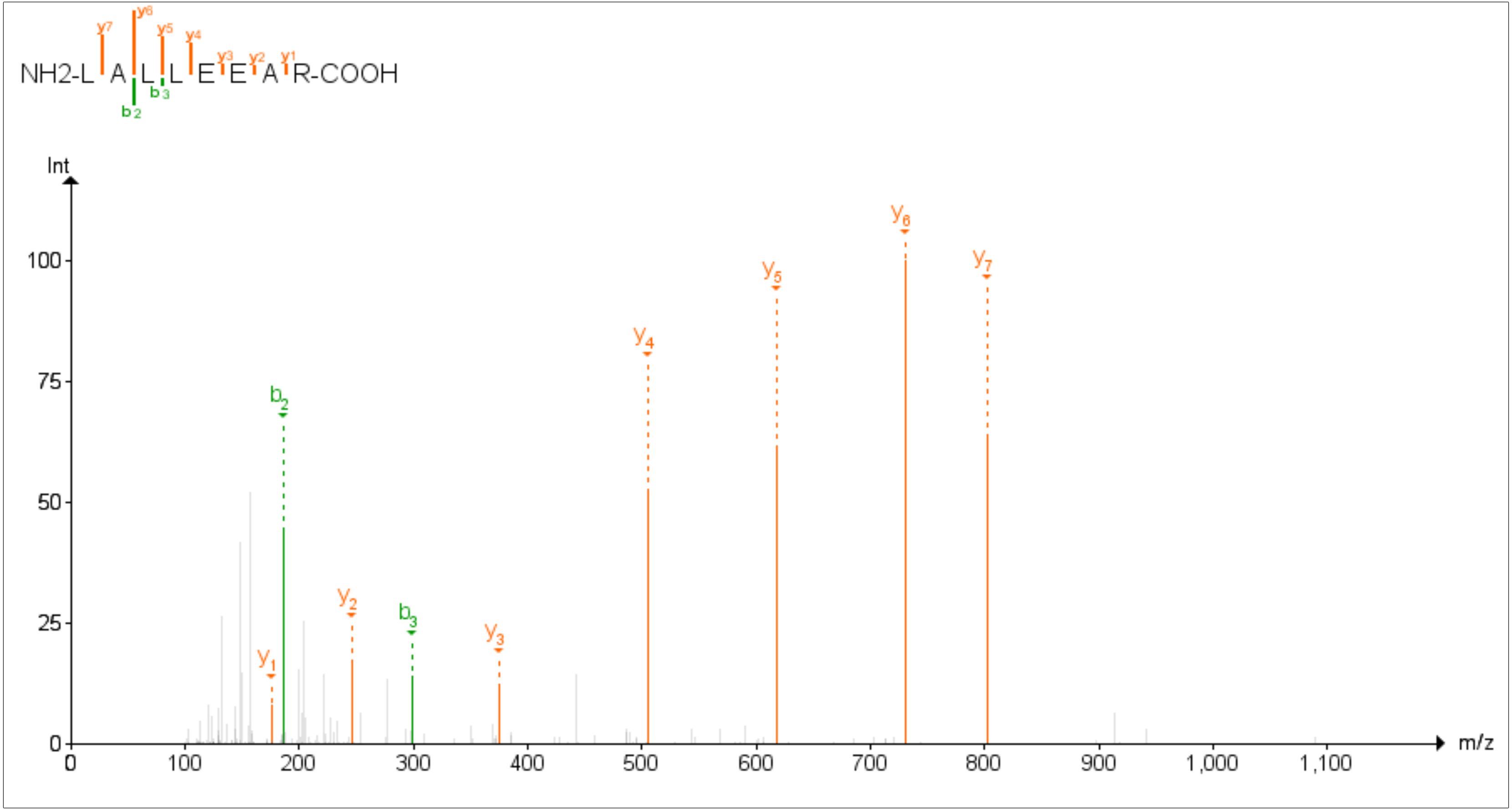
NH2-L A L L E E A R-COOH
y7
y6
y5
y4
y3
y2
y1
b2
b3
Int
m/z
0
25
50
75
100
100
200
300
400
500
600
700
800
900
1,000
1,100
y1
b2
y2
b3
y3
y4
y5
y6
y7

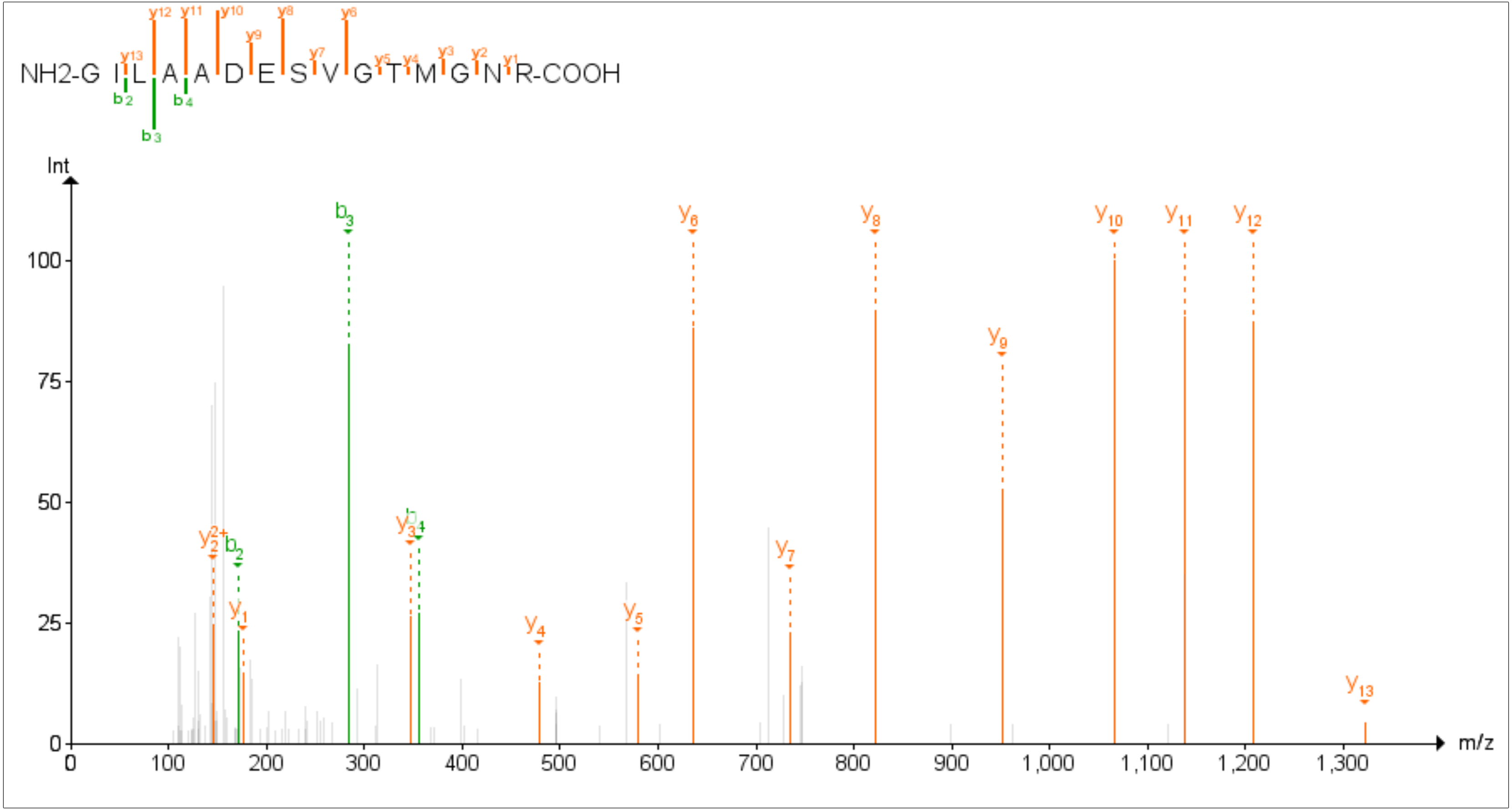

NH2-G I L A A D E S V G T M G N R-COOH
y13 y12 y11 y10 y9 y8 y7 y6 y5 y4 y3 y2 y1
b2 b3 b4
Int
100
75
50
25
0
m/z
0 100 200 300 400 500 600 700 800 900 1,000 1,100 1,200 1,300

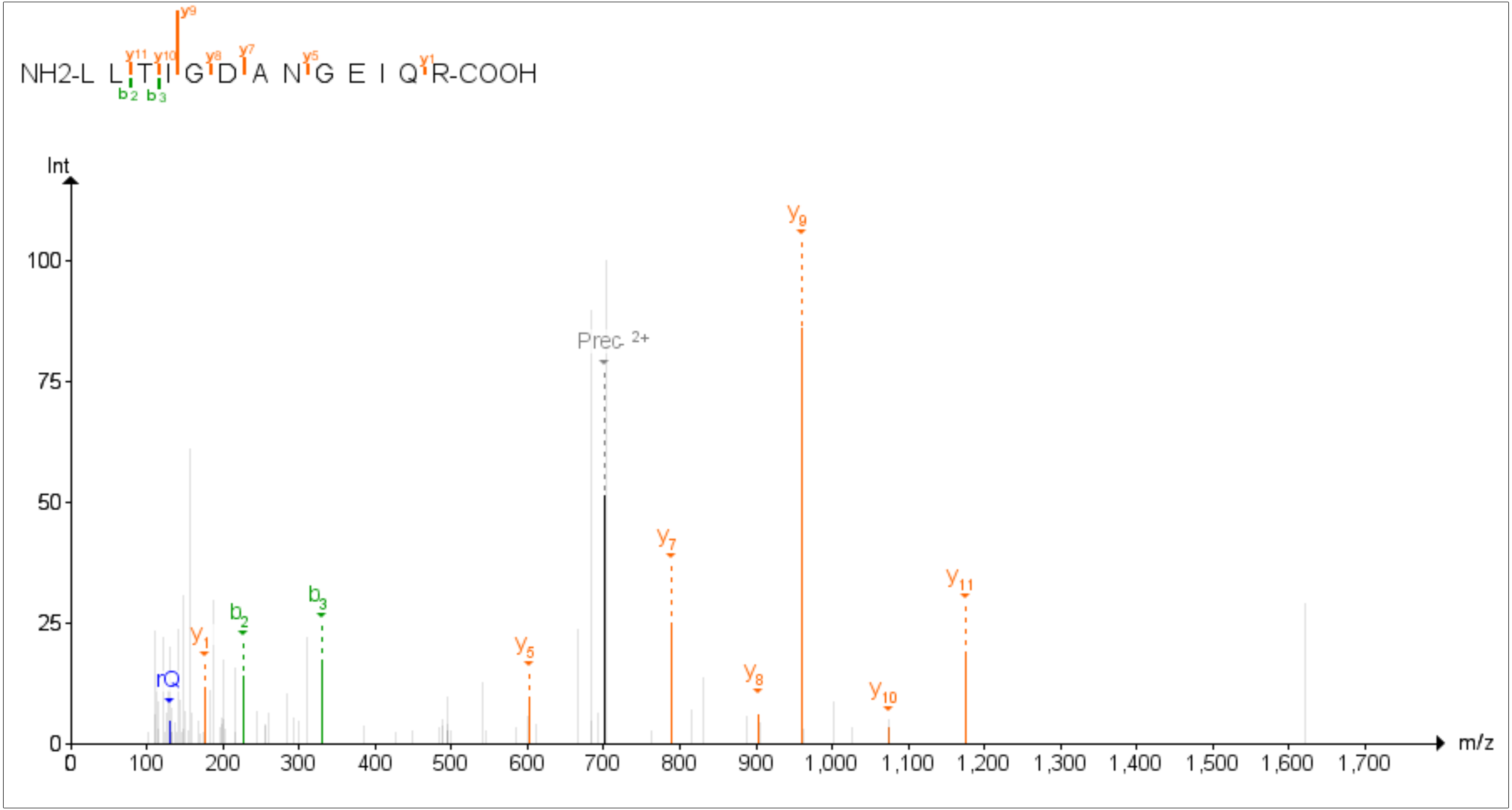

NH2-L L T I G D A N G E I Q R-COOH
y11 y10 y9 y8 y7 y5 y1
b2 b3
Int
m/z
Prec. 2+
rQ

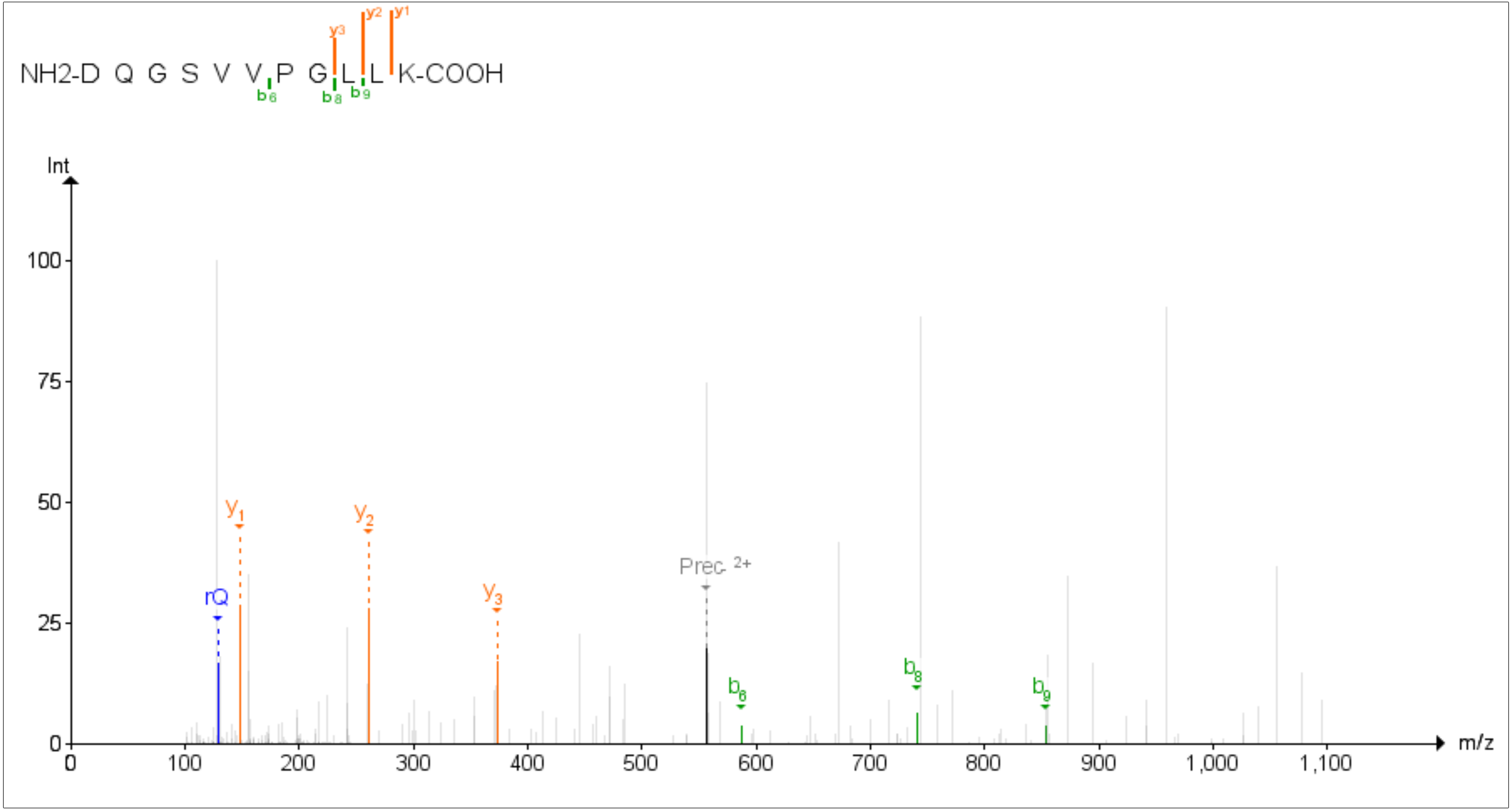
NH2-D Q G S V V P G L L K-COOH
y3
y2
y1
b6
b8
b9
Int
100
75
50
25
0
y1
y2
y3
rQ
Prec 2+
b6
b8
b9
0
100
200
300
400
500
600
700
800
900
1,000
1,100
m/z

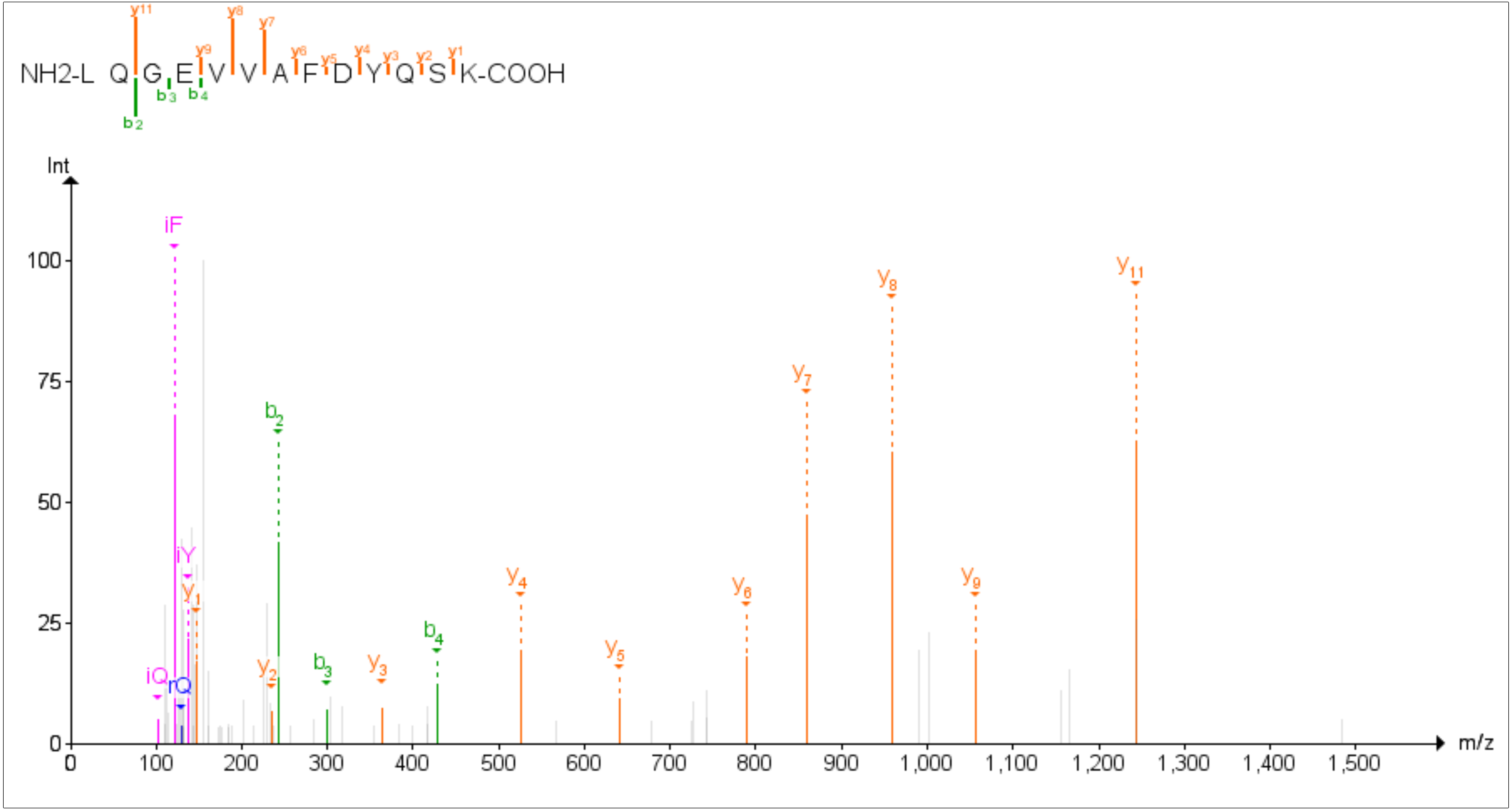

NH2-L Q G E V V A F D Y Q S K-COOH
y11 y9 y8 y7 y6 y5 y4 y3 y2 y1
b2 b3 b4
Int
m/z
iF
iY
iQ
rQ
y1 y2 y3 y4 y5 y6 y7 y8 y9 y11
b2 b3 b4
0
25
50
75
100
0 100 200 300 400 500 600 700 800 900 1,000 1,100 1,200 1,300 1,400 1,500

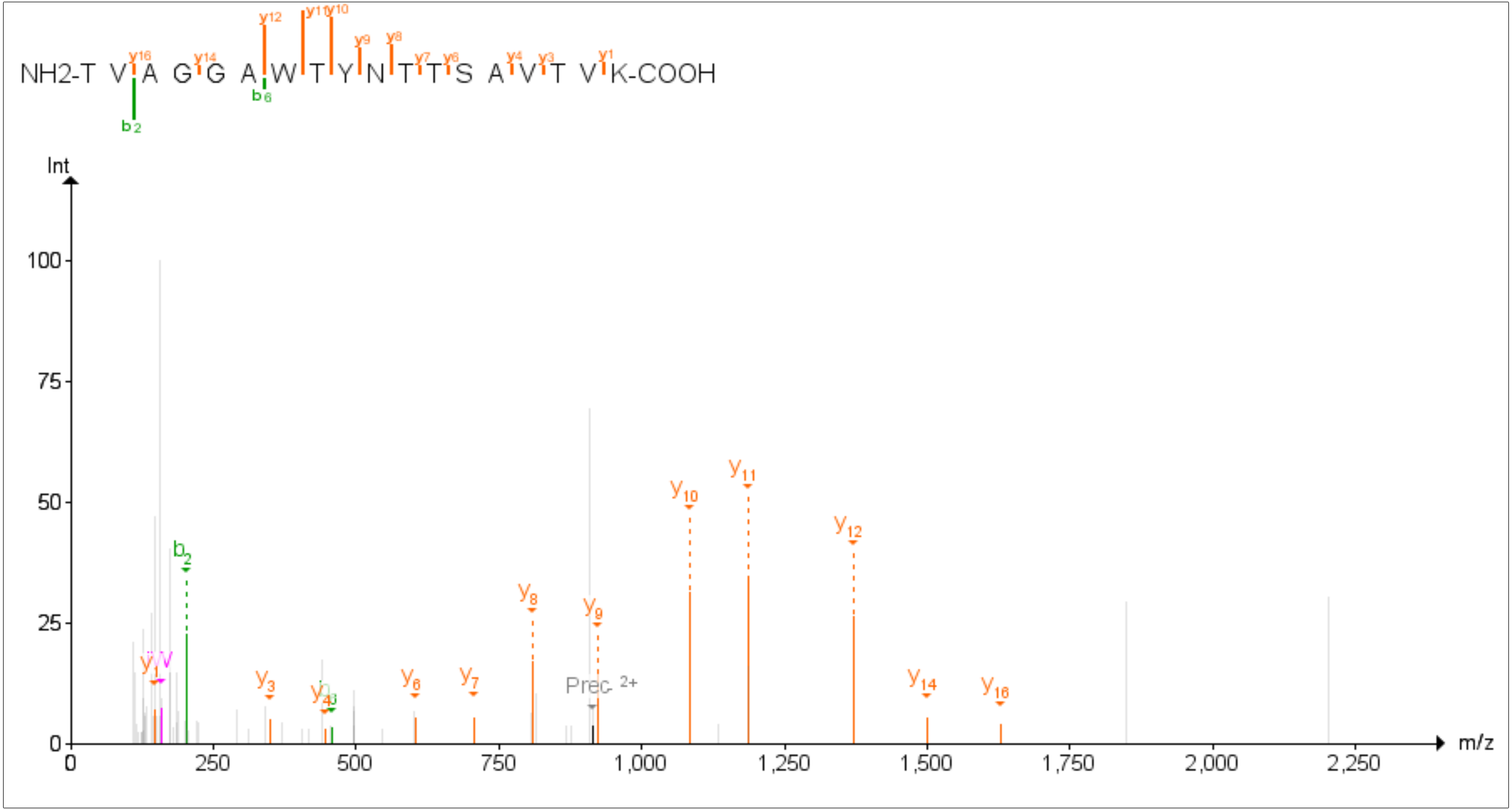
NH2-T V A G G A W T Y N T T S A V T V K-COOH
Int
100
75
50
25
0
m/z
0
250
500
750
1,000
1,250
1,500
1,750
2,000
2,250
b2
y10
y11
y12
y8
y9
y3
y6
y7
y14
y16
Prec- 2+

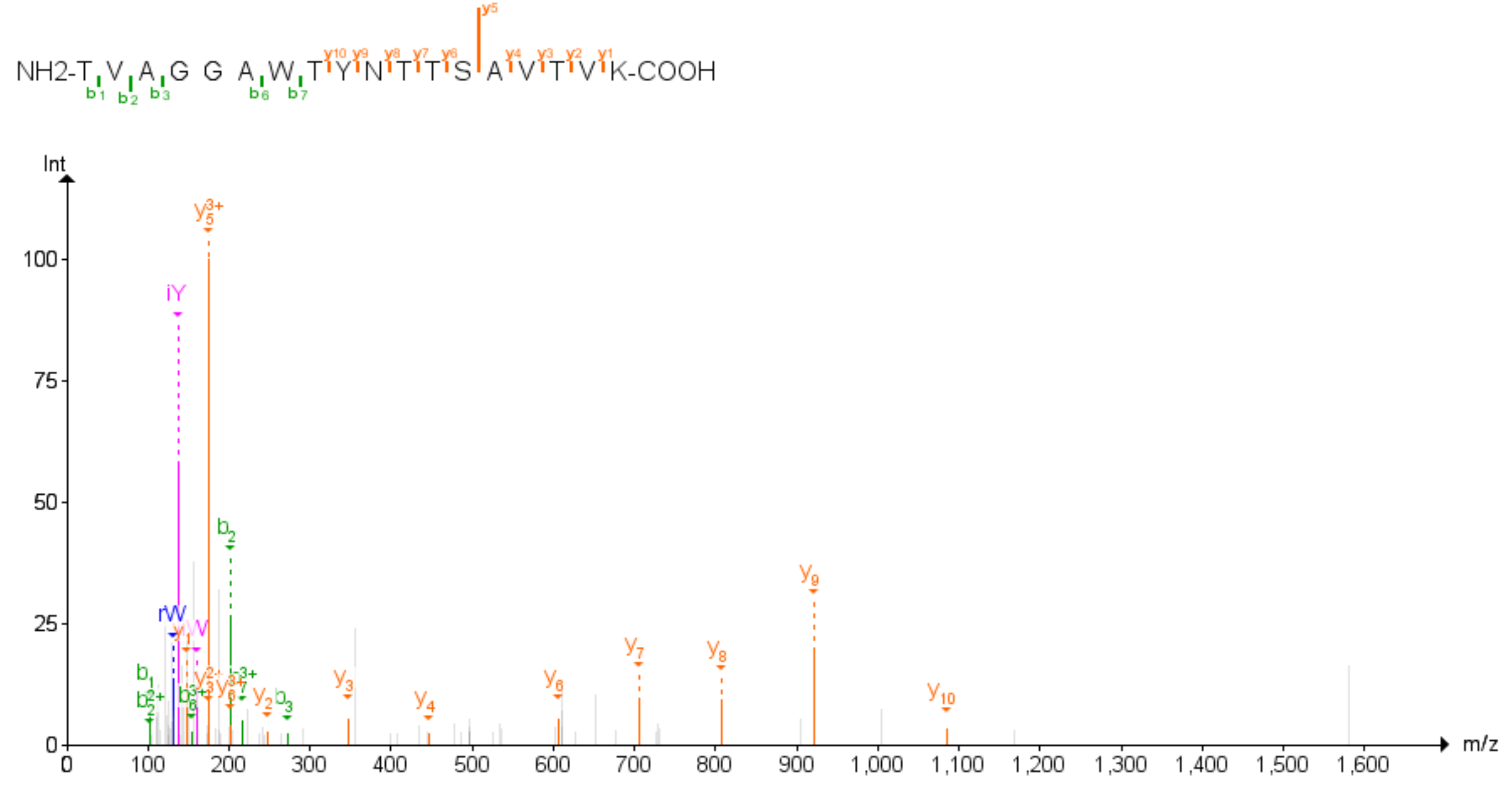
NH2-T V A G G A W T Y N T T S A V T V K-COOH
b1 b2 b3 b6 b7
y10 y9 y8 y7 y6 y5 y4 y3 y2 y1
Int
m/z
0
25
50
75
100
0 100 200 300 400 500 600 700 800 900 1,000 1,100 1,200 1,300 1,400 1,500 1,600
y5 3+
iY
b2
rW
y1
iW
y9
y7
y8
y6
y10
y3
y4
y2
b3
b1

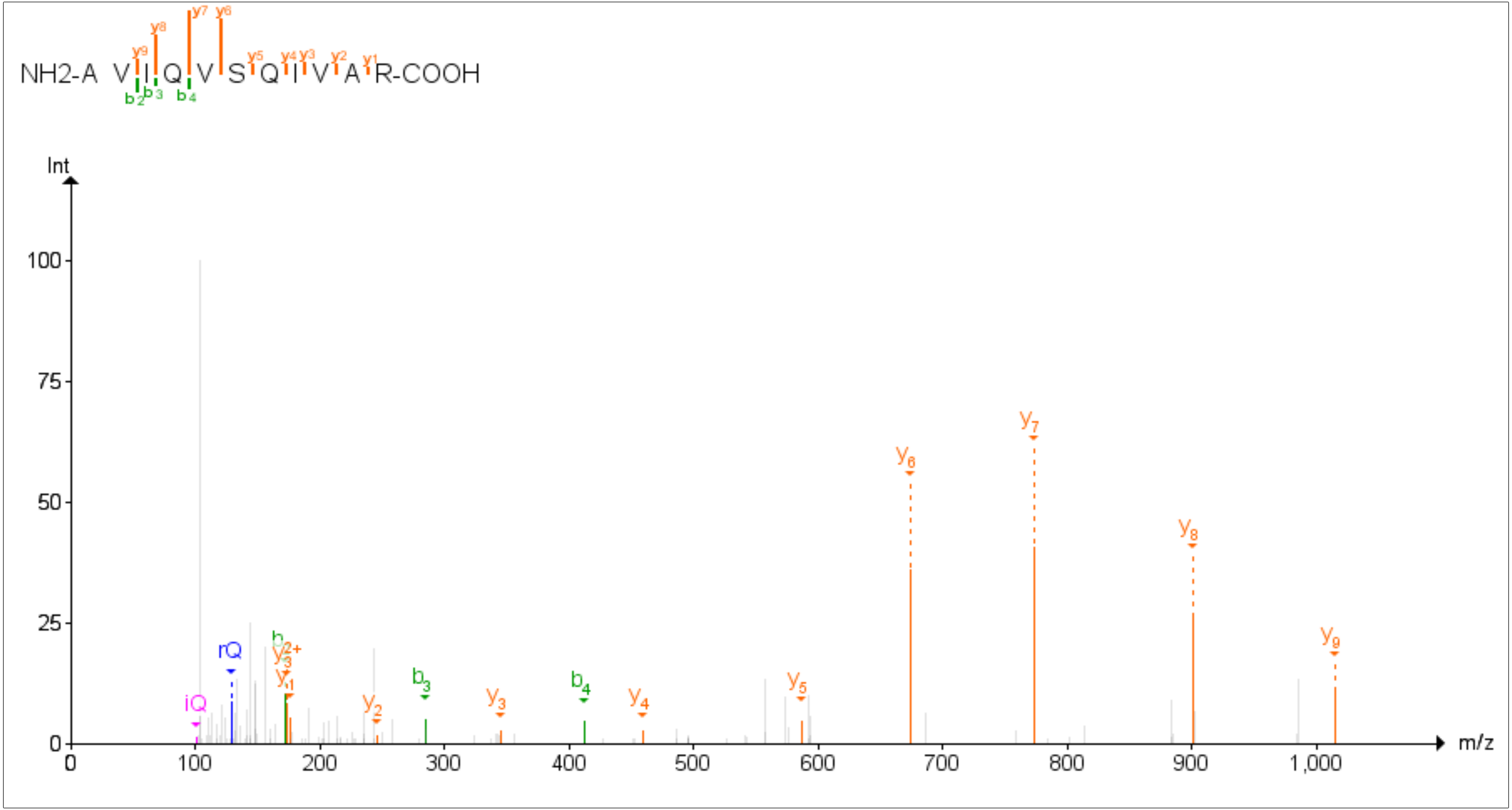

NH2-A V I Q V S Q I V A R-COOH
y9 y8 y7 y6 y5 y4 y3 y2 y1
b2 b3 b4
Int
100
75
50
25
0
m/z
0 100 200 300 400 500 600 700 800 900 1,000
iQ
rQ
b2
y3 2+
y1
y2
b3
y3
b4
y4
y5
y6
y7
y8
y9

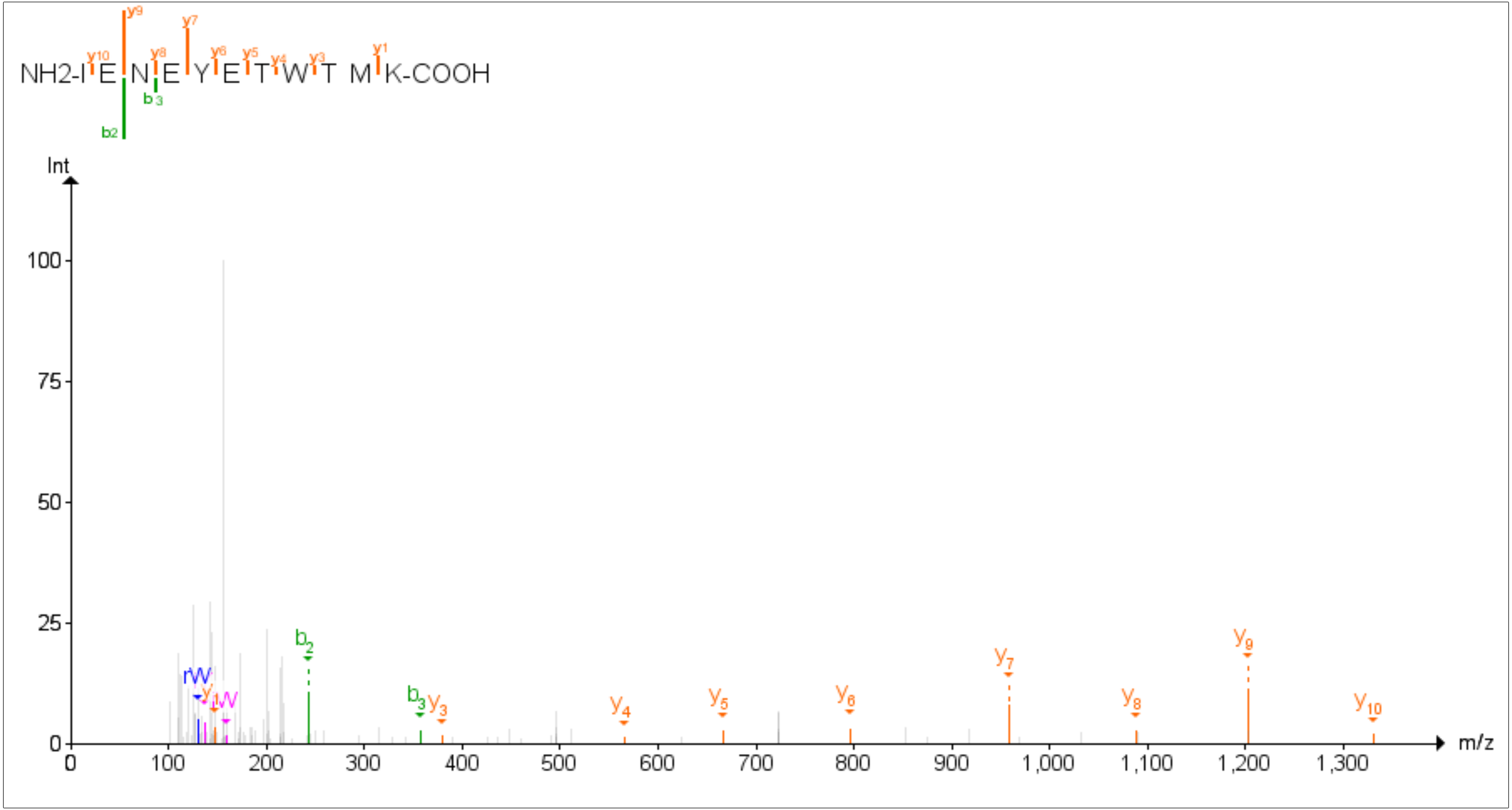

NH2-I E N E Y E T W T M K-COOH
y10 y9 y8 y7 y6 y5 y4 y3 y1
b2 b3
Int
100
75
50
25
0
rW
y1
iW
b2
b3
y3
y4
y5
y6
y7
y8
y9
y10
0 100 200 300 400 500 600 700 800 900 1,000 1,100 1,200 1,300
m/z

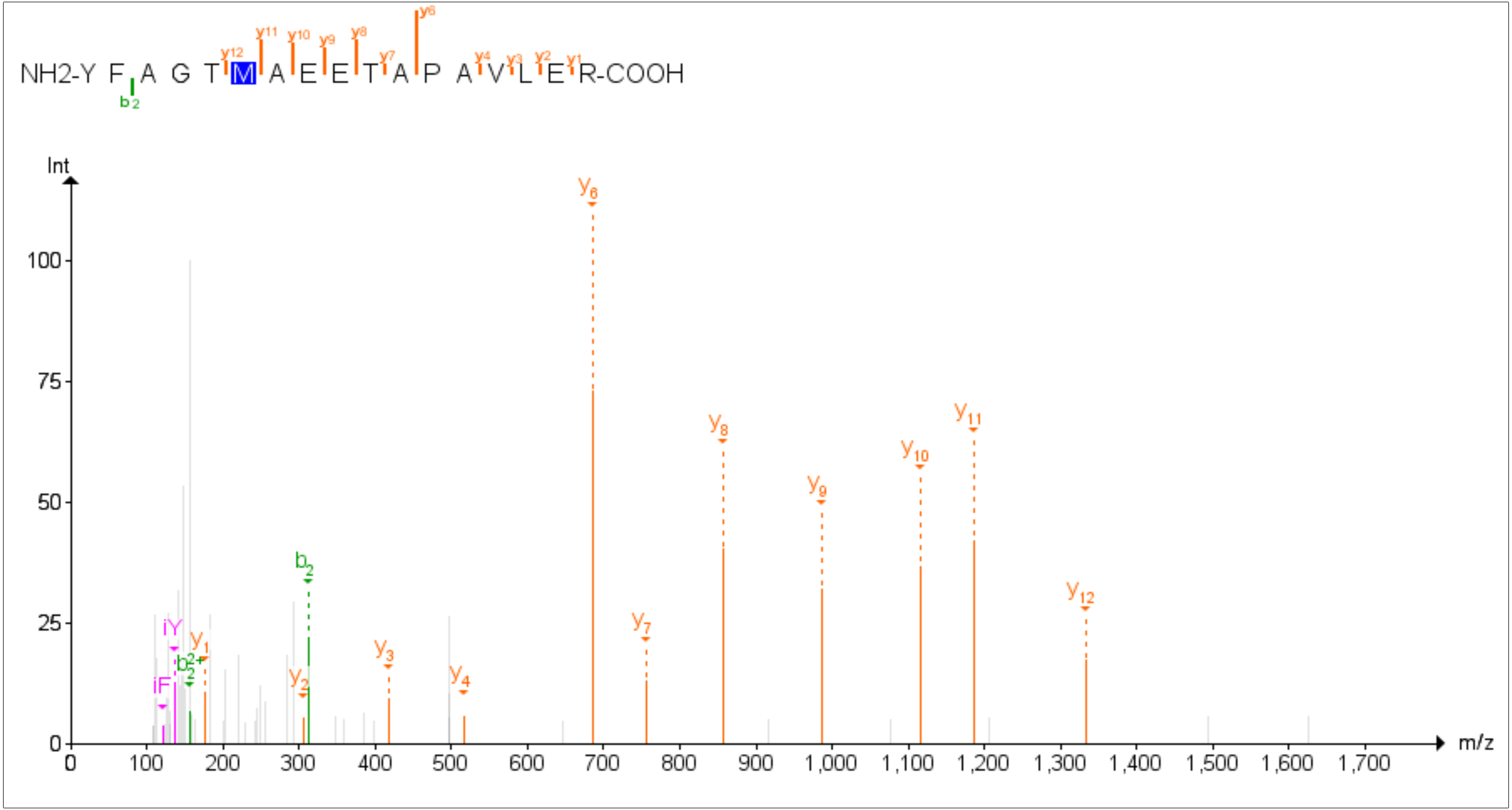
NH2-Y F A G T M A E E T A P A V L E R-COOH
b2
y12
y11
y10
y9
y8
y7
y6
y4
y3
y2
y1
Int
m/z
0
25
50
75
100
0
100
200
300
400
500
600
700
800
900
1,000
1,100
1,200
1,300
1,400
1,500
1,600
1,700
iY
iF
b2 2+
b2
y1
y2
y3
y4
y6
y7
y8
y9
y10
y11
y12

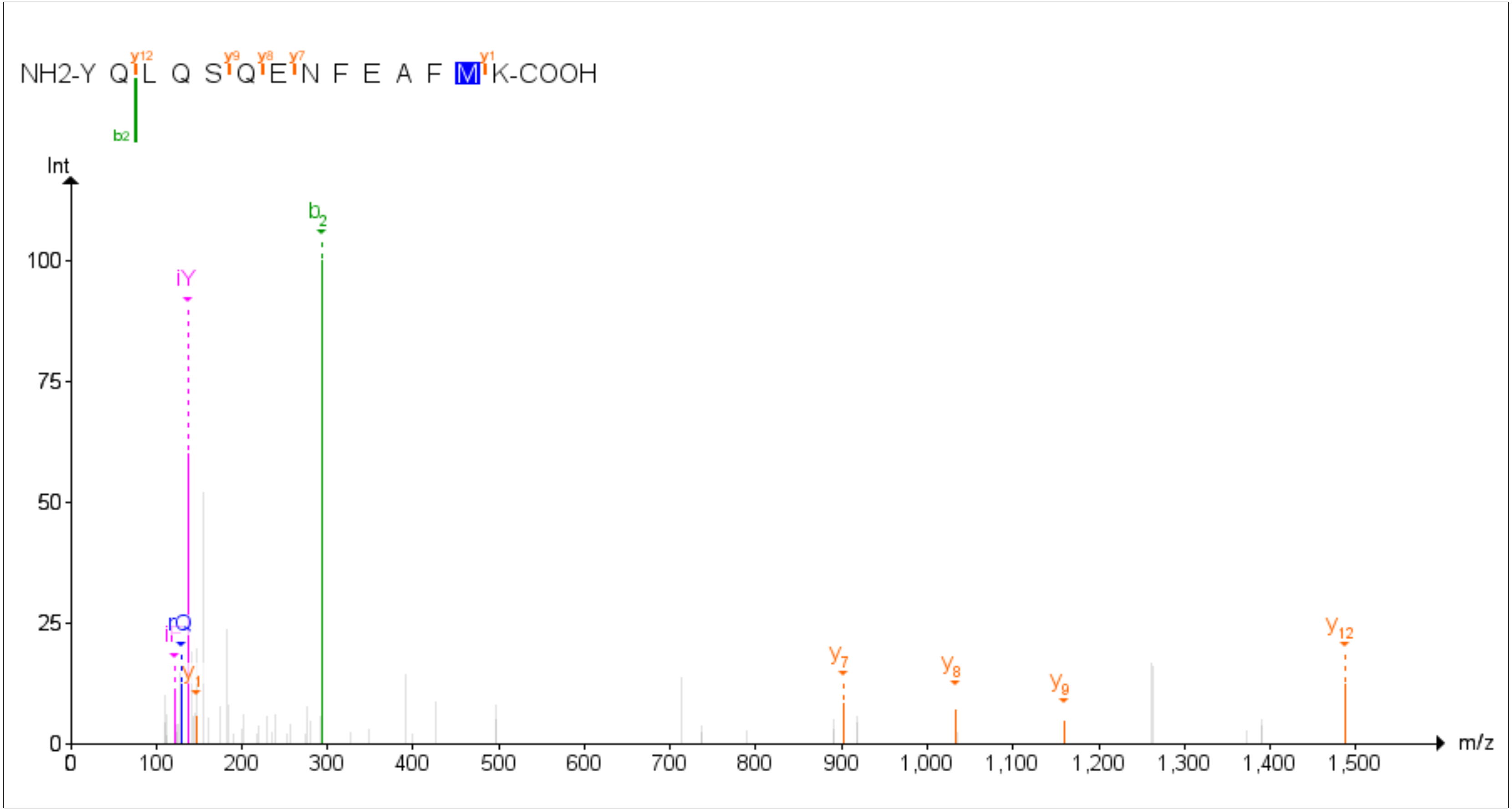
NH2-Y Q L Q S Q E N F E A F M K-COOH
y12
y9
y8
y7
y1
b2
Int
100
75
50
25
0
b2
iY
rQ
y1
y7
y8
y9
y12
0
100
200
300
400
500
600
700
800
900
1,000
1,100
1,200
1,300
1,400
1,500
m/z

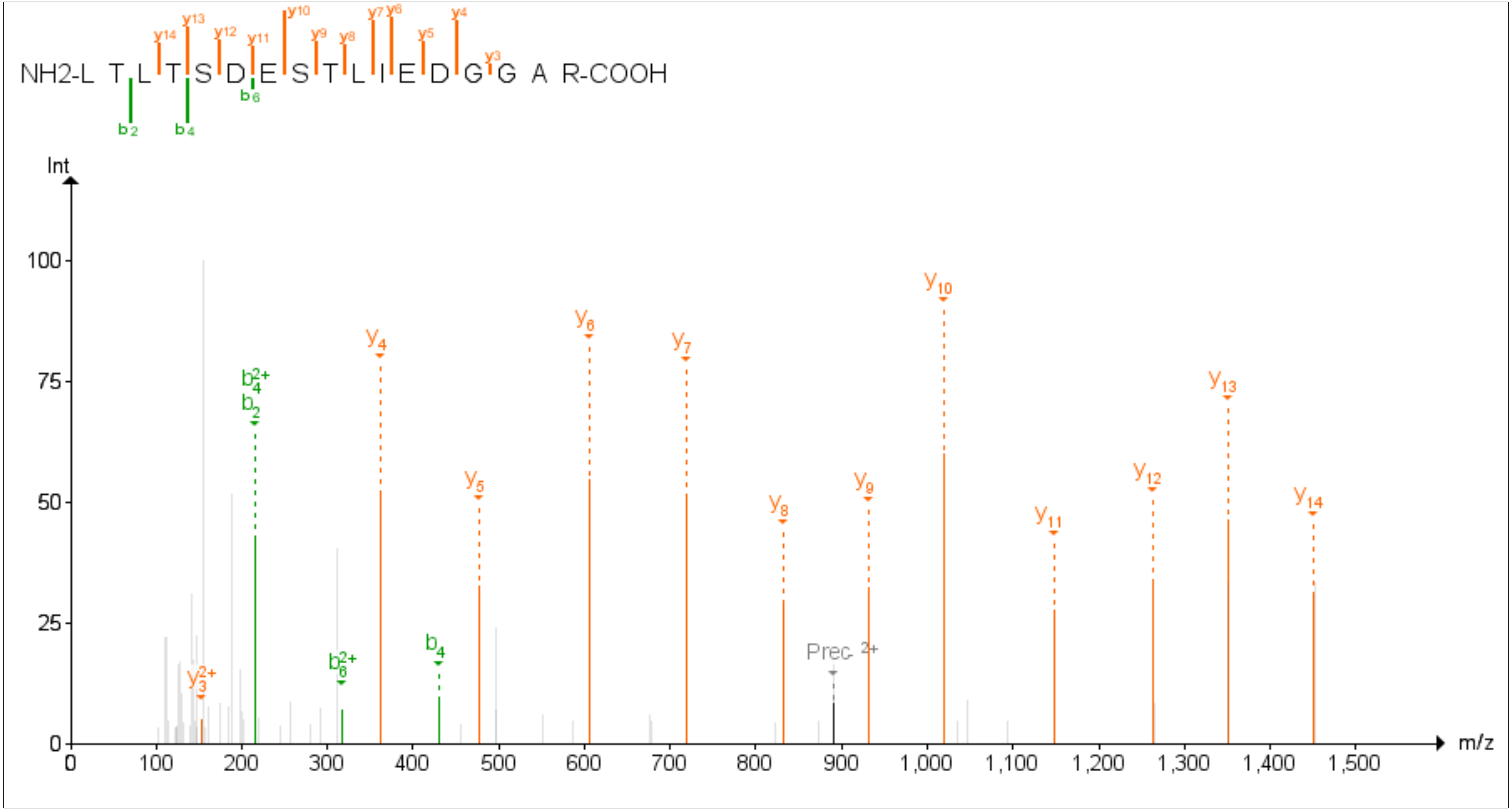

NH2-L T L T S D E S T L I E D G G A R-COOH
y14 y13 y12 y11 y10 y9 y8 y7 y6 y5 y4 y3
b2 b4 b6
Int
100
75
50
25
0
m/z
0 100 200 300 400 500 600 700 800 900 1,000 1,100 1,200 1,300 1,400 1,500
b_4^{2+}
b_2
y_4
y_6
y_7
y_{10}
y_{13}
y_5
y_8
y_9
y_{11}
y_{12}
y_{14}
y_3^{2+}
b_6^{2+}
b_4
Prec 2+

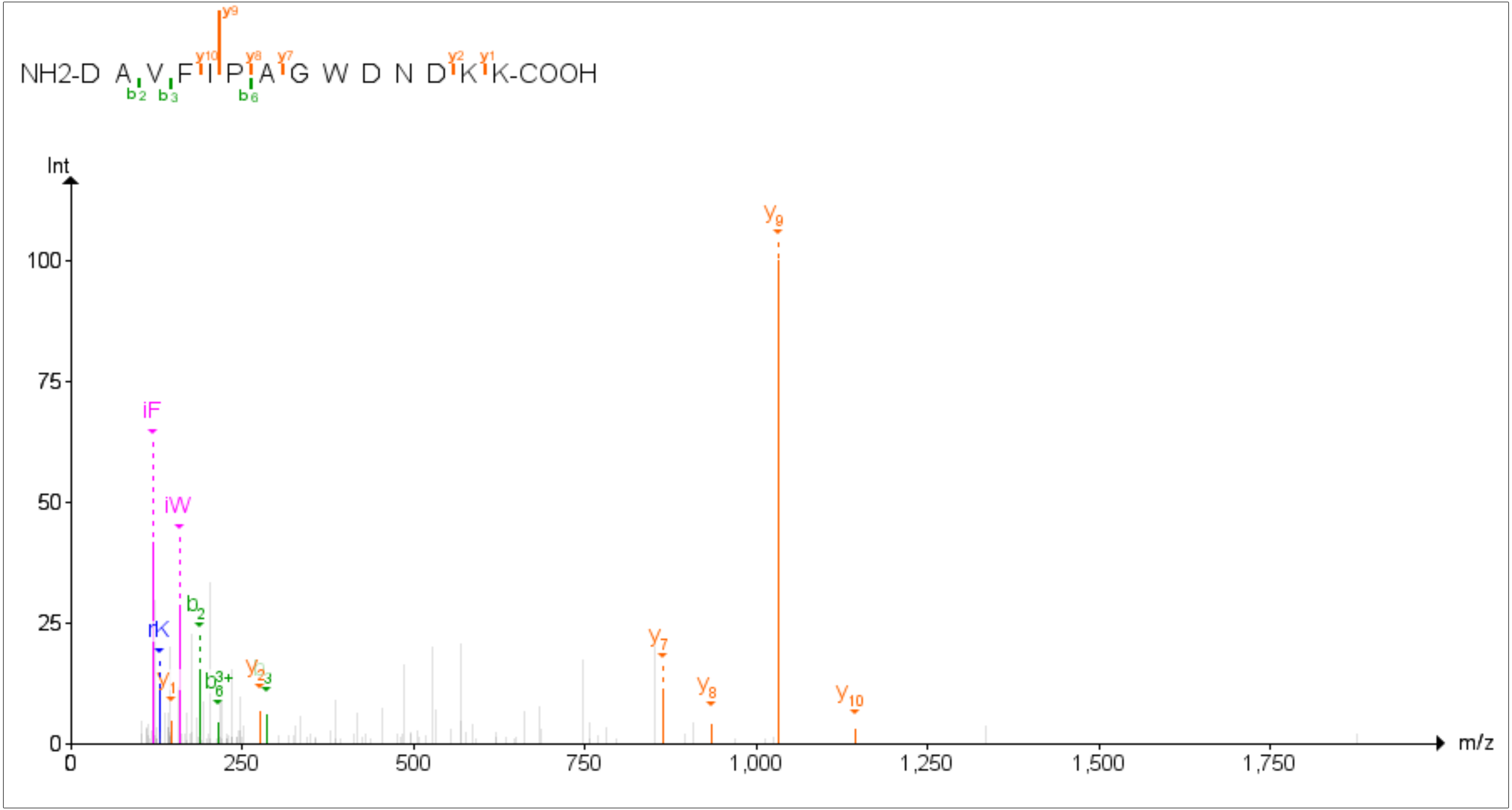

NH2-D A V F I P A G W D N D K K-COOH
y9
y10
y8
y7
y2
y1
b2
b3
b6
Int
100
75
50
25
0
iF
iW
rK
b2
y1
y9
y7
y8
y10
0
250
500
750
1,000
1,250
1,500
1,750
m/z

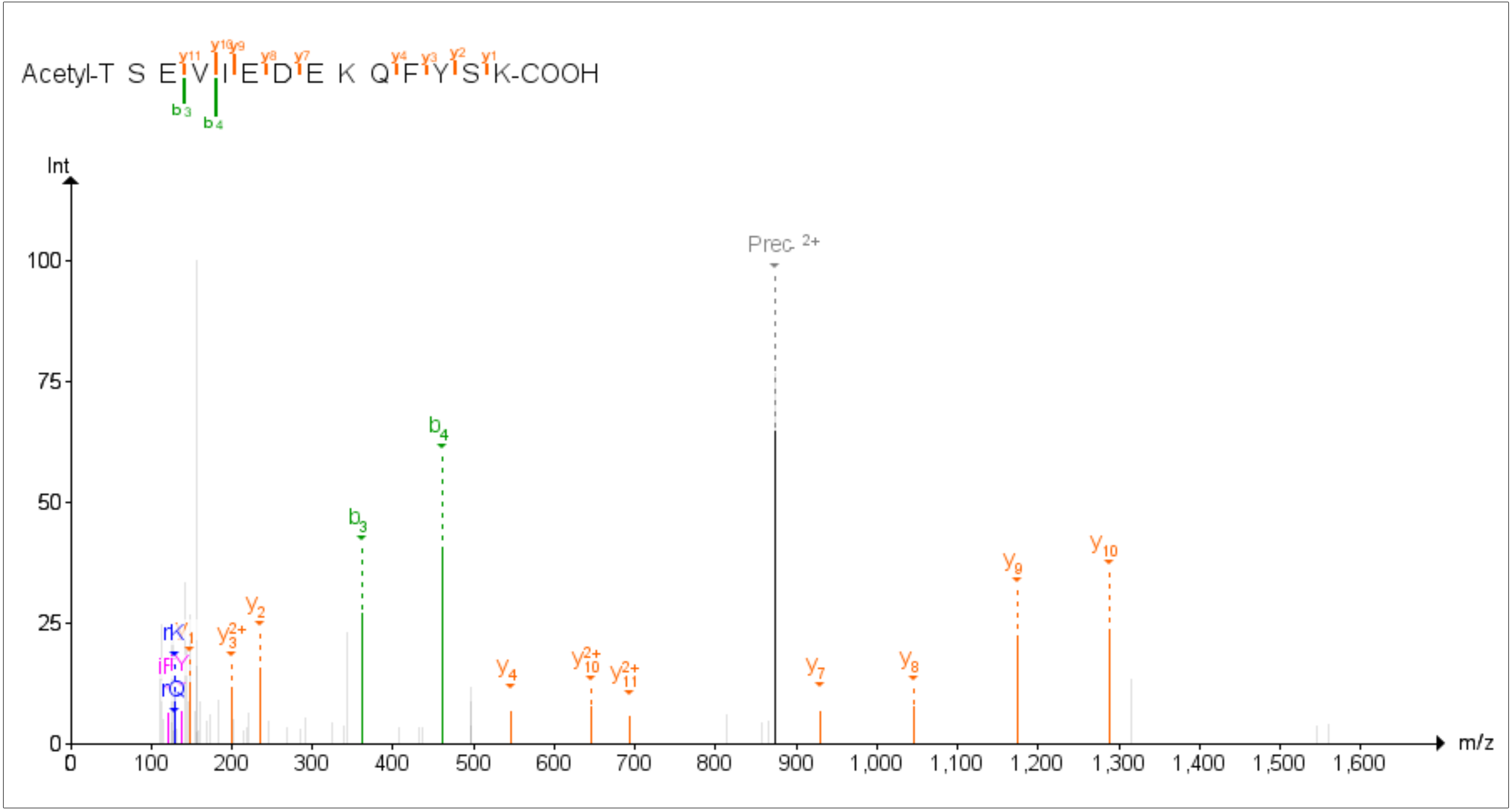

Acetyl-T S E V I E D E K Q F Y S K-COOH
y11 y10 y9 y8 y7 y4 y3 y2 y1
b3 b4
Int
100
75
50
25
0
Prec. 2+
b4
b3
y10
y9
y2
y3 2+
y1
rK
iF
Y
rQ
y4
y10 2+
y11 2+
y7
y8
0 100 200 300 400 500 600 700 800 900 1,000 1,100 1,200 1,300 1,400 1,500 1,600
m/z

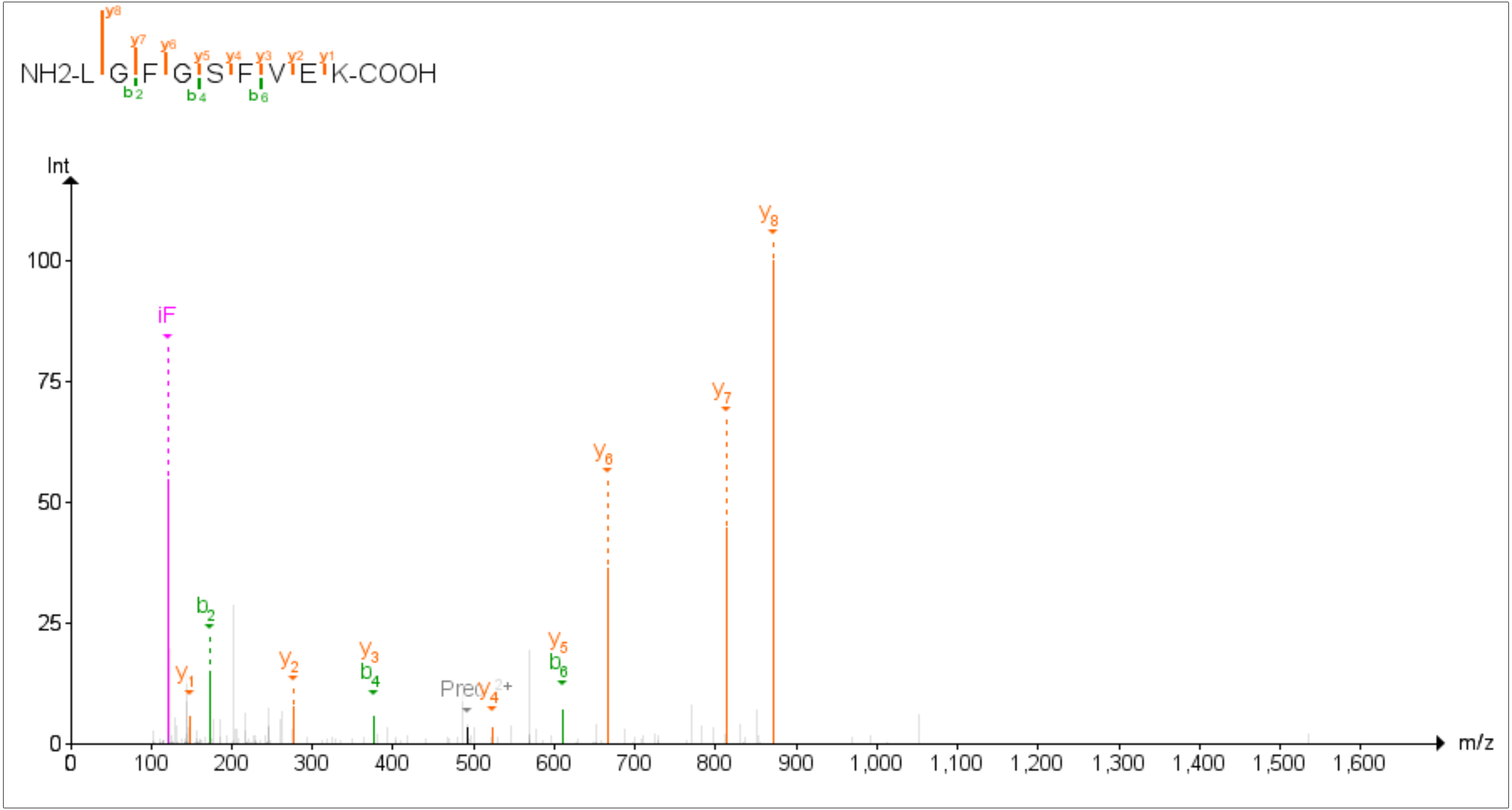

NH2-L G F G S F V E K-COOH
y8 y7 y6 y5 y4 y3 y2 y1
b2 b4 b6
Int
m/z
iF
b2
y1
y2
y3
b4
Prec2+
y4
y5
b6
y6
y7
y8
0
25
50
75
100
0
100
200
300
400
500
600
700
800
900
1,000
1,100
1,200
1,300
1,400
1,500
1,600

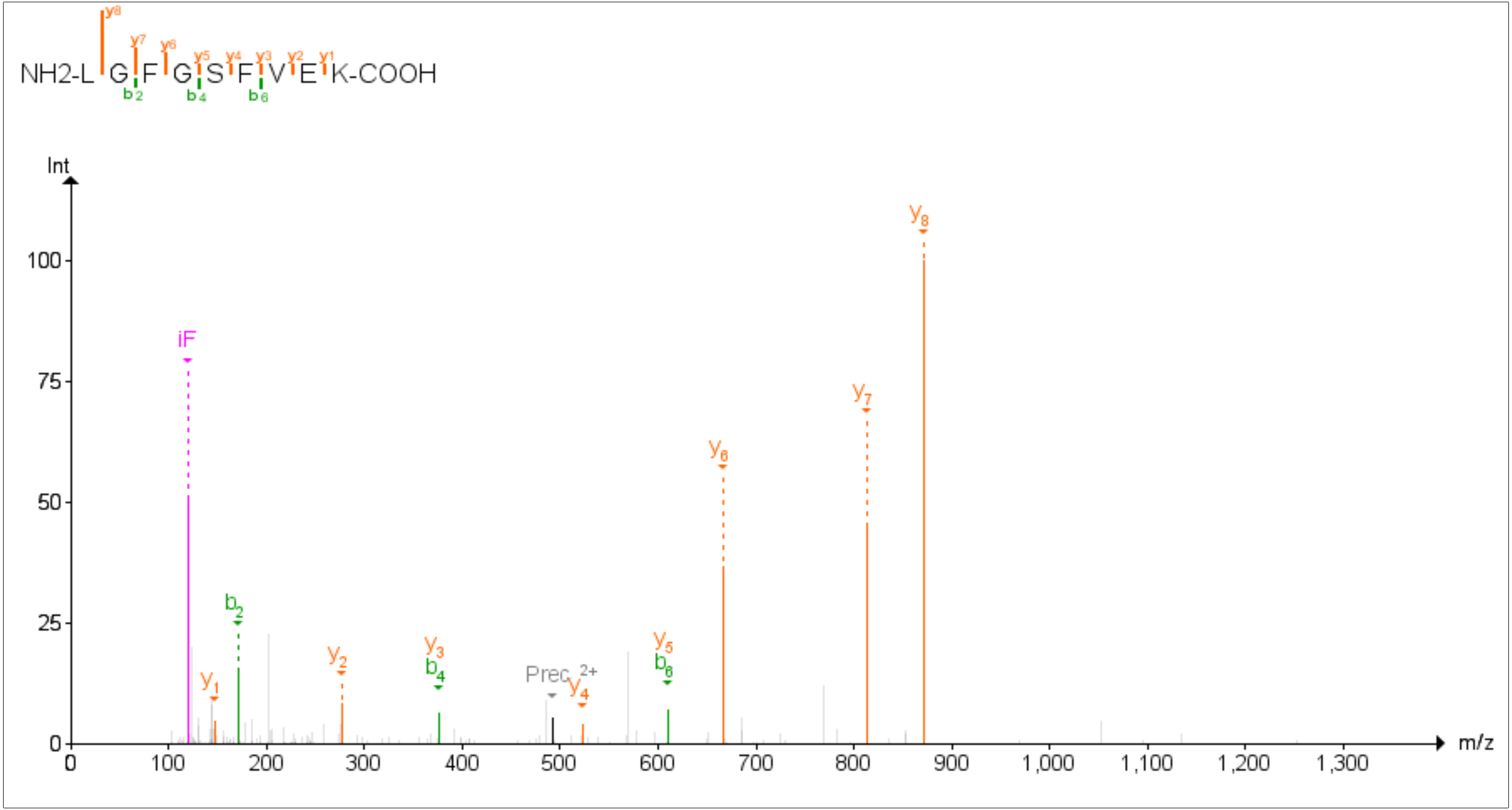

NH2-LGFGSFVEK-COOH
y8
y7
y6
y5
y4
y3
y2
y1
b2
b4
b6
Int
100
75
50
25
0
iF
y8
y7
y6
y5
b6
b2
y2
y3
b4
y1
Prec 2+
y4
0
100
200
300
400
500
600
700
800
900
1,000
1,100
1,200
1,300
m/z

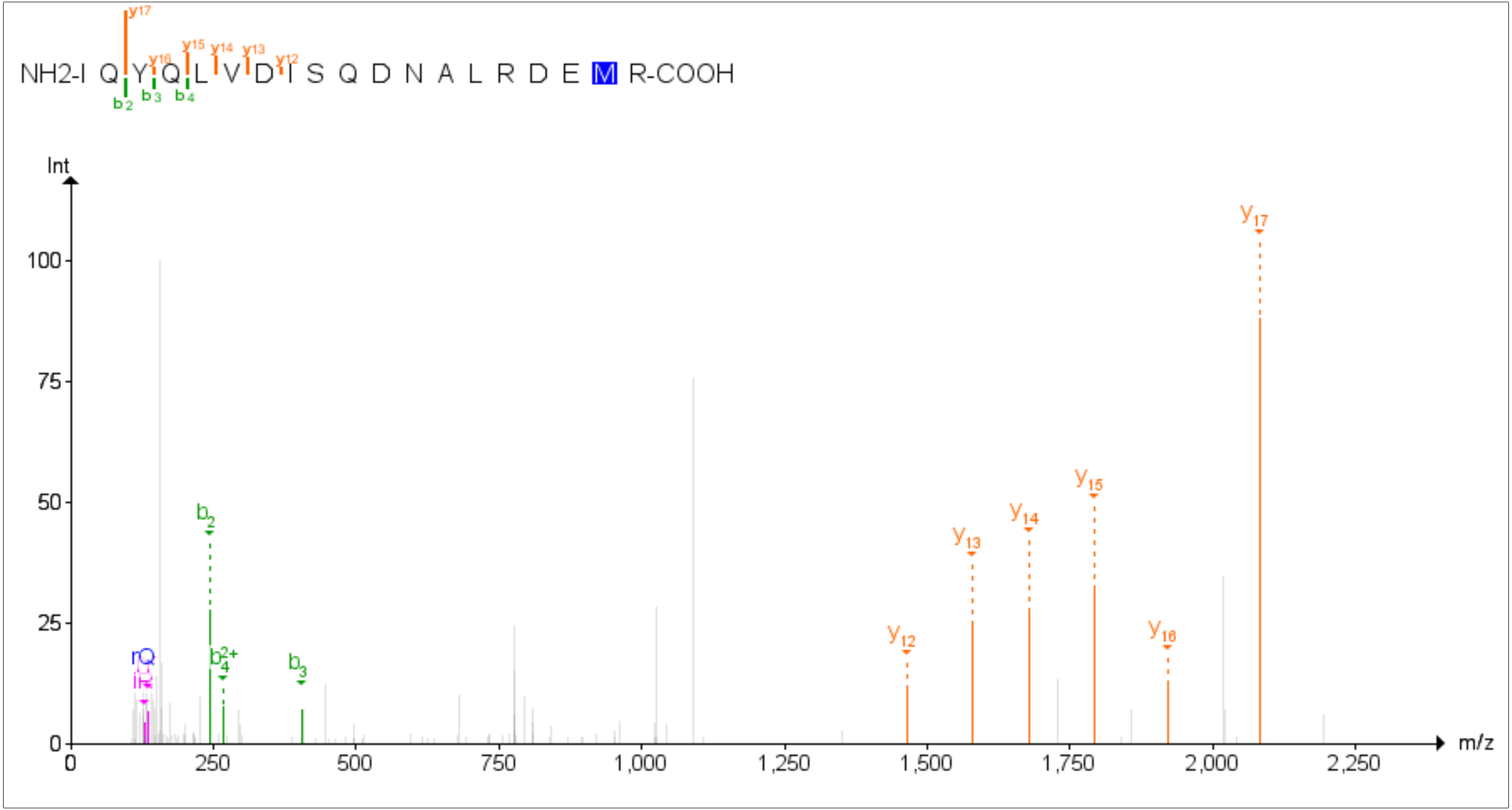

NH2-I Q Y Q L V D I S Q D N A L R D E M R-COOH
y17 y16 y15 y14 y13 y12
b2 b3 b4
Int
m/z
y17
y15
y14
y13
y12
y16
b2
b4 2+
b3
0
25
50
75
100
0
250
500
750
1,000
1,250
1,500
1,750
2,000
2,250

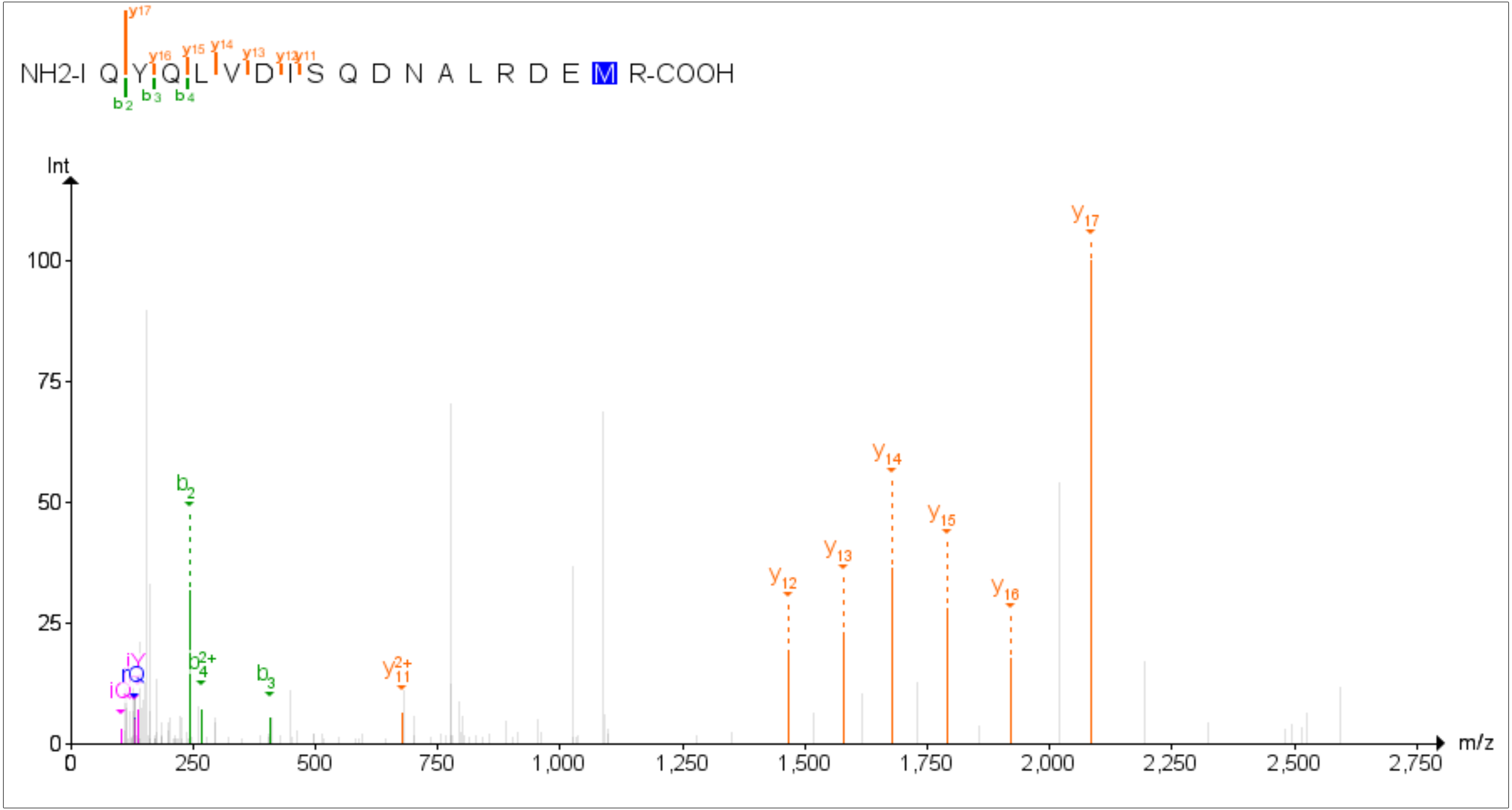

NH2-I Q Y Q L V D I S Q D N A L R D E M R-COOH
y17 y16 y15 y14 y13 y12 y11
b2 b3 b4
Int
m/z
0 25 50 75 100
0 250 500 750 1,000 1,250 1,500 1,750 2,000 2,250 2,500 2,750
y17 y14 y15 y13 y12 y16 b2 b4 2+ b3 y11 2+ iY rQ iQ

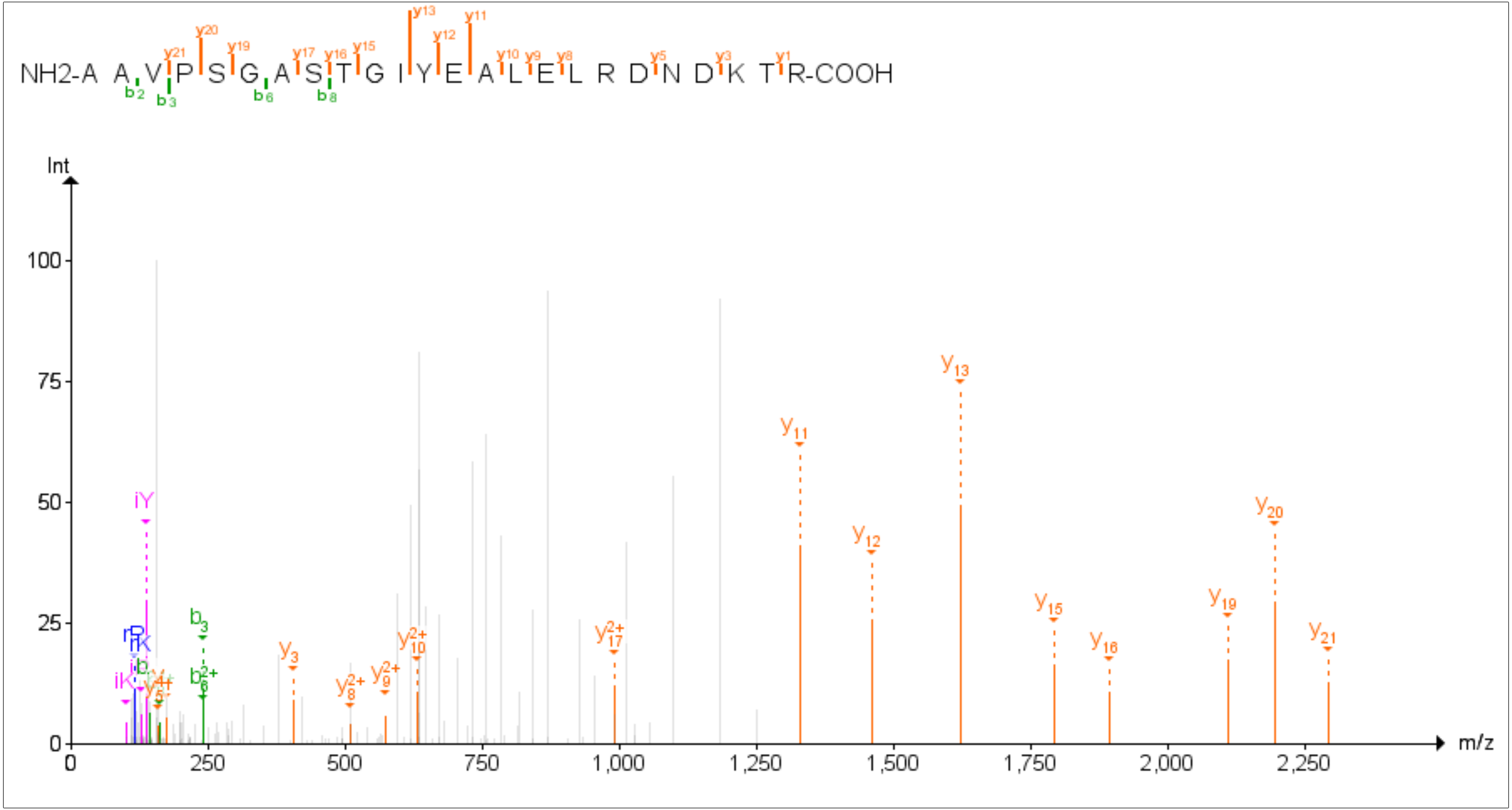

NH2-A A V P S G A S T G I Y E A L E L R D N D K T R-COOH
y21
y20
y19
y17
y16
y15
y13
y12
y11
y10
y9
y8
y5
y3
y1
b2
b3
b6
b8
Int
m/z
0
25
50
75
100
250
500
750
1,000
1,250
1,500
1,750
2,000
2,250
iY
iK
b3
b6 2+
y3
y8 2+
y9 2+
y10 2+
y17 2+
y11
y12
y13
y15
y16
y19
y20
y21

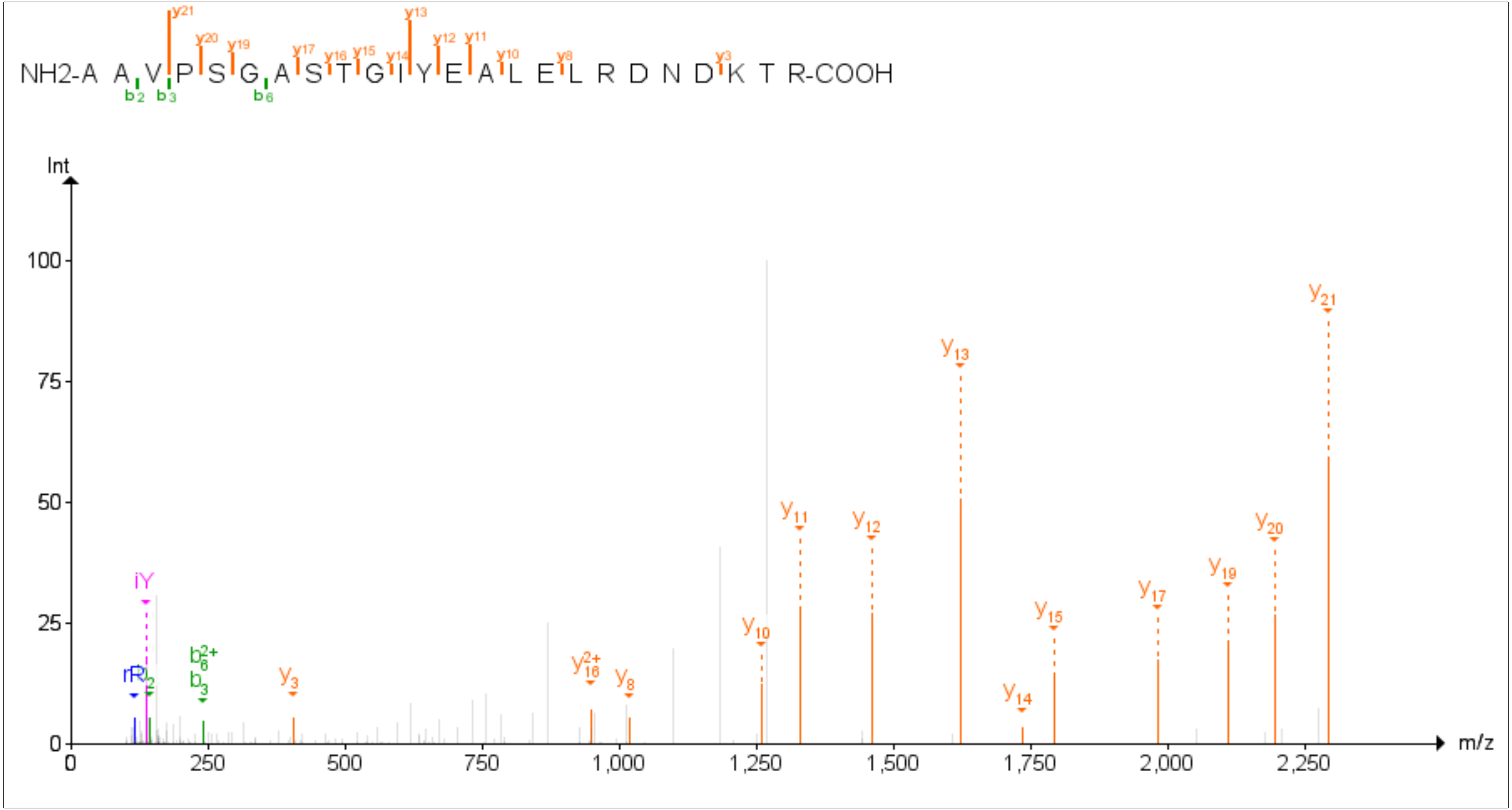

NH2-A A V P S G A S T G I Y E A L E L R D N D K T R-COOH
y21 y20 y19 y17 y16 y15 y14 y13 y12 y11 y10 y8 y3
b2 b3 b6
Int
m/z
0
25
50
75
100
250
500
750
1,000
1,250
1,500
1,750
2,000
2,250
iY
rR2
b6 2+
b3
y3
y16 2+
y8
y10
y11
y12
y13
y14
y15
y17
y19
y20
y21

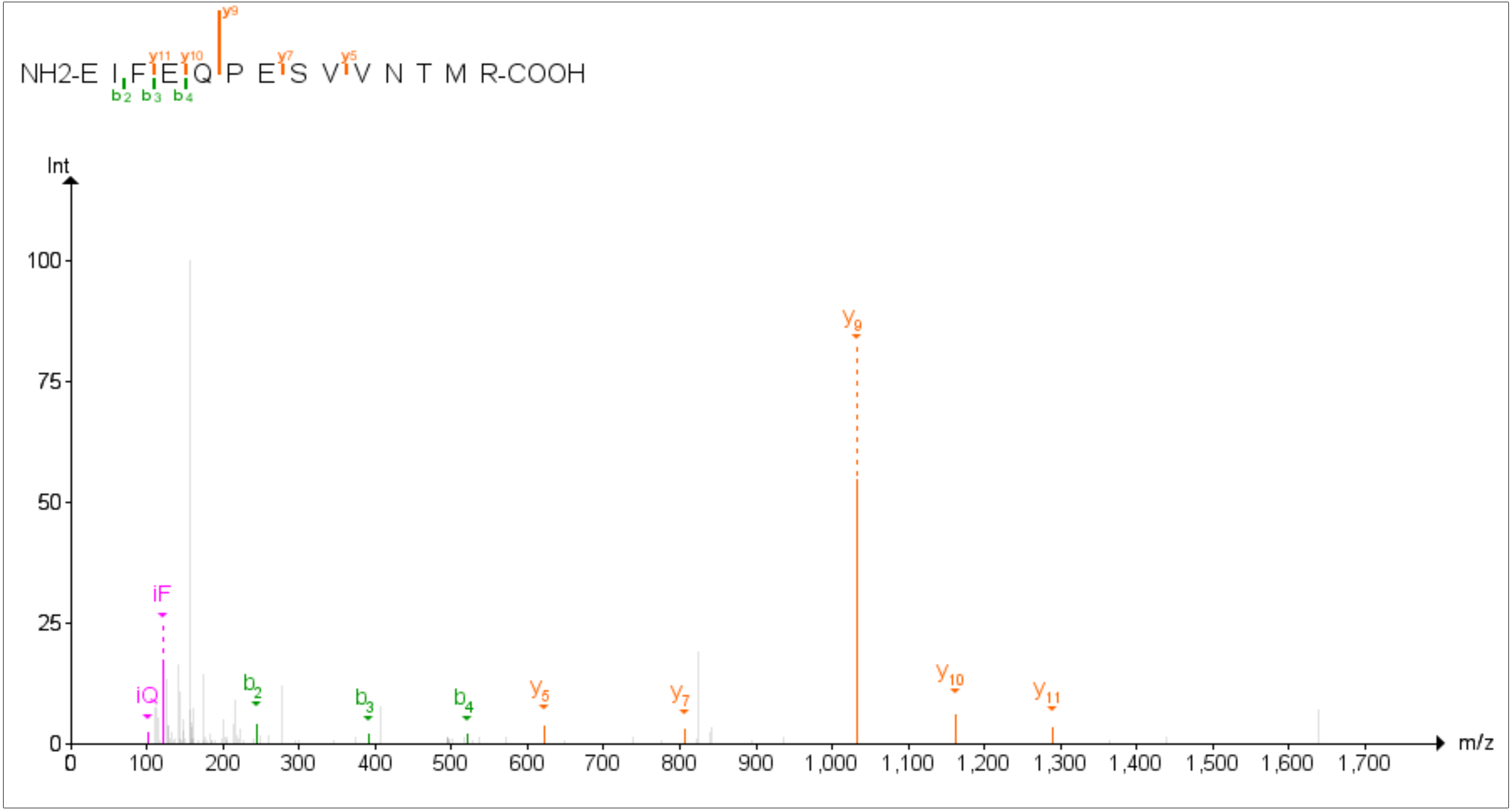
NH2-E I F E Q P E S V V N T M R-COOH
y9
y11
y10
y7
y5
b2
b3
b4
Int
100
75
50
25
0
iF
iQ
b2
b3
b4
y5
y7
y9
y10
y11
m/z
0
100
200
300
400
500
600
700
800
900
1,000
1,100
1,200
1,300
1,400
1,500
1,600
1,700

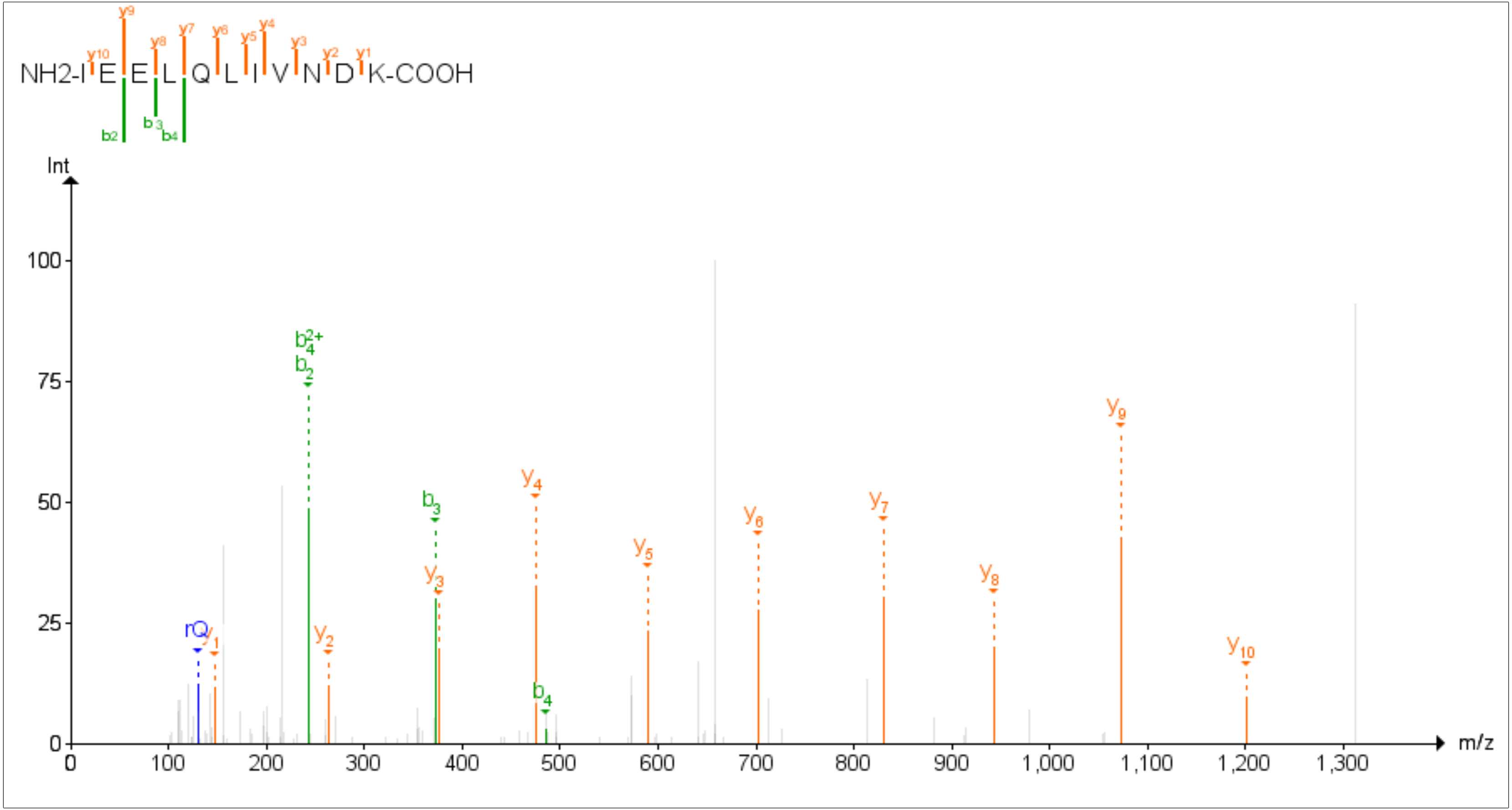

NH2-I E E L Q L I V N D K-COOH
y10 y9 y8 y7 y6 y5 y4 y3 y2 y1
b2 b3 b4
Int
m/z
rQ

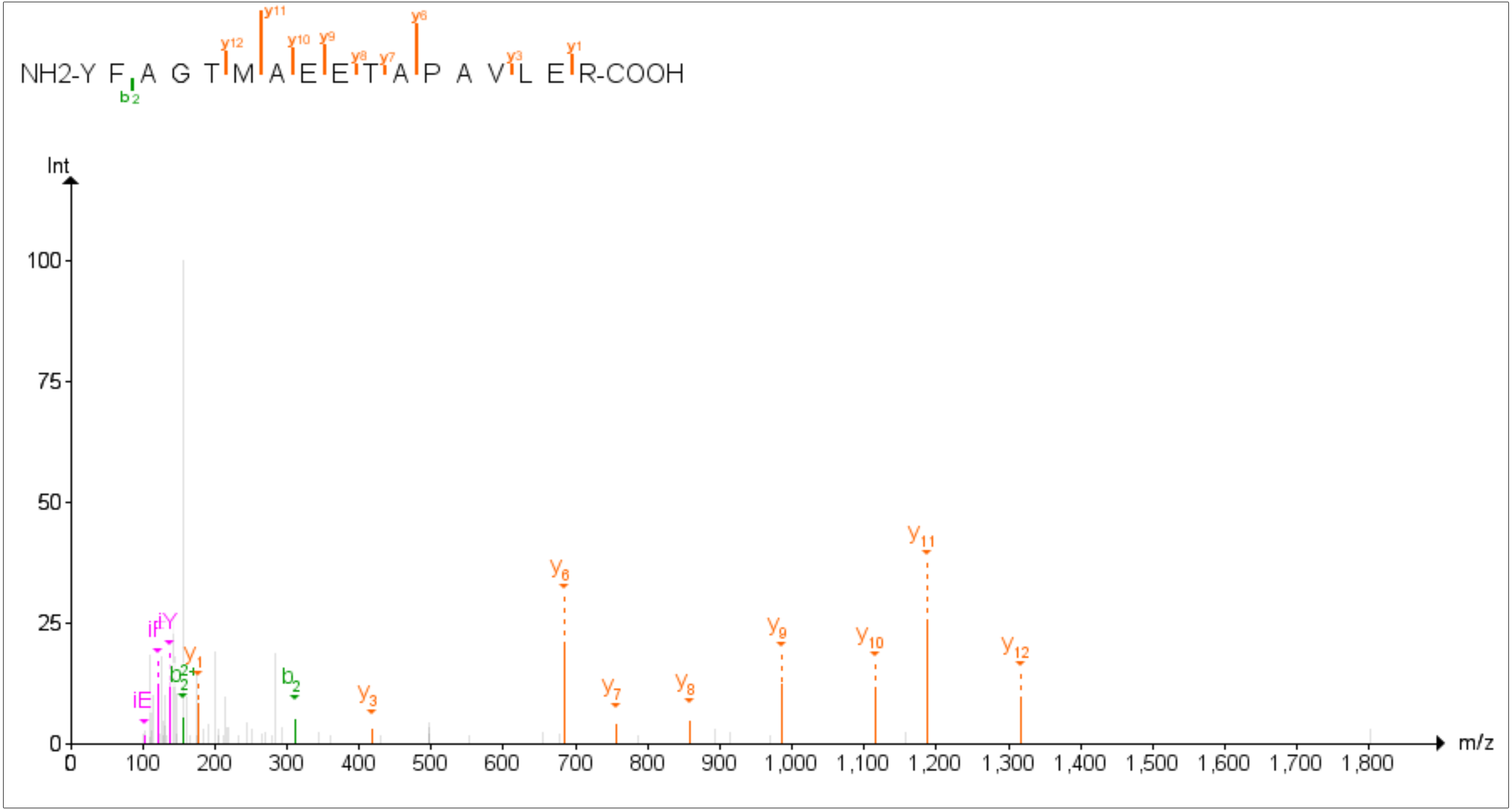

NH2-Y F A G T M A E E T A P A V L E R-COOH
b2
y12
y11
y10
y9
y8
y7
y6
y3
y1
Int
100
75
50
25
0
iF
iY
iE
y1
b2 2+
b2
y3
y6
y7
y8
y9
y10
y11
y12
0
100
200
300
400
500
600
700
800
900
1,000
1,100
1,200
1,300
1,400
1,500
1,600
1,700
1,800
m/z

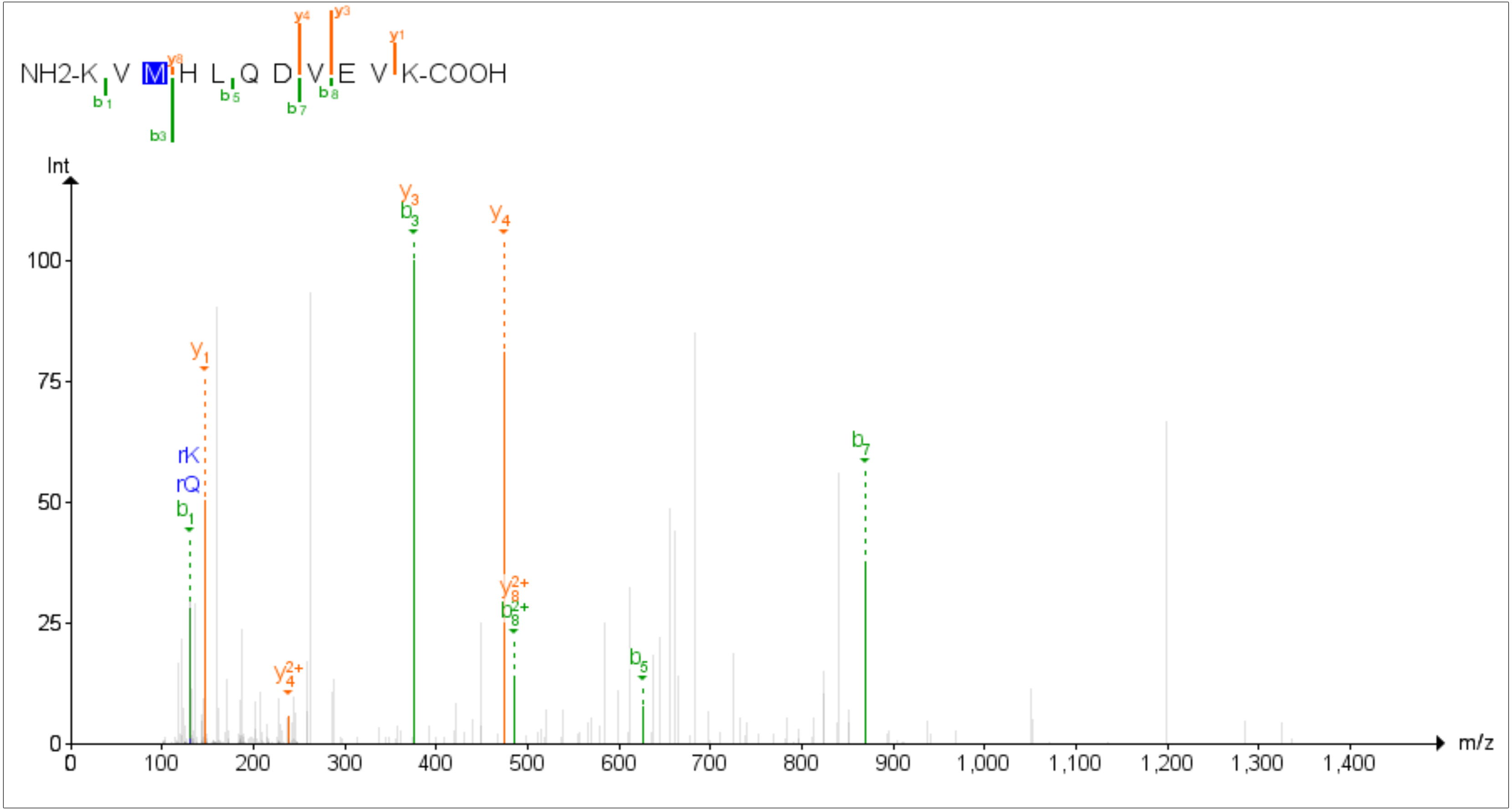

NH2-K V M H L Q D V E V K-COOH
y8
y4
y3
y1
b1
b3
b5
b7
b8
Int
100
75
50
25
0
y3
b3
y4
y1
rK
rQ
b1
b7
y8 2+
b8 2+
b5
y4 2+
m/z
0
100
200
300
400
500
600
700
800
900
1,000
1,100
1,200
1,300
1,400

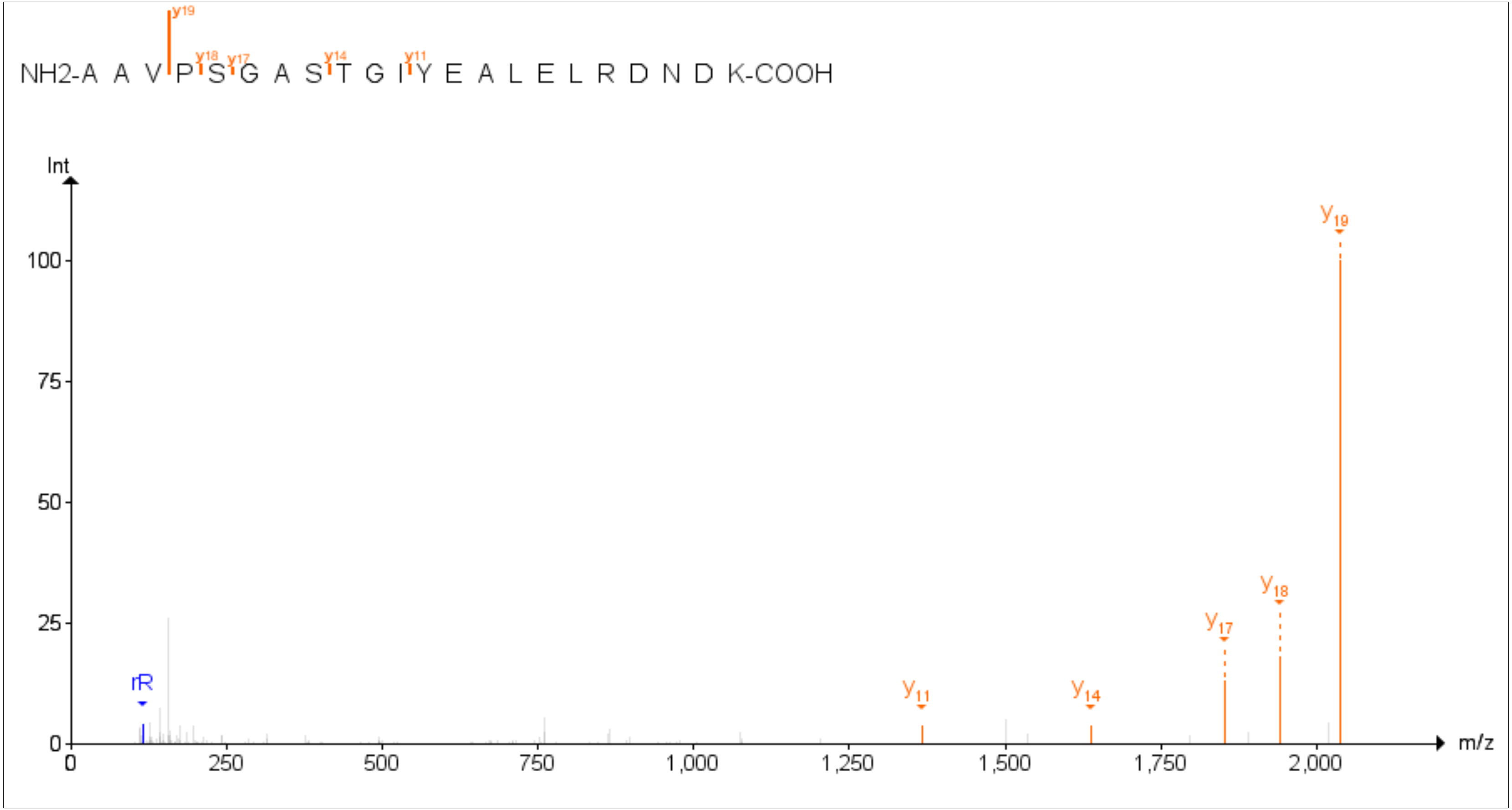

NH2-A A V P S G A S T G I Y E A L E L R D N D K-COOH
y19
y18
y17
y14
y11
Int
100
75
50
25
0
rR
m/z
0
250
500
750
1,000
1,250
1,500
1,750
2,000

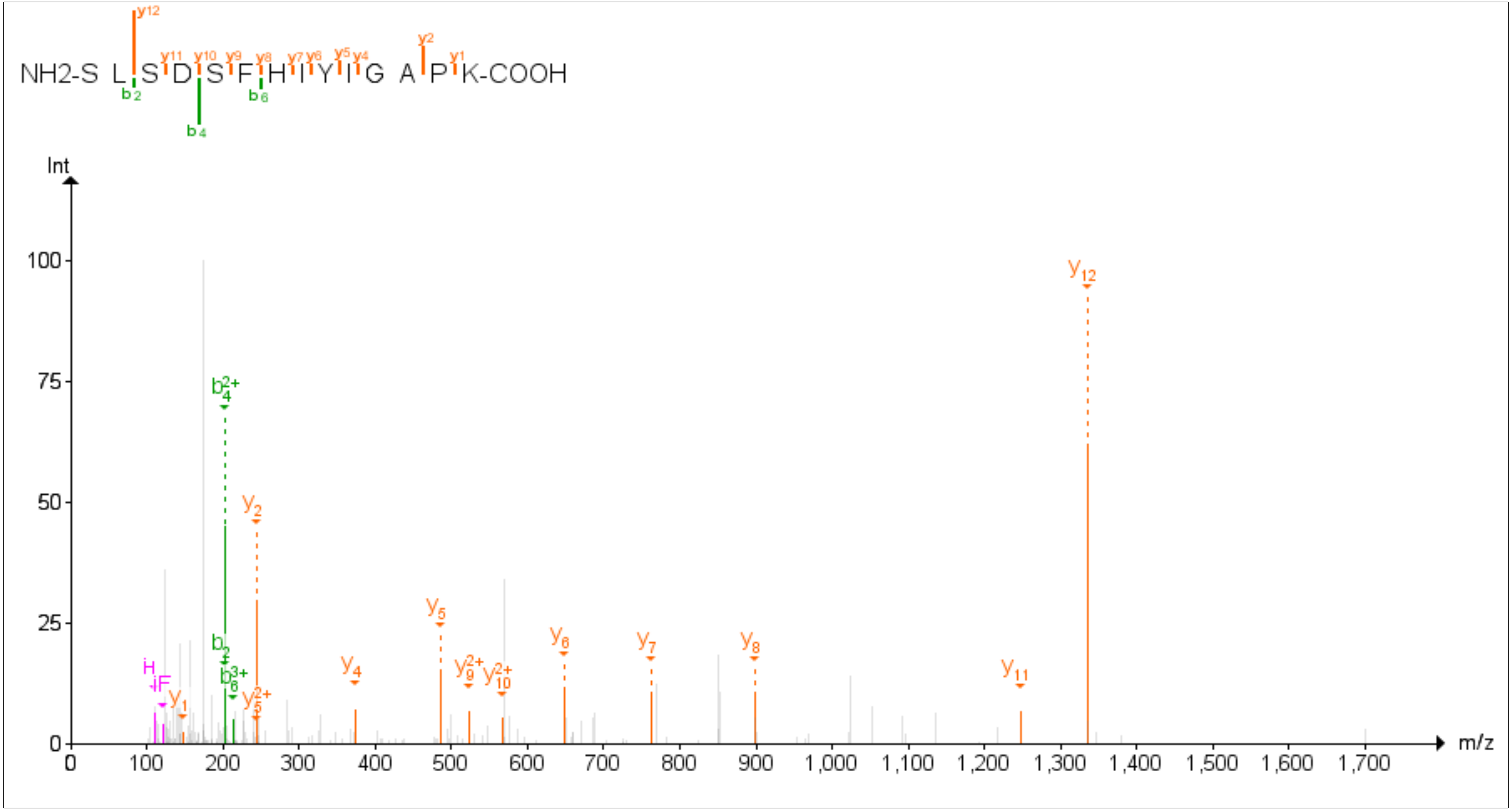

NH2-S L S D S F H I Y I G A P K-COOH
y12 y11 y10 y9 y8 y7 y6 y5 y4 y2 y1
b2 b4 b6
Int
m/z

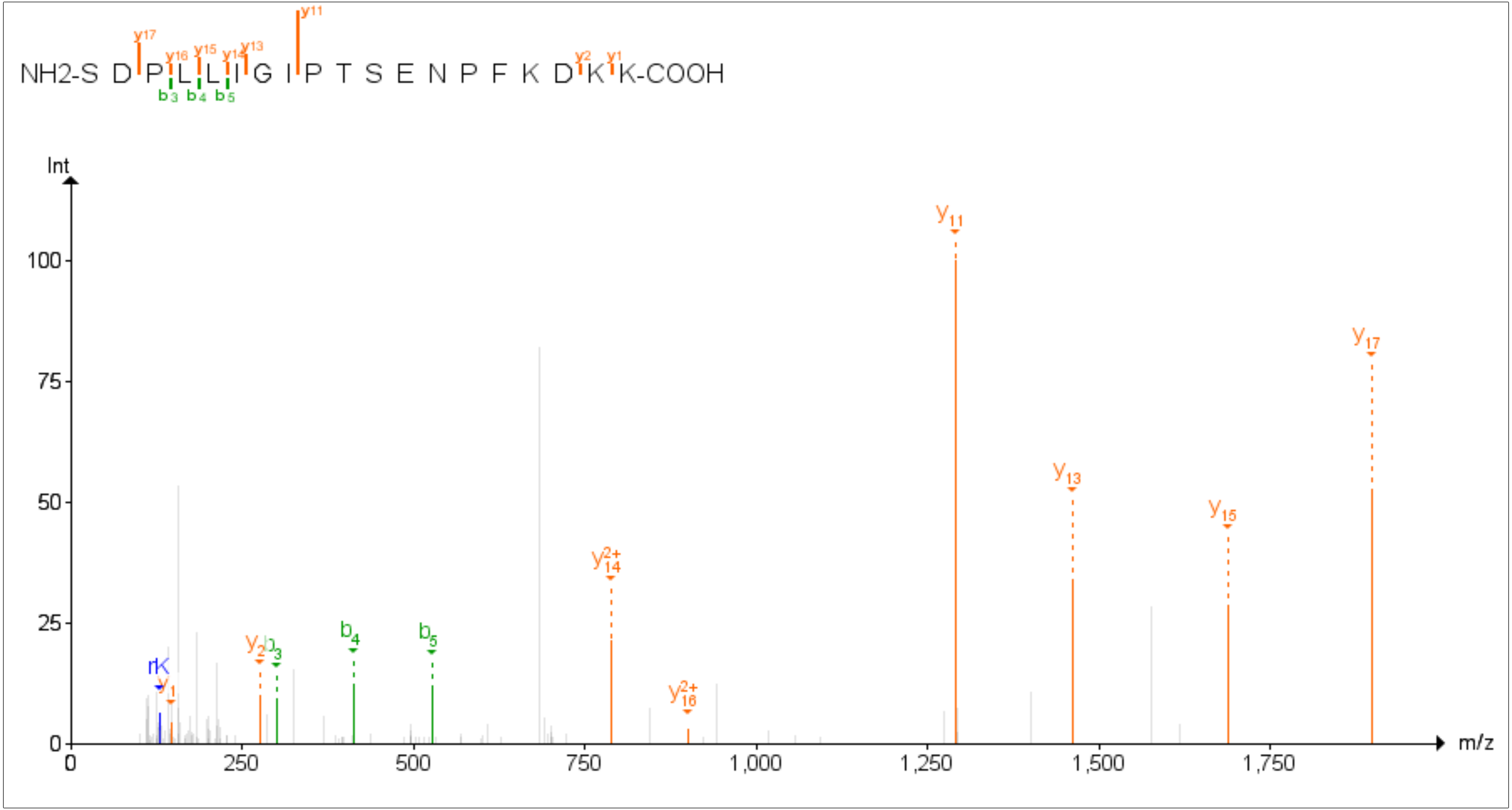

NH2-S D P L L I G I P T S E N P F K D K K-COOH
y17
y16
y15
y14
y13
y11
y2
y1
b3
b4
b5
Int
100
75
50
25
0
m/z
250
500
750
1,000
1,250
1,500
1,750
y11
y17
y13
y15
y14 2+
y16 2+
y2
b3
b4
b5
rK
y1

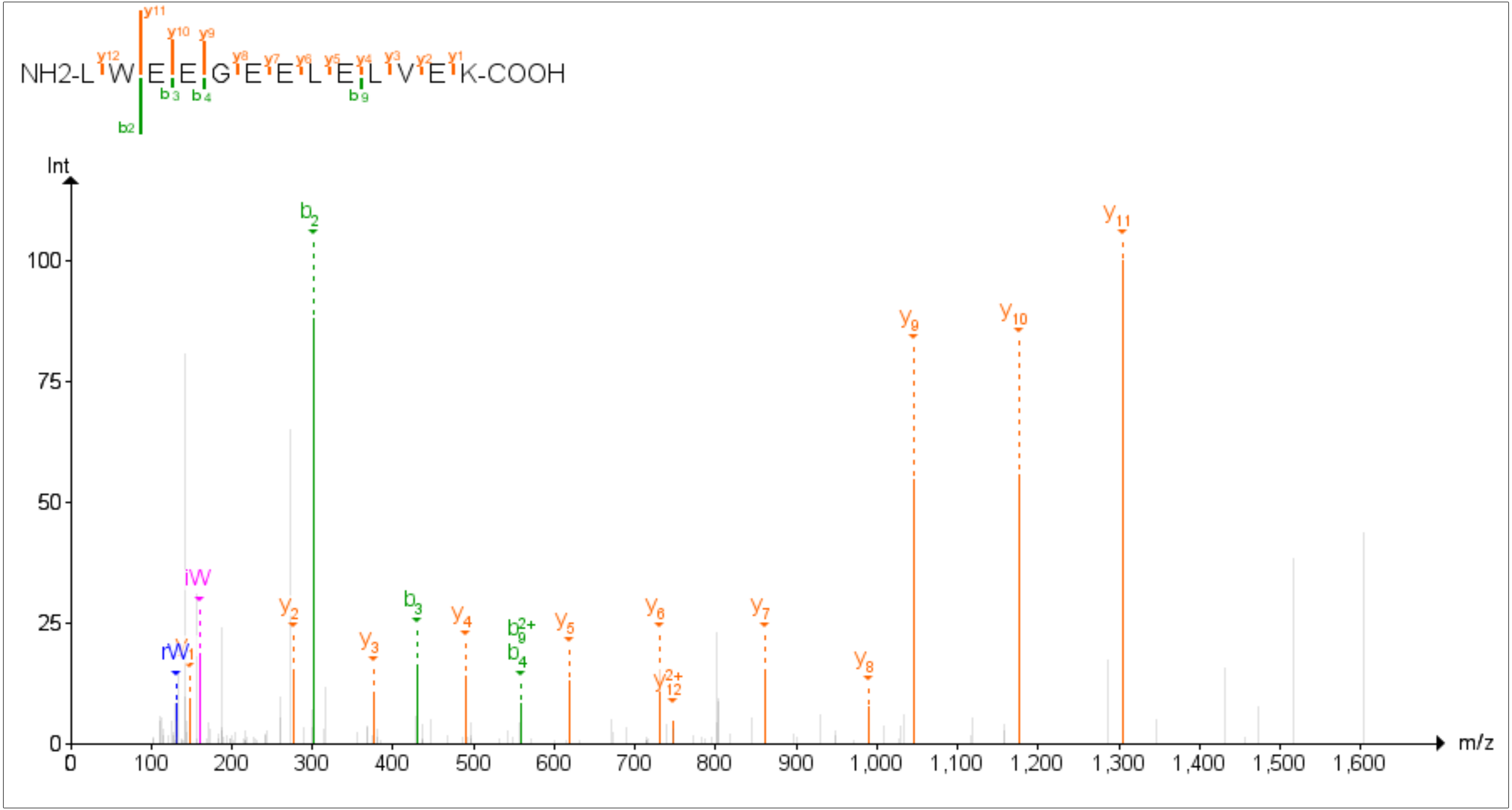

NH2-L W E E G E E L E L V E K-COOH
y12 y11 y10 y9 y8 y7 y6 y5 y4 y3 y2 y1
b2 b3 b4 b9
Int
m/z
b2
y11
y9
y10
iW
rW
y1
y2
y3
b3
y4
b9 2+
b4
y5
y6
y12 2+
y7
y8
0
25
50
75
100
0
100
200
300
400
500
600
700
800
900
1,000
1,100
1,200
1,300
1,400
1,500
1,600

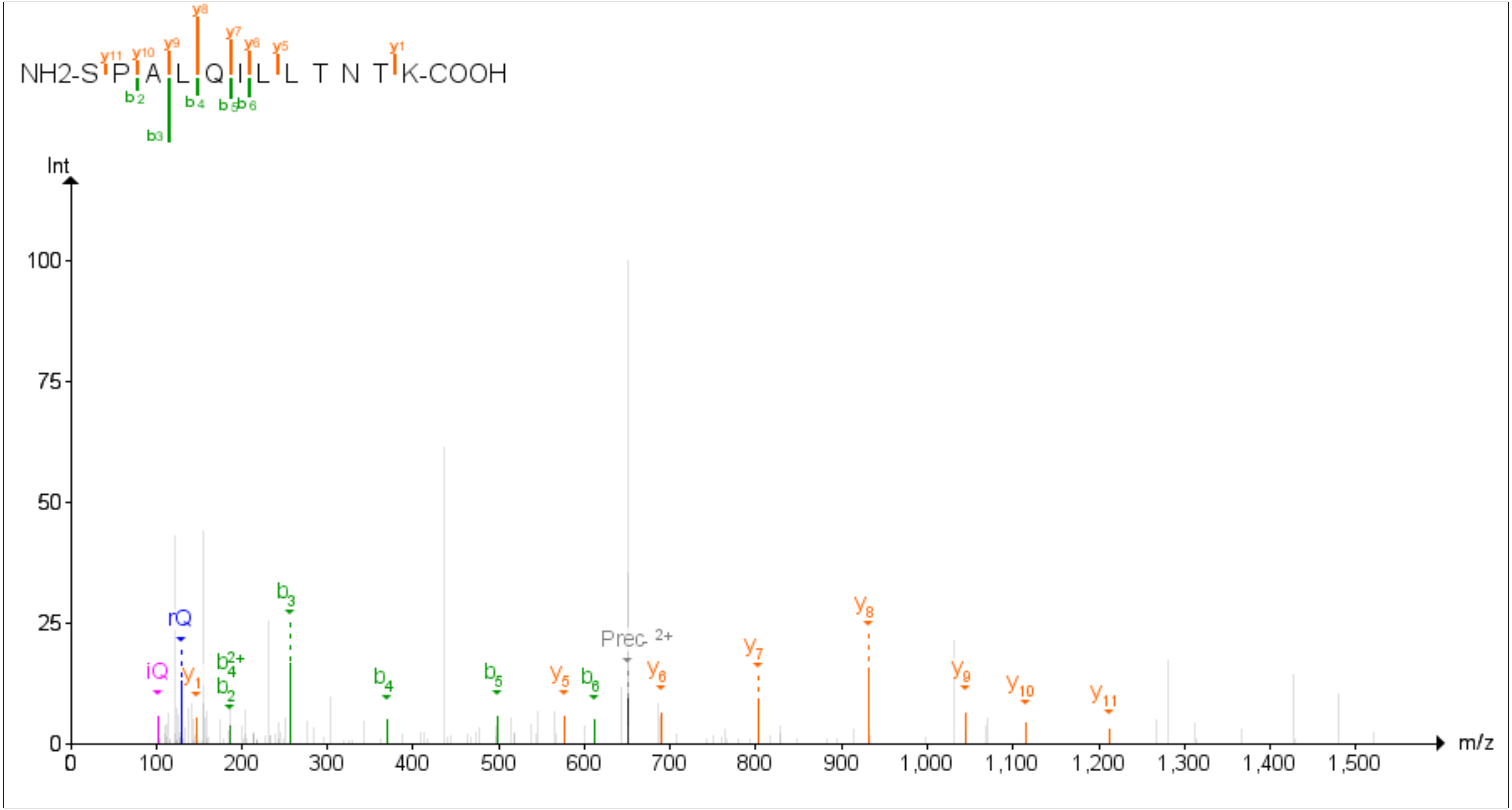

NH2-S P A L Q I L L T N T K-COOH
y11 y10 y9 y8 y7 y6 y5 y1
b2 b3 b4 b5 b6
Int
100
75
50
25
0
iQ
rQ
y1
b2
b4 2+
b3
b4
b5
y5
b6
Prec 2+
y6
y7
y8
y9
y10
y11
0 100 200 300 400 500 600 700 800 900 1,000 1,100 1,200 1,300 1,400 1,500
m/z

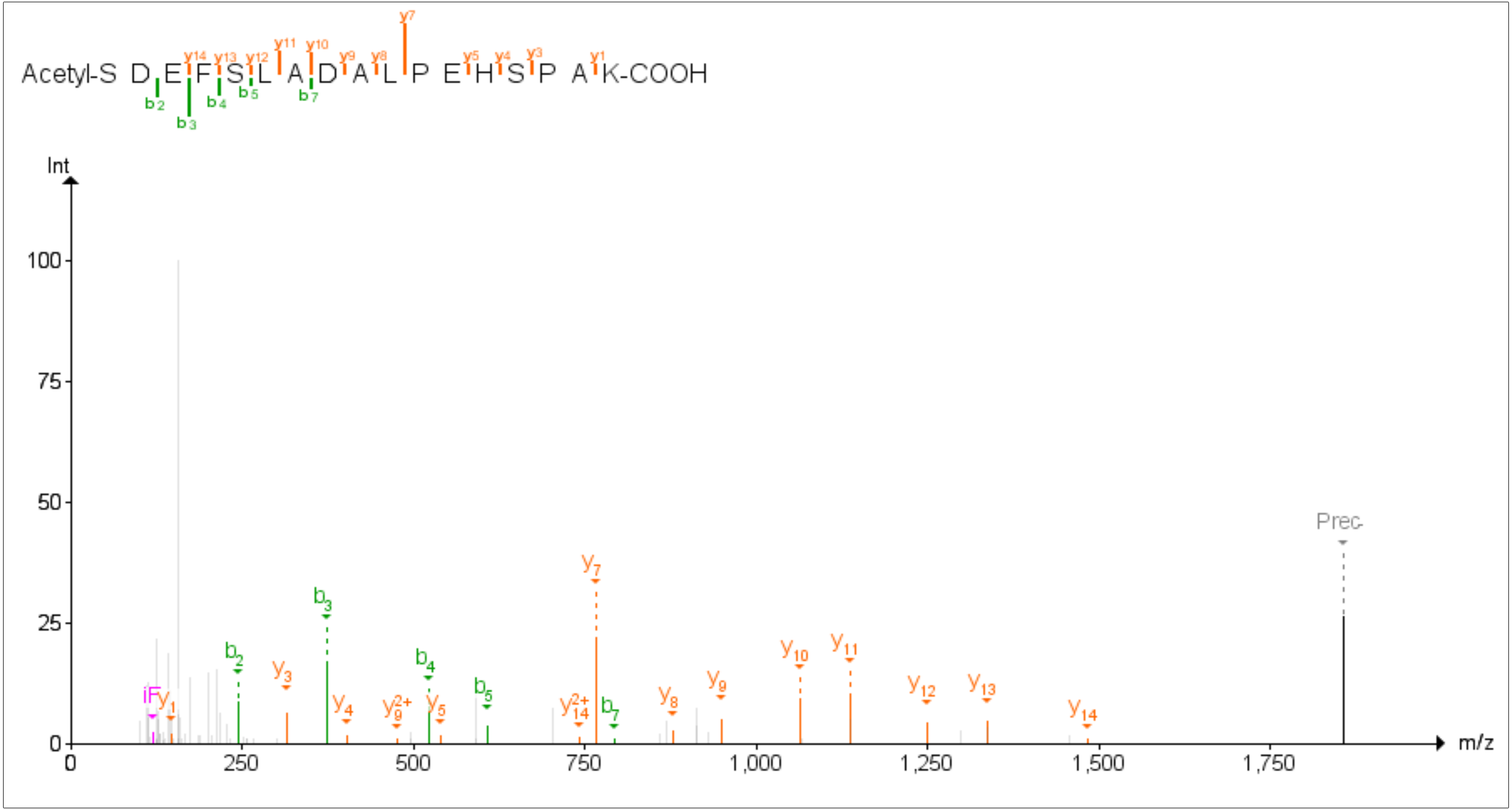

Acetyl-S D E F S L A D A L P E H S P A K-COOH
Int
m/z
Prec

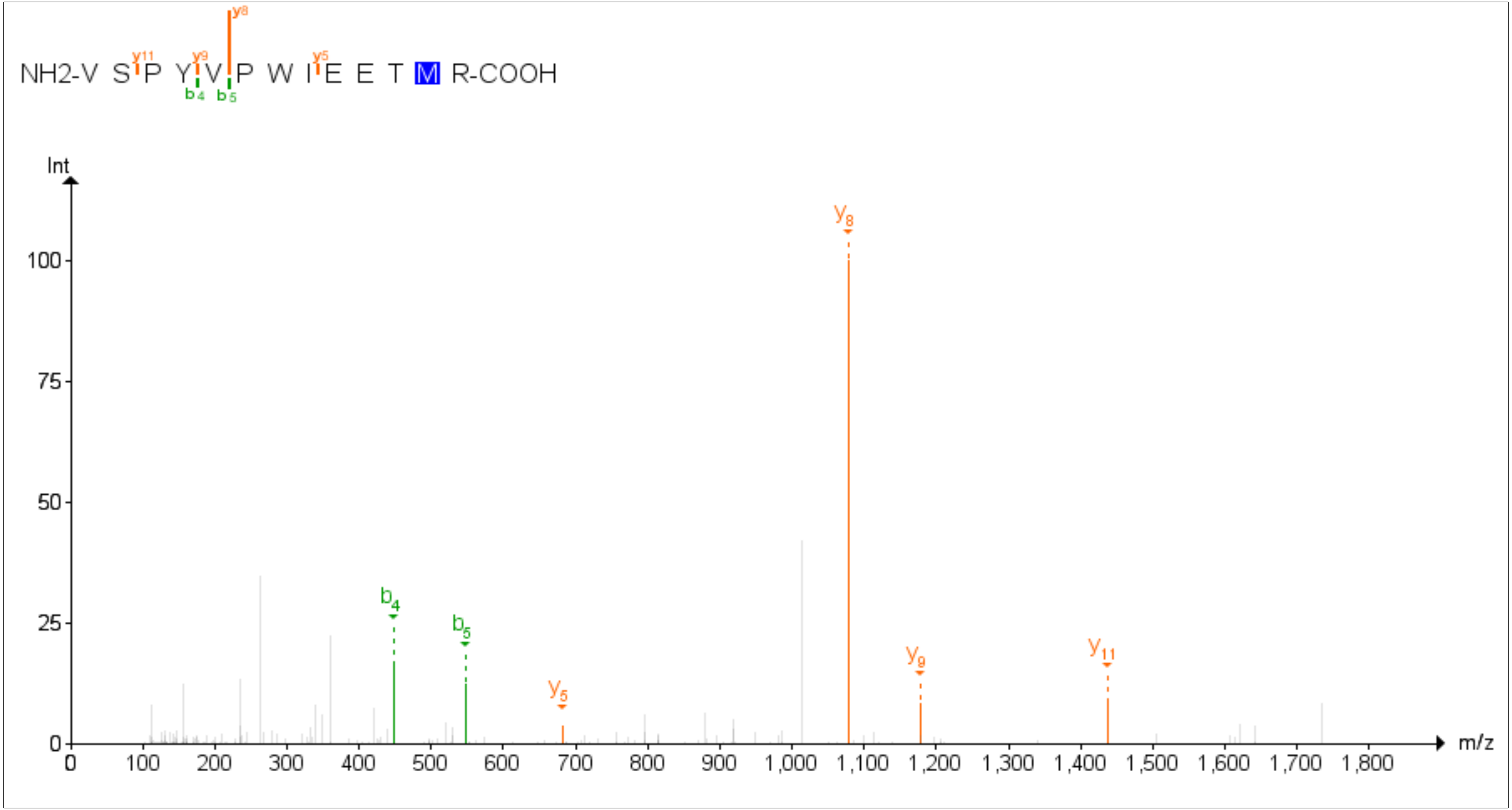
NH2-V S P Y V P W I E E T M R-COOH
y11
y9
y8
y5
b4
b5
Int
100
75
50
25
0
m/z
0
100
200
300
400
500
600
700
800
900
1,000
1,100
1,200
1,300
1,400
1,500
1,600
1,700
1,800
y8
y9
y11
y5
b4
b5

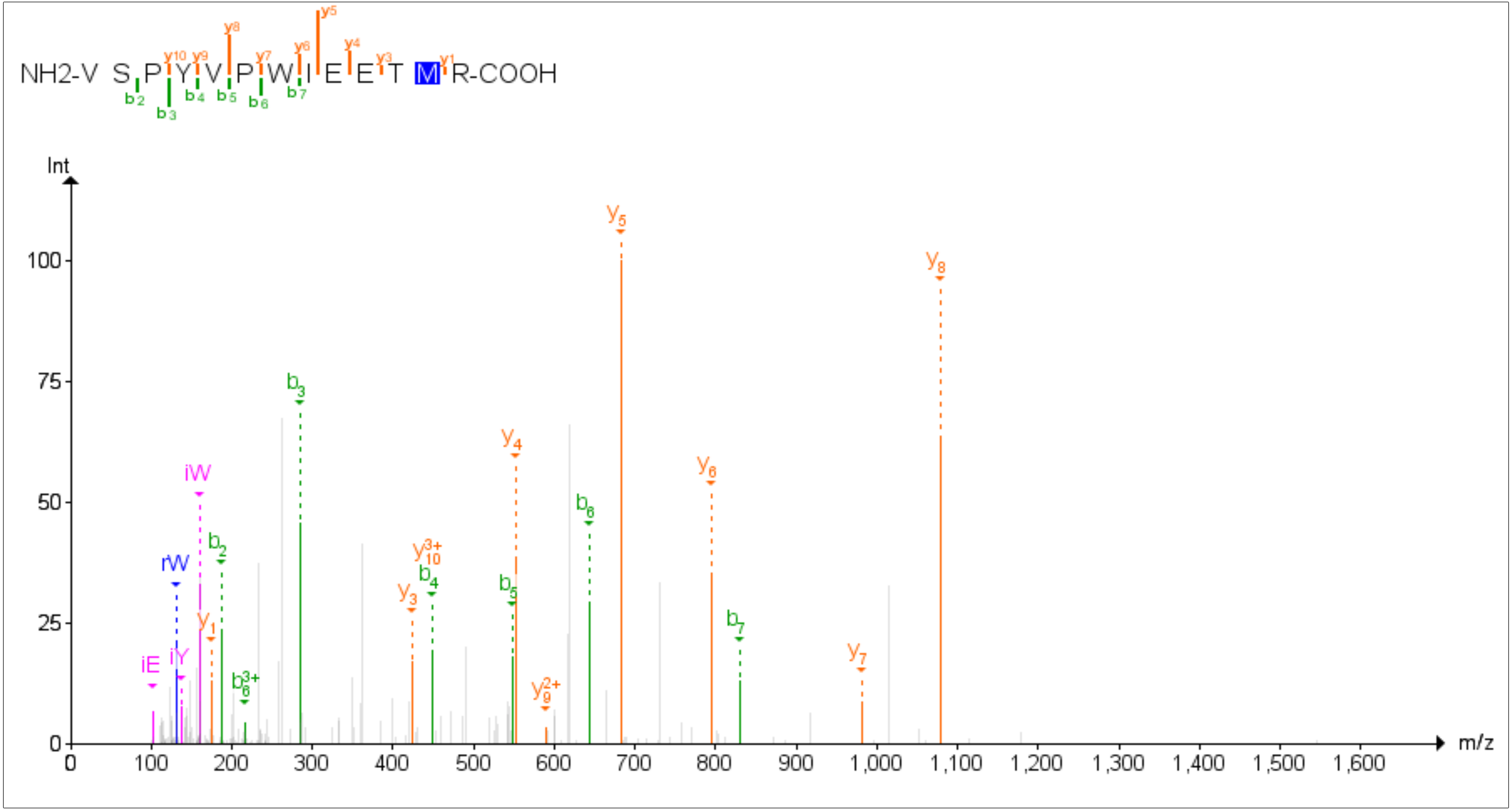

NH2-V S P Y V P W I E E T M R-COOH
y10 y9 y8 y7 y6 y5 y4 y3 y1
b2 b3 b4 b5 b6 b7
Int
m/z
0
25
50
75
100
0
100
200
300
400
500
600
700
800
900
1,000
1,100
1,200
1,300
1,400
1,500
1,600
iE
iY
rW
iW
y1
b2
b6 3+
b3
y3
y10 3+
b4
y4
b5
y9 2+
b6
y5
y6
b7
y7
y8

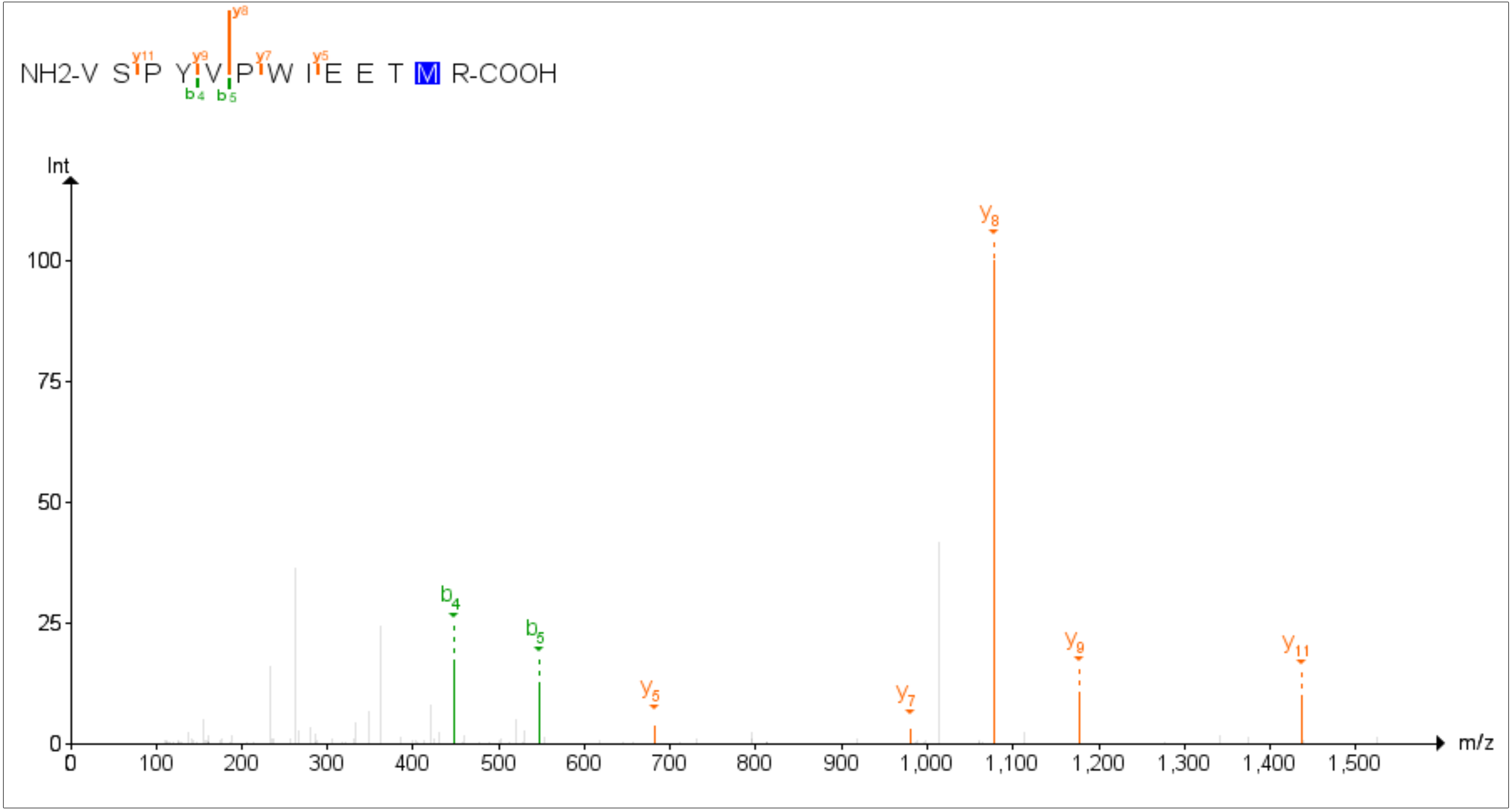
NH2-V S P Y V P W I E E T M R-COOH
y11 y9 y8 y7 y5
b4 b5
Int
m/z
y8
y9
y11
y5
y7
b4
b5
0
25
50
75
100
0
100
200
300
400
500
600
700
800
900
1,000
1,100
1,200
1,300
1,400
1,500

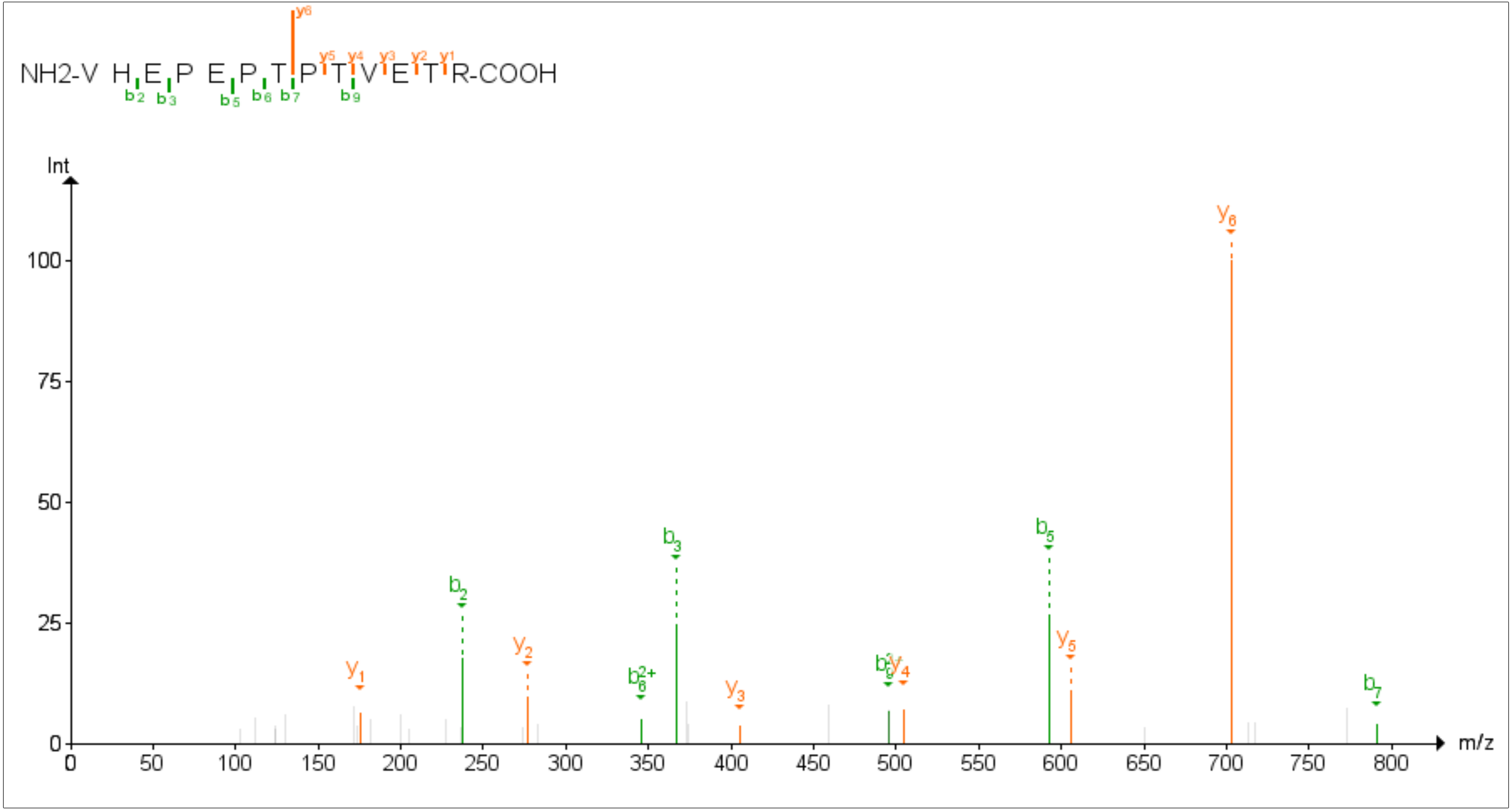

NH2-V H E P E P T P T V E T R-COOH
y6 y5 y4 y3 y2 y1
b2 b3 b5 b6 b7 b9
Int
0
25
50
75
100
m/z
0 50 100 150 200 250 300 350 400 450 500 550 600 650 700 750 800
y1 b2 y2 b3 y3 y4 b5 y5 y6 b7

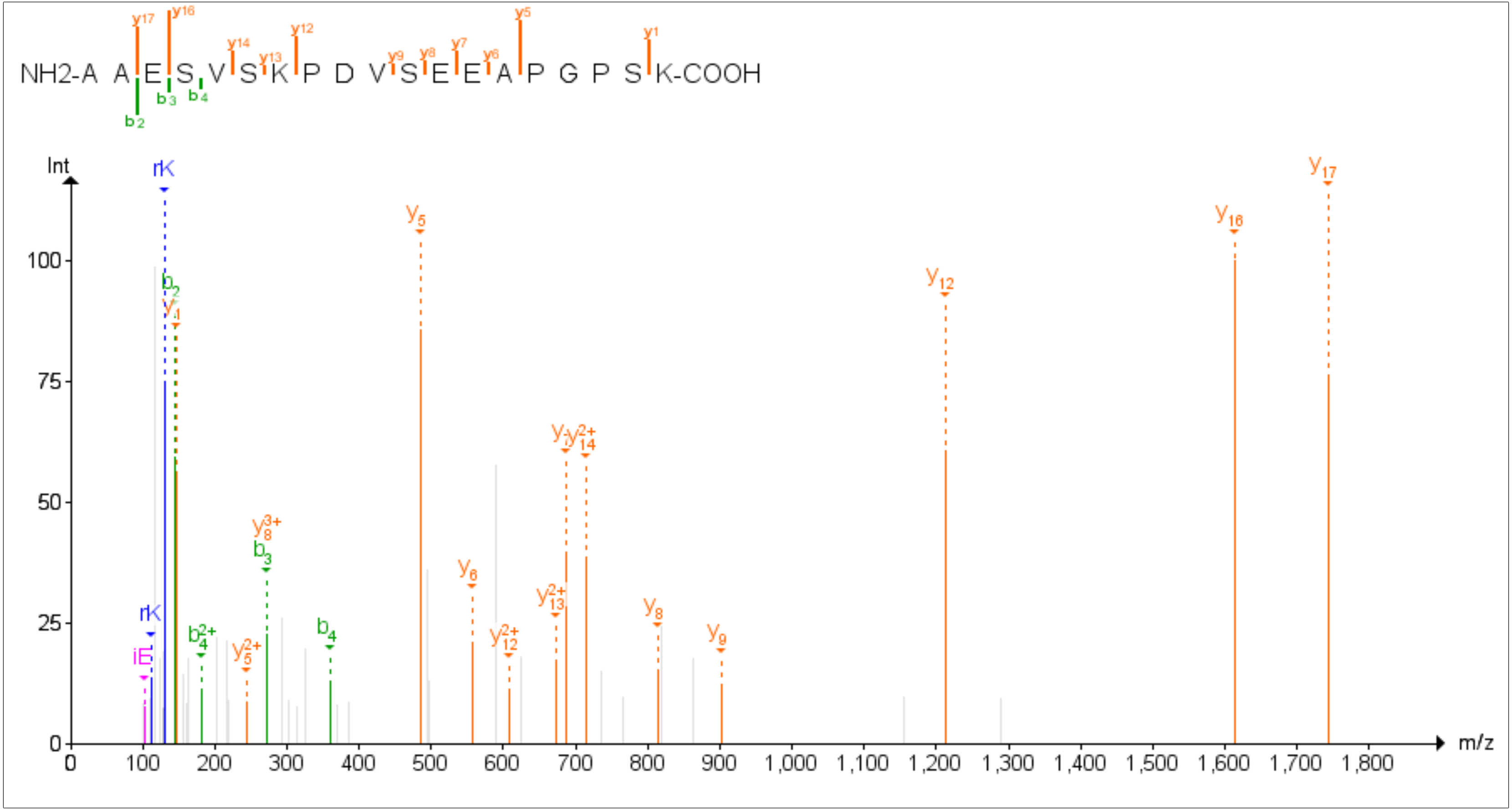

NH2-A A E S V S K P D V S E E A P G P S K-COOH
Int
m/z

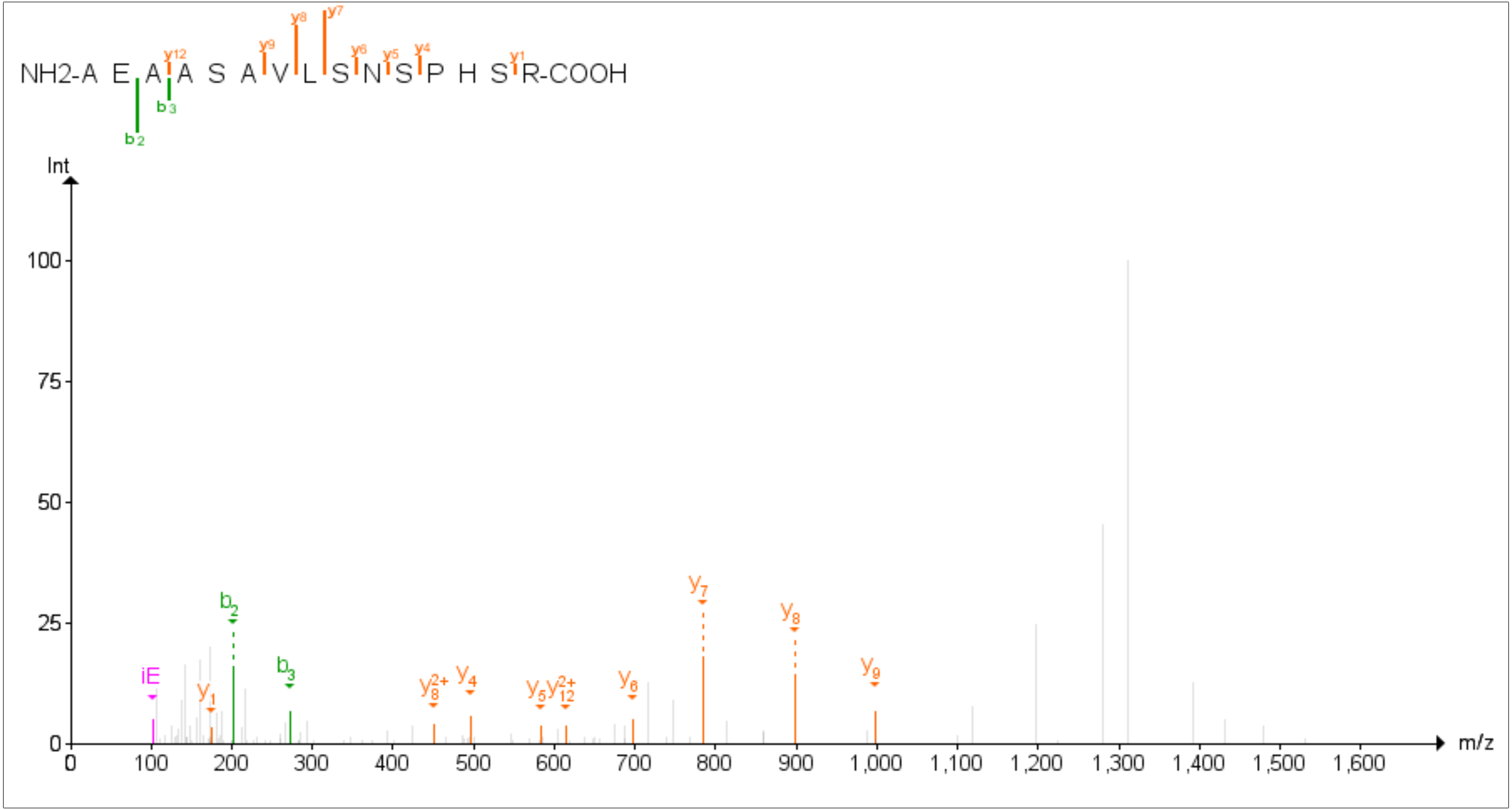

NH2-A E A A S A V L S N S P H S R-COOH
y12 y9 y8 y7 y6 y5 y4 y1
b2 b3
Int
m/z
iE
y1
b2
b3
y8 2+
y4
y5
y12 2+
y6
y7
y8
y9

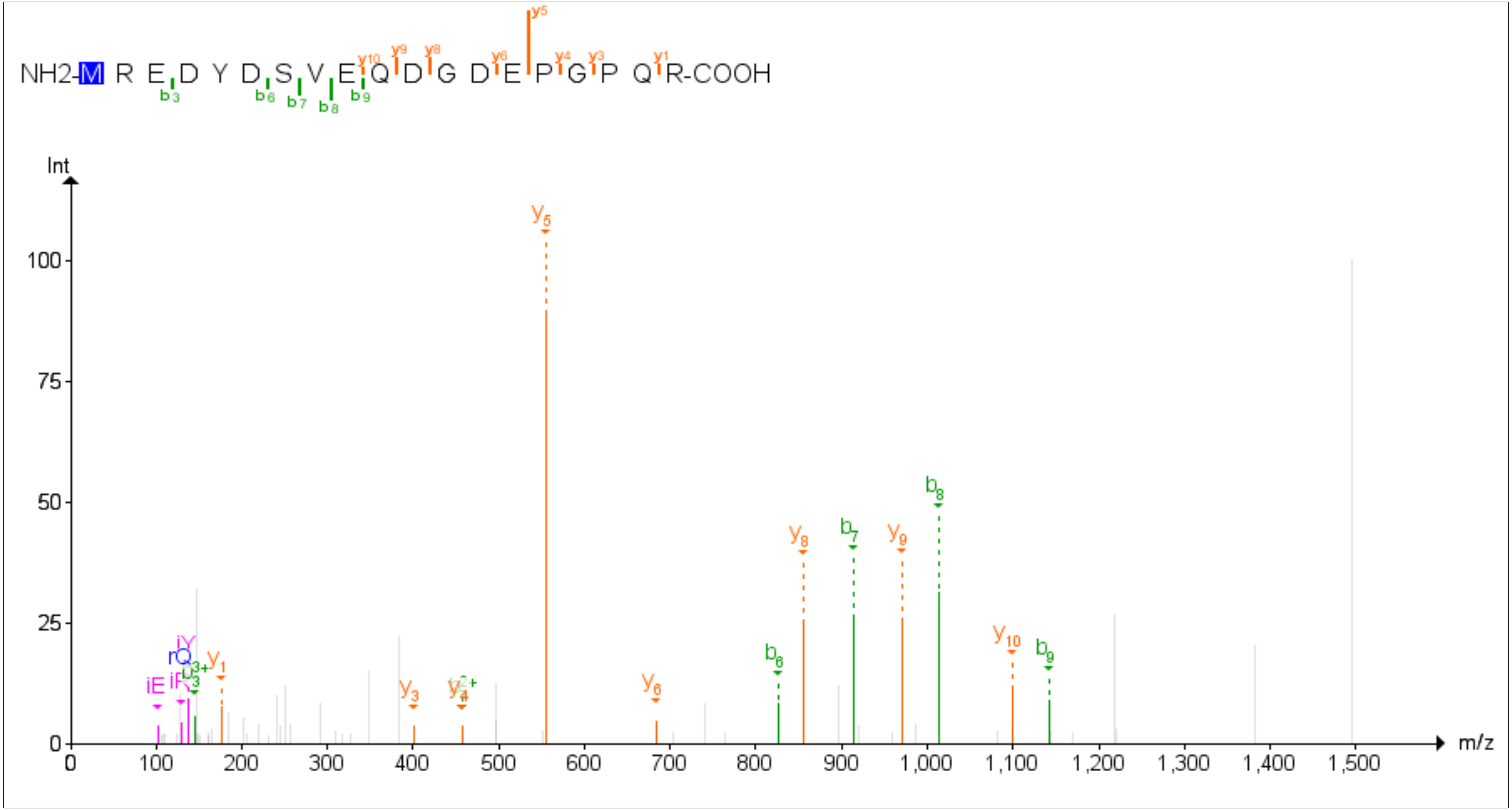

NH2-M R E D Y D S V E Q D G D E P G P Q R-COOH
b3 b6 b7 b8 b9
y10 y9 y8 y6 y5 y4 y3 y1
Int
m/z
0
25
50
75
100
0
100
200
300
400
500
600
700
800
900
1,000
1,100
1,200
1,300
1,400
1,500
y5
y8
b7
y9
b8
y10
b9
b6
y6
y3
y4
y1
iE
iR
rQ
iY

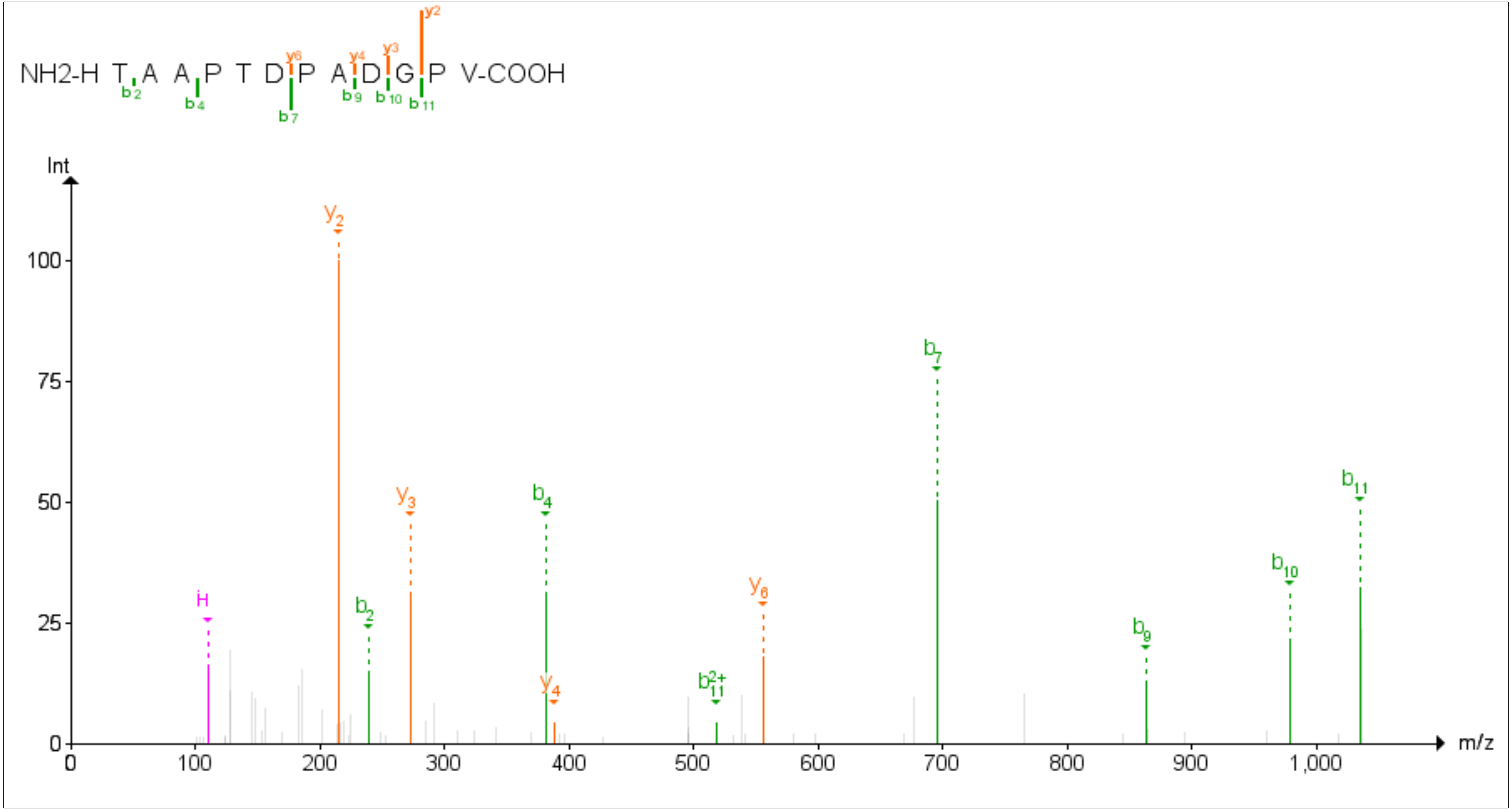

NH2-H T A A P T D P A D G P V-COOH
b2
b4
b7
b9
b10
b11
y6
y4
y3
y2
Int
m/z
0
25
50
75
100
0
100
200
300
400
500
600
700
800
900
1,000
H
y2
b2
y3
b4
y4
b11 2+
y6
b7
b9
b10
b11

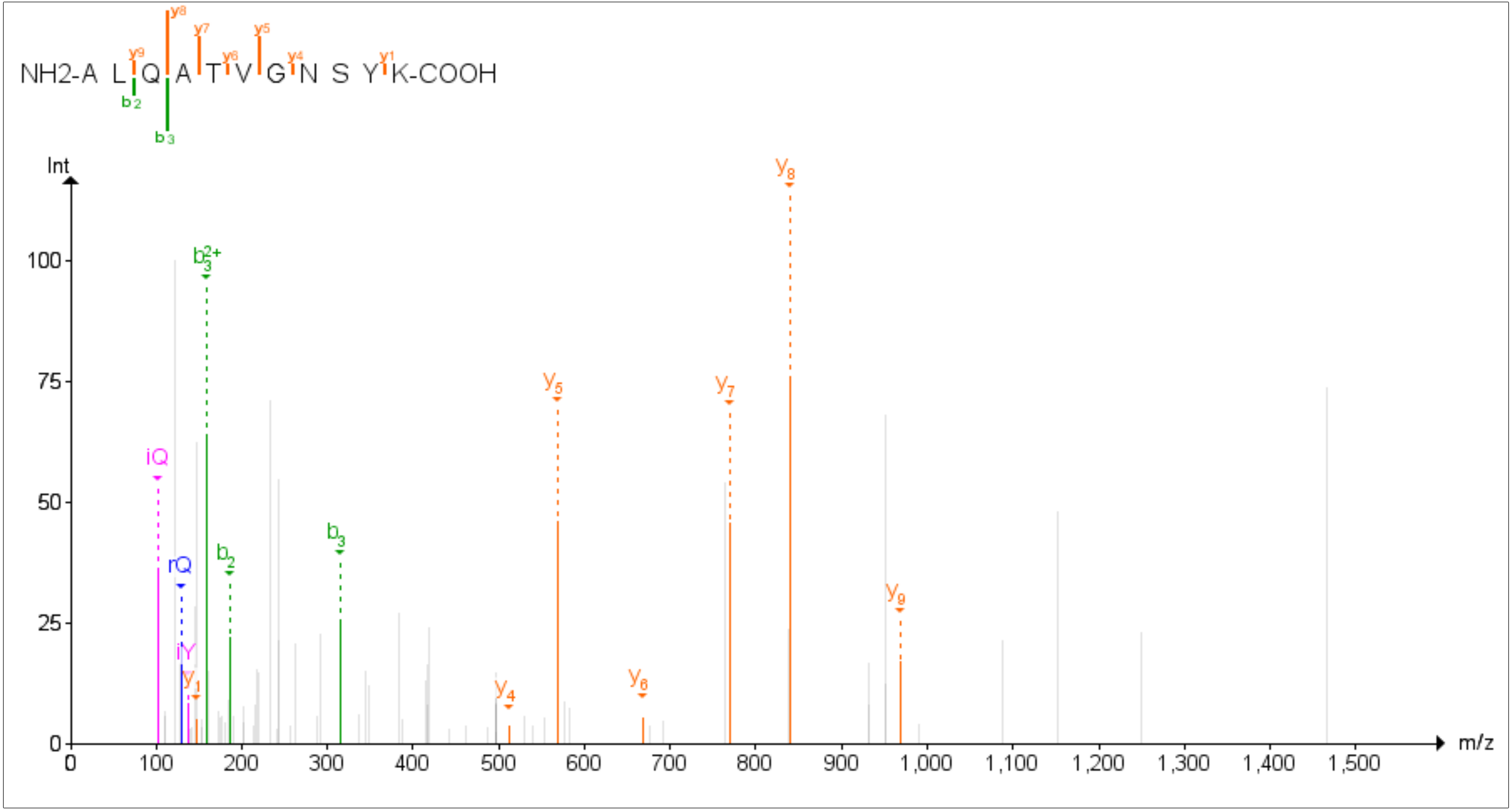
NH2-A L Q A T V G N S Y K-COOH
y9 y8 y7 y6 y5 y4 y1
b2 b3
Int
m/z
iQ
rQ
iY
y1
b2
b3
b_3^{2+}
y4
y5
y6
y7
y8
y9
0
25
50
75
100
0 100 200 300 400 500 600 700 800 900 1,000 1,100 1,200 1,300 1,400 1,500

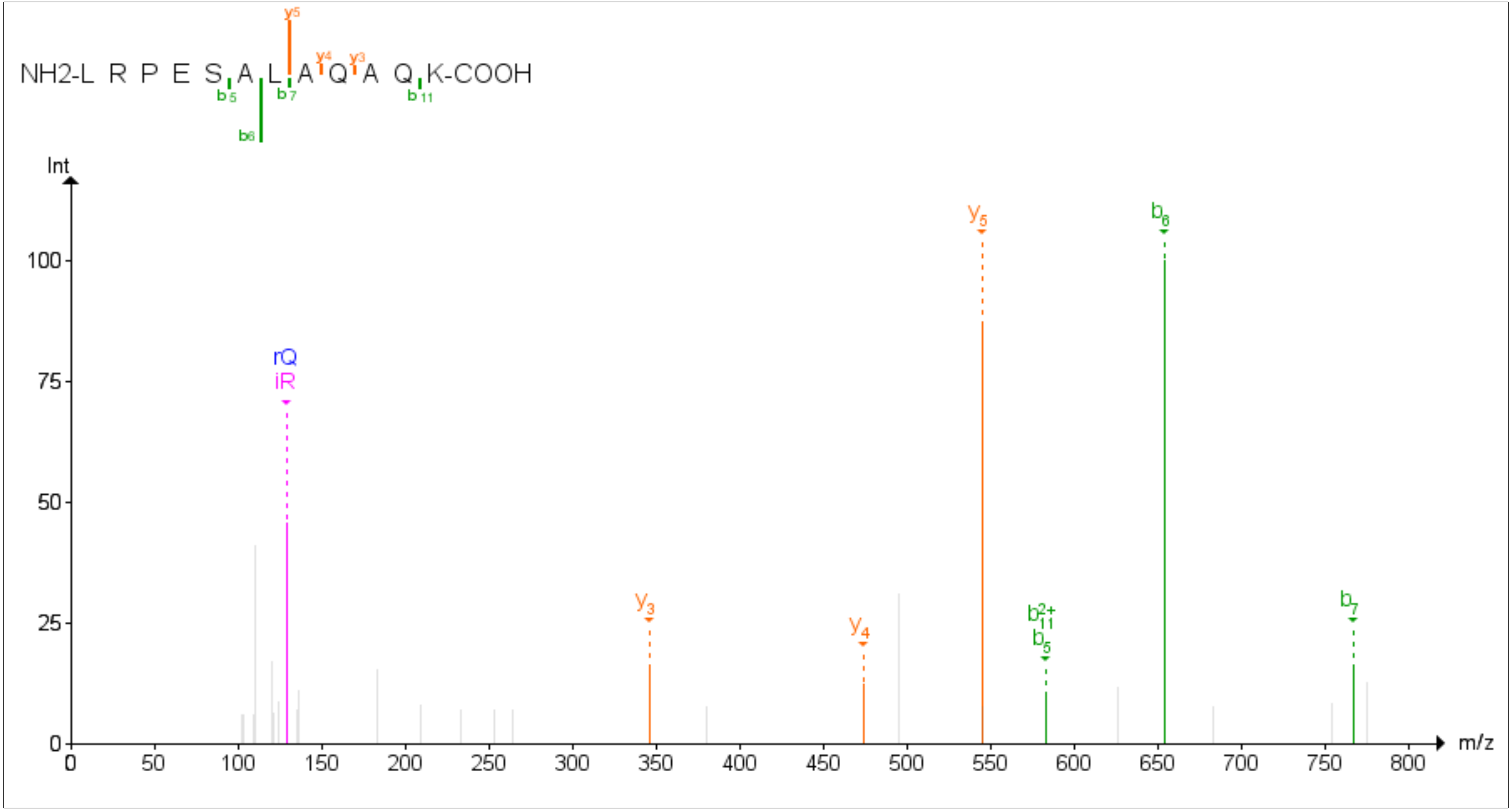

NH2-L R P E S A L A Q A Q K-COOH
y5
y4
y3
b5
b7
b11
b6
Int
100
75
50
25
0
rQ
iR
y5
b6
y3
y4
b11 2+
b5
b7
0
50
100
150
200
250
300
350
400
450
500
550
600
650
700
750
800
m/z

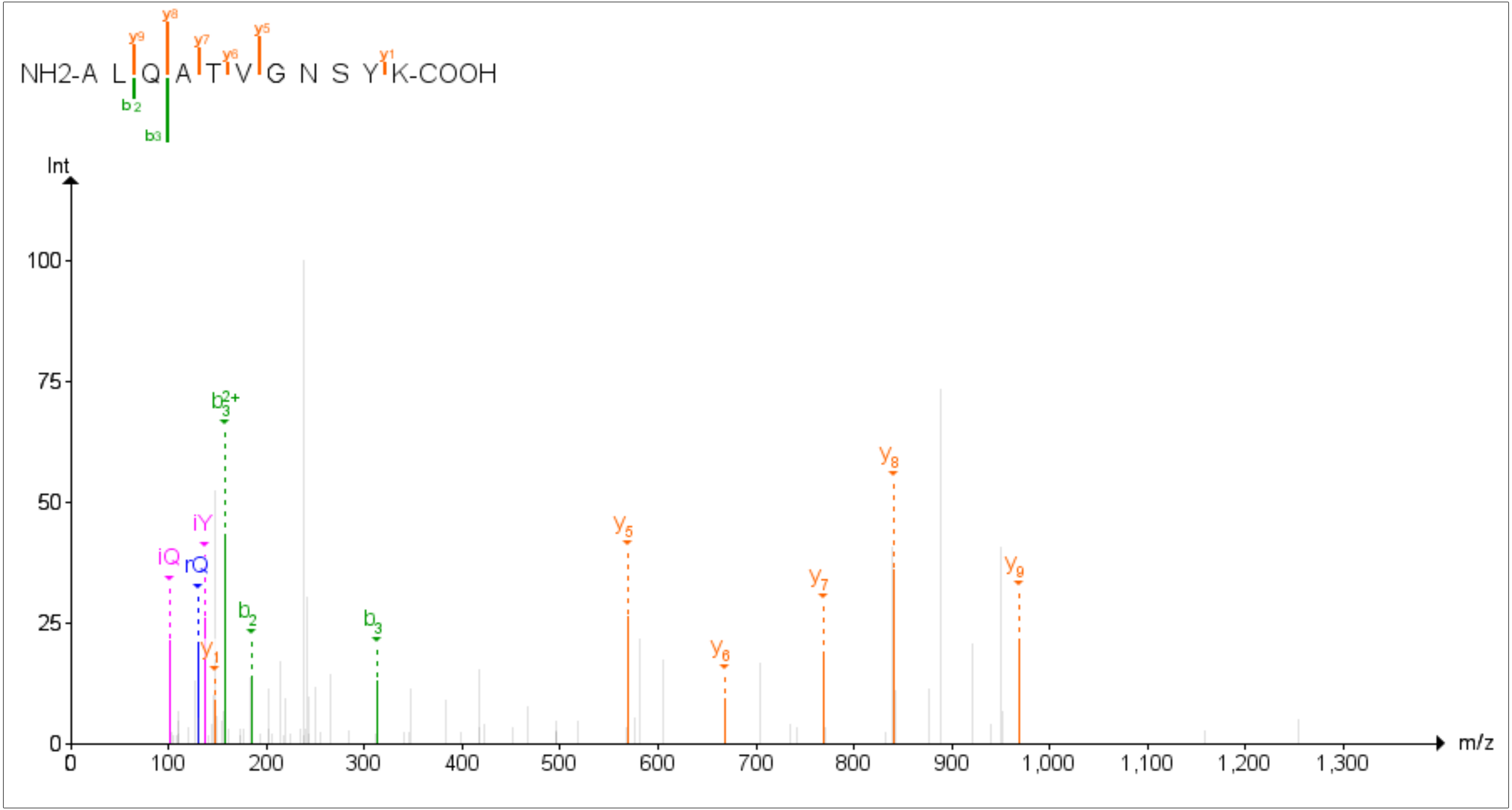

NH2-A L Q A T V G N S Y K-COOH
y9 y8 y7 y6 y5 y1
b2 b3
Int
100
75
50
25
0
iQ
rQ
iY
b_3^{2+}
y_1
b_2
b_3
y_5
y_6
y_7
y_8
y_9
0 100 200 300 400 500 600 700 800 900 1,000 1,100 1,200 1,300
m/z

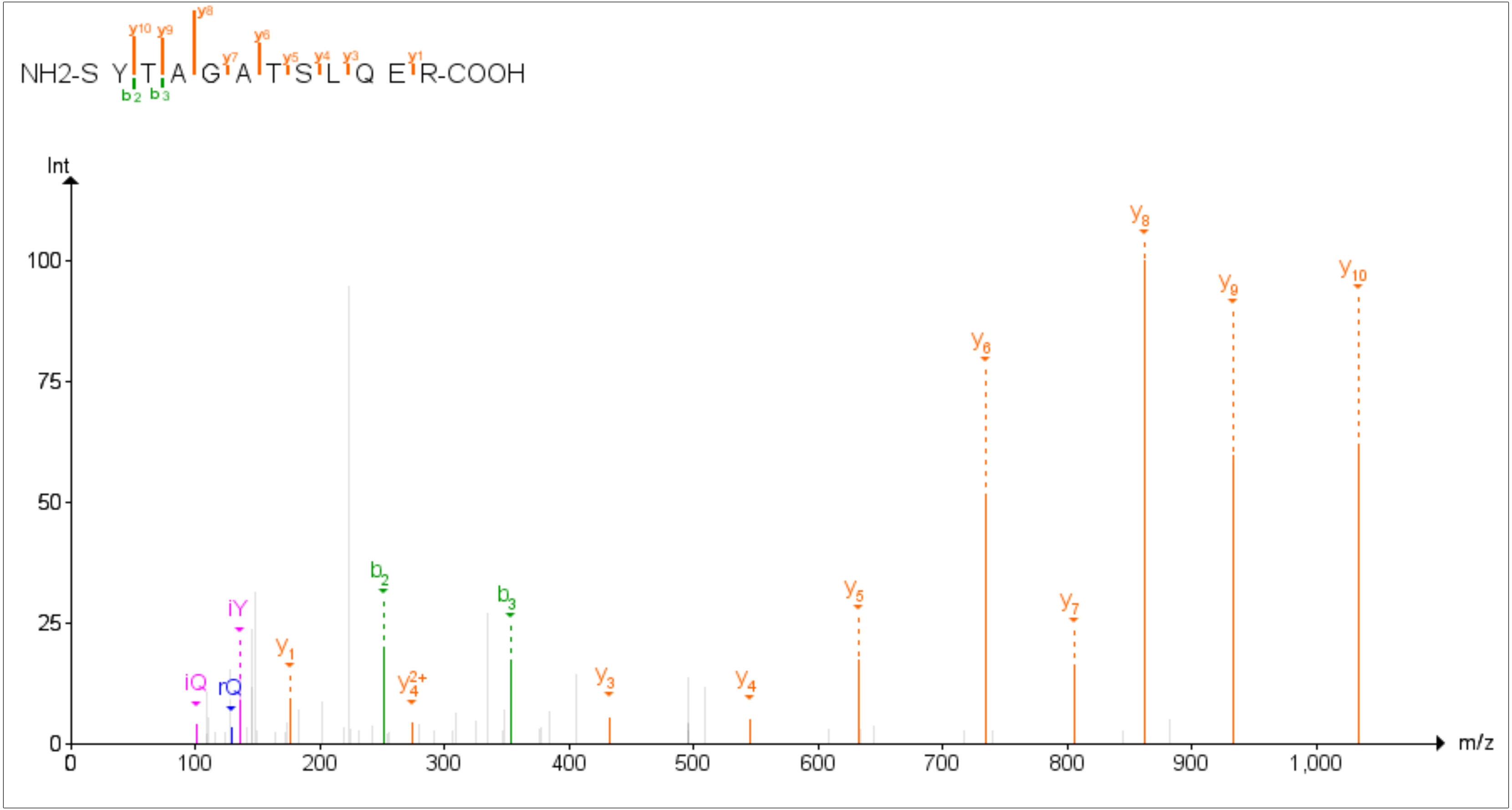

NH2-S Y T A G A T S L Q E R-COOH
y10 y9 y8 y7 y6 y5 y4 y3 y1
b2 b3
Int
m/z
0
25
50
75
100
0
100
200
300
400
500
600
700
800
900
1,000
iQ
rQ
iY
y1
b2
y4 2+
b3
y3
y4
y5
y6
y7
y8
y9
y10

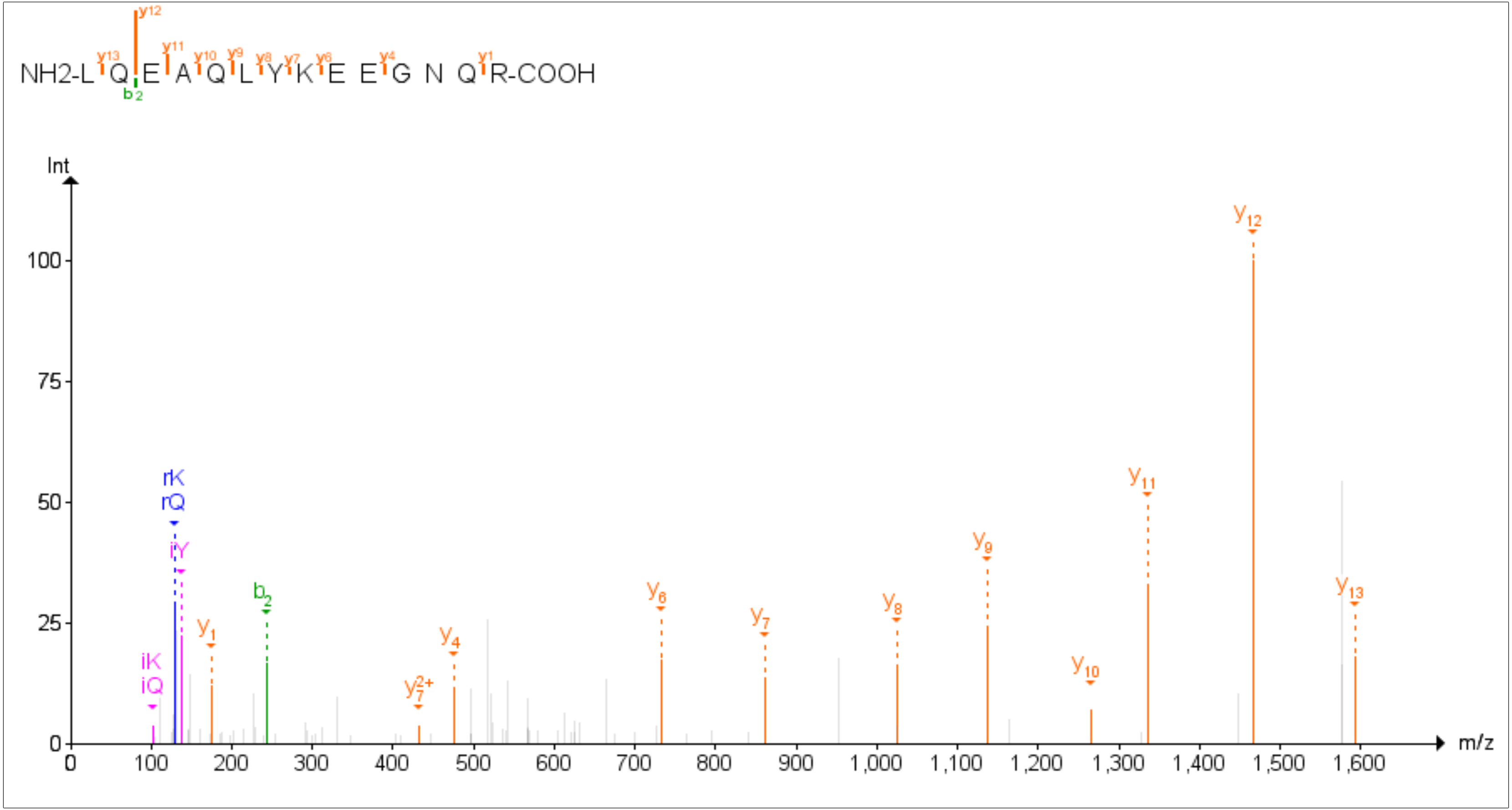

NH2-L Q E A Q L Y K E E G N Q R-COOH
y13 y12 y11 y10 y9 y8 y7 y6 y4 y1
b2
Int
100
75
50
25
0
rK
rQ
iY
iK
iQ
y1
b2
y_7^{2+}
y4
y6
y7
y8
y9
y10
y11
y12
y13
0 100 200 300 400 500 600 700 800 900 1,000 1,100 1,200 1,300 1,400 1,500 1,600
m/z

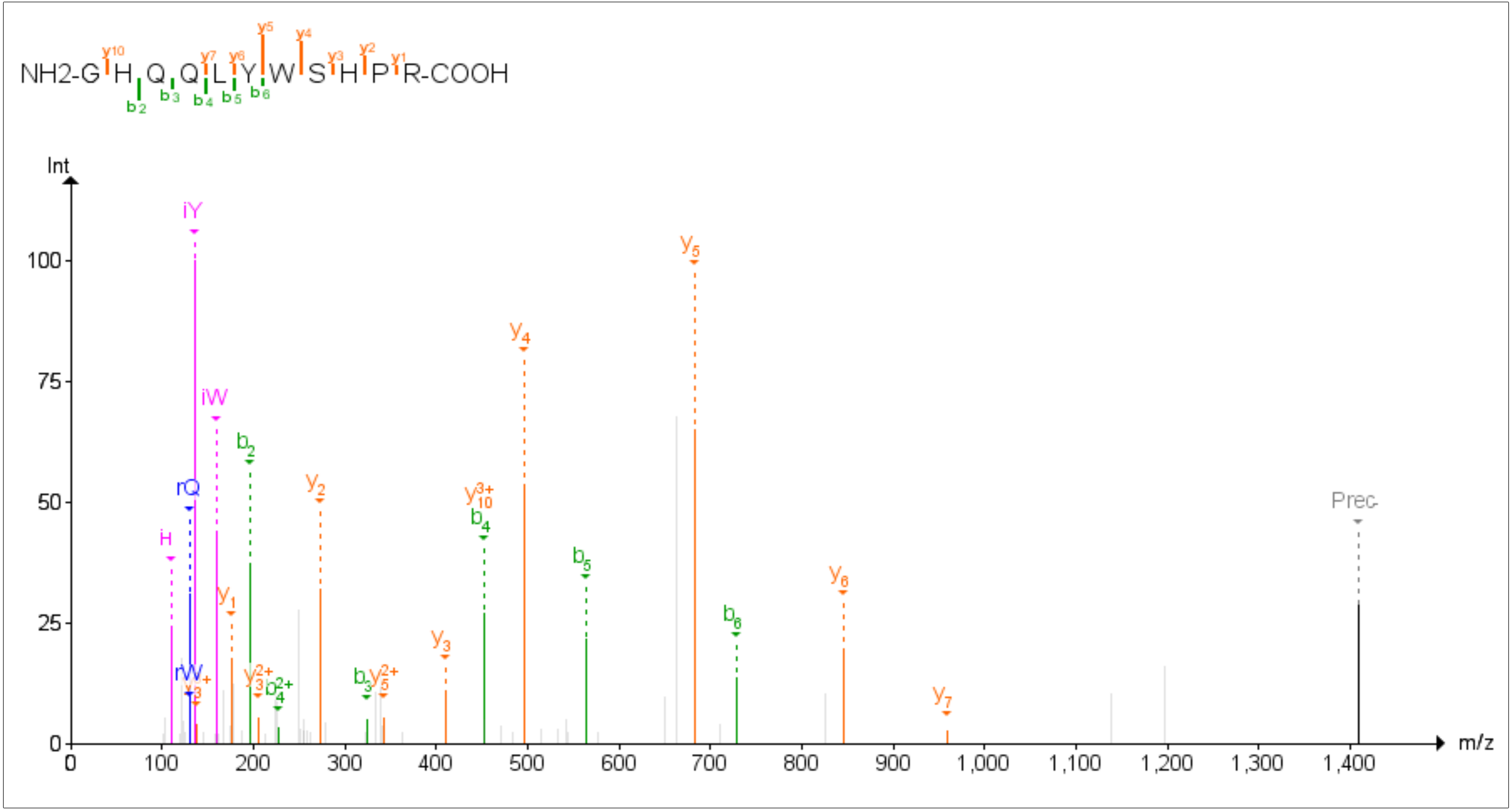
NH2-GHQQLYWSHPR-COOH
Int
m/z
iY
iW
iH
rQ
rW
Prec

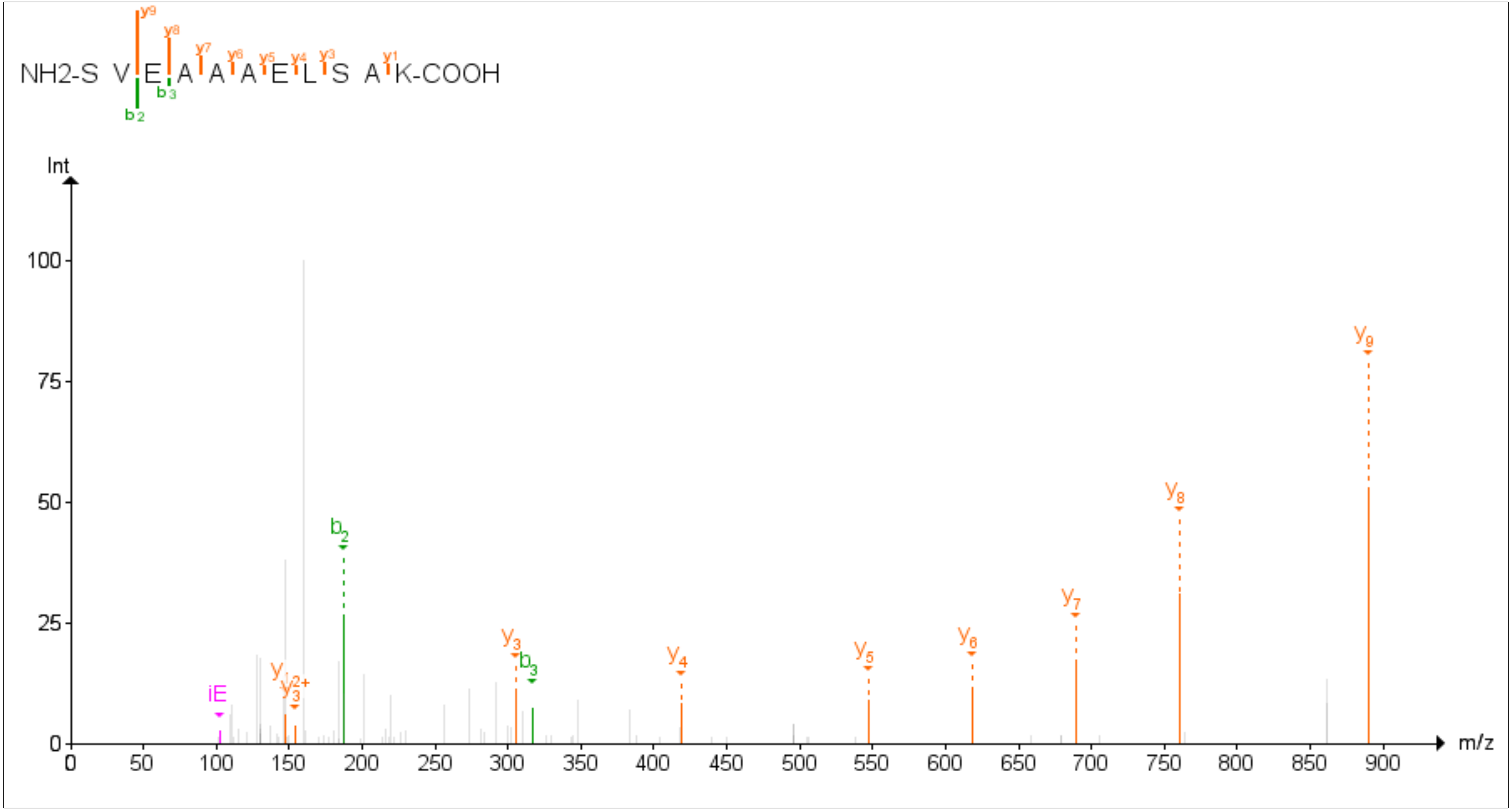

NH2-S V E A A A E L S A K-COOH
y9 y8 y7 y6 y5 y4 y3 y1
b2 b3
Int
100
75
50
25
0
m/z
0 50 100 150 200 250 300 350 400 450 500 550 600 650 700 750 800 850 900
iE
y1
y3 2+
b2
y3
b3
y4
y5
y6
y7
y8
y9

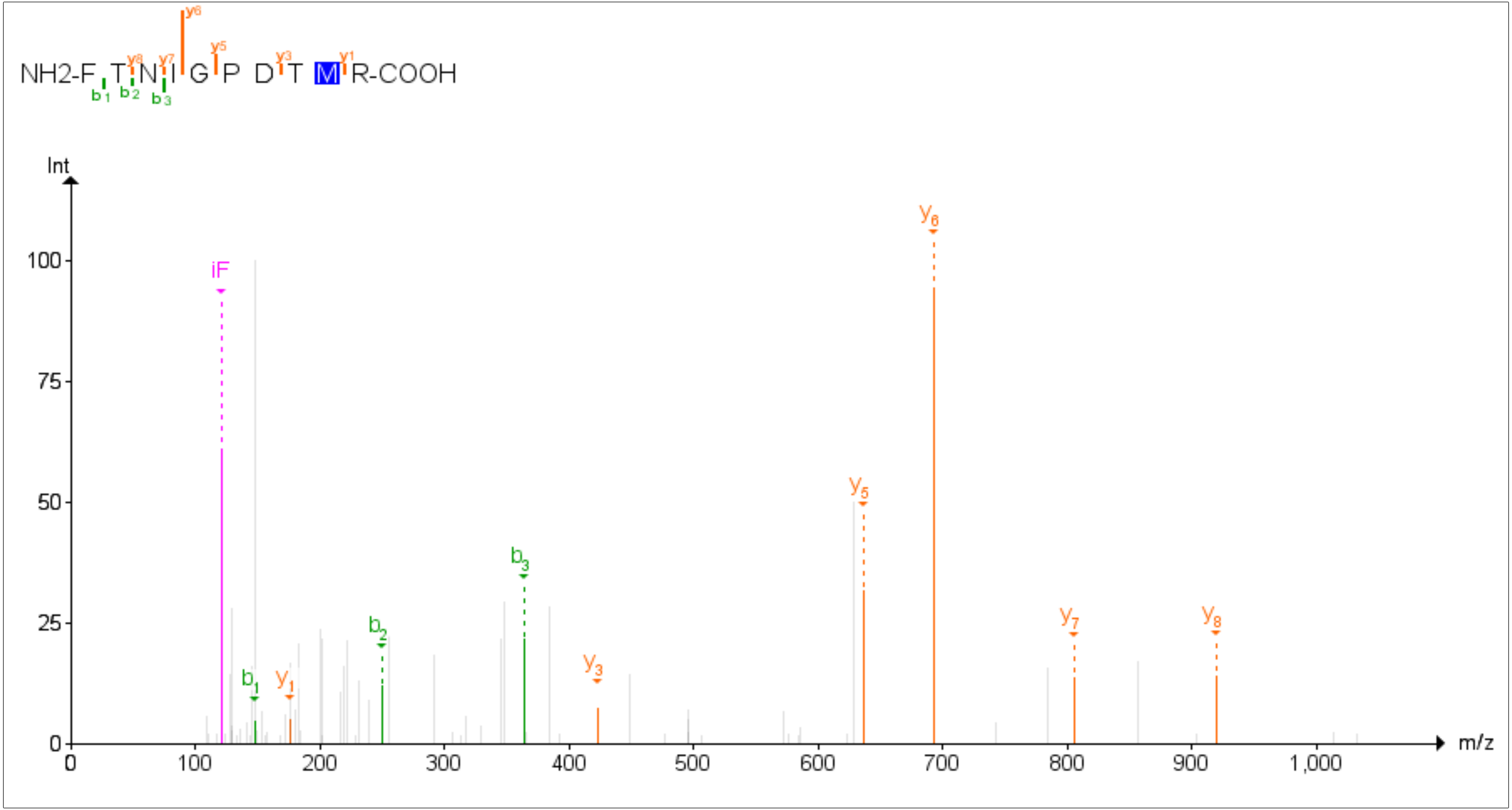

NH2-F T N I G P D T M R-COOH
b1 b2 b3
y8 y7 y6 y5 y3 y1
Int
100
75
50
25
0
iF
b1 y1 b2 b3 y3 y5 y6 y7 y8
0 100 200 300 400 500 600 700 800 900 1,000
m/z

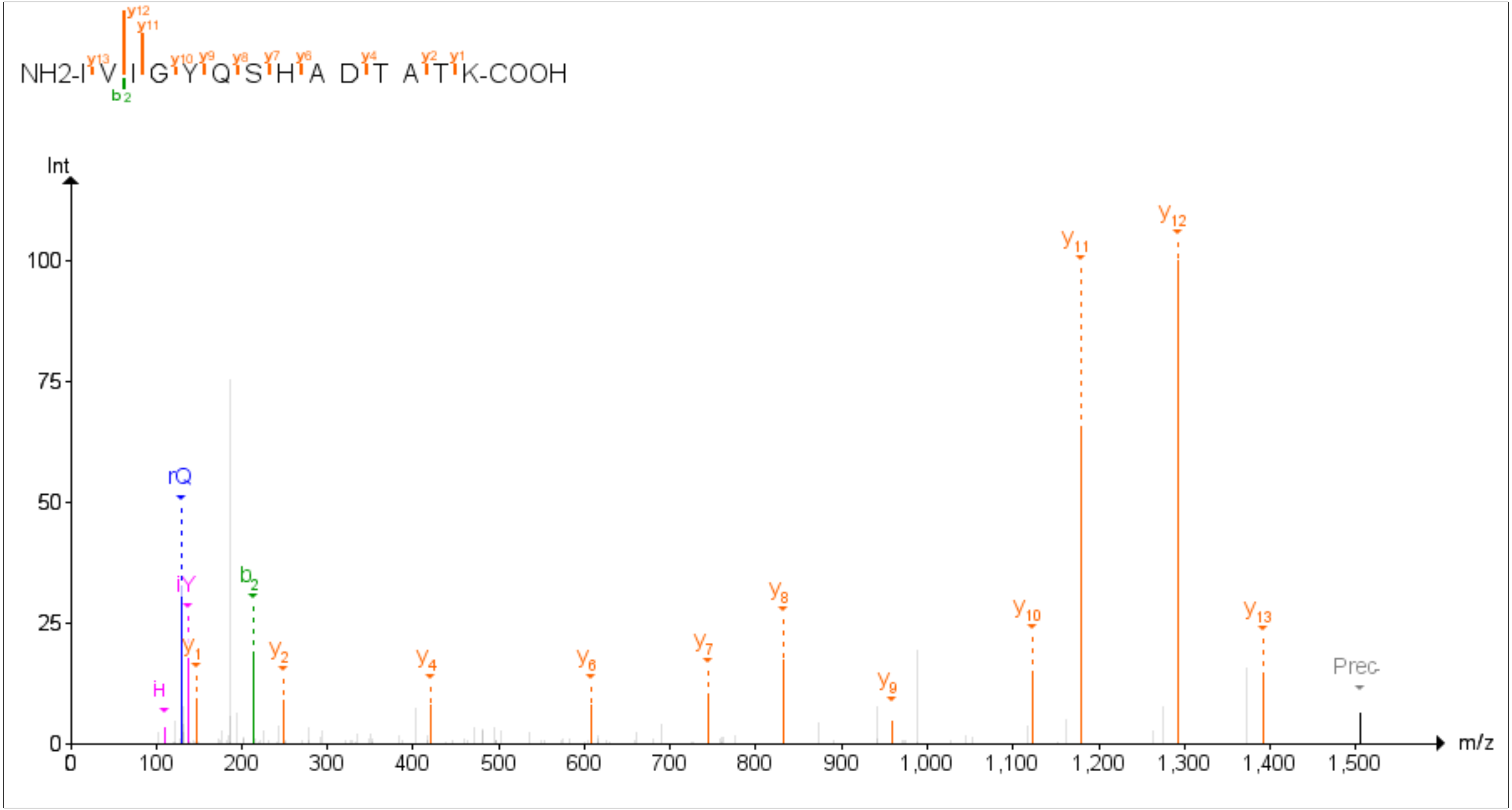

NH2-IVIGYQSHADTATK-COOH
y13 y12 y11 y10 y9 y8 y7 y6 y4 y2 y1
b2
Int
m/z
rQ
iY
iH
Prec.

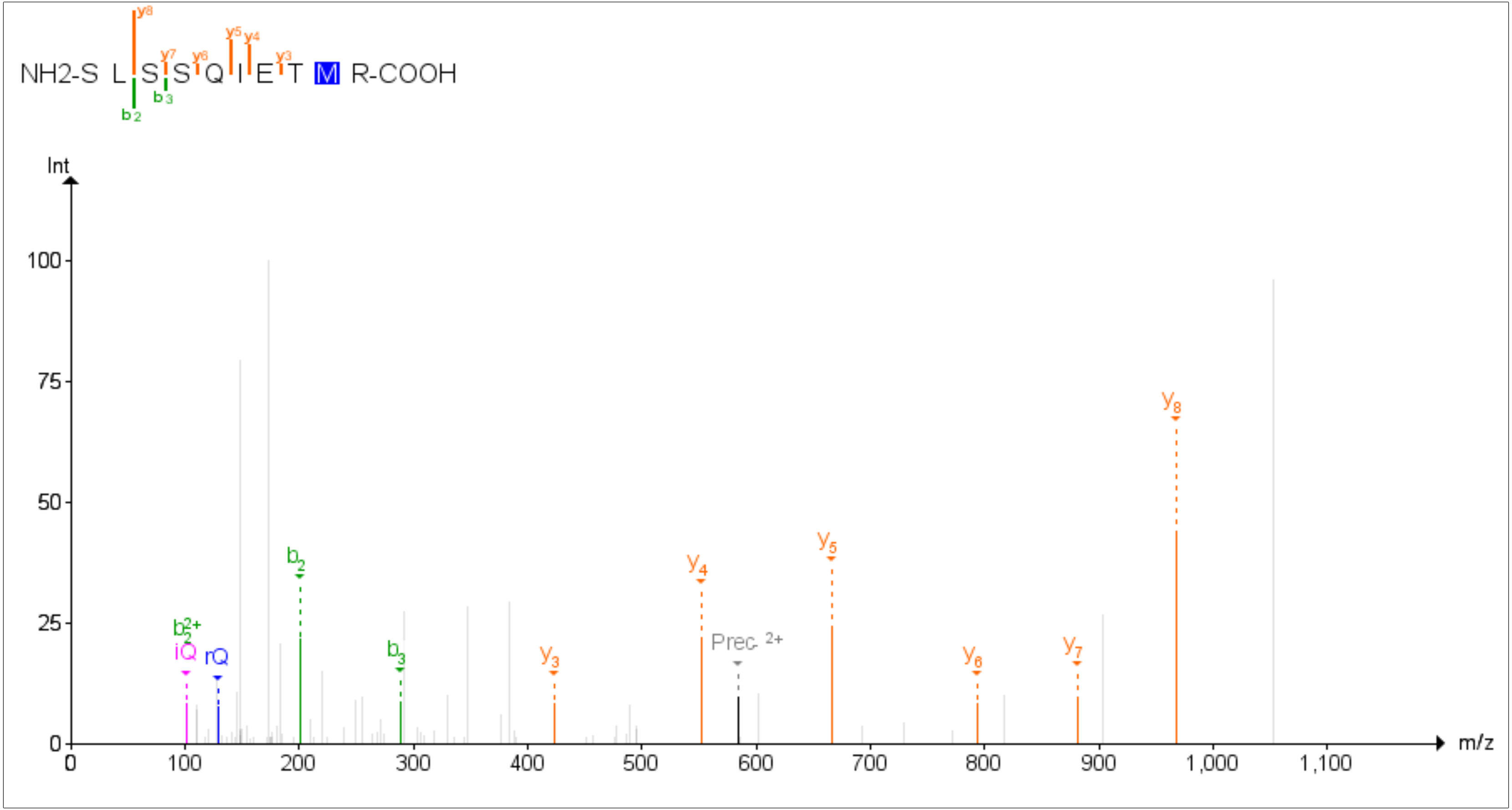

NH2-S L S S Q I E T M R-COOH
y8
y7
y6
y5
y4
y3
b2
b3
Int
100
75
50
25
0
b2
b2 2+
iQ
rQ
b3
y3
y4
Prec 2+
y5
y6
y7
y8
0
100
200
300
400
500
600
700
800
900
1,000
1,100
m/z

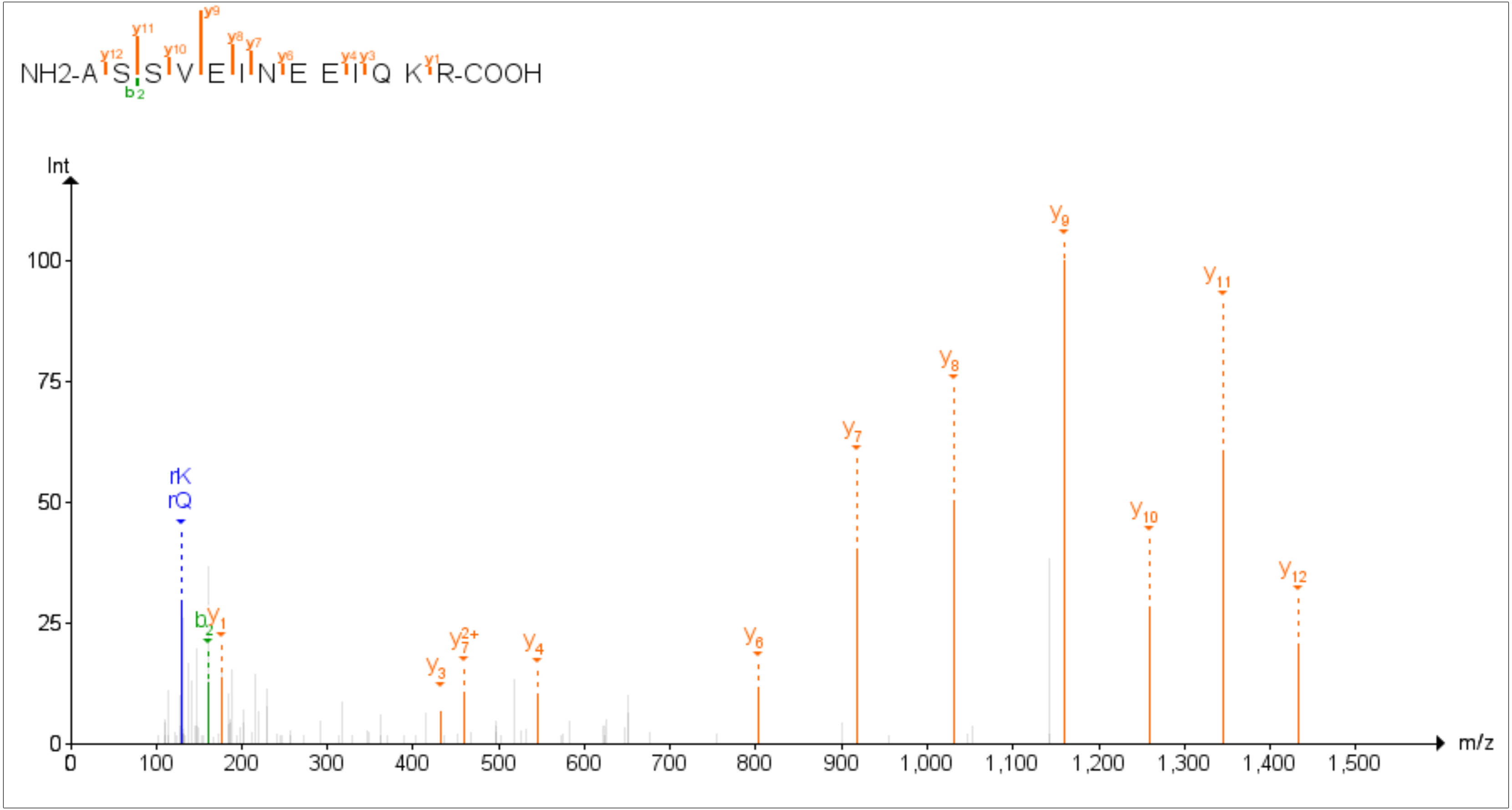

NH2-A S S V E I N E E I Q K R-COOH
y12 y11 y10 y9 y8 y7 y6 y4 y3 y1
b2
Int
m/z
rK
rQ
b2
y1
y3
y7 2+
y4
y6
y7
y8
y9
y10
y11
y12
0
25
50
75
100
0
100
200
300
400
500
600
700
800
900
1,000
1,100
1,200
1,300
1,400
1,500

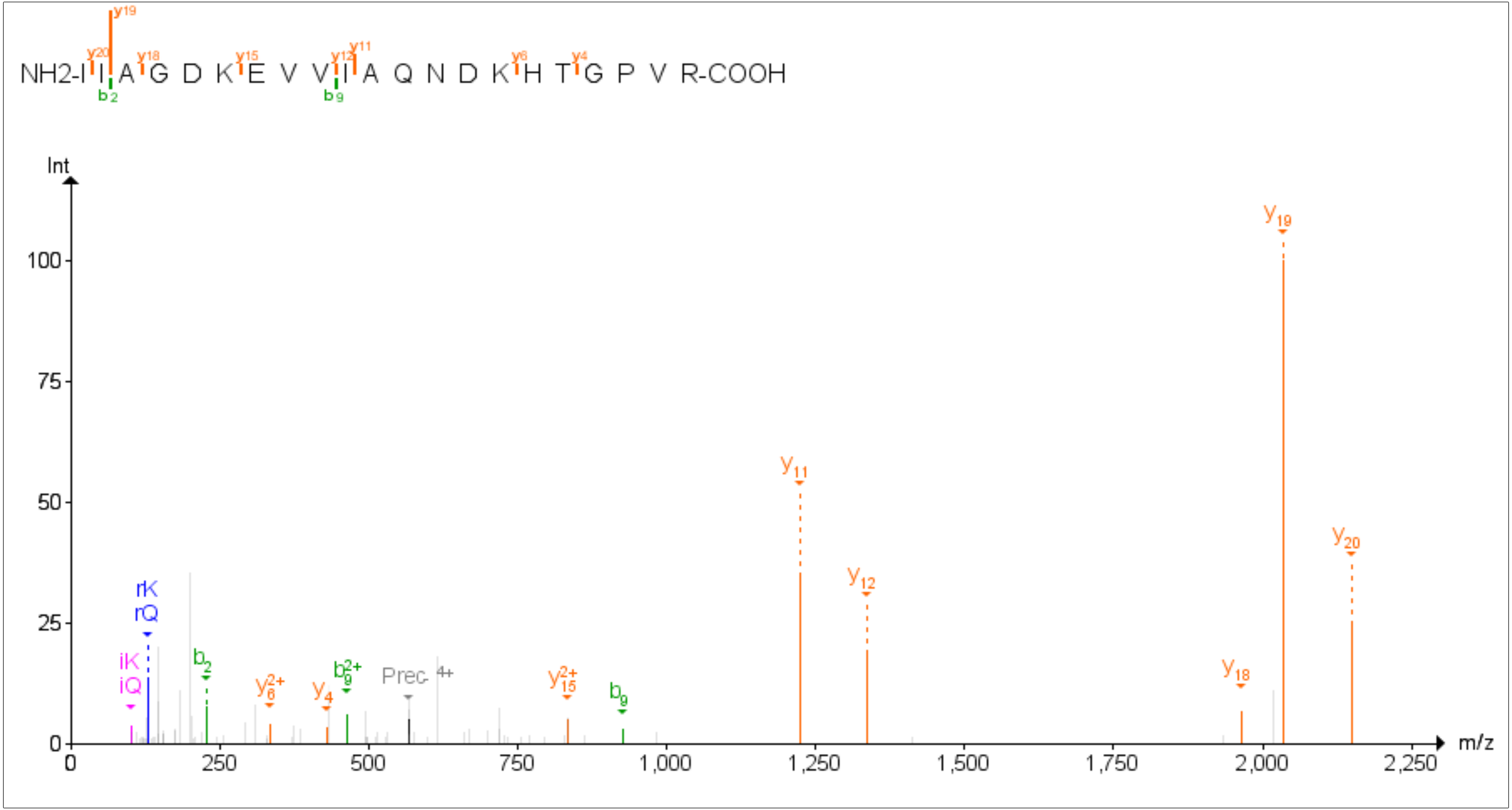

NH2-I I A G D K E V V I A Q N D K H T G P V R-COOH
y19
y20
y18
y15
y12
y11
y6
y4
b2
b9
Int
100
75
50
25
0
rK
rQ
iK
iQ
b2
y6 2+
y4
b9 2+
Prec 4+
y15 2+
b9
y11
y12
y18
y19
y20
0
250
500
750
1,000
1,250
1,500
1,750
2,000
2,250
m/z

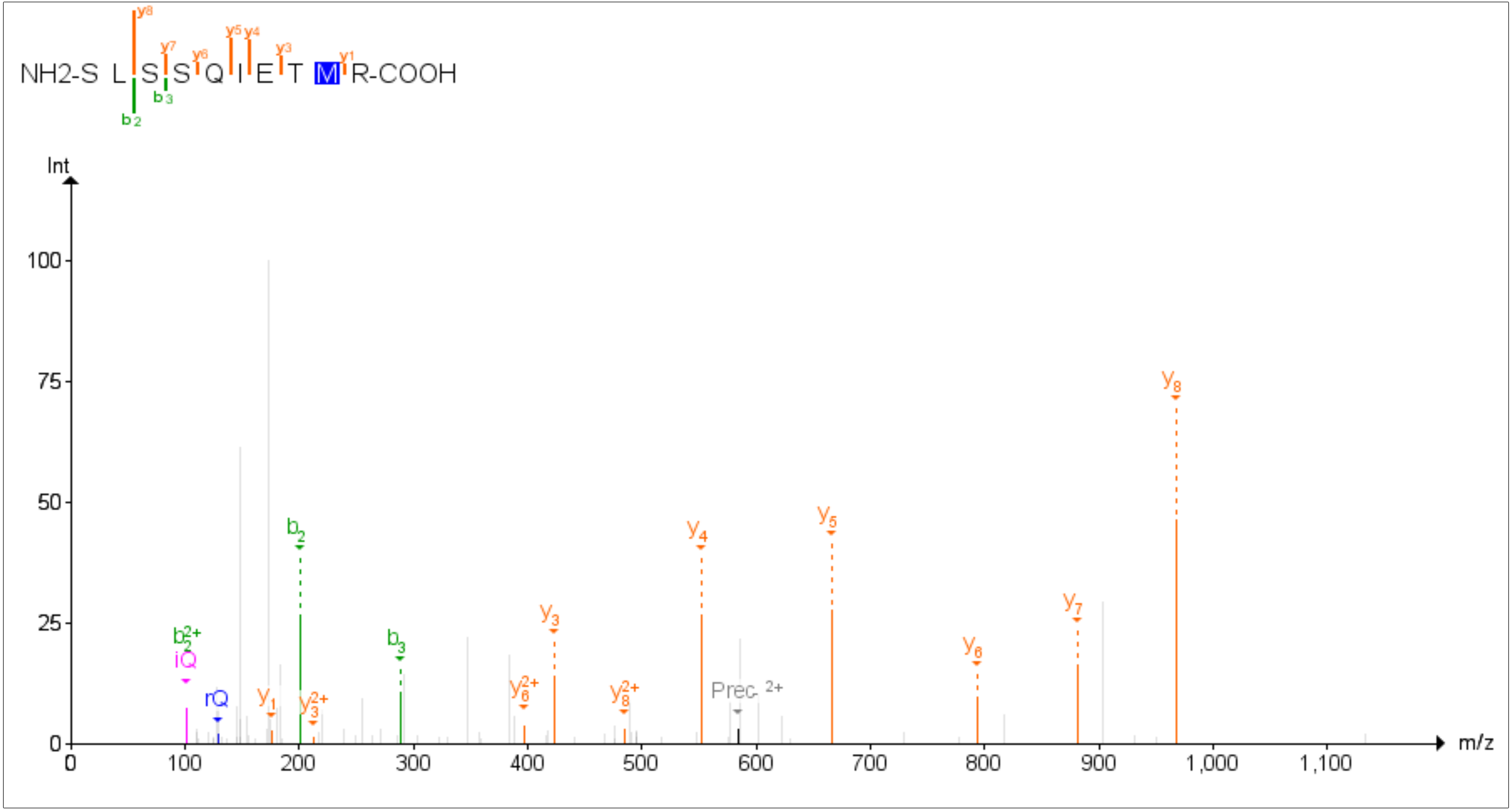

NH2-S L S S Q I E T M R-COOH
y8 y7 y6 y5 y4 y3 y1
b2 b3
Int
100
75
50
25
0
m/z
0 100 200 300 400 500 600 700 800 900 1,000 1,100
y8
y5
b2
y4
y7
y3
y6
b3
b2 2+
iQ
rQ
y1
y3 2+
y6 2+
y8 2+
Prec 2+

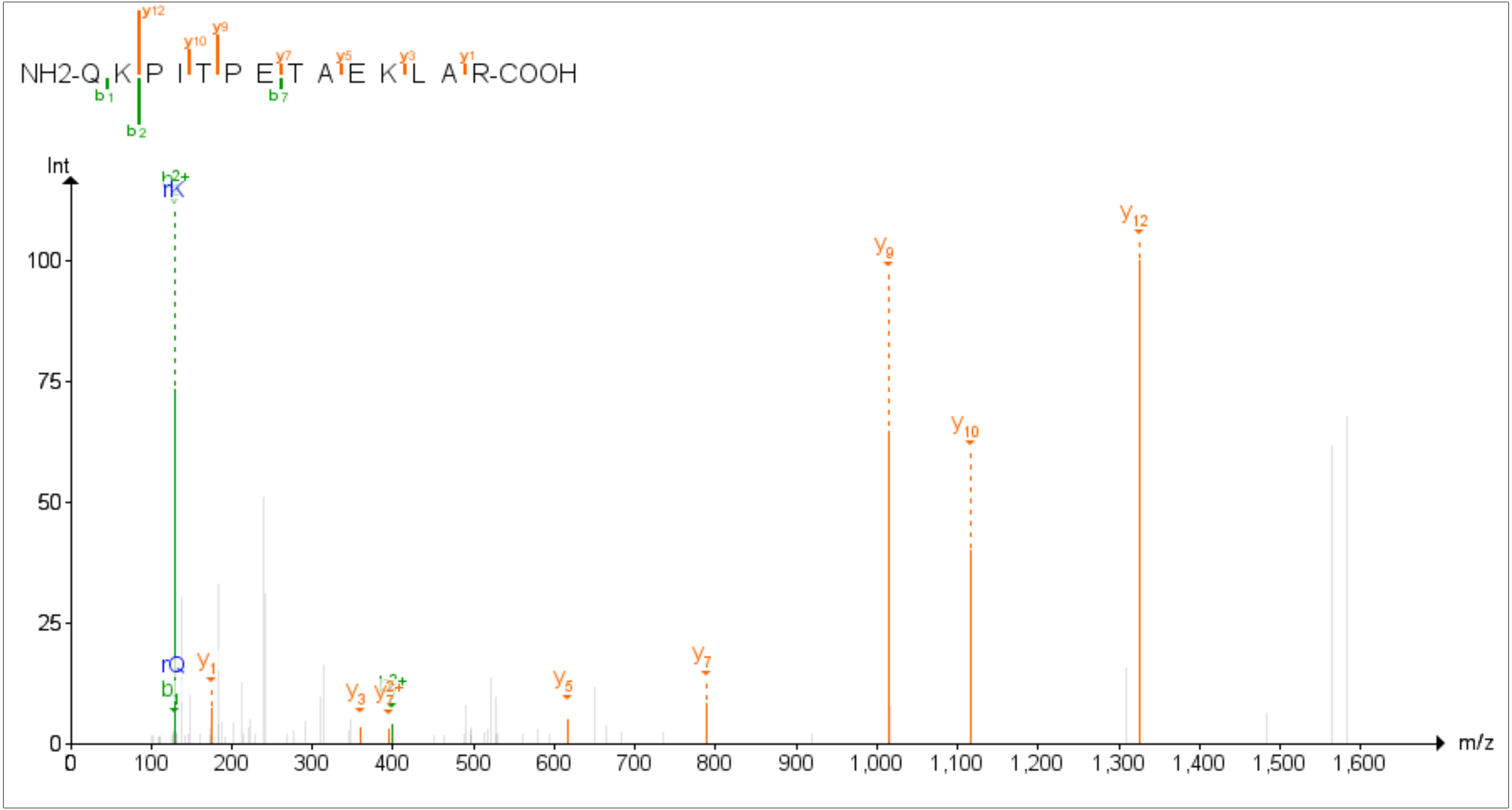

NH2-Q K P I T P E T A E K L A R-COOH
y12
y10
y9
y7
y5
y3
y1
b1
b2
b7
Int
rK
rQ
y12
y9
y10
y1
y3
y7
y5
y7
m/z
0
25
50
75
100
0
100
200
300
400
500
600
700
800
900
1,000
1,100
1,200
1,300
1,400
1,500
1,600

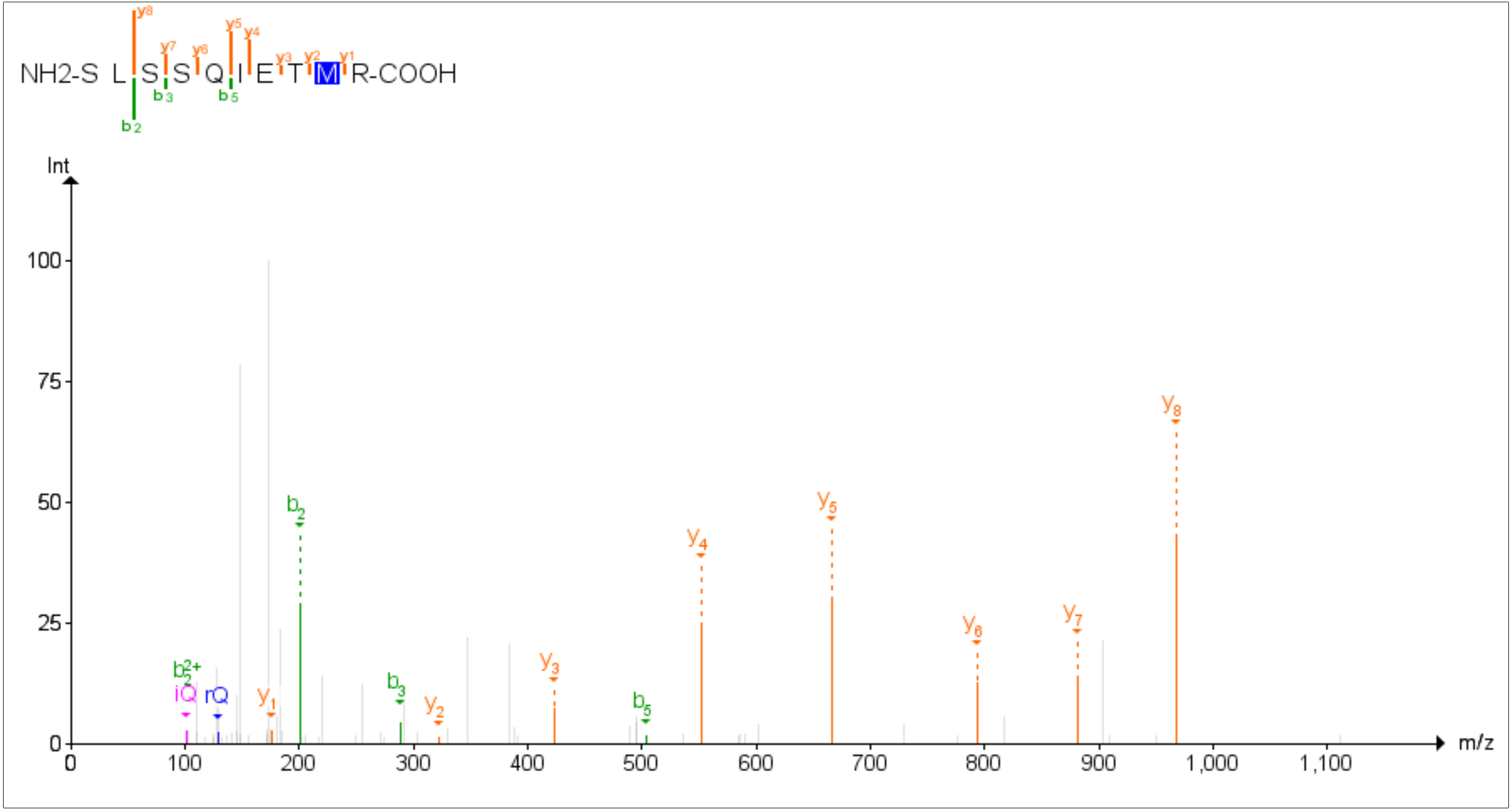
NH2-S L S S Q I E T M R-COOH
y8
y7
y6
y5
y4
y3
y2
y1
b 2
b 3
b 5
Int
100
75
50
25
0
m/z
0
100
200
300
400
500
600
700
800
900
1,000
1,100
b2
b2 2+
iQ
rQ
y1
b3
y2
y3
b5
y4
y5
y6
y7
y8

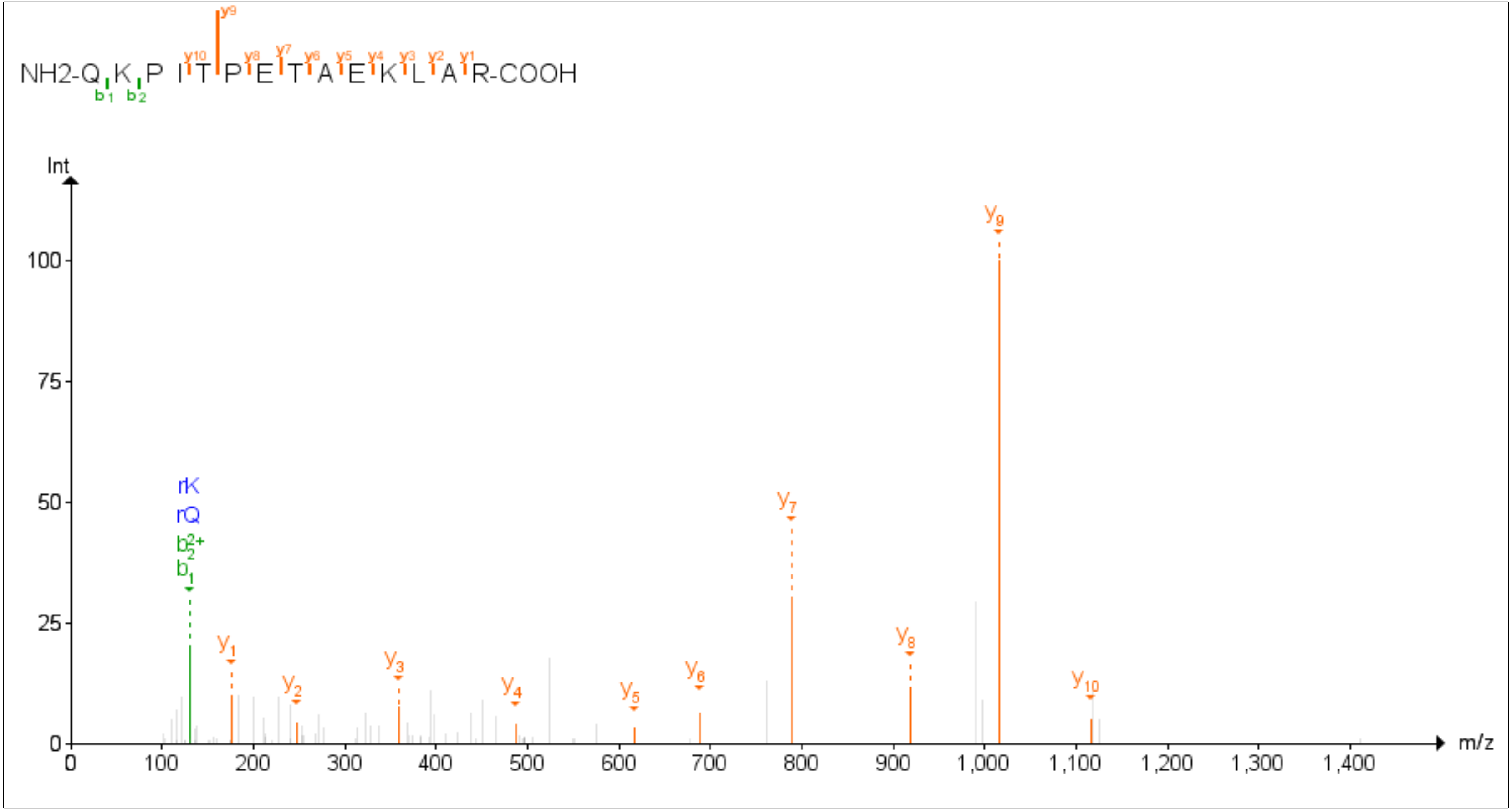
NH2-Q K P I T P E T A E K L A R-COOH
y9
y10
y8
y7
y6
y5
y4
y3
y2
y1
b1
b2
Int
100
75
50
25
0
rK
rQ
b2 2+
b1
y1
y2
y3
y4
y5
y6
y7
y8
y9
y10
0
100
200
300
400
500
600
700
800
900
1,000
1,100
1,200
1,300
1,400
m/z

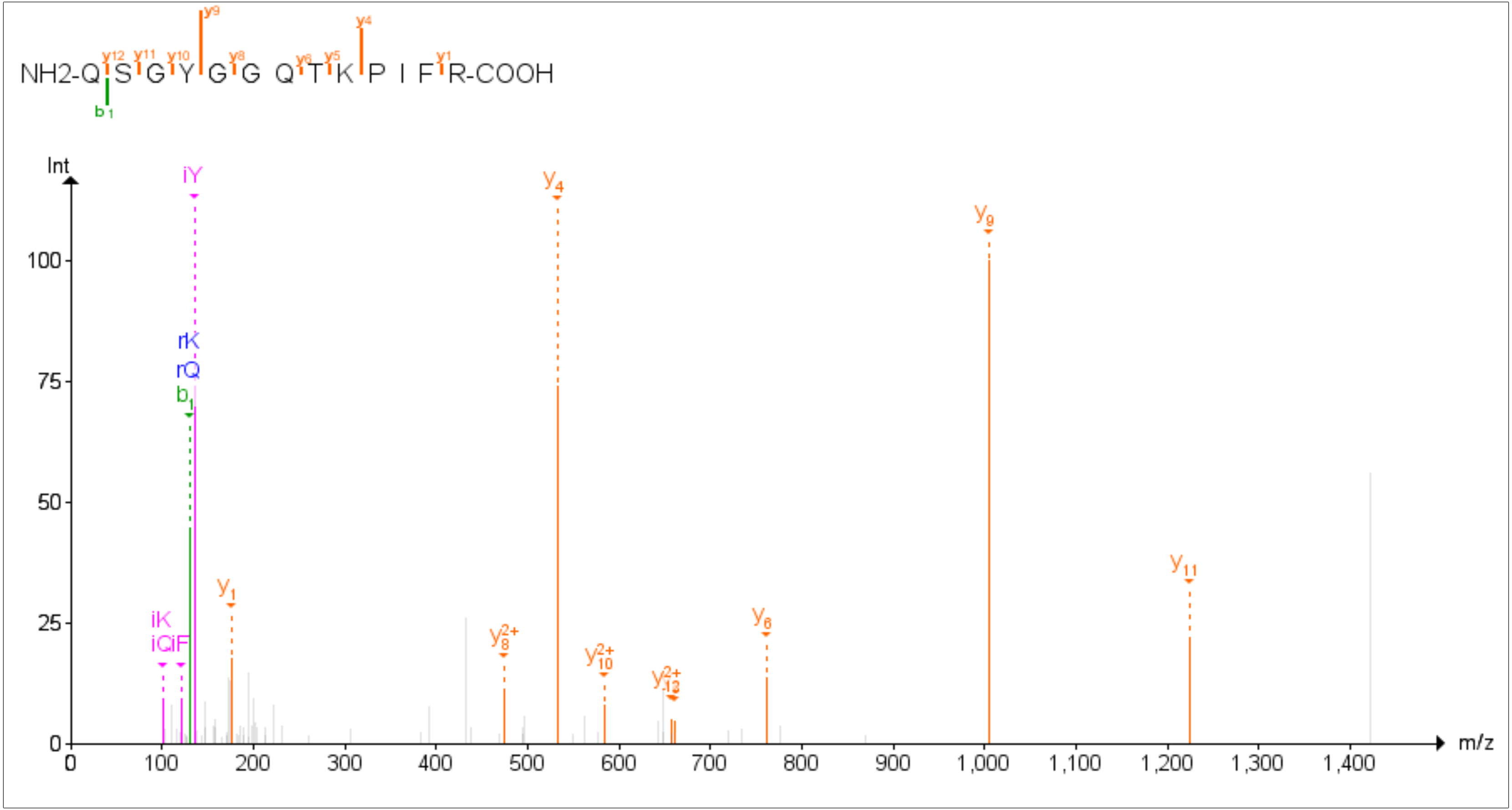
NH2-Q S G Y G G Q T K P I F R-COOH
y12 y11 y10 y9 y8 y6 y5 y4 y1
b1
Int
m/z
iY
y4
y9
rK
rQ
b1
y1
iK
iQiF
y8 2+
y10 2+
y13 2+
y6
y11
0
25
50
75
100
0
100
200
300
400
500
600
700
800
900
1,000
1,100
1,200
1,300
1,400

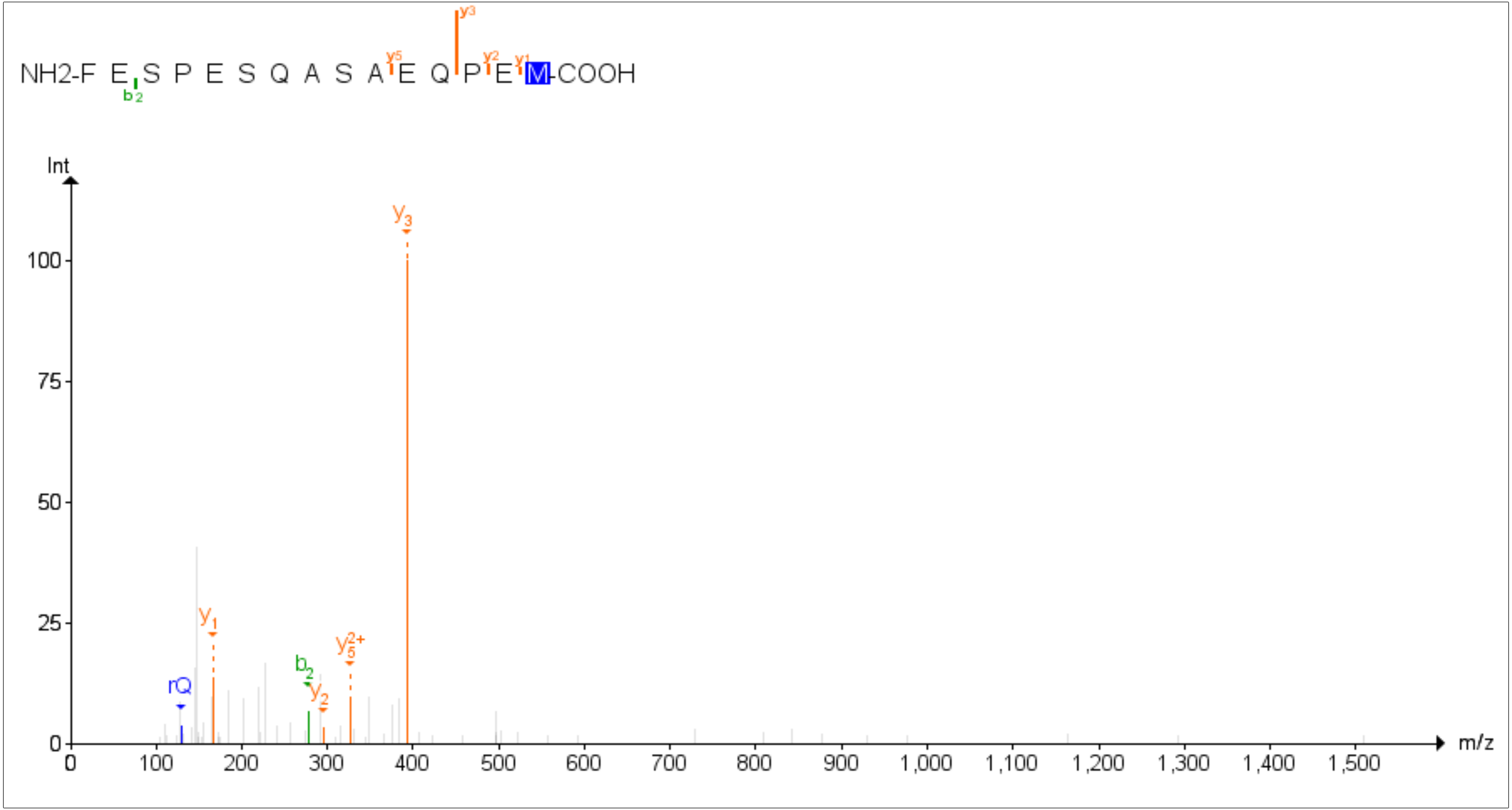

NH2-F E S P E S Q A S A E Q P E M-COOH
b2
y5
y3
y2
y1
Int
100
75
50
25
0
y3
y1
rQ
b2
y2
y5 2+
m/z
0
100
200
300
400
500
600
700
800
900
1,000
1,100
1,200
1,300
1,400
1,500

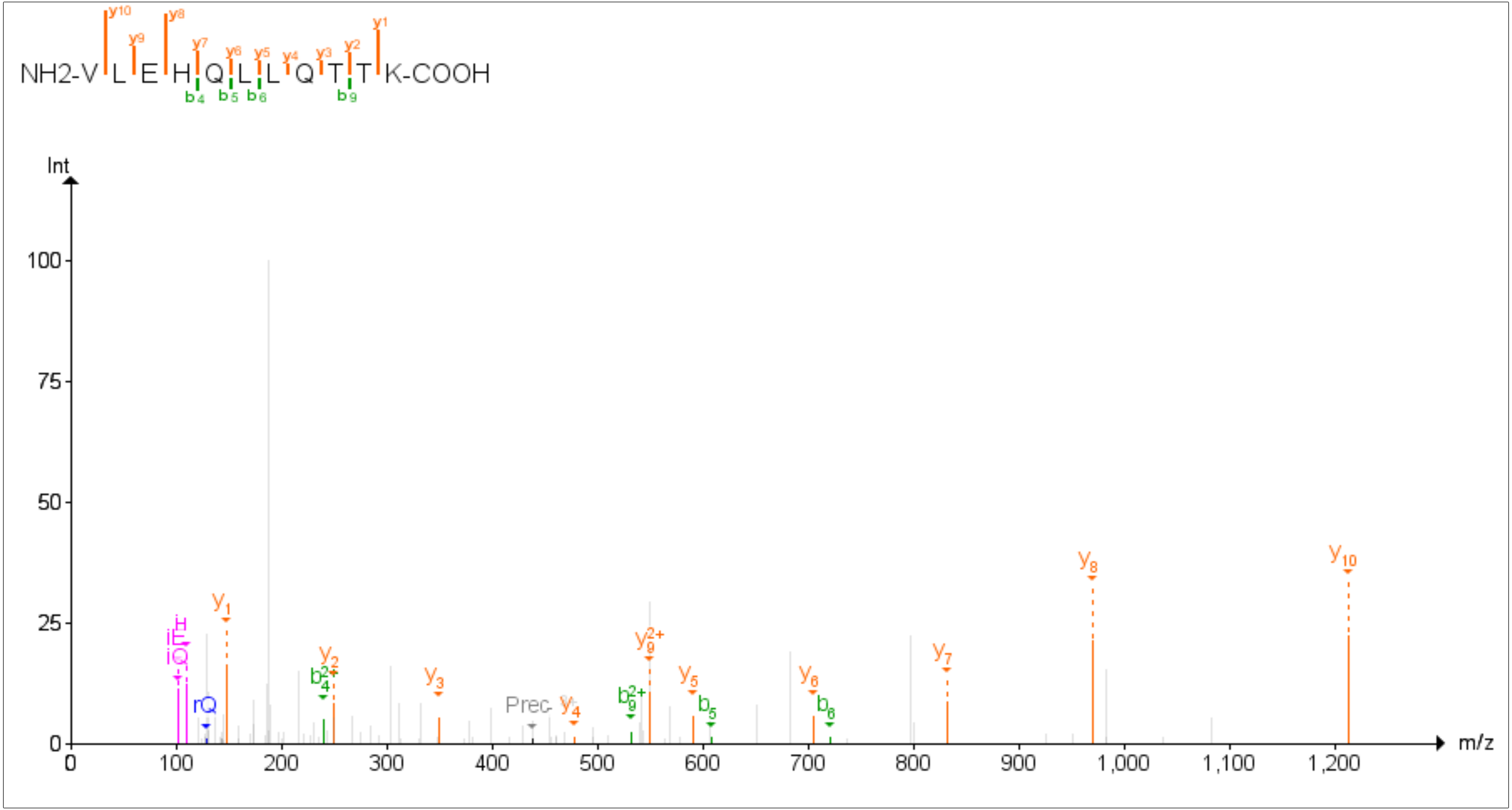

NH2-V L E H Q L L Q T T K-COOH
y10 y9 y8 y7 y6 y5 y4 y3 y2 y1
b4 b5 b6 b9
Int
100
75
50
25
0
m/z
0
100
200
300
400
500
600
700
800
900
1,000
1,100
1,200
iH
iE
iQ
rQ
y1
y2
b4 2+
y3
Prec-
y4
b9 2+
y9 2+
y5
b5
y6
b6
y7
y8
y10

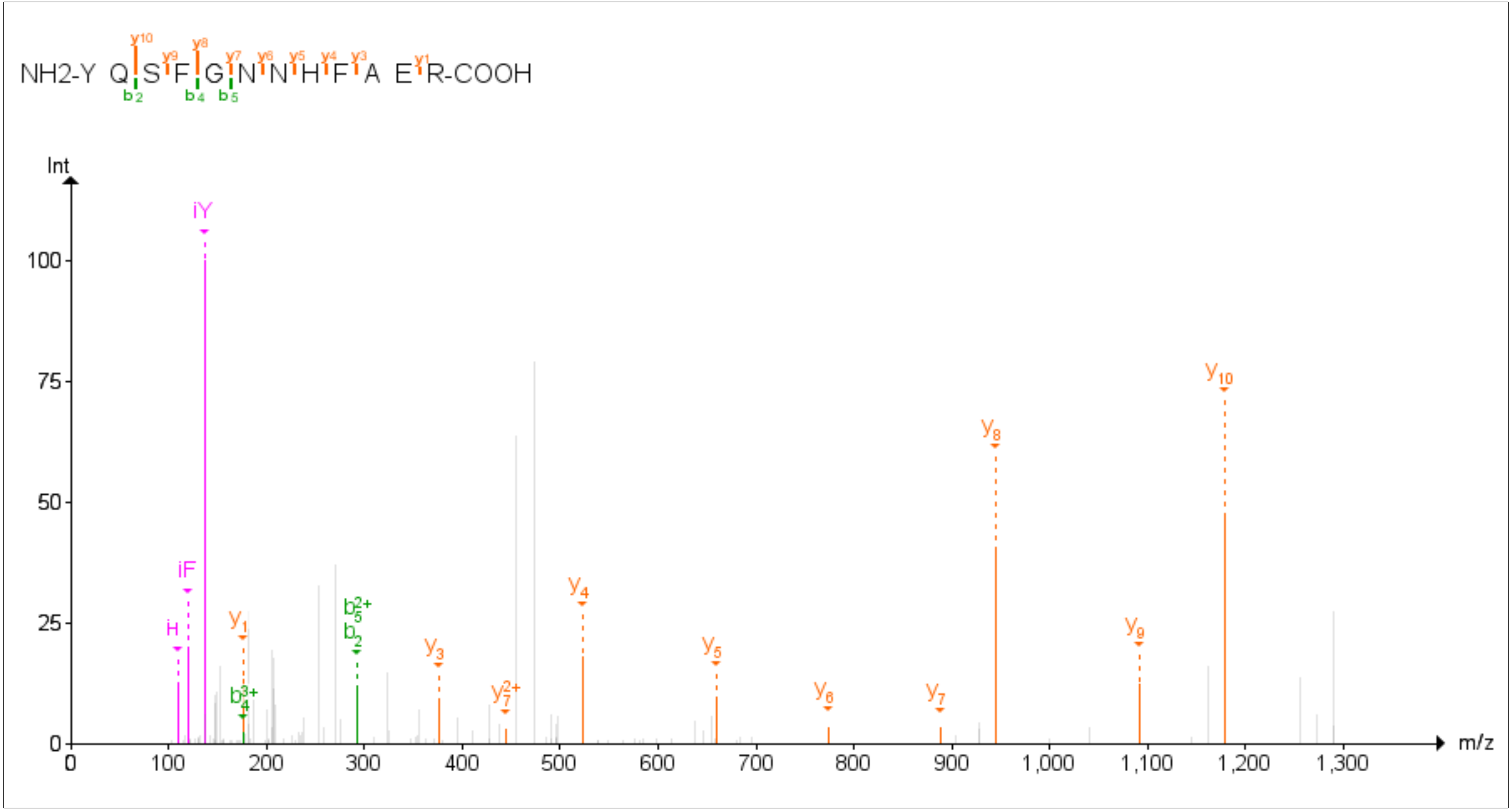
NH2-Y Q S F G N N H F A E R-COOH
y10 y9 y8 y7 y6 y5 y4 y3 y1
b2 b4 b5
Int
m/z
0
25
50
75
100
0
100
200
300
400
500
600
700
800
900
1,000
1,100
1,200
1,300
iY
iF
iH
y1
b4 3+
b5 2+
b2
y3
y7 2+
y4
y5
y6
y7
y8
y9
y10

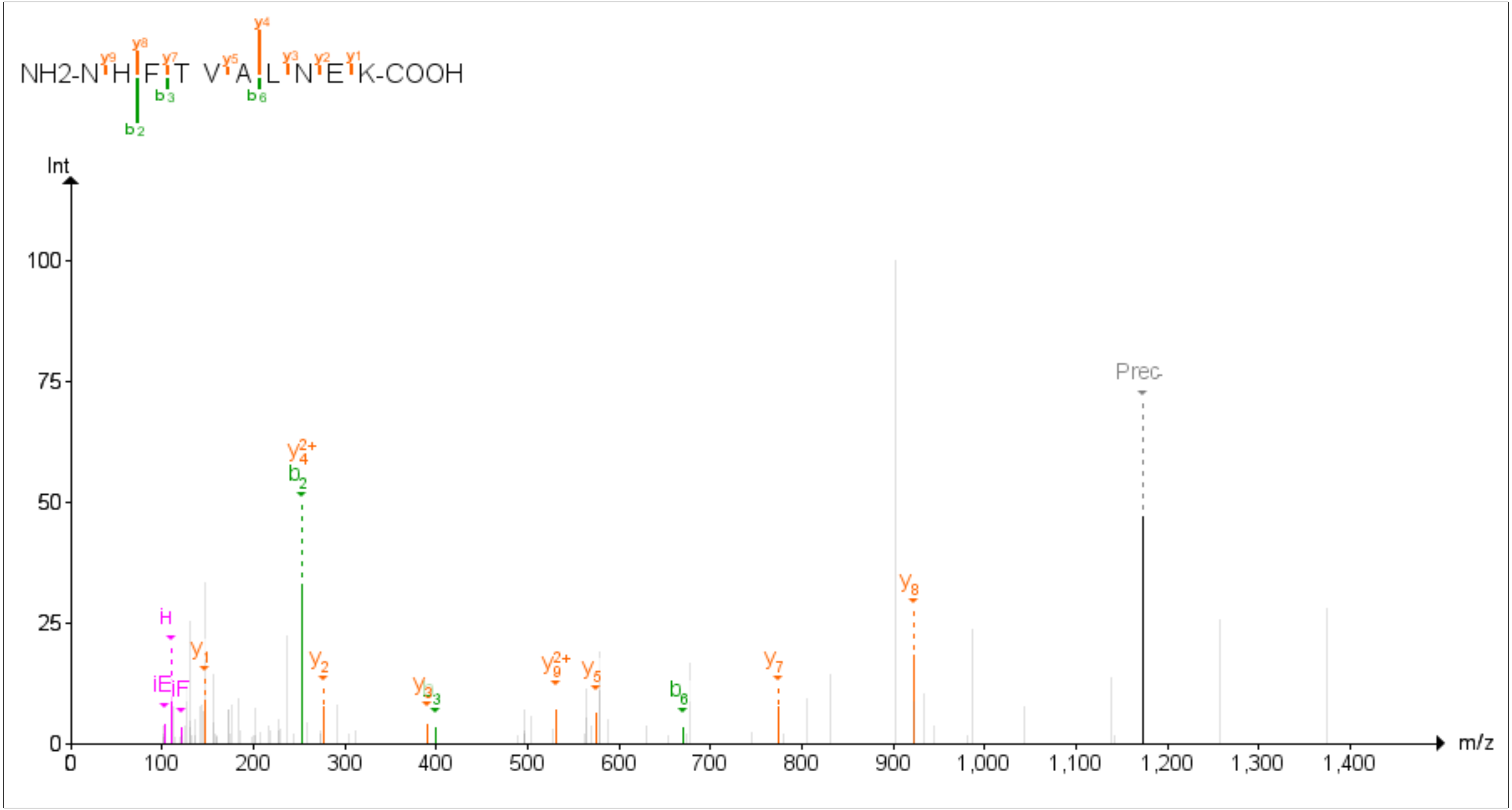

NH2-N H F T V A L N E K-COOH
y9 y8 y7 y5 y4 y3 y2 y1
b2 b3 b6
Int
m/z
Prec

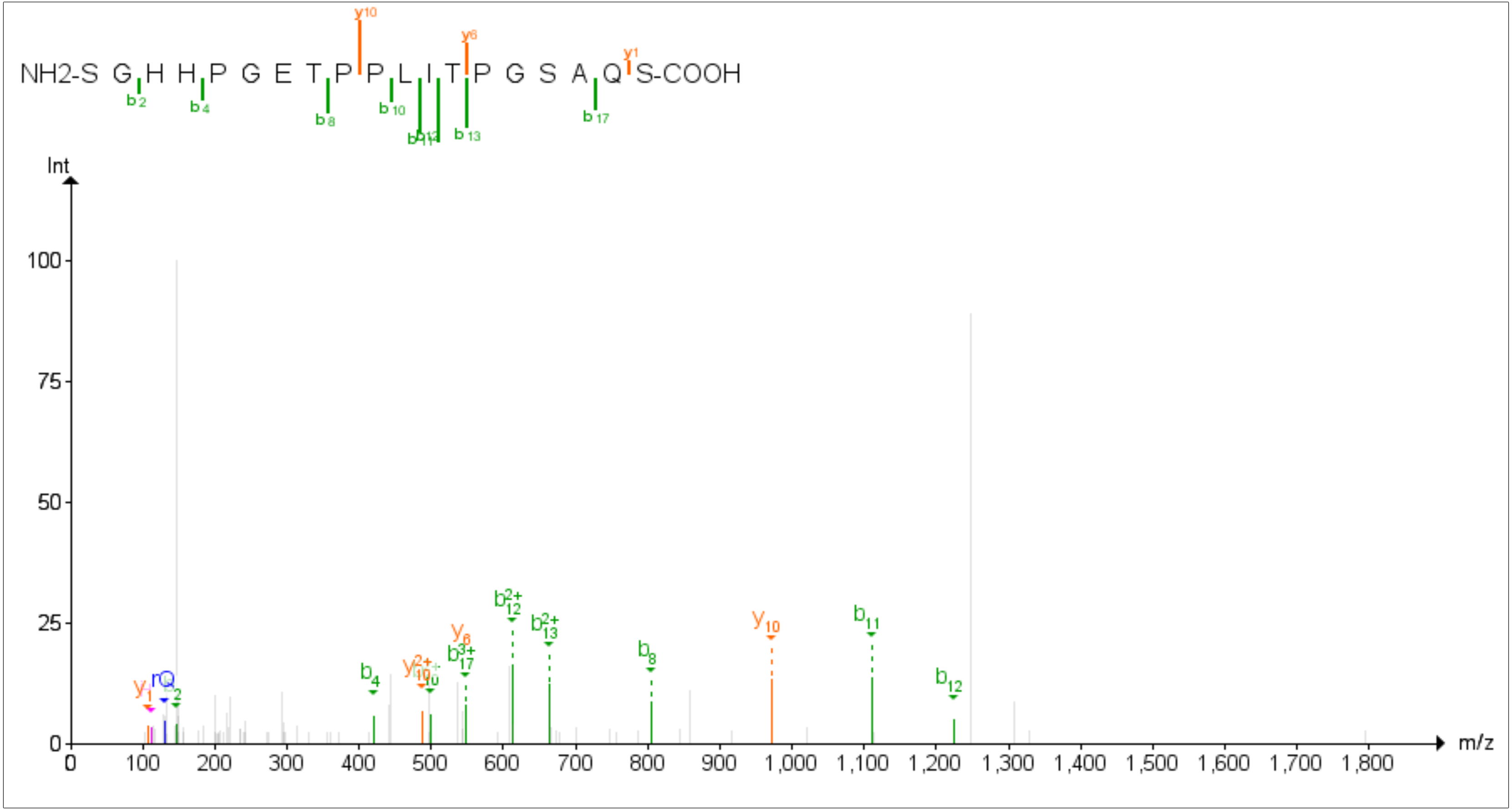

NH2-S G H H P G E T P P L I T P G S A Q S-COOH
y10
y6
y1
b2
b4
b8
b10
b11
b12
b13
b17
Int
100
75
50
25
0
m/z
0
100
200
300
400
500
600
700
800
900
1,000
1,100
1,200
1,300
1,400
1,500
1,600
1,700
1,800
y1
rQ
b2
b4
y_{10}^{2+}
b10
y6
b_{17}^{3+}
b_{12}^{2+}
b_{13}^{2+}
b8
y10
b11
b12

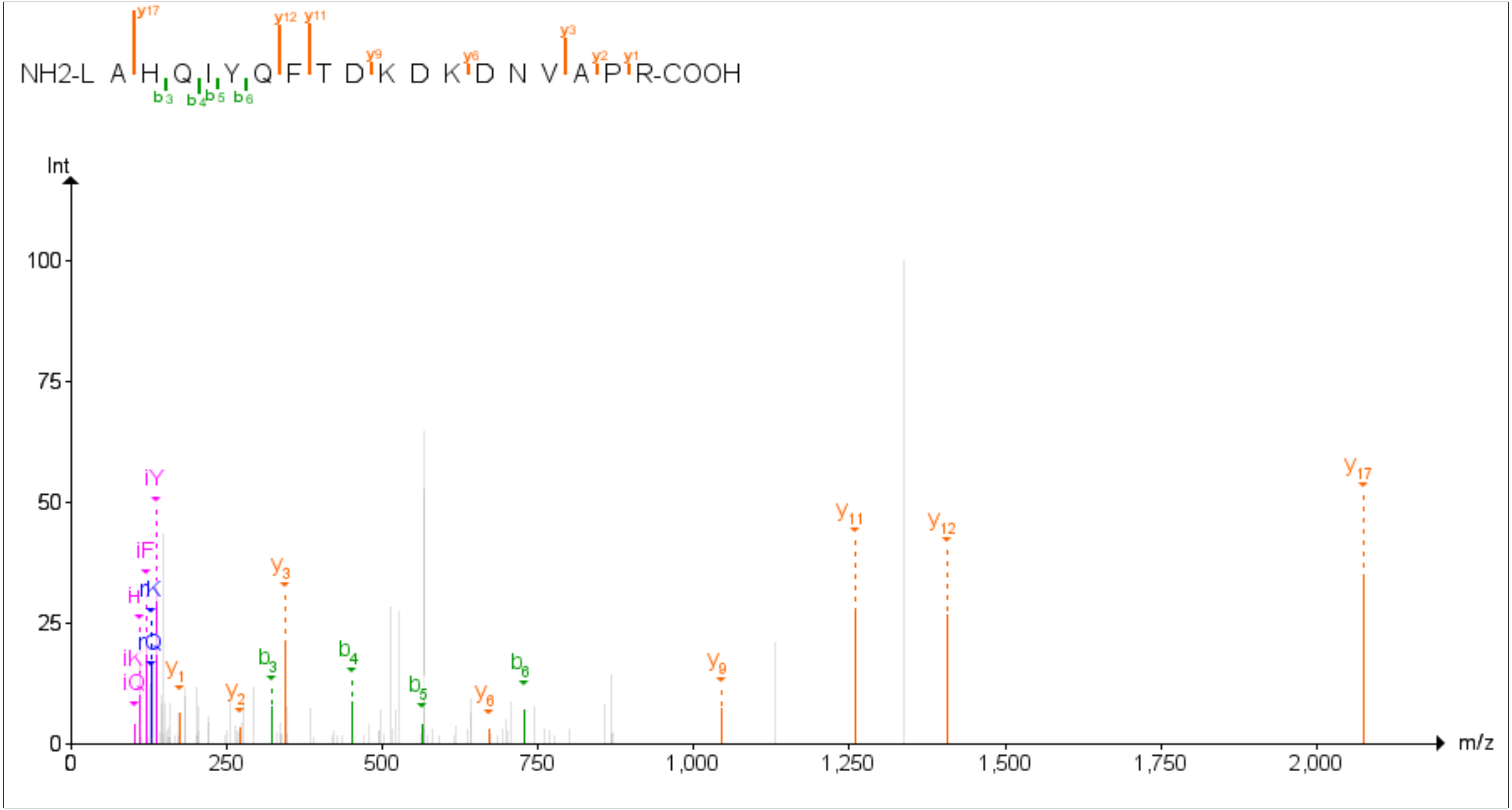
NH2-L A H Q I Y Q F T D K D K D N V A P R-COOH
y17
y12
y11
y9
y6
y3
y2
y1
b3
b4
b5
b6
Int
100
75
50
25
0
iY
iF
iH
rK
rQ
iK
iQ
y1
y2
y3
b3
b4
b5
y6
b6
y9
y11
y12
y17
0
250
500
750
1,000
1,250
1,500
1,750
2,000
m/z

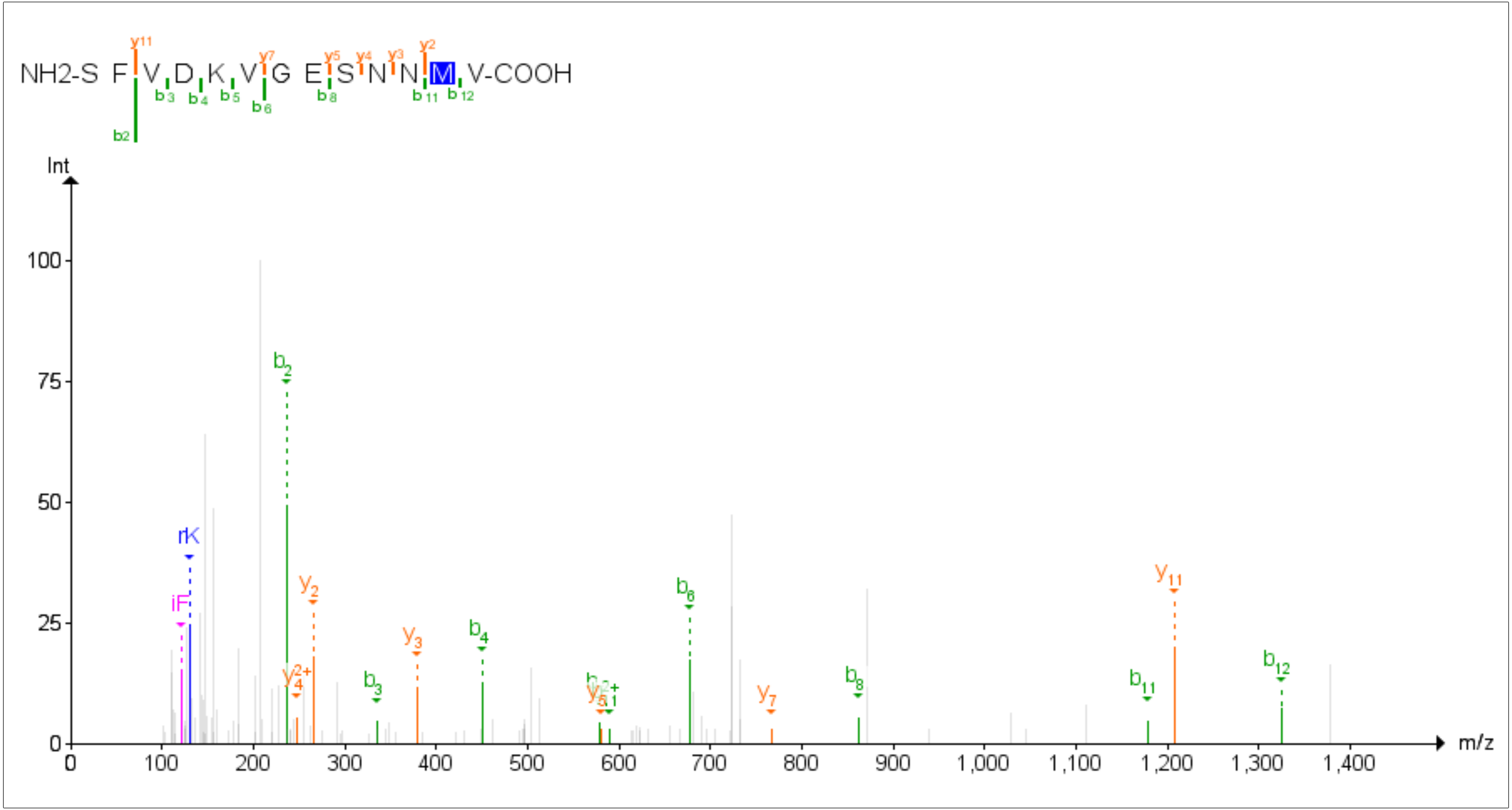

NH2-S F V D K V G E S N N M V-COOH
y11 y7 y5 y4 y3 y2
b2 b3 b4 b5 b6 b8 b11 b12
Int
100
75
50
25
0
rK
iF
b2
y2
y3
b4
b3
b6
y7
b8
b11
y11
b12
m/z
0 100 200 300 400 500 600 700 800 900 1,000 1,100 1,200 1,300 1,400

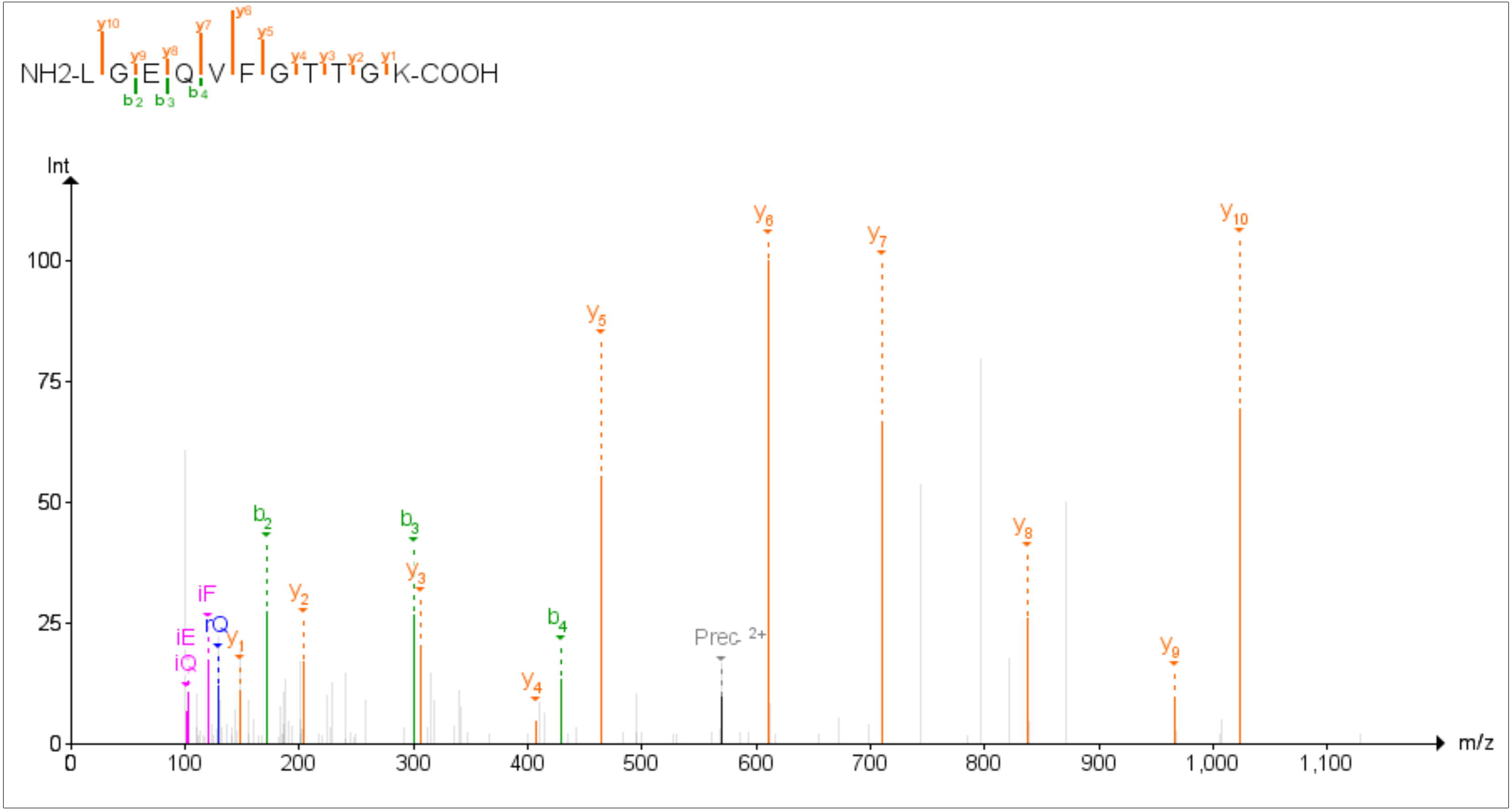

NH2-L G E Q V F G T T G K-COOH
y10 y9 y8 y7 y6 y5 y4 y3 y2 y1
b2 b3 b4
Int
m/z
0
25
50
75
100
0
100
200
300
400
500
600
700
800
900
1,000
1,100
y1
y2
y3
y4
y5
y6
y7
y8
y9
y10
b2
b3
b4
iE
iQ
iF
rQ
Prec 2+

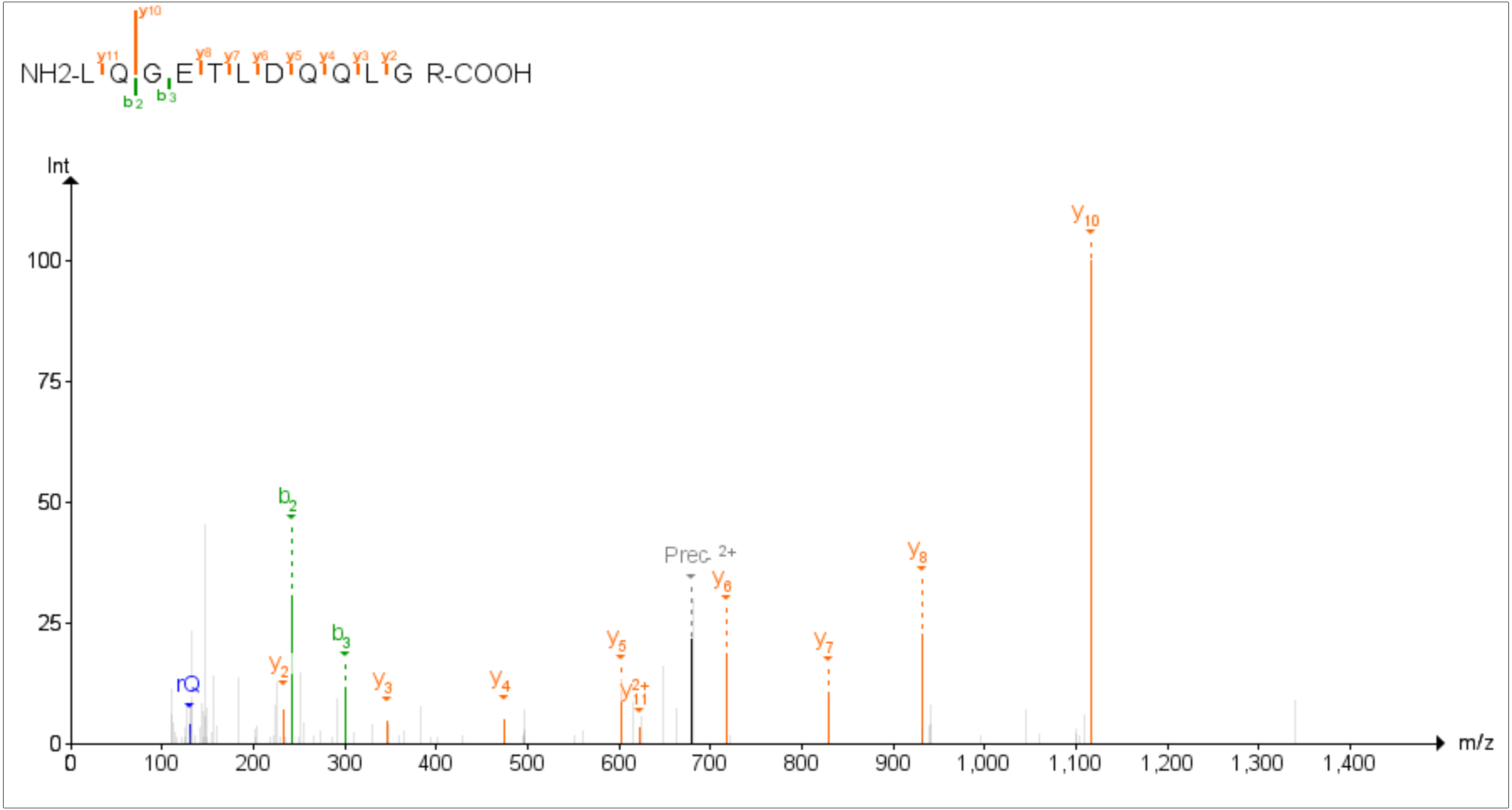

NH2-L Q G E T L D Q Q L G R-COOH
y10
y11
y8
y7
y6
y5
y4
y3
y2
b2
b3
Int
m/z
Prec 2+
rQ
0
25
50
75
100
0
100
200
300
400
500
600
700
800
900
1,000
1,100
1,200
1,300
1,400

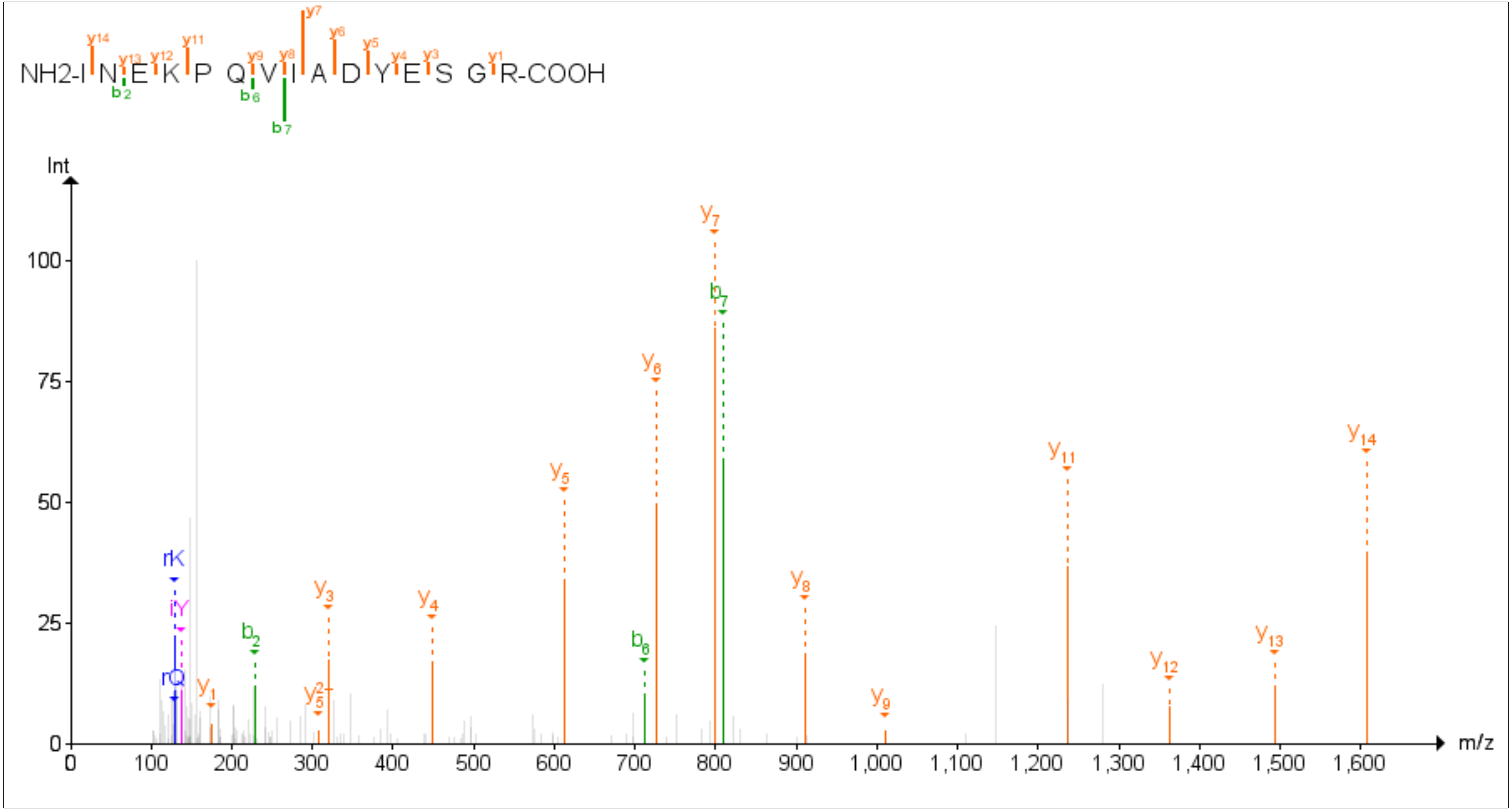

NH2-I N E K P Q V I A D Y E S G R-COOH
y14 y13 y12 y11 y9 y8 y7 y6 y5 y4 y3 y1
b2 b6 b7
Int
100
75
50
25
0
m/z
0 100 200 300 400 500 600 700 800 900 1,000 1,100 1,200 1,300 1,400 1,500 1,600
rK
iY
rQ
y1
b2
y3
y5 2+
y4
y5
b6
y6
y7
b7
y8
y9
y11
y12
y13
y14

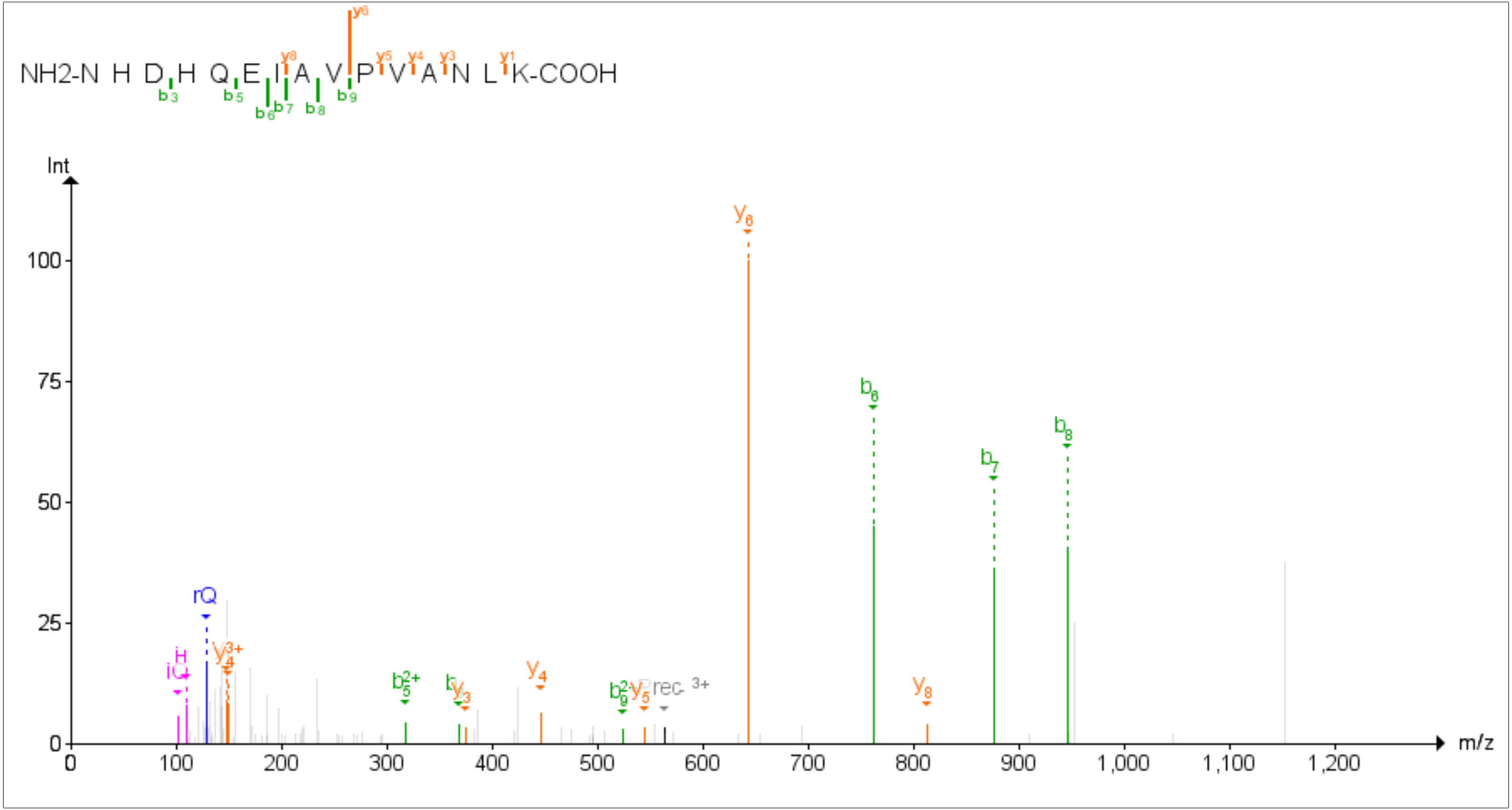
NH2-N H D H Q E I A V P V A N L K-COOH
Int
m/z
y6
b6
b7
b8
rQ
y8
y4
y5
y3
Prec

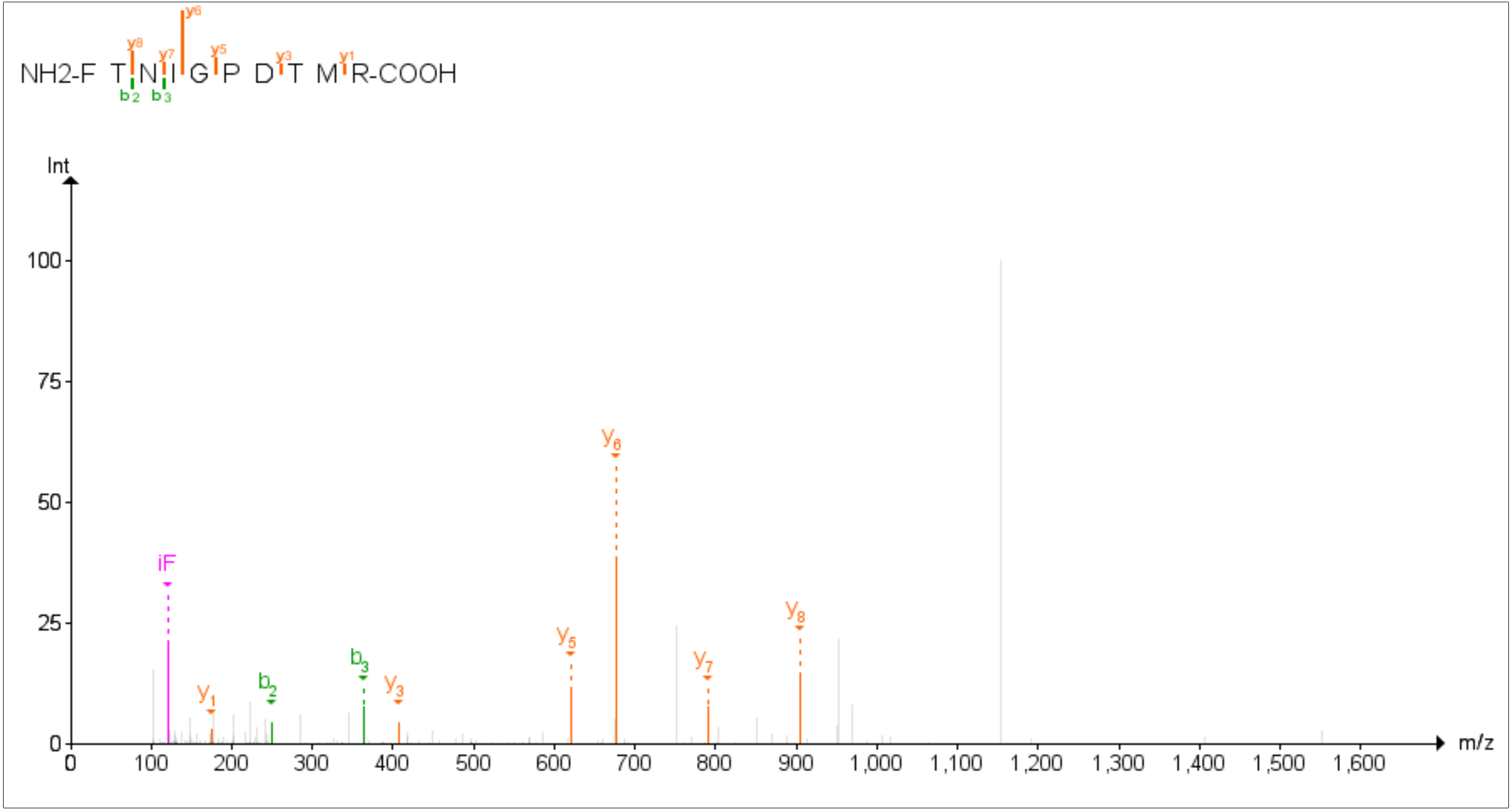
NH2-F T N I G P D T M R-COOH
y8
y7
y6
y5
y3
y1
b2
b3
Int
100
75
50
25
0
iF
y1
b2
b3
y3
y5
y6
y7
y8
0
100
200
300
400
500
600
700
800
900
1,000
1,100
1,200
1,300
1,400
1,500
1,600
m/z

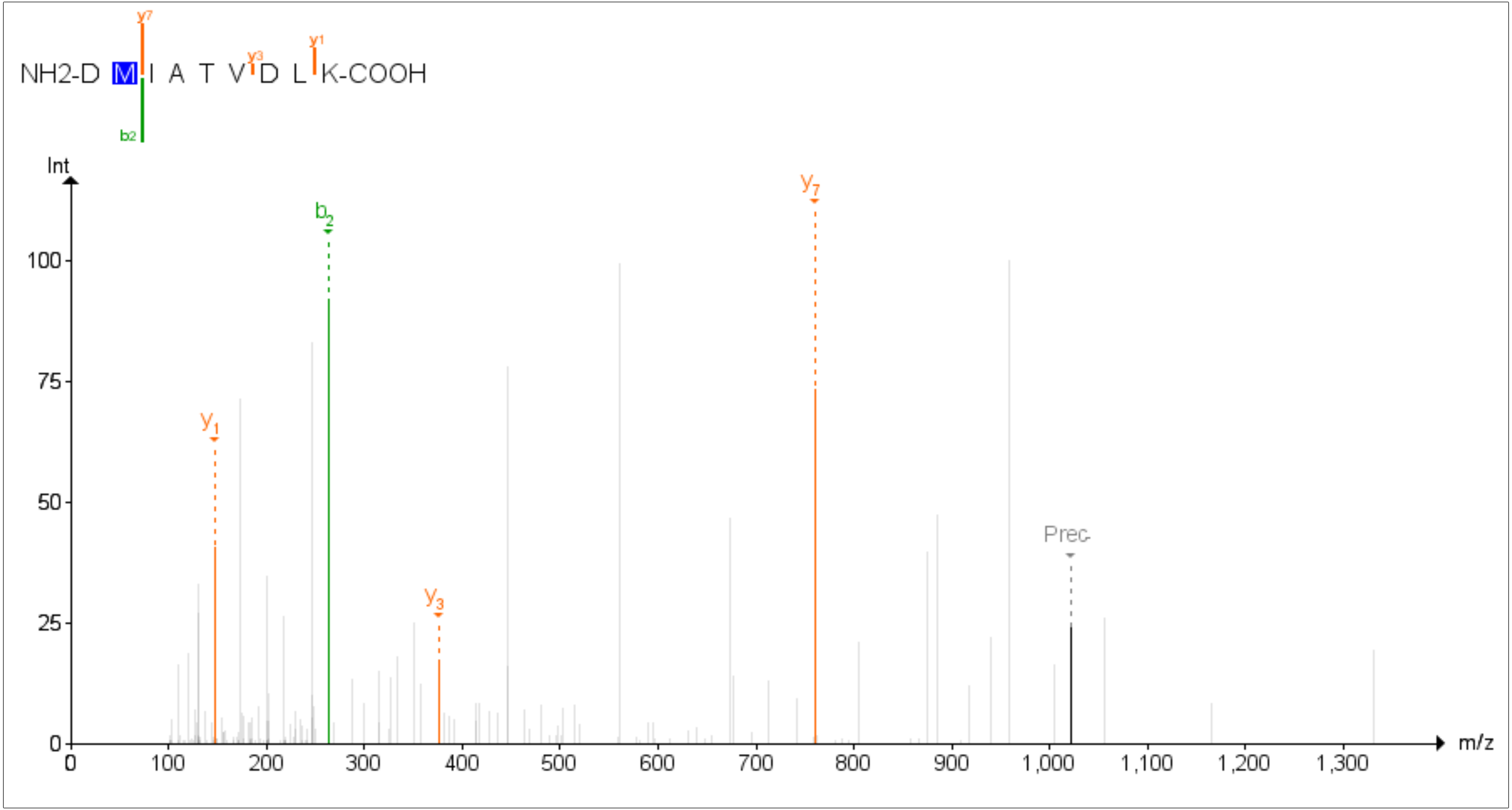

NH2-D M I A T V D L K-COOH
y7
y3
y1
b2
Int
100
75
50
25
0
y7
b2
y1
y3
Prec
m/z
0
100
200
300
400
500
600
700
800
900
1,000
1,100
1,200
1,300

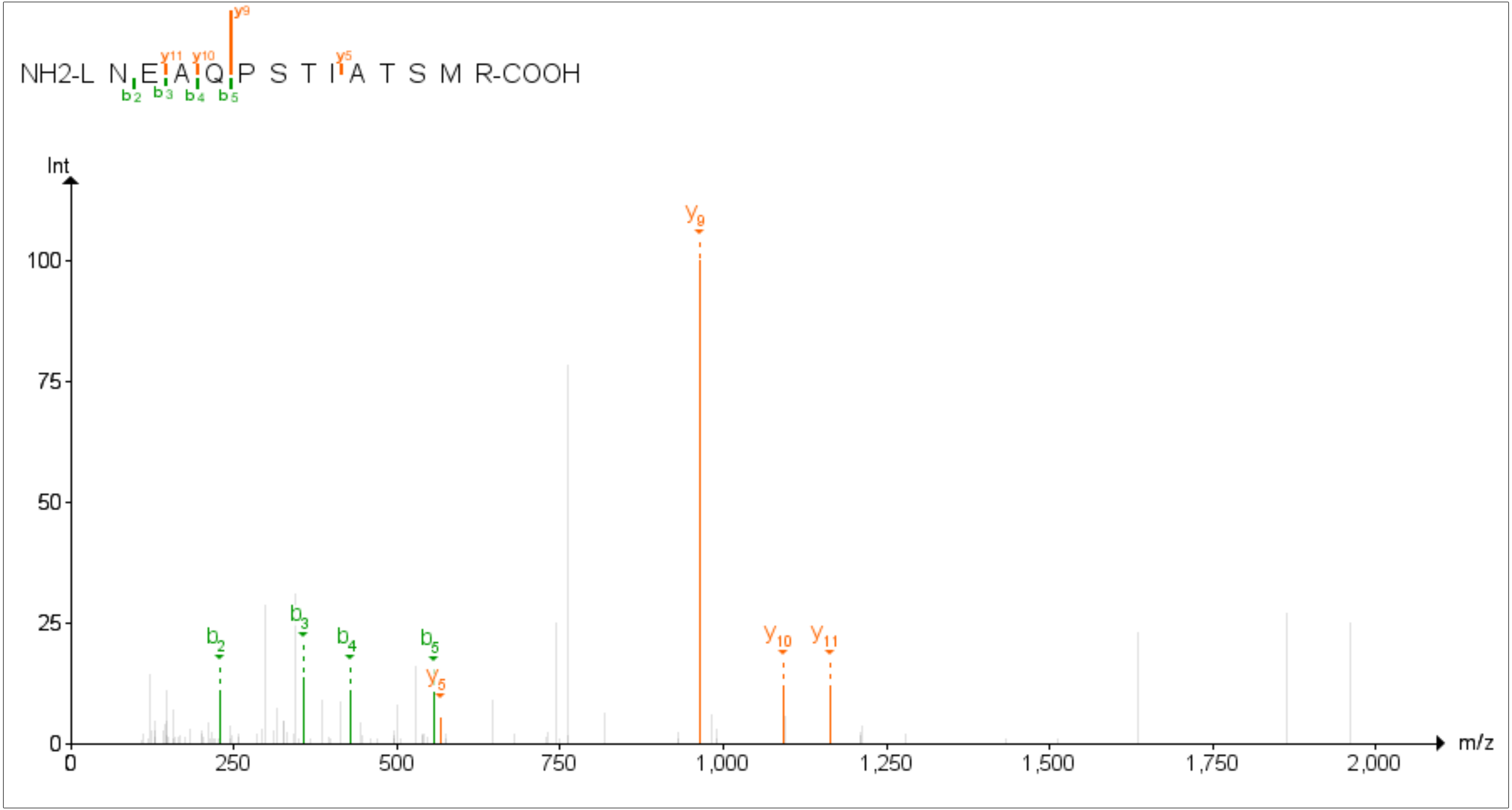

NH2-L N E A Q P S T I A T S M R-COOH
y9
y11
y10
y5
b2
b3
b4
b5
Int
100
75
50
25
0
y_9
b_2
b_3
b_4
b_5
y_5
y_{10}
y_{11}
0
250
500
750
1,000
1,250
1,500
1,750
2,000
m/z

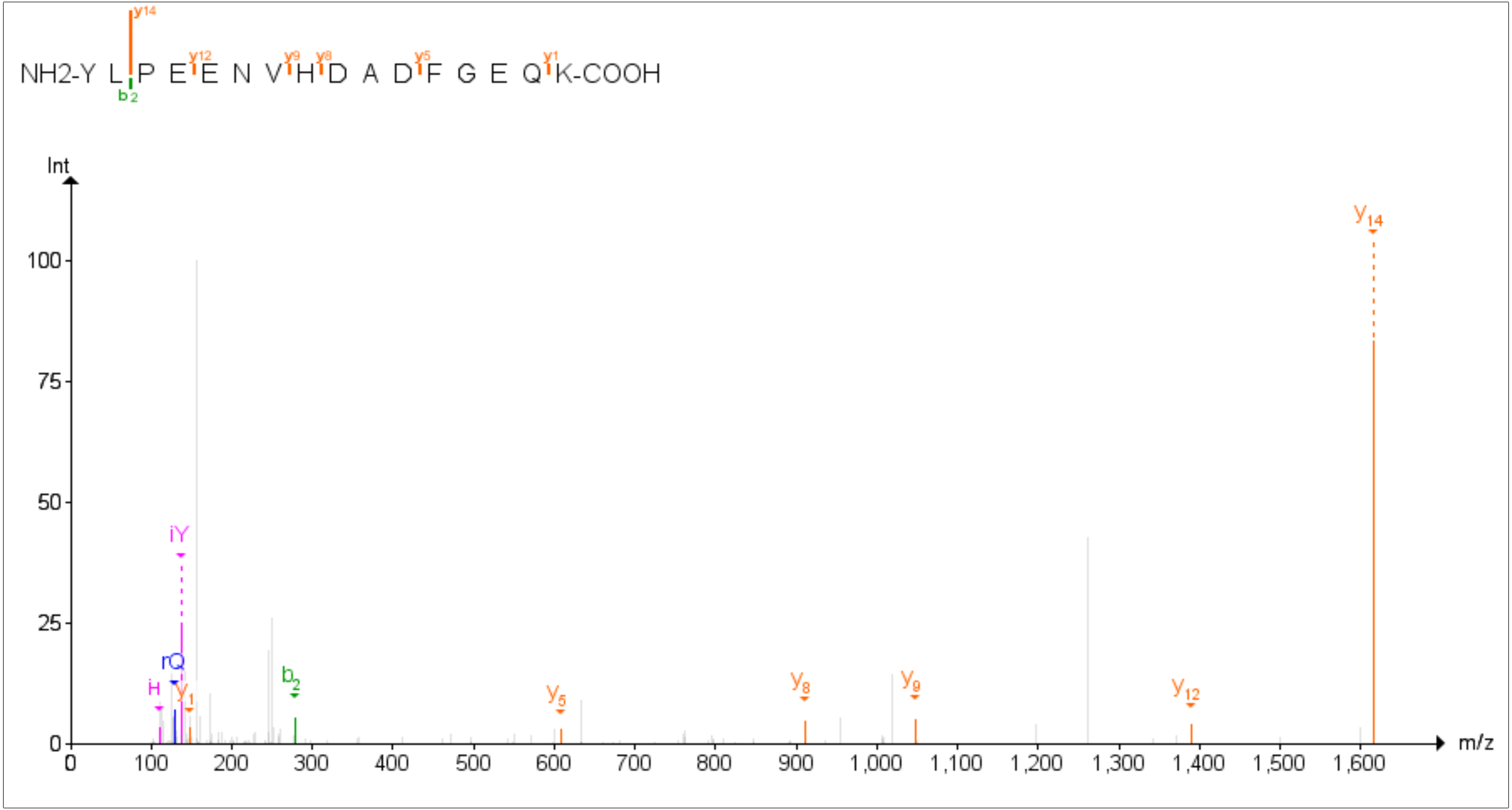

NH2-Y L P E E N V H D A D F G E Q K-COOH
y14
y12
y9
y8
y5
y1
b2
Int
m/z
iY
rQ
iH

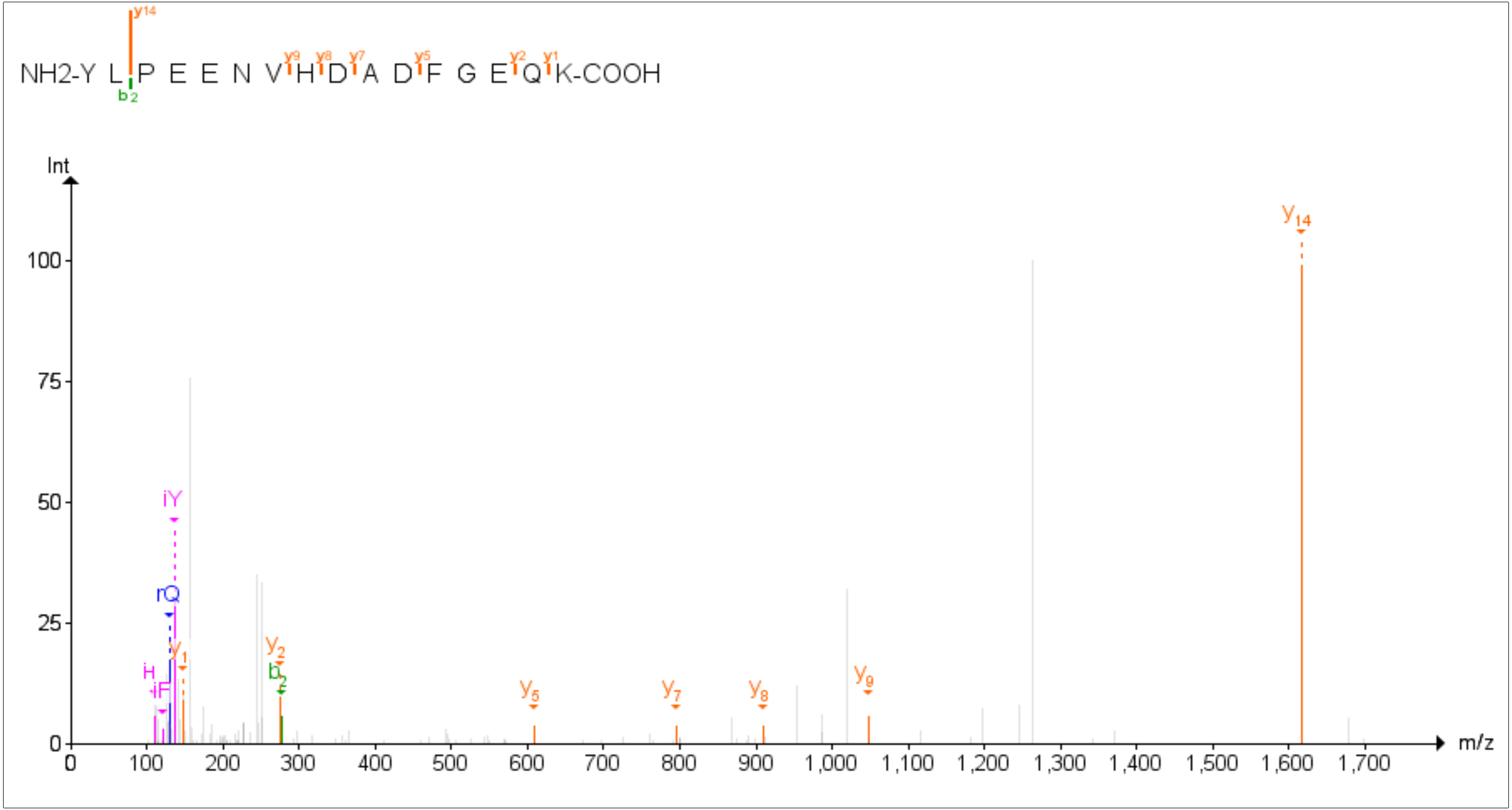

NH2-Y L P E E N V H D A D F G E Q K-COOH
y14
y9
y8
y7
y5
y2
y1
b2
Int
100
75
50
25
0
iY
rQ
y1
iH
iF
y2
b2
y5
y7
y8
y9
y14
0
100
200
300
400
500
600
700
800
900
1,000
1,100
1,200
1,300
1,400
1,500
1,600
1,700
m/z

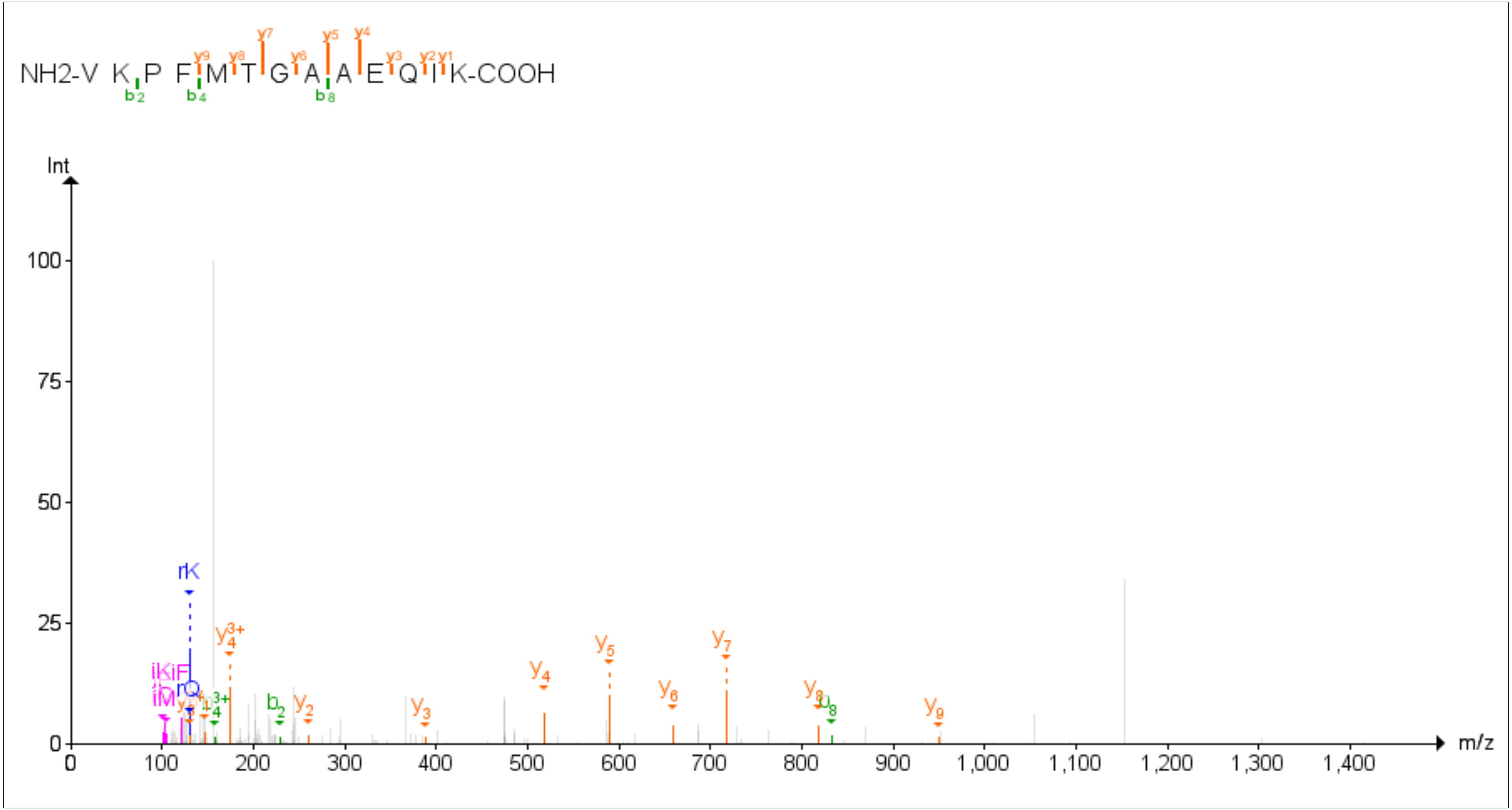

NH2-V K P F M T G A A E Q I K-COOH
b2
b4
b8
y9
y8
y7
y6
y5
y4
y3
y2
y1
Int
100
75
50
25
0
rK
rQ
iKiF
iM
y4 3+
b2
y2
y3
y4
y5
y6
y7
y8
b8
y9
m/z
0
100
200
300
400
500
600
700
800
900
1,000
1,100
1,200
1,300
1,400

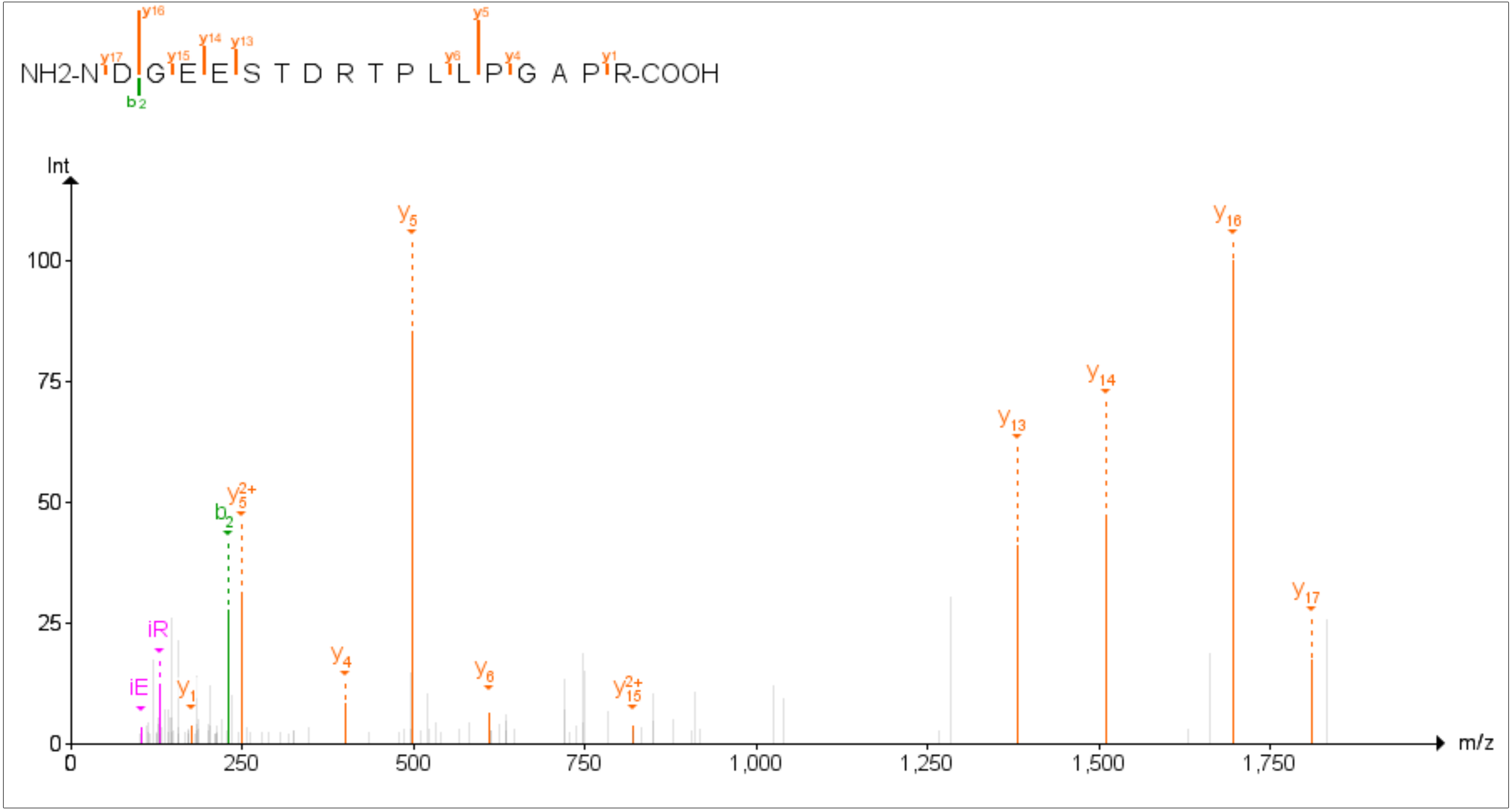
NH2-N D G E E S T D R T P L L P G A P R-COOH
y17 y16 y15 y14 y13 y6 y5 y4 y1
b2
Int
100
75
50
25
0
y5
y16
y14
y13
y5 2+
b2
iR
iE
y1
y4
y6
y15 2+
y17
0
250
500
750
1,000
1,250
1,500
1,750
m/z

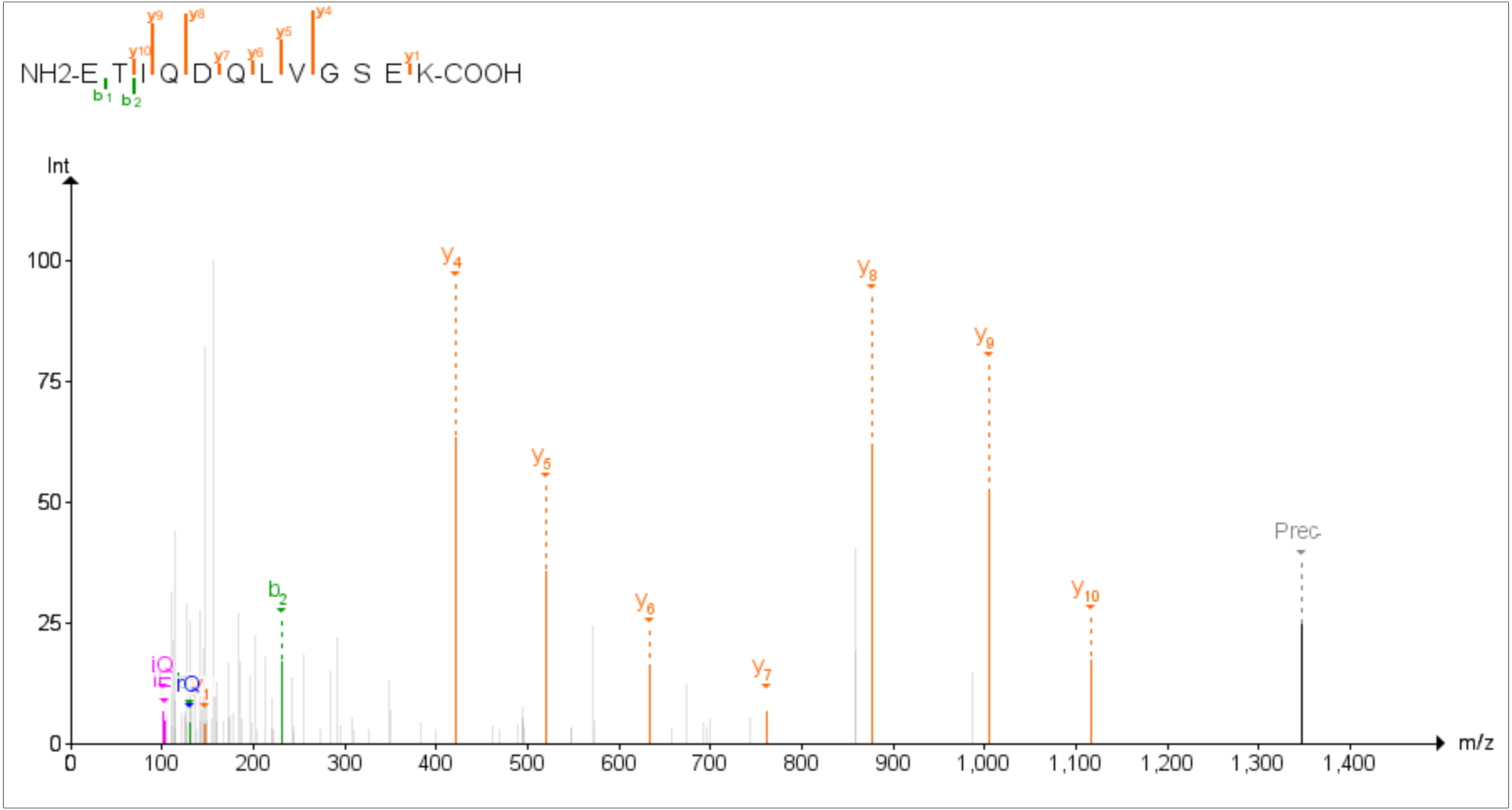

NH2-E T I Q D Q L V G S E K-COOH
b1
b2
y10
y9
y8
y7
y6
y5
y4
y1
Int
100
75
50
25
0
y4
y8
y9
y5
y6
y7
y10
b2
Prec
iQ
iE
rQ
0
100
200
300
400
500
600
700
800
900
1,000
1,100
1,200
1,300
1,400
m/z

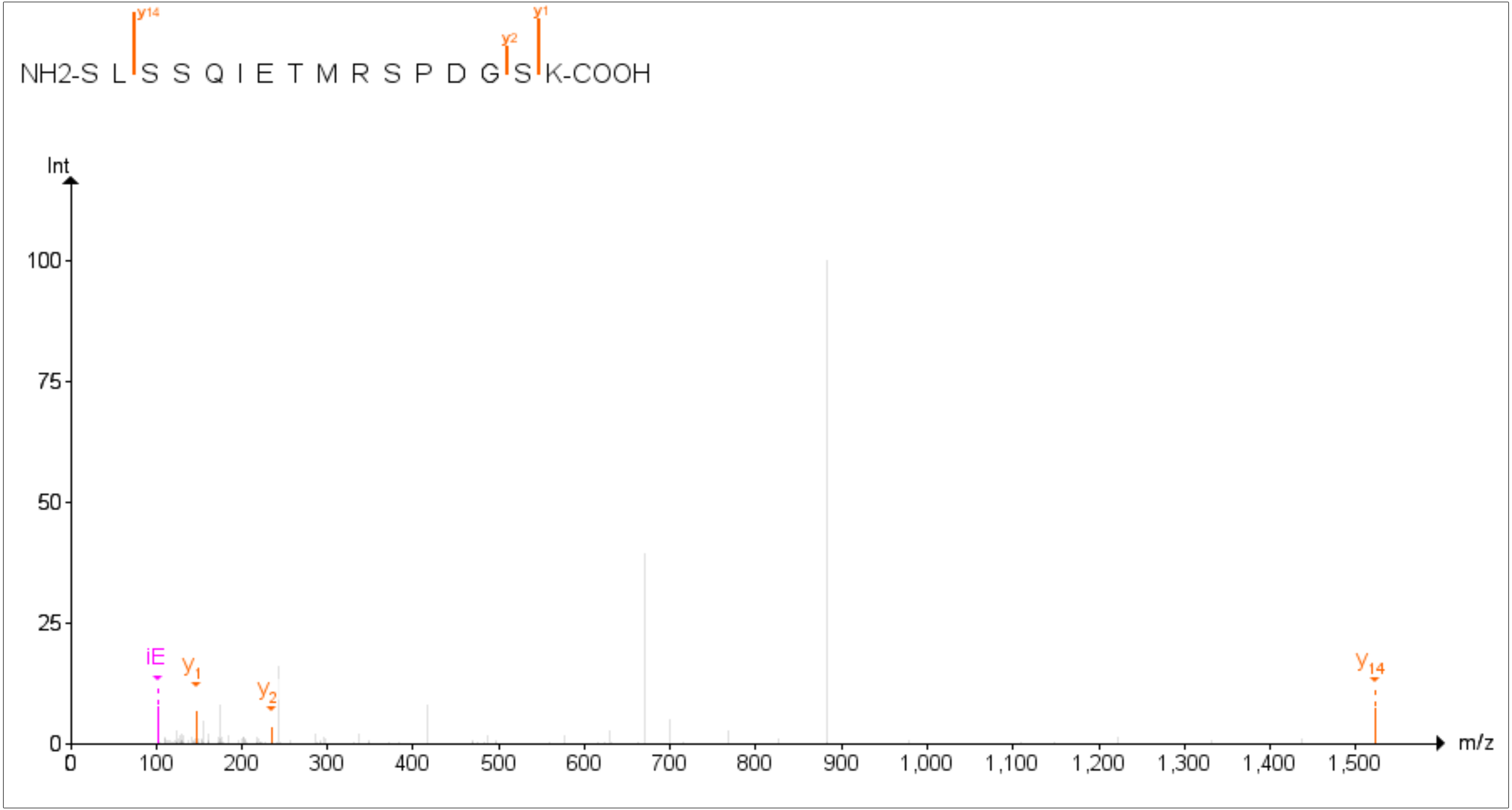

NH2-S L S S Q I E T M R S P D G S K-COOH
y14
y2
y1
Int
100
75
50
25
0
iE
y1
y2
y14
m/z
0
100
200
300
400
500
600
700
800
900
1,000
1,100
1,200
1,300
1,400
1,500

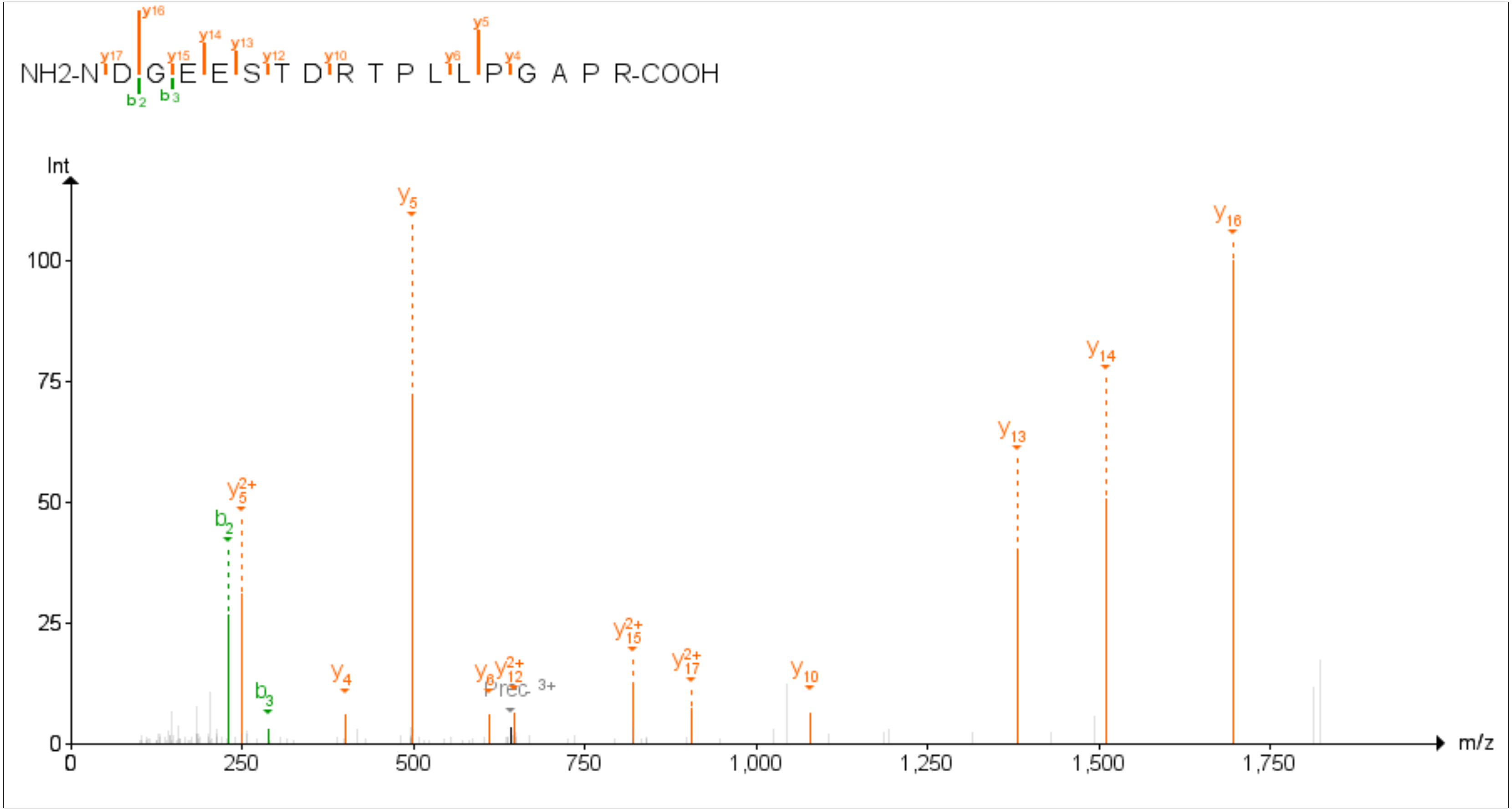

NH2-N D G E E S T D R T P L L P G A P R-COOH
y17 y16 y15 y14 y13 y12 y10 y6 y5 y4
b2 b3
Int
100
75
50
25
0
250
500
750
1,000
1,250
1,500
1,750
m/z
y5
y16
y14
y13
y5 2+
b2
b3
y4
y6
y12 2+
Prec. 3+
y15 2+
y17 2+
y10

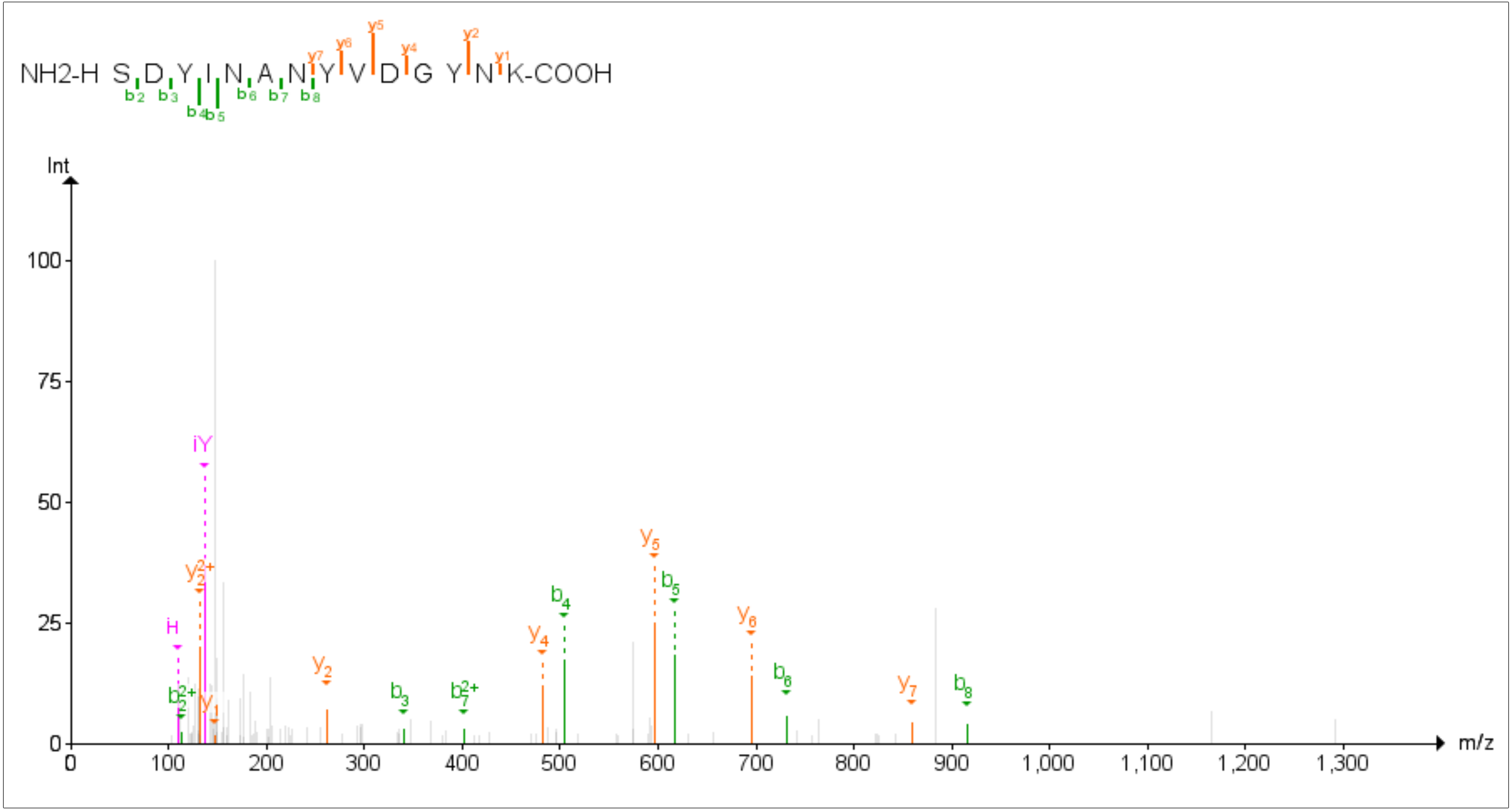

NH2-H S D Y I N A N Y V D G Y N K-COOH
b2 b3 b4 b5 b6 b7 b8
y7 y6 y5 y4 y2 y1
Int
100
75
50
25
0
iY
iH
y2 2+
b2 2+
y1
y2
b3
b7 2+
y4
b4
y5
b5
y6
b6
y7
b8
0 100 200 300 400 500 600 700 800 900 1,000 1,100 1,200 1,300
m/z

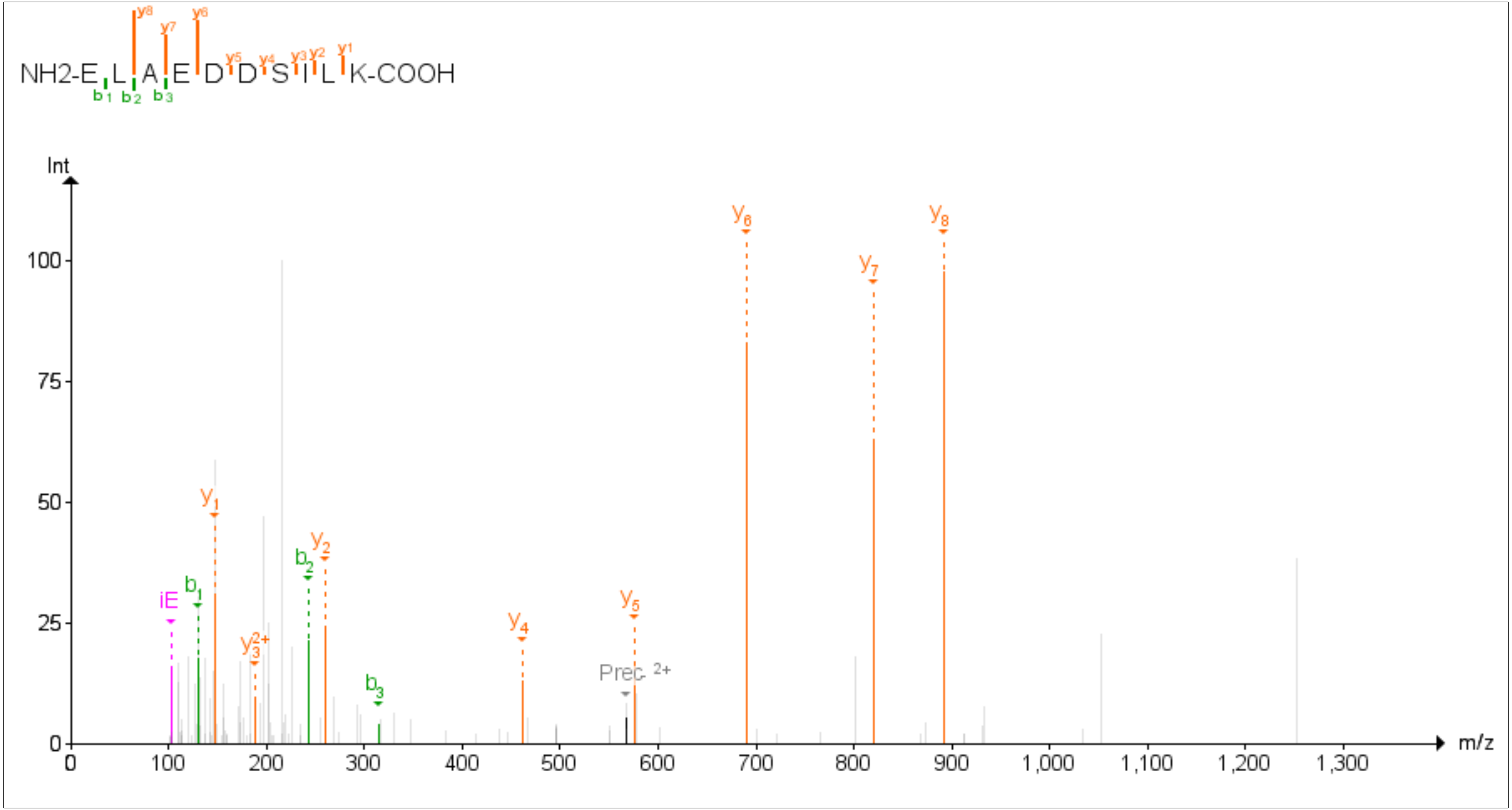

NH2-E L A E D D S I L K-COOH
y8 y7 y6 y5 y4 y3 y2 y1
b1 b2 b3
Int
100
75
50
25
0
iE
b1
y1
y_3^{2+}
b2
y2
b3
y4
Prec 2+
y5
y6
y7
y8
0 100 200 300 400 500 600 700 800 900 1,000 1,100 1,200 1,300
m/z

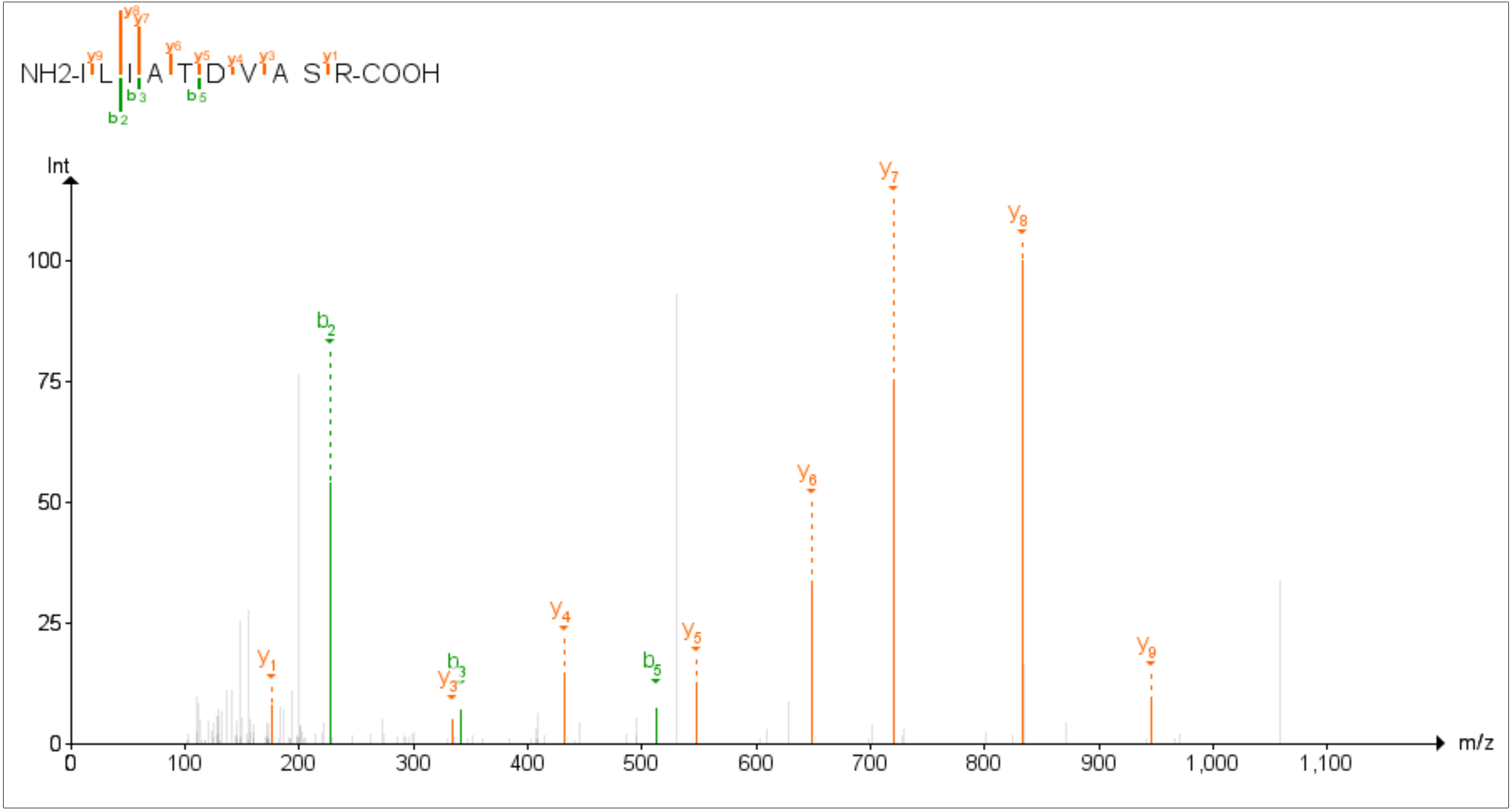

NH2-I L I A T D V A S R-COOH
y9 y8 y7 y6 y5 y4 y3 y1
b2 b3 b5
Int
100
75
50
25
0
m/z
0 100 200 300 400 500 600 700 800 900 1,000 1,100
b2 y1 y3 b3 y4 b5 y5 y6 y7 y8 y9

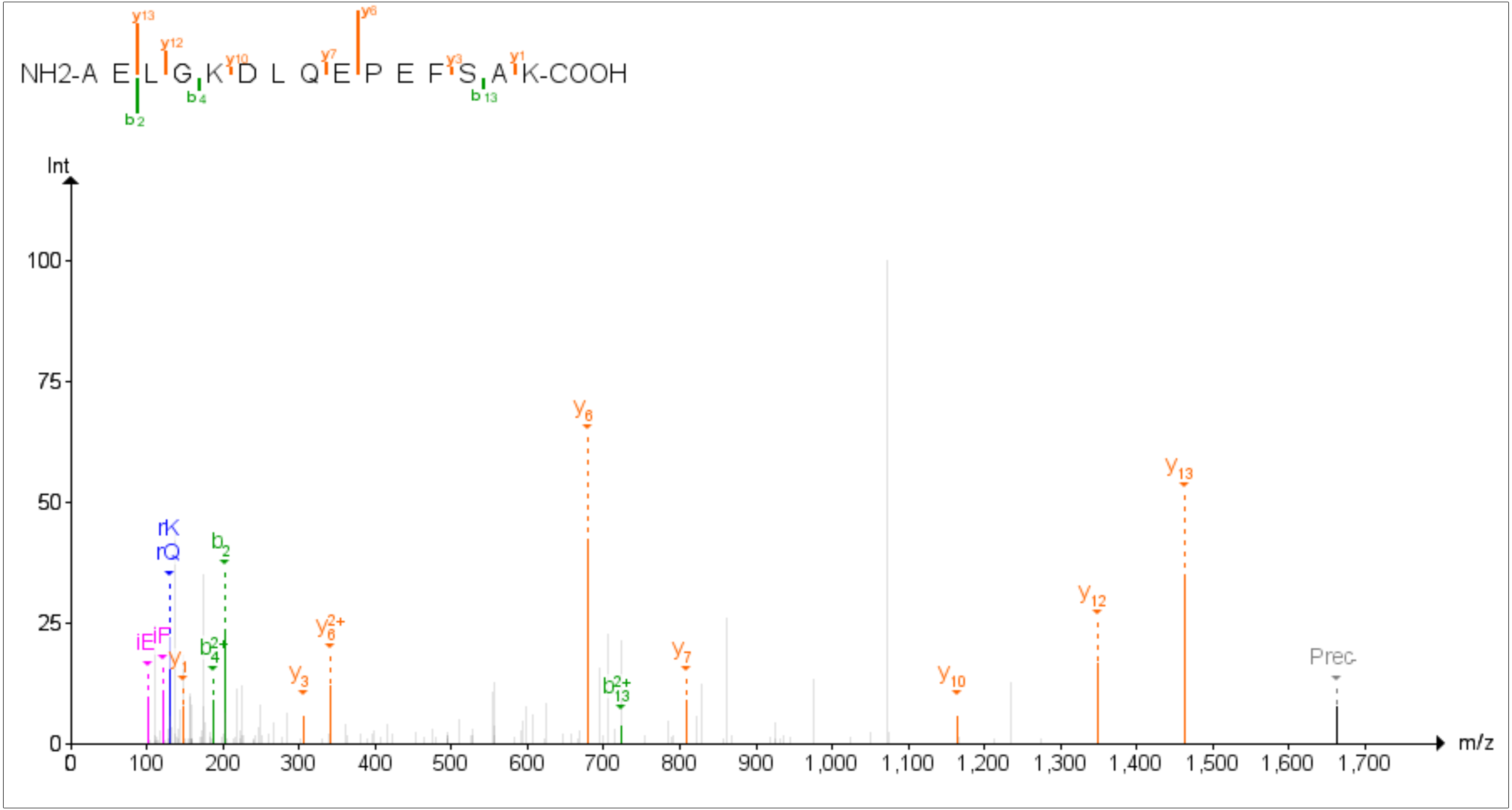

NH2-A E L G K D L Q E P E F S A K-COOH
y13
y12
y10
y7
y6
y3
y1
b2
b4
b13
Int
100
75
50
25
0
rK
rQ
iE
iP
y1
b2
b4 2+
y3
y6 2+
y6
b13 2+
y7
y10
y12
y13
Prec
0
100
200
300
400
500
600
700
800
900
1,000
1,100
1,200
1,300
1,400
1,500
1,600
1,700
m/z

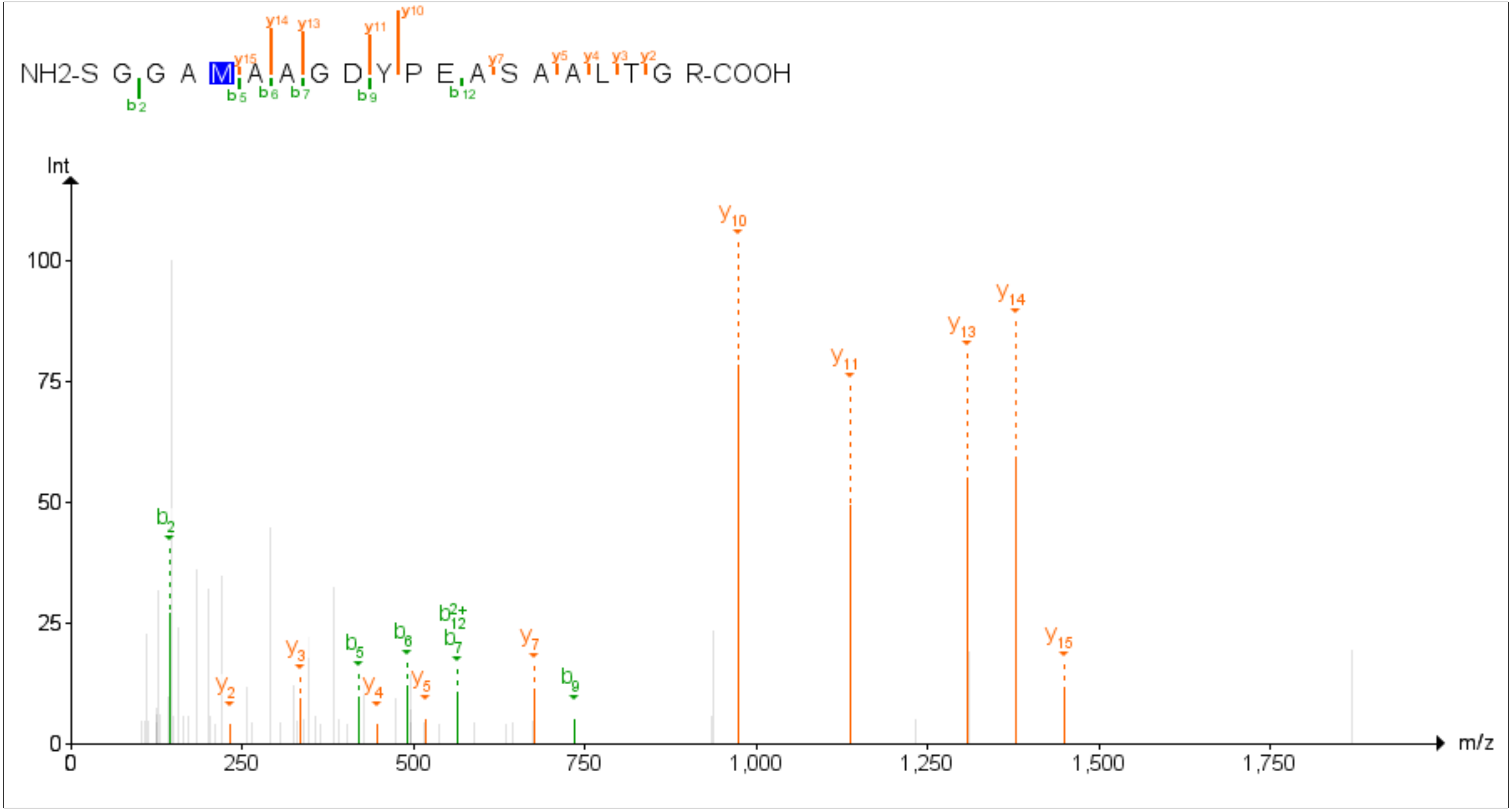

NH2-S G G A M A A G D Y P E A S A A L T G R-COOH
y14 y13 y11 y10 y15 y7 y5 y4 y3 y2
b2 b5 b6 b7 b9 b12
Int
m/z
0
25
50
75
100
250
500
750
1,000
1,250
1,500
1,750
y10
y11
y13
y14
y15
b2
y2
y3
b5
y4
b6
y5
b12 2+
b7
y7
b9

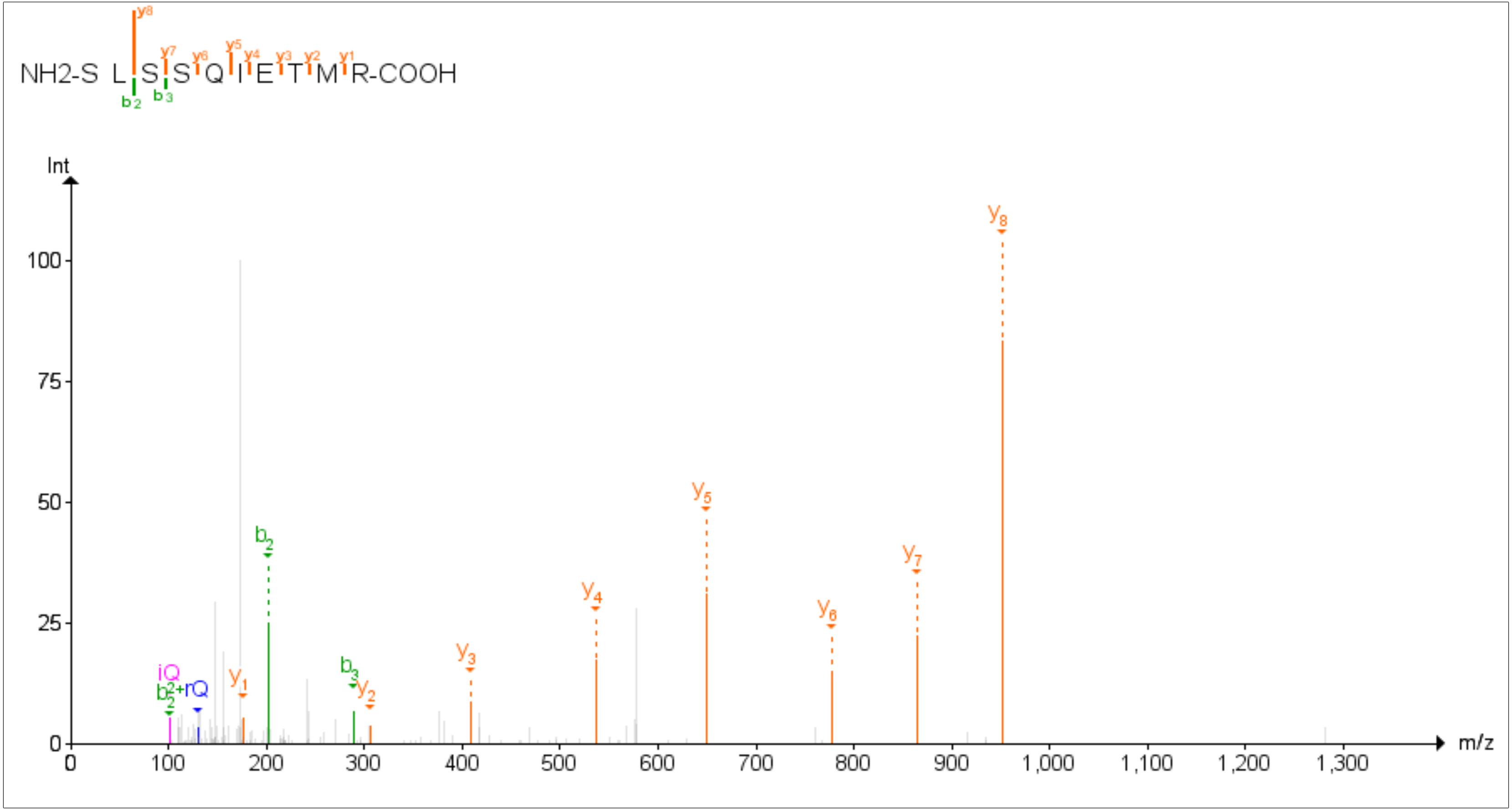

NH2-S L S S Q I E T M R-COOH
y8
y7
y6
y5
y4
y3
y2
y1
b2
b3
Int
100
75
50
25
0
m/z
0
100
200
300
400
500
600
700
800
900
1,000
1,100
1,200
1,300
iQ
b2 2+
rQ
y1
b2
b3
y2
y3
y4
y5
y6
y7
y8

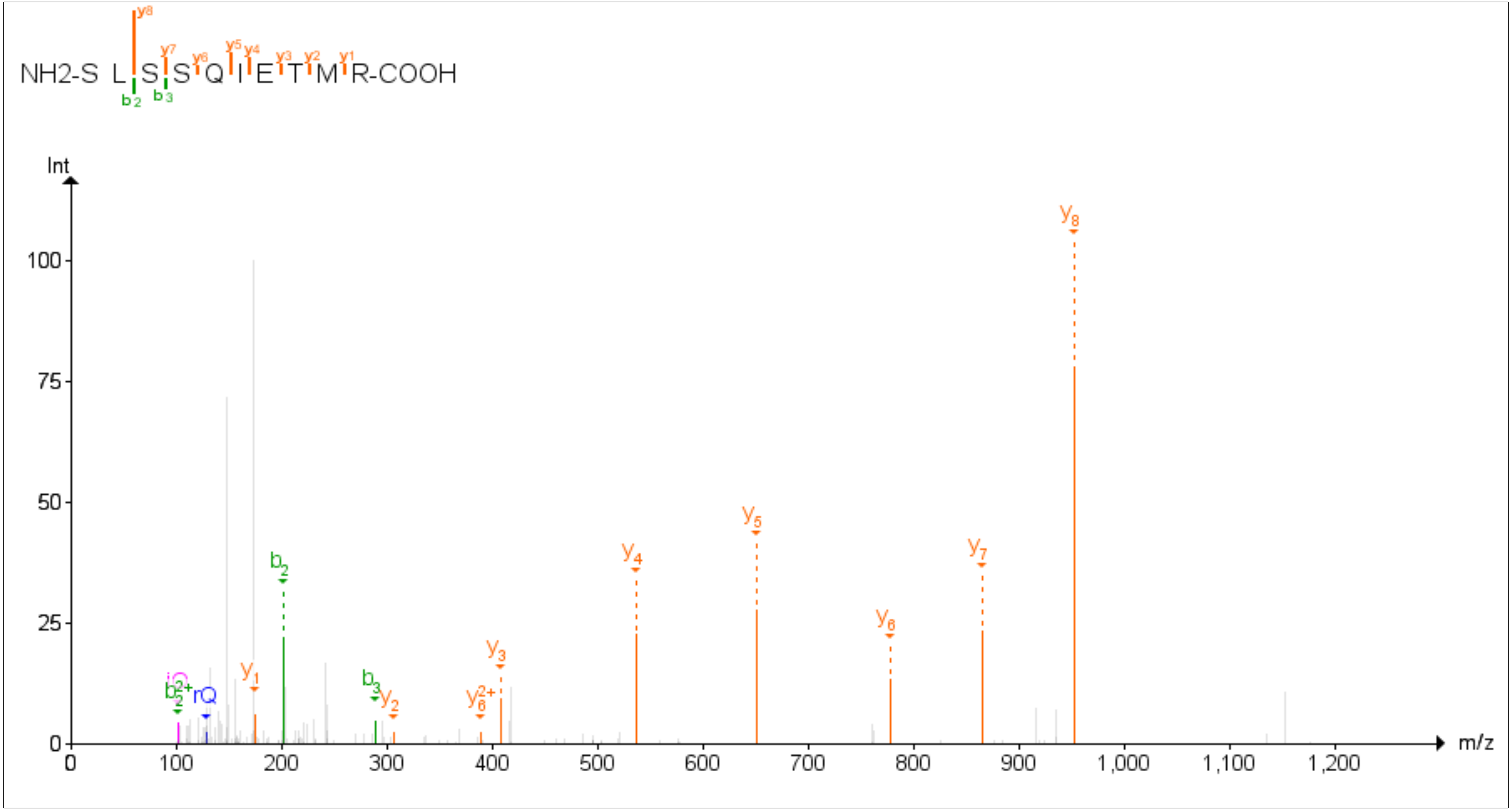

NH2-S L S S Q I E T M R-COOH
y8
y7
y6
y5
y4
y3
y2
y1
b2
b3
Int
100
75
50
25
0
m/z
0
100
200
300
400
500
600
700
800
900
1,000
1,100
1,200
y8
b2
y4
y5
y7
y6
y3
y1
b3
y2
y6 2+
b2 2+
rQ
iQ

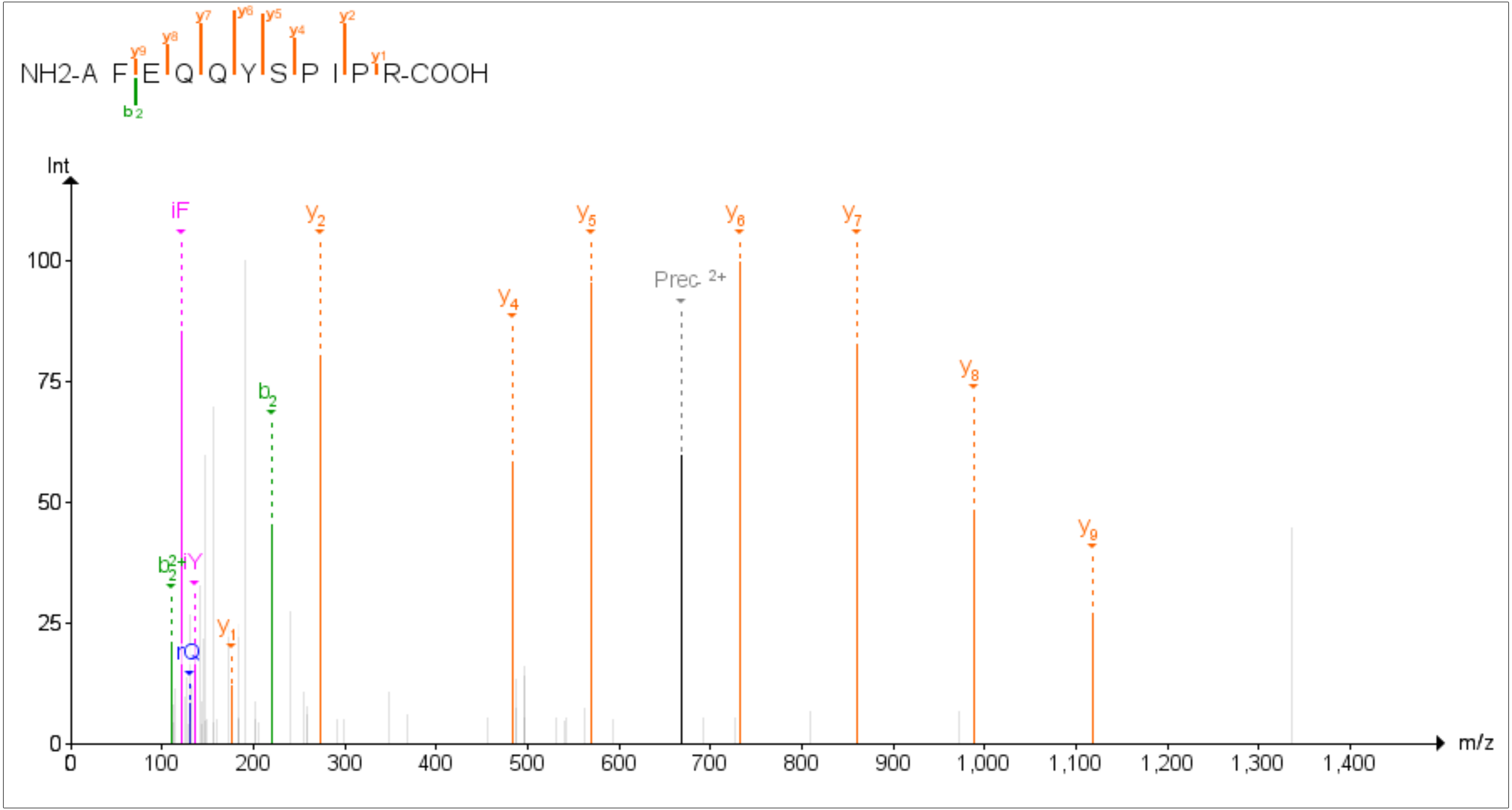

NH2-A F E Q Q Y S P I P R-COOH
y9 y8 y7 y6 y5 y4 y2 y1
b2
Int
m/z
iF
y2
y5
y6
y7
y4
Prec- 2+
b2
y8
y9
b2 2+
iY
y1
rQ

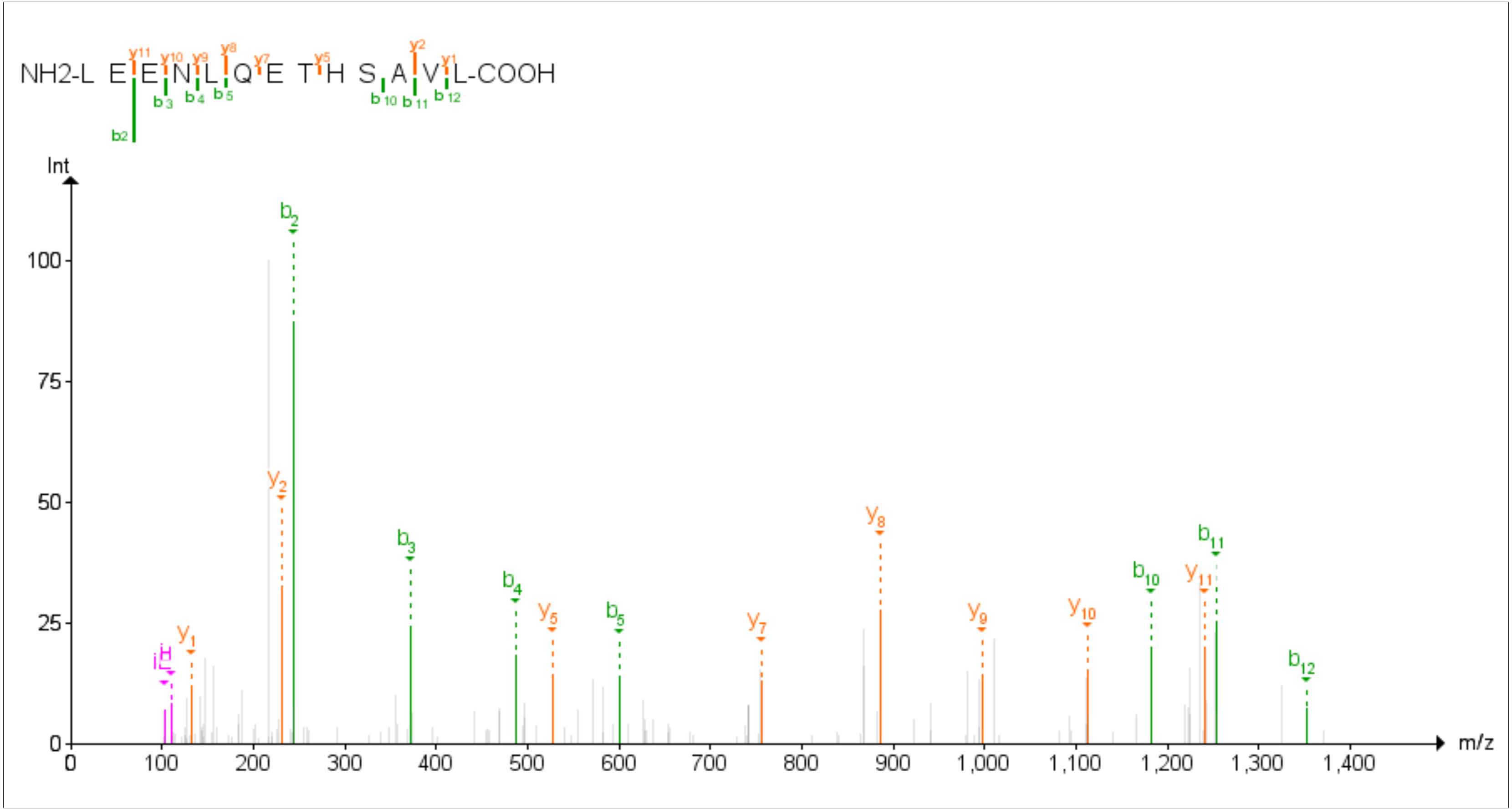

NH2-L E E N L Q E T H S A V L-COOH
y11 y10 y9 y8 y7 y5 y2 y1
b2 b3 b4 b5 b10 b11 b12
Int
m/z
0
25
50
75
100
0
100
200
300
400
500
600
700
800
900
1,000
1,100
1,200
1,300
1,400
b2
y2
b3
b4
y5
b5
y7
y8
y9
y10
b10
y11
b11
b12
y1
iL
iH

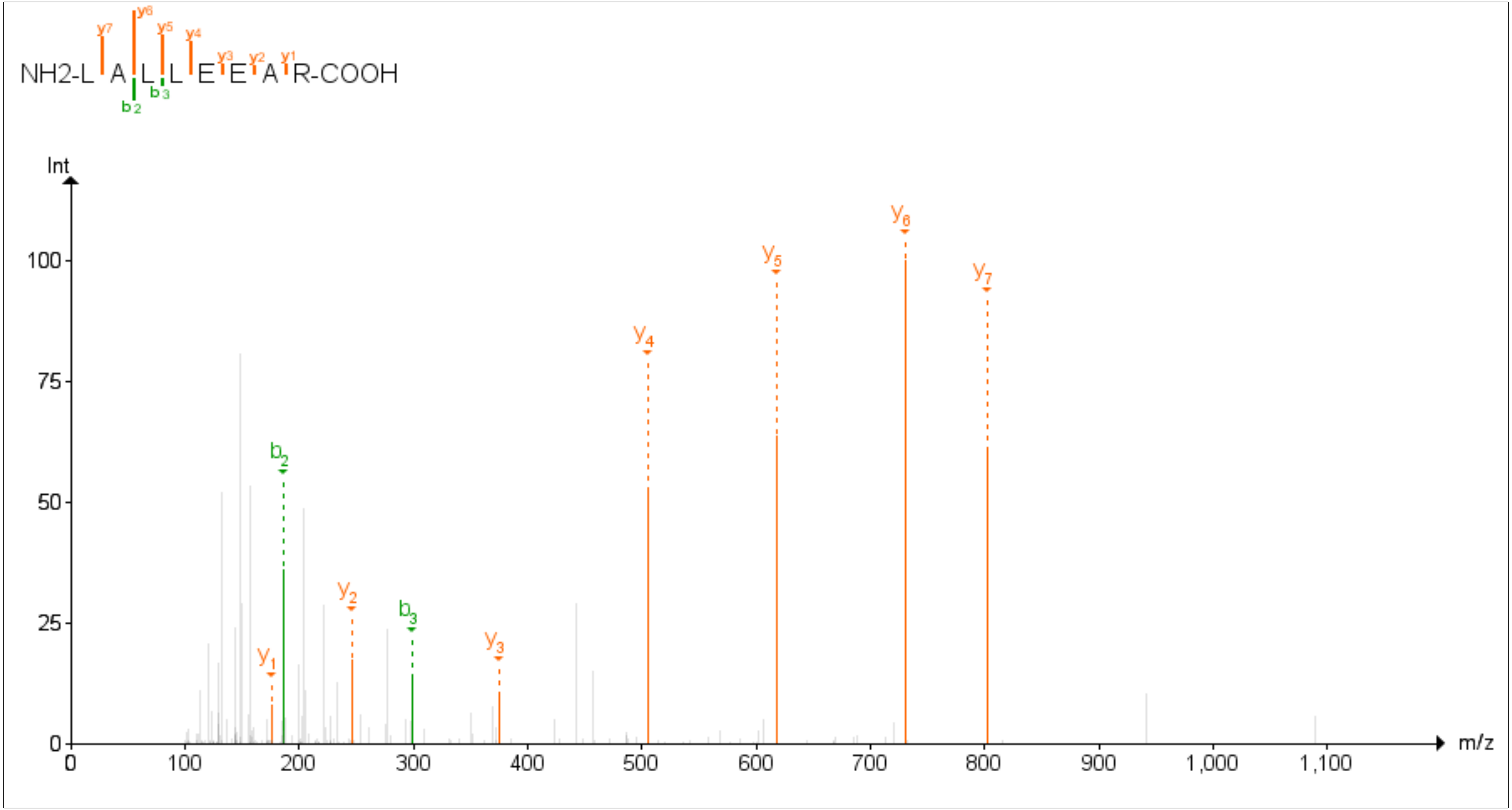
NH2-L A L L E E A R-COOH
y7 y6 y5 y4 y3 y2 y1
b2 b3
Int
m/z
0
25
50
75
100
0
100
200
300
400
500
600
700
800
900
1,000
1,100
b2
b3
y1
y2
y3
y4
y5
y6
y7

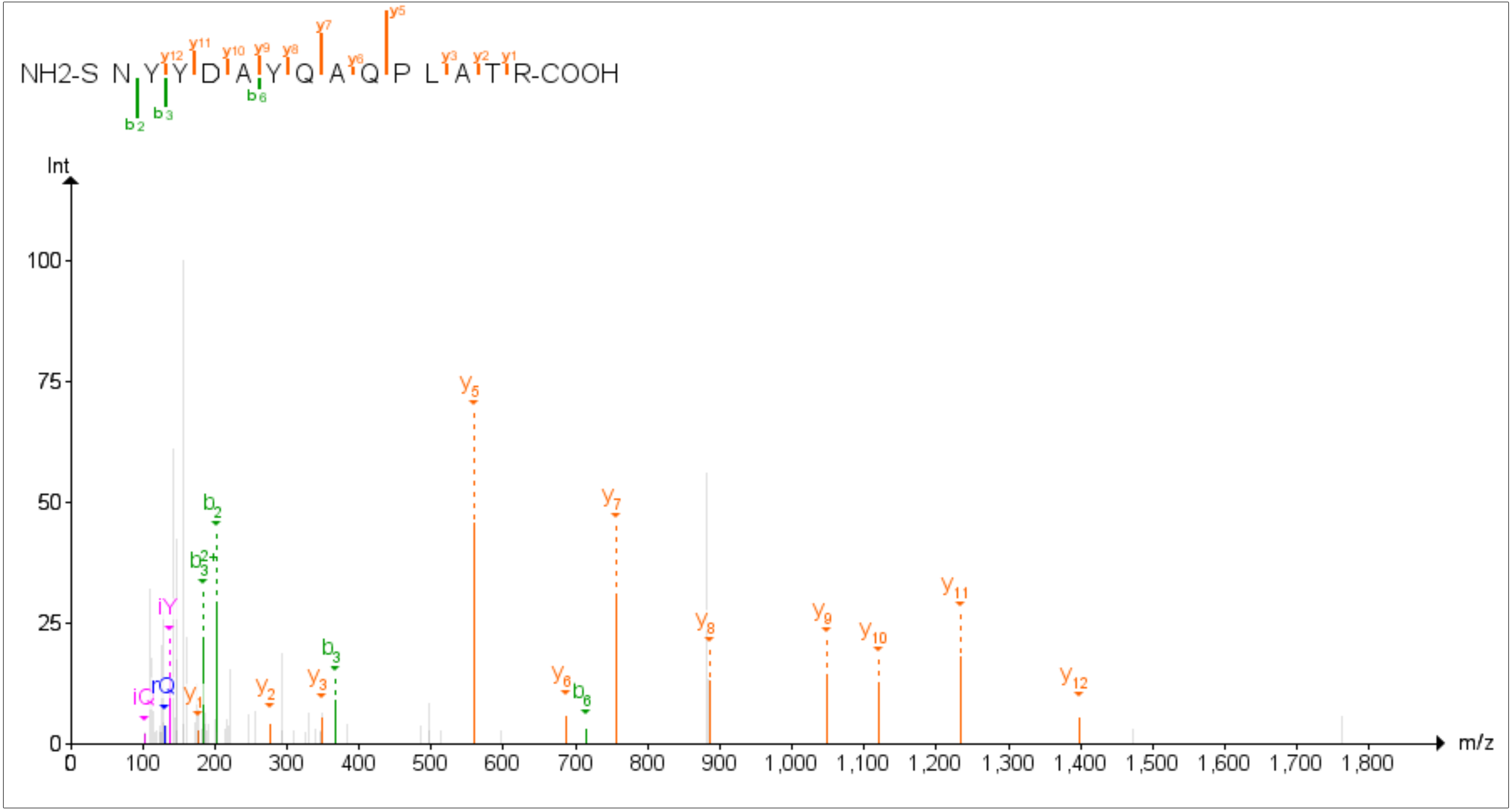

NH2-S N Y Y D A Y Q A Q P L A T R-COOH
b2 b3 b6
y12 y11 y10 y9 y8 y7 y6 y5 y3 y2 y1
Int
m/z
0 25 50 75 100
0 100 200 300 400 500 600 700 800 900 1,000 1,100 1,200 1,300 1,400 1,500 1,600 1,700 1,800
iQ rQ iY y1 b3 2+ b2 y2 y3 b3 y5 y6 b6 y7 y8 y9 y10 y11 y12

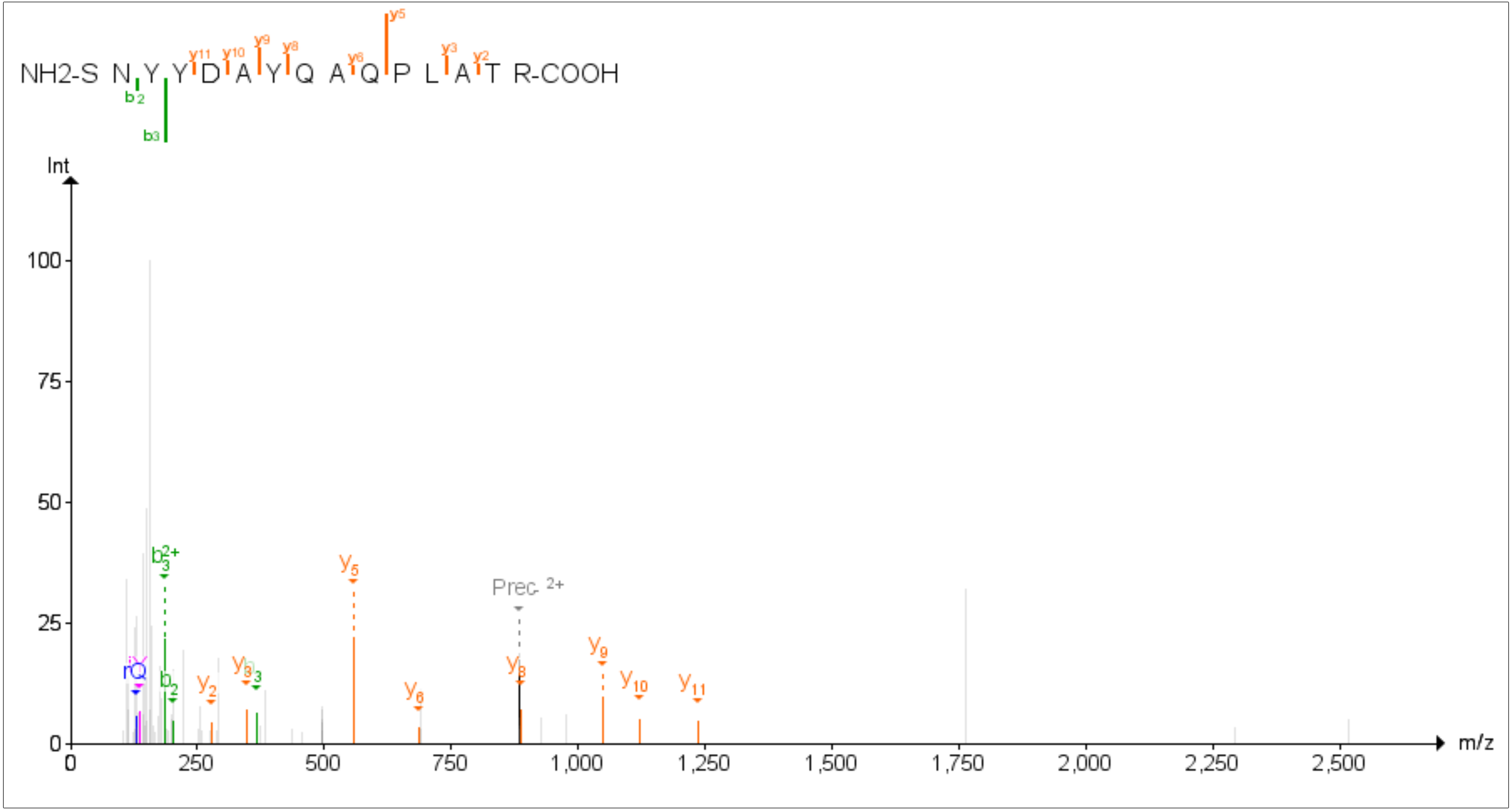
NH2-S N Y Y D A Y Q A Q P L A T R-COOH
y11 y10 y9 y8 y6 y5 y3 y2
b2 b3
Int
100
75
50
25
0
Prec 2+
m/z
0 250 500 750 1,000 1,250 1,500 1,750 2,000 2,250 2,500

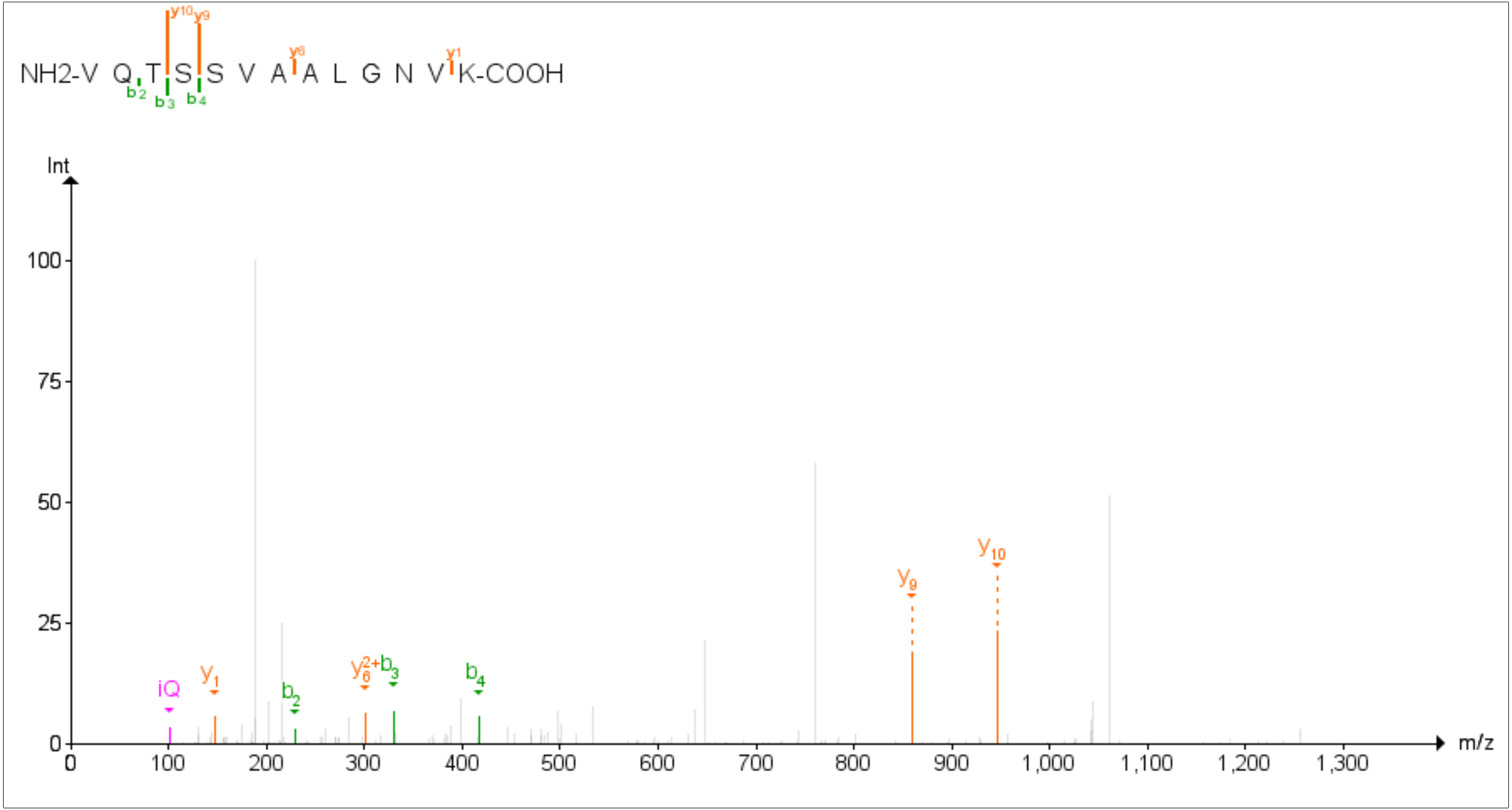

NH2-V Q T S S V A A L G N V K-COOH
y10 y9 y6 y1
b2 b3 b4
Int
100
75
50
25
0
iQ
y1
b2
y6 2+ b3
b4
y9
y10
0 100 200 300 400 500 600 700 800 900 1,000 1,100 1,200 1,300
m/z

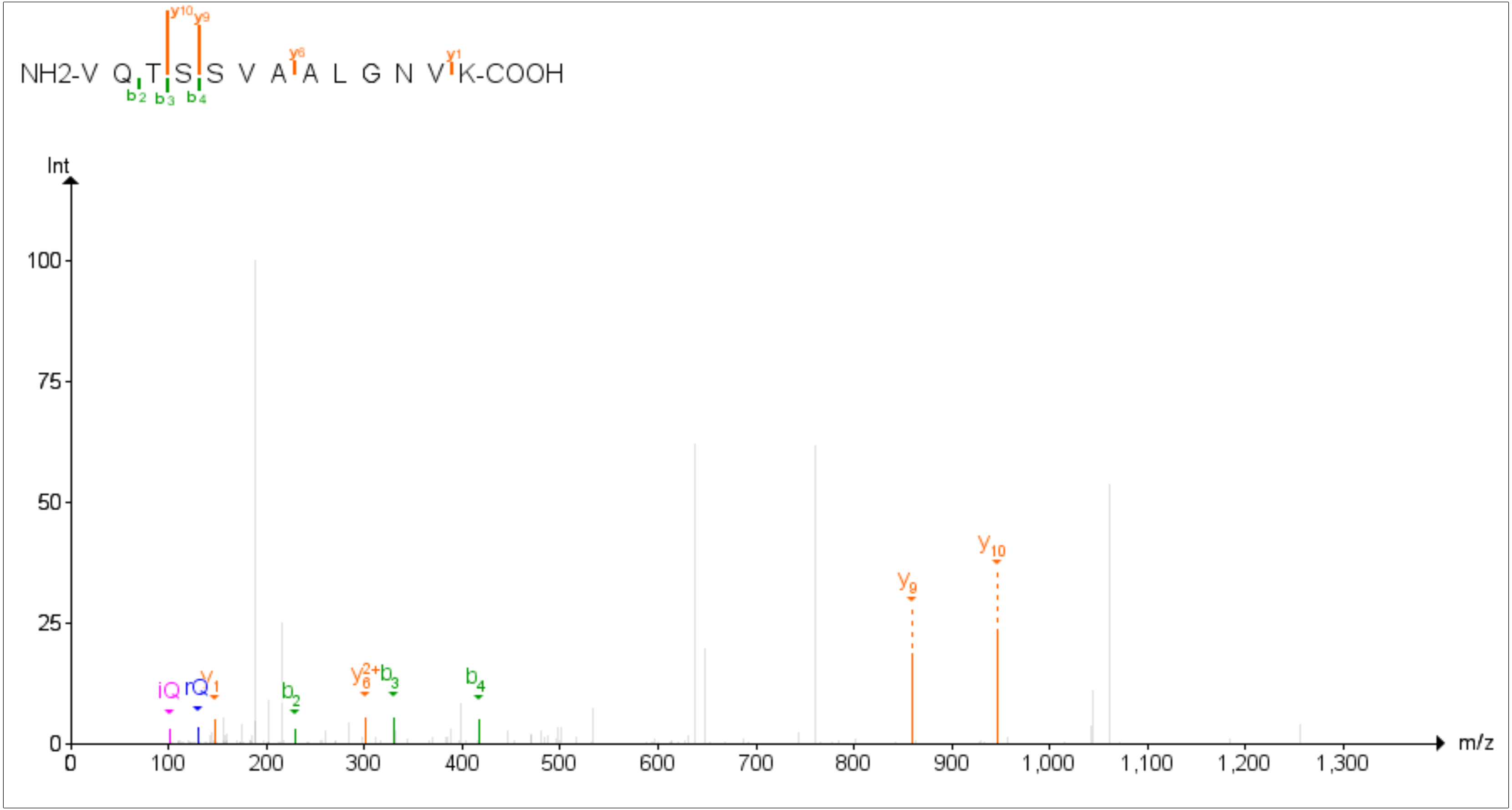

NH2-V Q T S S V A A L G N V K-COOH
y10 y9 y6 y1
b2 b3 b4
Int
100
75
50
25
0
iQ
rQ
y1
b2
y6 2+ b3
b4
y9
y10
0 100 200 300 400 500 600 700 800 900 1,000 1,100 1,200 1,300
m/z

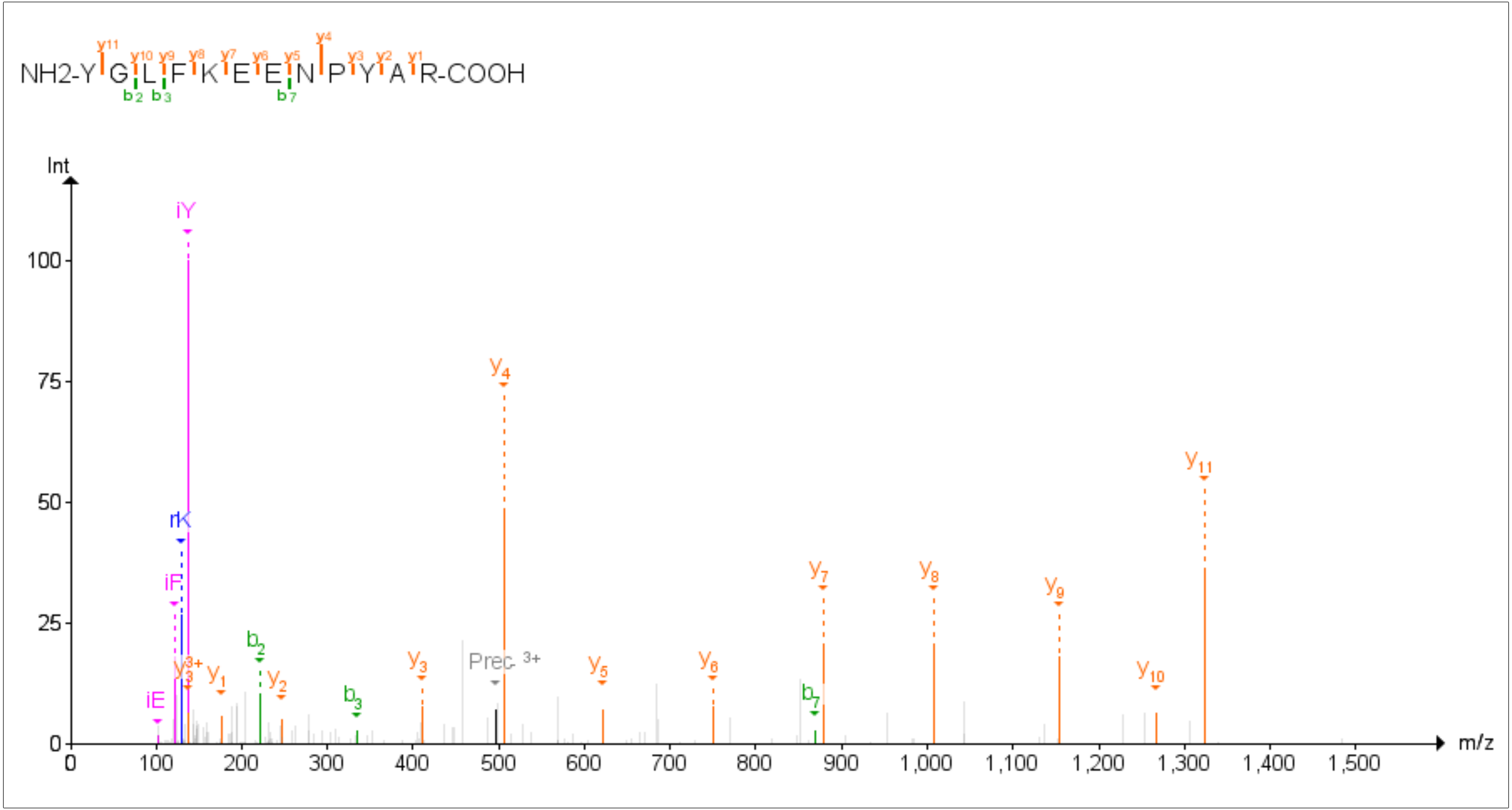

NH2-Y G L F K E E N P Y A R-COOH
y11 y10 y9 y8 y7 y6 y5 y4 y3 y2 y1
b2 b3 b7
Int
iY
rK
iF
iE
y4
y11
y7
y8
y9
y10
b2
b3
b7
y1
y2
y3
y3 3+
Prec 3+
y5
y6
0
25
50
75
100
0 100 200 300 400 500 600 700 800 900 1,000 1,100 1,200 1,300 1,400 1,500
m/z

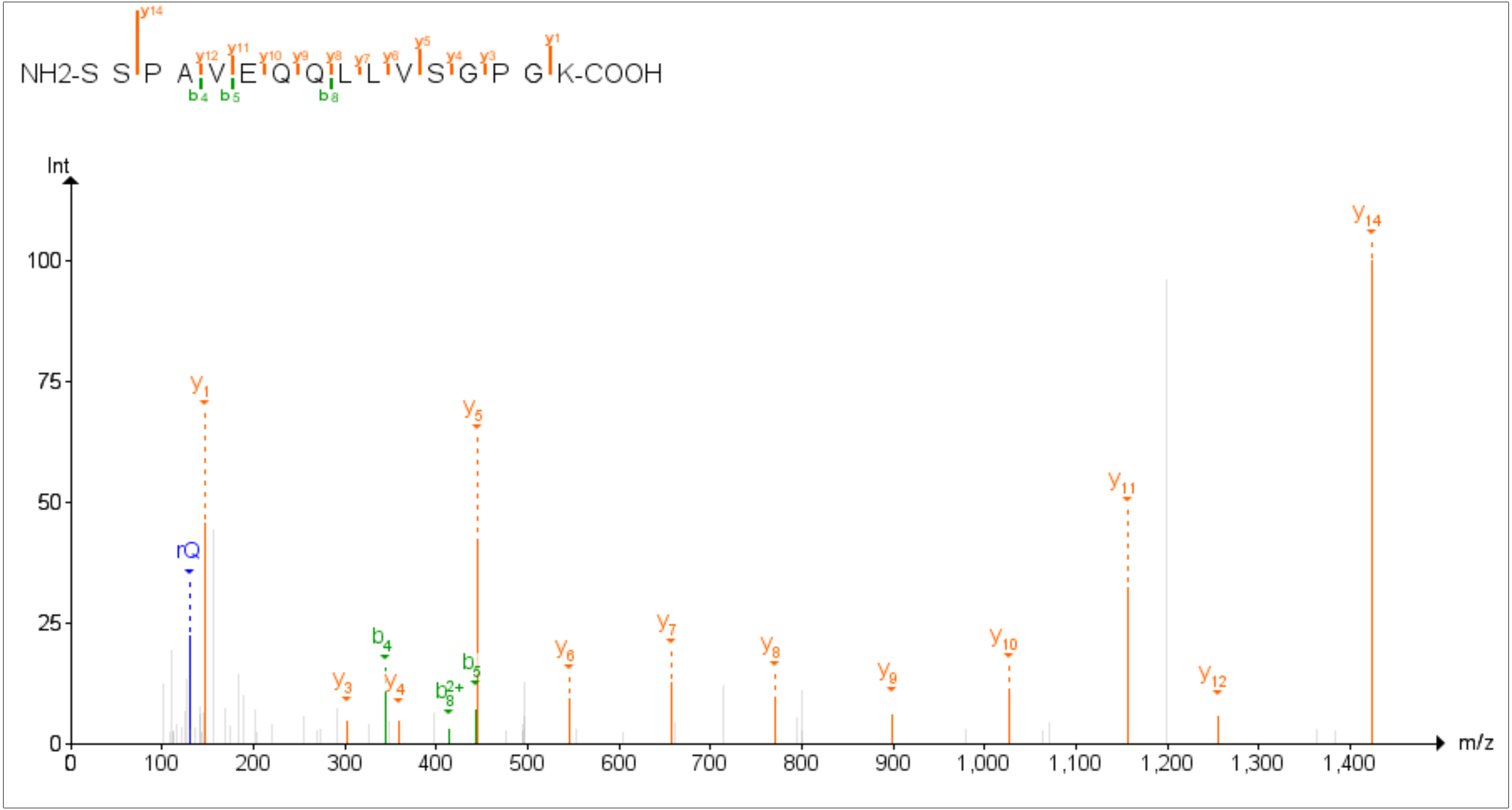

NH2-S S P A V E Q Q L L V S G P G K-COOH
y14
y12
y11
y10
y9
y8
y7
y6
y5
y4
y3
y1
b4
b5
b8
Int
m/z
rQ

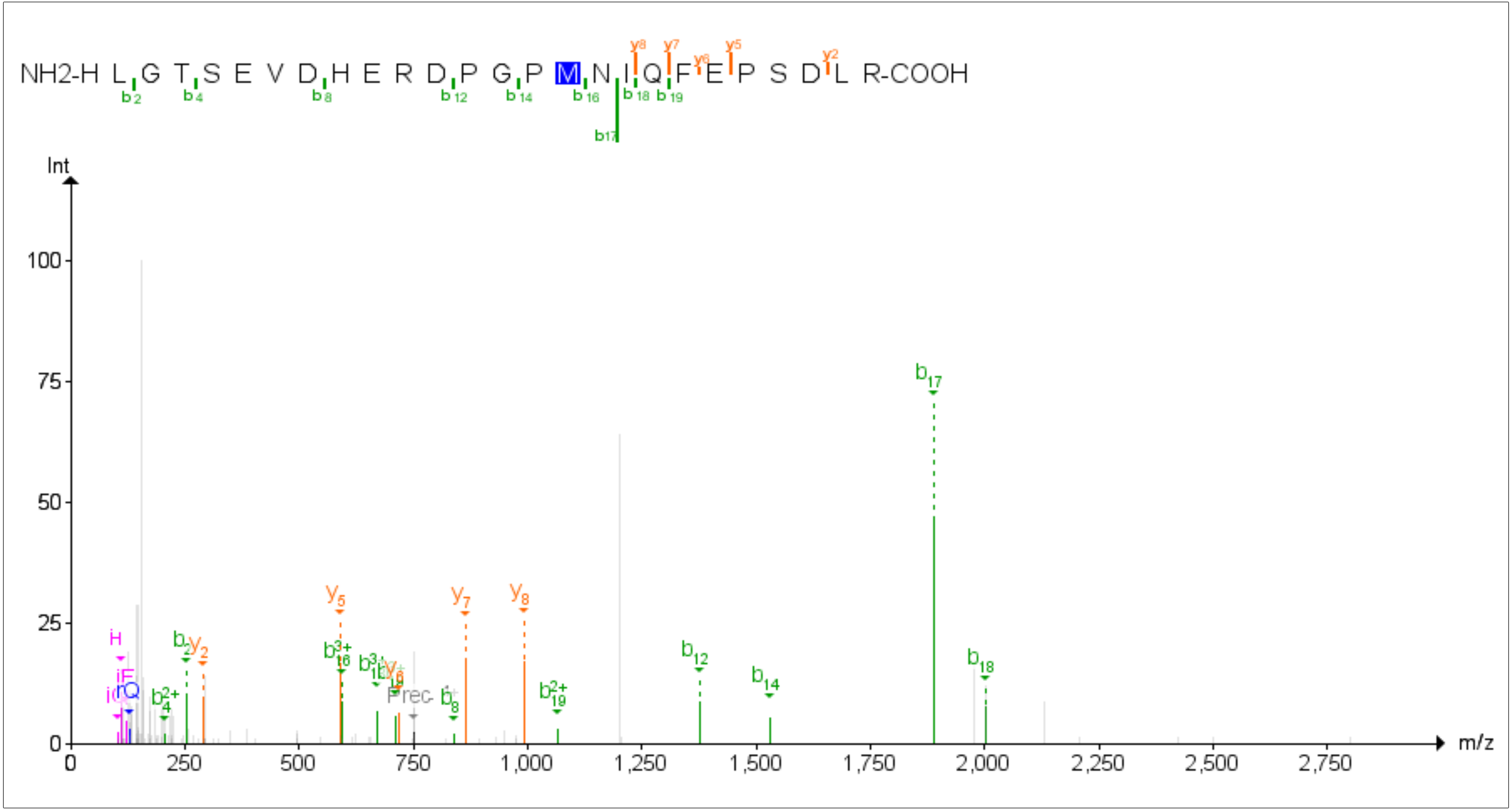

NH2-H L G T S E V D H E R D P G P M N I Q F E P S D L R-COOH
b2
b4
b8
b12
b14
b16
b17
b18
b19
y8
y7
y6
y5
y2
Int
100
75
50
25
0
m/z
0
250
500
750
1,000
1,250
1,500
1,750
2,000
2,250
2,500
2,750
y5
y7
y8
b17
b18
b12
b14
b2
y2
Prec

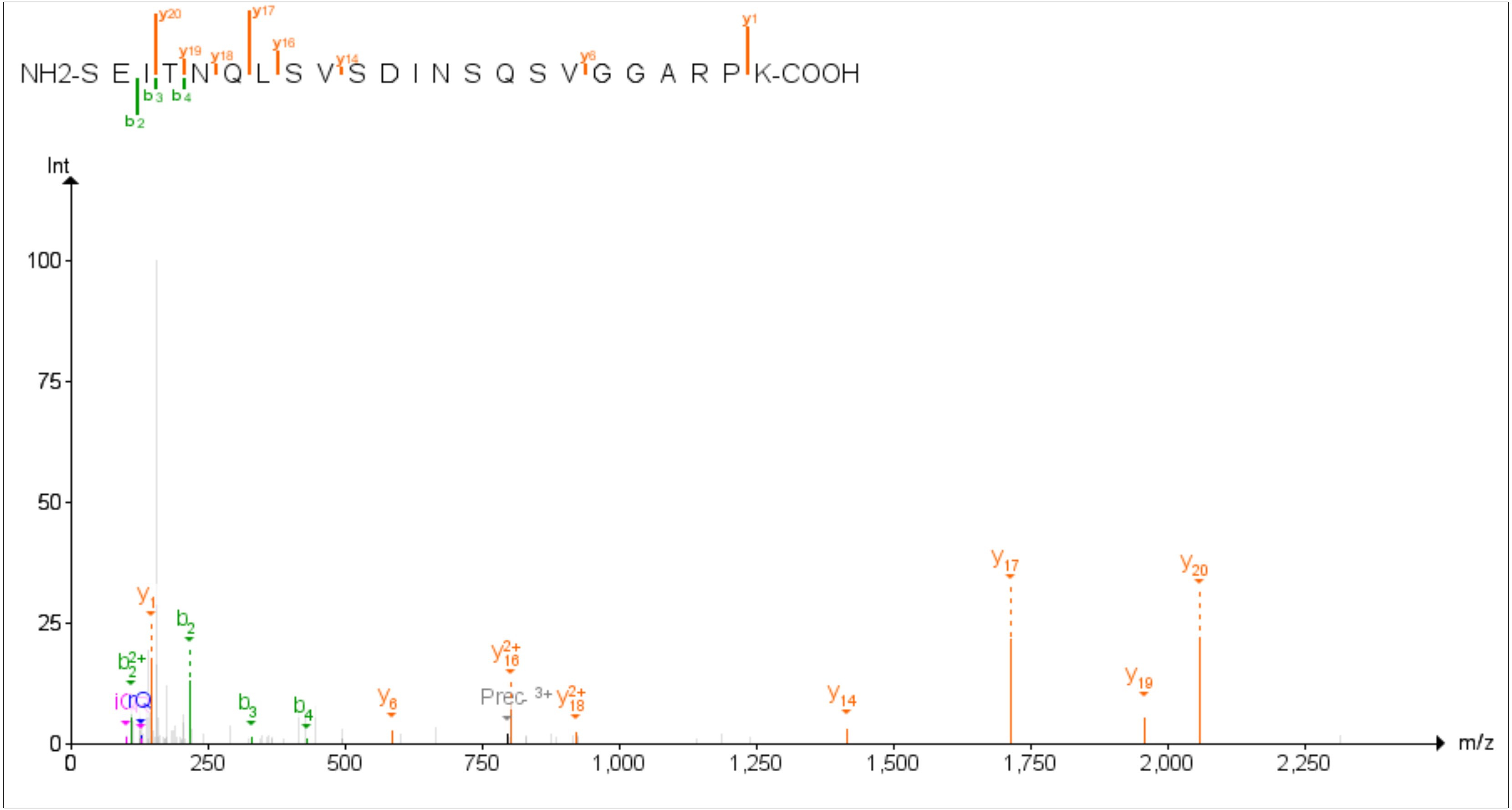

NH2-S E I T N Q L S V S D I N S Q S V G G A R P K-COOH
y20
y19
y18
y17
y16
y14
y6
y1
b2
b3
b4
Int
100
75
50
25
0
y1
b2
b2 2+
y6
y16 2+
Prec- 3+
y18 2+
y14
y17
y19
y20
b3
b4
0
250
500
750
1,000
1,250
1,500
1,750
2,000
2,250
m/z

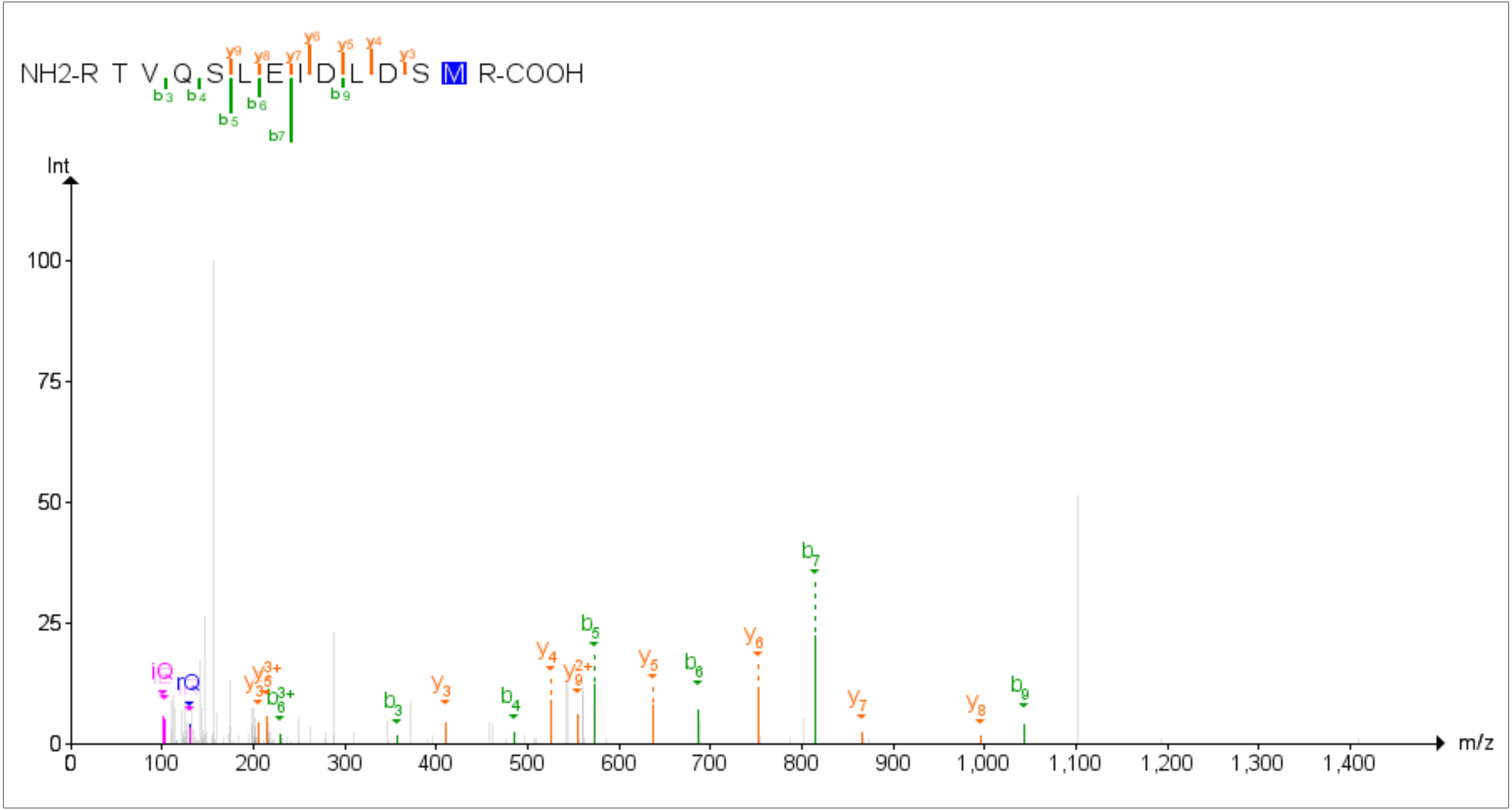
NH2-R T V Q S L E I D L D S M R-COOH
Int
m/z
0
25
50
75
100
0
100
200
300
400
500
600
700
800
900
1,000
1,100
1,200
1,300
1,400
b7
b5
b6
b9
b3
b4
y3
y4
y5
y6
y7
y8
y9
iQ
rQ

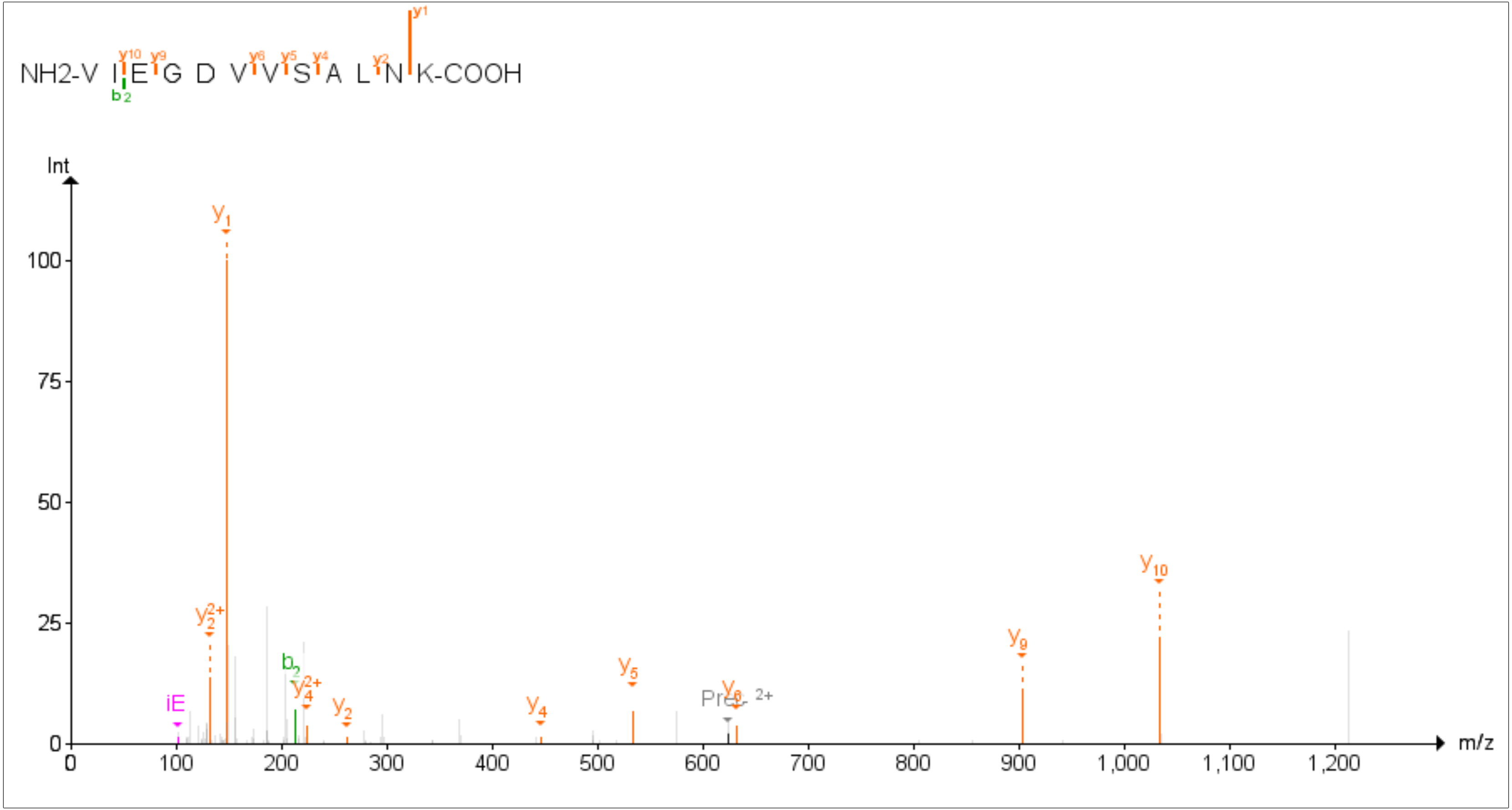

NH2-V I E G D V V S A L N K-COOH
y10 y9 y6 y5 y4 y2 y1
b2
Int
100
75
50
25
0
y1
y2 2+
b2
y4 2+
iE
y2
y4
y5
Pre- 2+
y6
y9
y10
0 100 200 300 400 500 600 700 800 900 1,000 1,100 1,200
m/z

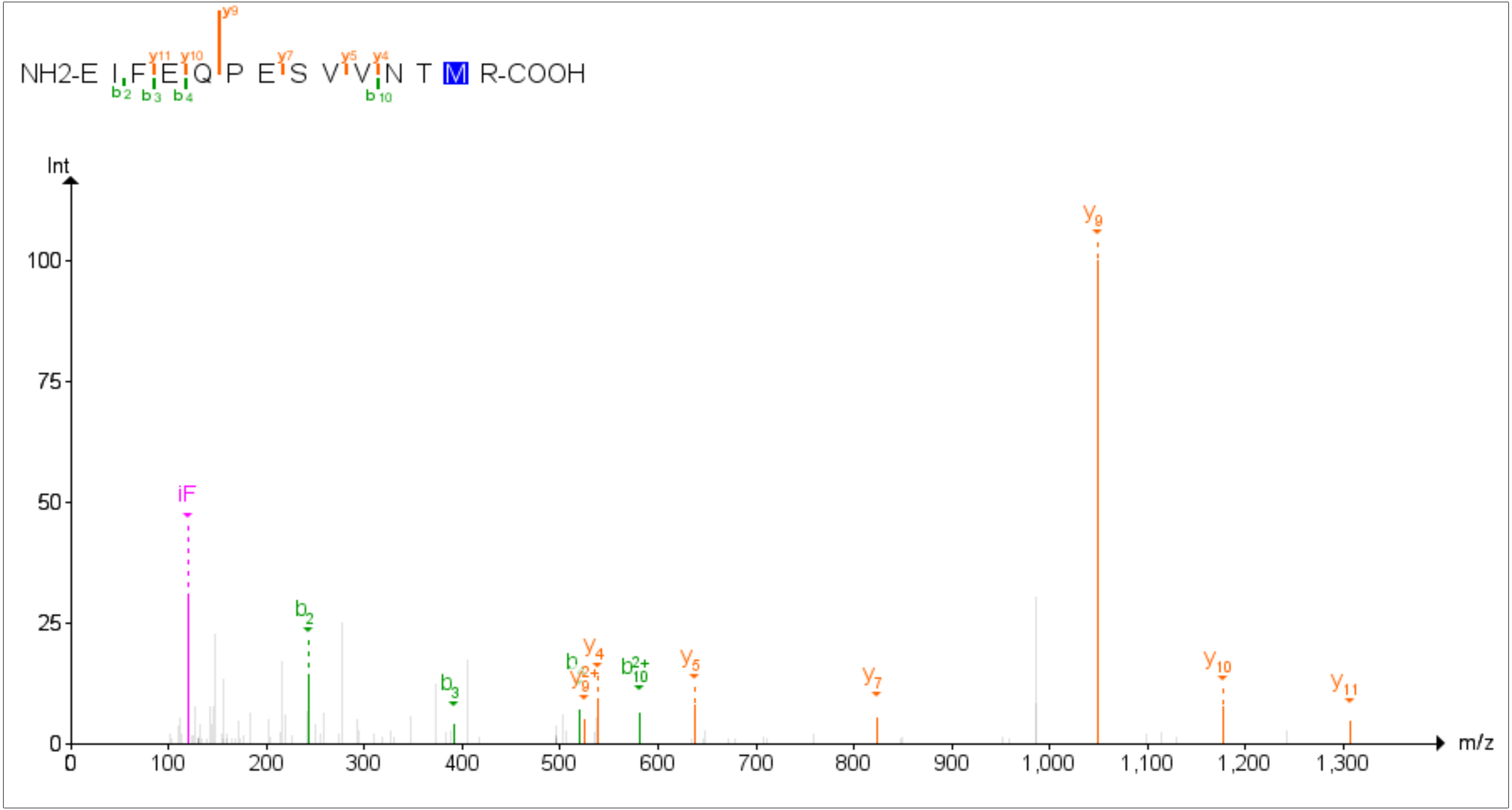
NH2-E I F E Q P E S V V N T M R-COOH
y9
y11
y10
y7
y5
y4
b2
b3
b4
b10
Int
100
75
50
25
0
iF
b2
b3
y4
b10 2+
y9 2+
y5
y7
y9
y10
y11
m/z
0
100
200
300
400
500
600
700
800
900
1,000
1,100
1,200
1,300

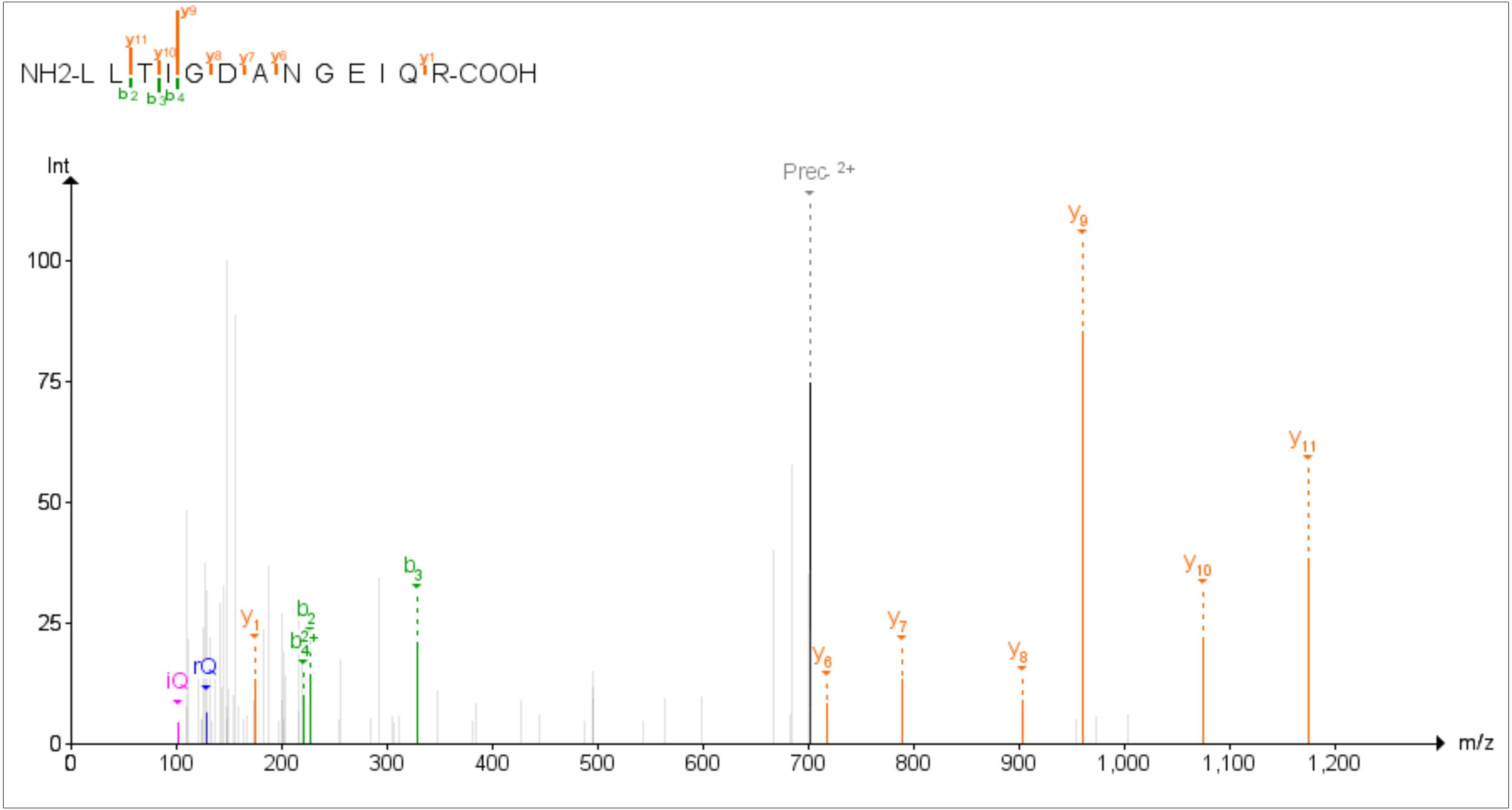

NH2-L L T I G D A N G E I Q R-COOH
y11 y10 y9 y8 y7 y6 y1
b2 b3 b4
Int
m/z
Prec 2+
y9
y11
y10
y7
y8
y6
y1
b3
b2
b4 2+
rQ
iQ
0
25
50
75
100
0
100
200
300
400
500
600
700
800
900
1,000
1,100
1,200

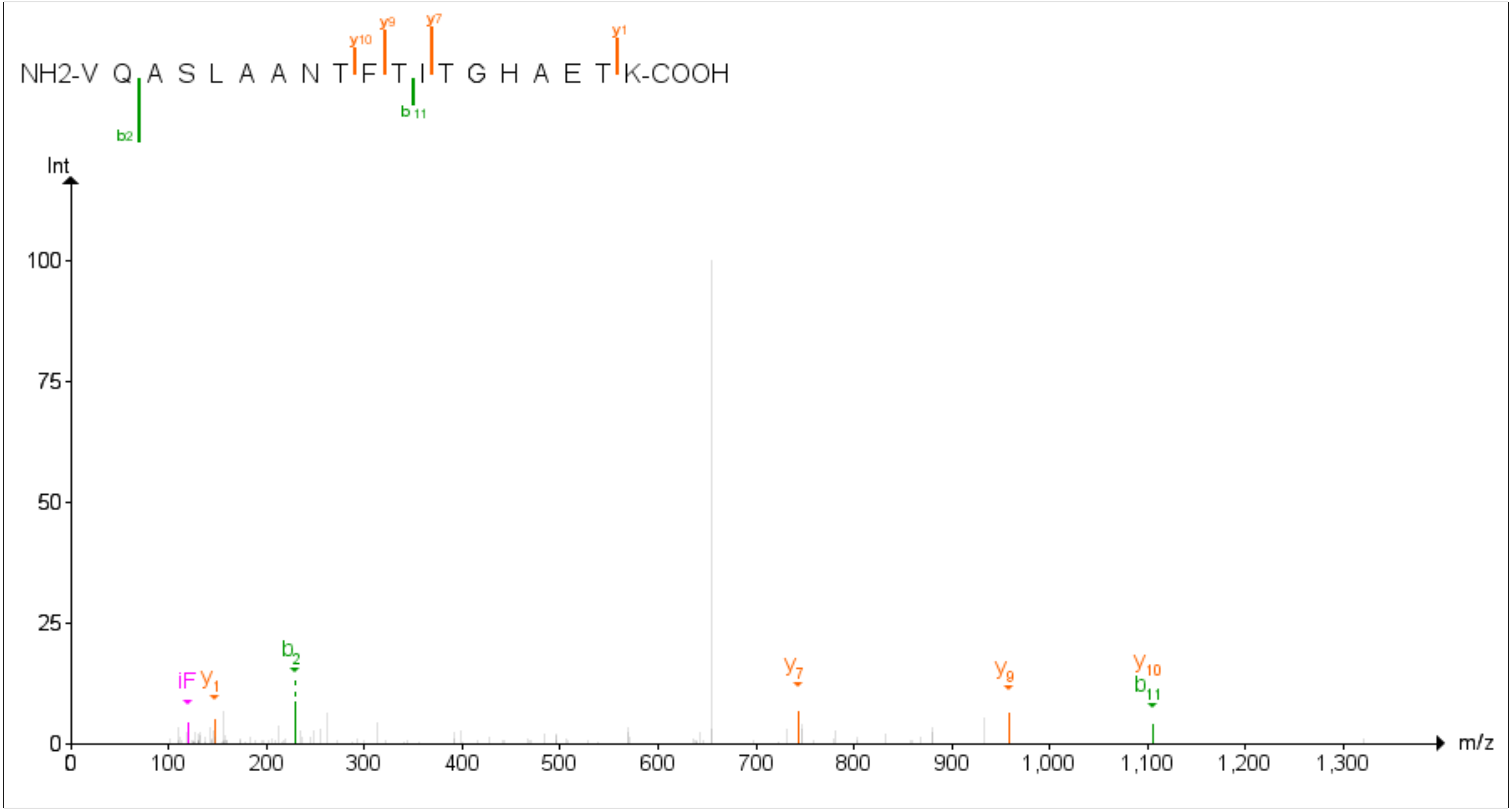

y10
y9
y7
y1
NH2-V Q A S L A A N T F T I T G H A E T K-COOH
b2
b 11
Int
100
75
50
25
0
iF
y1
b2
y7
y9
y10
b11
0
100
200
300
400
500
600
700
800
900
1,000
1,100
1,200
1,300
m/z

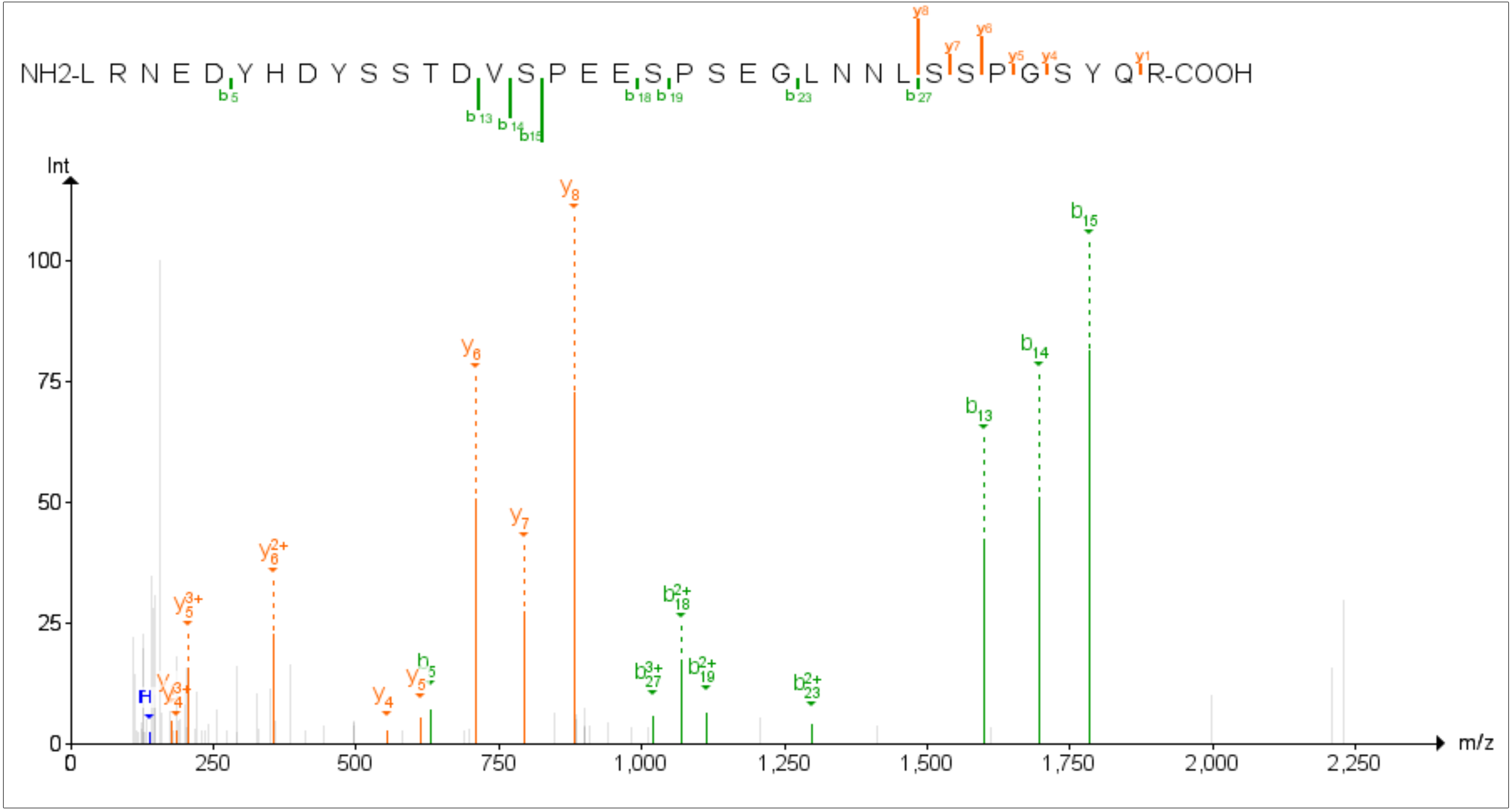

NH2-L R N E D Y H D Y S S T D V S P E E S P S E G L N N L S S P G S Y Q R-COOH
b5
b13
b14
b15
b18
b19
b23
b27
y8
y7
y6
y5
y4
y1
Int
m/z
0
25
50
75
100
0
250
500
750
1,000
1,250
1,500
1,750
2,000
2,250

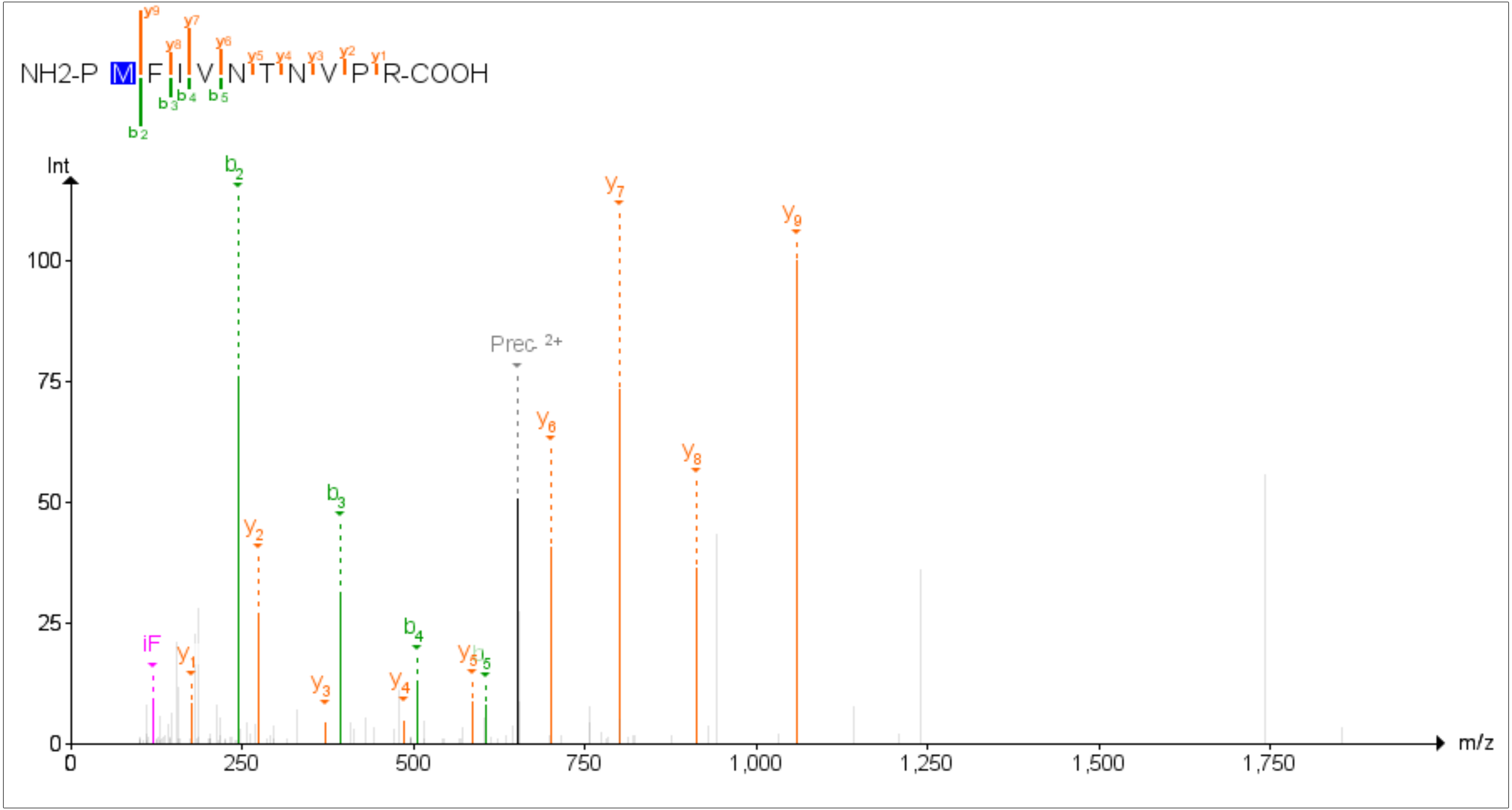

NH2-P M F I V N T N V P R-COOH
y9 y8 y7 y6 y5 y4 y3 y2 y1
b2 b3 b4 b5
Int
100
75
50
25
0
m/z
0
250
500
750
1,000
1,250
1,500
1,750
Prec 2+
iF

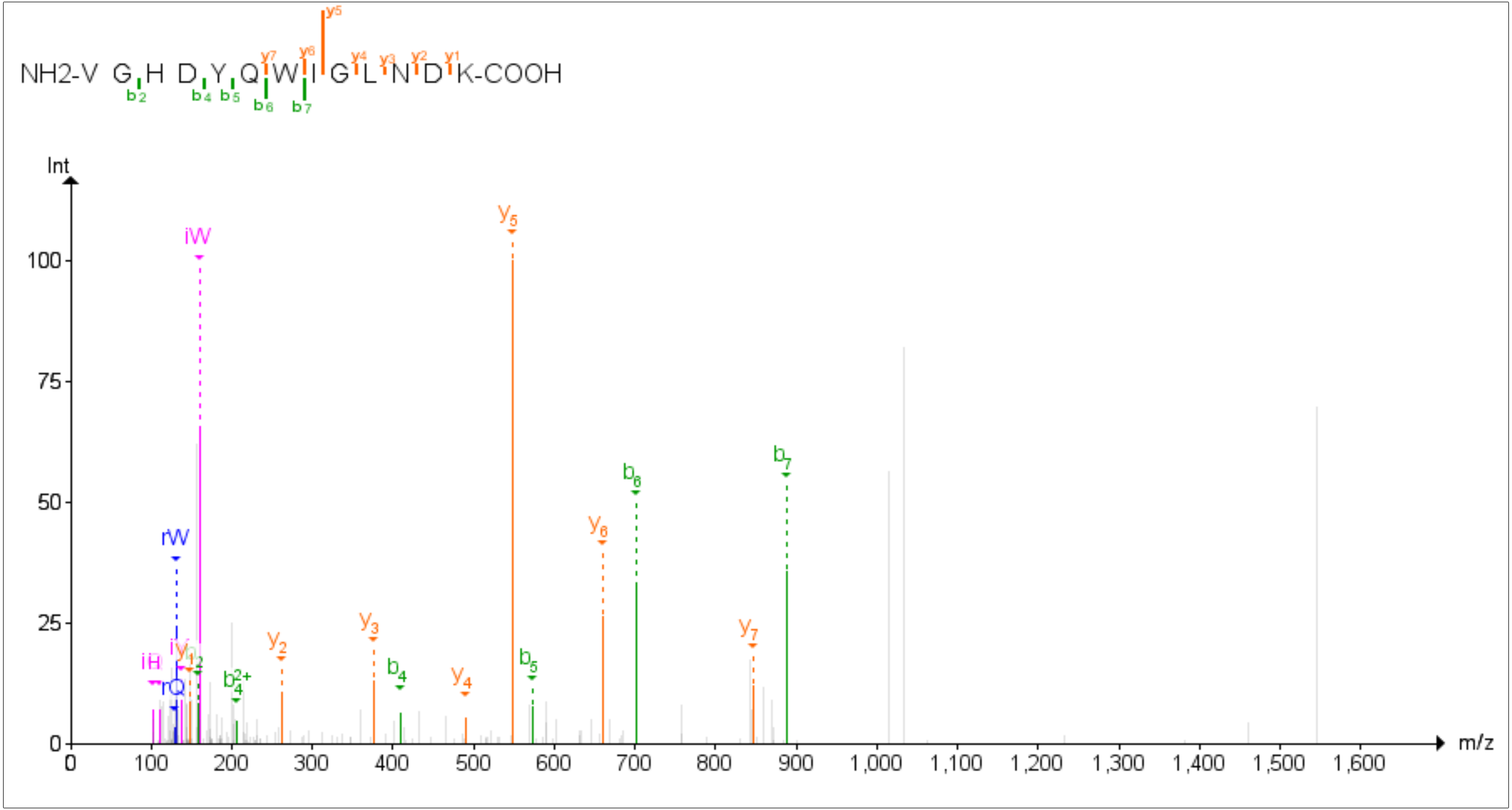

NH2-V G H D Y Q W I G L N D K-COOH
b2 b4 b5 b6 b7
y7 y6 y5 y4 y3 y2 y1
Int
m/z
iW
rW
iH
rQ
iY
y1
b4 2+
y2
y3
b4
y4
y5
b5
y6
b6
y7
b7

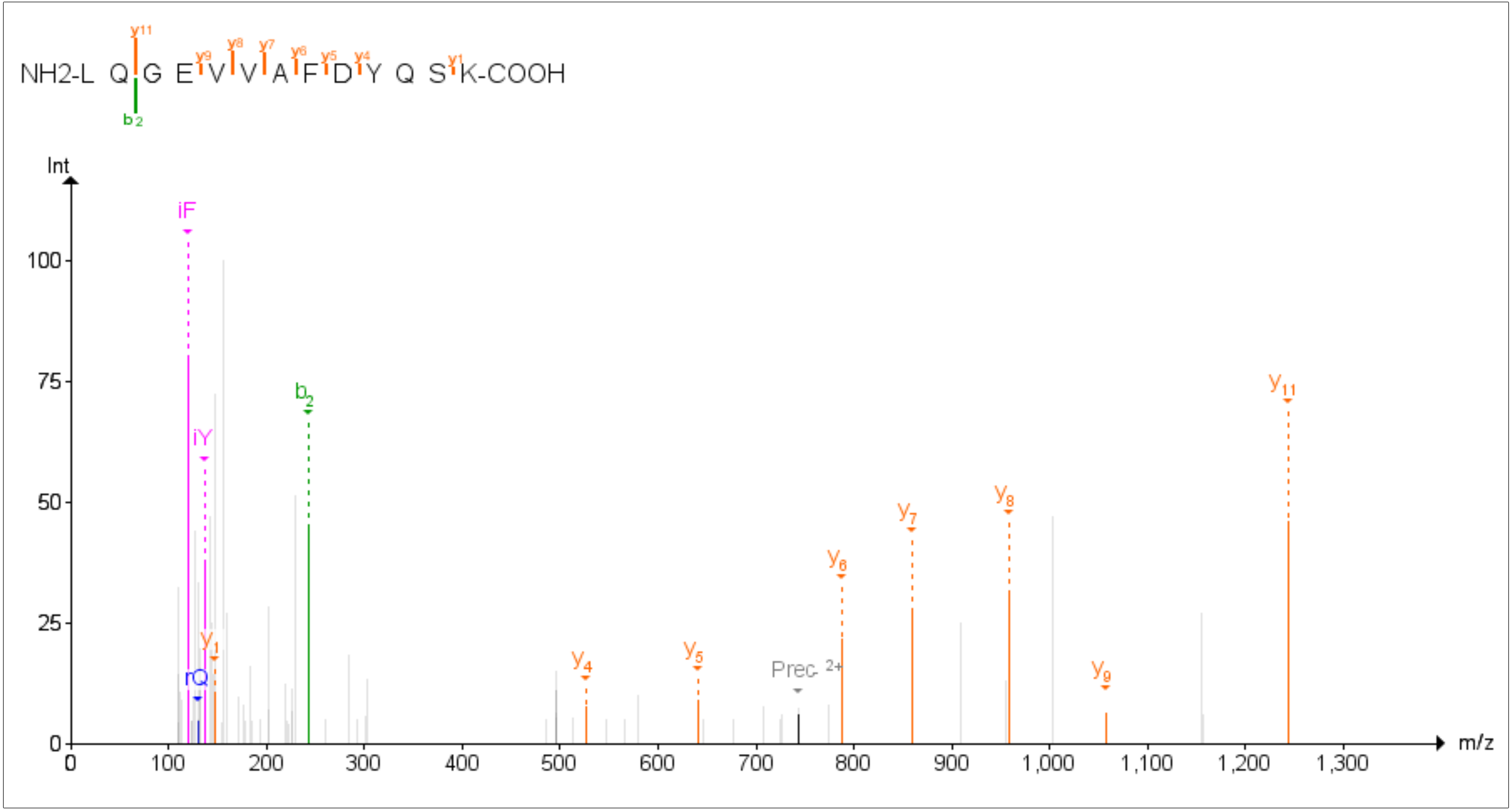

NH2-L Q G E V V A F D Y Q S K-COOH
y11
y9
y8
y7
y6
y5
y4
y1
b2
Int
m/z
iF
iY
rQ
y1
b2
y4
y5
Prec 2+
y6
y7
y8
y9
y11
0
25
50
75
100
0
100
200
300
400
500
600
700
800
900
1,000
1,100
1,200
1,300

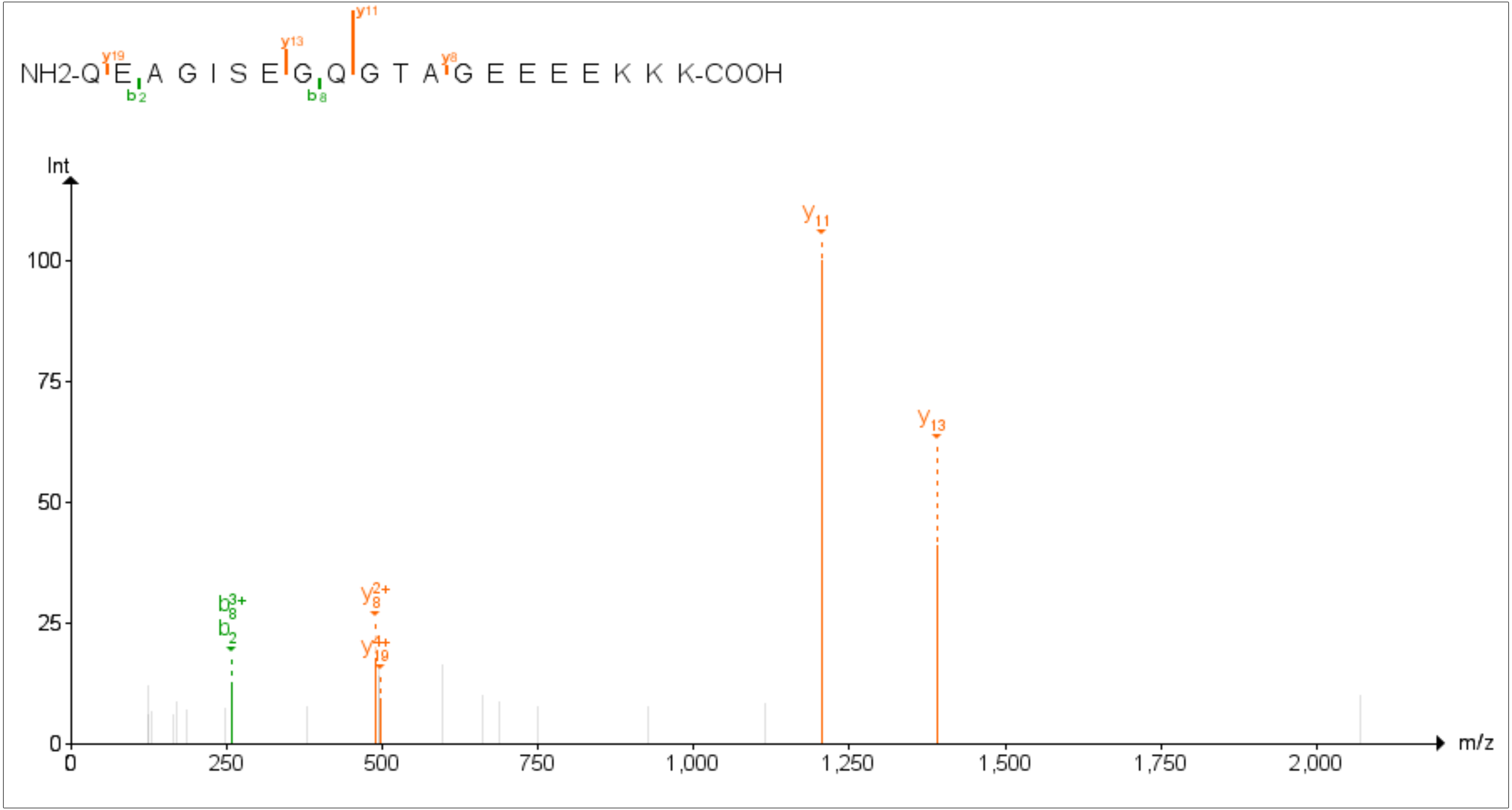
NH2-Q E A G I S E G Q G T A G E E E E K K K-COOH
y19
y13
y11
y8
b2
b8
Int
m/z
100
75
50
25
0
250
500
750
1,000
1,250
1,500
1,750
2,000
y11
y13
b8 3+
b2
y8 2+
y19 4+

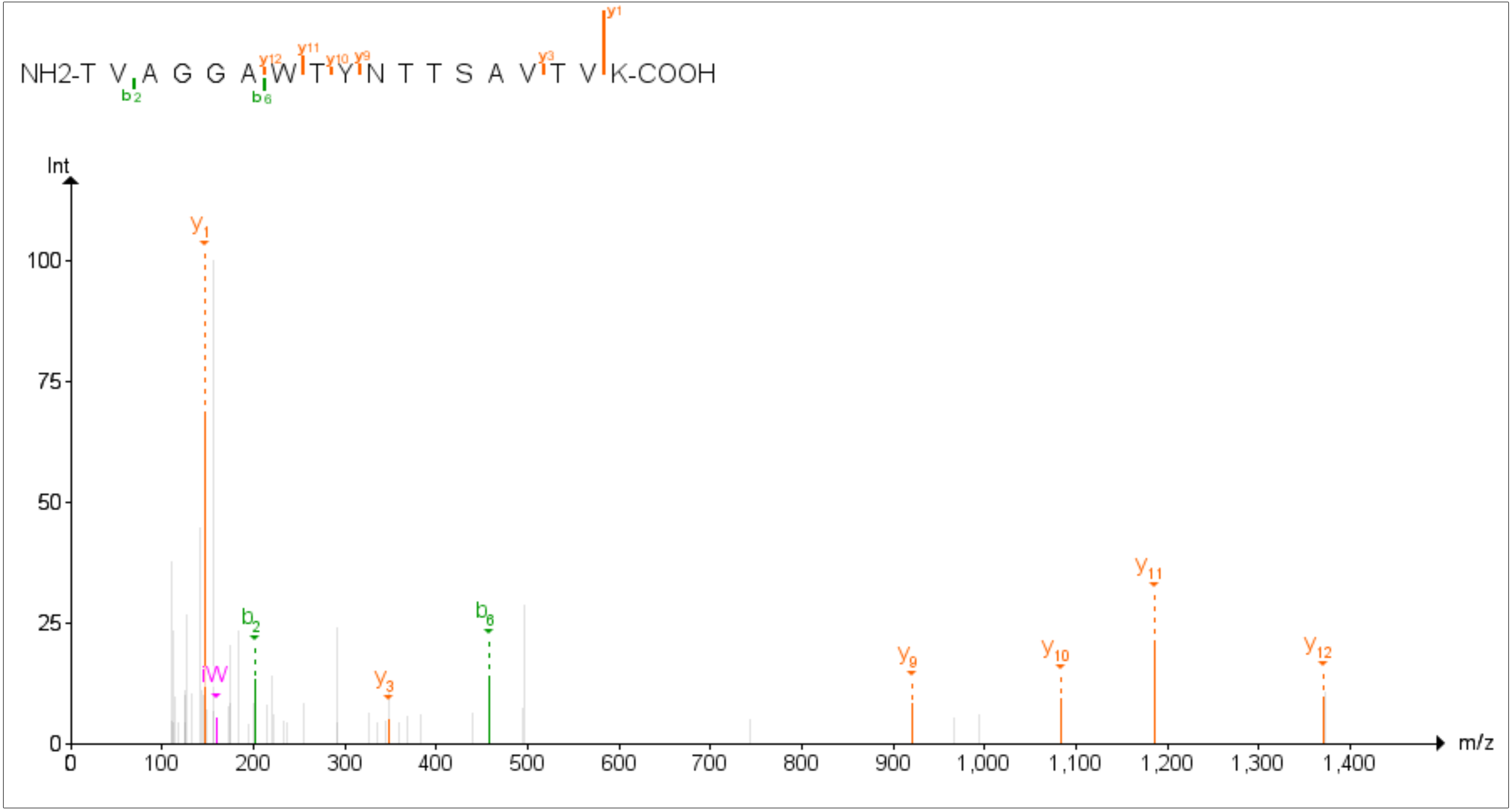

NH2-T V A G G A W T Y N T T S A V T V K-COOH
b2
b6
y12
y11
y10
y9
y3
y1
Int
100
75
50
25
0
y1
iW
b2
b6
y3
y9
y10
y11
y12
0
100
200
300
400
500
600
700
800
900
1,000
1,100
1,200
1,300
1,400
m/z

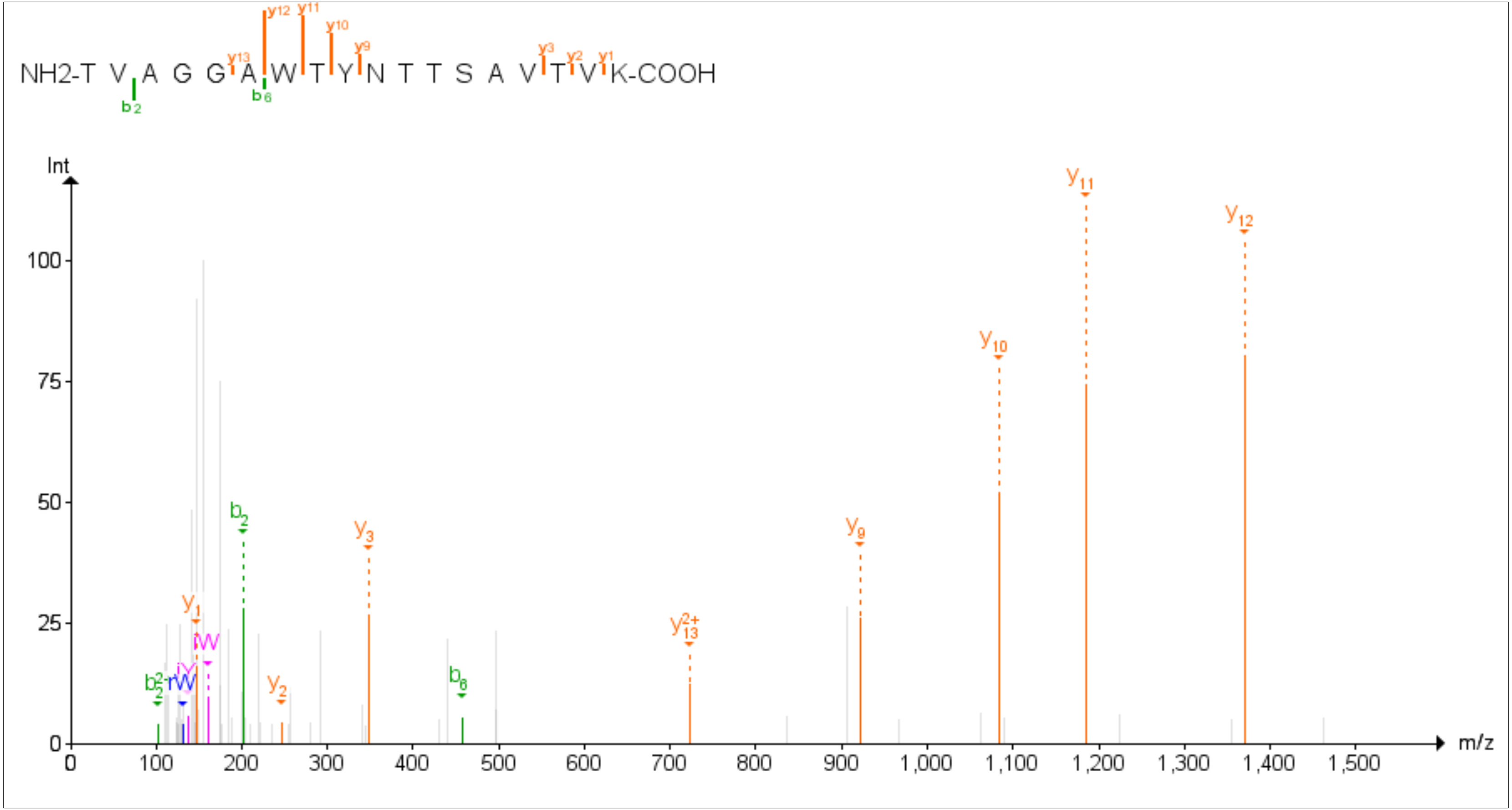

NH2-T V A G G A W T Y N T T S A V T V K-COOH
b2
b6
y13
y12
y11
y10
y9
y3
y2
y1
Int
100
75
50
25
0
m/z
0
100
200
300
400
500
600
700
800
900
1,000
1,100
1,200
1,300
1,400
1,500

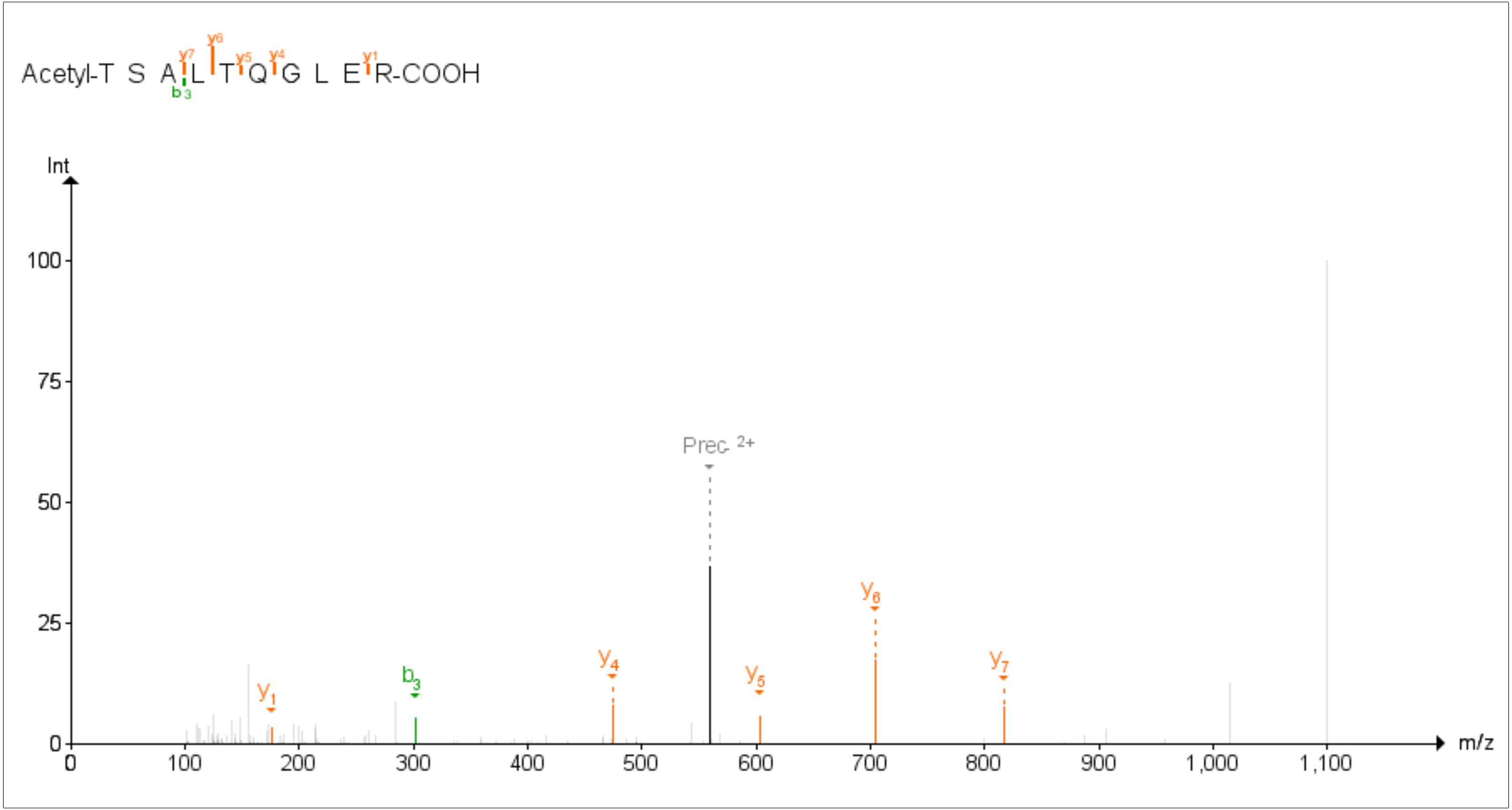

Acetyl-T S A L T Q G L E R-COOH
y7
y6
y5
y4
y1
b3
Int
100
75
50
25
0
Prec 2+
y1
b3
y4
y5
y6
y7
0
100
200
300
400
500
600
700
800
900
1,000
1,100
m/z

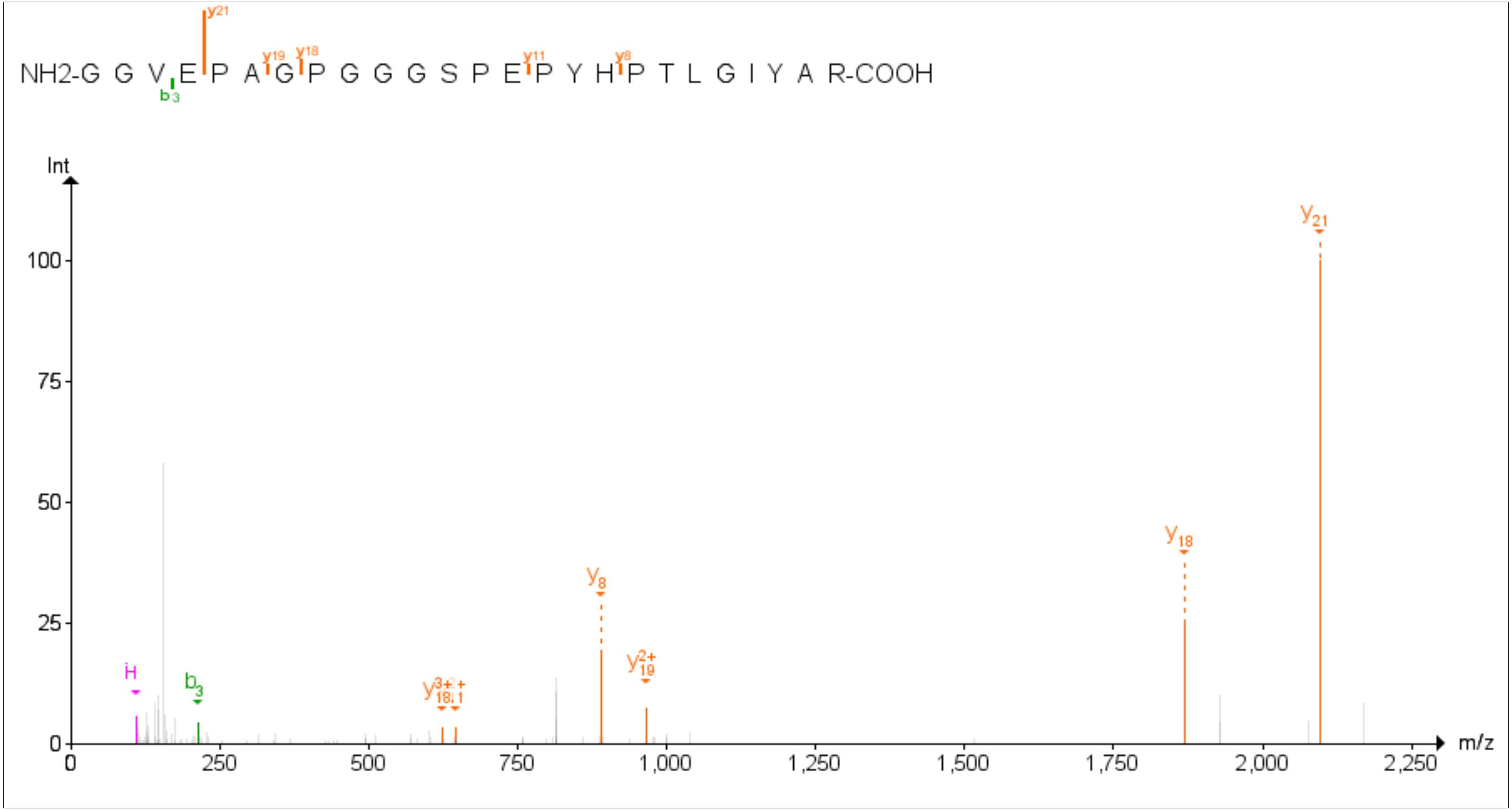
NH2-G G V E P A G P G G G S P E P Y H P T L G I Y A R-COOH
y21
y19
y18
y11
y8
b3
Int
m/z
100
75
50
25
0
250
500
750
1,000
1,250
1,500
1,750
2,000
2,250
H
y21
y18
y8
y19 2+

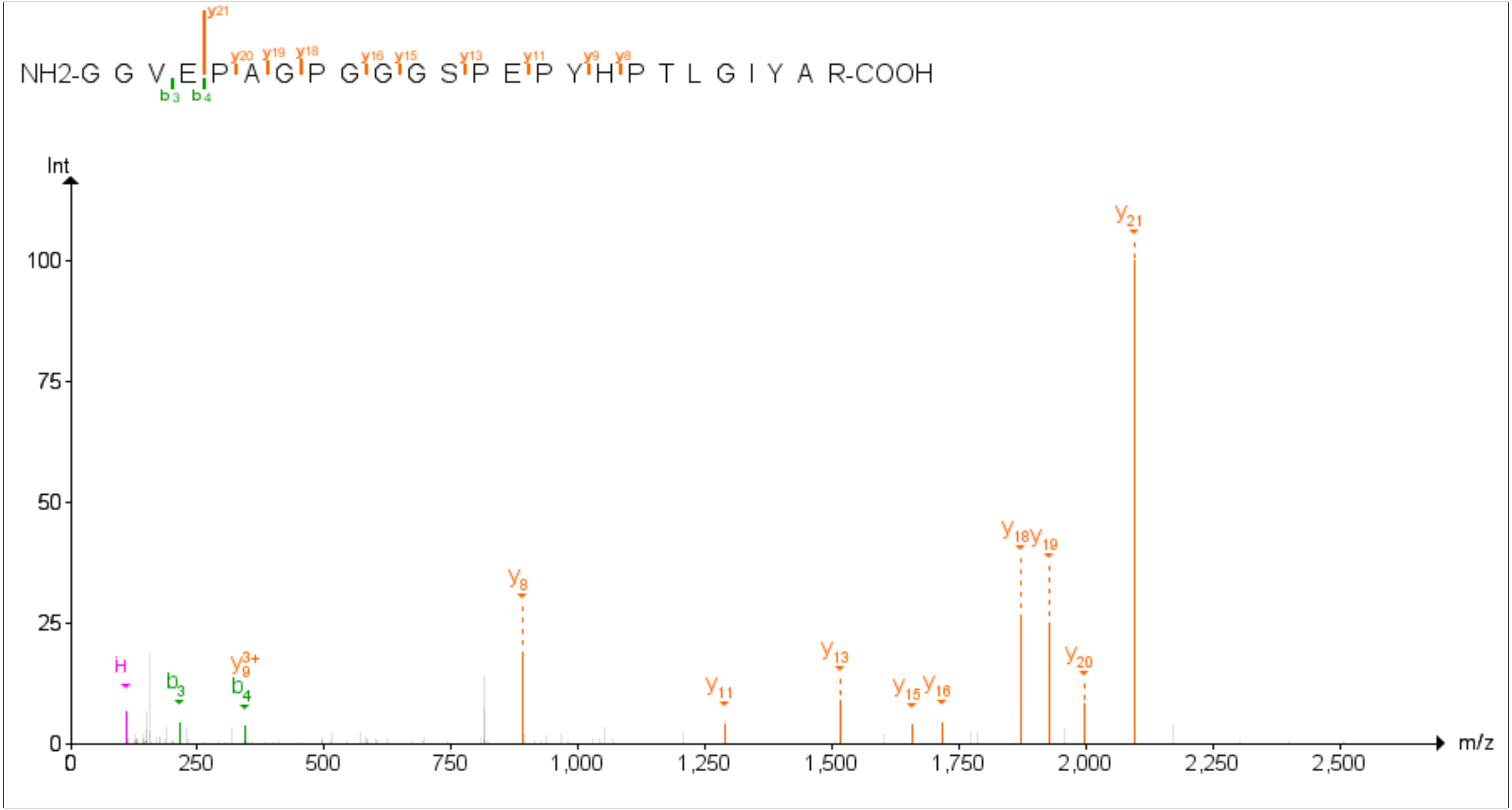

NH2-G G V E P A G P G G G S P E P Y H P T L G I Y A R-COOH
y21
y20
y19
y18
y16
y15
y13
y11
y9
y8
b3
b4
Int
100
75
50
25
0
m/z
0
250
500
750
1,000
1,250
1,500
1,750
2,000
2,250
2,500
H
b3
b4
y9 3+
y8
y11
y13
y15
y16
y18
y19
y20
y21

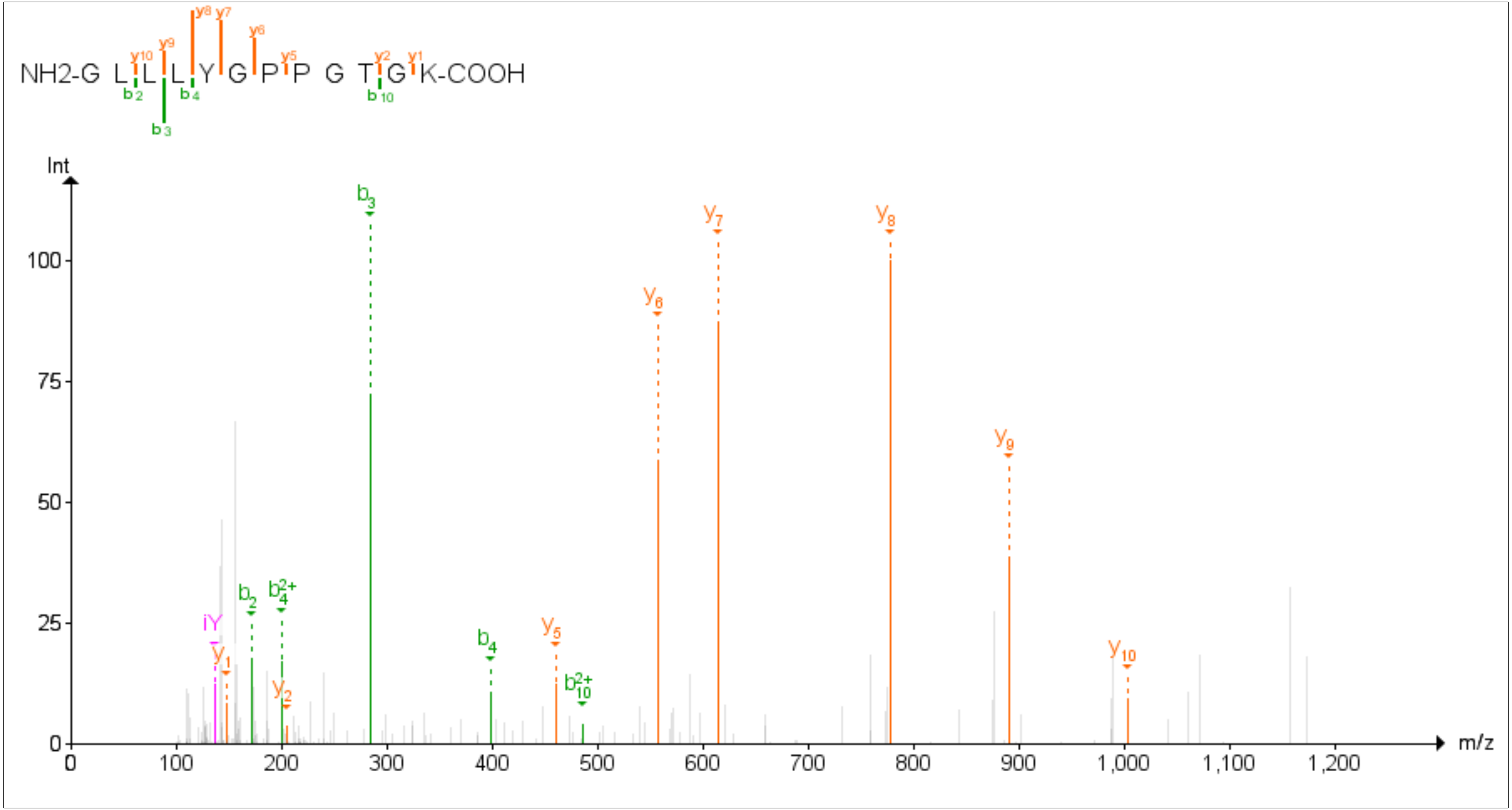

NH2-G L L L Y G P P G T G K-COOH
y10 y9 y8 y7 y6 y5 y2 y1
b2 b3 b4 b10
Int
m/z
b3 y7 y8 y6 y9 y10 y5 b4 b2 b4 2+ b10 2+ iY y1 y2
0 25 50 75 100
0 100 200 300 400 500 600 700 800 900 1,000 1,100 1,200

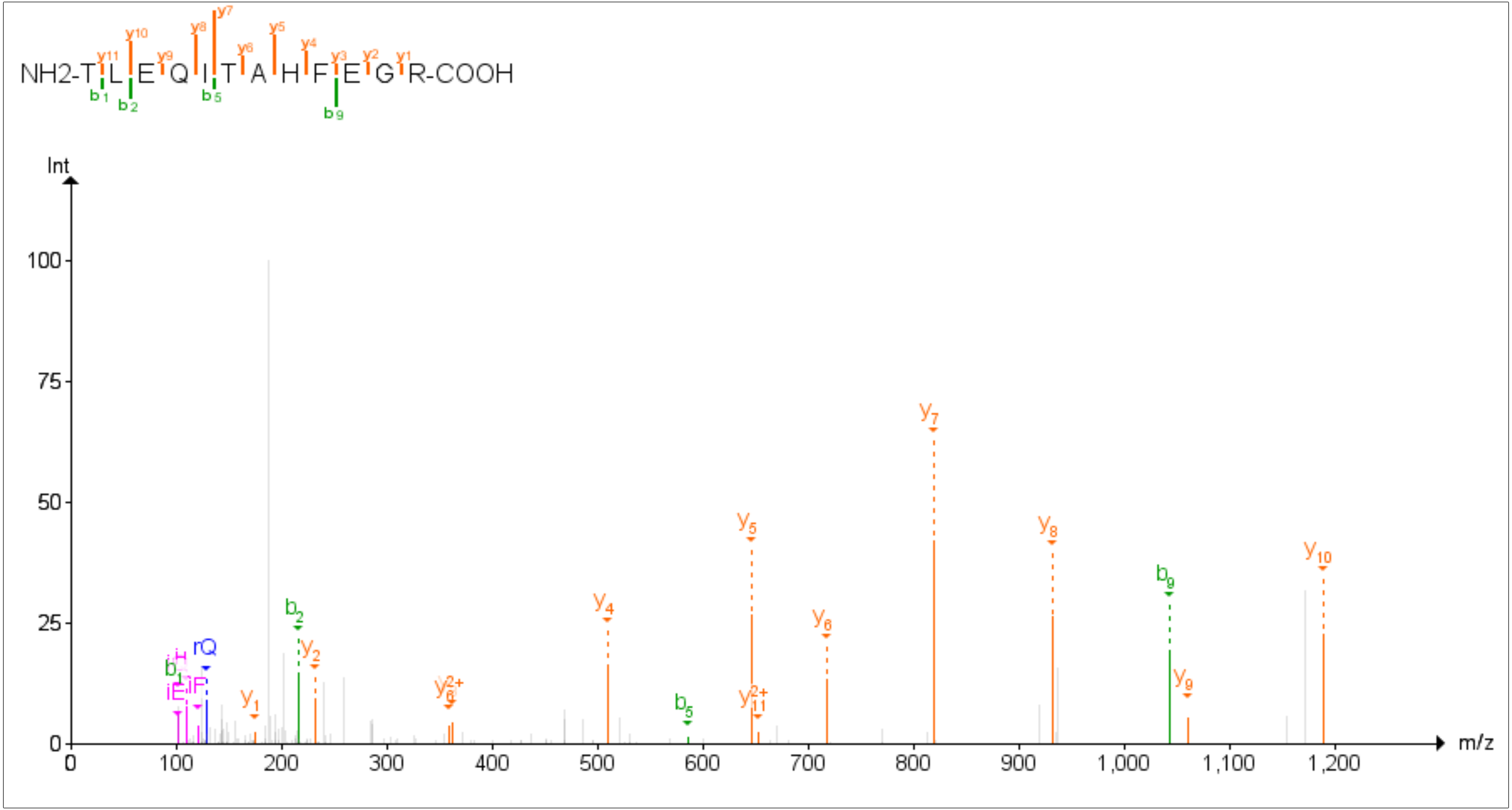

NH2-T L E Q I T A H F E G R-COOH
y11 y10 y9 y8 y7 y6 y5 y4 y3 y2 y1
b1 b2 b5 b9
Int
100
75
50
25
0
m/z
0 100 200 300 400 500 600 700 800 900 1,000 1,100 1,200
y7
y5
y8
b9
y10
y4
b2
y6
y2
rQ
y9
y1
b5

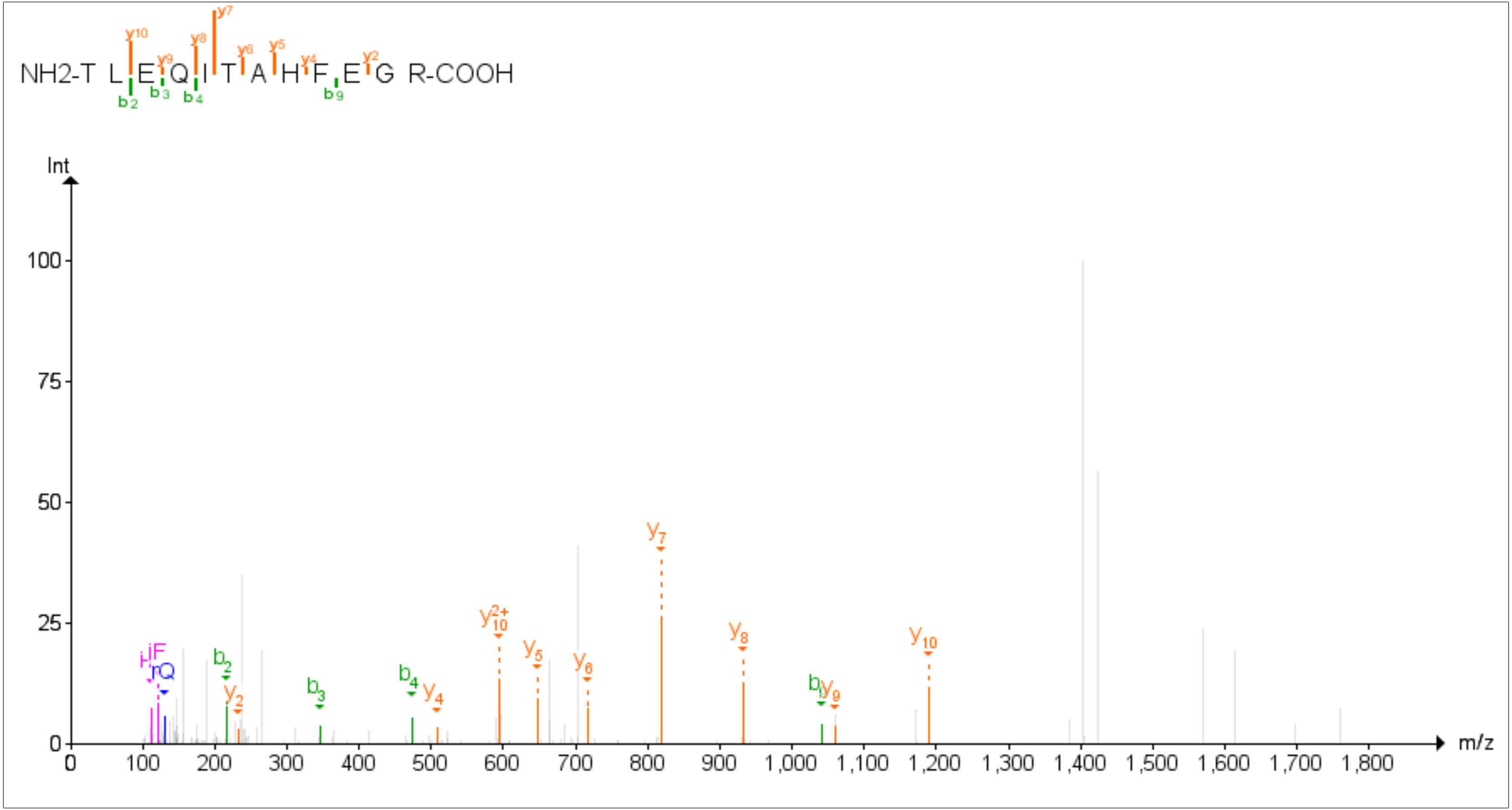

NH2-T L E Q I T A H F E G R-COOH
y10 y9 y8 y7 y6 y5 y4 y2
b2 b3 b4 b9
Int
100
75
50
25
0
m/z
0 100 200 300 400 500 600 700 800 900 1,000 1,100 1,200 1,300 1,400 1,500 1,600 1,700 1,800
iF
rQ
b2
y2
b3
b4
y4
y10 2+
y5
y6
y7
y8
b9
y9
y10

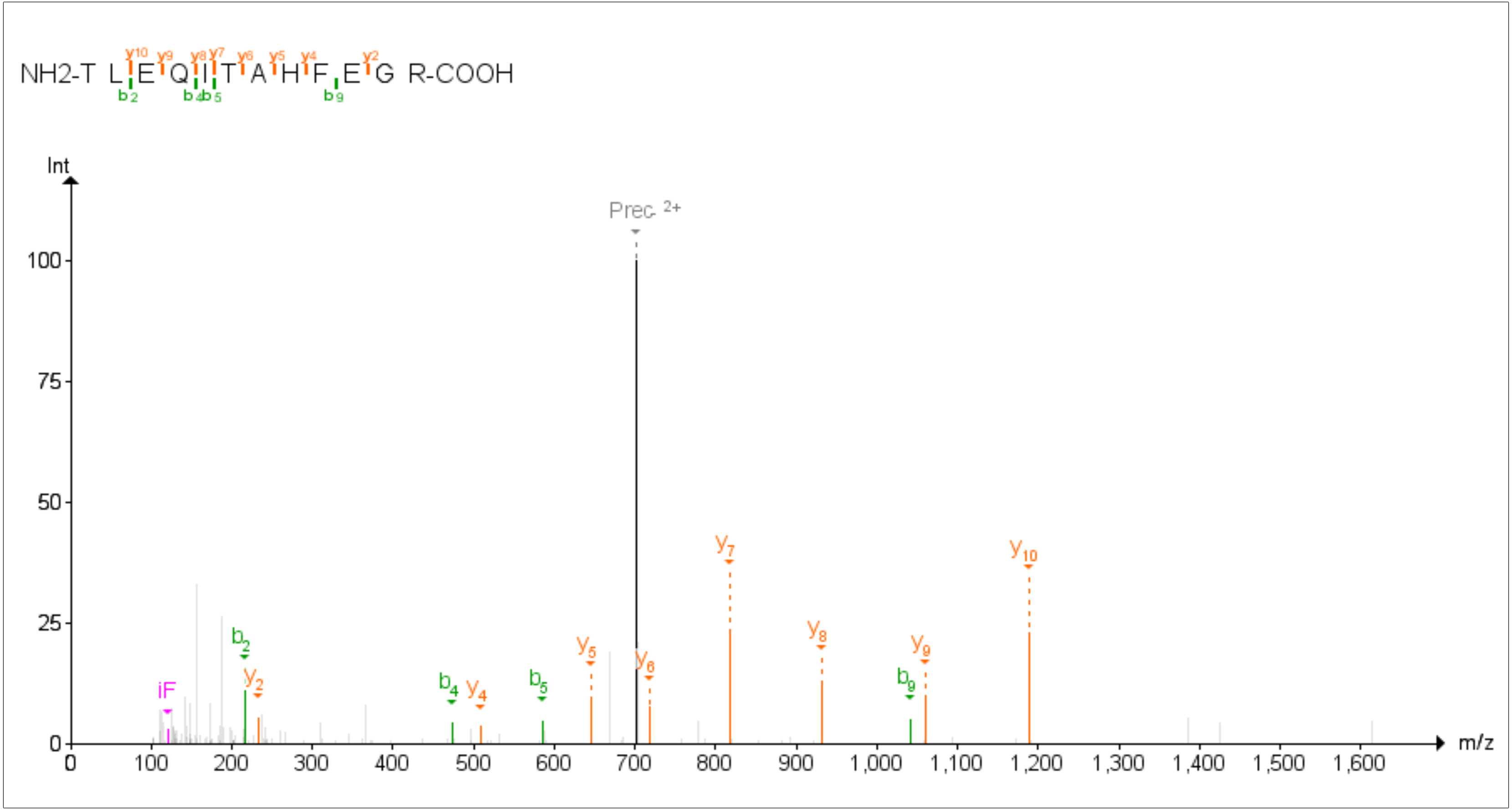
NH2-T L E Q I I T A H F E G R-COOH
y10 y9 y8 y7 y6 y5 y4 y2
b2 b4 b5 b9
Int
Prec 2+
100
75
50
25
0
iF
b2
y2
b4
y4
b5
y5
y6
y7
y8
b9
y9
y10
0 100 200 300 400 500 600 700 800 900 1,000 1,100 1,200 1,300 1,400 1,500 1,600
m/z

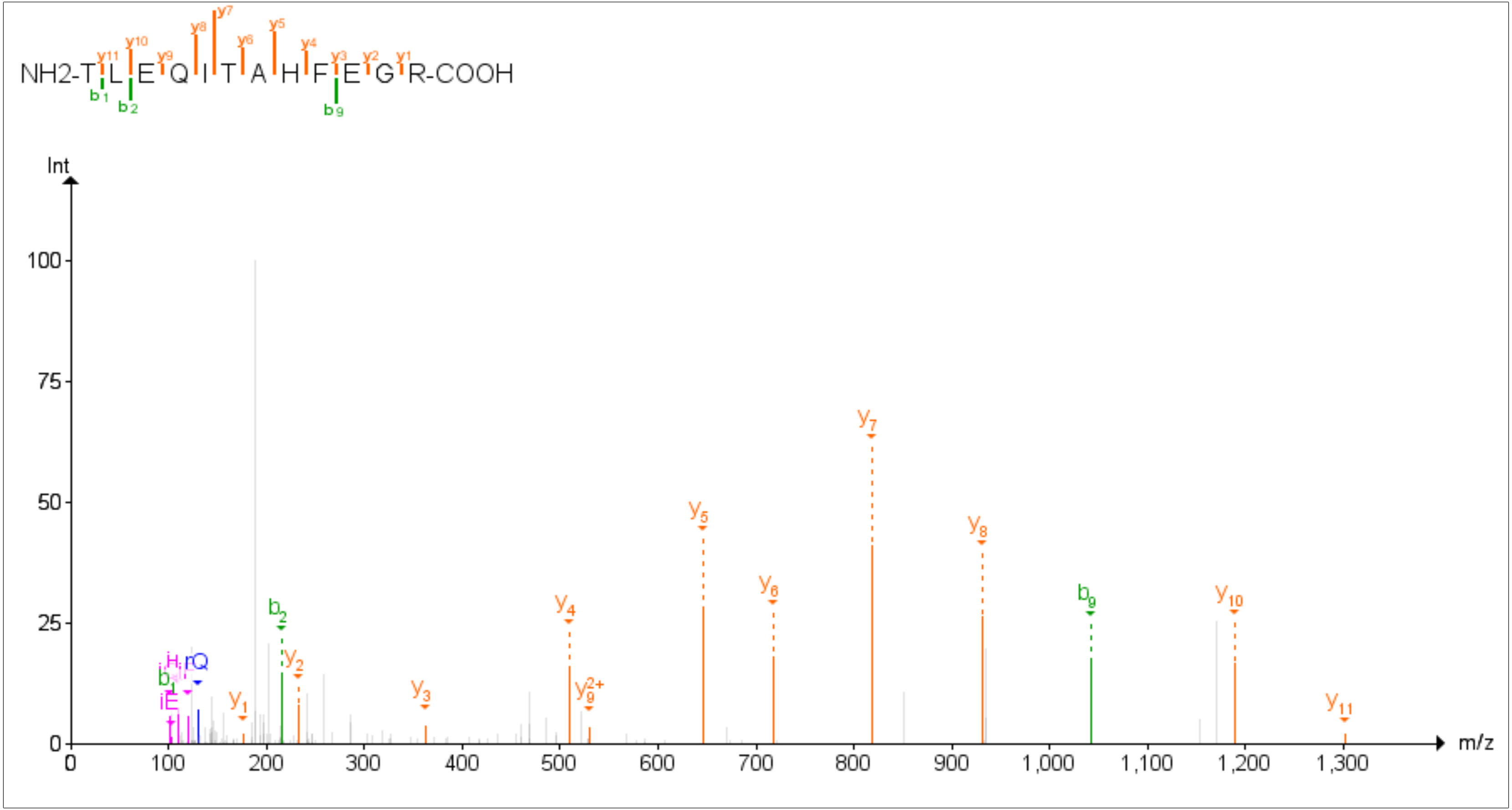
NH2-T L E Q I T A H F E G R-COOH
y11 y10 y9 y8 y7 y6 y5 y4 y3 y2 y1
b1 b2 b9
Int
100
75
50
25
0
m/z
0 100 200 300 400 500 600 700 800 900 1,000 1,100 1,200 1,300
y7
y5
y8
y6
b9
y10
y4
b2
y2
rQ
b1
iE
y1
y3
y_9^{2+}
y11

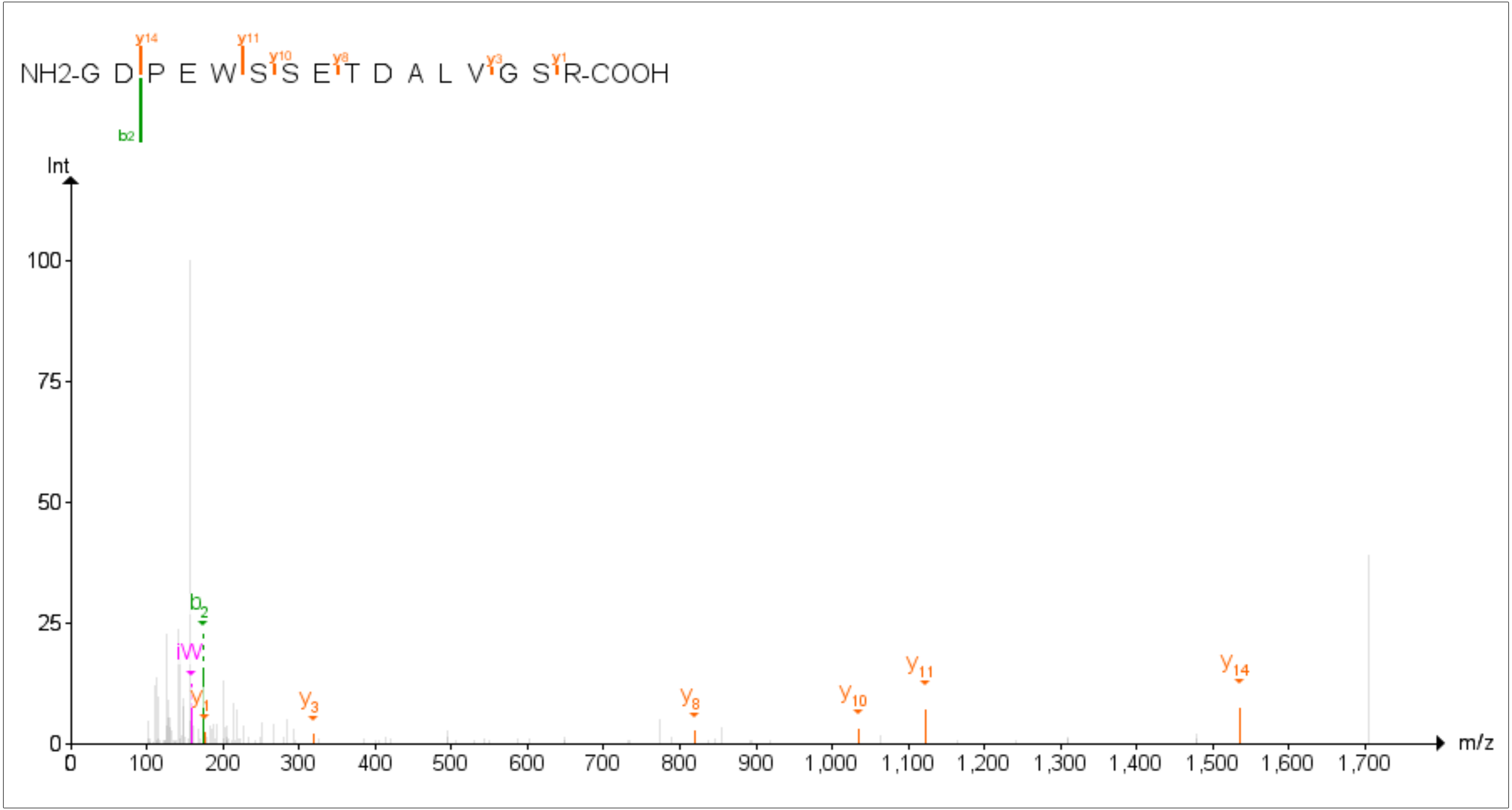
NH2-G D P E W S S E T D A L V G S R-COOH
y14
y11
y10
y8
y3
y1
b2
Int
m/z
b2
iW
y1
y3
y8
y10
y11
y14
0
25
50
75
100
0
100
200
300
400
500
600
700
800
900
1,000
1,100
1,200
1,300
1,400
1,500
1,600
1,700

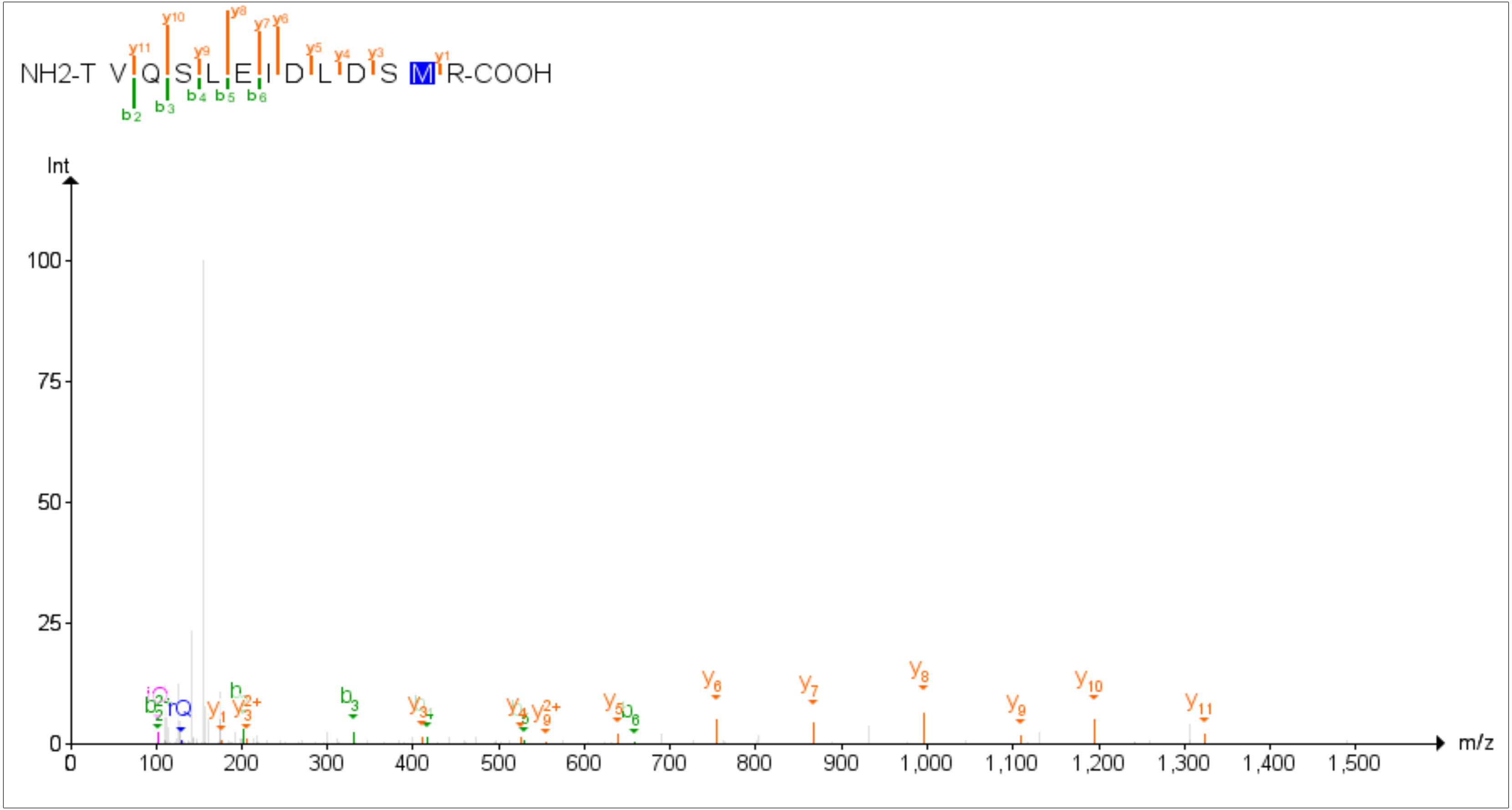
NH2-T V Q S L E I D L D S M R-COOH
Int
100
75
50
25
0
m/z
0
100
200
300
400
500
600
700
800
900
1,000
1,100
1,200
1,300
1,400
1,500

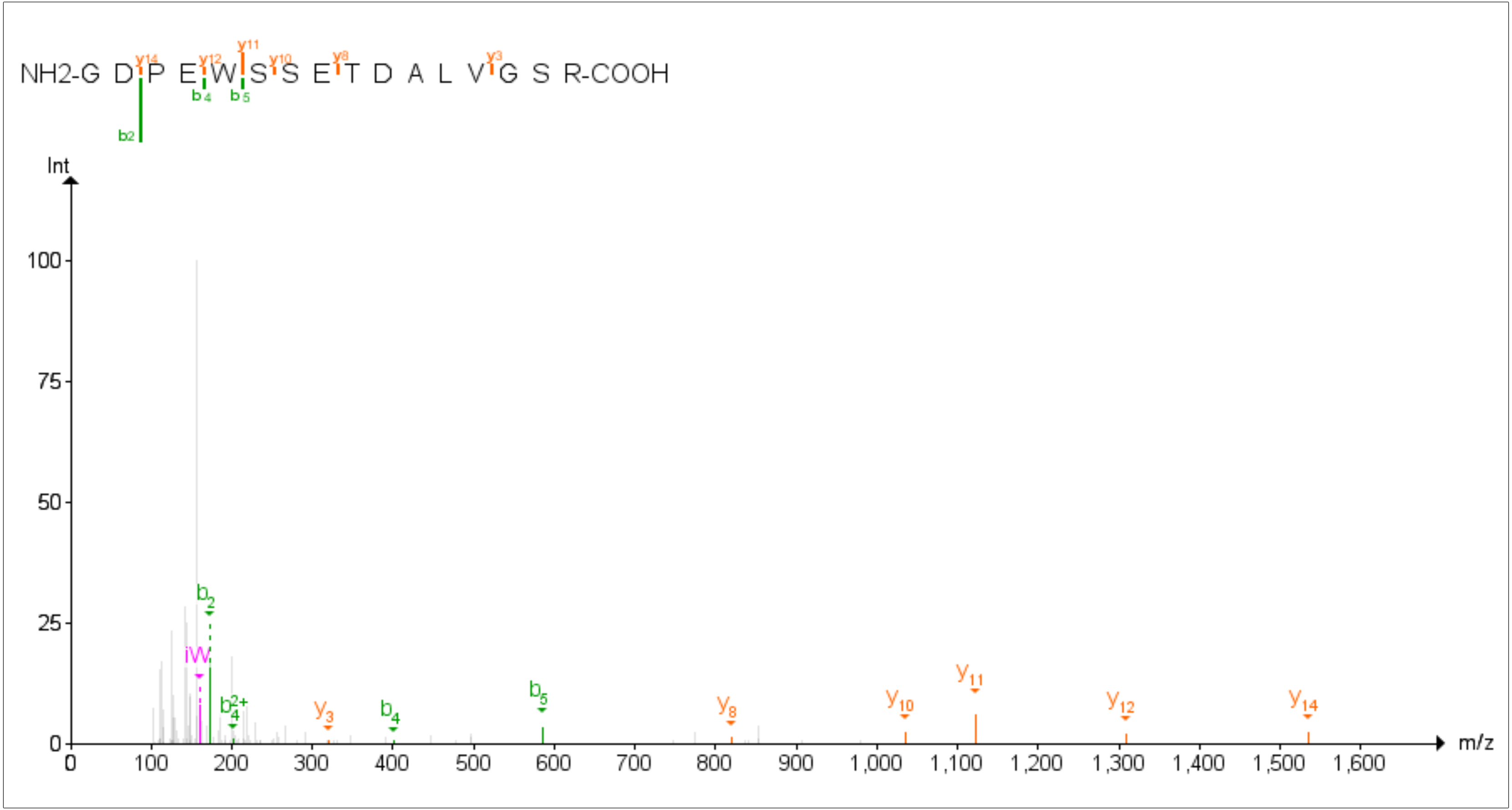
NH2-G D P E W S S E T D A L V G S R-COOH
y14 y12 y11 y10 y8 y3
b2 b4 b5
Int
m/z
b2
iW
b$_4^{2+}$
y3
b4
b5
y8
y10
y11
y12
y14

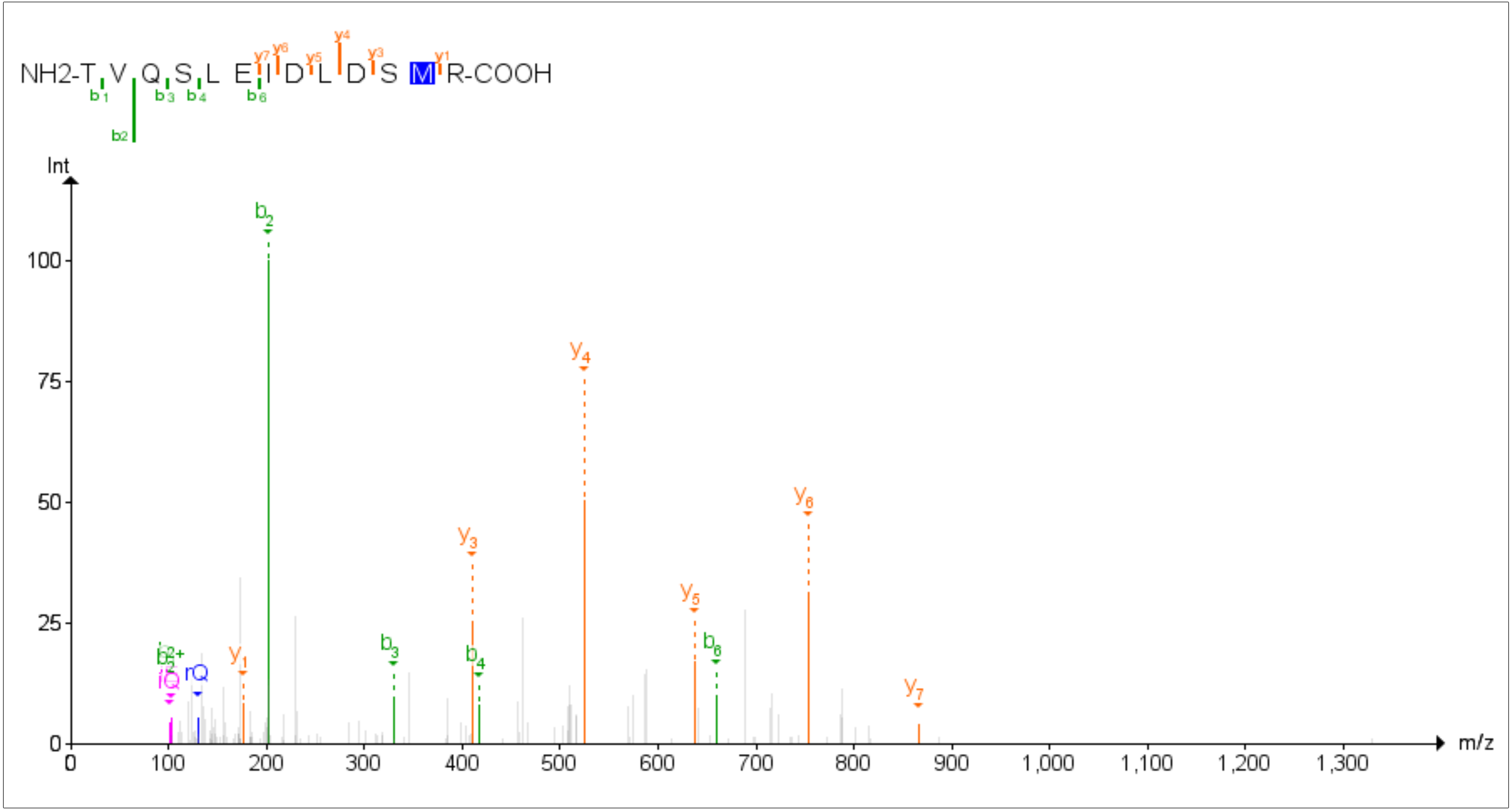
NH2-T V Q S L E I D L D S M R-COOH
b1 b2 b3 b4 b6
y7 y6 y5 y4 y3 y1
Int
100
75
50
25
0
b2
y4
y3
y6
y5
b6
b3
b4
y1
y7
rQ
iQ
m/z
0 100 200 300 400 500 600 700 800 900 1,000 1,100 1,200 1,300

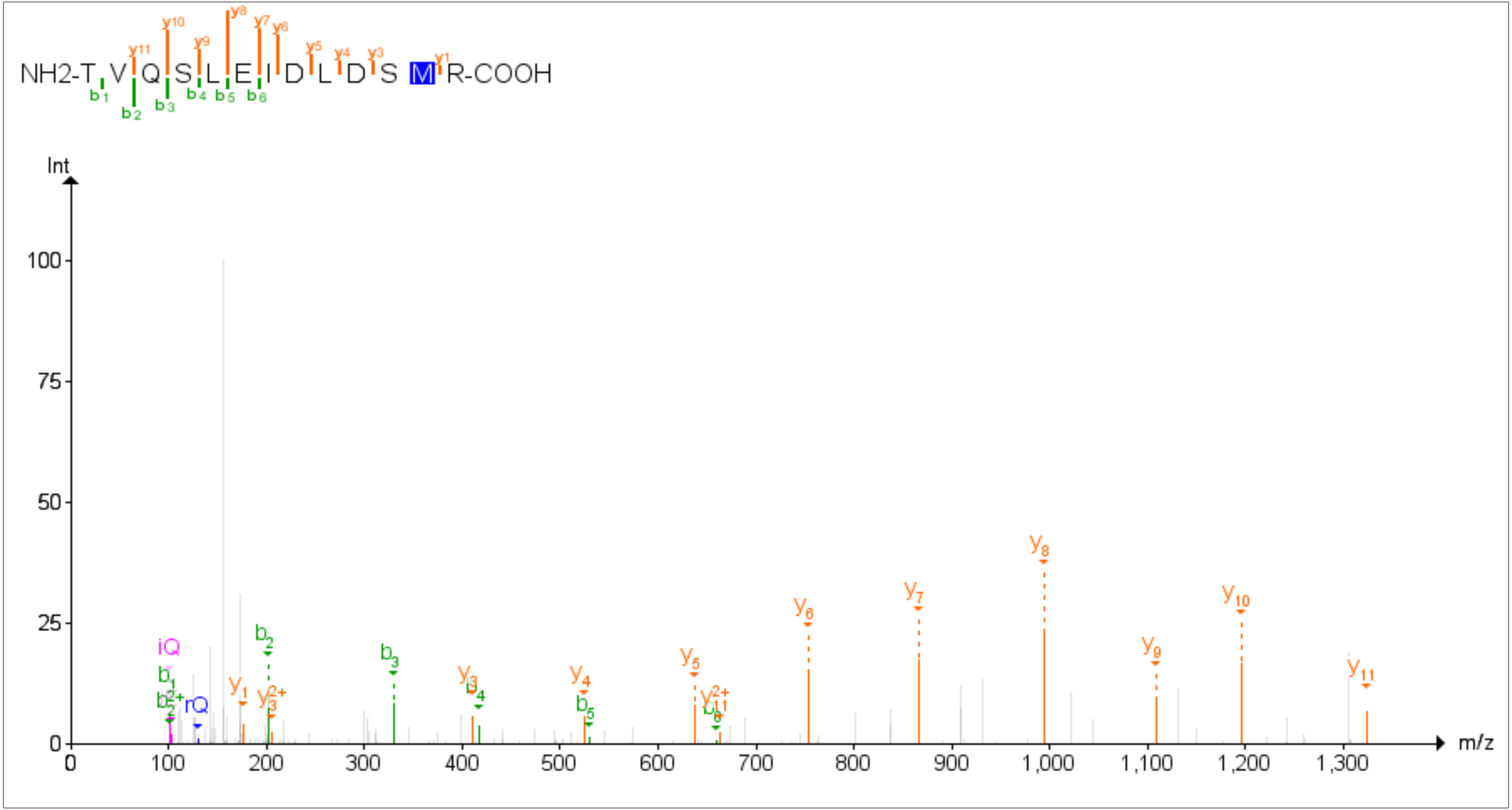

NH2-T V Q S L E I D L D S M R-COOH
Int
100
75
50
25
0
m/z
0
100
200
300
400
500
600
700
800
900
1,000
1,100
1,200
1,300
iQ
rQ

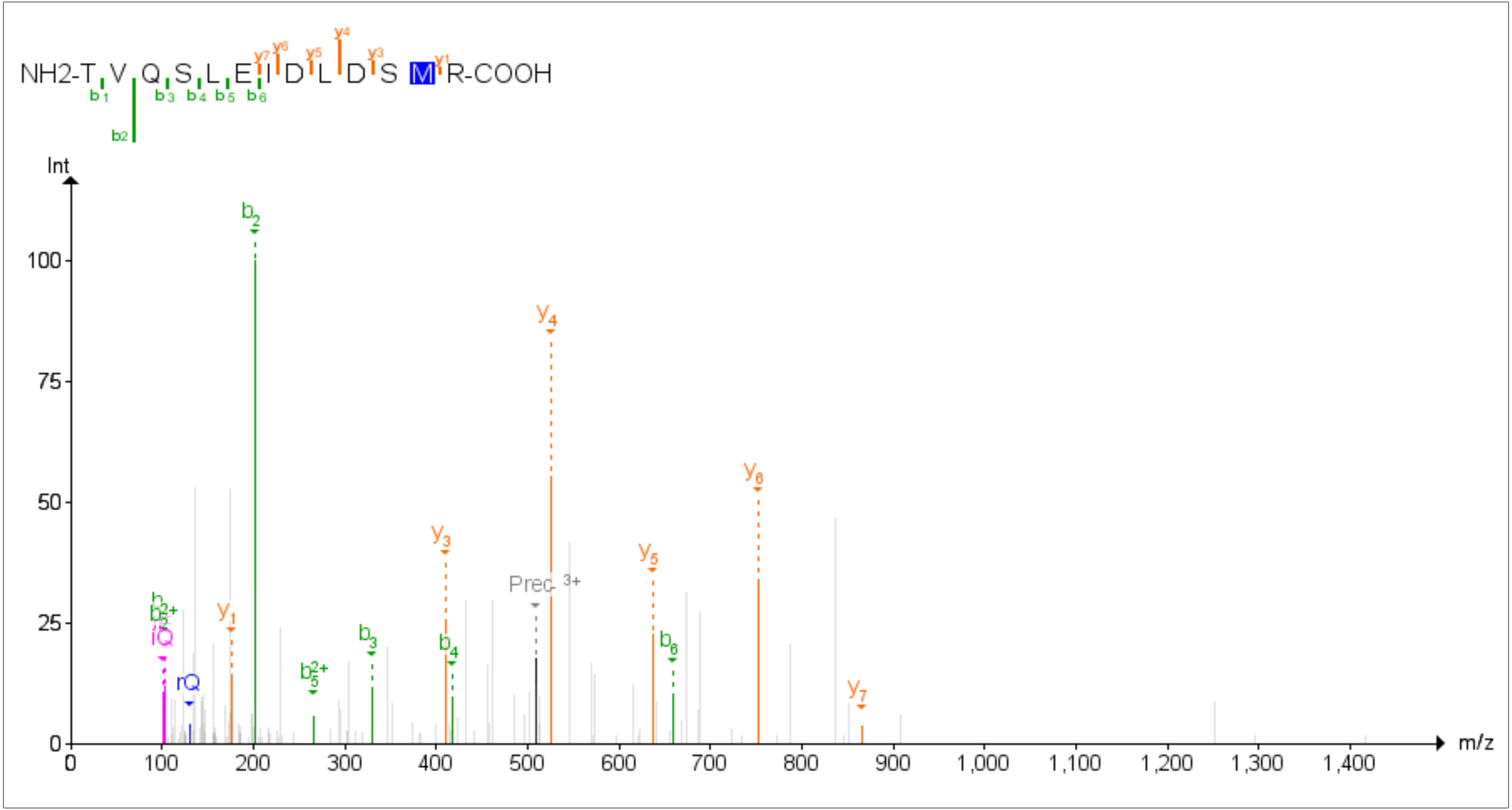

NH2-T V Q S L E I D L D S M R-COOH
b1 b2 b3 b4 b5 b6
y7 y6 y5 y4 y3 y1
Int
m/z
b2
y4
y6
y3
y5
y1
b3
b4
b6
y7
rQ
iQ
Prec 3+

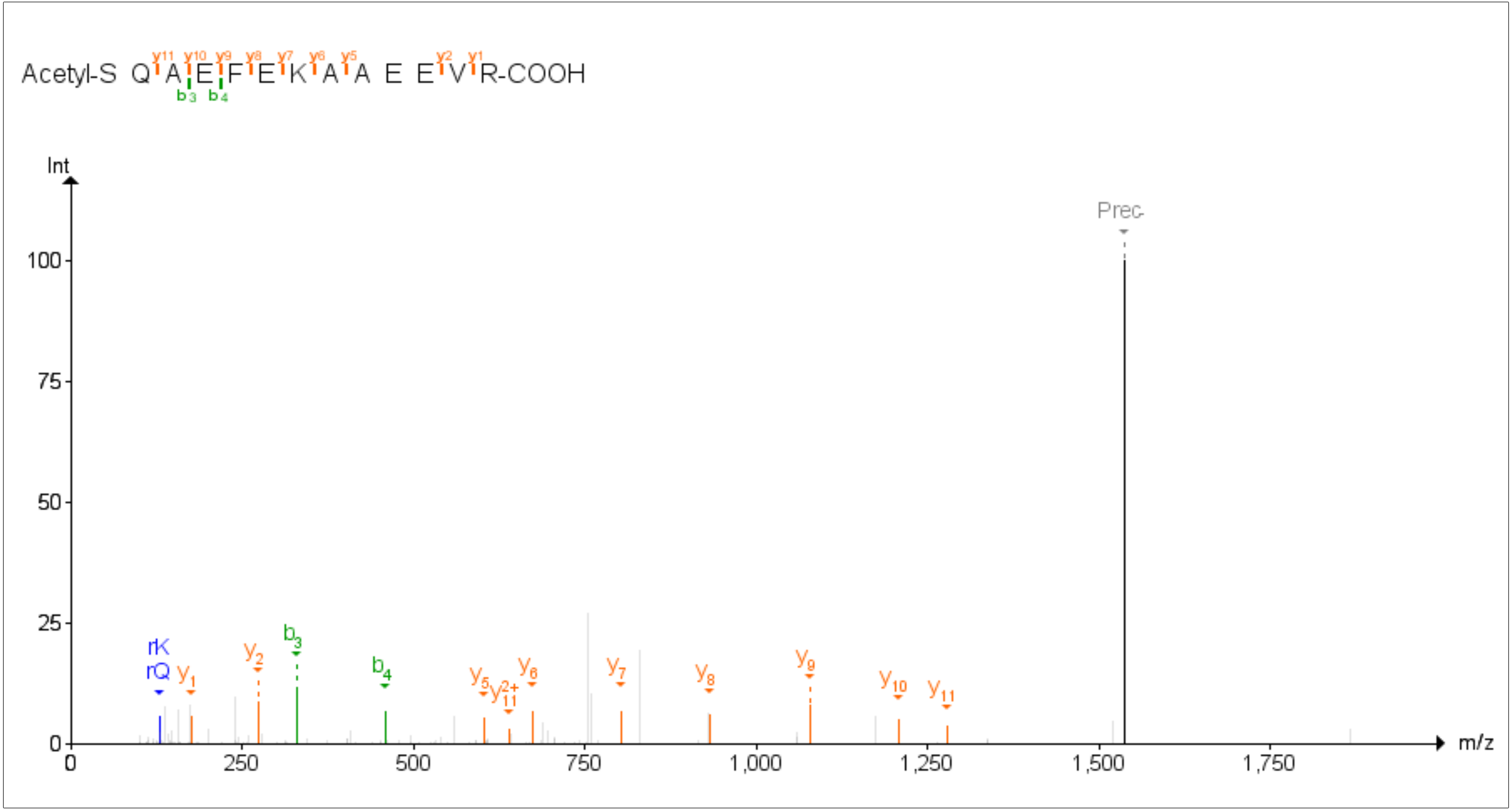

Acetyl-S Q A E F E K A A E E V R-COOH
y11 y10 y9 y8 y7 y6 y5 y2 y1
b3 b4
Int
100
75
50
25
0
Prec
rK
rQ
y1
y2
b3
b4
y5
y11 2+
y6
y7
y8
y9
y10
y11
0
250
500
750
1,000
1,250
1,500
1,750
m/z

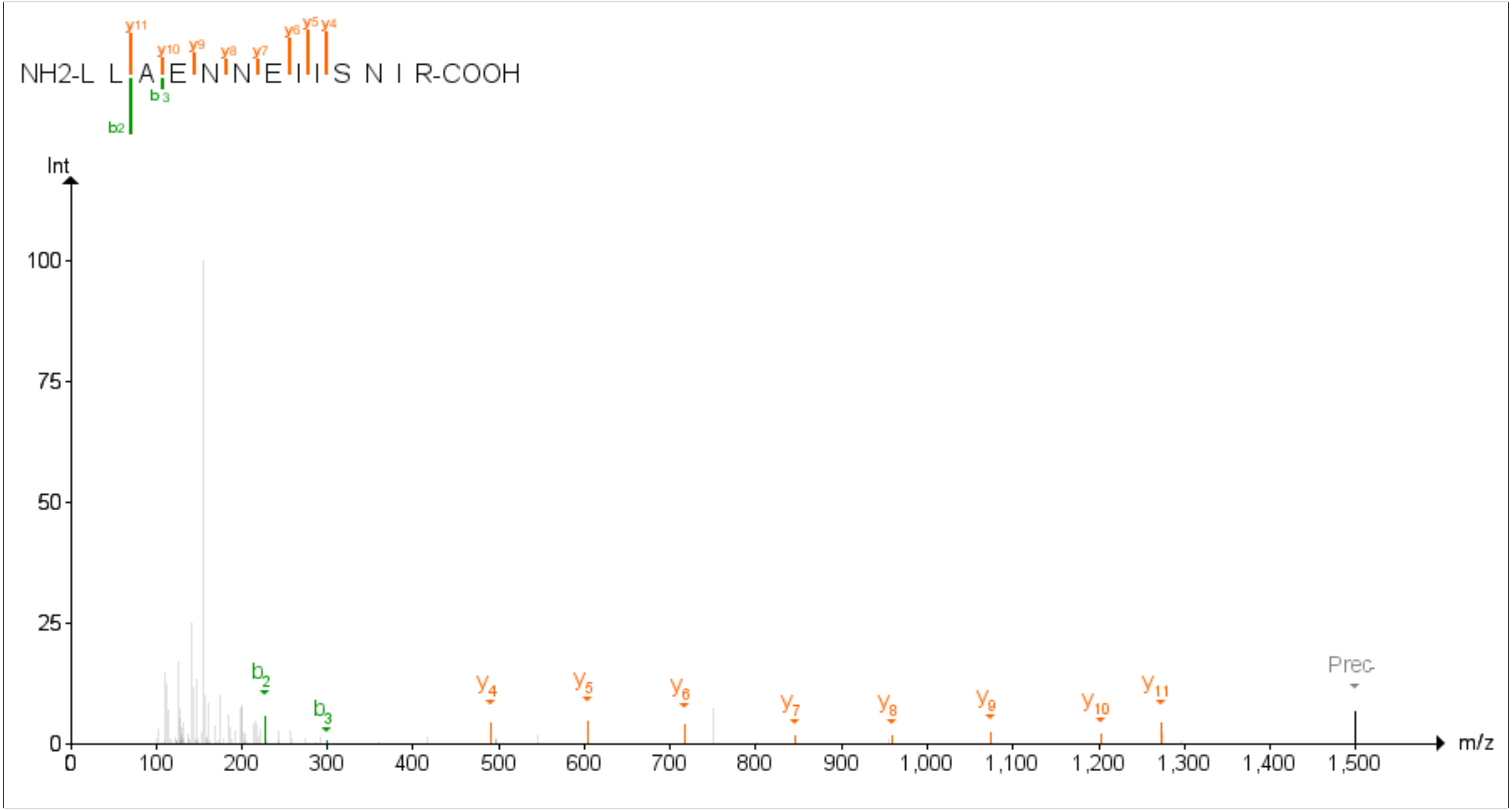

NH2-L L A E N N E I I S N I R-COOH
y11
y10
y9
y8
y7
y6
y5
y4
b2
b3
Int
100
75
50
25
0
b2
b3
y4
y5
y6
y7
y8
y9
y10
y11
Prec
0
100
200
300
400
500
600
700
800
900
1,000
1,100
1,200
1,300
1,400
1,500
m/z

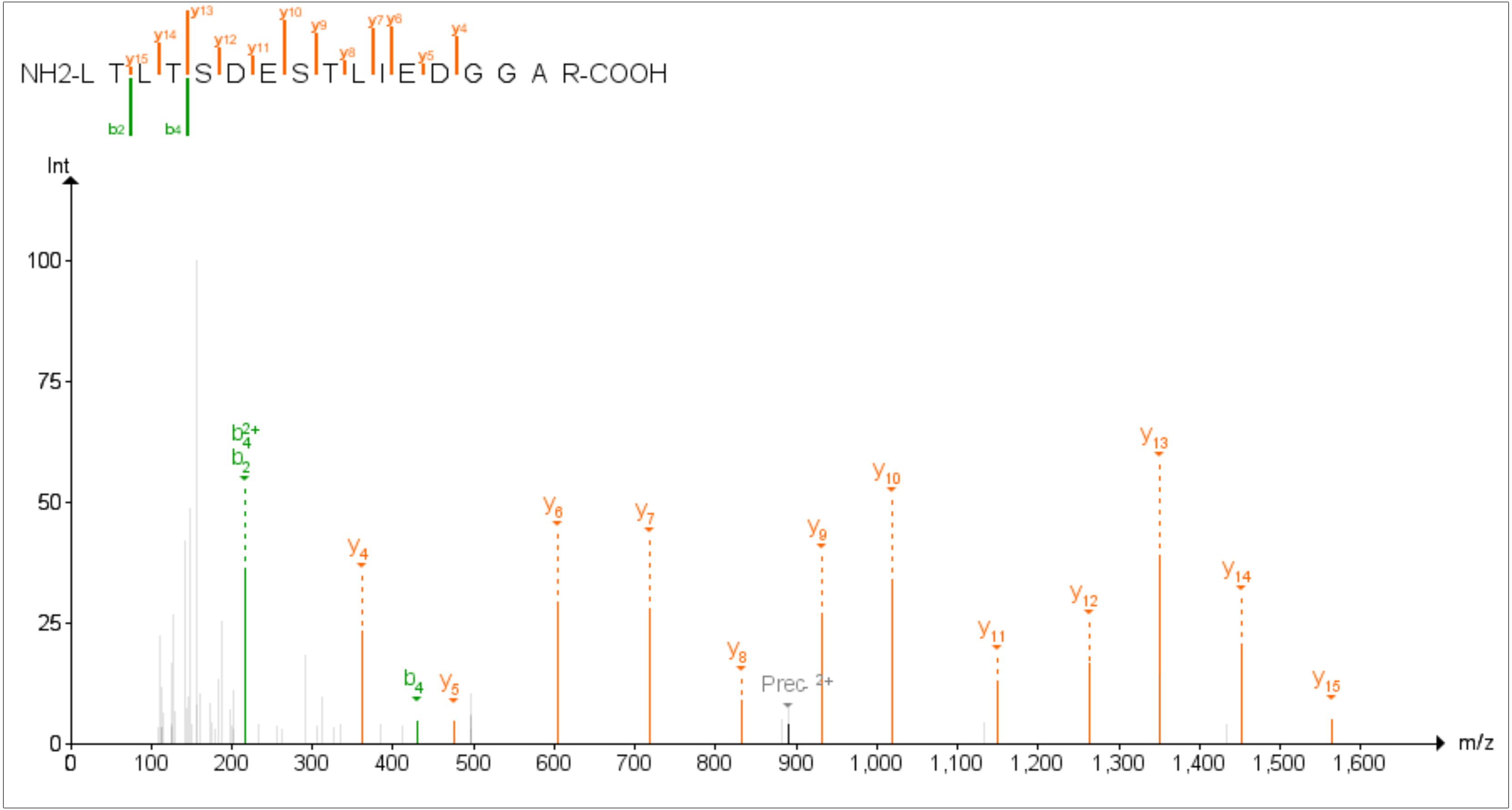

NH2-L T L T S D E S T L I E D G G A R-COOH
y15 y14 y13 y12 y11 y10 y9 y8 y7 y6 y5 y4
b2 b4
Int
m/z
Prec. 2+

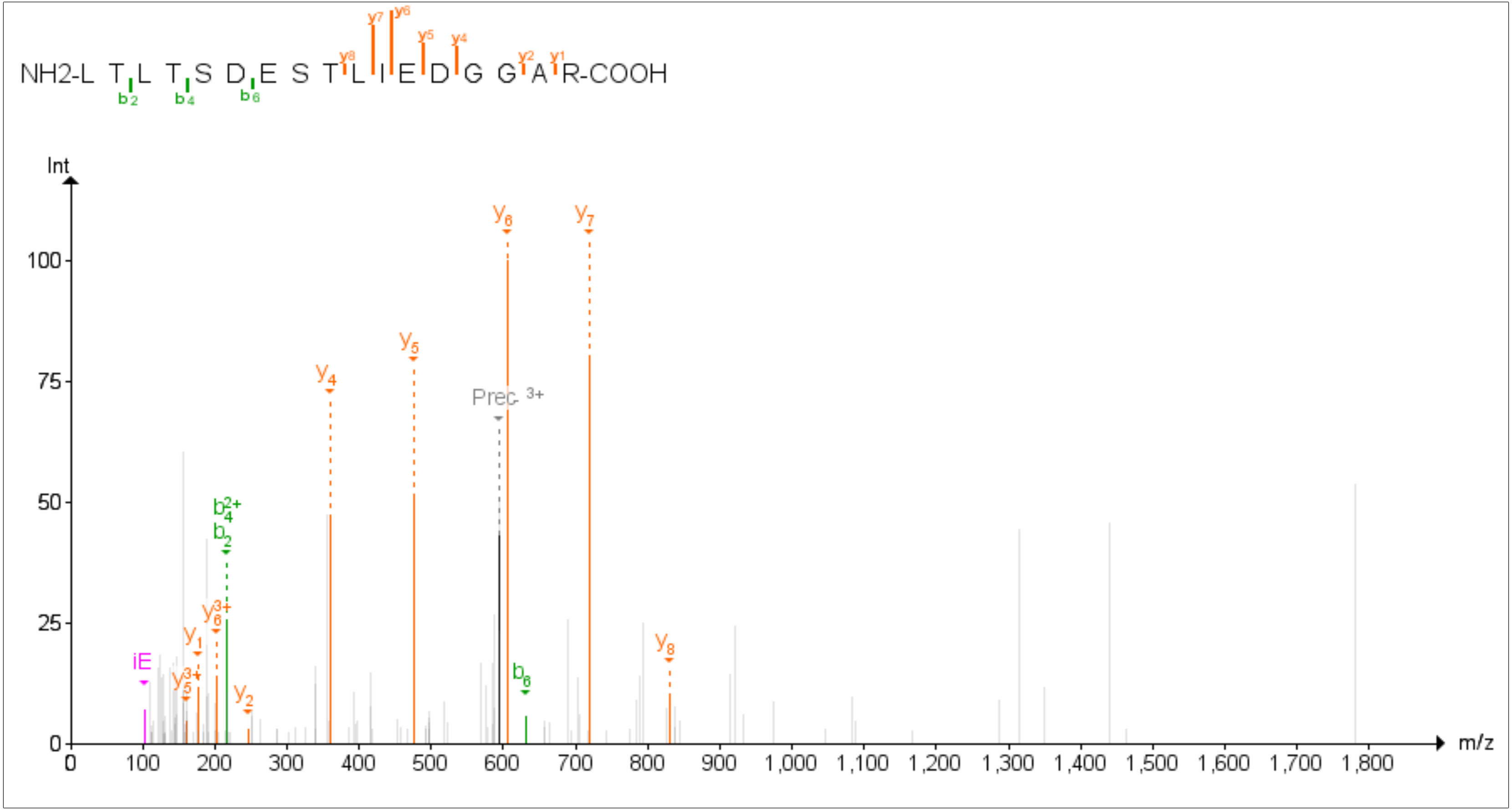
NH2-L T L T S D E S T L I E D G G A R-COOH
b2
b4
b6
y8
y7
y6
y5
y4
y2
y1
Int
m/z
Prec. 3+
iE
y_1
y_2
y_4
y_5
y_6
y_7
y_8
y_5^{3+}
y_6^{3+}
b_2
b_4^{2+}
b_6
0
25
50
75
100
0
100
200
300
400
500
600
700
800
900
1,000
1,100
1,200
1,300
1,400
1,500
1,600
1,700
1,800

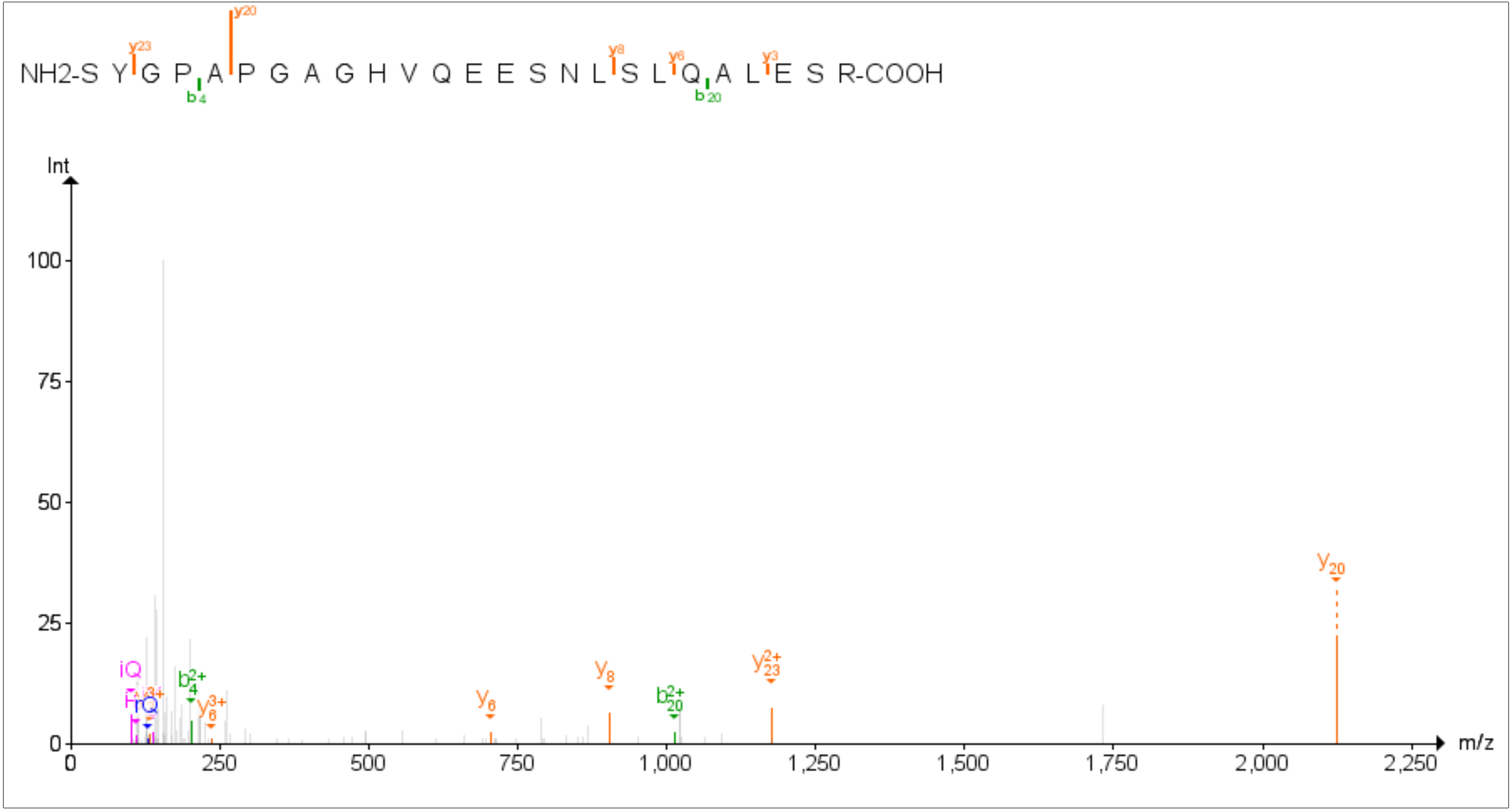

NH2-S Y G P A P G A G H V Q E E S N L S L Q A L E S R-COOH
y23
y20
y8
y6
y3
b4
b20
Int
100
75
50
25
0
m/z
0
250
500
750
1,000
1,250
1,500
1,750
2,000
2,250
iQ
y20
y8
y6
b4 2+
y6 3+
b20 2+
y23 2+

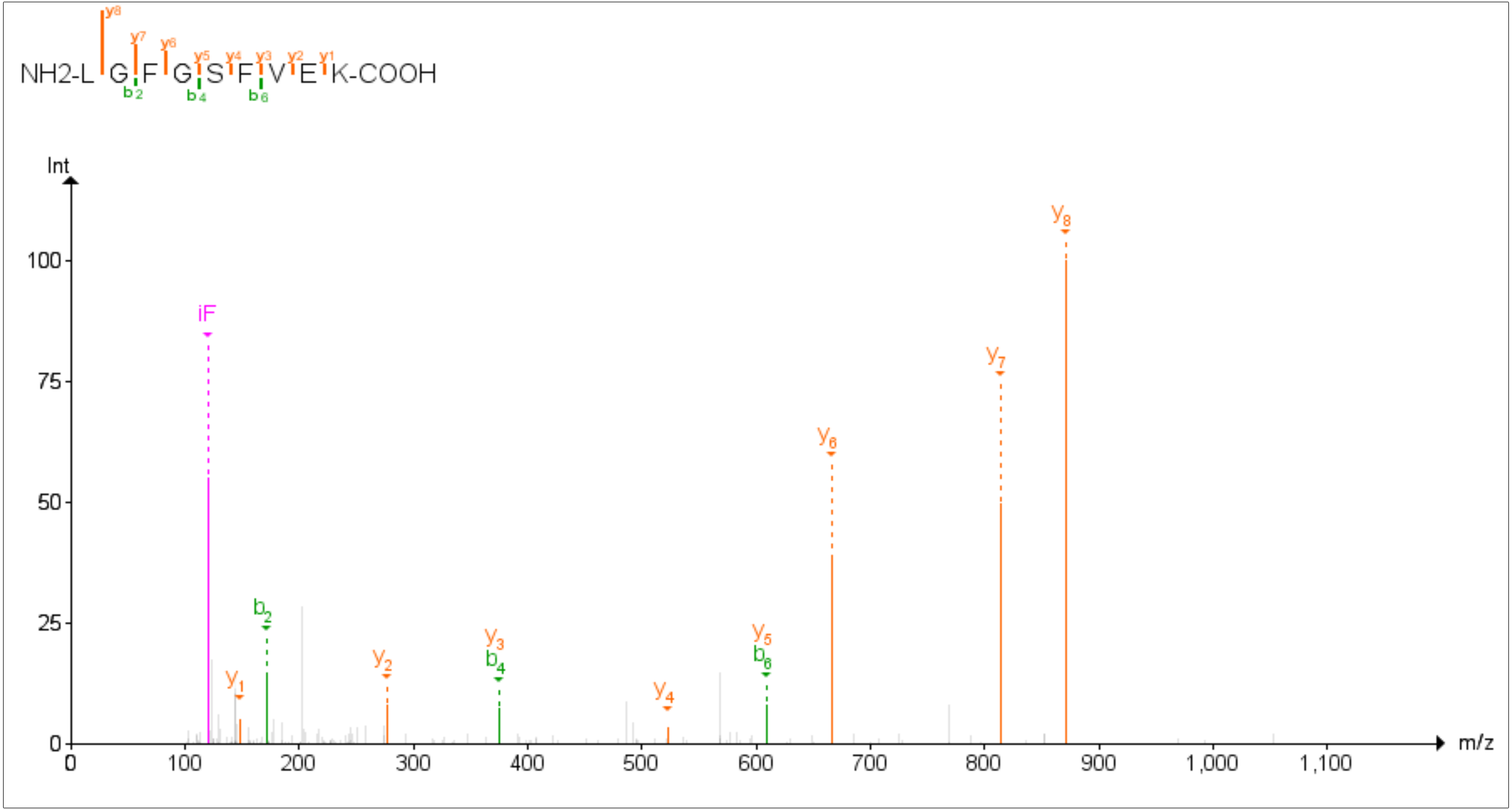

NH2-L G F G S F V E K-COOH
y8 y7 y6 y5 y4 y3 y2 y1
b2 b4 b6
Int
100
75
50
25
0
iF
y1
b2
y2
y3
b4
y4
y5
b6
y6
y7
y8
0 100 200 300 400 500 600 700 800 900 1,000 1,100
m/z

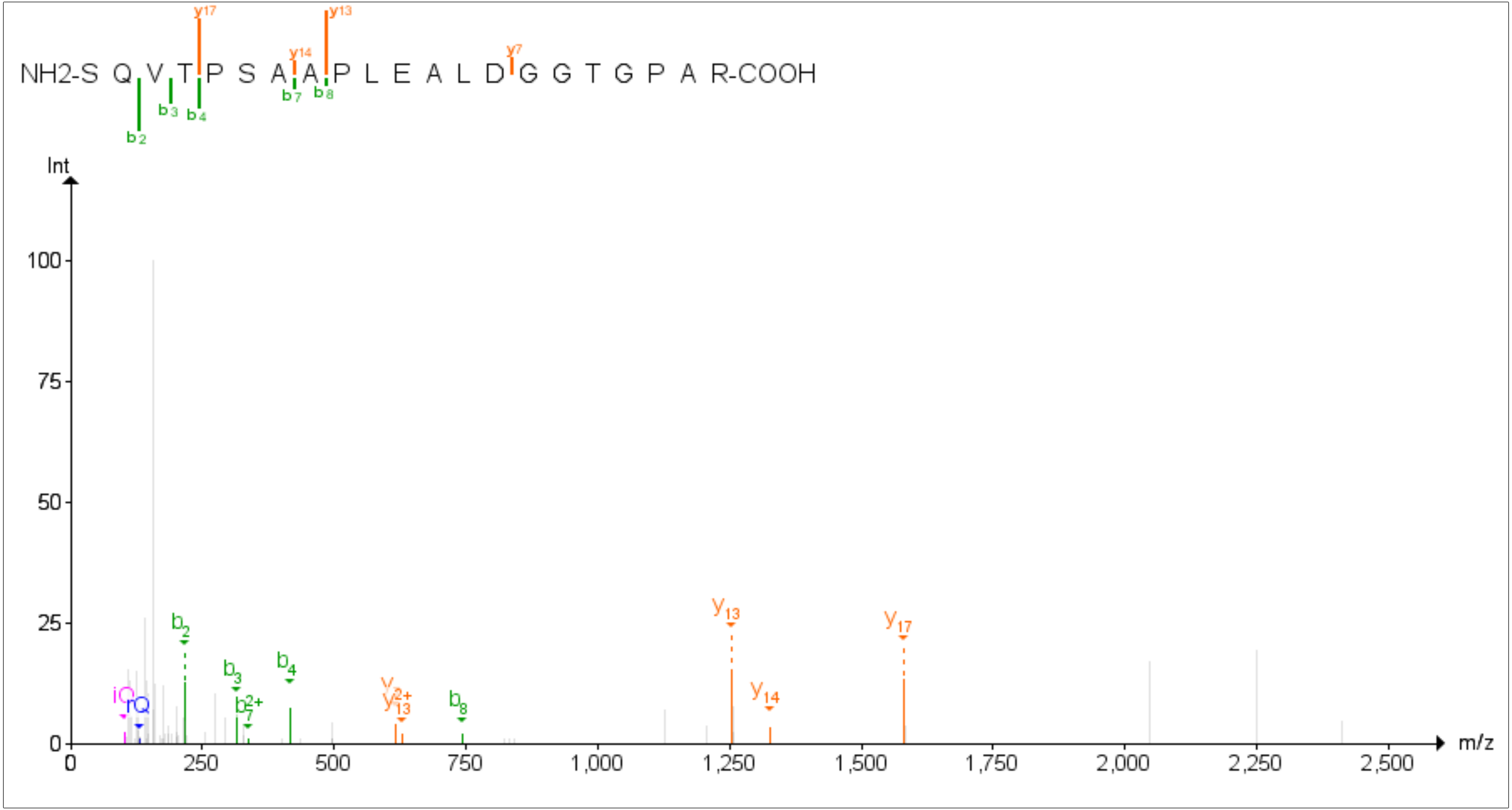

NH2-S Q V T P S A A P L E A L D G G T G P A R-COOH
y17
y13
y14
y7
b2
b3
b4
b7
b8
Int
100
75
50
25
0
250
500
750
1,000
1,250
1,500
1,750
2,000
2,250
2,500
m/z
iQ
rQ
b2
b3
b4
b7 2+
y13 2+
b8
y13
y14
y17

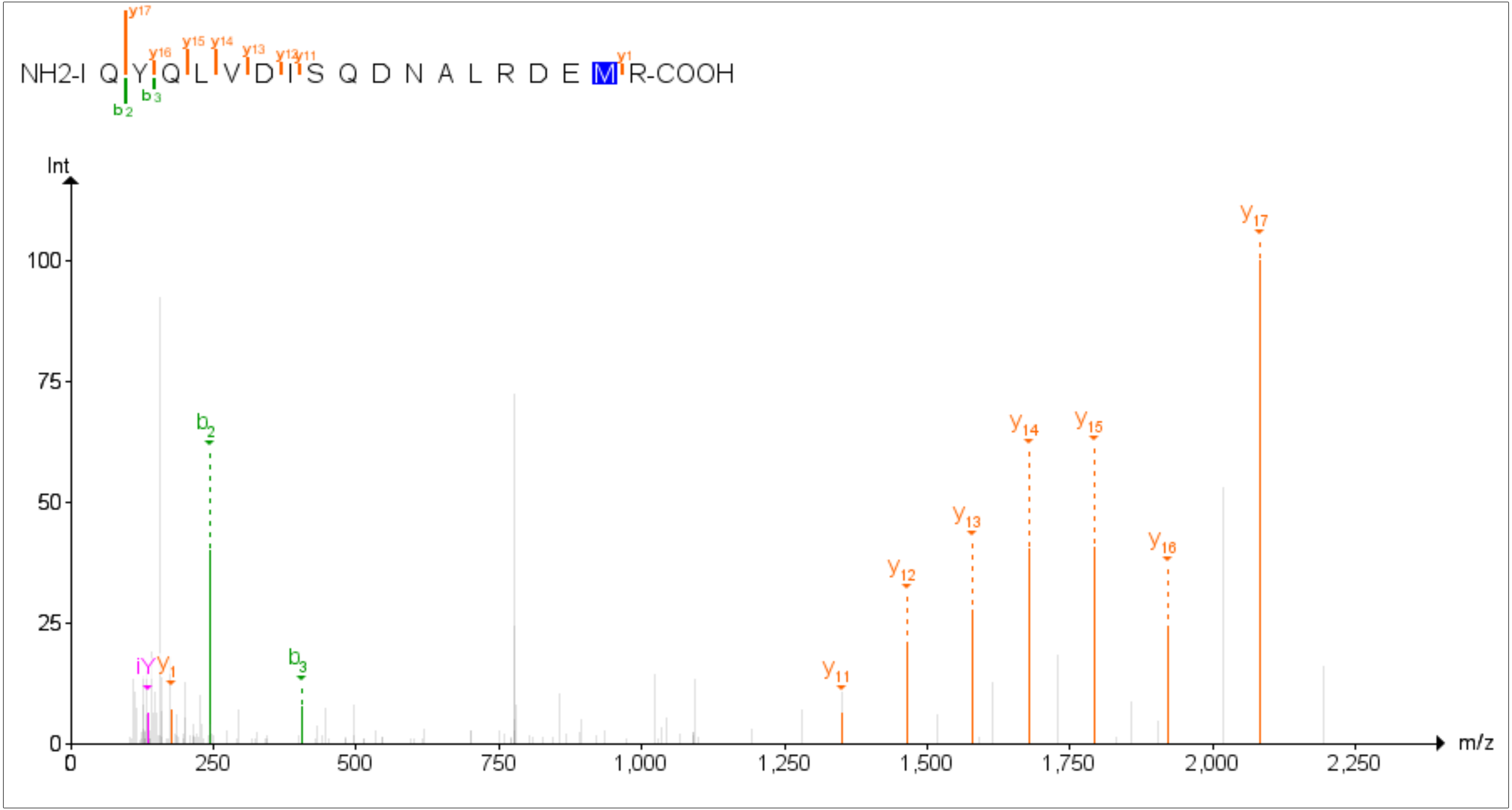

NH2-I Q Y Q L V D I S Q D N A L R D E M R-COOH
y17
y16
y15
y14
y13
y12
y11
y1
b2
b3
Int
100
75
50
25
0
m/z
0
250
500
750
1,000
1,250
1,500
1,750
2,000
2,250
iY

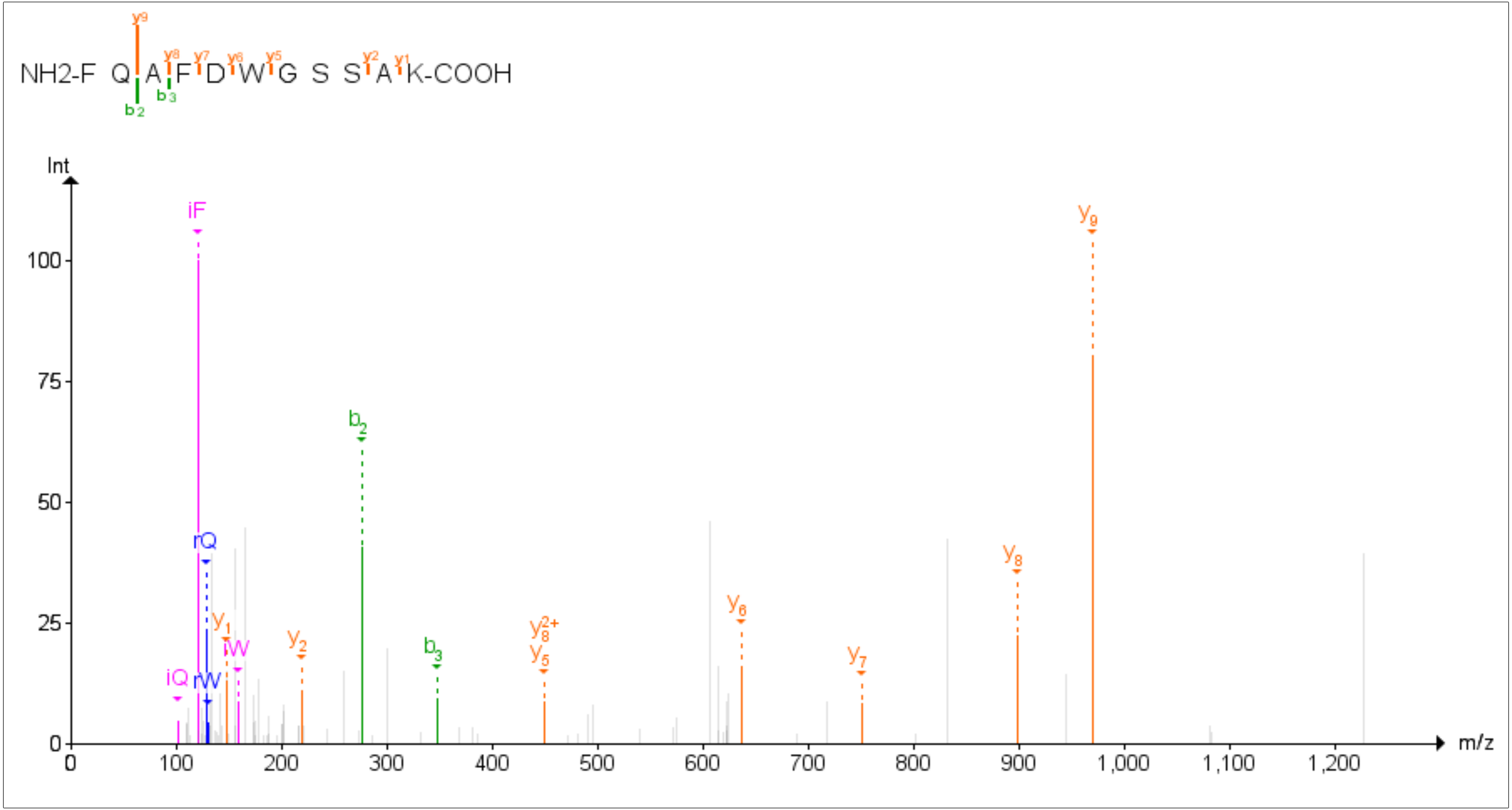

NH2-F Q A F D W G S S A K-COOH
y9
y8
y7
y6
y5
y2
y1
b2
b3
Int
100
75
50
25
0
iF
rQ
y1
iW
iQ
rW
y2
b2
b3
y8 2+
y5
y6
y7
y8
y9
0
100
200
300
400
500
600
700
800
900
1,000
1,100
1,200
m/z

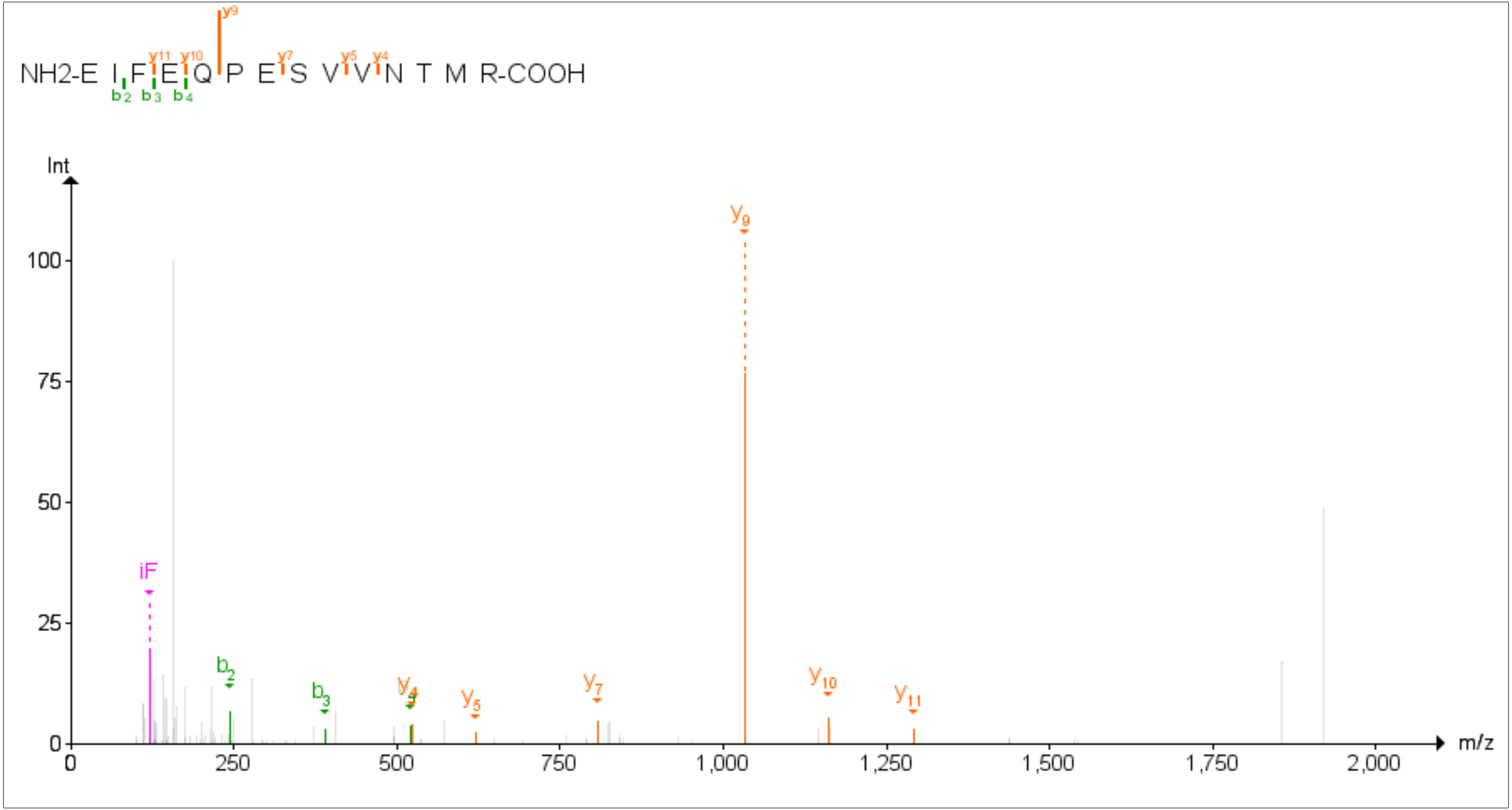

NH2-E I F E Q P E S V V N T M R-COOH
y9
y11
y10
y7
y5
y4
b2
b3
b4
Int
100
75
50
25
0
y9
iF
b2
b3
y4
y5
y7
y10
y11
0
250
500
750
1,000
1,250
1,500
1,750
2,000
m/z

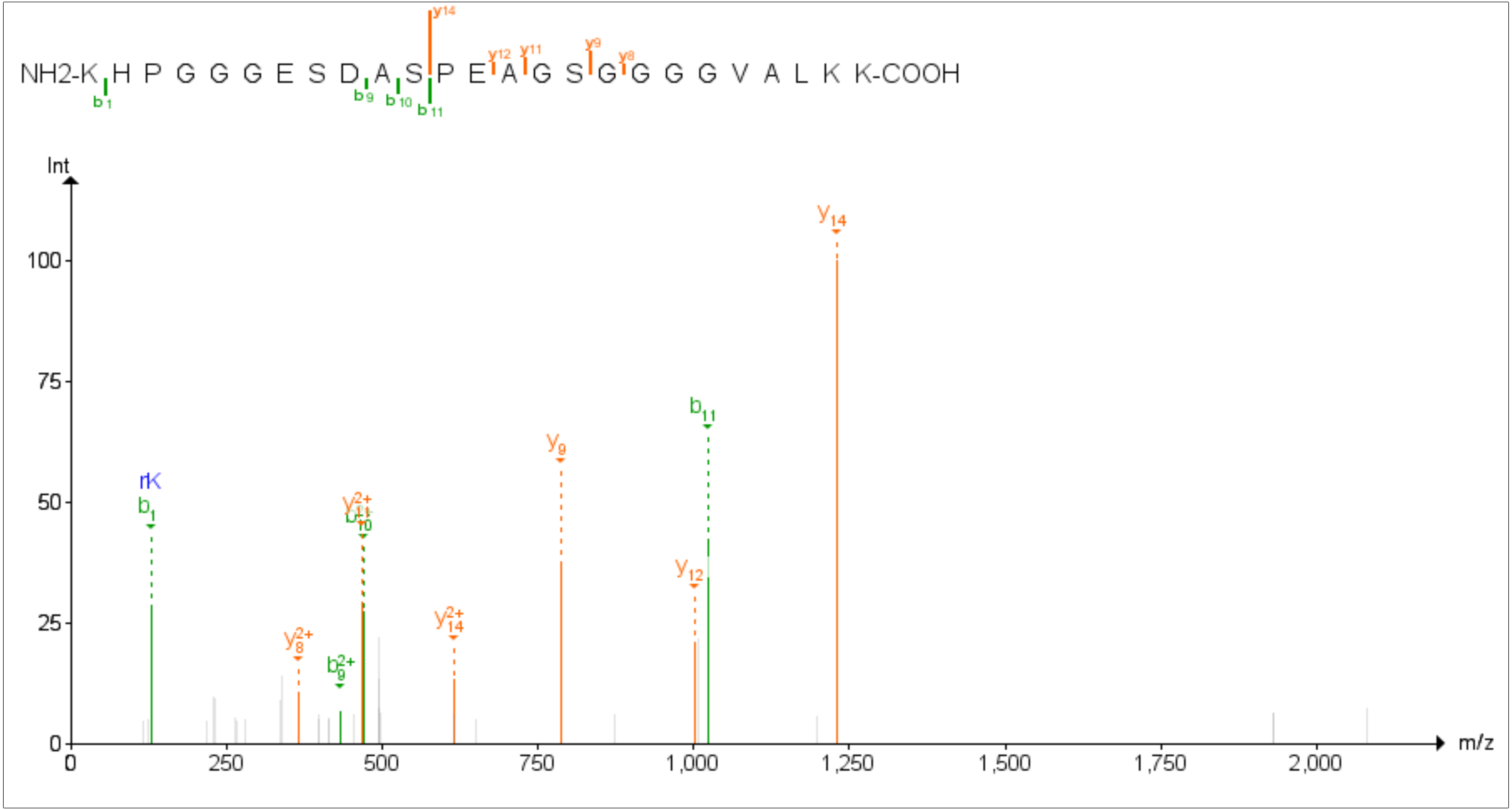
NH2-K H P G G G E S D A S P E A G S G G G G V A L K K-COOH
b1
b9
b10
b11
y14
y12
y11
y9
y8
Int
m/z
0
25
50
75
100
250
500
750
1,000
1,250
1,500
1,750
2,000
y14
b11
y9
rK
b1
y11 2+
b10
y12
y14 2+
y8 2+
b9 2+

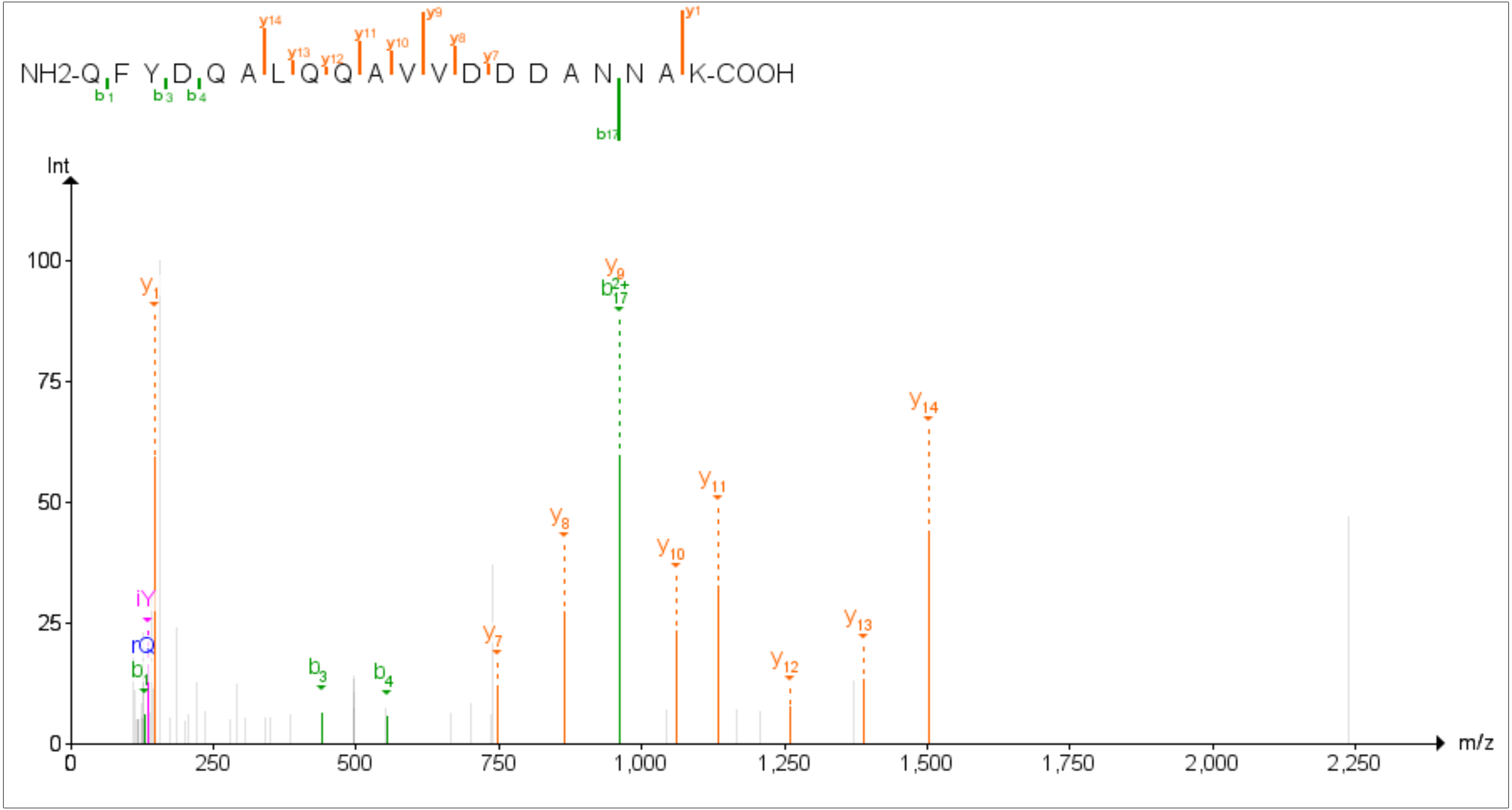
NH2-Q F Y D Q A L Q Q A V V D D D A N N A K-COOH
y14 y13 y12 y11 y10 y9 y8 y7 y1
b1 b3 b4 b17
Int
100
75
50
25
0
0
250
500
750
1,000
1,250
1,500
1,750
2,000
2,250
m/z
y1
y9
b17 2+
y14
y8
y11
y10
y13
y12
y7
b3
b4
iY
rQ
b1

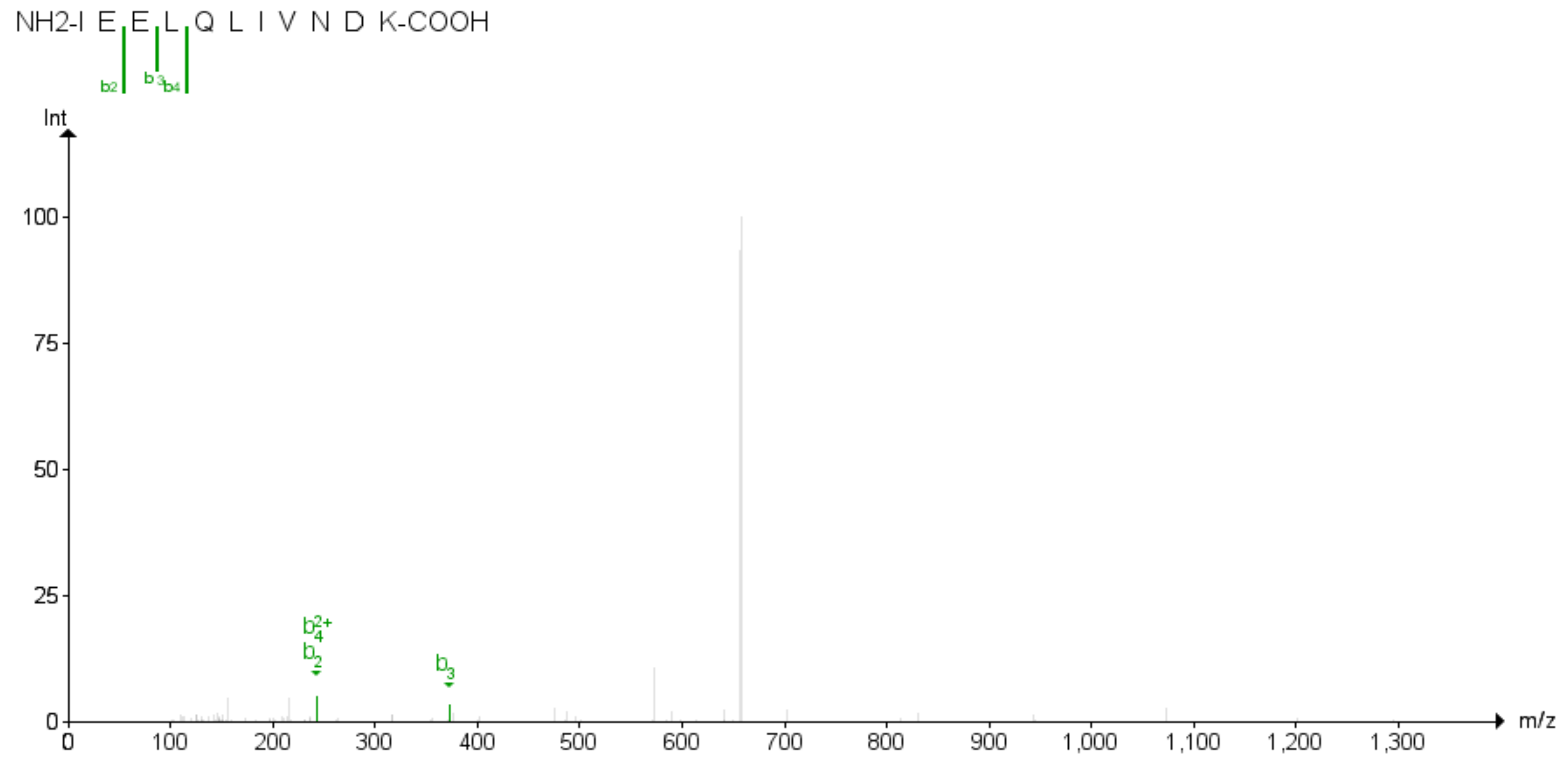

NH2-I E E L Q L I V N D K-COOH
b2
b3
b4
Int
100
75
50
25
0
b_4^{2+}
b_2
b_3
0
100
200
300
400
500
600
700
800
900
1,000
1,100
1,200
1,300
m/z

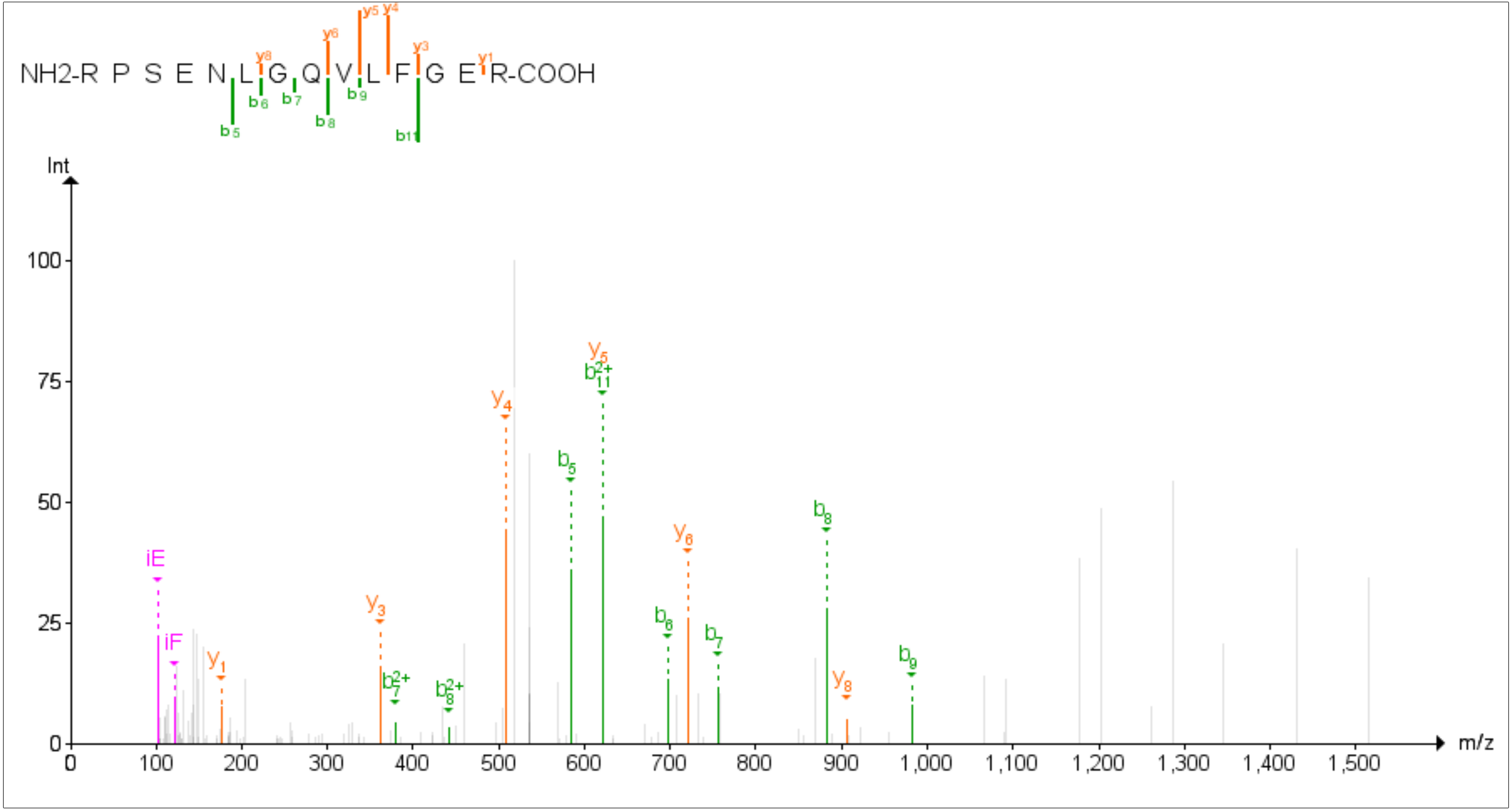

NH2-R P S E N L G Q V L F G E R-COOH
y8 y6 y5 y4 y3 y1
b5 b6 b7 b8 b9 b11
Int
100
75
50
25
0
m/z
0 100 200 300 400 500 600 700 800 900 1,000 1,100 1,200 1,300 1,400 1,500
iE
iF
y1
y3
y4
y5
y6
y8
b5
b6
b7
b8
b9
b7 2+
b8 2+
b11 2+

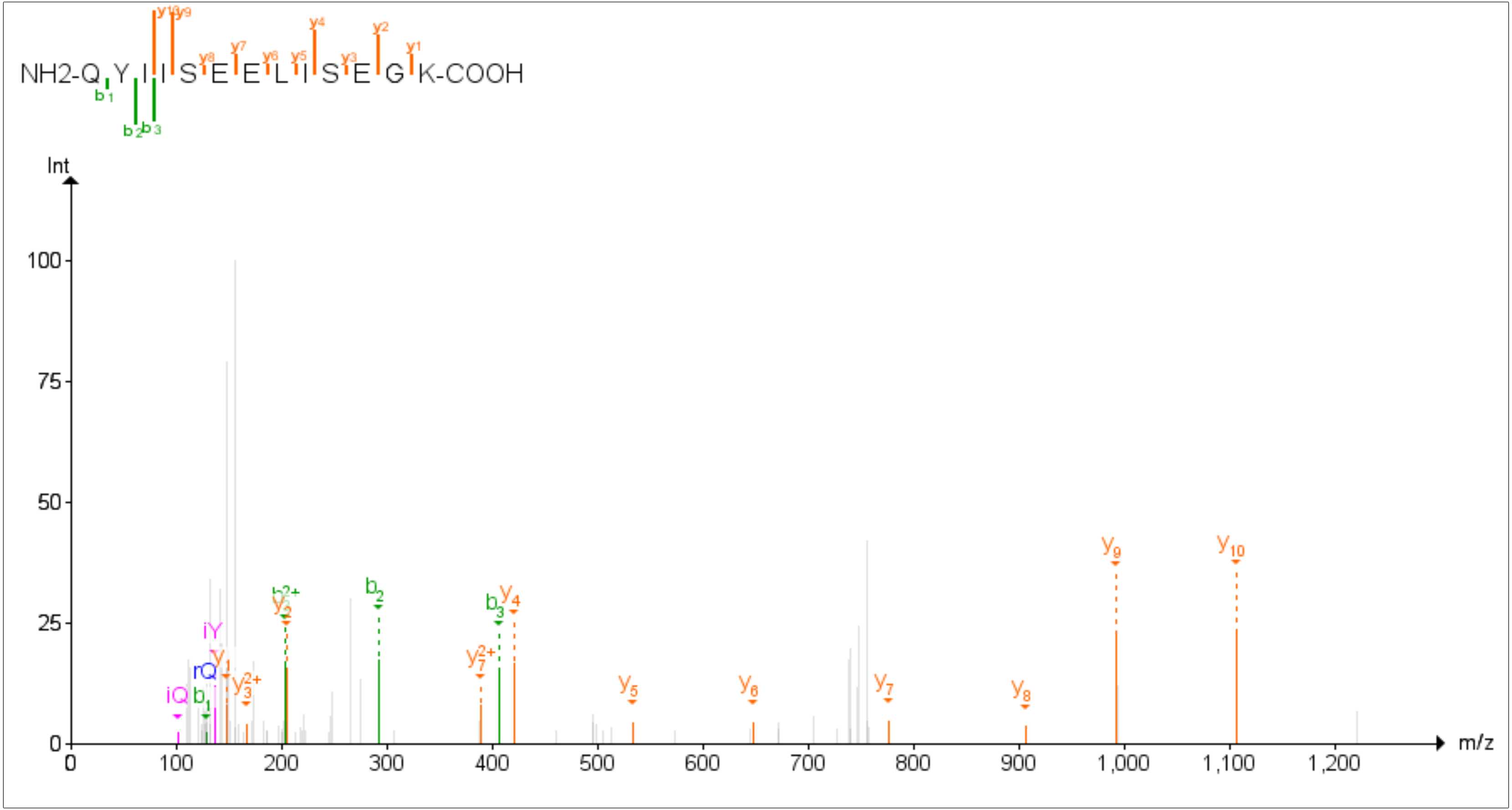

NH2-Q Y I I S E E L I S E G K-COOH
y10 y9 y8 y7 y6 y5 y4 y3 y2 y1
b1 b2 b3
Int
100
75
50
25
0
iQ
b1
rQ
iY
y1
y3 2+
y2
b2
b3
y4
y7 2+
y5
y6
y7
y8
y9
y10
0
100
200
300
400
500
600
700
800
900
1,000
1,100
1,200
m/z

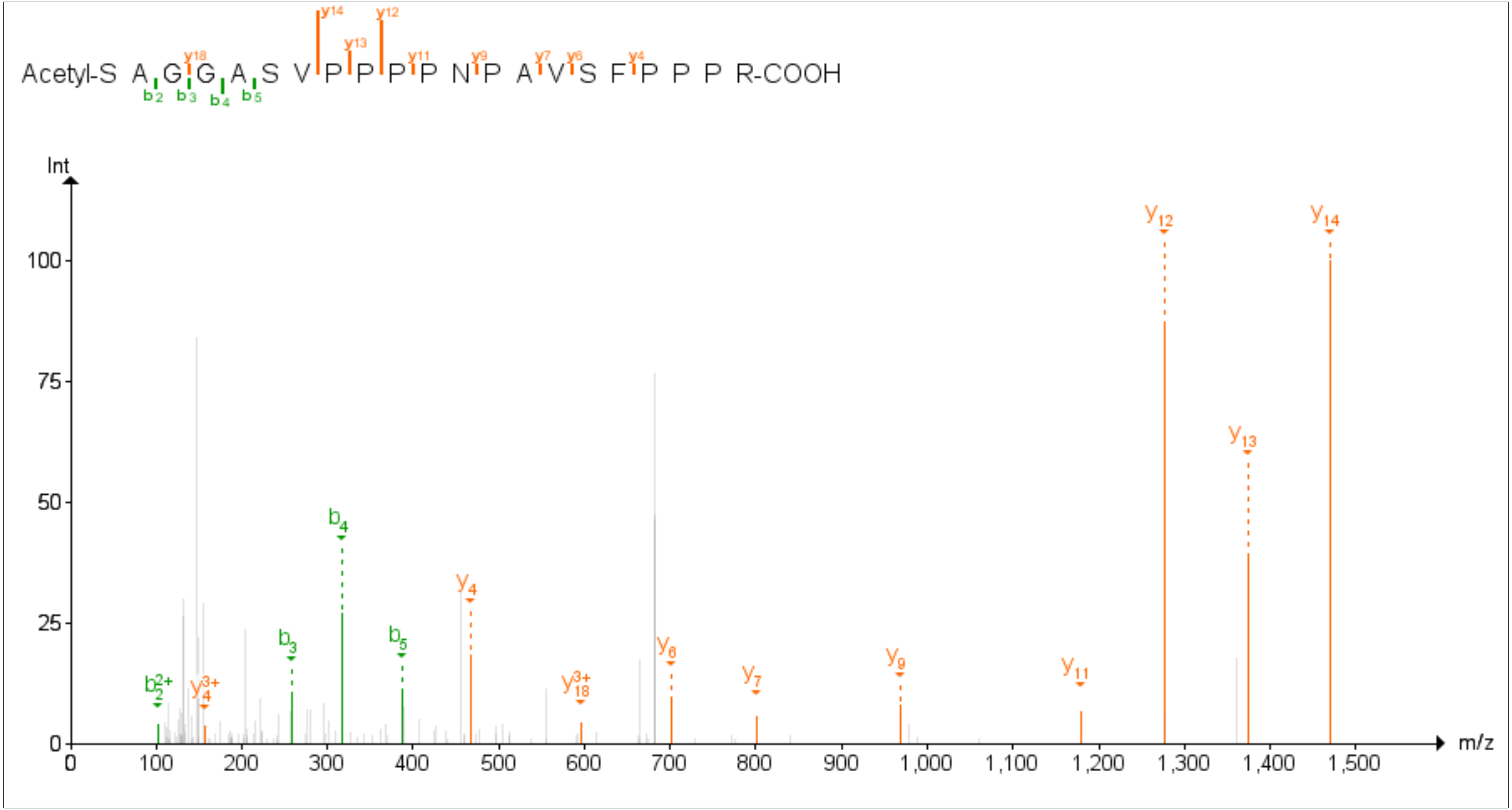

Acetyl-S A G G A S V P P P P N P A V S F P P P R-COOH
Int
m/z

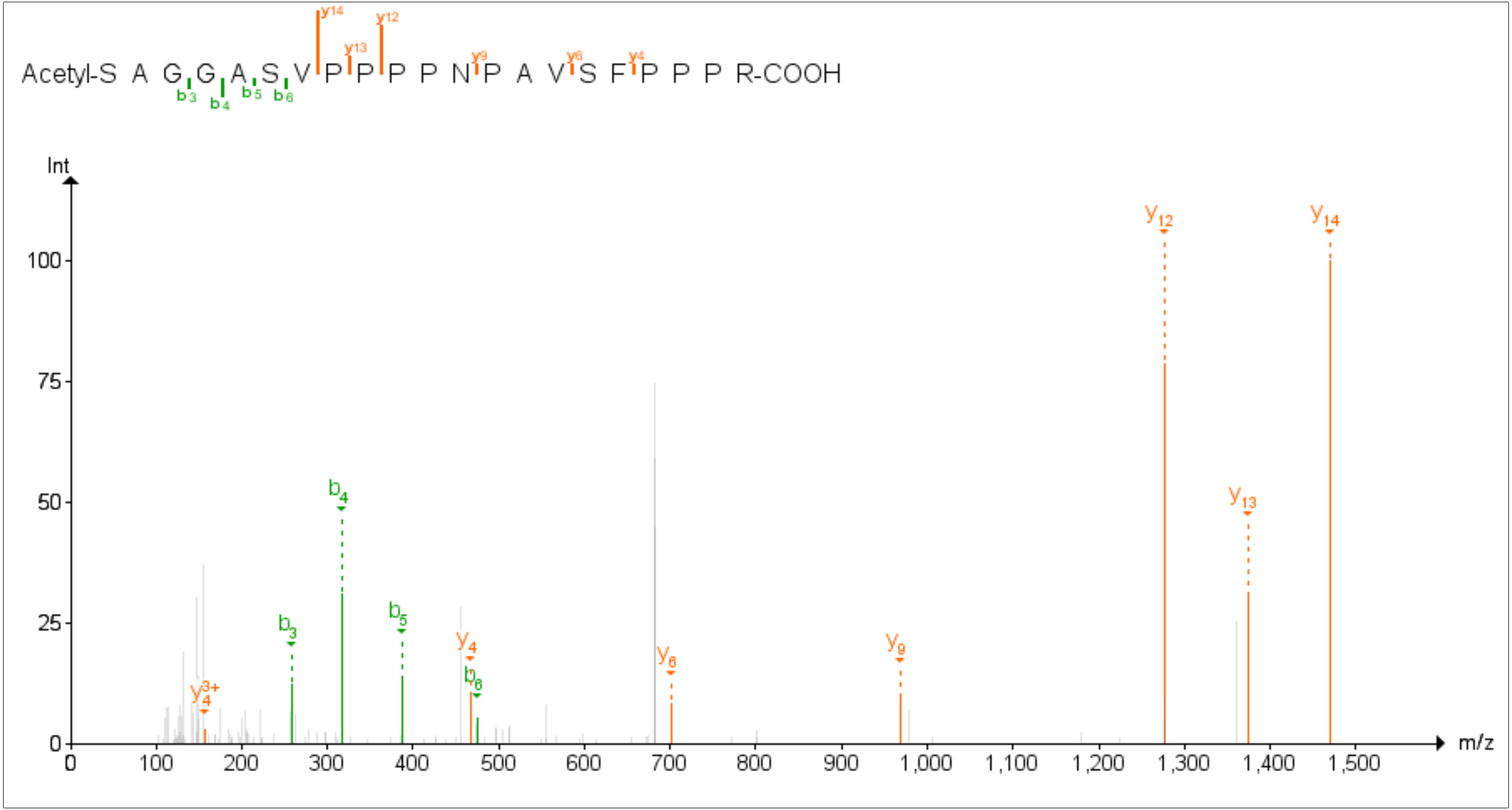
Acetyl-S A G G A S V P P P P N P A V S F P P P R-COOH
Int
m/z
b3
b4
b5
b6
y4
y6
y9
y12
y13
y14
y4 3+

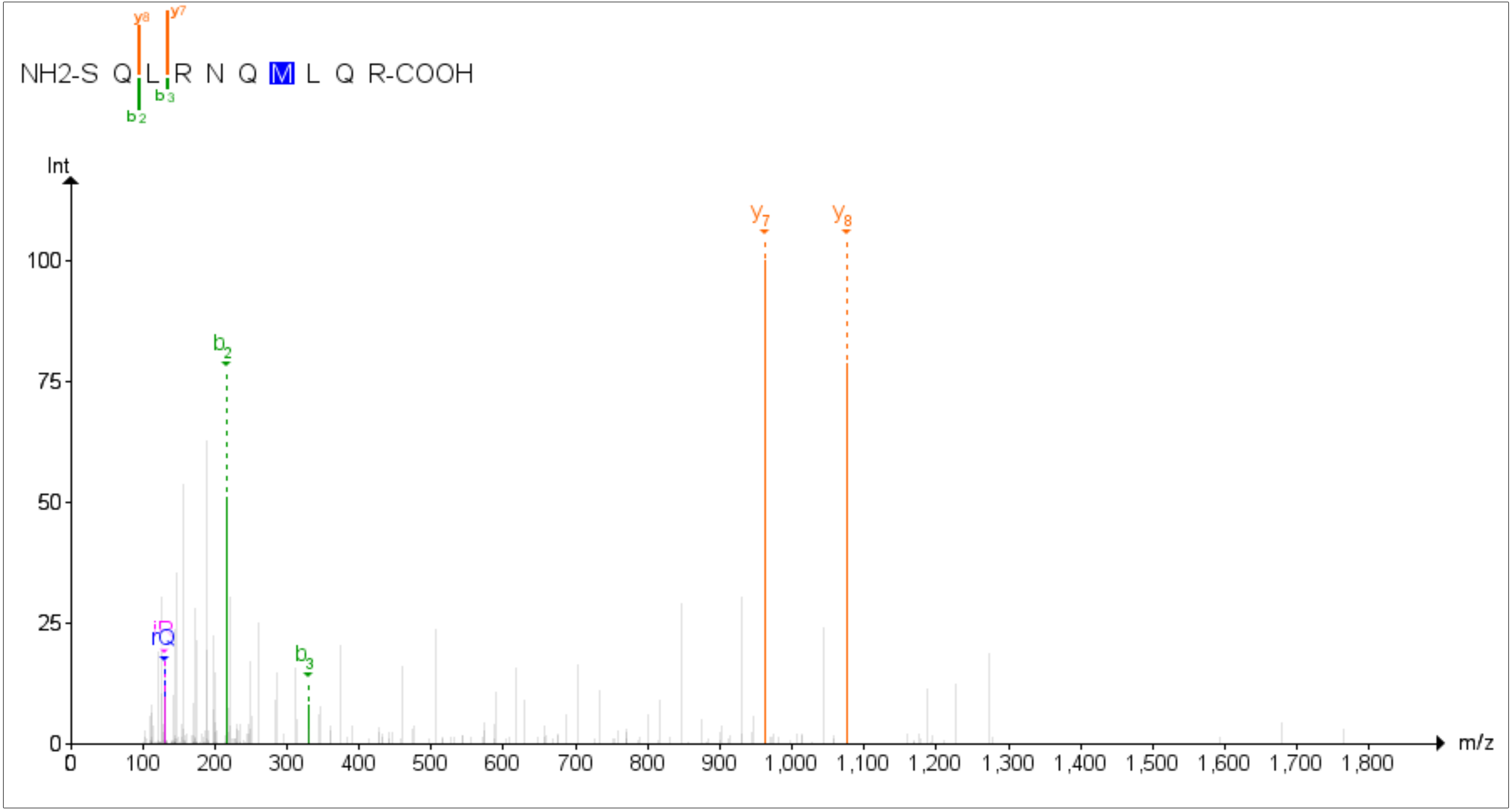

NH2-S Q L R N Q M L Q R-COOH
y8
y7
b2
b3
Int
100
75
50
25
0
y7
y8
b2
b3
iP
rQ
m/z
0
100
200
300
400
500
600
700
800
900
1,000
1,100
1,200
1,300
1,400
1,500
1,600
1,700
1,800

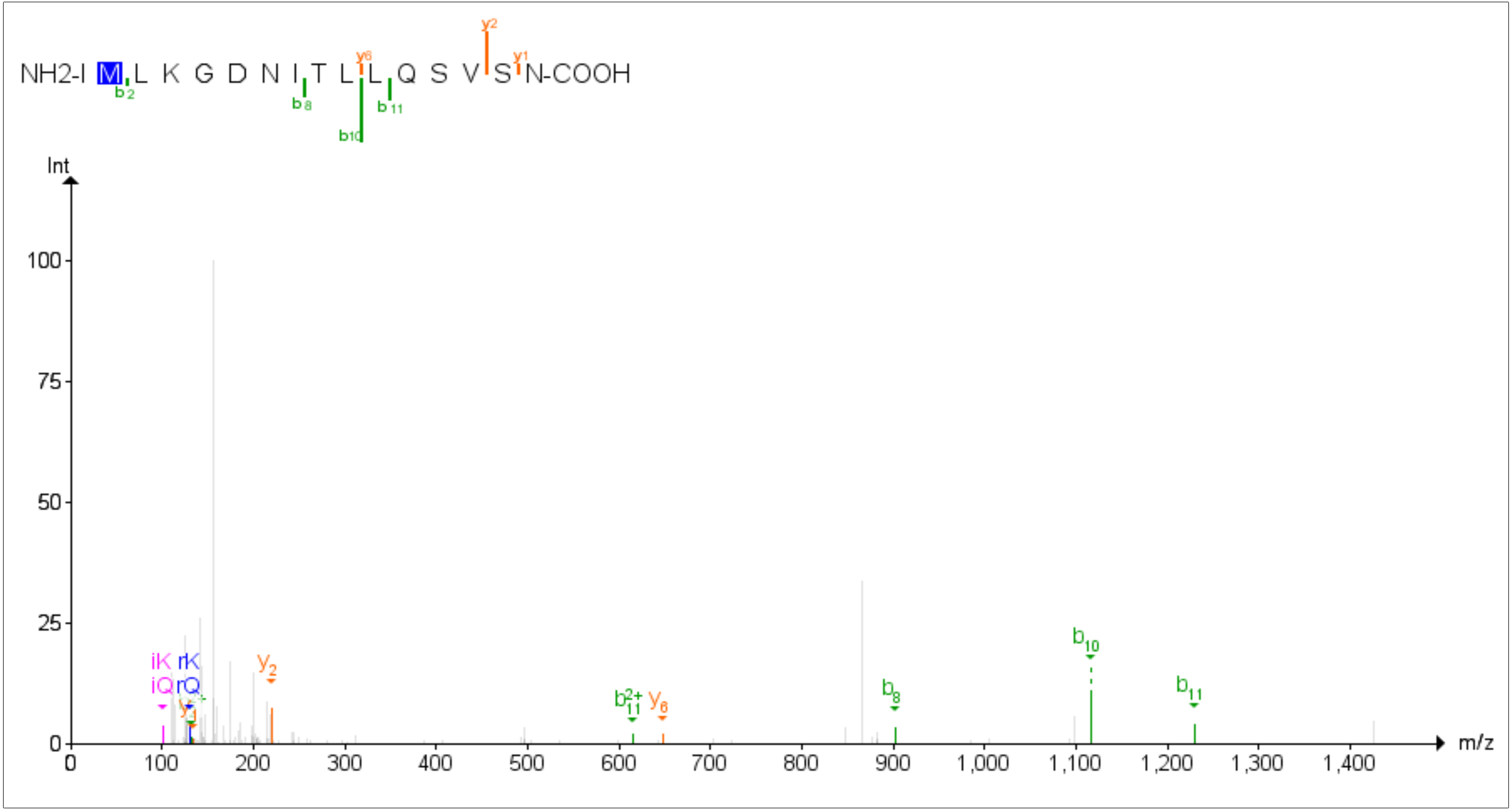

NH2-I M L K G D N I T L L Q S V S N-COOH
b2
b8
b10
b11
y6
y2
y1
Int
100
75
50
25
0
iK rK
iQ rQ
y2
y1
b11 2+
y6
b8
b10
b11
0
100
200
300
400
500
600
700
800
900
1,000
1,100
1,200
1,300
1,400
m/z

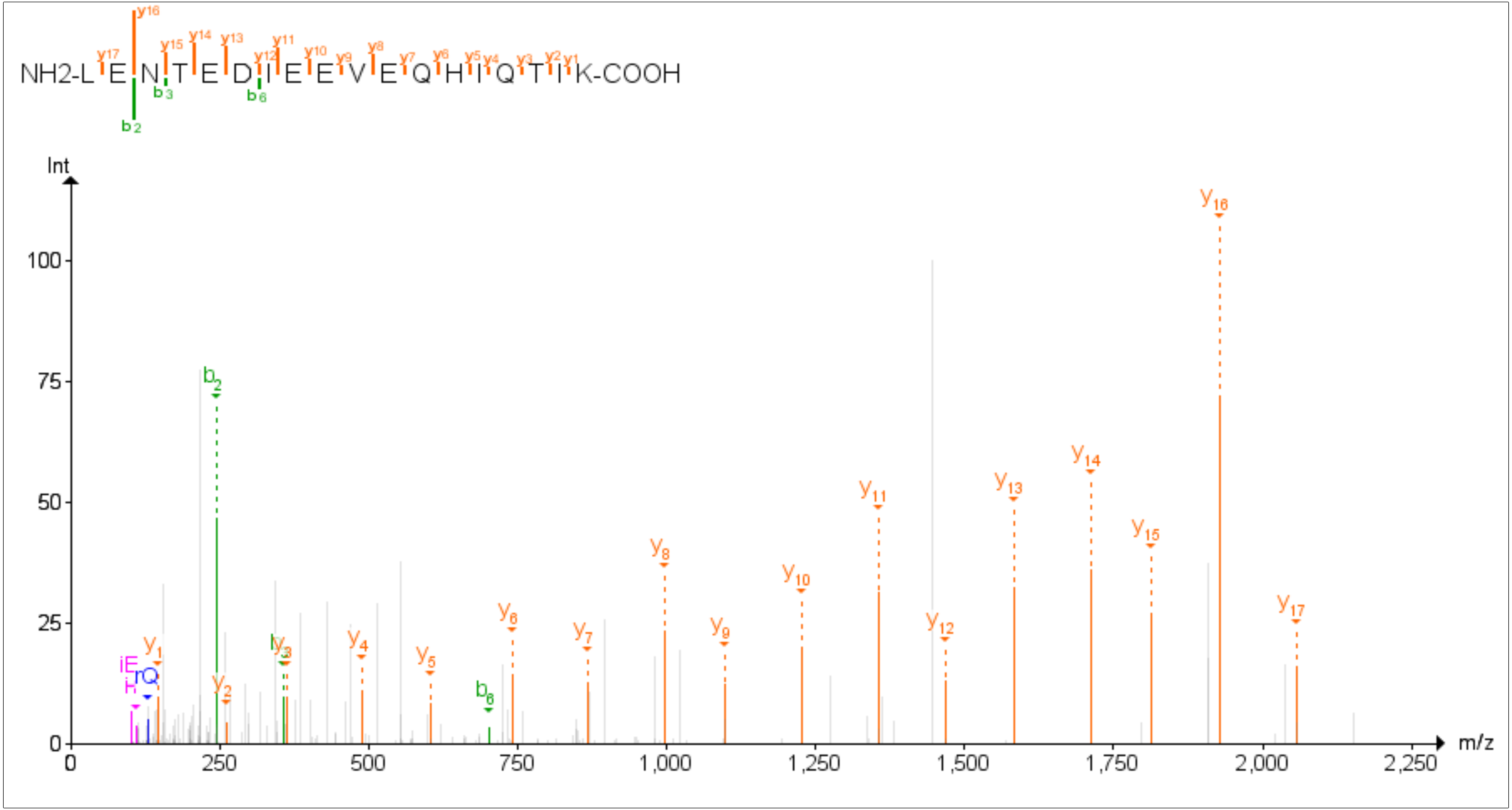
NH2-LENTEDIEEVEQHIQTIK-COOH
Int
m/z
0
25
50
75
100
250
500
750
1,000
1,250
1,500
1,750
2,000
2,250
b2
b3
b6
y1
y2
y3
y4
y5
y6
y7
y8
y9
y10
y11
y12
y13
y14
y15
y16
y17
iE
rQ
H
I

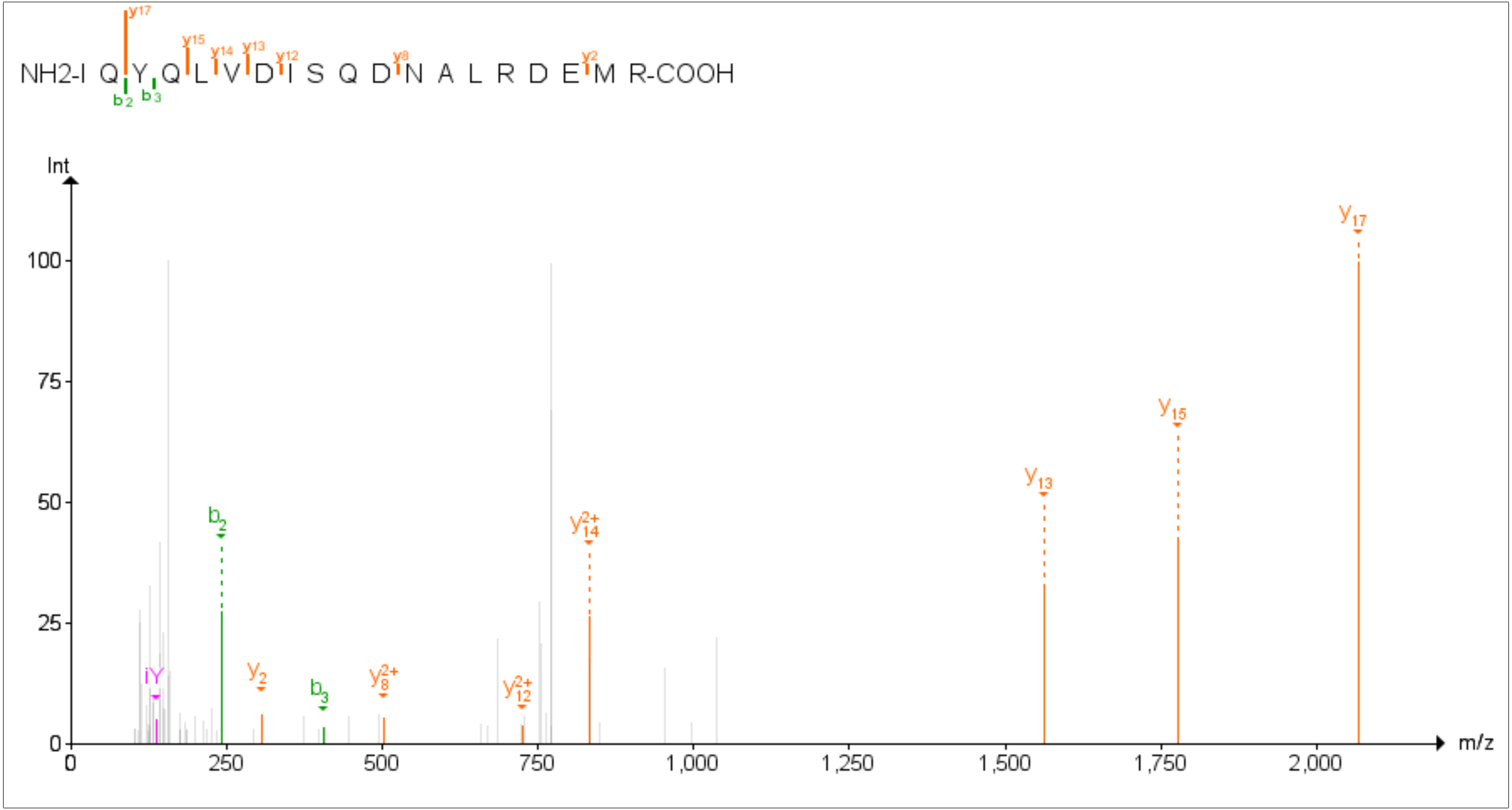

NH2-I Q Y Q L V D I S Q D N A L R D E M R-COOH
y17
y15
y14
y13
y12
y8
y2
b2
b3
Int
100
75
50
25
0
m/z
0
250
500
750
1,000
1,250
1,500
1,750
2,000
iY
y2
b3
y_8^{2+}
y_{12}^{2+}
y_{14}^{2+}
y13
y15
y17

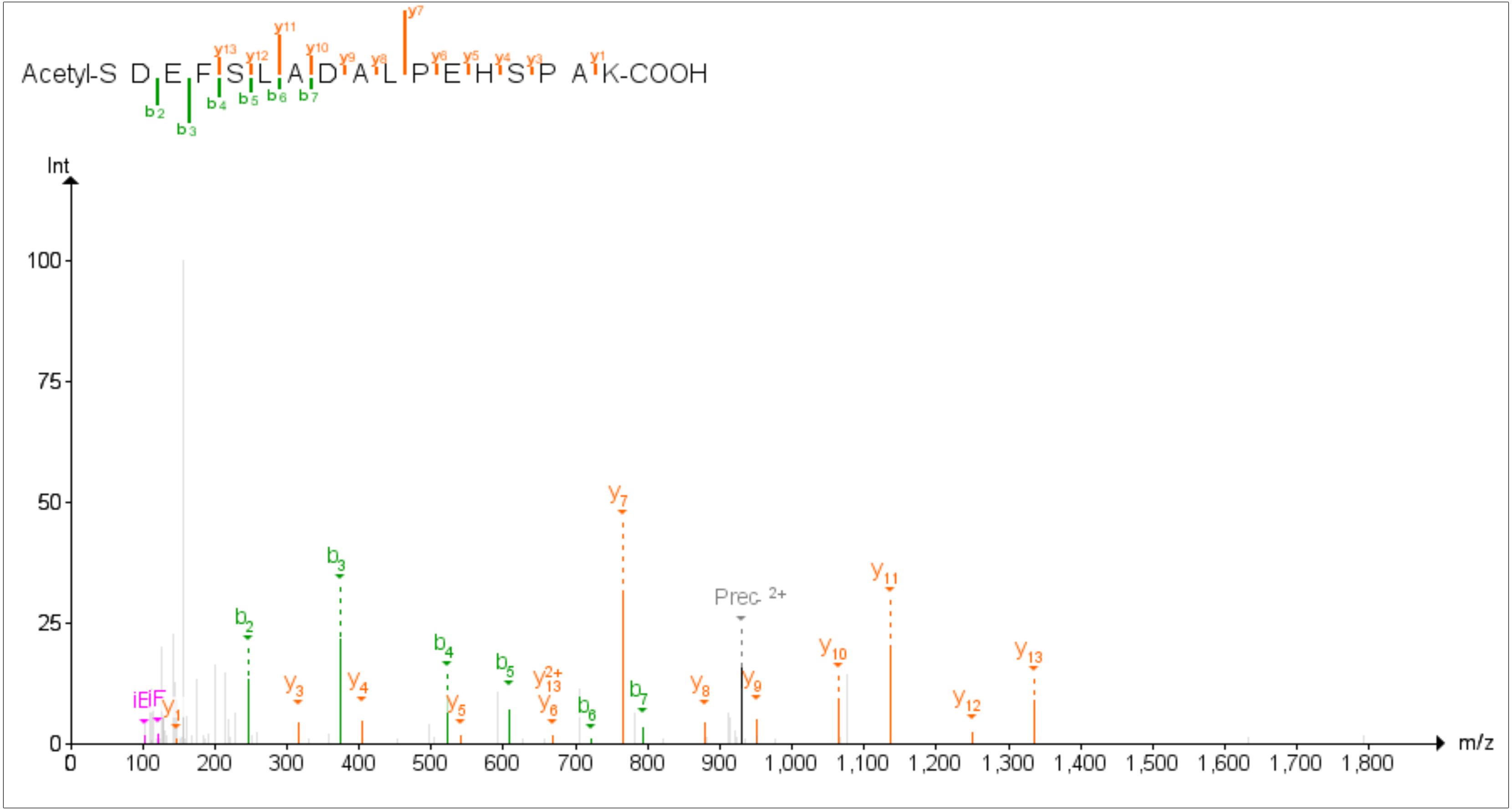

Acetyl-S D E F S L A D A L P E H S P A K-COOH
y13 y12 y11 y10 y9 y8 y7 y6 y5 y4 y3 y1
b2 b3 b4 b5 b6 b7
Int
100
75
50
25
0
iEiF
y1
b2
y3
b3
y4
b4
y5
b5
y13 2+
y6
b6
y7
b7
y8
Prec 2+
y9
y10
y11
y12
y13
0
100
200
300
400
500
600
700
800
900
1,000
1,100
1,200
1,300
1,400
1,500
1,600
1,700
1,800
m/z

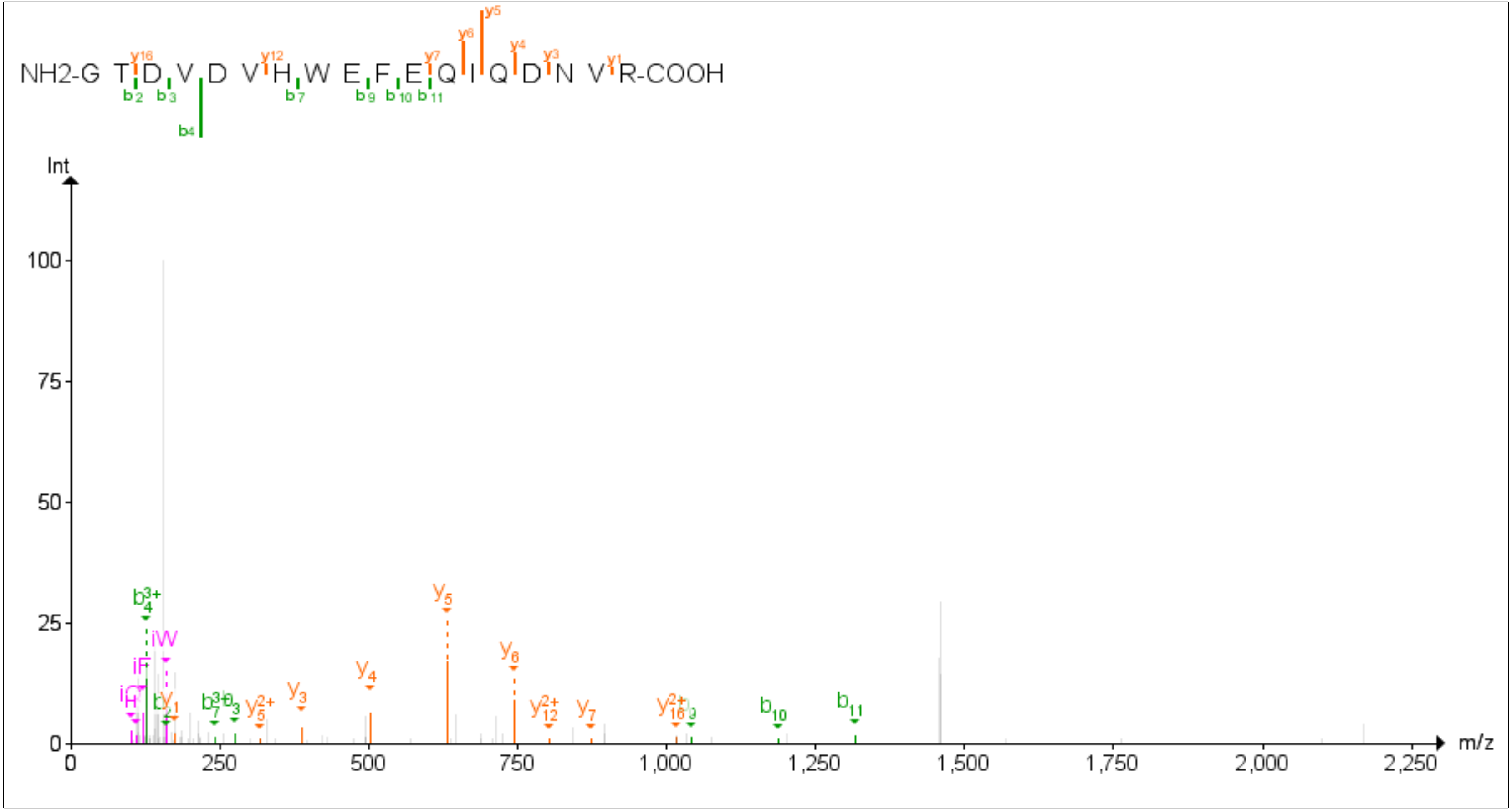

NH2-G T D V D V H W E F E Q I Q D N V R-COOH
y16 y12 y7 y6 y5 y4 y3 y1
b2 b3 b4 b7 b9 b10 b11
Int
100
75
50
25
0
m/z
0
250
500
750
1,000
1,250
1,500
1,750
2,000
2,250
y5
y6
y4
y3
y7
b10
b11
iW
iF

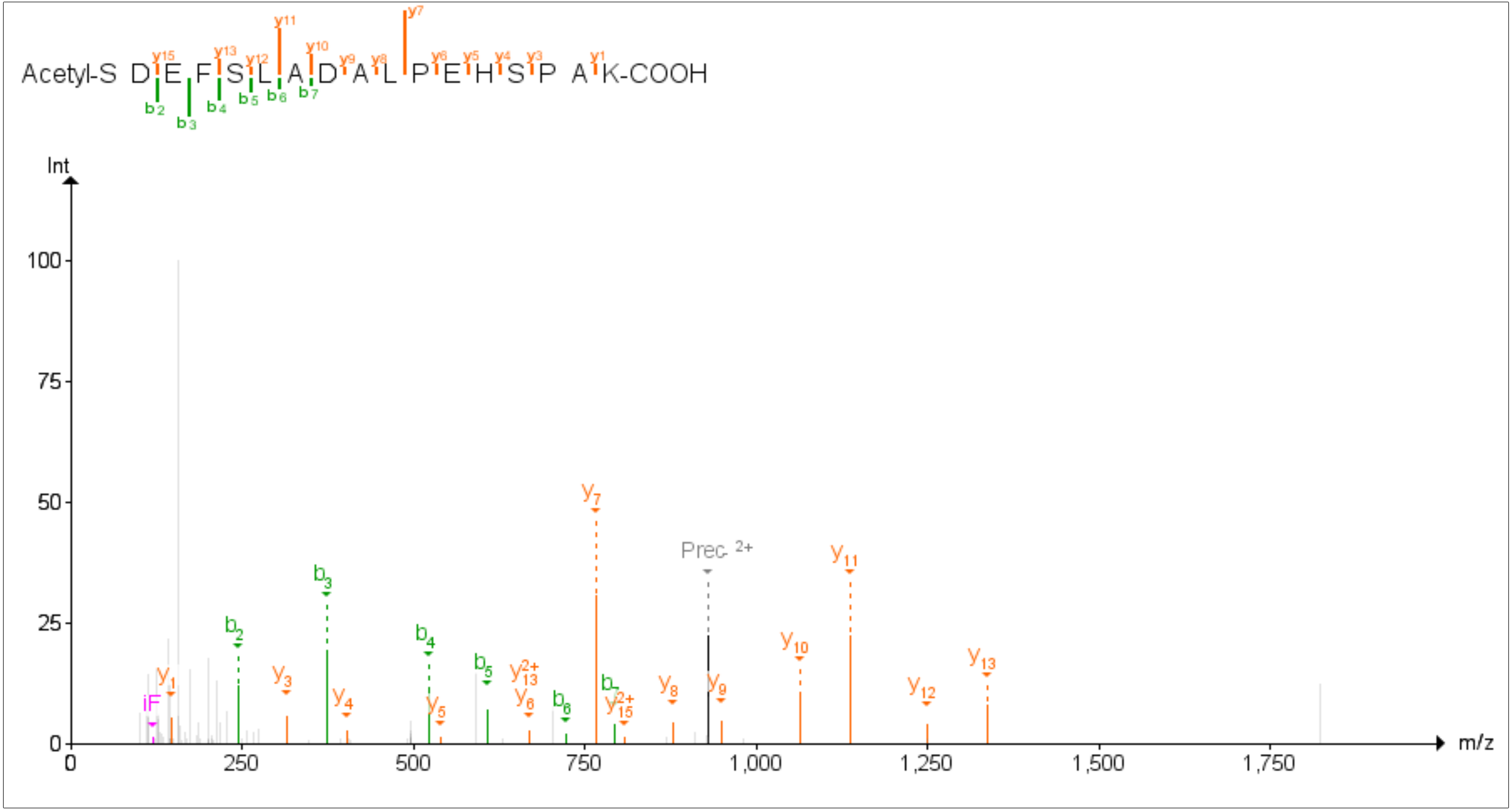

Acetyl-S D E F S L A D A L P E H S P A K-COOH
y15 y13 y12 y11 y10 y9 y8 y7 y6 y5 y4 y3 y1
b2 b3 b4 b5 b6 b7
Int
100
75
50
25
0
m/z
0
250
500
750
1,000
1,250
1,500
1,750
iF
y1
b2
y3
b3
y4
b4
y5
b5
y13 2+
y6
b6
y7
b7
y15 2+
y8
Prec. 2+
y9
y10
y11
y12
y13

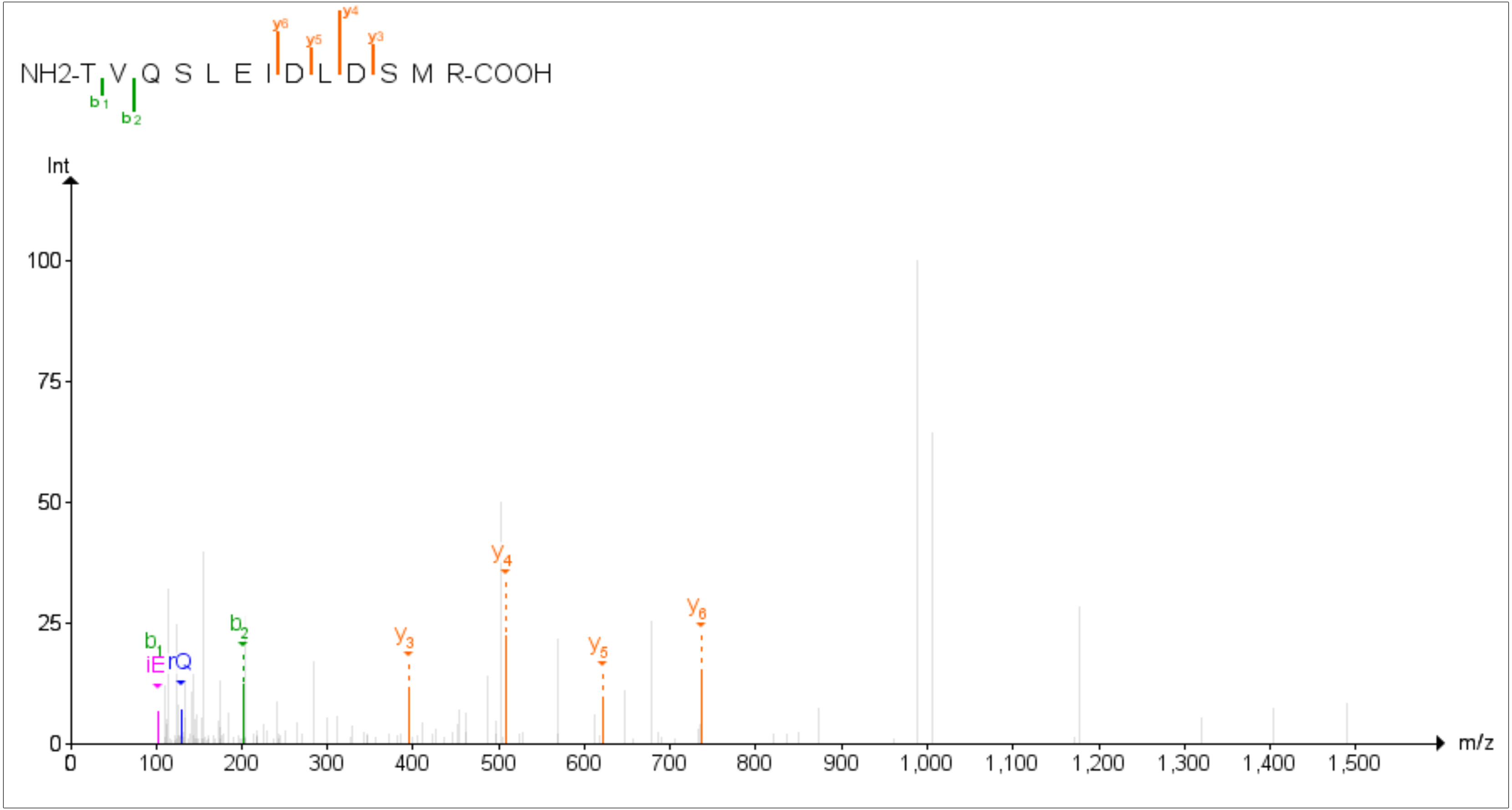
NH2-T V Q S L E I D L D S M R-COOH
b1
b2
y6
y5
y4
y3
Int
100
75
50
25
0
b1
iE
rQ
b2
y3
y4
y5
y6
m/z
0
100
200
300
400
500
600
700
800
900
1,000
1,100
1,200
1,300
1,400
1,500

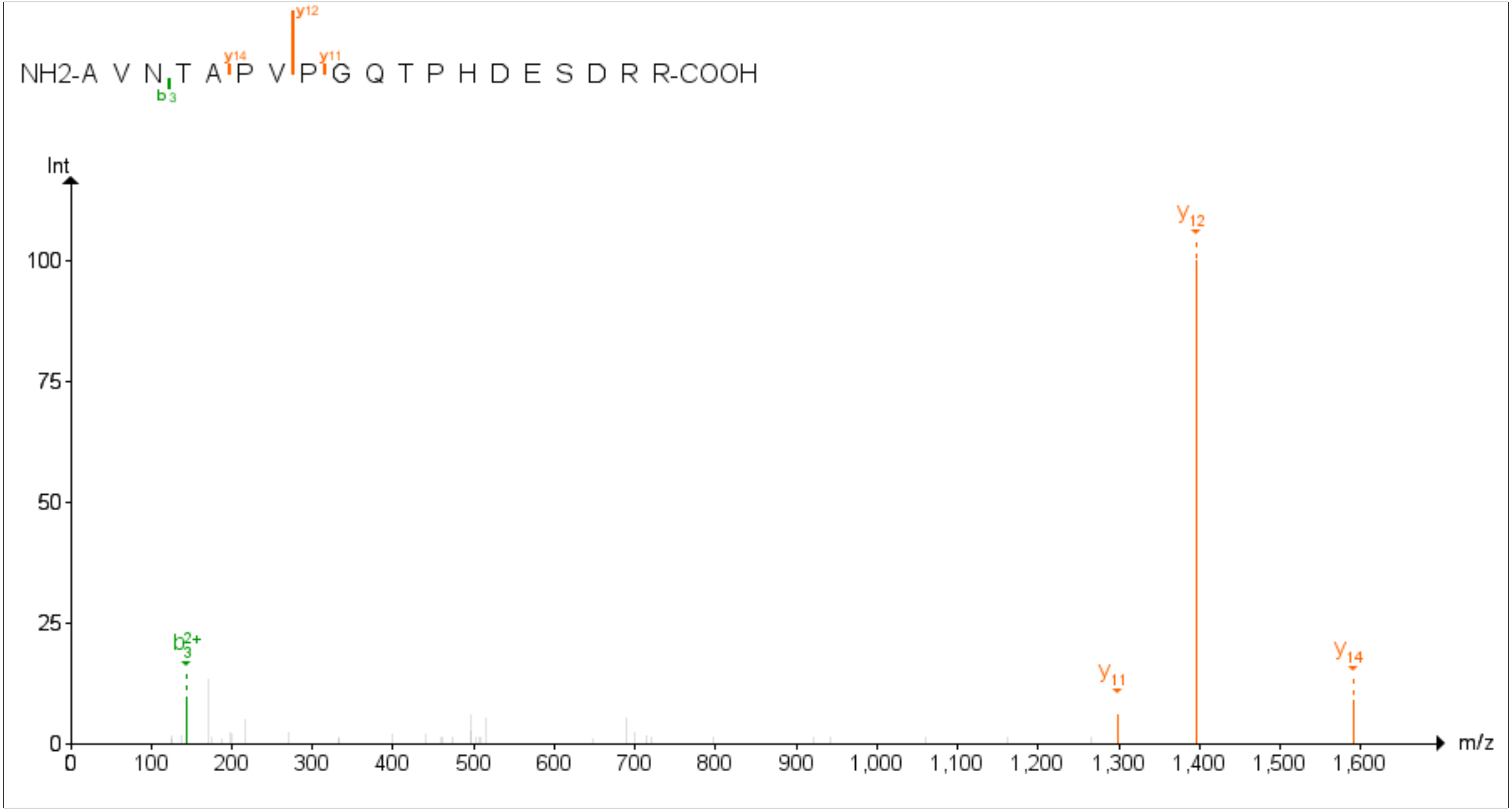

NH2-A V N T A P V P G Q T P H D E S D R R-COOH
b3
y14
y12
y11
Int
100
75
50
25
0
b_3^{2+}
y_{11}
y_{12}
y_{14}
0
100
200
300
400
500
600
700
800
900
1,000
1,100
1,200
1,300
1,400
1,500
1,600
m/z

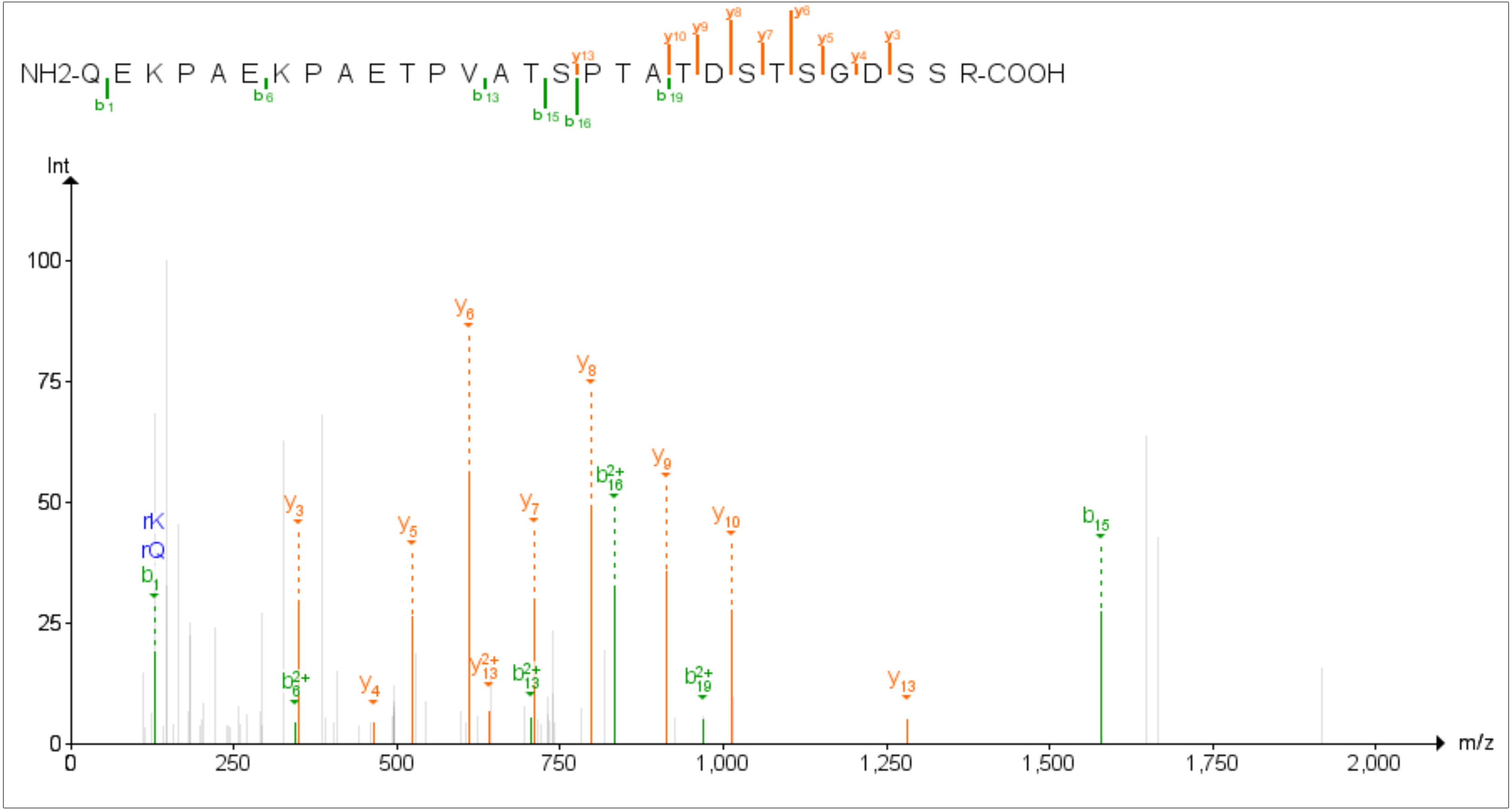

NH2-Q E K P A E K P A E T P V A T S P T A T D S T S G D S S R-COOH
b 1
b 6
b 13
b 15
b 16
b 19
y13
y10
y9
y8
y7
y6
y5
y4
y3
Int
100
75
50
25
0
rK
rQ
b1
y3
b6 2+
y4
y5
y6
y13 2+
b13 2+
y7
y8
b16 2+
y9
b19 2+
y10
y13
b15
m/z
0
250
500
750
1,000
1,250
1,500
1,750
2,000

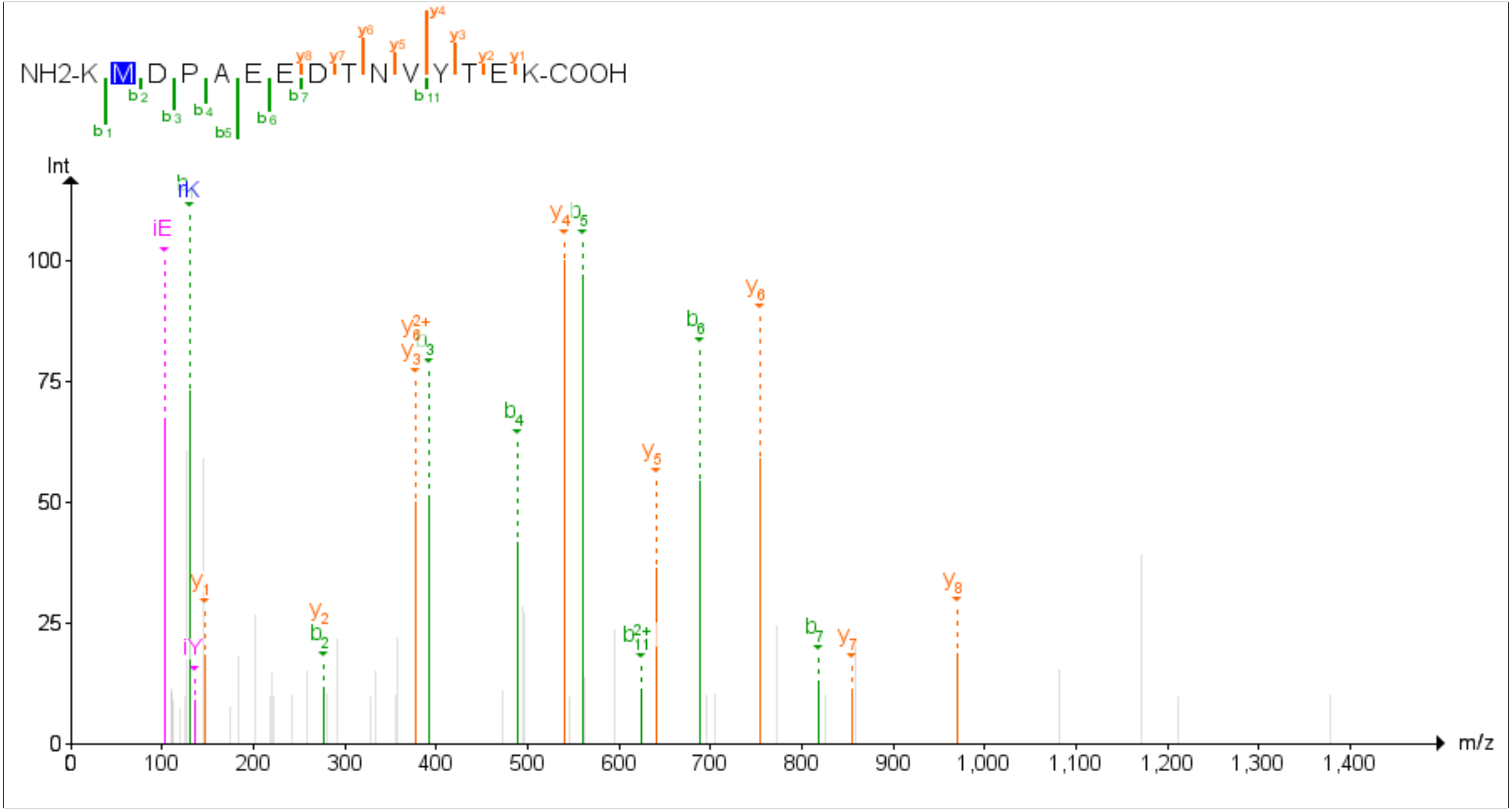

NH2-K M D P A E E D T N V Y T E K-COOH
b1 b2 b3 b4 b5 b6 b7 b11
y8 y7 y6 y5 y4 y3 y2 y1
Int
m/z
iE
iK
iY

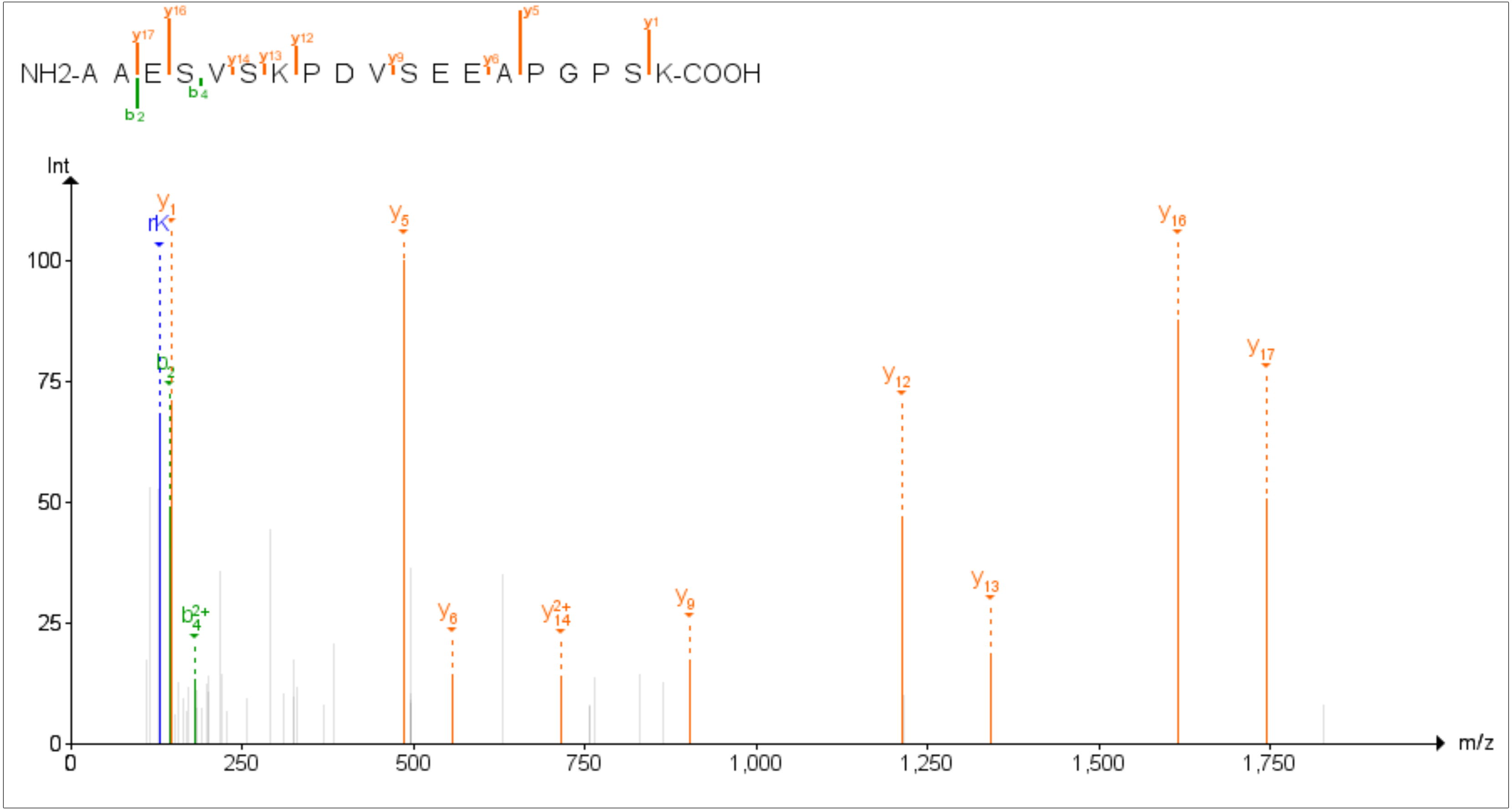

NH2-A A E S V S K P D V S E E A P G P S K-COOH
y17 y16 y14 y13 y12 y9 y6 y5 y1
b2 b4
Int
m/z
y1
rK
y5
y16
y17
y12
y13
b2
b_4^{2+}
y6
y_{14}^{2+}
y9
0
25
50
75
100
0
250
500
750
1,000
1,250
1,500
1,750

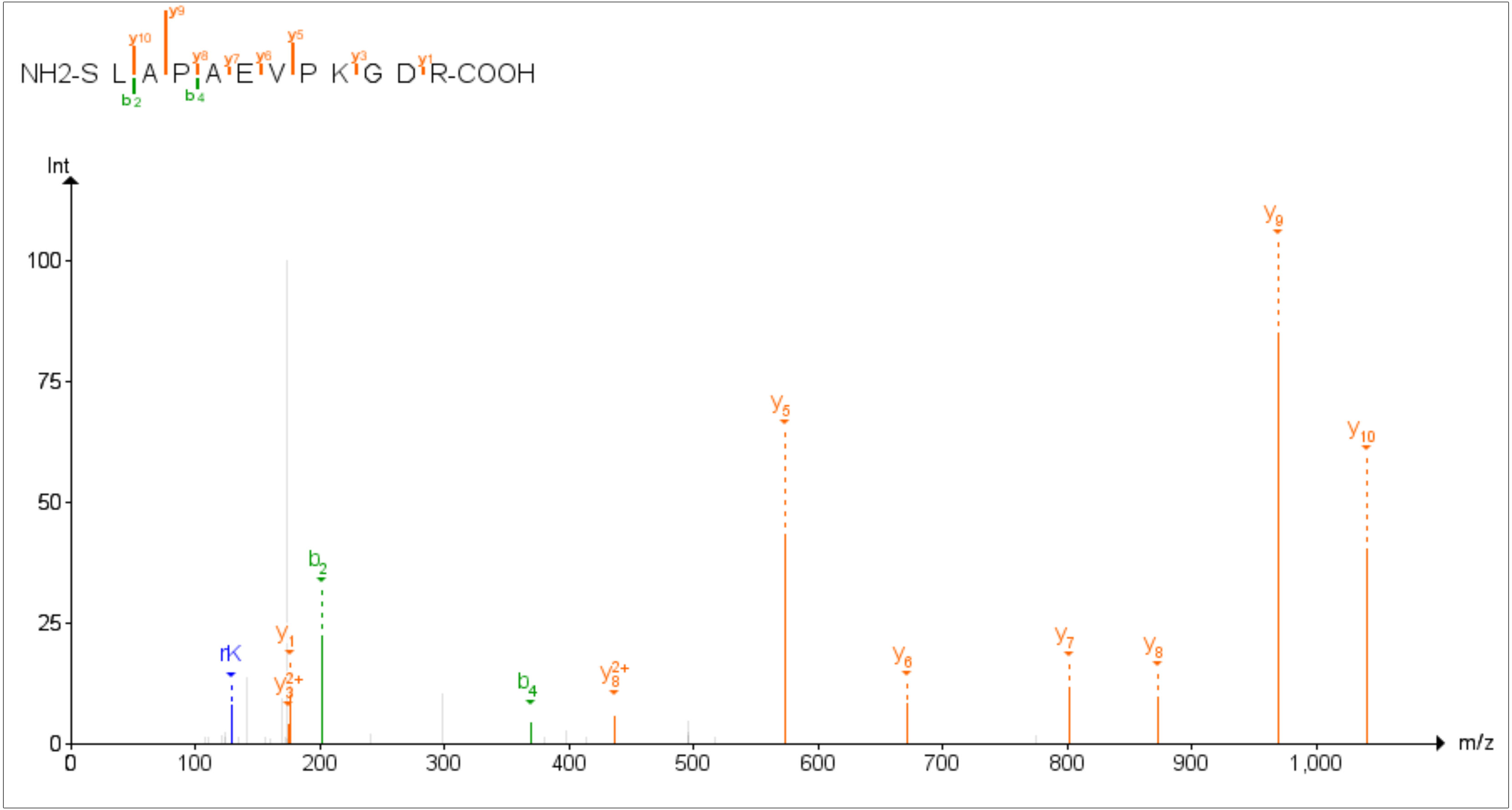

NH2-S L A P A E V P K G D R-COOH
y10
y9
y8
y7
y6
y5
y3
y1
b2
b4
Int
100
75
50
25
0
m/z
0
100
200
300
400
500
600
700
800
900
1,000
rK
y1
y3 2+
b2
b4
y8 2+
y5
y6
y7
y8
y9
y10

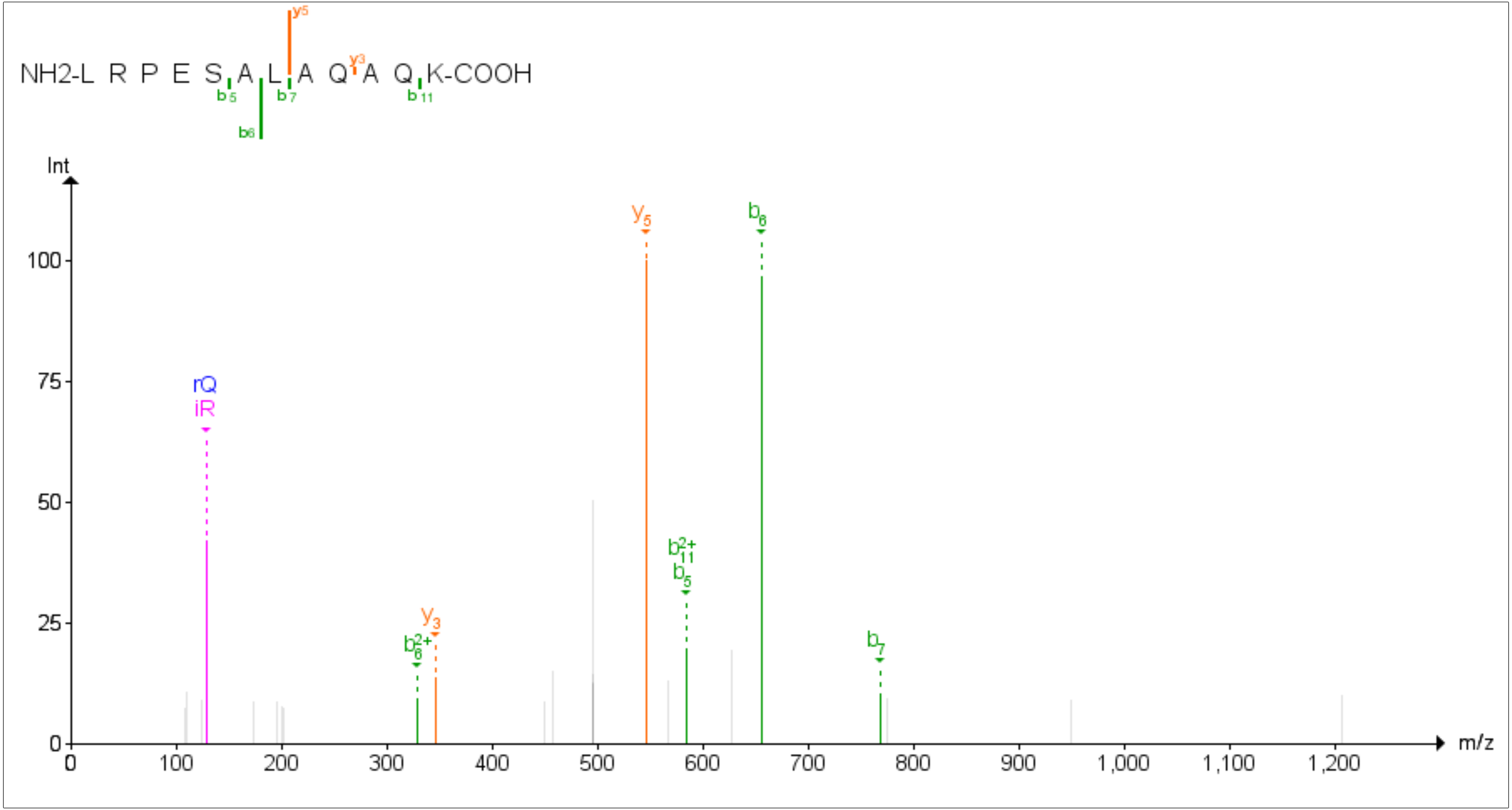

NH2-L R P E S A L A Q A Q K-COOH
b5 b6 b7 b11 y5 y3
Int
100
75
50
25
0
rQ
iR
y5
b6
b11 2+
b5
y3
b6 2+
b7
0 100 200 300 400 500 600 700 800 900 1,000 1,100 1,200
m/z

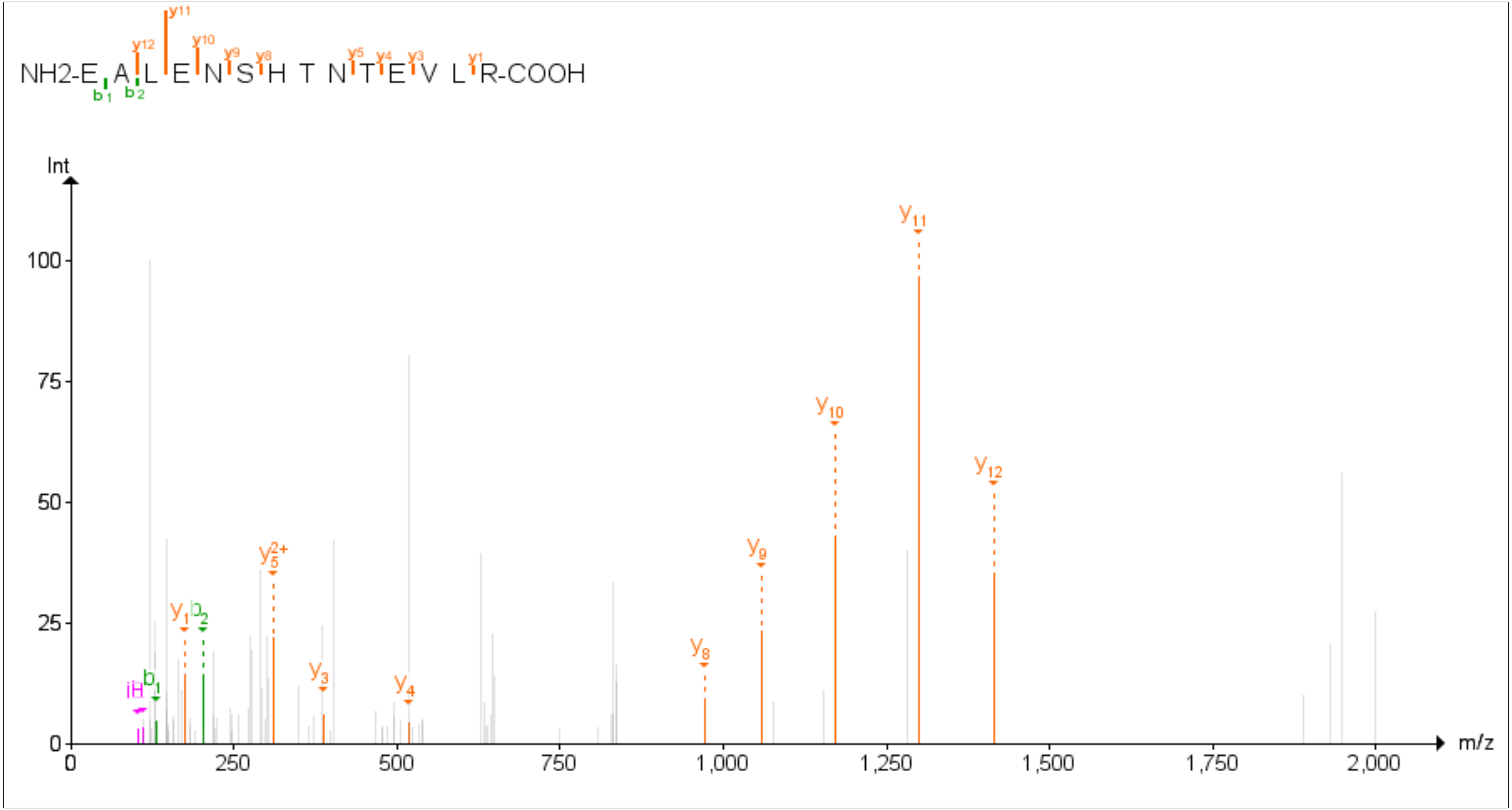

NH2-E A L E N S H T N T E V L R-COOH
b1
b2
y12
y11
y10
y9
y8
y5
y4
y3
y1
Int
m/z
0
25
50
75
100
250
500
750
1,000
1,250
1,500
1,750
2,000
iH
y5 2+

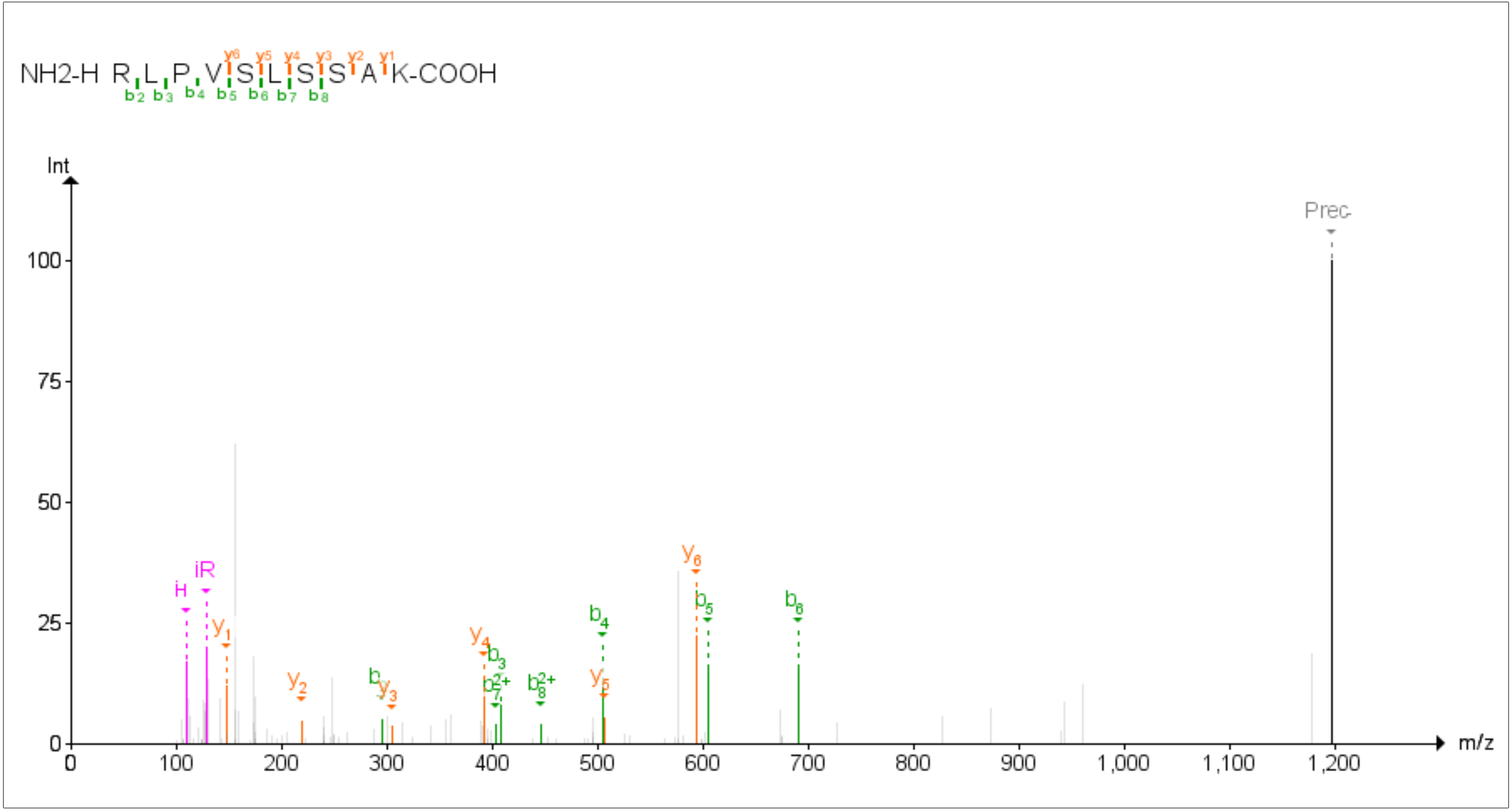

NH2-H R L P V S L S S A K-COOH
y6 y5 y4 y3 y2 y1
b2 b3 b4 b5 b6 b7 b8
Int
Prec
100
75
50
25
0
iR
H
y1
y2
y3
y4
y5
y6
b3
b4
b5
b6
b7 2+
b8 2+
m/z
0
100
200
300
400
500
600
700
800
900
1,000
1,100
1,200

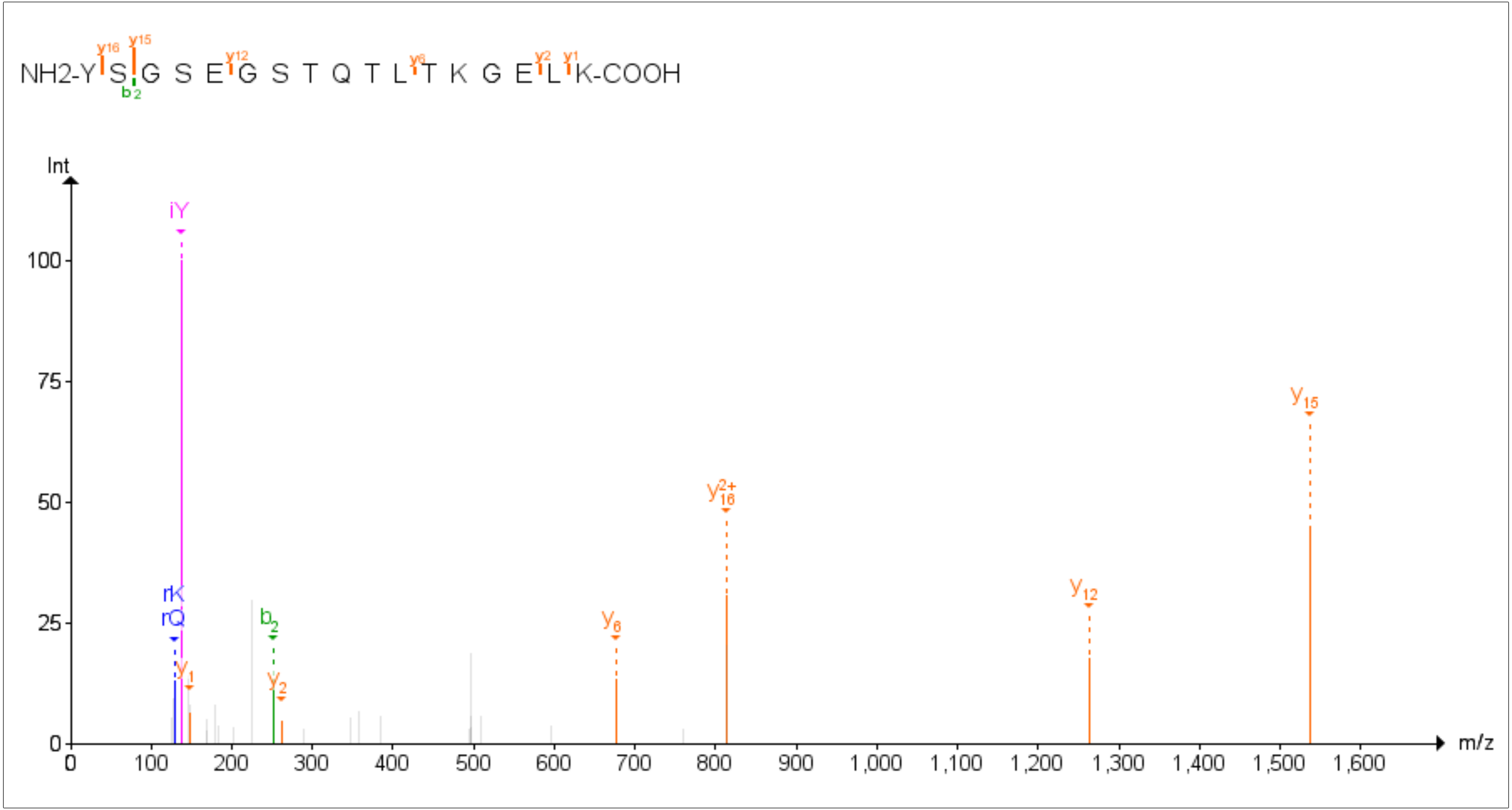

NH2-Y S G S E G S T Q T L T K G E L K-COOH
y16
y15
b2
y12
y6
y2
y1
Int
iY
100
75
50
25
0
rK
rQ
b2
y1
y2
y6
y16 2+
y12
y15
0
100
200
300
400
500
600
700
800
900
1,000
1,100
1,200
1,300
1,400
1,500
1,600
m/z

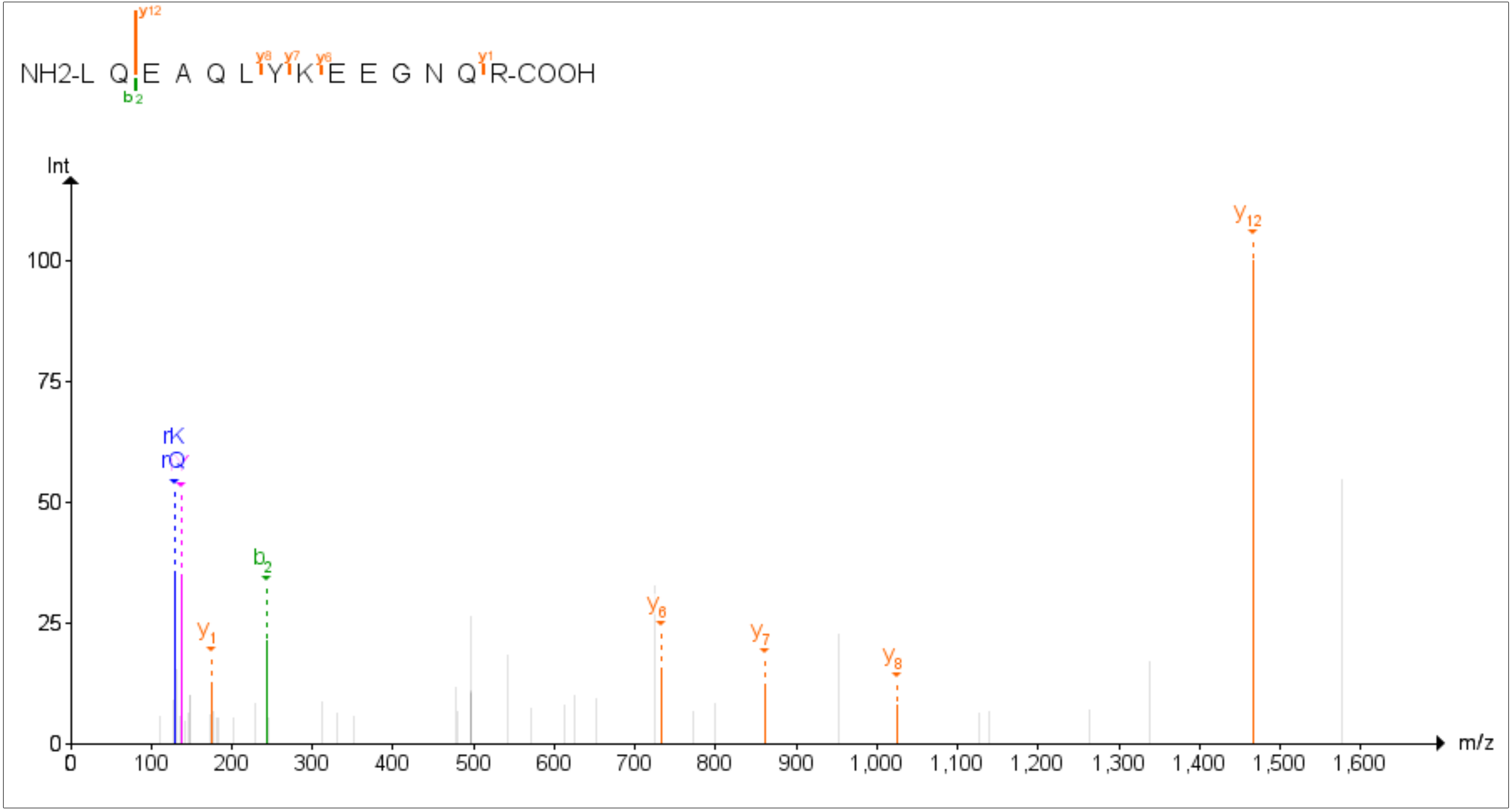

NH2-L Q E A Q L Y K E E G N Q R-COOH
y12
y8
y7
y6
y1
b2
Int
100
75
50
25
0
rK
rQ
b2
y1
y6
y7
y8
y12
0
100
200
300
400
500
600
700
800
900
1,000
1,100
1,200
1,300
1,400
1,500
1,600
m/z

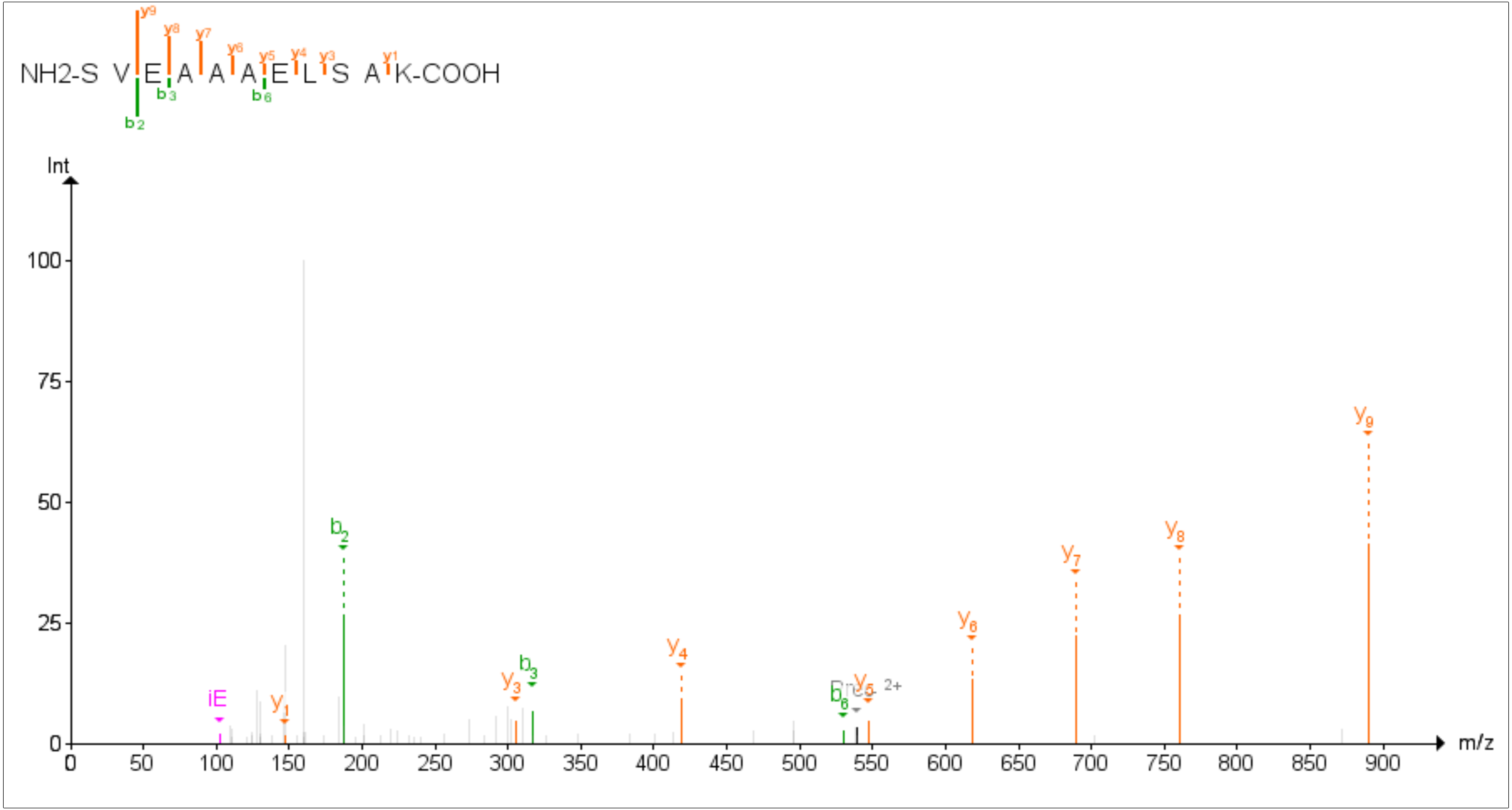

NH2-S V E A A A E L S A K-COOH
y9
y8
y7
y6
y5
y4
y3
y1
b2
b3
b6
Int
100
75
50
25
0
m/z
0
50
100
150
200
250
300
350
400
450
500
550
600
650
700
750
800
850
900
iE
y1
b2
y3
b3
y4
b6
y5
2+
y6
y7
y8
y9

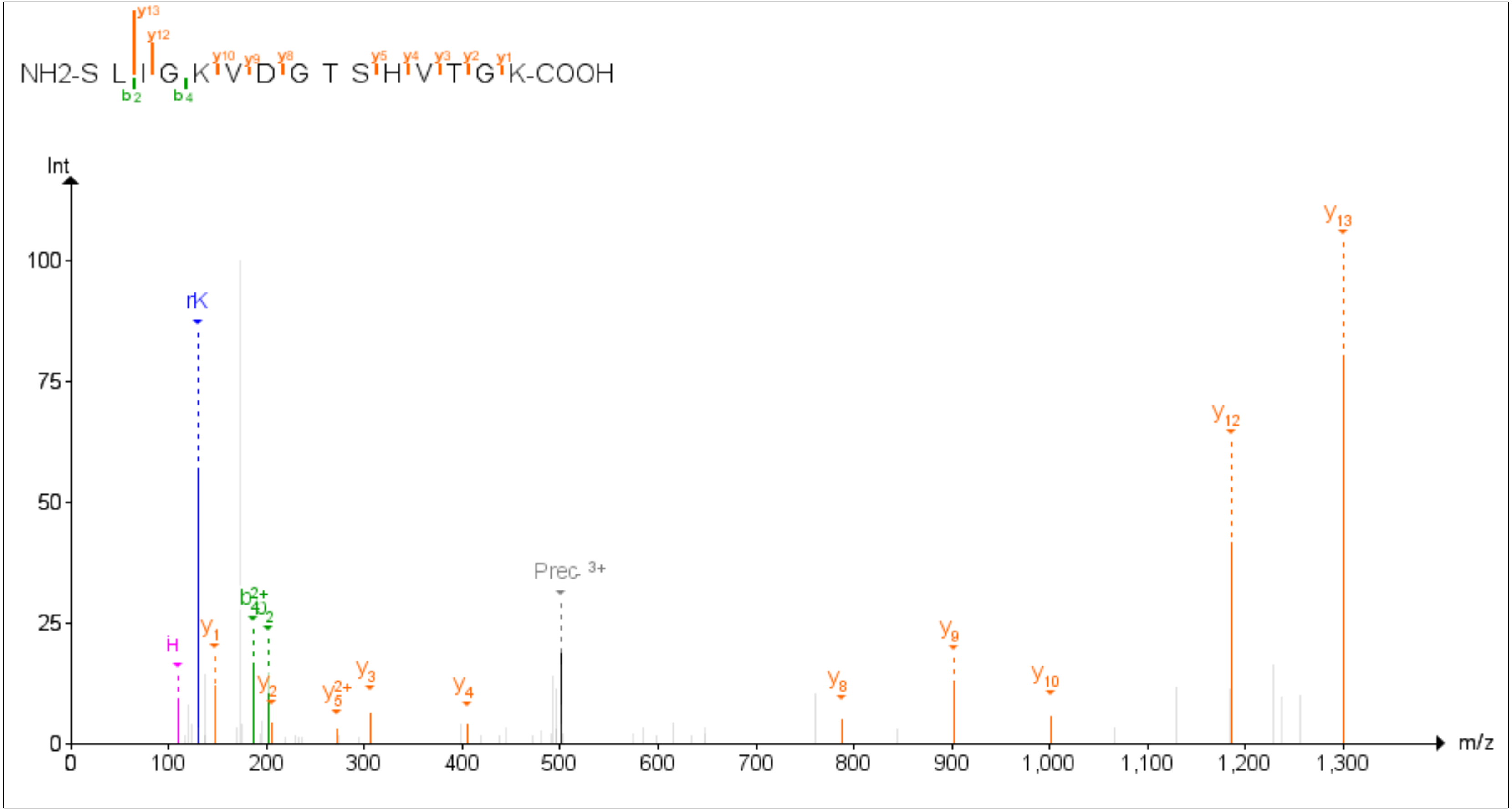

NH2-S L I G K V D G T S H V T G K-COOH
y13
y12
y10
y9
y8
y5
y4
y3
y2
y1
b2
b4
Int
m/z
100
75
50
25
0
0
100
200
300
400
500
600
700
800
900
1,000
1,100
1,200
1,300
iK
iH
y1
b4 2+
b2
y2
y5 2+
y3
y4
Prec 3+
y8
y9
y10
y12
y13

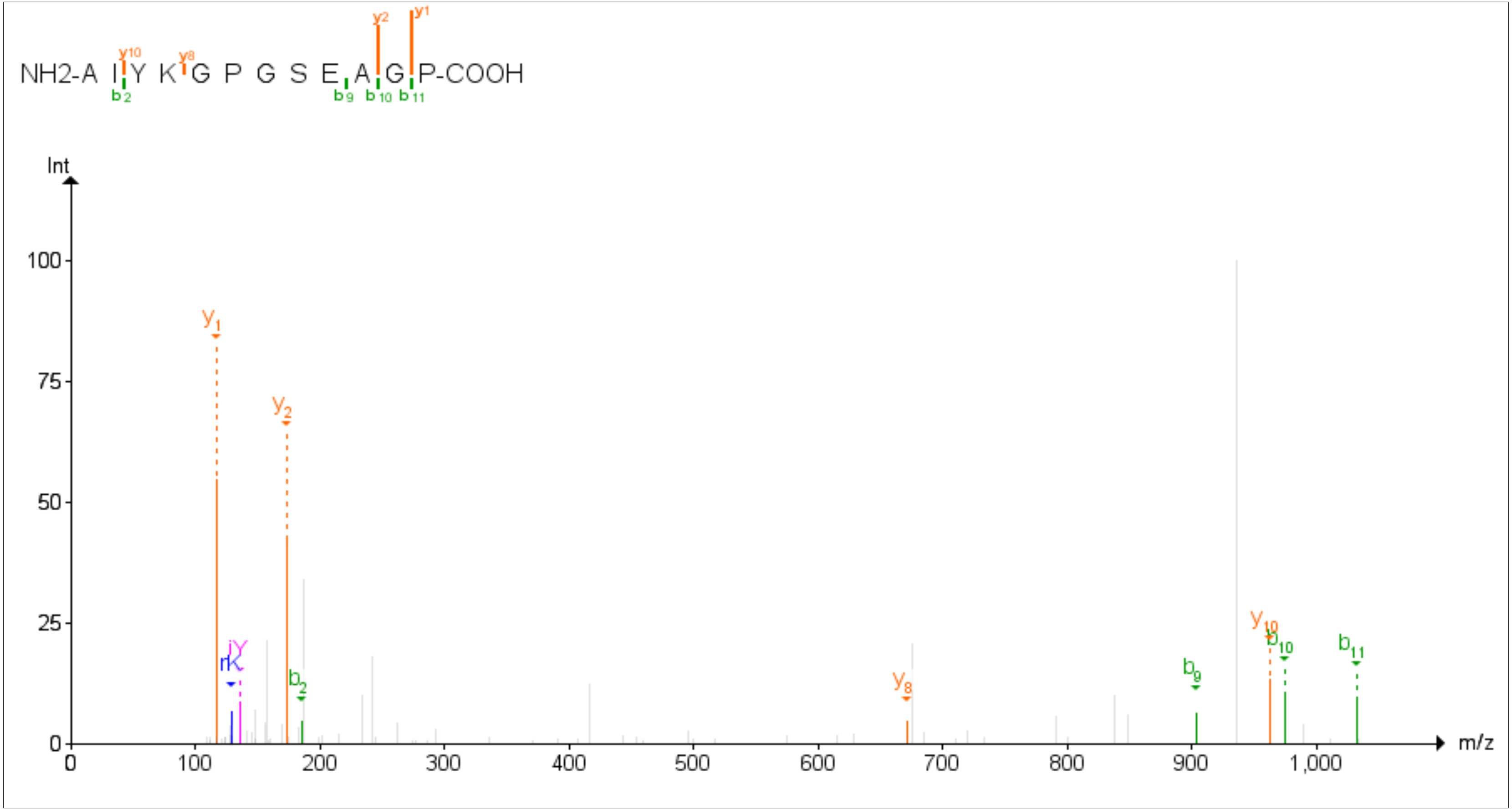

NH2-A I Y K G P G S E A G P-COOH
y10
y8
y2
y1
b2
b9
b10
b11
Int
100
75
50
25
0
y1
y2
rK
iY
b2
y8
b9
y10
b10
b11
0
100
200
300
400
500
600
700
800
900
1,000
m/z

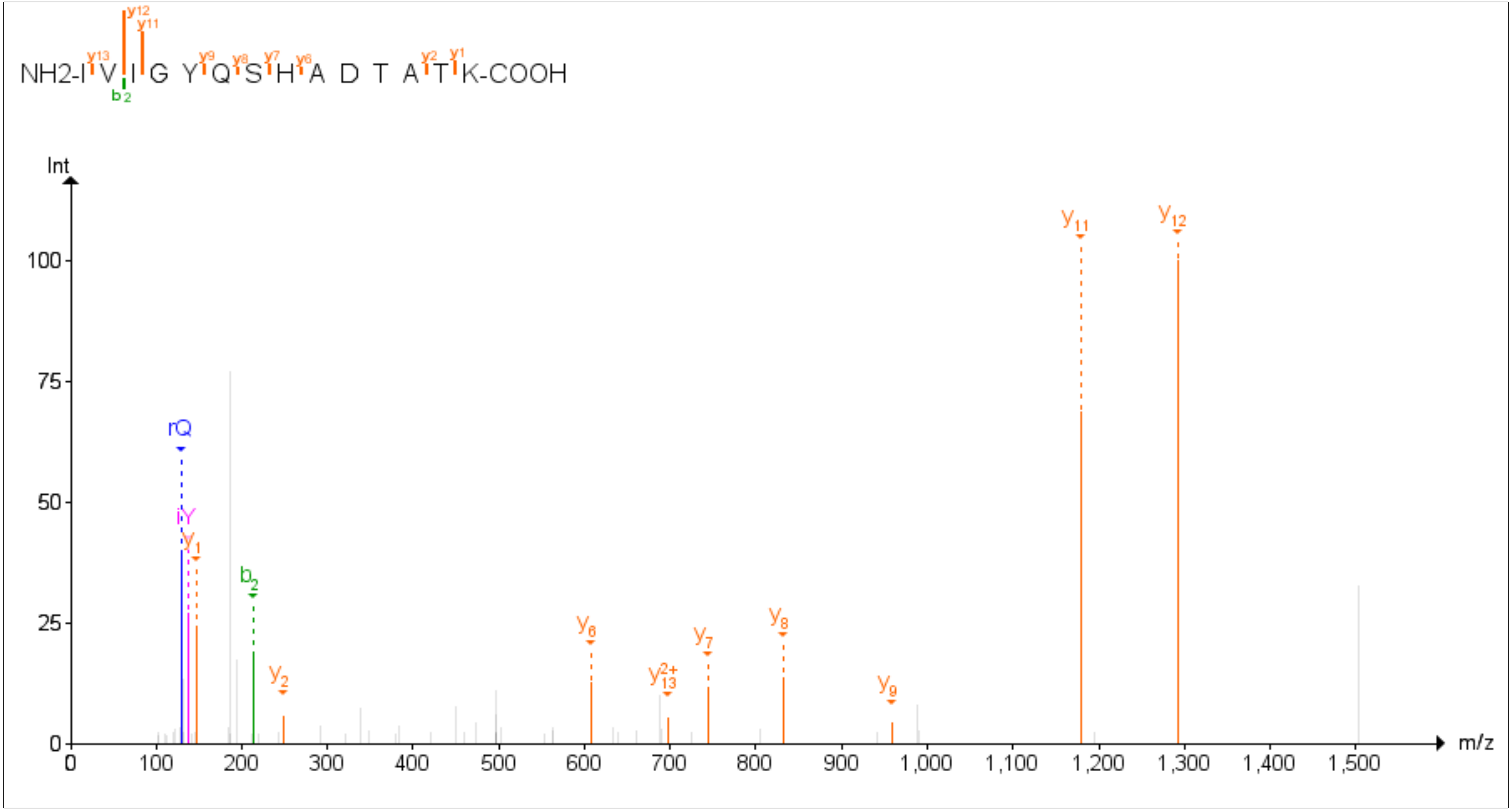

NH2-I V I G Y Q S H A D T A T K-COOH
y12
y11
y13
y9
y8
y7
y6
y2
y1
b2
Int
m/z
rQ
iY
y1
b2
y2
y6
y13 2+
y7
y8
y9
y11
y12
0
25
50
75
100
0
100
200
300
400
500
600
700
800
900
1,000
1,100
1,200
1,300
1,400
1,500

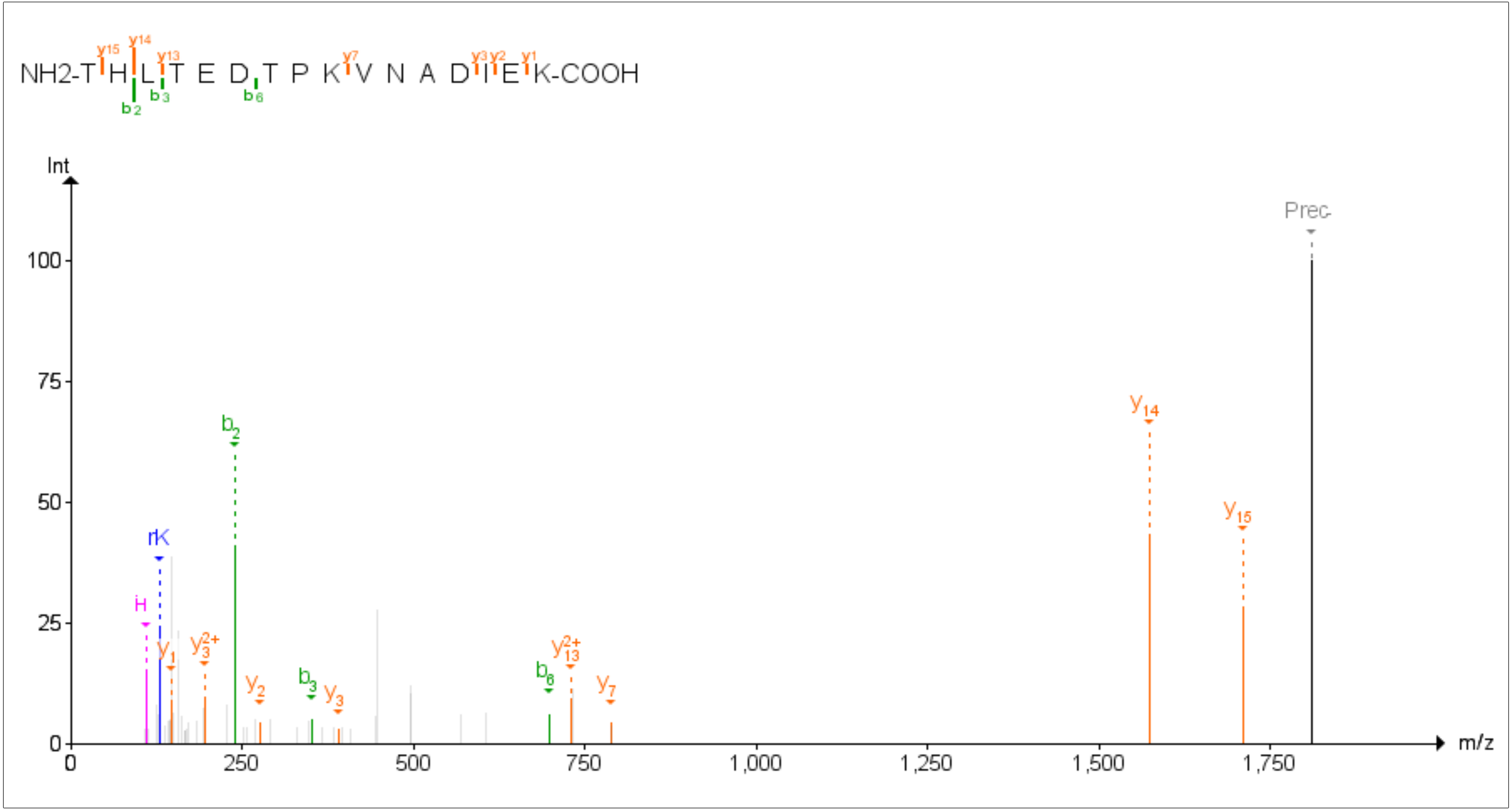

NH2-T H L T E D T P K V N A D I E K-COOH
y15 y14 y13 y7 y3 y2 y1
b2 b3 b6
Int
m/z
Prec
100
75
50
25
0
250
500
750
1,000
1,250
1,500
1,750
rK
H
y1
y3 2+
b2
y2
b3
y3
b6
y13 2+
y7
y14
y15

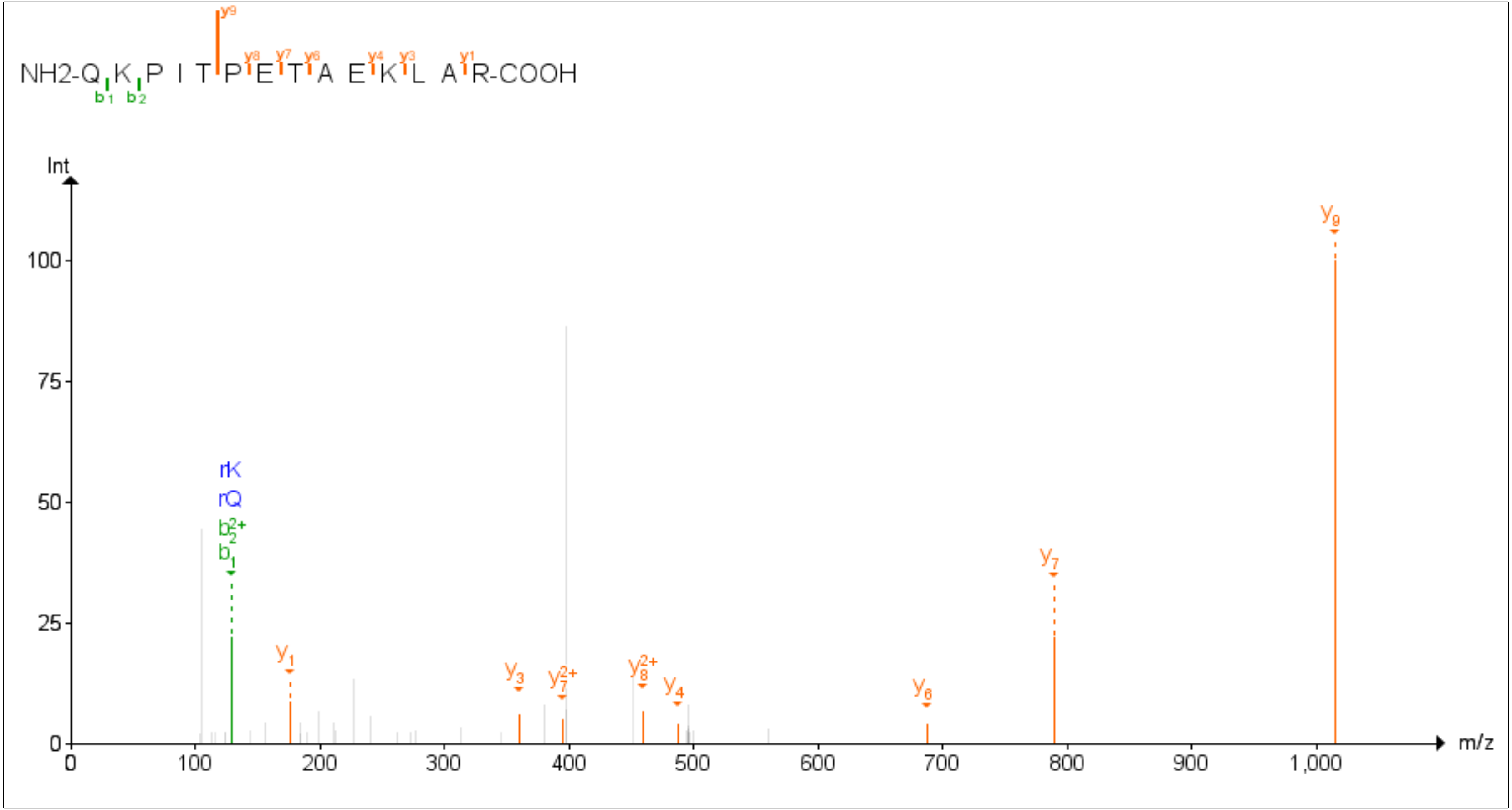

NH2-Q K P I T P E T A E K L A R-COOH
y9 y8 y7 y6 y4 y3 y1
b1 b2
Int
100
75
50
25
0
rK
rQ
b2 2+
b1
y1
y3
y7 2+
y8 2+
y4
y6
y7
y9
0 100 200 300 400 500 600 700 800 900 1,000
m/z

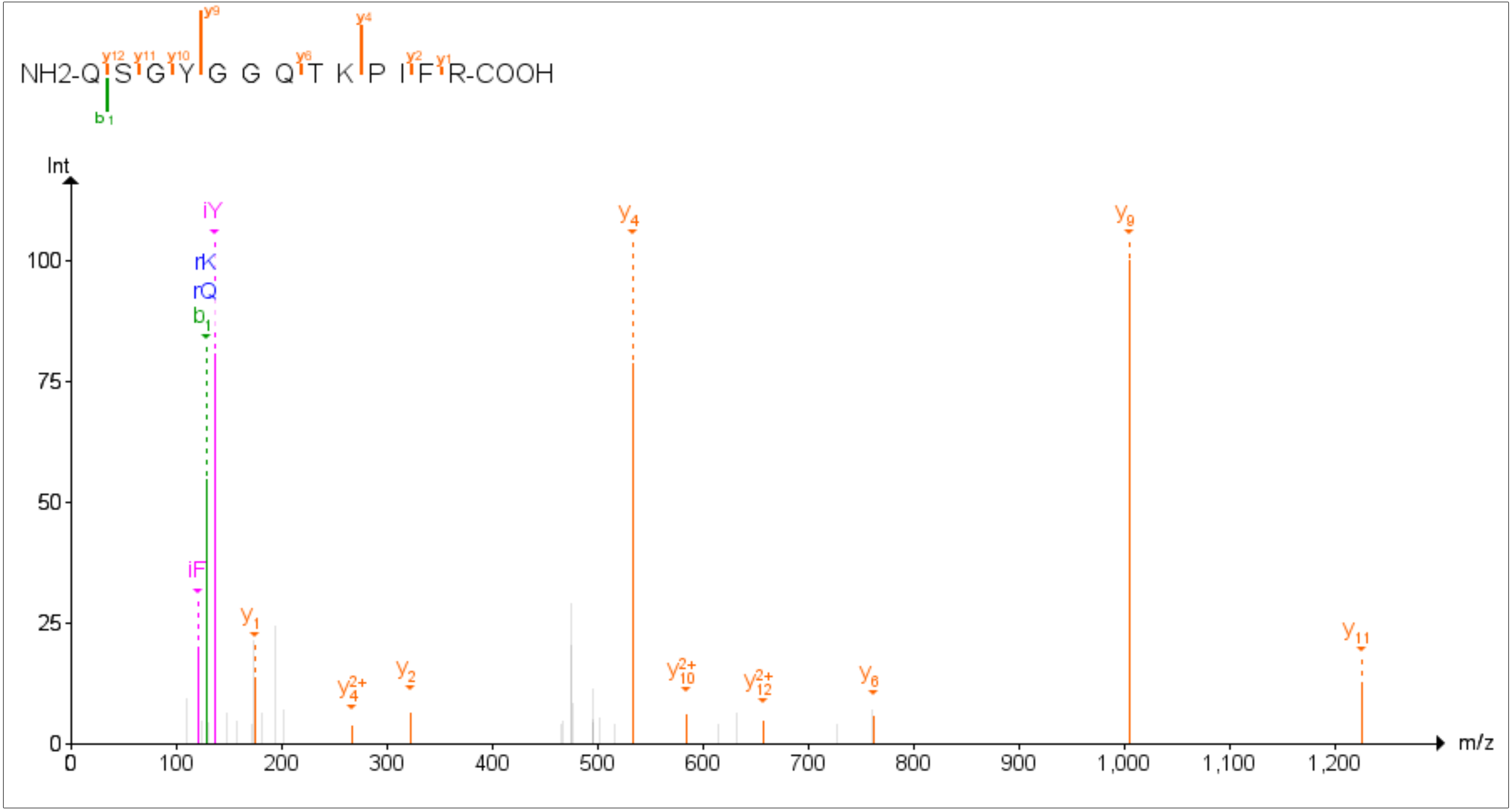

NH2-Q S G Y G G Q T K P I F R-COOH
y12 y11 y10 y9 y6 y4 y2 y1
b 1
Int
iY
rK
rQ
b1
iF
y1
y4
y9
y11
y2
y4 2+
y10 2+
y12 2+
y6
m/z
0
25
50
75
100
0
100
200
300
400
500
600
700
800
900
1,000
1,100
1,200

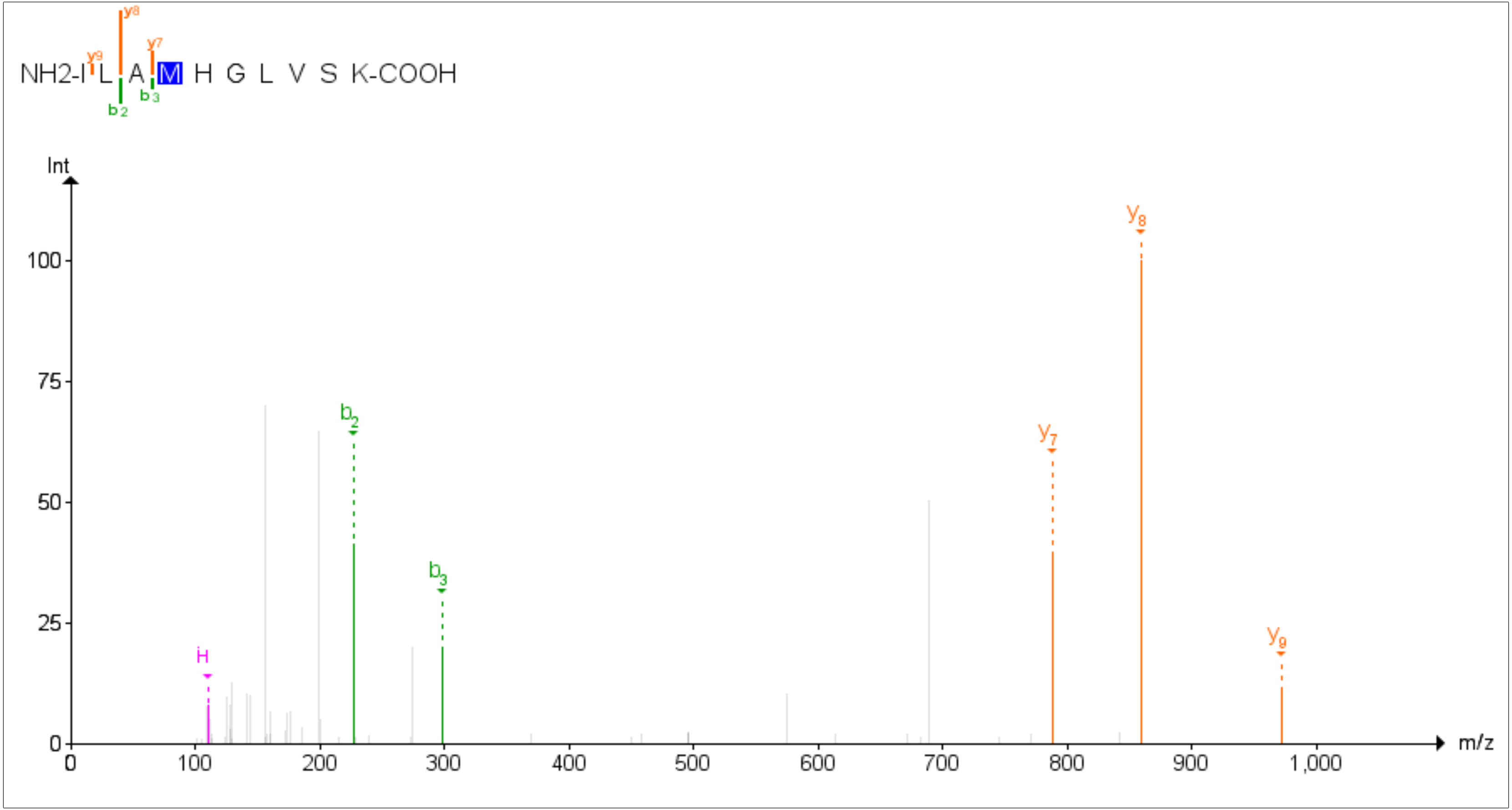

NH2-I L A M H G L V S K-COOH
y9
y8
y7
b2
b3
Int
100
75
50
25
0
H
b2
b3
y7
y8
y9
0
100
200
300
400
500
600
700
800
900
1,000
m/z

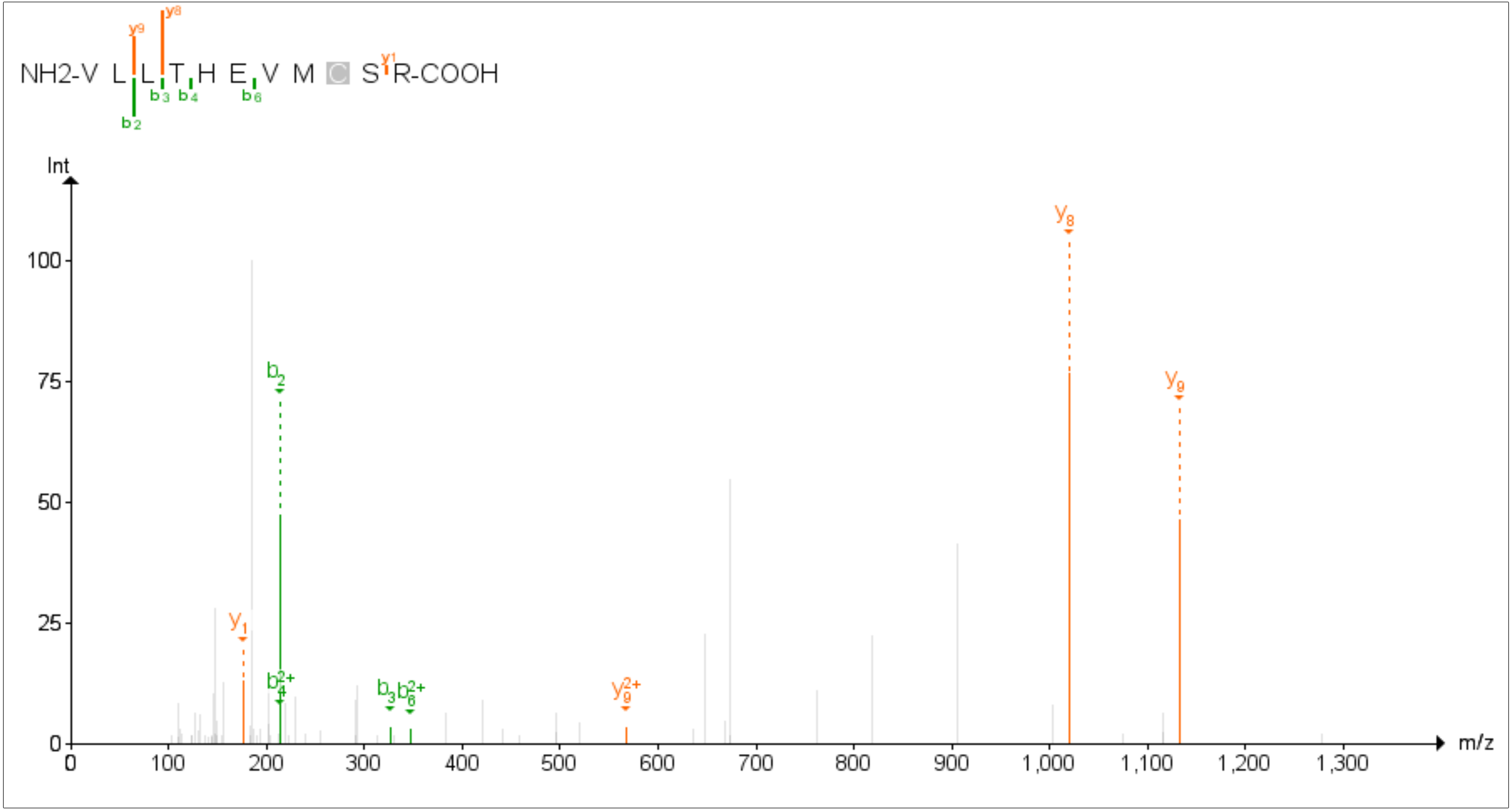

NH2-V L L T H E V M C S R-COOH
y9
y8
y1
b2
b3
b4
b6
Int
100
75
50
25
0
y8
y9
b2
y1
b4 2+
b3
b6 2+
y9 2+
0
100
200
300
400
500
600
700
800
900
1,000
1,100
1,200
1,300
m/z

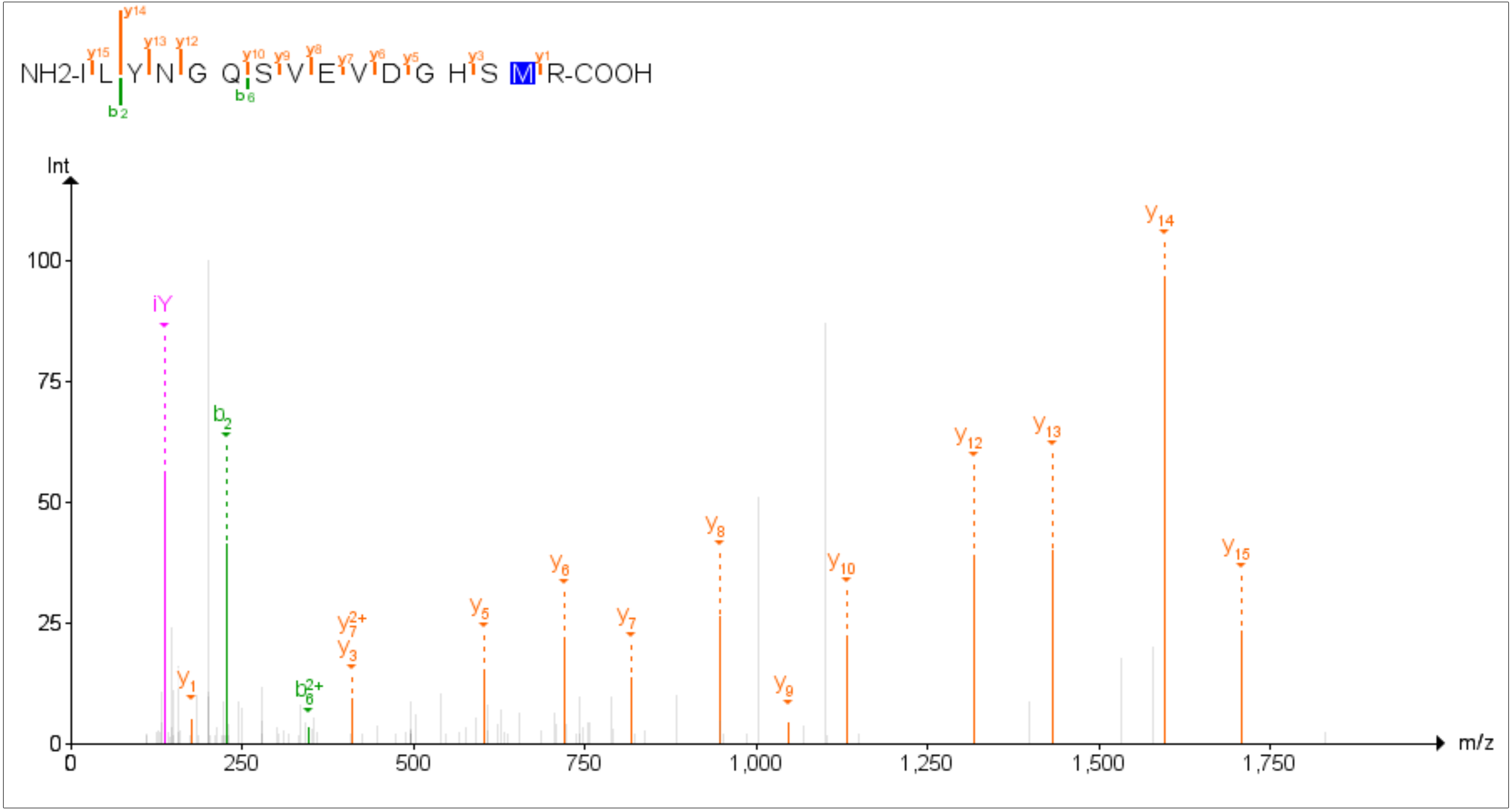

NH2-I L Y N G Q S V E V D G H S M R-COOH
y15 y14 y13 y12 y10 y9 y8 y7 y6 y5 y3 y1
b2 b6
Int
100
75
50
25
0
iY
b2
y1
b6 2+
y7 2+
y3
y5
y6
y7
y8
y9
y10
y12
y13
y14
y15
0
250
500
750
1,000
1,250
1,500
1,750
m/z

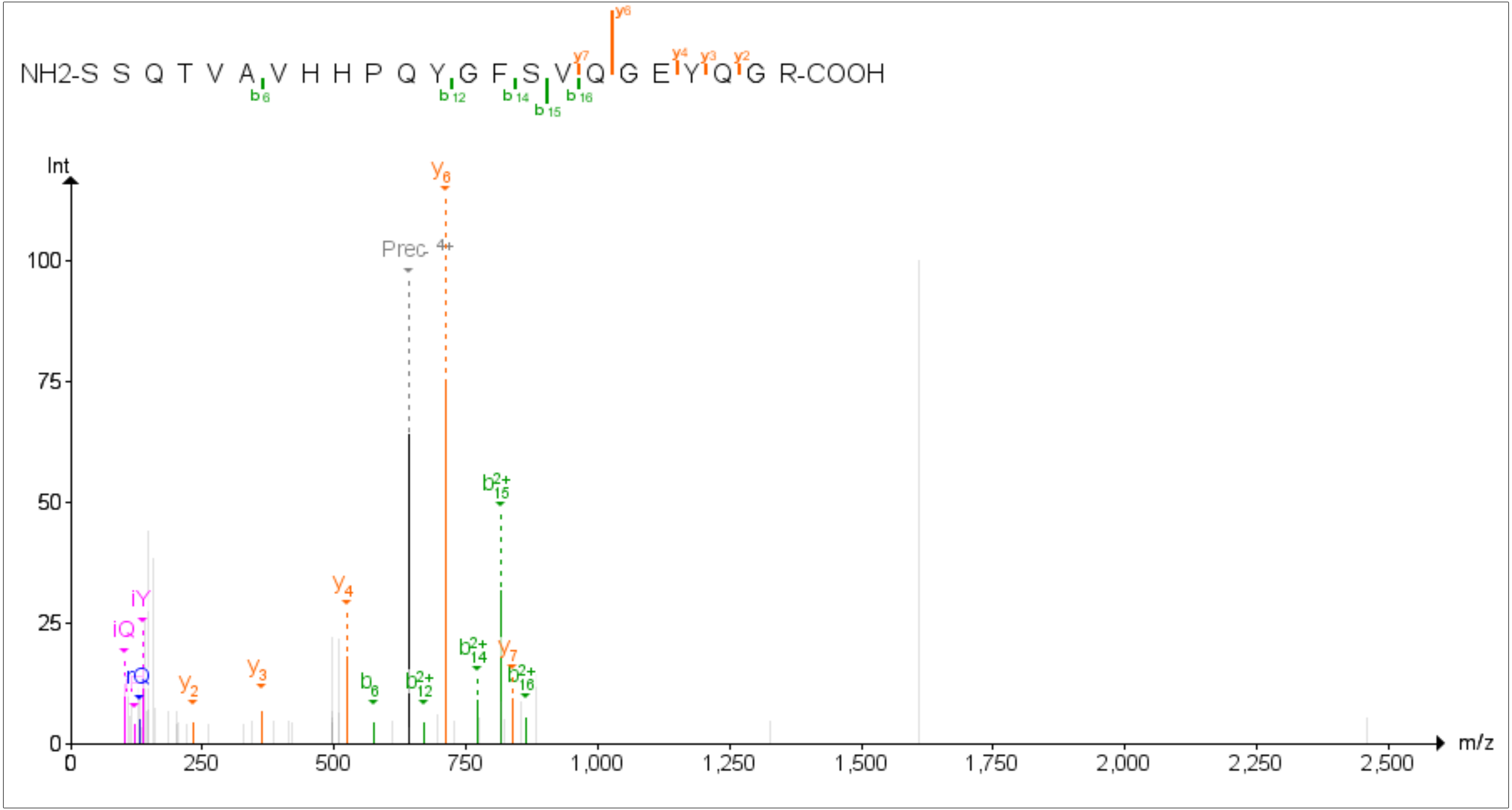

NH2-S S Q T V A V H H P Q Y G F S V Q G E Y Q G R-COOH
b6
b12
b14
b15
b16
y7
y6
y4
y3
y2
Int
Prec 4+
y6
b2+ 15
y4
iY
iQ
rQ
y2
y3
b6
b2+ 12
b2+ 14
y7
b2+ 16
100
75
50
25
0
0
250
500
750
1,000
1,250
1,500
1,750
2,000
2,250
2,500
m/z

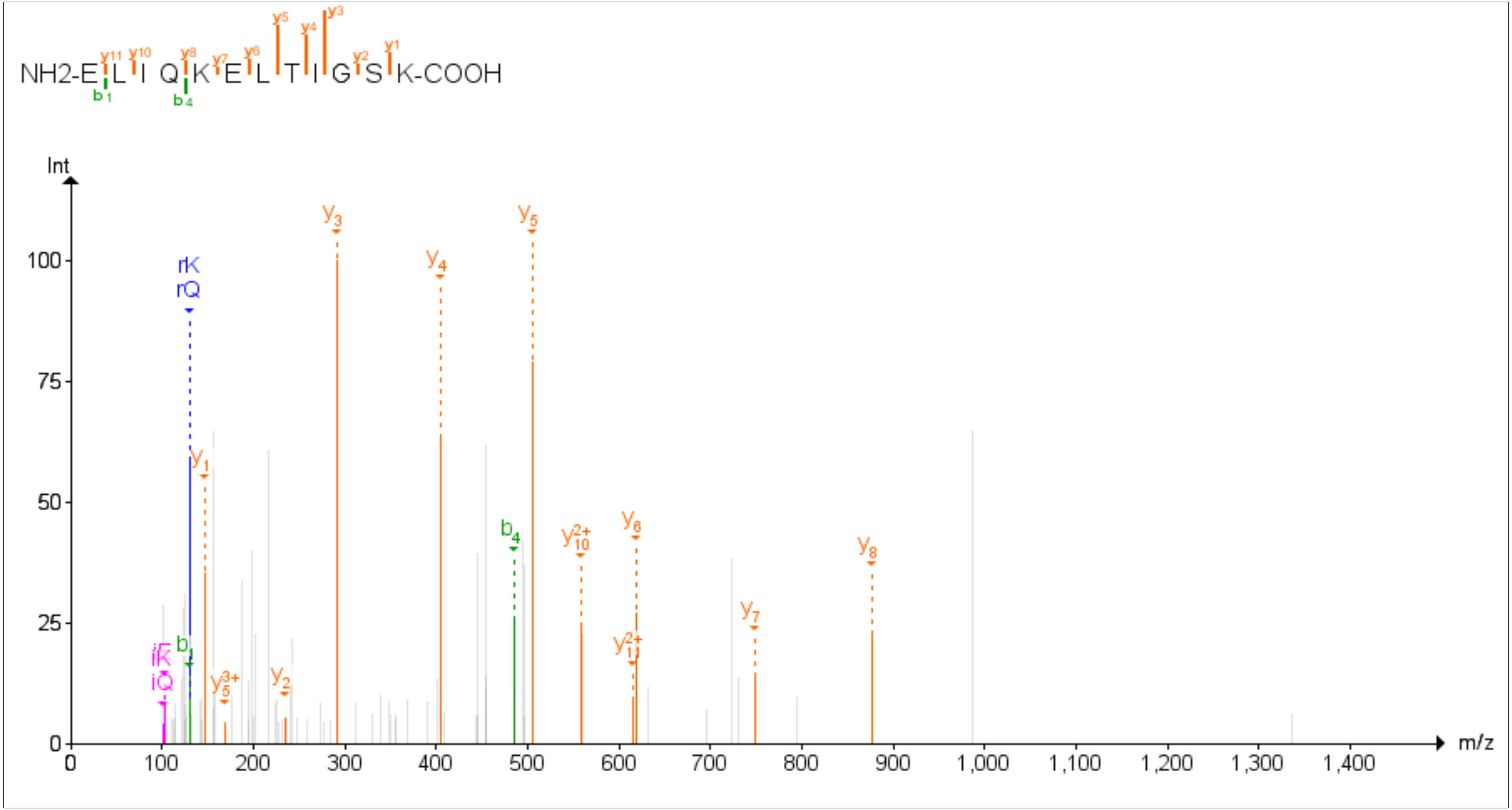

NH2-E L I Q K E L T I G S K-COOH
y11 y10 y8 y7 y6 y5 y4 y3 y2 y1
b1 b4
Int
100
75
50
25
0
rK
rQ
y1
y3
y4
y5
b4
y_{10}^{2+}
y6
y8
y7
y_{11}^{2+}
iK
iQ
b1
y_5^{3+}
y2
m/z
0 100 200 300 400 500 600 700 800 900 1,000 1,100 1,200 1,300 1,400

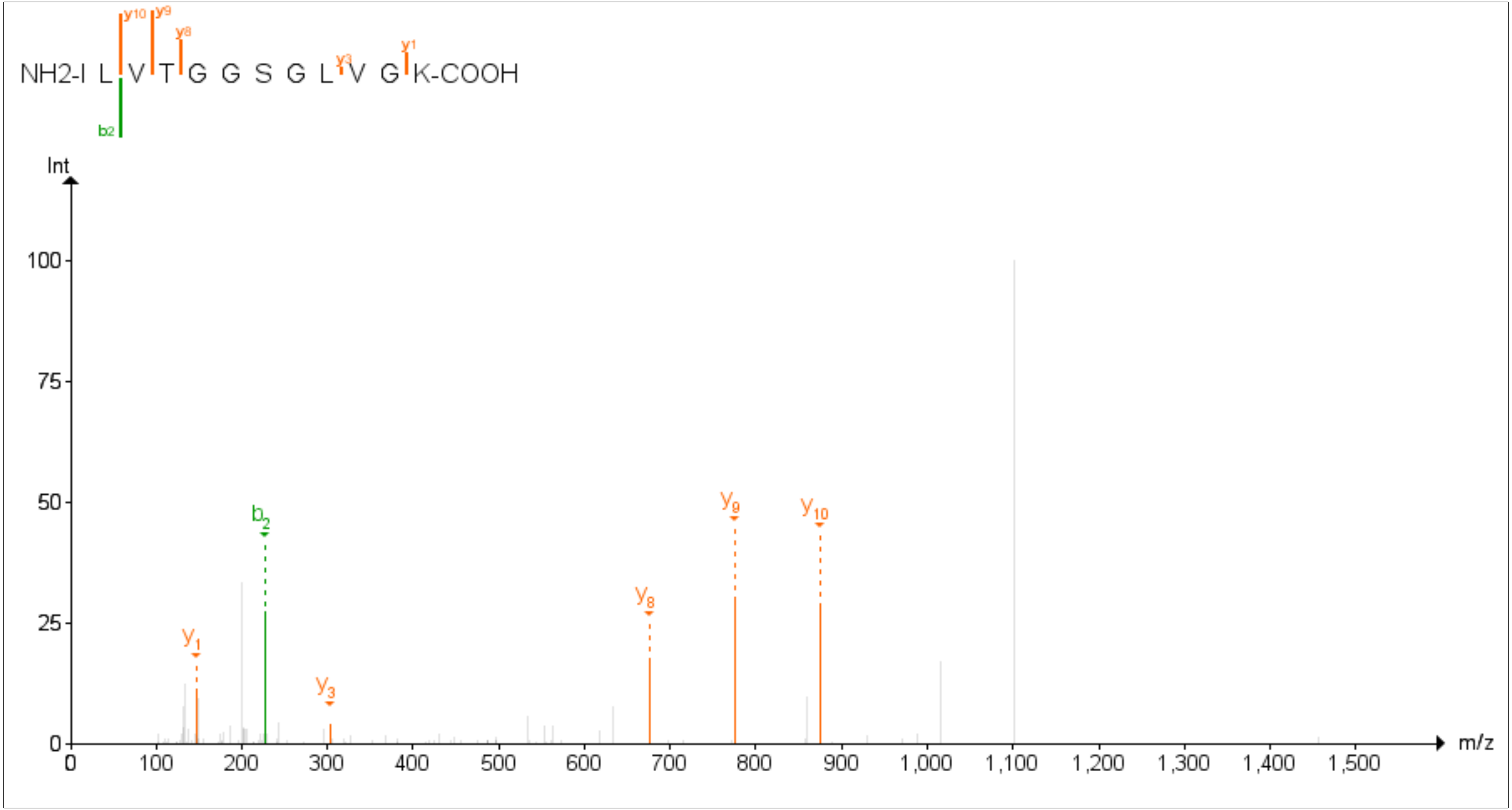
NH2-I L V T G G S G L V G K-COOH
y10
y9
y8
y3
y1
b2
Int
100
75
50
25
0
b2
y1
y3
y8
y9
y10
0
100
200
300
400
500
600
700
800
900
1,000
1,100
1,200
1,300
1,400
1,500
m/z

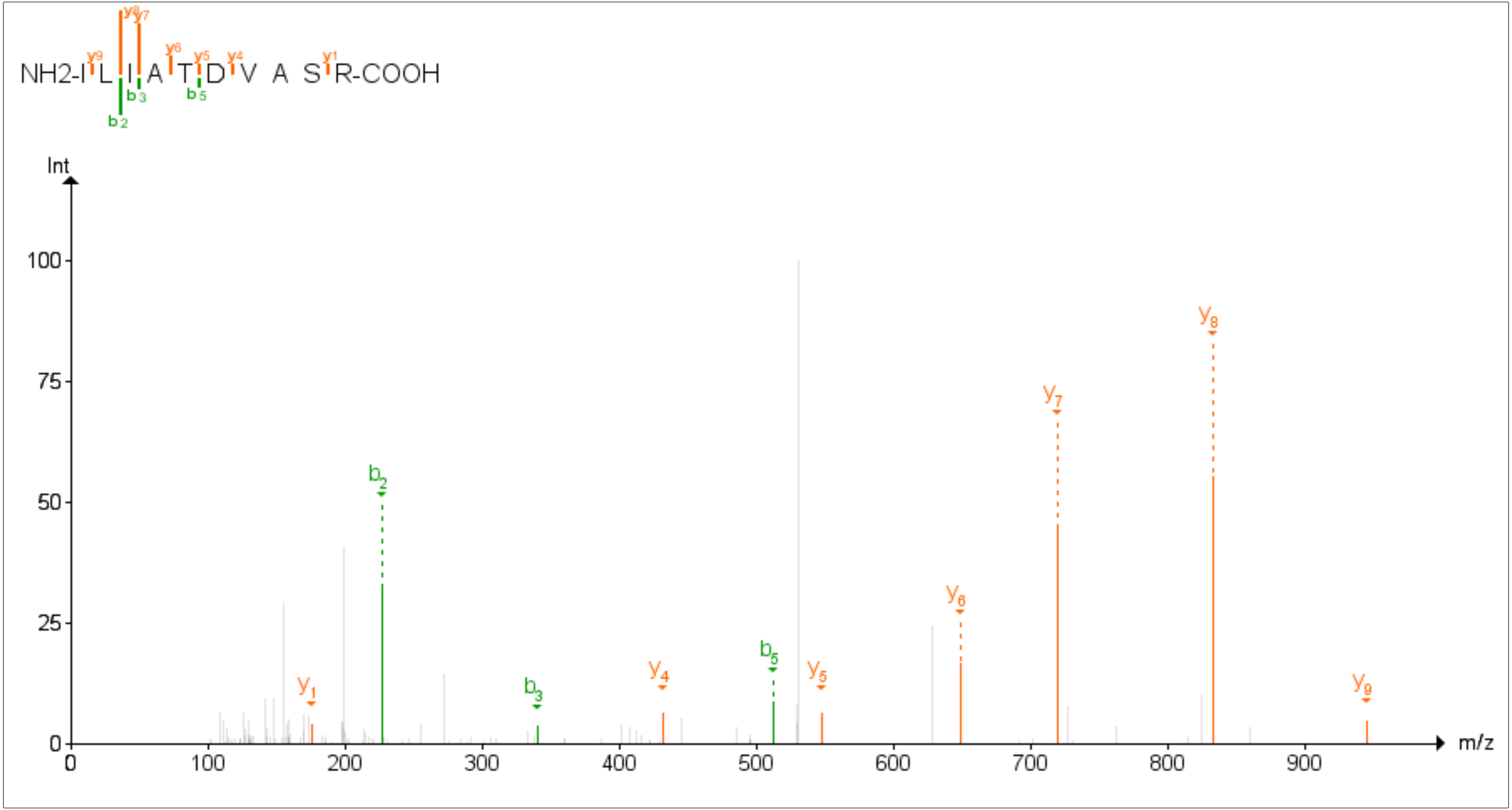

NH2-I L I A T D V A S R-COOH
y9 y8 y7 y6 y5 y4 y1
b2 b3 b5
Int
100
75
50
25
0
m/z
0
100
200
300
400
500
600
700
800
900
b_2
b_3
b_5
y_1
y_4
y_5
y_6
y_7
y_8
y_9

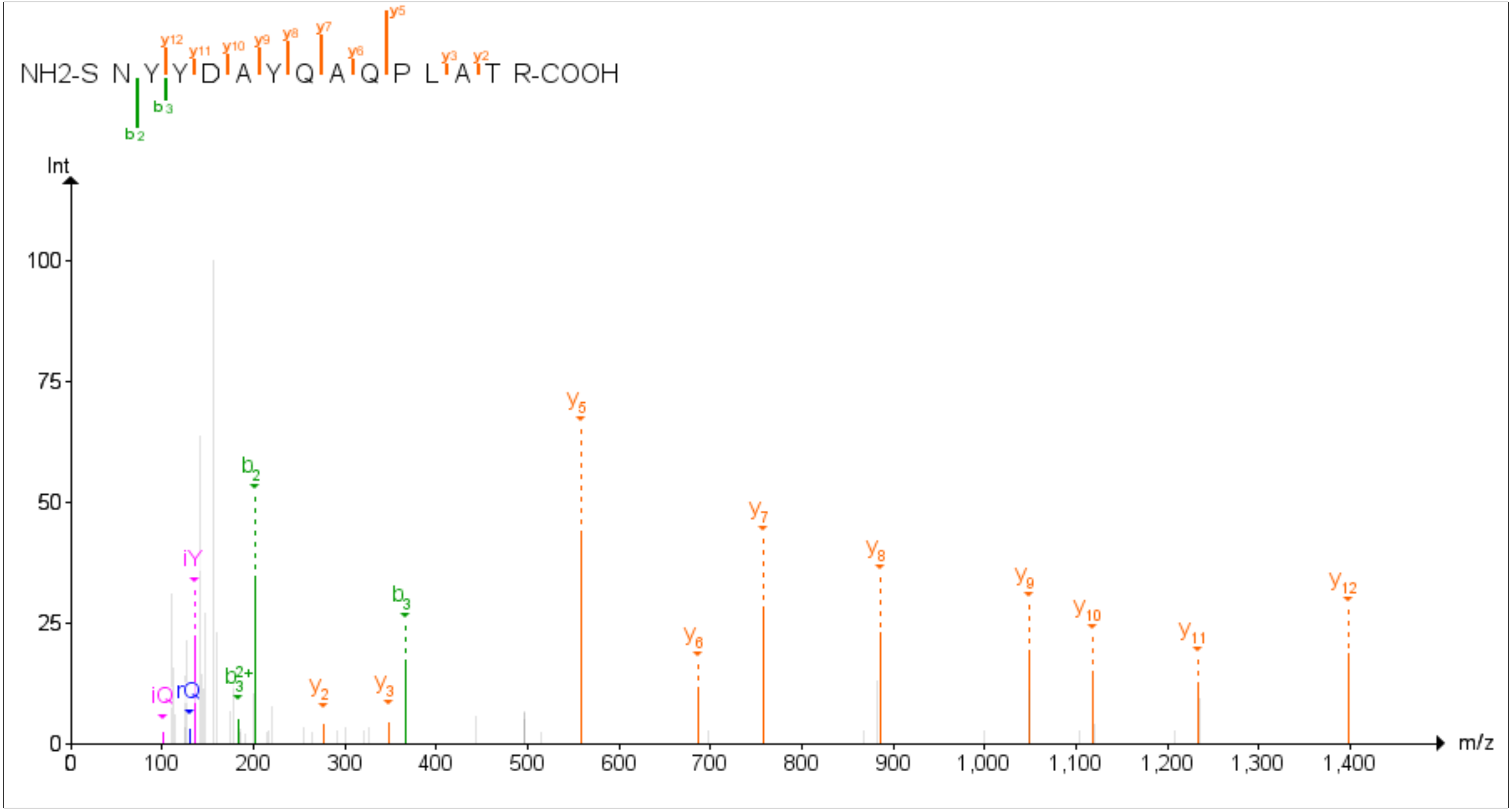

NH2-S N Y Y D A Y Q A Q P L A T R-COOH
y12 y11 y10 y9 y8 y7 y6 y5 y3 y2
b2 b3
Int
100
75
50
25
0
m/z
0 100 200 300 400 500 600 700 800 900 1,000 1,100 1,200 1,300 1,400
y5 y7 y8 y9 y10 y11 y12 y6 y2 y3
b2 b3
iY iQ rQ

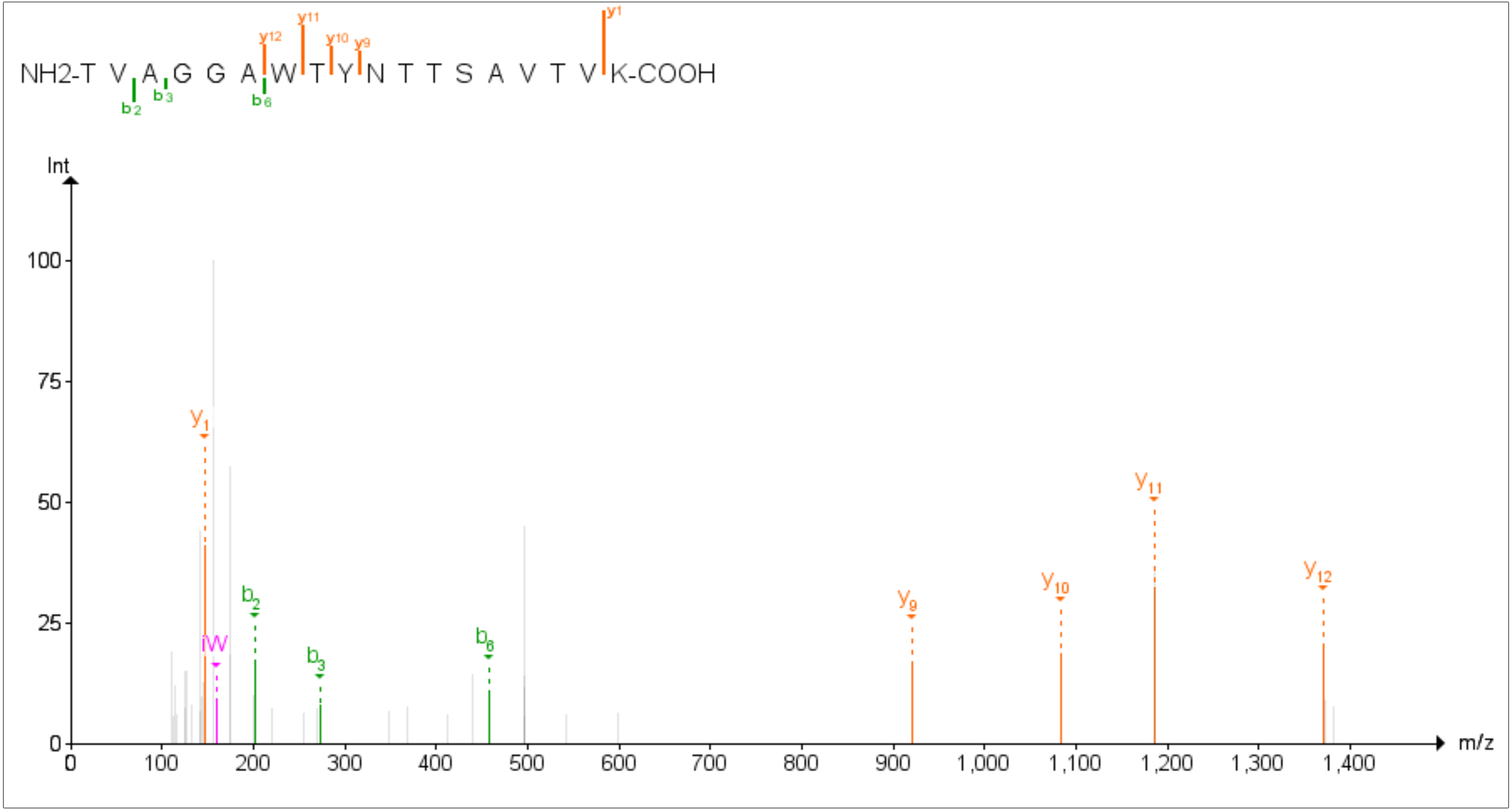

NH2-T V A G G A W T Y N T T S A V T V K-COOH
b2
b3
b6
y12
y11
y10
y9
y1
Int
m/z
0
25
50
75
100
0
100
200
300
400
500
600
700
800
900
1,000
1,100
1,200
1,300
1,400
iW

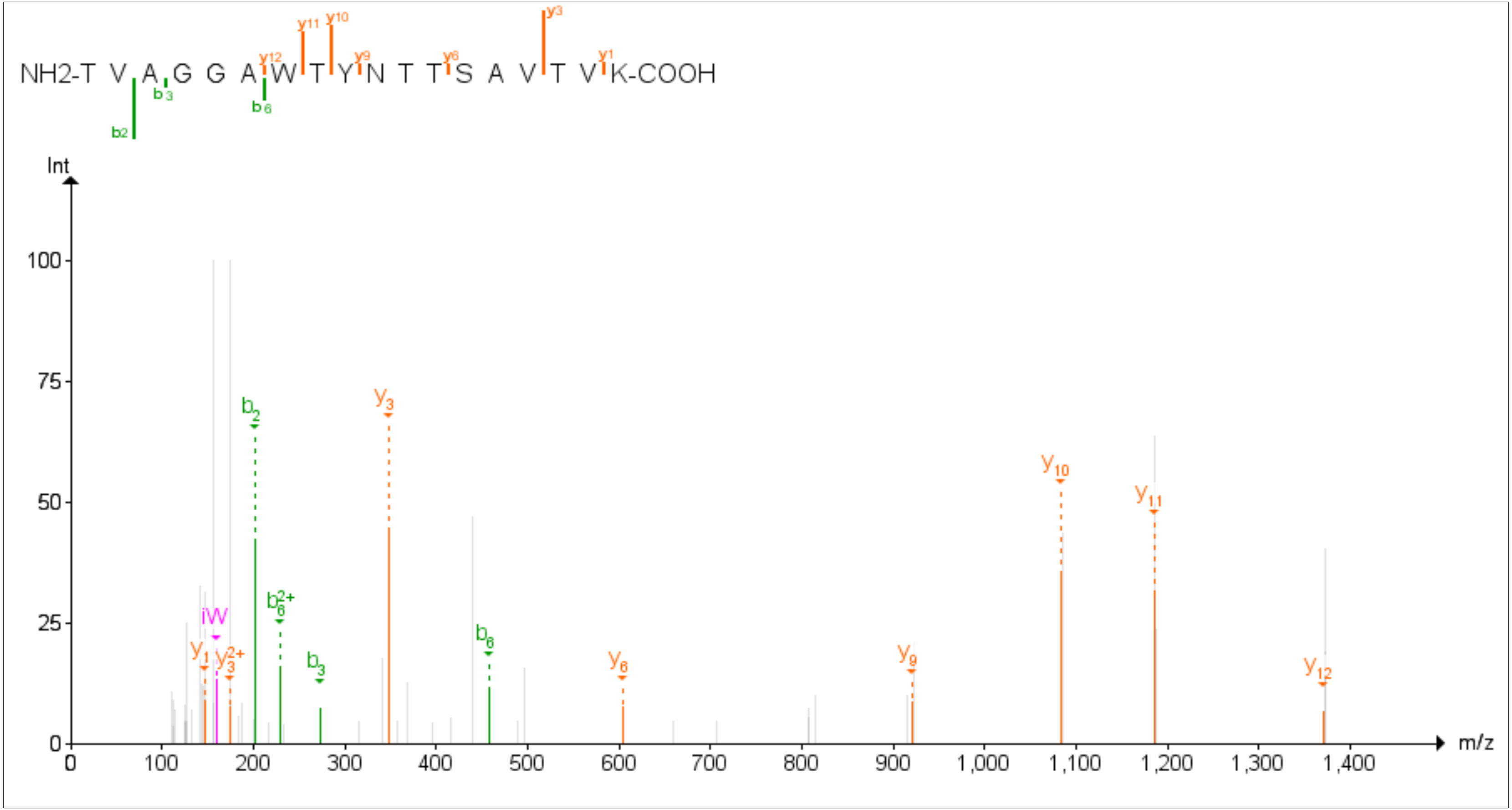
NH2-T V A G G A W T Y N T T S A V T V K-COOH
b2
b3
b6
y12
y11
y10
y9
y6
y3
y1
Int
100
75
50
25
0
m/z
0
100
200
300
400
500
600
700
800
900
1,000
1,100
1,200
1,300
1,400
iW
y1
y3 2+
b2
b6 2+
b3
y3
b6
y6
y9
y10
y11
y12

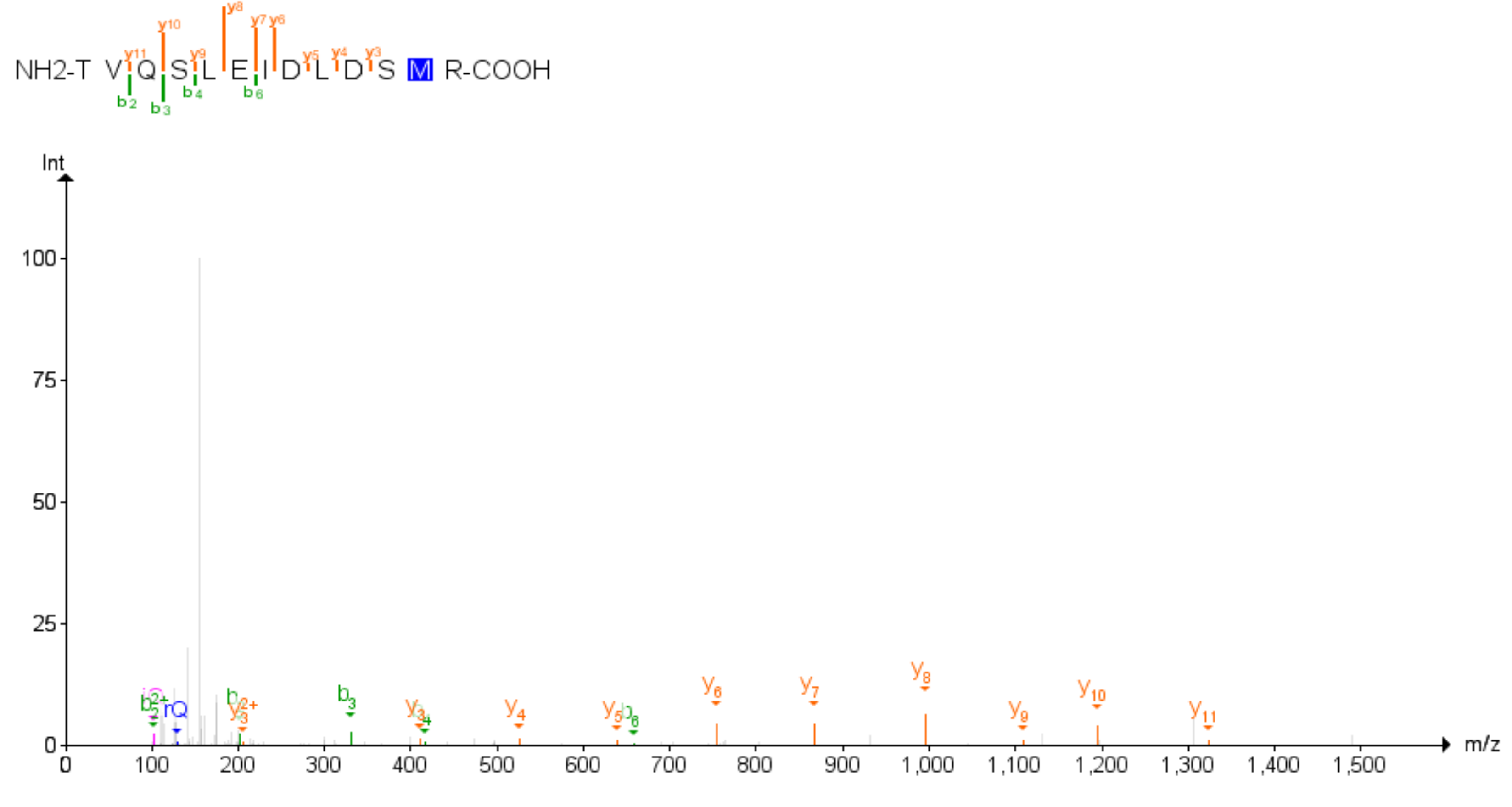

NH2-T V Q S L E I D L D S M R-COOH
y11 y10 y9 y8 y7 y6 y5 y4 y3
b2 b3 b4 b6
Int
100
75
50
25
0
m/z
0 100 200 300 400 500 600 700 800 900 1,000 1,100 1,200 1,300 1,400 1,500
rQ
b3
y4
y6
y7
y8
y9
y10
y11

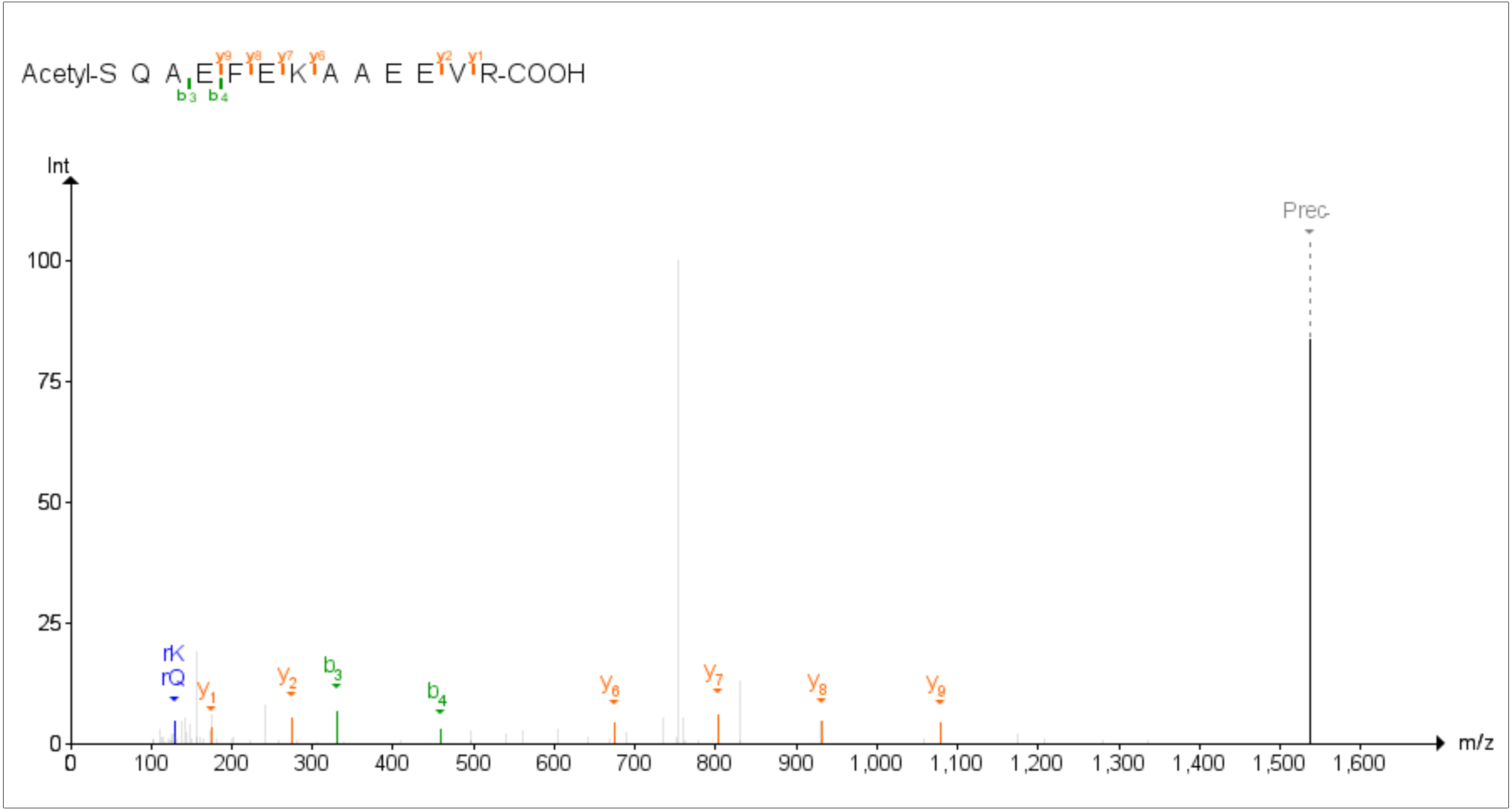

Acetyl-S Q A E F E K A A E E V R-COOH
y9 y8 y7 y6 y2 y1
b3 b4
Int
Prec
100
75
50
25
0
rK
rQ
y1
y2
b3
b4
y6
y7
y8
y9
0
100
200
300
400
500
600
700
800
900
1,000
1,100
1,200
1,300
1,400
1,500
1,600
m/z

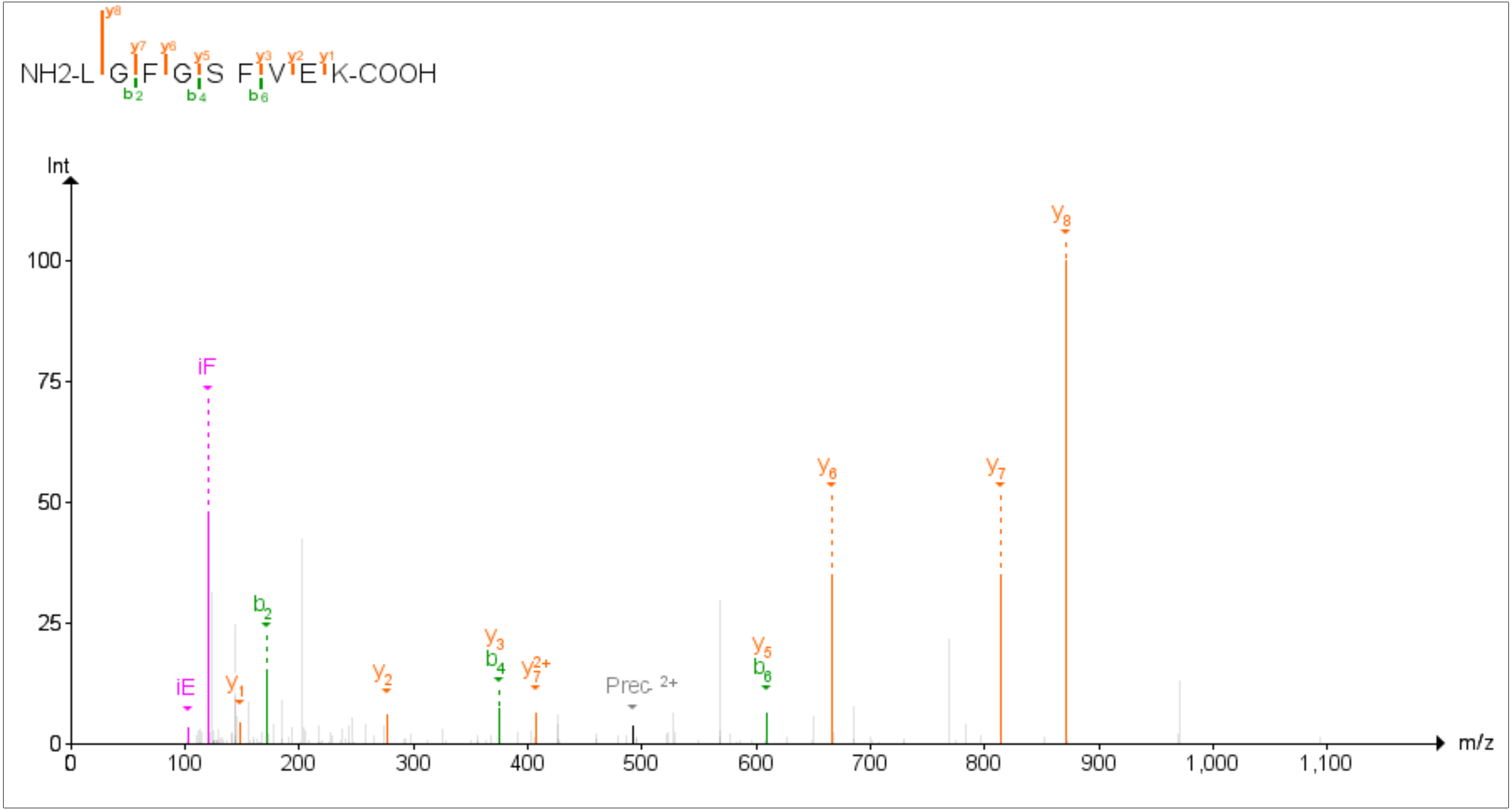

NH2-L G F G S F V E K-COOH
y8 y7 y6 y5 y3 y2 y1
b2 b4 b6
Int
m/z
iF
iE
y1
b2
y2
y3
b4
y7 2+
Prec 2+
y5
b6
y6
y7
y8
0
25
50
75
100
0
100
200
300
400
500
600
700
800
900
1,000
1,100

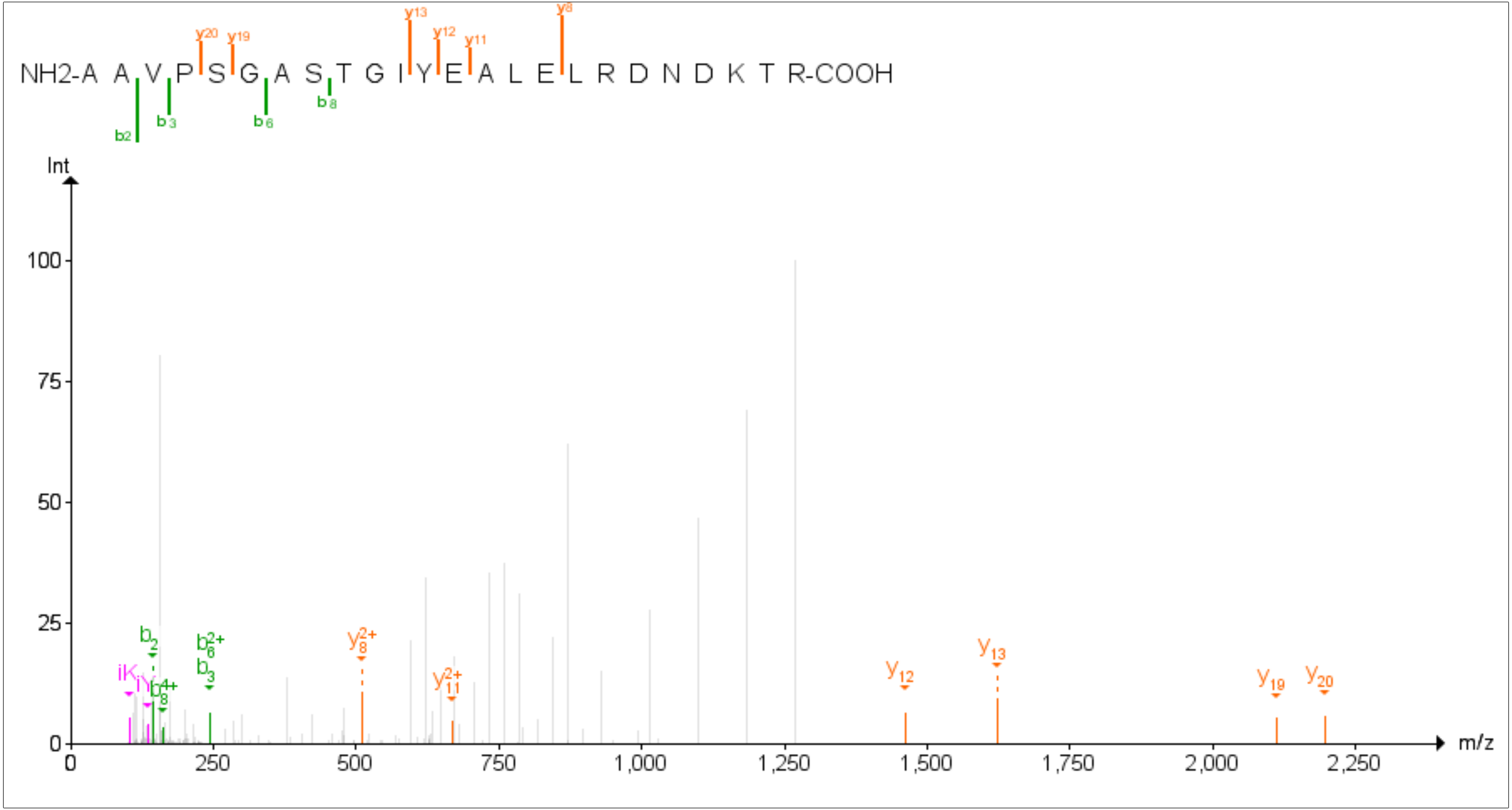

NH2-A A V P S G A S T G I Y E A L E L R D N D K T R-COOH
y20 y19 y13 y12 y11 y8
b2 b3 b6 b8
Int
100
75
50
25
0
m/z
0
250
500
750
1,000
1,250
1,500
1,750
2,000
2,250
iK
iY
b2
b6 2+
b3
b8 4+
y8 2+
y11 2+
y12
y13
y19
y20

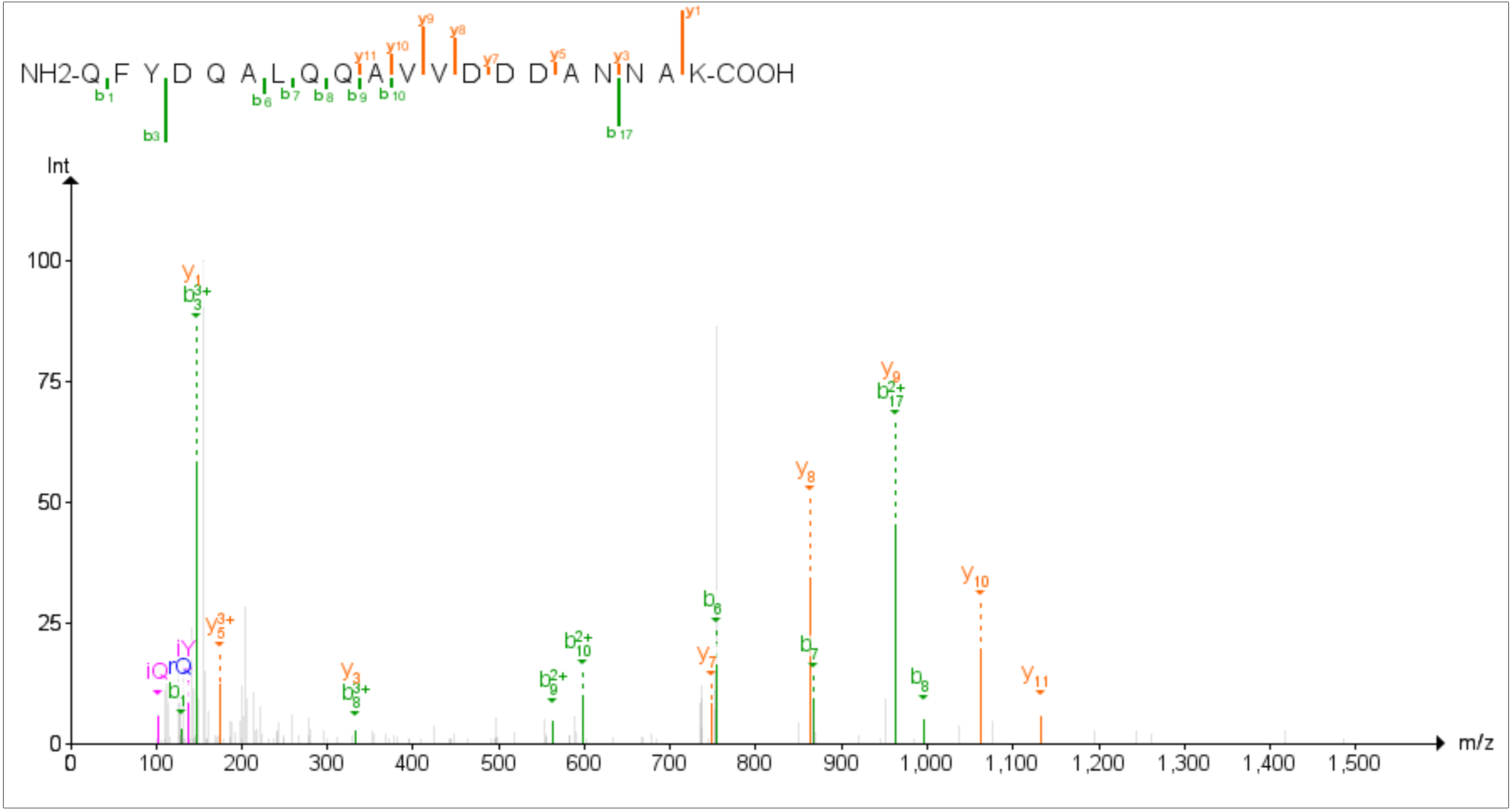
NH2-Q F Y D Q A L Q Q A V V D D D A N N A K-COOH
Int
m/z

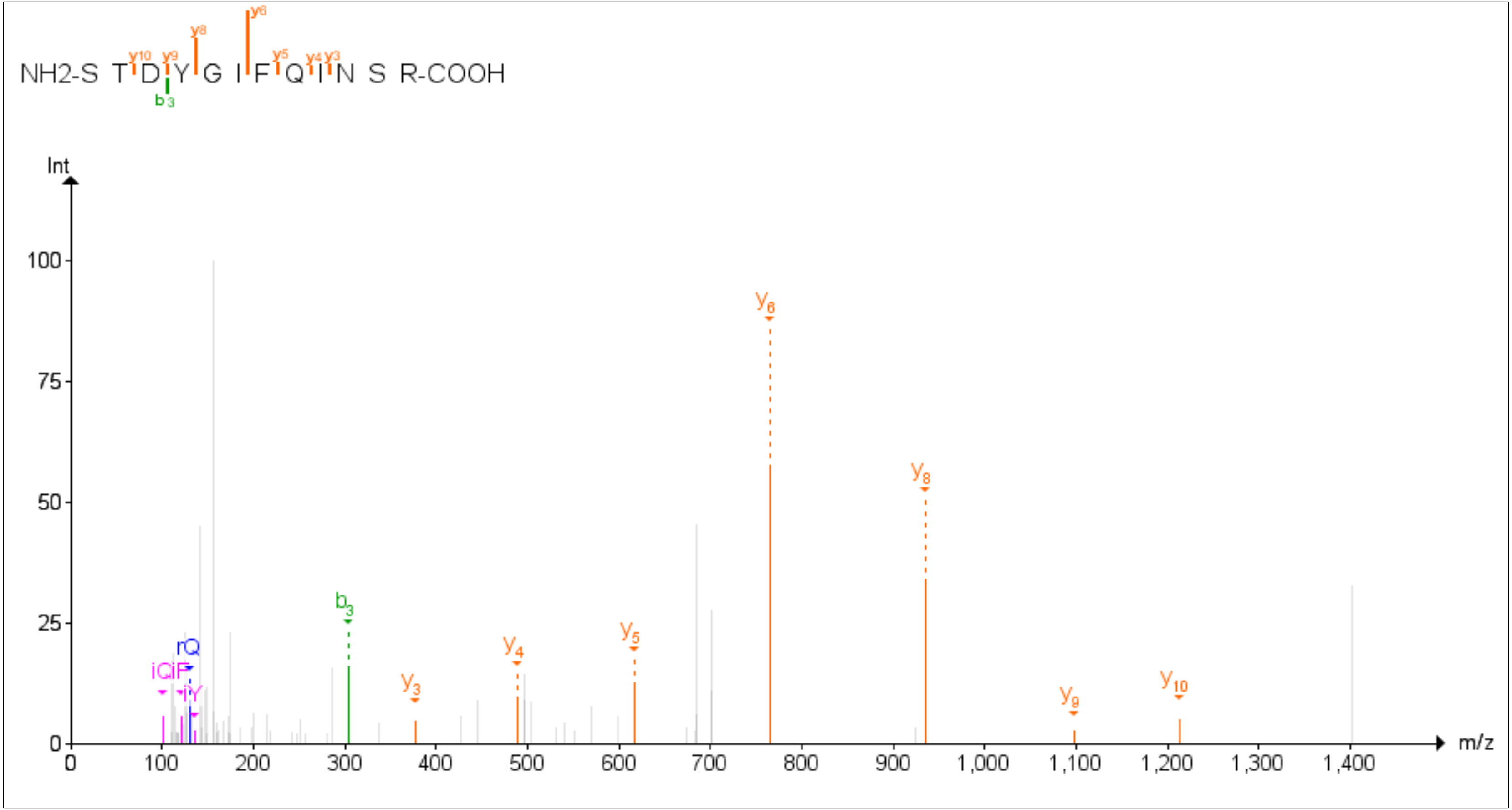
NH2-S T D Y G I F Q I N S R-COOH
y10 y9 y8 y6 y5 y4 y3
b3
Int
100
75
50
25
0
m/z
0 100 200 300 400 500 600 700 800 900 1,000 1,100 1,200 1,300 1,400
y6
y8
b3
y3
y4
y5
y9
y10
rQ
iQiF
iY

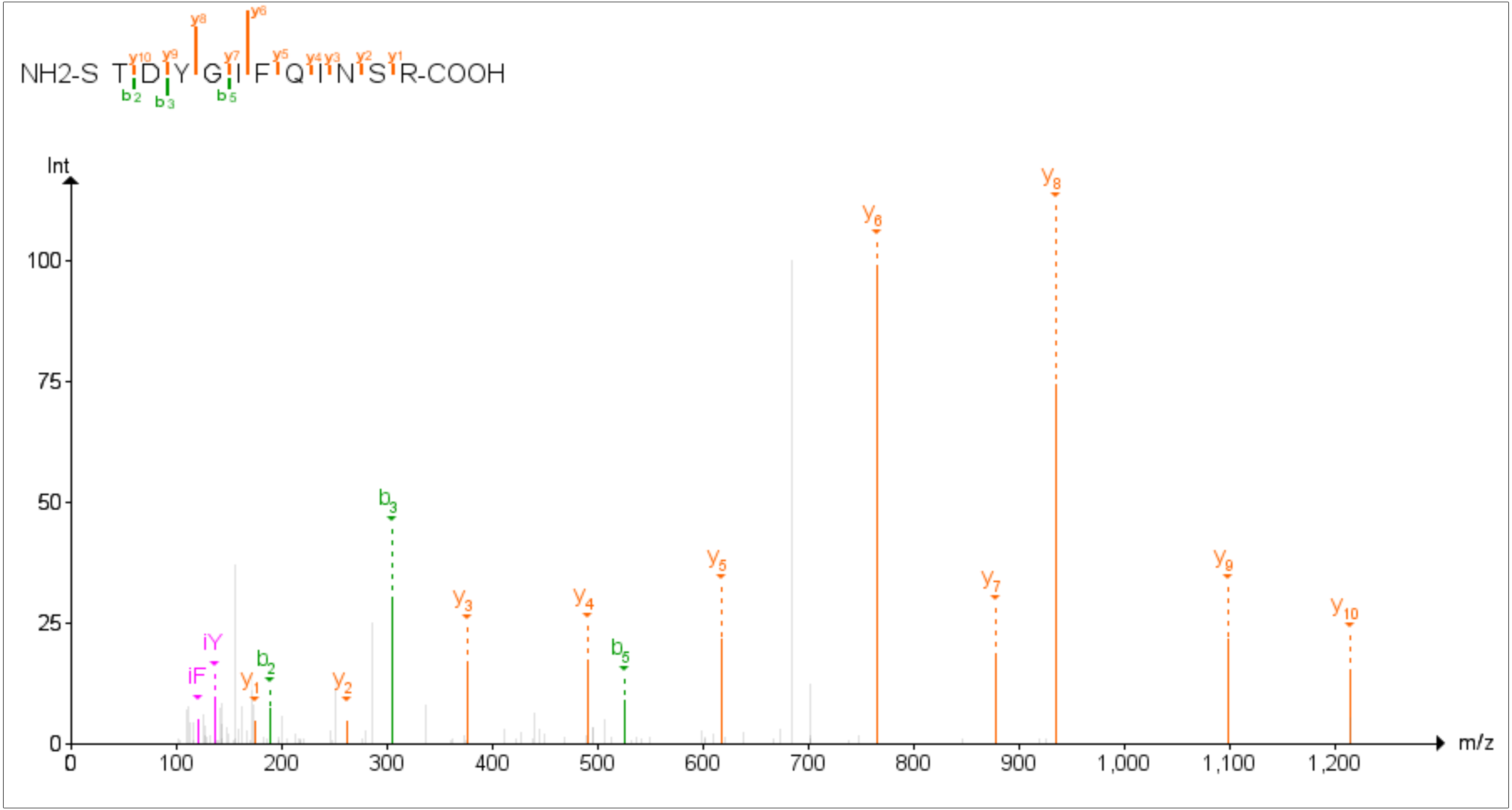

NH2-S T D Y G I F Q I N S R-COOH
y10 y9 y8 y7 y6 y5 y4 y3 y2 y1
b2 b3 b5
Int
100
75
50
25
0
0 100 200 300 400 500 600 700 800 900 1,000 1,100 1,200
m/z
iY
iF
y1
b2
y2
b3
y3
y4
b5
y5
y6
y7
y8
y9
y10

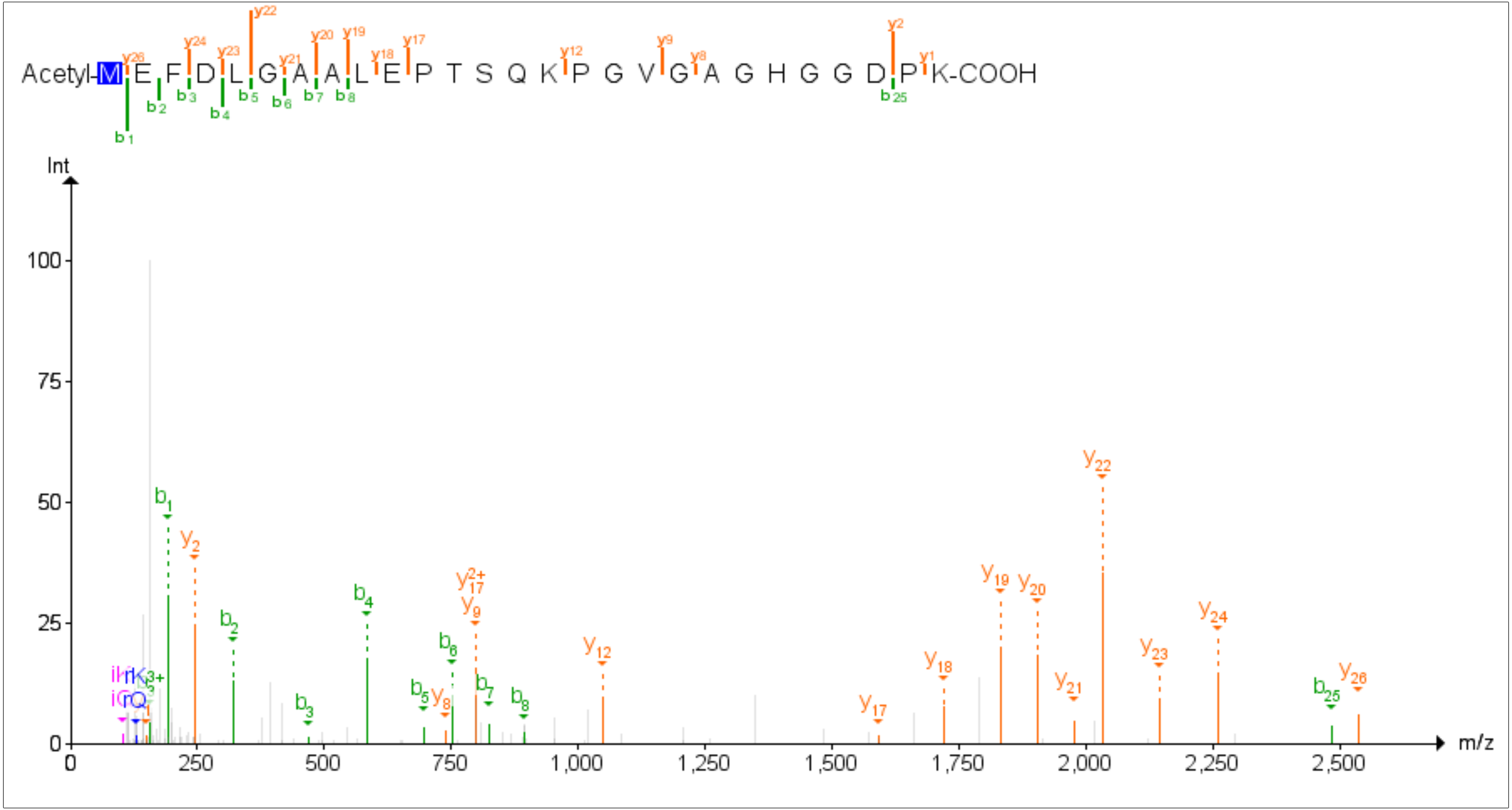

Acetyl-M E F D L G A A L E P T S Q K P G V G A G H G G D P K-COOH
Int
m/z

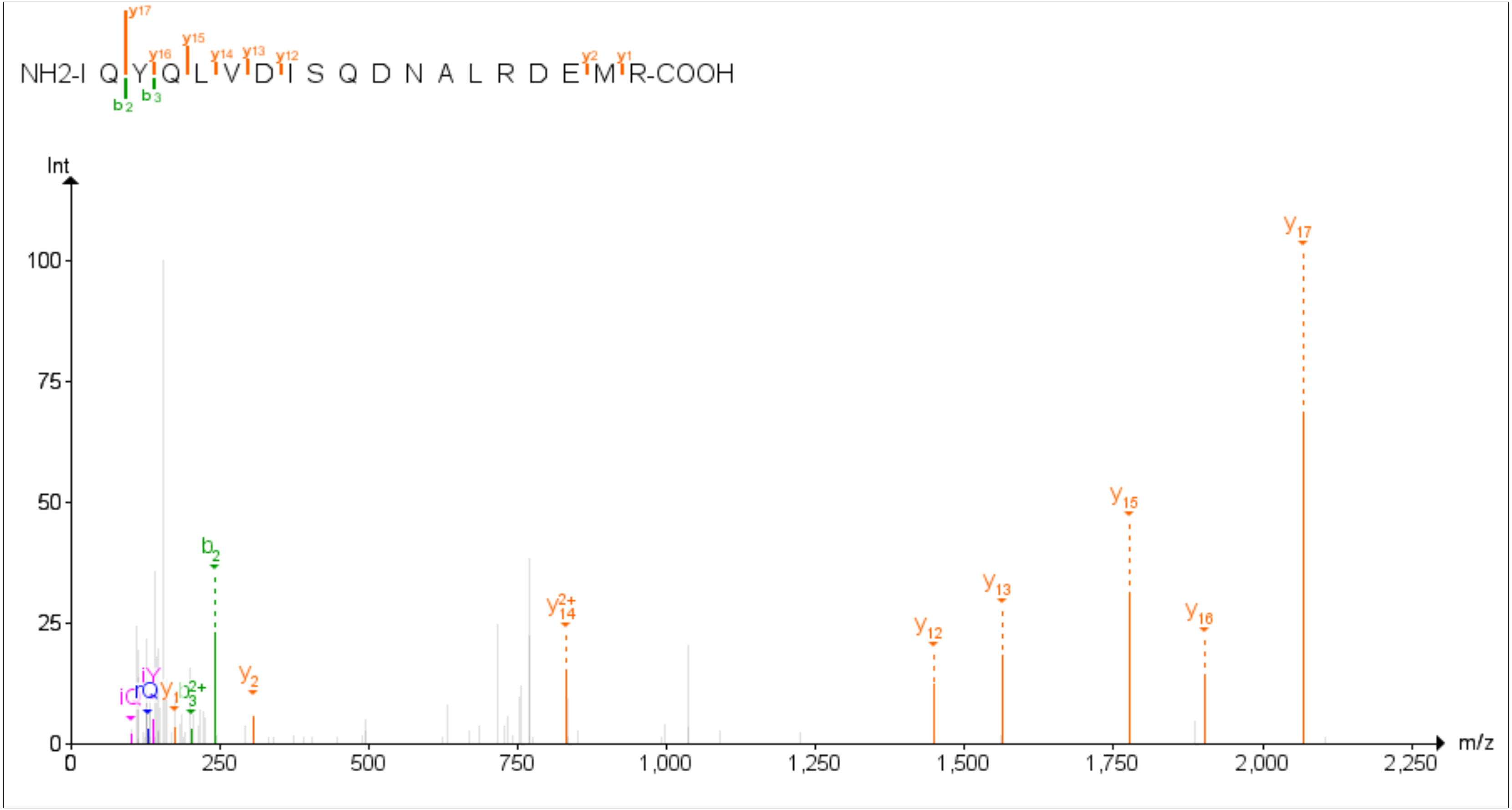

NH2-I Q Y Q L V D I S Q D N A L R D E M R-COOH
y17
y16
y15
y14
y13
y12
y2
y1
b2
b3
Int
100
75
50
25
0
m/z
0
250
500
750
1,000
1,250
1,500
1,750
2,000
2,250
y17
y15
y13
y12
y16
y14 2+
b2
y2
y1
b3 2+
iQ
iY
rQ

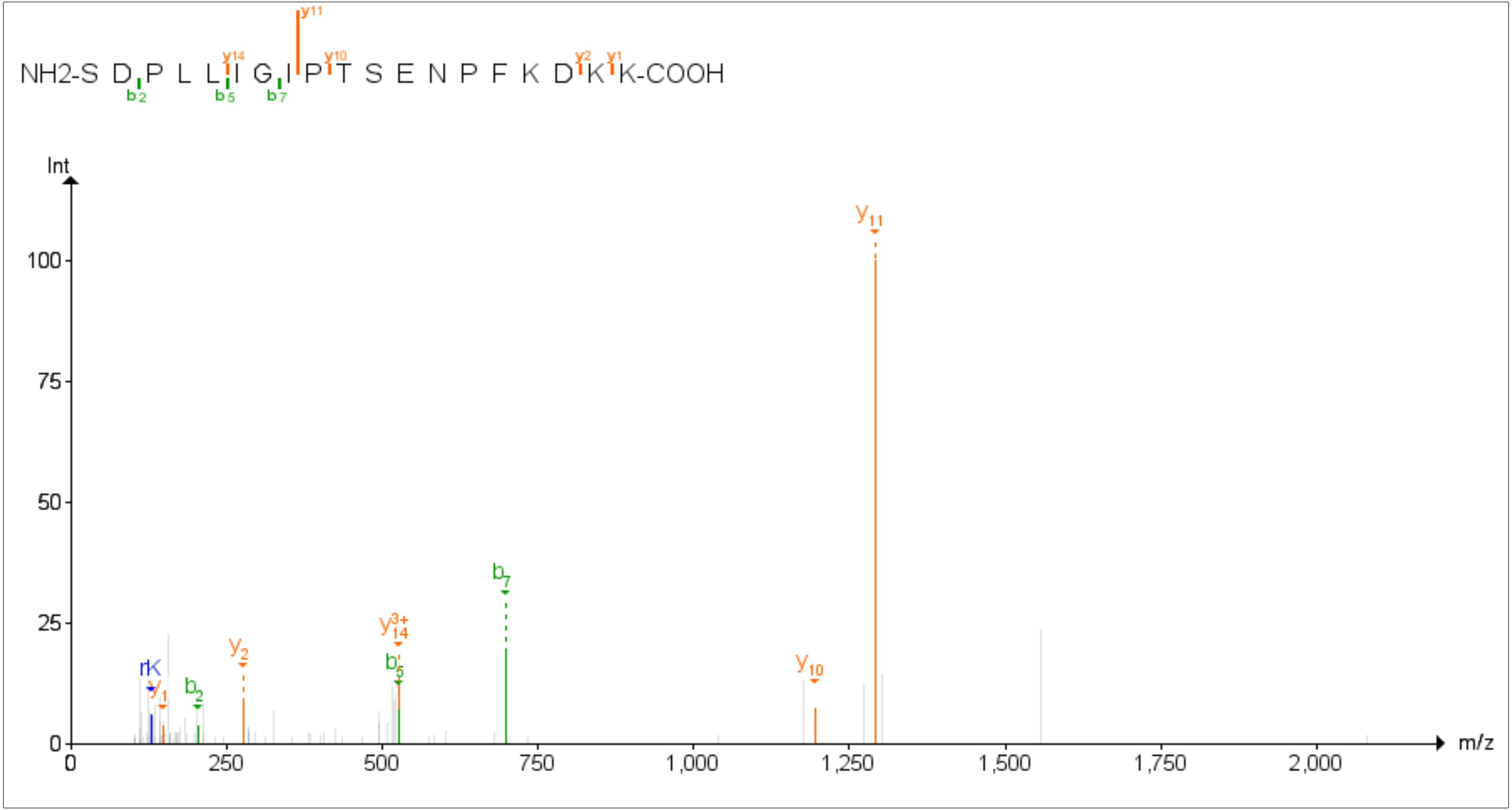

NH2-S D P L L I G I P T S E N P F K D K K-COOH
y11
y14
y10
y2
y1
b2
b5
b7
Int
100
75
50
25
0
rK
y1
b2
y2
y14 3+
b5
b7
y10
y11
0
250
500
750
1,000
1,250
1,500
1,750
2,000
m/z

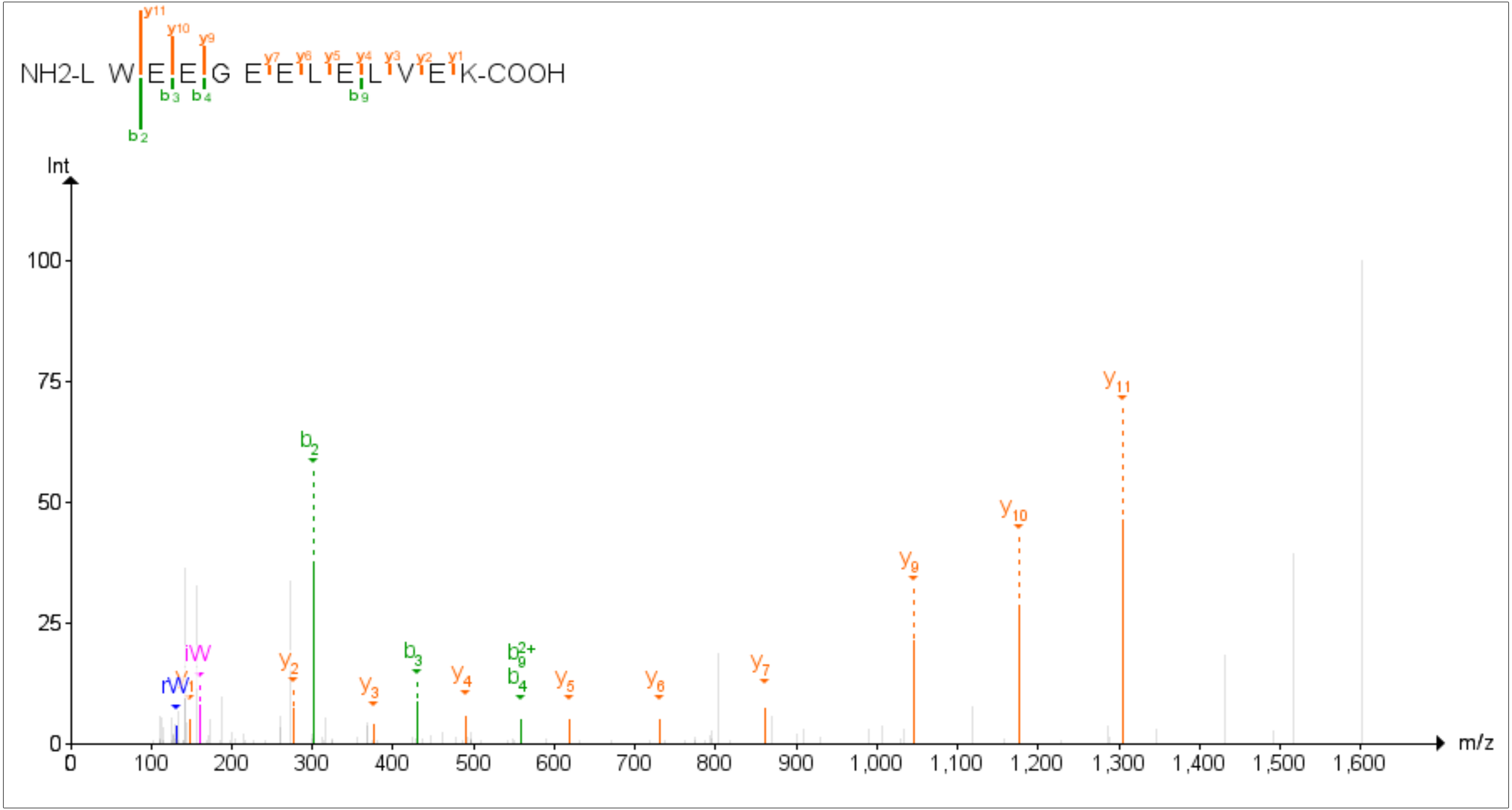

NH2-L W E E G E E L E L V E K-COOH
y11
y10
y9
y7
y6
y5
y4
y3
y2
y1
b2
b3
b4
b9
Int
m/z
0
25
50
75
100
100
200
300
400
500
600
700
800
900
1,000
1,100
1,200
1,300
1,400
1,500
1,600
rW
iW
b2
b3
b4
y2
y3
y4
y5
y6
y7
y9
y10
y11

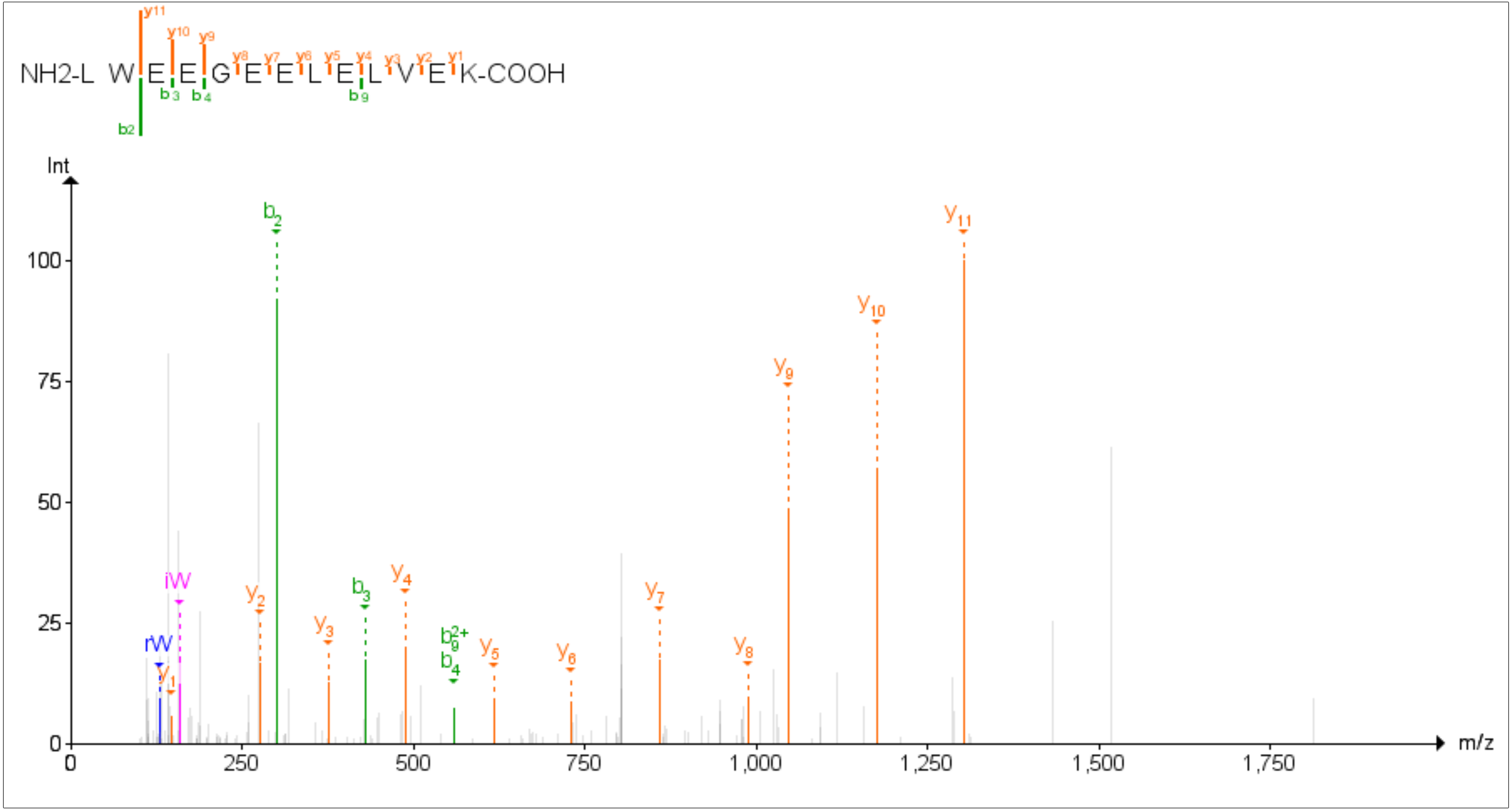
NH2-L W E E G E E L E L V E K-COOH
y11 y10 y9 y8 y7 y6 y5 y4 y3 y2 y1
b2 b3 b4 b9
Int
100
75
50
25
0
0 250 500 750 1,000 1,250 1,500 1,750
m/z
b2
y11
y10
y9
y7
y8
y6
y5
b9 2+
b4
y4
b3
y3
y2
iW
rW
y1

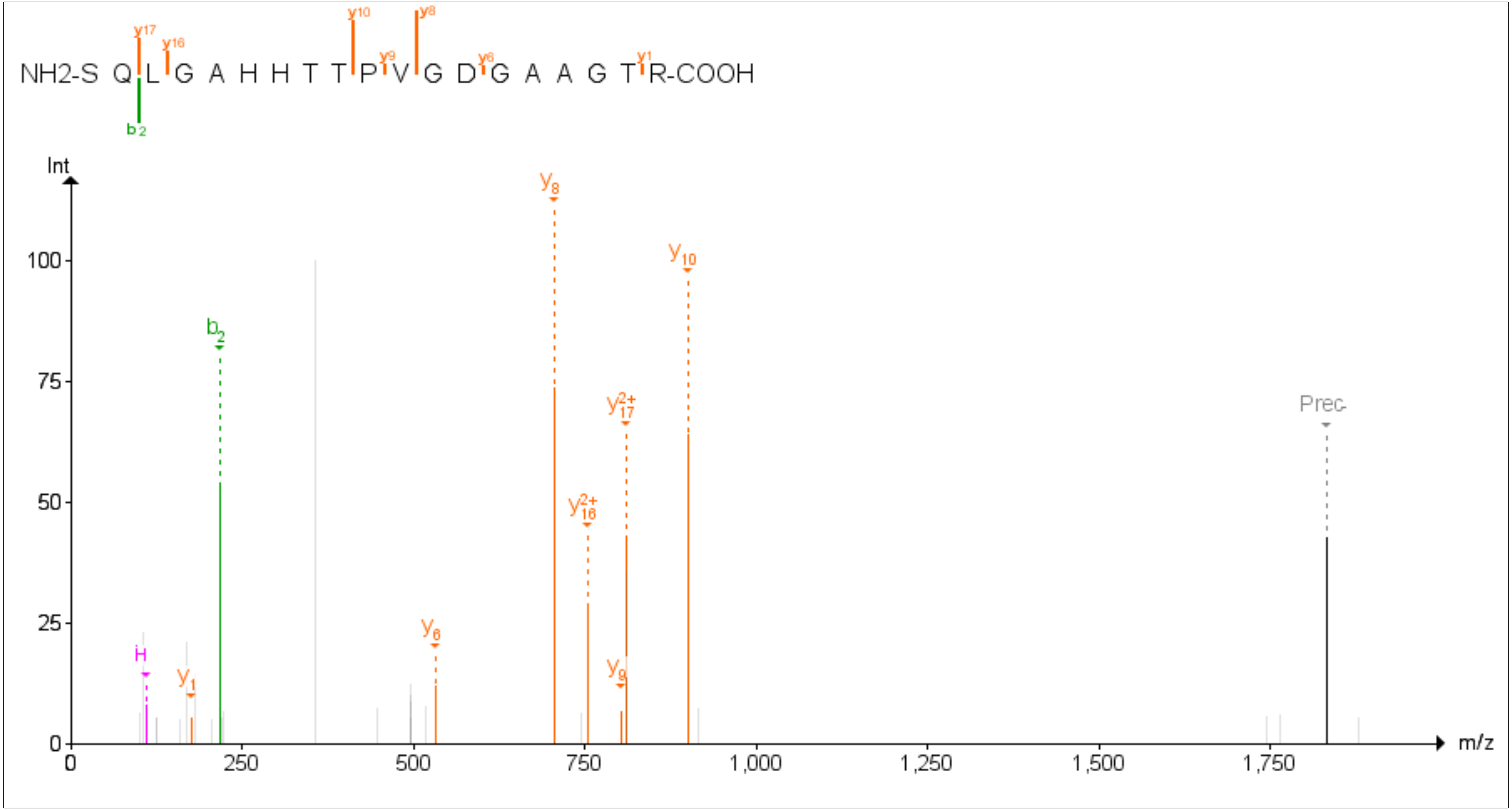
NH2-S Q L G A H H T T P V G D G A A G T R-COOH
y17
y16
y10
y9
y8
y6
y1
b2
Int
m/z
100
75
50
25
0
250
500
750
1,000
1,250
1,500
1,750
y8
y10
b2
y17 2+
y16 2+
Prec.
y6
y9
y1
H

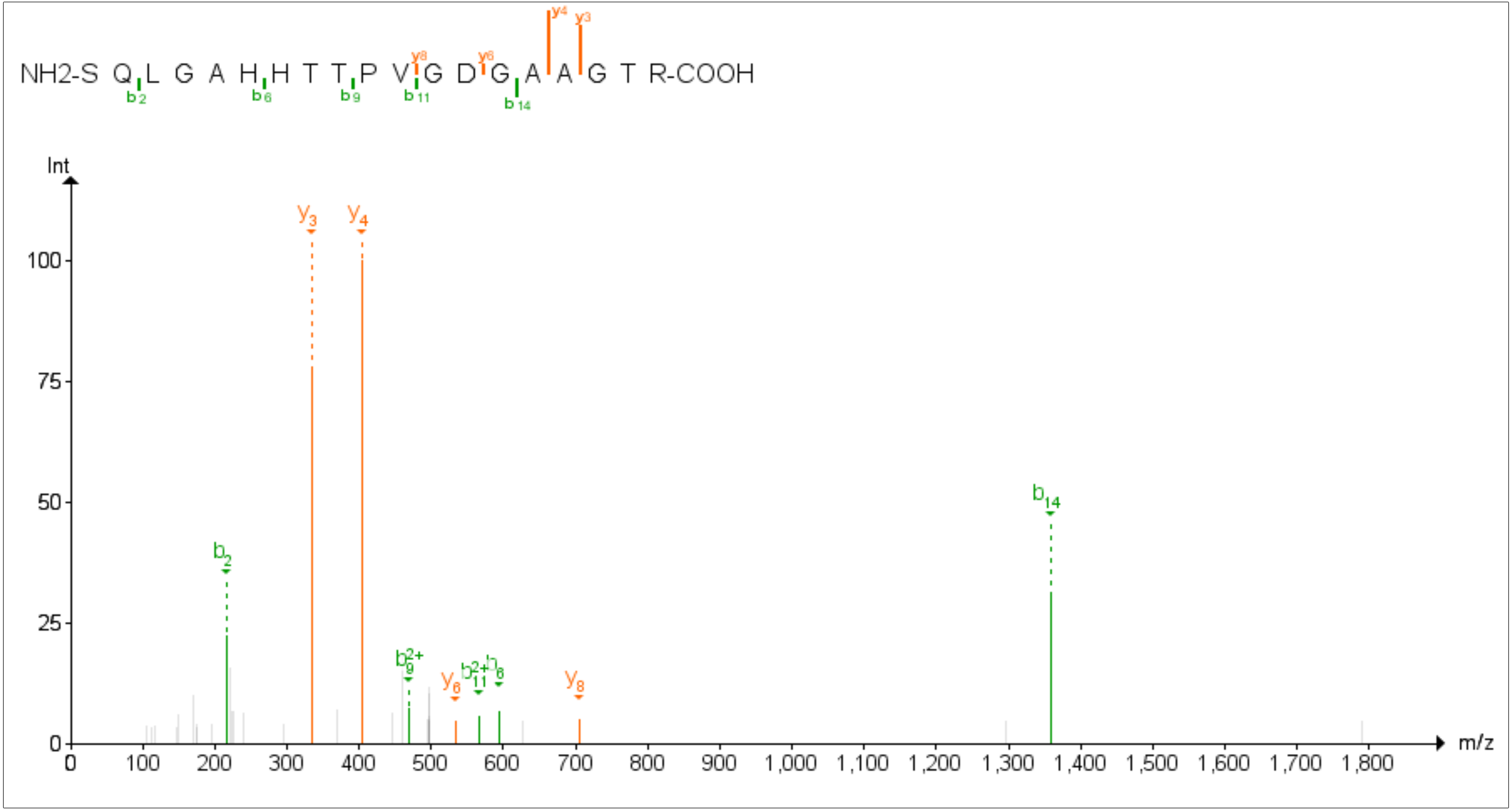

NH2-S Q L G A H H T T P V G D G A A G T R-COOH
y4 y3 y8 y6
b2 b6 b9 b11 b14
Int
100
75
50
25
0
y3 y4 b2 b14 b9 2+ y6 b11 2+ b6 y8
0 100 200 300 400 500 600 700 800 900 1,000 1,100 1,200 1,300 1,400 1,500 1,600 1,700 1,800
m/z

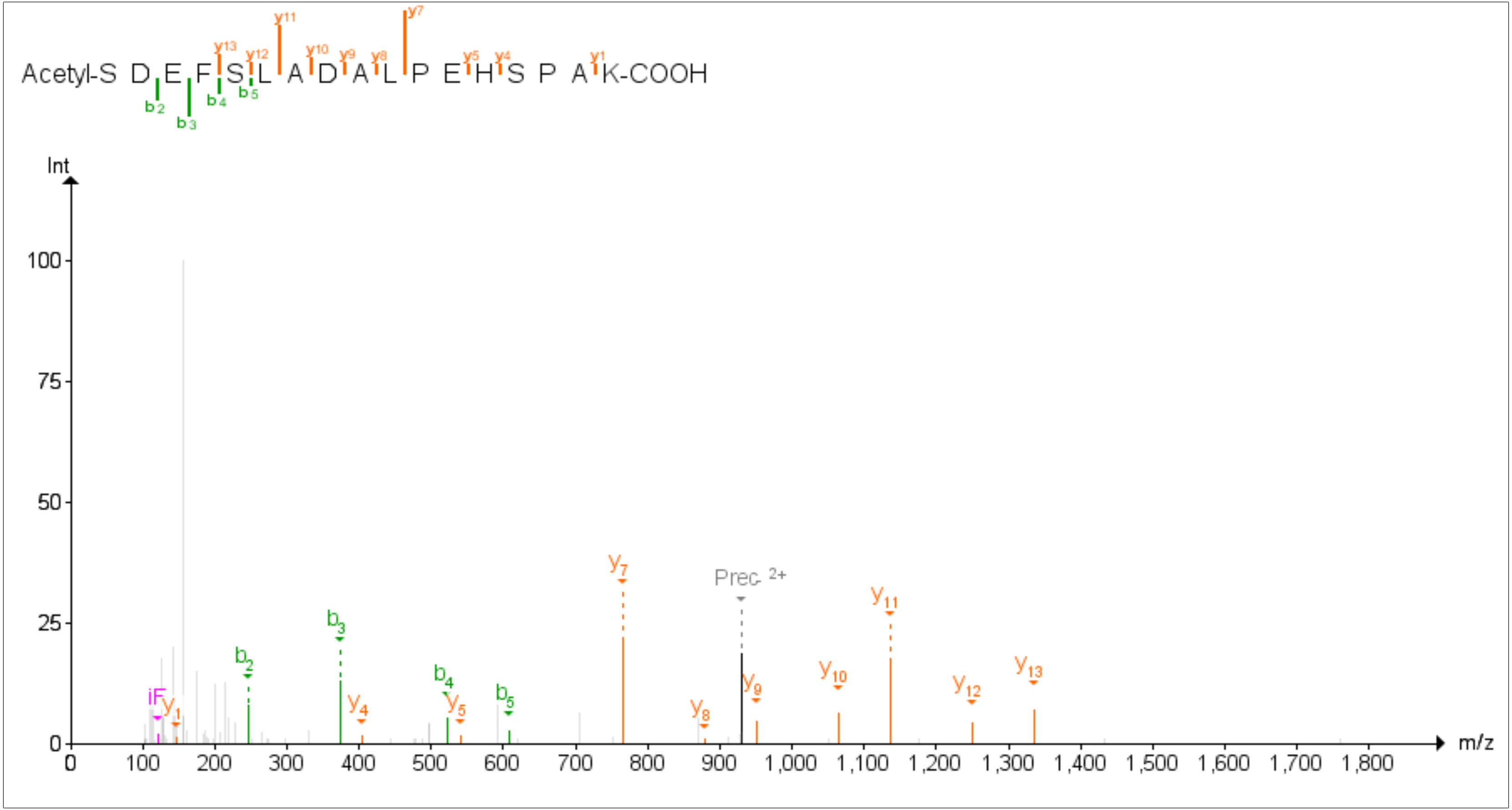

Acetyl-S D E F S L A D A L P E H S P A K-COOH
y11 y7 y13 y12 y10 y9 y8 y5 y4 y1
b2 b3 b4 b5
Int
100
75
50
25
0
iF
y1 y4 y5 y7 y8 y9 y10 y11 y12 y13
b2 b3 b4 b5
Prec 2+
0 100 200 300 400 500 600 700 800 900 1,000 1,100 1,200 1,300 1,400 1,500 1,600 1,700 1,800
m/z

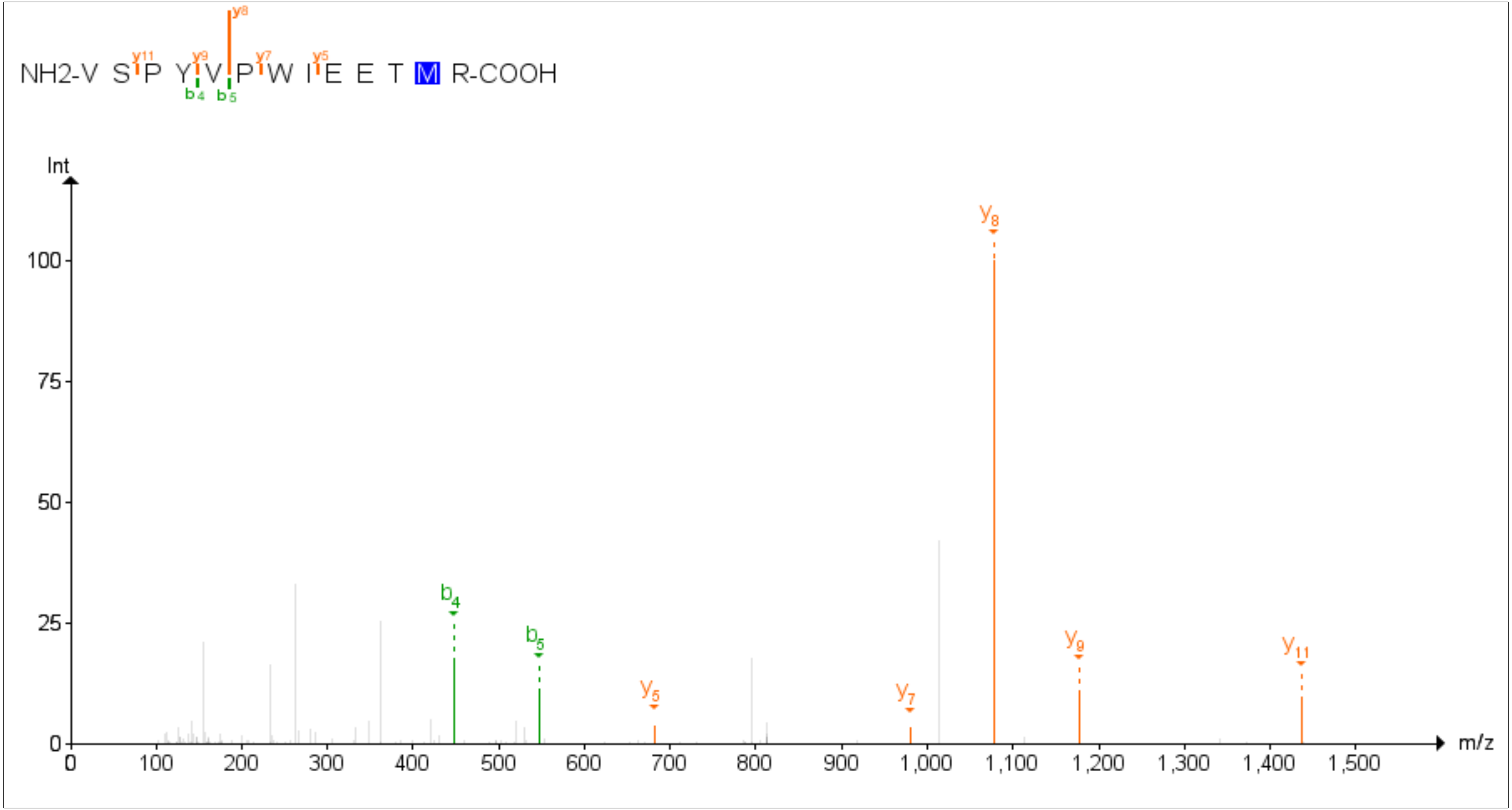

NH2-V S P Y V P W I E E T M R-COOH
y11 y9 y8 y7 y5
b4 b5
Int
100
75
50
25
0
m/z
0 100 200 300 400 500 600 700 800 900 1,000 1,100 1,200 1,300 1,400 1,500
b4 b5 y5 y7 y8 y9 y11

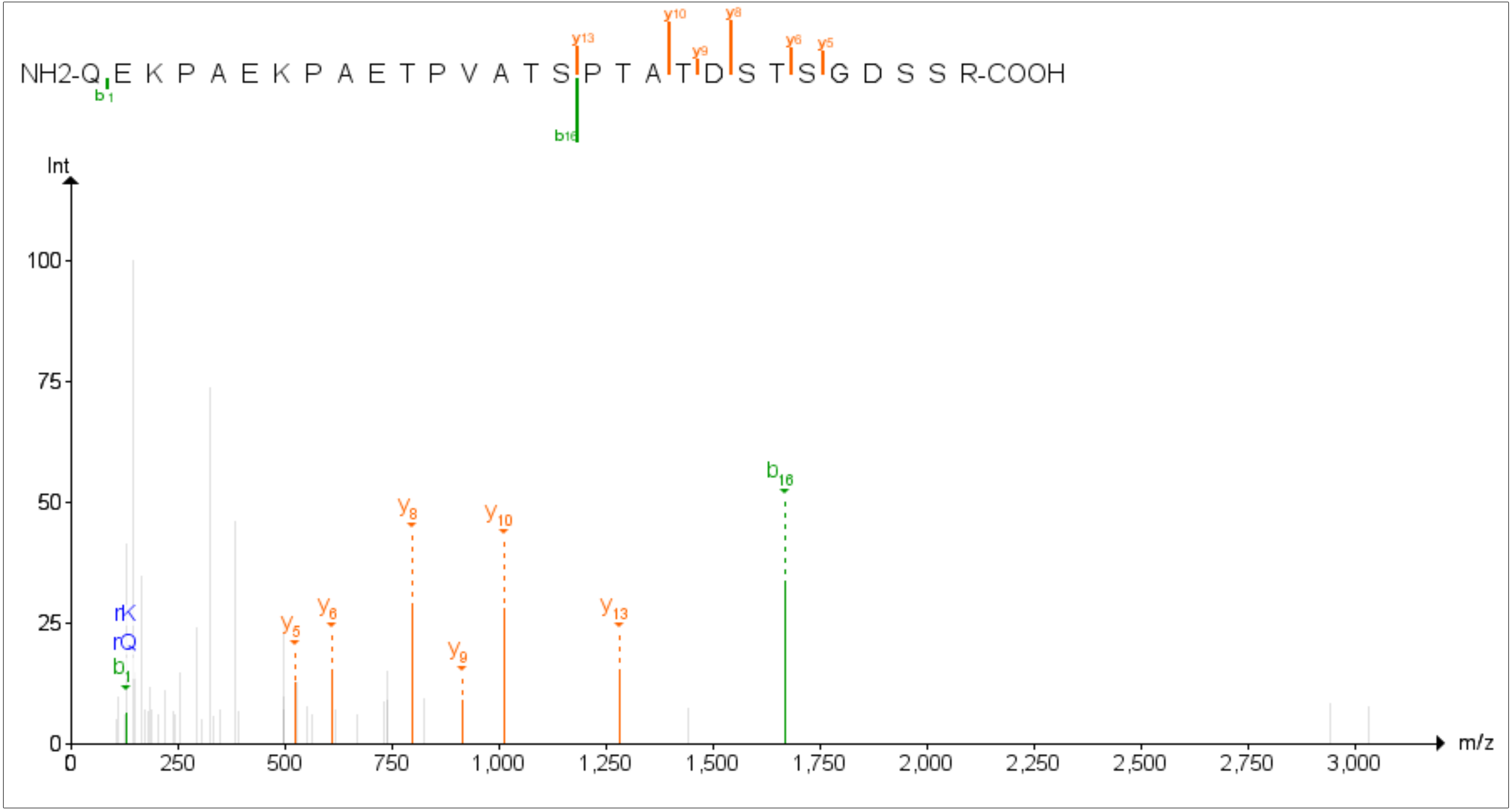

NH2-Q E K P A E K P A E T P V A T S P T A T D S T S G D S S R-COOH
b1
b16
y13
y10
y9
y8
y6
y5
Int
100
75
50
25
0
m/z
0
250
500
750
1,000
1,250
1,500
1,750
2,000
2,250
2,500
2,750
3,000
rK
rQ
b1
y5
y6
y8
y9
y10
y13
b16

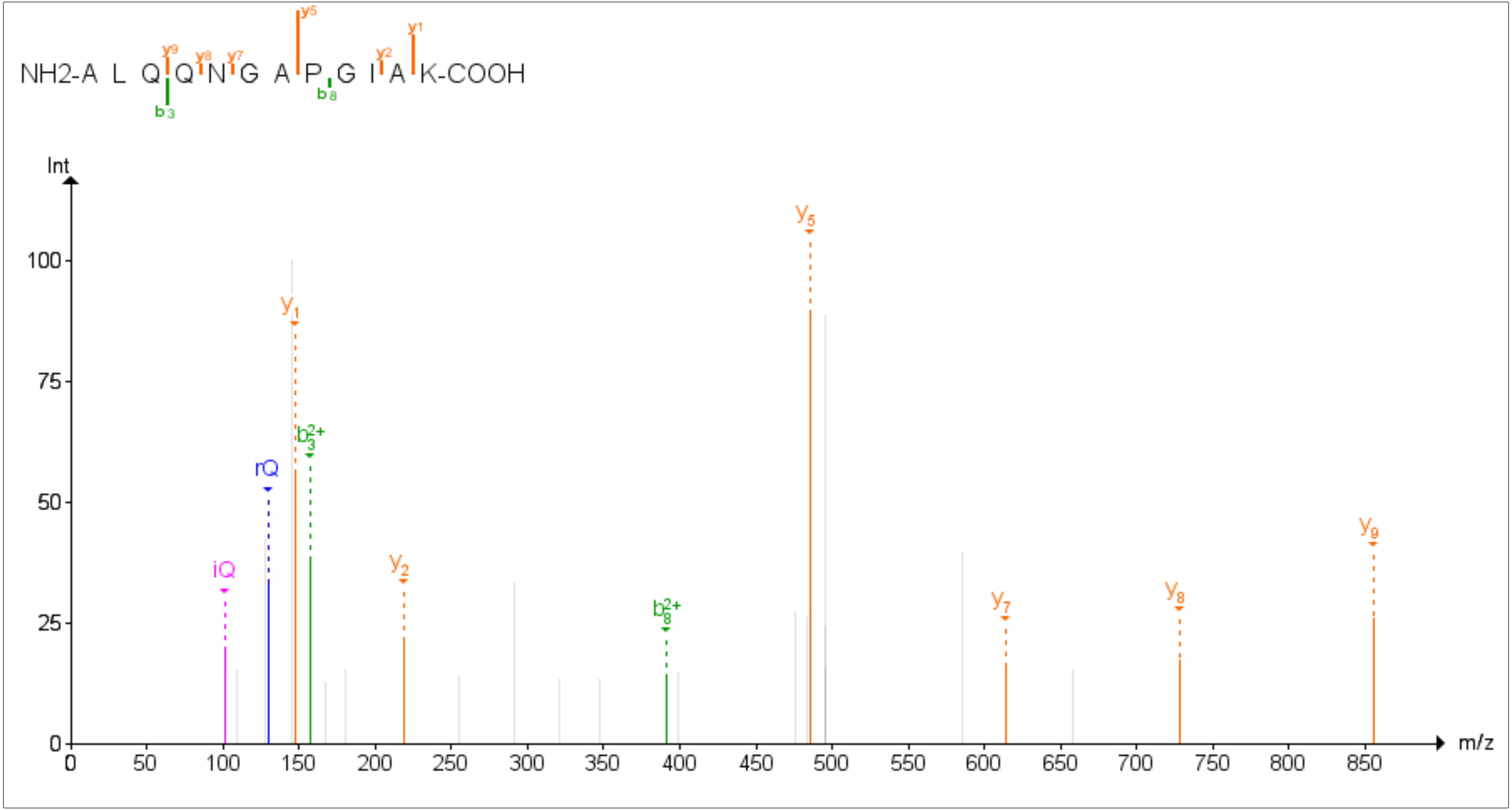

NH2-A L Q Q N G A P G I A K-COOH
y9 y8 y7 y5 y2 y1
b3 b8
Int
100
75
50
25
0
m/z
0 50 100 150 200 250 300 350 400 450 500 550 600 650 700 750 800 850
iQ
rQ
y1
b_3^{2+}
y2
b_8^{2+}
y5
y7
y8
y9

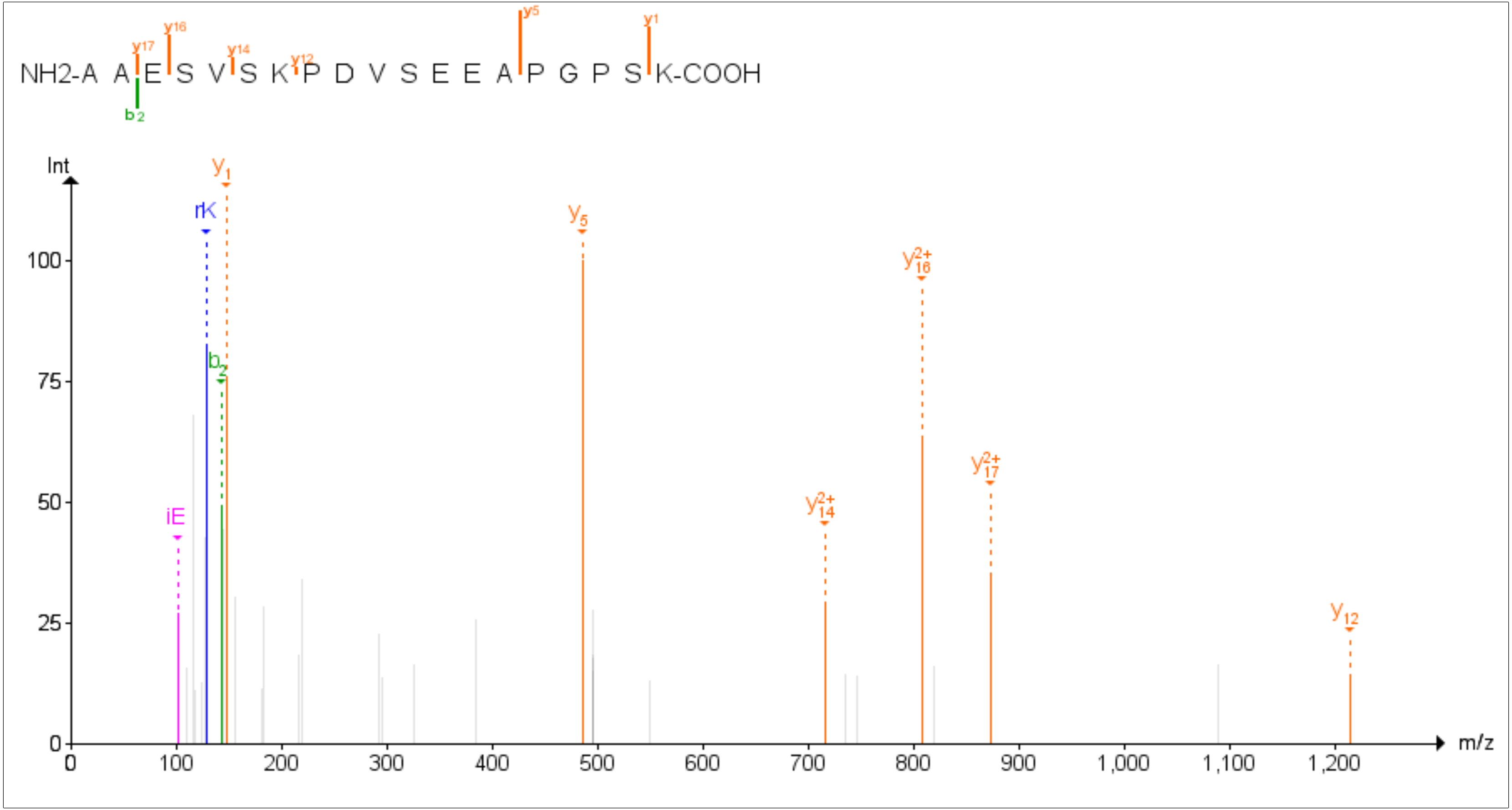

NH2-A A E S V S K P D V S E E A P G P S K-COOH
y17
y16
y14
y12
y5
y1
b2
Int
y1
rK
y5
b2
iE
y_{16}^{2+}
y_{17}^{2+}
y_{14}^{2+}
y_{12}
100
75
50
25
0
0
100
200
300
400
500
600
700
800
900
1,000
1,100
1,200
m/z

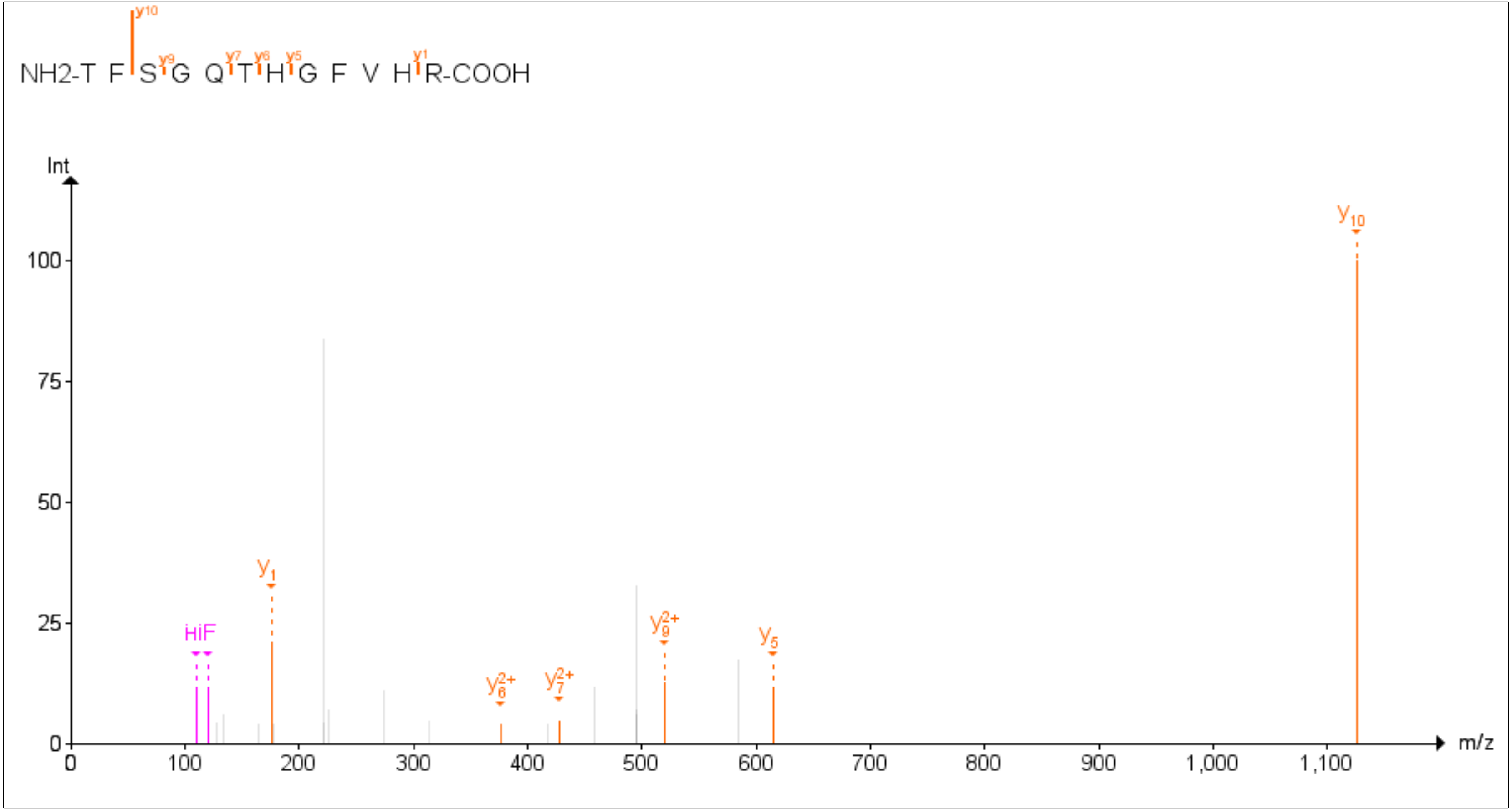

NH2-T F S G Q T H G F V H R-COOH
y10
y9
y7
y6
y5
y1
Int
100
75
50
25
0
m/z
0
100
200
300
400
500
600
700
800
900
1,000
1,100
HiF
y1
y6 2+
y7 2+
y9 2+
y5
y10

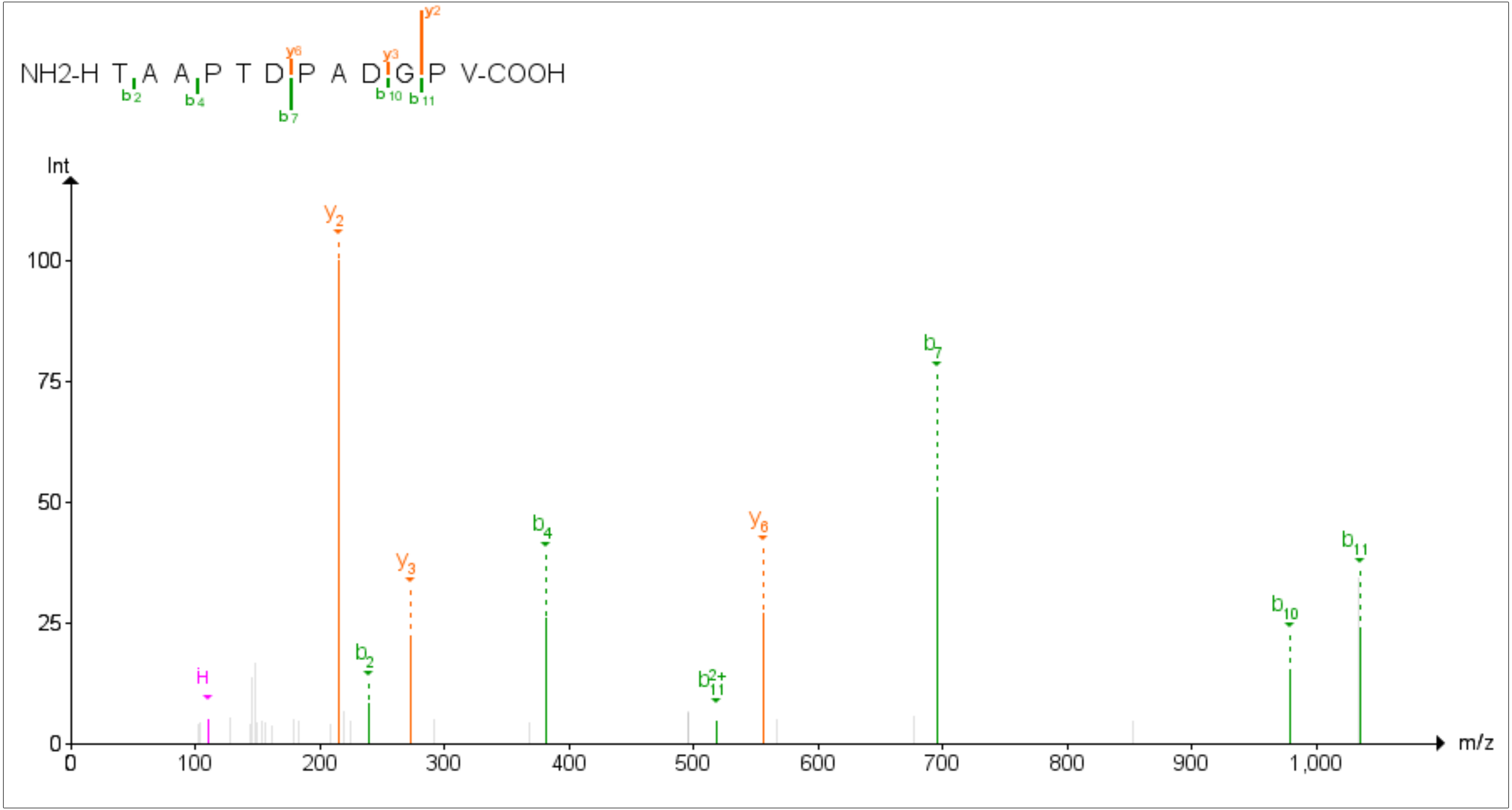

NH2-H T A A P T D P A D G P V-COOH
b2 b4 b7 b10 b11
y6 y3 y2
Int
100
75
50
25
0
y_2
y_3
b_2
b_4
b_{11}^{2+}
y_6
b_7
b_{10}
b_{11}
H
0 100 200 300 400 500 600 700 800 900 1,000
m/z

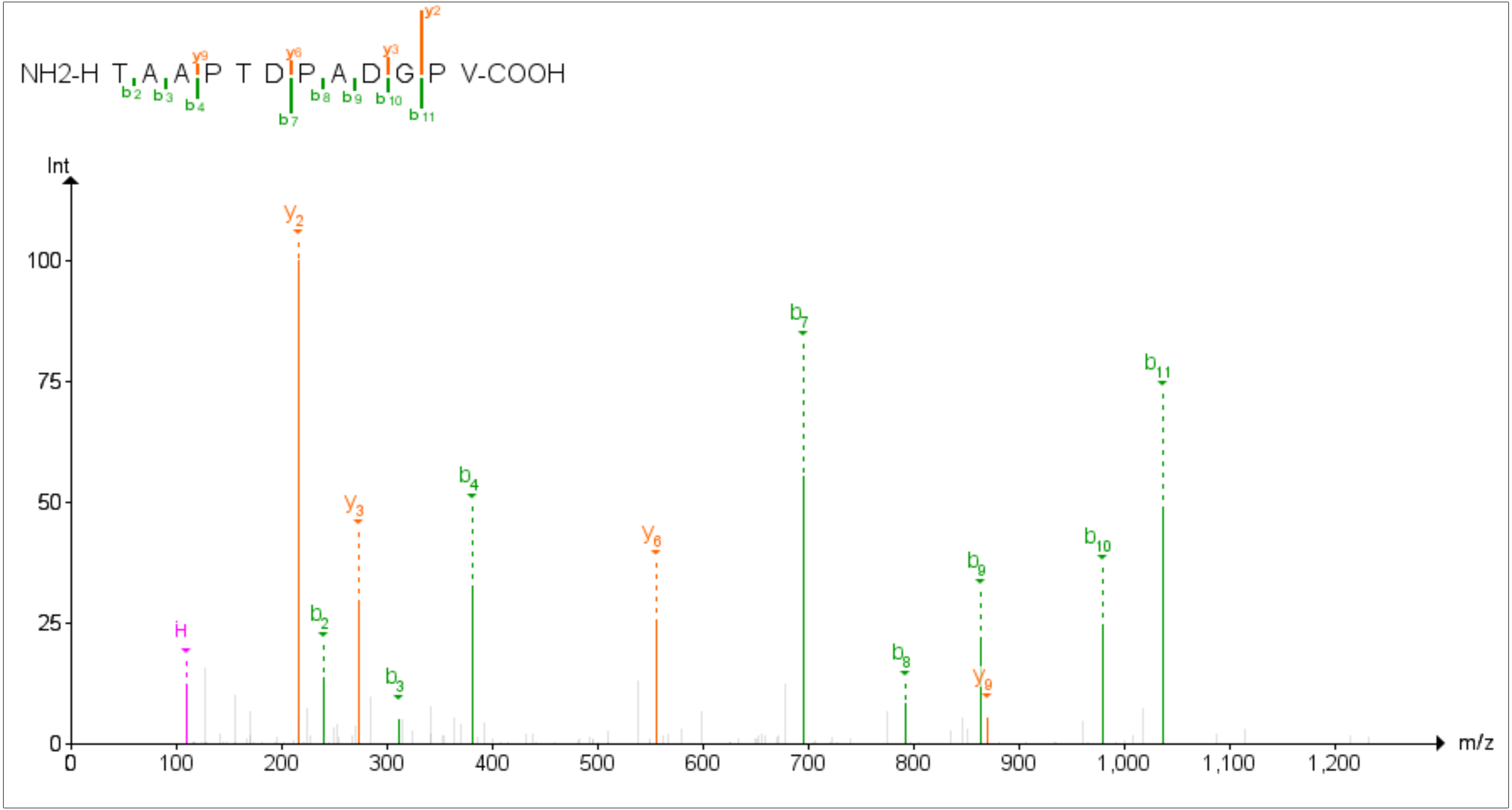

NH2-H T A A P T D P A D G P V-COOH
y9 y6 y3 y2
b2 b3 b4 b7 b8 b9 b10 b11
Int
100
75
50
25
0
y2
y3
y6
y9
H
b2
b3
b4
b7
b8
b9
b10
b11
0
100
200
300
400
500
600
700
800
900
1,000
1,100
1,200
m/z

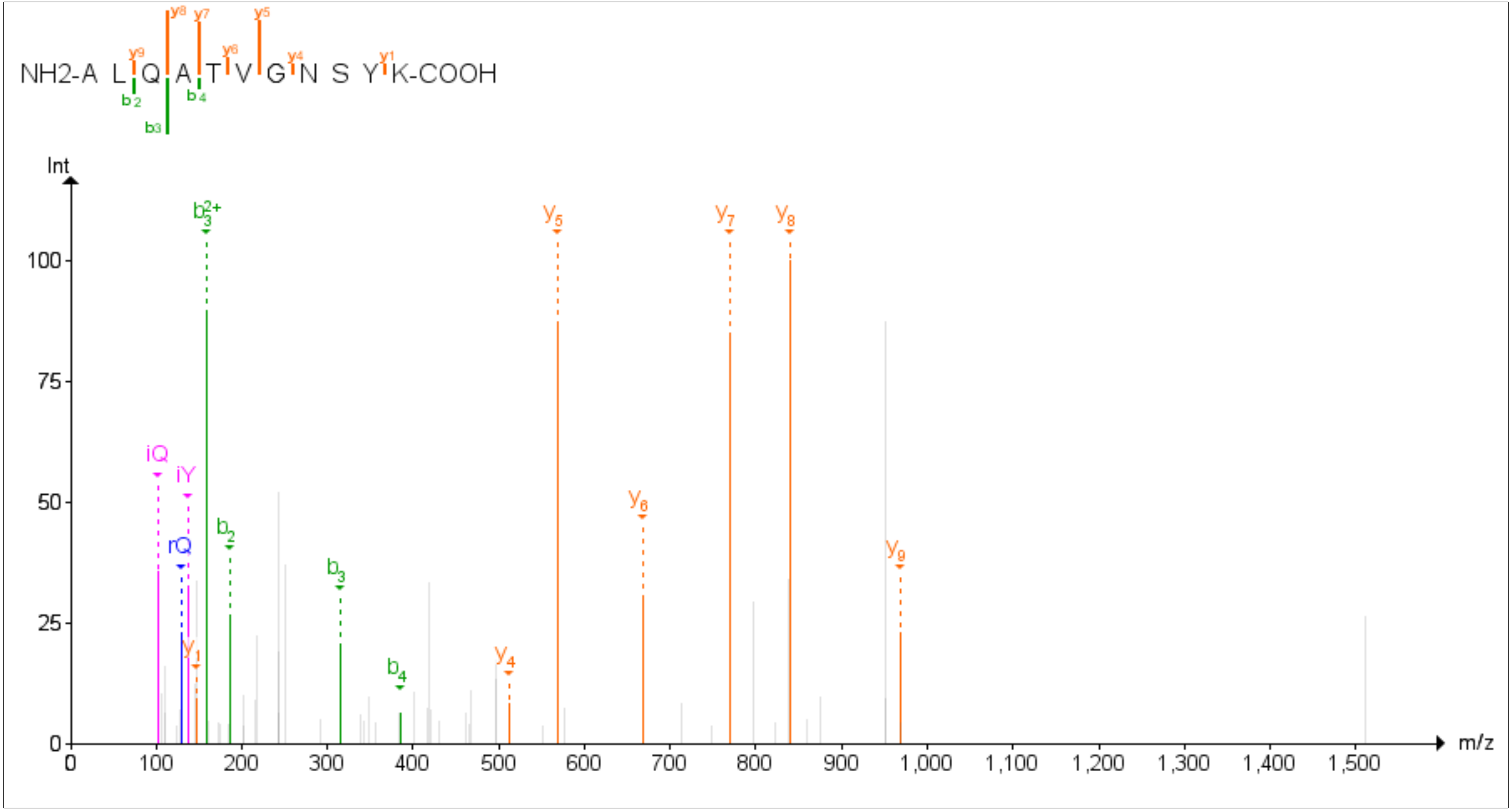

NH2-A L Q A T V G N S Y K-COOH
y9 y8 y7 y6 y5 y4 y1
b2 b3 b4
Int
m/z
iQ
iY
rQ
b_3^{2+}
b_2
b_3
b_4
y_1
y_4
y_5
y_6
y_7
y_8
y_9
0
25
50
75
100
0 100 200 300 400 500 600 700 800 900 1,000 1,100 1,200 1,300 1,400 1,500

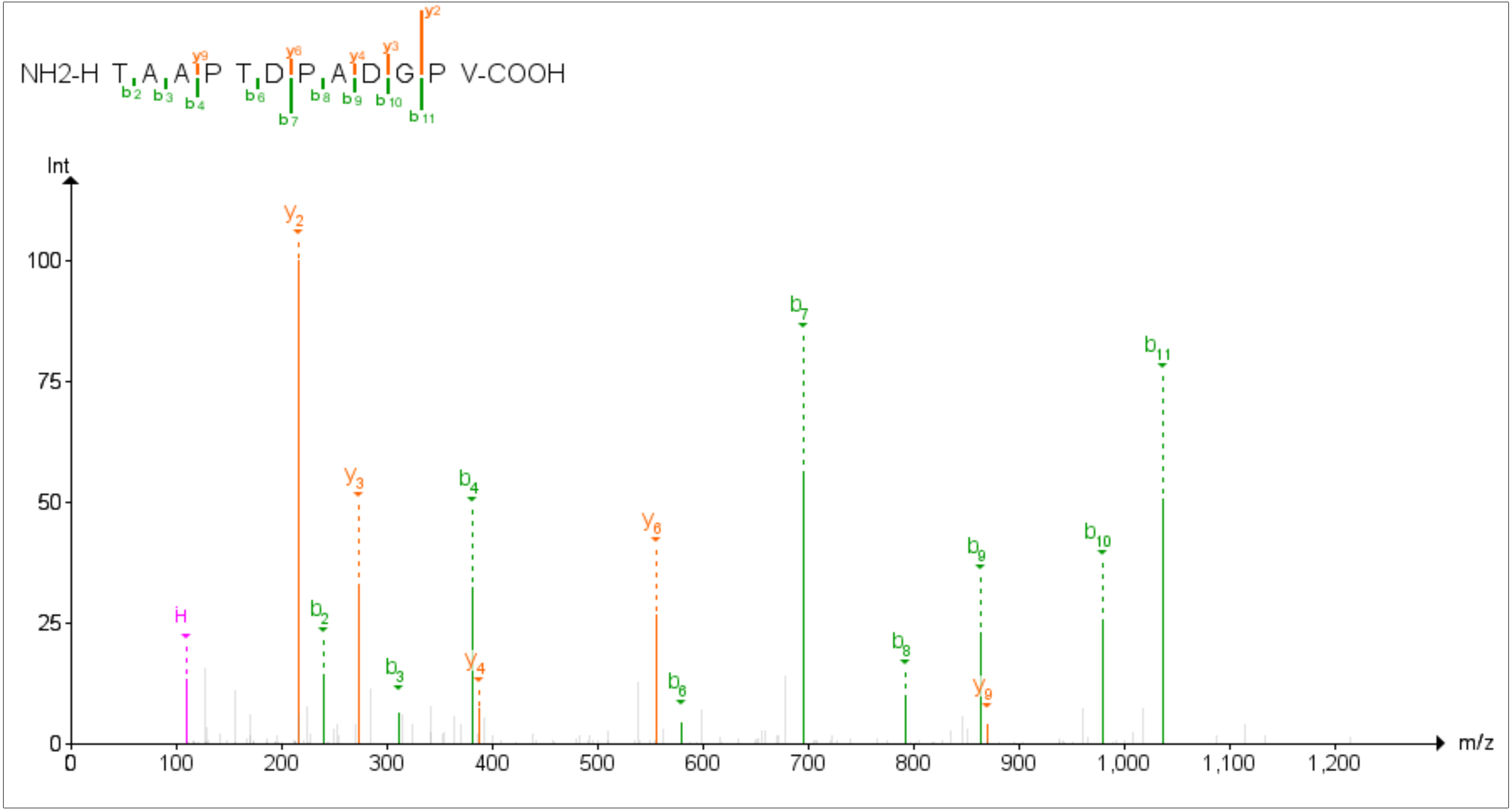
NH2-H T A A P T D P A D G P V-COOH
y9 y6 y4 y3 y2
b2 b3 b4 b6 b7 b8 b9 b10 b11
Int
100
75
50
25
0
H
y2
y3
y4
y6
y9
b2
b3
b4
b6
b7
b8
b9
b10
b11
0 100 200 300 400 500 600 700 800 900 1,000 1,100 1,200
m/z

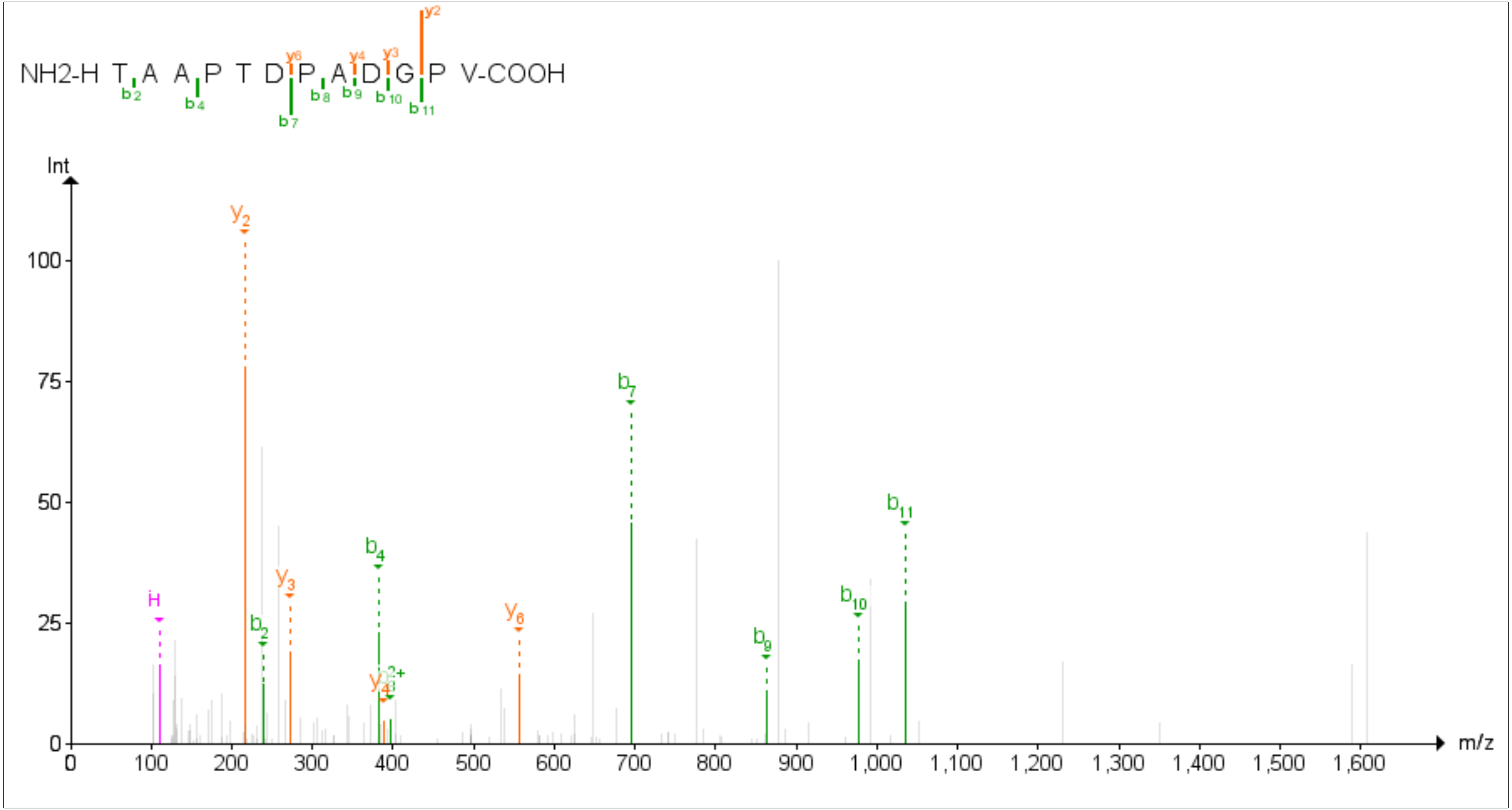

NH2-H T A A P T D P A D G P V-COOH
y6 y4 y3 y2
b2 b4 b7 b8 b9 b10 b11
Int
100
75
50
25
0
y2
y3
y4
y6
b2
b4
b7
b9
b10
b11
H
m/z
0 100 200 300 400 500 600 700 800 900 1,000 1,100 1,200 1,300 1,400 1,500 1,600

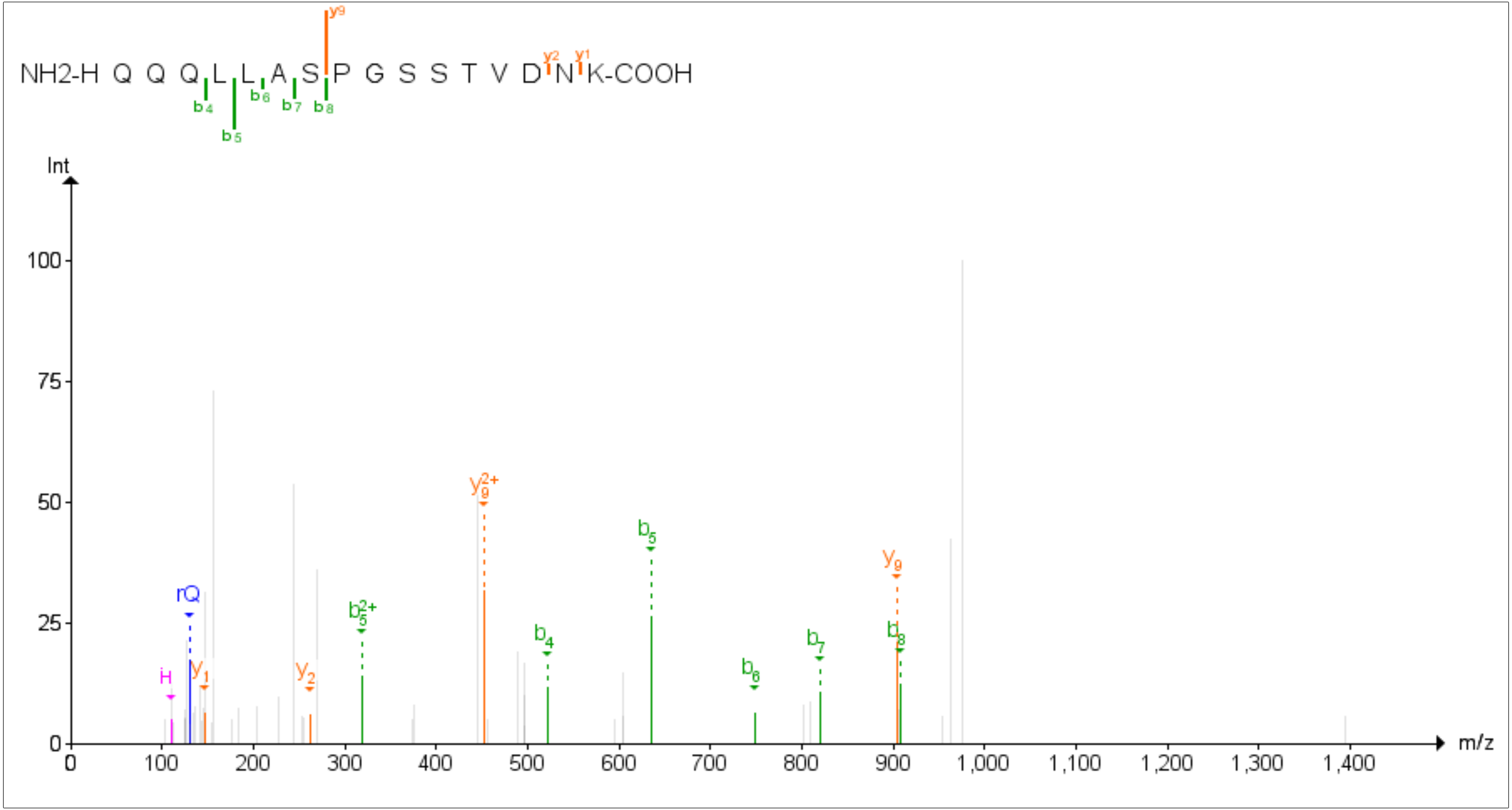

NH2-H Q Q Q L L A S P G S S T V D N K-COOH
y9
y2
y1
b4
b5
b6
b7
b8
Int
100
75
50
25
0
m/z
0
100
200
300
400
500
600
700
800
900
1,000
1,100
1,200
1,300
1,400
rQ
H
y1
y2
b5 2+
y9 2+
b4
b5
b6
b7
y9
b8

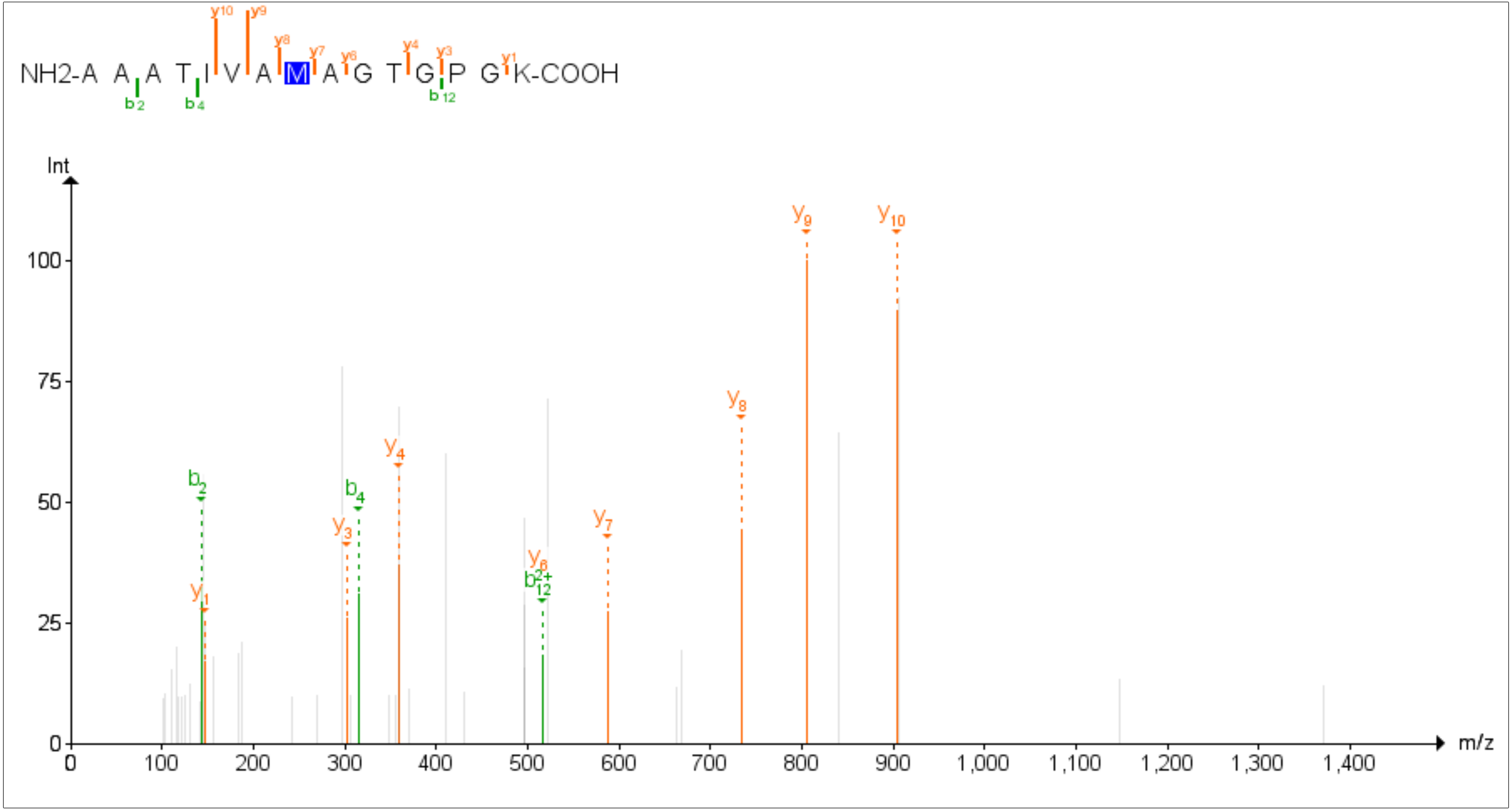

NH2-A A A T I V A M A G T G P G K-COOH
y10 y9 y8 y7 y6 y4 y3 y1
b2 b4 b12
Int
100
75
50
25
0
m/z
0 100 200 300 400 500 600 700 800 900 1,000 1,100 1,200 1,300 1,400
y9 y10 y8 y4 b2 b4 y3 y7 y6 b2+12 y1

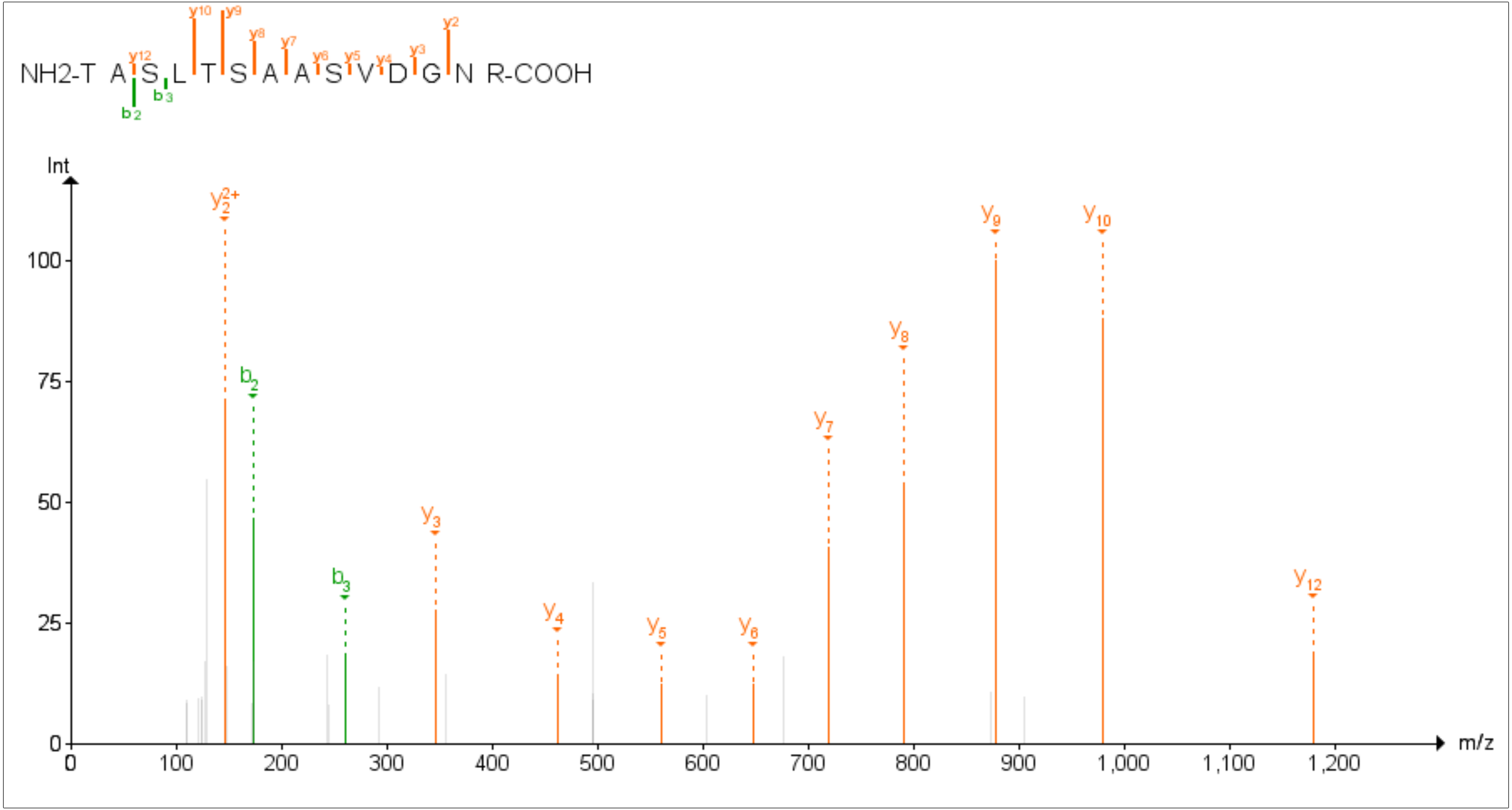
NH2-T A S L T S A A S V D G N R-COOH
y12 y10 y9 y8 y7 y6 y5 y4 y3 y2
b2 b3
Int
m/z
0
25
50
75
100
0
100
200
300
400
500
600
700
800
900
1,000
1,100
1,200
y2 2+
b2
b3
y3
y4
y5
y6
y7
y8
y9
y10
y12

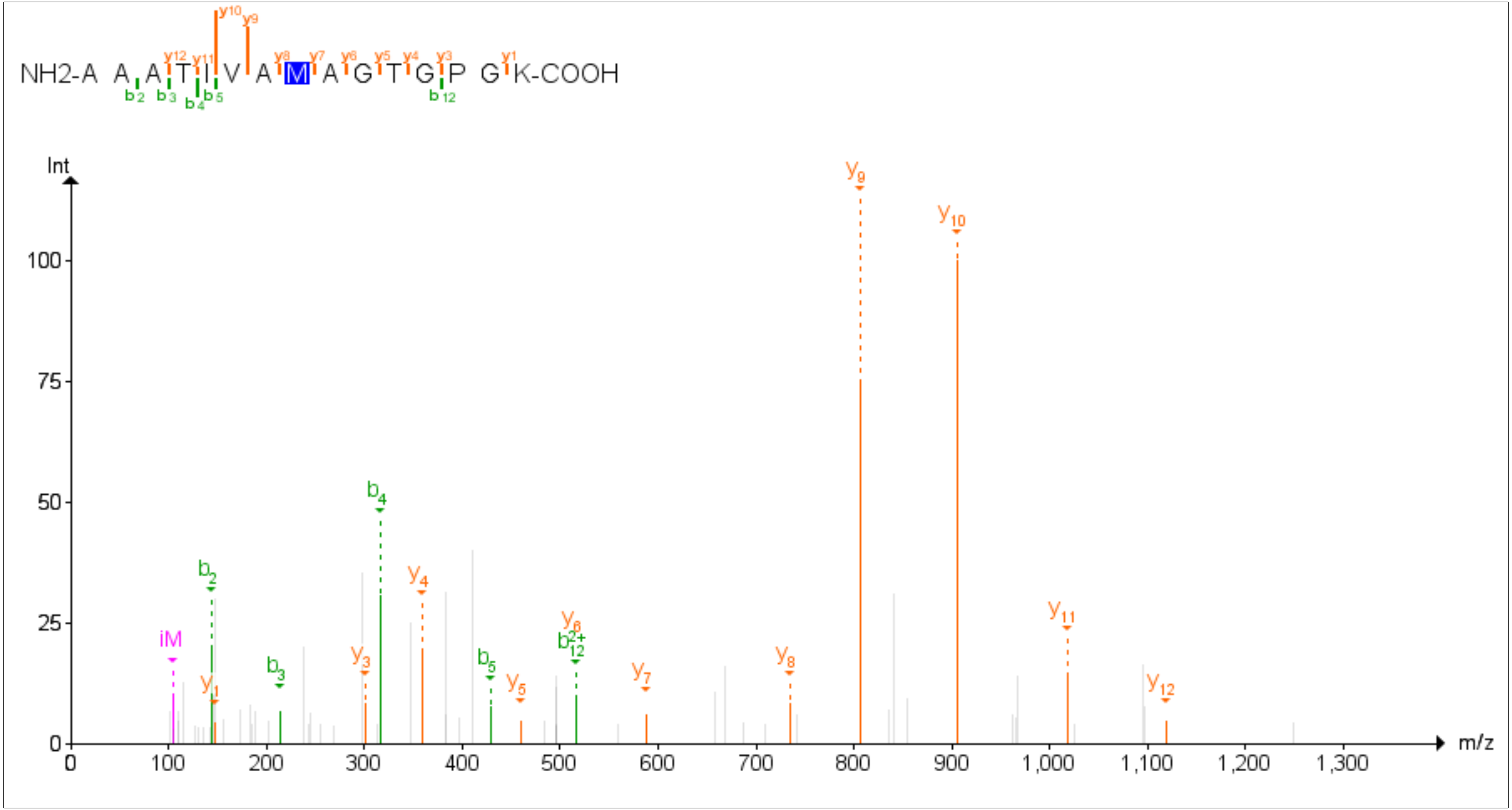

NH2-A A A T I V A M A G T G P G K-COOH
y10 y9 y12 y11 y8 y7 y6 y5 y4 y3 y1
b2 b3 b4 b5 b12
Int
100
75
50
25
0
m/z
0 100 200 300 400 500 600 700 800 900 1,000 1,100 1,200 1,300
iM
y1
b2
b3
y3
b4
y4
b5
y5
y6
b12 2+
y7
y8
y9
y10
y11
y12

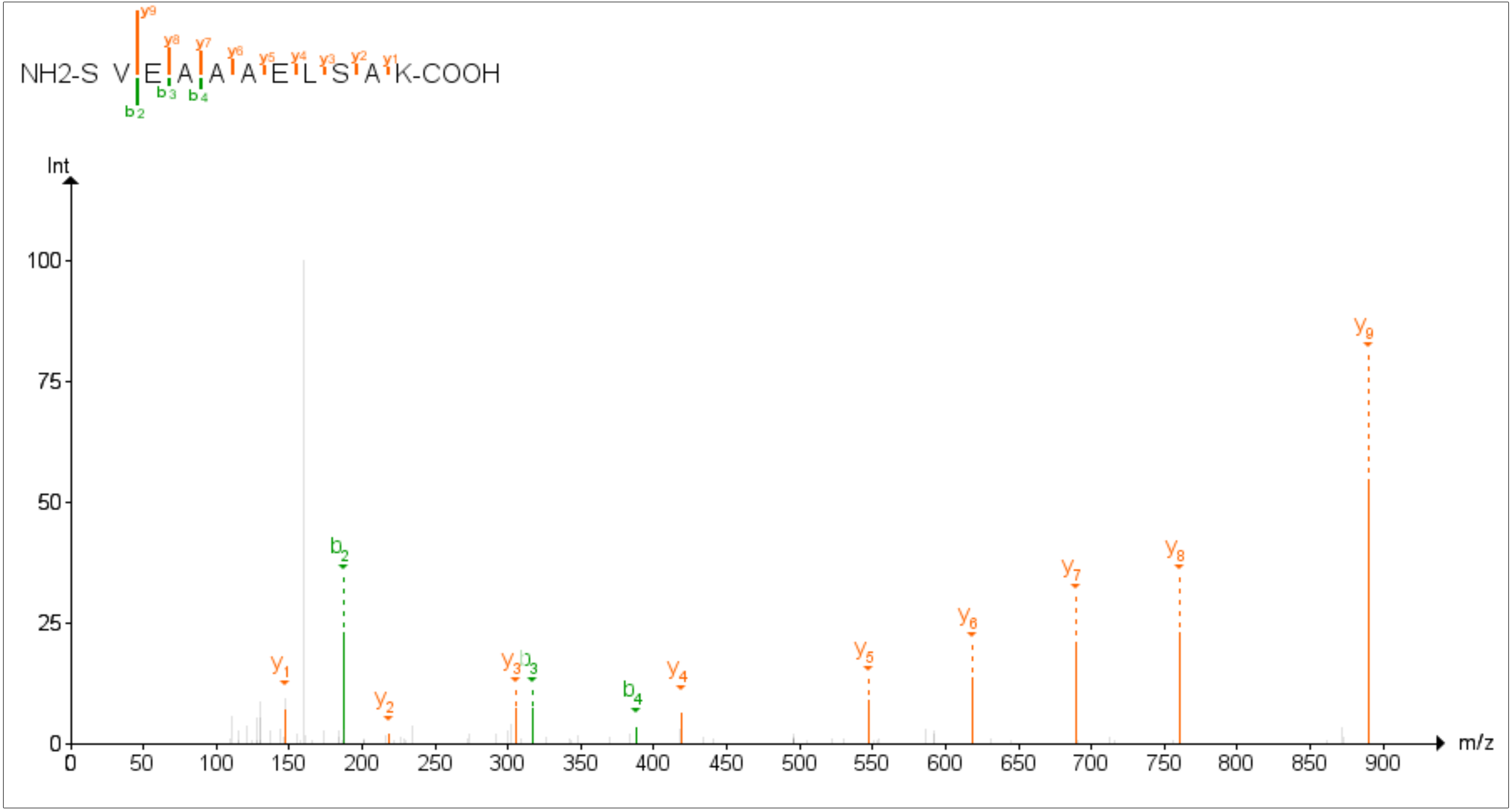

NH2-S V E A A A E L S A K-COOH
y9 y8 y7 y6 y5 y4 y3 y2 y1
b2 b3 b4
Int
100
75
50
25
0
m/z
0 50 100 150 200 250 300 350 400 450 500 550 600 650 700 750 800 850 900
y1
b2
y2
y3
b3
b4
y4
y5
y6
y7
y8
y9

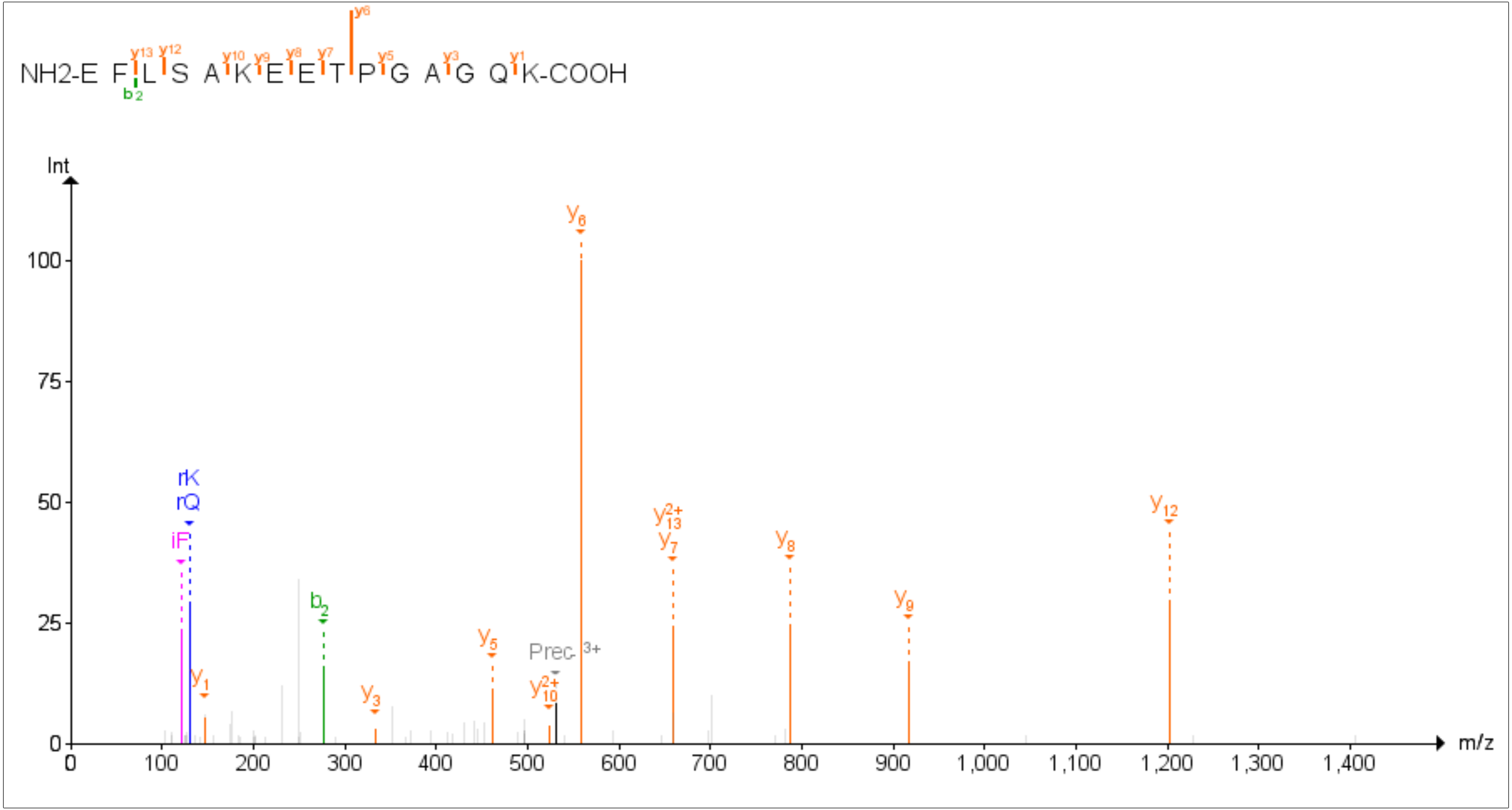

NH2-E F L S A K E E T P G A G Q K-COOH
Int
m/z
y6
y13
y12
y10
y9
y8
y7
y5
y3
y1
b2
rK
rQ
iF
Prec- 3+

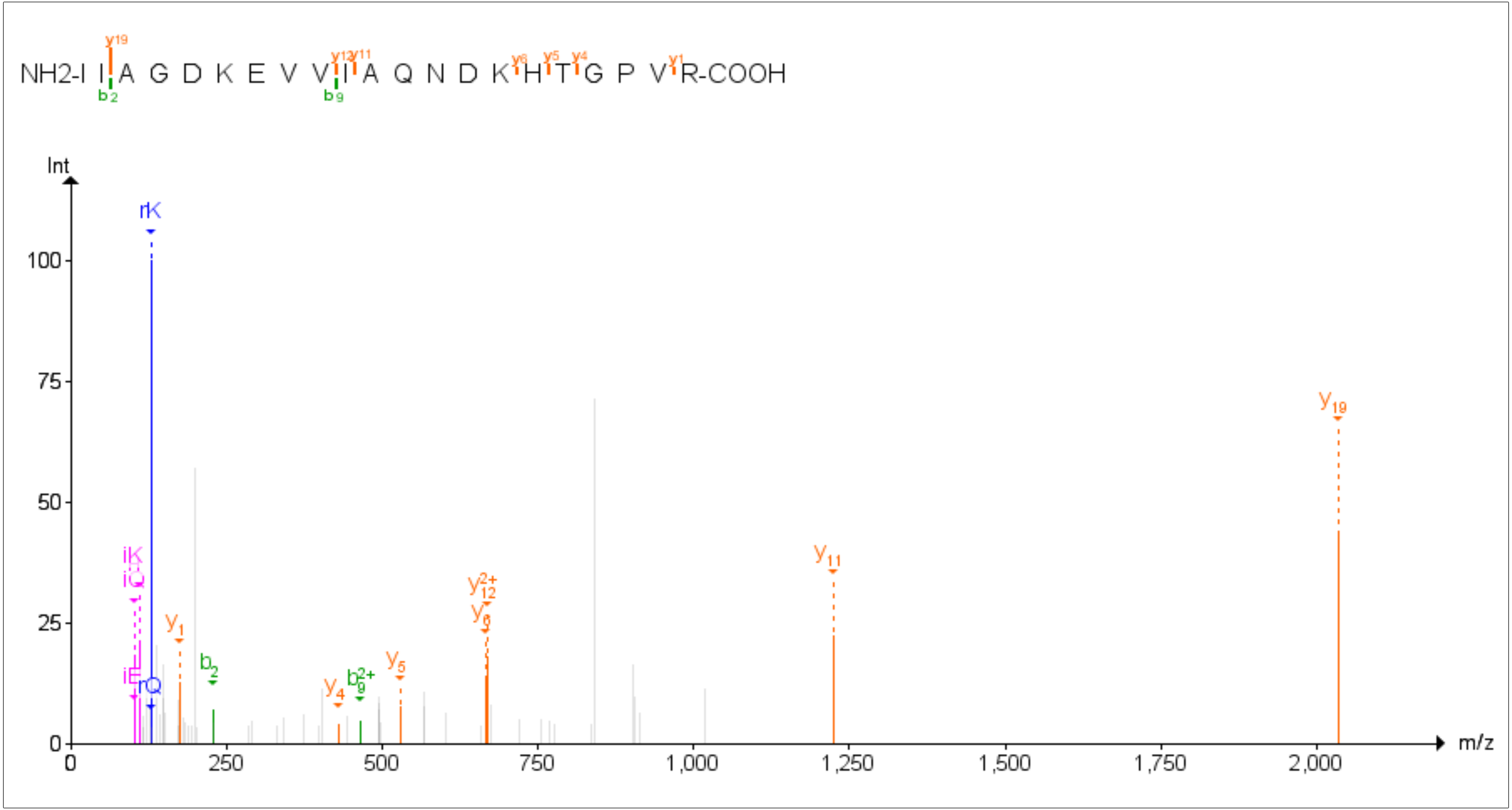

NH2-I I A G D K E V V I A Q N D K H T G P V R-COOH
y19
y12
y11
y6
y5
y4
y1
b2
b9
Int
100
75
50
25
0
rK
iK
iQ
iE
rQ
y1
b2
y4
b9 2+
y5
y12 2+
y6
y11
y19
0
250
500
750
1,000
1,250
1,500
1,750
2,000
m/z

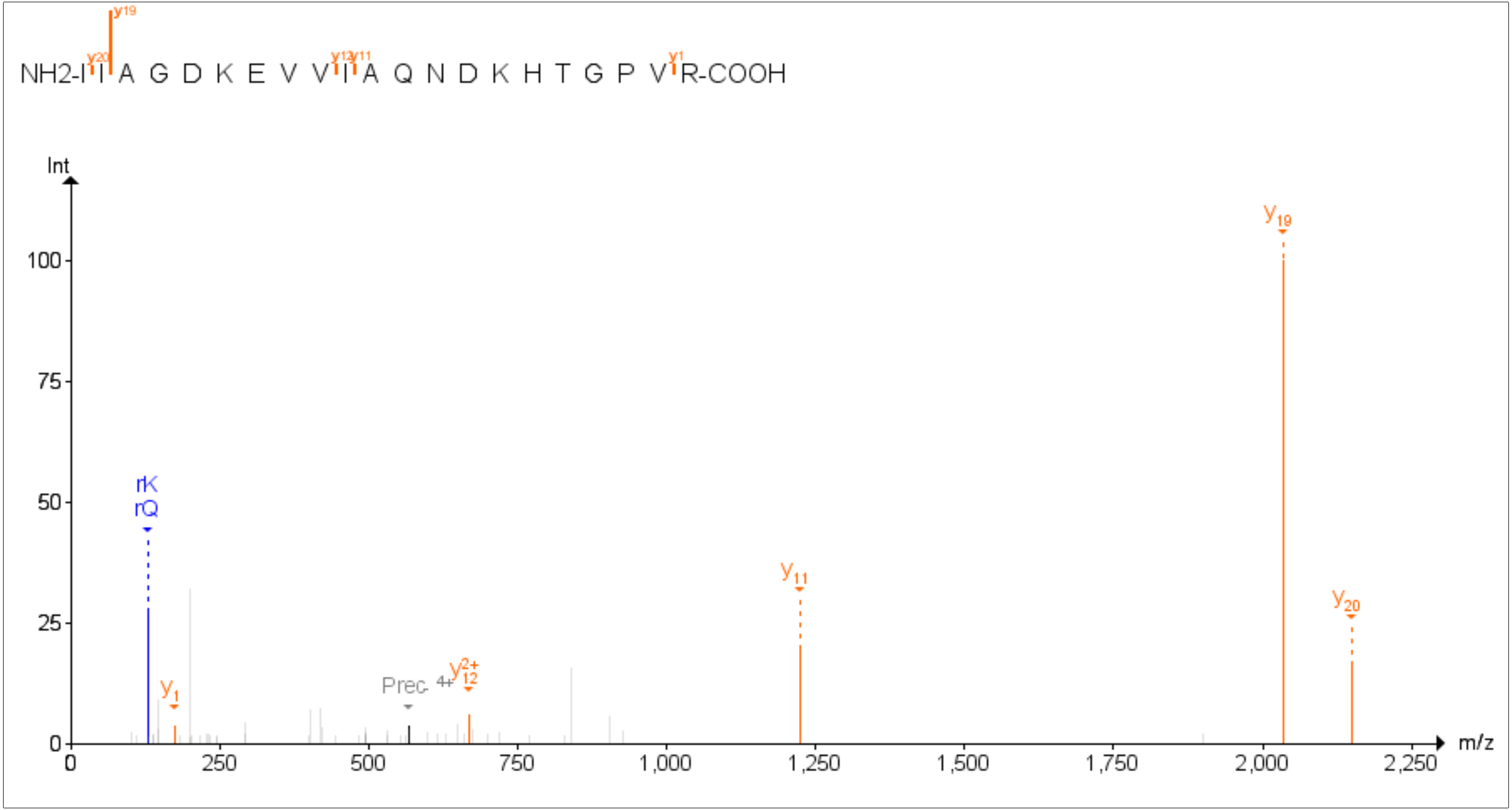

NH2-I I A G D K E V V I A Q N D K H T G P V R-COOH
y19
y20
y12
y11
y1
Int
100
75
50
25
0
rK
rQ
y1
Prec 4+
y12 2+
y11
y19
y20
0
250
500
750
1,000
1,250
1,500
1,750
2,000
2,250
m/z

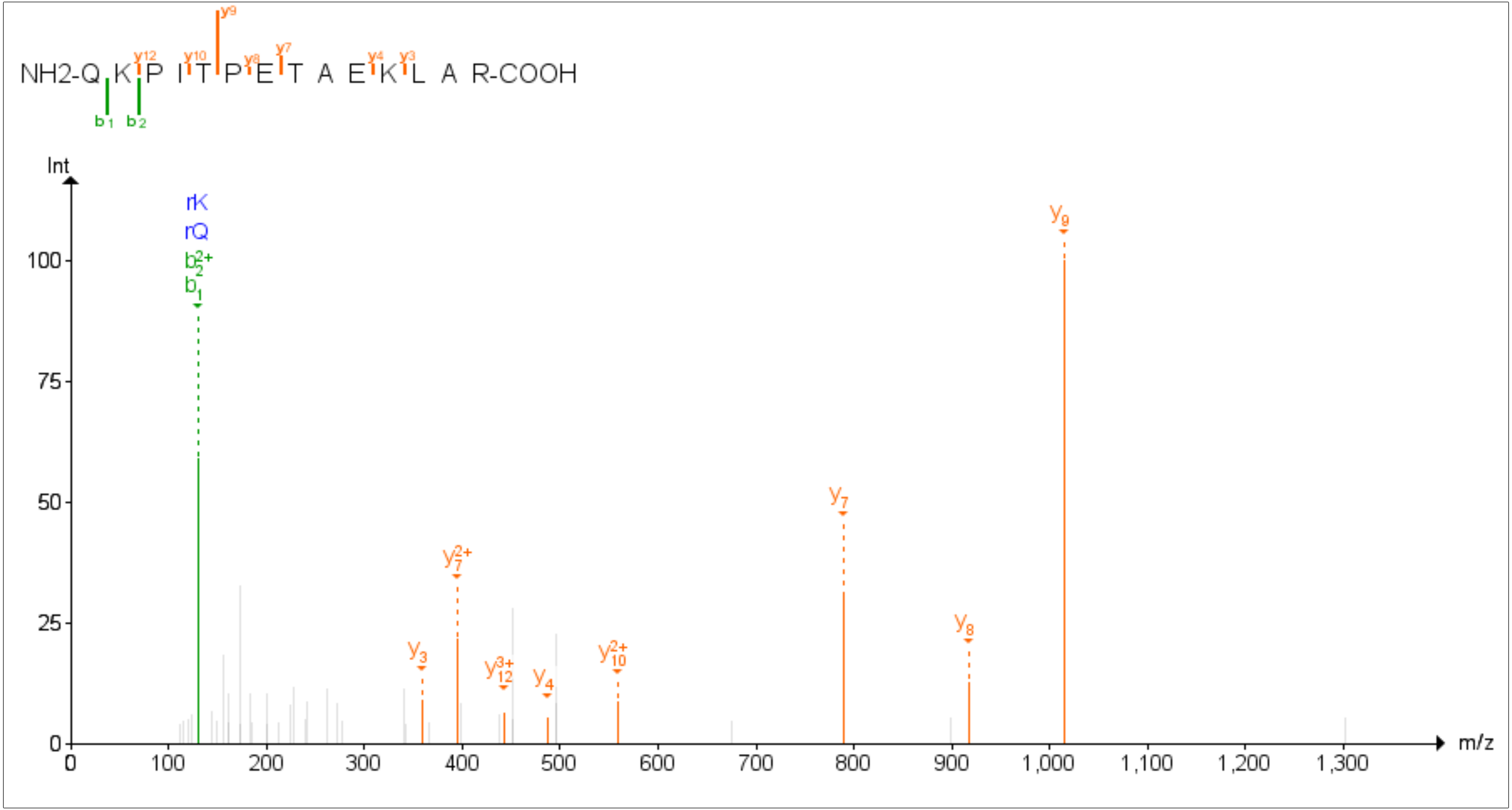

NH2-Q K P I T P E T A E K L A R-COOH
y9
y12
y10
y8
y7
y4
y3
b1
b2
Int
rK
rQ
b2 2+
b1
y9
y7
y7 2+
y3
y12 3+
y4
y10 2+
y8
100
75
50
25
0
0
100
200
300
400
500
600
700
800
900
1,000
1,100
1,200
1,300
m/z

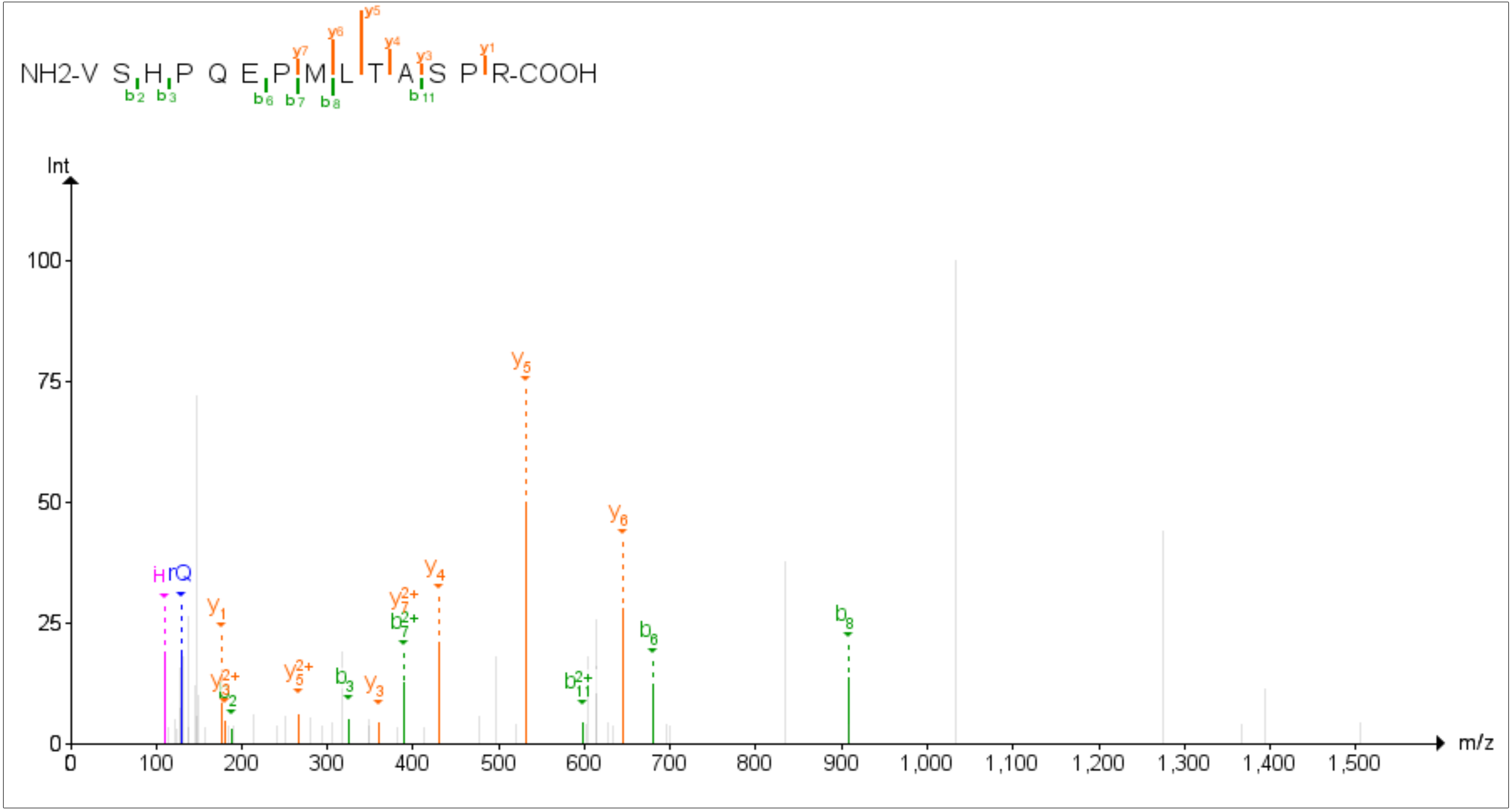
NH2-V S H P Q E P M L T A S P R-COOH
y5 y6 y7 y4 y3 y1
b2 b3 b6 b7 b8 b11
Int
100
75
50
25
0
m/z
0 100 200 300 400 500 600 700 800 900 1,000 1,100 1,200 1,300 1,400 1,500
iH
rQ
y1
y3 2+
b2
y5 2+
b3
y3
y7 2+
b7 2+
y4
y5
b11 2+
y6
b6
b8

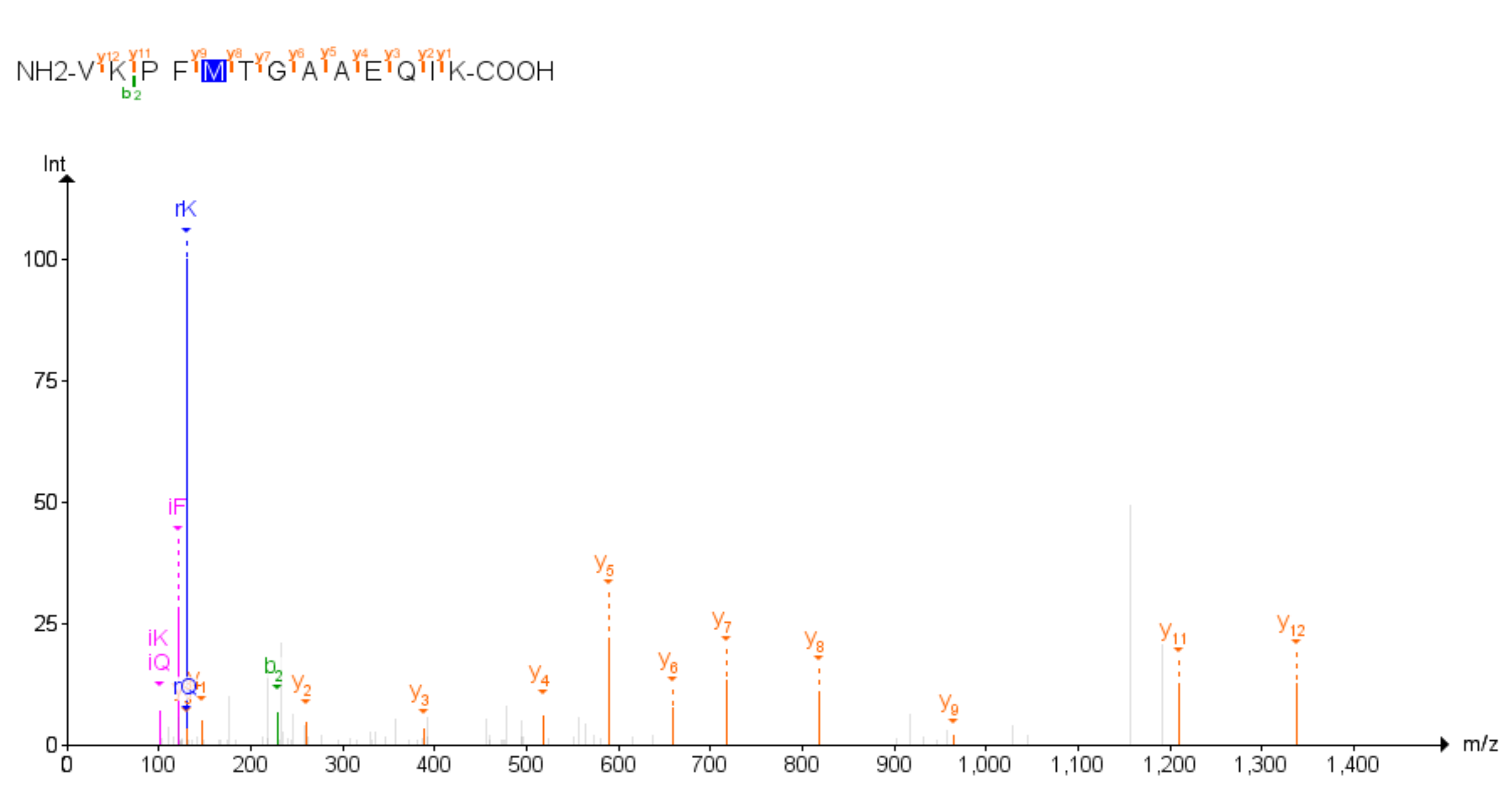

NH2-VKPFMTGAAEQIK-COOH
y12 y11 y9 y8 y7 y6 y5 y4 y3 y2 y1
b2
Int
rK
iF
iK
iQ
rQ
y1
b2
y2
y3
y4
y5
y6
y7
y8
y9
y11
y12
100
75
50
25
0
0 100 200 300 400 500 600 700 800 900 1,000 1,100 1,200 1,300 1,400
m/z

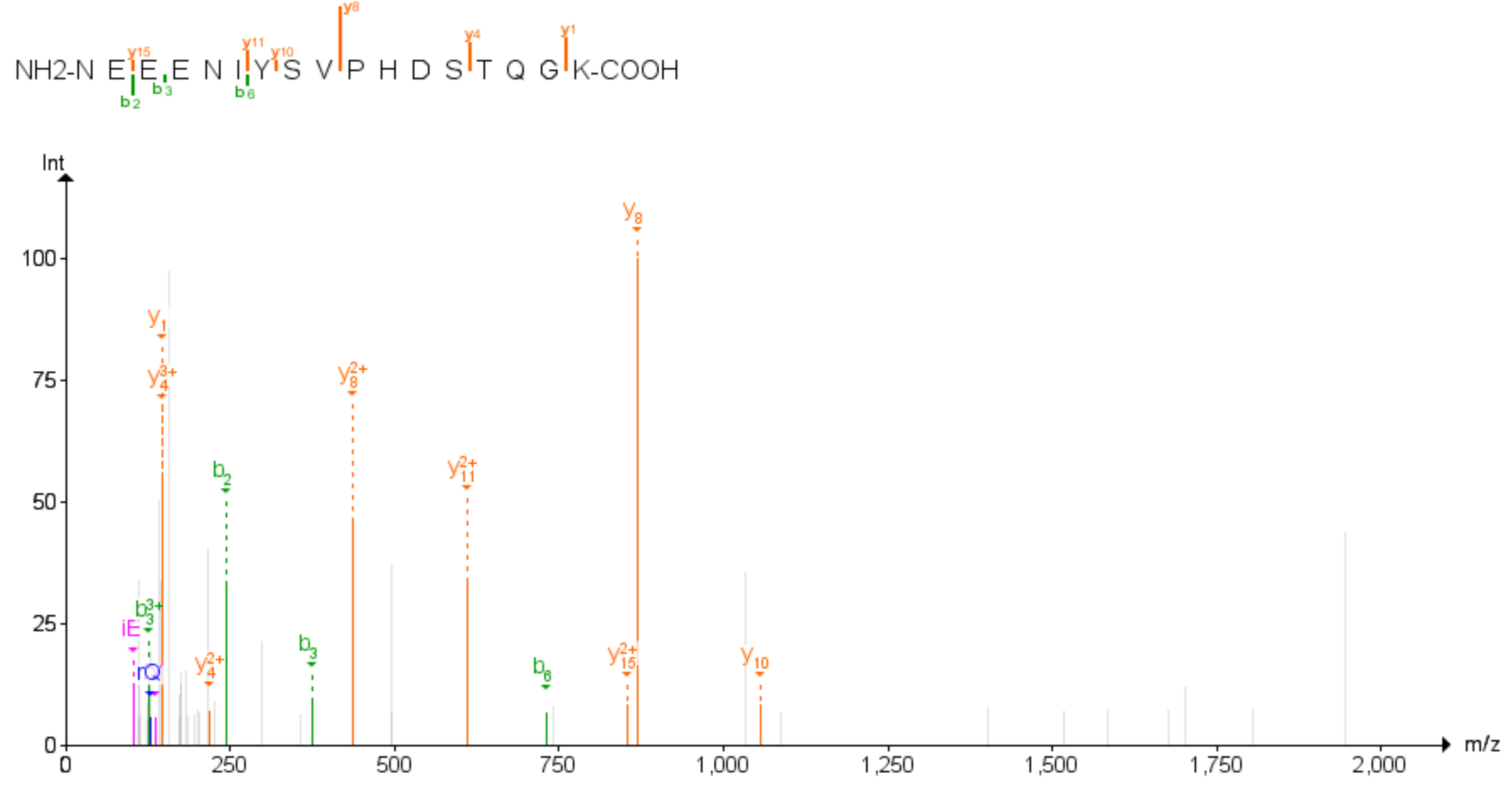

NH2-N E E E N I Y S V P H D S T Q G K-COOH
y15 y11 y10 y8 y4 y1
b2 b3 b6
Int
m/z
0
25
50
75
100
250
500
750
1,000
1,250
1,500
1,750
2,000
y8
y1
y4 3+
y8 2+
b2
y11 2+
b3 3+
iE
rQ
y4 2+
b3
b6
y15 2+
y10

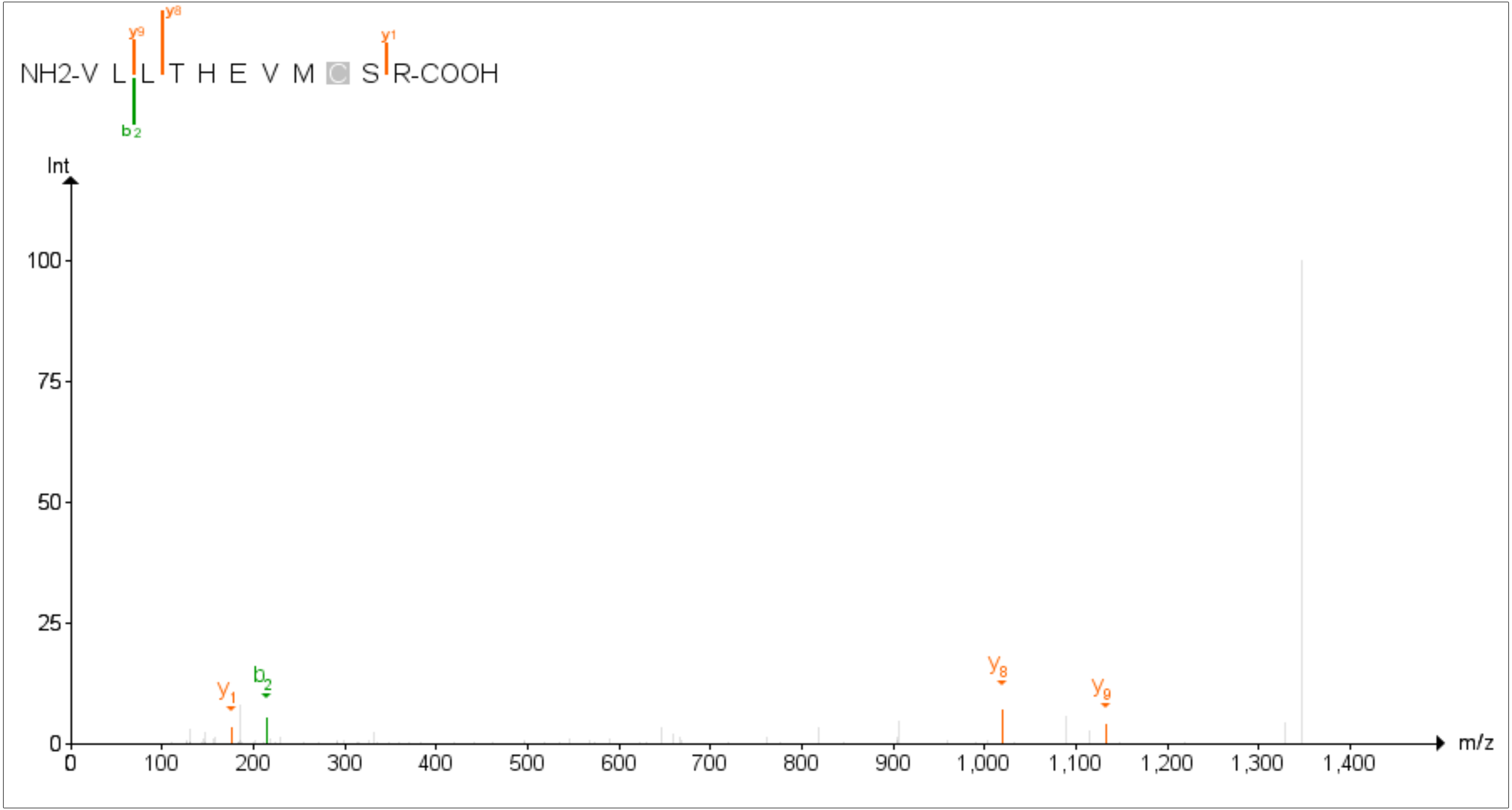
NH2-V L L T H E V M C S R-COOH
y9
y8
y1
b2
Int
100
75
50
25
0
y1
b2
y8
y9
0
100
200
300
400
500
600
700
800
900
1,000
1,100
1,200
1,300
1,400
m/z

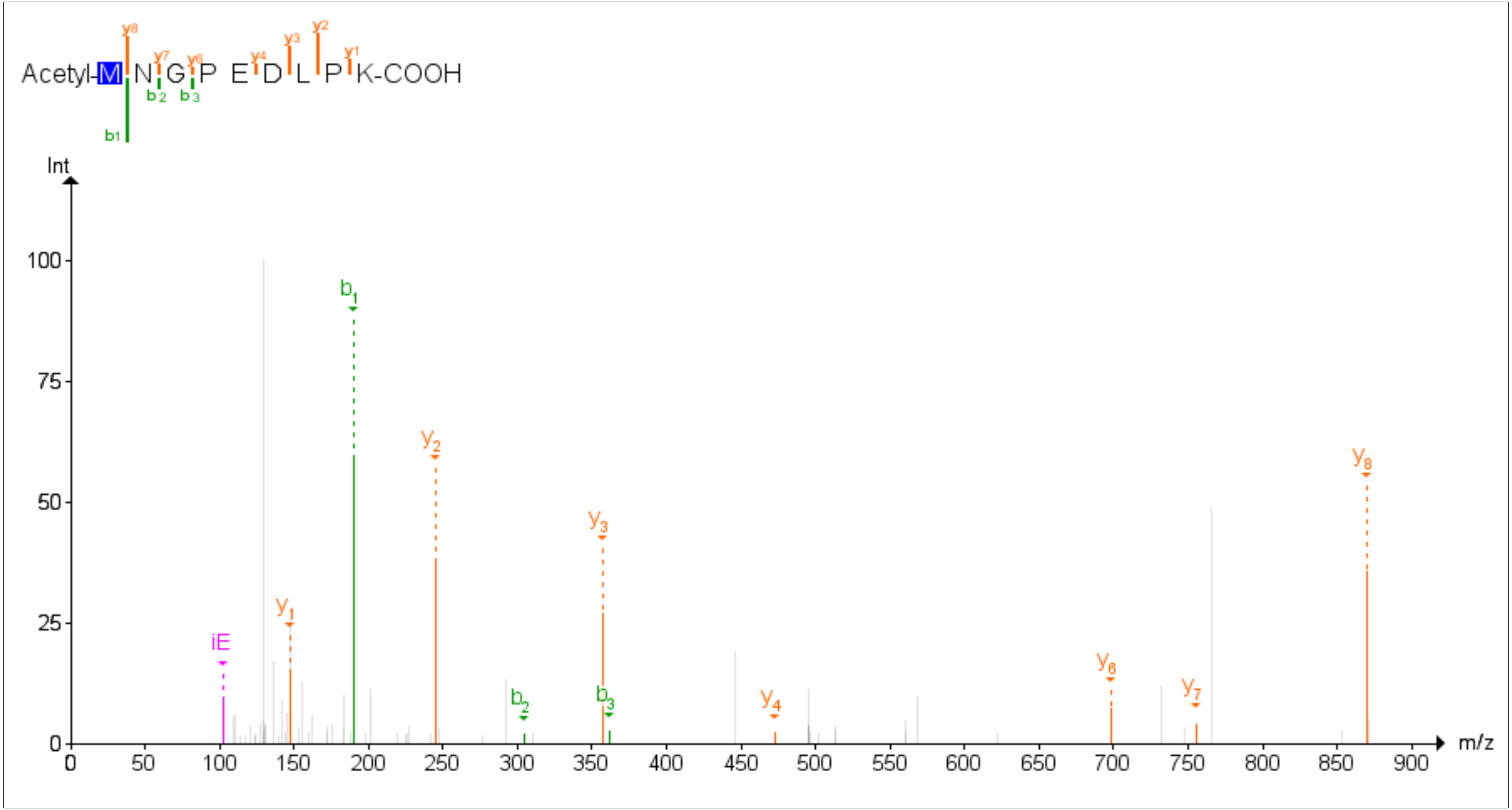

Acetyl-M N G P E D L P K-COOH
y8 y7 y6 y4 y3 y2 y1
b1 b2 b3
Int
100
75
50
25
0
m/z
0 50 100 150 200 250 300 350 400 450 500 550 600 650 700 750 800 850 900
iE
y1
b1
y2
b2
y3
b3
y4
y6
y7
y8

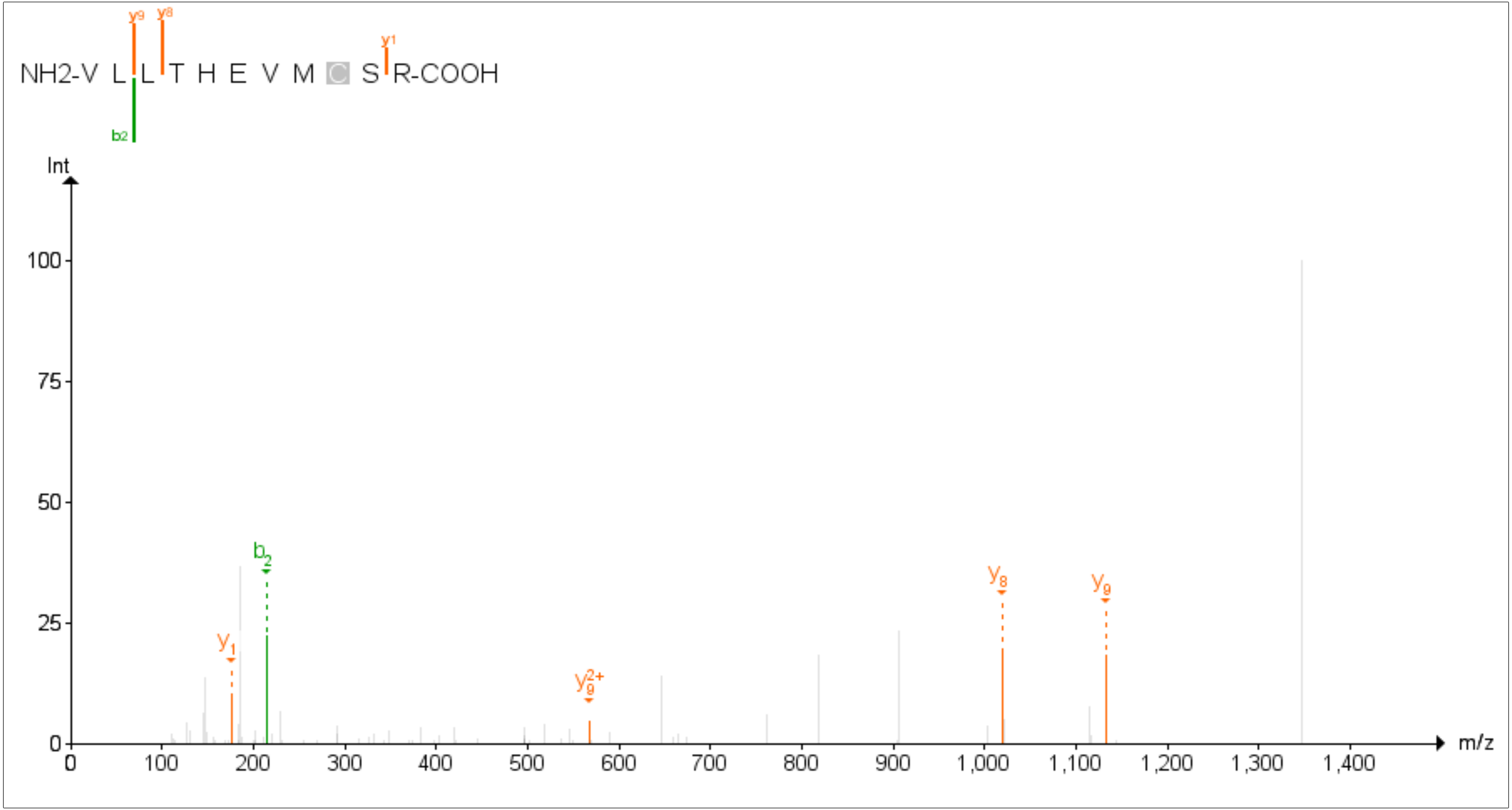

NH2-V L L T H E V M C S R-COOH
y9
y8
y1
b2
Int
100
75
50
25
0
b2
y1
y9 2+
y8
y9
0
100
200
300
400
500
600
700
800
900
1,000
1,100
1,200
1,300
1,400
m/z

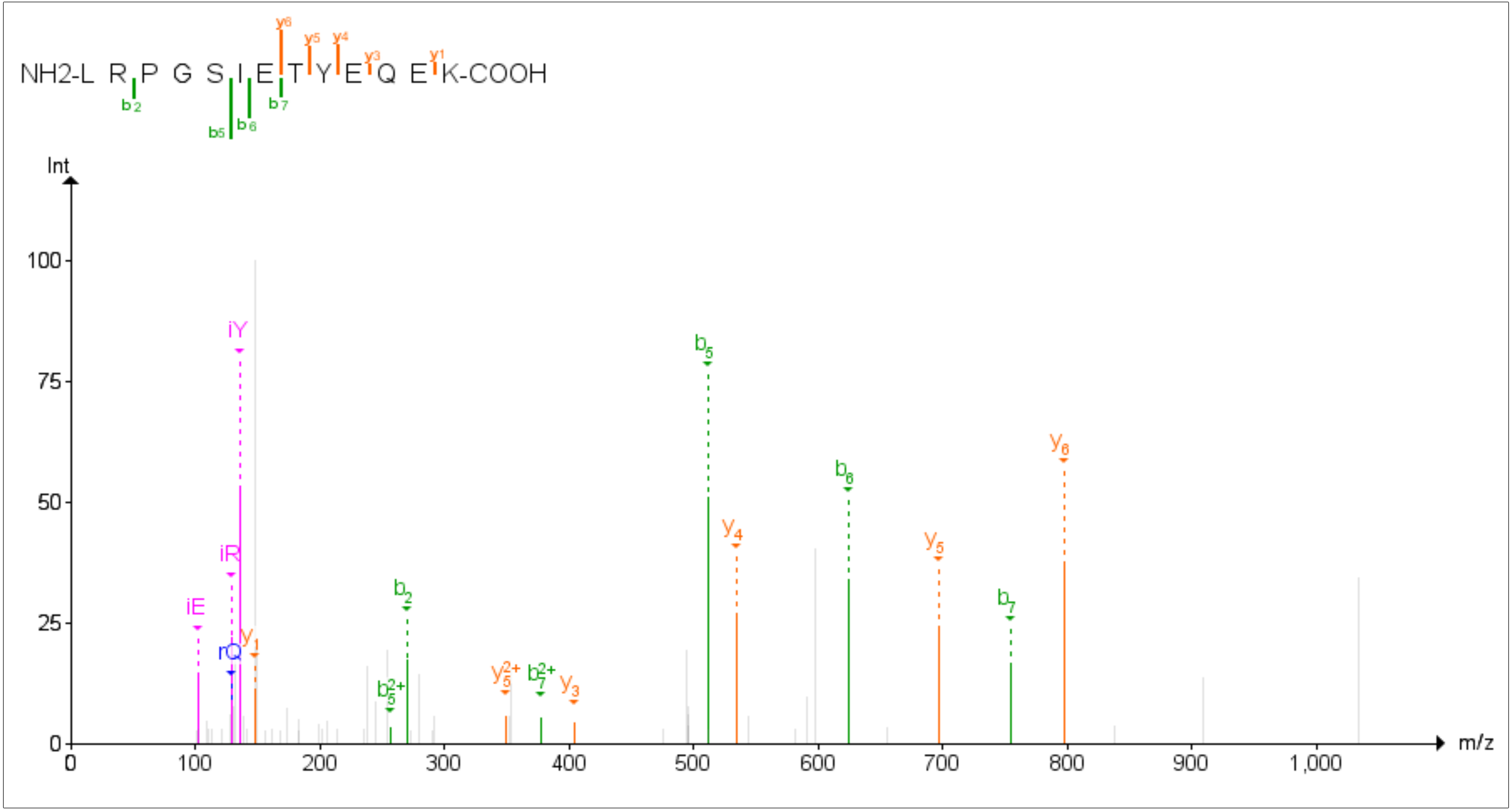

NH2-L R P G S I E T Y E Q E K-COOH
y6 y5 y4 y3 y1
b2 b5 b6 b7
Int
100
75
50
25
0
iY
iR
iE
rQ
y1
b2
b_5^{2+}
y_5^{2+}
b_7^{2+}
y3
b5
y4
b6
y5
b7
y6
0
100
200
300
400
500
600
700
800
900
1,000
m/z

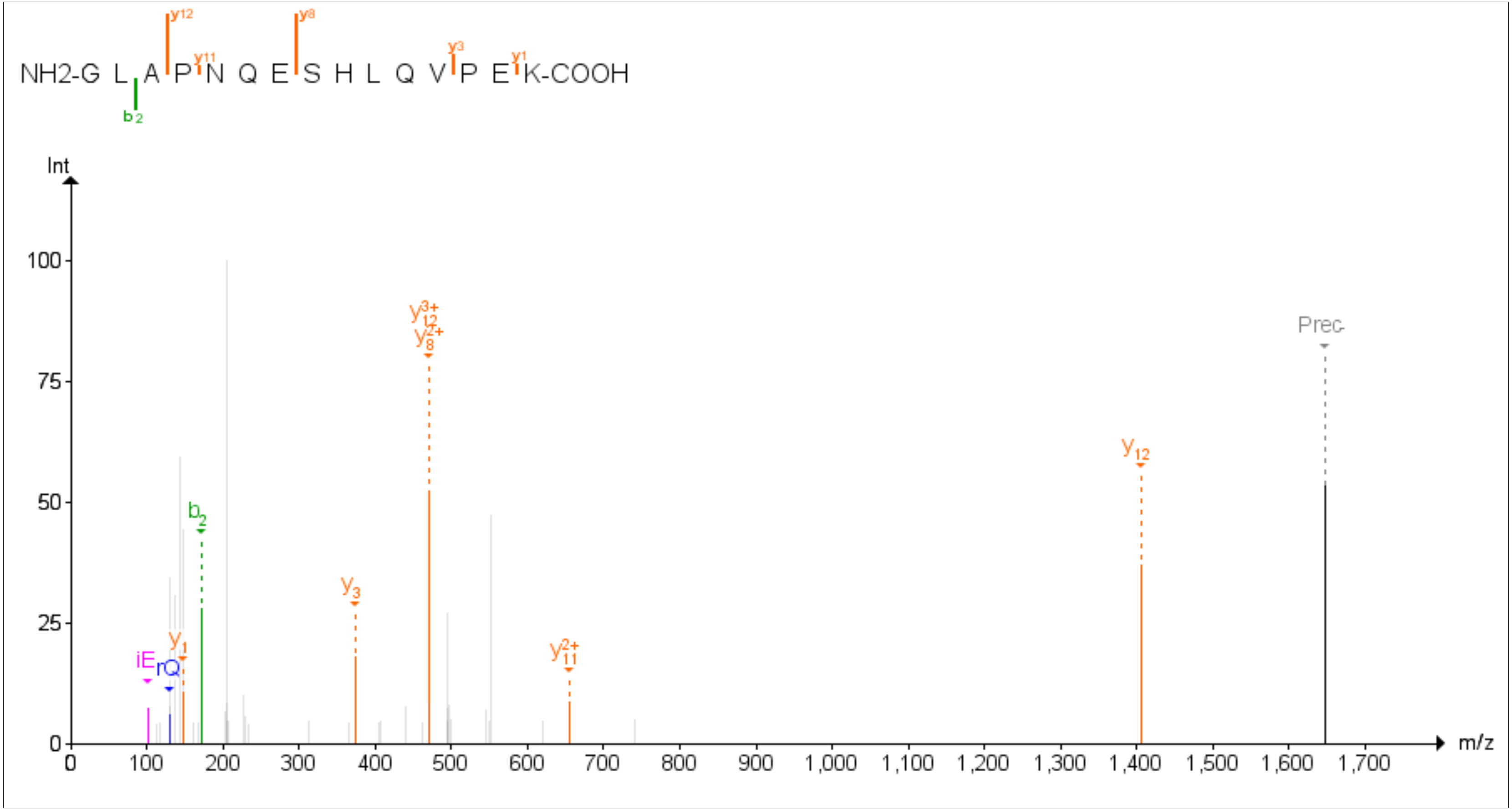

NH2-G L A P N Q E S H L Q V P E K-COOH
y12
y11
y8
y3
y1
b2
Int
m/z
100
75
50
25
0
0
100
200
300
400
500
600
700
800
900
1,000
1,100
1,200
1,300
1,400
1,500
1,600
1,700
Prec
y_{12}^{3+}
y_8^{2+}
y_{12}
b_2
y_3
y_1
iE
rQ
y_{11}^{2+}

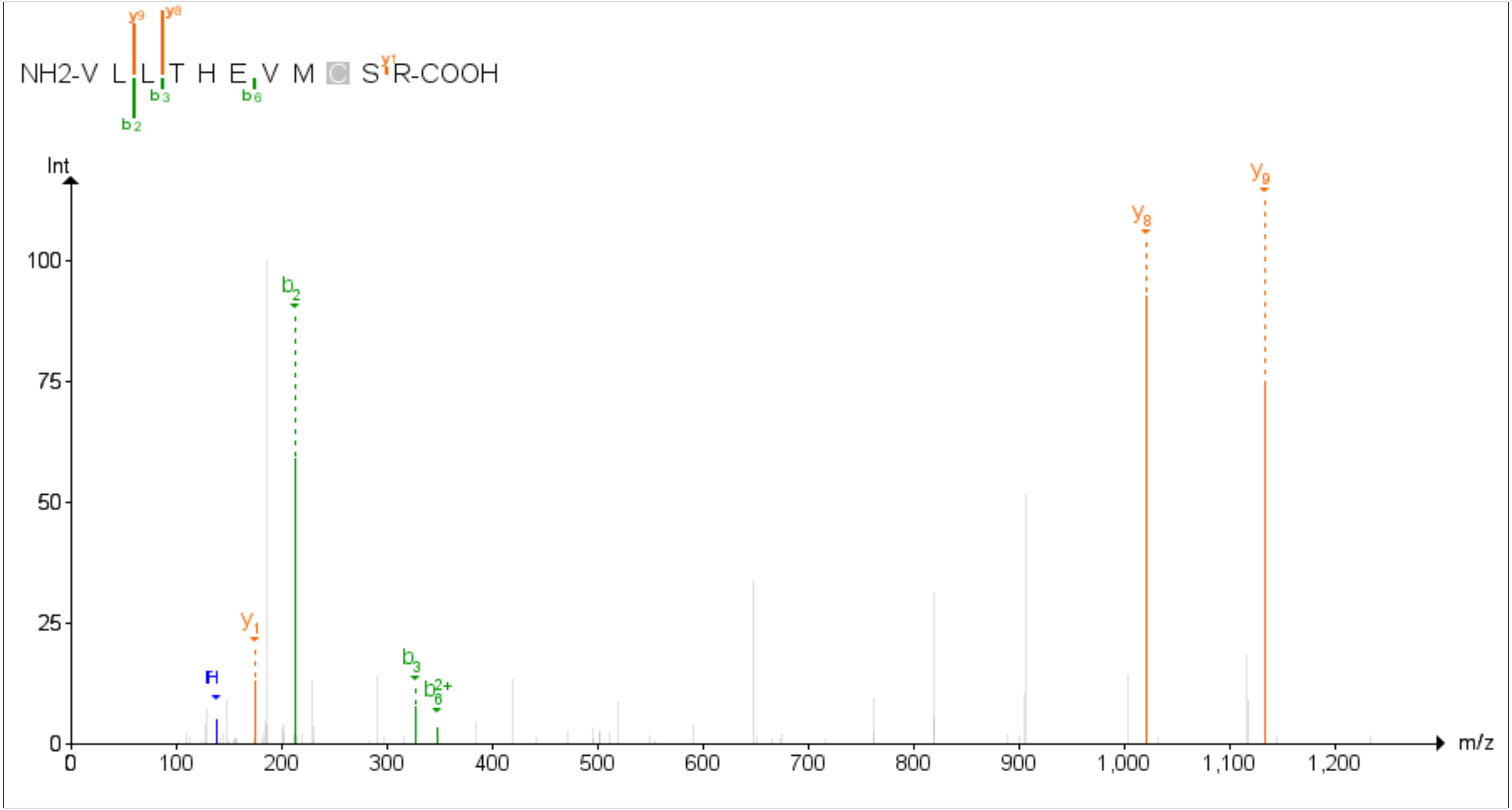

NH2-V L L T H E V M C S R-COOH
y9
y8
y1
b2
b3
b6
Int
100
75
50
25
0
y9
y8
b2
y1
IH
b3
b6 2+
0
100
200
300
400
500
600
700
800
900
1,000
1,100
1,200
m/z

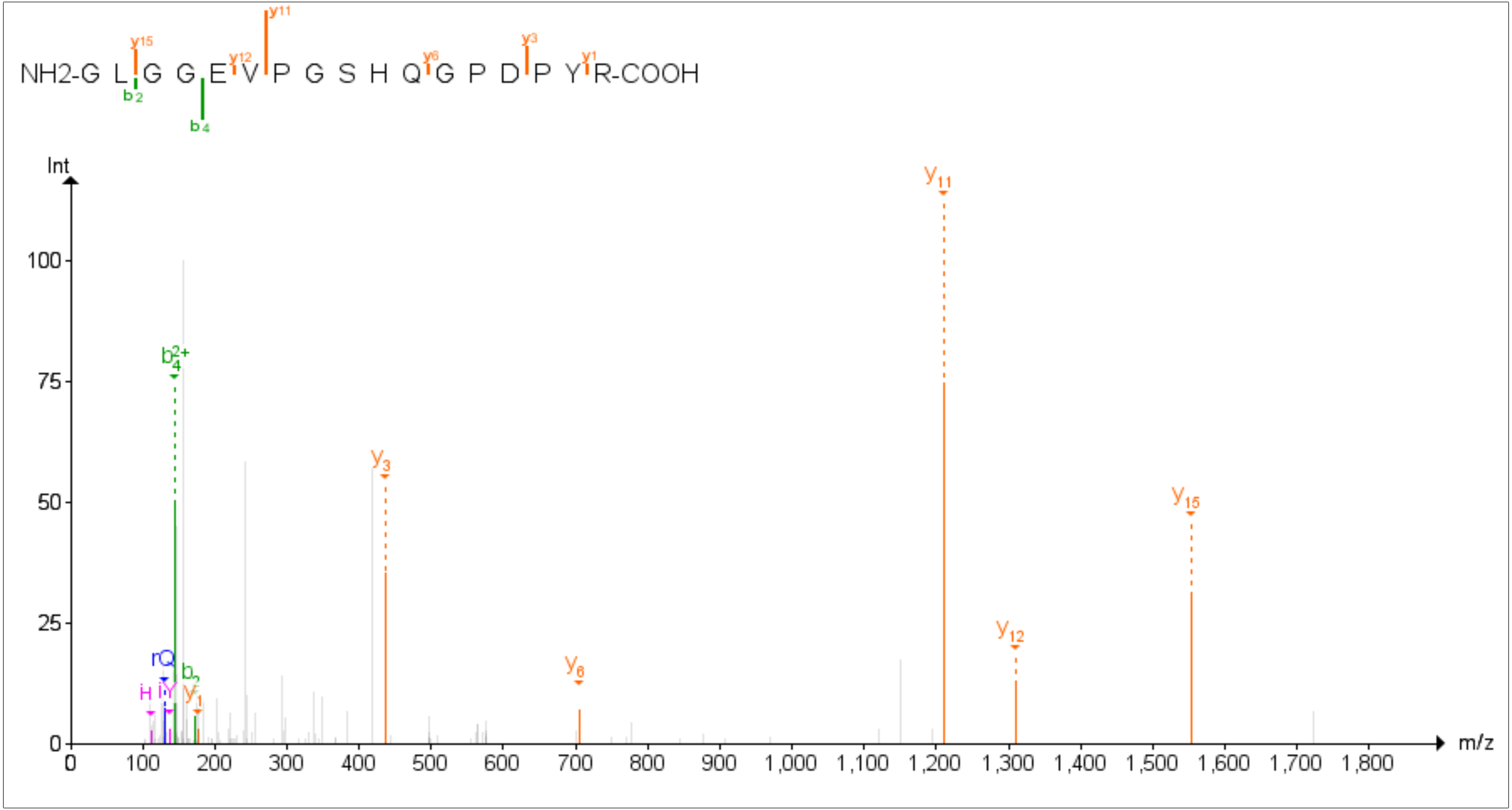
NH2-G L G G E V P G S H Q G P D P Y R-COOH
y15
y12
y11
y6
y3
y1
b2
b4
Int
100
75
50
25
0
m/z
0
100
200
300
400
500
600
700
800
900
1,000
1,100
1,200
1,300
1,400
1,500
1,600
1,700
1,800
b4 2+
y3
y11
y15
y12
y6
rQ
b2
iH
iY
y1

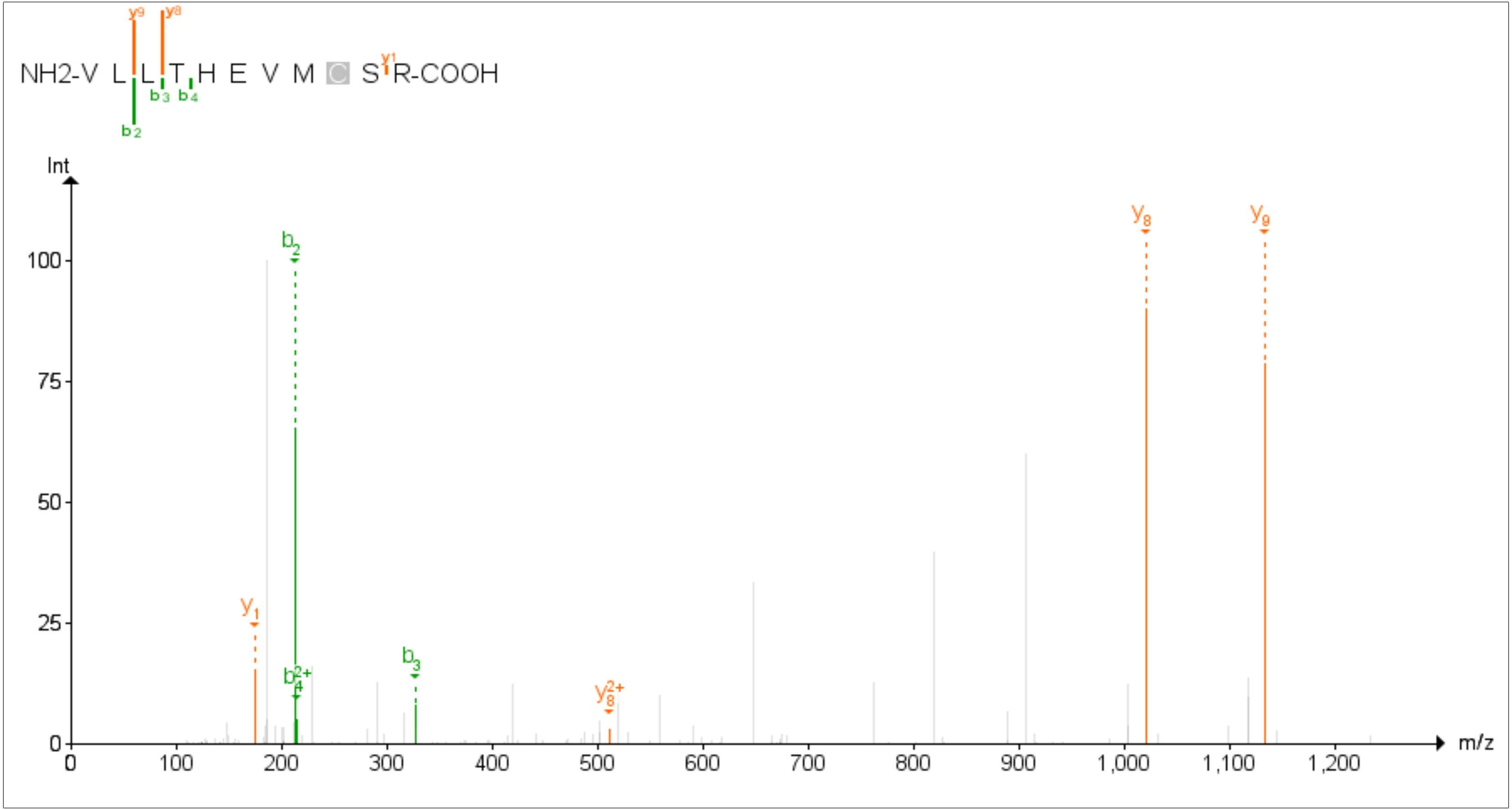

NH2-V L L T H E V M C S R-COOH
y9
y8
y1
b2
b3
b4
Int
100
75
50
25
0
b2
y8
y9
y1
b4 2+
b3
y8 2+
0
100
200
300
400
500
600
700
800
900
1,000
1,100
1,200
m/z

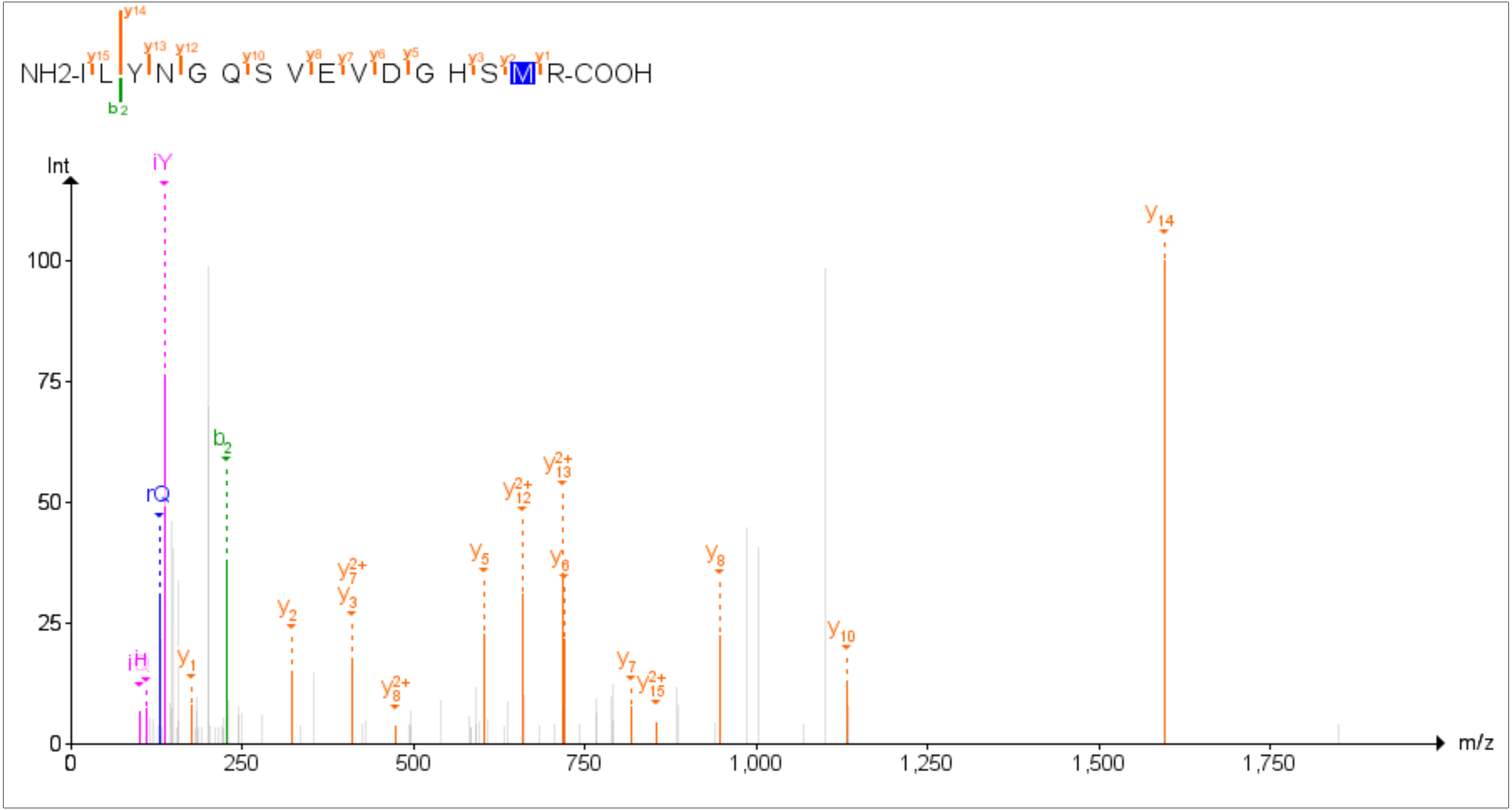

NH2-I L Y N G Q S V E V D G H S M R-COOH
Int
m/z
0
25
50
75
100
250
500
750
1,000
1,250
1,500
1,750
iY
rQ
iH
b2
y1
y2
y3
y5
y6
y7
y8
y10
y14
y2+7
y2+8
y2+12
y2+13
y2+15

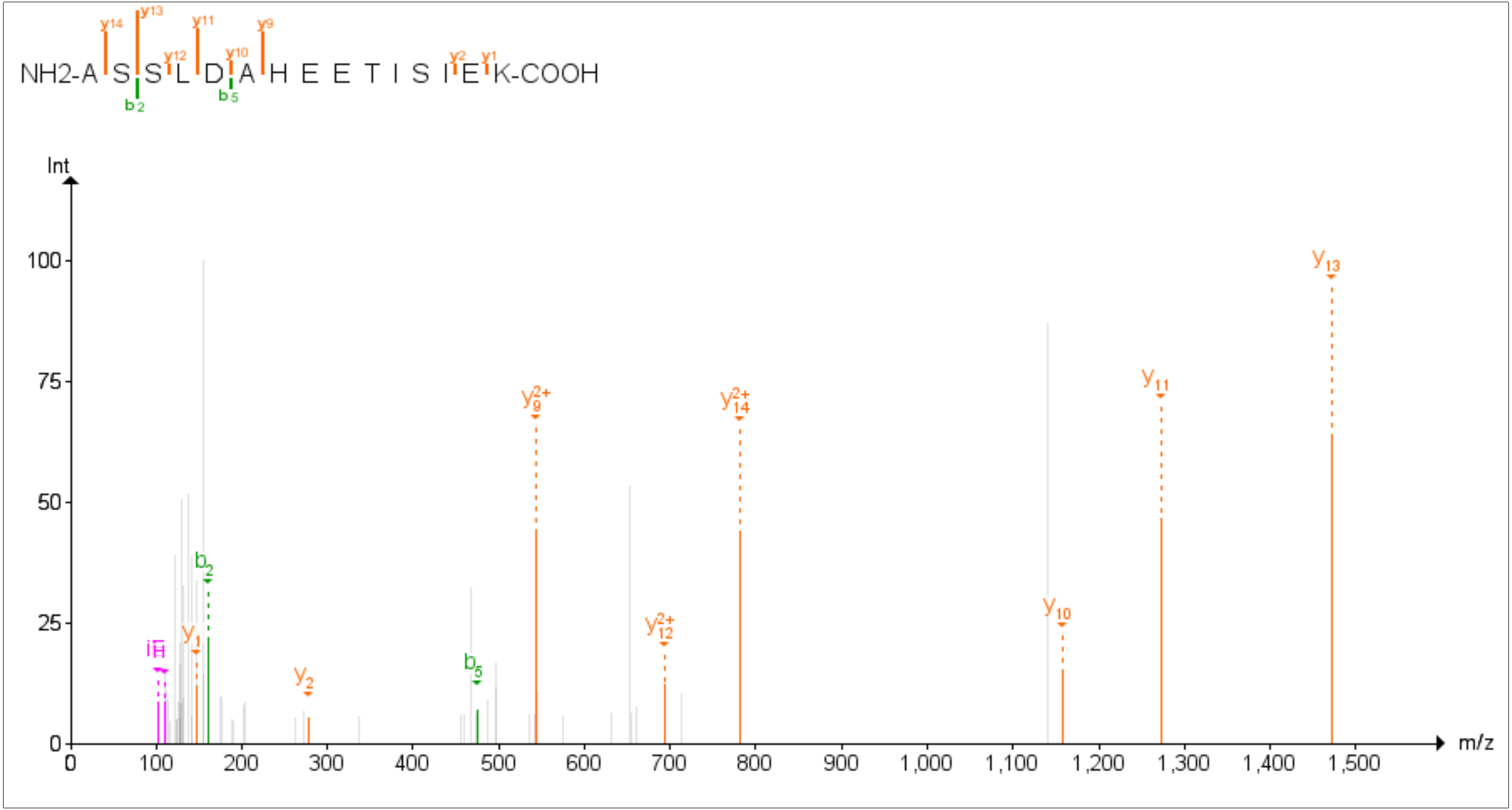

NH2-A S S L D A H E E T I S I E K-COOH
y14
y13
y12
y11
y10
y9
y2
y1
b2
b5
Int
100
75
50
25
0
m/z
0
100
200
300
400
500
600
700
800
900
1,000
1,100
1,200
1,300
1,400
1,500
y13
y11
y9 2+
y14 2+
y12 2+
y10
b2
y1
y2
b5
iH

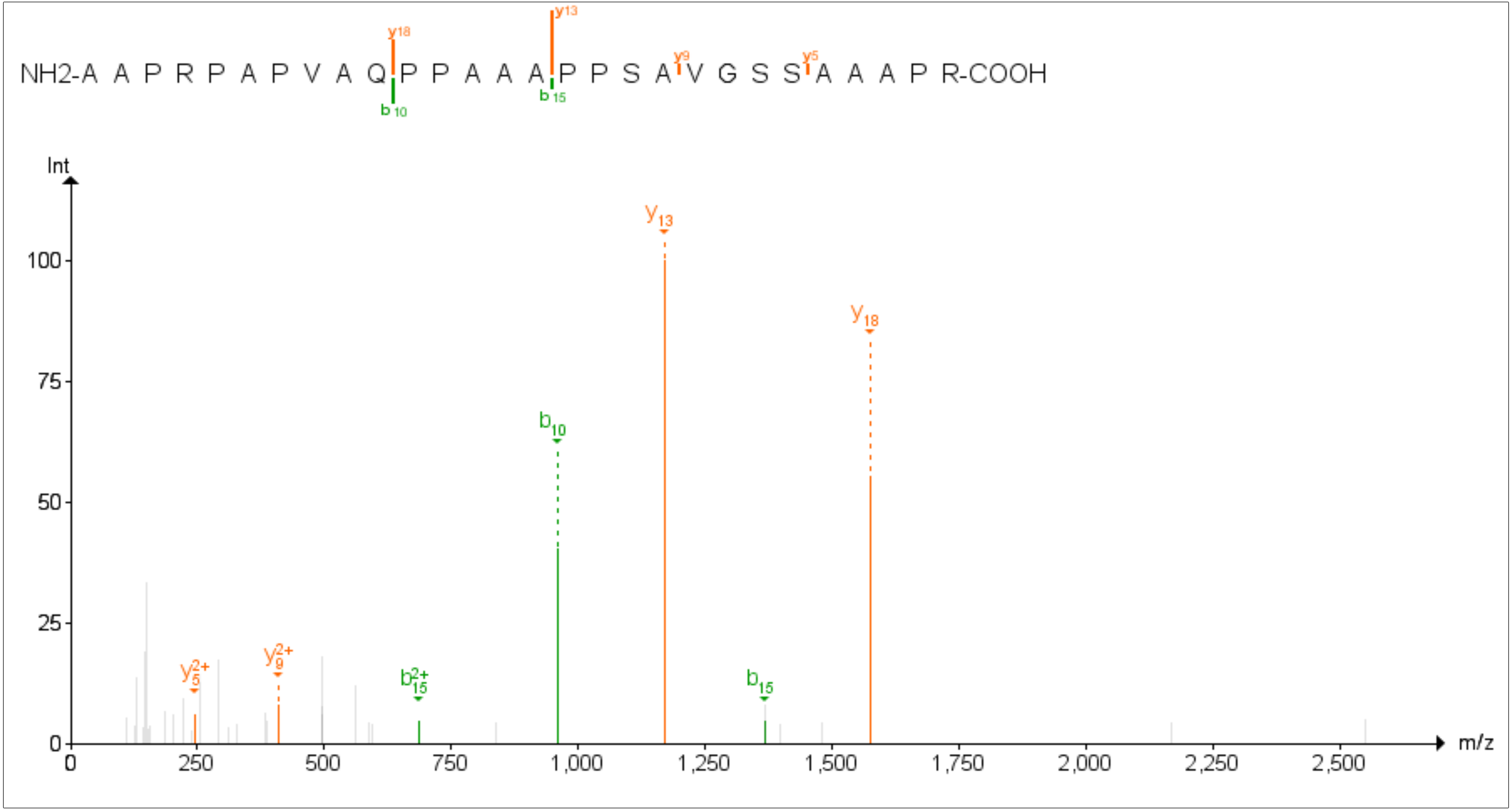

NH2-A A P R P A P V A Q P P A A A P P S A V G S S A A A P R-COOH
y18
y13
y9
y5
b 10
b 15
Int
100
75
50
25
0
y13
y18
b10
y5 2+
y9 2+
b15 2+
b15
0
250
500
750
1,000
1,250
1,500
1,750
2,000
2,250
2,500
m/z

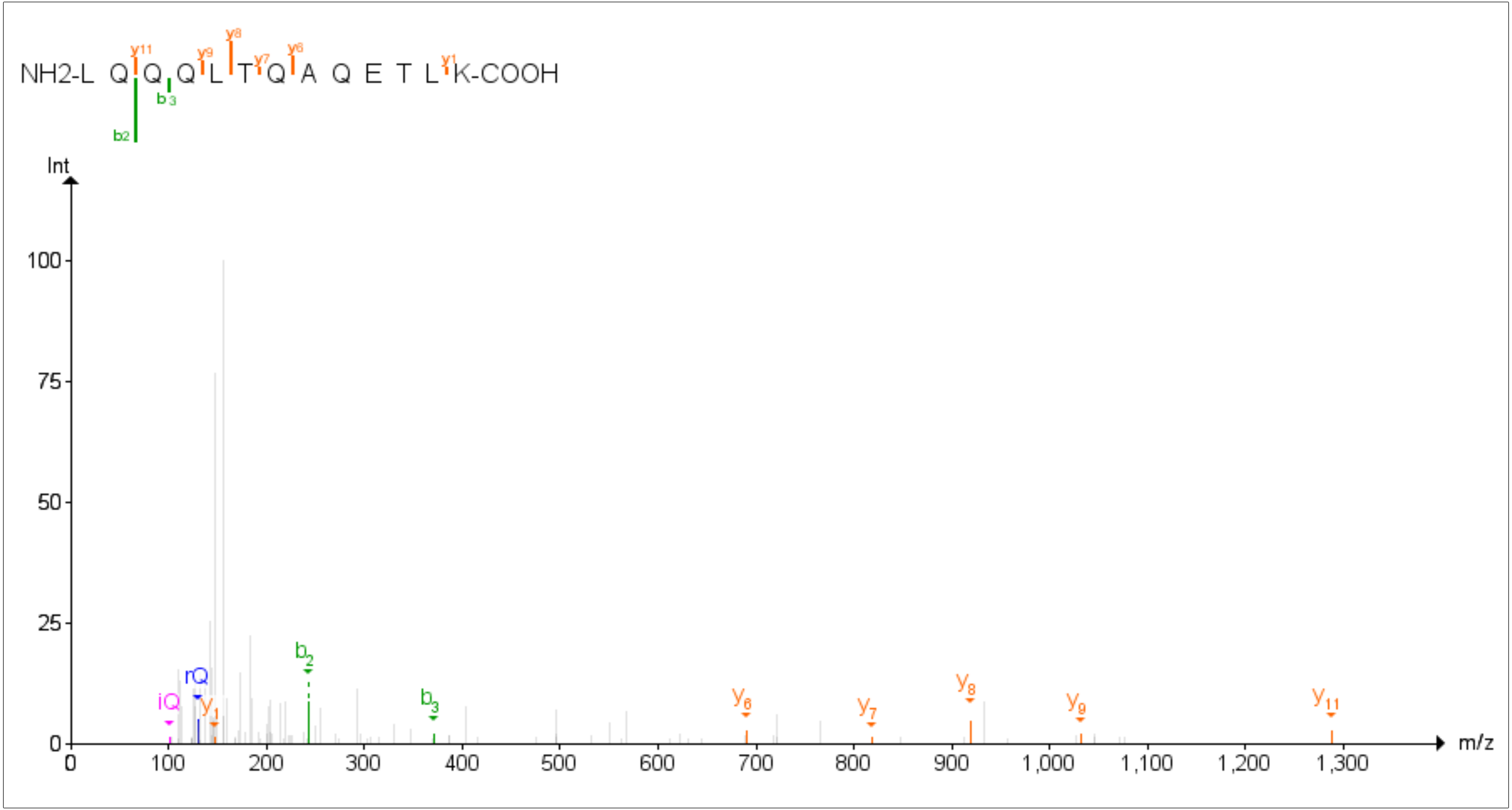

NH2-L Q Q Q L T Q A Q E T L K-COOH
y11
y9
y8
y7
y6
y1
b2
b3
Int
100
75
50
25
0
m/z
0
100
200
300
400
500
600
700
800
900
1,000
1,100
1,200
1,300
iQ
rQ
y1
b2
b3
y6
y7
y8
y9
y11

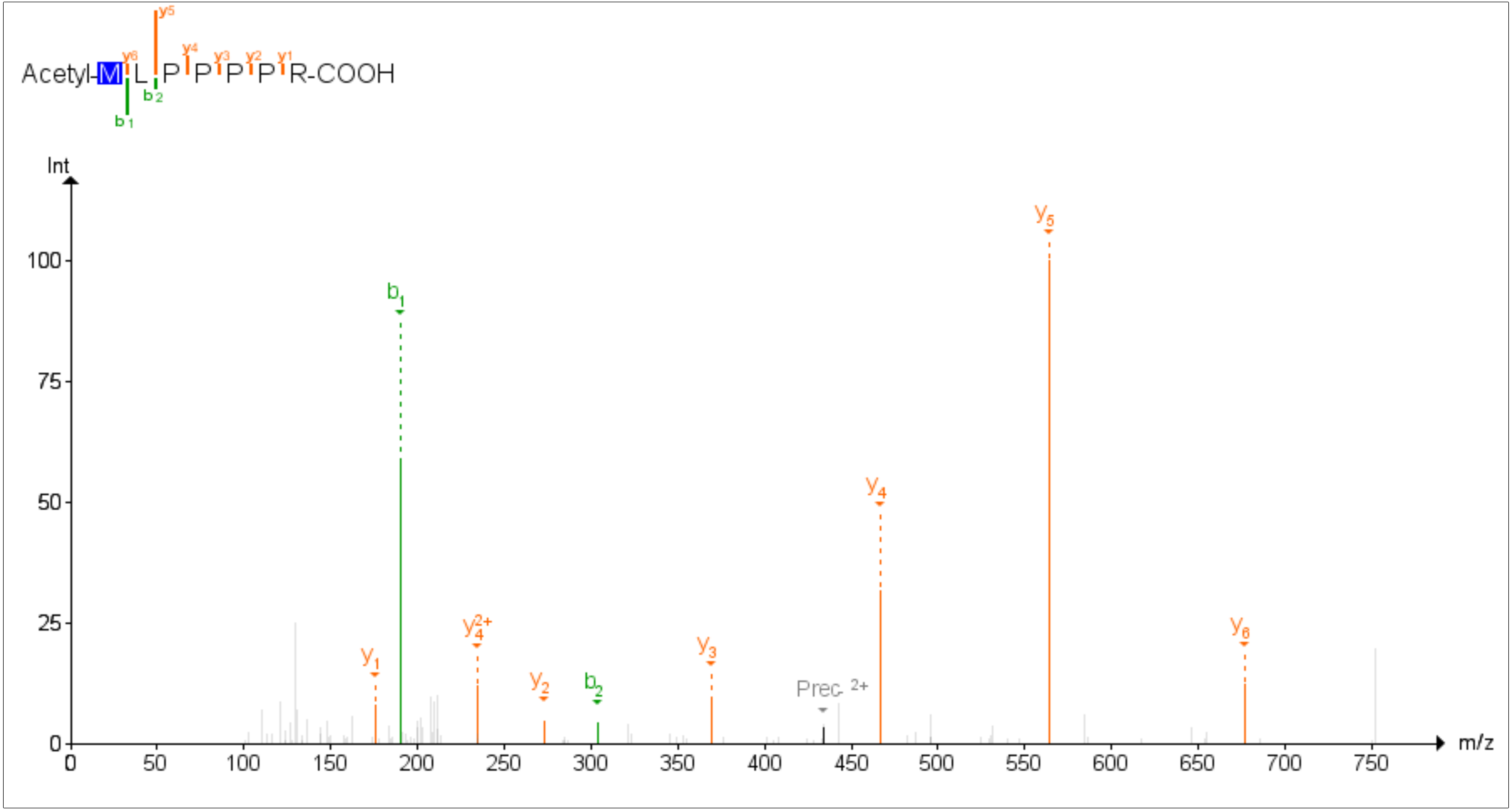

Acetyl-M L P P P P R-COOH
y6
y5
y4
y3
y2
y1
b1
b2
Int
100
75
50
25
0
m/z
0
50
100
150
200
250
300
350
400
450
500
550
600
650
700
750
b1
y1
y4 2+
y2
b2
y3
Prec. 2+
y4
y5
y6

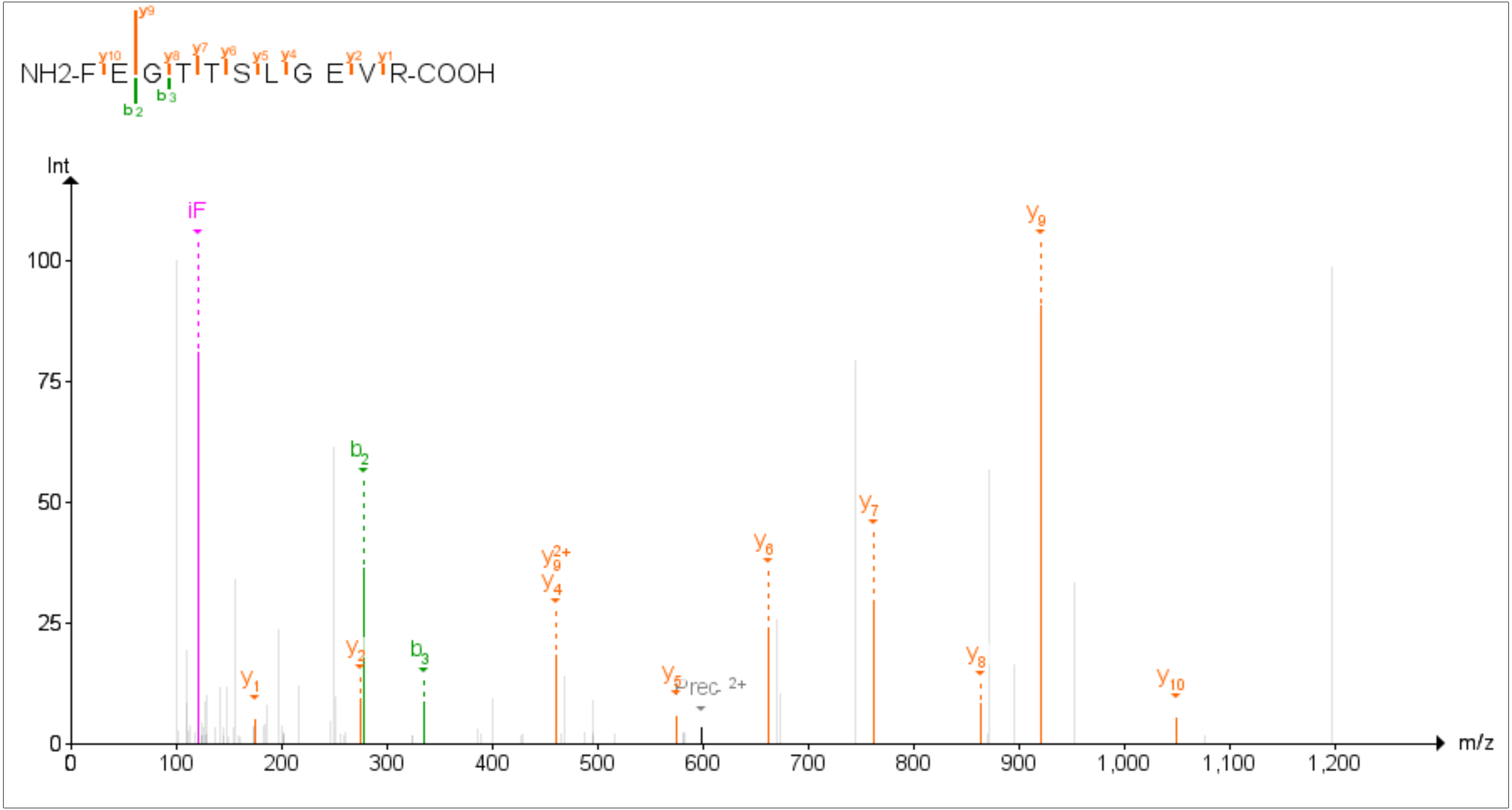

NH2-F E G T T S L G E V R-COOH
y9
y10
y8
y7
y6
y5
y4
y2
y1
b2
b3
Int
iF
y9
b2
y7
y6
y9 2+
y4
y2
b3
y1
y5
Prec. 2+
y8
y10
100
75
50
25
0
0
100
200
300
400
500
600
700
800
900
1,000
1,100
1,200
m/z

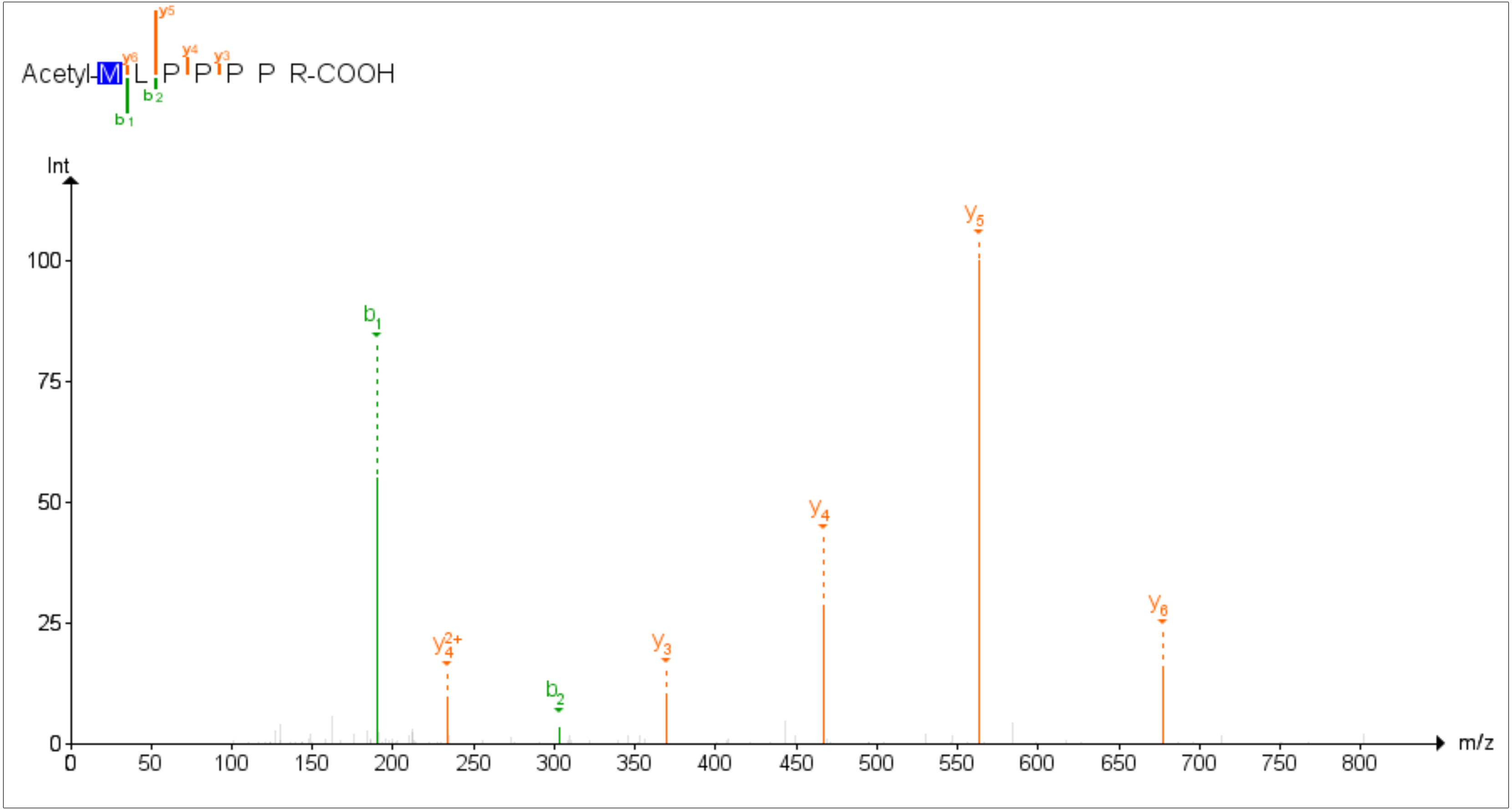

Acetyl-M L P P P P R-COOH
y5
y6
y4
y3
b1
b2
Int
100
75
50
25
0
b1
y5
y4
y6
y4 2+
b2
y3
m/z
0
50
100
150
200
250
300
350
400
450
500
550
600
650
700
750
800

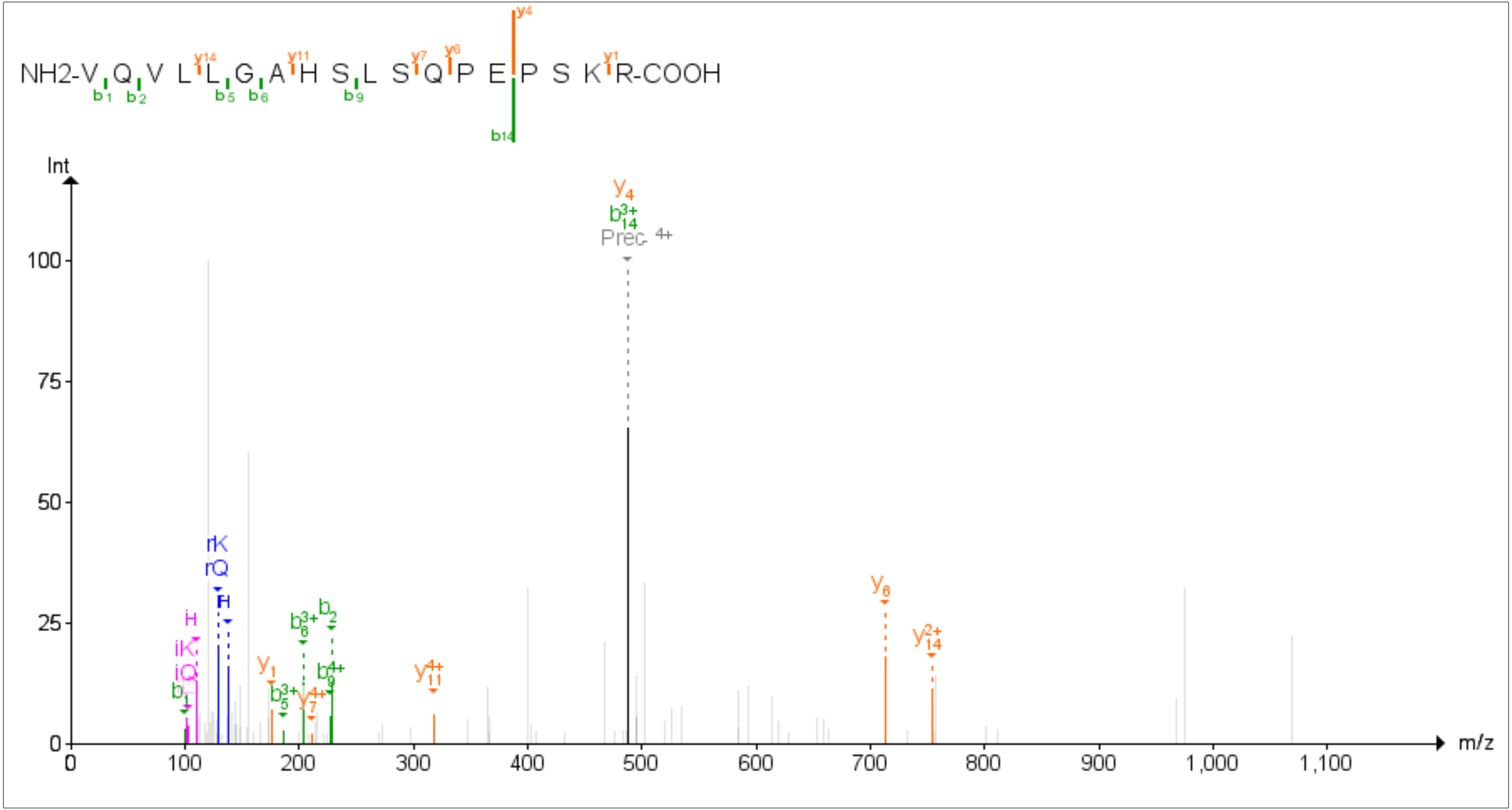

NH2-V Q V L L G A H S L S Q P E P S K R-COOH
b1 b2 b5 b6 b9 b14
y14 y11 y7 y6 y4 y1
Int
m/z
0
25
50
75
100
0
100
200
300
400
500
600
700
800
900
1,000
1,100
y4
b14 3+
Prec 4+
rK
rQ
rH
iH
iK
iQ
b1
y1
b5 3+
b6 3+
b2
b9 4+
y7 4+
y11 4+
y6
y14 2+

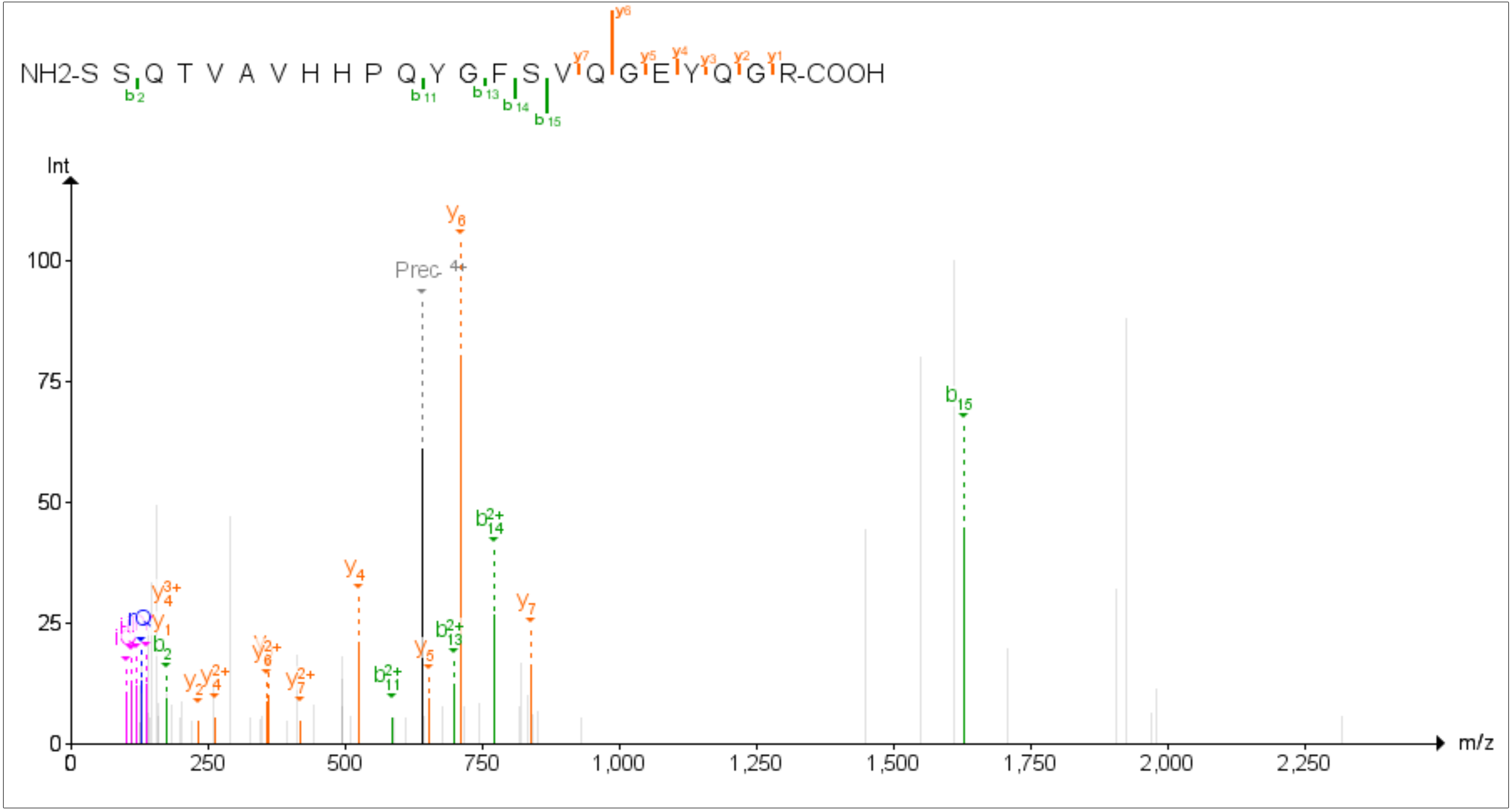
NH2-S S Q T V A V H H P Q Y G F S V Q G E Y Q G R-COOH
b2
b11
b13
b14
b15
y7
y6
y5
y4
y3
y2
y1
Int
100
75
50
25
0
Prec 4+
y6
b15
b2+14
y7
y4
b2+13
y5
b2+11
y2+7
y2+6
y2
y2+4
y3+4
y1
b2
rQ
0
250
500
750
1,000
1,250
1,500
1,750
2,000
2,250
m/z

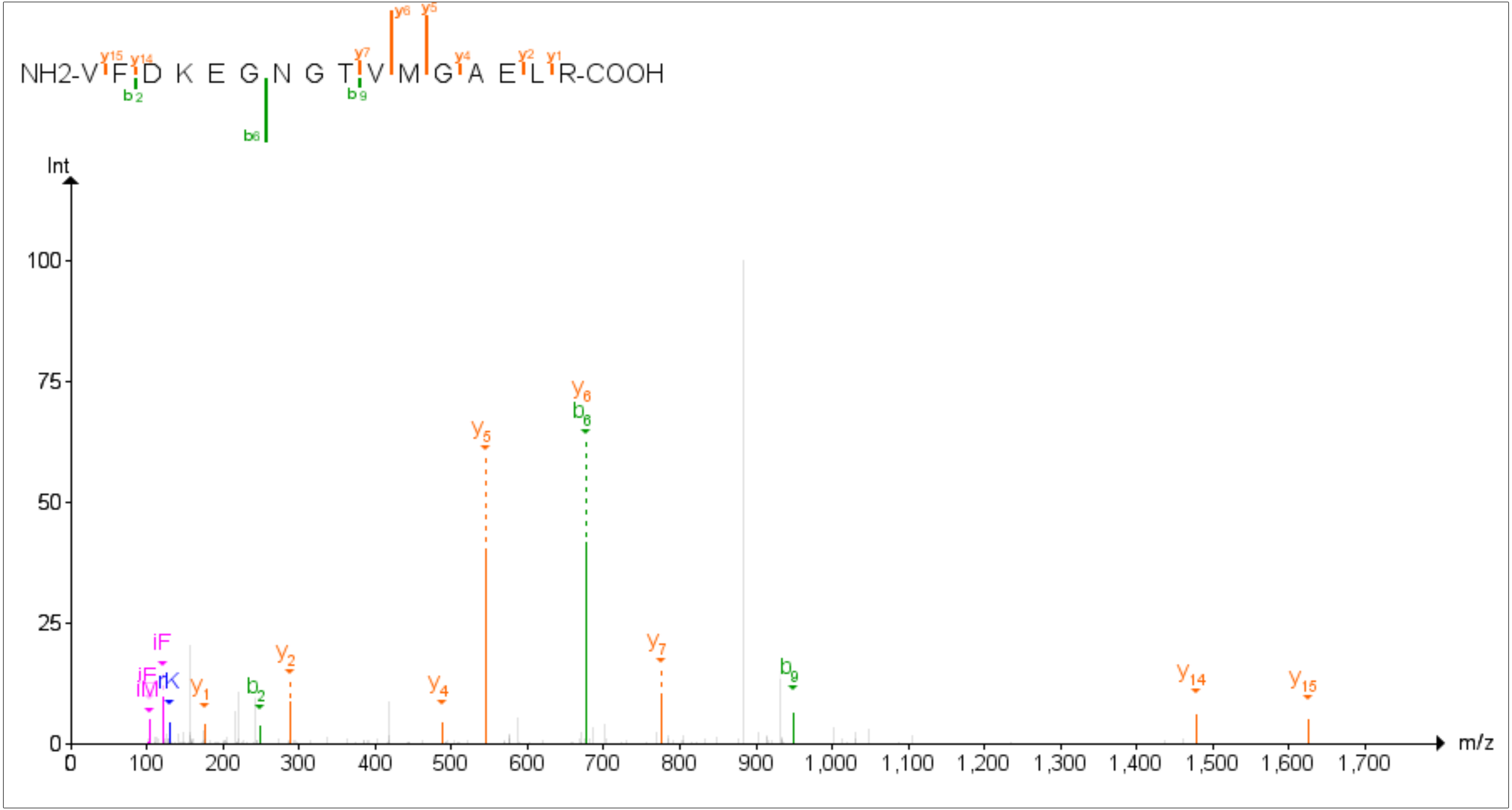

NH2-V F D K E G N G T V M G A E L R-COOH
y15 y14 y7 y6 y5 y4 y2 y1
b2 b6 b9
Int
m/z
y5
y6
b6
y7
b9
y14
y15
iF
iF
rK
iM
y1
b2
y2
y4

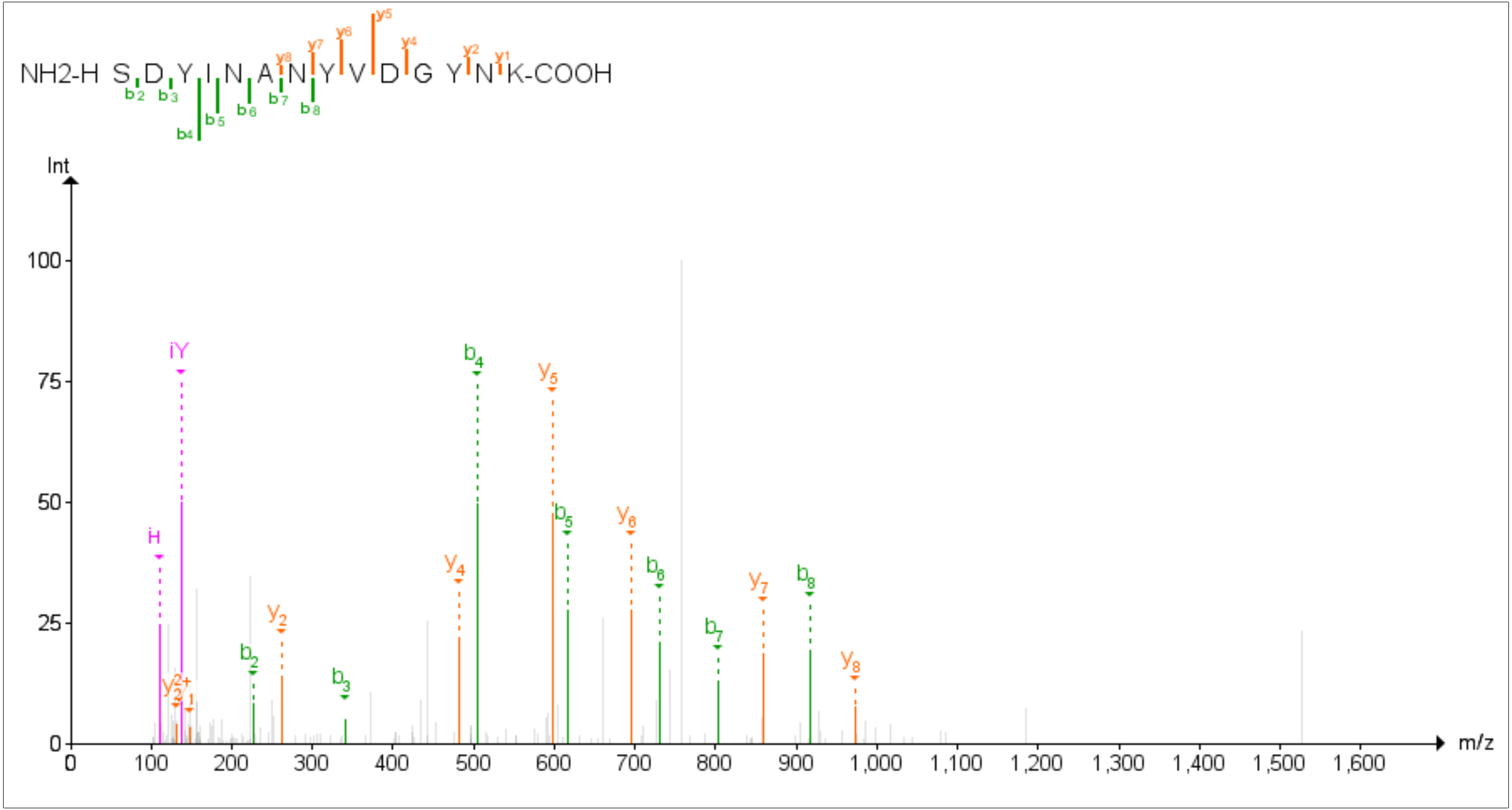

NH2-H S D Y I N A N Y V D G Y N K-COOH
b2 b3 b4 b5 b6 b7 b8
y8 y7 y6 y5 y4 y2 y1
Int
m/z
100
75
50
25
0
iY
iH
b4
y5
b5
y6
b6
y4
y2
b2
b3
b7
y7
b8
y8
0 100 200 300 400 500 600 700 800 900 1,000 1,100 1,200 1,300 1,400 1,500 1,600

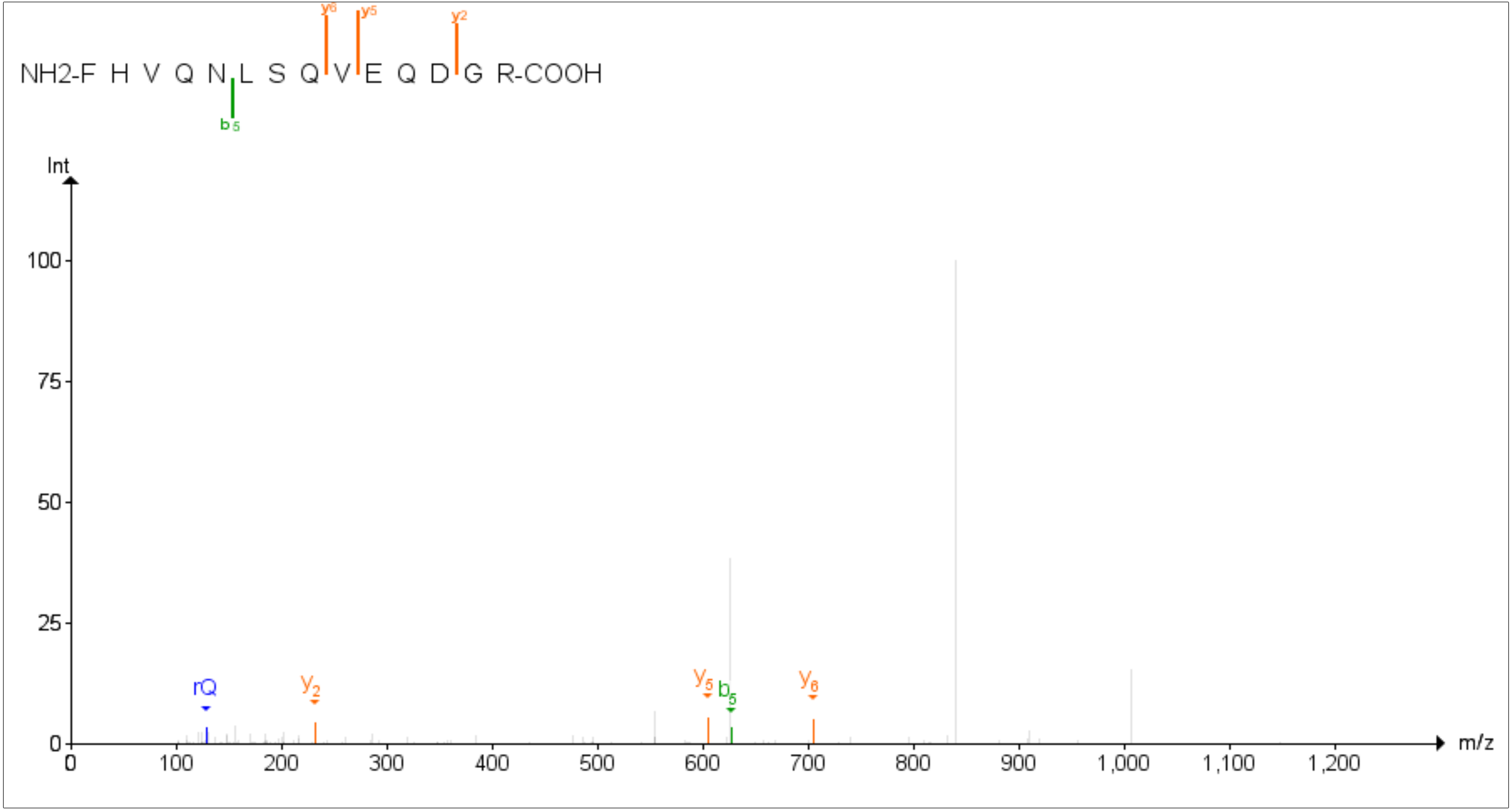

NH2-F H V Q N L S Q V E Q D G R-COOH
y6
y5
y2
b5
Int
100
75
50
25
0
rQ
y2
y5
b5
y6
0
100
200
300
400
500
600
700
800
900
1,000
1,100
1,200
m/z

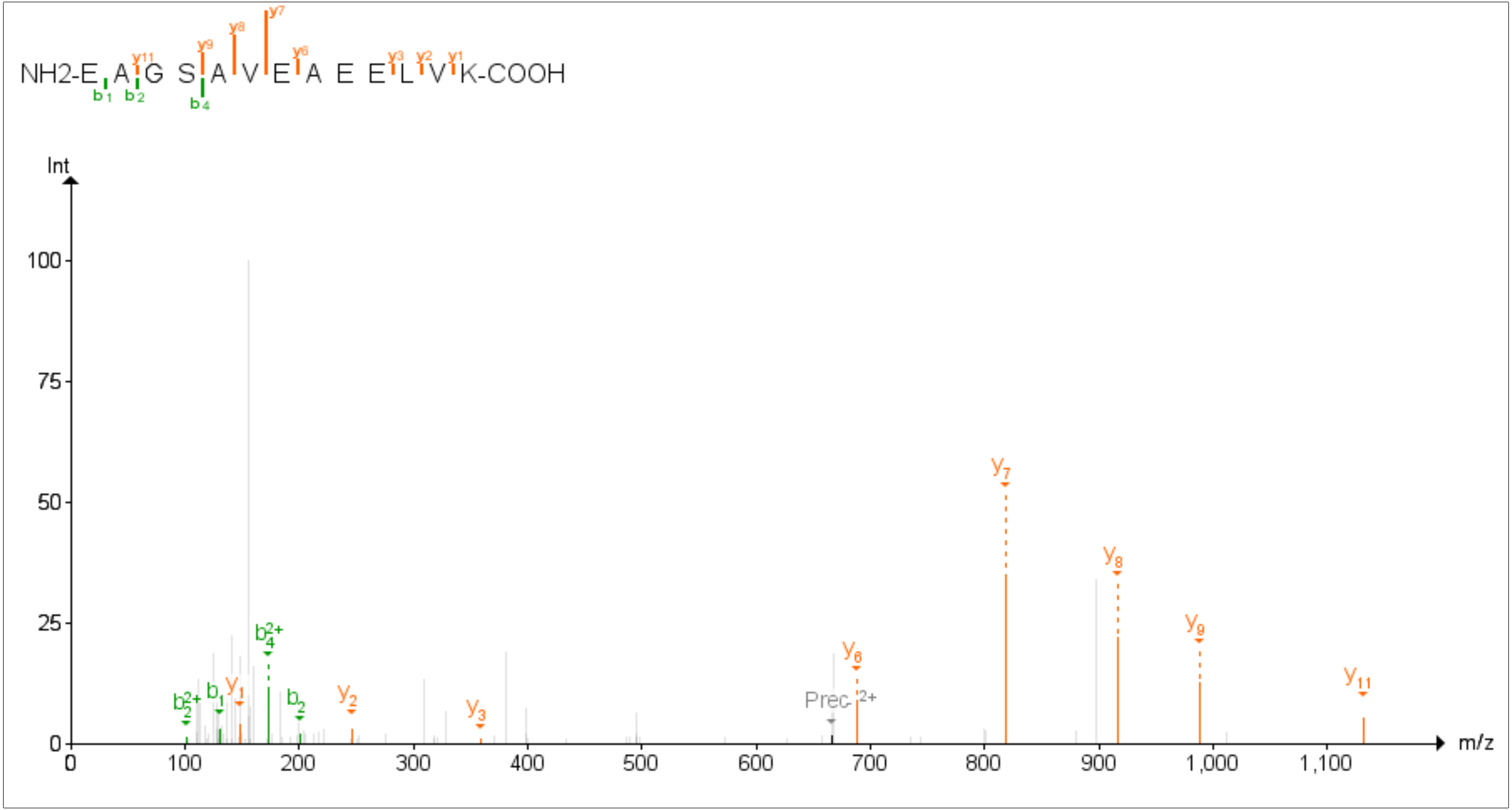

NH2-E A G S A V E A E E L V K-COOH
b1 b2 b4
y11 y9 y8 y7 y6 y3 y2 y1
Int
100
75
50
25
0
m/z
0
100
200
300
400
500
600
700
800
900
1,000
1,100
b$_2^{2+}$
b1
y1
b$_4^{2+}$
b2
y2
y3
Prec 2+
y6
y7
y8
y9
y11

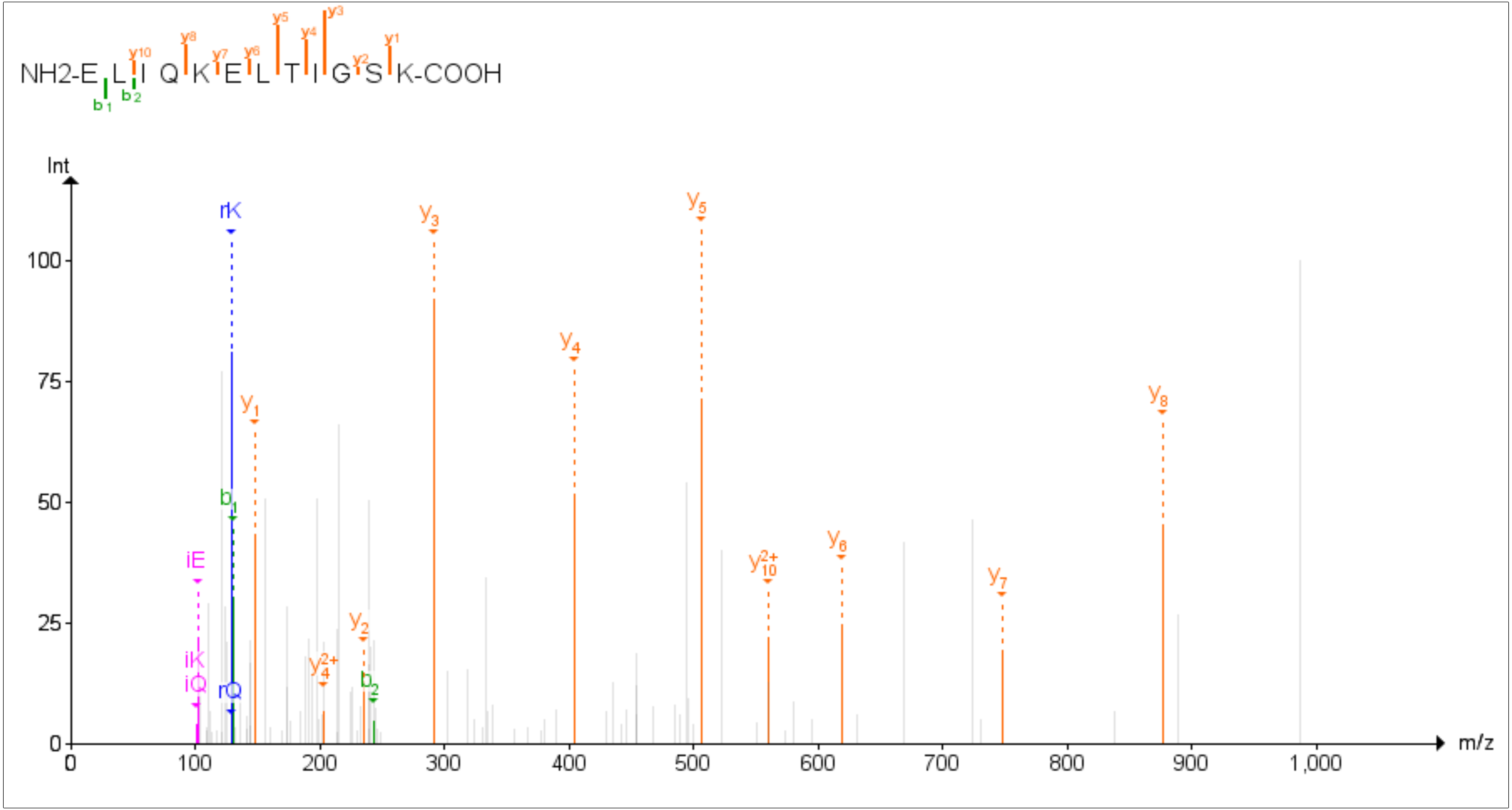

NH2-E L I Q K E L T I G S K-COOH
y10
y8
y7
y6
y5
y4
y3
y2
y1
b1
b2
Int
100
75
50
25
0
rK
y3
y5
y4
y1
b1
iE
iK
iQ
rQ
y4 2+
y2
b2
y10 2+
y6
y7
y8
m/z
0
100
200
300
400
500
600
700
800
900
1,000

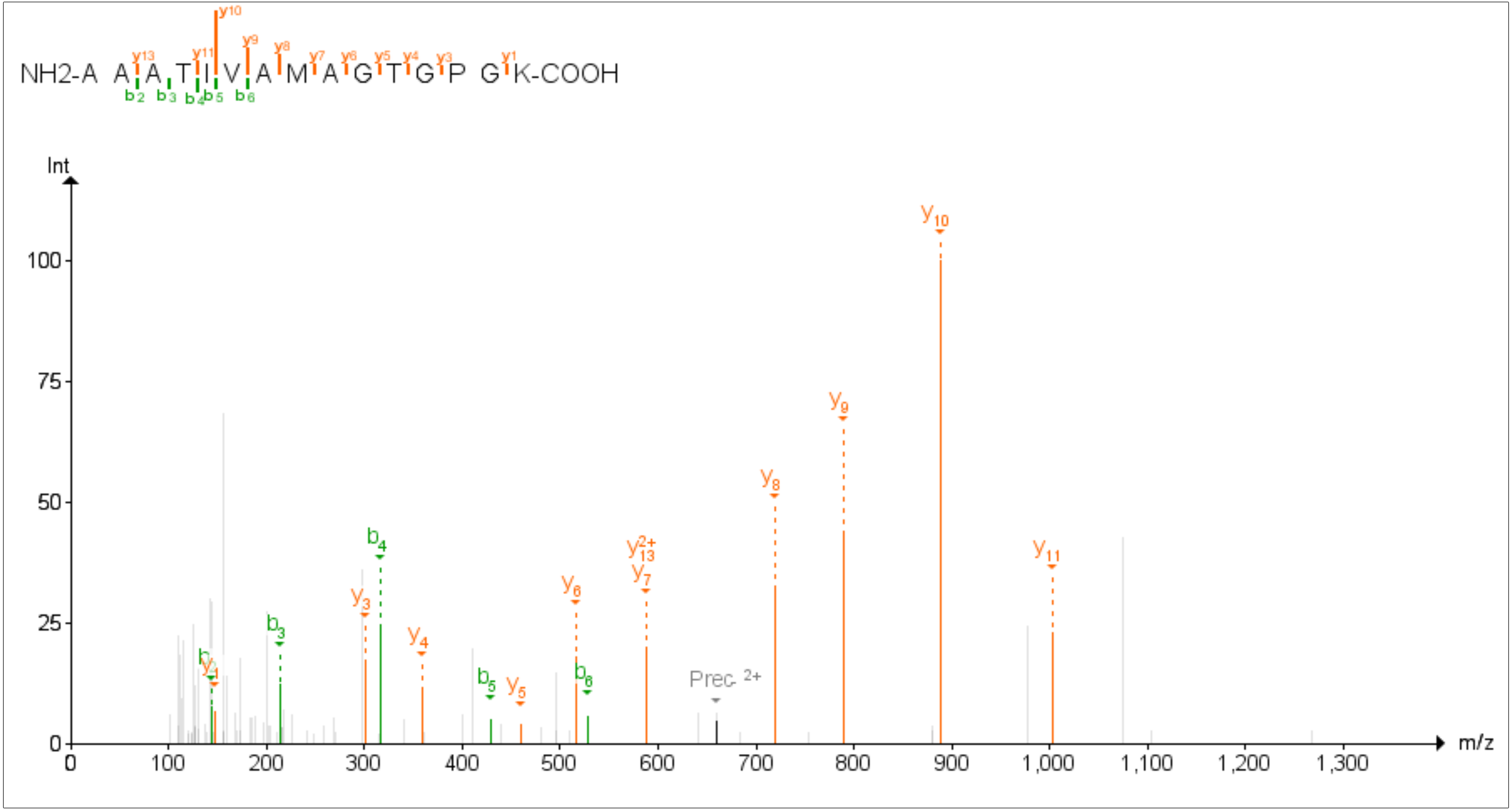

NH2-A A A T I V A M A G T G P G K-COOH
y13 y11 y10 y9 y8 y7 y6 y5 y4 y3 y1
b2 b3 b4 b5 b6
Int
100
75
50
25
0
y10
y9
y8
y11
b4
y3
y13 2+
y7
y6
b3
y4
b5
y5
b6
Prec. 2+
0 100 200 300 400 500 600 700 800 900 1,000 1,100 1,200 1,300
m/z

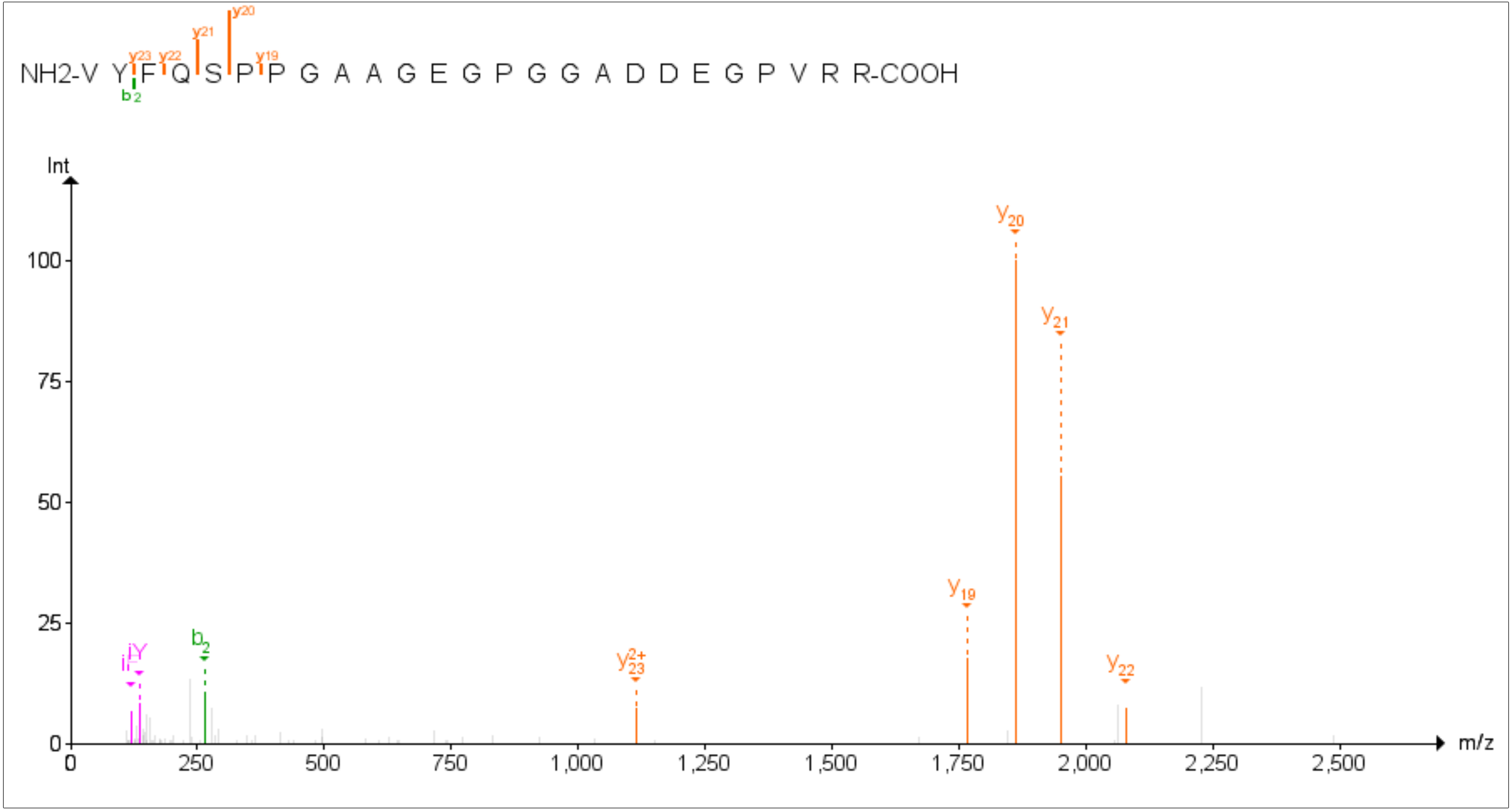

NH2-V Y F Q S P P G A A G E G P G G A D D E G P V R R-COOH
y23
y22
y21
y20
y19
b2
Int
100
75
50
25
0
iF
iY
b2
y23 2+
y19
y20
y21
y22
0
250
500
750
1,000
1,250
1,500
1,750
2,000
2,250
2,500
m/z

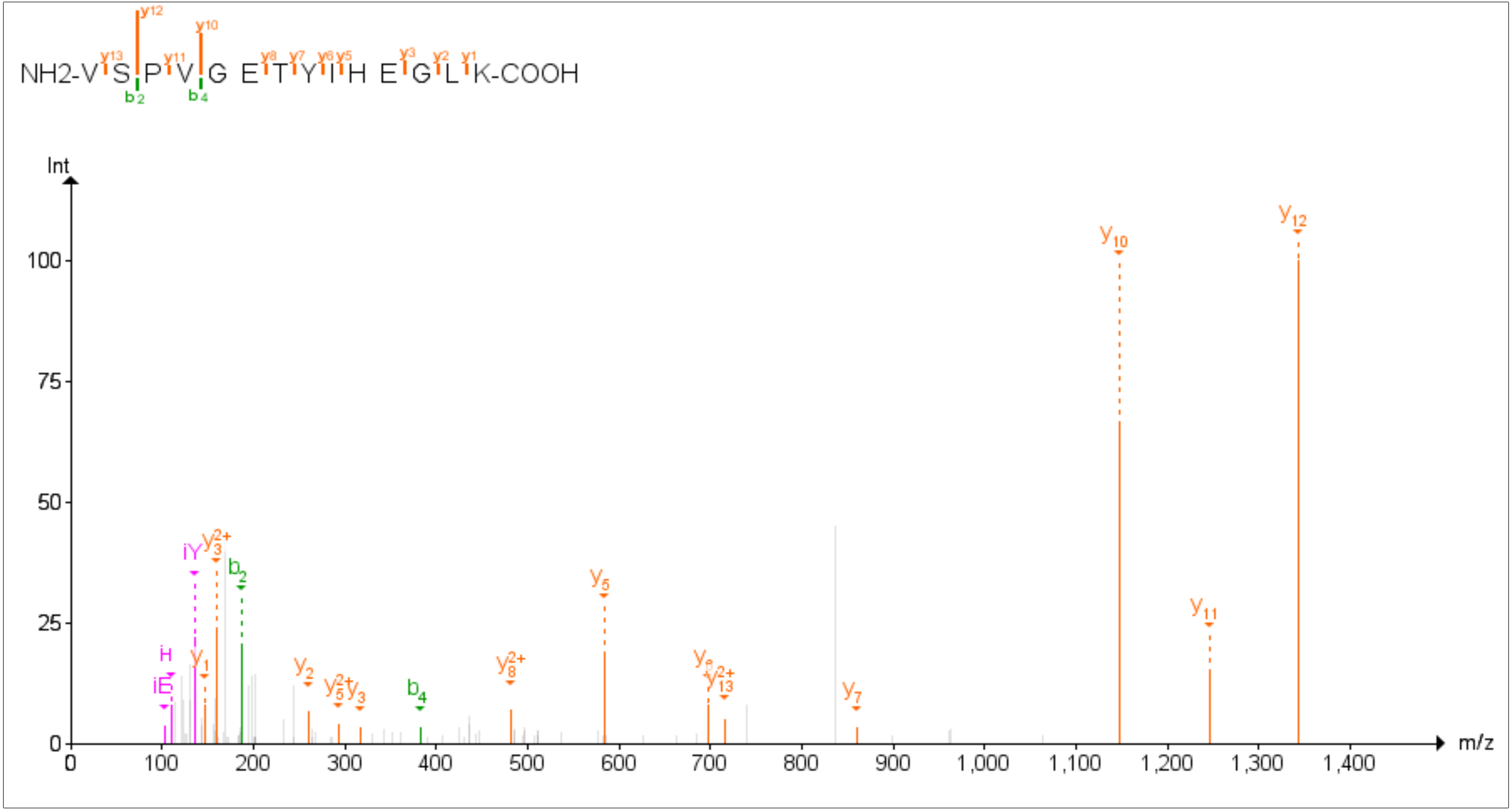
NH2-V S P V G E T Y I H E G L K-COOH
y13 y12 y11 y10 y8 y7 y6 y5 y3 y2 y1
b2 b4
Int
m/z
iY
y3 2+
b2
y5
y10
y12
y11
iH
iE
y1
y2
y5 2+
y3
b4
y8 2+
y6
y13 2+
y7
0
25
50
75
100
0
100
200
300
400
500
600
700
800
900
1,000
1,100
1,200
1,300
1,400

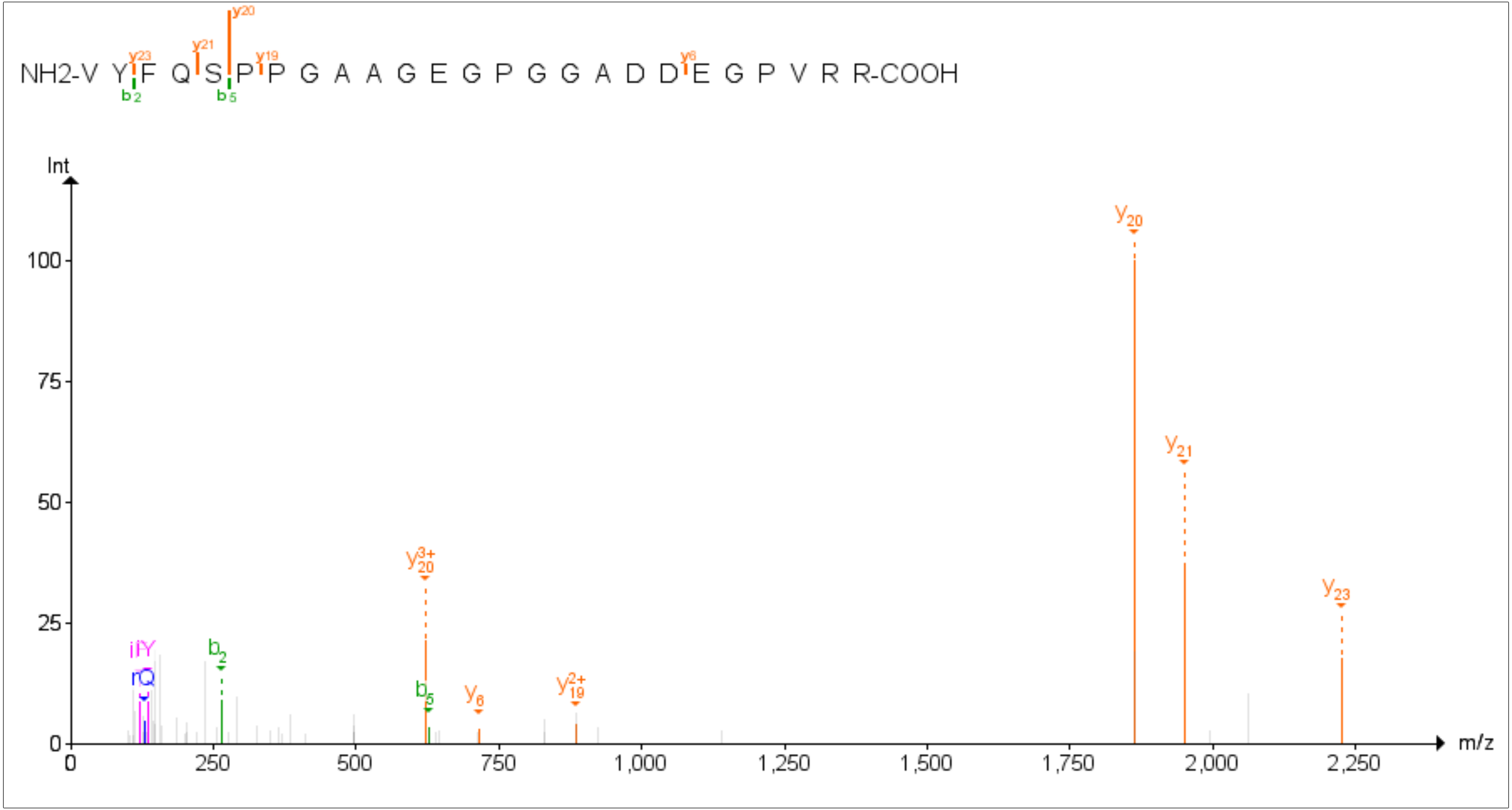

NH2-V Y F Q S P P G A A G E G P G G A D D E G P V R R-COOH
y23
y21
y20
y19
y6
b2
b5
Int
100
75
50
25
0
m/z
0
250
500
750
1,000
1,250
1,500
1,750
2,000
2,250
y20
y21
y23
y20 3+
y19 2+
y6
b2
b5
iY
rQ

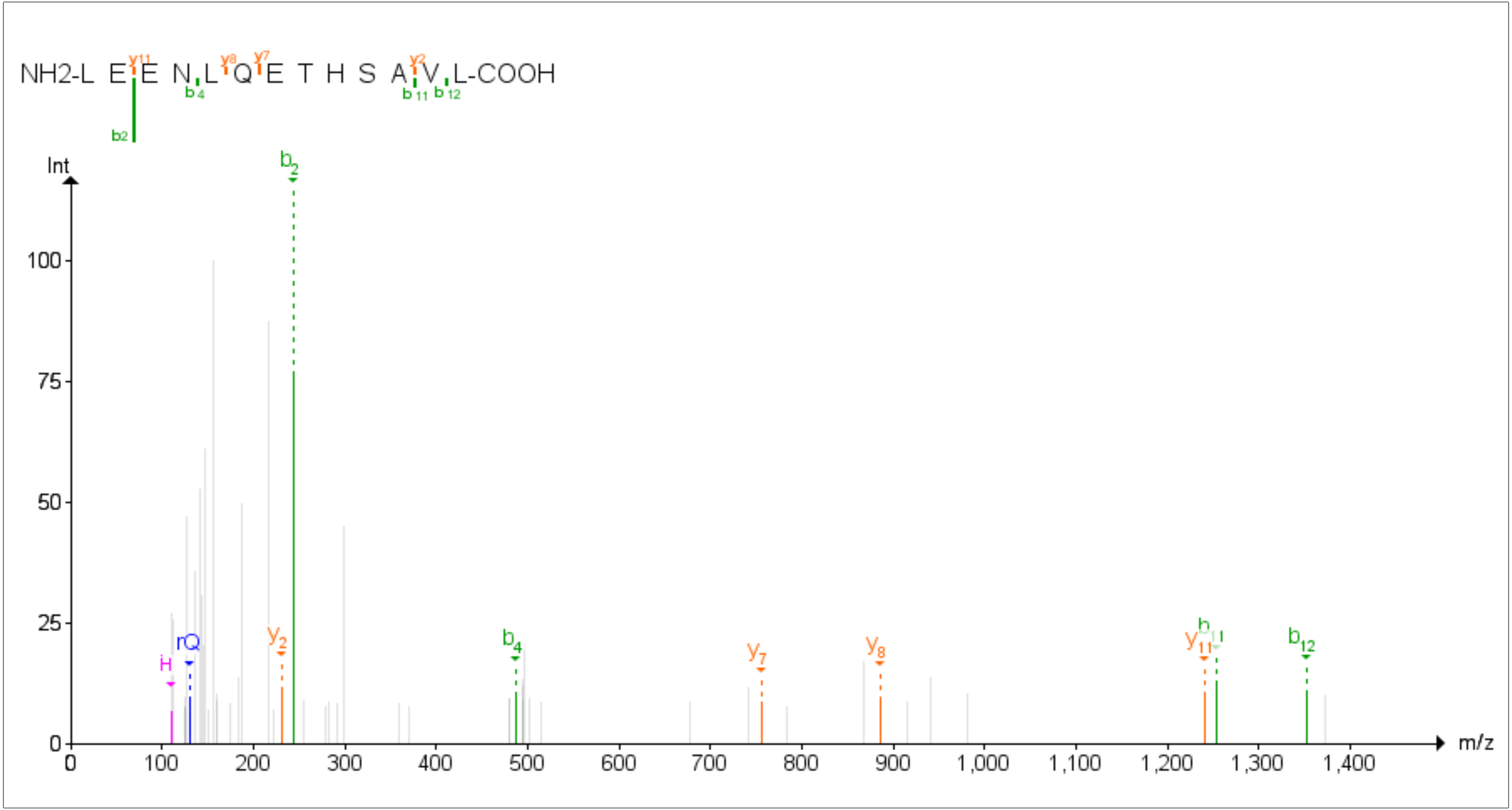

NH2-L E E N L Q E T H S A V L-COOH
y11
y8
y7
y2
b2
b4
b11
b12
Int
m/z
0
25
50
75
100
0
100
200
300
400
500
600
700
800
900
1,000
1,100
1,200
1,300
1,400
iH
rQ
y2
b2
b4
y7
y8
y11
b11
b12

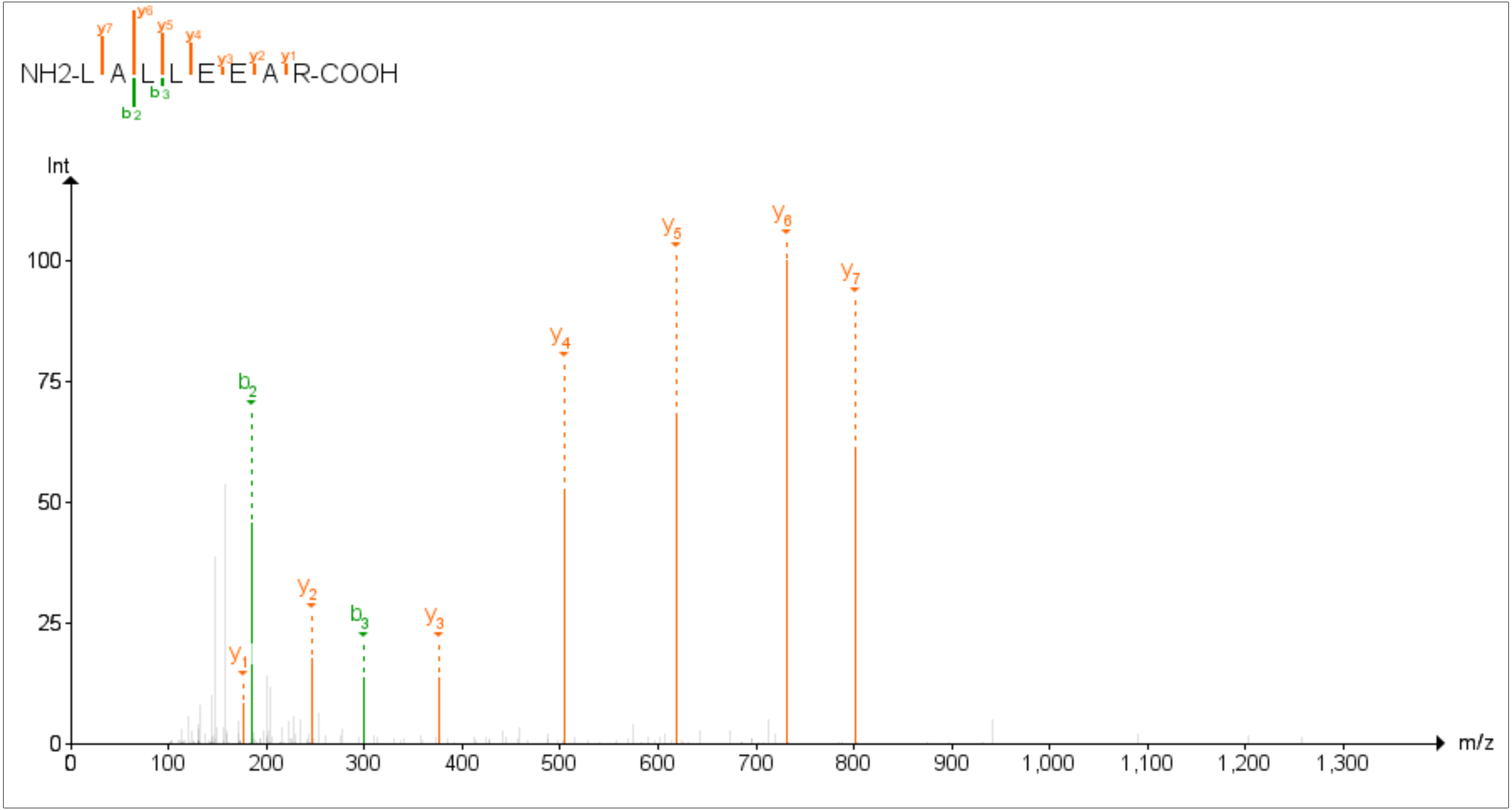

NH2-L A L L E E A R-COOH
y7 y6 y5 y4 y3 y2 y1
b2 b3
Int
100
75
50
25
0
m/z
0 100 200 300 400 500 600 700 800 900 1,000 1,100 1,200 1,300
y1 b2 y2 b3 y3 y4 y5 y6 y7

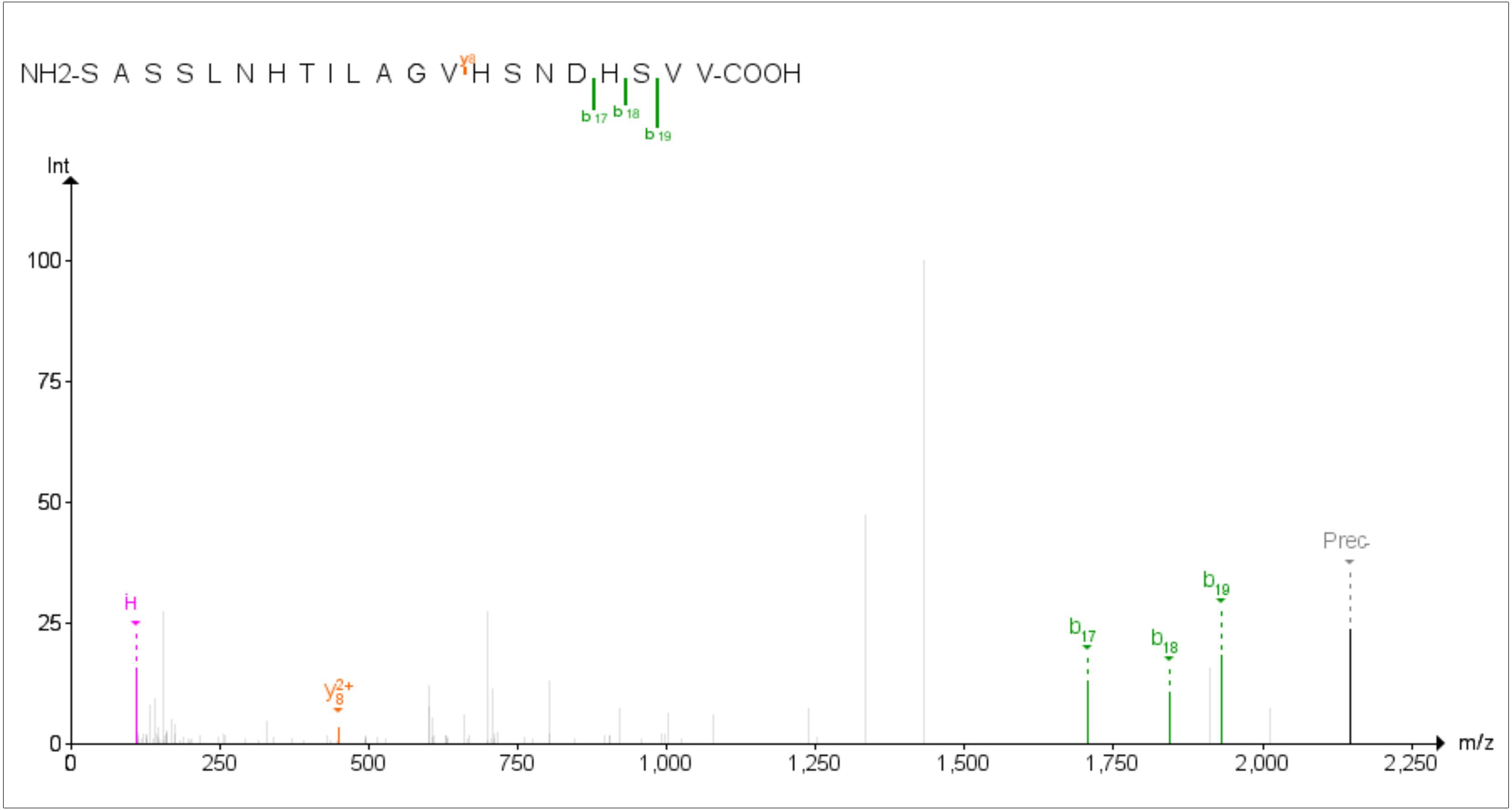

NH2-S A S S L N H T I L A G V H S N D H S V V-COOH
y8
b 17
b 18
b 19
Int
100
75
50
25
0
m/z
0
250
500
750
1,000
1,250
1,500
1,750
2,000
2,250
H
y8 2+
b17
b18
b19
Prec

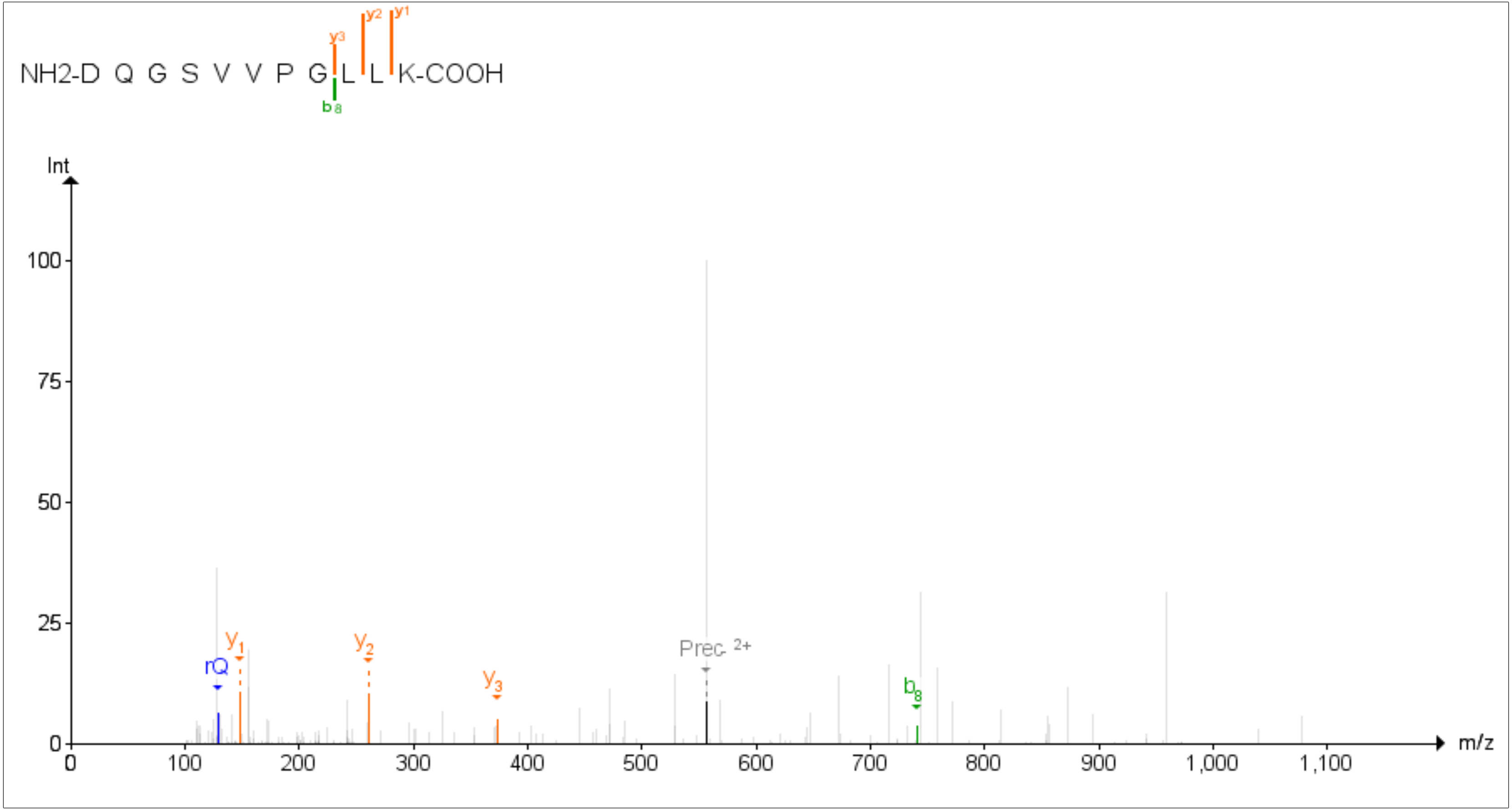

NH2-D Q G S V V P G L L K-COOH
y3 y2 y1
b8
Int
100
75
50
25
0
rQ
y1
y2
y3
Prec 2+
b8
0 100 200 300 400 500 600 700 800 900 1,000 1,100
m/z

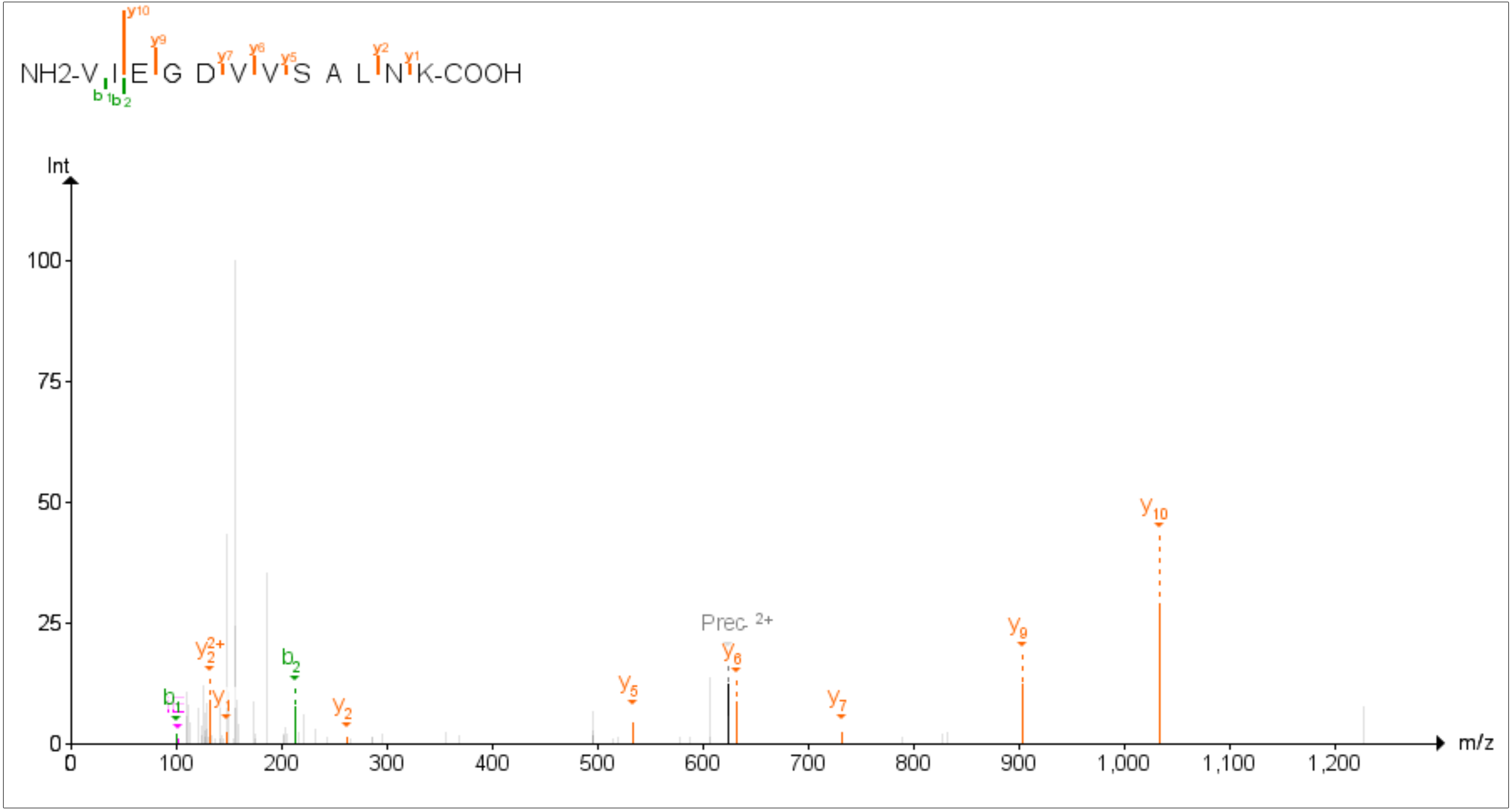

NH2-V I E G D V V S A L N K-COOH
y10
y9
y7
y6
y5
y2
y1
b1
b2
Int
100
75
50
25
0
y10
y9
Prec 2+
y6
y7
y5
y2
y2 2+
y1
b1
b2
0
100
200
300
400
500
600
700
800
900
1,000
1,100
1,200
m/z

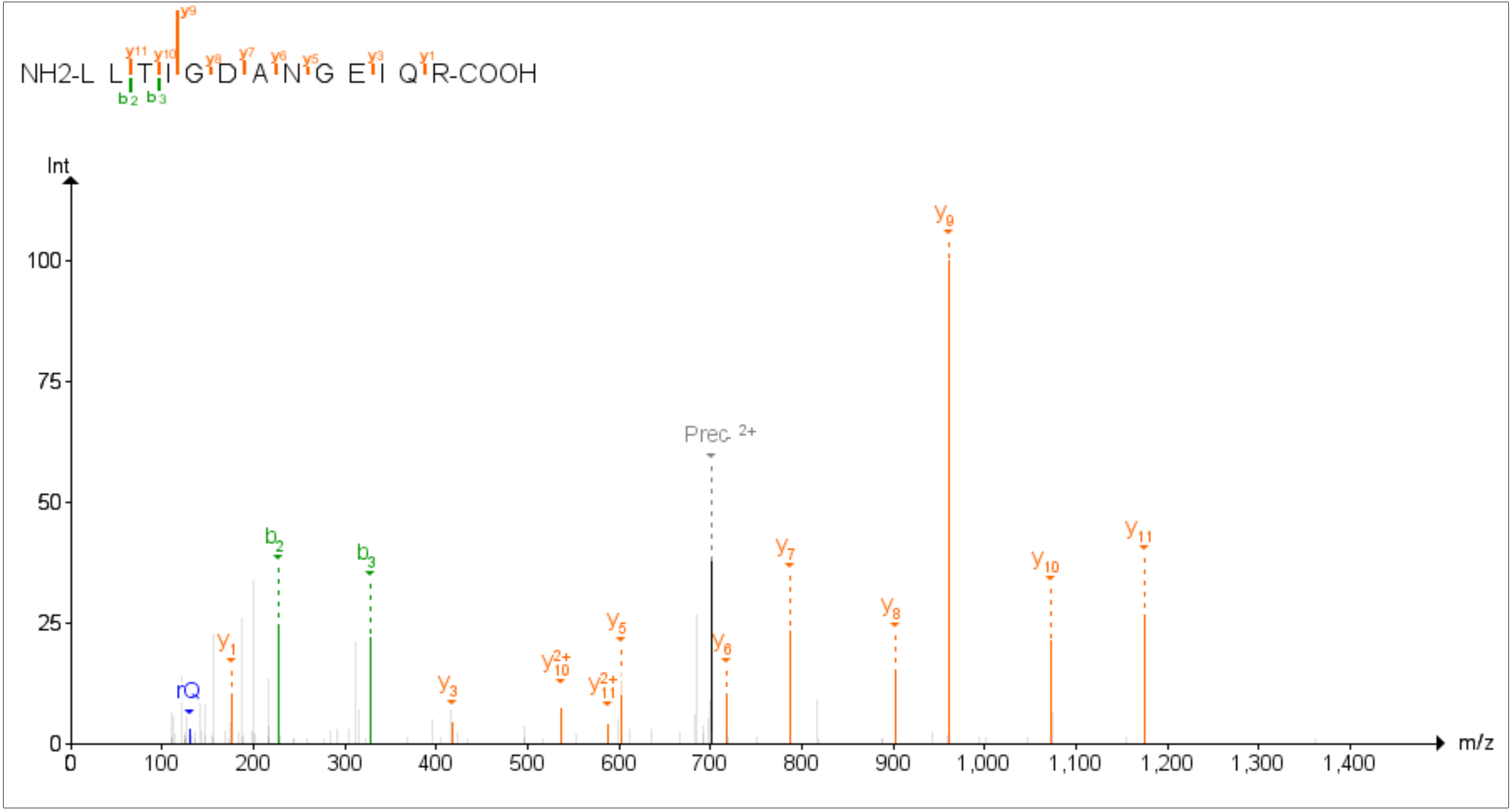

NH2-L L T I G D A N G E I Q R-COOH
y11 y10 y9 y8 y7 y6 y5 y3 y1
b2 b3
Int
m/z
0
25
50
75
100
0 100 200 300 400 500 600 700 800 900 1,000 1,100 1,200 1,300 1,400
rQ
y1
b2
b3
y3
y10 2+
y11 2+
y5
Prec 2+
y6
y7
y8
y9
y10
y11

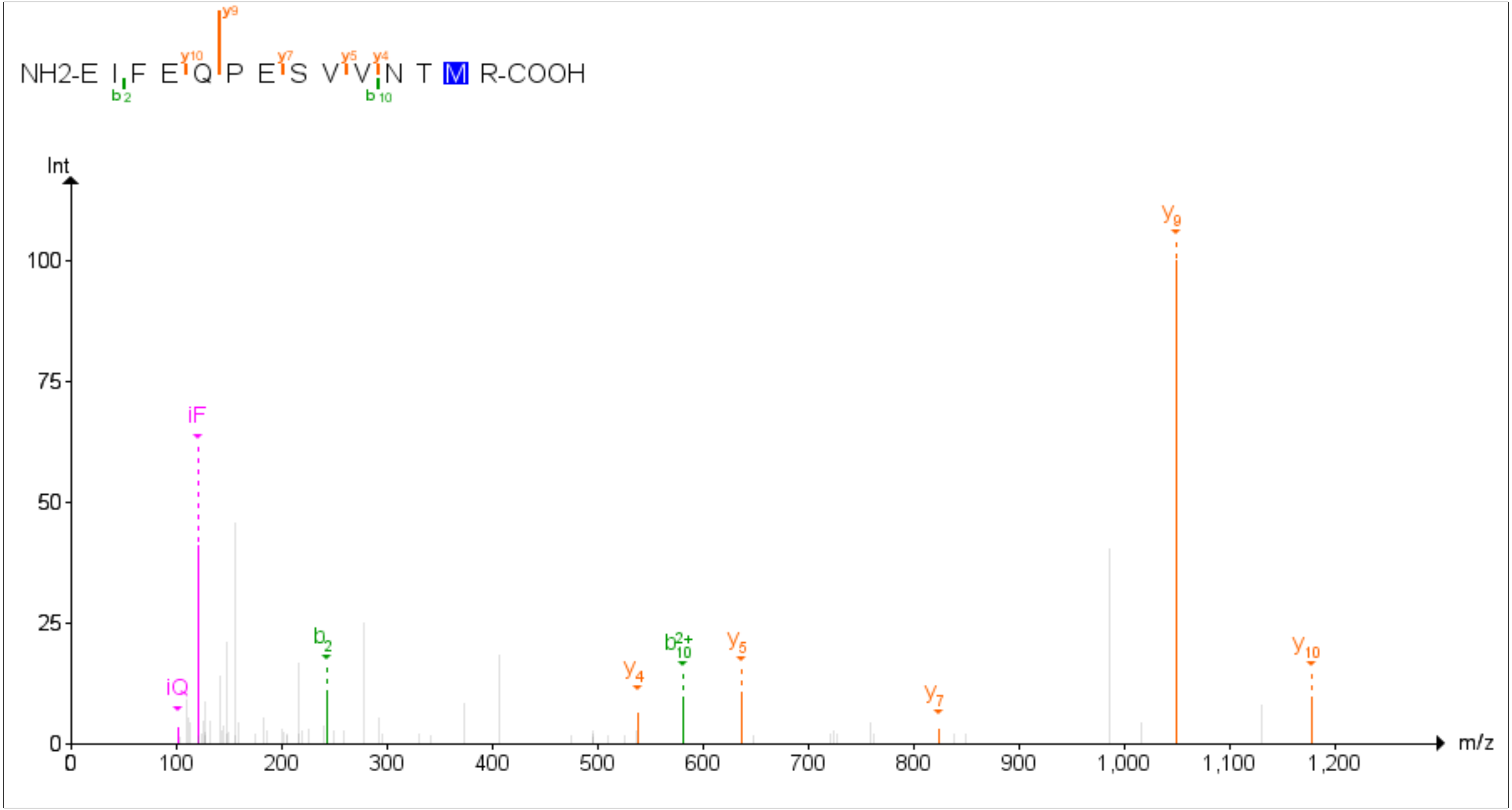
NH2-E I F E Q P E S V V N T M R-COOH
y9
y10
y7
y5
y4
b2
b10
Int
100
75
50
25
0
iF
iQ
b2
y4
b10 2+
y5
y7
y9
y10
0
100
200
300
400
500
600
700
800
900
1,000
1,100
1,200
m/z

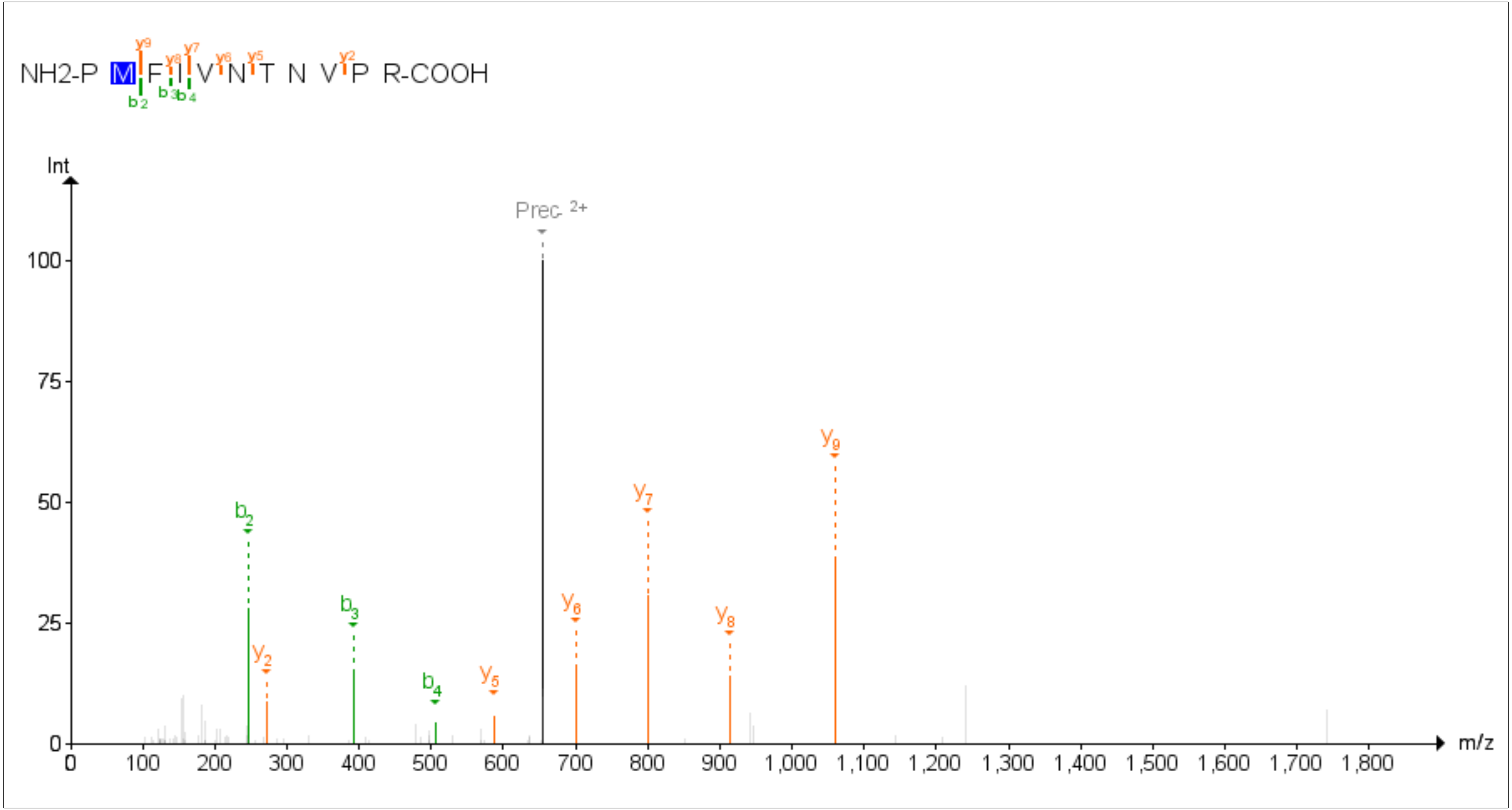
NH2-P M F I I V N T N V P R-COOH
y9 y8 y7 y6 y5 y2
b2 b3 b4
Int
Prec 2+
100
75
50
25
0
b2
y2
b3
b4
y5
y6
y7
y8
y9
0 100 200 300 400 500 600 700 800 900 1,000 1,100 1,200 1,300 1,400 1,500 1,600 1,700 1,800
m/z

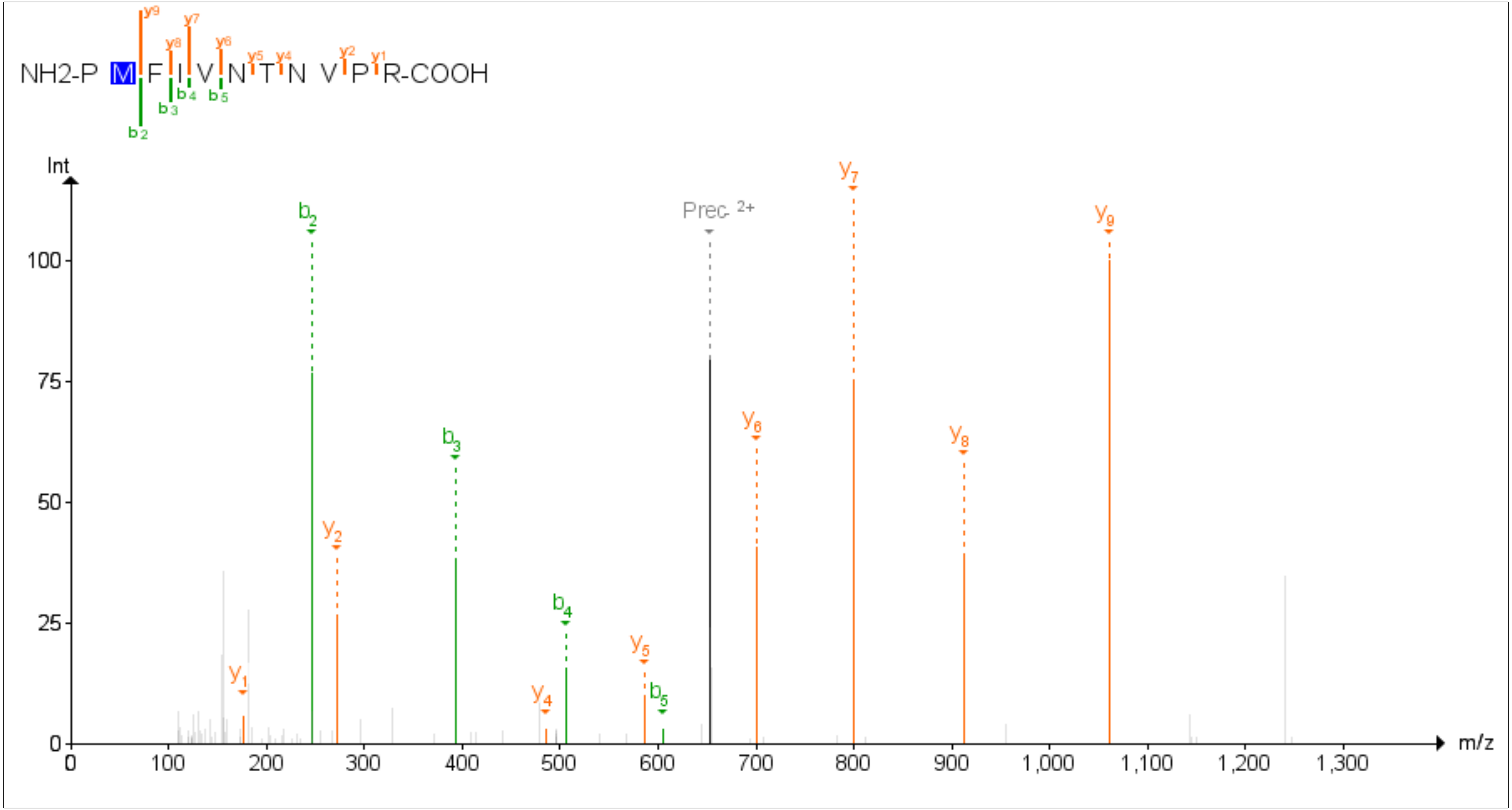

NH2-P M F I V N T N V P R-COOH
y9 y8 y7 y6 y5 y4 y2 y1
b2 b3 b4 b5
Int
m/z
0 25 50 75 100
0 100 200 300 400 500 600 700 800 900 1,000 1,100 1,200 1,300
b2 b3 b4 b5
y1 y2 y4 y5 y6 y7 y8 y9
Prec 2+

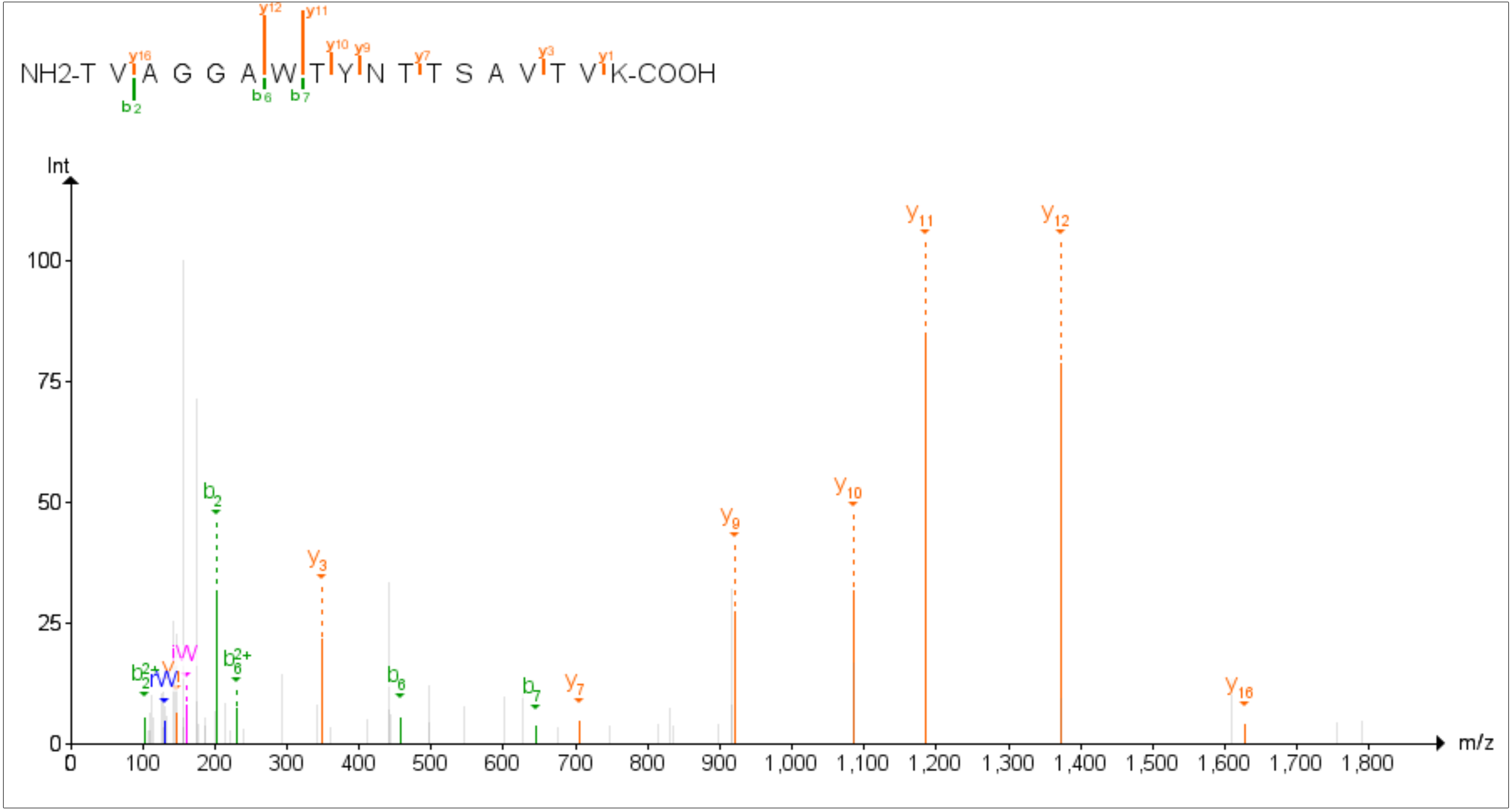

NH2-T V A G G A W T Y N T T S A V T V K-COOH
y16 y12 y11 y10 y9 y7 y3 y1
b2 b6 b7
Int
100
75
50
25
0
m/z
0 100 200 300 400 500 600 700 800 900 1,000 1,100 1,200 1,300 1,400 1,500 1,600 1,700 1,800
y11
y12
y10
y9
y3
b2
b6
b7
y7
y16
iW
rW

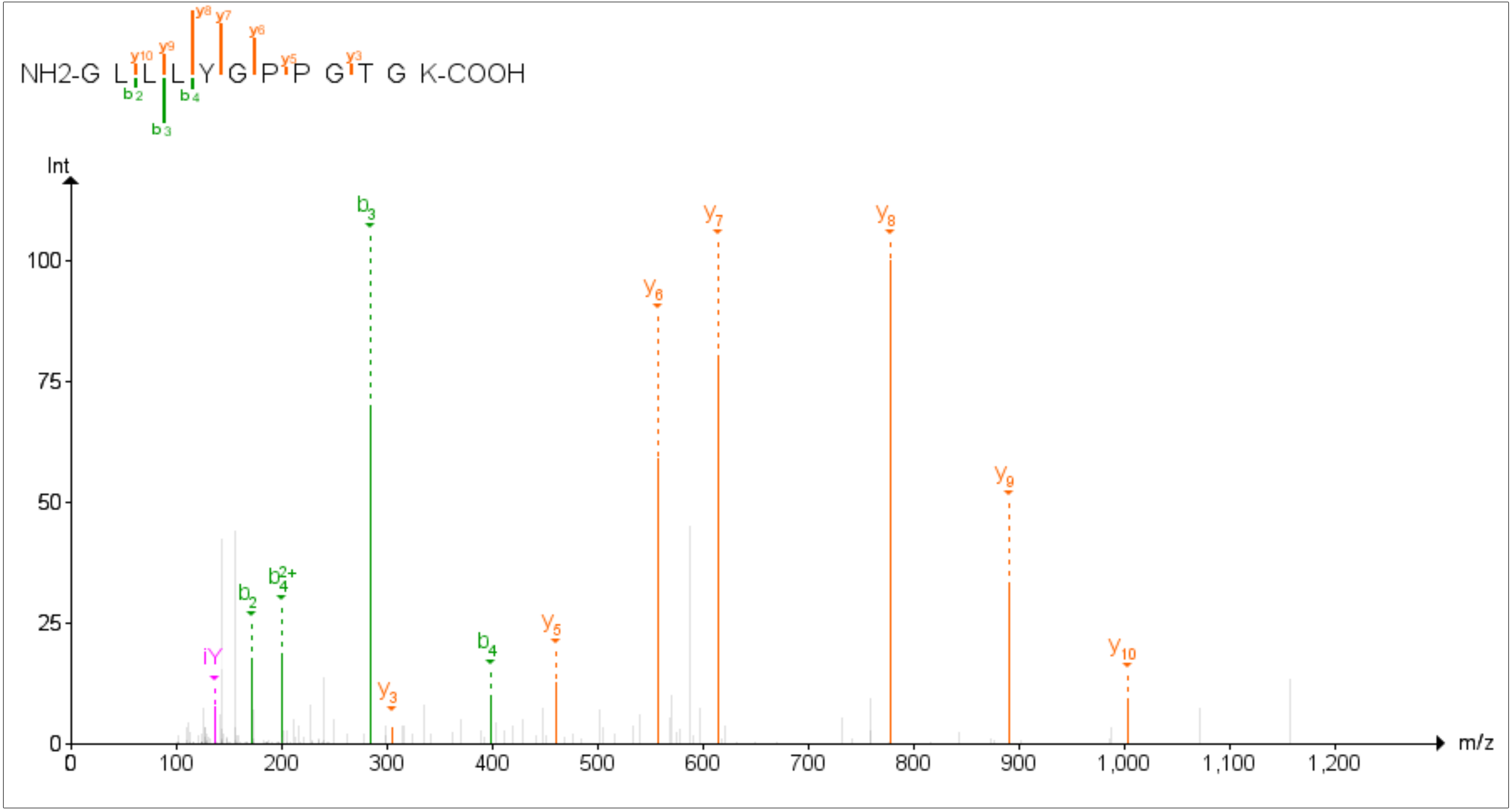

NH2-G L L L Y G P P G T G K-COOH
y10 y9 y8 y7 y6 y5 y3
b2 b3 b4
Int
100
75
50
25
0
iY
b2
b4 2+
b3
y3
b4
y5
y6
y7
y8
y9
y10
0 100 200 300 400 500 600 700 800 900 1,000 1,100 1,200
m/z

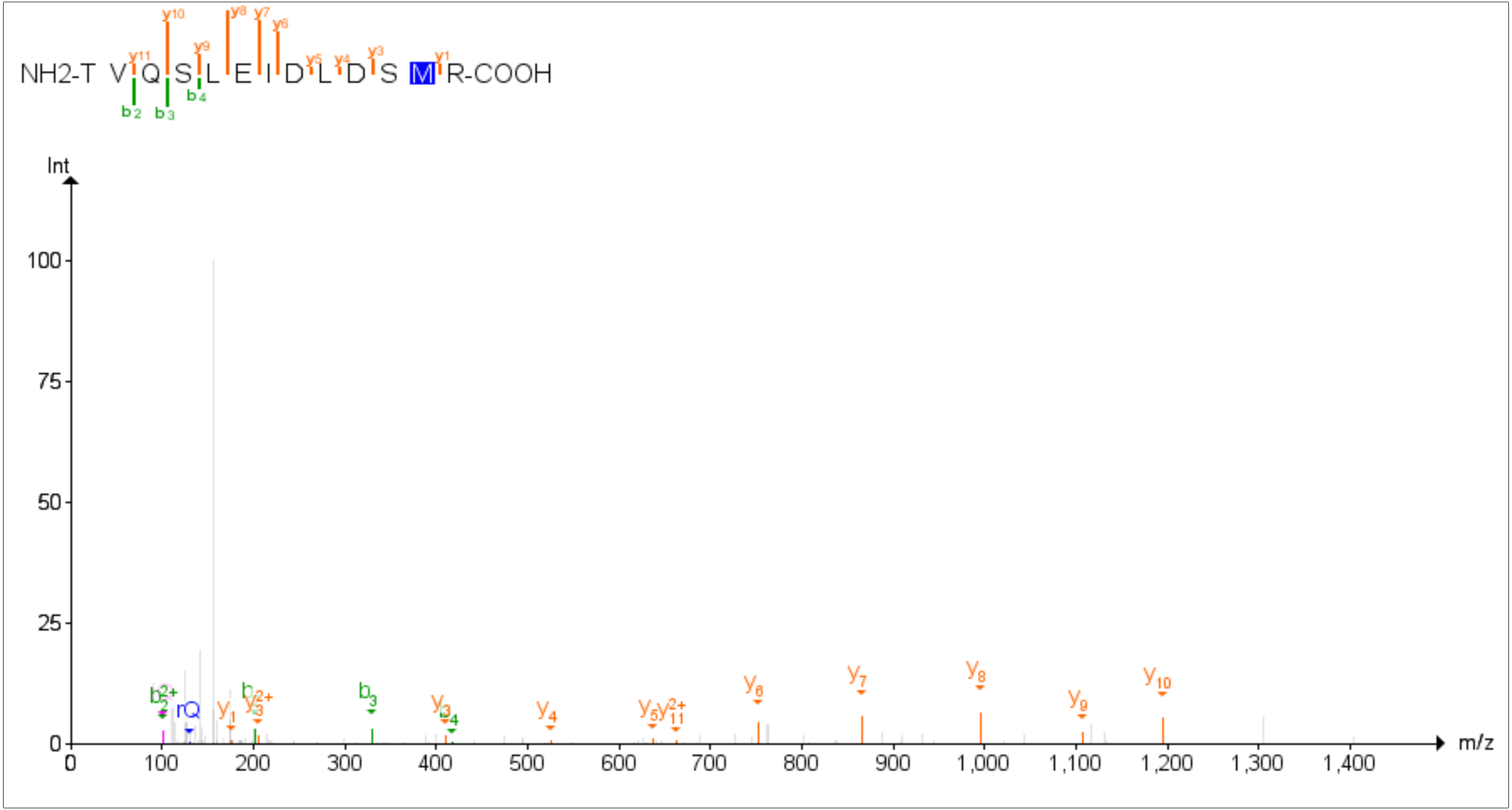

NH2-T V Q S L E I D L D S M R-COOH
y11 y10 y9 y8 y7 y6 y5 y4 y3 y1
b2 b3 b4
Int
100
75
50
25
0
m/z
0 100 200 300 400 500 600 700 800 900 1,000 1,100 1,200 1,300 1,400
rQ

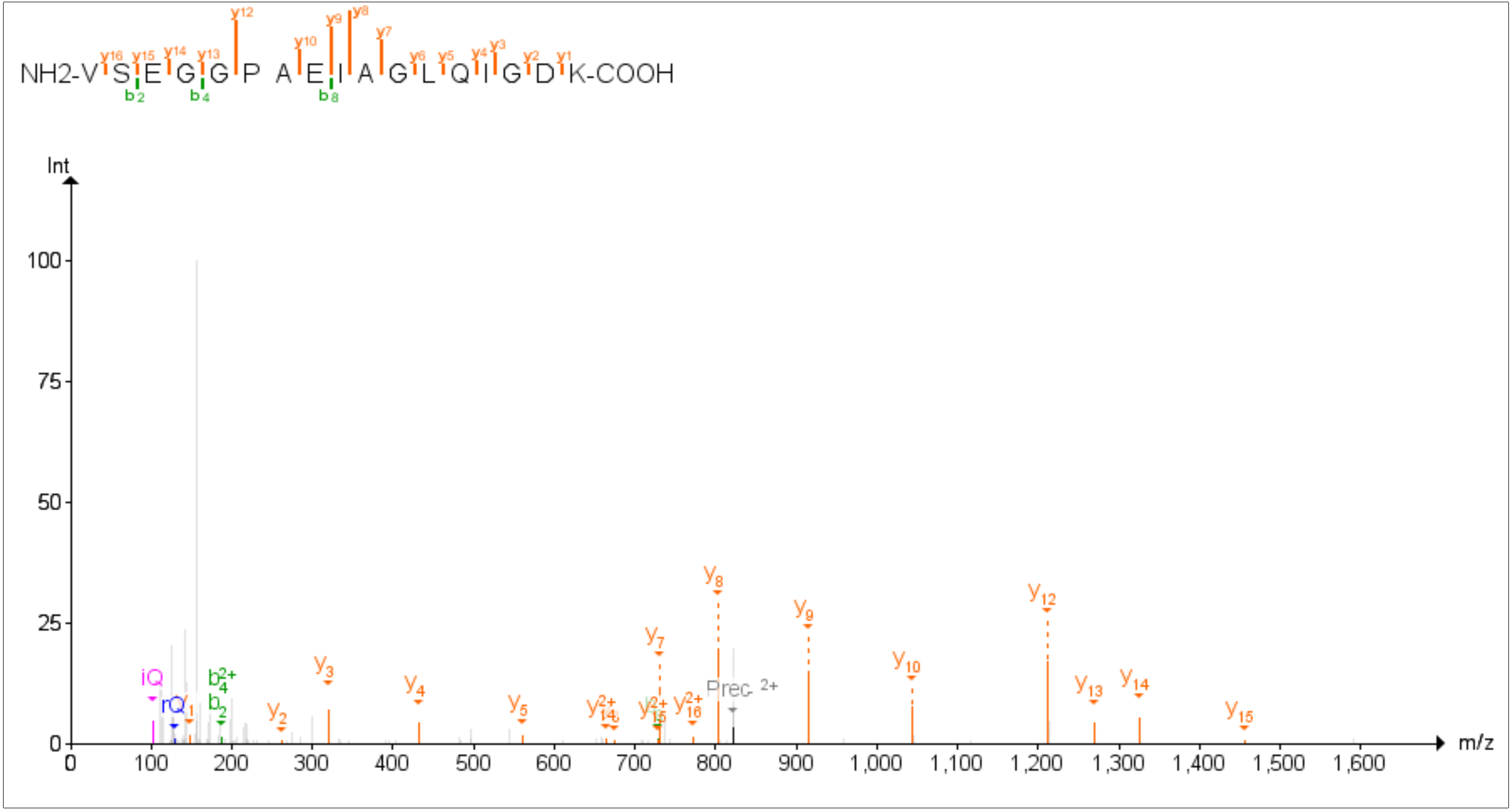

NH2-V S E G G P A E I A G L Q I G D K-COOH
Int
100
75
50
25
0
m/z
0
100
200
300
400
500
600
700
800
900
1,000
1,100
1,200
1,300
1,400
1,500
1,600
Prec. 2+

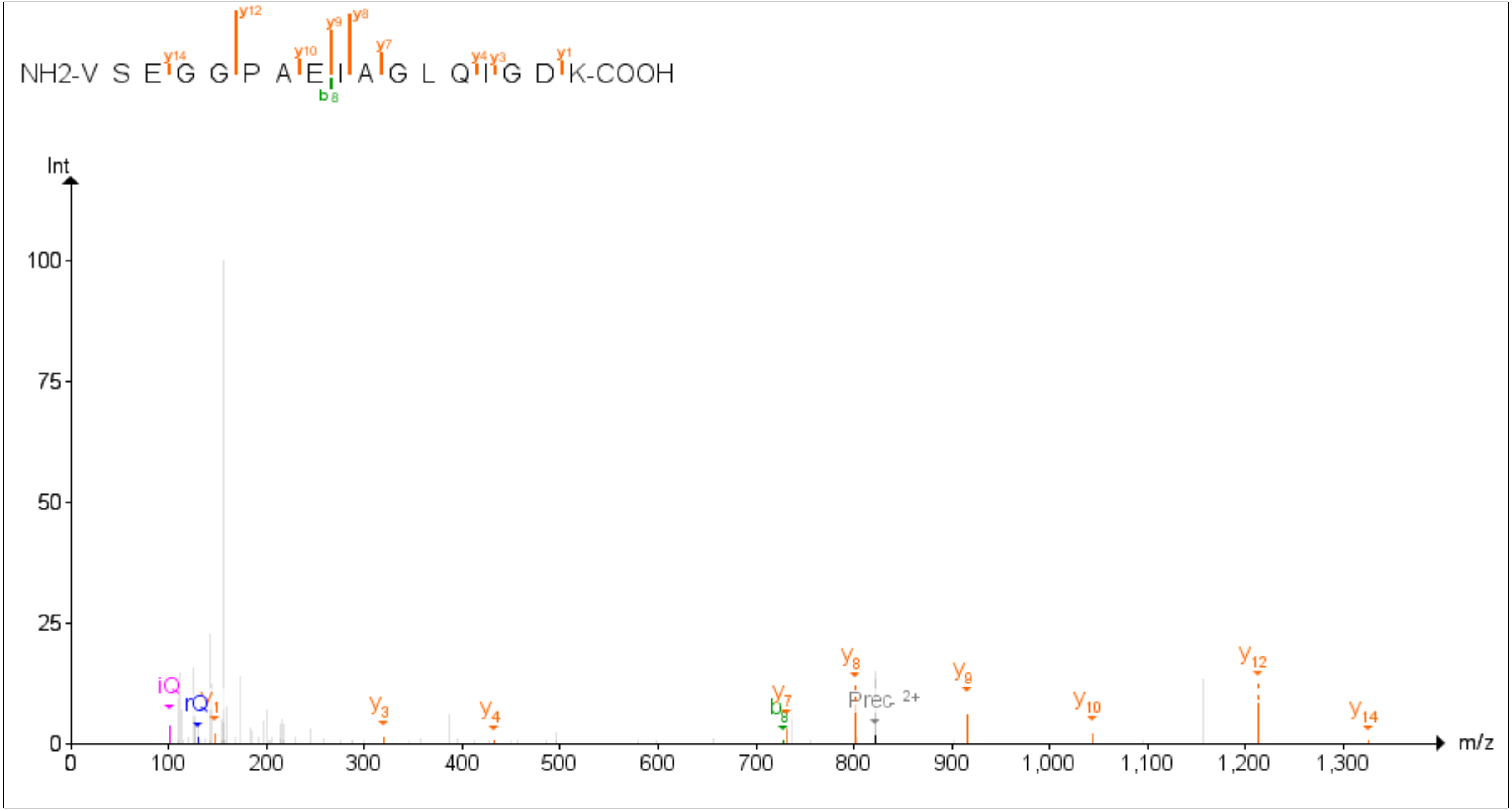

NH2-V S E G G P A E I A G L Q I G D K-COOH
y14 y12 y10 y9 y8 y7 y4 y3 y1
b8
Int
100
75
50
25
0
iQ
rQ
y1
y3
y4
b8
y7
y8
Prec 2+
y9
y10
y12
y14
0 100 200 300 400 500 600 700 800 900 1,000 1,100 1,200 1,300
m/z

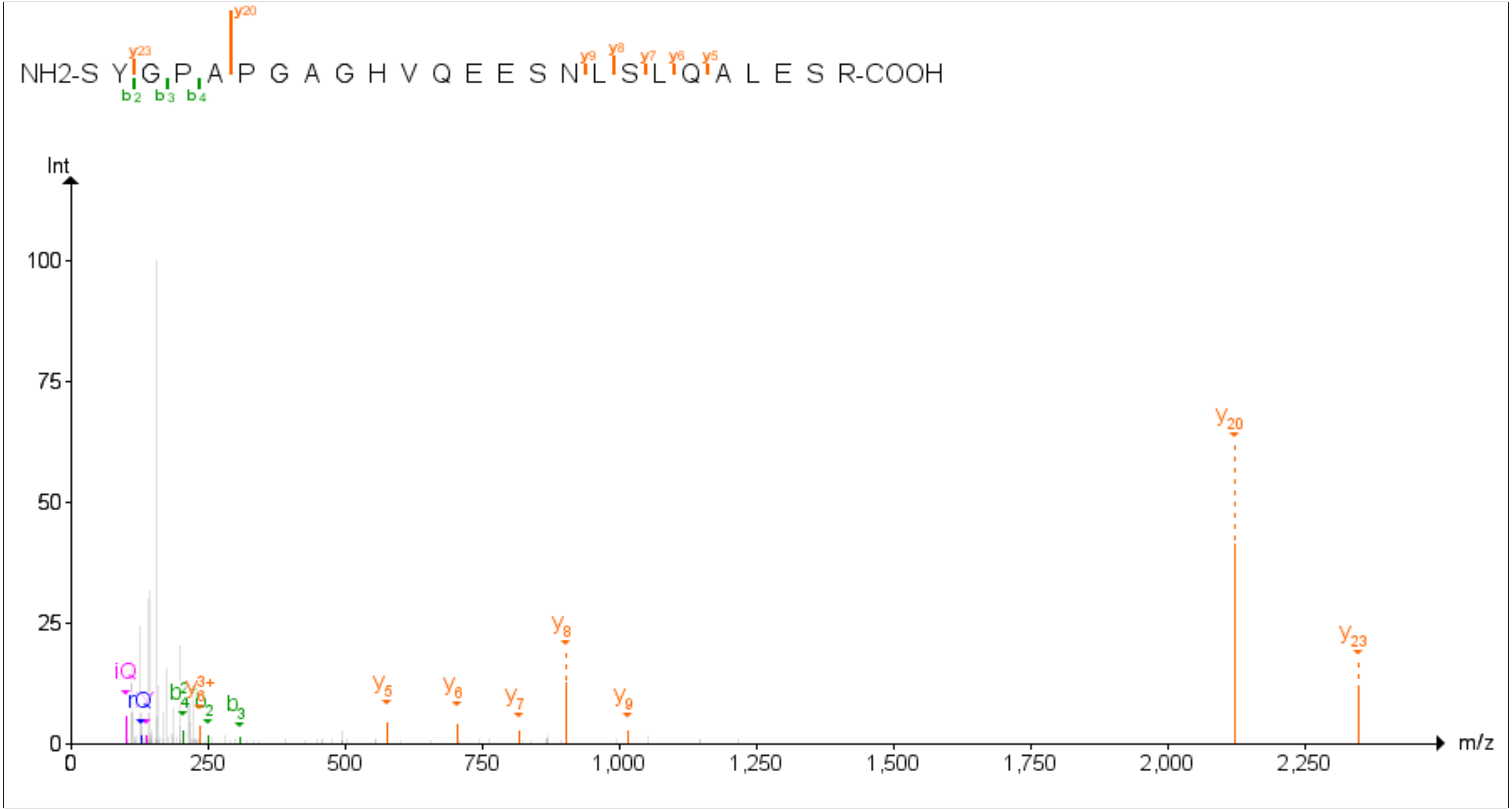

NH2-S Y G P A P G A G H V Q E E S N L S L Q A L E S R-COOH
Int
100
75
50
25
0
0
250
500
750
1,000
1,250
1,500
1,750
2,000
2,250
m/z
y20
y23
y8
y5
y6
y7
y9
iQ
rQ
b3

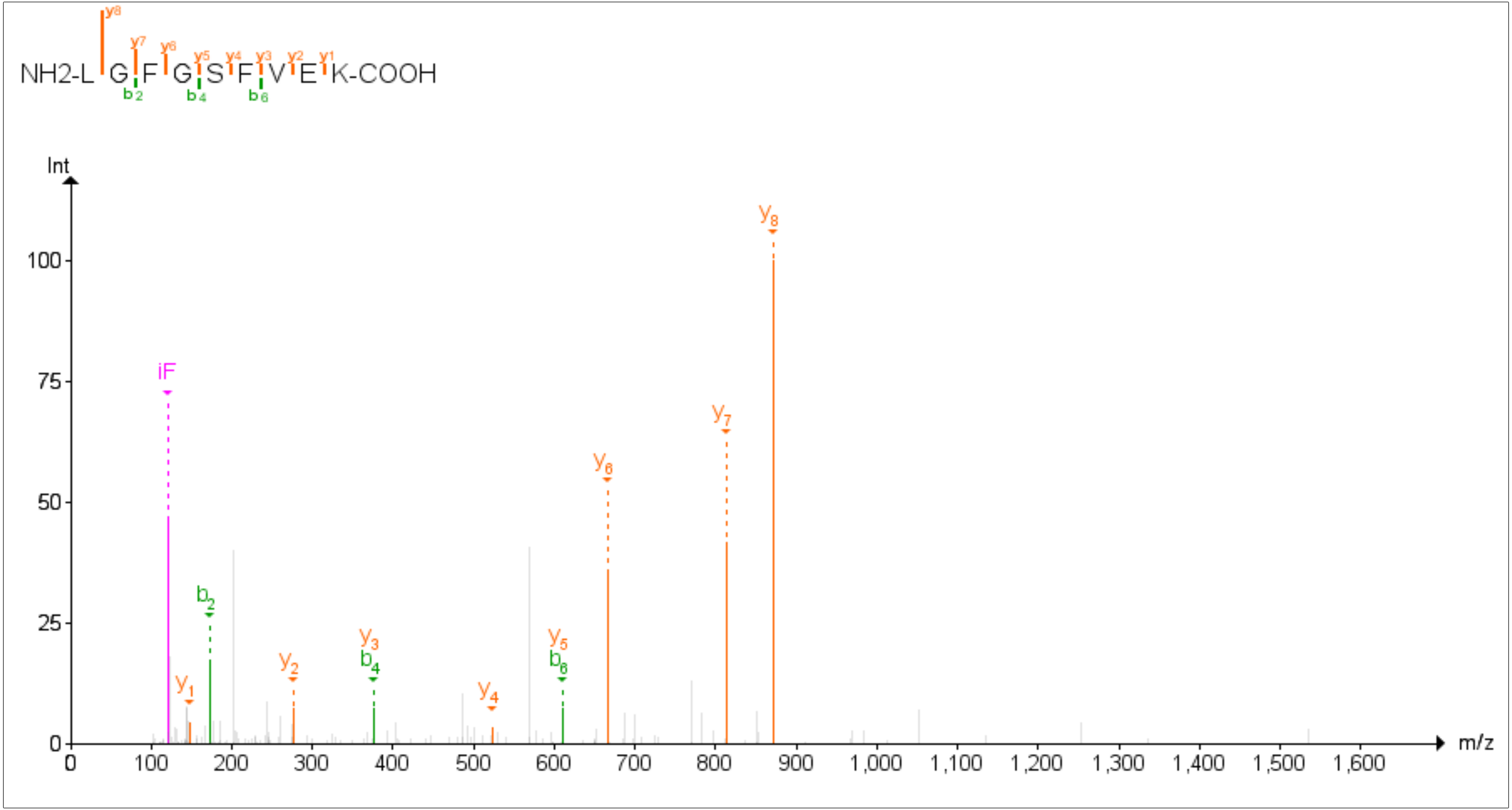

NH2-L G F G S F V E K-COOH
y8
y7
y6
y5
y4
y3
y2
y1
b2
b4
b6
Int
m/z
iF
y1
b2
y2
y3
b4
y4
y5
b6
y6
y7
y8
0
25
50
75
100
0
100
200
300
400
500
600
700
800
900
1,000
1,100
1,200
1,300
1,400
1,500
1,600

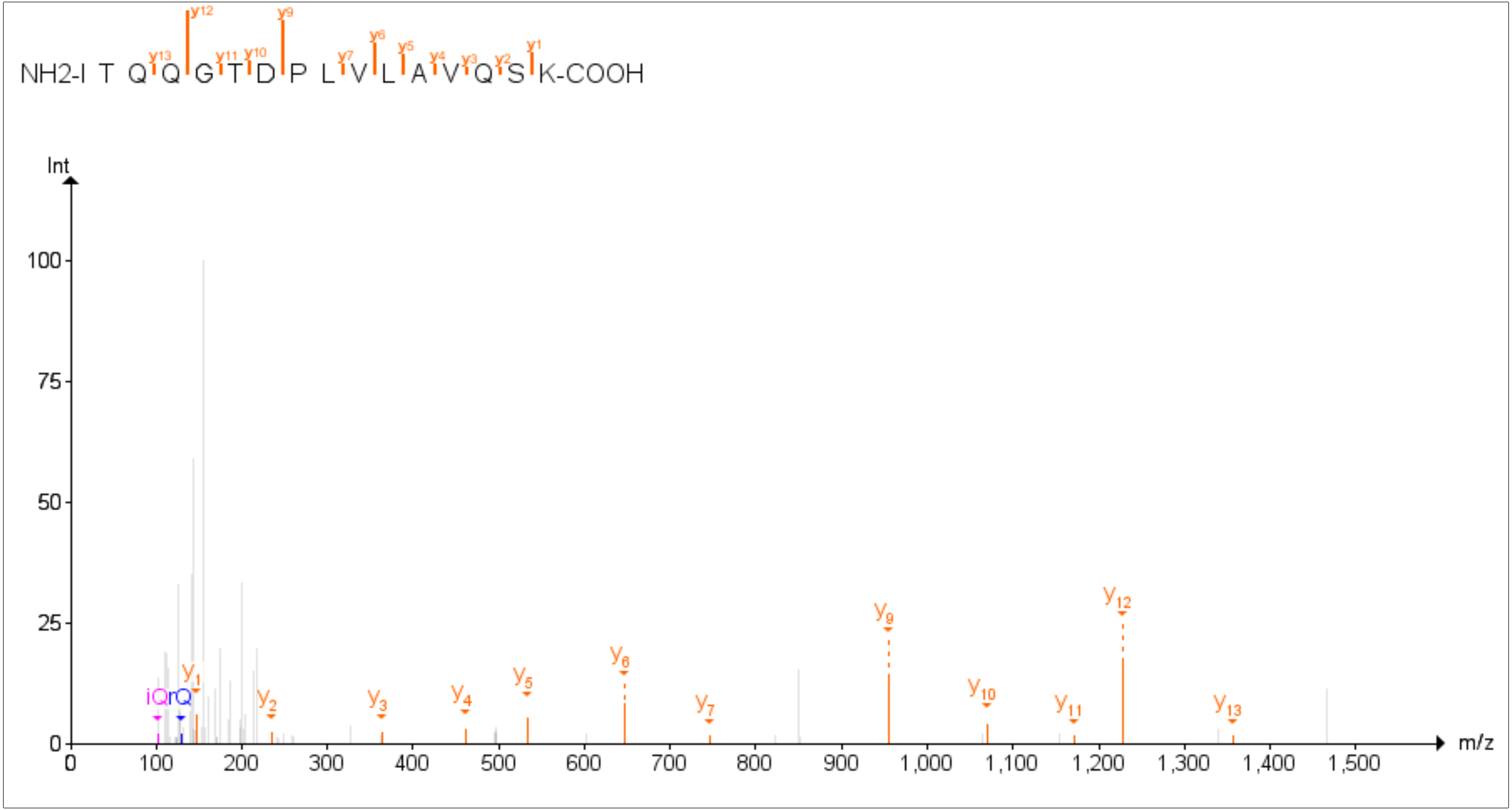
NH2-I T Q Q G T D P L V L A V Q S K-COOH
y13 y12 y11 y10 y9 y7 y6 y5 y4 y3 y2 y1
Int
100
75
50
25
0
iQ rQ
y1 y2 y3 y4 y5 y6 y7 y9 y10 y11 y12 y13
0 100 200 300 400 500 600 700 800 900 1,000 1,100 1,200 1,300 1,400 1,500
m/z

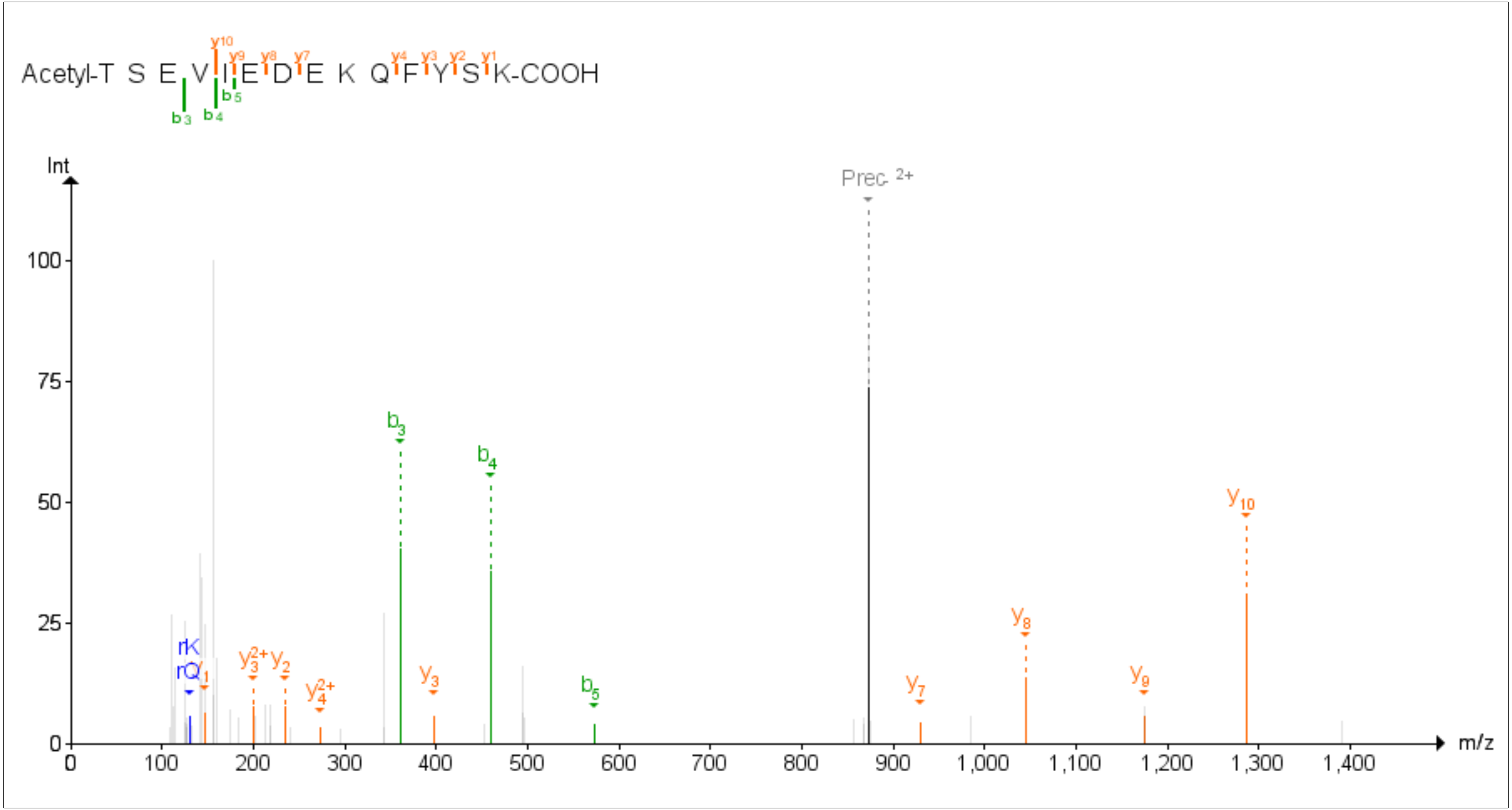

Acetyl-T S E V I E D E K Q F Y S K-COOH
y10 y9 y8 y7 y4 y3 y2 y1
b3 b4 b5
Int
Prec 2+
b3
b4
y10
y8
y9
y7
b5
y3
rK
rQ
y1
y3 2+
y2
y4 2+
100
75
50
25
0
0 100 200 300 400 500 600 700 800 900 1,000 1,100 1,200 1,300 1,400
m/z

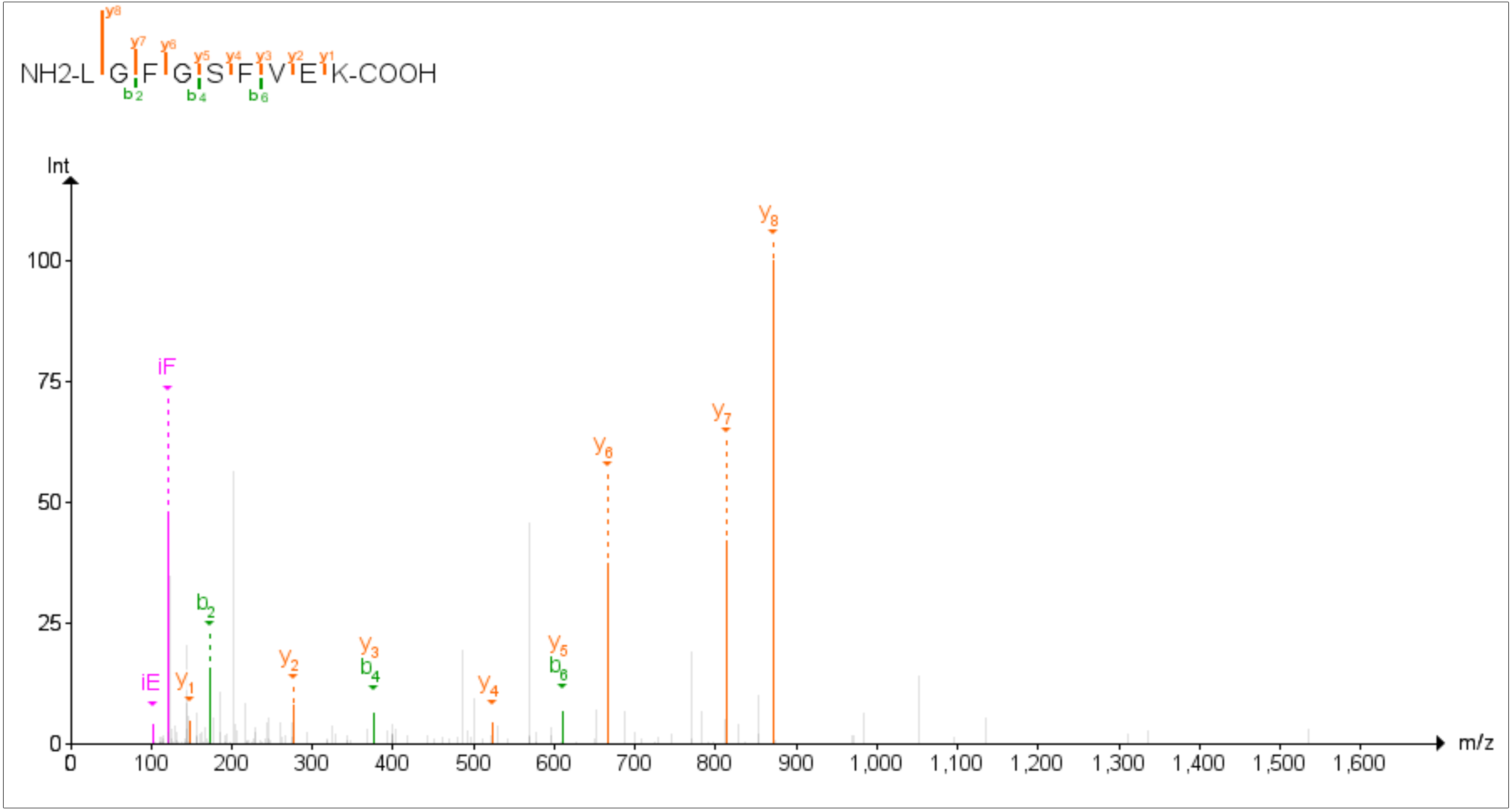
NH2-L G F G S F V E K-COOH
y8 y7 y6 y5 y4 y3 y2 y1
b2 b4 b6
Int
m/z
iF
iE
y1
b2
y2
y3
b4
y4
y5
b6
y6
y7
y8
0
25
50
75
100
0 100 200 300 400 500 600 700 800 900 1,000 1,100 1,200 1,300 1,400 1,500 1,600

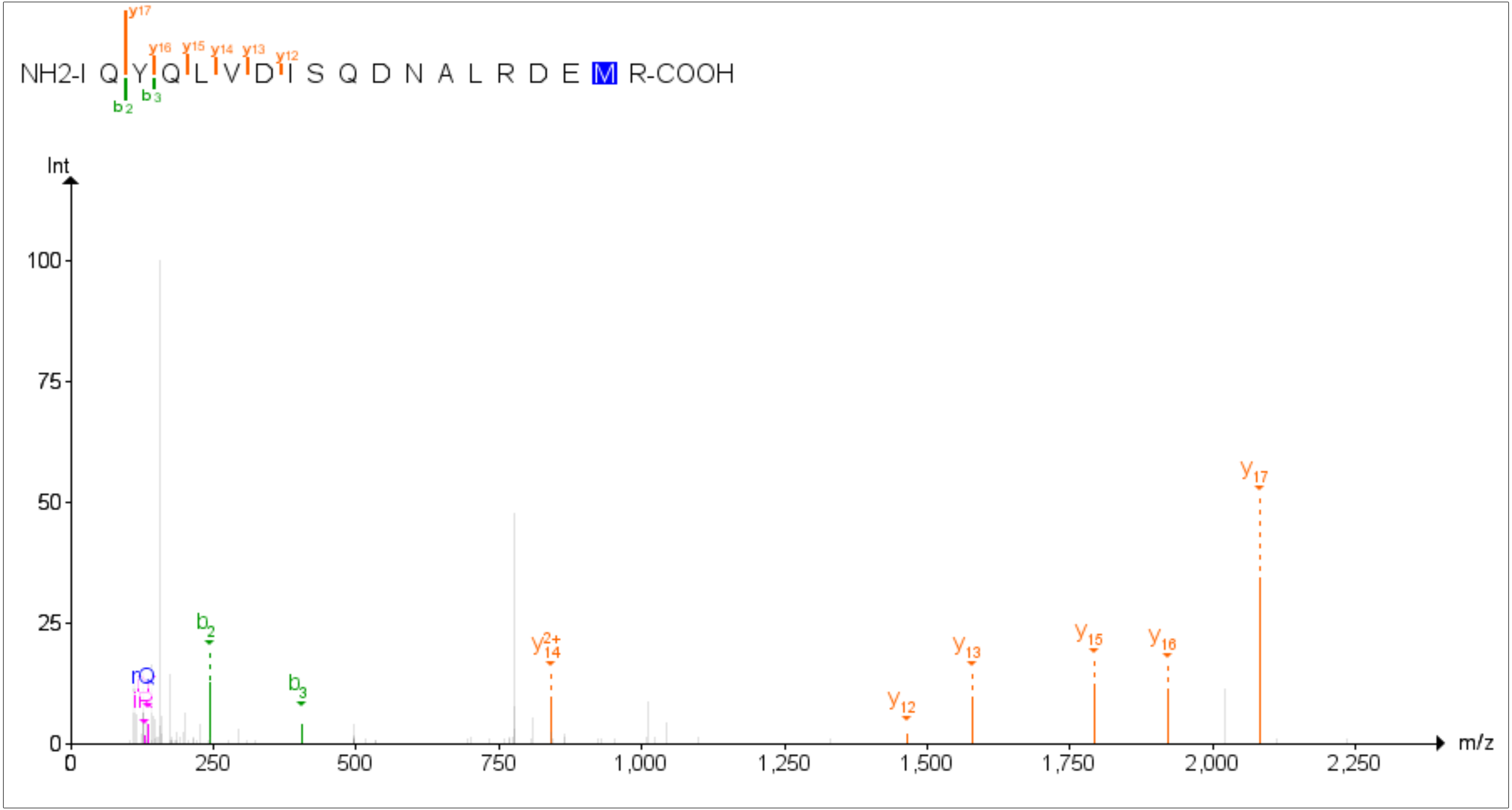

NH2-I Q Y Q L V D I S Q D N A L R D E M R-COOH
y17
y16
y15
y14
y13
y12
b2
b3
Int
100
75
50
25
0
m/z
0
250
500
750
1,000
1,250
1,500
1,750
2,000
2,250
nQ
b2
b3
y14 2+
y12
y13
y15
y16
y17

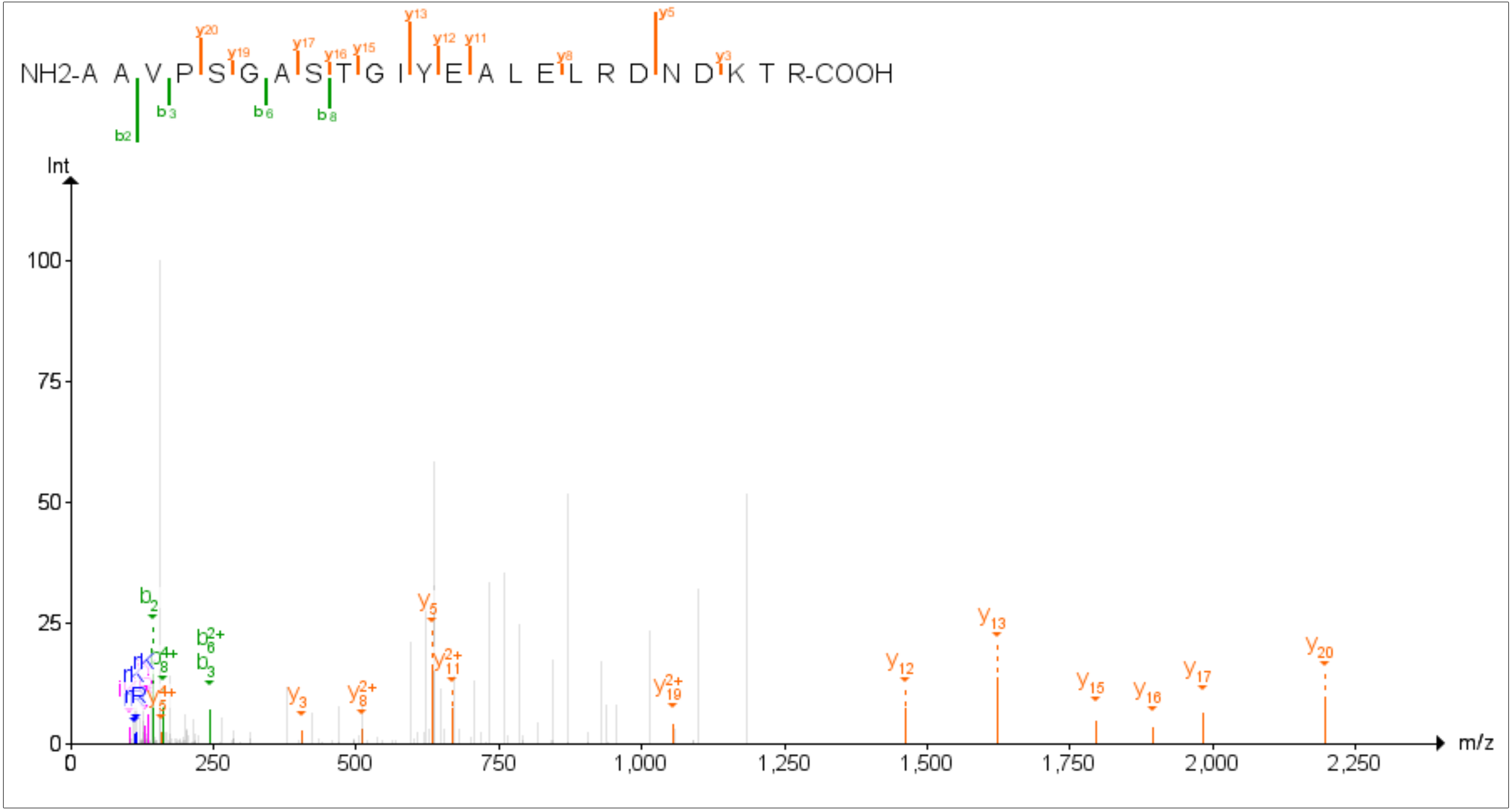

NH2-A A V P S G A S T G I Y E A L E L R D N D K T R-COOH
y20
y19
y17
y16
y15
y13
y12
y11
y8
y5
y3
b2
b3
b6
b8
Int
100
75
50
25
0
m/z
0
250
500
750
1,000
1,250
1,500
1,750
2,000
2,250

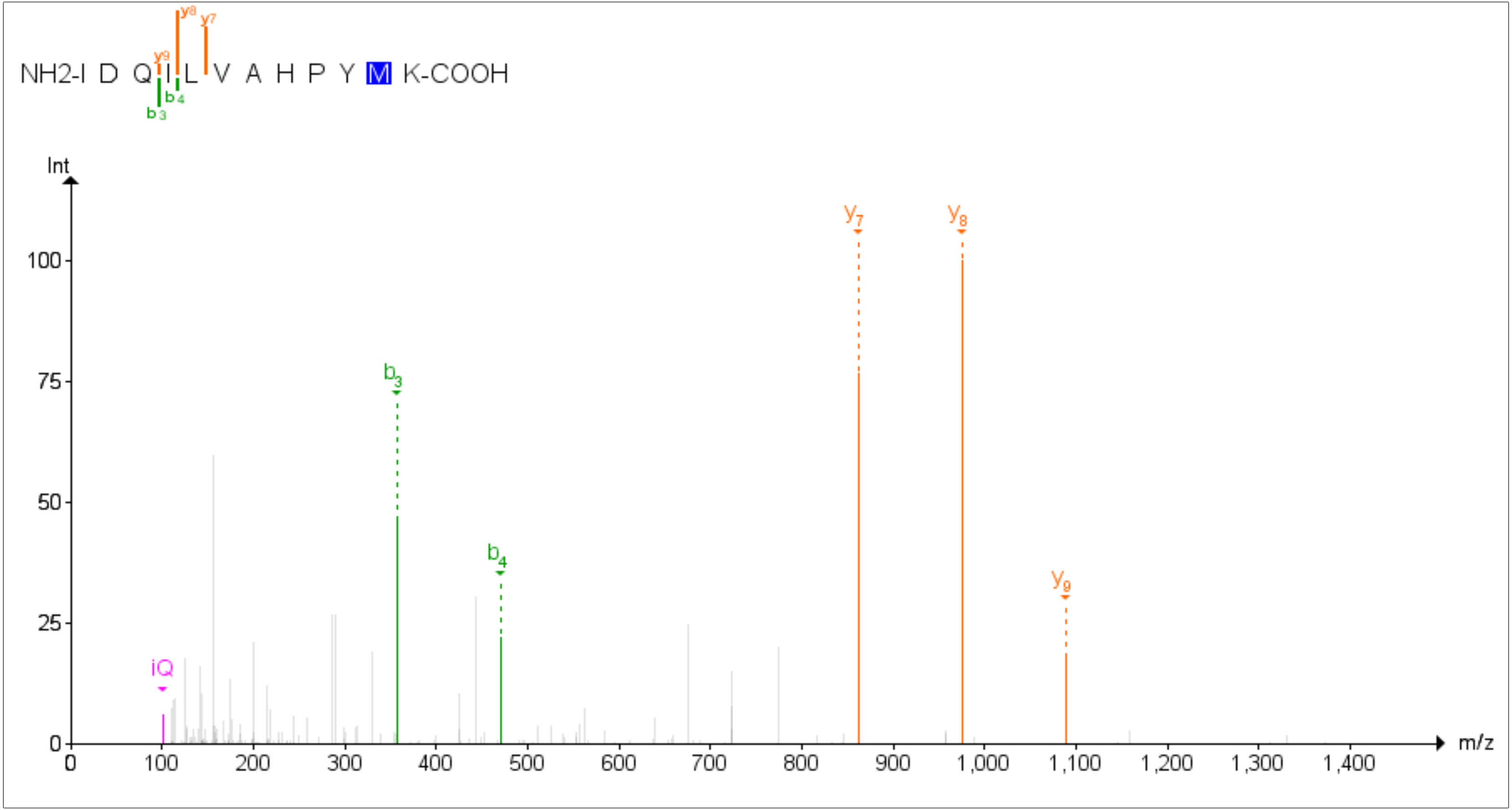

NH2-I D Q I L V A H P Y M K-COOH
y8
y7
y9
b3
b4
Int
100
75
50
25
0
iQ
b3
b4
y7
y8
y9
0
100
200
300
400
500
600
700
800
900
1,000
1,100
1,200
1,300
1,400
m/z

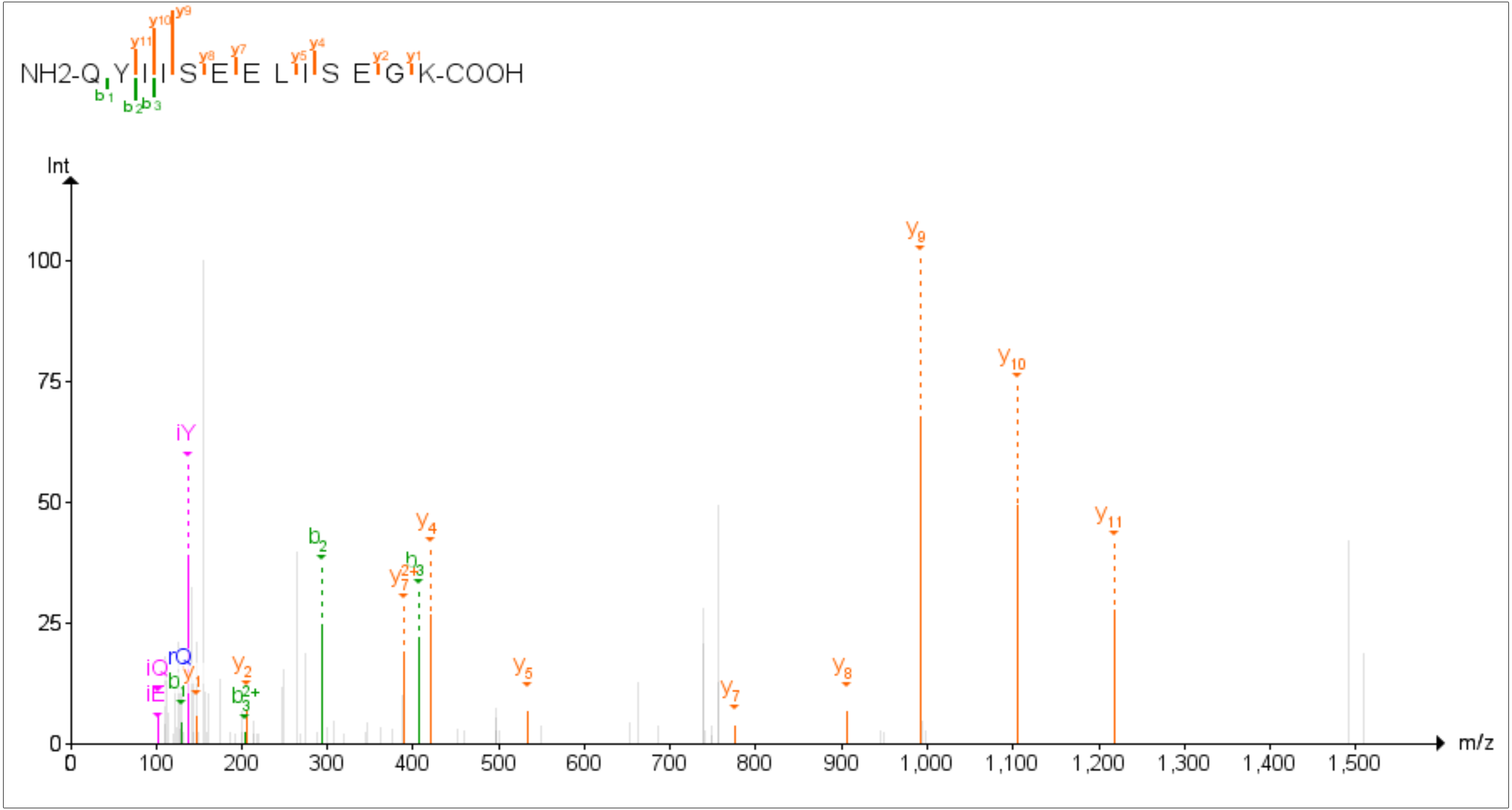

NH2-Q Y I I S E E L I S E G K-COOH
Int
m/z
y9
y10
y11
y4
y2+ 7
b2
b3
y2
y5
y7
y8
iY
iQ
iE
rQ
b1
y1
b2+ 3

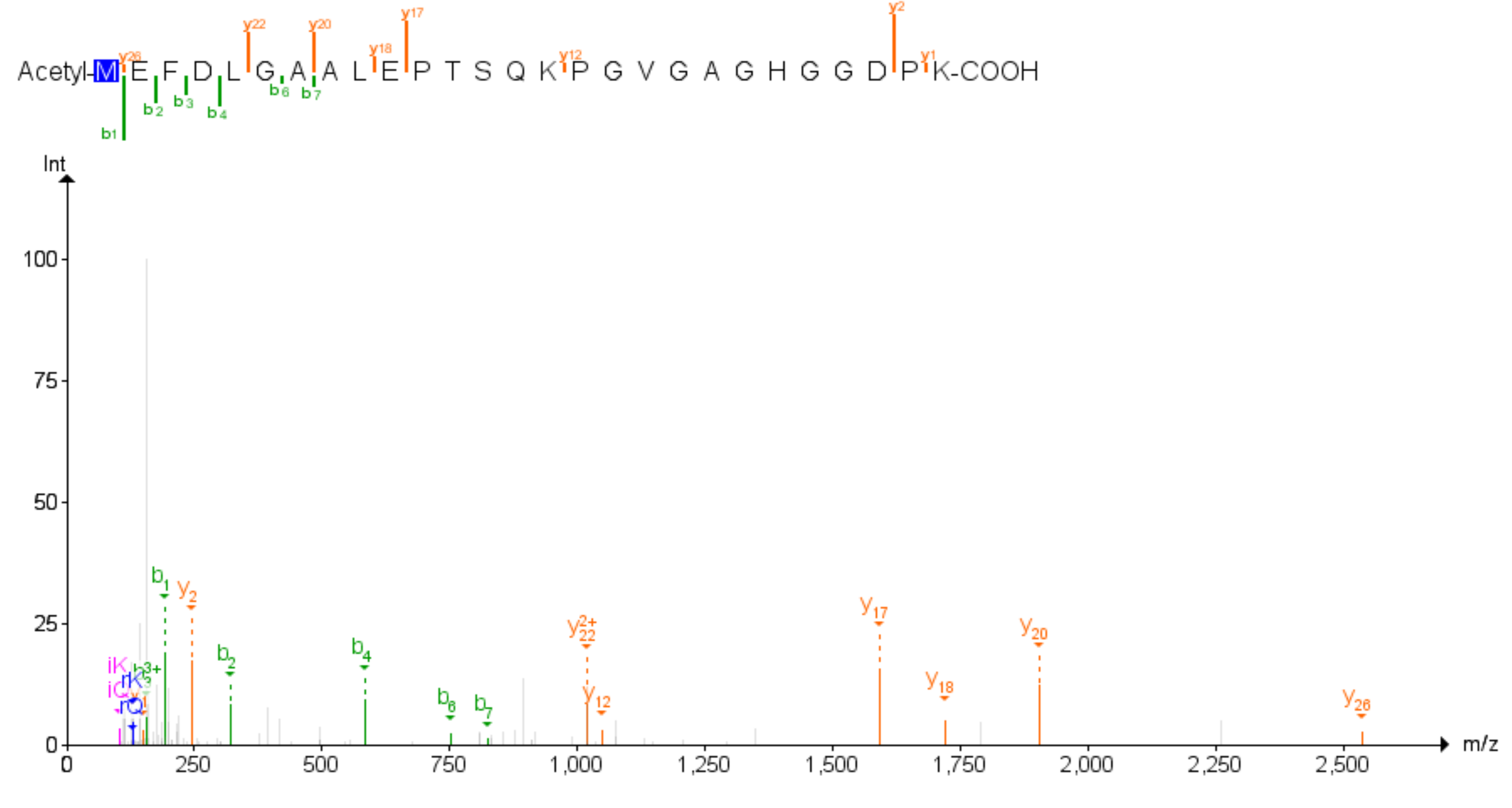

Acetyl-M E F D L G A A L E P T S Q K P G V G A G H G G D P K-COOH
Int
m/z

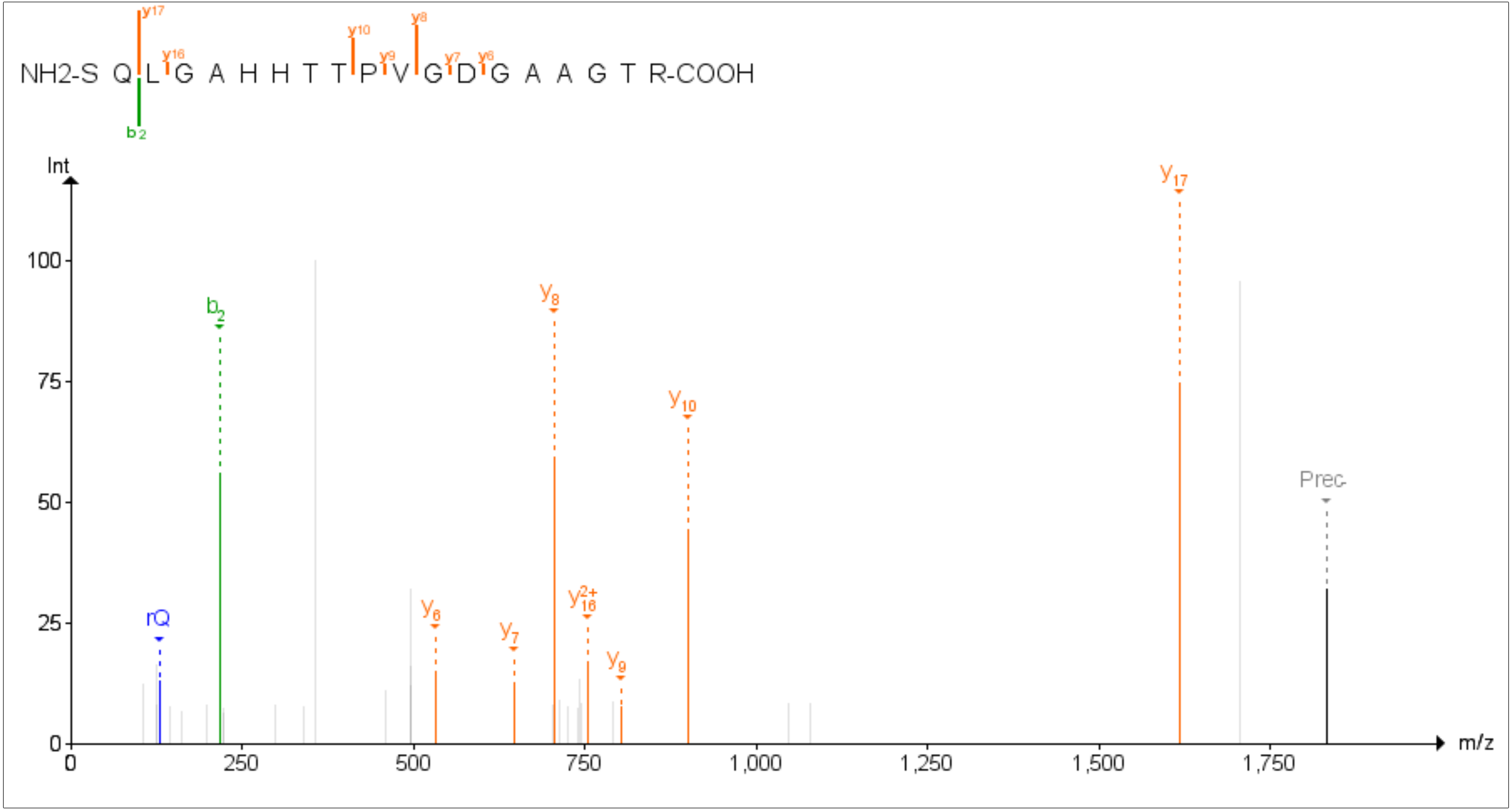

NH2-S Q L G A H H T T P V G D G A A G T R-COOH
y17
y16
y10
y9
y8
y7
y6
b2
Int
100
75
50
25
0
b2
y8
y10
y17
Prec.
rQ
y6
y7
y16 2+
y9
0
250
500
750
1,000
1,250
1,500
1,750
m/z

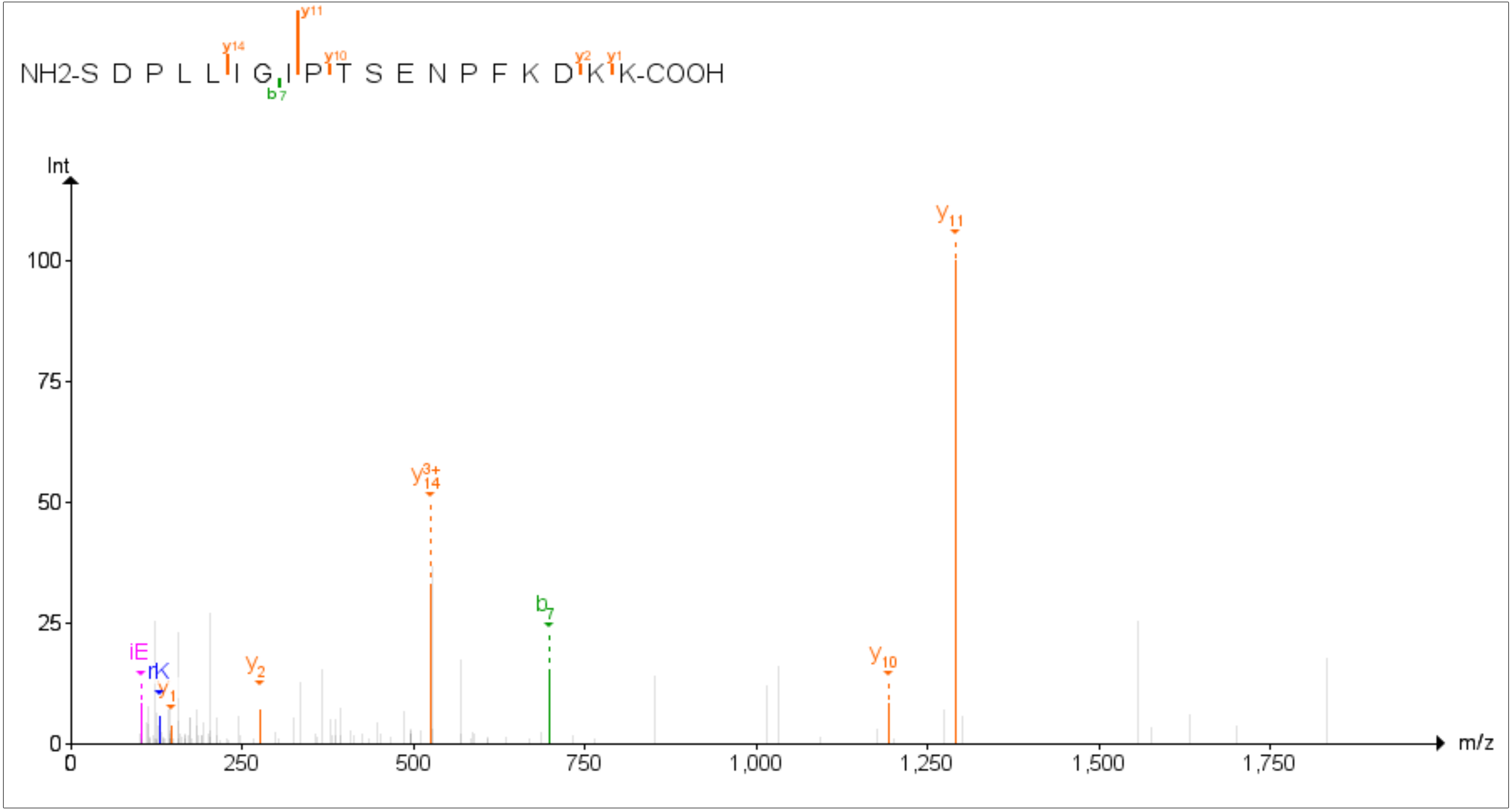

NH2-S D P L L I G I P T S E N P F K D K K-COOH
y14
y11
y10
y2
y1
b7
Int
100
75
50
25
0
iE
rK
y1
y2
y14 3+
b7
y10
y11
0
250
500
750
1,000
1,250
1,500
1,750
m/z

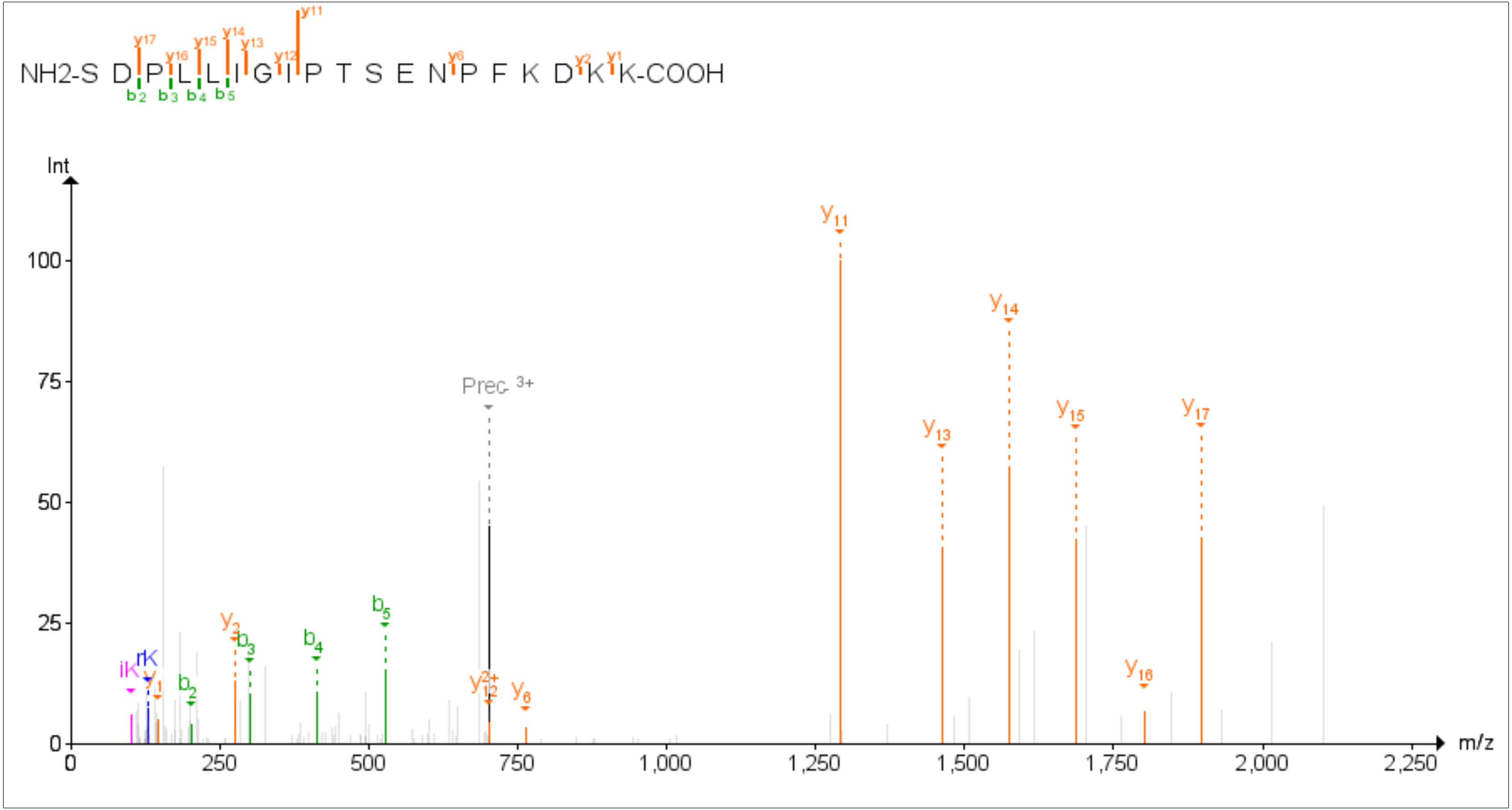

NH2-S D P L L I G I P T S E N P F K D K K-COOH
y17 y16 y15 y14 y13 y12 y11 y6 y2 y1
b2 b3 b4 b5
Int
100
75
50
25
0
Prec- 3+
y11
y14
y13
y15
y17
y16
y2
b3
b4
b5
b2
y1
iK
rK
y_{12}^{2+}
y6
0
250
500
750
1,000
1,250
1,500
1,750
2,000
2,250
m/z

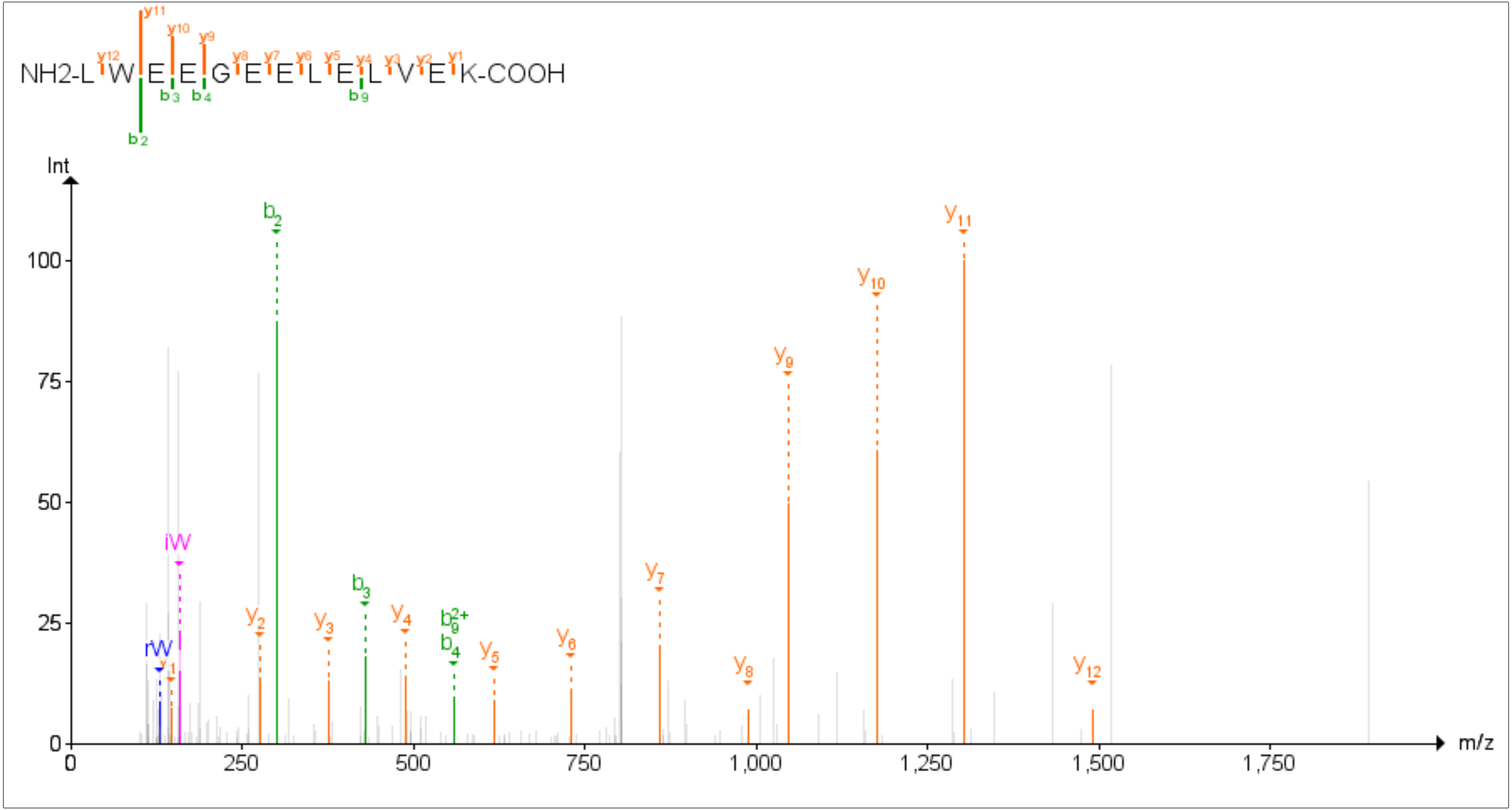

NH2-L W E E G E E L E L V E K-COOH
y12 y11 y10 y9 y8 y7 y6 y5 y4 y3 y2 y1
b2 b3 b4 b9
Int
100
75
50
25
0
250
500
750
1,000
1,250
1,500
1,750
m/z
b2
y11
y10
y9
iW
rW
y1
y2
y3
b3
y4
b9 2+
b4
y5
y6
y7
y8
y12

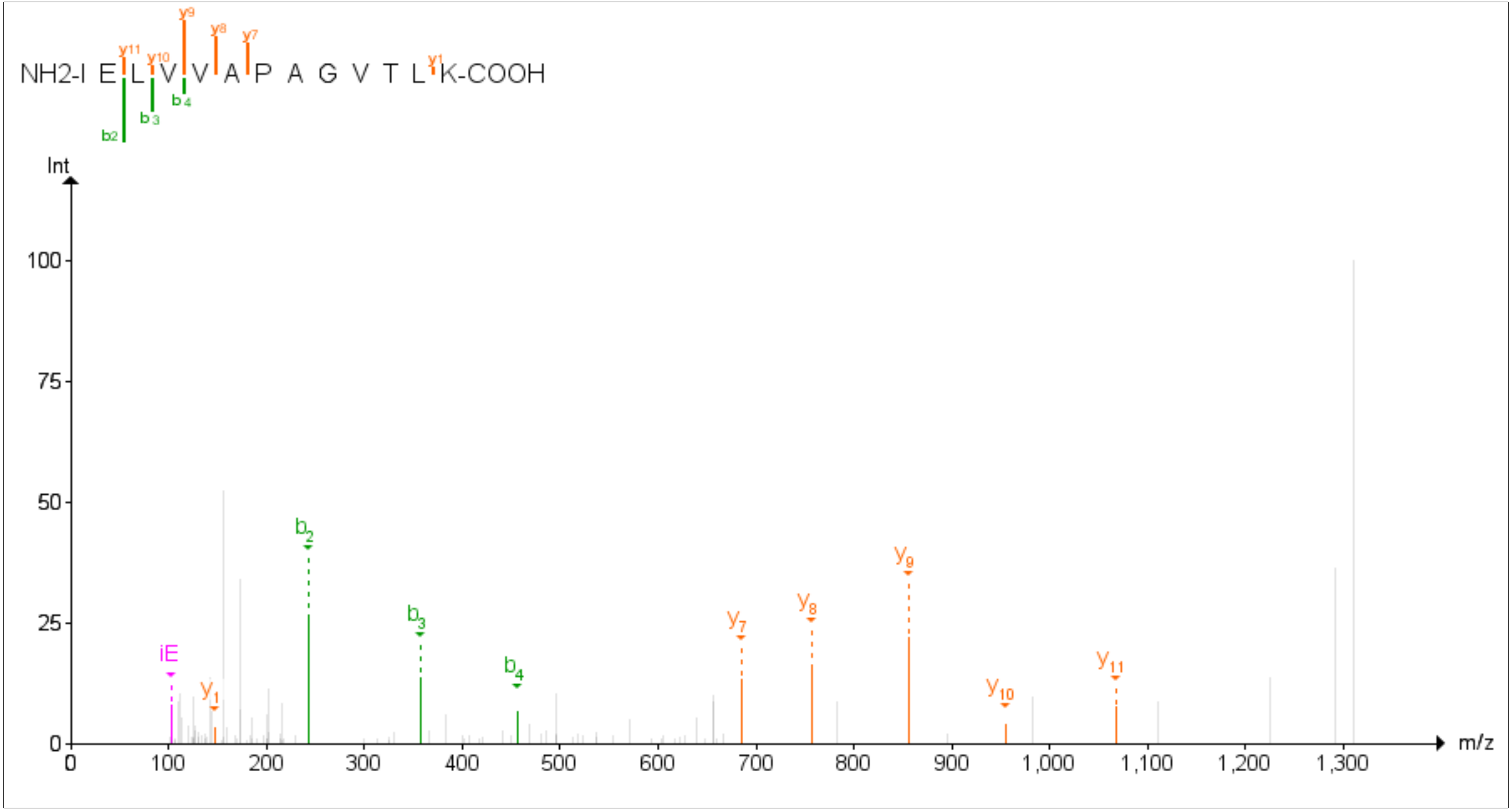
NH2-I E L V V A P A G V T L K-COOH
y11 y10 y9 y8 y7 y1
b2 b3 b4
Int
100
75
50
25
0
iE
y1
b2
b3
b4
y7
y8
y9
y10
y11
0 100 200 300 400 500 600 700 800 900 1,000 1,100 1,200 1,300
m/z

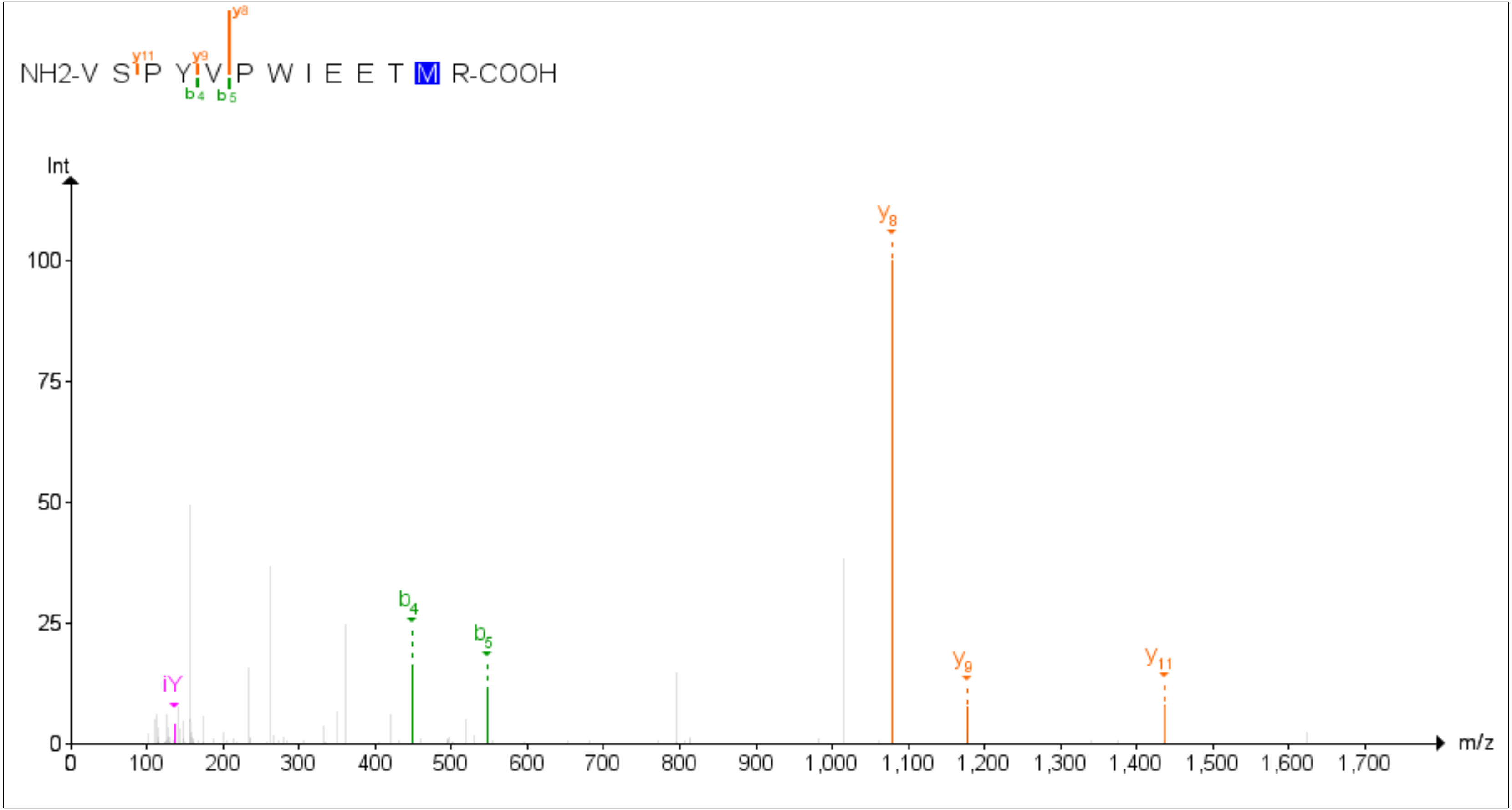

NH2-V S P Y V P W I E E T M R-COOH
y11
y9
y8
b4
b5
Int
100
75
50
25
0
y8
y9
y11
b4
b5
iY
0
100
200
300
400
500
600
700
800
900
1,000
1,100
1,200
1,300
1,400
1,500
1,600
1,700
m/z

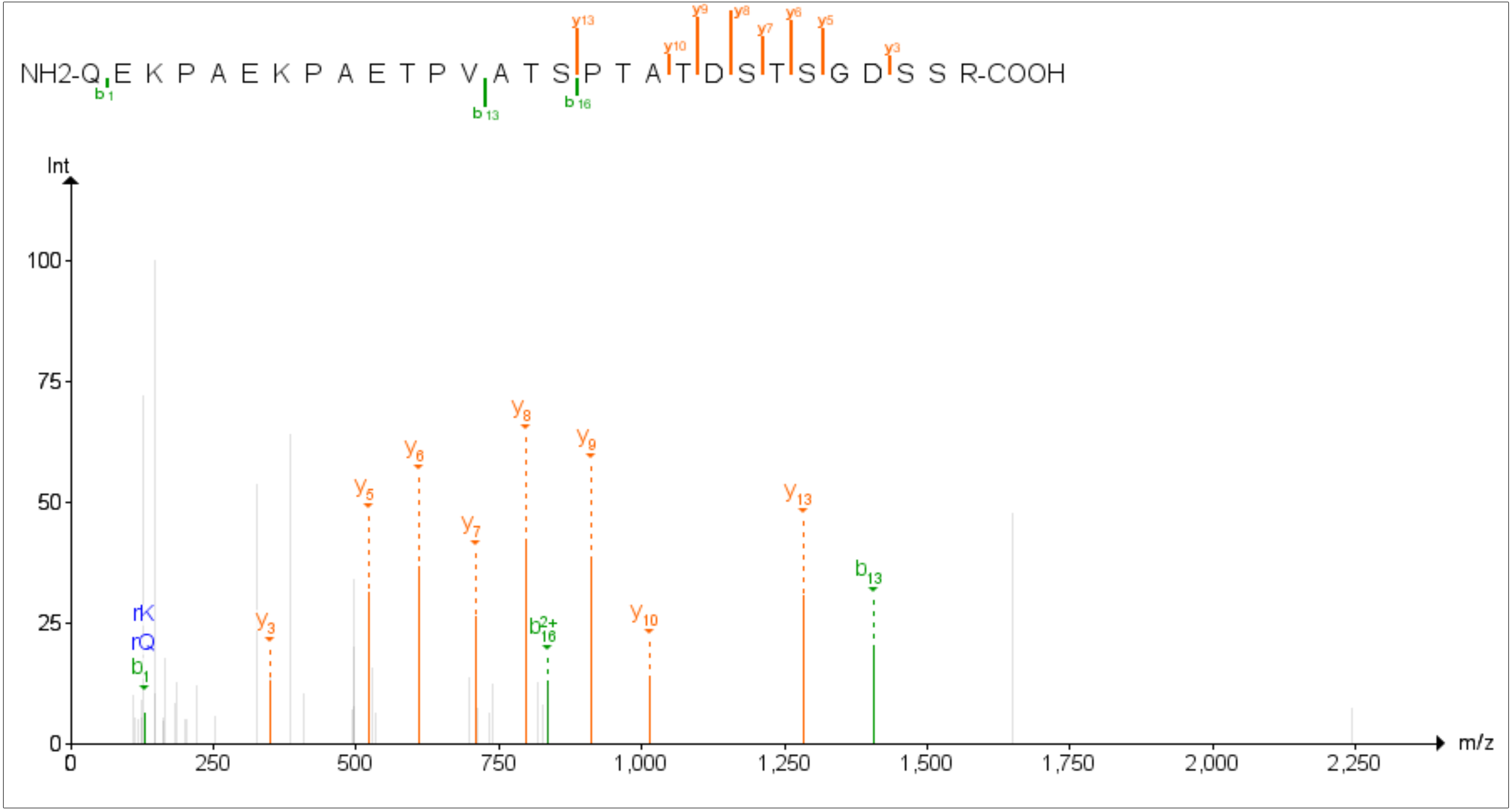
NH2-Q E K P A E K P A E T P V A T S P T A T D S T S G D S S R-COOH
b1
b13
b16
y13
y10
y9
y8
y7
y6
y5
y3
Int
100
75
50
25
0
rK
rQ
b1
y3
y5
y6
y7
y8
y9
b16 2+
y10
y13
b13
0
250
500
750
1,000
1,250
1,500
1,750
2,000
2,250
m/z

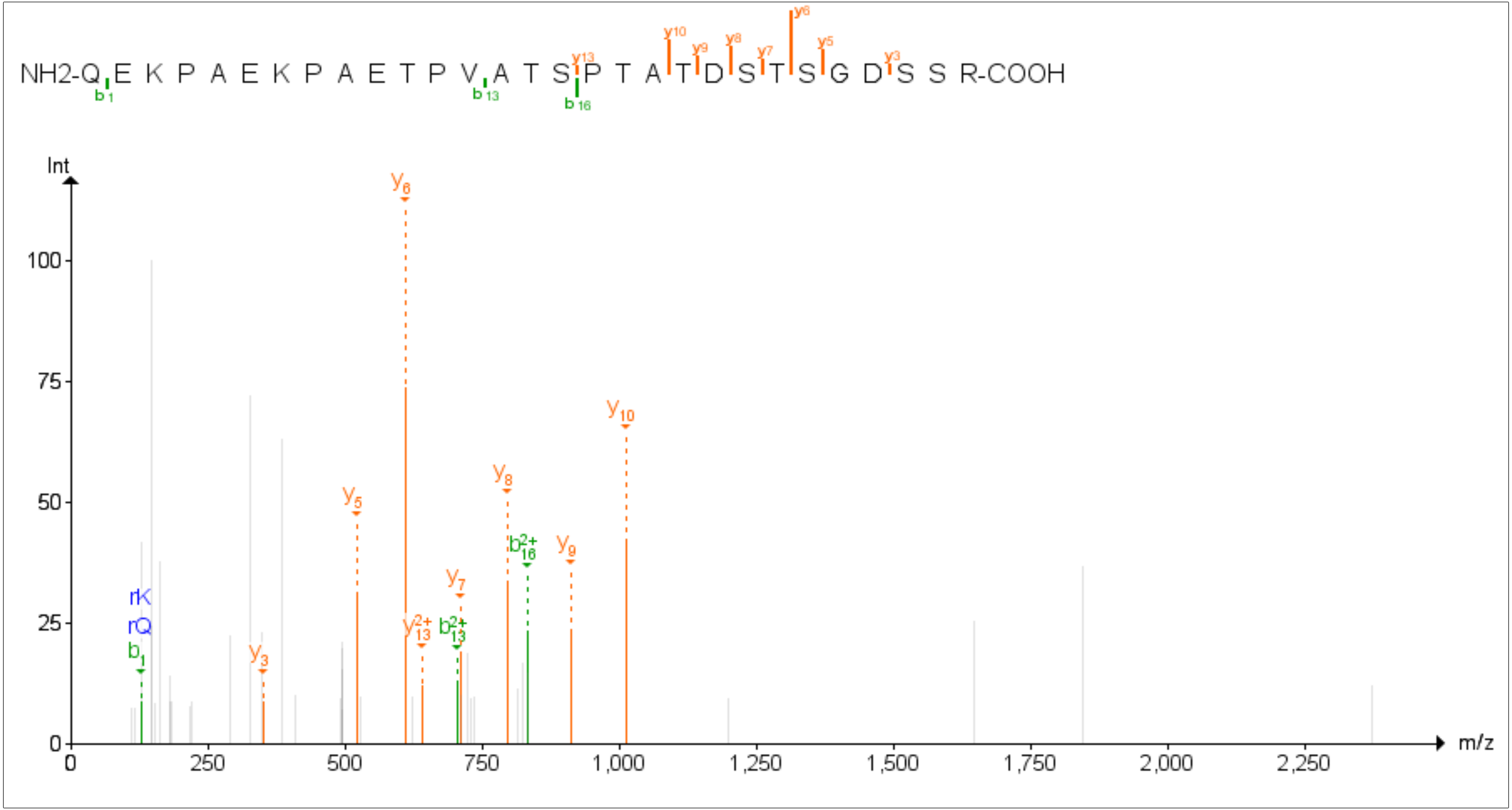

NH2-Q E K P A E K P A E T P V A T S P T A T D S T S G D S S R-COOH
b1
b13
b16
y13
y10
y9
y8
y7
y6
y5
y3
Int
100
75
50
25
0
0
250
500
750
1,000
1,250
1,500
1,750
2,000
2,250
m/z
rK
rQ
b1
y3
y5
y6
y13 2+
b13 2+
y7
y8
b16 2+
y9
y10

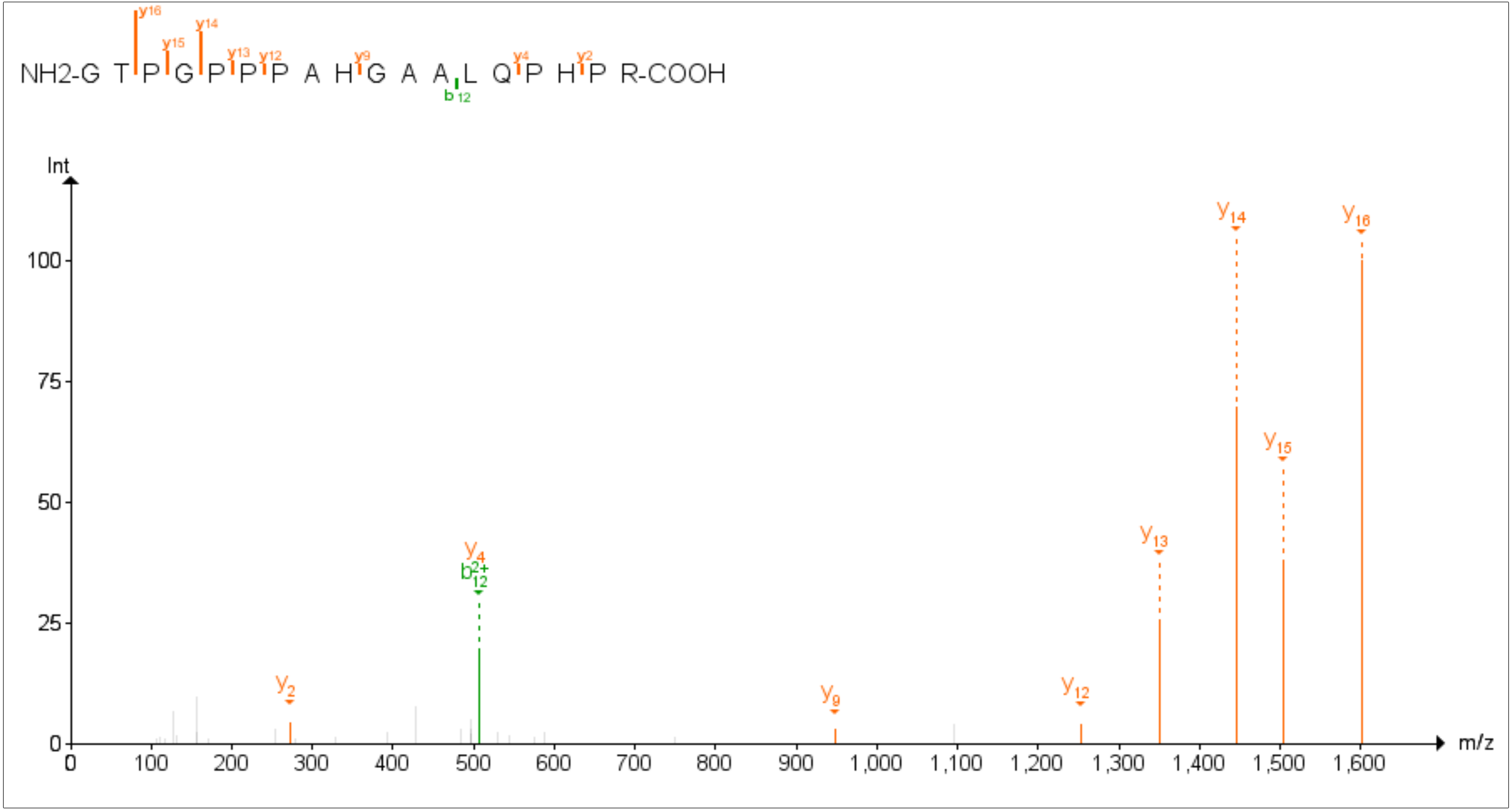
NH2-G T P G P P P A H G A A L Q P H P R-COOH
y16
y15
y14
y13
y12
y9
y4
y2
b12
Int
100
75
50
25
0
m/z
0
100
200
300
400
500
600
700
800
900
1,000
1,100
1,200
1,300
1,400
1,500
1,600
y2
y4
b12 2+
y9
y12
y13
y14
y15
y16

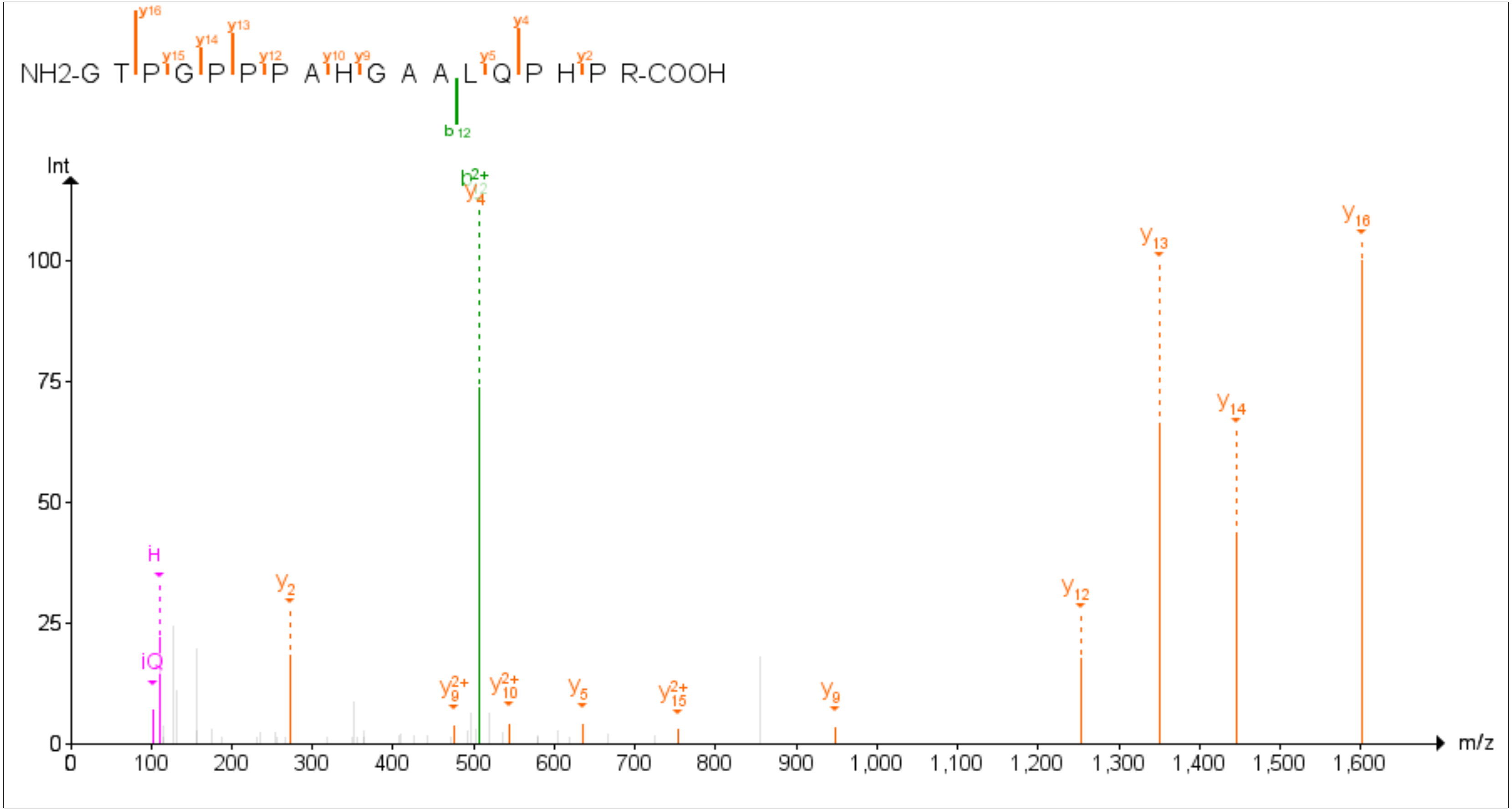

NH2-G T P G P P P A H G A A L Q P H P R-COOH
y16
y15
y14
y13
y12
y10
y9
y5
y4
y2
b 12
Int
m/z
0
25
50
75
100
0
100
200
300
400
500
600
700
800
900
1,000
1,100
1,200
1,300
1,400
1,500
1,600
iH
iQ
y_2
b_{12}^{2+}
y_4
y_9^{2+}
y_{10}^{2+}
y_5
y_{15}^{2+}
y_9
y_{12}
y_{13}
y_{14}
y_{16}

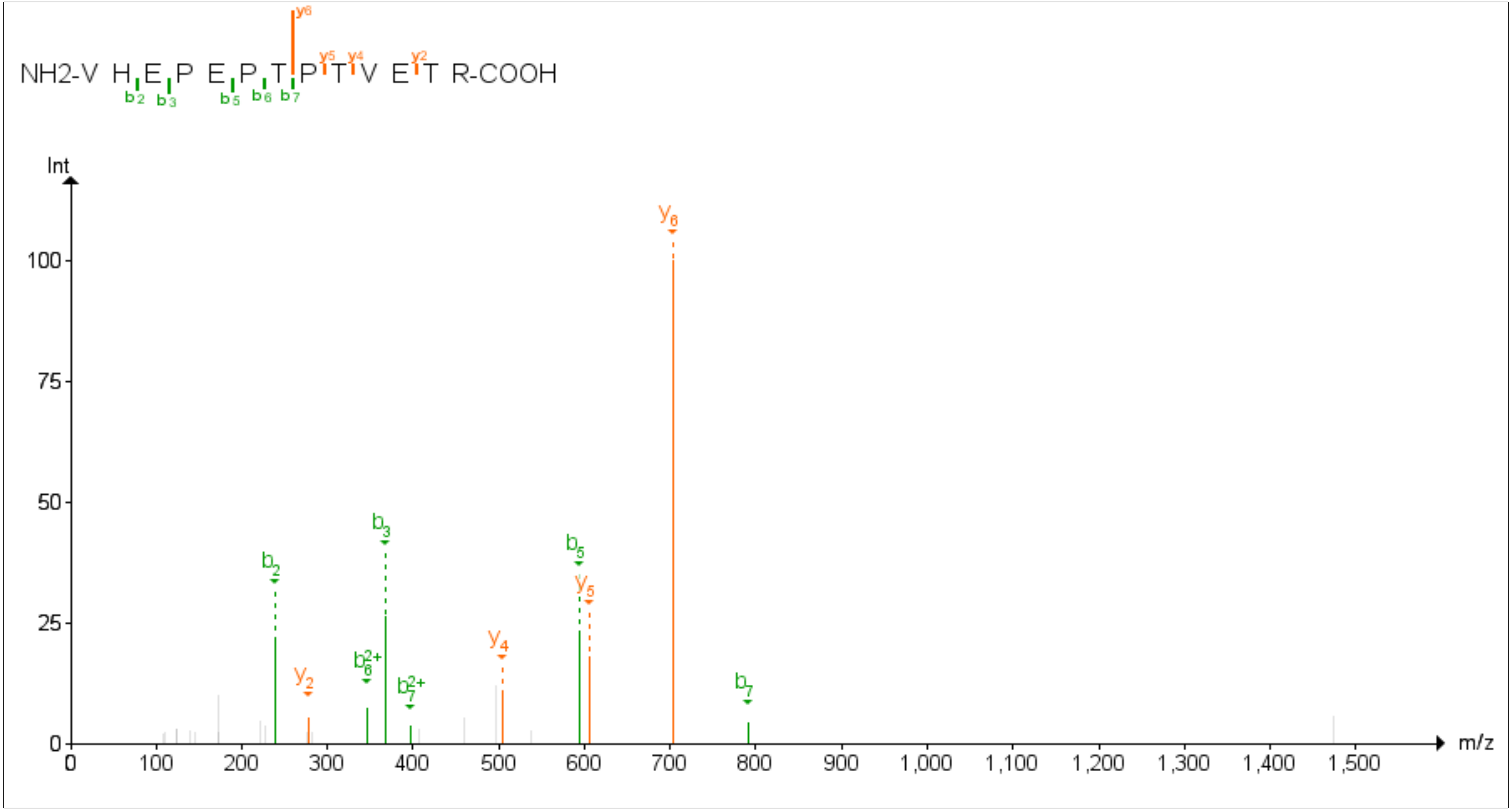

NH2-V H E P E P T P T V E T R-COOH
y6
y5
y4
y2
b2
b3
b5
b6
b7
Int
100
75
50
25
0
m/z
0
100
200
300
400
500
600
700
800
900
1,000
1,100
1,200
1,300
1,400
1,500
y6
b3
b5
y5
b2
y4
b_6^{2+}
b_7^{2+}
y2
b7

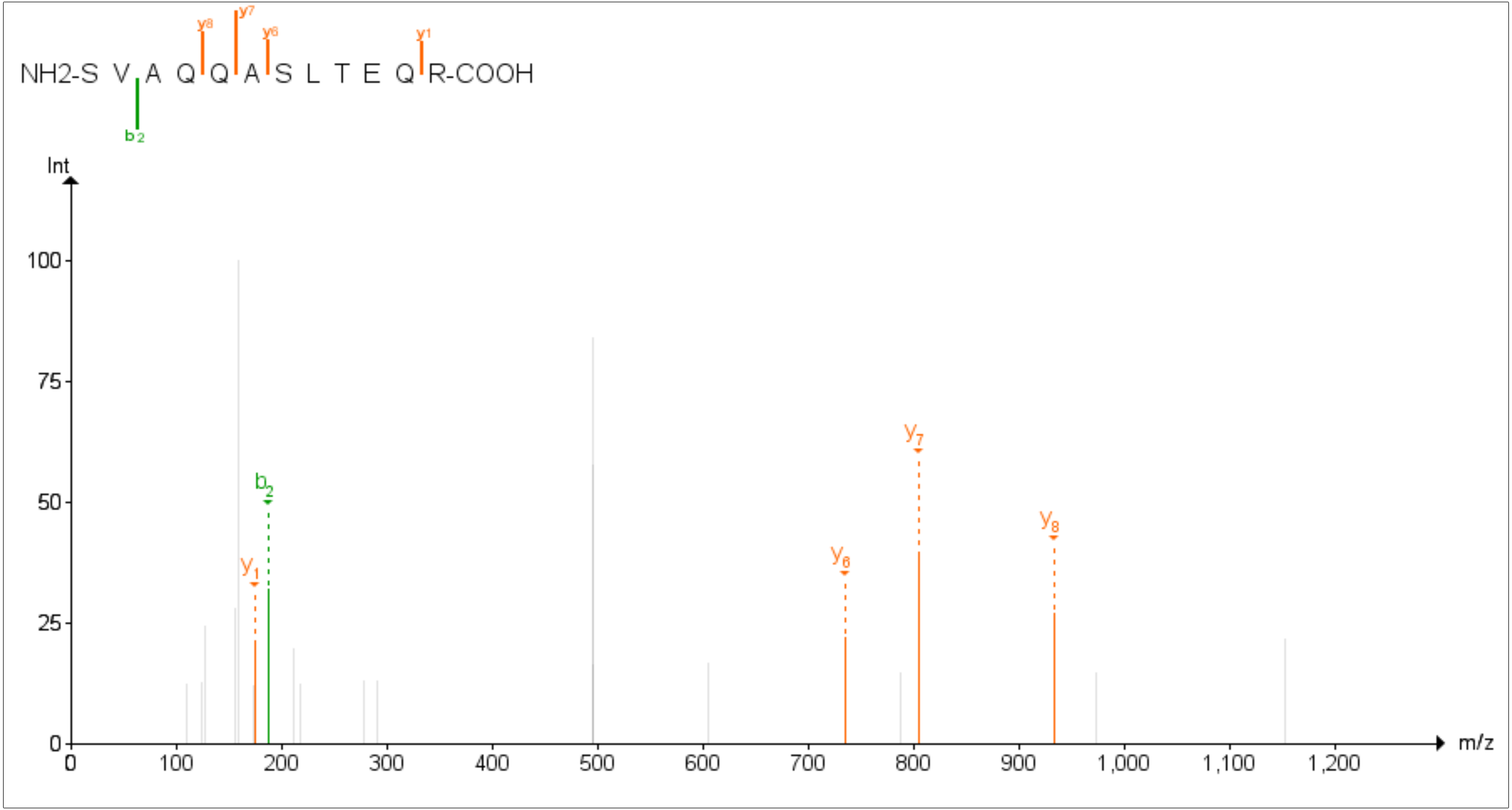
NH2-S V A Q Q A S L T E Q R-COOH
y8
y7
y6
y1
b2
Int
100
75
50
25
0
0
100
200
300
400
500
600
700
800
900
1,000
1,100
1,200
m/z
b2
y1
y6
y7
y8

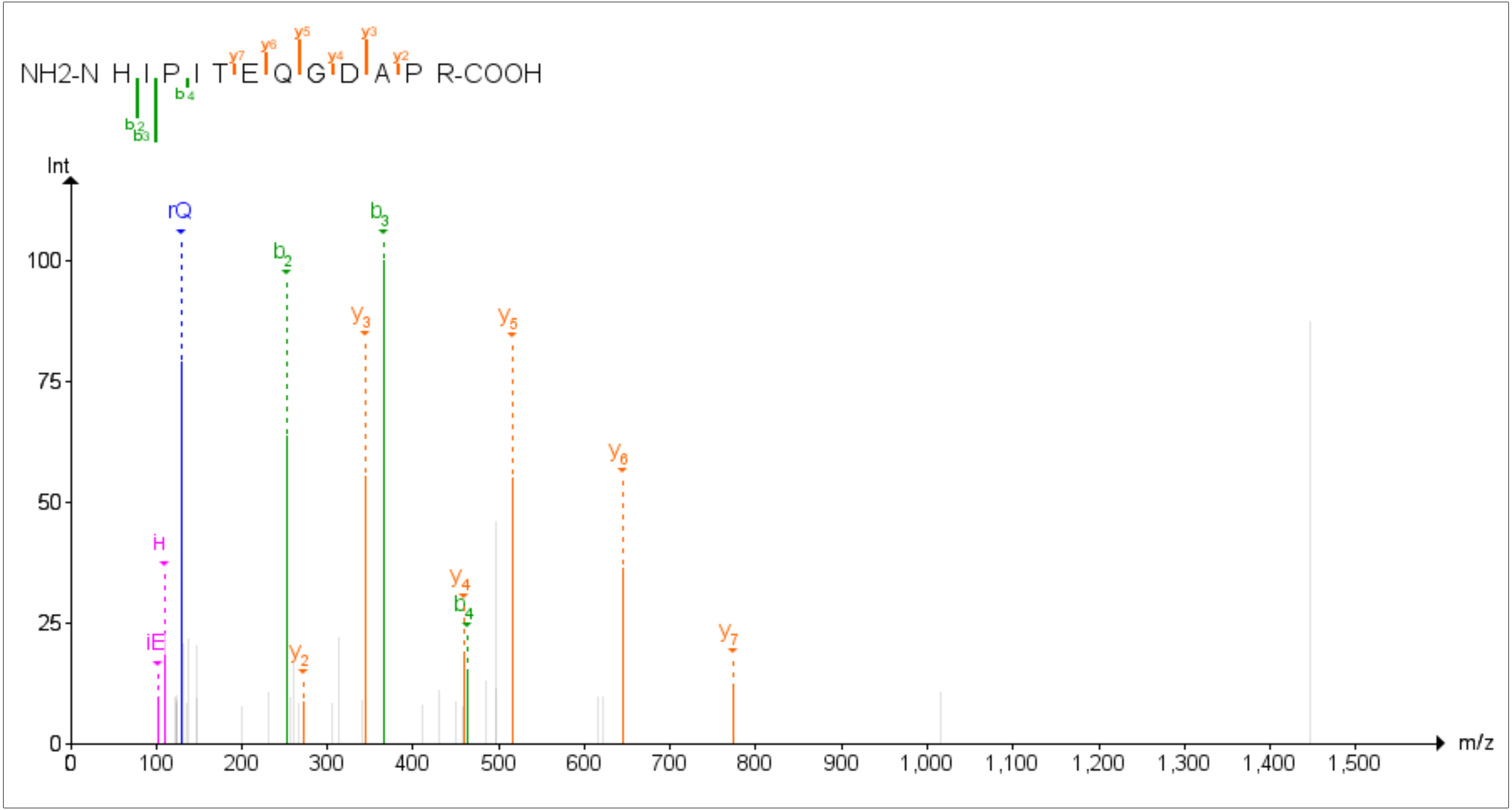

NH2-N H I P I T E Q G D A P R-COOH
b2
b3
b4
y7
y6
y5
y4
y3
y2
Int
100
75
50
25
0
rQ
b3
b2
y3
y5
y6
iH
iE
y2
y4
b4
y7
m/z
0
100
200
300
400
500
600
700
800
900
1,000
1,100
1,200
1,300
1,400
1,500

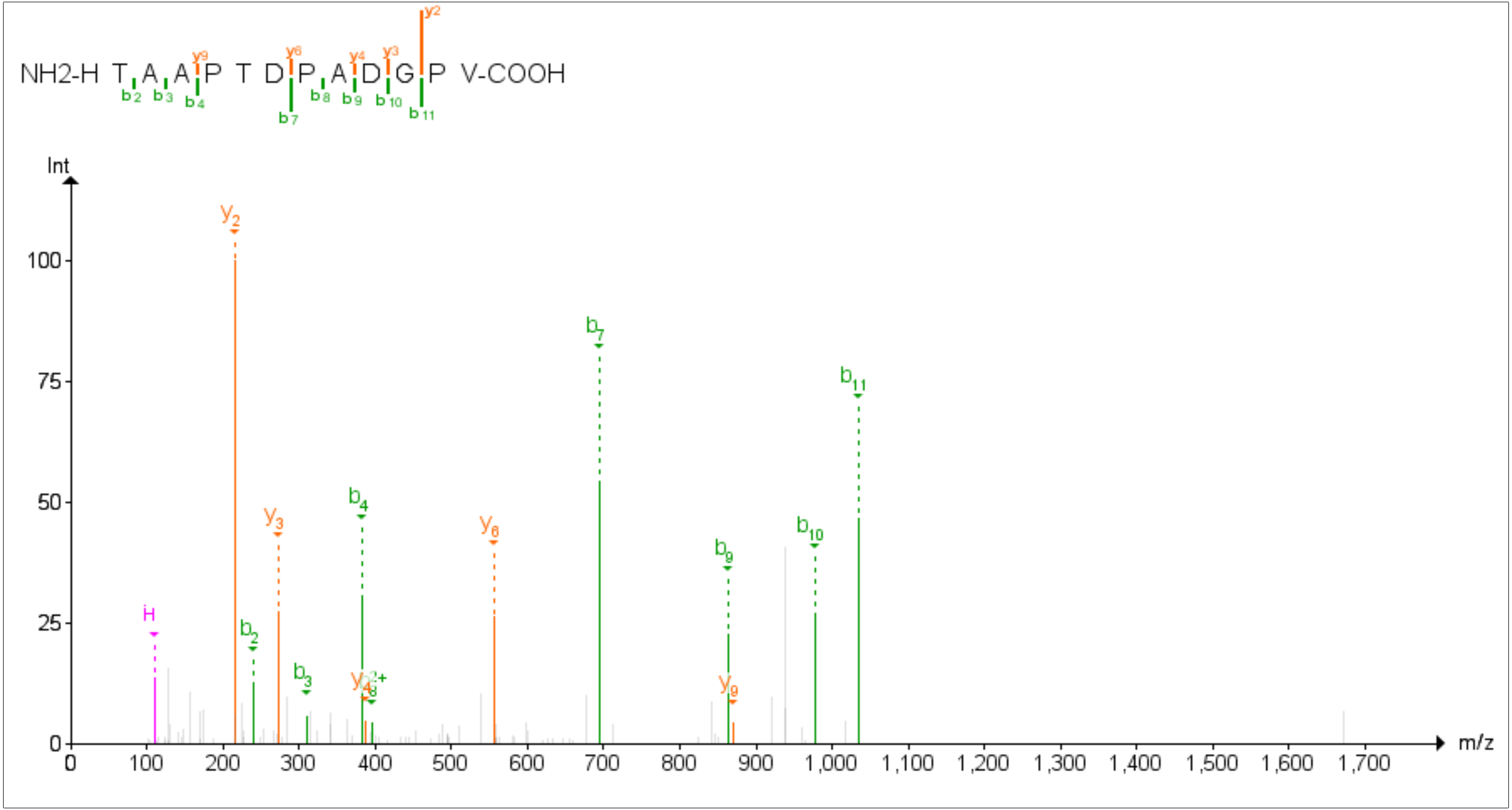
NH2-H T A A P T D P A D G P V-COOH
Int
m/z
y2
y3
y6
y9
b2
b3
b4
b7
b9
b10
b11

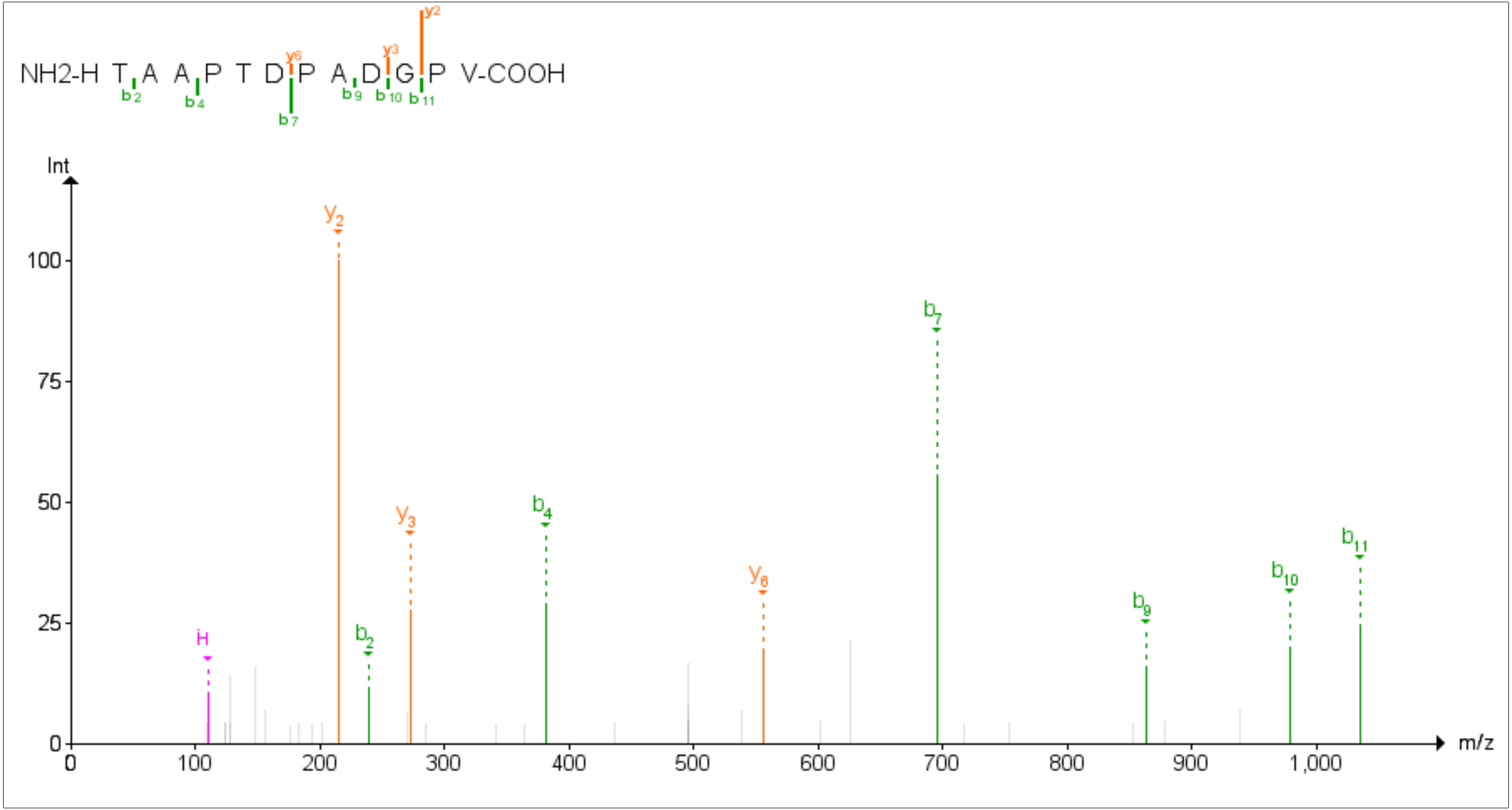

NH2-H T A A P T D P A D G P V-COOH
b2
b4
b7
b9
b10
b11
y6
y3
y2
Int
100
75
50
25
0
H
y2
b2
y3
b4
y6
b7
b9
b10
b11
0
100
200
300
400
500
600
700
800
900
1,000
m/z

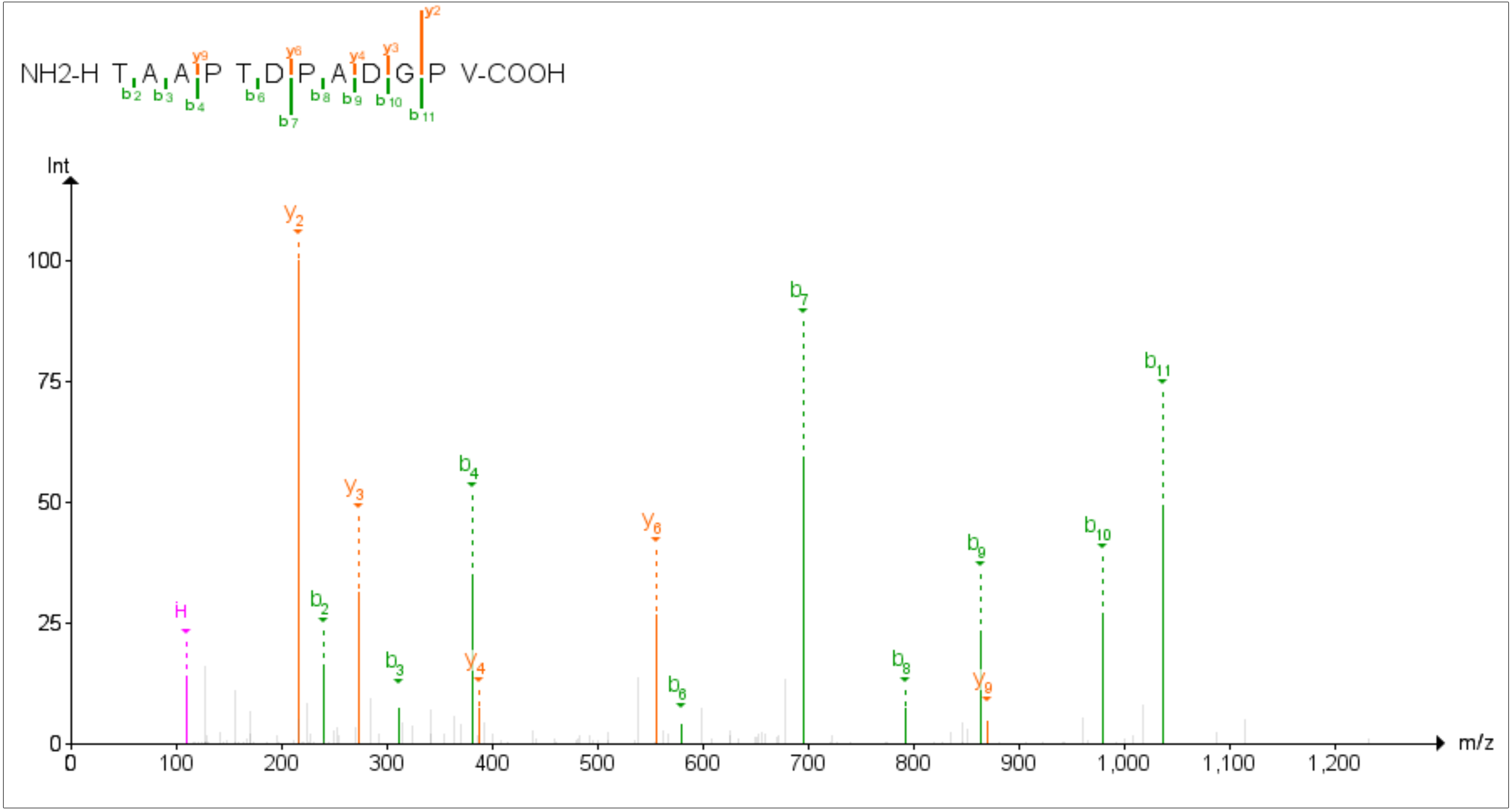
NH2-H T A A P T D P A D G P V-COOH
b2 b3 b4 b6 b7 b8 b9 b10 b11
y9 y6 y4 y3 y2
Int
100
75
50
25
0
m/z
0
100
200
300
400
500
600
700
800
900
1,000
1,100
1,200
y2
y3
y4
y6
y9
b2
b3
b4
b6
b7
b8
b9
b10
b11
H

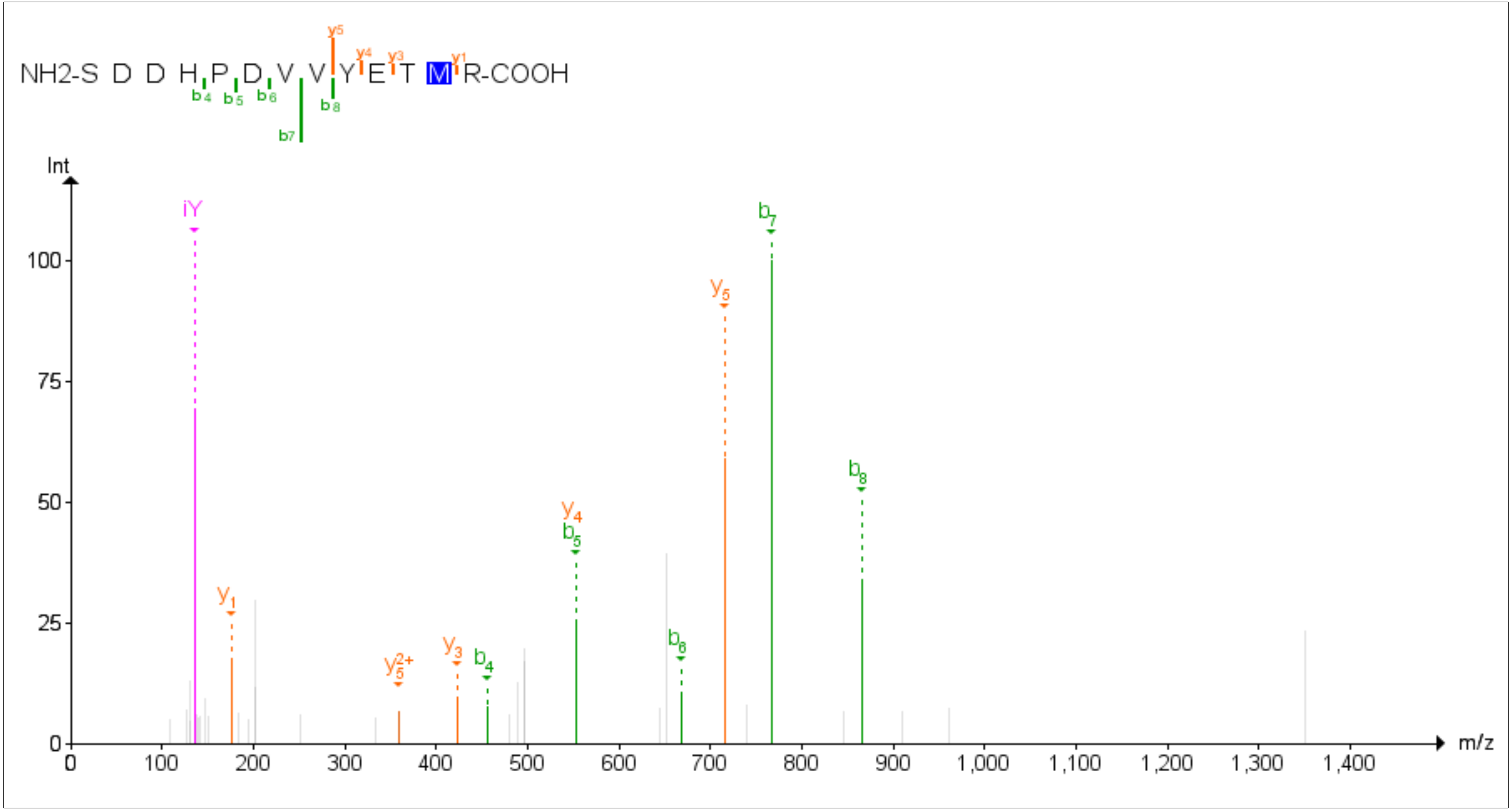

NH2-S D D H P D V V Y E T M R-COOH
y5
y4
y3
y1
b4
b5
b6
b7
b8
Int
iY
b7
y5
b8
y4
b5
y1
y5 2+
y3
b4
b6
100
75
50
25
0
0
100
200
300
400
500
600
700
800
900
1,000
1,100
1,200
1,300
1,400
m/z

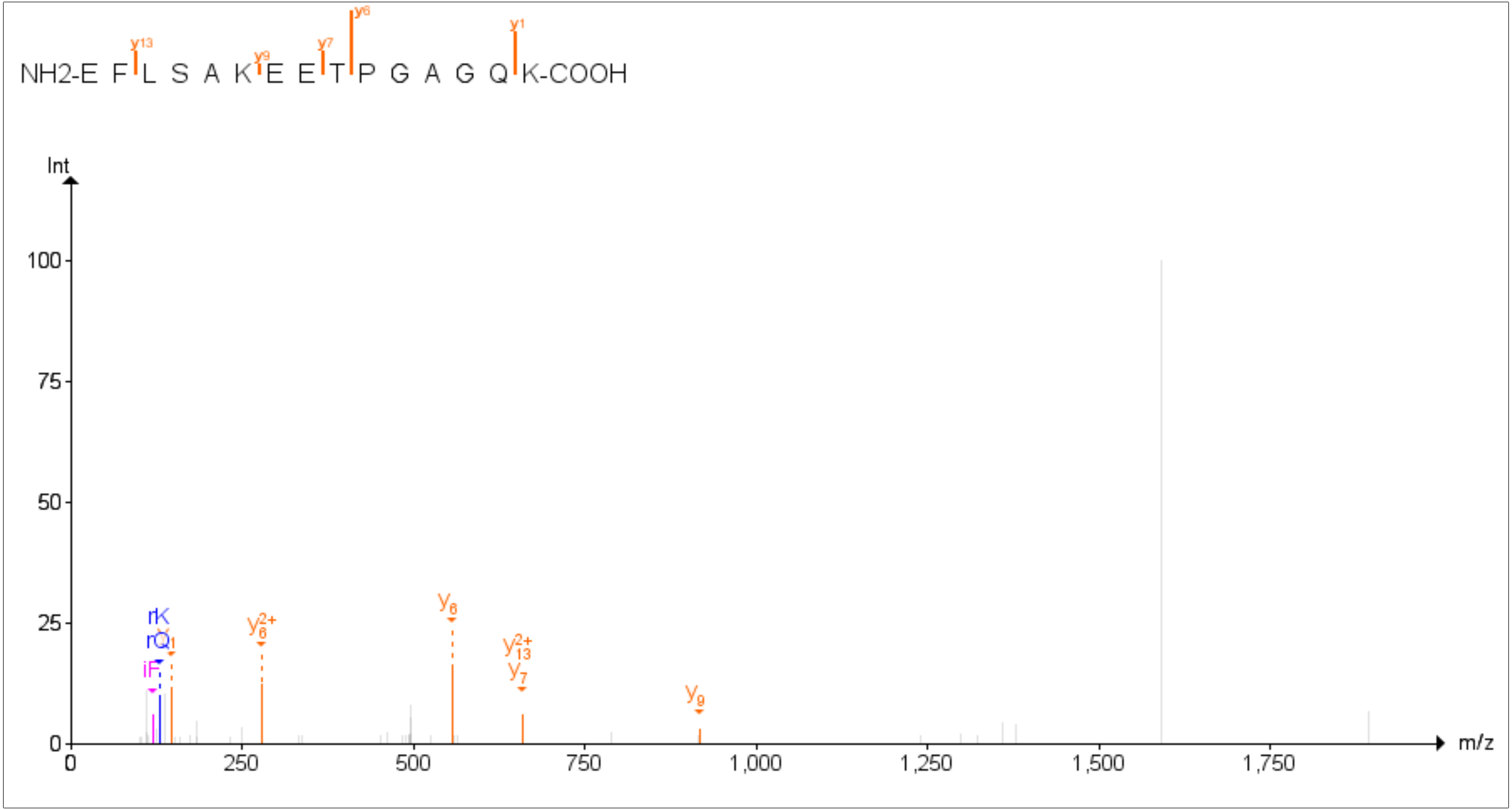

NH2-E F L S A K E E T P G A G Q K-COOH
y13
y9
y7
y8
y1
Int
100
75
50
25
0
rK
rQ
iF
y1
y6 2+
y6
y13 2+
y7
y9
m/z
0
250
500
750
1,000
1,250
1,500
1,750

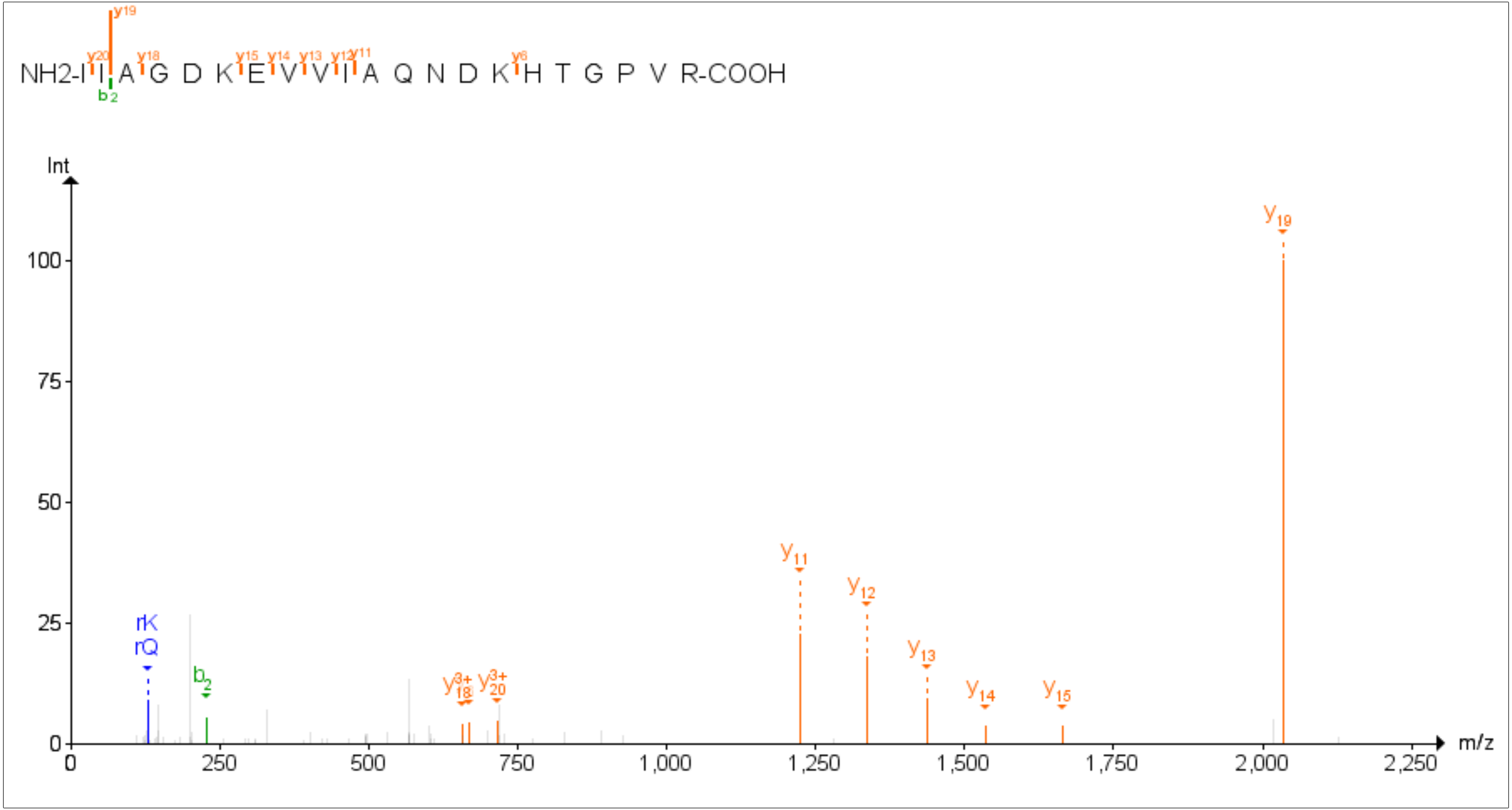

NH2-I I A G D K E V V I A Q N D K H T G P V R-COOH
y20
y19
y18
y15
y14
y13
y12
y11
y6
b2
Int
100
75
50
25
0
rK
rQ
b2
y3+ 18
y3+ 20
y11
y12
y13
y14
y15
y19
0
250
500
750
1,000
1,250
1,500
1,750
2,000
2,250
m/z

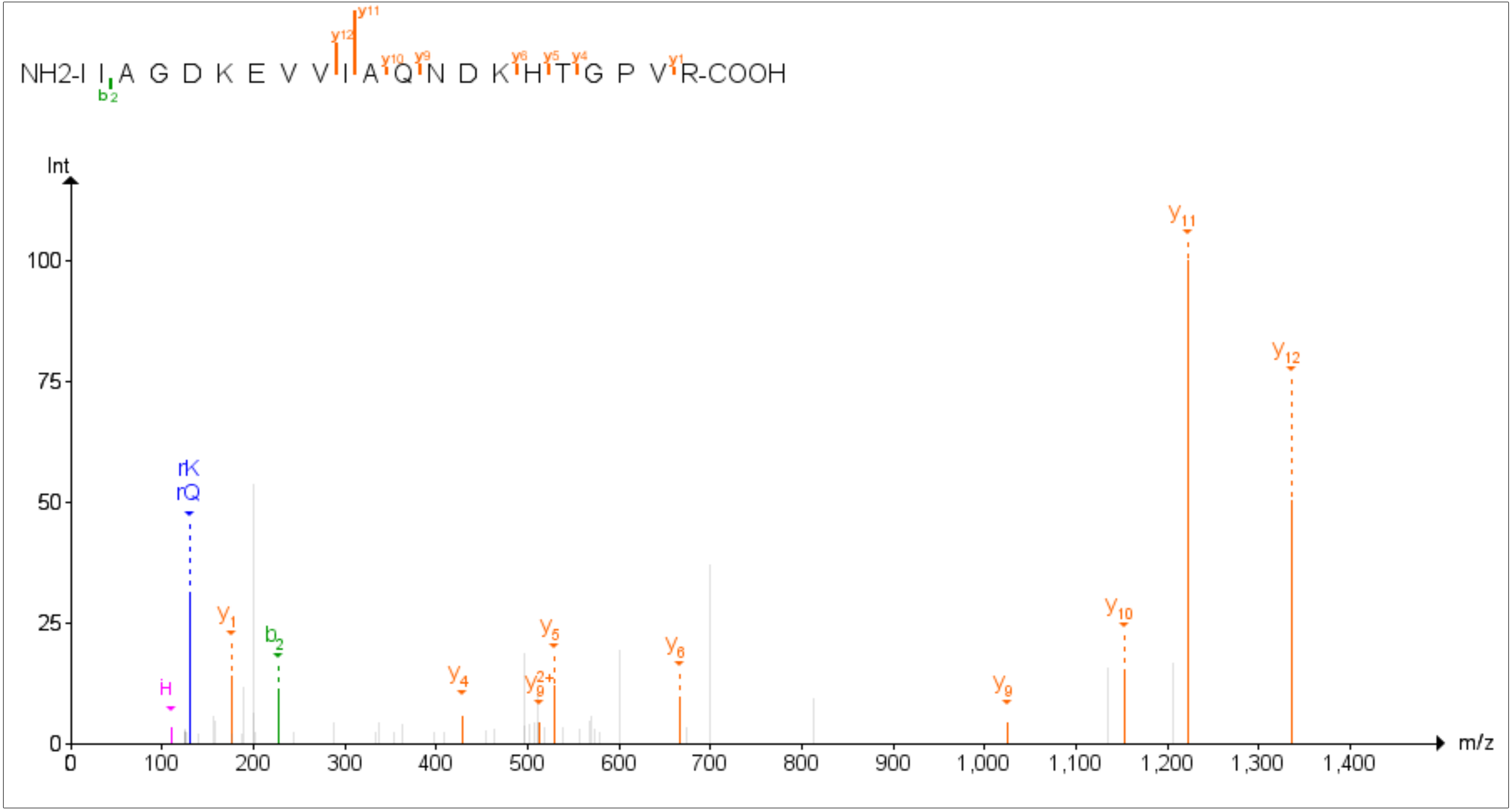

NH2-I I A G D K E V V I A Q N D K H T G P V R-COOH
b2
y12
y11
y10
y9
y6
y5
y4
y1
Int
100
75
50
25
0
rK
rQ
iH
y1
b2
y4
y9 2+
y5
y6
y9
y10
y11
y12
0
100
200
300
400
500
600
700
800
900
1,000
1,100
1,200
1,300
1,400
m/z

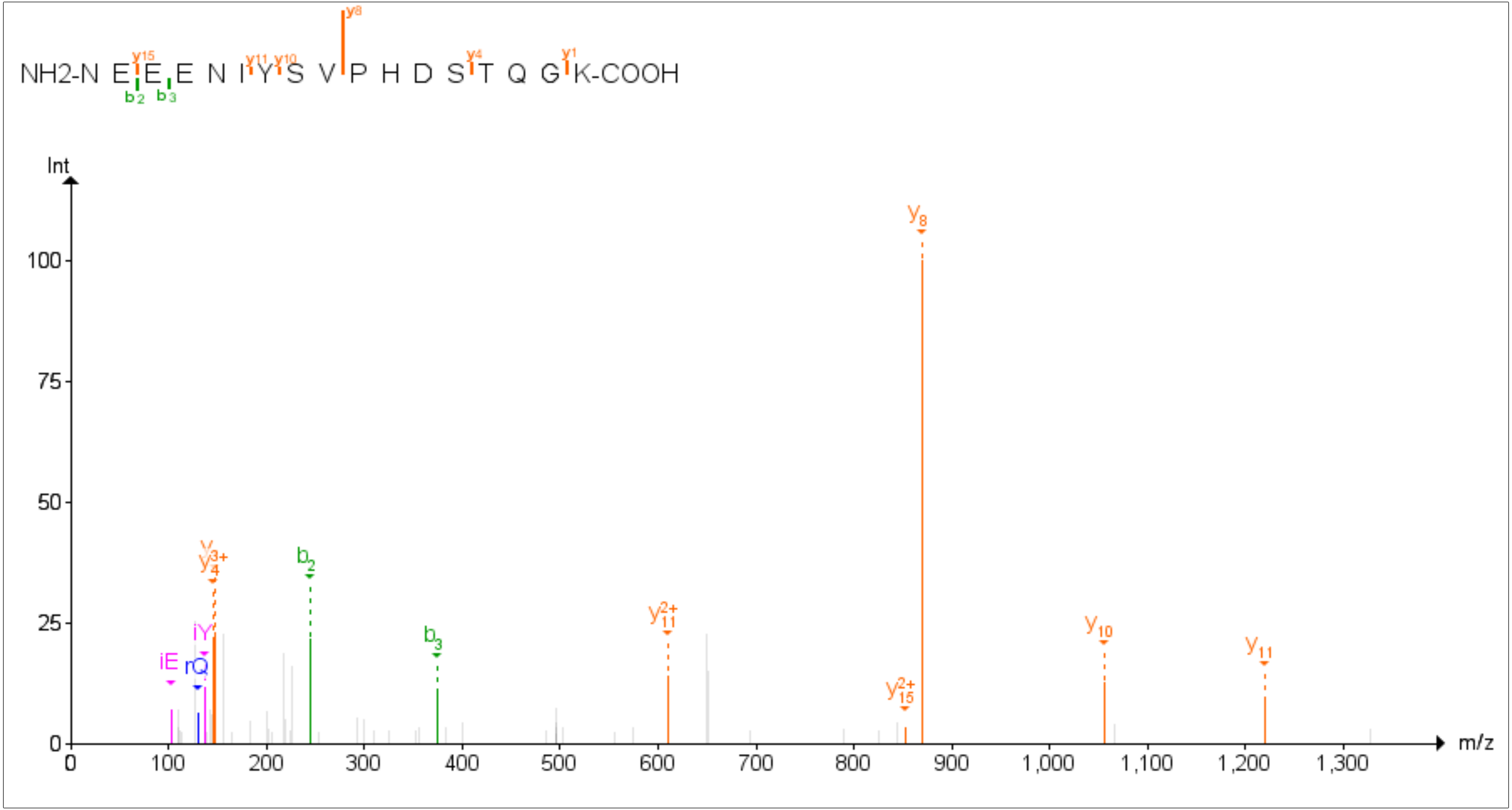

NH2-N E E E N I Y S V P H D S T Q G K-COOH
y15
y11
y10
y8
y4
y1
b2
b3
Int
100
75
50
25
0
m/z
0
100
200
300
400
500
600
700
800
900
1,000
1,100
1,200
1,300
iE
rQ
iY
y4
y3+
b2
b3
y2+11
y2+15
y8
y10
y11

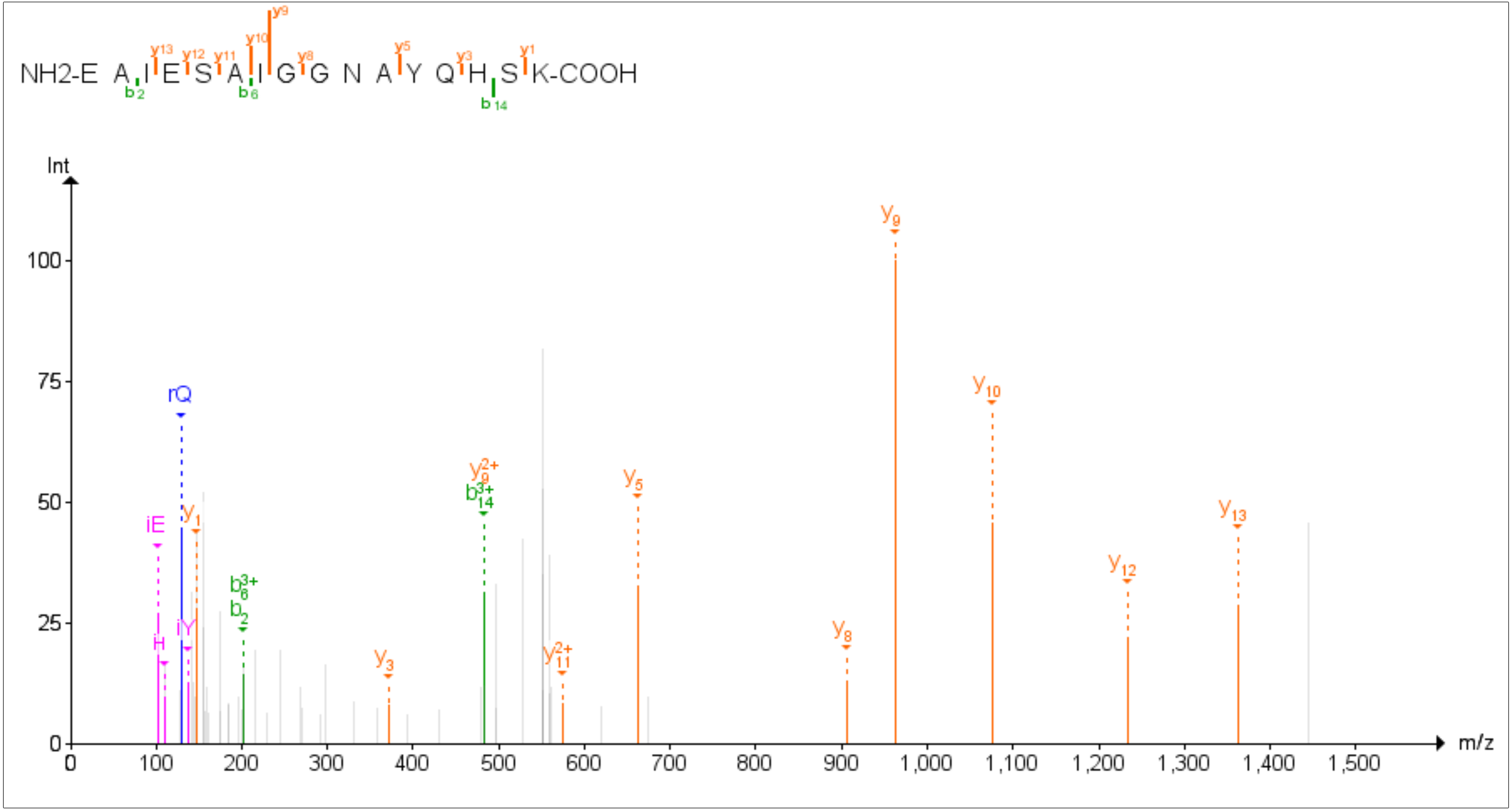

NH2-E A I E S A I G G N A Y Q H S K-COOH
y13 y12 y11 y10 y9 y8 y5 y3 y1
b2 b6 b14
Int
100
75
50
25
0
rQ
iE
y1
iH
iY
b6 3+
b2
y9 2+
b14 3+
y3
y11 2+
y5
y8
y9
y10
y12
y13
0 100 200 300 400 500 600 700 800 900 1,000 1,100 1,200 1,300 1,400 1,500
m/z

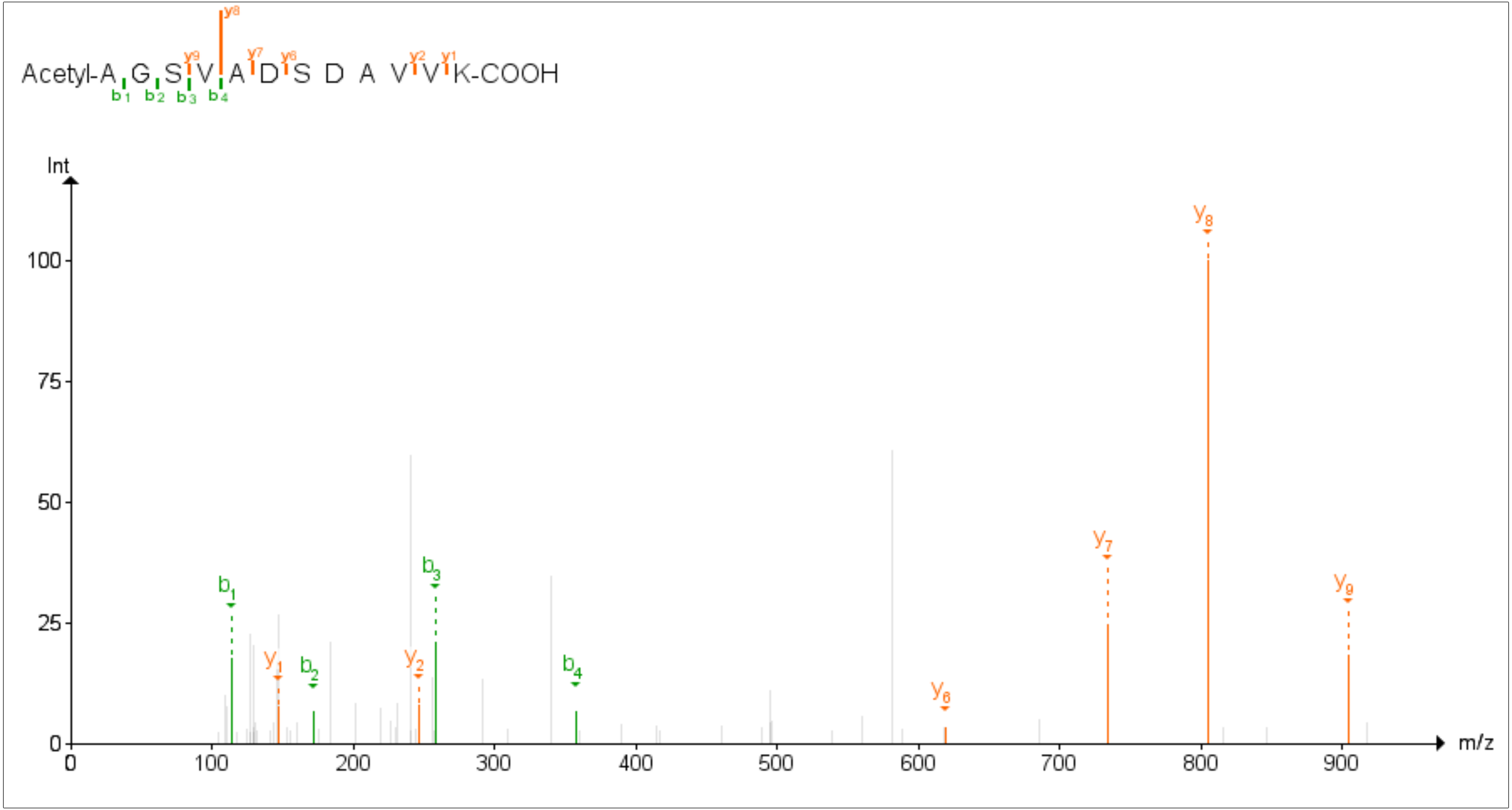

Acetyl-A G S V A D S D A V V K-COOH
y8 y9 y7 y6 y2 y1
b1 b2 b3 b4
Int
100
75
50
25
0
m/z
0 100 200 300 400 500 600 700 800 900
y8 y7 y9 b1 y1 b2 y2 b3 b4 y6

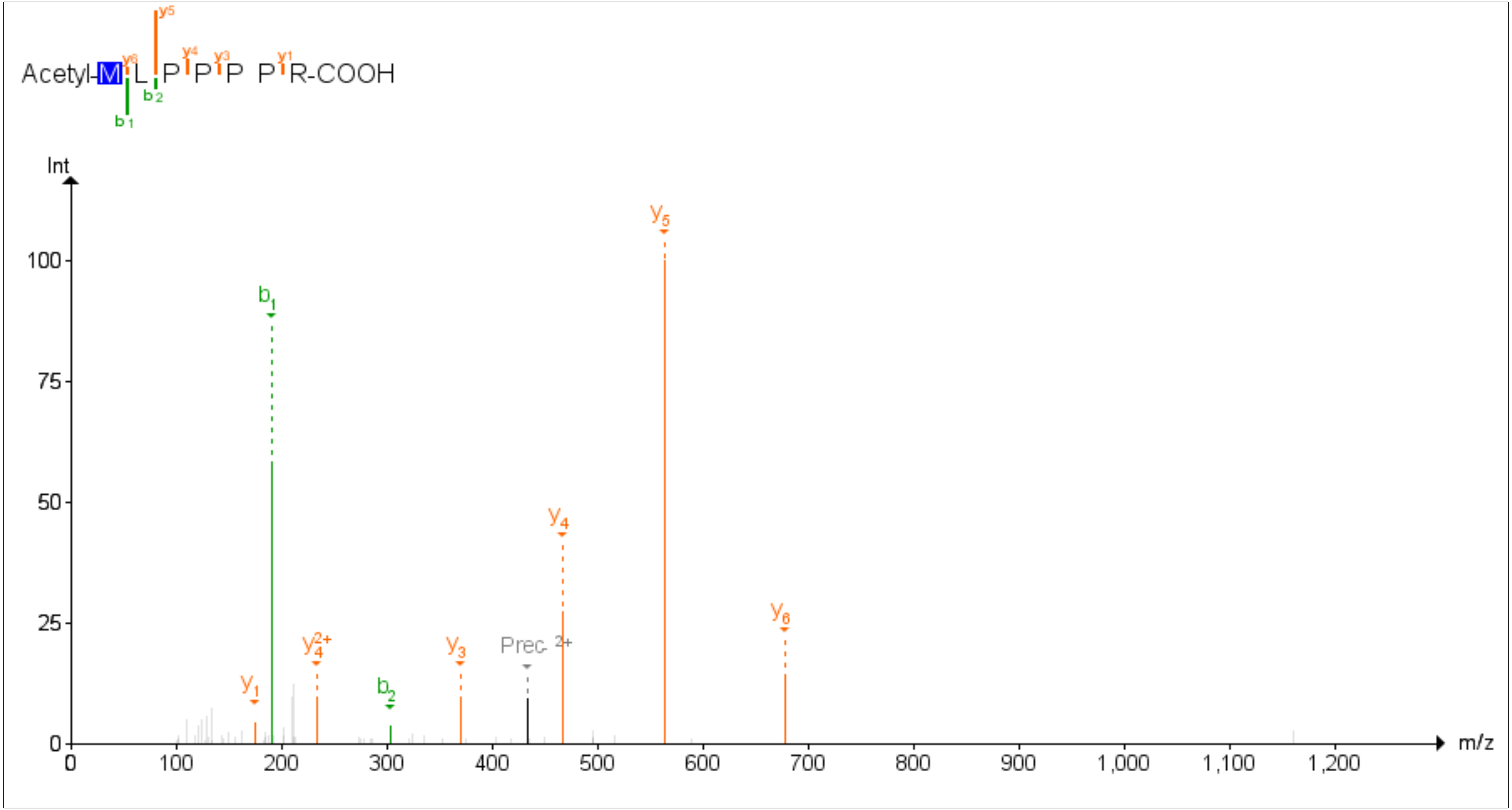
Acetyl-M L P P P P R-COOH
y6 y5 y4 y3 y1
b1 b2
Int
100
75
50
25
0
b1
y5
y4
y6
y1
y4 2+
b2
y3
Prec 2+
0 100 200 300 400 500 600 700 800 900 1,000 1,100 1,200
m/z

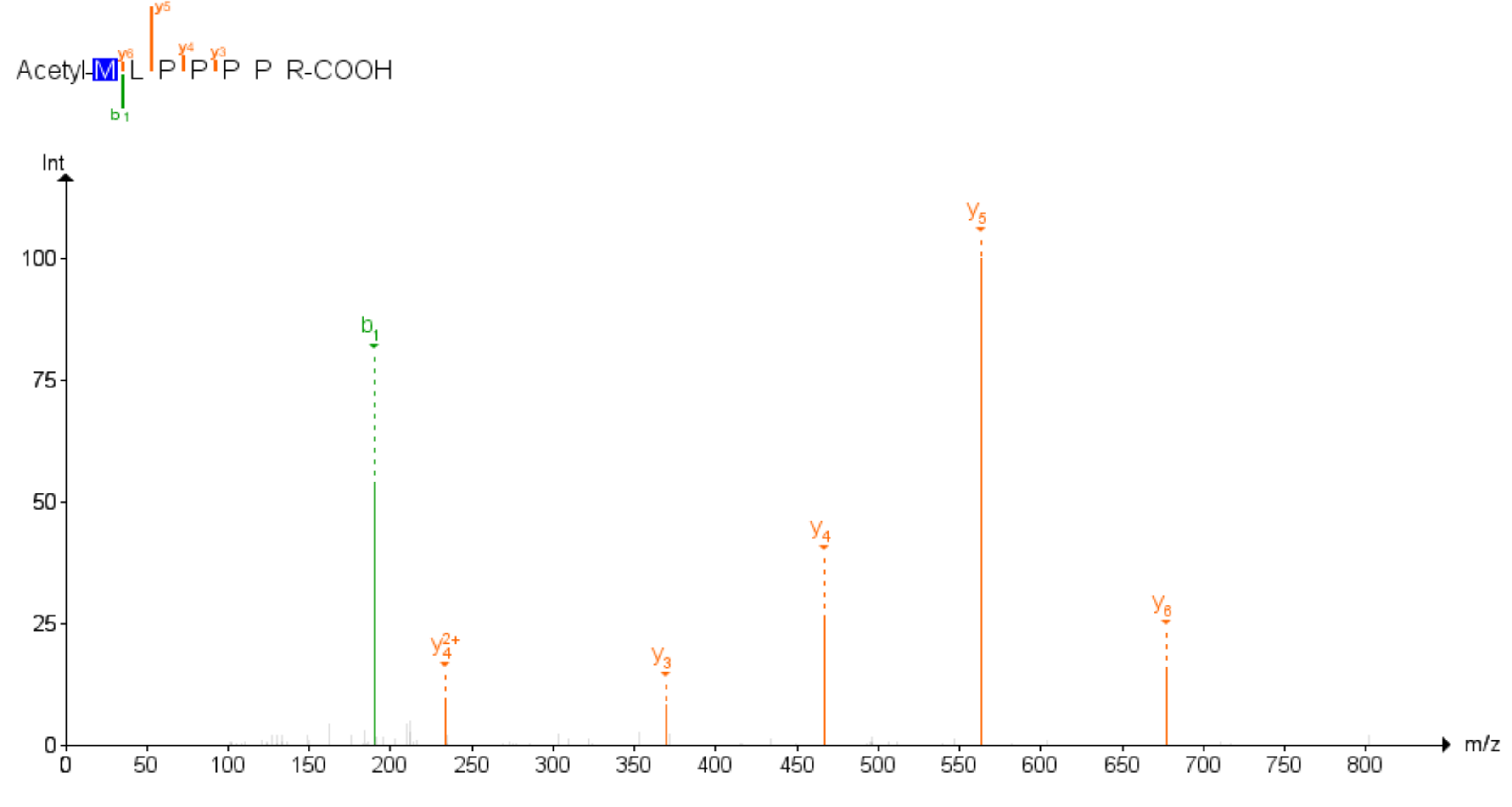

Acetyl-M L P P P P R-COOH
y5
y6
y4
y3
b1
Int
100
75
50
25
0
b1
y4 2+
y3
y4
y5
y6
0
50
100
150
200
250
300
350
400
450
500
550
600
650
700
750
800
m/z

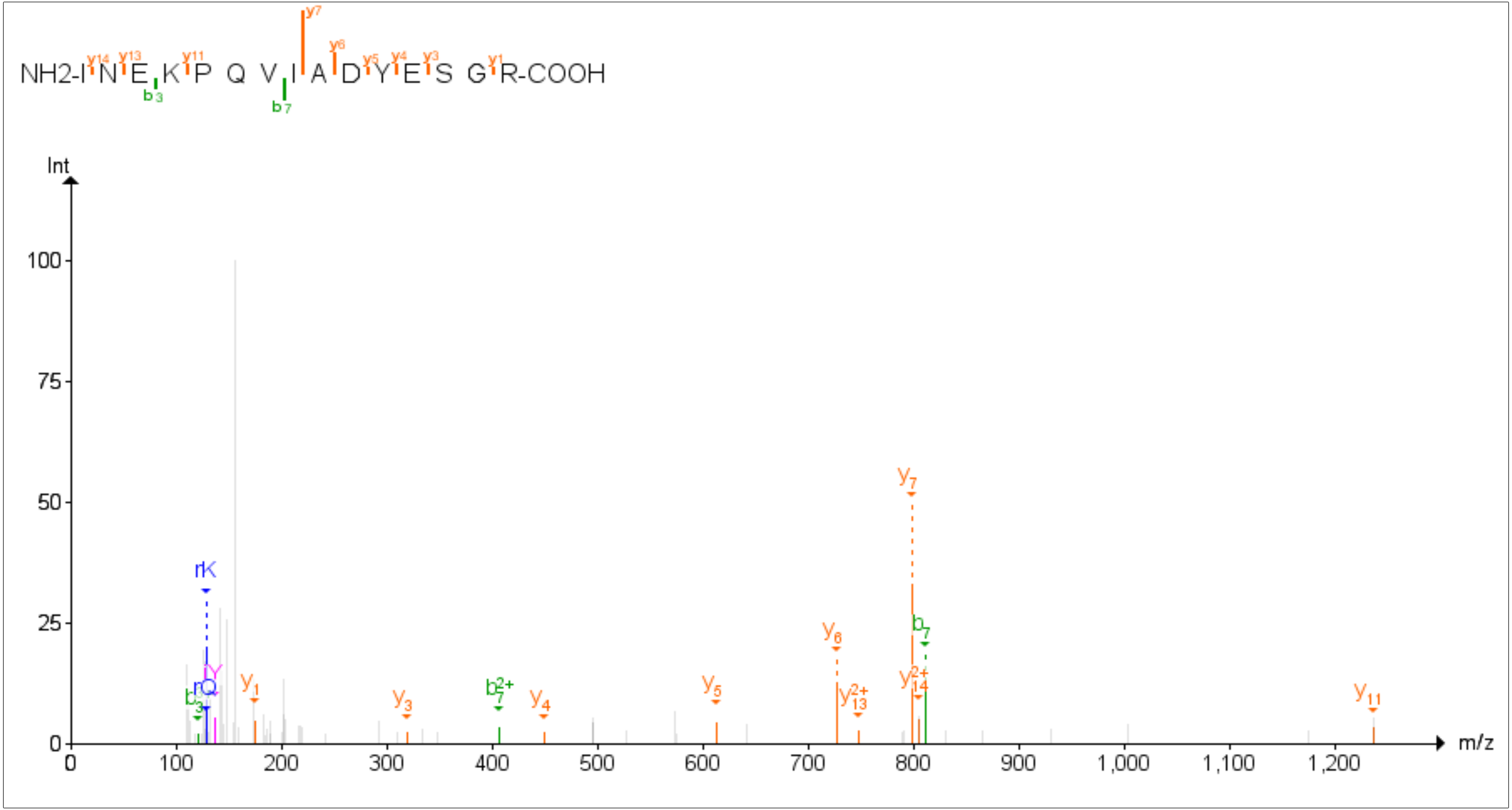

NH2-I N E K P Q V I A D Y E S G R-COOH
y14
y13
y11
y7
y6
y5
y4
y3
y1
b3
b7
Int
100
75
50
25
0
rK
IY
rQ
y1
y3
y4
y5
y6
y7
b7
y11
m/z
0
100
200
300
400
500
600
700
800
900
1,000
1,100
1,200

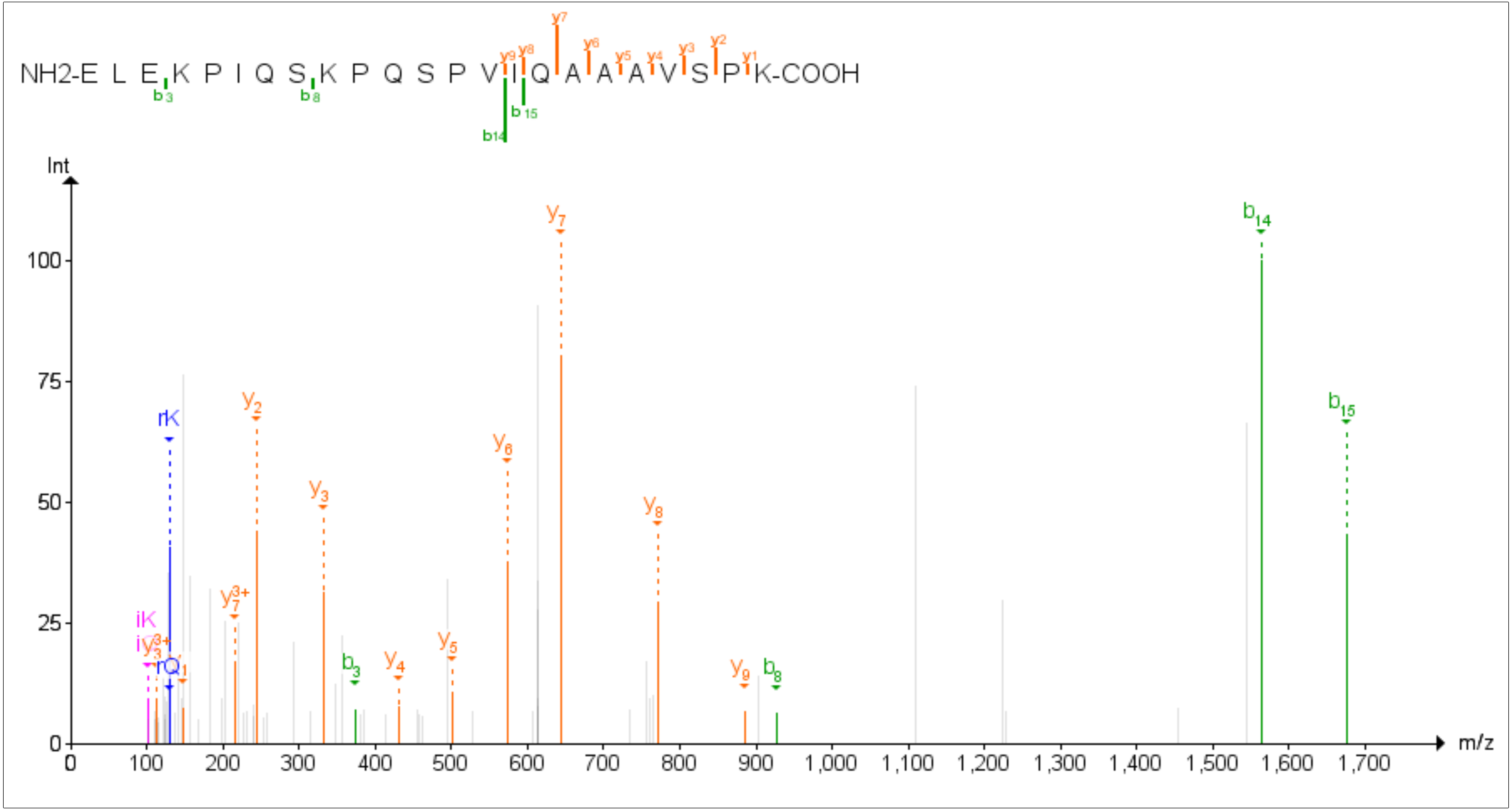
NH2-E L E K P I Q S K P Q S P V I Q A A A V S P K-COOH
b3
b8
b14
b15
y9
y8
y7
y6
y5
y4
y3
y2
y1
Int
m/z
rK
iK
rQ
b3
b8
b14
b15
y1
y2
y3
y4
y5
y6
y7
y8
y9
y3 3+
y7 3+
0
25
50
75
100
0
100
200
300
400
500
600
700
800
900
1,000
1,100
1,200
1,300
1,400
1,500
1,600
1,700

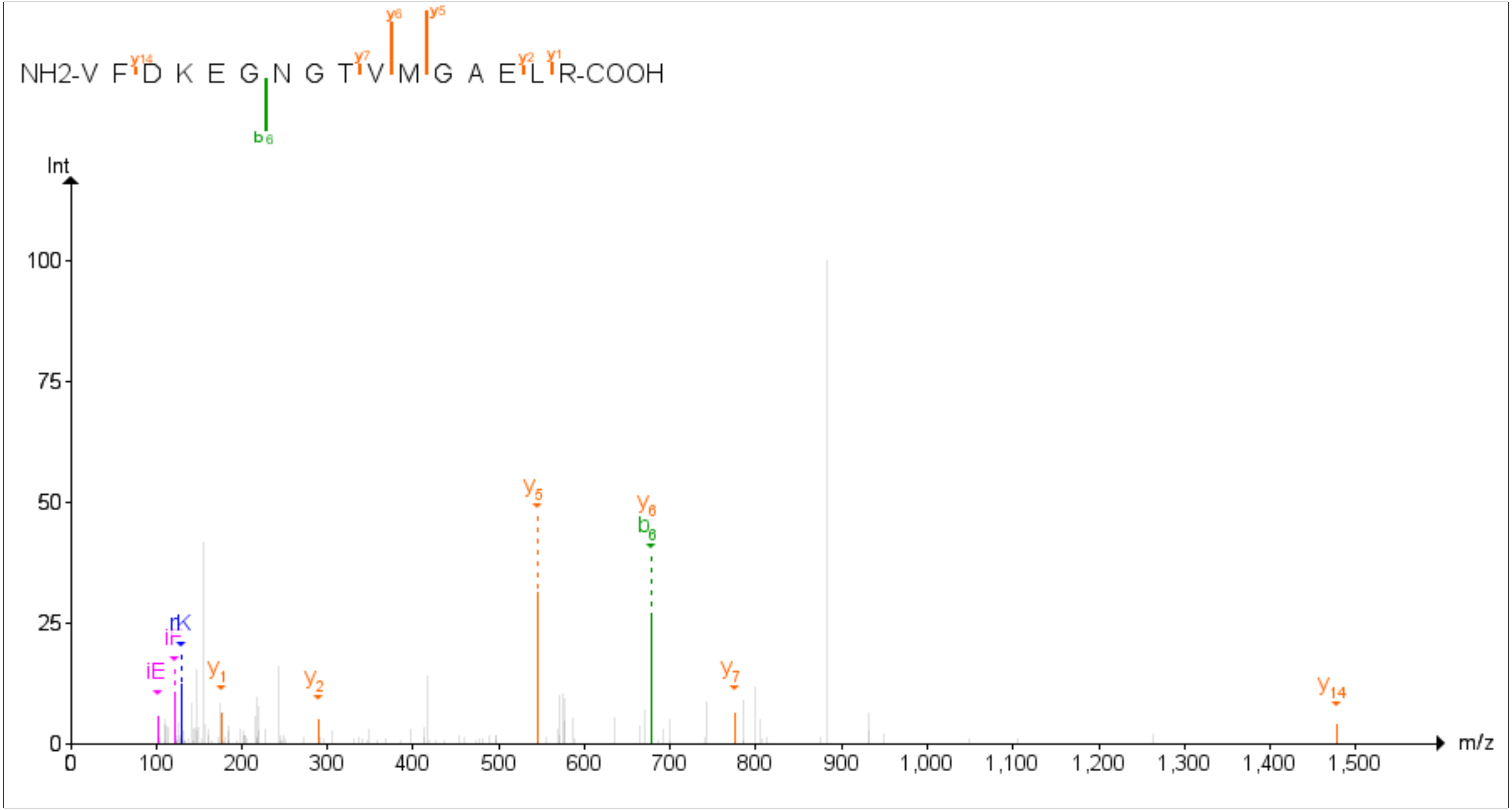
NH2-V F D K E G N G T V M G A E L R-COOH
y14
y7
y6
y5
y2
y1
b6
Int
100
75
50
25
0
m/z
0
100
200
300
400
500
600
700
800
900
1,000
1,100
1,200
1,300
1,400
1,500
iE
iF
rK
y1
y2
y5
y6
b6
y7
y14

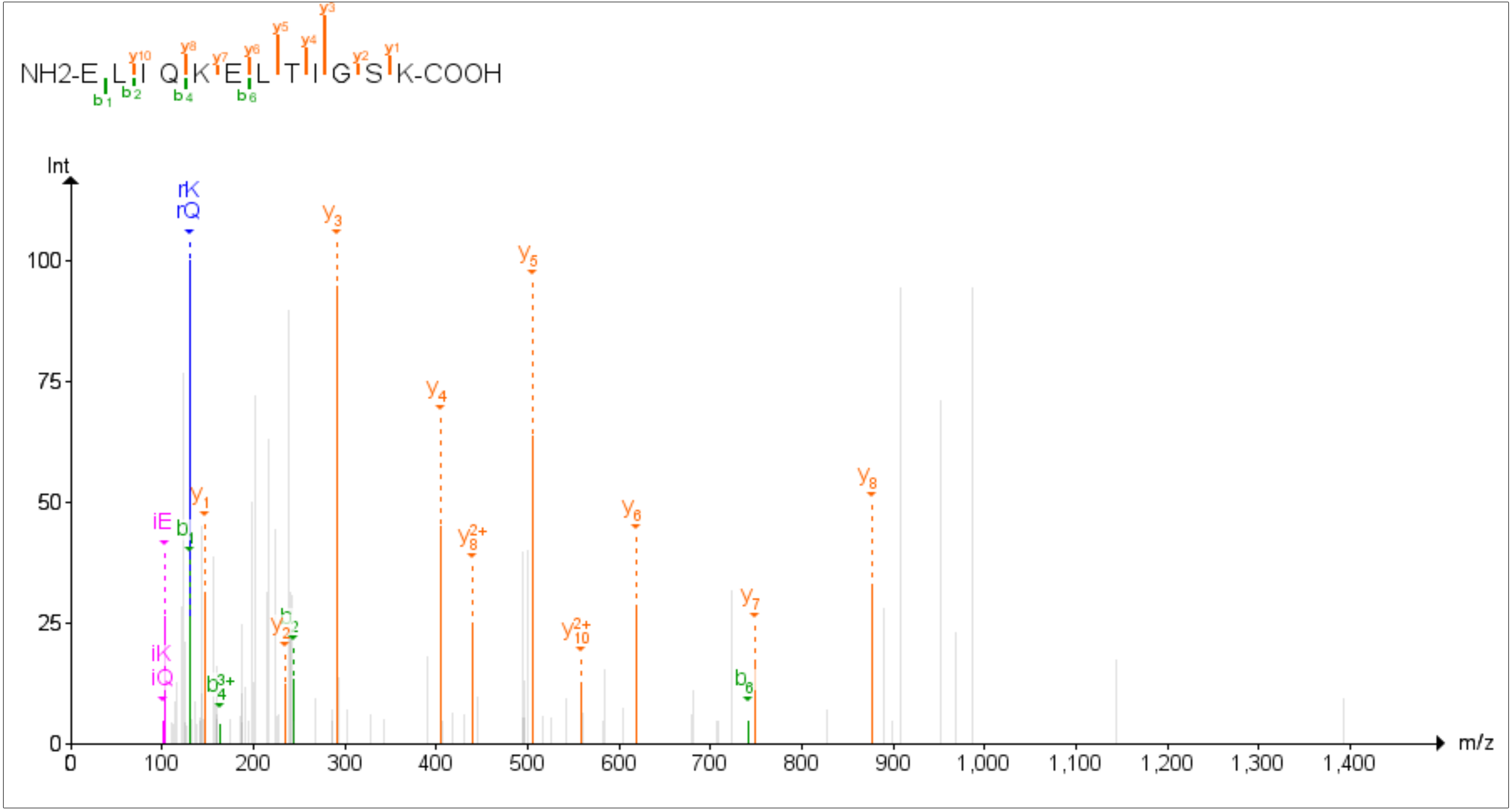

NH2-E L I Q K E L T I G S K-COOH
b1 b2 b4 b6
y10 y8 y7 y6 y5 y4 y3 y2 y1
Int
rK
rQ
y3
y5
y4
y8
y6
y7
iE
b1
y1
iK
iQ
b4 3+
y2
b2
y8 2+
y10 2+
b6
100
75
50
25
0
0
100
200
300
400
500
600
700
800
900
1,000
1,100
1,200
1,300
1,400
m/z

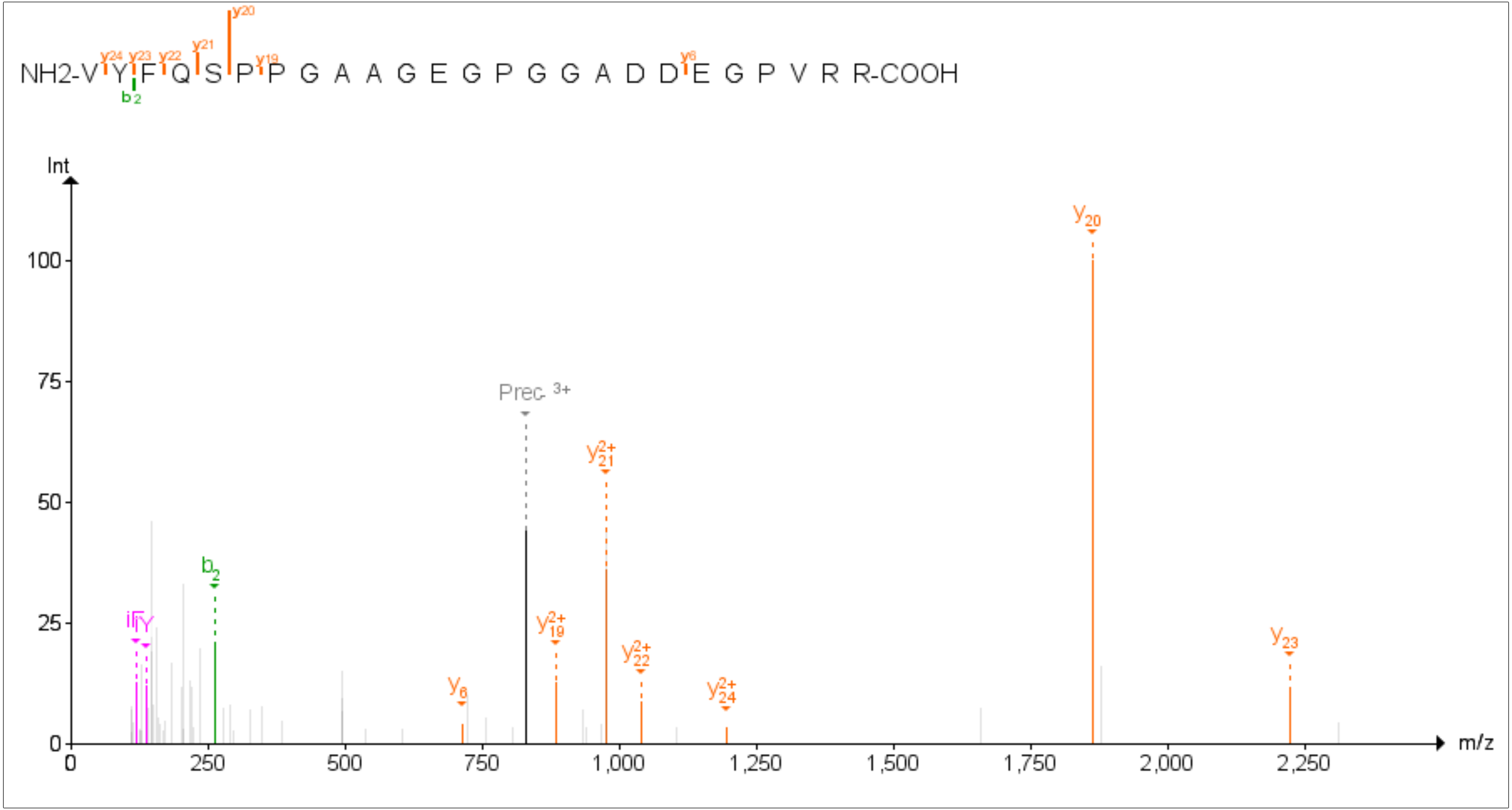

NH2-V Y F Q S P P G A A G E G P G G A D D E G P V R R-COOH
y24 y23 y22 y21 y20 y19 y6
b2
Int
100
75
50
25
0
Prec 3+
y20
y21 2+
y19 2+
y22 2+
y24 2+
y23
y6
b2
iF
iY
0
250
500
750
1,000
1,250
1,500
1,750
2,000
2,250
m/z

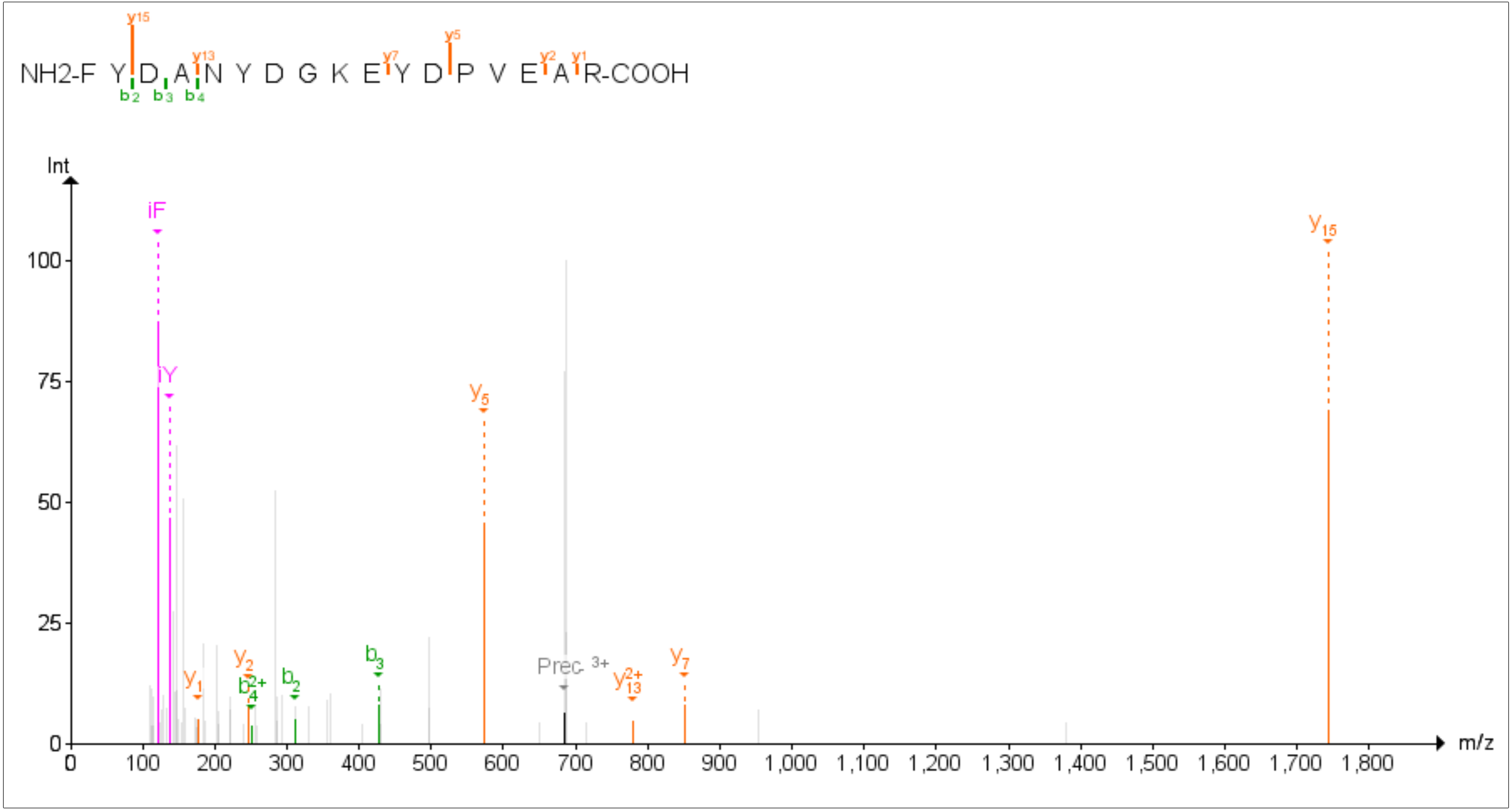

NH2-F Y D A N Y D G K E Y D P V E A R-COOH
y15
y13
y7
y5
y2
y1
b2
b3
b4
Int
iF
iY
y15
y5
y2
y1
b4 2+
b2
b3
Prec- 3+
y13 2+
y7
m/z

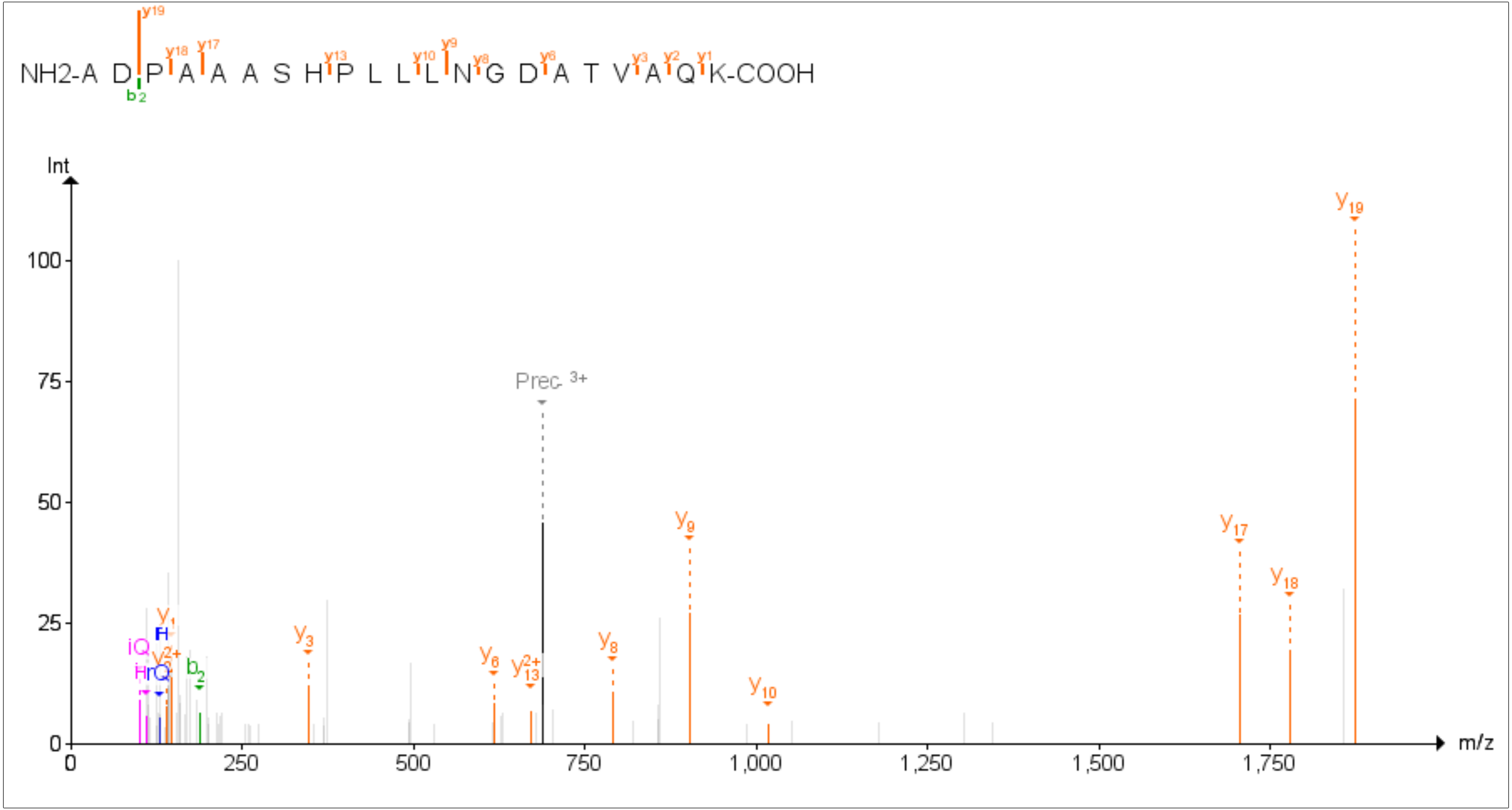

NH2-A D P A A A S H P L L L N G D A T V A Q K-COOH
y19
y18
y17
y13
y10
y9
y8
y6
y3
y2
y1
b2
Int
Prec 3+
m/z
0
25
50
75
100
250
500
750
1,000
1,250
1,500
1,750

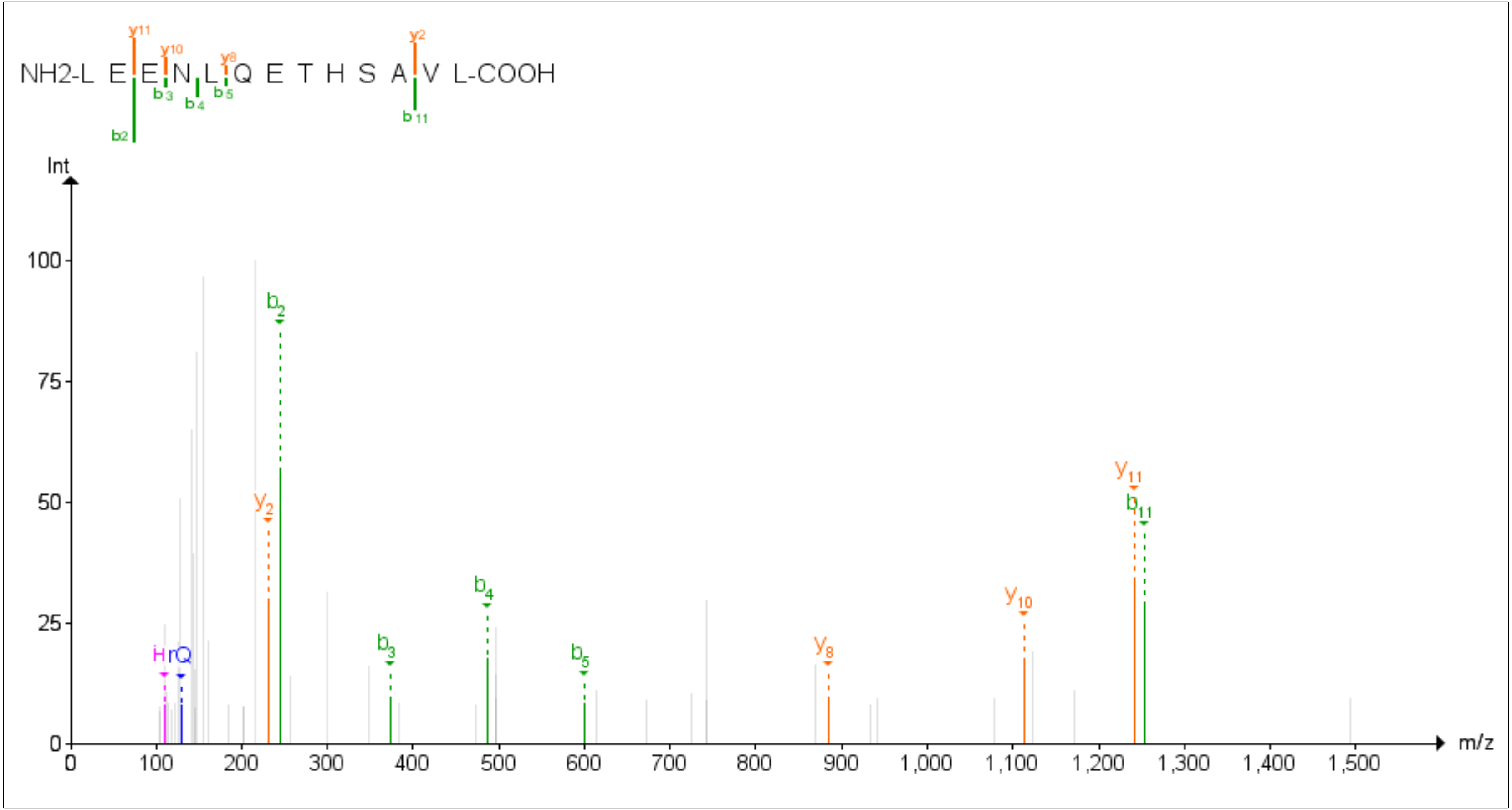

NH2-L E E N L Q E T H S A V L-COOH
y11
y10
y8
y2
b2
b3
b4
b5
b11
Int
100
75
50
25
0
m/z
0
100
200
300
400
500
600
700
800
900
1,000
1,100
1,200
1,300
1,400
1,500
b2
y2
b3
b4
b5
y8
y10
y11
b11
iH
rQ

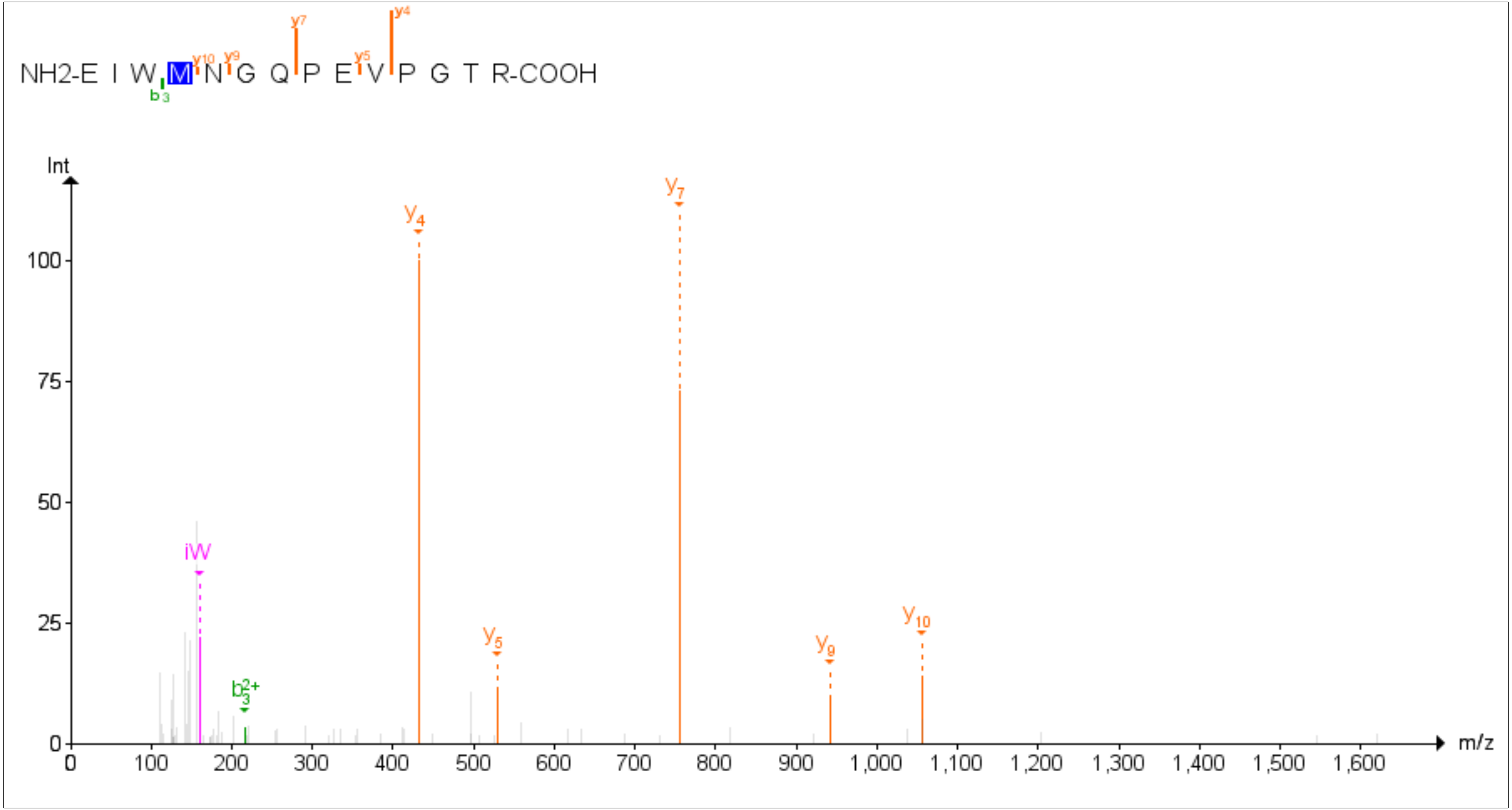

NH2-E I W M N G Q P E V P G T R-COOH
b3
y10
y9
y7
y5
y4
Int
100
75
50
25
0
iW
b3 2+
y4
y5
y7
y9
y10
0
100
200
300
400
500
600
700
800
900
1,000
1,100
1,200
1,300
1,400
1,500
1,600
m/z

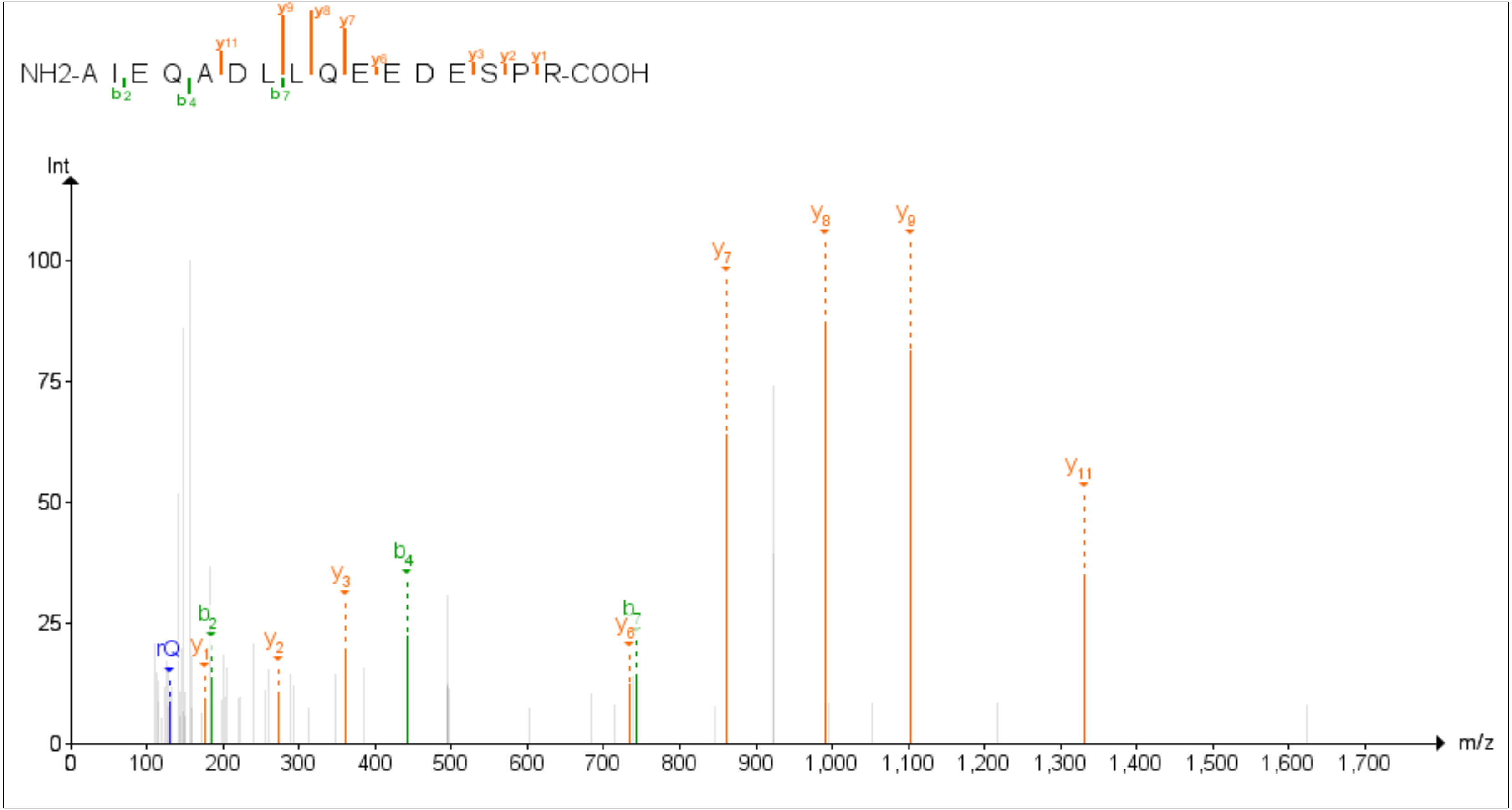

NH2-A I E Q A D L L Q E E D E S P R-COOH
y11 y9 y8 y7 y6 y3 y2 y1
b2 b4 b7
Int
100
75
50
25
0
rQ y1 b2 y2 y3 b4 y6 b7 y7 y8 y9 y11
0 100 200 300 400 500 600 700 800 900 1,000 1,100 1,200 1,300 1,400 1,500 1,600 1,700
m/z

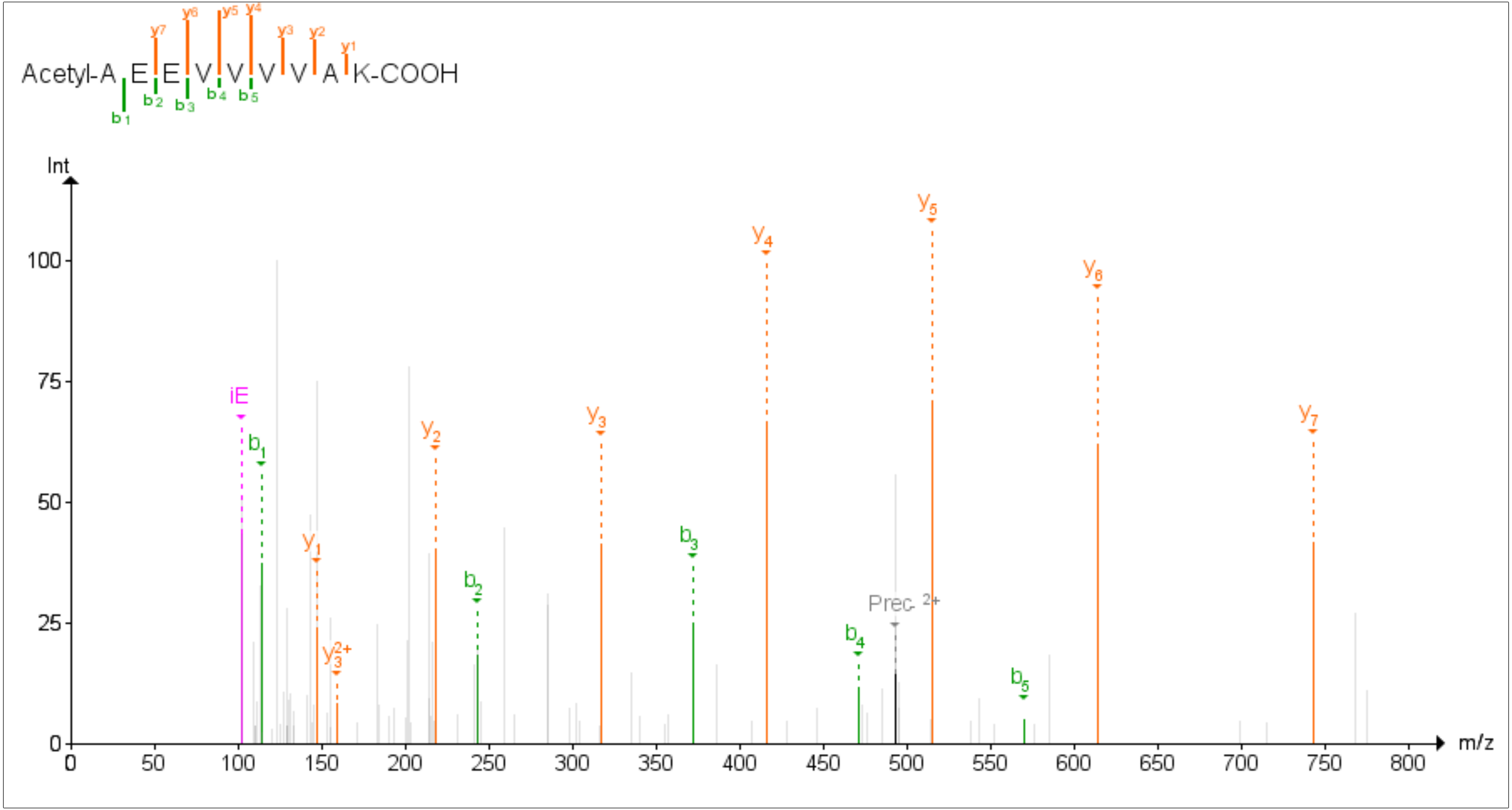

Acetyl-A E E V V V V A K-COOH
y7 y6 y5 y4 y3 y2 y1
b1 b2 b3 b4 b5
Int
100
75
50
25
0
iE
b1
y1
y3 2+
y2
b2
y3
b3
y4
Prec 2+
b4
y5
b5
y6
y7
0 50 100 150 200 250 300 350 400 450 500 550 600 650 700 750 800
m/z

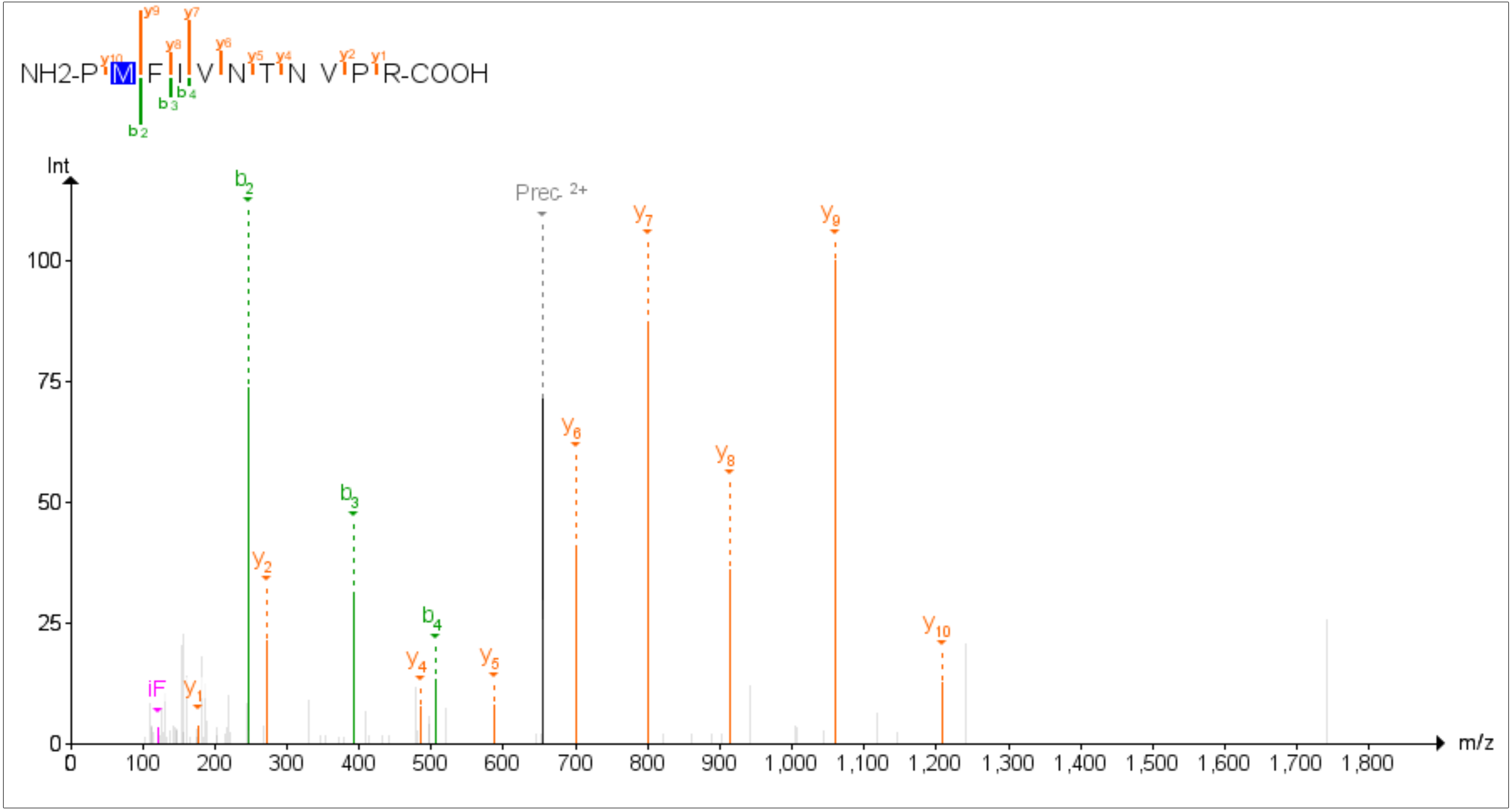

NH2-P M F I V N T N V P R-COOH
y10 y9 y8 y7 y6 y5 y4 y2 y1
b2 b3 b4
Int
Prec 2+
m/z
0
25
50
75
100
0
100
200
300
400
500
600
700
800
900
1,000
1,100
1,200
1,300
1,400
1,500
1,600
1,700
1,800
iF

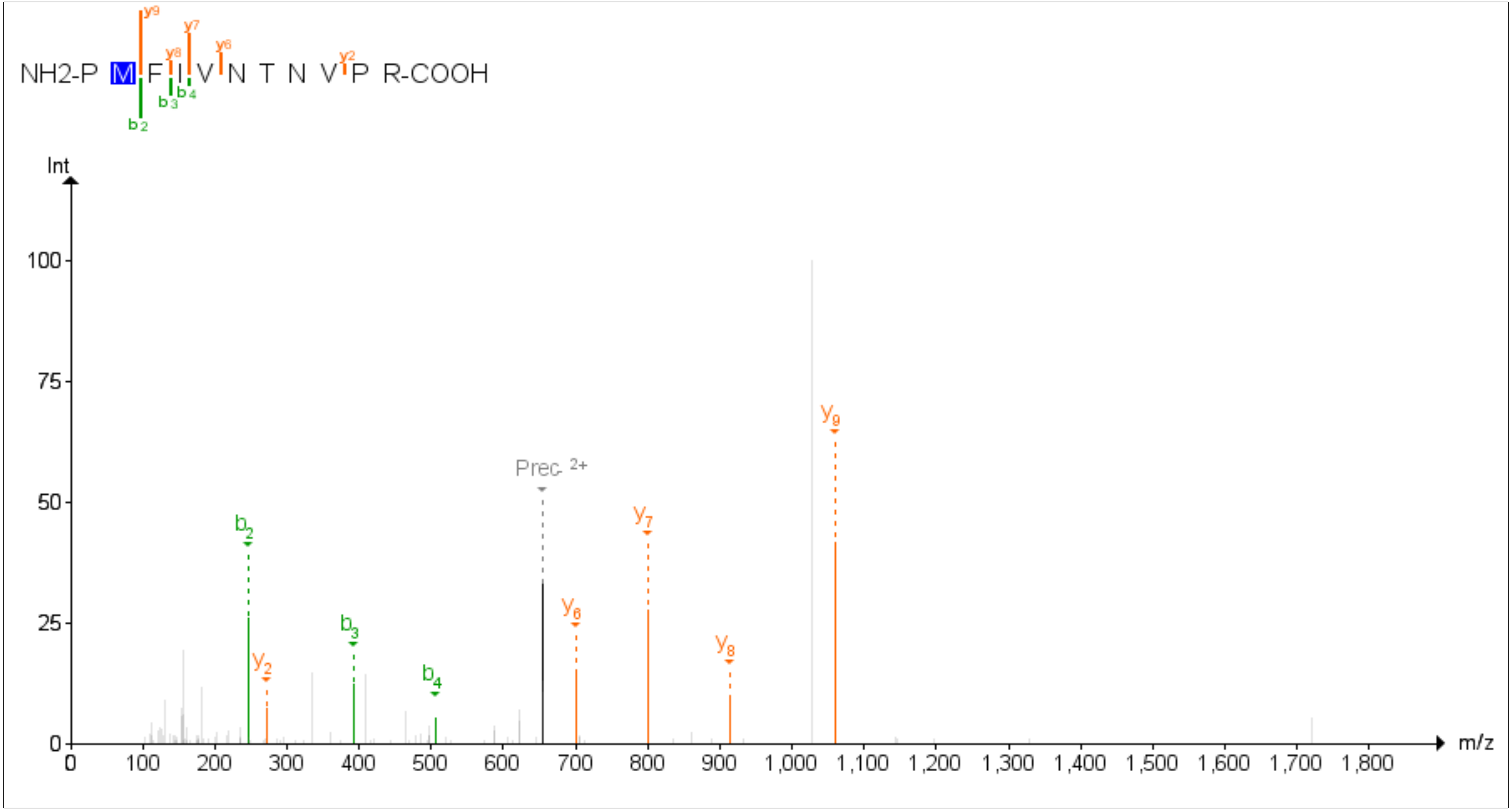

NH2-P M F I V N T N V P R-COOH
y9
y7
y8
y6
y2
b2
b3
b4
Int
100
75
50
25
0
Prec 2+
b2
y2
b3
b4
y6
y7
y8
y9
0
100
200
300
400
500
600
700
800
900
1,000
1,100
1,200
1,300
1,400
1,500
1,600
1,700
1,800
m/z

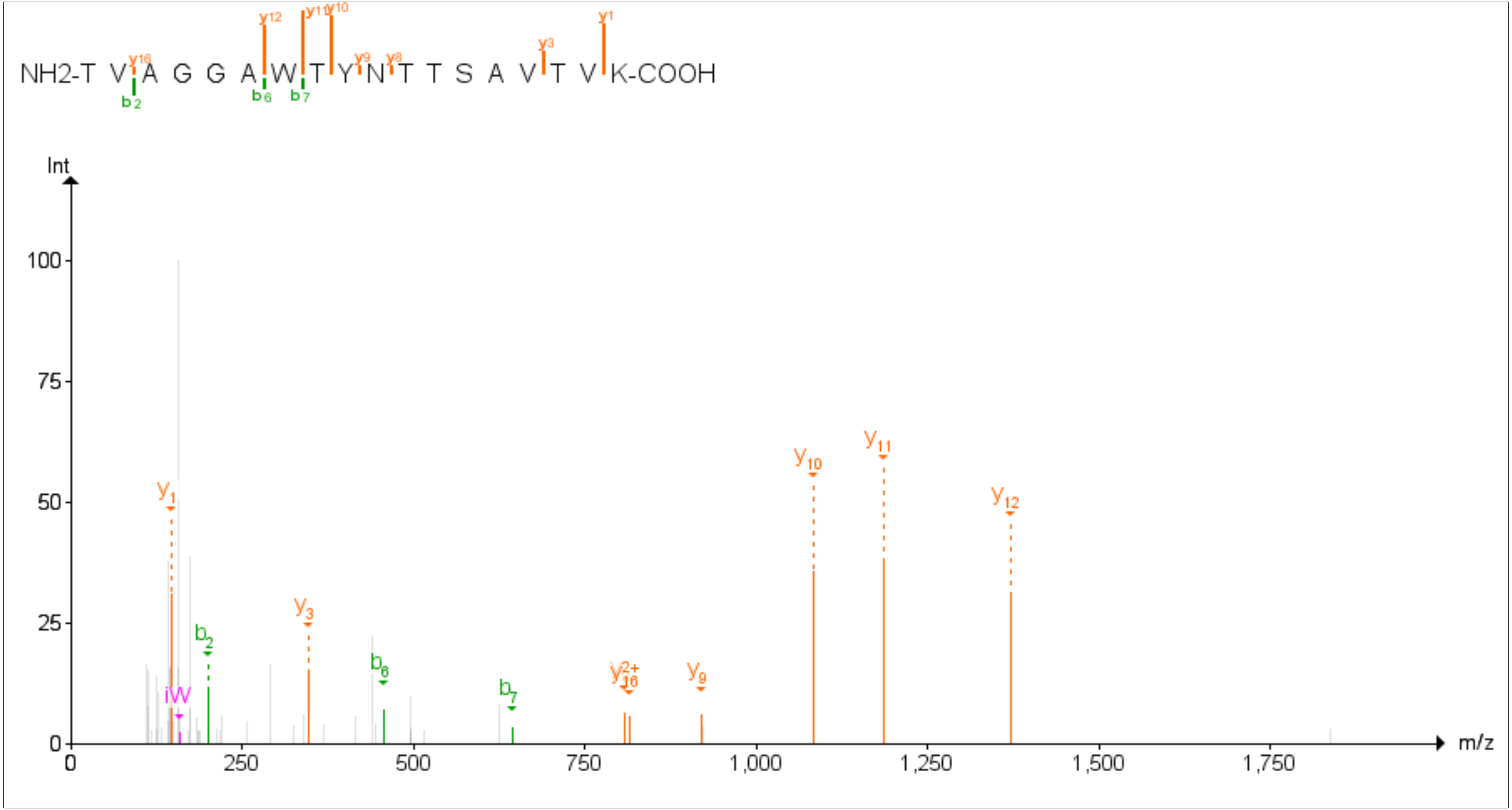

NH2-T V A G G A W T Y N T T S A V T V K-COOH
y16
y12
y11
y10
y9
y8
y3
y1
b2
b6
b7
Int
100
75
50
25
0
y1
iW
b2
y3
b6
b7
y16 2+
y9
y10
y11
y12
0
250
500
750
1,000
1,250
1,500
1,750
m/z

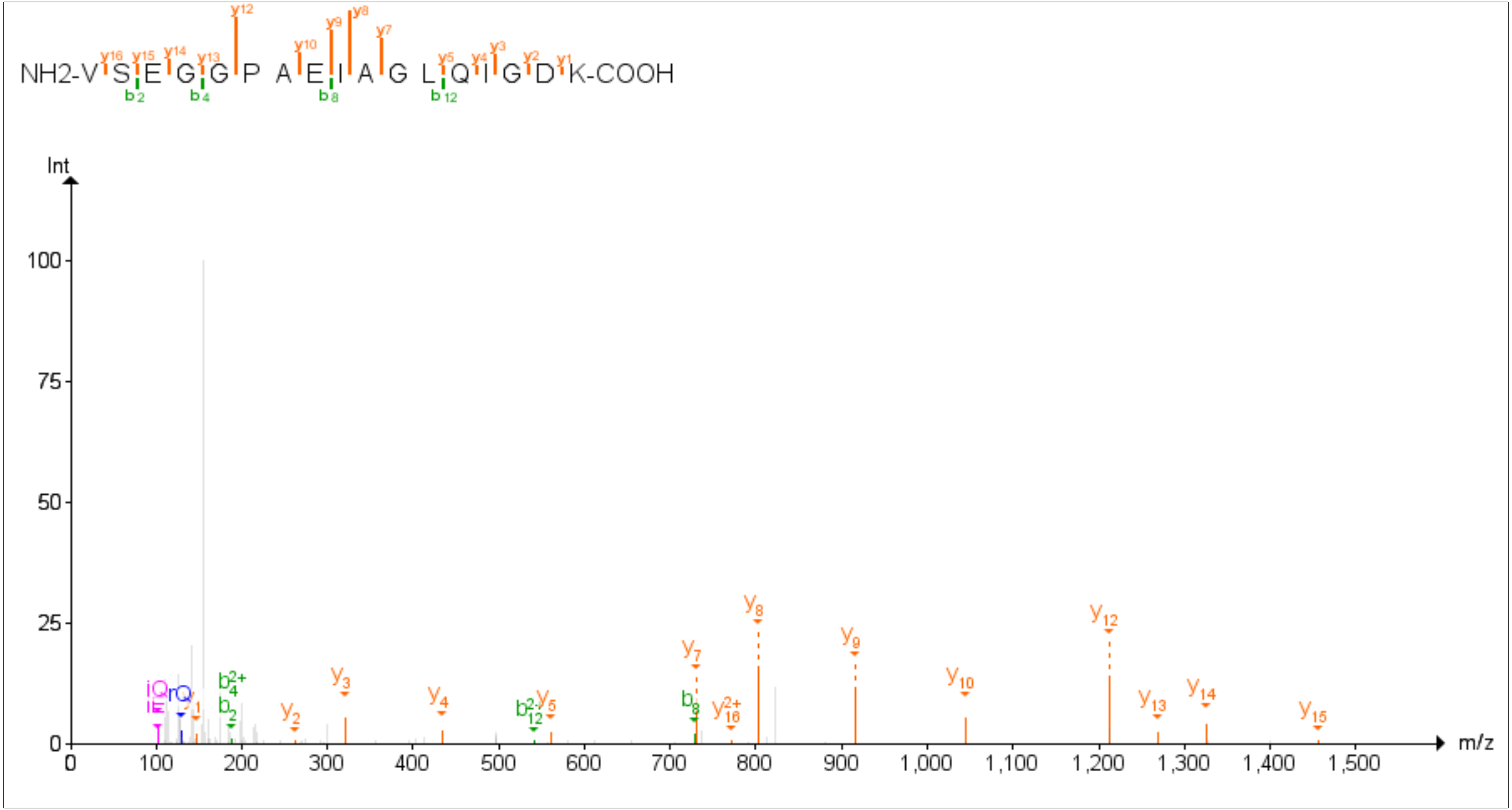

NH2-V S E G G P A E I A G L Q I G D K-COOH
Int
m/z

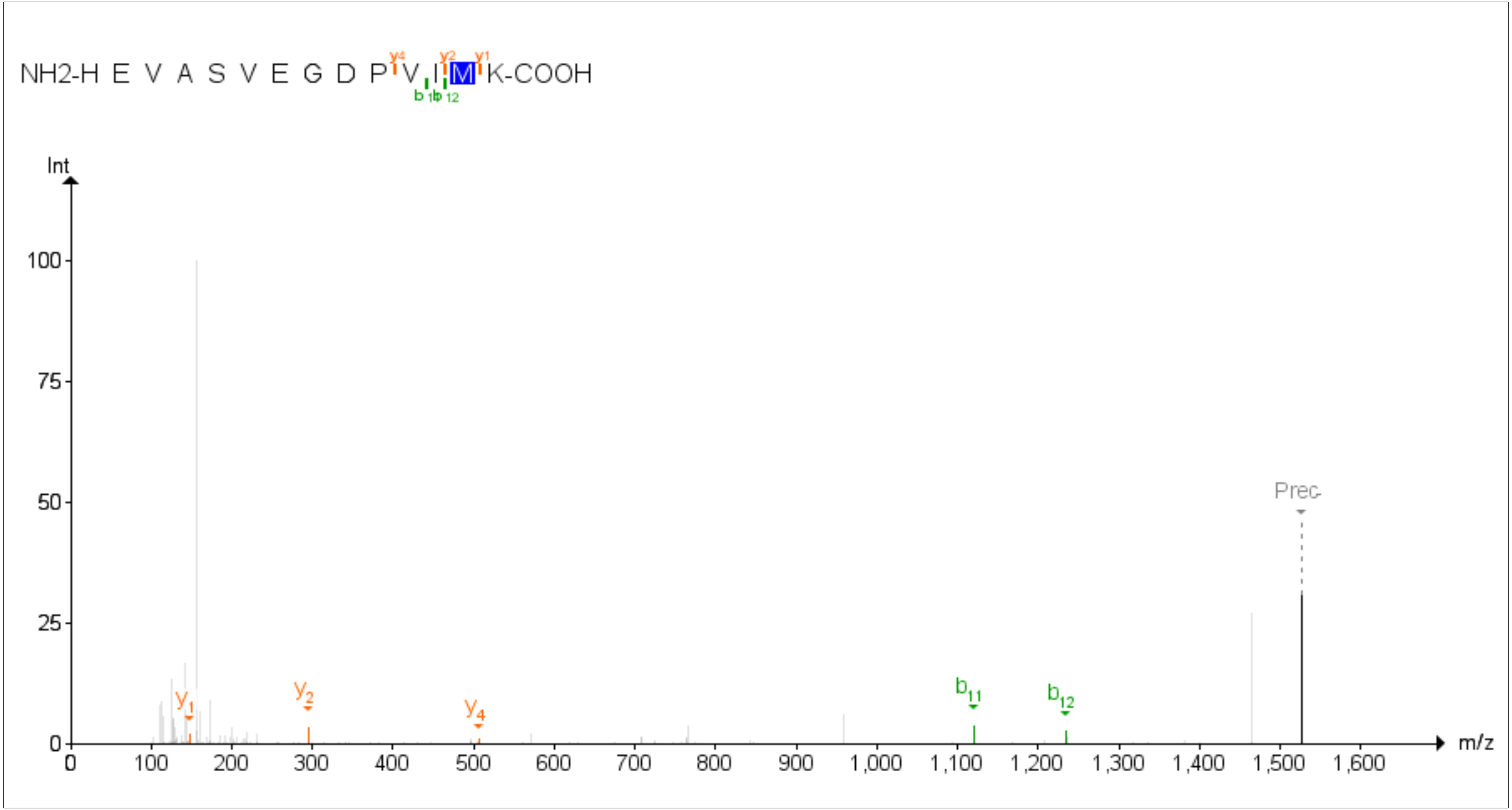

NH2-H E V A S V E G D P V I M K-COOH
y4
y2
y1
b 11
b 12
Int
100
75
50
25
0
Prec
y1
y2
y4
b11
b12
0
100
200
300
400
500
600
700
800
900
1,000
1,100
1,200
1,300
1,400
1,500
1,600
m/z

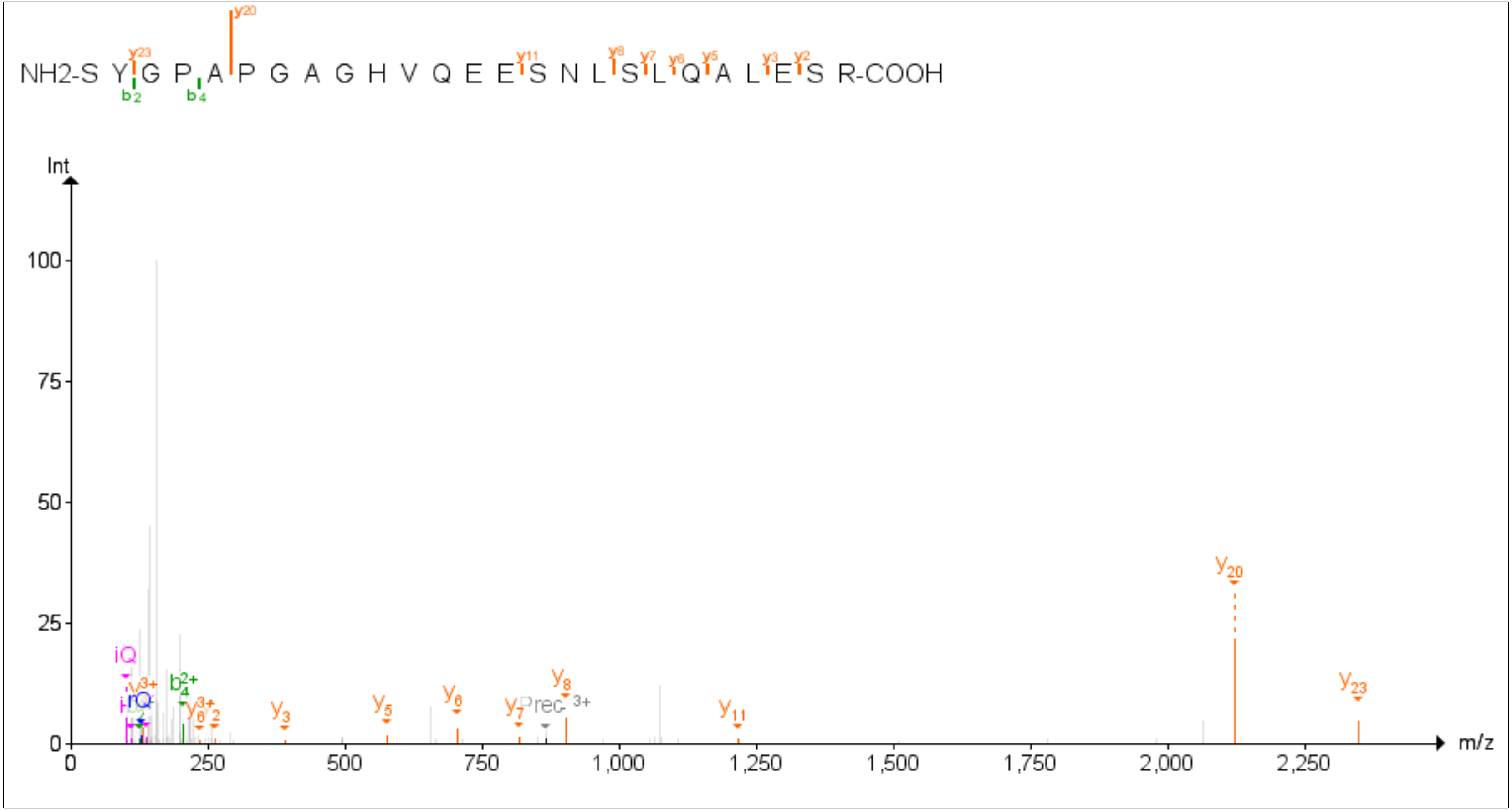
NH2-S Y G P A P G A G H V Q E E S N L S L Q A L E S R-COOH
y20
y23
y11
y8
y7
y6
y5
y3
y2
b2
b4
Int
100
75
50
25
0
iQ
b4 2+
y3
y5
y6
y7
Prec 3+
y8
y11
y20
y23
0
250
500
750
1,000
1,250
1,500
1,750
2,000
2,250
m/z

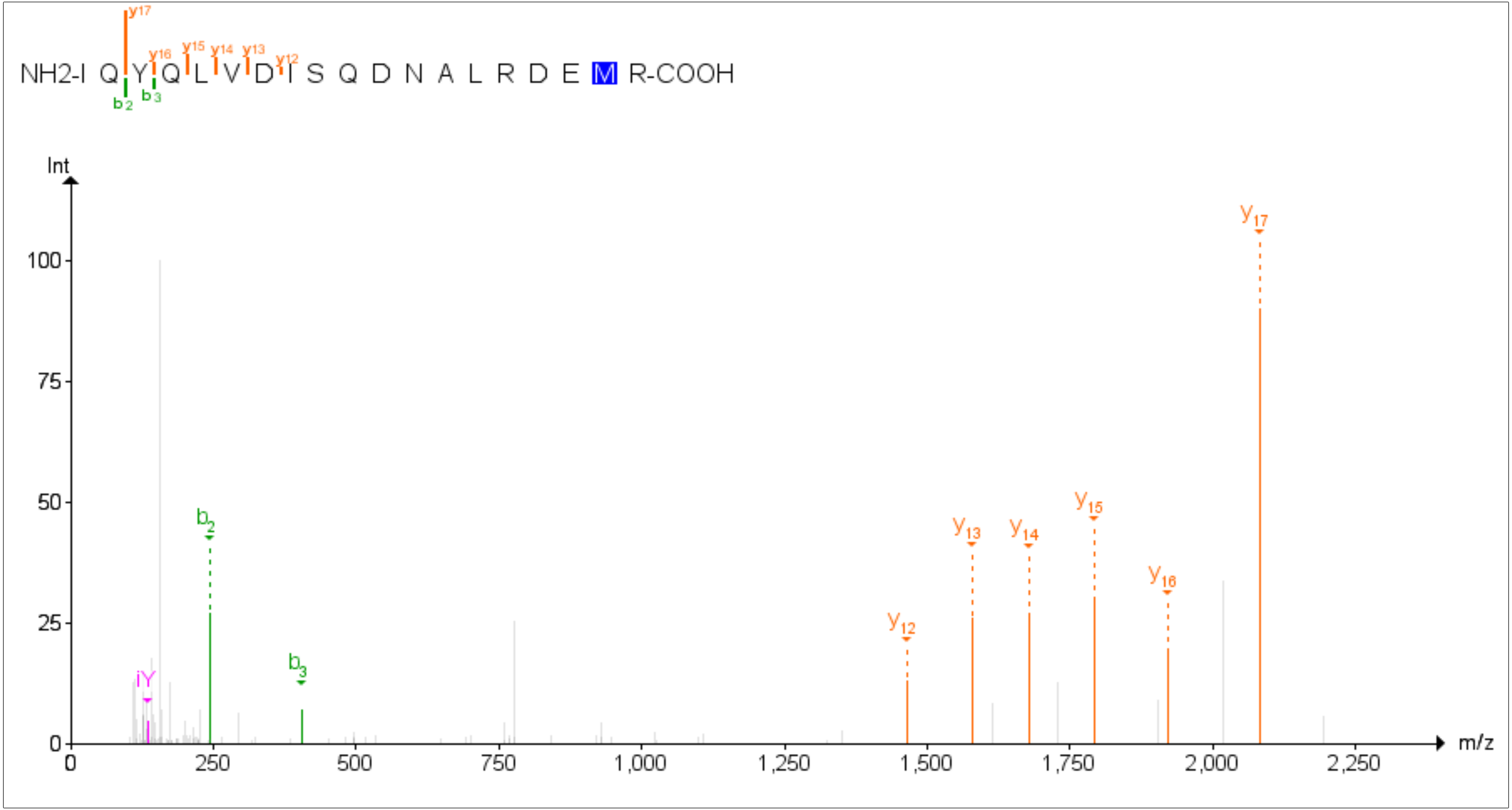

NH2-I Q Y Q L V D I S Q D N A L R D E M R-COOH
y17
y16
y15
y14
y13
y12
b2
b3
Int
100
75
50
25
0
iY
y17
y16
y15
y14
y13
y12
b2
b3
0
250
500
750
1,000
1,250
1,500
1,750
2,000
2,250
m/z

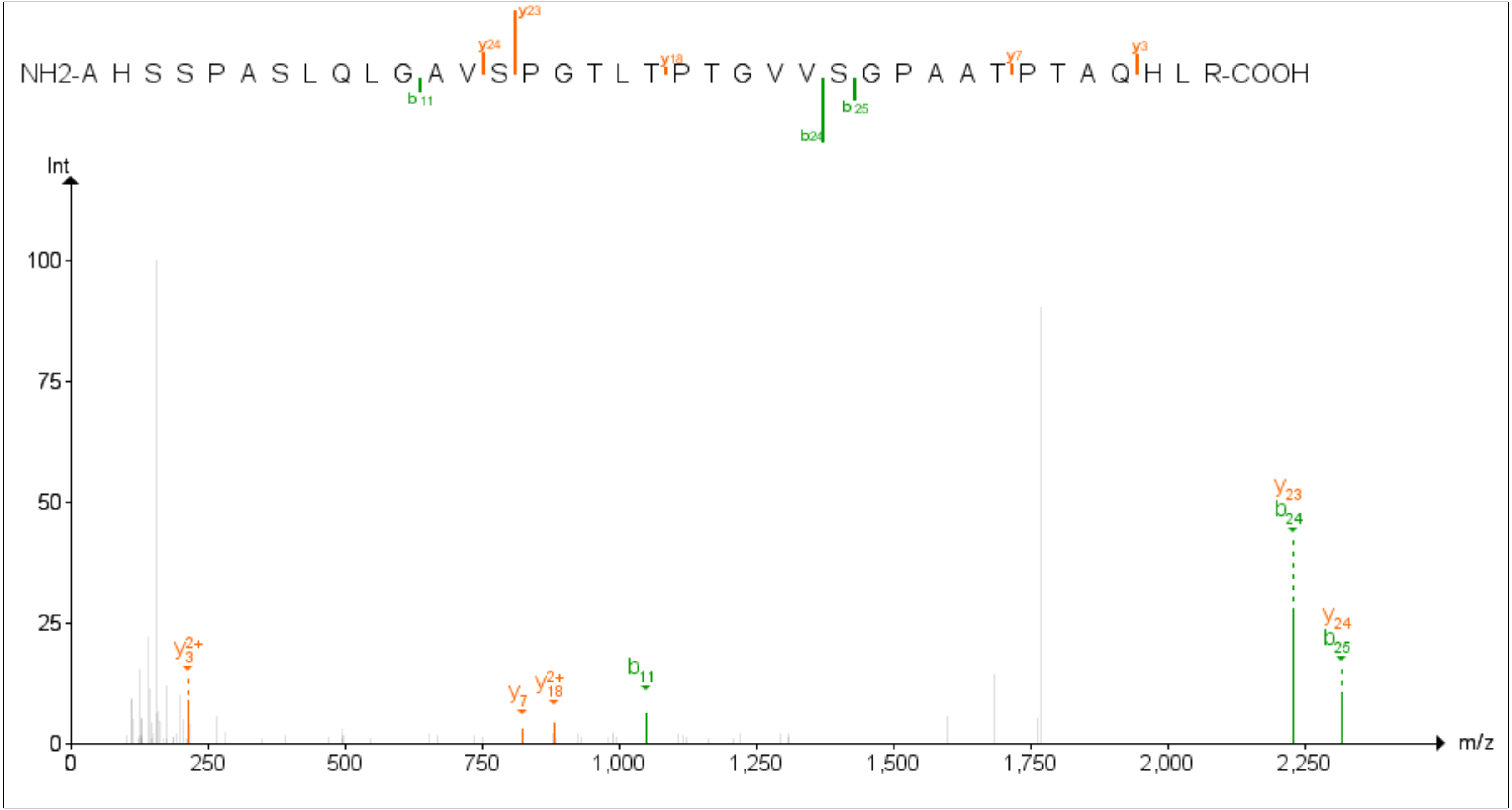

NH2-A H S S P A S L Q L G A V S P G T L T P T G V V S G P A A T P T A Q H L R-COOH
y23
y24
y18
y7
y3
b11
b24
b25
Int
100
75
50
25
0
250
500
750
1,000
1,250
1,500
1,750
2,000
2,250
m/z
y3 2+
y7
y18 2+
b11
y23
b24
y24
b25

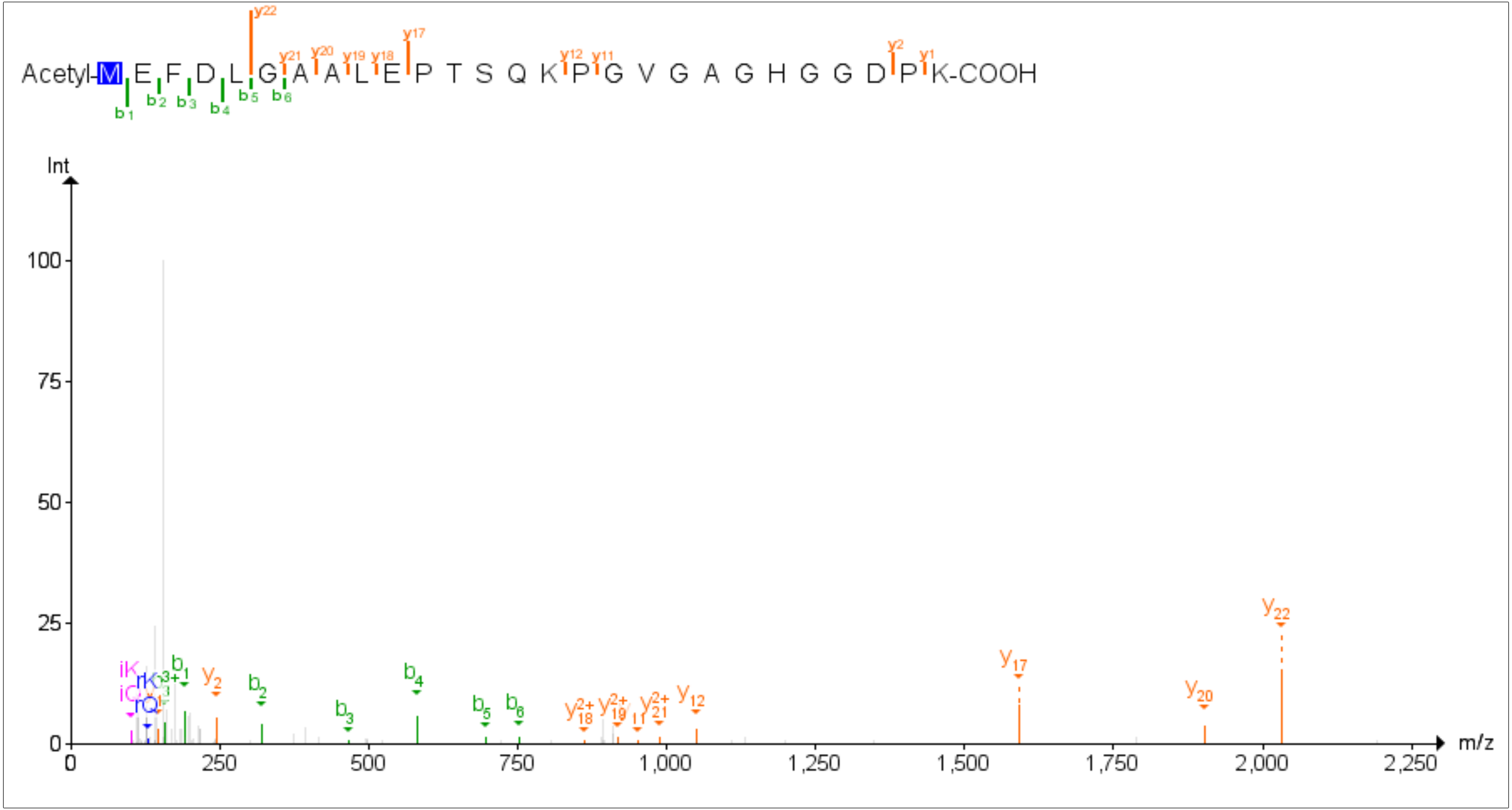

Acetyl-M E F D L G A A L E P T S Q K P G V G A G H G G D P K-COOH
Int
m/z
y22
y17
y20
y12
b4
b2
b3
b5
b6
b1
y2

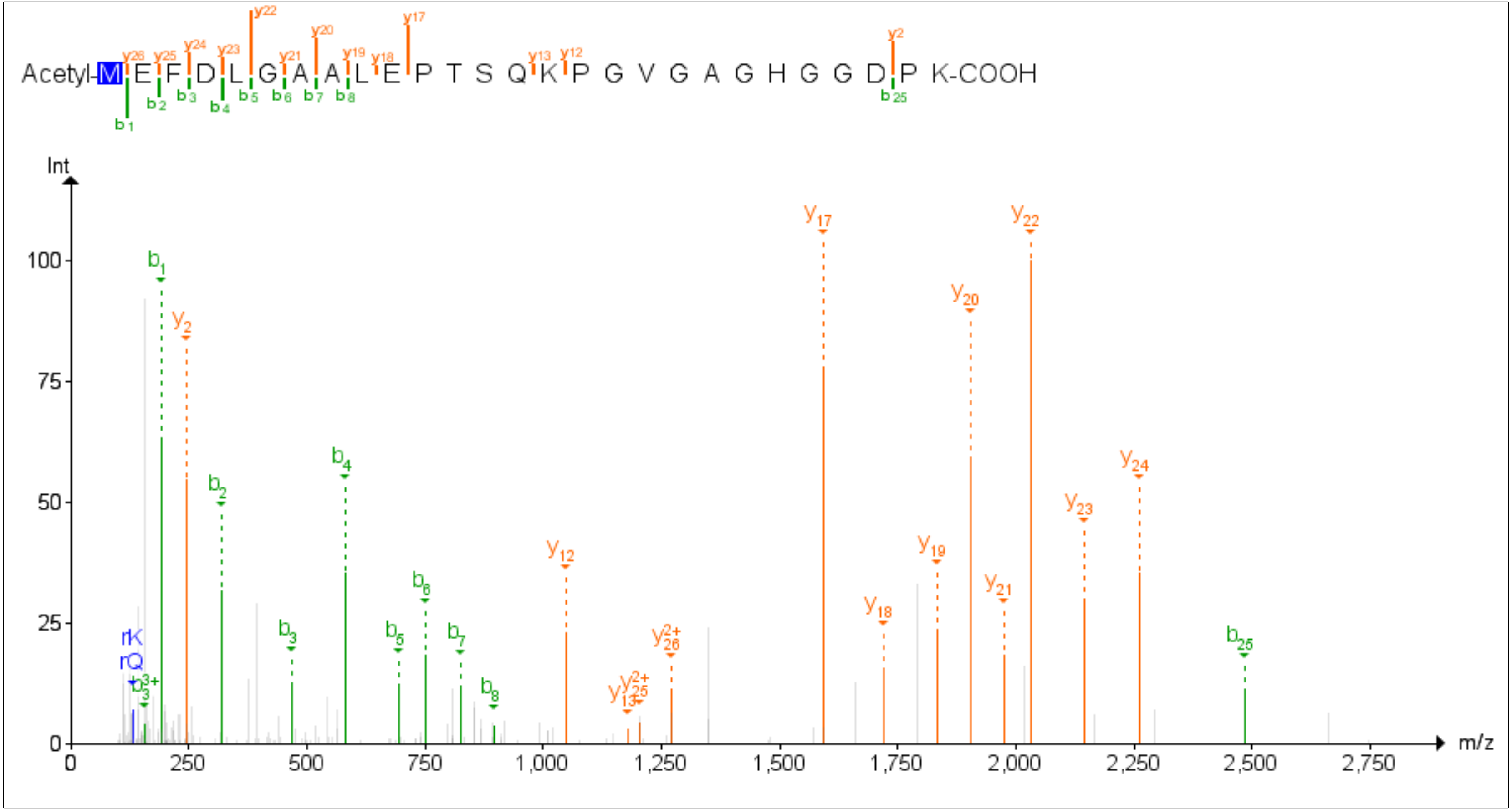

Acetyl-M E F D L G A A L E P T S Q K P G V G A G H G G D P K-COOH
Int
m/z

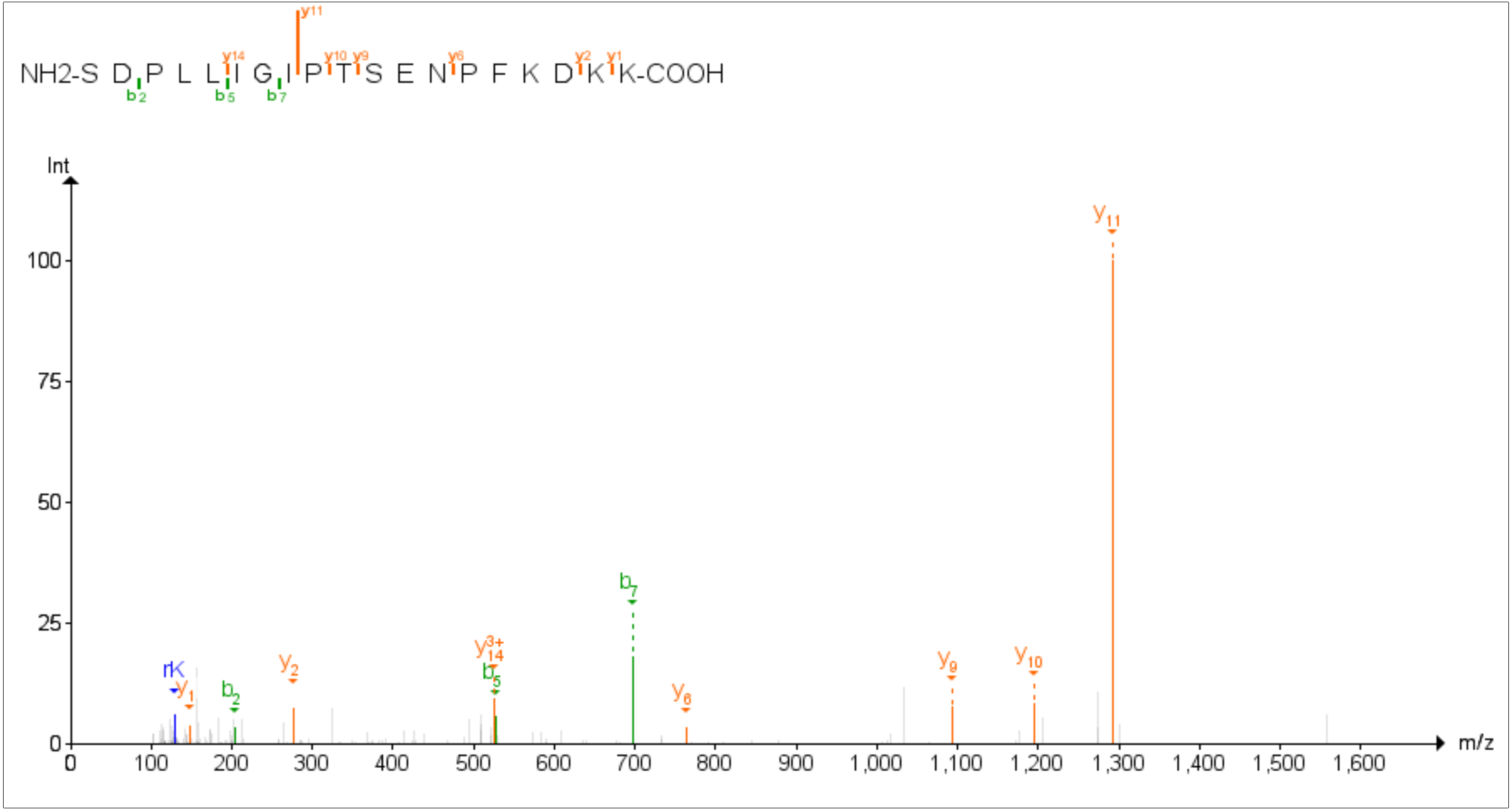

NH2-S D P L L I G I P T S E N P F K D K K-COOH
Int
m/z
y11
y10
y9
y6
y2
y1
b7
b5
b2
rK
0
25
50
75
100
0
100
200
300
400
500
600
700
800
900
1,000
1,100
1,200
1,300
1,400
1,500
1,600

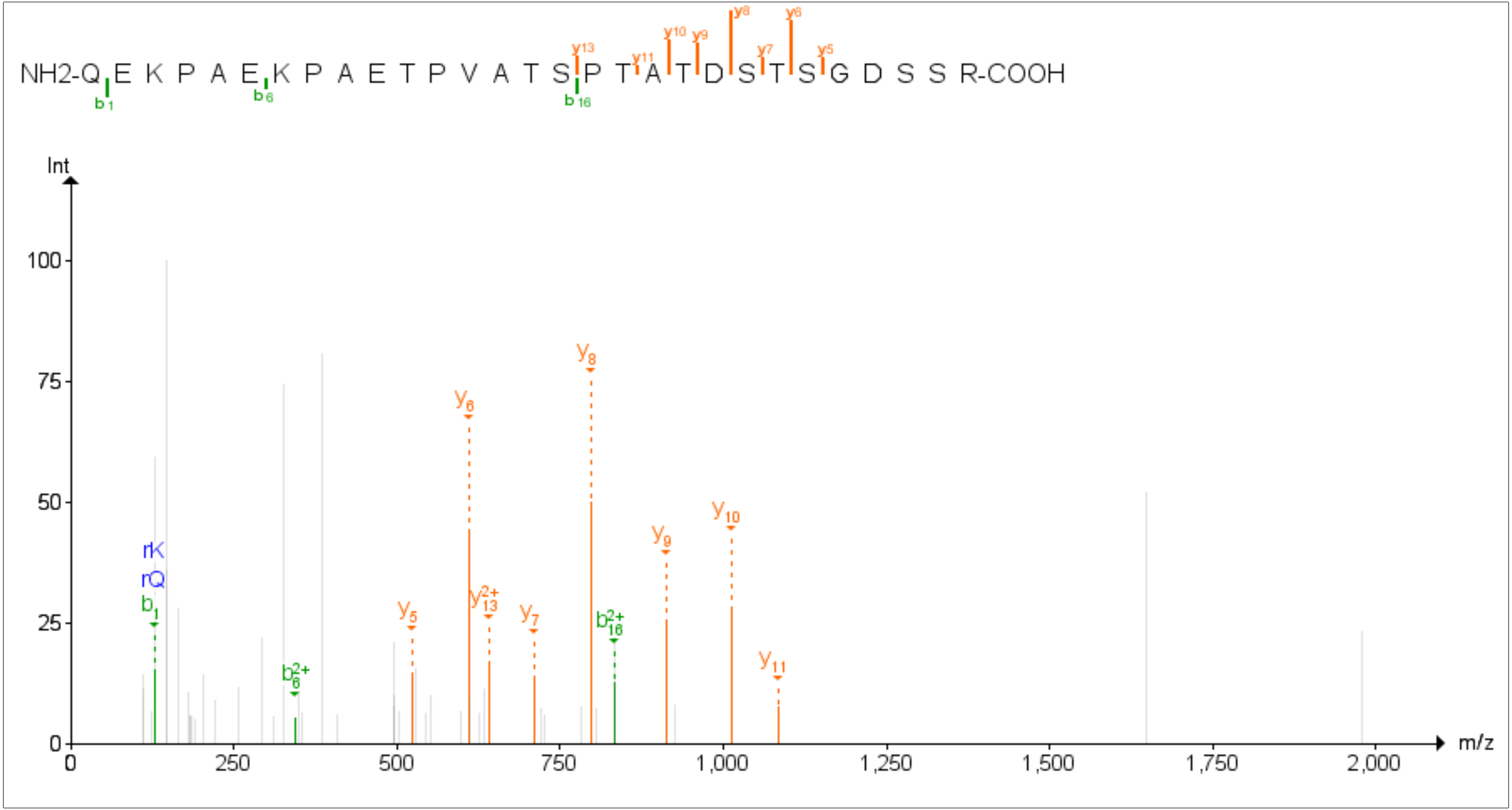

NH2-Q E K P A E K P A E T P V A T S P T A T D S T S G D S S R-COOH
b1
b6
b16
y13
y11
y10
y9
y8
y7
y6
y5
Int
100
75
50
25
0
rK
rQ
b1
b6 2+
y5
y6
y13 2+
y7
y8
b16 2+
y9
y10
y11
0
250
500
750
1,000
1,250
1,500
1,750
2,000
m/z

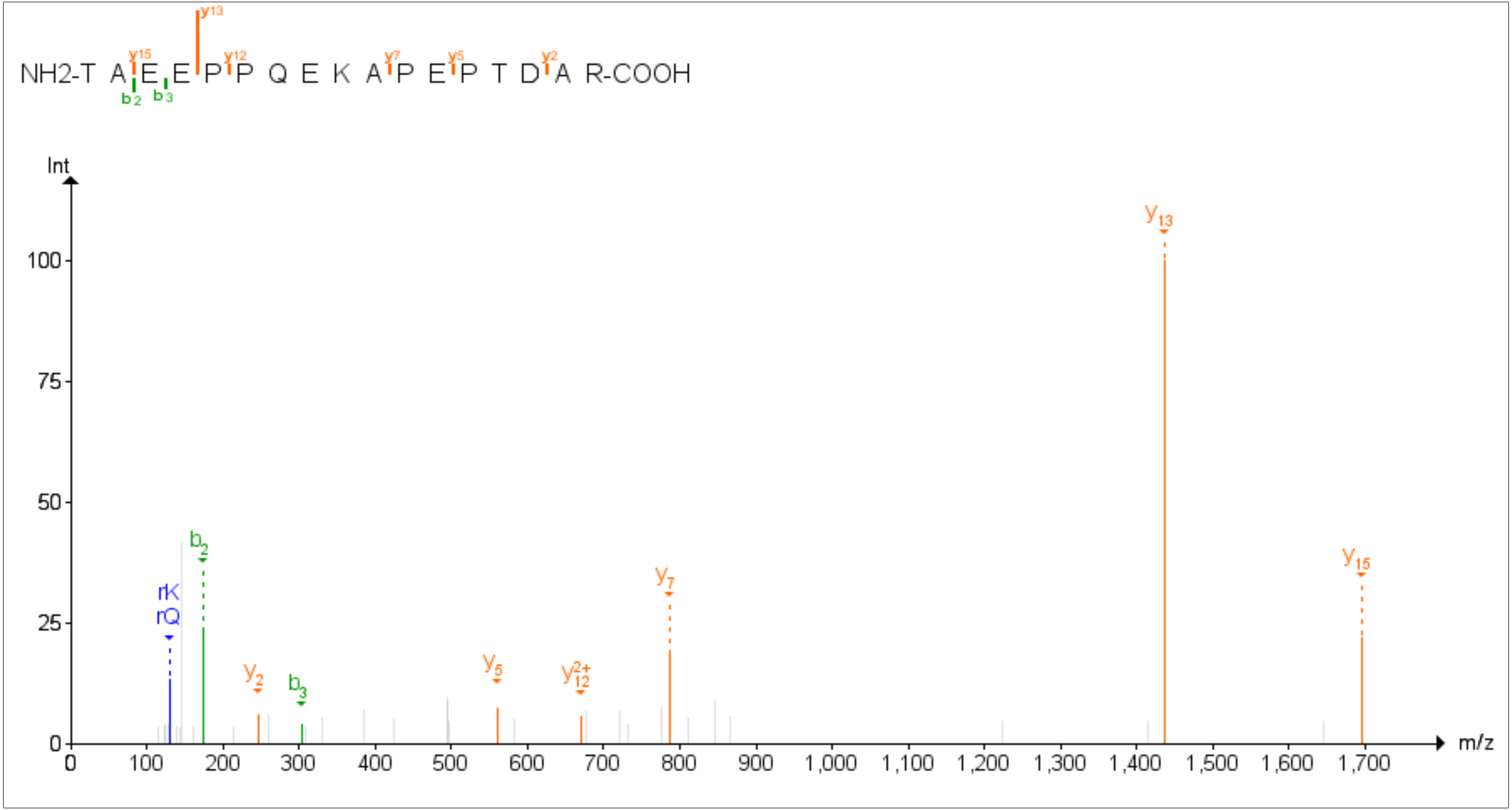

NH2-T A E E P P Q E K A P E P T D A R-COOH
y15
y13
y12
y7
y5
y2
b2
b3
Int
100
75
50
25
0
rK
rQ
b2
y2
b3
y5
y12 2+
y7
y13
y15
0
100
200
300
400
500
600
700
800
900
1,000
1,100
1,200
1,300
1,400
1,500
1,600
1,700
m/z

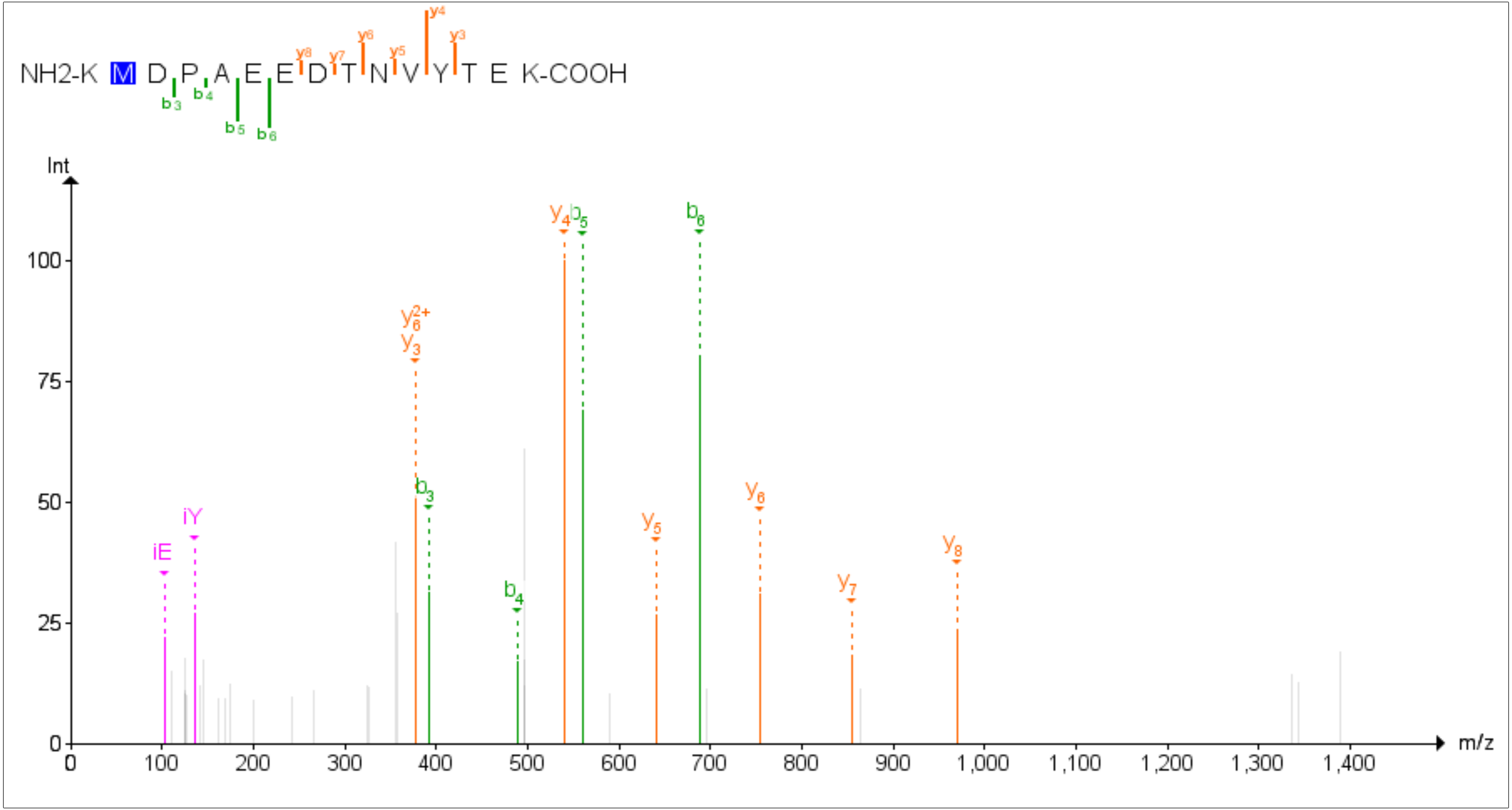

NH2-K M D P A E E D T N V Y T E K-COOH
b3 b4 b5 b6
y8 y7 y6 y5 y4 y3
Int
100
75
50
25
0
m/z
0 100 200 300 400 500 600 700 800 900 1,000 1,100 1,200 1,300 1,400
iE
iY
y6 2+
y3
b3
b4
y4
b5
y5
b6
y6
y7
y8

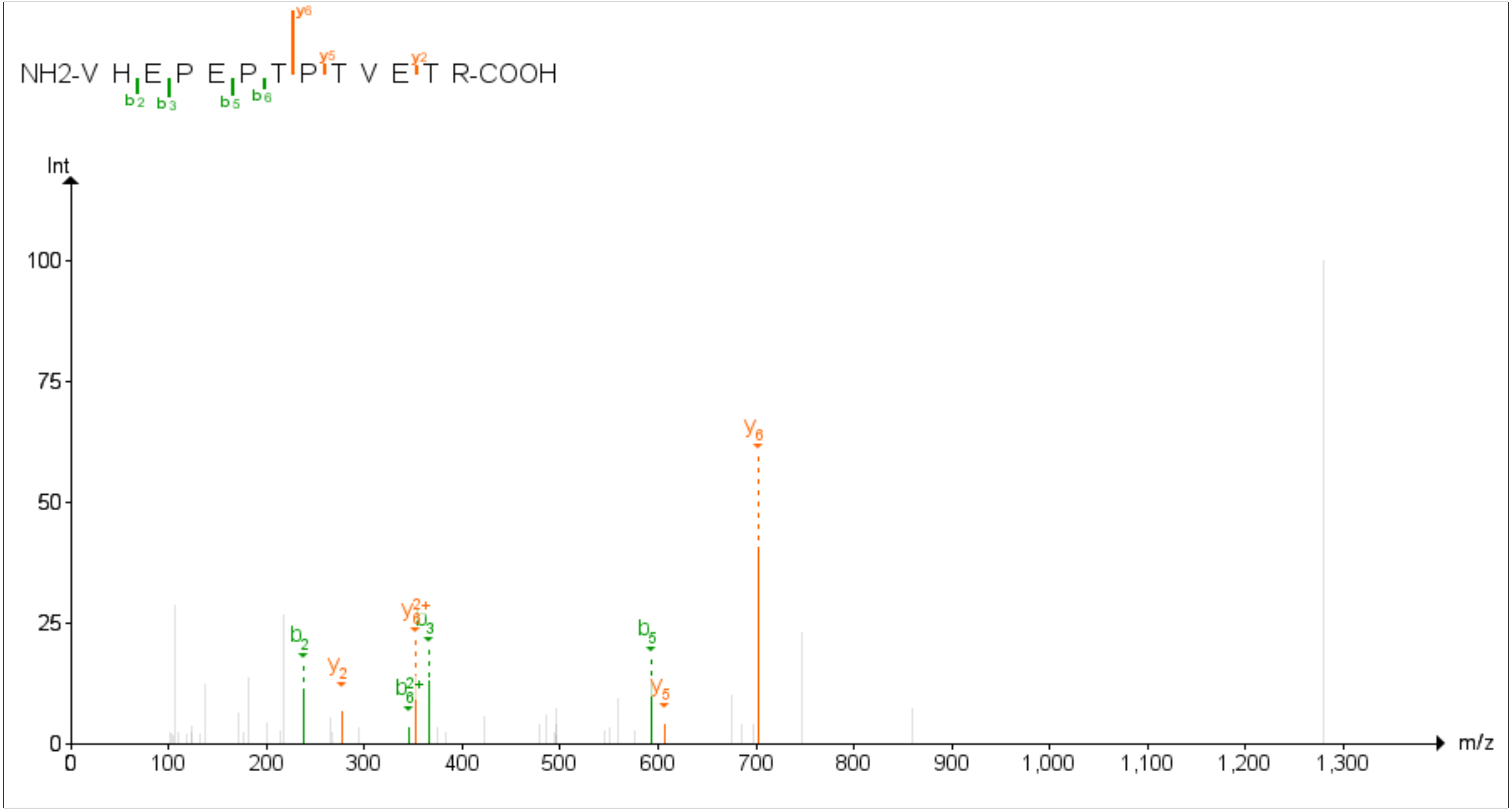

NH2-V H E P E P T P T V E T R-COOH
y6
y5
y2
b2
b3
b5
b6
Int
100
75
50
25
0
m/z
0
100
200
300
400
500
600
700
800
900
1,000
1,100
1,200
1,300

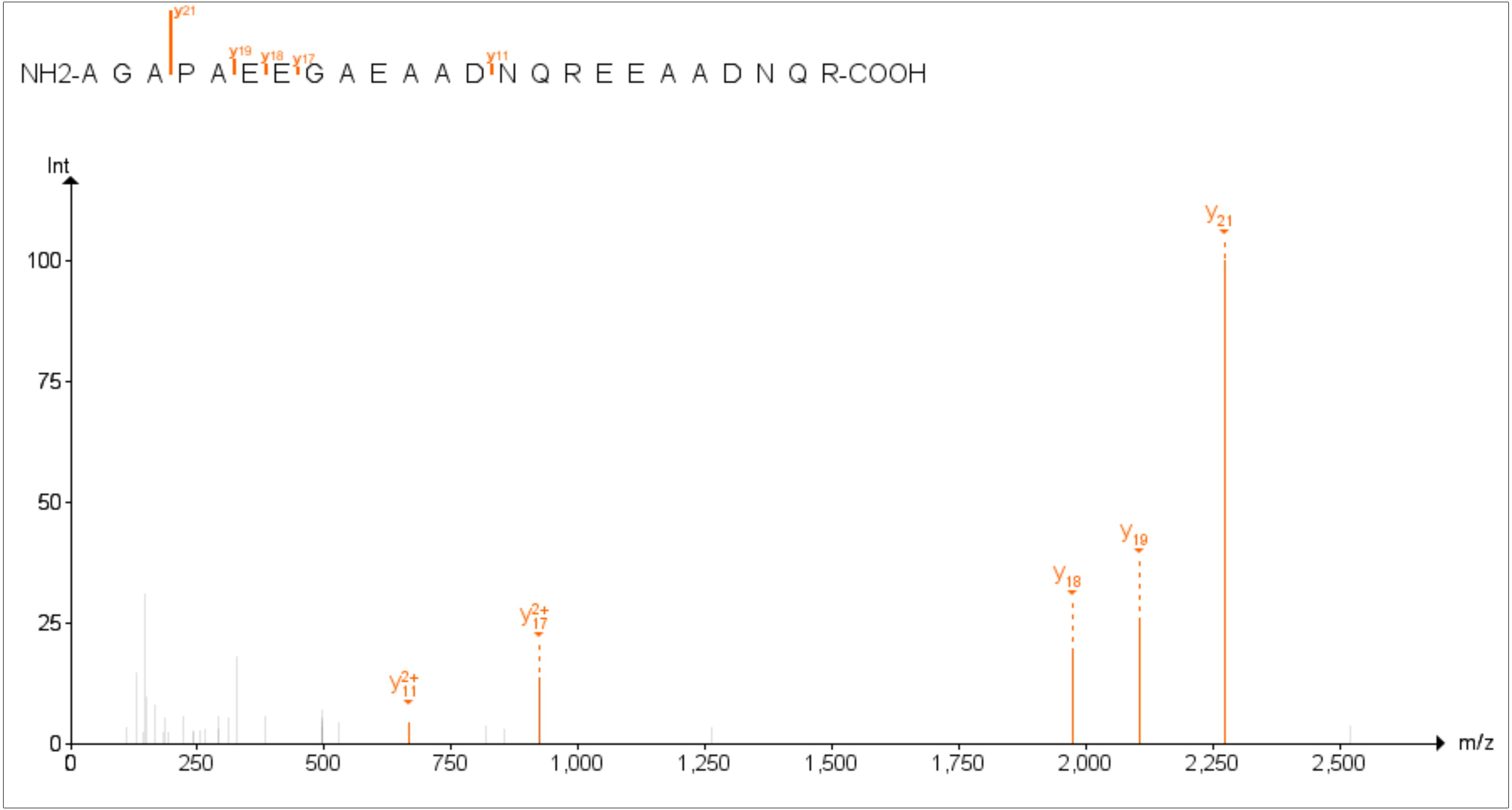

NH2-A G A P A E E G A E A A D N Q R E E A A D N Q R-COOH
y21
y19
y18
y17
y11
Int
100
75
50
25
0
m/z
0
250
500
750
1,000
1,250
1,500
1,750
2,000
2,250
2,500
y_{21}
y_{19}
y_{18}
y_{17}^{2+}
y_{11}^{2+}

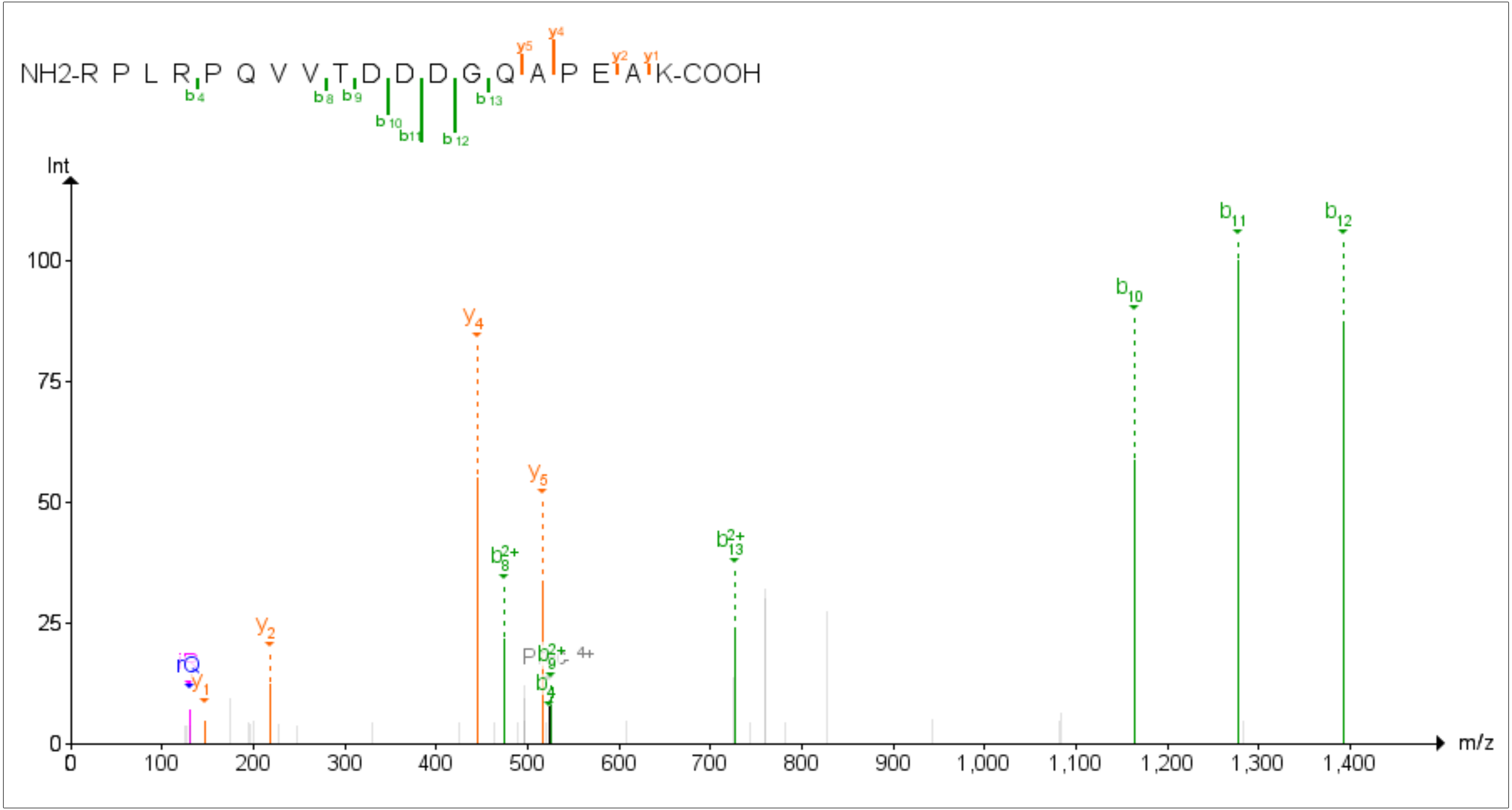

NH2-R P L R P Q V V T D D D G Q A P E A K-COOH
b4
b8
b9
b10
b11
b12
b13
y5
y4
y2
y1
Int
100
75
50
25
0
m/z
0
100
200
300
400
500
600
700
800
900
1,000
1,100
1,200
1,300
1,400
b11
b12
b10
y4
y5
b8 2+
b13 2+
y2
y1
rQ
b9 2+
b4

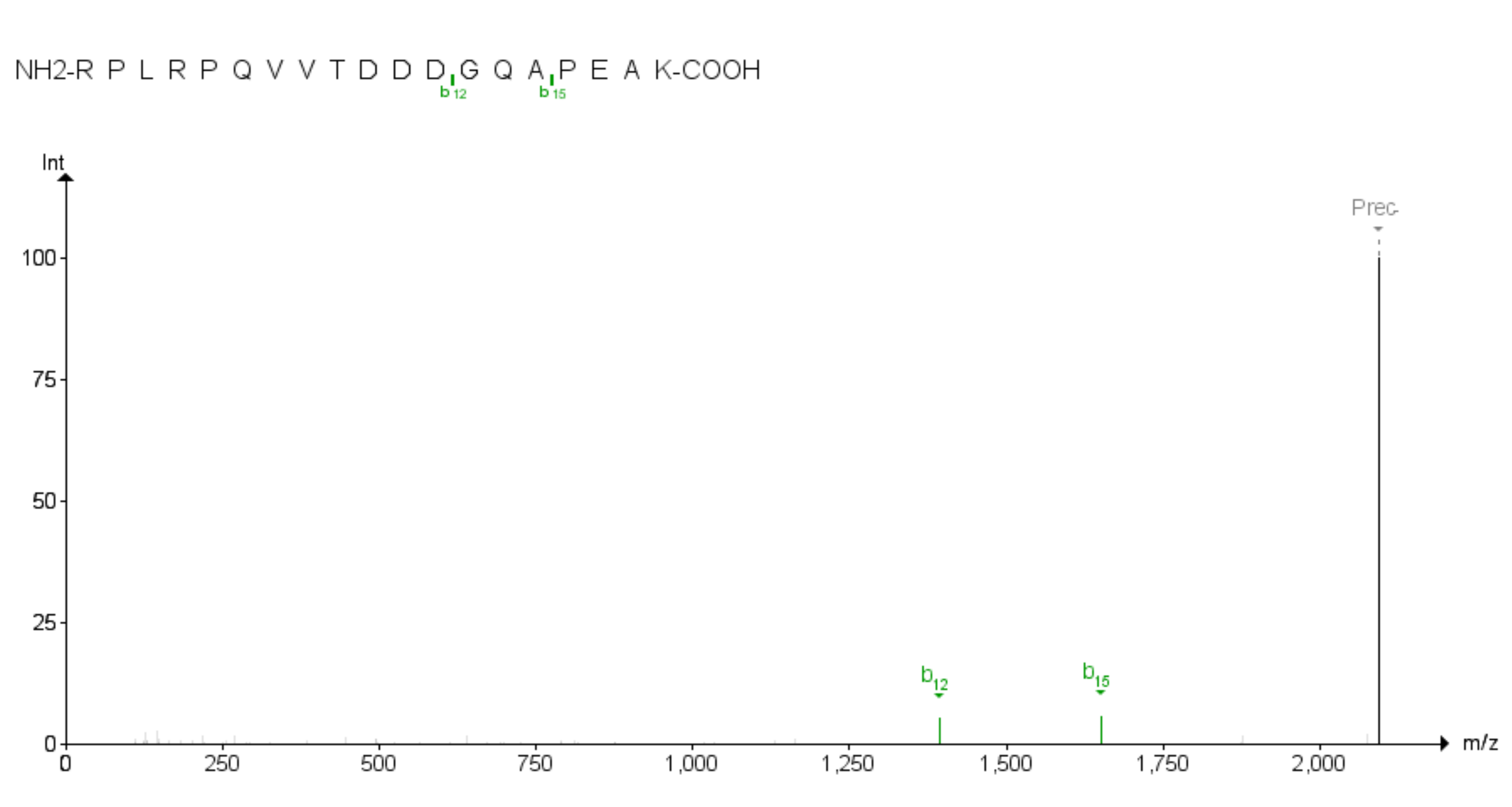

NH2-R P L R P Q V V T D D D G Q A P E A K-COOH
b 12
b 15
Int
100
75
50
25
0
Prec
b12
b15
0
250
500
750
1,000
1,250
1,500
1,750
2,000
m/z

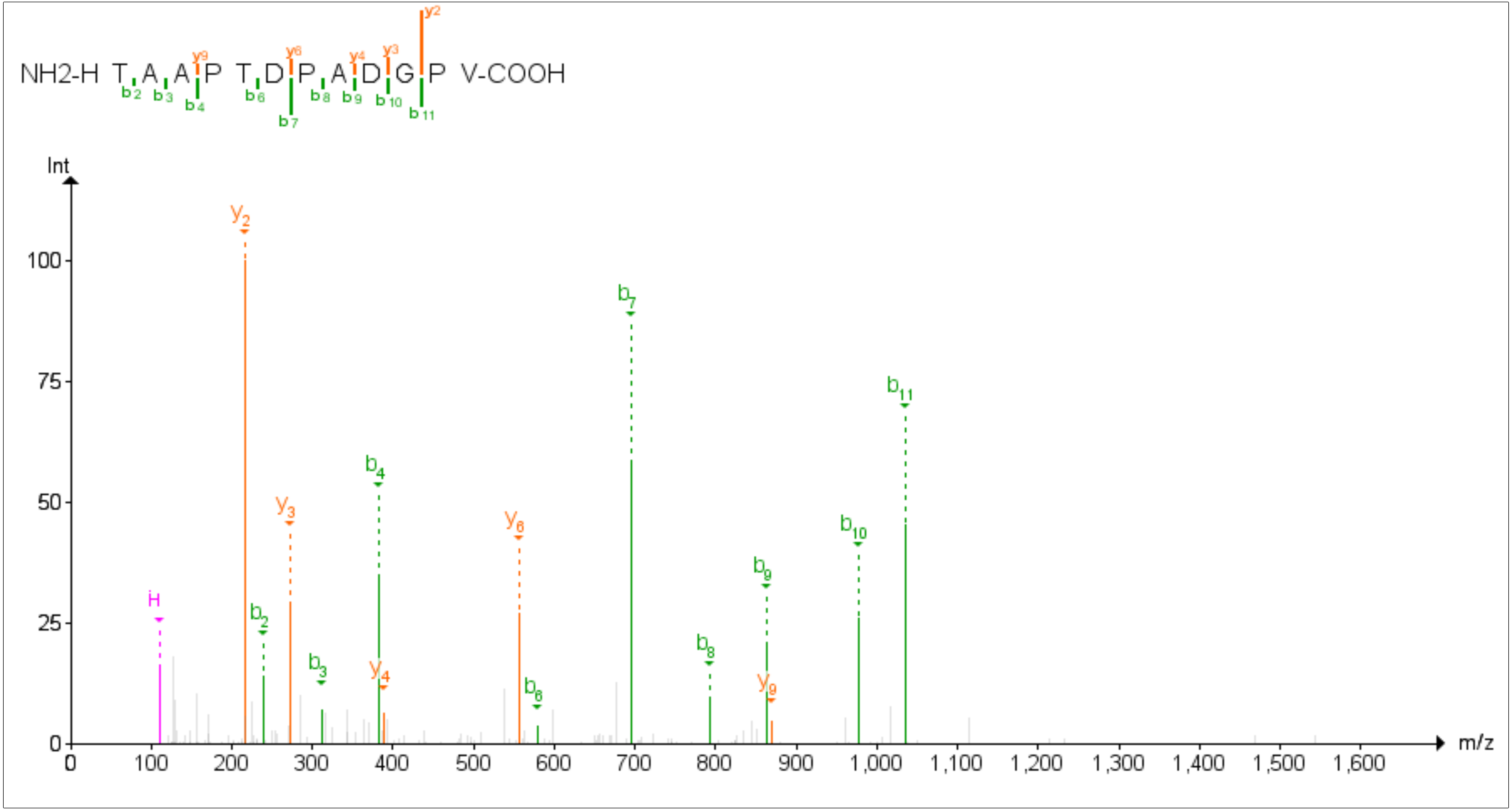

NH2-H T A A P T D P A D G P V-COOH
y9 y6 y4 y3 y2
b2 b3 b4 b6 b7 b8 b9 b10 b11
Int
m/z
0
25
50
75
100
0
100
200
300
400
500
600
700
800
900
1,000
1,100
1,200
1,300
1,400
1,500
1,600
H
y2
y3
y4
y6
y9
b2
b3
b4
b6
b7
b8
b9
b10
b11

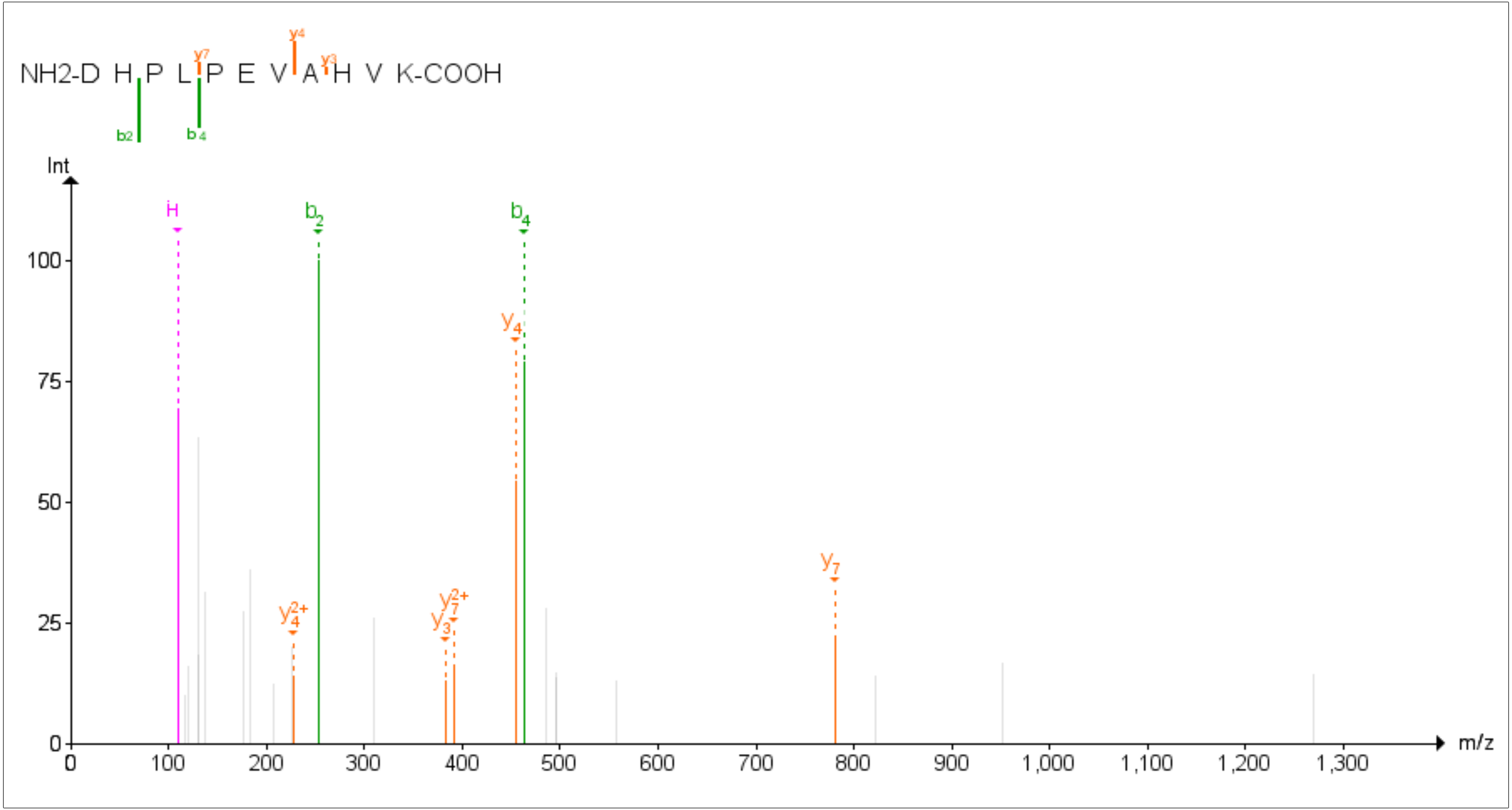
NH2-D H P L P E V A H V K-COOH
b2
b4
y7
y4
y3
Int
m/z
H
b_2
b_4
y_4
y_4^{2+}
y_3
y_7^{2+}
y_7
0
25
50
75
100
0
100
200
300
400
500
600
700
800
900
1,000
1,100
1,200
1,300

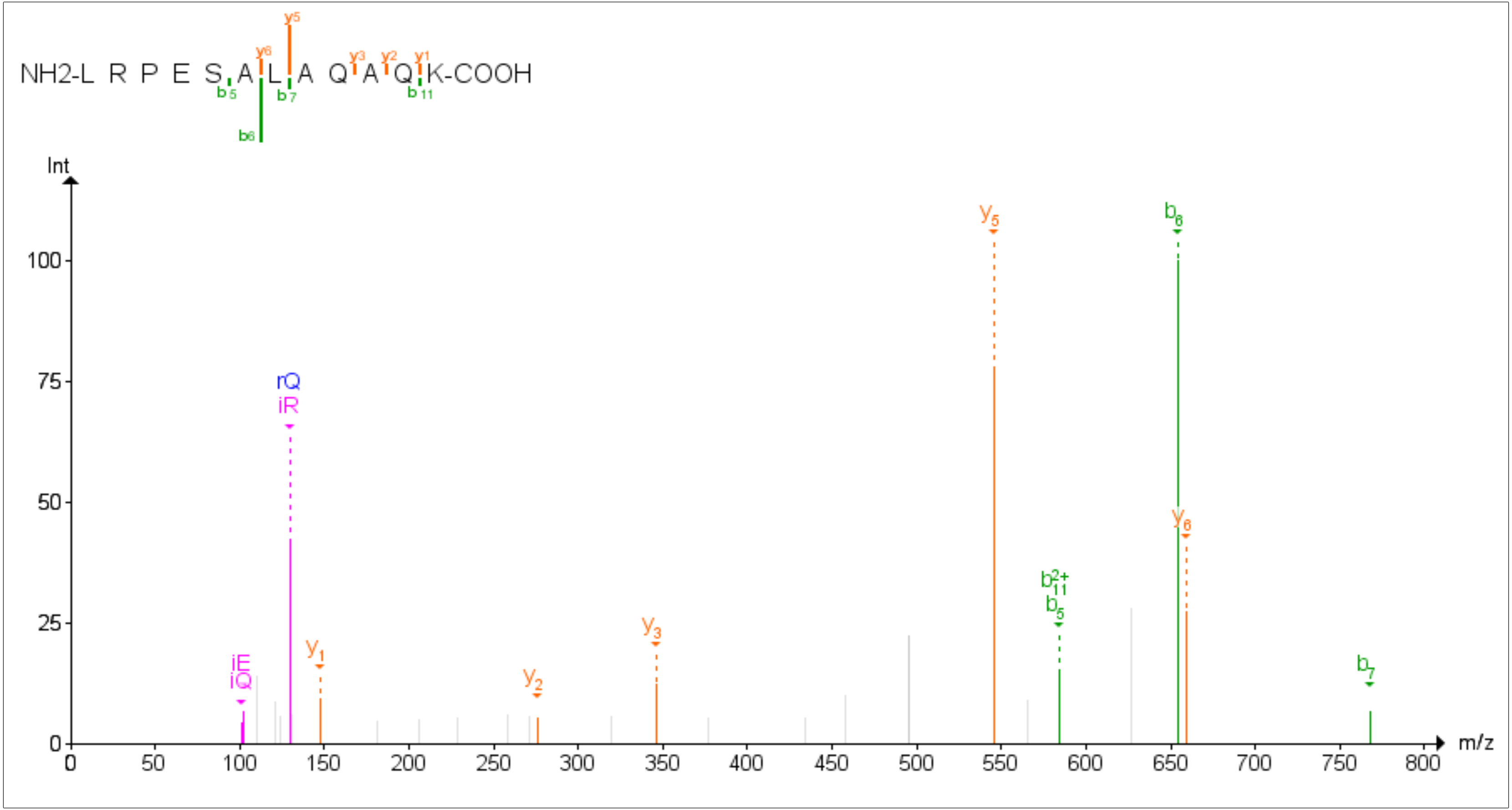

NH2-L R P E S A L A Q A Q K-COOH
y5
y6
y3
y2
y1
b5
b7
b11
b6
Int
100
75
50
25
0
rQ
iR
iE
iQ
y1
y2
y3
y5
b6
y6
b11 2+
b5
b7
0
50
100
150
200
250
300
350
400
450
500
550
600
650
700
750
800
m/z

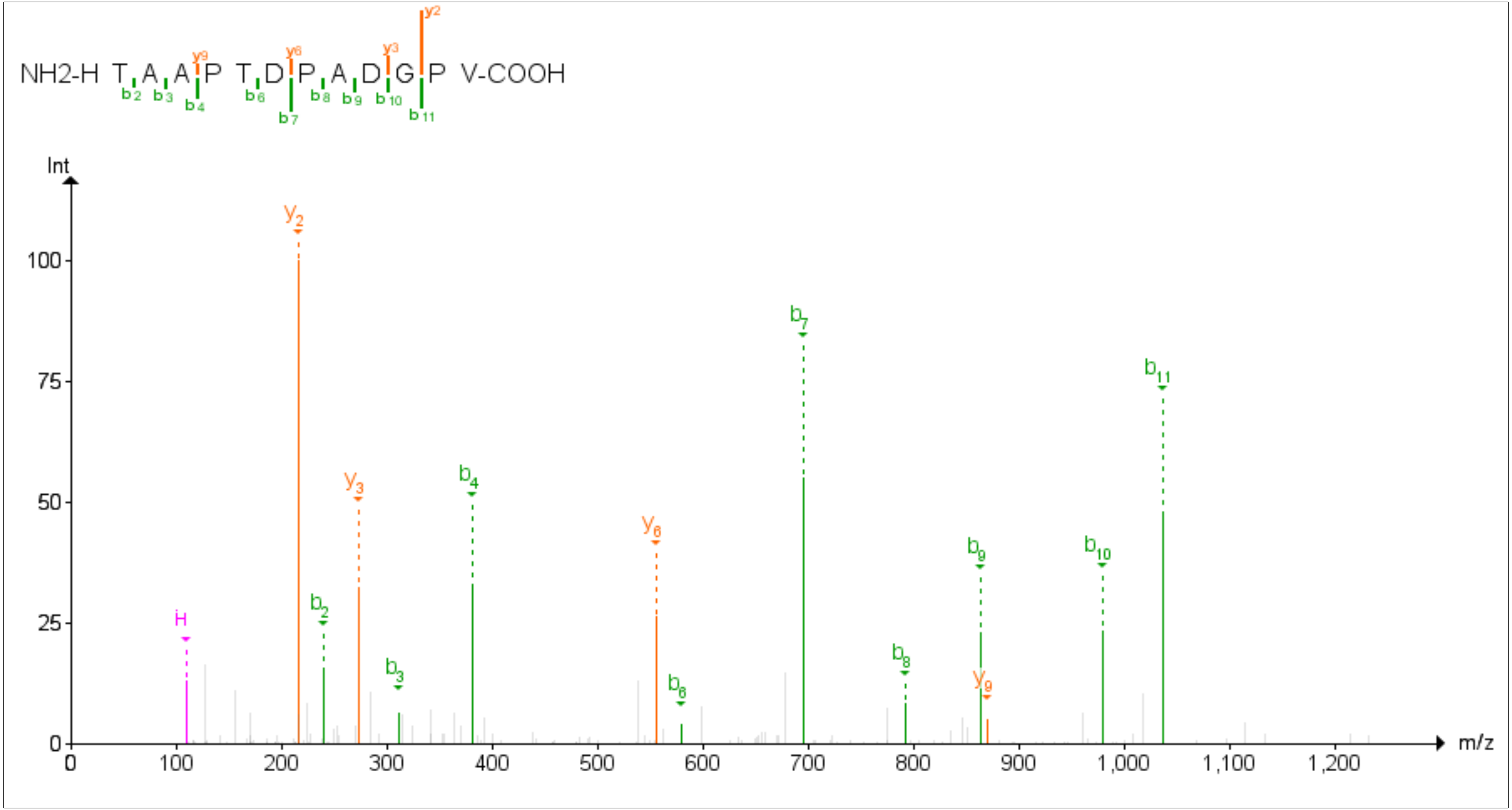
NH2-H T A A P T D P A D G P V-COOH
Int
m/z
y2
y3
y6
y9
b2
b3
b4
b6
b7
b8
b9
b10
b11
H
0
25
50
75
100
0
100
200
300
400
500
600
700
800
900
1,000
1,100
1,200

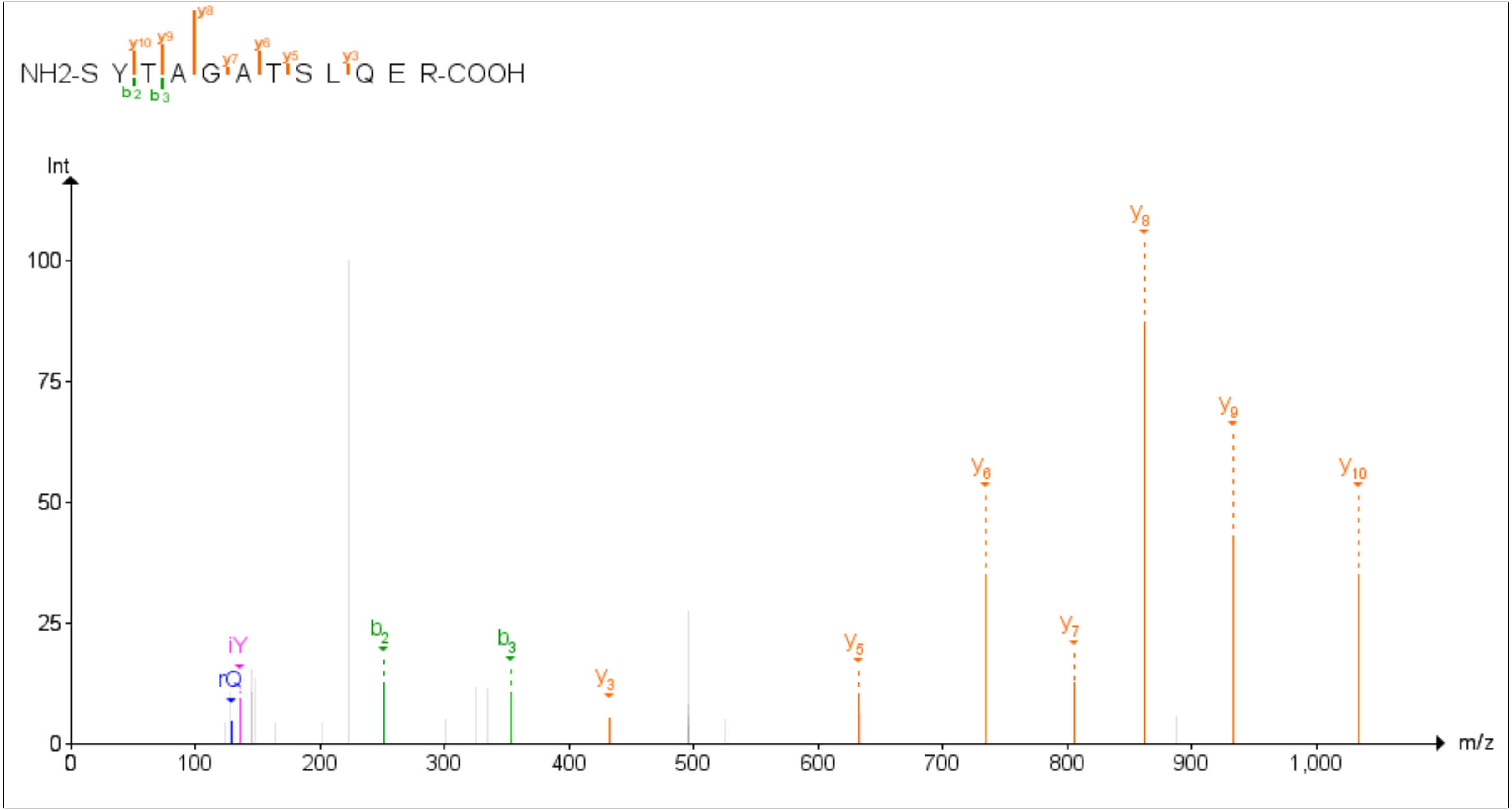
NH2-S Y T A G A T S L Q E R-COOH
y10 y9 y8 y7 y6 y5 y3
b2 b3
Int
m/z
0
25
50
75
100
0
100
200
300
400
500
600
700
800
900
1,000
iY
rQ
b2
b3
y3
y5
y6
y7
y8
y9
y10

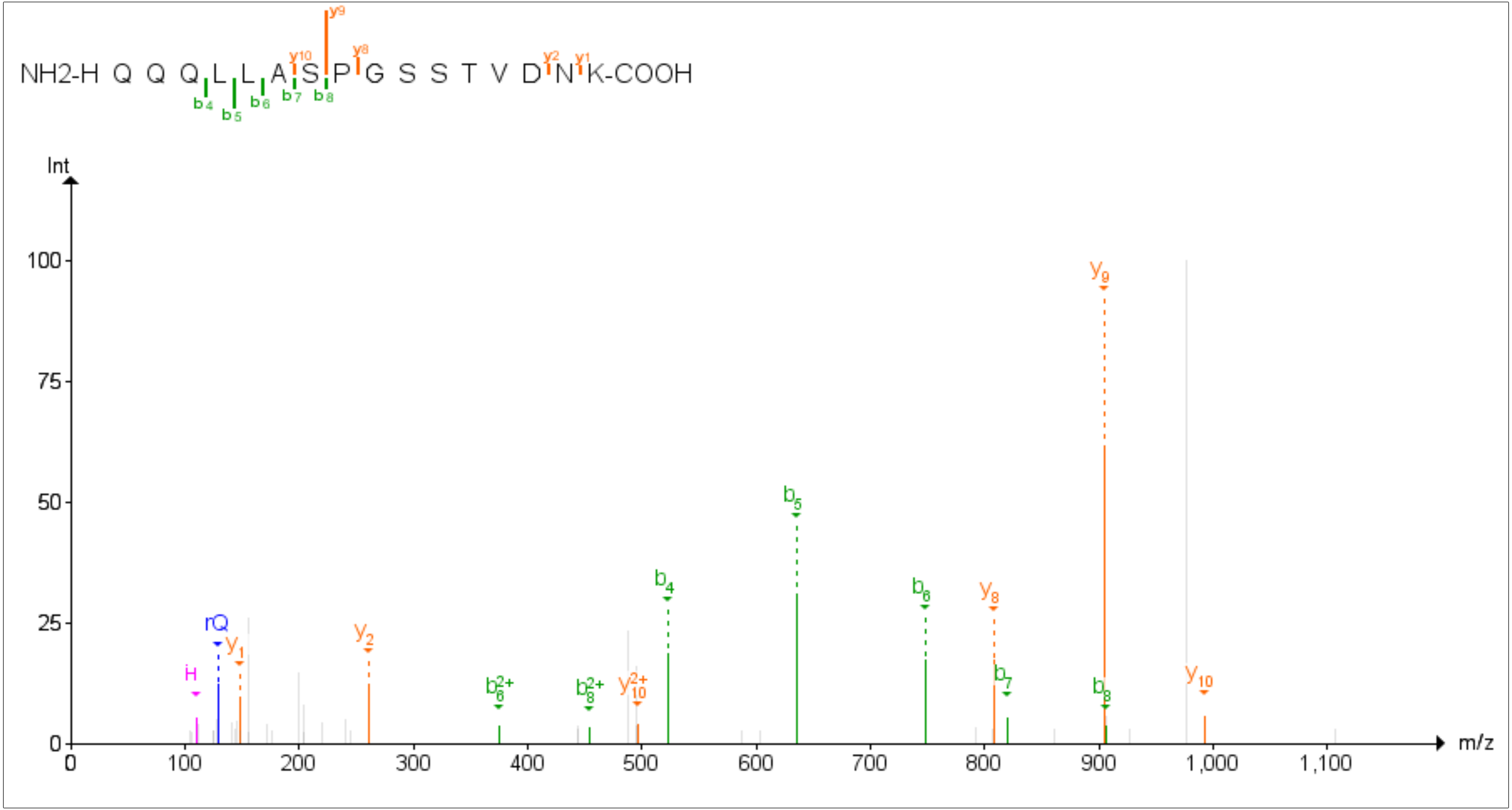

NH2-H Q Q Q L L A S P G S S T V D N K-COOH
y9
y10
y8
y2
y1
b4
b5
b6
b7
b8
Int
100
75
50
25
0
m/z
0
100
200
300
400
500
600
700
800
900
1,000
1,100
H
rQ
y1
y2
b6 2+
b8 2+
y10 2+
b4
b5
b6
y8
b7
y9
b8
y10

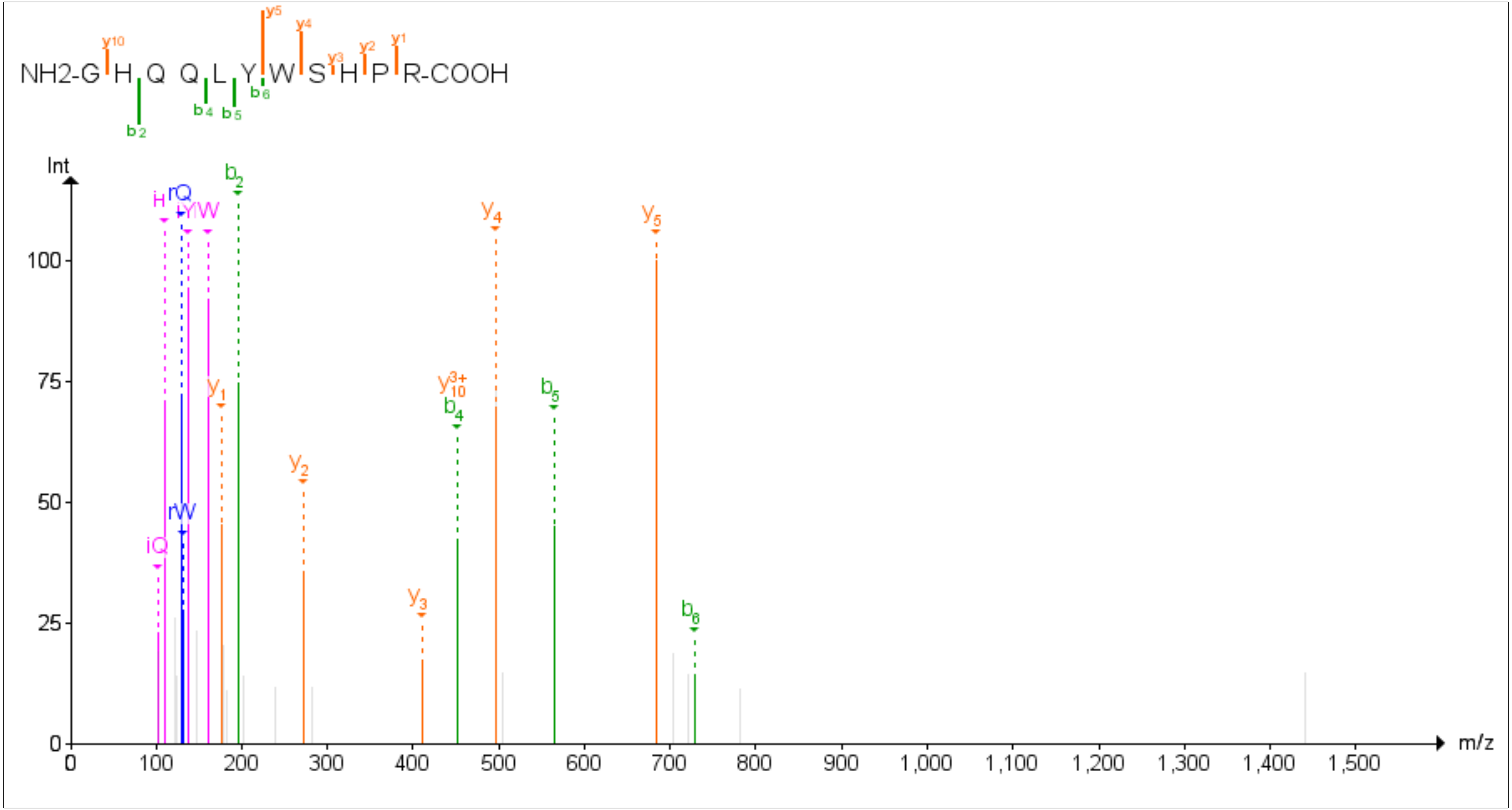

NH2-G H Q Q L Y W S H P R-COOH
y10
y5
y4
y3
y2
y1
b2
b4
b5
b6
Int
iH
rQ
iY
iW
b2
y4
y5
y1
y10 3+
b4
b5
y2
rW
iQ
y3
b6
100
75
50
25
0
0
100
200
300
400
500
600
700
800
900
1,000
1,100
1,200
1,300
1,400
1,500
m/z

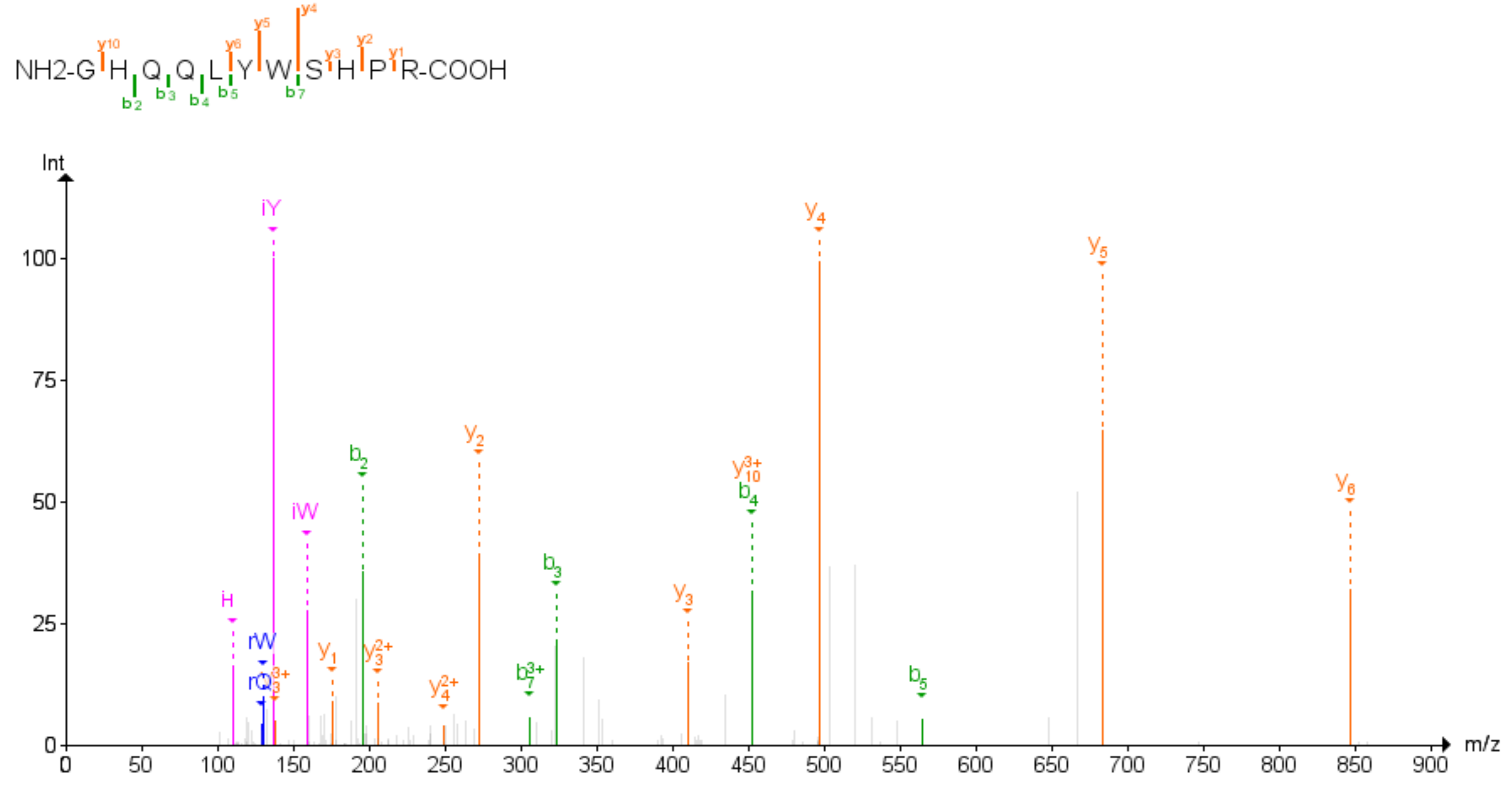

NH2-G H Q Q L Y W S H P R-COOH
y10 y6 y5 y4 y3 y2 y1
b2 b3 b4 b5 b7
Int
m/z
iY
iW
iH
rW
rQ
y4
y5
y6
y2
b2
y10 3+
b4
b3
y3
y1
y3 2+
y4 2+
b7 3+
b5
y3 3+
0
25
50
75
100
0 50 100 150 200 250 300 350 400 450 500 550 600 650 700 750 800 850 900

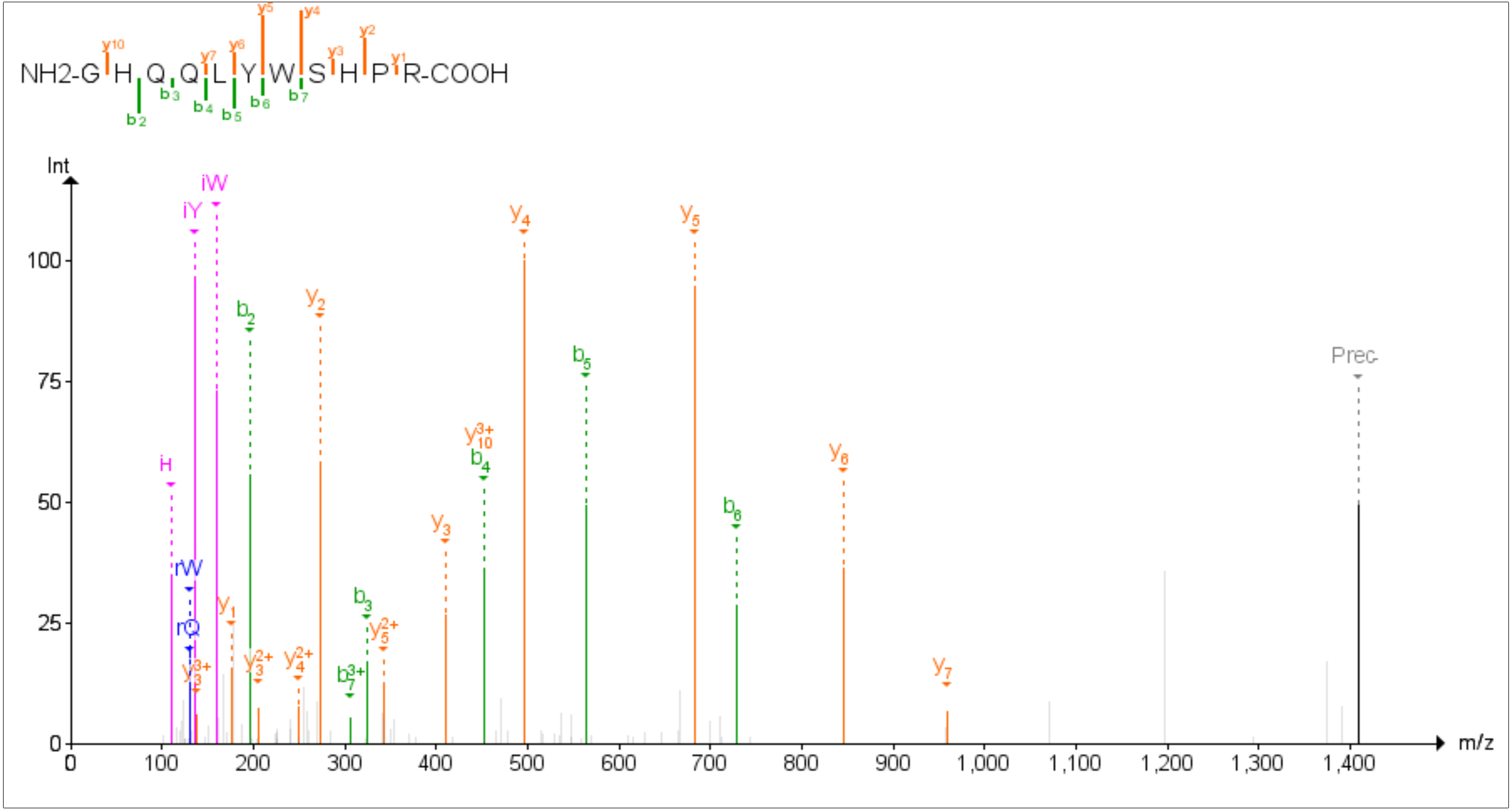

NH2-G H Q Q L Y W S H P R-COOH
y10 y7 y6 y5 y4 y3 y2 y1
b2 b3 b4 b5 b6 b7
Int
iW
iY
y4
y5
b2
y2
b5
Prec
y10 3+
b4
iH
y6
b6
y3
rW
y1
b3
rQ
y5 2+
y3 3+
y3 2+
y4 2+
b7 3+
y7
m/z
0
25
50
75
100
0 100 200 300 400 500 600 700 800 900 1,000 1,100 1,200 1,300 1,400

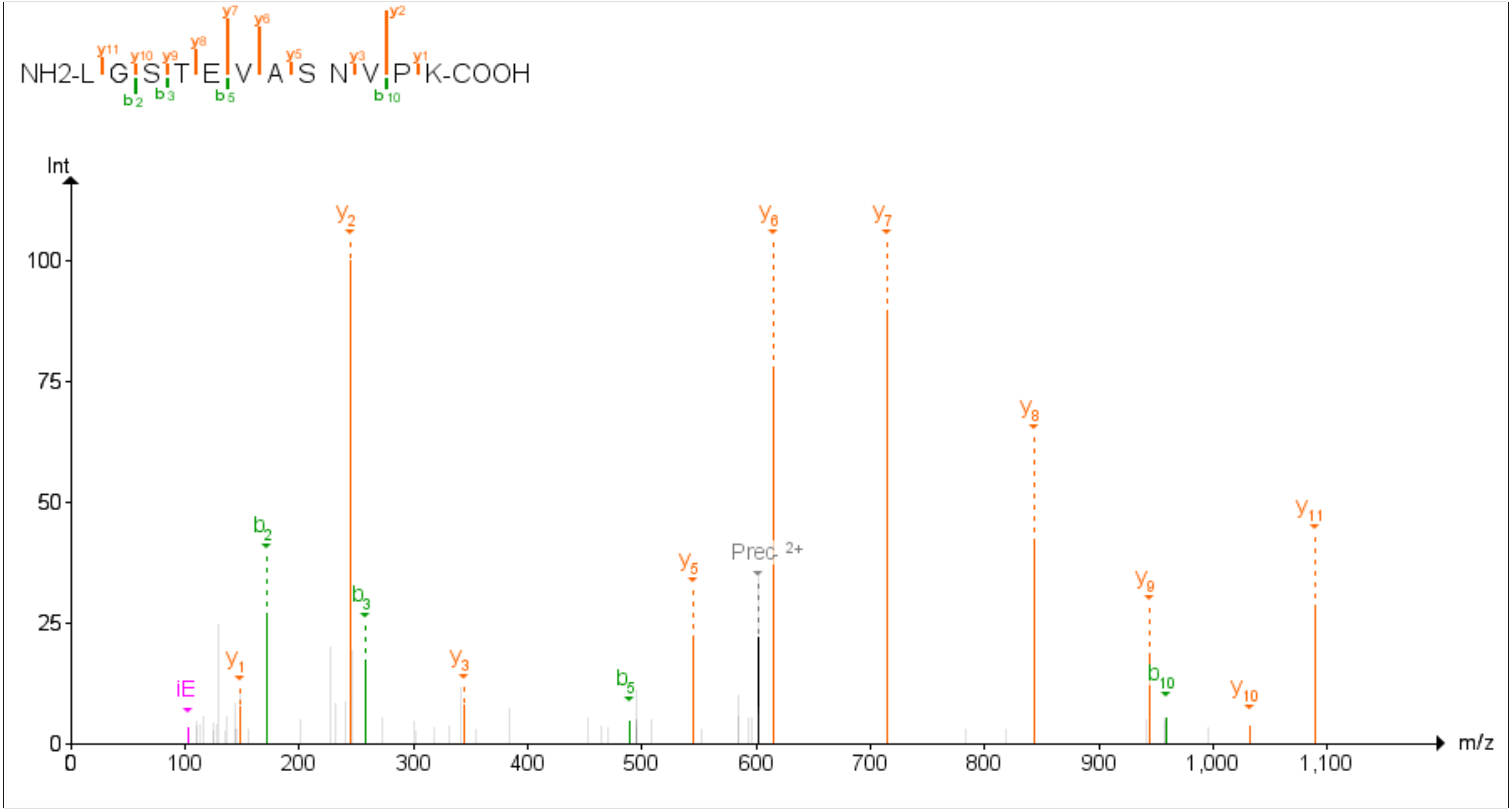
NH2-L G S T E V A S N V P K-COOH
y11 y10 y9 y8 y7 y6 y5 y3 y2 y1
b2 b3 b5 b10
Int
100
75
50
25
0
y2
y6
y7
y8
y11
b2
Prec 2+
y5
y9
b3
y1
y3
iE
b5
b10
y10
0 100 200 300 400 500 600 700 800 900 1,000 1,100
m/z

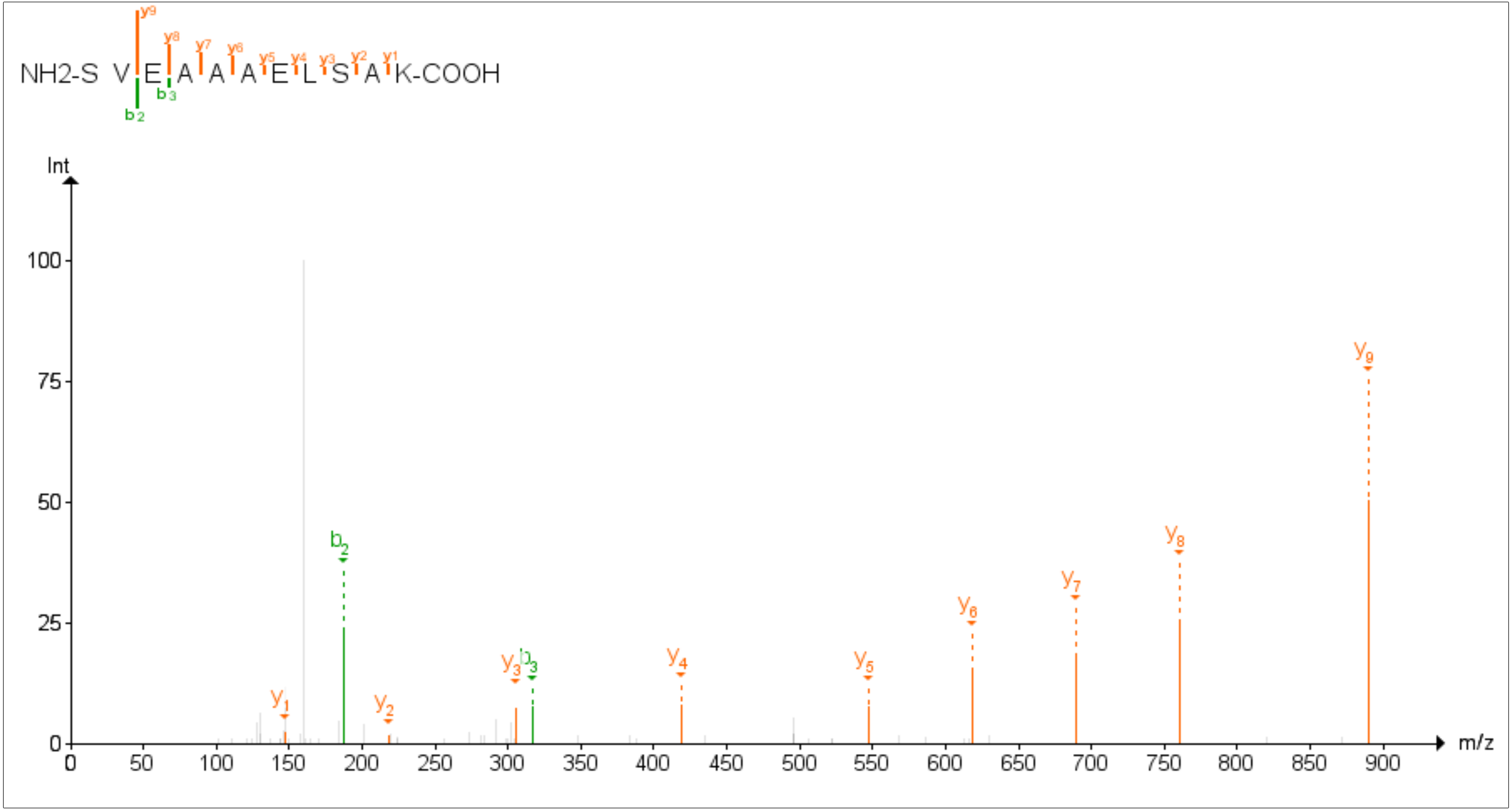
NH2-S V E A A A E L S A K-COOH
y9 y8 y7 y6 y5 y4 y3 y2 y1
b2 b3
Int
100
75
50
25
0
m/z
0 50 100 150 200 250 300 350 400 450 500 550 600 650 700 750 800 850 900
y1 b2 y2 y3 b3 y4 y5 y6 y7 y8 y9

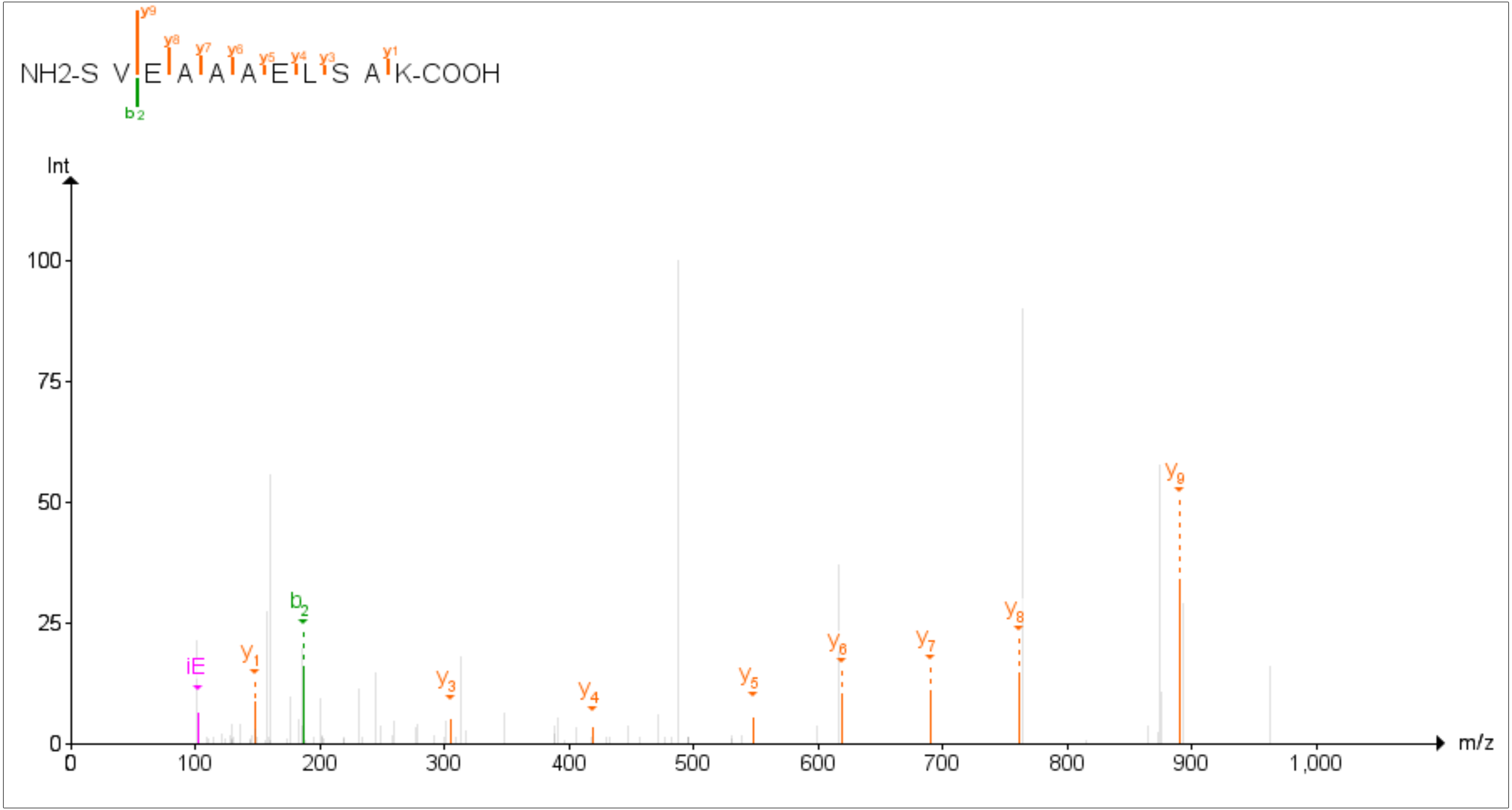

NH2-S V E A A A E L S A K-COOH
y9 y8 y7 y6 y5 y4 y3 y1
b2
Int
100
75
50
25
0
m/z
0 100 200 300 400 500 600 700 800 900 1,000
iE
y1
b2
y3
y4
y5
y6
y7
y8
y9

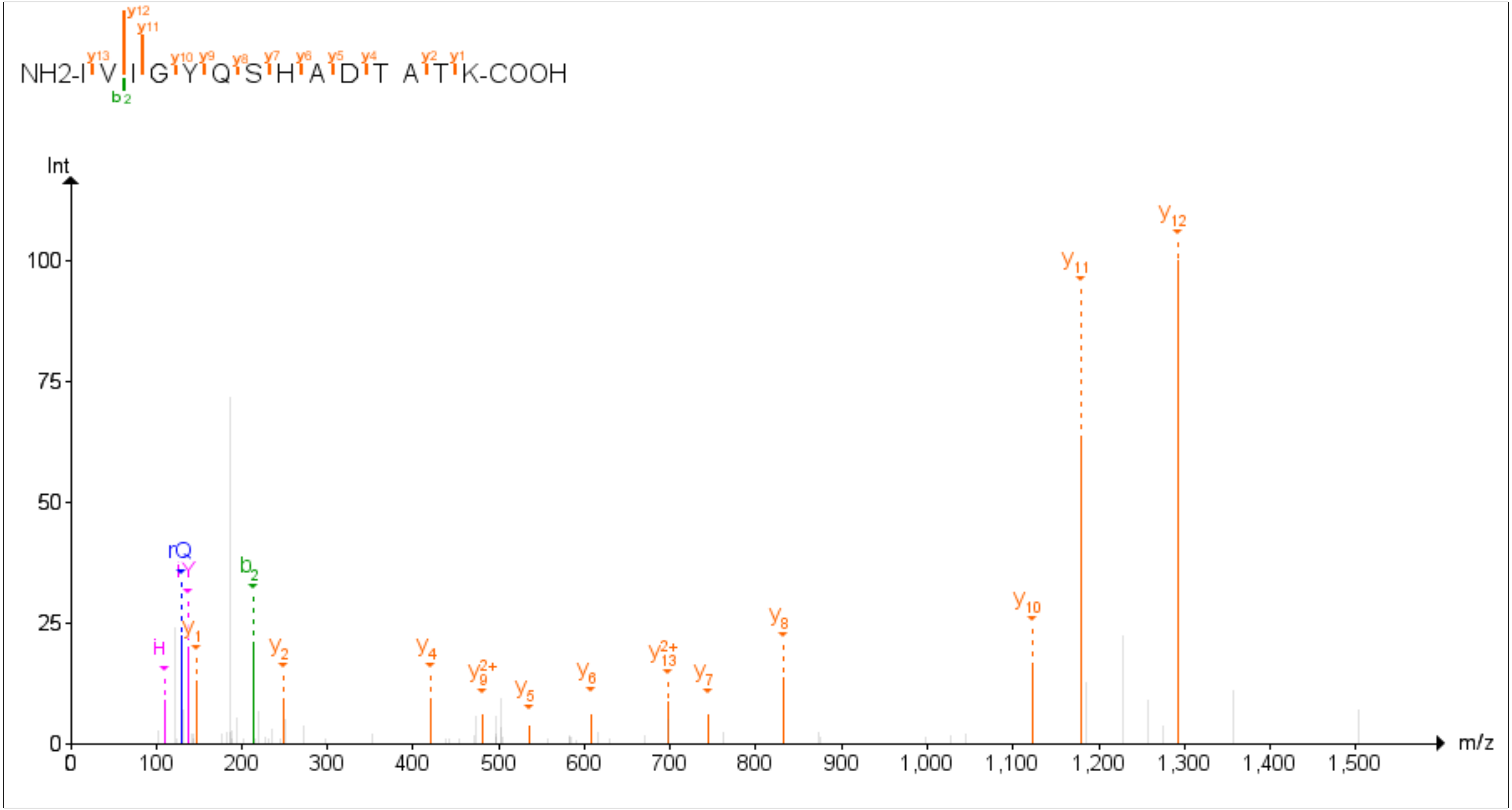

NH2-IVIGYQSHADTATK-COOH
y13 y12 y11 y10 y9 y8 y7 y6 y5 y4 y2 y1
b2
Int
100
75
50
25
0
rQ
Y
H
b2
y1
y2
y4
y9 2+
y5
y6
y13 2+
y7
y8
y10
y11
y12
m/z
0 100 200 300 400 500 600 700 800 900 1,000 1,100 1,200 1,300 1,400 1,500

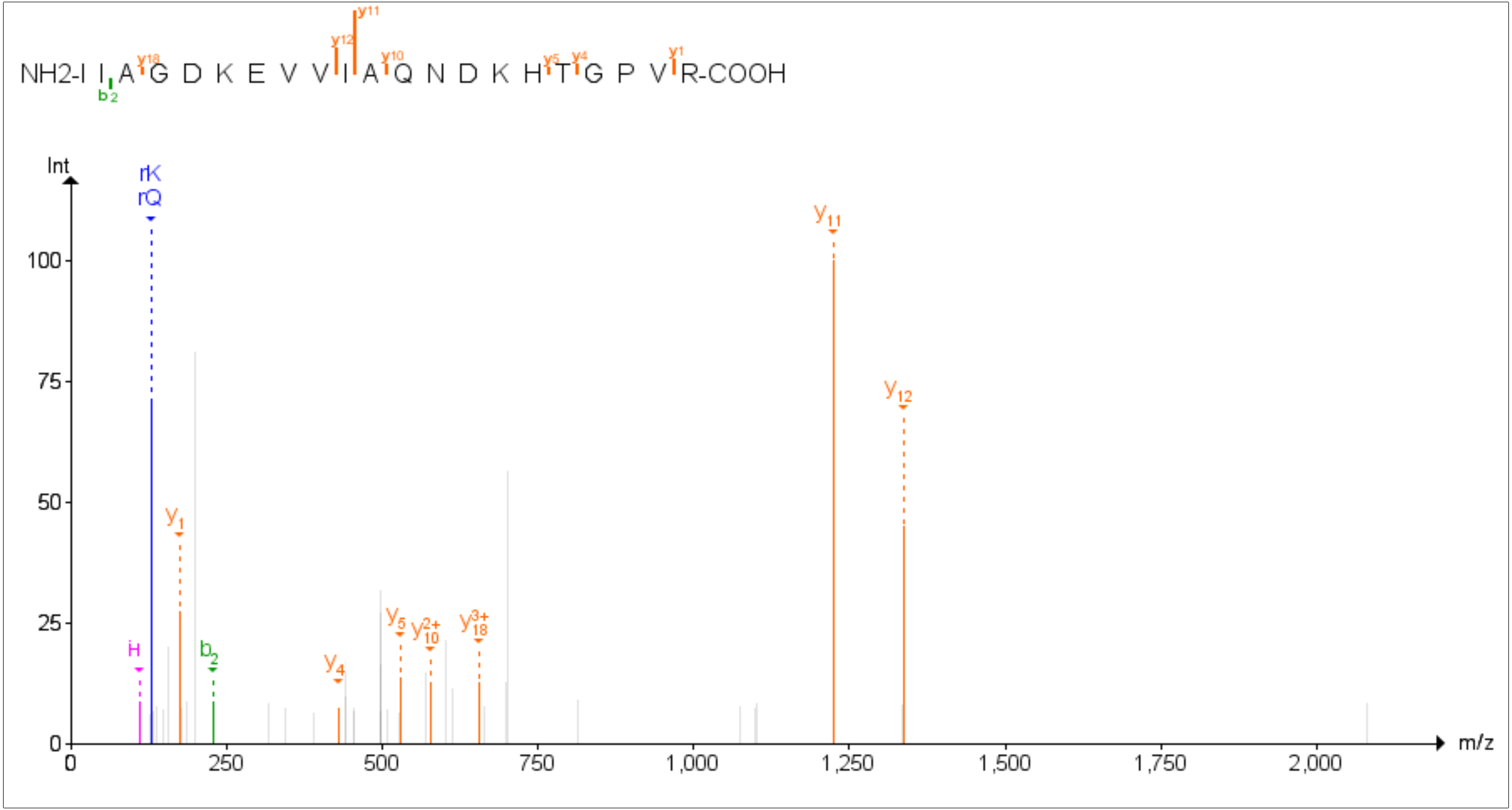

NH2-I I A G D K E V V I A Q N D K H T G P V R-COOH
b2
y18
y12
y11
y10
y5
y4
y1
Int
rK
rQ
H
y1
b2
y4
y5
y10 2+
y18 3+
y11
y12
0
25
50
75
100
0
250
500
750
1,000
1,250
1,500
1,750
2,000
m/z

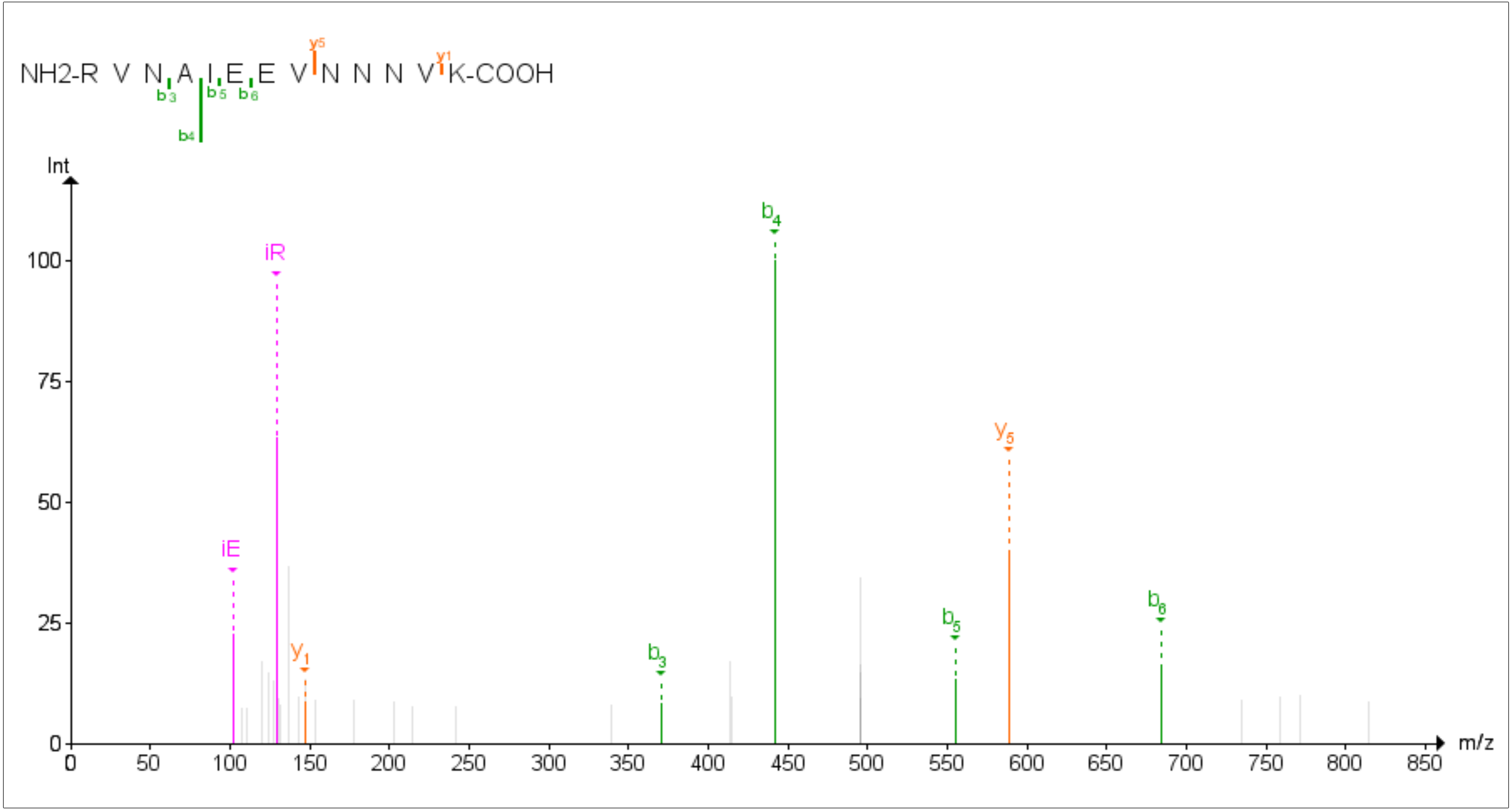

NH2-R V N A I E E V N N N V K-COOH
b3
b4
b5
b6
y5
y1
Int
100
75
50
25
0
iR
iE
y1
b3
b4
b5
y5
b6
0 50 100 150 200 250 300 350 400 450 500 550 600 650 700 750 800 850
m/z

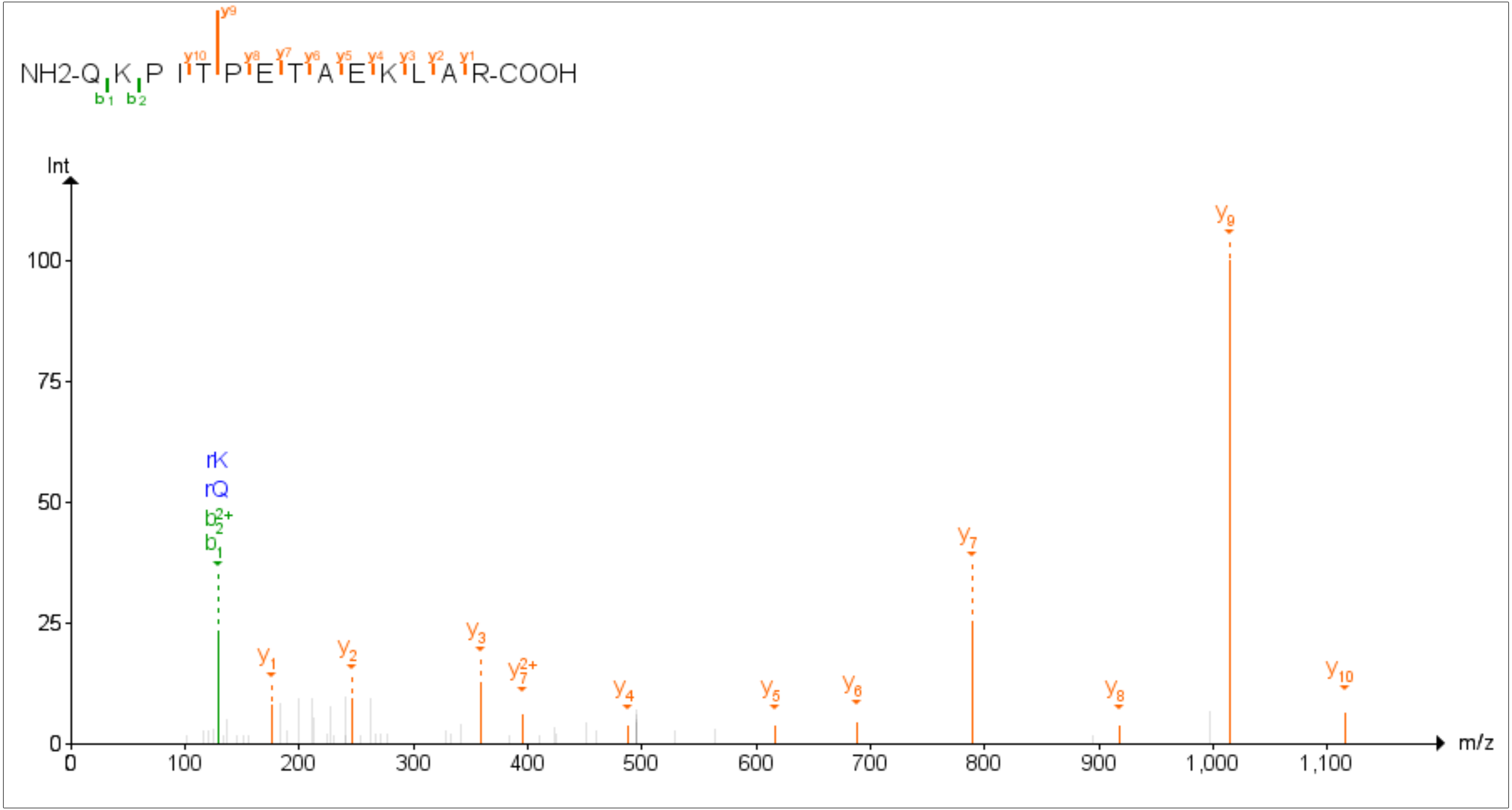

NH2-QKPITPETAEKLAR-COOH
y9
y10
y8
y7
y6
y5
y4
y3
y2
y1
b1
b2
Int
100
75
50
25
0
rK
rQ
b_2^{2+}
b_1
y_1
y_2
y_3
y_7^{2+}
y_4
y_5
y_6
y_7
y_8
y_9
y_{10}
0
100
200
300
400
500
600
700
800
900
1,000
1,100
m/z

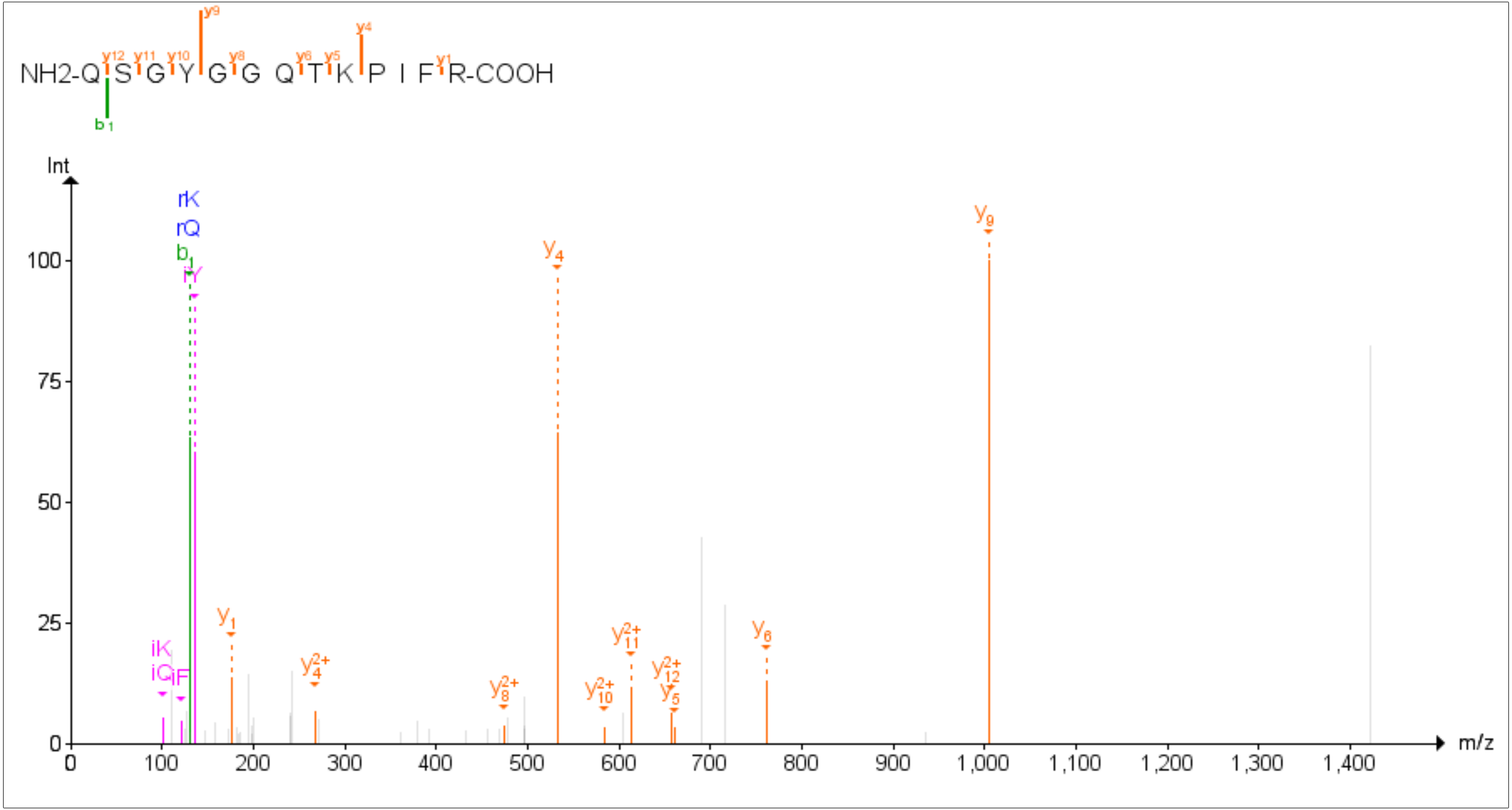

NH2-Q S G Y G G Q T K P I F R-COOH
y12 y11 y10 y9 y8 y6 y5 y4 y1
b1
Int
100
75
50
25
0
rK
rQ
b1
iY
y4
y9
y1
iK
iQ
iF
y4 2+
y8 2+
y10 2+
y11 2+
y12 2+
y5
y6
0
100
200
300
400
500
600
700
800
900
1,000
1,100
1,200
1,300
1,400
m/z

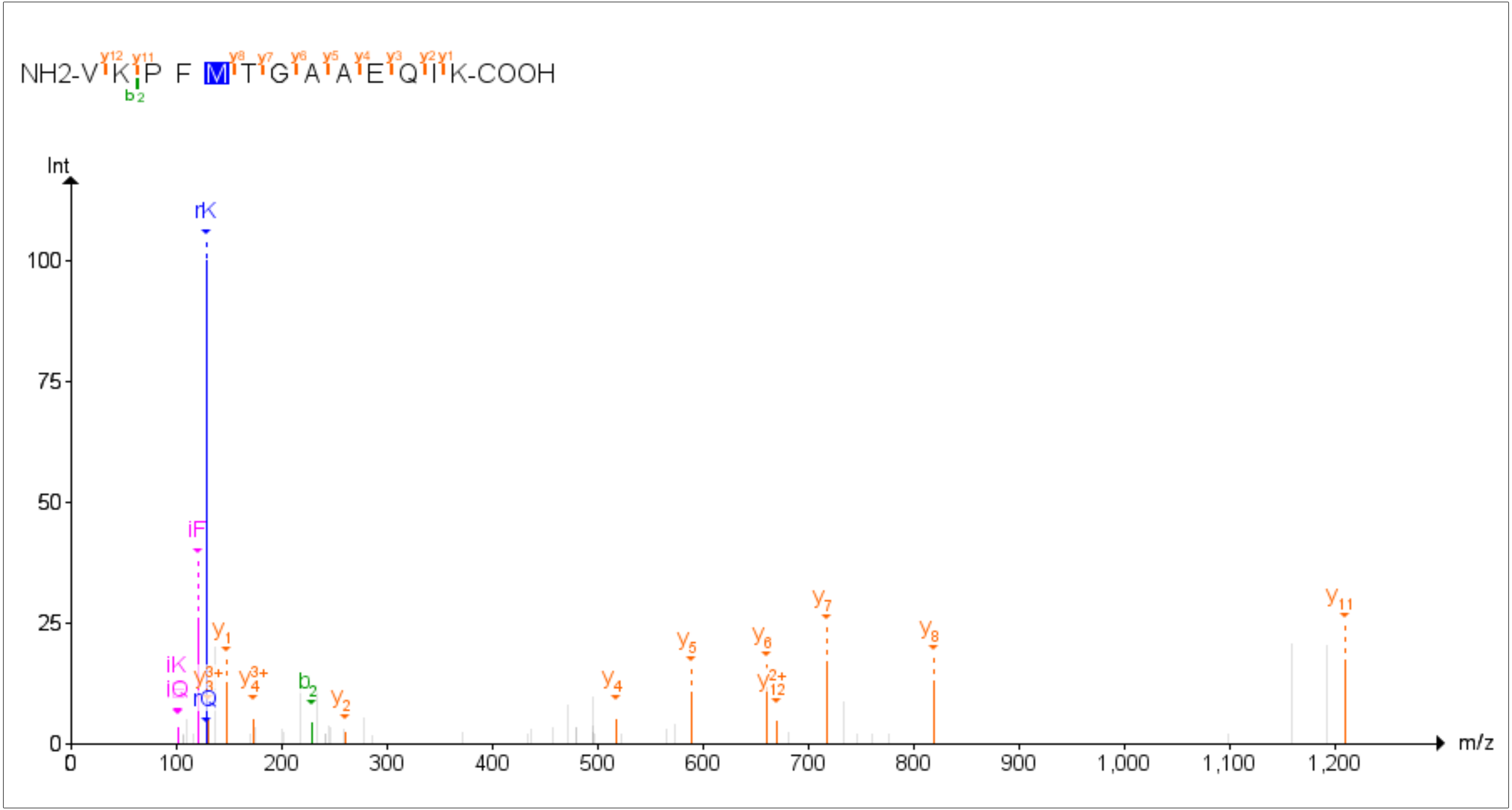

NH2-V K P F M T G A A E Q I K-COOH
y12 y11 y8 y7 y6 y5 y4 y3 y2 y1
b2
Int
100
75
50
25
0
rK
iF
iK
iQ
rQ
y1
y3 3+
y4 3+
b2
y2
y4
y5
y6
y12 2+
y7
y8
y11
0
100
200
300
400
500
600
700
800
900
1,000
1,100
1,200
m/z

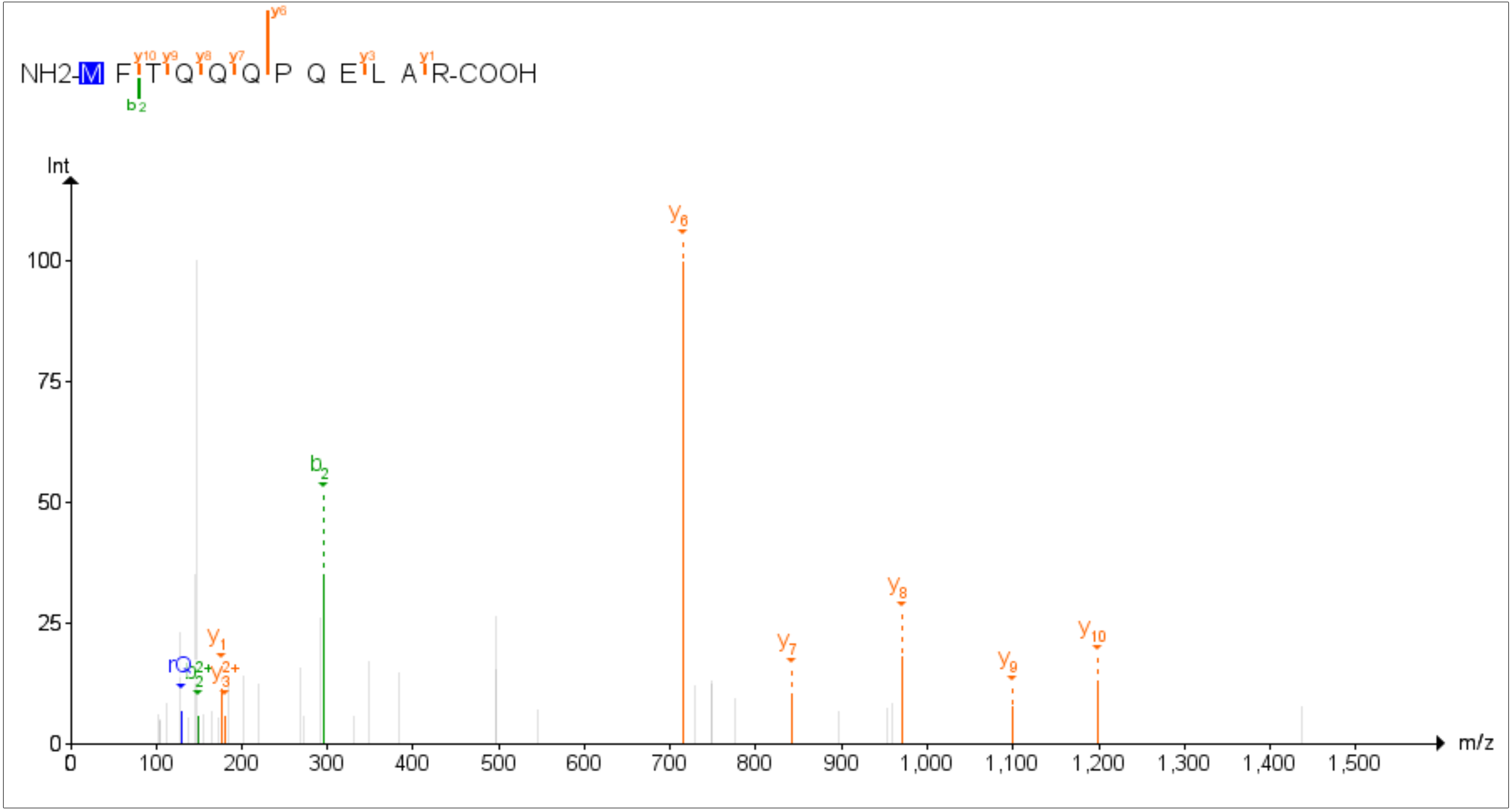

NH2-M F T Q Q Q P Q E L A R-COOH
y10 y9 y8 y7 y6 y3 y1
b2
Int
100
75
50
25
0
y6
b2
y8
y10
y7
y9
y1
rQ
m/z
0 100 200 300 400 500 600 700 800 900 1,000 1,100 1,200 1,300 1,400 1,500

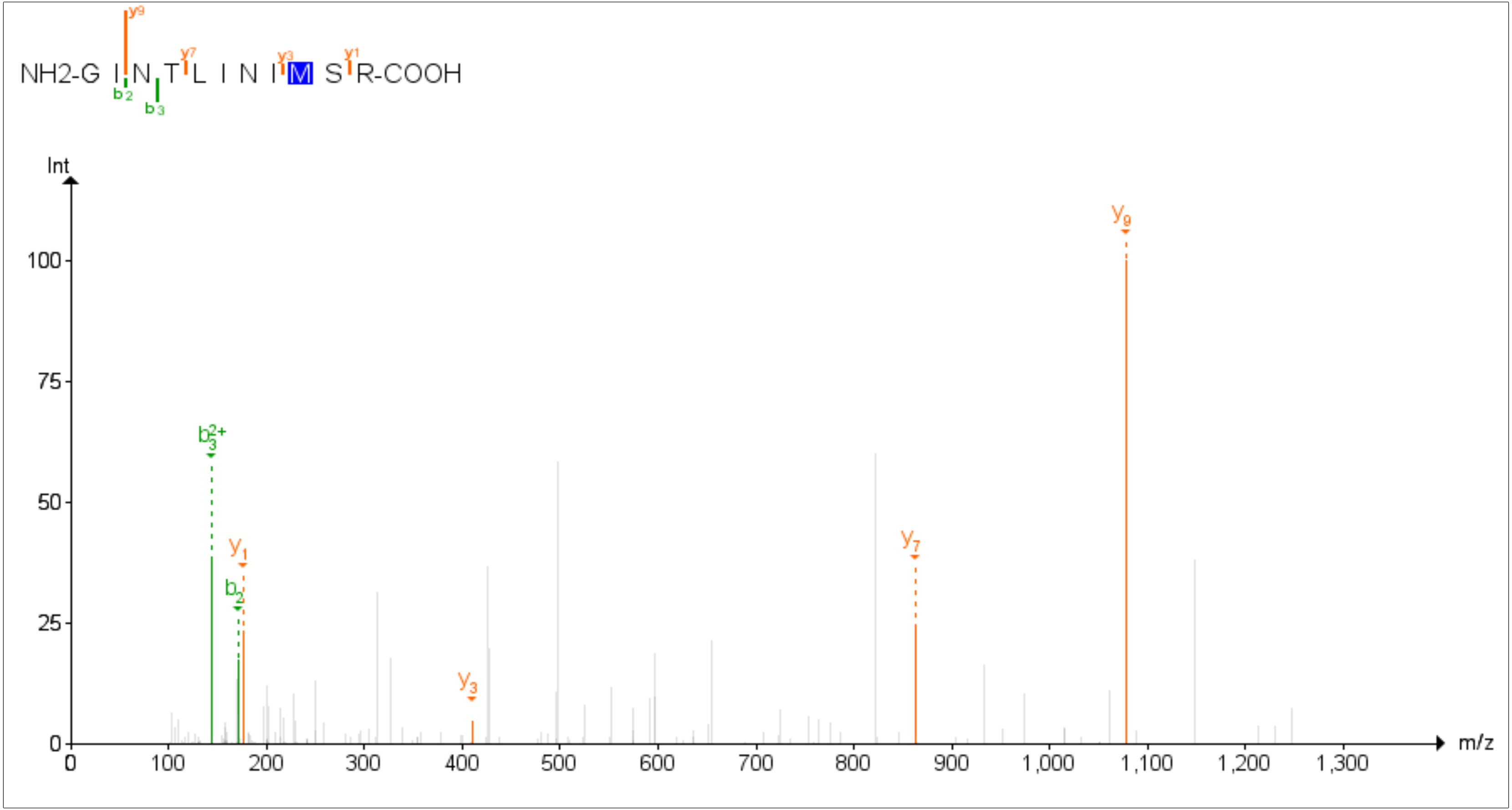

NH2-G I N T L I N I M S R-COOH
y9
y7
y3
y1
b2
b3
Int
100
75
50
25
0
b_3^{2+}
y_1
b_2
y_3
y_7
y_9
0
100
200
300
400
500
600
700
800
900
1,000
1,100
1,200
1,300
m/z

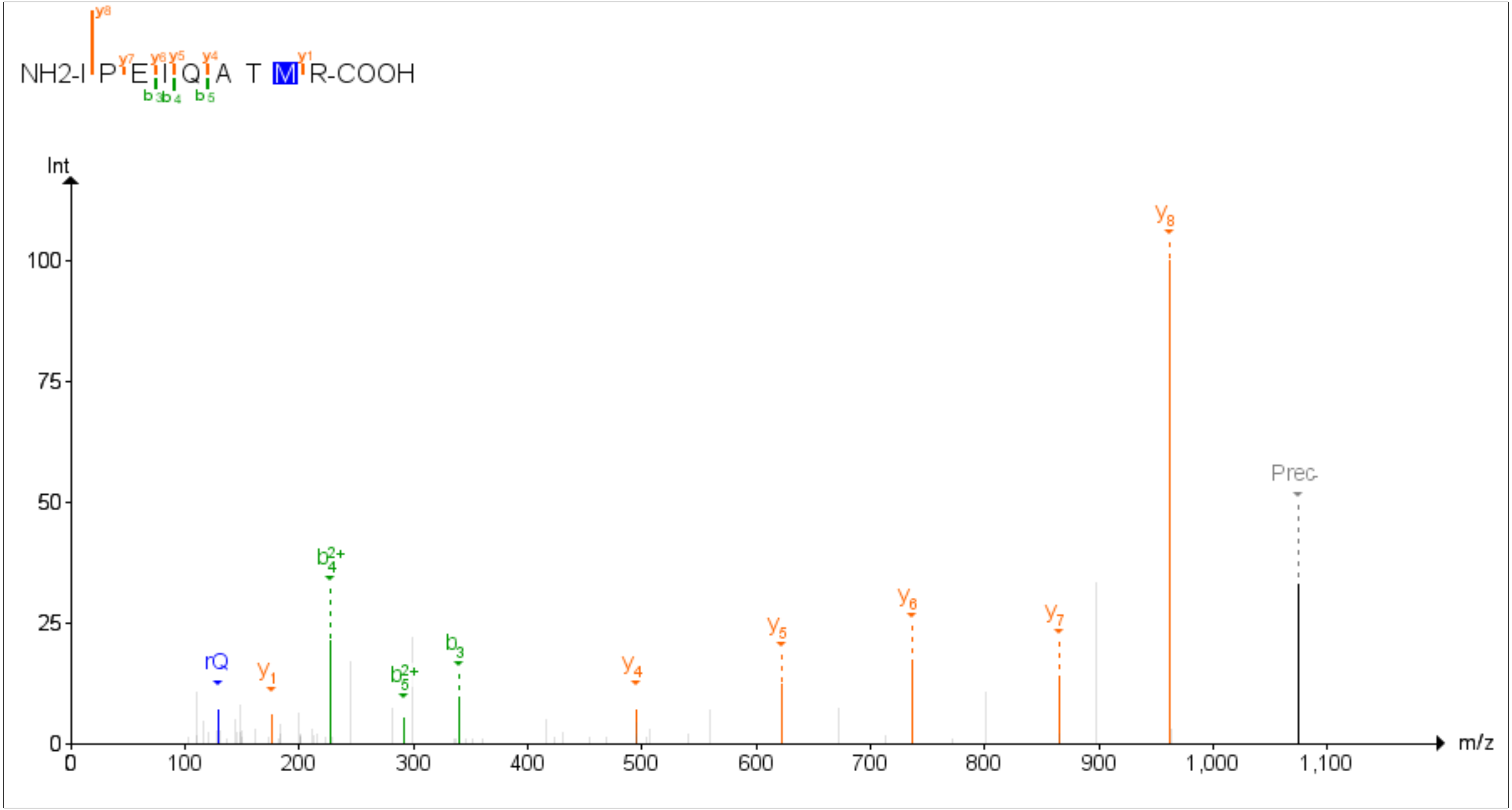

NH2-I P E I I Q A T M R-COOH
y8 y7 y6 y5 y4 y1
b3 b4 b5
Int
100
75
50
25
0
rQ
y1
b4 2+
b5 2+
b3
y4
y5
y6
y7
y8
Prec
0 100 200 300 400 500 600 700 800 900 1,000 1,100
m/z

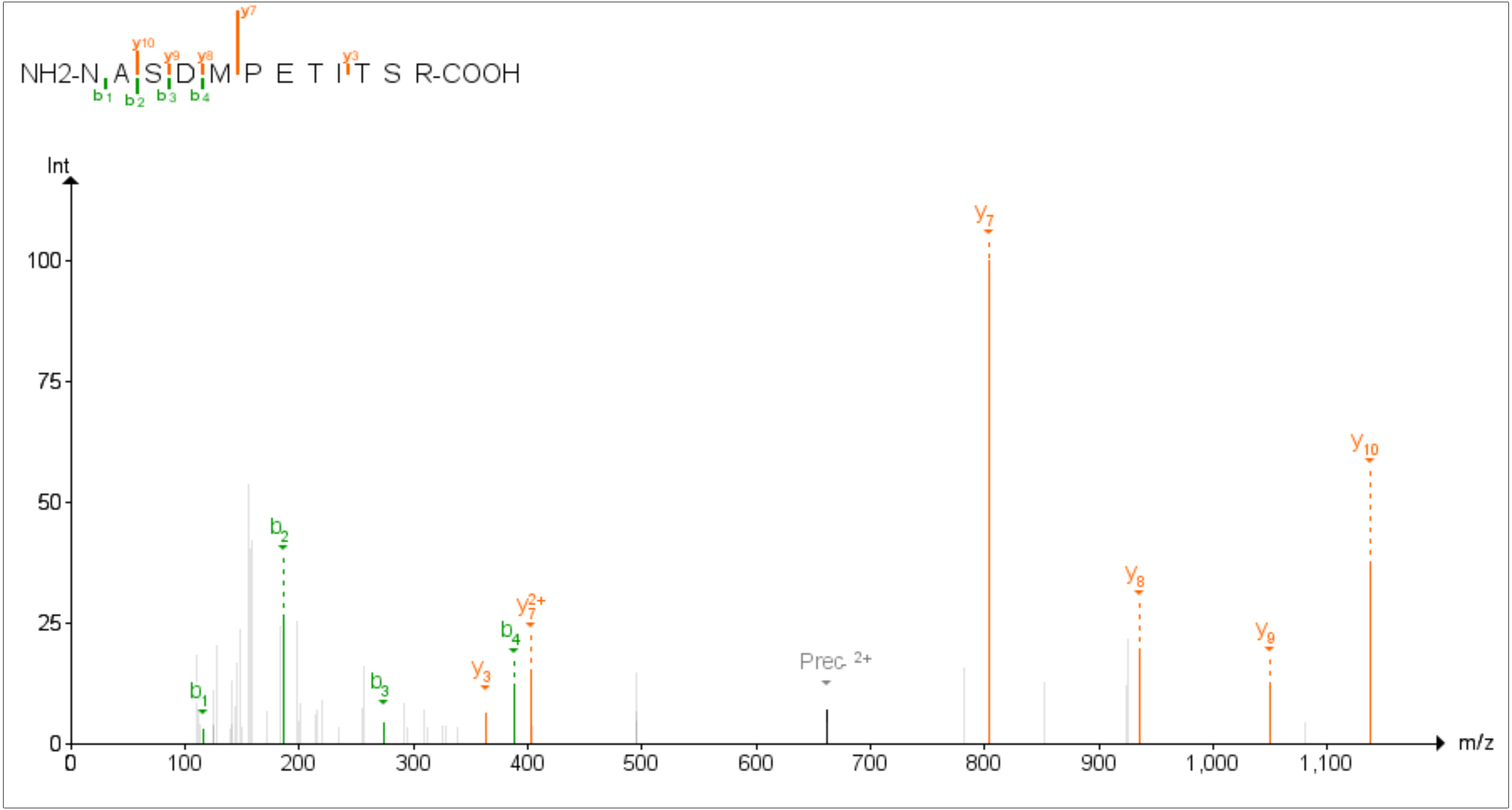
NH2-N A S D M P E T I T S R-COOH
y10 y9 y8 y7 y3
b1 b2 b3 b4
Int
100
75
50
25
0
m/z
0
100
200
300
400
500
600
700
800
900
1,000
1,100
b_1
b_2
b_3
b_4
y_3
y_7^{2+}
Prec. 2+
y_7
y_8
y_9
y_{10}

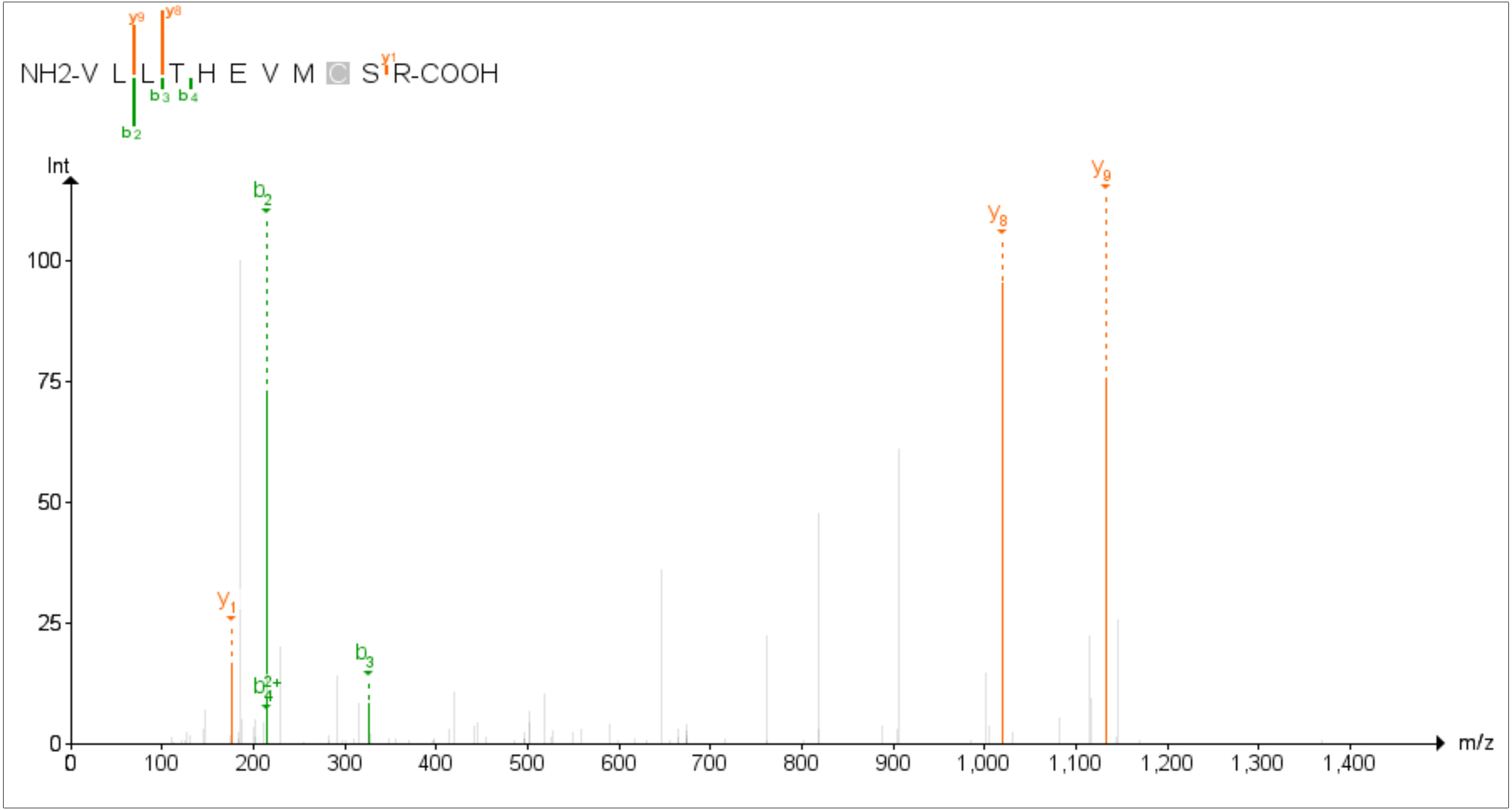
NH2-V L L T H E V M C S R-COOH
y9 y8 y1
b2 b3 b4
Int
100
75
50
25
0
b2
y1
b3
b$_4^{2+}$
y8
y9
0 100 200 300 400 500 600 700 800 900 1,000 1,100 1,200 1,300 1,400
m/z

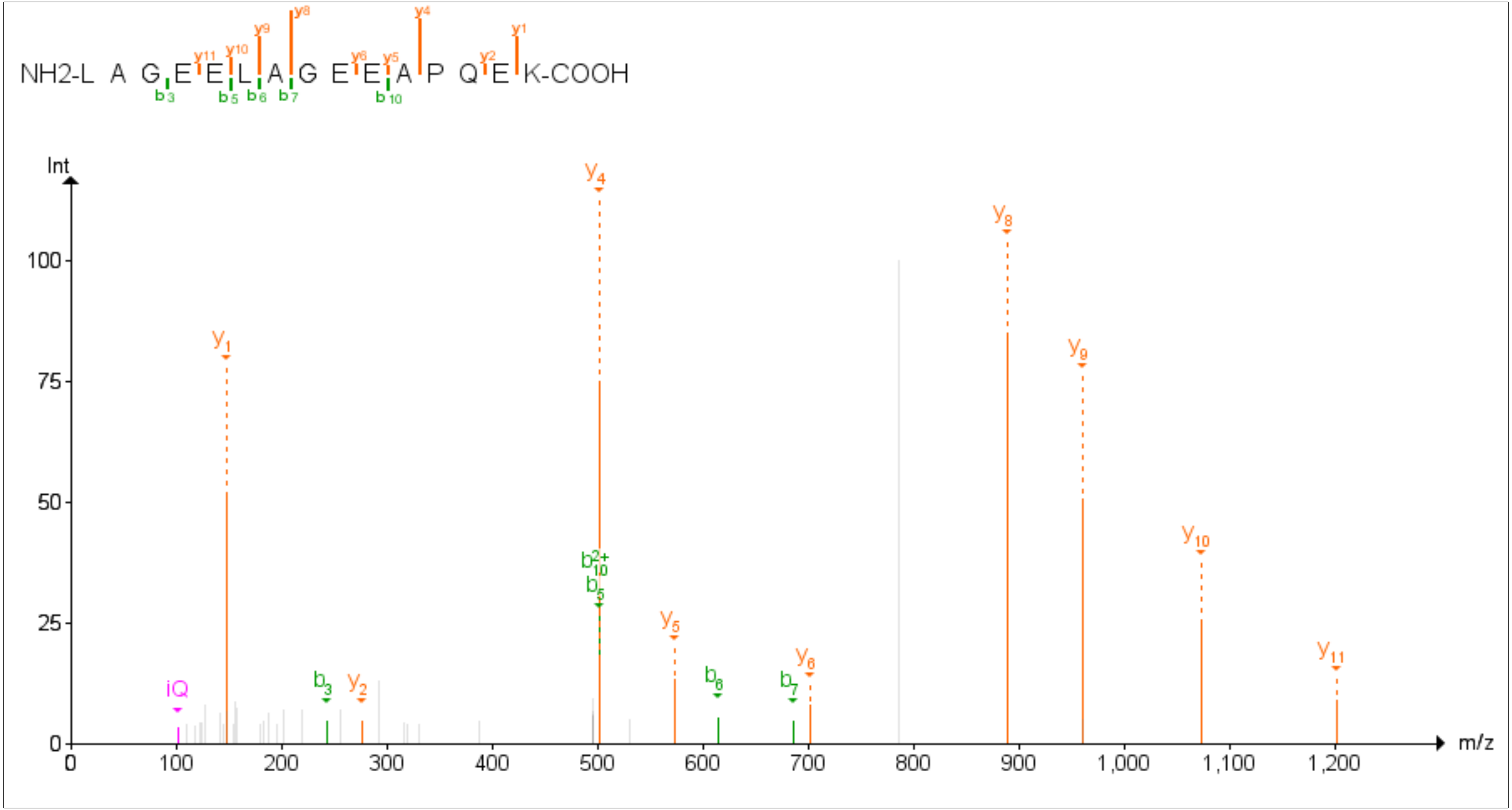

NH2-L A G E E L A G E E A P Q E K-COOH
Int
m/z
y1
y2
y4
y5
y6
y8
y9
y10
y11
b3
b5
b6
b7
b10
iQ

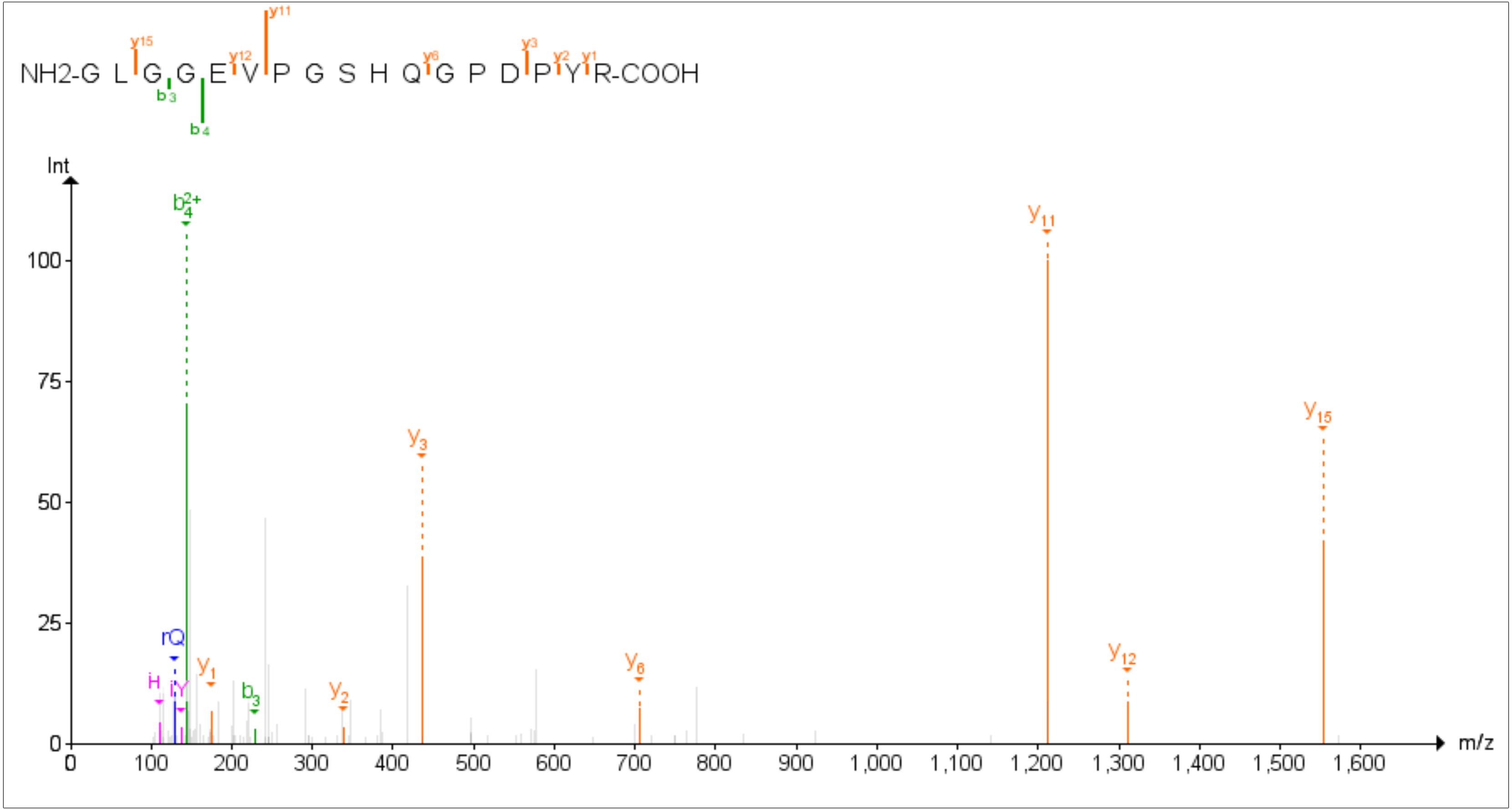
NH2-G L G G E V P G S H Q G P D P Y R-COOH
y15
y11
y12
y6
y3
y2
y1
b3
b4
Int
100
75
50
25
0
m/z
0
100
200
300
400
500
600
700
800
900
1,000
1,100
1,200
1,300
1,400
1,500
1,600
b4 2+
y11
y15
y3
rQ
y1
H
iY
b3
y2
y6
y12

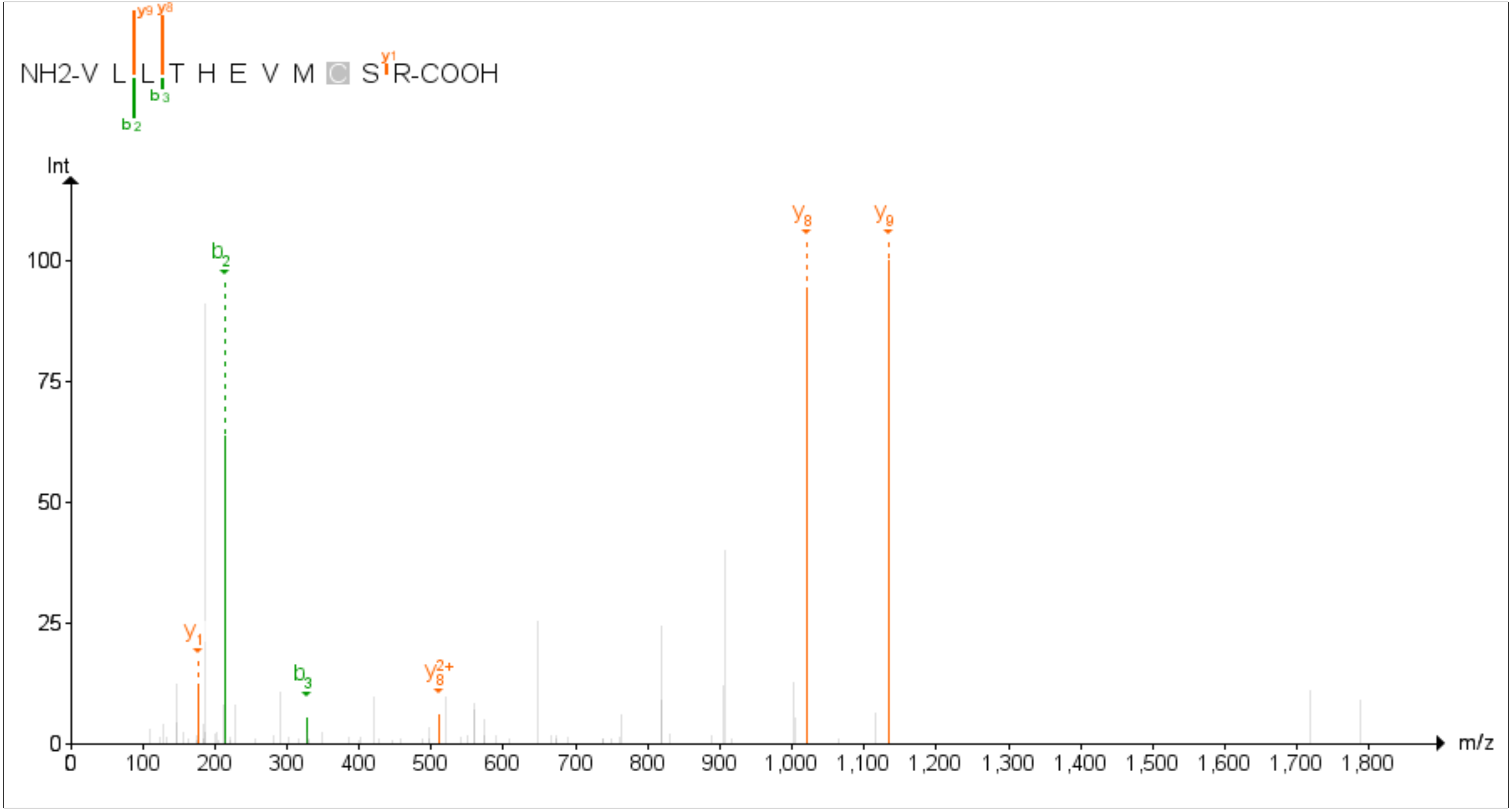

NH2-V L L T H E V M C S R-COOH
y9 y8 y1
b2 b3
Int
100
75
50
25
0
b2
y1
b3
y8 2+
y8
y9
0 100 200 300 400 500 600 700 800 900 1,000 1,100 1,200 1,300 1,400 1,500 1,600 1,700 1,800
m/z

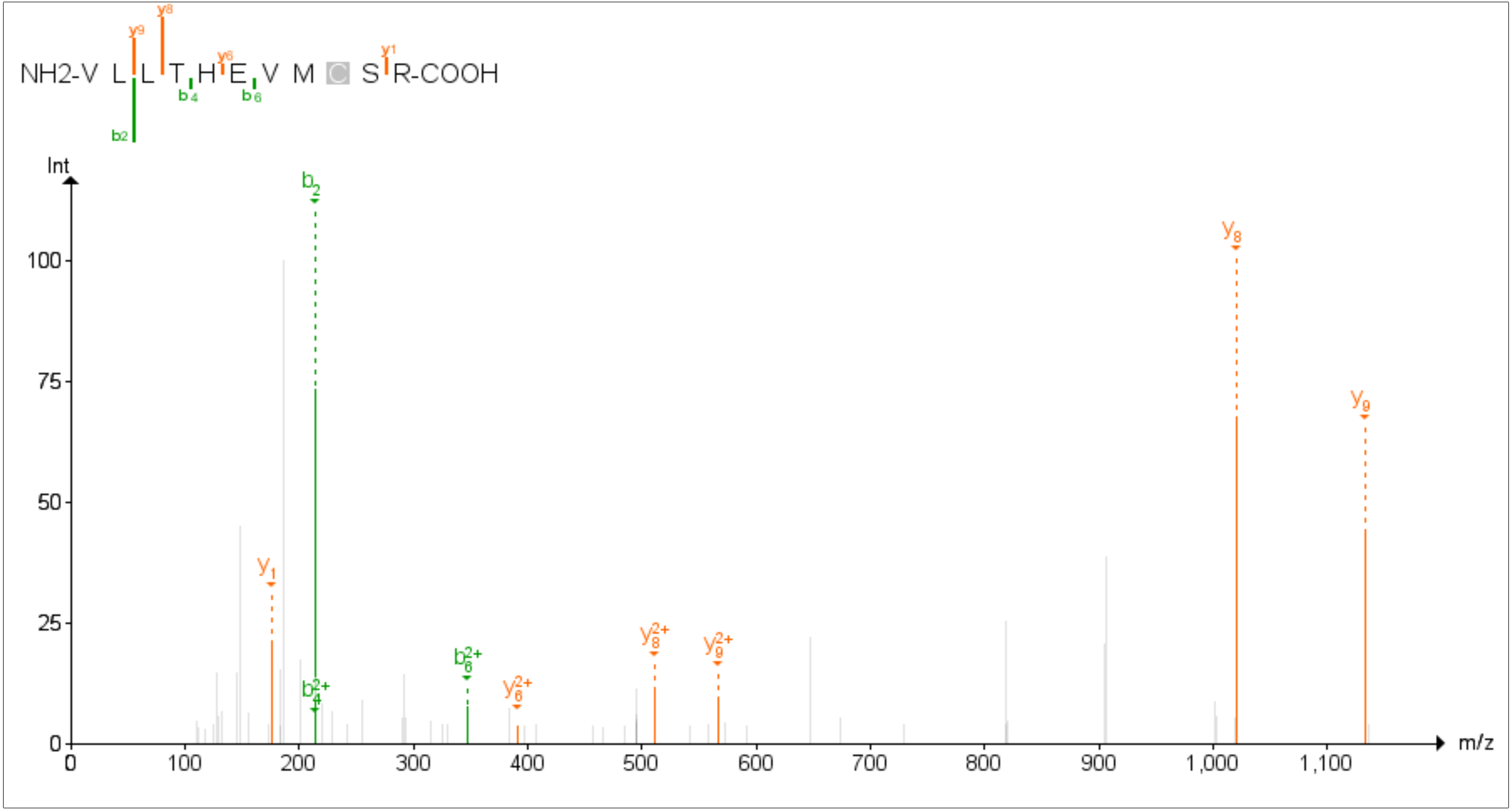
NH2-V L L T H E V M C S R-COOH
y9
y8
y6
y1
b2
b4
b6
Int
100
75
50
25
0
b2
y8
y9
y1
b4 2+
b6 2+
y6 2+
y8 2+
y9 2+
0
100
200
300
400
500
600
700
800
900
1,000
1,100
m/z

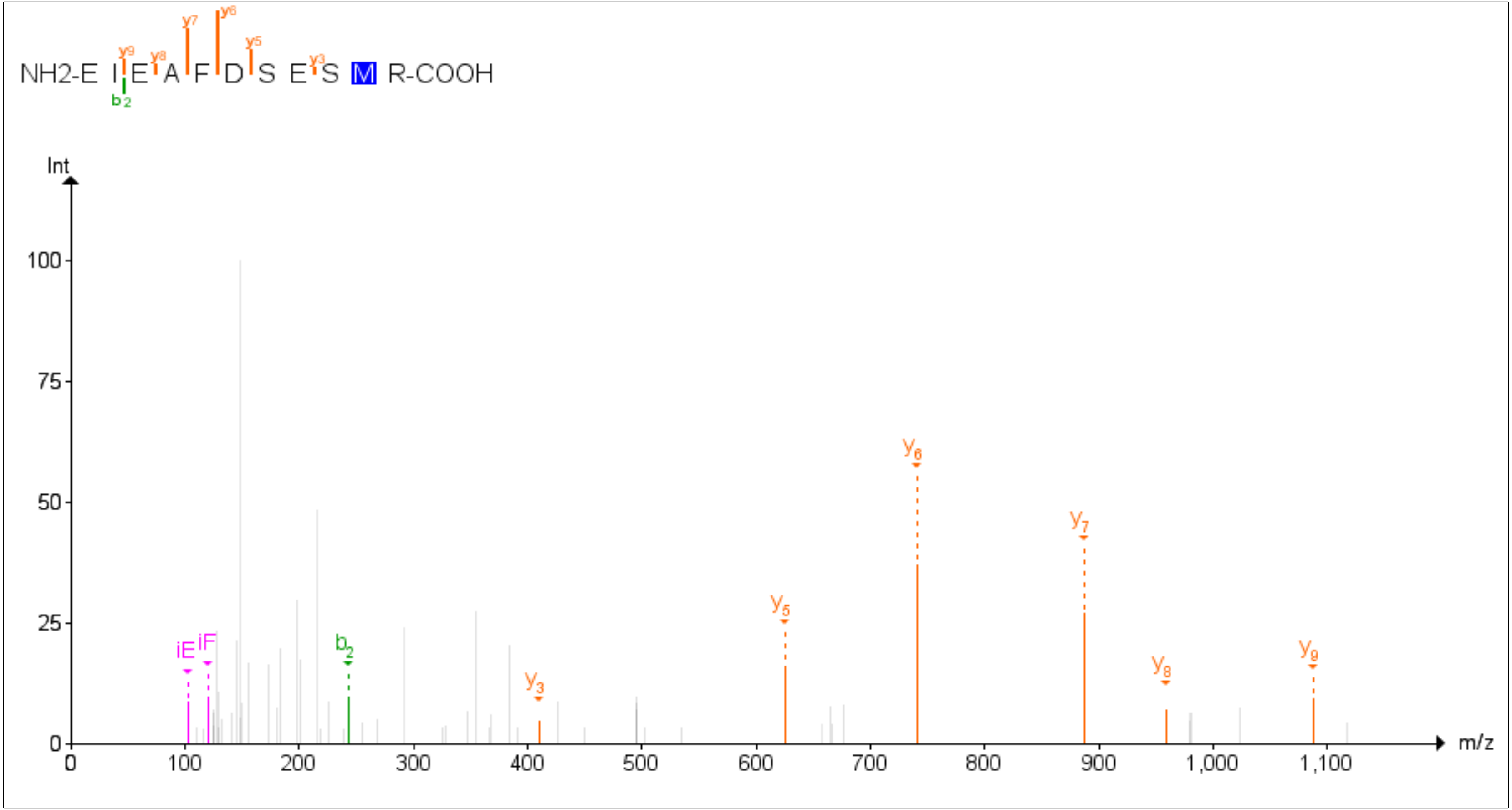

NH2-E I E A F D S E S M R-COOH
y9
y8
y7
y6
y5
y3
b2
Int
100
75
50
25
0
iE
iF
b2
y3
y5
y6
y7
y8
y9
0
100
200
300
400
500
600
700
800
900
1,000
1,100
m/z

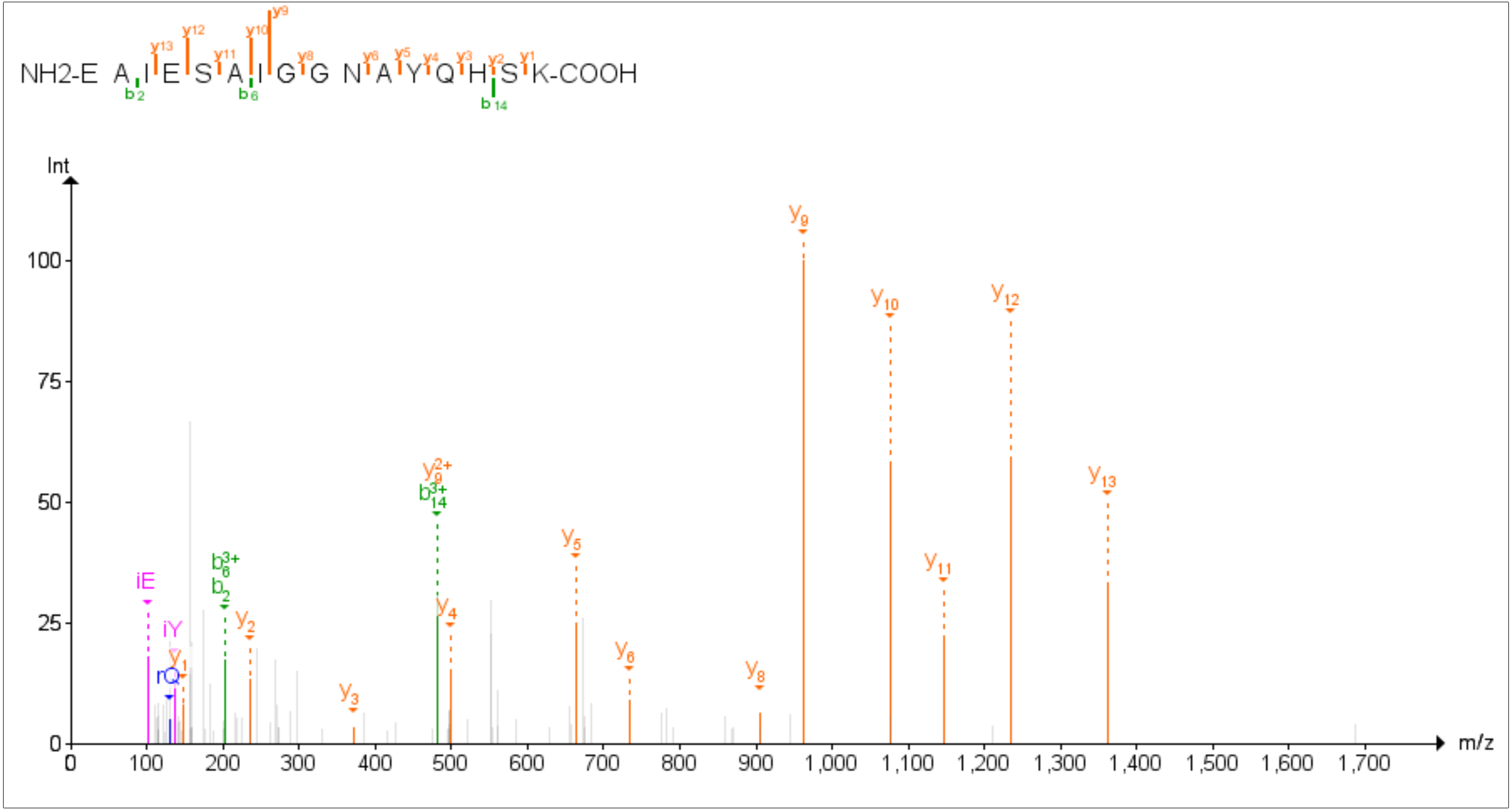

NH2-E A I E S A I G G N A Y Q H S K-COOH
Int
m/z
0
25
50
75
100
0
100
200
300
400
500
600
700
800
900
1,000
1,100
1,200
1,300
1,400
1,500
1,600
1,700

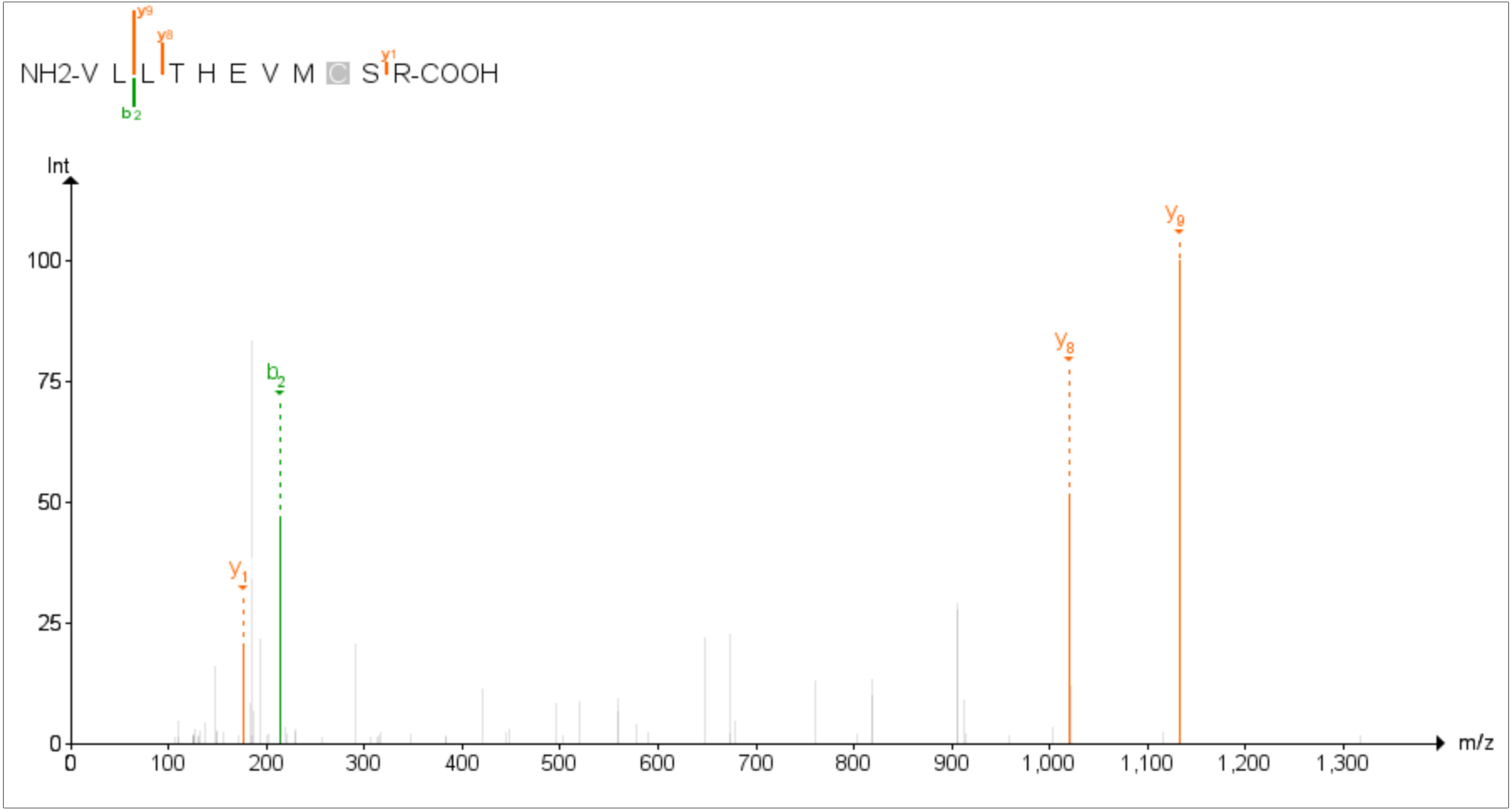

NH2-V L L T H E V M C S R-COOH
y9
y8
y1
b2
Int
100
75
50
25
0
0
100
200
300
400
500
600
700
800
900
1,000
1,100
1,200
1,300
m/z
y1
b2
y8
y9

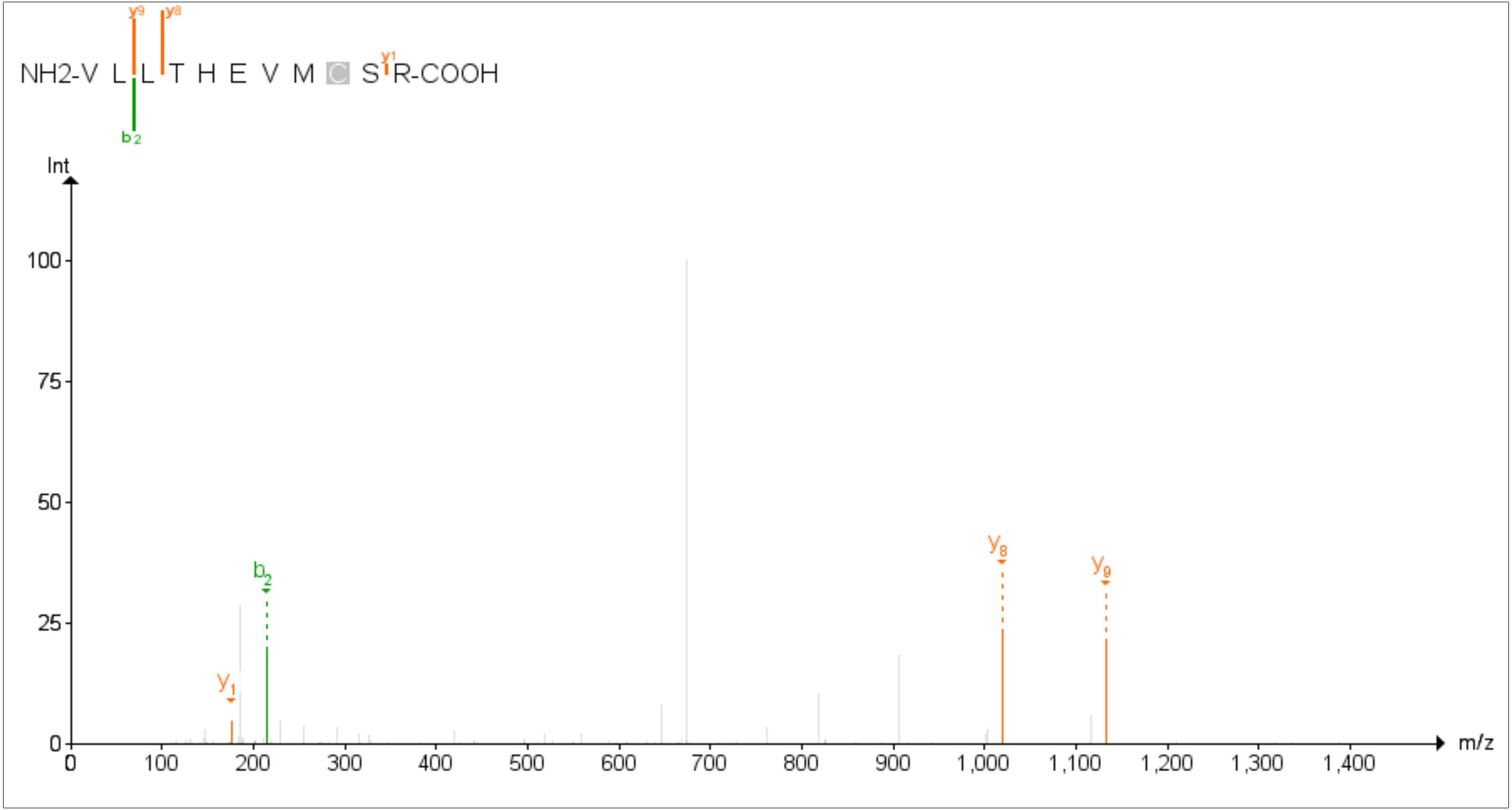

NH2-V L L T H E V M C S R-COOH
y9
y8
y1
b2
Int
100
75
50
25
0
0
100
200
300
400
500
600
700
800
900
1,000
1,100
1,200
1,300
1,400
m/z
y1
b2
y8
y9

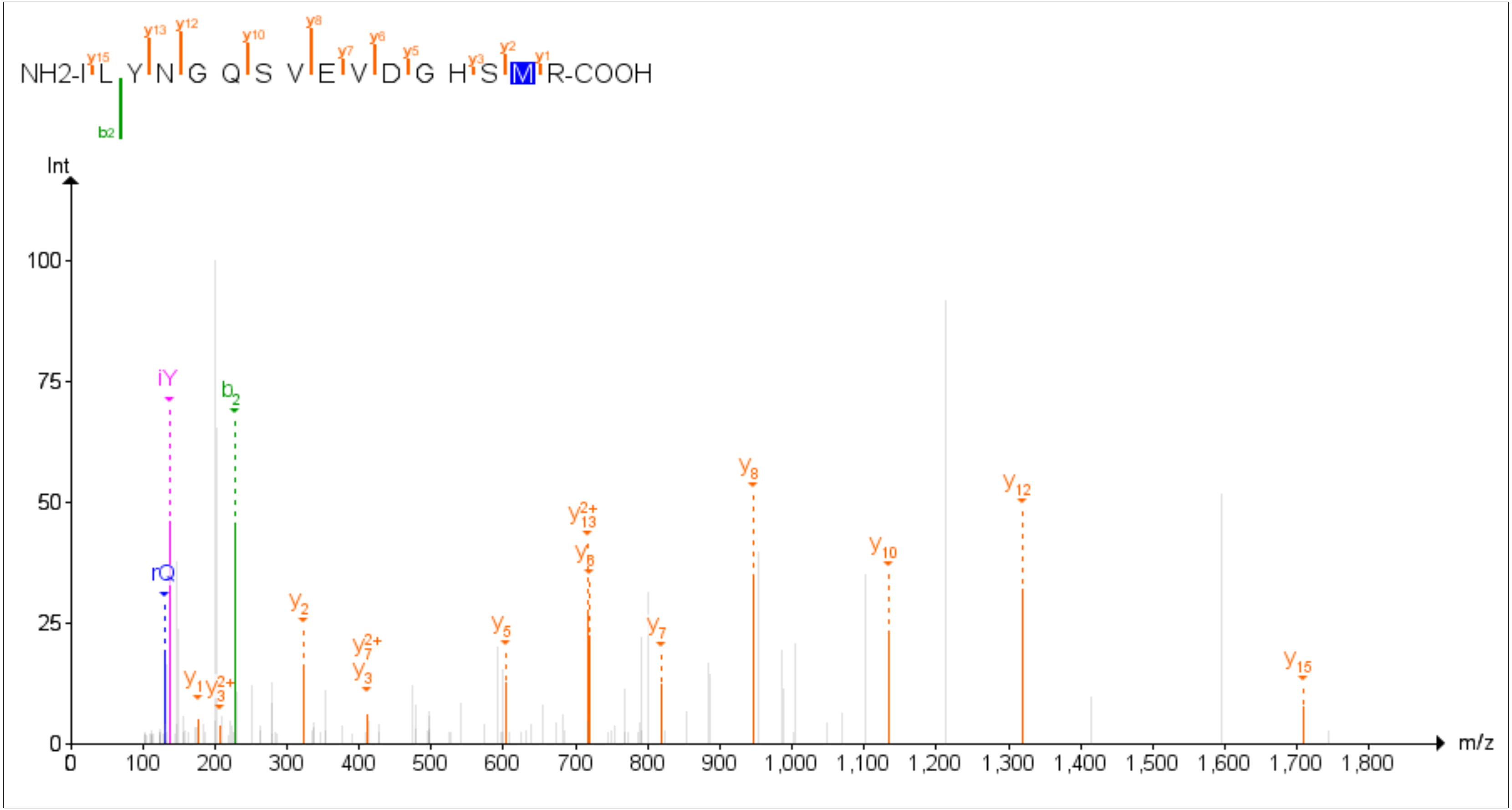

NH2-I L Y N G Q S V E V D G H S M R-COOH
y15 y13 y12 y10 y8 y7 y6 y5 y3 y2 y1
b2
Int
100
75
50
25
0
iY
b2
rQ
y1
y3 2+
y2
y7 2+
y3
y5
y13 2+
y6
y7
y8
y10
y12
y15
0 100 200 300 400 500 600 700 800 900 1,000 1,100 1,200 1,300 1,400 1,500 1,600 1,700 1,800
m/z

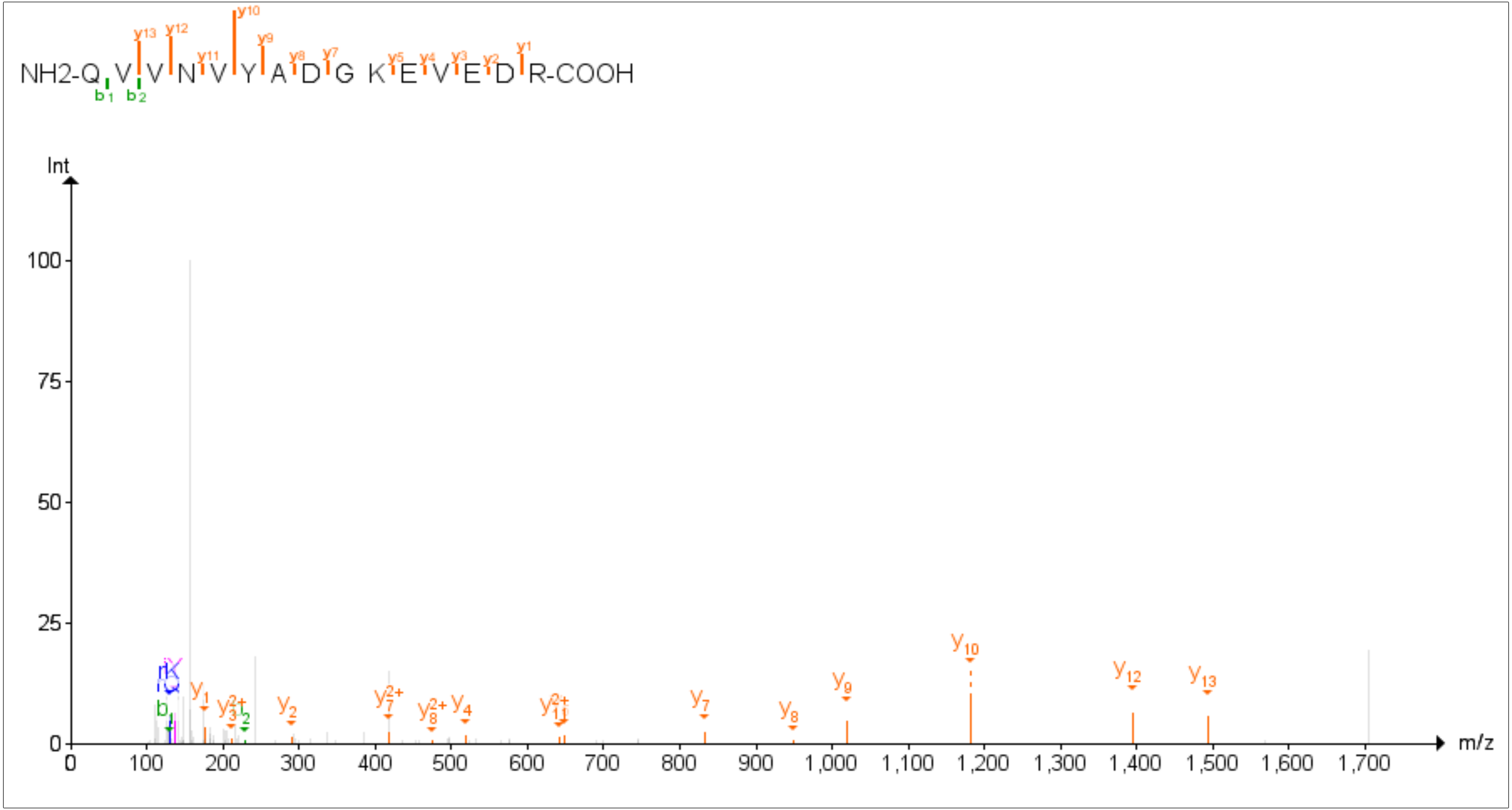
NH2-Q V V N V Y A D G K E V E D R-COOH
Int
100
75
50
25
0
m/z
0
100
200
300
400
500
600
700
800
900
1,000
1,100
1,200
1,300
1,400
1,500
1,600
1,700

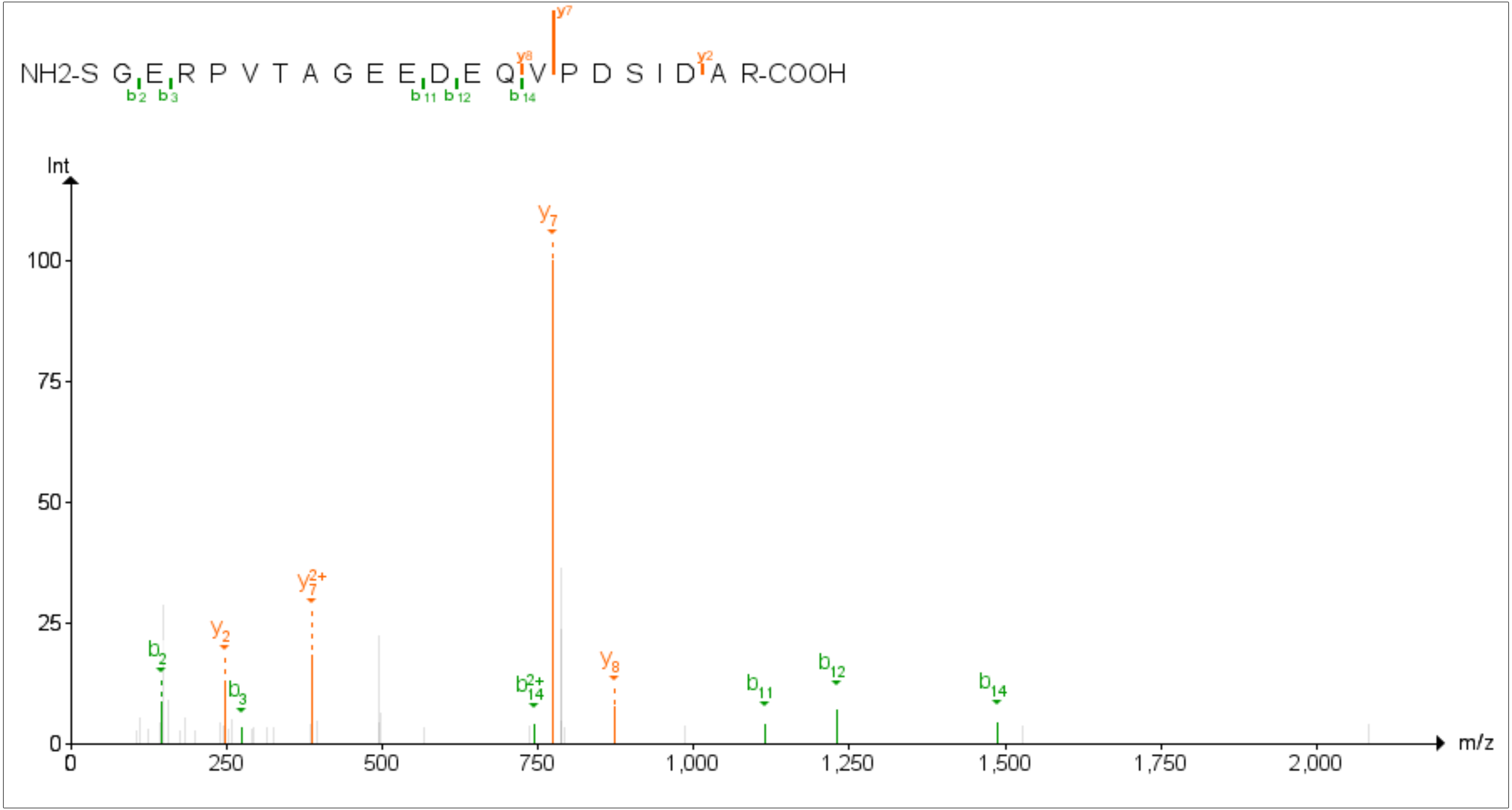

NH2-S G E R P V T A G E E D E Q V P D S I D A R-COOH
b2 b3 b11 b12 b14
y8 y7 y2
Int
100
75
50
25
0
y7
y7 2+
y2
b2
b3
b14 2+
y8
b11
b12
b14
0 250 500 750 1,000 1,250 1,500 1,750 2,000
m/z

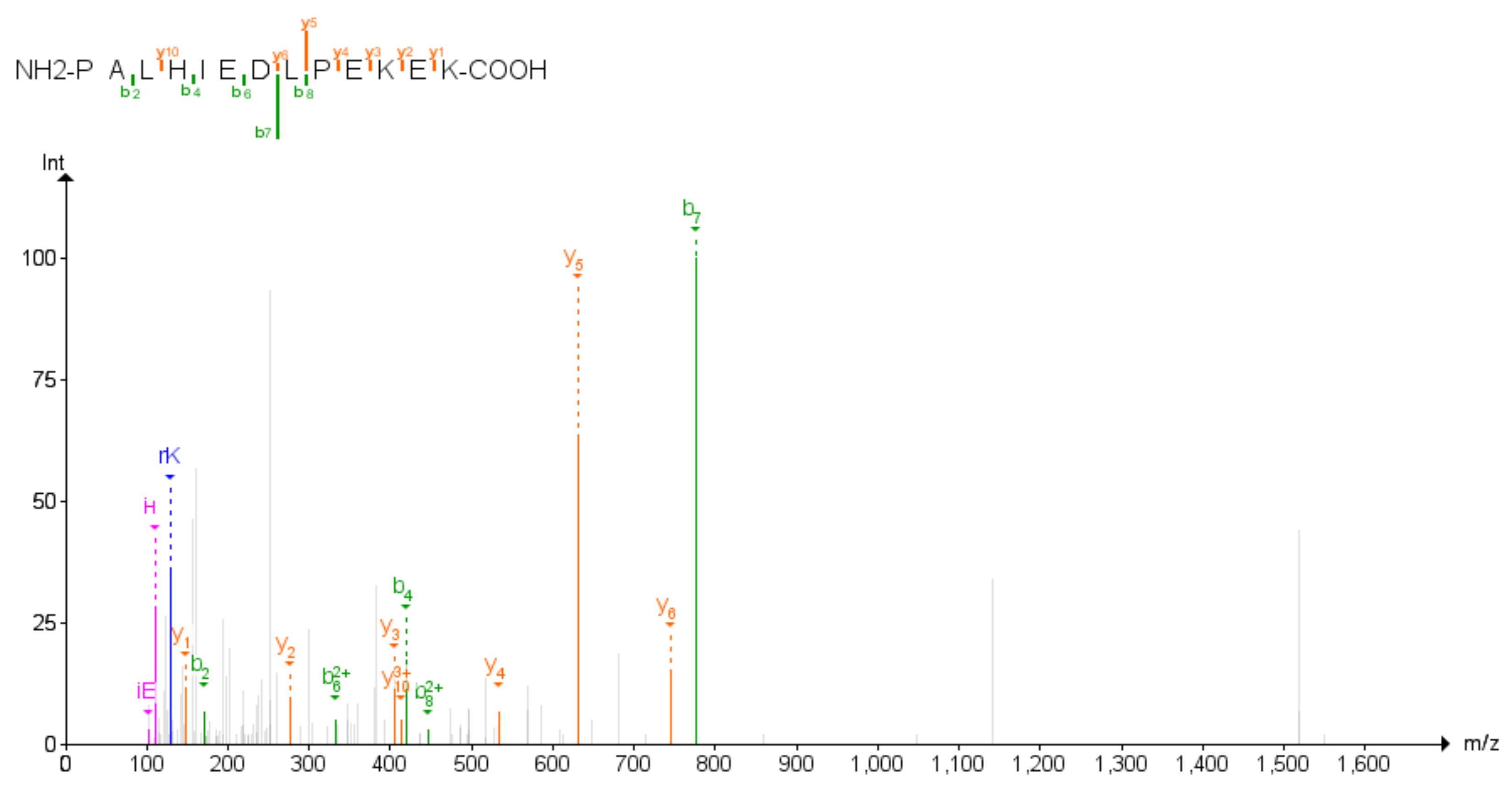

NH2-P A L H I E D L P E K E K-COOH
y10 y6 y5 y4 y3 y2 y1
b2 b4 b6 b7 b8
Int
m/z
rK
iH
iE
y1 b2 y2 b6 2+ y3 y10 3+ b4 b8 2+ y4 y5 y6 b7
0 25 50 75 100
0 100 200 300 400 500 600 700 800 900 1,000 1,100 1,200 1,300 1,400 1,500 1,600

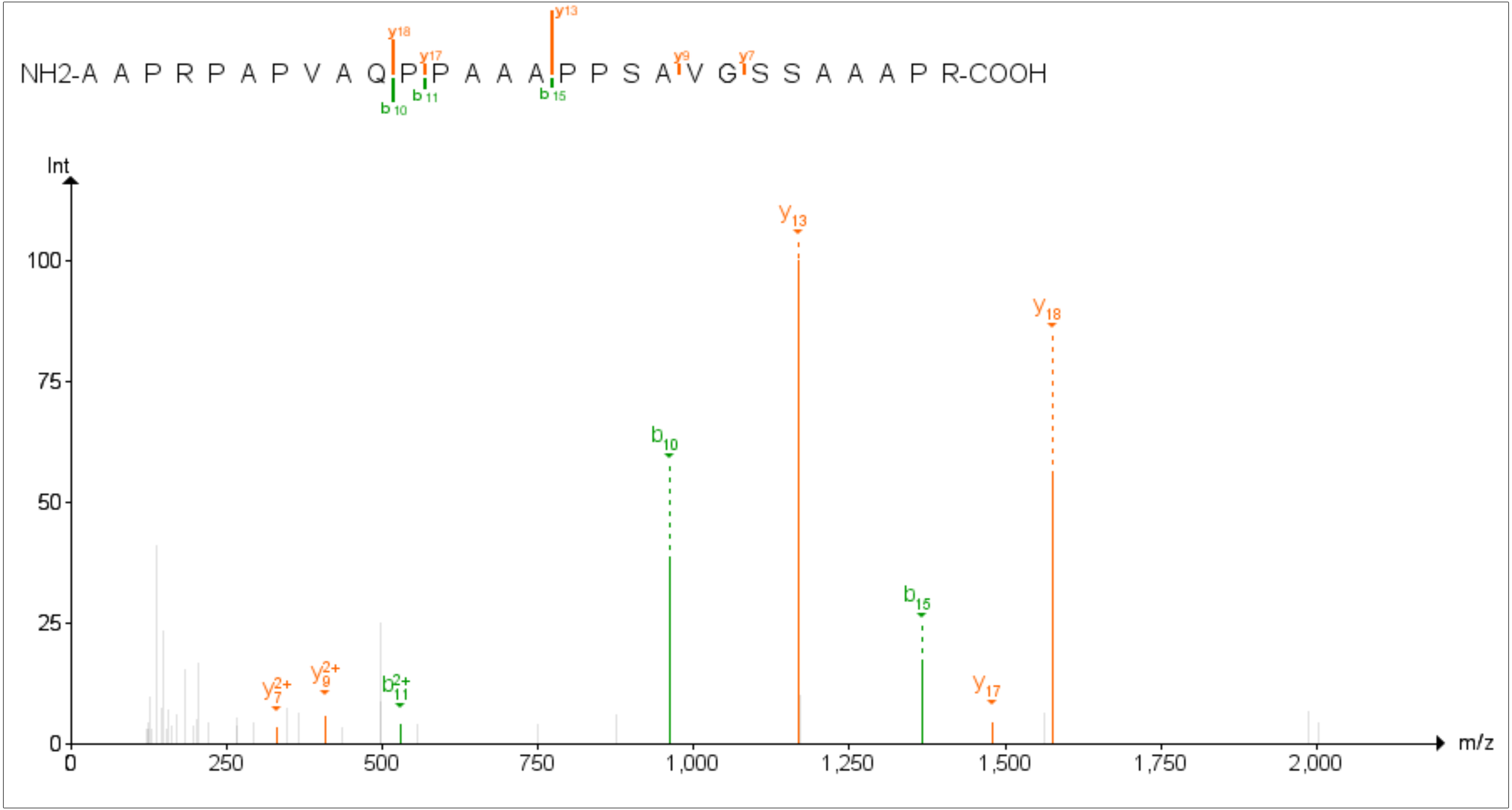

NH2-A A P R P A P V A Q P P A A A P P S A V G S S A A A P R-COOH
y18
y17
y13
y9
y7
b 10
b 11
b 15
Int
100
75
50
25
0
y13
y18
b10
b15
y17
y7 2+
y9 2+
b11 2+
0
250
500
750
1,000
1,250
1,500
1,750
2,000
m/z

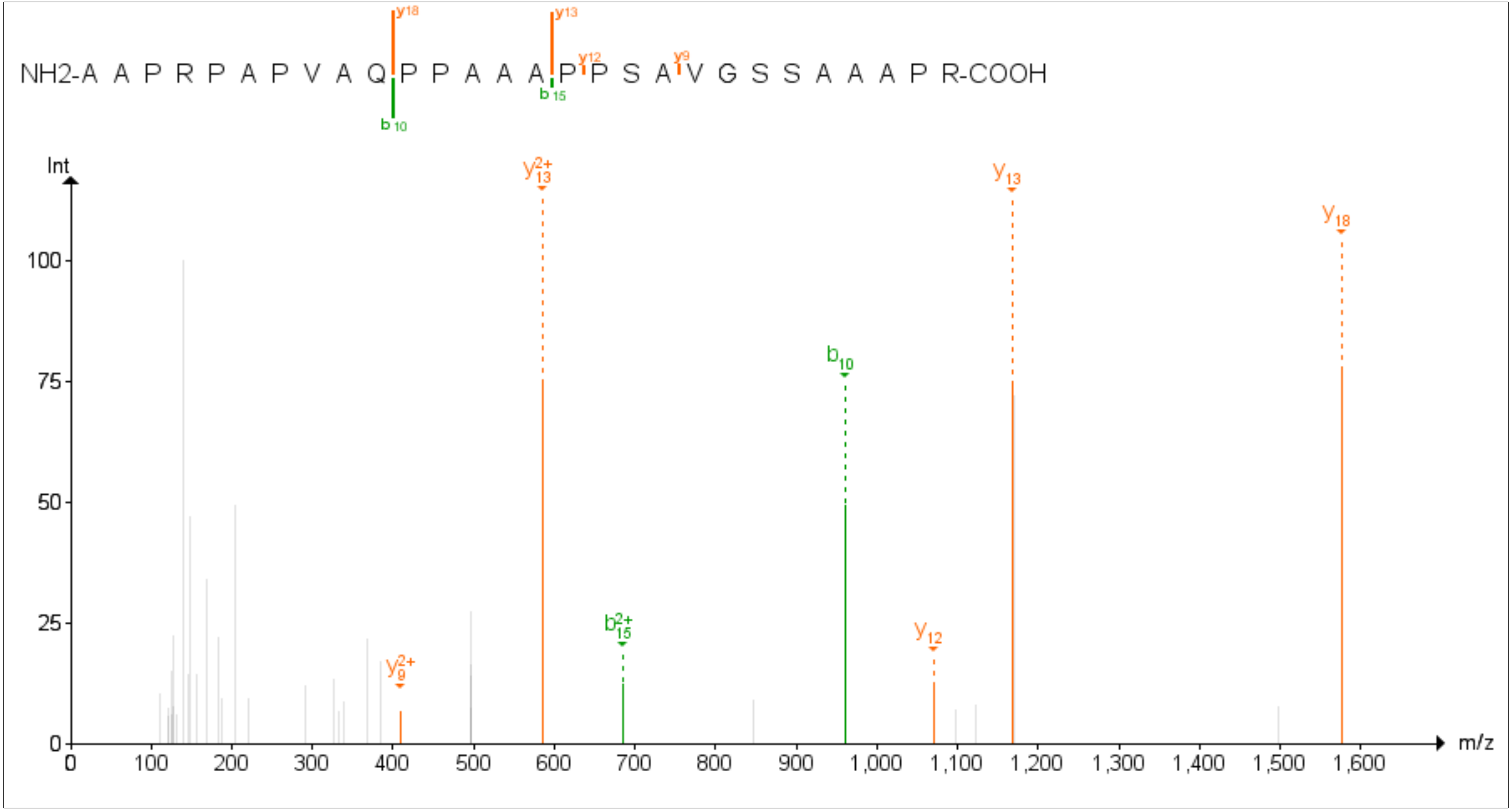

NH2-A A P R P A P V A Q P P A A A P P S A V G S S A A A P R-COOH
y18
y13
y12
y9
b 10
b 15
Int
m/z
y13 2+
y13
y18
b10
b15 2+
y12
y9 2+

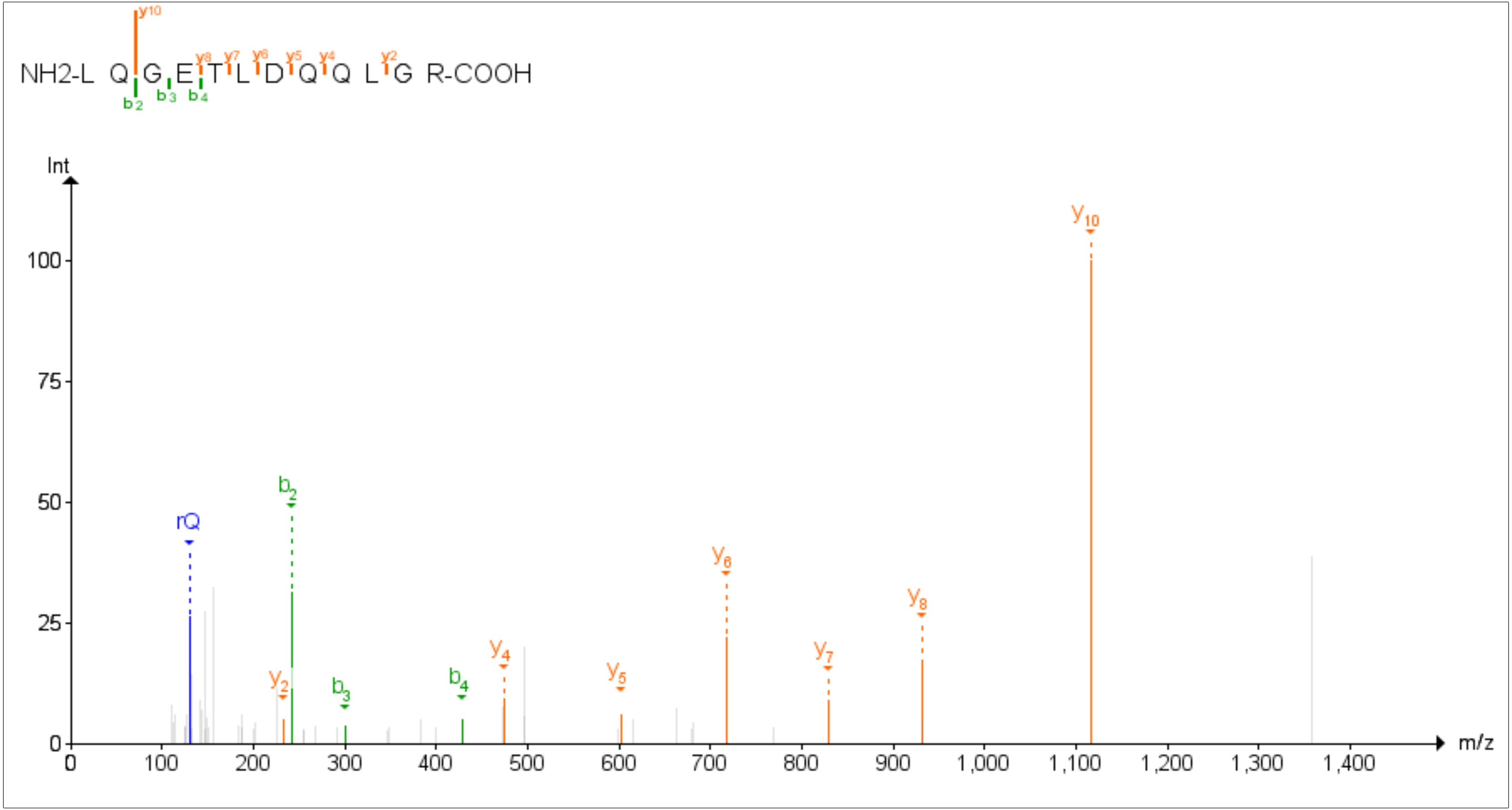

NH2-L Q G E T L D Q Q L G R-COOH
y10
y8
y7
y6
y5
y4
y2
b2
b3
b4
Int
100
75
50
25
0
rQ
b2
y2
b3
b4
y4
y5
y6
y7
y8
y10
0
100
200
300
400
500
600
700
800
900
1,000
1,100
1,200
1,300
1,400
m/z

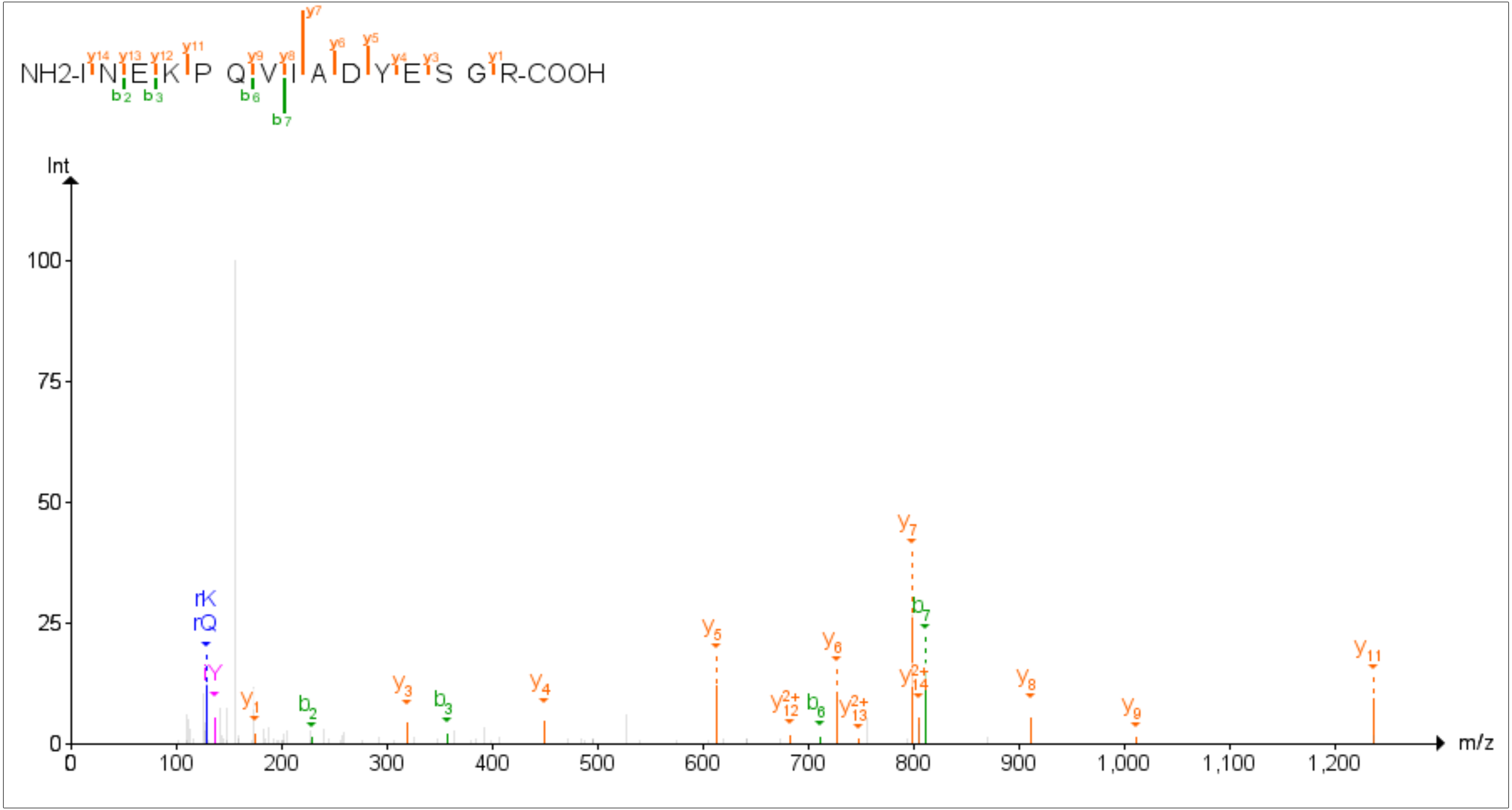

NH2-I N E K P Q V I A D Y E S G R-COOH
y14 y13 y12 y11 y9 y8 y7 y6 y5 y4 y3 y1
b2 b3 b6 b7
Int
m/z
rK
rQ
rY

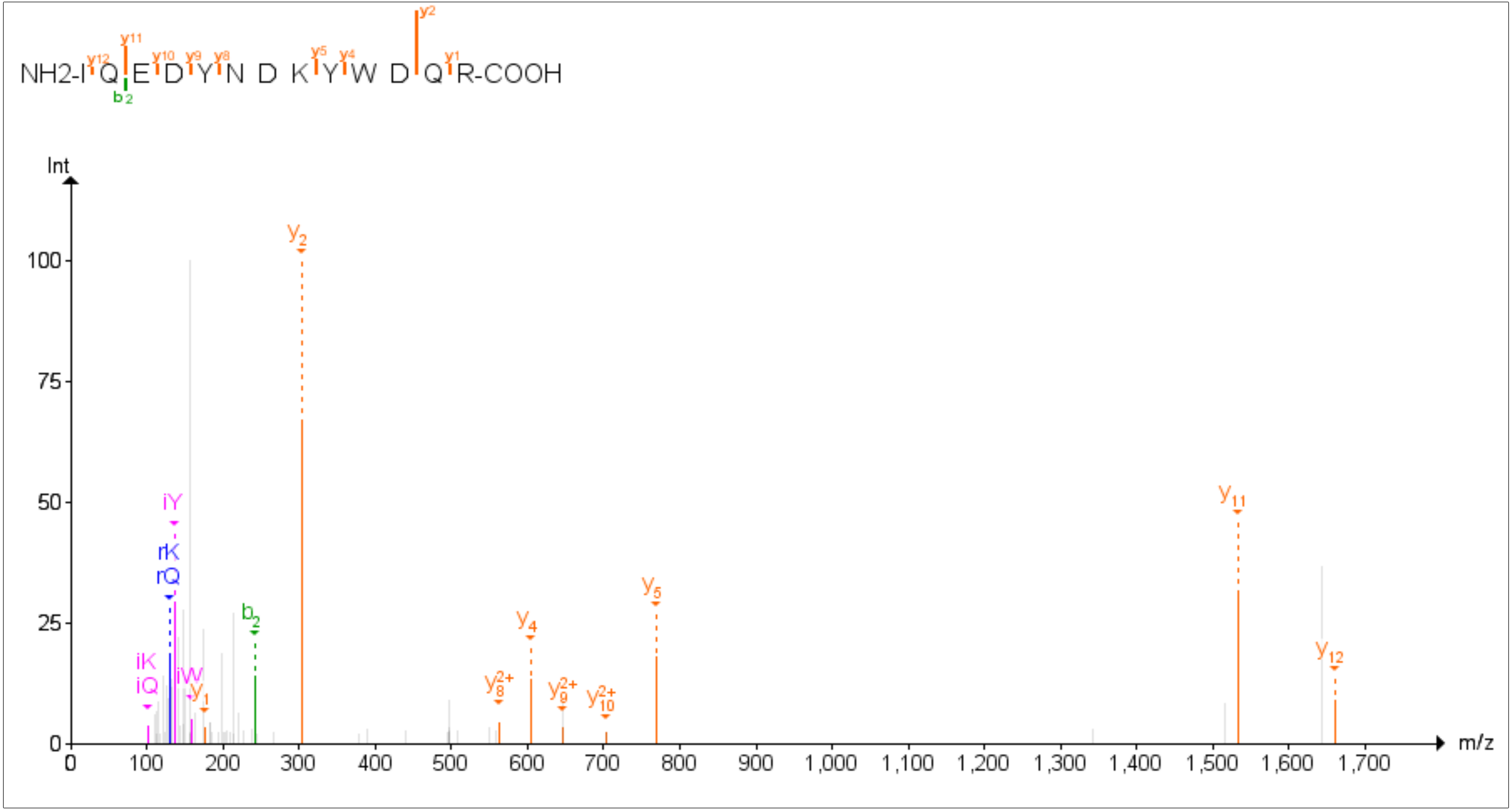

NH2-I Q E D Y N D K Y W D Q R-COOH
y12 y11 y10 y9 y8 y5 y4 y2 y1 b2
Int
100
75
50
25
0
y2
iY
rK
rQ
b2
iK
iQ
iW
y1
y4
y5
y11
y12
0 100 200 300 400 500 600 700 800 900 1,000 1,100 1,200 1,300 1,400 1,500 1,600 1,700
m/z

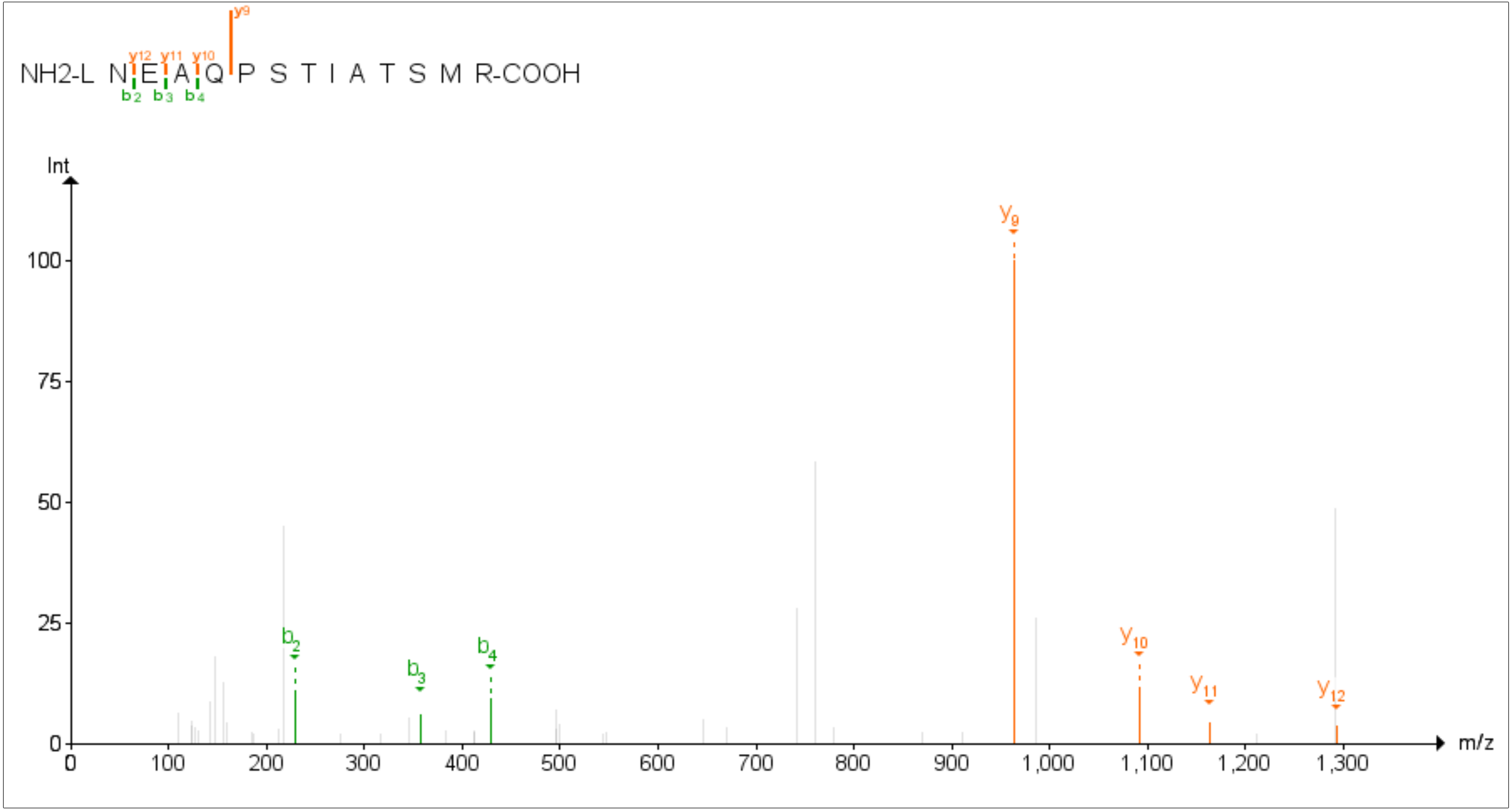

NH2-L N E A Q P S T I A T S M R-COOH
y12 y11 y10 y9
b2 b3 b4
Int
100
75
50
25
0
0 100 200 300 400 500 600 700 800 900 1,000 1,100 1,200 1,300
m/z
y9
y10
y11
y12
b2
b3
b4

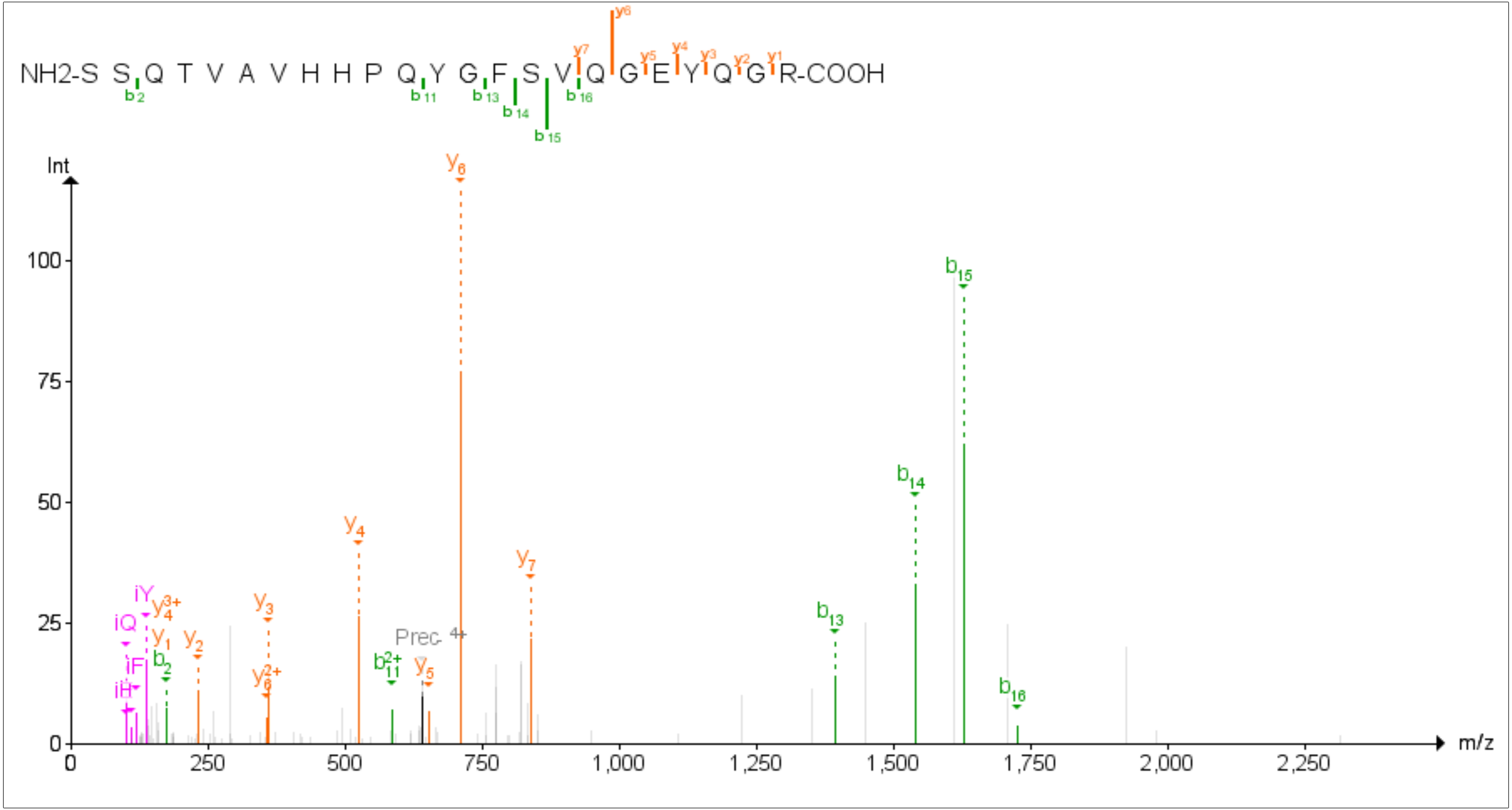

NH2-S S Q T V A V H H P Q Y G F S V Q G E Y Q G R-COOH
b2
b11
b13
b14
b15
b16
y8
y7
y6
y5
y4
y3
y2
y1
Int
100
75
50
25
0
m/z
0
250
500
750
1,000
1,250
1,500
1,750
2,000
2,250
y6
b15
b14
y4
y7
b13
b16
iY
y4 3+
iQ
y1
y2
y3
b2
iF
iH
y6 2+
b11 2+
y5
Prec 4+

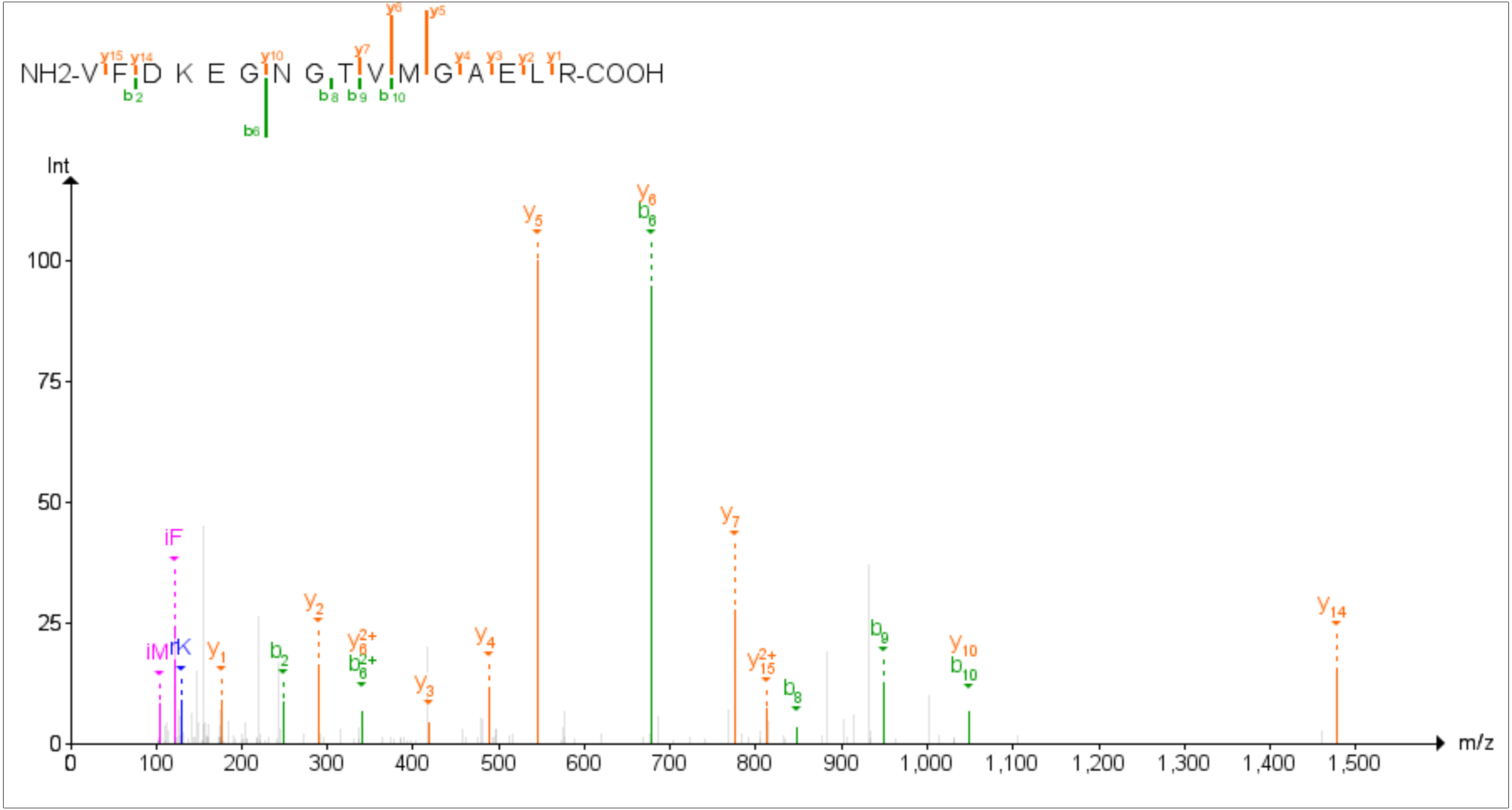

NH2-V F D K E G N G T V M G A E L R-COOH
y15 y14 y10 y7 y6 y5 y4 y3 y2 y1
b2 b6 b8 b9 b10
Int
m/z
y5
y6
b6
y7
y14
iF
iM
rK
y1
b2
y2
y6 2+
b6 2+
y3
y4
y15 2+
b8
b9
y10
b10
0
25
50
75
100
0
100
200
300
400
500
600
700
800
900
1,000
1,100
1,200
1,300
1,400
1,500

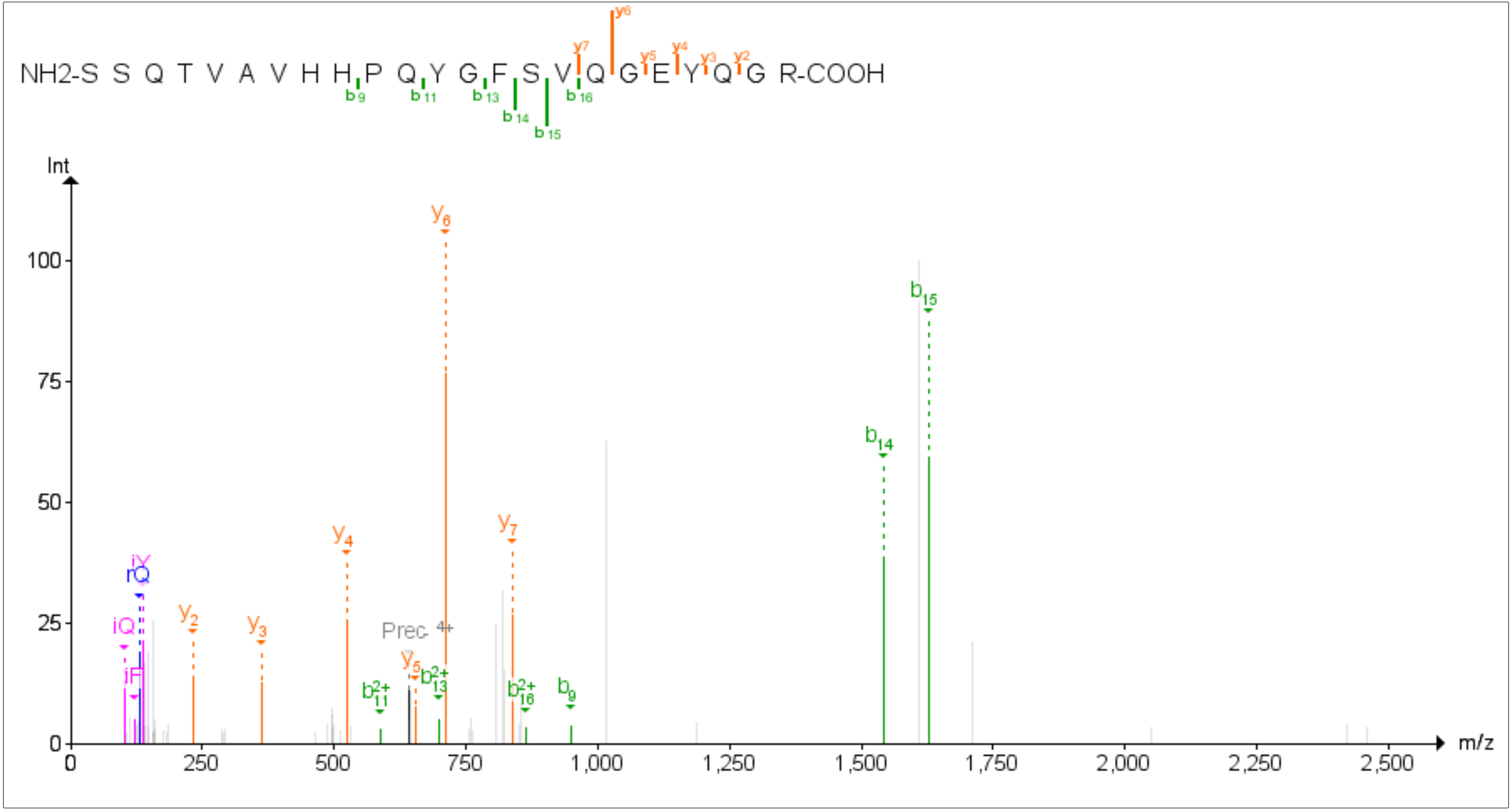

NH2-S S Q T V A V H H P Q Y G F S V Q G E Y Q G R-COOH
b9 b11 b13 b14 b15 b16
y7 y6 y5 y4 y3 y2
Int
m/z
y6
b15
b14
y4
y7
iY
rQ
iQ
y2
y3
Prec 4+
y5
iF
b2+ 11
b2+ 13
b2+ 16
b9
0 25 50 75 100
0 250 500 750 1,000 1,250 1,500 1,750 2,000 2,250 2,500

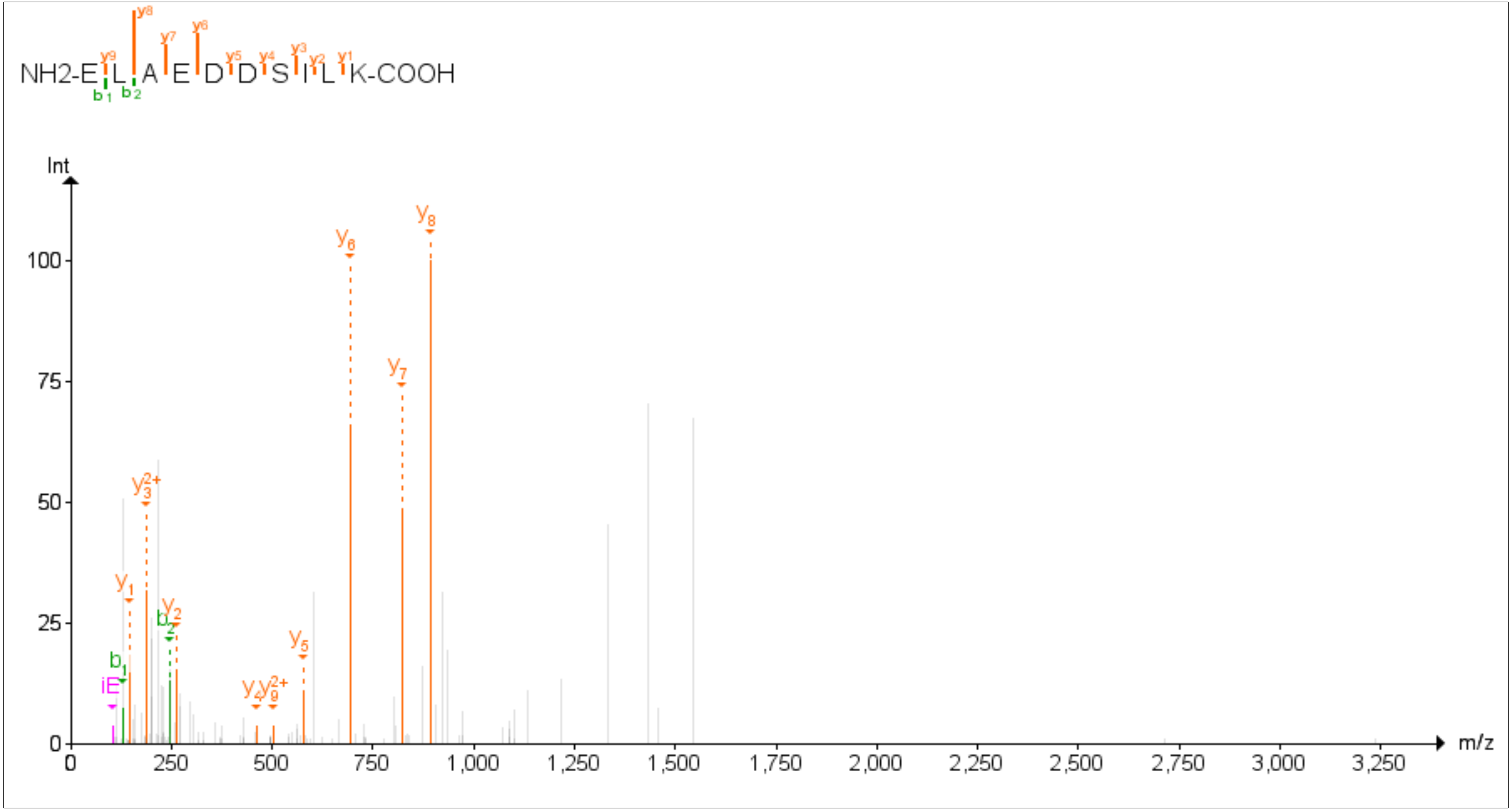

NH2-E L A E D D S I L K-COOH
y9 y8 y7 y6 y5 y4 y3 y2 y1
b1 b2
Int
100
75
50
25
0
0 250 500 750 1,000 1,250 1,500 1,750 2,000 2,250 2,500 2,750 3,000 3,250
m/z
y8
y6
y7
y3 2+
y1
y2
b2
y5
b1
iE
y4 y9 2+

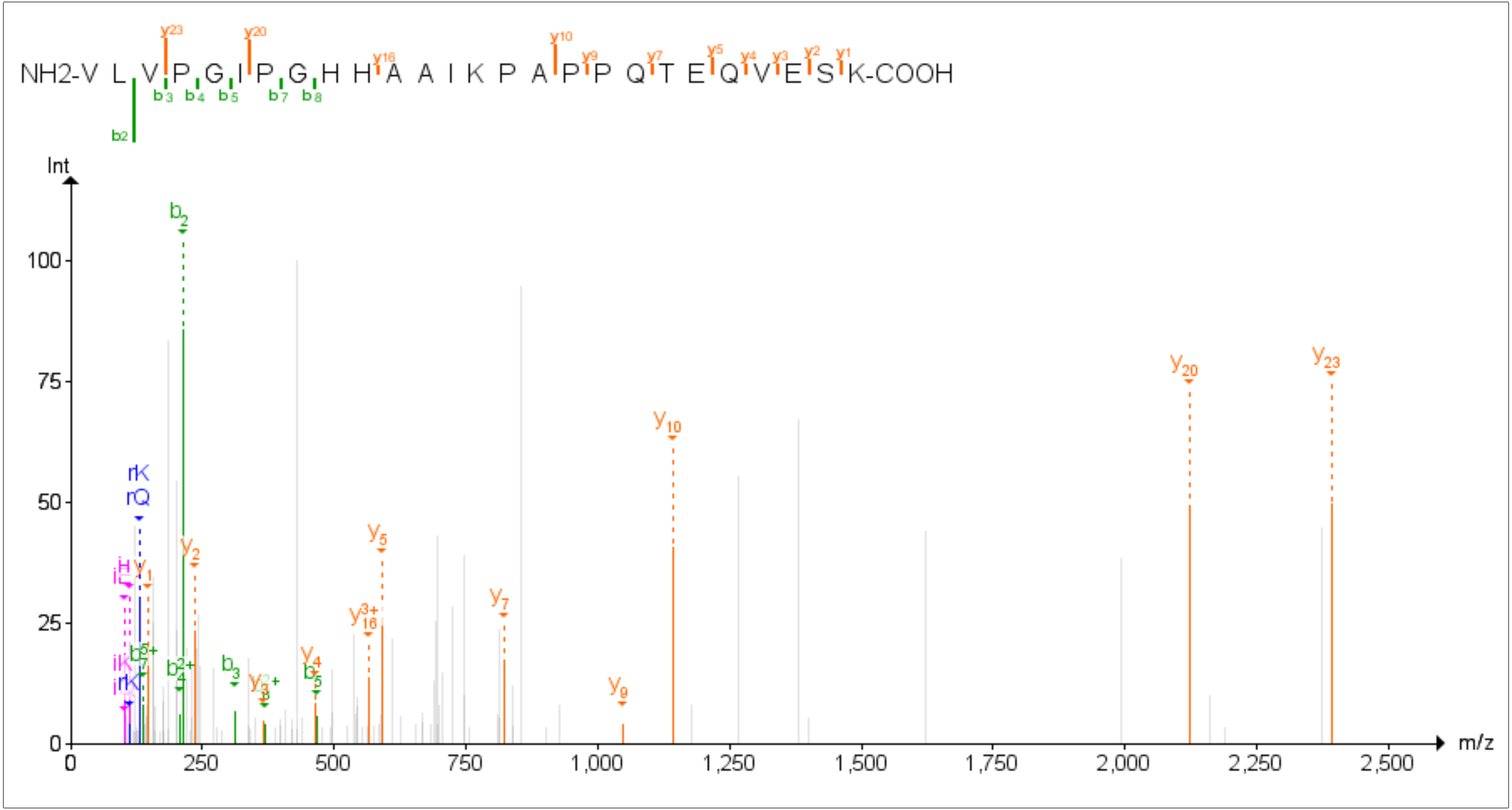
NH2-V L V P G I P G H H A A I K P A P P Q T E Q V E S K-COOH
b2
b3
b4
b5
b7
b8
y23
y20
y16
y10
y9
y7
y5
y4
y3
y2
y1
Int
m/z
0
25
50
75
100
250
500
750
1,000
1,250
1,500
1,750
2,000
2,250
2,500
rK
rQ
y10
y20
y23
y5
y7
y9
y2
y4
b3
b5

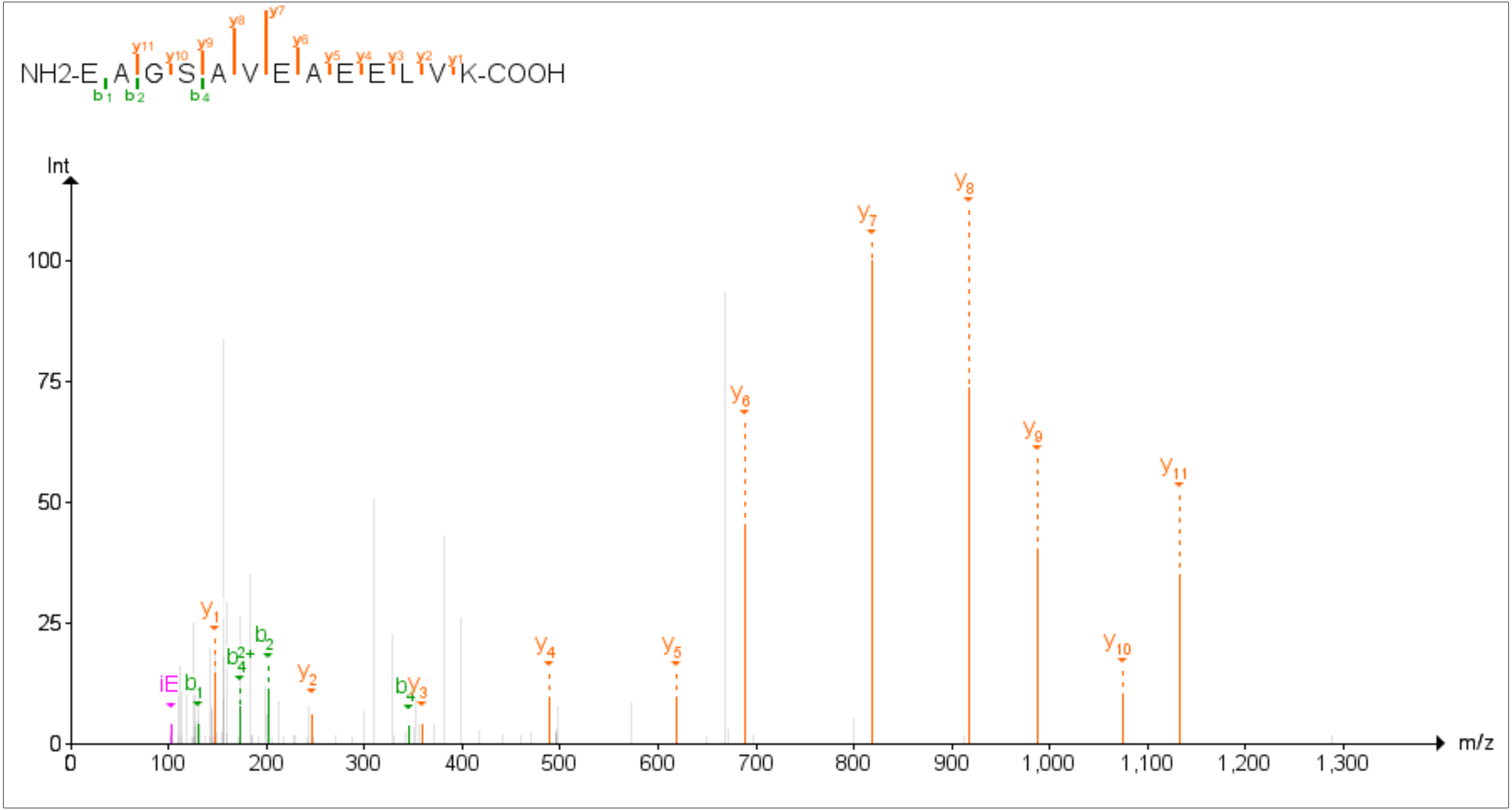

NH2-E A G S A V E A E E L V K-COOH
y11 y10 y9 y8 y7 y6 y5 y4 y3 y2 y1
b1 b2 b4
Int
100
75
50
25
0
m/z
0 100 200 300 400 500 600 700 800 900 1,000 1,100 1,200 1,300
iE b1 y1 b4 2+ b2 y2 b4 + y3 y4 y5 y6 y7 y8 y9 y10 y11

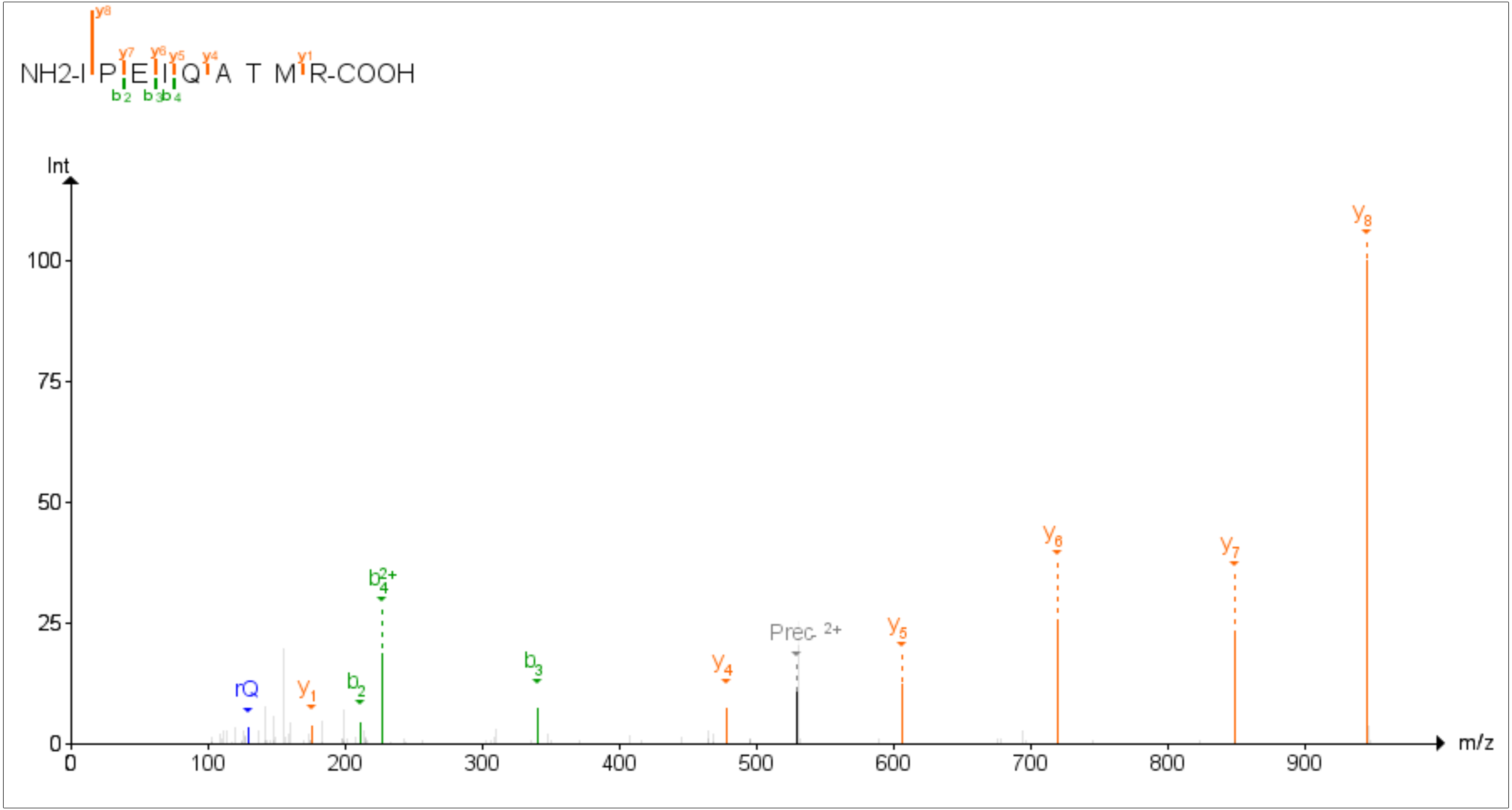

NH2-I P E I Q A T M R-COOH
y8 y7 y6 y5 y4 y1
b2 b3 b4
Int
100
75
50
25
0
m/z
0 100 200 300 400 500 600 700 800 900
rQ
y1
b2
b4 2+
b3
y4
Prec- 2+
y5
y6
y7
y8

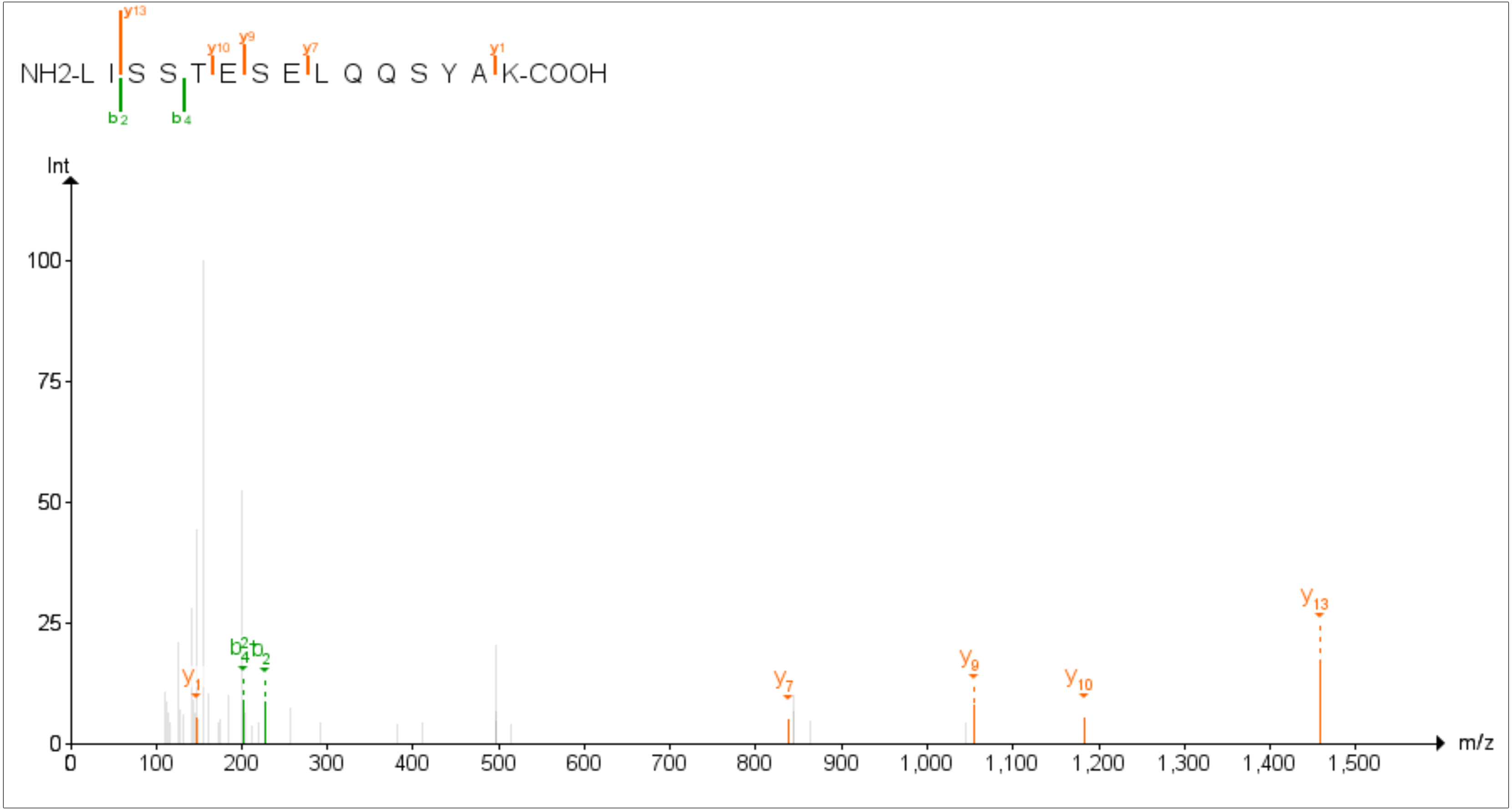

NH2-L I S S T E S E L Q Q S Y A K-COOH
y13
y10
y9
y7
y1
b2
b4
Int
100
75
50
25
0
b4 2+
b2
y1
y7
y9
y10
y13
0
100
200
300
400
500
600
700
800
900
1,000
1,100
1,200
1,300
1,400
1,500
m/z

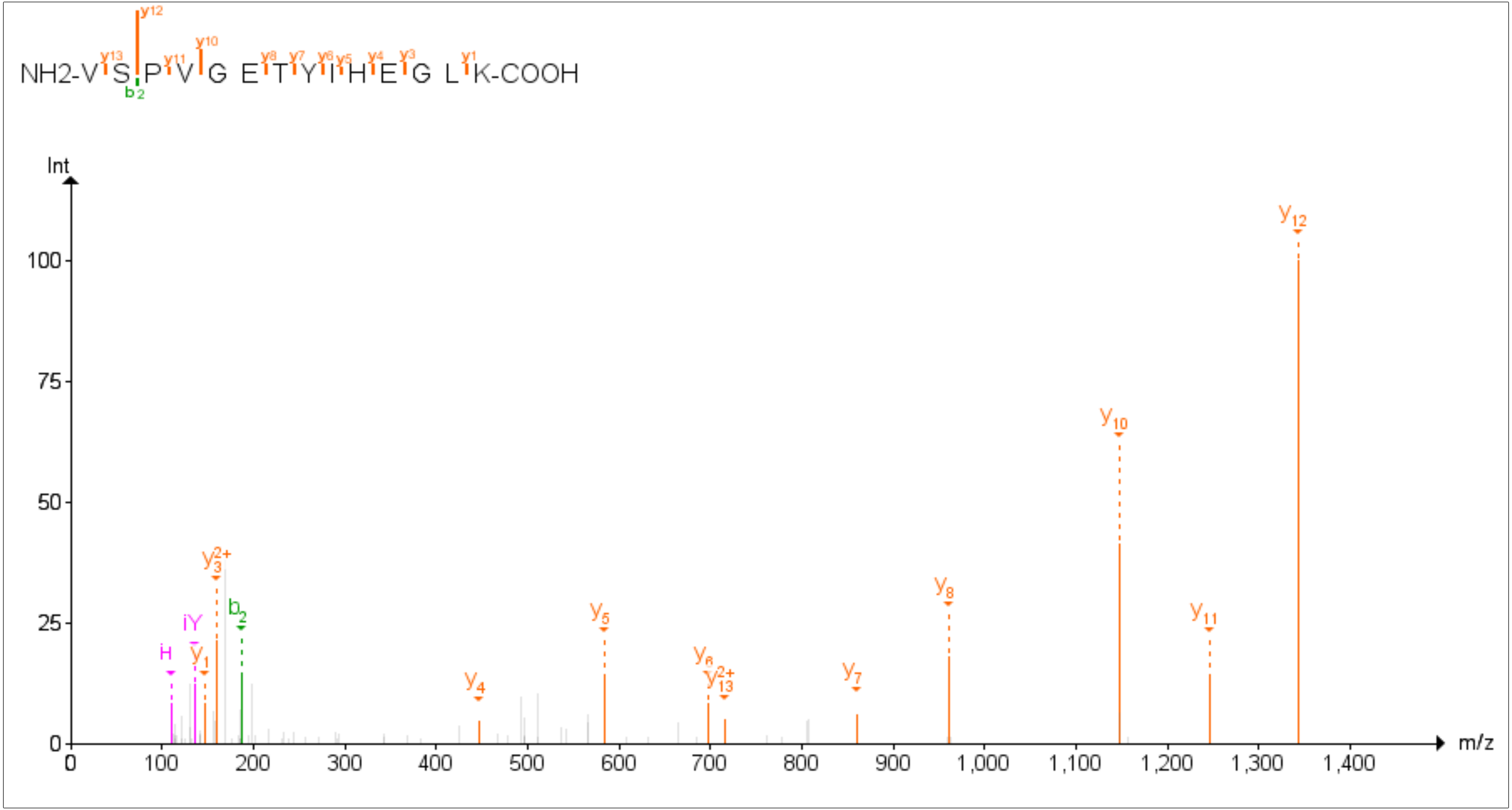

NH2-V S P V G E T Y I H E G L K-COOH
y13
y12
y11
y10
y8
y7
y6
y5
y4
y3
y1
b2
Int
100
75
50
25
0
m/z
0
100
200
300
400
500
600
700
800
900
1,000
1,100
1,200
1,300
1,400
y12
y10
y8
y11
y5
y6
y13 2+
y7
y4
y3 2+
b2
iY
iH
y1

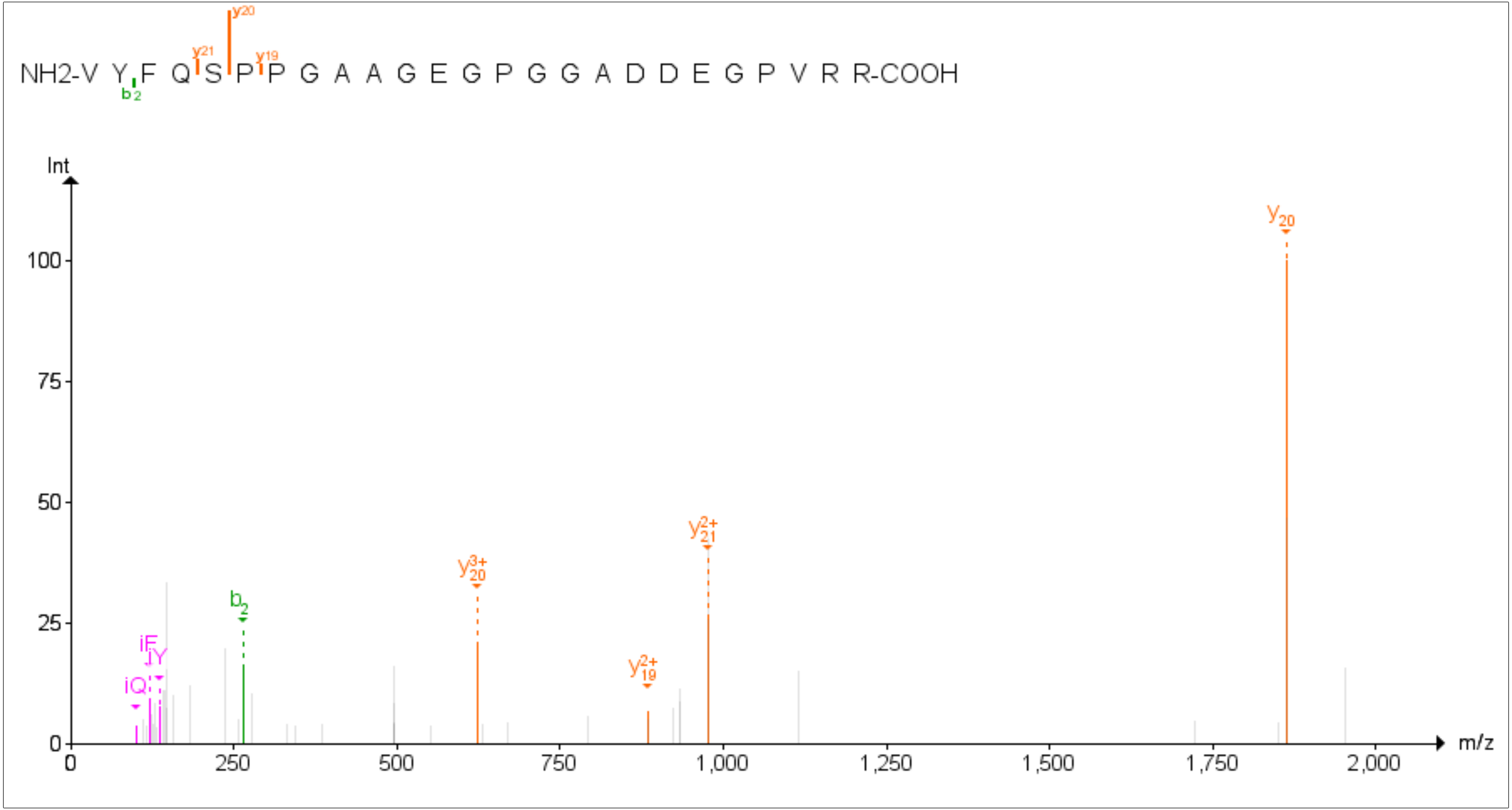

NH2-V Y F Q S P P G A A G E G P G G A D D E G P V R R-COOH
b2
y21
y20
y19
Int
100
75
50
25
0
iQ
iF
iY
b2
y20 3+
y19 2+
y21 2+
y20
0
250
500
750
1,000
1,250
1,500
1,750
2,000
m/z

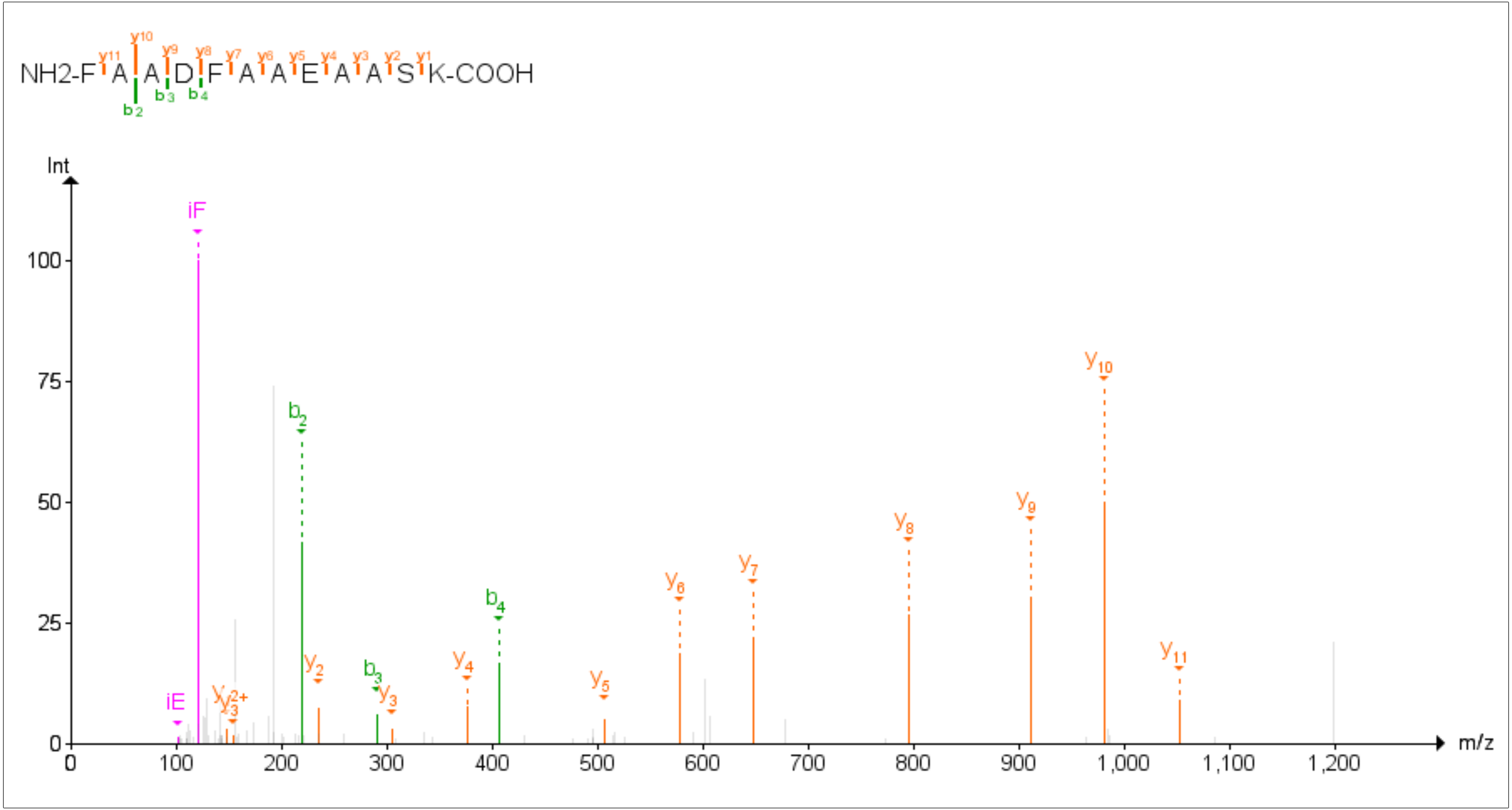

NH2-FAADFAAEAASK-COOH
y11 y10 y9 y8 y7 y6 y5 y4 y3 y2 y1
b2 b3 b4
Int
m/z
0
25
50
75
100
0 100 200 300 400 500 600 700 800 900 1,000 1,100 1,200
iF
iE
b2
b3
b4
y2
y3
y3 2+
y4
y5
y6
y7
y8
y9
y10
y11

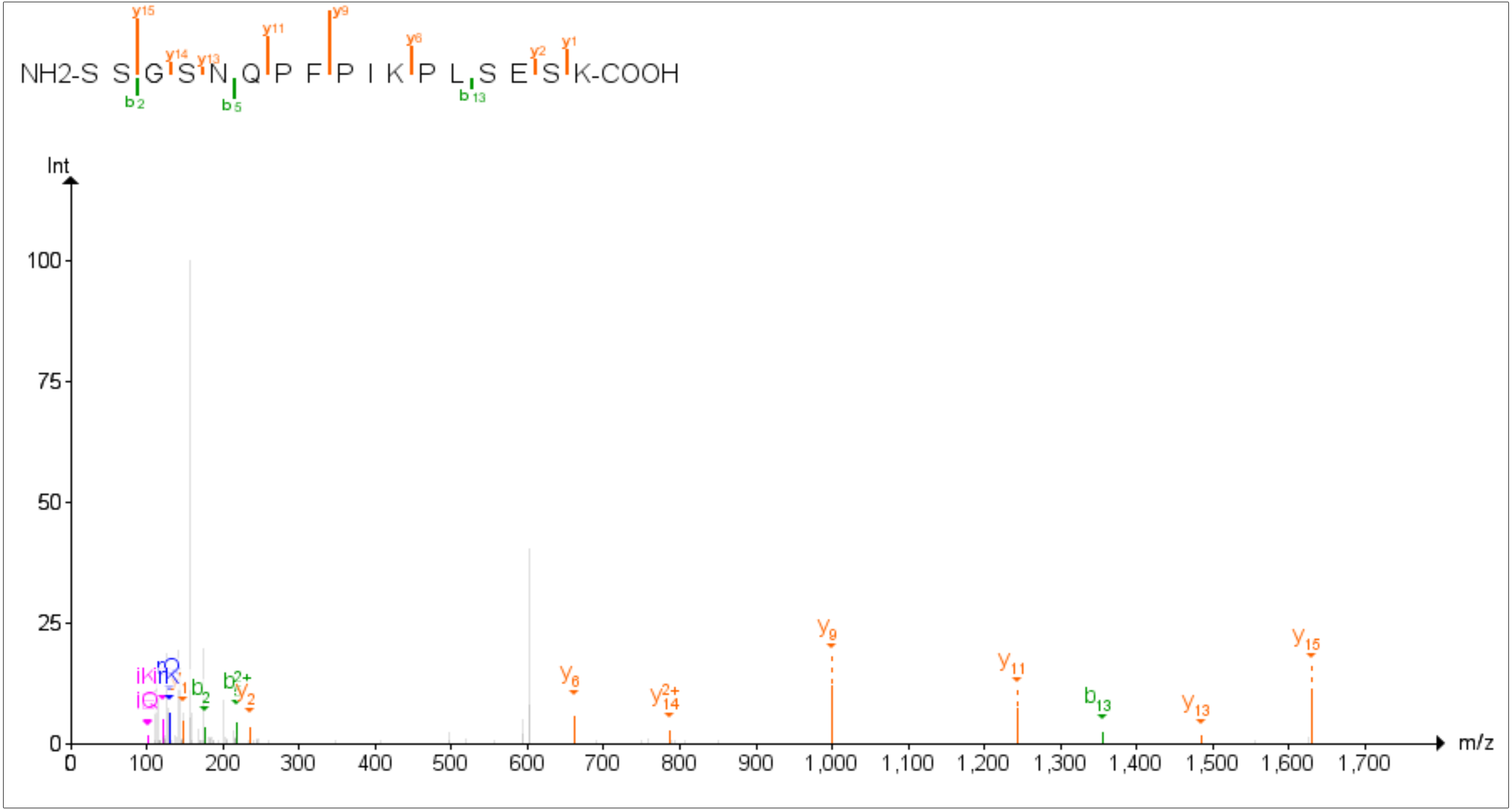

NH2-S S G S N Q P F P I K P L S E S K-COOH
y15
y14
y13
y11
y9
y6
y2
y1
b2
b5
b13
Int
100
75
50
25
0
m/z
0
100
200
300
400
500
600
700
800
900
1,000
1,100
1,200
1,300
1,400
1,500
1,600
1,700
y9
y11
y15
y6
y14 2+
b13
y13
b2
b2+
y2
iK
iQ

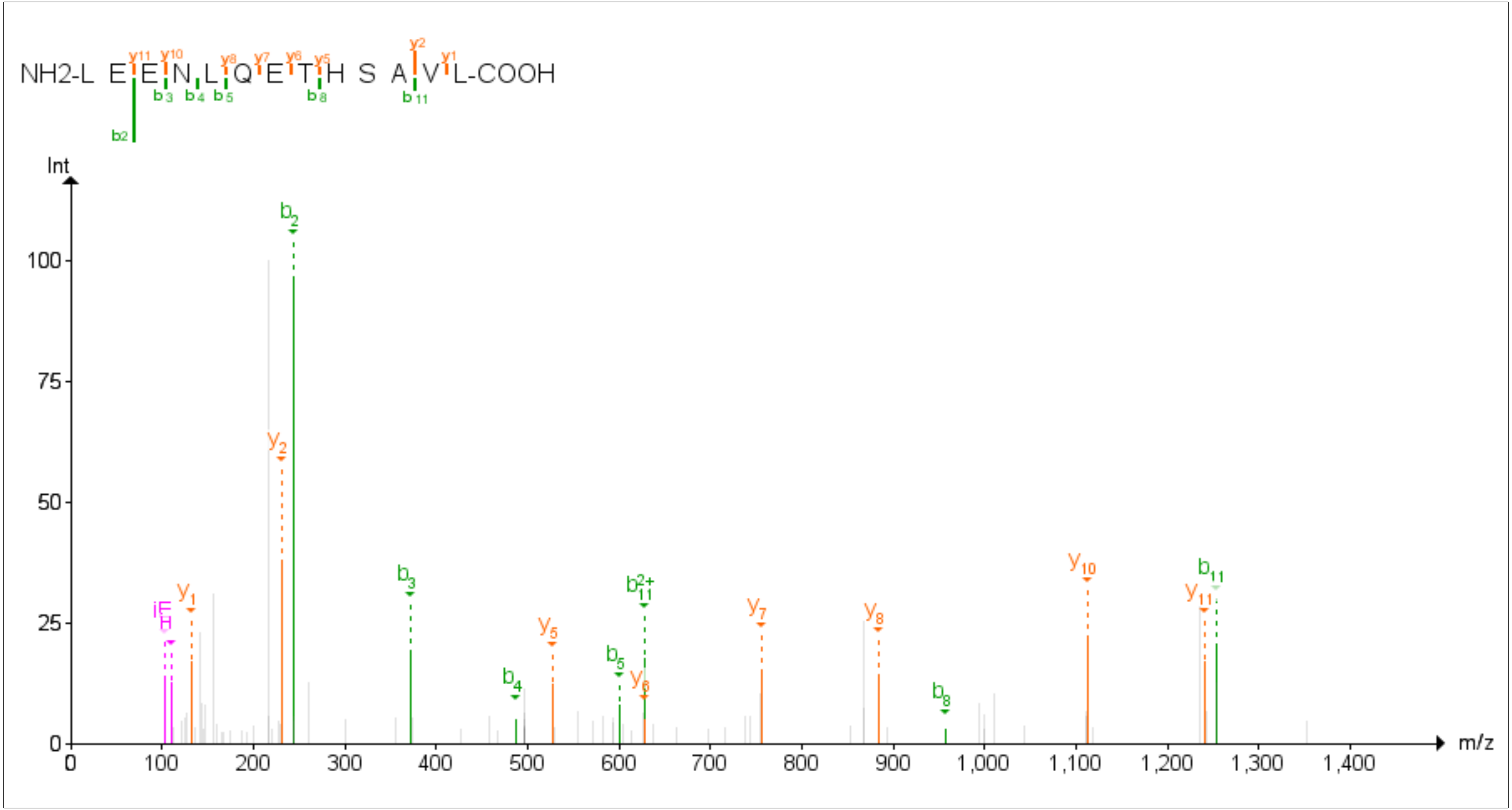
NH2-L E E N L Q E T H S A V L-COOH
y11 y10 y8 y7 y6 y5 y2 y1
b2 b3 b4 b5 b8 b11
Int
100
75
50
25
0
b2
y2
b3
b4
y5
b5
b11 2+
y6
y7
y8
b8
y10
y11
b11
y1
iF H
0 100 200 300 400 500 600 700 800 900 1,000 1,100 1,200 1,300 1,400
m/z

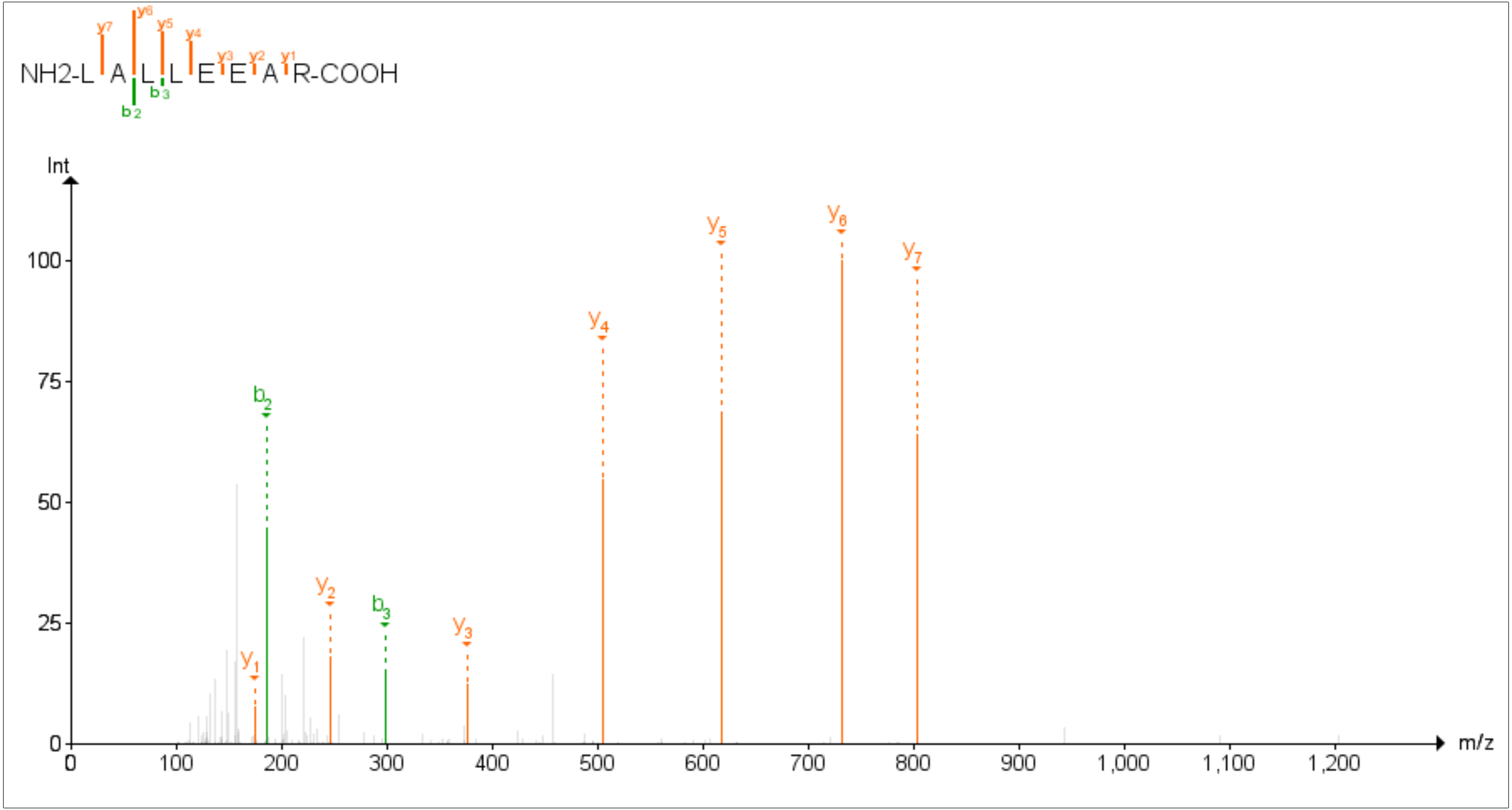

NH2-L A L L E E A R-COOH
y7 y6 y5 y4 y3 y2 y1
b2 b3
Int
100
75
50
25
0
m/z
0 100 200 300 400 500 600 700 800 900 1,000 1,100 1,200
b_2 b_3 y_1 y_2 y_3 y_4 y_5 y_6 y_7

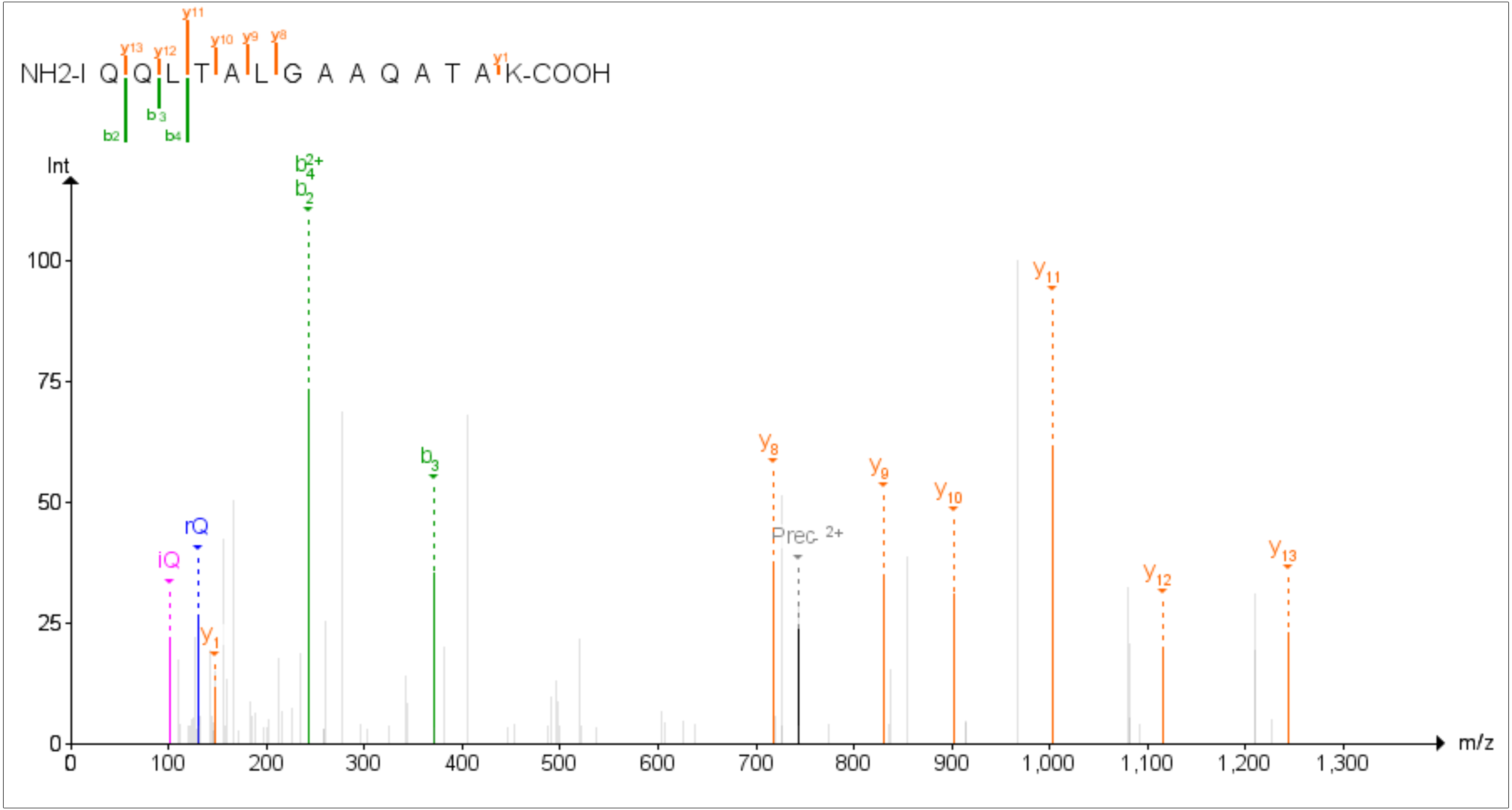
NH2-I Q Q L T A L G A A Q A T A K-COOH
y13 y12 y11 y10 y9 y8 y1
b2 b3 b4
Int
100
75
50
25
0
b$_4^{2+}$
b$_2$
b$_3$
iQ
rQ
y$_1$
y$_8$
Prec. 2+
y$_9$
y$_{10}$
y$_{11}$
y$_{12}$
y$_{13}$
0 100 200 300 400 500 600 700 800 900 1,000 1,100 1,200 1,300
m/z

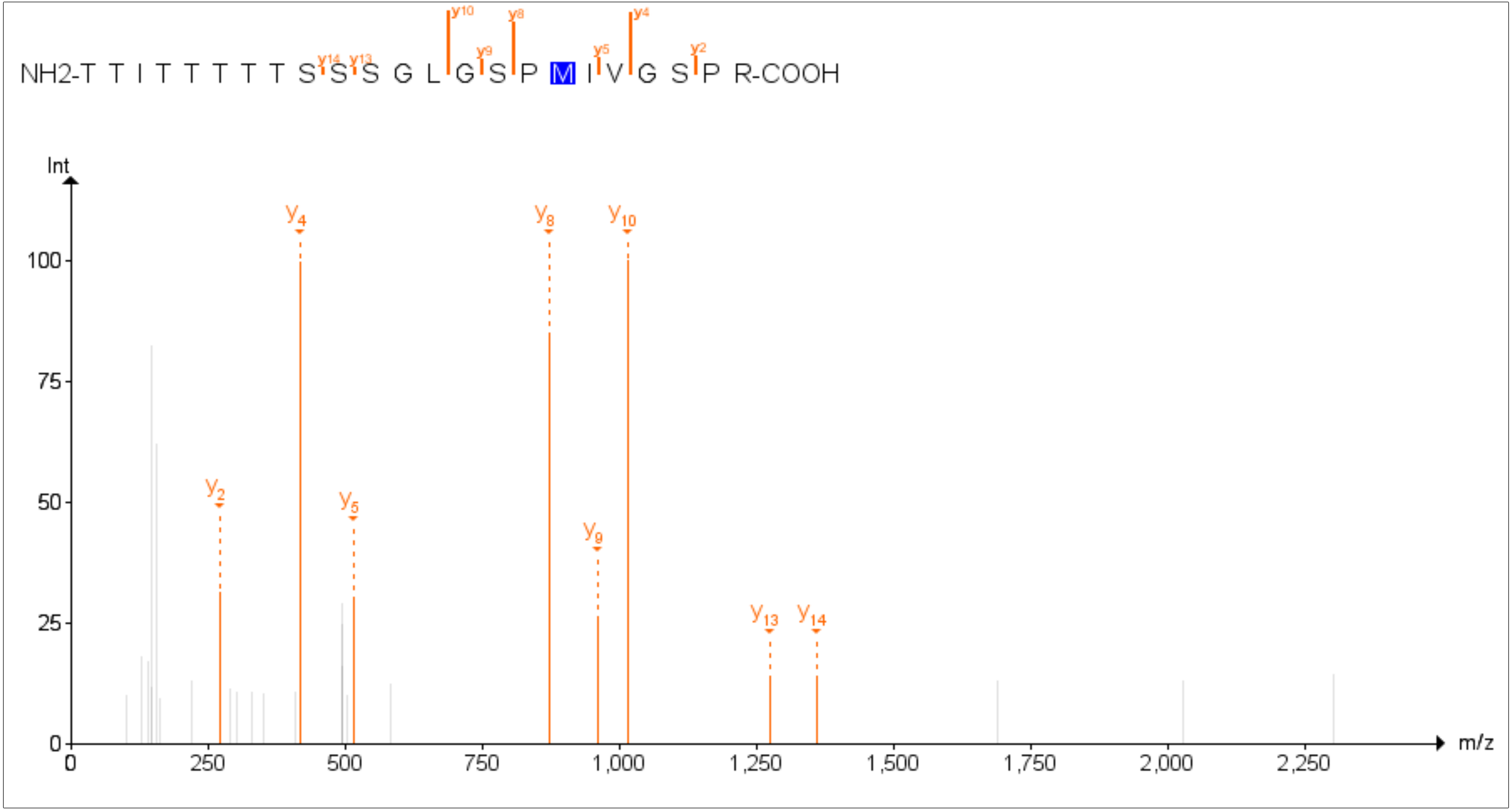
NH2-T T I T T T T T S S S G L G S P M I V G S P R-COOH
y14
y13
y10
y9
y8
y5
y4
y2
Int
m/z
y2
y4
y5
y8
y9
y10
y13
y14
0
25
50
75
100
250
500
750
1,000
1,250
1,500
1,750
2,000
2,250

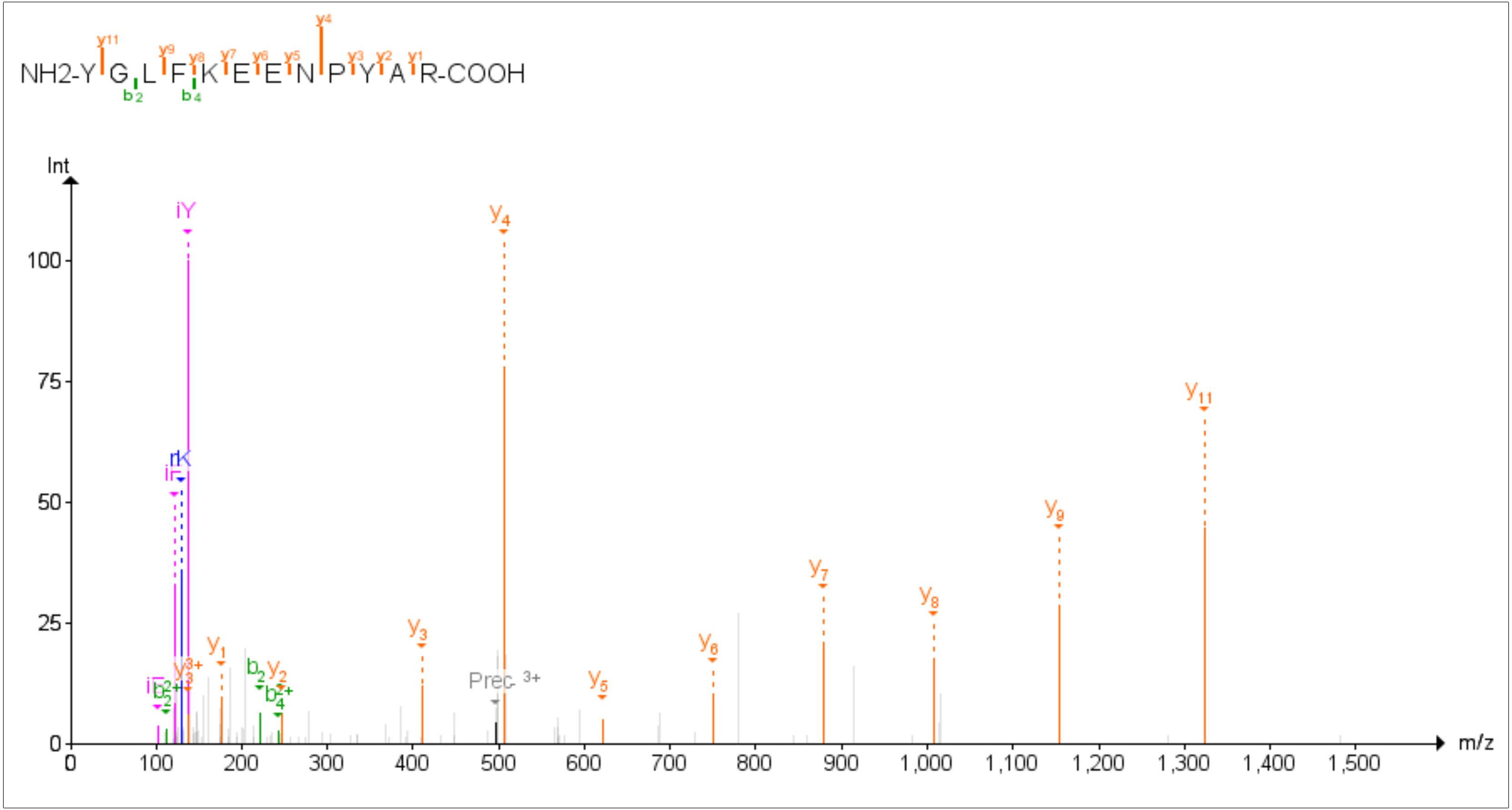
NH2-Y G L F K E E N P Y A R-COOH
y11 y9 y8 y7 y6 y5 y4 y3 y2 y1
b2 b4
Int
iY
y4
y11
y9
y7
y8
y3
y6
y5
y1
y2
b2
rK
iF
Prec 3+
0
25
50
75
100
0 100 200 300 400 500 600 700 800 900 1,000 1,100 1,200 1,300 1,400 1,500
m/z

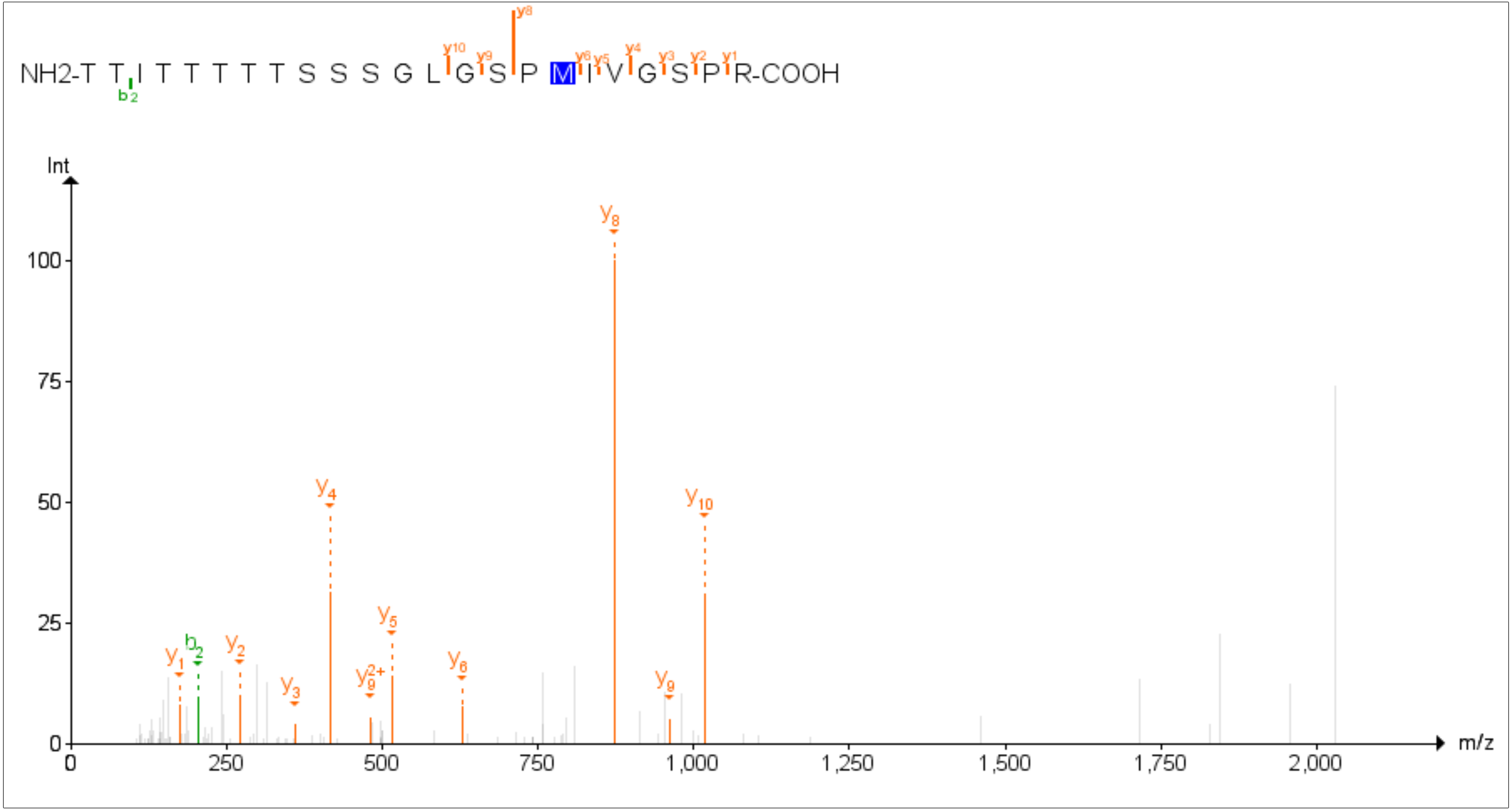

NH2-T T I T T T T T S S S G L G S P M I V G S P R-COOH
b2
y10 y9 y8 y6 y5 y4 y3 y2 y1
Int
100
75
50
25
0
y1
b2
y2
y3
y4
y9 2+
y5
y6
y8
y9
y10
0
250
500
750
1,000
1,250
1,500
1,750
2,000
m/z

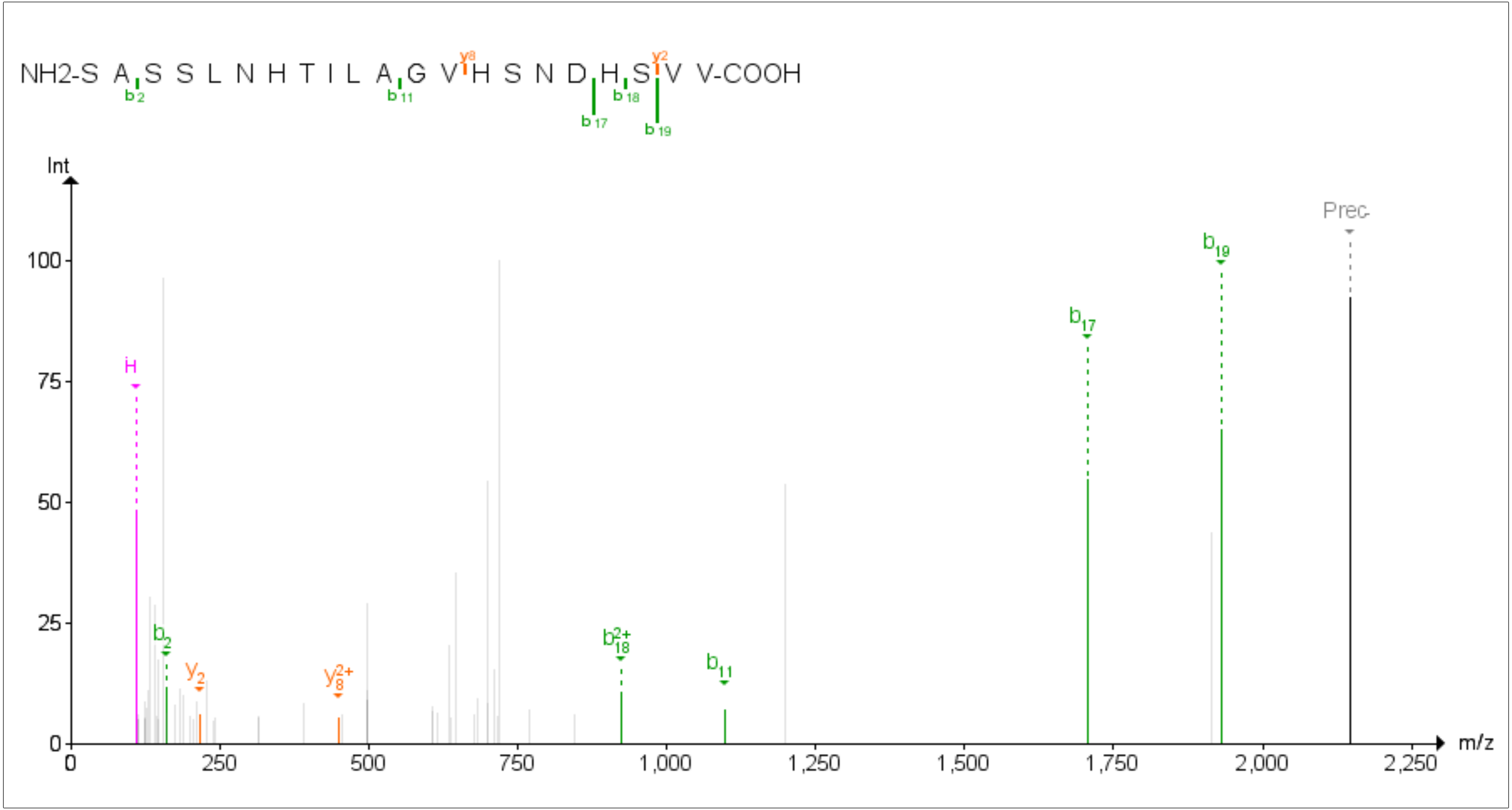

NH2-S A S S L N H T I L A G V H S N D H S V V-COOH
b2
b11
y8
b17
b18
y2
b19
Int
m/z
Prec
b19
b17
H
b2
y2
y2+8
b2+18
b11
0
25
50
75
100
0
250
500
750
1,000
1,250
1,500
1,750
2,000
2,250

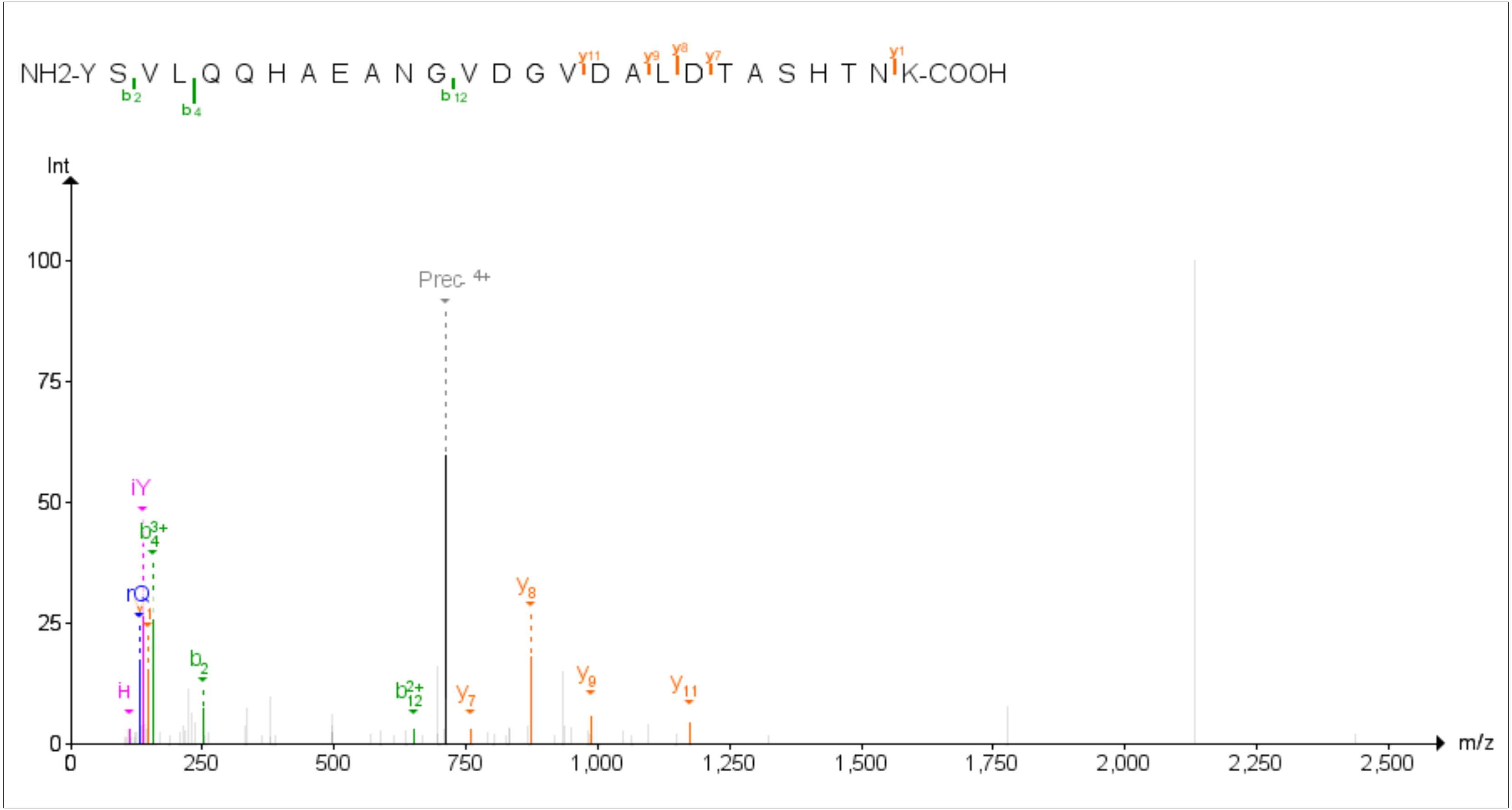

NH2-Y S V L Q Q H A E A N G V D G V D A L D T A S H T N K-COOH
b2
b4
b12
y11
y9
y8
y7
y1
Int
100
75
50
25
0
Prec- 4+
iY
b4 3+
rQ
y1
b2
iH
b12 2+
y7
y8
y9
y11
0
250
500
750
1,000
1,250
1,500
1,750
2,000
2,250
2,500
m/z

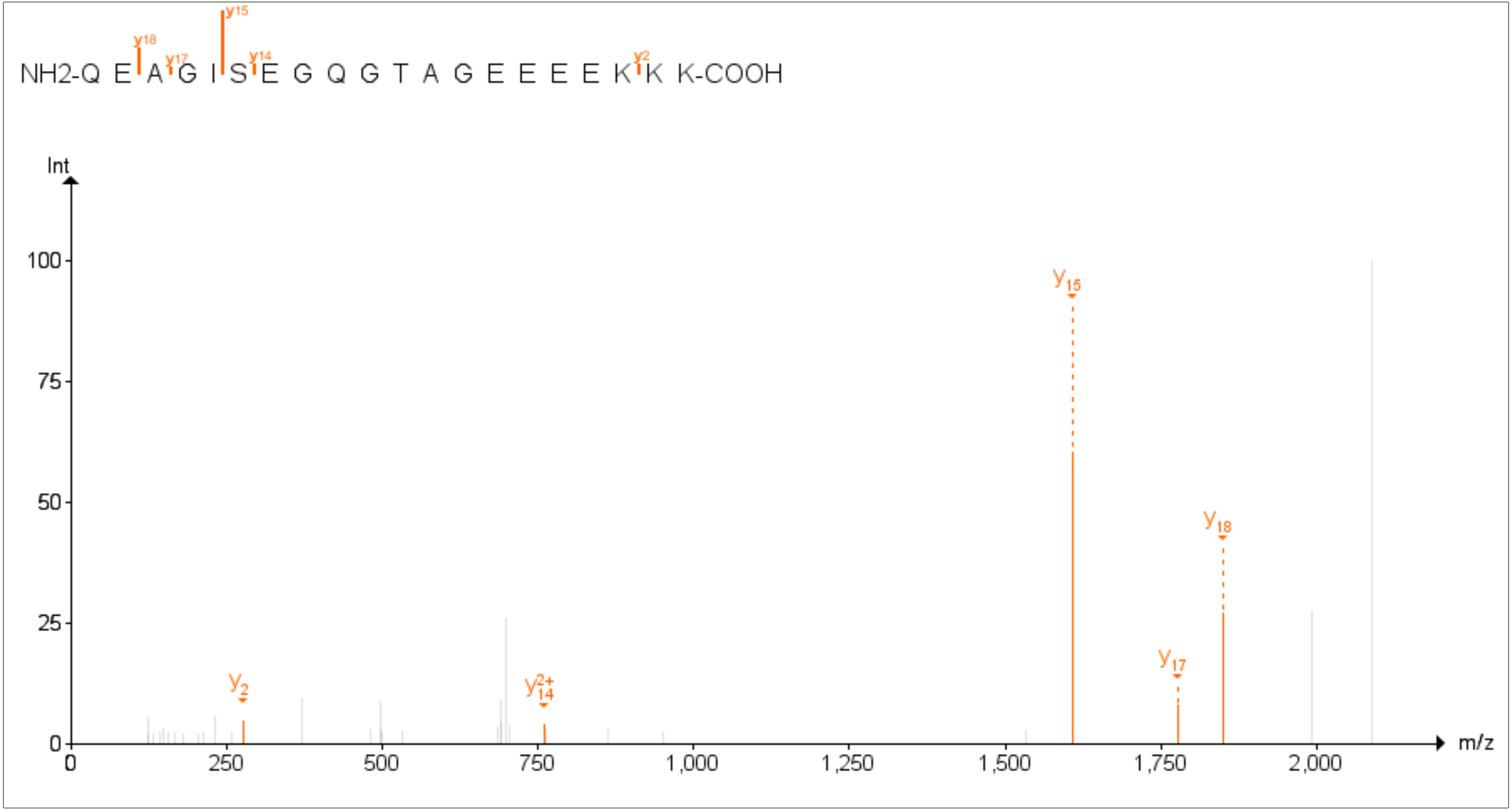
NH2-Q E A G I S E G Q G T A G E E E E K K K-COOH
y18
y17
y15
y14
y2
Int
100
75
50
25
0
y15
y18
y17
y2
y14 2+
0
250
500
750
1,000
1,250
1,500
1,750
2,000
m/z

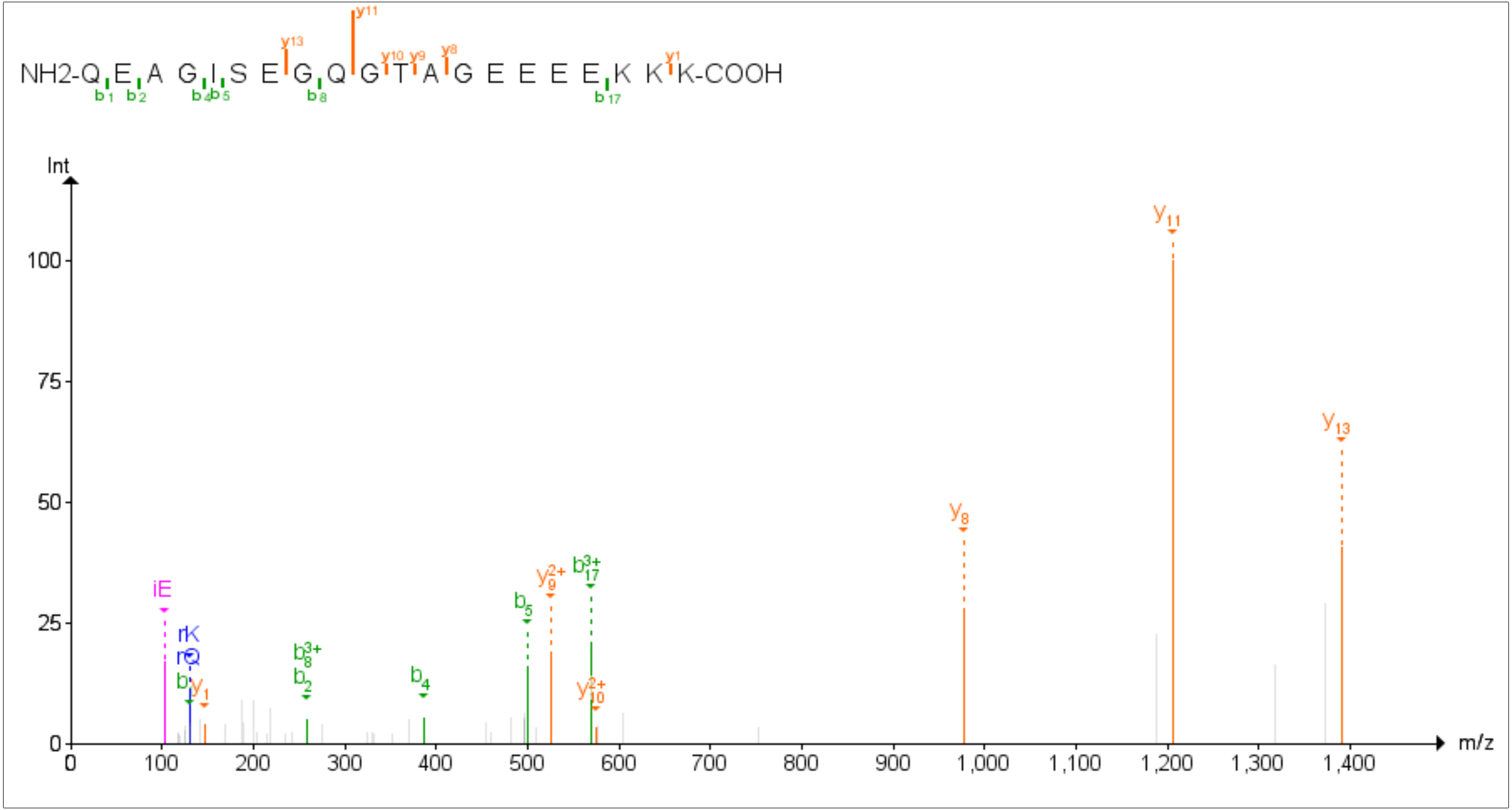

NH2-Q E A G I S E G Q G T A G E E E E K K K-COOH
b1 b2 b4 b5 b8 b17
y13 y11 y10 y9 y8 y1
Int
m/z
iE
rK
rQ
y11
y13
y8
b5
y9 2+
b17 3+
b8 3+
b2
b4
y1
y10 2+

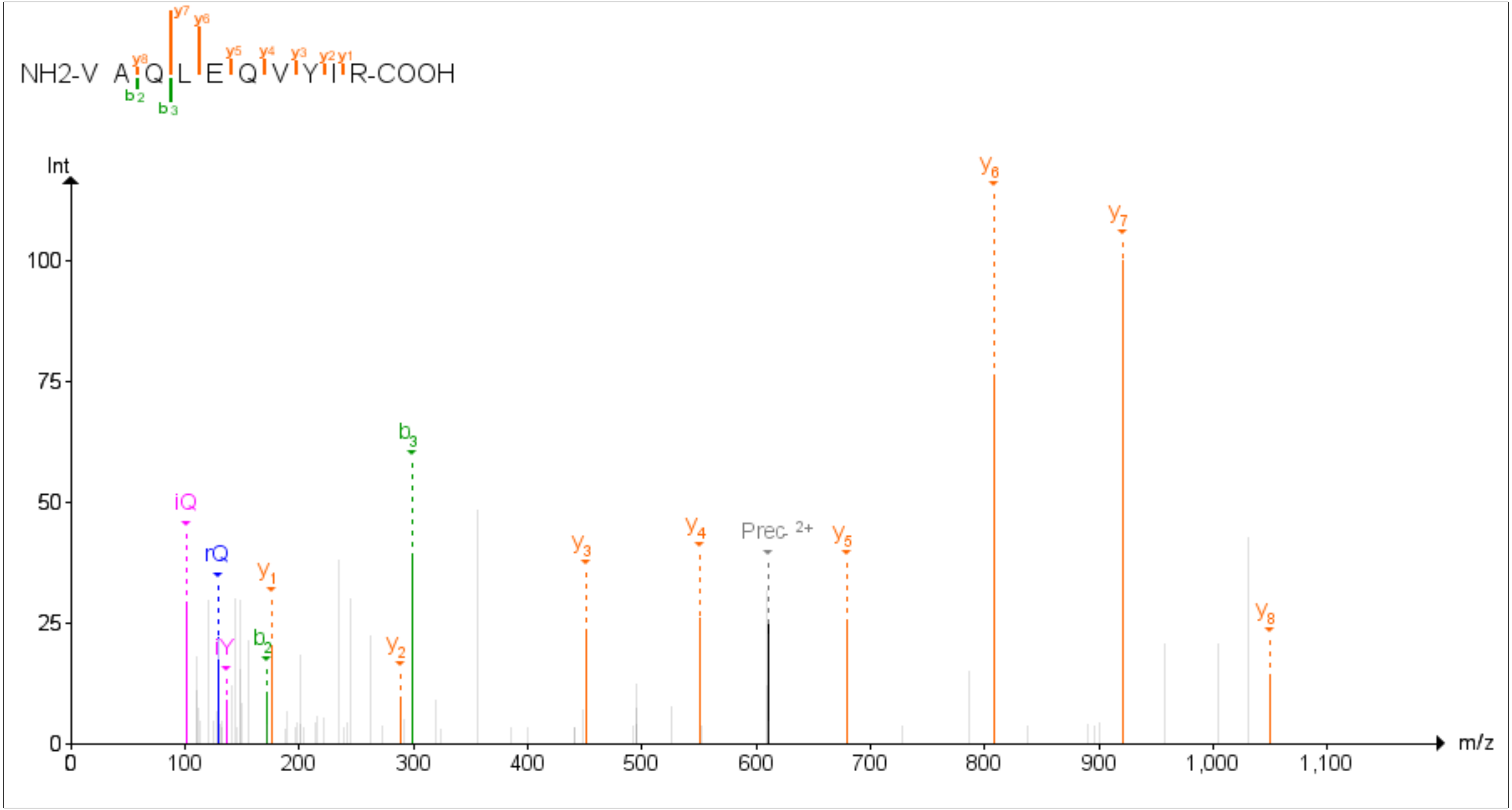

NH2-V A Q L E Q V Y I R-COOH
y7 y6 y8 y5 y4 y3 y2 y1
b2 b3
Int
100
75
50
25
0
iQ
rQ
iY
y1
b2
y2
b3
y3
y4
Prec 2+
y5
y6
y7
y8
0 100 200 300 400 500 600 700 800 900 1,000 1,100
m/z

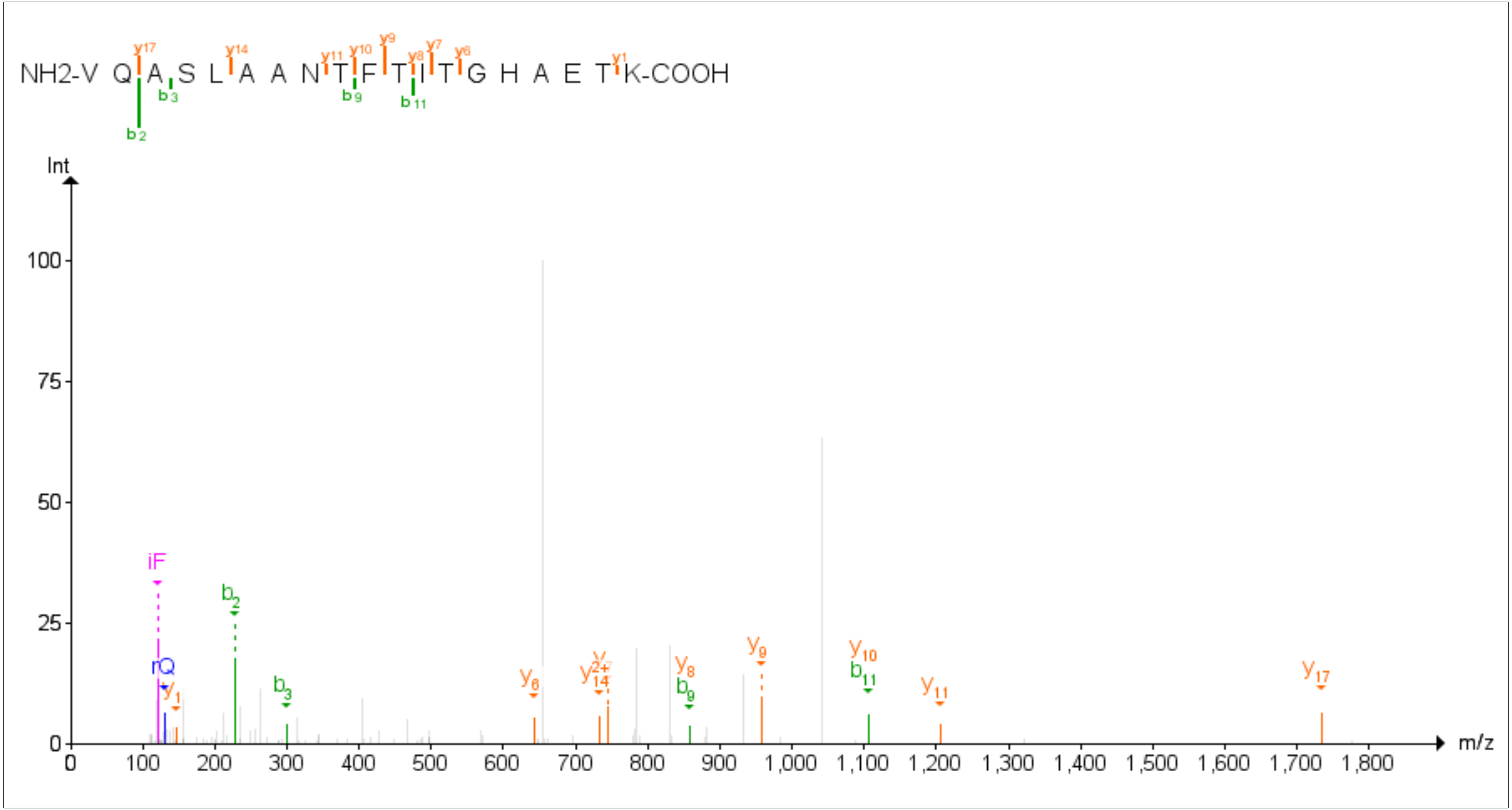

NH2-V Q A S L A A N T F T I T G H A E T K-COOH
y17 y14 y11 y10 y9 y8 y7 y6 y1
b2 b3 b9 b11
Int
100
75
50
25
0
iF
rQ
y1
b2
b3
y6
y14 2+
y8
b9
y9
y10
b11
y11
y17
0 100 200 300 400 500 600 700 800 900 1,000 1,100 1,200 1,300 1,400 1,500 1,600 1,700 1,800
m/z

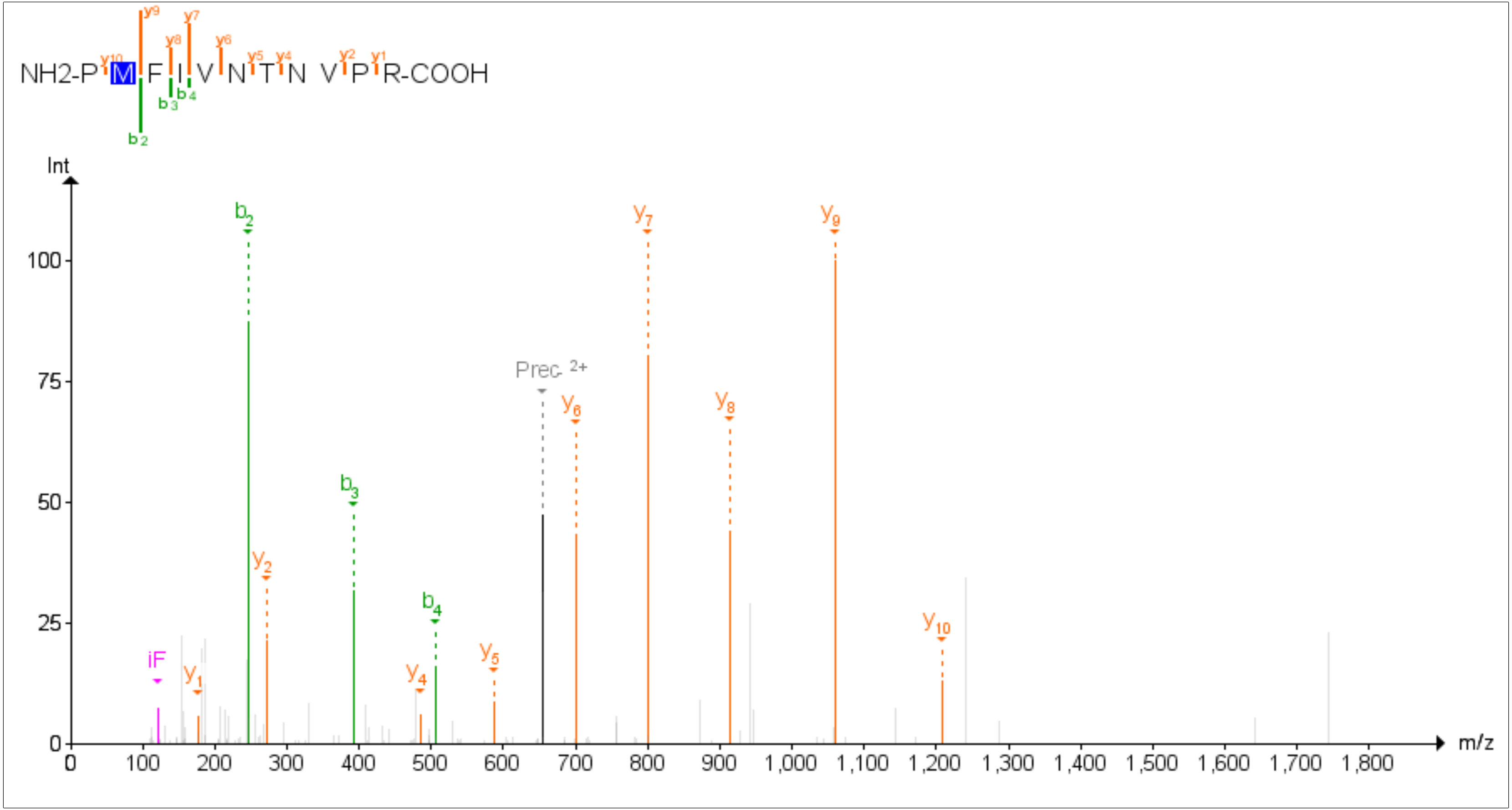

NH2-P M F I V N T N V P R-COOH
y10 y9 y8 y7 y6 y5 y4 y2 y1
b2 b3 b4
Int
m/z
b2
y7
y9
Prec 2+
y6
y8
b3
y2
b4
y10
y5
iF
y1
y4
0
25
50
75
100
0 100 200 300 400 500 600 700 800 900 1,000 1,100 1,200 1,300 1,400 1,500 1,600 1,700 1,800

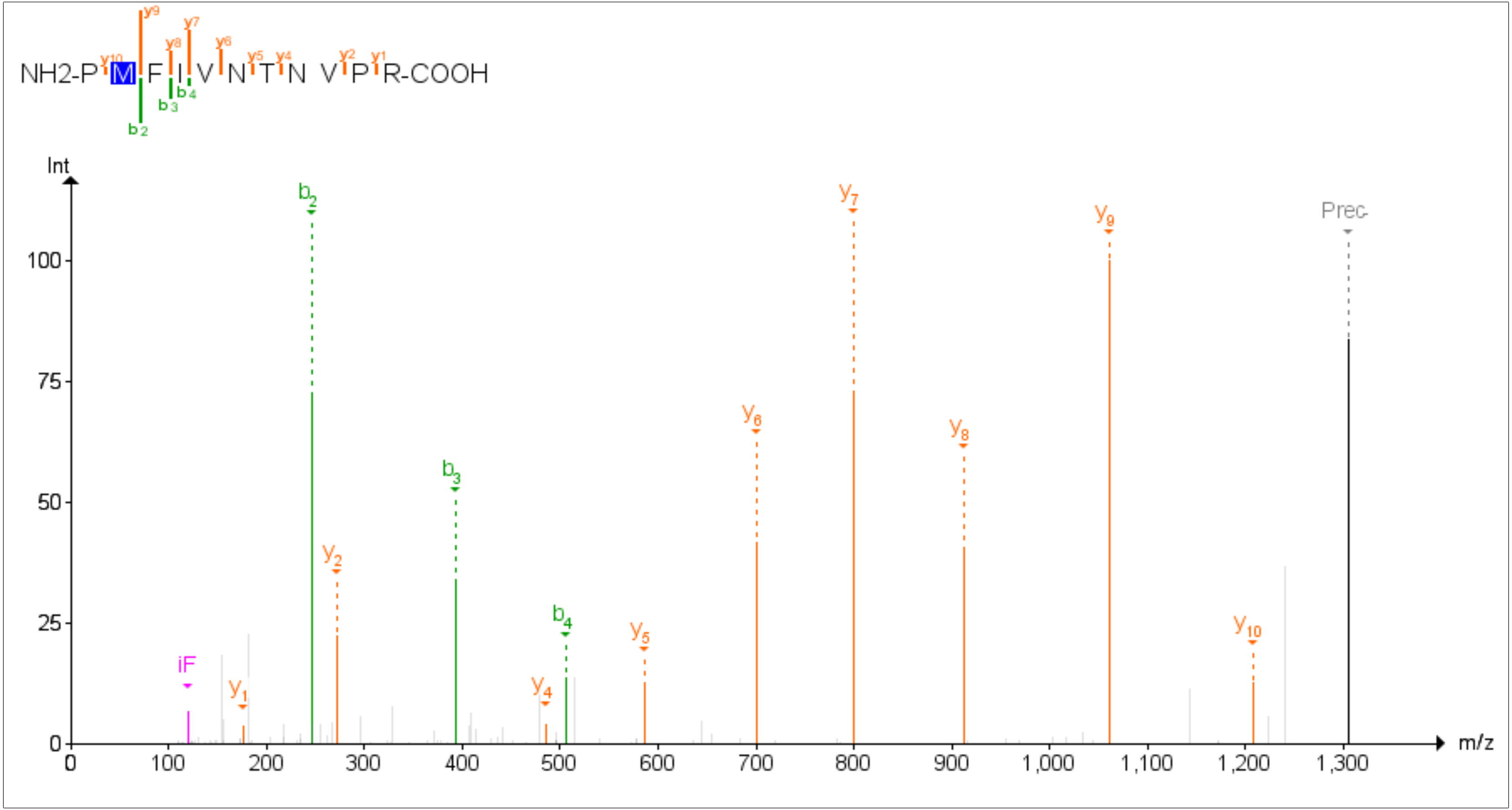
NH2-P M F I V N T N V P R-COOH
y10 y9 y8 y7 y6 y5 y4 y2 y1
b2 b3 b4
Int
m/z
Prec.
b2 b3 b4
y1 y2 y4 y5 y6 y7 y8 y9 y10
iF
0
25
50
75
100
0
100
200
300
400
500
600
700
800
900
1,000
1,100
1,200
1,300

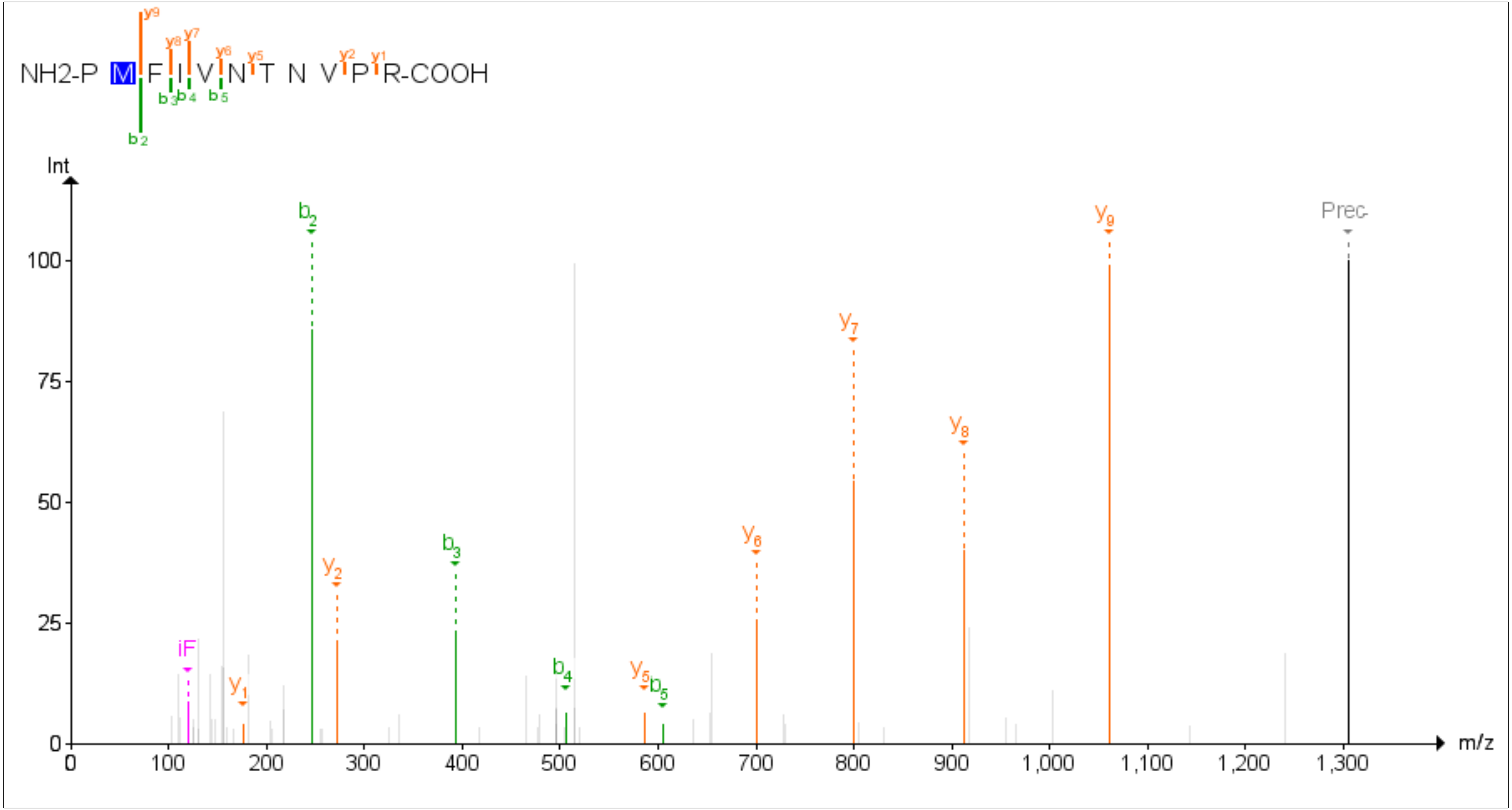

NH2-P M F I V N T N V P R-COOH
y9 y8 y7 y6 y5 y2 y1
b2 b3 b4 b5
Int
100
75
50
25
0
b2
y9
Prec
y7
y8
y6
b3
y2
iF
y1
b4
y5
b5
0 100 200 300 400 500 600 700 800 900 1,000 1,100 1,200 1,300
m/z

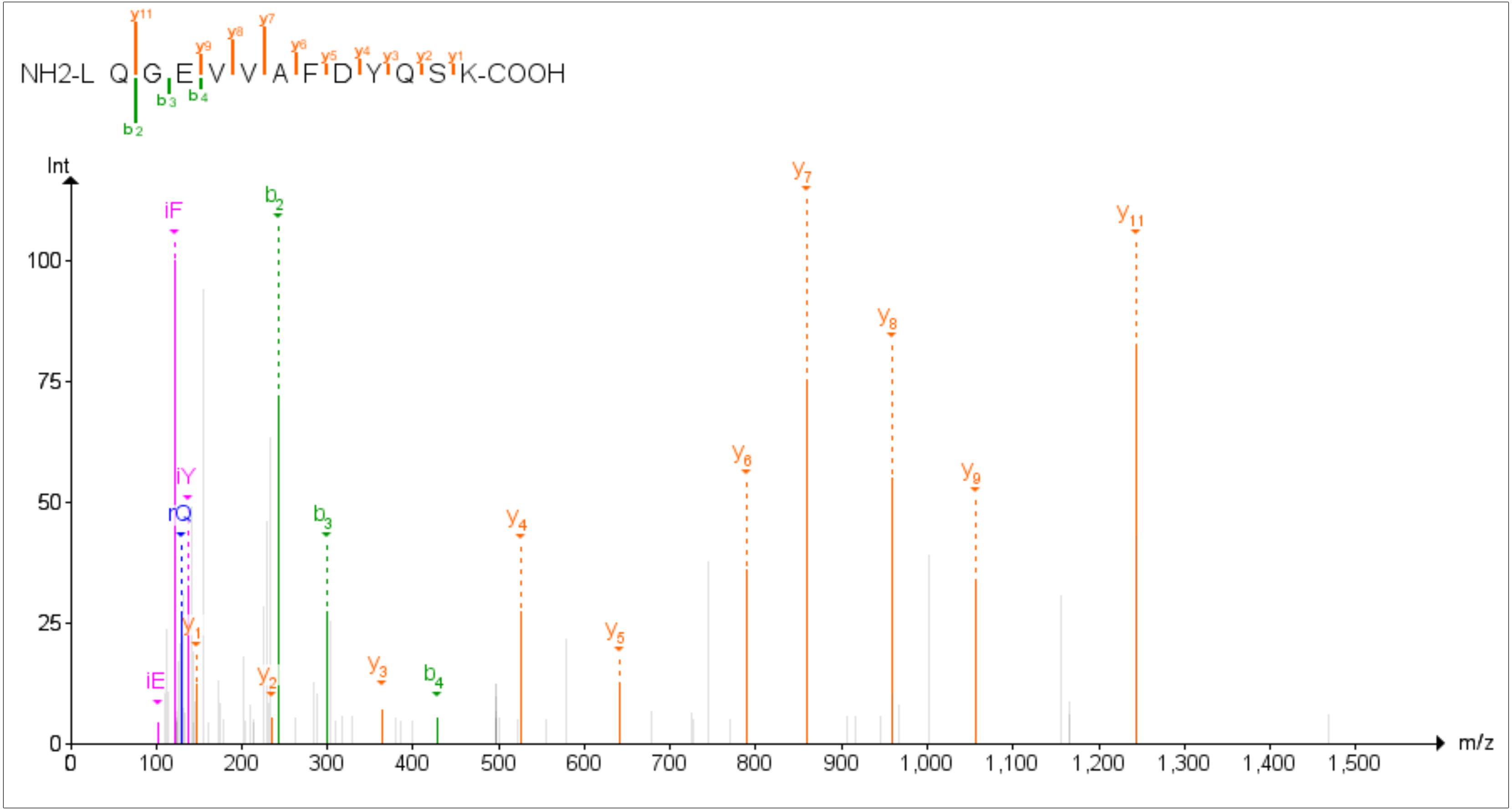

NH2-L Q G E V V A F D Y Q S K-COOH
y11 y9 y8 y7 y6 y5 y4 y3 y2 y1
b2 b3 b4
Int
m/z
iF
b2
y7
y11
y8
y6
y9
iY
rQ
b3
y4
y1
y5
iE
y2
y3
b4
0
25
50
75
100
0
100
200
300
400
500
600
700
800
900
1,000
1,100
1,200
1,300
1,400
1,500

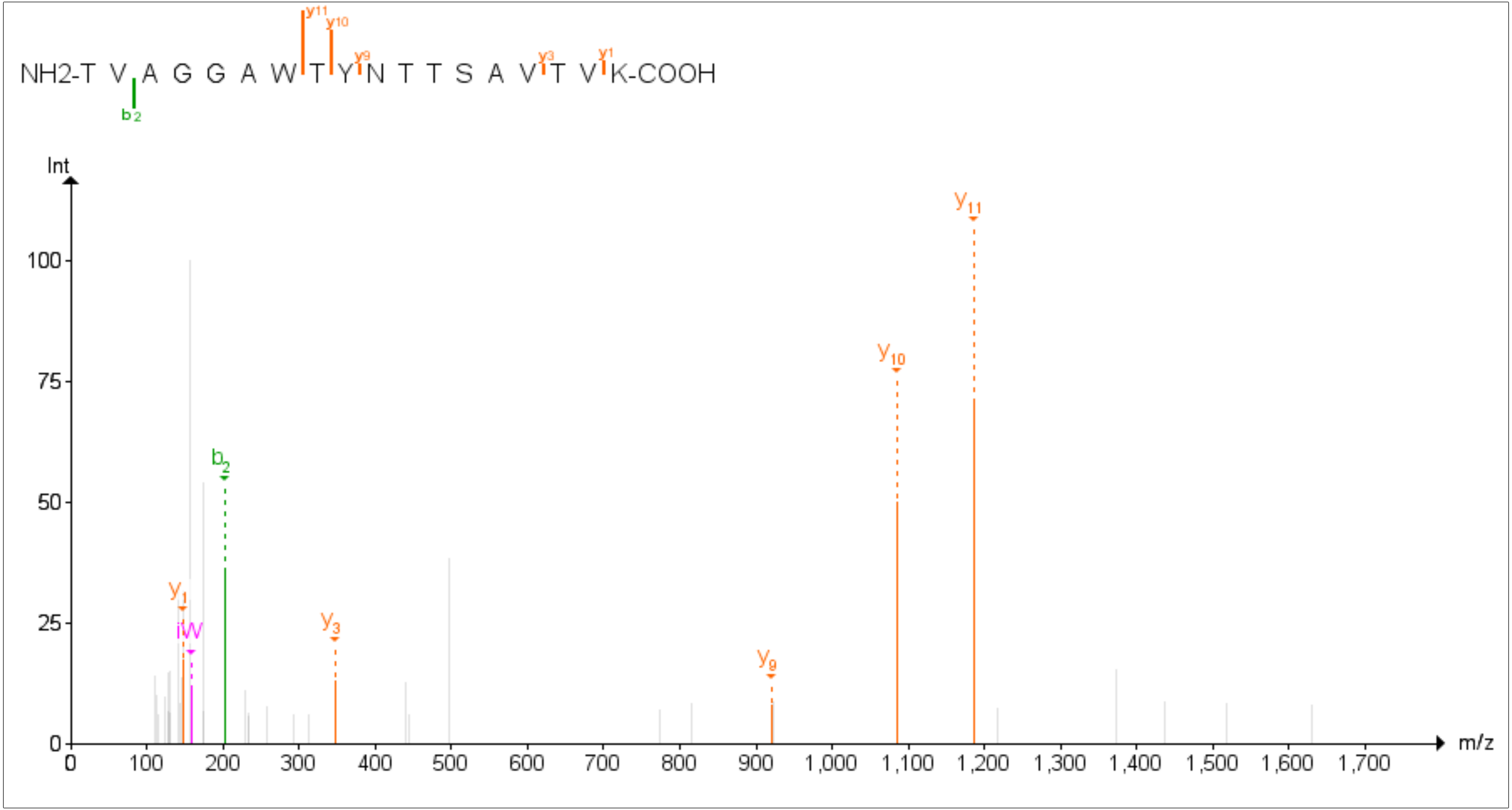

NH2-T V A G G A W T Y N T T S A V T V K-COOH
b2
y11
y10
y9
y3
y1
Int
100
75
50
25
0
m/z
0
100
200
300
400
500
600
700
800
900
1,000
1,100
1,200
1,300
1,400
1,500
1,600
1,700
iW

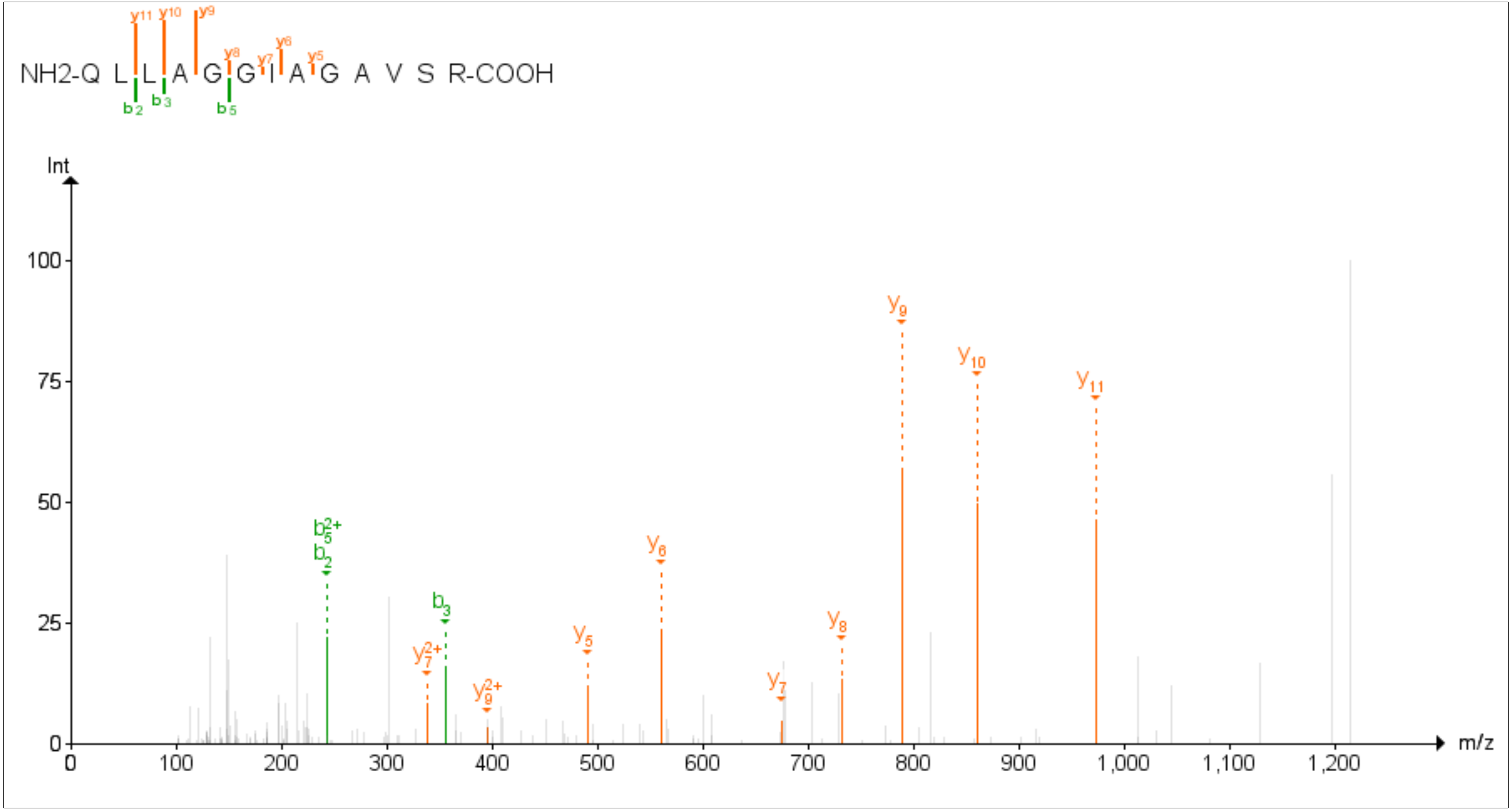

NH2-Q L L A G G I A G A V S R-COOH
y11 y10 y9 y8 y7 y6 y5
b2 b3 b5
Int
100
75
50
25
0
m/z
0 100 200 300 400 500 600 700 800 900 1,000 1,100 1,200
b5 2+
b2
b3
y7 2+
y9 2+
y5
y6
y7
y8
y9
y10
y11

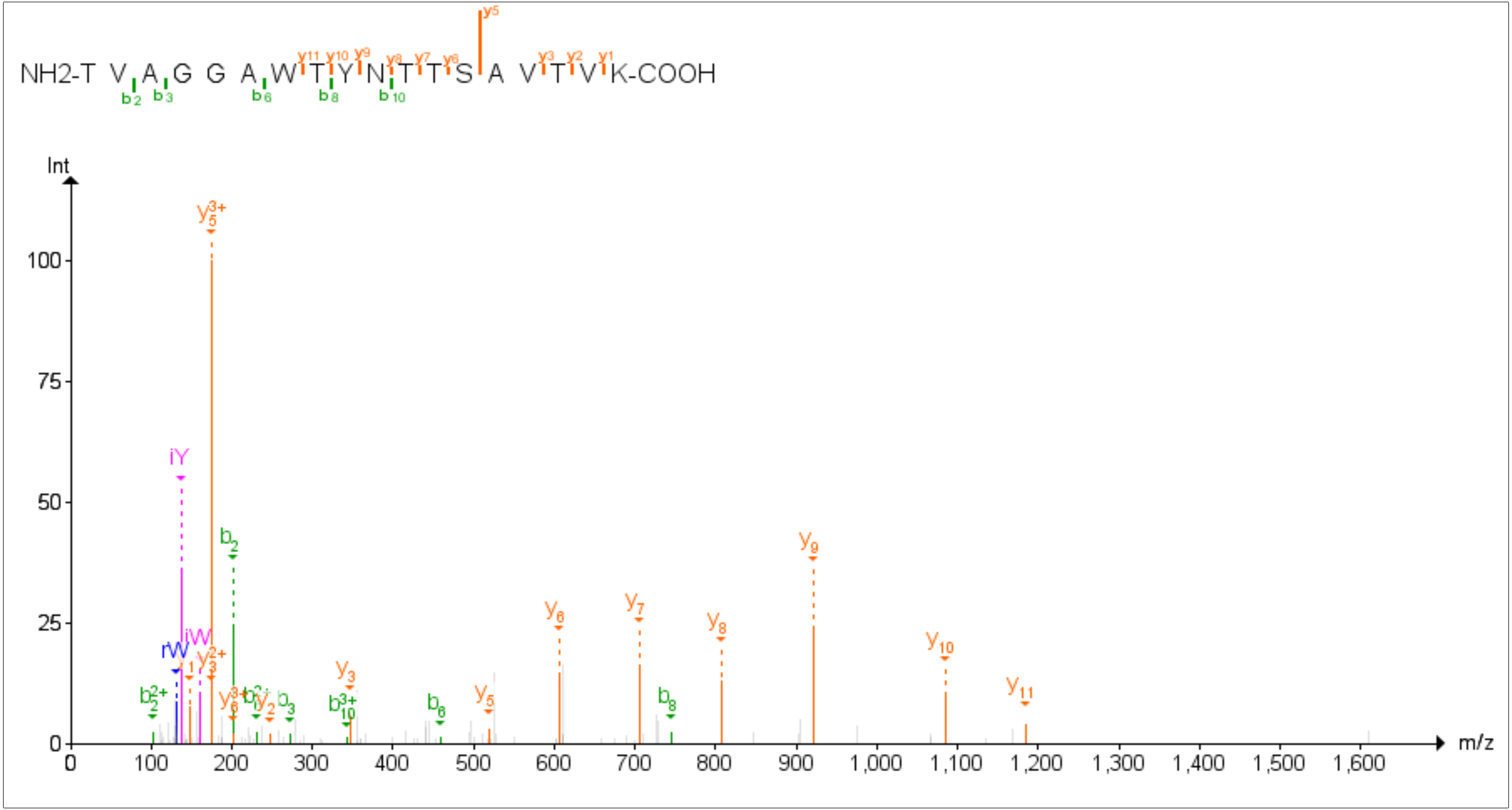
NH2-T V A G G A W T Y N T T S A V T V K-COOH
b2 b3 b6 b8 b10
y11 y10 y9 y8 y7 y6 y5 y3 y2 y1
Int
m/z
0
25
50
75
100
0
100
200
300
400
500
600
700
800
900
1,000
1,100
1,200
1,300
1,400
1,500
1,600
y5 3+
iY
b2
y9
y6
y7
y8
y10
y11
rW
iW
y3 2+
y3
b2 2+
b3
b10 3+
b6
y5
b8

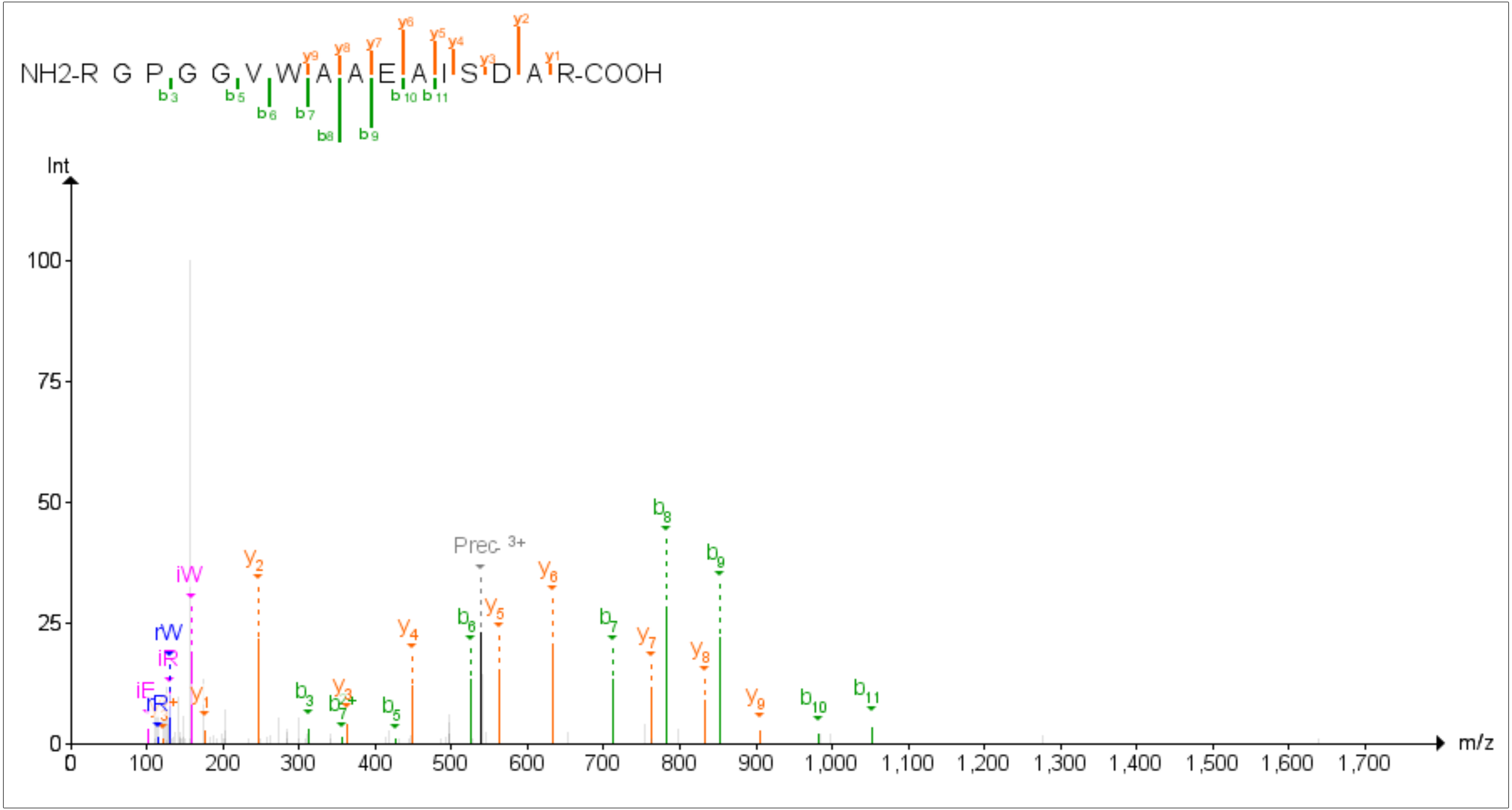

NH2-R G P G G V W A A E A I S D A R-COOH
Int
m/z
Prec 3+
iW
rW
iR
iF

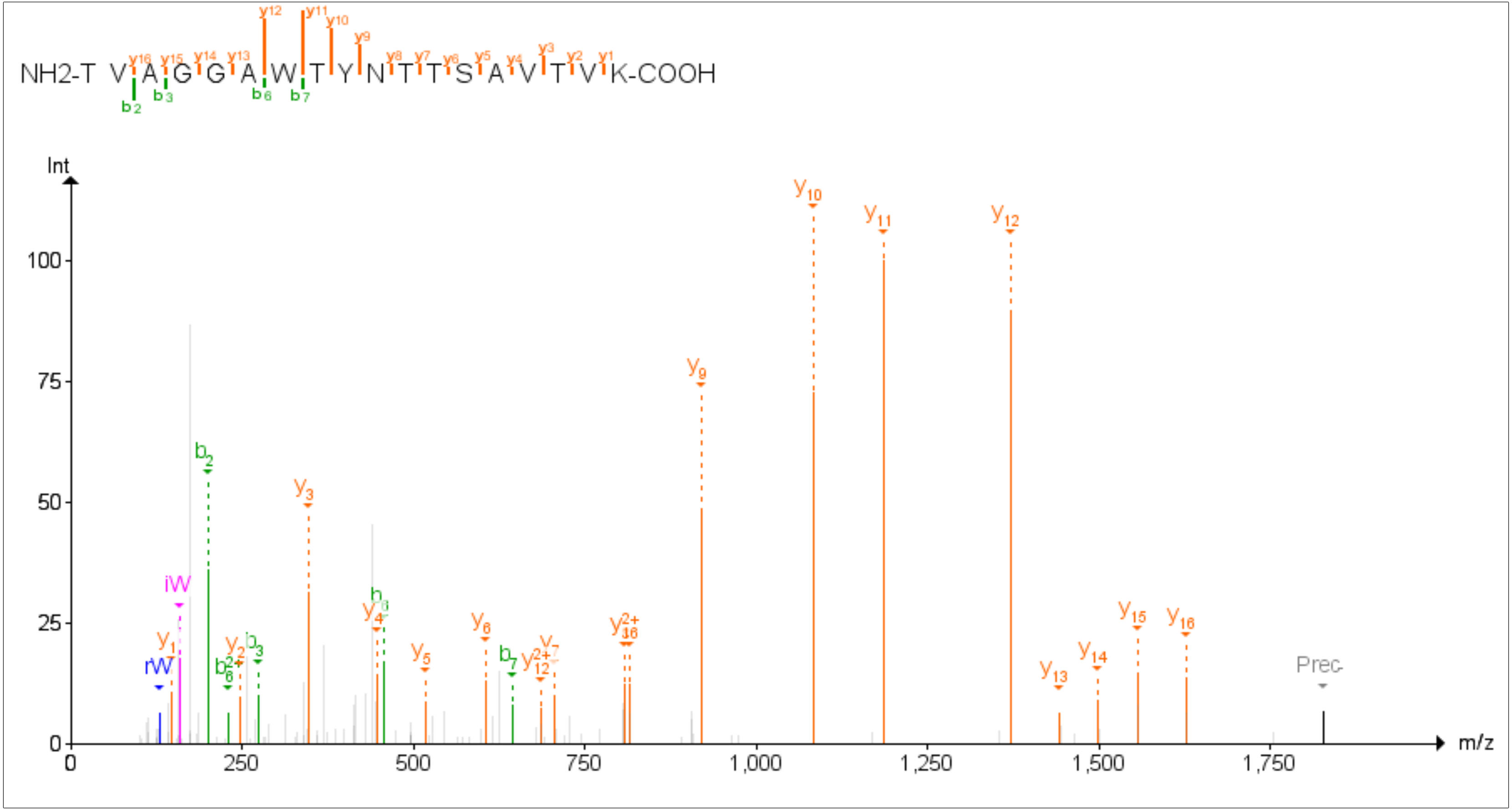
NH2-T V A G G A W T Y N T T S A V T V K-COOH
Int
m/z
Prec
iW
rW
0
25
50
75
100
250
500
750
1,000
1,250
1,500
1,750

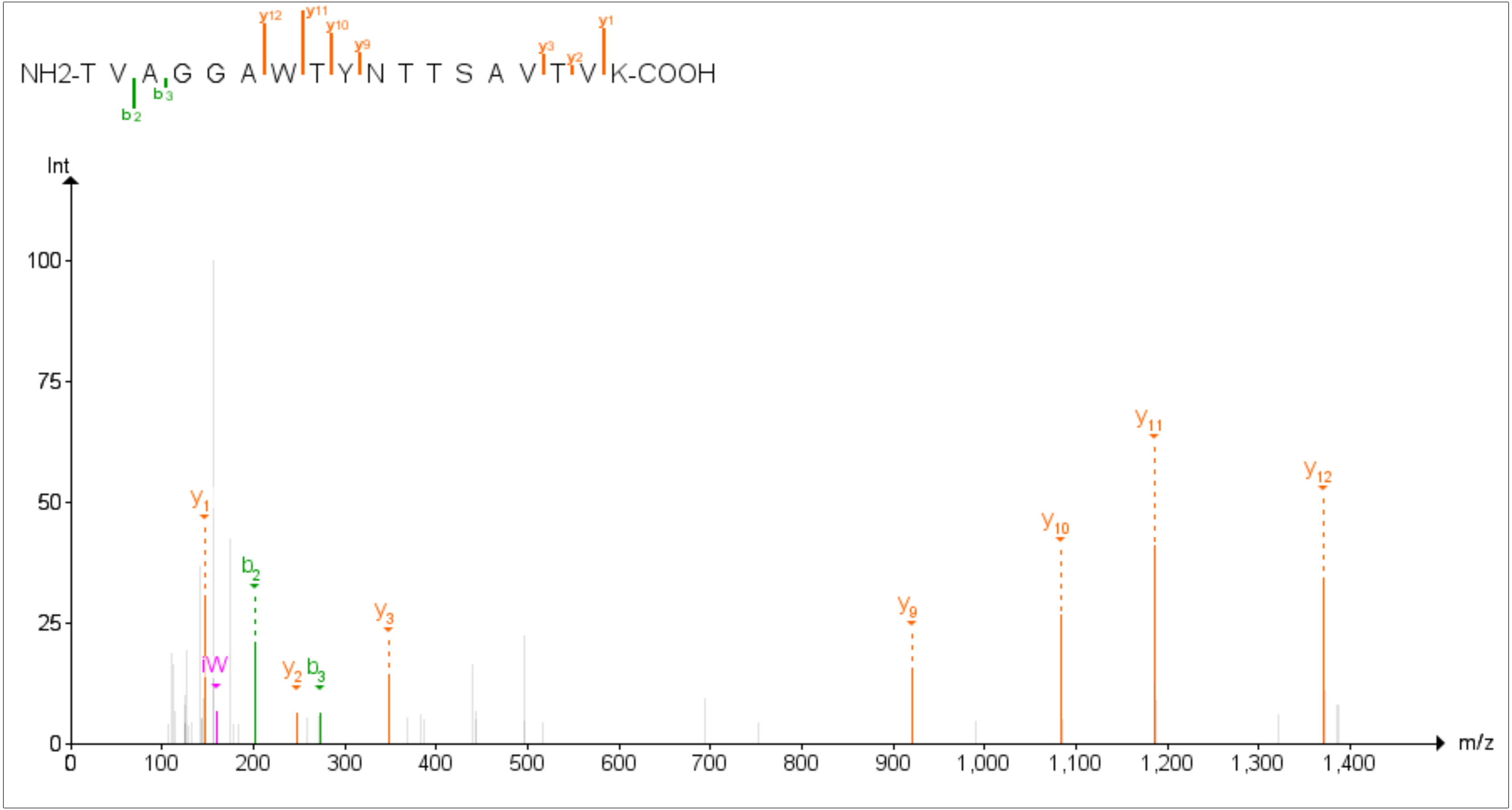

NH2-T V A G G A W T Y N T T S A V T V K-COOH
y12
y11
y10
y9
y3
y2
y1
b2
b3
Int
100
75
50
25
0
y1
iW
b2
y2
b3
y3
y9
y10
y11
y12
0
100
200
300
400
500
600
700
800
900
1,000
1,100
1,200
1,300
1,400
m/z

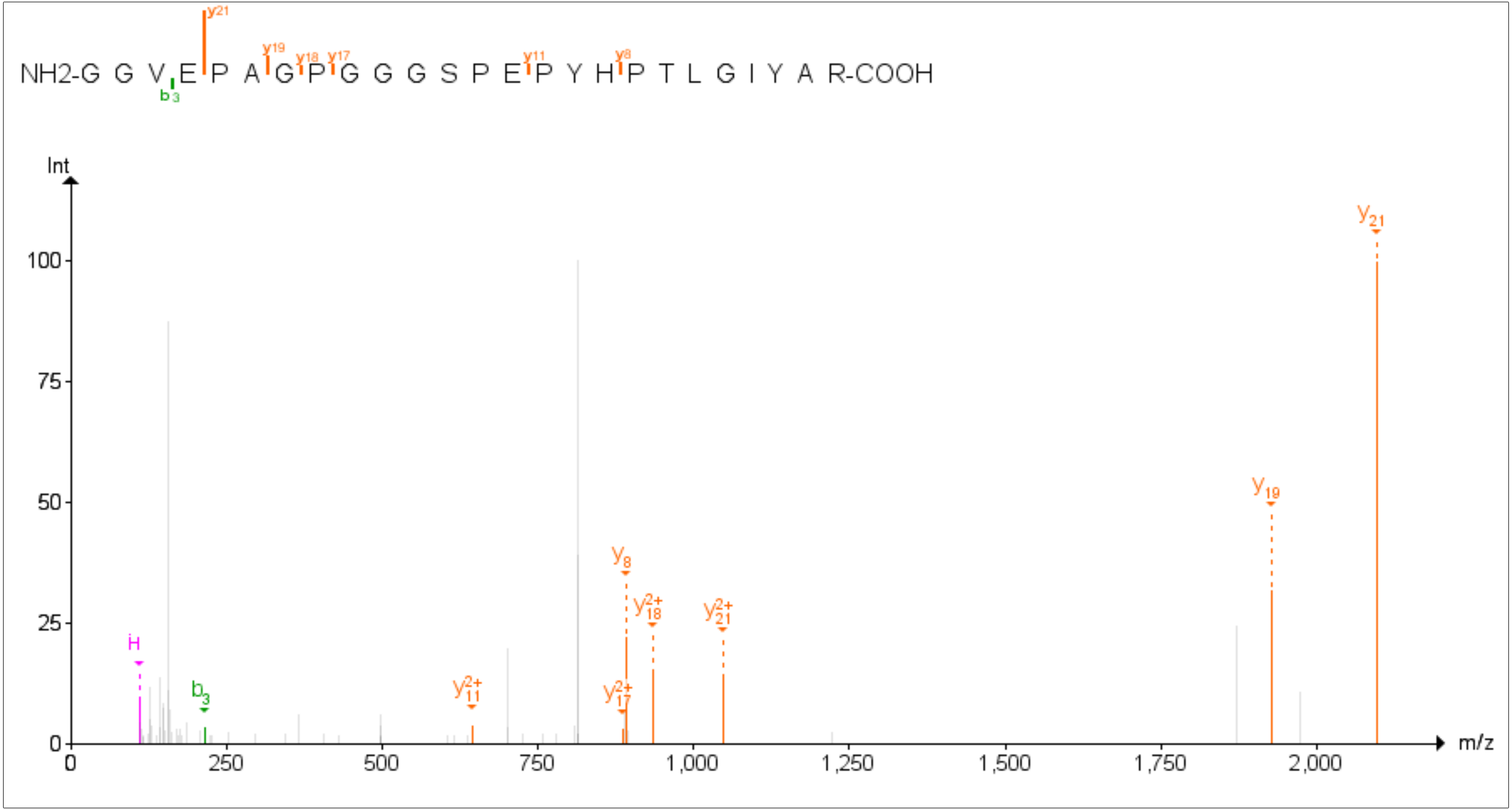

NH2-G G V E P A G P G G G S P E P Y H P T L G I Y A R-COOH
y21
y19
y18
y17
y11
y8
b3
Int
100
75
50
25
0
m/z
0
250
500
750
1,000
1,250
1,500
1,750
2,000
H
b3
y11 2+
y17 2+
y8
y18 2+
y21 2+
y19
y21

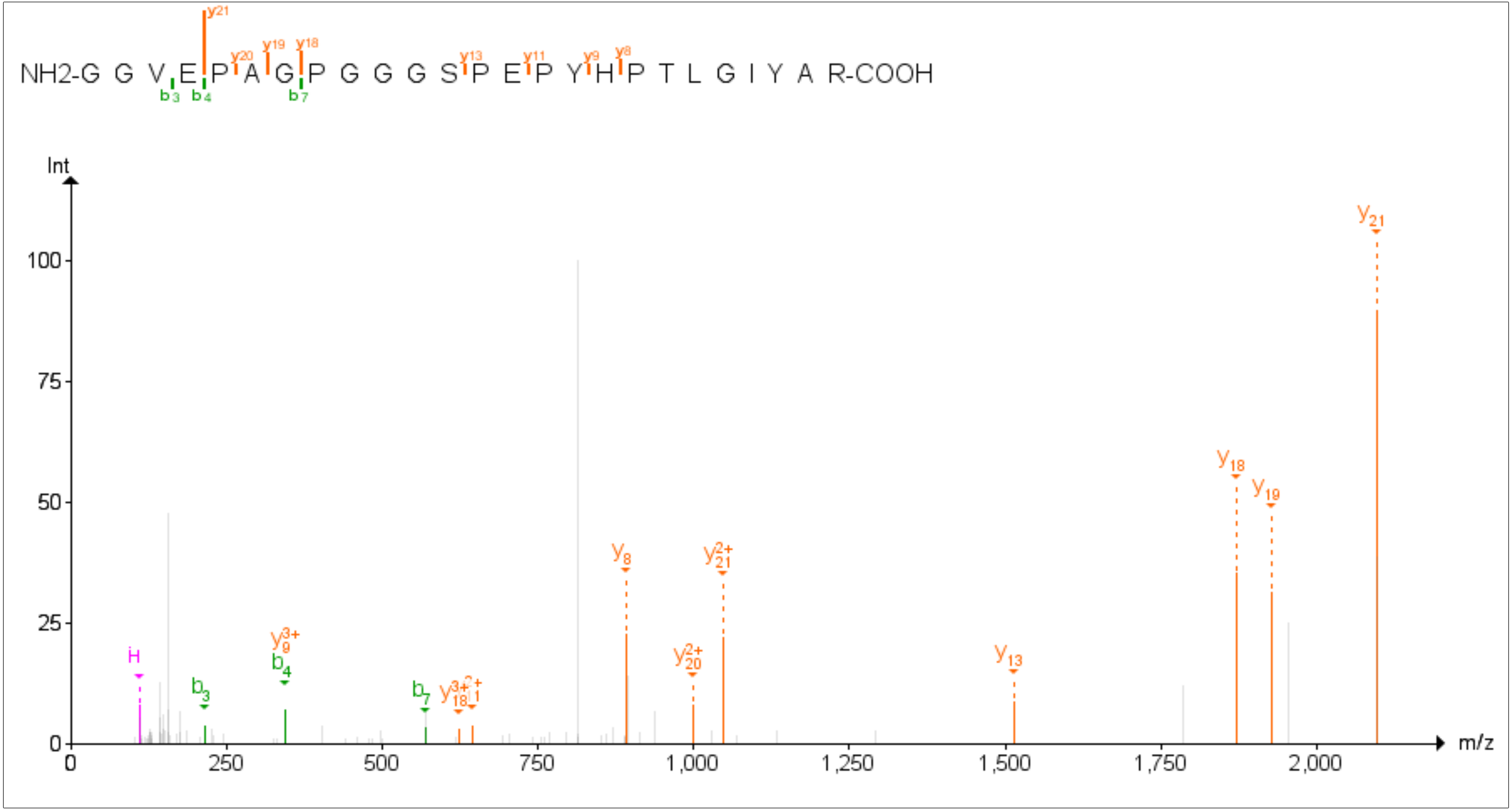
NH2-G G V E P A G P G G G S P E P Y H P T L G I Y A R-COOH
Int
m/z
0
25
50
75
100
250
500
750
1,000
1,250
1,500
1,750
2,000

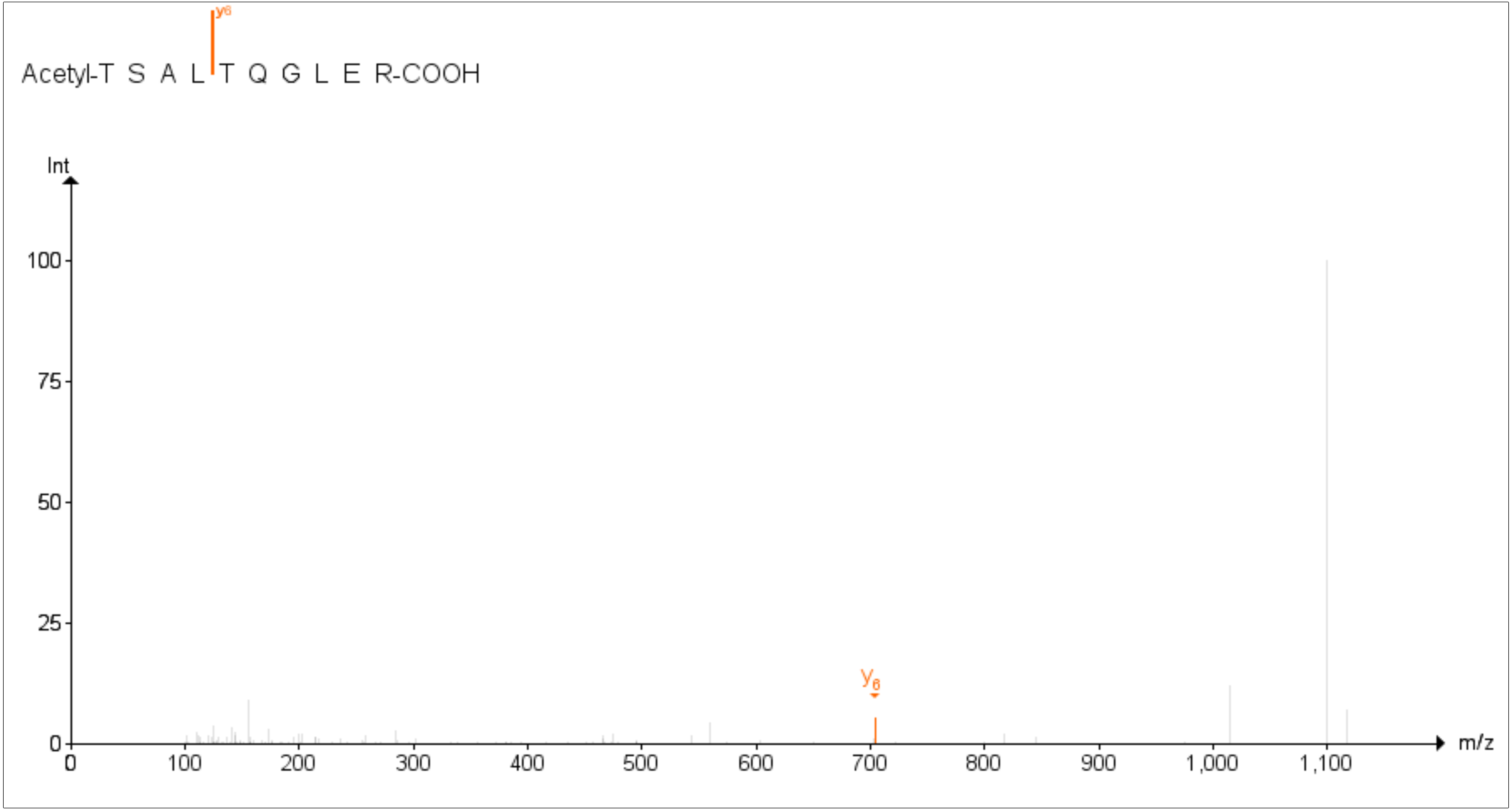

y6
Acetyl-T S A L T Q G L E R-COOH
Int
100
75
50
25
0
y6
0
100
200
300
400
500
600
700
800
900
1,000
1,100
m/z

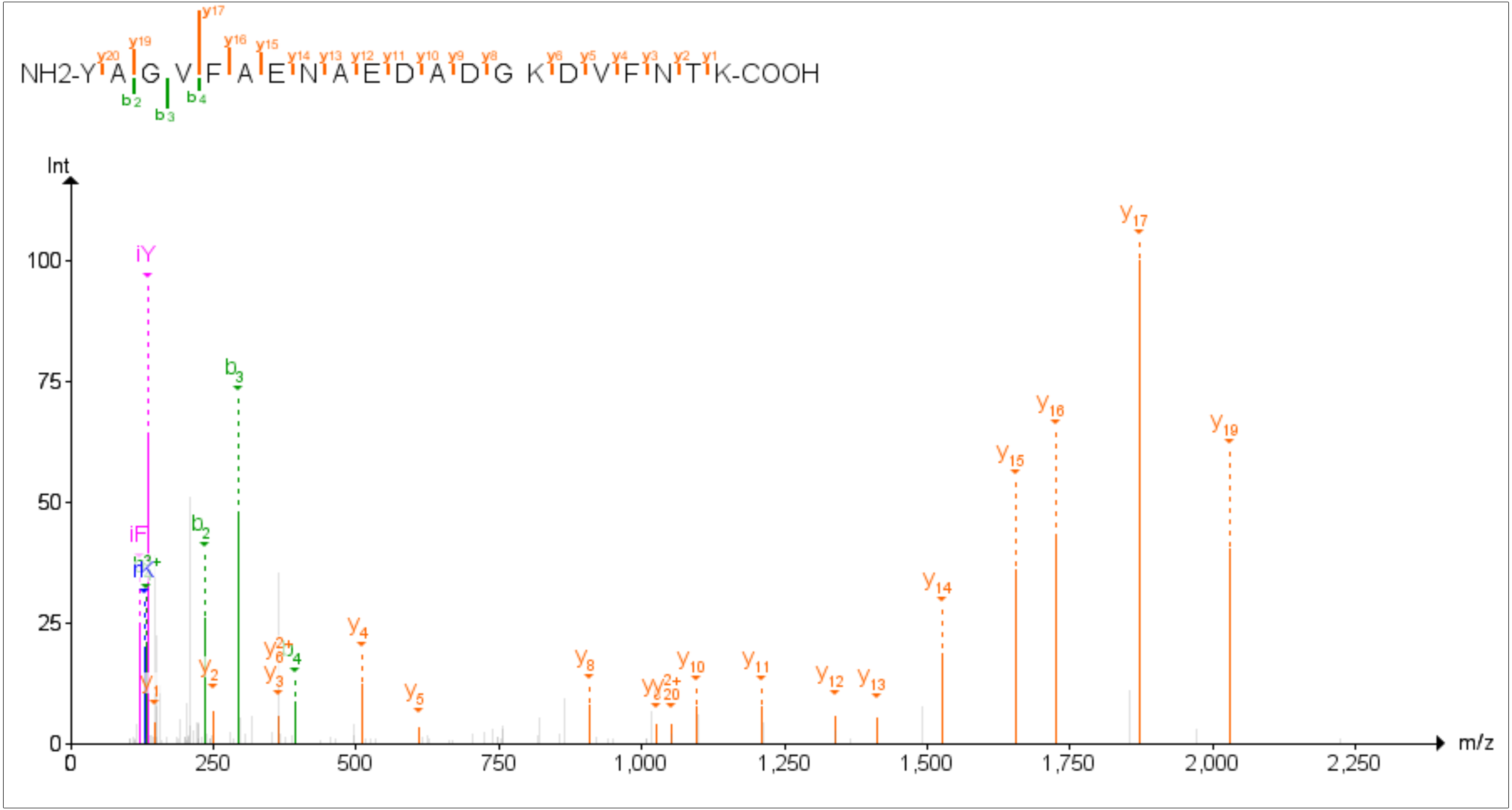

NH2-Y A G V F A E N A E D A D G K D V F N T K-COOH
y20 y19 y17 y16 y15 y14 y13 y12 y11 y10 y9 y8 y6 y5 y4 y3 y2 y1
b2 b3 b4
Int
m/z
0
25
50
75
100
0
250
500
750
1,000
1,250
1,500
1,750
2,000
2,250
iY
iF
iK
b3
b2
b4
y1
y2
y3
y4
y5
y8
y10
y11
y12
y13
y14
y15
y16
y17
y19

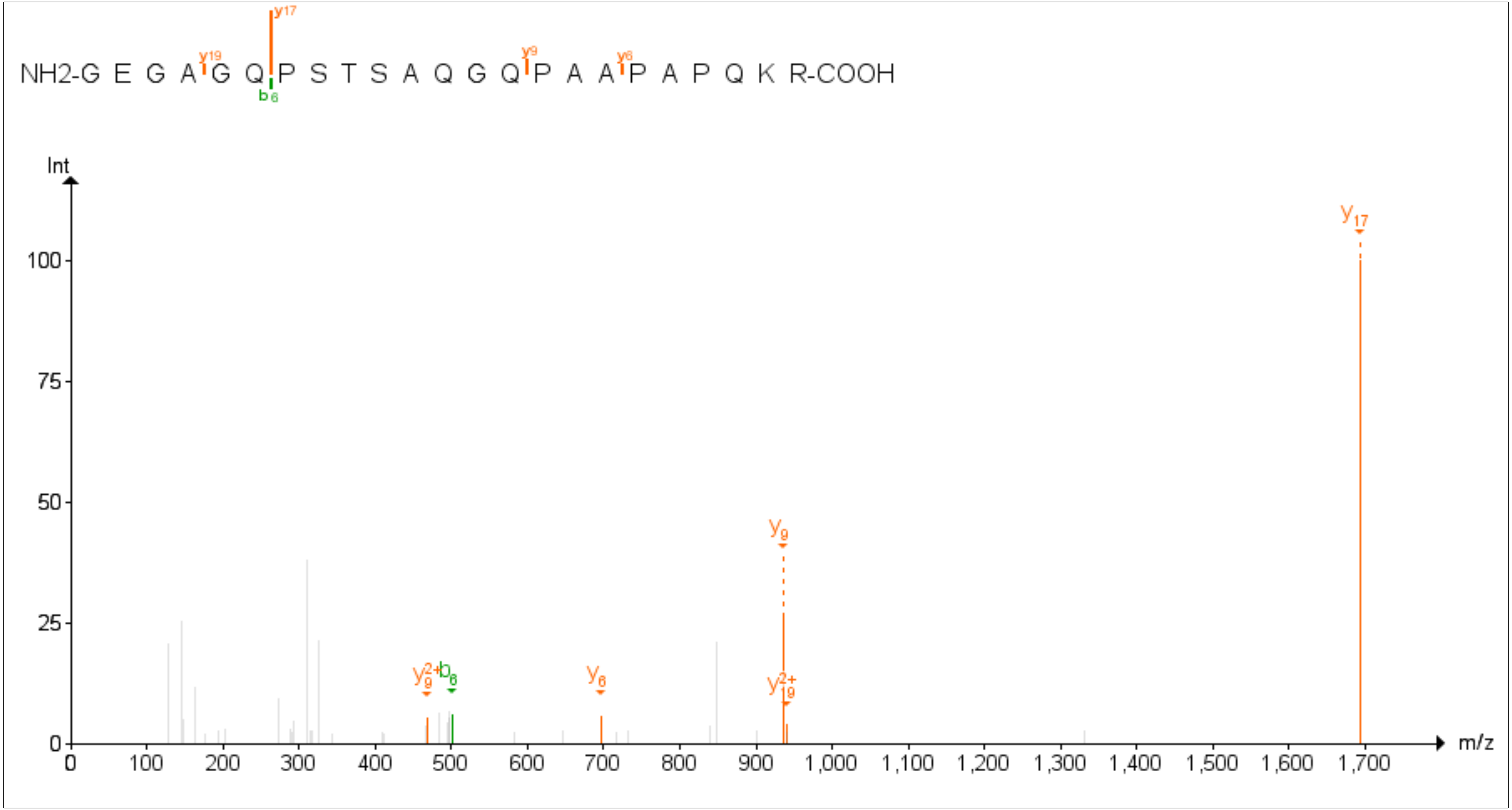

NH2-G E G A G Q P S T S A Q G Q P A A P A P Q K R-COOH
y19
y17
b6
y9
y6
Int
100
75
50
25
0
y17
y9
y9 2+
b6
y6
y19 2+
m/z
0
100
200
300
400
500
600
700
800
900
1,000
1,100
1,200
1,300
1,400
1,500
1,600
1,700

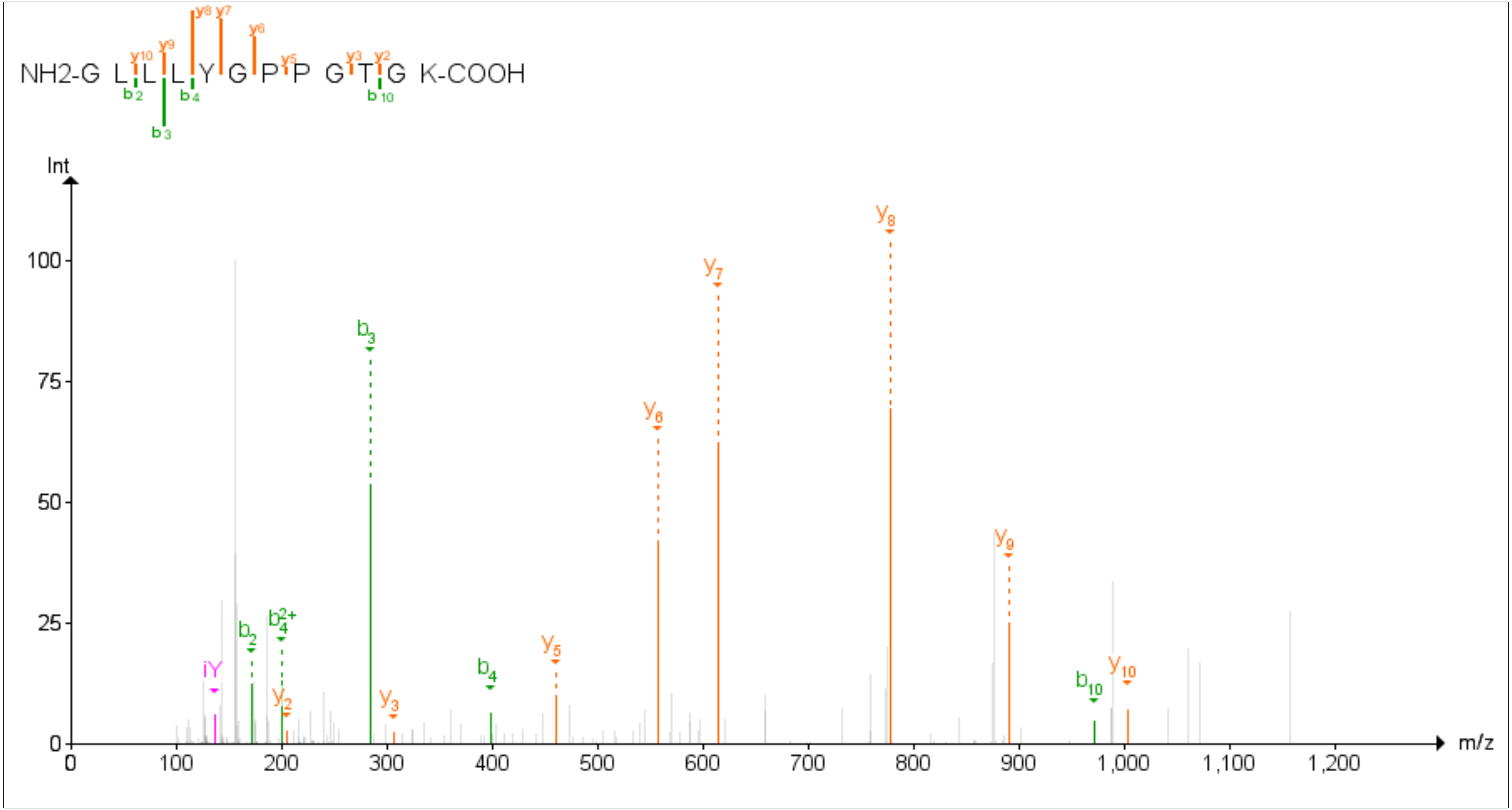

NH2-G L L L Y G P P G T G K-COOH
y10 y9 y8 y7 y6 y5 y3 y2
b2 b3 b4 b10
Int
100
75
50
25
0
m/z
0 100 200 300 400 500 600 700 800 900 1,000 1,100 1,200
iY
b2
b4 2+
y2
b3
y3
b4
y5
y6
y7
y8
y9
b10
y10

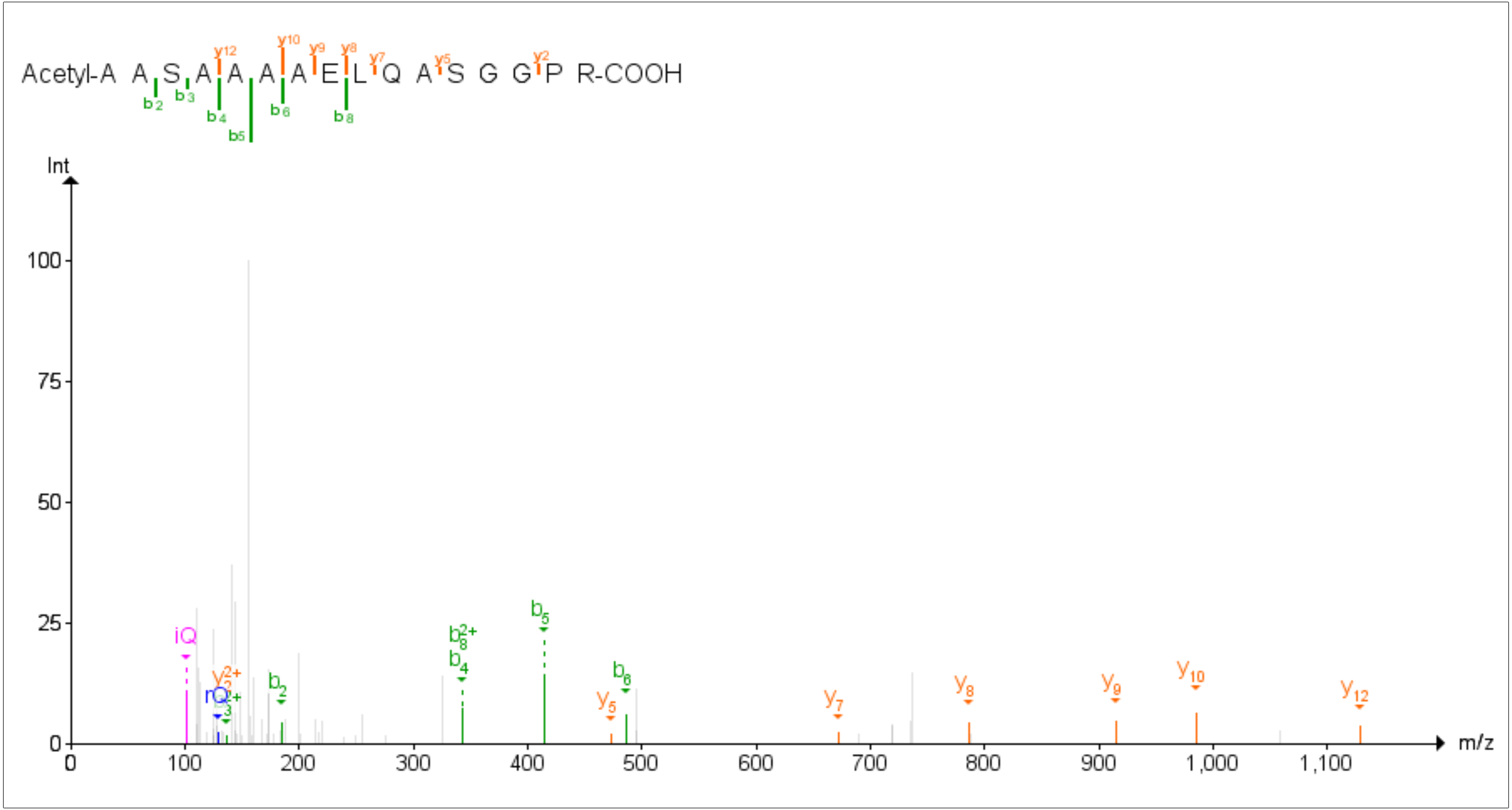

Acetyl-A A S A A A A E L Q A S G G P R-COOH
y12 y10 y9 y8 y7 y5 y2
b2 b3 b4 b5 b6 b8
Int
100
75
50
25
0
iQ
rQ
y2 2+
b3 2+
b2
b8 2+
b4
b5
b6
y5
y7
y8
y9
y10
y12
0 100 200 300 400 500 600 700 800 900 1,000 1,100
m/z

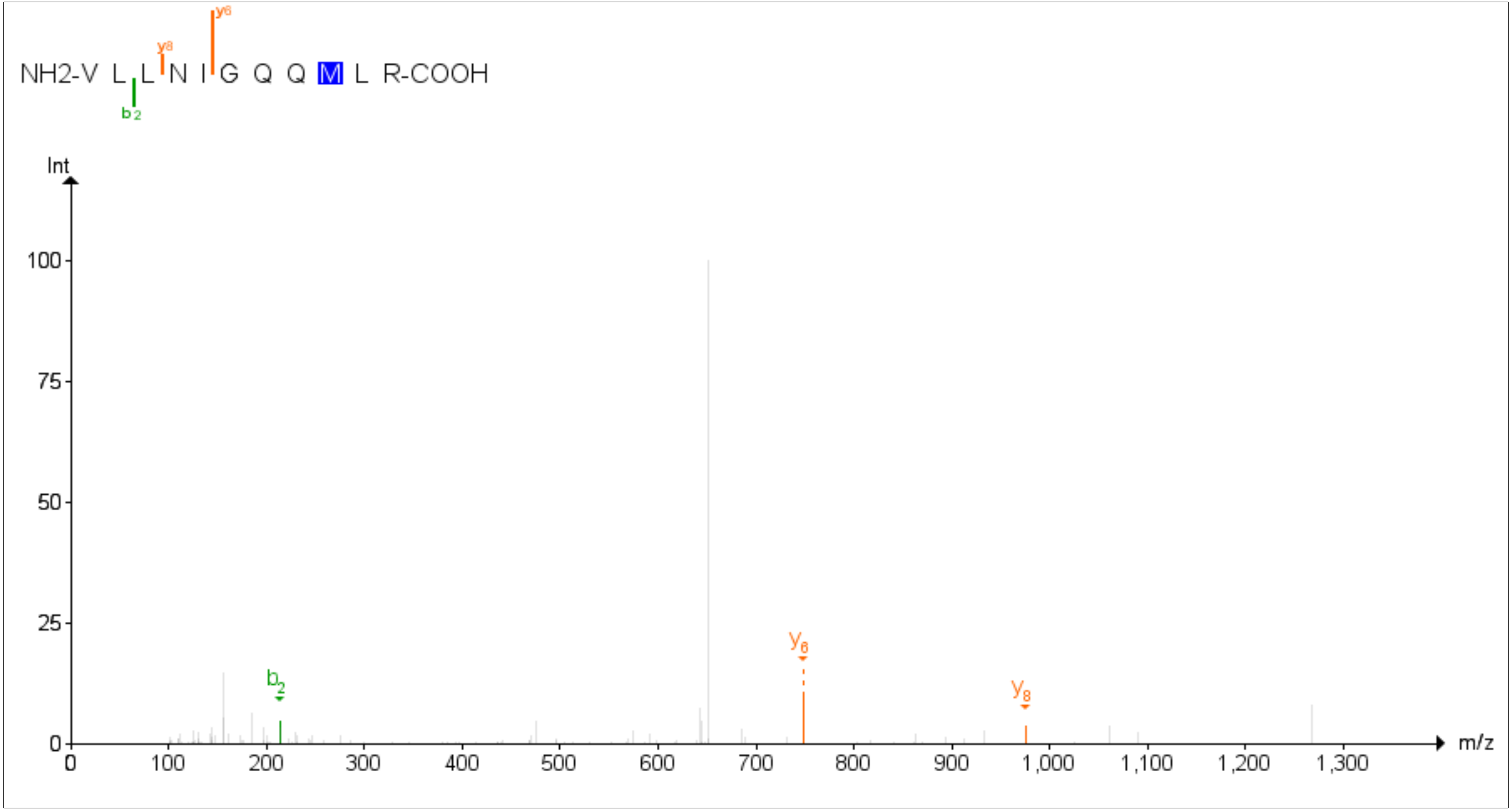

NH2-V L L N I G Q Q M L R-COOH
y6
y8
b 2
Int
100
75
50
25
0
b2
y6
y8
m/z
0
100
200
300
400
500
600
700
800
900
1,000
1,100
1,200
1,300

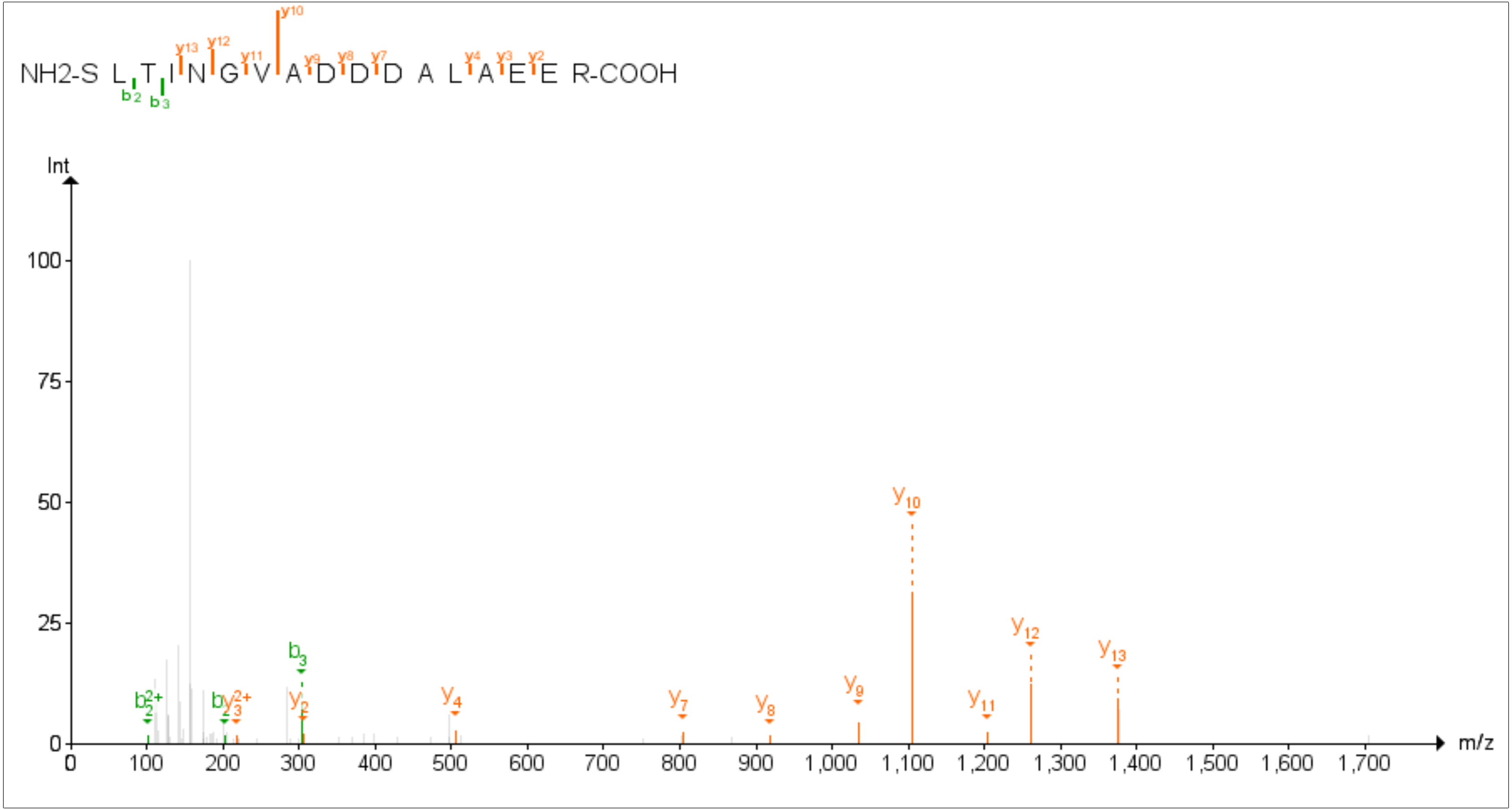

NH2-S L T I N G V A D D D A L A E E R-COOH
b2
b3
y13
y12
y11
y10
y9
y8
y7
y4
y3
y2
Int
100
75
50
25
0
m/z
0
100
200
300
400
500
600
700
800
900
1,000
1,100
1,200
1,300
1,400
1,500
1,600
1,700

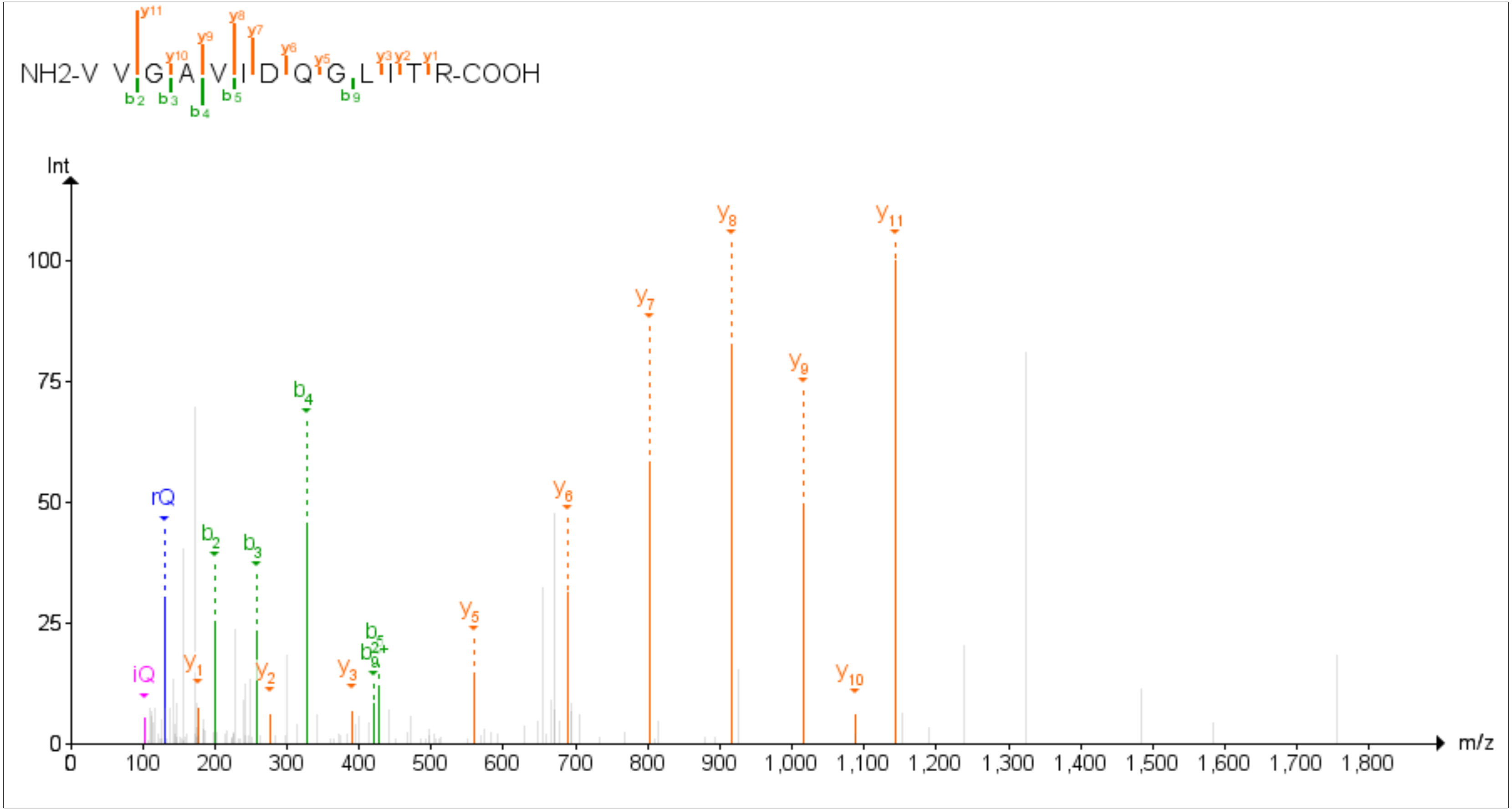

NH2-V V G A V I D Q G L I T R-COOH
y11 y10 y9 y8 y7 y6 y5 y3 y2 y1
b2 b3 b4 b5 b9
Int
m/z
0
25
50
75
100
0
100
200
300
400
500
600
700
800
900
1,000
1,100
1,200
1,300
1,400
1,500
1,600
1,700
1,800
iQ
rQ
y1
b2
b3
y2
b4
y3
b5
b9 2+
y5
y6
y7
y8
y9
y10
y11

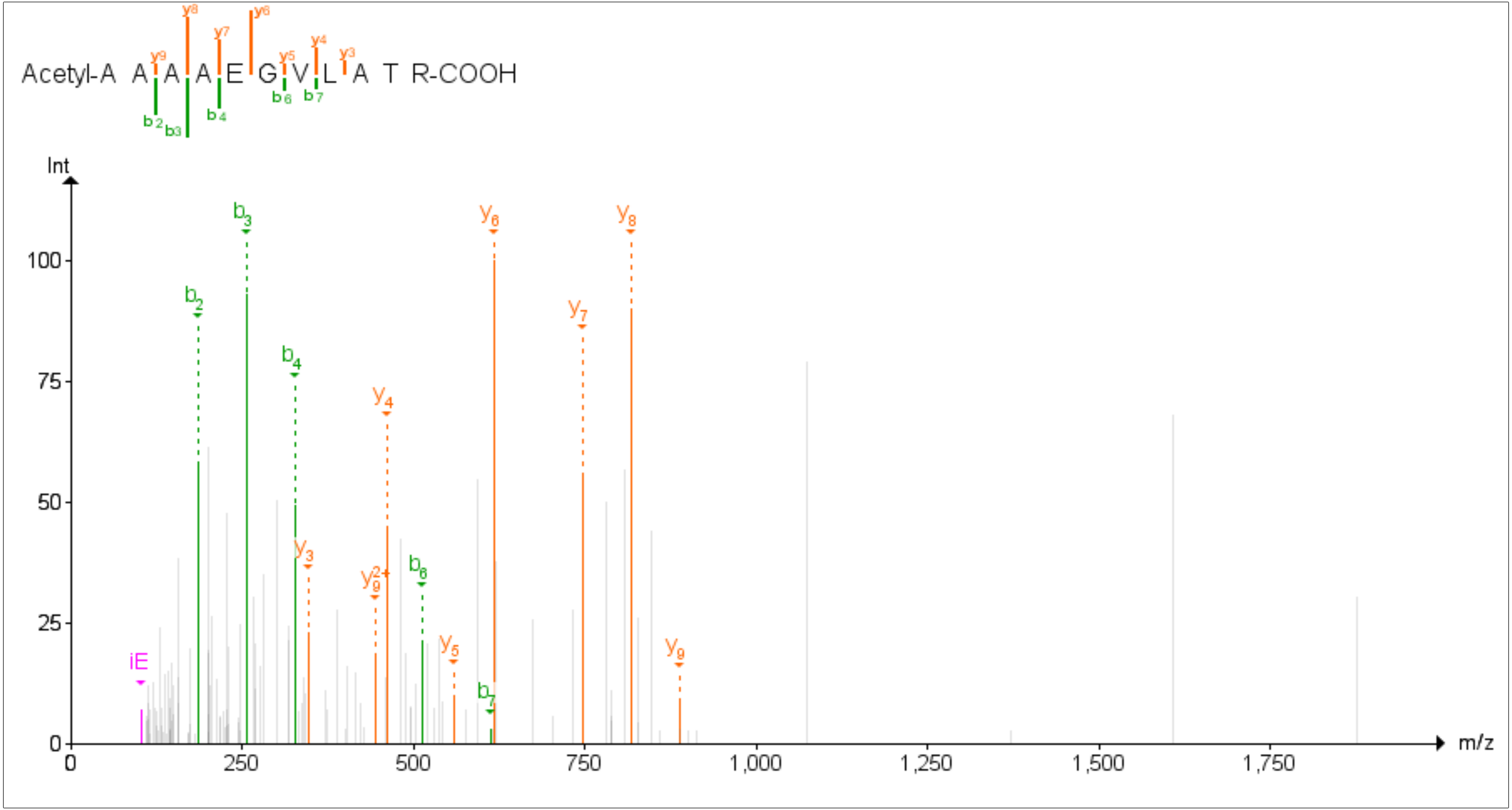

Acetyl-A A A A E G V L A T R-COOH
Int
m/z

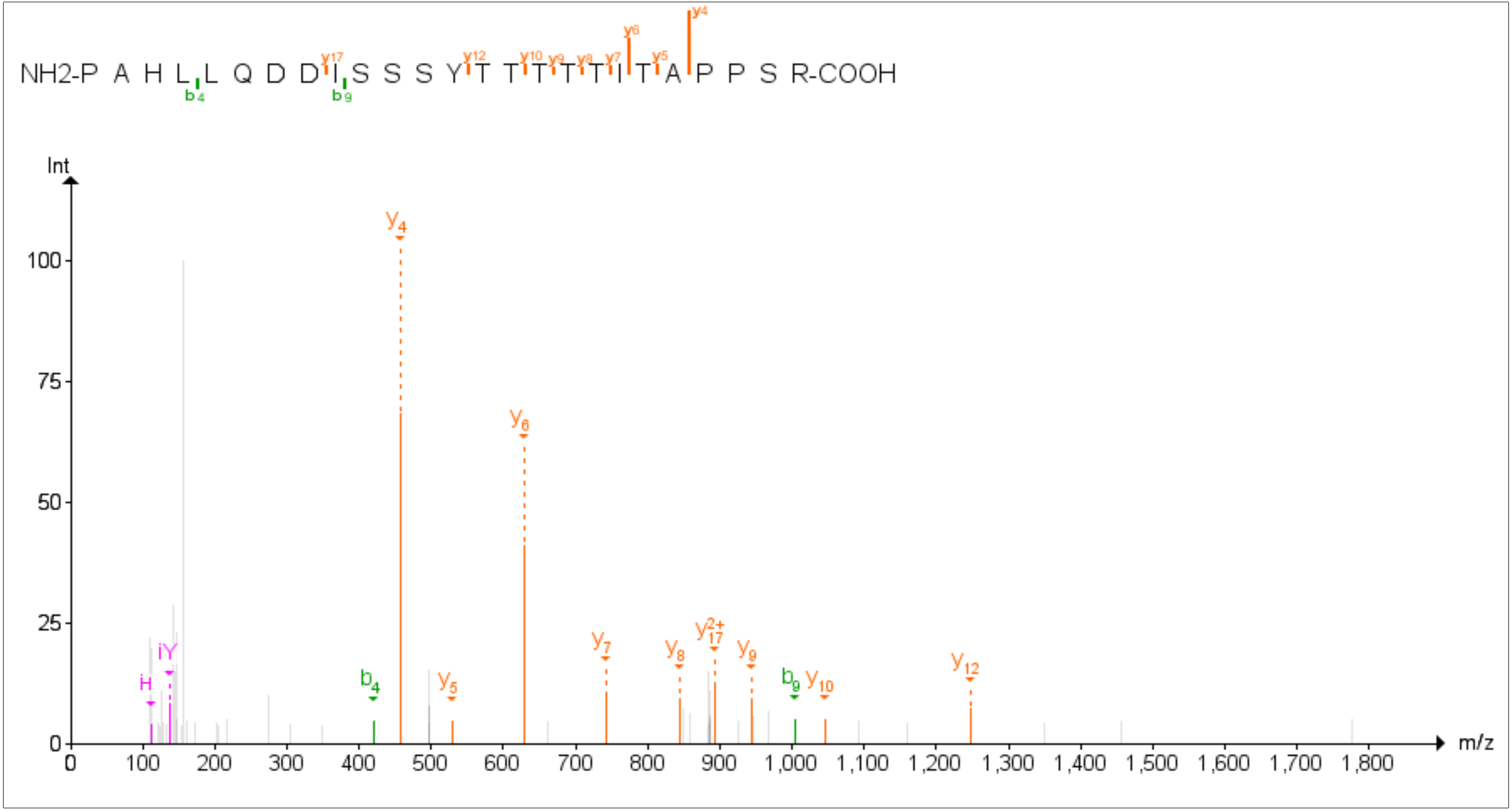

NH2-P A H L L Q D D I S S S Y T T T T T I T A P P S R-COOH
Int
m/z
y4
y6
y5
y7
y8
y17 2+
y9
b4
b9
y10
y12
H
iY

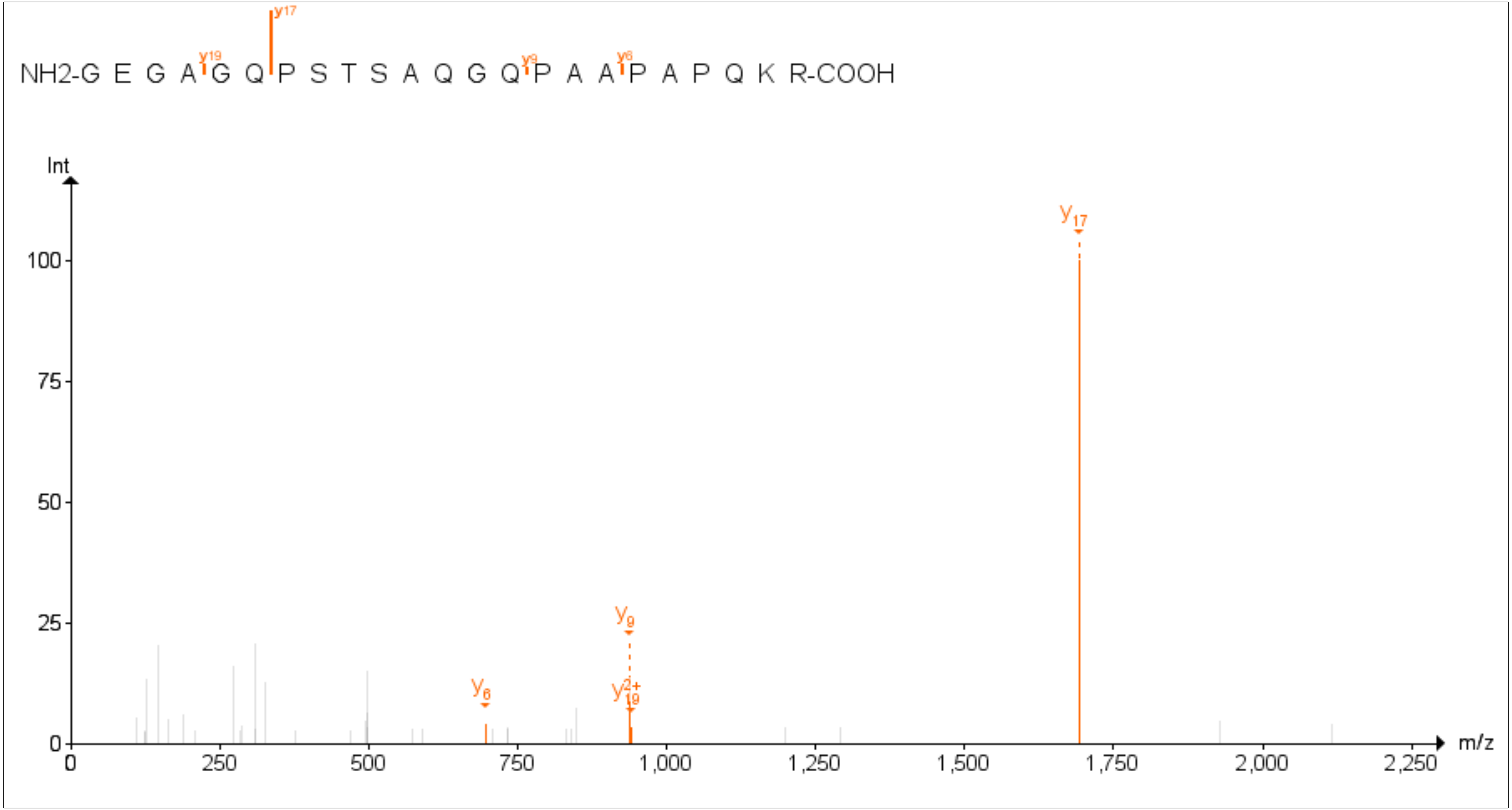

NH2-G E G A G Q P S T S A Q G G Q P A A P A P Q K R-COOH
y19
y17
y9
y6
Int
100
75
50
25
0
y17
y9
y6
y19 2+
0
250
500
750
1,000
1,250
1,500
1,750
2,000
2,250
m/z

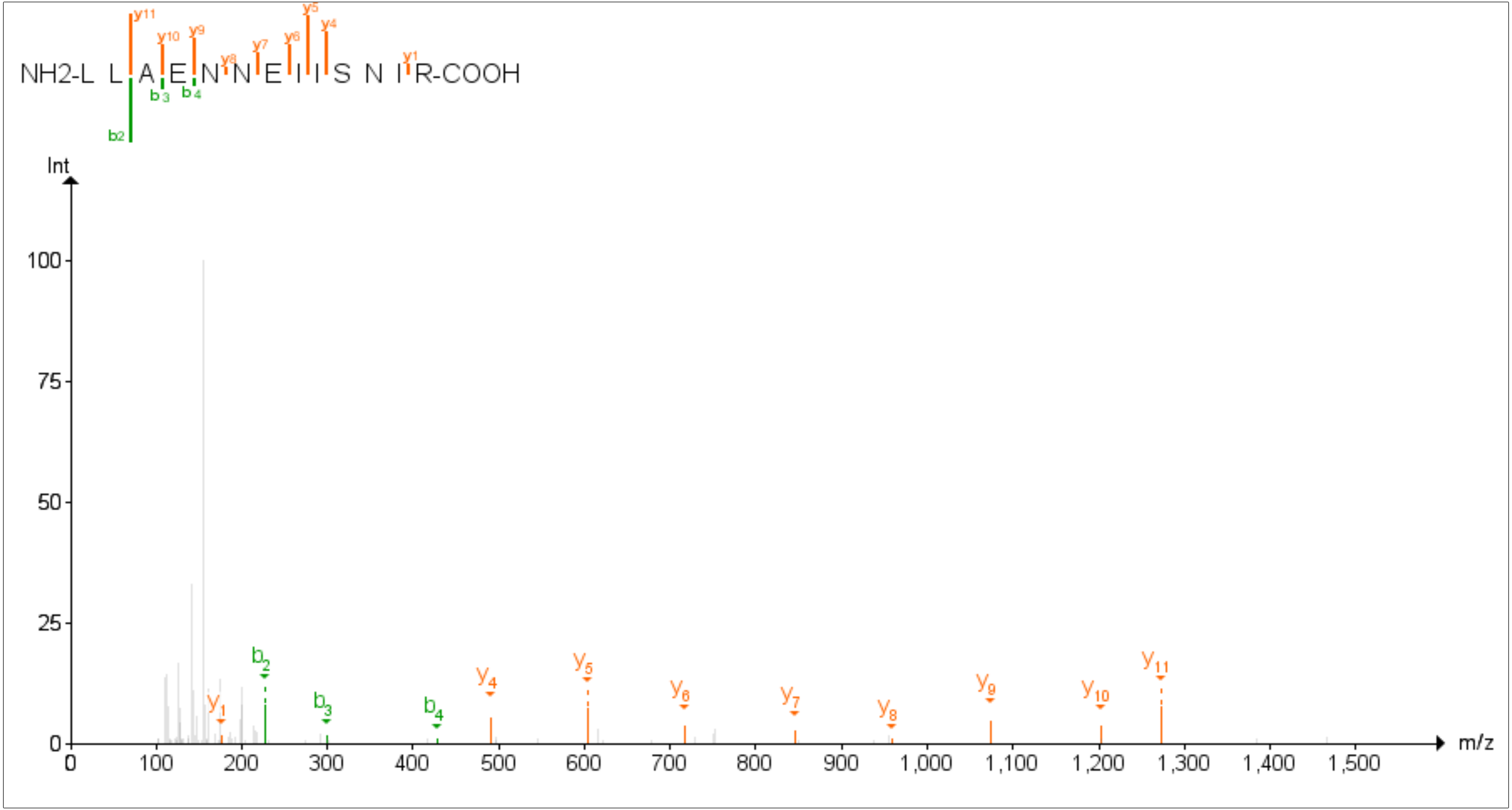
NH2-L L A E N N E I I S N I R-COOH
y11
y10
y9
y8
y7
y6
y5
y4
y1
b2
b3
b4
Int
100
75
50
25
0
b2
b3
b4
y1
y4
y5
y6
y7
y8
y9
y10
y11
0
100
200
300
400
500
600
700
800
900
1,000
1,100
1,200
1,300
1,400
1,500
m/z

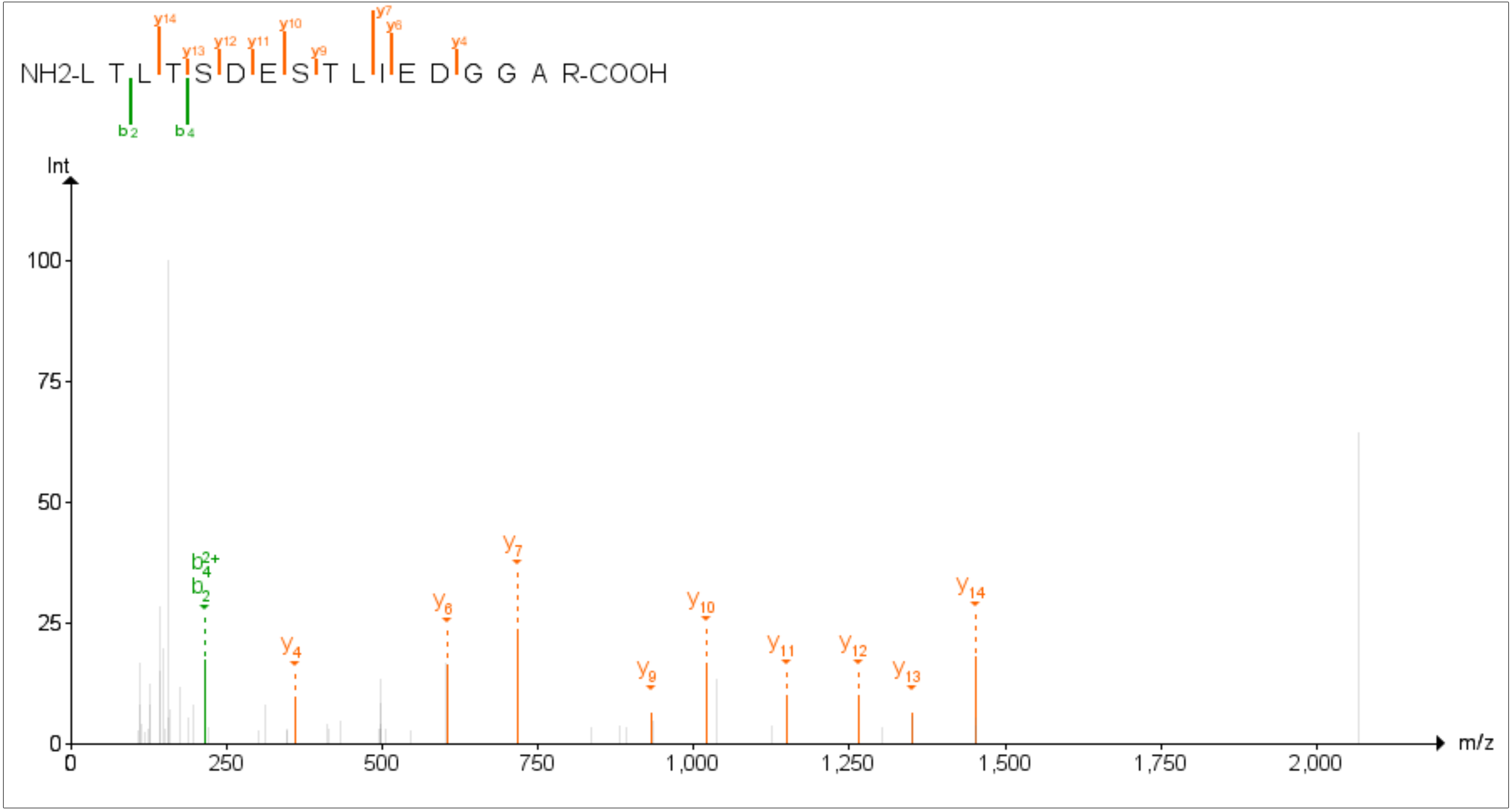

NH2-L T L T S D E S T L I E D G G A R-COOH
y14 y13 y12 y11 y10 y9 y7 y6 y4
b2 b4
Int
100
75
50
25
0
m/z
0 250 500 750 1,000 1,250 1,500 1,750 2,000
b_4^{2+}
b_2
y_4 y_6 y_7 y_9 y_{10} y_{11} y_{12} y_{13} y_{14}

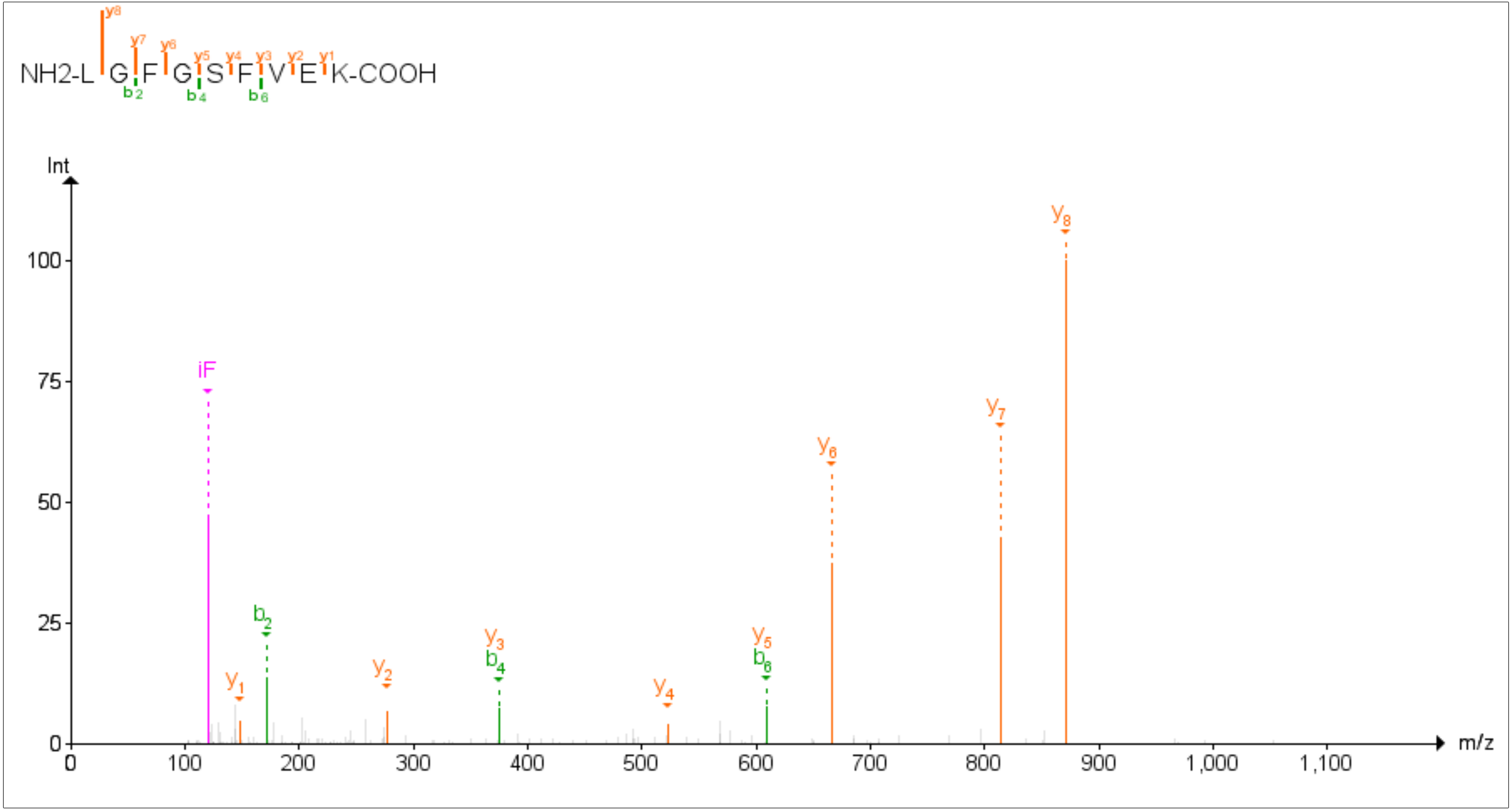
NH2-L G F G S F V E K-COOH
y8 y7 y6 y5 y4 y3 y2 y1
b2 b4 b6
Int
100
75
50
25
0
iF
y1
b2
y2
y3
b4
y4
y5
b6
y6
y7
y8
0 100 200 300 400 500 600 700 800 900 1,000 1,100
m/z

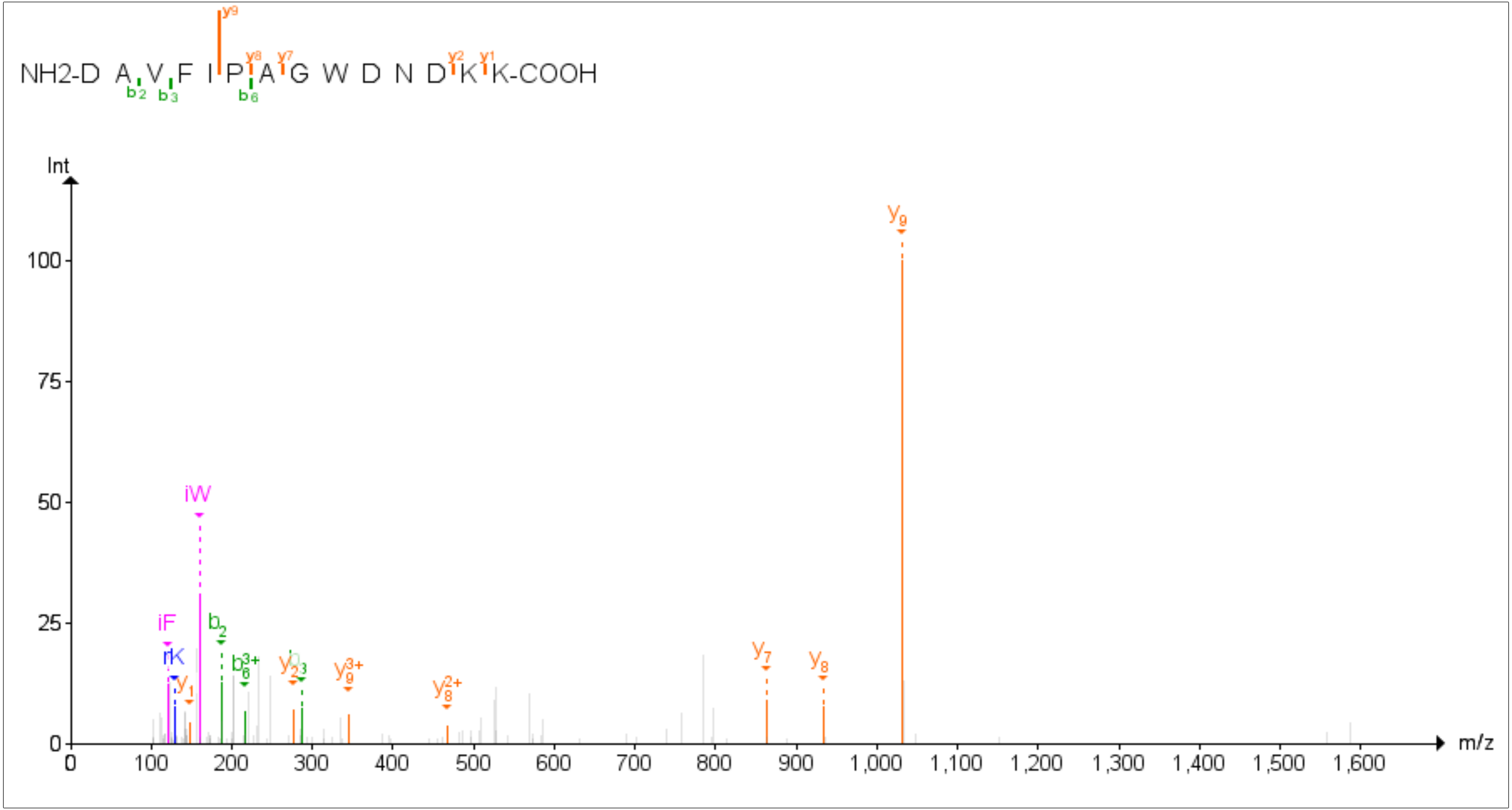
NH2-D A V F I P A G W D N D K K-COOH
y9
y8
y7
y2
y1
b2
b3
b6
Int
m/z
y9
iW
iF
rK
y1
b2
b3
y2
y9 3+
y8 2+
y7
y8
0
25
50
75
100
0
100
200
300
400
500
600
700
800
900
1,000
1,100
1,200
1,300
1,400
1,500
1,600

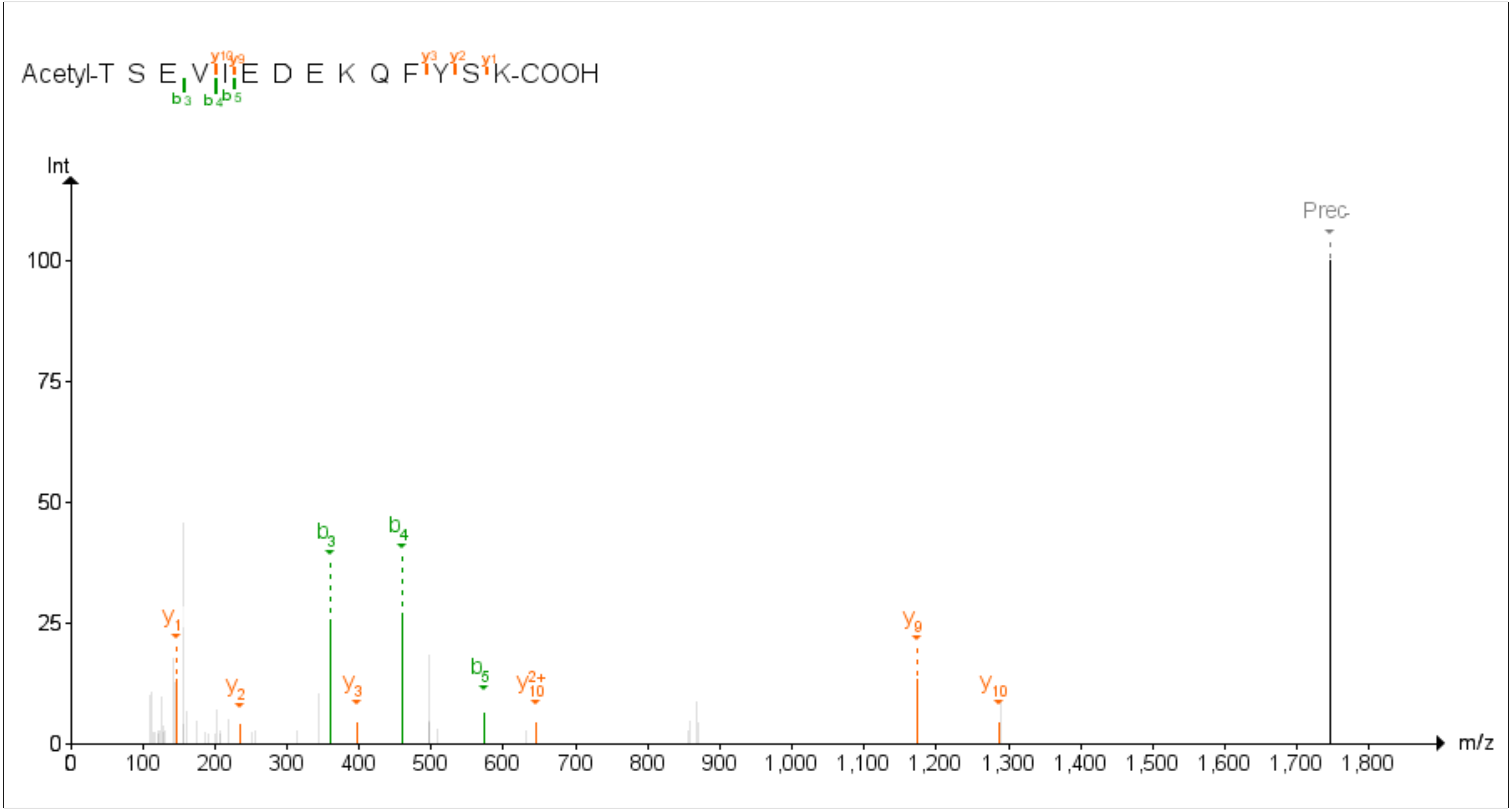

Acetyl-T S E V I I E D E K Q F Y S K-COOH
y10 y9 y3 y2 y1
b3 b4 b5
Int
m/z
Prec.
b3
b4
b5
y1
y2
y3
y9
y10
y2+ 10
0
25
50
75
100
0
100
200
300
400
500
600
700
800
900
1,000
1,100
1,200
1,300
1,400
1,500
1,600
1,700
1,800

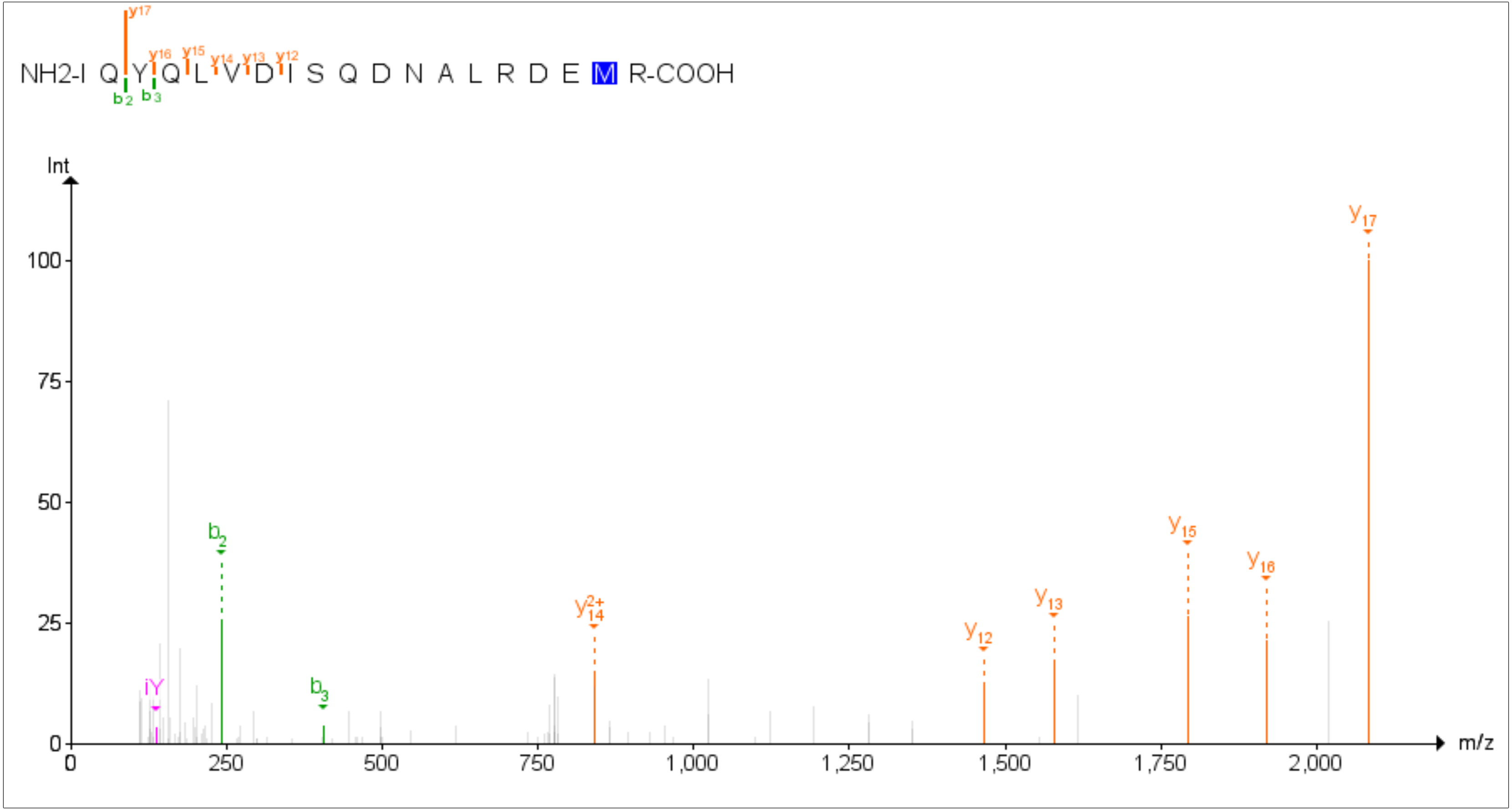

NH2-I Q Y Q L V D I S Q D N A L R D E M R-COOH
y17
y16
y15
y14
y13
y12
b2
b3
Int
100
75
50
25
0
0
250
500
750
1,000
1,250
1,500
1,750
2,000
m/z
iY
b2
b3
y14 2+
y12
y13
y15
y16
y17

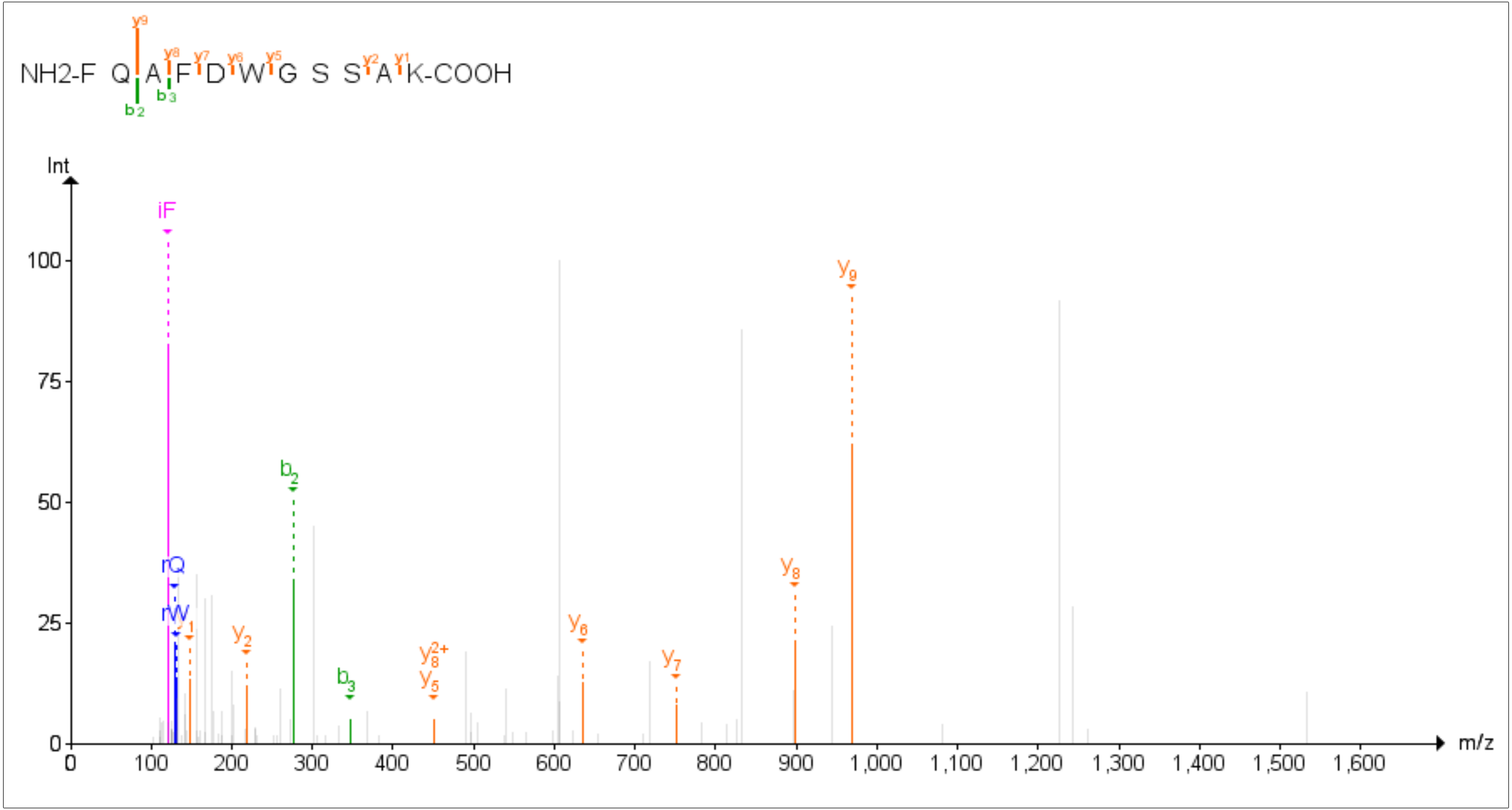

NH2-F Q A F D W G S S A K-COOH
y9 y8 y7 y6 y5 y2 y1
b2 b3
Int
iF
b2
rQ
rW
y2
b3
y8 2+
y5
y6
y7
y8
y9
0
25
50
75
100
0 100 200 300 400 500 600 700 800 900 1,000 1,100 1,200 1,300 1,400 1,500 1,600
m/z

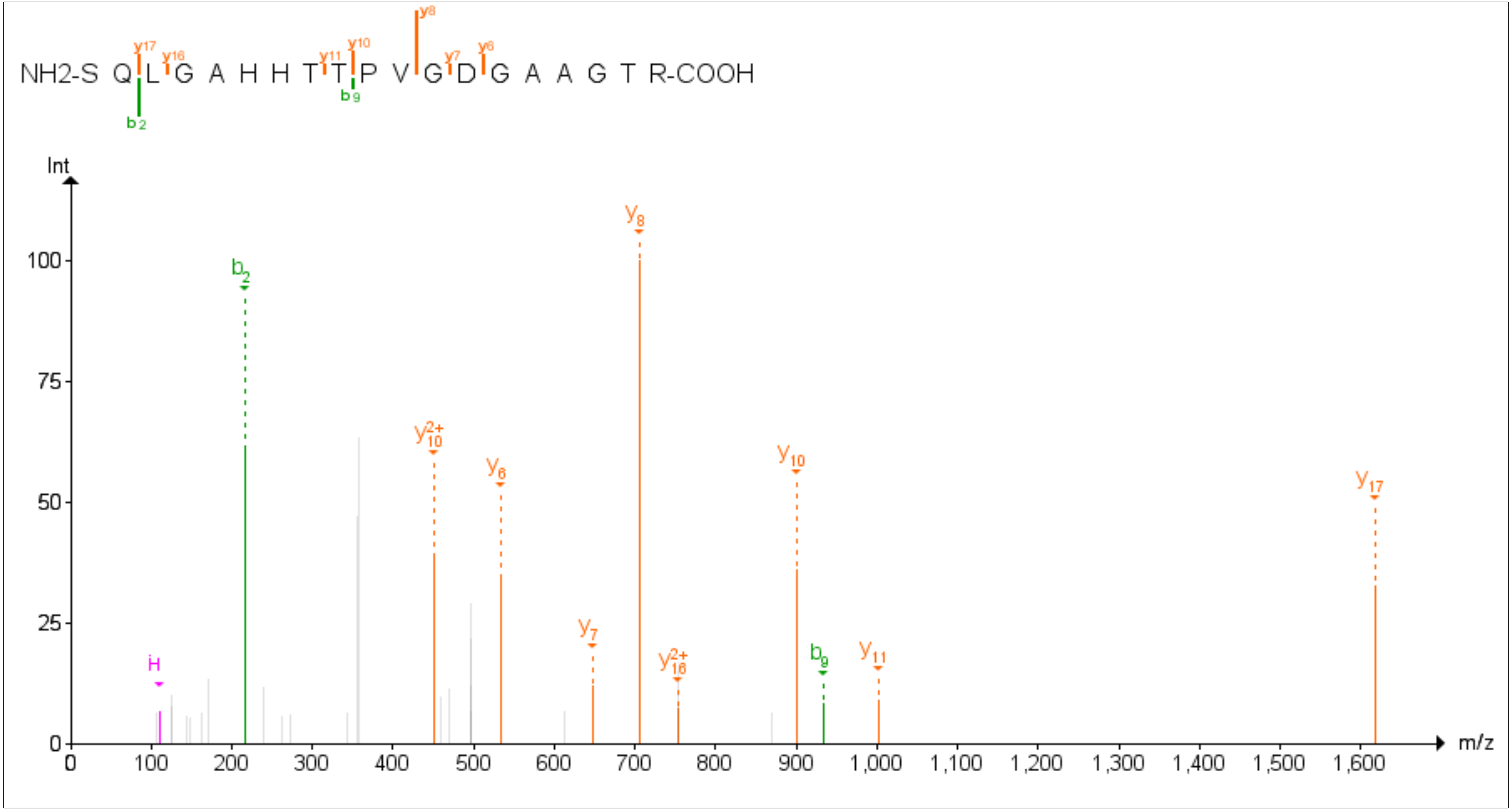

NH2-S Q L G A H H T T P V G D G A A G T R-COOH
y17 y16 y11 y10 y8 y7 y6
b2 b9
Int
100
75
50
25
0
b2
y8
y10 2+
y6
y10
y17
H
y7
y16 2+
b9
y11
0 100 200 300 400 500 600 700 800 900 1,000 1,100 1,200 1,300 1,400 1,500 1,600
m/z

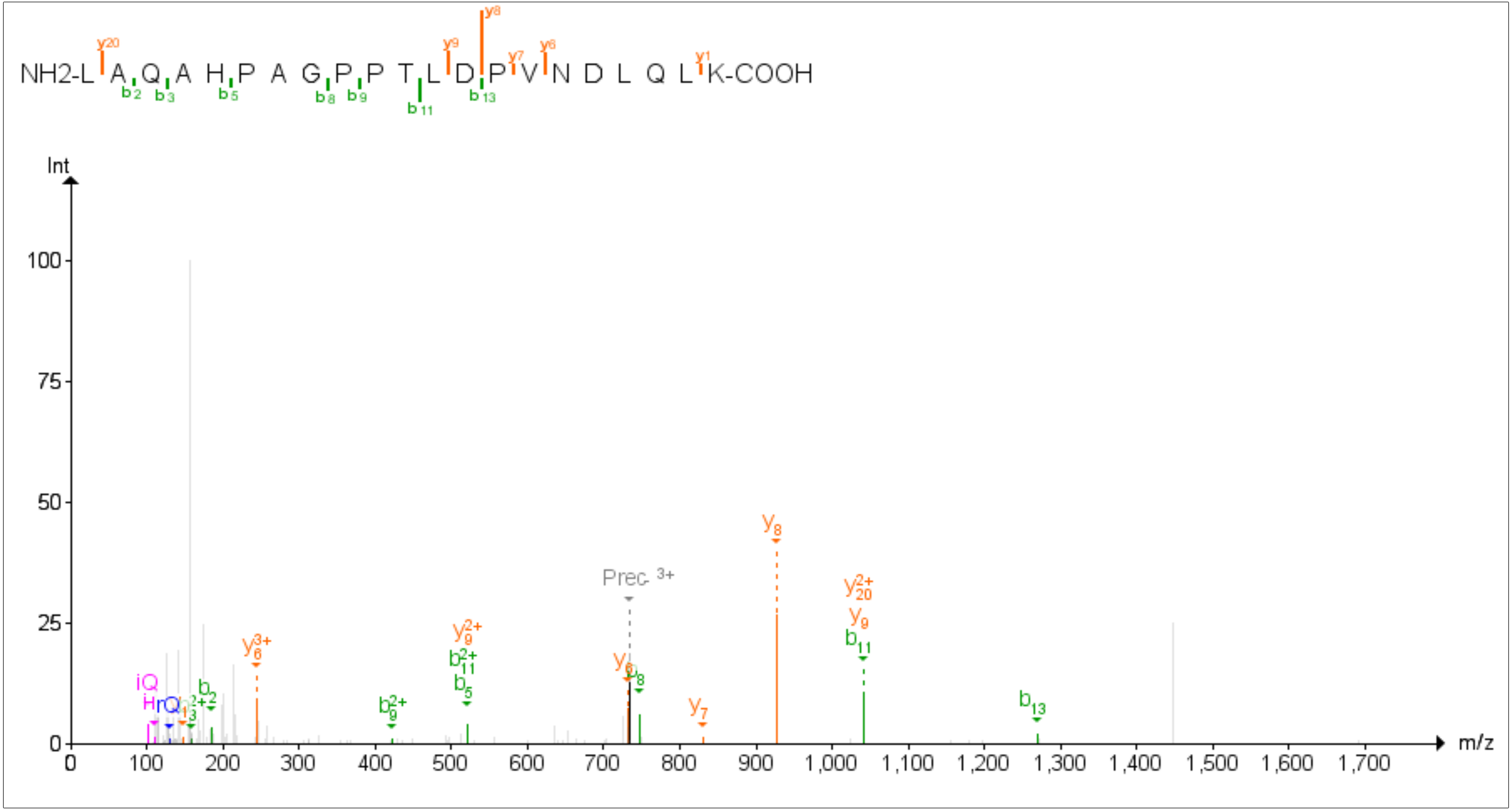

NH2-L A Q A H P A G P P T L D P V N D L Q L K-COOH
y20 y9 y8 y7 y6 y1
b2 b3 b5 b8 b9 b11 b13
Int
m/z
Prec. 3+
y8
y20 2+
y9
b11
b13
y7
y6
b8
y9 2+
b11 2+
b5
b9 2+
y6 3+
b2
iQ

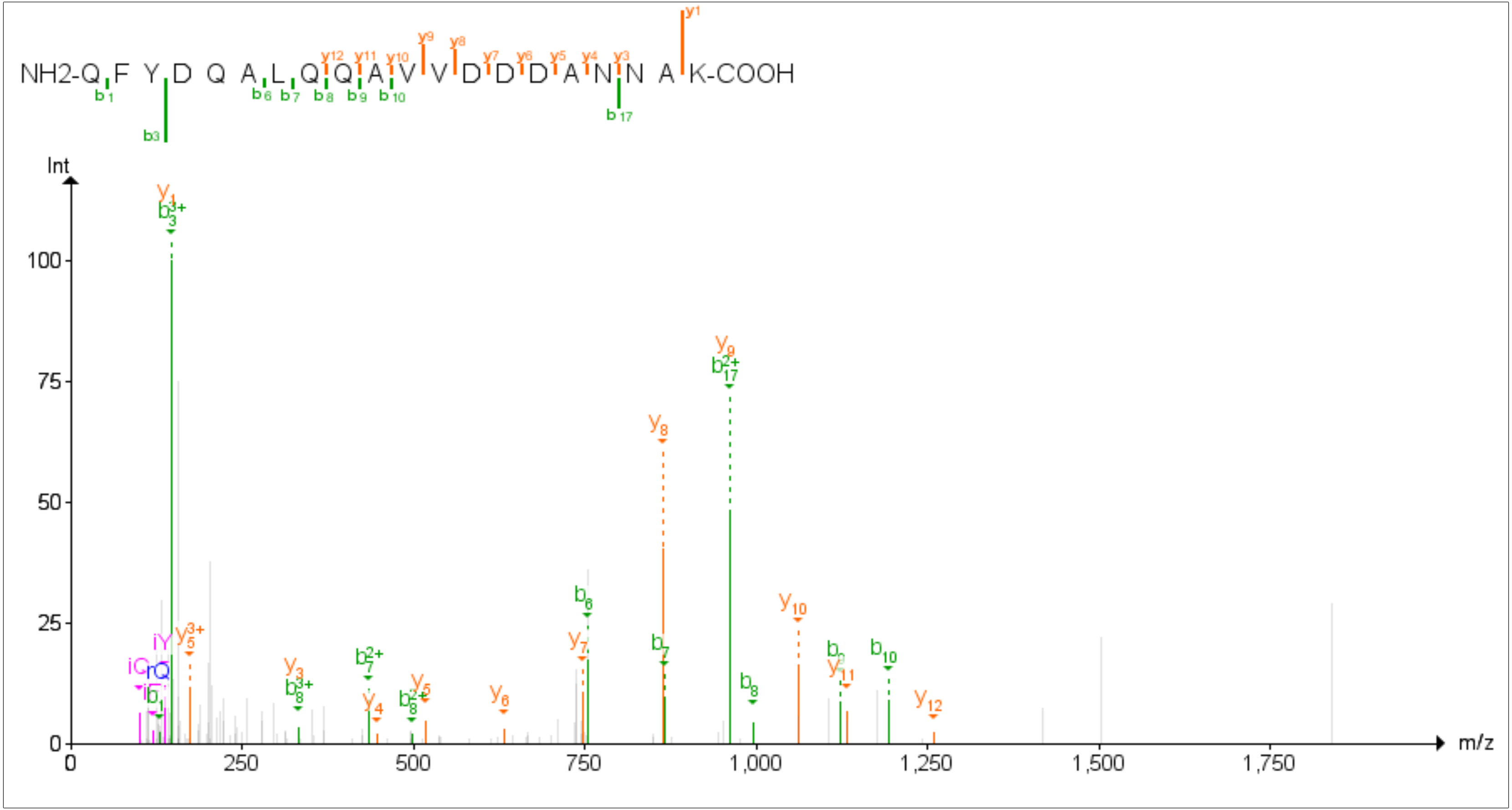
NH2-Q F Y D Q A L Q Q A V V D D D A N N A K-COOH
Int
m/z
0
25
50
75
100
250
500
750
1,000
1,250
1,500
1,750

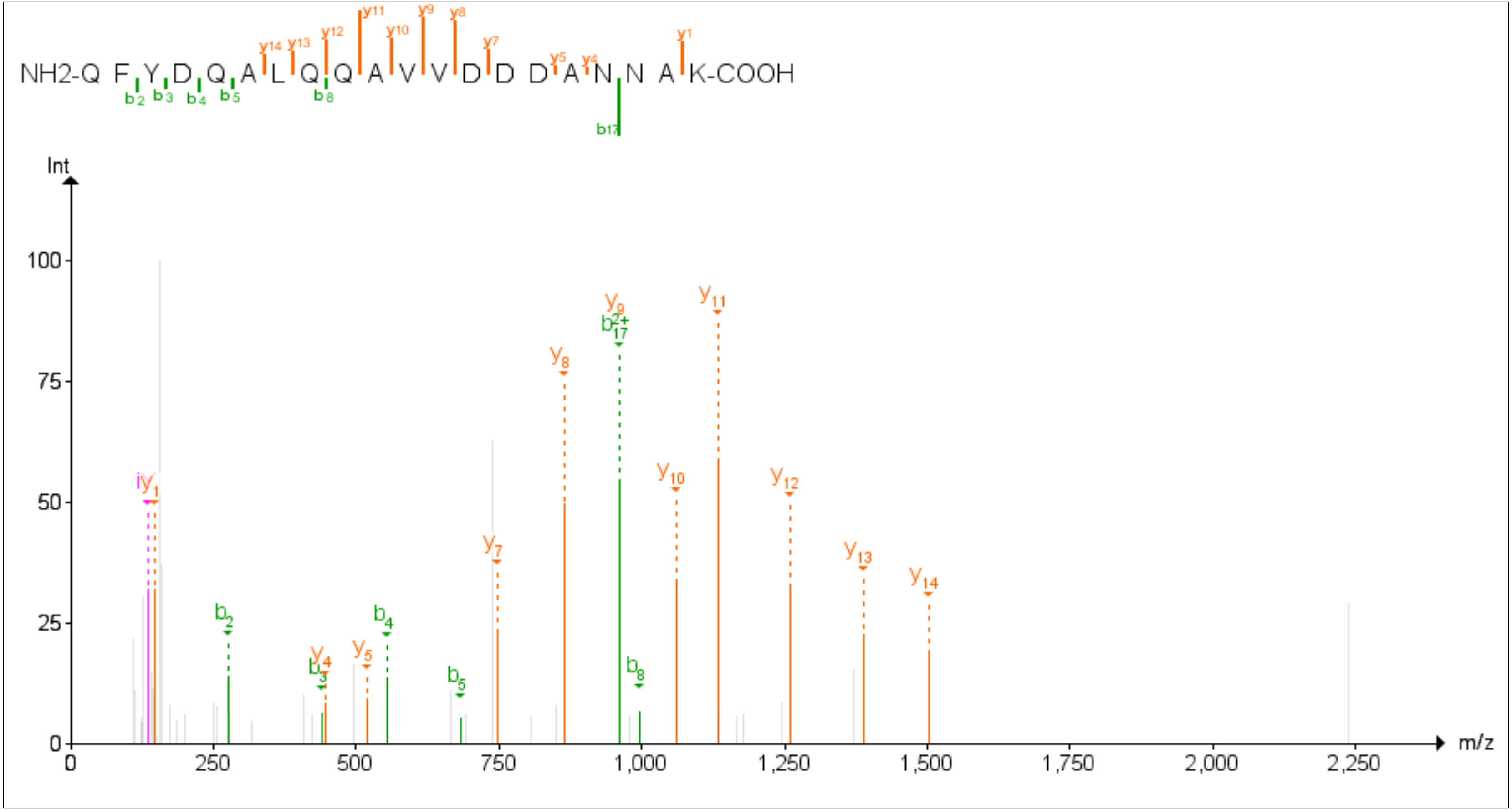

NH2-Q F Y D Q A L Q Q A V V D D D A N N A K-COOH
b2 b3 b4 b5 b8 b17
y14 y13 y12 y11 y10 y9 y8 y7 y5 y4 y1
Int
100
75
50
25
0
m/z
0
250
500
750
1,000
1,250
1,500
1,750
2,000
2,250
iy1
y1
b2
b3
y4
y5
b4
b5
y7
y8
y9
b2+ 17
b8
y10
y11
y12
y13
y14

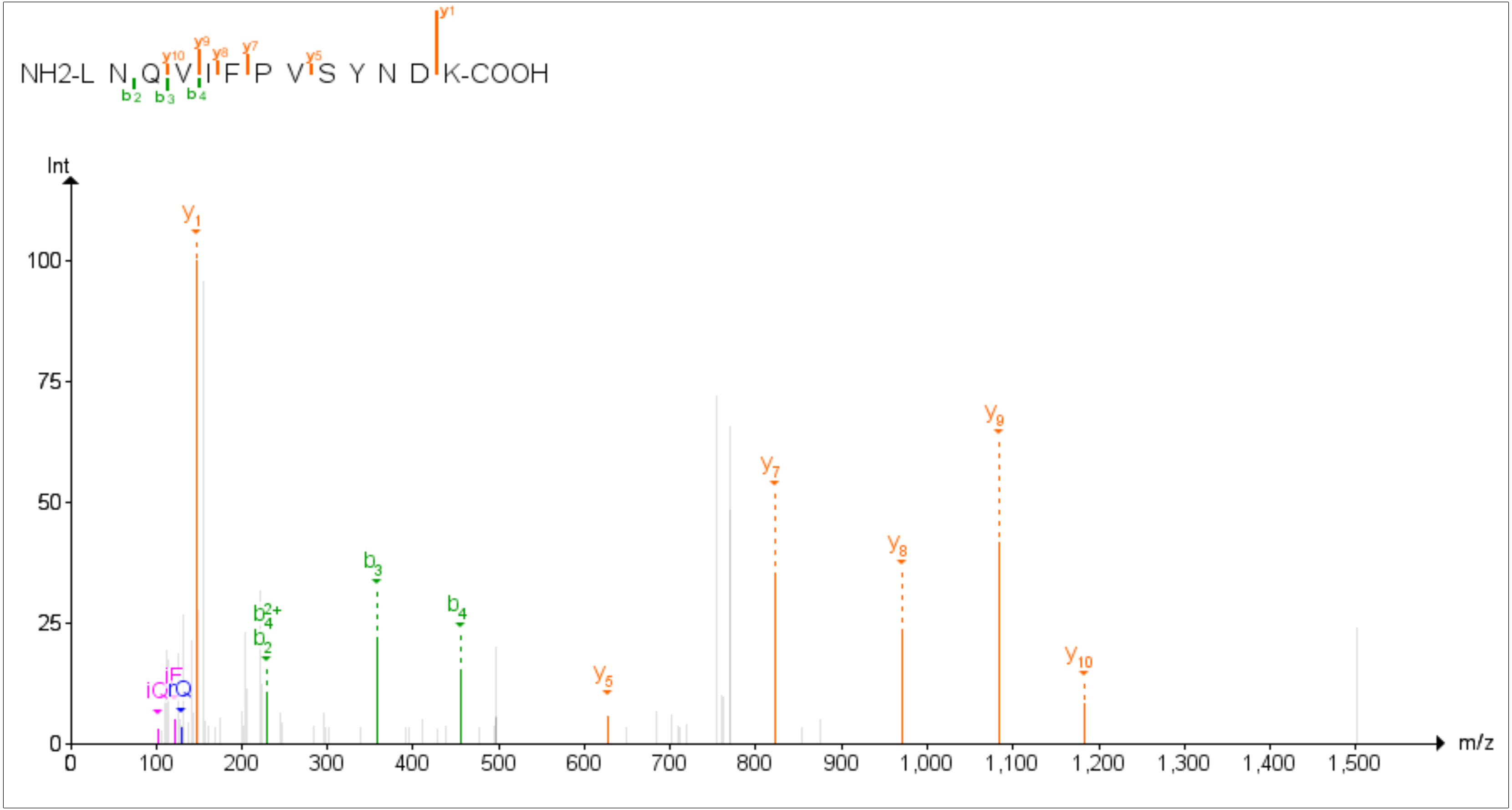
NH2-L N Q V I F P V S Y N D K-COOH
Int
m/z

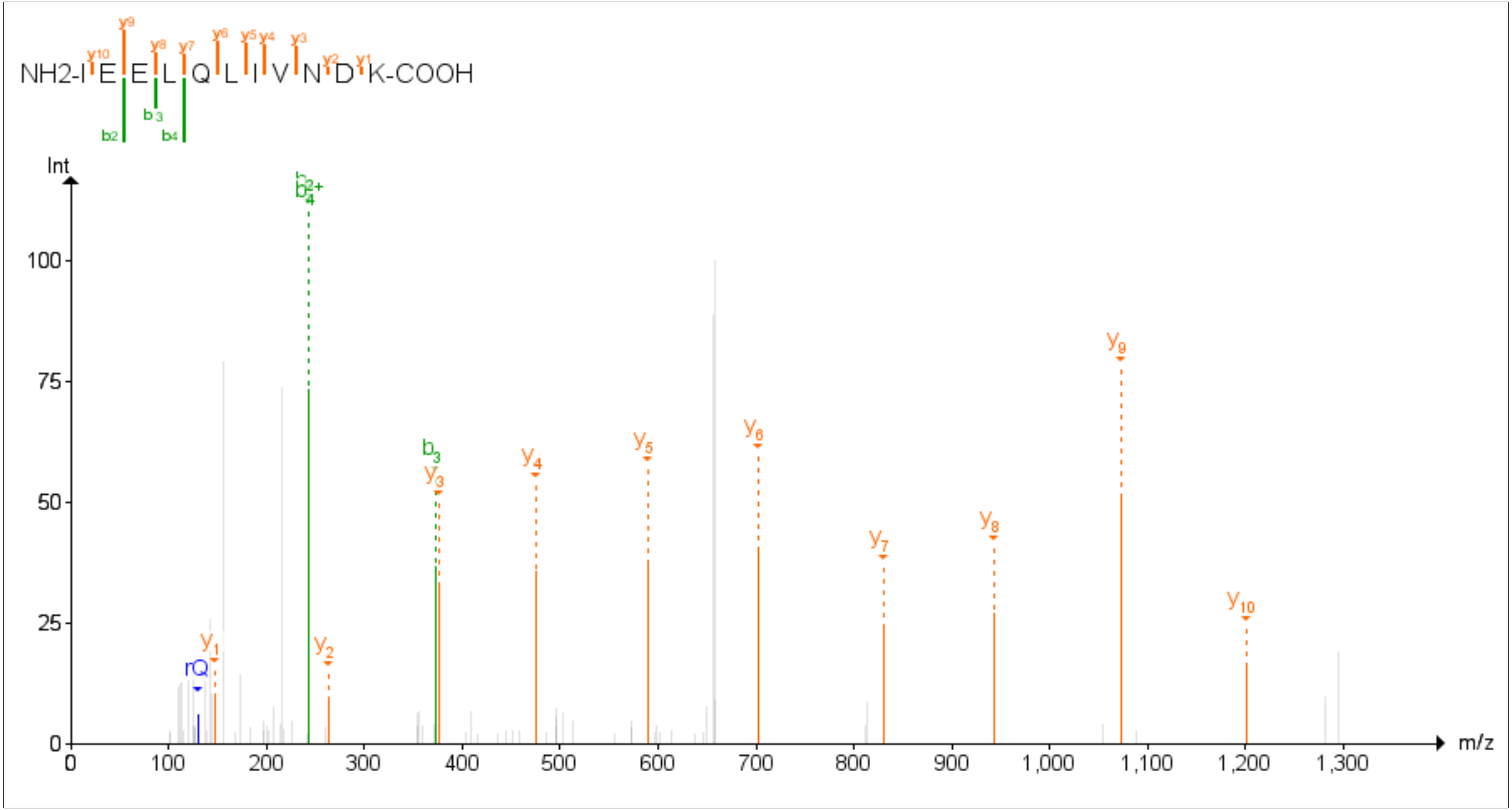

NH2-I E E L Q L I V N D K-COOH
y10 y9 y8 y7 y6 y5 y4 y3 y2 y1
b2 b3 b4
Int
m/z
b4 2+
b3
y1 y2 y3 y4 y5 y6 y7 y8 y9 y10
rQ
0
25
50
75
100
0
100
200
300
400
500
600
700
800
900
1,000
1,100
1,200
1,300

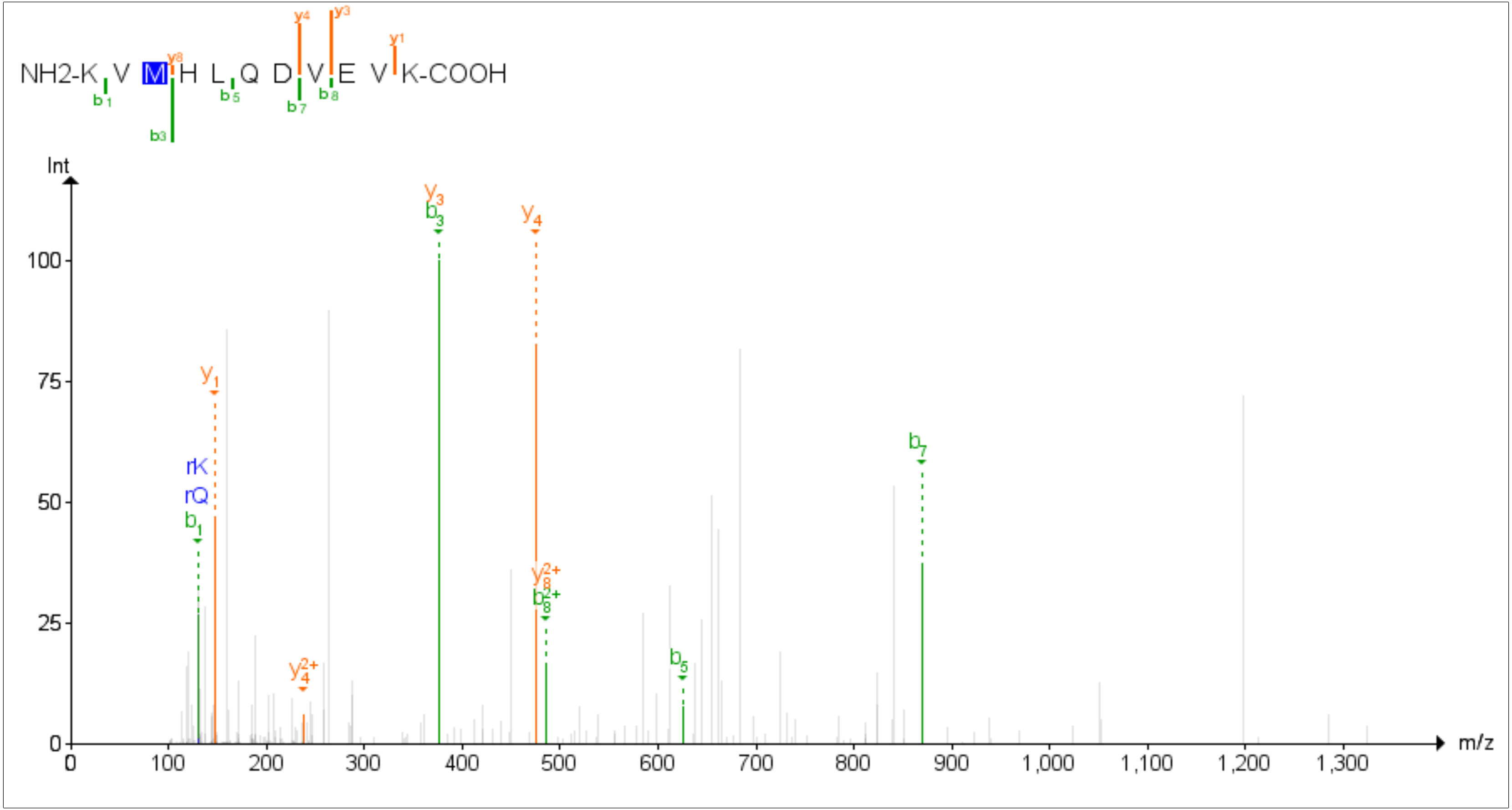

NH2-K V M H L Q D V E V K-COOH
y8
y4
y3
y1
b1
b3
b5
b7
b8
Int
y3
b3
y4
y1
rK
rQ
b1
b7
y4 2+
y8 2+
b8 2+
b5
100
75
50
25
0
0
100
200
300
400
500
600
700
800
900
1,000
1,100
1,200
1,300
m/z

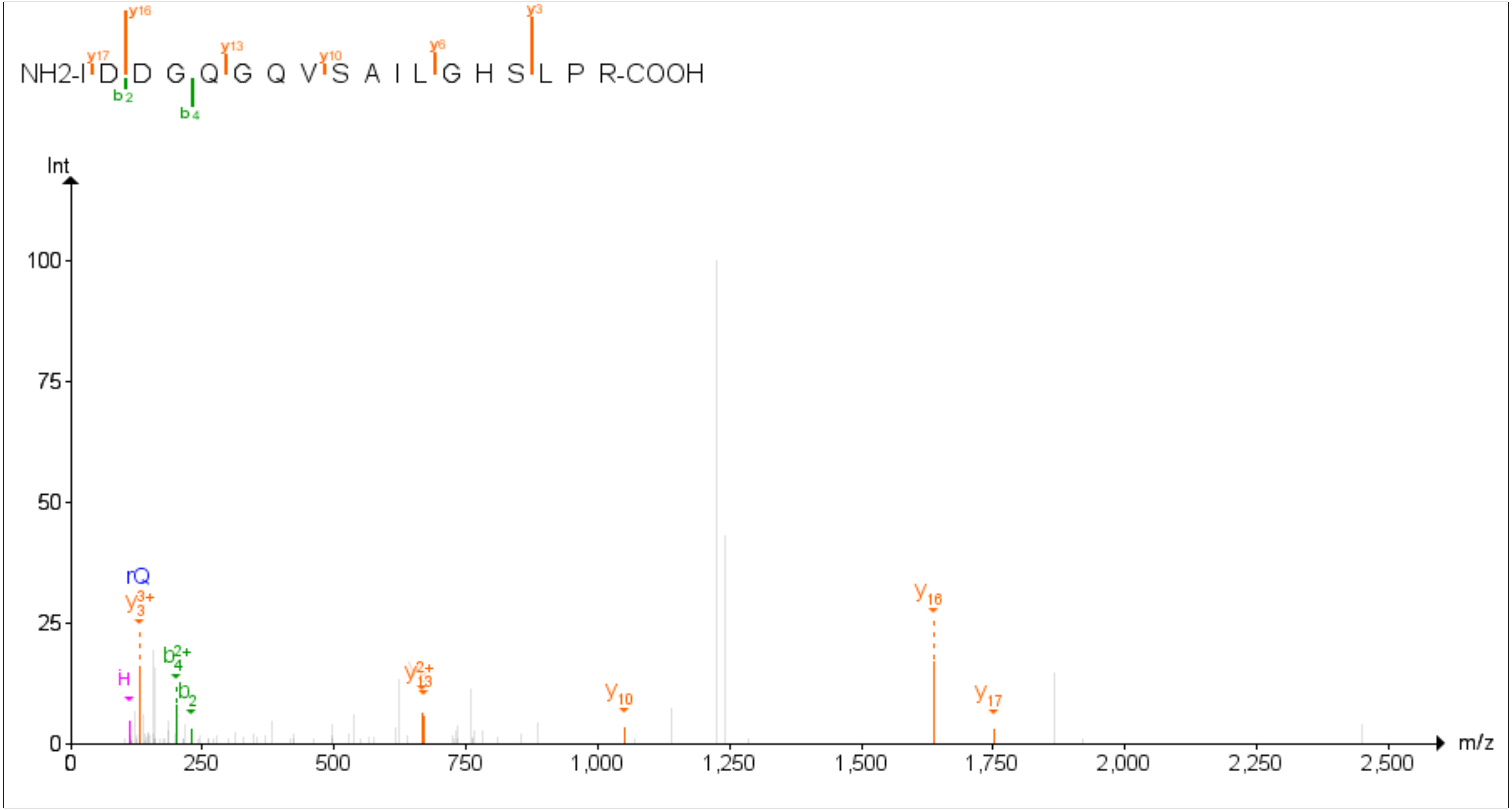

NH2-I D D G Q G Q V S A I L G H S L P R-COOH
y17 y16 y13 y10 y6 y3
b2 b4
Int
100
75
50
25
0
rQ
y3 3+
H
b4 2+
b2
y13 2+
y10
y16
y17
0 250 500 750 1,000 1,250 1,500 1,750 2,000 2,250 2,500
m/z

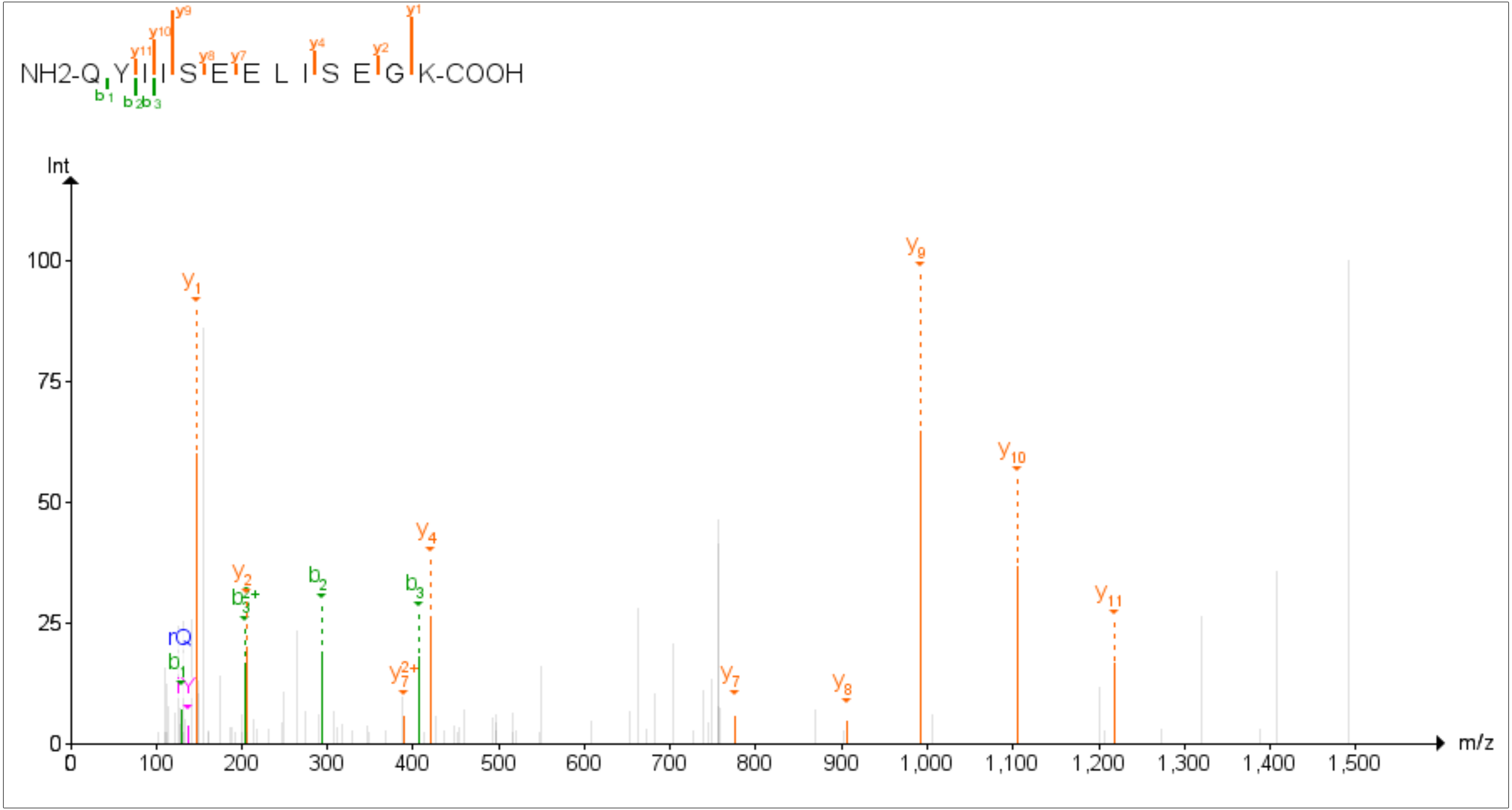

NH2-Q Y I I S E E L I S E G K-COOH
y1
y2
y4
y7
y8
y9
y10
y11
b1
b2
b3
Int
m/z
rQ
iY

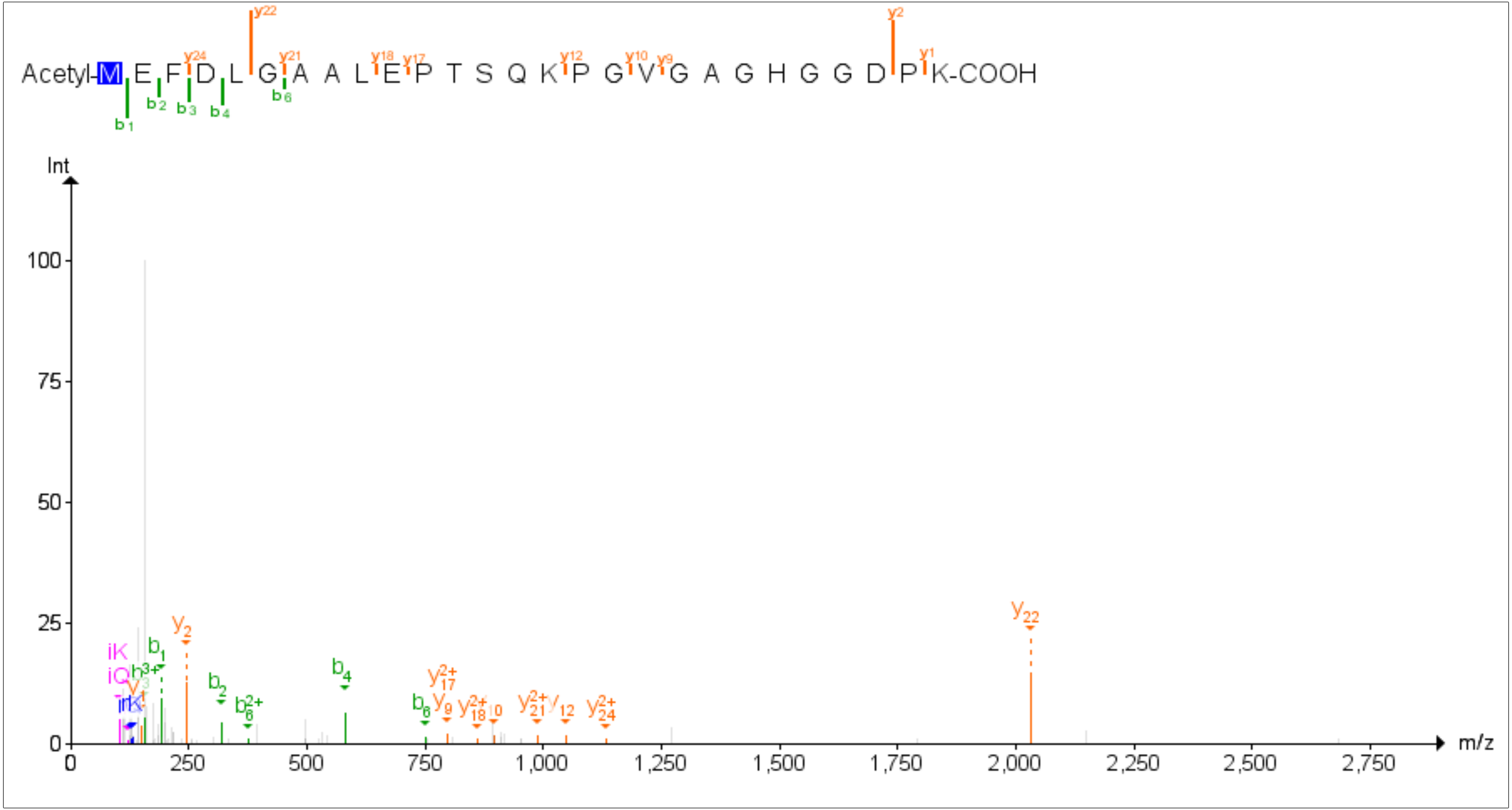
Acetyl-M E F D L G A A L E P T S Q K P G V G A G H G G D P K-COOH
Int
m/z
0
25
50
75
100
250
500
750
1,000
1,250
1,500
1,750
2,000
2,250
2,500
2,750

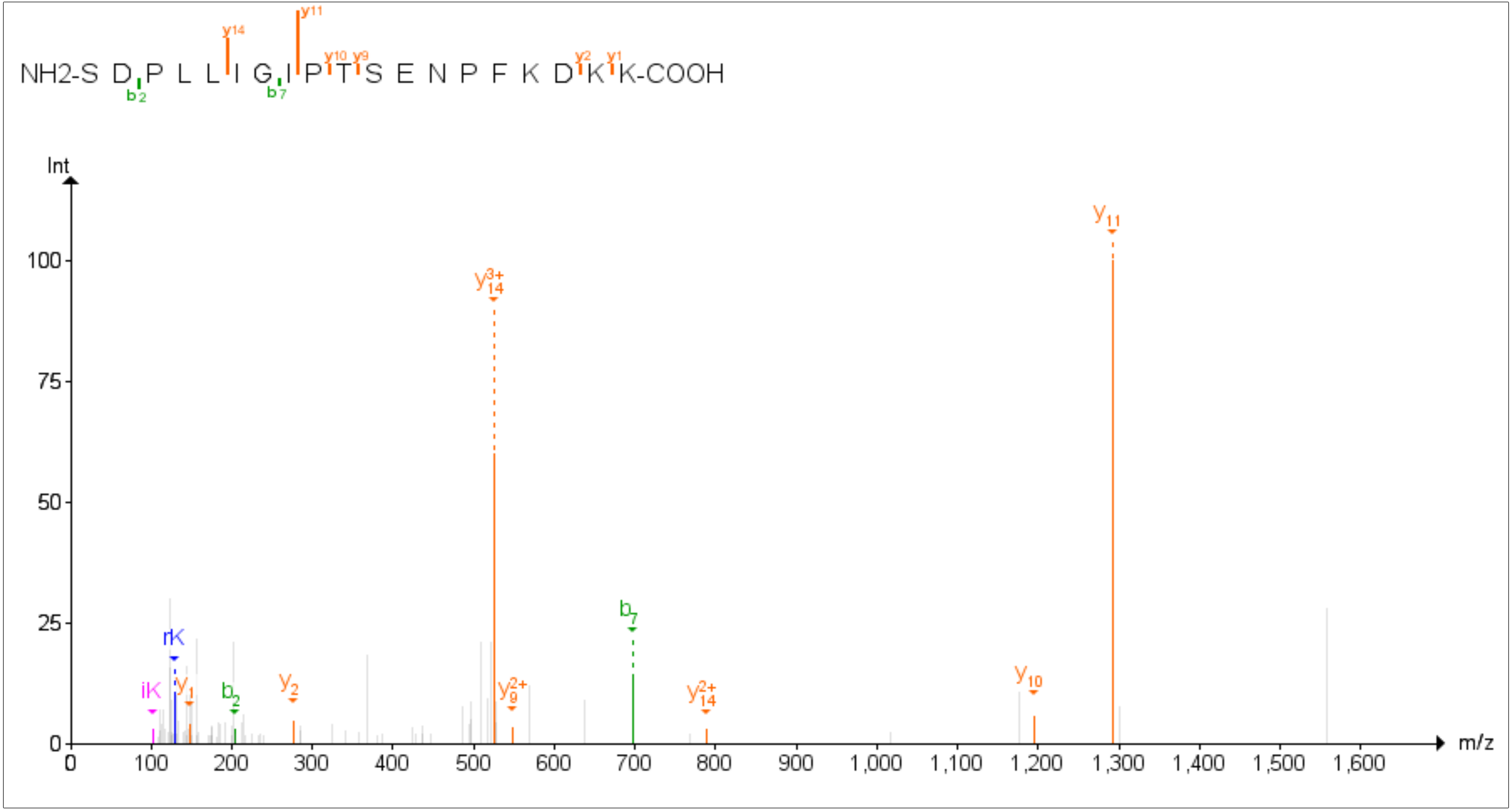

NH2-S D P L L I G I P T S E N P F K D K K-COOH
b2
b7
y14
y11
y10
y9
y2
y1
Int
100
75
50
25
0
y11
y14 3+
b7
rK
iK
y1
b2
y2
y9 2+
y14 2+
y10
m/z
0
100
200
300
400
500
600
700
800
900
1,000
1,100
1,200
1,300
1,400
1,500
1,600

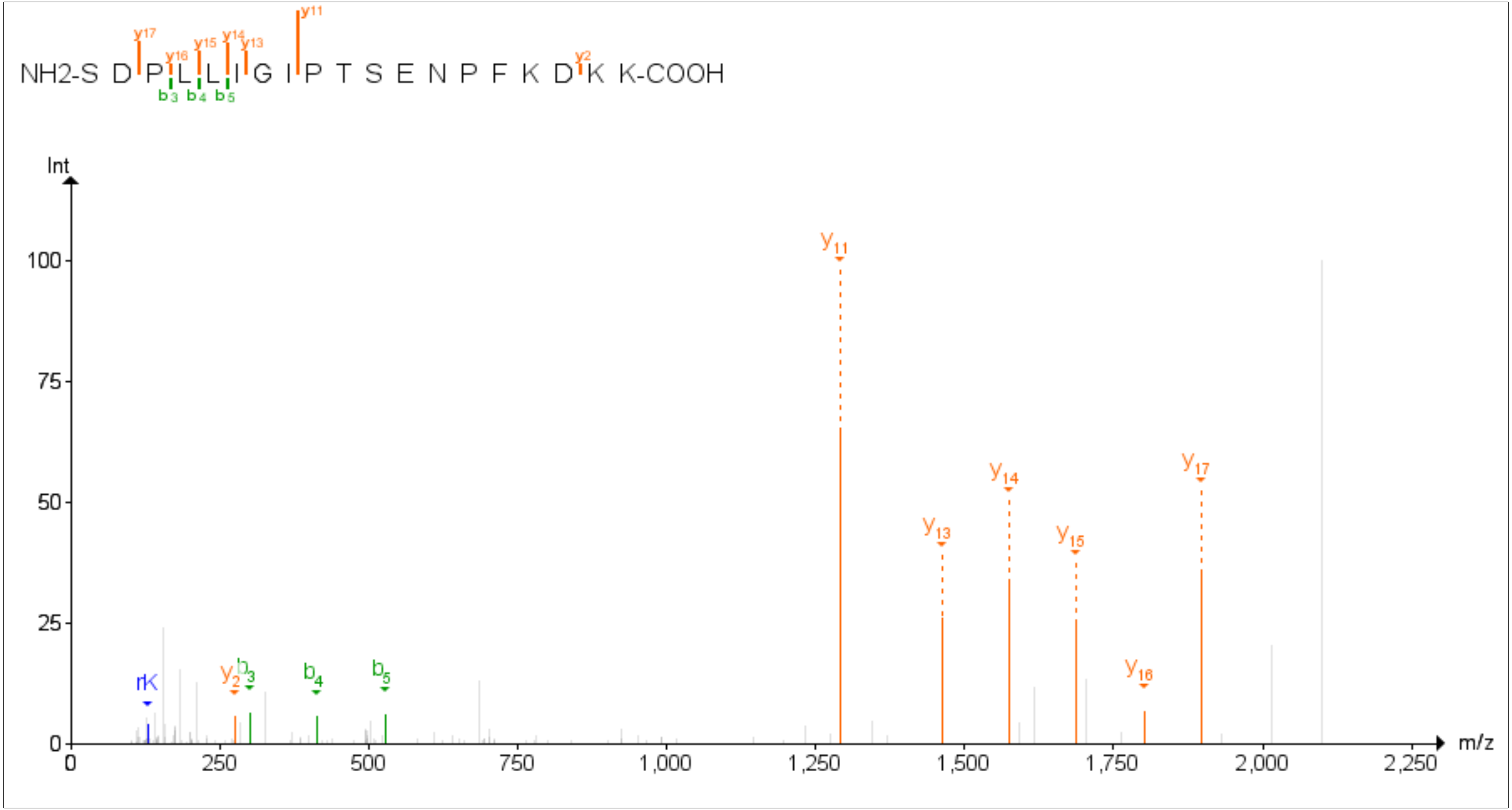
NH2-S D P L L I G I P T S E N P F K D K K-COOH
y17 y16 y15 y14 y13 y11 y2
b3 b4 b5
Int
100
75
50
25
0
y11
y13
y14
y15
y16
y17
rK
y2
b3
b4
b5
0
250
500
750
1,000
1,250
1,500
1,750
2,000
2,250
m/z

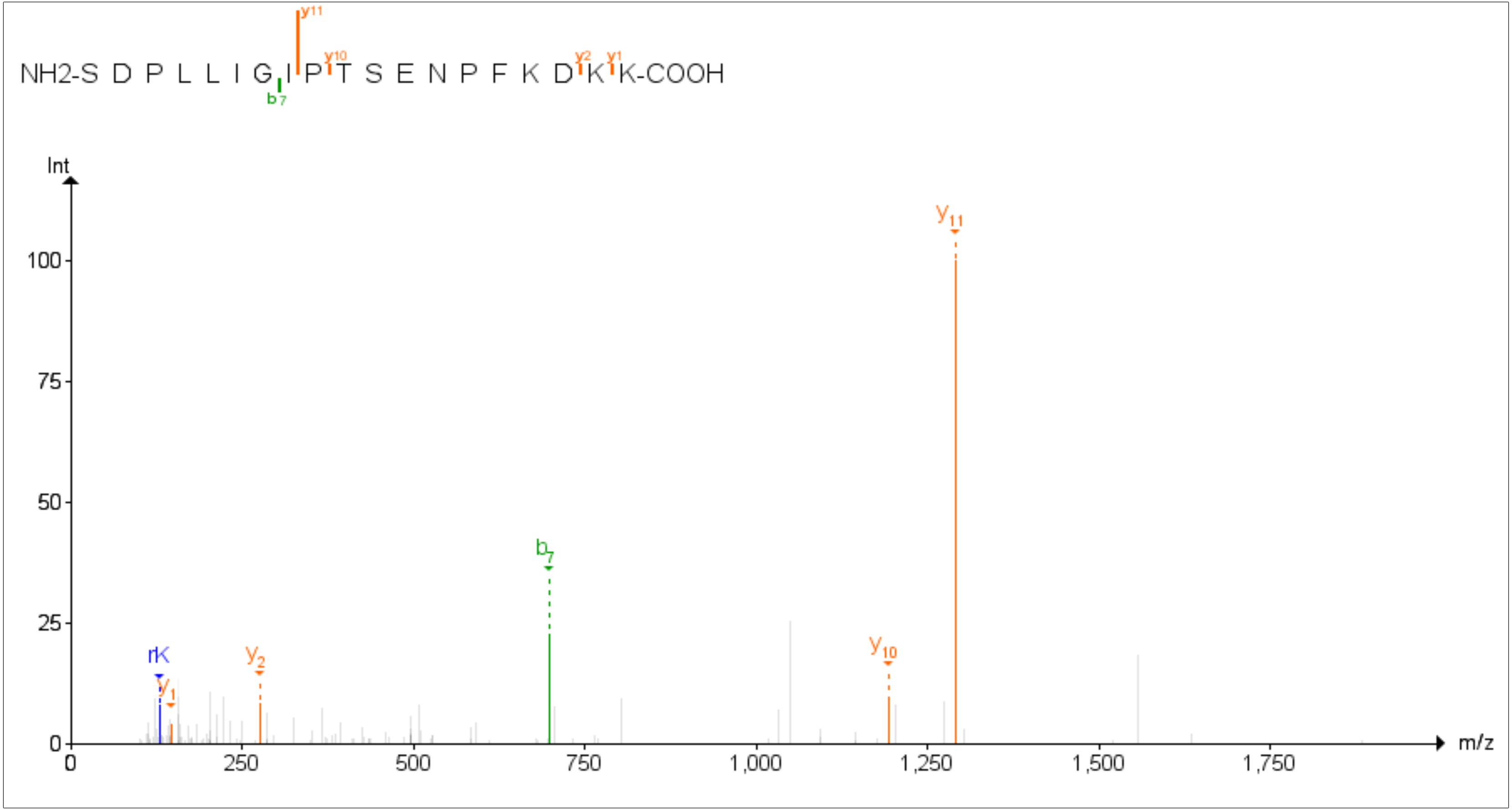

NH2-S D P L L I G I P T S E N P F K D K K-COOH
y11
y10
y2
y1
b7
Int
100
75
50
25
0
rK
y1
y2
b7
y10
y11
0
250
500
750
1,000
1,250
1,500
1,750
m/z

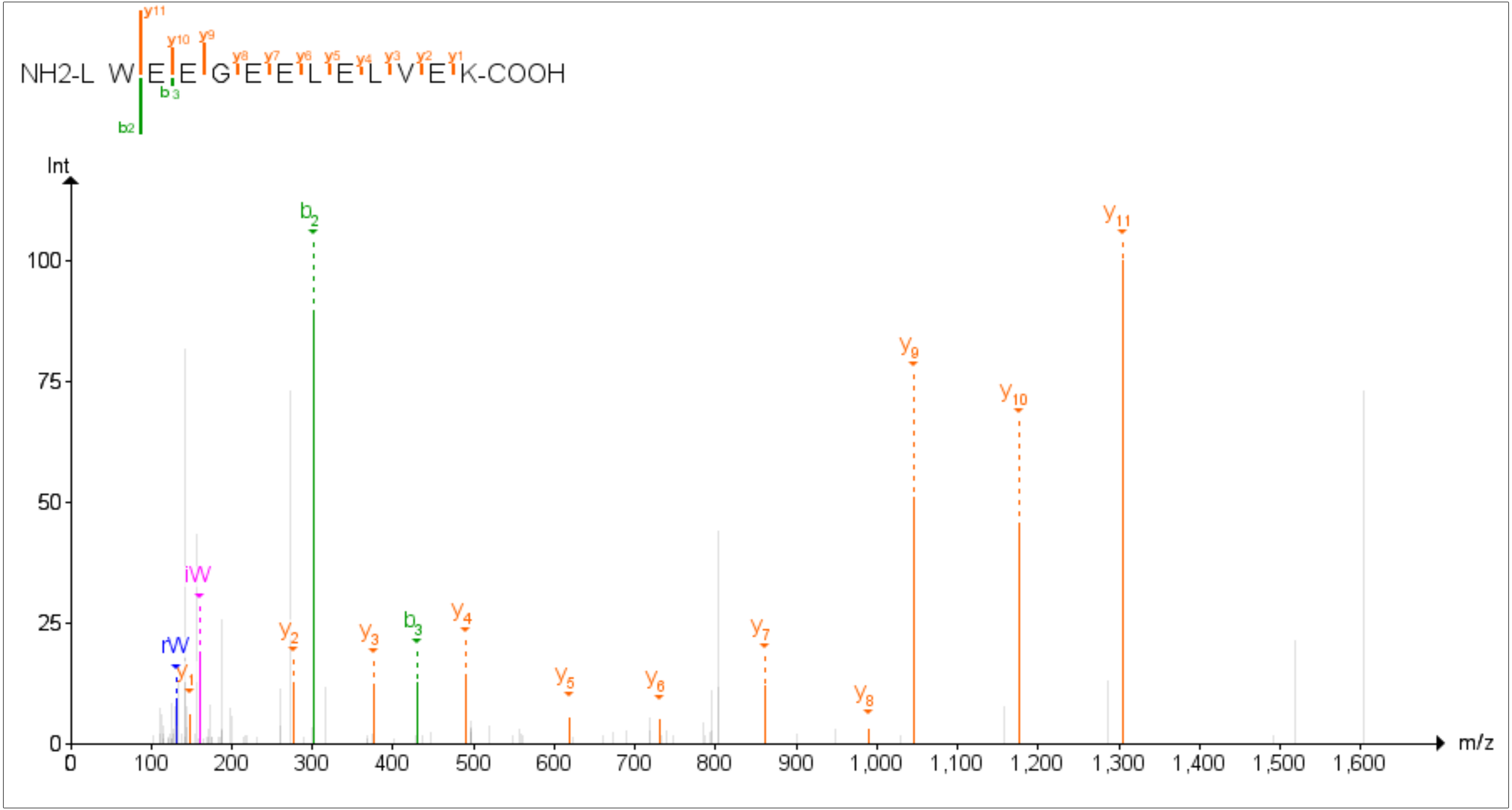

NH2-L W E E G E E L E L V E K-COOH
y11 y10 y9 y8 y7 y6 y5 y4 y3 y2 y1
b2 b3
Int
m/z
b2
y11
y9
y10
iW
rW
y1
y2
y3
b3
y4
y5
y6
y7
y8
0
25
50
75
100
0
100
200
300
400
500
600
700
800
900
1,000
1,100
1,200
1,300
1,400
1,500
1,600

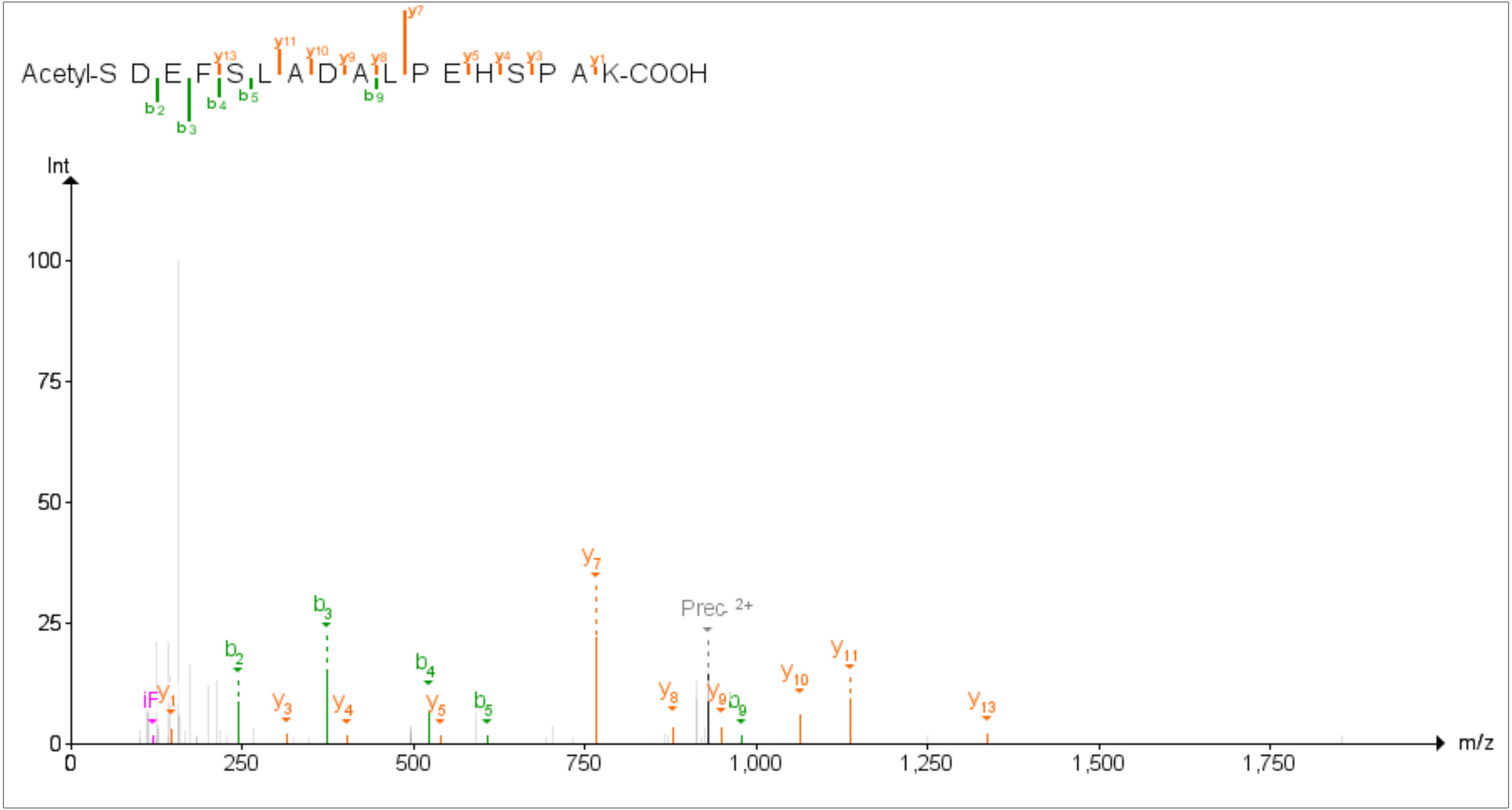

Acetyl-S D E F S L A D A L P E H S P A K-COOH
b2
b3
b4
b5
b9
y13
y11
y10
y9
y8
y7
y5
y4
y3
y1
Int
100
75
50
25
0
Prec 2+
iF
m/z
250
500
750
1,000
1,250
1,500
1,750

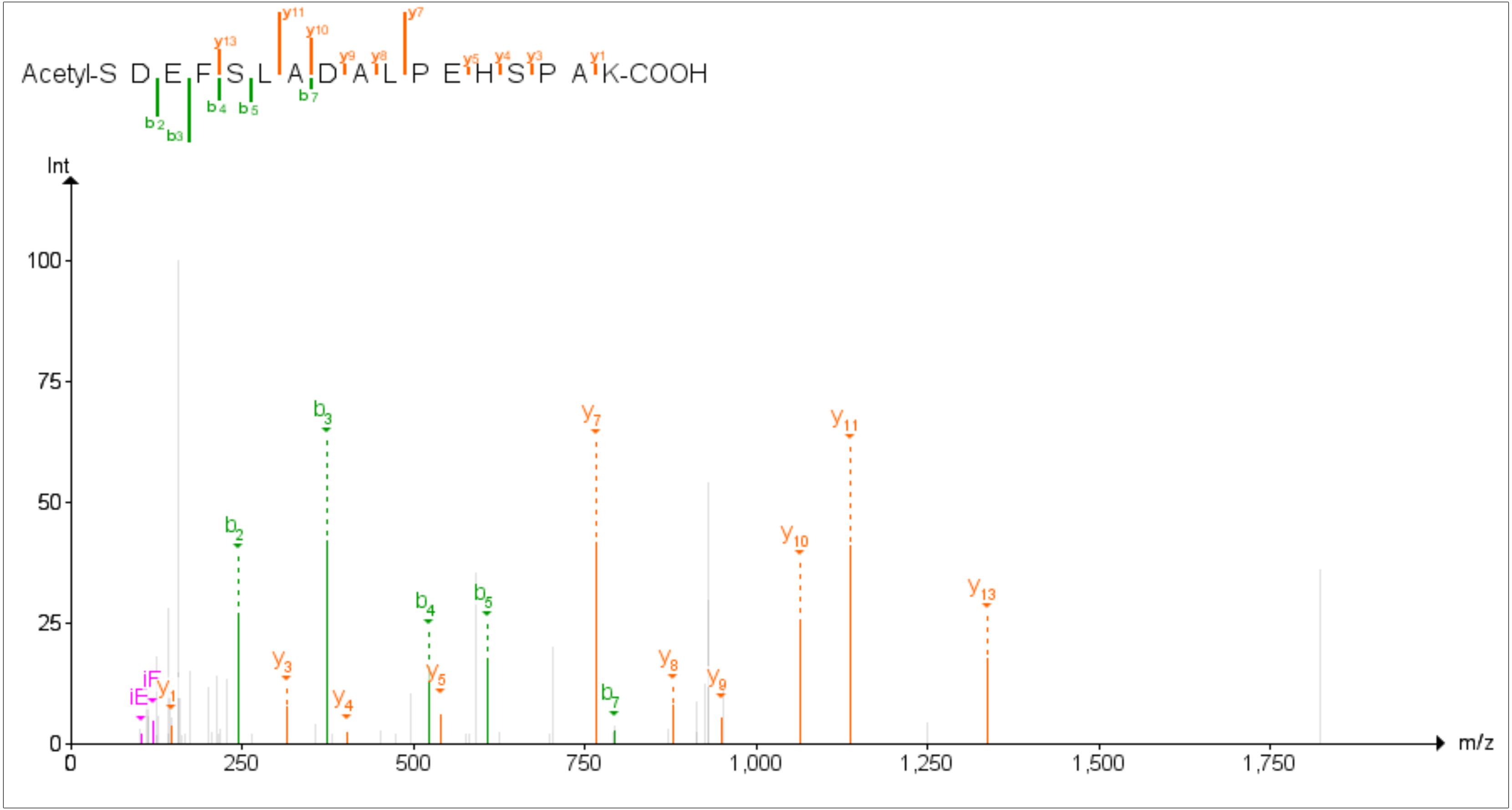

Acetyl-S D E F S L A D A L P E H S P A K-COOH
y13 y11 y10 y9 y8 y7 y5 y4 y3 y1
b2 b3 b4 b5 b7
Int
100
75
50
25
0
b2 b3 b4 b5 b7
y1 y3 y4 y5 y7 y8 y9 y10 y11 y13
iE iF
0 250 500 750 1,000 1,250 1,500 1,750
m/z

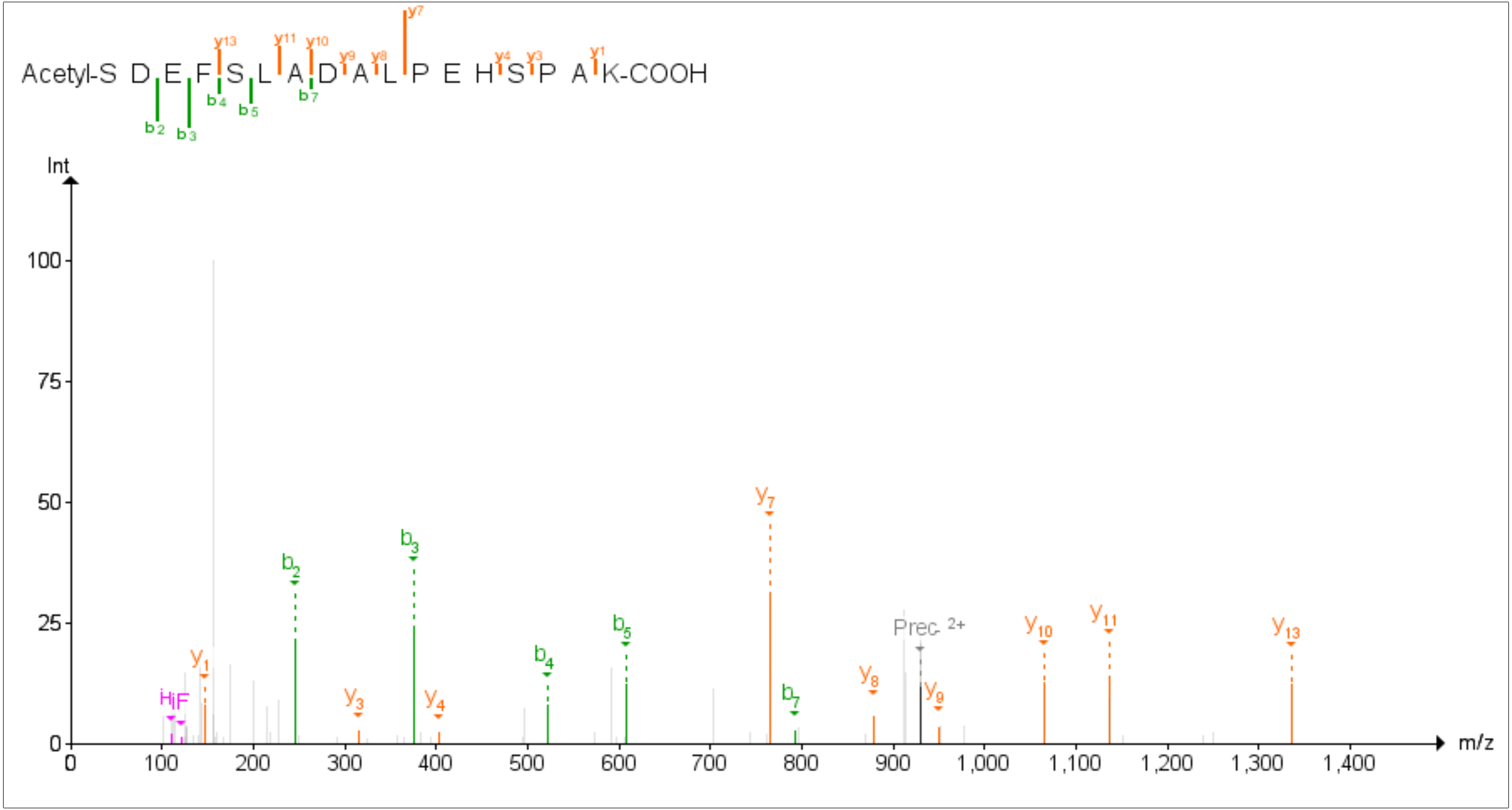

Acetyl-S D E F S L A D A L P E H S P A K-COOH
y13
y11
y10
y9
y8
y7
y4
y3
y1
b2
b3
b4
b5
b7
Int
100
75
50
25
0
m/z
0
100
200
300
400
500
600
700
800
900
1,000
1,100
1,200
1,300
1,400
Prec- 2+
HiF

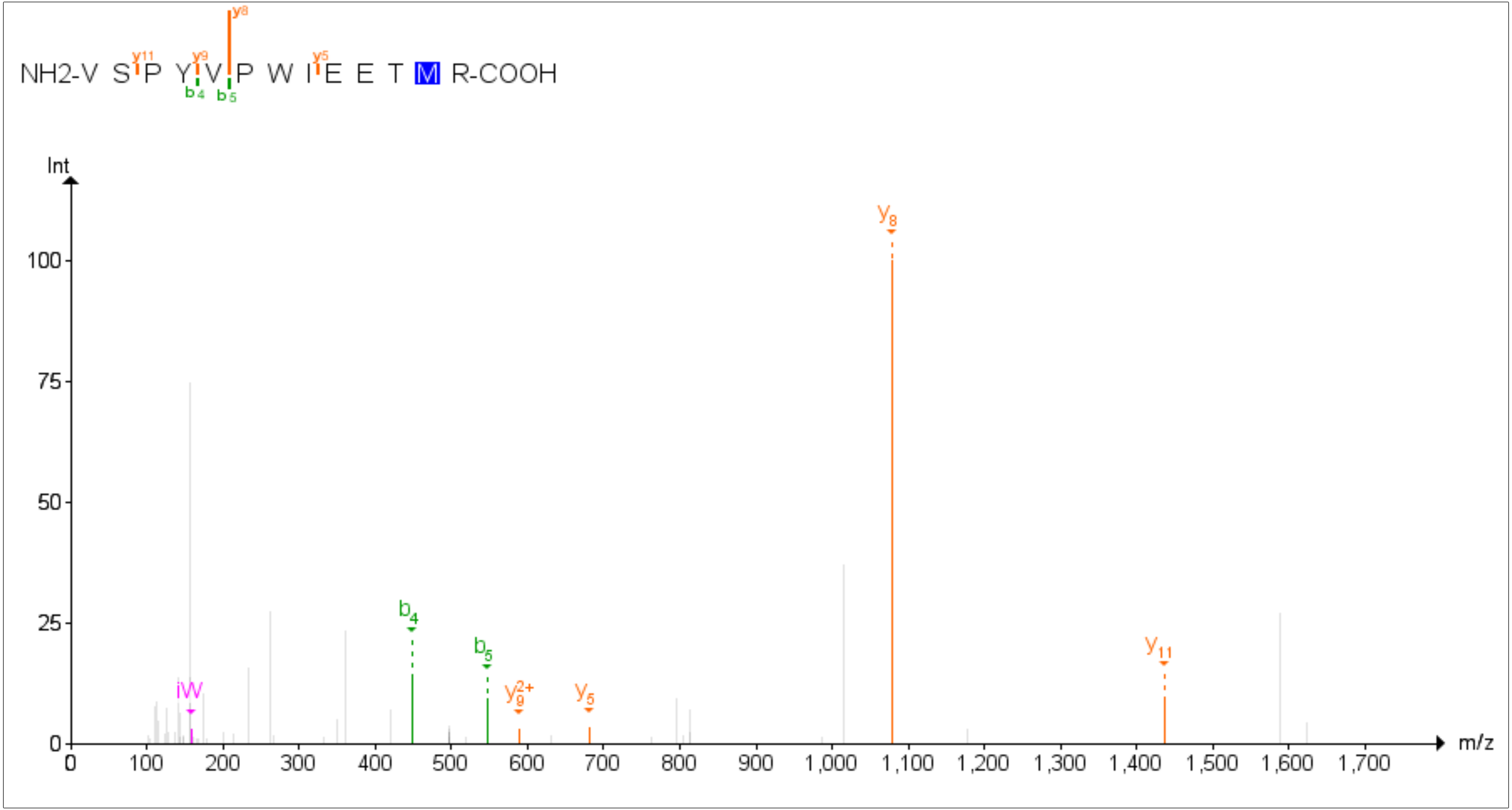

NH2-V S P Y V P W I E E T M R-COOH
y11
y9
y8
y5
b4
b5
Int
m/z
y8
y11
b4
b5
y9 2+
y5
iW
0
25
50
75
100
0
100
200
300
400
500
600
700
800
900
1,000
1,100
1,200
1,300
1,400
1,500
1,600
1,700

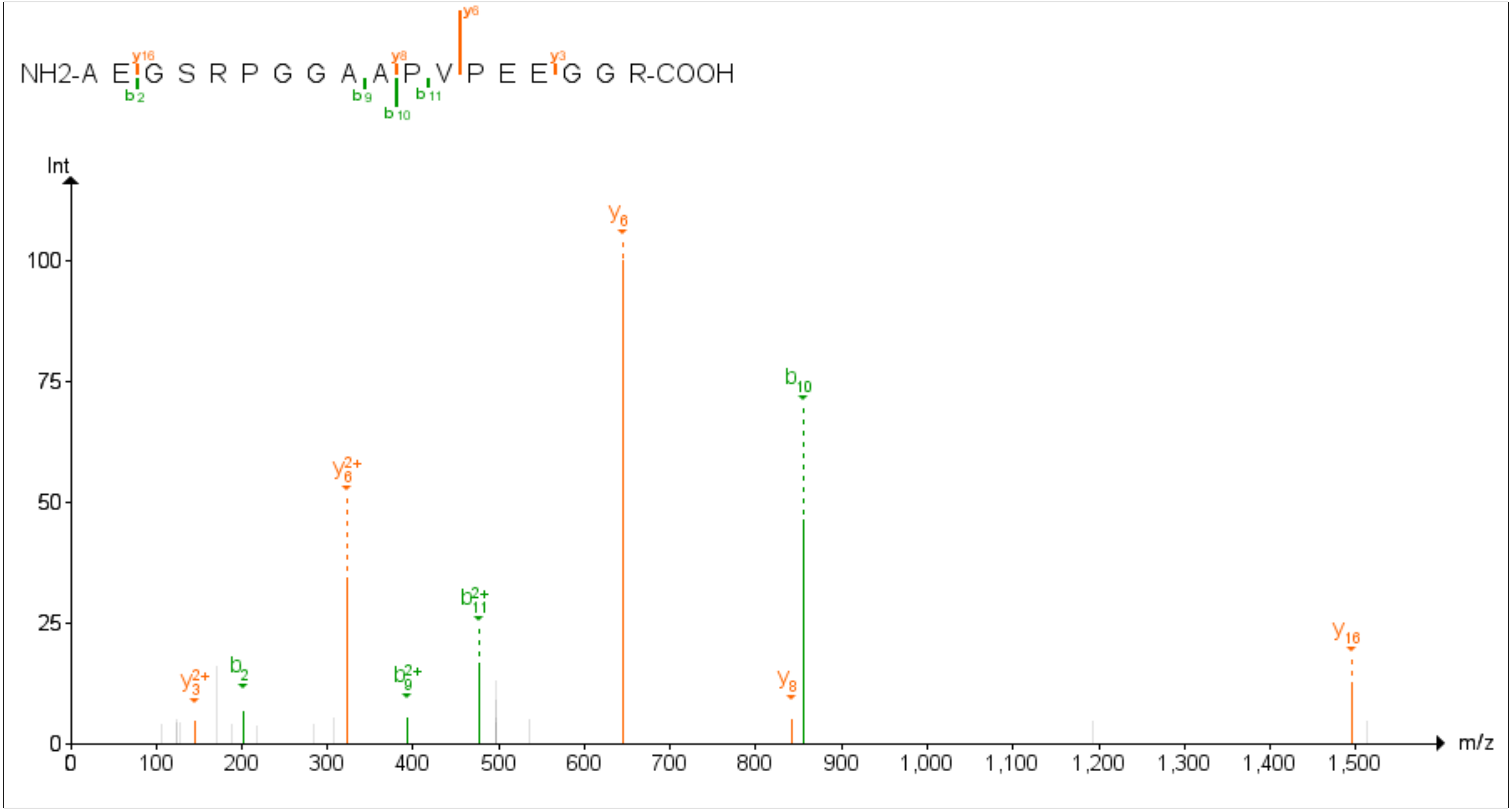
NH2-A E G S R P G G A A P V P E E G G R-COOH
y16
y8
y6
y3
b2
b9
b10
b11
Int
100
75
50
25
0
y6
b10
y6 2+
b11 2+
y3 2+
b2
b9 2+
y8
y16
m/z
0
100
200
300
400
500
600
700
800
900
1,000
1,100
1,200
1,300
1,400
1,500

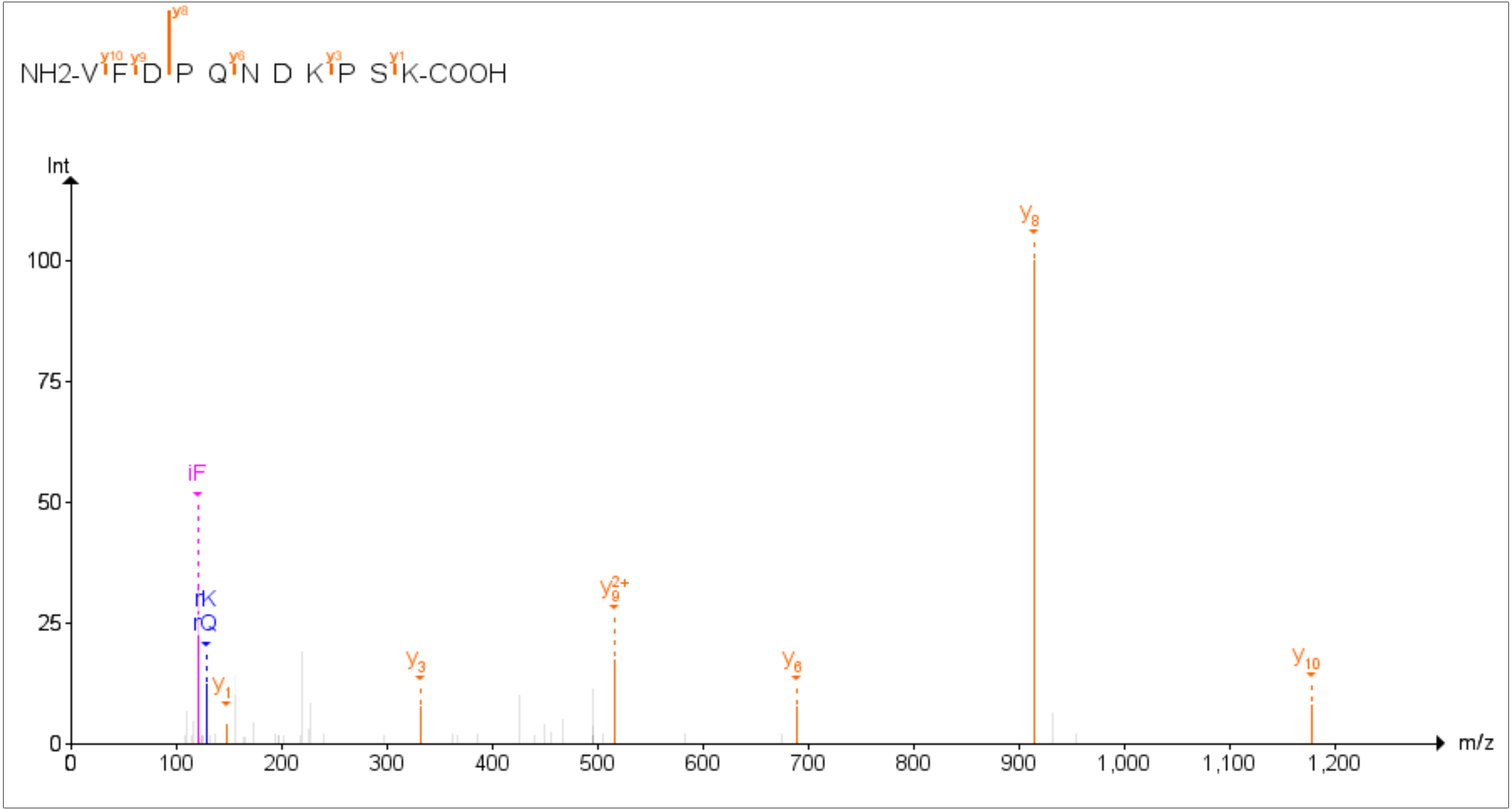

NH2-V F D P Q N D K P S K-COOH
y10 y9 y8 y6 y3 y1
Int
100
75
50
25
0
iF
rK
rQ
y1
y3
y9 2+
y6
y8
y10
0 100 200 300 400 500 600 700 800 900 1,000 1,100 1,200
m/z

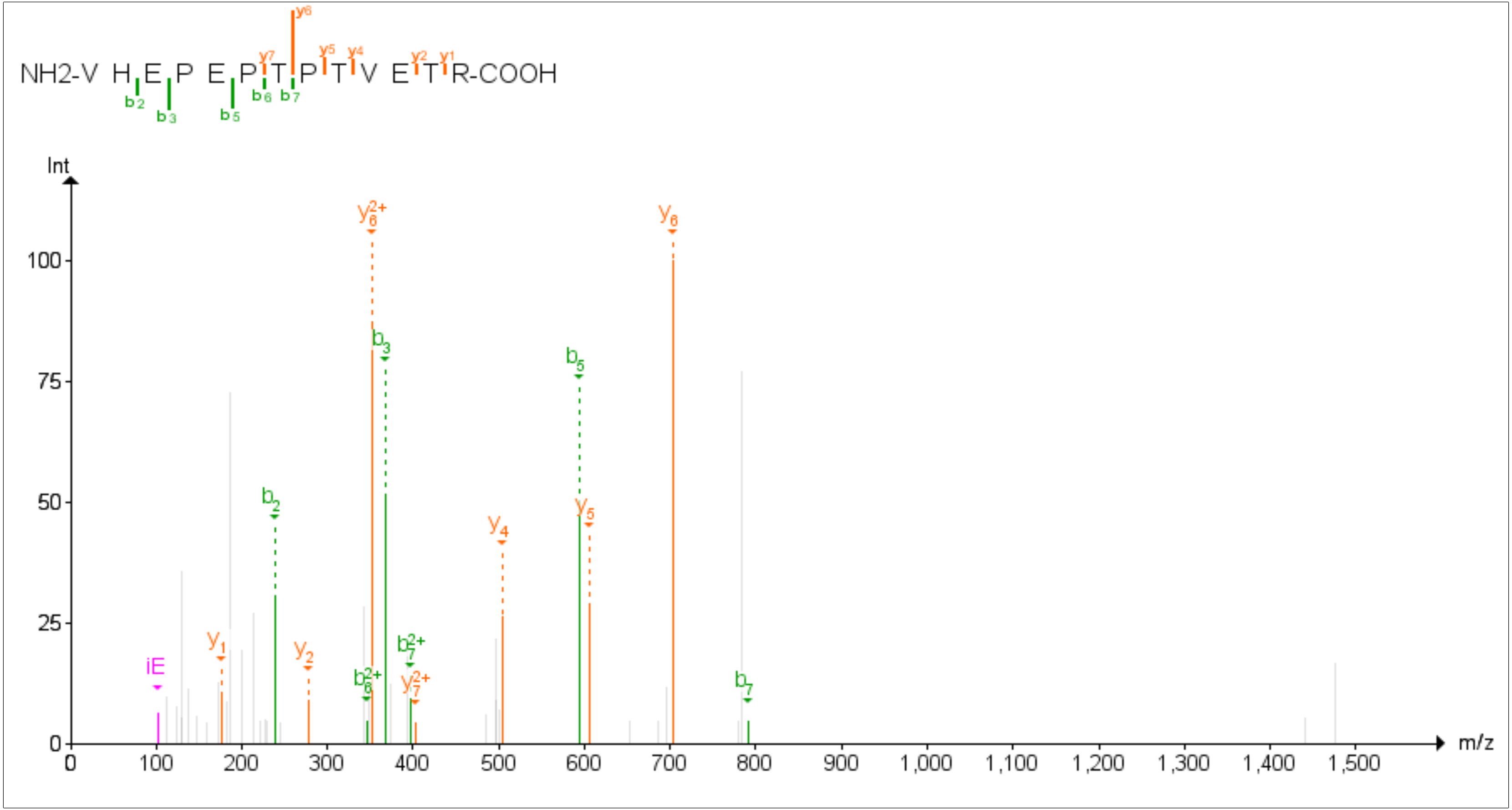

NH2-V H E P E P T P T V E T R-COOH
Int
m/z
0
25
50
75
100
100
200
300
400
500
600
700
800
900
1,000
1,100
1,200
1,300
1,400
1,500
iE

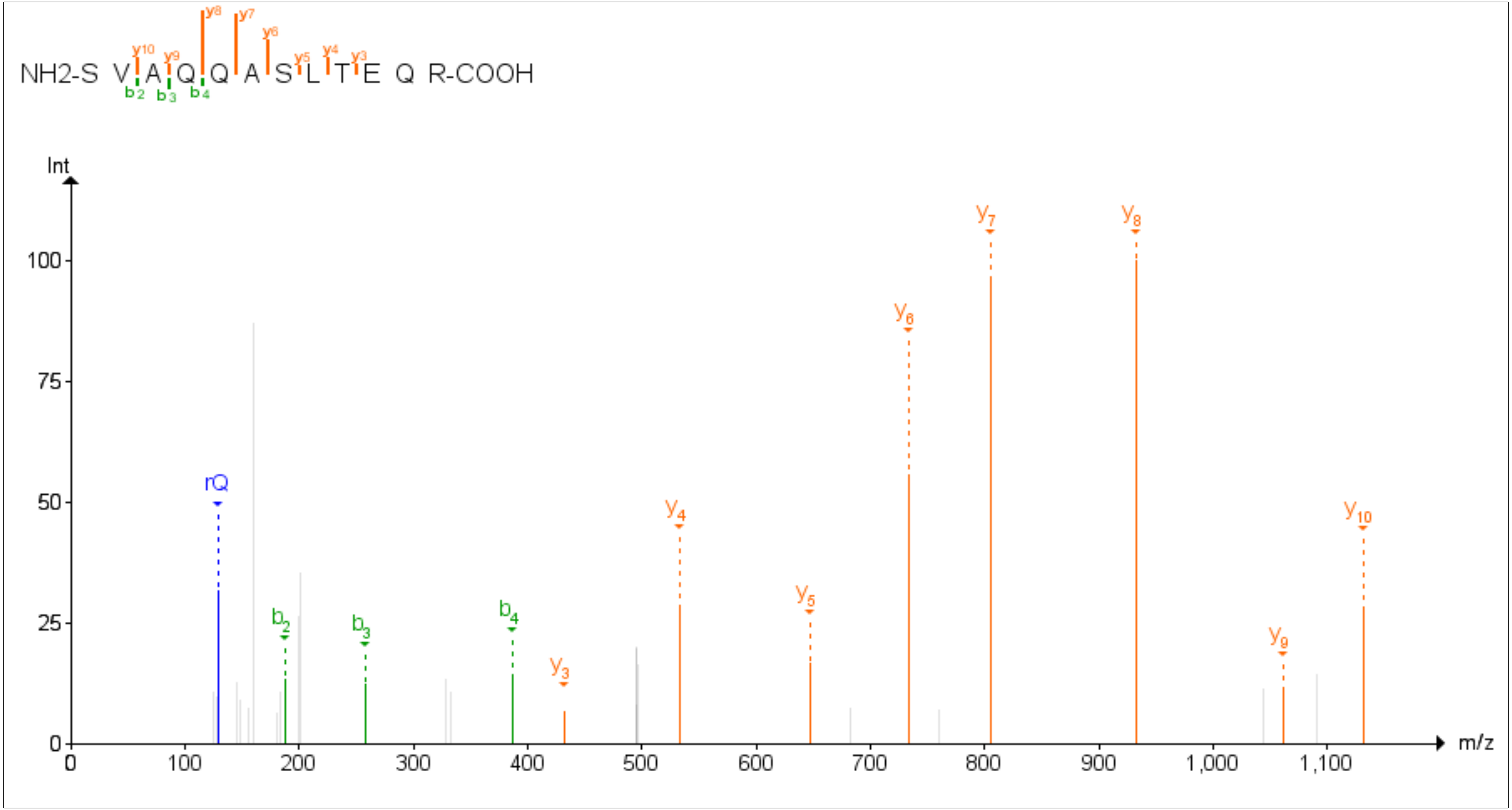
NH2-S V A Q Q A S L T E Q R-COOH
y10 y9 y8 y7 y6 y5 y4 y3
b2 b3 b4
Int
100
75
50
25
0
rQ
b2
b3
b4
y3
y4
y5
y6
y7
y8
y9
y10
0 100 200 300 400 500 600 700 800 900 1,000 1,100
m/z

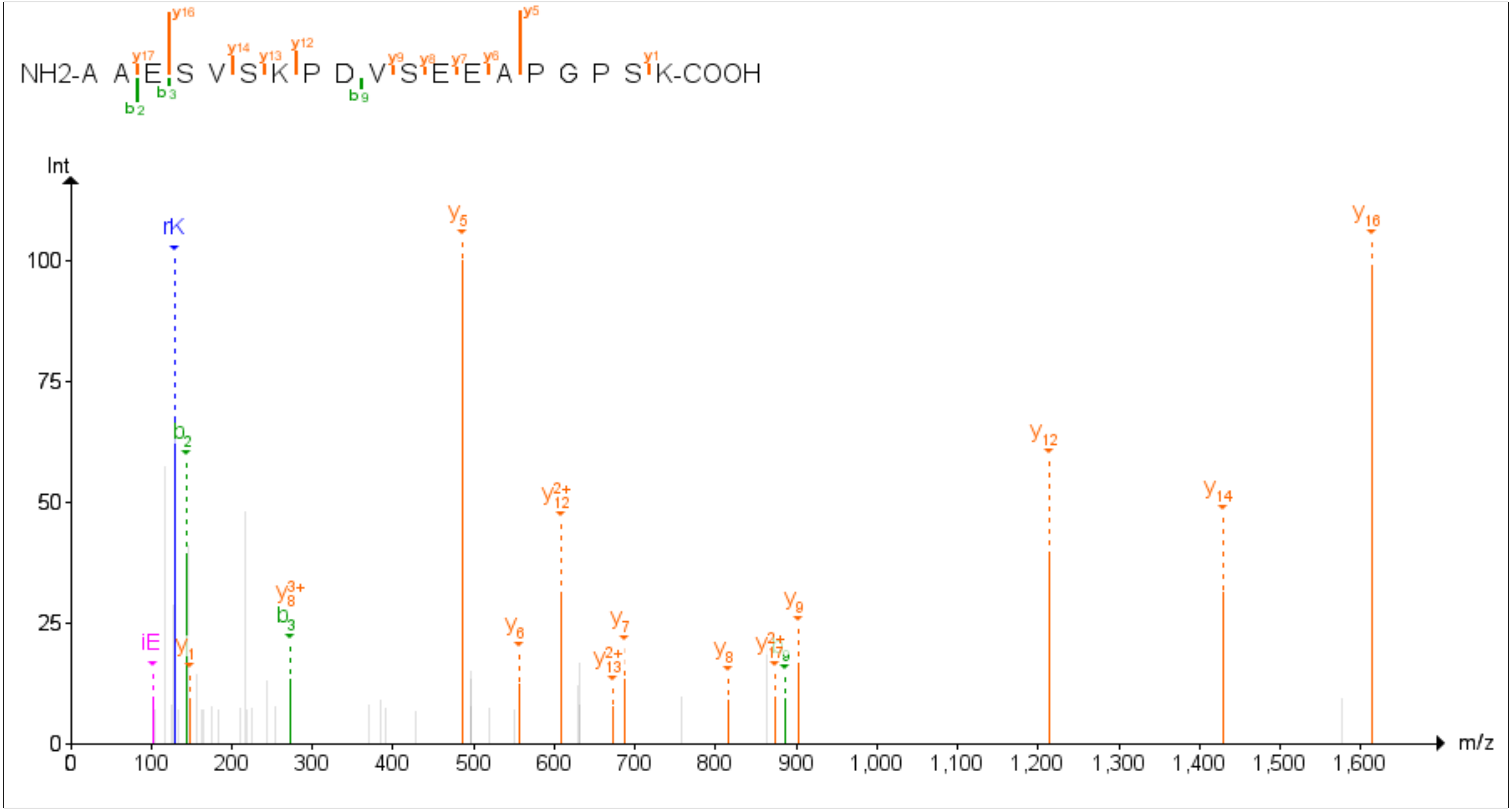

NH2-A A E S V S K P D V S E E A P G P S K-COOH
y16 y17 y14 y13 y12 y9 y8 y7 y6 y5 y1
b2 b3 b9
Int
m/z
rK
y5
y16
b2
y12
y14
y12 2+
y8 3+
b3
iE
y1
y6
y7
y13 2+
y8
y17 2+
y9
0 25 50 75 100
0 100 200 300 400 500 600 700 800 900 1,000 1,100 1,200 1,300 1,400 1,500 1,600

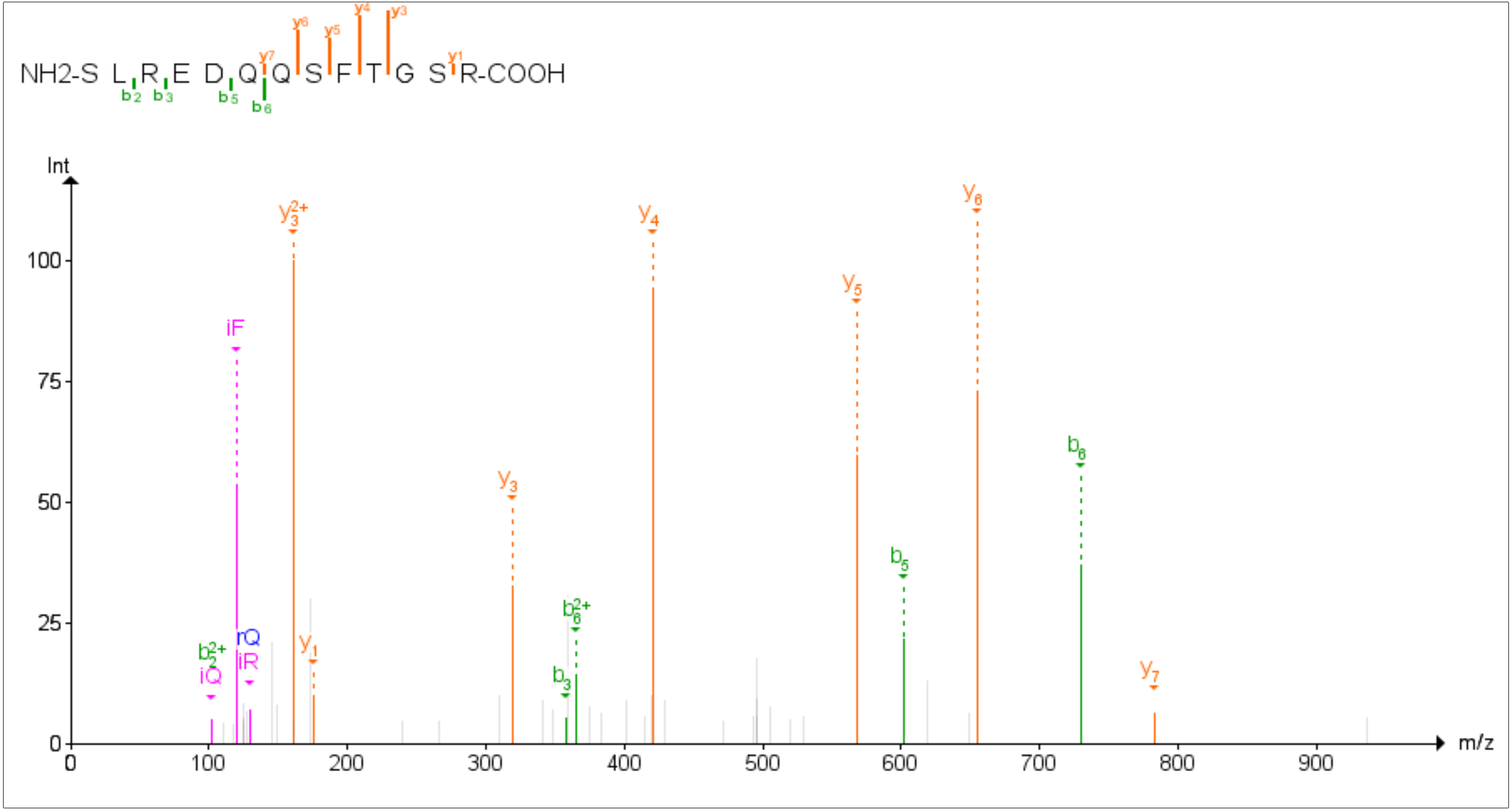

NH2-S L R E D Q Q S F T G S R-COOH
Int
m/z
iF
iQ
iR
rQ
100
75
50
25
0
0
100
200
300
400
500
600
700
800
900

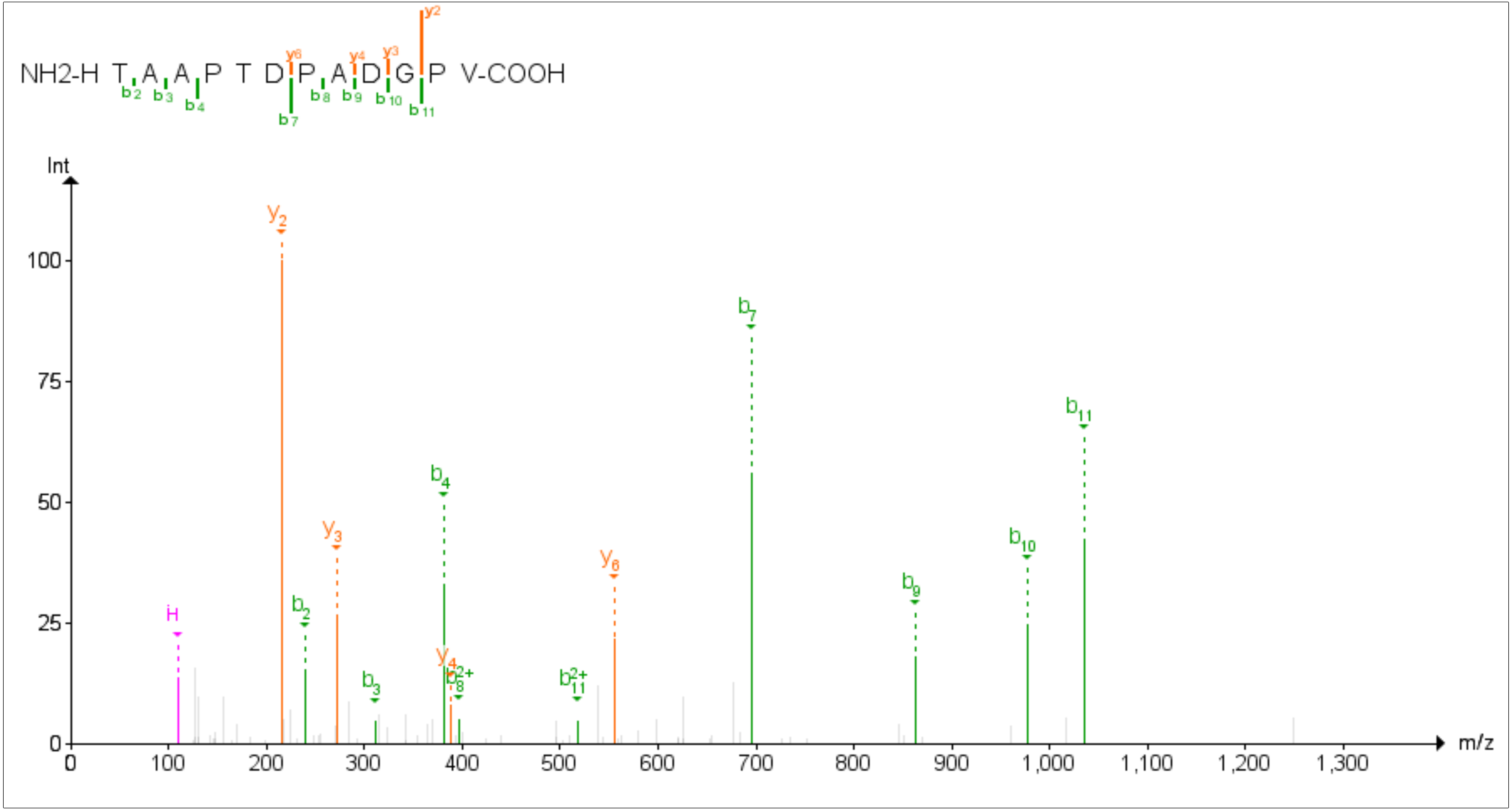

NH2-H T A A P T D P A D G P V-COOH
b2 b3 b4 b7 b8 b9 b10 b11
y6 y4 y3 y2
Int
100
75
50
25
0
y2
b7
b11
b4
y3
y6
b10
b9
b2
H
b3
y4
b8 2+
b11 2+
0 100 200 300 400 500 600 700 800 900 1,000 1,100 1,200 1,300
m/z

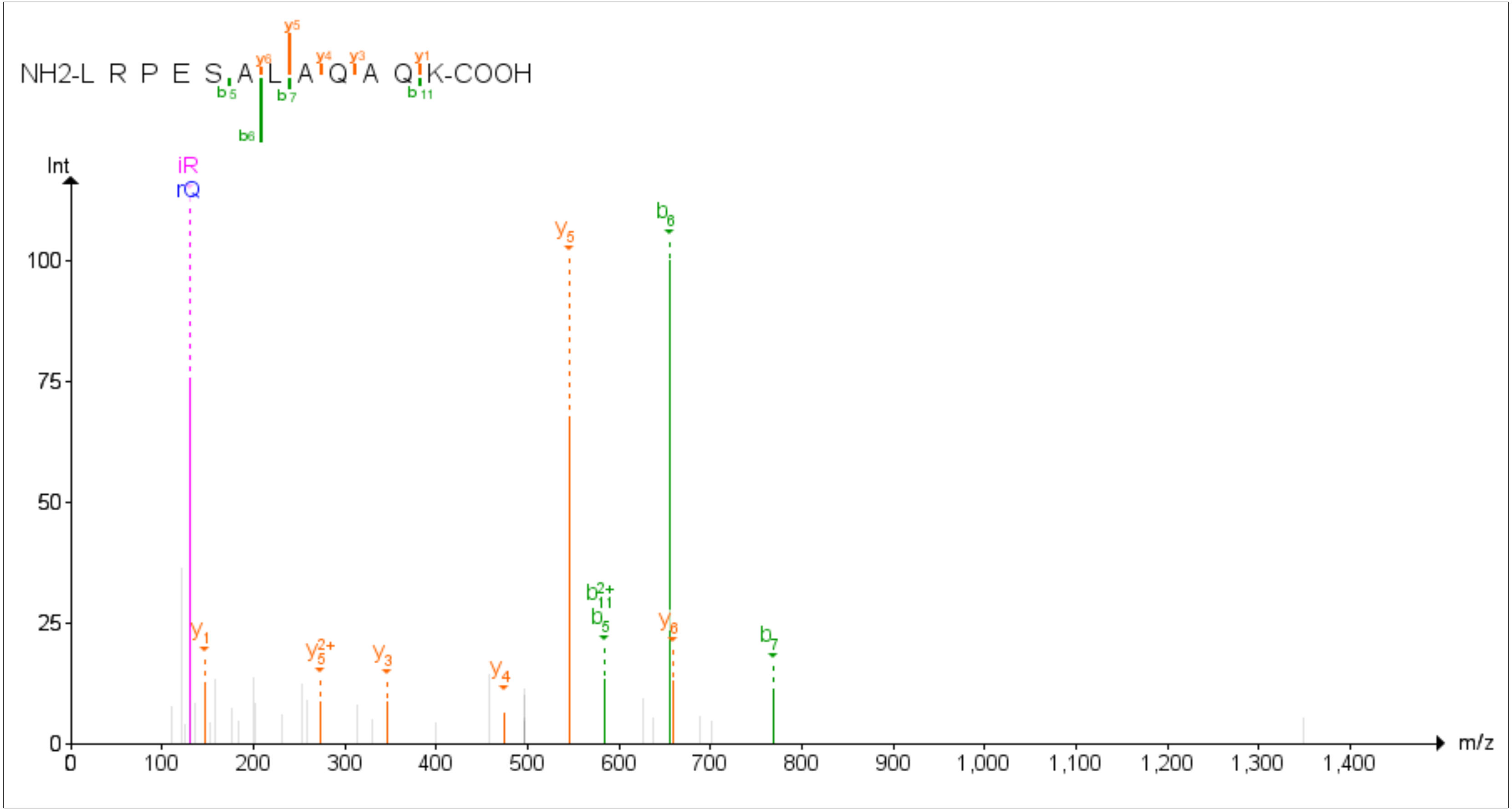
NH2-L R P E S A L A Q A Q K-COOH
b5
b6
b7
b11
y6
y5
y4
y3
y1
Int
iR
rQ
100
75
50
25
0
y1
y5 2+
y3
y4
y5
b11 2+
b5
b6
y6
b7
0
100
200
300
400
500
600
700
800
900
1,000
1,100
1,200
1,300
1,400
m/z

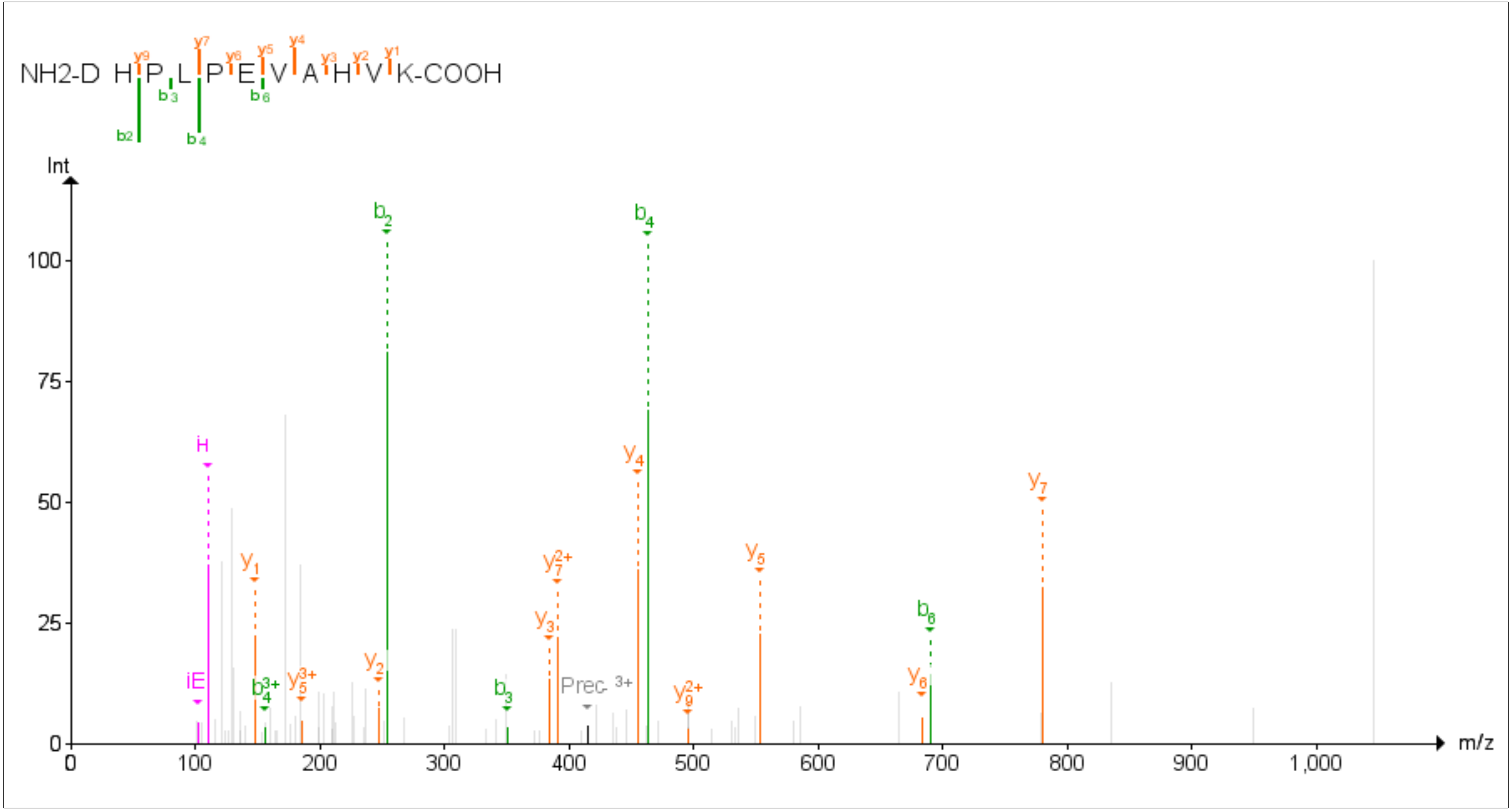

NH2-D H P L P E V A H V K-COOH
y9 y7 y6 y5 y4 y3 y2 y1
b2 b3 b4 b6
Int
m/z
b2
b4
iH
y1
y4
y7
y7 2+
y3
y5
b6
iE
b4 3+
y5 3+
y2
b3
Prec 3+
y9 2+
y6
0
25
50
75
100
0
100
200
300
400
500
600
700
800
900
1,000

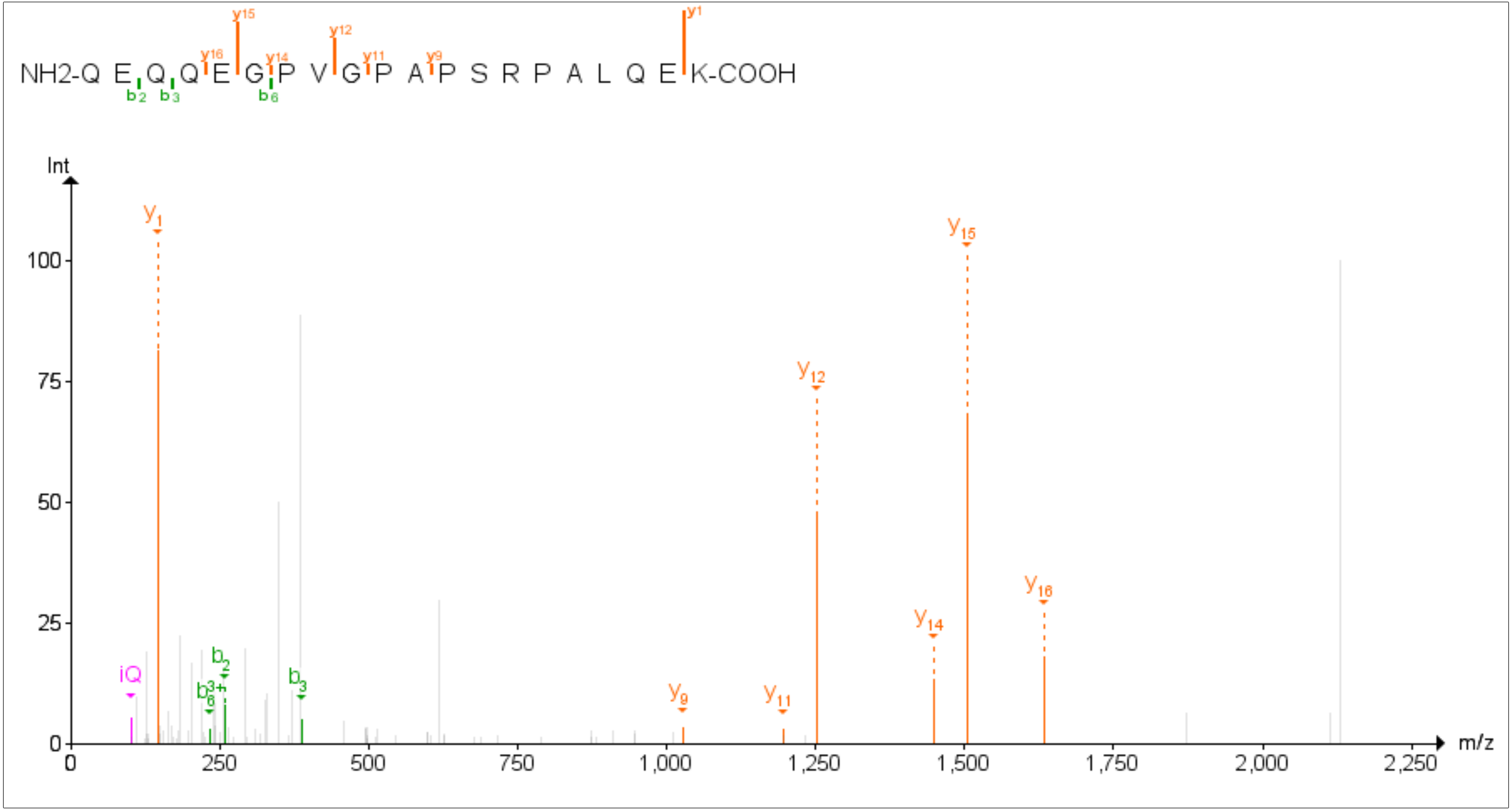

NH2-Q E Q Q E G P V G P A P S R P A L Q E K-COOH
y15
y16
y14
y12
y11
y9
y1
b2
b3
b6
Int
100
75
50
25
0
y1
y15
y12
y16
y14
y9
y11
iQ
b2
b6 3+
b3
0
250
500
750
1,000
1,250
1,500
1,750
2,000
2,250
m/z

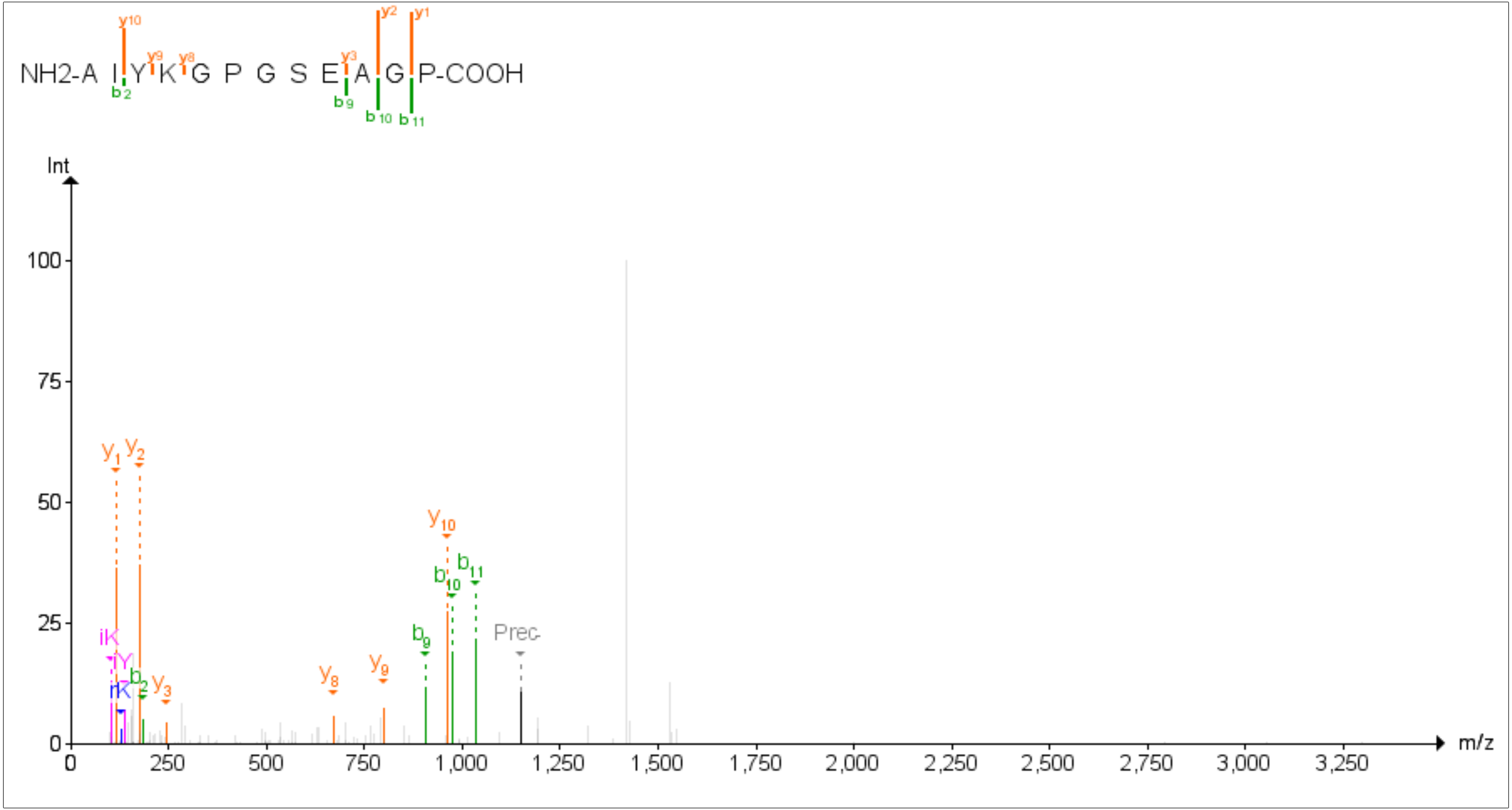

NH2-A I Y K G P G S E A G P-COOH
y10 y9 y8 y3 y2 y1
b2 b9 b10 b11
Int
100
75
50
25
0
y1 y2 y10 b10 b11 iK iY b2 y3 rK y8 y9 b9 Prec
0 250 500 750 1,000 1,250 1,500 1,750 2,000 2,250 2,500 2,750 3,000 3,250
m/z

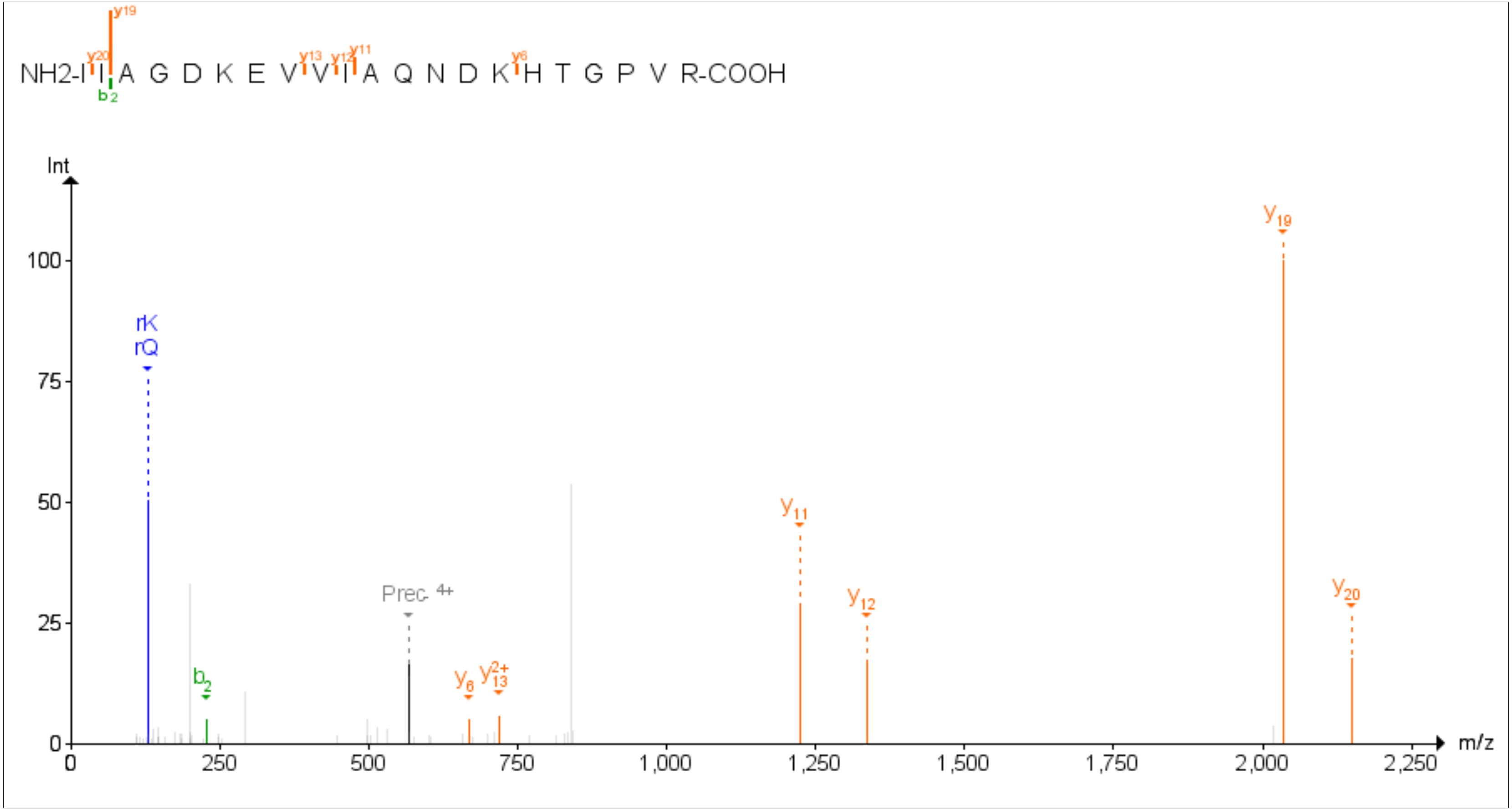

NH2-I I A G D K E V V I A Q N D K H T G P V R-COOH
y19
y20
y13
y12
y11
y6
b2
Int
100
75
50
25
0
rK
rQ
b2
Prec 4+
y6
y13 2+
y11
y12
y19
y20
0
250
500
750
1,000
1,250
1,500
1,750
2,000
2,250
m/z

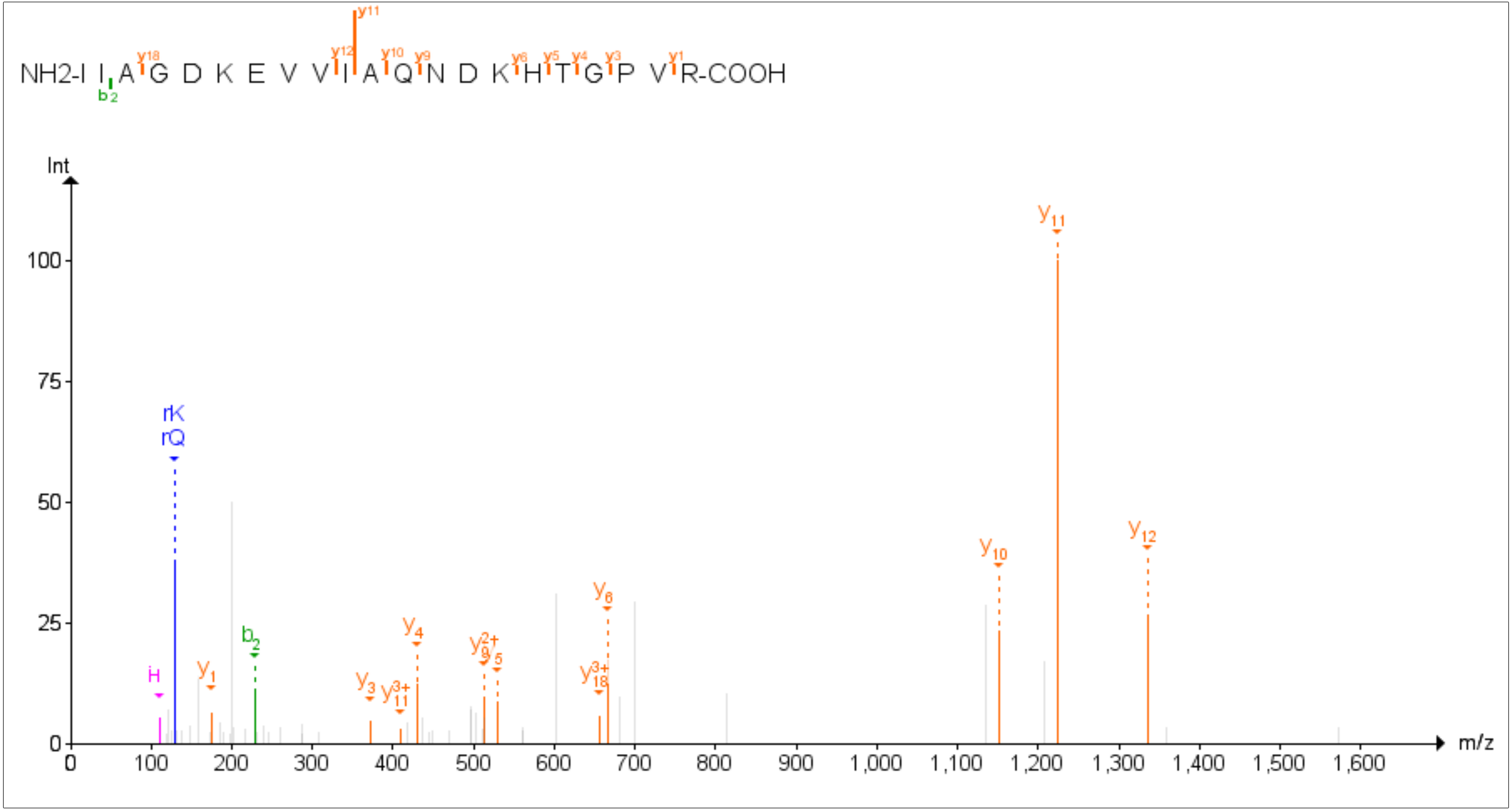

NH2-I I A G D K E V V I A Q N D K H T G P V R-COOH
b2
y18 y12 y11 y10 y9 y6 y5 y4 y3 y1
Int
m/z
0
25
50
75
100
0
100
200
300
400
500
600
700
800
900
1,000
1,100
1,200
1,300
1,400
1,500
1,600
rK
rQ
iH
y1
b2
y3
y11 3+
y4
y9 2+
y5
y18 3+
y6
y10
y11
y12

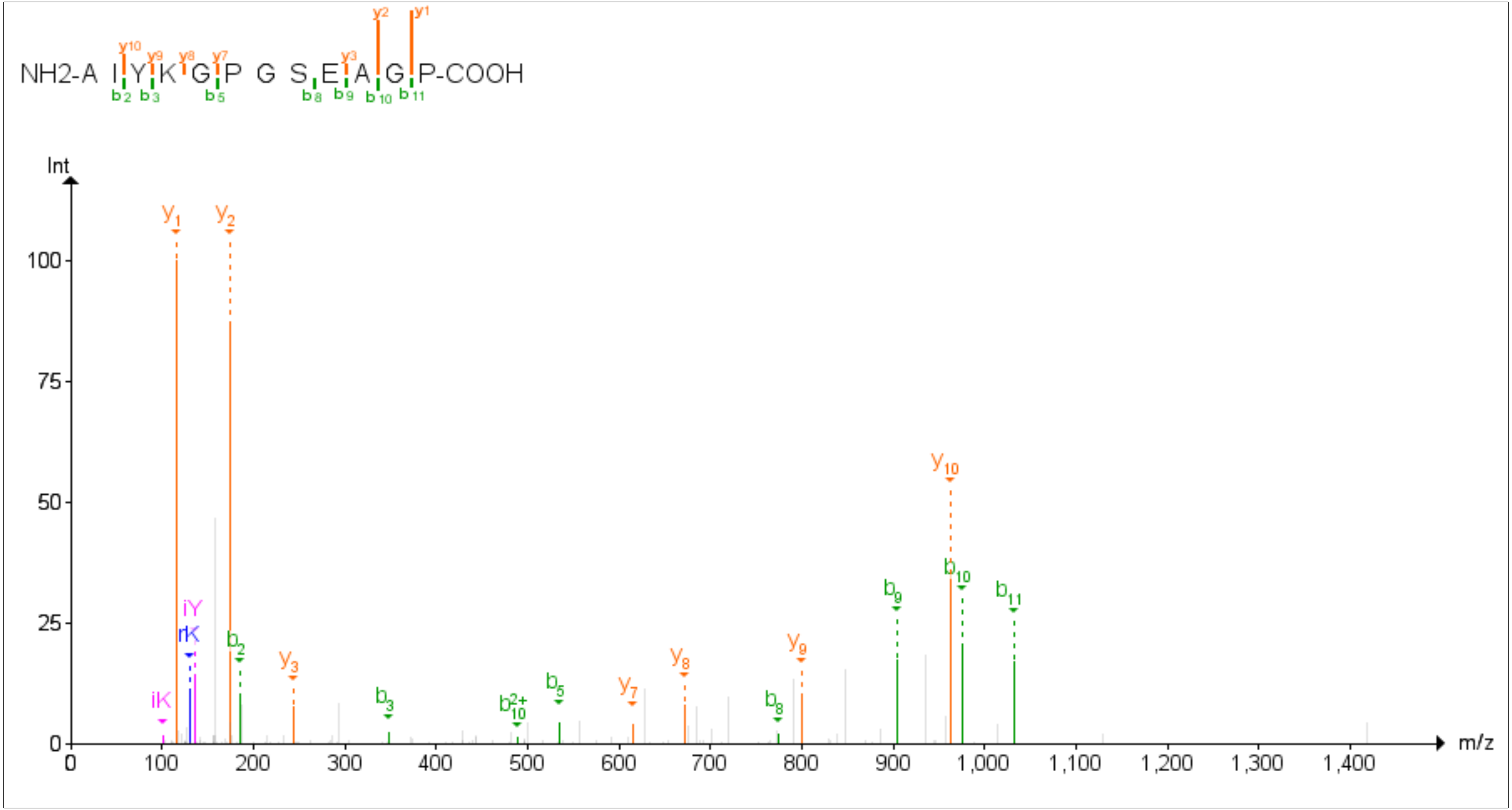

NH2-A I Y K G P G S E A G P-COOH
Int
m/z
y1
y2
y3
y7
y8
y9
y10
b2
b3
b5
b8
b9
b10
b11
b10 2+
iK
iY
rK
0
25
50
75
100
0
100
200
300
400
500
600
700
800
900
1,000
1,100
1,200
1,300
1,400

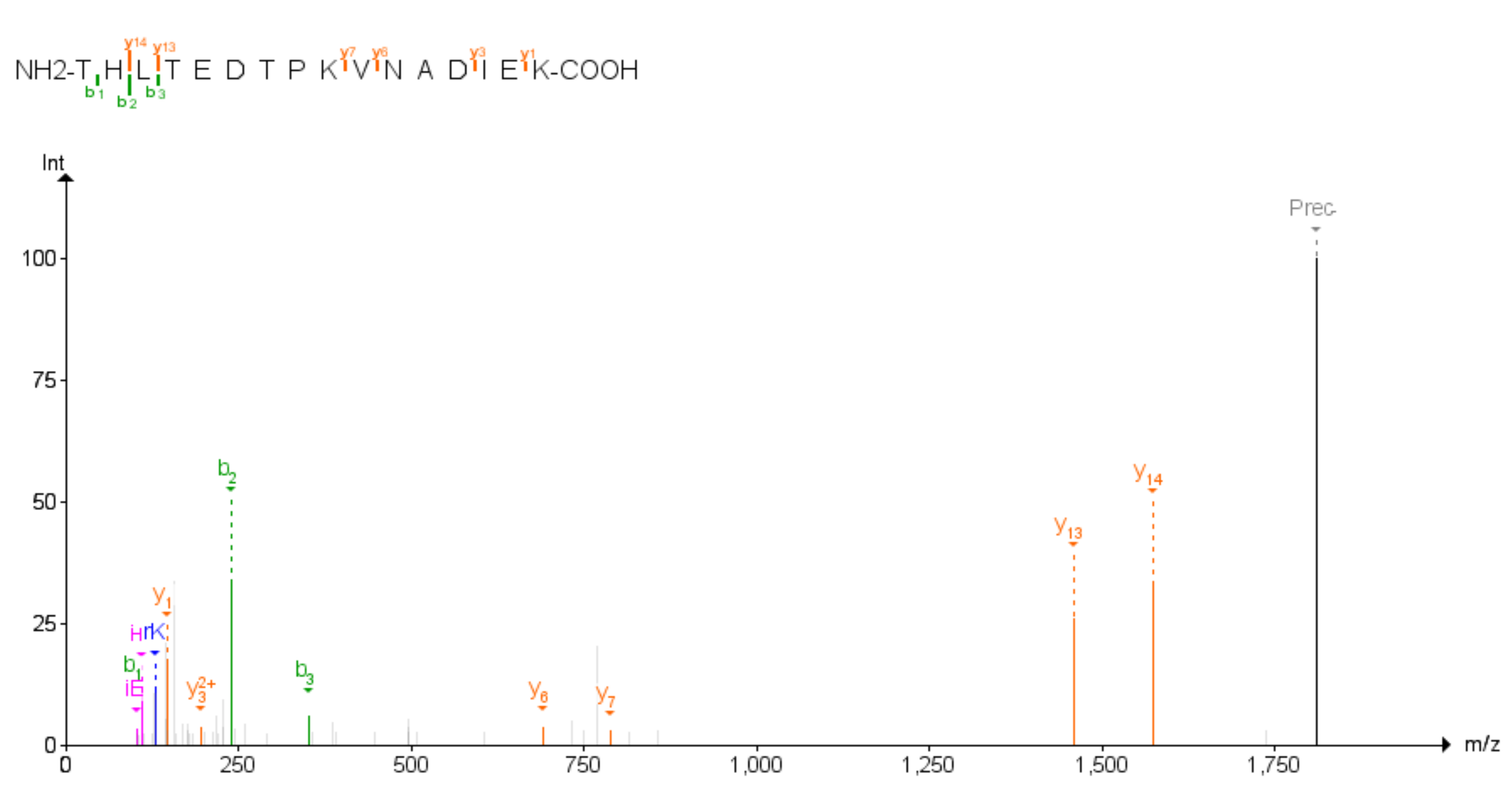

NH2-T H L T E D T P K V N A D I E K-COOH
y14 y13 y7 y6 y3 y1
b1 b2 b3
Int
100
75
50
25
0
Prec
b2
y13
y14
y1
b1
b3
y6
y7
0
250
500
750
1,000
1,250
1,500
1,750
m/z

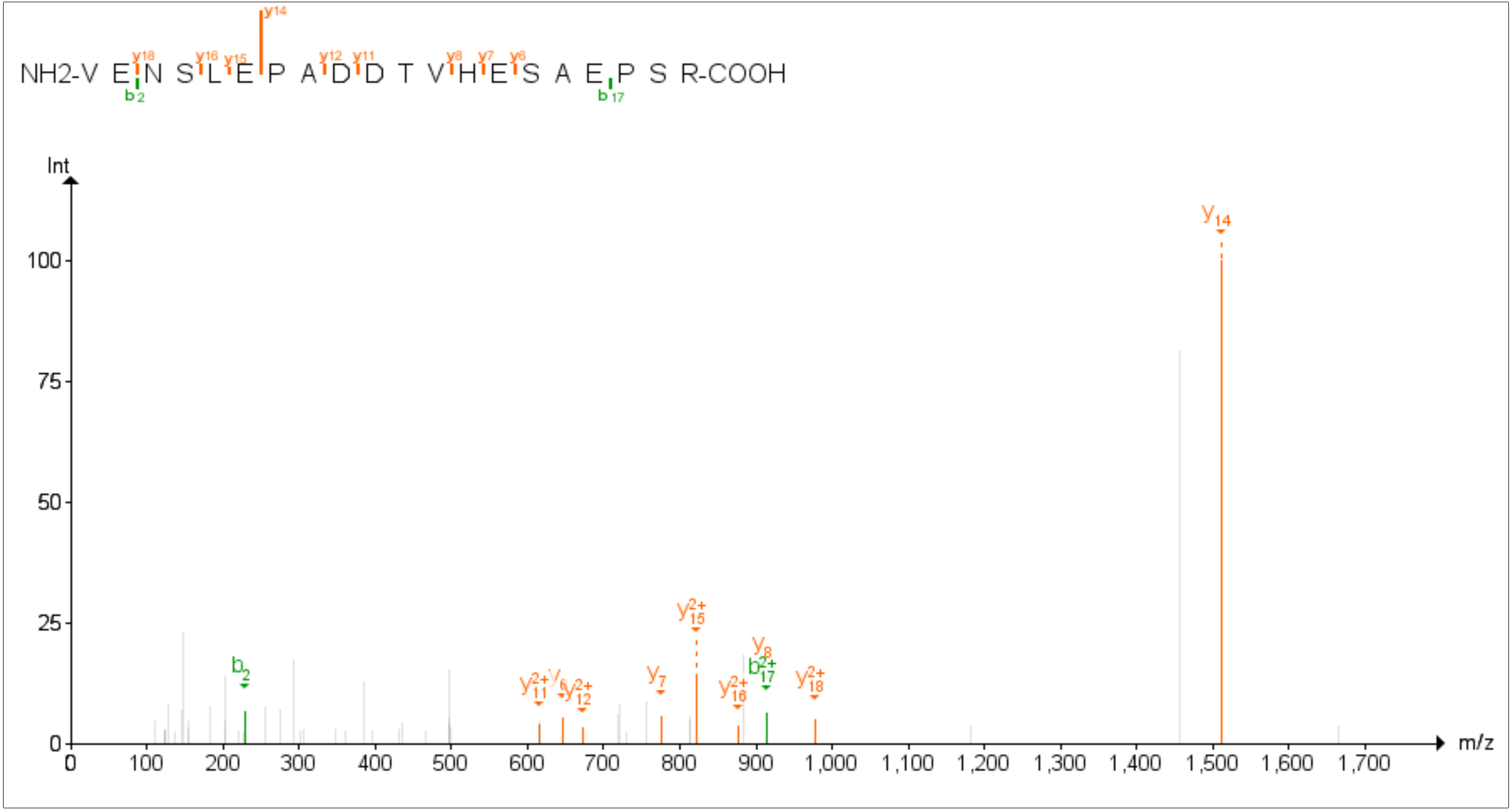

NH2-V E N S L E P A D D T V H E S A E P S R-COOH
y18 y16 y15 y14 y12 y11 y8 y7 y6
b2 b17
Int
100
75
50
25
0
m/z
0 100 200 300 400 500 600 700 800 900 1,000 1,100 1,200 1,300 1,400 1,500 1,600 1,700

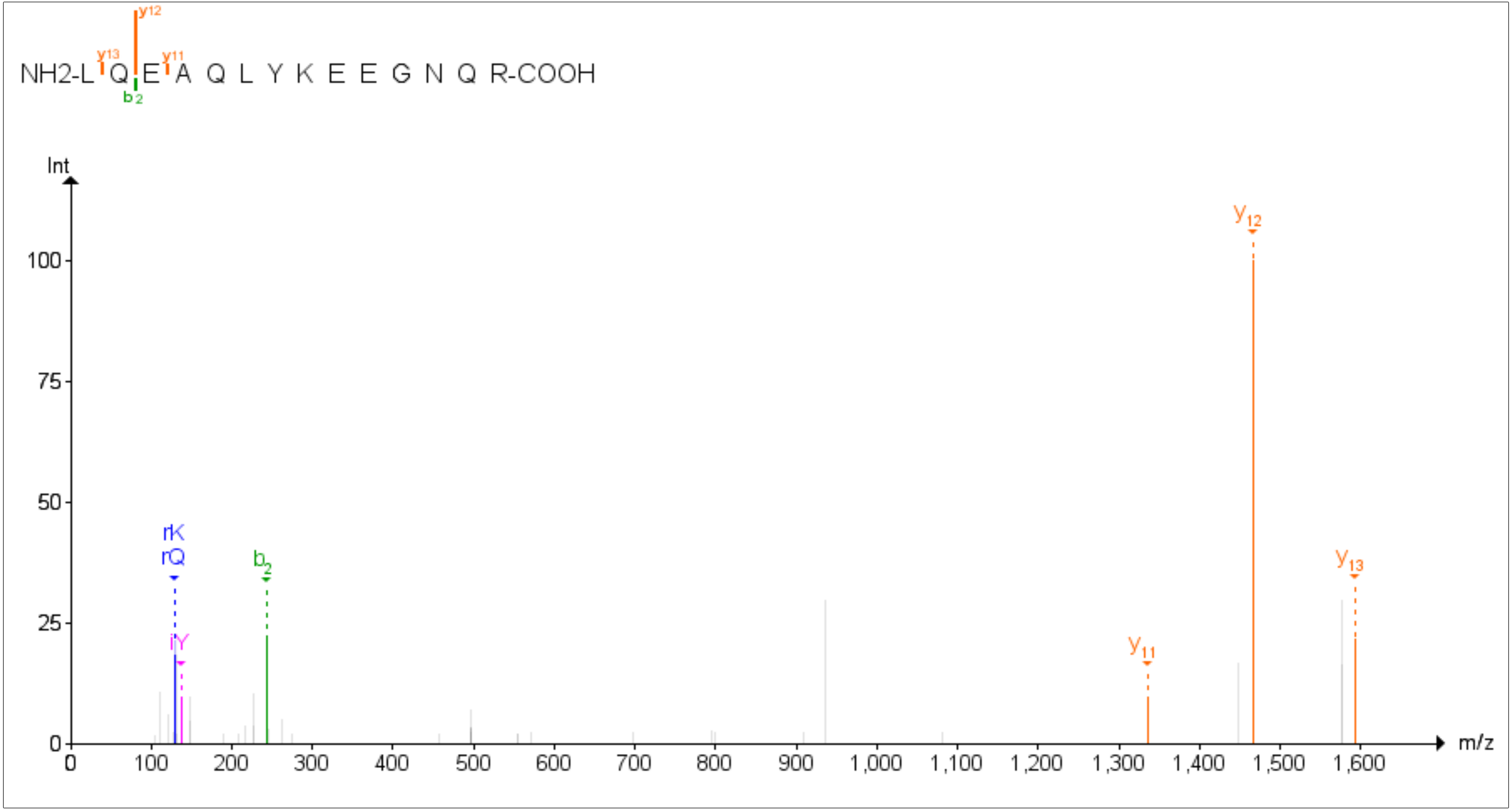

NH2-L Q E A Q L Y K E E G N Q R-COOH
y12
y13
y11
b2
Int
100
75
50
25
0
rK
rQ
iY
b2
y11
y12
y13
m/z
0 100 200 300 400 500 600 700 800 900 1,000 1,100 1,200 1,300 1,400 1,500 1,600

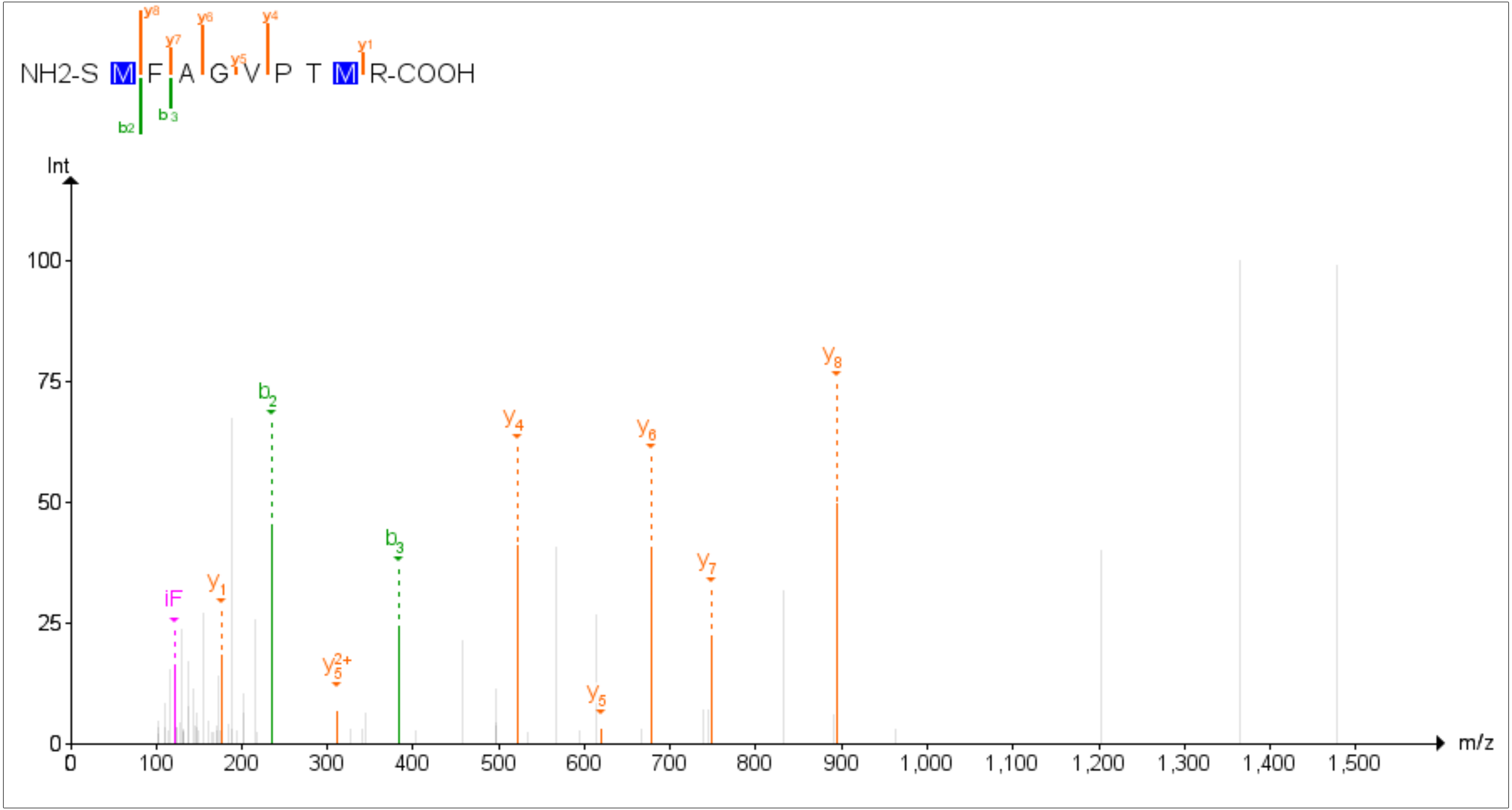
NH2-S M F A G V P T M R-COOH
y8
y7
y6
y5
y4
y1
b2
b3
Int
100
75
50
25
0
b2
b3
y1
iF
y5 2+
y4
y5
y6
y7
y8
0
100
200
300
400
500
600
700
800
900
1,000
1,100
1,200
1,300
1,400
1,500
m/z

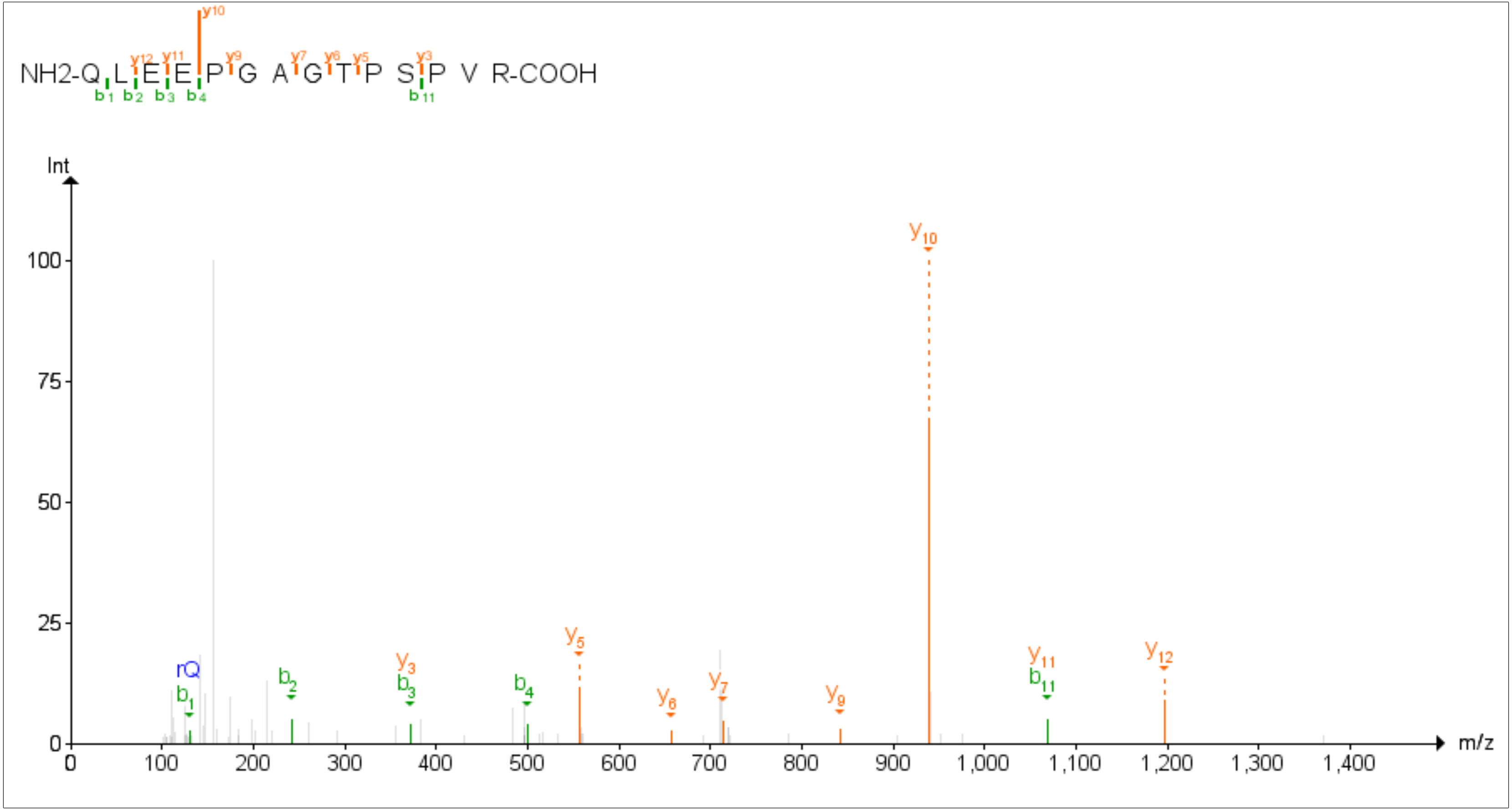

NH2-Q L E E P G A G T P S P V R-COOH
y10
y12
y11
y9
y7
y6
y5
y3
b1
b2
b3
b4
b11
Int
100
75
50
25
0
rQ
m/z
0
100
200
300
400
500
600
700
800
900
1,000
1,100
1,200
1,300
1,400

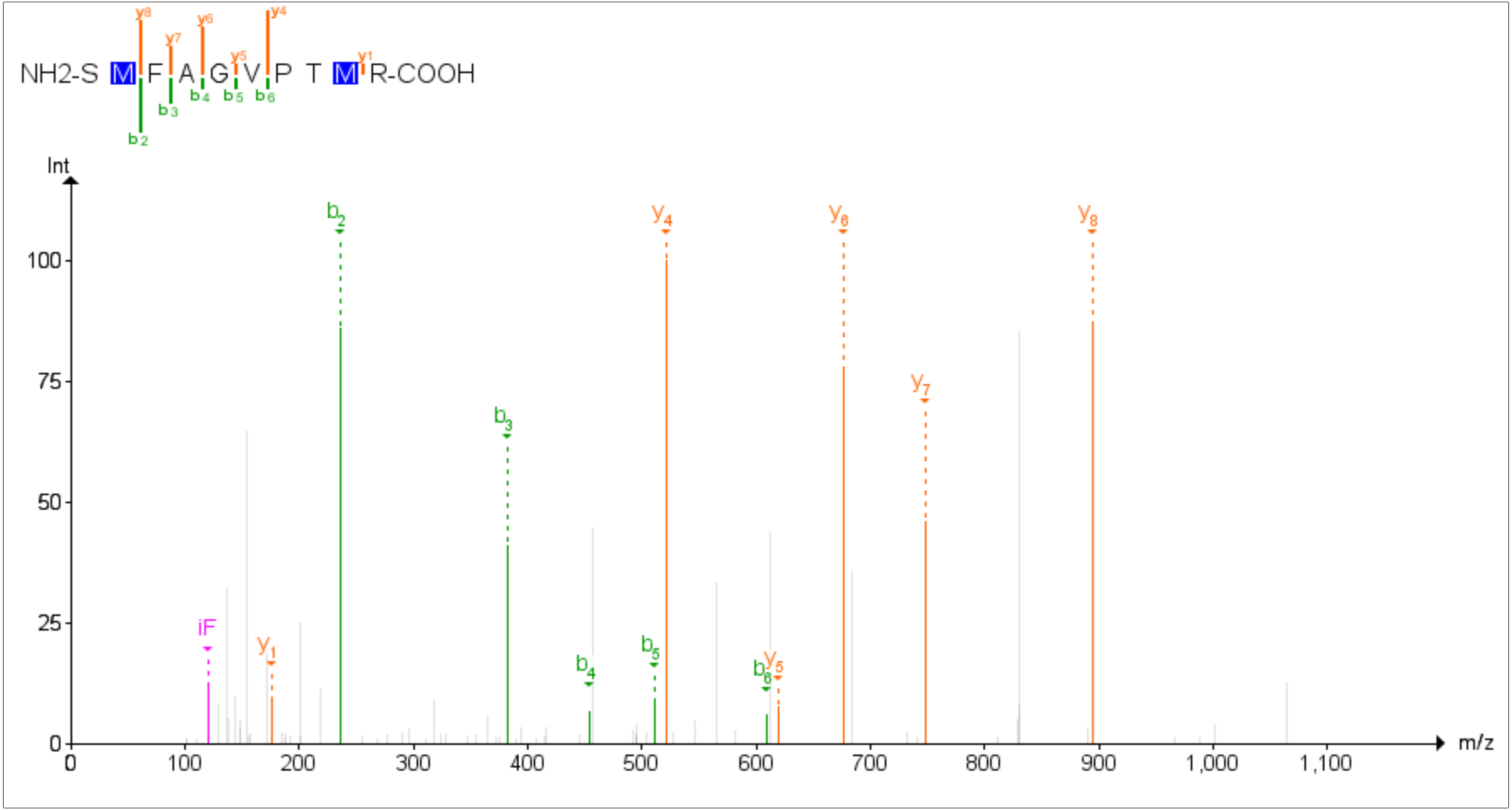

NH2-S M F A G V P T M R-COOH
y8
y7
y6
y5
y4
y1
b2
b3
b4
b5
b6
Int
100
75
50
25
0
b2
b3
b4
b5
b6
y1
y4
y5
y6
y7
y8
iF
m/z
0
100
200
300
400
500
600
700
800
900
1,000
1,100

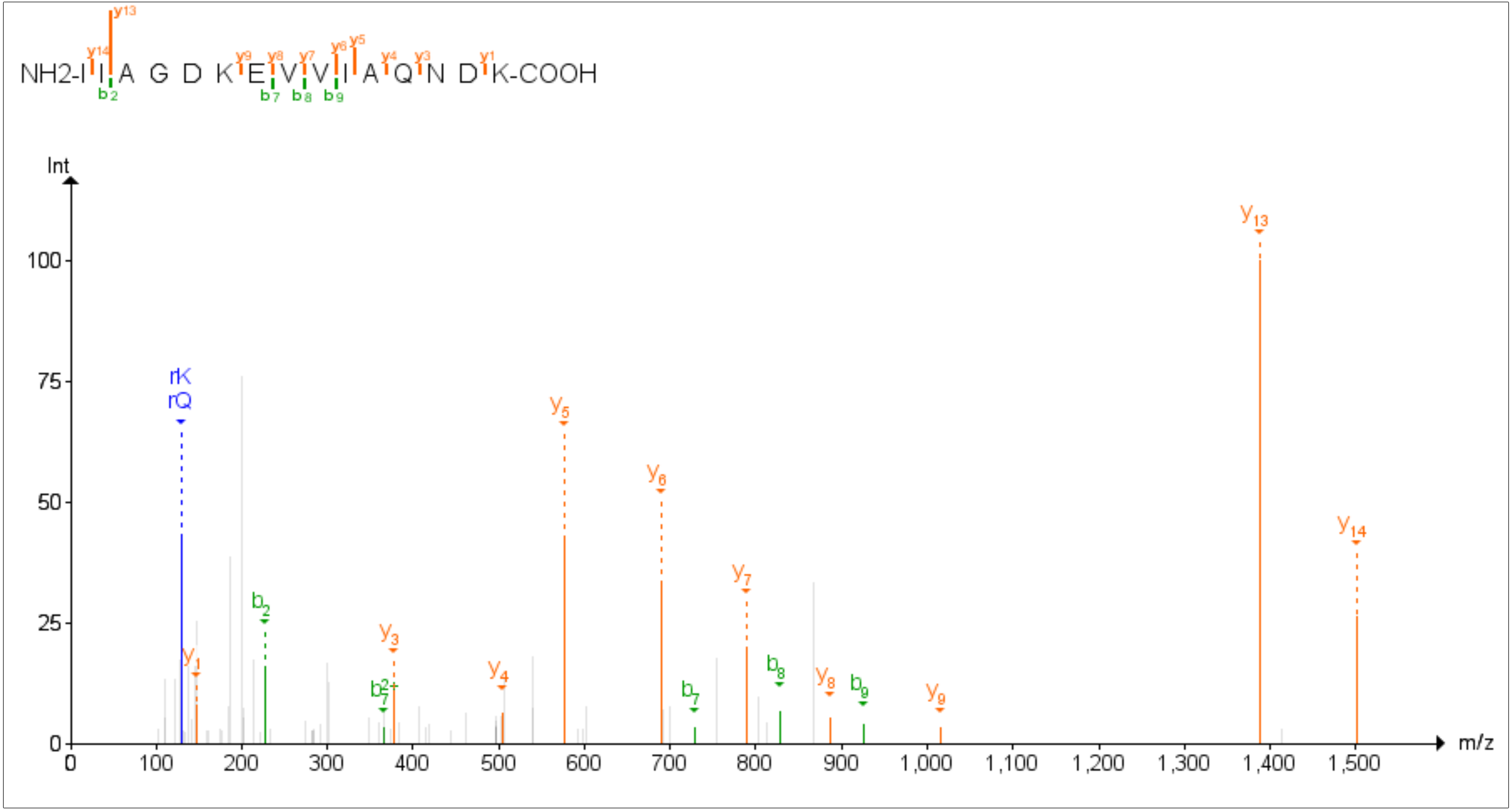

NH2-I I A G D K E V V I A Q N D K-COOH
y13
y14
y9 y8 y7 y6 y5 y4 y3 y1
b2 b7 b8 b9
Int
m/z
rK
rQ
y13
y14
y5
y6
y7
b2
y3
y1
y4
b7 2+
b7
b8
y8
b9
y9
0
25
50
75
100
0
100
200
300
400
500
600
700
800
900
1,000
1,100
1,200
1,300
1,400
1,500

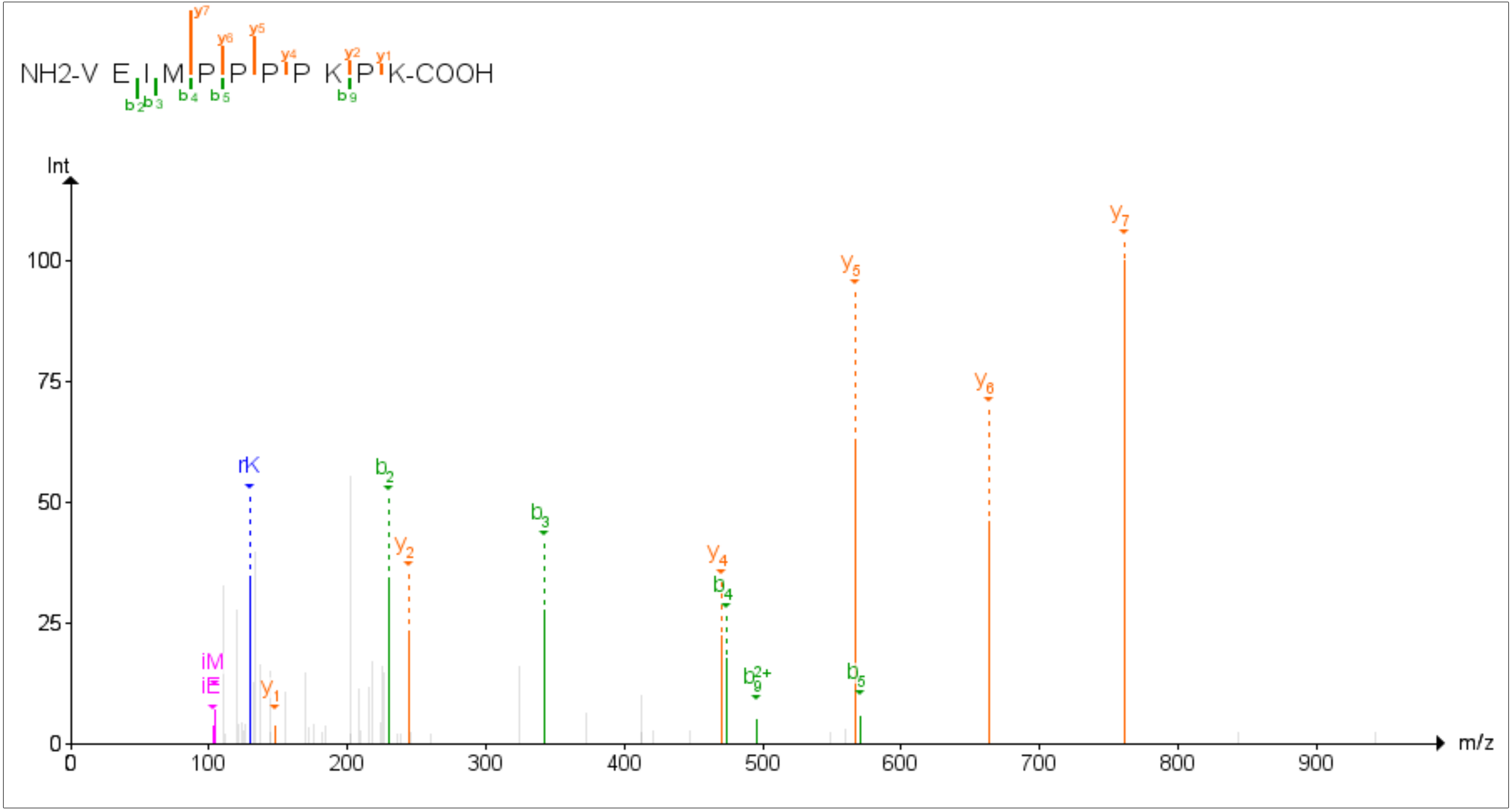

NH2-V E I M P P P P K P K-COOH
y7
y6
y5
y4
y2
y1
b2
b3
b4
b5
b9
Int
100
75
50
25
0
rK
b2
y2
b3
y4
b4
y5
y6
y7
iM
iE
y1
b9 2+
b5
0
100
200
300
400
500
600
700
800
900
m/z

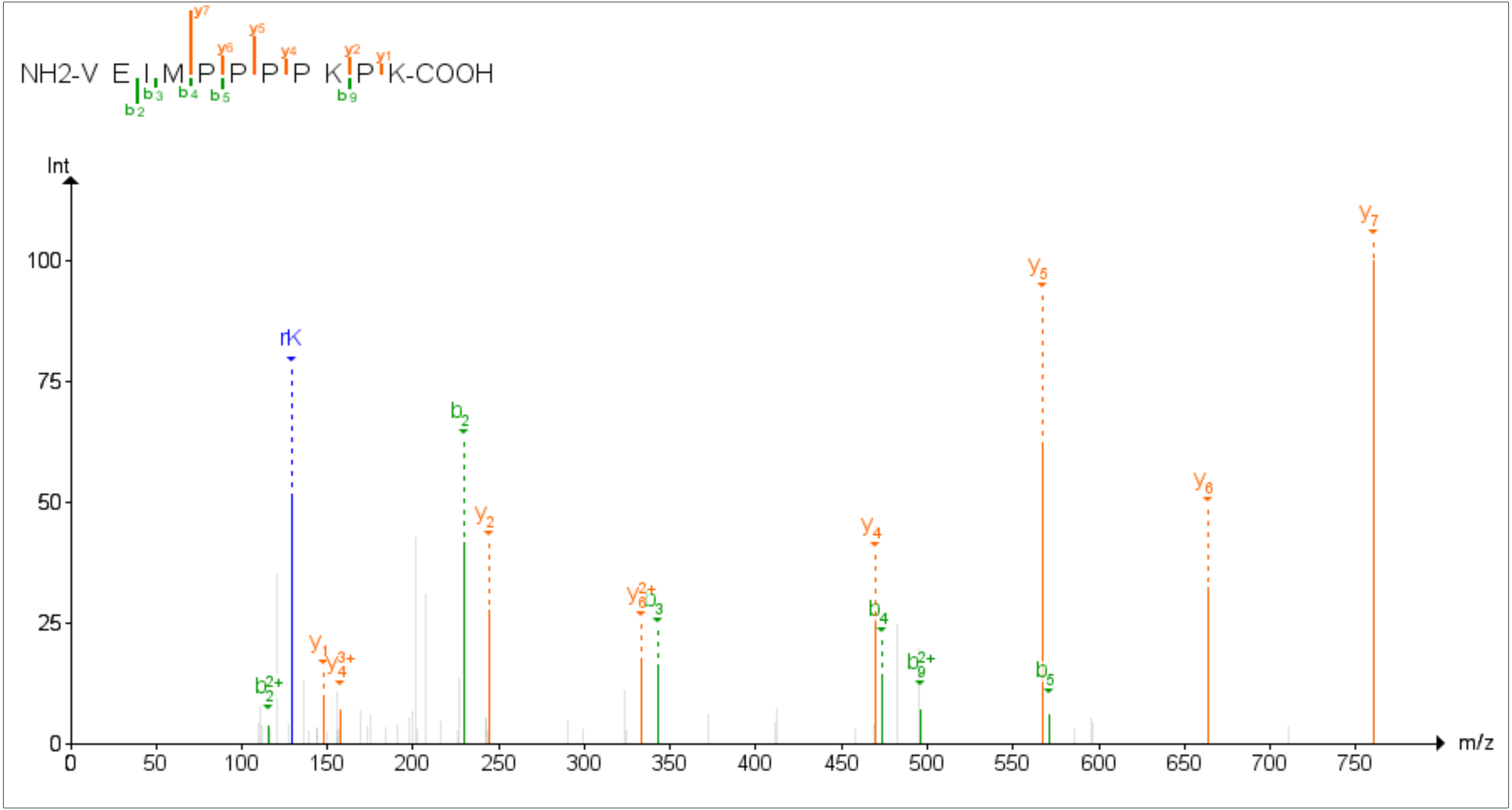

NH2-V E I M P P P P K P K-COOH
y7
y6
y5
y4
y2
y1
b2
b3
b4
b5
b9
Int
m/z
0
25
50
75
100
0
50
100
150
200
250
300
350
400
450
500
550
600
650
700
750
rK
b2
y2
y1
y4 3+
b2 2+
y6 2+
b3
y4
b4
b9 2+
y5
b5
y6
y7

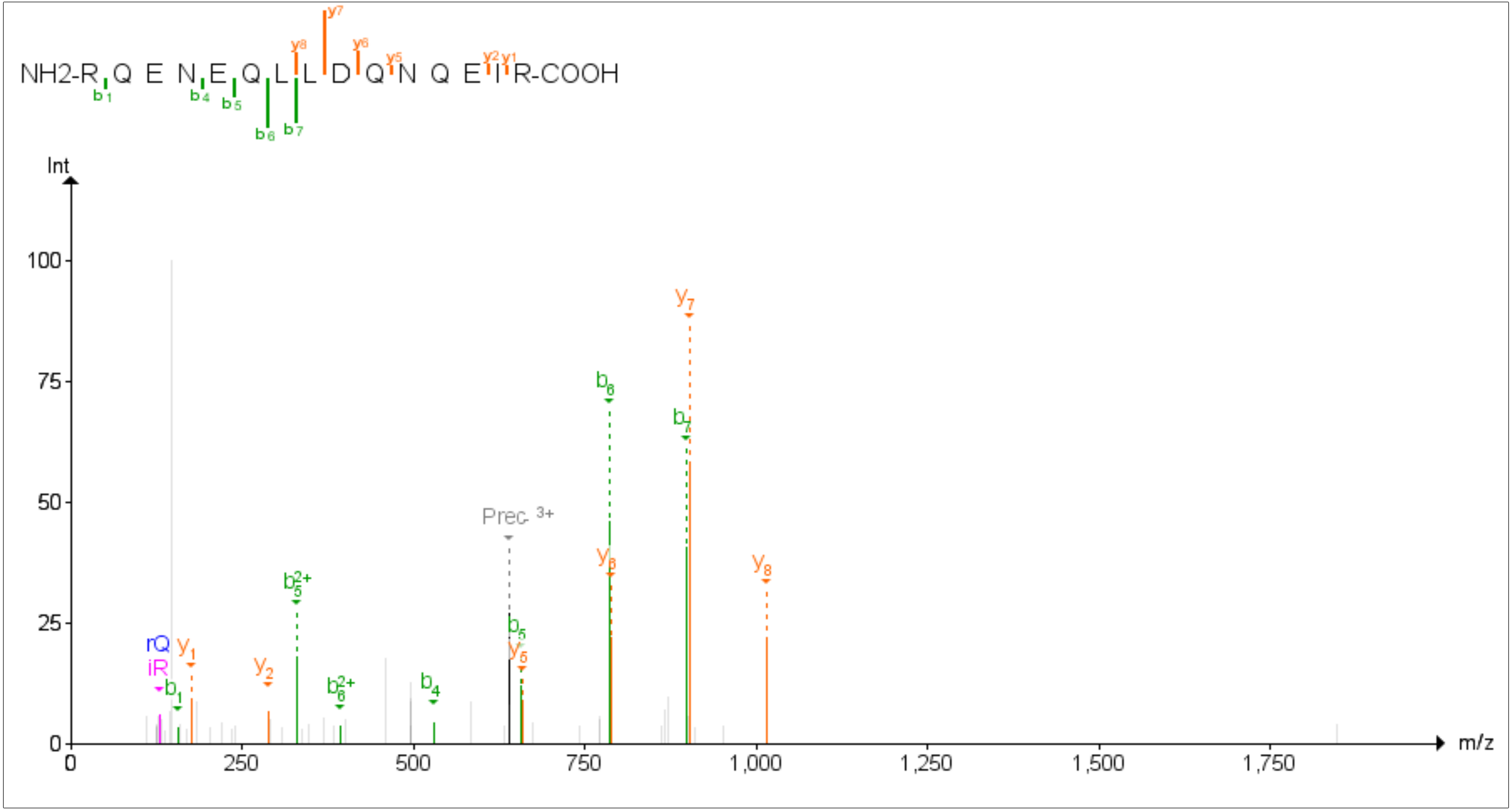
NH2-R Q E N E Q L L D Q N Q E I R-COOH
Int
m/z
Prec 3+

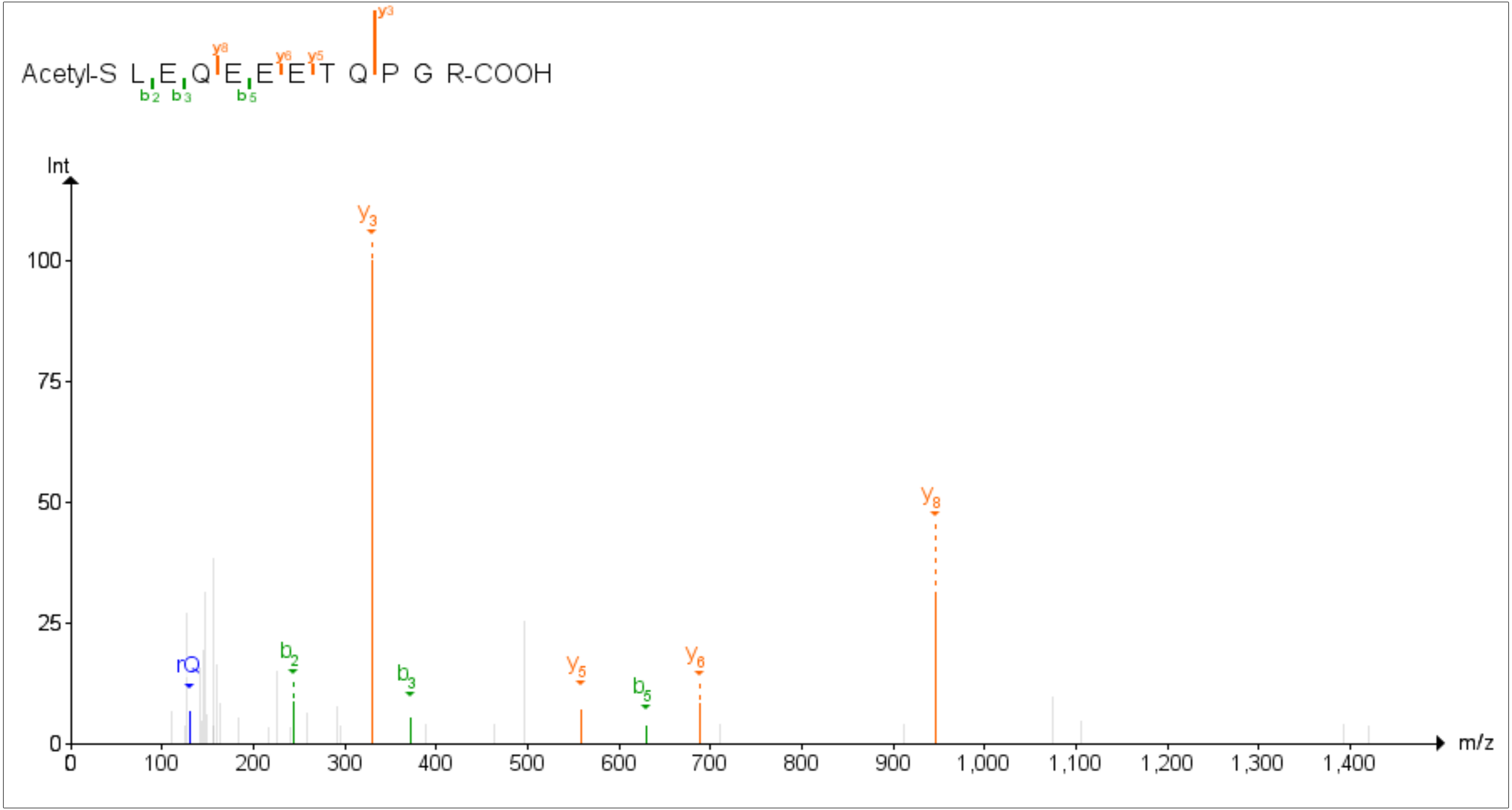

Acetyl-S L E Q E E E T Q P G R-COOH
y3
y8
y6
y5
b2
b3
b5
Int
100
75
50
25
0
y3
y8
rQ
b2
b3
y5
b5
y6
0
100
200
300
400
500
600
700
800
900
1,000
1,100
1,200
1,300
1,400
m/z

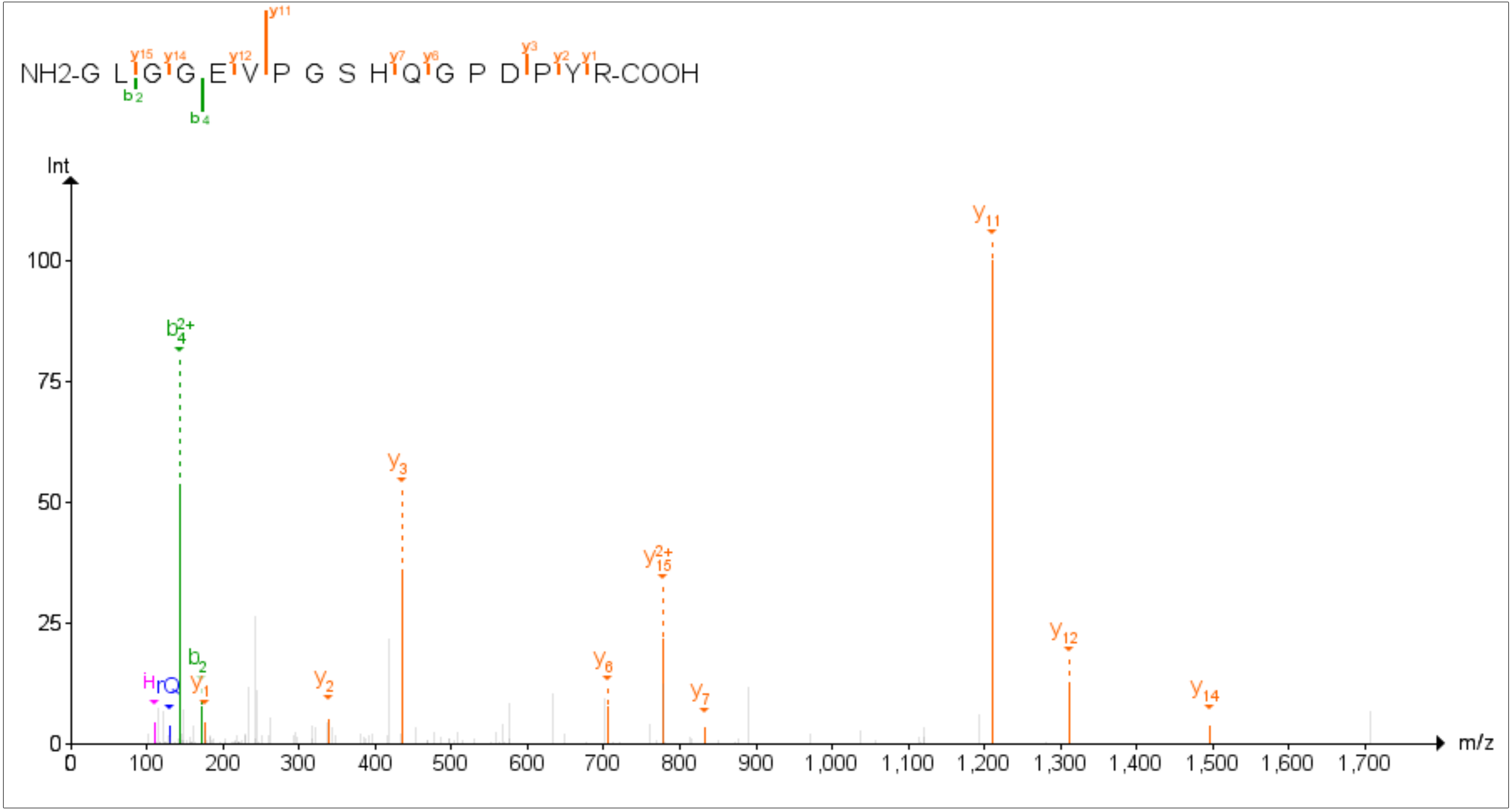

NH2-G L G G E V P G S H Q G P D P Y R-COOH
y15 y14 y12 y11 y7 y6 y3 y2 y1
b2 b4
Int
m/z
b4 2+
y3
y11
y15 2+
y12
y14
y6
y7
y2
y1
b2
H
rQ

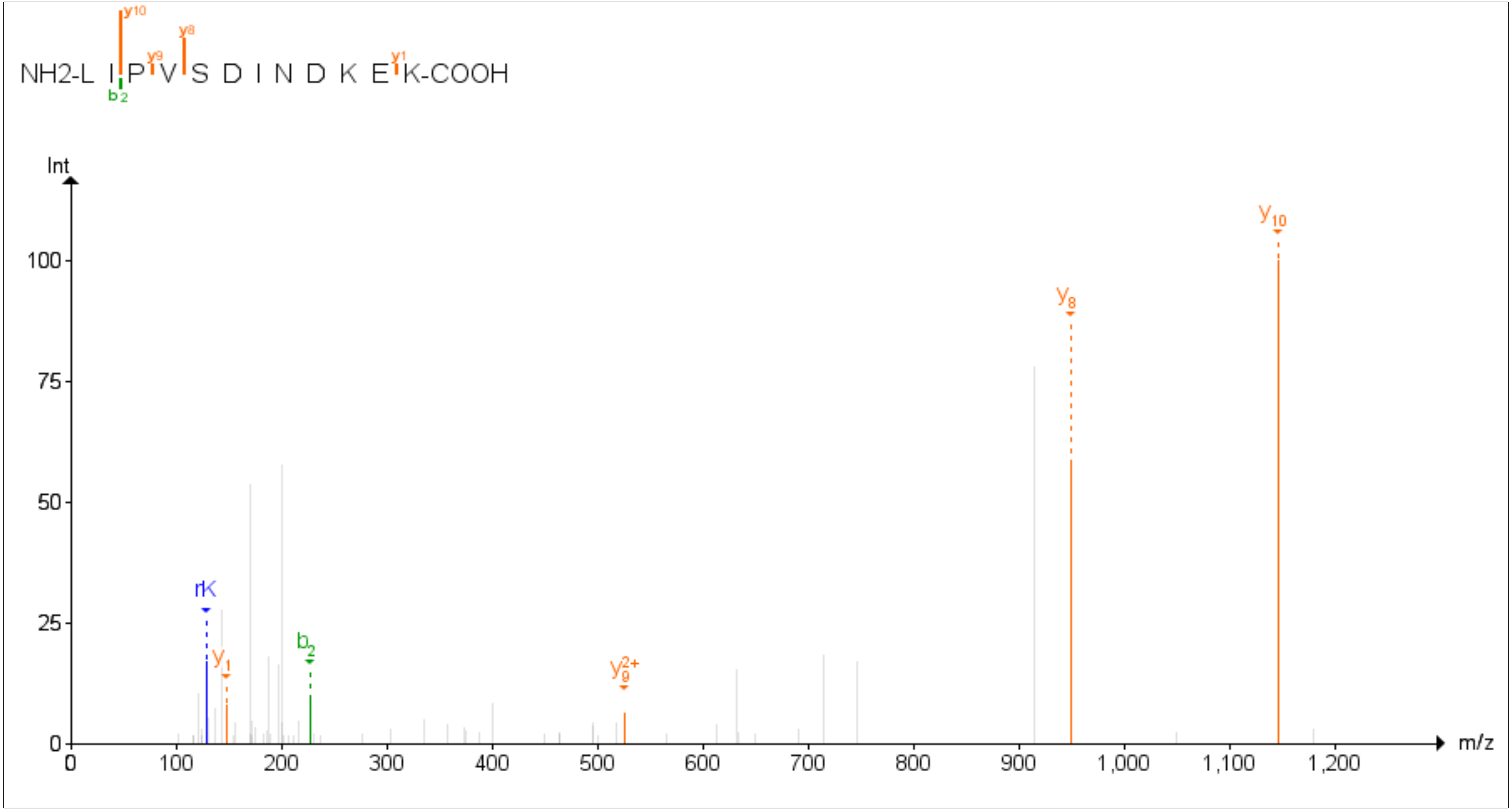

NH2-L I P V S D I N D K E K-COOH
y10
y9
y8
y1
b2
Int
100
75
50
25
0
rK
y1
b2
y9 2+
y8
y10
0
100
200
300
400
500
600
700
800
900
1,000
1,100
1,200
m/z

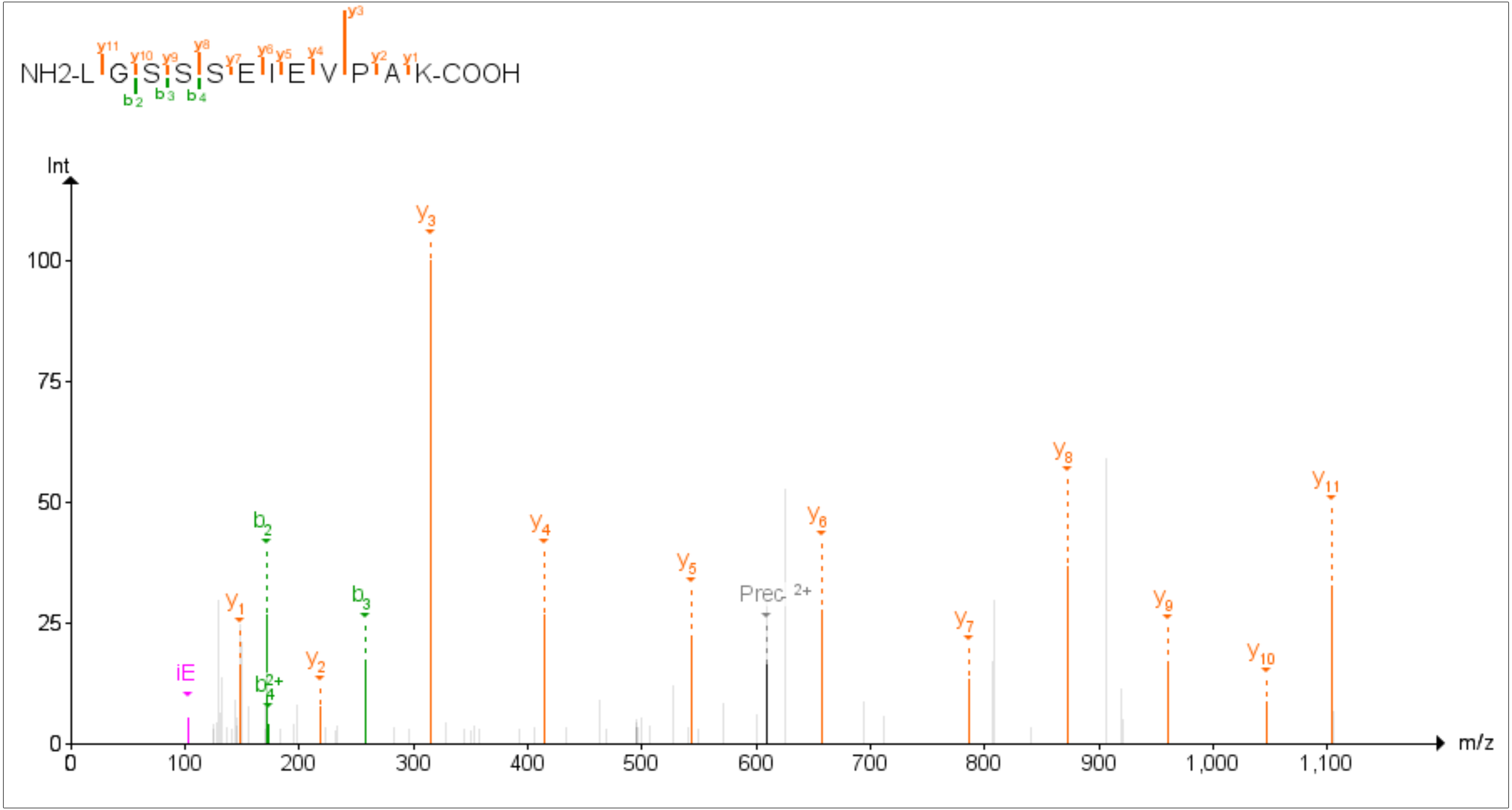

NH2-L G S S S E I E V P A K-COOH
y11 y10 y9 y8 y7 y6 y5 y4 y3 y2 y1
b2 b3 b4
Int
100
75
50
25
0
y3
y4
y5
y6
y7
y8
y9
y10
y11
y1
y2
b2
b3
iE
Prec 2+
0
100
200
300
400
500
600
700
800
900
1,000
1,100
m/z

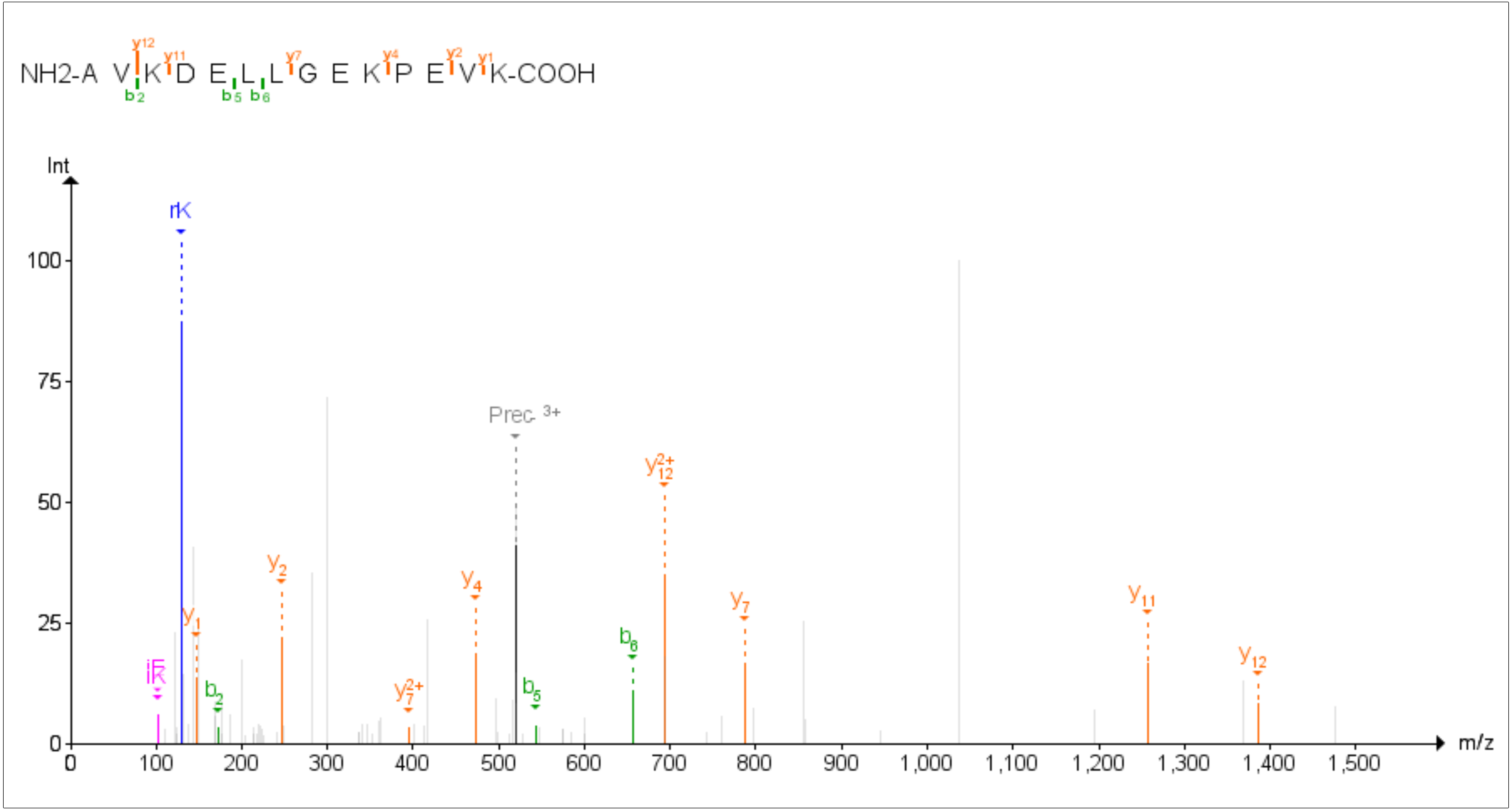

NH2-A V K D E L L G E K P E V K-COOH
y12 y11 y7 y4 y2 y1
b2 b5 b6
Int
m/z
rK
iF
iK
Prec 3+
y1
y2
b2
y7 2+
y4
b5
b6
y12 2+
y7
y11
y12
0
25
50
75
100
0
100
200
300
400
500
600
700
800
900
1,000
1,100
1,200
1,300
1,400
1,500

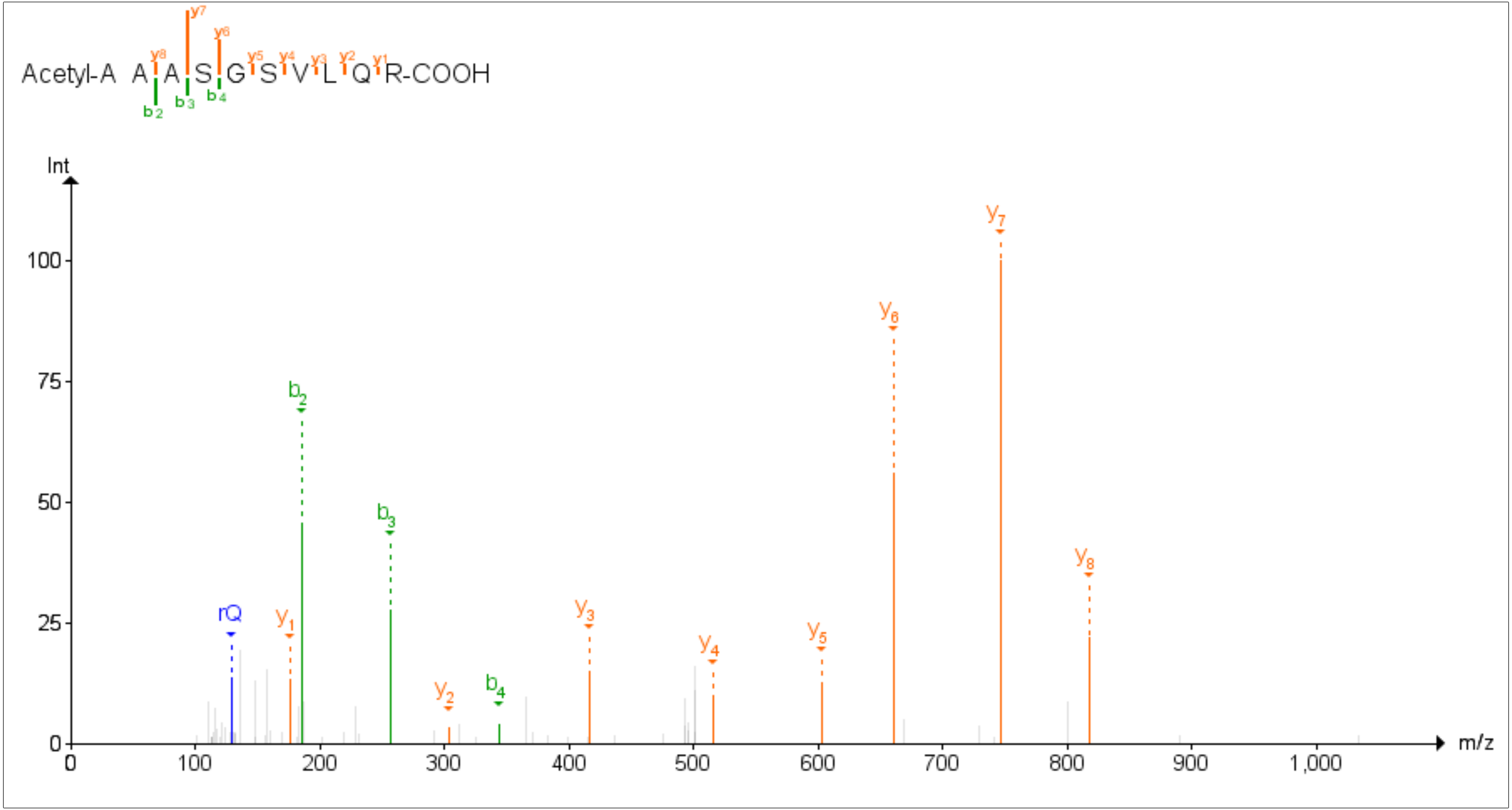

Acetyl-A A A S G S V L Q R-COOH
y7 y6 y8 y5 y4 y3 y2 y1
b2 b3 b4
Int
100
75
50
25
0
rQ
y1
b2
b3
y2
b4
y3
y4
y5
y6
y7
y8
0 100 200 300 400 500 600 700 800 900 1,000
m/z

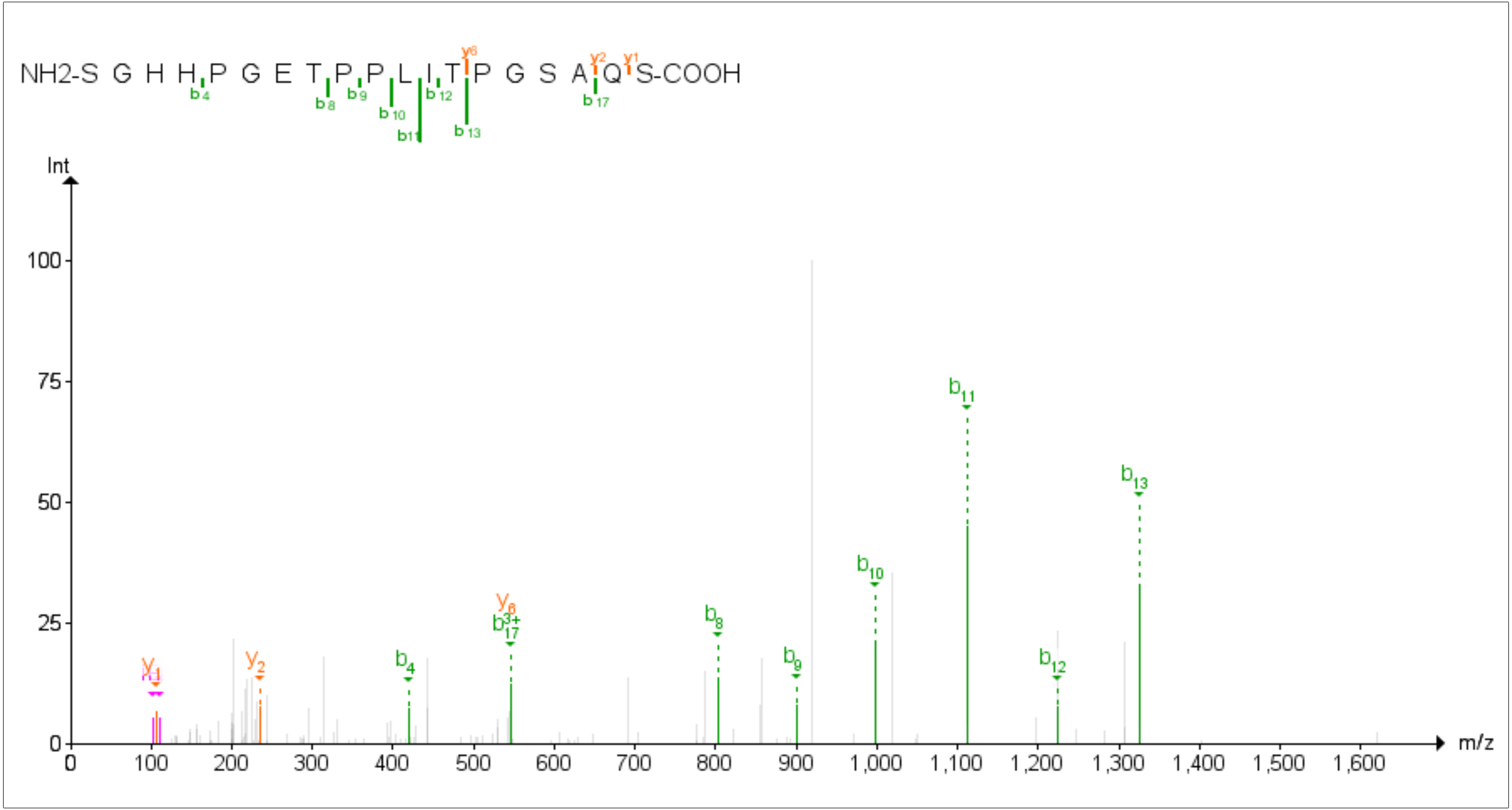

NH2-S G H H P G E T P P L I T P G S A Q S-COOH
b4 b8 b9 b10 b11 b12 b13 b17 y6 y2 y1
Int
100
75
50
25
0
m/z
0 100 200 300 400 500 600 700 800 900 1,000 1,100 1,200 1,300 1,400 1,500 1,600

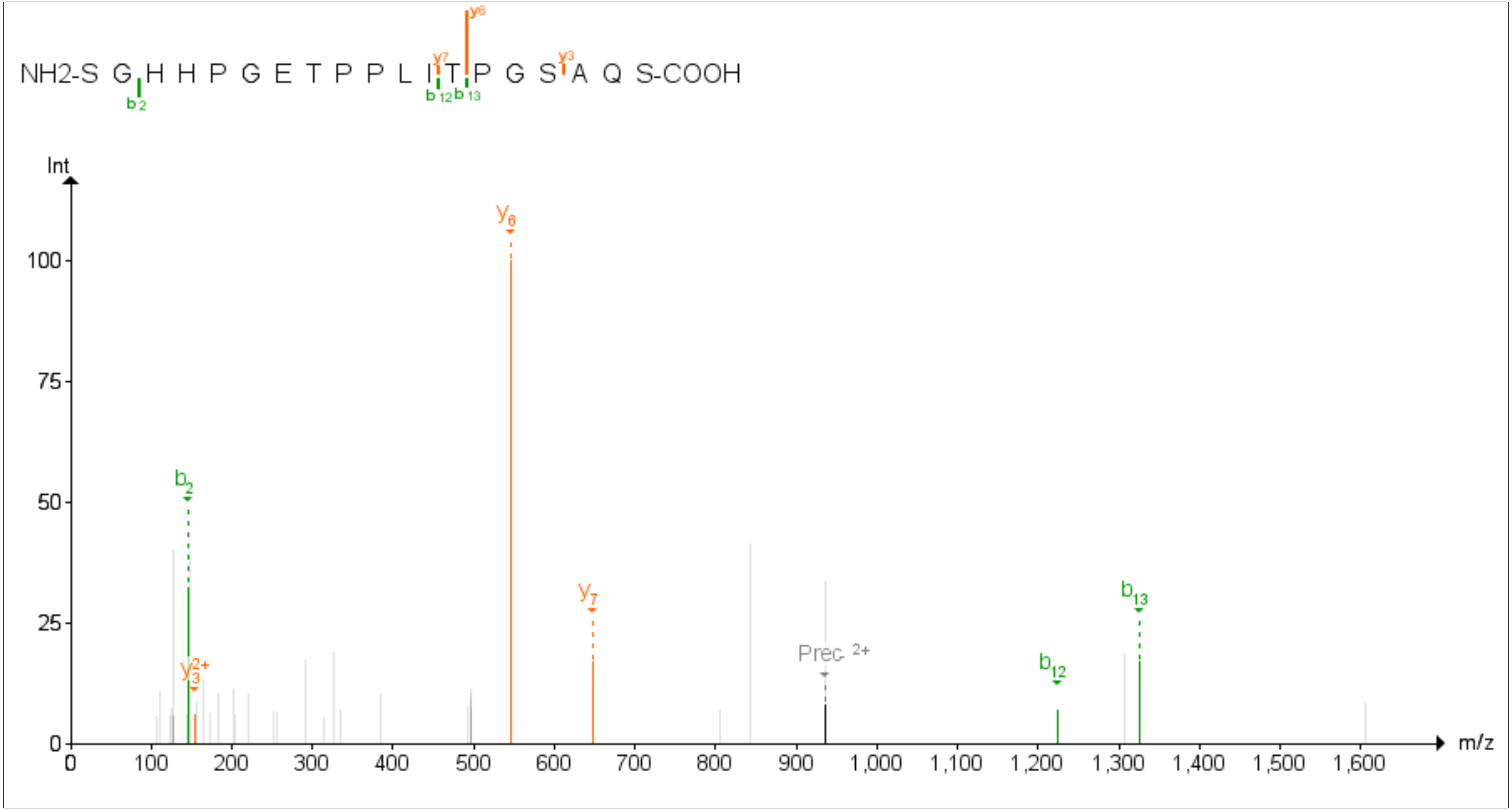

NH2-S G H H P G E T P P L I T P G S A Q S-COOH
b2
b12
b13
y7
y6
y3
Int
100
75
50
25
0
b2
y6
y7
y3 2+
Prec. 2+
b12
b13
m/z
0
100
200
300
400
500
600
700
800
900
1,000
1,100
1,200
1,300
1,400
1,500
1,600

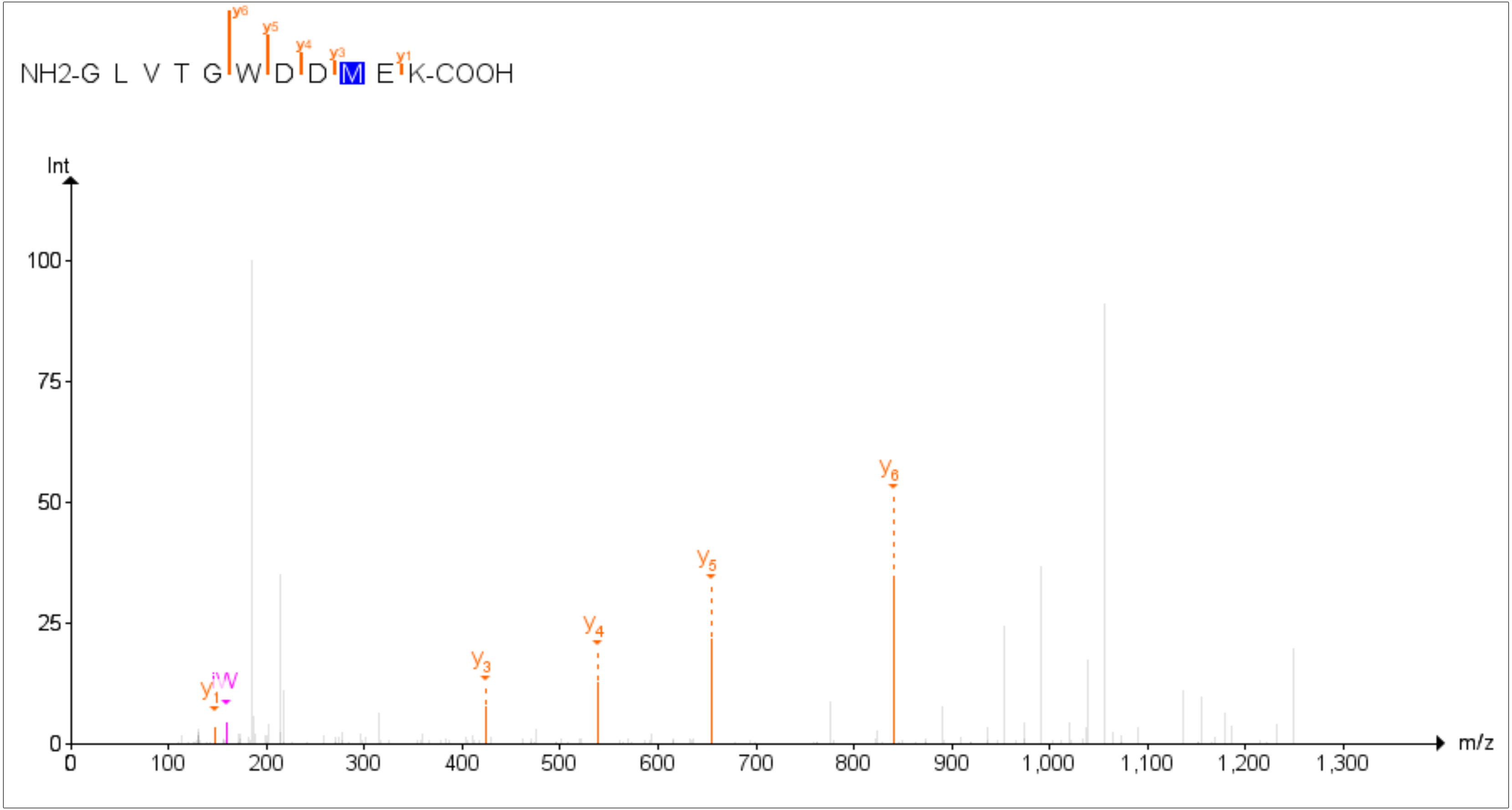

NH2-G L V T G W D D M E K-COOH
y6
y5
y4
y3
y1
Int
100
75
50
25
0
m/z
0
100
200
300
400
500
600
700
800
900
1,000
1,100
1,200
1,300
y1
iW
y3
y4
y5
y6

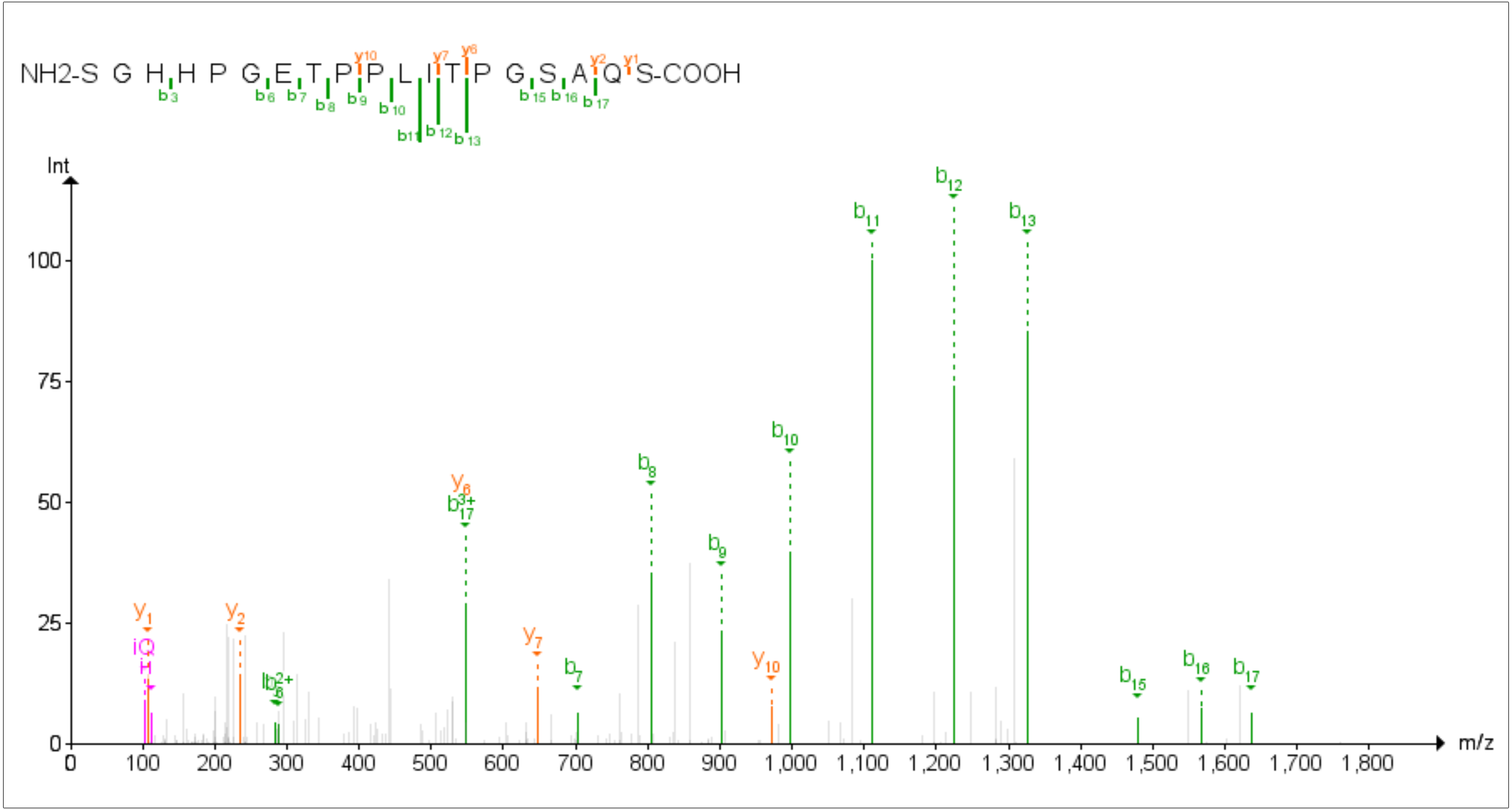
NH2-S G H H P G E T P P L I T P G S A Q S-COOH
Int
m/z
100
75
50
25
0
100
200
300
400
500
600
700
800
900
1,000
1,100
1,200
1,300
1,400
1,500
1,600
1,700
1,800

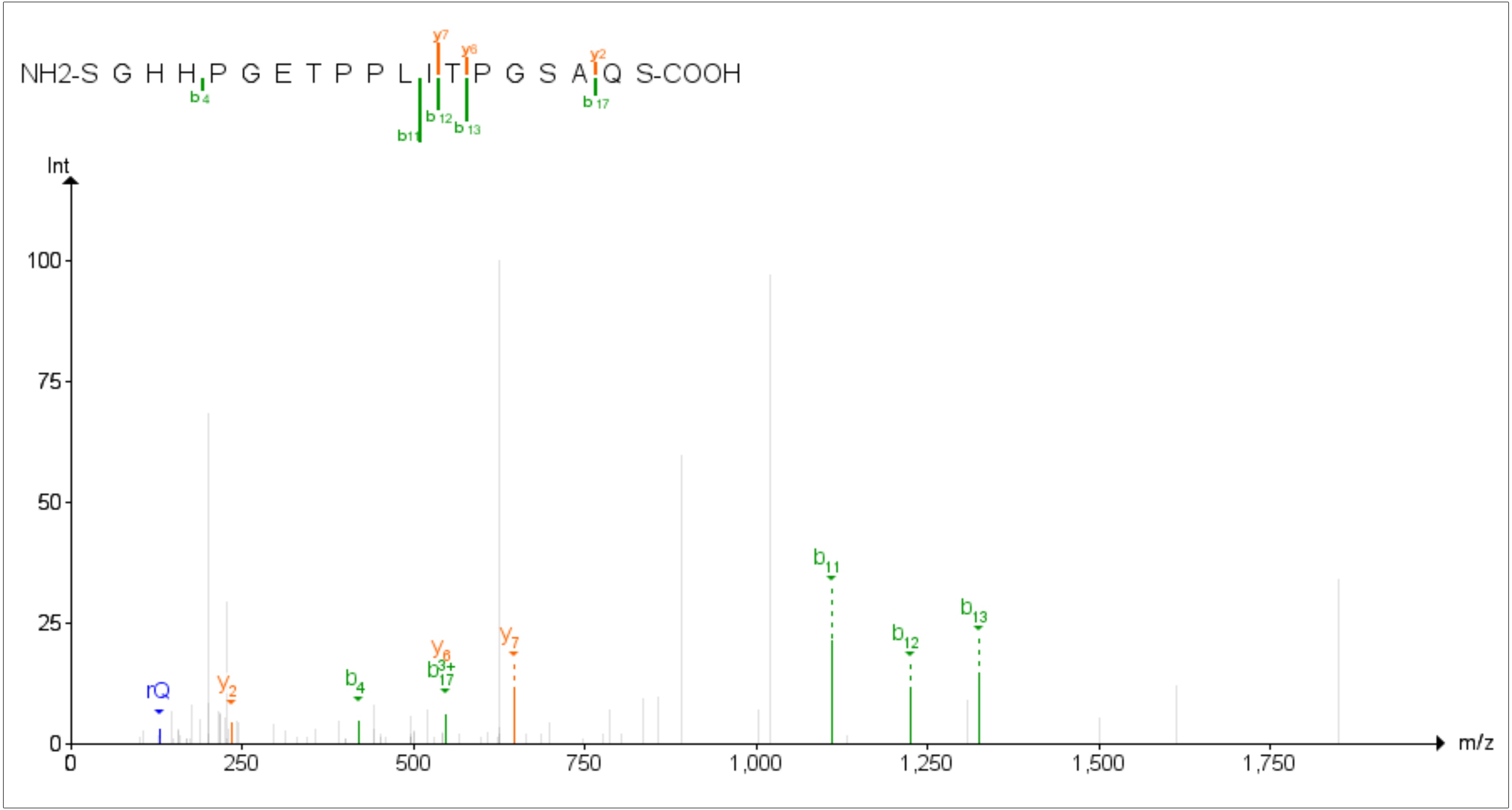

NH2-S G H H P G E T P P L I T P G S A Q S-COOH
y7 y6 y2
b4 b11 b12 b13 b17
Int
100
75
50
25
0
rQ
y2
b4
y6
b17 3+
y7
b11
b12
b13
0 250 500 750 1,000 1,250 1,500 1,750
m/z

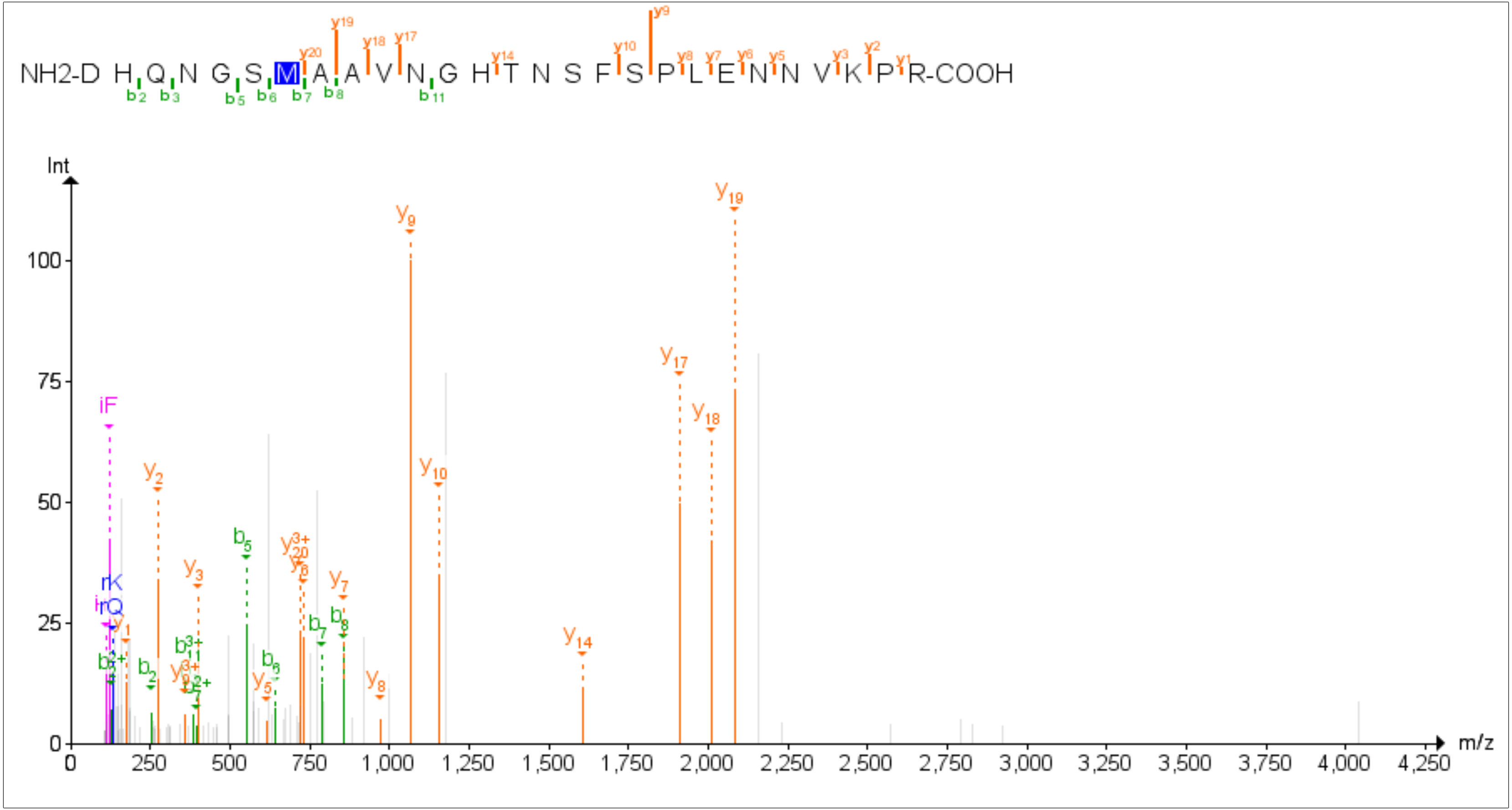

NH2-D H Q N G S M A A V N G H T N S F S P L E N N V K P R-COOH
b2 b3 b5 b6 b7 b8 b11
y20 y19 y18 y17 y14 y10 y9 y8 y7 y6 y5 y3 y2 y1
Int
m/z
0
25
50
75
100
250
500
750
1,000
1,250
1,500
1,750
2,000
2,250
2,500
2,750
3,000
3,250
3,500
3,750
4,000
4,250

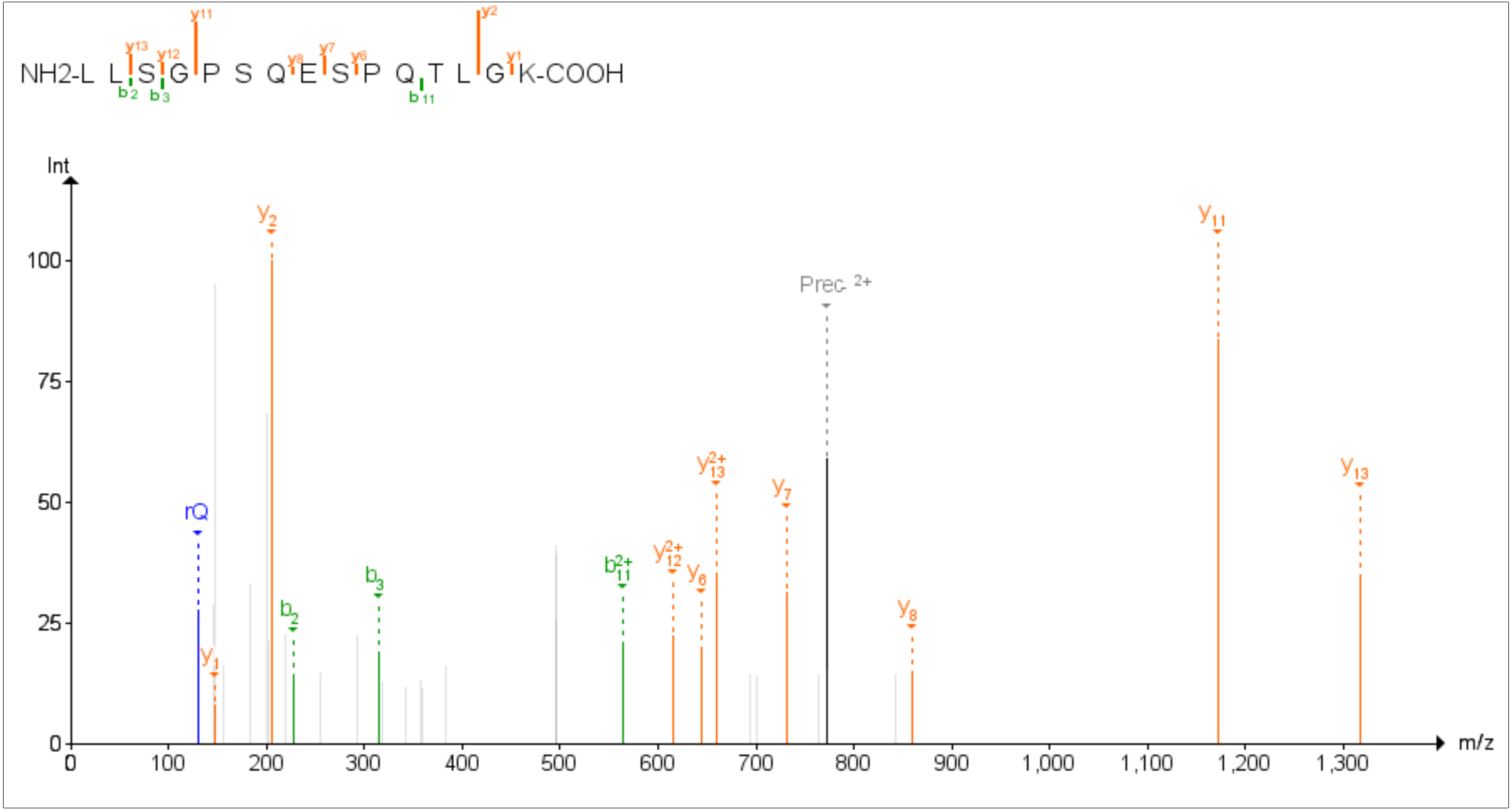

NH2-L L S G P S Q E S P Q T L G K-COOH
y13 y12 y11 y8 y7 y6 y2 y1
b2 b3 b11
Int
m/z
y2
y11
Prec 2+
rQ
y1
b2
b3
b11 2+
y12 2+
y6
y13 2+
y7
y8
y13

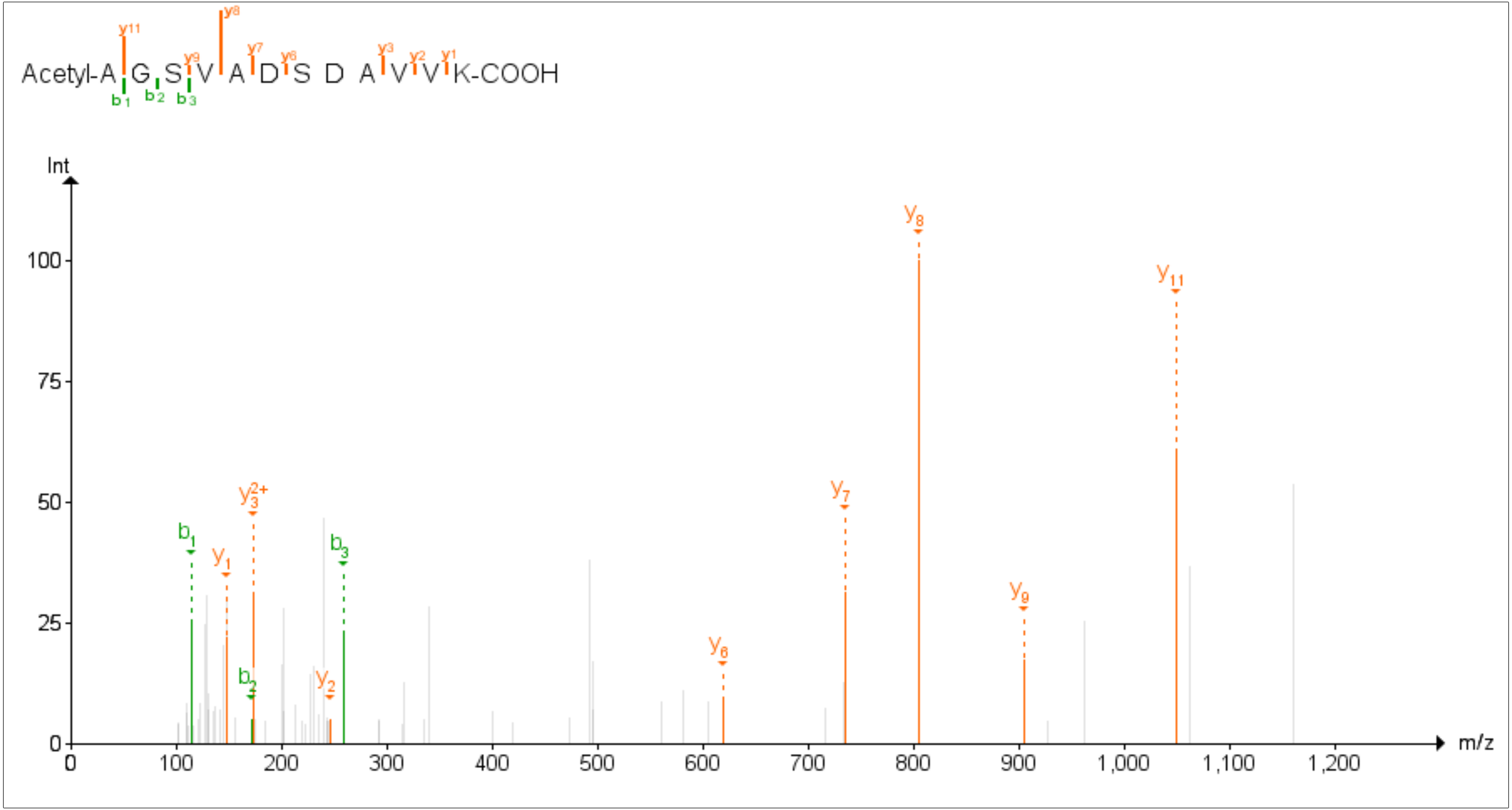
Acetyl-A G S V A D S D A V V K-COOH
y11
y9
y8
y7
y6
y3
y2
y1
b1
b2
b3
Int
100
75
50
25
0
b1
y1
y3 2+
b2
y2
b3
y6
y7
y8
y9
y11
0
100
200
300
400
500
600
700
800
900
1,000
1,100
1,200
m/z

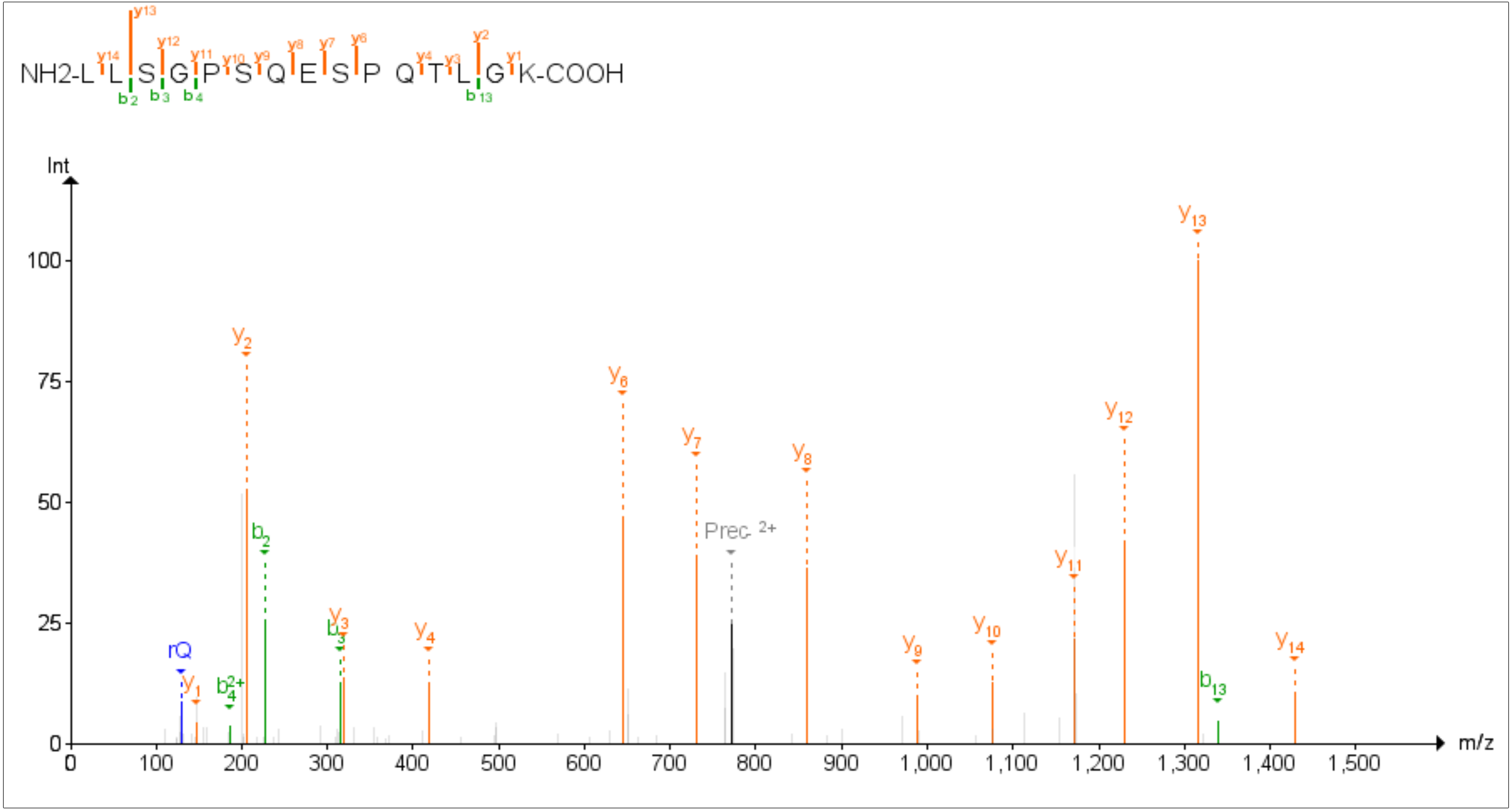

NH2-L L S G P S Q E S P Q T L G K-COOH
y14 y13 y12 y11 y10 y9 y8 y7 y6 y4 y3 y2 y1
b2 b3 b4 b13
Int
100
75
50
25
0
rQ
Prec 2+
m/z
0 100 200 300 400 500 600 700 800 900 1,000 1,100 1,200 1,300 1,400 1,500

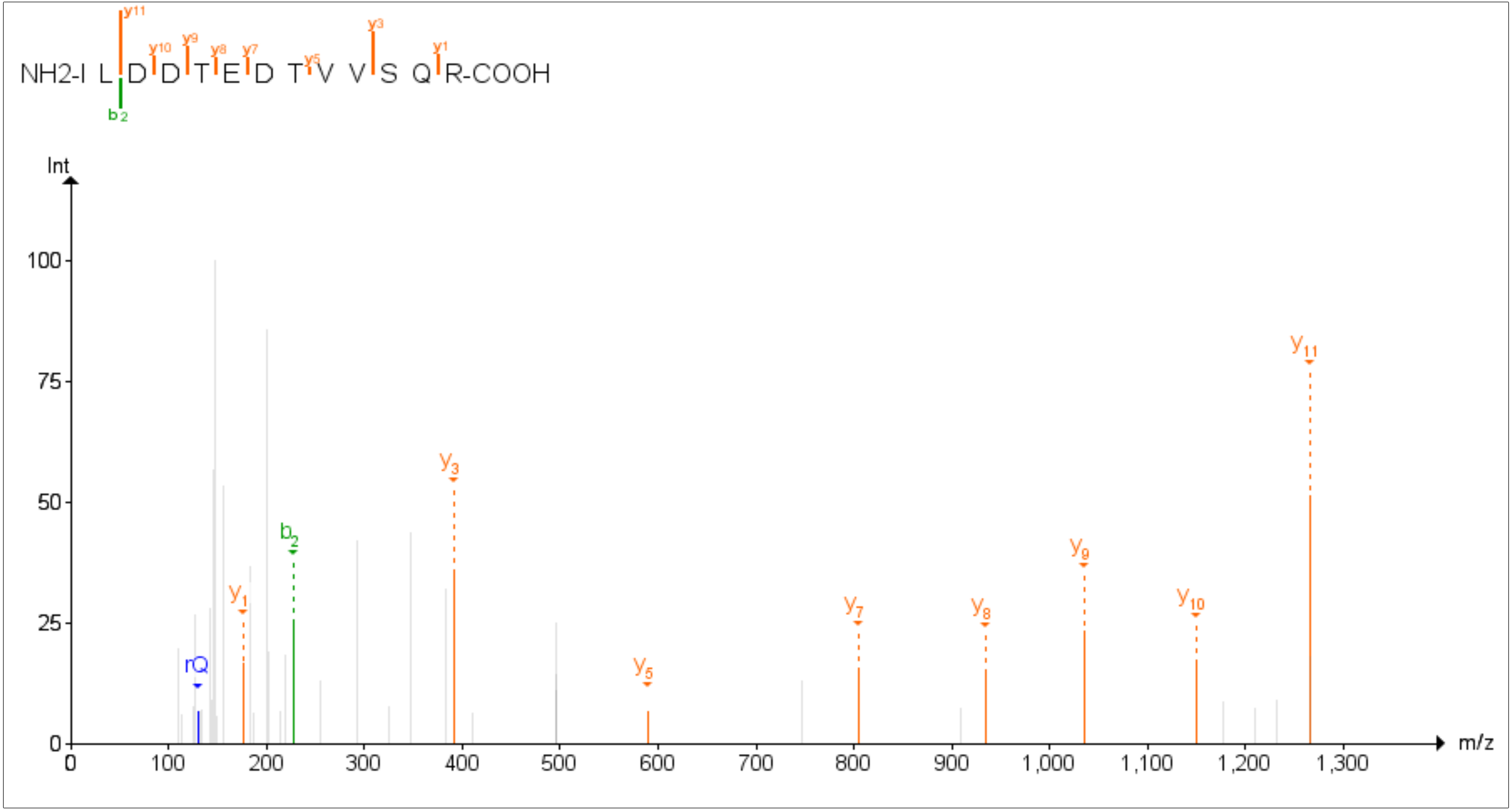

NH2-I L D D T E D T V V S Q R-COOH
y11 y10 y9 y8 y7 y5 y3 y1
b2
Int
100
75
50
25
0
0 100 200 300 400 500 600 700 800 900 1,000 1,100 1,200 1,300
m/z
rQ
y1
b2
y3
y5
y7
y8
y9
y10
y11

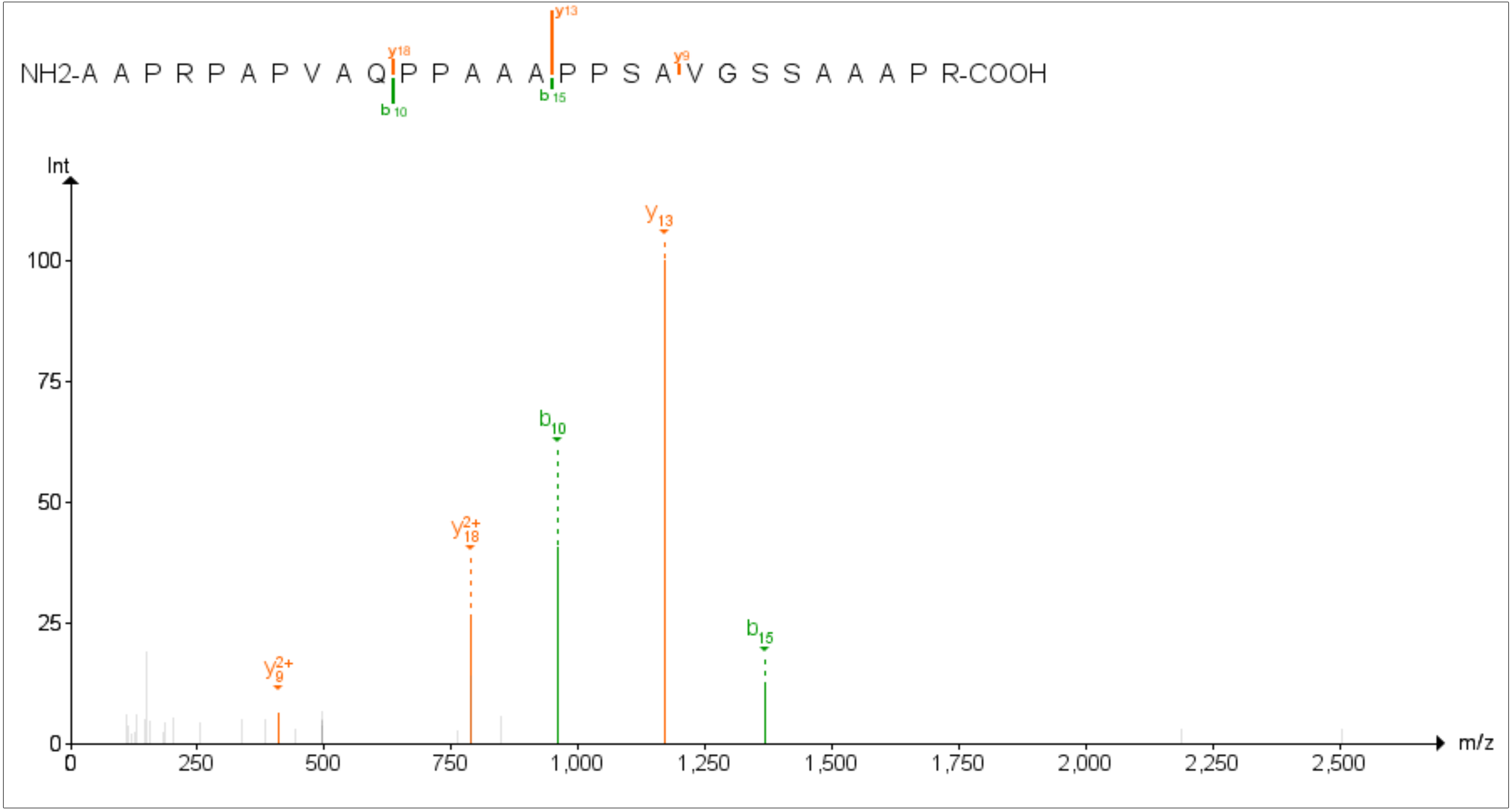
NH2-A A P R P A P V A Q P P A A A P P S A V G S S A A A P R-COOH
y18
y13
y9
b 10
b 15
Int
y13
b10
y18 2+
b15
y9 2+
100
75
50
25
0
0
250
500
750
1,000
1,250
1,500
1,750
2,000
2,250
2,500
m/z

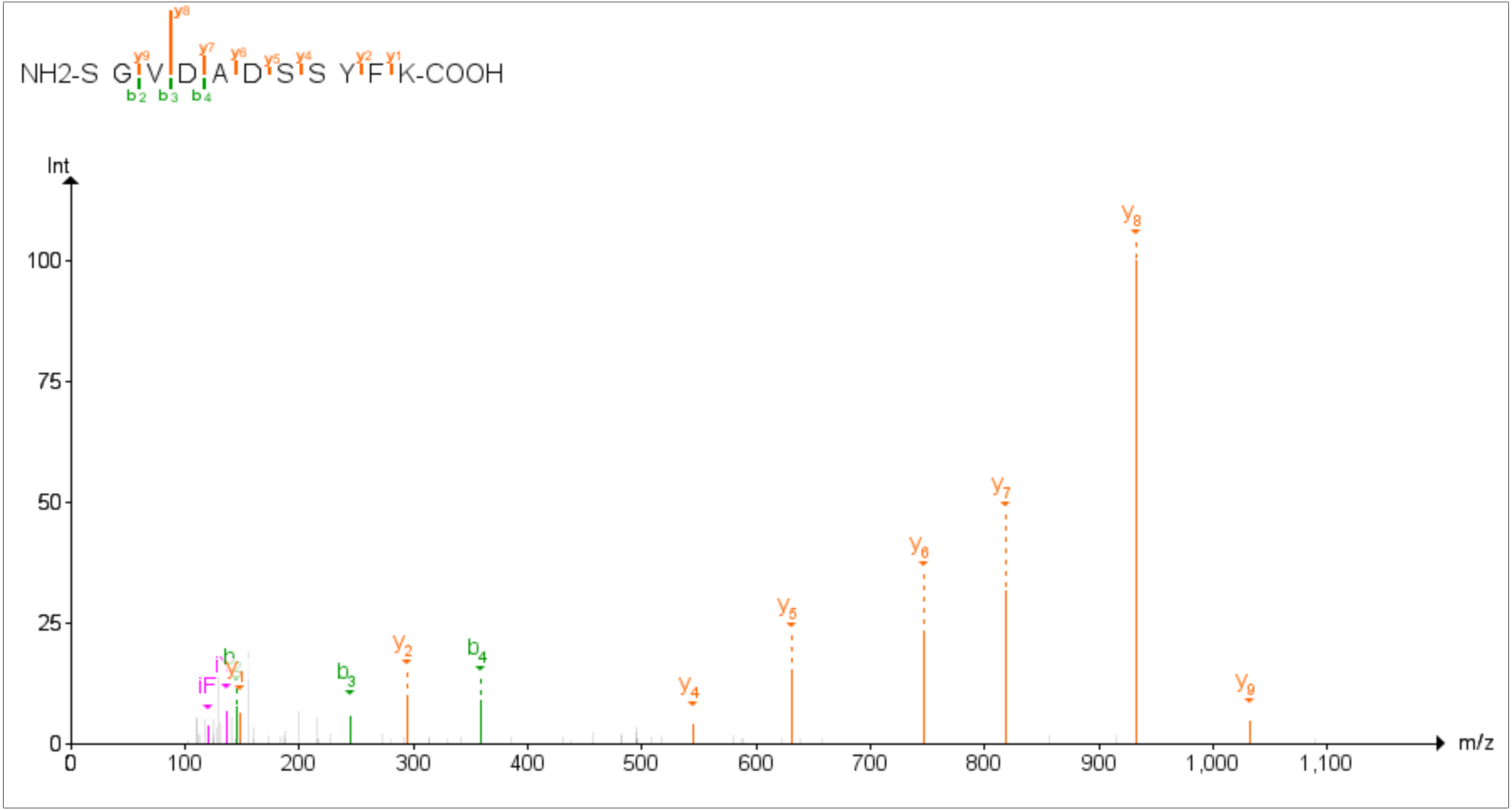
NH2-S G V D A D S S Y F K-COOH
y9 y8 y7 y6 y5 y4 y2 y1
b2 b3 b4
Int
100
75
50
25
0
m/z
0
100
200
300
400
500
600
700
800
900
1,000
1,100
y8
y7
y6
y5
y4
y2
y9
b4
b3
iF

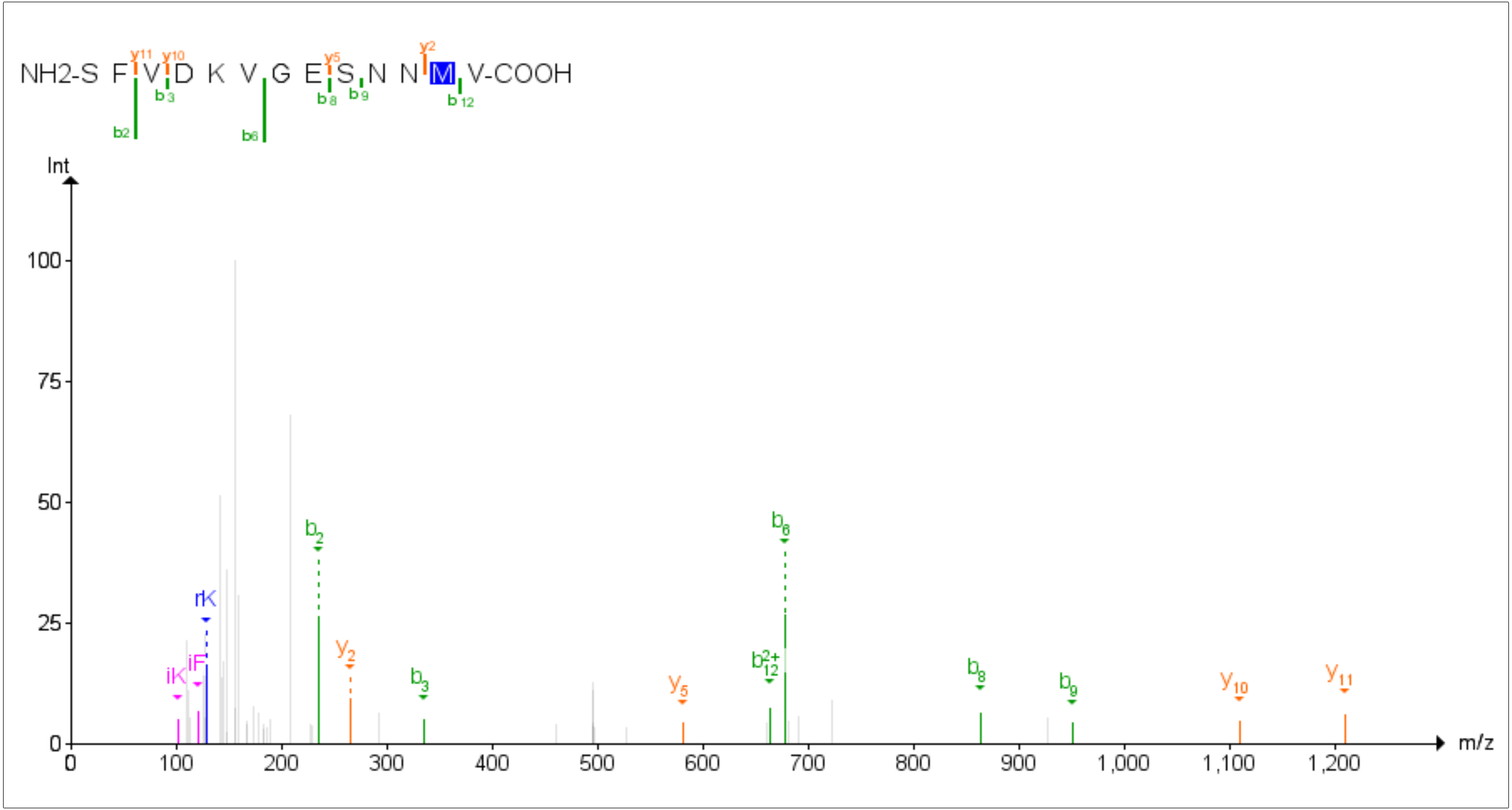

NH2-S F V D K V G E S N N M V-COOH
y11 y10 y5 y2
b2 b3 b6 b8 b9 b12
Int
100
75
50
25
0
rK
iK
iF
b2
y2
b3
b6
b12 2+
y5
b8
b9
y10
y11
0 100 200 300 400 500 600 700 800 900 1,000 1,100 1,200
m/z

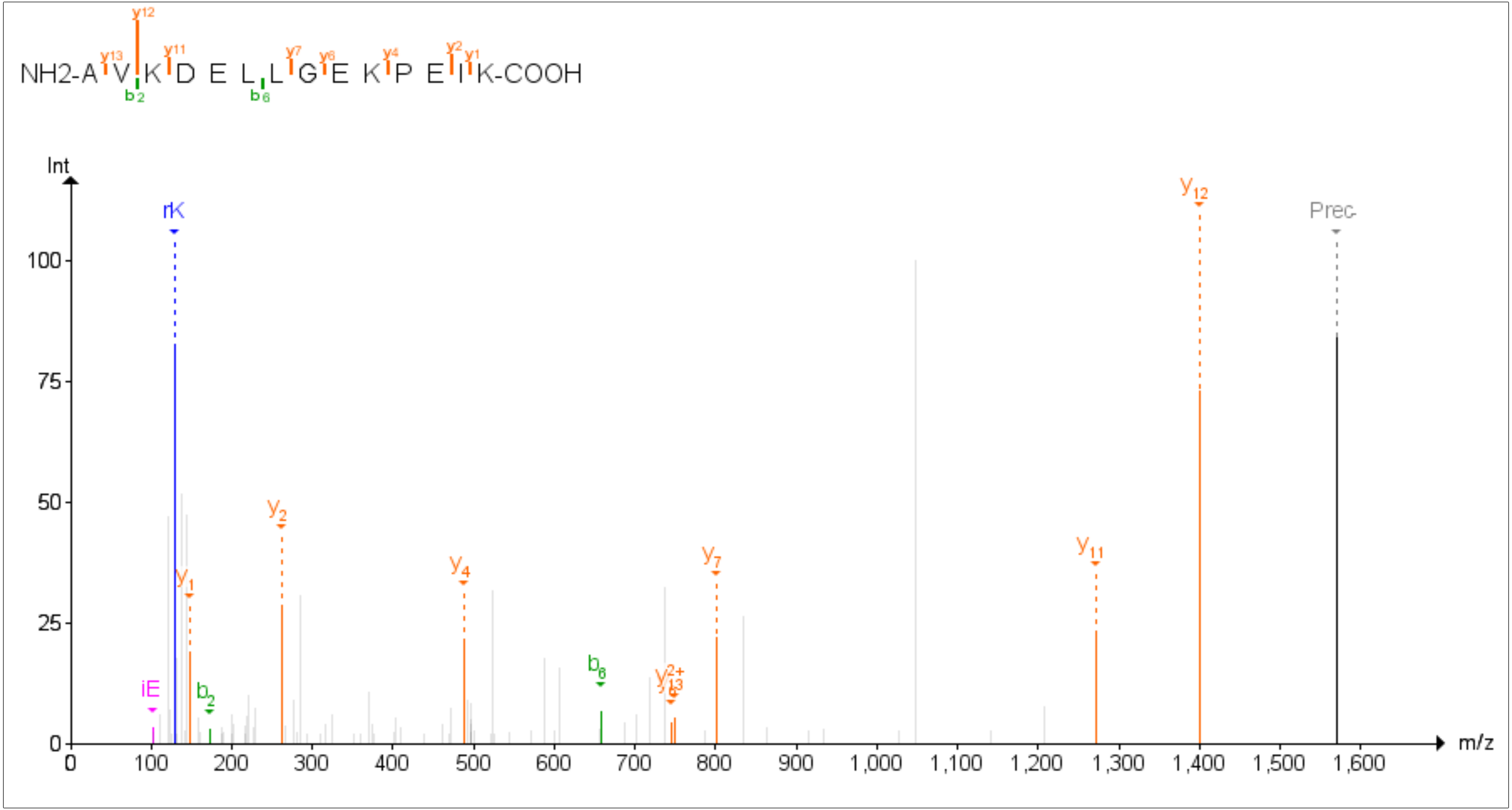

NH2-A V K D E L L G E K P E I K-COOH
y13 y12 y11 y7 y6 y4 y2 y1
b2 b6
Int
m/z
rK
Prec
iE
y1 y2 y4 y7 y11 y12
b2 b6
y13 2+
y6
0 25 50 75 100
0 100 200 300 400 500 600 700 800 900 1,000 1,100 1,200 1,300 1,400 1,500 1,600

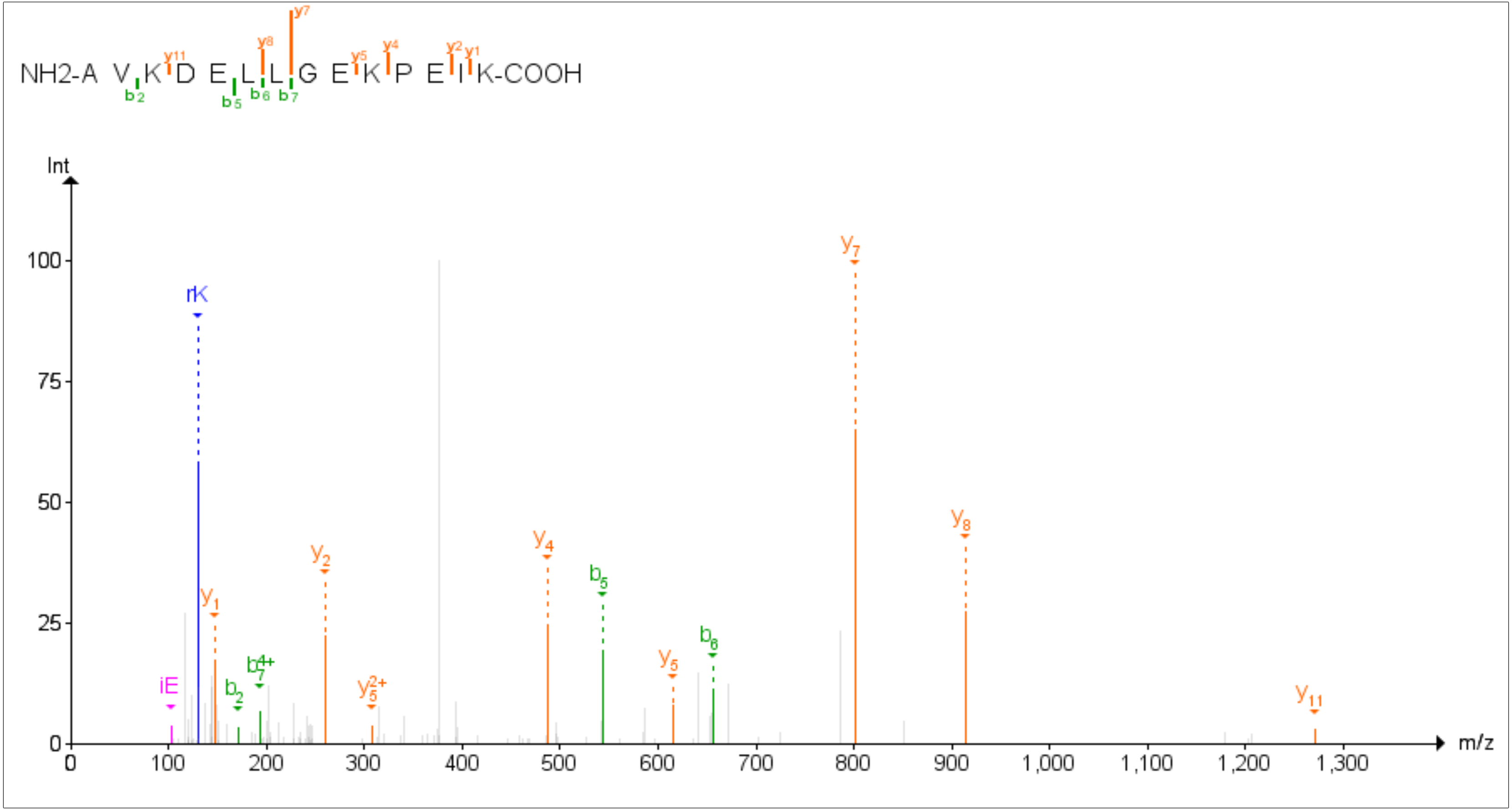

NH2-A V K D E L L G E K P E I K-COOH
y11 y8 y7 y5 y4 y2 y1
b2 b5 b6 b7
Int
100
75
50
25
0
rK
y7
y8
y4
y2
b5
y1
b6
y5
b_7^{4+}
iE
b_2
y_5^{2+}
y11
m/z
0 100 200 300 400 500 600 700 800 900 1,000 1,100 1,200 1,300

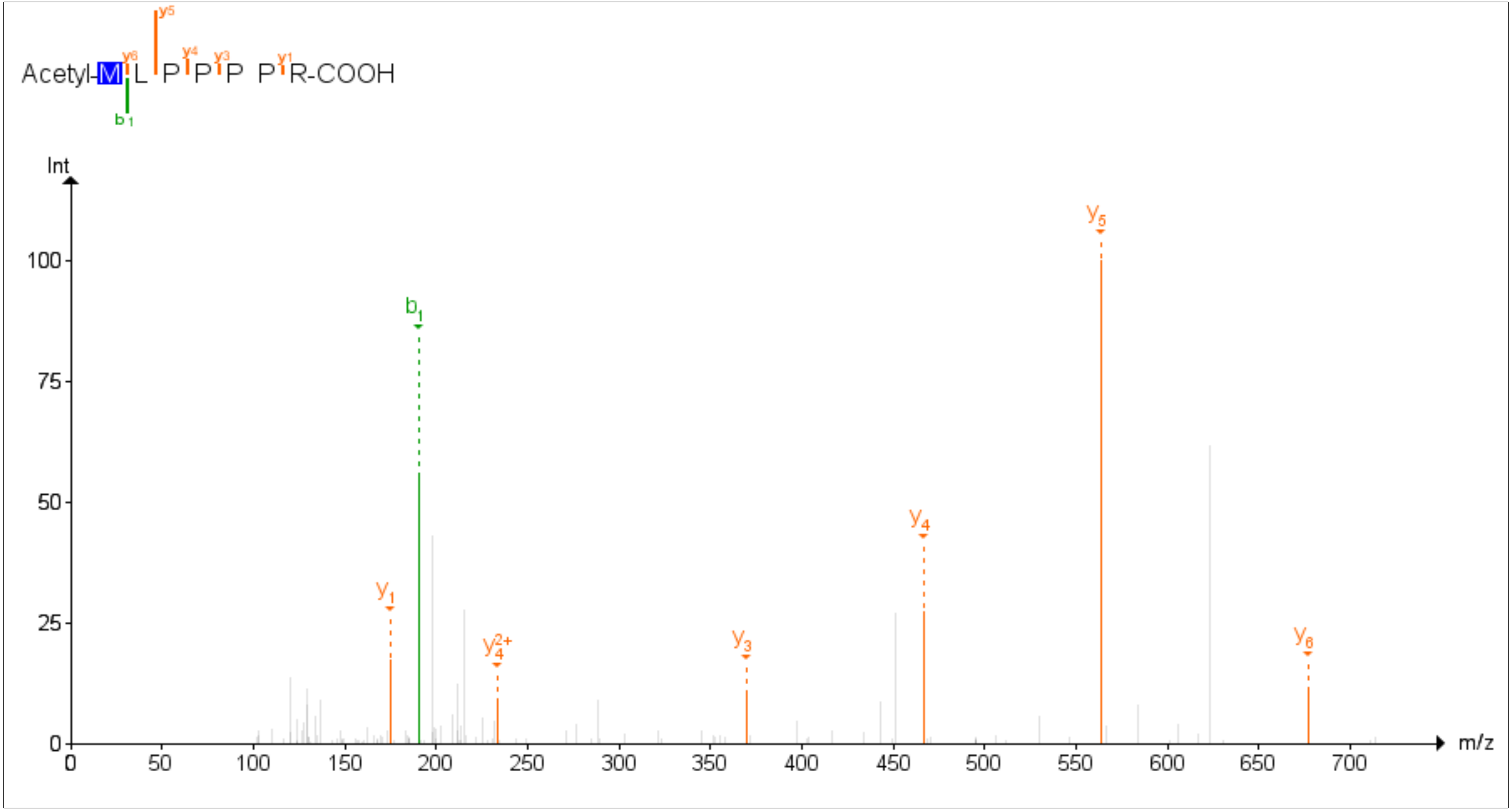

Acetyl-M L P P P P R-COOH
y6 y5 y4 y3 y1
b1
Int
100
75
50
25
0
b1
y1
y4 2+
y3
y4
y5
y6
0 50 100 150 200 250 300 350 400 450 500 550 600 650 700
m/z

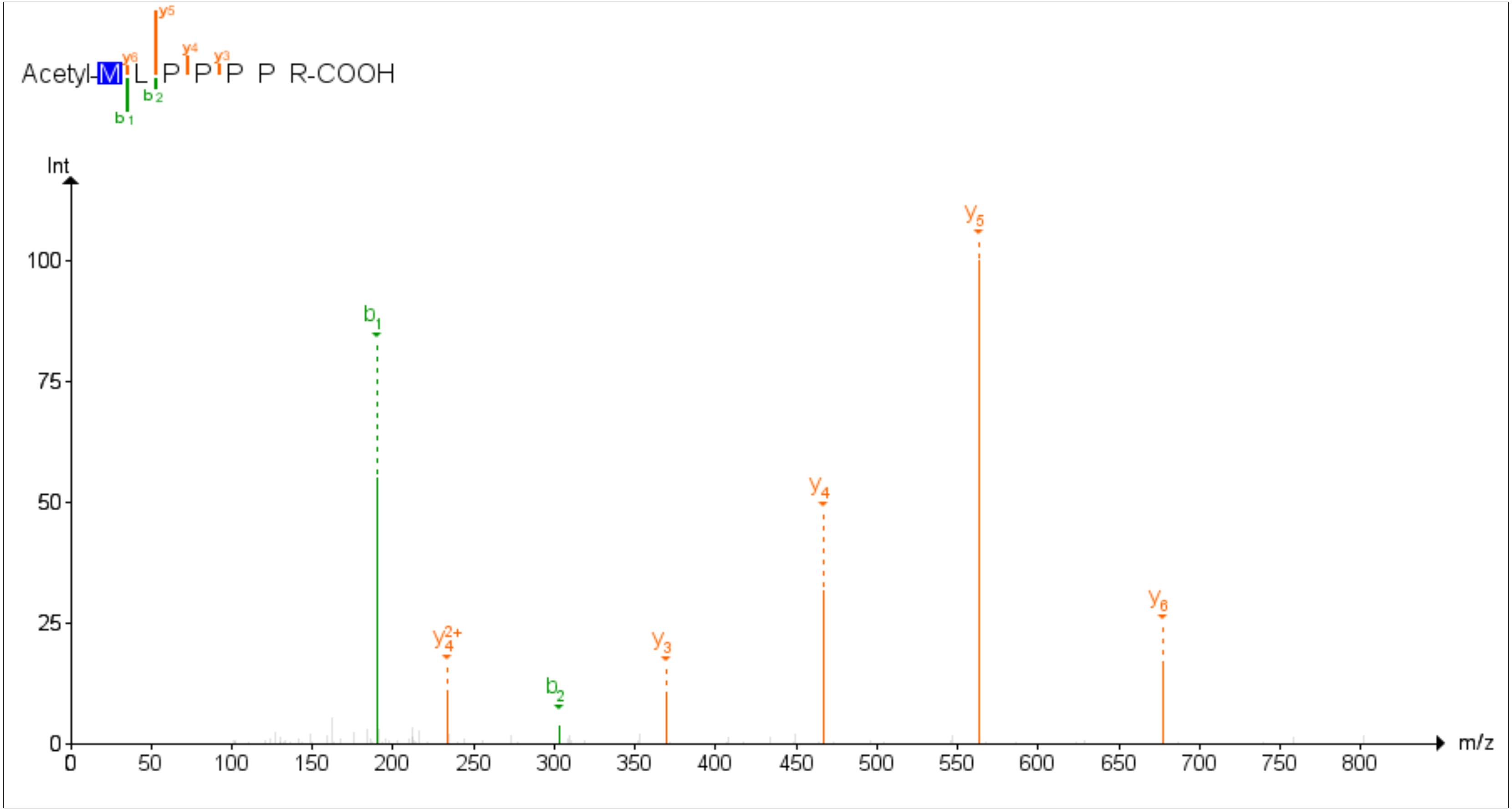
Acetyl-M L P P P P R-COOH
y5
y6
y4
y3
b 1
b 2
Int
100
75
50
25
0
b1
y4 2+
b2
y3
y4
y5
y6
0
50
100
150
200
250
300
350
400
450
500
550
600
650
700
750
800
m/z

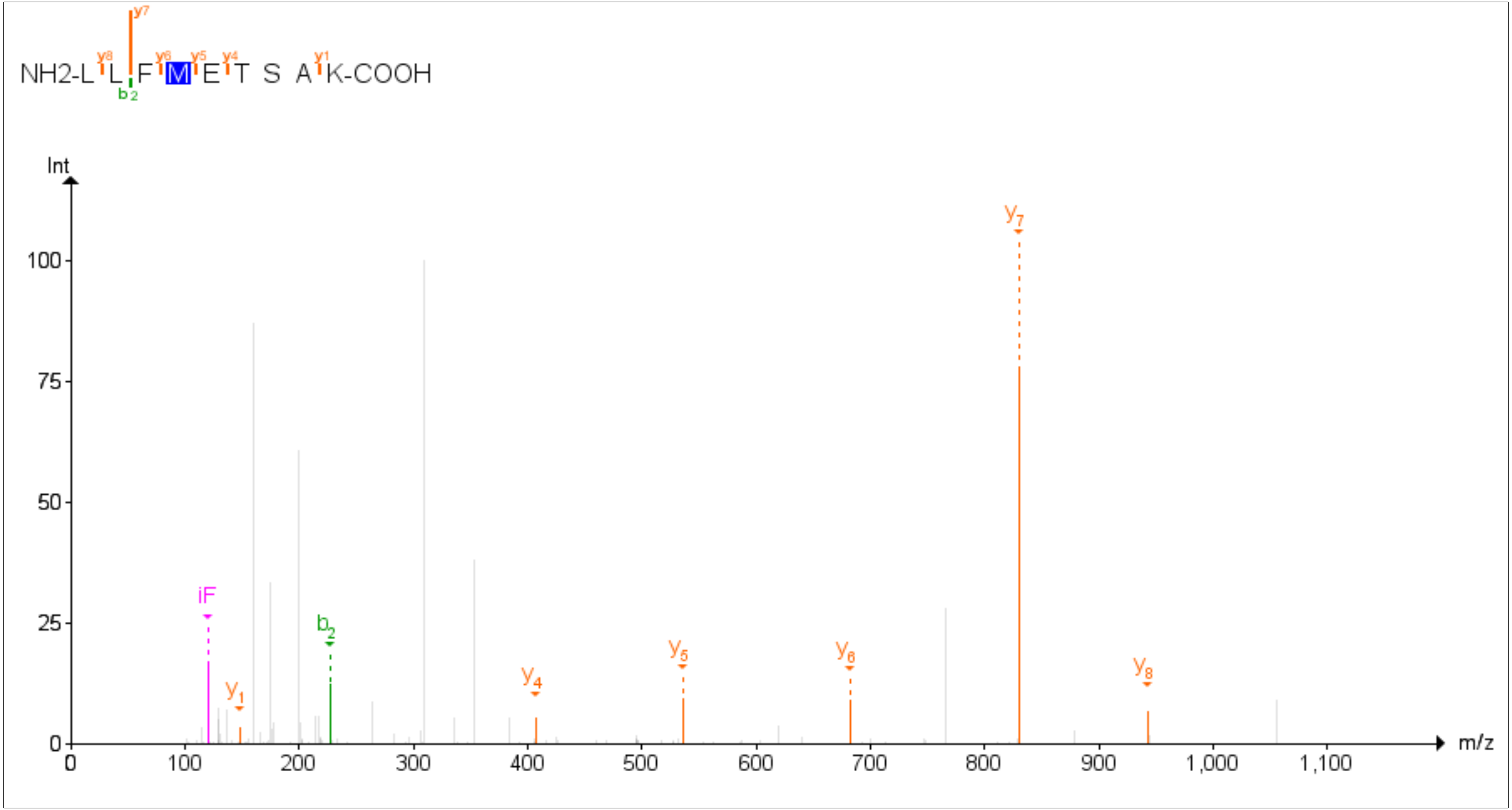
NH2-L L F M E T S A K-COOH
y8 y7 y6 y5 y4 y1
b2
Int
100
75
50
25
0
iF
y1
b2
y4
y5
y6
y7
y8
0 100 200 300 400 500 600 700 800 900 1,000 1,100
m/z

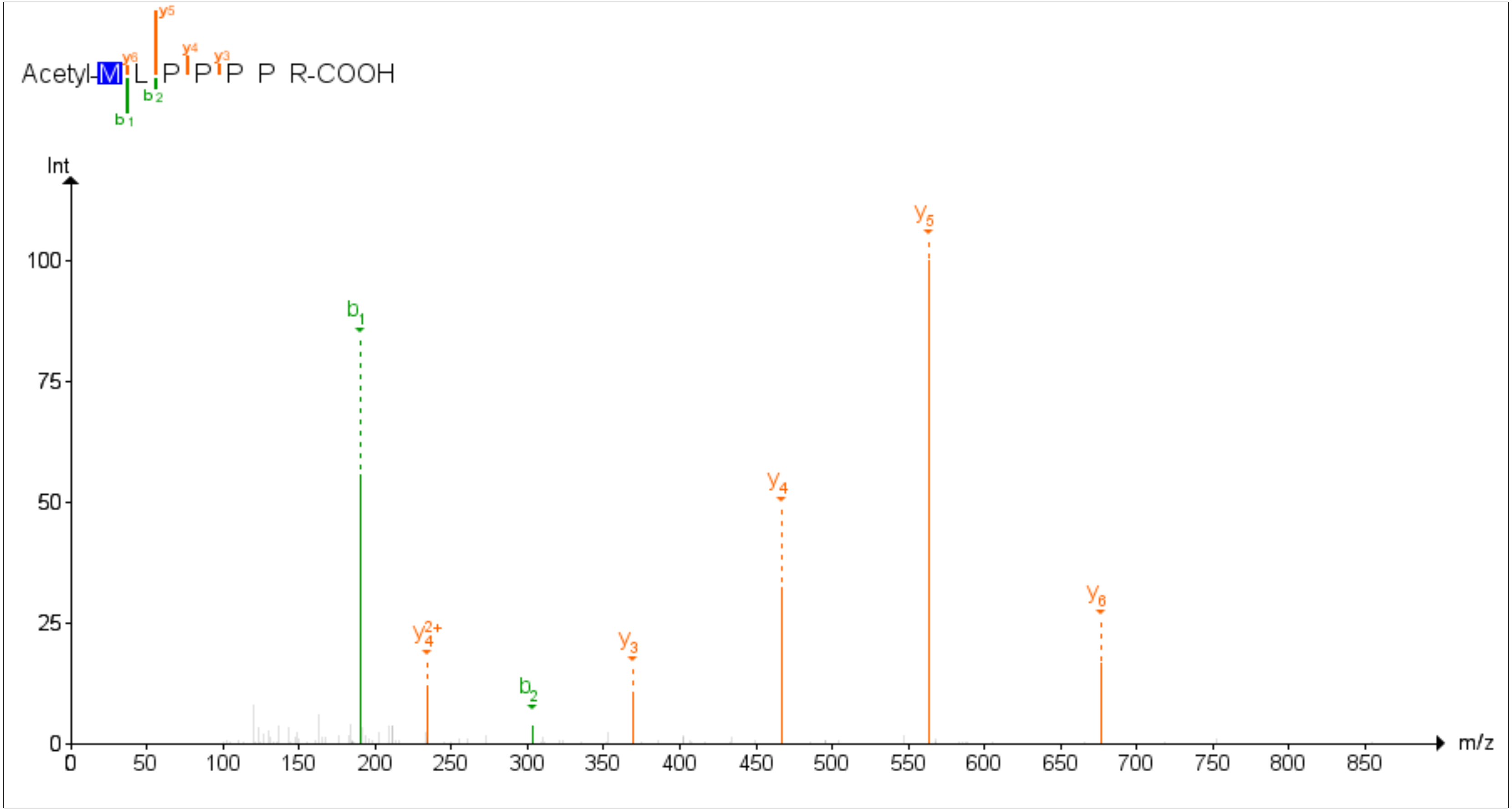
Acetyl-M L P P P P R-COOH
y5
y6
y4
y3
b 1
b 2
Int
100
75
50
25
0
b1
y4 2+
b2
y3
y4
y5
y6
m/z
0
50
100
150
200
250
300
350
400
450
500
550
600
650
700
750
800
850

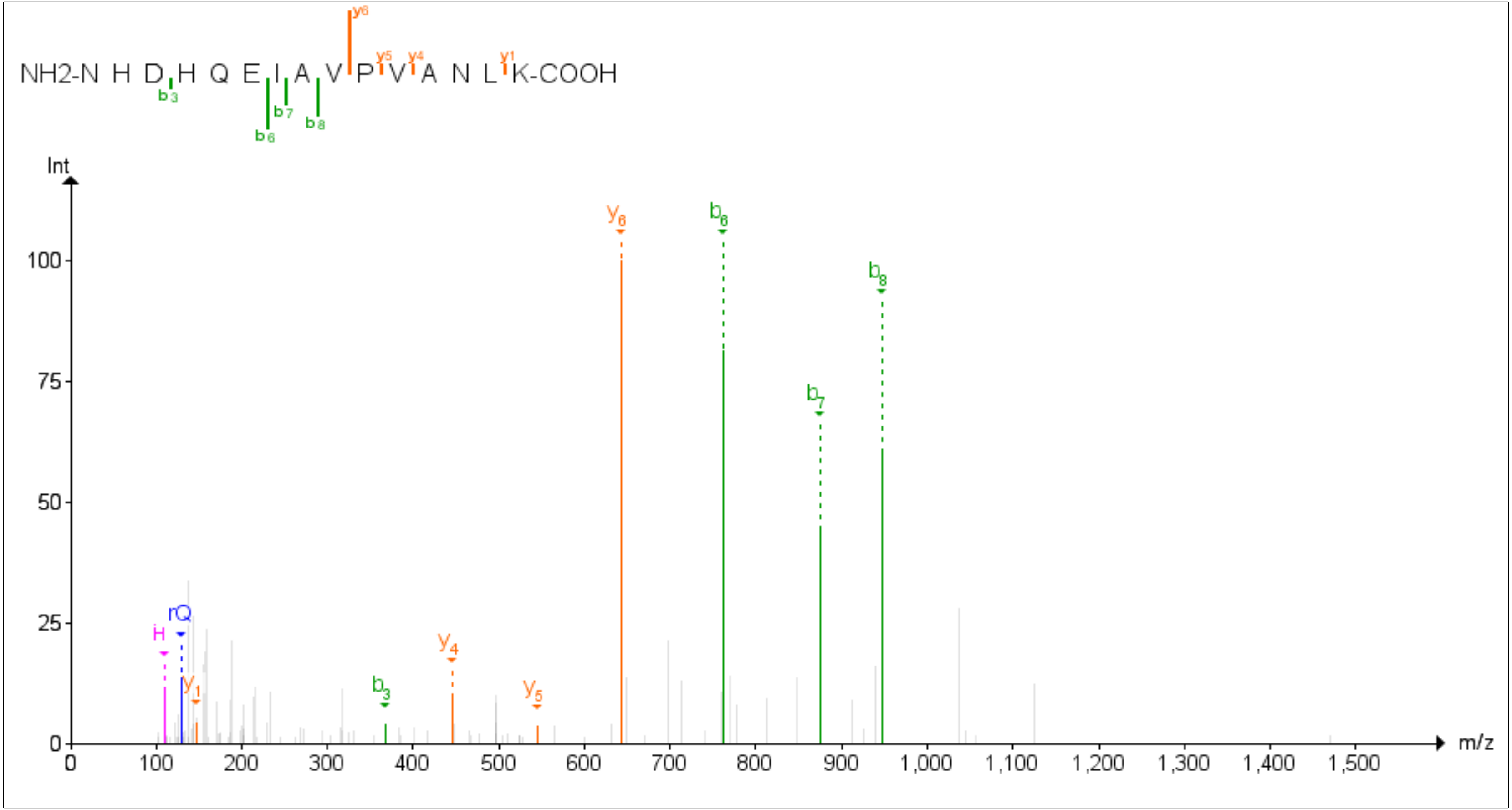
NH2-N H D H Q E I A V P V A N L K-COOH
y6
y5
y4
y1
b3
b7
b6
b8
Int
m/z
y6
b6
b8
b7
rQ
H
y1
b3
y4
y5
0
25
50
75
100
0
100
200
300
400
500
600
700
800
900
1,000
1,100
1,200
1,300
1,400
1,500

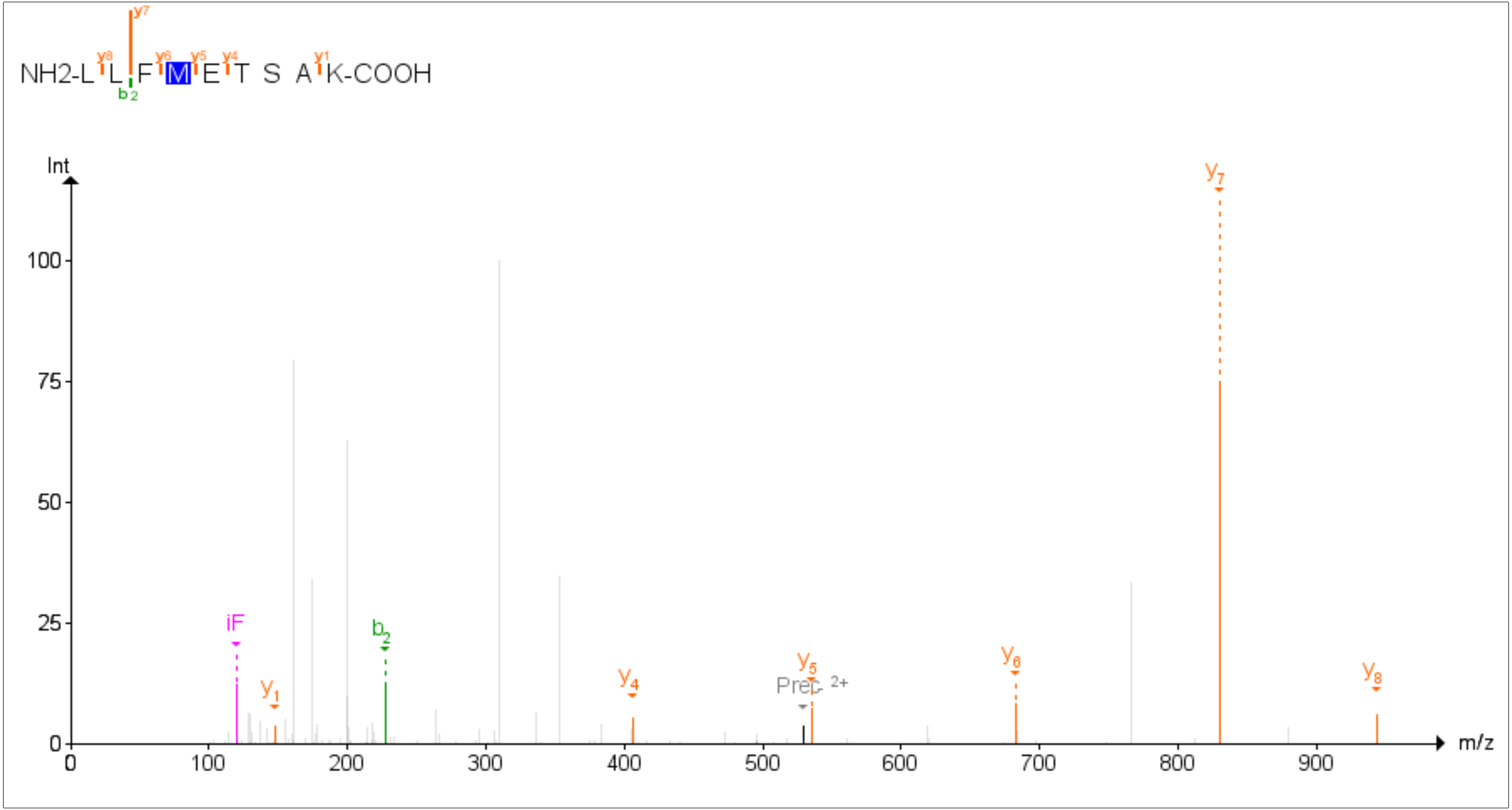

NH2-L L F M E T S A K-COOH
y8 y7 y6 y5 y4 y1 b2
Int
100
75
50
25
0
iF
y1
b2
y4
Prec 2+
y5
y6
y7
y8
0 100 200 300 400 500 600 700 800 900
m/z

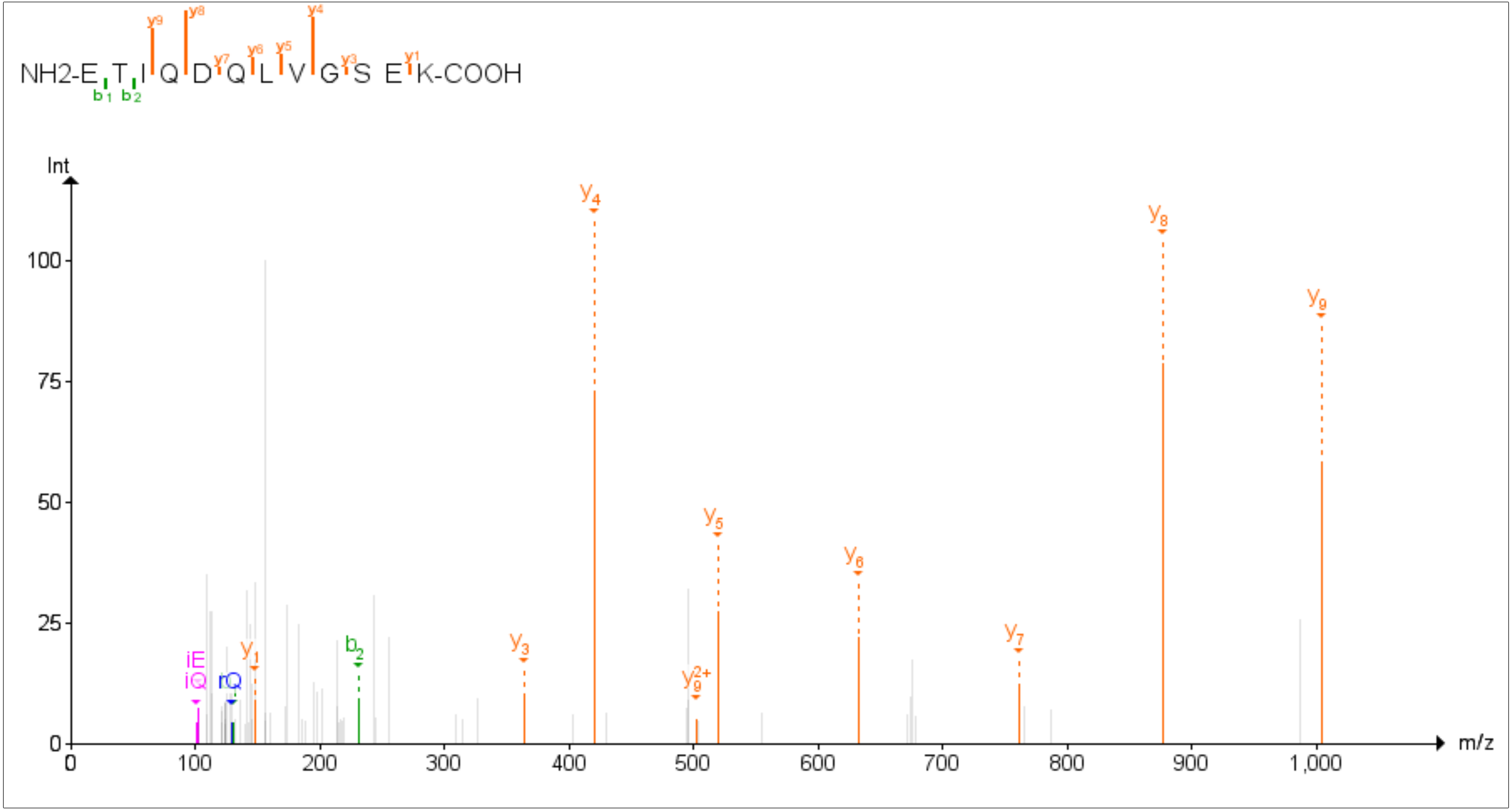
NH2-E T I Q D Q L V G S E K-COOH
y9 y8 y7 y6 y5 y4 y3 y1
b1 b2
Int
100
75
50
25
0
m/z
0 100 200 300 400 500 600 700 800 900 1,000
y4
y8
y9
y5
y6
y7
y3
y1
b2
iE
iQ
rQ
y9 2+

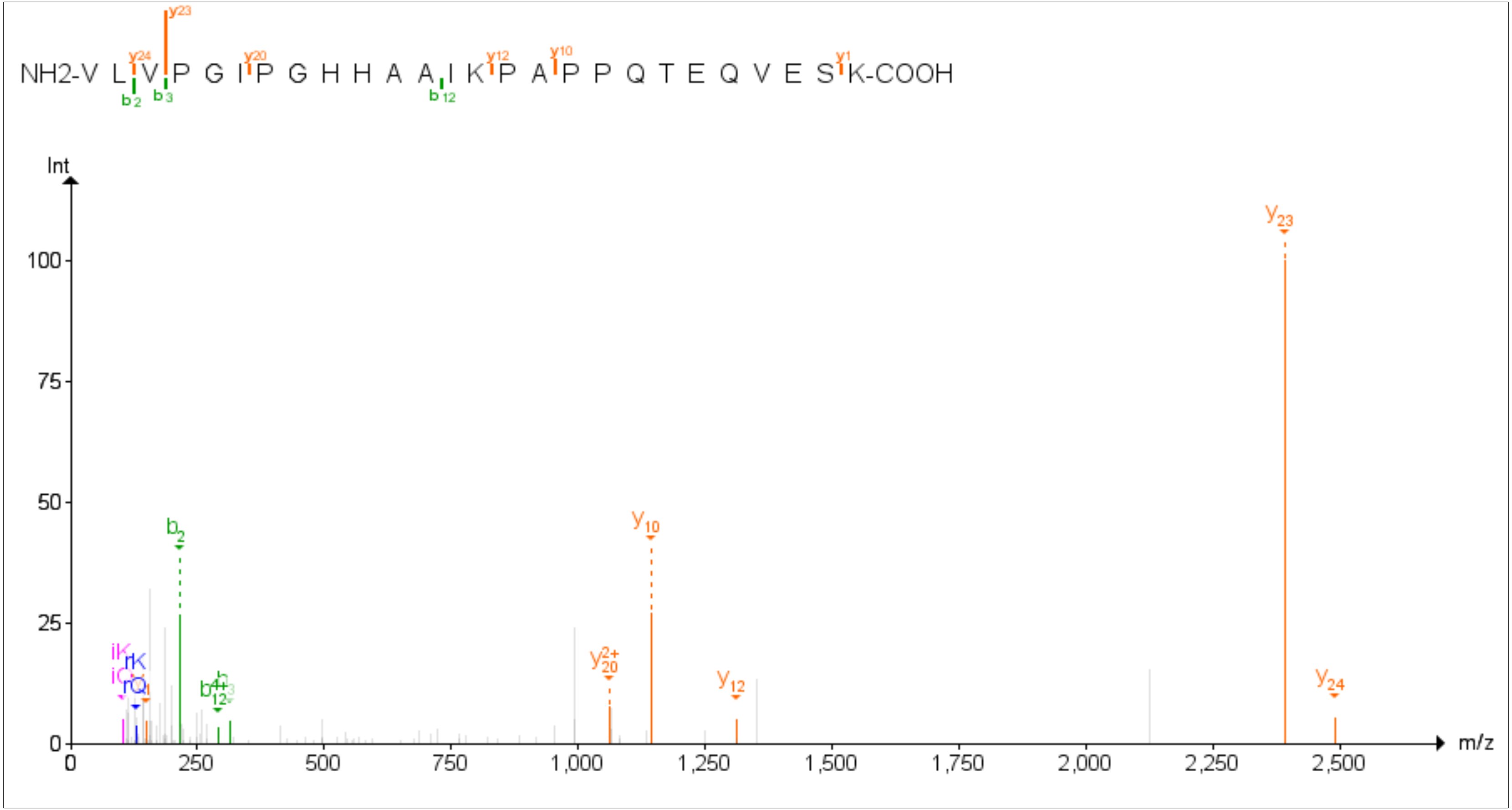
NH2-V L V P G I P G H H A A I K P A P P Q T E Q V E S K-COOH
y24
y23
y20
y12
y10
y1
b2
b3
b12
Int
100
75
50
25
0
y23
y10
b2
y20 2+
y12
y24
iK
rK
rQ
0
250
500
750
1,000
1,250
1,500
1,750
2,000
2,250
2,500
m/z

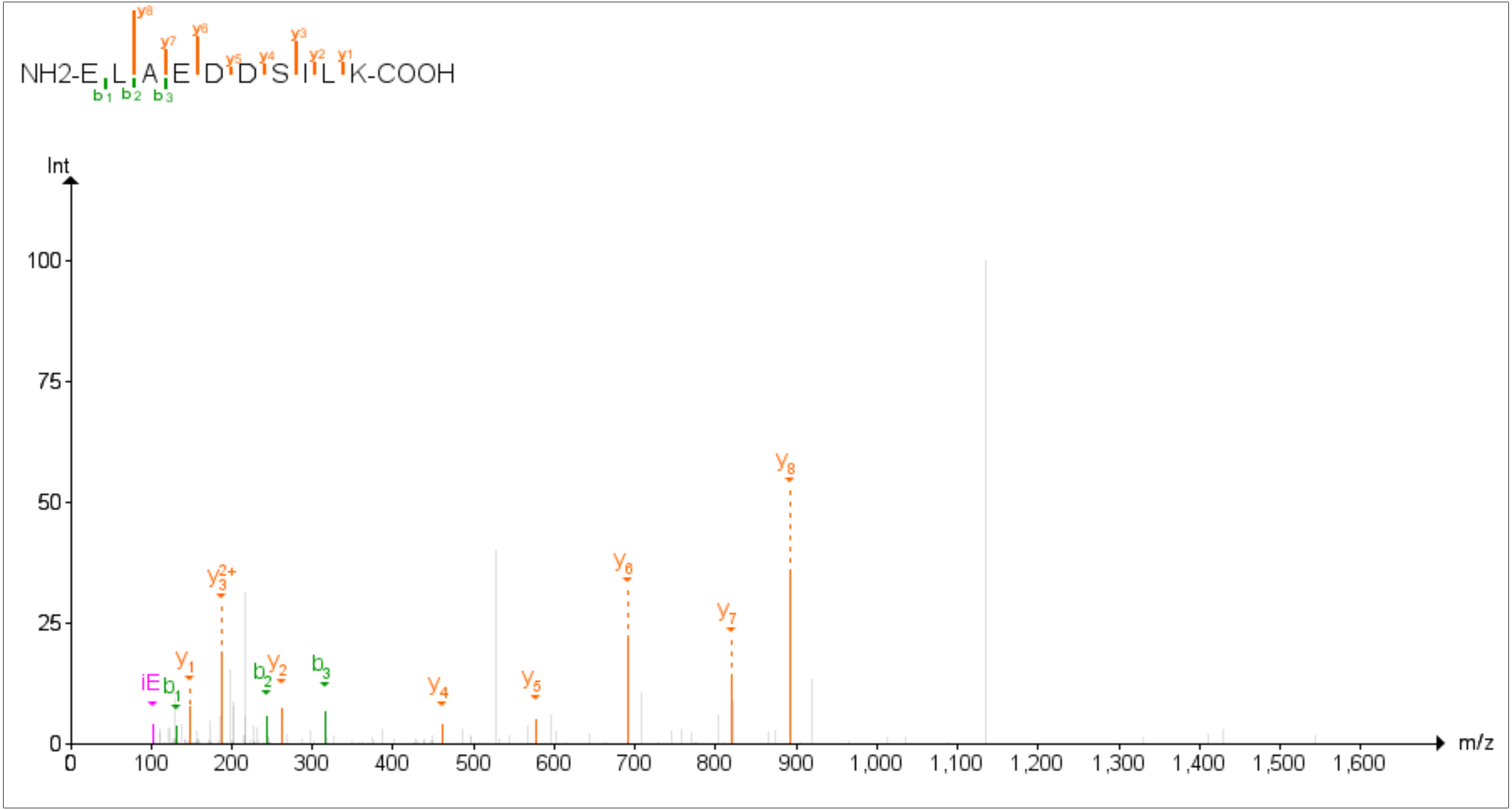

NH2-E L A E D D S I L K-COOH
b1 b2 b3
y8 y7 y6 y5 y4 y3 y2 y1
Int
m/z
iE

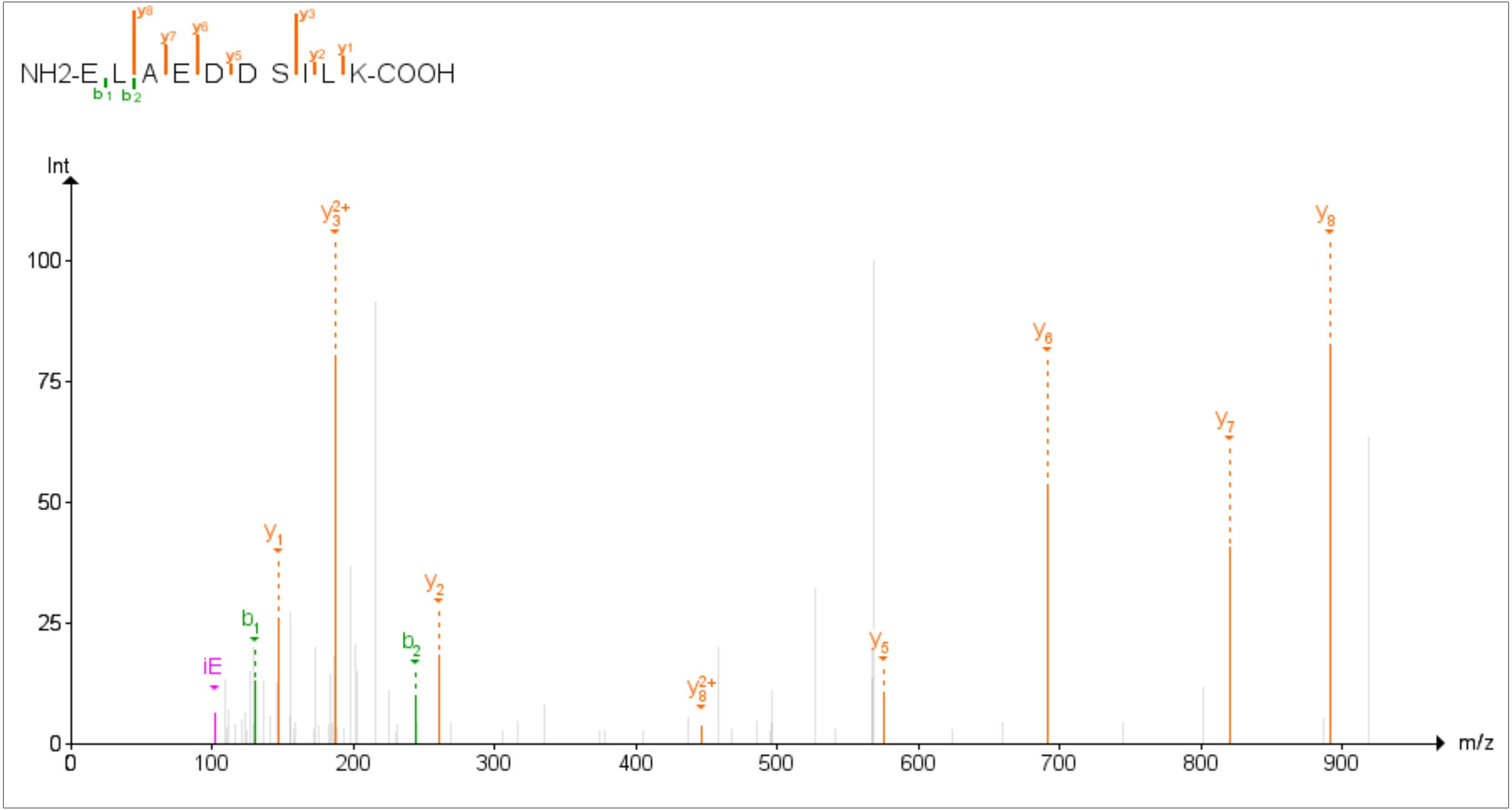

NH2-E L A E D D S I L K-COOH
b1 b2
y8 y7 y6 y5 y3 y2 y1
Int
m/z
0
25
50
75
100
0
100
200
300
400
500
600
700
800
900
iE
b1
b2
y1
y2
y3 2+
y5
y6
y7
y8
y8 2+

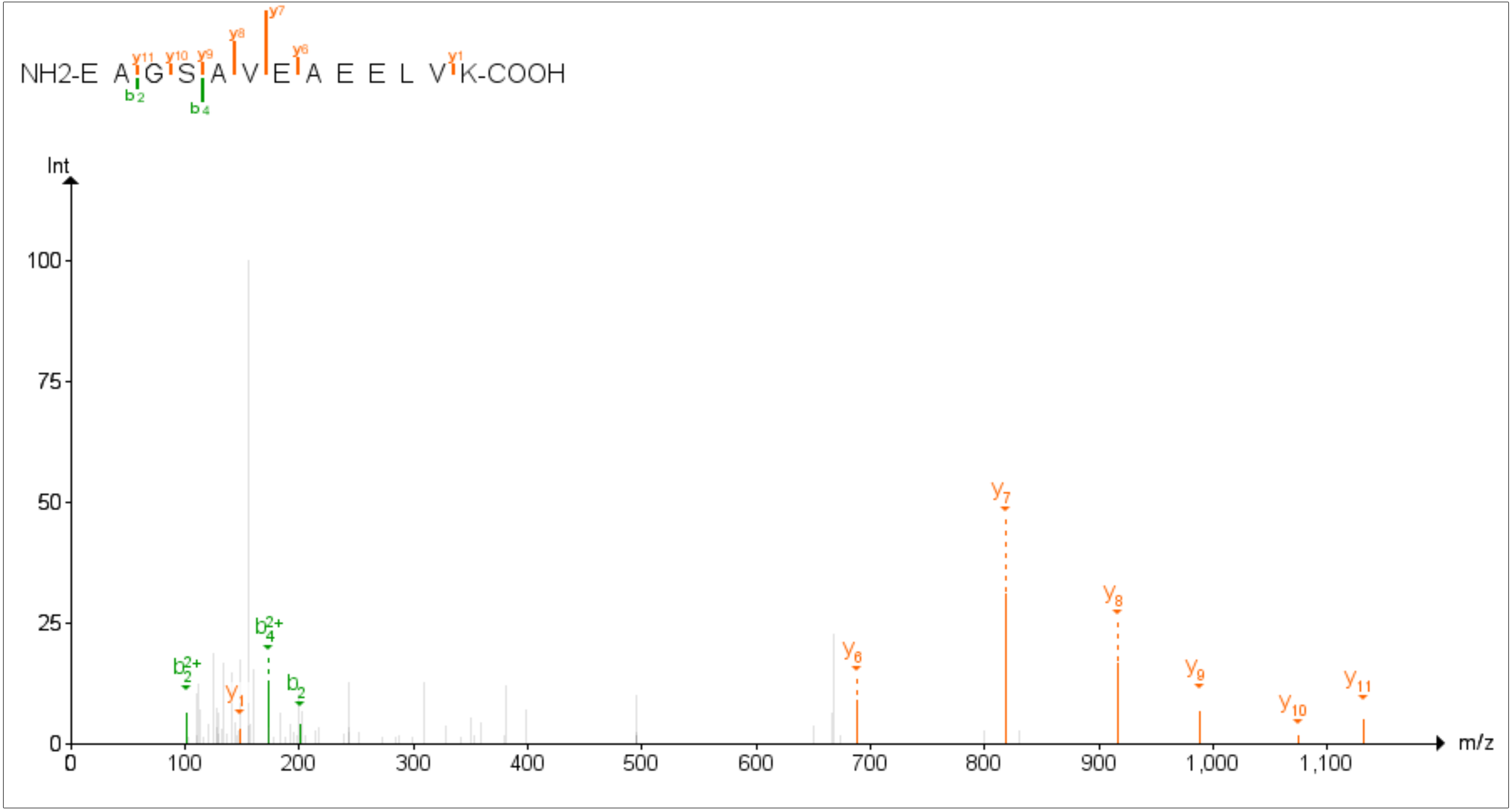

NH2-E A G S A V E A E E L V K-COOH
y11 y10 y9 y8 y7 y6 y1
b2 b4
Int
100
75
50
25
0
0 100 200 300 400 500 600 700 800 900 1,000 1,100
m/z
b2 2+
y1
b4 2+
b2
y6
y7
y8
y9
y10
y11

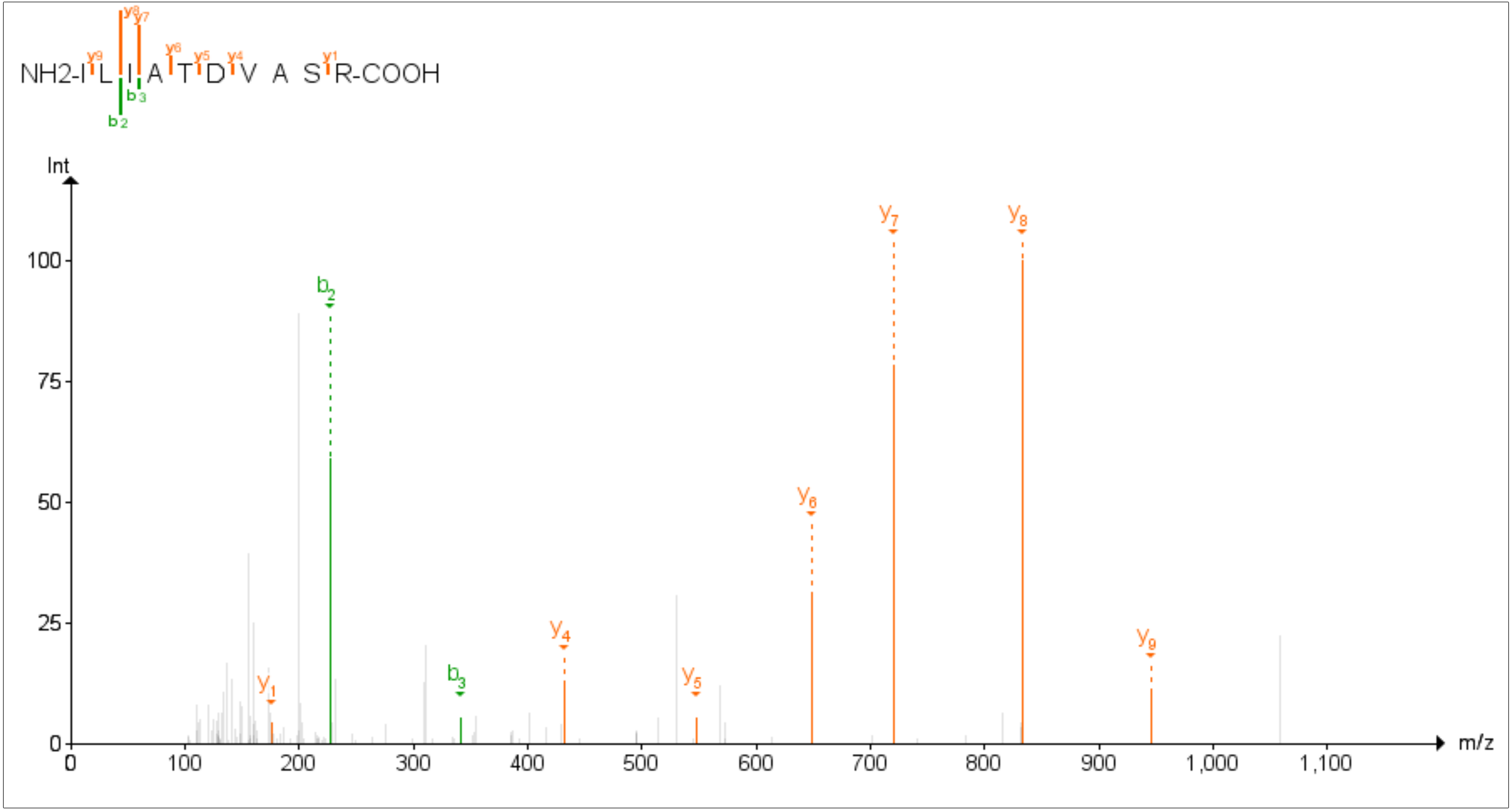

NH2-I L I A T D V A S R-COOH
y9 y8 y7 y6 y5 y4 y1
b2 b3
Int
100
75
50
25
0
0 100 200 300 400 500 600 700 800 900 1,000 1,100
m/z
b2 b3 y1 y4 y5 y6 y7 y8 y9

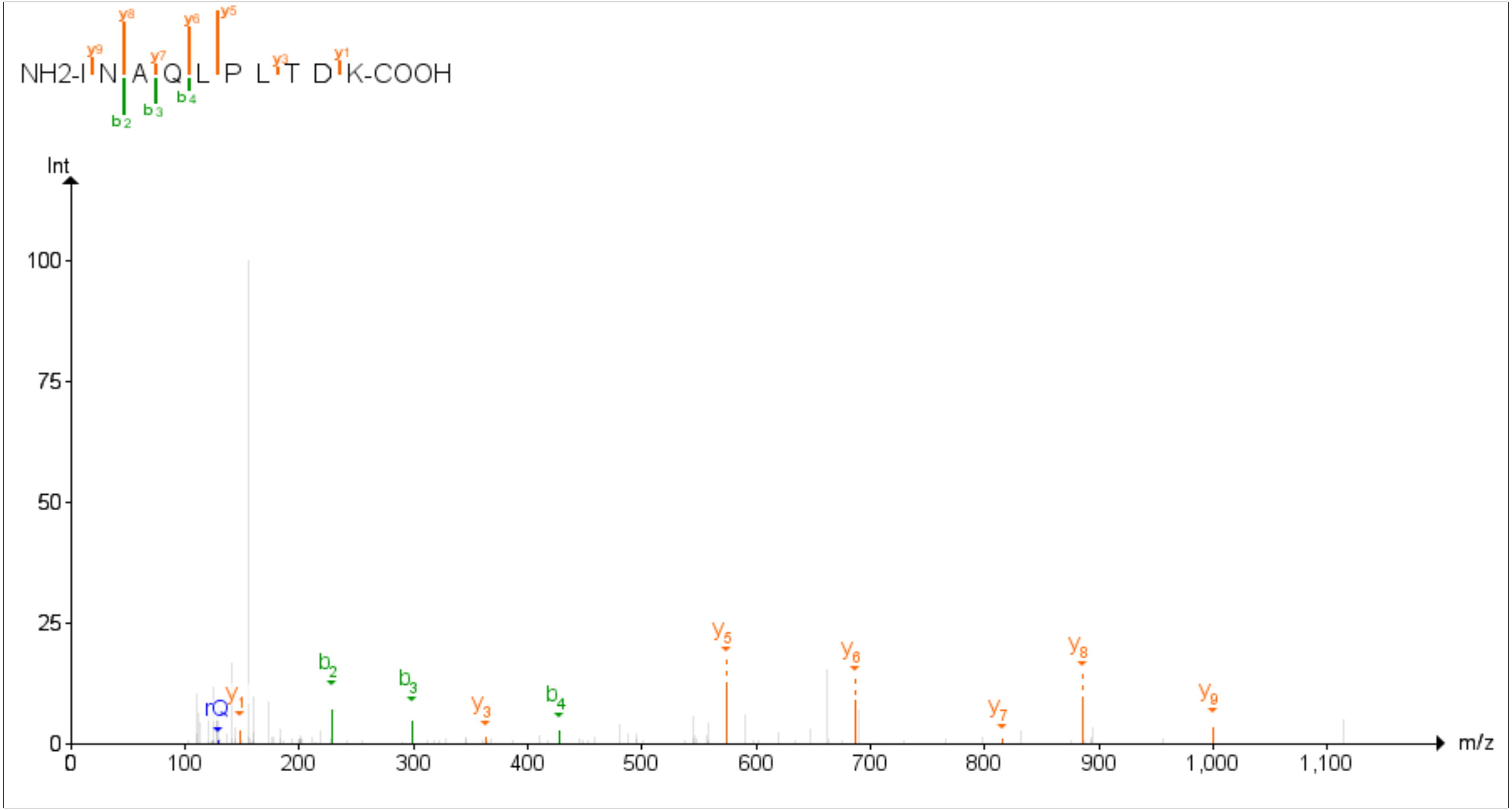

NH2-I N A Q L P L T D K-COOH
y9 y8 y7 y6 y5 y3 y1
b2 b3 b4
Int
100
75
50
25
0
m/z
0 100 200 300 400 500 600 700 800 900 1,000 1,100
rQ y1 b2 b3 y3 b4 y5 y6 y7 y8 y9

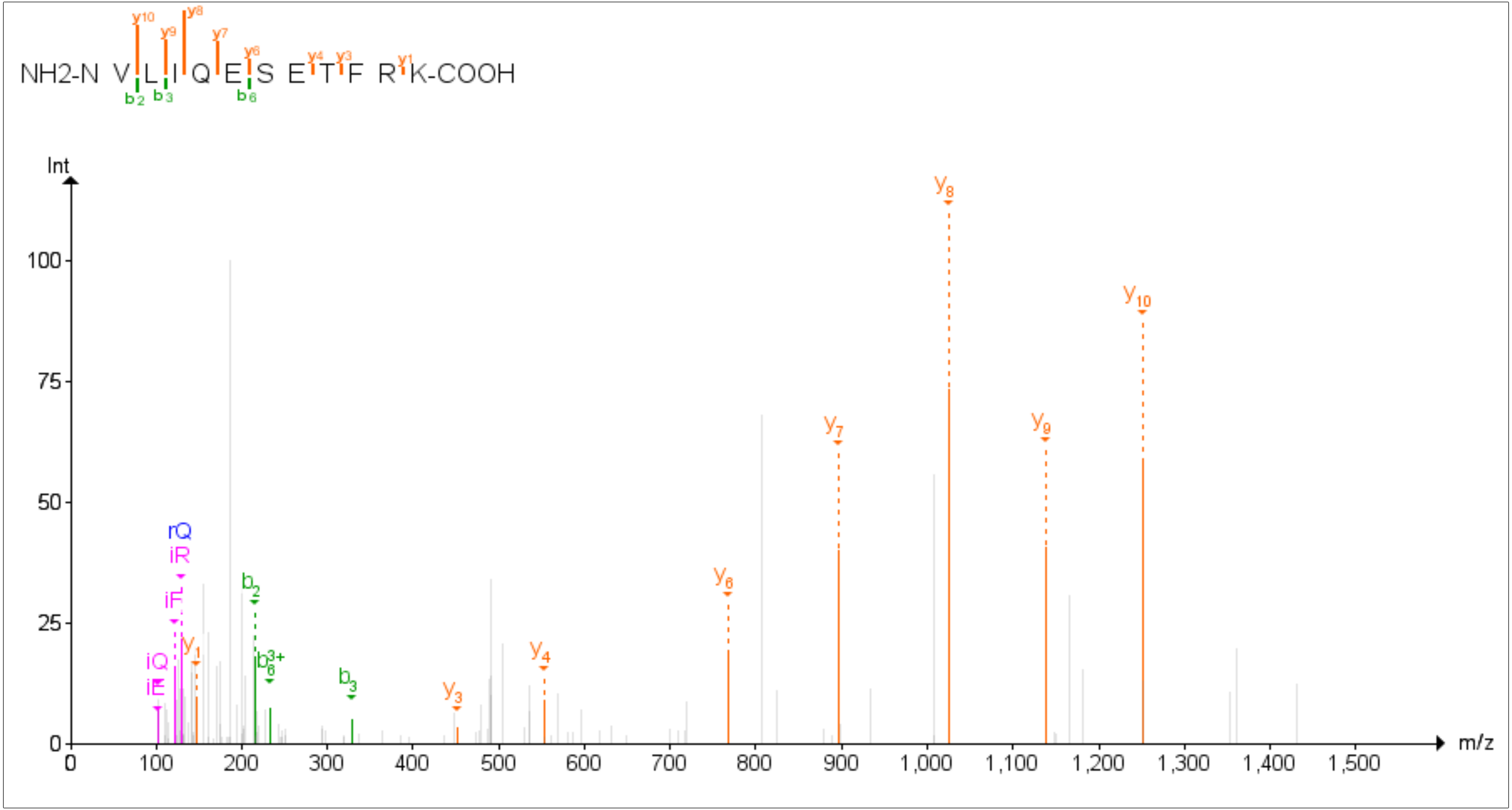

NH2-N V L I Q E S E T F R K-COOH
y10 y9 y8 y7 y6 y4 y3 y1
b2 b3 b6
Int
m/z
y8
y10
y7
y9
y6
y4
y3
y1
b2
b3
b6 3+
rQ
iR
iF
iQ
iE
0
25
50
75
100
0
100
200
300
400
500
600
700
800
900
1,000
1,100
1,200
1,300
1,400
1,500

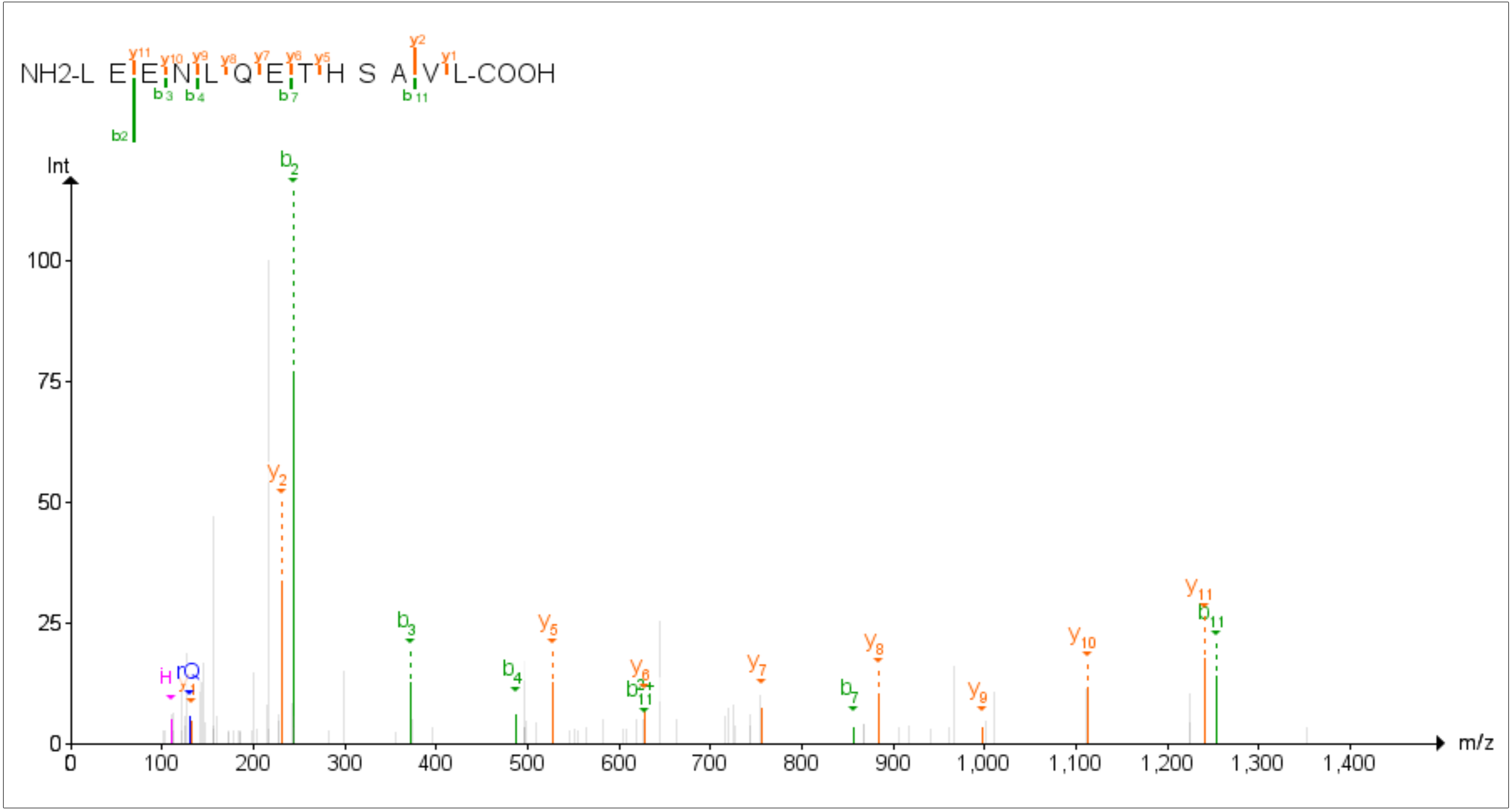

NH2-L E E N L Q E T H S A V L-COOH
Int
m/z

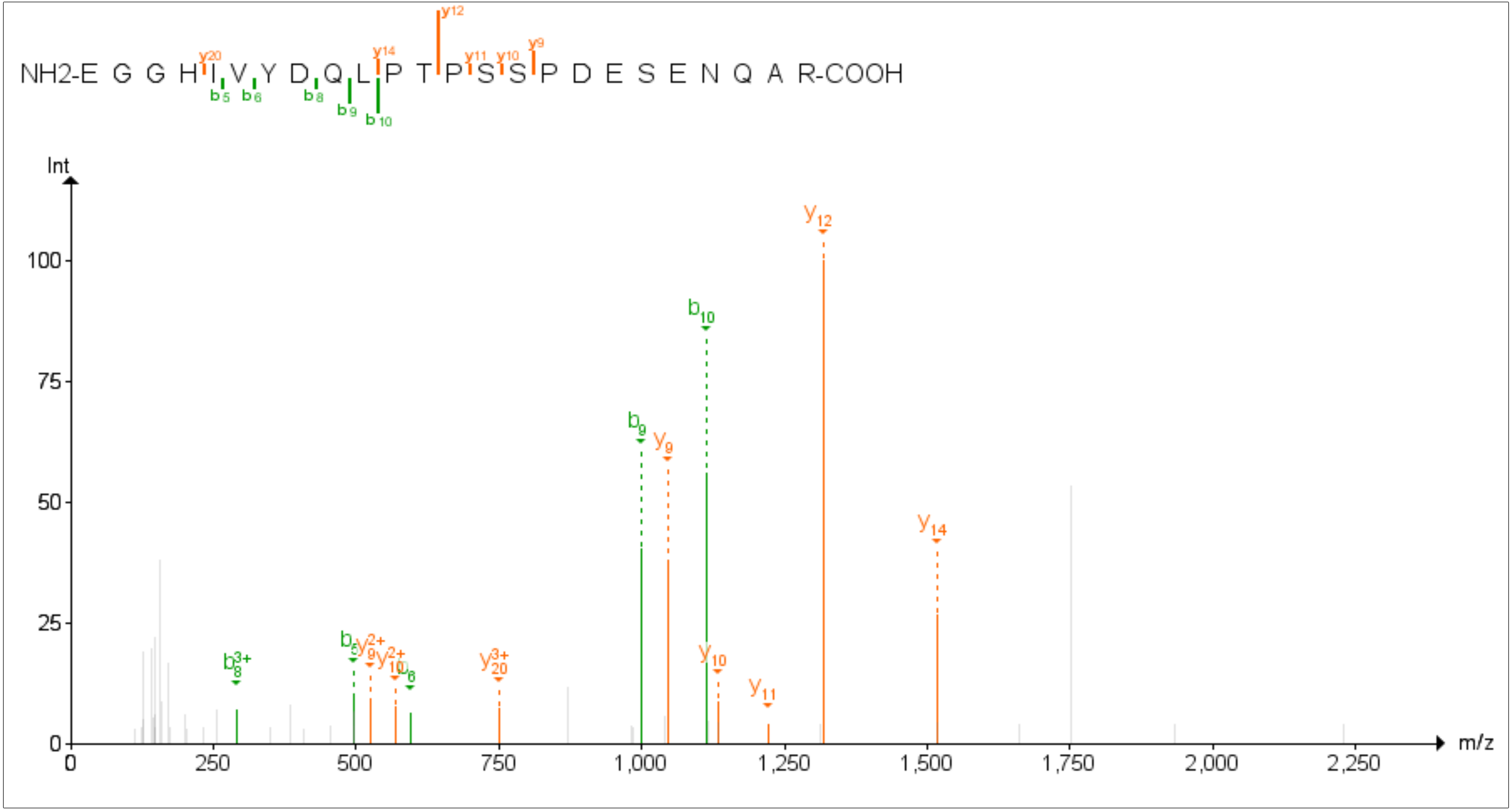

NH2-E G G H I V Y D Q L P T P S S P D E S E N Q A R-COOH
y20 y14 y12 y11 y10 y9
b5 b6 b8 b9 b10
Int
m/z
0
25
50
75
100
0
250
500
750
1,000
1,250
1,500
1,750
2,000
2,250
y12
b10
b9
y9
y14
b8 3+
b5
y9 2+
y10 2+
b6
y20 3+
y10
y11

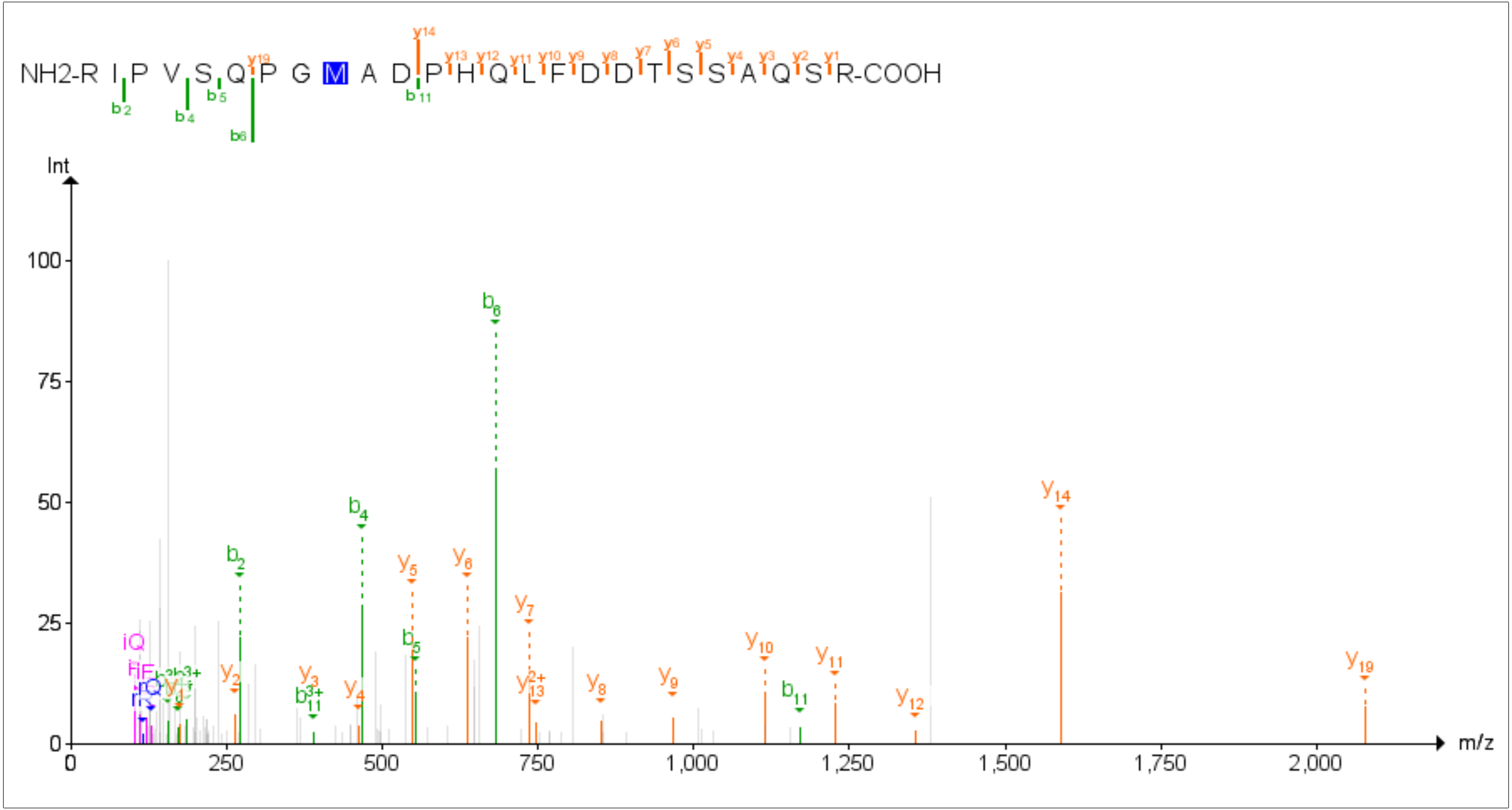

NH2-R I P V S Q P G M A D P H Q L F D D T S S A Q S R-COOH
Int
m/z
0
25
50
75
100
250
500
750
1,000
1,250
1,500
1,750
2,000
b2
b4
b5
b6
b11
y1
y2
y3
y4
y5
y6
y7
y8
y9
y10
y11
y12
y13
y14
y19
iQ

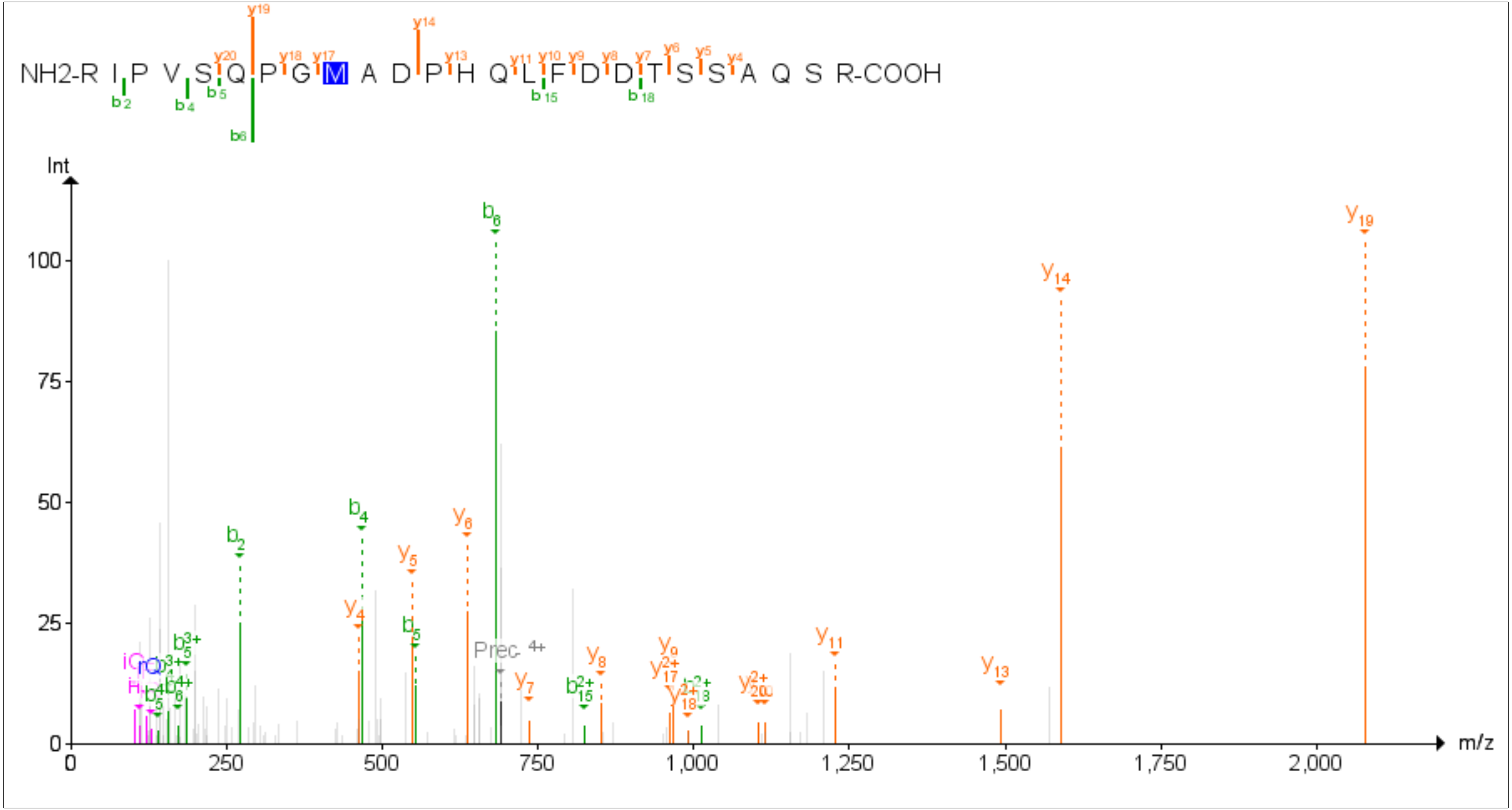

NH2-R I P V S Q P G M A D P H Q L F D D T S S A Q S R-COOH
Int
m/z
Prec 4+

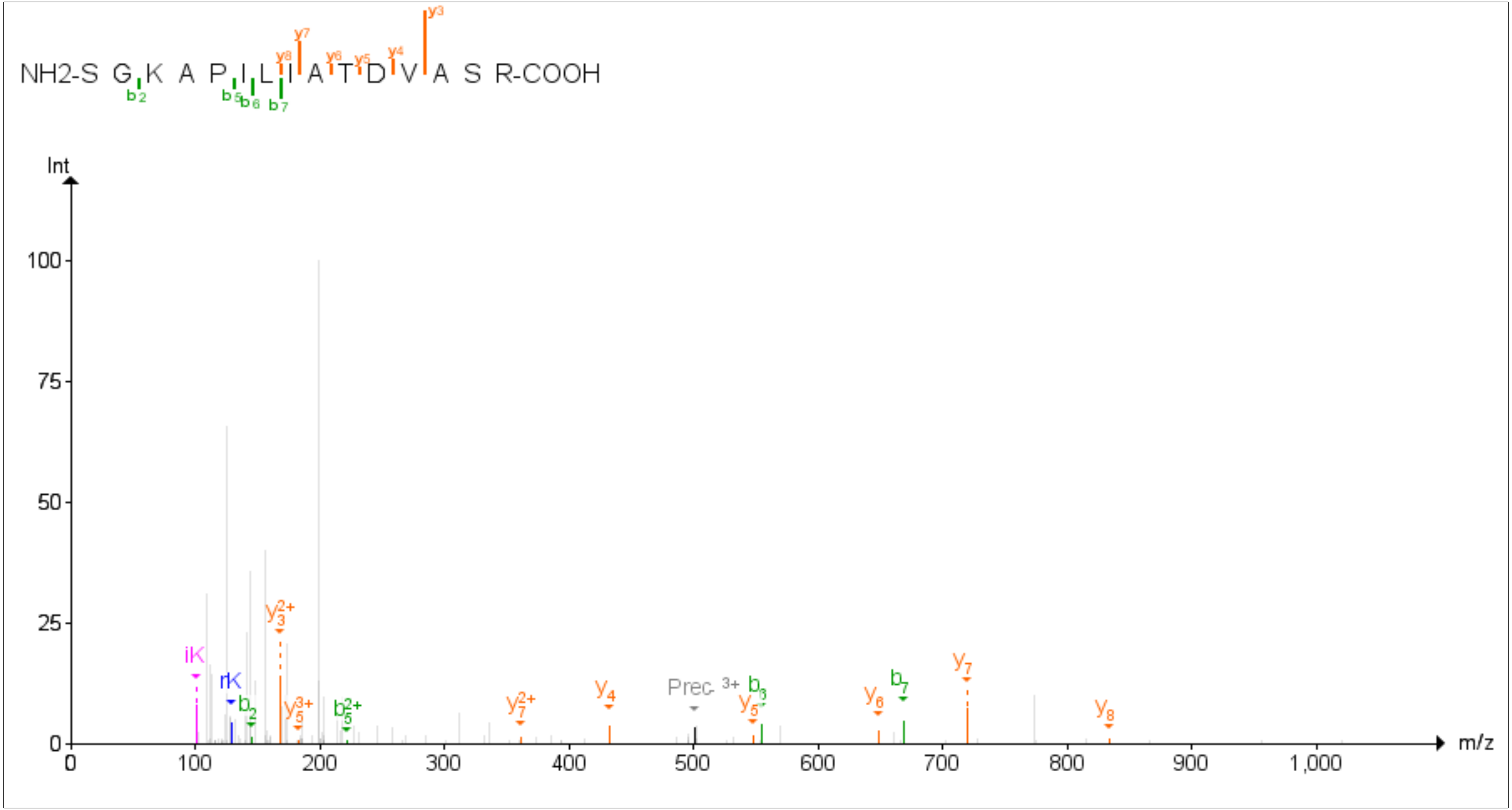

NH2-S G K A P I L I A T D V A S R-COOH
Int
m/z
iK
rK
Prec 3+

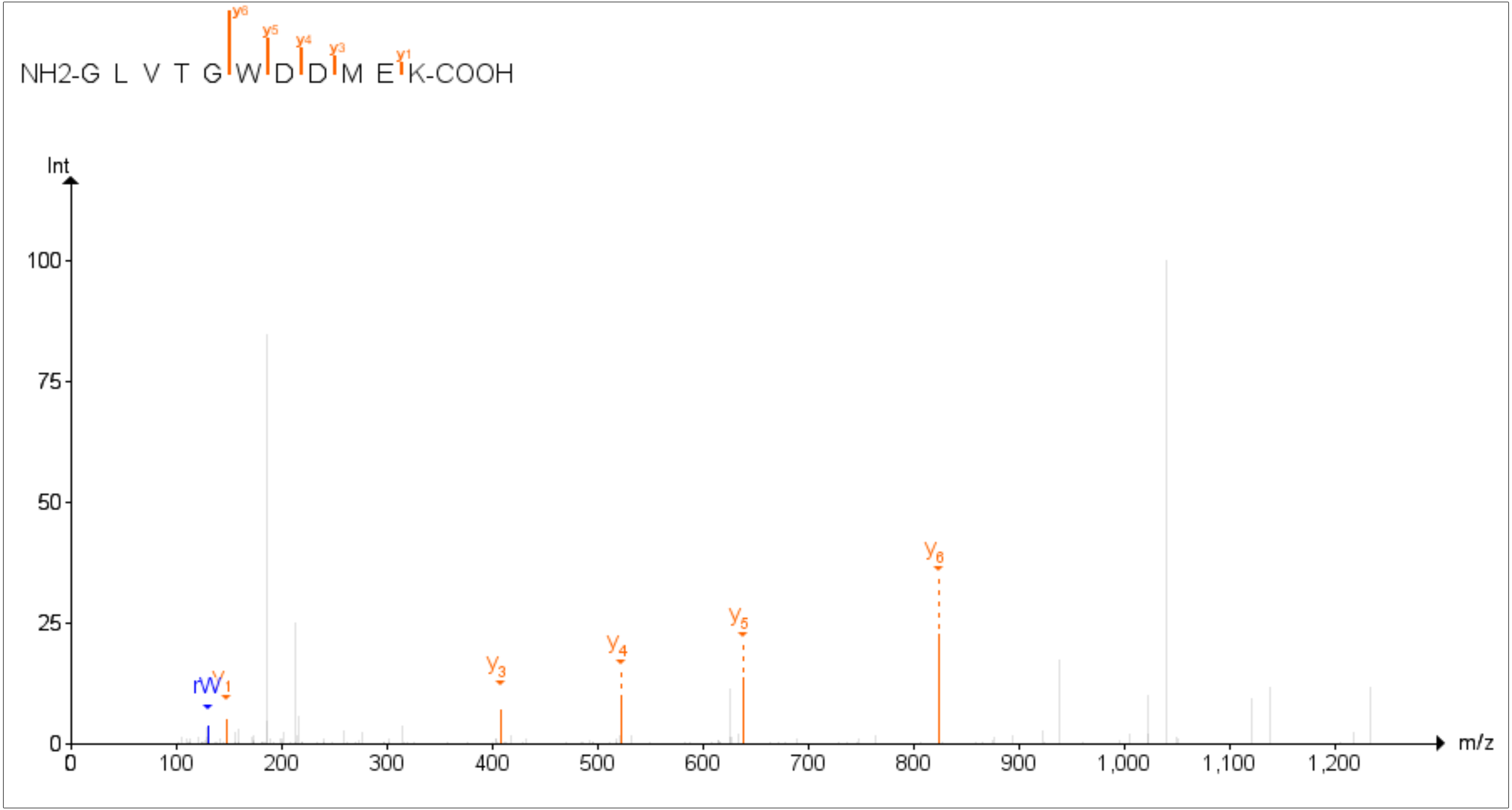

NH2-G L V T G W D D M E K-COOH
y6
y5
y4
y3
y1
Int
100
75
50
25
0
rW
y1
y3
y4
y5
y6
0
100
200
300
400
500
600
700
800
900
1,000
1,100
1,200
m/z

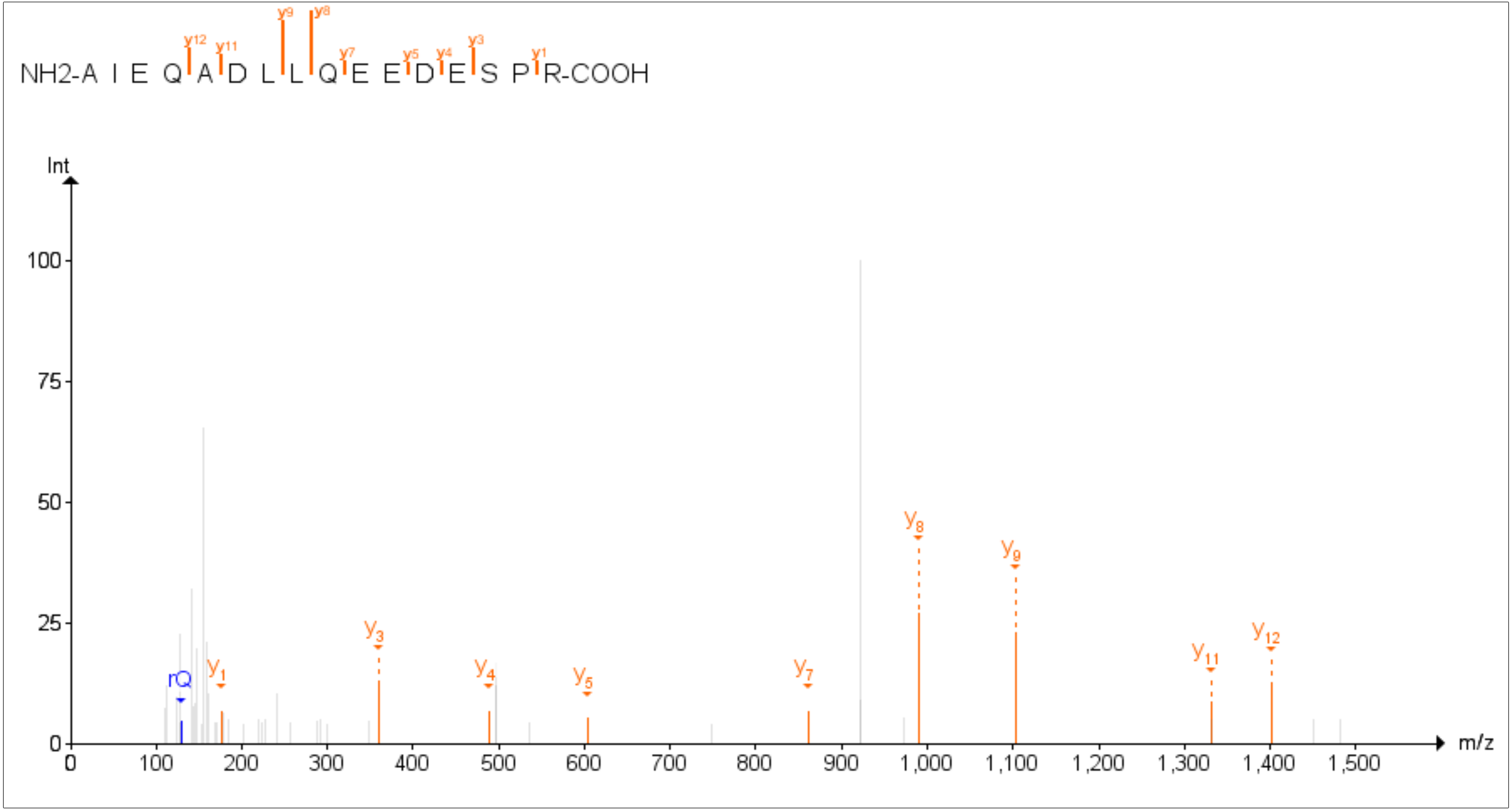

NH2-A I E Q A D L L Q E E D E S P R-COOH
y12
y11
y9
y8
y7
y5
y4
y3
y1
Int
100
75
50
25
0
rQ
y1
y3
y4
y5
y7
y8
y9
y11
y12
0
100
200
300
400
500
600
700
800
900
1,000
1,100
1,200
1,300
1,400
1,500
m/z

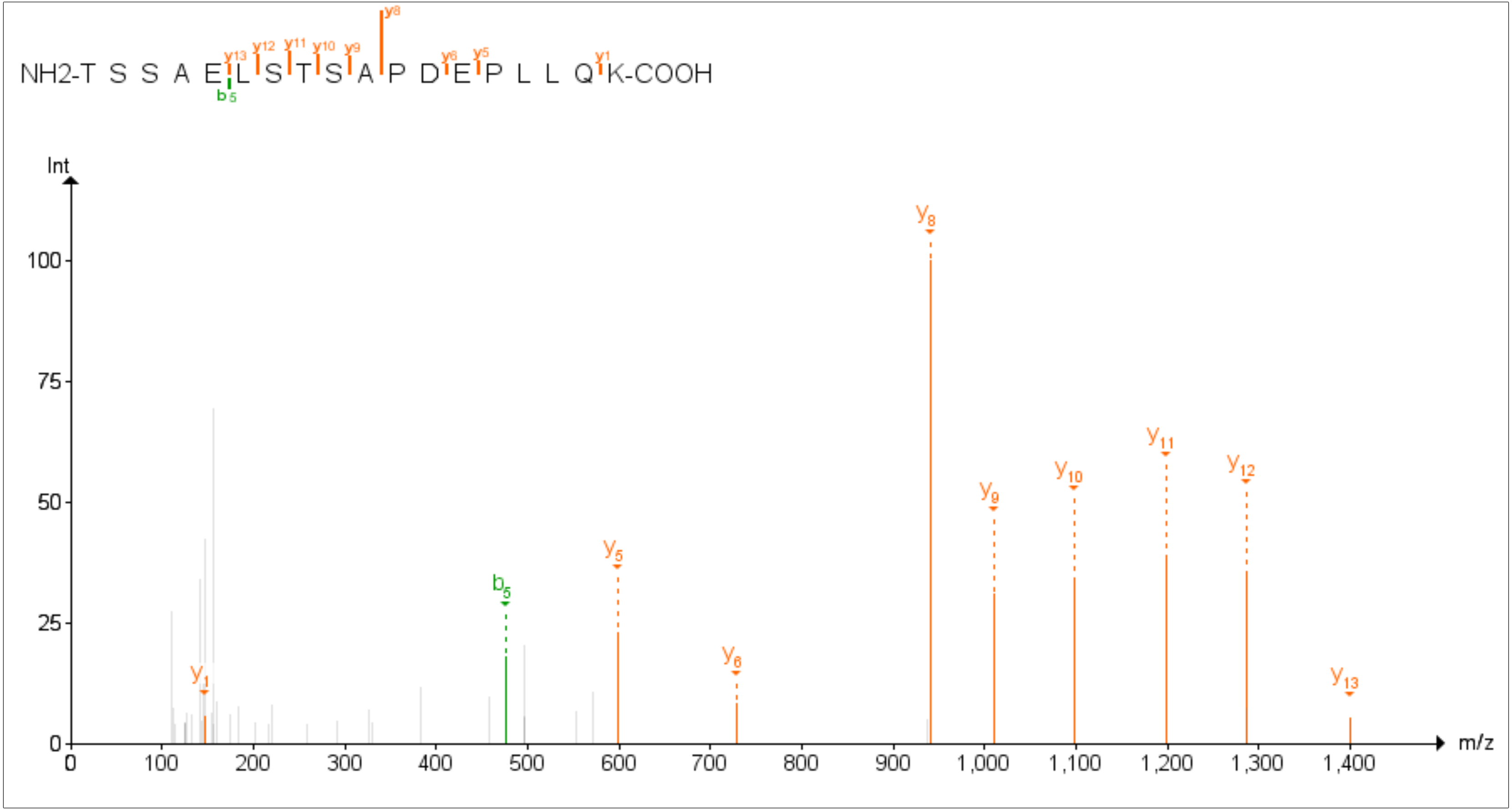

NH2-T S S A E L S T S A P D E P L L Q K-COOH
y13 y12 y11 y10 y9 y8 y6 y5 y1
b5
Int
100
75
50
25
0
y8
y9
y10
y11
y12
y5
b5
y6
y1
y13
0 100 200 300 400 500 600 700 800 900 1,000 1,100 1,200 1,300 1,400
m/z

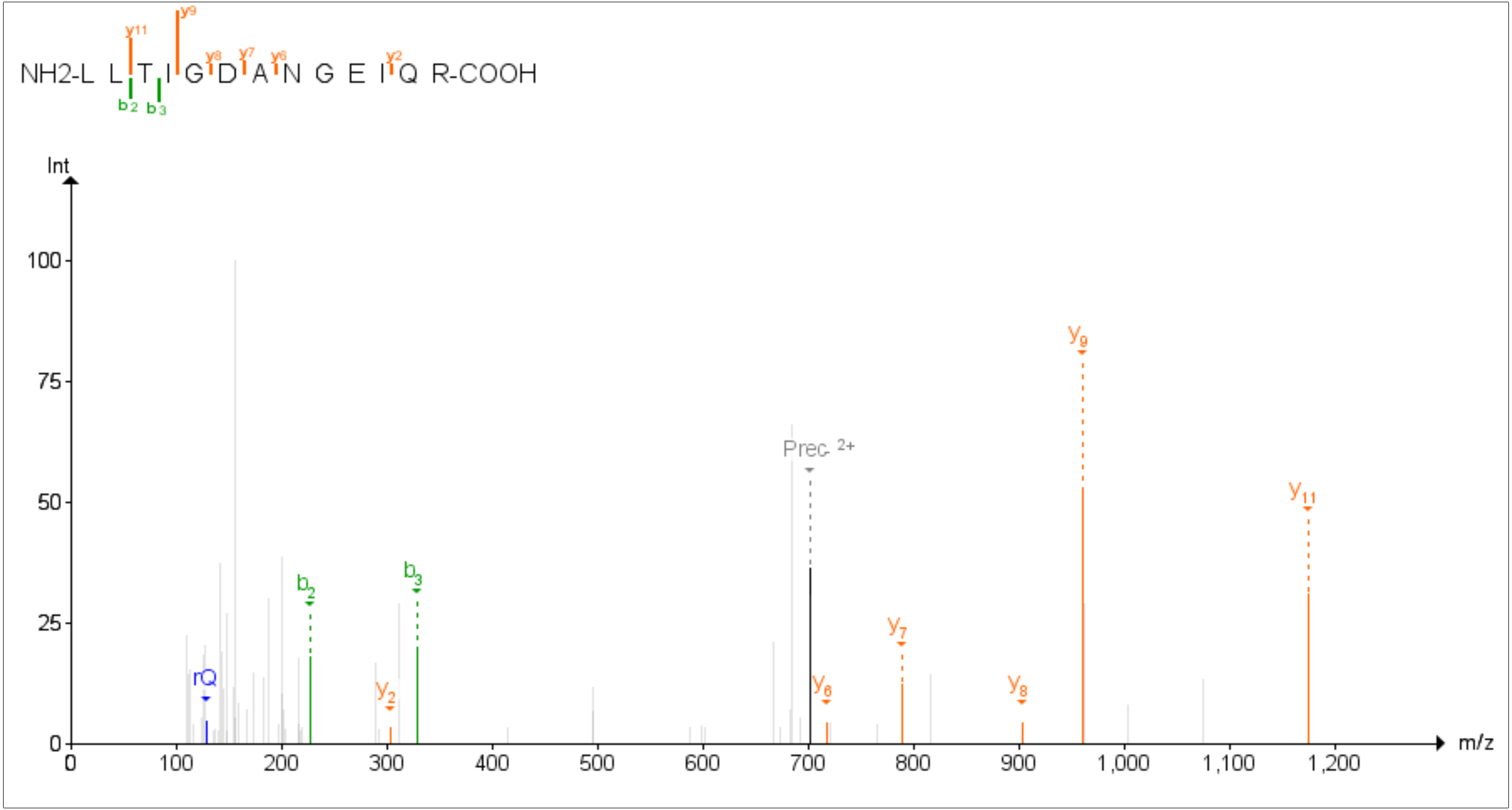

NH2-L L T I G D A N G E I Q R-COOH
y11 y9 y8 y7 y6 y2
b2 b3
Int
m/z
0
25
50
75
100
0
100
200
300
400
500
600
700
800
900
1,000
1,100
1,200
rQ
b2
y2
b3
Prec 2+
y6
y7
y8
y9
y11

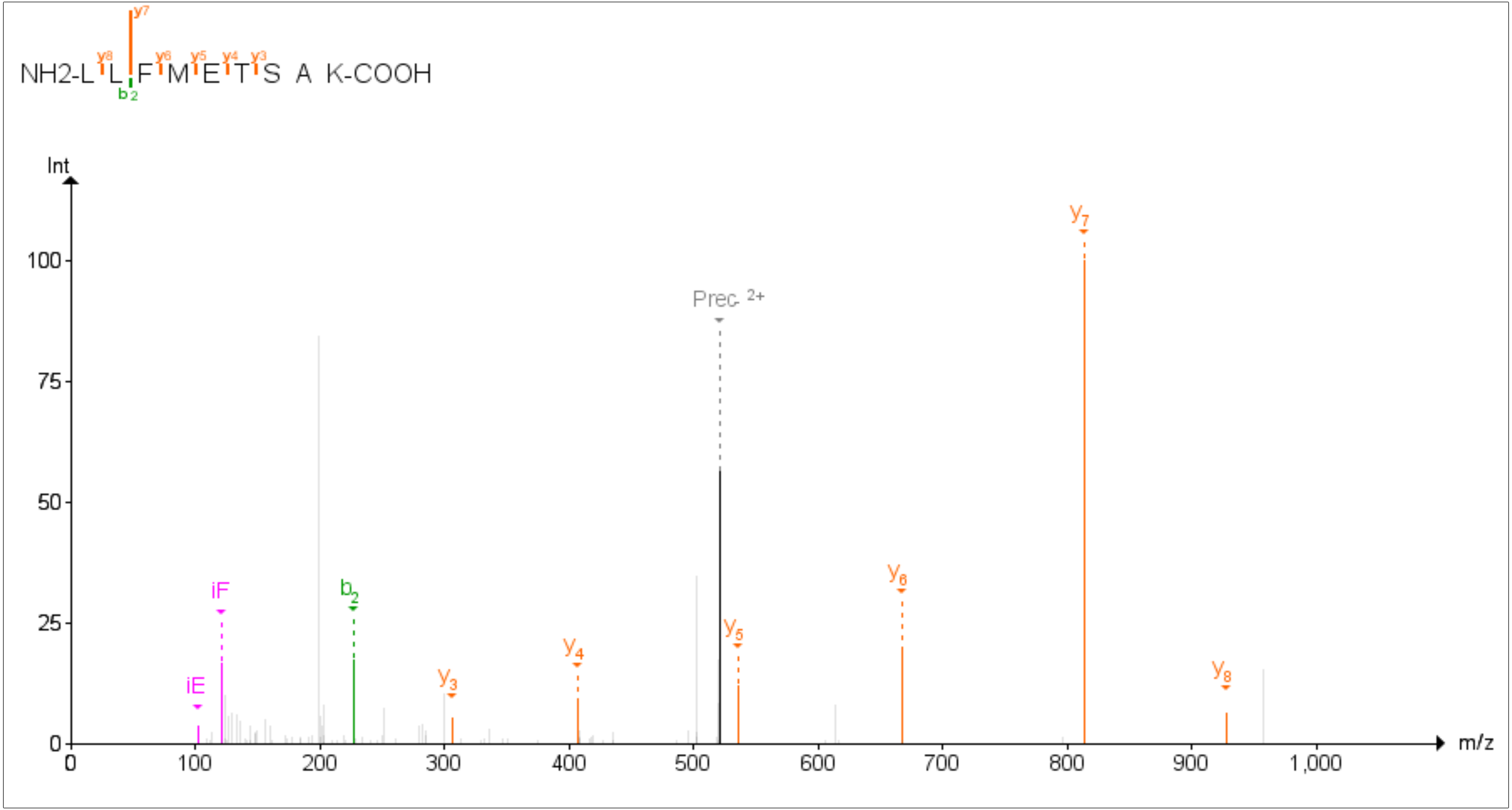

NH2-L L F M E T S A K-COOH
y8
y7
y6
y5
y4
y3
b2
Int
100
75
50
25
0
Prec 2+
y7
y6
y5
y4
y3
y8
iF
iE
b2
0
100
200
300
400
500
600
700
800
900
1,000
m/z

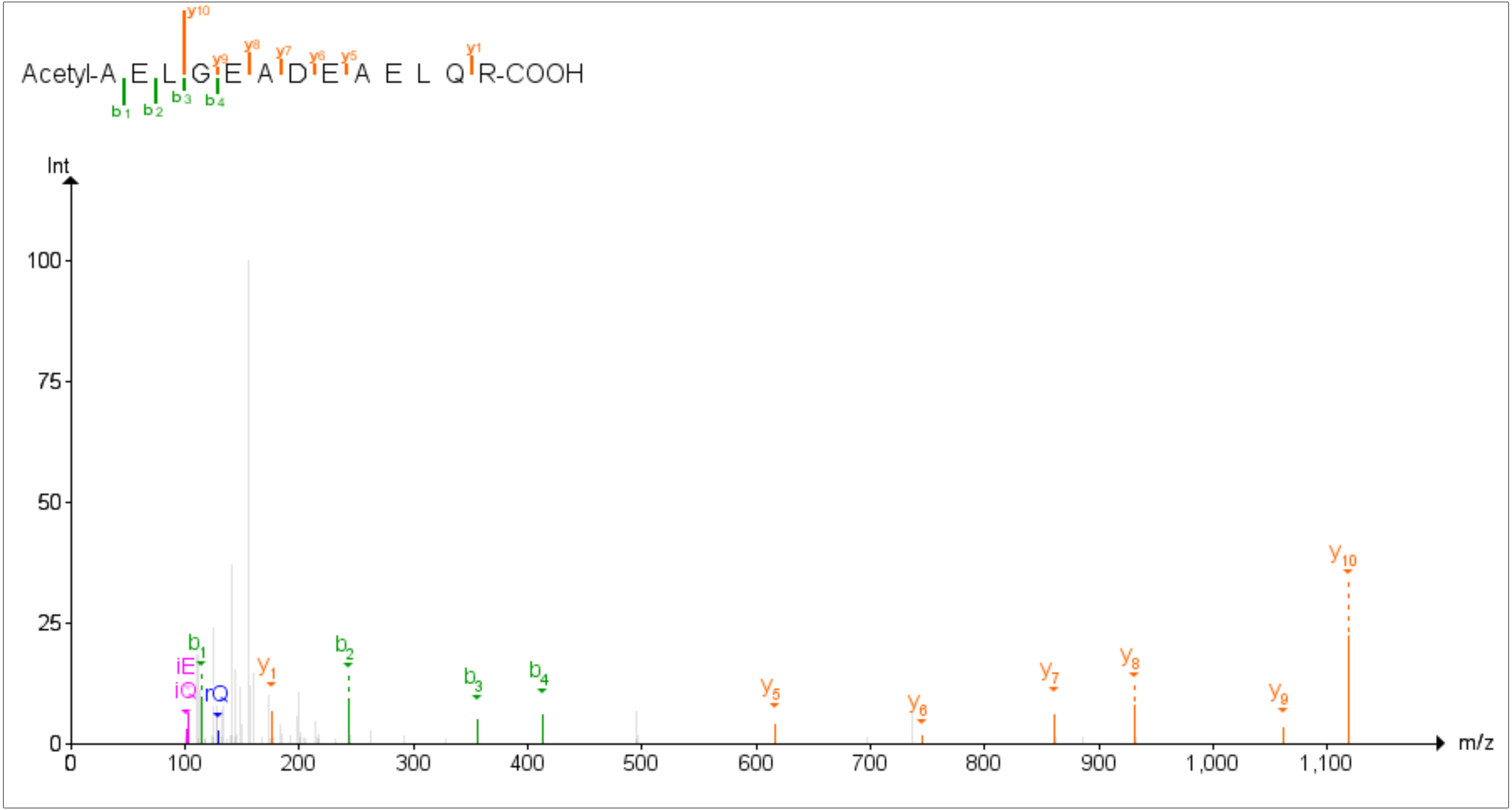

Acetyl-A E L G E A D E A E L Q R-COOH
b1 b2 b3 b4
y10 y9 y8 y7 y6 y5 y1
Int
100
75
50
25
0
b1
iE
iQ
rQ
y1
b2
b3
b4
y5
y6
y7
y8
y9
y10
0 100 200 300 400 500 600 700 800 900 1,000 1,100
m/z

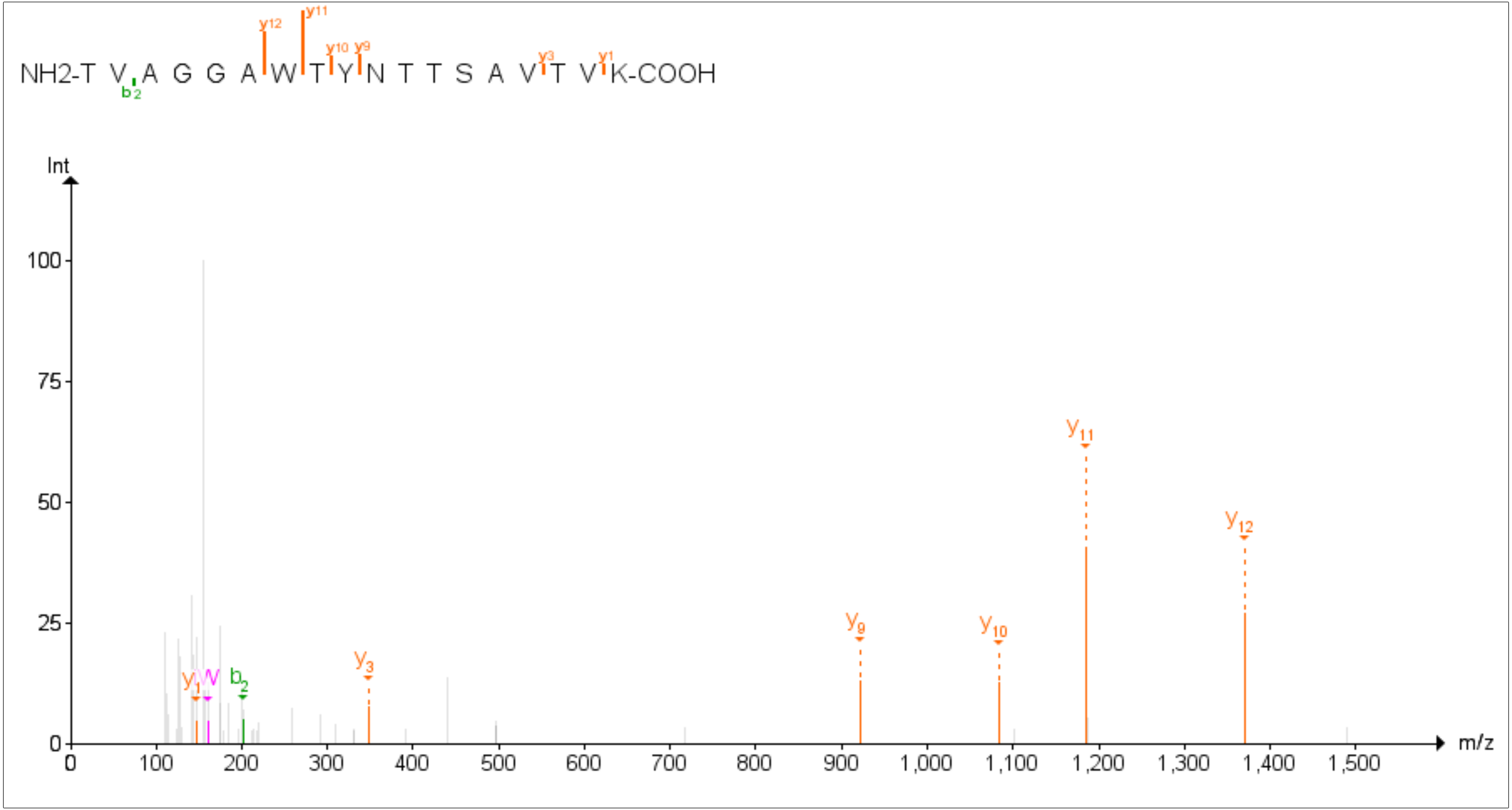

NH2-T V A G G A W T Y N T T S A V T V K-COOH
b2
y12
y11
y10
y9
y3
y1
Int
100
75
50
25
0
y1
W
b2
y3
y9
y10
y11
y12
0
100
200
300
400
500
600
700
800
900
1,000
1,100
1,200
1,300
1,400
1,500
m/z

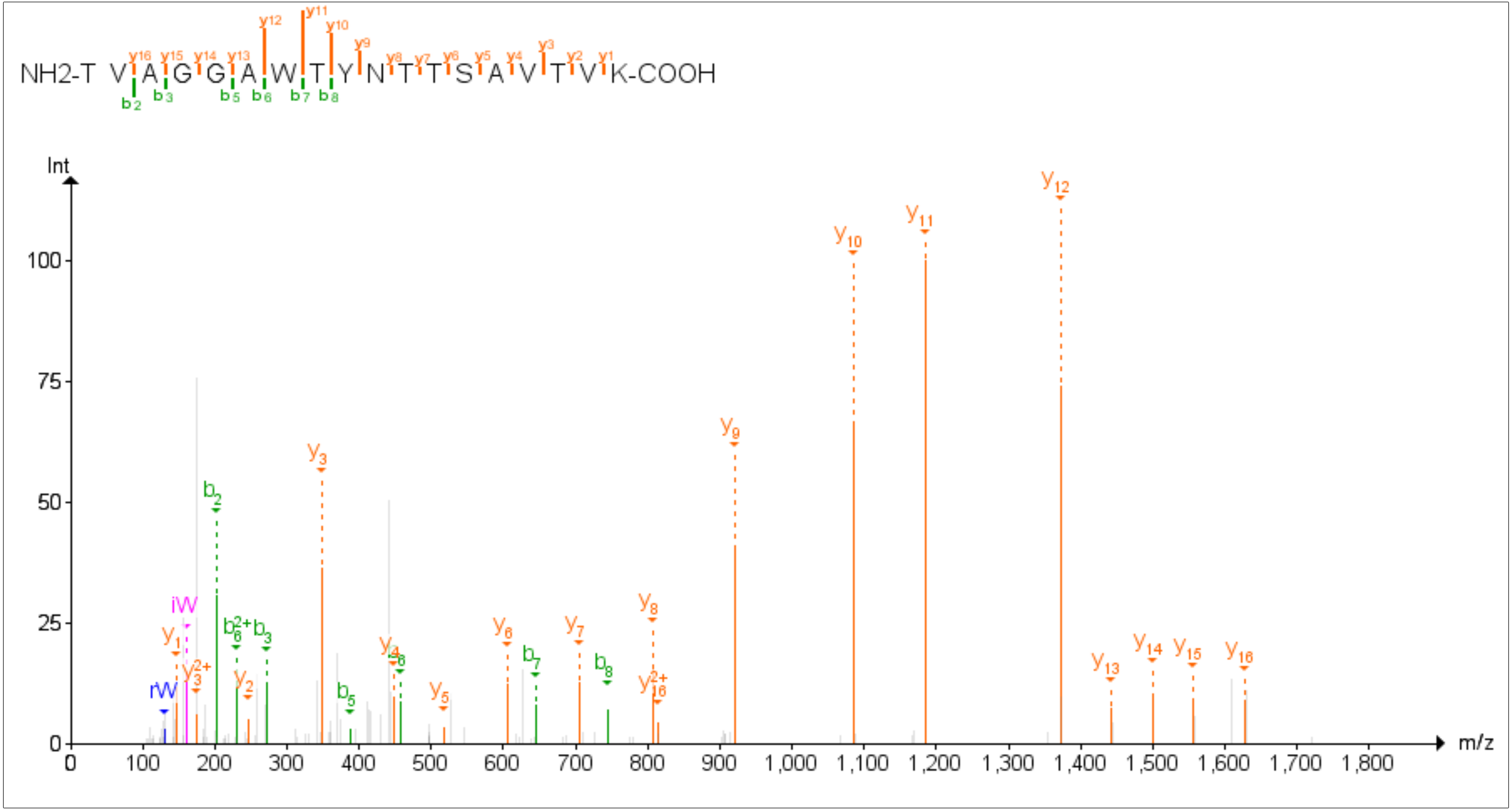

NH2-T V A G G A W T Y N T T S A V T V K-COOH
y16 y15 y14 y13 y12 y11 y10 y9 y8 y7 y6 y5 y4 y3 y2 y1
b2 b3 b5 b6 b7 b8
Int
100
75
50
25
0
m/z
0 100 200 300 400 500 600 700 800 900 1,000 1,100 1,200 1,300 1,400 1,500 1,600 1,700 1,800

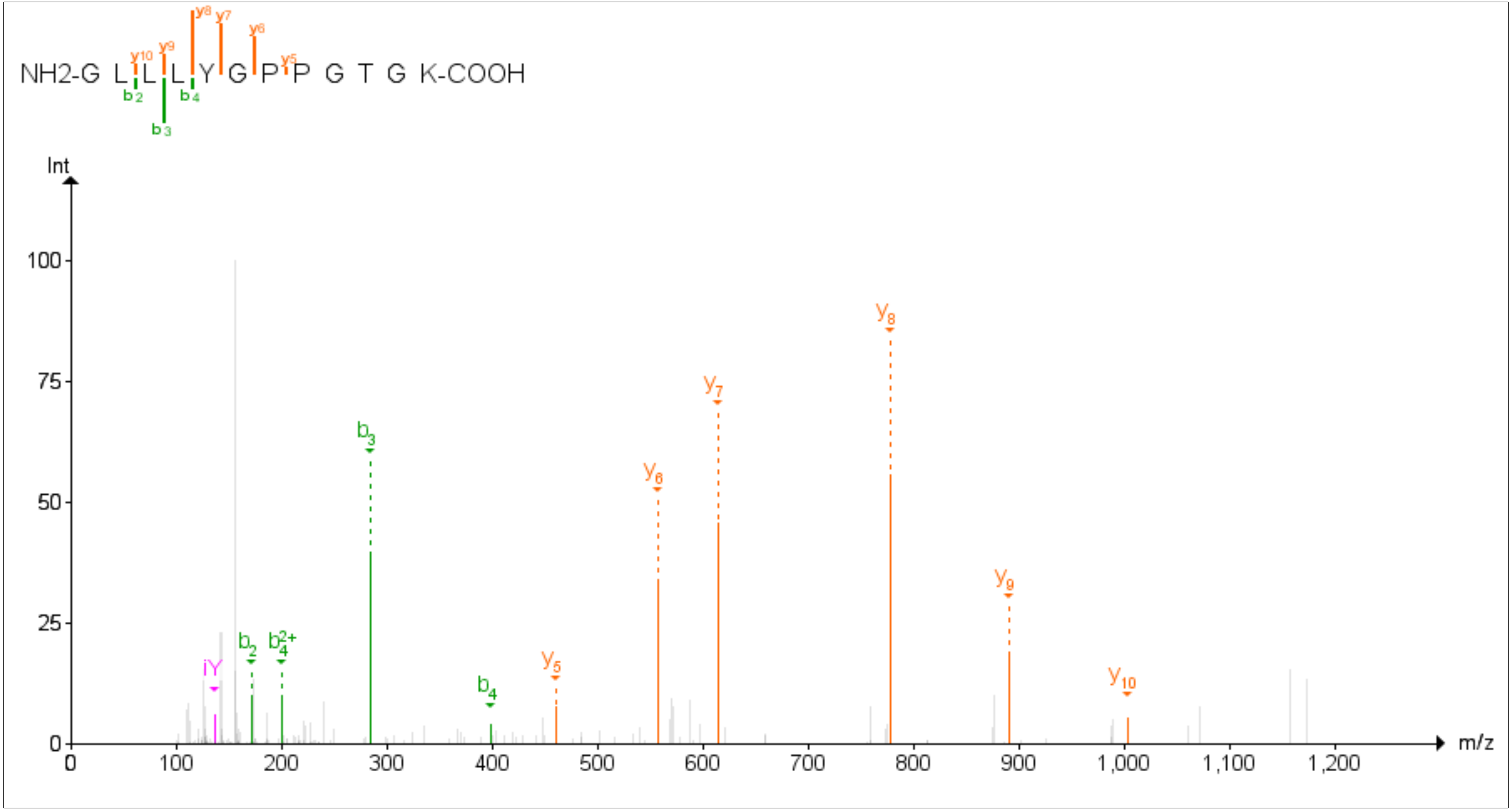

NH2-G L L L Y G P P G T G K-COOH
y10 y9 y8 y7 y6 y5
b2 b3 b4
Int
100
75
50
25
0
m/z
0 100 200 300 400 500 600 700 800 900 1,000 1,100 1,200
iY
b2
b4 2+
b3
b4
y5
y6
y7
y8
y9
y10

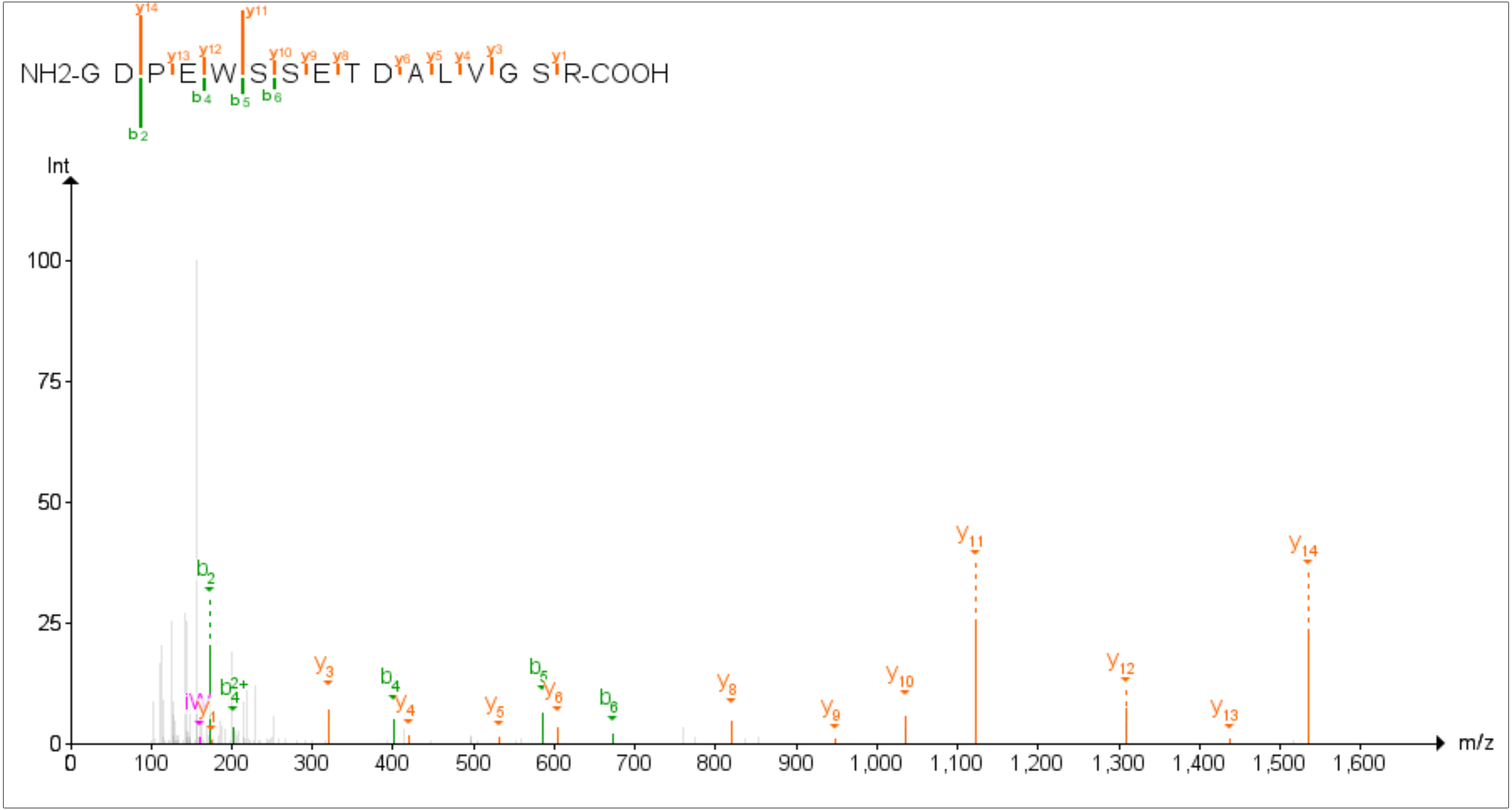

NH2-G D P E W S S E T D A L V G S R-COOH
y14
y13
y12
y11
y10
y9
y8
y6
y5
y4
y3
y1
b2
b4
b5
b6
Int
100
75
50
25
0
m/z
0
100
200
300
400
500
600
700
800
900
1,000
1,100
1,200
1,300
1,400
1,500
1,600

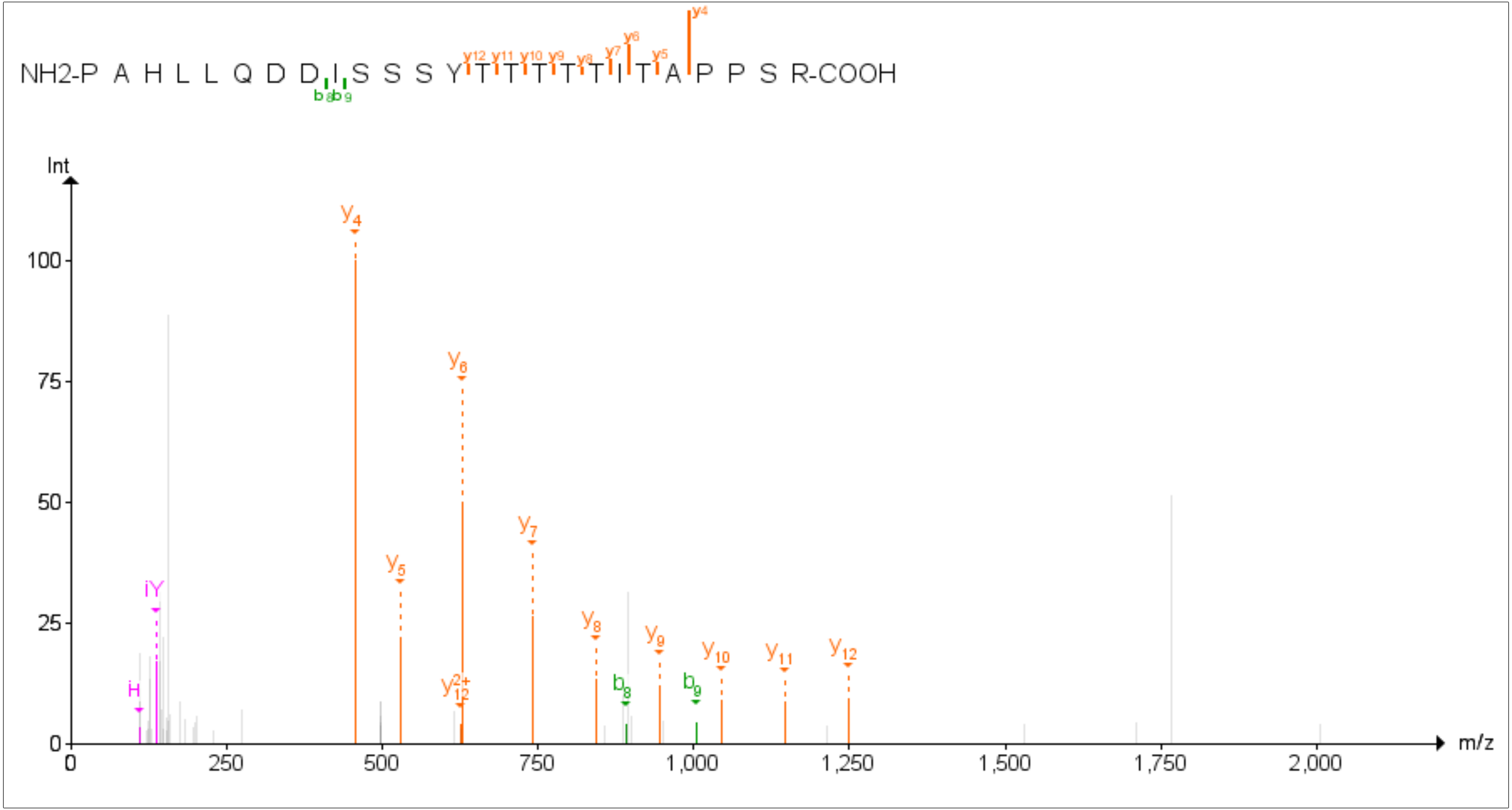

NH2-P A H L L Q D D I S S S Y T T T T T T I T A P P S R-COOH
y12 y11 y10 y9 y8 y7 y6 y5 y4
b8 b9
Int
100
75
50
25
0
y4
y6
y7
y5
iY
H
y8
y9
y10
y11
y12
b8
b9
y_{12}^{2+}
0
250
500
750
1,000
1,250
1,500
1,750
2,000
m/z

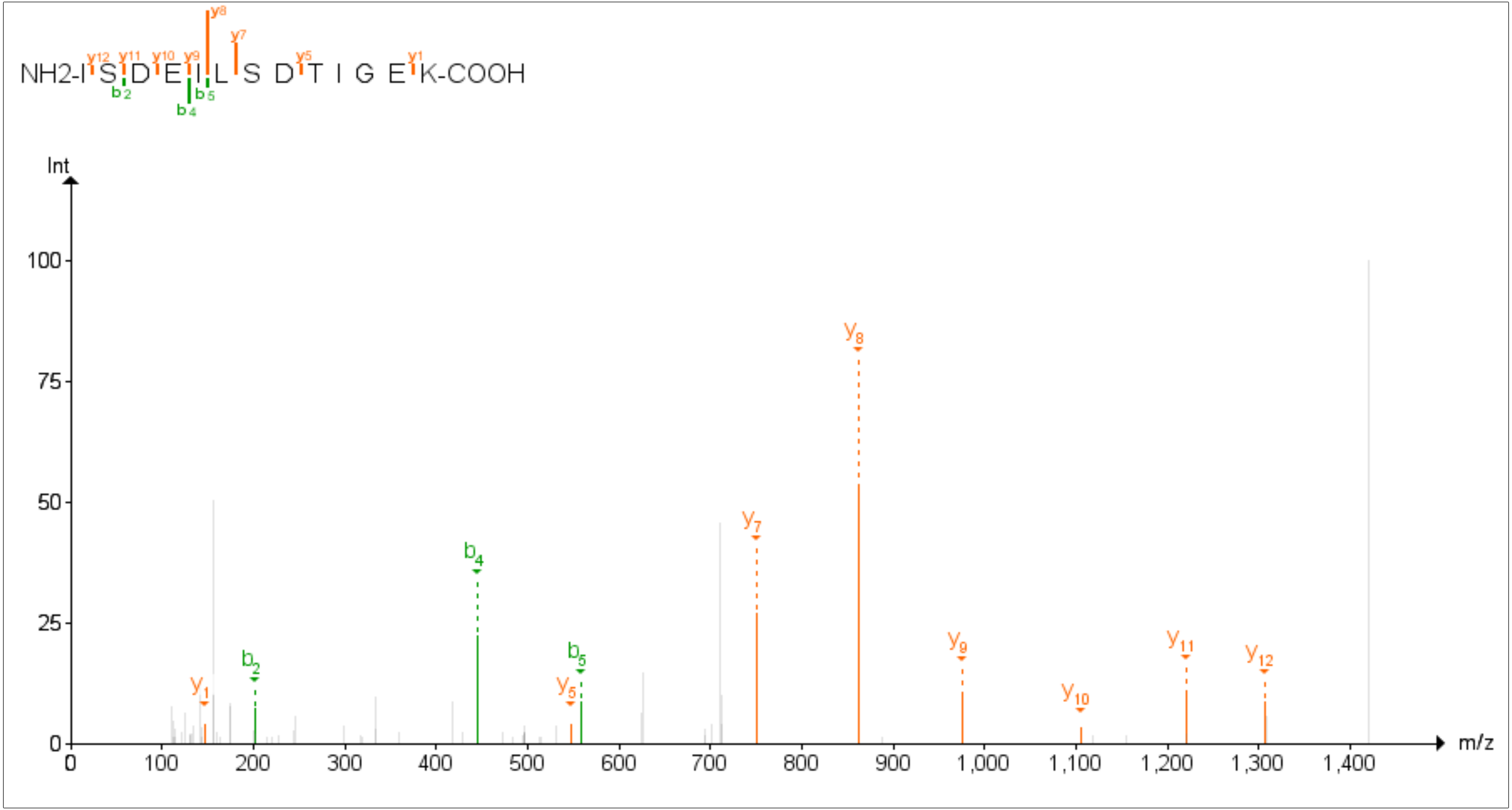
NH2-I S D E I I L S D T I G E K-COOH
y12 y11 y10 y9 y8 y7 y5 y1
b2 b4 b5
Int
m/z
100
75
50
25
0
0 100 200 300 400 500 600 700 800 900 1,000 1,100 1,200 1,300 1,400
y1 b2 b4 y5 b5 y7 y8 y9 y10 y11 y12

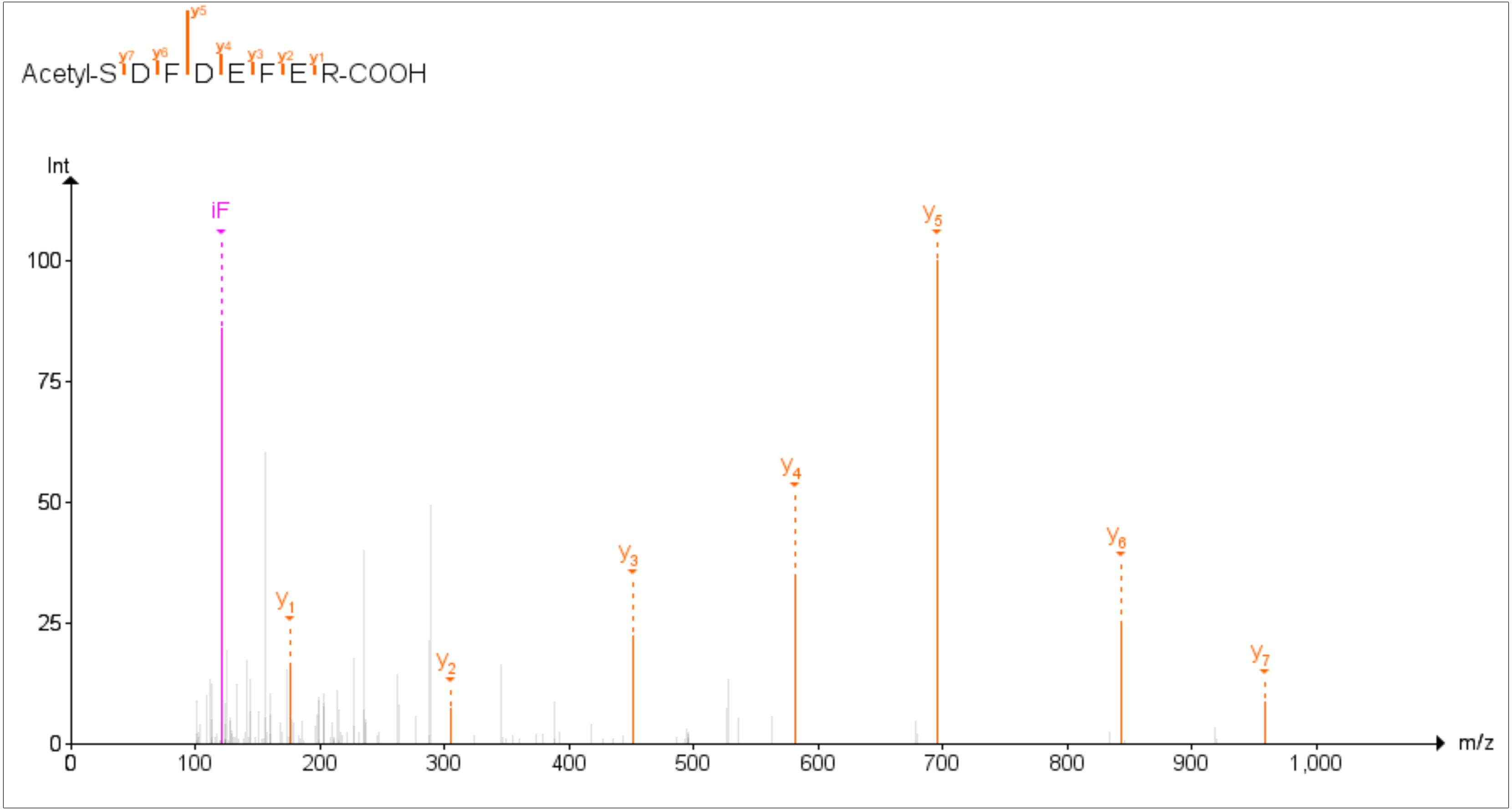

Acetyl-S D F D E F E R-COOH
y7 y6 y5 y4 y3 y2 y1
Int
iF
y1 y2 y3 y4 y5 y6 y7
0 25 50 75 100
0 100 200 300 400 500 600 700 800 900 1,000
m/z

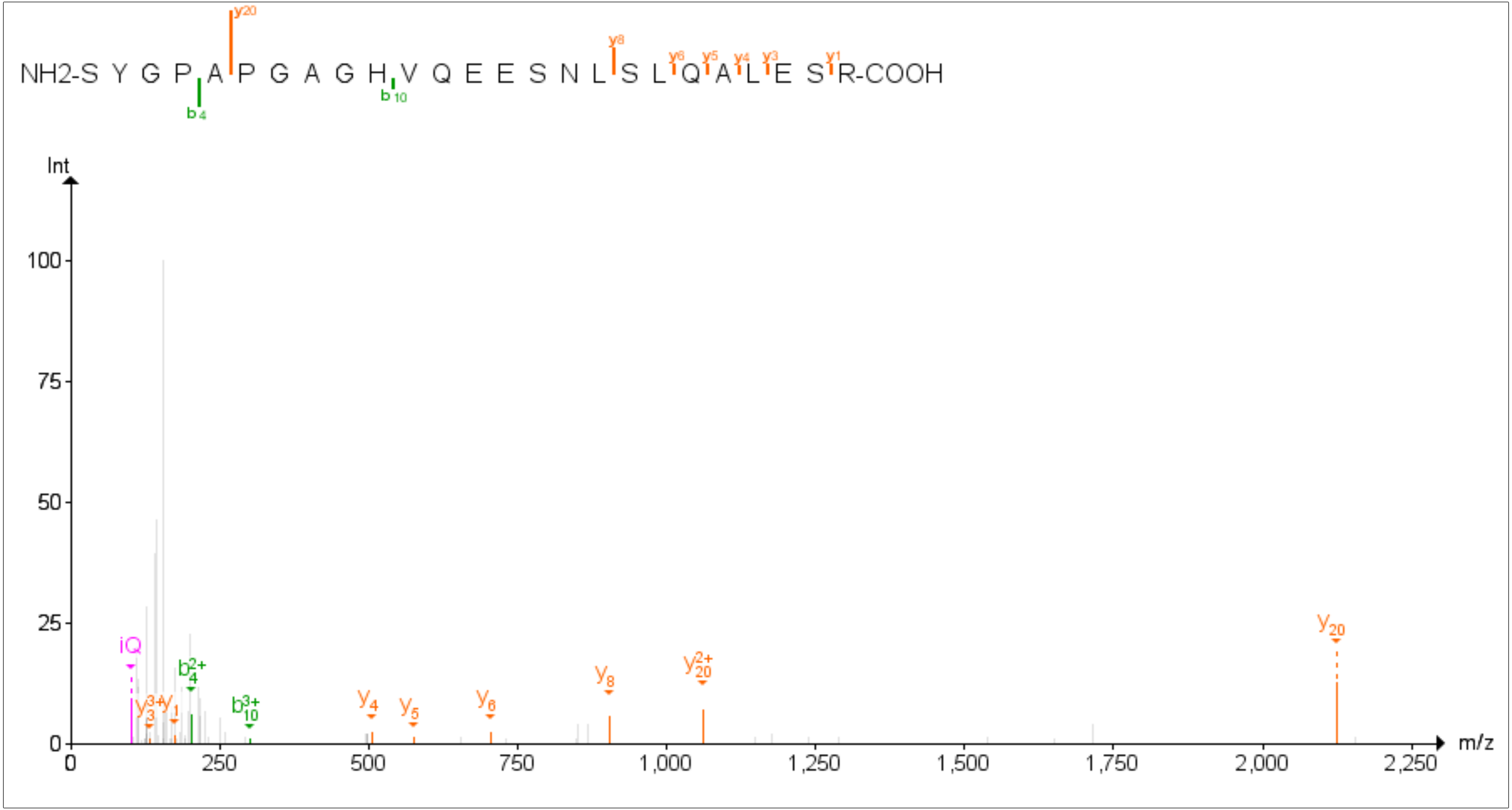

NH2-S Y G P A P G A G H V Q E E S N L S L Q A L E S R-COOH
y20
y8
y6
y5
y4
y3
y1
b4
b10
Int
100
75
50
25
0
iQ
b4 2+
y3 3+
y1
b10 3+
y4
y5
y6
y8
y20 2+
y20
0
250
500
750
1,000
1,250
1,500
1,750
2,000
2,250
m/z

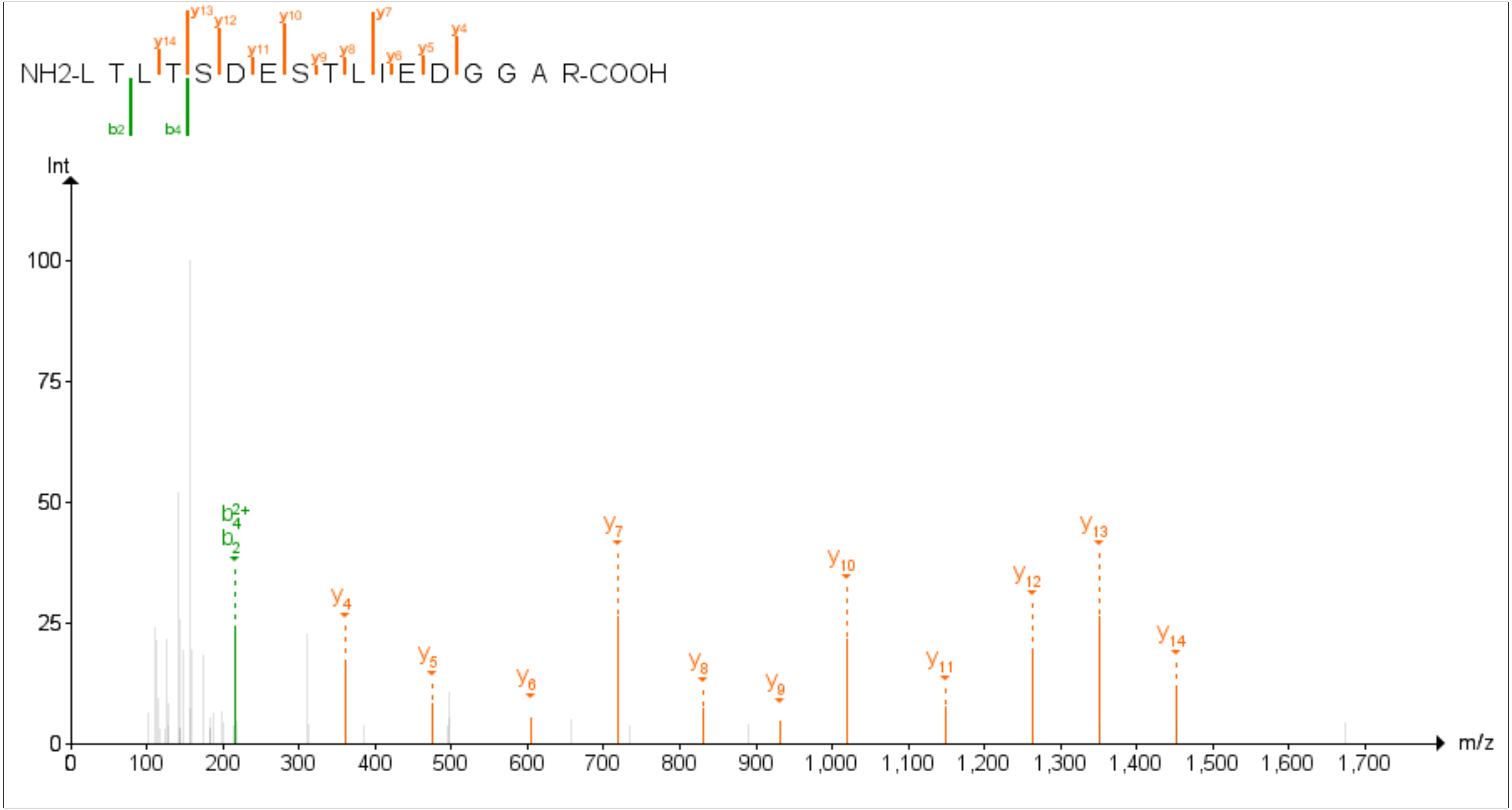

NH2-L T L T S D E S T L I E D G G A R-COOH
y14 y13 y12 y11 y10 y9 y8 y7 y6 y5 y4
b2 b4
Int
100
75
50
25
0
m/z
0 100 200 300 400 500 600 700 800 900 1,000 1,100 1,200 1,300 1,400 1,500 1,600 1,700
b4 2+
b2
y4
y5
y6
y7
y8
y9
y10
y11
y12
y13
y14

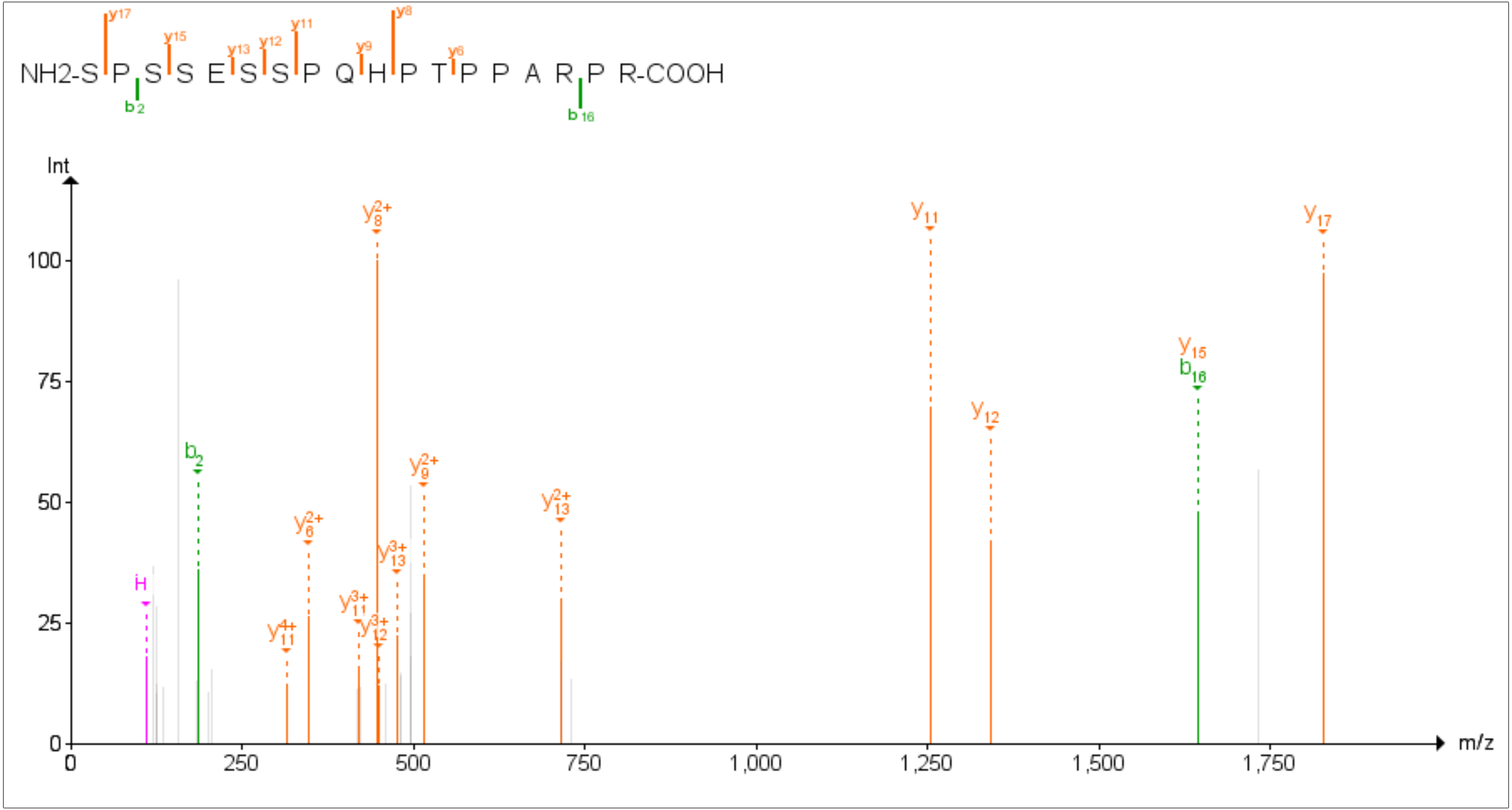

NH2-S P S S E S S P Q H P T P P A R P R-COOH
y17 y15 y13 y12 y11 y9 y8 y6
b 2 b 16
Int
m/z
0 25 50 75 100
0 250 500 750 1,000 1,250 1,500 1,750

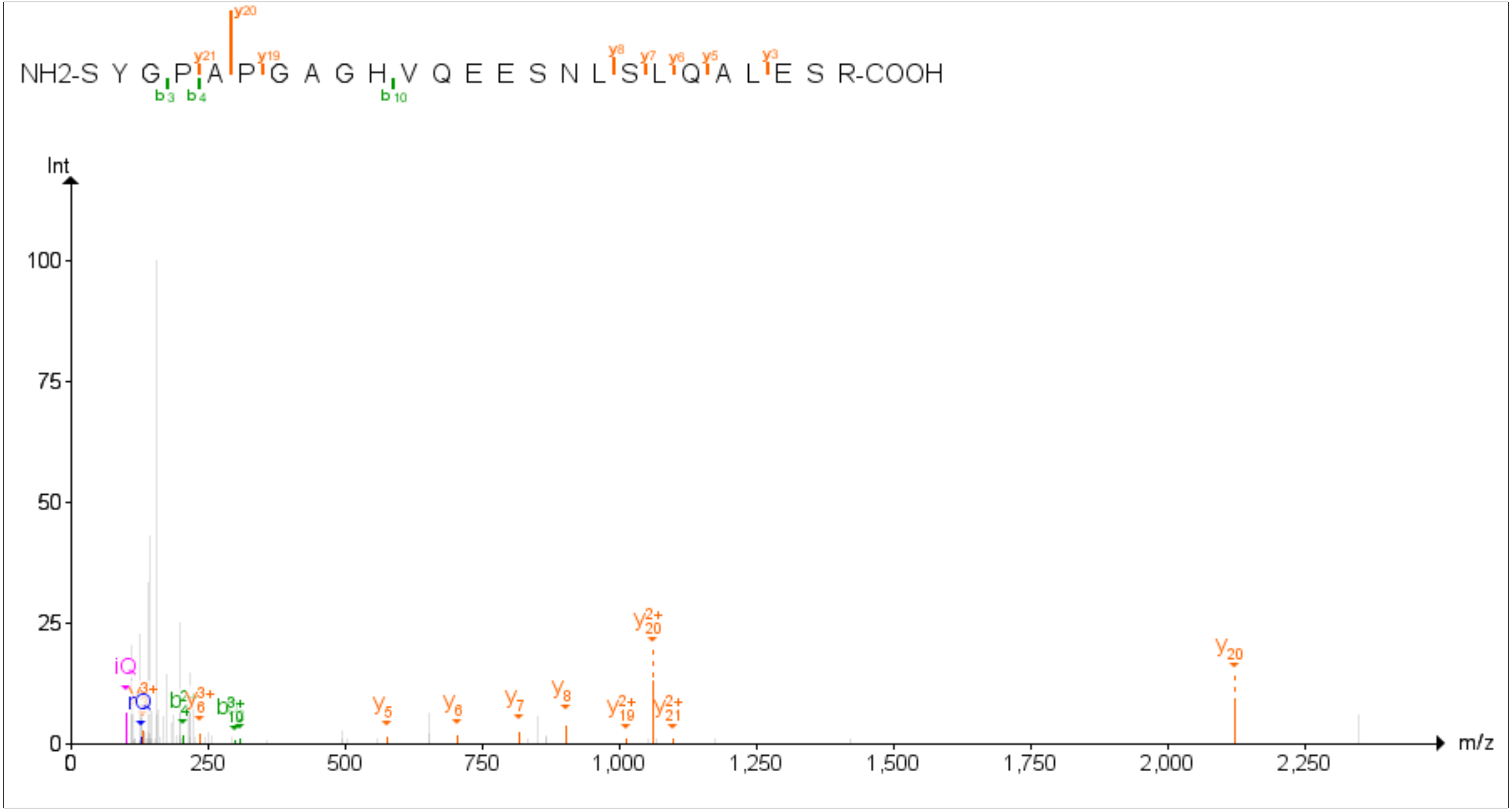

NH2-S Y G P A P G A G H V Q E E S N L S L Q A L E S R-COOH
y20
y21
y19
y8
y7
y6
y5
y3
b3
b4
b10
Int
100
75
50
25
0
iQ
rQ
y20 2+
y20
y5
y6
y7
y8
y19 2+
y21 2+
0
250
500
750
1,000
1,250
1,500
1,750
2,000
2,250
m/z

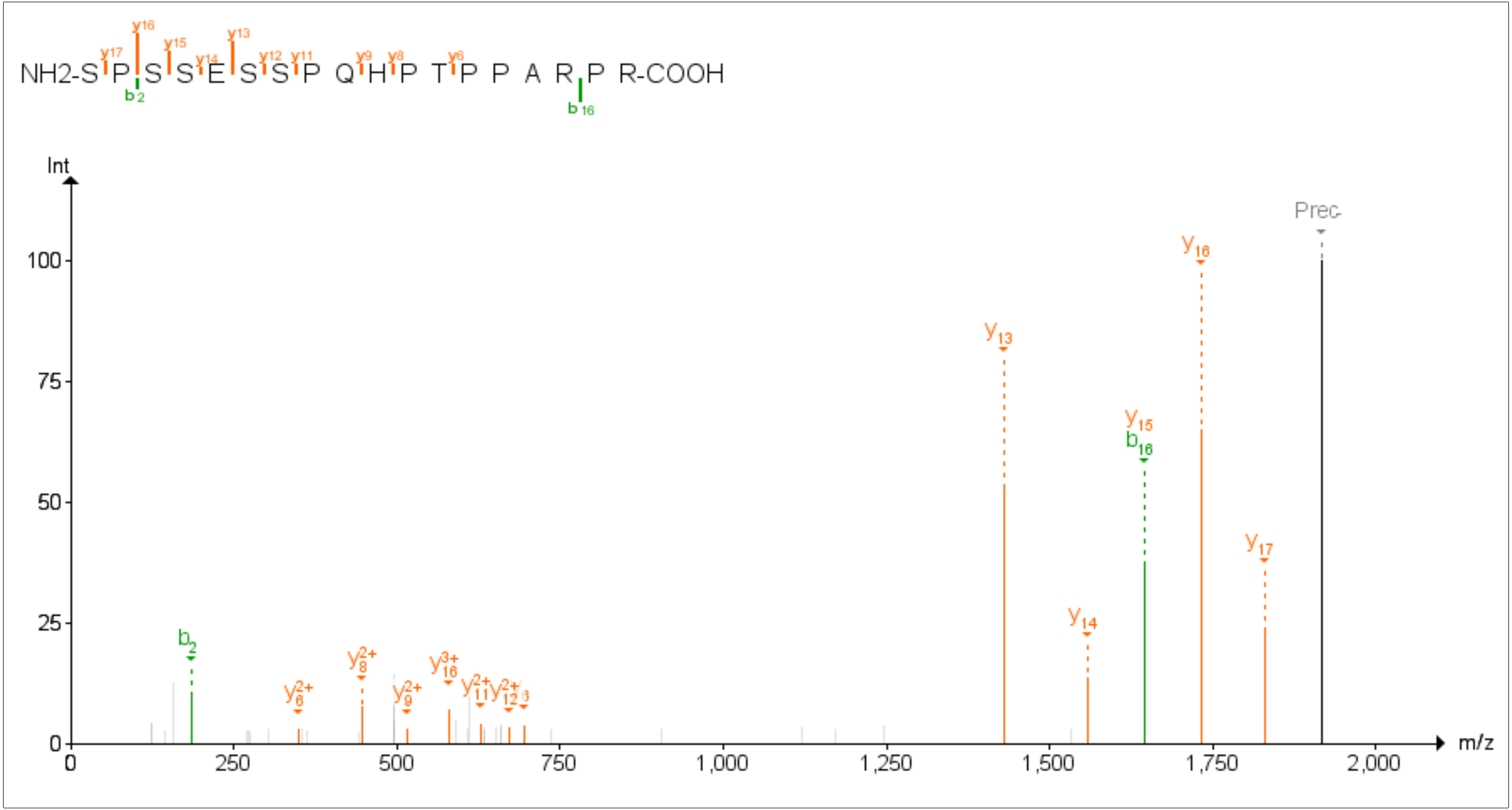

NH2-S P S S E S S P Q H P T P P A R P R-COOH
y17 y16 y15 y14 y13 y12 y11 y9 y8 y6
b2 b16
Int
100
75
50
25
0
m/z
0
250
500
750
1,000
1,250
1,500
1,750
2,000
Prec.
y16
y13
y15
b16
y17
y14
b2
y8 2+
y6 2+
y9 2+
y16 3+
y11 2+
y12 2+

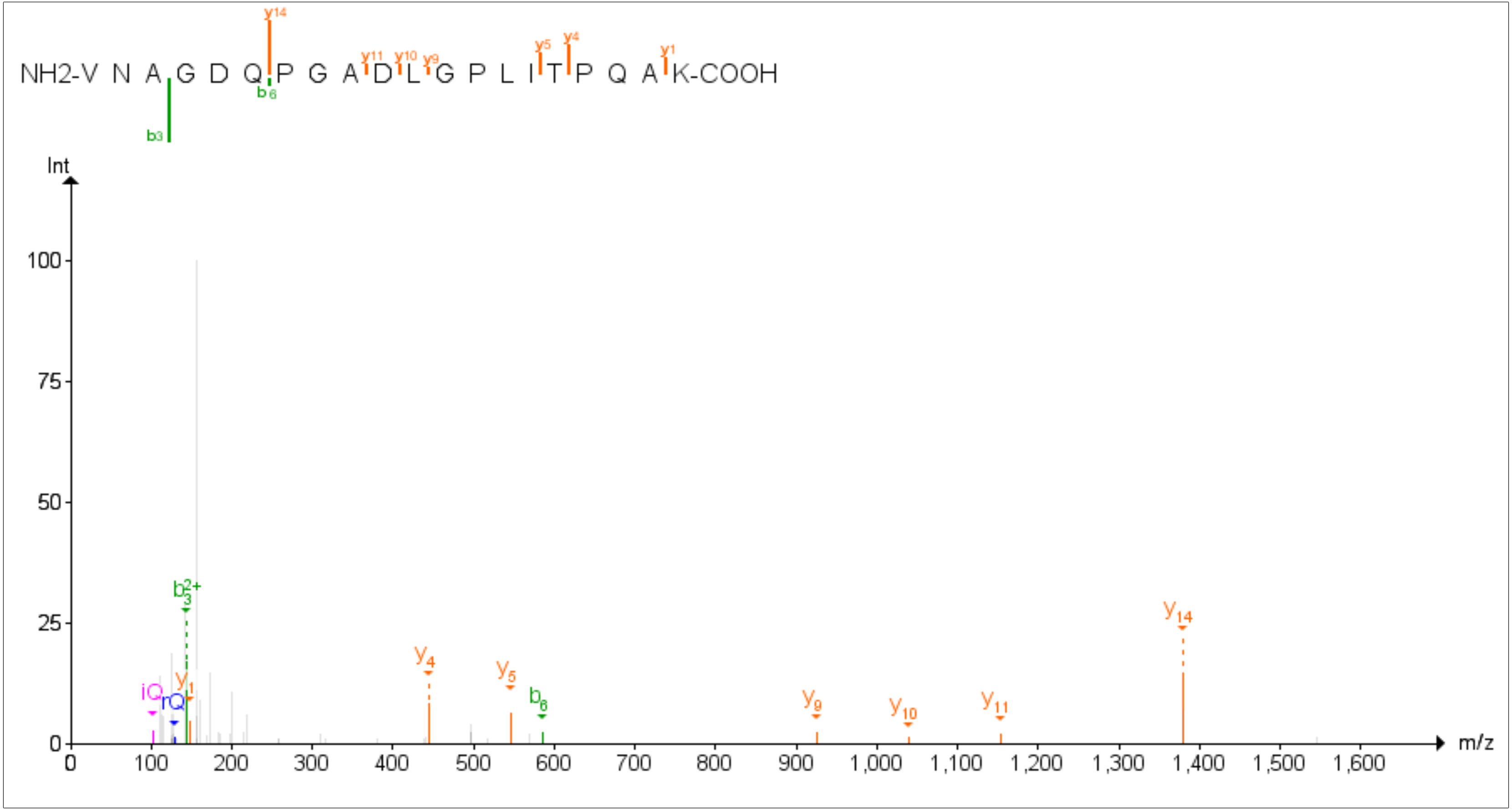
NH2-V N A G D Q P G A D L G P L I T P Q A K-COOH
y14
y11 y10 y9
y5 y4
y1
b3
b6
Int
100
75
50
25
0
b_3^{2+}
iQ
rQ
y_1
y_4
y_5
b_6
y_9
y_{10}
y_{11}
y_{14}
0 100 200 300 400 500 600 700 800 900 1,000 1,100 1,200 1,300 1,400 1,500 1,600
m/z

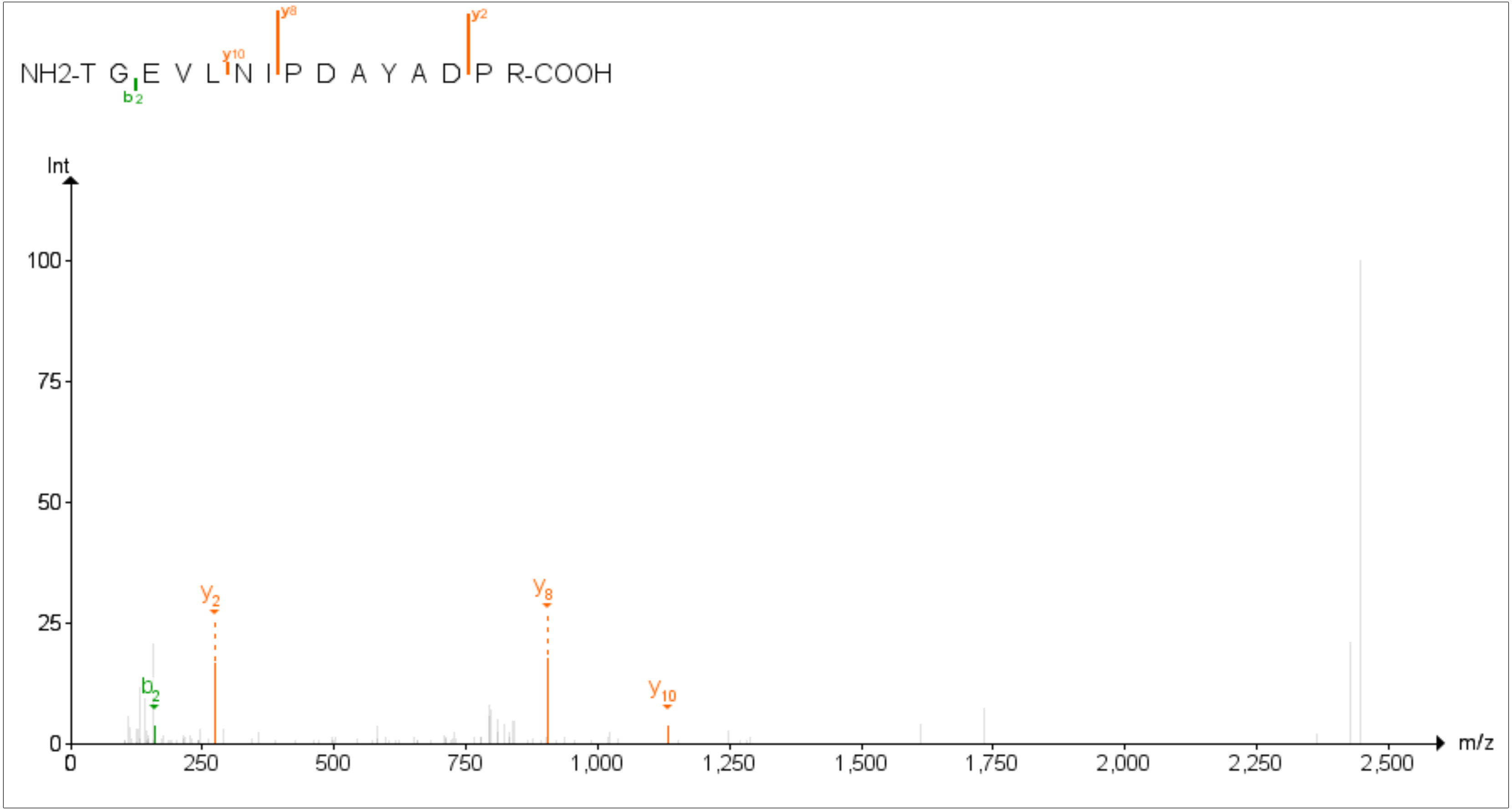

NH2-T G E V L N I P D A Y A D P R-COOH
b2
y10
y8
y2
Int
100
75
50
25
0
y2
y8
b2
y10
0
250
500
750
1,000
1,250
1,500
1,750
2,000
2,250
2,500
m/z

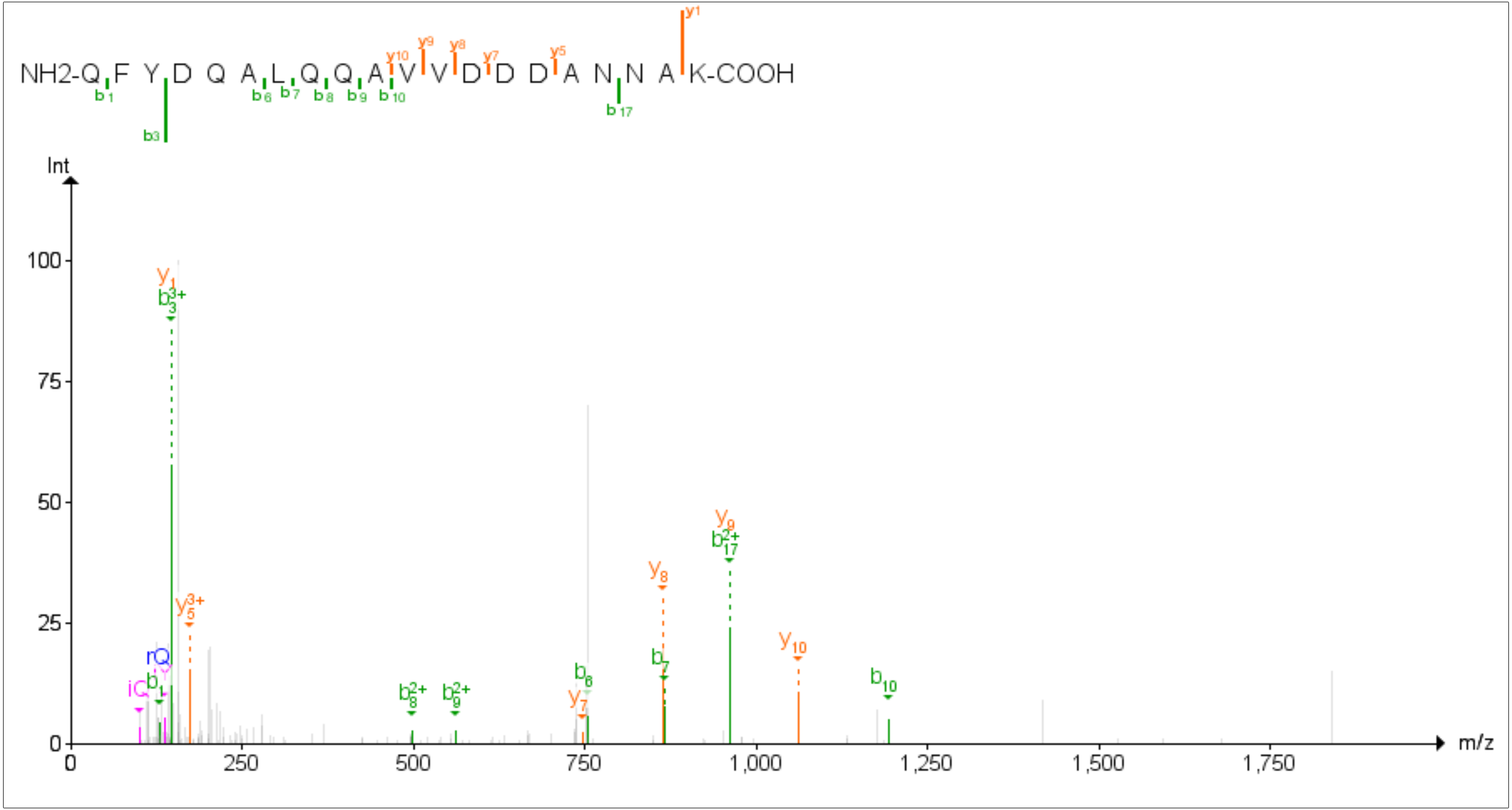

NH2-Q F Y D Q A L Q Q A V V D D D A N N A K-COOH
b1 b3 b6 b7 b8 b9 b10 b17
y10 y9 y8 y7 y5 y1
Int
m/z
0
25
50
75
100
250
500
750
1,000
1,250
1,500
1,750
y1
b3 3+
y5 3+
rQ
iQ
b1
b8 2+
b9 2+
b6
y7
b7
y8
y9
b17 2+
y10
b10

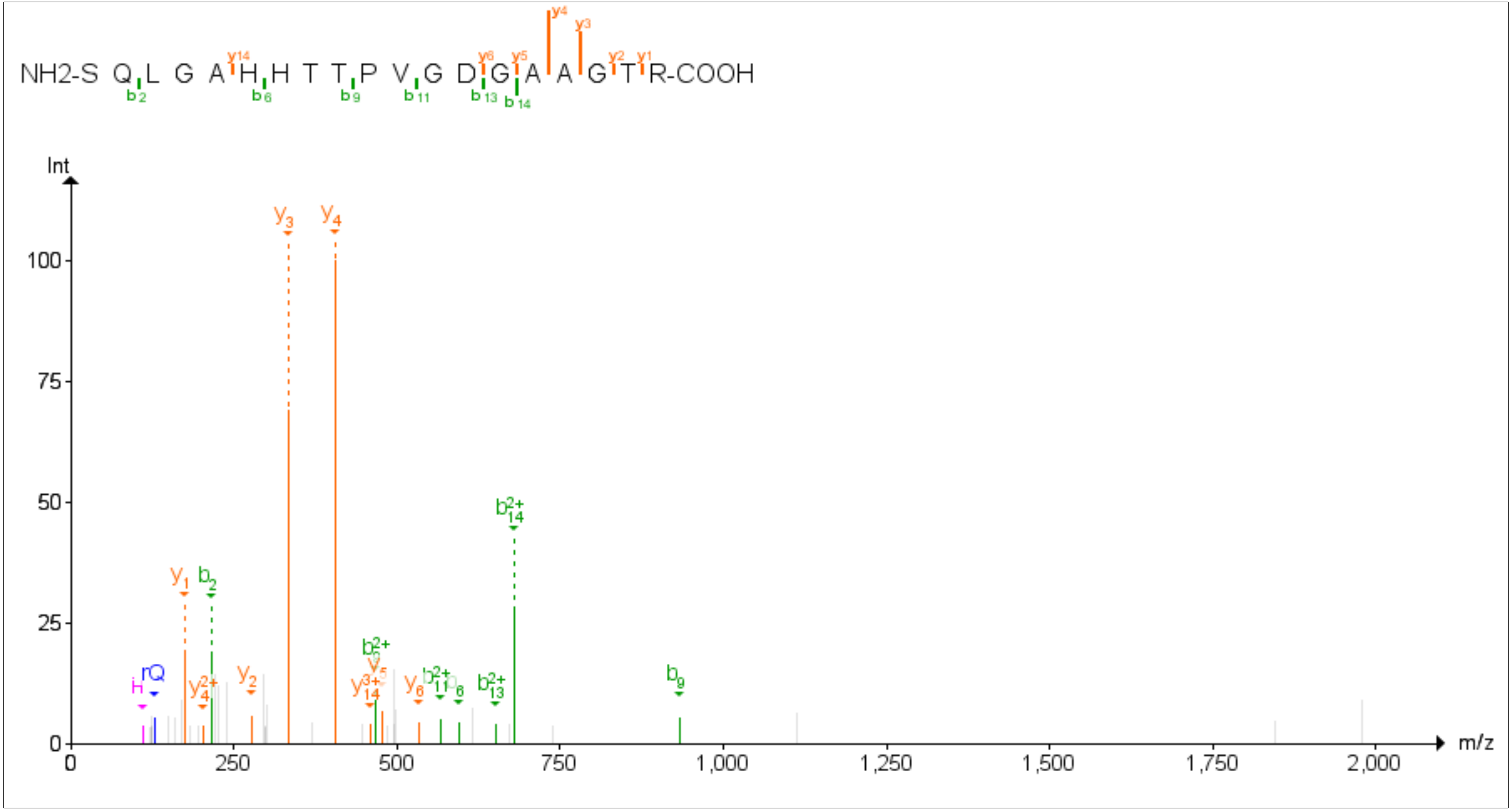
NH2-S Q L G A H H T T P V G D G A A G T R-COOH
Int
m/z
y3
y4
b$^{2+}_{14}$
y1
b2
rQ
H
y$^{2+}_{4}$
y2
b$^{2+}_{9}$
y$^{3+}_{14}$
y6
b$^{2+}_{11}$
b$^{2+}_{13}$
b9
0
25
50
75
100
250
500
750
1,000
1,250
1,500
1,750
2,000

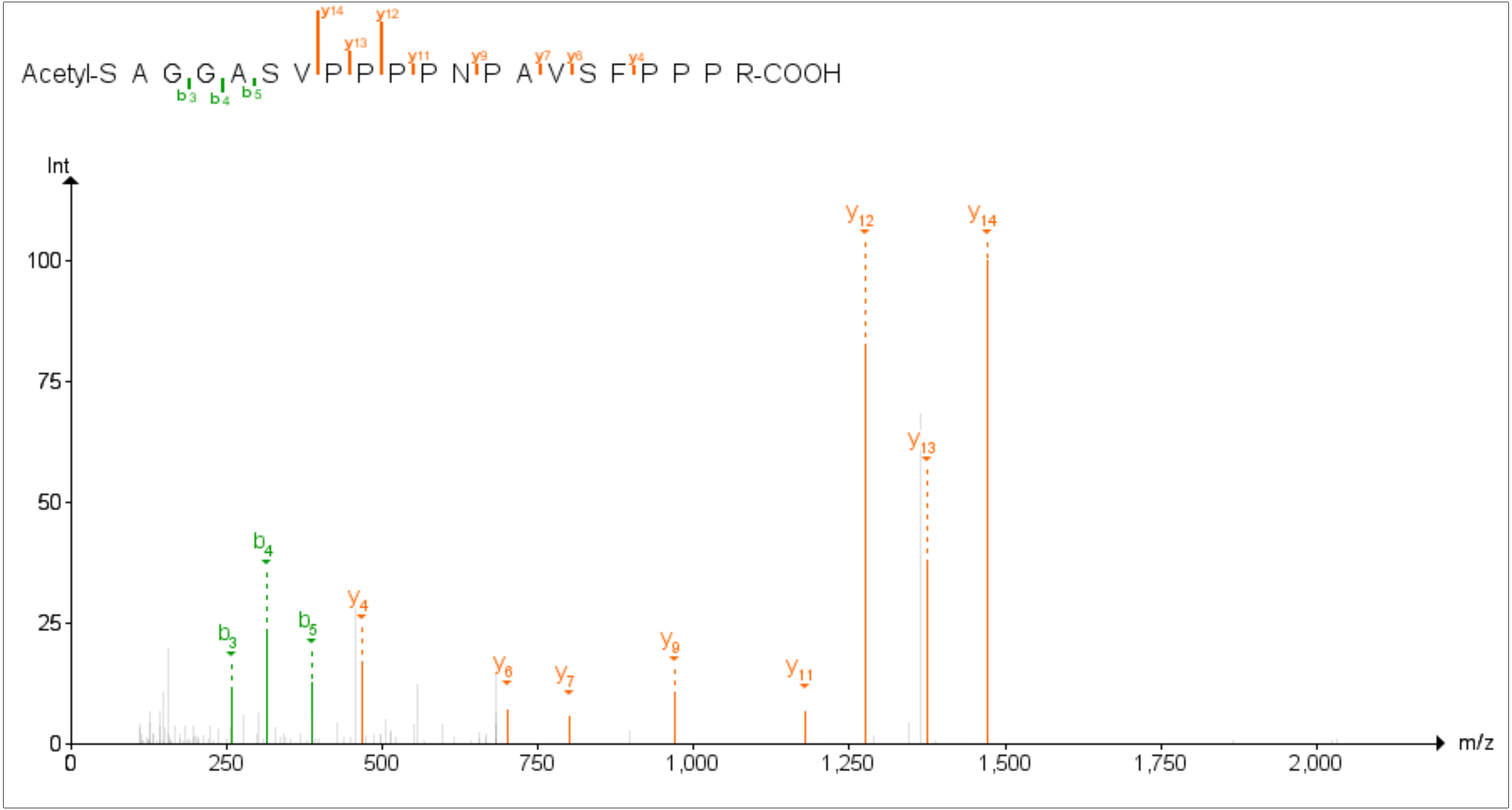

Acetyl-S A G G A S V P P P P N P A V S F P P P R-COOH
b3
b4
b5
y14
y13
y12
y11
y9
y7
y6
y4
Int
100
75
50
25
0
m/z
0
250
500
750
1,000
1,250
1,500
1,750
2,000

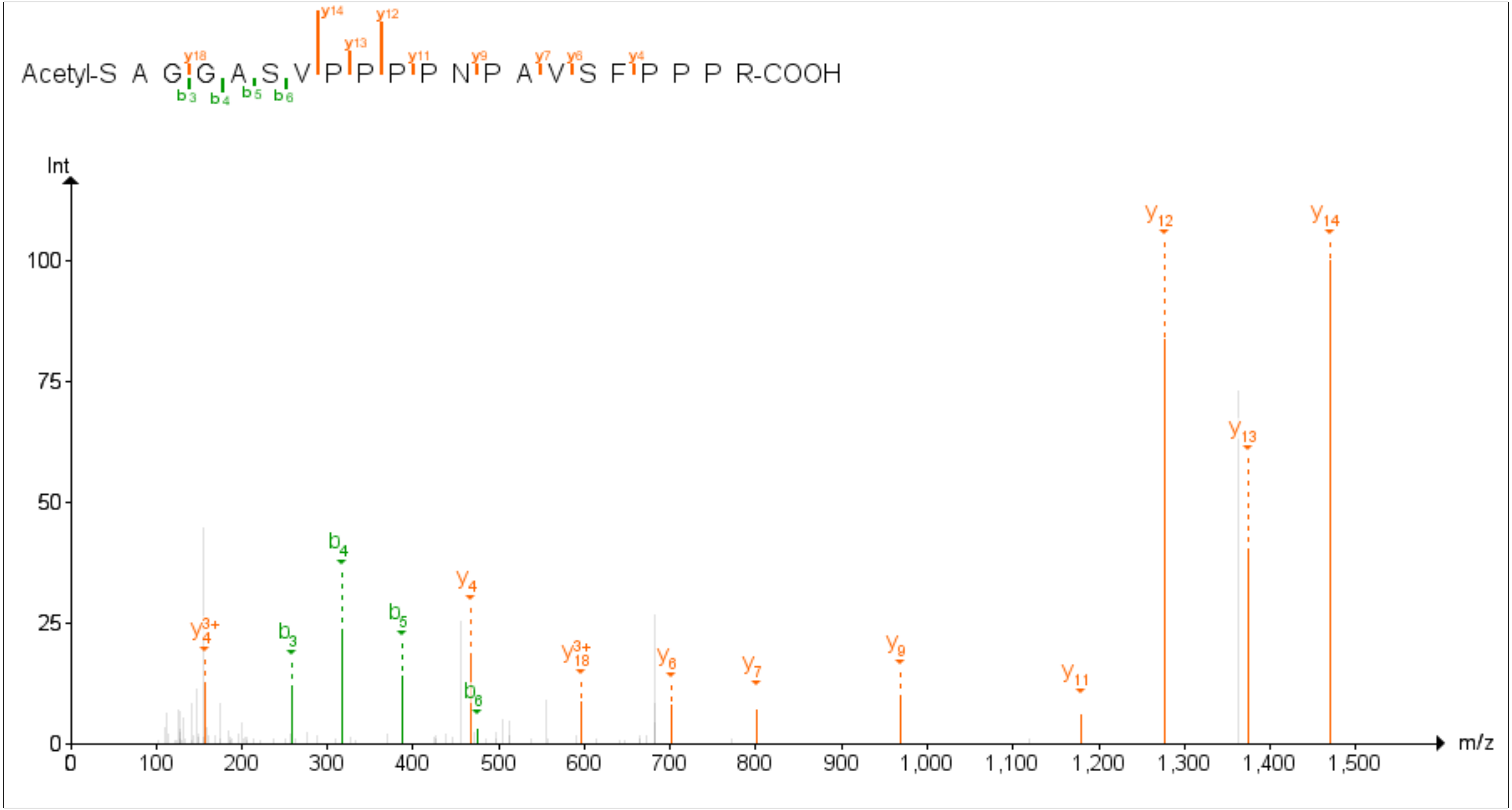

Acetyl-S A G G A S V P P P P N P A V S F P P P R-COOH
y18 y14 y13 y12 y11 y9 y7 y6 y4
b3 b4 b5 b6
Int
100
75
50
25
0
m/z
0 100 200 300 400 500 600 700 800 900 1,000 1,100 1,200 1,300 1,400 1,500
y12 y14 y13 b4 y4 b5 y4 3+ b3 y18 3+ y6 y7 y9 y11 b6

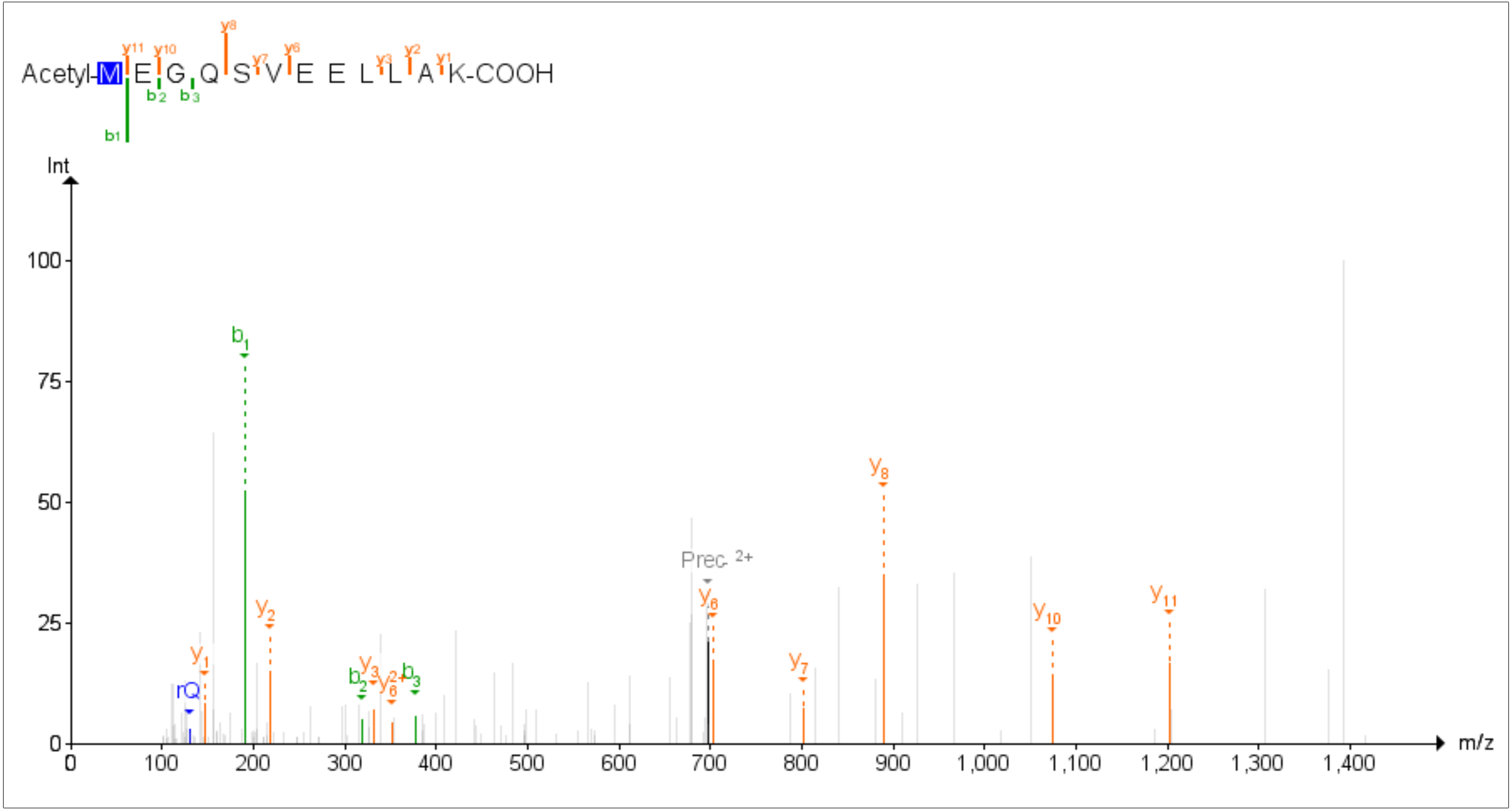

Acetyl-M E G Q S V E E L L A K-COOH
y11 y10 y8 y7 y6 y3 y2 y1
b1 b2 b3
Int
100
75
50
25
0
b1
y8
Prec 2+
y6
y2
y1
rQ
b2
y3
y6 2+
b3
y7
y10
y11
0 100 200 300 400 500 600 700 800 900 1,000 1,100 1,200 1,300 1,400
m/z

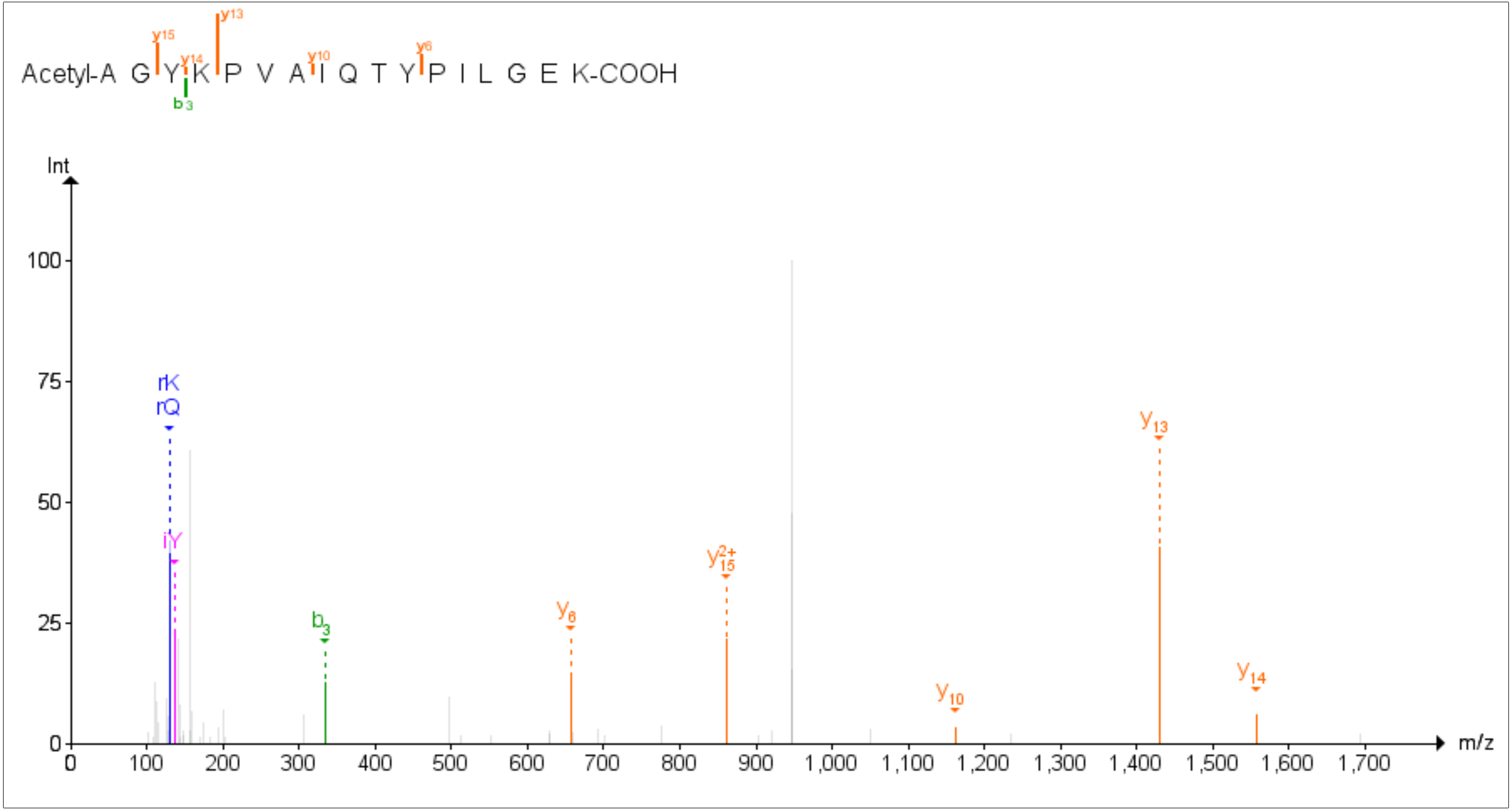

Acetyl-A G Y K P V A I Q T Y P I L G E K-COOH
y15
y14
y13
y10
y6
b3
Int
100
75
50
25
0
rK
rQ
iY
b3
y6
y15 2+
y10
y13
y14
0
100
200
300
400
500
600
700
800
900
1,000
1,100
1,200
1,300
1,400
1,500
1,600
1,700
m/z

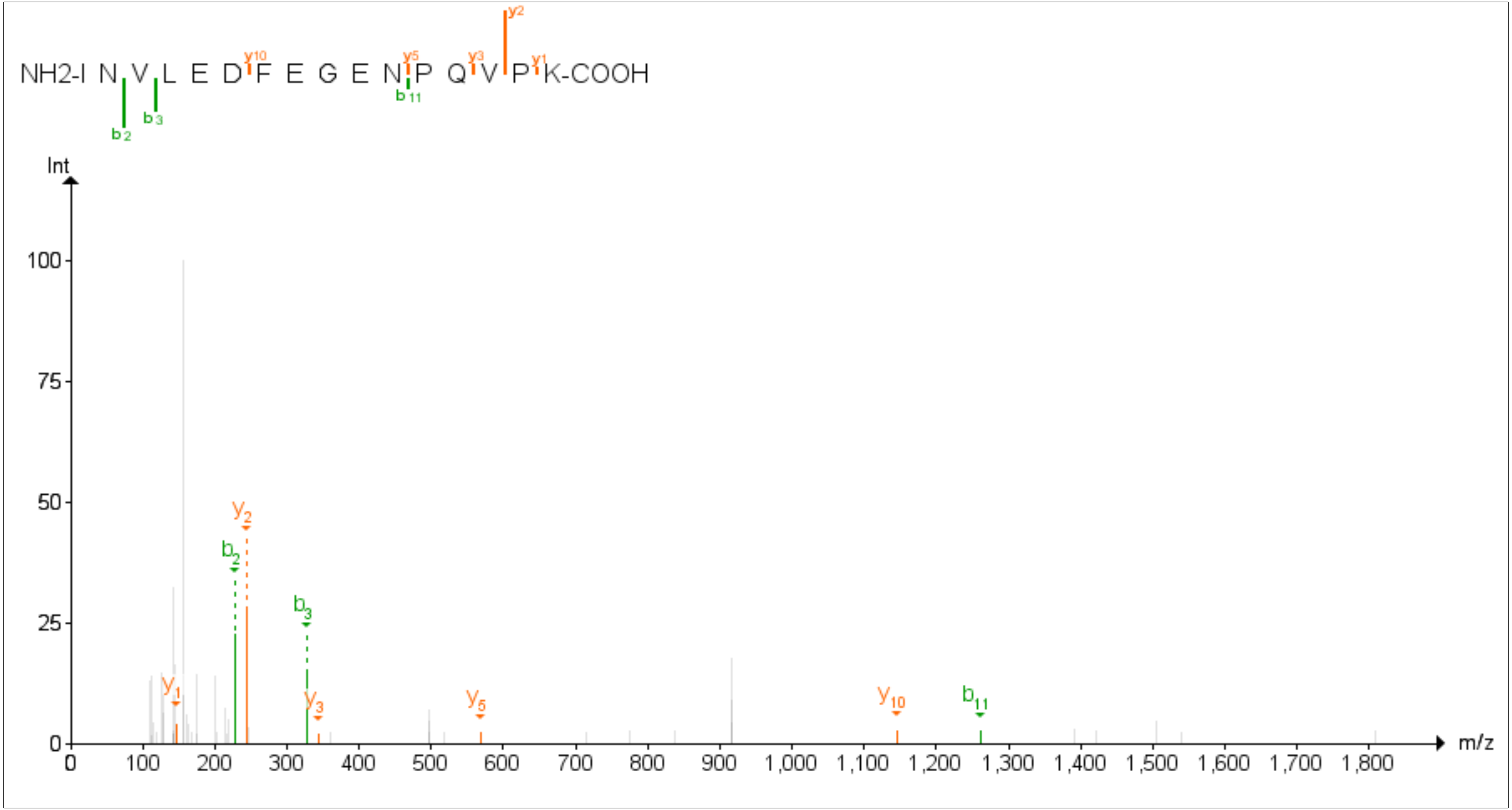

NH2-I N V L E D F E G E N P Q V P K-COOH
y10
y5
y3
y2
y1
b2
b3
b11
Int
100
75
50
25
0
y1
b2
y2
b3
y3
y5
y10
b11
0
100
200
300
400
500
600
700
800
900
1,000
1,100
1,200
1,300
1,400
1,500
1,600
1,700
1,800
m/z

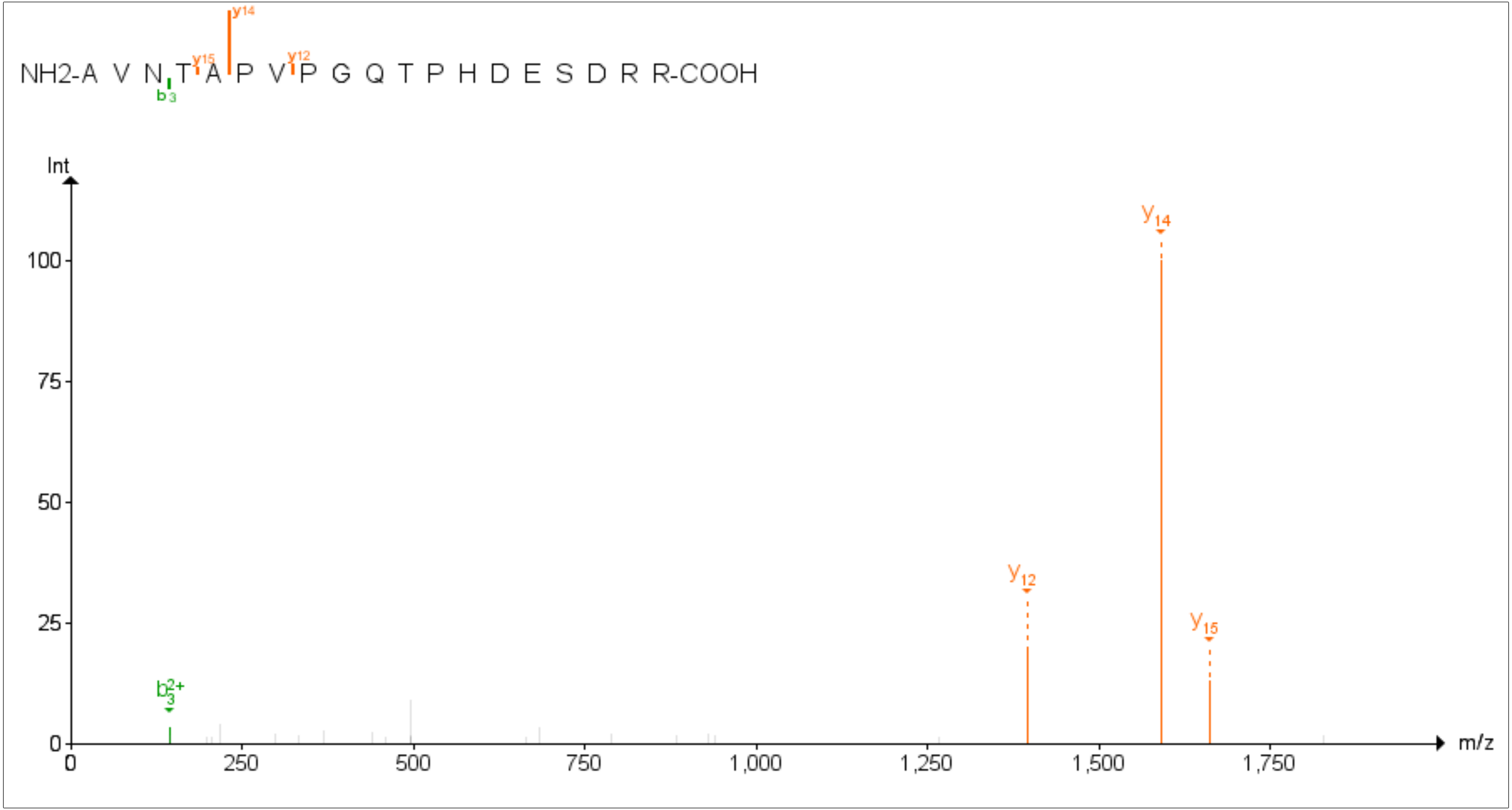

y14
y15
y12
NH2-A V N T A P V P G Q T P H D E S D R R-COOH
b3
Int
100
75
50
25
0
y14
y12
y15
b3 2+
0
250
500
750
1,000
1,250
1,500
1,750
m/z

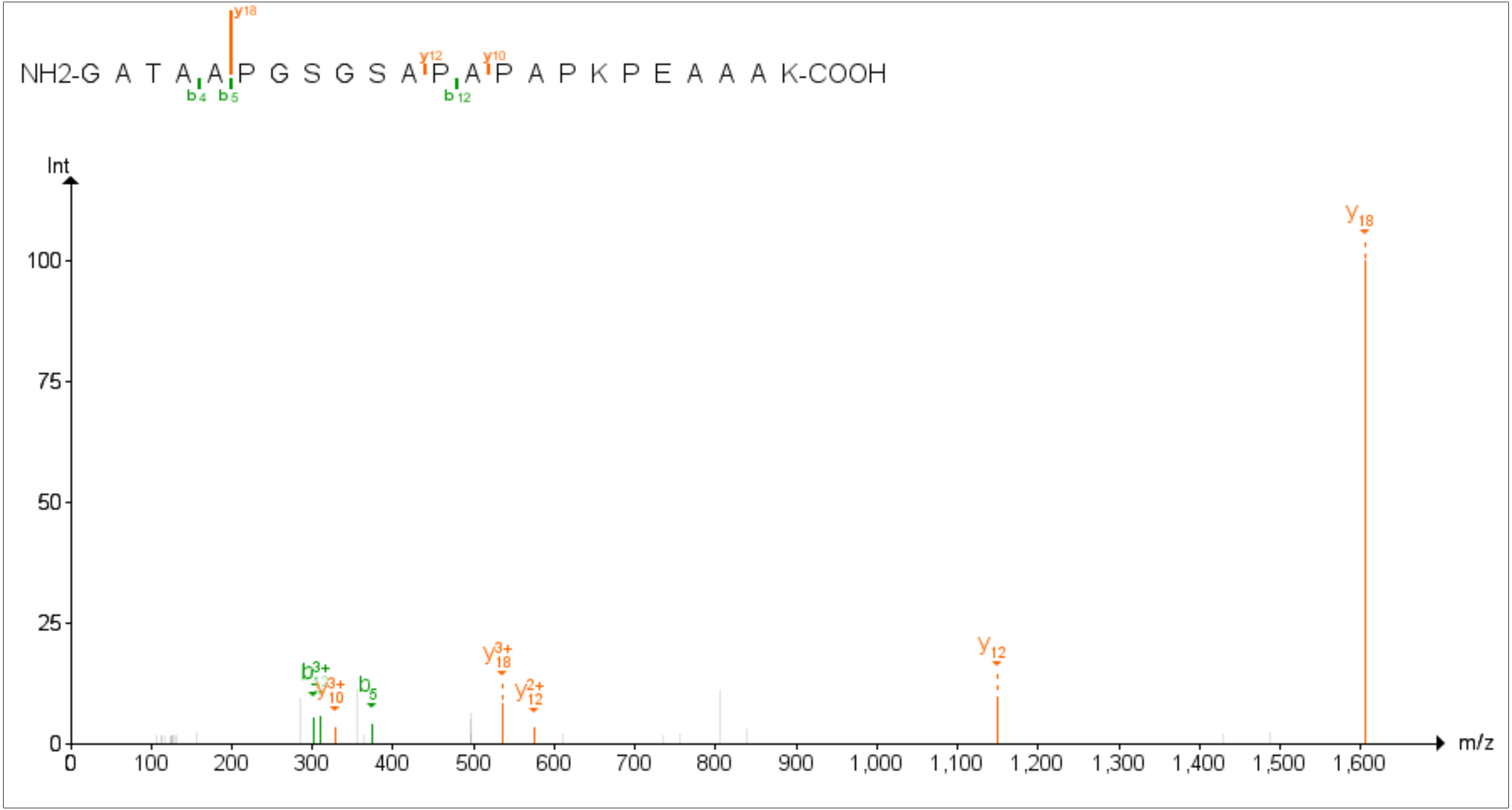

NH2-G A T A A P G S G S A P A P A P K P E A A A K-COOH
y18
y12
y10
b4
b5
b12
Int
100
75
50
25
0
m/z
0
100
200
300
400
500
600
700
800
900
1,000
1,100
1,200
1,300
1,400
1,500
1,600
y18
y12
y18 3+
y12 2+
b5
y10 3+
b12 3+

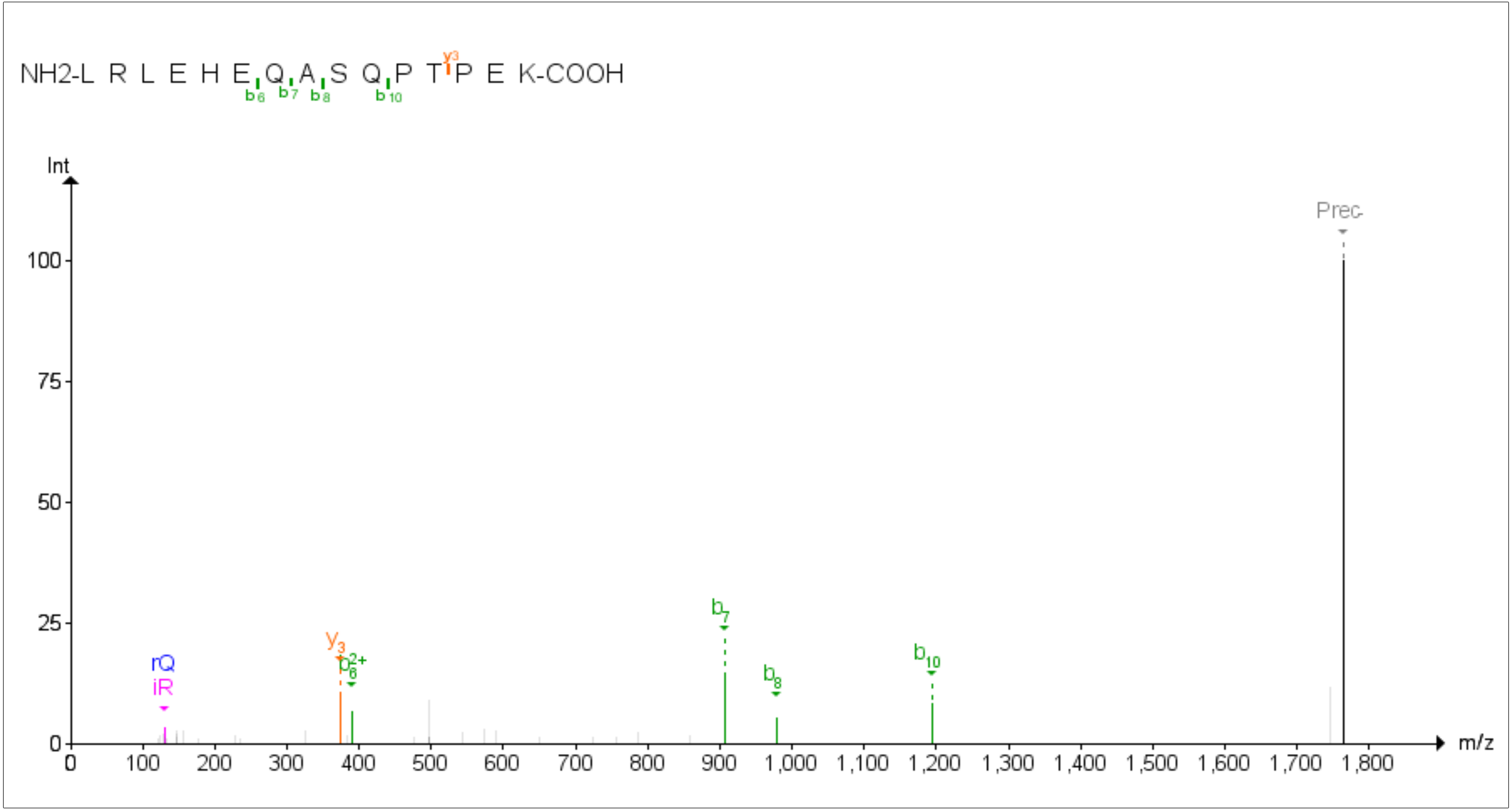

NH2-L R L E H E Q A S Q P T P E K-COOH
b6 b7 b8 b10
y3
Int
100
75
50
25
0
m/z
0 100 200 300 400 500 600 700 800 900 1,000 1,100 1,200 1,300 1,400 1,500 1,600 1,700 1,800
Prec
rQ
iR
y3
b6 2+
b7
b8
b10

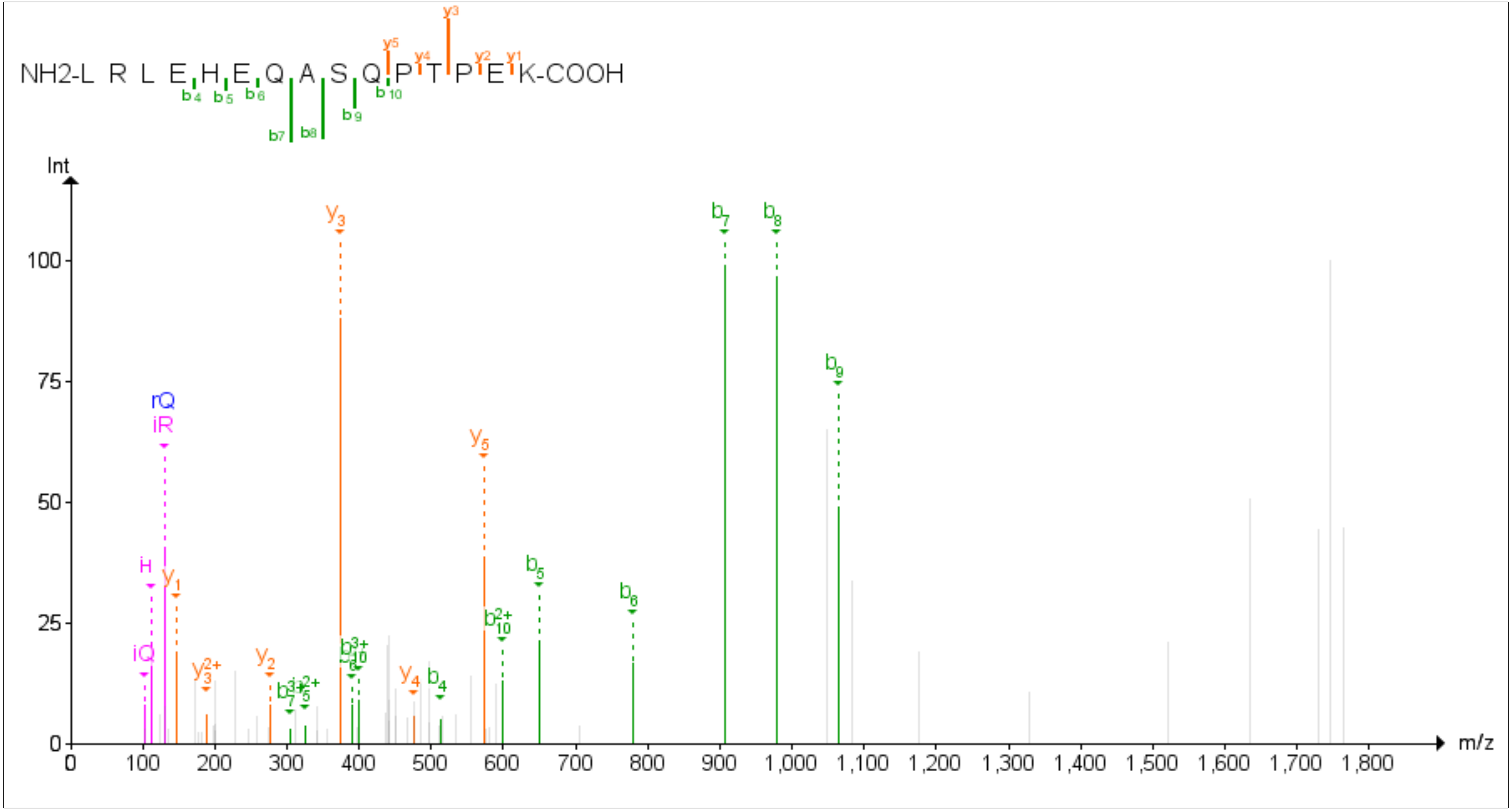
NH2-L R L E H E Q A S Q P T P E K-COOH
b4 b5 b6 b7 b8 b9 b10
y5 y4 y3 y2 y1
Int
m/z
y3
b7
b8
b9
y5
rQ
iR
iH
y1
iQ
b5
b6
y2
y4
b4
0
25
50
75
100
0
100
200
300
400
500
600
700
800
900
1,000
1,100
1,200
1,300
1,400
1,500
1,600
1,700
1,800

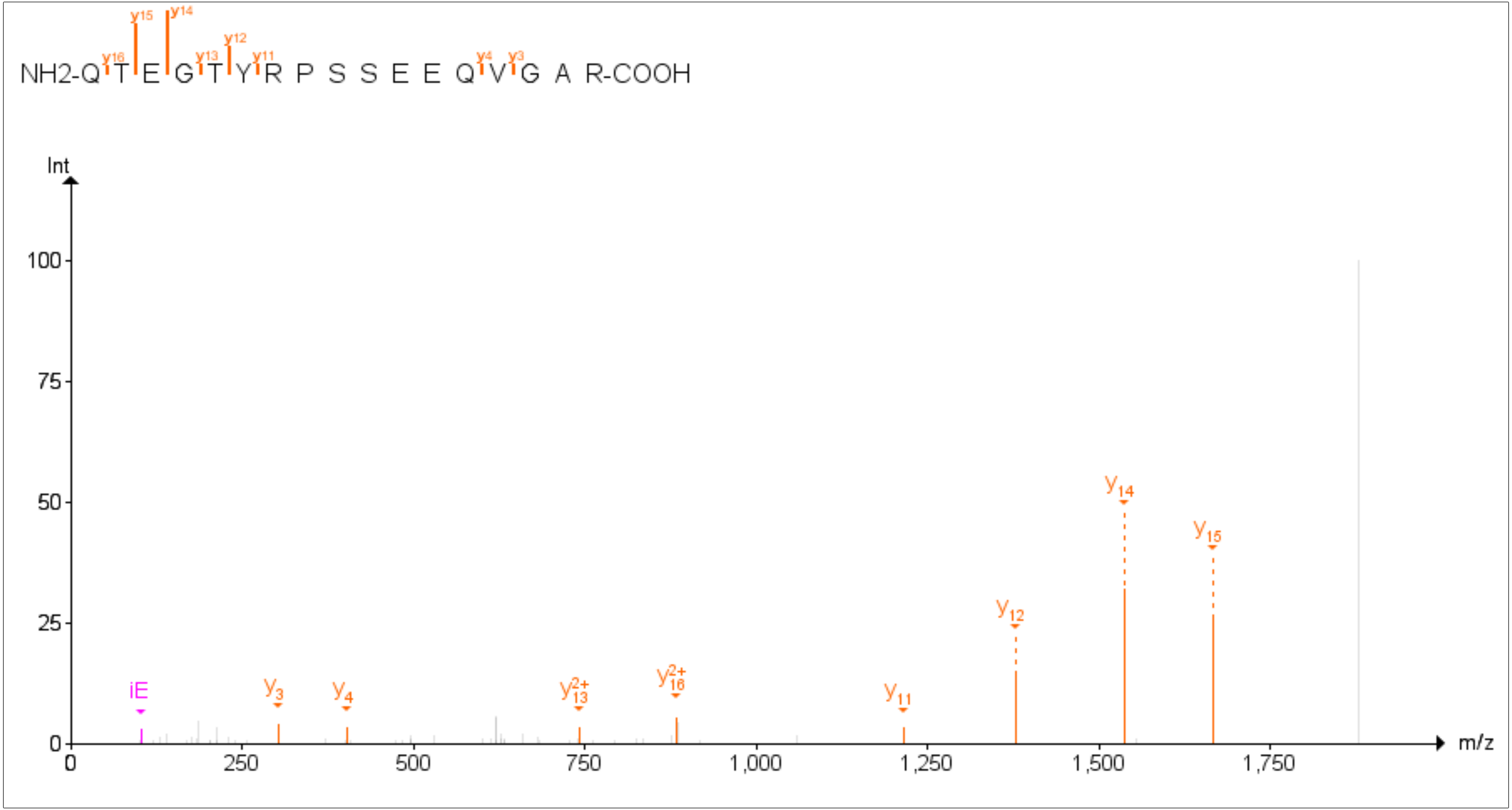

NH2-Q T E G T Y R P S S E E Q V G A R-COOH
y16
y15
y14
y13
y12
y11
y4
y3
Int
100
75
50
25
0
m/z
0
250
500
750
1,000
1,250
1,500
1,750
iE
y3
y4
y13 2+
y16 2+
y11
y12
y14
y15

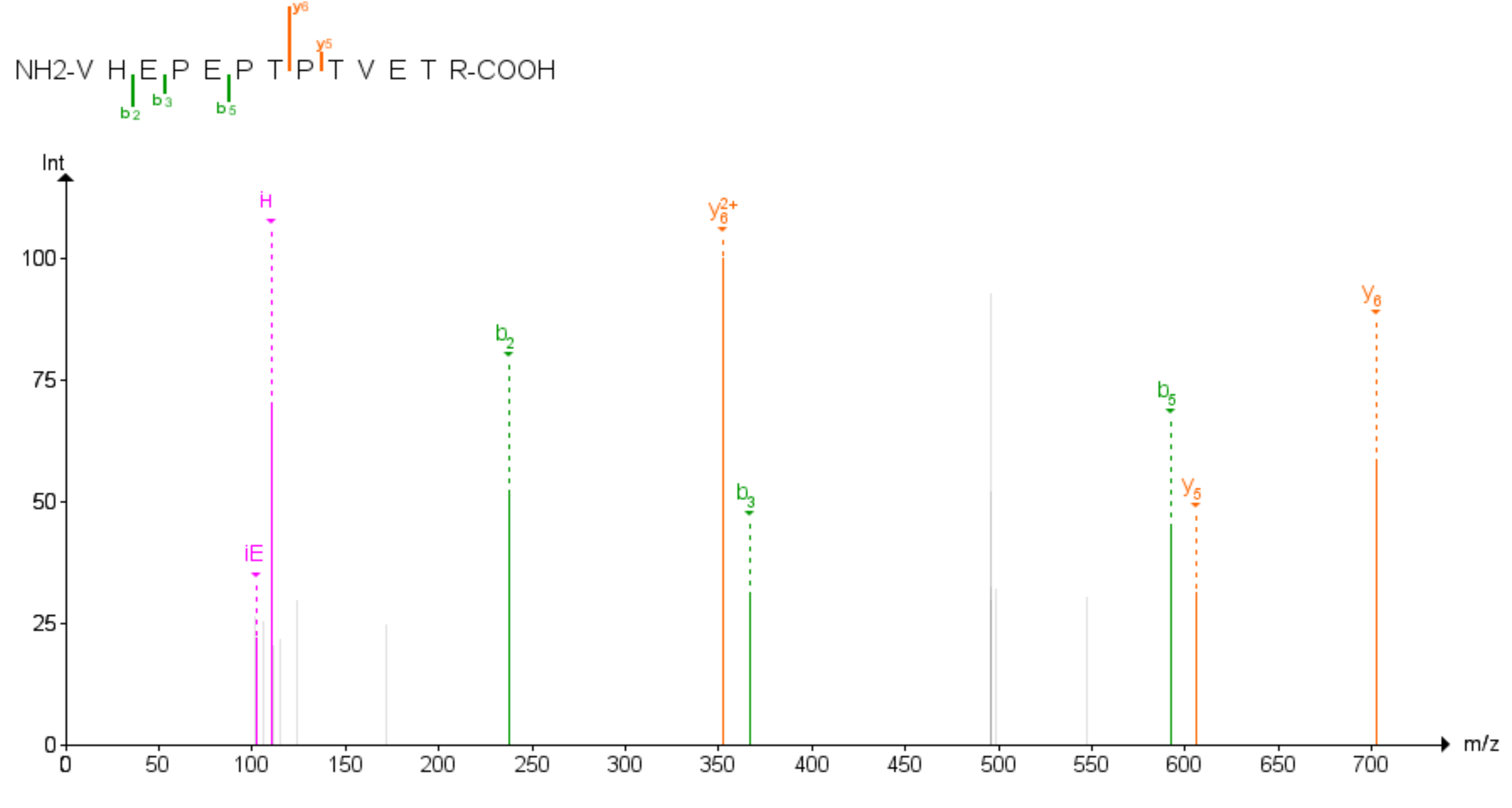

NH2-V H E P E P T P T V E T R-COOH
b2
b3
b5
y6
y5
Int
iH
iE
b2
y6 2+
b3
b5
y5
y6
100
75
50
25
0
0
50
100
150
200
250
300
350
400
450
500
550
600
650
700
m/z

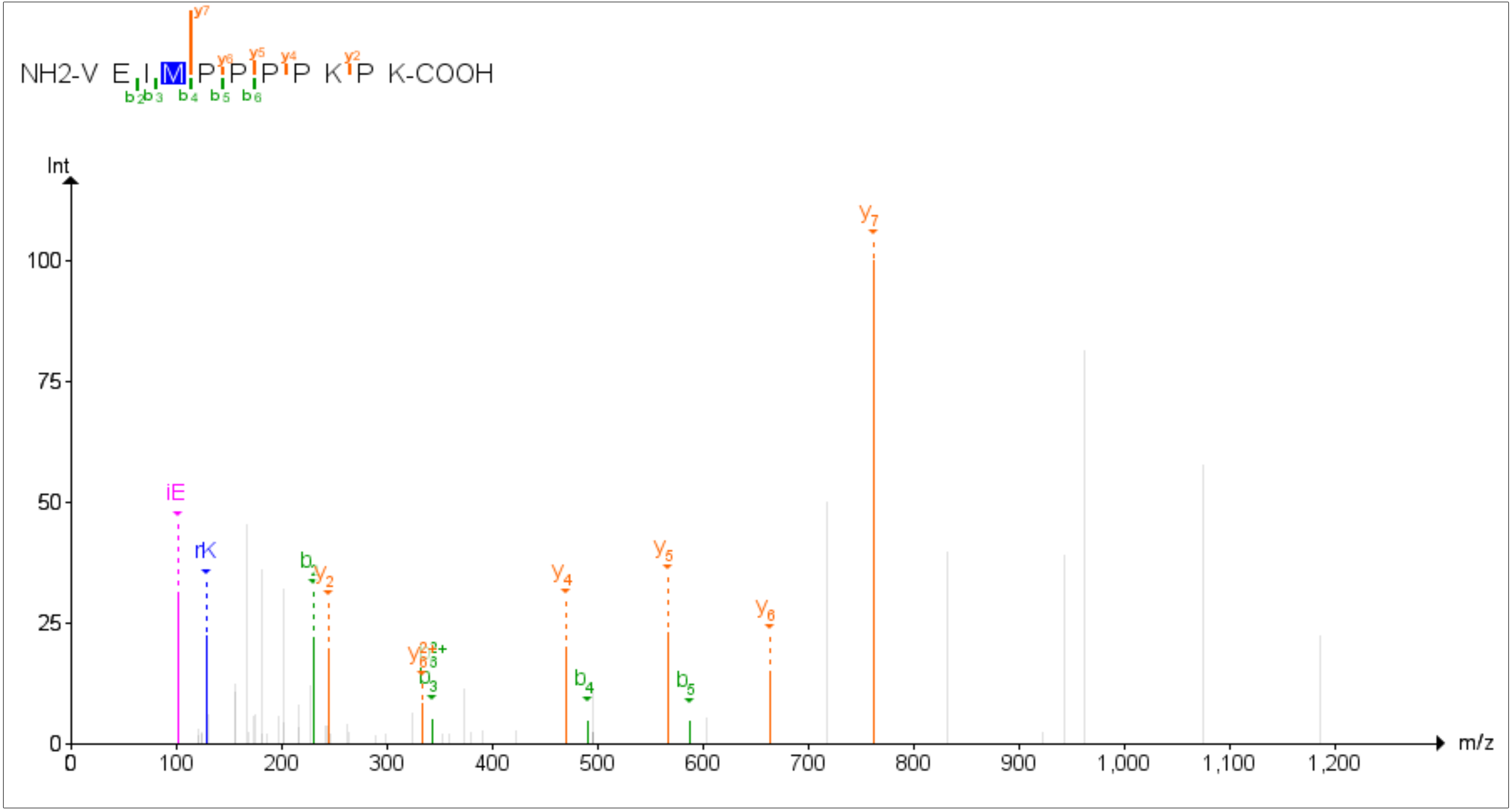

NH2-V E I M P P P P K P K-COOH
Int
m/z
y7
y6
y5
y4
y2
b2
b3
b4
b5
b6
iE
rK

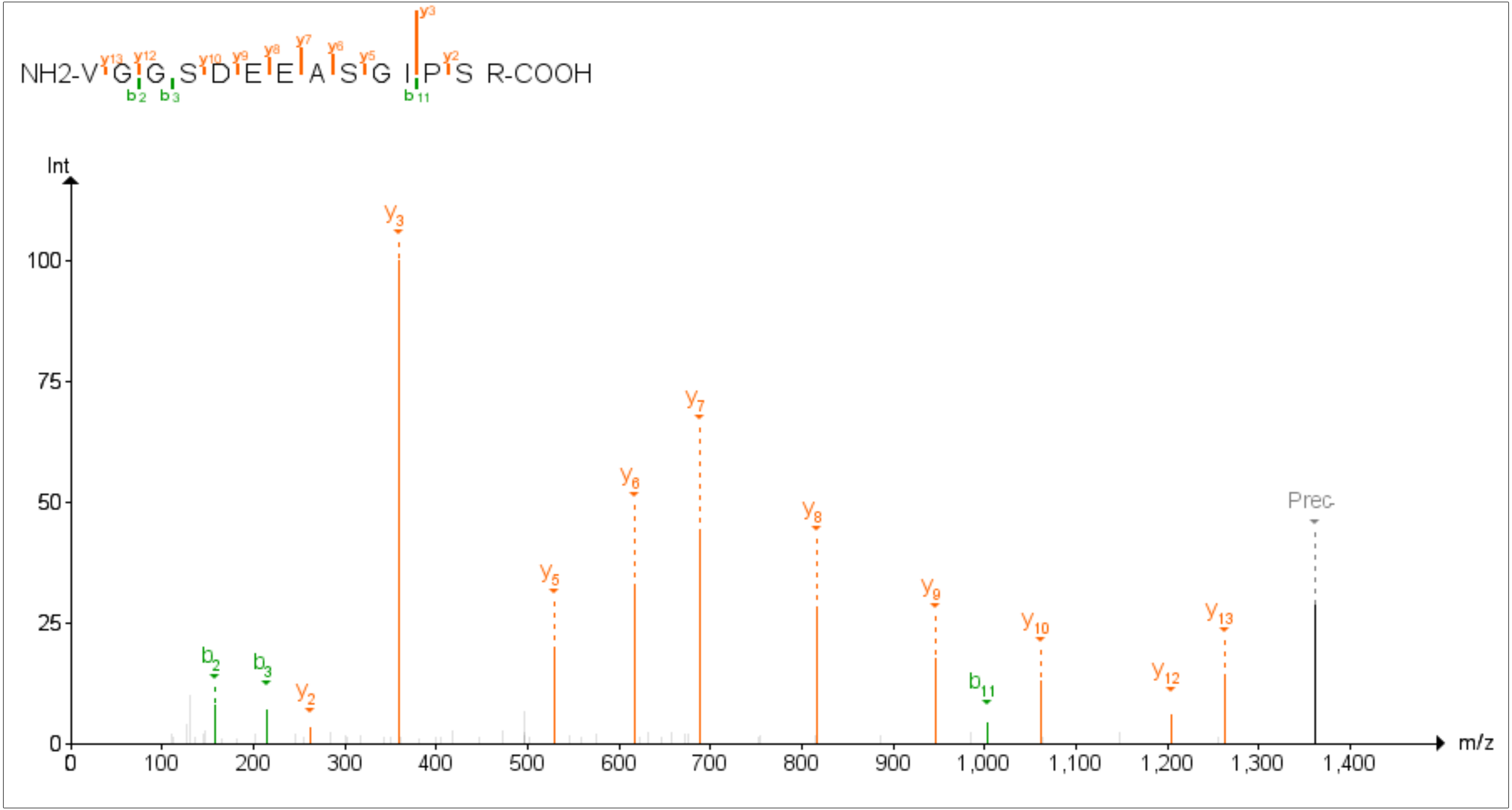

NH2-V G G S D E E A S G I P S R-COOH
y13 y12 y10 y9 y8 y7 y6 y5 y3 y2
b2 b3 b11
Int
m/z
0
25
50
75
100
0 100 200 300 400 500 600 700 800 900 1,000 1,100 1,200 1,300 1,400
y3
y7
y6
y8
Prec
y5
y9
y10
y13
b2
b3
y2
b11
y12

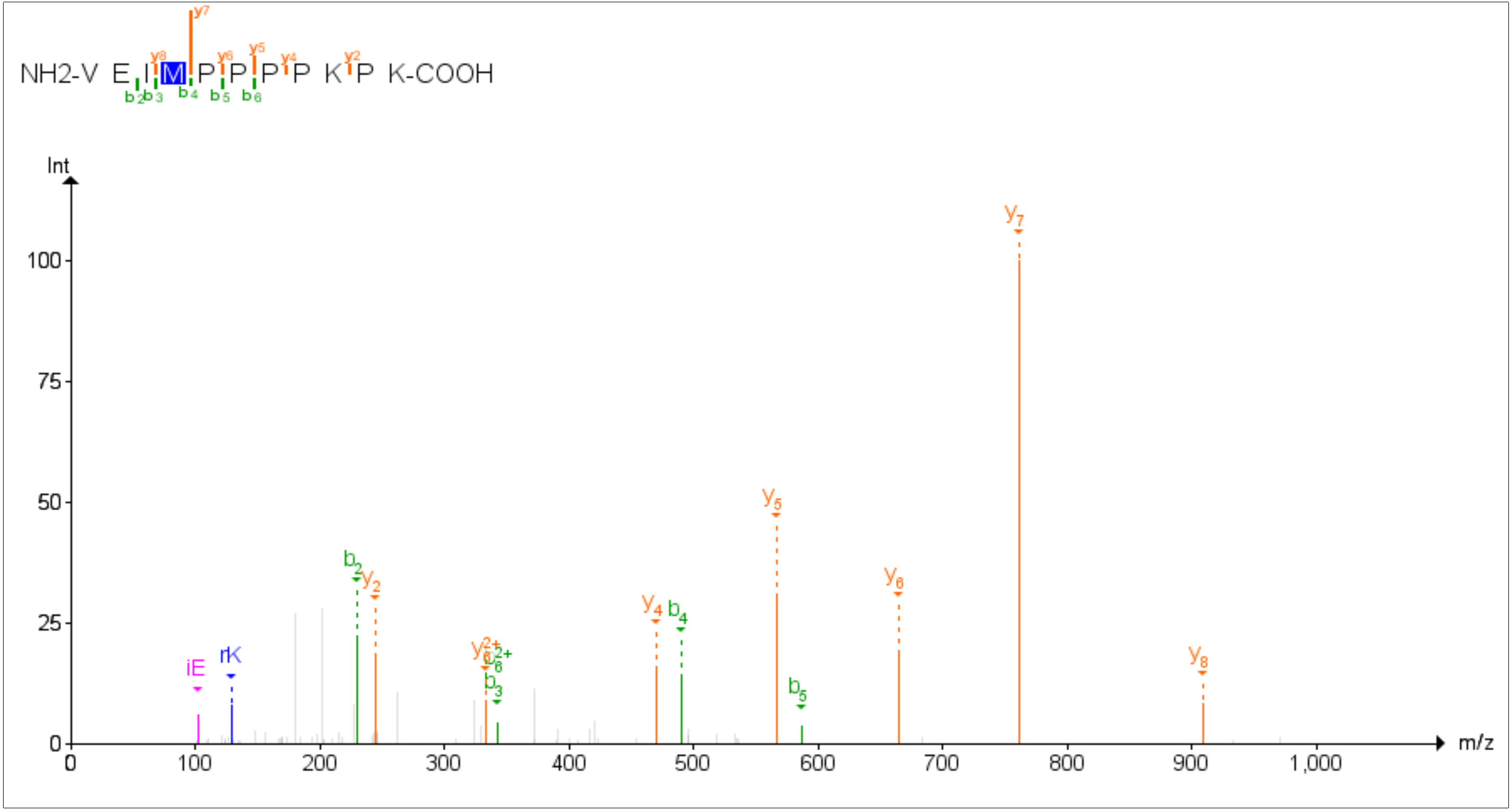

NH2-V E I M P P P P K P K-COOH
y7
y8
y6
y5
y4
y2
b2
b3
b4
b5
b6
Int
100
75
50
25
0
iE
rK
b2
y2
y6 2+
b6 2+
b3
y4
b4
y5
b5
y6
y7
y8
0
100
200
300
400
500
600
700
800
900
1,000
m/z

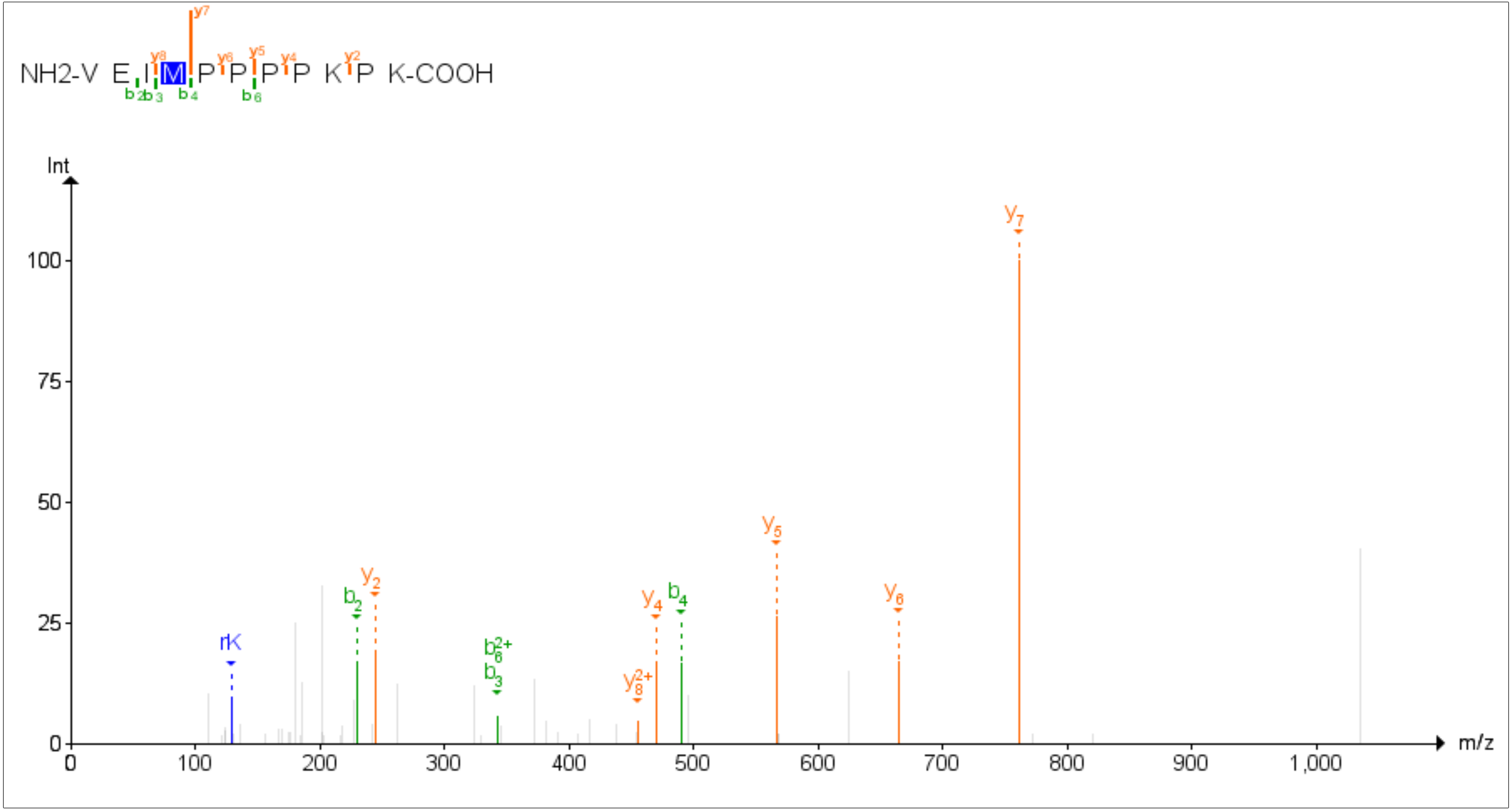

NH2-V E I M P P P P K P K-COOH
y7
y8
y6
y5
y4
y2
b2
b3
b4
b6
Int
100
75
50
25
0
y7
y5
y2
b2
y4
b4
y6
rK
b6 2+
b3
y8 2+
0
100
200
300
400
500
600
700
800
900
1,000
m/z

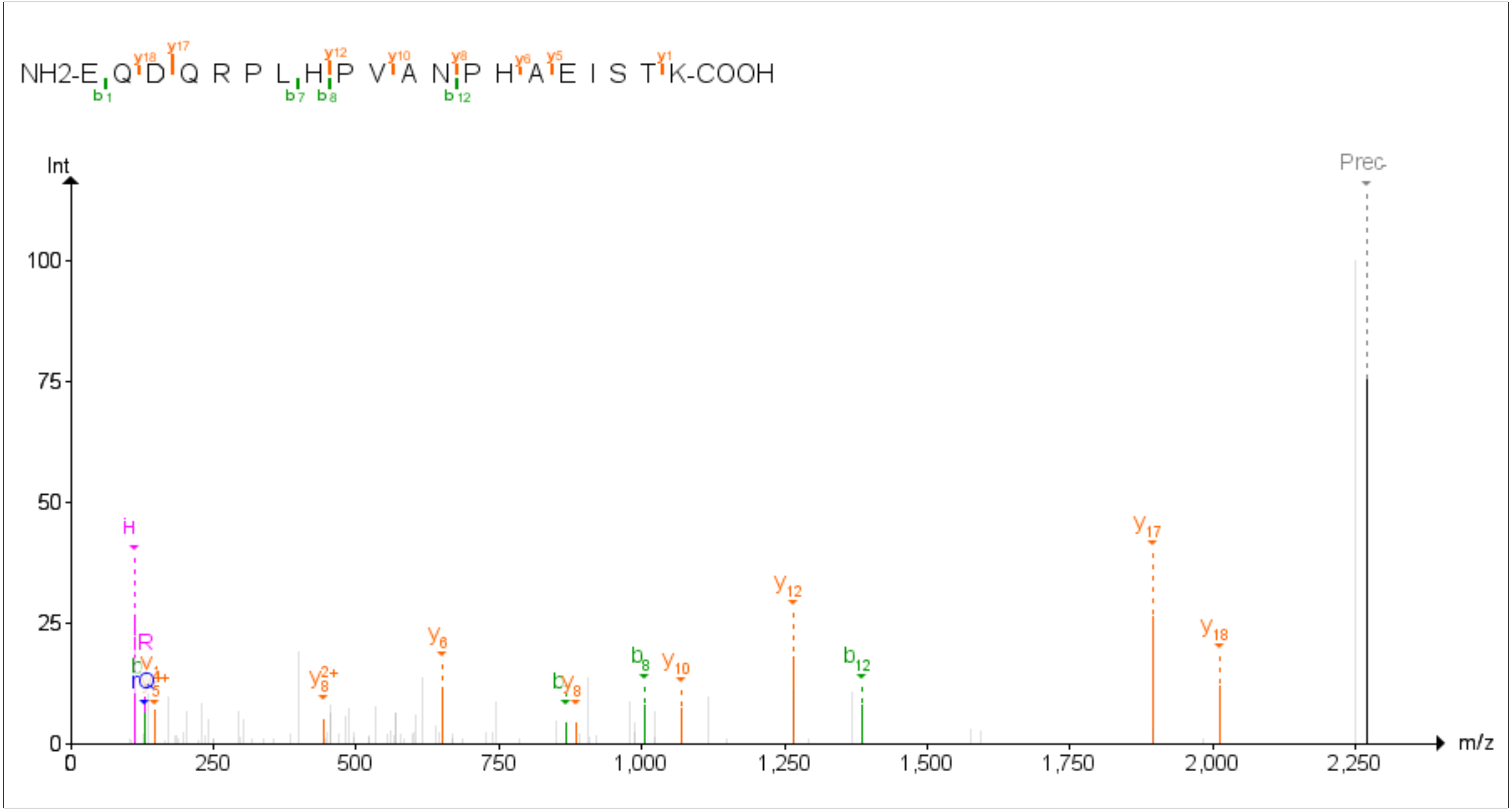

NH2-E Q D Q R P L H P V A N P H A E I S T K-COOH
Int
m/z
Prec
y17
y18
y12
y10
y6
b12
b8
y8
y_8^{2+}

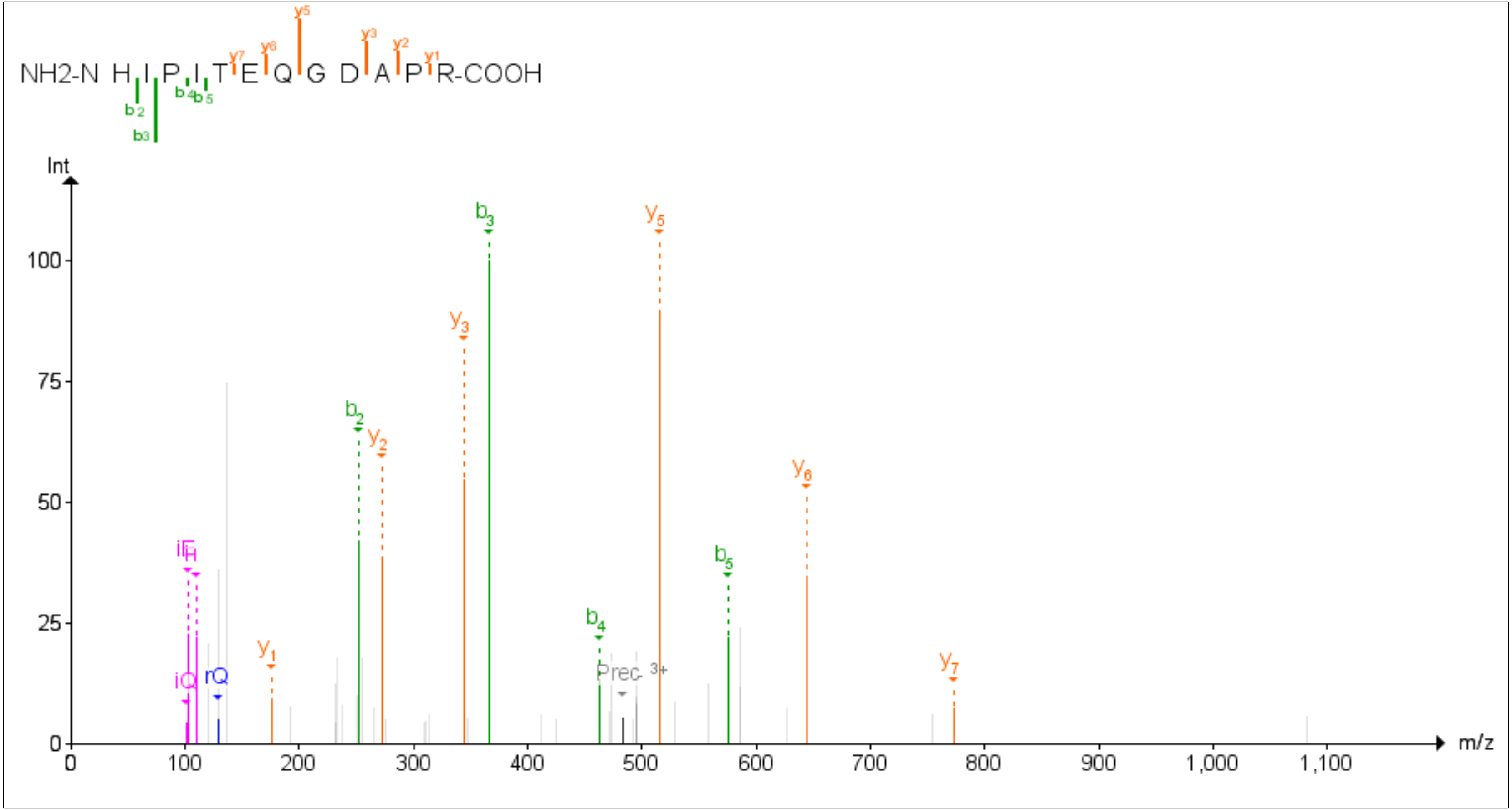

NH2-N H I P I T E Q G D A P R-COOH
y7 y6 y5 y3 y2 y1
b2 b3 b4 b5
Int
m/z
0
25
50
75
100
0
100
200
300
400
500
600
700
800
900
1,000
1,100
b3
y5
y3
b2
y2
y6
b5
b4
y1
y7
iF
iH
iQ
rQ
Prec 3+

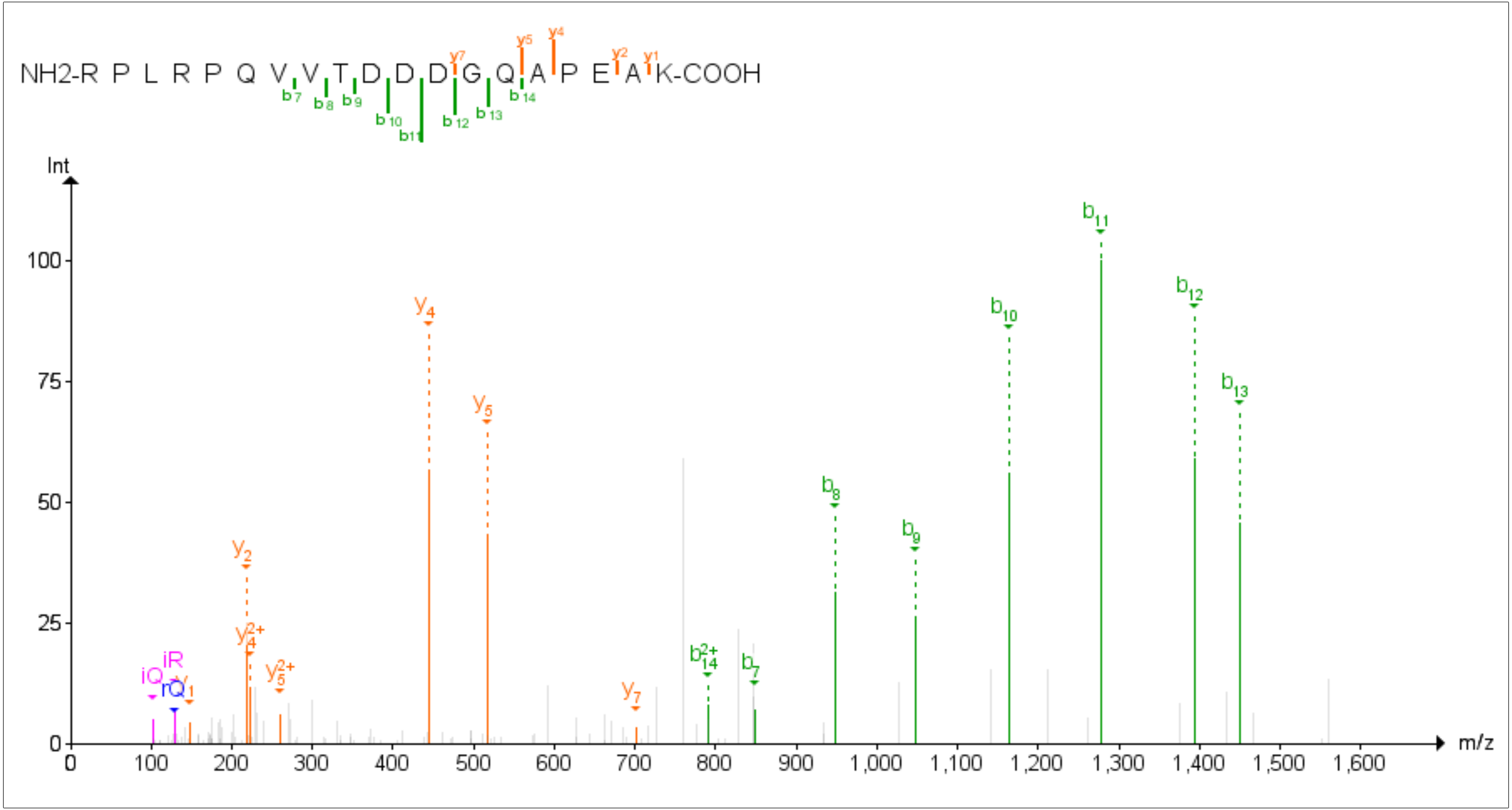

NH2-R P L R P Q V V T D D D G Q A P E A K-COOH
y7 y5 y4 y2 y1
b7 b8 b9 b10 b11 b12 b13 b14
Int
100
75
50
25
0
0 100 200 300 400 500 600 700 800 900 1,000 1,100 1,200 1,300 1,400 1,500 1,600
m/z
iQ iR rQ y1 y2 y4 y5 y7
b7 b8 b9 b10 b11 b12 b13

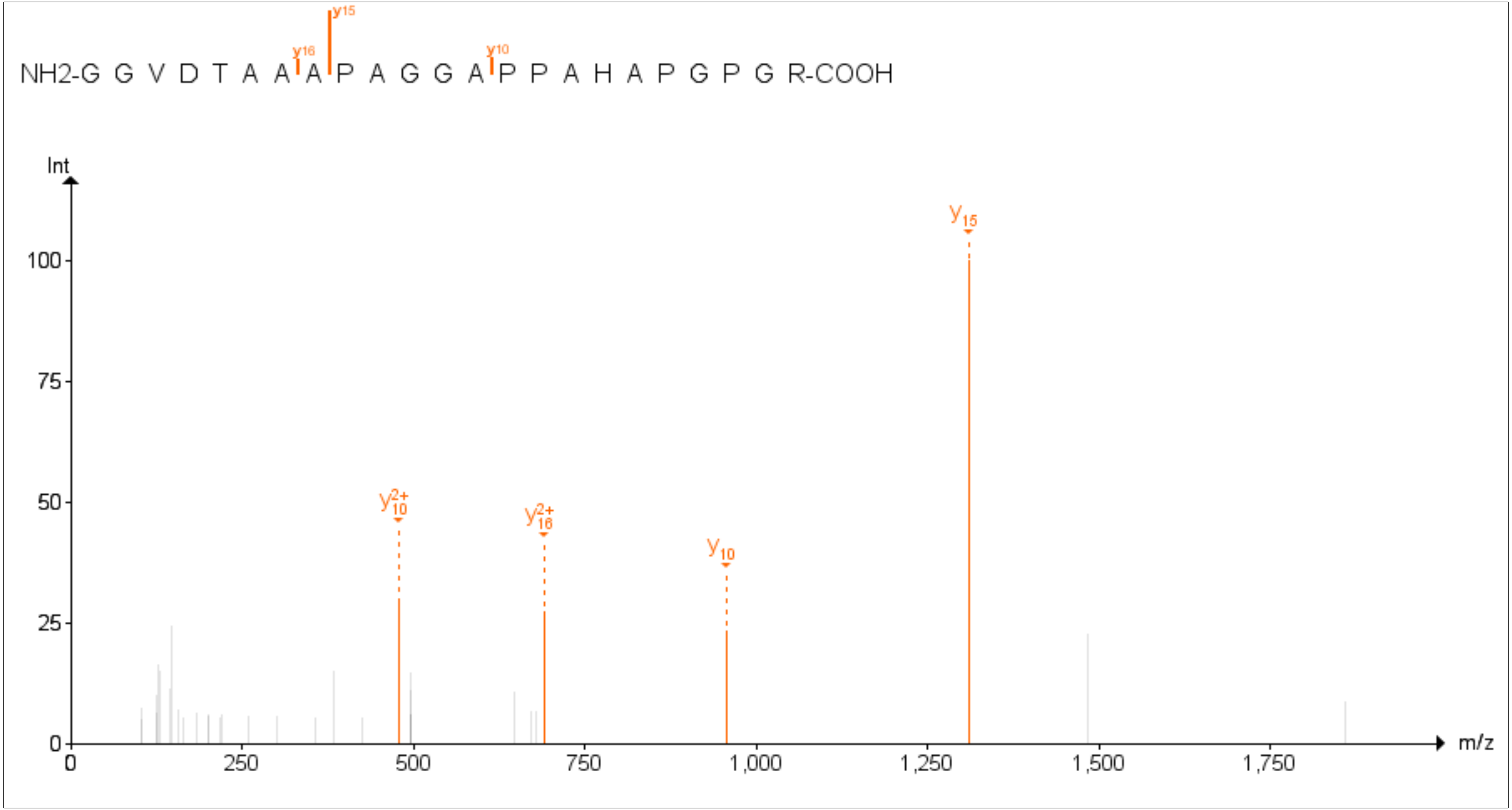

NH2-G G V D T A A A P A G G A P P A H A P G P G R-COOH
y16
y15
y10
Int
100
75
50
25
0
y15
y10 2+
y16 2+
y10
0
250
500
750
1,000
1,250
1,500
1,750
m/z

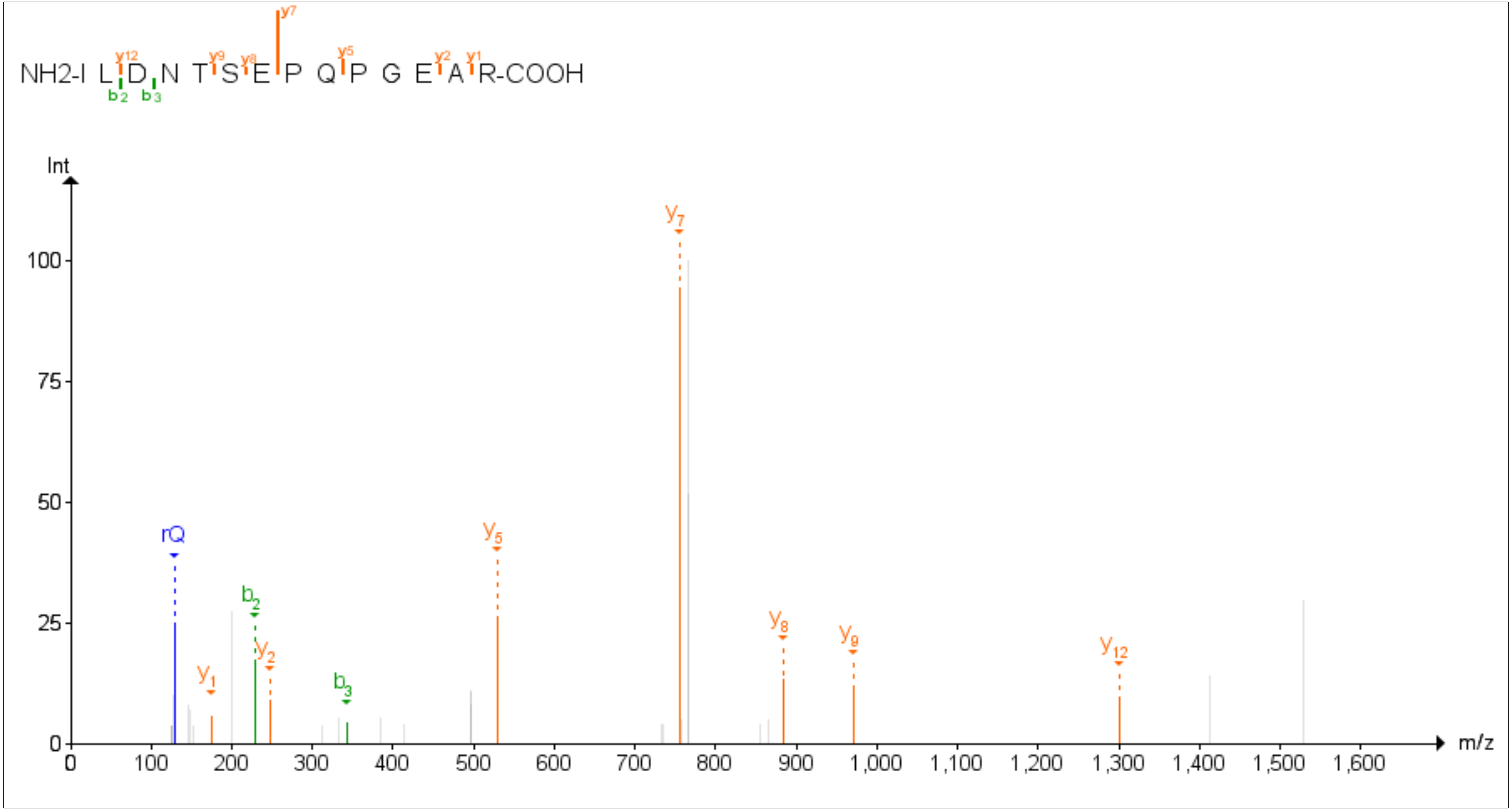

NH2-I L D N T S E P Q P G E A R-COOH
y12 y9 y8 y7 y5 y2 y1
b2 b3
Int
m/z
y7
y5
rQ
b2
y2
y1
b3
y8
y9
y12
0
25
50
75
100
100
200
300
400
500
600
700
800
900
1,000
1,100
1,200
1,300
1,400
1,500
1,600

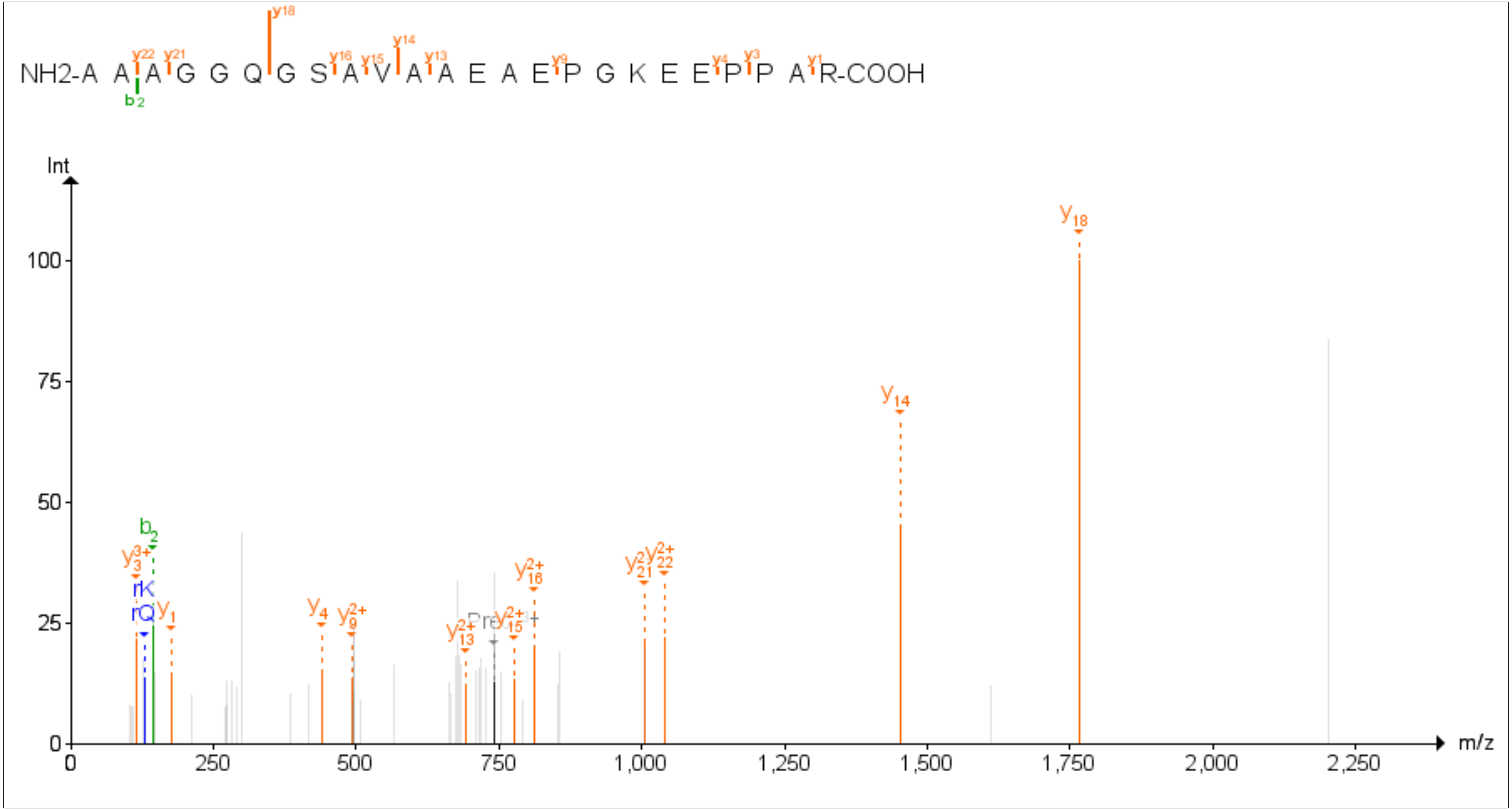

NH2-A A A G G Q G S A V A A E A E P G K E E P P A R-COOH
y22
y21
y18
y16
y15
y14
y13
y9
y4
y3
y1
b2
Int
100
75
50
25
0
m/z
0
250
500
750
1,000
1,250
1,500
1,750
2,000
2,250

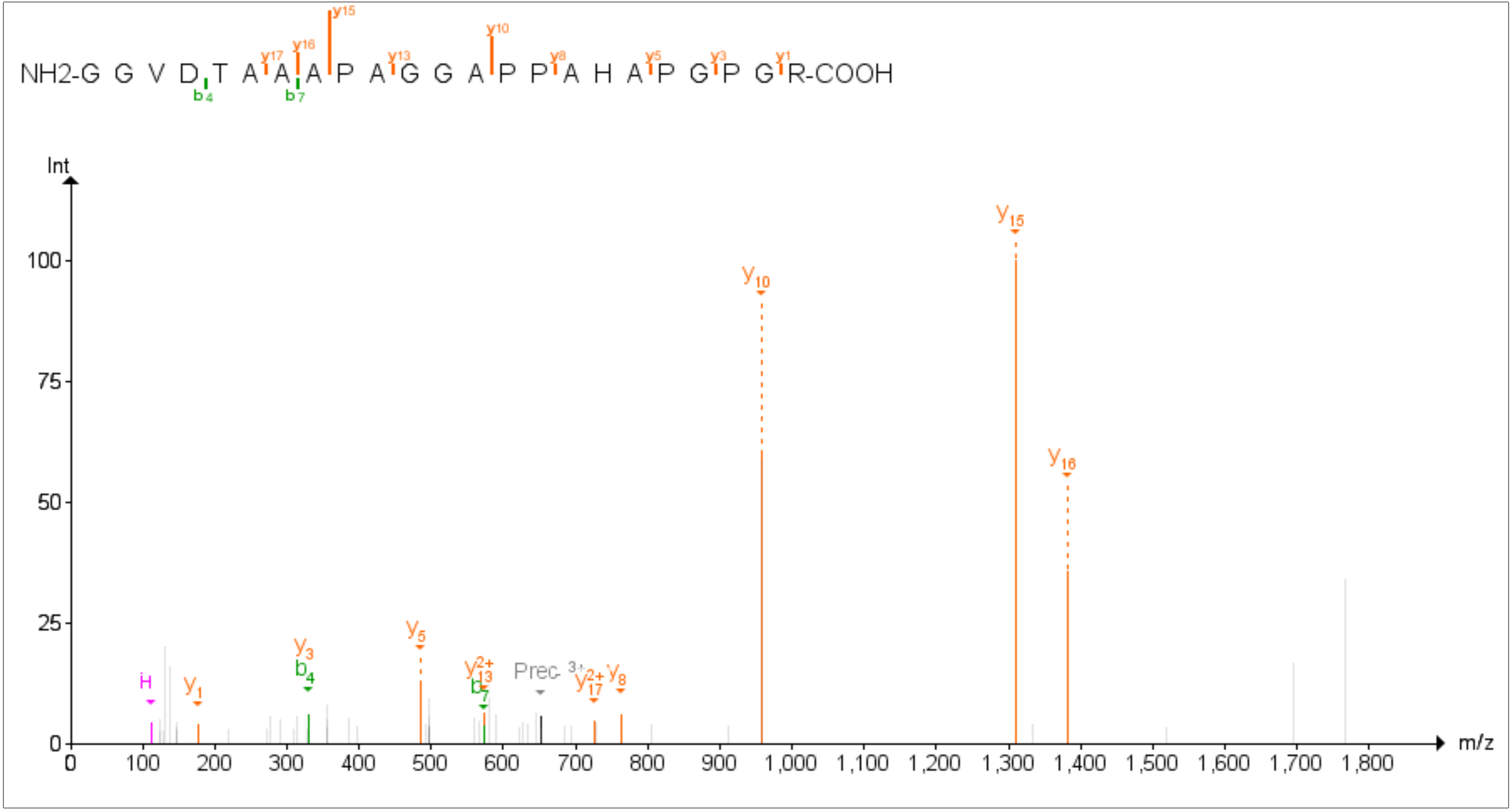

NH2-G G V D T A A A P A G G A P P A H A P G P G R-COOH
y15
y10
y17
y16
y13
y8
y5
y3
y1
b4
b7
Int
100
75
50
25
0
y15
y10
y16
y5
y3
b4
y1
H
y13 2+
b7
Prec 3+
y17 2+
y8
0
100
200
300
400
500
600
700
800
900
1,000
1,100
1,200
1,300
1,400
1,500
1,600
1,700
1,800
m/z

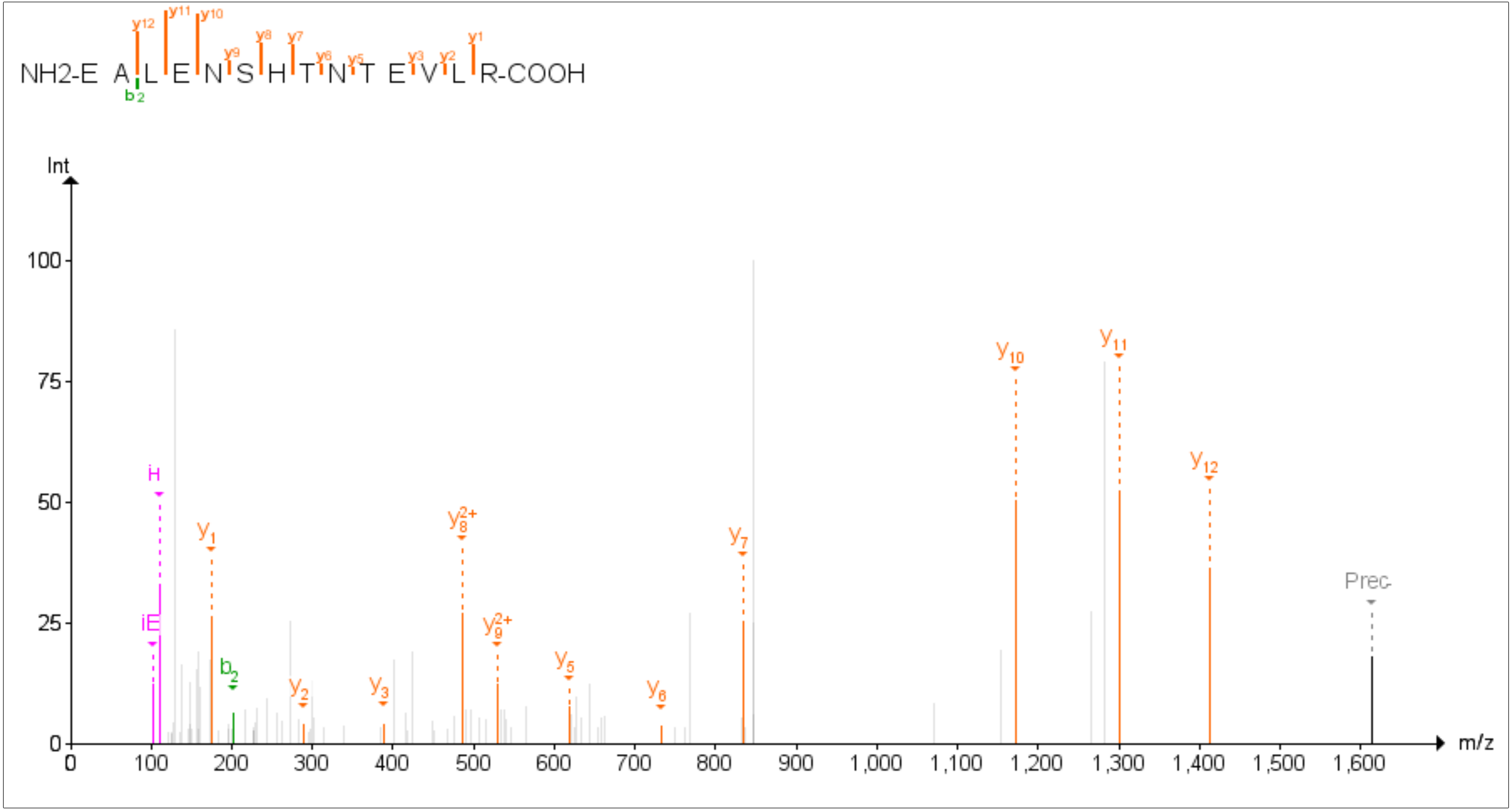

NH2-E A L E N S H T N T E V L R-COOH
y12 y11 y10 y9 y8 y7 y6 y5 y3 y2 y1
b2
Int
100
75
50
25
0
iH
iE
y1
b2
y2
y3
y8 2+
y9 2+
y5
y6
y7
y10
y11
y12
Prec
0
100
200
300
400
500
600
700
800
900
1,000
1,100
1,200
1,300
1,400
1,500
1,600
m/z

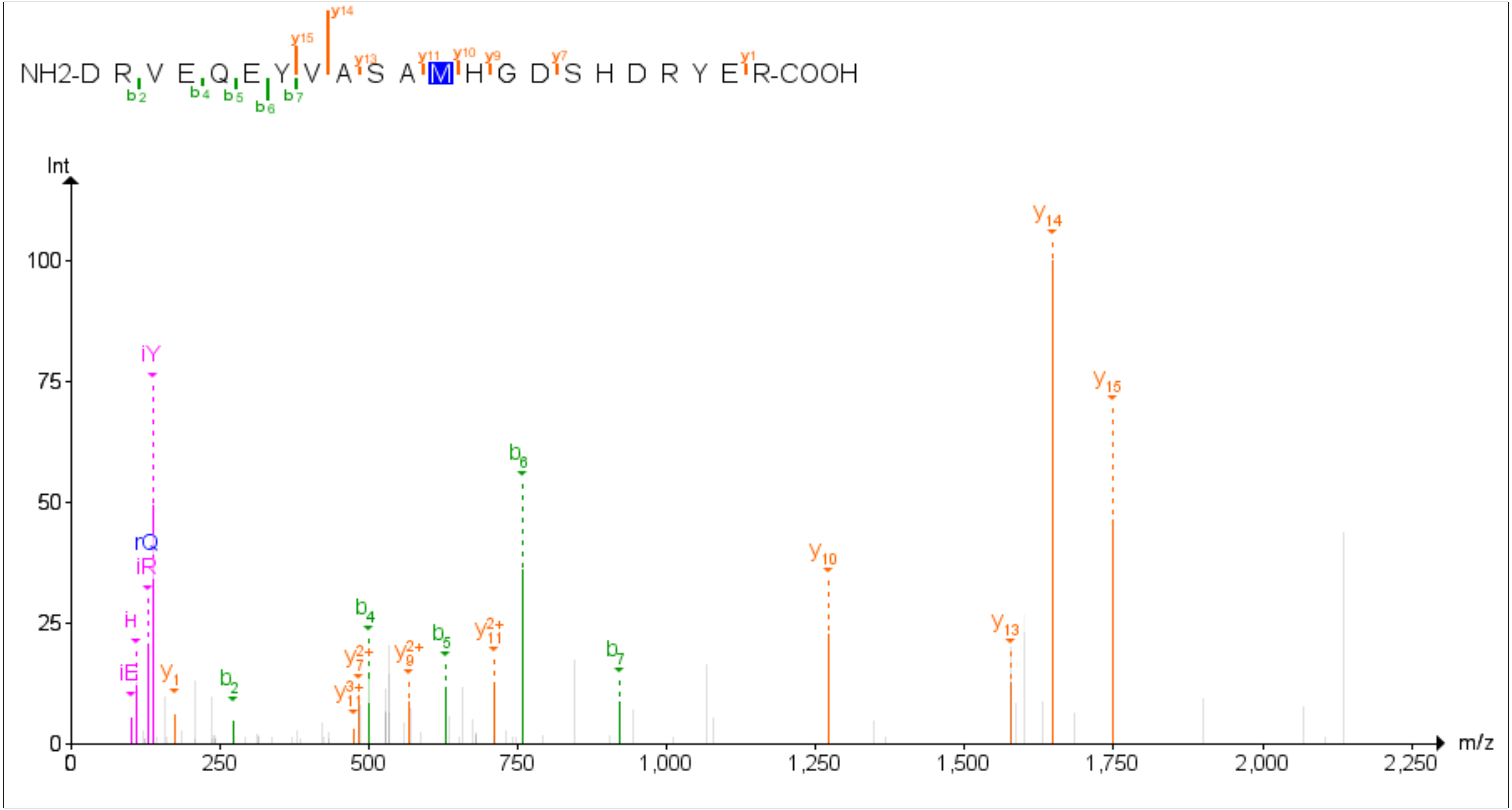

NH2-D R V E Q E Y V A S A M H G D S H D R Y E R-COOH
b2 b4 b5 b6 b7
y15 y14 y13 y11 y10 y9 y7 y1
Int
m/z
iY
rQ
iR
iH
iE
y1
b2
b4
b5
b6
b7
y10
y13
y14
y15
0
25
50
75
100
0
250
500
750
1,000
1,250
1,500
1,750
2,000
2,250

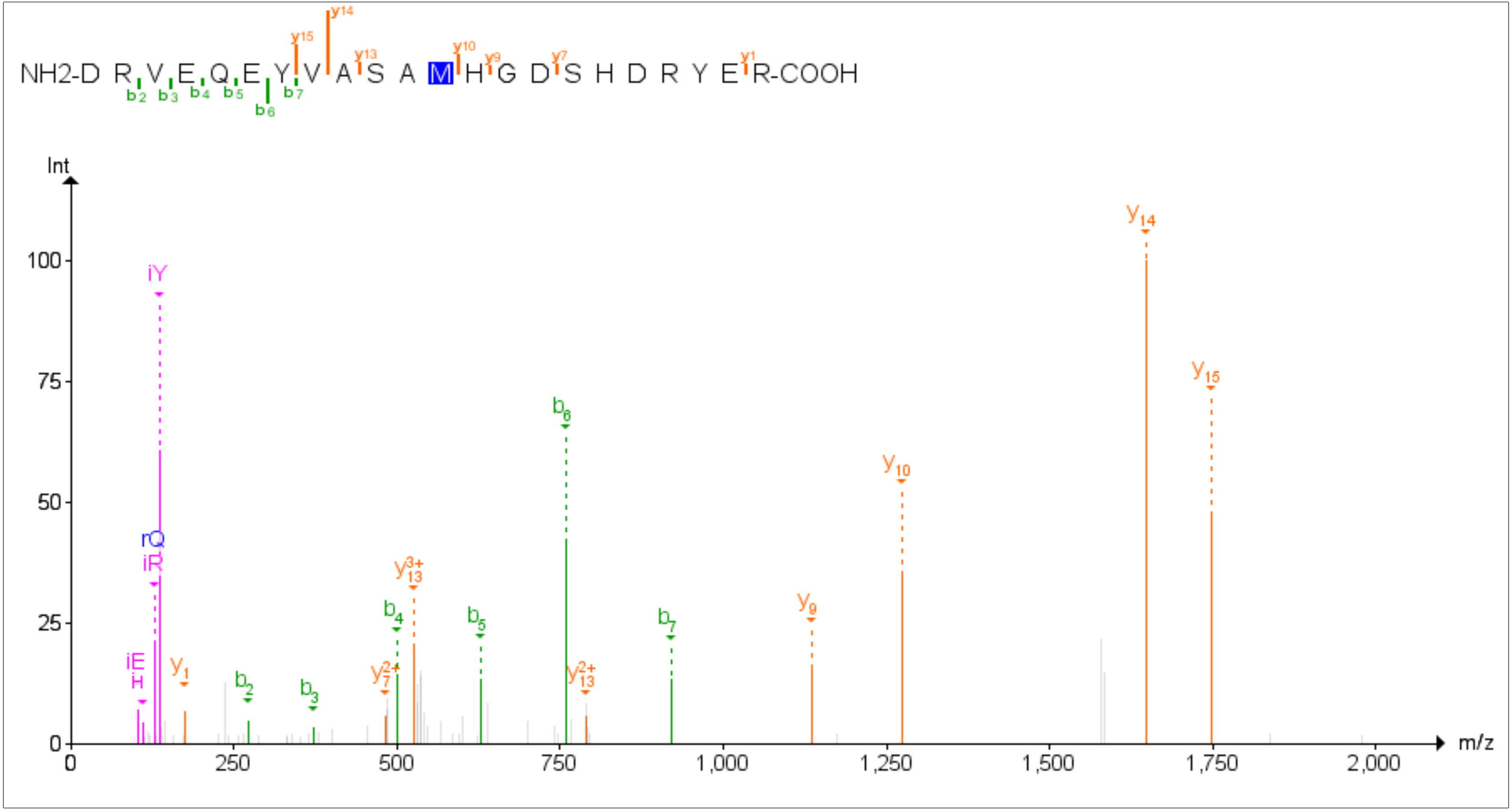
NH2-D R V E Q E Y V A S A M H G D S H D R Y E R-COOH
Int
m/z
0
25
50
75
100
250
500
750
1,000
1,250
1,500
1,750
2,000
iY
rQ
iR
iE
iH
y1
b2
b3
y7 2+
b4
y13 3+
b5
b6
y13 2+
b7
y9
y10
y14
y15

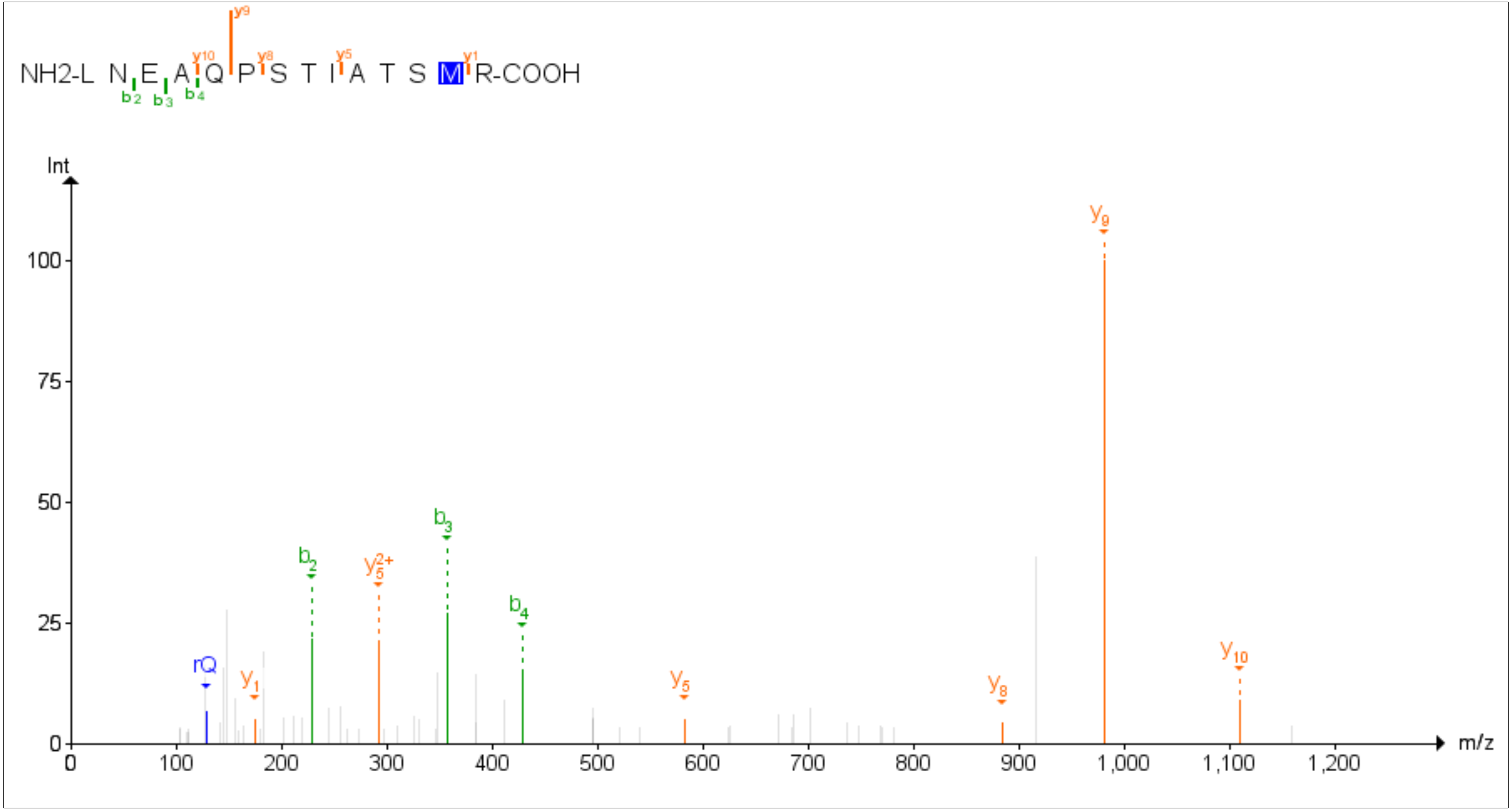
NH2-L N E A Q P S T I A T S M R-COOH
b2
b3
b4
y10
y9
y8
y5
y1
Int
m/z
0
25
50
75
100
100
200
300
400
500
600
700
800
900
1,000
1,100
1,200
rQ
y1
b2
y5 2+
b3
b4
y5
y8
y9
y10

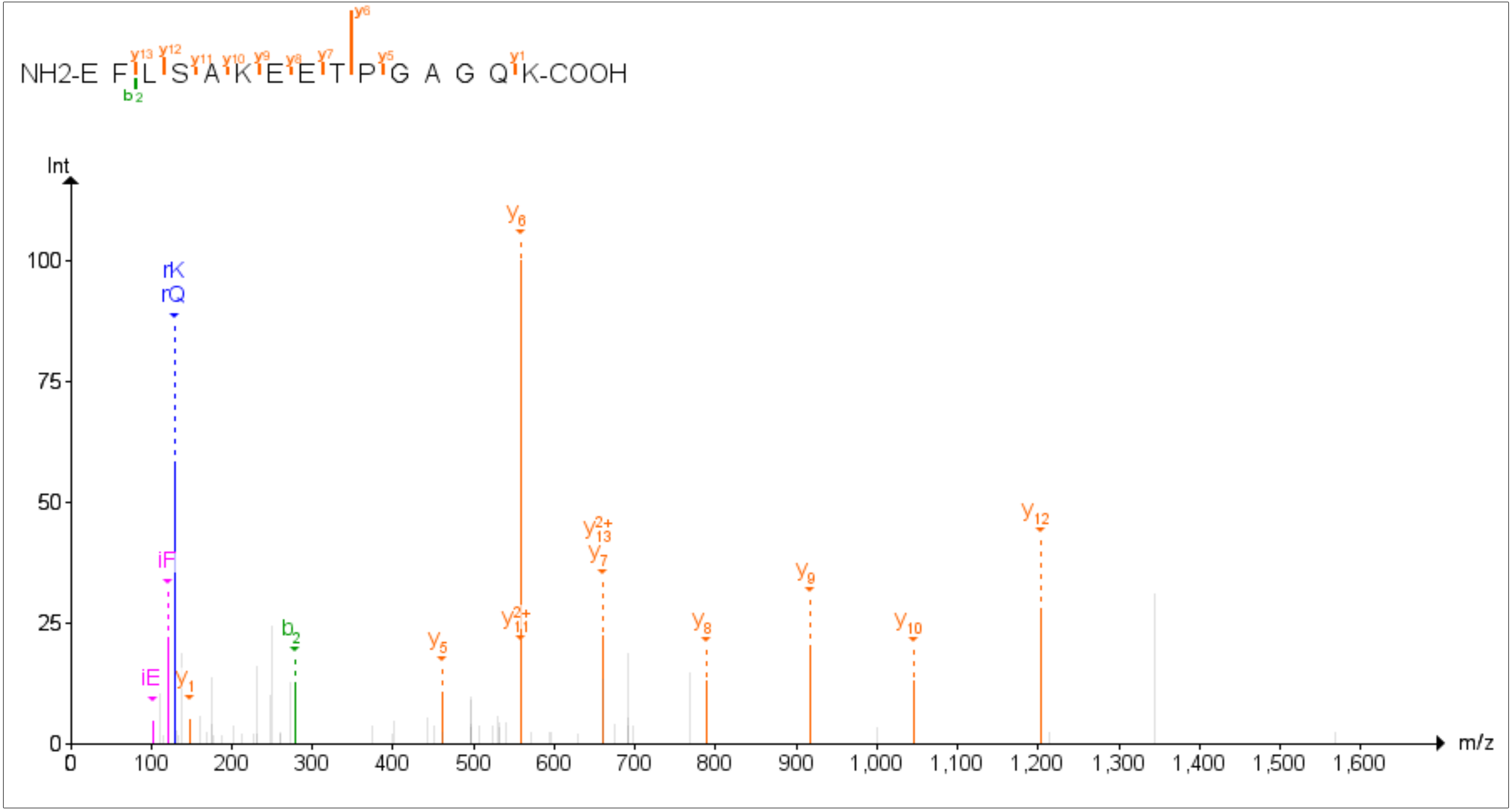

NH2-E F L S A K E E T P G A G Q K-COOH
y13 y12 y11 y10 y9 y8 y7 y6 y5 y1
b2
Int
m/z
rK
rQ
iF
iE
y1
b2
y5
y6
y11 2+
y13 2+
y7
y8
y9
y10
y12
0
25
50
75
100
0
100
200
300
400
500
600
700
800
900
1,000
1,100
1,200
1,300
1,400
1,500
1,600

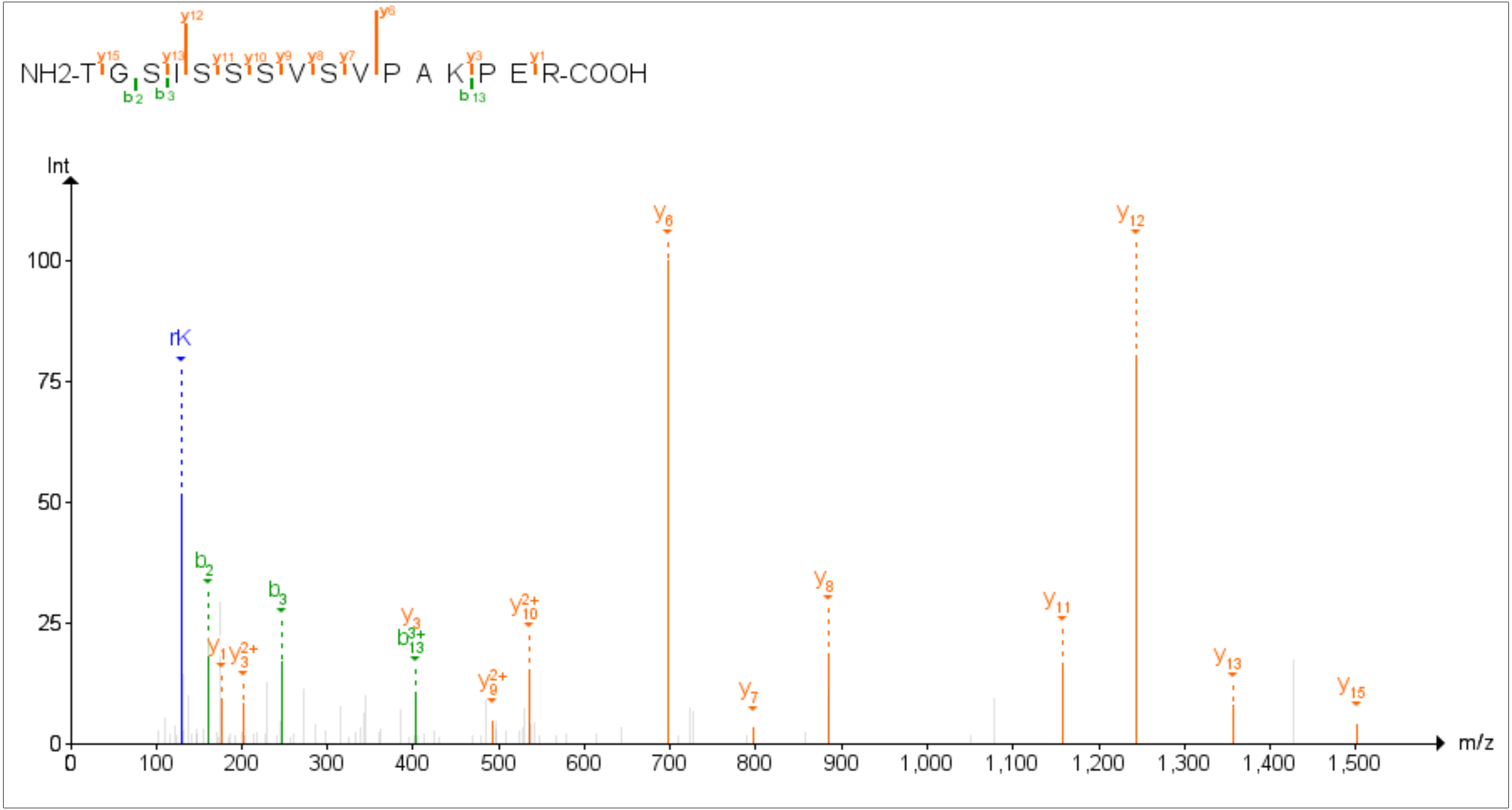

NH2-T G S I I S S S V S V P A K P E R-COOH
y15 y13 y12 y11 y10 y9 y8 y7 y6 y3 y1
b2 b3 b13
Int
m/z
0
25
50
75
100
100
200
300
400
500
600
700
800
900
1,000
1,100
1,200
1,300
1,400
1,500
rK
b2
b3
y1
y3 2+
y3
b13 3+
y9 2+
y10 2+
y6
y7
y8
y11
y12
y13
y15

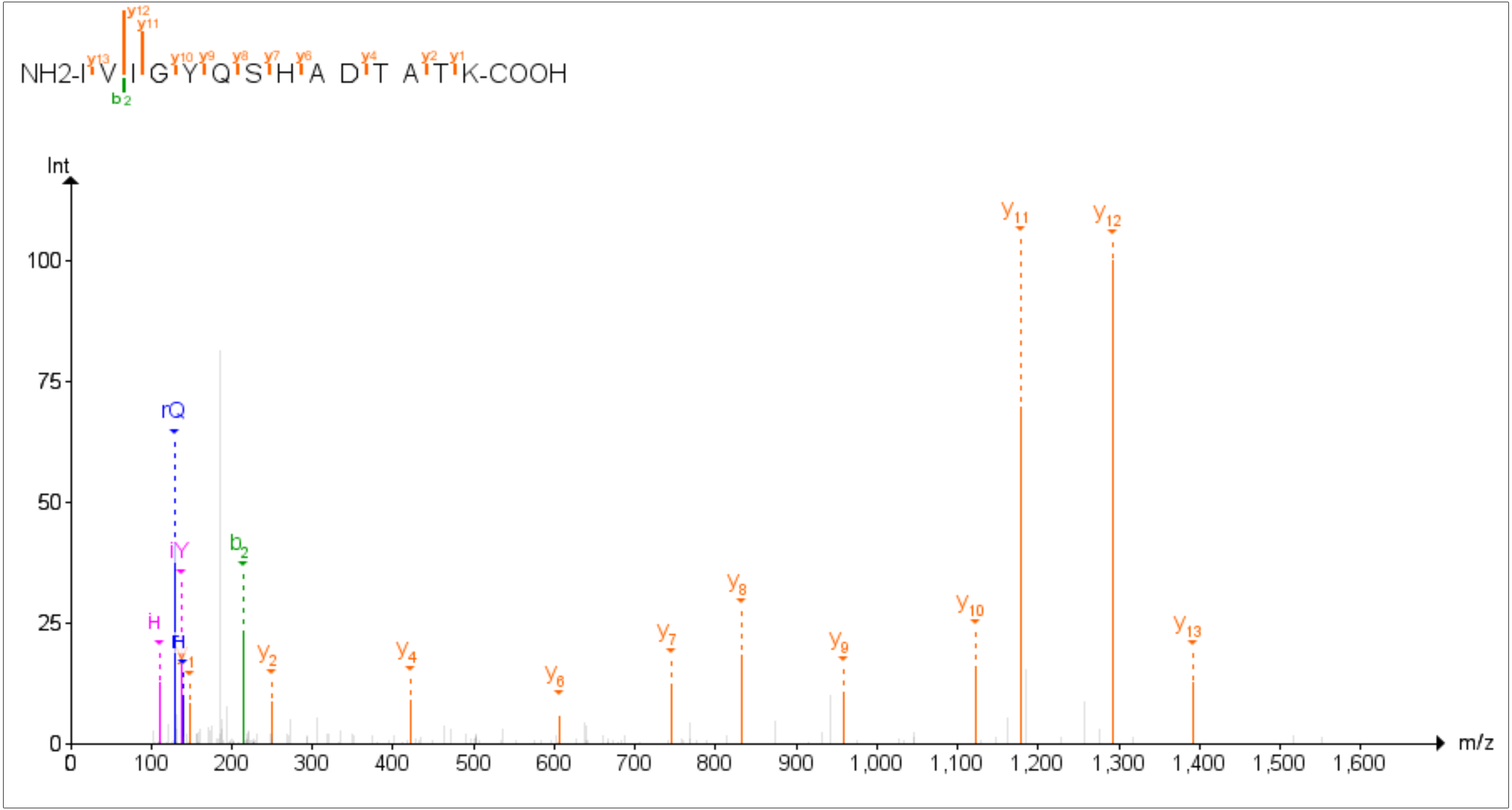

NH2-IVIGYQSHADTATK-COOH
y13 y12 y11 y10 y9 y8 y7 y6 y4 y2 y1
b2
Int
m/z
rQ
iY
iH
b2
y1
y2
y4
y6
y7
y8
y9
y10
y11
y12
y13

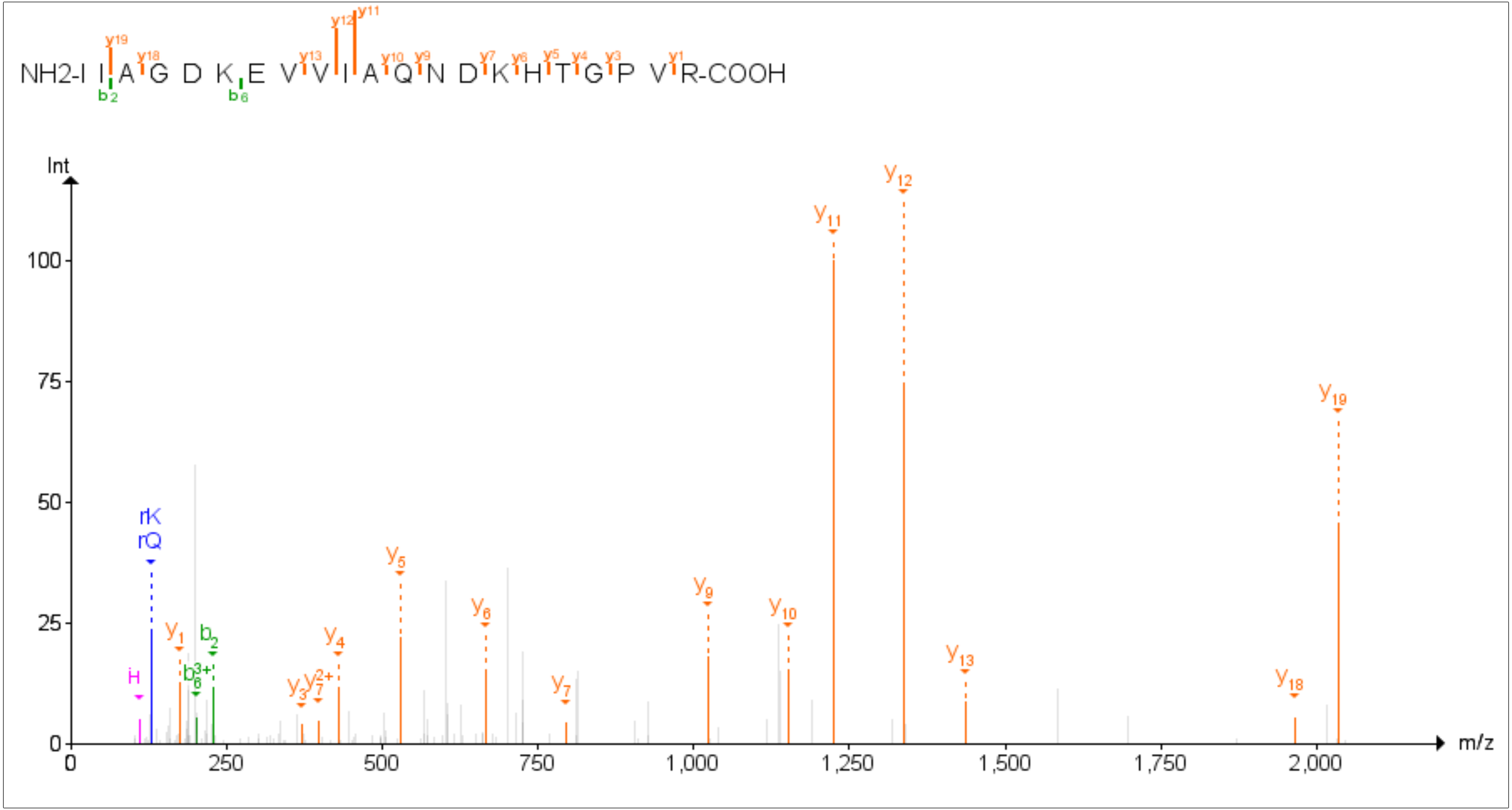

NH2-I I A G D K E V V I A Q N D K H T G P V R-COOH
Int
m/z
rK
rQ
y1
y4
y5
y6
y7
y9
y10
y11
y12
y13
y18
y19
b2

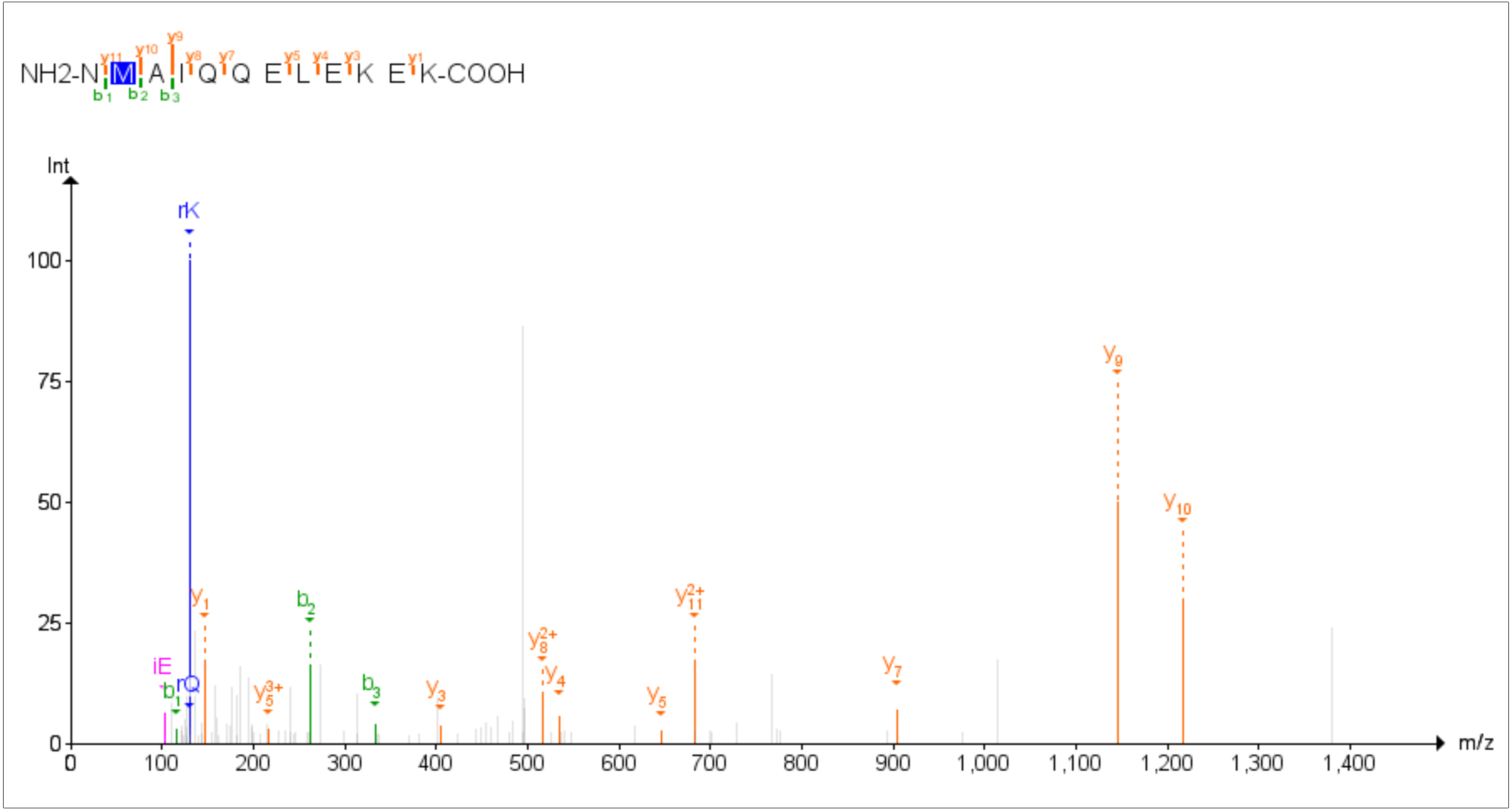

NH2-N M A I Q Q E L E K E K-COOH
y11 y10 y9 y8 y7 y5 y4 y3 y1
b1 b2 b3
Int
rK
100
75
50
25
0
y9
y10
y1
b2
y11 2+
y8 2+
iE
rQ
b1
y5 3+
b3
y3
y4
y5
y7
0 100 200 300 400 500 600 700 800 900 1,000 1,100 1,200 1,300 1,400
m/z

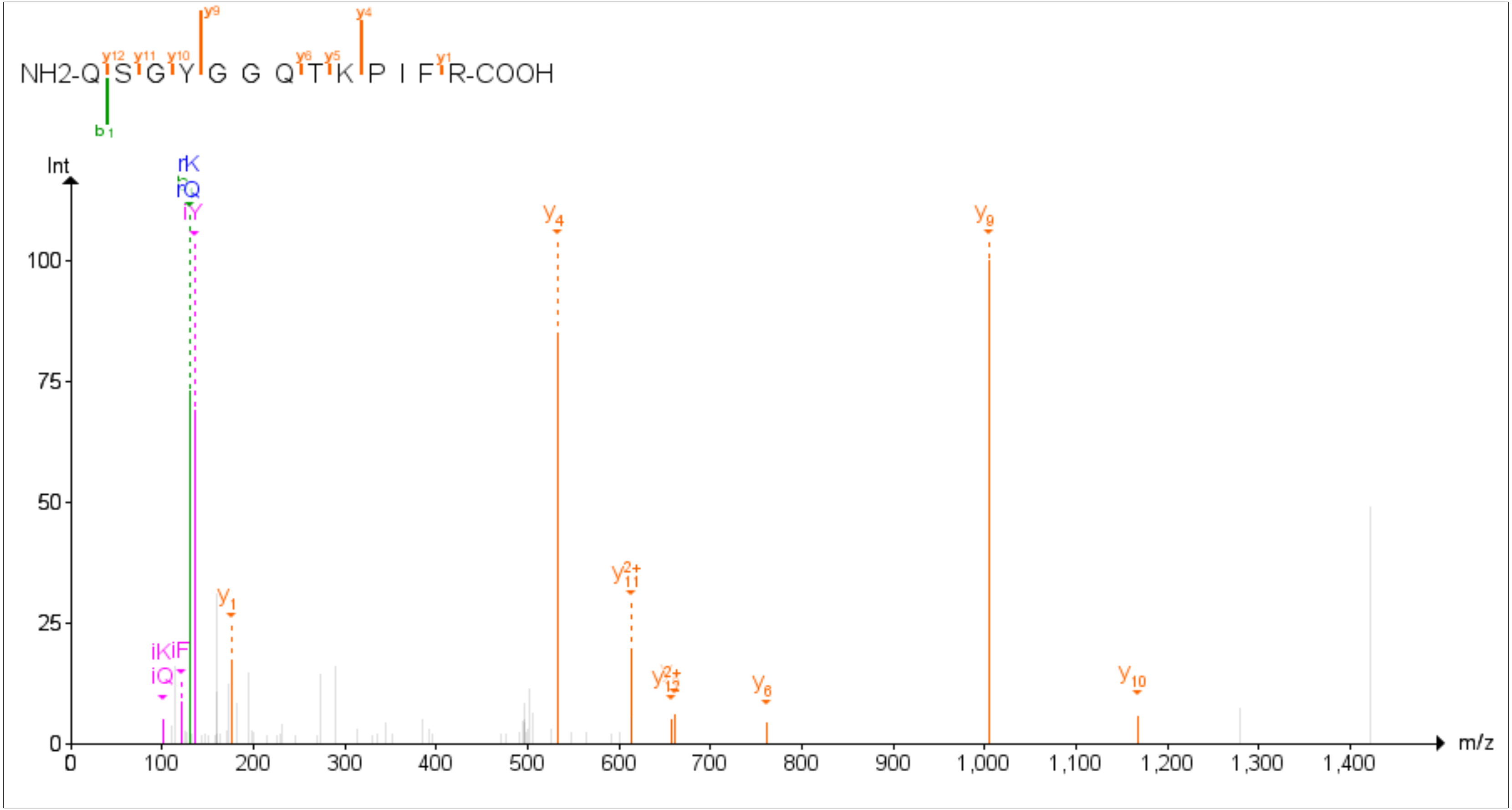
NH2-Q S G Y G G Q T K P I F R-COOH
y12 y11 y10 y9 y6 y5 y4 y1
b1
Int
rK
rQ
iY
y4
y9
100
75
50
25
0
y1
iKiF
iQ
y11 2+
y12 2+
y6
y10
0 100 200 300 400 500 600 700 800 900 1,000 1,100 1,200 1,300 1,400
m/z

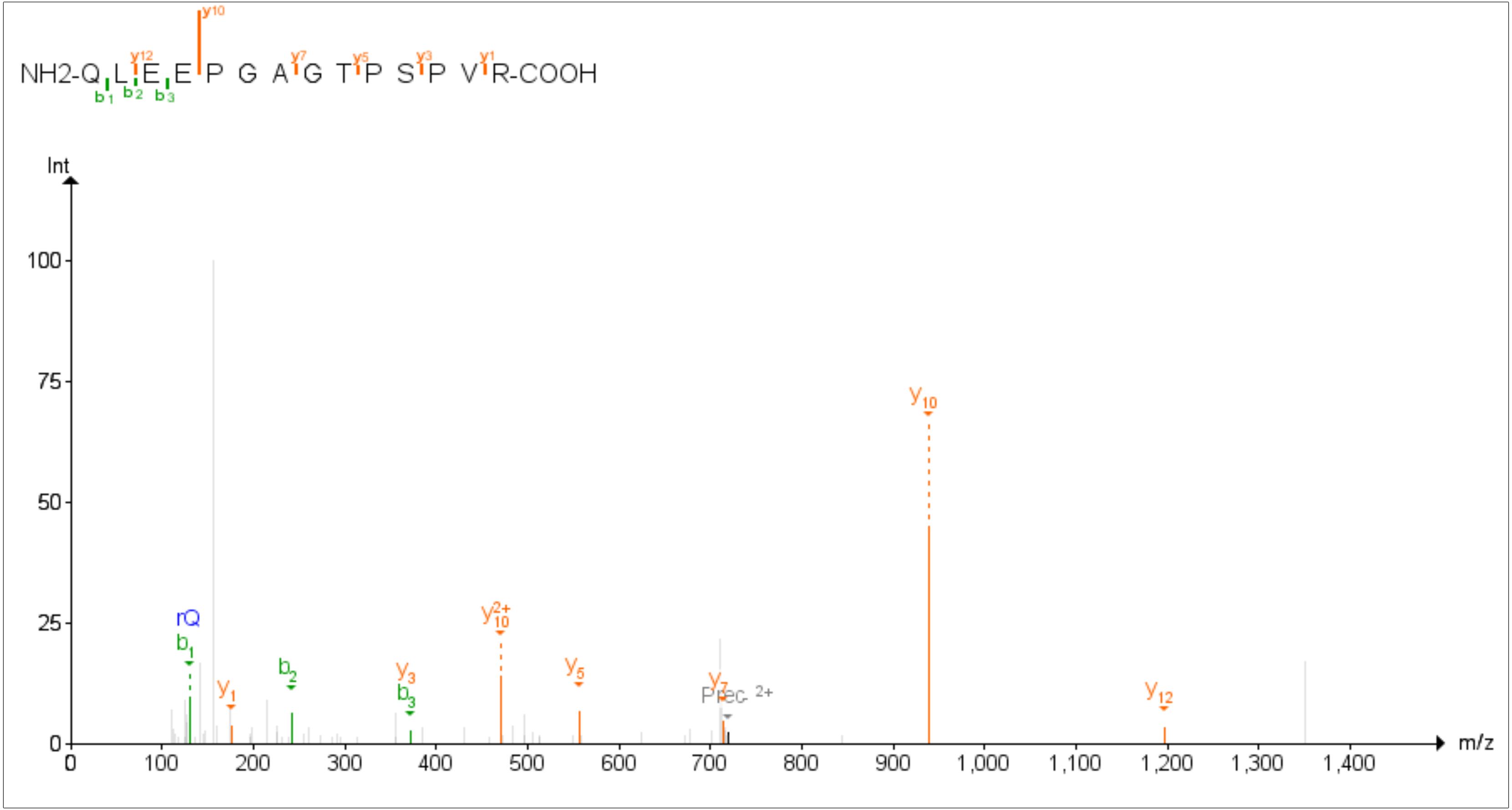

NH2-Q L E E P G A G T P S P V R-COOH
y10
y12
y7
y5
y3
y1
b1
b2
b3
Int
100
75
50
25
0
rQ
b1
y1
b2
y3
b3
y10 2+
y5
y7
Prec- 2+
y10
y12
0
100
200
300
400
500
600
700
800
900
1,000
1,100
1,200
1,300
1,400
m/z

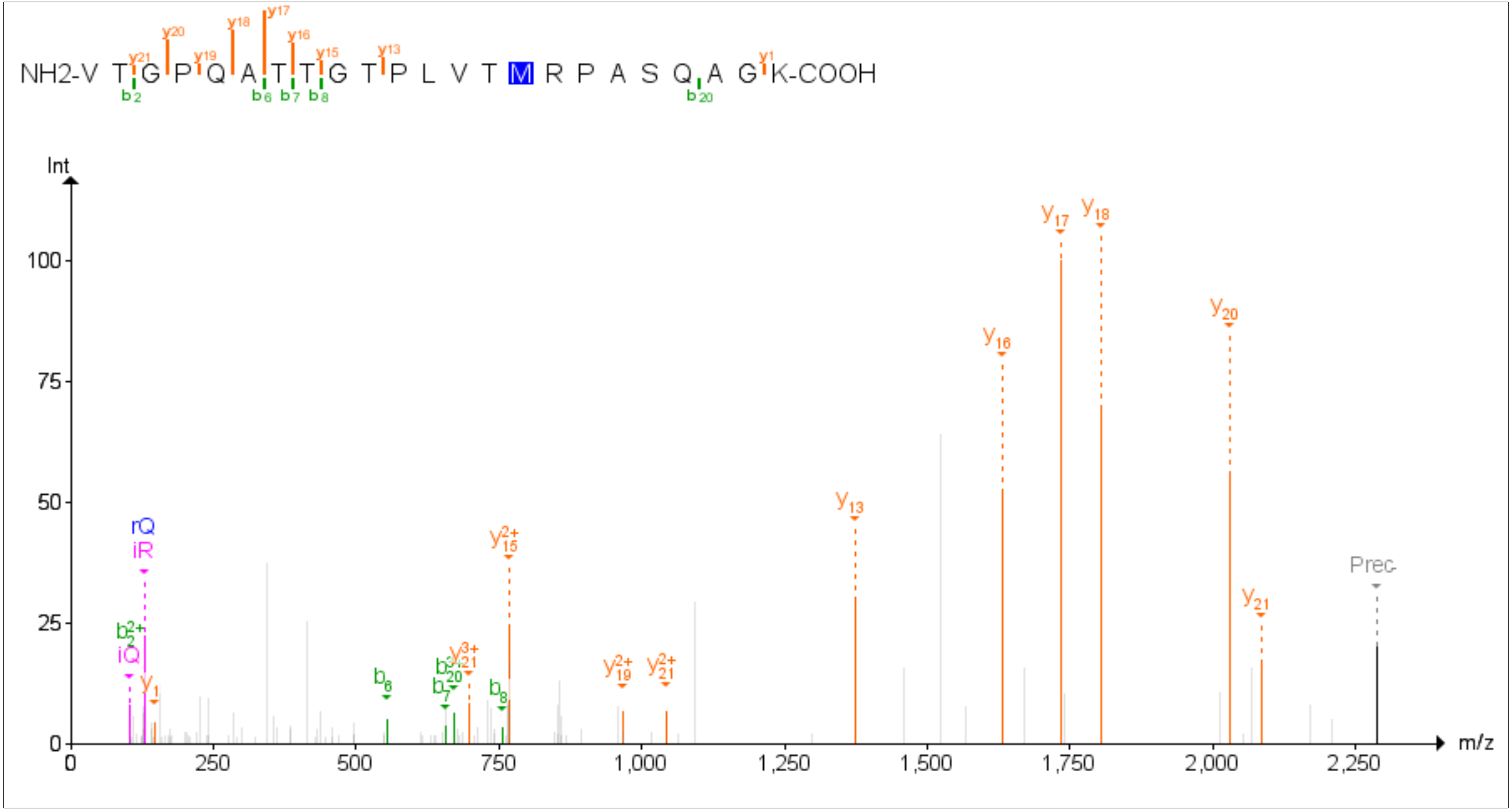

NH2-V T G P Q A T T G T P L V T M R P A S Q A G K-COOH
Int
m/z
Prec

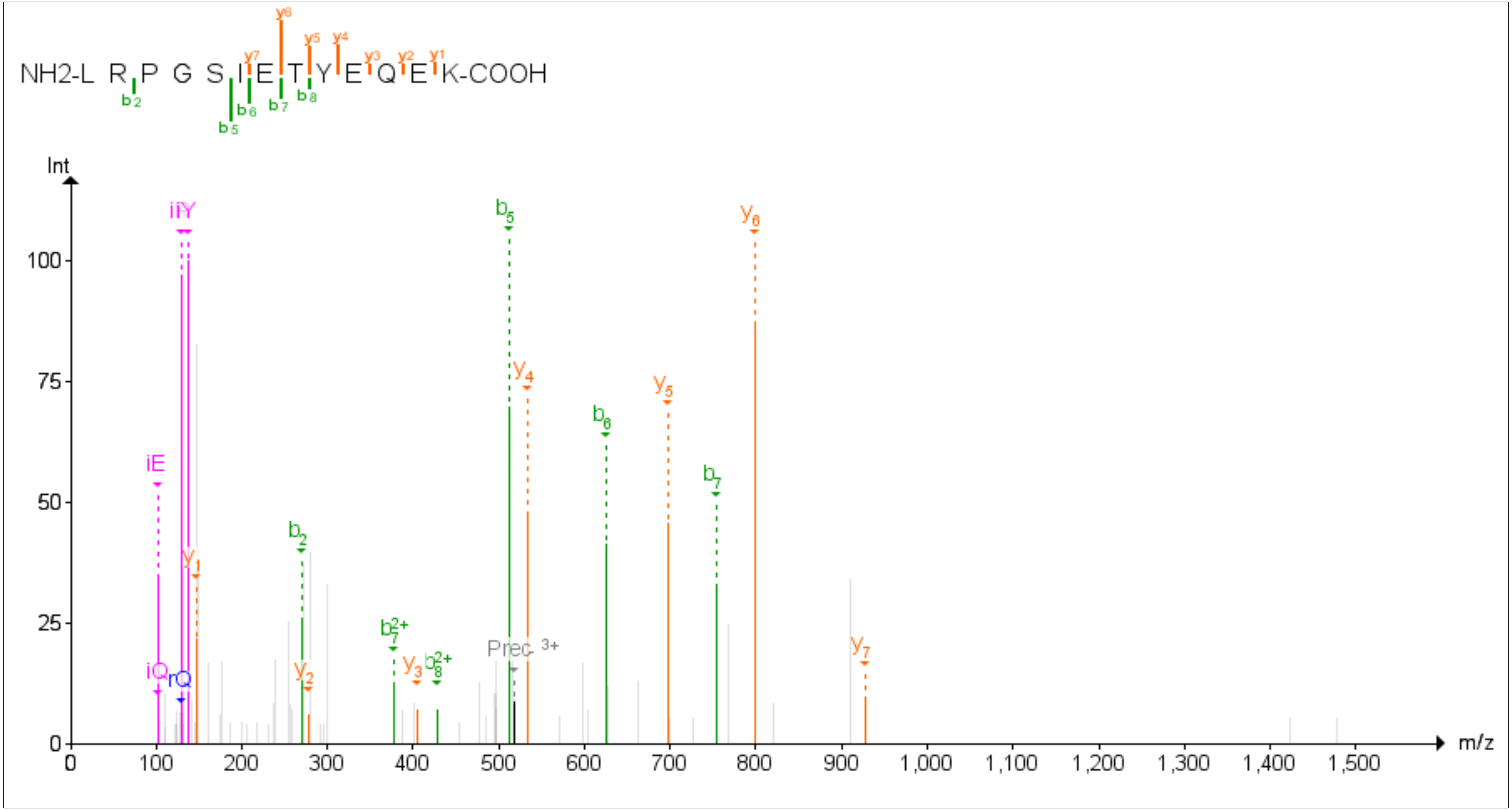
NH2-L R P G S I E T Y E Q E K-COOH
Int
m/z
Prec 3+

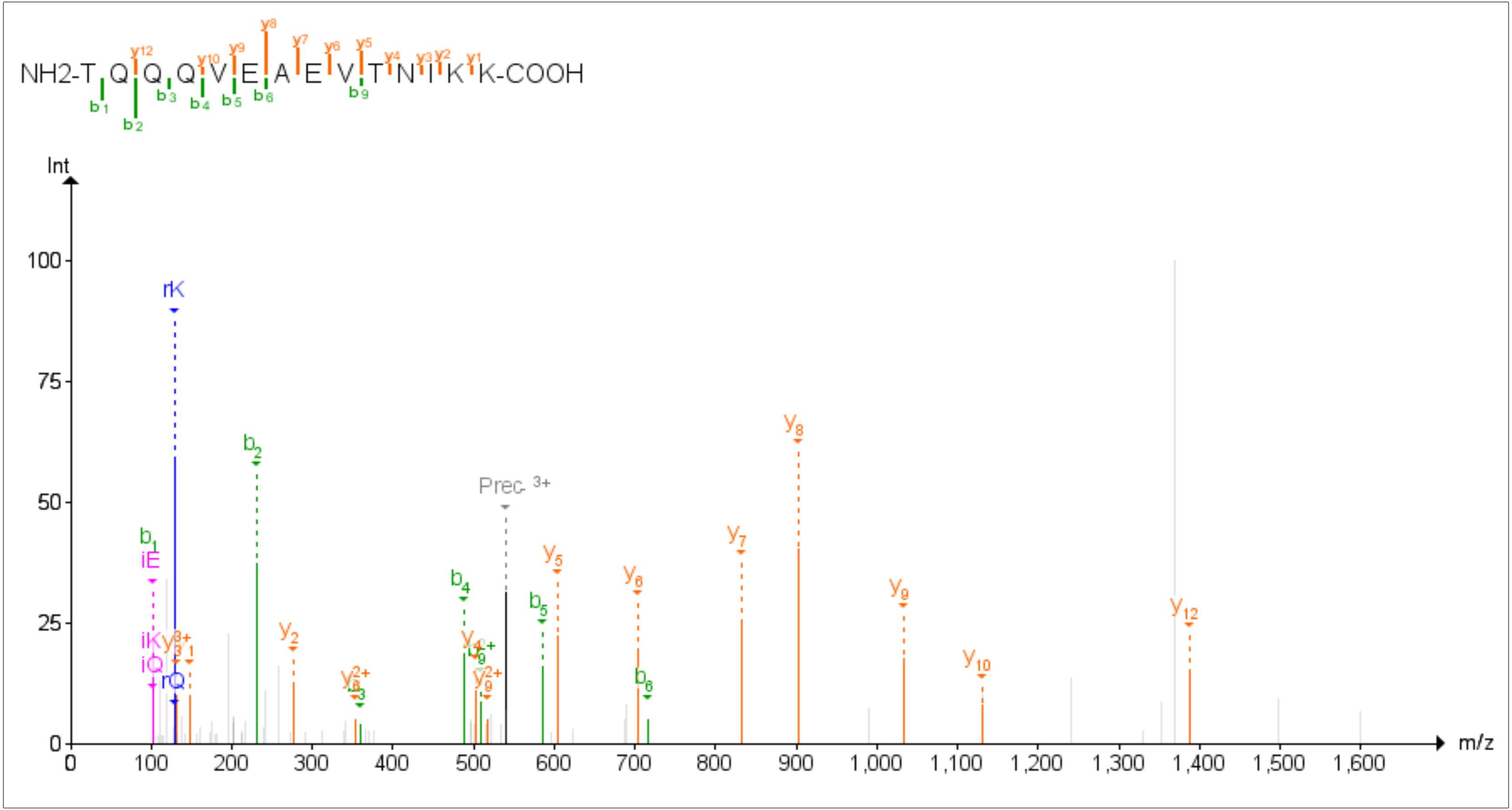

NH2-T Q Q Q V E A E V T N I K K-COOH
Int
m/z
rK
b2
Prec 3+
y8
y7
y5
y6
b4
b5
y9
y12
y10
b1
iE
y2
b6
0
25
50
75
100
0
100
200
300
400
500
600
700
800
900
1,000
1,100
1,200
1,300
1,400
1,500
1,600

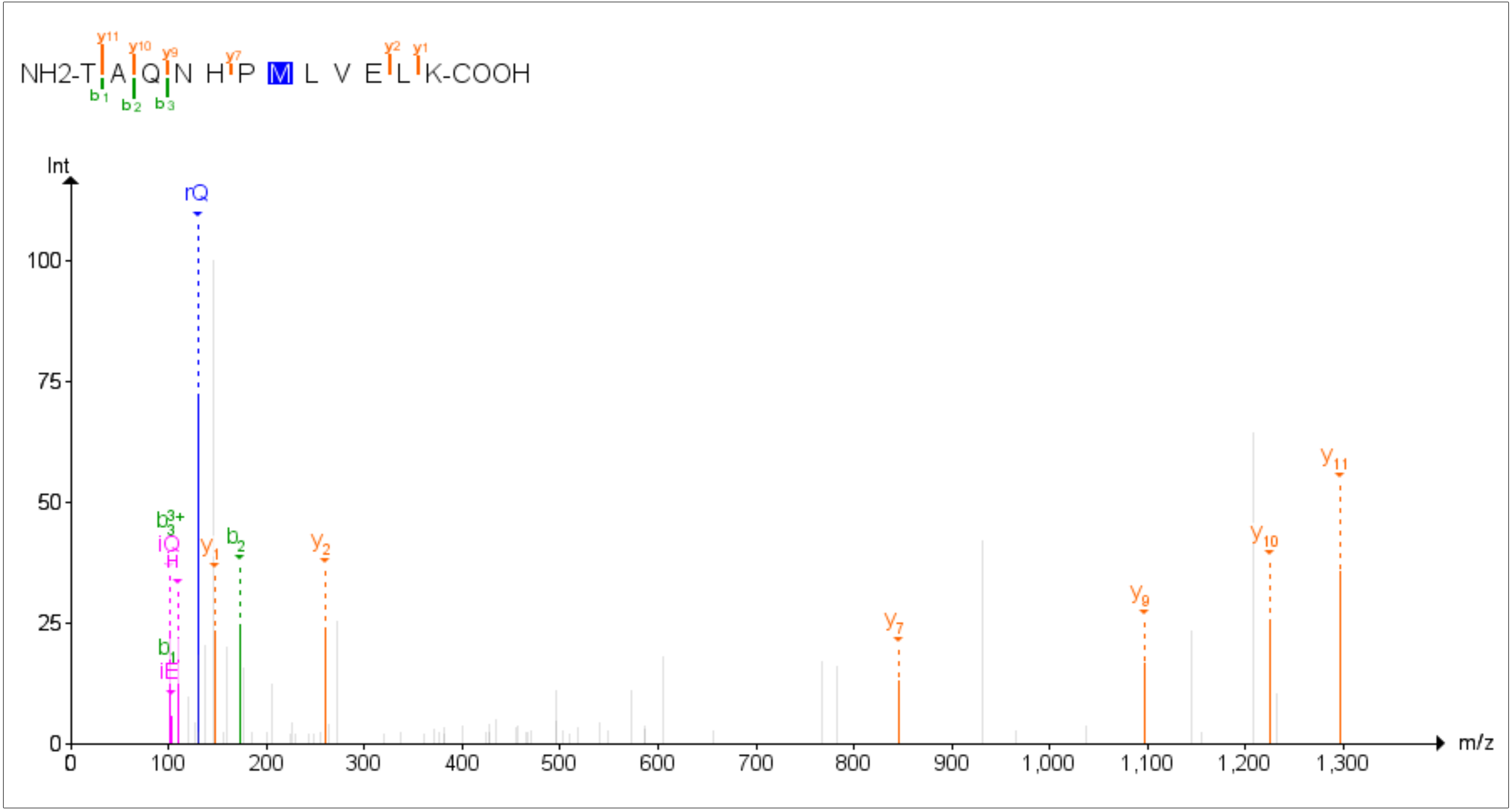

NH2-T A Q N H P M L V E L K-COOH
y11 y10 y9 y7 y2 y1
b1 b2 b3
Int
rQ
b3 3+
iQ
-H
y1
b2
y2
b1
iE
y7
y9
y10
y11
100
75
50
25
0
0 100 200 300 400 500 600 700 800 900 1,000 1,100 1,200 1,300
m/z

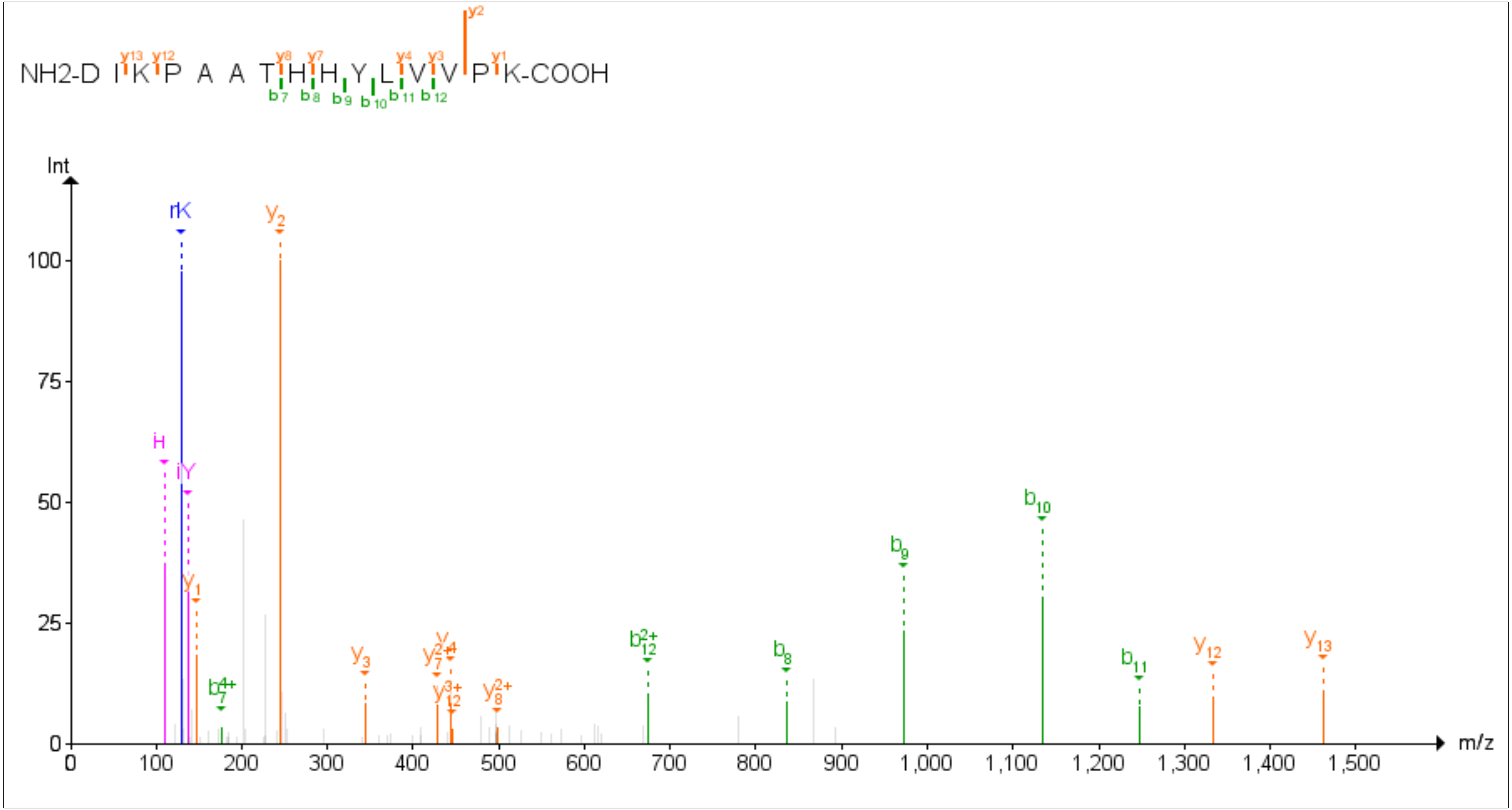

NH2-D I K P A A T H H Y L V V P K-COOH
y13 y12 y8 y7 y4 y3 y2 y1
b7 b8 b9 b10 b11 b12
Int
m/z
rK
y2
iH
iY
y1
b7 4+
y3
y7 2+
y4
y12 3+
y8 2+
b12 2+
b8
b9
b10
b11
y12
y13
0
25
50
75
100
0
100
200
300
400
500
600
700
800
900
1,000
1,100
1,200
1,300
1,400
1,500

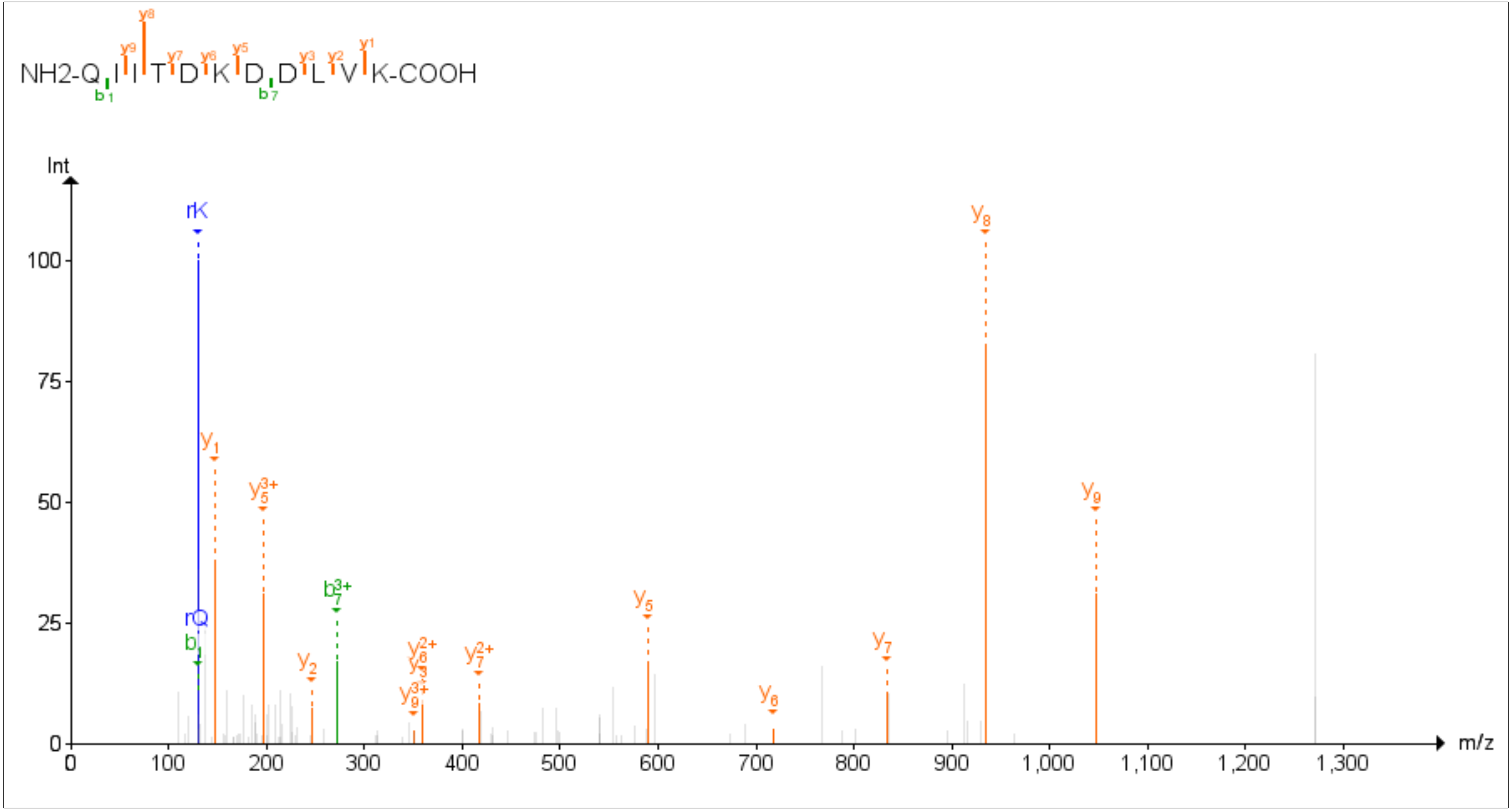
NH2-QIITDKDDLVK-COOH
y8
y9
y7
y6
y5
y3
y2
y1
b1
b7
Int
rK
rQ
100
75
50
25
0
y1
y5 3+
b7 3+
y2
y6 2+
y3
y9 3+
y7 2+
y5
y6
y7
y8
y9
b1
m/z
0
100
200
300
400
500
600
700
800
900
1,000
1,100
1,200
1,300

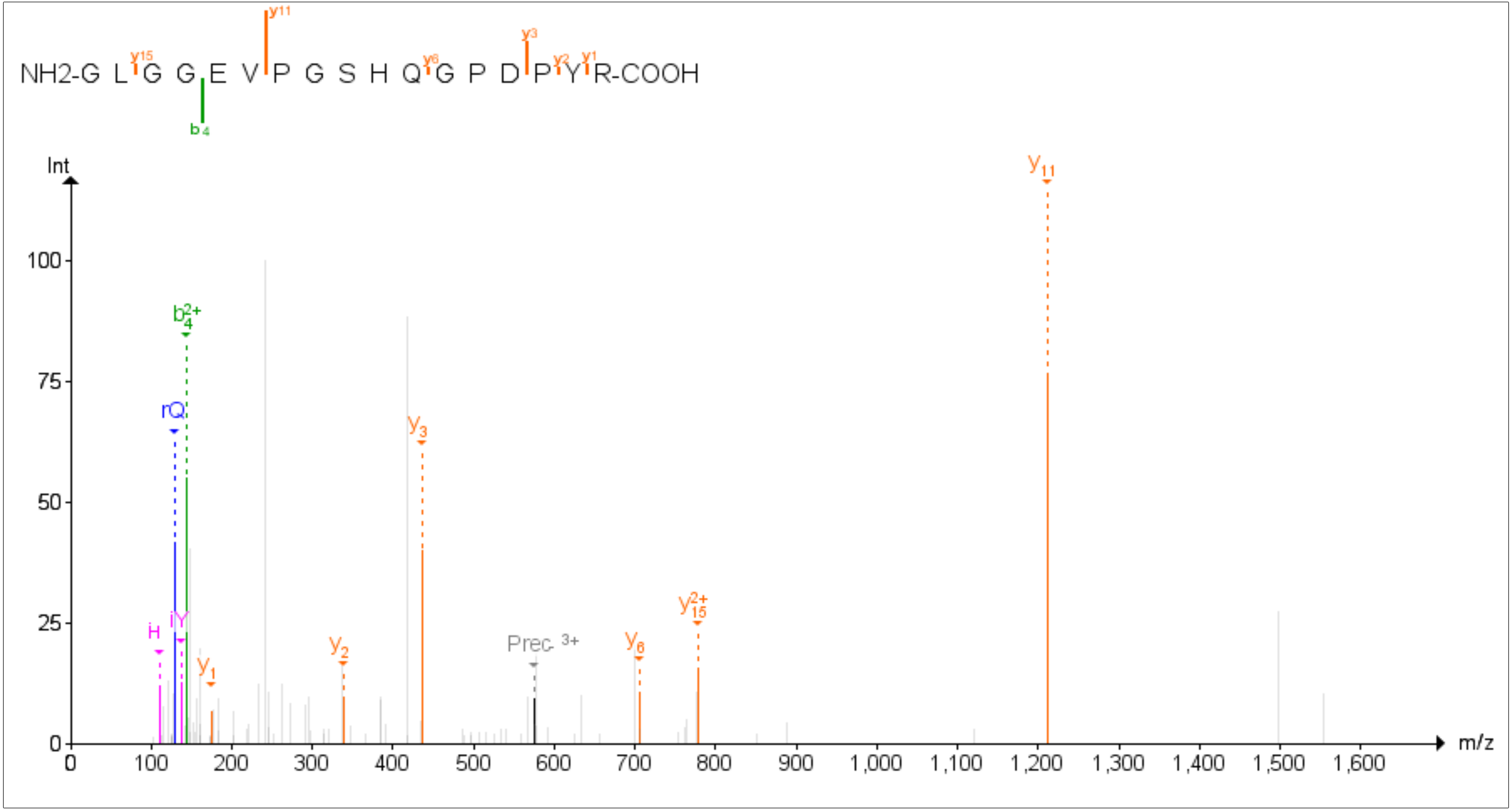
NH2-G L G G E V P G S H Q G P D P Y R-COOH
y15
y11
y6
y3
y2
y1
b4
Int
m/z
y11
b4 2+
rQ
y3
iH
iY
y1
y2
Prec 3+
y6
y15 2+
0
25
50
75
100
0
100
200
300
400
500
600
700
800
900
1,000
1,100
1,200
1,300
1,400
1,500
1,600

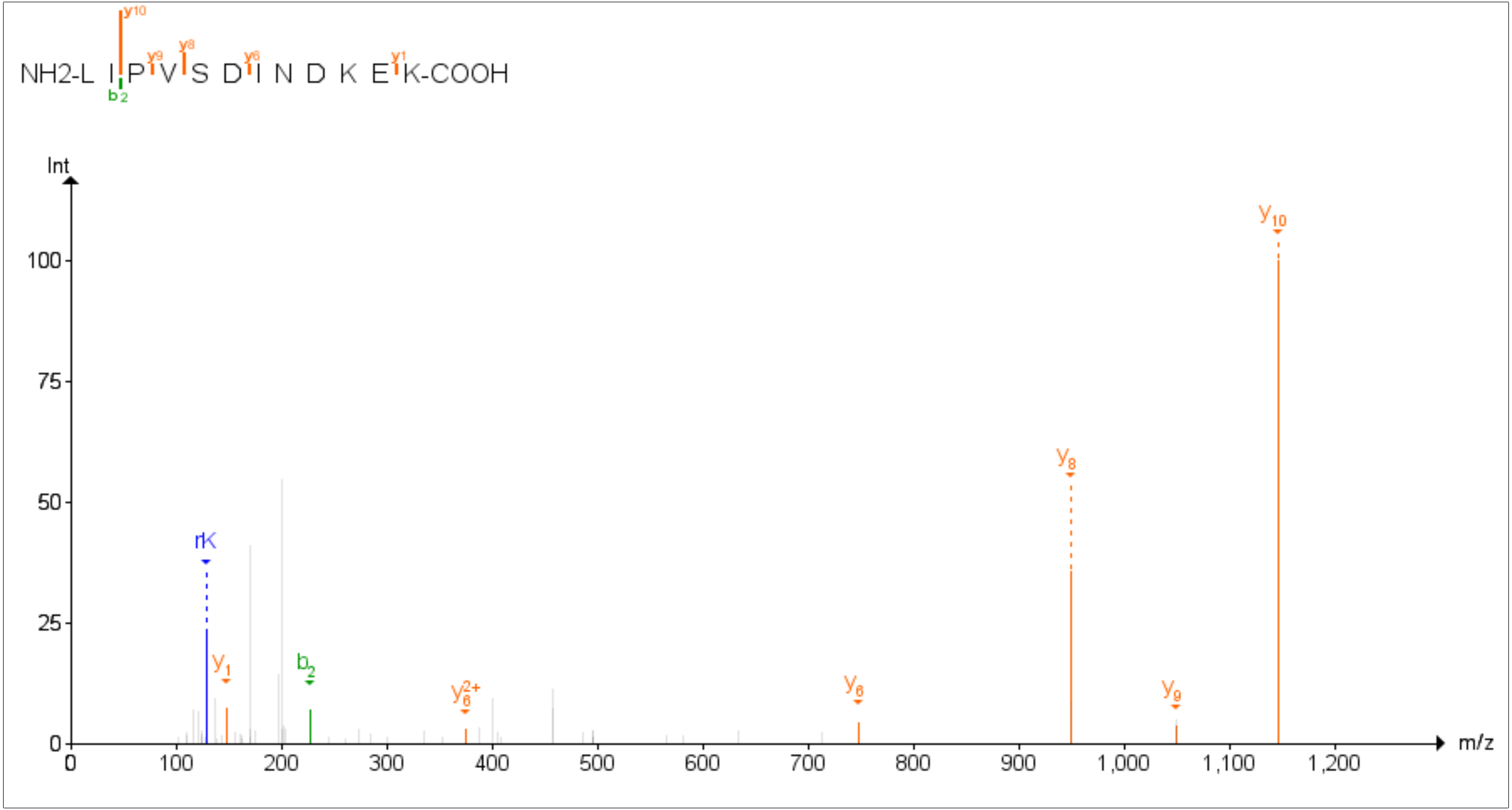

NH2-L I P V S D I N D K E K-COOH
y10
y9
y8
y6
y1
b2
Int
100
75
50
25
0
rK
y10
y8
y1
b2
y6 2+
y6
y9
0
100
200
300
400
500
600
700
800
900
1,000
1,100
1,200
m/z

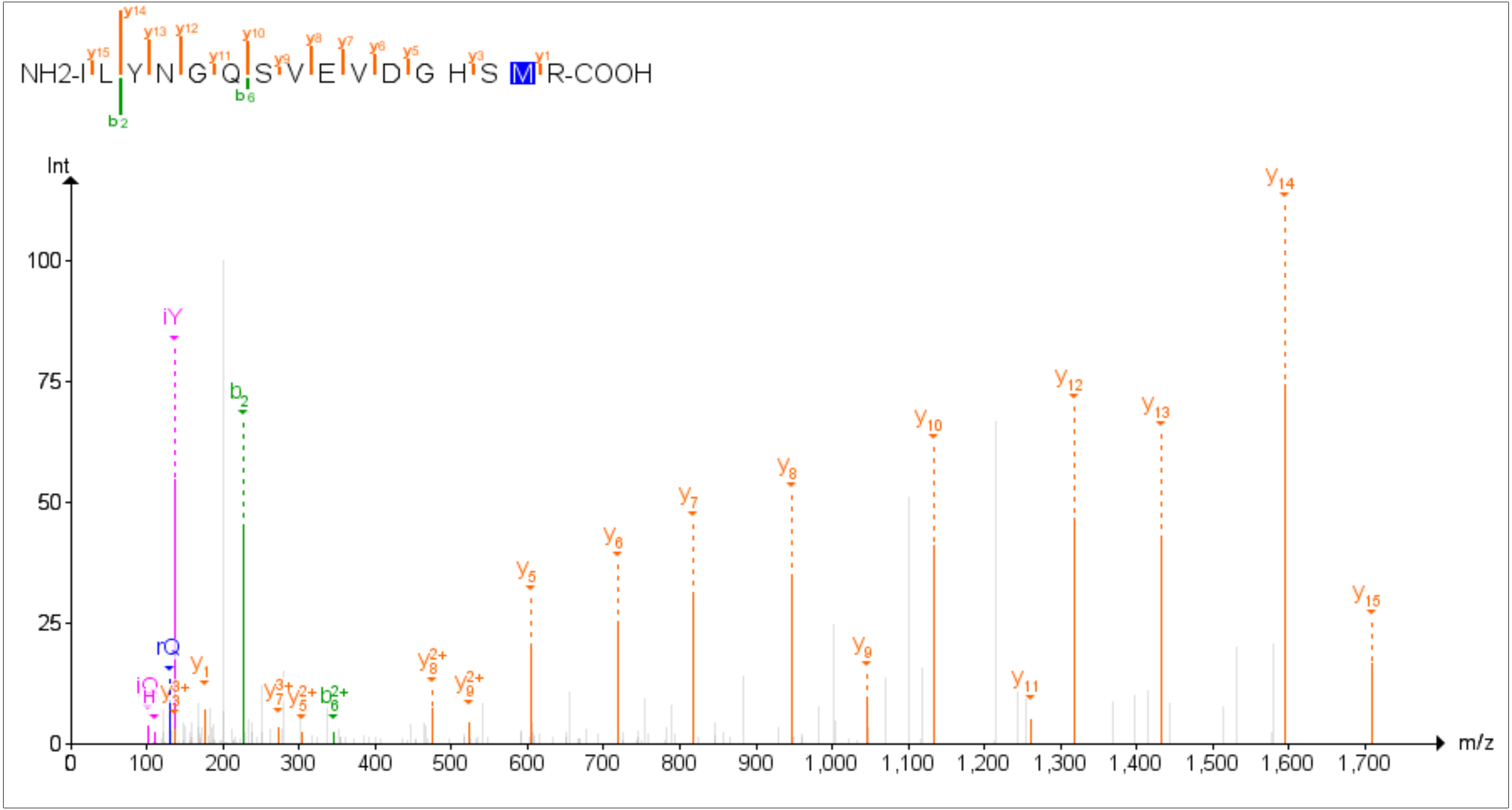

NH2-I L Y N G Q S V E V D G H S M R-COOH
Int
m/z
0
25
50
75
100
0
100
200
300
400
500
600
700
800
900
1,000
1,100
1,200
1,300
1,400
1,500
1,600
1,700

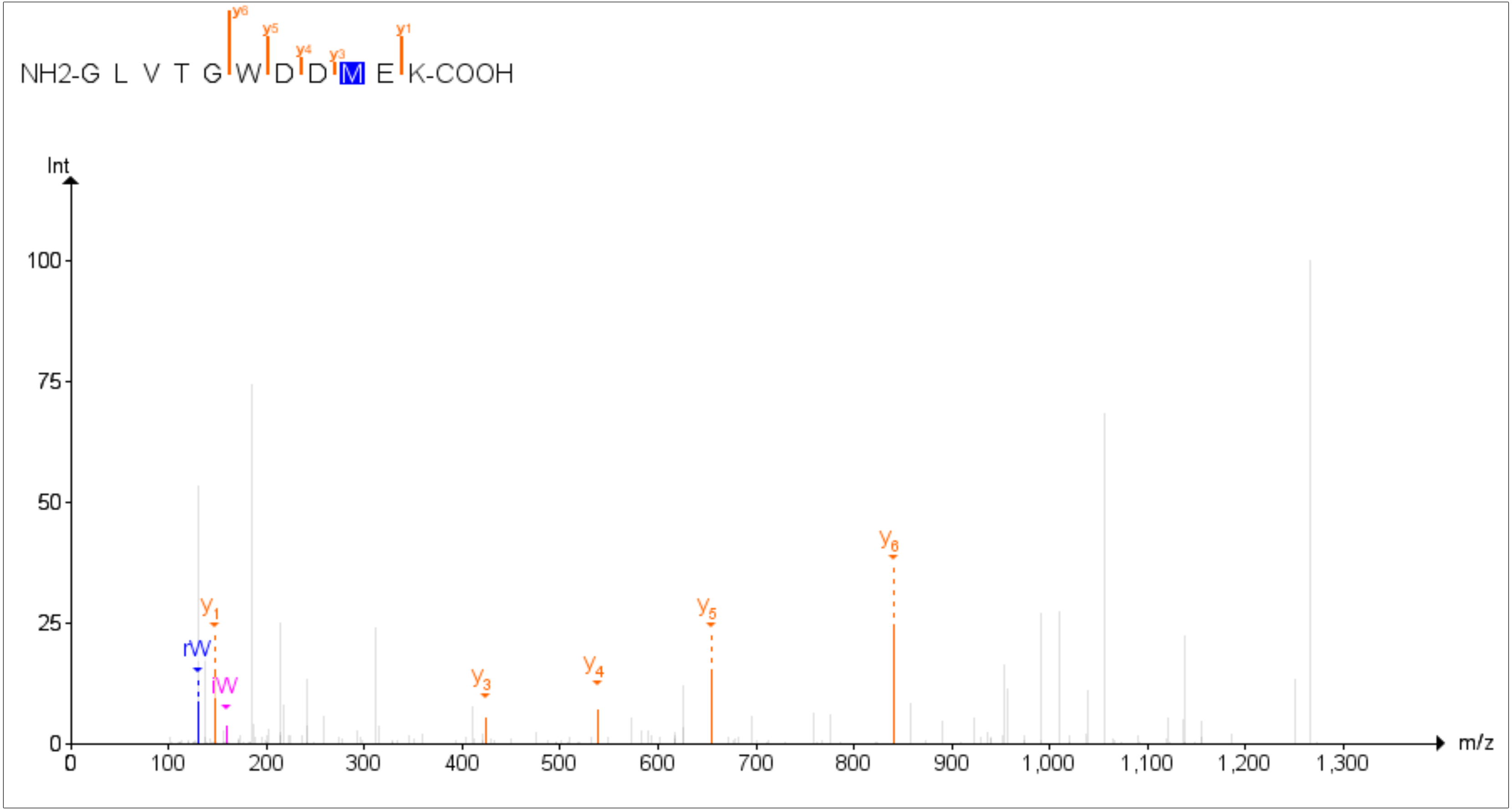

NH2-G L V T G W D D M E K-COOH
y6
y5
y4
y3
y1
Int
100
75
50
25
0
m/z
0
100
200
300
400
500
600
700
800
900
1,000
1,100
1,200
1,300
rW
iW
y_1
y_3
y_4
y_5
y_6

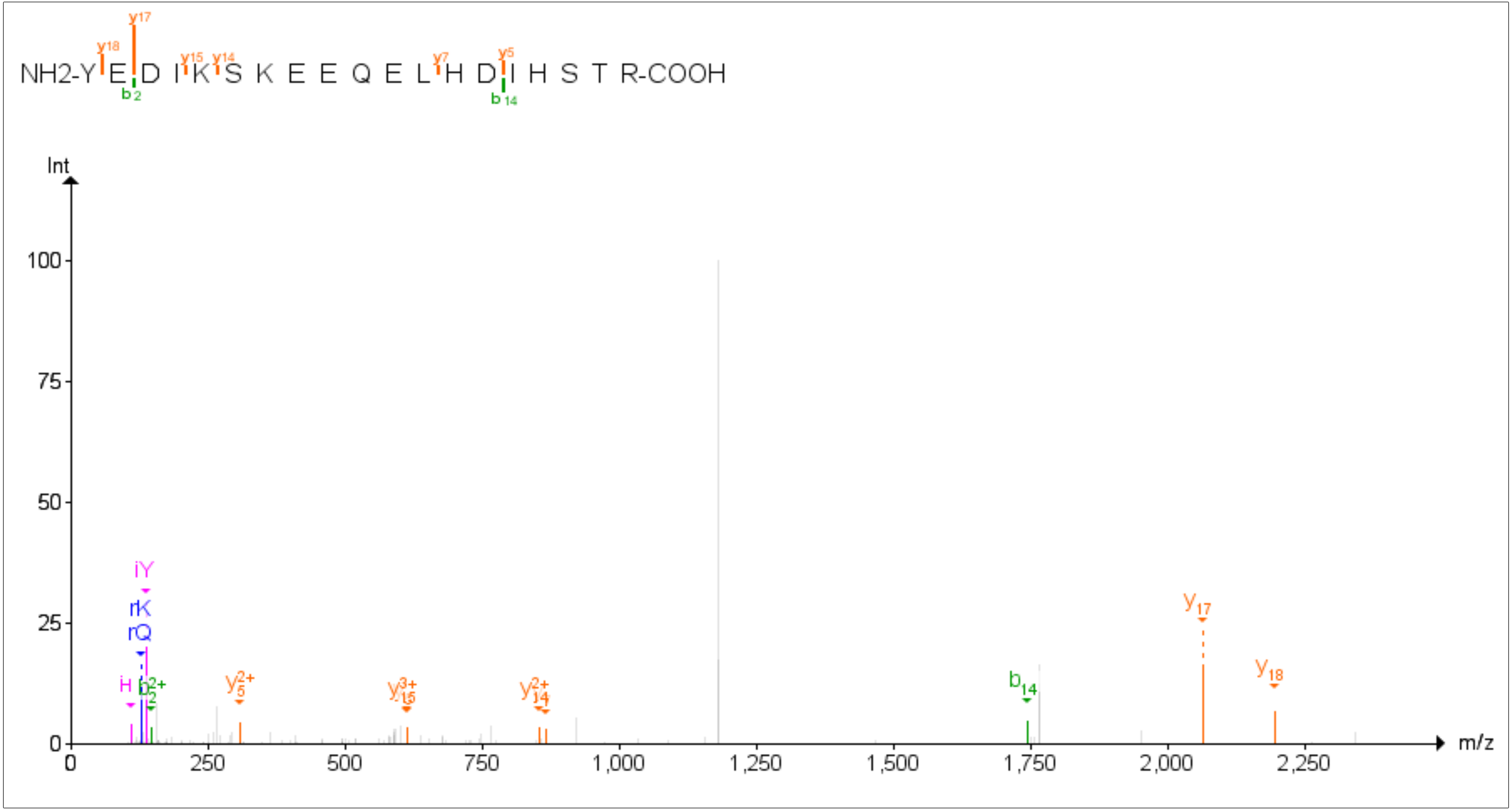

NH2-Y E D I K S K E E Q E L H D I H S T R-COOH
y17
y18
y15
y14
y7
y5
b2
b14
Int
100
75
50
25
0
iY
rK
rQ
iH
b2 2+
y5 2+
y15 3+
y14 2+
b14
y17
y18
0
250
500
750
1,000
1,250
1,500
1,750
2,000
2,250
m/z

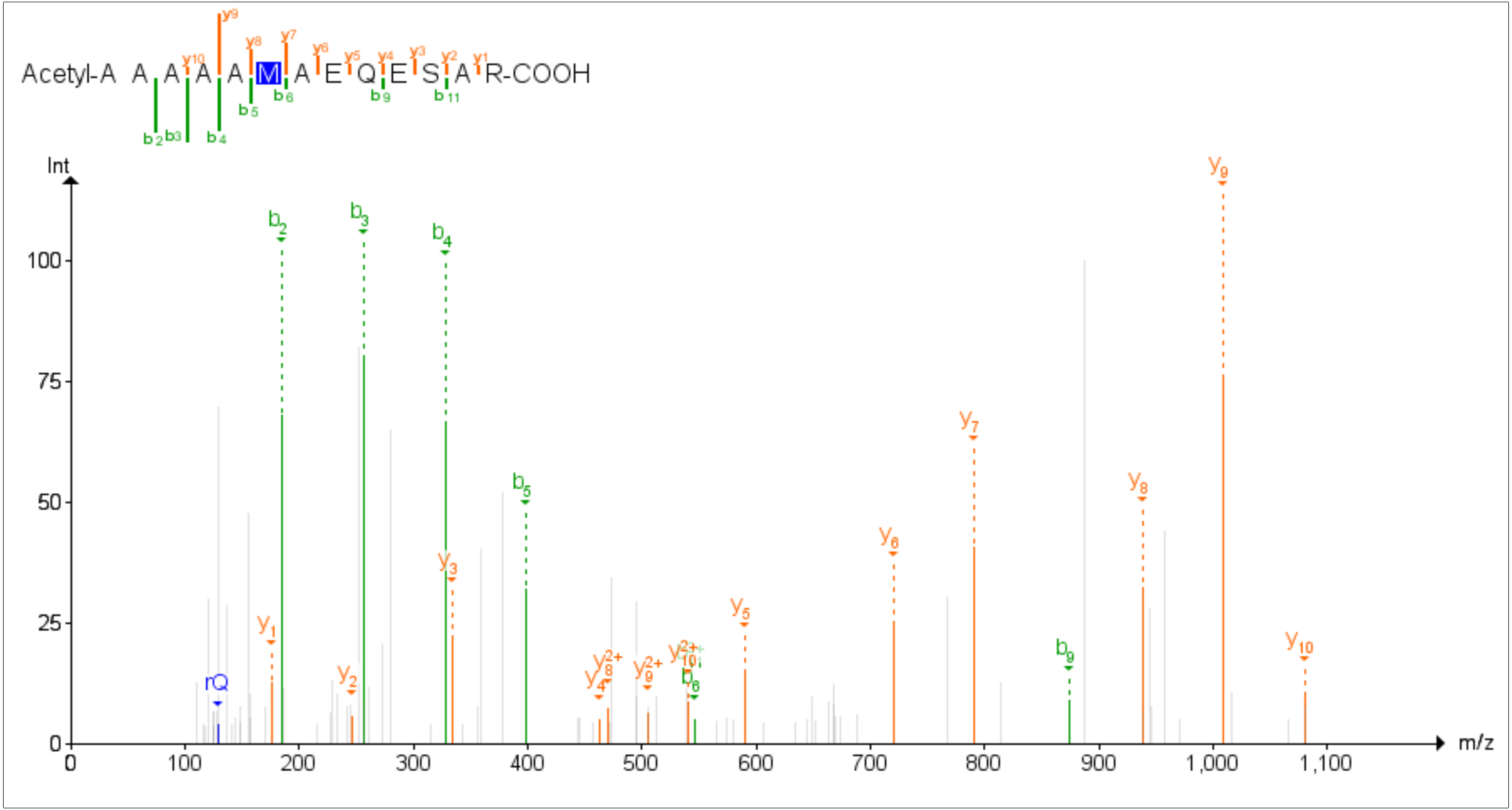
Acetyl-A A A A A M A E Q E S A R-COOH
Int
m/z
0
25
50
75
100
100
200
300
400
500
600
700
800
900
1,000
1,100
b2
b3
b4
b5
b9
y1
y2
y3
y5
y6
y7
y8
y9
y10
rQ

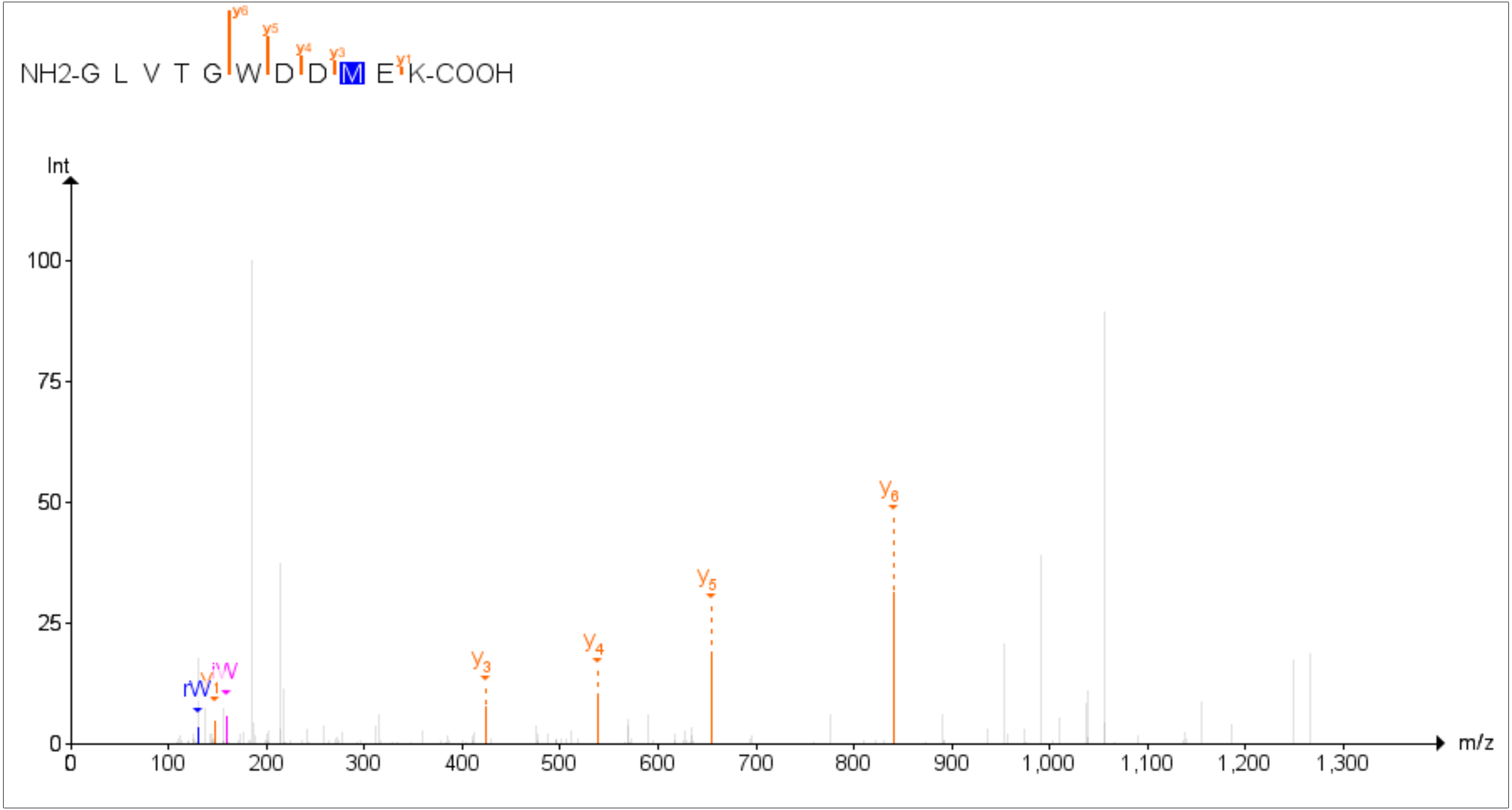

NH2-G L V T G W D D M E K-COOH
y6
y5
y4
y3
y1
Int
100
75
50
25
0
y6
y5
y4
y3
rW
y1
iW
0
100
200
300
400
500
600
700
800
900
1,000
1,100
1,200
1,300
m/z

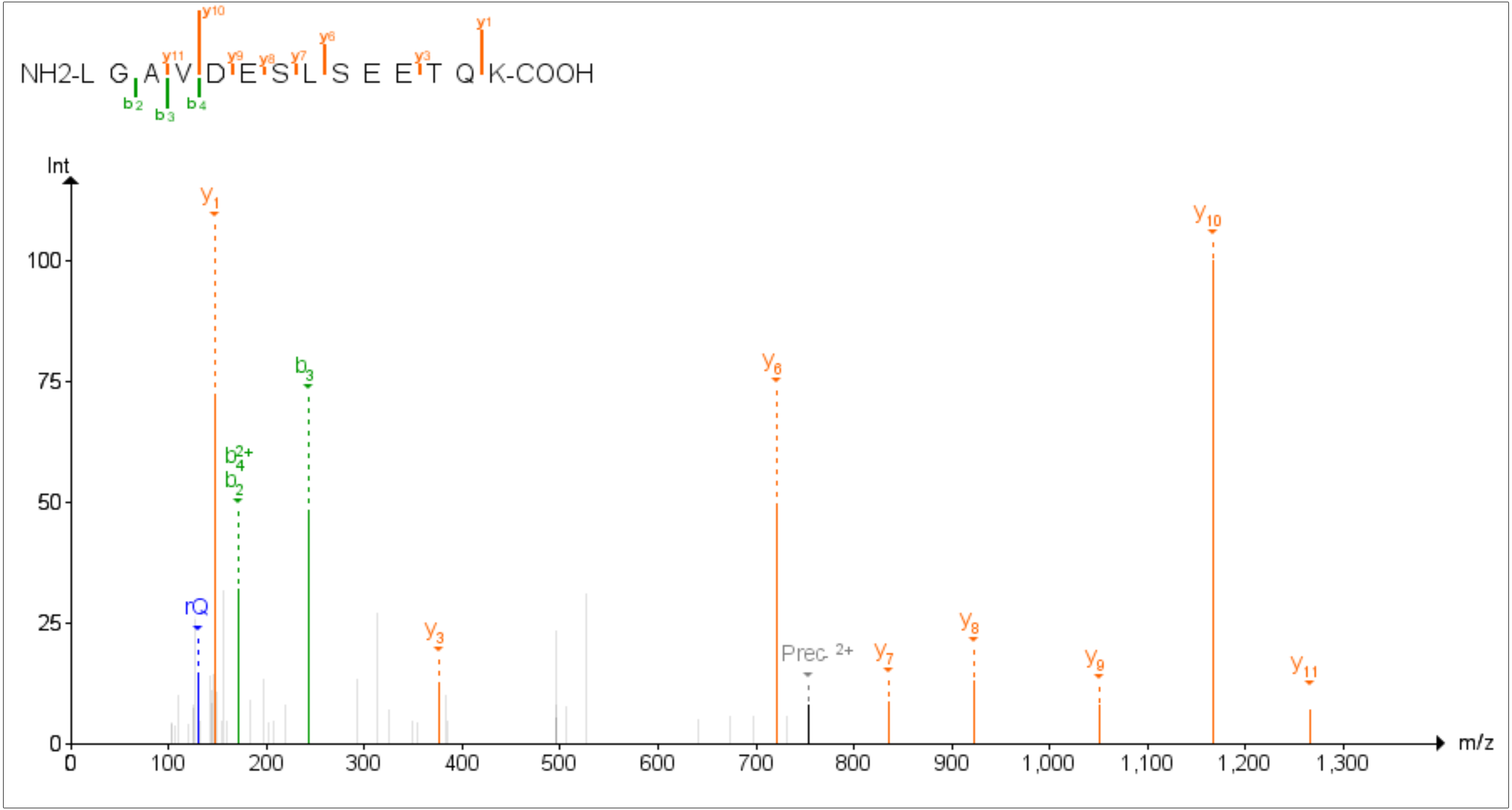

NH2-L G A V D E S L S E E T Q K-COOH
y10
y11
y9
y8
y7
y6
y3
y1
b2
b3
b4
Int
m/z
0
25
50
75
100
0
100
200
300
400
500
600
700
800
900
1,000
1,100
1,200
1,300
y1
y10
b3
y6
b4 2+
b2
rQ
y3
Prec. 2+
y7
y8
y9
y11

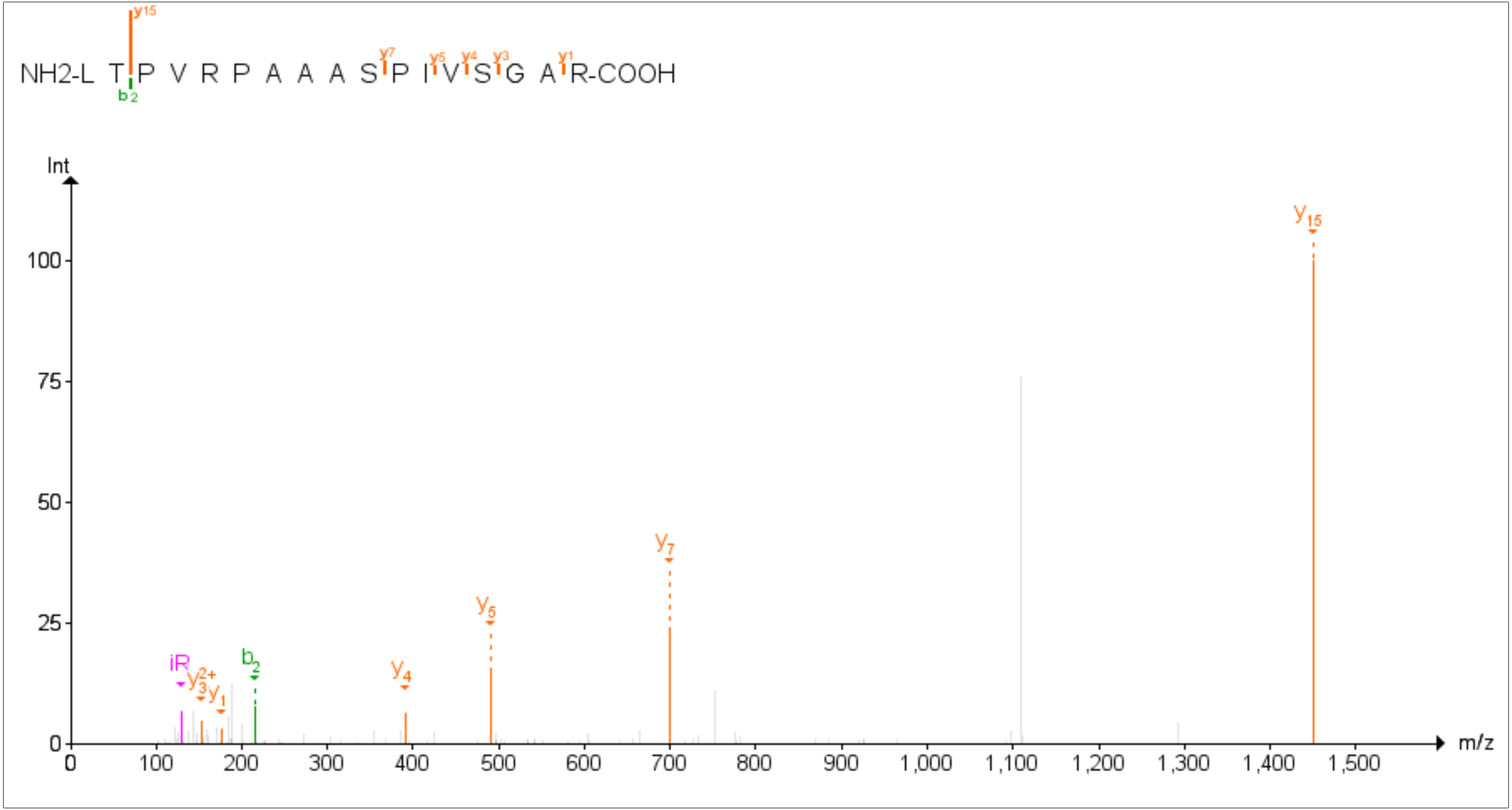

NH2-L T P V R P A A A S P I V S G A R-COOH
y15
y7
y5
y4
y3
y1
b2
Int
m/z
iR
y3 2+
y1
b2
y4
y5
y7
y15

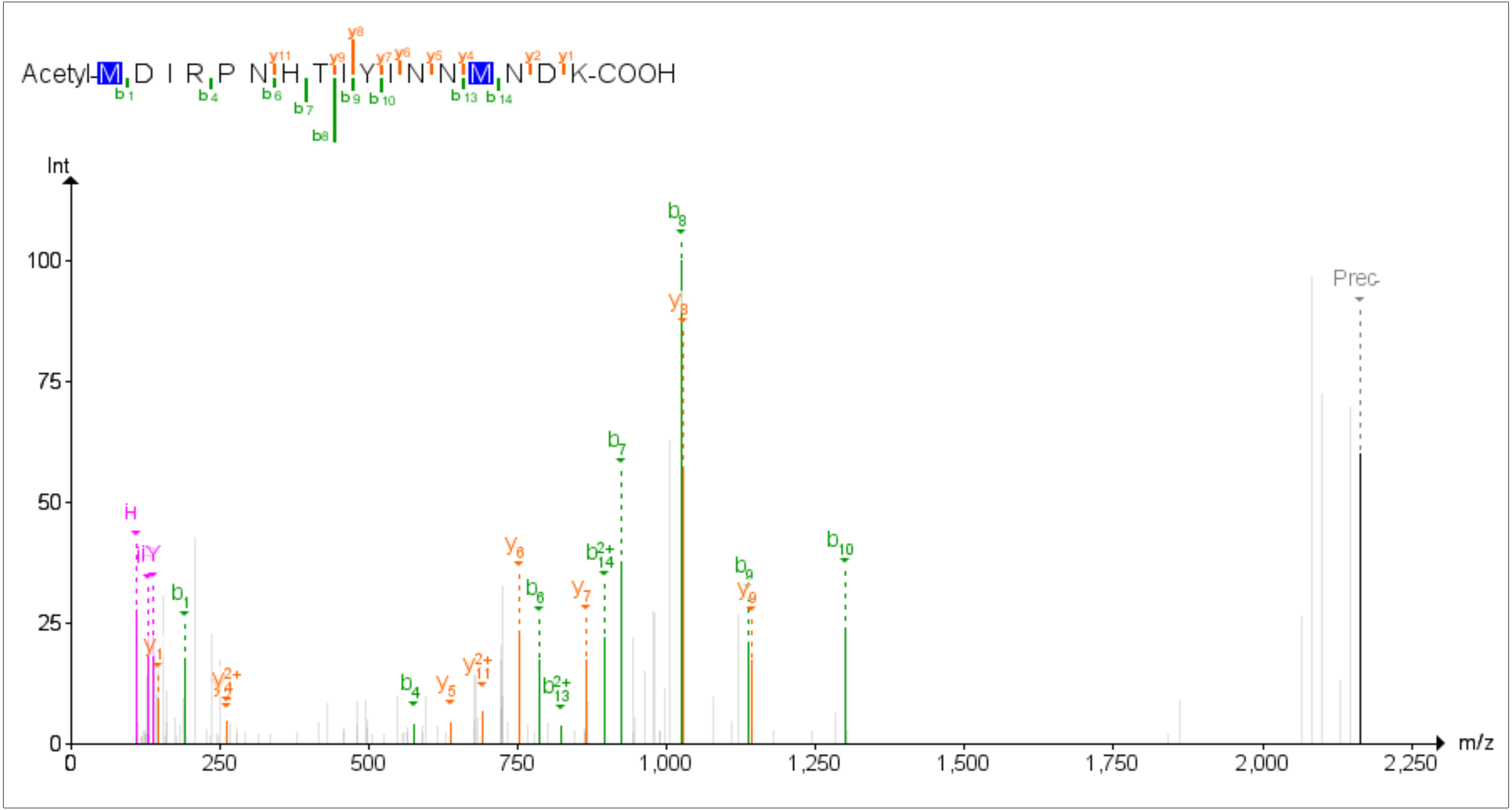

Acetyl-M D I R P N H T I Y I N N M N D K-COOH
Int
m/z
Prec
b8
y8
b7
b10
y6
b6
y7
b9
y9
b1
y1
b4
y5
0
25
50
75
100
250
500
750
1,000
1,250
1,500
1,750
2,000
2,250

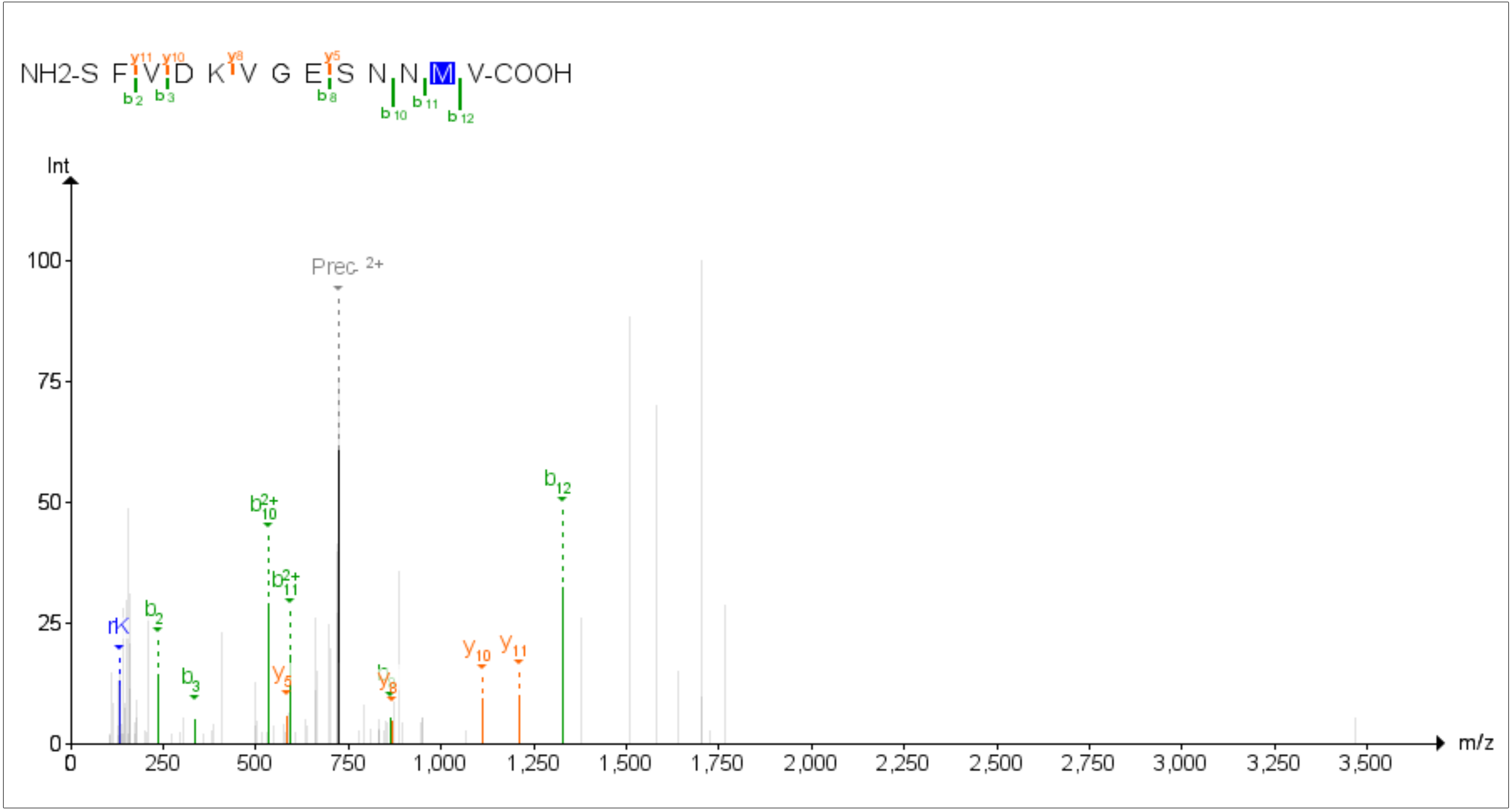

NH2-S F I V D K V G E S N N M V-COOH
y11 y10 y8 y5
b2 b3 b8 b10 b11 b12
Int
100
75
50
25
0
Prec 2+
rK
b2
b3
b10 2+
b11 2+
y5
y8
y10
y11
b12
0 250 500 750 1,000 1,250 1,500 1,750 2,000 2,250 2,500 2,750 3,000 3,250 3,500
m/z

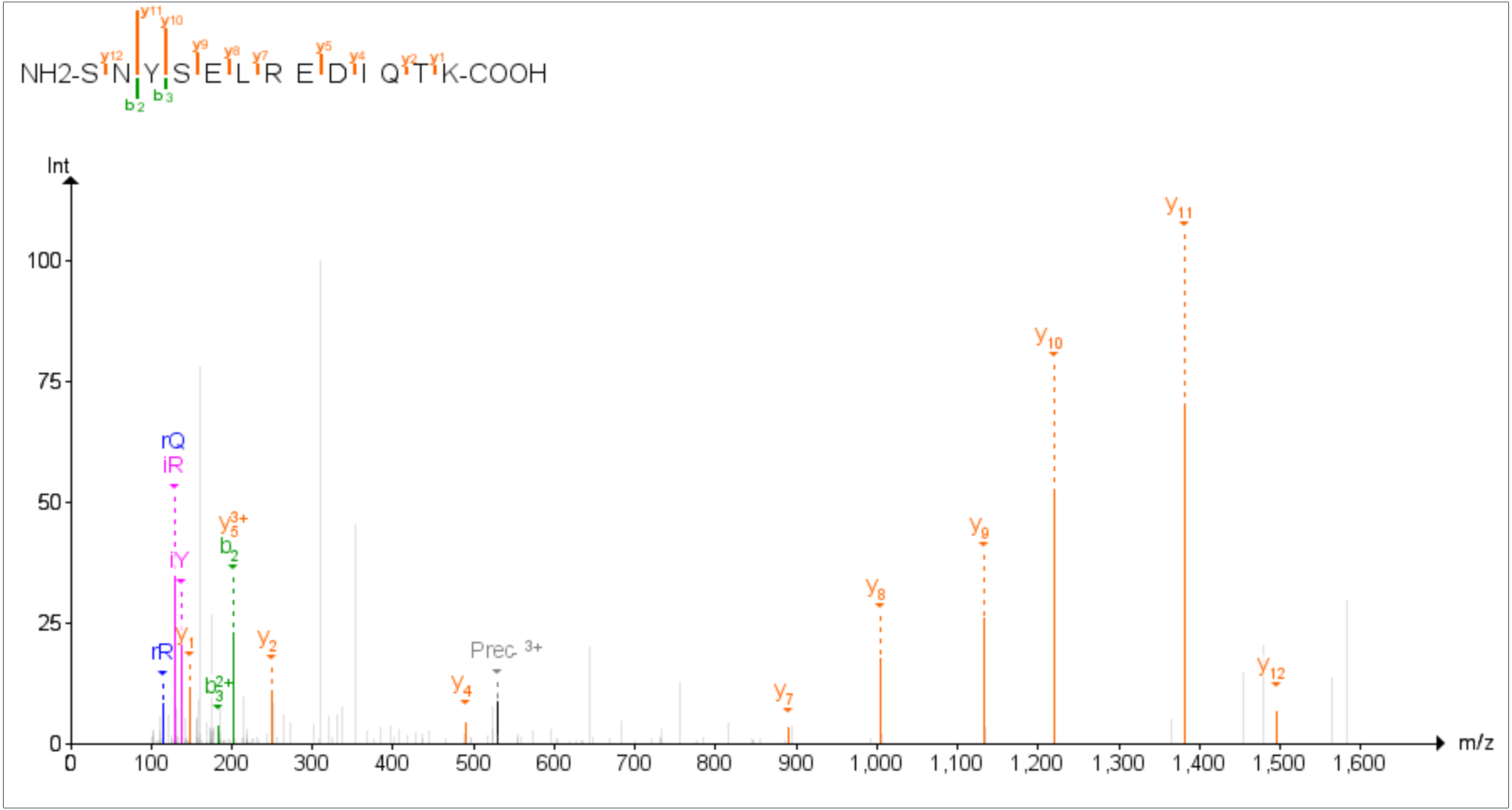

NH2-S N Y S E L R E D I Q T K-COOH
y11 y10 y12 y9 y8 y7 y5 y4 y2 y1
b2 b3
Int
m/z
rQ
iR
iY
rR
Prec 3+
0
25
50
75
100
0
100
200
300
400
500
600
700
800
900
1,000
1,100
1,200
1,300
1,400
1,500
1,600

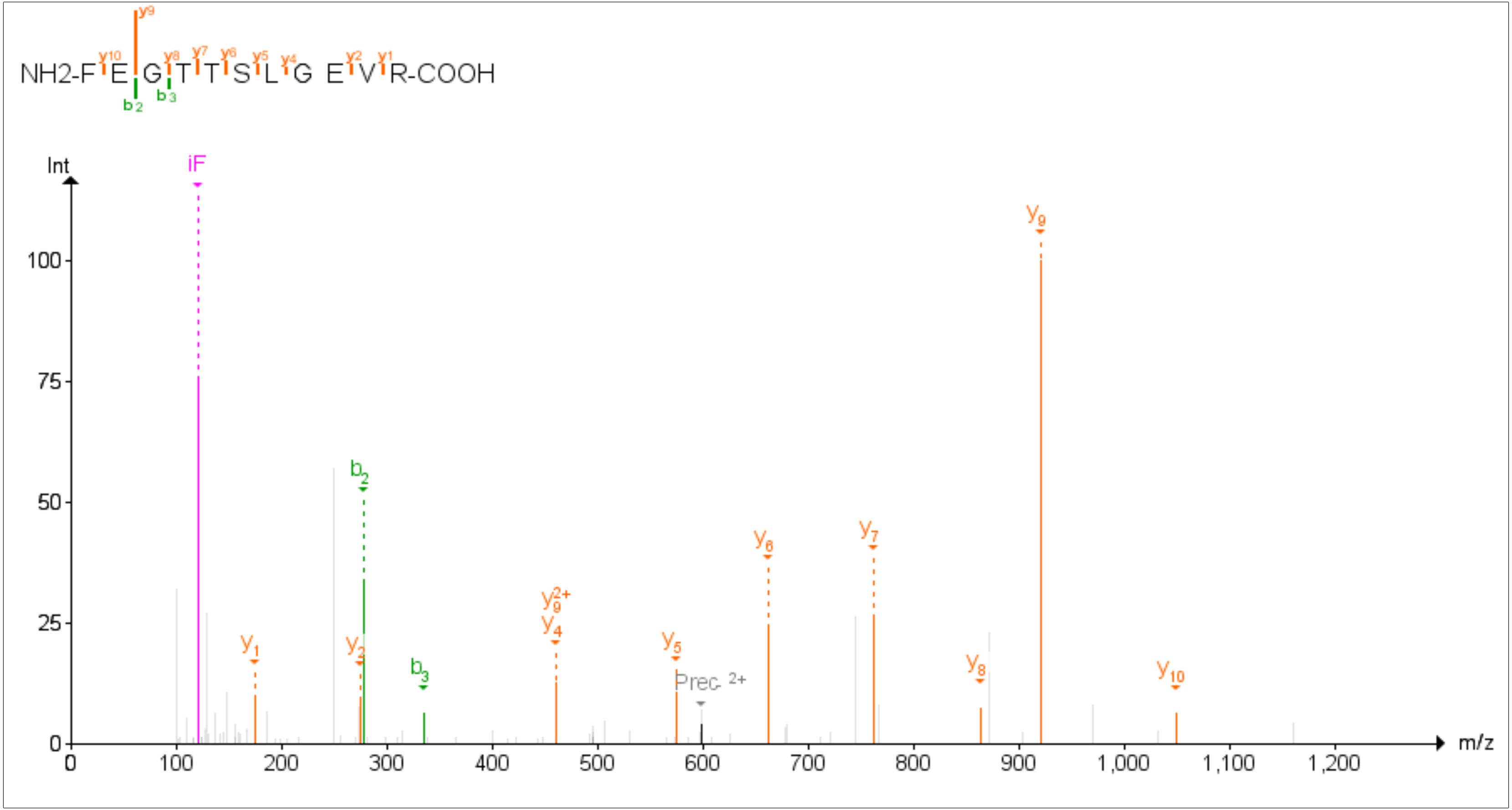

NH2-F E G T T S L G E V R-COOH
y9
y10
y8
y7
y6
y5
y4
y2
y1
b2
b3
Int
iF
y9
100
75
50
25
0
b2
y6
y7
y9 2+
y4
y1
y2
b3
y5
Prec 2+
y8
y10
0
100
200
300
400
500
600
700
800
900
1,000
1,100
1,200
m/z

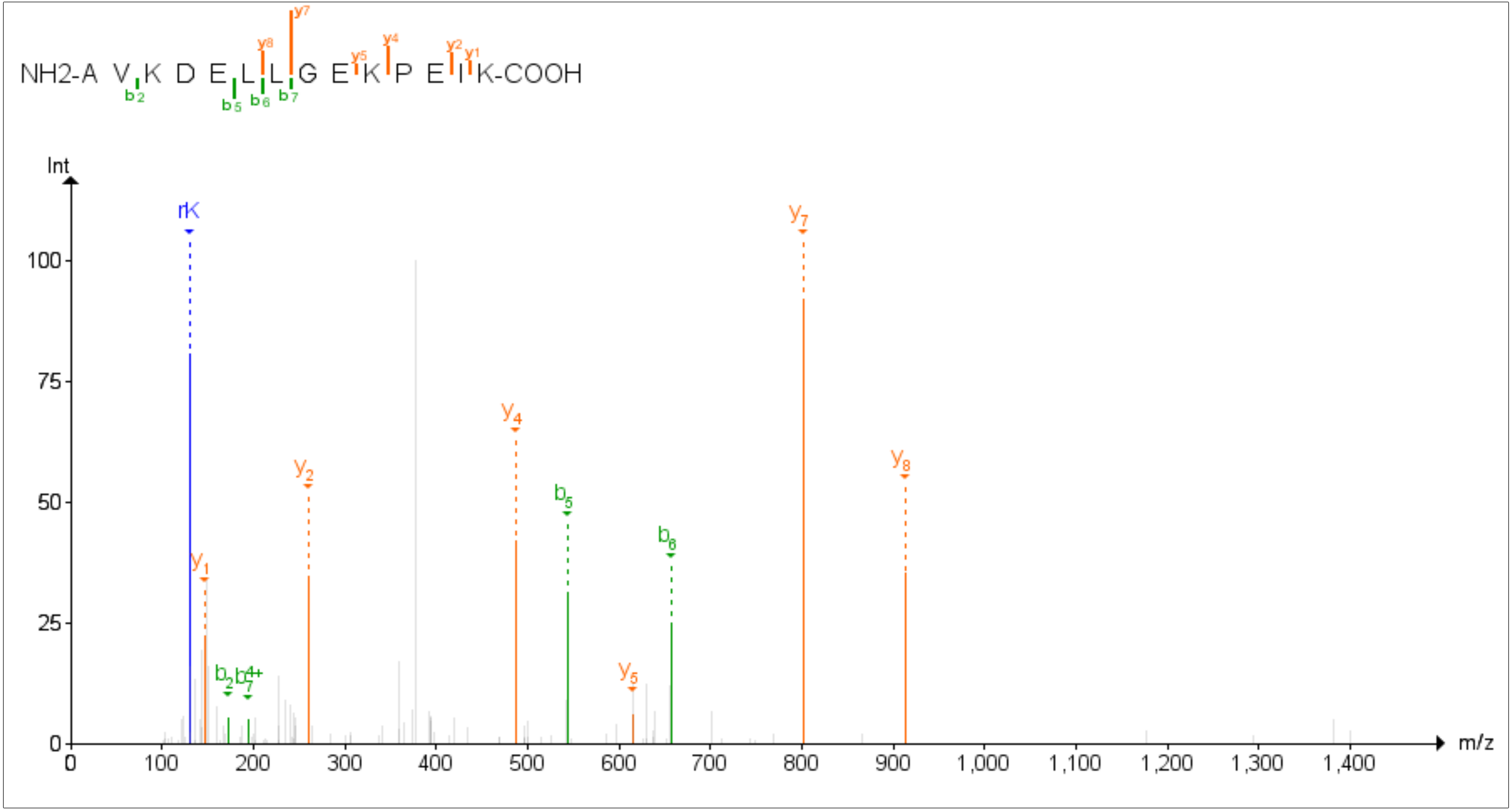
NH2-A V K D E L L G E K P E I K-COOH
b2 b5 b6 b7
y8 y7 y5 y4 y2 y1
Int
rK
y7
y4
y2
b5
b6
y8
y1
y5
b2
b7
b4++
100
75
50
25
0
0 100 200 300 400 500 600 700 800 900 1,000 1,100 1,200 1,300 1,400
m/z

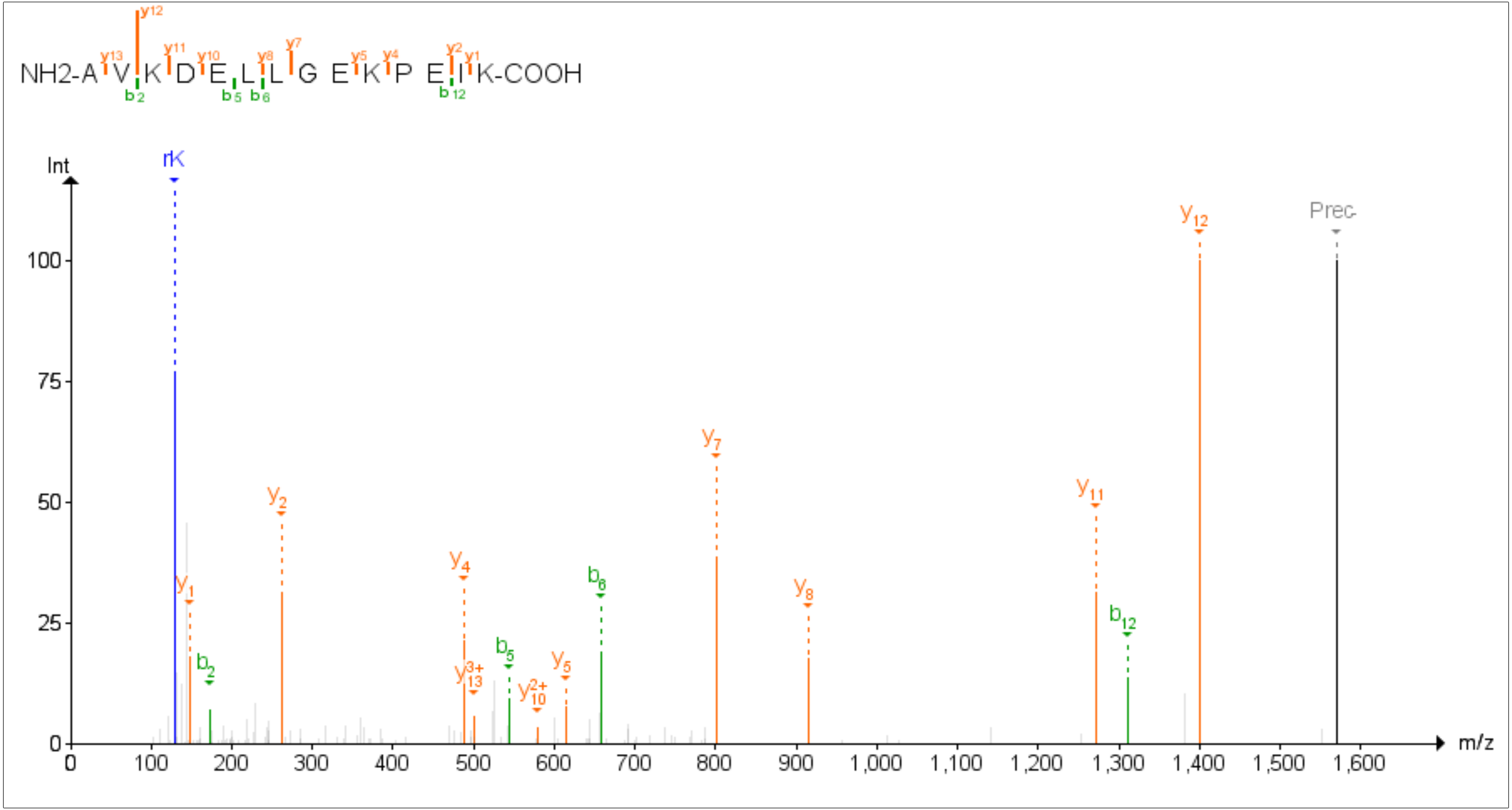

NH2-A V K D E L L G E K P E I K-COOH
y13 y12 y11 y10 y8 y7 y5 y4 y2 y1
b2 b5 b6 b12
Int
rK
Prec
y12
y7
y11
y2
y4
b6
y8
y1
b12
b5
b2
y5
y13 3+
y10 2+
0
25
50
75
100
0 100 200 300 400 500 600 700 800 900 1,000 1,100 1,200 1,300 1,400 1,500 1,600
m/z

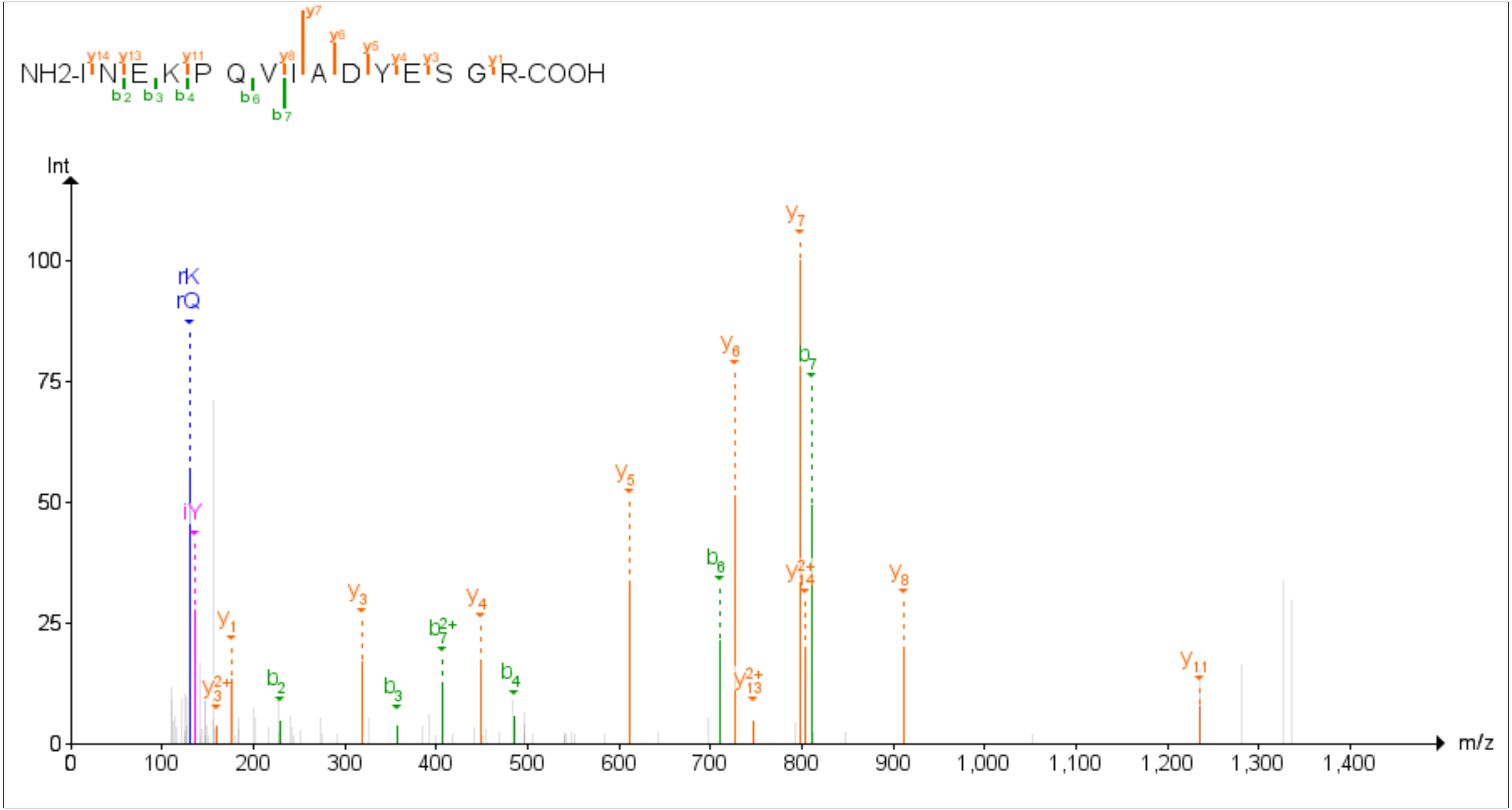
NH2-I N E K P Q V I A D Y E S G R-COOH
y14 y13 y11 y8 y7 y6 y5 y4 y3 y1
b2 b3 b4 b6 b7
Int
m/z
0
25
50
75
100
100
200
300
400
500
600
700
800
900
1,000
1,100
1,200
1,300
1,400
rK
rQ
iY
y1
y3
y4
y5
y6
y7
y8
y11
b2
b3
b4
b6
b7
y3 2+
b7 2+
y13 2+
y14 2+

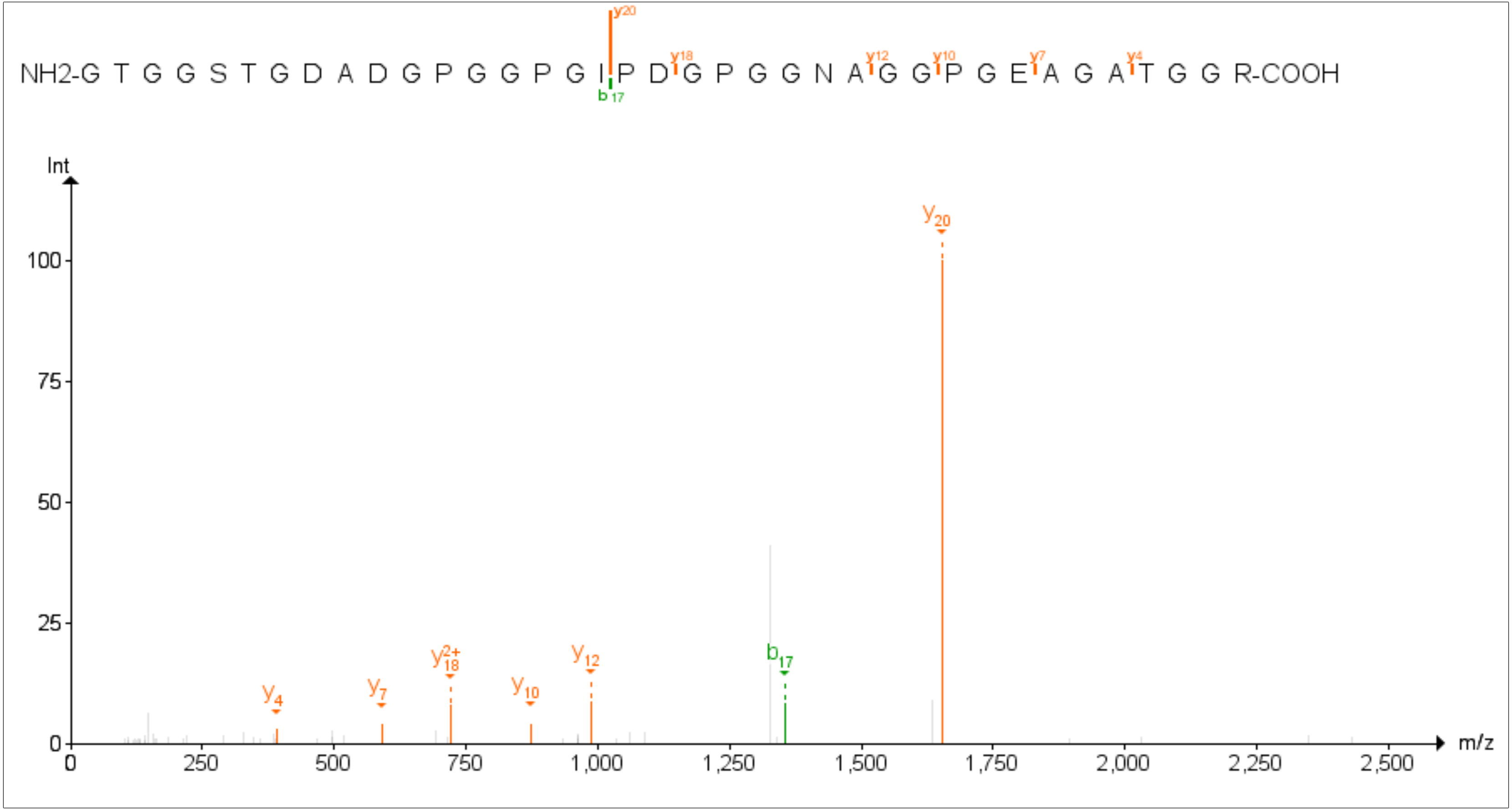

NH2-G T G G S T G D A D G P G G P G I P D G P G G N A G G P G E A G A T G G R-COOH
y20
y18
y12
y10
y7
y4
b 17
Int
100
75
50
25
0
y20
y4
y7
y18 2+
y10
y12
b17
0
250
500
750
1,000
1,250
1,500
1,750
2,000
2,250
2,500
m/z

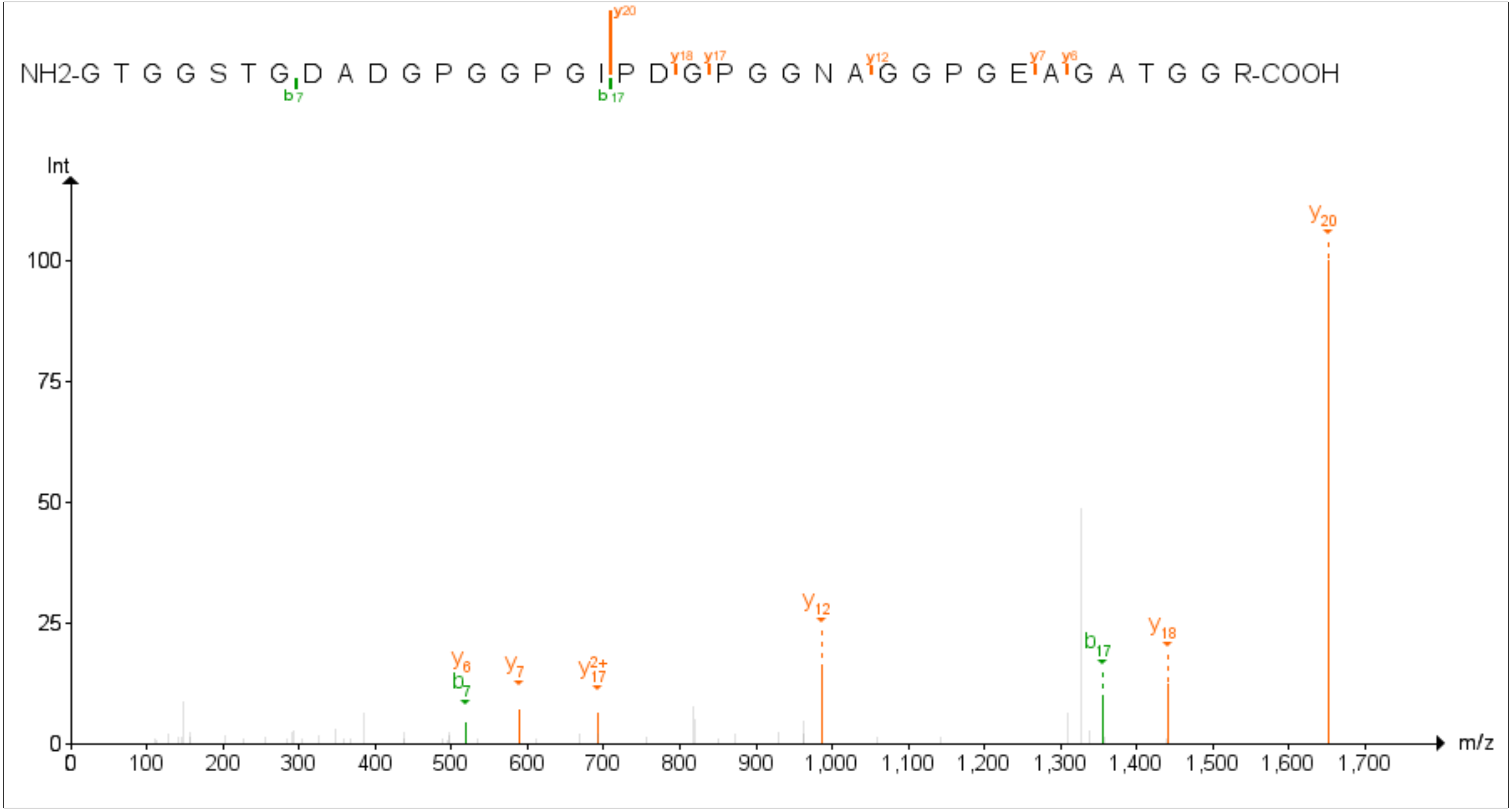

NH2-G T G G S T G D A D G P G G P G I P D G P G G N A G G P G E A G A T G G R-COOH
b7
b17
y20
y18
y17
y12
y7
y6
Int
100
75
50
25
0
m/z
0
100
200
300
400
500
600
700
800
900
1,000
1,100
1,200
1,300
1,400
1,500
1,600
1,700
y20
y12
y18
b17
y6
b7
y7
y17 2+

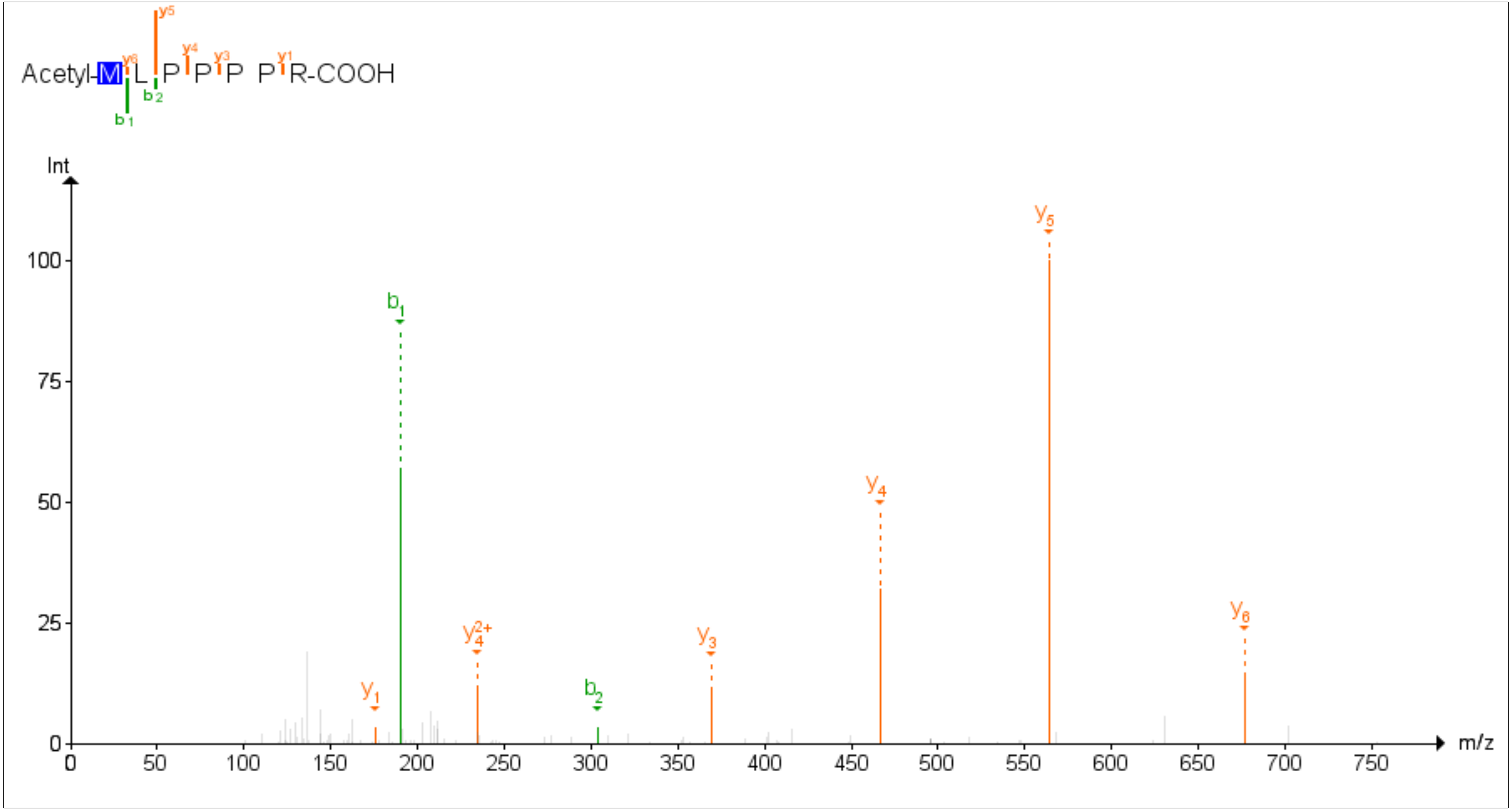

Acetyl-M L P P P P R-COOH
y6 y5 y4 y3 y1
b 1 b 2
Int
100
75
50
25
0
b1
y1
y4 2+
b2
y3
y4
y5
y6
0 50 100 150 200 250 300 350 400 450 500 550 600 650 700 750
m/z

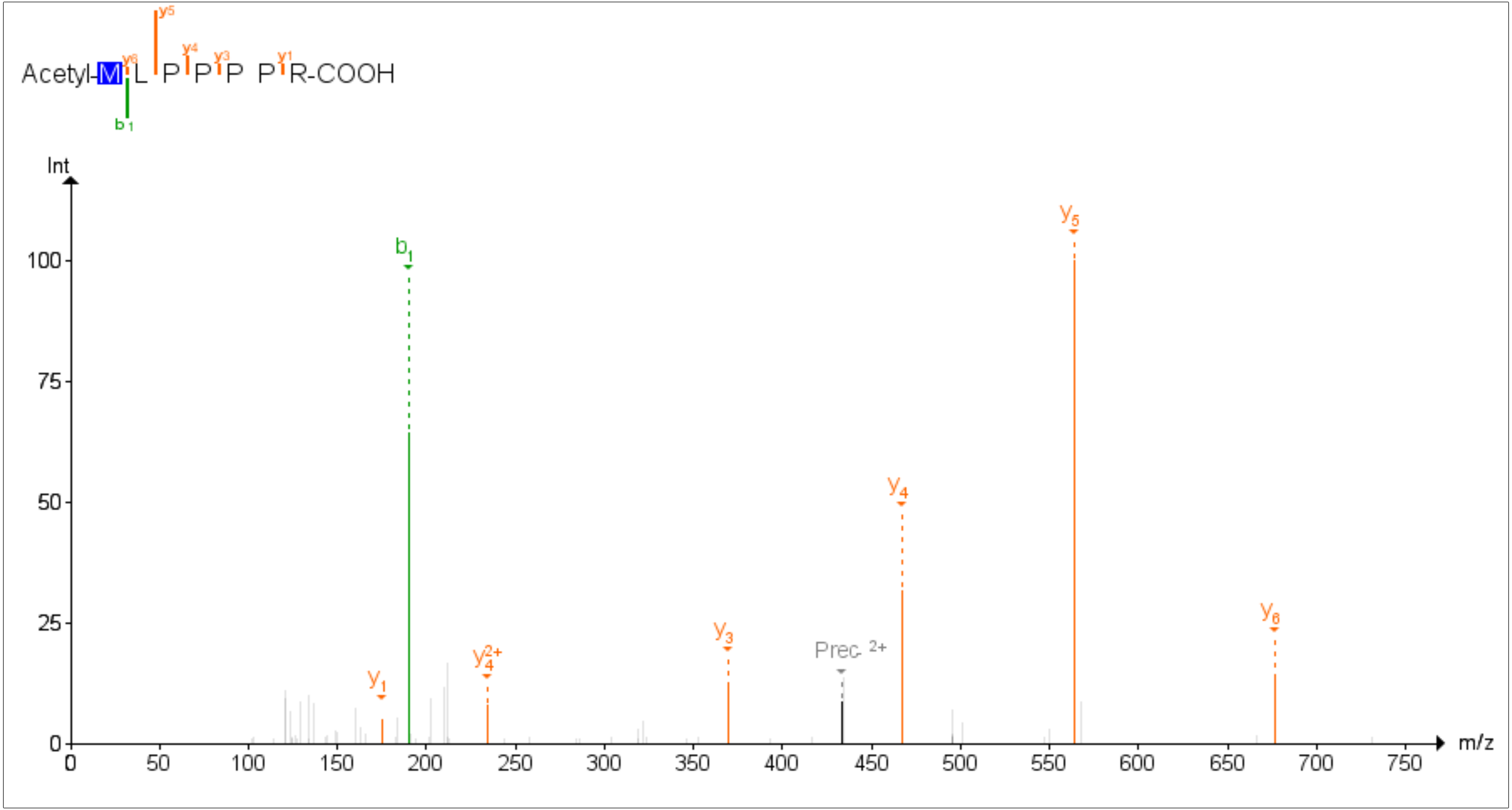

Acetyl-M L P P P P R-COOH
y6
y5
y4
y3
y1
b 1
Int
b1
y5
y4
y1
y_4^{2+}
y3
Prec 2+
y6
m/z
0
25
50
75
100
0
50
100
150
200
250
300
350
400
450
500
550
600
650
700
750

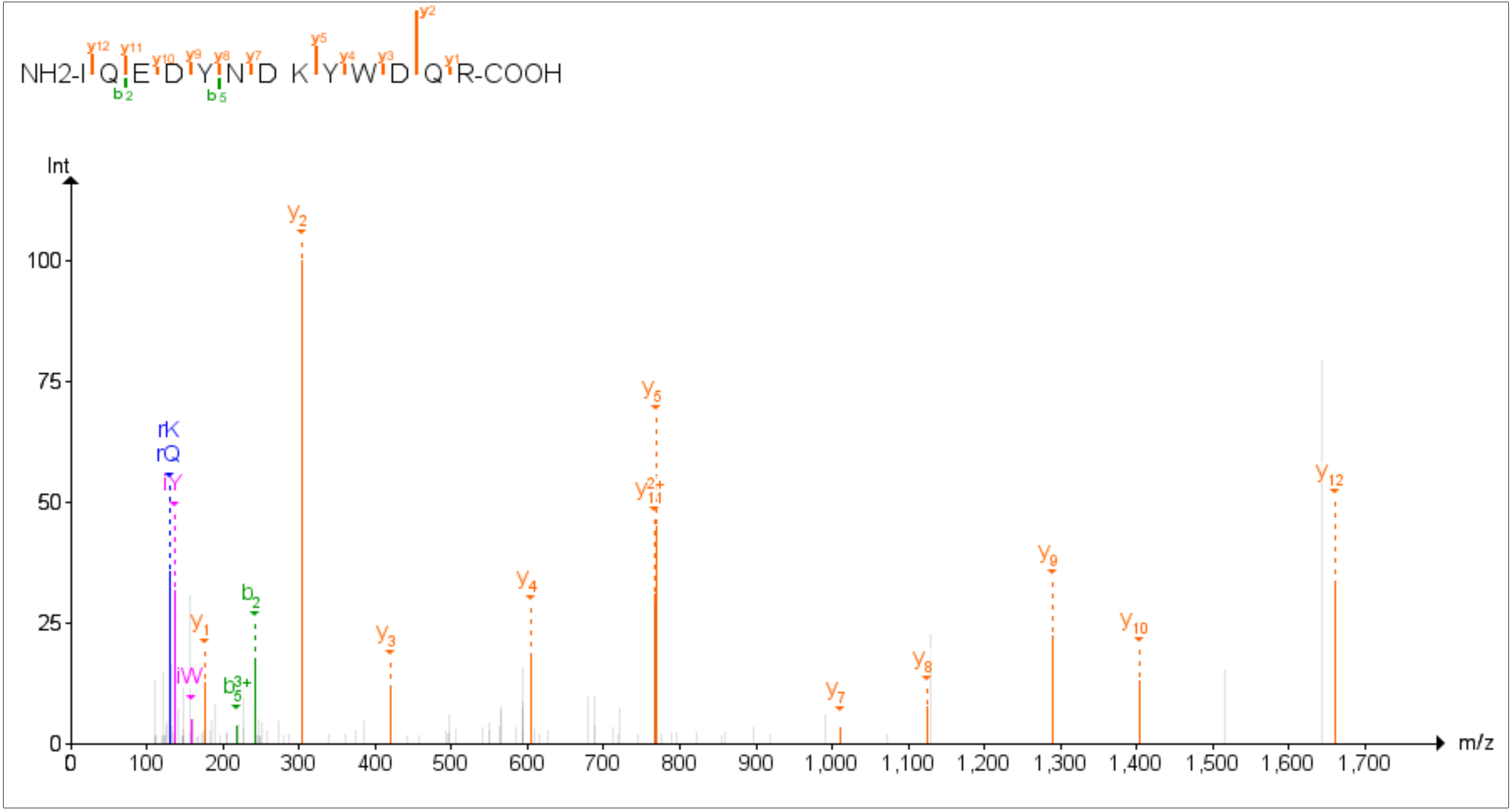

NH2-I Q E D Y N D K Y W D Q R-COOH
y12 y11 y10 y9 y8 y7 y5 y4 y3 y2 y1
b2 b5
Int
100
75
50
25
0
rK
rQ
iY
iW
y1
b2
b5 3+
y2
y3
y4
y5
y11 2+
y7
y8
y9
y10
y12
0 100 200 300 400 500 600 700 800 900 1,000 1,100 1,200 1,300 1,400 1,500 1,600 1,700
m/z

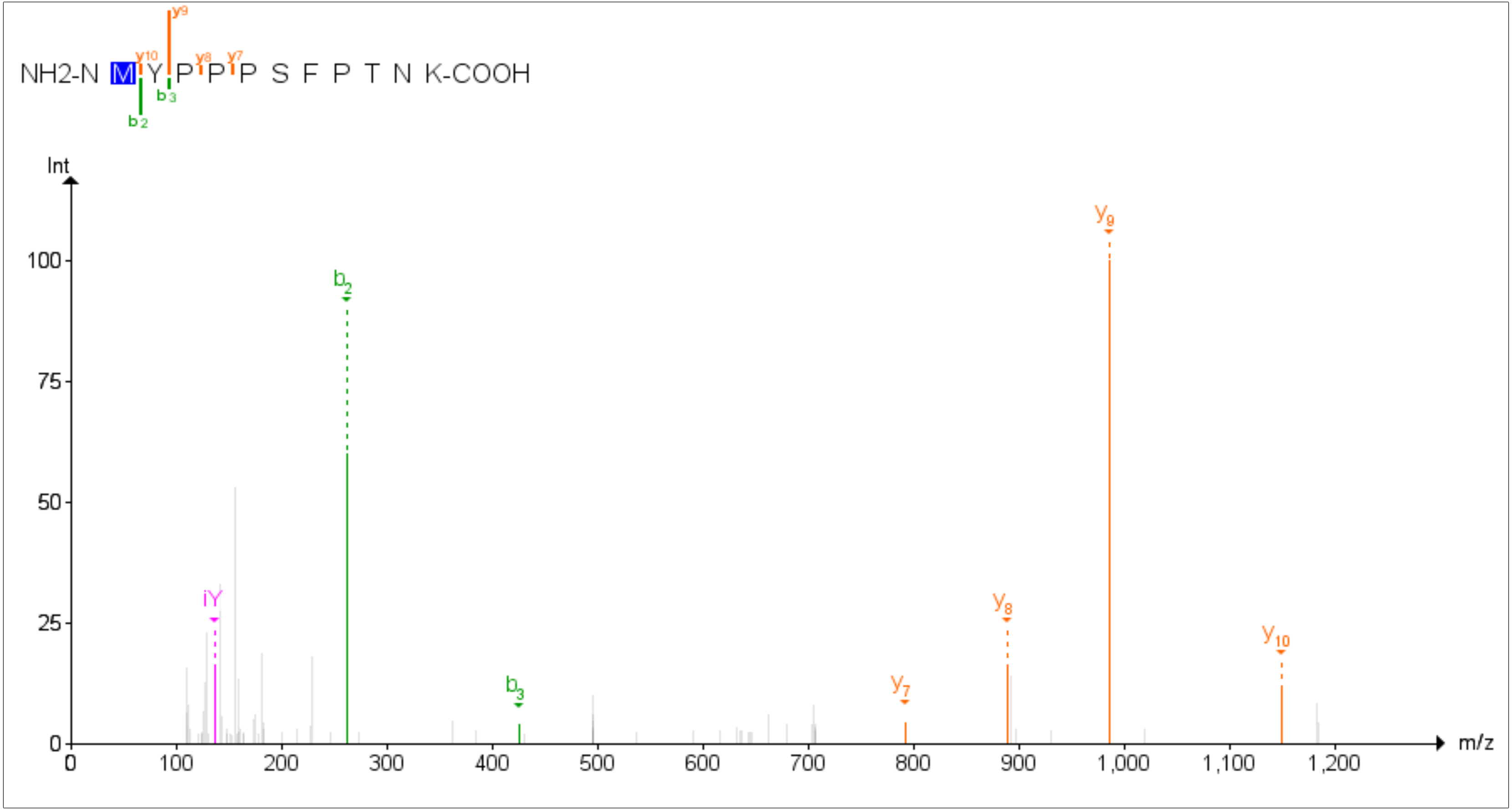

NH2-N M Y P P P S F P T N K-COOH
y9
y10
y8
y7
b 2
b 3
Int
100
75
50
25
0
b2
b3
iY
y7
y8
y9
y10
0
100
200
300
400
500
600
700
800
900
1,000
1,100
1,200
m/z

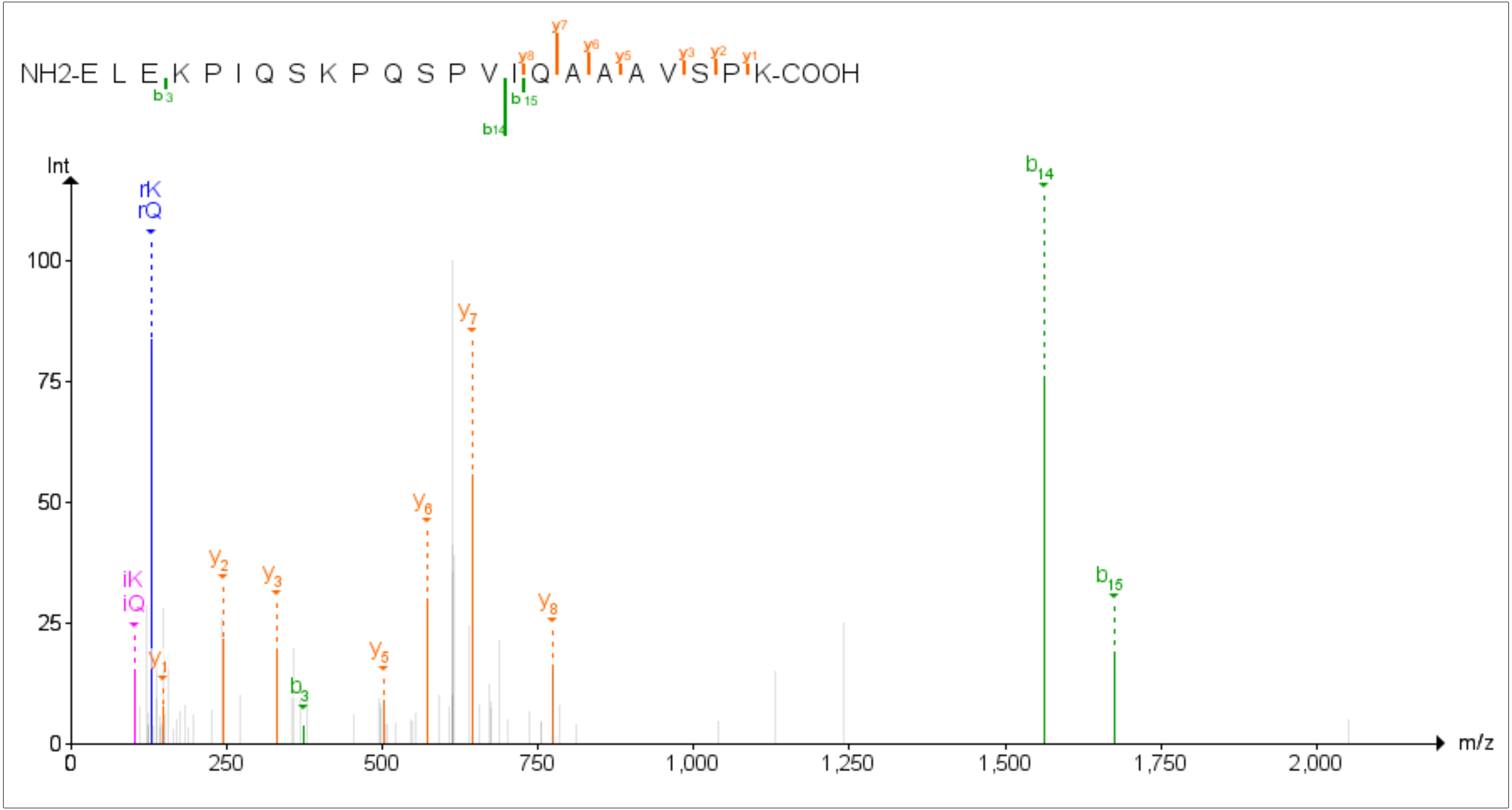

NH2-E L E K P I Q S K P Q S P V I Q A A A V S P K-COOH
b3
b14
b15
y8
y7
y6
y5
y3
y2
y1
Int
rK
rQ
iK
iQ
100
75
50
25
0
0
250
500
750
1,000
1,250
1,500
1,750
2,000
m/z

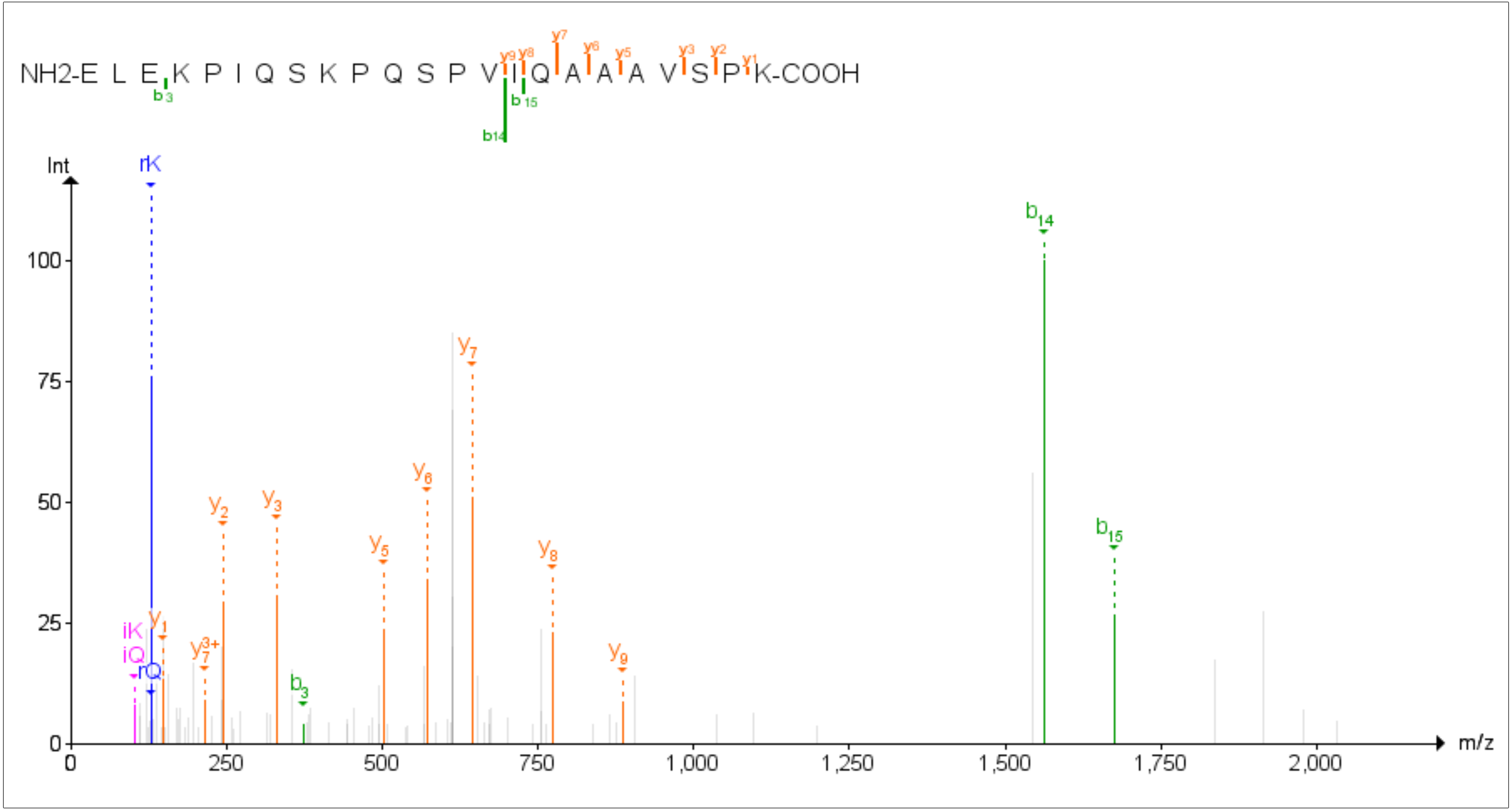

NH2-E L E K P I Q S K P Q S P V I Q A A A V S P K-COOH
b3
b14
b15
y9
y8
y7
y6
y5
y3
y2
y1
Int
rK
b14
b15
y7
y6
y2
y3
y5
y8
y9
y1
iK
iQ
rQ
y7 3+
b3
100
75
50
25
0
0
250
500
750
1,000
1,250
1,500
1,750
2,000
m/z

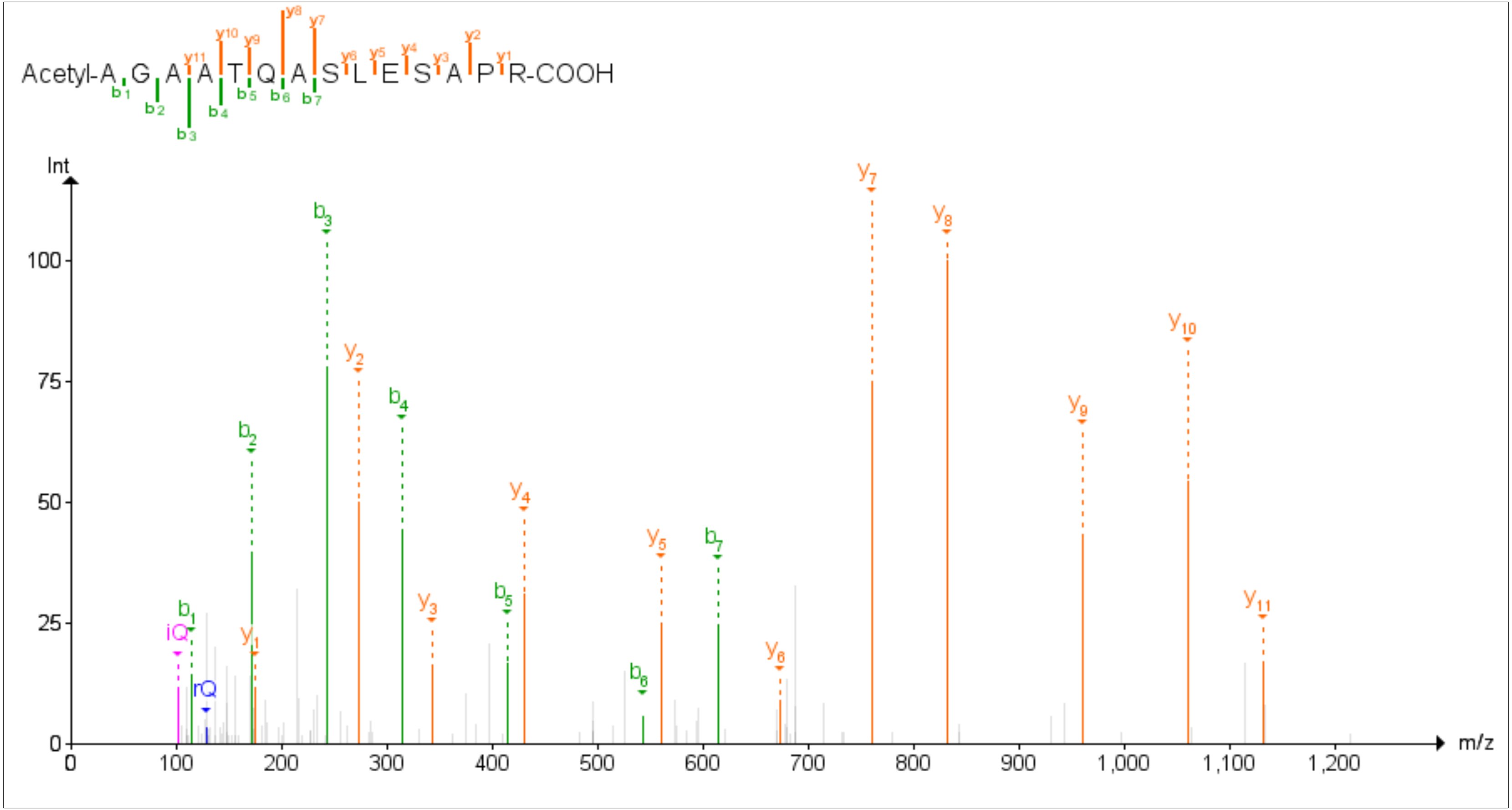

Acetyl-A G A A T Q A S L E S A P R-COOH
b1 b2 b3 b4 b5 b6 b7
y11 y10 y9 y8 y7 y6 y5 y4 y3 y2 y1
Int
m/z
0 25 50 75 100
0 100 200 300 400 500 600 700 800 900 1,000 1,100 1,200
iQ rQ b1 y1 b2 b3 y2 b4 y3 b5 y4 b6 y5 b7 y6 y7 y8 y9 y10 y11

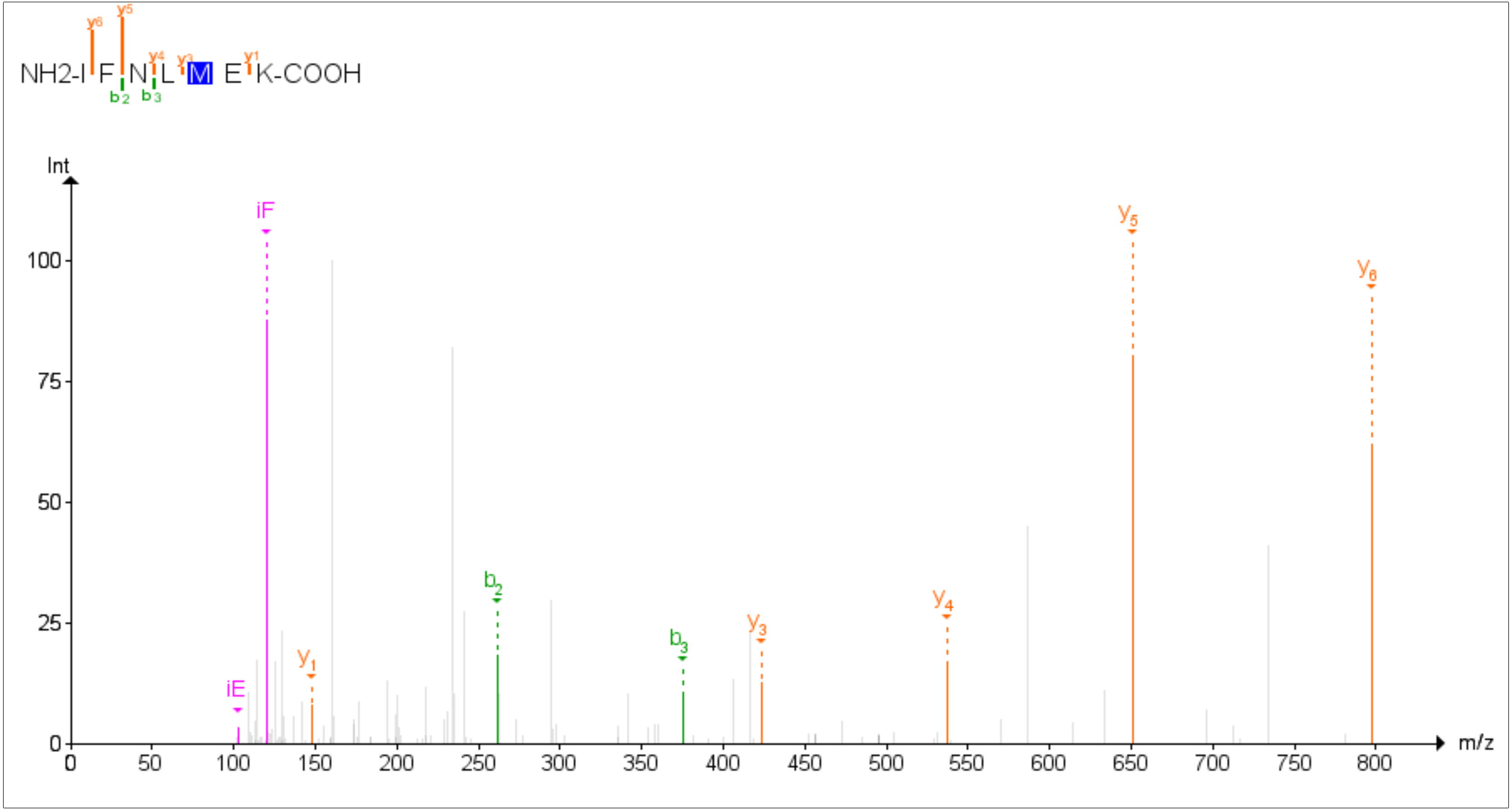

NH2-I F N L M E K-COOH
y6 y5 y4 y3 y1
b2 b3
Int
m/z
iF
iE
y1
b2
b3
y3
y4
y5
y6
0
25
50
75
100
0 50 100 150 200 250 300 350 400 450 500 550 600 650 700 750 800

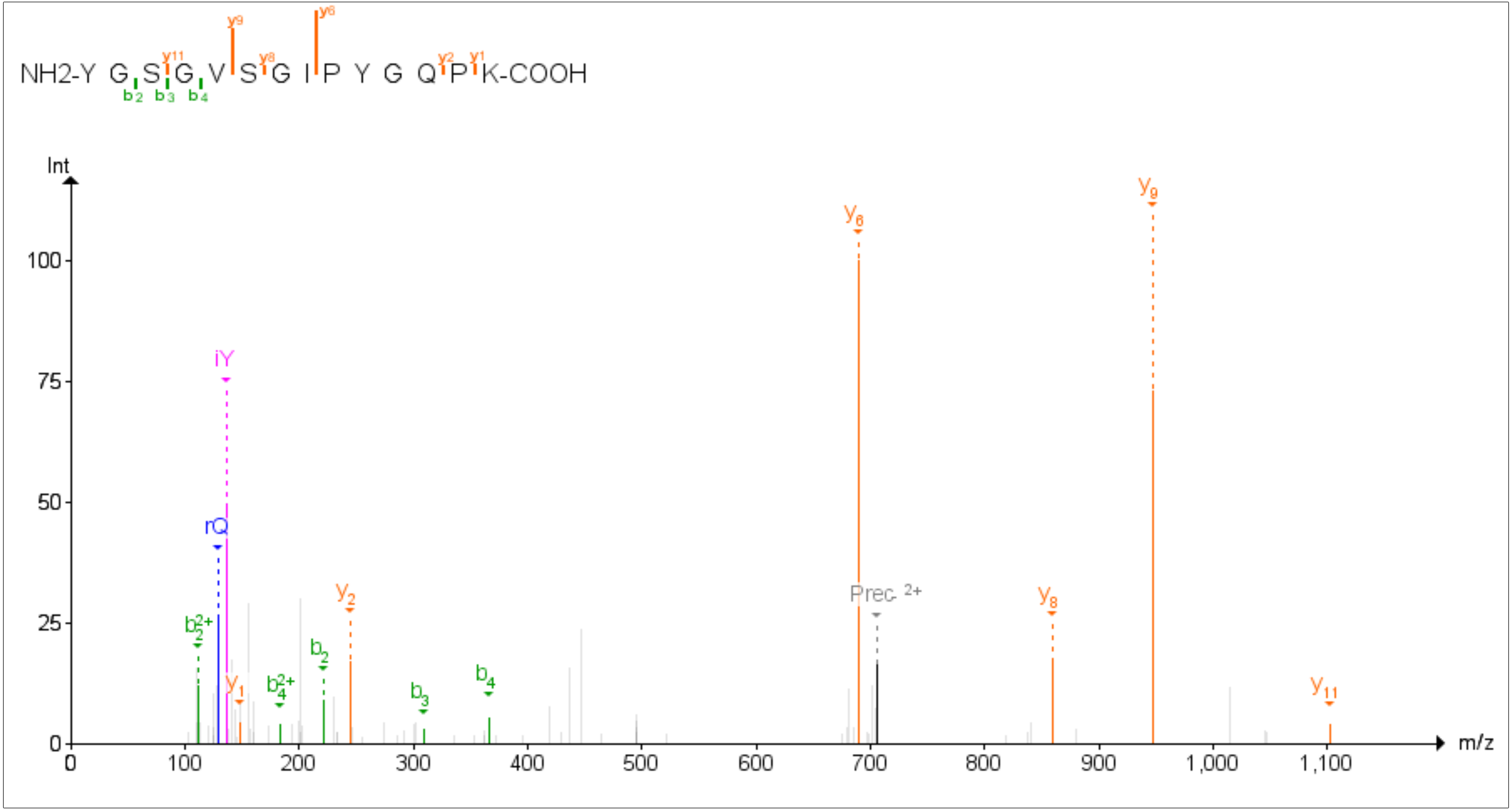
NH2-Y G S G V S G I P Y G Q P K-COOH
y11
y9
y8
y6
y2
y1
b2
b3
b4
Int
100
75
50
25
0
iY
rQ
b2 2+
y1
b4 2+
b2
y2
b3
b4
y6
Prec 2+
y8
y9
y11
0
100
200
300
400
500
600
700
800
900
1,000
1,100
m/z

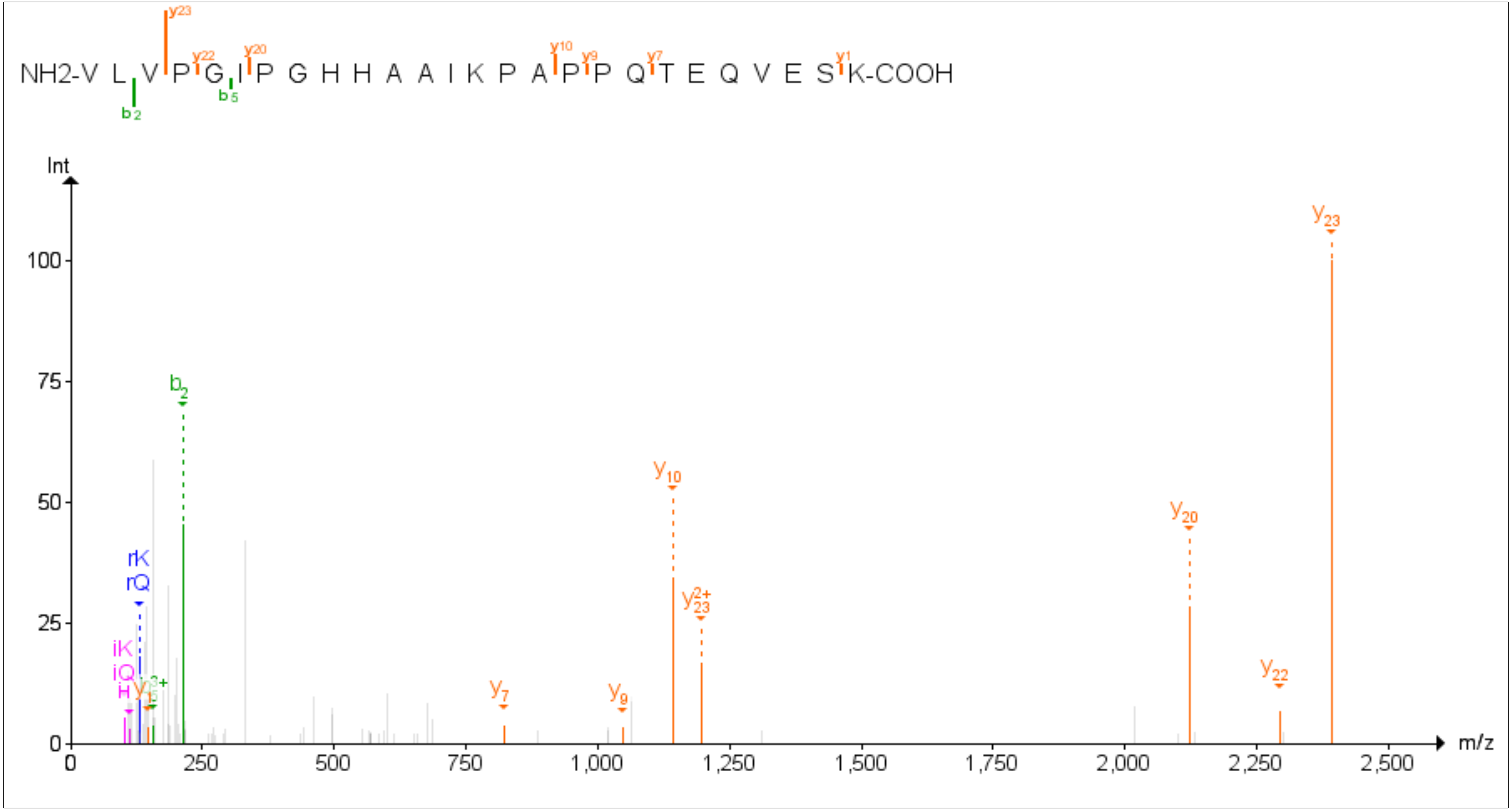

NH2-V L V P G I P G H H A A I K P A P P Q T E Q V E S K-COOH
y23
y22
y20
y10
y9
y7
y1
b2
b5
Int
m/z
0
25
50
75
100
250
500
750
1,000
1,250
1,500
1,750
2,000
2,250
2,500
b2
rK
rQ
iK
iQ
H
y10
y23 2+
y20
y22
y23
y7
y9

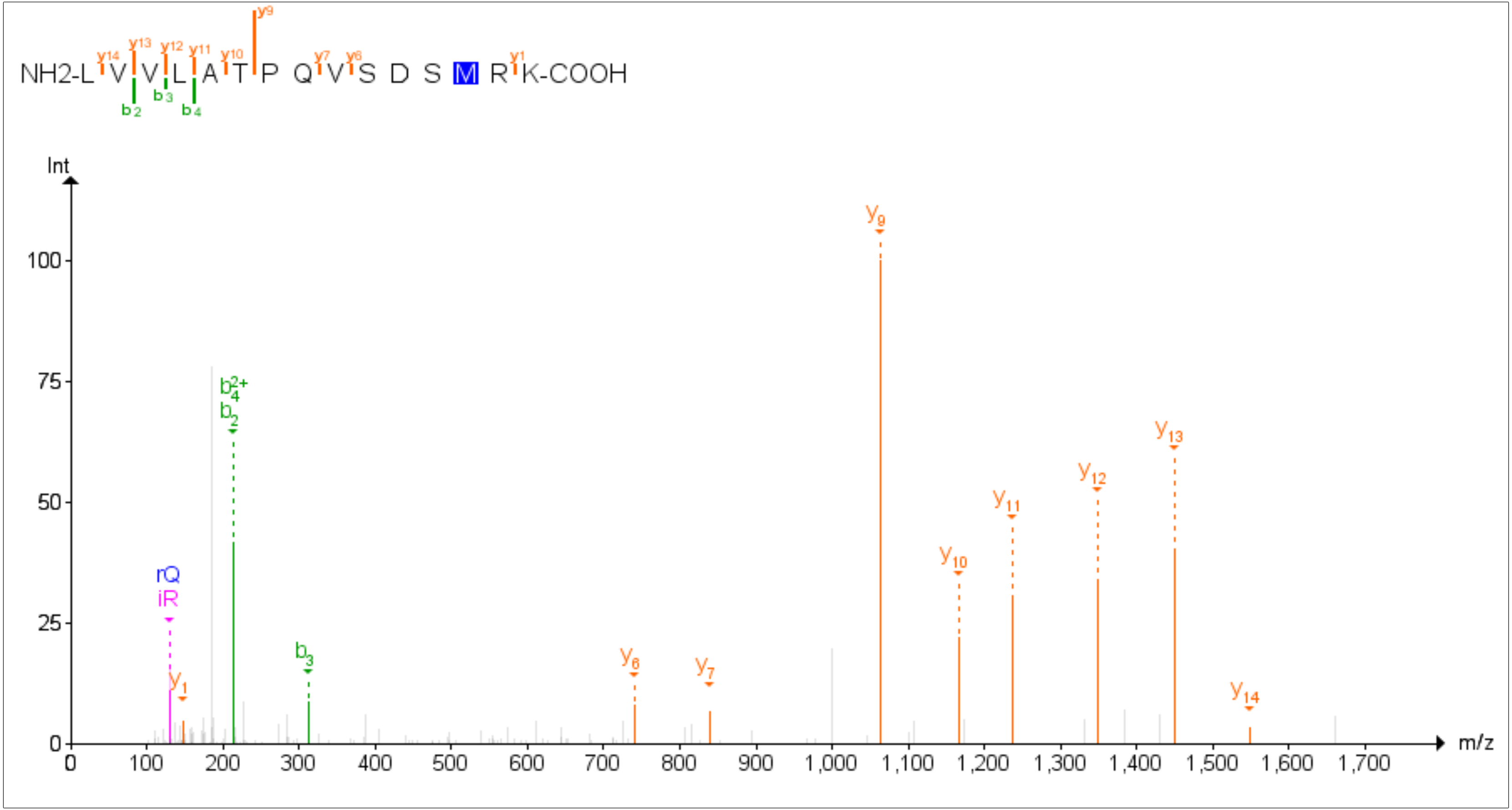

NH2-L V V L A T P Q V S D S M R K-COOH
y14 y13 y12 y11 y10 y9 y7 y6 y1
b2 b3 b4
Int
100
75
50
25
0
rQ
iR
y1
b4 2+
b2
b3
y6
y7
y9
y10
y11
y12
y13
y14
0 100 200 300 400 500 600 700 800 900 1,000 1,100 1,200 1,300 1,400 1,500 1,600 1,700
m/z

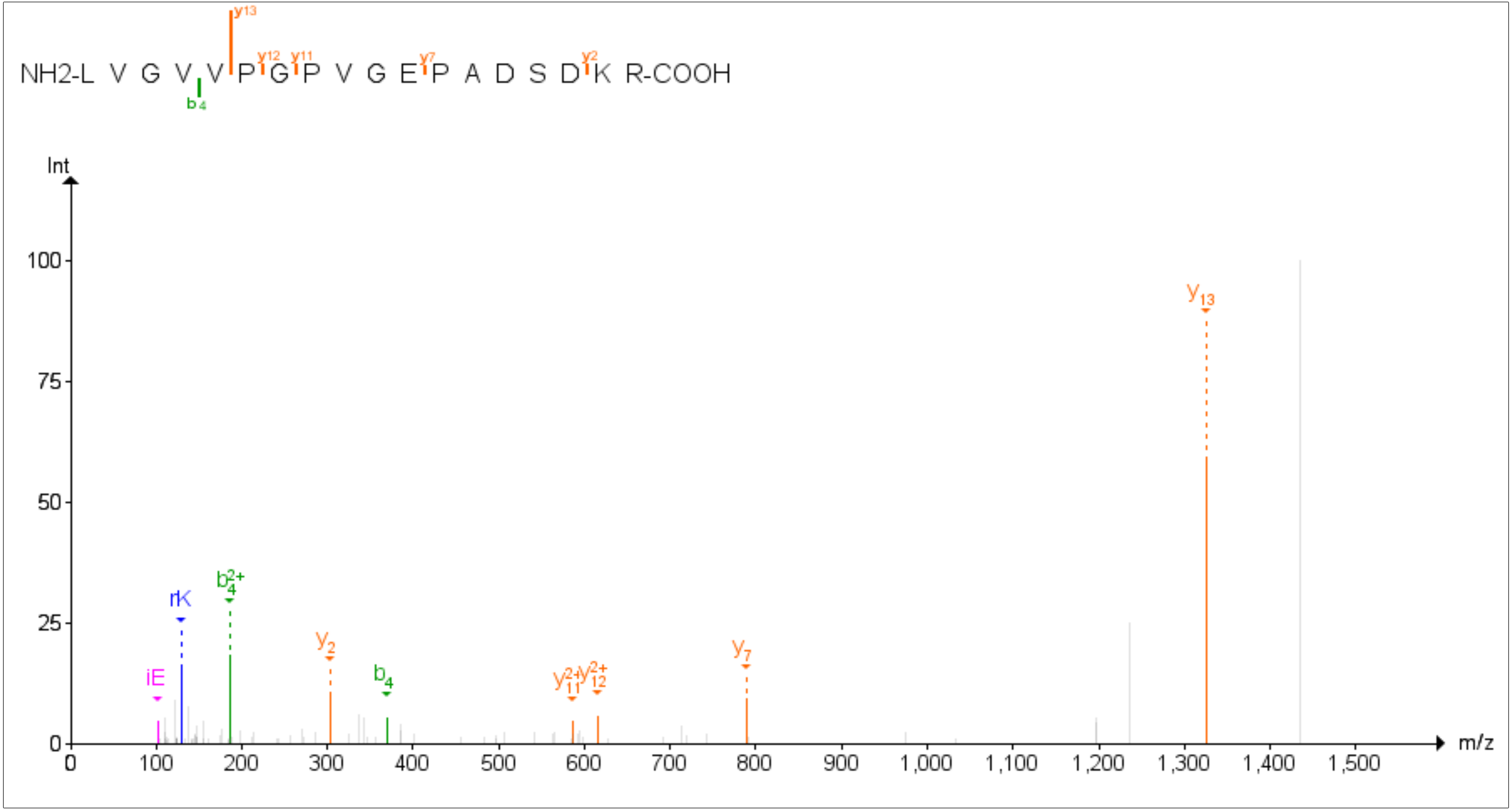

NH2-L V G V V P G P V G E P A D S D K R-COOH
y13
y12
y11
y7
y2
b4
Int
100
75
50
25
0
y13
rK
b4 2+
iE
y2
b4
y11 2+
y12 2+
y7
0
100
200
300
400
500
600
700
800
900
1,000
1,100
1,200
1,300
1,400
1,500
m/z

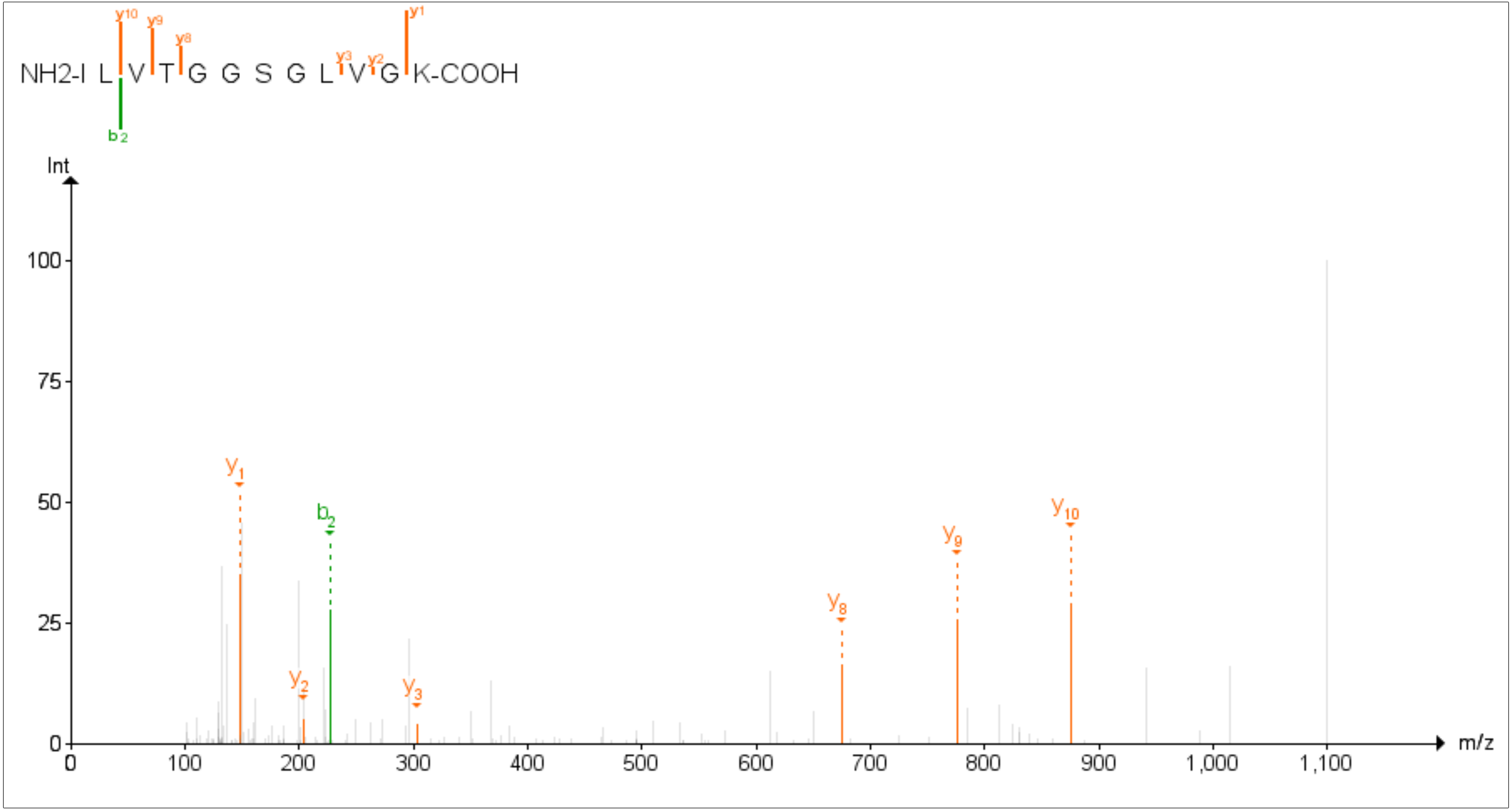

NH2-I L V T G G S G L V G K-COOH
y10
y9
y8
y3
y2
y1
b2
Int
100
75
50
25
0
0
100
200
300
400
500
600
700
800
900
1,000
1,100
m/z
y1
b2
y2
y3
y8
y9
y10

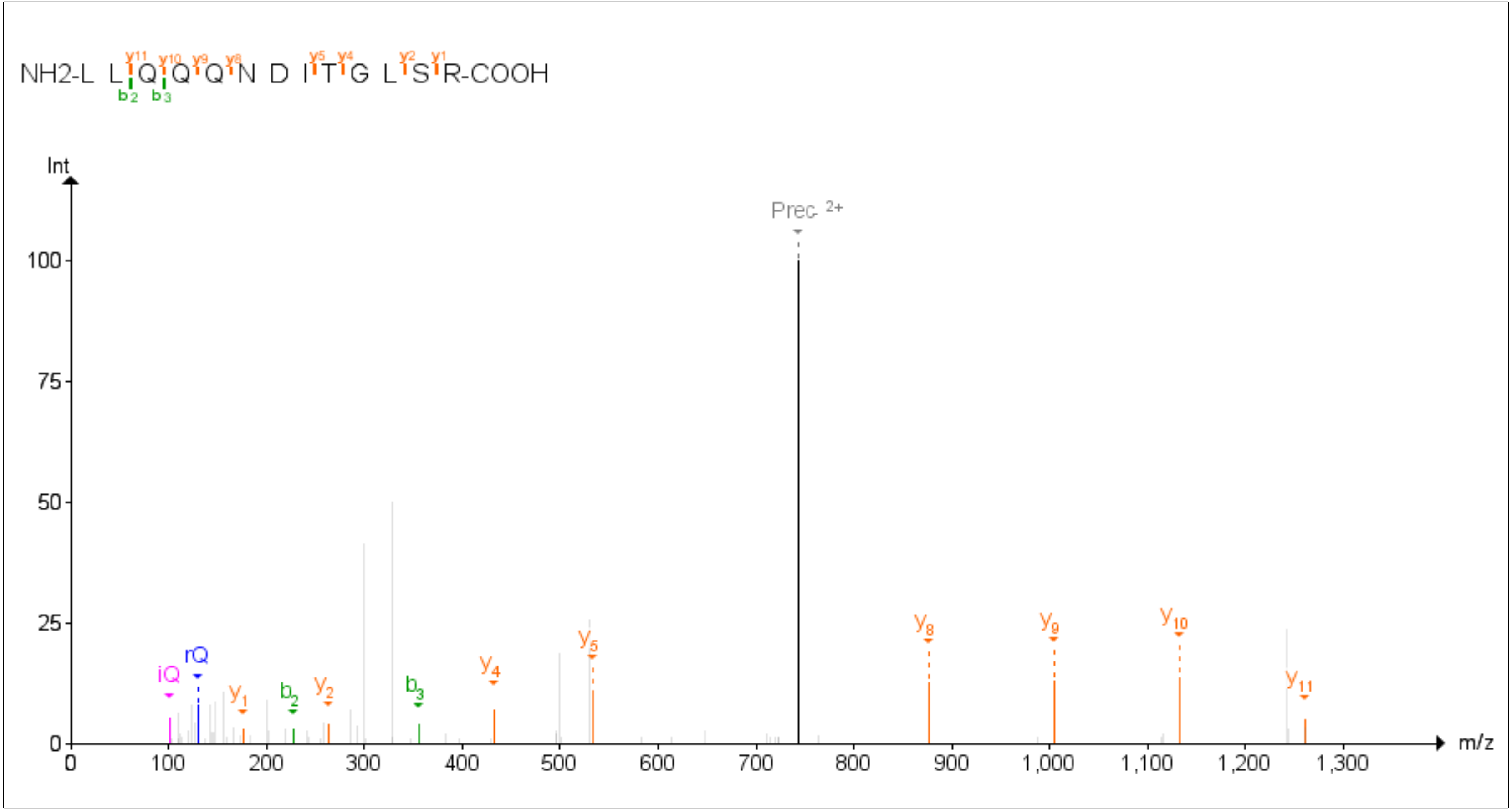
NH2-L L Q Q Q N D I T G L S R-COOH
y11 y10 y9 y8 y5 y4 y2 y1
b2 b3
Int
100
75
50
25
0
Prec 2+
iQ
rQ
y1
b2
y2
b3
y4
y5
y8
y9
y10
y11
0
100
200
300
400
500
600
700
800
900
1,000
1,100
1,200
1,300
m/z

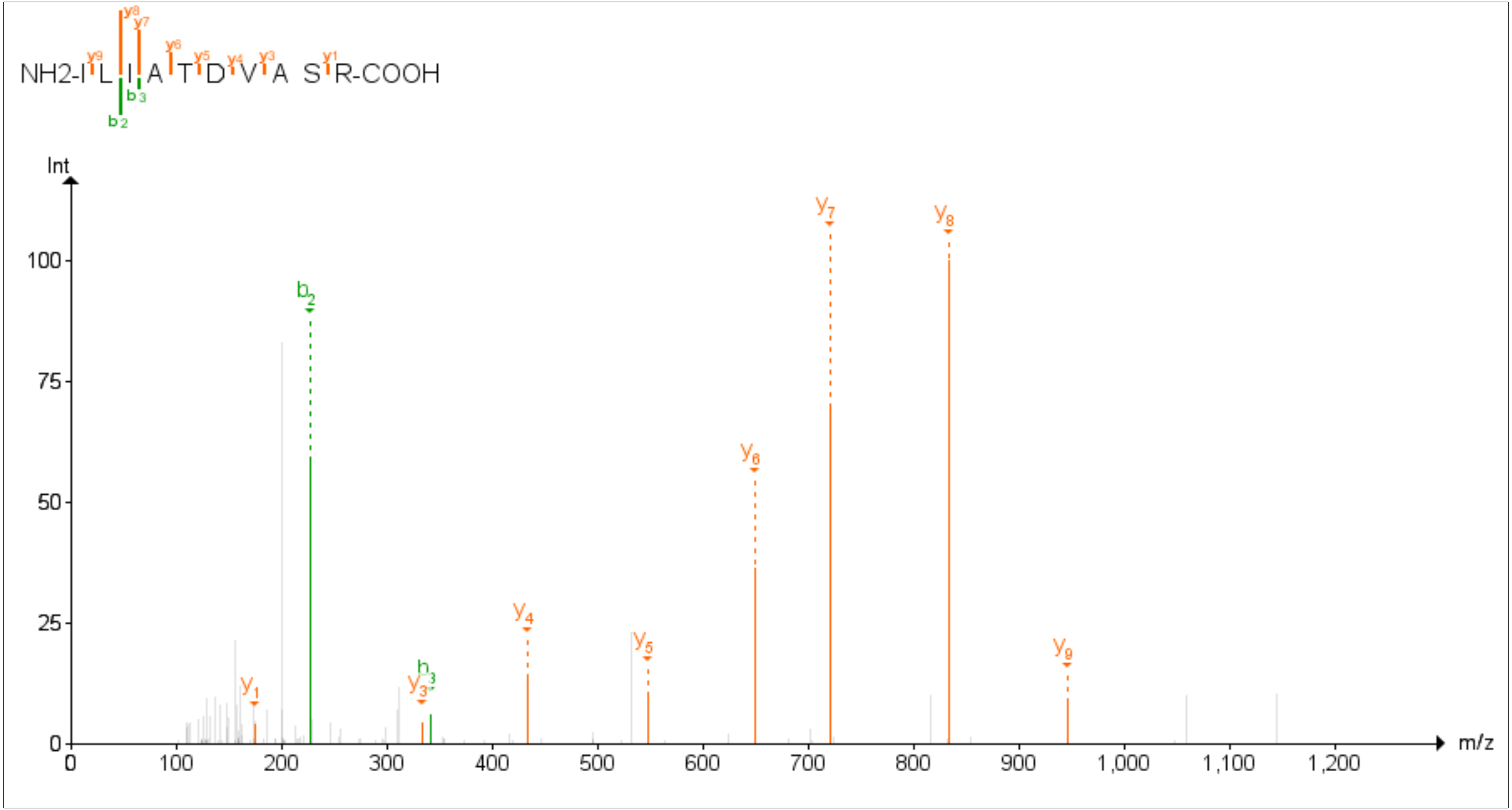

NH2-I L I A T D V A S R-COOH
y9 y8 y7 y6 y5 y4 y3 y1
b2 b3
Int
100
75
50
25
0
m/z
0
100
200
300
400
500
600
700
800
900
1,000
1,100
1,200
b2
y1
y3
b3
y4
y5
y6
y7
y8
y9

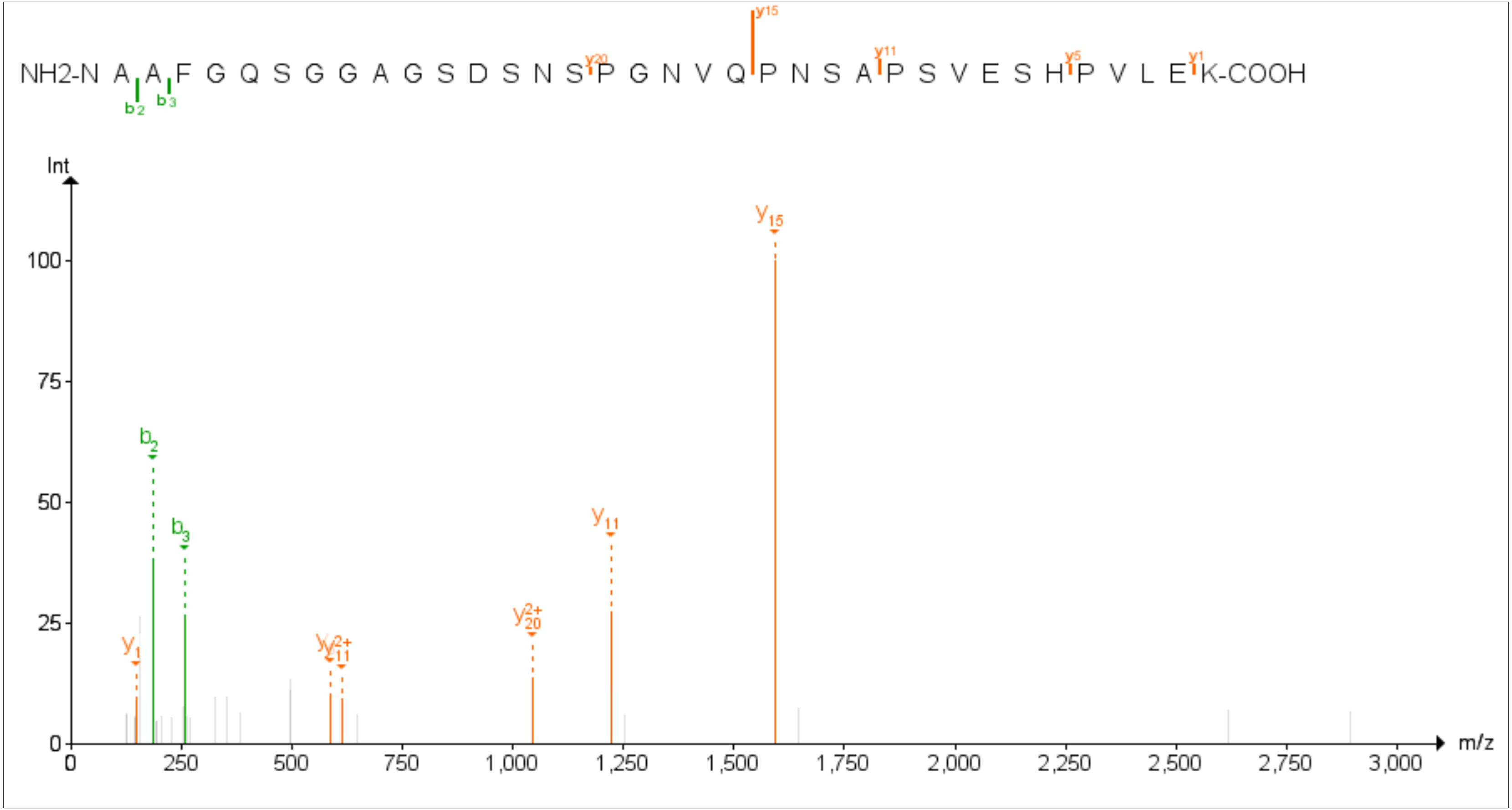

NH2-N A A F G Q S G G A G S D S N S P G N V Q P N S A P S V E S H P V L E K-COOH
b2
b3
y20
y15
y11
y5
y1
Int
100
75
50
25
0
b2
b3
y1
y11
y2+
y2+ 20
y11
y15
m/z
0
250
500
750
1,000
1,250
1,500
1,750
2,000
2,250
2,500
2,750
3,000

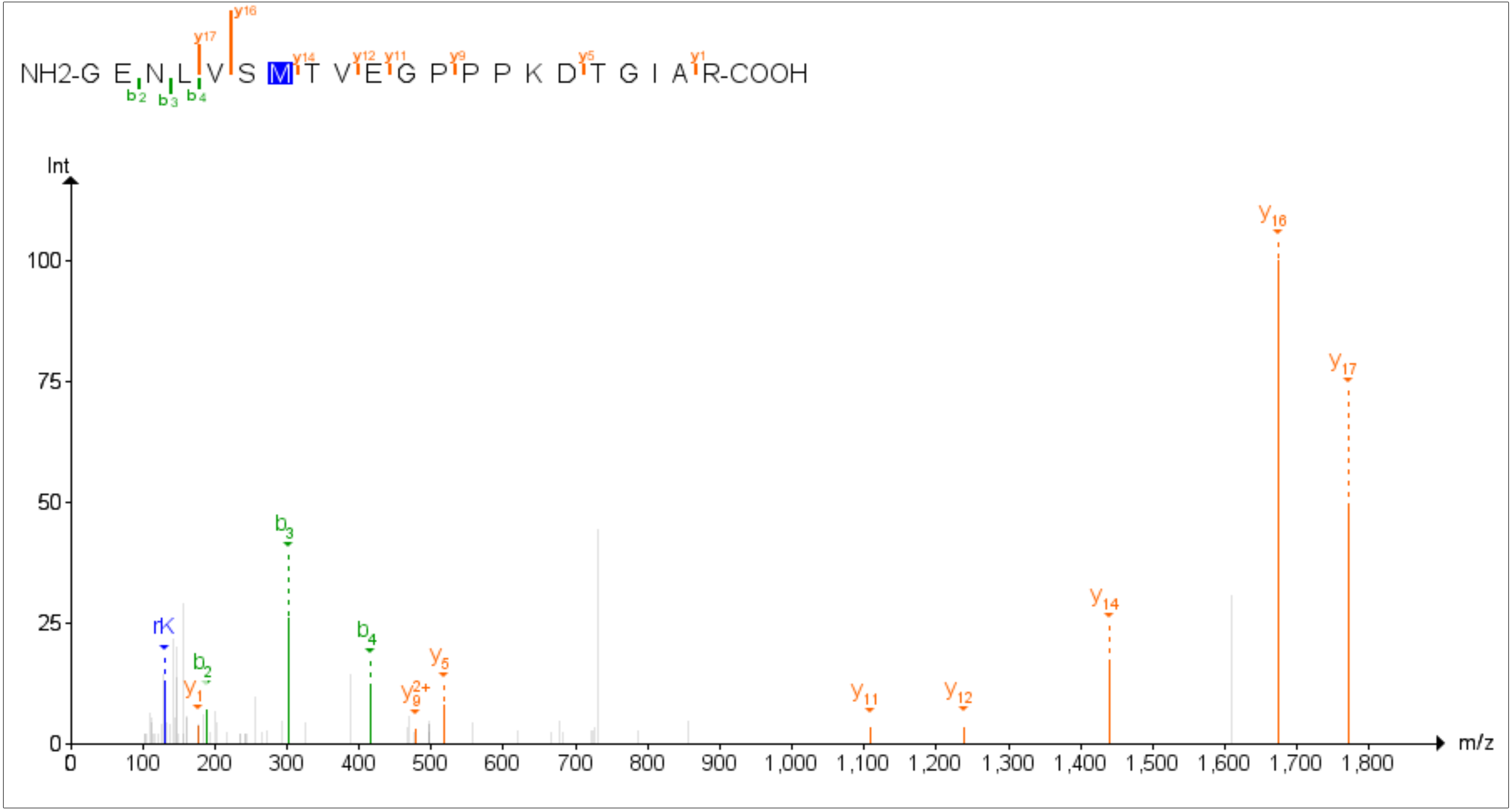

NH2-G E N L V S M T V E G P P P K D T G I A R-COOH
b2
b3
b4
y17
y16
y14
y12
y11
y9
y5
y1
Int
100
75
50
25
0
m/z
0
100
200
300
400
500
600
700
800
900
1,000
1,100
1,200
1,300
1,400
1,500
1,600
1,700
1,800
rK
y1
b2
b3
b4
y9 2+
y5
y11
y12
y14
y16
y17

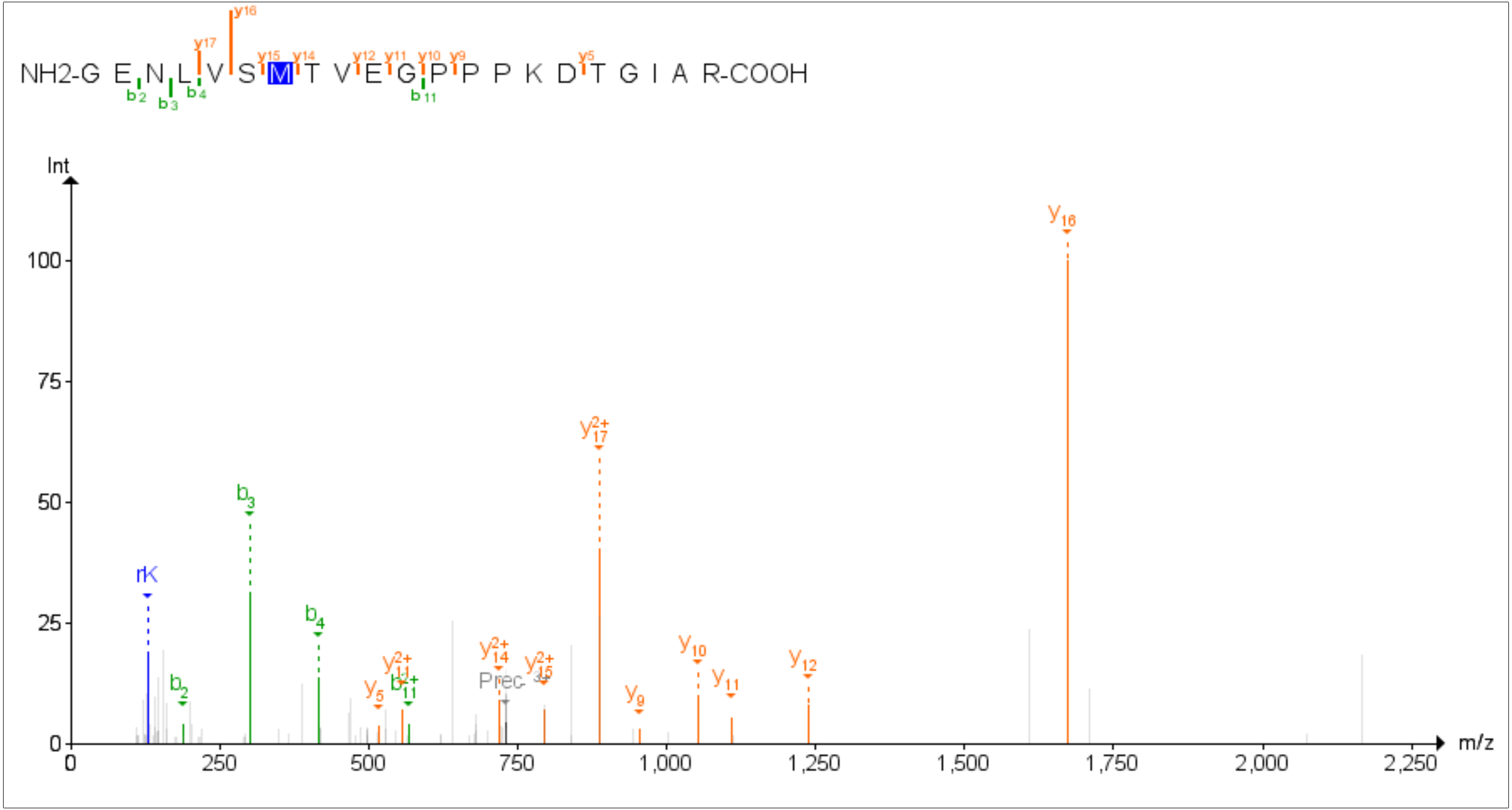

NH2-G E N L V S M T V E G P P P K D T G I A R-COOH
y16
y17
y15
y14
y12
y11
y10
y9
y5
b2
b3
b4
b11
Int
100
75
50
25
0
m/z
0
250
500
750
1,000
1,250
1,500
1,750
2,000
2,250

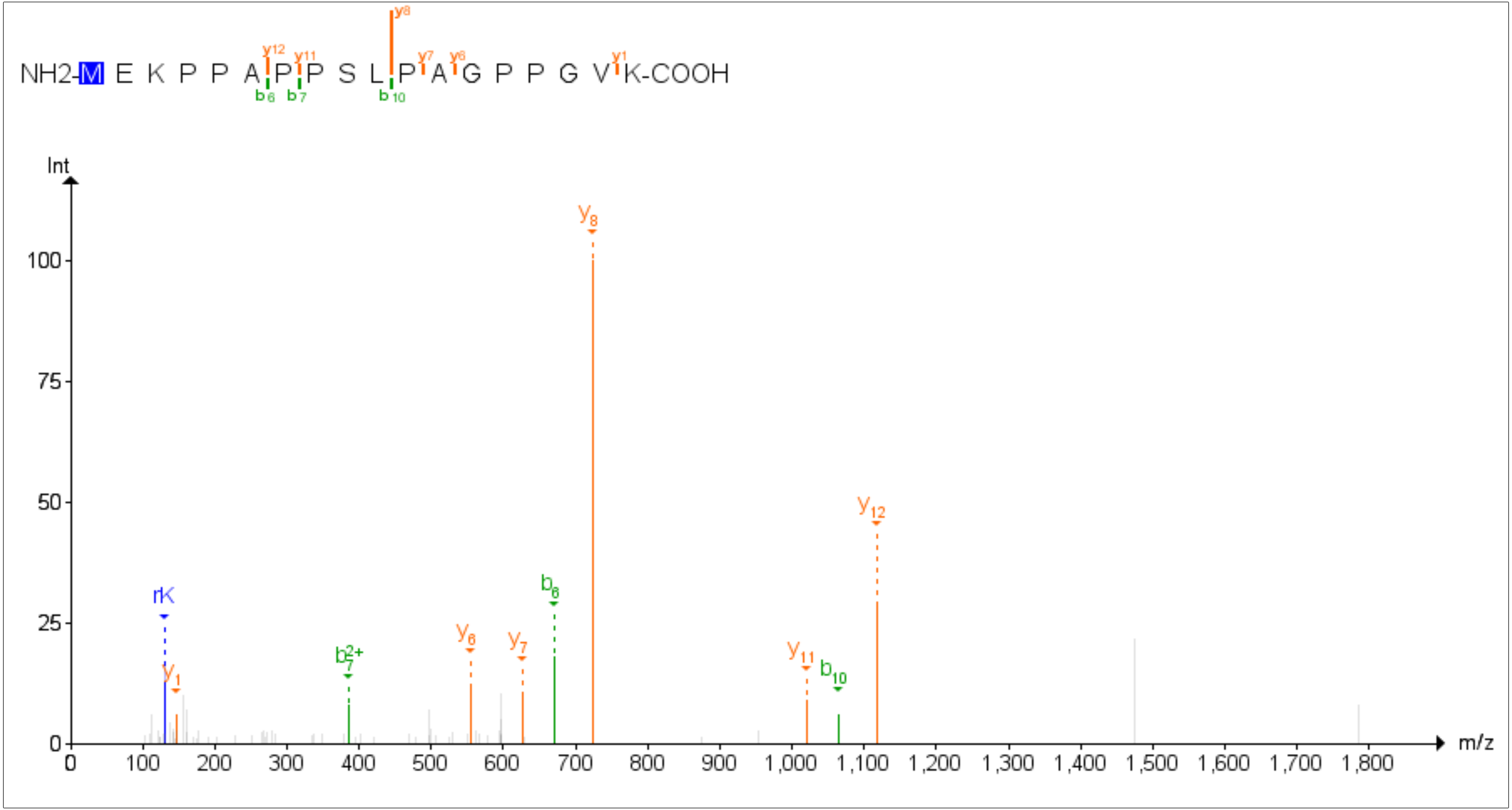

NH2-M E K P P A P P S L P A G P P G V K-COOH
y12 y11 y8 y7 y6 y1
b6 b7 b10
Int
100
75
50
25
0
y8
y12
rK
y1
b7 2+
y6
y7
b6
y11
b10
0 100 200 300 400 500 600 700 800 900 1,000 1,100 1,200 1,300 1,400 1,500 1,600 1,700 1,800
m/z

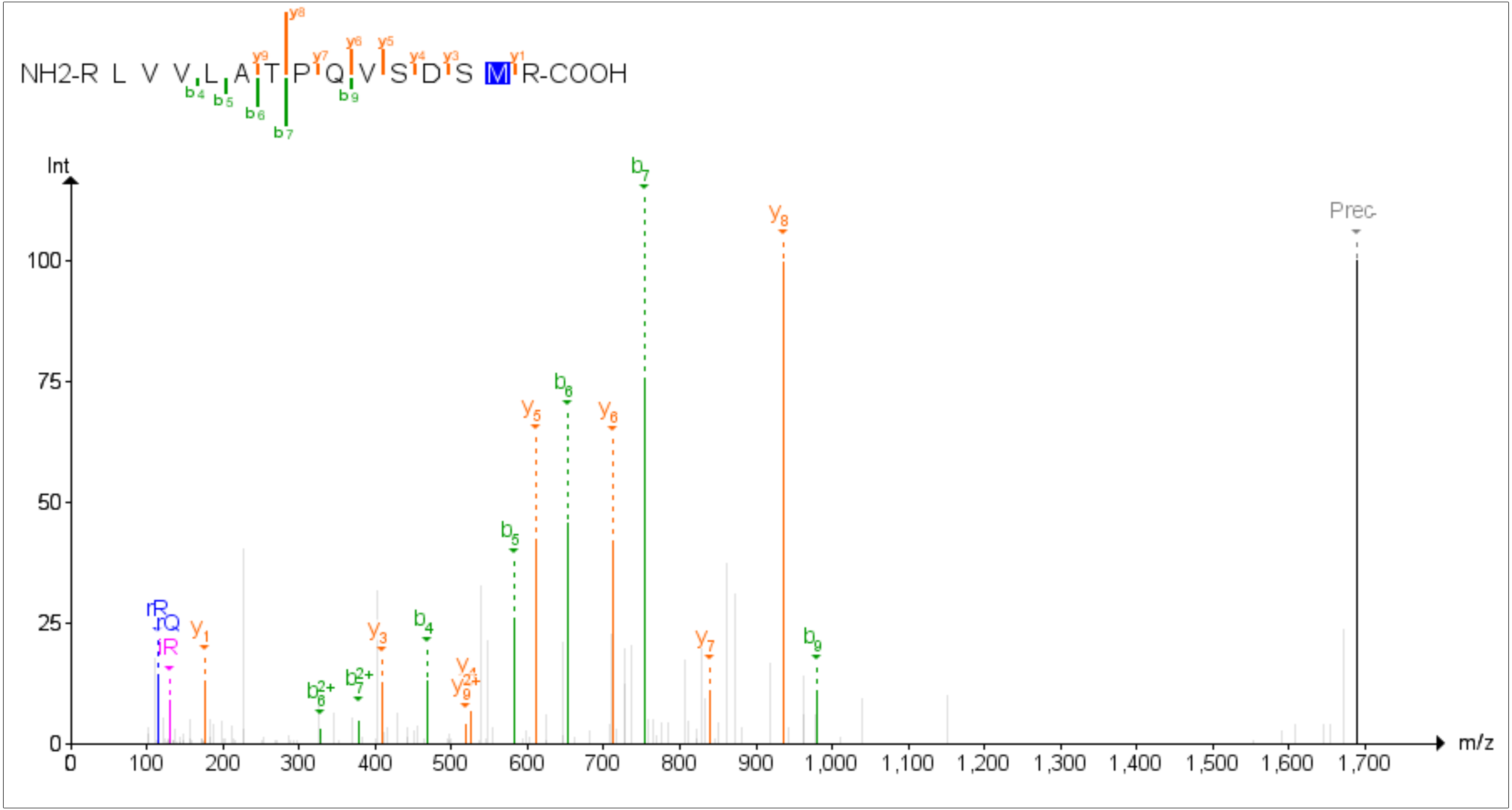

NH2-R L V V L A T P Q V S D S M R-COOH
Int
m/z
Prec.
b7
y8
b6
y5
y6
b5
y1
y3
b4
b9
y7

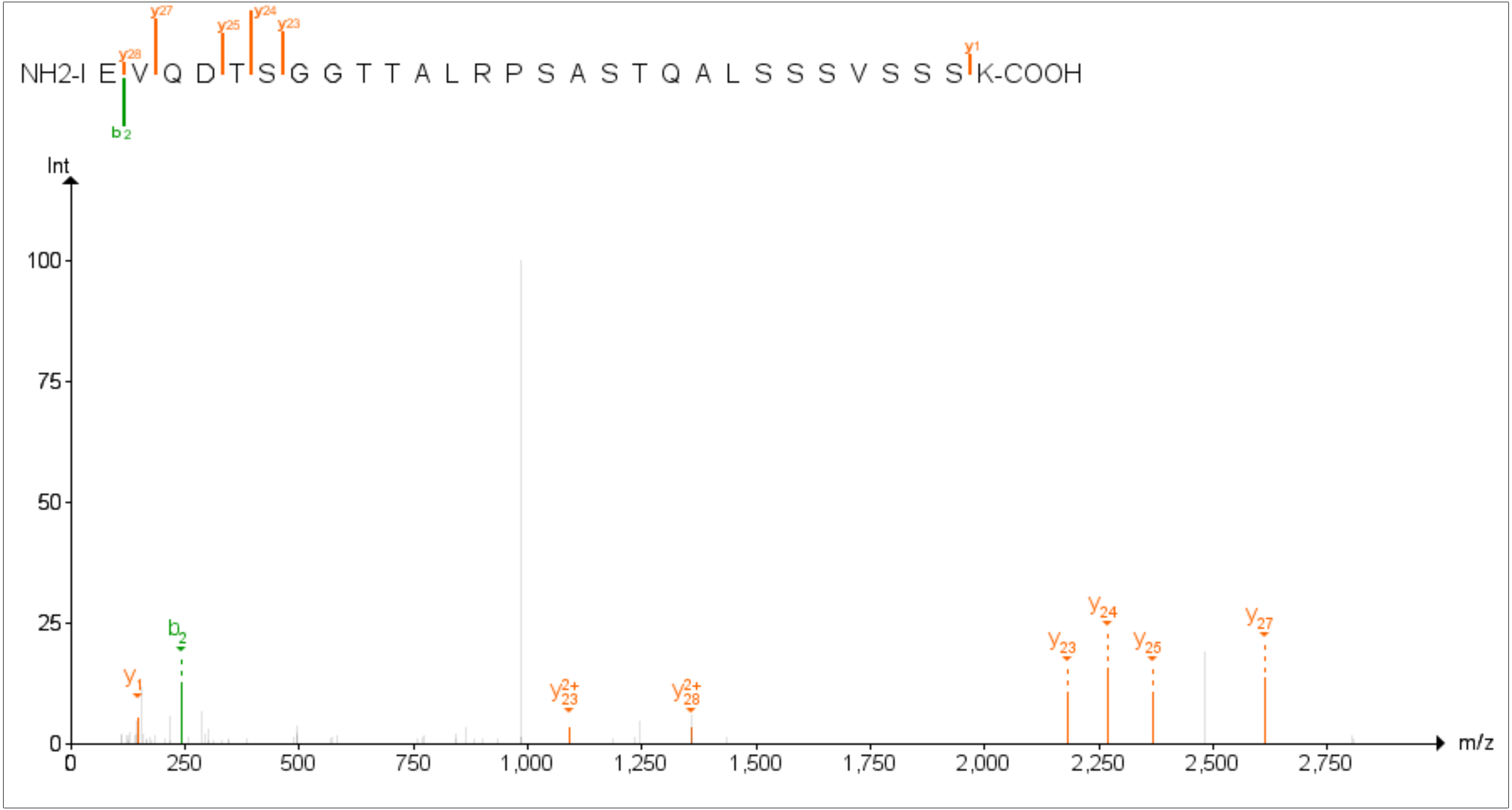

NH2-I E V Q D T S G G T T A L R P S A S T Q A L S S S V S S S K-COOH
y28 y27 y25 y24 y23 y1
b 2
Int
100
75
50
25
0
0 250 500 750 1,000 1,250 1,500 1,750 2,000 2,250 2,500 2,750
m/z
y1
b2
y23 2+
y28 2+
y23
y24
y25
y27

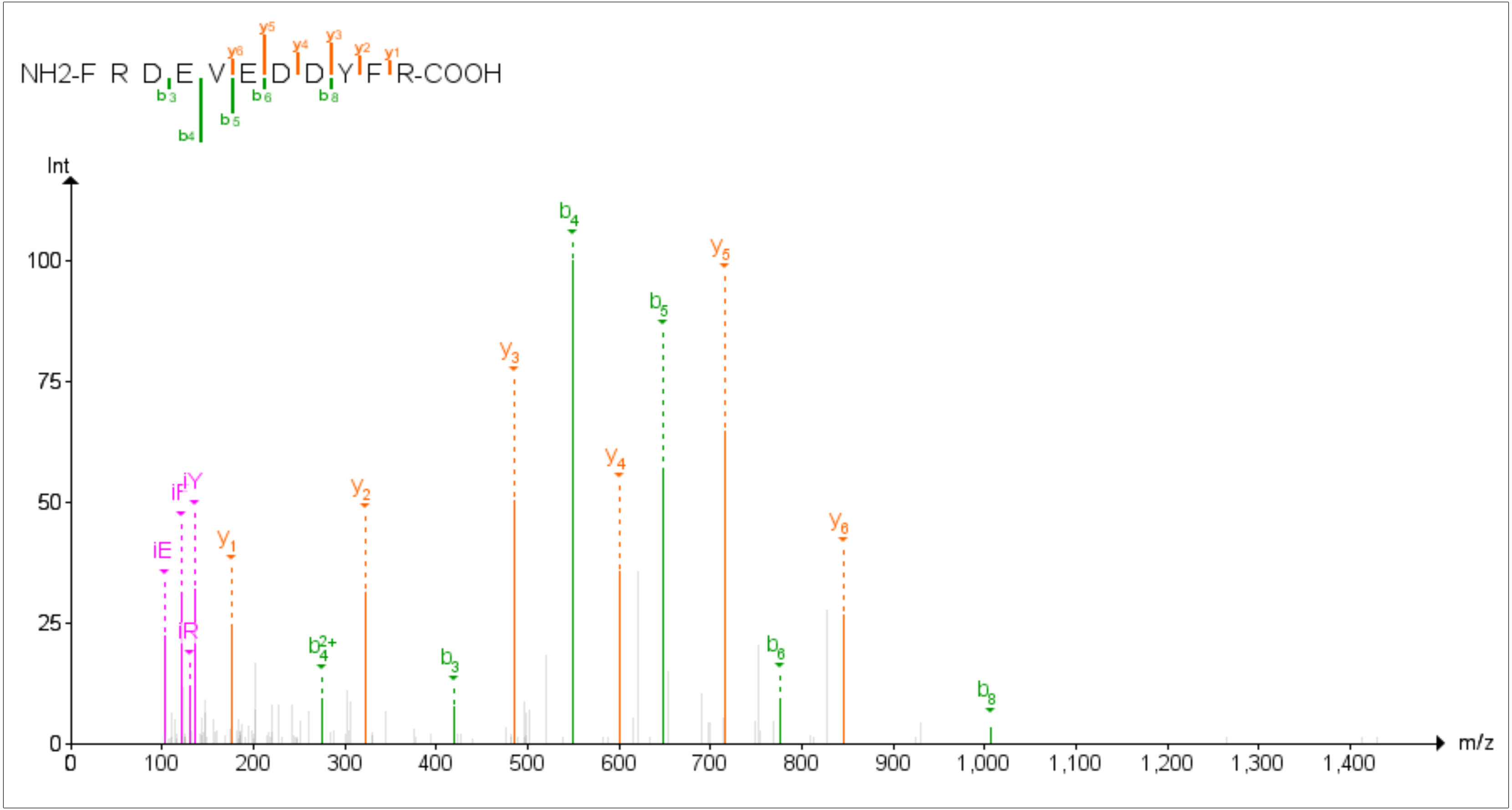

NH2-F R D E V E D D Y F R-COOH
Int
m/z
b4
y5
b5
y3
y4
y2
iY
iF
y6
iE
y1
iR
b4 2+
b3
b6
b8

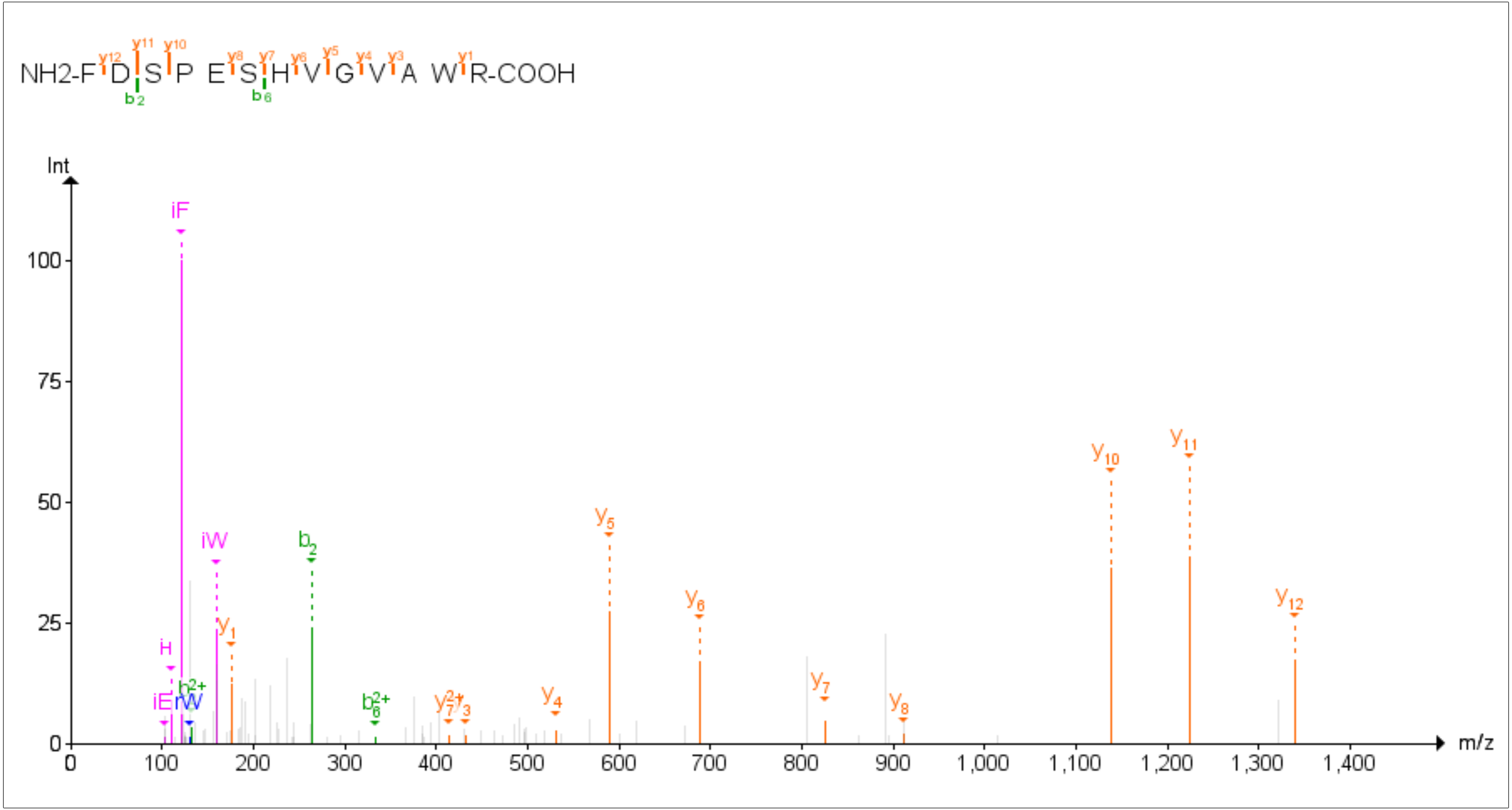

NH2-F D S P E S H V G V A W R-COOH
y12 y11 y10 y8 y7 y6 y5 y4 y3 y1
b2 b6
Int
100
75
50
25
0
iF
iW
b2
y5
y6
y10
y11
y12
y1
iH
iE
rW
y7
y8
y4
0
100
200
300
400
500
600
700
800
900
1,000
1,100
1,200
1,300
1,400
m/z

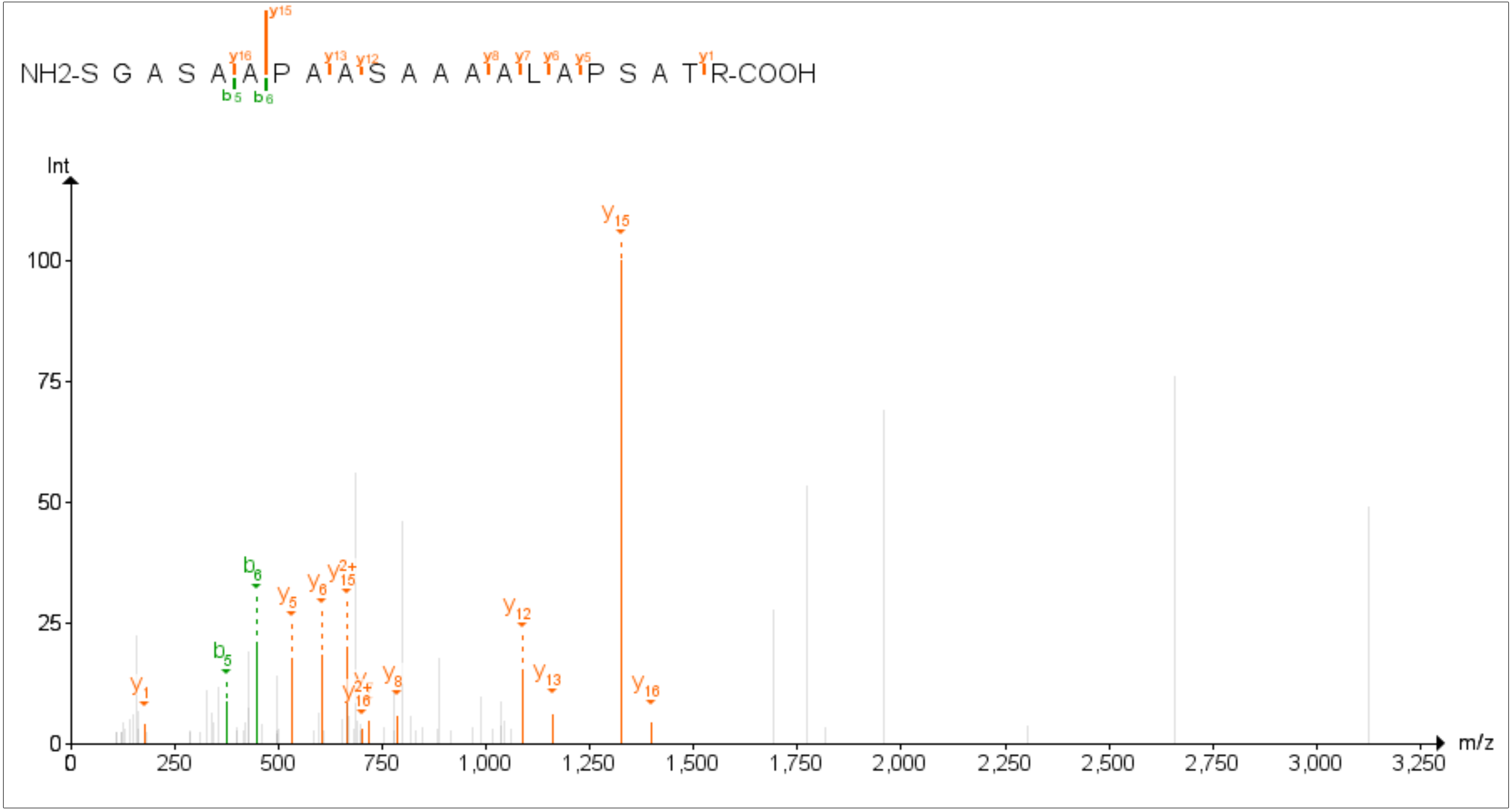

NH2-S G A S A A P A A S A A A A L A P S A T R-COOH
y15
y16
y13
y12
y8
y7
y6
y5
y1
b5
b6
Int
100
75
50
25
0
y15
b6
y5
y6
y^{2+}_{15}
y12
b5
y1
y^{2+}_{16}
y8
y13
y16
0
250
500
750
1,000
1,250
1,500
1,750
2,000
2,250
2,500
2,750
3,000
3,250
m/z

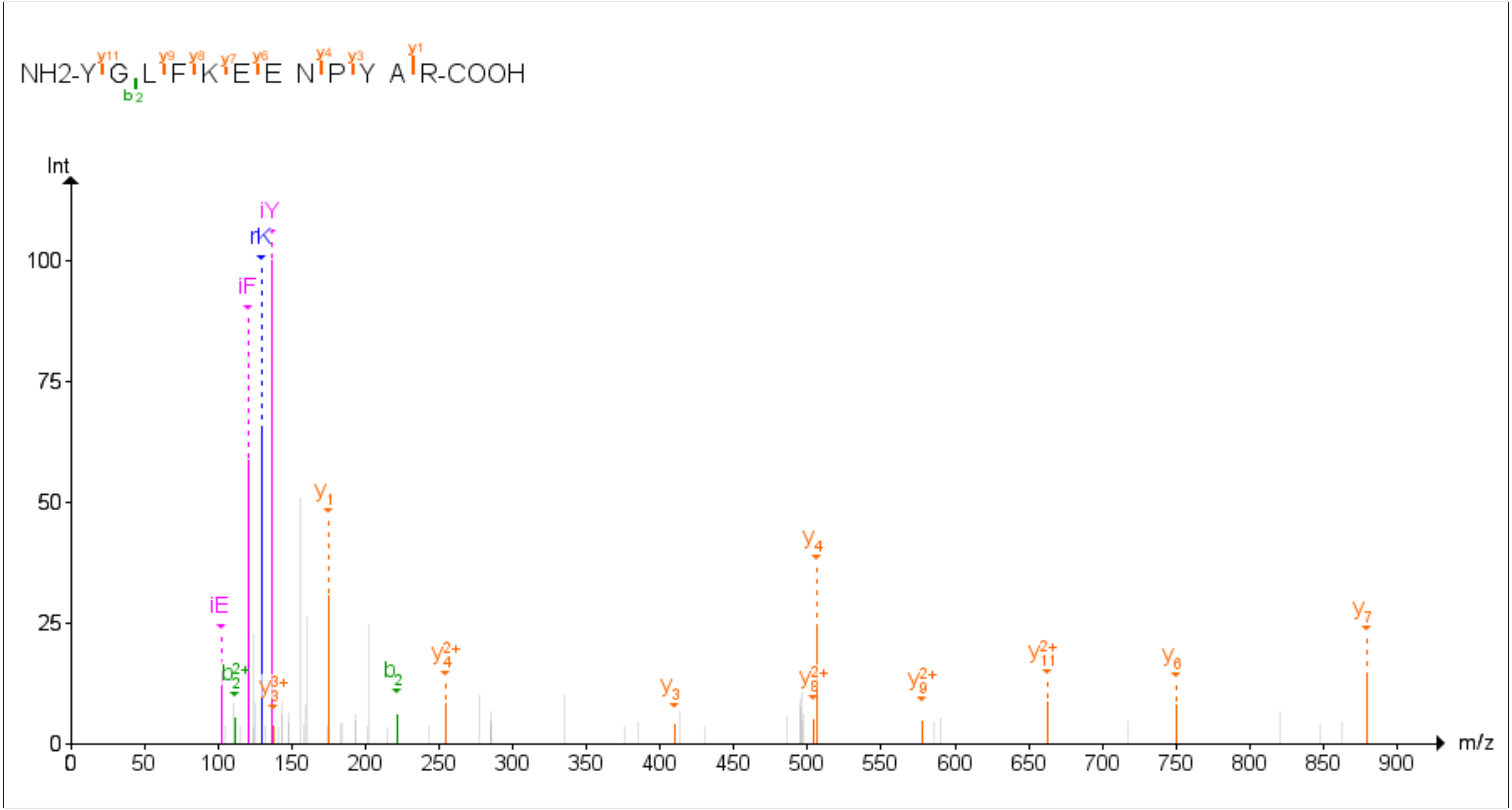

NH2-Y G L F K E E N P Y A R-COOH
Int
m/z
iY
rK
iF
iE
y1
y4
y7
y6
y3
b2
y2+4
y2+8
y2+9
y2+11
b2+2
y3+3

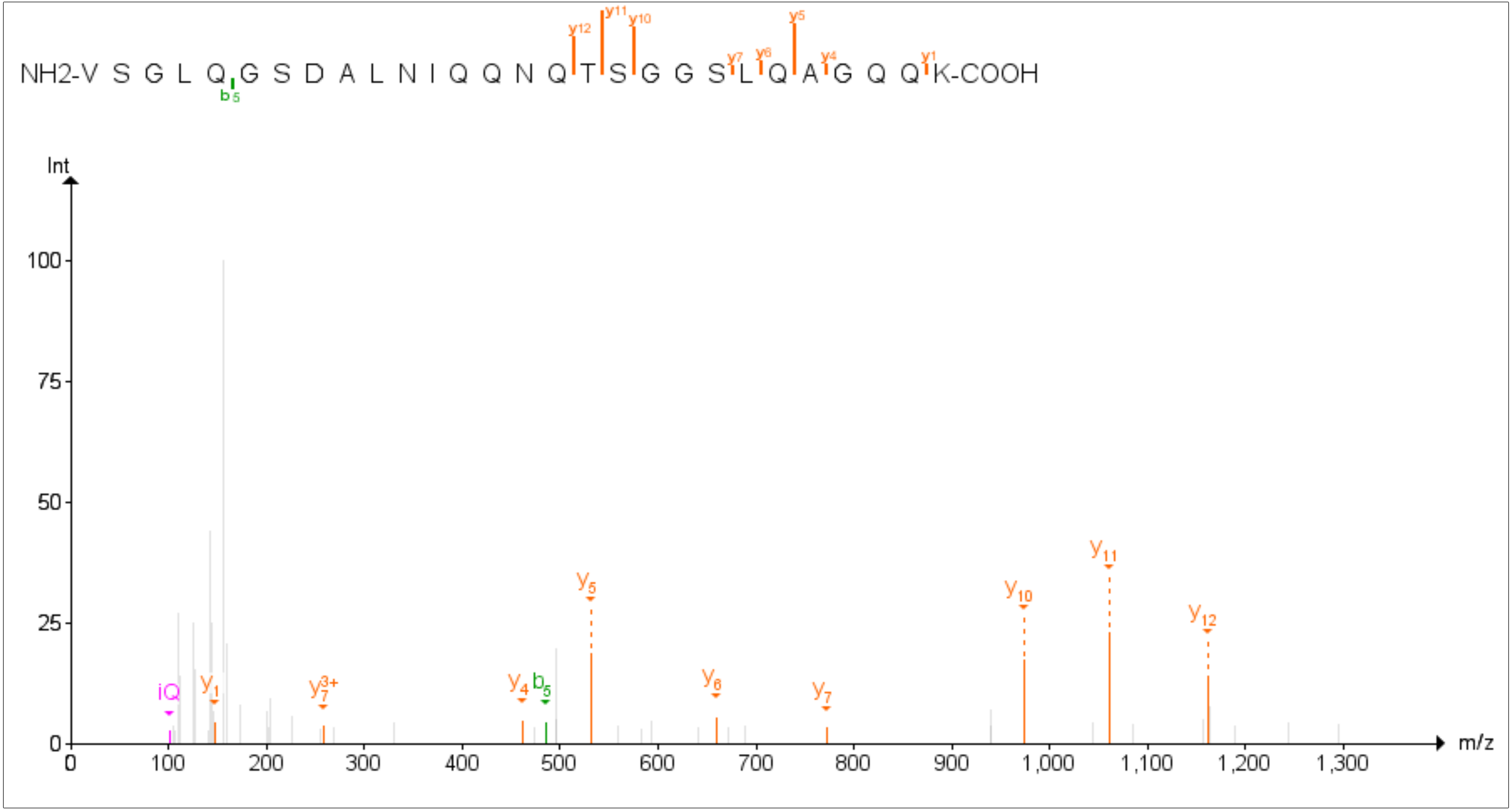

NH2-V S G L Q G S D A L N I Q Q N Q T S G G S L Q A G Q Q K-COOH
b5
y12 y11 y10 y7 y6 y5 y4 y1
Int
100
75
50
25
0
iQ
y1
y7 3+
y4
b5
y5
y6
y7
y10
y11
y12
0 100 200 300 400 500 600 700 800 900 1,000 1,100 1,200 1,300
m/z

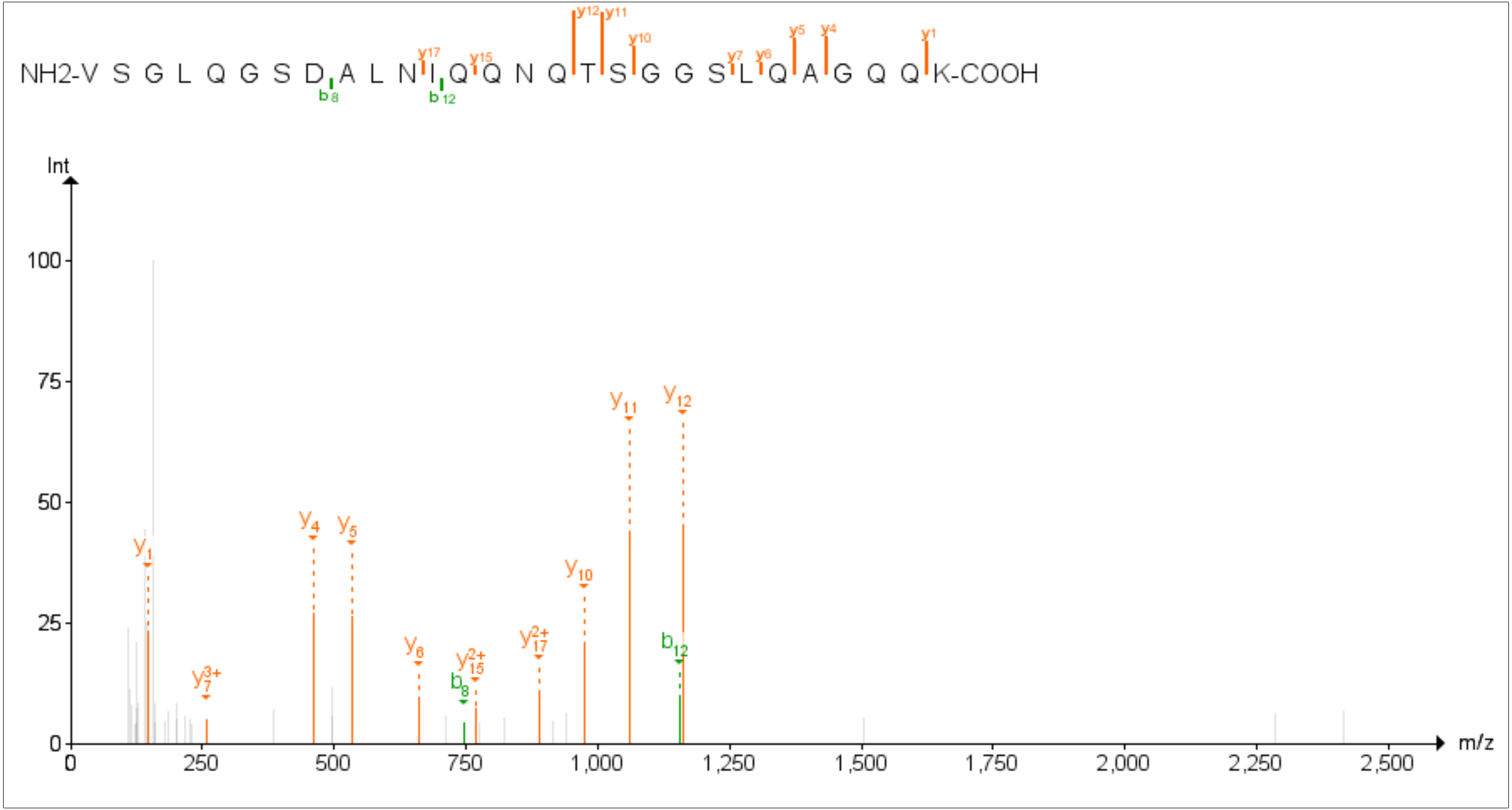
NH2-V S G L Q G S D A L N I Q Q N Q T S G G S L Q A G Q Q K-COOH
y12 y11 y10 y17 y15 y7 y6 y5 y4 y1
b8 b12
Int
m/z
0
25
50
75
100
250
500
750
1,000
1,250
1,500
1,750
2,000
2,250
2,500
y1 y7 3+ y4 y5 y6 b8 y15 2+ y17 2+ y10 y11 y12 b12

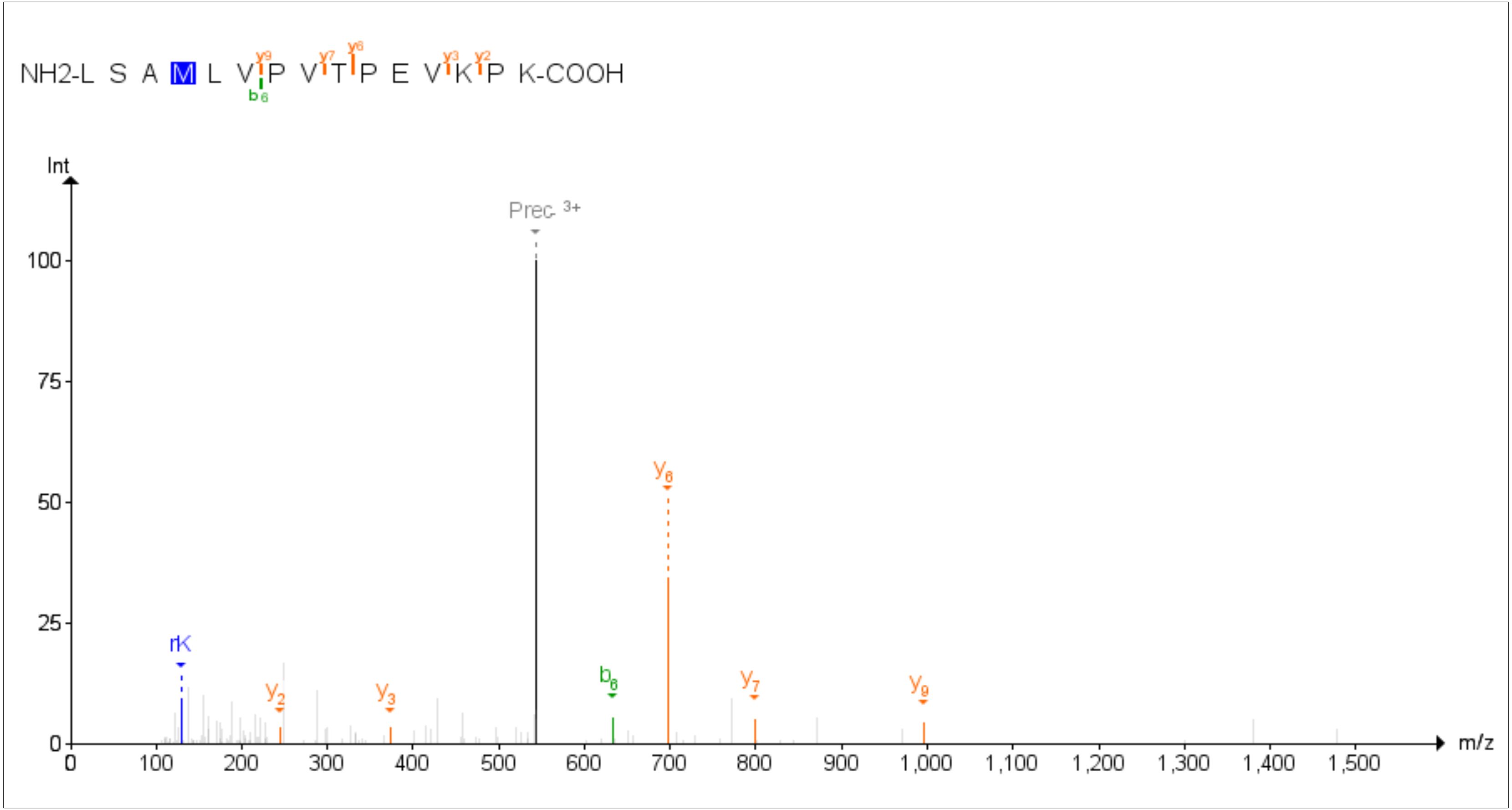

NH2-L S A M L V P V T P E V K P K-COOH
y9
y7
y6
y3
y2
b6
Int
Prec 3+
y6
rK
y2
y3
b6
y7
y9
100
75
50
25
0
0
100
200
300
400
500
600
700
800
900
1,000
1,100
1,200
1,300
1,400
1,500
m/z

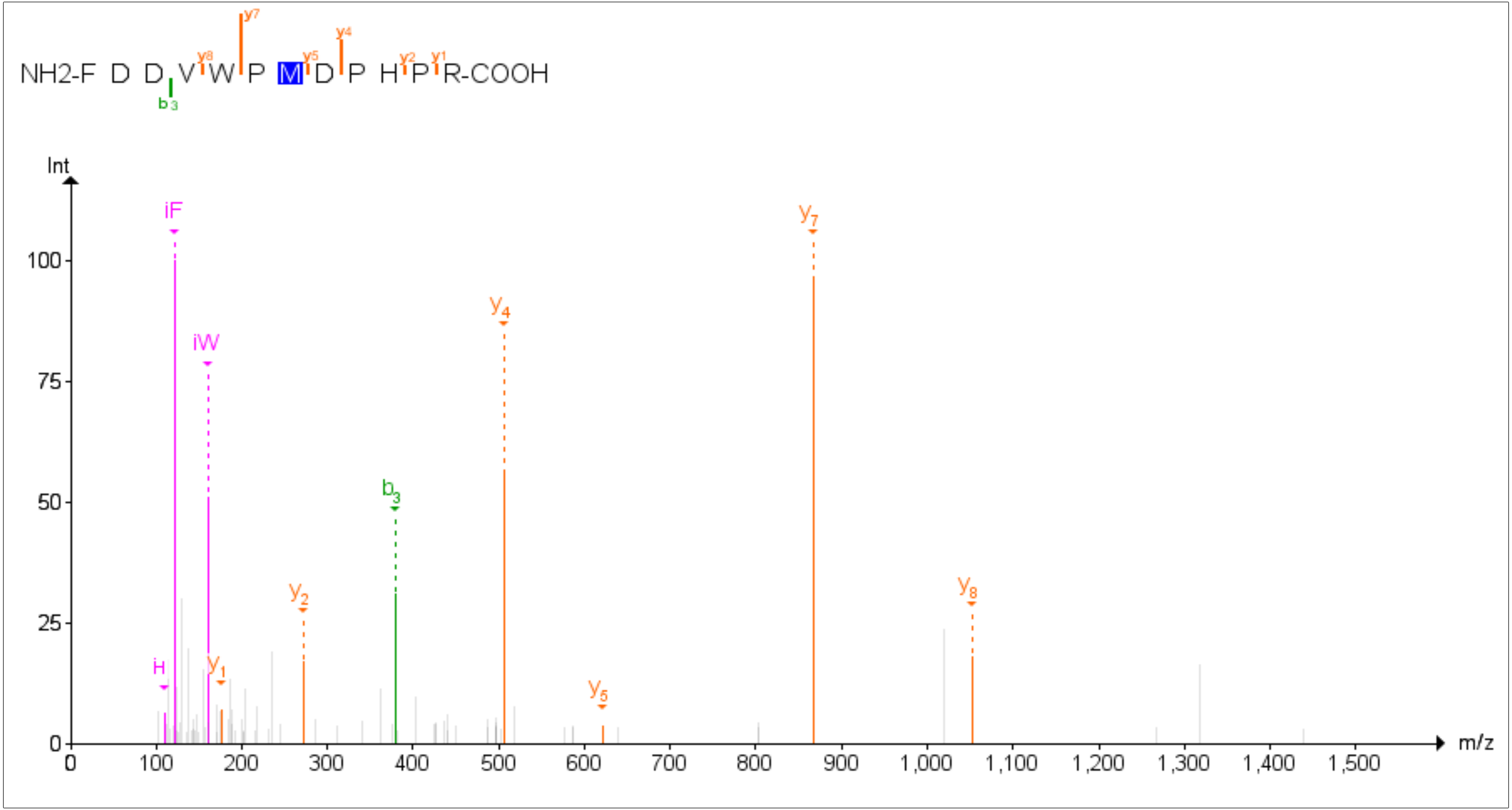

NH2-F D D V W P M D P H P R-COOH
b3
y8 y7 y5 y4 y2 y1
Int
iF
iW
iH
y1
y2
b3
y4
y5
y7
y8
100
75
50
25
0
0 100 200 300 400 500 600 700 800 900 1,000 1,100 1,200 1,300 1,400 1,500
m/z

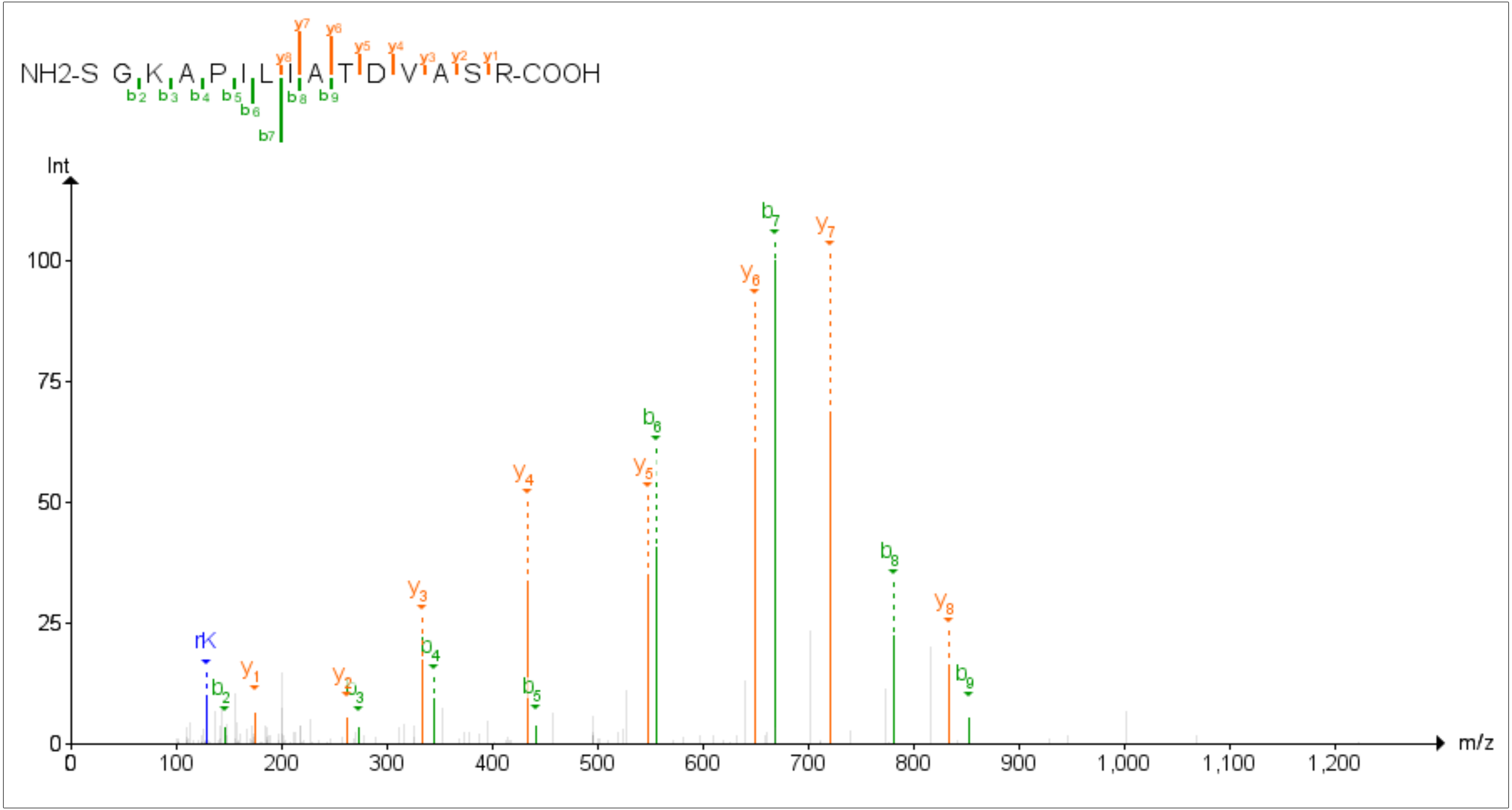

NH2-S G K A P I L I A T D V A S R-COOH
y7 y6 y5 y4 y3 y2 y1 y8
b2 b3 b4 b5 b6 b7 b8 b9
Int
100
75
50
25
0
rK
y1 y2 y3 y4 y5 y6 y7 y8
b2 b3 b4 b5 b6 b7 b8 b9
0 100 200 300 400 500 600 700 800 900 1,000 1,100 1,200
m/z

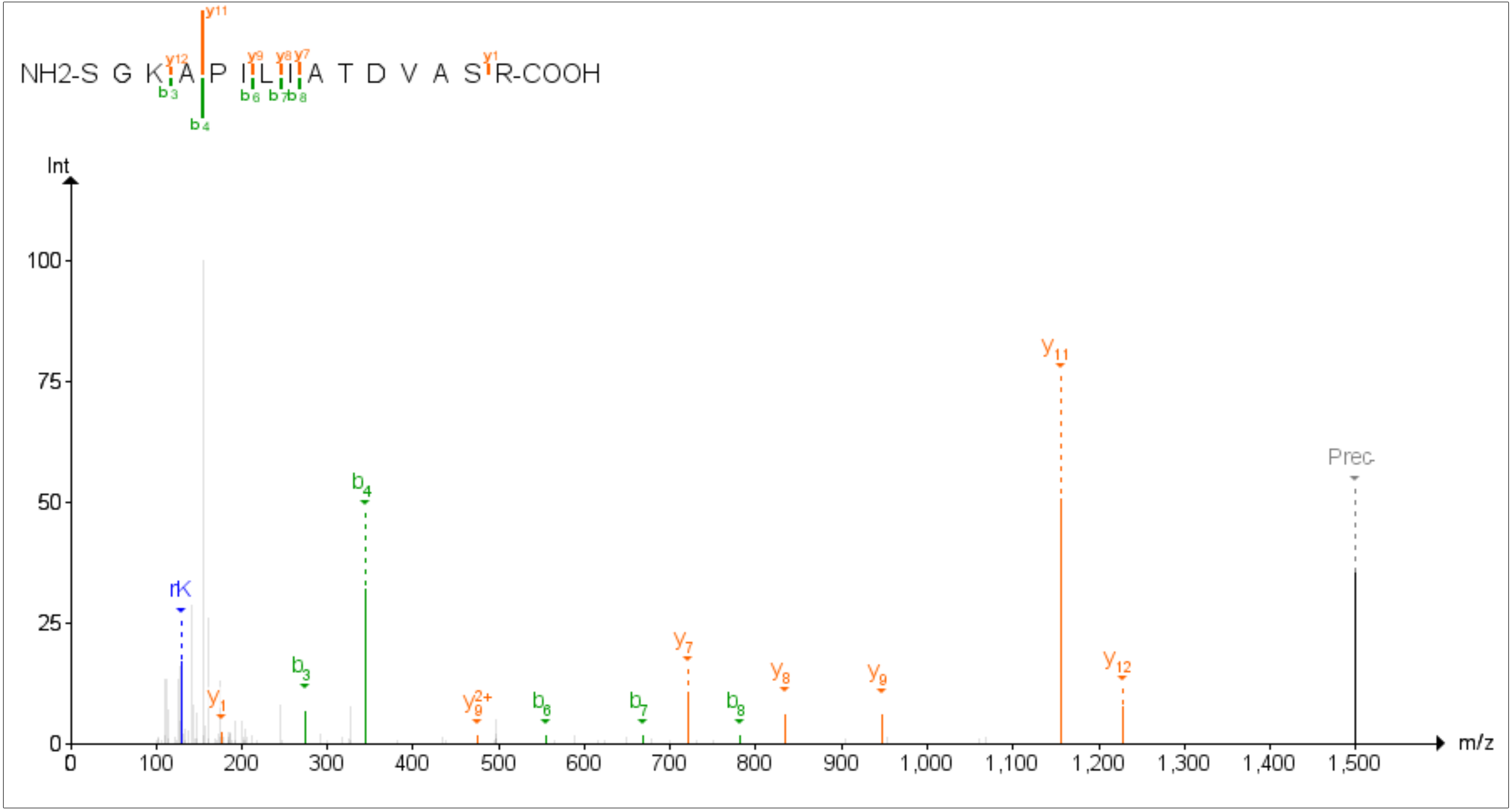

NH2-S G K A P I L I A T D V A S R-COOH
y11
y12
y9
y8
y7
y1
b3
b4
b6
b7
b8
Int
100
75
50
25
0
rK
y1
b3
b4
y9 2+
b6
b7
y7
b8
y8
y9
y11
y12
Prec
0
100
200
300
400
500
600
700
800
900
1,000
1,100
1,200
1,300
1,400
1,500
m/z

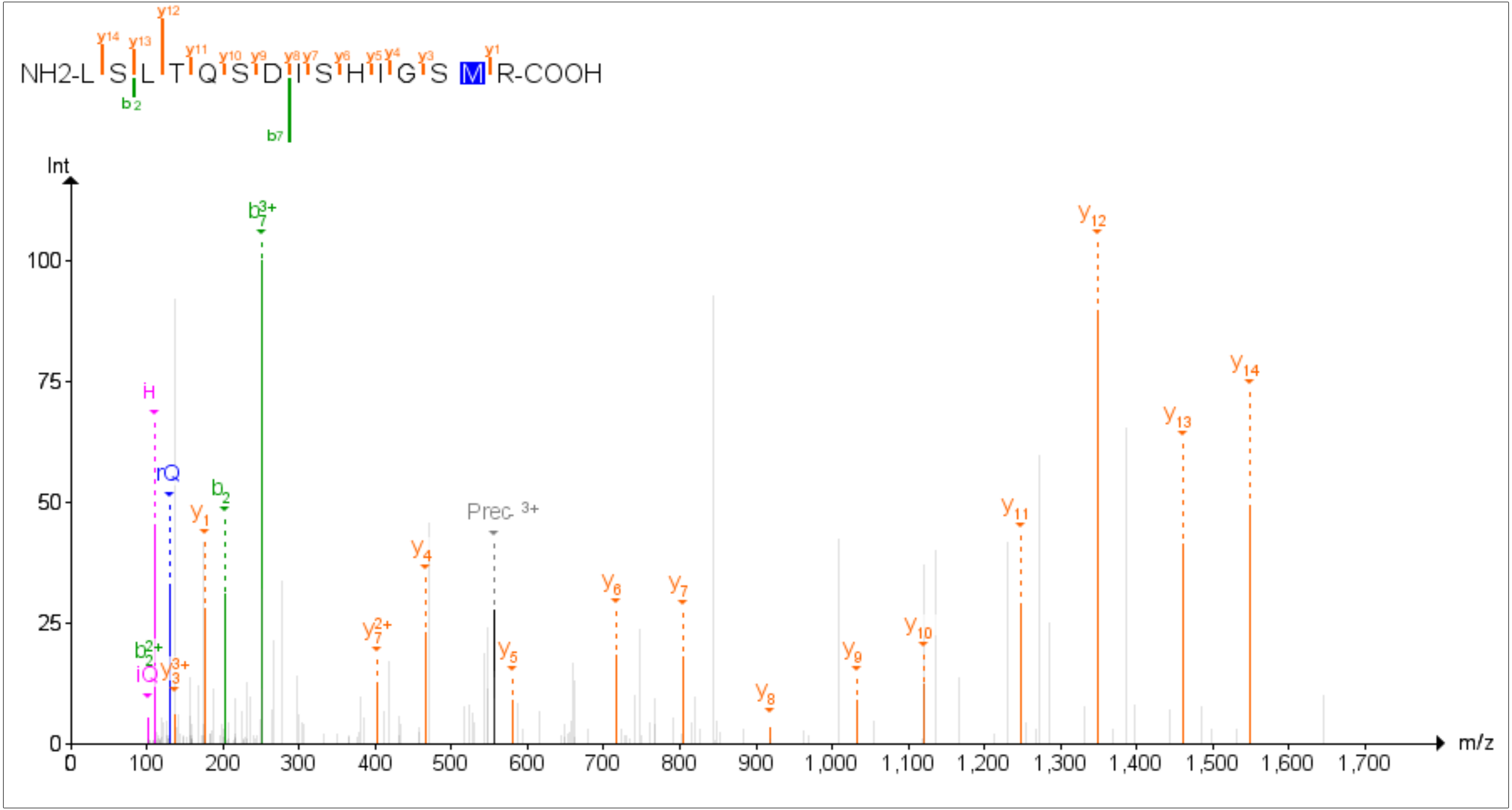

NH2-L S L T Q S D I I S H I G S M R-COOH
y14 y13 y12 y11 y10 y9 y8 y7 y6 y5 y4 y3 y1
b2 b7
Int
100
75
50
25
0
b7 3+
y12
y14
y13
y11
iH
rQ
b2
y1
Prec 3+
y4
y6
y7
y7 2+
y5
y10
y9
y8
b2 2+
iQ
y3 3+
0 100 200 300 400 500 600 700 800 900 1,000 1,100 1,200 1,300 1,400 1,500 1,600 1,700
m/z

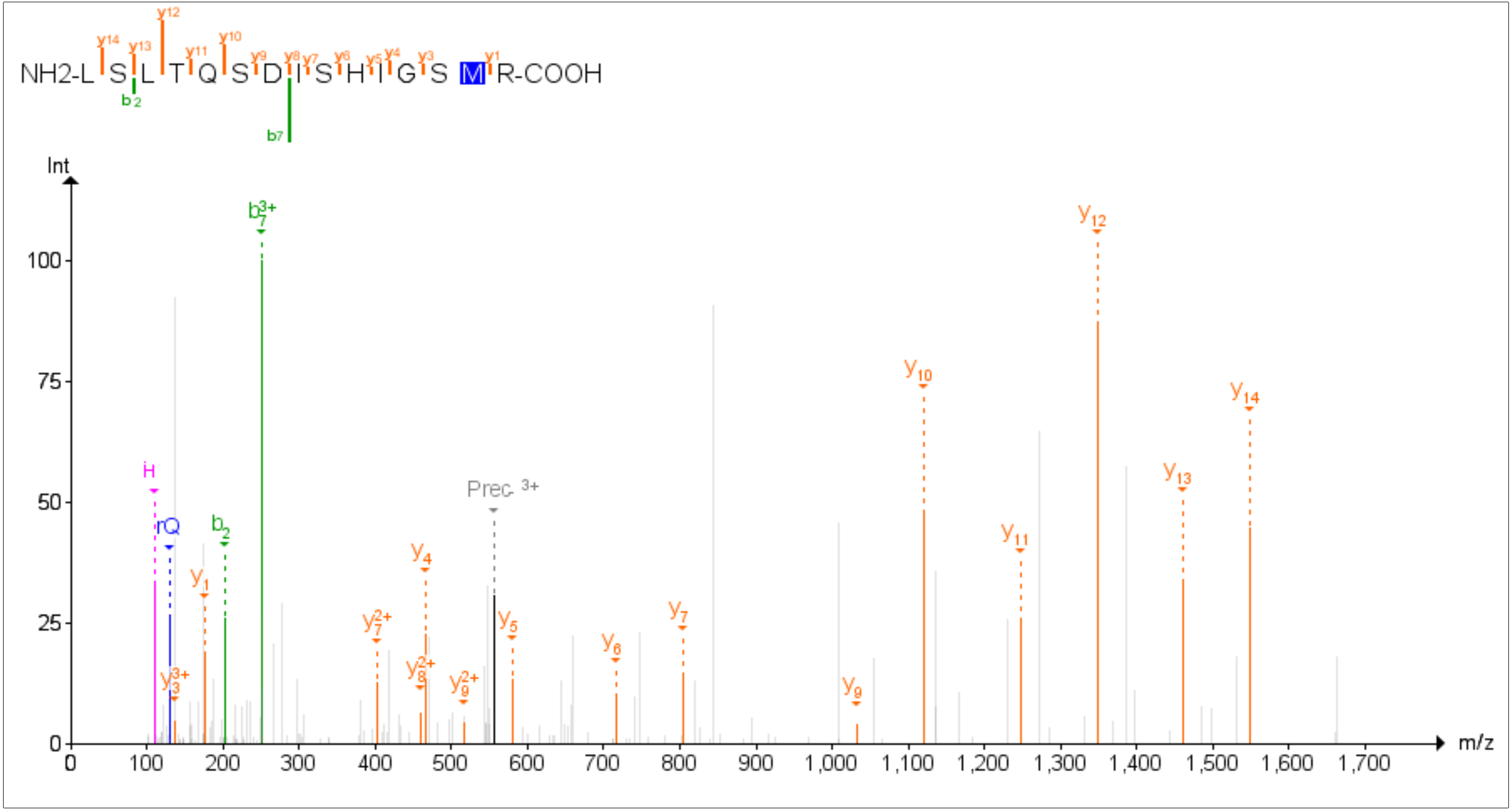

NH2-L S L T Q S D I S H I G S M R-COOH
y14 y13 y12 y11 y10 y9 y8 y7 y6 y5 y4 y3 y1
b2 b7
Int
m/z
Prec 3+

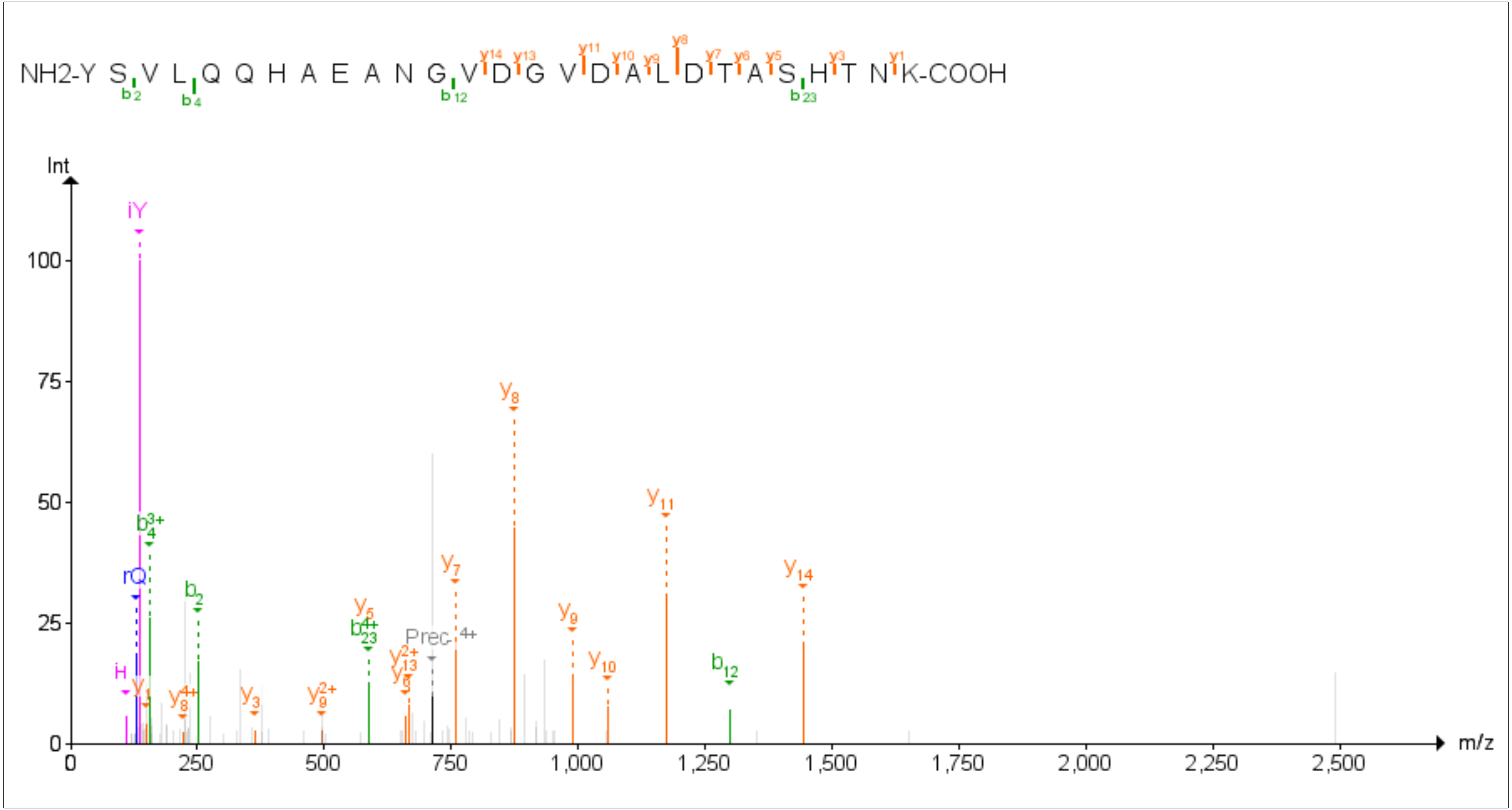
NH2-Y S V L Q Q H A E A N G V D G V D A L D T A S H T N K-COOH
Int
m/z
iY
y8
y11
y7
y14
y9
y10
b12
b2
rQ
iH
Prec 4+
0
25
50
75
100
250
500
750
1,000
1,250
1,500
1,750
2,000
2,250
2,500

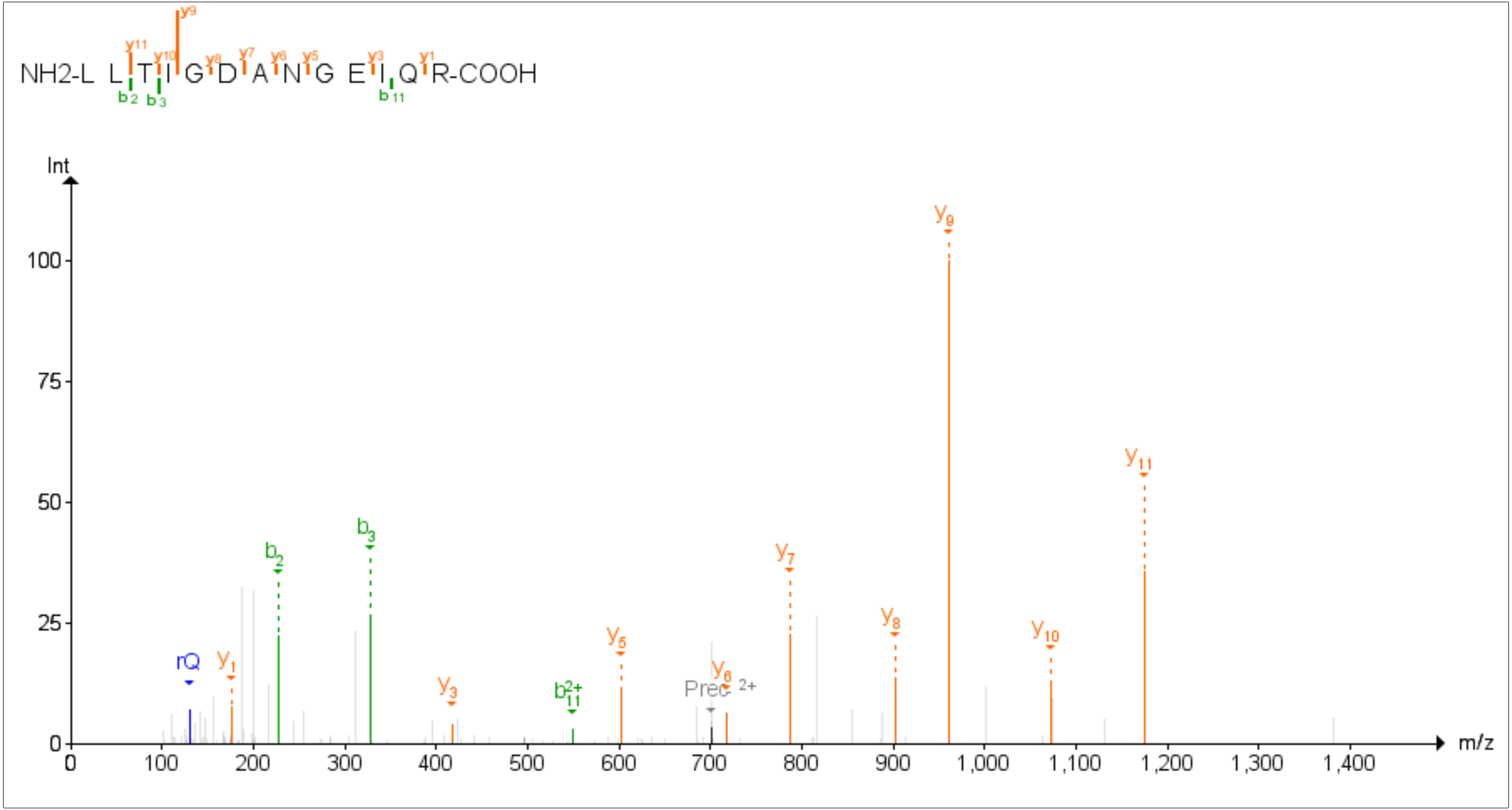

NH2-L L T I G D A N G E I Q R-COOH
y9
y11
y10
y8
y7
y6
y5
y3
y1
b2
b3
b11
Int
100
75
50
25
0
m/z
0
100
200
300
400
500
600
700
800
900
1,000
1,100
1,200
1,300
1,400
rQ
y1
b2
b3
y3
b11 2+
y5
Prec 2+
y6
y7
y8
y9
y10
y11

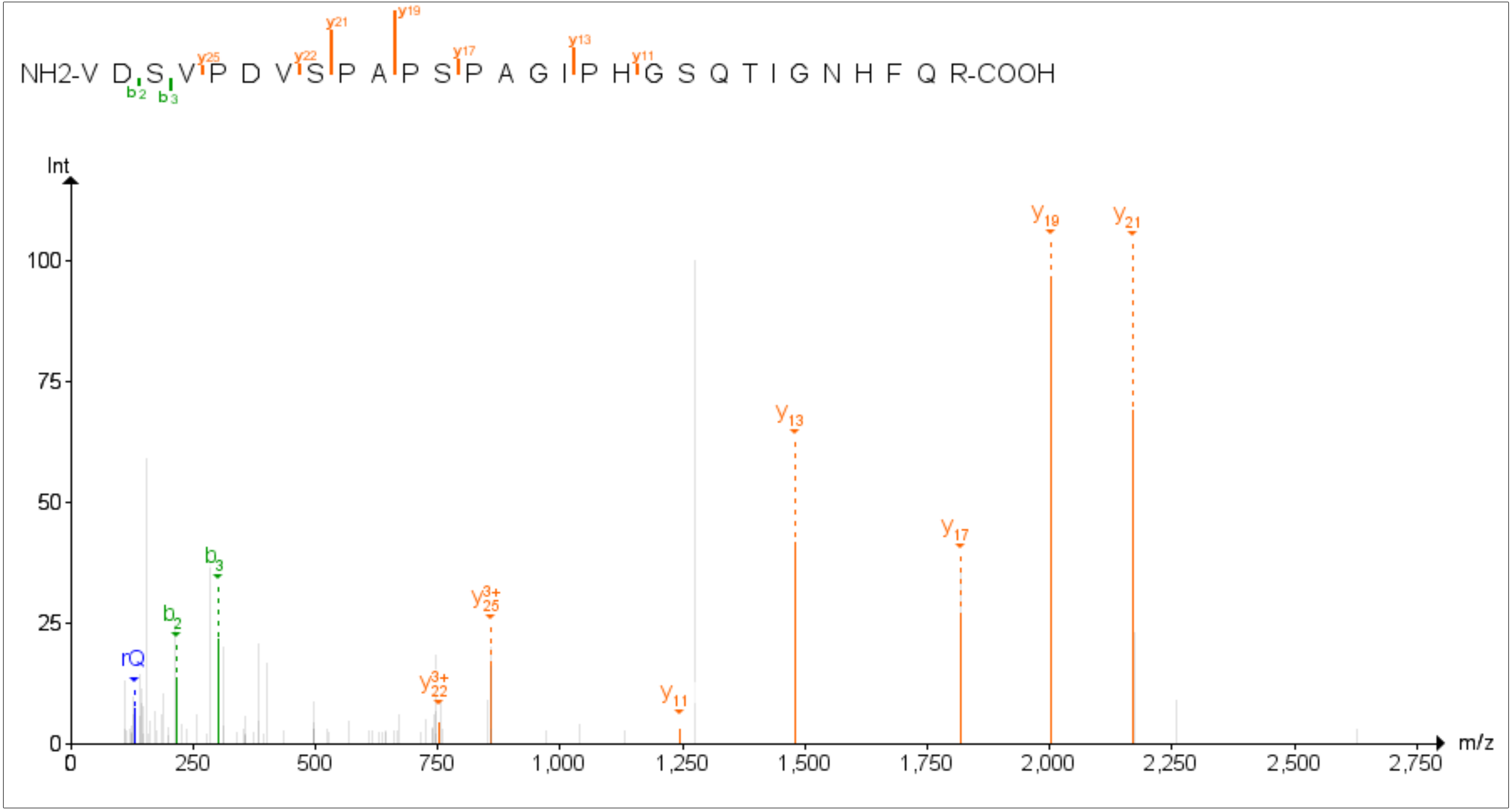

NH2-V D S V P D V S P A P S P A G I P H G S Q T I G N H F Q R-COOH
b2
b3
y25
y22
y21
y19
y17
y13
y11
Int
m/z
rQ
b2
b3
y22 3+
y25 3+
y11
y13
y17
y19
y21
0
25
50
75
100
0
250
500
750
1,000
1,250
1,500
1,750
2,000
2,250
2,500
2,750

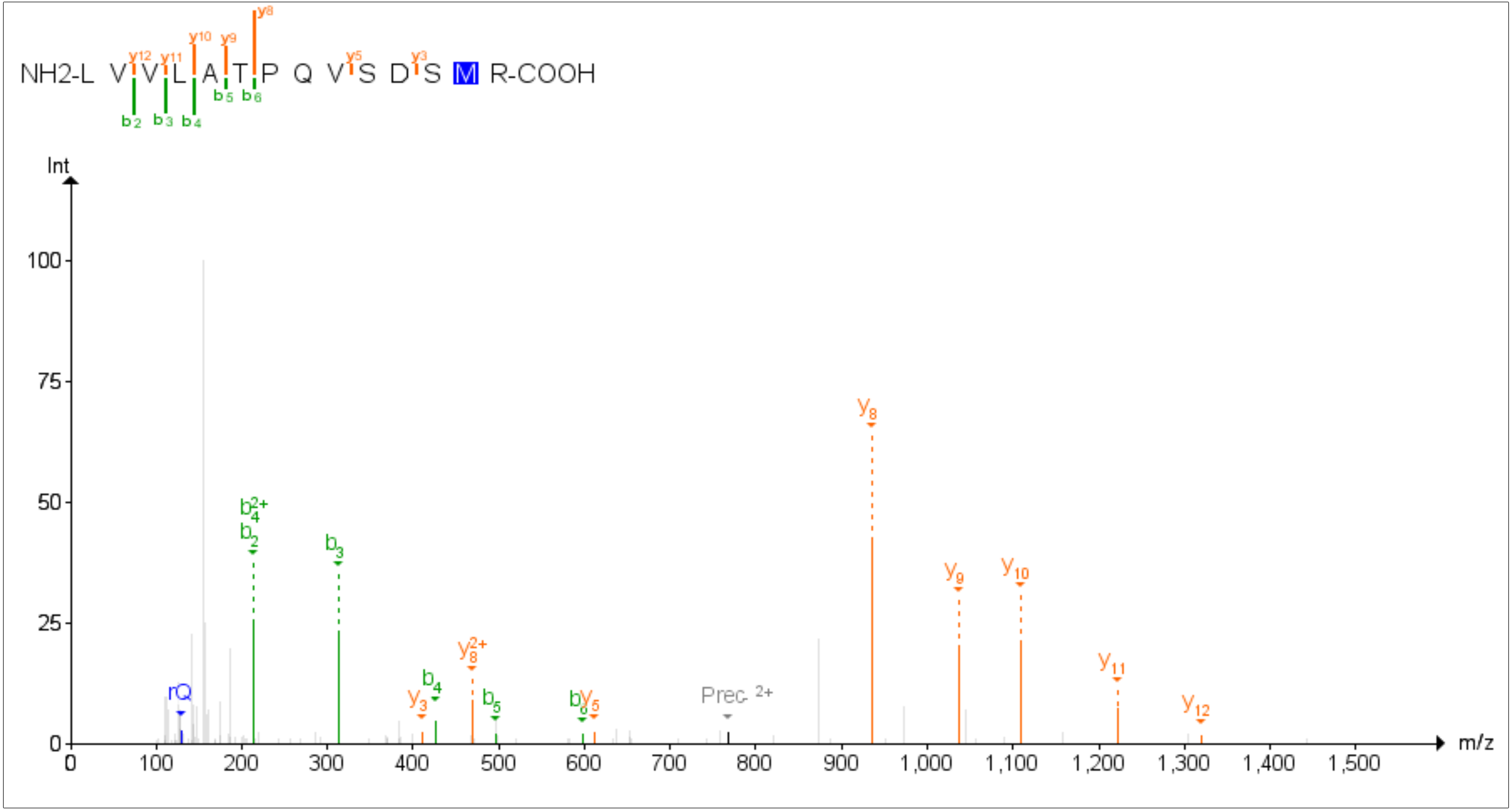
NH2-L V V L A T P Q V S D S M R-COOH
y12 y11 y10 y9 y8 y5 y3
b2 b3 b4 b5 b6
Int
100
75
50
25
0
rQ
b4 2+
b2
b3
y3
b4
y8 2+
b5
b6
y5
Prec 2+
y8
y9
y10
y11
y12
0 100 200 300 400 500 600 700 800 900 1,000 1,100 1,200 1,300 1,400 1,500
m/z

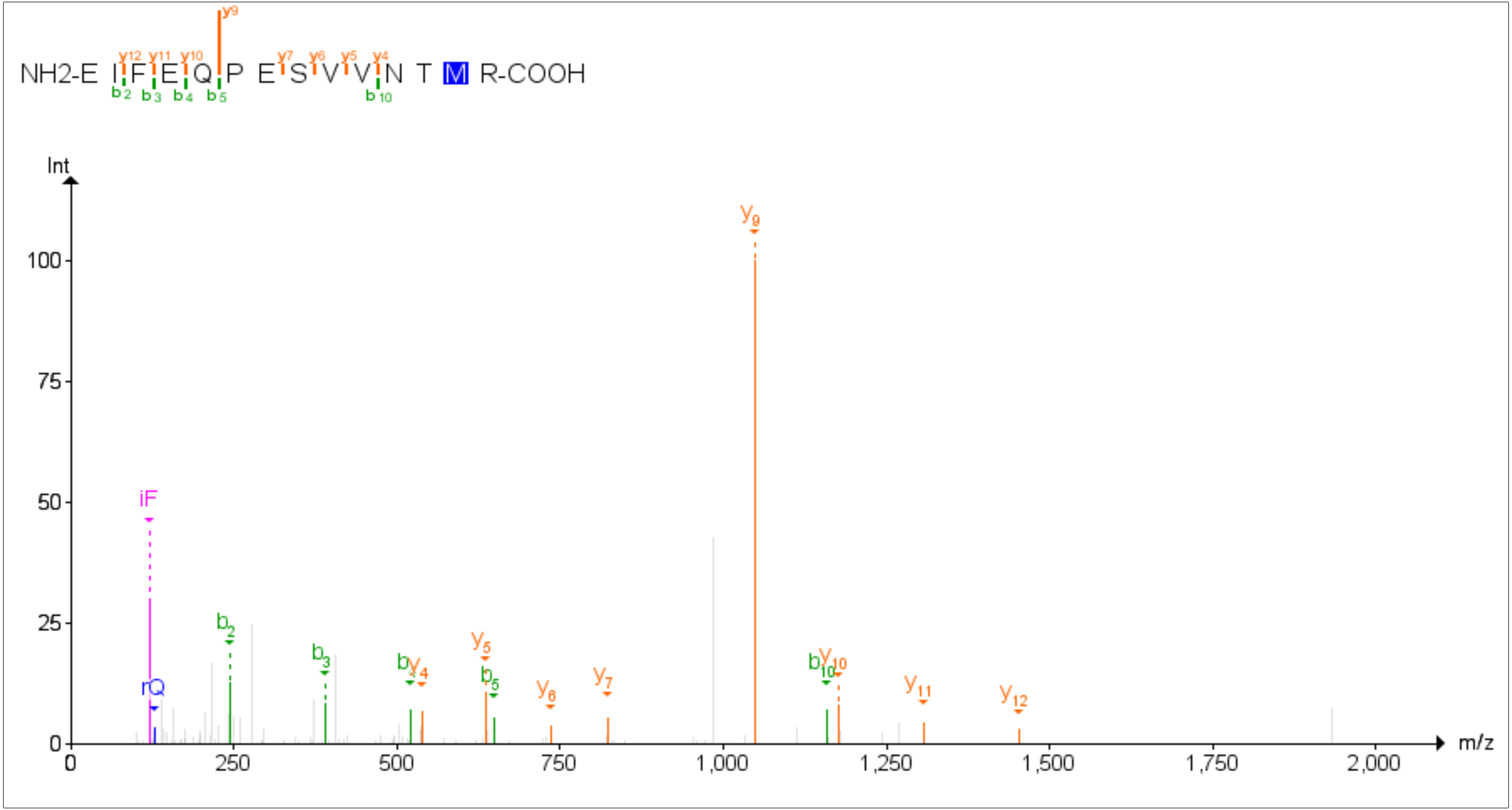
NH2-E I F E Q P E S V V N T M R-COOH
Int
100
75
50
25
0
0
250
500
750
1,000
1,250
1,500
1,750
2,000
m/z
y9
iF
rQ
b2
b3
b4
y4
y5
b5
y6
y7
b10
y10
y11
y12

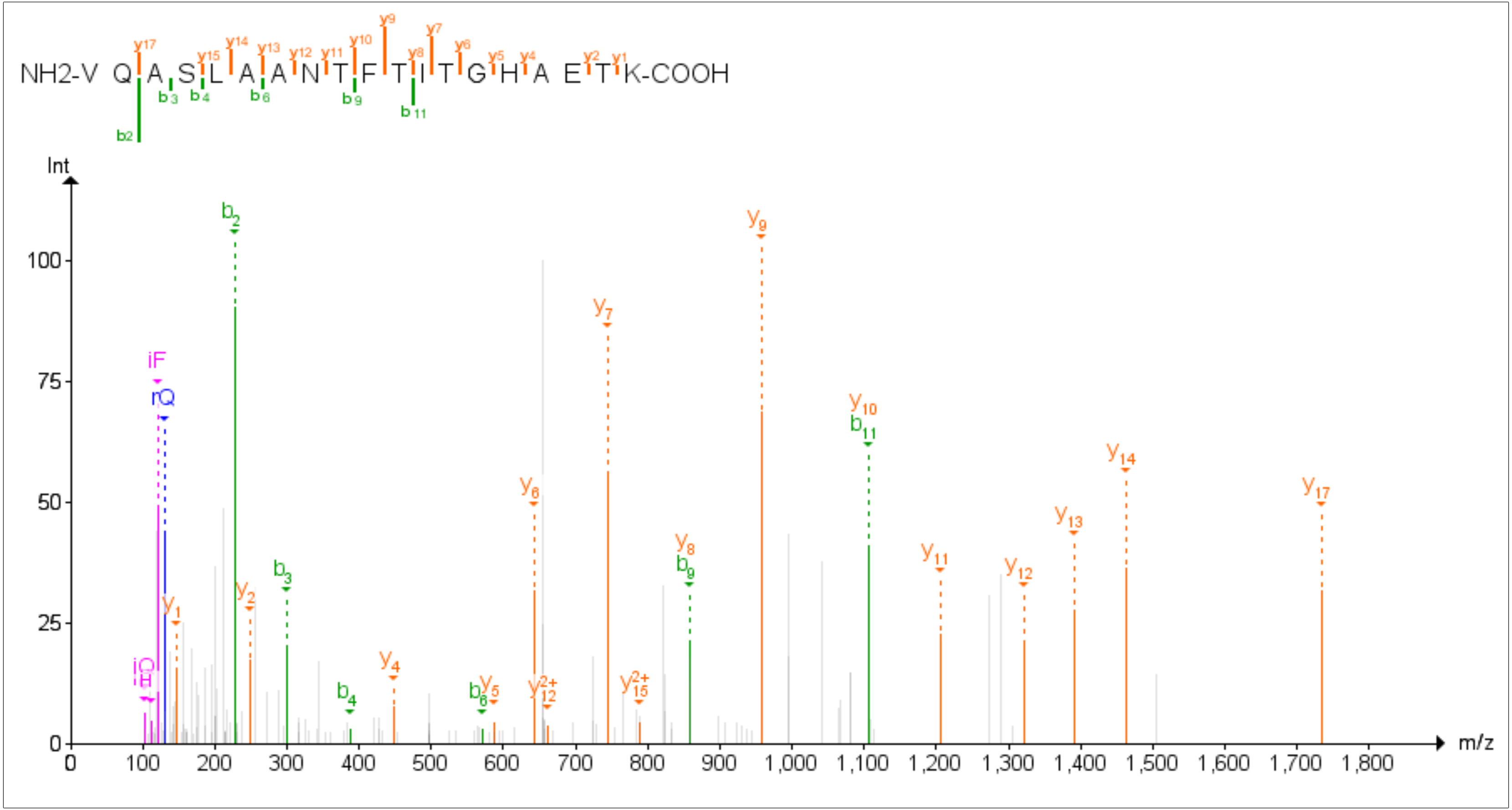
NH2-V Q A S L A A N T F T I T G H A E T K-COOH
Int
m/z
b2
y9
y7
iF
rQ
y10
b11
y14
y17
y6
y13
y8
b9
y11
y12
b3
y2
y1
y4
b4
b6
y5
y_{12}^{2+}
y_{15}^{2+}
0
25
50
75
100
0
100
200
300
400
500
600
700
800
900
1,000
1,100
1,200
1,300
1,400
1,500
1,600
1,700
1,800

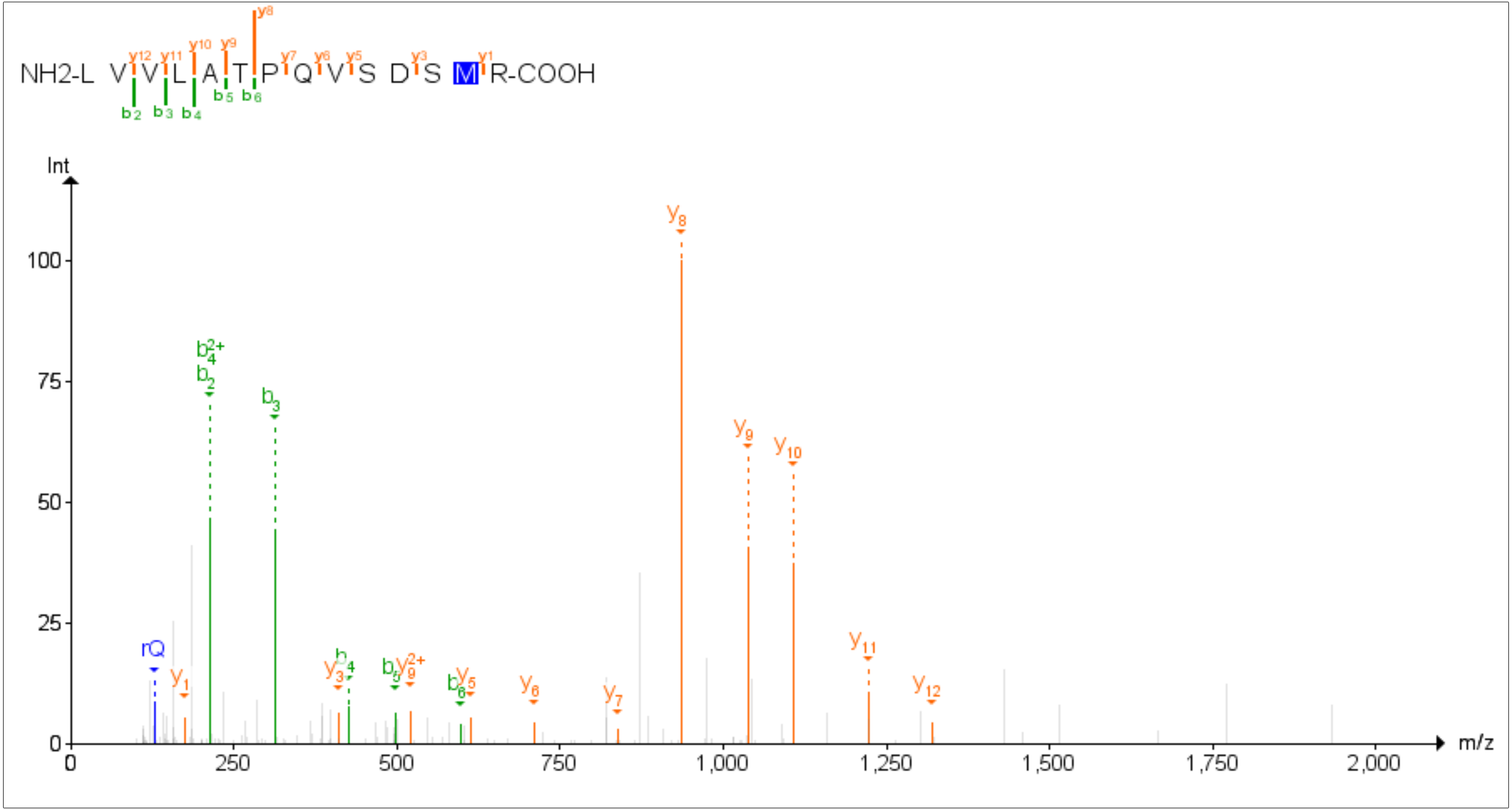

NH2-L V V L A T P Q V S D S M R-COOH
Int
m/z
100
75
50
25
0
250
500
750
1,000
1,250
1,500
1,750
2,000

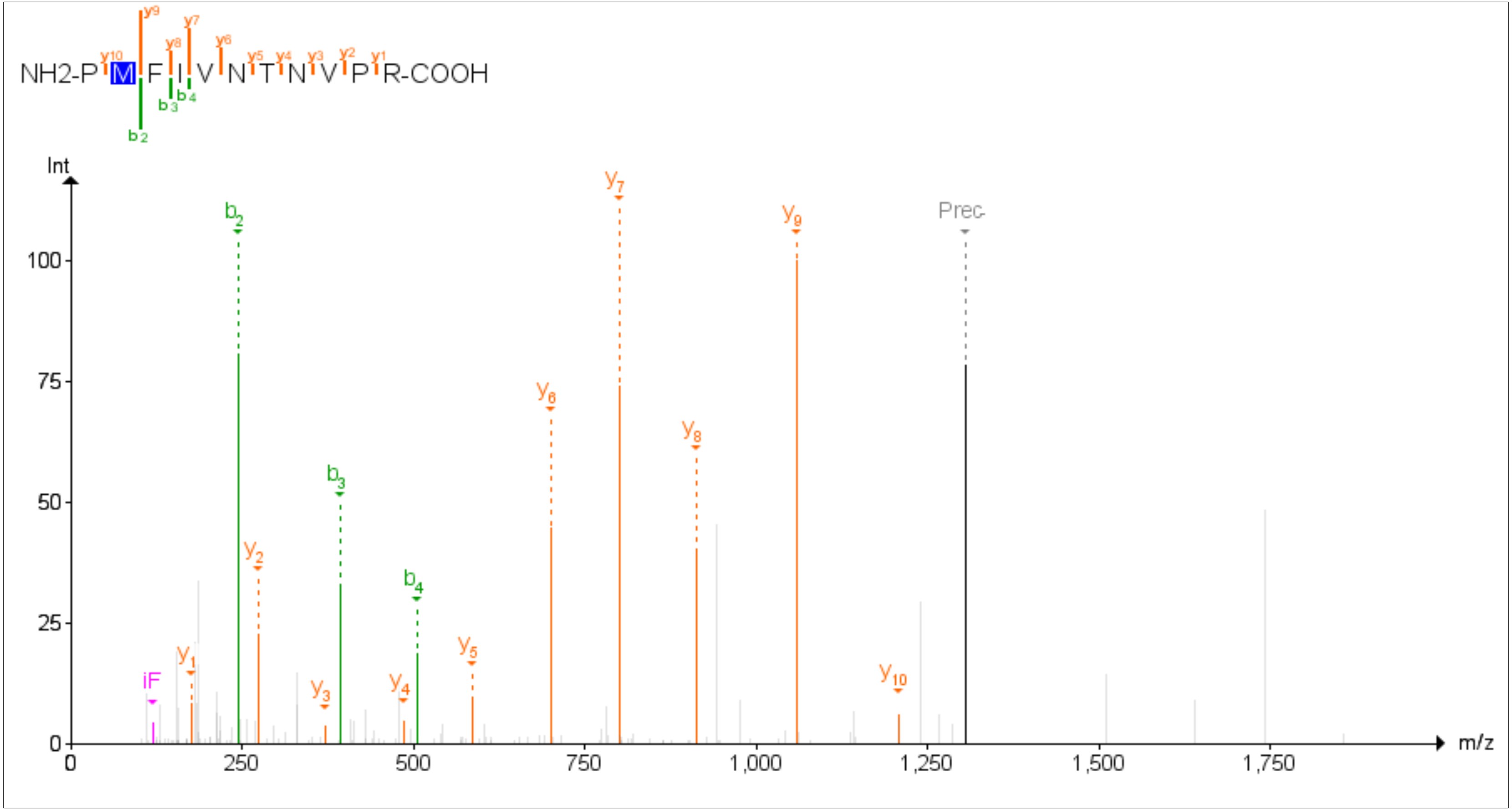

NH2-P M F I V N T N V P R-COOH
y10 y9 y8 y7 y6 y5 y4 y3 y2 y1
b2 b3 b4
Int
m/z
0
25
50
75
100
0
250
500
750
1,000
1,250
1,500
1,750
b2
b3
b4
y1
y2
y3
y4
y5
y6
y7
y8
y9
y10
iF
Prec

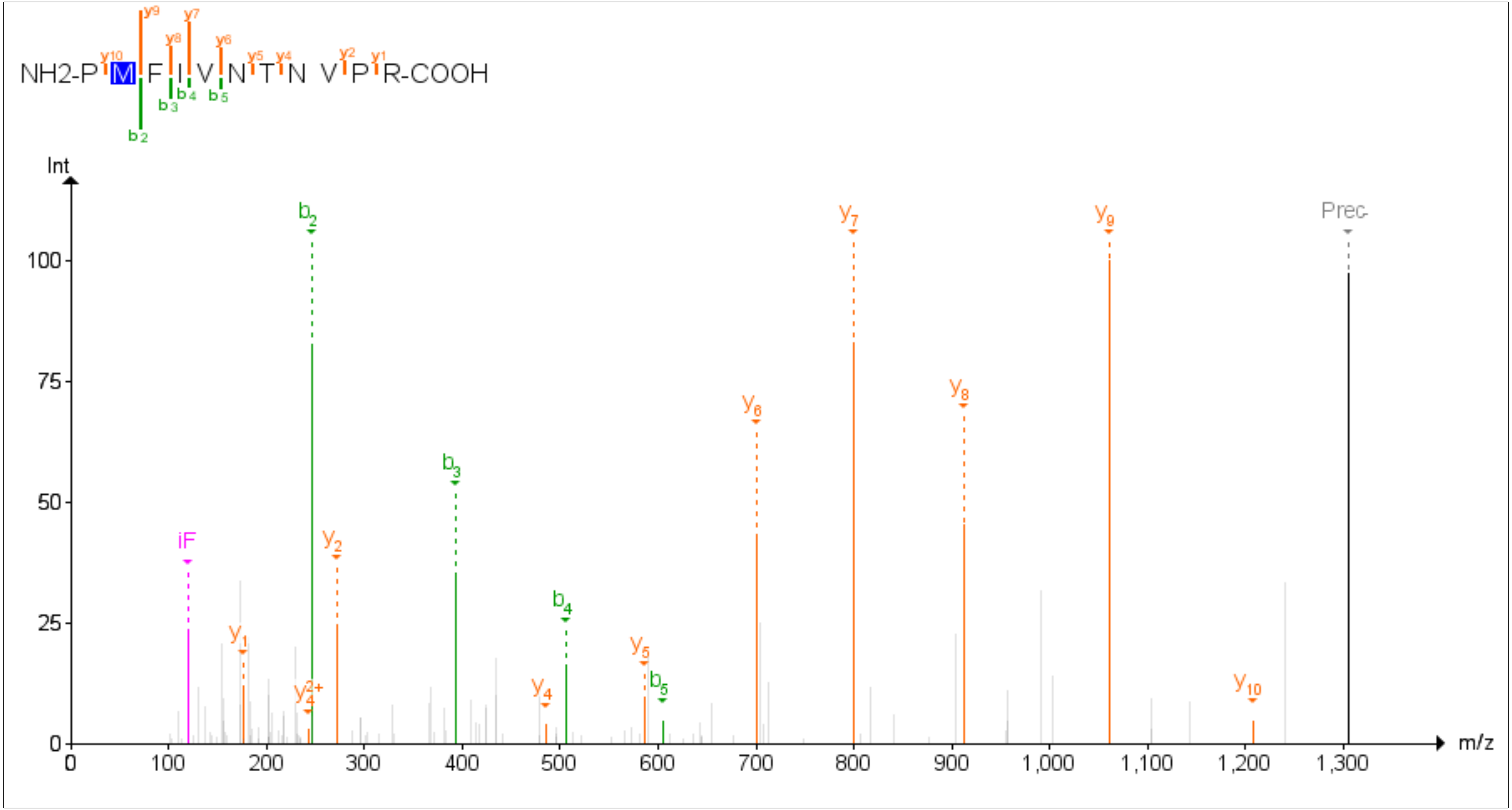

NH2-P M F I V N T N V P R-COOH
y10 y9 y8 y7 y6 y5 y4 y2 y1
b2 b3 b4 b5
Int
m/z
0
25
50
75
100
0
100
200
300
400
500
600
700
800
900
1,000
1,100
1,200
1,300
iF
y1
y_4^{2+}
b2
y2
b3
y4
b4
y5
b5
y6
y7
y8
y9
y10
Prec.

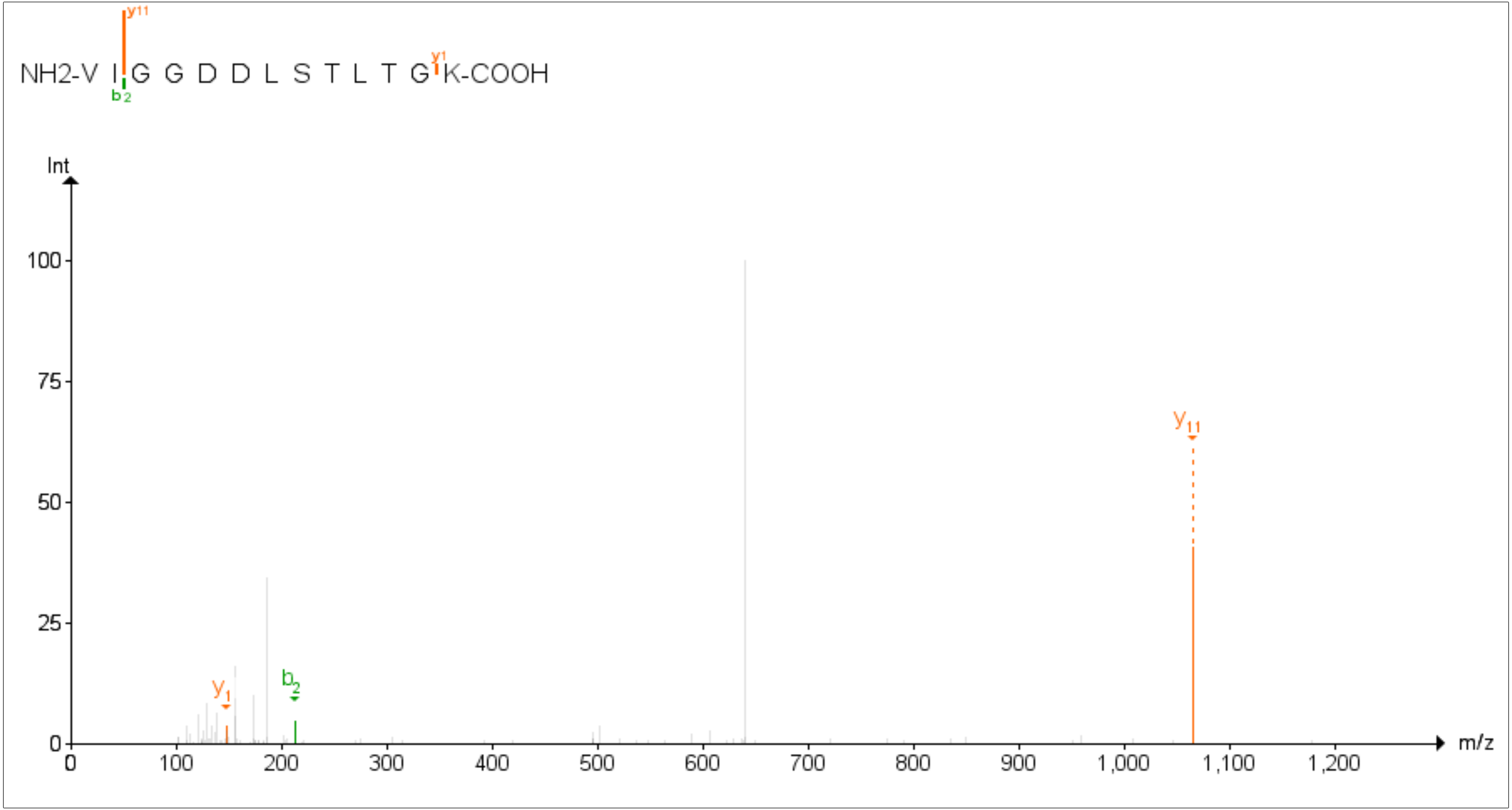

NH2-V I G G D D L S T L T G K-COOH
y11
b2
y1
Int
100
75
50
25
0
y11
y1
b2
0
100
200
300
400
500
600
700
800
900
1,000
1,100
1,200
m/z

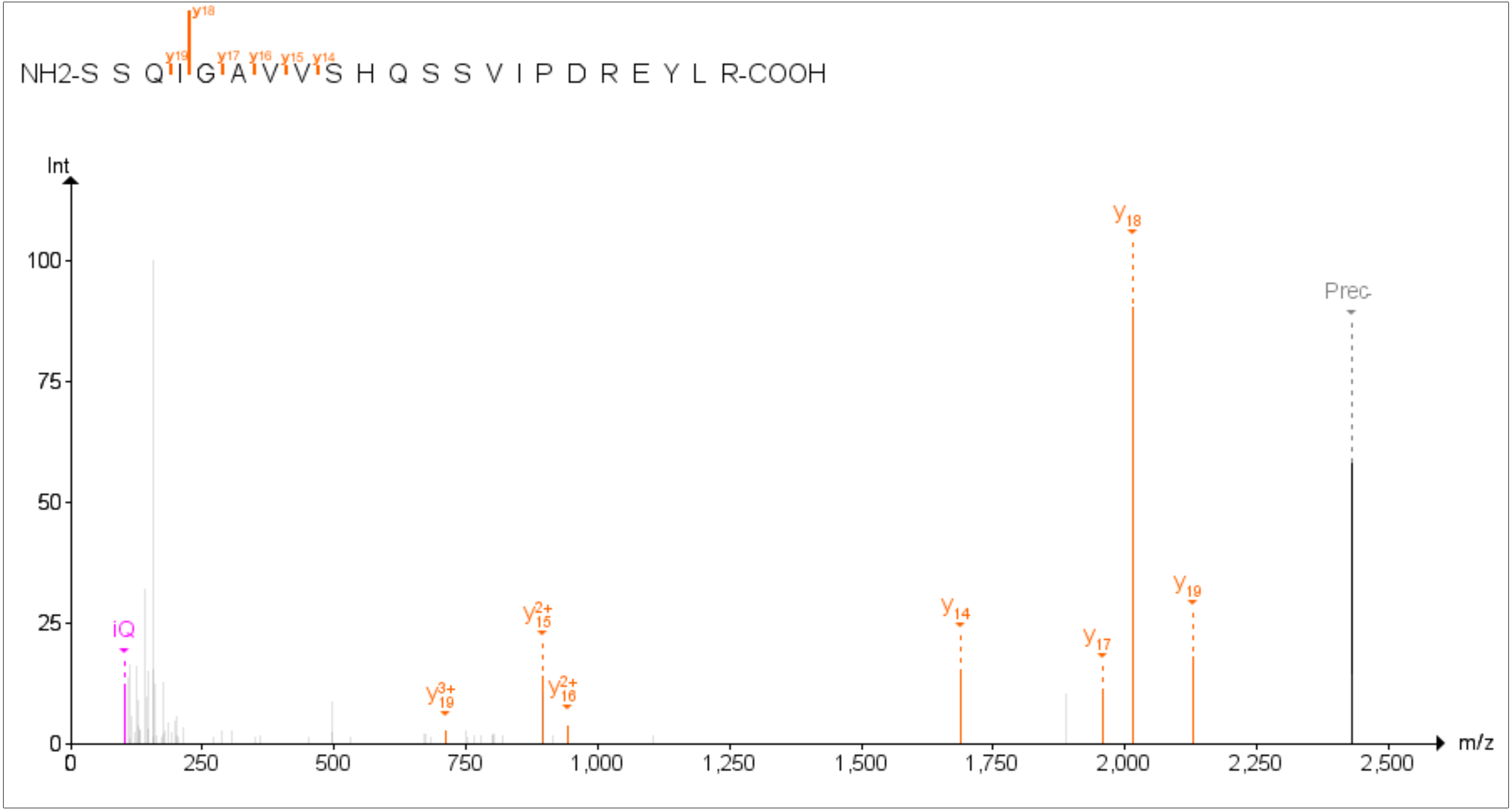

NH2-S S Q I G A V V S H Q S S V I P D R E Y L R-COOH
y18
y19
y17
y16
y15
y14
Int
100
75
50
25
0
iQ
y_{19}^{3+}
y_{15}^{2+}
y_{16}^{2+}
y_{14}
y_{17}
y_{18}
y_{19}
Prec
0
250
500
750
1,000
1,250
1,500
1,750
2,000
2,250
2,500
m/z

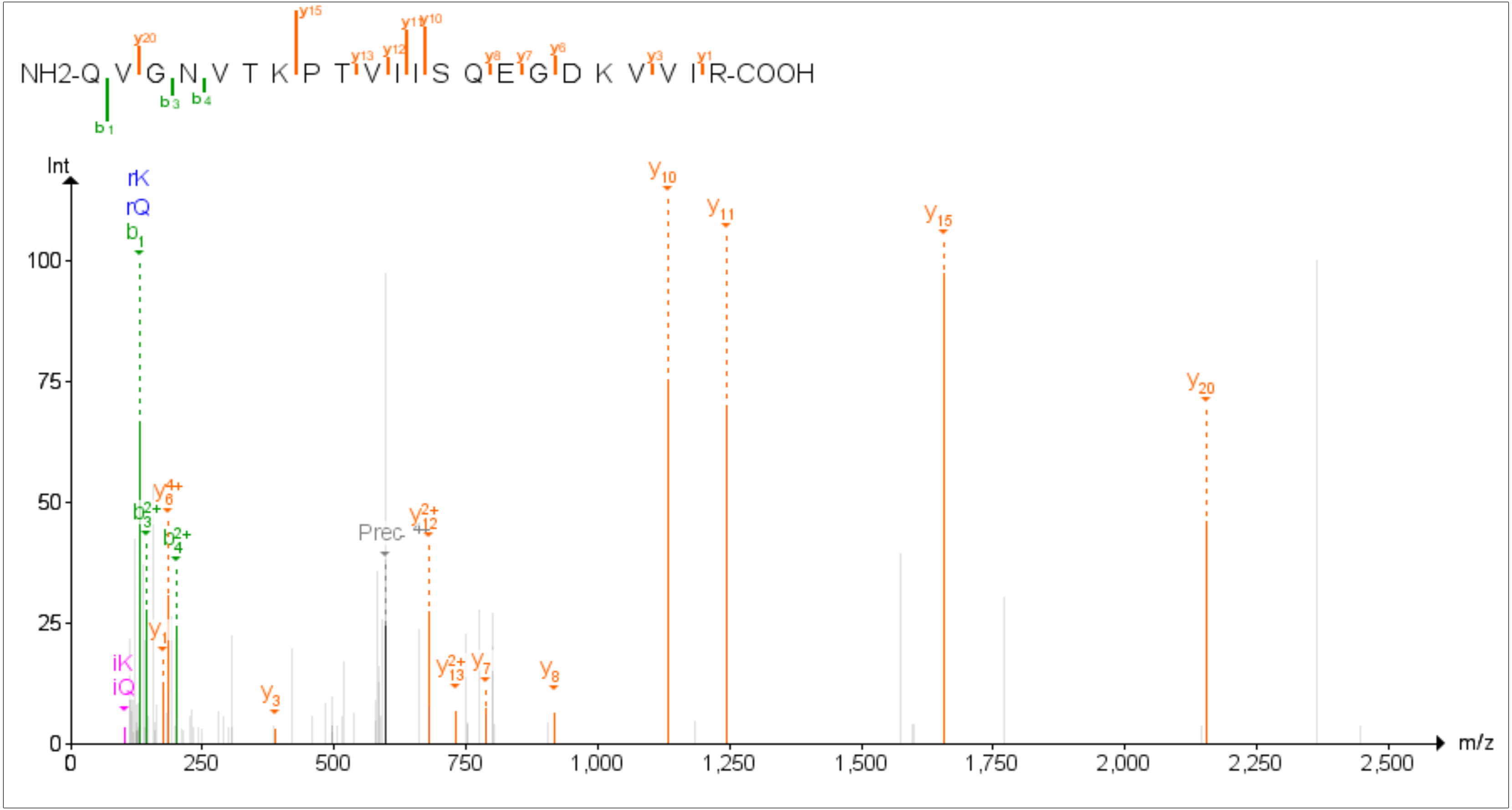

NH2-Q V G N V T K P T V I I S Q E G D K V V I R-COOH
b1 b3 b4
y20 y15 y13 y12 y11 y10 y8 y7 y6 y3 y1
Int
m/z
rK
rQ
b1
y10
y11
y15
y20
Prec ++

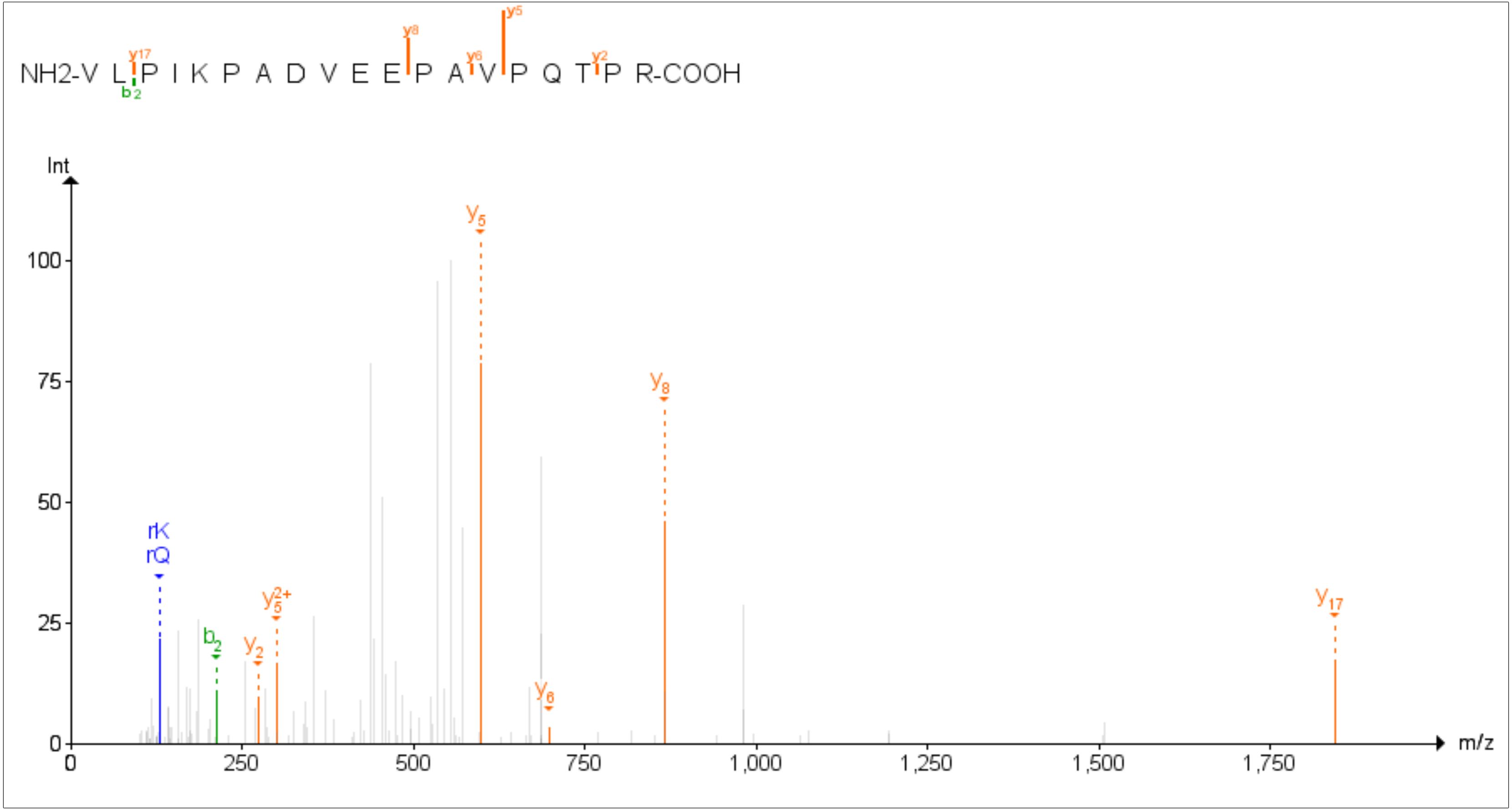
NH2-V L P I K P A D V E E P A V P Q T P R-COOH
y17
b2
y8
y6
y5
y2
Int
100
75
50
25
0
rK
rQ
b2
y2
y5 2+
y5
y6
y8
y17
0
250
500
750
1,000
1,250
1,500
1,750
m/z

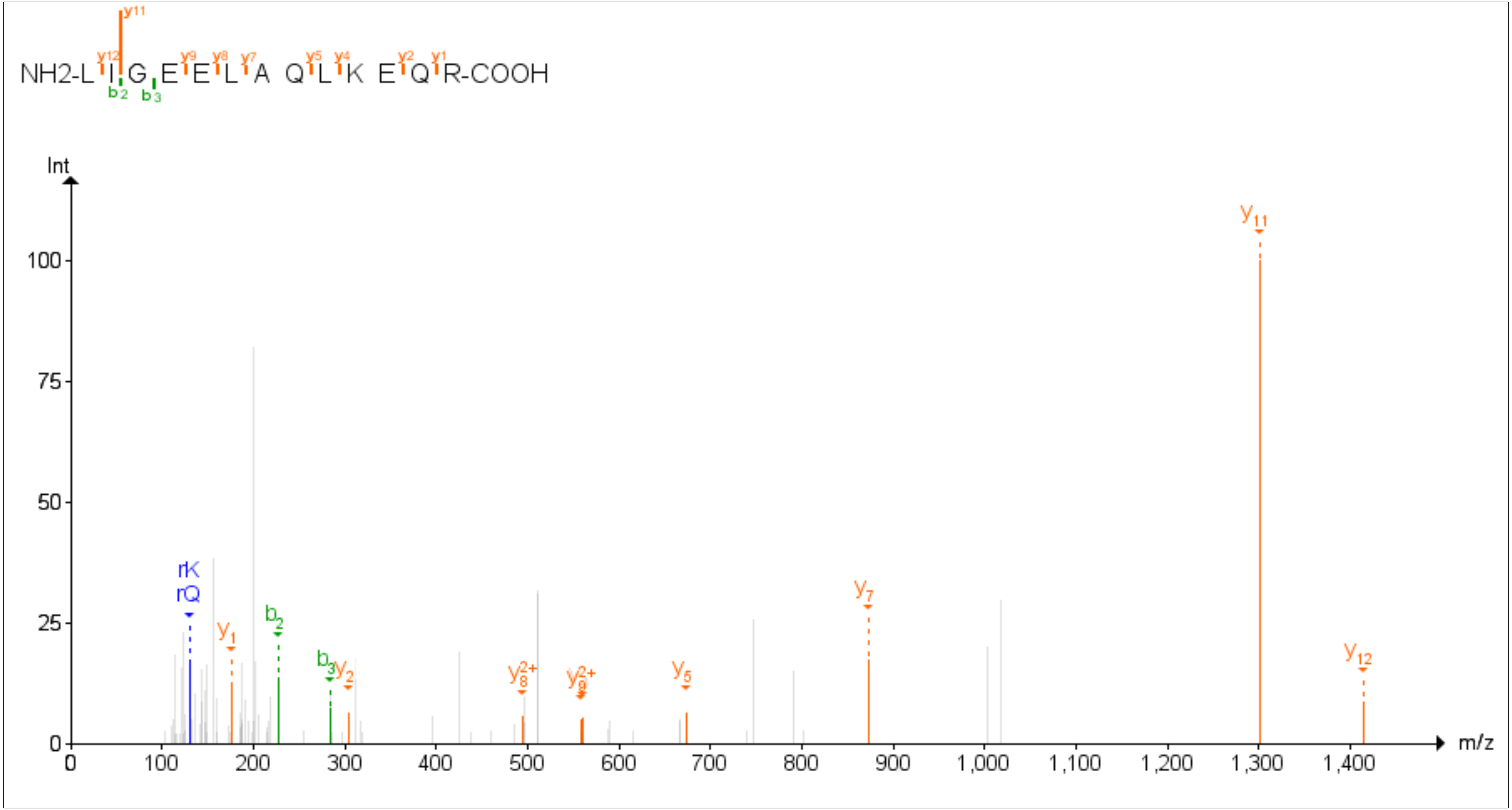

NH2-L I G E E L A Q L K E Q R-COOH
y11
y12
y9
y8
y7
y5
y4
y2
y1
b2
b3
Int
100
75
50
25
0
rK
rQ
y1
b2
b3
y2
y8 2+
y9 2+
y5
y7
y11
y12
0
100
200
300
400
500
600
700
800
900
1,000
1,100
1,200
1,300
1,400
m/z

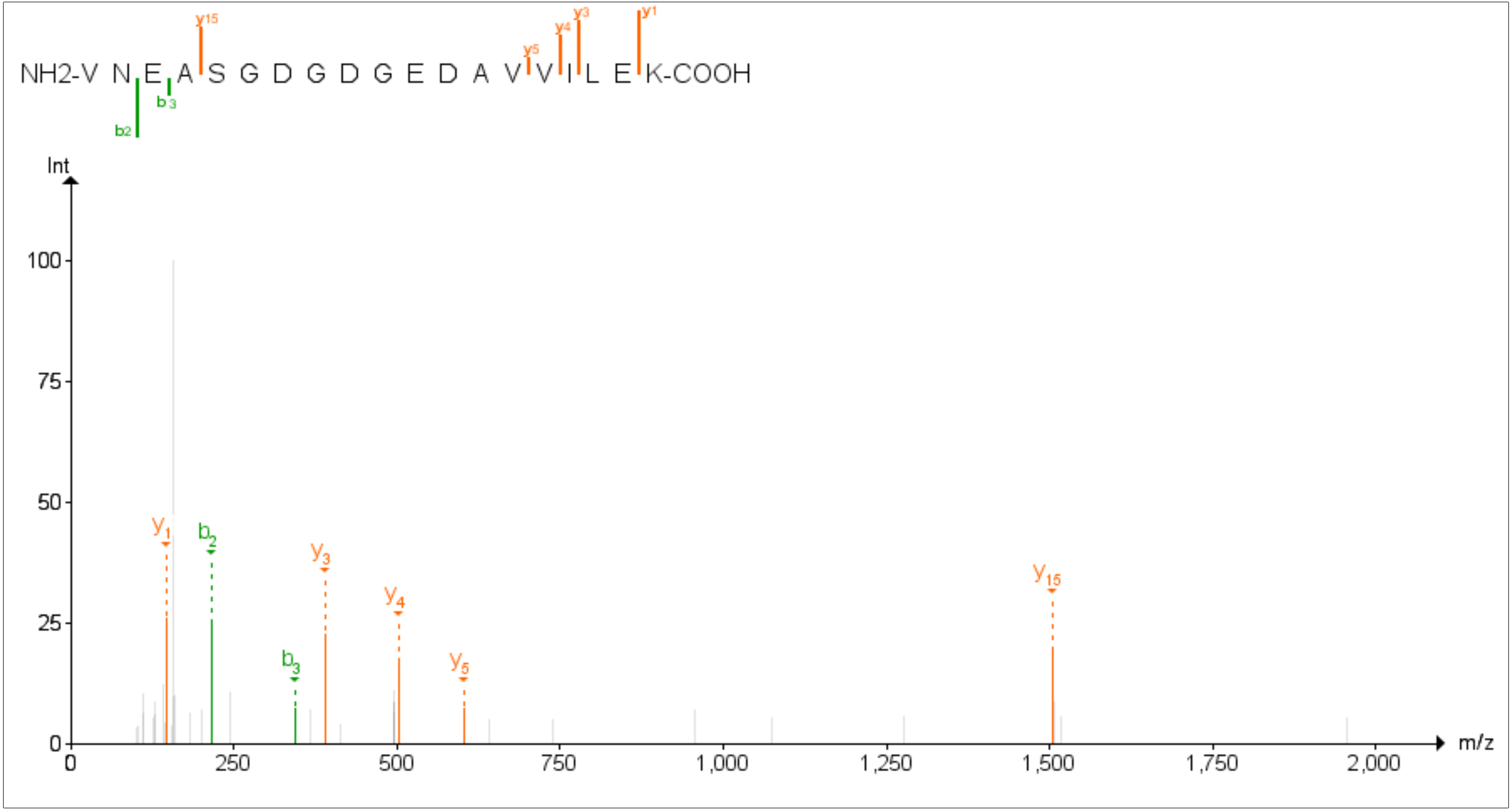

NH2-V N E A S G D G D G E D A V V I L E K-COOH
y15
y5
y4
y3
y1
b2
b3
Int
100
75
50
25
0
y1
b2
y3
y4
b3
y5
y15
0
250
500
750
1,000
1,250
1,500
1,750
2,000
m/z

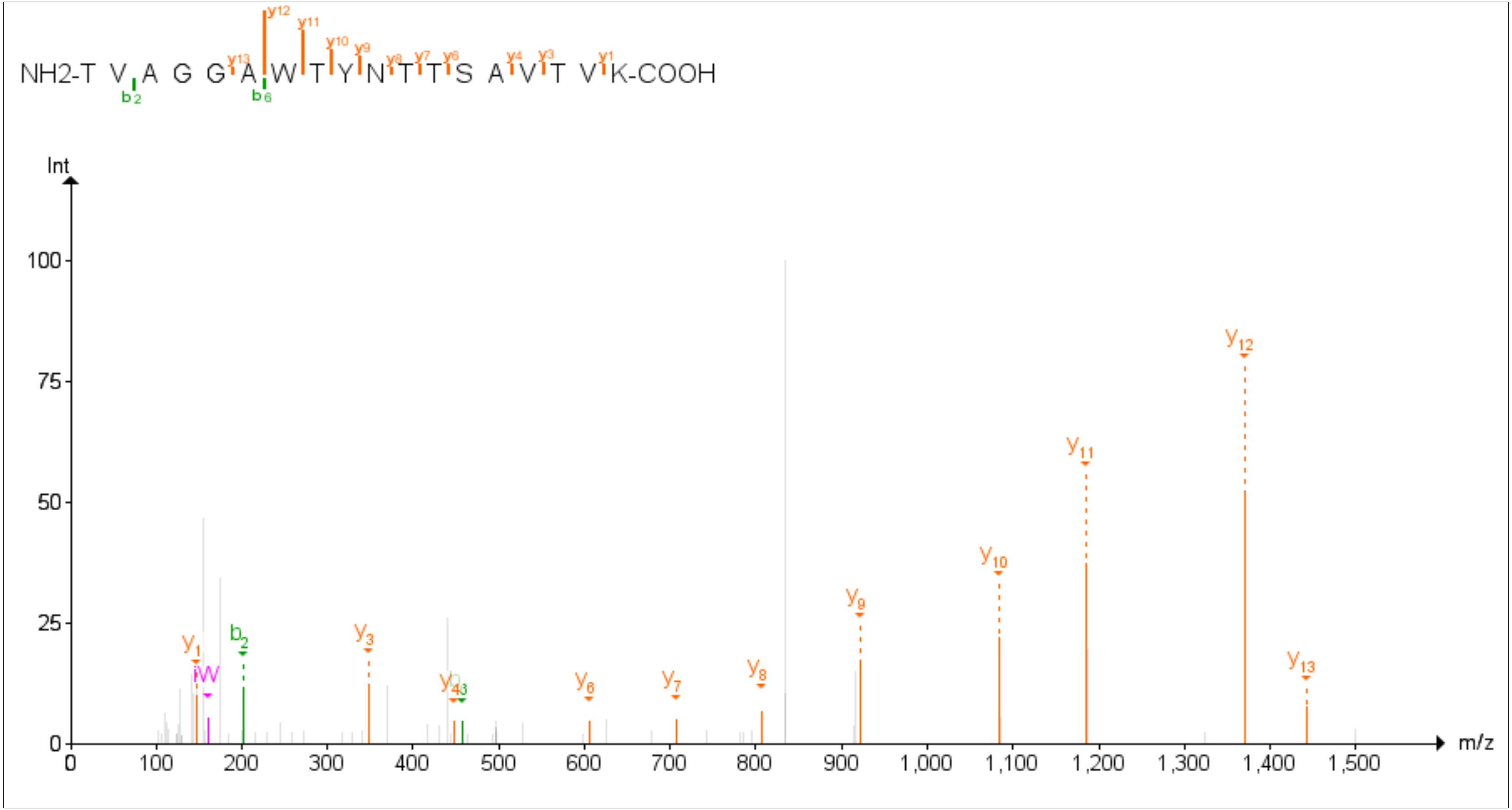

NH2-T V A G G A W T Y N T T S A V T V K-COOH
y12 y11 y10 y9 y8 y7 y6 y4 y3 y1 y13
b2 b6
Int
100
75
50
25
0
0 100 200 300 400 500 600 700 800 900 1,000 1,100 1,200 1,300 1,400 1,500
m/z
y1 iW b2 y3 y4 y6 y7 y8 y9 y10 y11 y12 y13

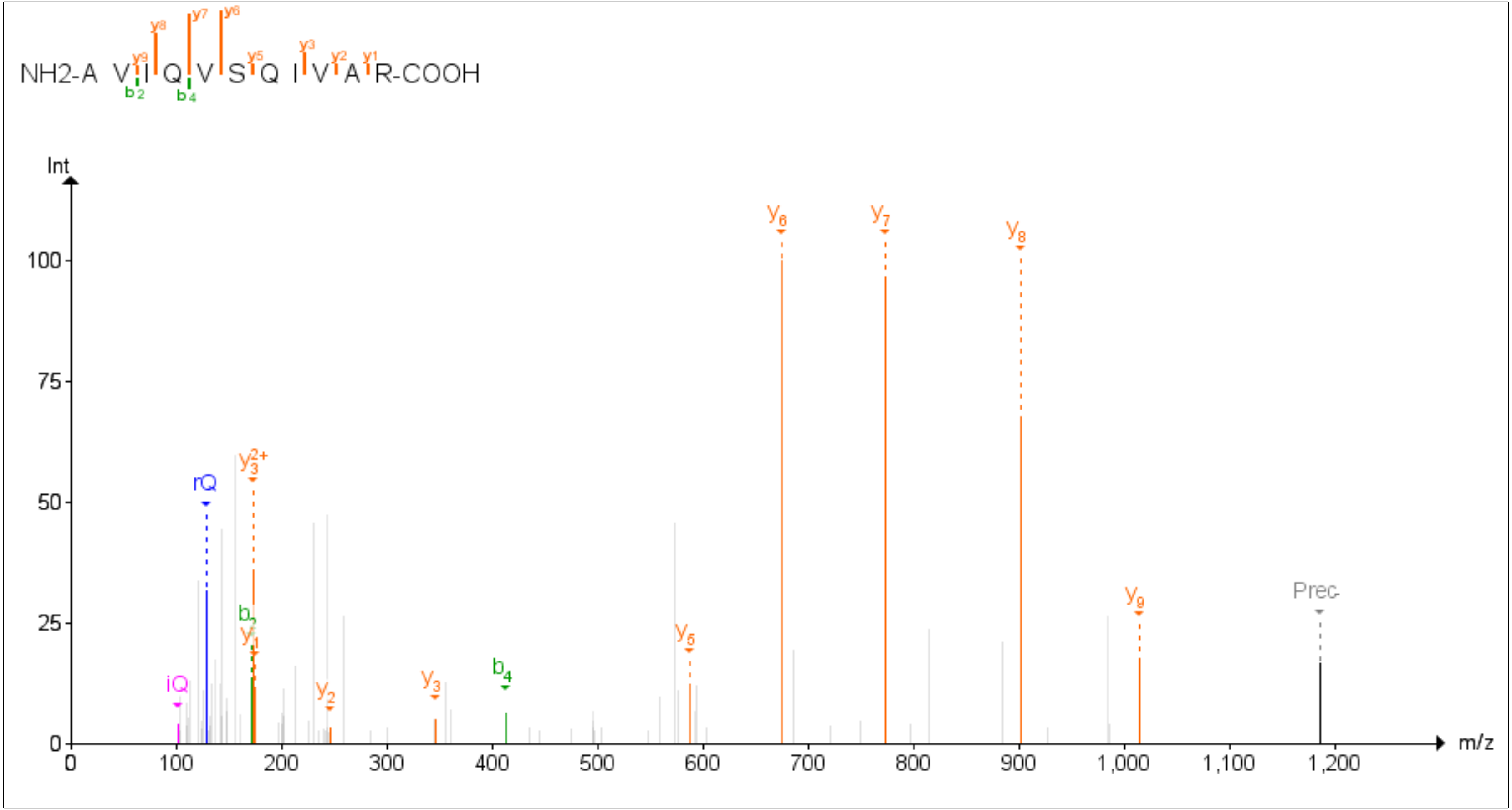

NH2-A V I Q V S Q I V A R-COOH
Int
m/z
Prec-

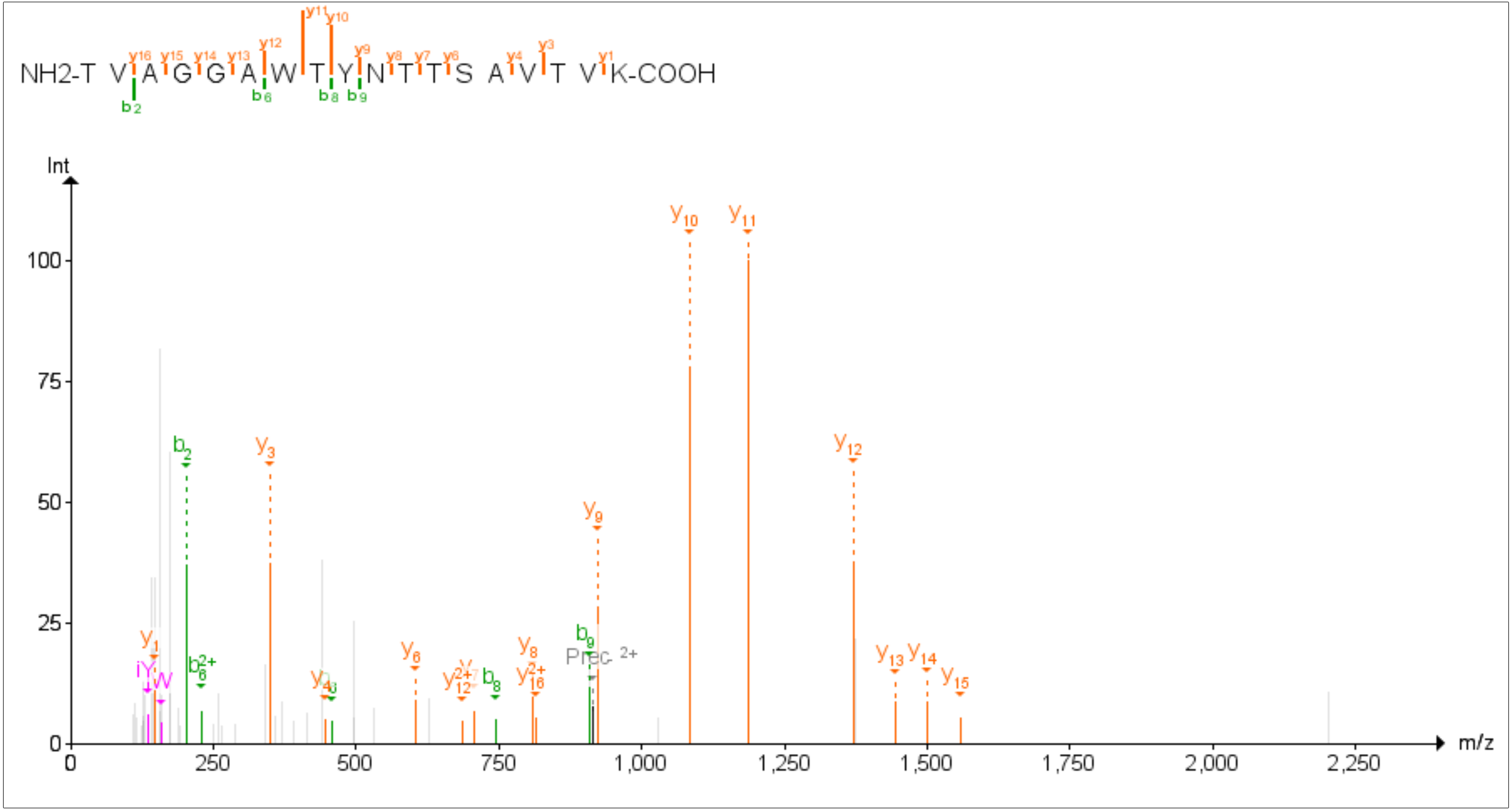

NH2-T V A G G A W T Y N T T S A V T V K-COOH
y16 y15 y14 y13 y12 y11 y10 y9 y8 y7 y6 y4 y3 y1
b2 b6 b8 b9
Int
100
75
50
25
0
0 250 500 750 1,000 1,250 1,500 1,750 2,000 2,250
m/z
y10 y11 y12 y3 b2 y9 y1 iY W b6 2+ y4 y6 y8 b8 b9 Prec- 2+ y13 y14 y15 y12 2+ y16 2+

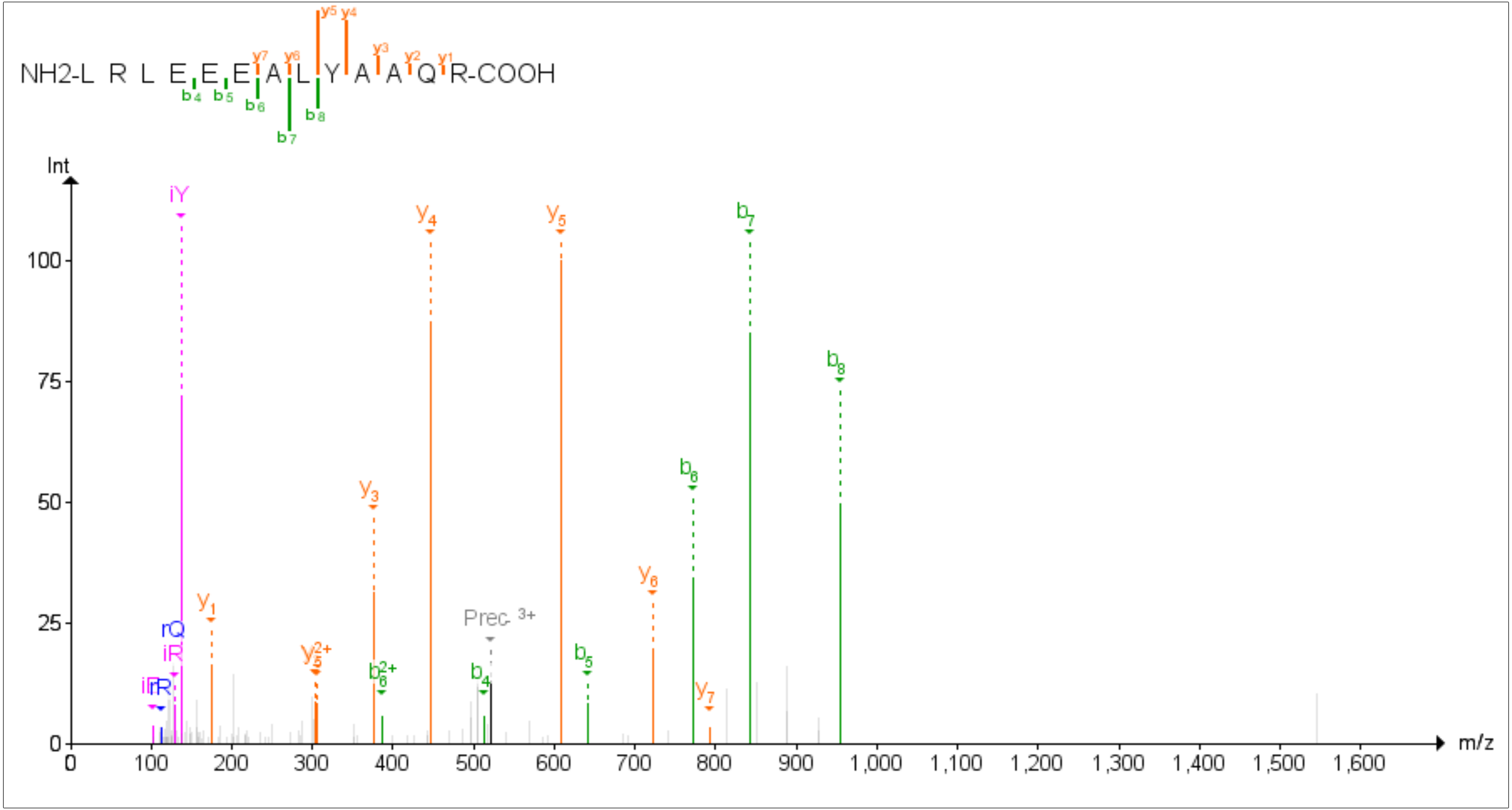
NH2-L R L E E E A L Y A A Q R-COOH
y7 y6 y5 y4 y3 y2 y1
b4 b5 b6 b7 b8
Int
iY
y4
y5
b7
b8
b6
y3
y6
y1
rQ
iR
iR
y5 2+
b6 2+
Prec 3+
b4
b5
y7
0
25
50
75
100
0 100 200 300 400 500 600 700 800 900 1,000 1,100 1,200 1,300 1,400 1,500 1,600
m/z

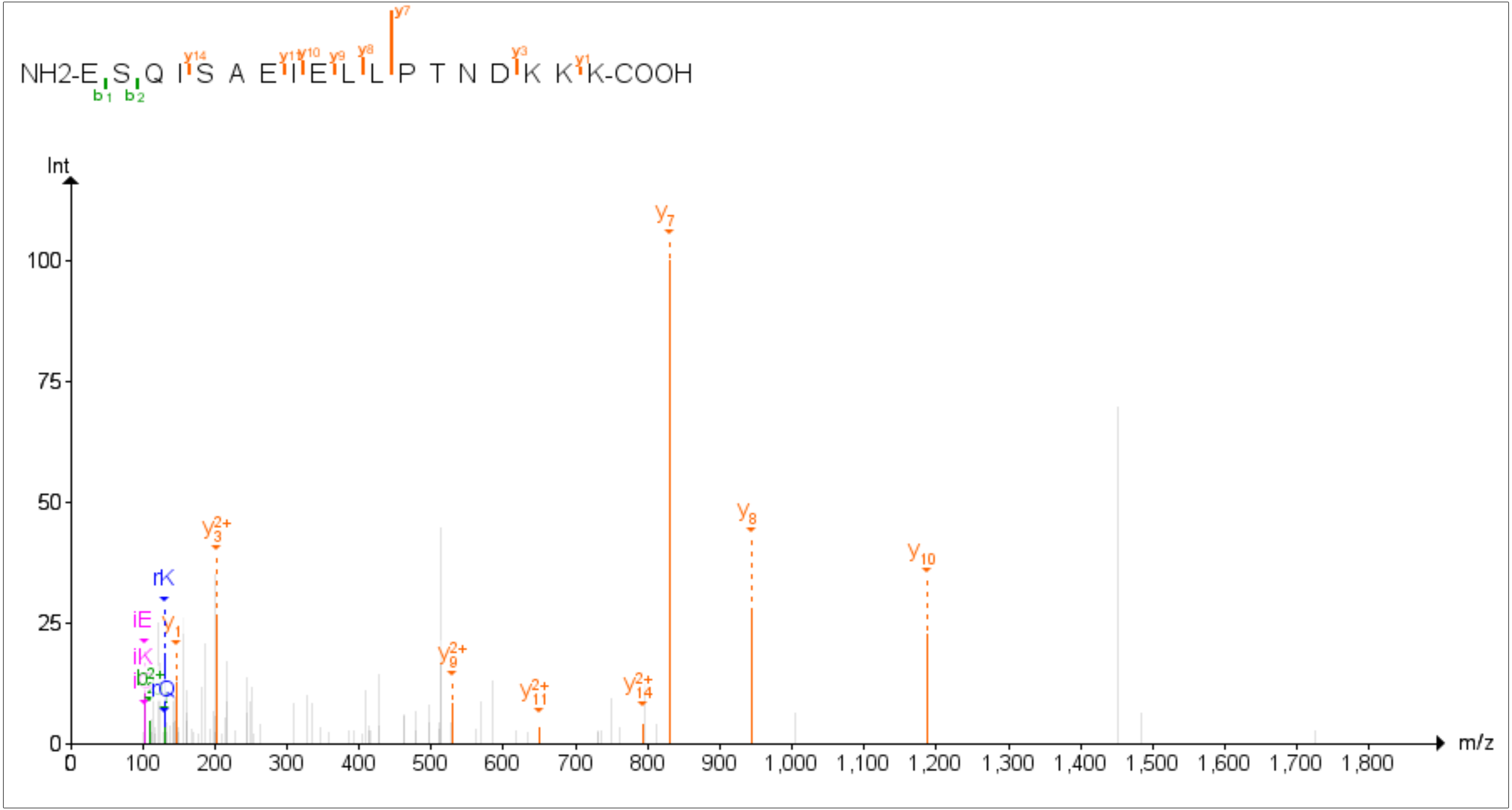

NH2-E S Q I S A E I E L L P T N D K K K-COOH
b1
b2
y14
y11
y10
y9
y8
y7
y3
y1
Int
m/z
y7
y8
y10
y3 2+
y9 2+
y11 2+
y14 2+
y1
rK
iE
iK
rQ
0
25
50
75
100
0
100
200
300
400
500
600
700
800
900
1,000
1,100
1,200
1,300
1,400
1,500
1,600
1,700
1,800

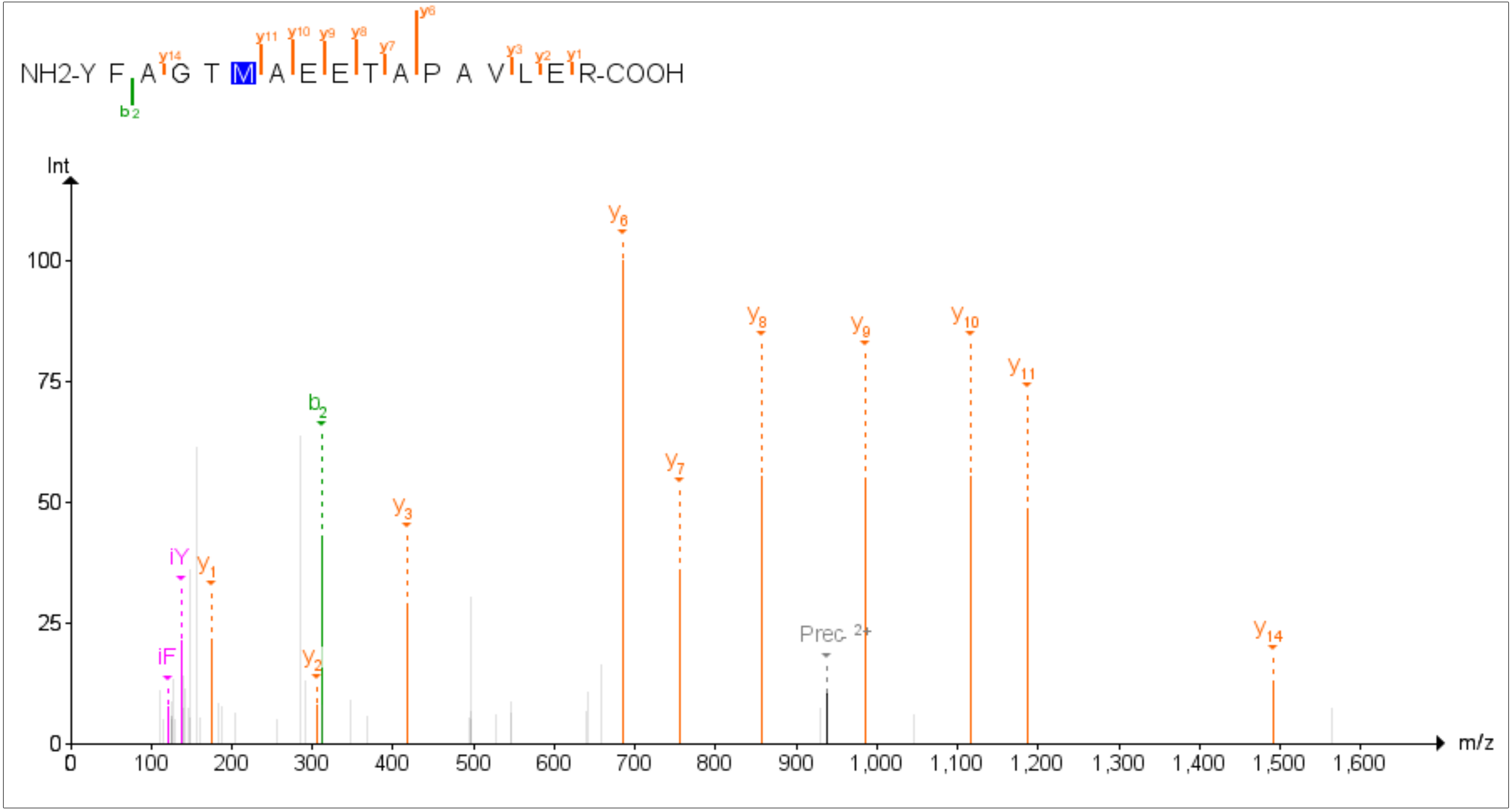

NH2-Y F A G T M A E E T A P A V L E R-COOH
b2
y14 y11 y10 y9 y8 y7 y6 y3 y2 y1
Int
m/z
Prec. 2+
iF
iY

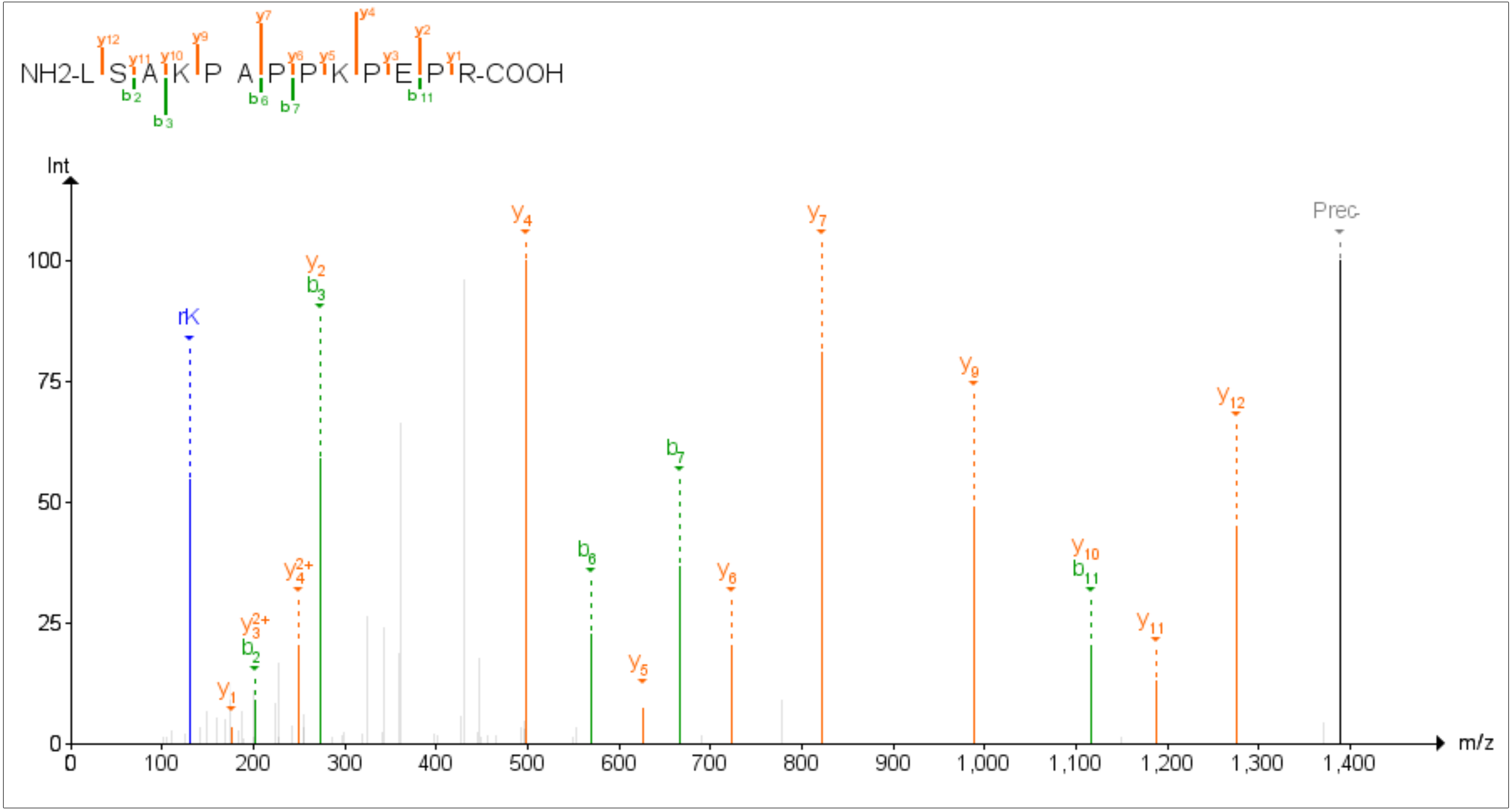

NH2-L S A K P A P P K P E P R-COOH
Int
m/z
Prec.
rK
y4
y7
y2
b3
y9
y12
b7
b6
y10
b11
y11
y6
y5
y1
y3 2+
b2
y4 2+
0
25
50
75
100
0
100
200
300
400
500
600
700
800
900
1,000
1,100
1,200
1,300
1,400

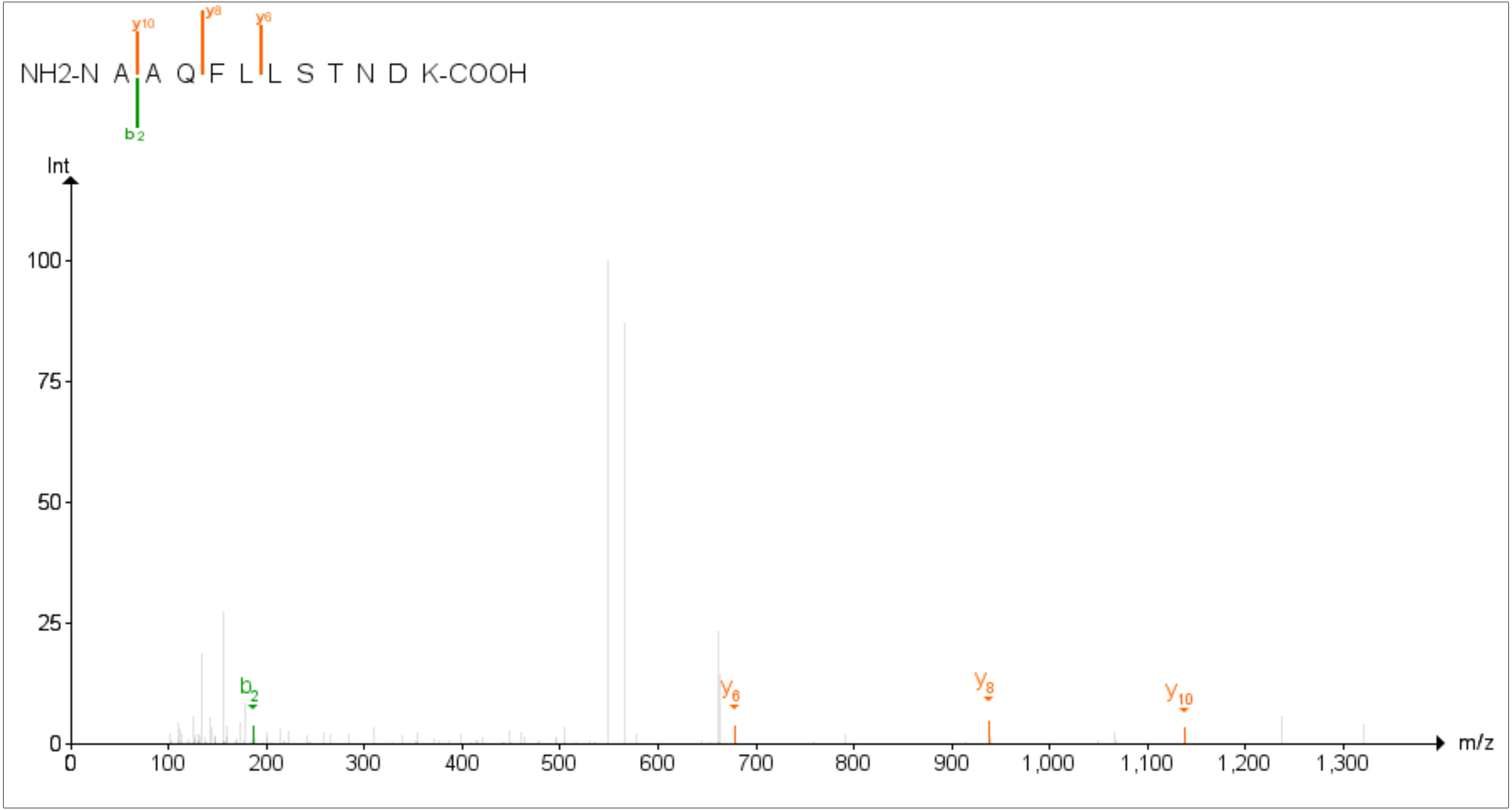

NH2-N A A Q F L L S T N D K-COOH
y10
y8
y6
b2
Int
100
75
50
25
0
m/z
0
100
200
300
400
500
600
700
800
900
1,000
1,100
1,200
1,300
b2
y6
y8
y10

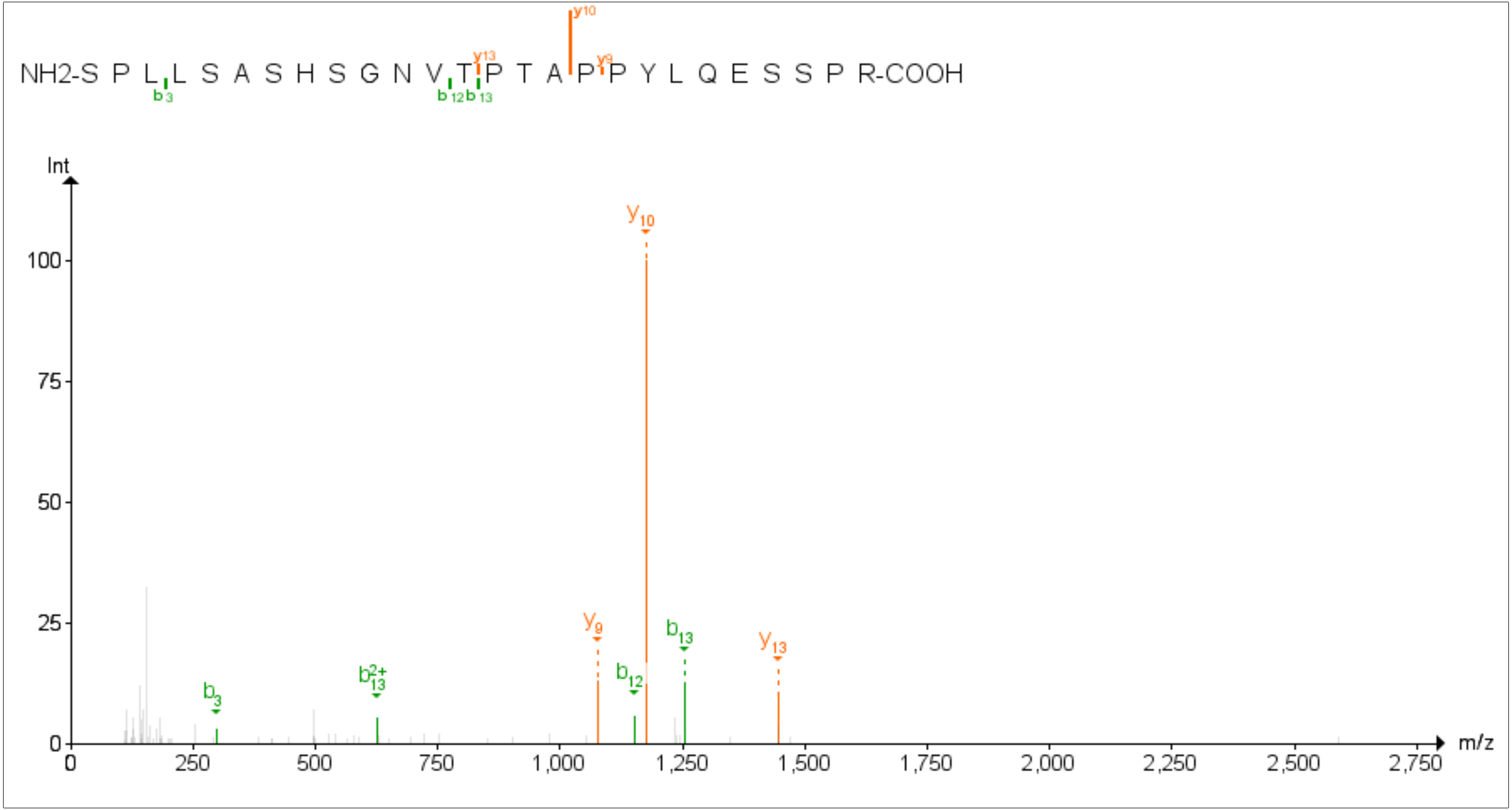

NH2-S P L L S A S H S G N V T P T A P P Y L Q E S S P R-COOH
y10
y13
y9
b3
b12
b13
Int
100
75
50
25
0
m/z
0
250
500
750
1,000
1,250
1,500
1,750
2,000
2,250
2,500
2,750
y10
y9
b13
y13
b12
b13 2+
b3

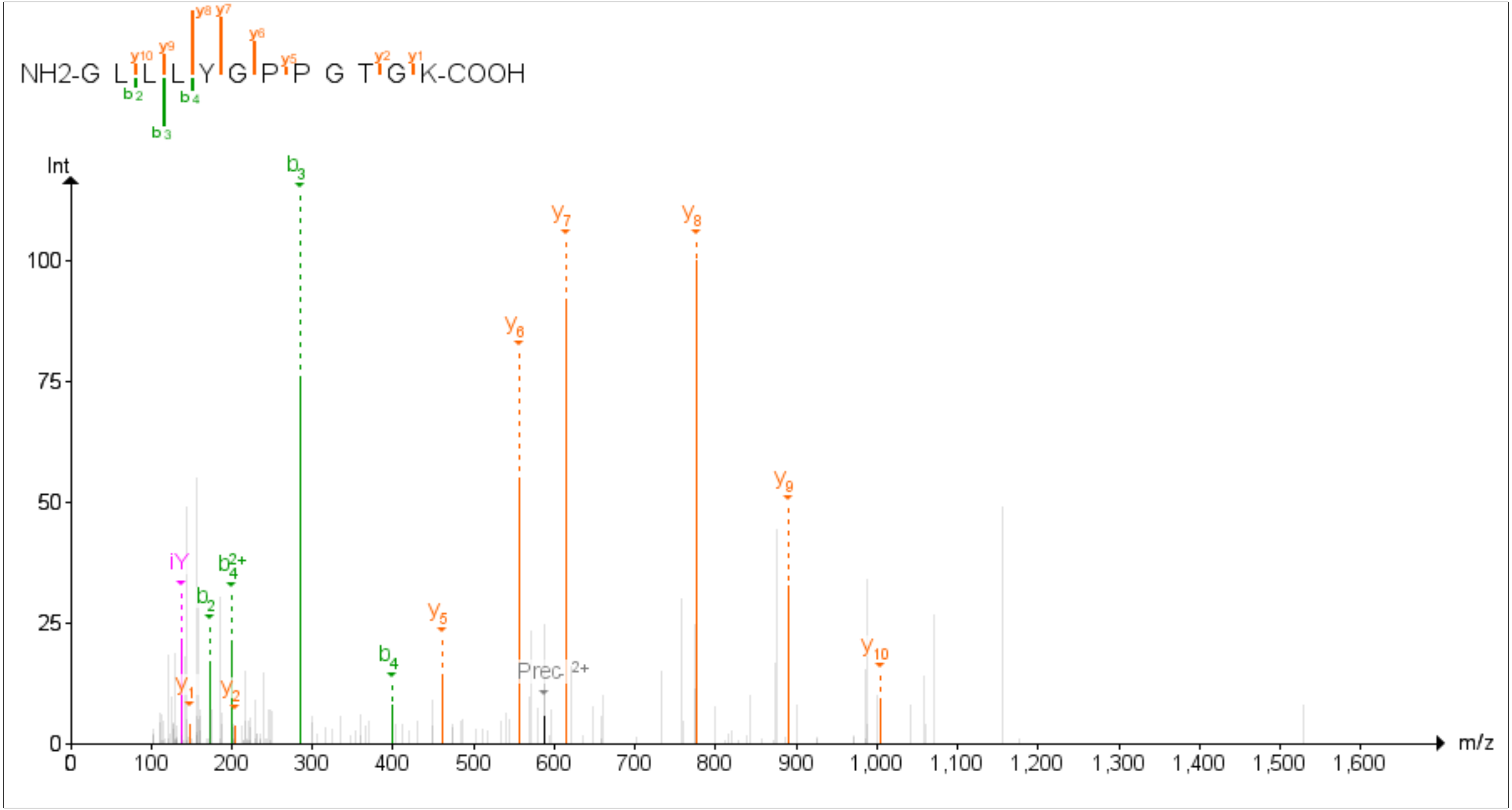

NH2-G L L L Y G P P G T G K-COOH
y10 y9 y8 y7 y6 y5 y2 y1
b2 b3 b4
Int
m/z
b3
y7
y8
y6
y9
iY
b4 2+
b2
y5
b4
y1
y2
Prec. 2+
y10
0
25
50
75
100
0
100
200
300
400
500
600
700
800
900
1,000
1,100
1,200
1,300
1,400
1,500
1,600

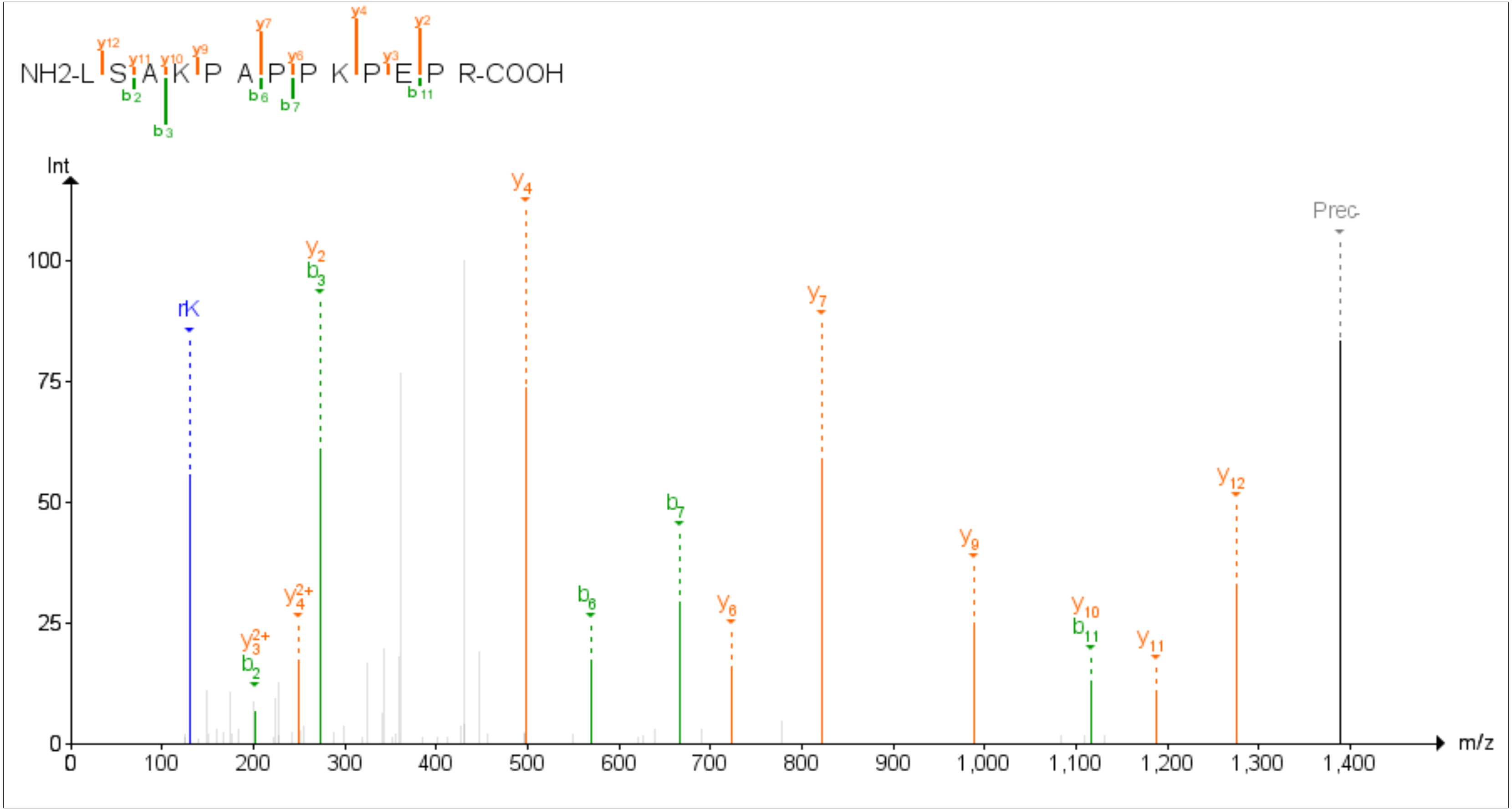

NH2-L S A K P A P P K P E P R-COOH
y12
y11
y10
y9
y7
y6
y4
y3
y2
b2
b3
b6
b7
b11
Int
100
75
50
25
0
rK
Prec
m/z
0
100
200
300
400
500
600
700
800
900
1,000
1,100
1,200
1,300
1,400

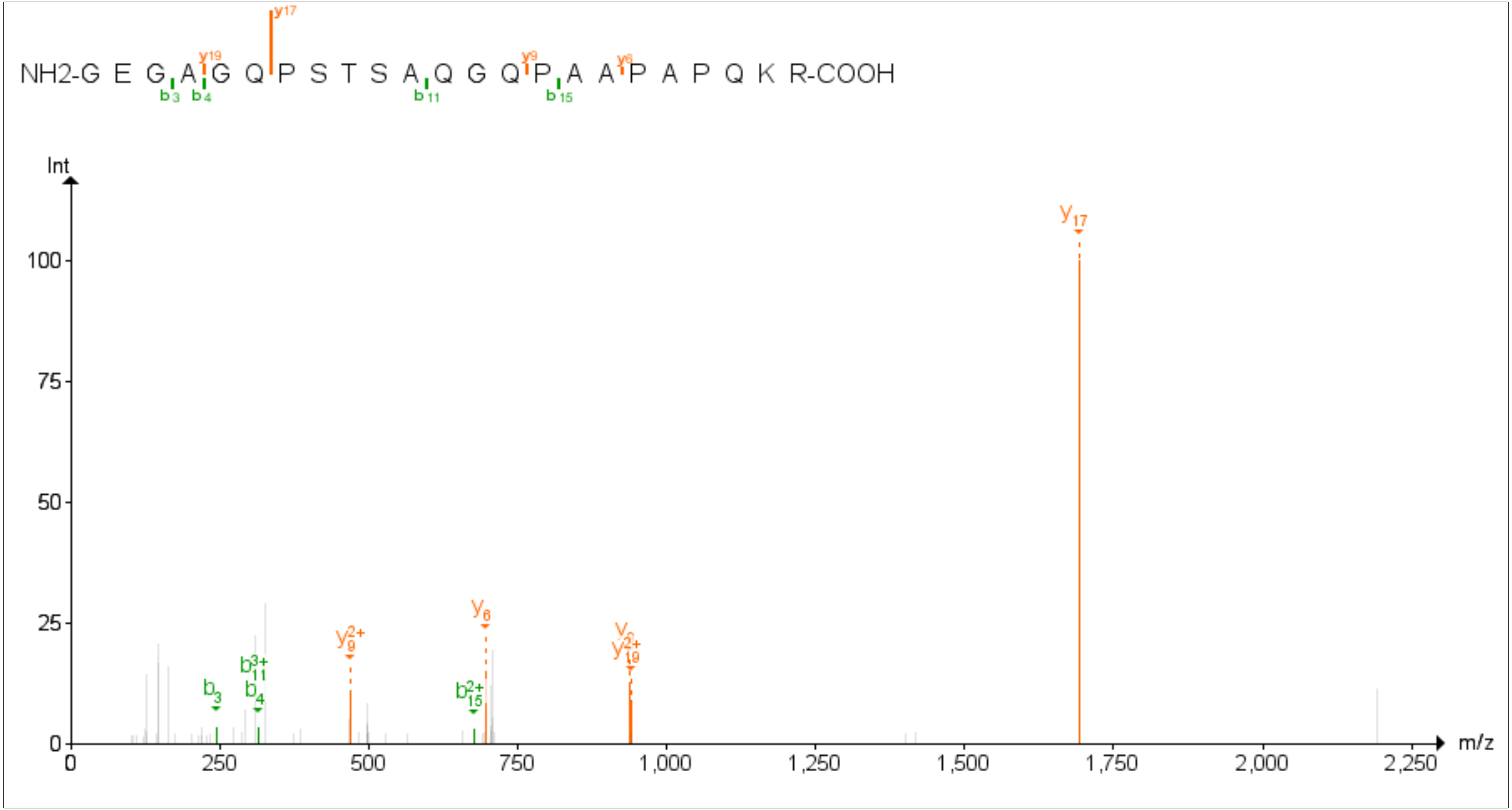
NH2-G E G A G Q P S T S A Q G Q P A A P A P Q K R-COOH
y17
y19
y9
y6
b3
b4
b11
b15
Int
100
75
50
25
0
m/z
0
250
500
750
1,000
1,250
1,500
1,750
2,000
2,250

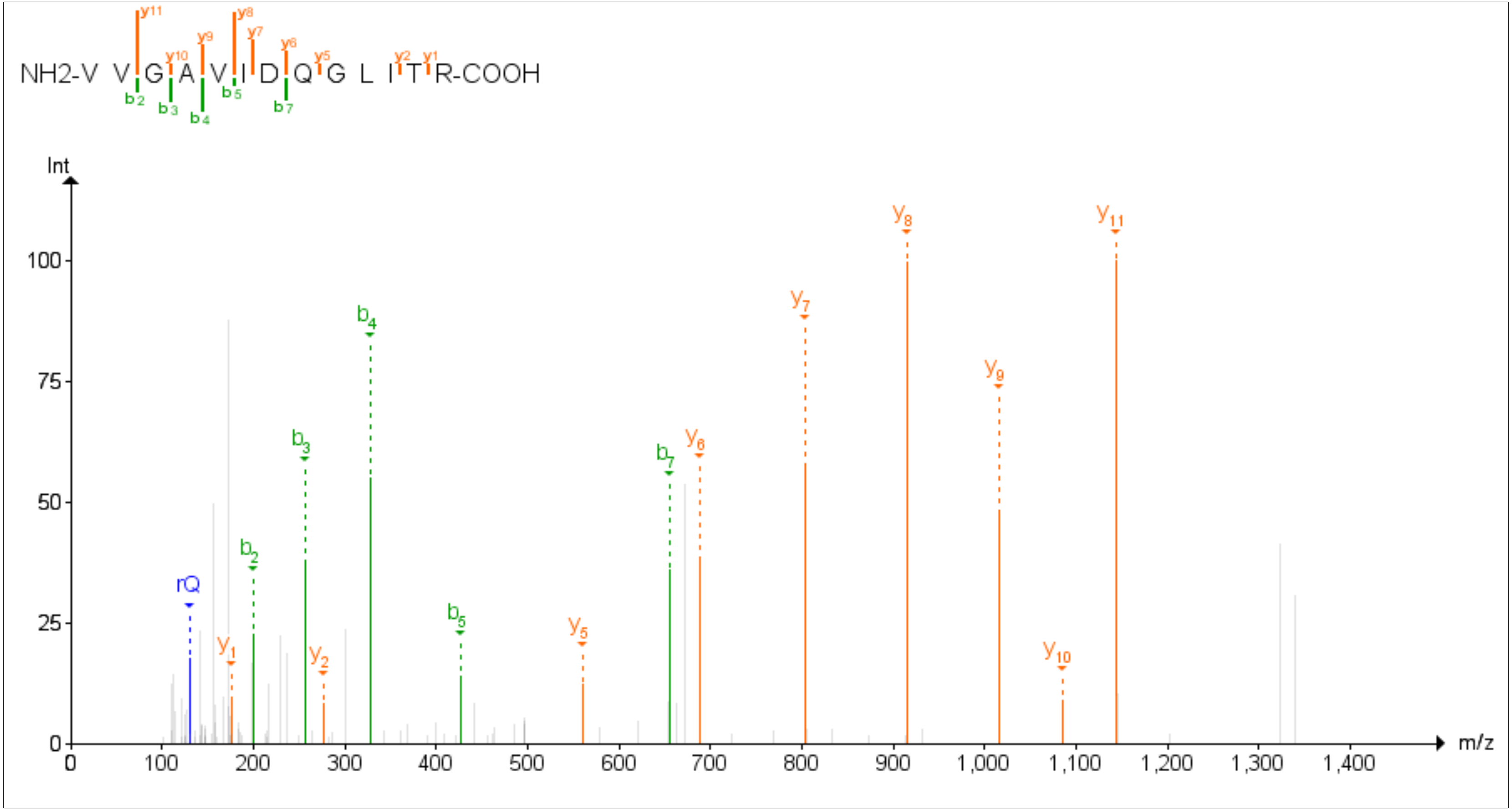

NH2-V V G A V I D Q G L I T R-COOH
y11 y10 y9 y8 y7 y6 y5 y2 y1
b2 b3 b4 b5 b7
Int
100
75
50
25
0
rQ
y1
b2
b3
y2
b4
b5
y5
b7
y6
y7
y8
y9
y10
y11
0
100
200
300
400
500
600
700
800
900
1,000
1,100
1,200
1,300
1,400
m/z

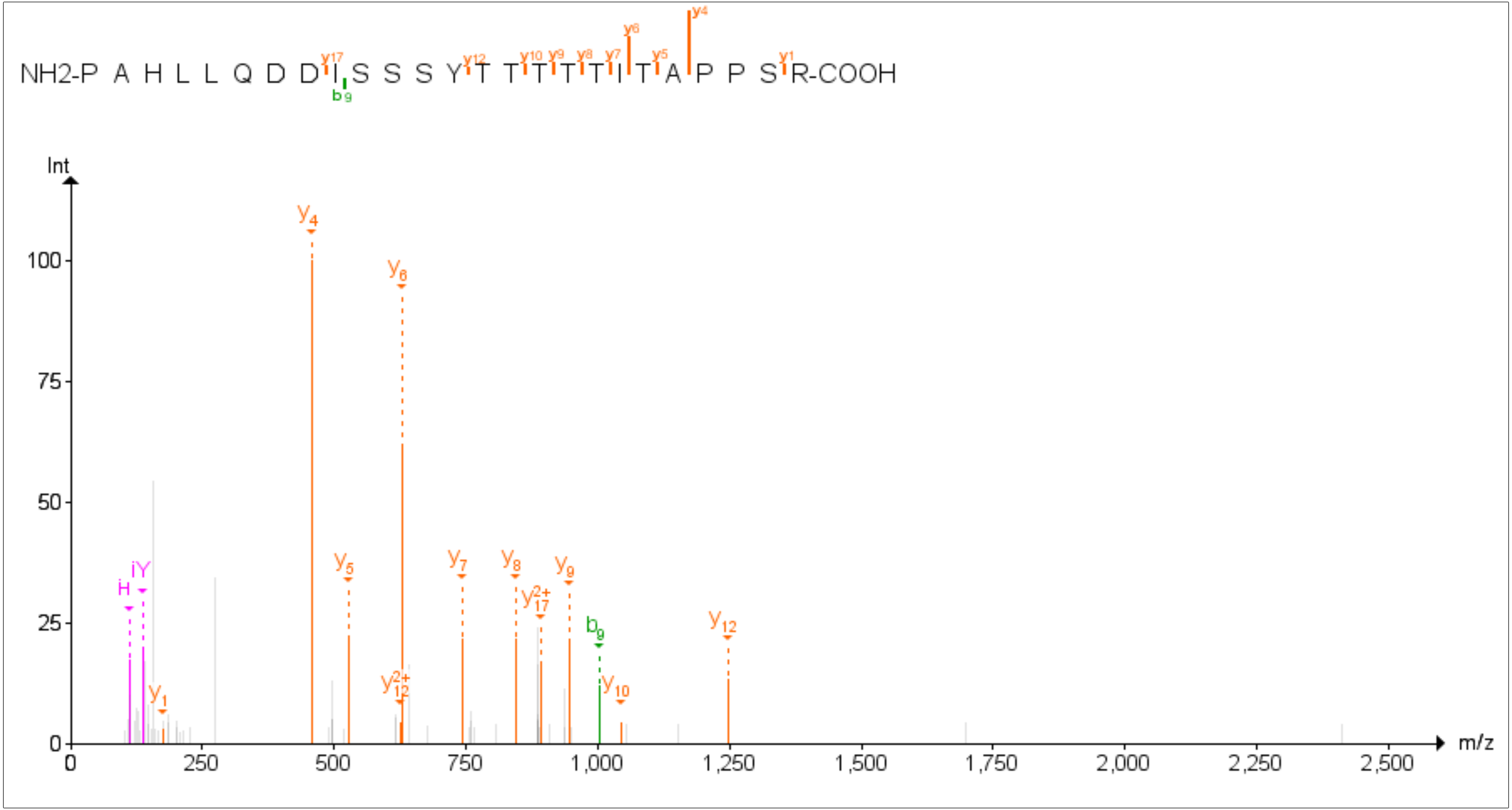

NH2-P A H L L Q D D I S S S Y T T T T T I T A P P S R-COOH
y17 y12 y10 y9 y8 y7 y6 y5 y4 y1
b9
Int
m/z
100
75
50
25
0
0 250 500 750 1,000 1,250 1,500 1,750 2,000 2,250 2,500
iH iY y1 y4 y5 y12 2+ y6 y7 y8 y17 2+ y9 b9 y10 y12

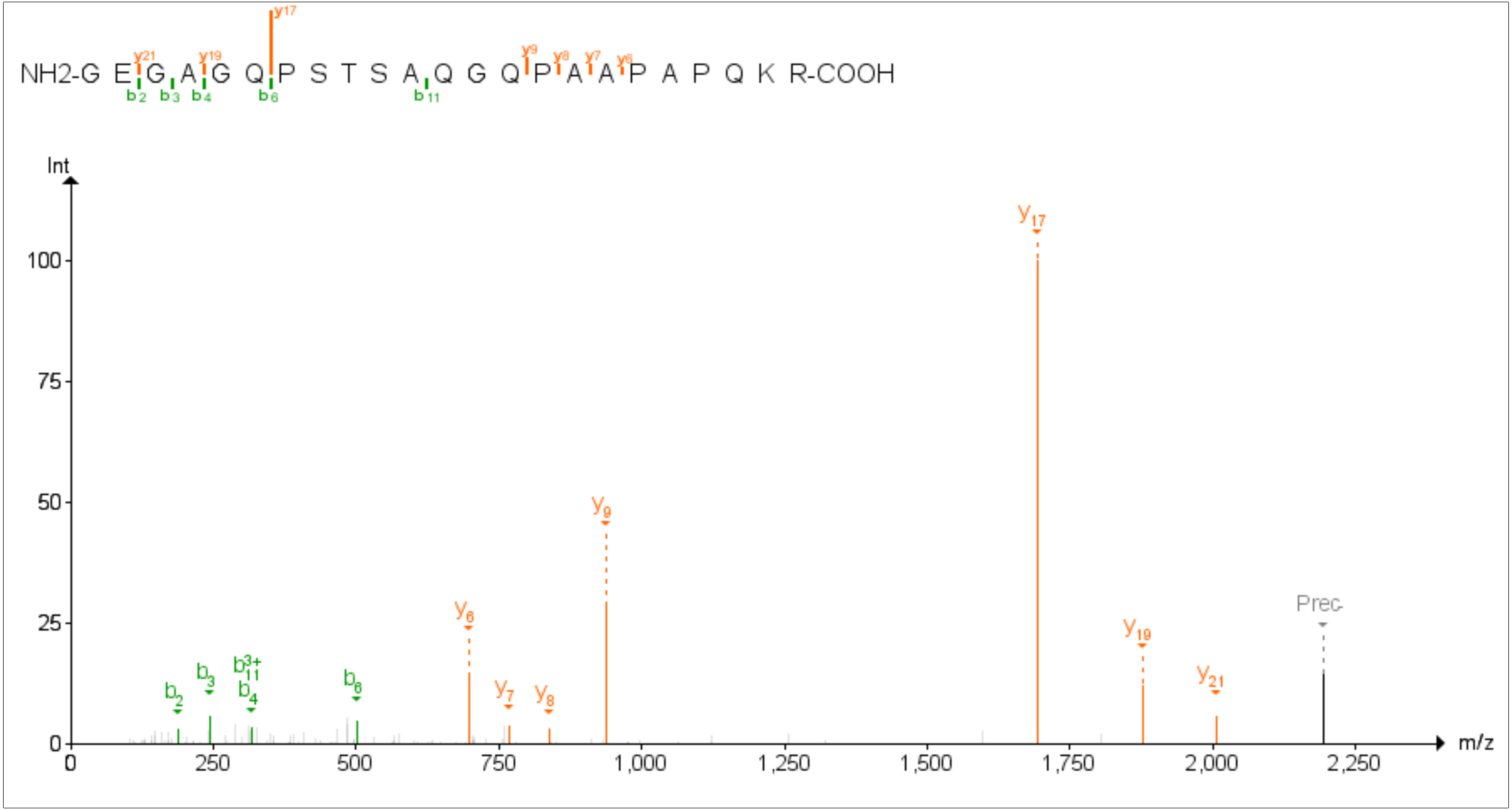

NH2-G E G A G Q P S T S A Q G G Q P A A P A P Q K R-COOH
y17
y21
y19
y9
y8
y7
y6
b2
b3
b4
b6
b11
Int
100
75
50
25
0
y17
y9
y6
y7
y8
y19
y21
Prec
b2
b3
b11 3+
b4
b6
0
250
500
750
1,000
1,250
1,500
1,750
2,000
2,250
m/z

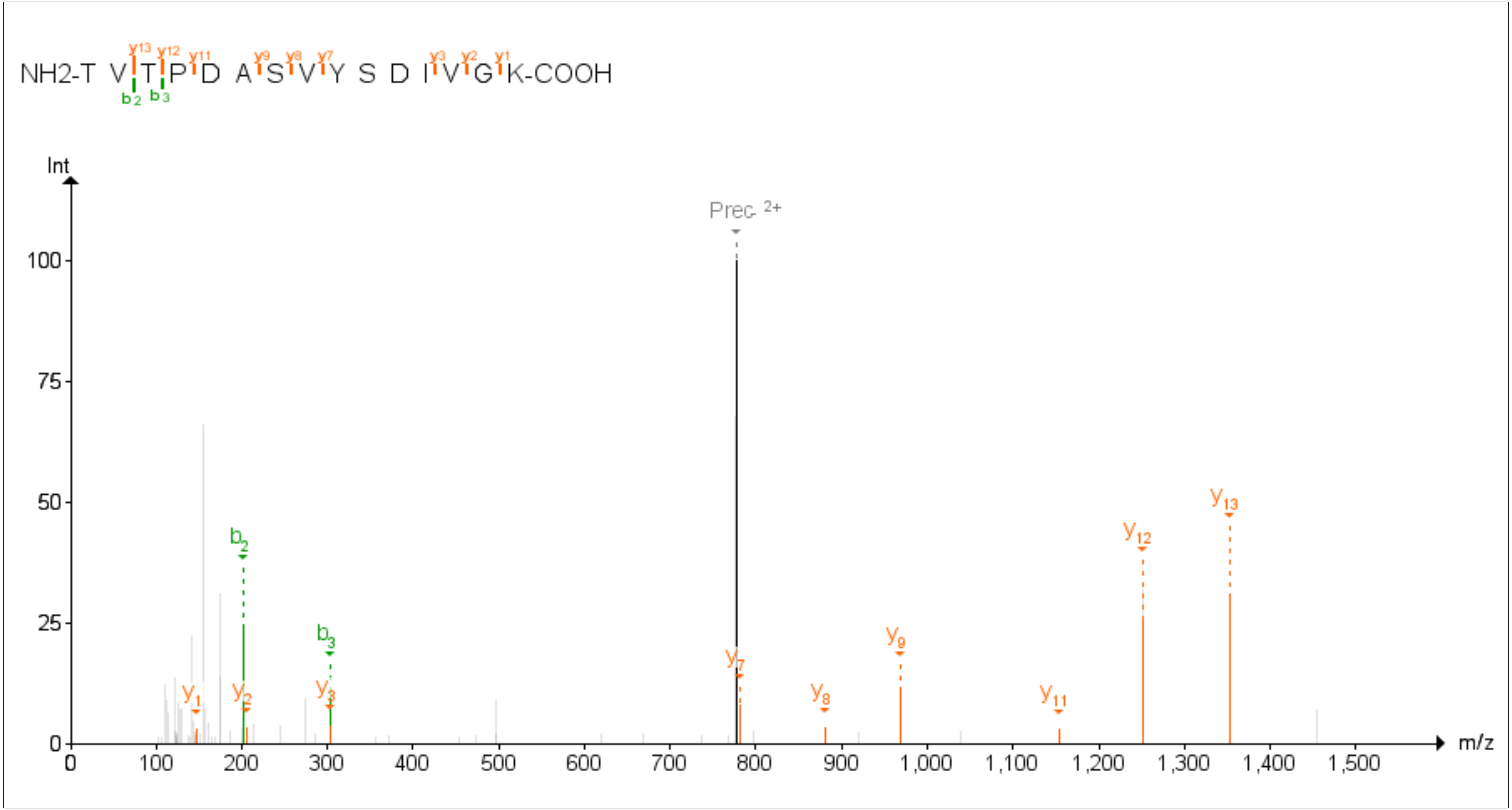

NH2-T V T P D A S V Y S D I V G K-COOH
y13 y12 y11 y9 y8 y7 y3 y2 y1
b2 b3
Int
100
75
50
25
0
Prec 2+
b2
b3
y1
y2
y3
y7
y8
y9
y11
y12
y13
0
100
200
300
400
500
600
700
800
900
1,000
1,100
1,200
1,300
1,400
1,500
m/z

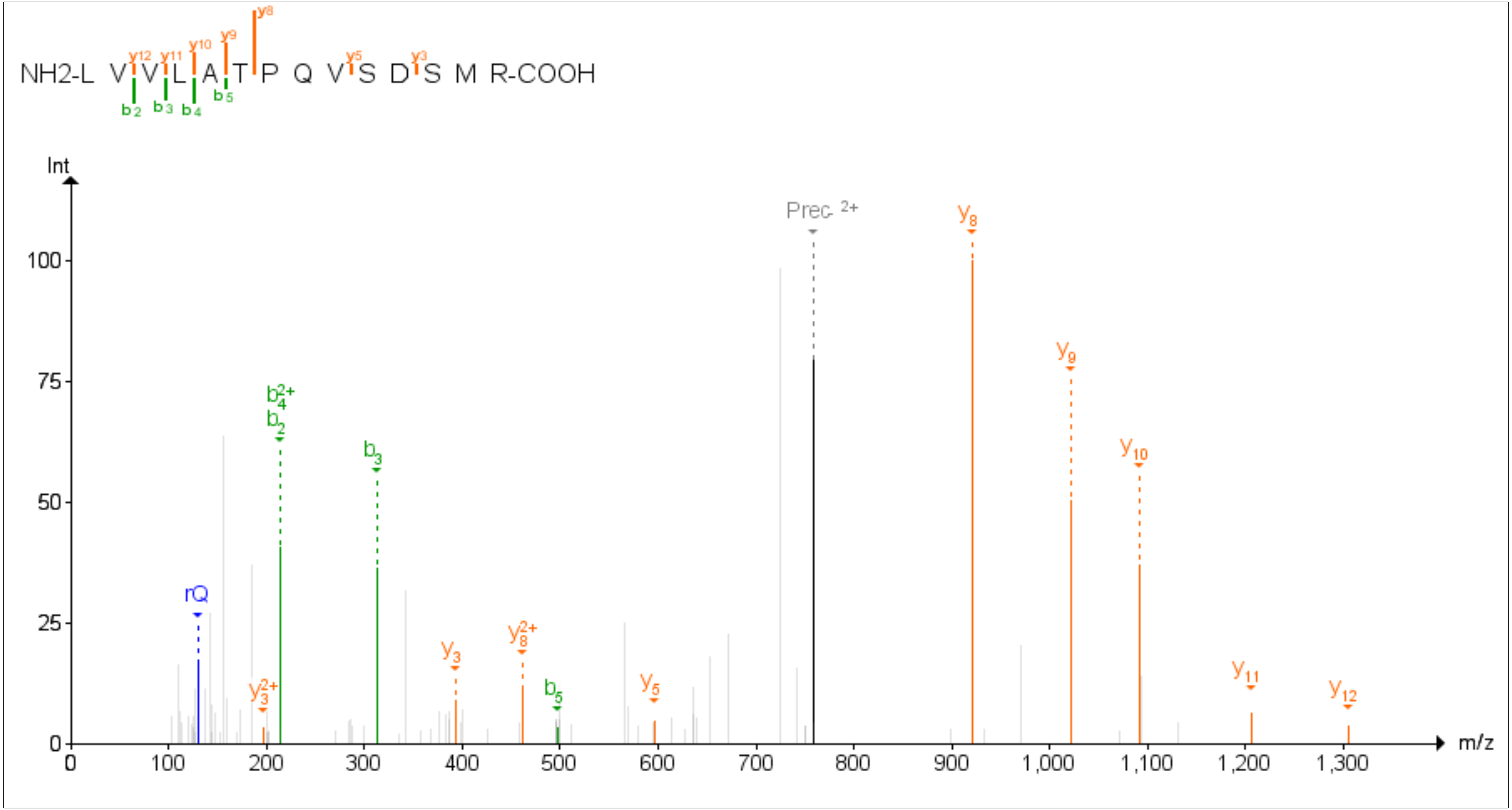
NH2-L V V L A T P Q V S D S M R-COOH
y12 y11 y10 y9 y8 y5 y3
b2 b3 b4 b5
Int
m/z
Prec 2+
y8
y9
y10
y11
y12
b4 2+
b2
b3
rQ
y3 2+
y3
y8 2+
b5
y5
0
25
50
75
100
0
100
200
300
400
500
600
700
800
900
1,000
1,100
1,200
1,300

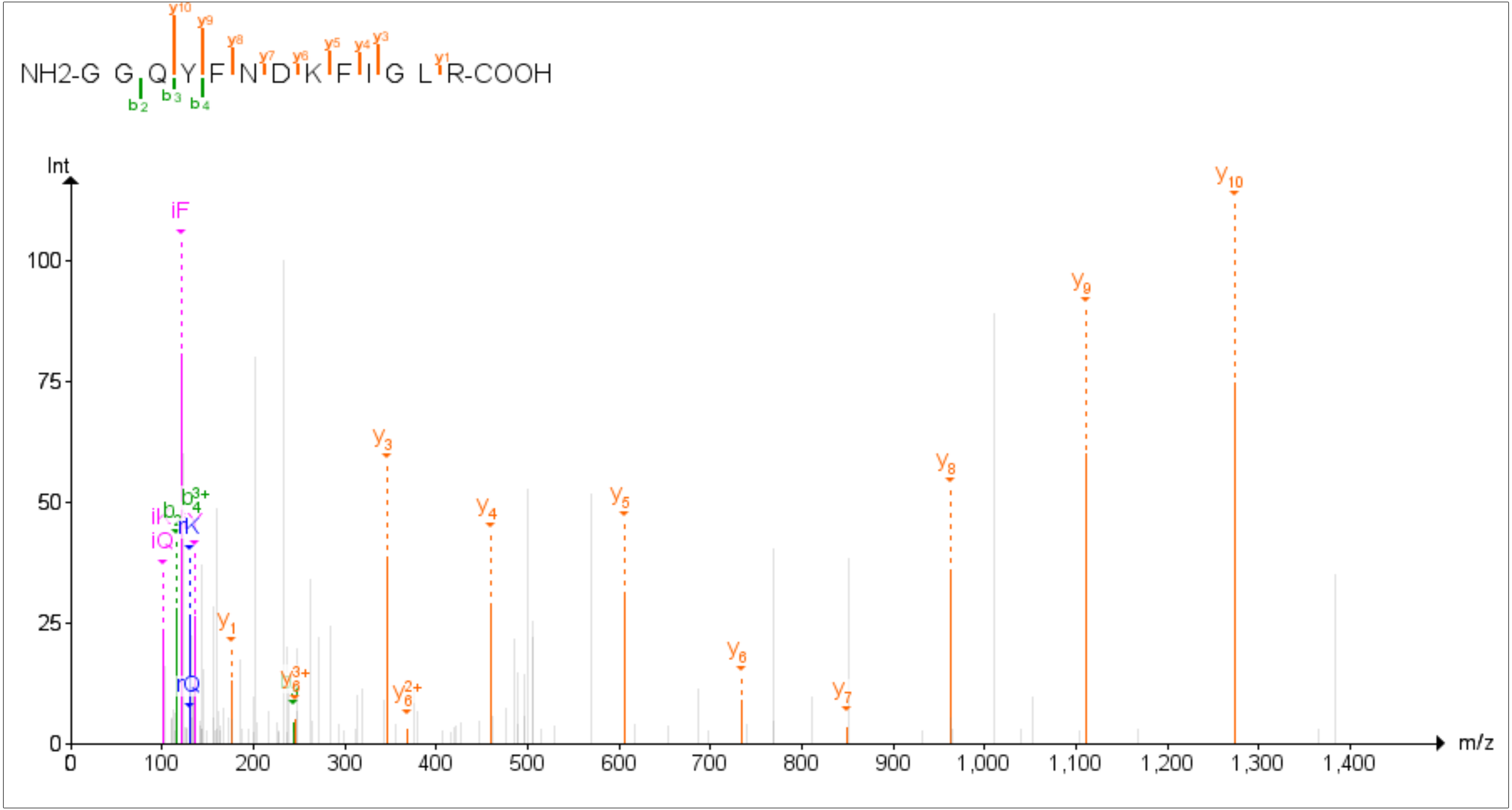

NH2-G G Q Y F N D K F I G L R-COOH
Int
m/z
iF
iQ
rK
rQ
y1
y3
y4
y5
y6
y7
y8
y9
y10

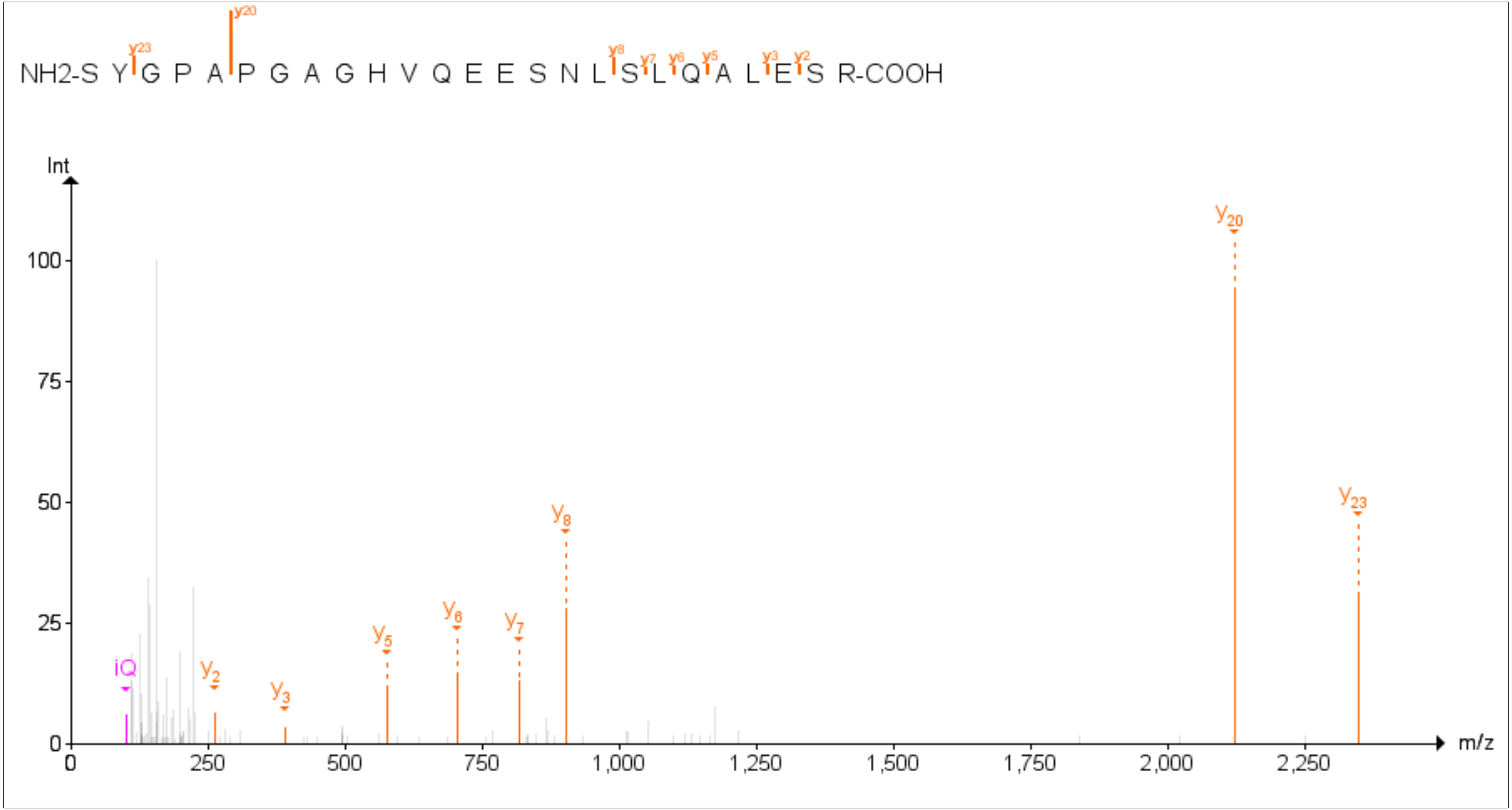

NH2-S Y G P A P G A G H V Q E E S N L S L Q A L E S R-COOH
y23
y20
y8
y7
y6
y5
y3
y2
Int
100
75
50
25
0
iQ
y_2
y_3
y_5
y_6
y_7
y_8
y_{20}
y_{23}
0
250
500
750
1,000
1,250
1,500
1,750
2,000
2,250
m/z

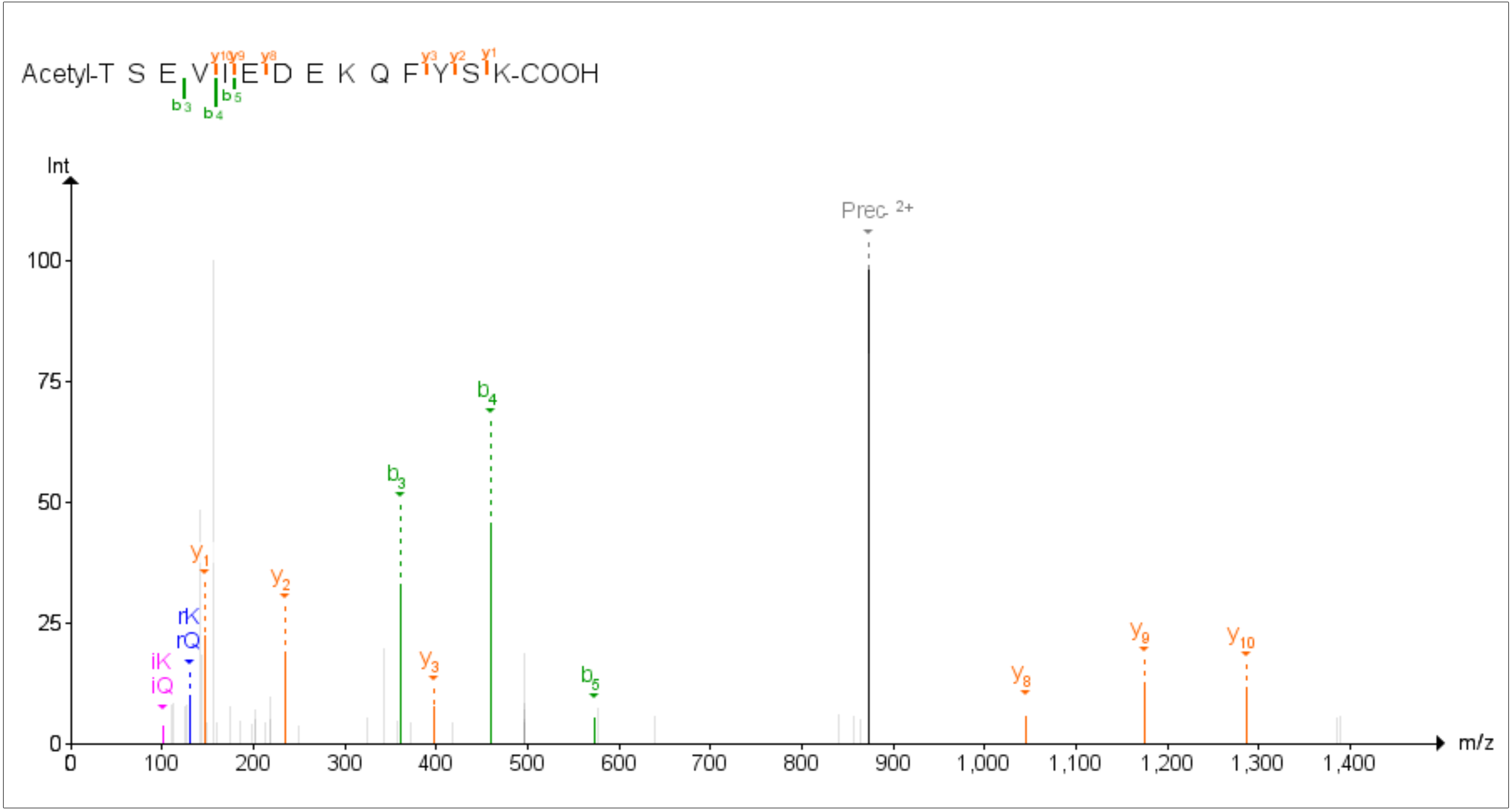

Acetyl-T S E V I E D E K Q F Y S K-COOH
y10 y9 y8 y3 y2 y1
b3 b4 b5
Int
Prec. 2+
100
75
50
25
0
b4
b3
y1
y2
rK
rQ
iK
iQ
y3
b5
y8
y9
y10
0 100 200 300 400 500 600 700 800 900 1,000 1,100 1,200 1,300 1,400
m/z

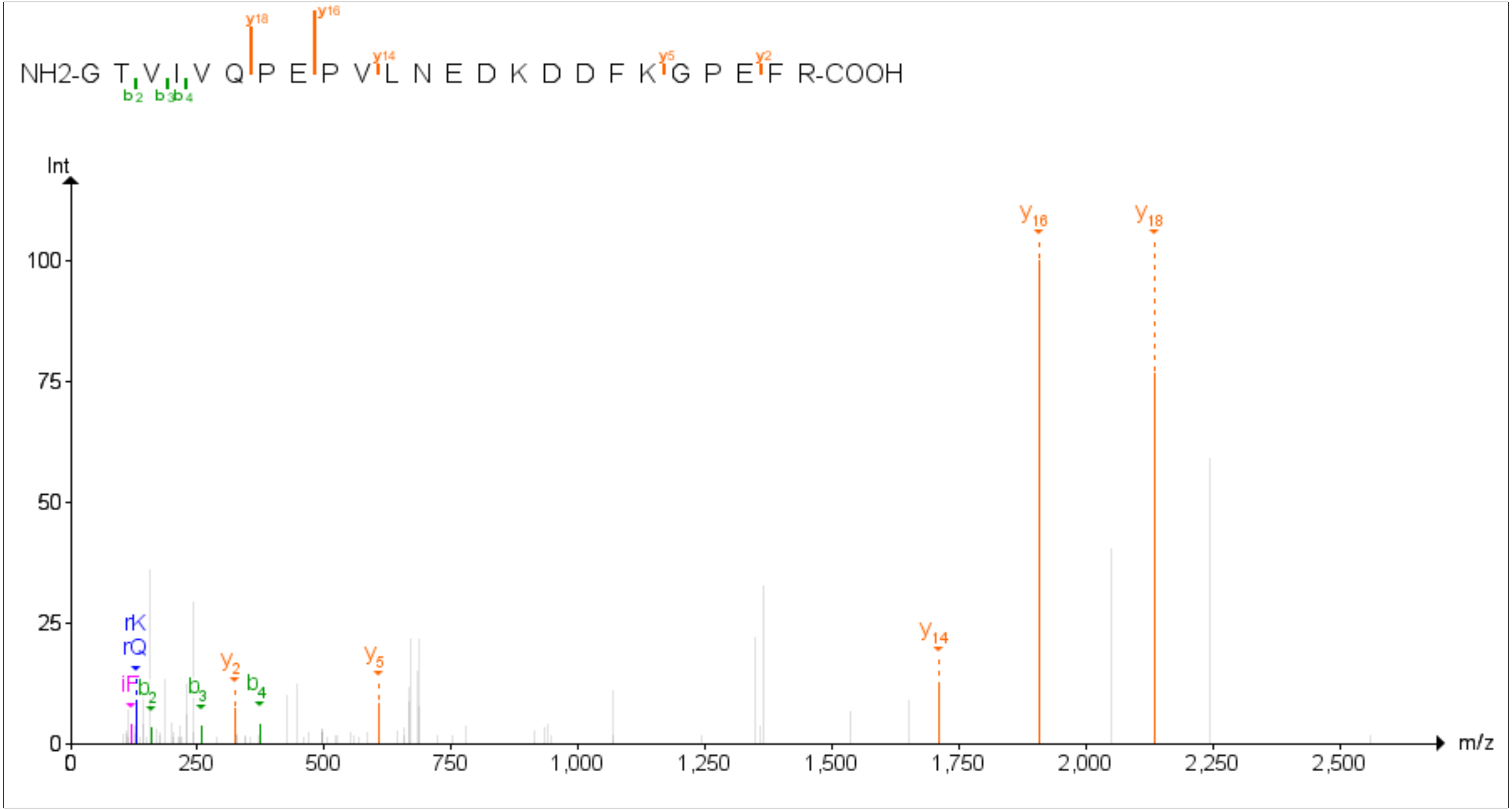

NH2-G T V I V Q P E P V L N E D K D D F K G P E F R-COOH
b2 b3 b4
y18 y16 y14 y5 y2
Int
100
75
50
25
0
m/z
0 250 500 750 1,000 1,250 1,500 1,750 2,000 2,250 2,500
rK
rQ
iF
b2
b3
y2
b4
y5
y14
y16
y18

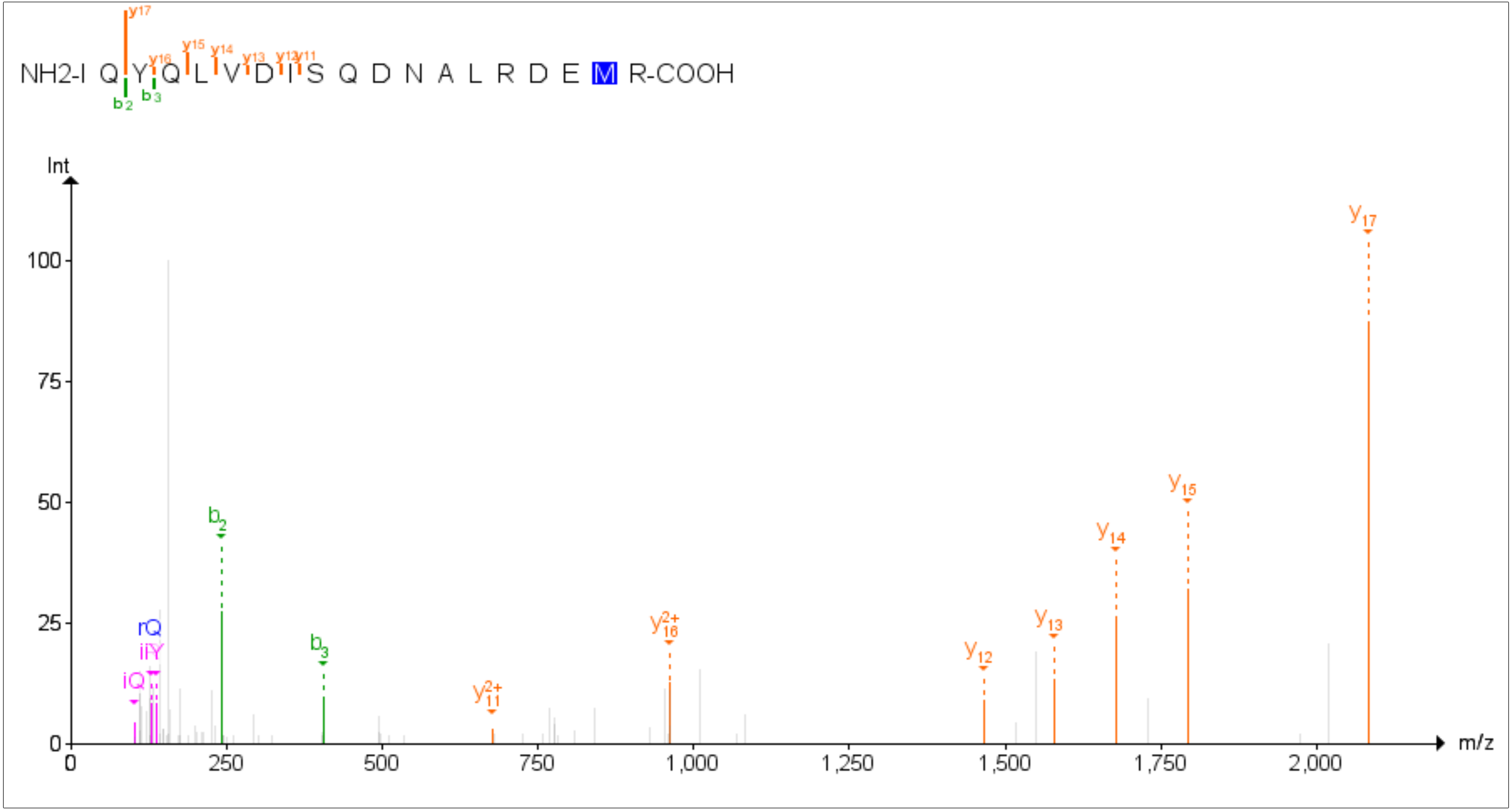

NH2-I Q Y Q L V D I S Q D N A L R D E M R-COOH
y17
y16
y15
y14
y13
y12
y11
b2
b3
Int
m/z
0
25
50
75
100
250
500
750
1,000
1,250
1,500
1,750
2,000
rQ
iiY
iQ
y11 2+
y16 2+

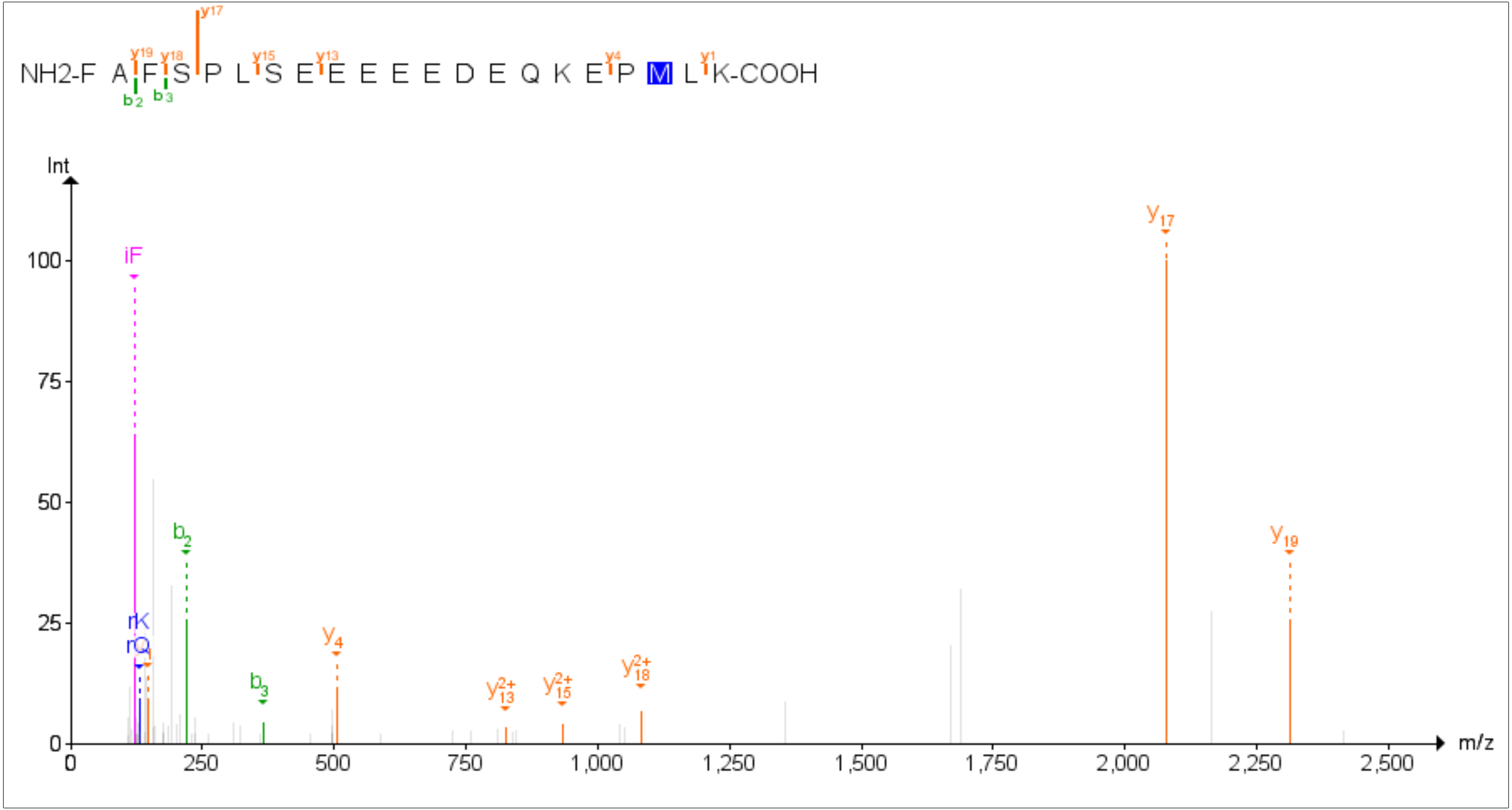

NH2-F A F S P L S E E E E E D E Q K E P M L K-COOH
y17
y19
y18
y15
y13
y4
y1
b2
b3
Int
100
75
50
25
0
iF
b2
rK
rQ
y4
b3
y13 2+
y15 2+
y18 2+
y17
y19
0
250
500
750
1,000
1,250
1,500
1,750
2,000
2,250
2,500
m/z

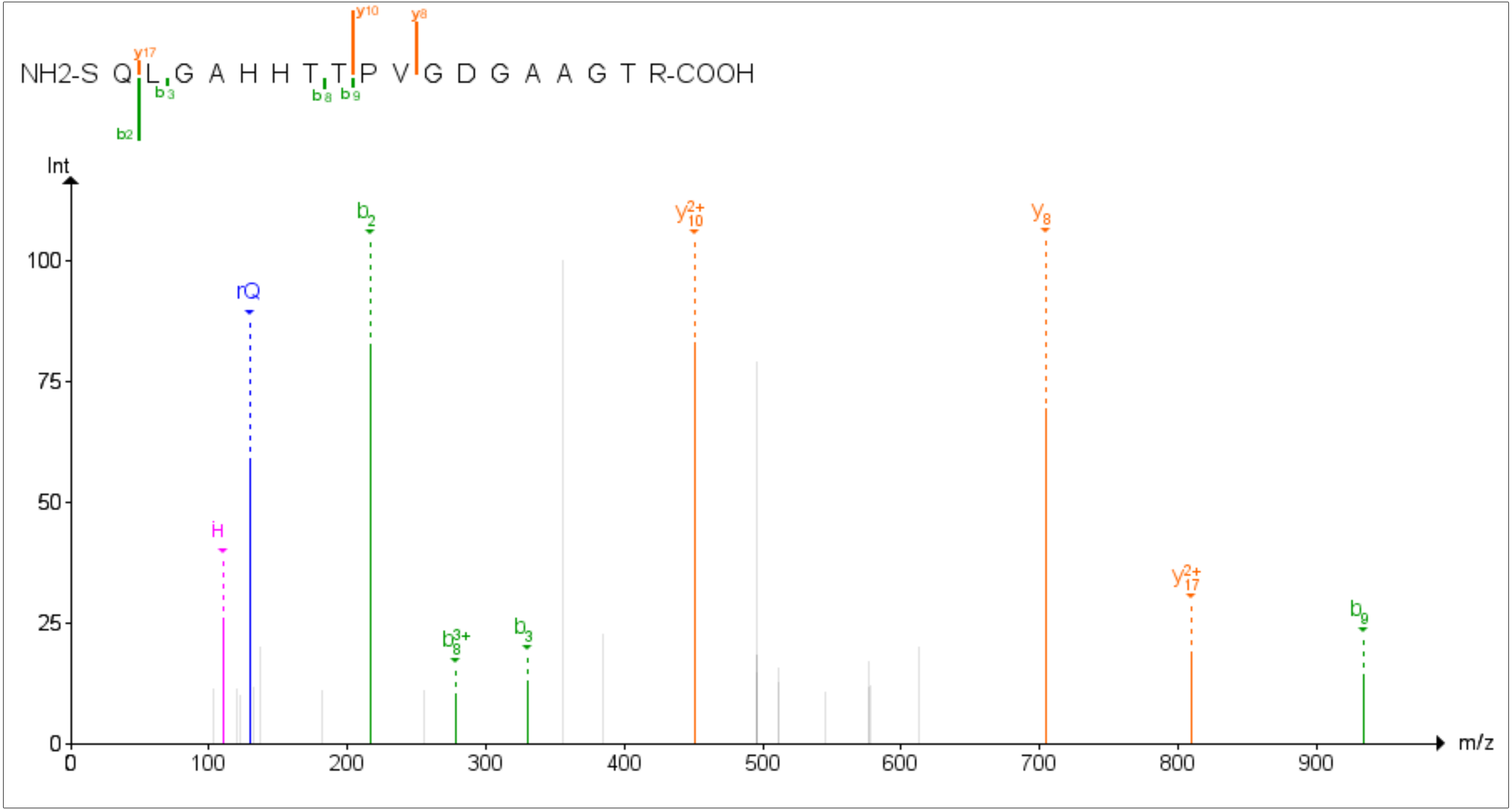

NH2-S Q L G A H H T T P V G D G A A G T R-COOH
y17
y10
y8
b2
b3
b8
b9
Int
100
75
50
25
0
m/z
0
100
200
300
400
500
600
700
800
900
H
rQ
b2
b8 3+
b3
y10 2+
y8
y17 2+
b9

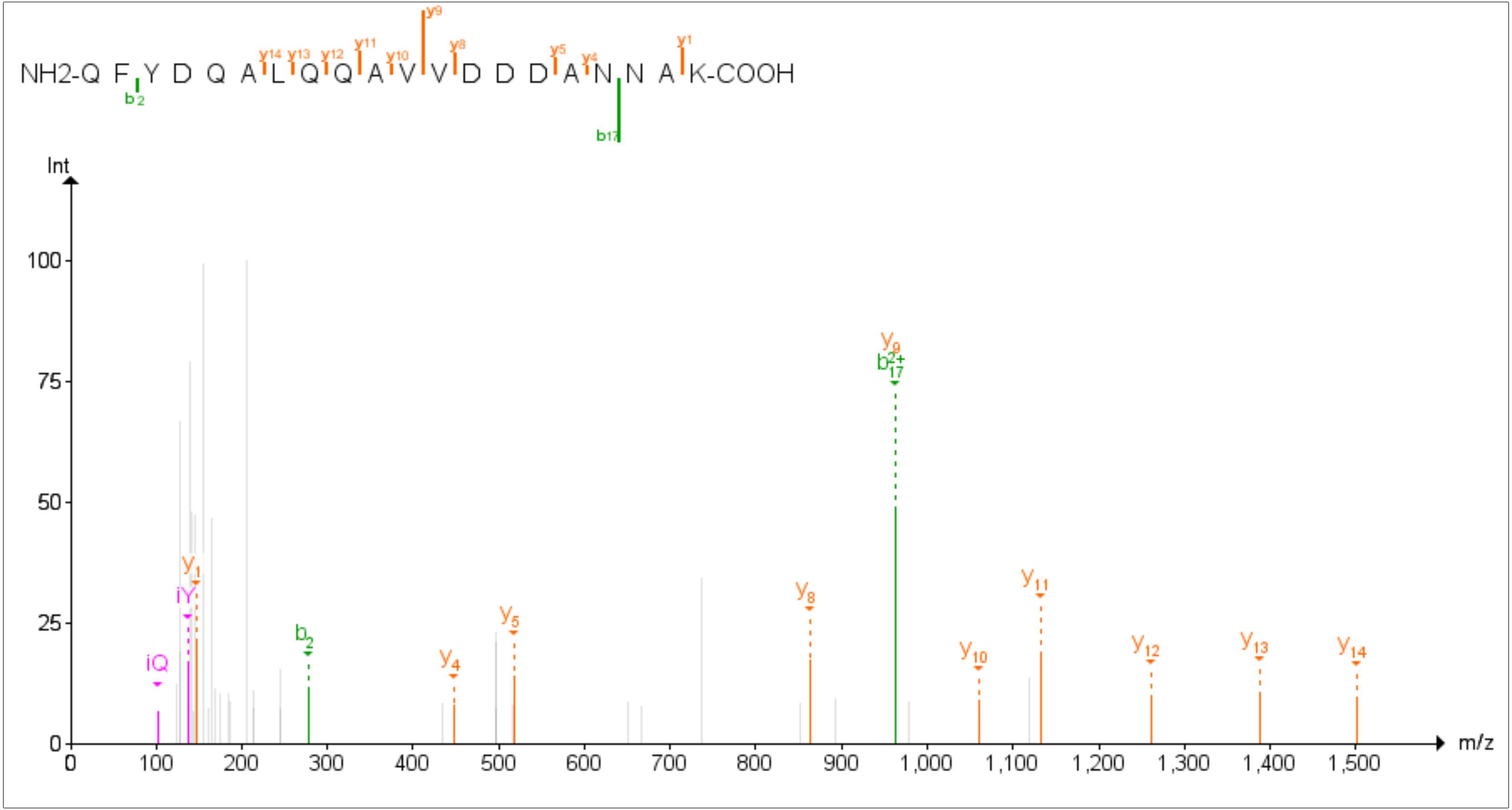

NH2-Q F Y D Q A L Q Q A V V D D D A N N A K-COOH
b2
b17
y14 y13 y12 y11 y10 y9 y8 y5 y4 y1
Int
m/z
iQ
iY
y1
b2
y4
y5
y8
y9
b17 2+
y10
y11
y12
y13
y14

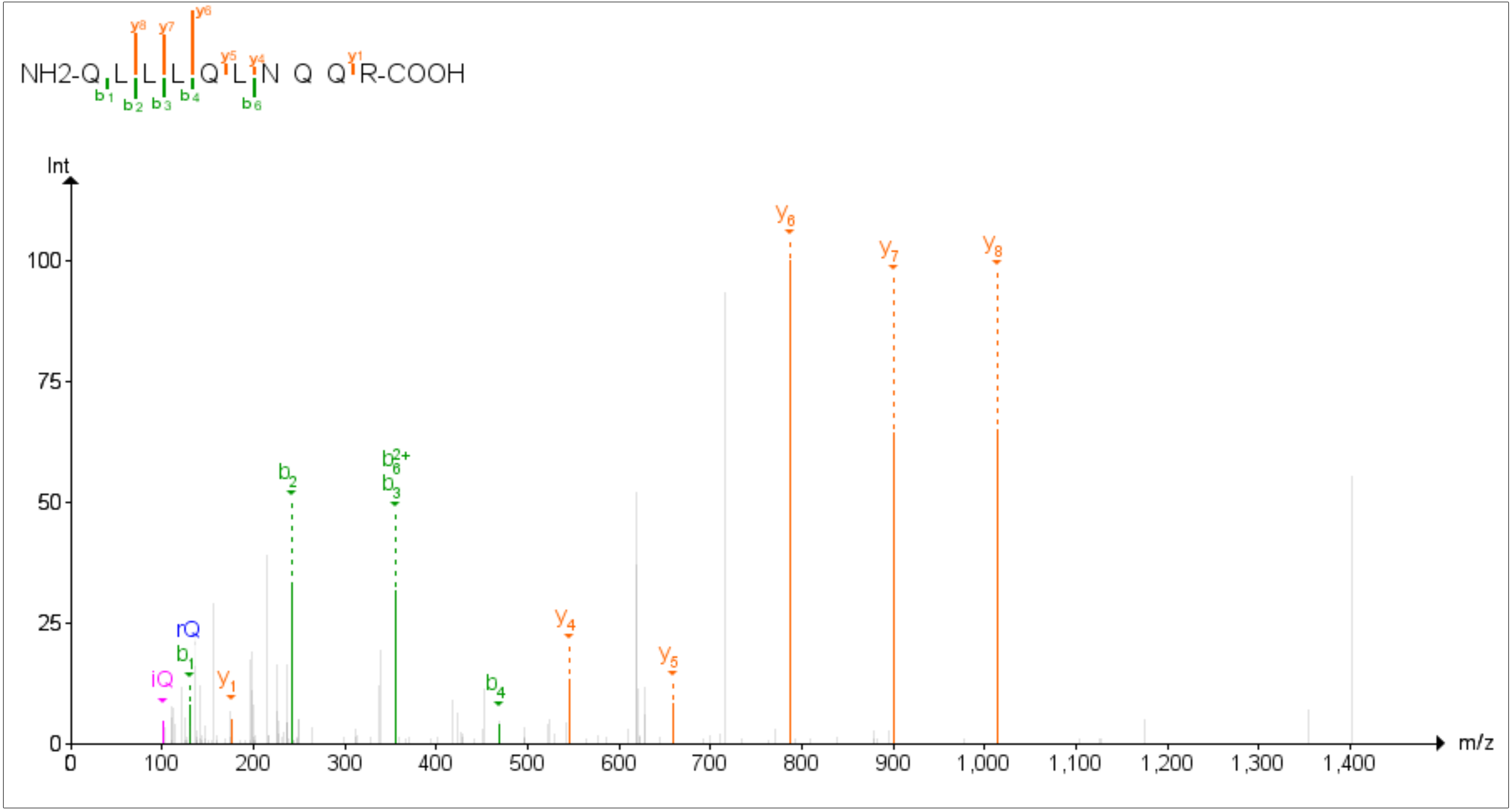
NH2-Q L L L L Q L N Q Q R-COOH
b1 b2 b3 b4 b6
y8 y7 y6 y5 y4 y1
Int
100
75
50
25
0
m/z
0 100 200 300 400 500 600 700 800 900 1,000 1,100 1,200 1,300 1,400
iQ
rQ
b1
y1
b2
b6 2+
b3
b4
y4
y5
y6
y7
y8

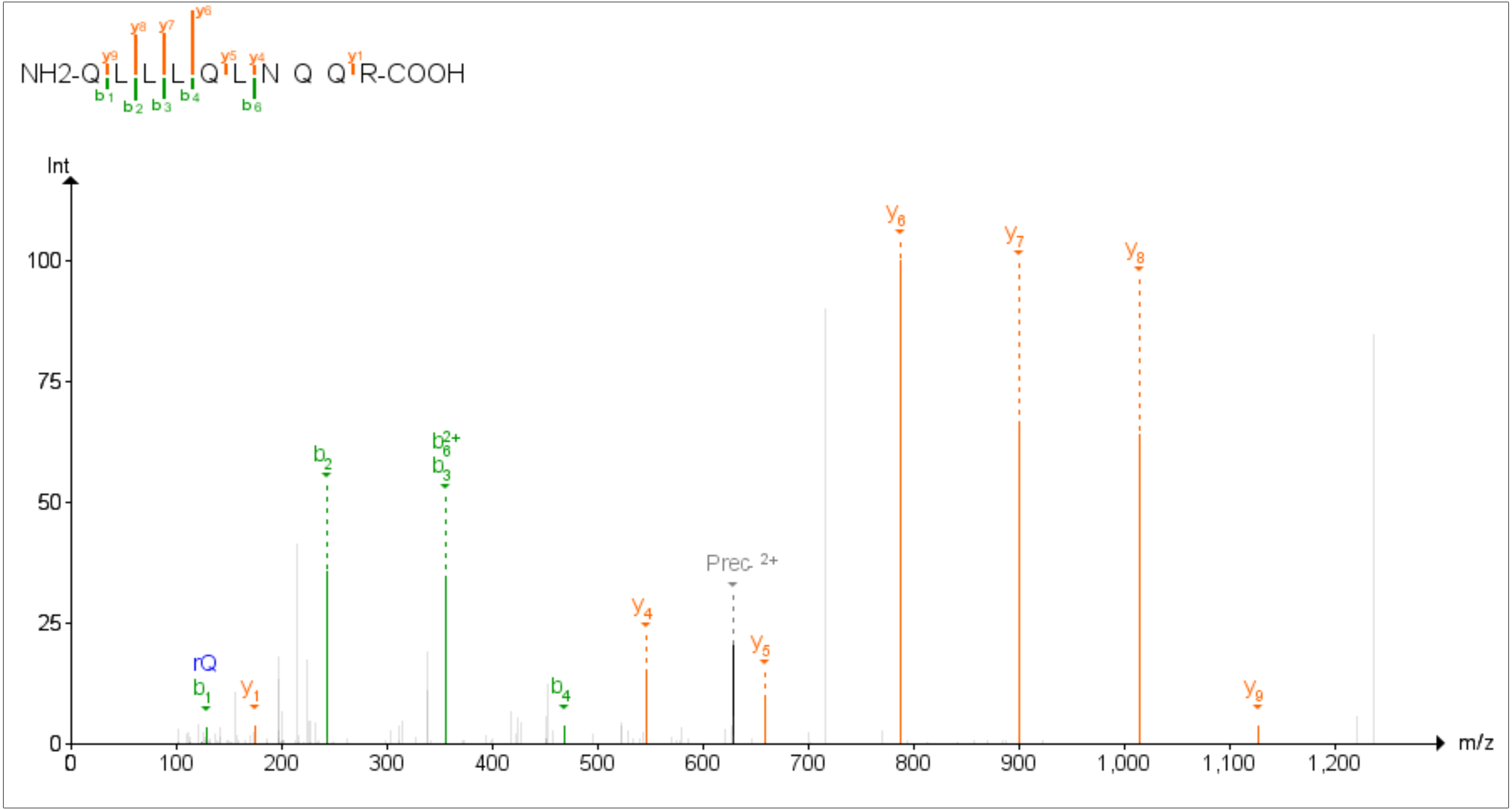

NH2-Q L L L Q L N Q Q R-COOH
y9 y8 y7 y6 y5 y4 y1
b1 b2 b3 b4 b6
Int
m/z
rQ
b1
y1
b2
b6 2+
b3
b4
y4
Prec. 2+
y5
y6
y7
y8
y9

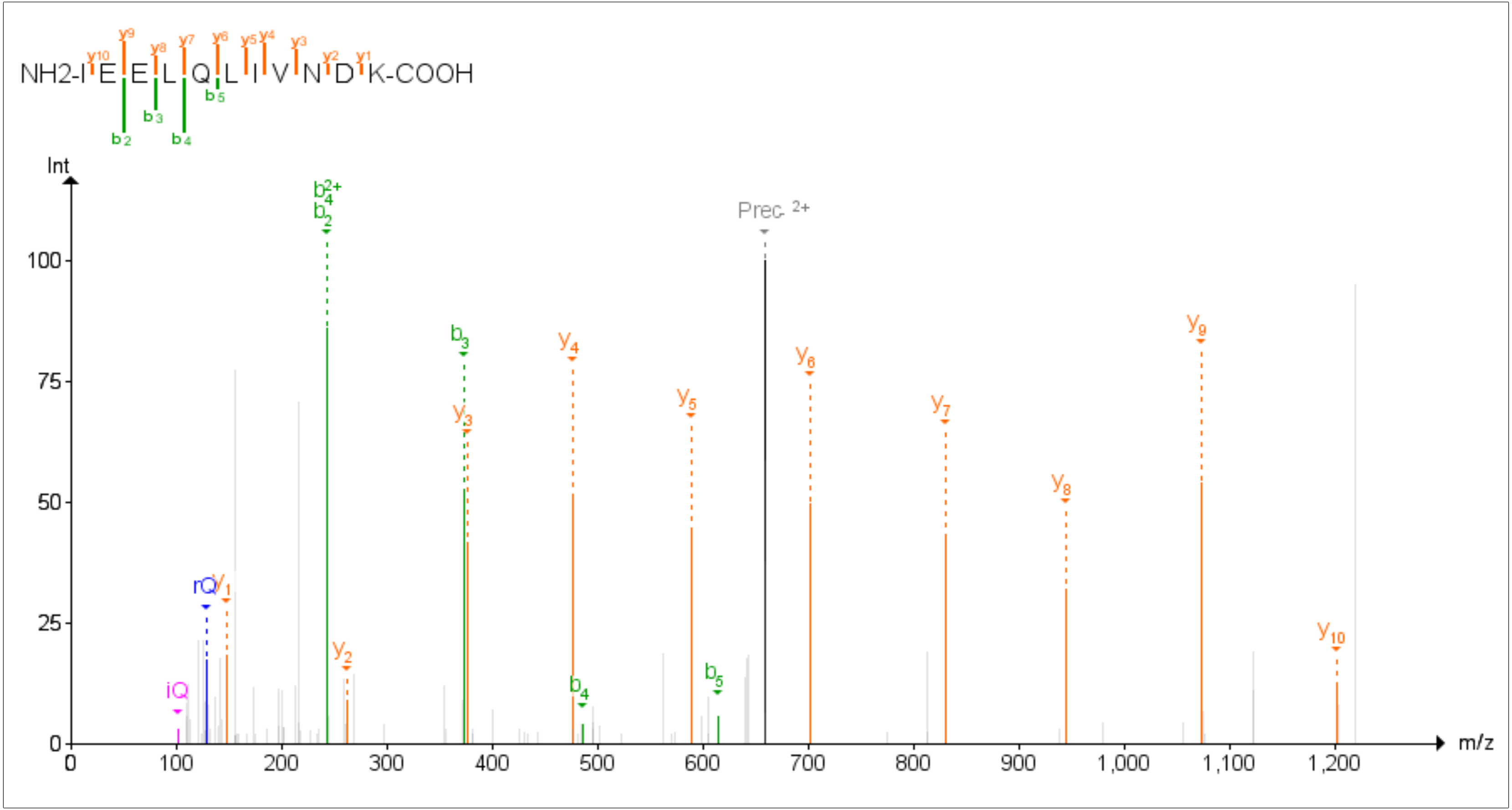

NH2-I E E L Q L I V N D K-COOH
y10 y9 y8 y7 y6 y5 y4 y3 y2 y1
b2 b3 b4 b5
Int
m/z
Prec 2+
b4 2+
b2
b3
y3
y4
y5
y6
y7
y8
y9
y10
y1
y2
rQ
iQ
b4
b5
0
25
50
75
100
0
100
200
300
400
500
600
700
800
900
1,000
1,100
1,200

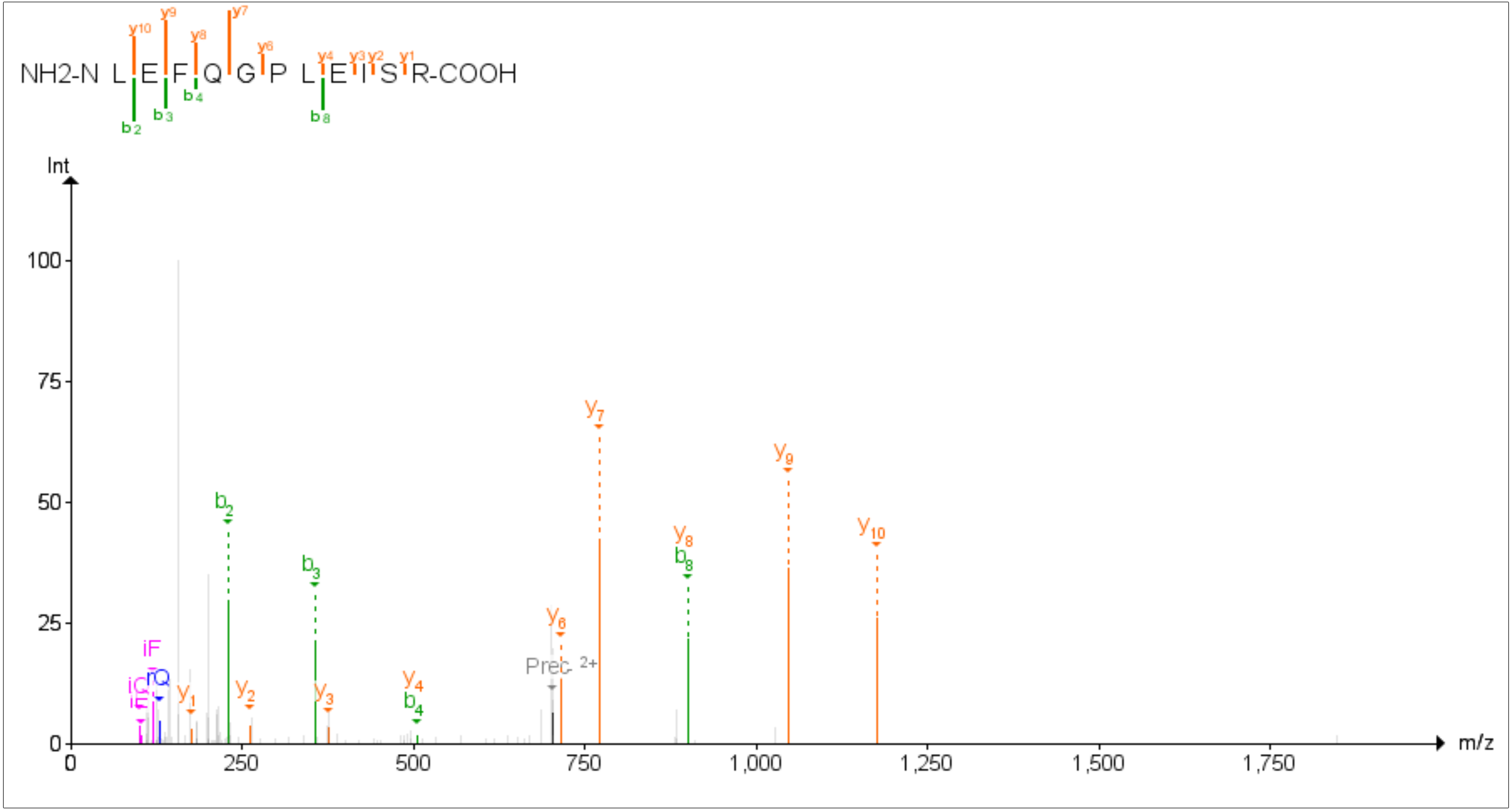

NH2-N L E F Q G P L E I S R-COOH
y10 y9 y8 y7 y6 y4 y3 y2 y1
b2 b3 b4 b8
Int
100
75
50
25
0
b2
b3
y7
y9
y8
b8
y10
y6
Prec 2+
iF
iQ
rQ
iE
y1
y2
y3
y4
b4
0
250
500
750
1,000
1,250
1,500
1,750
m/z

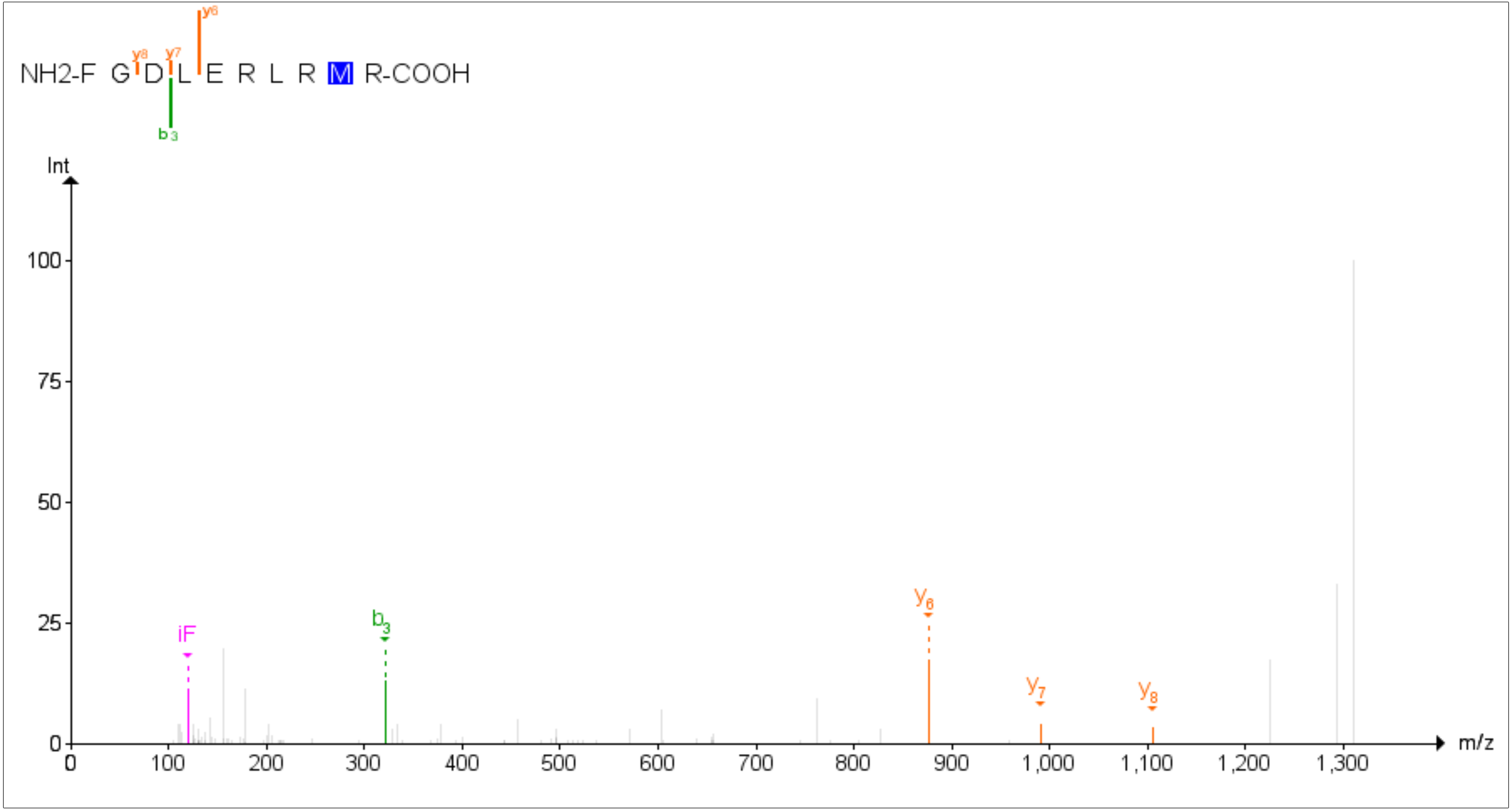

NH2-F G D L E R L R M R-COOH
y8
y7
y6
b3
Int
100
75
50
25
0
iF
b3
y6
y7
y8
m/z
0
100
200
300
400
500
600
700
800
900
1,000
1,100
1,200
1,300

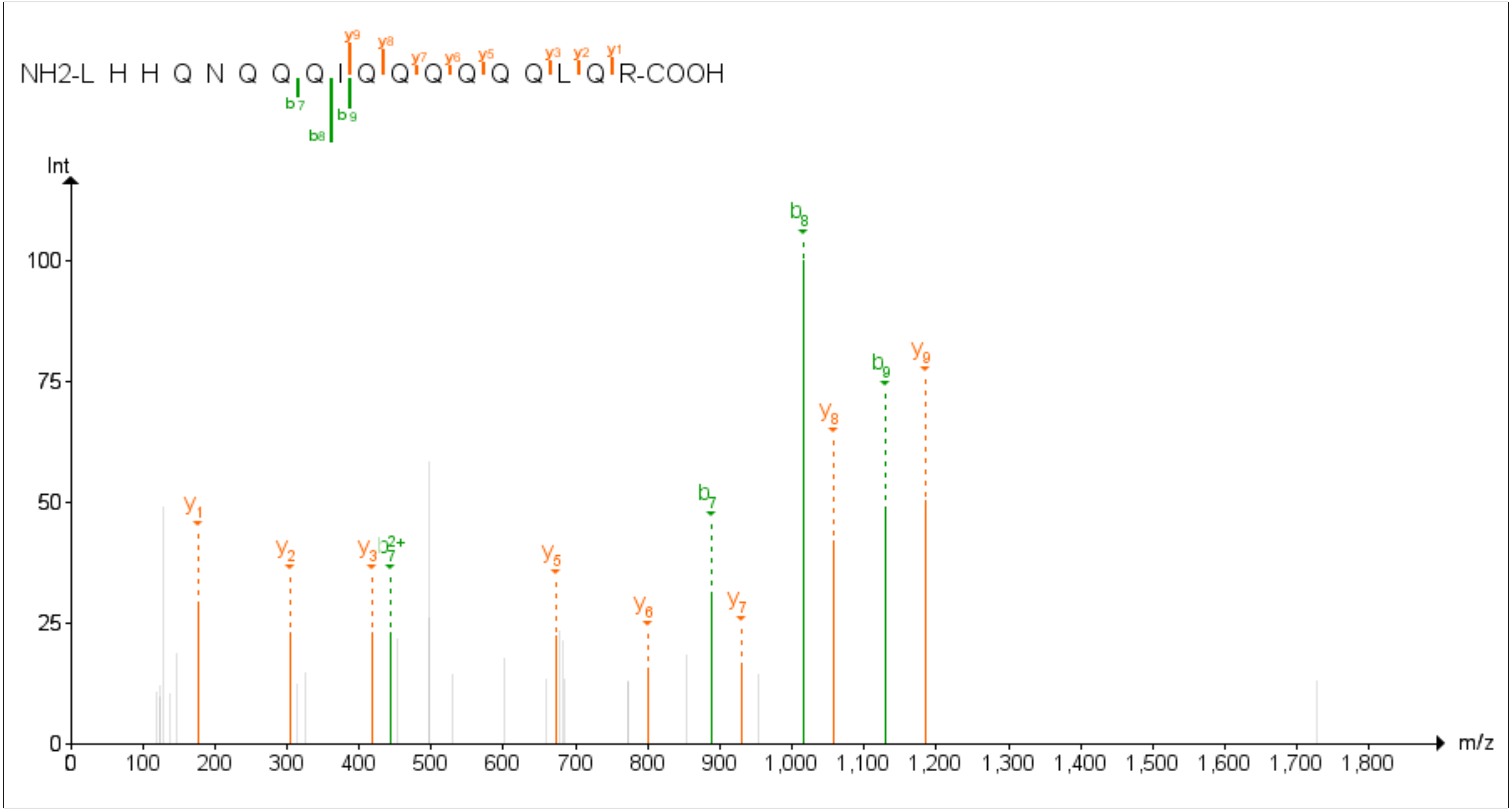

NH2-L H H Q N Q Q Q I Q Q Q Q Q Q L Q R-COOH
y9
y8
y7
y6
y5
y3
y2
y1
b7
b8
b9
Int
100
75
50
25
0
b8
b9
y9
y8
b7
y1
y2
y3
b7 2+
y5
y6
y7
m/z
0
100
200
300
400
500
600
700
800
900
1,000
1,100
1,200
1,300
1,400
1,500
1,600
1,700
1,800

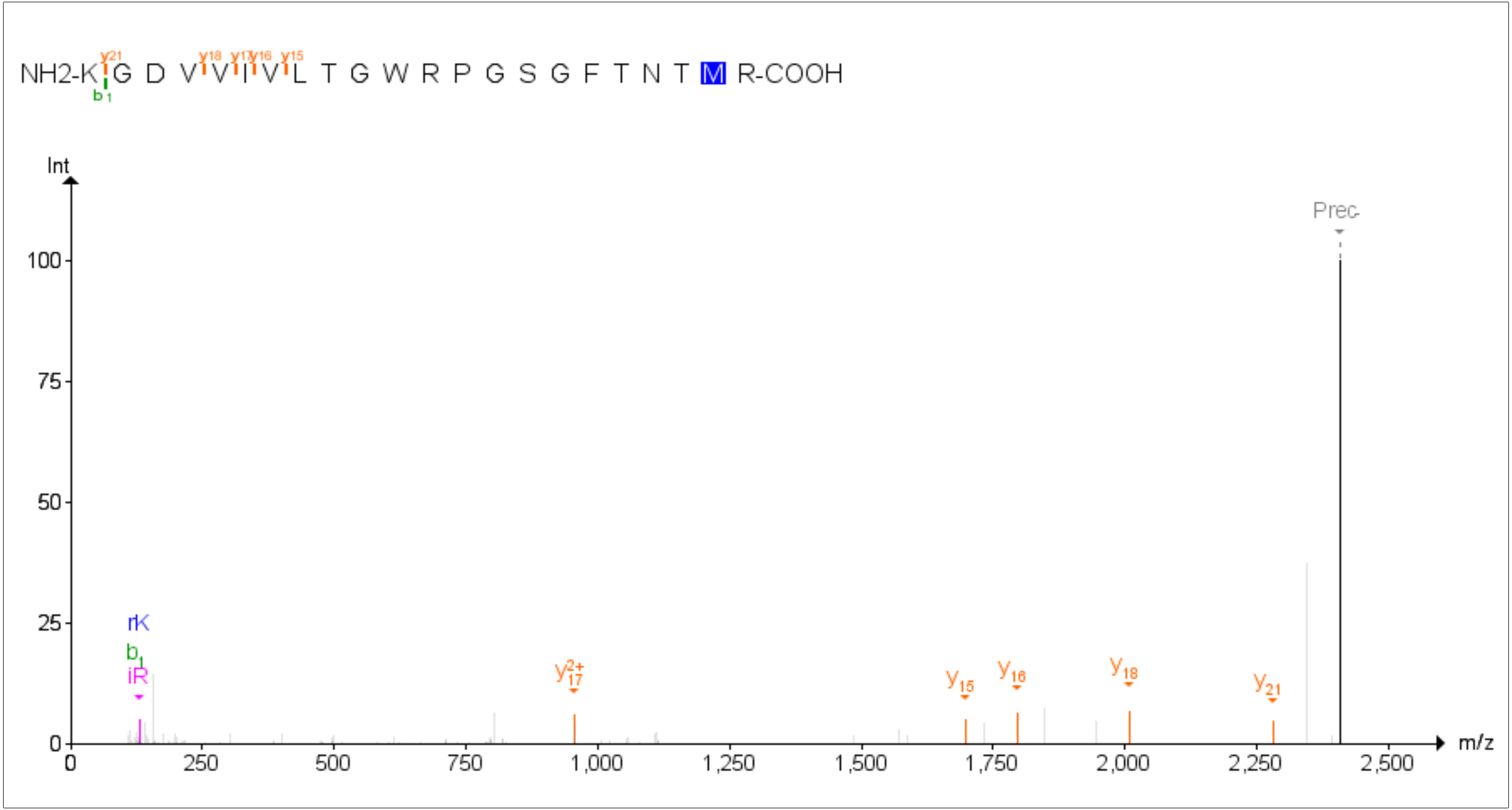

NH2-K G D V V I V L T G W R P G S G F T N T M R-COOH
y21 y18 y17 y16 y15
b1
Int
100
75
50
25
0
Prec
rK
b1
iR
y17 2+
y15
y16
y18
y21
0 250 500 750 1,000 1,250 1,500 1,750 2,000 2,250 2,500
m/z

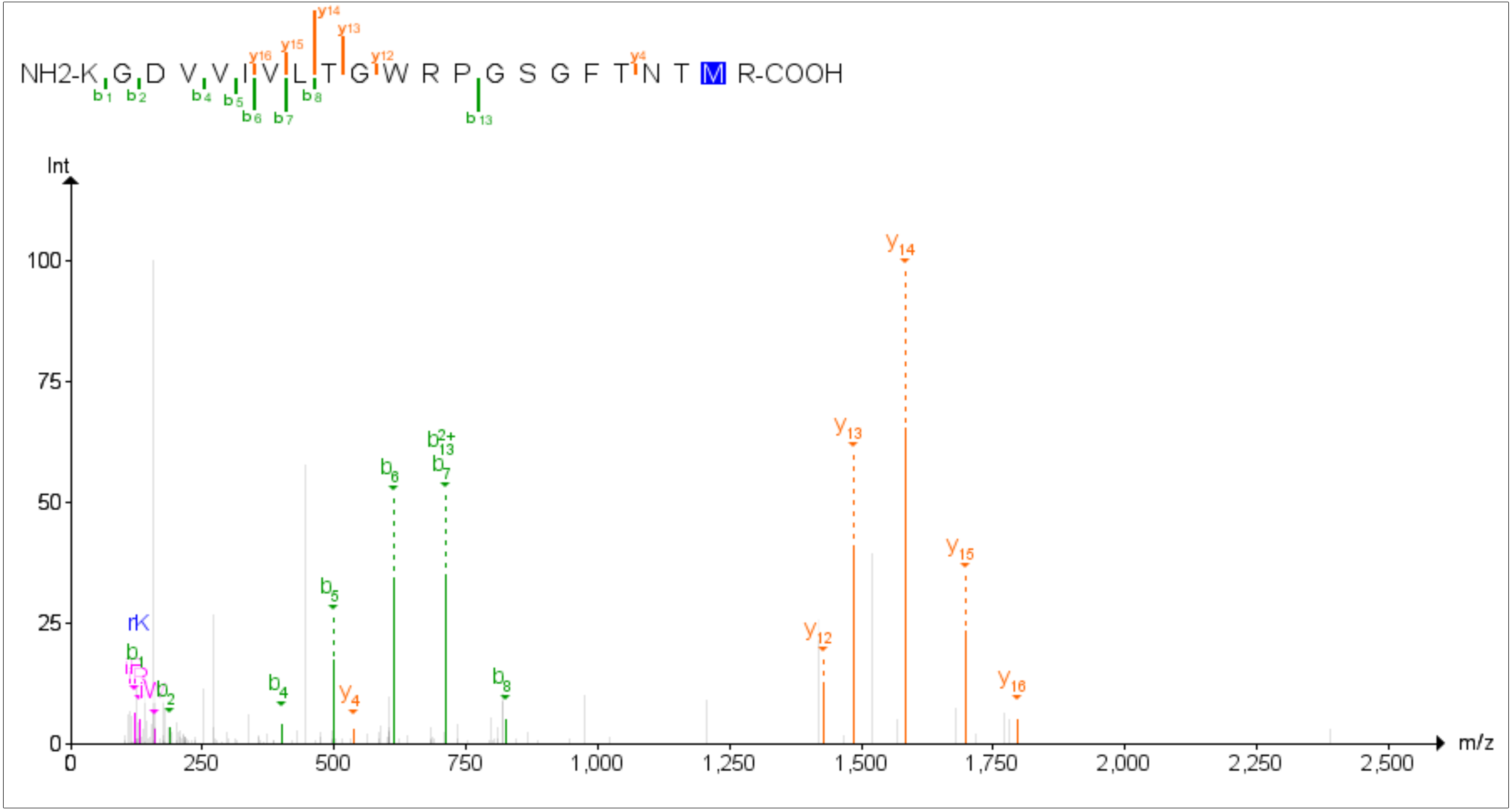
NH2-K G D V V I V L T G W R P G S G F T N T M R-COOH
b1 b2 b4 b5 b6 b7 b8 b13
y16 y15 y14 y13 y12 y4
Int
100
75
50
25
0
m/z
0 250 500 750 1,000 1,250 1,500 1,750 2,000 2,250 2,500
rK
b1
b2
b4
b5
b6
b7
b13 2+
b8
y4
y12
y13
y14
y15
y16

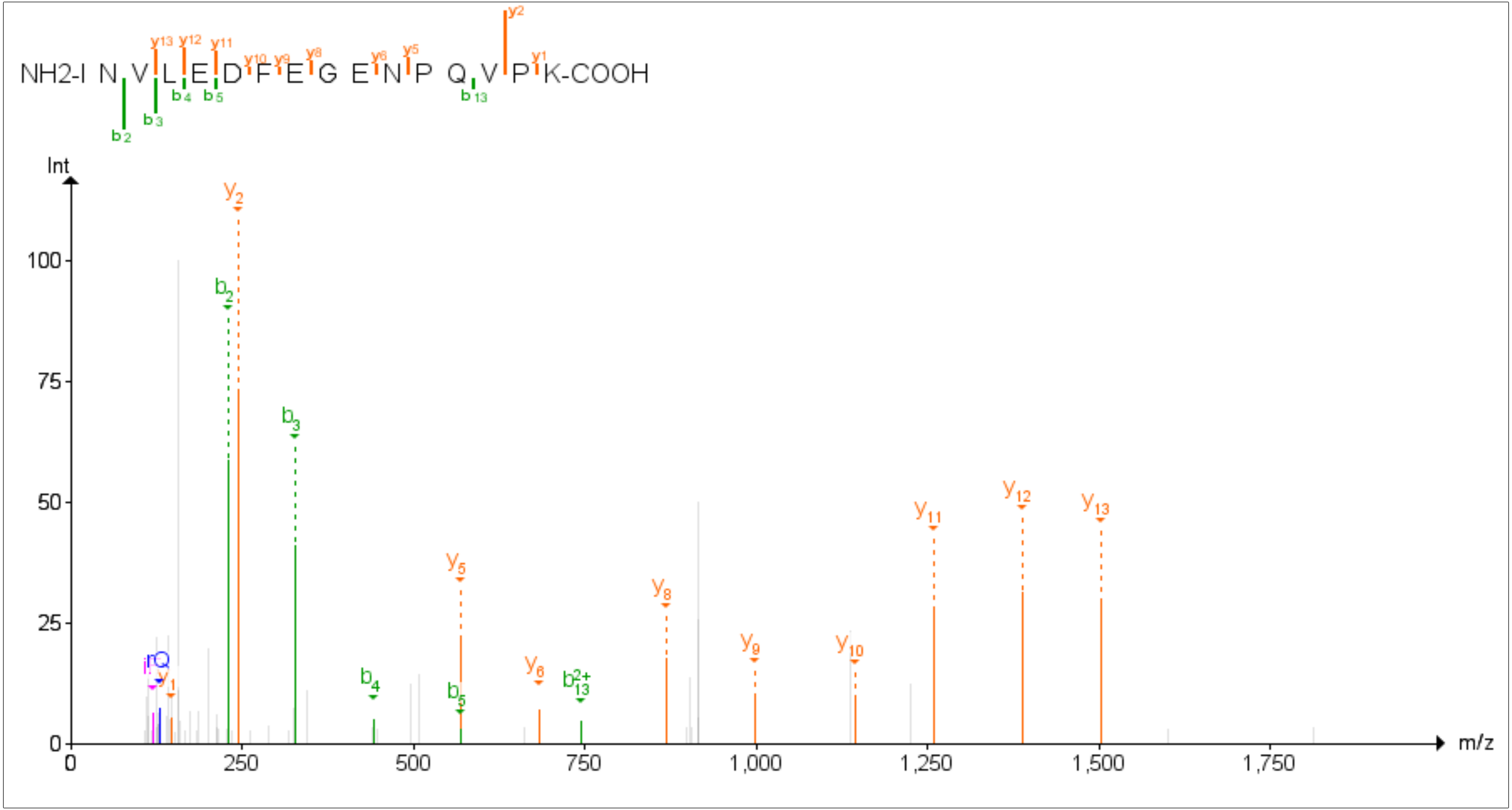

NH2-I N V L E D F E G E N P Q V P K-COOH
b2 b3 b4 b5 b13
y13 y12 y11 y10 y9 y8 y6 y5 y2 y1
Int
100
75
50
25
0
0
250
500
750
1,000
1,250
1,500
1,750
m/z
y2
b2
b3
y5
y8
y11
y12
y13
y9
y10
b4
b5
y6
b13 2+
y1
iQ

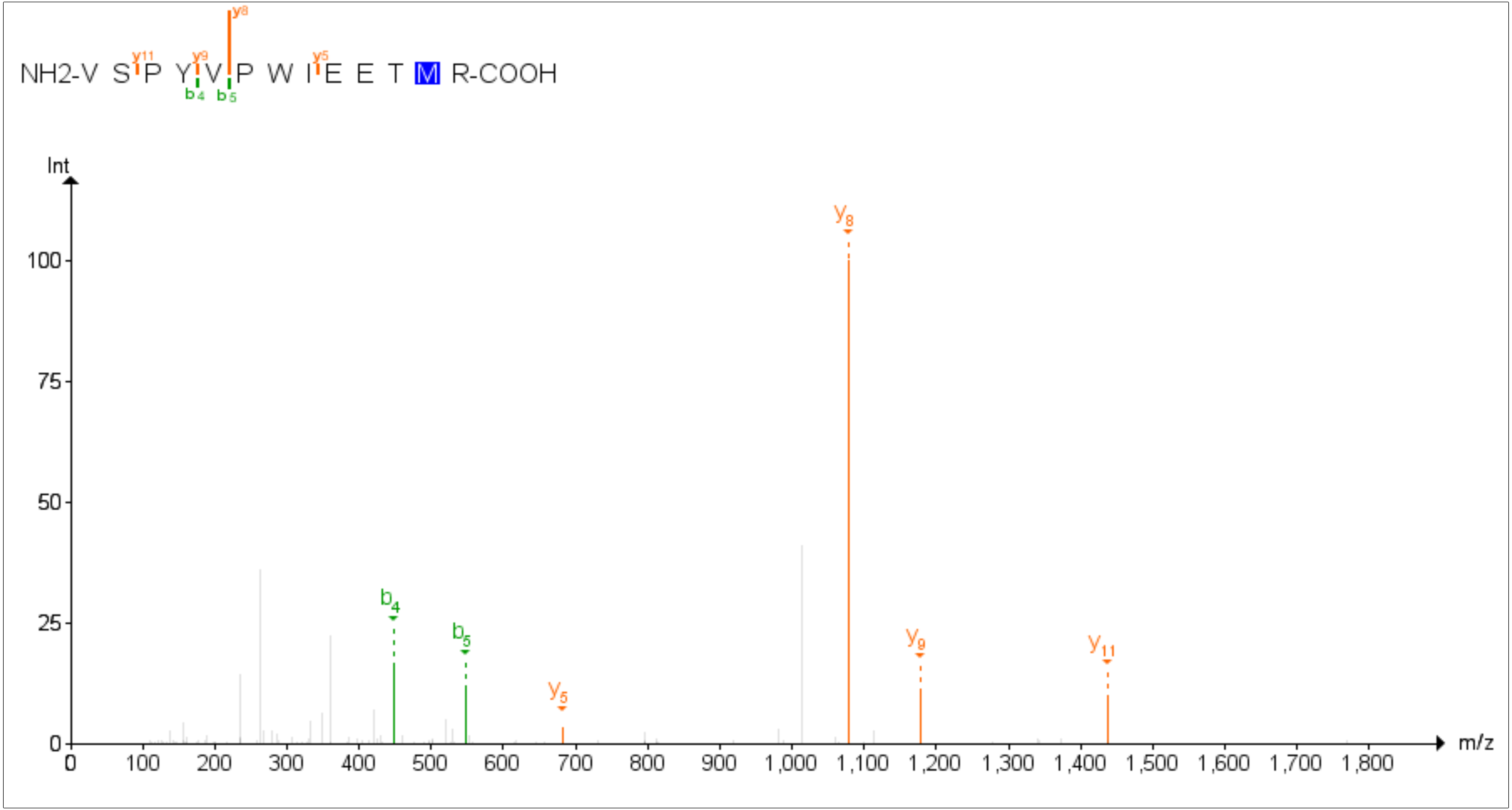

NH2-V S P Y V P W I E E T M R-COOH
y11 y9 y8 y5
b4 b5
Int
m/z
0 25 50 75 100
0 100 200 300 400 500 600 700 800 900 1,000 1,100 1,200 1,300 1,400 1,500 1,600 1,700 1,800
b4
b5
y5
y8
y9
y11

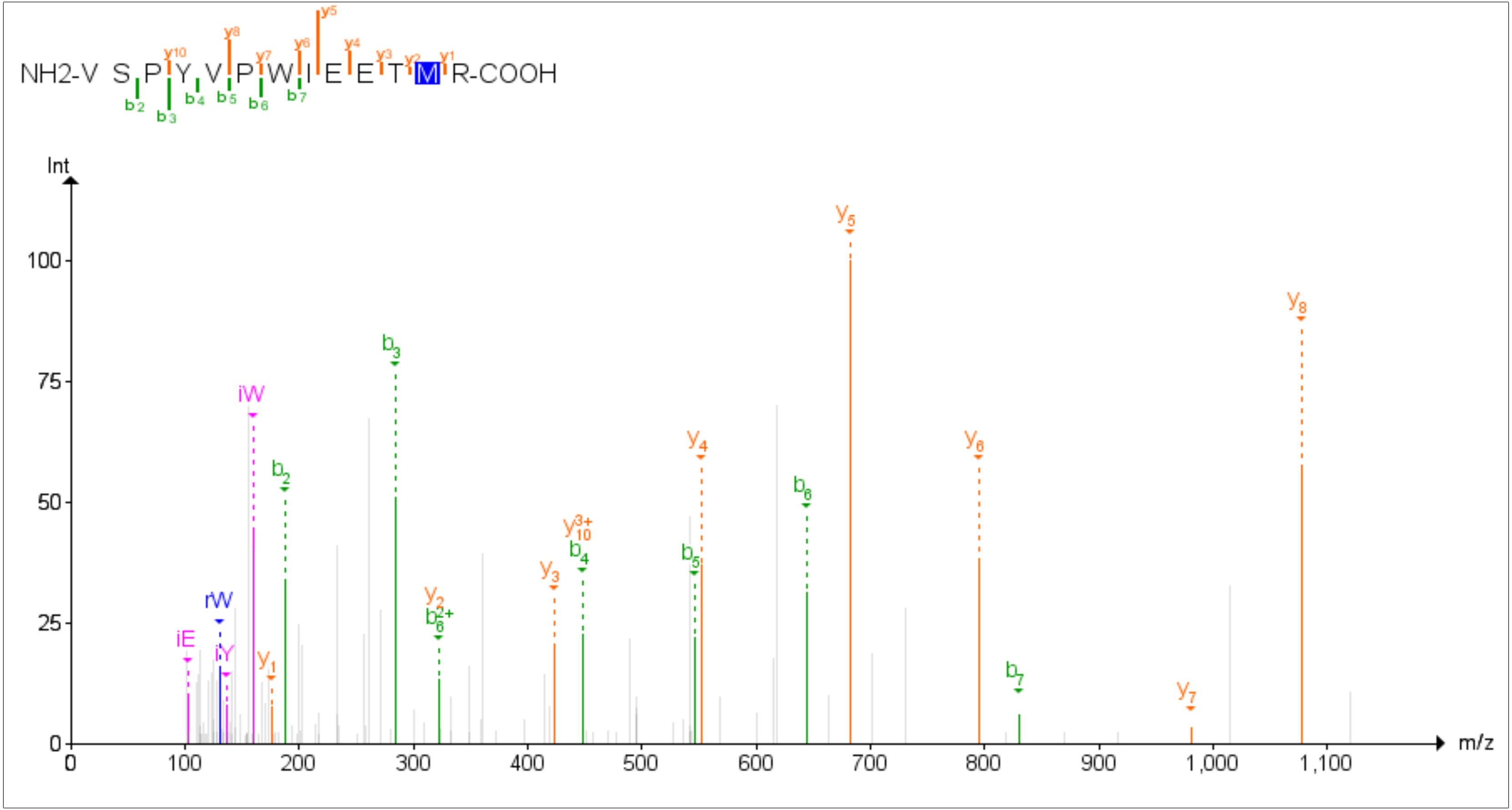

NH2-V S P Y V P W I E E T M R-COOH
y10 y8 y7 y6 y5 y4 y3 y2 y1
b2 b3 b4 b5 b6 b7
Int
m/z
0
25
50
75
100
0
100
200
300
400
500
600
700
800
900
1,000
1,100
iE
rW
iY
iW
y1
b2
b3
y2
b6 2+
y3
b4
y10 3+
b5
y4
b6
y5
y6
b7
y7
y8

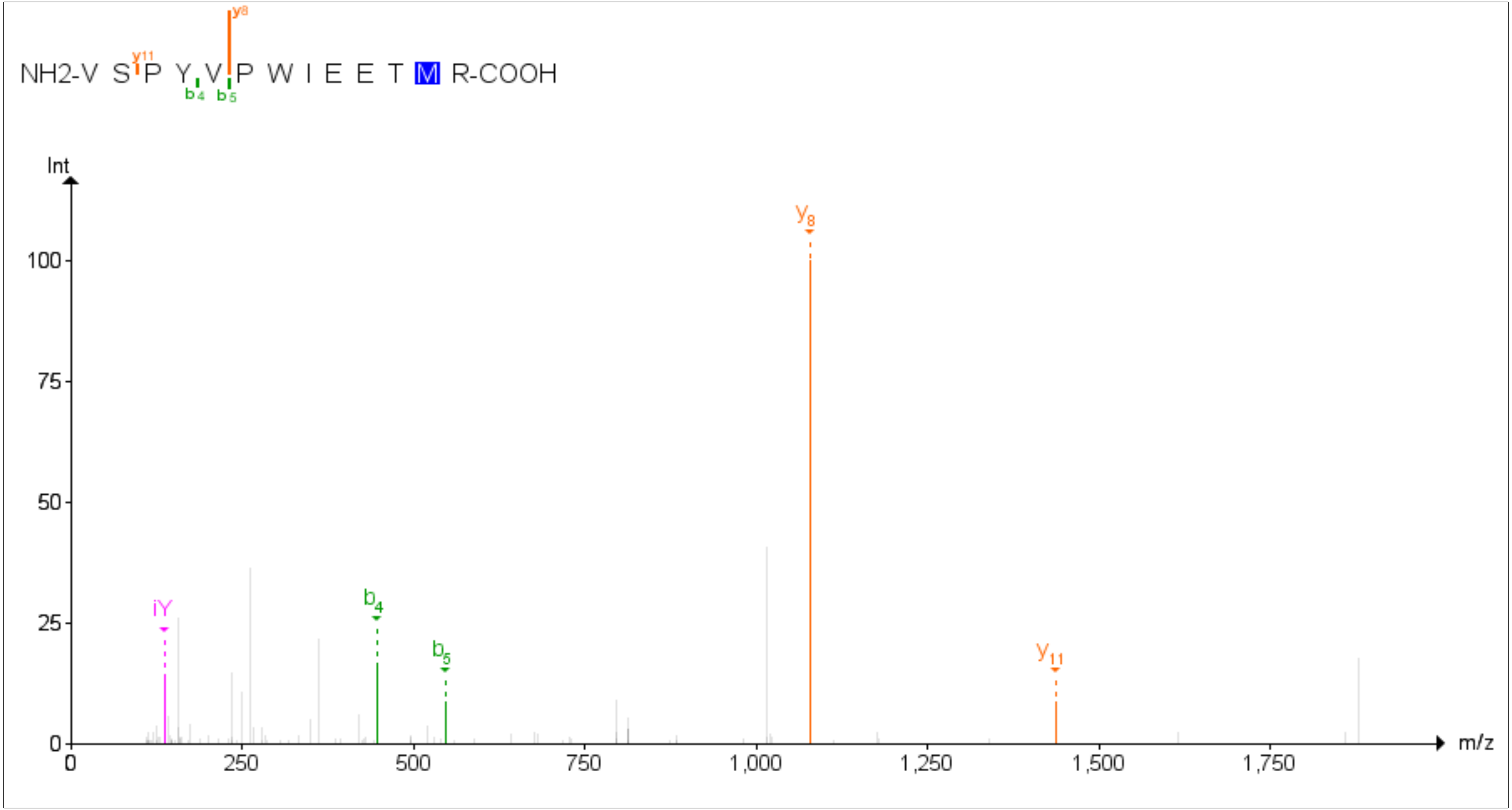

NH2-V S P Y V P W I E E T M R-COOH
y8
y11
b4
b5
Int
100
75
50
25
0
iY
b4
b5
y8
y11
0
250
500
750
1,000
1,250
1,500
1,750
m/z

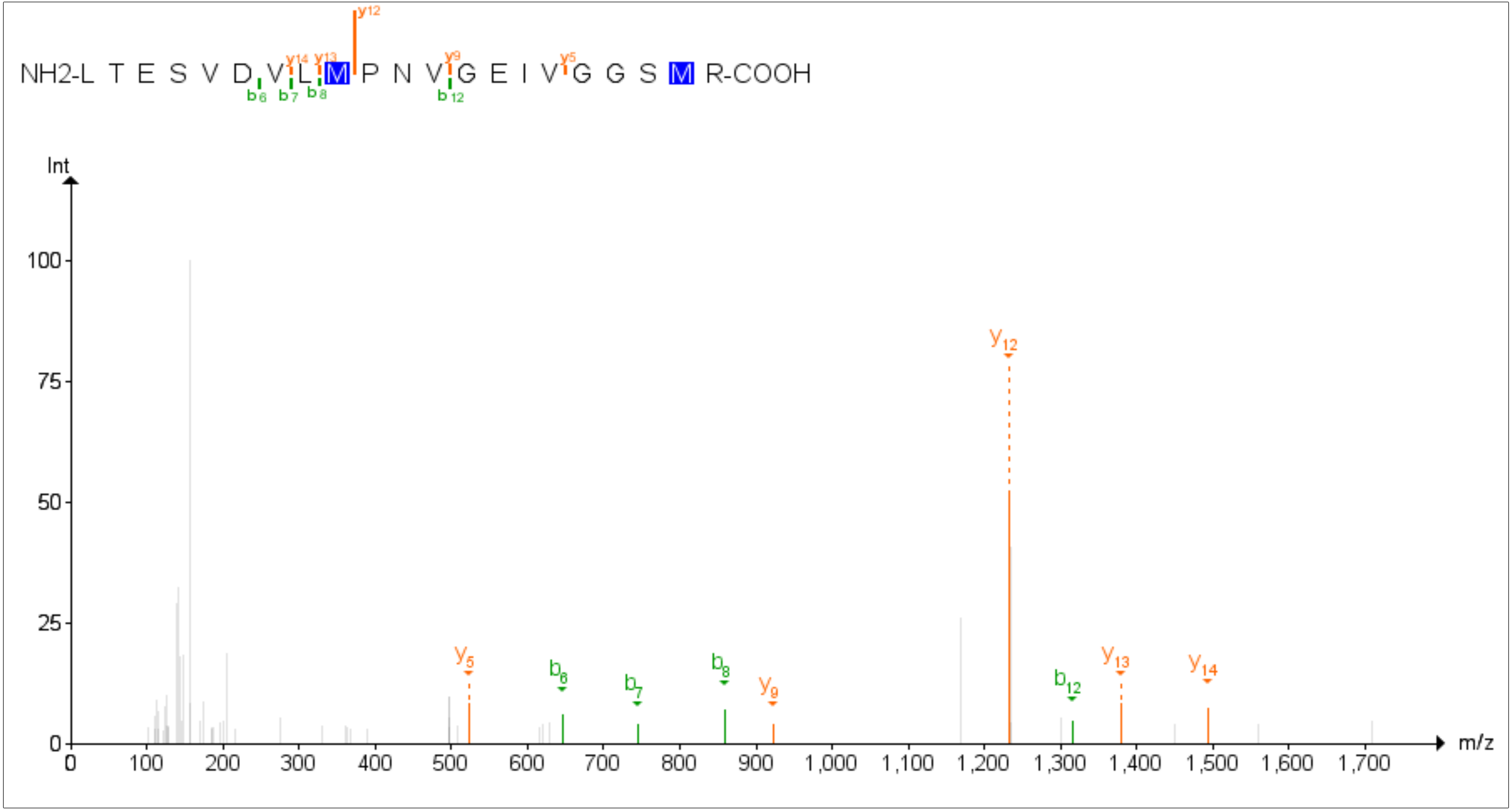

NH2-L T E S V D V L M P N V G E I V G G S M R-COOH
y12
y14
y13
y9
y5
b6
b7
b8
b12
Int
100
75
50
25
0
y12
y5
b6
b7
b8
y9
b12
y13
y14
0
100
200
300
400
500
600
700
800
900
1,000
1,100
1,200
1,300
1,400
1,500
1,600
1,700
m/z

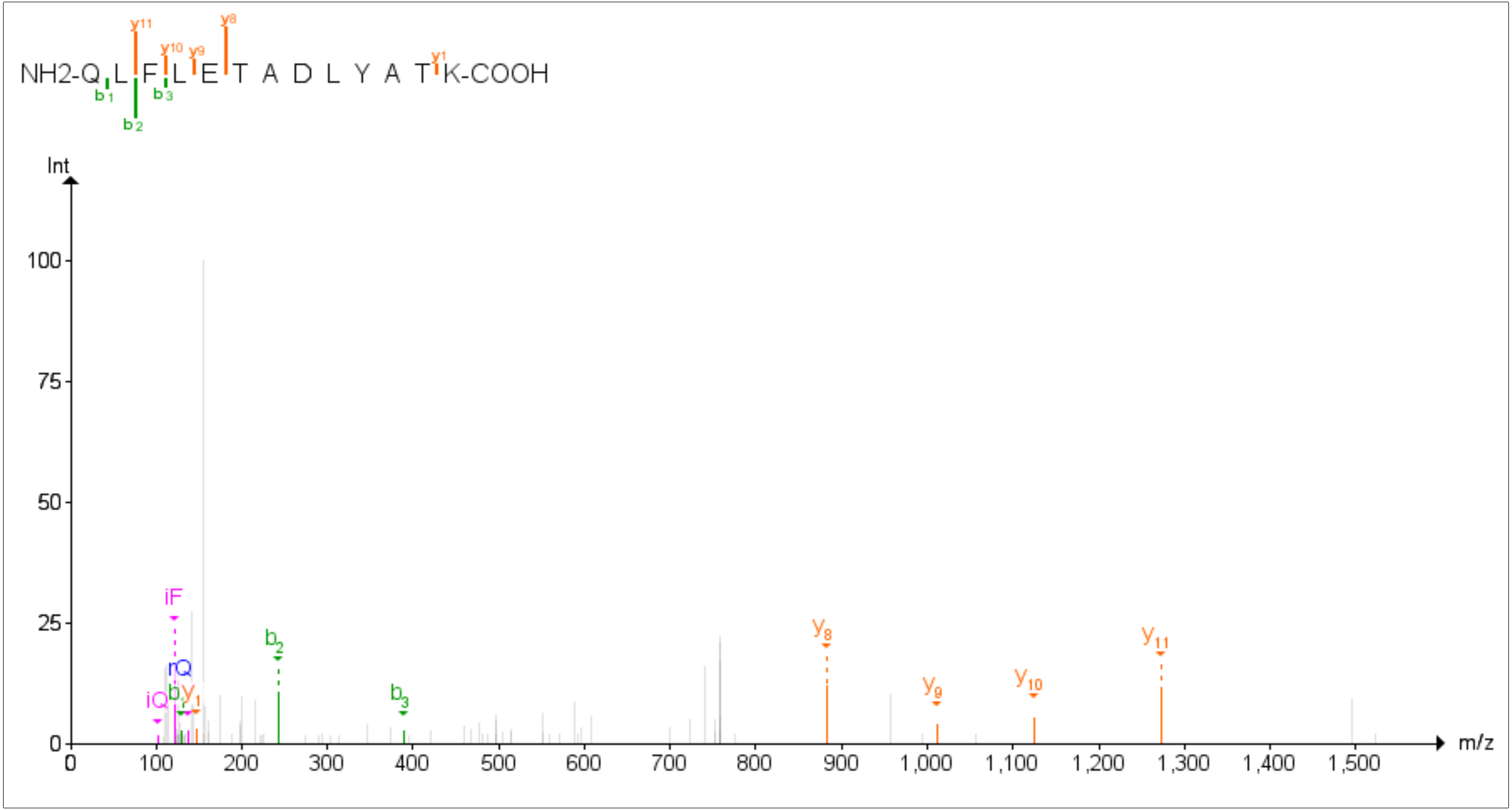

NH2-Q L F L E T A D L Y A T K-COOH
y11
y10
y9
y8
y1
b1
b2
b3
Int
100
75
50
25
0
iF
rQ
iQ
b1
y1
b2
b3
y8
y9
y10
y11
0
100
200
300
400
500
600
700
800
900
1,000
1,100
1,200
1,300
1,400
1,500
m/z

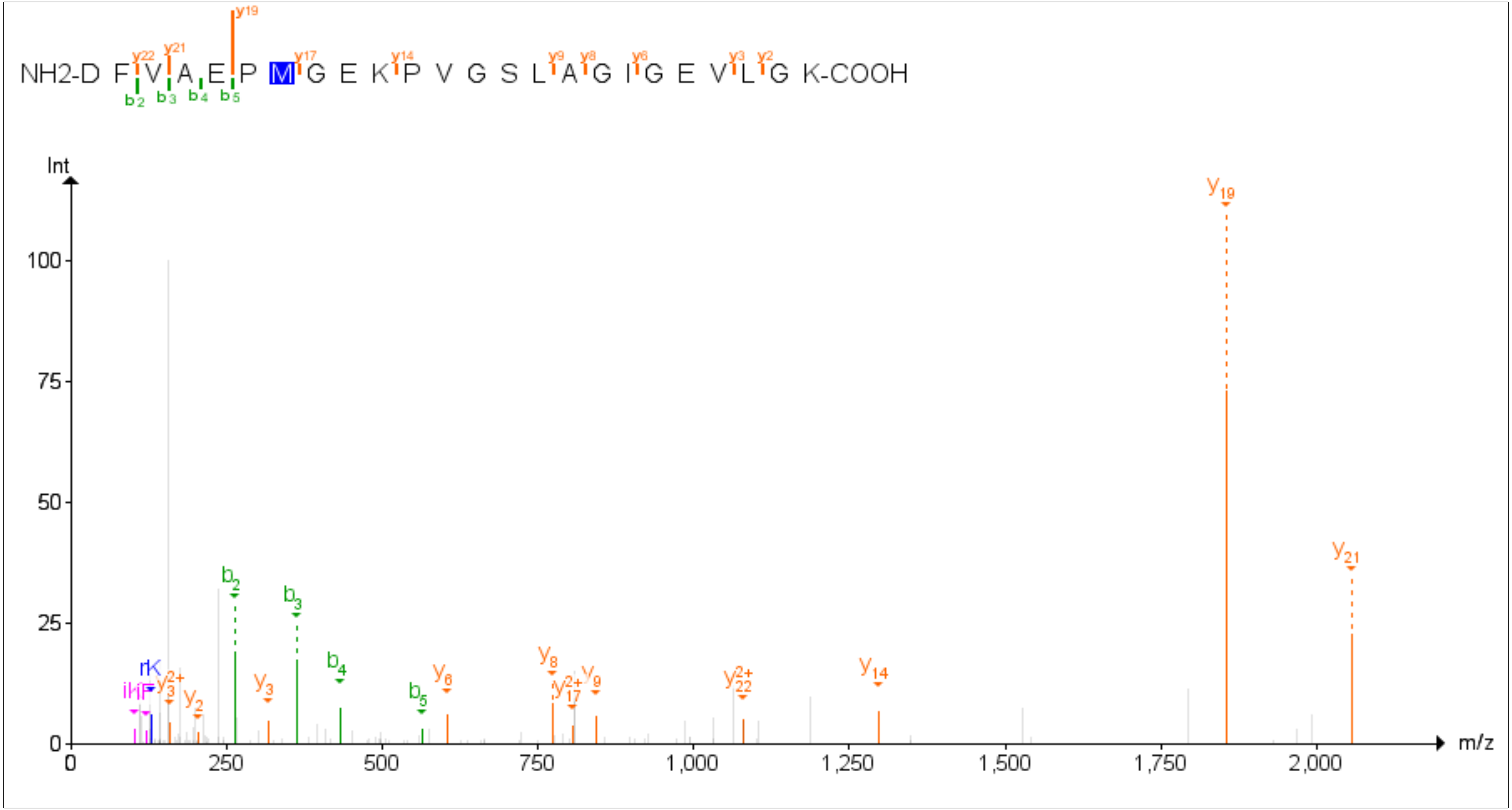

NH2-D F V A E P M G E K P V G S L A G I G E V L G K-COOH
y19
y22
y21
y17
y14
y9
y8
y6
y3
y2
b2
b3
b4
b5
Int
100
75
50
25
0
y19
y21
b2
b3
b4
b5
rK
iF
y3
y2
y6
y8
y9
y14
m/z
0
250
500
750
1,000
1,250
1,500
1,750
2,000

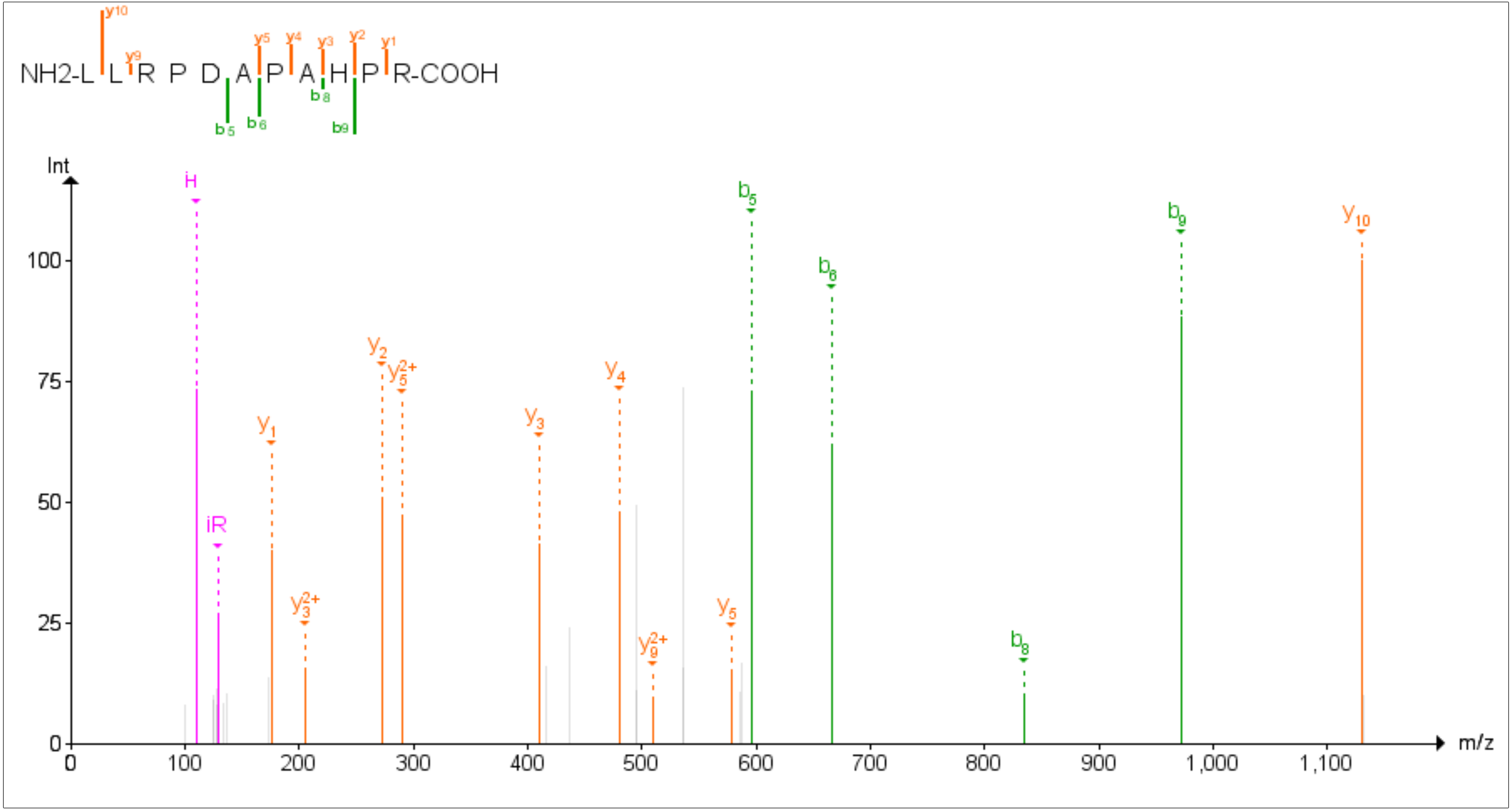
NH2-L L R P D A P A H P R-COOH
y10 y9 y5 y4 y3 y2 y1
b5 b6 b8 b9
Int
m/z
iH
iR
y1 y2 y3 y4 y5 y10
y2+3 y2+5 y2+9
b5 b6 b8 b9
0 25 50 75 100
0 100 200 300 400 500 600 700 800 900 1,000 1,100

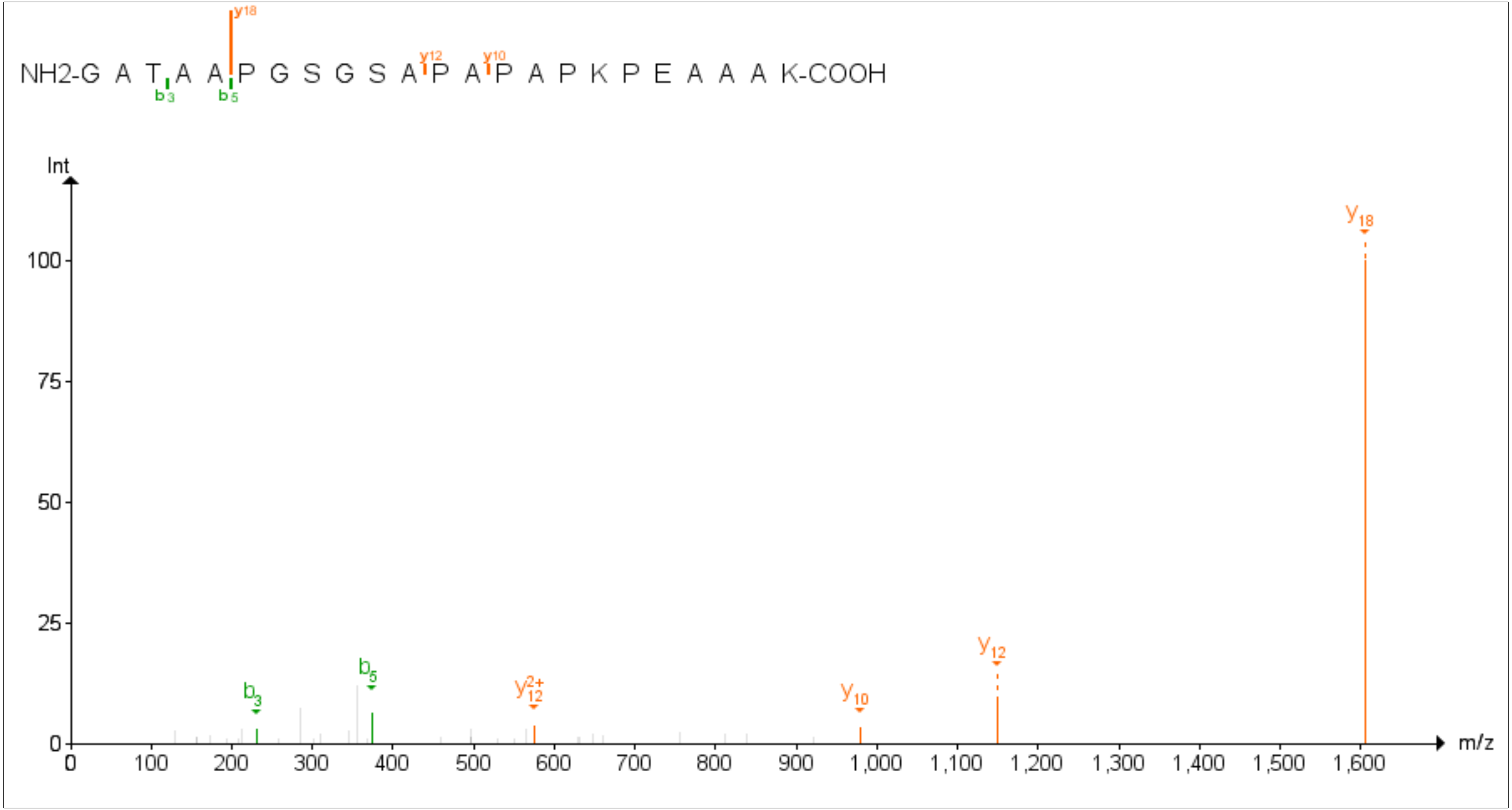

NH2-G A T A A P G S G S A P A P A P K P E A A A K-COOH
y18
y12
y10
b3
b5
Int
100
75
50
25
0
y18
y12
y10
y12 2+
b3
b5
m/z
0
100
200
300
400
500
600
700
800
900
1,000
1,100
1,200
1,300
1,400
1,500
1,600

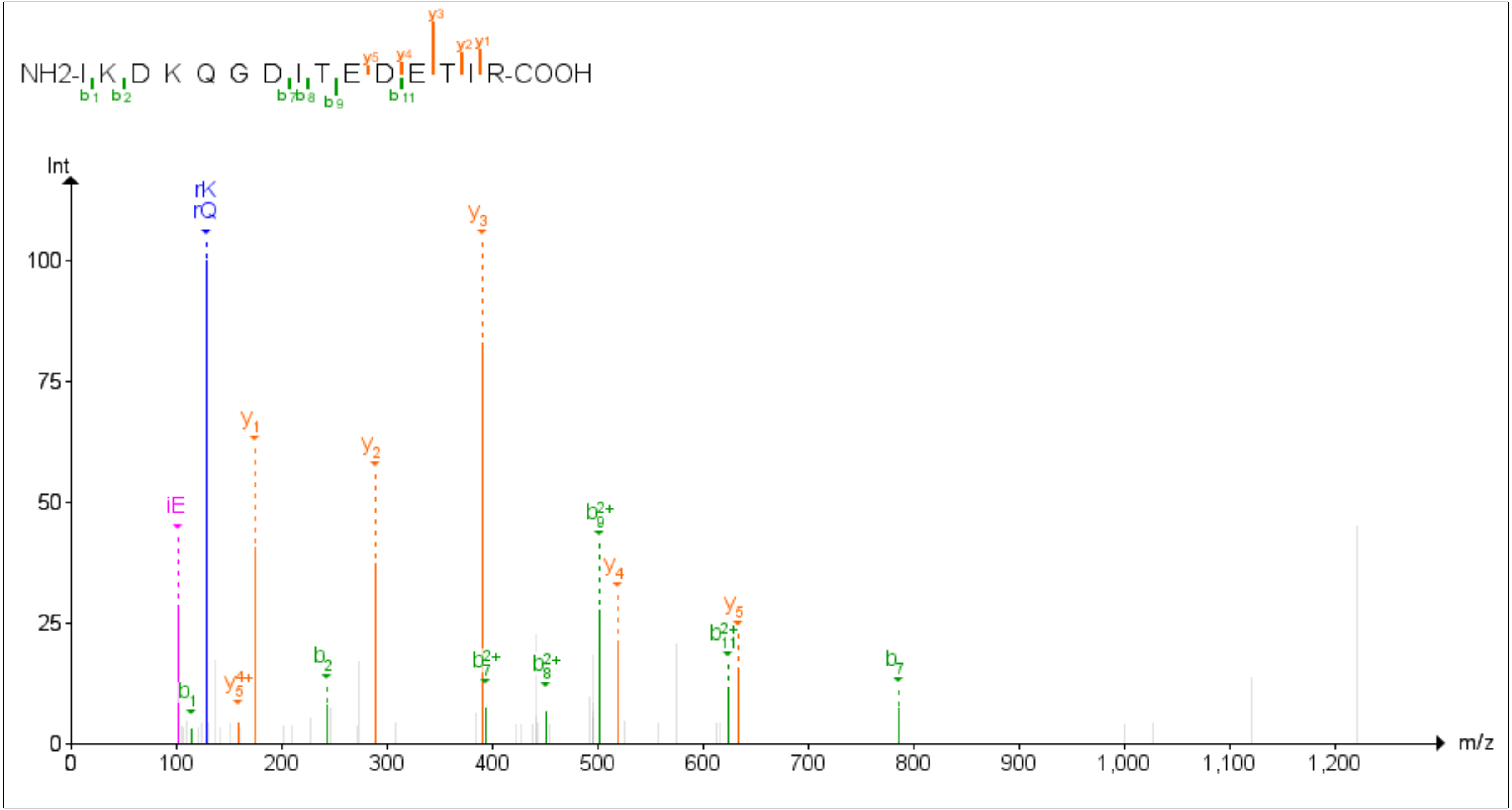

NH2-I K D K Q G D I T E D E T I R-COOH
b1 b2 b7 b8 b9 b11
y5 y4 y3 y2 y1
Int
m/z
rK
rQ
iE
b1
y5 4+
y1
b2
y2
y3
b7 2+
b8 2+
b9 2+
y4
b11 2+
y5
b7
0
25
50
75
100
0
100
200
300
400
500
600
700
800
900
1,000
1,100
1,200

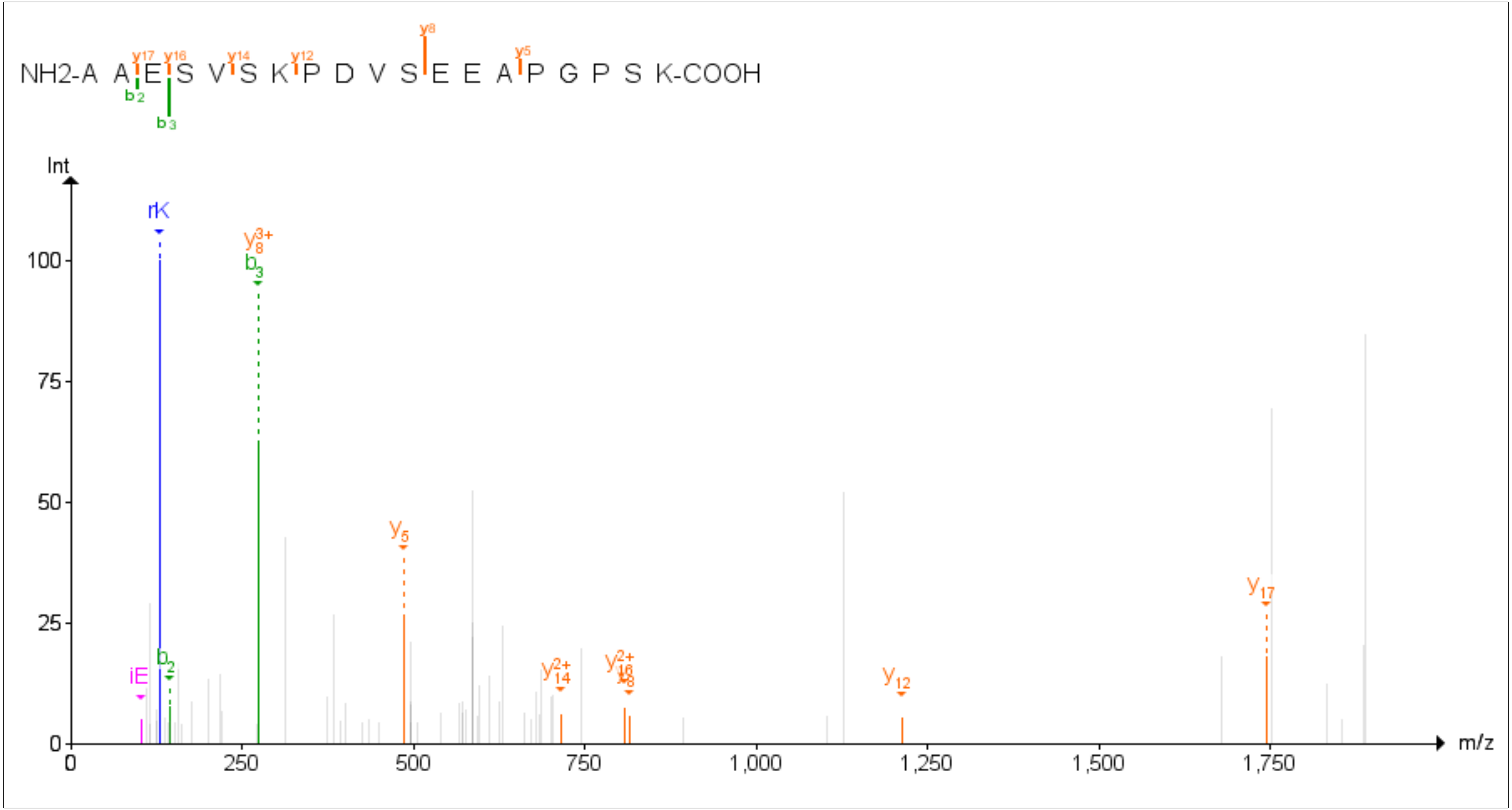

NH2-A A E S V S K P D V S E E A P G P S K-COOH
y17 y16 y14 y12 y8 y5
b2 b3
Int
rK
iE
b2
b3
y5
y17
y12
100
75
50
25
0
250
500
750
1,000
1,250
1,500
1,750
m/z

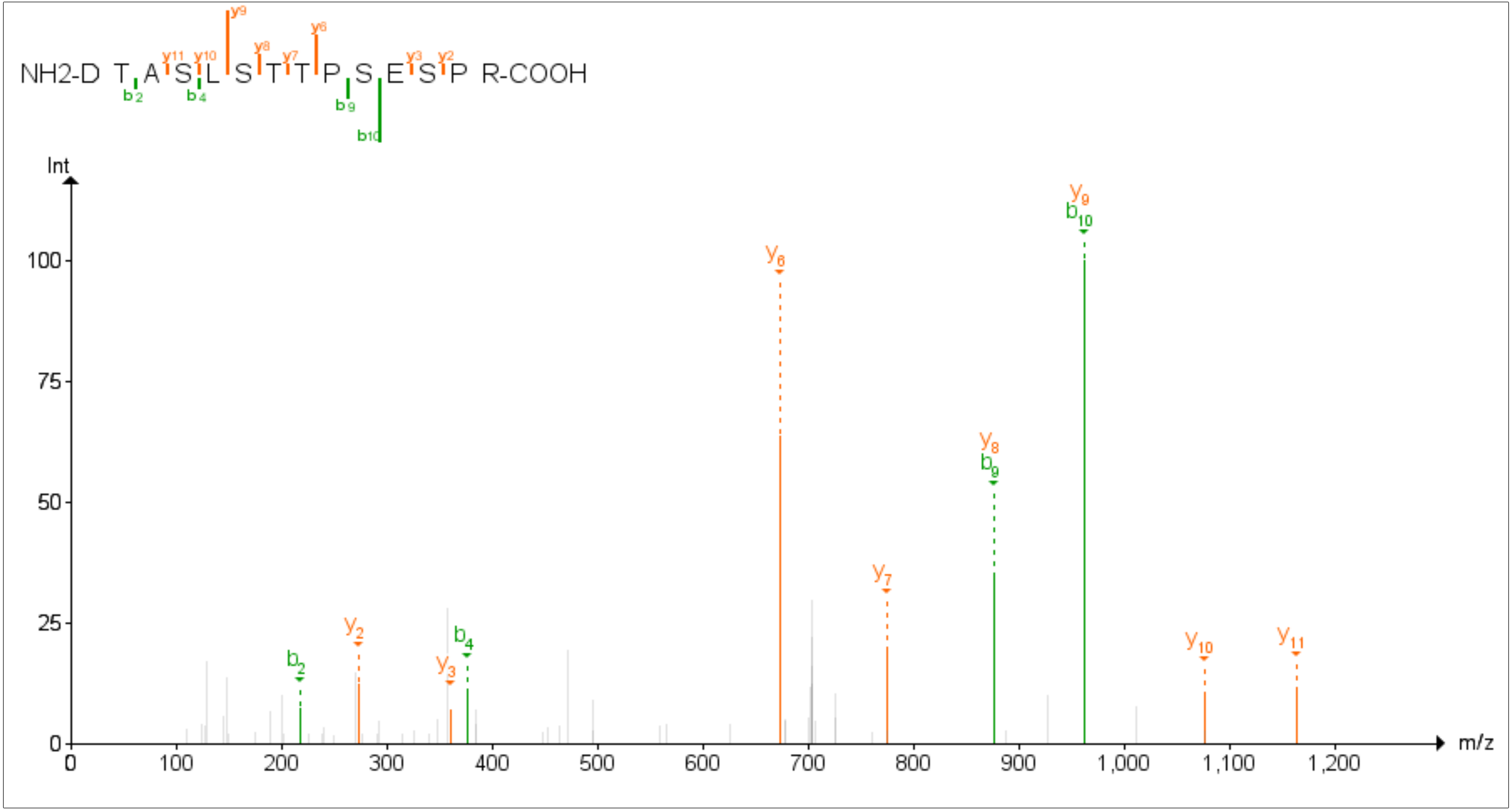

NH2-D T A S L S T T P S E S P R-COOH
b2
b4
b9
b10
y11
y10
y9
y8
y7
y6
y3
y2
Int
m/z
0
25
50
75
100
0
100
200
300
400
500
600
700
800
900
1,000
1,100
1,200

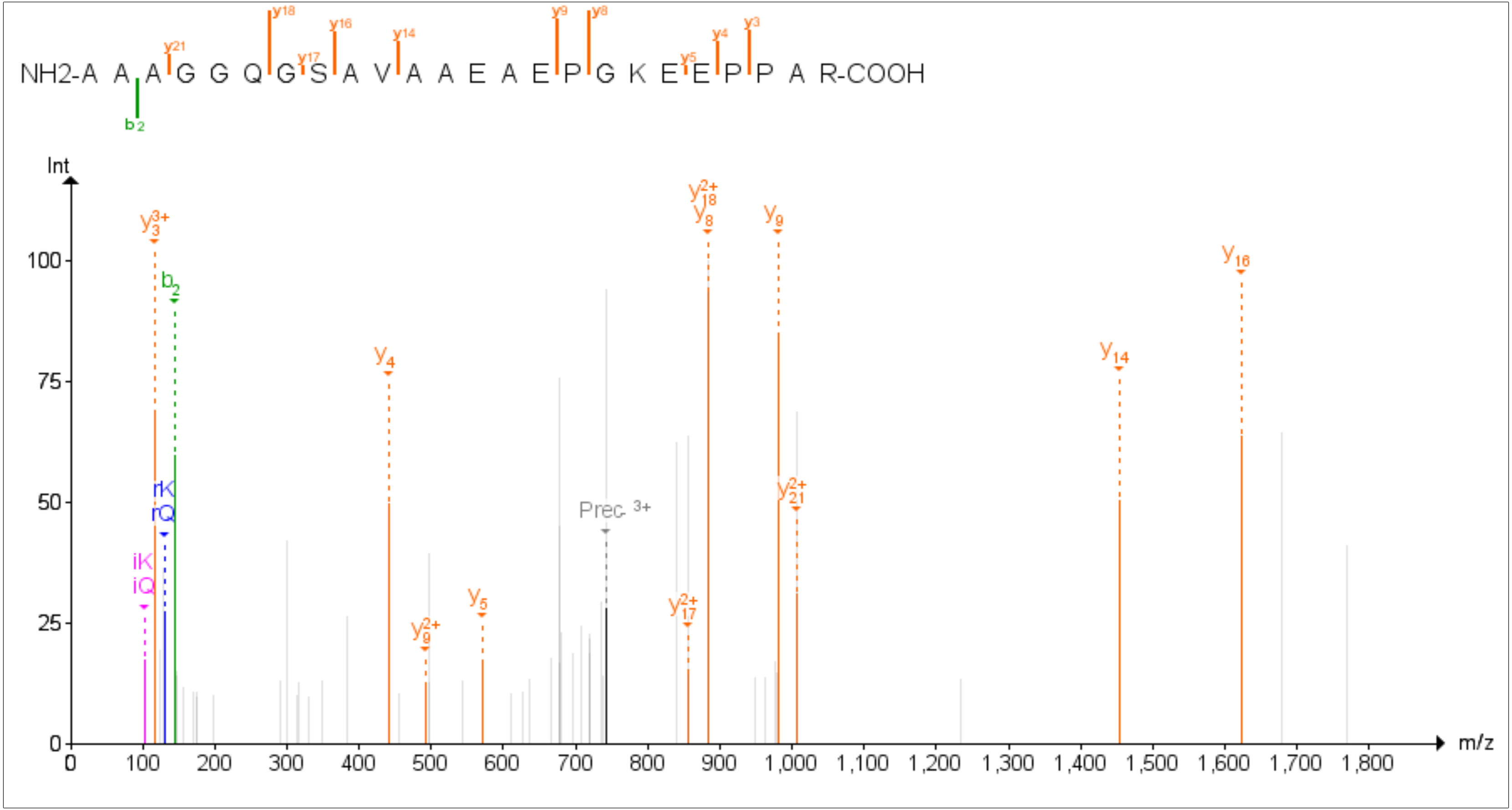

NH2-A A A G G Q G S A V A A E A E P G K E E P P A R-COOH
b2
y21 y18 y17 y16 y14 y9 y8 y5 y4 y3
Int
m/z
Prec 3+

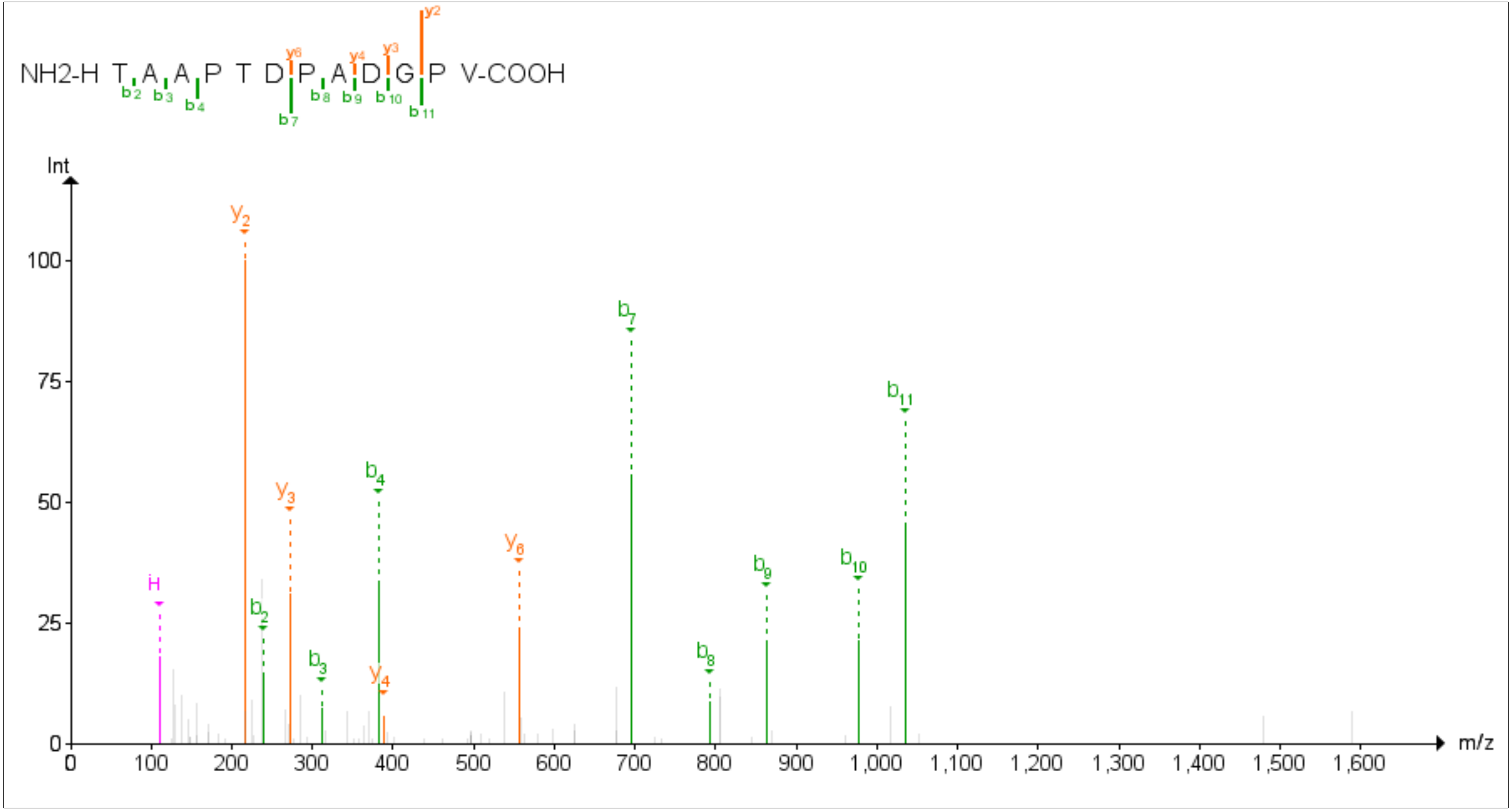

NH2-H T A A P T D P A D G P V-COOH
b2 b3 b4 b7 b8 b9 b10 b11
y6 y4 y3 y2
Int
m/z
0
25
50
75
100
0 100 200 300 400 500 600 700 800 900 1,000 1,100 1,200 1,300 1,400 1,500 1,600
H
y2
y3
y4
y6
b2
b3
b4
b7
b8
b9
b10
b11

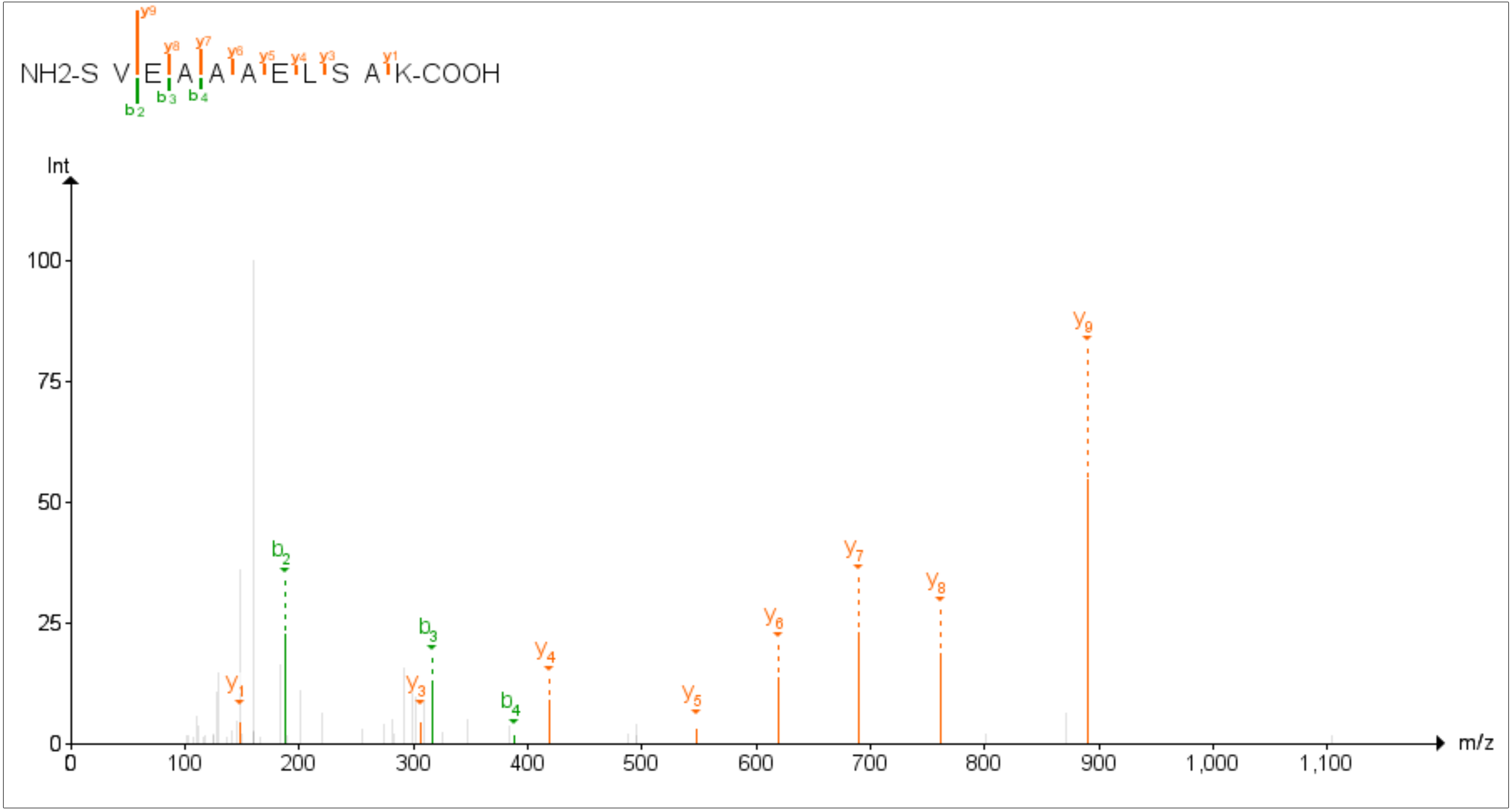

NH2-S V E A A A E L S A K-COOH
y9 y8 y7 y6 y5 y4 y3 y1
b2 b3 b4
Int
100
75
50
25
0
m/z
0 100 200 300 400 500 600 700 800 900 1,000 1,100
y_1 b_2 y_3 b_3 b_4 y_4 y_5 y_6 y_7 y_8 y_9

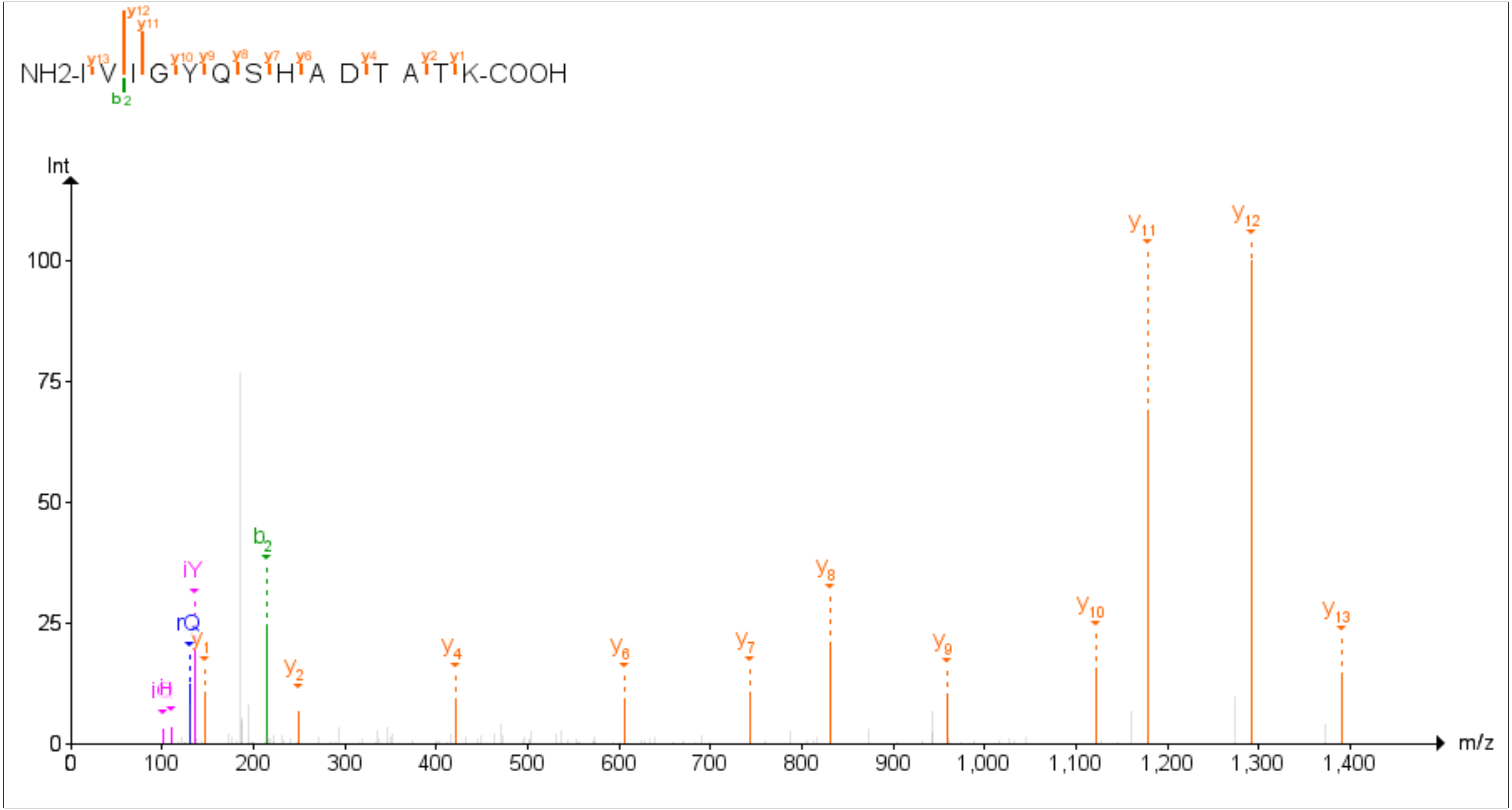
NH2-I V I G Y Q S H A D T A T K-COOH
y13 y12 y11 y10 y9 y8 y7 y6 y4 y2 y1
b2
Int
100
75
50
25
0
m/z
0 100 200 300 400 500 600 700 800 900 1,000 1,100 1,200 1,300 1,400
y11 y12 b2 iY rQ y1 y2 y4 y6 y7 y8 y9 y10 y13 iH

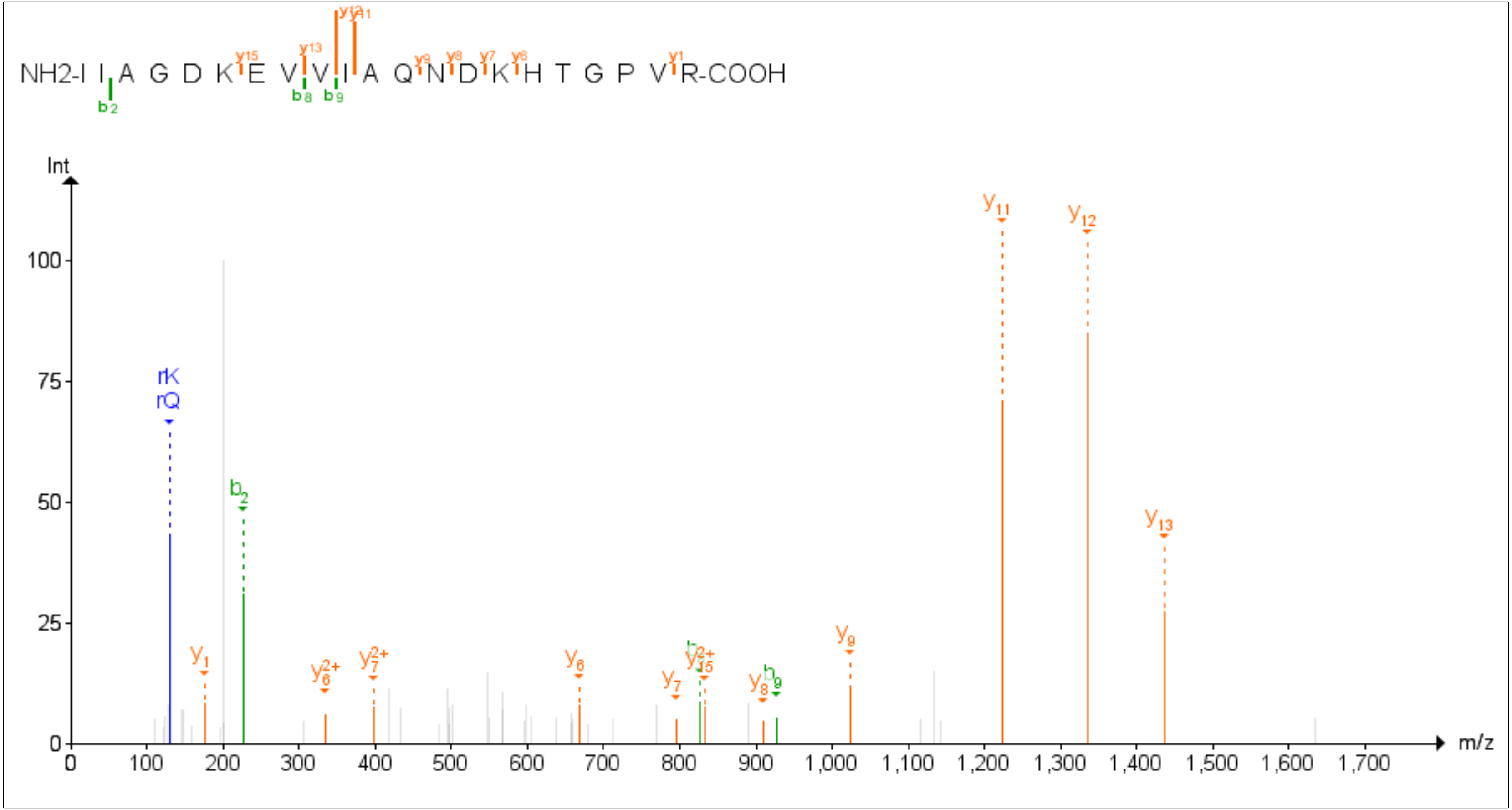

NH2-I I A G D K E V V I A Q N D K H T G P V R-COOH
Int
m/z

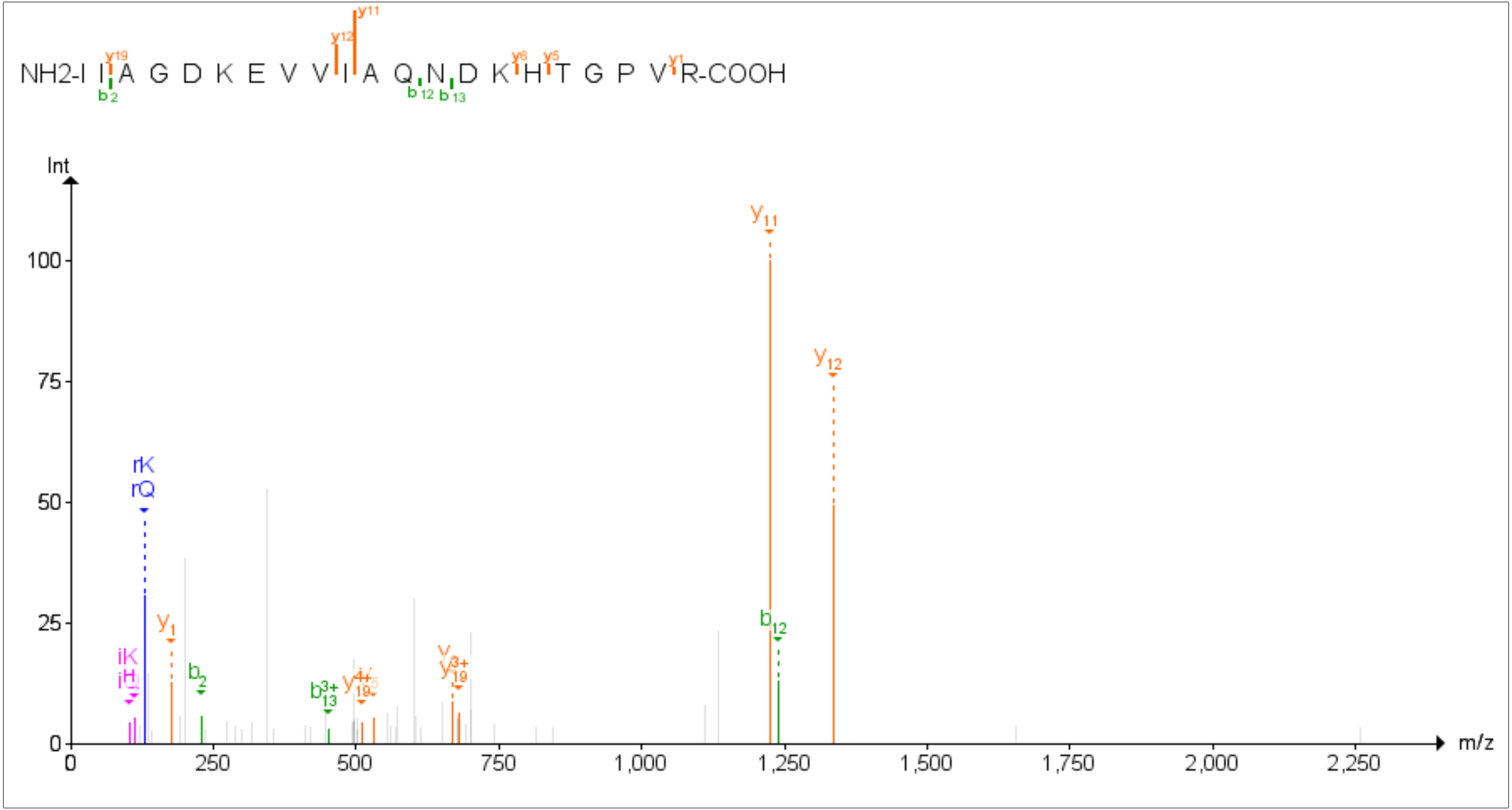

NH2-I I A G D K E V V I A Q N D K H T G P V R-COOH
y19
y12
y11
y6
y5
y1
b 2
b 12
b 13
Int
m/z
0
25
50
75
100
250
500
750
1,000
1,250
1,500
1,750
2,000
2,250
y11
y12
rK
rQ
y1
iK
iH
b2
b12

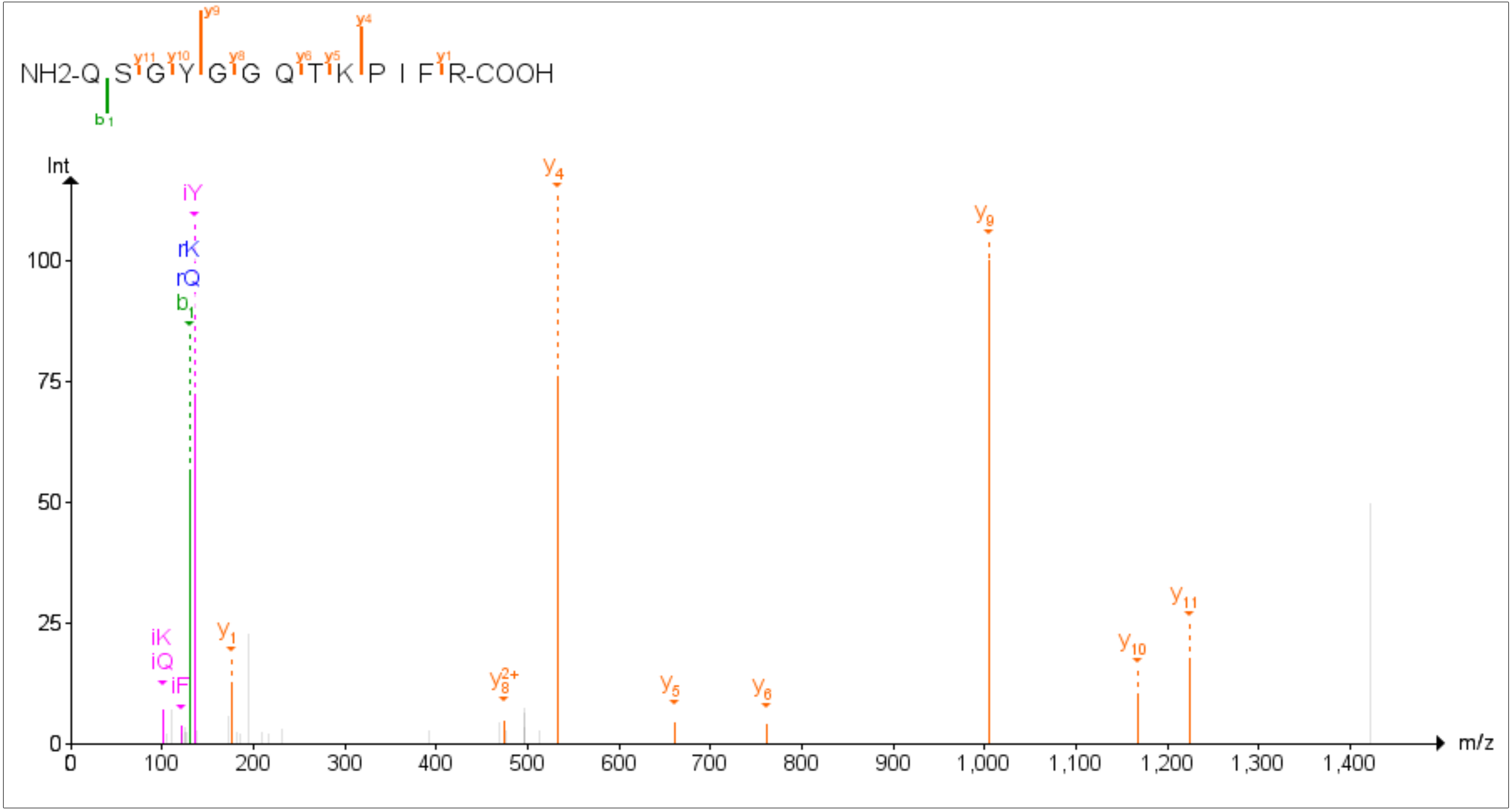

NH2-Q S G Y G G Q T K P I F R-COOH
b1
y11 y10 y9 y8 y6 y5 y4 y1
Int
m/z
iY
rK
rQ
b1
y4
y9
iK
iQ
iF
y1
y_8^{2+}
y5
y6
y10
y11
0
25
50
75
100
0
100
200
300
400
500
600
700
800
900
1,000
1,100
1,200
1,300
1,400

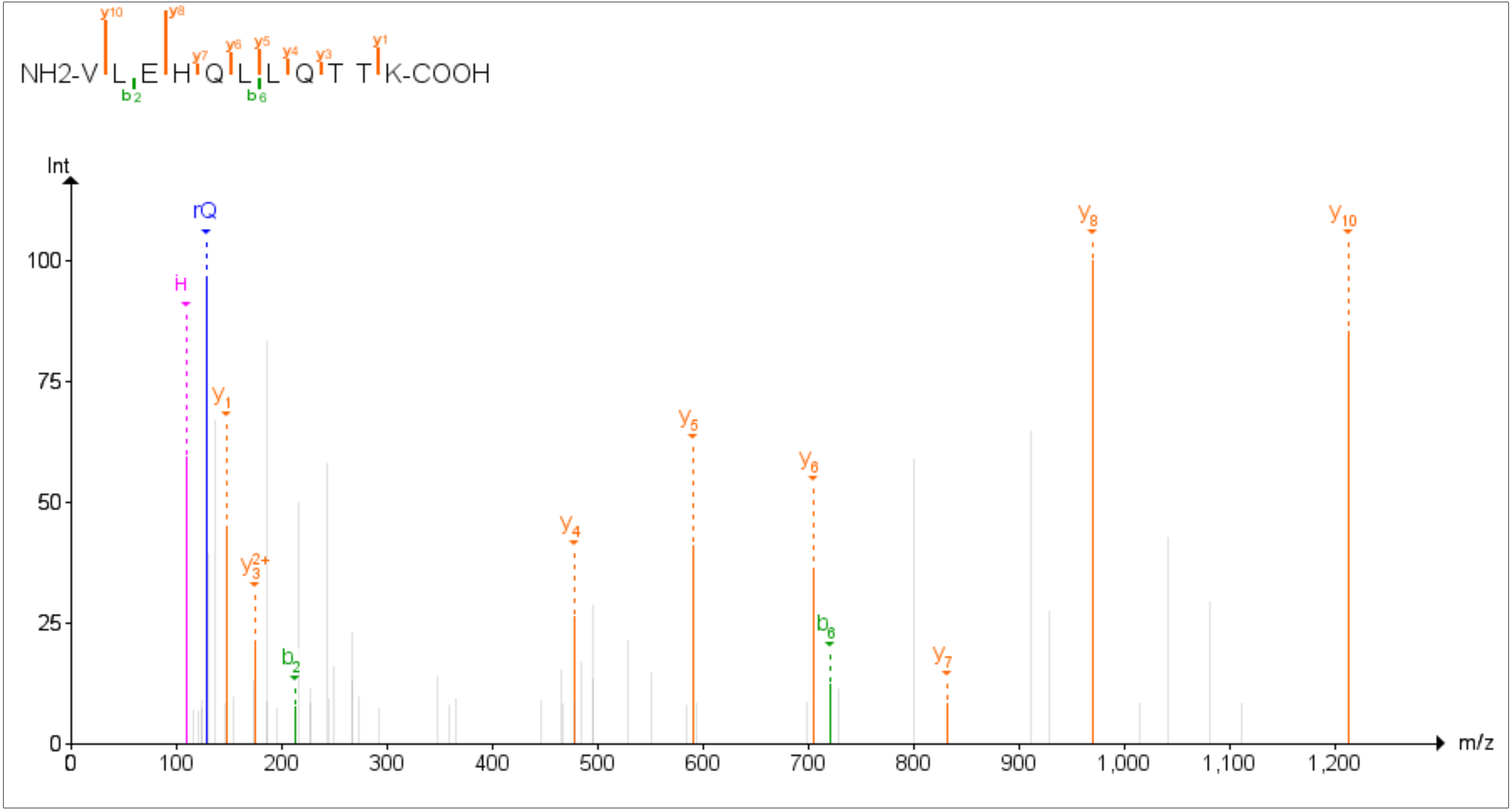

NH2-V L E H Q L L L Q T T K-COOH
y10
y8
y7
y6
y5
y4
y3
y1
b2
b6
Int
100
75
50
25
0
rQ
H
y1
y3 2+
b2
y4
y5
y6
b6
y7
y8
y10
0
100
200
300
400
500
600
700
800
900
1,000
1,100
1,200
m/z

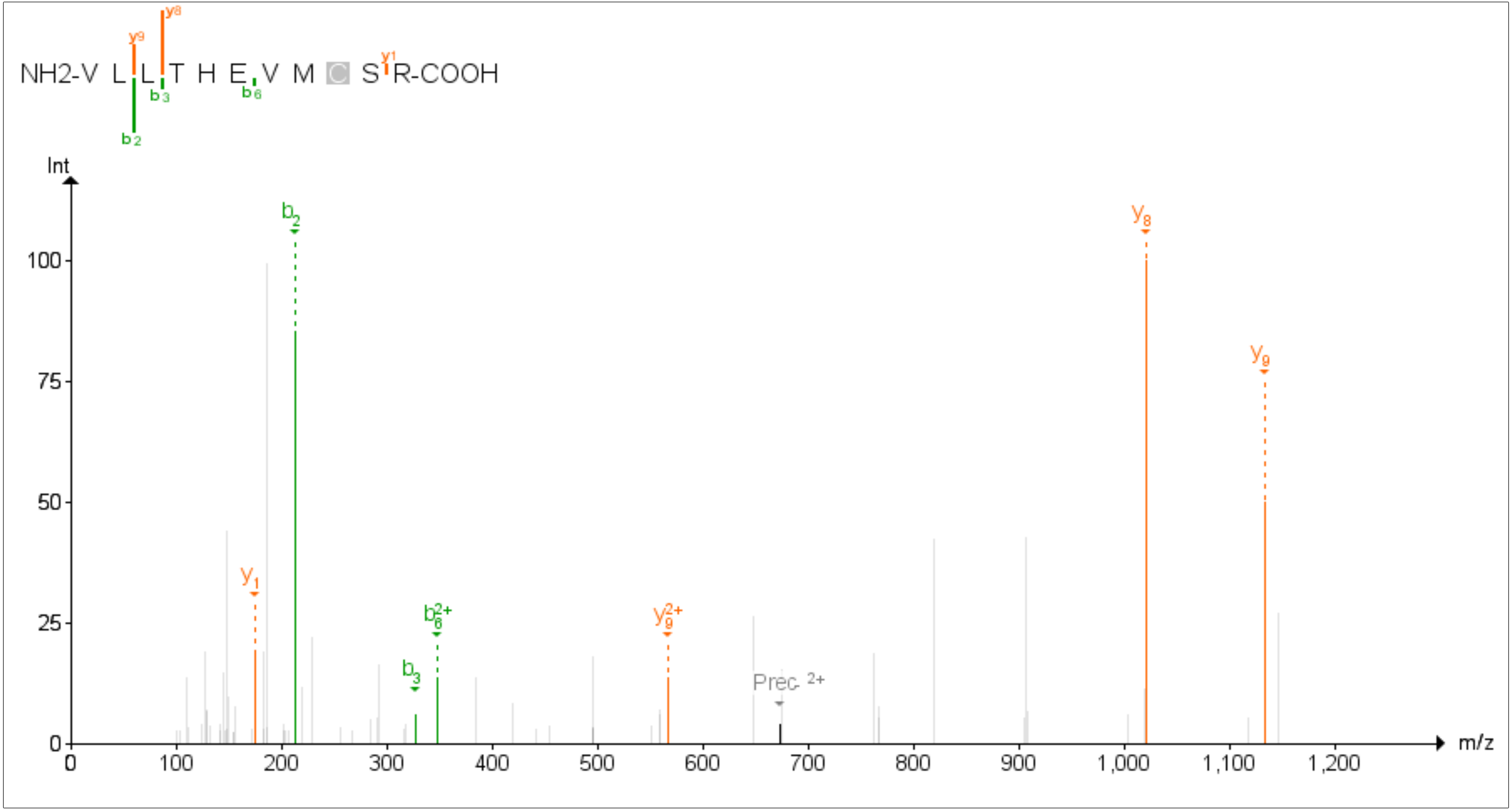

NH2-V L L T H E V M C S R-COOH
y9
y8
y1
b2
b3
b6
Int
100
75
50
25
0
b2
y8
y9
y1
b6 2+
y9 2+
b3
Prec 2+
0
100
200
300
400
500
600
700
800
900
1,000
1,100
1,200
m/z

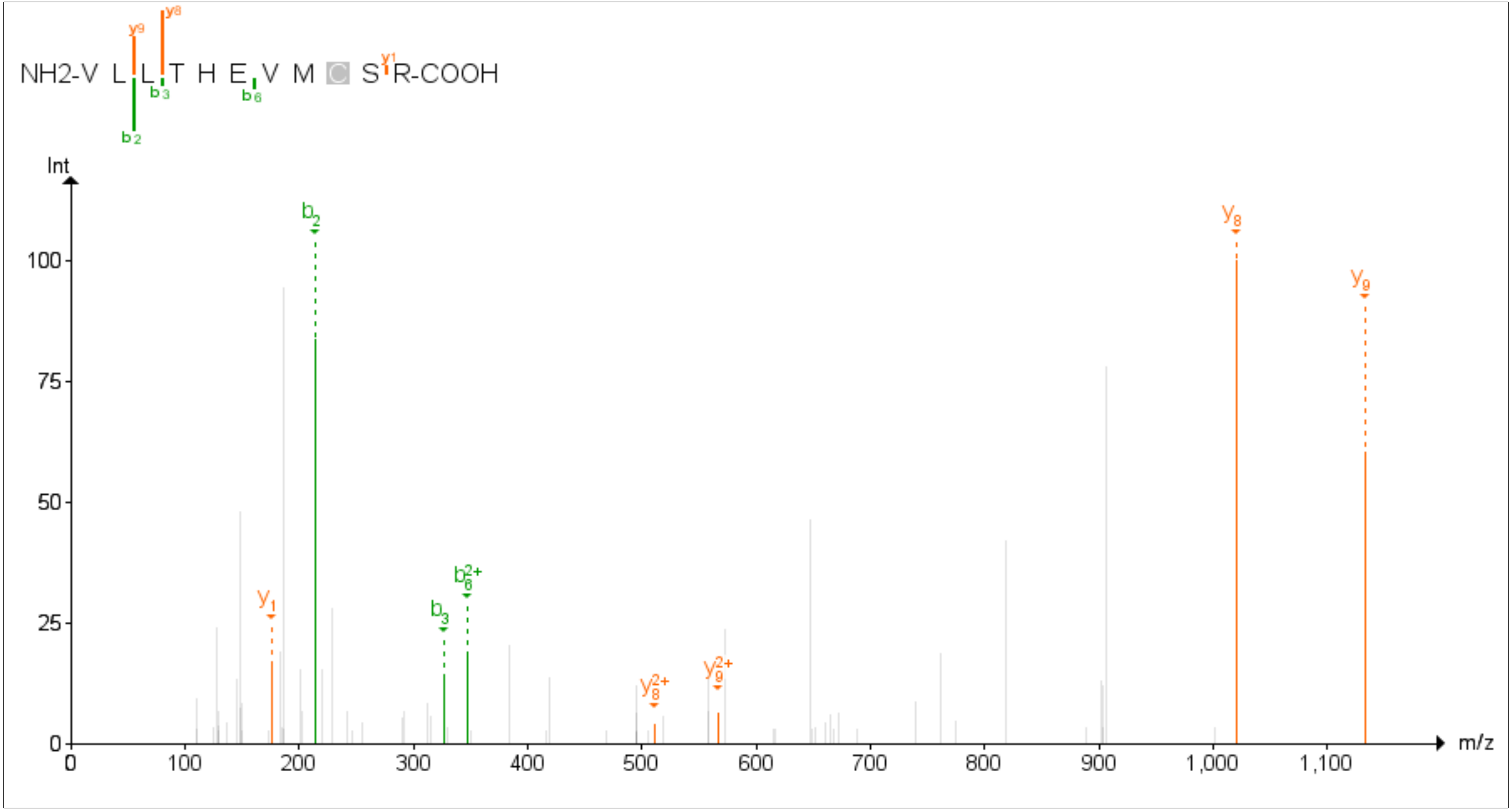

NH2-V L L T H E V M C S R-COOH
y9
y8
y1
b2
b3
b6
Int
100
75
50
25
0
b2
y8
y9
y1
b3
b6 2+
y8 2+
y9 2+
0
100
200
300
400
500
600
700
800
900
1,000
1,100
m/z

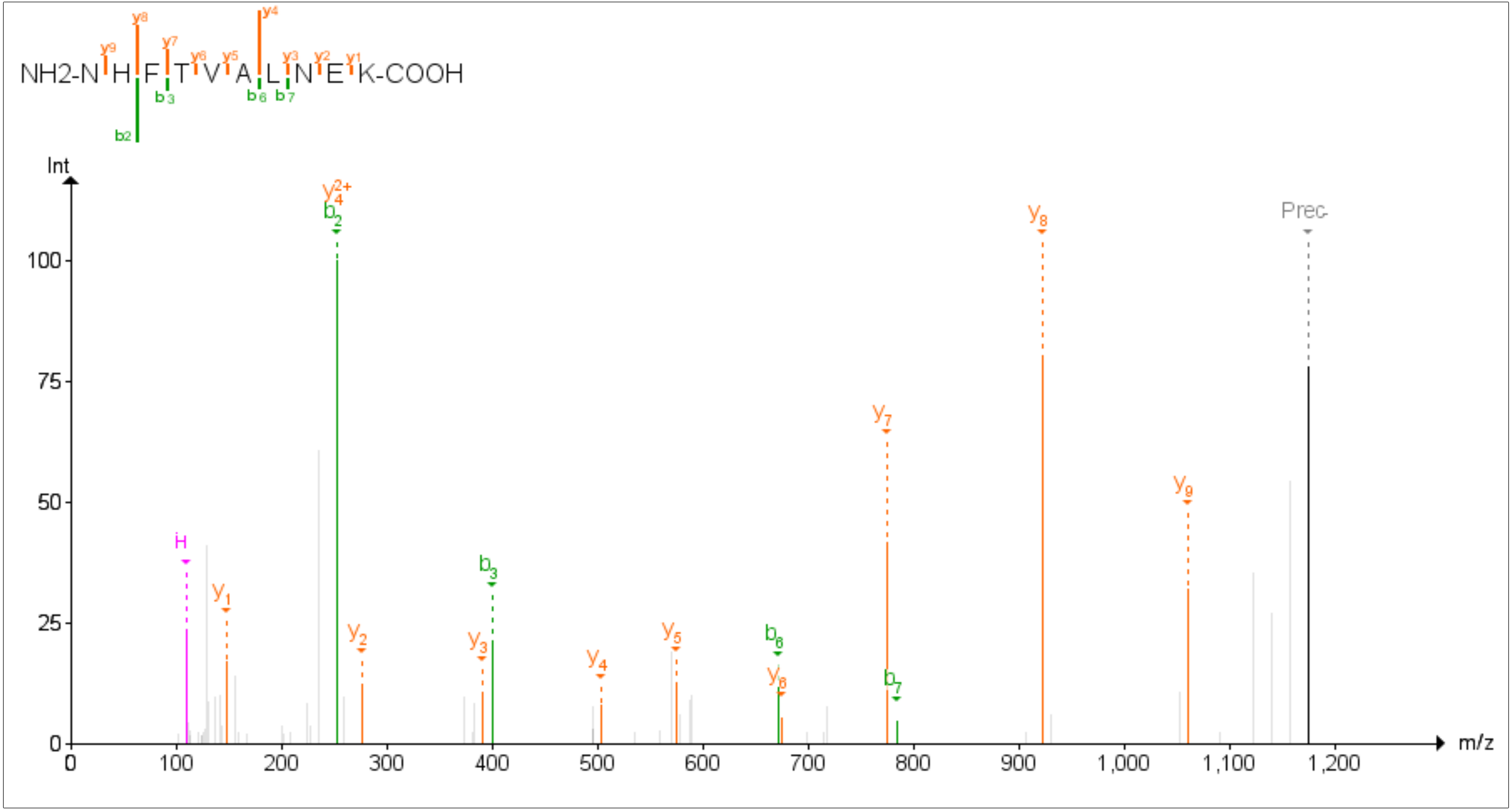

NH2-N H F T V A L N E K-COOH
y9 y8 y7 y6 y5 y4 y3 y2 y1
b2 b3 b6 b7
Int
100
75
50
25
0
y4 2+
b2
y8
Prec
y7
y9
H
y1
b3
y2
y3
y4
y5
b6
y6
b7
0
100
200
300
400
500
600
700
800
900
1,000
1,100
1,200
m/z

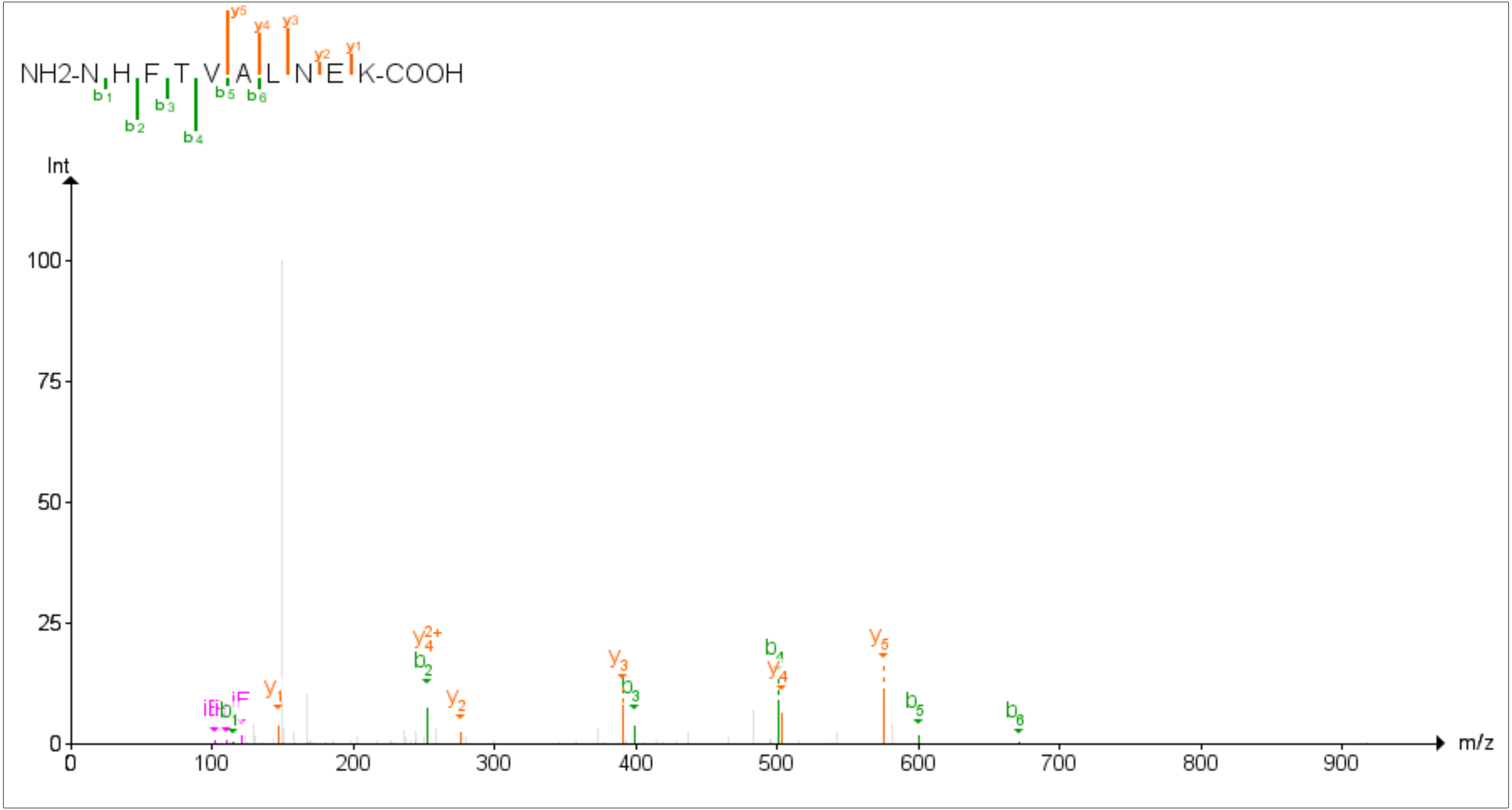

NH2-N H F T V A L N E K-COOH
Int
m/z

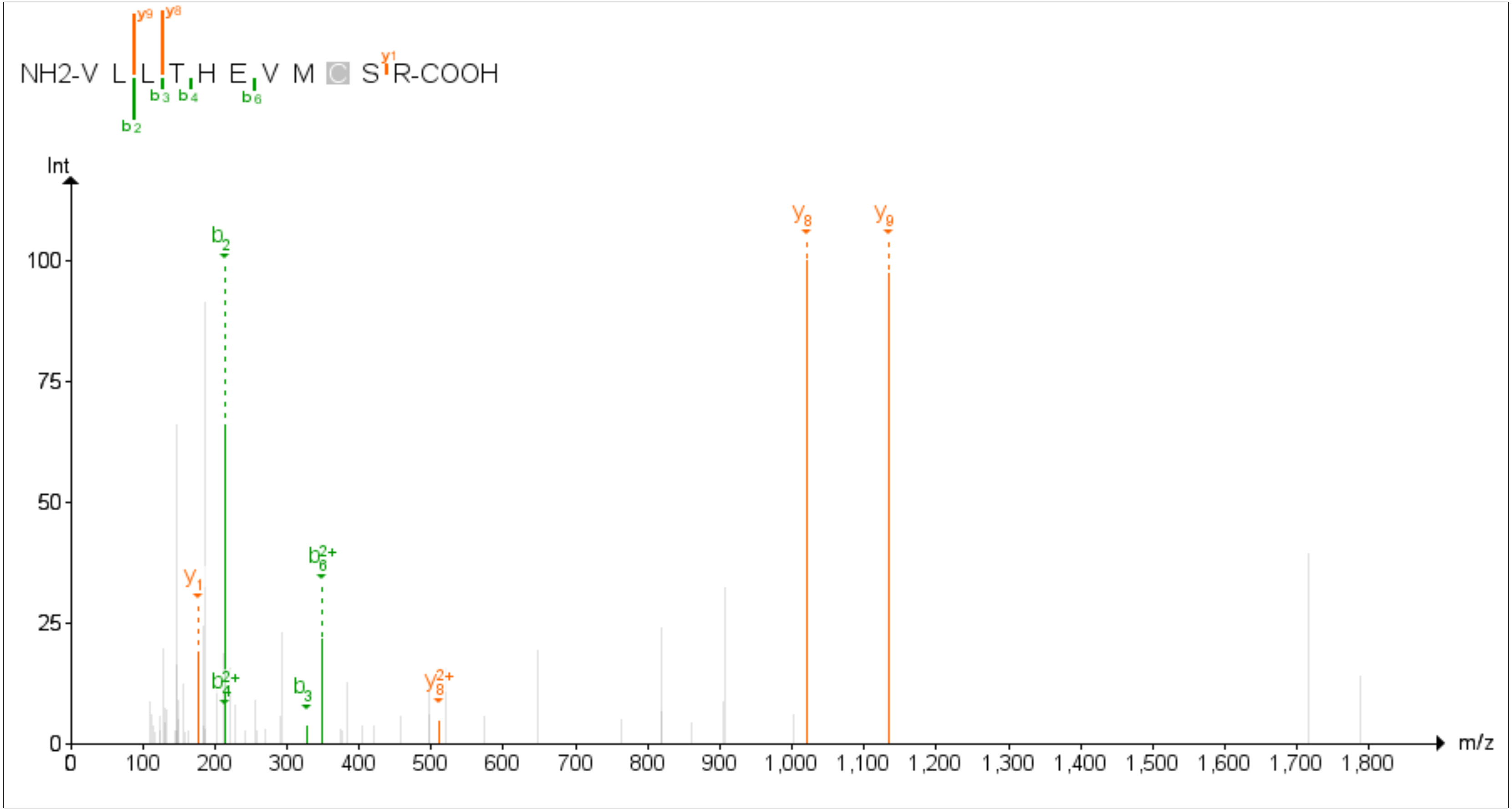

NH2-V L L T H E V M C S R-COOH
y9
y8
y1
b2
b3
b4
b6
Int
100
75
50
25
0
y8
y9
b2
y1
b6 2+
b4 2+
b3
y8 2+
0
100
200
300
400
500
600
700
800
900
1,000
1,100
1,200
1,300
1,400
1,500
1,600
1,700
1,800
m/z

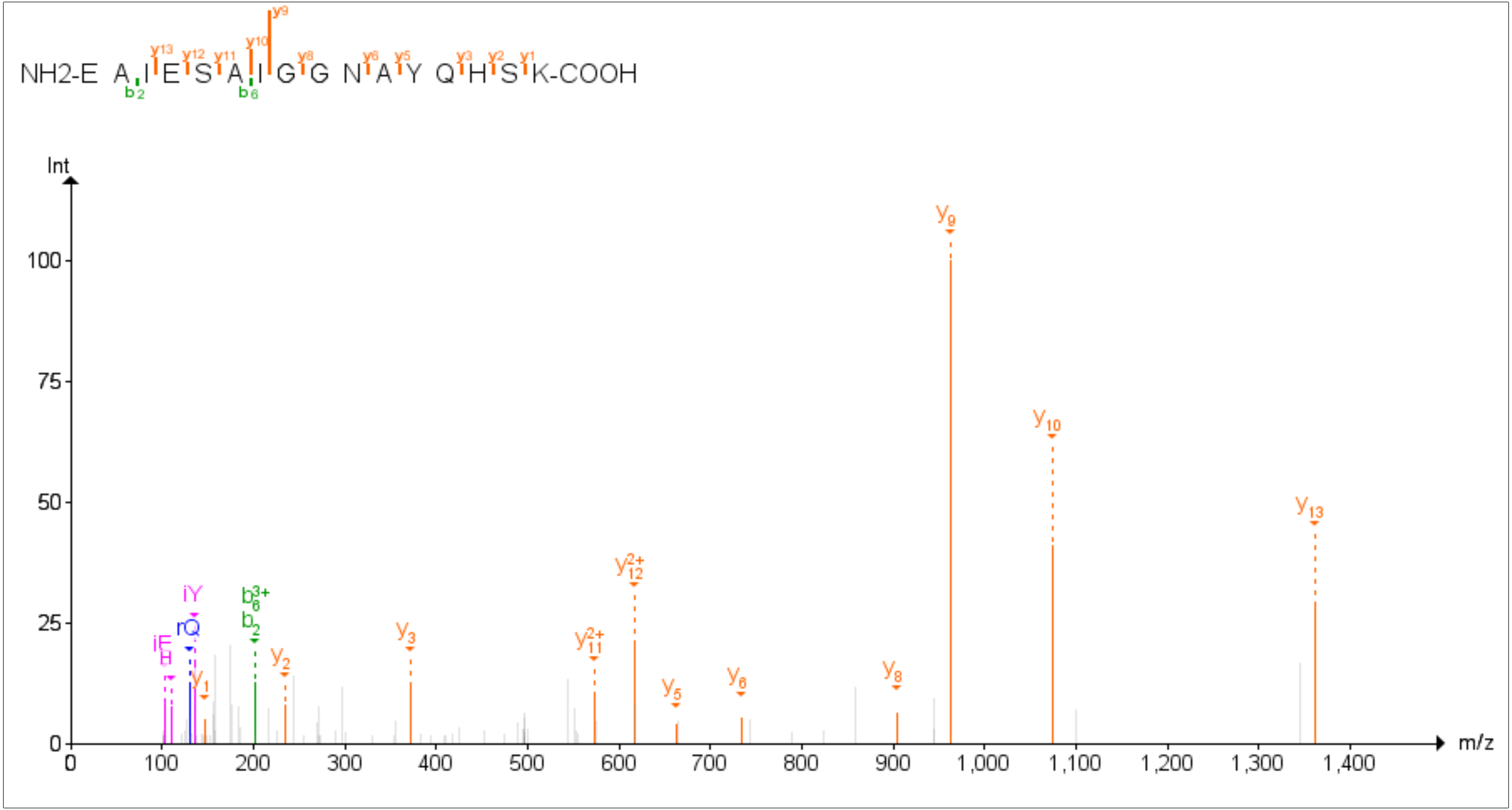

NH2-E A I E S A I G G N A Y Q H S K-COOH
y13 y12 y11 y10 y9 y8 y6 y5 y3 y2 y1
b2 b6
Int
m/z
y9
y10
y13
y12 2+
y11 2+
y3
y2
y1
y5
y6
y8
b6 3+
b2
iY
rQ
iF

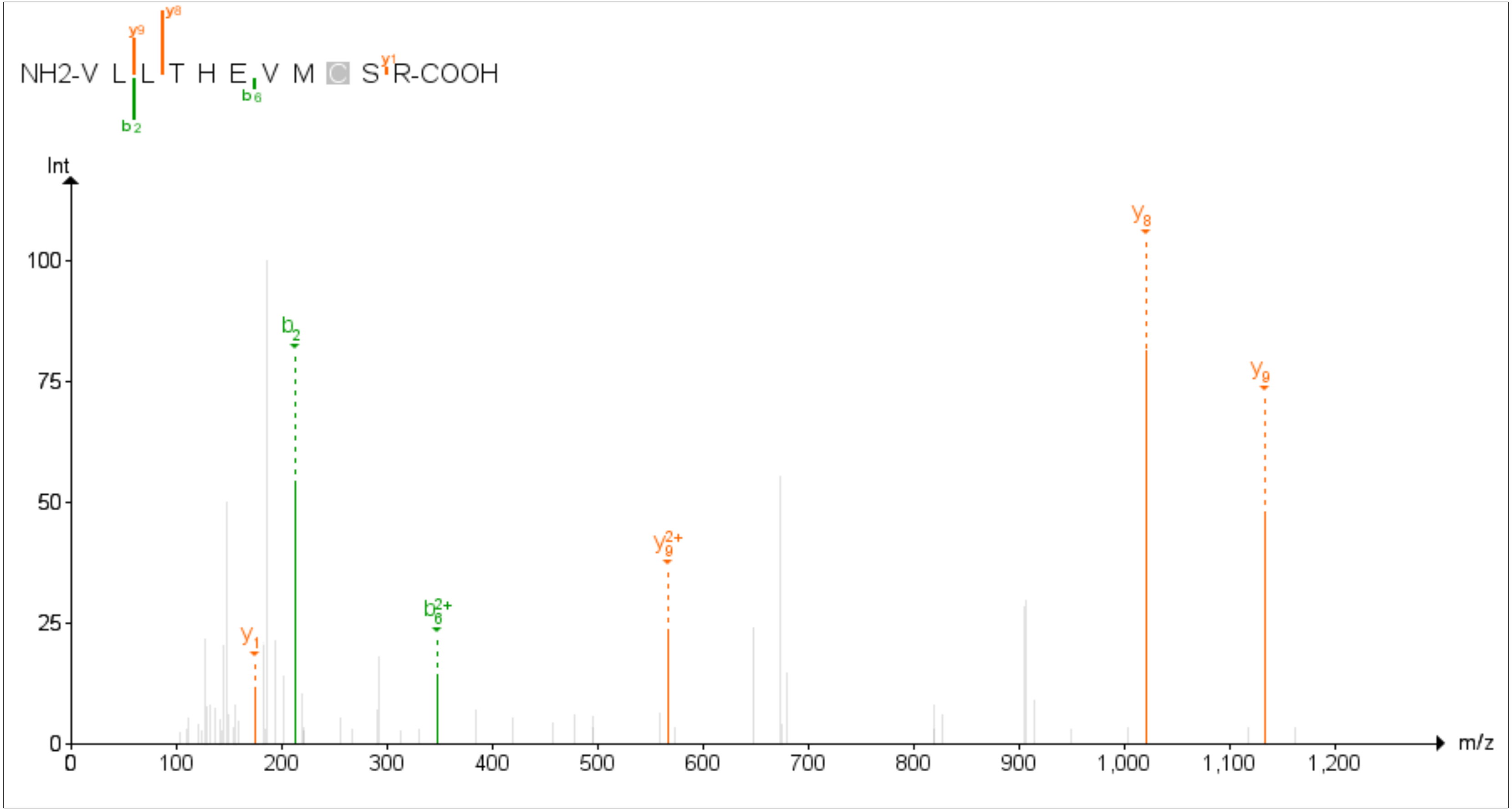

NH2-V L L T H E V M C S R-COOH
y9
y8
y1
b2
b6
Int
100
75
50
25
0
m/z
0
100
200
300
400
500
600
700
800
900
1,000
1,100
1,200
y8
y9
b2
y1
b6 2+
y9 2+

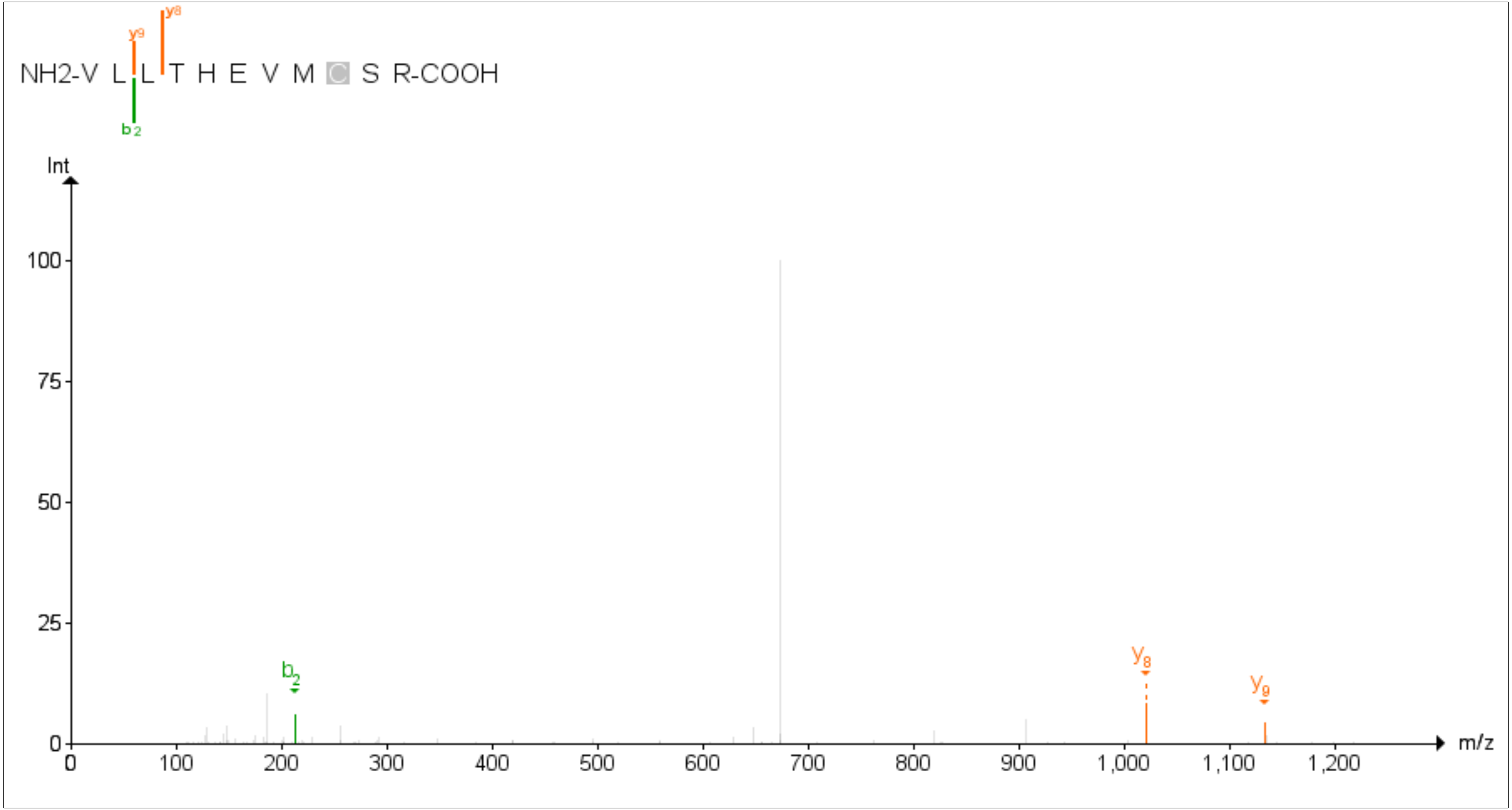

NH2-V L L T H E V M C S R-COOH
y9
y8
b2
Int
100
75
50
25
0
b2
y8
y9
0
100
200
300
400
500
600
700
800
900
1,000
1,100
1,200
m/z

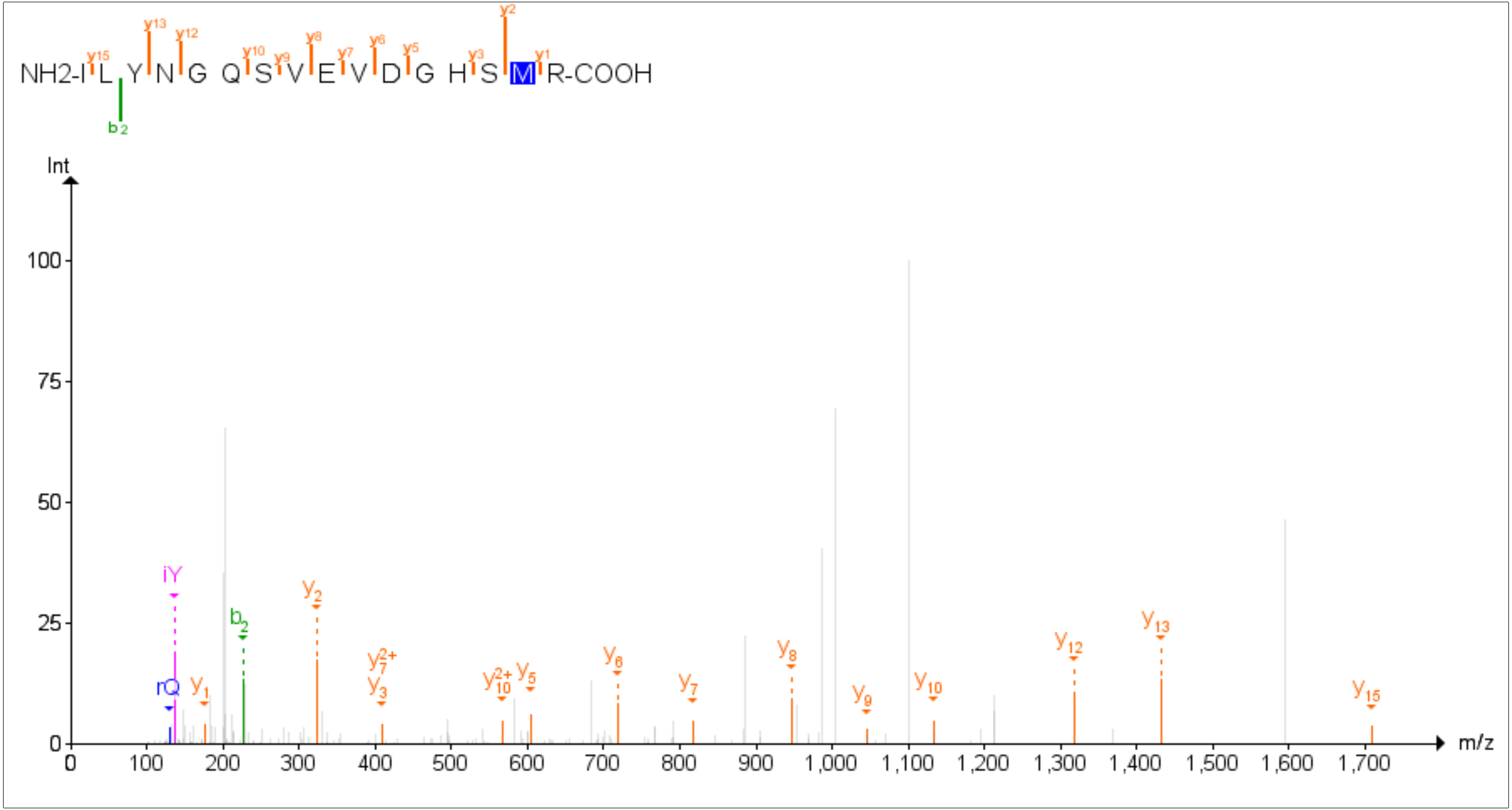
NH2-I L Y N G Q S V E V D G H S M R-COOH
Int
m/z
iY
rQ
b2
y1
y2
y3
y5
y6
y7
y8
y9
y10
y12
y13
y15
y7 2+
y10 2+

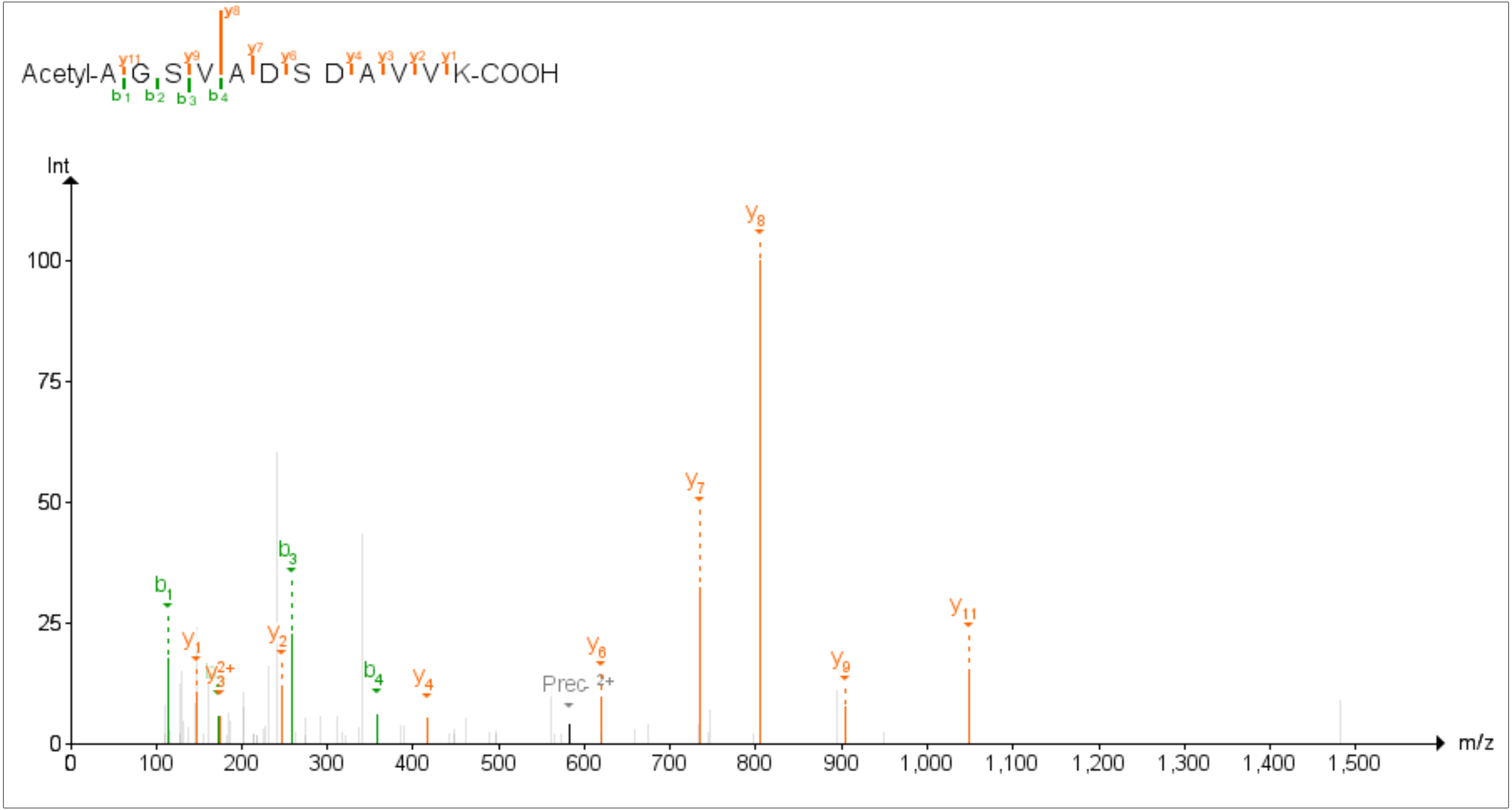
Acetyl-A G S V A D S D A V V K-COOH
b1 b2 b3 b4
y11 y9 y8 y7 y6 y4 y3 y2 y1
Int
m/z
b1
y1
y3 2+
y2
b3
b4
y4
Prec- 2+
y6
y7
y8
y9
y11

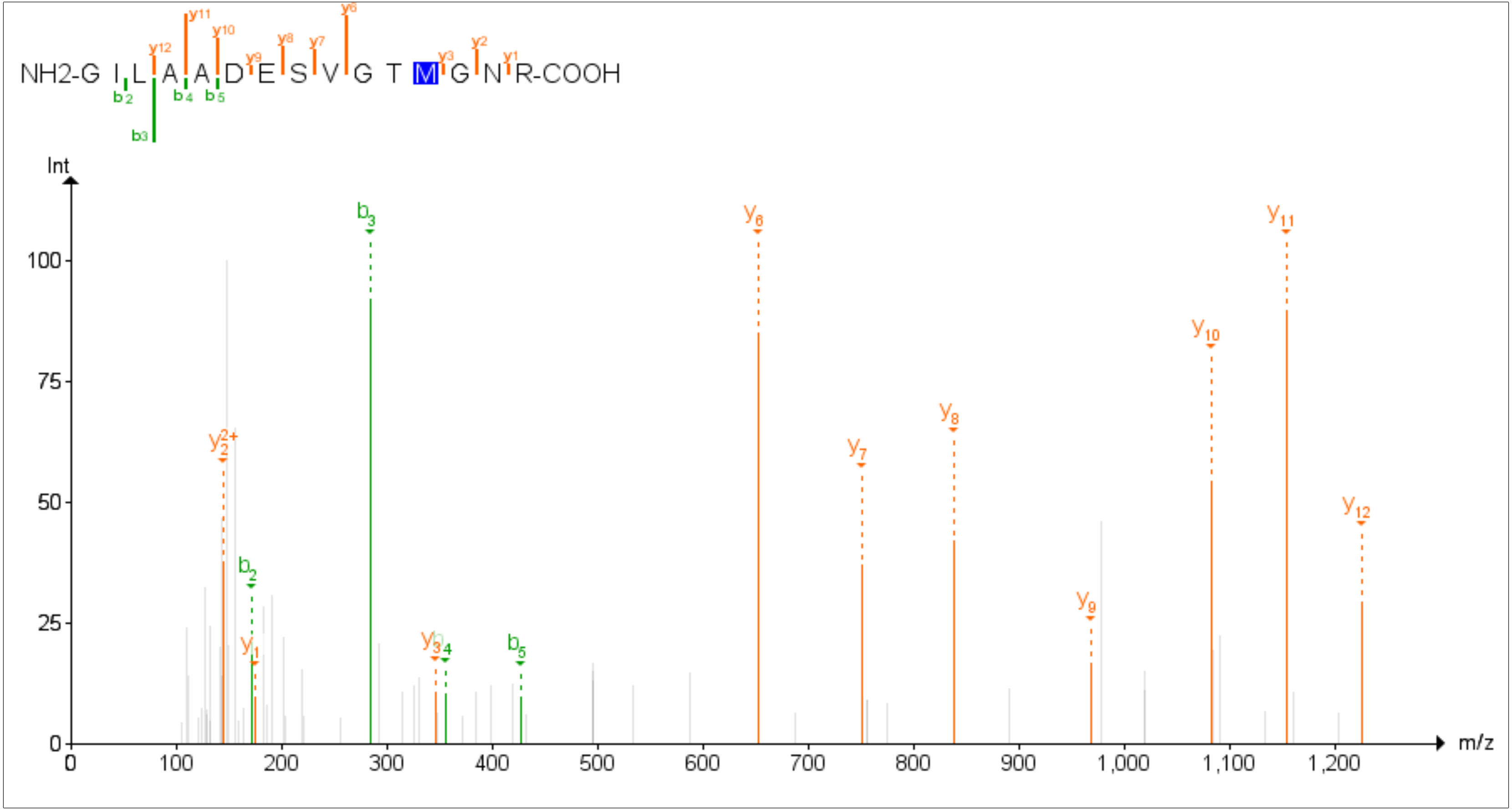

NH2-G I L A A D E S V G T M G N R-COOH
y12 y11 y10 y9 y8 y7 y6 y3 y2 y1
b2 b3 b4 b5
Int
100
75
50
25
0
m/z
0
100
200
300
400
500
600
700
800
900
1,000
1,100
1,200

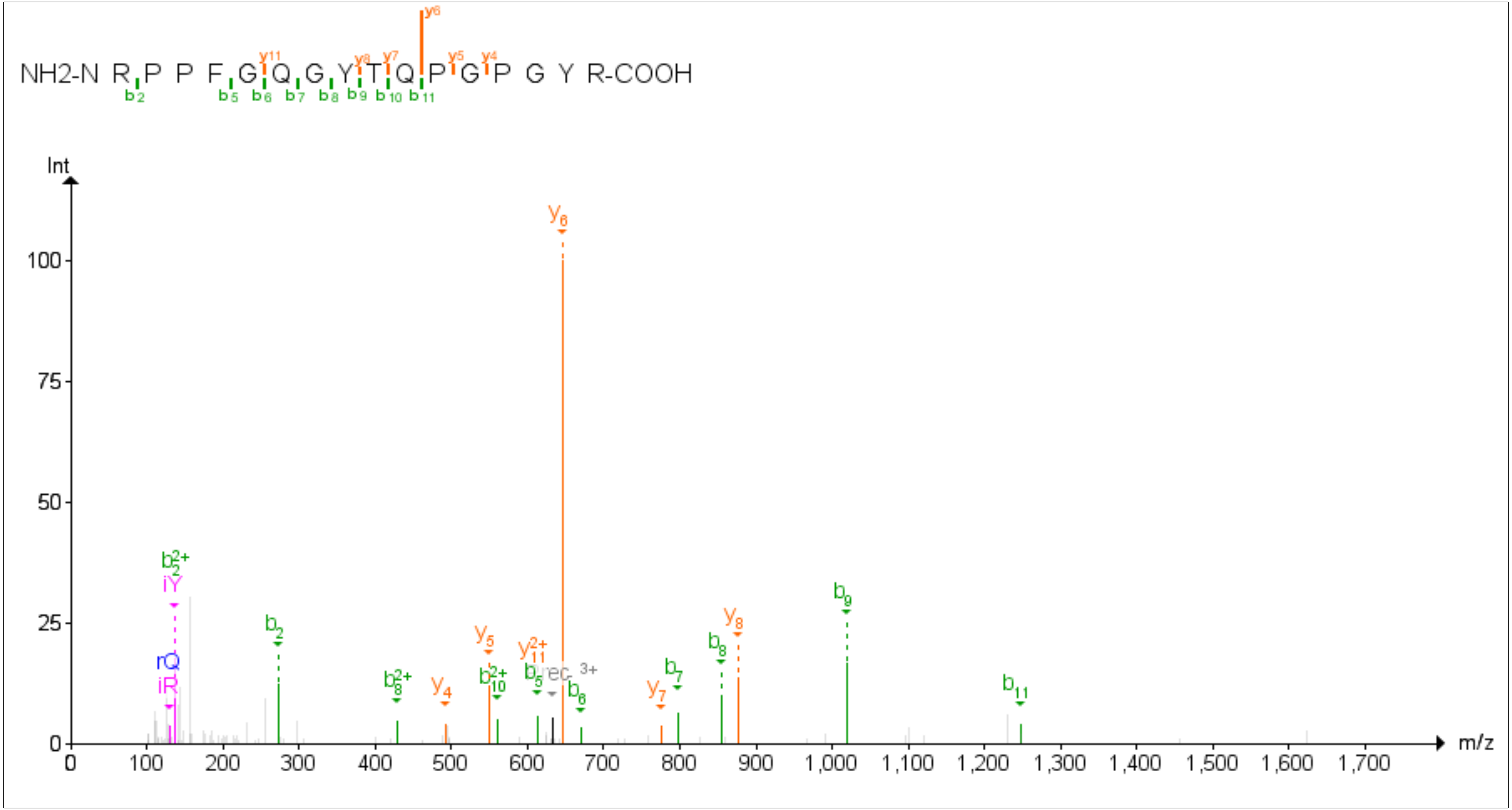

NH2-N R P P F G Q G Y T Q P G P G Y R-COOH
y11 y8 y7 y6 y5 y4
b2 b5 b6 b7 b8 b9 b10 b11
Int
m/z
0
25
50
75
100
0
100
200
300
400
500
600
700
800
900
1,000
1,100
1,200
1,300
1,400
1,500
1,600
1,700
b2 2+
iY
rQ
iR
b2
b8 2+
y4
y5
b10 2+
y11 2+
b5
Prec. 3+
b6
y6
y7
b7
b8
y8
b9
b11

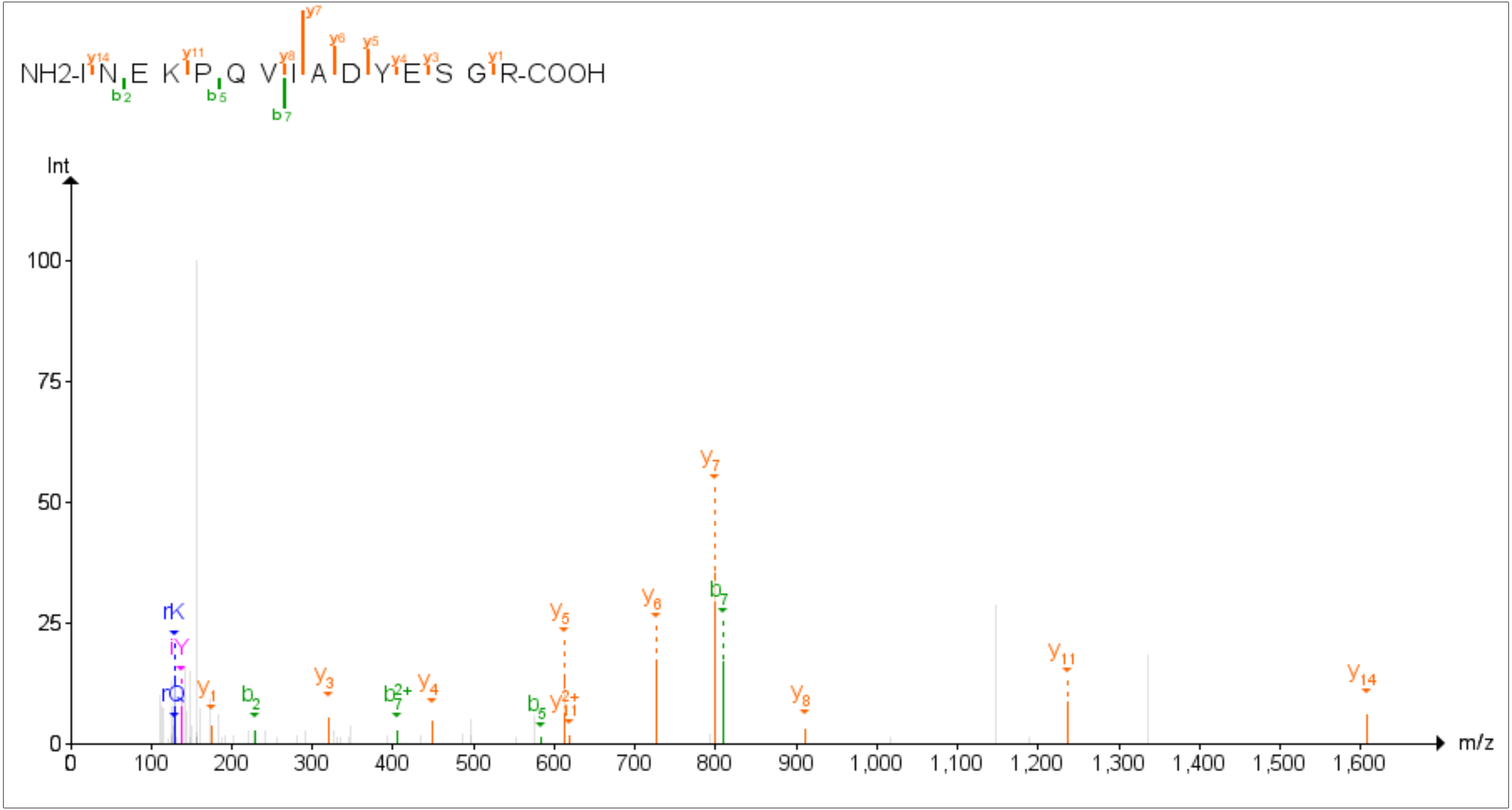

NH2-I N E K P Q V I A D Y E S G R-COOH
y14 y11 y8 y7 y6 y5 y4 y3 y1
b2 b5 b7
Int
m/z
rK
iY
rQ
y1
b2
y3
b7 2+
y4
b5
y11 2+
y5
y6
y7
b7
y8
y11
y14

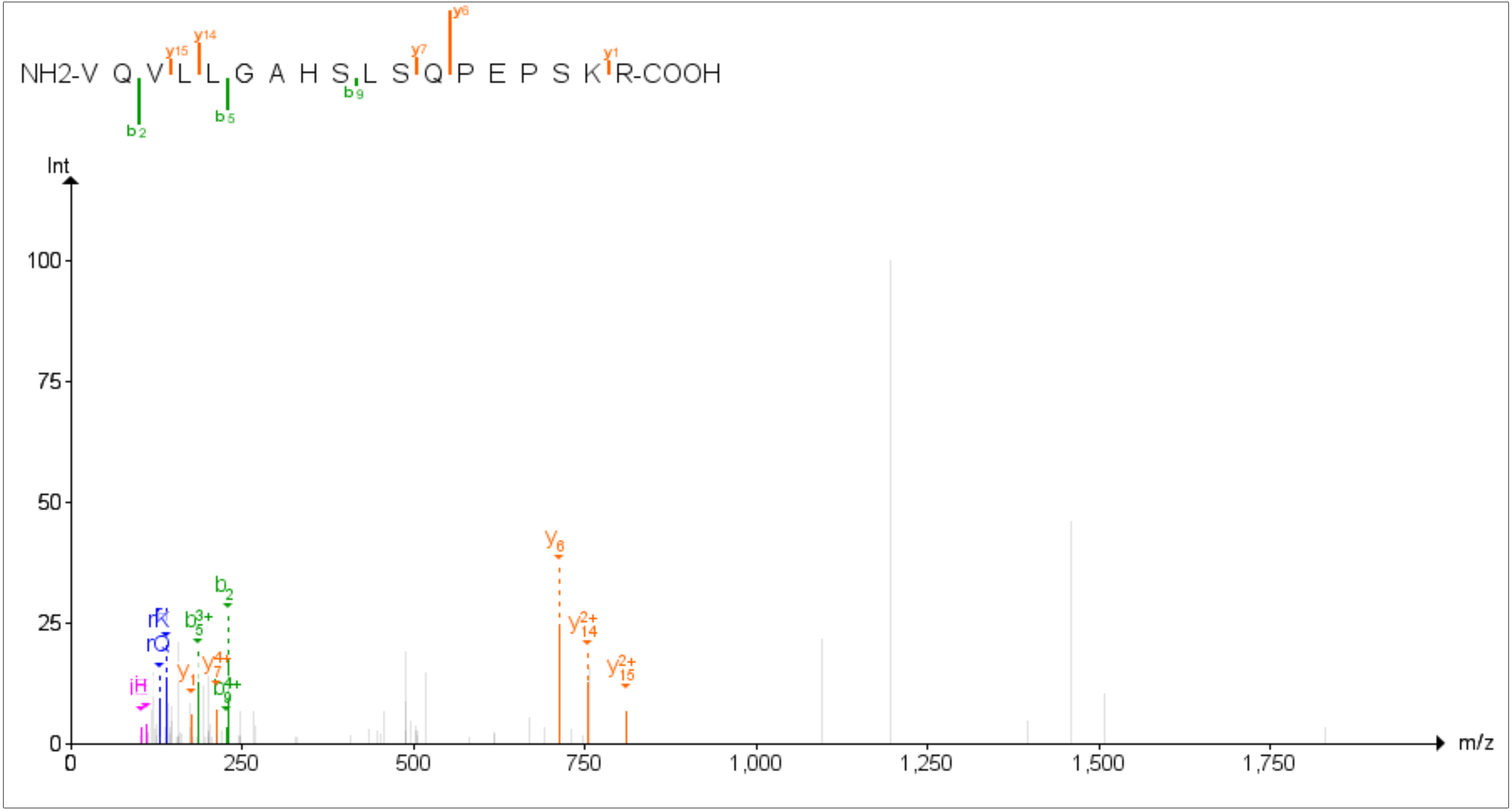
NH2-V Q V L L G A H S L S Q P E P S K R-COOH
y15
y14
y6
y7
y1
b2
b5
b9
Int
100
75
50
25
0
m/z
0
250
500
750
1,000
1,250
1,500
1,750
y6
b2
y2+ 14
y2+ 15
b3+ 5
y4+ 7
b4+ 9
y1
rK
rQ
iH

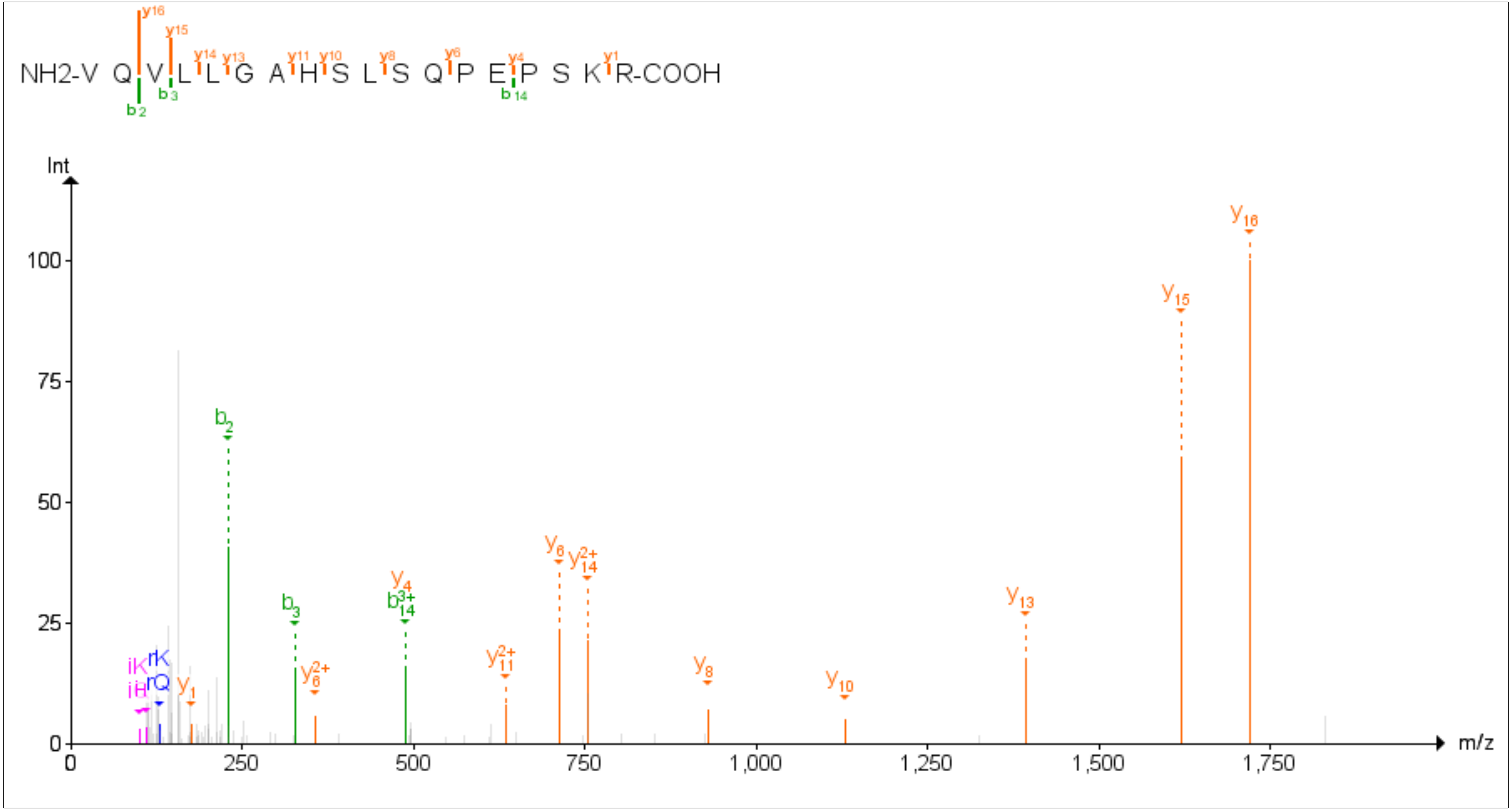

NH2-V Q V L L G A H S L S Q P E P S K R-COOH
y16
y15
y14
y13
y11
y10
y8
y6
y4
y1
b 2
b 3
b 14
Int
m/z
0
25
50
75
100
250
500
750
1,000
1,250
1,500
1,750
iK
rK
iH
rQ
y1
b2
b3
y6 2+
y4
b14 3+
y11 2+
y6
y14 2+
y8
y10
y13
y15
y16

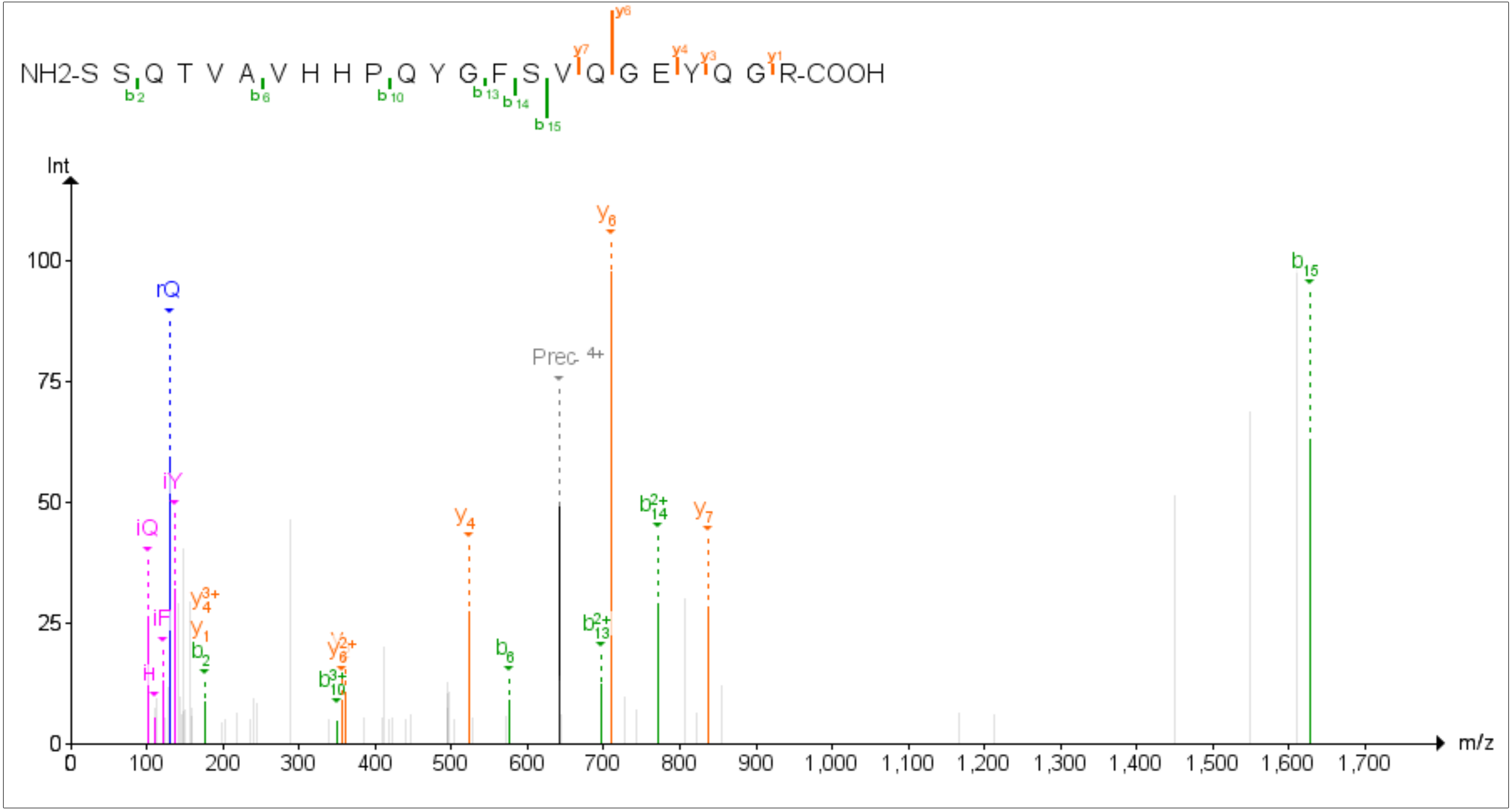

NH2-S S Q T V A V H H P Q Y G F S V Q G E Y Q G R-COOH
b2 b6 b10 b13 b14 b15
y7 y6 y4 y3 y1
Int
100
75
50
25
0
m/z
0 100 200 300 400 500 600 700 800 900 1,000 1,100 1,200 1,300 1,400 1,500 1,600 1,700
rQ
iY
iQ
iF
iH
y4 3+
y1
b2
b10 3+
y6 2+
y4
Prec 4+
y6
b13 2+
b6
b14 2+
y7
b15

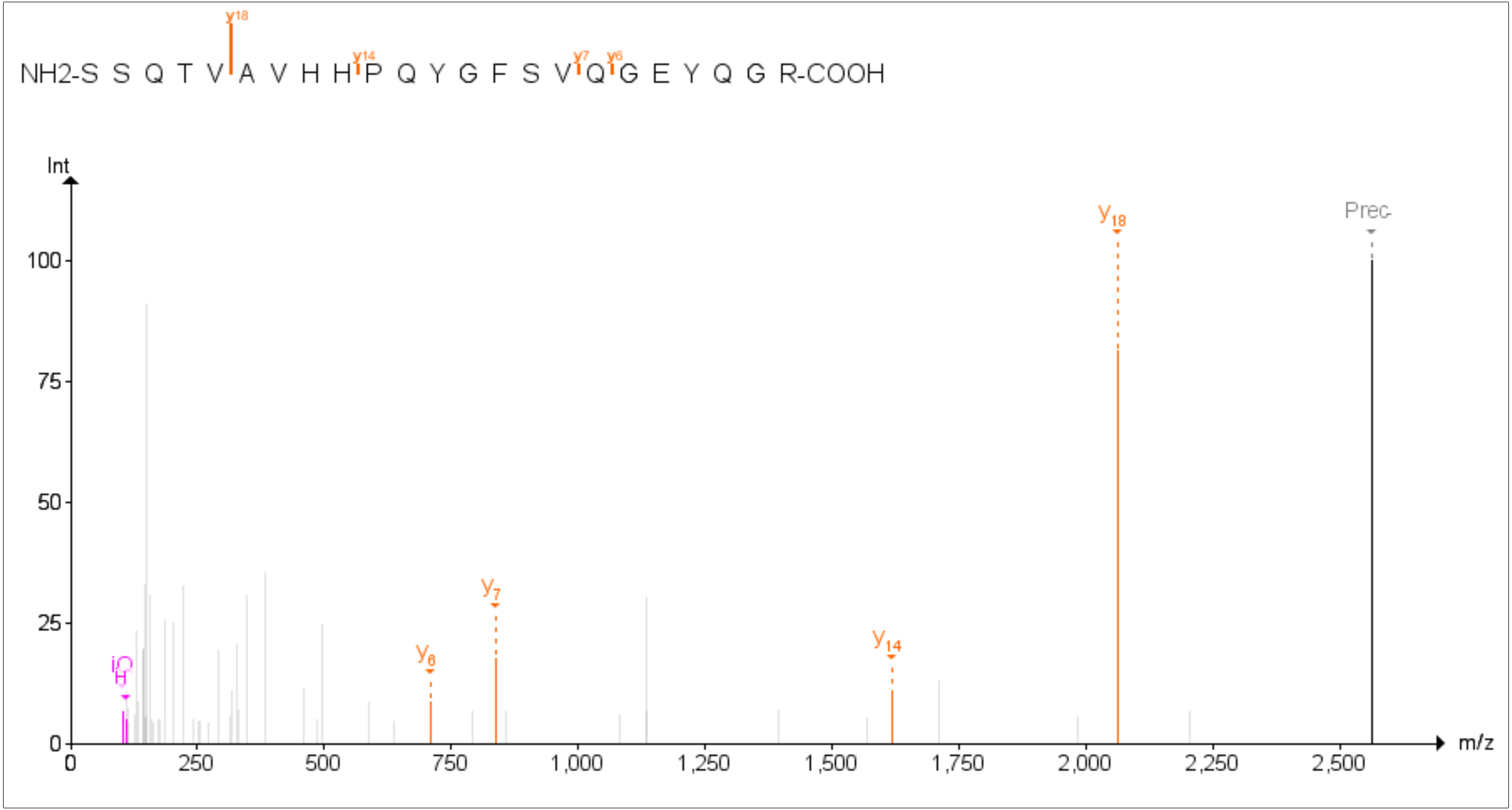

NH2-S S Q T V A V H H P Q Y G F S V Q G E Y Q G R-COOH
y18
y14
y7
y6
Int
100
75
50
25
0
Prec
y_{18}
y_7
y_6
y_{14}
0
250
500
750
1,000
1,250
1,500
1,750
2,000
2,250
2,500
m/z

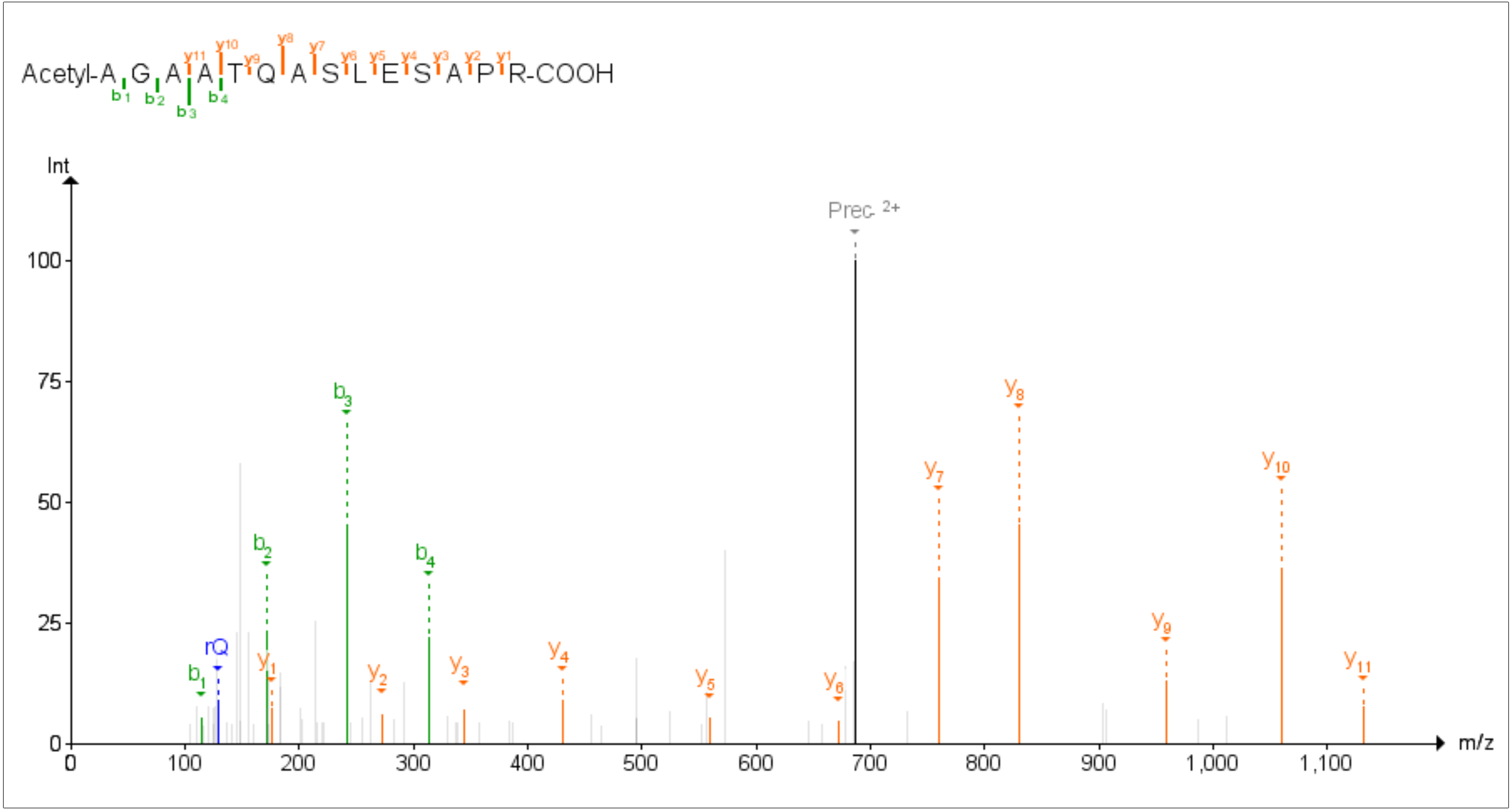

Acetyl-A G A A T Q A S L E S A P R-COOH
b1 b2 b3 b4
y11 y10 y9 y8 y7 y6 y5 y4 y3 y2 y1
Int
Prec 2+
b3
y8
y7
y10
b2
b4
y9
rQ
b1
y1
y2
y3
y4
y5
y6
y11
m/z

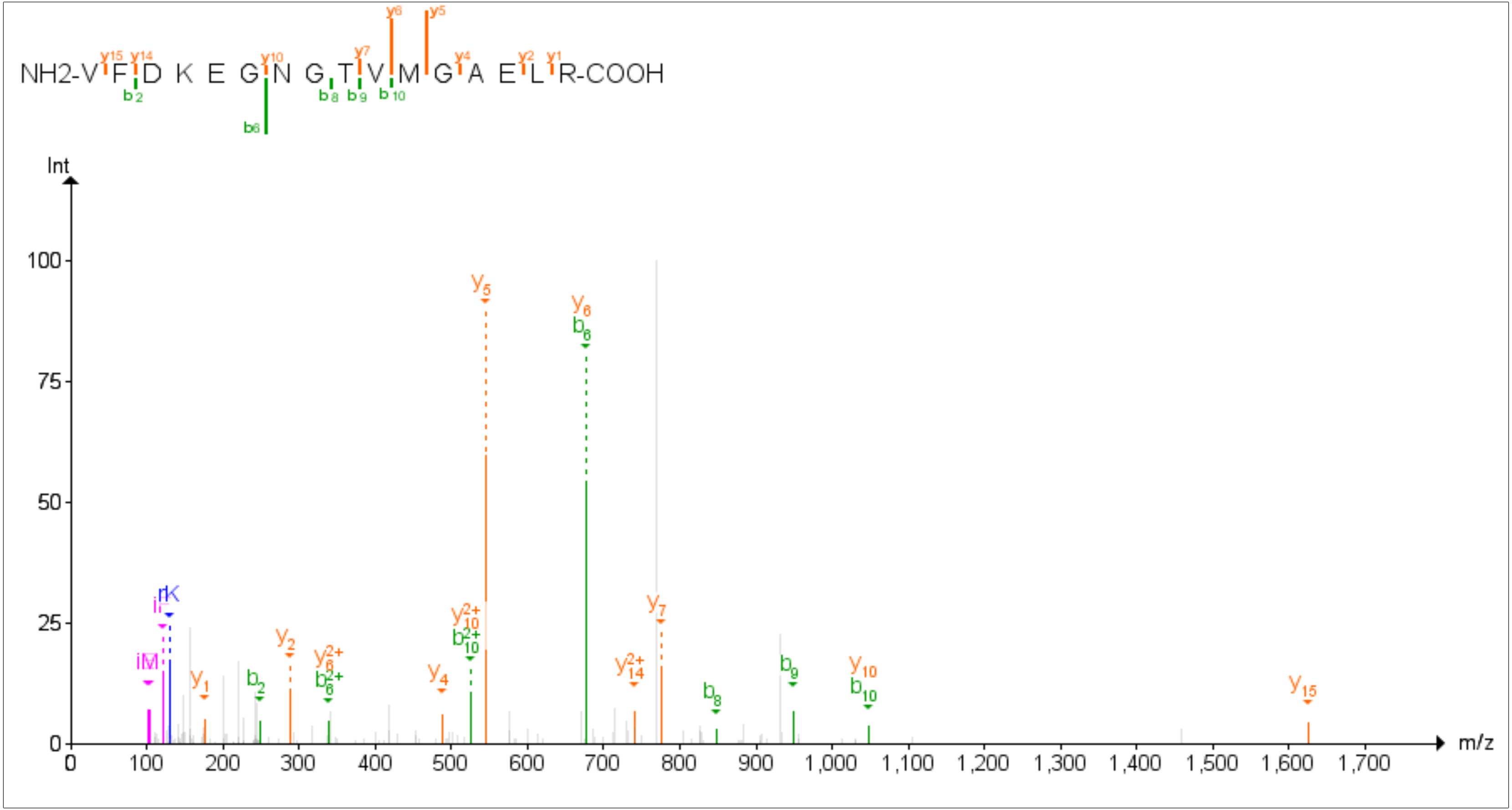

NH2-V F D K E G N G T V M G A E L R-COOH
y15 y14 y10 y7 y6 y5 y4 y2 y1
b2 b6 b8 b9 b10
Int
m/z
y5
y6
b6
y7
y2
y1
b2
iM
iF
rK
y10
b10
y4
y14
b8
b9
y15
0
25
50
75
100
0
100
200
300
400
500
600
700
800
900
1,000
1,100
1,200
1,300
1,400
1,500
1,600
1,700

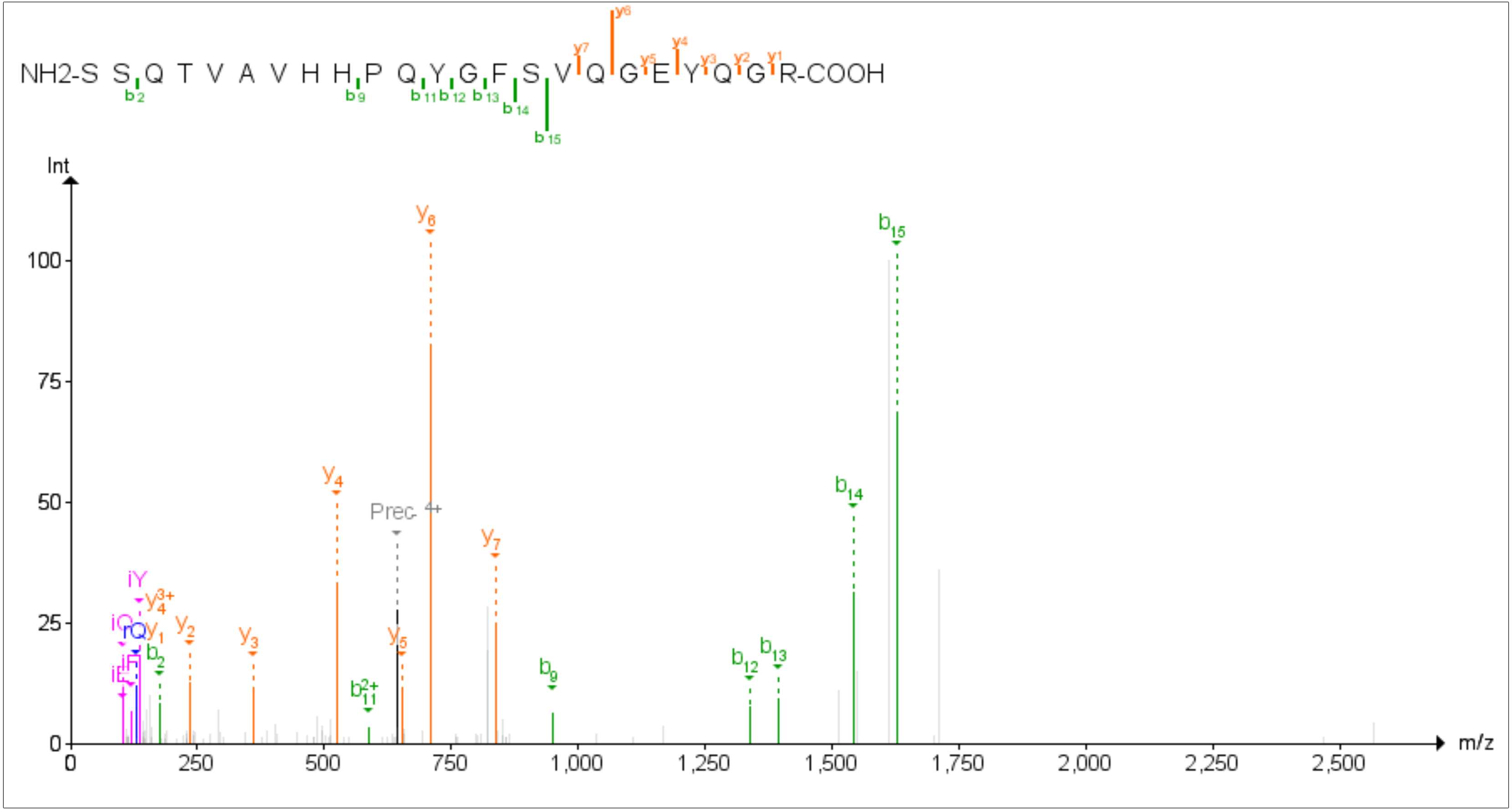

NH2-S S Q T V A V H H P Q Y G F S V Q G E Y Q G R-COOH
b2
b9
b11
b12
b13
b14
b15
y7
y6
y5
y4
y3
y2
y1
Int
m/z
Prec 4+

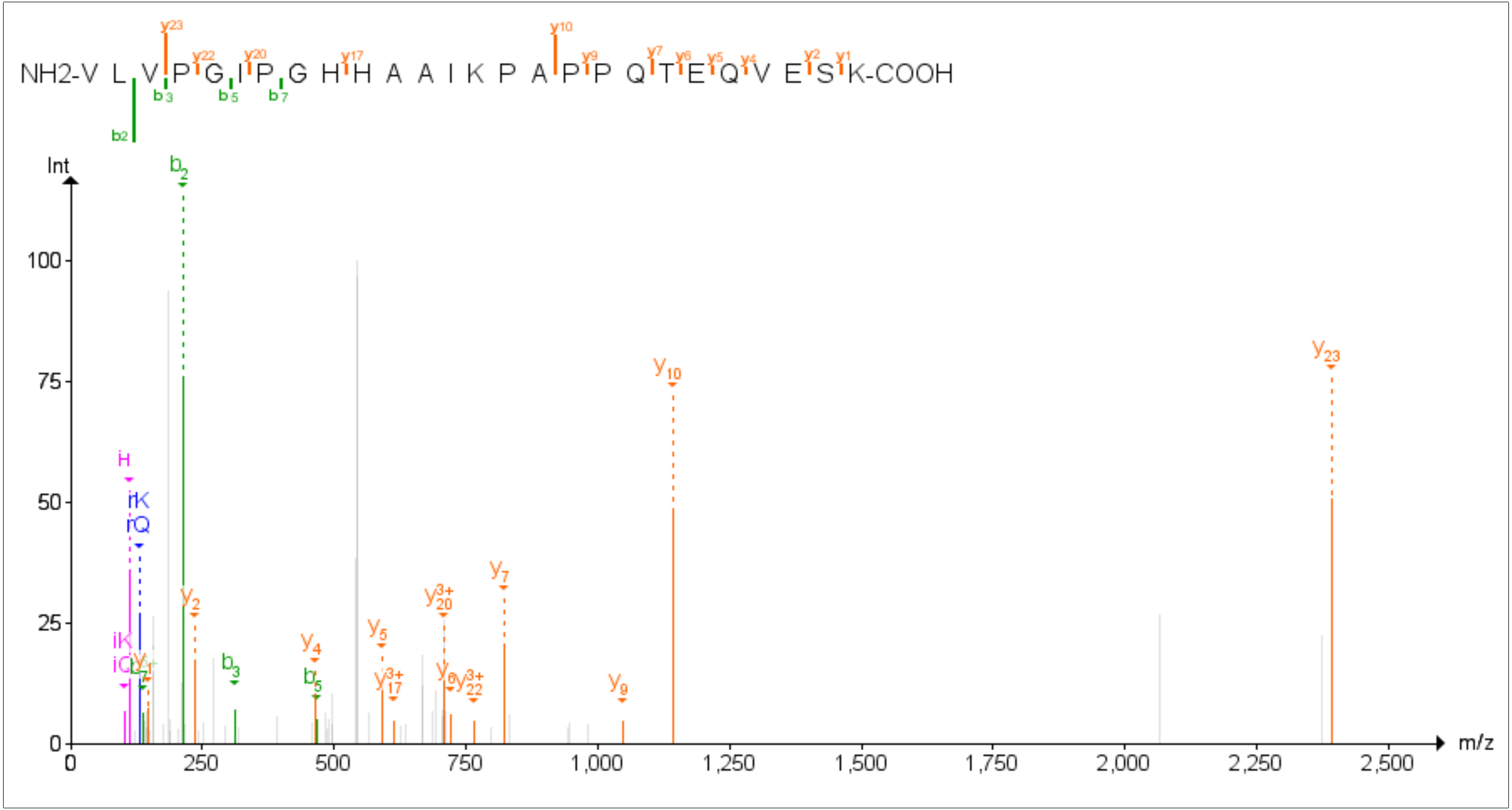

NH2-V L V P G I P G H H A A I K P A P P Q T E Q V E S K-COOH
y23
y22
y20
y17
y10
y9
y7
y6
y5
y4
y2
y1
b2
b3
b5
b7
Int
100
75
50
25
0
m/z
0
250
500
750
1,000
1,250
1,500
1,750
2,000
2,250
2,500
iH
rK
rQ
iK
iQ

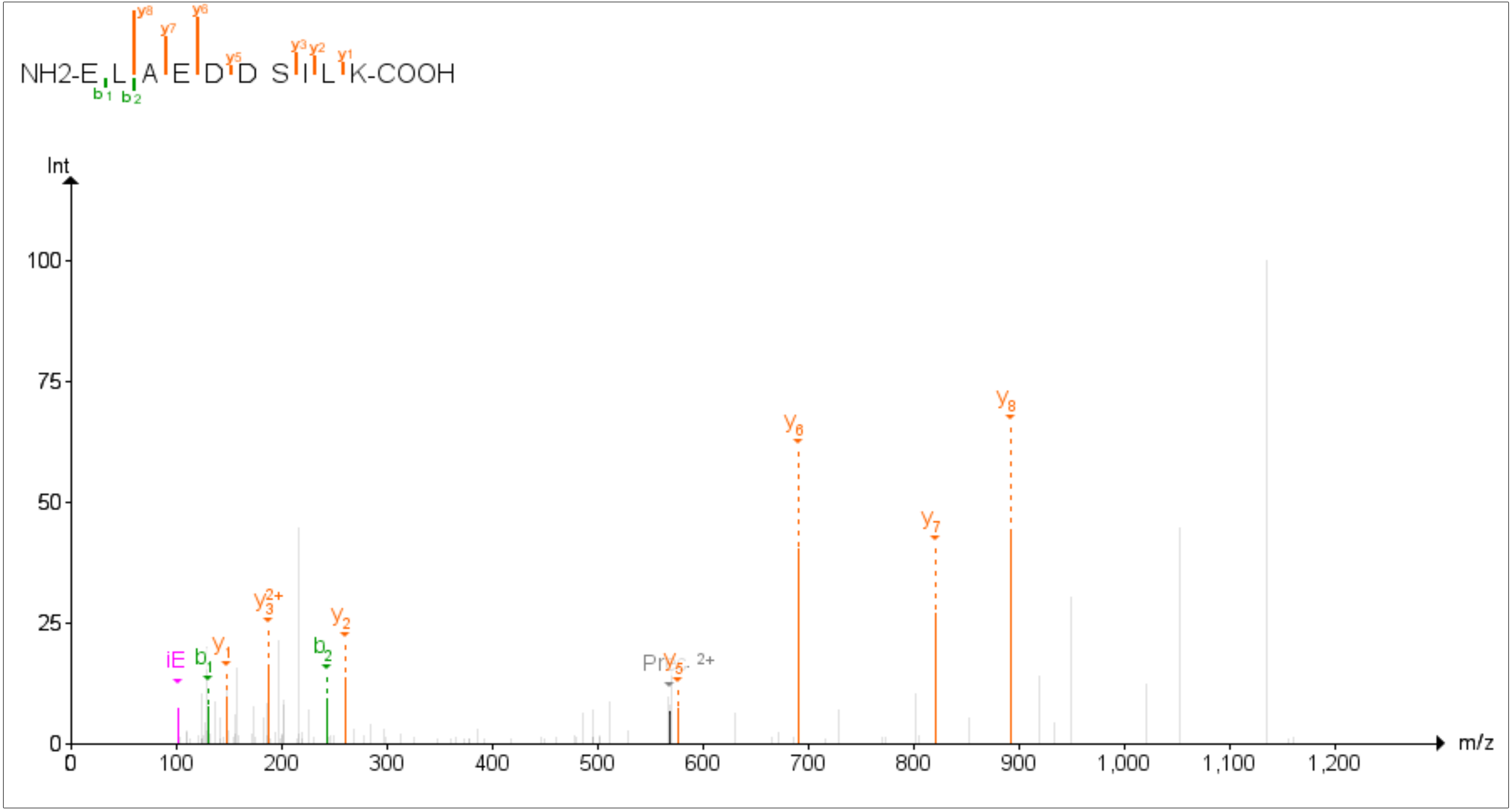

NH2-E L A E D D S I L K-COOH
y8 y7 y6 y5 y3 y2 y1
b1 b2
Int
m/z
0
25
50
75
100
0
100
200
300
400
500
600
700
800
900
1,000
1,100
1,200
iE
b_1
y_1
y_3^{2+}
b_2
y_2
Pr
y_5
2+
y_6
y_7
y_8

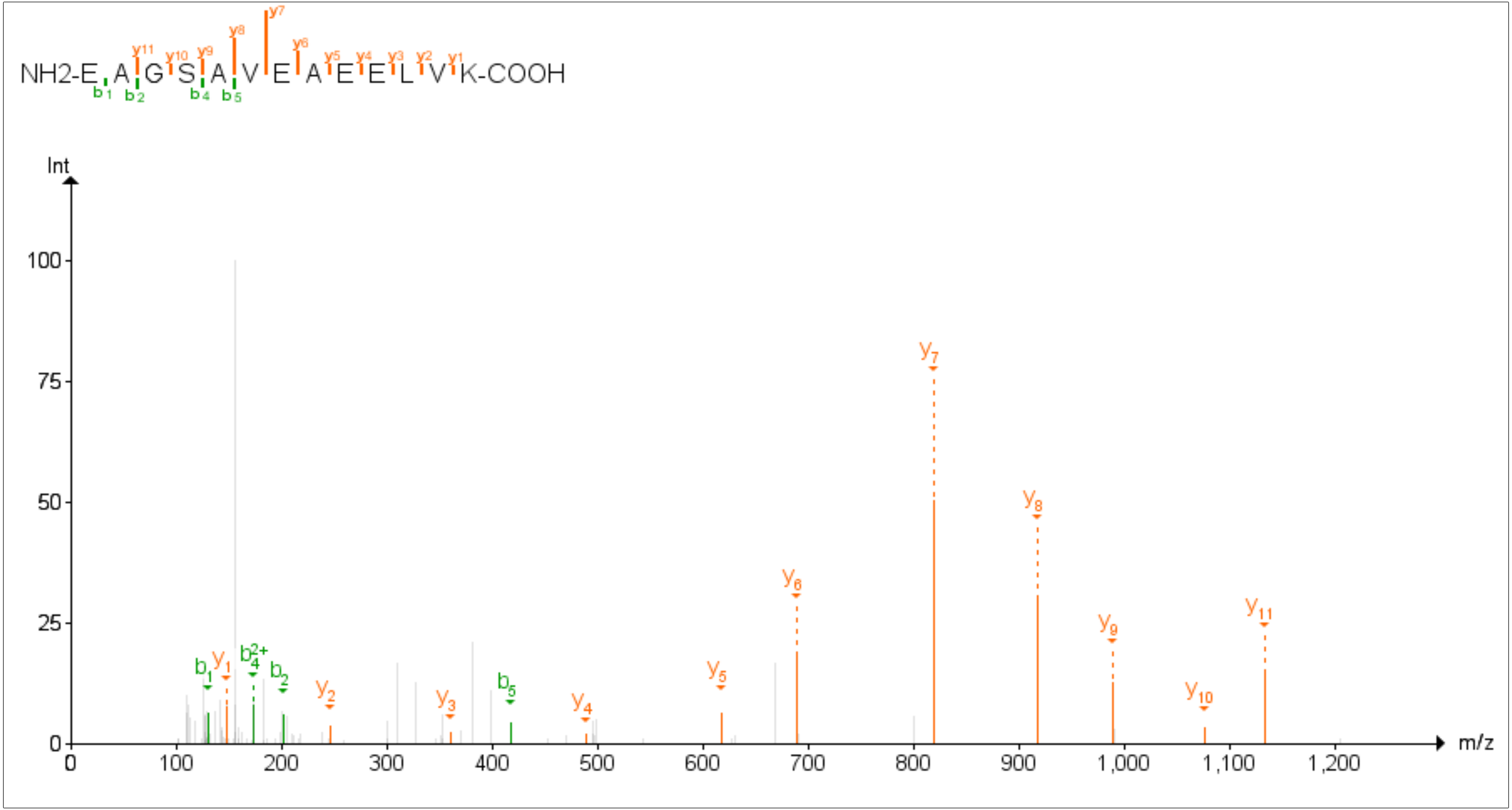
NH2-EAGSAVEAEELVK-COOH
Int
m/z
y7
y8
y6
y9
y11
y10
y5
y4
y3
y2
y1
b1
b2
b4
b5
b4 2+
0
25
50
75
100
0
100
200
300
400
500
600
700
800
900
1,000
1,100
1,200

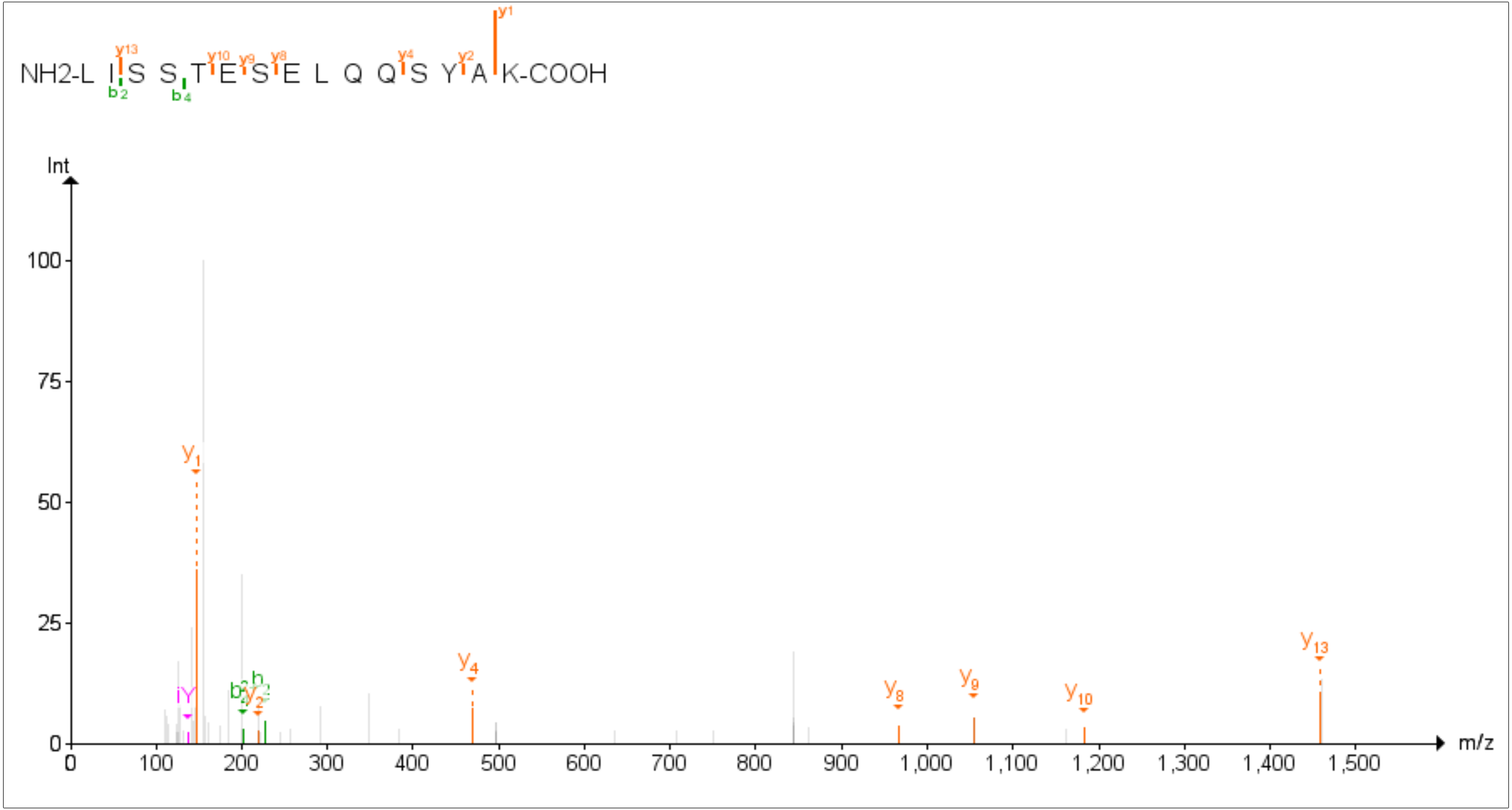

NH2-L I S S T E S E L Q Q S Y A K-COOH
y13 y10 y9 y8 y4 y2 y1
b2 b4
Int
100
75
50
25
0
y1
iY
y4
y8
y9
y10
y13
0 100 200 300 400 500 600 700 800 900 1,000 1,100 1,200 1,300 1,400 1,500
m/z

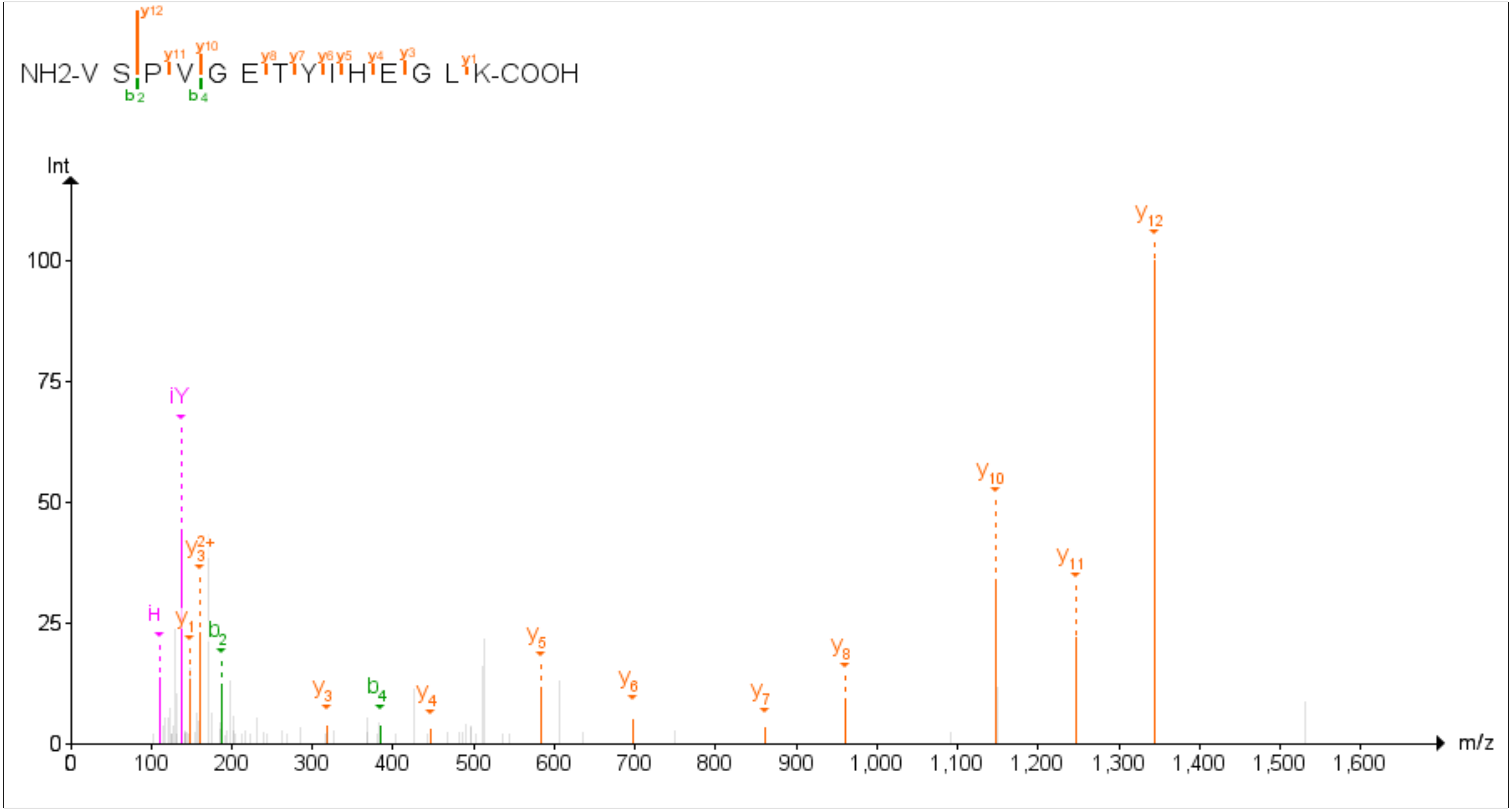
NH2-V S P V G E T Y I H E G L K-COOH
y12
y11
y10
y8
y7
y6
y5
y4
y3
y1
b2
b4
Int
100
75
50
25
0
iY
iH
y_3^{2+}
y_1
b_2
y_3
b_4
y_4
y_5
y_6
y_7
y_8
y_{10}
y_{11}
y_{12}
m/z
0
100
200
300
400
500
600
700
800
900
1,000
1,100
1,200
1,300
1,400
1,500
1,600

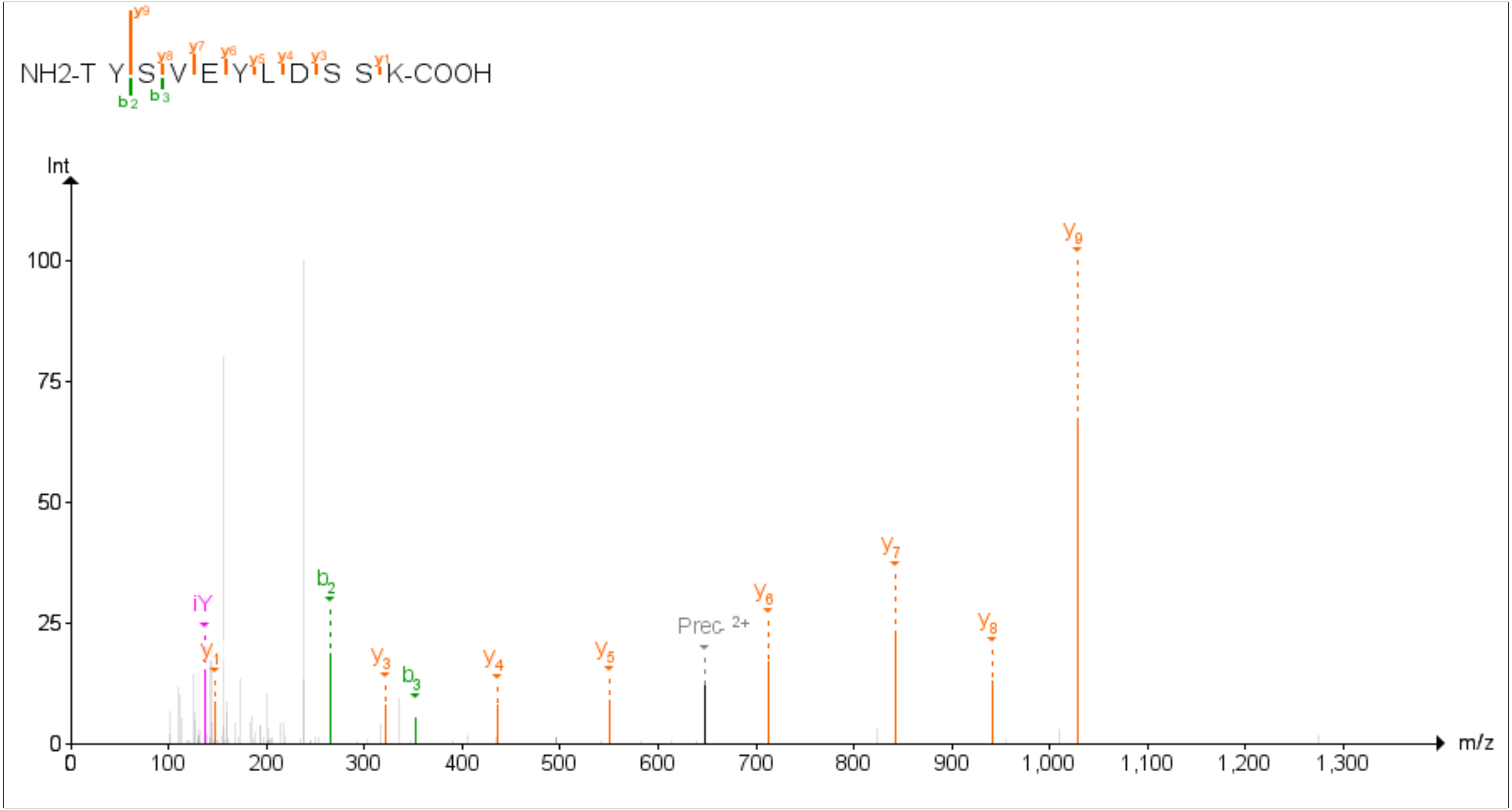

NH2-T Y S V E Y L D S S K-COOH
y9 y8 y7 y6 y5 y4 y3 y1
b2 b3
Int
100
75
50
25
0
iY
y1
b2
y3
b3
y4
y5
Prec 2+
y6
y7
y8
y9
0 100 200 300 400 500 600 700 800 900 1,000 1,100 1,200 1,300
m/z

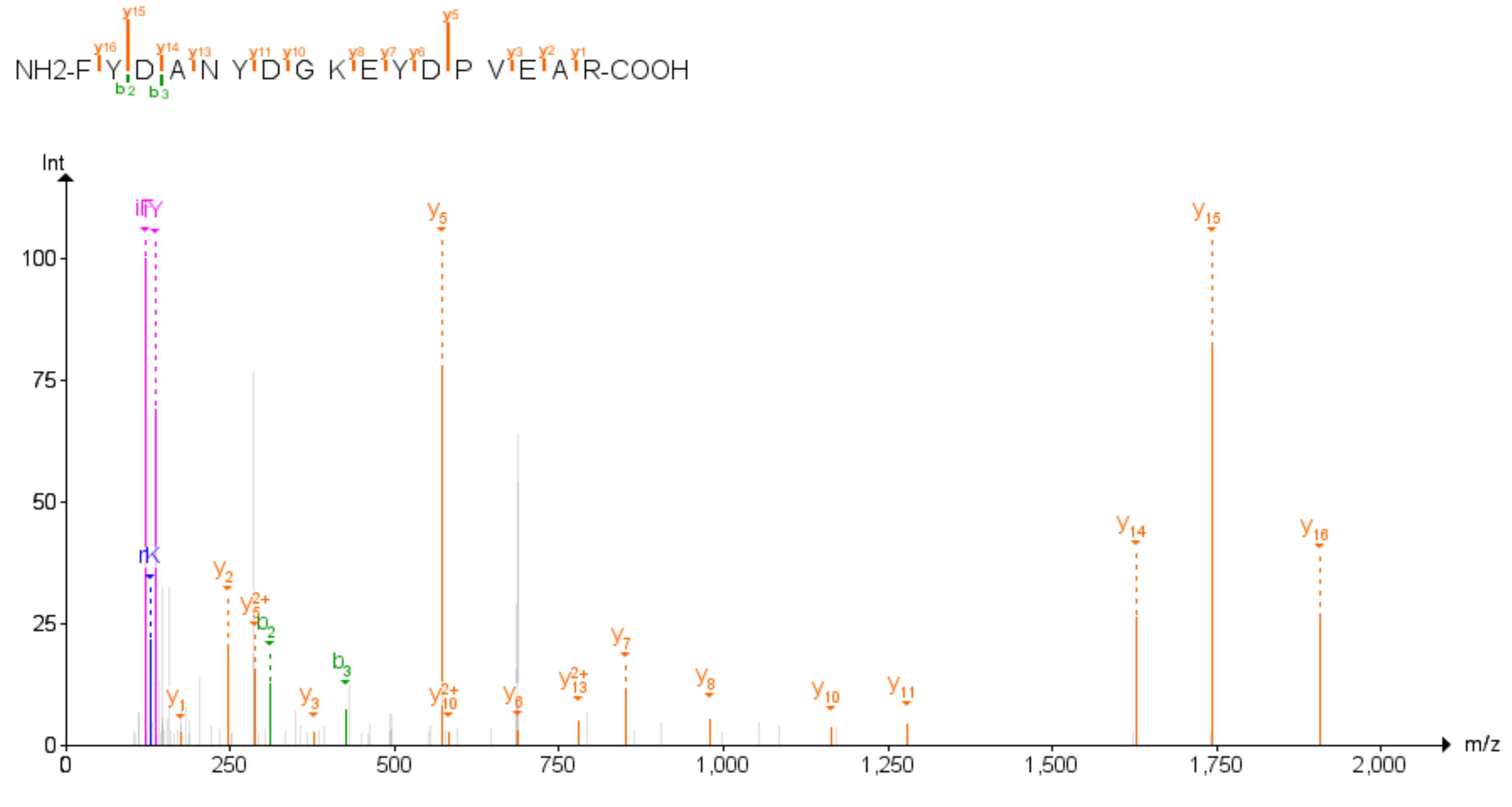

NH2-F Y D A N Y D G K E Y D P V E A R-COOH
y16 y15 y14 y13 y11 y10 y8 y7 y6 y5 y3 y2 y1
b2 b3
Int
iF iY
rK
y1 y2 y3 y5 y6 y7 y8 y10 y11 y14 y15 y16
y5 2+
y10 2+
y13 2+
b2 b3
0
25
50
75
100
0 250 500 750 1,000 1,250 1,500 1,750 2,000
m/z

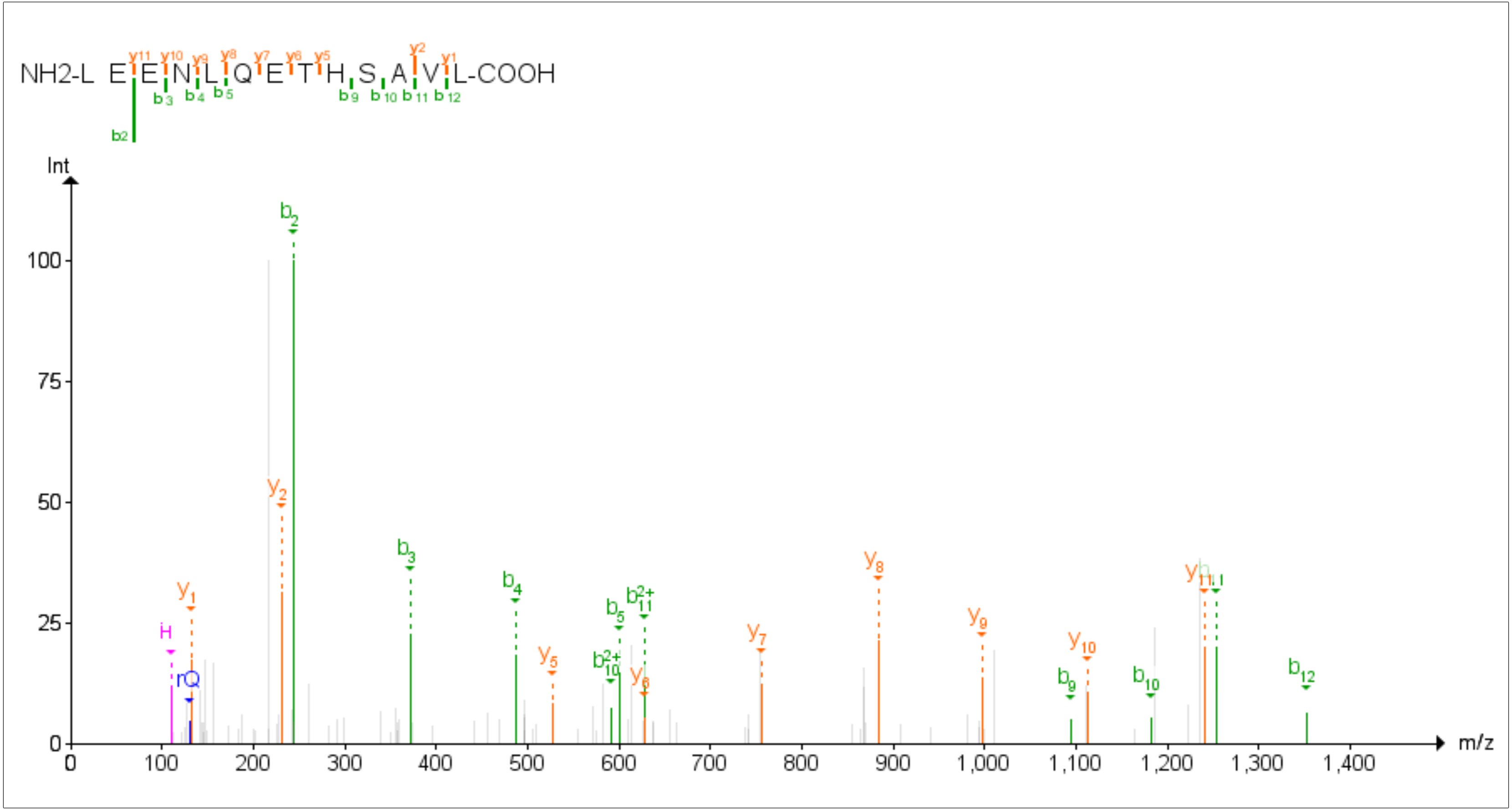

NH2-L E E N L Q E T H S A V L-COOH
y11 y10 y9 y8 y7 y6 y5 y2 y1
b2 b3 b4 b5 b9 b10 b11 b12
Int
100
75
50
25
0
m/z
0 100 200 300 400 500 600 700 800 900 1,000 1,100 1,200 1,300 1,400
b2
y2
y1
iH
rQ
b3
b4
y5
b5
b11 2+
b10 2+
y6
y7
y8
y9
y10
b9
b10
y11
b12

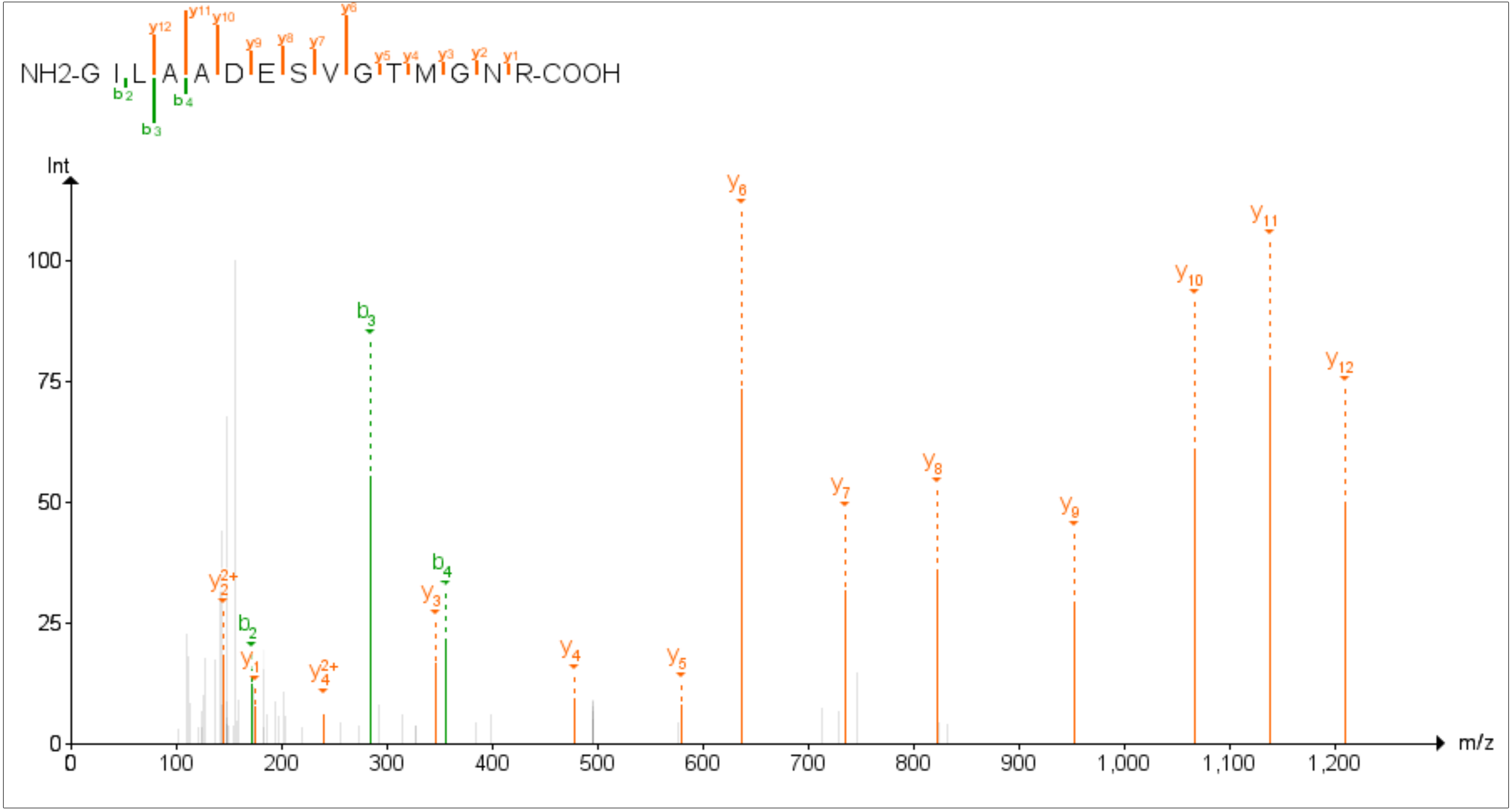
NH2-G I L A A D E S V G T M G N R-COOH
y12 y11 y10 y9 y8 y7 y6 y5 y4 y3 y2 y1
b2 b3 b4
Int
m/z
y6
y11
y10
y12
b3
y8
y7
y9
b4
y3
y2 2+
b2
y1
y4 2+
y4
y5
0
25
50
75
100
0 100 200 300 400 500 600 700 800 900 1,000 1,100 1,200

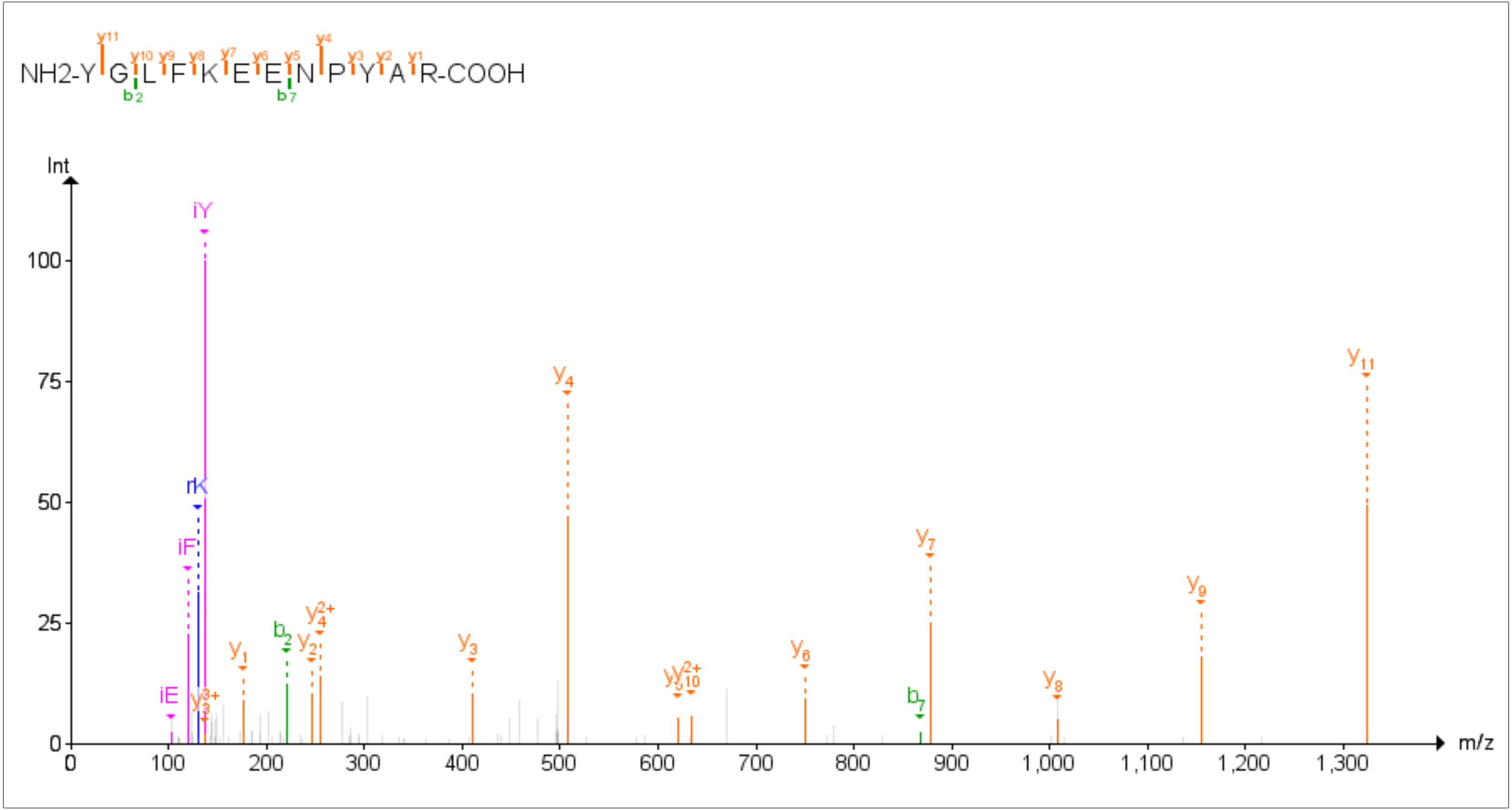

NH2-YGILFKEEINPYAR-COOH
Int
m/z
iY
rK
iF
iE
y4
y11
y7
y9
y1
b2
y2
y3
y6
y8
b7

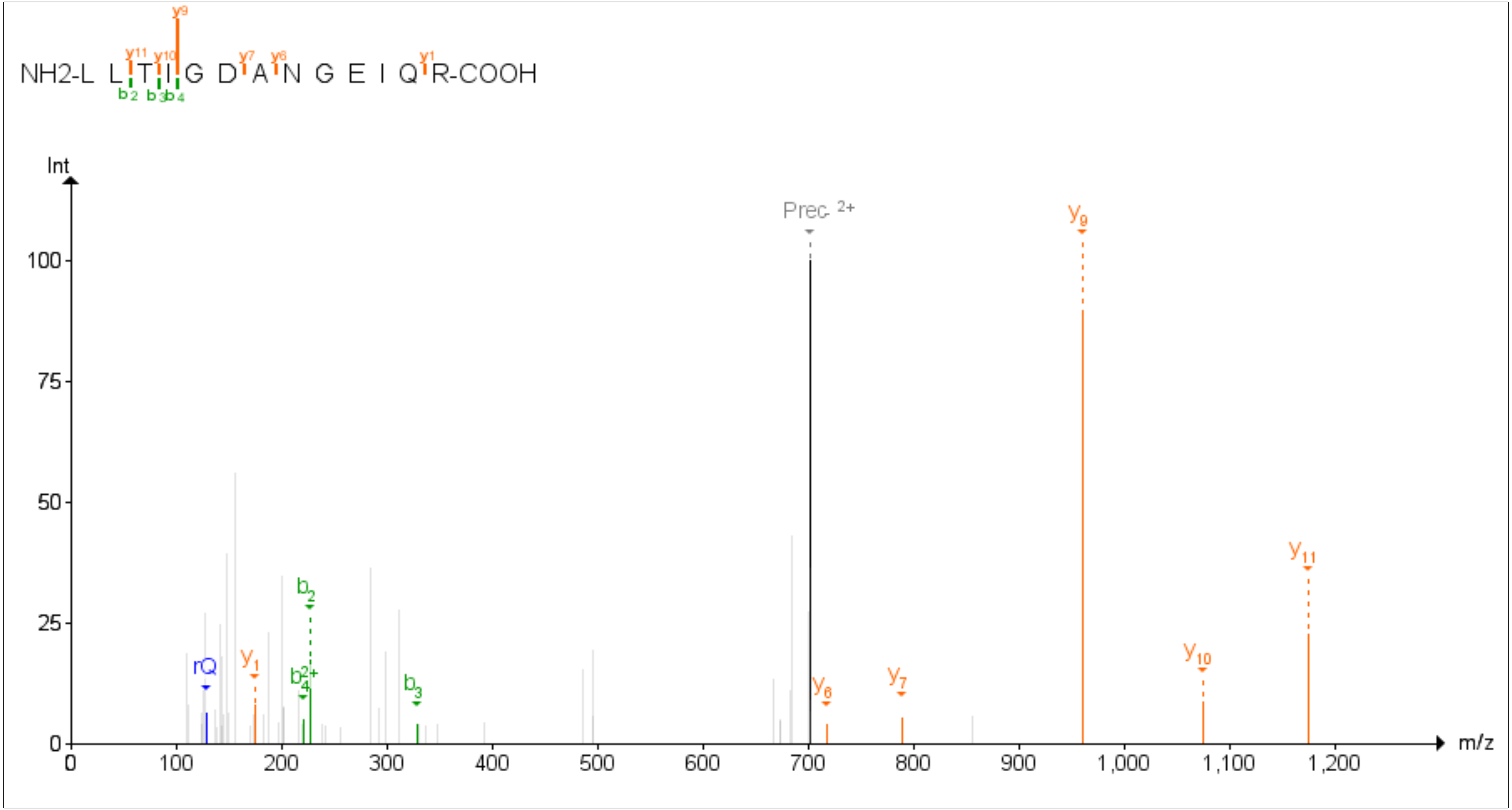

NH2-L L T I G D A N G E I Q R-COOH
y11 y10 y9 y7 y6 y1
b2 b3 b4
Int
Prec 2+
y9
y11
y10
y7
y6
b2
b4 2+
b3
y1
rQ
m/z
0
25
50
75
100
0
100
200
300
400
500
600
700
800
900
1,000
1,100
1,200

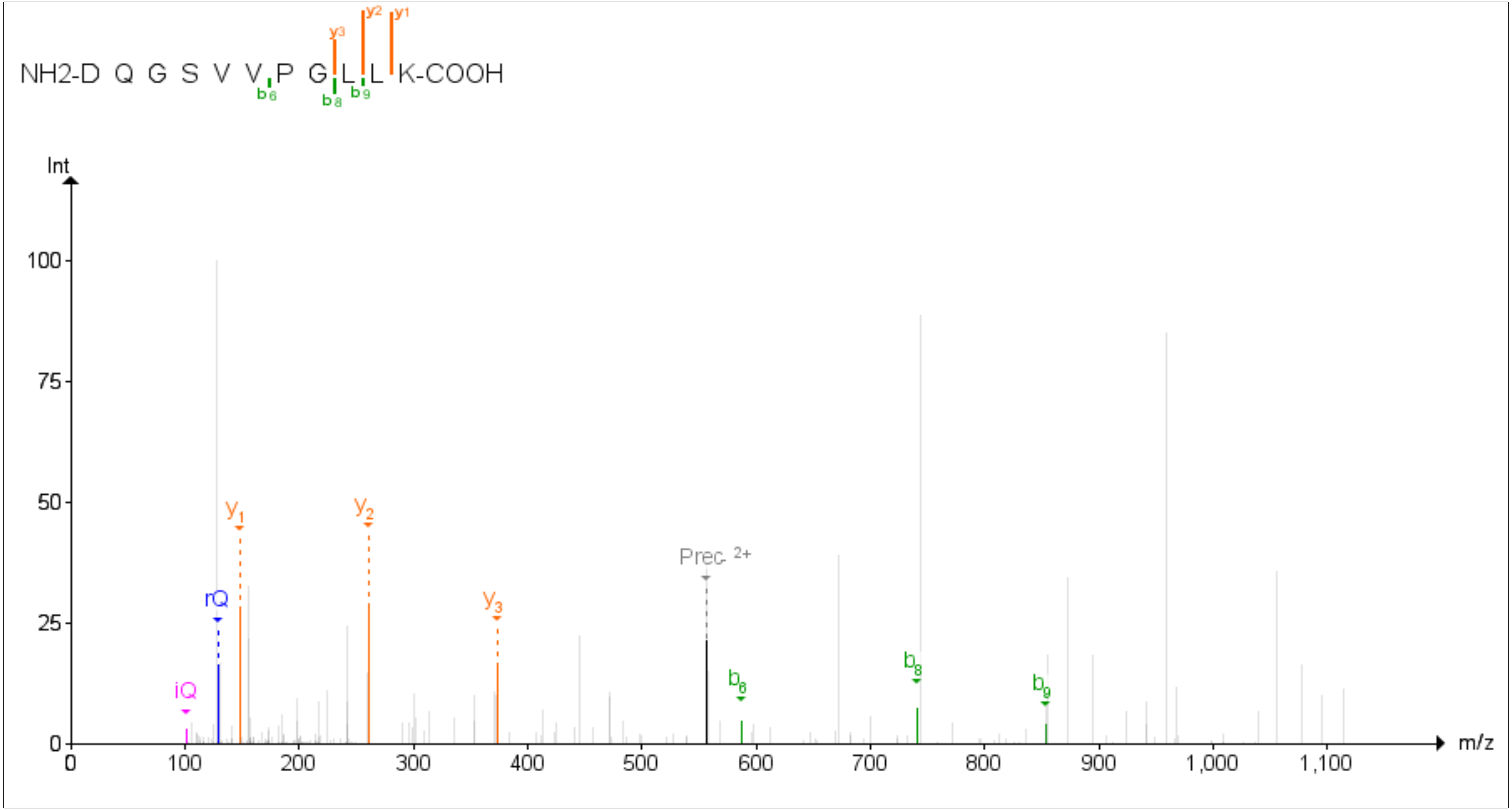

NH2-D Q G S V V P G L L K-COOH
y3
y2
y1
b6
b8
b9
Int
m/z
y1
y2
y3
rQ
iQ
Prec 2+
b6
b8
b9

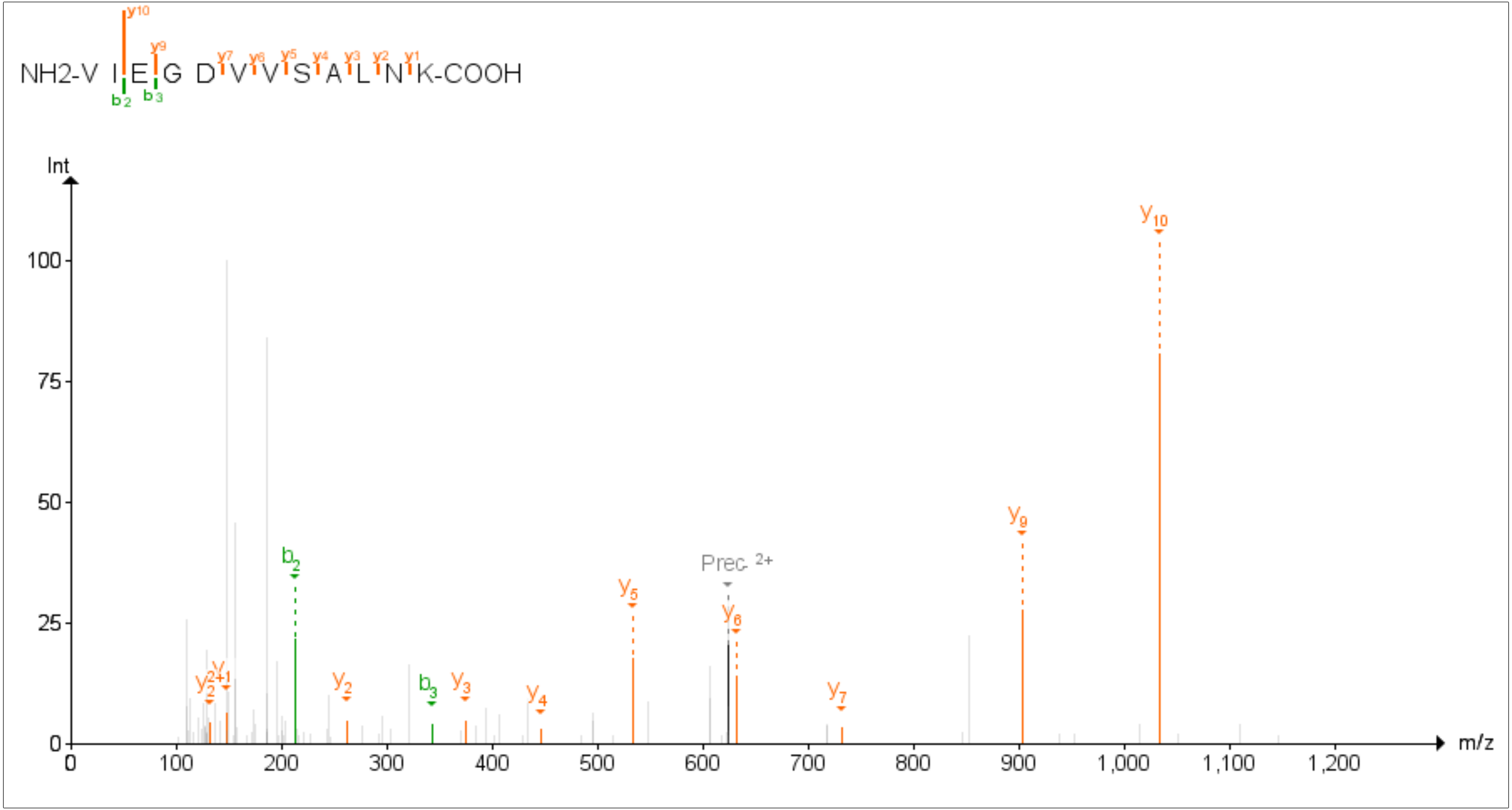

NH2-V I E G D V V S A L N K-COOH
y10 y9 y7 y6 y5 y4 y3 y2 y1
b2 b3
Int
100
75
50
25
0
m/z
0
100
200
300
400
500
600
700
800
900
1,000
1,100
1,200
y10
y9
Prec 2+
y6
y5
b2
b3
y3
y2
y4
y7
y1

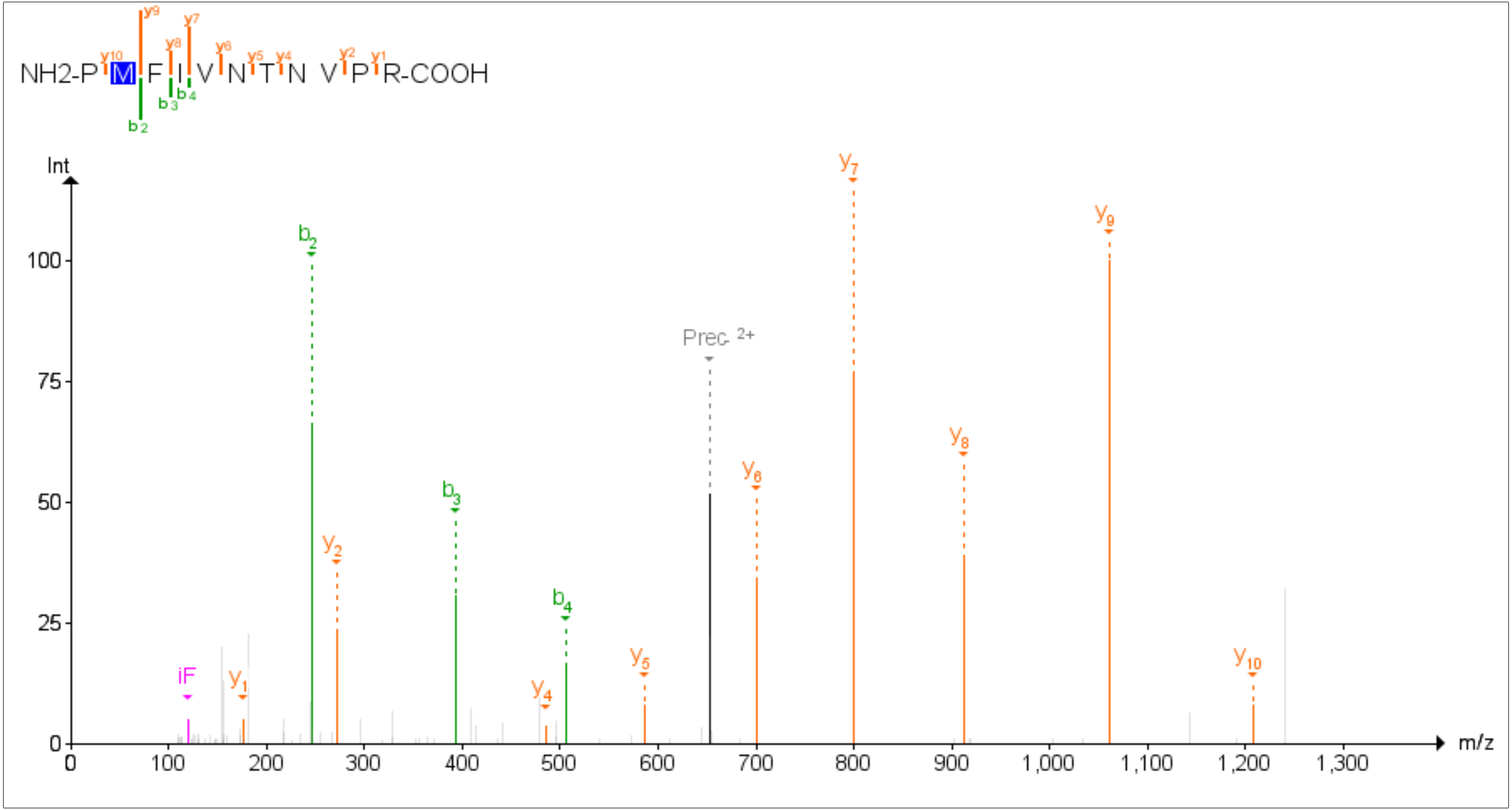

NH2-P M F I V N T N V P R-COOH
y10 y9 y8 y7 y6 y5 y4 y2 y1
b2 b3 b4
Int
100
75
50
25
0
m/z
0 100 200 300 400 500 600 700 800 900 1,000 1,100 1,200 1,300
b2 b3 b4
y1 y2 y4 y5 y6 y7 y8 y9 y10
iF
Prec 2+

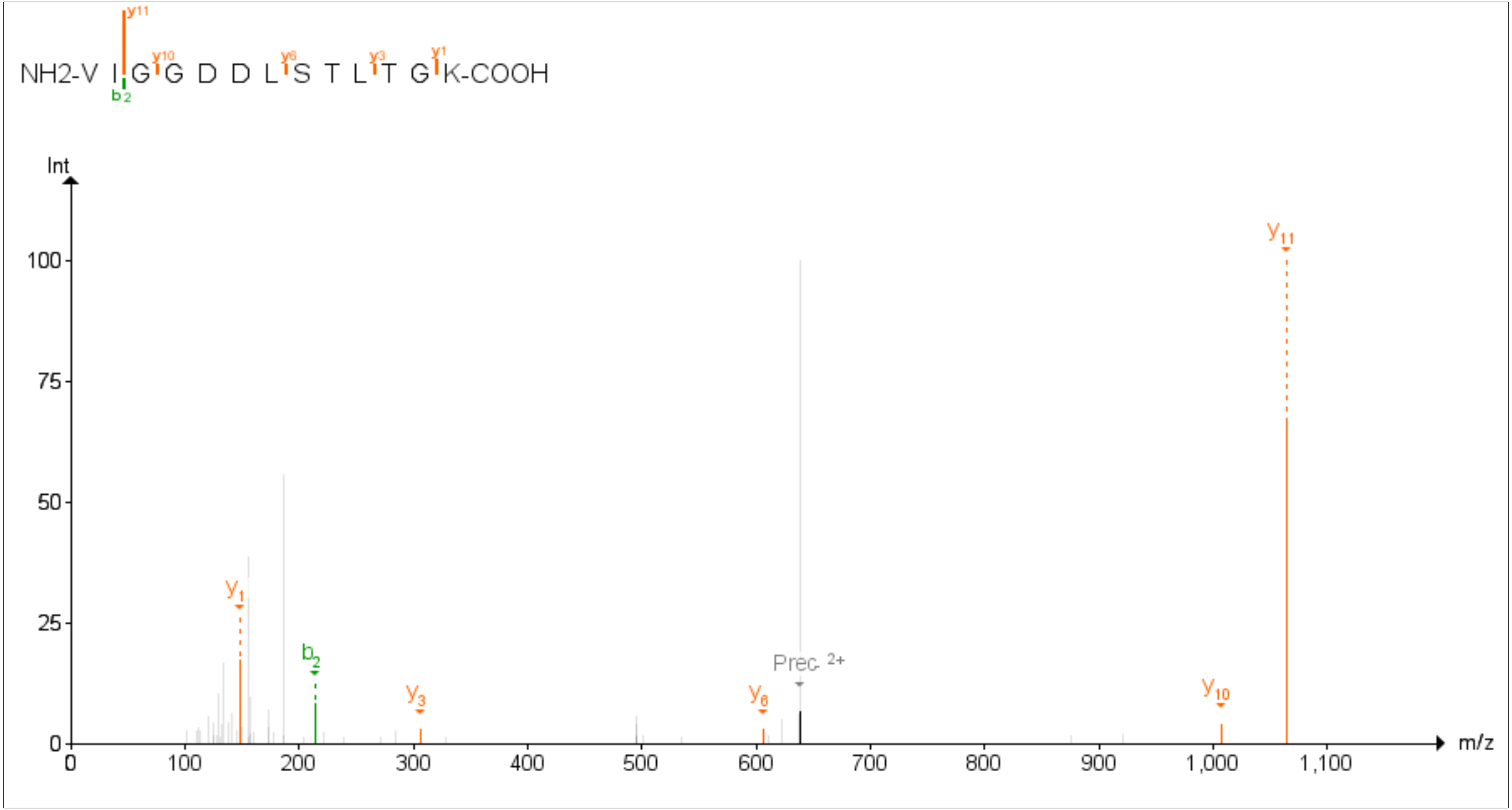

NH2-V I G G D D L S T L T G K-COOH
y11
y10
y6
y3
y1
b2
Int
100
75
50
25
0
y11
y1
b2
y3
Prec. 2+
y6
y10
0
100
200
300
400
500
600
700
800
900
1,000
1,100
m/z

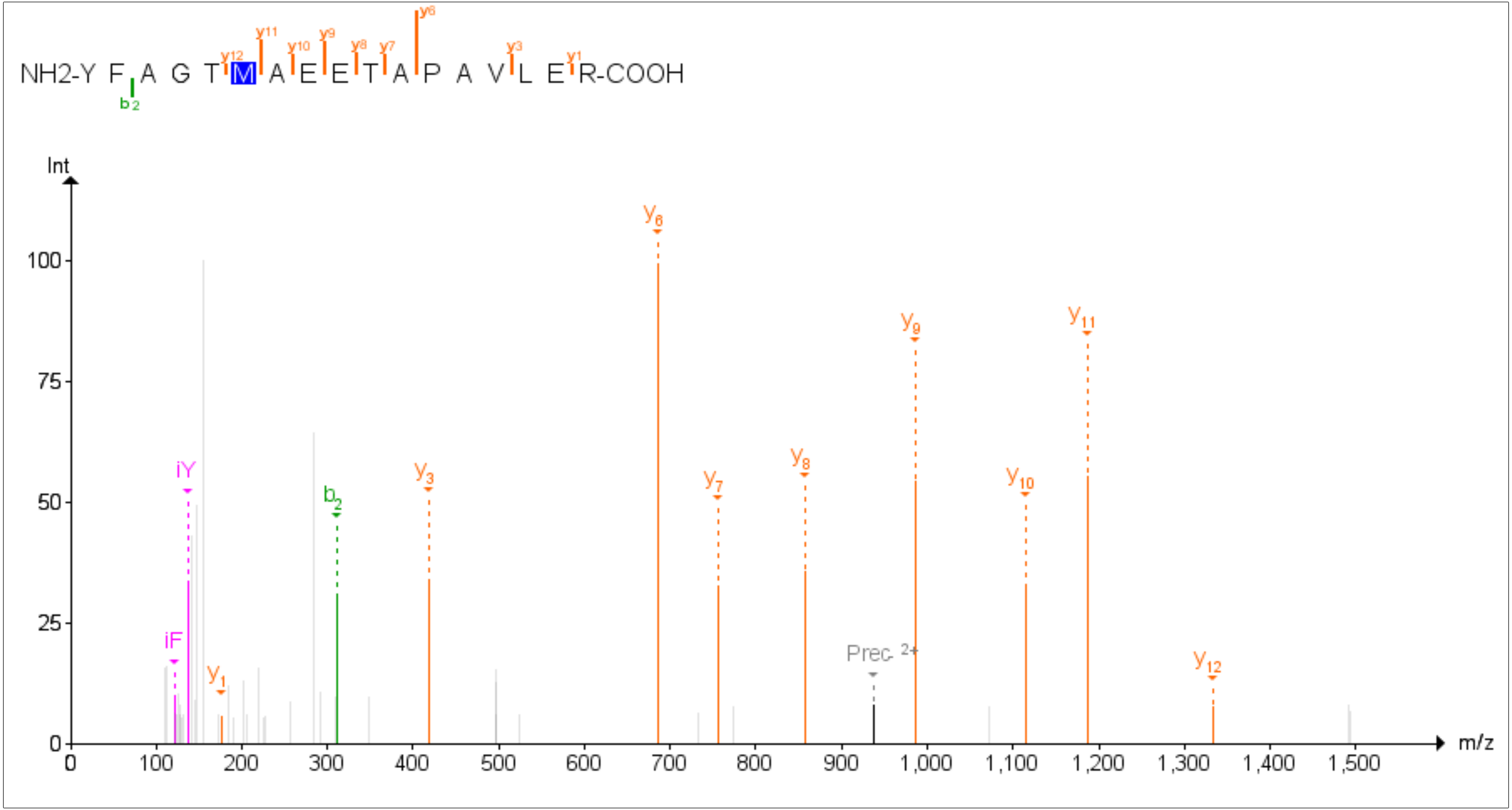

NH2-Y F A G T M A E E T A P A V L E R-COOH
b2
y12
y11
y10
y9
y8
y7
y6
y3
y1
Int
100
75
50
25
0
iY
iF
y1
b2
y3
y6
y7
y8
y9
y10
y11
y12
Prec 2+
0
100
200
300
400
500
600
700
800
900
1,000
1,100
1,200
1,300
1,400
1,500
m/z

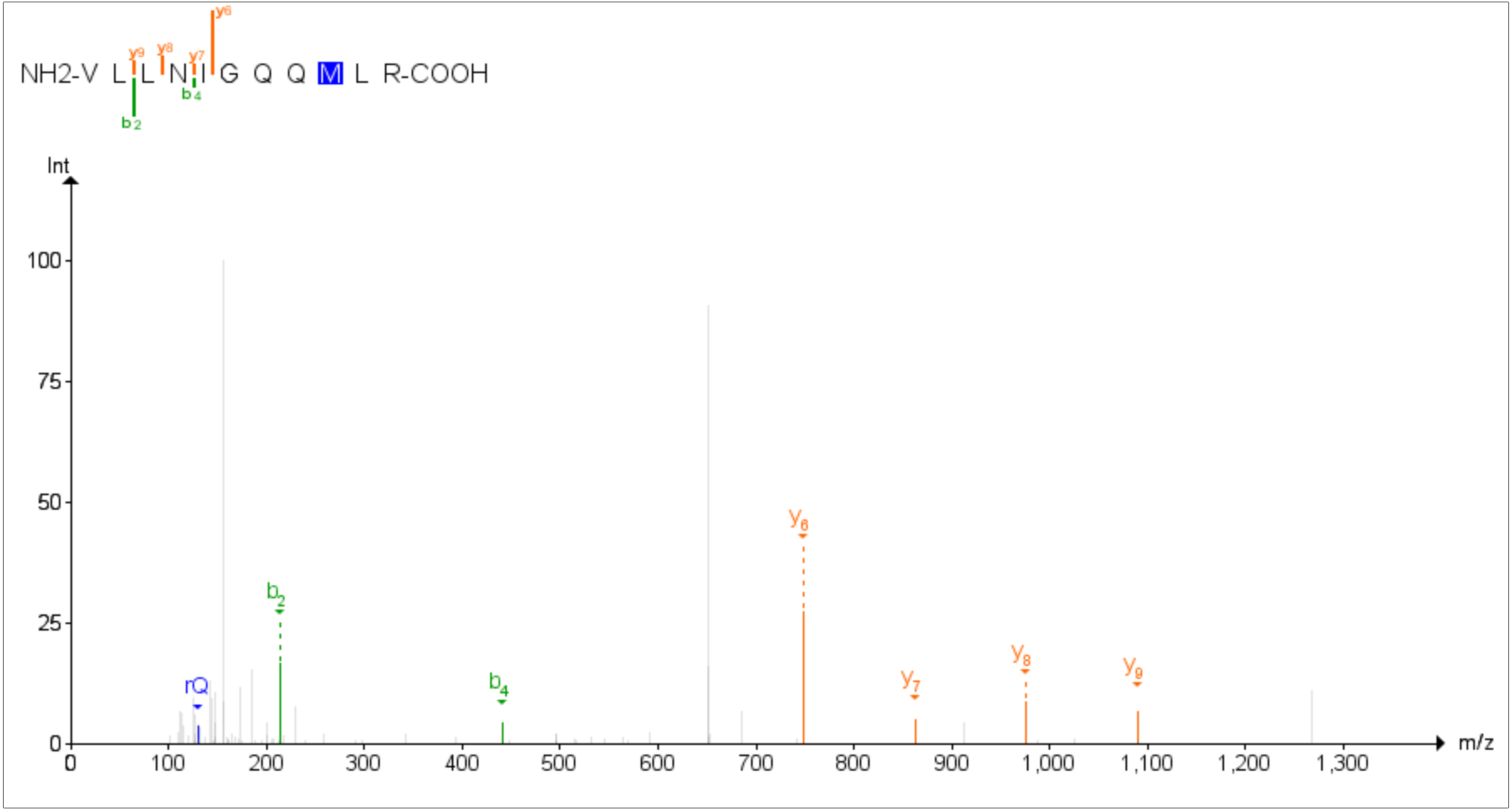

NH2-V L L N I G Q Q M L R-COOH
y9 y8 y7 y6
b2 b4
Int
100
75
50
25
0
rQ
b2
b4
y6
y7
y8
y9
0 100 200 300 400 500 600 700 800 900 1,000 1,100 1,200 1,300
m/z

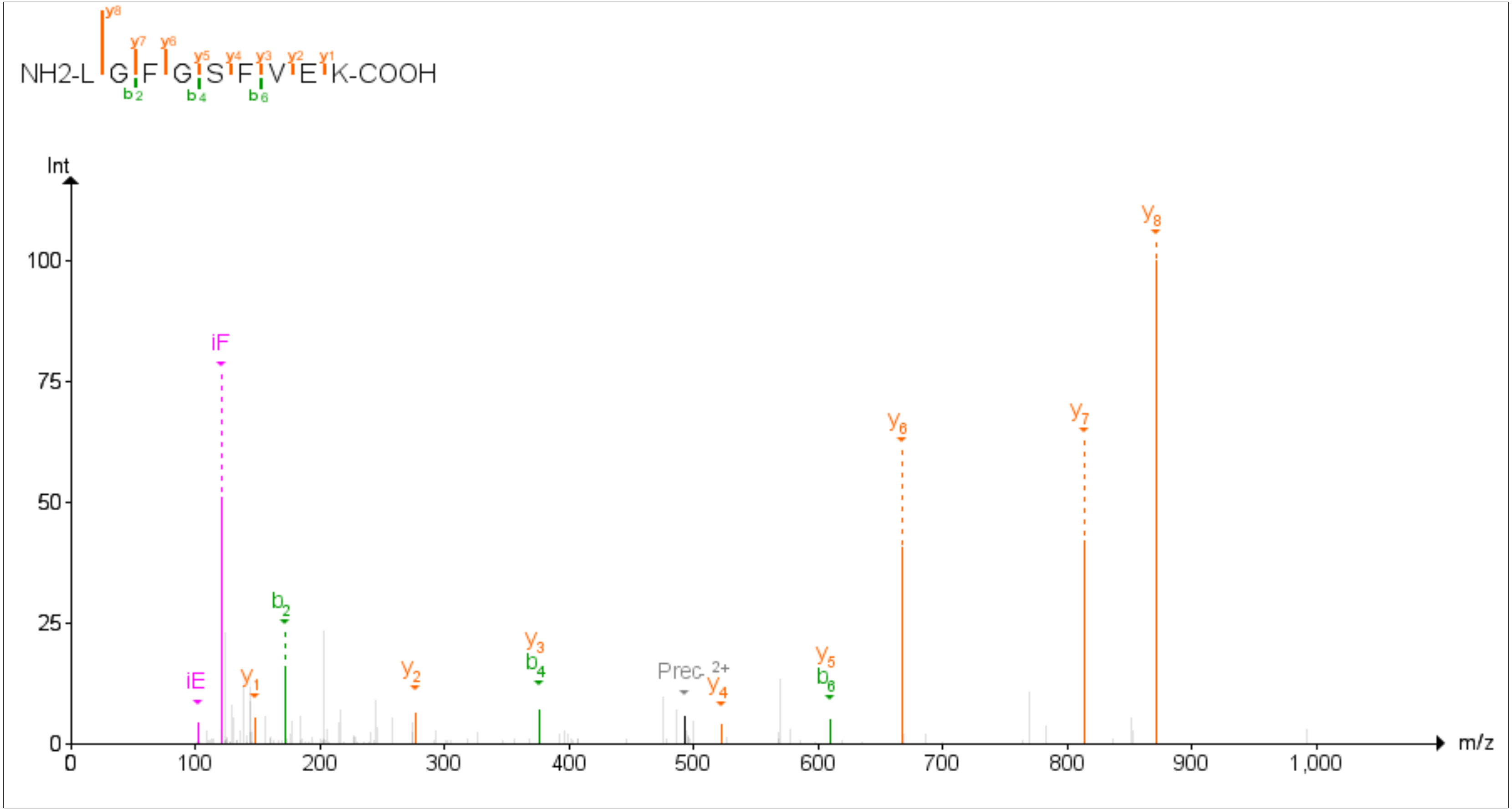

NH2-L G F G S F V E K-COOH
y8 y7 y6 y5 y4 y3 y2 y1
b2 b4 b6
Int
100
75
50
25
0
iF
iE
y1
b2
y2
y3
b4
Prec 2+
y4
y5
b6
y6
y7
y8
0 100 200 300 400 500 600 700 800 900 1,000
m/z

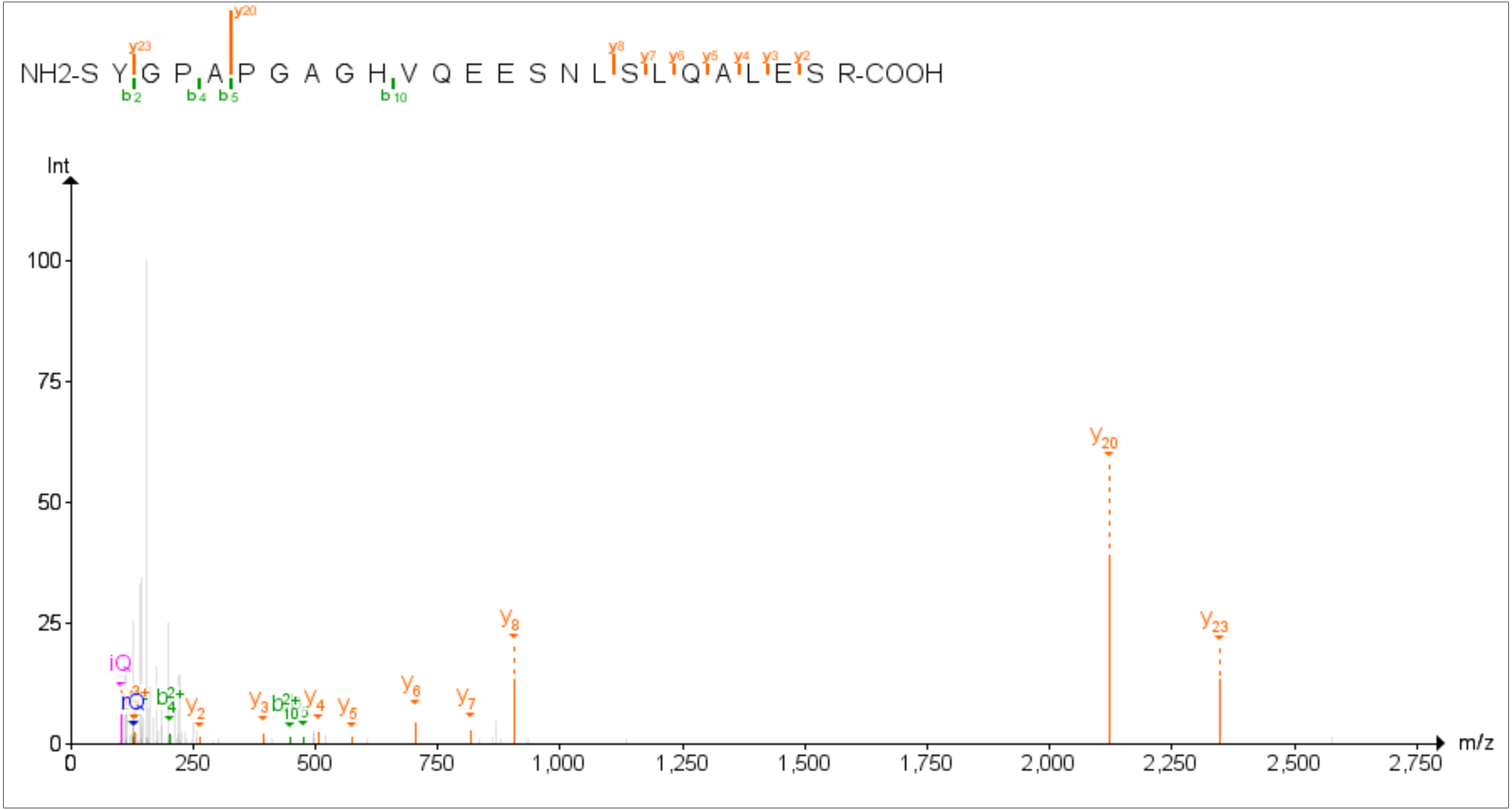

NH2-S Y G P A P G A G H V Q E E S N L S L Q A L E S R-COOH
y23
y20
y8
y7
y6
y5
y4
y3
y2
b2
b4
b5
b10
Int
100
75
50
25
0
m/z
0
250
500
750
1,000
1,250
1,500
1,750
2,000
2,250
2,500
2,750
iQ
rQ
y2
y3
y4
y5
y6
y7
y8
y20
y23

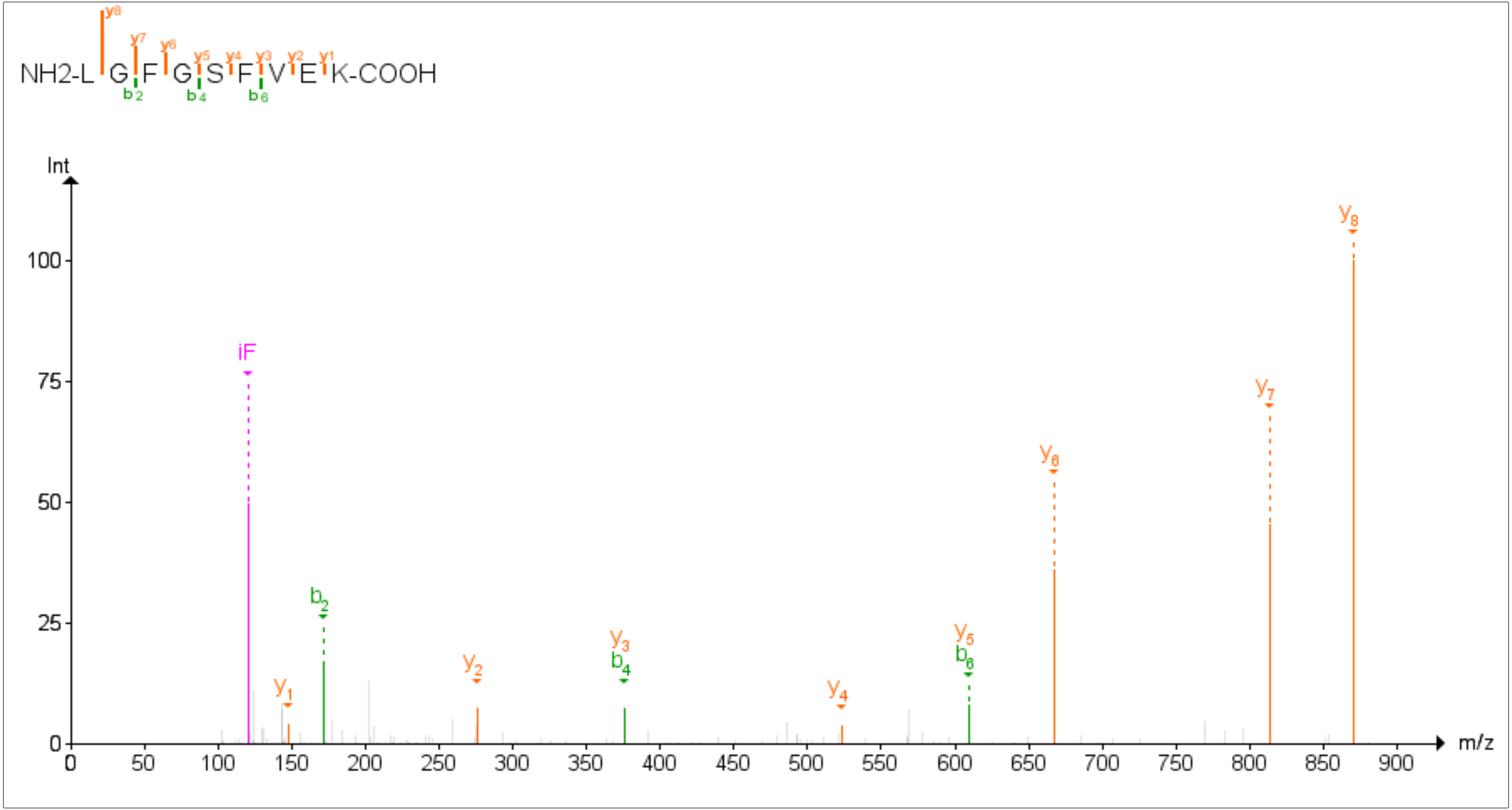
NH2-L G F G S F V E K-COOH
y8 y7 y6 y5 y4 y3 y2 y1
b2 b4 b6
Int
m/z
iF
y1 b2 y2 y3 b4 y4 y5 b6 y6 y7 y8
0 25 50 75 100
0 50 100 150 200 250 300 350 400 450 500 550 600 650 700 750 800 850 900

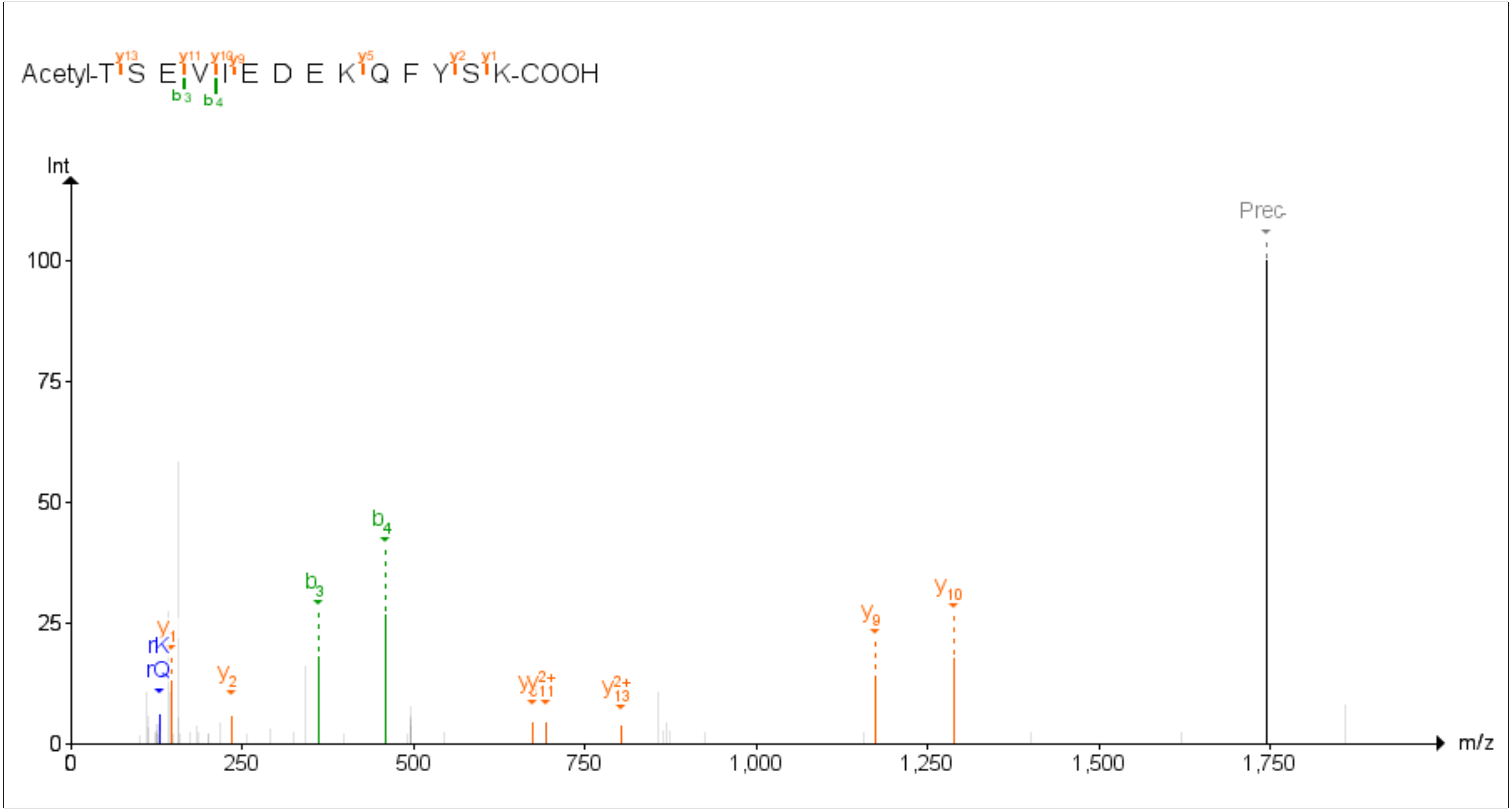

Acetyl-T S E V I E D E K Q F Y S K-COOH
y13
y11
y10
y9
y5
y2
y1
b3
b4
Int
100
75
50
25
0
Prec
b4
b3
y10
y9
y1
rK
rQ
y2
y11 2+
y13 2+
0
250
500
750
1,000
1,250
1,500
1,750
m/z

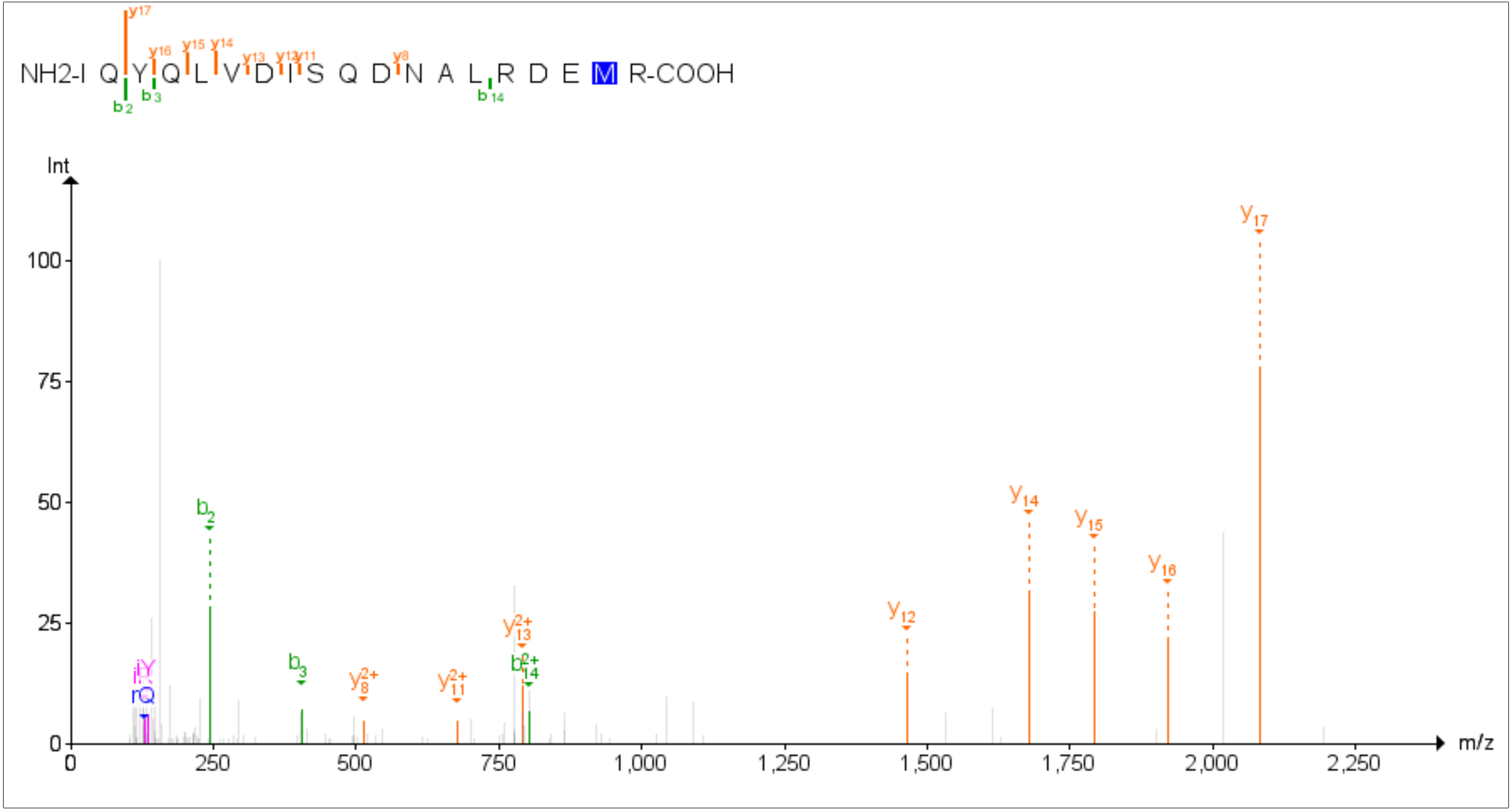
NH2-I Q Y Q L V D I S Q D N A L R D E M R-COOH
Int
m/z
0
25
50
75
100
0
250
500
750
1,000
1,250
1,500
1,750
2,000
2,250

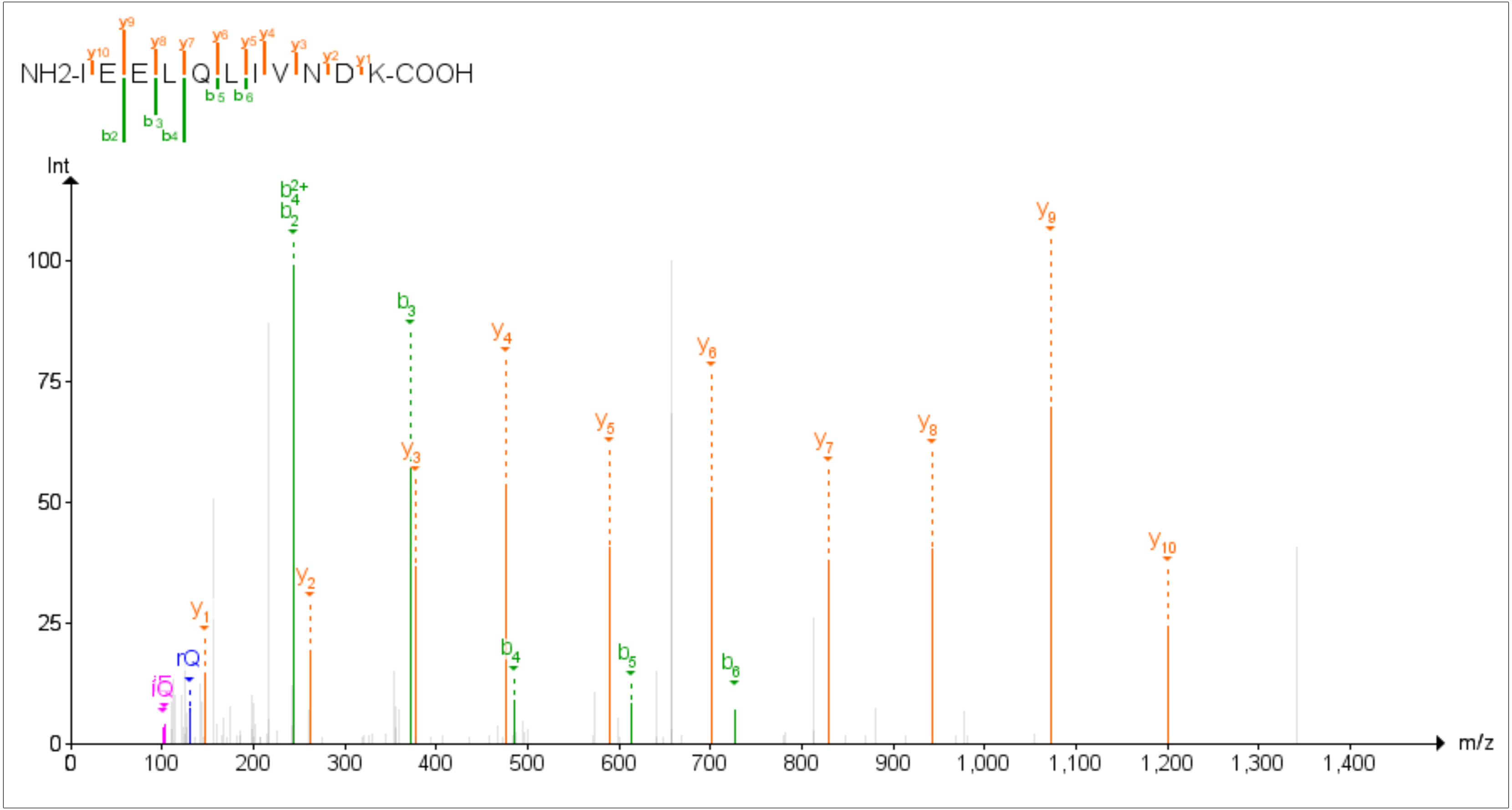

NH2-I E E L Q L I V N D K-COOH
y10 y9 y8 y7 y6 y5 y4 y3 y2 y1
b2 b3 b4 b5 b6
Int
m/z
0
25
50
75
100
0
100
200
300
400
500
600
700
800
900
1,000
1,100
1,200
1,300
1,400
iQ
rQ
y1
y2
b4 2+
b2
b3
y3
y4
b4
y5
b5
y6
b6
y7
y8
y9
y10

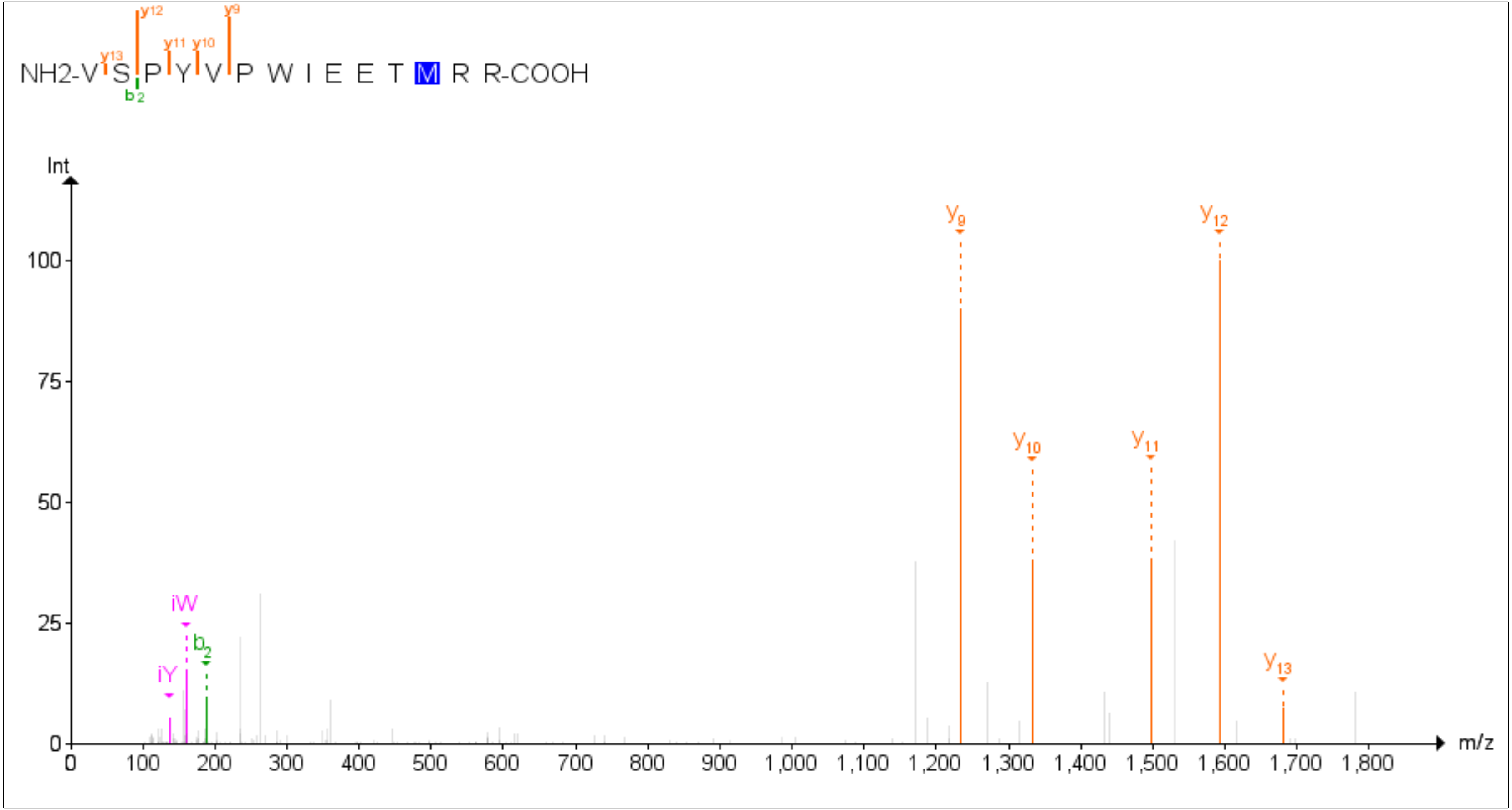

NH2-V S P Y V P W I E E T M R R-COOH
y13
y12
y11
y10
y9
b2
Int
100
75
50
25
0
y9
y10
y11
y12
y13
iW
iY
b2
m/z
0
100
200
300
400
500
600
700
800
900
1,000
1,100
1,200
1,300
1,400
1,500
1,600
1,700
1,800

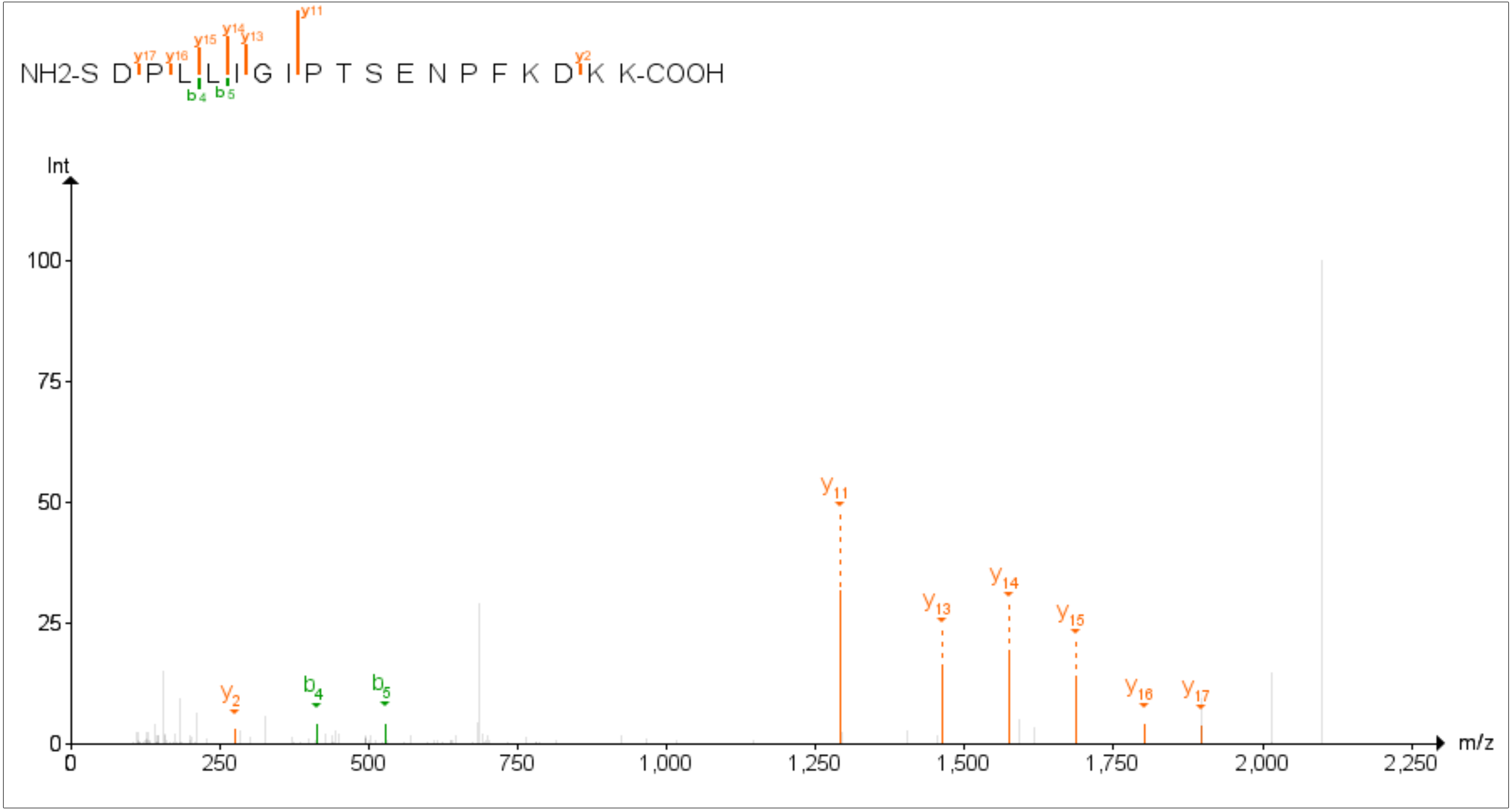

NH2-S D P L L I G I P T S E N P F K D K K-COOH
y17 y16 y15 y14 y13 y11 y2
b4 b5
Int
100
75
50
25
0
y11
y13
y14
y15
y16
y17
y2
b4
b5
0
250
500
750
1,000
1,250
1,500
1,750
2,000
2,250
m/z

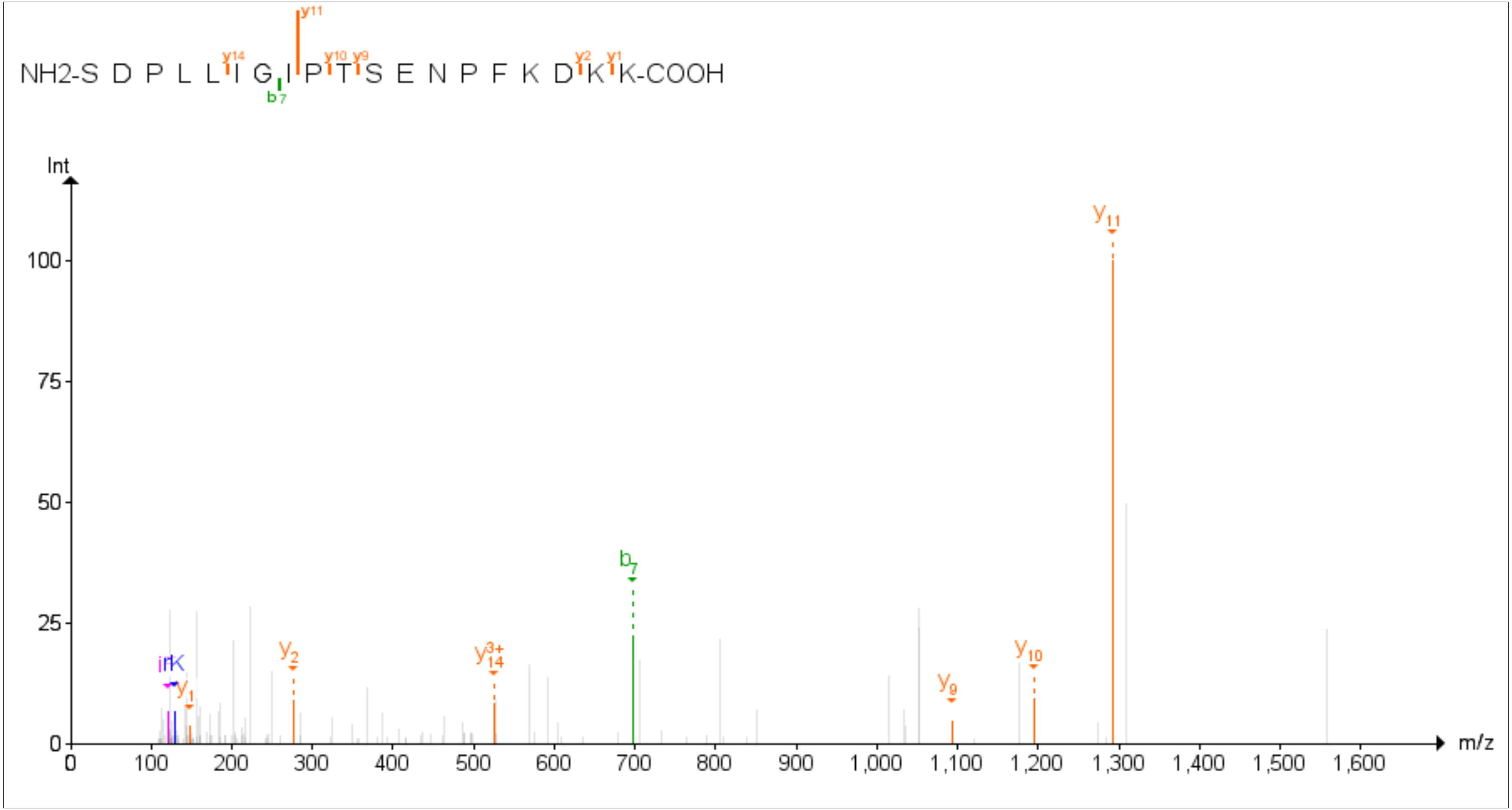

NH2-S D P L L I G I P T S E N P F K D K K-COOH
y14
b7
y11
y10
y9
y2
y1
Int
100
75
50
25
0
y11
b7
y2
y3+ 14
y9
y10
y1
m/z
0
100
200
300
400
500
600
700
800
900
1,000
1,100
1,200
1,300
1,400
1,500
1,600

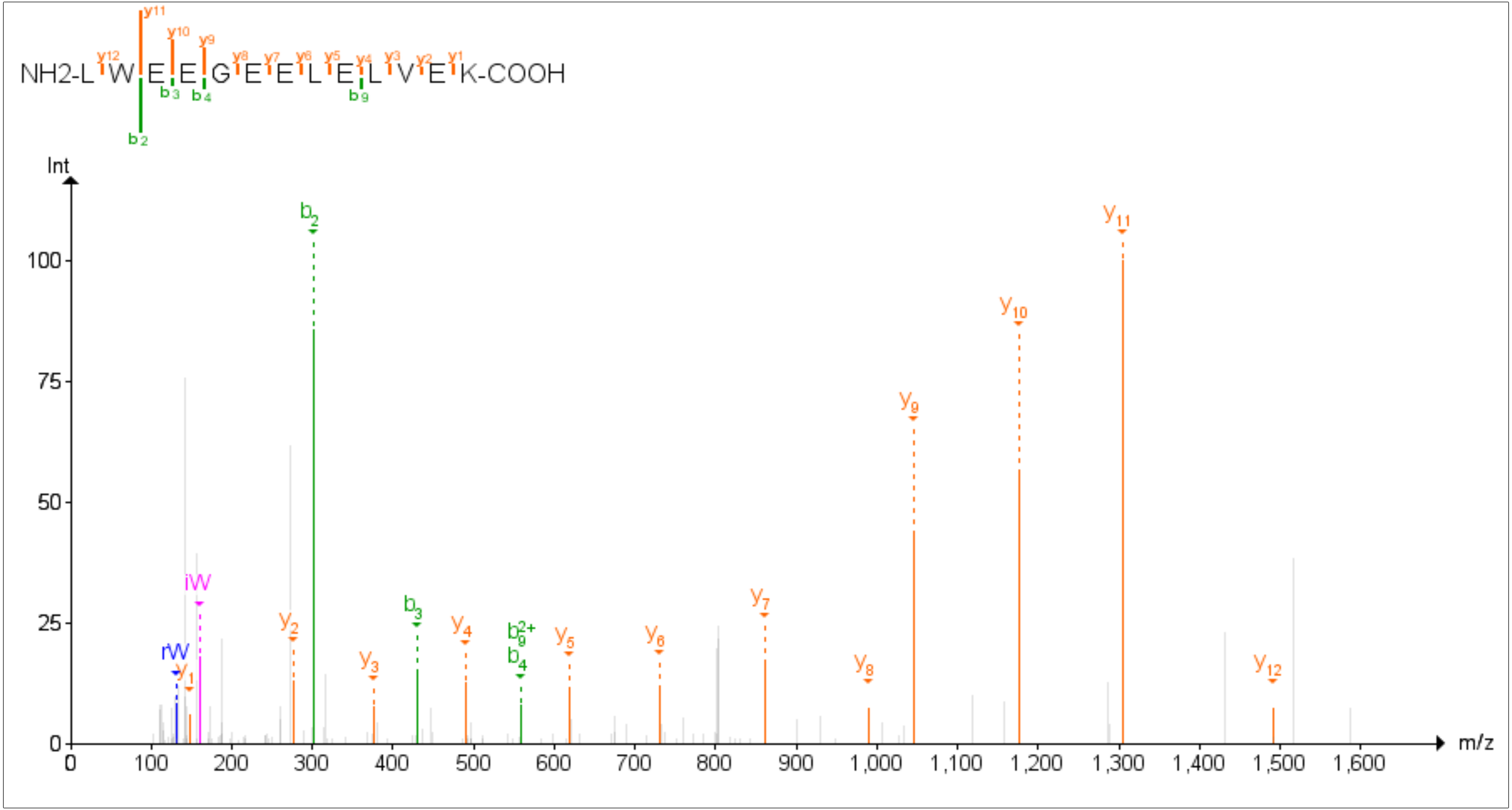

NH2-LWEEGEELELVEK-COOH
y12 y11 y10 y9 y8 y7 y6 y5 y4 y3 y2 y1
b2 b3 b4 b9
Int
100
75
50
25
0
m/z
0 100 200 300 400 500 600 700 800 900 1,000 1,100 1,200 1,300 1,400 1,500 1,600
b2
y11
y10
y9
iW
rW
y1
y2
y3
b3
y4
b9 2+
b4
y5
y6
y7
y8
y12

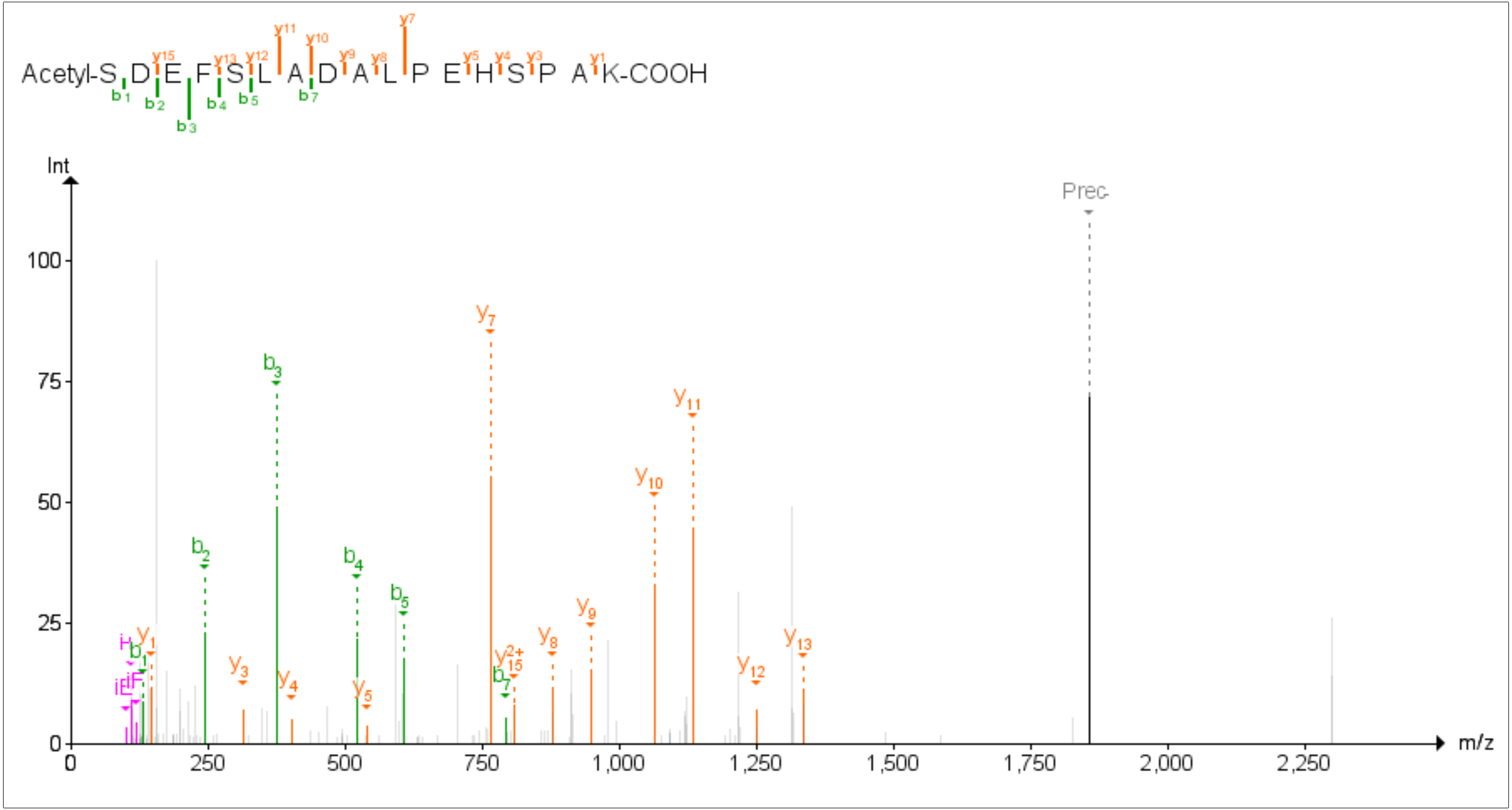

Acetyl-S D E F S L A D A L P E H S P A K-COOH
b1 b2 b3 b4 b5 b7
y15 y13 y12 y11 y10 y9 y8 y7 y5 y4 y3 y1
Int
Prec
100
75
50
25
0
0
250
500
750
1,000
1,250
1,500
1,750
2,000
2,250
m/z
y7
b3
y11
y10
b2
b4
b5
y9
y8
y13
y12
y3
y4
y5
y1
b1
y2+15
b7
iF
iE

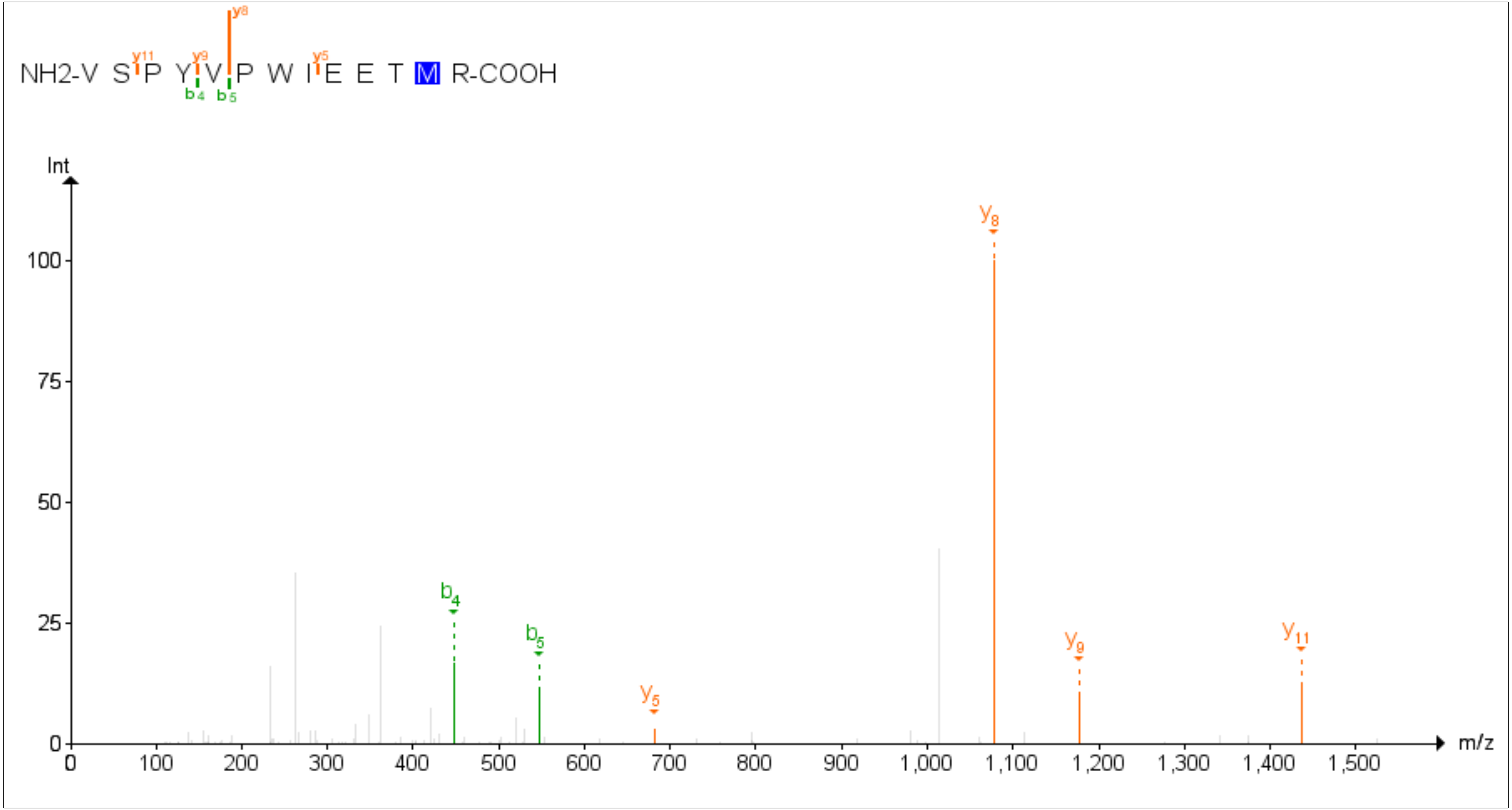

NH2-V S P Y V P W I E E T M R-COOH
y11 y9 y8 y5
b4 b5
Int
100
75
50
25
0
0 100 200 300 400 500 600 700 800 900 1,000 1,100 1,200 1,300 1,400 1,500
m/z
y8
b4
b5
y5
y9
y11

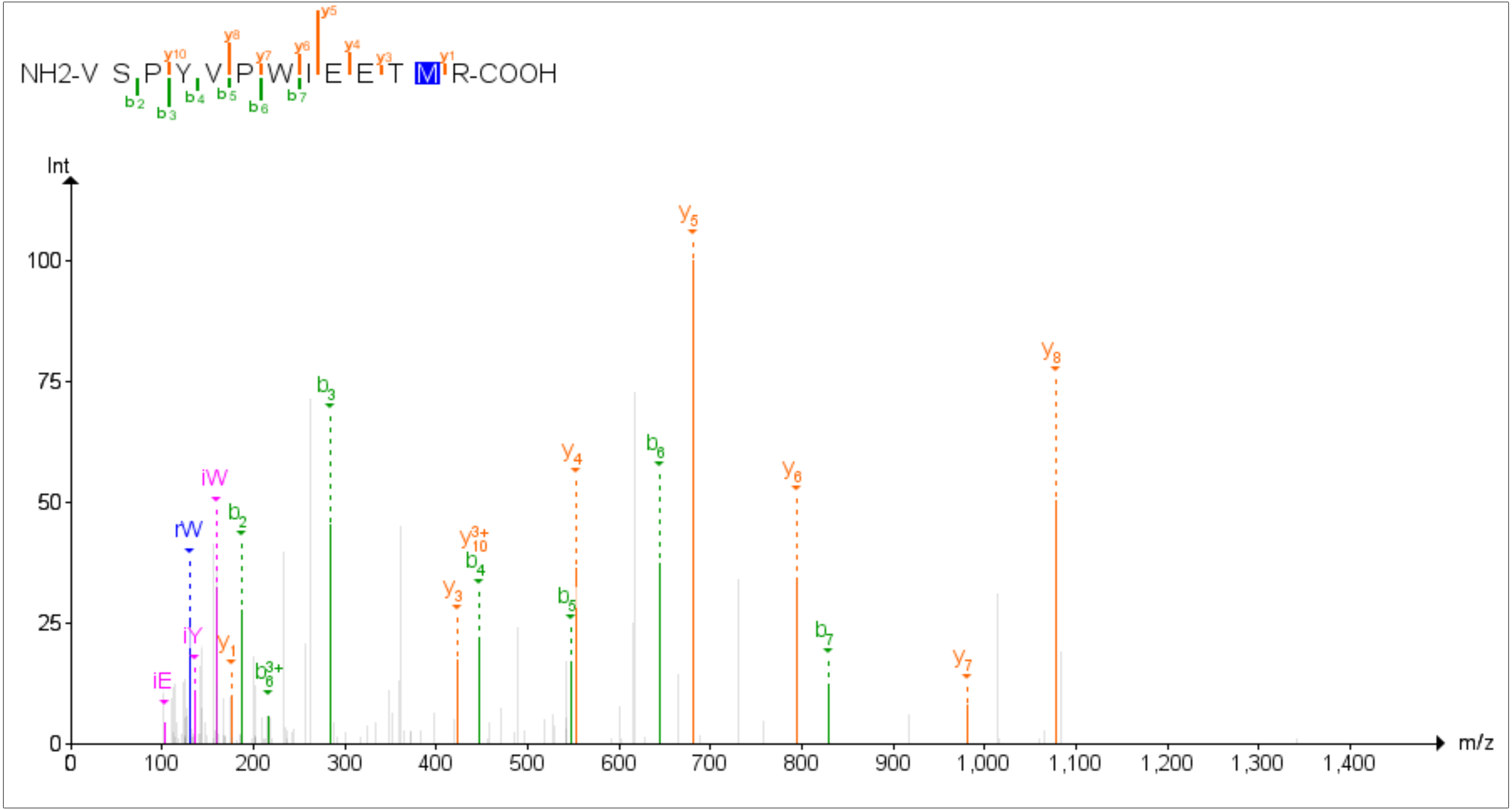
NH2-V S P Y V P W I E E T M R-COOH
b2 b3 b4 b5 b6 b7
y10 y8 y7 y6 y5 y4 y3 y1
Int
m/z
0
25
50
75
100
0
100
200
300
400
500
600
700
800
900
1,000
1,100
1,200
1,300
1,400
iE
iY
rW
iW
y1
b2
b6 3+
b3
y3
y10 3+
b4
b5
y4
b6
y5
y6
b7
y7
y8

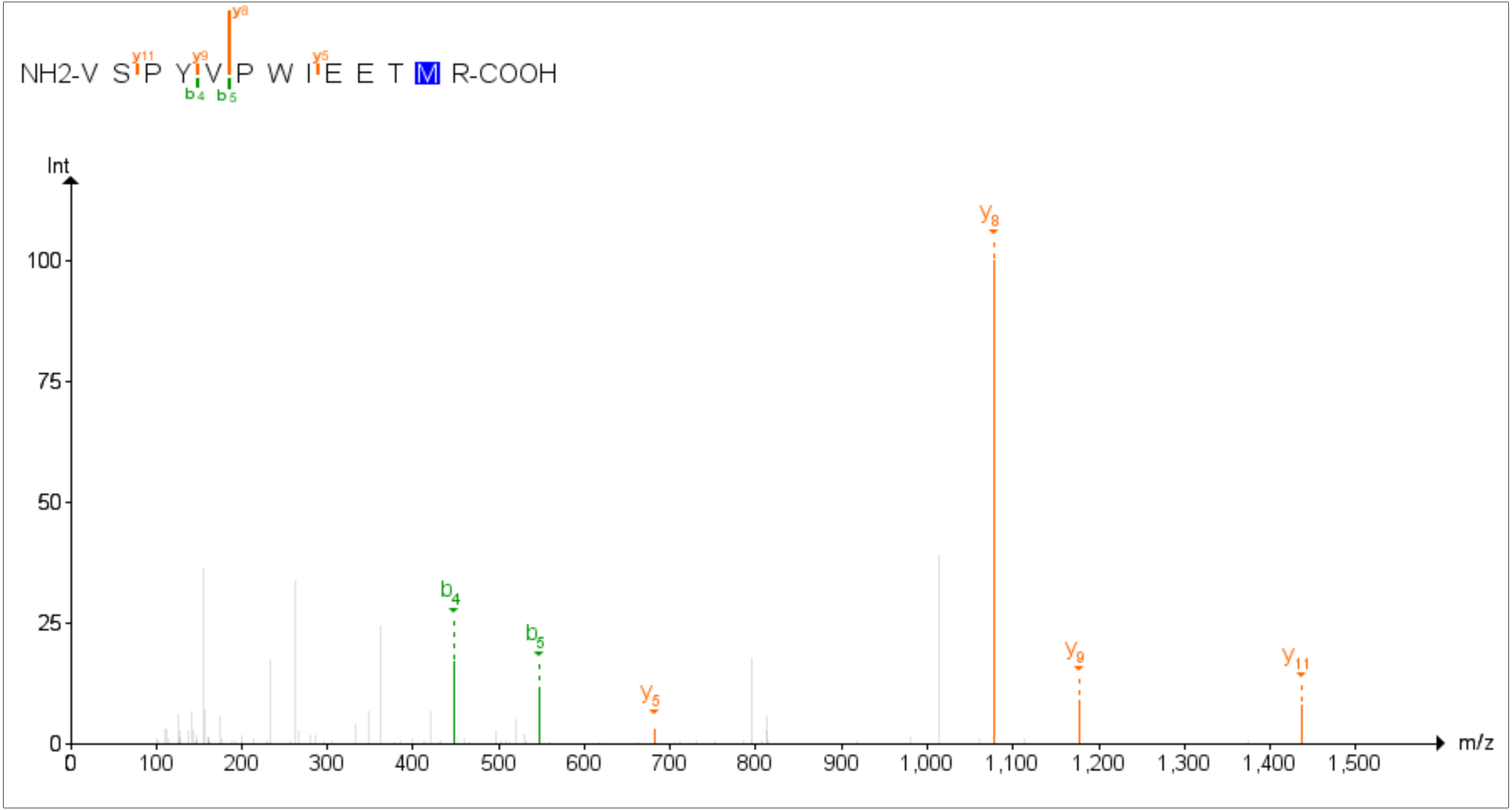

NH2-V S P Y V P W I E E T M R-COOH
y11
y9
y8
y5
b4
b5
Int
100
75
50
25
0
m/z
0
100
200
300
400
500
600
700
800
900
1,000
1,100
1,200
1,300
1,400
1,500
b4
b5
y5
y8
y9
y11

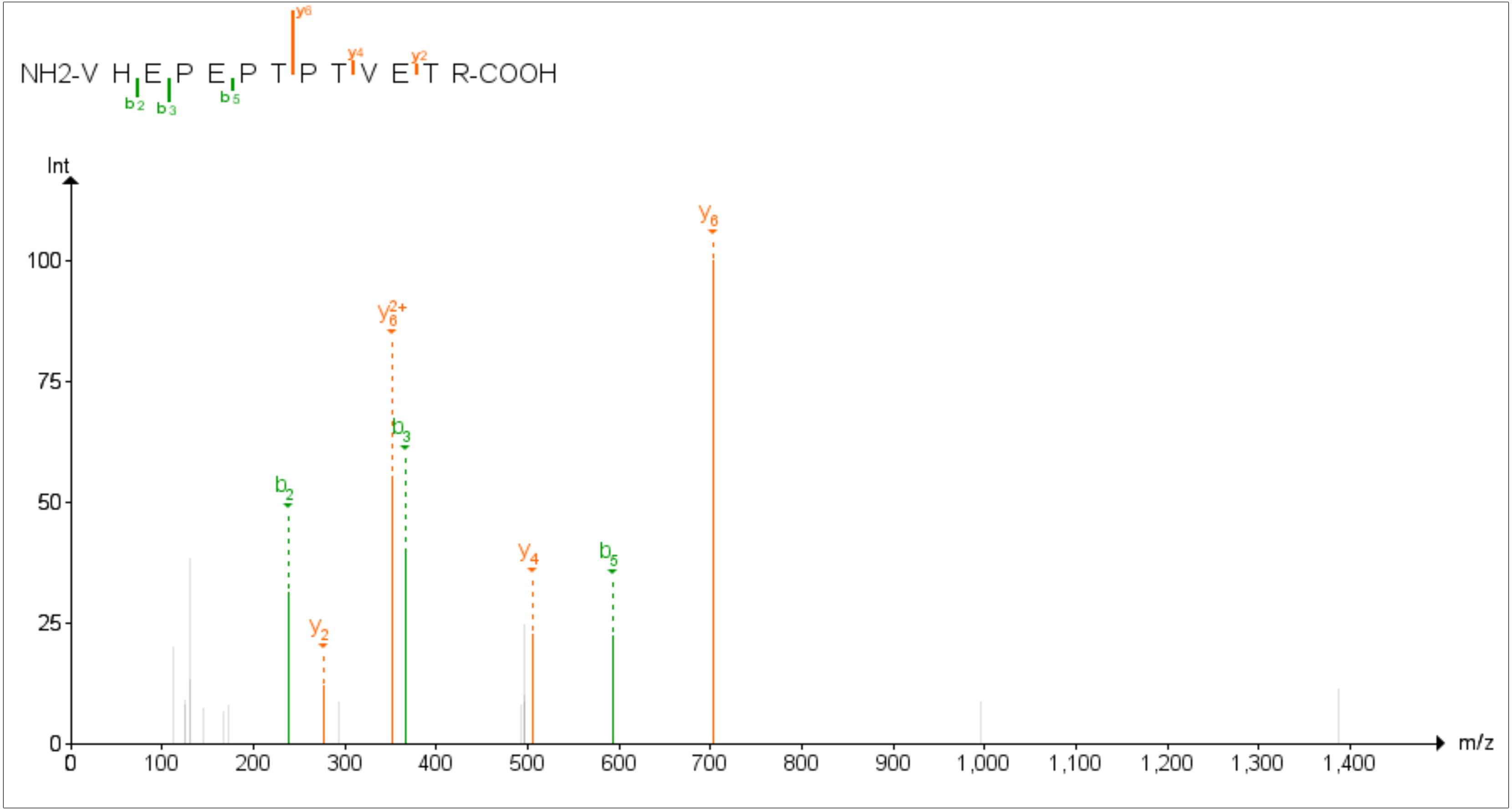

NH2-V H E P E P T P T V E T R-COOH
y6
y4
y2
b2
b3
b5
Int
100
75
50
25
0
y6
y6 2+
b3
b2
y2
y4
b5
m/z
0
100
200
300
400
500
600
700
800
900
1,000
1,100
1,200
1,300
1,400

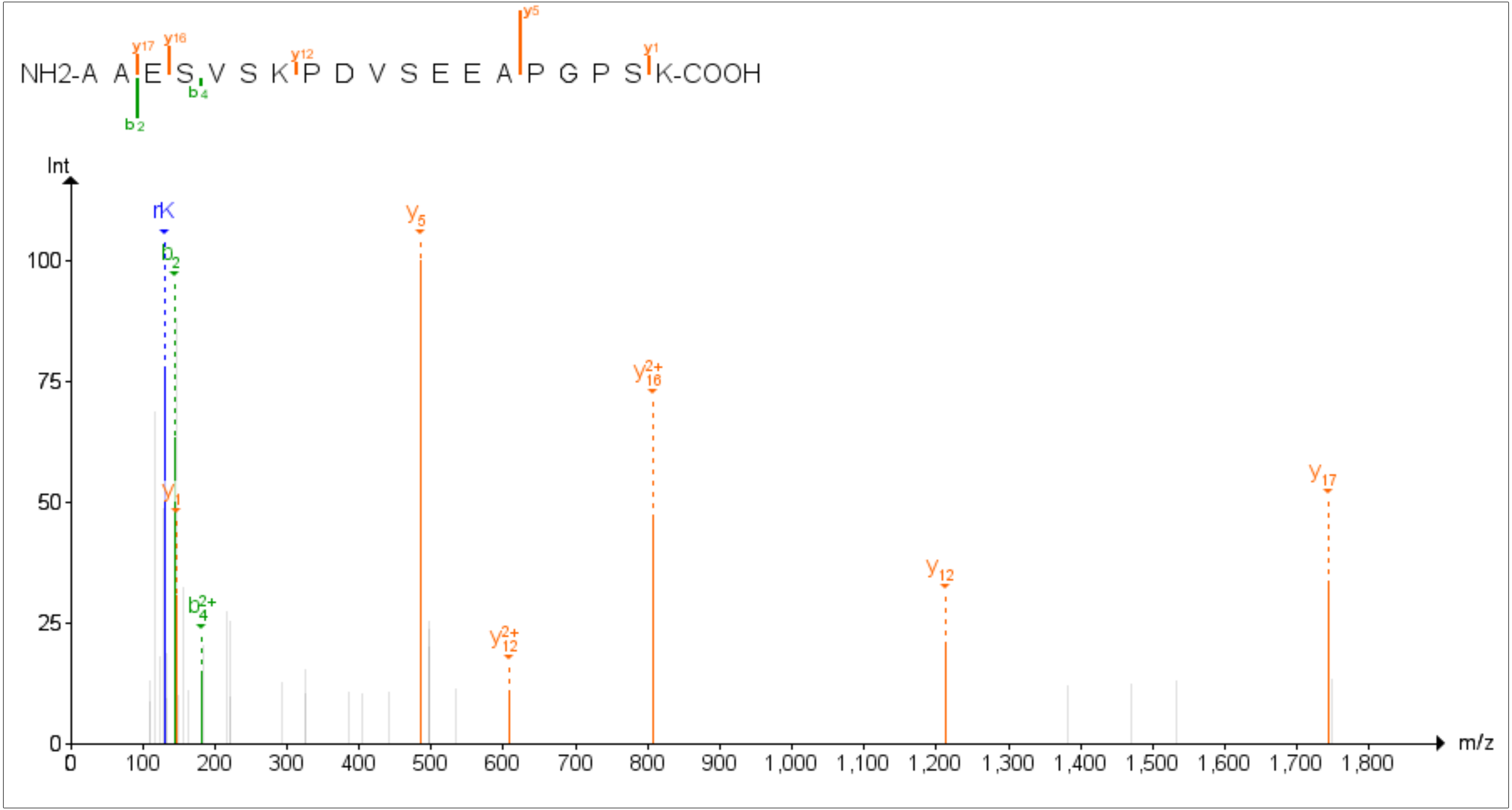

NH2-A A E S V S K P D V S E E A P G P S K-COOH
y17
y16
y12
y5
y1
b2
b4
Int
rK
b2
y5
y1
b_4^{2+}
y_{12}^{2+}
y_{16}^{2+}
y_{12}
y_{17}
100
75
50
25
0
0
100
200
300
400
500
600
700
800
900
1,000
1,100
1,200
1,300
1,400
1,500
1,600
1,700
1,800
m/z

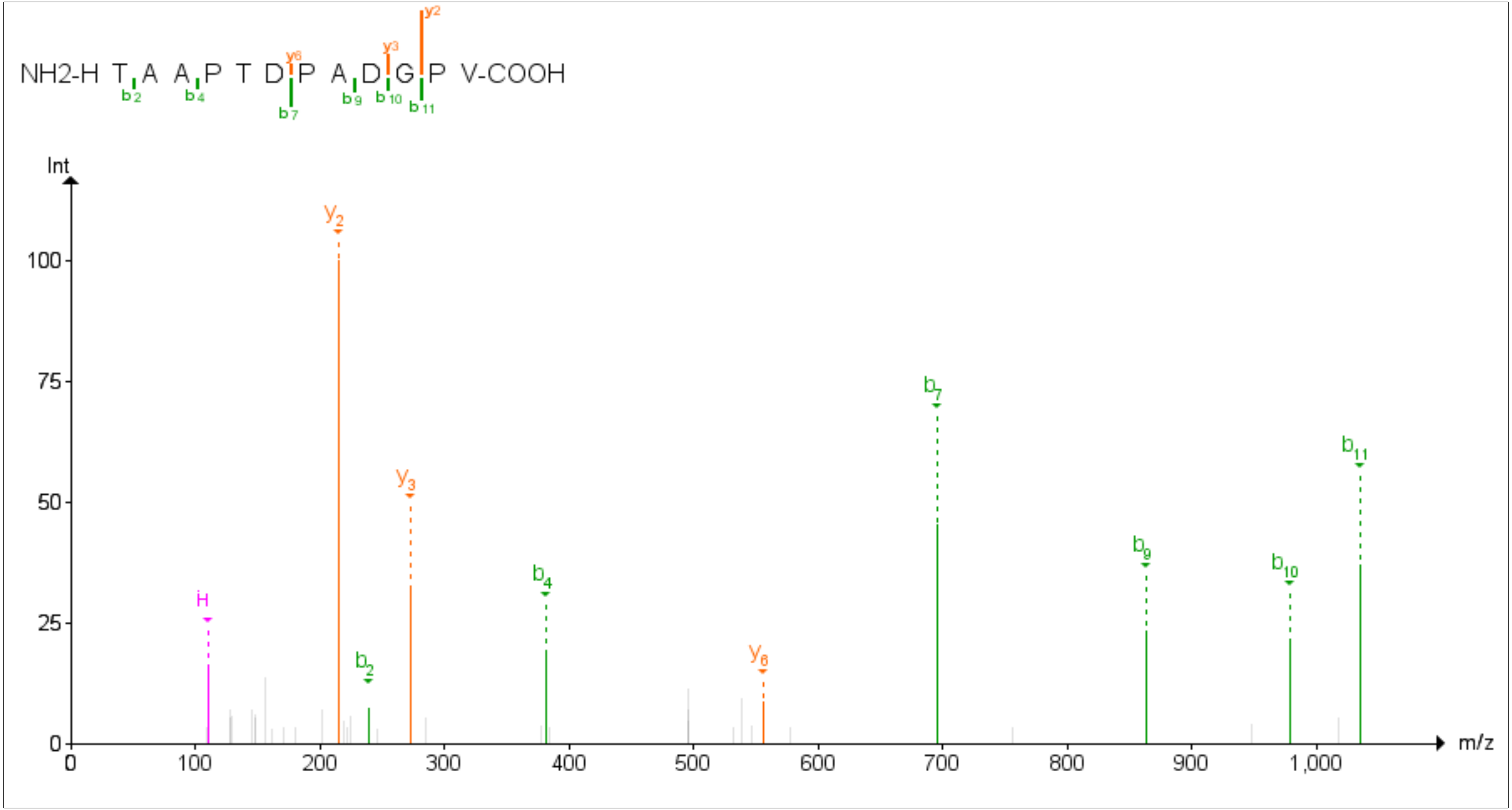

NH2-H T A A P T D P A D G P V-COOH
y6 y3 y2
b2 b4 b7 b9 b10 b11
Int
100
75
50
25
0
y2
y3
b2
b4
y6
b7
b9
b10
b11
H
0 100 200 300 400 500 600 700 800 900 1,000
m/z

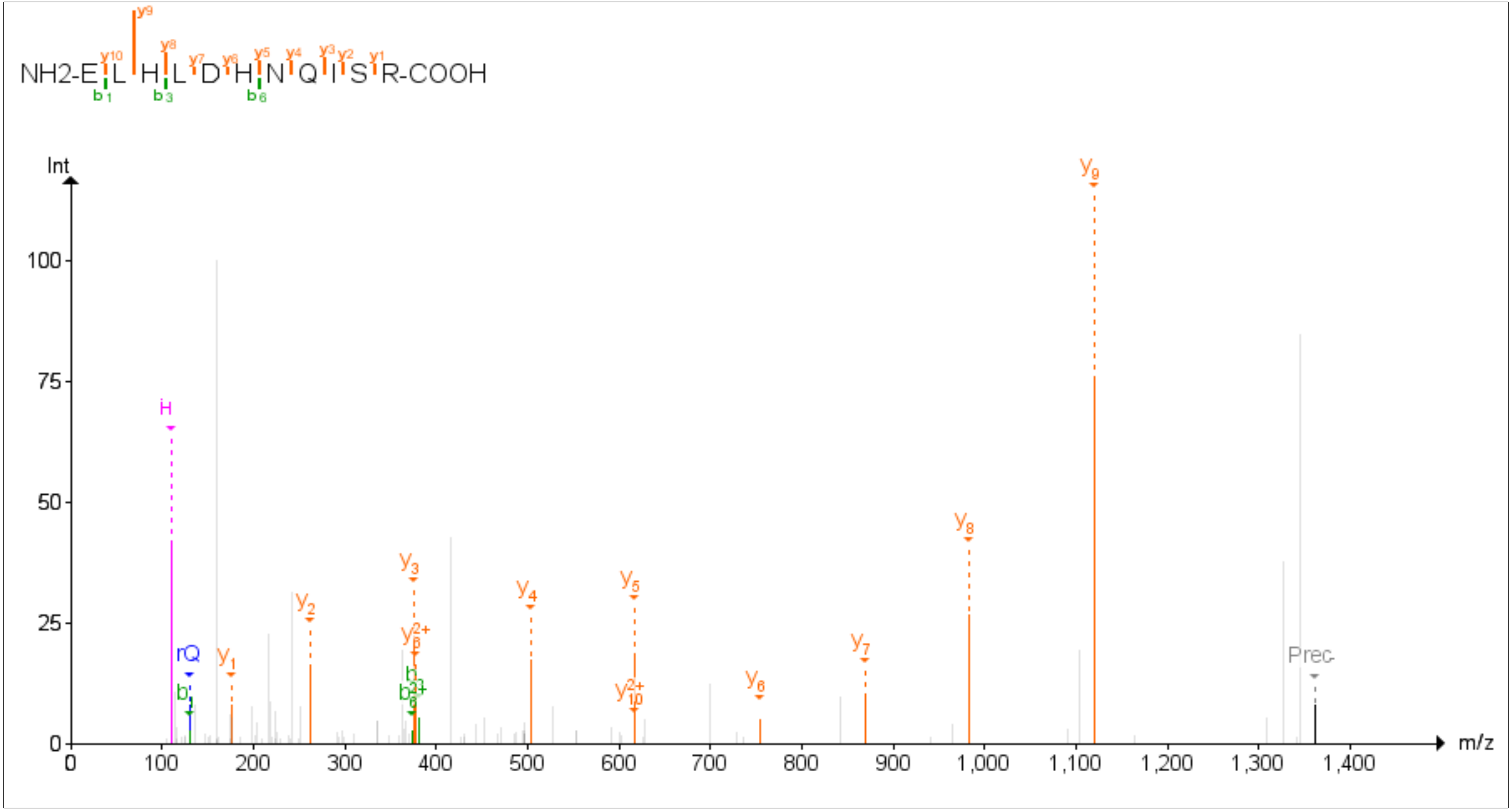
NH2-E L H L D H N Q I S R-COOH
y10 y9 y8 y7 y6 y5 y4 y3 y2 y1
b1 b3 b6
Int
100
75
50
25
0
m/z
0 100 200 300 400 500 600 700 800 900 1,000 1,100 1,200 1,300 1,400
y9
y8
y3
y4
y5
y2
H
rQ
y1
y7
y6
Prec

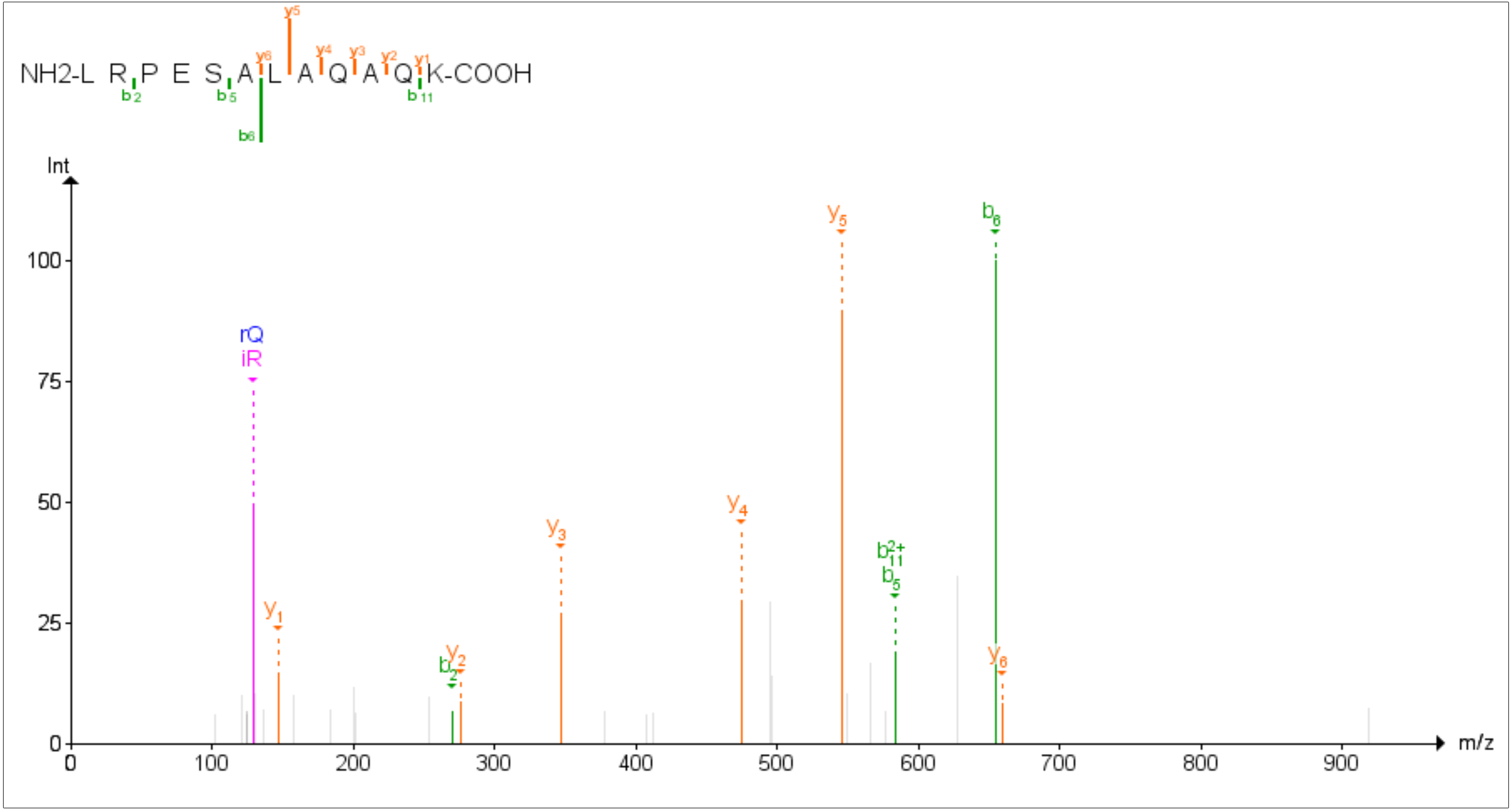

NH2-L R P E S A L A Q A Q K-COOH
y5
y6
y4
y3
y2
y1
b2
b5
b11
b6
Int
m/z
rQ
iR
y1
b2
y2
y3
y4
y5
b11 2+
b5
b6
y6
0
25
50
75
100
0
100
200
300
400
500
600
700
800
900

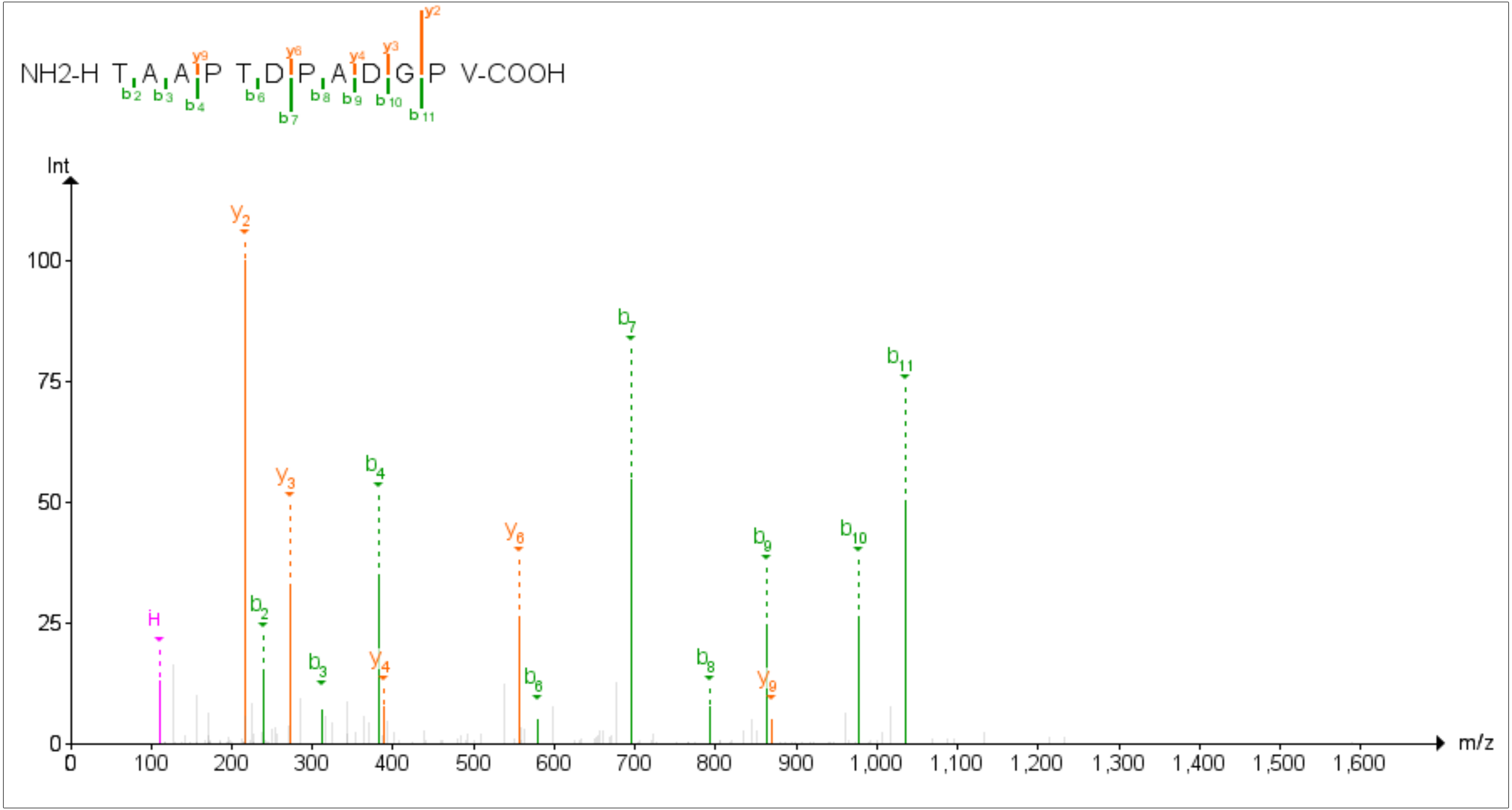

NH2-H T A A P T D P A D G P V-COOH
b2 b3 b4 b6 b7 b8 b9 b10 b11
y9 y6 y4 y3 y2
Int
100
75
50
25
0
0 100 200 300 400 500 600 700 800 900 1,000 1,100 1,200 1,300 1,400 1,500 1,600
m/z
H
y2
y3
b2
b3
b4
y4
y6
b6
b7
b8
b9
y9
b10
b11

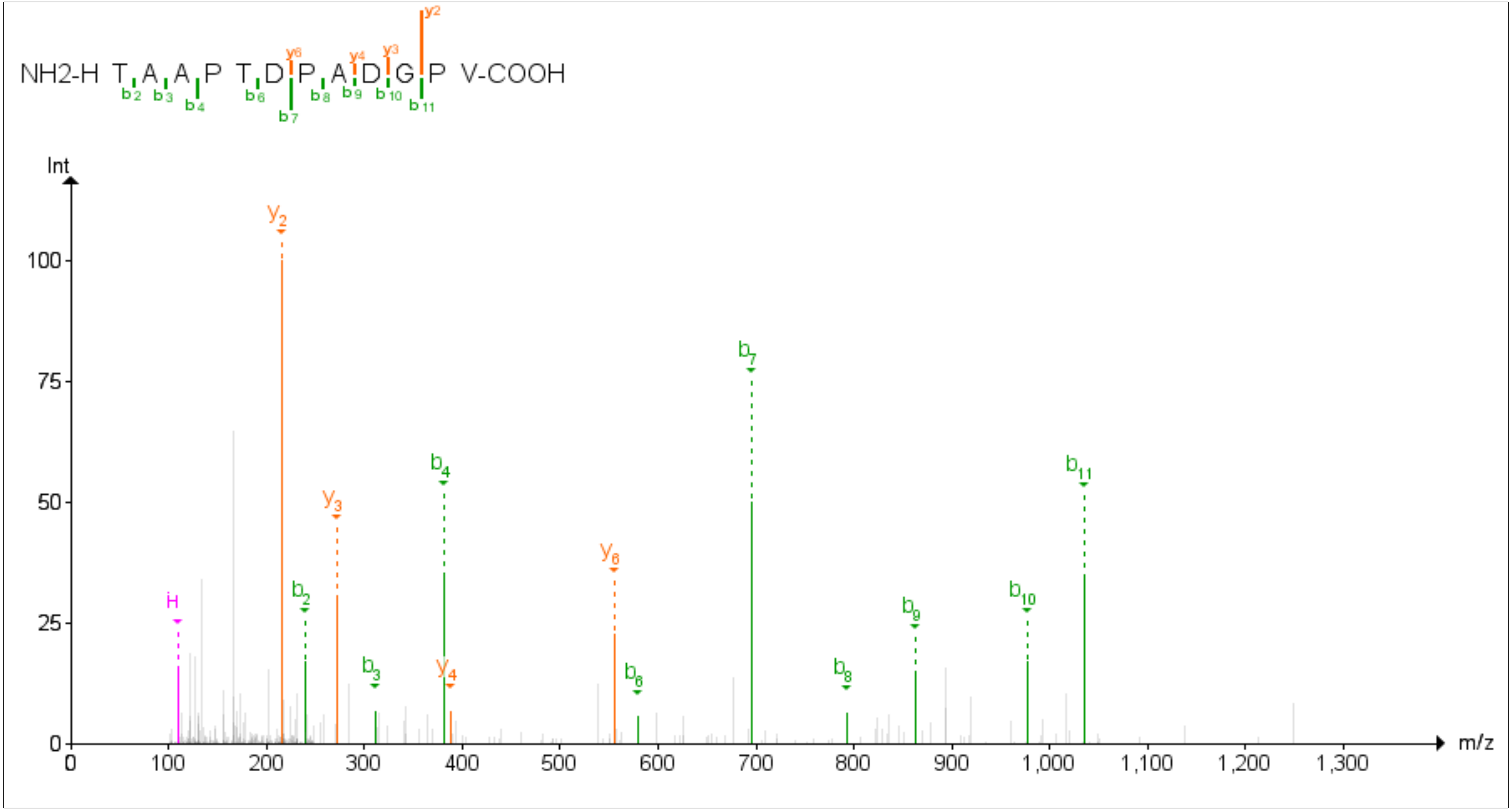
NH2-H T A A P T D P A D G P V-COOH
Int
m/z
0
25
50
75
100
100
200
300
400
500
600
700
800
900
1,000
1,100
1,200
1,300

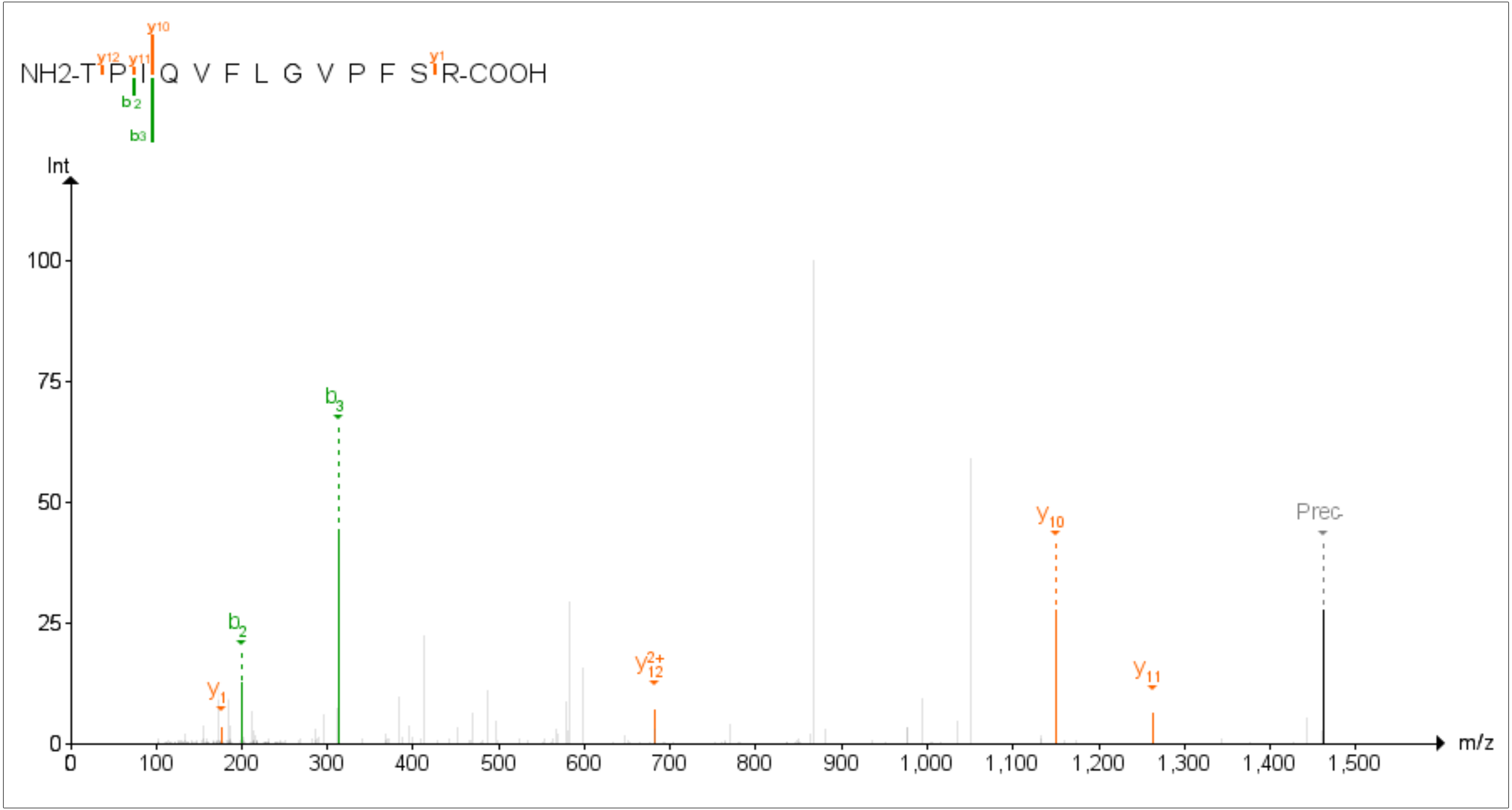

NH2-T P I Q V F L G V P F S R-COOH
Int
m/z
Prec.

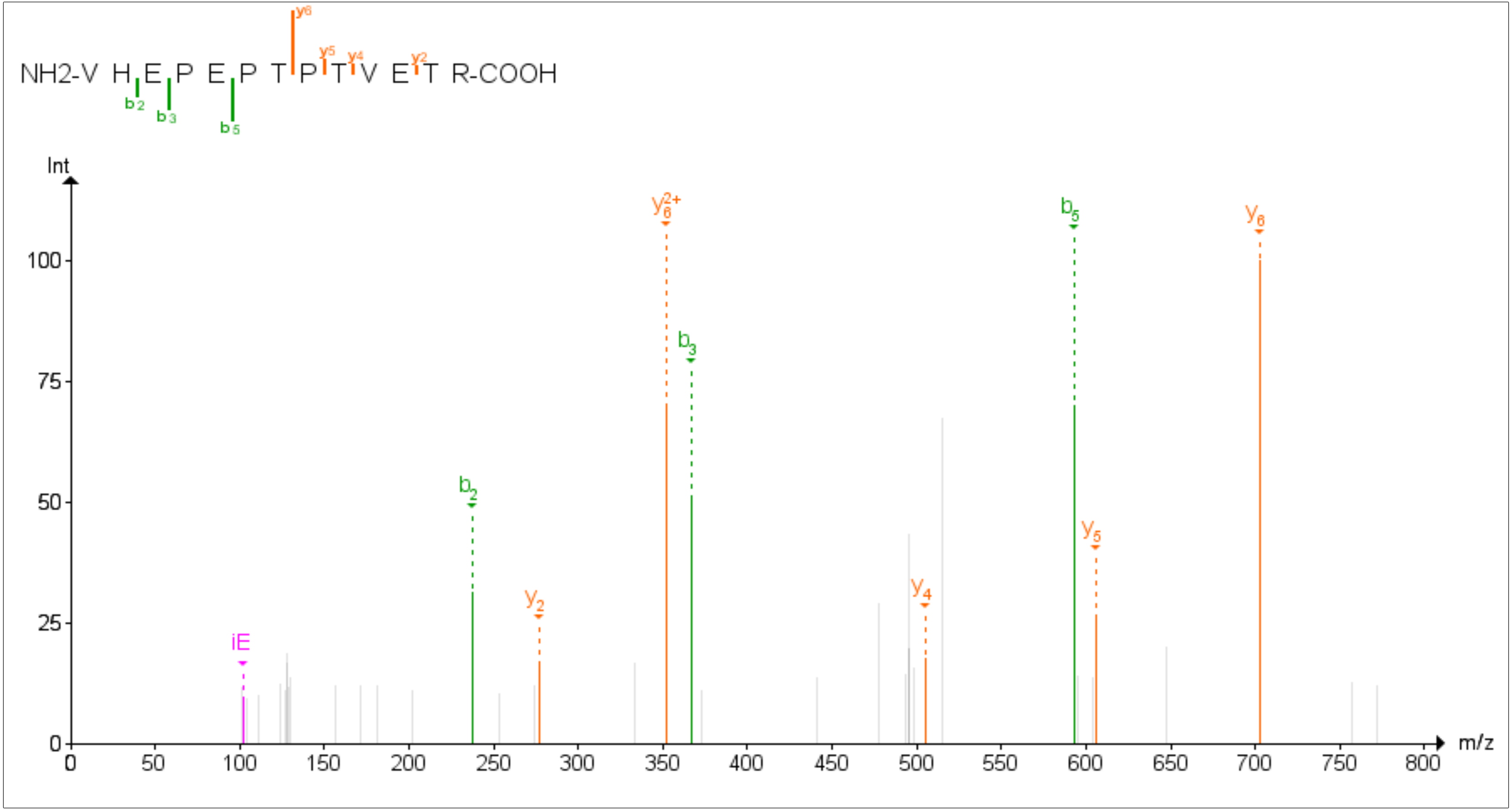
NH2-V H E P E P T P T V E T R-COOH
b2
b3
b5
y6
y5
y4
y2
Int
100
75
50
25
0
0
50
100
150
200
250
300
350
400
450
500
550
600
650
700
750
800
m/z
iE
b2
y2
y6 2+
b3
y4
b5
y5
y6

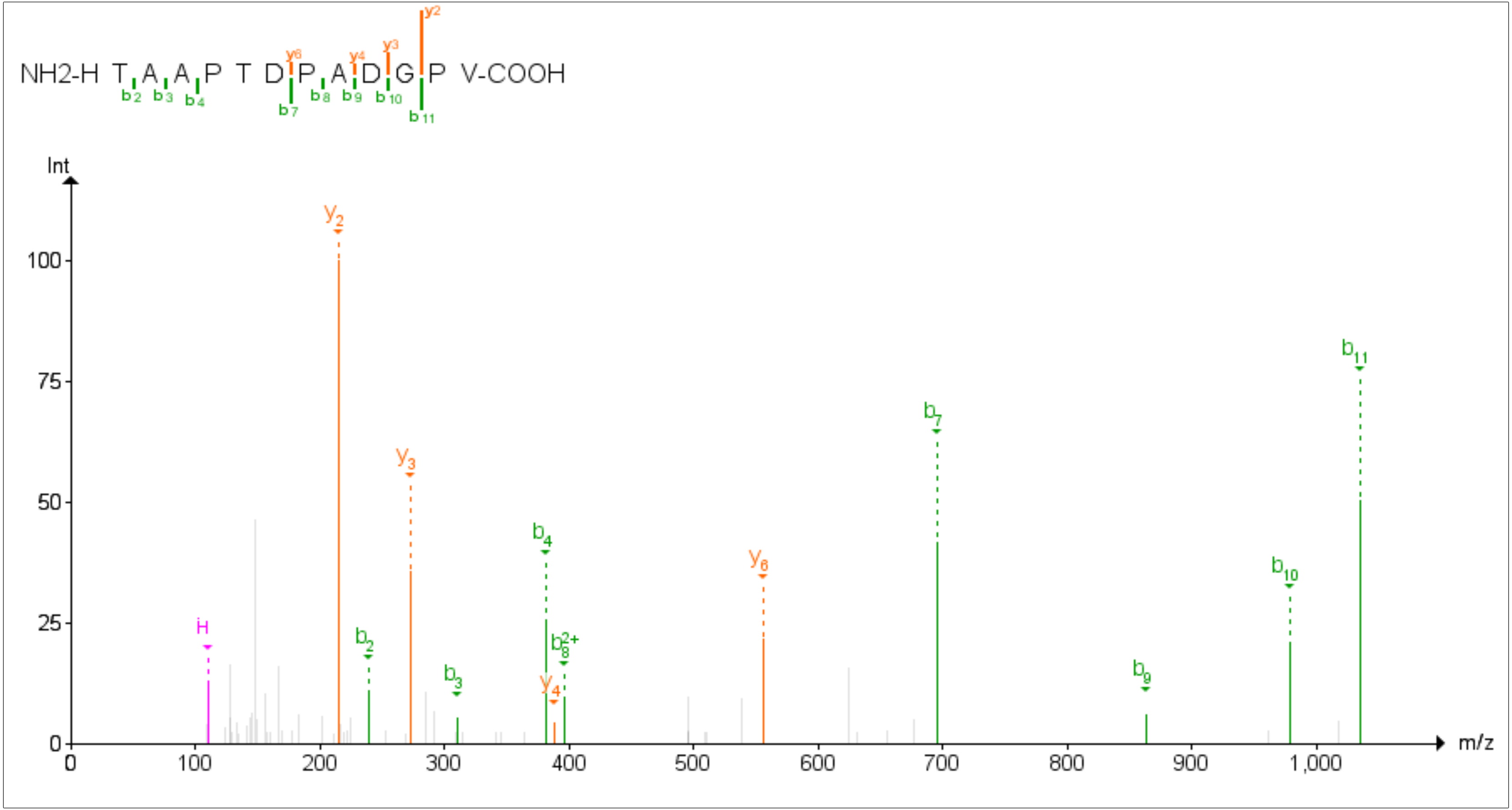

NH2-H T A A P T D P A D G P V-COOH
y6 y4 y3 y2
b2 b3 b4 b7 b8 b9 b10 b11
Int
100
75
50
25
0
m/z
0 100 200 300 400 500 600 700 800 900 1,000
y2
y3
b4
y6
b7
b11
b10
b9
H
b2
b3
y4
b8 2+

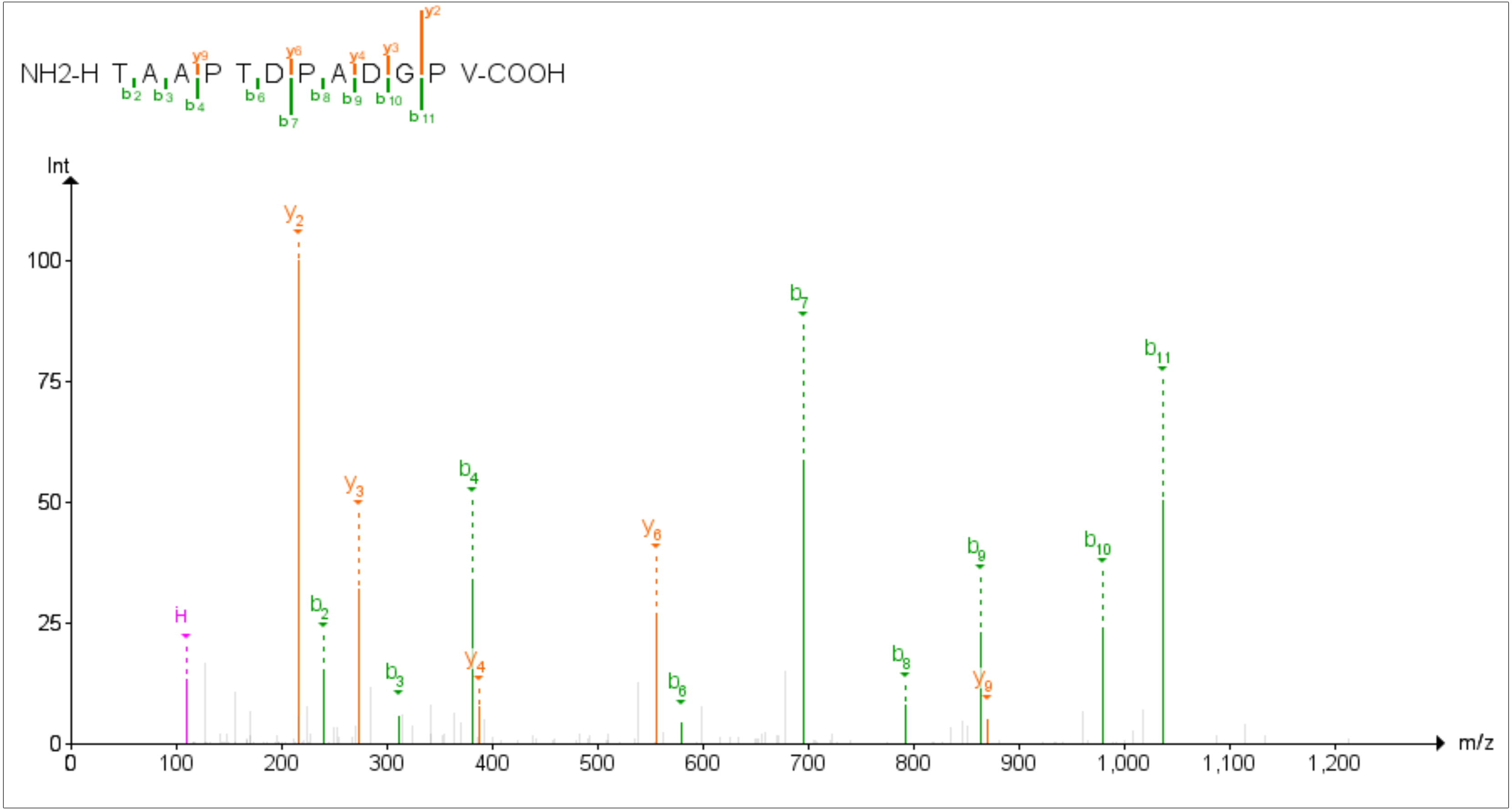

NH2-H T A A P T D P A D G P V-COOH
Int
m/z

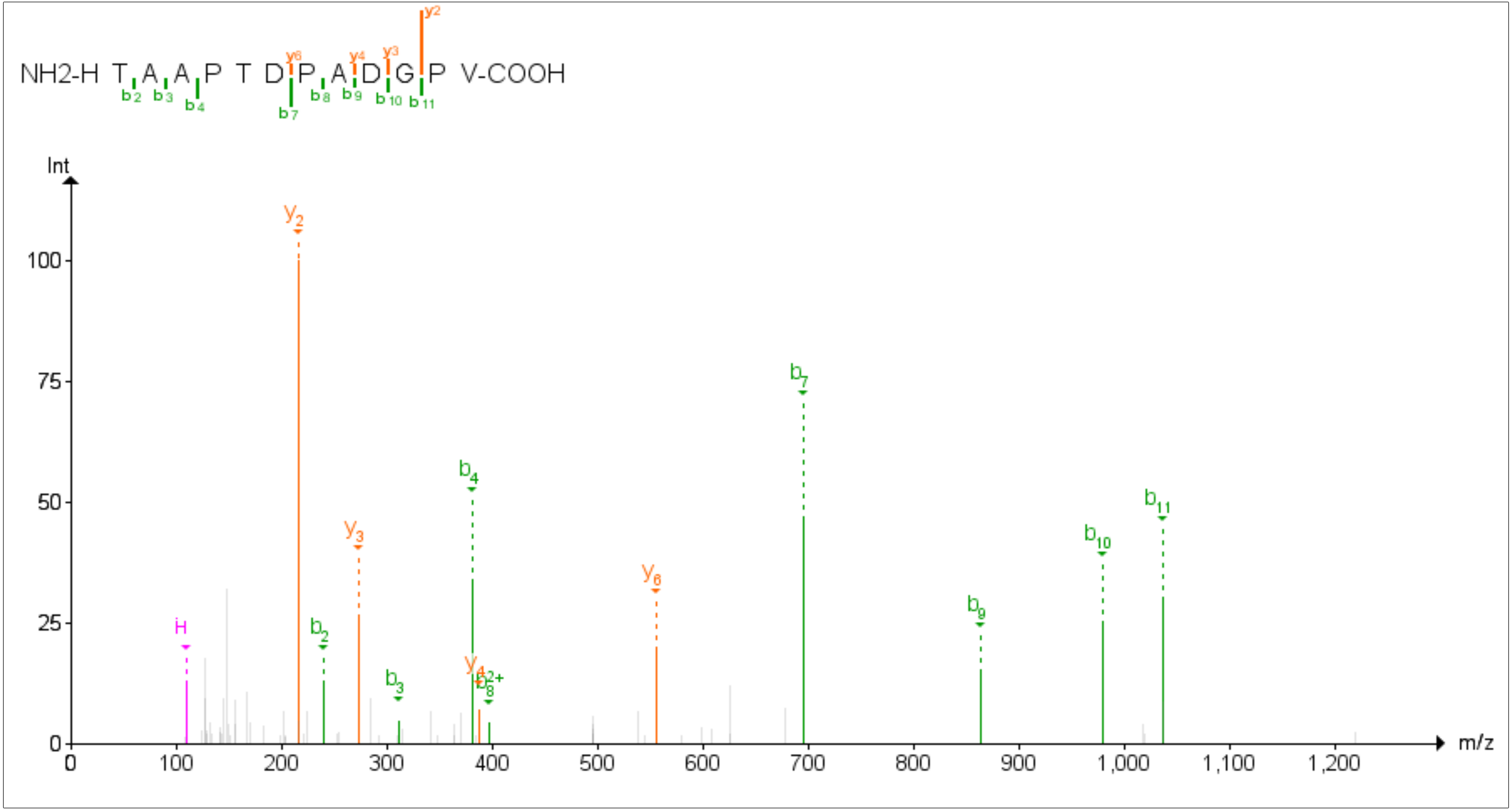
NH2-H T A A P T D P A D G P V-COOH
y6
y4
y3
y2
b2
b3
b4
b7
b8
b9
b10
b11
Int
100
75
50
25
0
H
y2
y3
y4
y6
b2
b3
b4
b7
b8 2+
b9
b10
b11
0
100
200
300
400
500
600
700
800
900
1,000
1,100
1,200
m/z

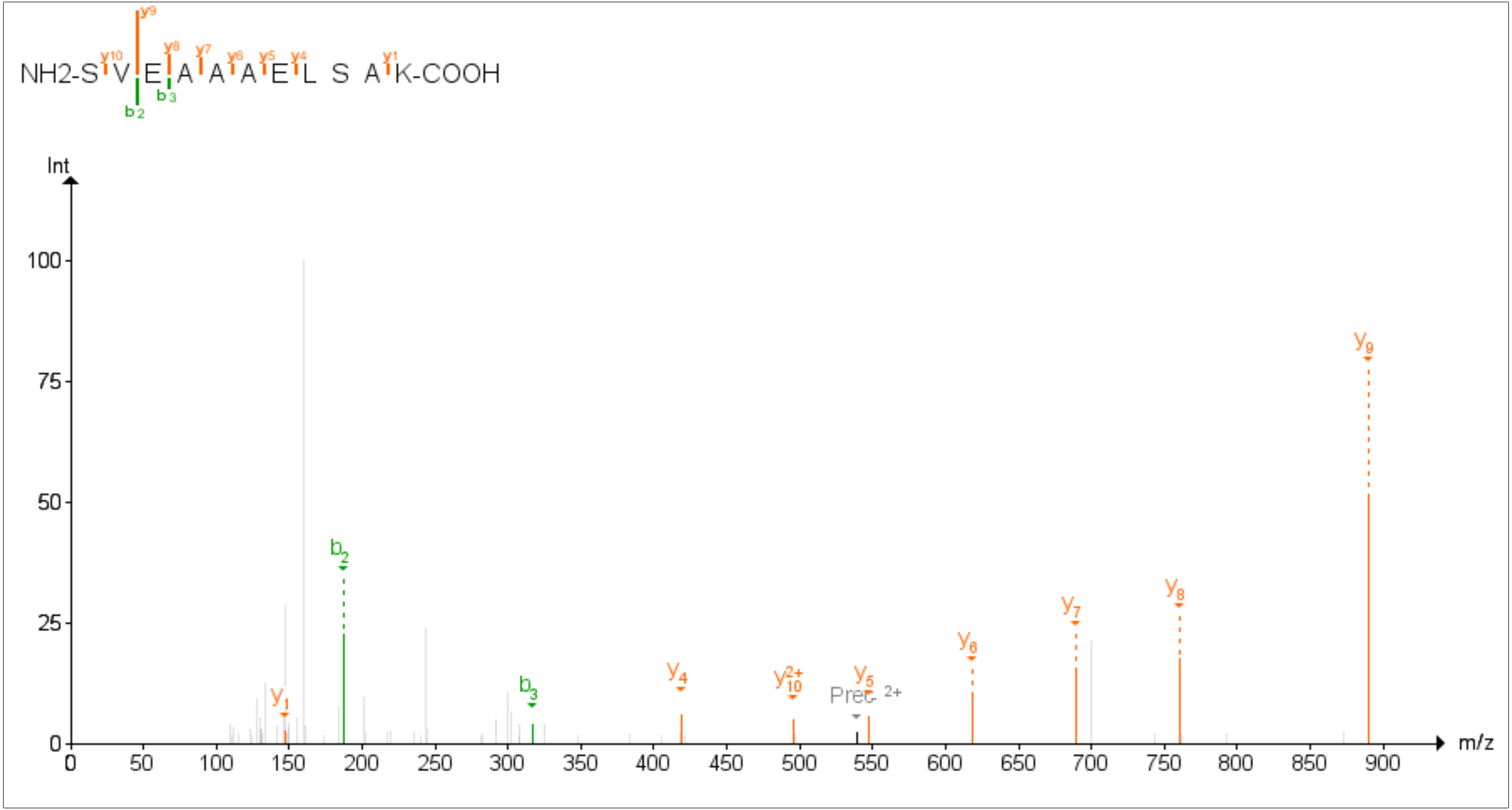

NH2-S V E A A A E L S A K-COOH
y10 y9 y8 y7 y6 y5 y4 y1
b2 b3
Int
m/z
y1
b2
b3
y4
y10 2+
Prec- 2+
y5
y6
y7
y8
y9

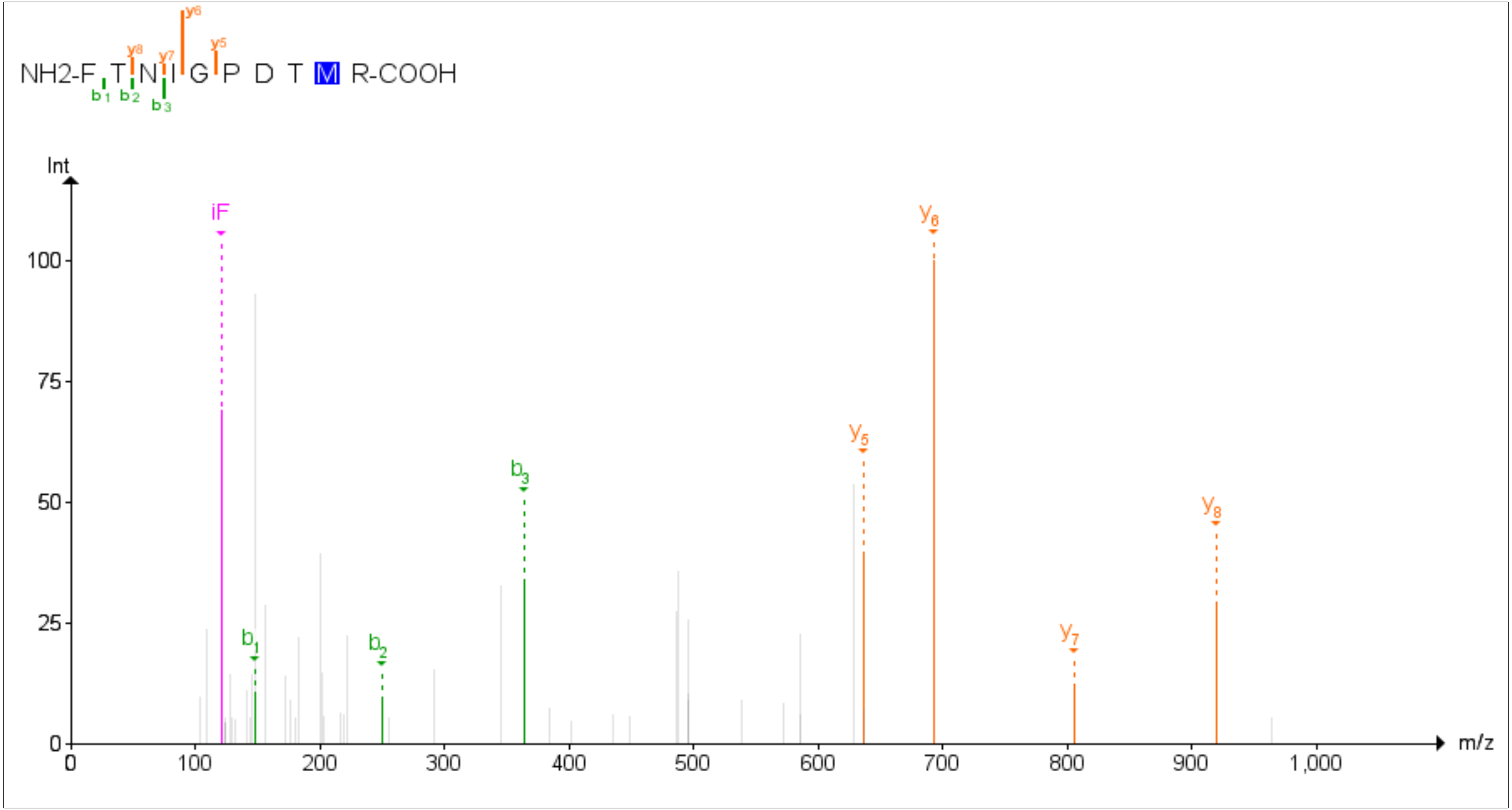

NH2-F T N I G P D T M R-COOH
y8
y7
y6
y5
b1
b2
b3
Int
iF
y6
y5
b3
y8
y7
b1
b2
100
75
50
25
0
0
100
200
300
400
500
600
700
800
900
1,000
m/z

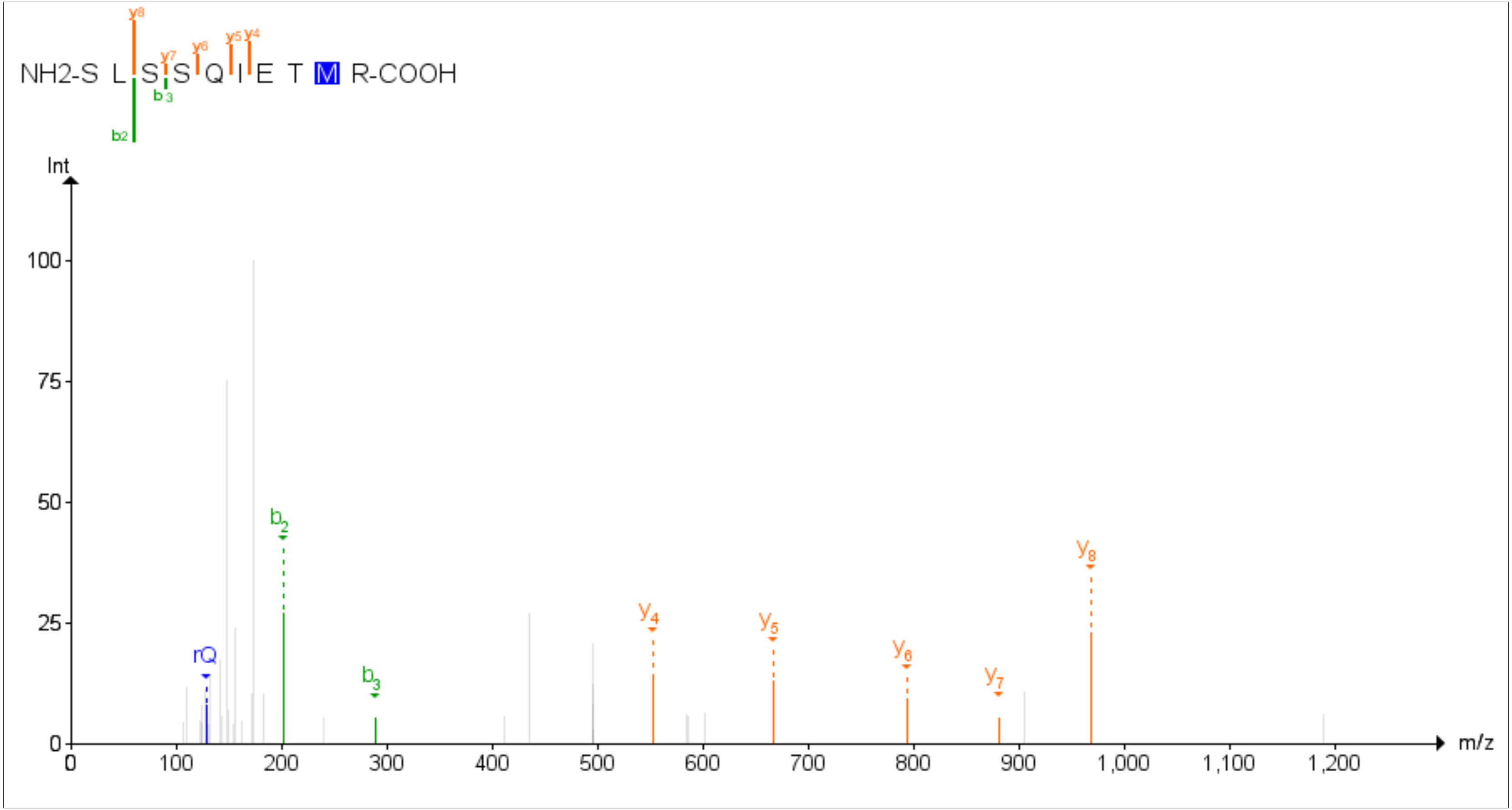

NH2-S L S S Q I E T M R-COOH
y8
y7
y6
y5
y4
b2
b3
Int
100
75
50
25
0
rQ
b2
b3
y4
y5
y6
y7
y8
0
100
200
300
400
500
600
700
800
900
1,000
1,100
1,200
m/z

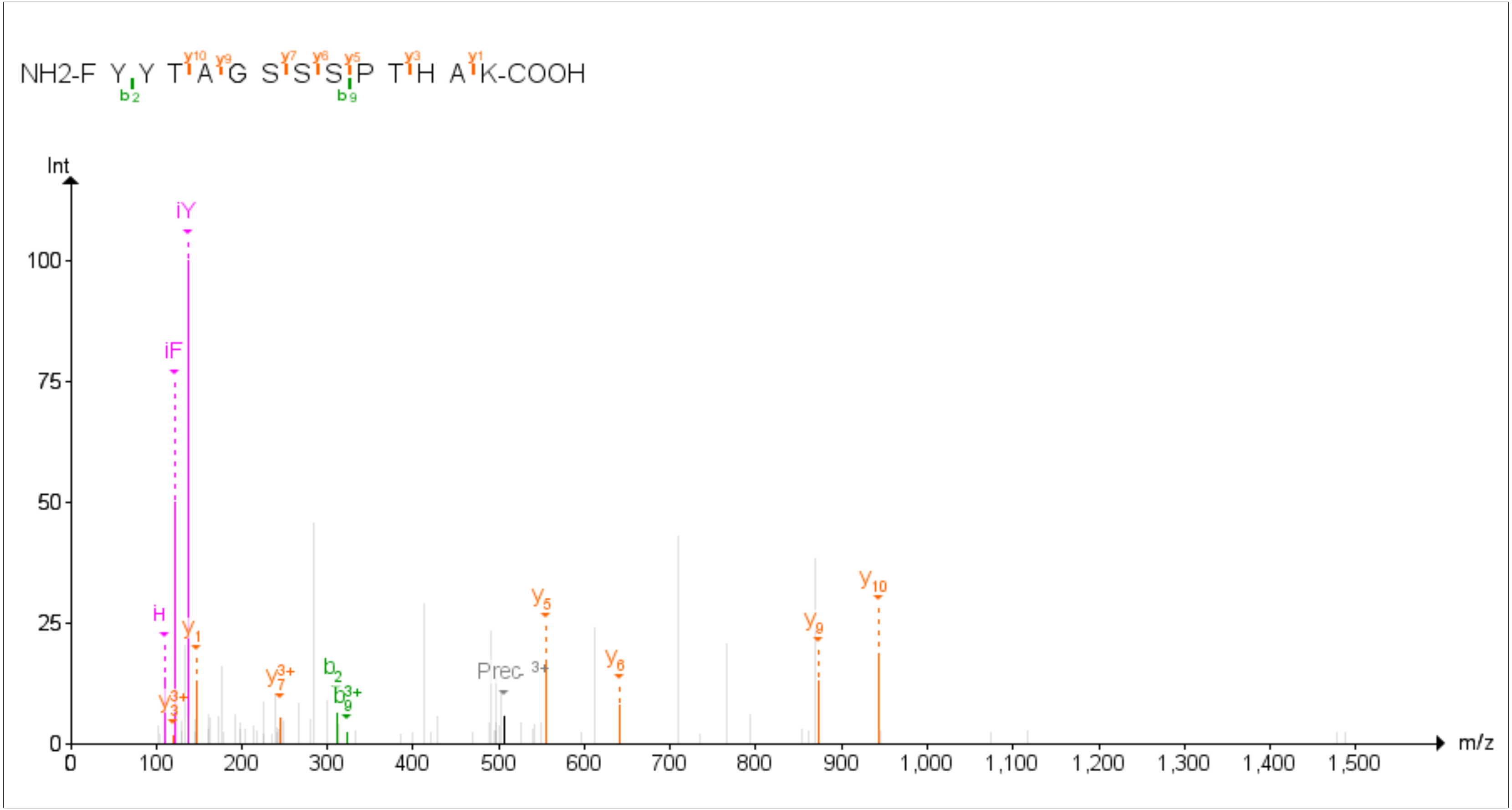

NH2-F Y Y T A G S S S P T H A K-COOH
y10 y9 y7 y6 y5 y3 y1
b2 b9
Int
100
75
50
25
0
iY
iF
iH
y1
y3 3+
y7 3+
b2
b9 3+
Prec 3+
y5
y6
y9
y10
0 100 200 300 400 500 600 700 800 900 1,000 1,100 1,200 1,300 1,400 1,500
m/z

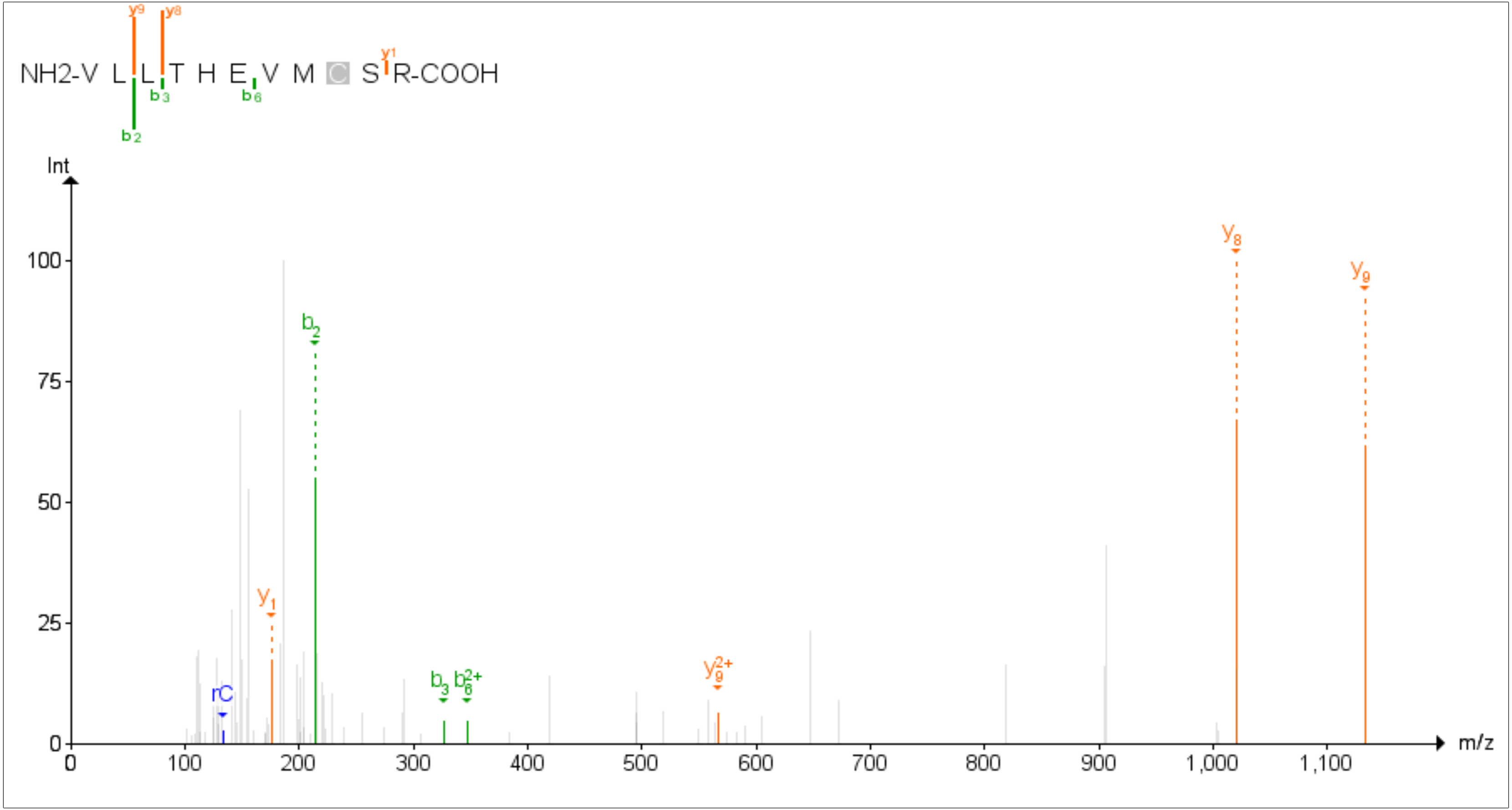

NH2-V L L T H E V M C S R-COOH
y9
y8
y1
b2
b3
b6
Int
100
75
50
25
0
y8
y9
b2
y1
rC
b3
b6 2+
y9 2+
0
100
200
300
400
500
600
700
800
900
1,000
1,100
m/z

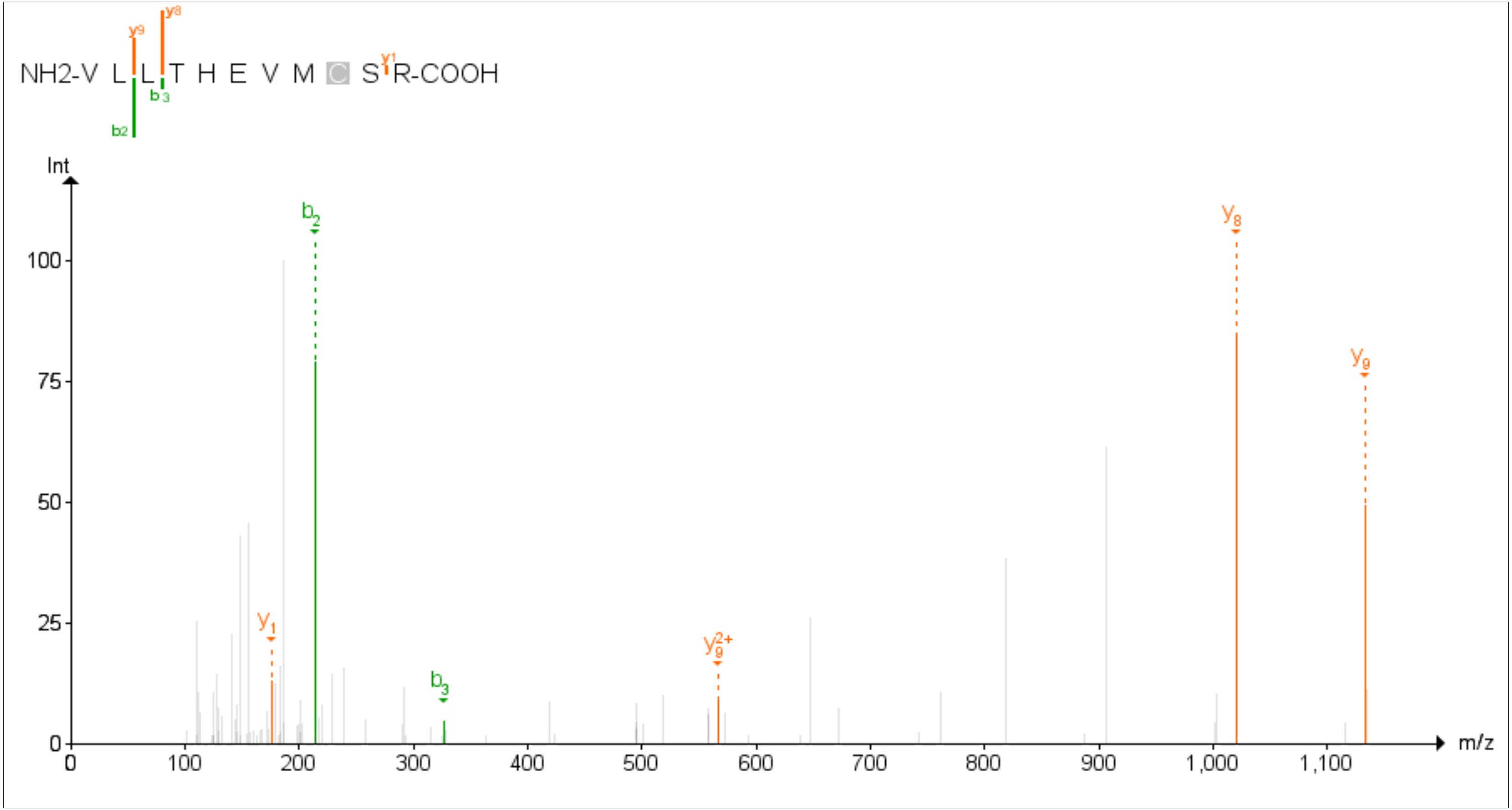
NH2-V L L T H E V M C S R-COOH
y9
y8
b2
b3
y1
Int
m/z
0
25
50
75
100
100
200
300
400
500
600
700
800
900
1,000
1,100
b2
b3
y1
y9 2+
y8
y9

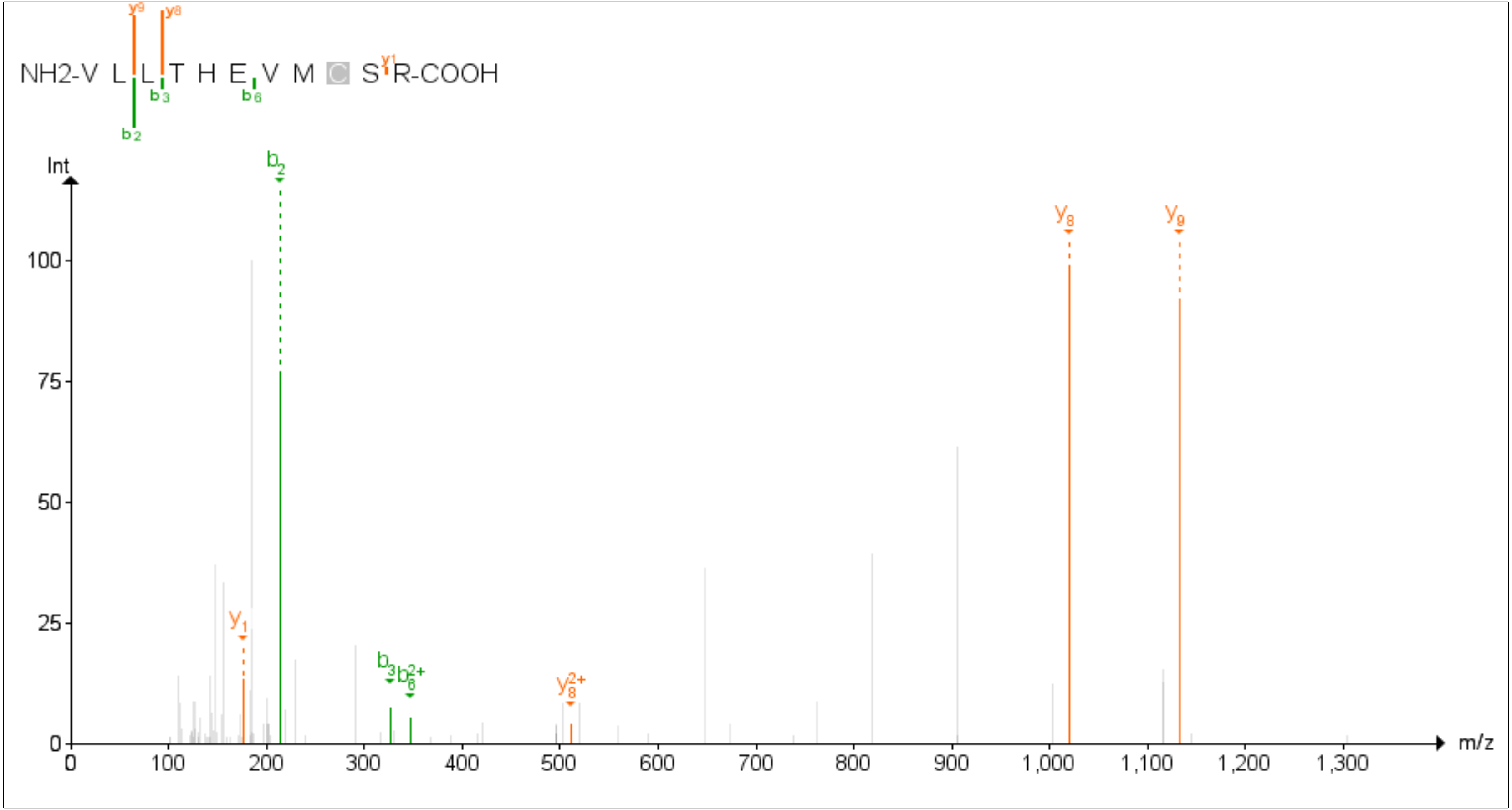

NH2-V L L T H E V M C S R-COOH
y9
y8
y1
b2
b3
b6
Int
m/z
100
75
50
25
0
0
100
200
300
400
500
600
700
800
900
1,000
1,100
1,200
1,300
y1
b3
b6 2+
y8 2+
y8
y9

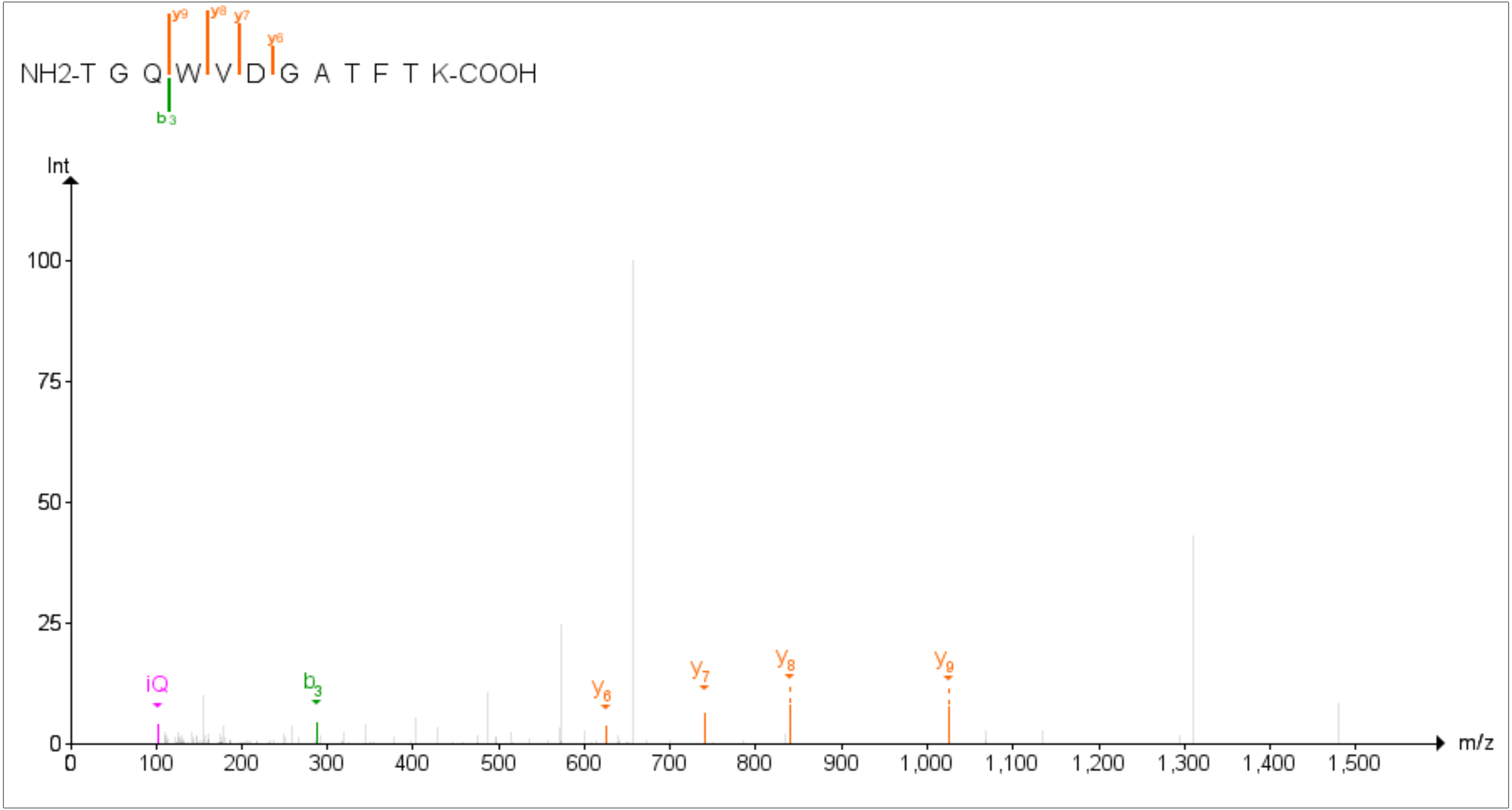

NH2-T G Q W V D G A T F T K-COOH
y9
y8
y7
y6
b3
Int
100
75
50
25
0
iQ
b3
y6
y7
y8
y9
0
100
200
300
400
500
600
700
800
900
1,000
1,100
1,200
1,300
1,400
1,500
m/z

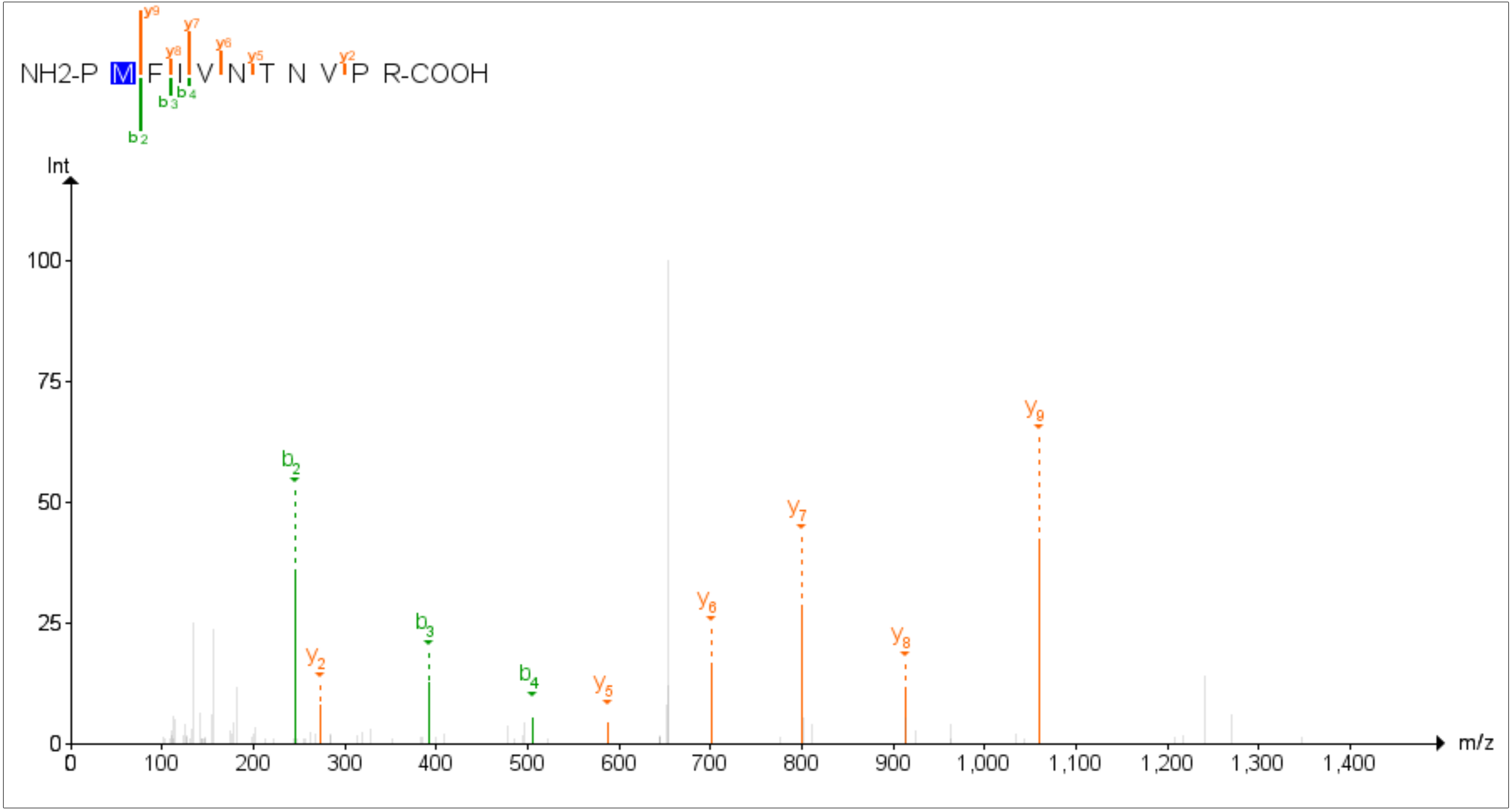

NH2-P M F I V N T N V P R-COOH
y9
y8
y7
y6
y5
y2
b2
b3
b4
Int
100
75
50
25
0
m/z
0
100
200
300
400
500
600
700
800
900
1,000
1,100
1,200
1,300
1,400
b2
y2
b3
b4
y5
y6
y7
y8
y9

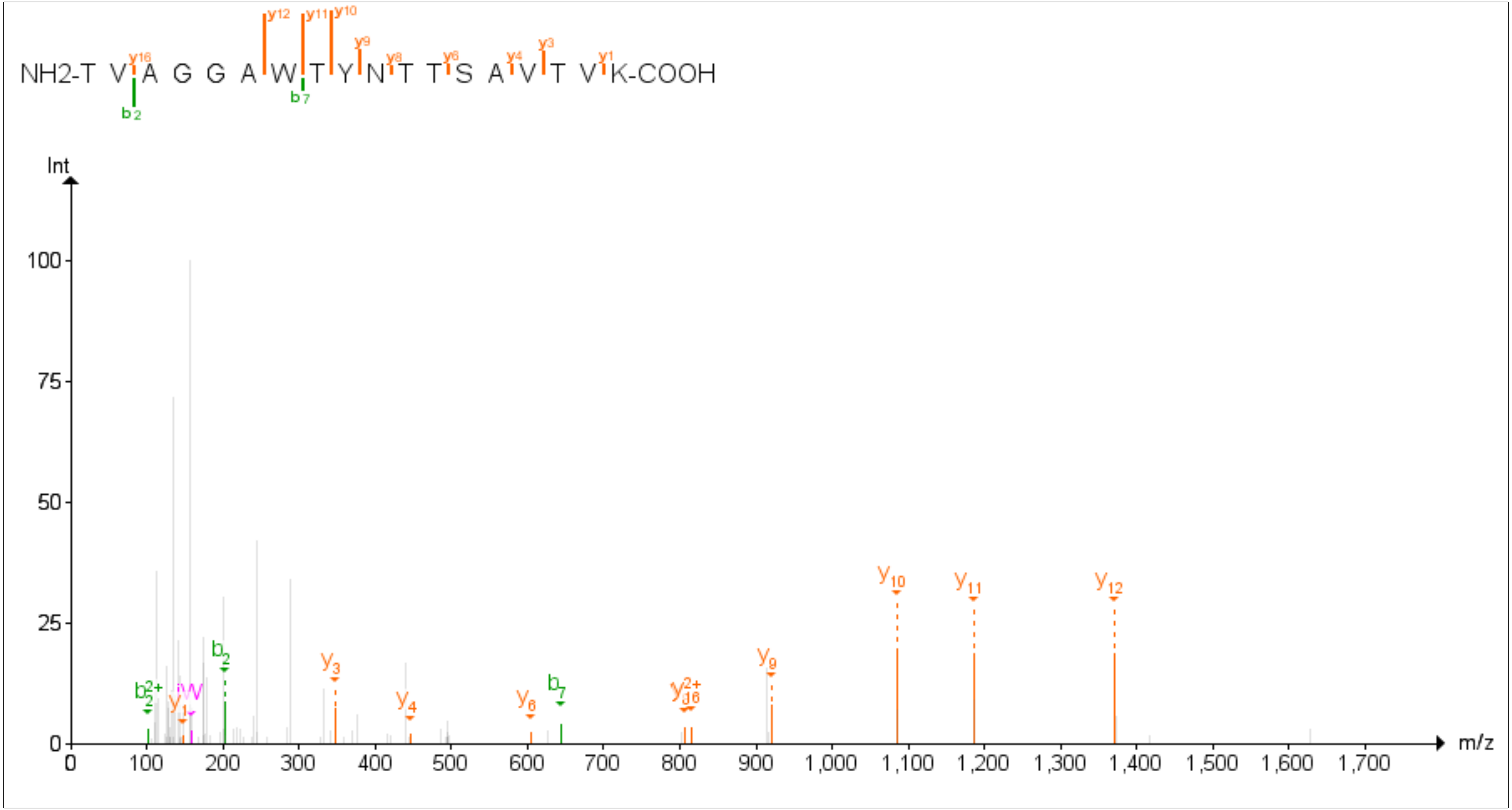
NH2-T V A G G A W T Y N T T S A V T V K-COOH
y16 y12 y11 y10 y9 y8 y6 y4 y3 y1
b2 b7
Int
100
75
50
25
0
m/z
0 100 200 300 400 500 600 700 800 900 1,000 1,100 1,200 1,300 1,400 1,500 1,600 1,700
b2
b2 2+
iW
y1
y3
y4
y6
b7
y16 2+
y9
y10
y11
y12

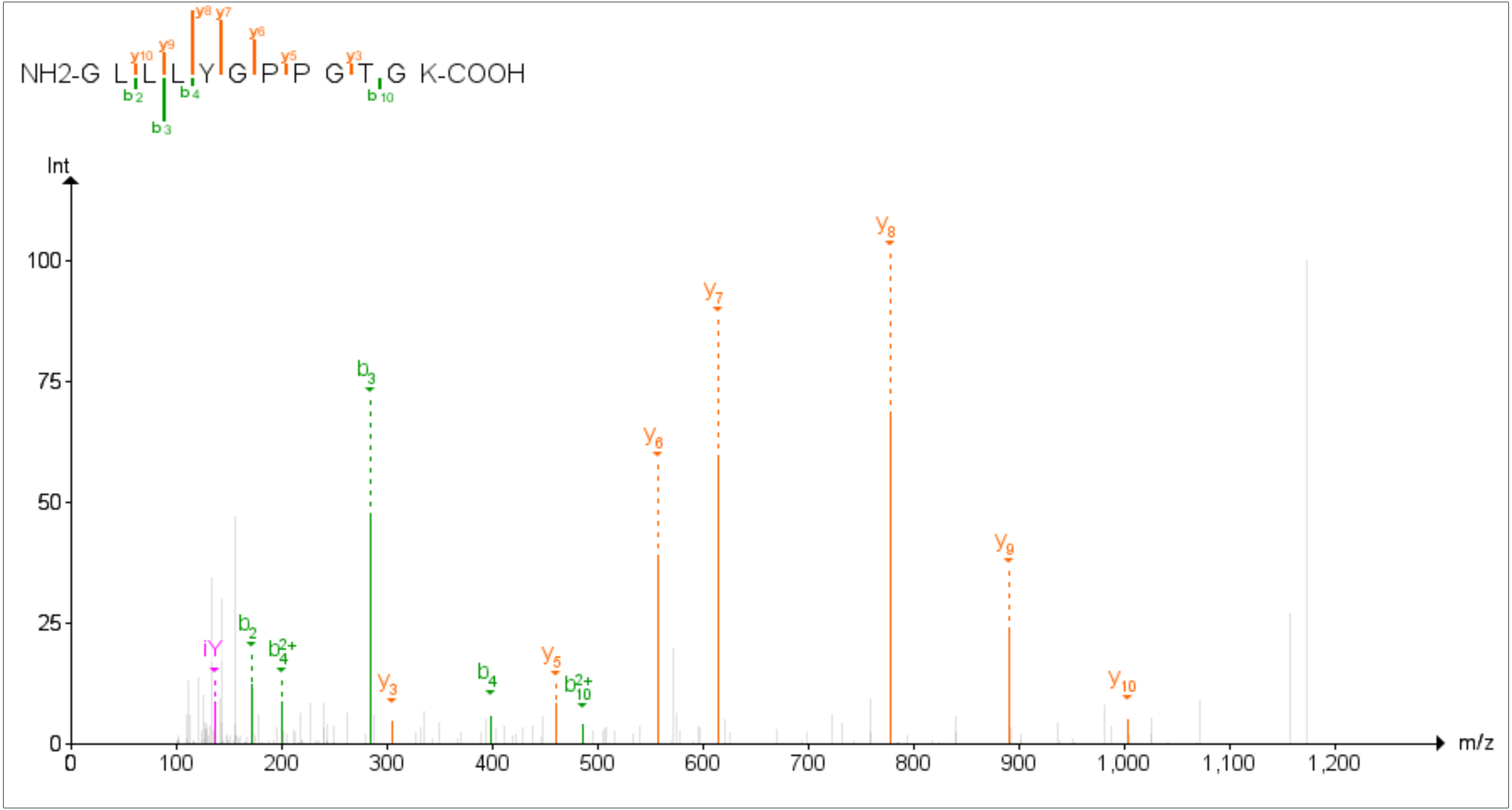

NH2-G L L L Y G P P G T G K-COOH
y10 y9 y8 y7 y6 y5 y3
b2 b3 b4 b10
Int
100
75
50
25
0
iY
b2
b4 2+
b3
y3
b4
y5
b10 2+
y6
y7
y8
y9
y10
0 100 200 300 400 500 600 700 800 900 1,000 1,100 1,200
m/z

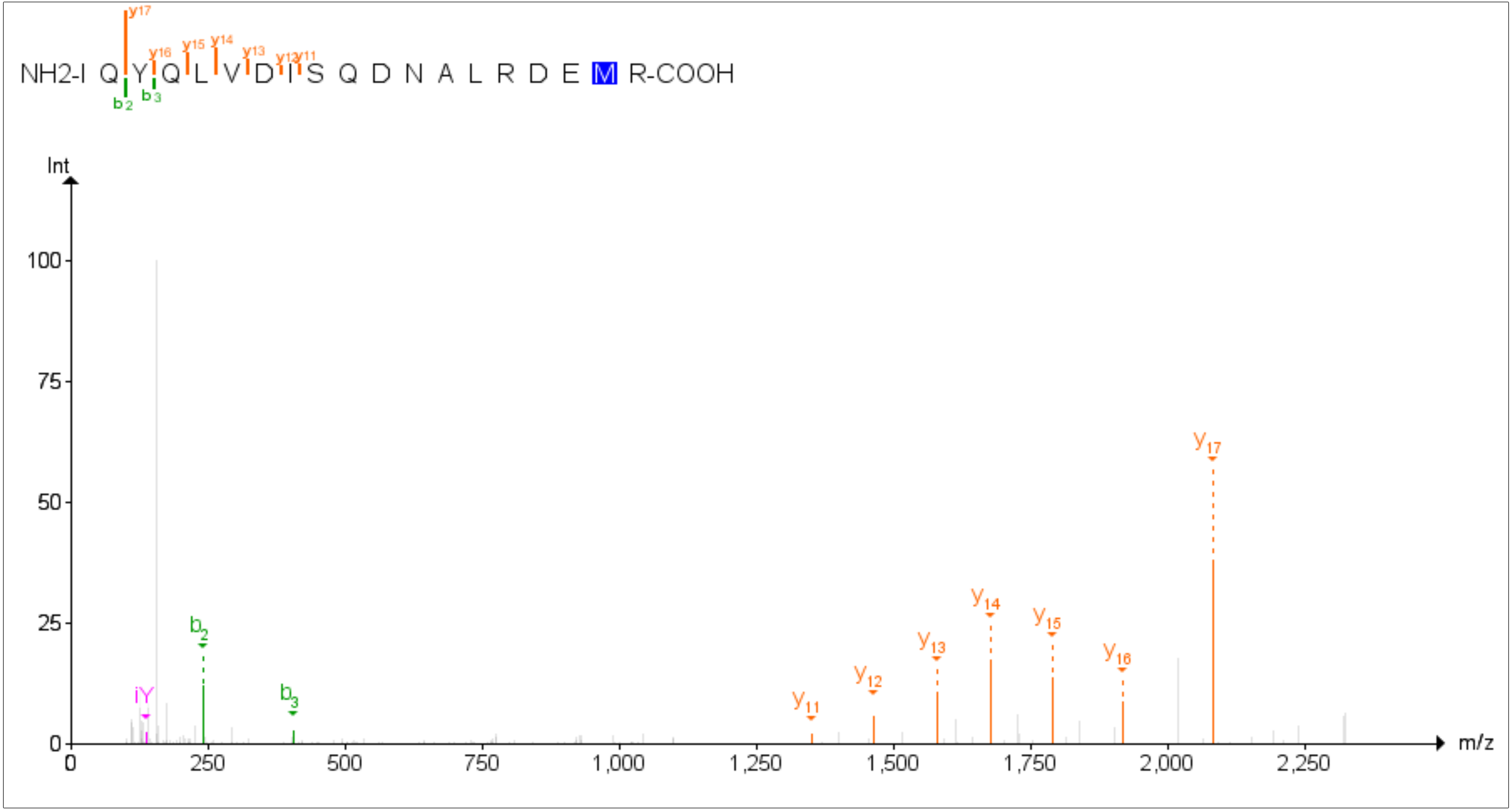
NH2-I Q Y Q L V D I S Q D N A L R D E M R-COOH
y17
y16
y15
y14
y13
y12
y11
b2
b3
Int
100
75
50
25
0
iY
m/z
0
250
500
750
1,000
1,250
1,500
1,750
2,000
2,250

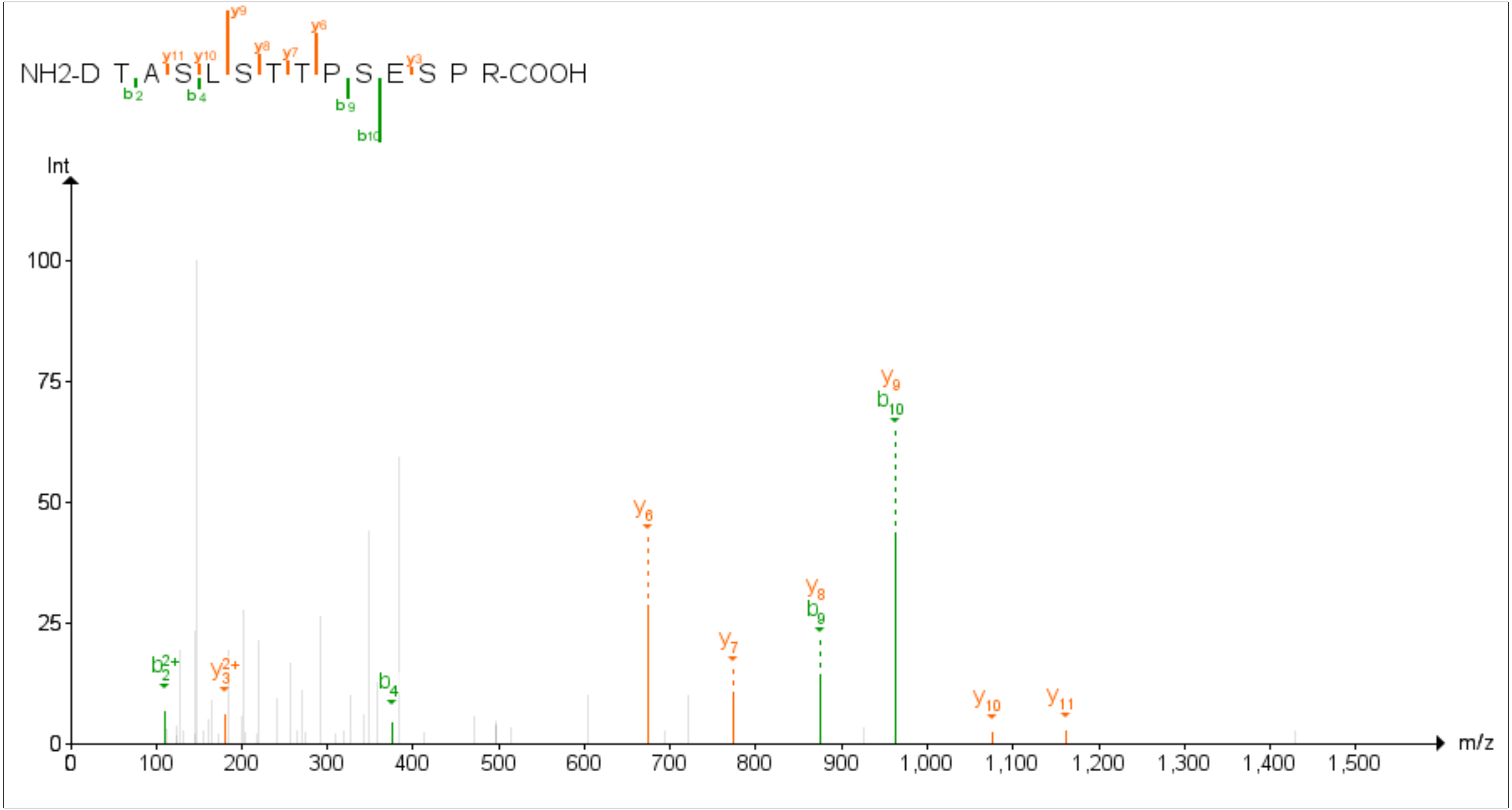

NH2-D T A S L S T T P S E S P R-COOH
y11 y10 y9 y8 y7 y6 y3
b2 b4 b9 b10
Int
100
75
50
25
0
m/z
0 100 200 300 400 500 600 700 800 900 1,000 1,100 1,200 1,300 1,400 1,500
b2 2+
y3 2+
b4
y6
y7
y8
b9
y9
b10
y10
y11

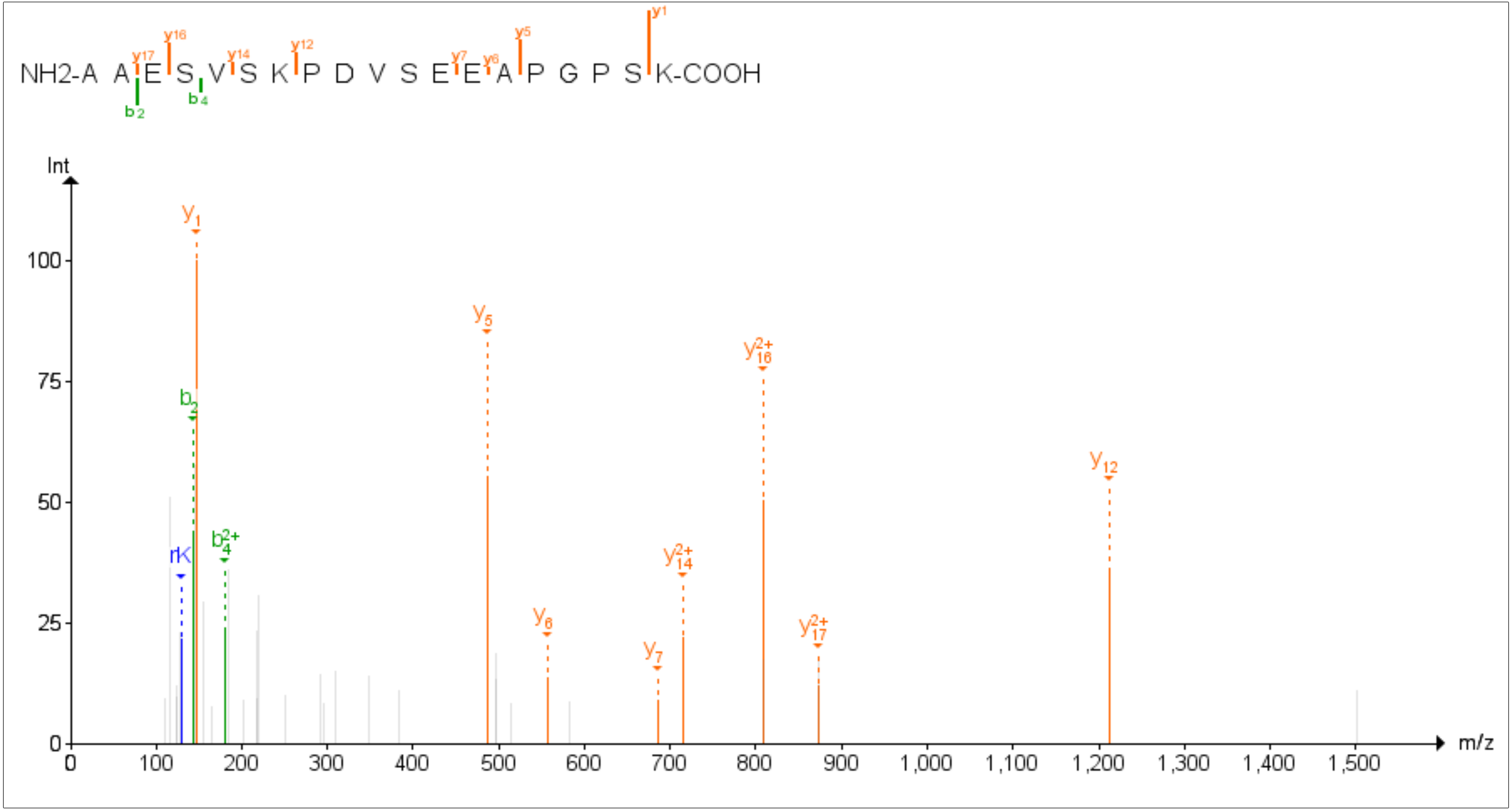

NH2-A A E S V S K P D V S E E A P G P S K-COOH
y1
y5
y6
y7
y12
y14
y16
y17
b2
b4
Int
m/z
y1
y5
y6
y7
y12
b2
b4 2+
y14 2+
y16 2+
y17 2+
rK
0
25
50
75
100
0
100
200
300
400
500
600
700
800
900
1,000
1,100
1,200
1,300
1,400
1,500

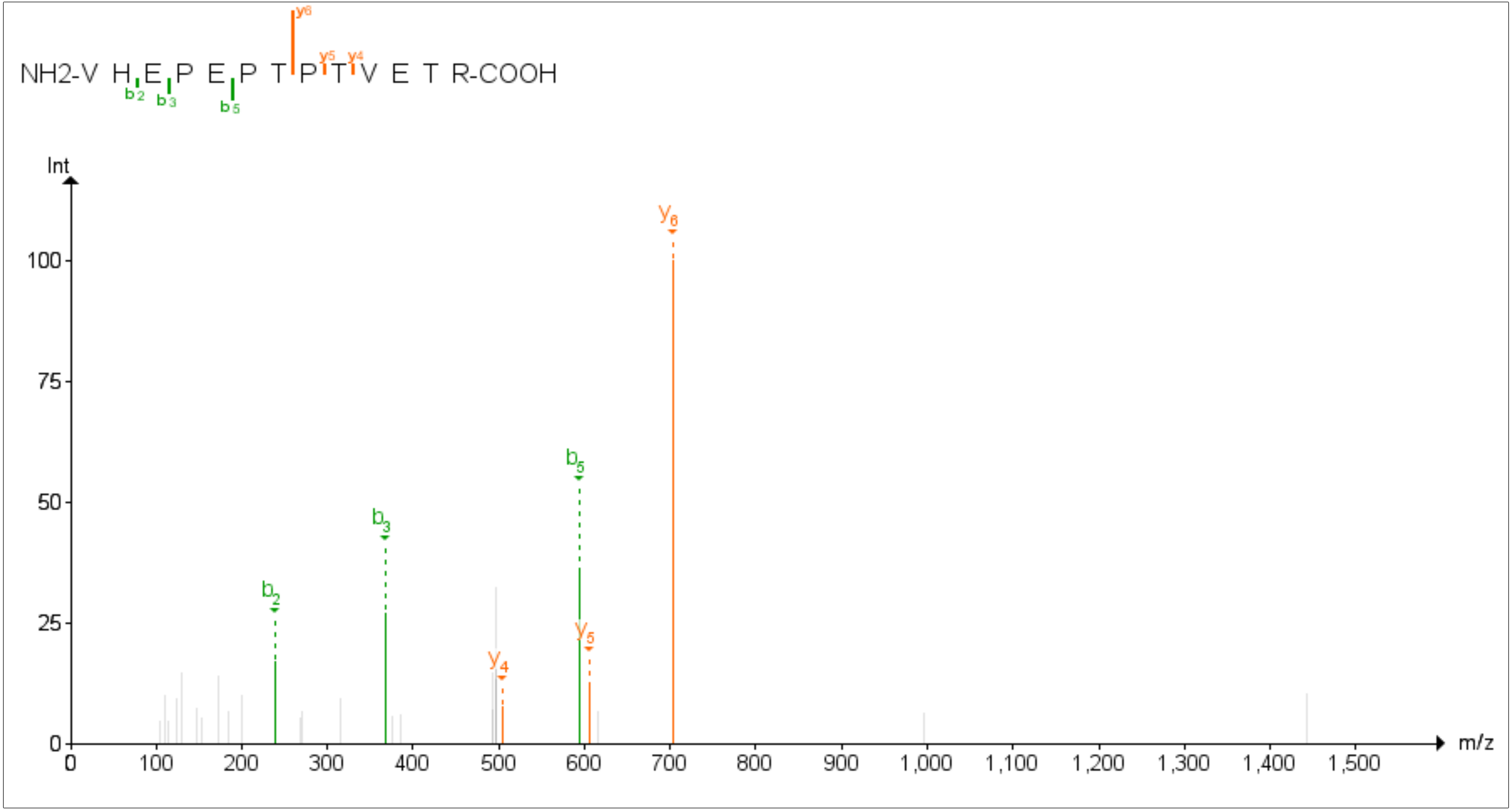

NH2-V H E P E P T P T V E T R-COOH
b2
b3
b5
y6
y5
y4
Int
100
75
50
25
0
m/z
0
100
200
300
400
500
600
700
800
900
1,000
1,100
1,200
1,300
1,400
1,500

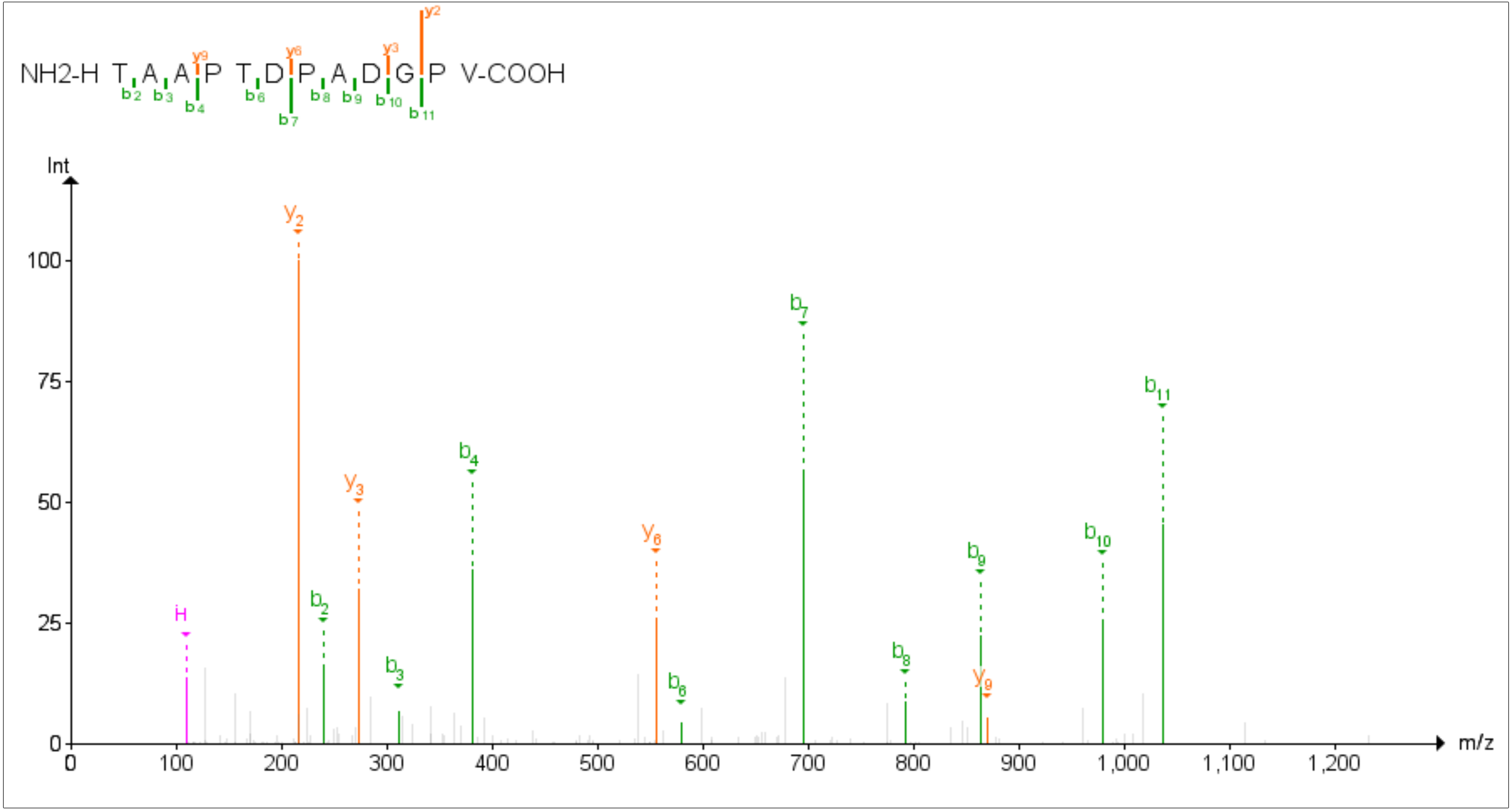

NH2-H T A A P T D P A D G P V-COOH
b2 b3 b4 b6 b7 b8 b9 b10 b11
y9 y6 y3 y2
Int
100
75
50
25
0
y2
y3
b2
b3
b4
y6
b6
b7
b8
b9
y9
b10
b11
H
0 100 200 300 400 500 600 700 800 900 1,000 1,100 1,200
m/z

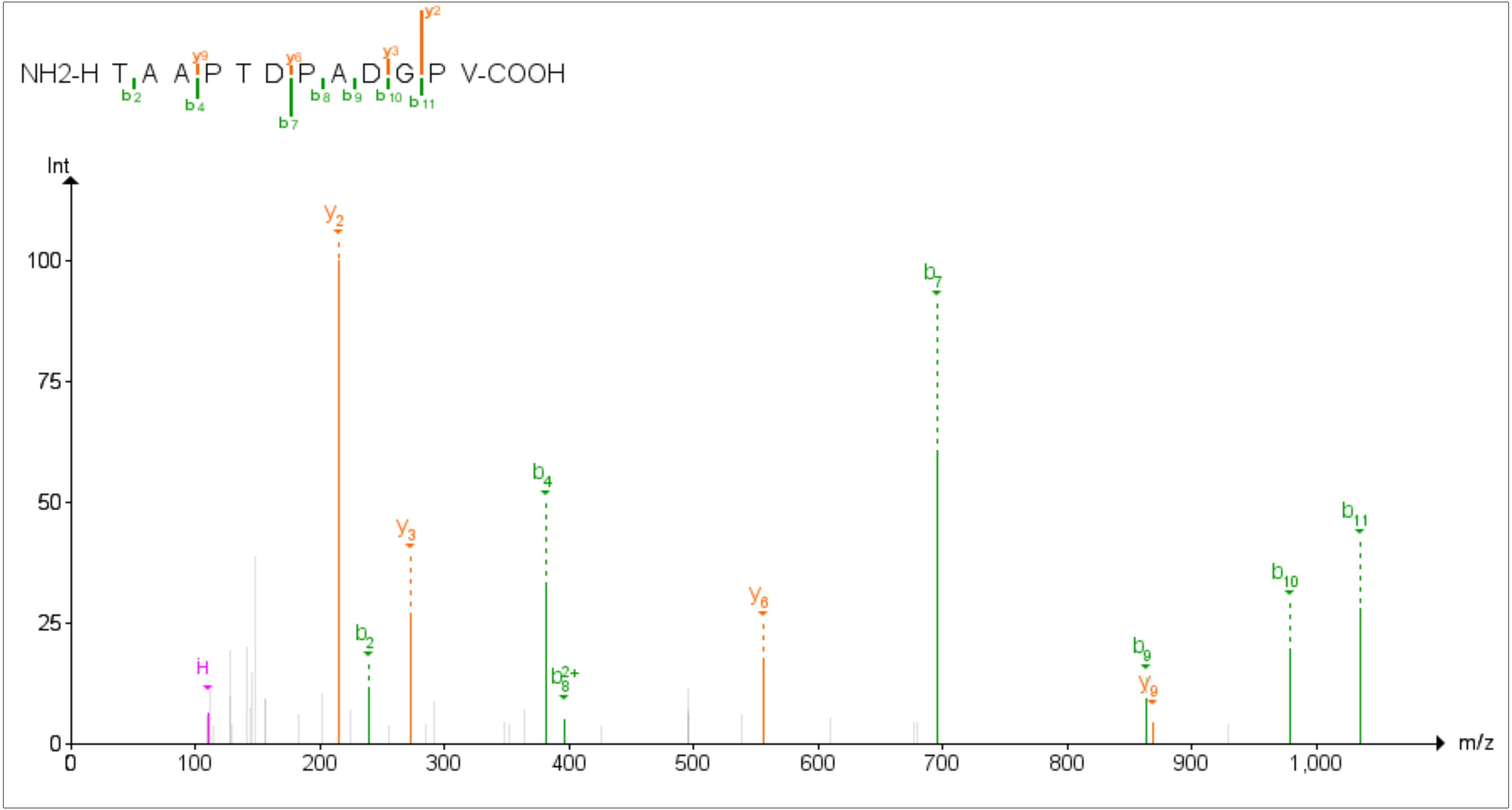

NH2-H T A A P T D P A D G P V-COOH
y9 y6 y3 y2
b2 b4 b7 b8 b9 b10 b11
Int
100
75
50
25
0
m/z
0 100 200 300 400 500 600 700 800 900 1,000
y2 y3 y6 y9
b2 b4 b7 b9 b10 b11
b8 2+
H

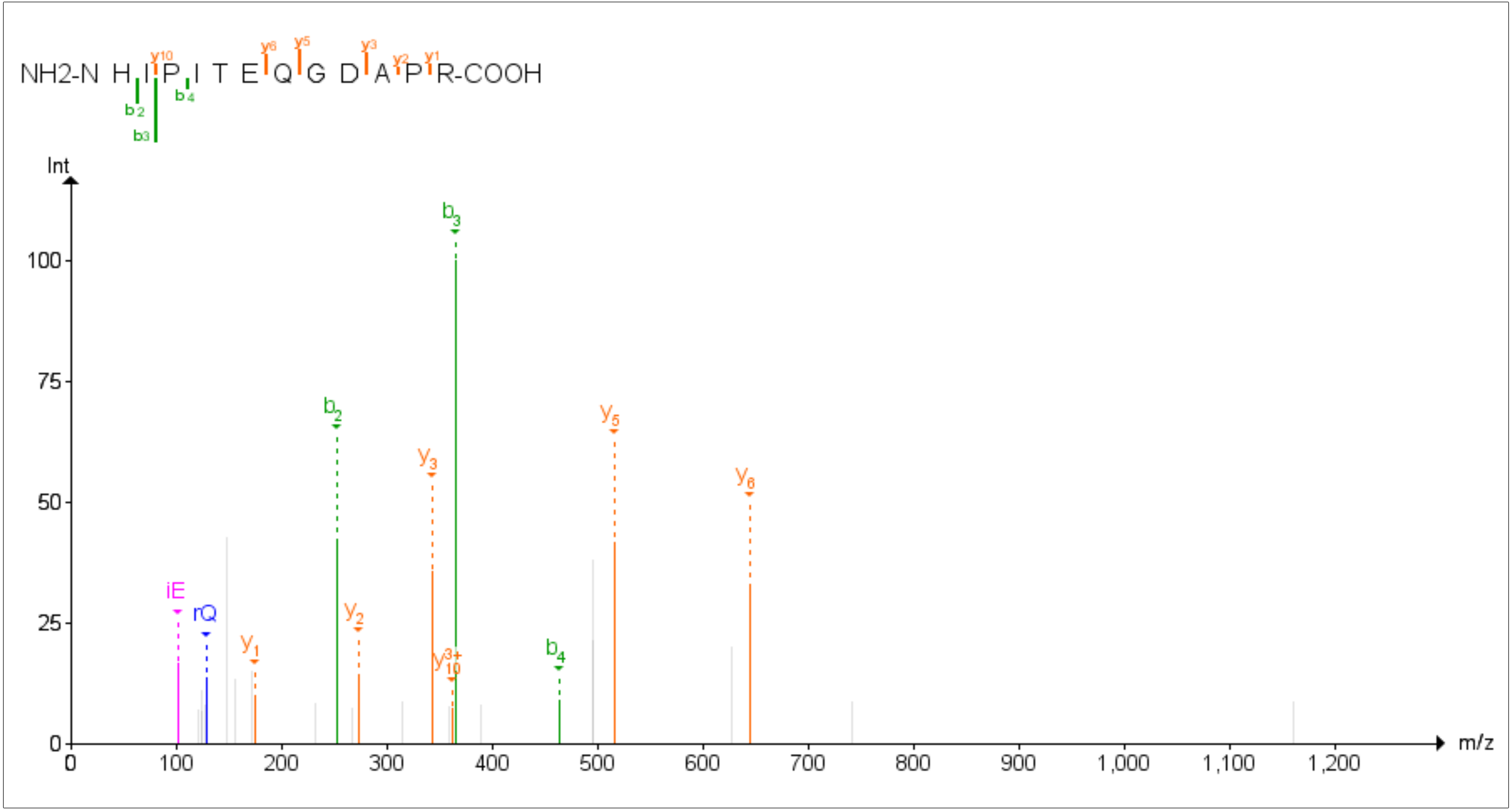

NH2-N H I P I T E Q G D A P R-COOH
y10 y6 y5 y3 y2 y1
b2 b3 b4
Int
100
75
50
25
0
b3
b2
y5
y3
y6
iE
rQ
y1
y2
y10 3+
b4
m/z
0 100 200 300 400 500 600 700 800 900 1,000 1,100 1,200

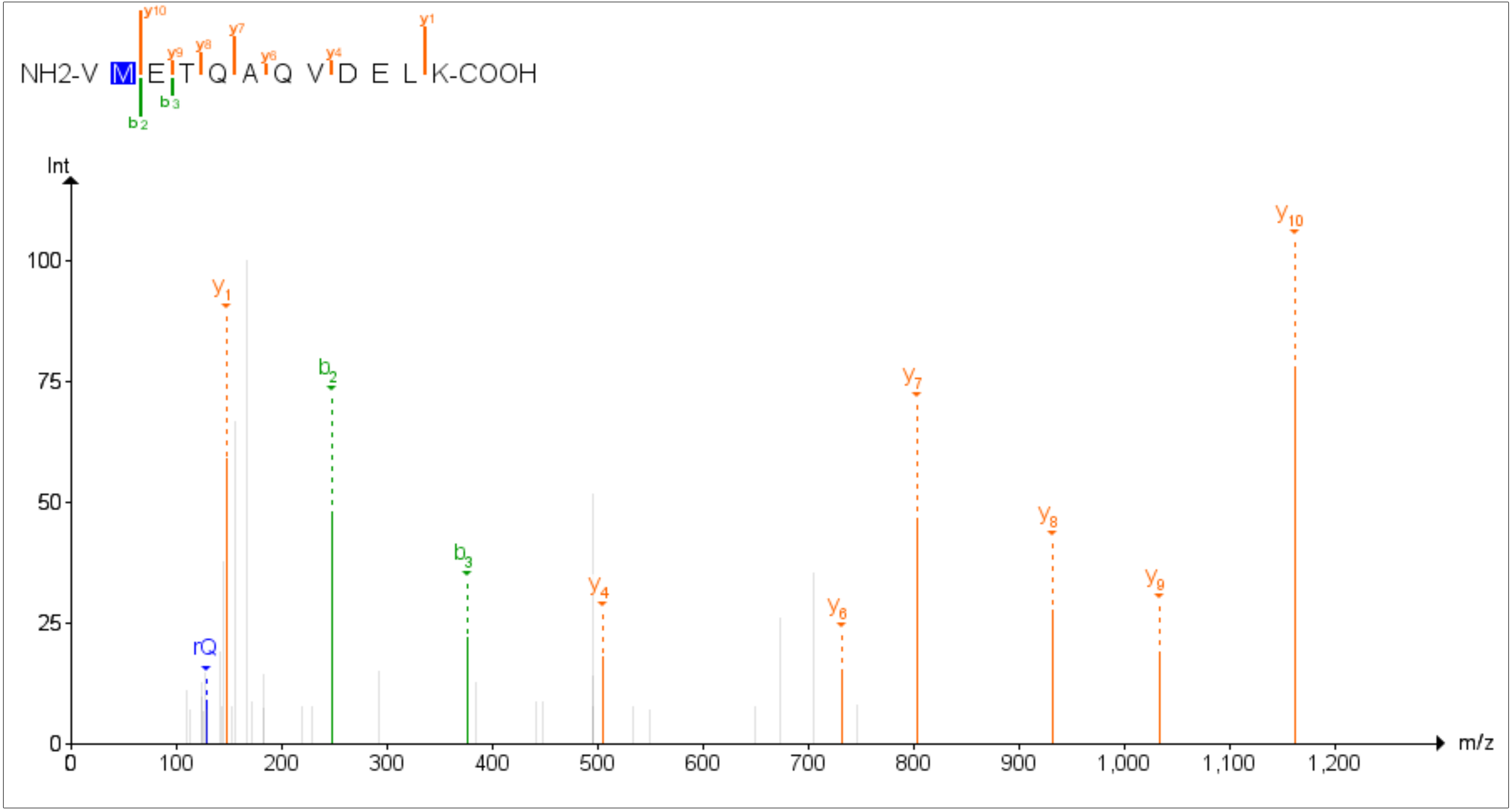

NH2-V M E T Q A Q V D E L K-COOH
y10
y9
y8
y7
y6
y4
y1
b 2
b 3
Int
m/z
y1
b2
b3
y4
y6
y7
y8
y9
y10
rQ
0
25
50
75
100
0
100
200
300
400
500
600
700
800
900
1,000
1,100
1,200

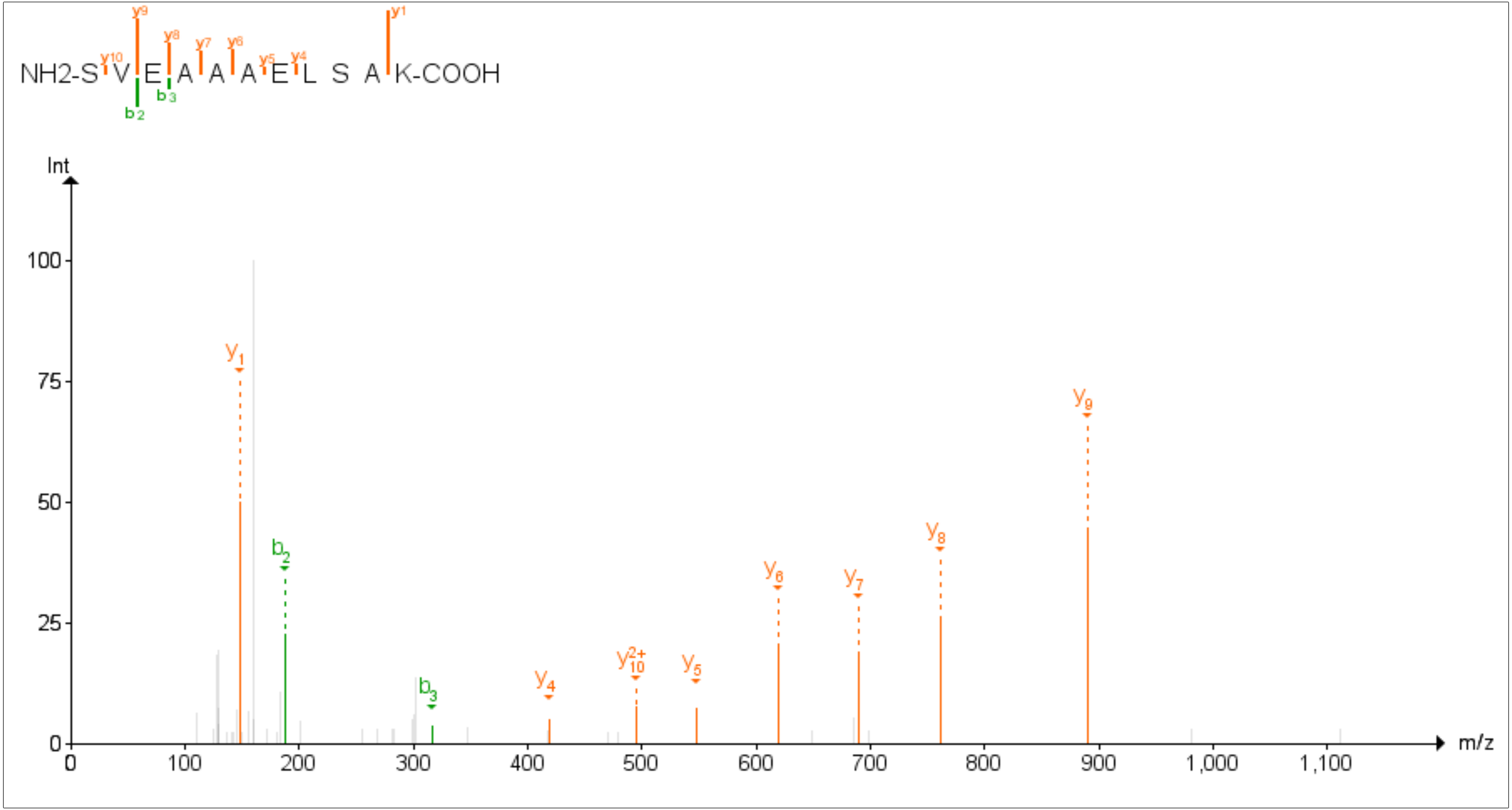

NH2-S V E A A A E L S A K-COOH
y10
y9
y8
y7
y6
y5
y4
y1
b2
b3
Int
100
75
50
25
0
y1
b2
b3
y4
y10 2+
y5
y6
y7
y8
y9
0
100
200
300
400
500
600
700
800
900
1,000
1,100
m/z

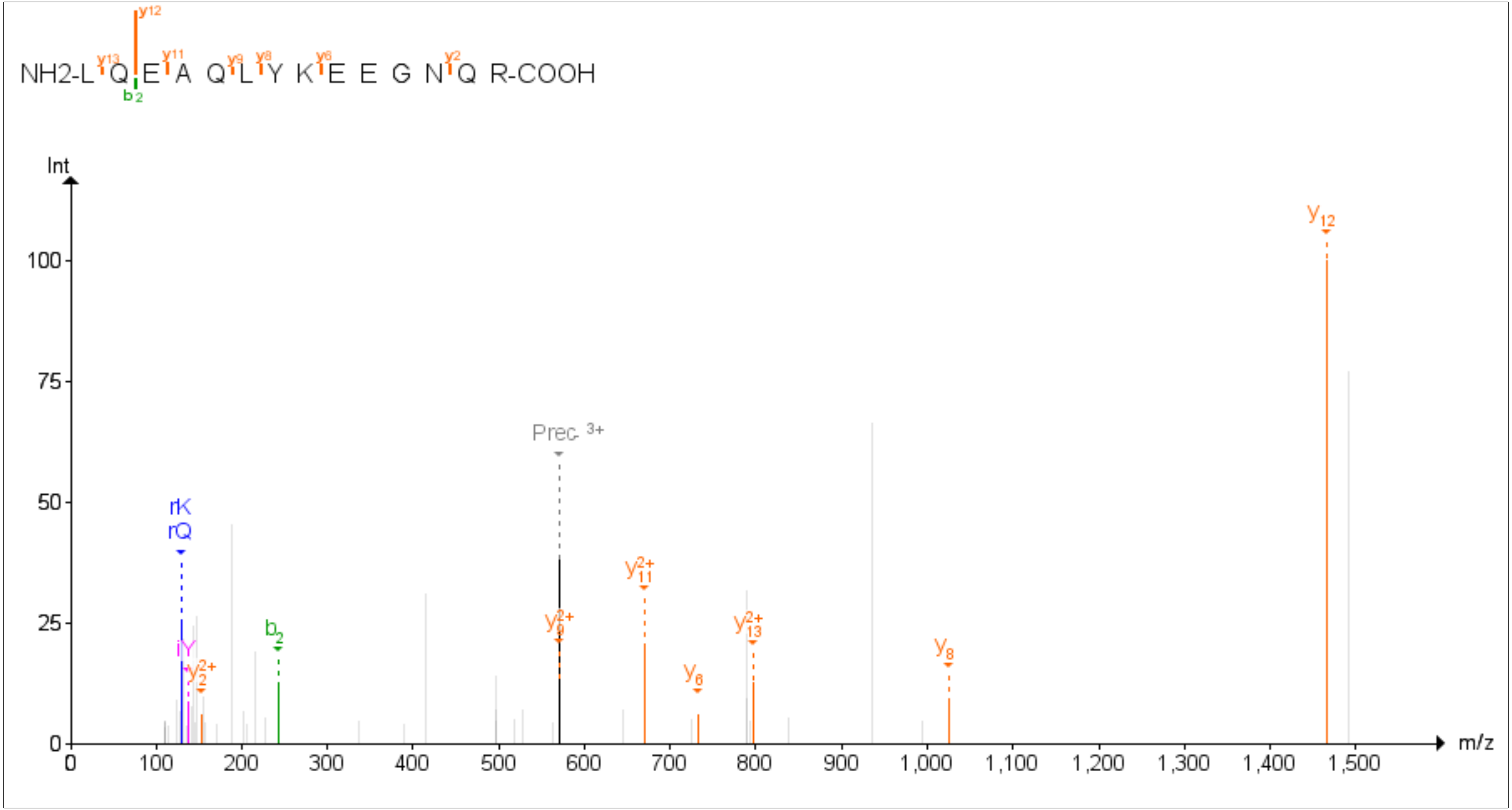

NH2-L Q E A Q L Y K E E G N Q R-COOH
y12
y13
y11
y9
y8
y6
y2
b2
Int
100
75
50
25
0
y12
Prec 3+
rK
rQ
iY
y2 2+
b2
y9 2+
y11 2+
y6
y13 2+
y8
0
100
200
300
400
500
600
700
800
900
1,000
1,100
1,200
1,300
1,400
1,500
m/z

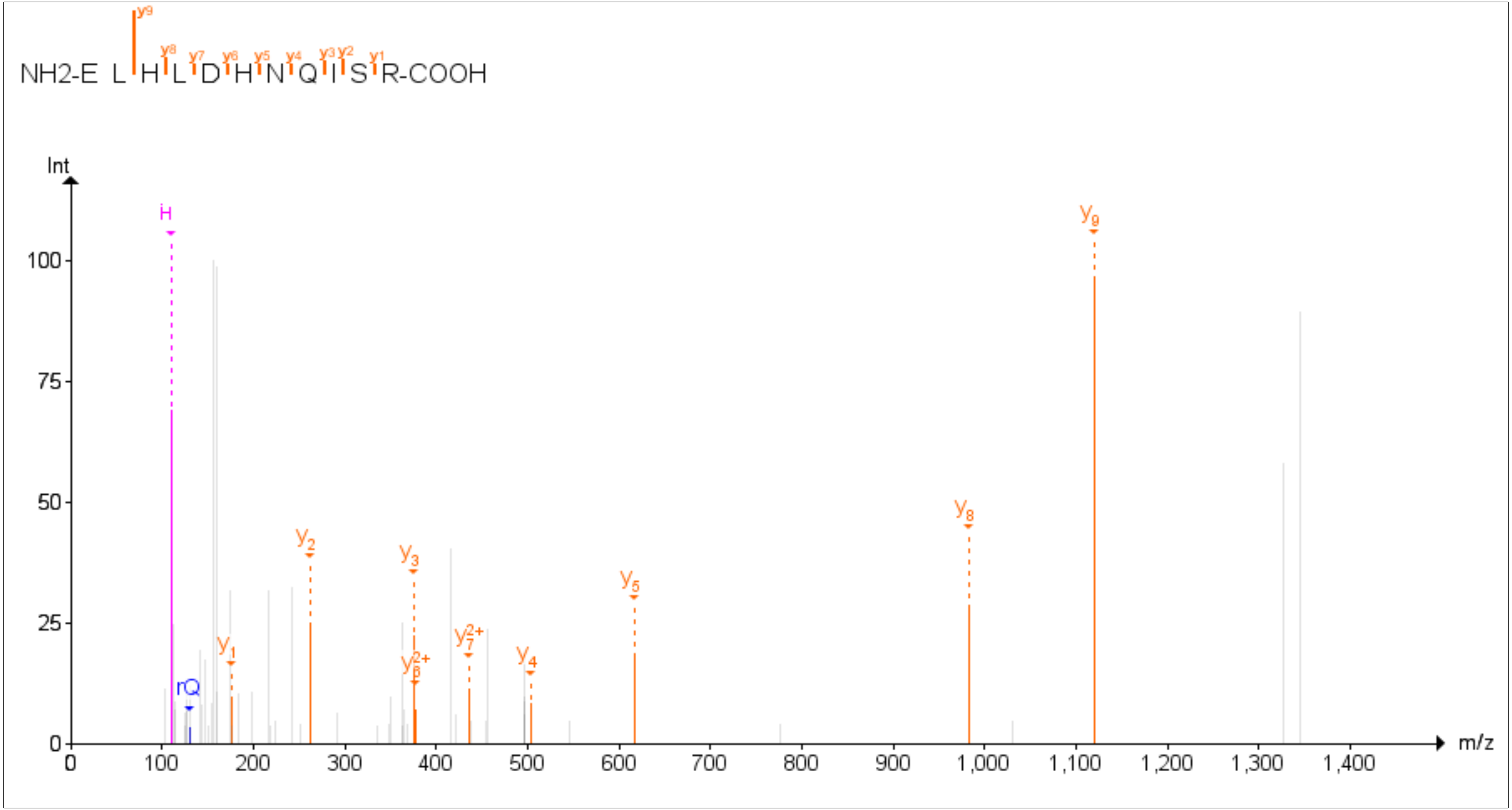
NH2-E L H L D H N Q I S R-COOH
y9 y8 y7 y6 y5 y4 y3 y2 y1
Int
m/z
0
25
50
75
100
0
100
200
300
400
500
600
700
800
900
1,000
1,100
1,200
1,300
1,400
iH
rQ
y1
y2
y3
y4
y5
y8
y9
y72+
y62+

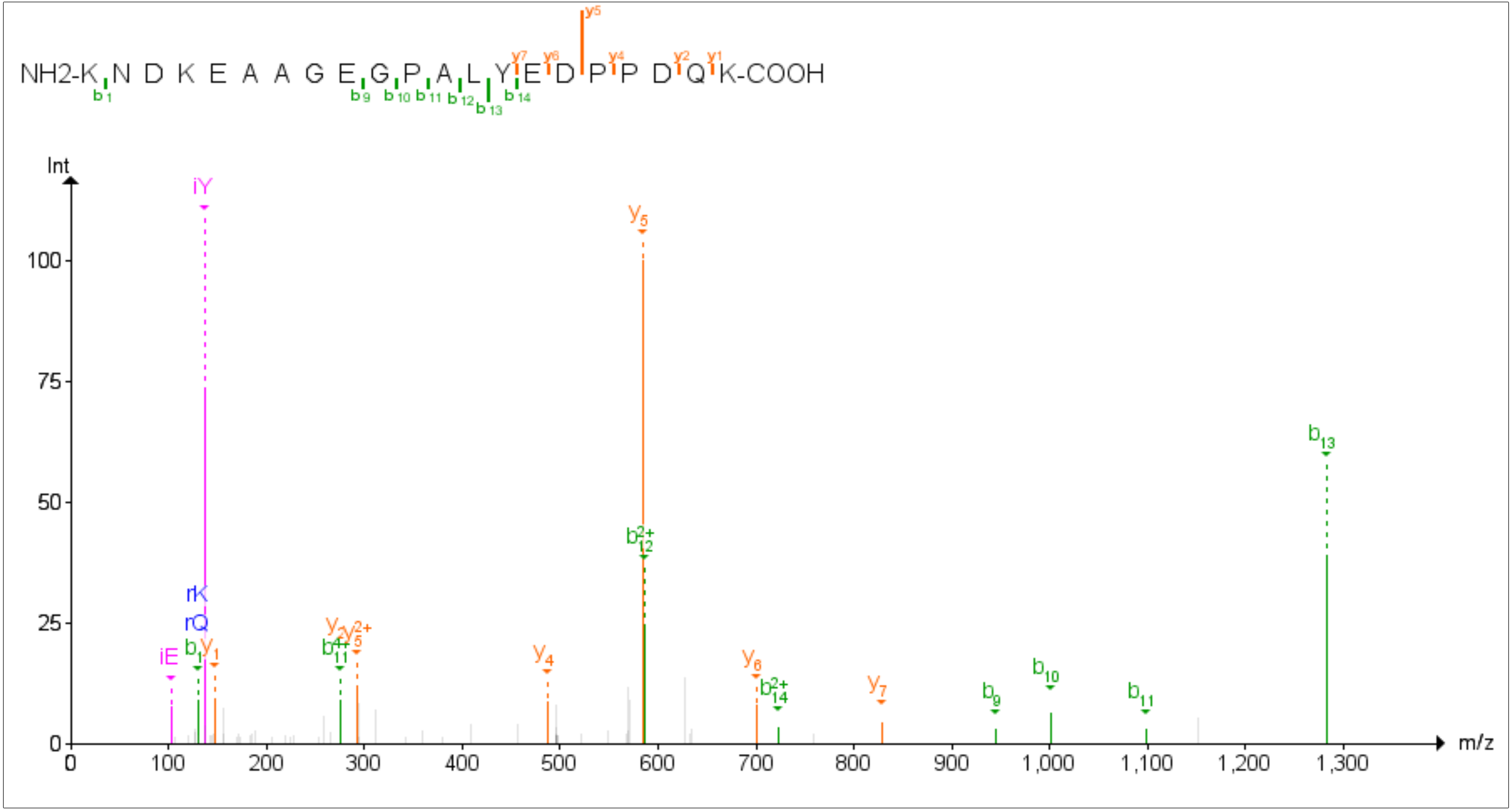

NH2-K N D K E A A G E G P A L Y E D P P D Q K-COOH
Int
m/z

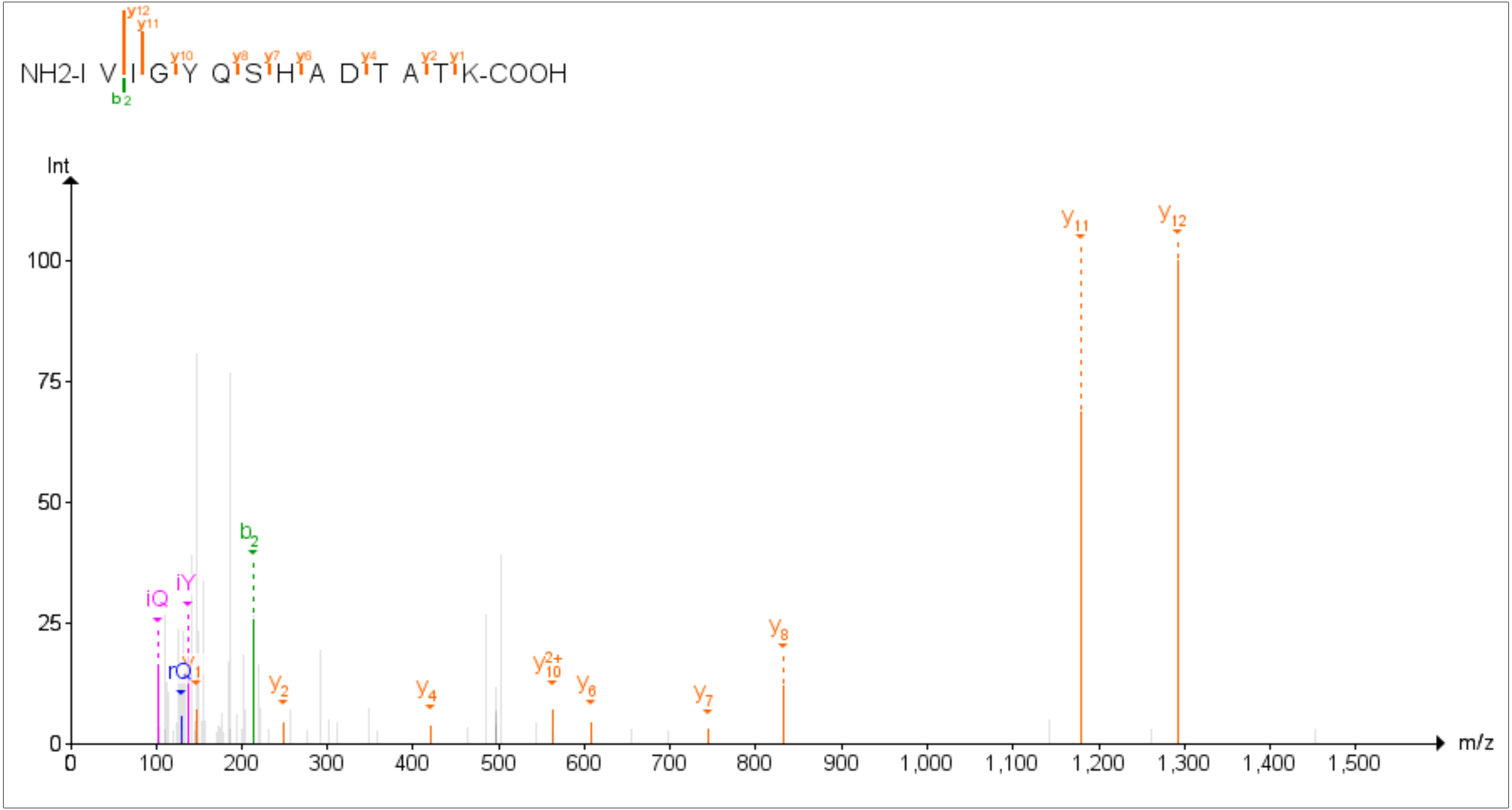

NH2-I V I G Y Q S H A D T A T K-COOH
y12
y11
y10
y8
y7
y6
y4
y2
y1
b2
Int
100
75
50
25
0
iQ
iY
rQ
y1
b2
y2
y4
y10 2+
y6
y7
y8
y11
y12
0
100
200
300
400
500
600
700
800
900
1,000
1,100
1,200
1,300
1,400
1,500
m/z

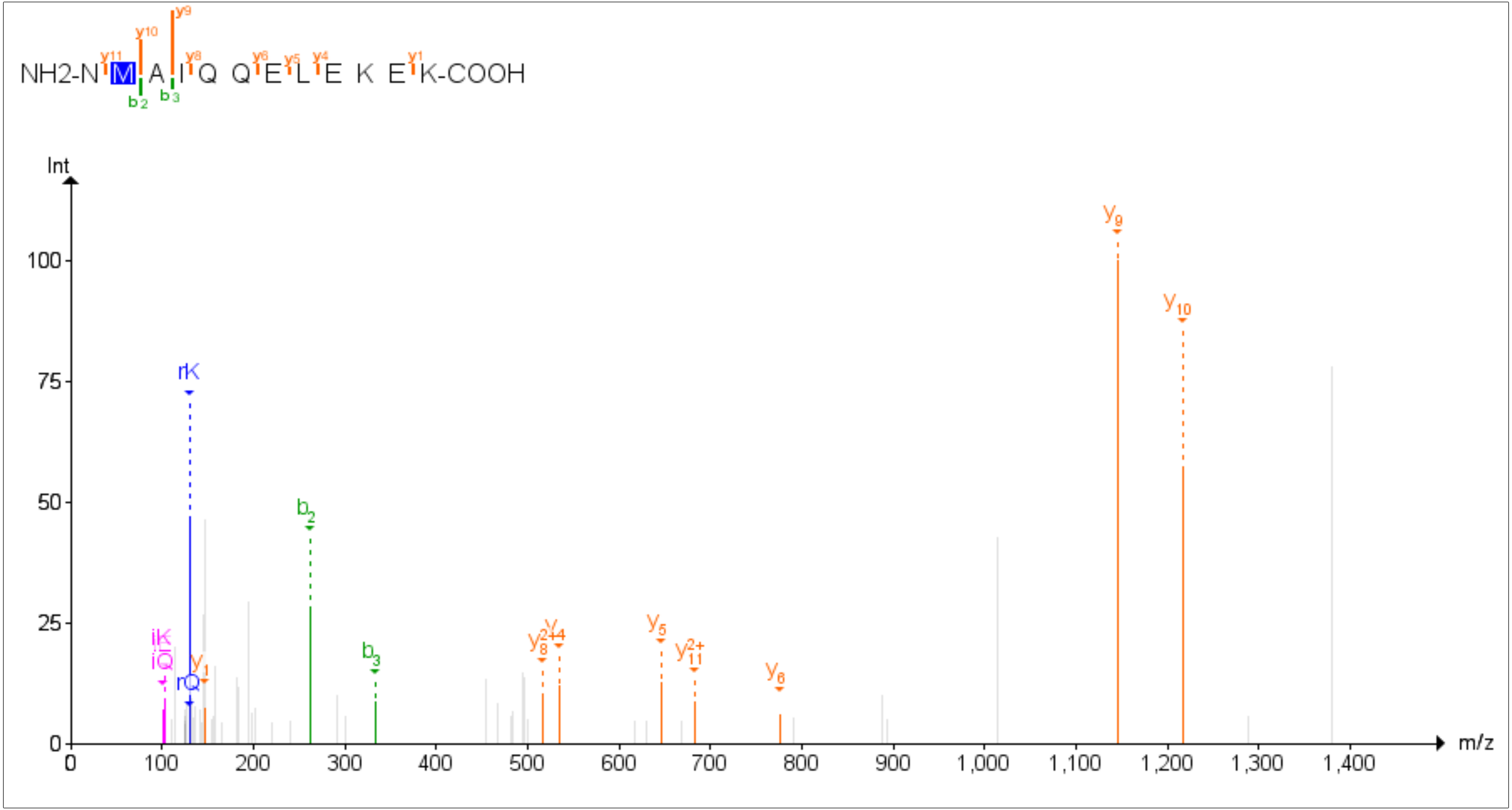

NH2-N M A I Q Q E L E K E K-COOH
y11
y10
y9
y8
y6
y5
y4
y1
b2
b3
Int
m/z
rK
rQ
iK
iQ
b2
b3
y1
y9
y10
y5
y6

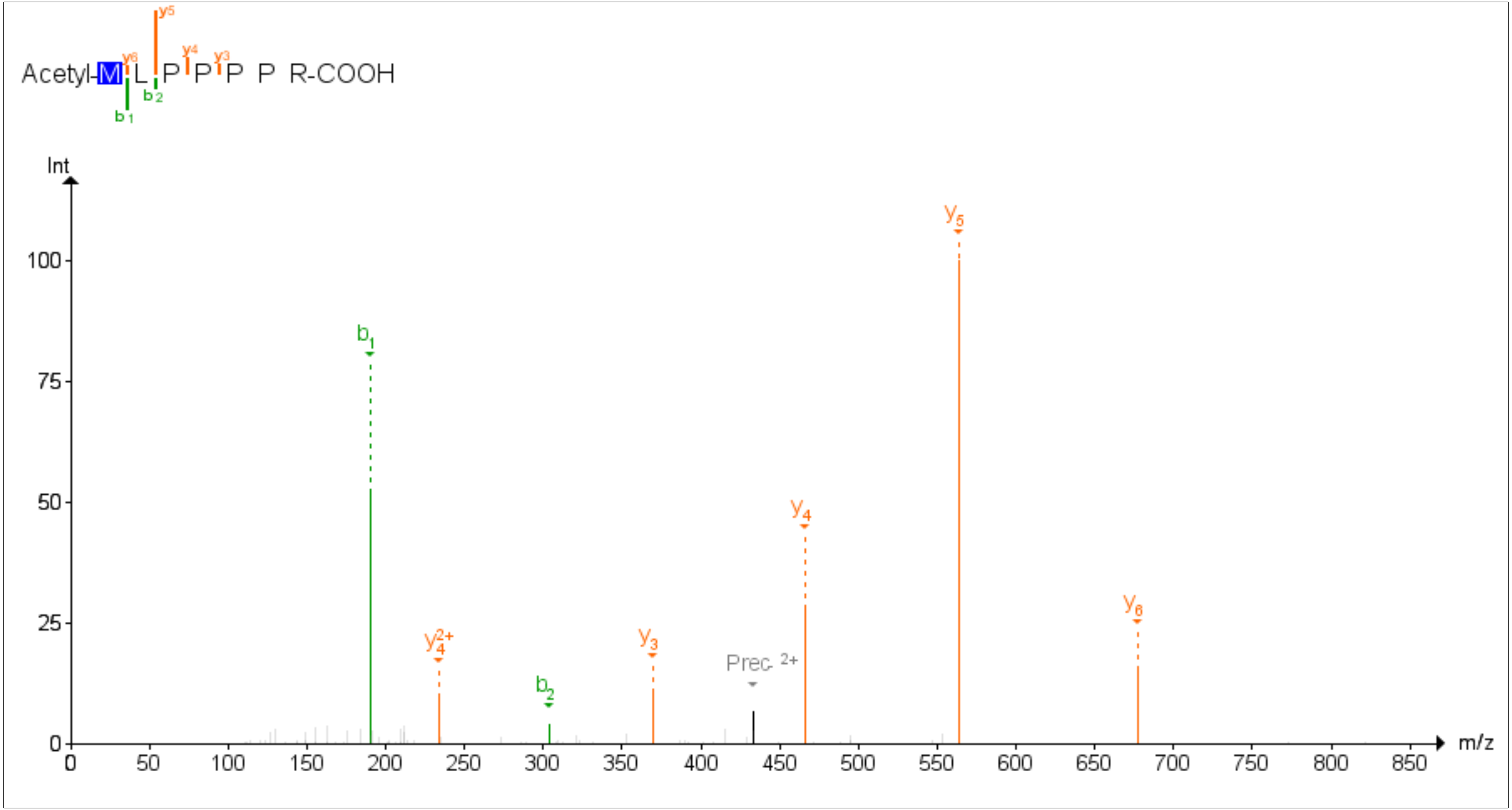

Acetyl-M L P P P P R-COOH
y5
y6
y4
y3
b 1
b 2
Int
100
75
50
25
0
y5
b1
y4
y6
y4 2+
b2
y3
Prec- 2+
0
50
100
150
200
250
300
350
400
450
500
550
600
650
700
750
800
850
m/z

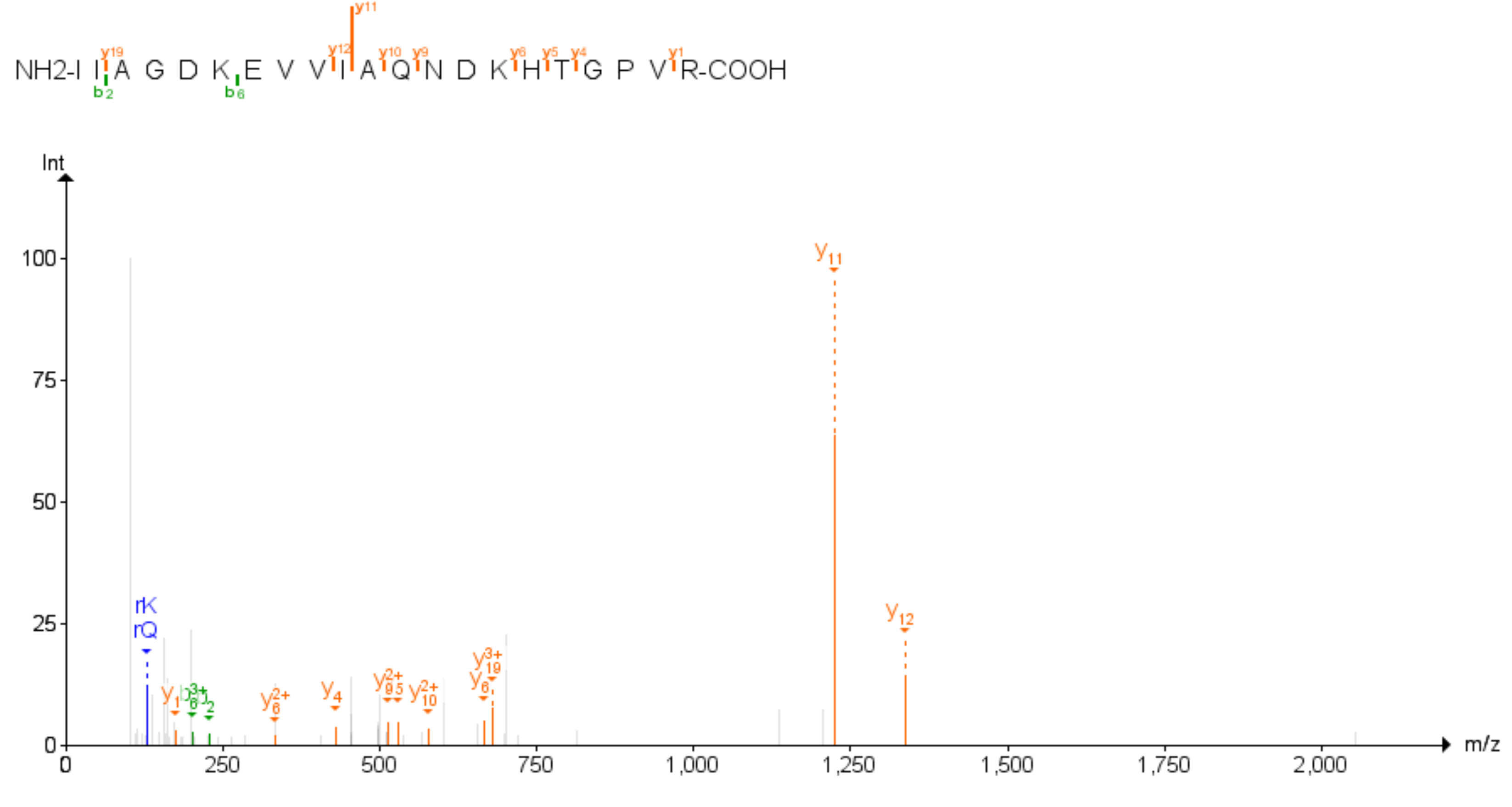

NH2-I I A G D K E V V I A Q N D K H T G P V R-COOH
y19
y12
y11
y10
y9
y6
y5
y4
y1
b2
b6
Int
100
75
50
25
0
rK
rQ
y_1
y_4
y_6^{2+}
y_{10}^{2+}
y_{19}^{3+}
y_6
y_{11}
y_{12}
0
250
500
750
1,000
1,250
1,500
1,750
2,000
m/z

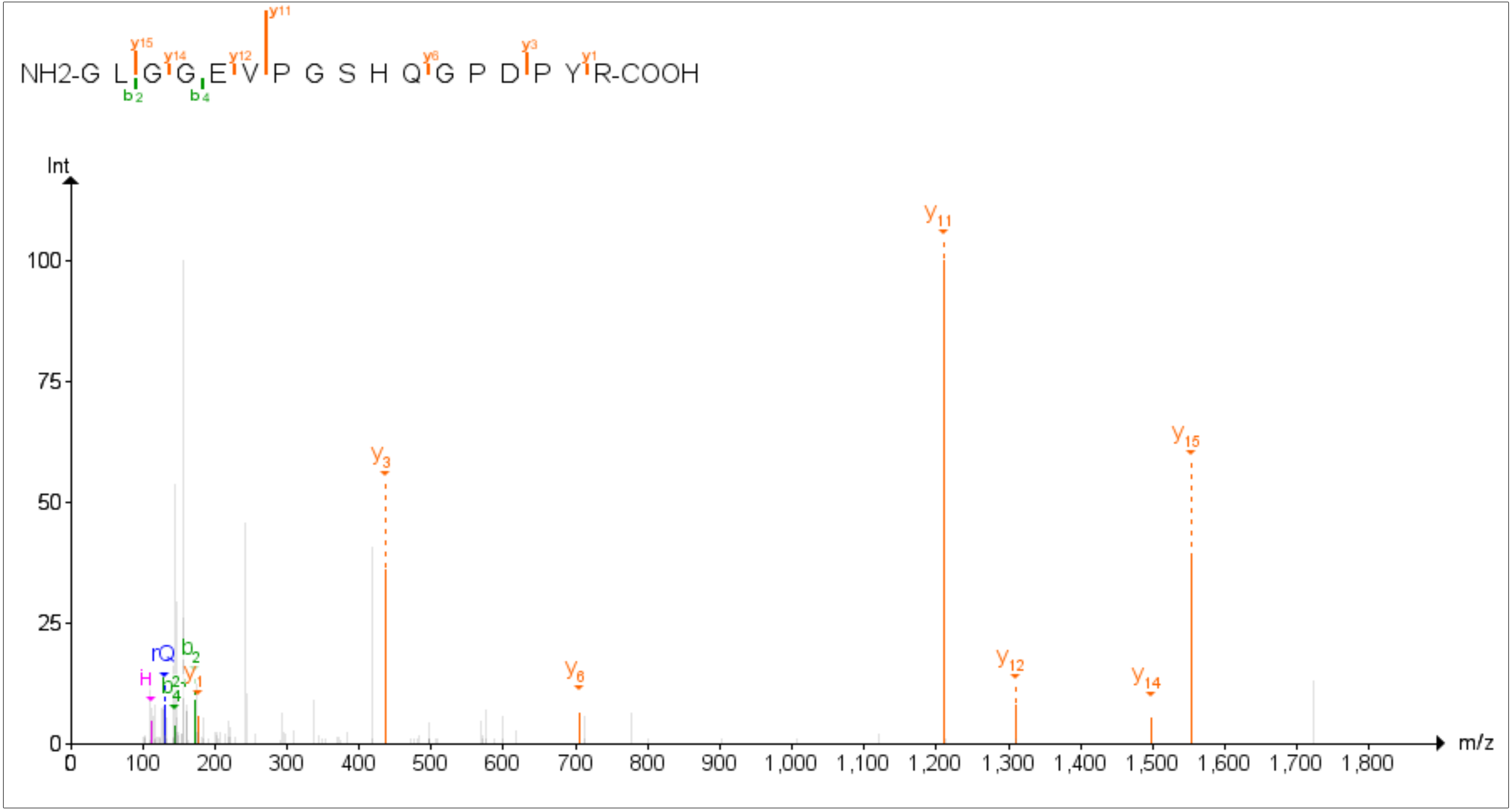

NH2-G L G G E V P G S H Q G P D P Y R-COOH
y15
y14
y12
y11
y6
y3
y1
b2
b4
Int
100
75
50
25
0
m/z
0
100
200
300
400
500
600
700
800
900
1,000
1,100
1,200
1,300
1,400
1,500
1,600
1,700
1,800
y11
y15
y3
y6
y12
y14
rQ
b2
H
b4
y1

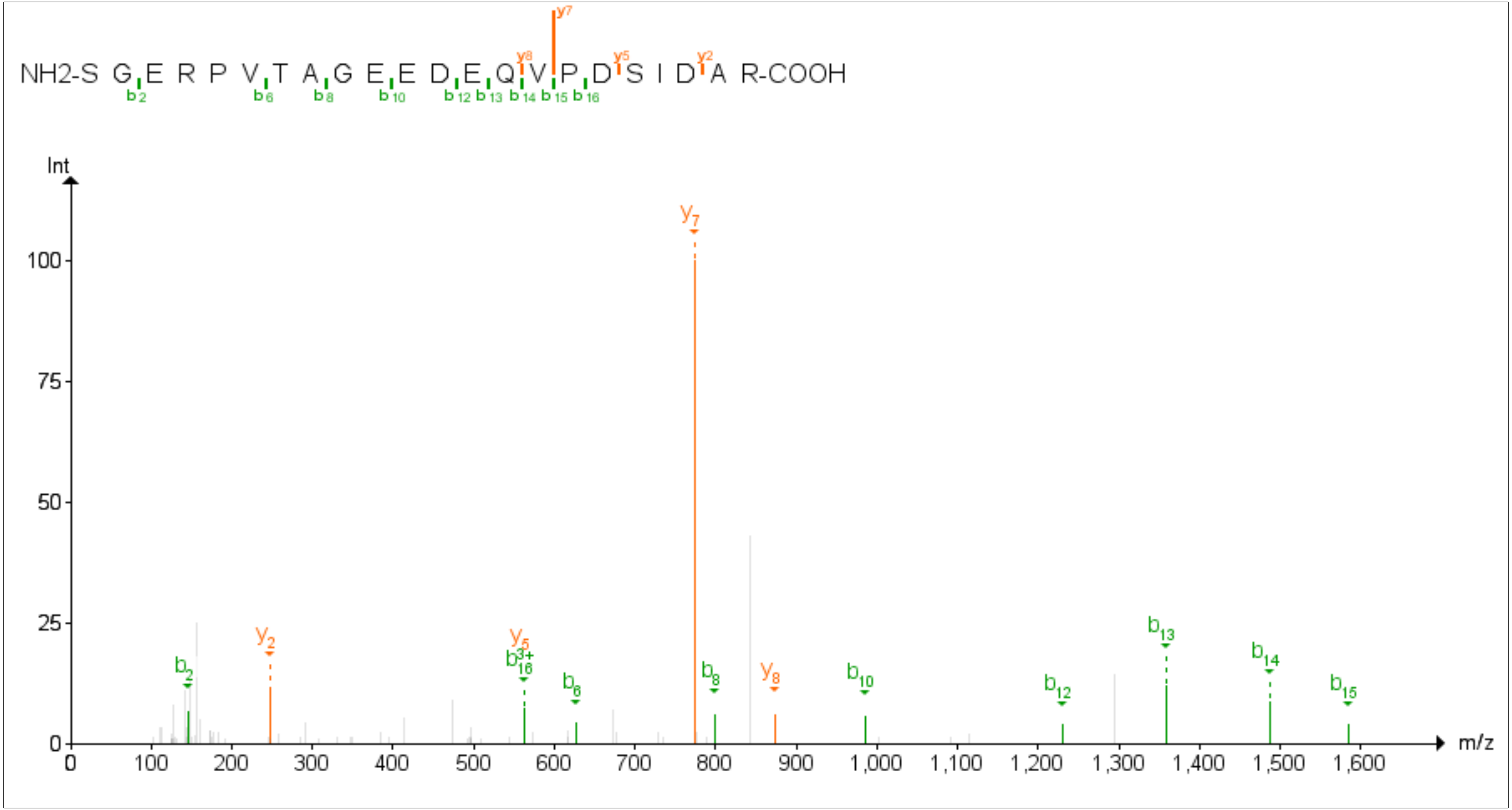

NH2-S G E R P V T A G E E D E Q V P D S I D A R-COOH
b2 b6 b8 b10 b12 b13 b14 b15 b16
y8 y7 y5 y2
Int
m/z
0
25
50
75
100
0 100 200 300 400 500 600 700 800 900 1,000 1,100 1,200 1,300 1,400 1,500 1,600
y7
y2
y5
b16 3+
b2
b6
b8
y8
b10
b12
b13
b14
b15

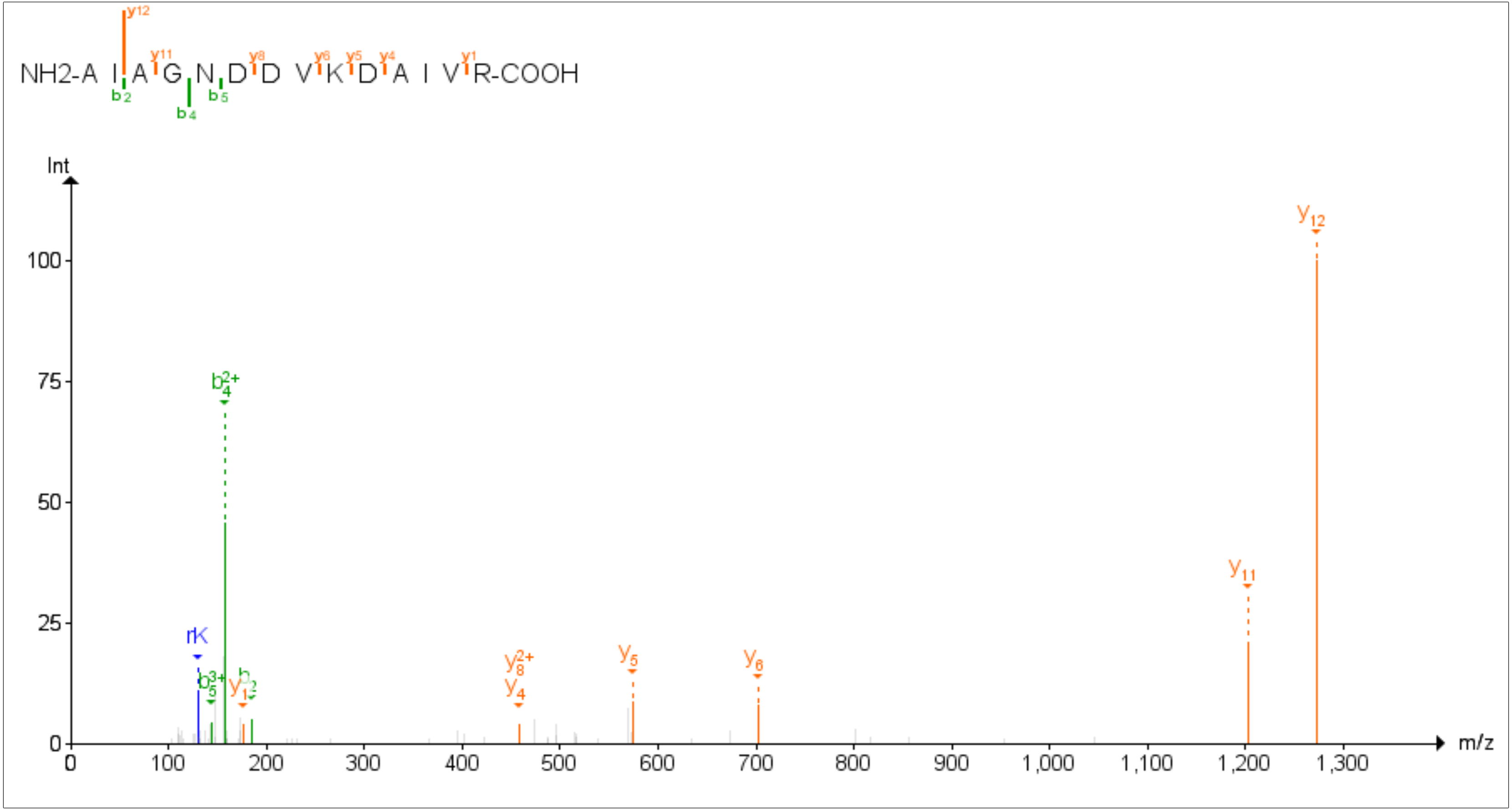

NH2-A I A G N D D V K D A I V R-COOH
y12
y11
y8
y6
y5
y4
y1
b2
b4
b5
Int
100
75
50
25
0
b_4^{2+}
rK
b_5^{3+}
y_1
b_2
y_8^{2+}
y_4
y_5
y_6
y_{11}
y_{12}
0
100
200
300
400
500
600
700
800
900
1,000
1,100
1,200
1,300
m/z

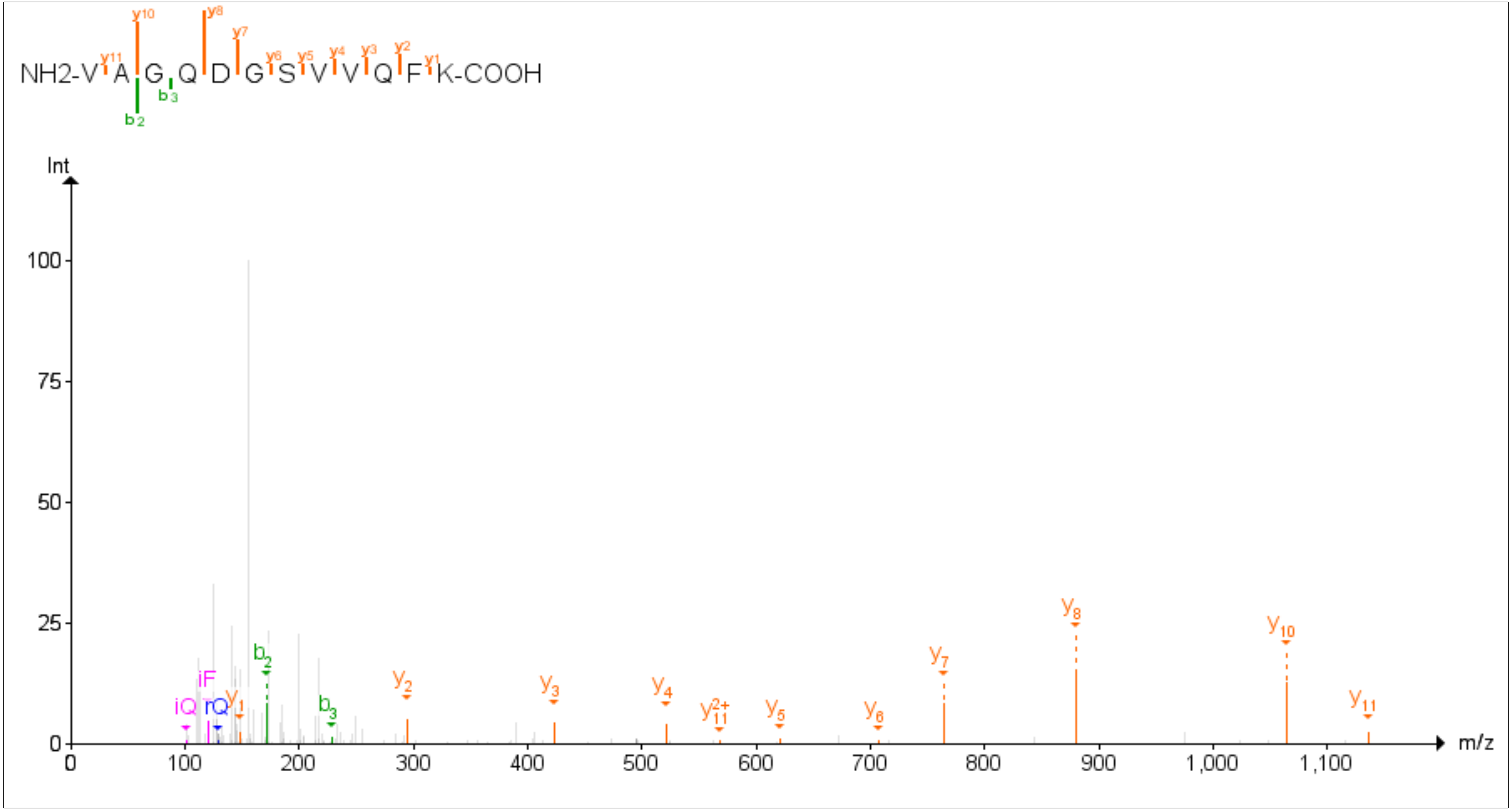

NH2-V A G Q D G S V V Q F K-COOH
y11 y10 y8 y7 y6 y5 y4 y3 y2 y1
b2 b3
Int
100
75
50
25
0
m/z
0 100 200 300 400 500 600 700 800 900 1,000 1,100
iQ iF rQ y1 b2 b3 y2 y3 y4 y11 2+ y5 y6 y7 y8 y10 y11

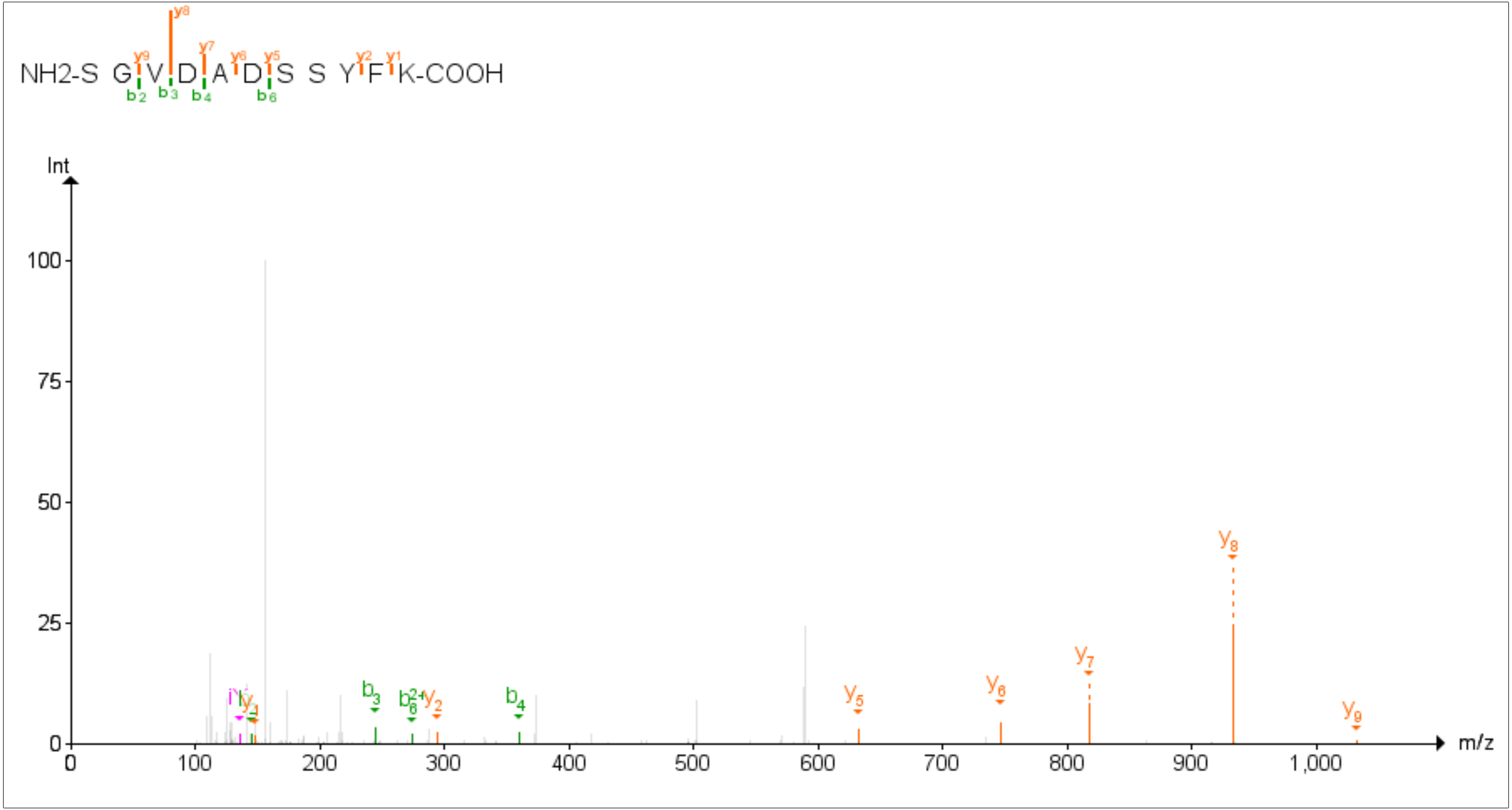

NH2-S G V D A D S S Y F K-COOH
y9 y8 y7 y6 y5 y2 y1
b2 b3 b4 b6
Int
100
75
50
25
0
y8
y7
y6
y5
y9
b3
b4
y2
m/z
0
100
200
300
400
500
600
700
800
900
1,000

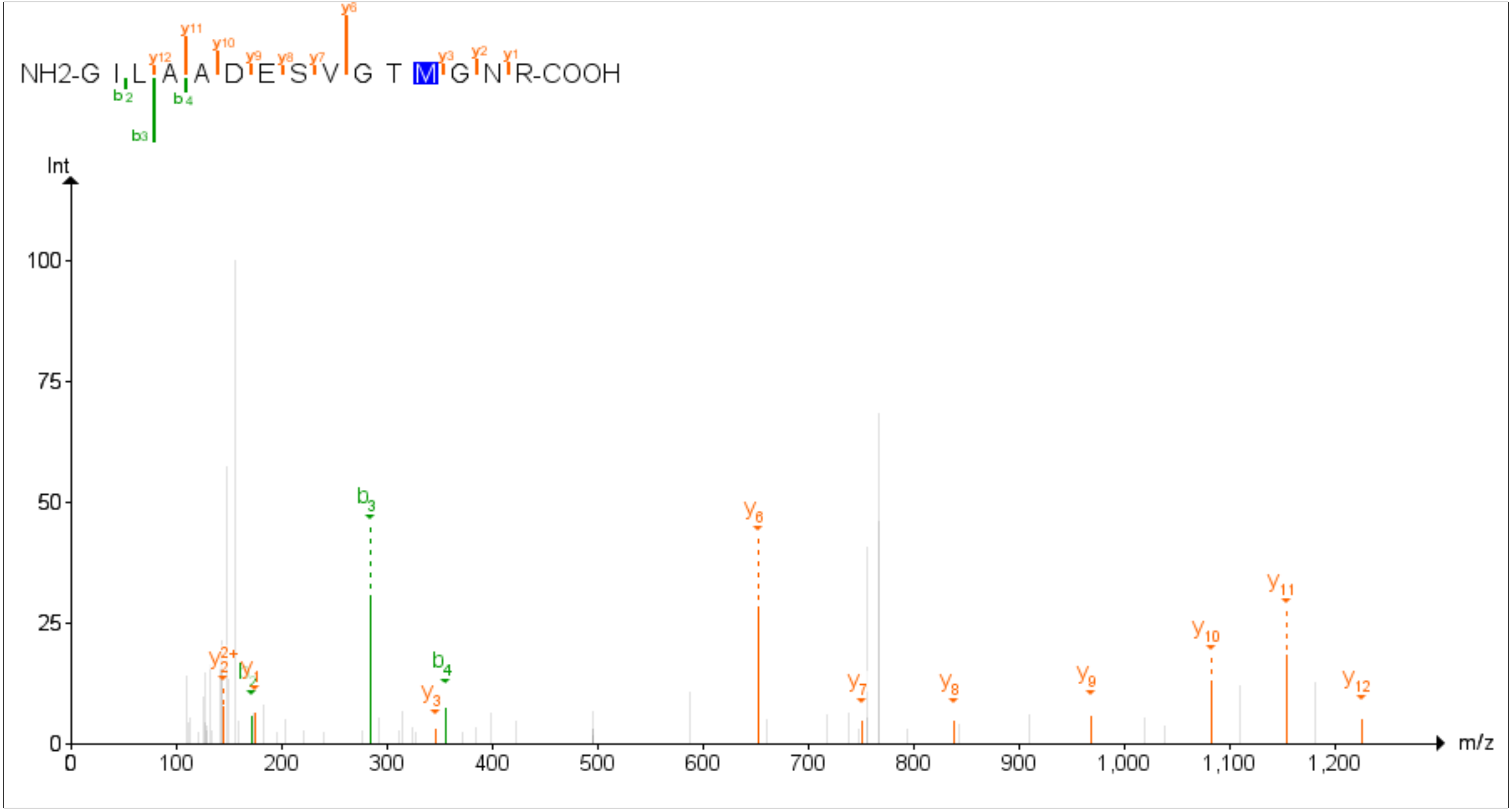

NH2-G I L A A D E S V G T M G N R-COOH
b2
b3
b4
y12
y11
y10
y9
y8
y7
y6
y3
y2
y1
Int
100
75
50
25
0
m/z
0
100
200
300
400
500
600
700
800
900
1,000
1,100
1,200

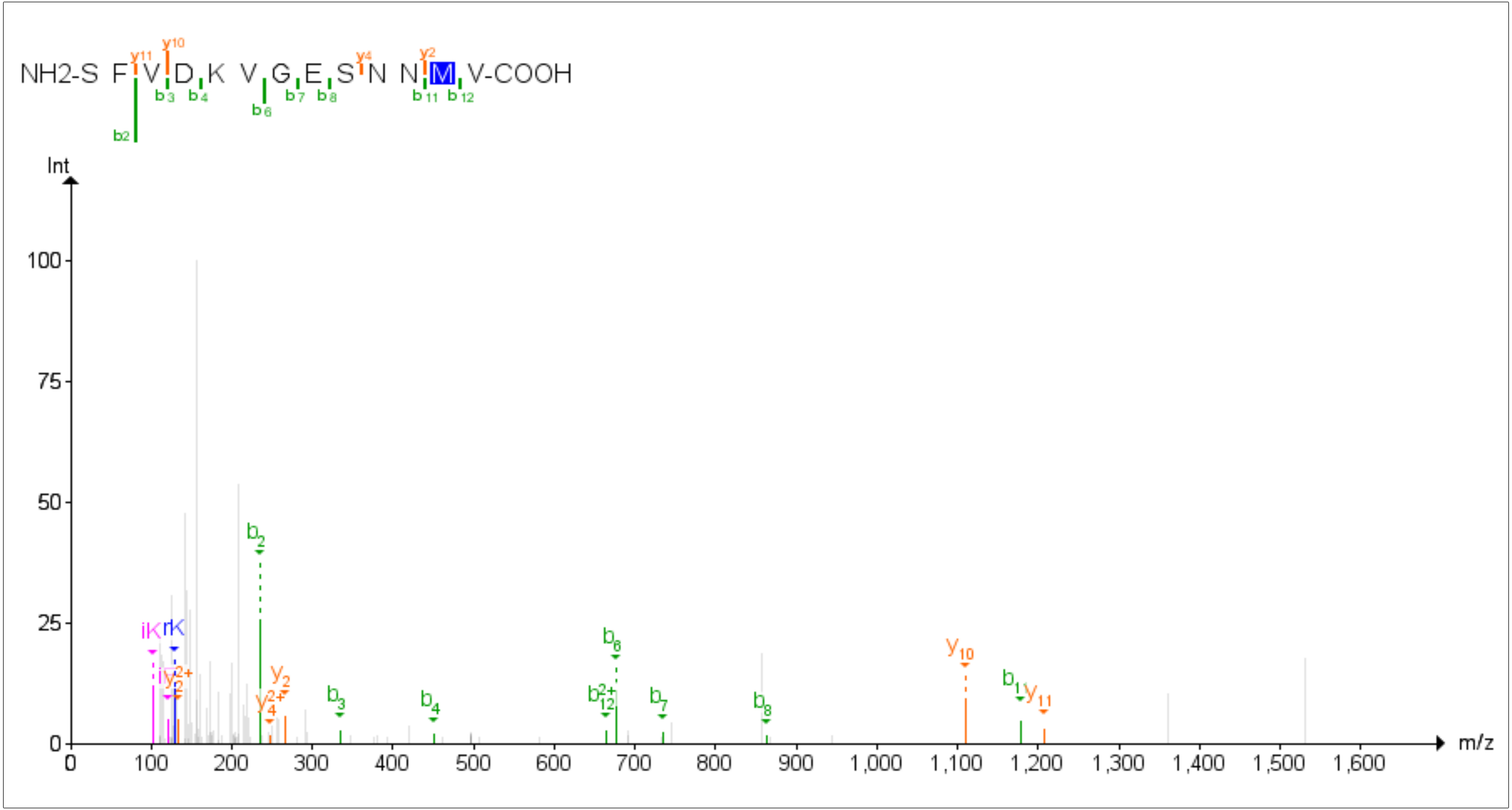

NH2-S F V D K V G E S N N M V-COOH
y11 y10 y4 y2
b2 b3 b4 b6 b7 b8 b11 b12
Int
100
75
50
25
0
m/z
0 100 200 300 400 500 600 700 800 900 1,000 1,100 1,200 1,300 1,400 1,500 1,600
iK rK
b2 y2 b3 b4 b6 b7 b8 y10 b11 y11

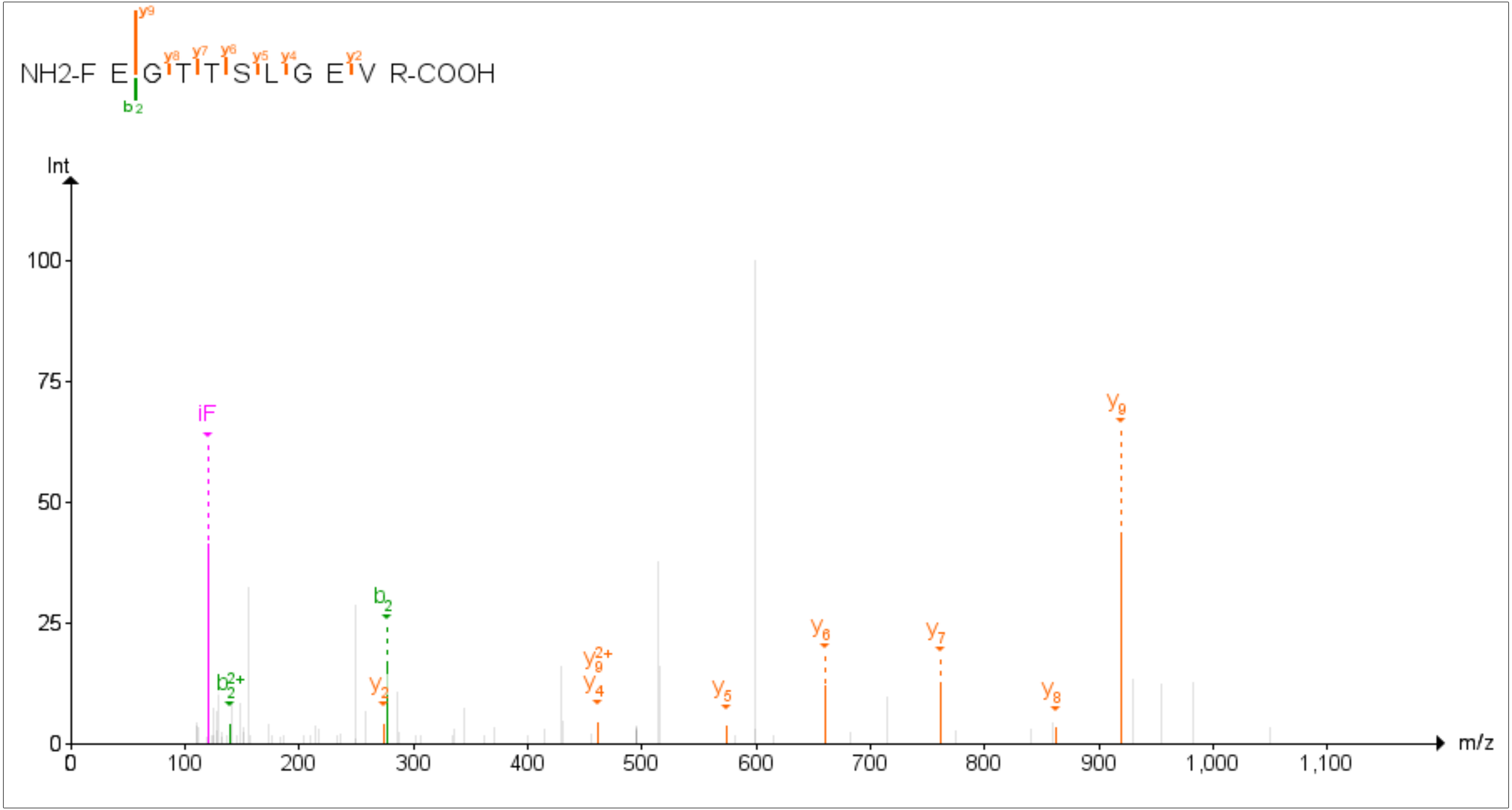
NH2-F E G T T S L G E V R-COOH
y9
y8
y7
y6
y5
y4
y2
b2
Int
100
75
50
25
0
iF
b2
b2 2+
y2
y9 2+
y4
y5
y6
y7
y8
y9
0
100
200
300
400
500
600
700
800
900
1,000
1,100
m/z

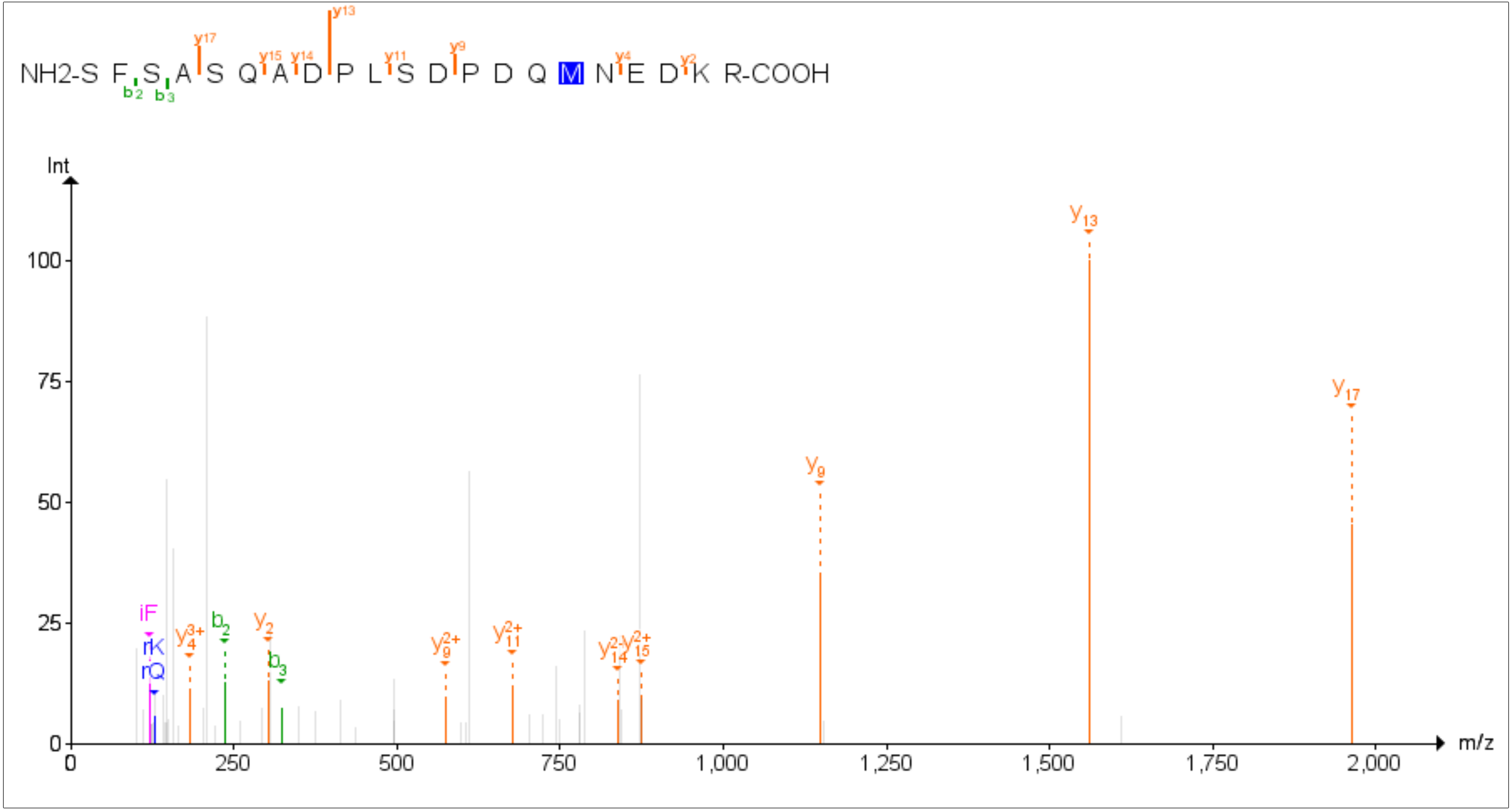

NH2-S F S A S Q A D P L S D P D Q M N E D K R-COOH
b2
b3
y17
y15
y14
y13
y11
y9
y4
y2
Int
100
75
50
25
0
iF
rK
rQ
y4 3+
b2
y2
b3
y9 2+
y11 2+
y14 2+
y15 2+
y9
y13
y17
0
250
500
750
1,000
1,250
1,500
1,750
2,000
m/z

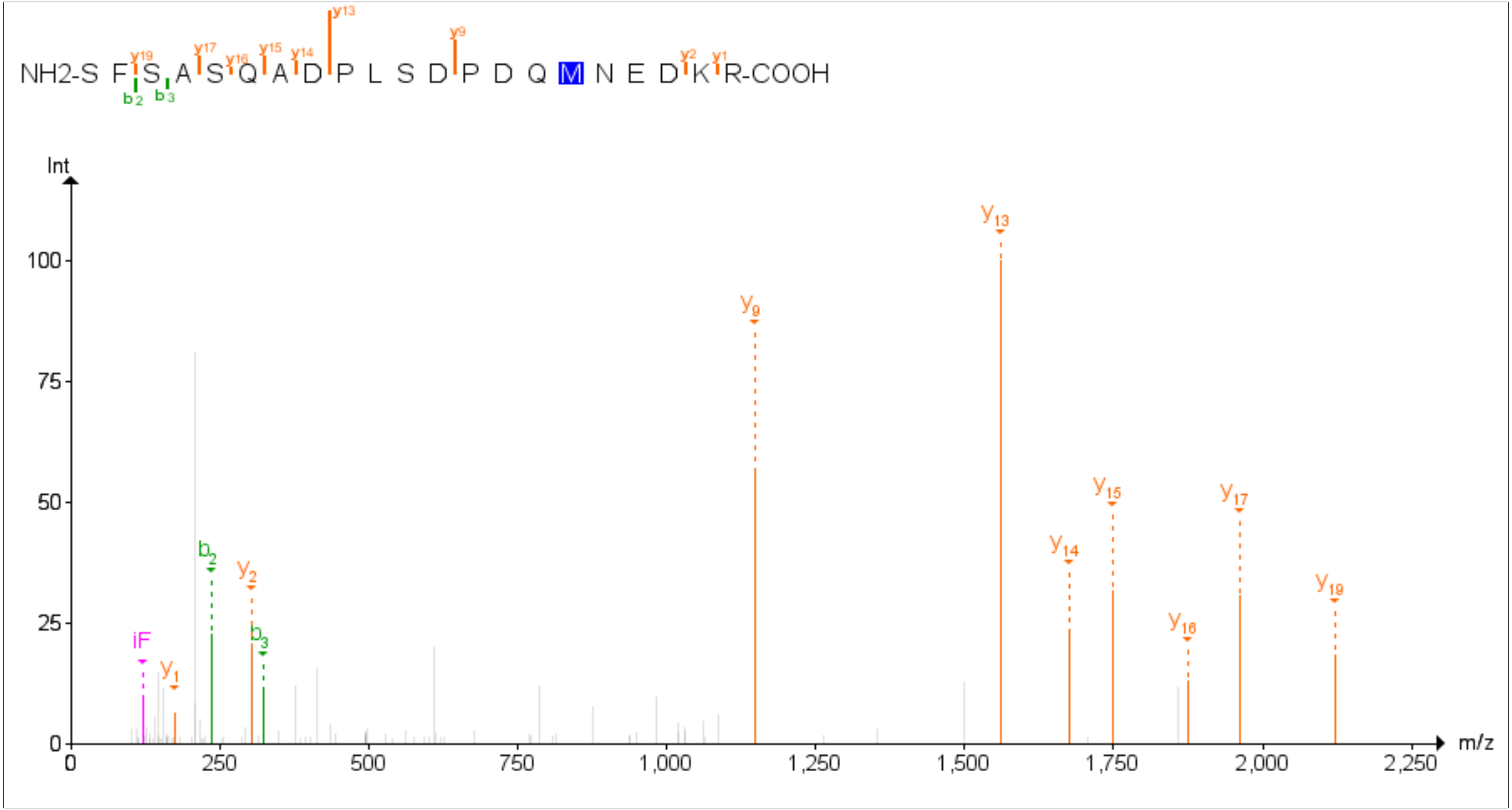

NH2-S F S A S Q A D P L S D P D Q M N E D K R-COOH
y19 y17 y16 y15 y14 y13 y9 y2 y1
b2 b3
Int
100
75
50
25
0
0 250 500 750 1,000 1,250 1,500 1,750 2,000 2,250
m/z
iF y1 b2 y2 b3 y9 y13 y14 y15 y16 y17 y19

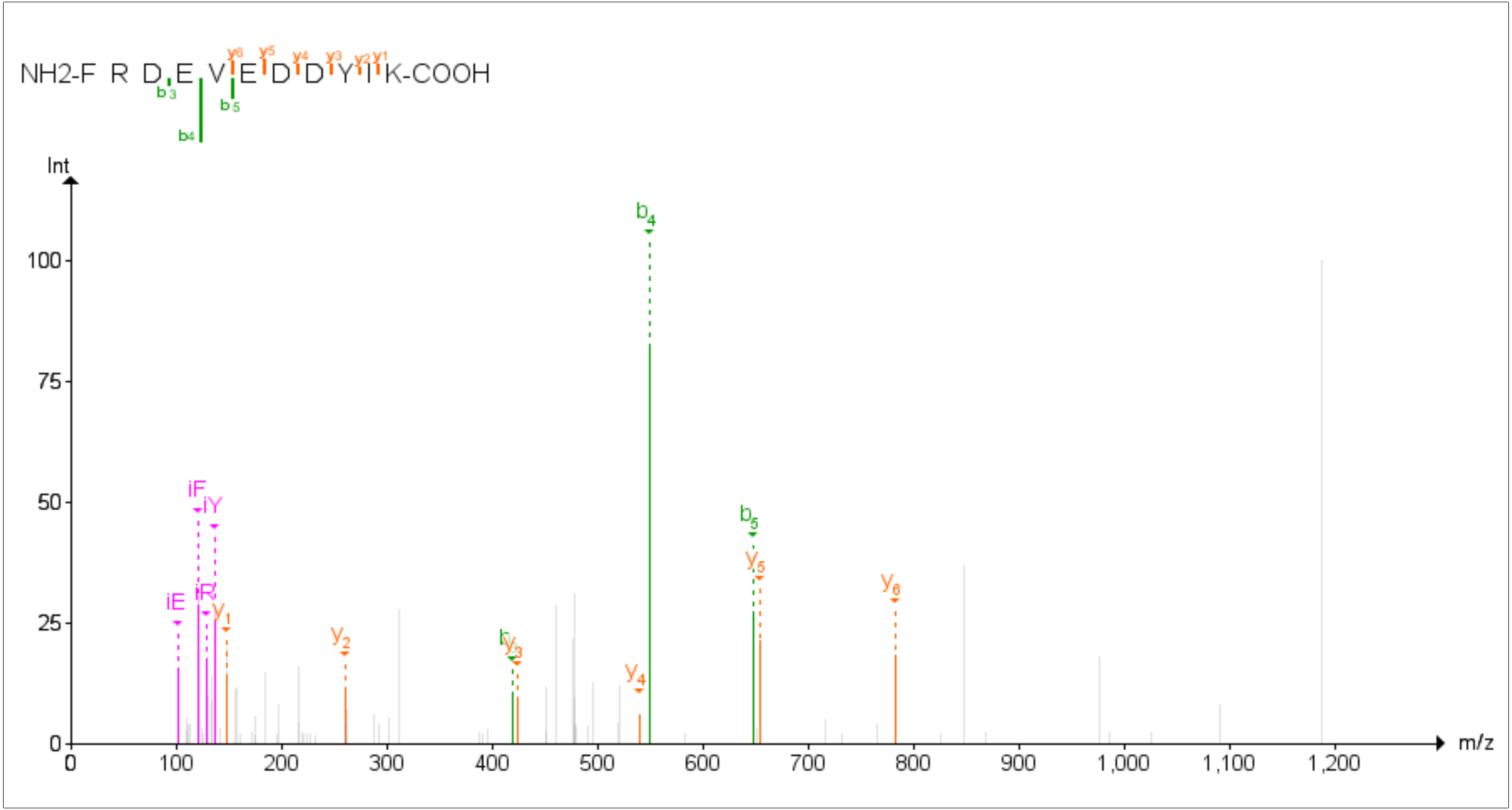
NH2-F R D E V E D D Y I K-COOH
b3
b4
b5
y6 y5 y4 y3 y2 y1
Int
m/z
b4
b5
y5
y6
iF
iY
iE
iR
y1
y2
y3
y4
0
25
50
75
100
0
100
200
300
400
500
600
700
800
900
1,000
1,100
1,200

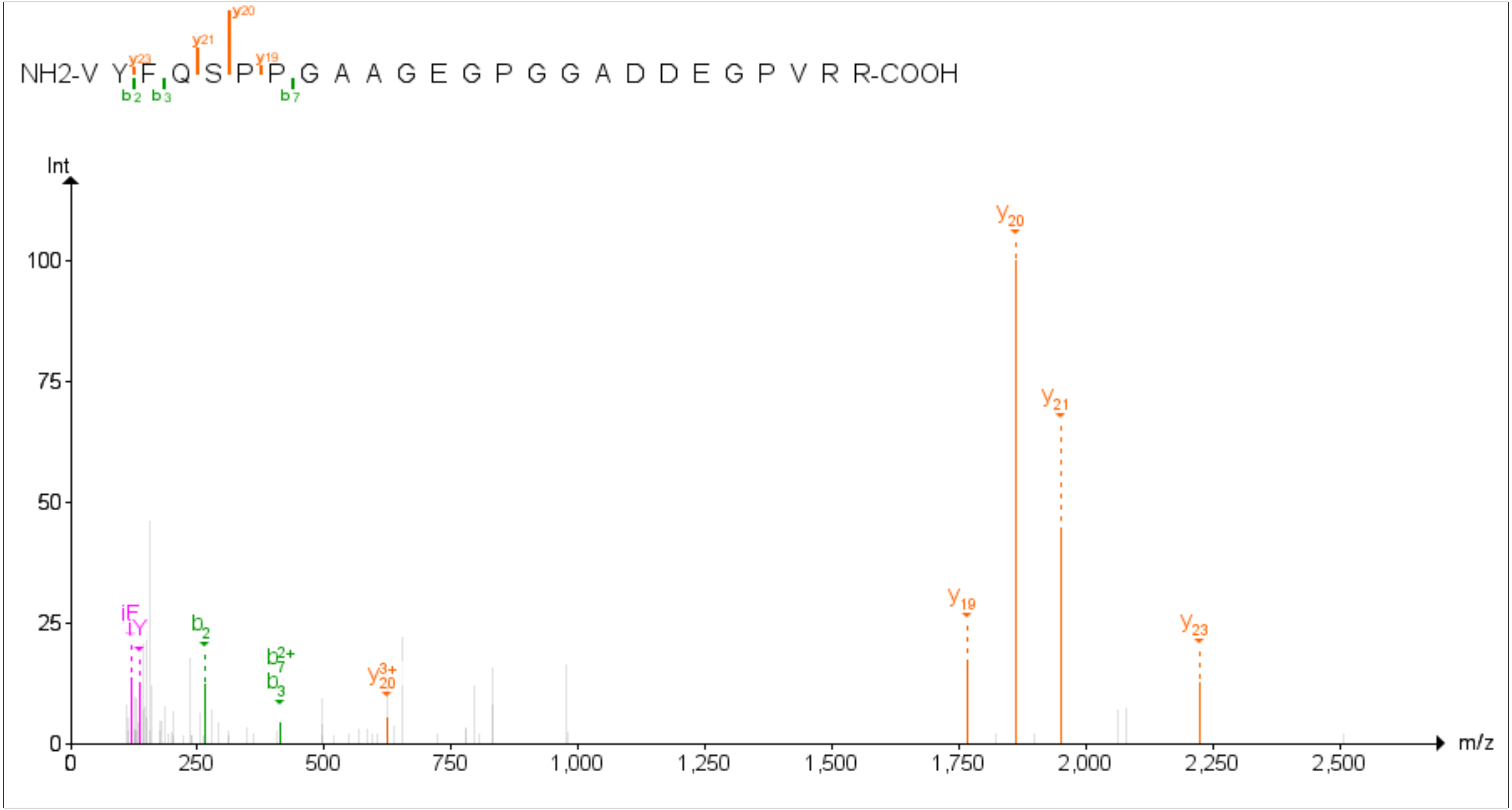

NH2-V Y F Q S P P G A A G E G P G G A D D E G P V R R-COOH
y23 y21 y20 y19
b2 b3 b7
Int
100
75
50
25
0
iF
iY
b2
b7 2+
b3
y20 3+
y19
y20
y21
y23
0
250
500
750
1,000
1,250
1,500
1,750
2,000
2,250
2,500
m/z

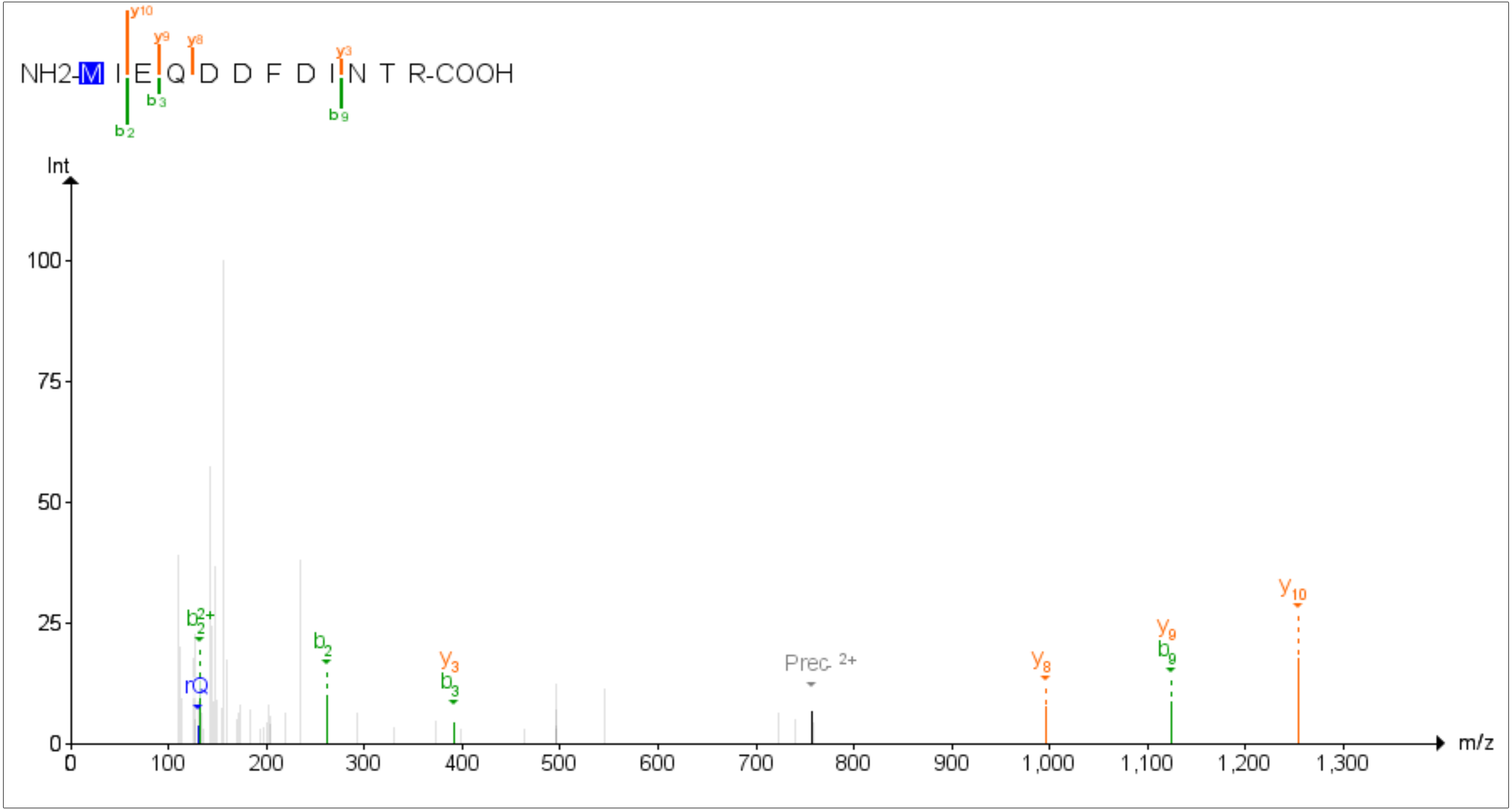
NH2-M I E Q D D F D I N T R-COOH
y10
y9
y8
y3
b2
b3
b9
Int
100
75
50
25
0
b2 2+
rQ
b2
y3
b3
Prec 2+
y8
y9
b9
y10
0
100
200
300
400
500
600
700
800
900
1,000
1,100
1,200
1,300
m/z

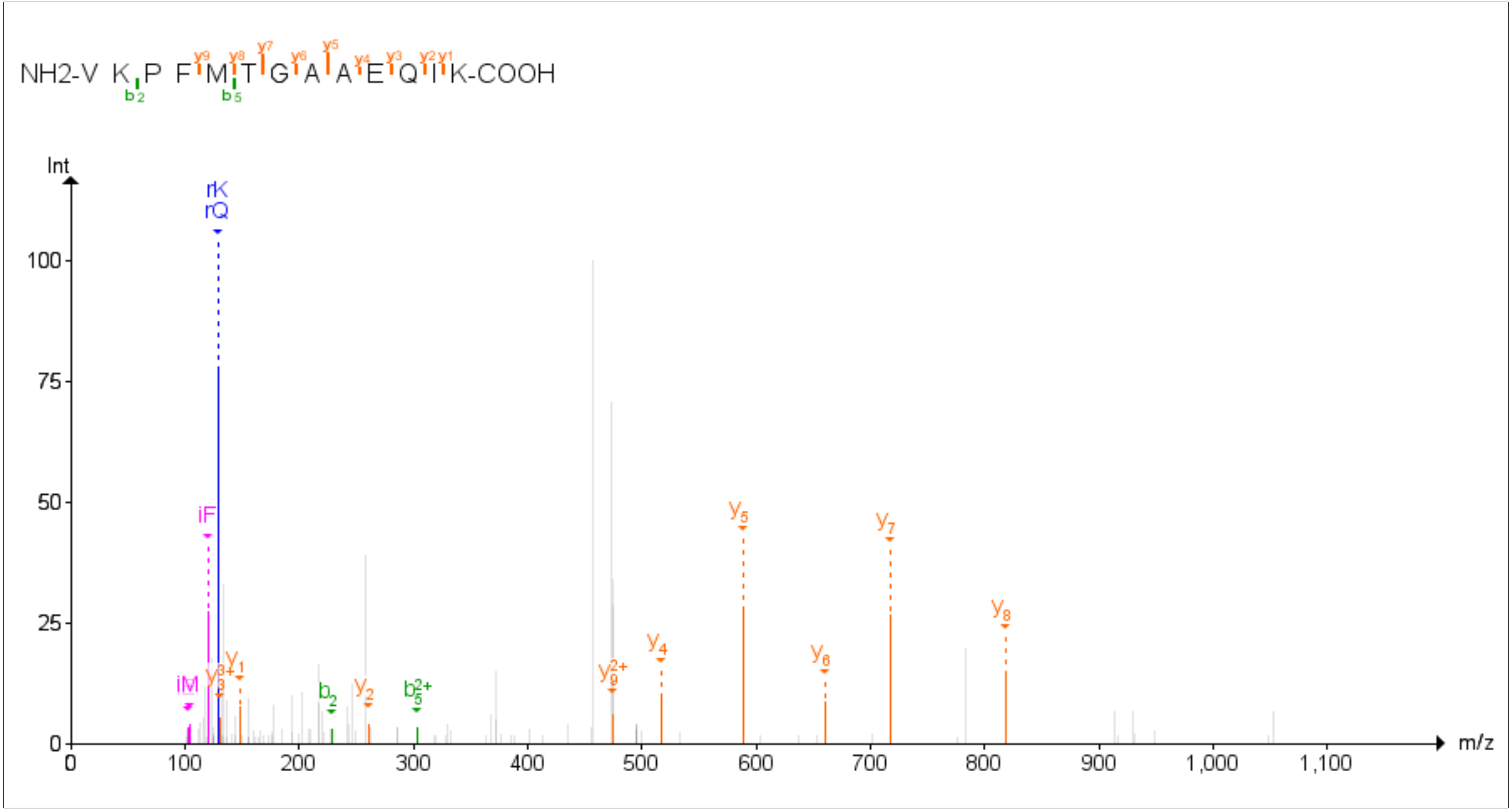
NH2-V K P F M T G A A E Q I K-COOH
y9 y8 y7 y6 y5 y4 y3 y2 y1
b2 b5
Int
m/z
rK
rQ
iF
iM
y1
b2
y2
b5 2+
y9 2+
y4
y5
y6
y7
y8
0
25
50
75
100
0
100
200
300
400
500
600
700
800
900
1,000
1,100

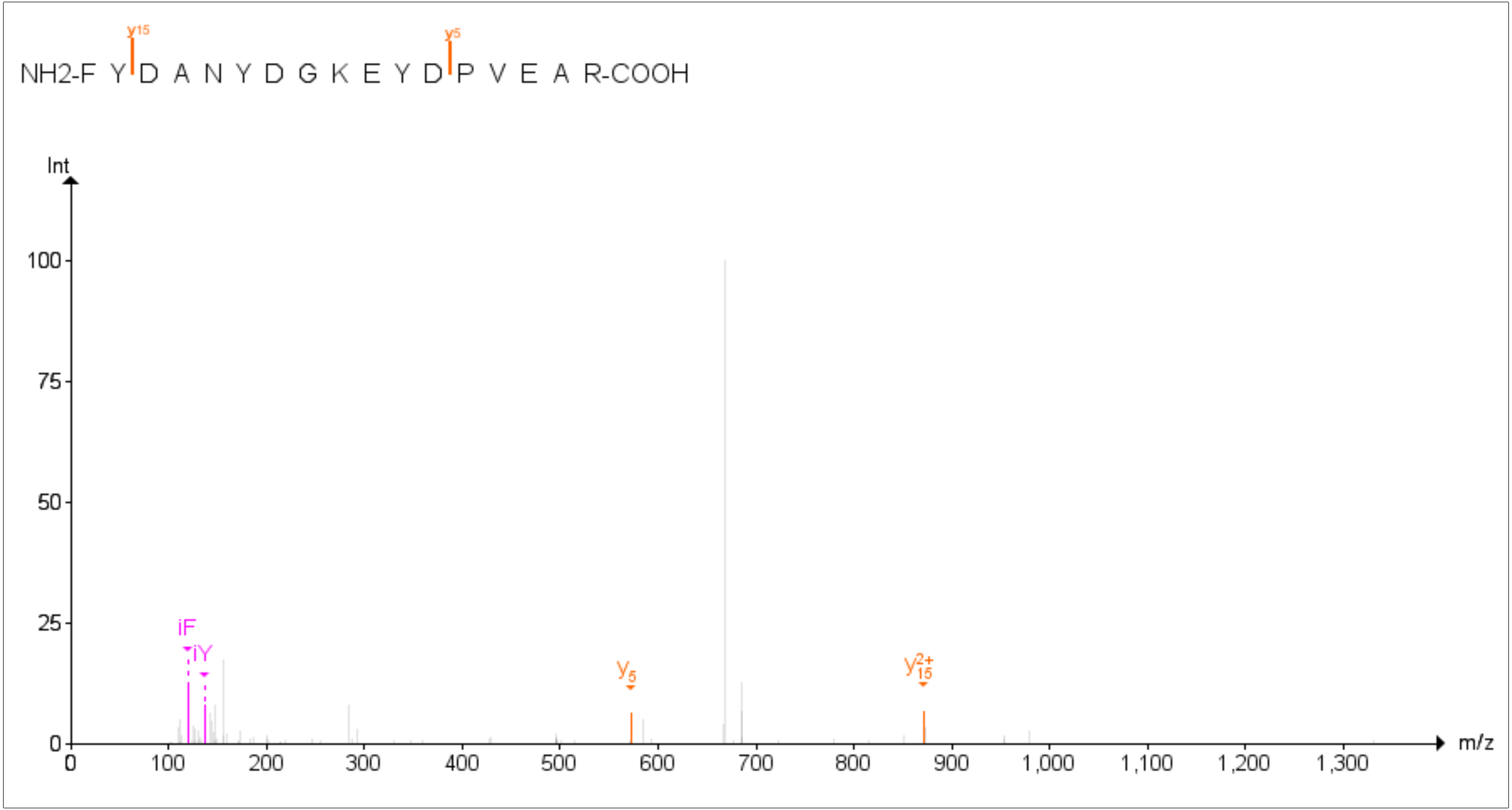

NH2-F Y D A N Y D G K E Y D P V E A R-COOH
y15
y5
Int
100
75
50
25
0
iF
iY
y5
y15 2+
0
100
200
300
400
500
600
700
800
900
1,000
1,100
1,200
1,300
m/z

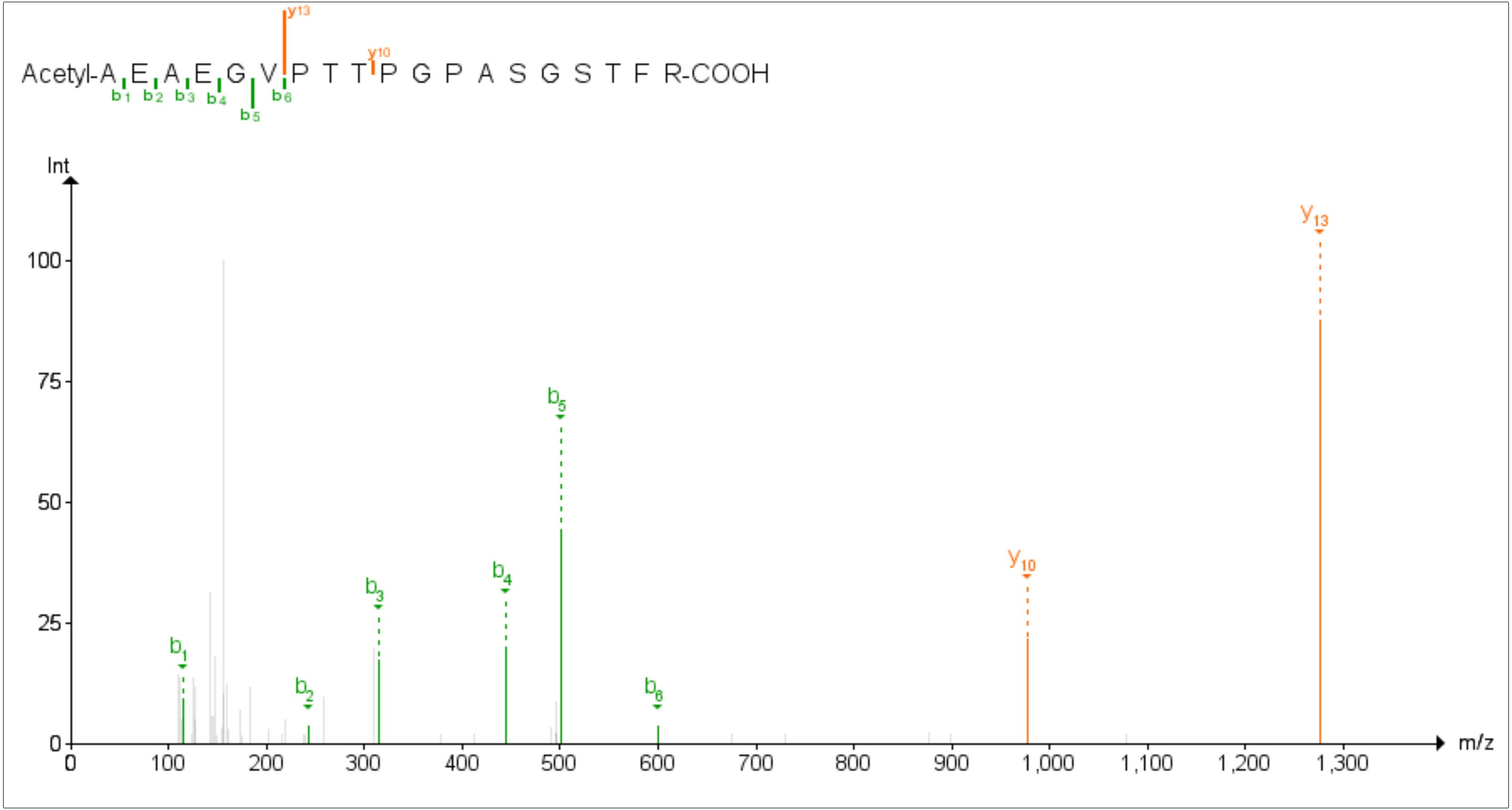

Acetyl-A E A E G V P T T P G P A S G S T F R-COOH
b1 b2 b3 b4 b5 b6
y13 y10
Int
100
75
50
25
0
0 100 200 300 400 500 600 700 800 900 1,000 1,100 1,200 1,300
m/z
b1 b2 b3 b4 b5 b6 y10 y13

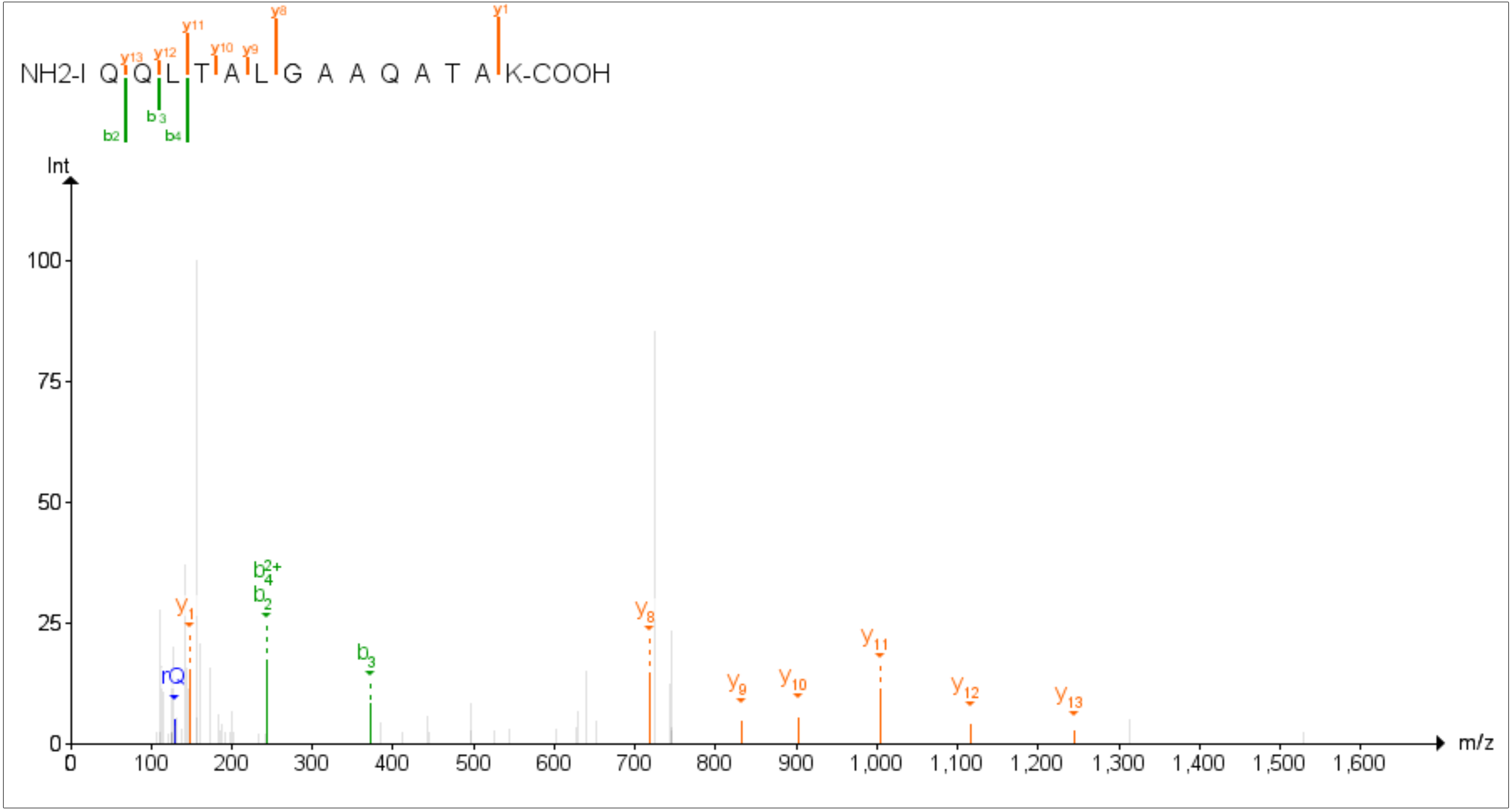

NH2-I Q Q L T A L G A A Q A T A K-COOH
y13 y12 y11 y10 y9 y8 y1
b2 b3 b4
Int
m/z
rQ
y_1
b_4^{2+}
b_2
b_3
y_8
y_9
y_{10}
y_{11}
y_{12}
y_{13}
0 25 50 75 100
0 100 200 300 400 500 600 700 800 900 1,000 1,100 1,200 1,300 1,400 1,500 1,600

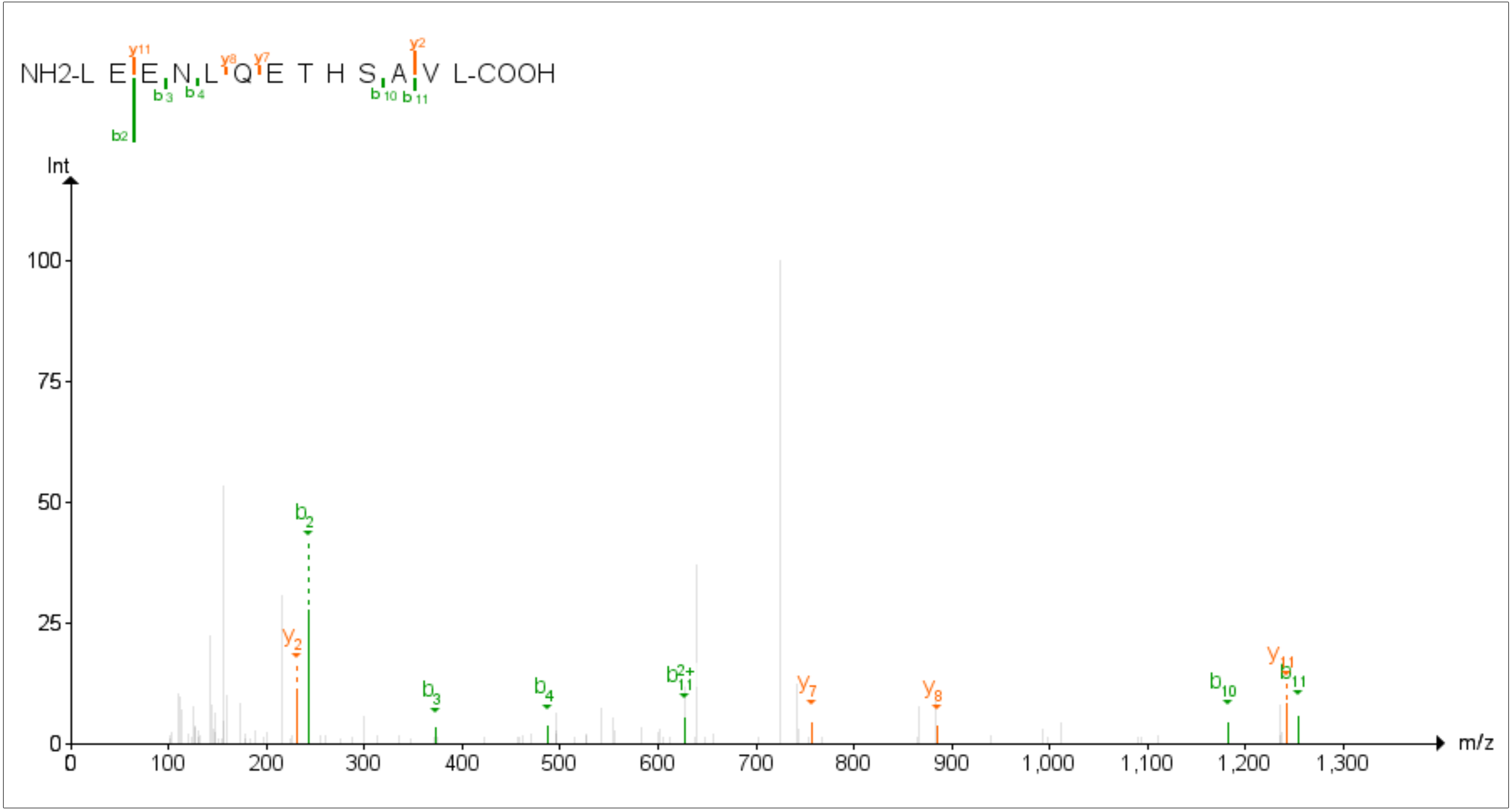

NH2-L E E N L Q E T H S A V L-COOH
y11 y8 y7 y2
b2 b3 b4 b10 b11
Int
100
75
50
25
0
b2
y2
b3
b4
b11 2+
y7
y8
b10
y11
b11
0
100
200
300
400
500
600
700
800
900
1,000
1,100
1,200
1,300
m/z

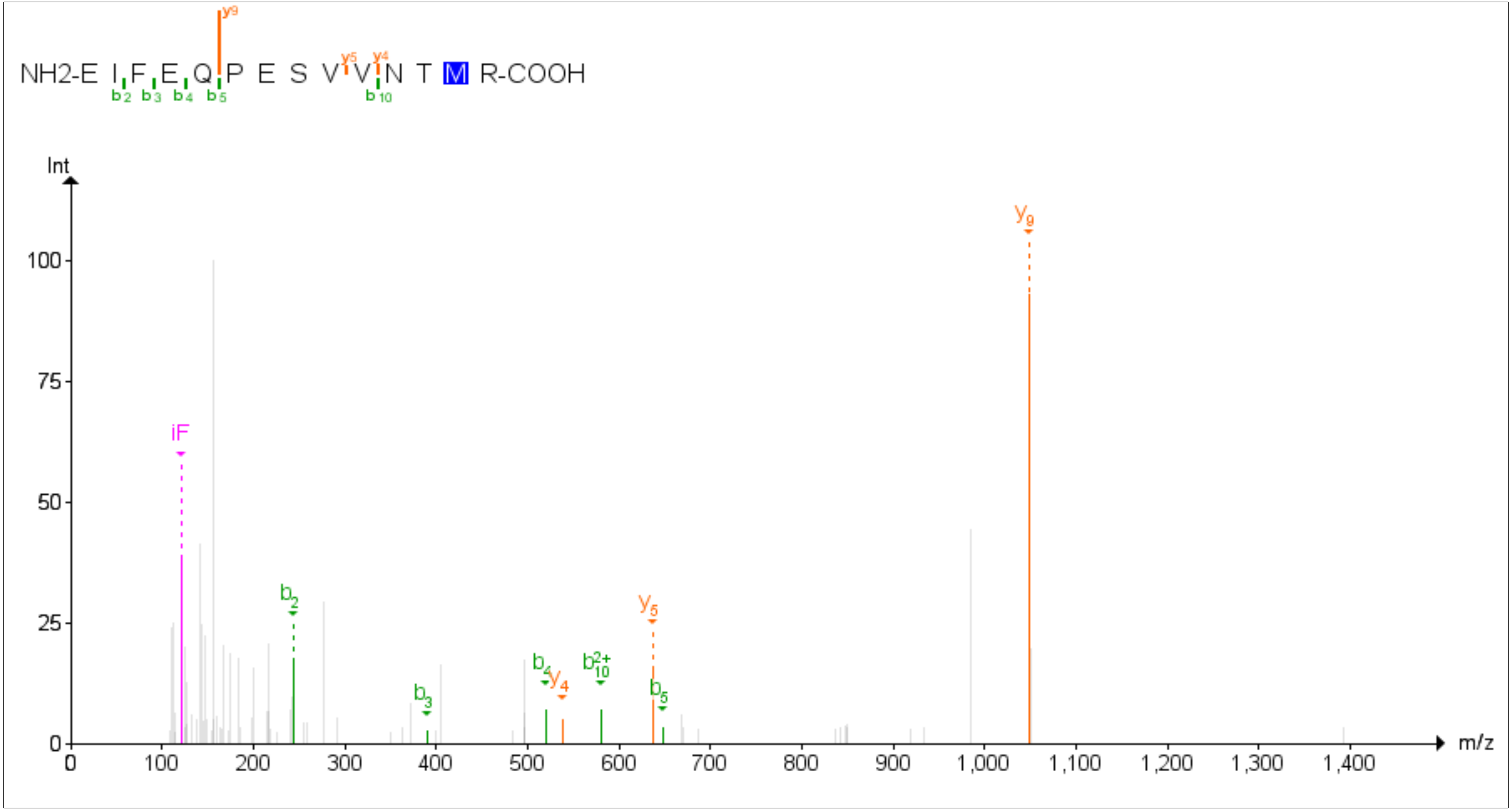
NH2-E I F E Q P E S V V N T M R-COOH
y9
y5
y4
b2
b3
b4
b5
b10
Int
iF
y9
b2
b3
b4
y4
b10 2+
y5
b5
m/z
0
25
50
75
100
0
100
200
300
400
500
600
700
800
900
1,000
1,100
1,200
1,300
1,400

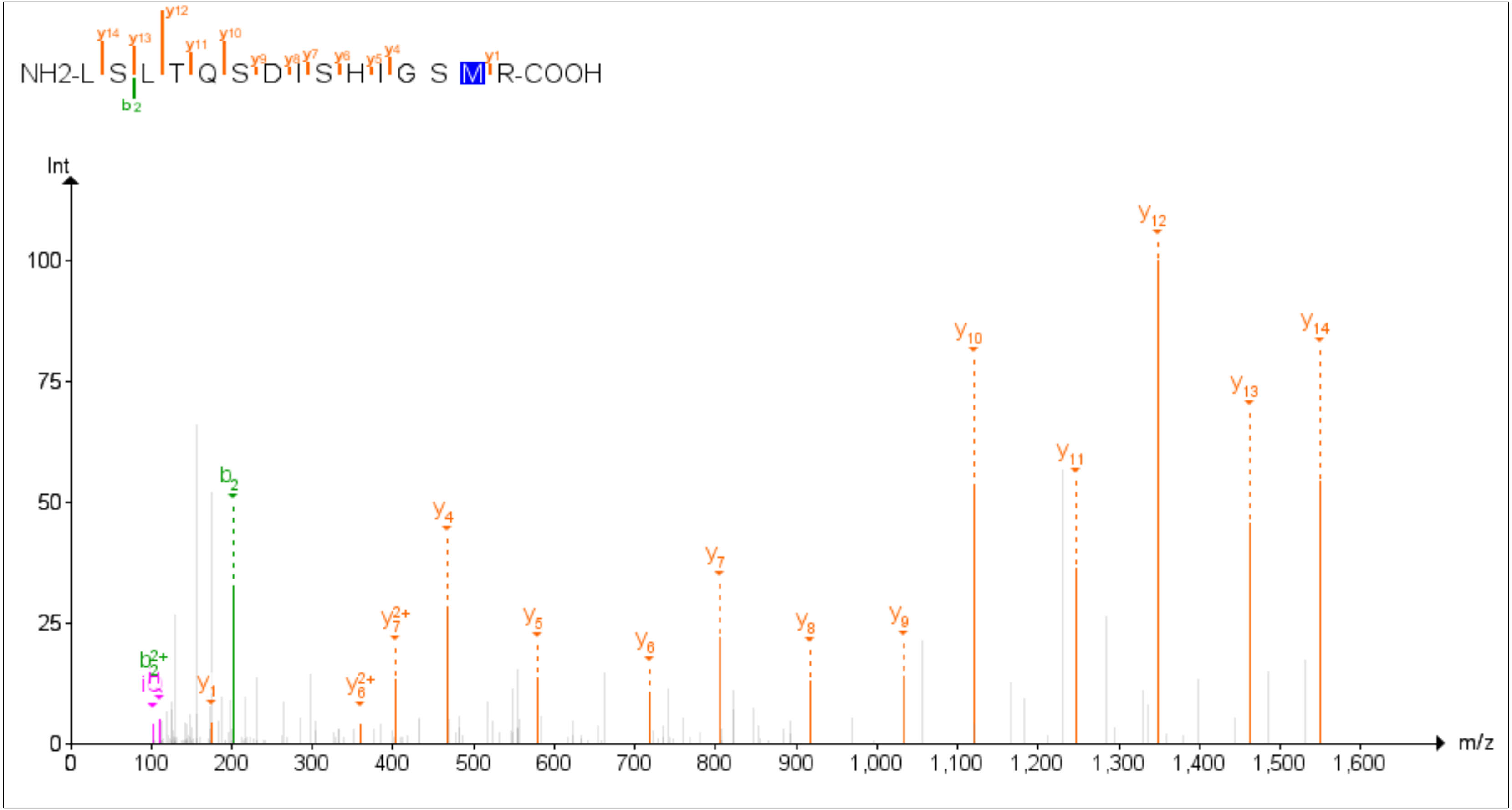

NH2-L S L T Q S D I S H I G S M R-COOH
y14 y13 y12 y11 y10 y9 y8 y7 y6 y5 y4 y1
b2
Int
100
75
50
25
0
m/z
0 100 200 300 400 500 600 700 800 900 1,000 1,100 1,200 1,300 1,400 1,500 1,600
y12
y10
y14
y13
y11
b2
y4
y7
y7 2+
y5
y6
y8
y9
y6 2+
y1
b2 2+

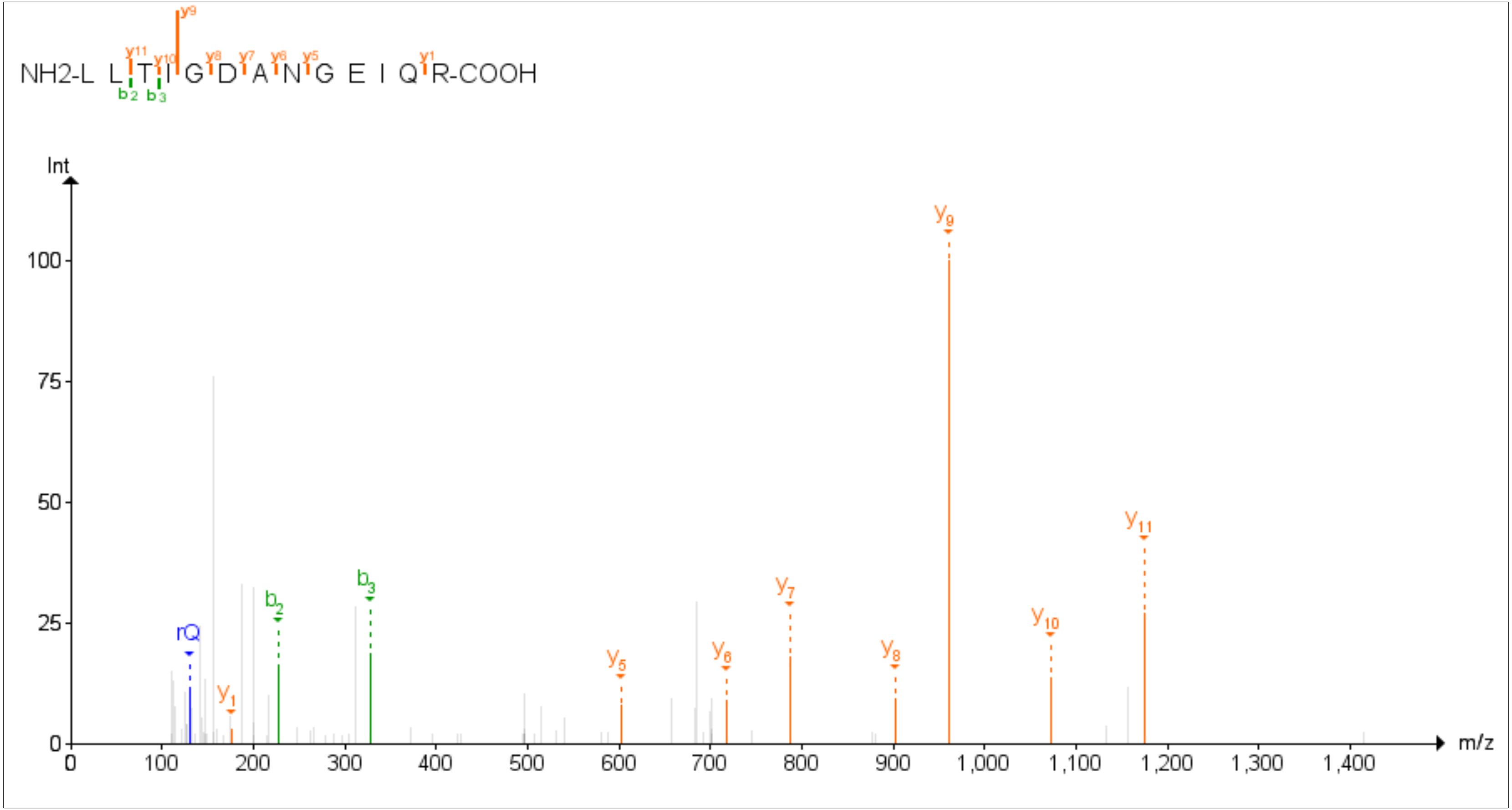

NH2-L L T I G D A N G E I Q R-COOH
y9
y11
y10
y8
y7
y6
y5
y1
b2
b3
Int
100
75
50
25
0
rQ
y1
b2
b3
y5
y6
y7
y8
y9
y10
y11
m/z
0
100
200
300
400
500
600
700
800
900
1,000
1,100
1,200
1,300
1,400

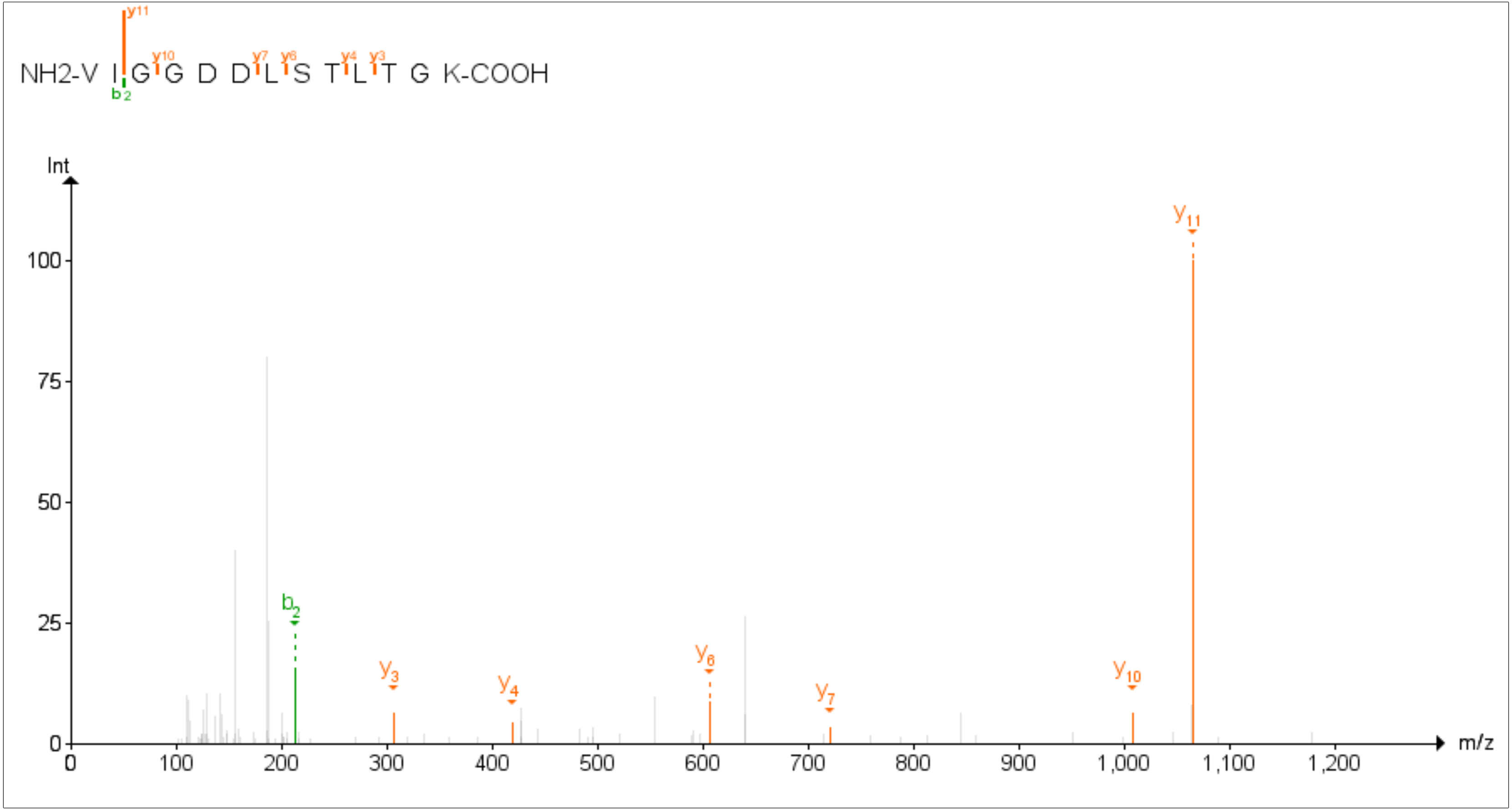

NH2-V I G G D D L S T L T G K-COOH
b2
y11
y10
y7
y6
y4
y3
Int
100
75
50
25
0
m/z
0
100
200
300
400
500
600
700
800
900
1,000
1,100
1,200

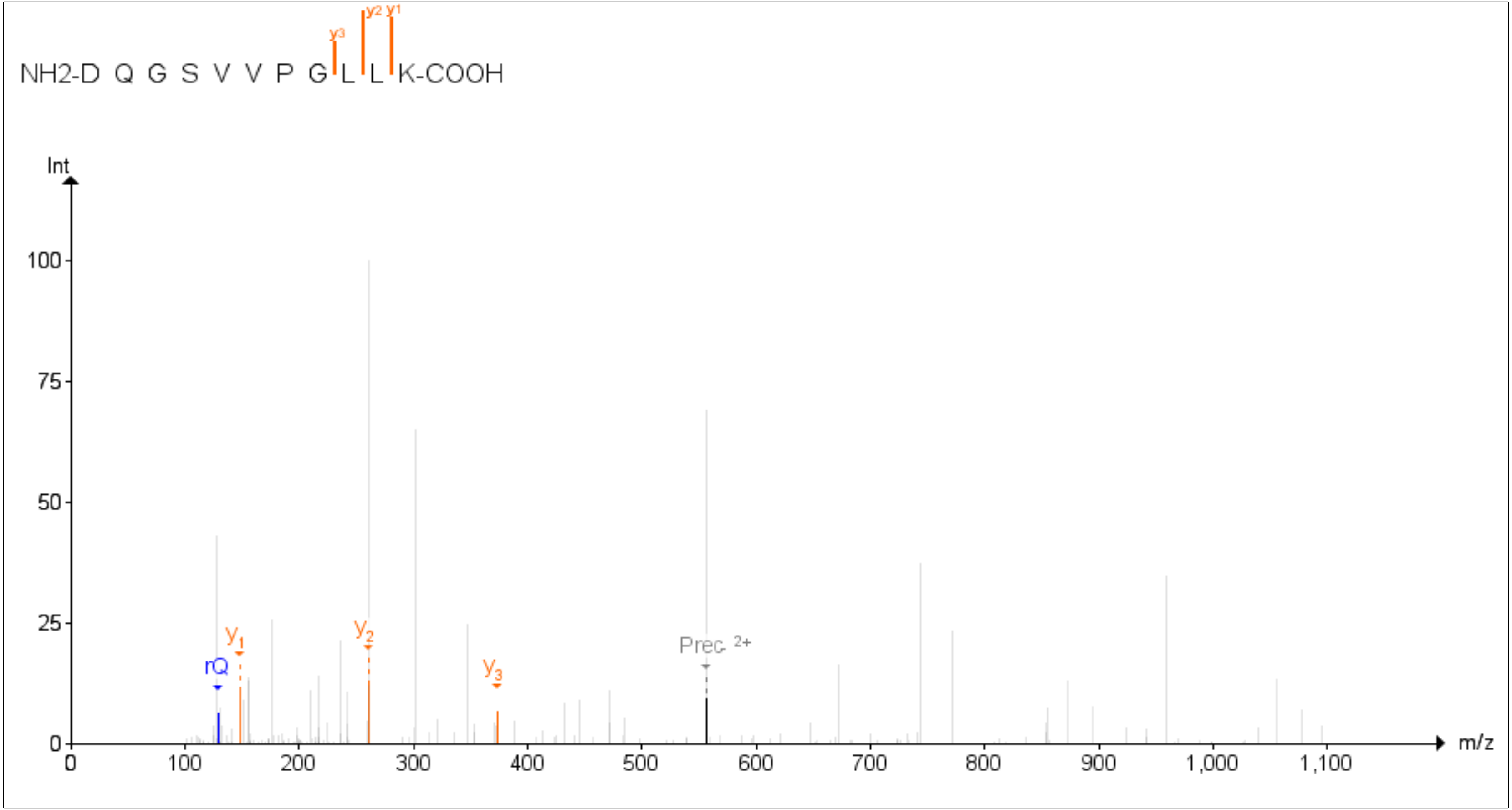

NH2-D Q G S V V P G L L K-COOH
y3 y2 y1
Int
100
75
50
25
0
rQ
y1
y2
y3
Prec. 2+
0 100 200 300 400 500 600 700 800 900 1,000 1,100
m/z

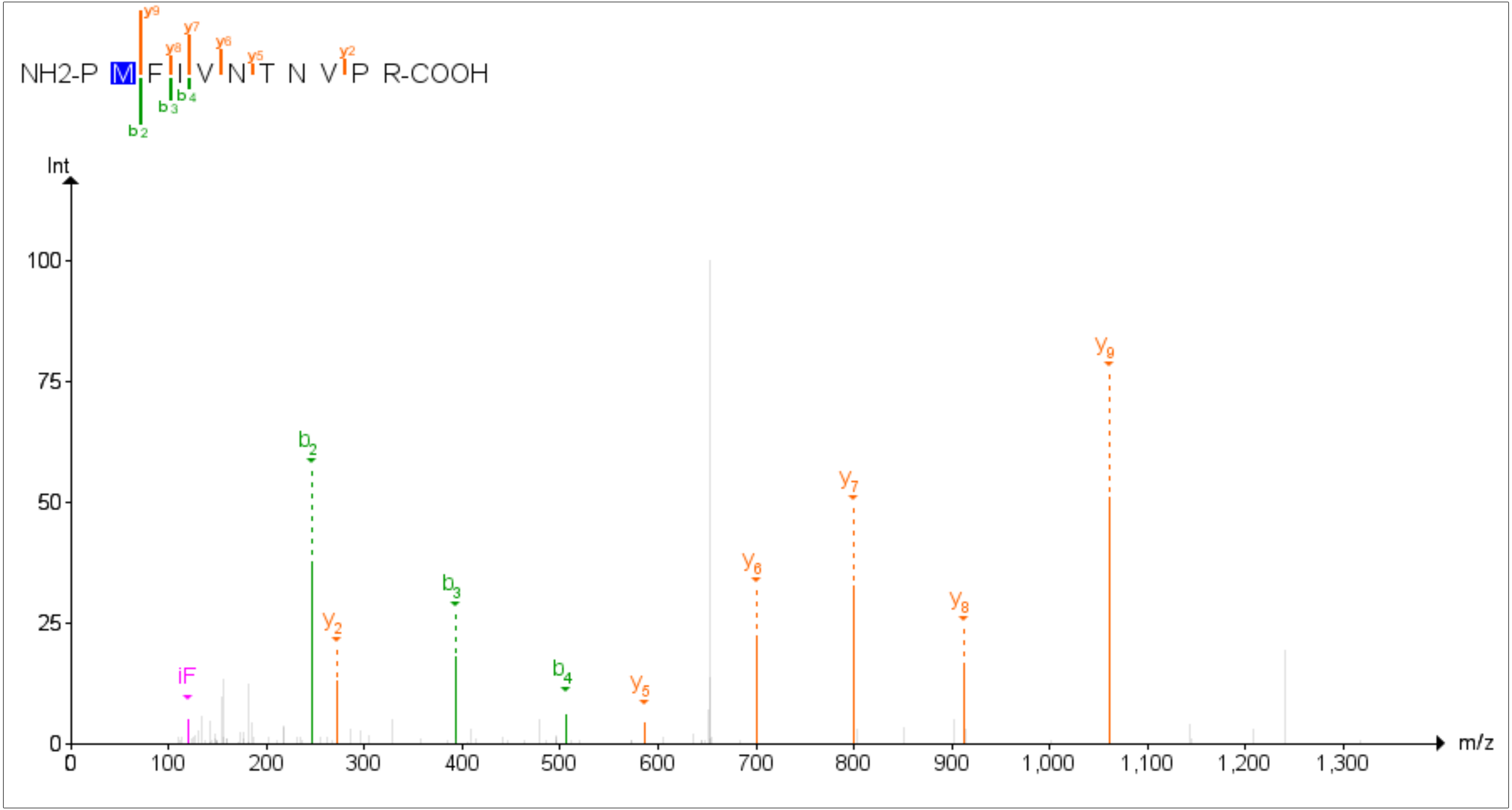

NH2-P M F I V N T N V P R-COOH
y9
y8
y7
y6
y5
y2
b2
b3
b4
Int
100
75
50
25
0
iF
b2
y2
b3
b4
y5
y6
y7
y8
y9
0
100
200
300
400
500
600
700
800
900
1,000
1,100
1,200
1,300
m/z

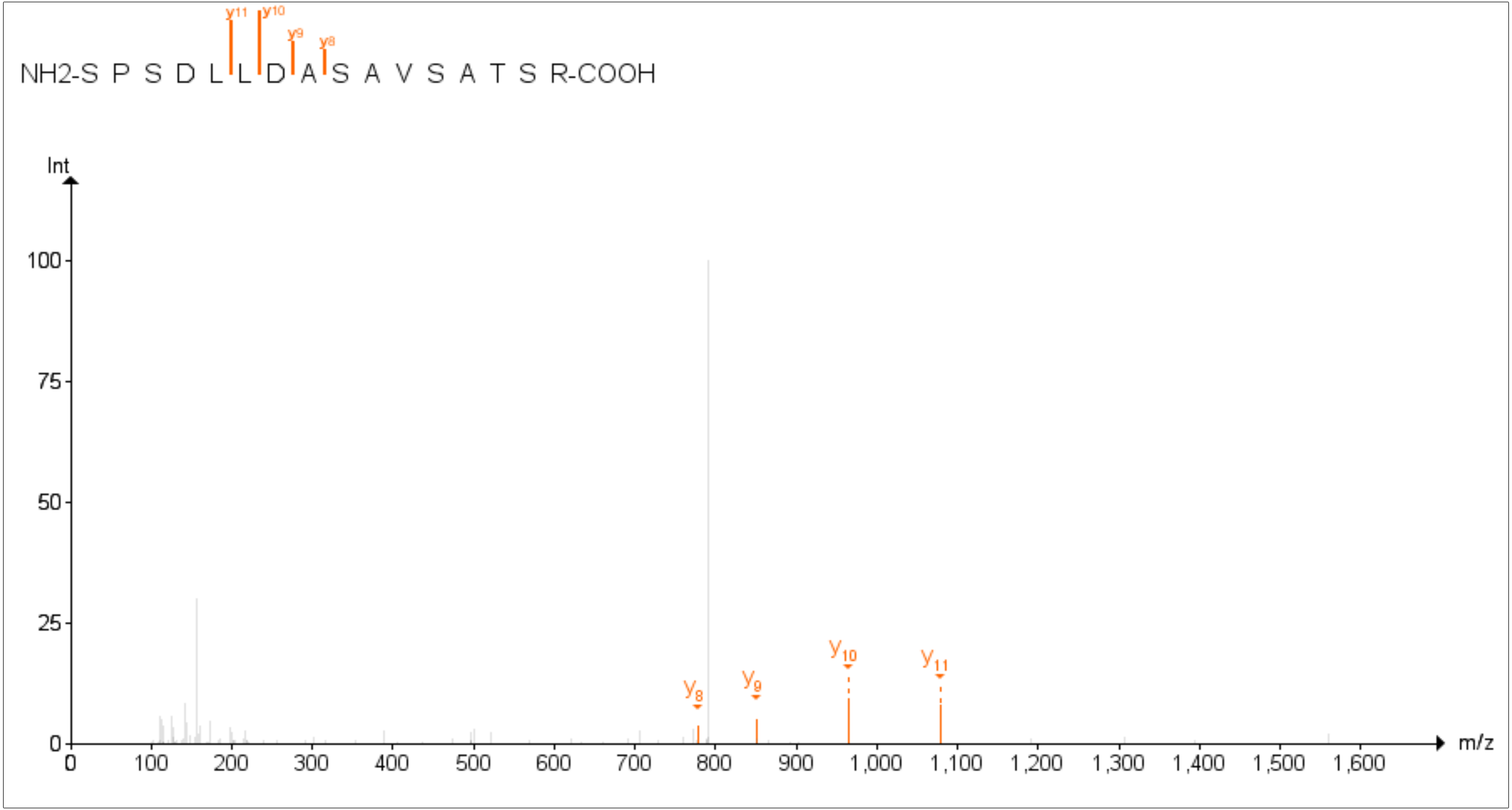

NH2-S P S D L L D A S A V S A T S R-COOH
y11 y10 y9 y8
Int
100
75
50
25
0
m/z
0 100 200 300 400 500 600 700 800 900 1,000 1,100 1,200 1,300 1,400 1,500 1,600
y8 y9 y10 y11

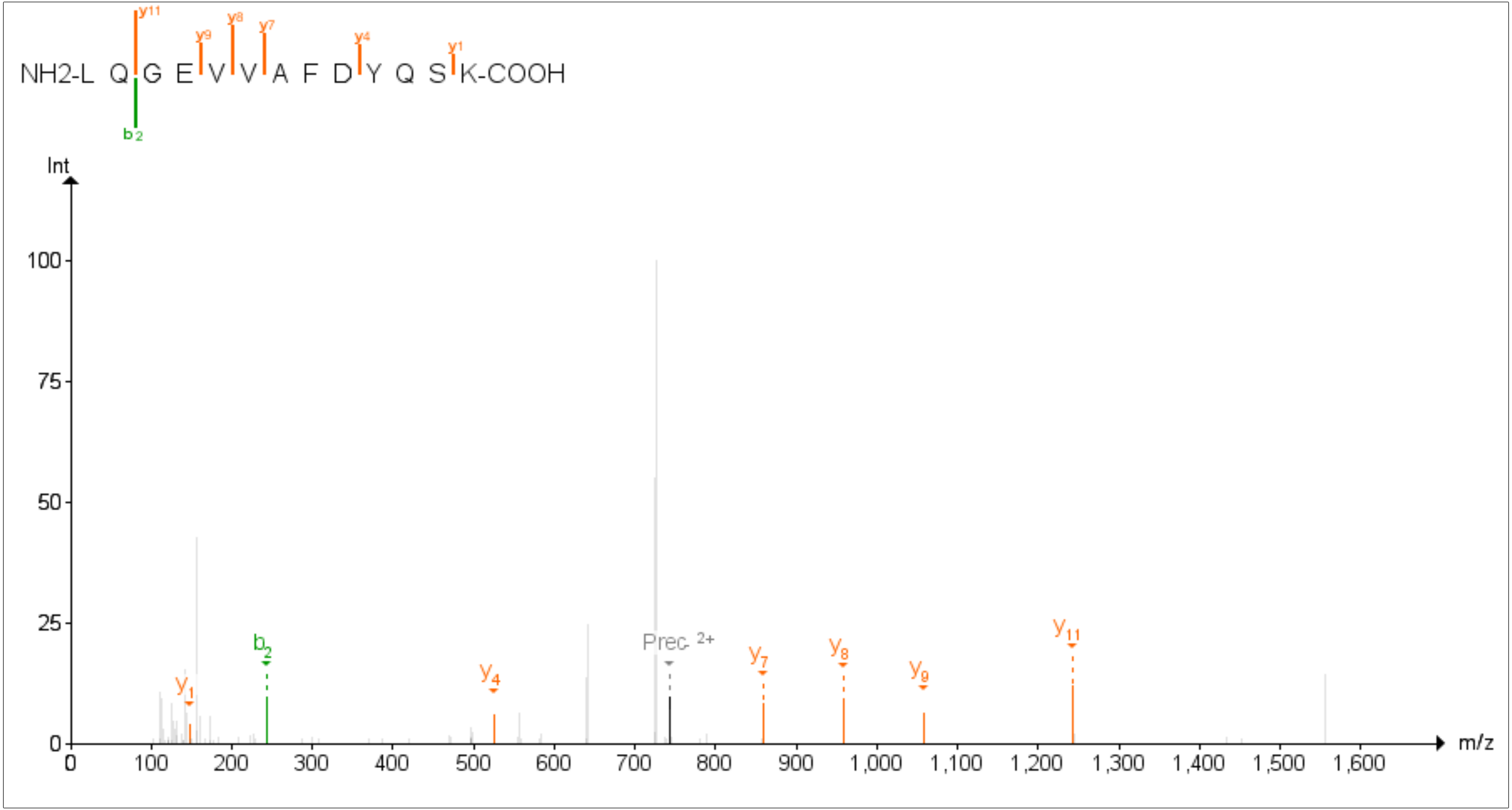

NH2-L Q G E V V A F D Y Q S K-COOH
y11
y9
y8
y7
y4
y1
b2
Int
100
75
50
25
0
b2
y1
y4
Prec 2+
y7
y8
y9
y11
m/z
0
100
200
300
400
500
600
700
800
900
1,000
1,100
1,200
1,300
1,400
1,500
1,600

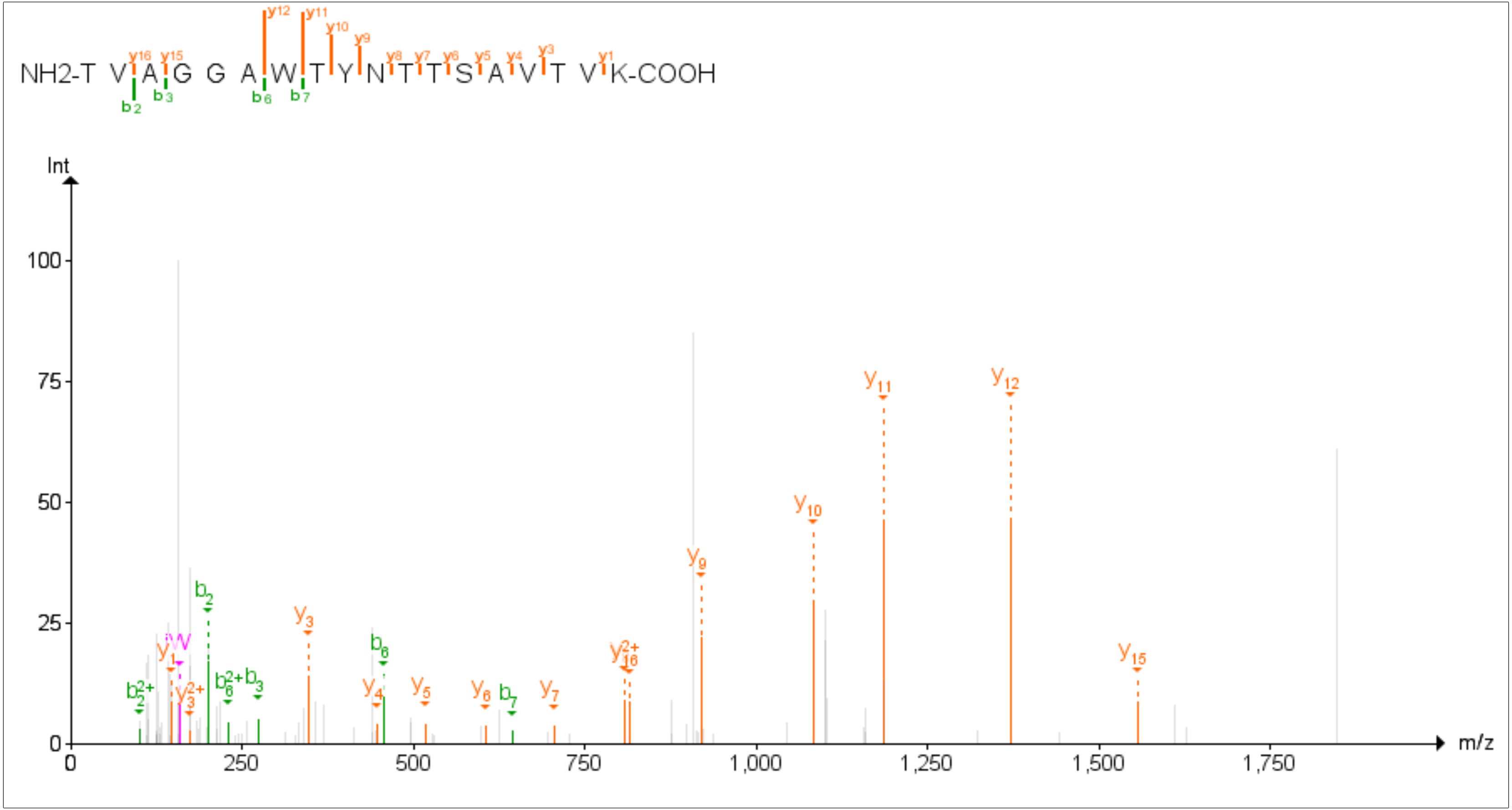
NH2-T V A G G A W T Y N T T S A V T V K-COOH
y16 y15 y12 y11 y10 y9 y8 y7 y6 y5 y4 y3 y1
b2 b3 b6 b7
Int
100
75
50
25
0
250
500
750
1,000
1,250
1,500
1,750
m/z
y11
y12
y10
y9
y15
b2
y3
b6
y1
b2+ 2
b2+ 6
b3
y2+ 3
y4
y5
y6
b7
y7
y2+ 16

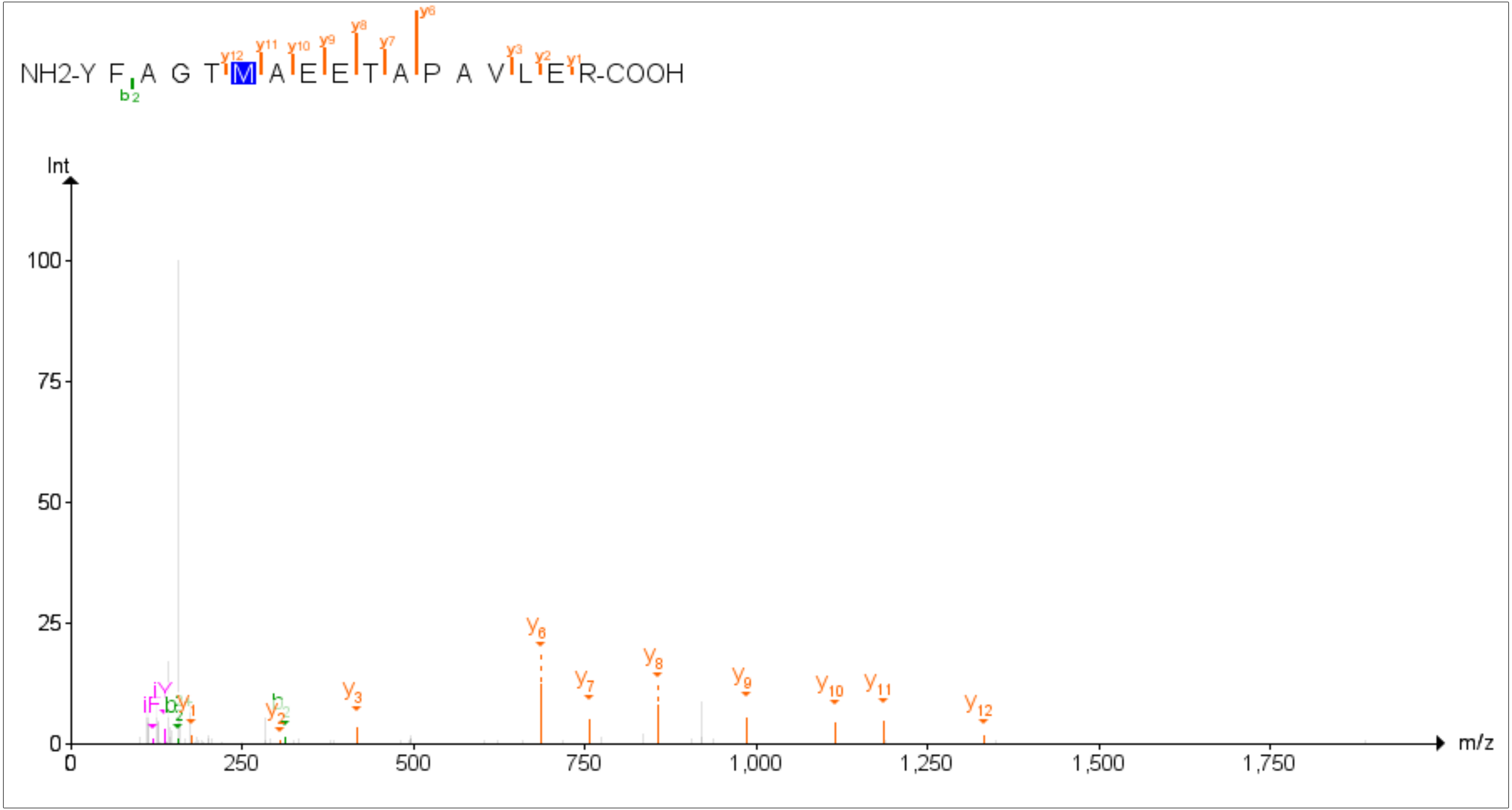

NH2-Y F A G T M A E E T A P A V L E R-COOH
b2
y12 y11 y10 y9 y8 y7 y6 y3 y2 y1
Int
100
75
50
25
0
250
500
750
1,000
1,250
1,500
1,750
m/z

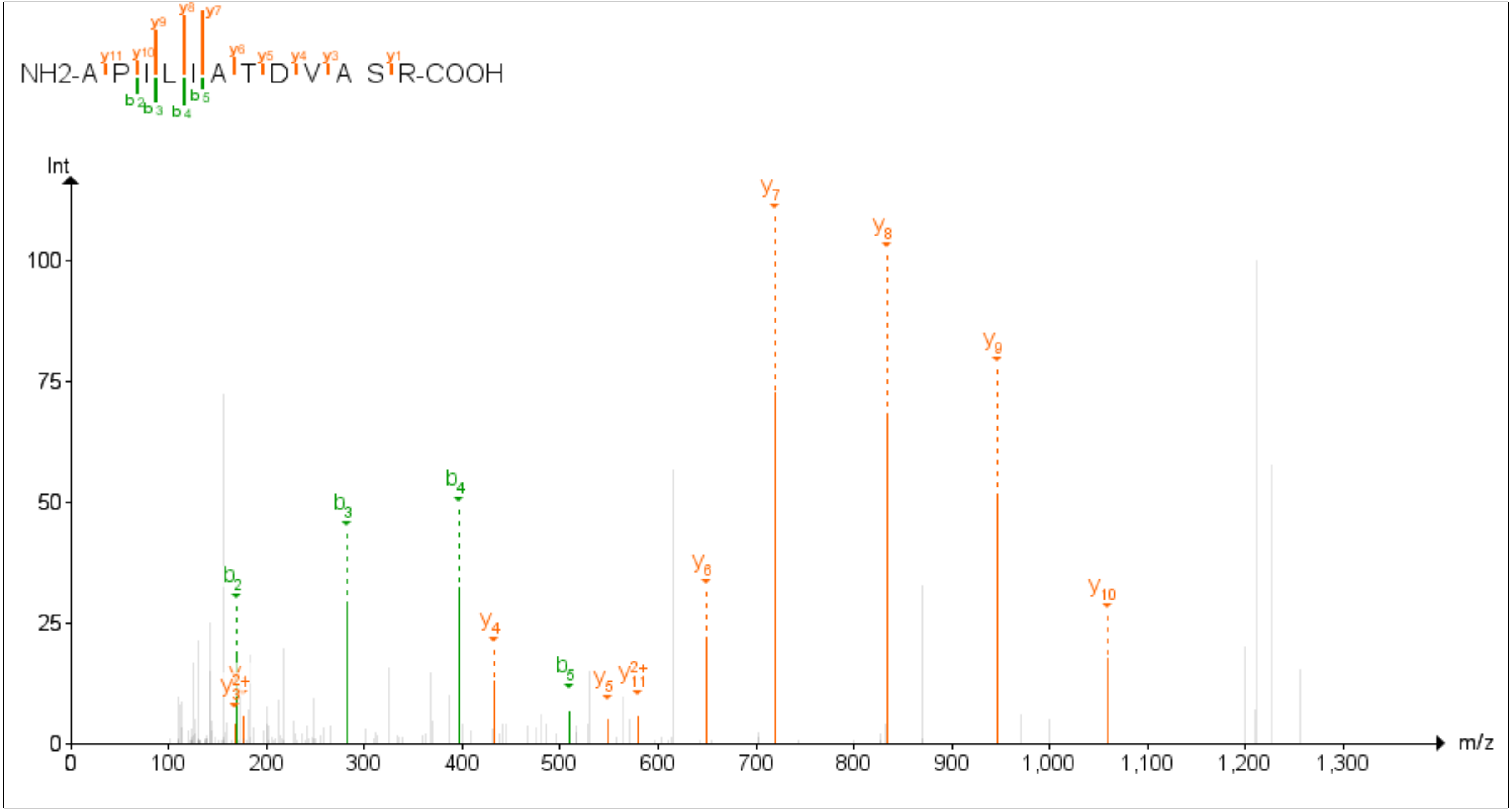

NH2-A P I L I A T D V A S R-COOH
Int
m/z

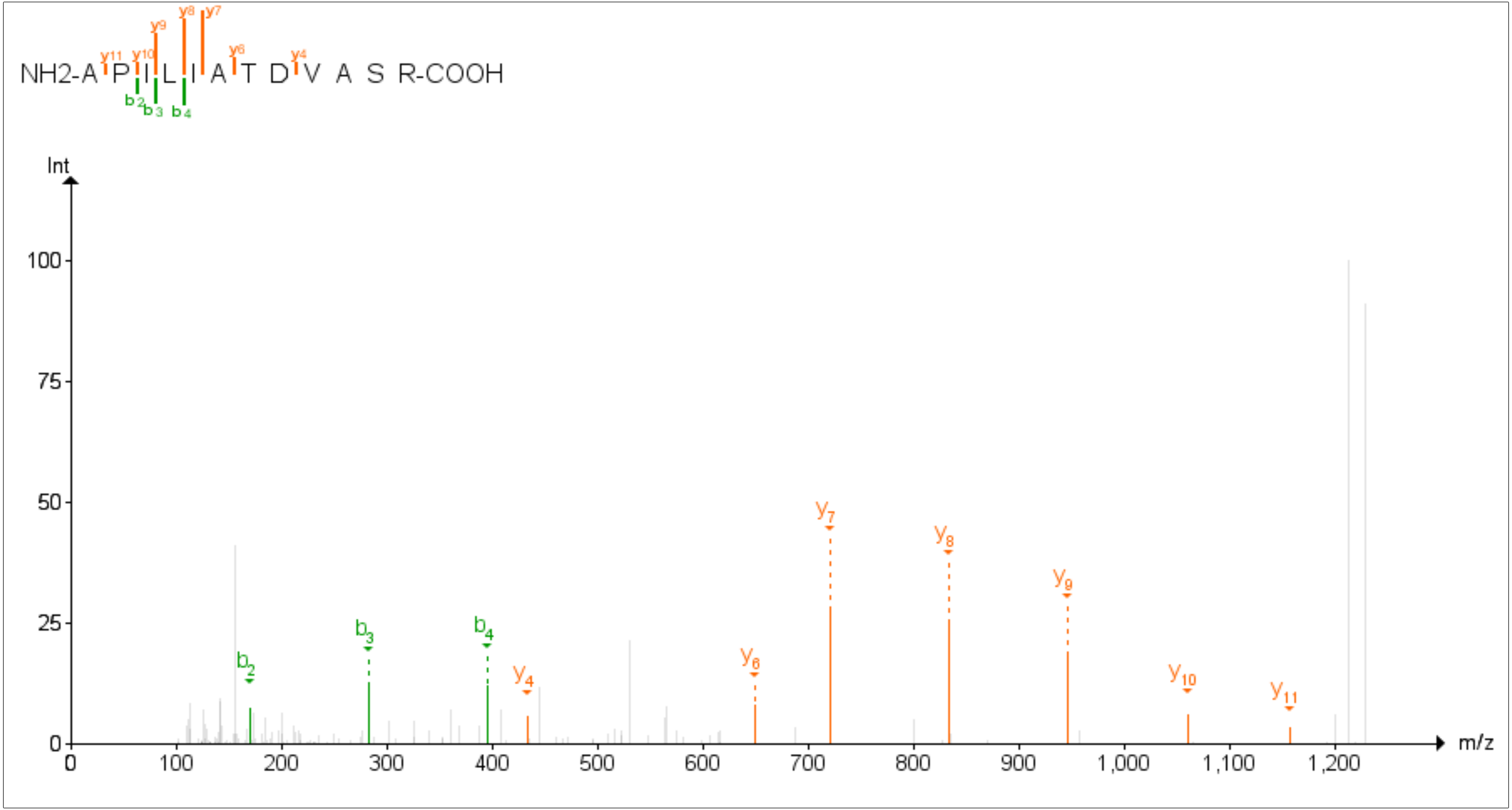

NH2-A P I L I A T D V A S R-COOH
y11
y10
y9
y8
y7
y6
y4
b2
b3
b4
Int
100
75
50
25
0
m/z
0
100
200
300
400
500
600
700
800
900
1,000
1,100
1,200
b2
b3
b4
y4
y6
y7
y8
y9
y10
y11

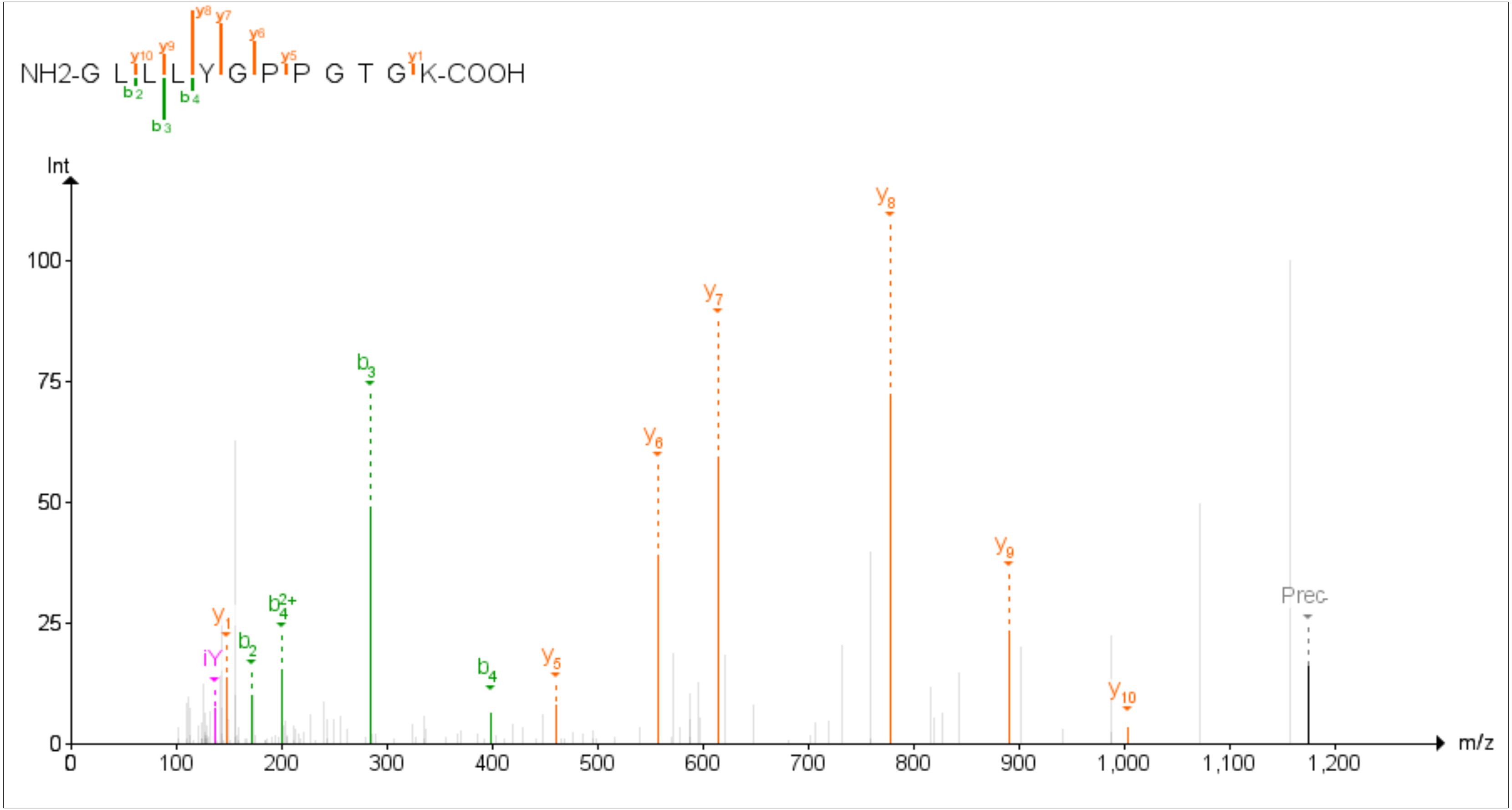

NH2-G L L L Y G P P G T G K-COOH
y10 y9 y8 y7 y6 y5 y1
b2 b3 b4
Int
100
75
50
25
0
y8
y7
b3
y6
y9
Prec
y1
b4 2+
iY
b2
b4
y5
y10
m/z
0 100 200 300 400 500 600 700 800 900 1,000 1,100 1,200

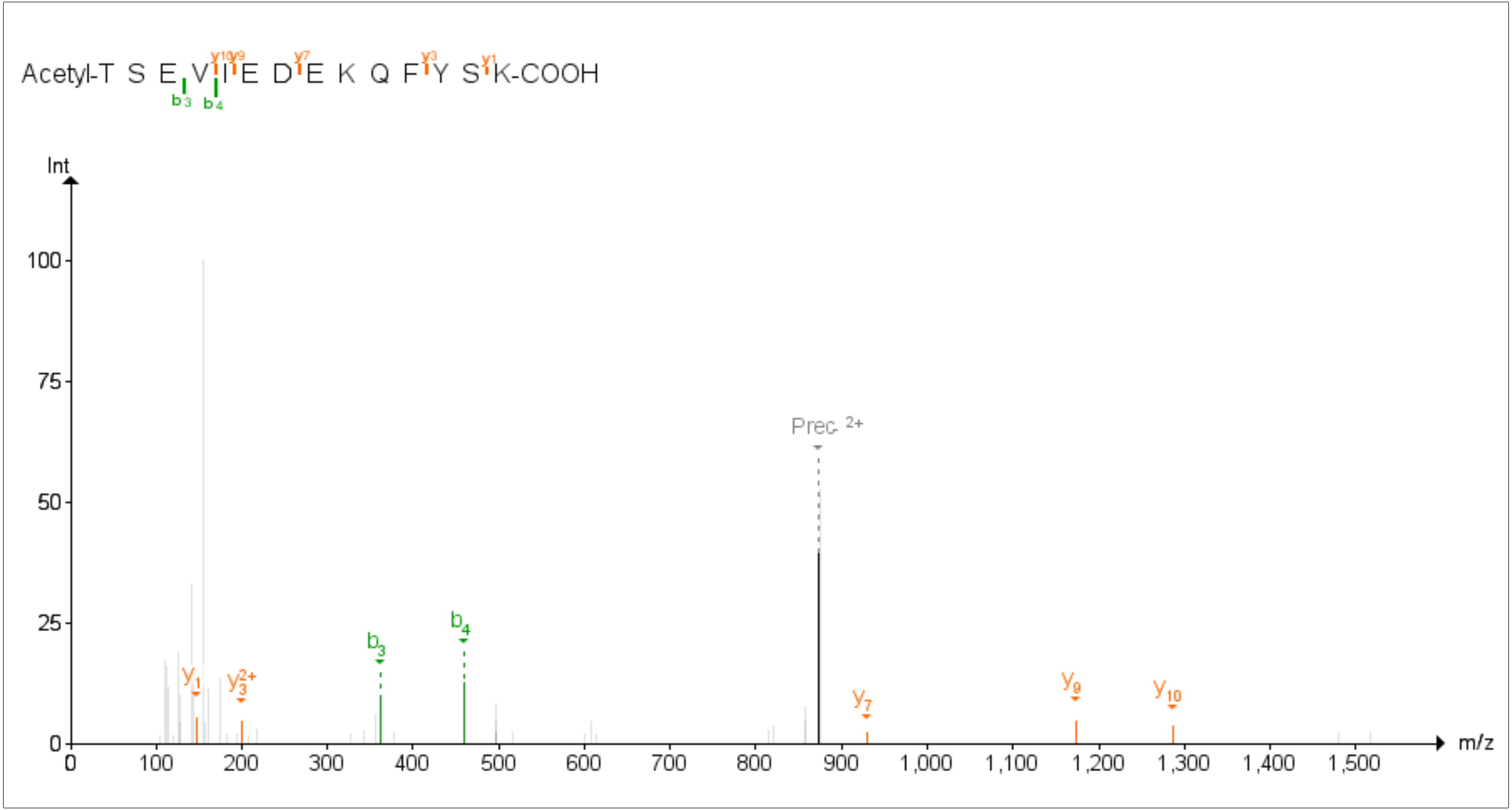
Acetyl-T S E V I E D E K Q F Y S K-COOH
y10 y9 y7 y3 y1
b3 b4
Int
100
75
50
25
0
Prec 2+
y1
y3 2+
b3
b4
y7
y9
y10
0 100 200 300 400 500 600 700 800 900 1,000 1,100 1,200 1,300 1,400 1,500
m/z

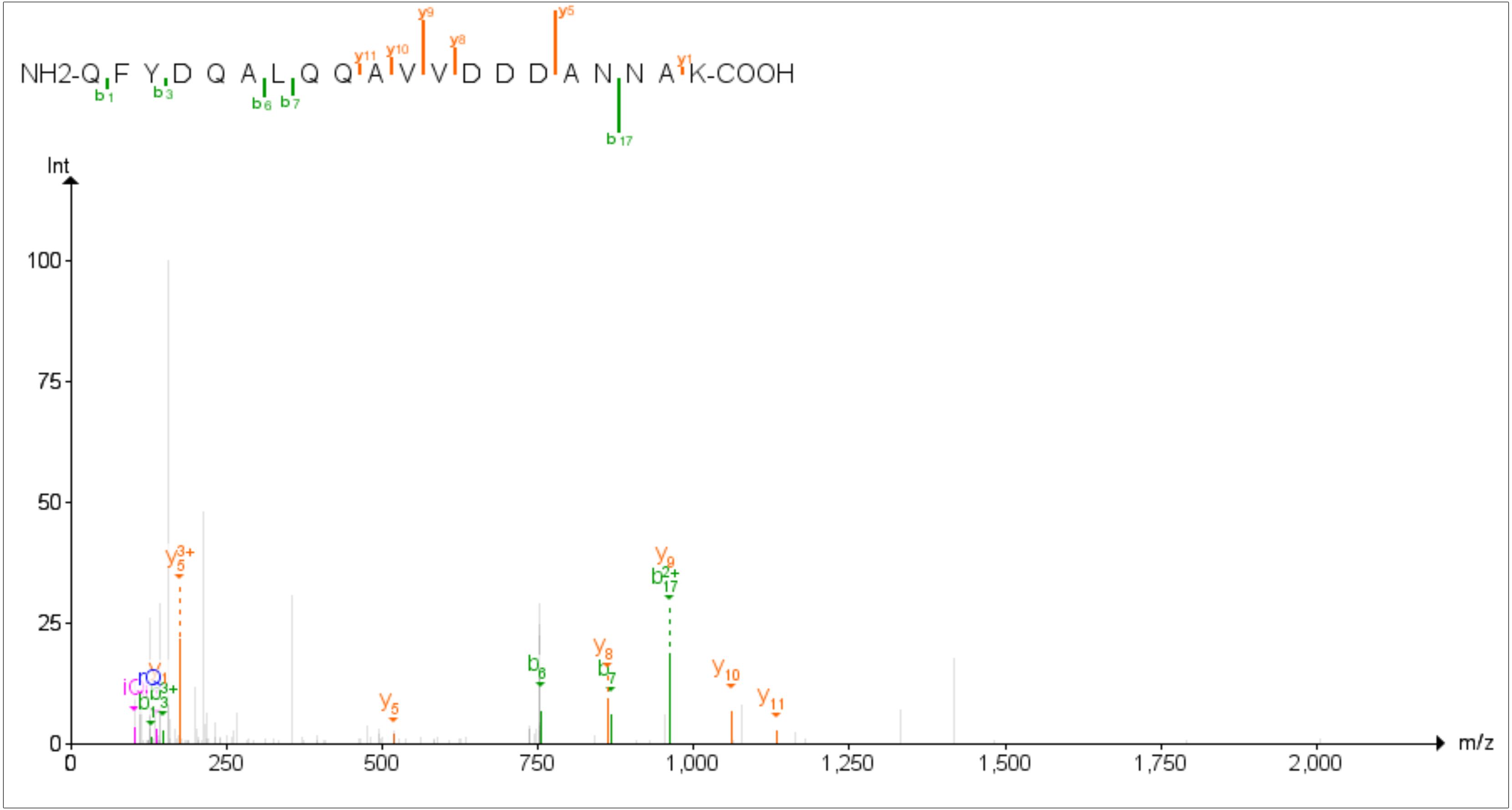
NH2-Q F Y D Q A L Q Q A V V D D D A N N A K-COOH
b1 b3 b6 b7 b17
y11 y10 y9 y8 y5 y1
Int
m/z
0
25
50
75
100
250
500
750
1,000
1,250
1,500
1,750
2,000

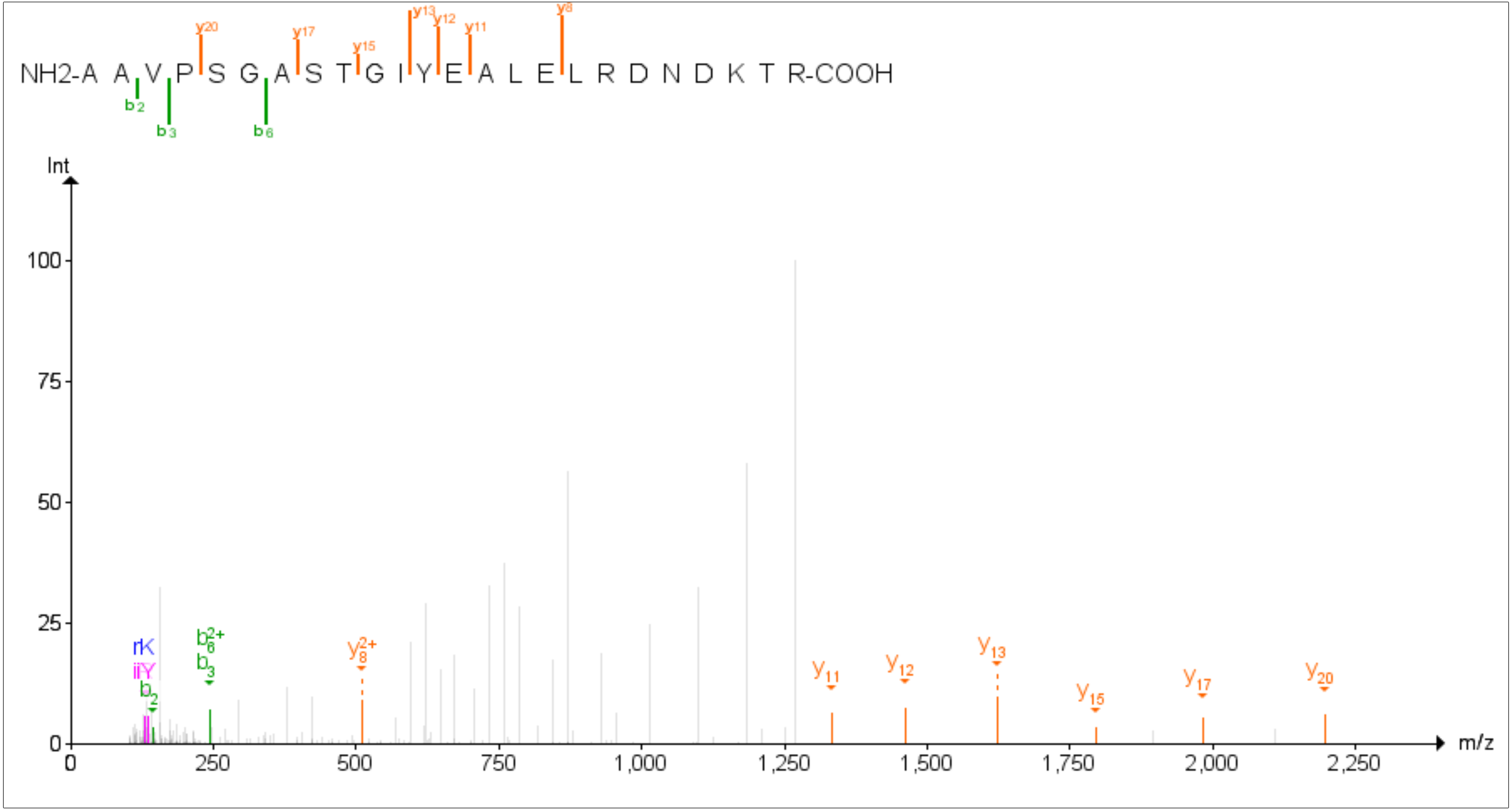

NH2-A A V P S G A S T G I Y E A L E L R D N D K T R-COOH
y20
y17
y15
y13
y12
y11
y8
b2
b3
b6
Int
100
75
50
25
0
rK
iiY
b2
b6 2+
b3
y8 2+
y11
y12
y13
y15
y17
y20
0
250
500
750
1,000
1,250
1,500
1,750
2,000
2,250
m/z

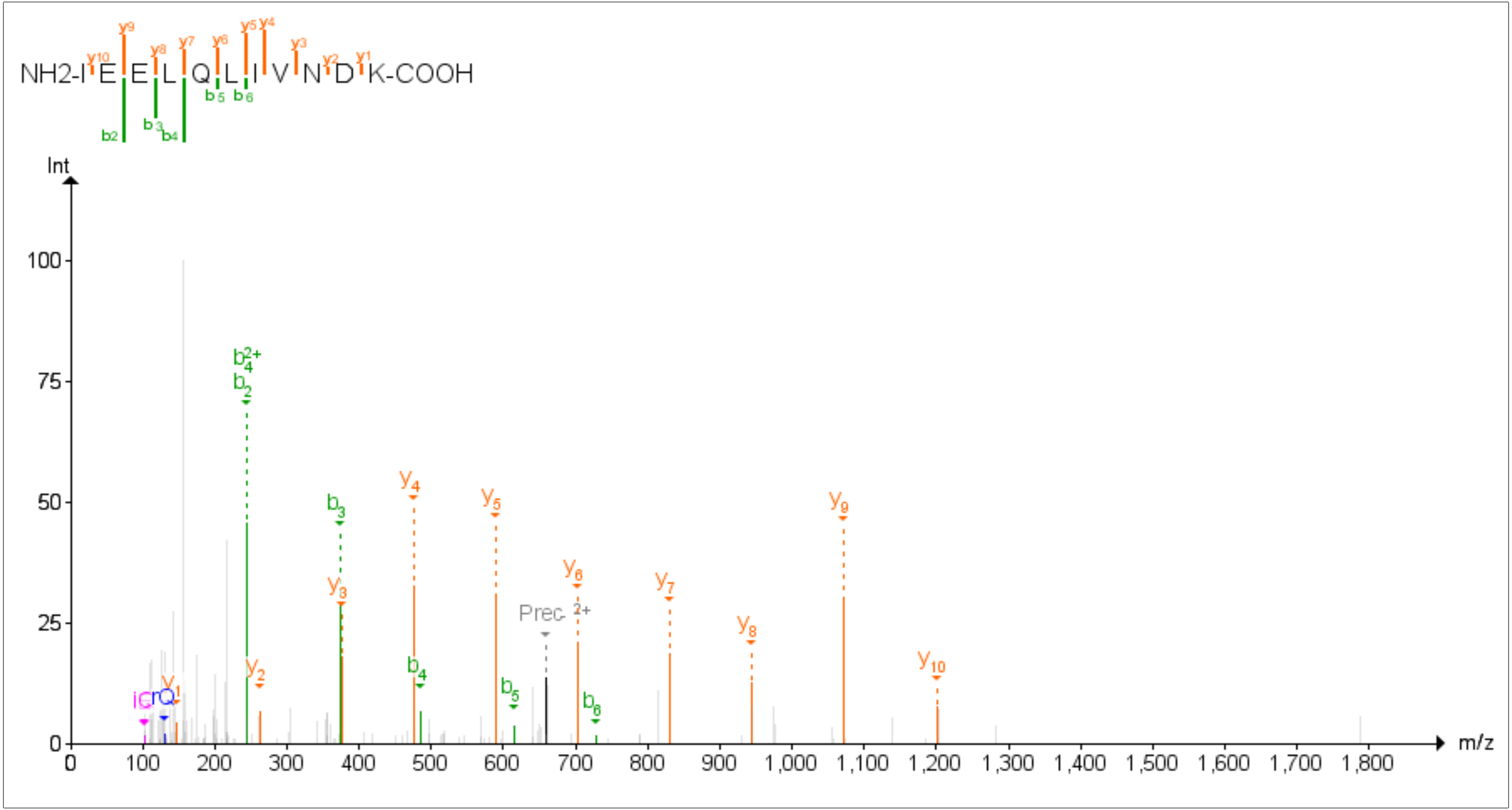

NH2-I E E L Q L I V N D K-COOH
y10 y9 y8 y7 y6 y5 y4 y3 y2 y1
b2 b3 b4 b5 b6
Int
100
75
50
25
0
b$_4^{2+}$
b$_2$
b$_3$
y$_4$
y$_5$
y$_9$
y$_3$
y$_6$
y$_7$
Prec 2+
y$_8$
y$_2$
b$_4$
b$_5$
b$_6$
y$_{10}$
y$_1$
iC
rQ
0 100 200 300 400 500 600 700 800 900 1,000 1,100 1,200 1,300 1,400 1,500 1,600 1,700 1,800
m/z

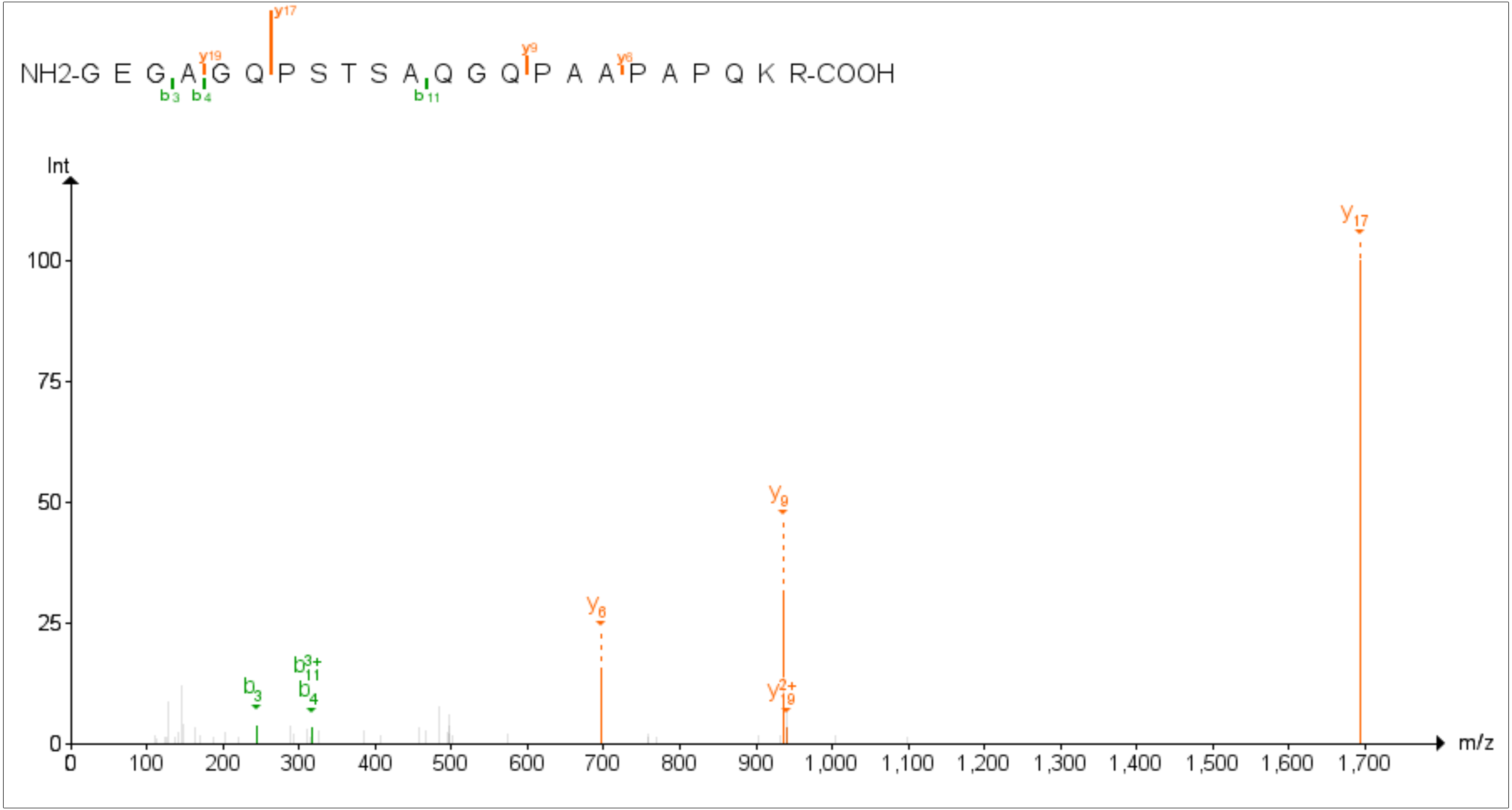

NH2-G E G A G Q P S T S A Q G G Q P A A P A P Q K R-COOH
y17
y19
y9
y6
b3
b4
b11
Int
m/z
0
25
50
75
100
0
100
200
300
400
500
600
700
800
900
1,000
1,100
1,200
1,300
1,400
1,500
1,600
1,700
y17
y9
y6
y19 2+
b3
b4
b11 3+

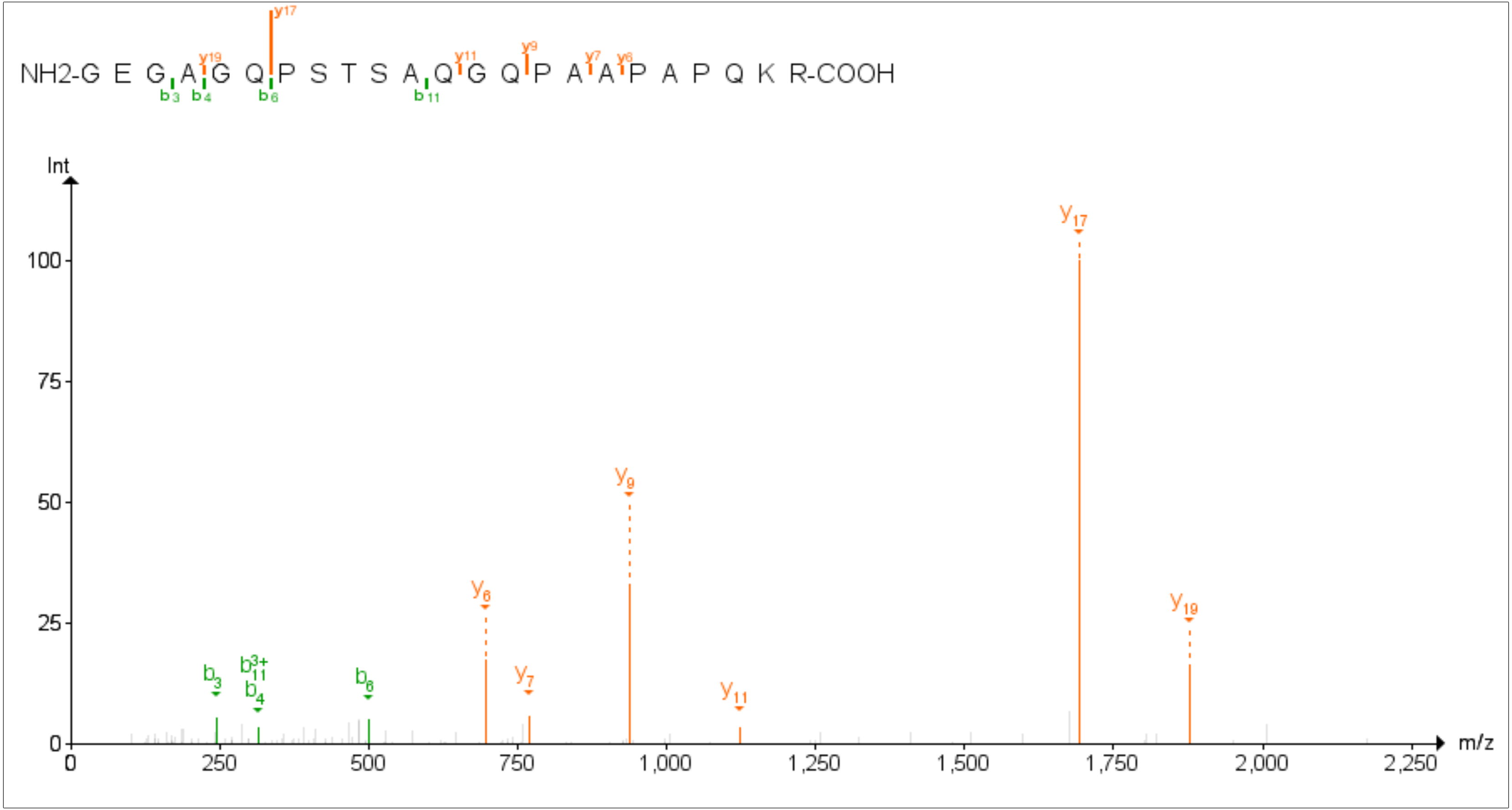
NH2-G E G A G Q P S T S A Q G Q P A A P A P Q K R-COOH
y17
y19
y11
y9
y7
y6
b3
b4
b6
b11
Int
100
75
50
25
0
m/z
0
250
500
750
1,000
1,250
1,500
1,750
2,000
2,250
y17
y9
y6
y19
y7
y11
b3
b11 3+
b4
b6

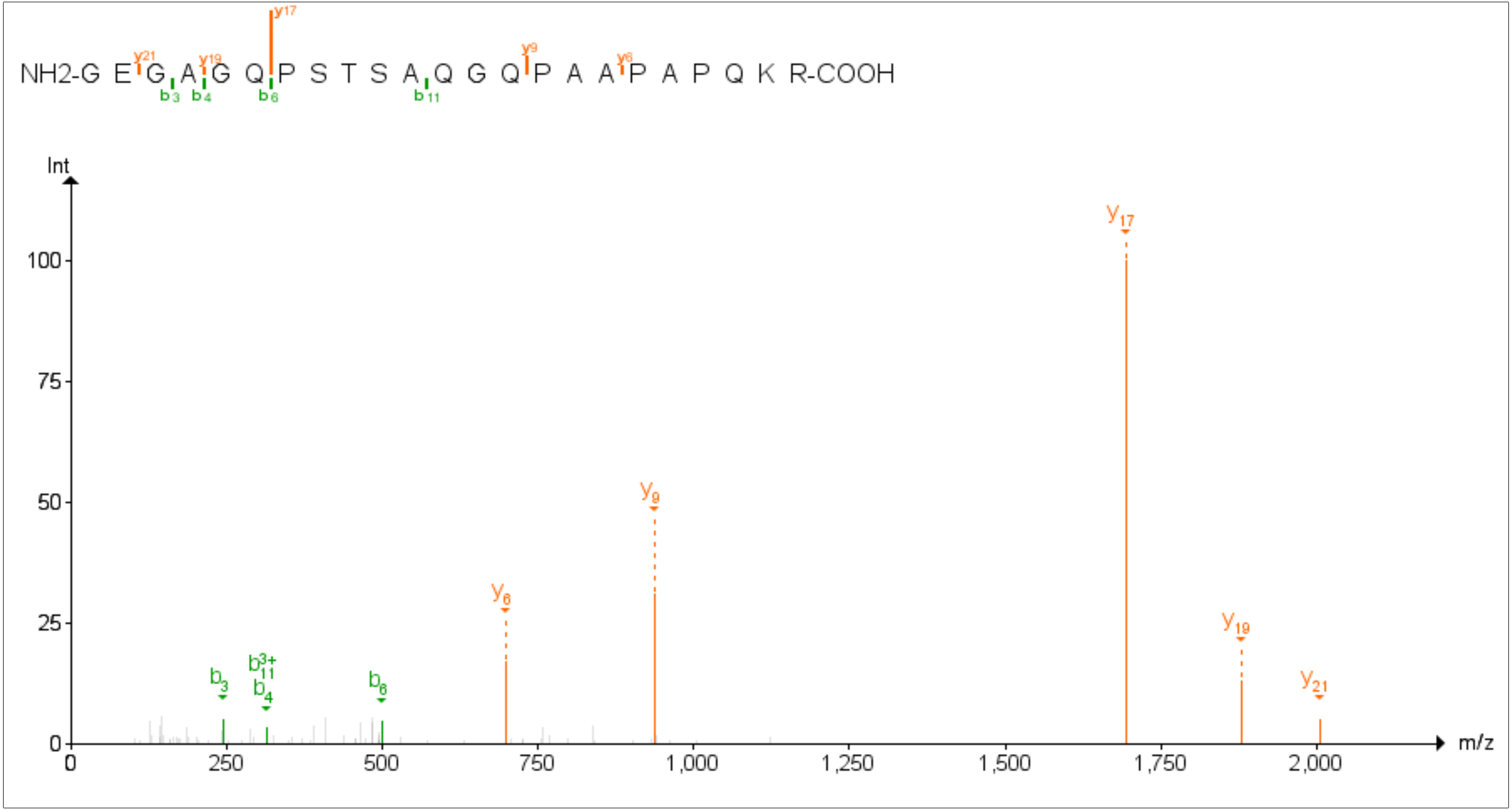

NH2-G E G A G Q P S T S A Q G Q P A A P A P Q K R-COOH
y21 y19 y17 y9 y6
b3 b4 b6 b11
Int
100
75
50
25
0
0
250
500
750
1,000
1,250
1,500
1,750
2,000
m/z
y17
y9
y6
y19
y21
b3
b11 3+
b4
b6

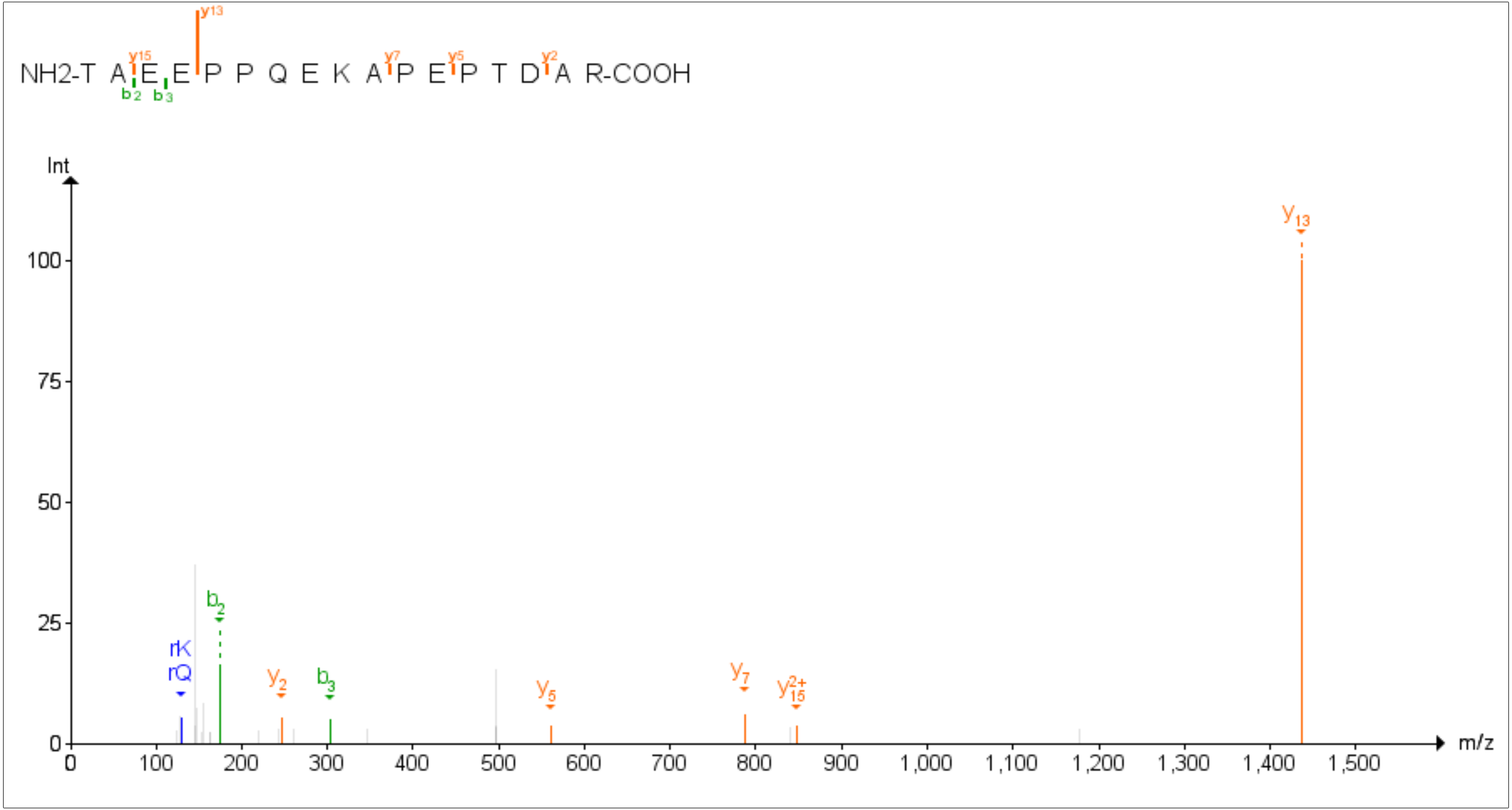

NH2-T A E E P P Q E K A P E P T D A R-COOH
y13
y15
y7
y5
y2
b2
b3
Int
100
75
50
25
0
y13
b2
rK
rQ
y2
b3
y5
y7
y2+15
m/z
0
100
200
300
400
500
600
700
800
900
1,000
1,100
1,200
1,300
1,400
1,500

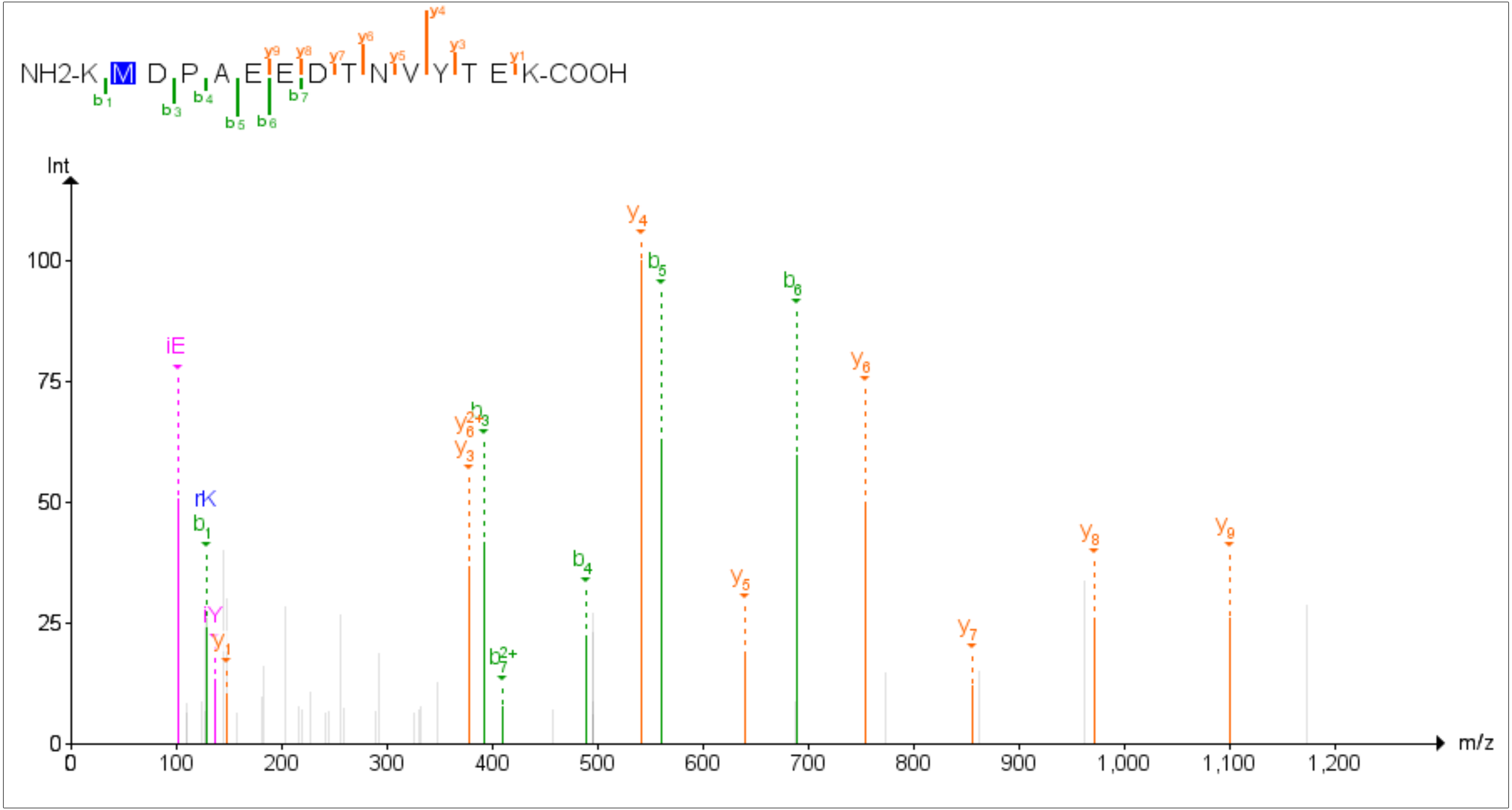

NH2-K M D P A E E D T N V Y T E K-COOH
b1 b3 b4 b5 b6 b7
y9 y8 y7 y6 y5 y4 y3 y1
Int
m/z
0
25
50
75
100
0
100
200
300
400
500
600
700
800
900
1,000
1,100
1,200
iE
rK
b1
iY
y1
y3
y6 2+
b3
b7 2+
b4
y4
b5
y5
b6
y6
y7
y8
y9

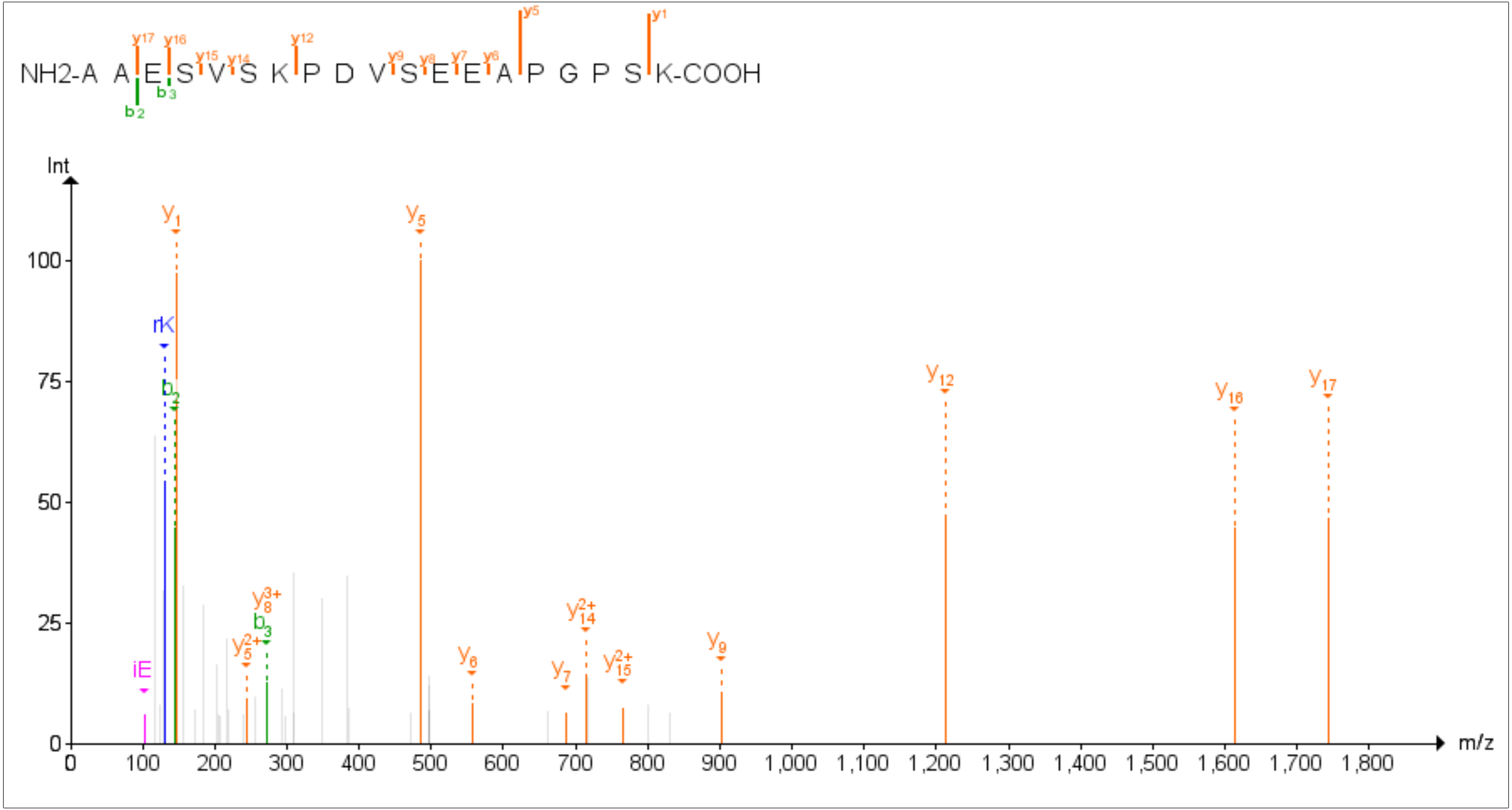

NH2-A A E S V S K P D V S E E A P G P S K-COOH
y17 y16 y15 y14 y12 y9 y8 y7 y6 y5 y1
b2 b3
Int
m/z
y1
y5
rK
b2
iE
y5 2+
y8 3+
b3
y6
y7
y14 2+
y15 2+
y9
y12
y16
y17
0
25
50
75
100
0 100 200 300 400 500 600 700 800 900 1,000 1,100 1,200 1,300 1,400 1,500 1,600 1,700 1,800

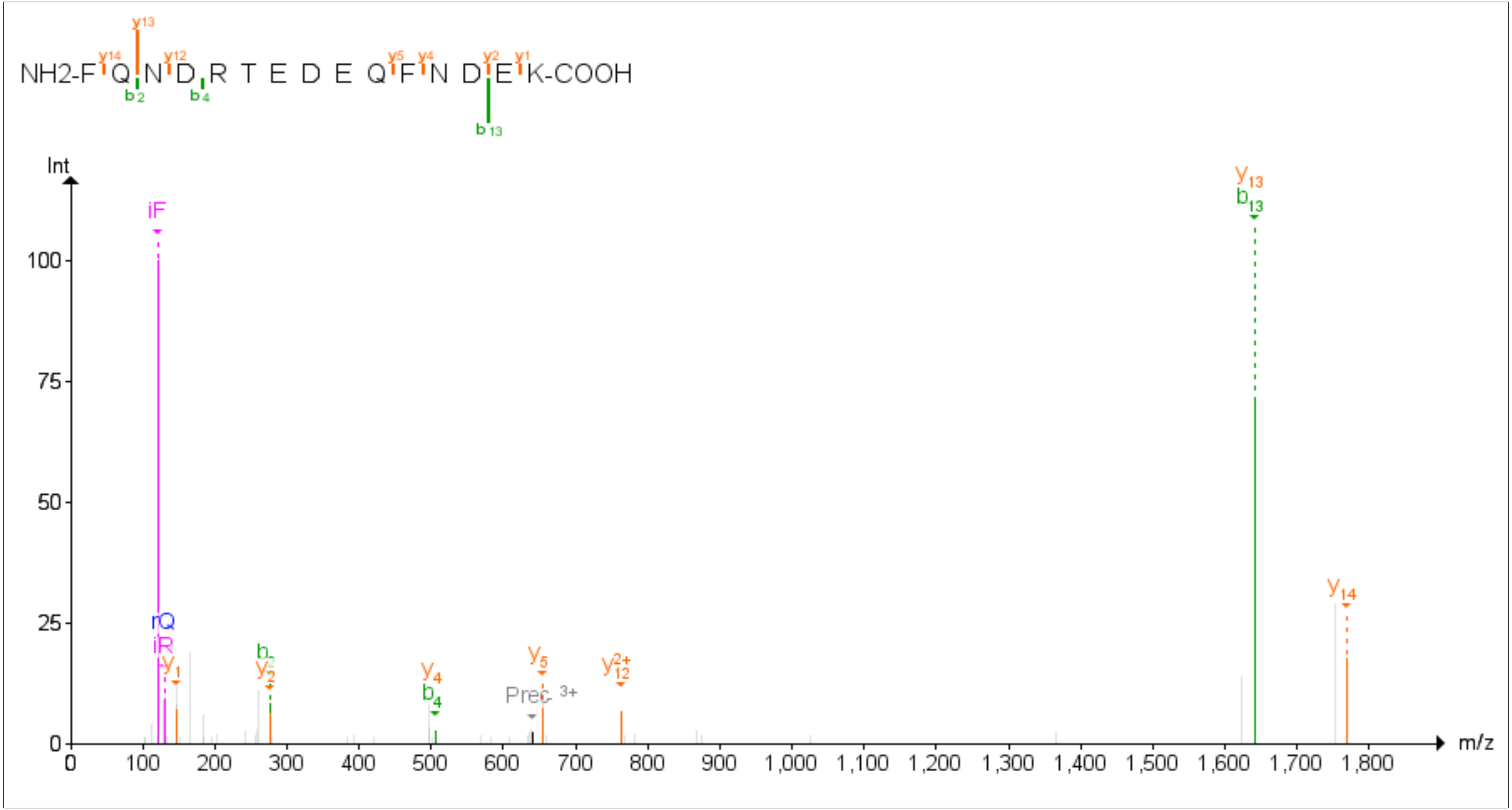

NH2-F Q N D R T E D E Q F N D E K-COOH
y14 y13 y12 y5 y4 y2 y1
b2 b4 b13
Int
iF
y13
b13
y14
rQ
iR
y1
b2
y2
y4
b4
Prec 3+
y5
y12 2+
0
25
50
75
100
0 100 200 300 400 500 600 700 800 900 1,000 1,100 1,200 1,300 1,400 1,500 1,600 1,700 1,800
m/z

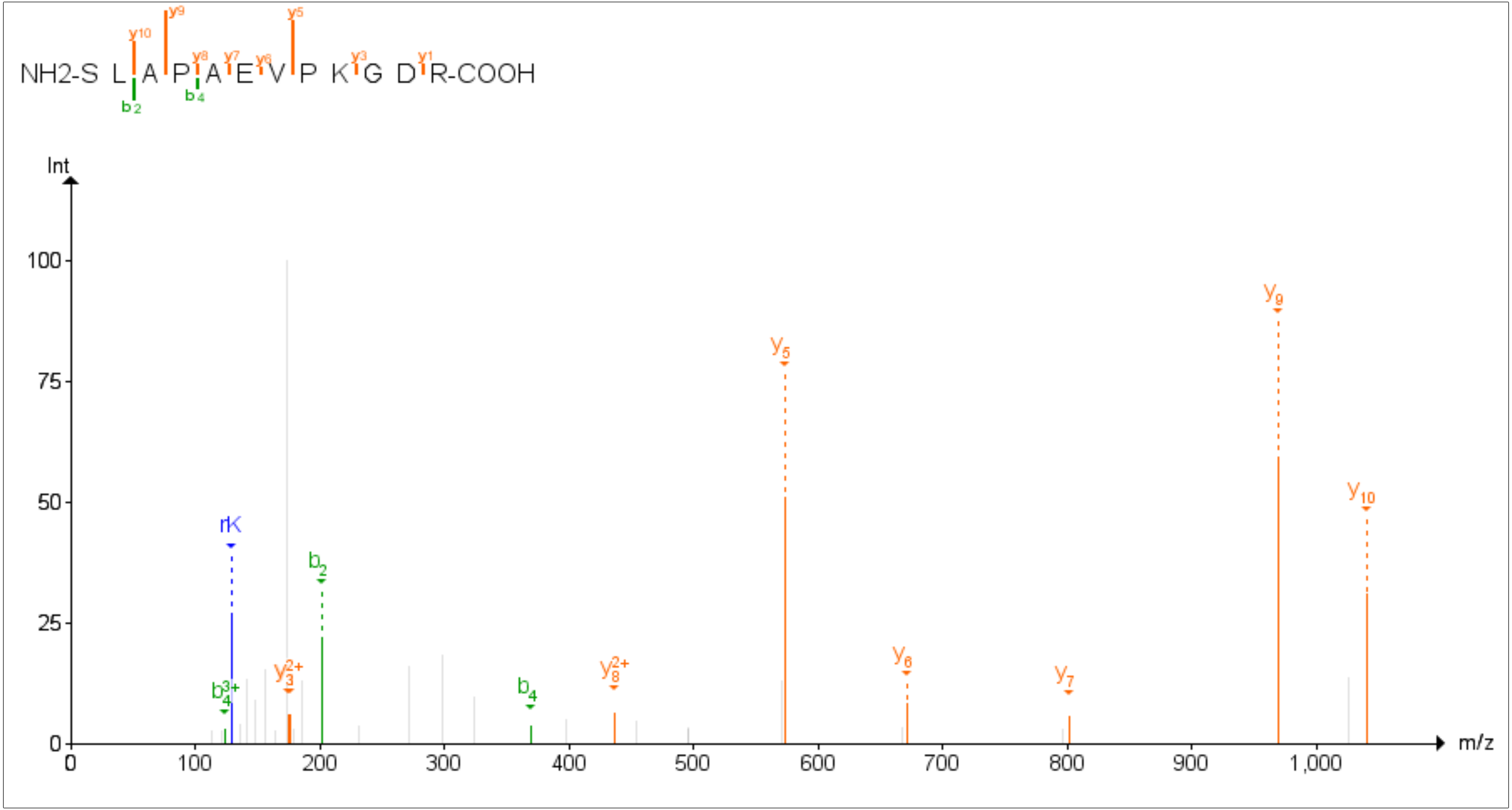

NH2-S L A P A E V P K G D R-COOH
y10
y9
y8
y7
y6
y5
y3
y1
b2
b4
Int
100
75
50
25
0
rK
b2
y3 2+
b4 3+
b4
y8 2+
y5
y6
y7
y9
y10
m/z
0
100
200
300
400
500
600
700
800
900
1,000

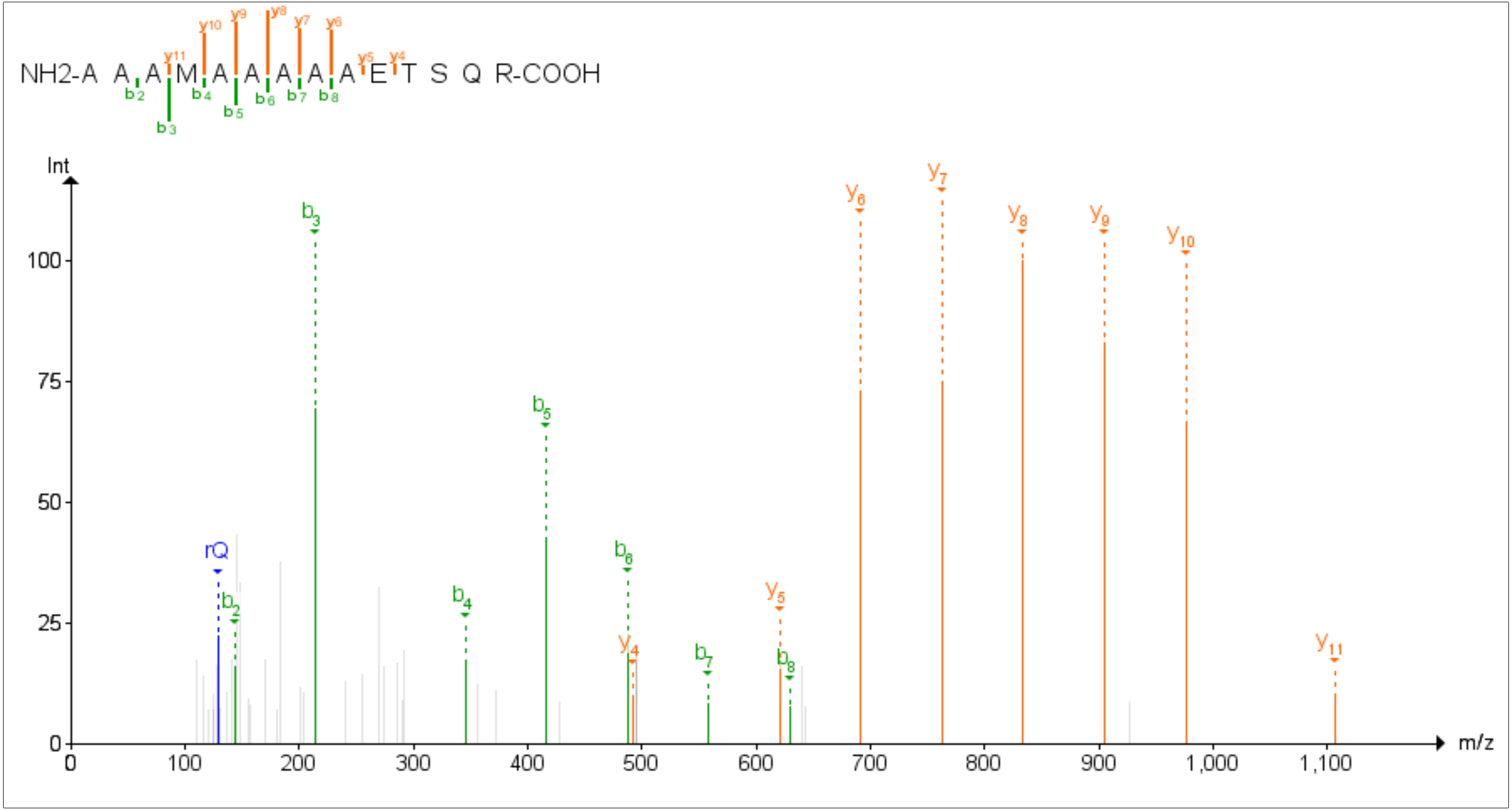

NH2-A A A M A A A A A E T S Q R-COOH
y11 y10 y9 y8 y7 y6 y5 y4
b2 b3 b4 b5 b6 b7 b8
Int
0
25
50
75
100
rQ
b2
b3
b4
b5
b6
y4
b7
y5
b8
y6
y7
y8
y9
y10
y11
0
100
200
300
400
500
600
700
800
900
1,000
1,100
m/z

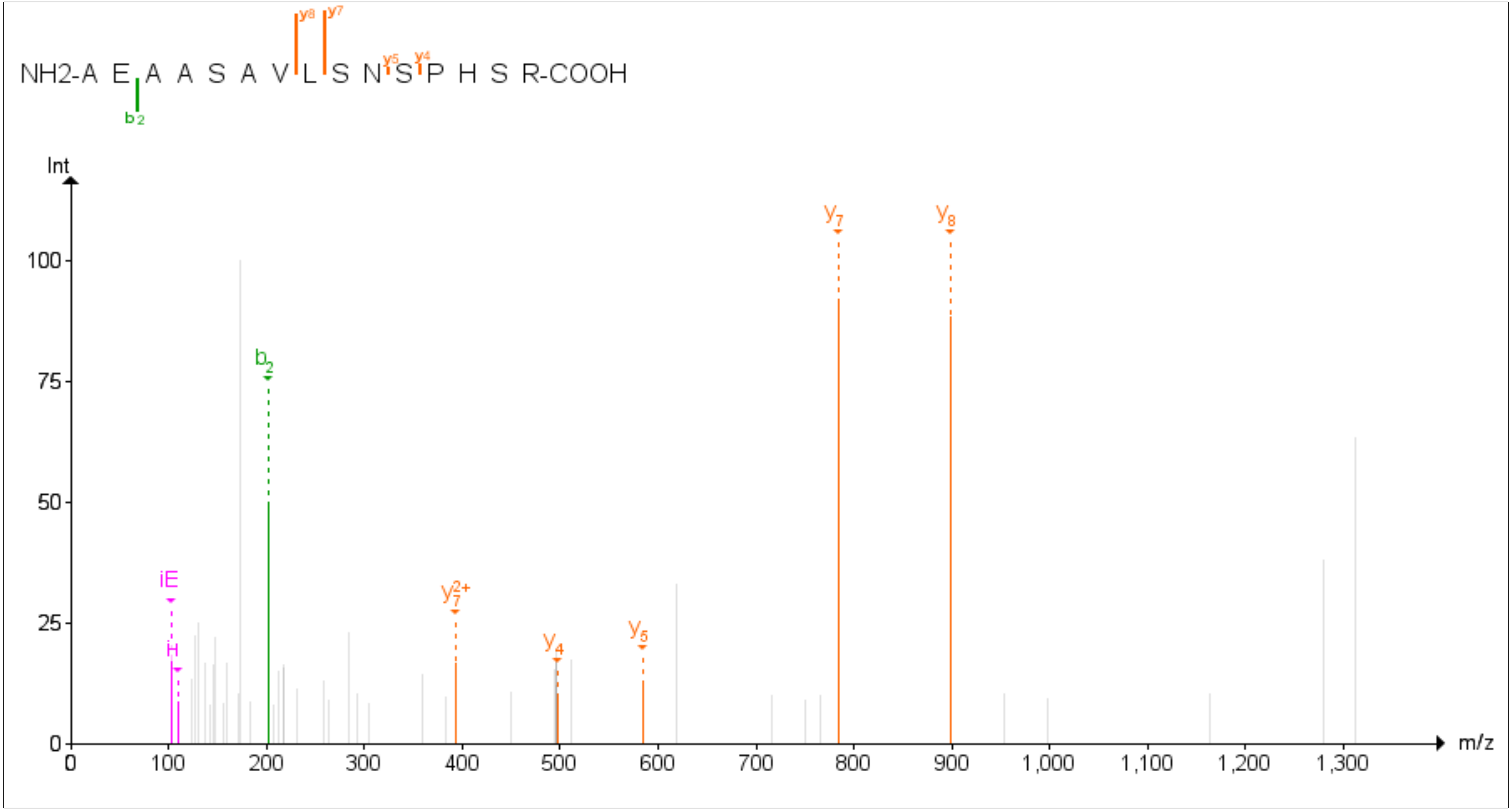

NH2-A E A A S A V L S N S P H S R-COOH
y8
y7
y5
y4
b2
Int
100
75
50
25
0
b2
y7
y8
iE
H
y7 2+
y4
y5
0
100
200
300
400
500
600
700
800
900
1,000
1,100
1,200
1,300
m/z

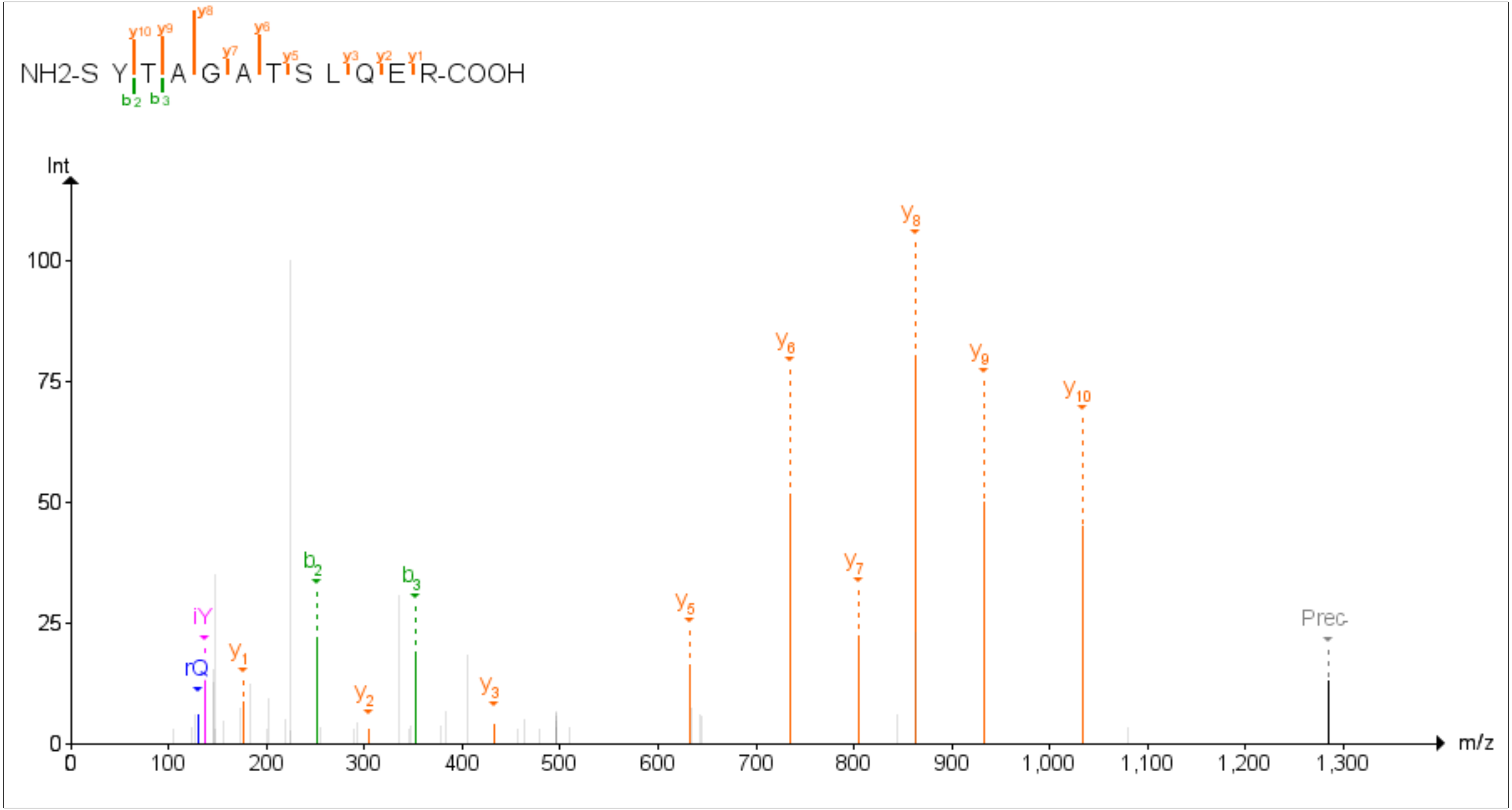

NH2-S Y T A G A T S L Q E R-COOH
y10 y9 y8 y7 y6 y5 y3 y2 y1
b2 b3
Int
100
75
50
25
0
m/z
0 100 200 300 400 500 600 700 800 900 1,000 1,100 1,200 1,300
y8
y6
y9
y10
y7
y5
b2
b3
iY
rQ
y1
y2
y3
Prec

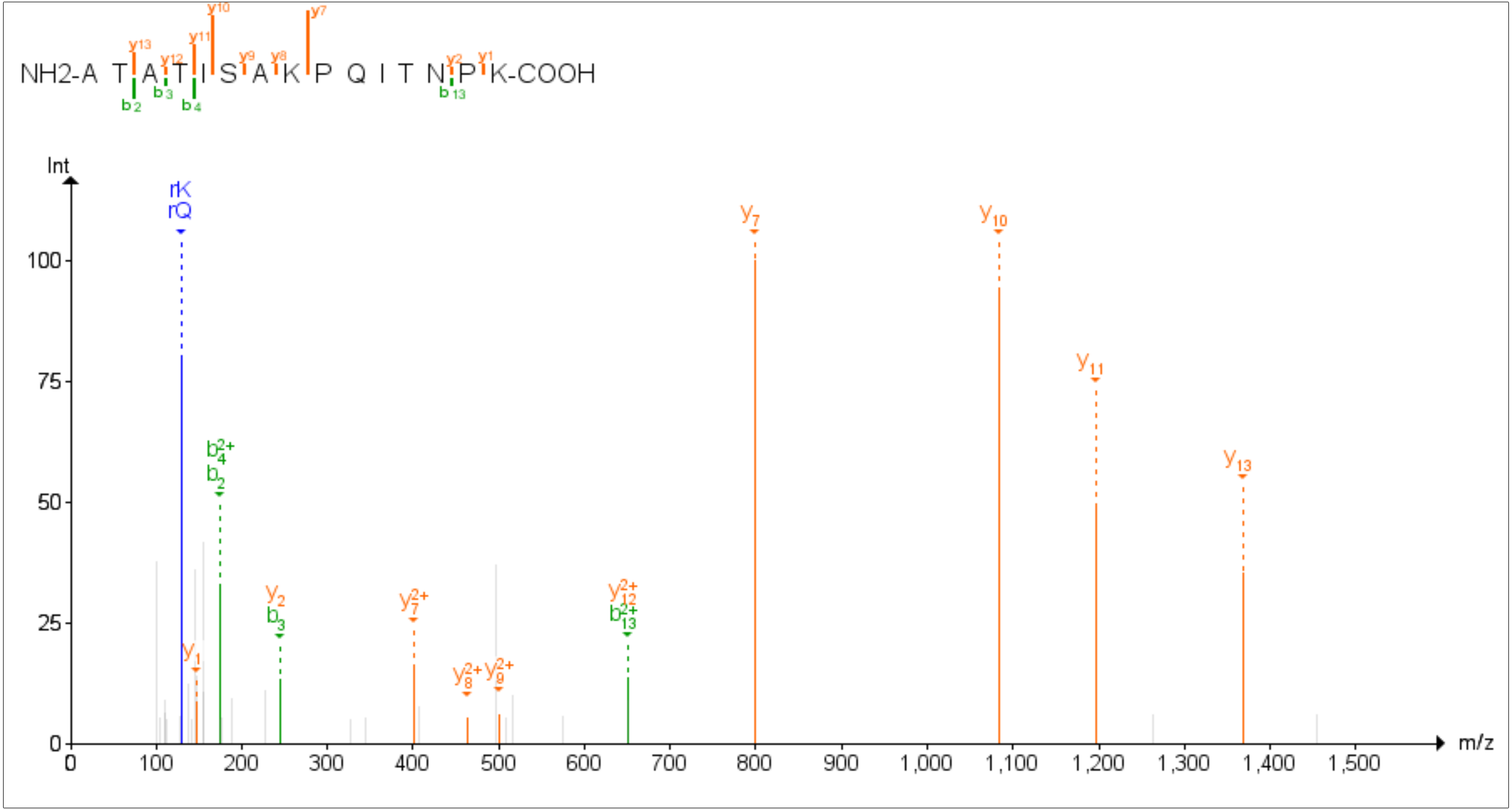

NH2-A T A T I S A K P Q I T N P K-COOH
y13
y12
y11
y10
y9
y8
y7
y2
y1
b2
b3
b4
b13
Int
rK
rQ
y7
y10
y11
y13
b4 2+
b2
y2
b3
y1
y7 2+
y8 2+
y9 2+
y12 2+
b13 2+
0
25
50
75
100
0
100
200
300
400
500
600
700
800
900
1,000
1,100
1,200
1,300
1,400
1,500
m/z

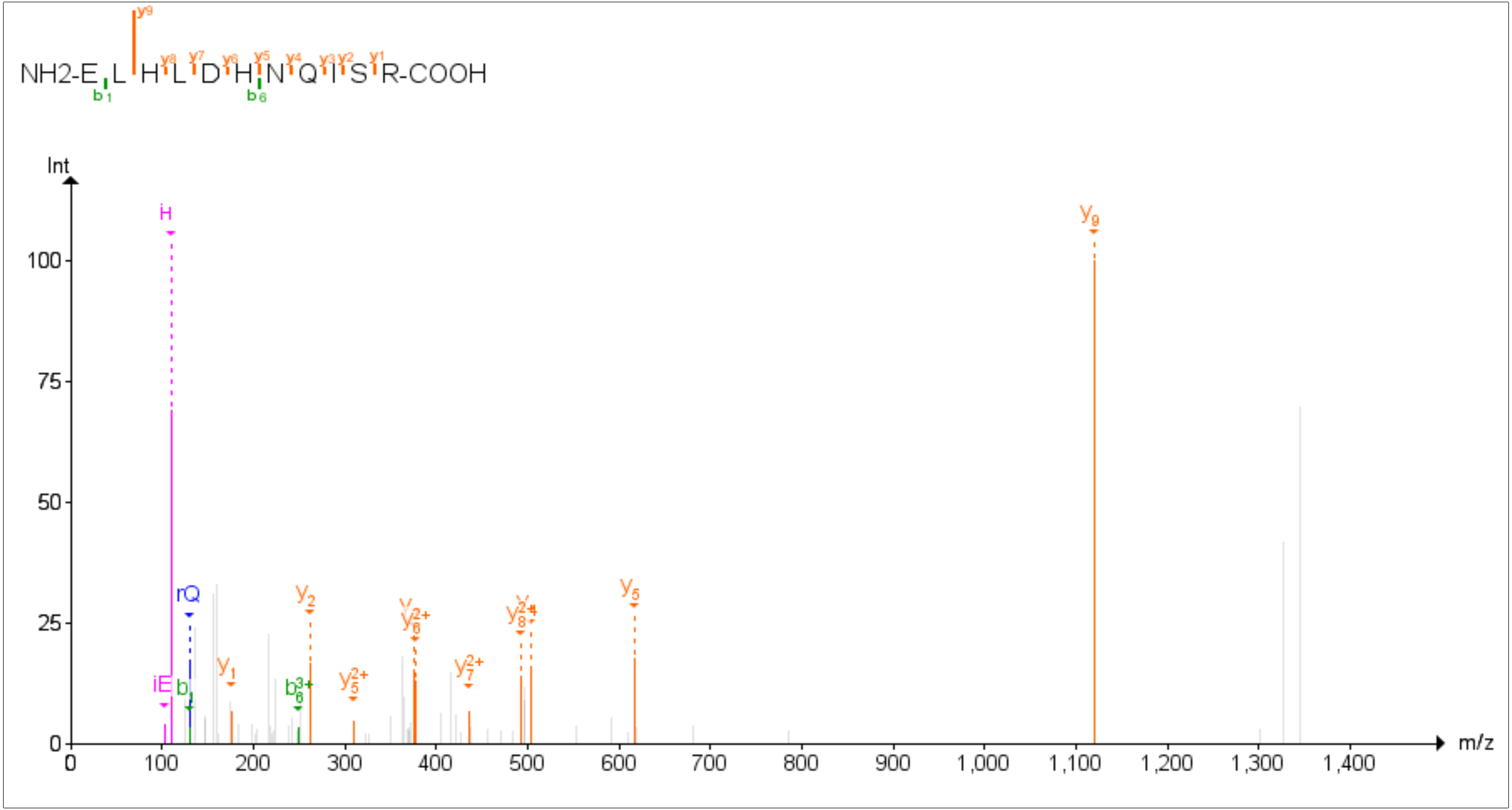
NH2-E L H L D H N Q I S R-COOH
y9 y8 y7 y6 y5 y4 y3 y2 y1
b1 b6
Int
100
75
50
25
0
iH
y9
rQ
y2
y5
iE
b1
y1
b6 3+
y5 2+
y6 2+
y7 2+
y8 2+
0 100 200 300 400 500 600 700 800 900 1,000 1,100 1,200 1,300 1,400
m/z

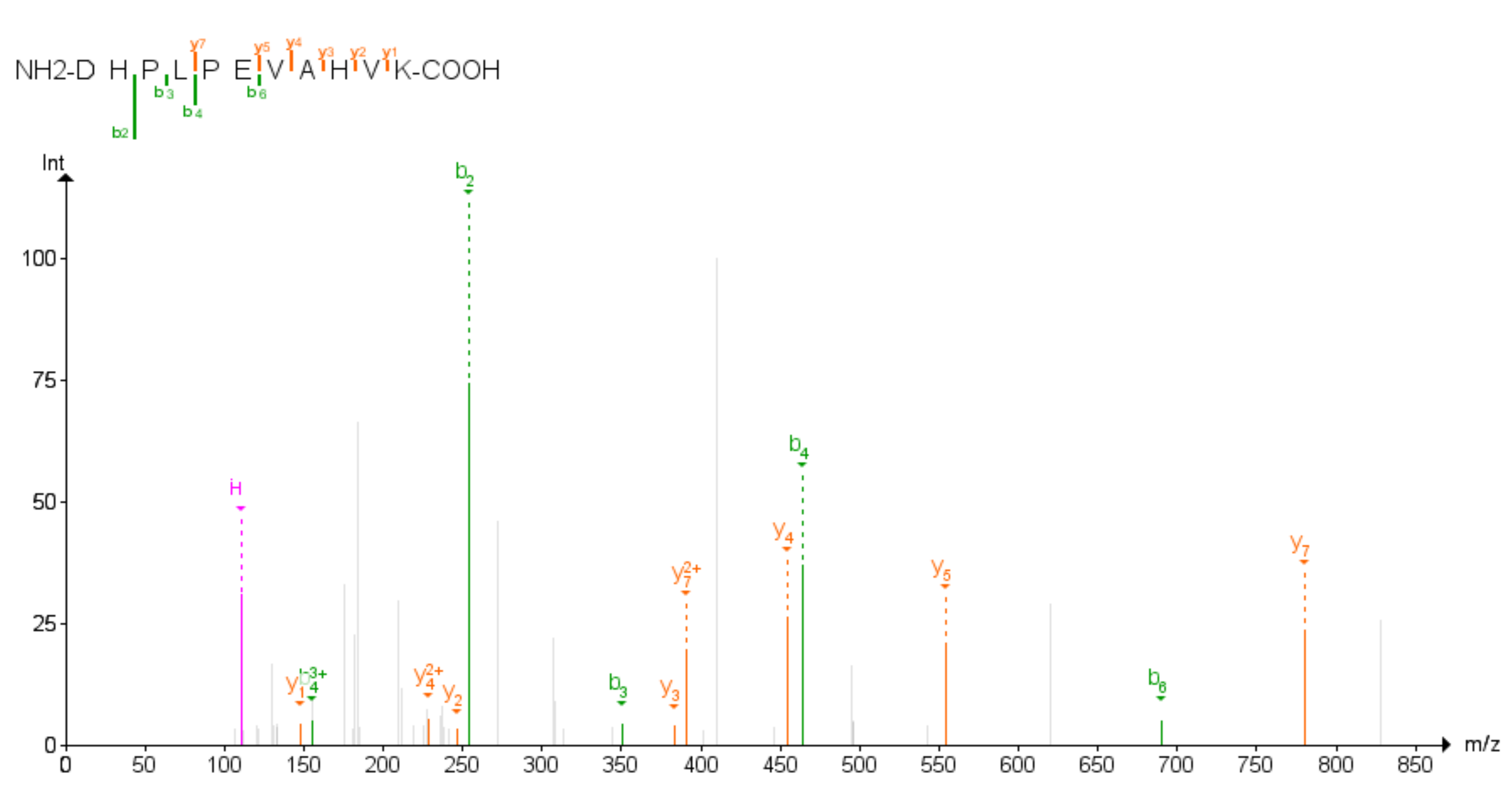
NH2-D H P L P E V A H V K-COOH
y7 y5 y4 y3 y2 y1
b2 b3 b4 b6
Int
100
75
50
25
0
m/z
0 50 100 150 200 250 300 350 400 450 500 550 600 650 700 750 800 850

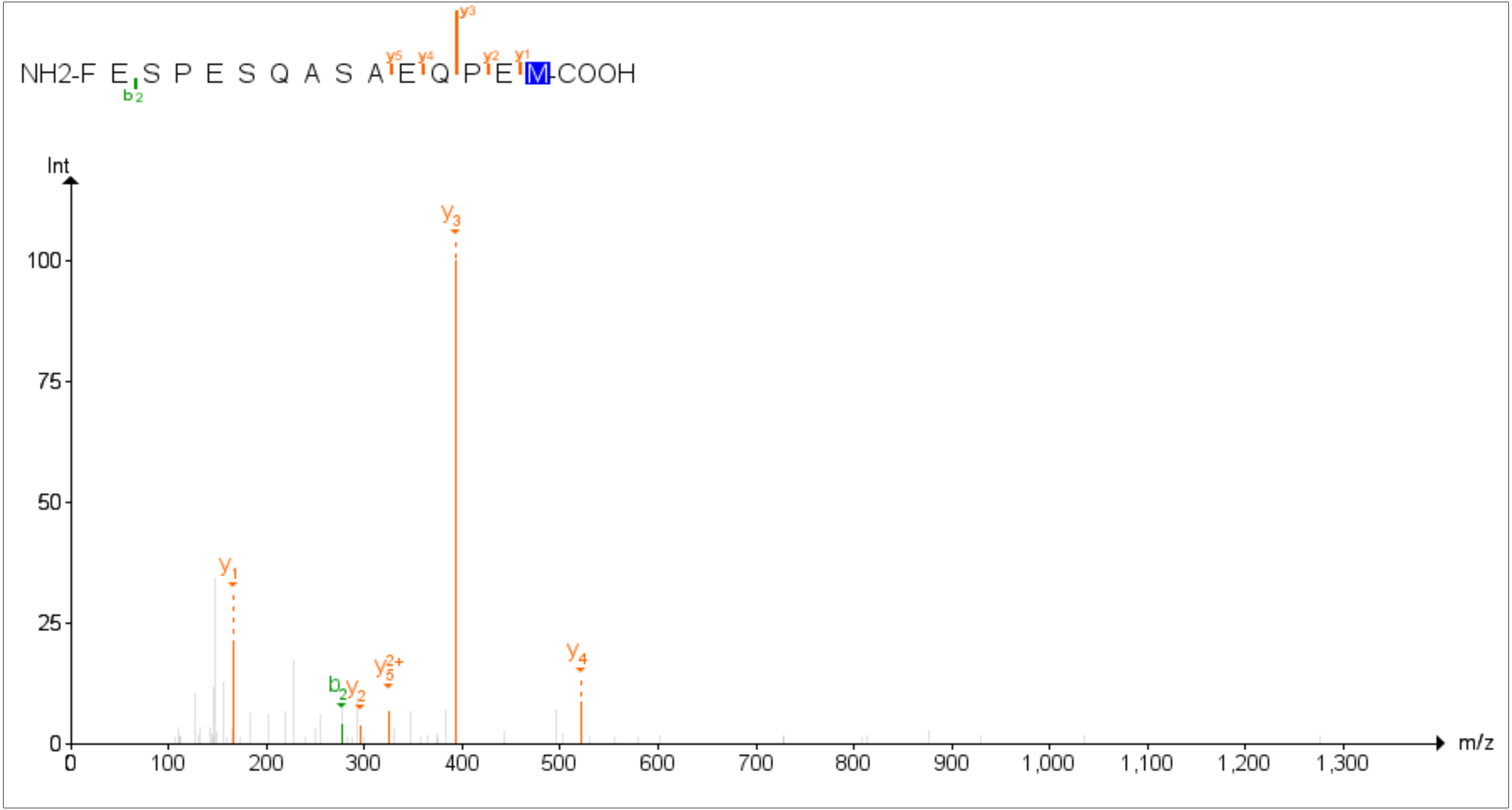

NH2-F E S P E S Q A S A E Q P E M-COOH
b2
y5 y4 y3 y2 y1
Int
100
75
50
25
0
y3
y1
b2 y2
y5 2+
y4
0 100 200 300 400 500 600 700 800 900 1,000 1,100 1,200 1,300
m/z

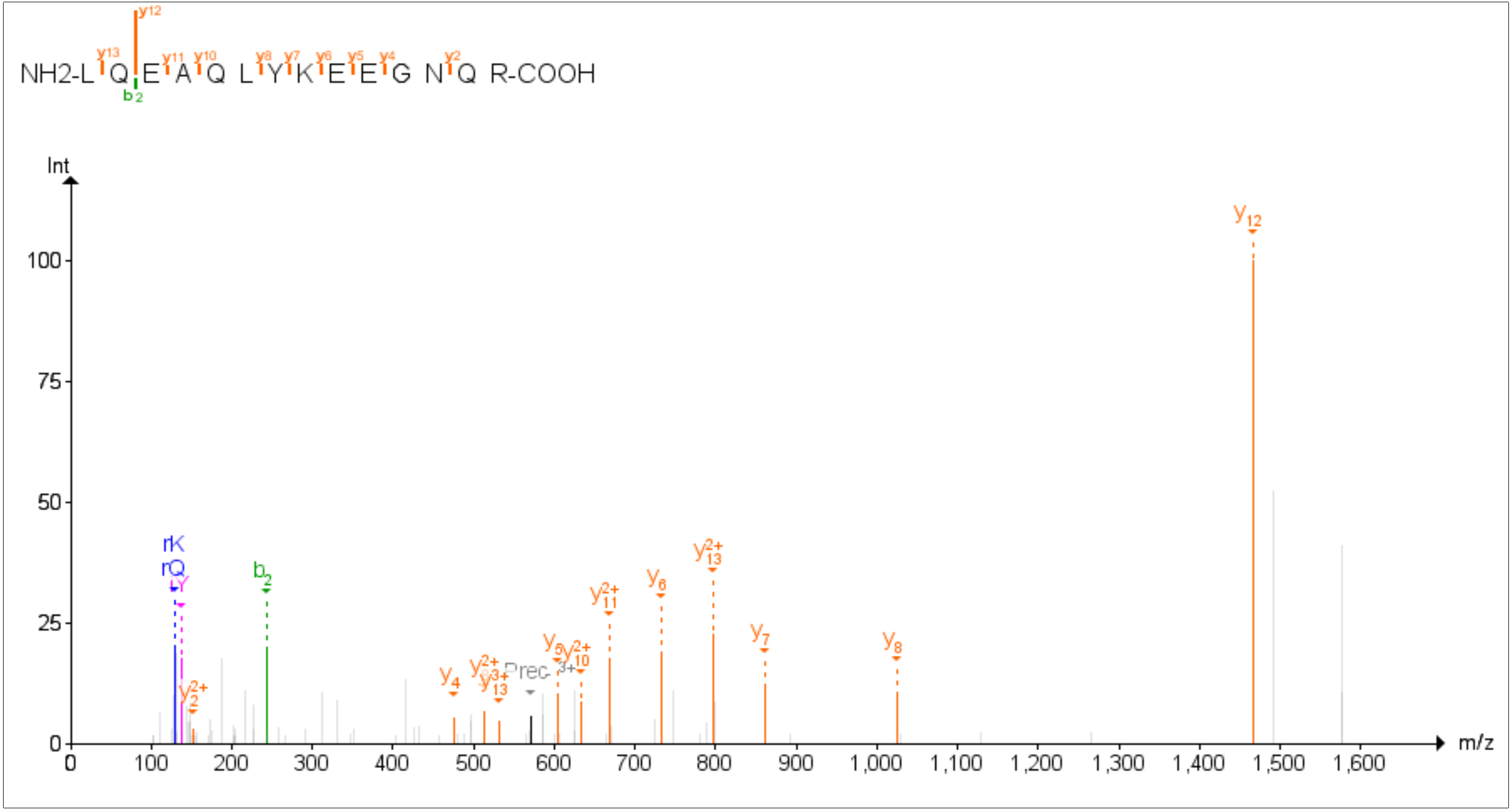

NH2-L Q E A Q L Y K E E G N Q R-COOH
y12
y13
y11
y10
y8
y7
y6
y5
y4
y2
b2
Int
100
75
50
25
0
m/z
rK
rQ
iY
b2
y4
y5
y6
y7
y8
y12
Prec

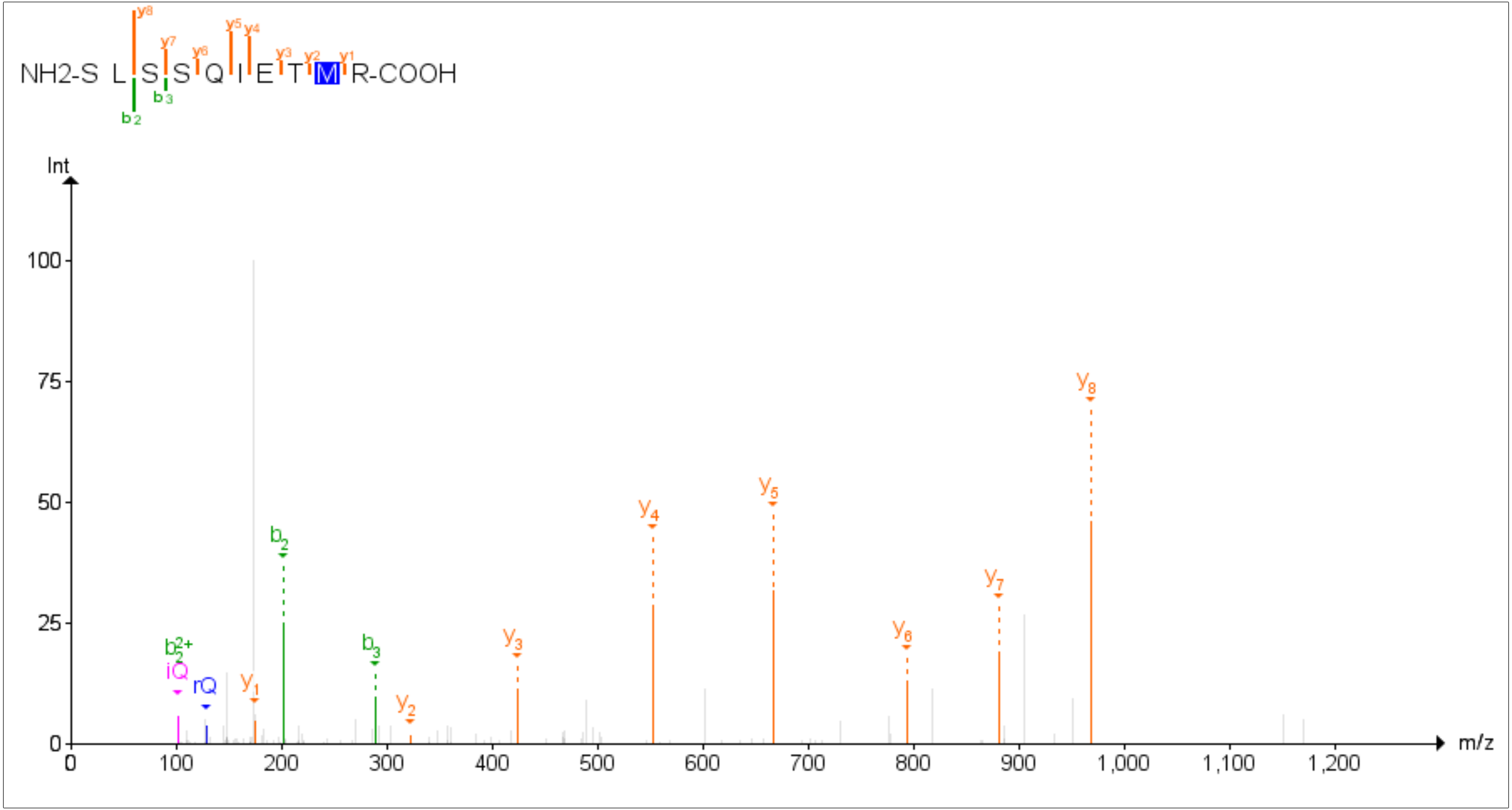

NH2-S L S S Q I E T M R-COOH
y8 y7 y6 y5 y4 y3 y2 y1
b2 b3
Int
100
75
50
25
0
m/z
0 100 200 300 400 500 600 700 800 900 1,000 1,100 1,200
b2 2+
iQ
rQ
y1
b2
b3
y2
y3
y4
y5
y6
y7
y8

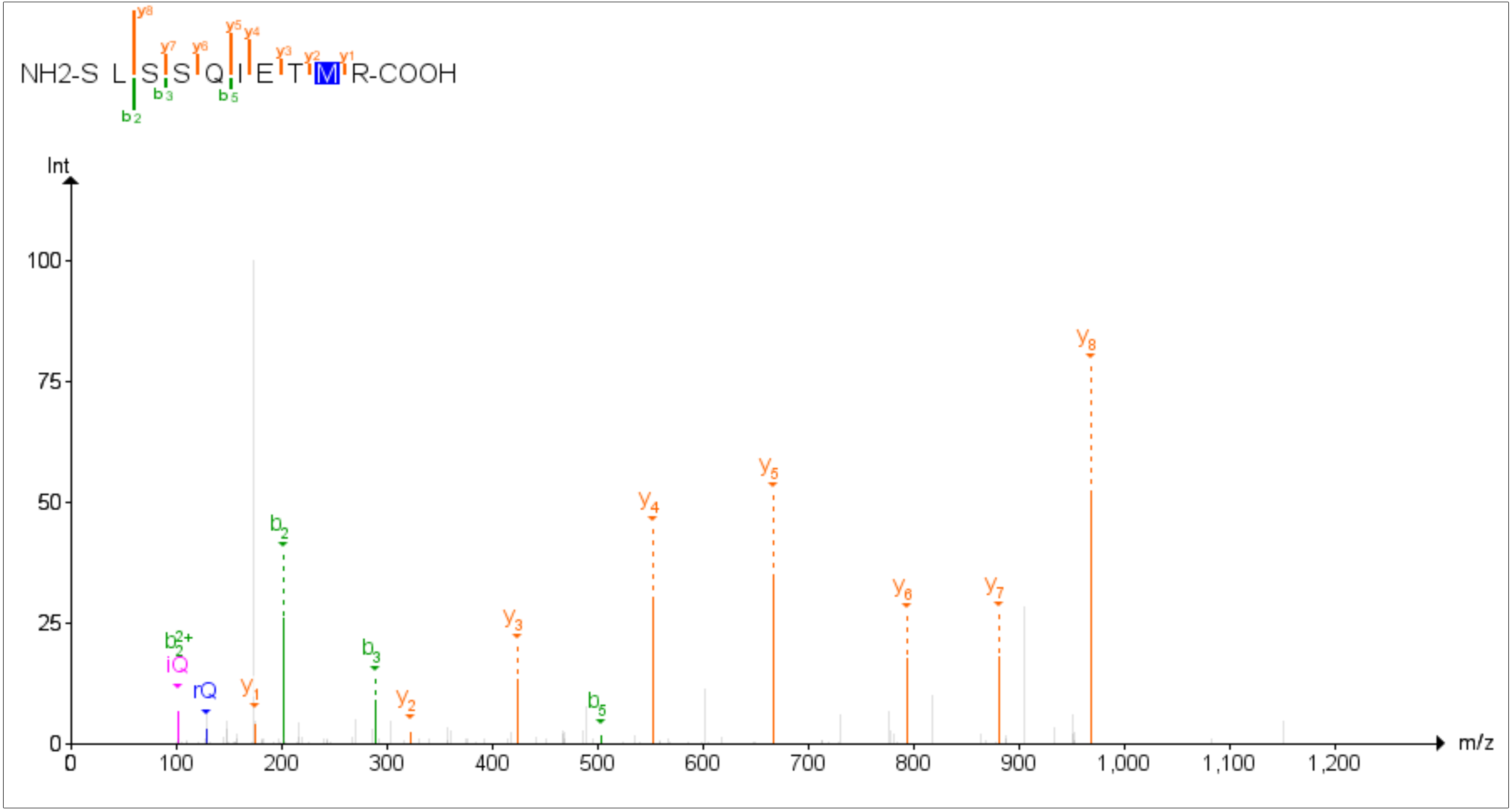
NH2-S L S S Q I E T M R-COOH
y8 y7 y6 y5 y4 y3 y2 y1
b2 b3 b5
Int
m/z
y8 y5 y4 b2 y6 y7 y3 b3 y2 b5 y1
b2 2+
iQ
rQ
0 25 50 75 100
0 100 200 300 400 500 600 700 800 900 1,000 1,100 1,200

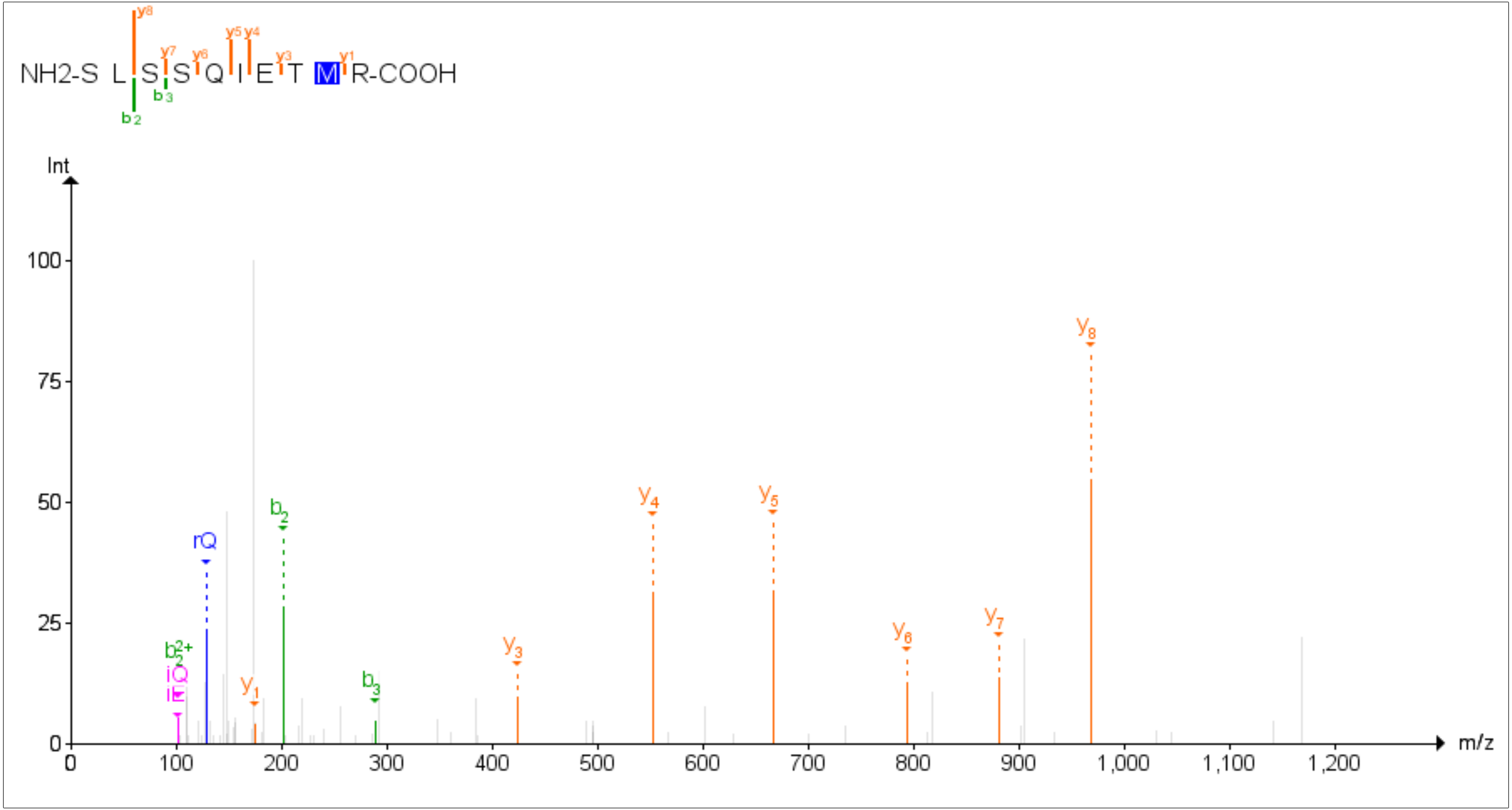

NH2-S L S S Q I E T M R-COOH
y8 y7 y6 y5 y4 y3 y1
b2 b3
Int
100
75
50
25
0
0 100 200 300 400 500 600 700 800 900 1,000 1,100 1,200
m/z
rQ
b2
b2 2+
iQ
iE
y1
b3
y3
y4
y5
y6
y7
y8

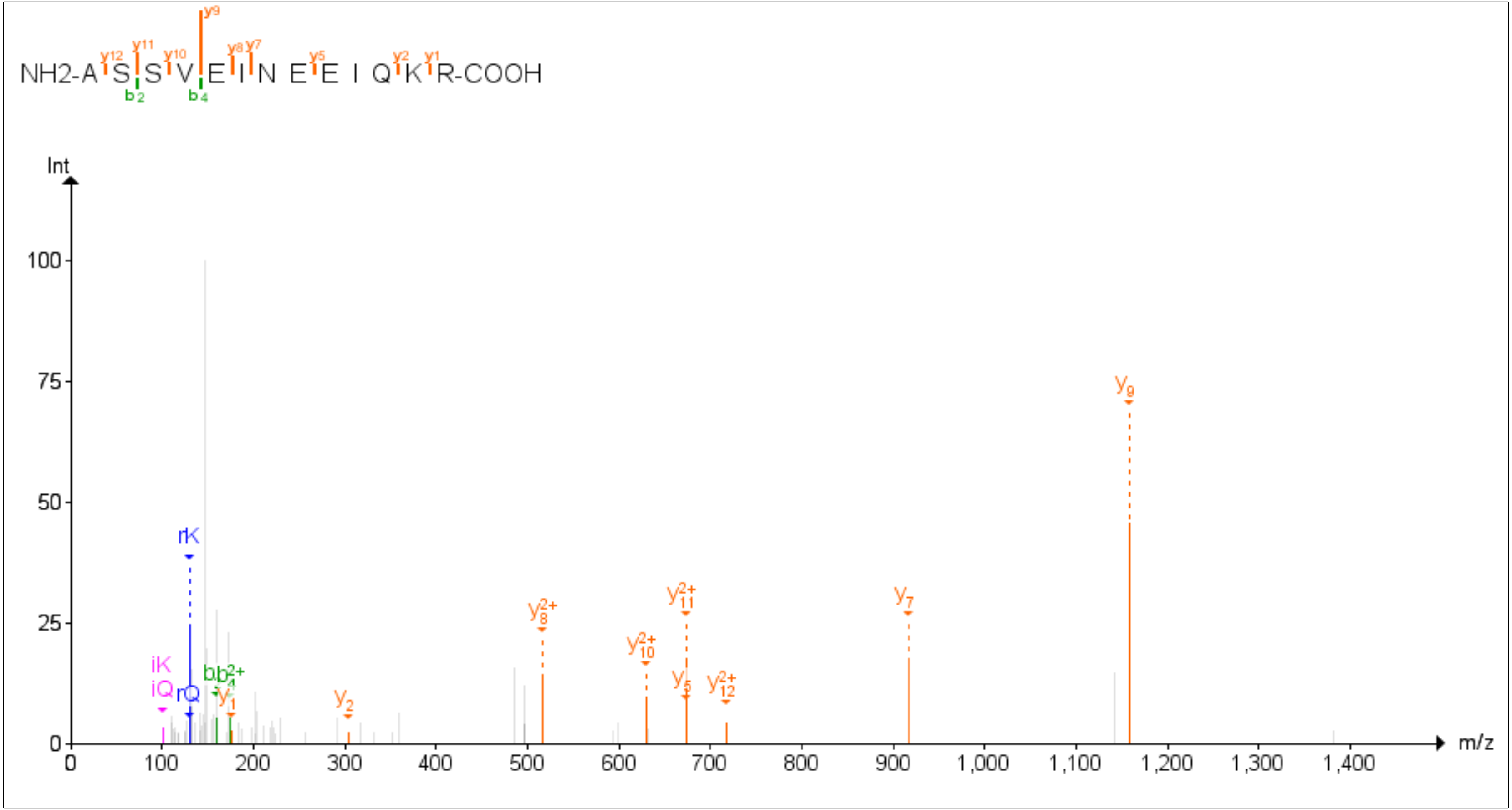
NH2-A S S V E I N E E I Q K R-COOH
Int
m/z
y9
y7
y2
y11
y10
y12
b.b2+
y1
iK
iQ
rQ
rK

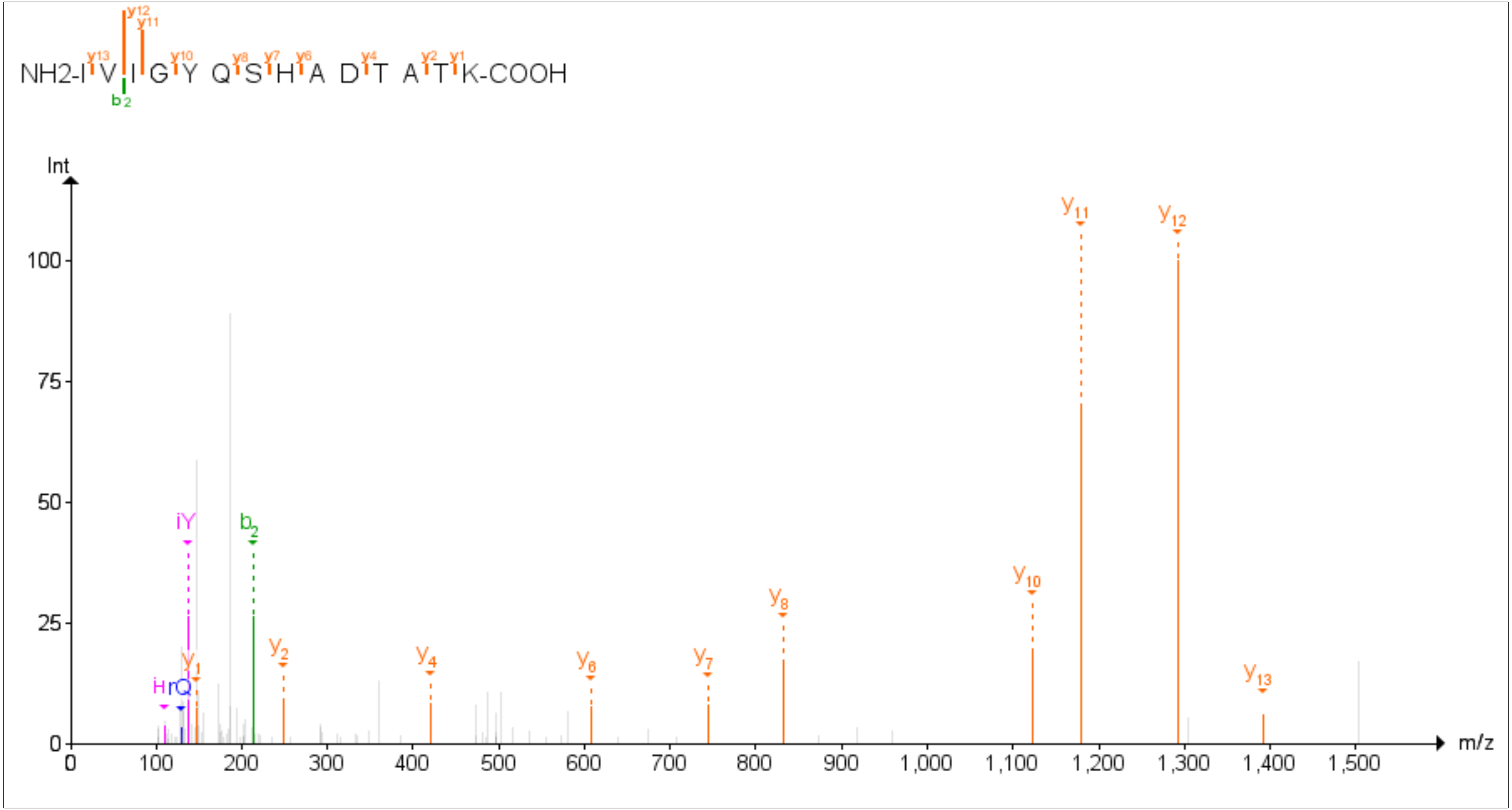

NH2-I V I G Y Q S H A D T A T K-COOH
y13
y12
y11
y10
y8
y7
y6
y4
y2
y1
b2
Int
100
75
50
25
0
iY
b2
y1
y2
y4
y6
y7
y8
y10
y11
y12
y13
iH
rQ
m/z
0
100
200
300
400
500
600
700
800
900
1,000
1,100
1,200
1,300
1,400
1,500

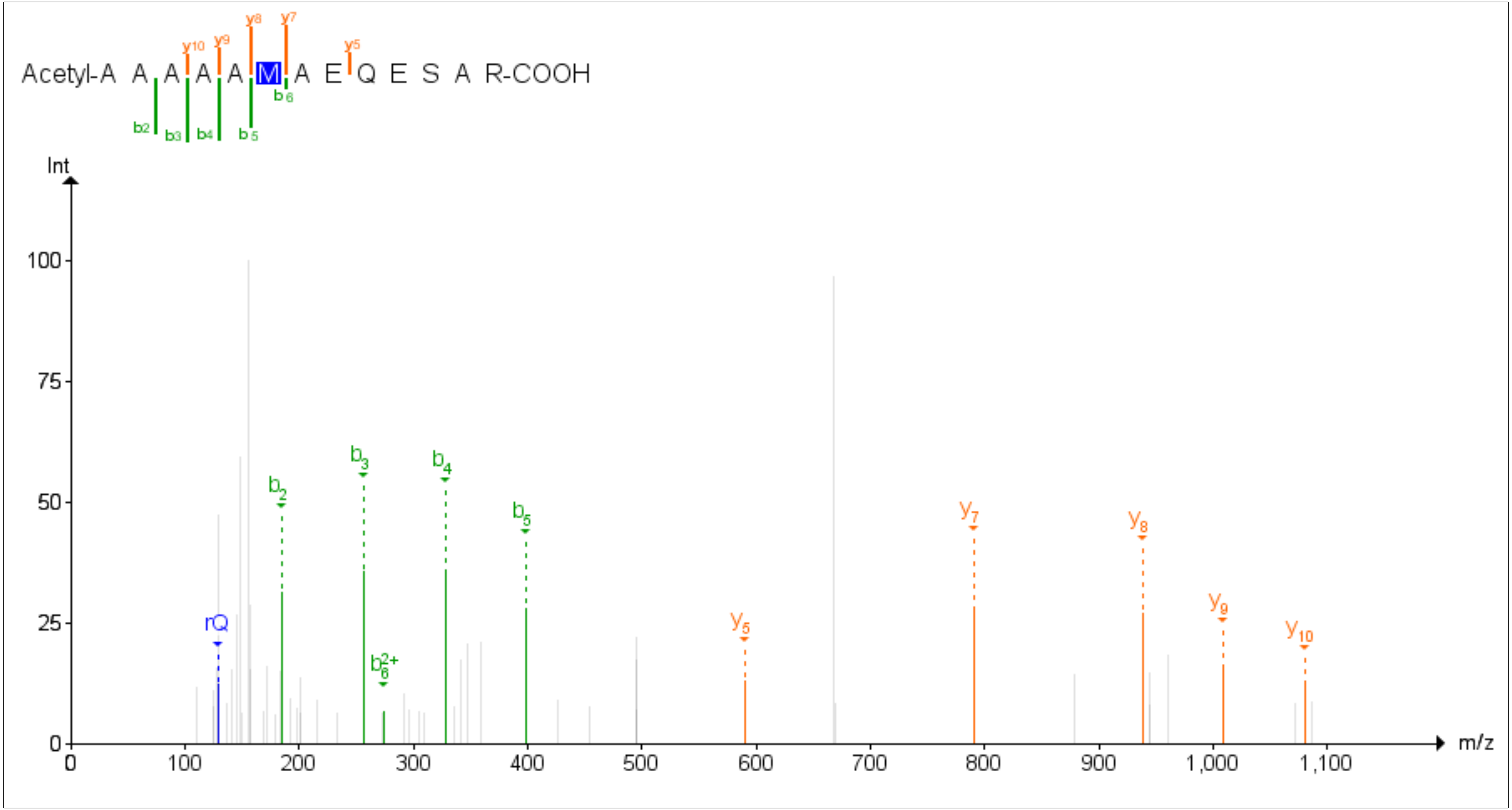

Acetyl-A A A A A M A E Q E S A R-COOH
y10 y9 y8 y7 y5
b2 b3 b4 b5 b6
Int
100
75
50
25
0
rQ
b2
b3
b4
b5
b6 2+
y5
y7
y8
y9
y10
0 100 200 300 400 500 600 700 800 900 1,000 1,100
m/z

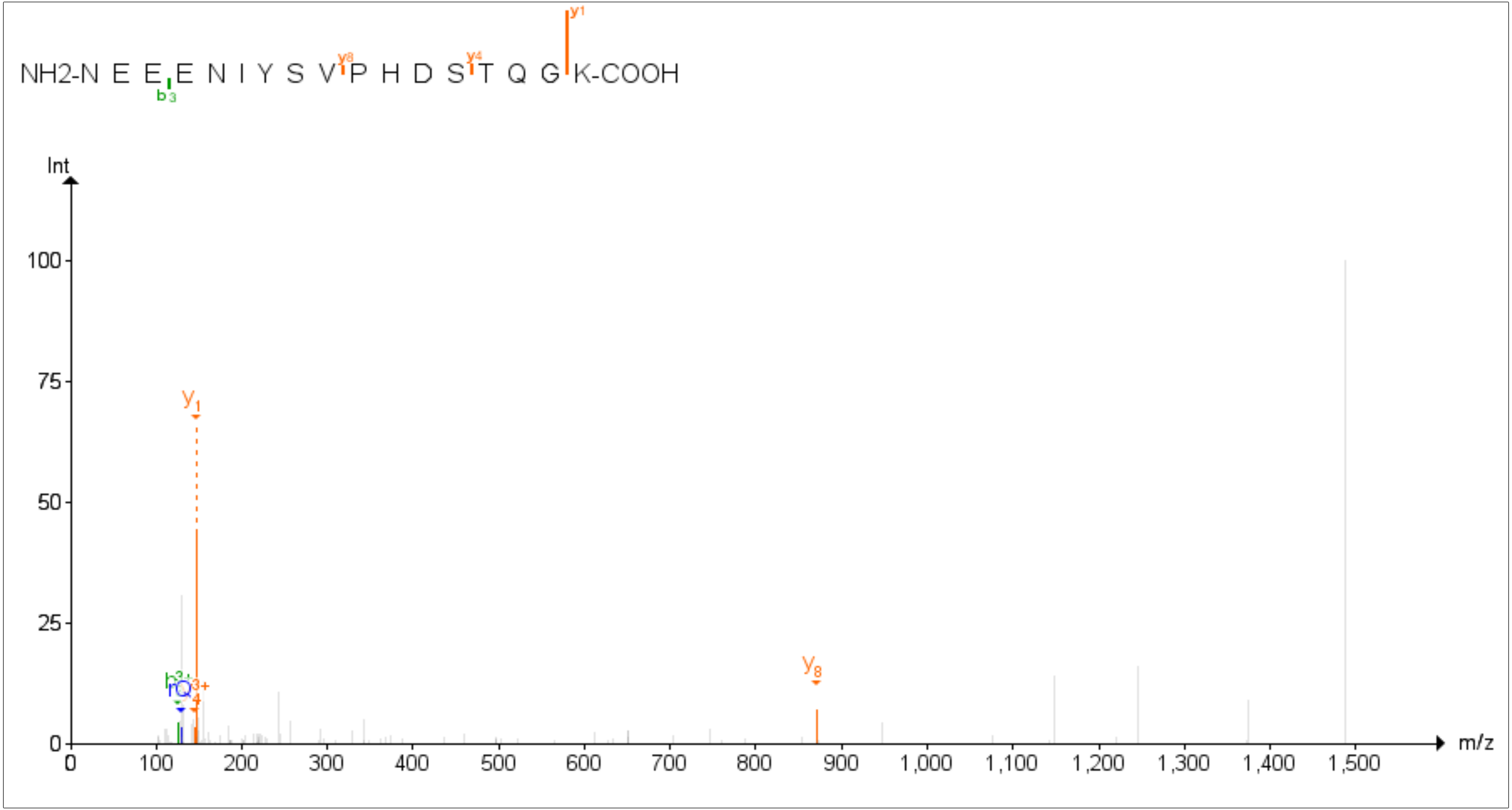

NH2-N E E E N I Y S V P H D S T Q G K-COOH
b3
y8
y4
y1
Int
100
75
50
25
0
y1
y8
m/z
0
100
200
300
400
500
600
700
800
900
1,000
1,100
1,200
1,300
1,400
1,500

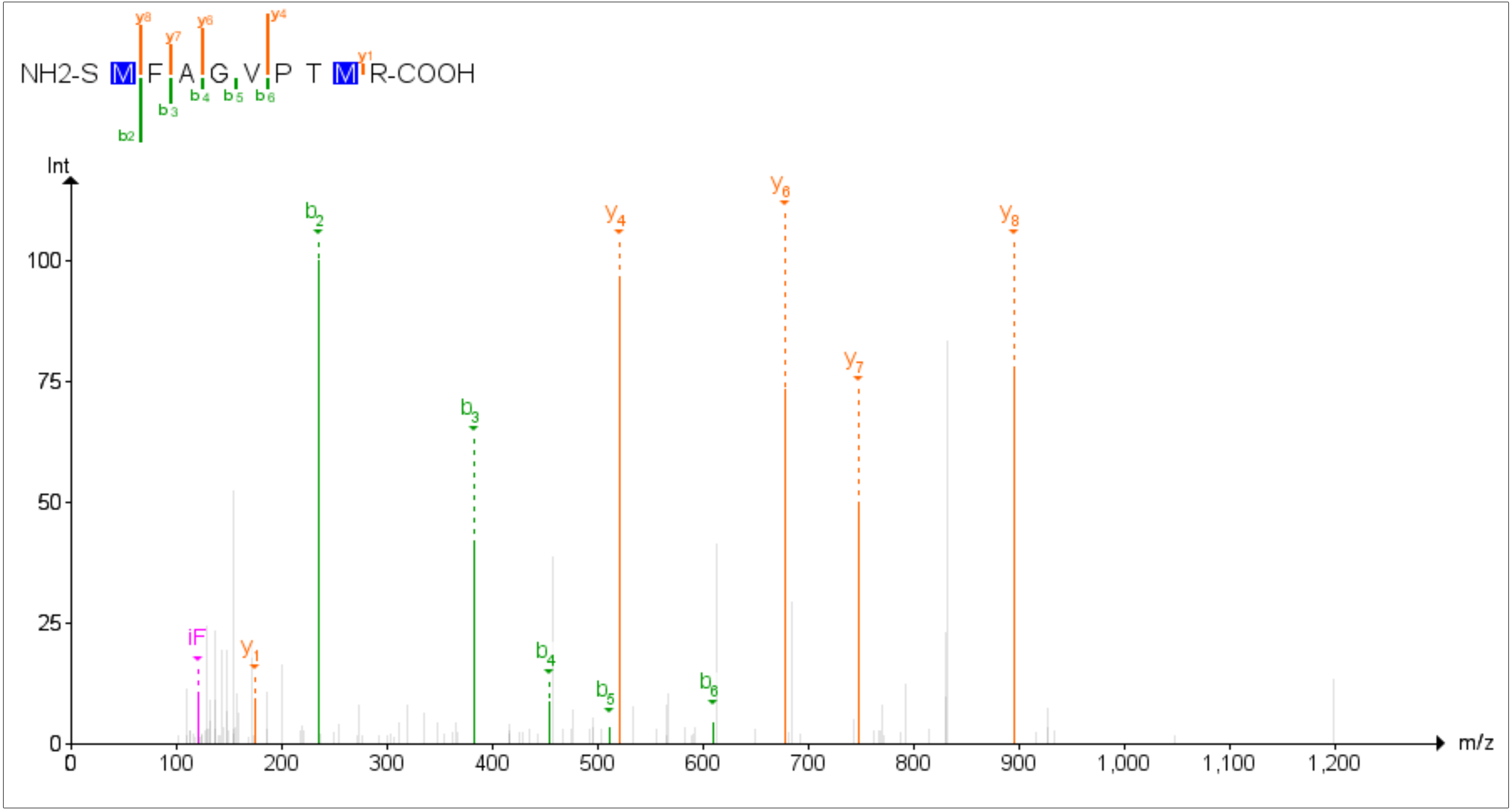

NH2-S M F A G V P T M R-COOH
y8
y7
y6
y4
y1
b2
b3
b4
b5
b6
Int
m/z
b2
b3
b4
b5
b6
y1
y4
y6
y7
y8
iF
0
25
50
75
100
0
100
200
300
400
500
600
700
800
900
1,000
1,100
1,200

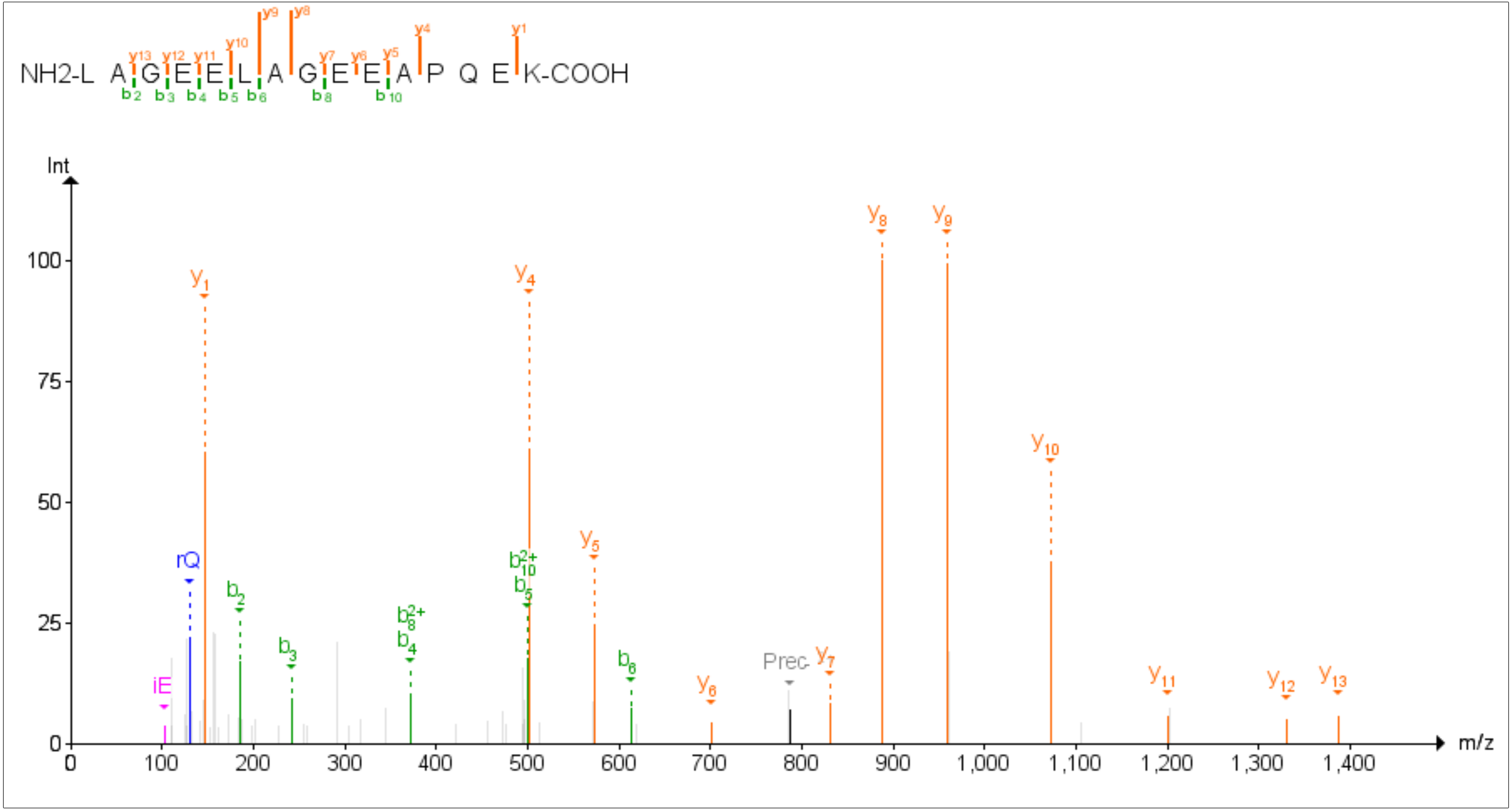

NH2-L A G E E L A G E E A P Q E K-COOH
y13 y12 y11 y10 y9 y8 y7 y6 y5 y4 y1
b2 b3 b4 b5 b6 b8 b10
Int
m/z
y1
y4
y8
y9
y10
y5
rQ
b2
b3
b8 2+
b4
b10 2+
b5
b6
iE
y6
Prec
y7
y11
y12
y13
0
25
50
75
100
0
100
200
300
400
500
600
700
800
900
1,000
1,100
1,200
1,300
1,400

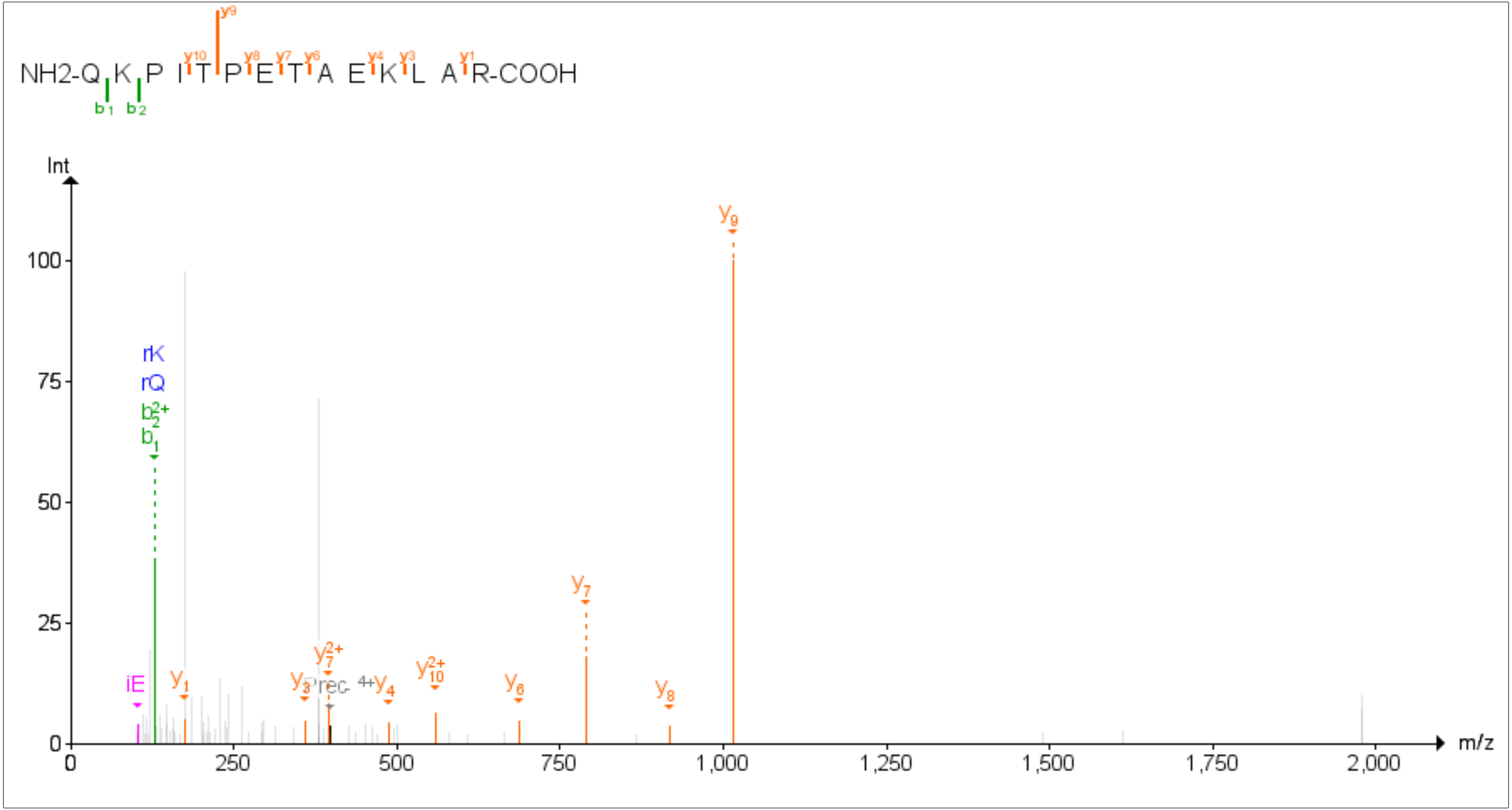

NH2-Q K P I T P E T A E K L A R-COOH
y9
y10
y8
y7
y6
y4
y3
y1
b1
b2
Int
100
75
50
25
0
rK
rQ
b_2^{2+}
b_1
iE
y_1
y_3
y_7^{2+}
Prec- 4+
y_4
y_{10}^{2+}
y_6
y_7
y_8
y_9
0
250
500
750
1,000
1,250
1,500
1,750
2,000
m/z

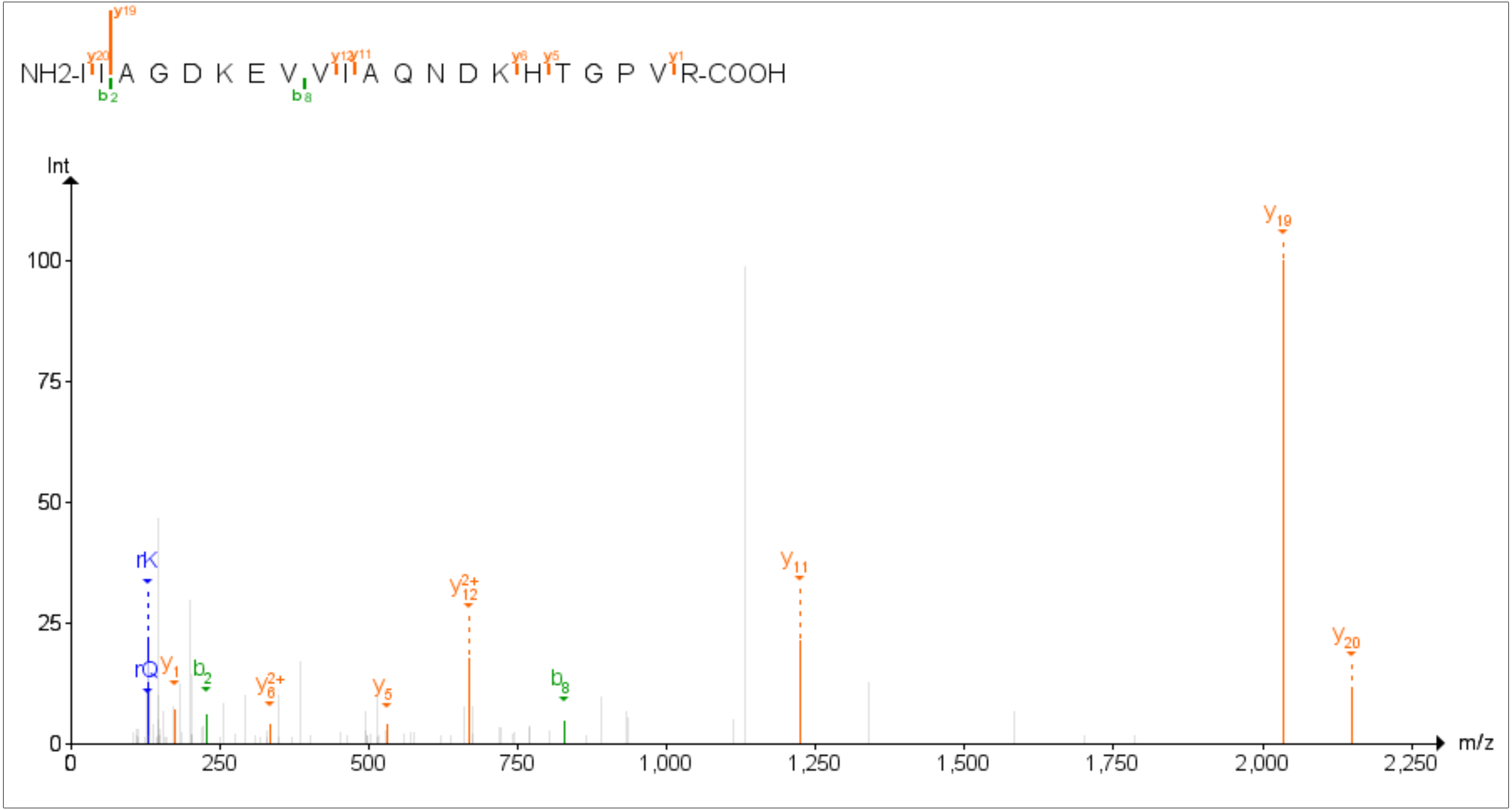

NH2-I I A G D K E V V I A Q N D K H T G P V R-COOH
y20
y19
y12
y11
y6
y5
y1
b2
b8
Int
100
75
50
25
0
m/z
0
250
500
750
1,000
1,250
1,500
1,750
2,000
2,250
rK
rQ
y1
b2
y6 2+
y5
y12 2+
b8
y11
y19
y20

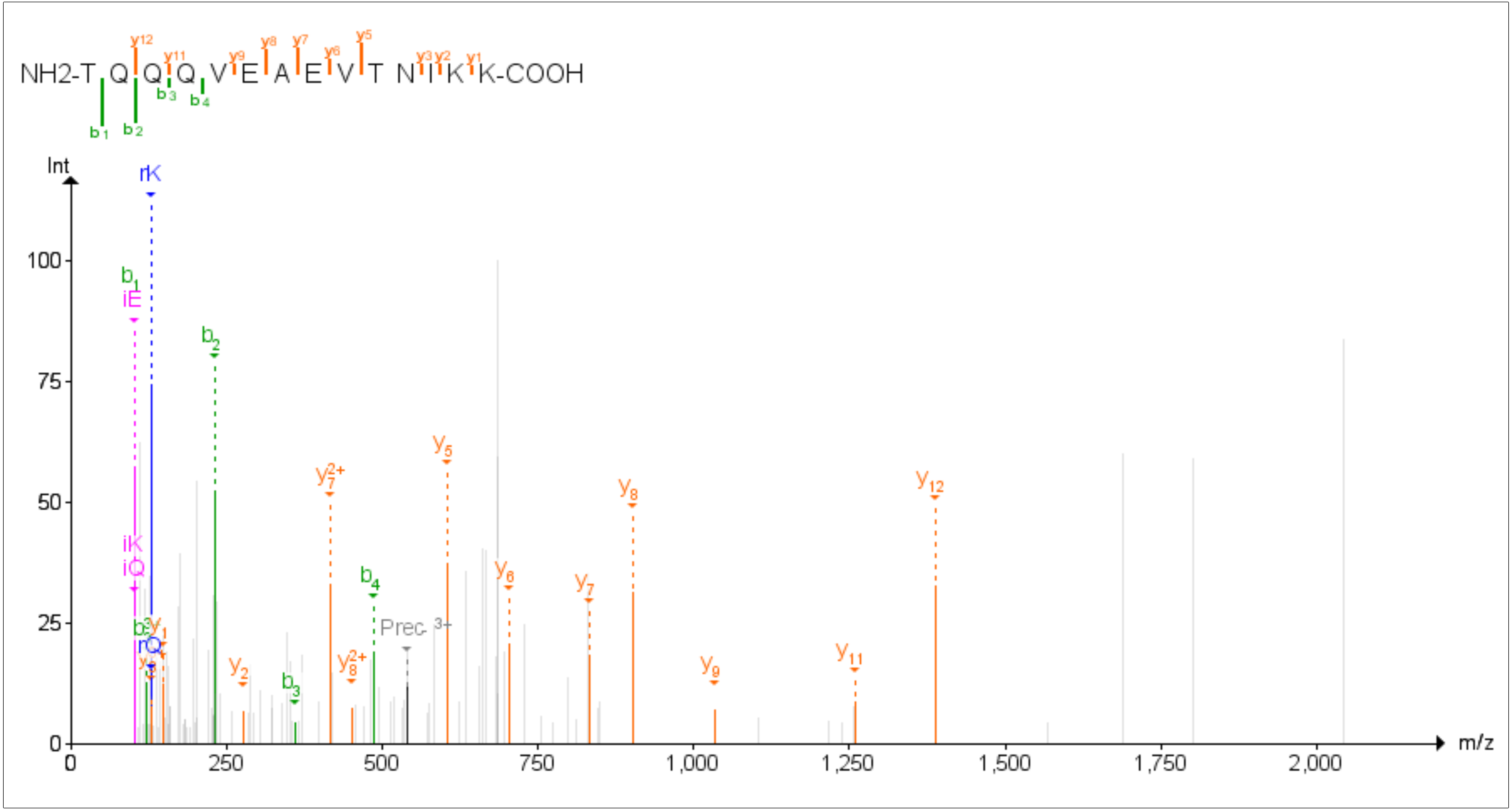

NH2-T Q Q Q V E A E V T N I K K-COOH
Int
m/z
rK
b1
iE
b2
iK
iQ
y5
y8
y12
b4
y6
y7
Prec 3+
y2
b3
y9
y11
rQ
y1
0
25
50
75
100
250
500
750
1,000
1,250
1,500
1,750
2,000

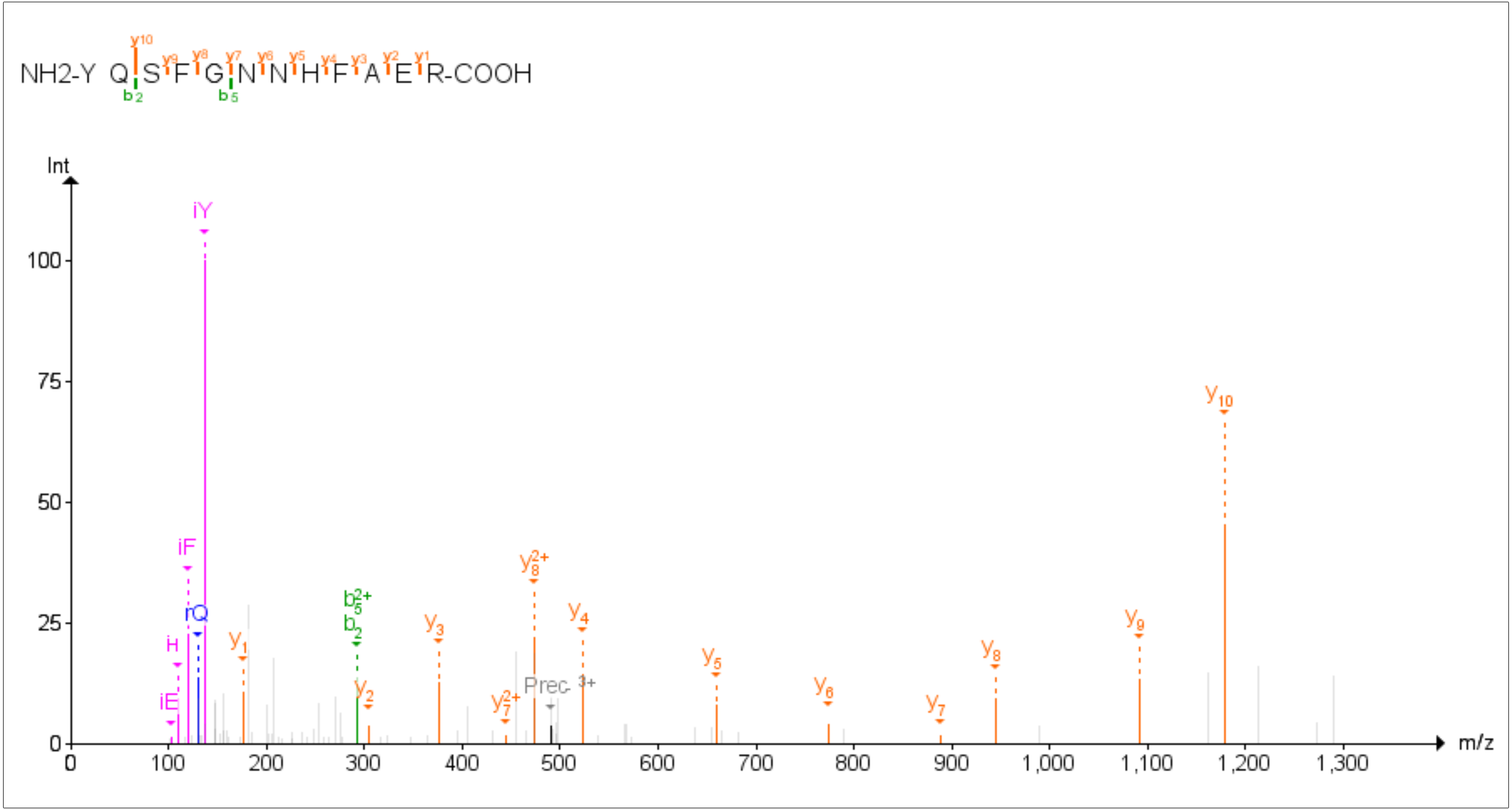
NH2-Y Q S F G N N H F A E R-COOH
y10 y9 y8 y7 y6 y5 y4 y3 y2 y1
b2 b5
Int
m/z
iY
iF
rQ
iH
iE
y1
y2
y3
y4
y5
y6
y7
y8
y9
y10
y8 2+
y7 2+
b5 2+
b2
Prec 3+
0
25
50
75
100
0
100
200
300
400
500
600
700
800
900
1,000
1,100
1,200
1,300

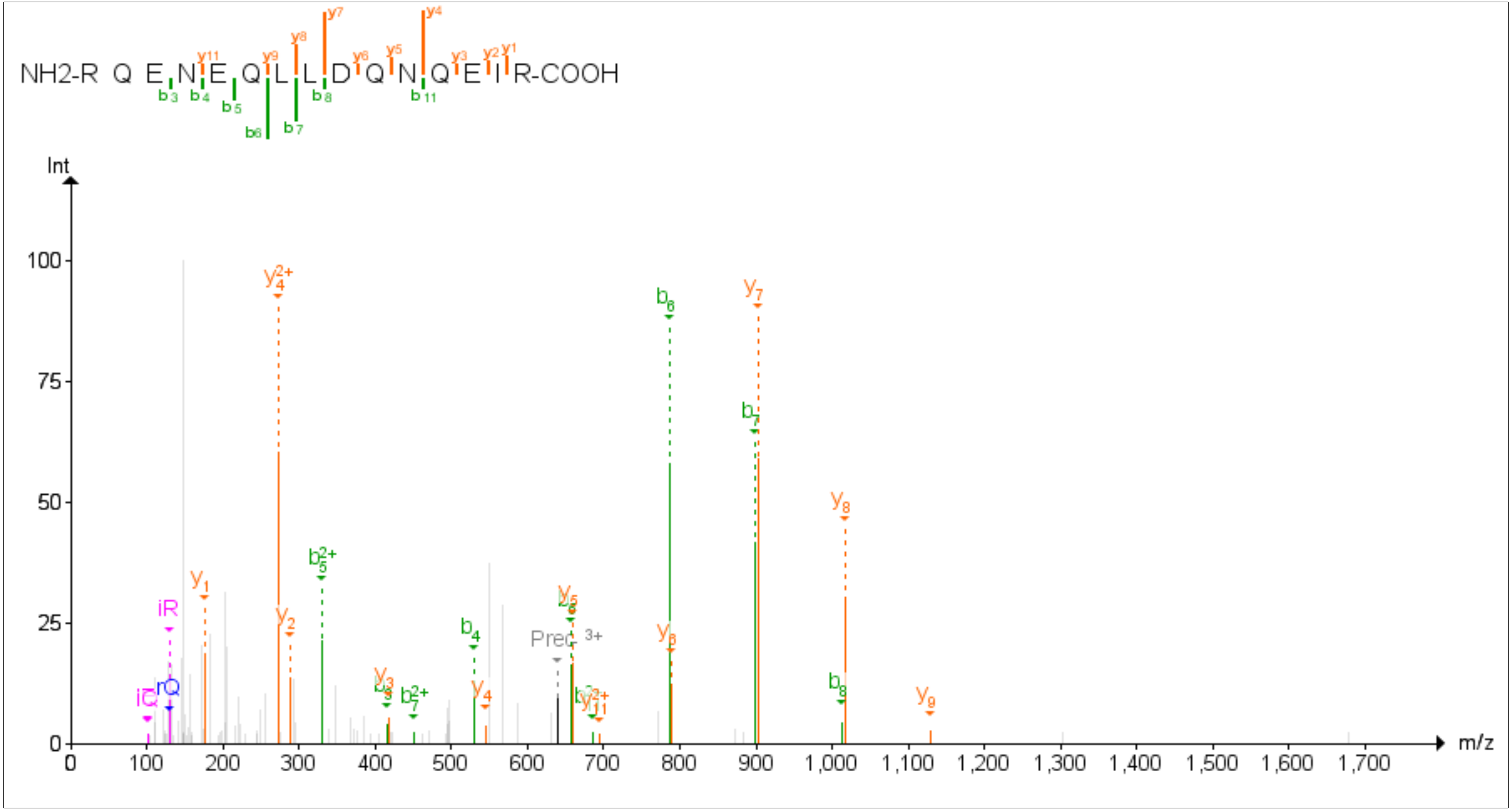
NH2-R Q E N E Q L L D Q N Q E I R-COOH
Int
m/z
Prec 3+

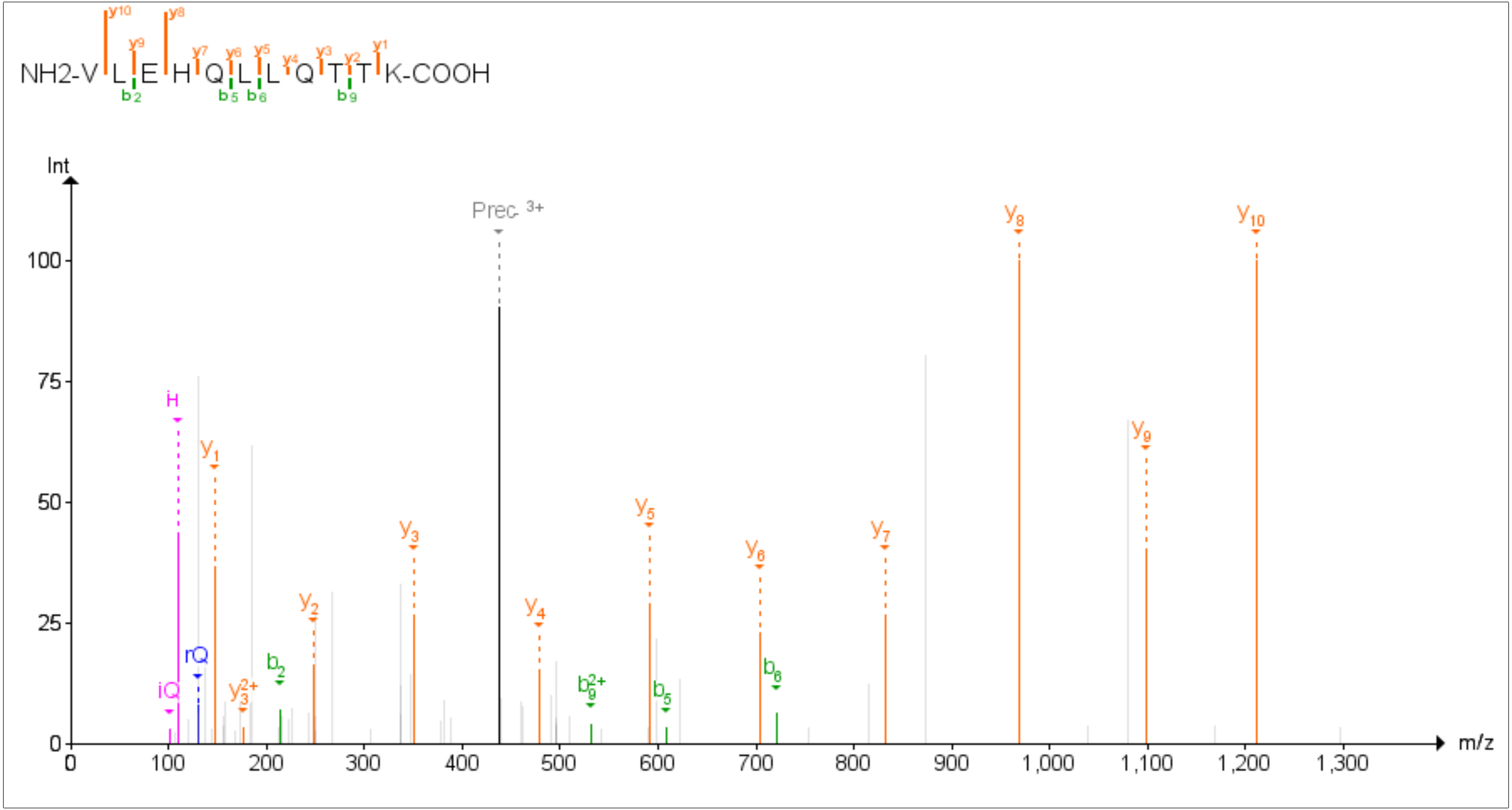

NH2-V L E H Q L L Q T T K-COOH
y10 y9 y8 y7 y6 y5 y4 y3 y2 y1
b2 b5 b6 b9
Int
m/z
Prec 3+
y8
y10
iH
y1
y9
y5
y3
y7
y6
y4
y2
rQ
b2
iQ
y3 2+
b9 2+
b5
b6
0
25
50
75
100
0
100
200
300
400
500
600
700
800
900
1,000
1,100
1,200
1,300

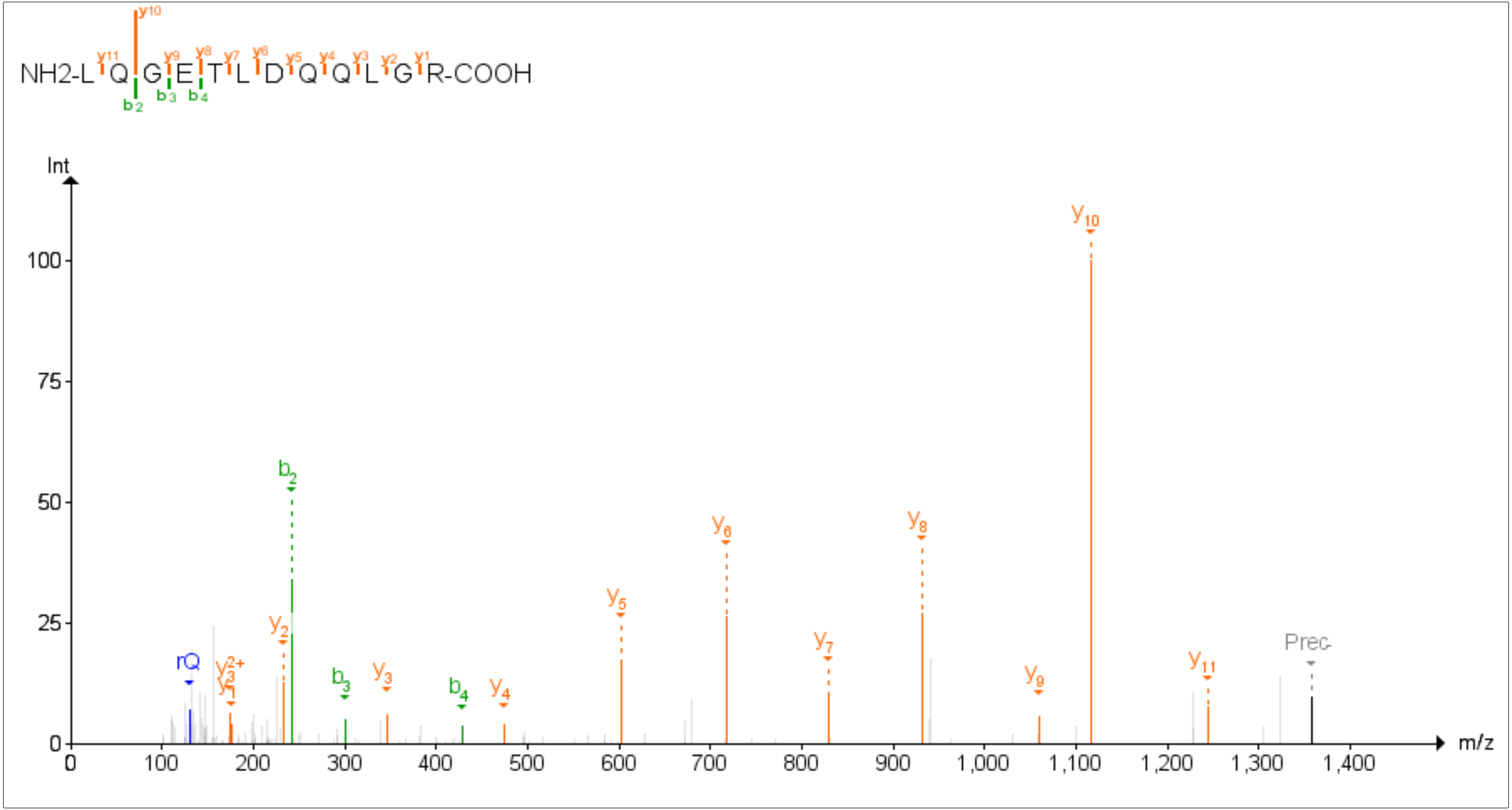

NH2-L Q G E T L D Q Q L G R-COOH
y11 y10 y9 y8 y7 y6 y5 y4 y3 y2 y1
b2 b3 b4
Int
100
75
50
25
0
rQ
y3 2+
y1
y2
b2
b3
y3
b4
y4
y5
y6
y7
y8
y9
y10
y11
Prec
0 100 200 300 400 500 600 700 800 900 1,000 1,100 1,200 1,300 1,400
m/z

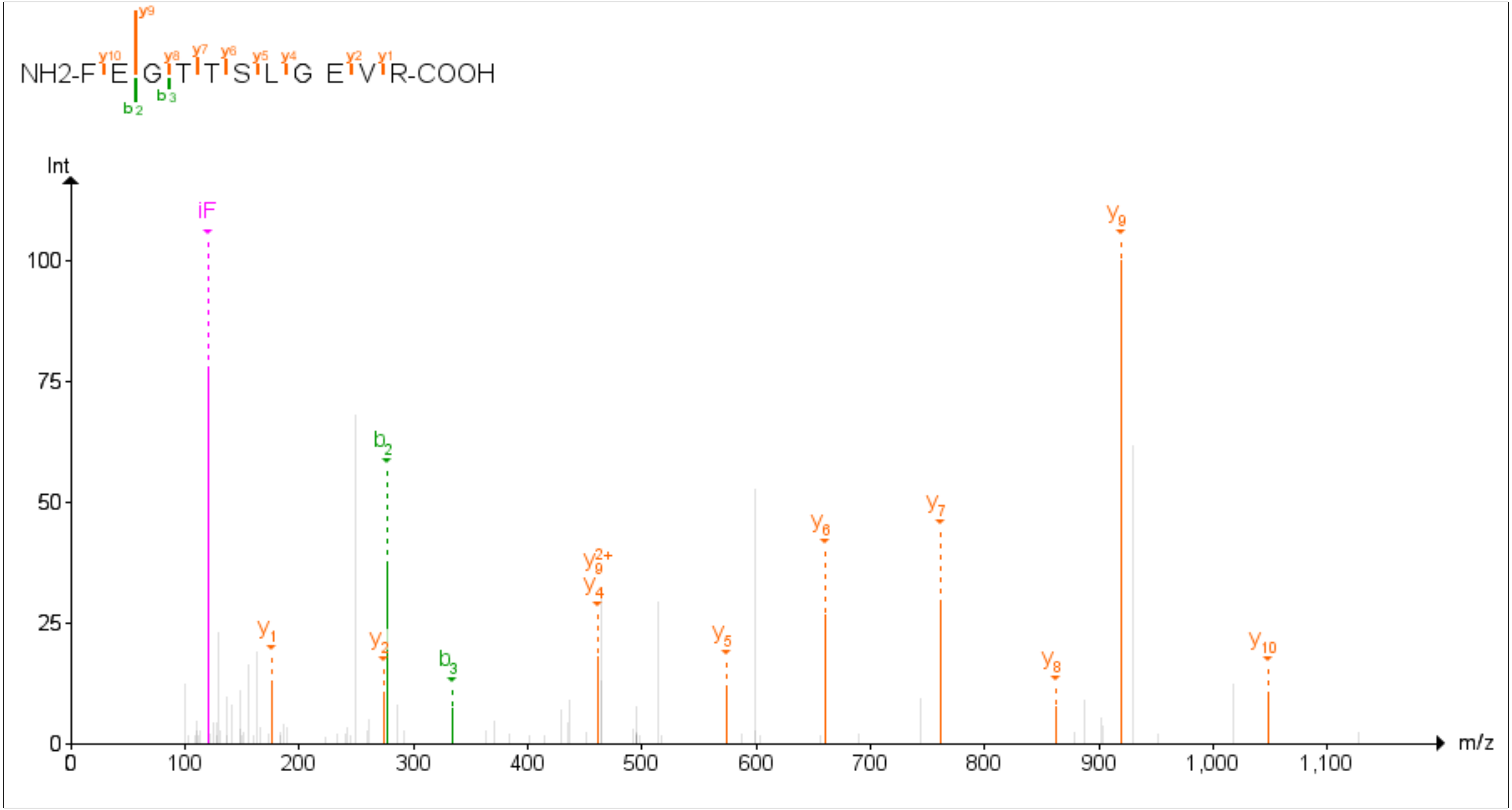

NH2-F E G T T S L G E V R-COOH
y10 y9 y8 y7 y6 y5 y4 y2 y1
b2 b3
Int
m/z
iF
y1
y2
b2
b3
y9 2+
y4
y5
y6
y7
y8
y9
y10
0
25
50
75
100
0
100
200
300
400
500
600
700
800
900
1,000
1,100

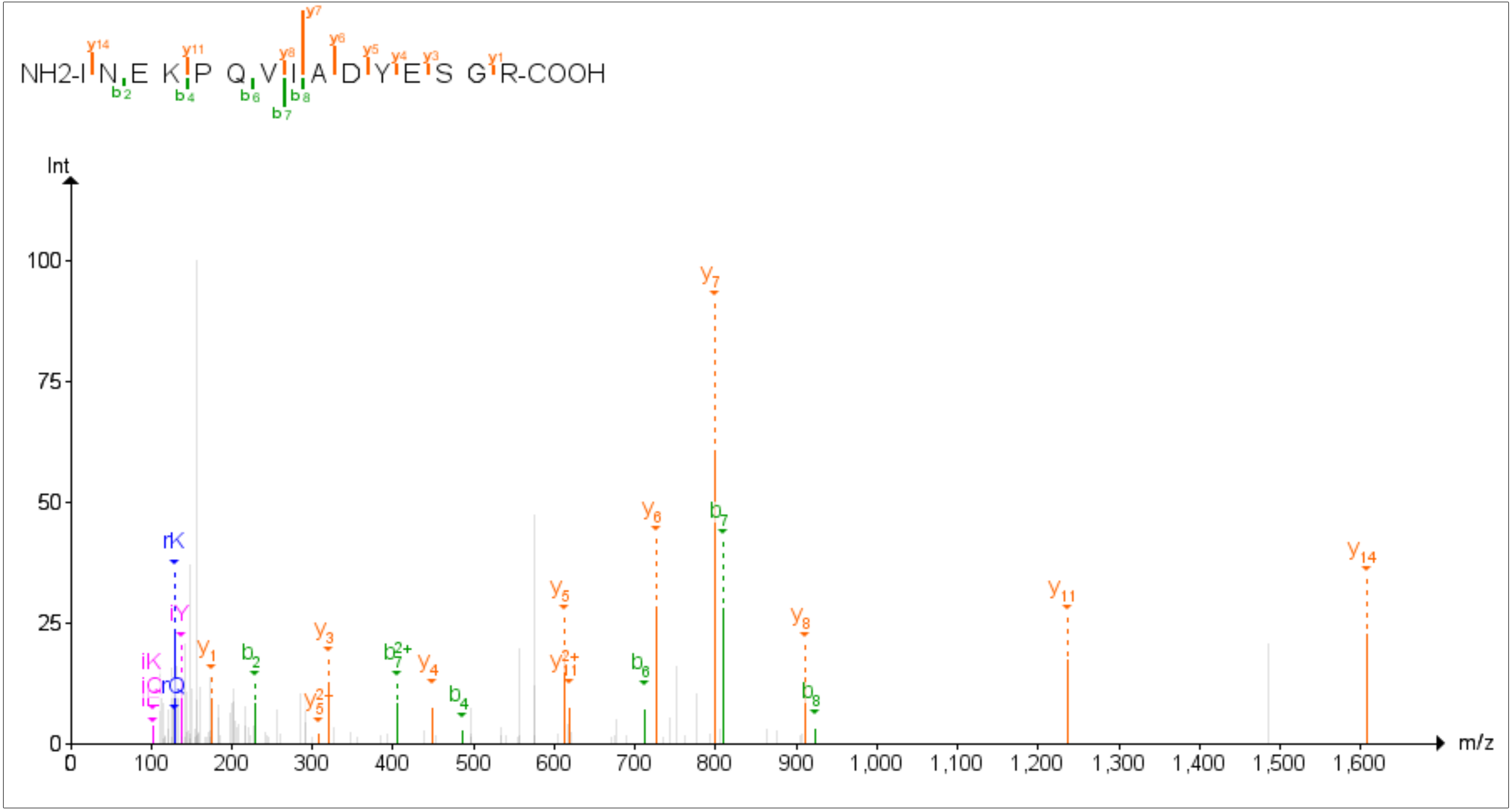
NH2-I N E K P Q V I A D Y E S G R-COOH
Int
m/z
0
25
50
75
100
0
100
200
300
400
500
600
700
800
900
1,000
1,100
1,200
1,300
1,400
1,500
1,600
y7
y6
b7
y14
y11
y8
b8
y5
y2+11
b6
y4
b4
b2+7
y3
y1
b2
y2+5
rK
iY
iK
iQ
rQ
iE

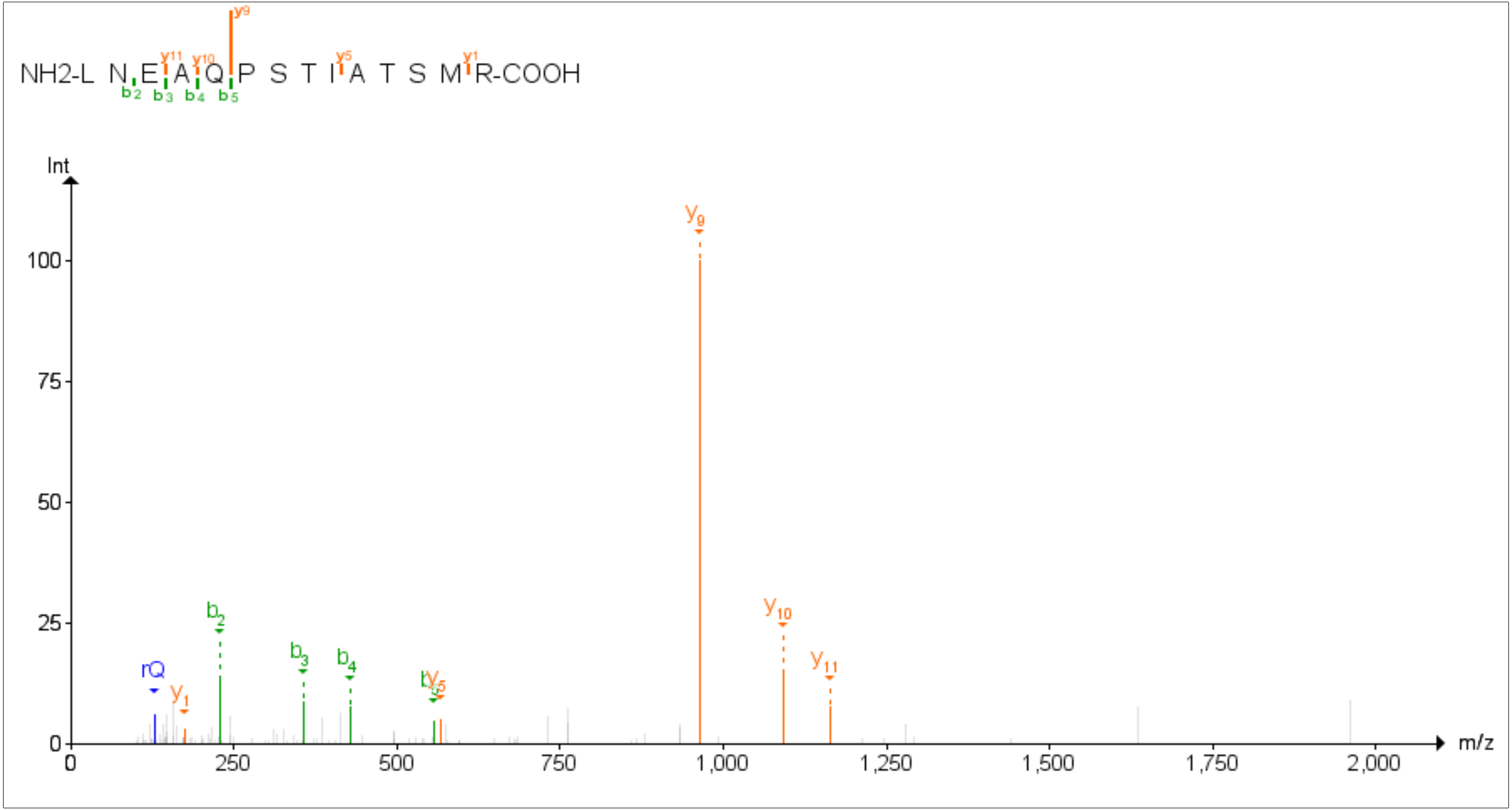

NH2-L N E A Q P S T I A T S M R-COOH
y9
y11
y10
y5
y1
b2
b3
b4
b5
Int
100
75
50
25
0
y9
y10
y11
b2
b3
b4
y5
rQ
y1
0
250
500
750
1,000
1,250
1,500
1,750
2,000
m/z

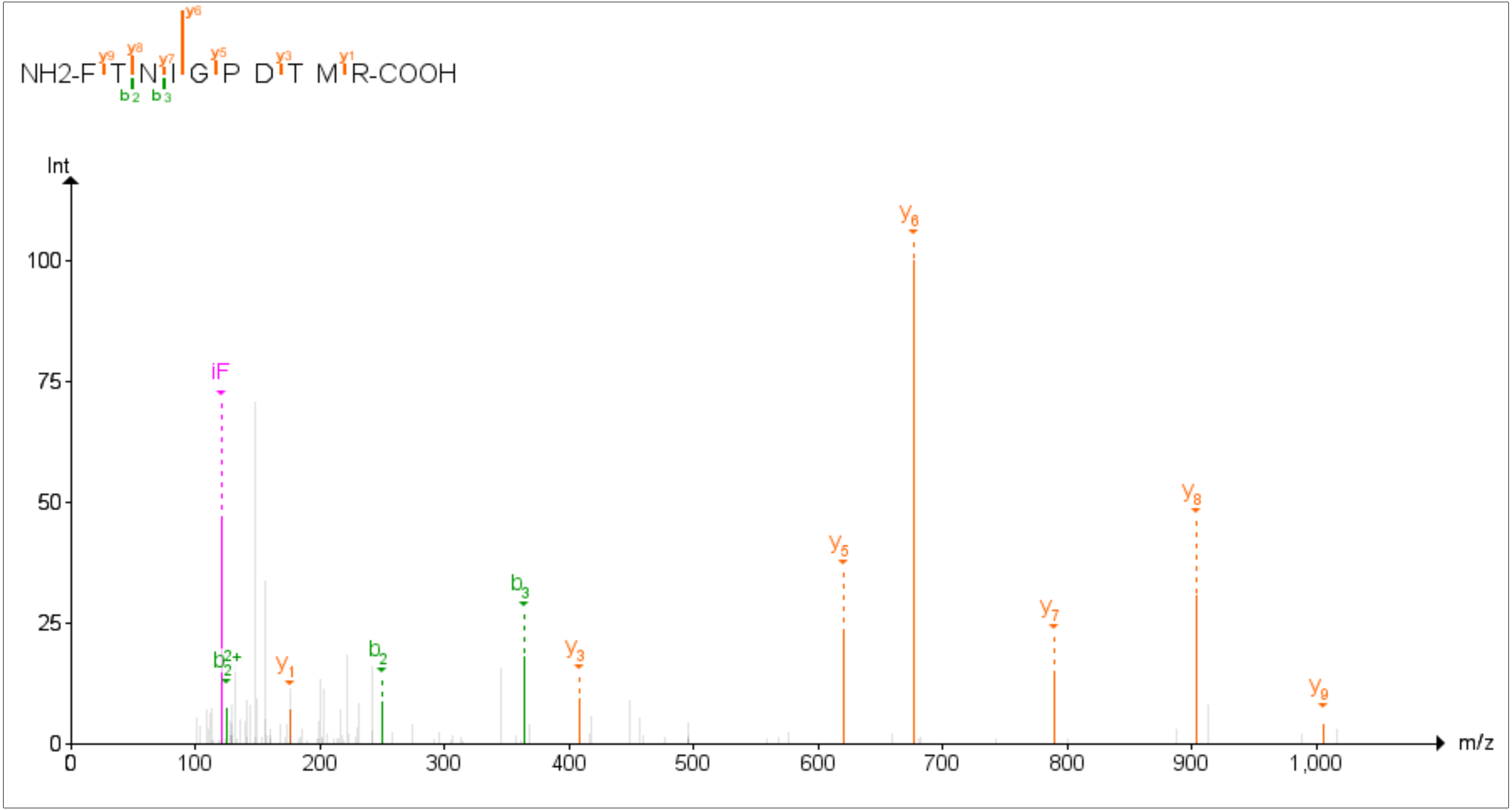

NH2-F T N I G P D T M R-COOH
y9 y8 y7 y6 y5 y3 y1
b2 b3
Int
m/z
0 25 50 75 100
0 100 200 300 400 500 600 700 800 900 1,000
iF
b_2^{2+}
y_1
b_2
b_3
y_3
y_5
y_6
y_7
y_8
y_9

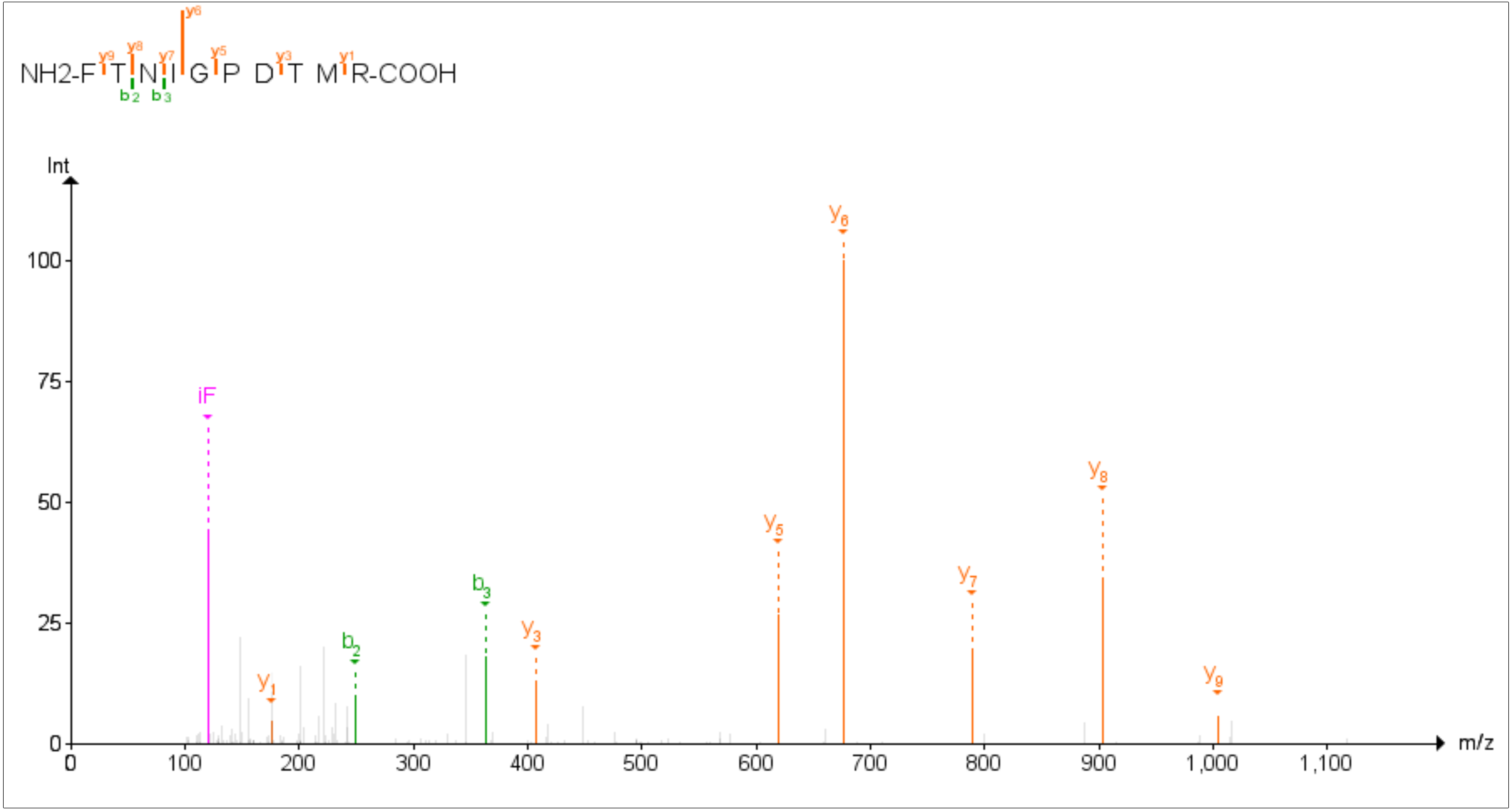

NH2-F T N I G P D T M R-COOH
y9 y8 y7 y6 y5 y3 y1
b2 b3
Int
100
75
50
25
0
iF
y1
b2
b3
y3
y5
y6
y7
y8
y9
0 100 200 300 400 500 600 700 800 900 1,000 1,100
m/z

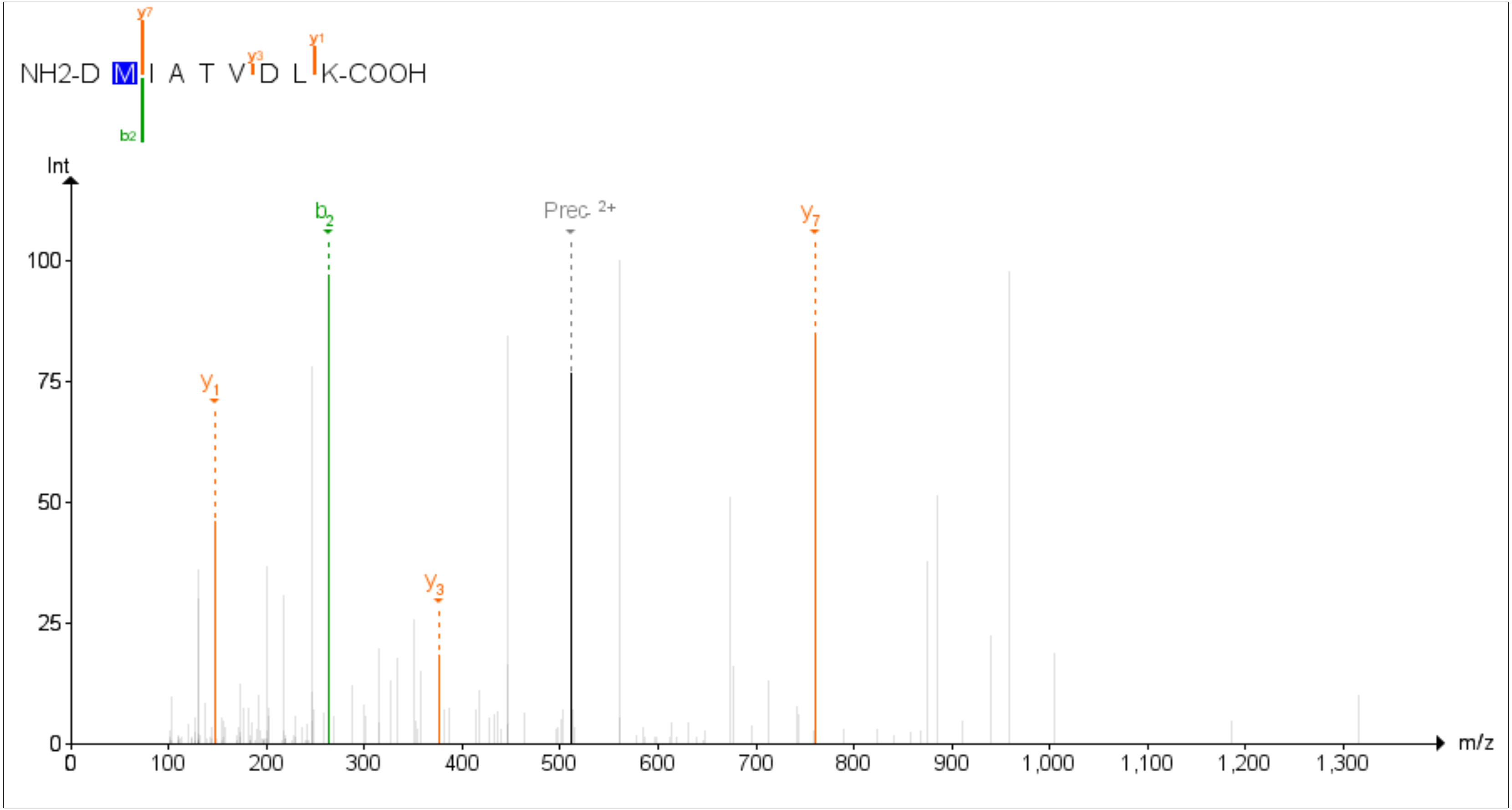

NH2-D M I A T V D L K-COOH
y7
y3
y1
b2
Int
b2
Prec 2+
y7
y1
y3
100
75
50
25
0
0
100
200
300
400
500
600
700
800
900
1,000
1,100
1,200
1,300
m/z

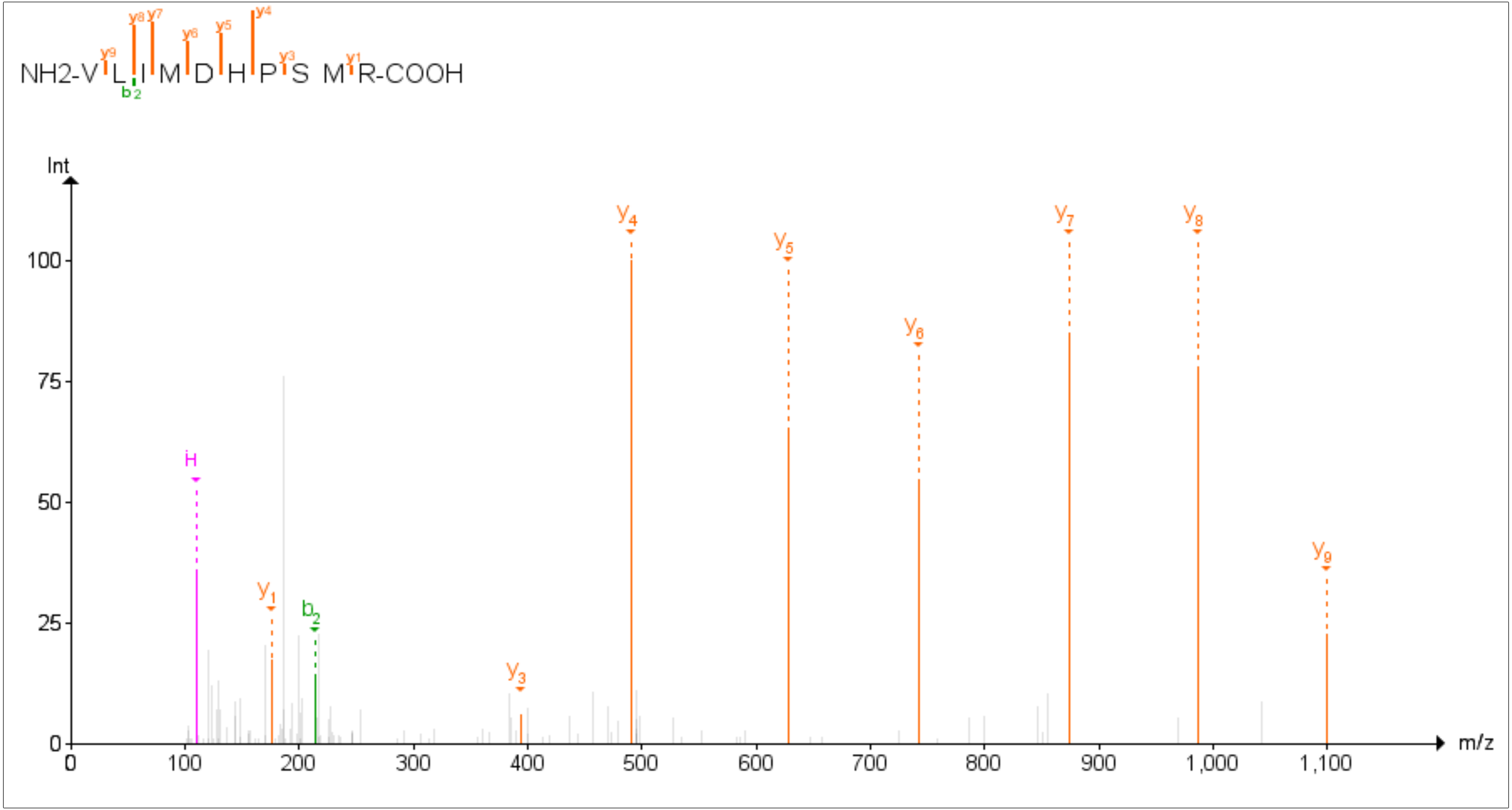
NH2-V L I M D H P S M R-COOH
y9 y8 y7 y6 y5 y4 y3 y1
b2
Int
100
75
50
25
0
m/z
0
100
200
300
400
500
600
700
800
900
1,000
1,100
y4
y5
y6
y7
y8
y9
H
y1
b2
y3

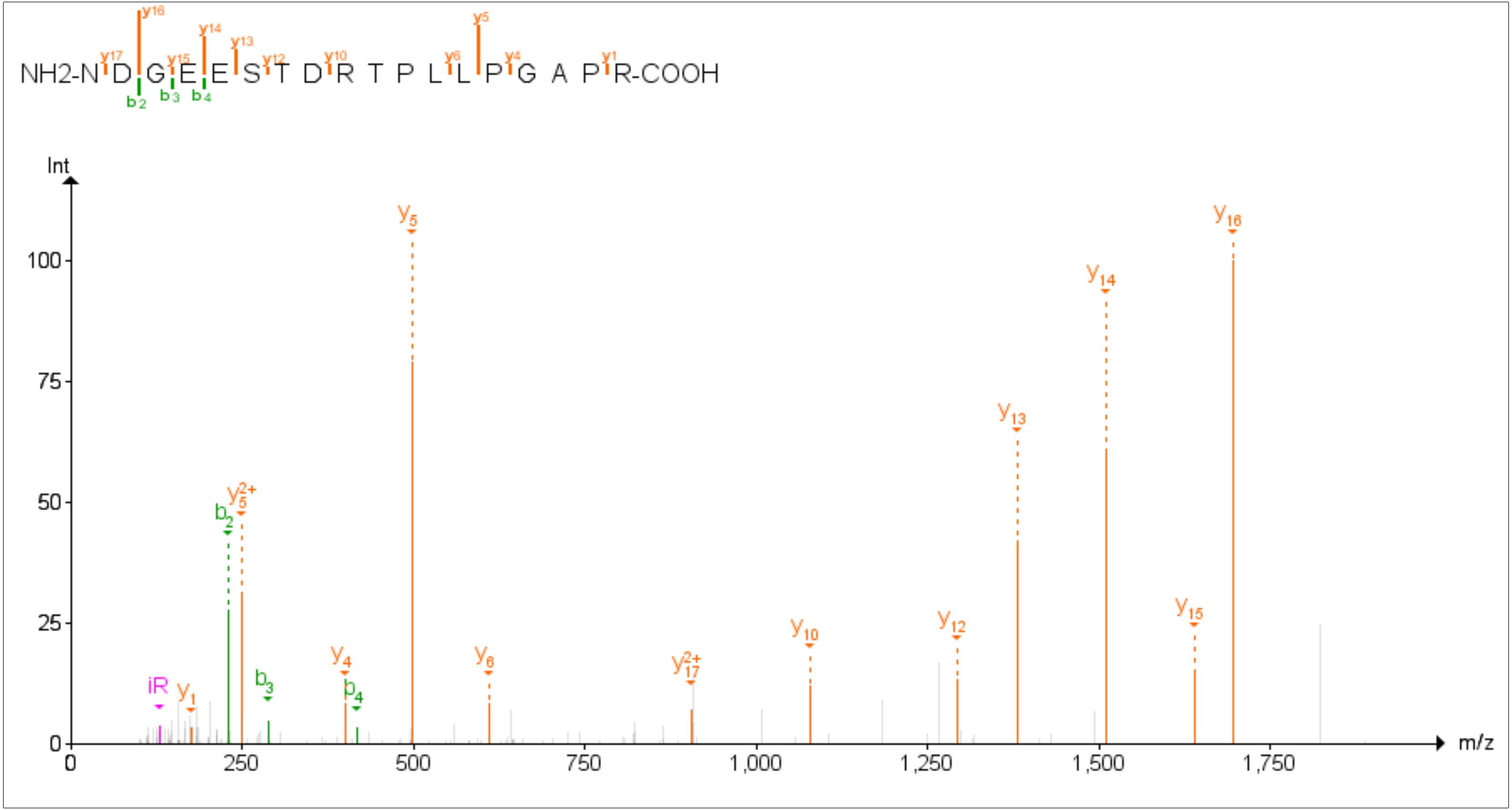

NH2-N D G E E S T D R T P L L P G A P R-COOH
y17 y16 y15 y14 y13 y12 y10 y6 y5 y4 y1
b2 b3 b4
Int
100
75
50
25
0
iR
y_1
b_2
y_5^{2+}
b_3
y_4
b_4
y_5
y_6
y_{17}^{2+}
y_{10}
y_{12}
y_{13}
y_{14}
y_{15}
y_{16}
0
250
500
750
1,000
1,250
1,500
1,750
m/z

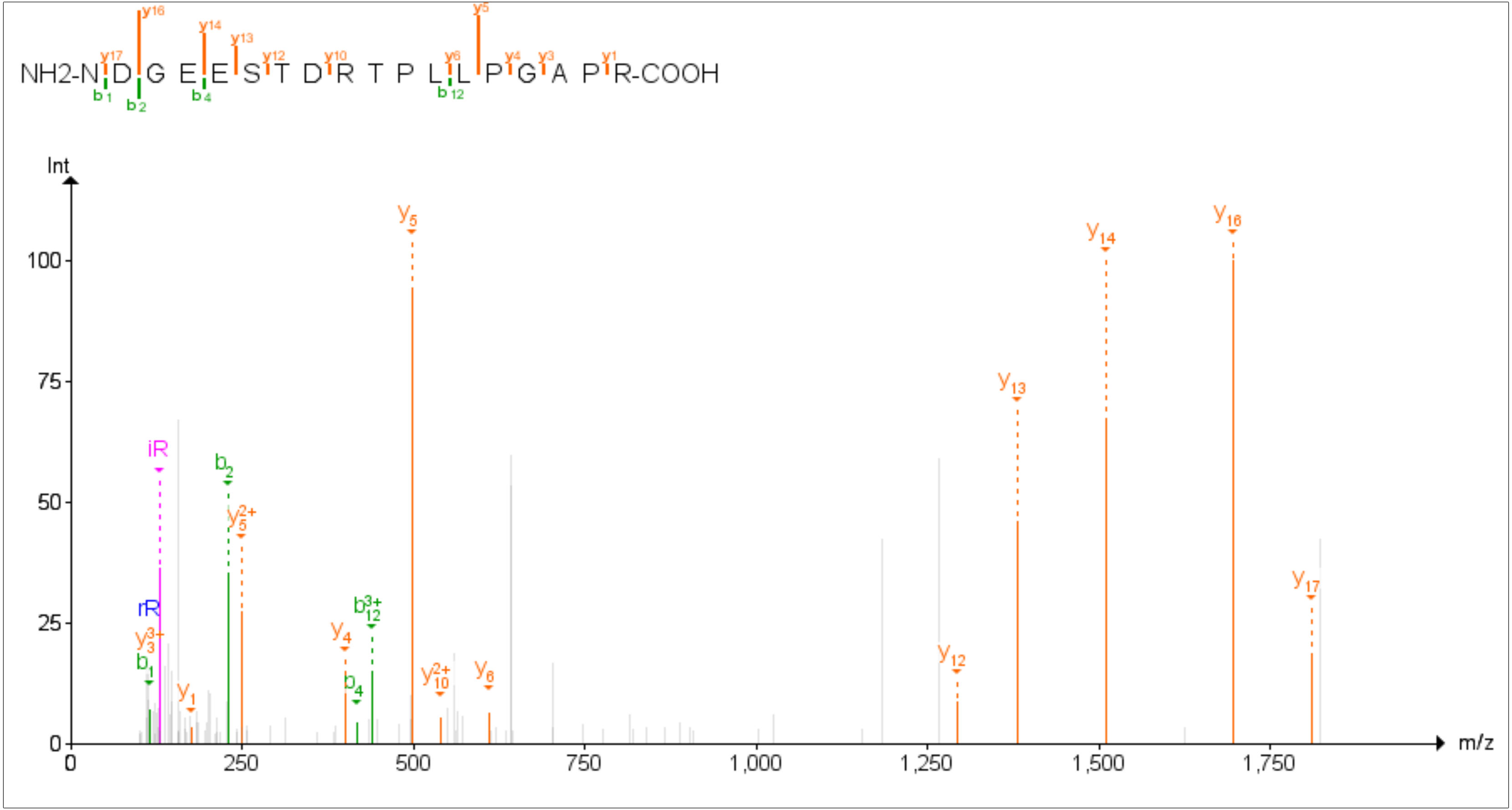

NH2-N D G E E S T D R T P L L P G A P R-COOH
Int
m/z
0
25
50
75
100
250
500
750
1,000
1,250
1,500
1,750

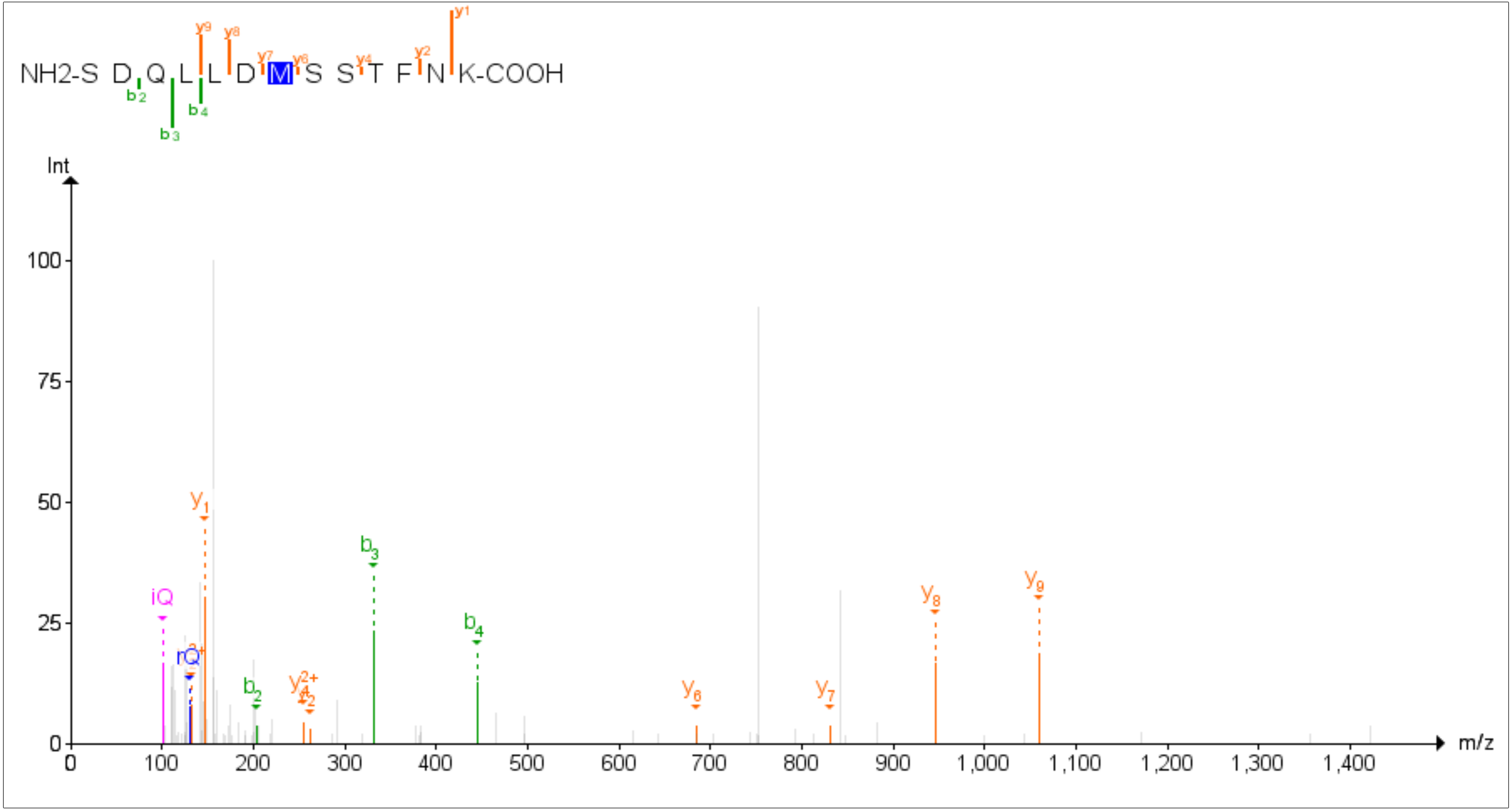

NH2-S D Q L L D M S S T F N K-COOH
y9 y8 y7 y6 y4 y2 y1
b2 b3 b4
Int
100
75
50
25
0
iQ
y1
b3
b4
y8
y9
y6
y7
b2
m/z
0
100
200
300
400
500
600
700
800
900
1,000
1,100
1,200
1,300
1,400

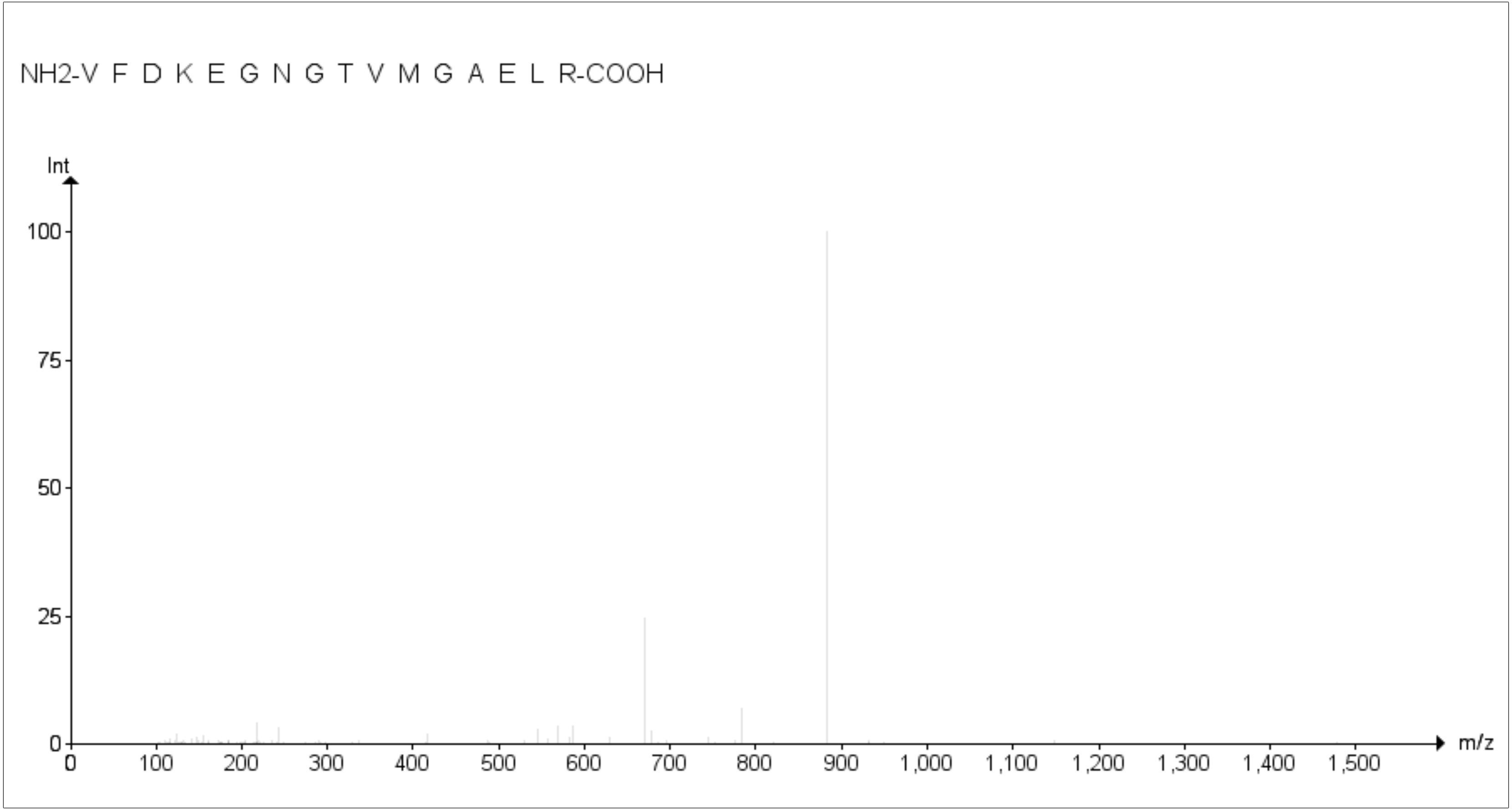

NH2-V F D K E G N G T V M G A E L R-COOH
Int
100
75
50
25
0
m/z
0
100
200
300
400
500
600
700
800
900
1,000
1,100
1,200
1,300
1,400
1,500

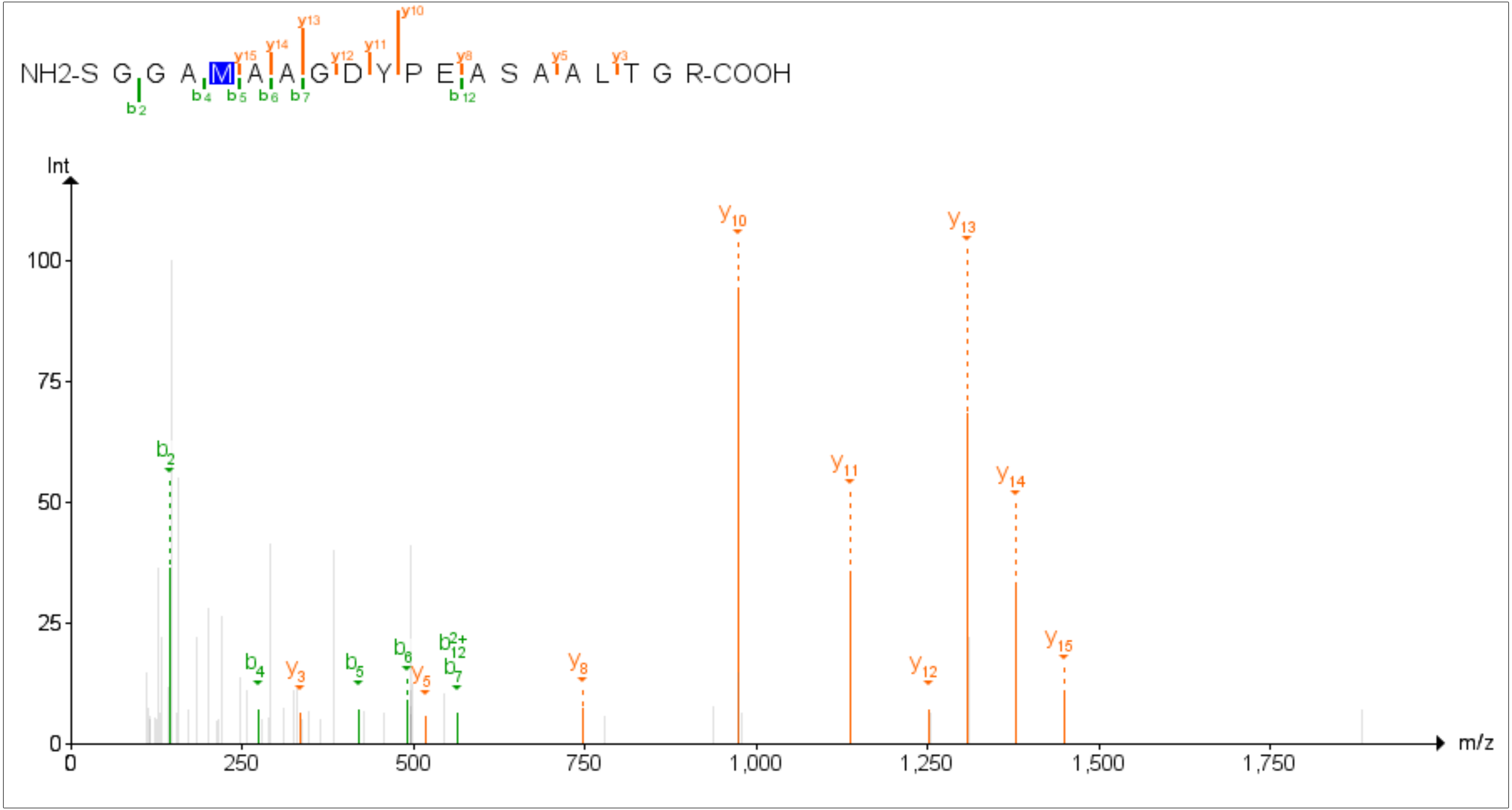

NH2-S G G A M A A G D Y P E A S A A L T G R-COOH
Int
m/z
y10
y13
y11
y14
y15
y12
y8
y5
y3
b2
b4
b5
b6
b7
b12
b_{12}^{2+}
0
25
50
75
100
250
500
750
1,000
1,250
1,500
1,750

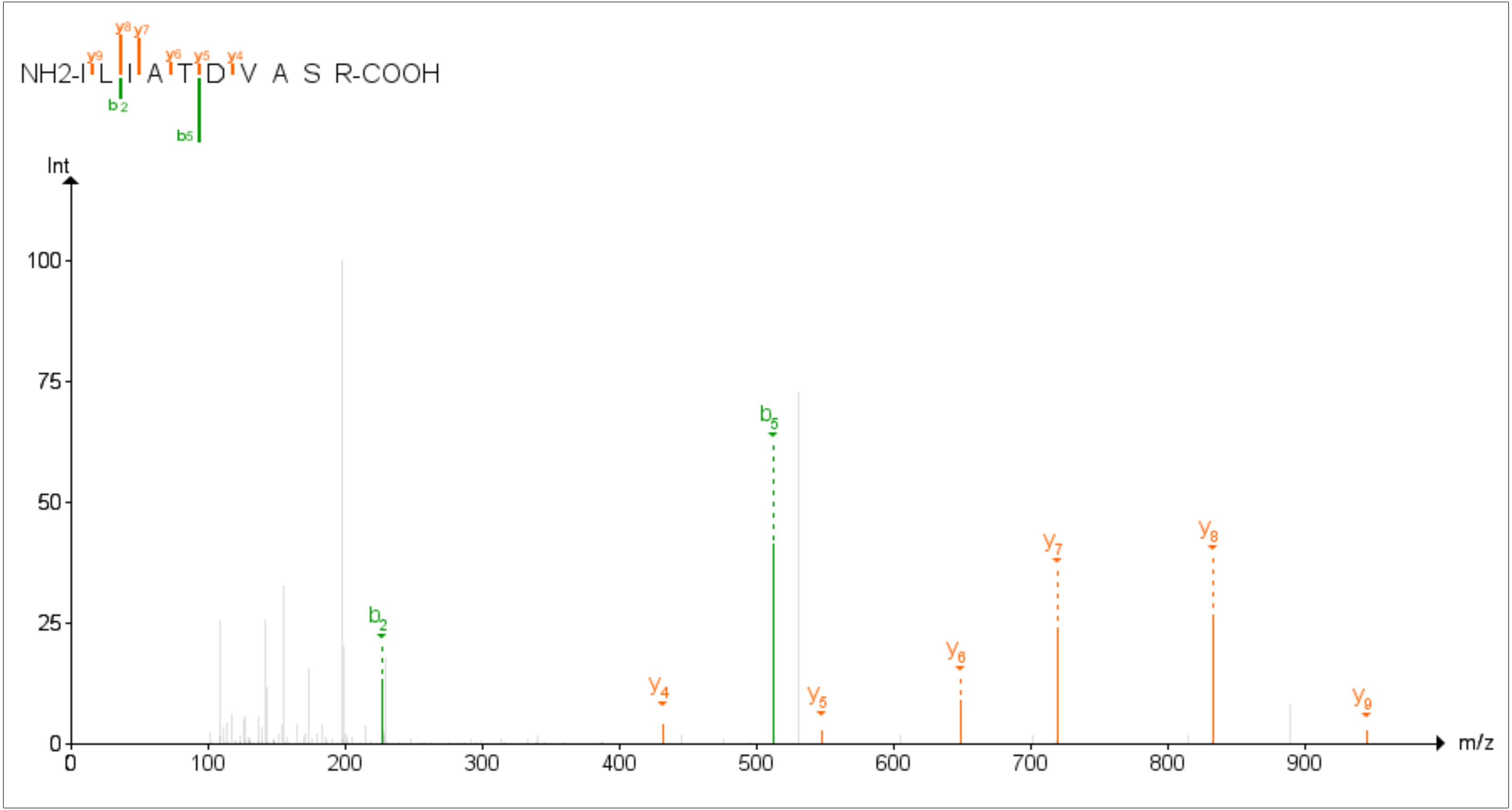

NH2-I L I A T D V A S R-COOH
y9
y8
y7
y6
y5
y4
b 2
b5
Int
100
75
50
25
0
b2
b5
y4
y5
y6
y7
y8
y9
0
100
200
300
400
500
600
700
800
900
m/z

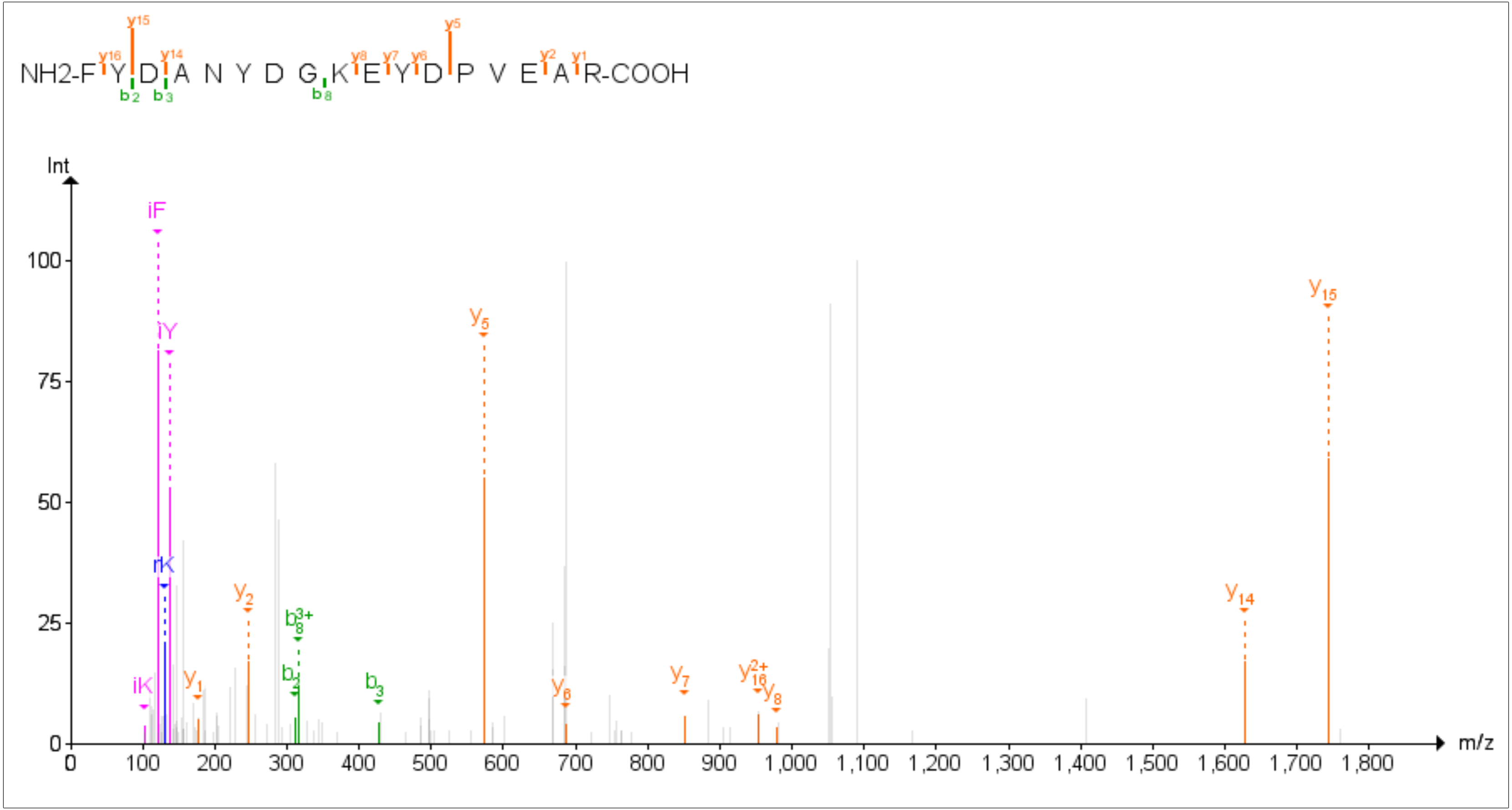

NH2-F Y D A N Y D G K E Y D P V E A R-COOH
y16 y15 y14 y8 y7 y6 y5 y2 y1
b2 b3 b8
Int
100
75
50
25
0
iF
iY
rK
iK
y1
y2
b_8^{3+}
b_2
b_3
y_5
y_6
y_7
y_{16}^{2+}
y_8
y_{14}
y_{15}
0 100 200 300 400 500 600 700 800 900 1,000 1,100 1,200 1,300 1,400 1,500 1,600 1,700 1,800
m/z

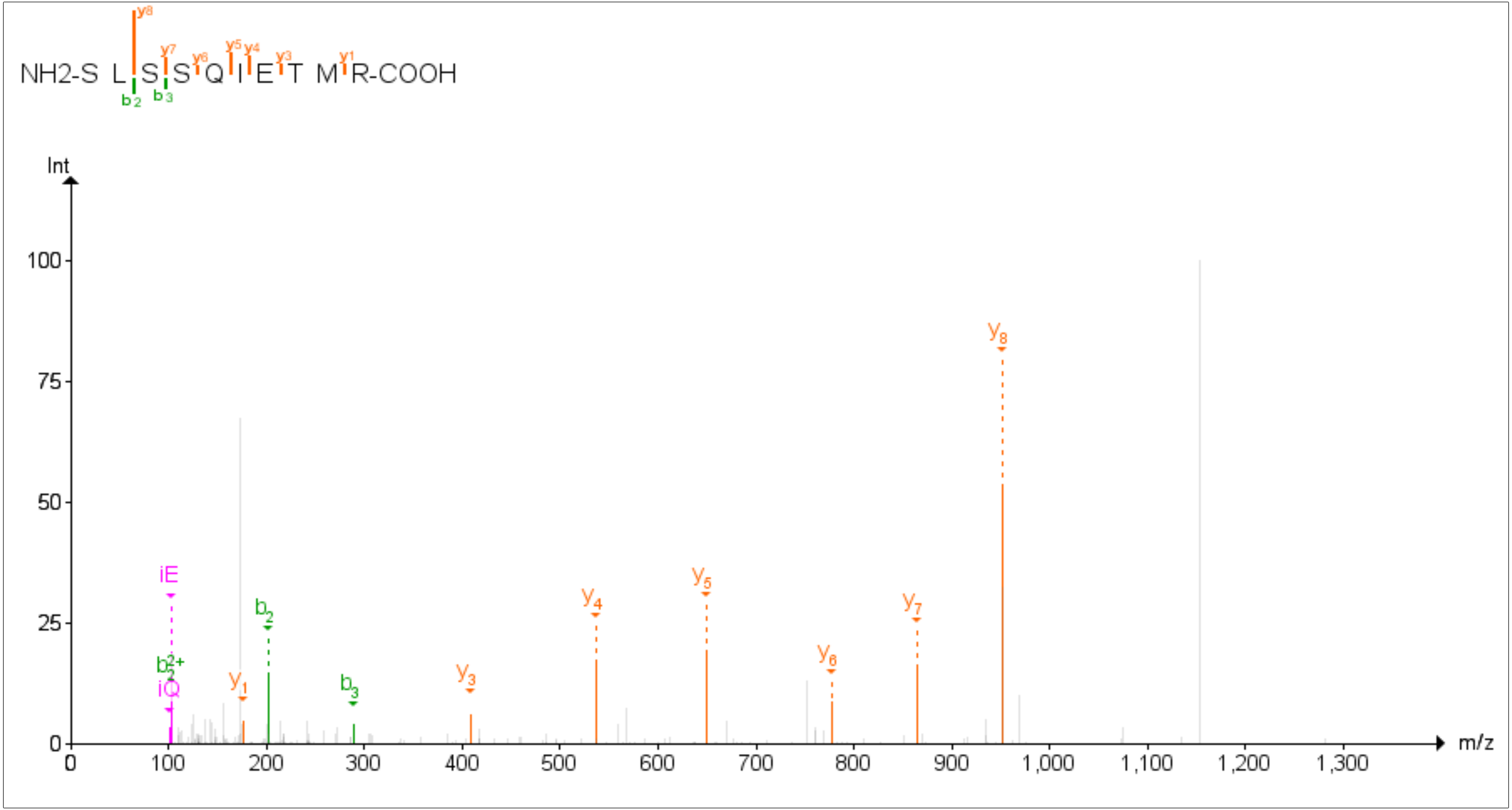

NH2-S L S S Q I E T M R-COOH
y8 y7 y6 y5 y4 y3 y1
b2 b3
Int
100
75
50
25
0
iE
b2
b2 2+
iQ
y1
b3
y3
y4
y5
y6
y7
y8
0
100
200
300
400
500
600
700
800
900
1,000
1,100
1,200
1,300
m/z

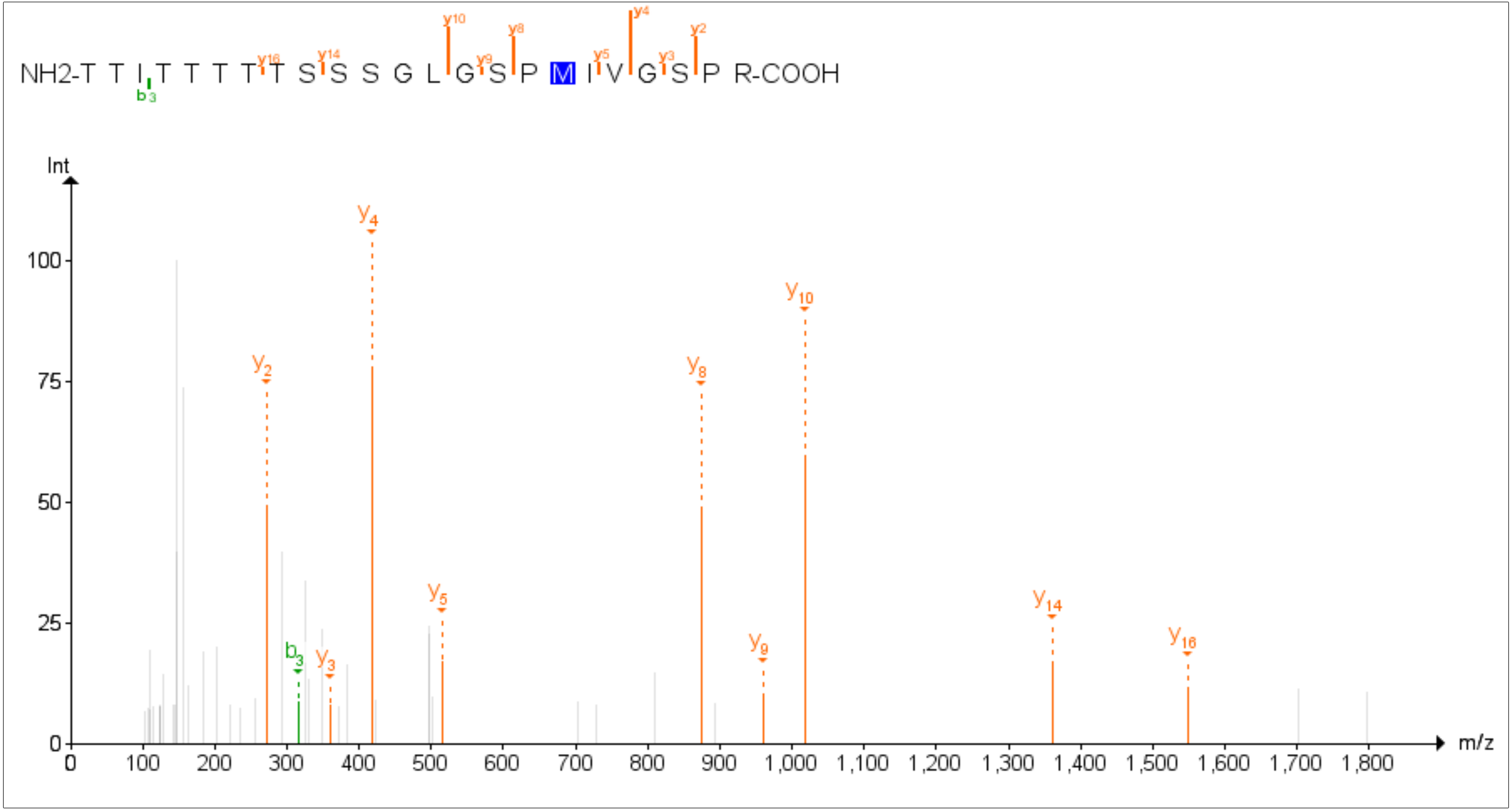

NH2-T T I T T T T T S S S G L G S P M I V G S P R-COOH
b3
y16
y14
y10
y9
y8
y5
y4
y3
y2
Int
m/z
100
75
50
25
0
0
100
200
300
400
500
600
700
800
900
1,000
1,100
1,200
1,300
1,400
1,500
1,600
1,700
1,800

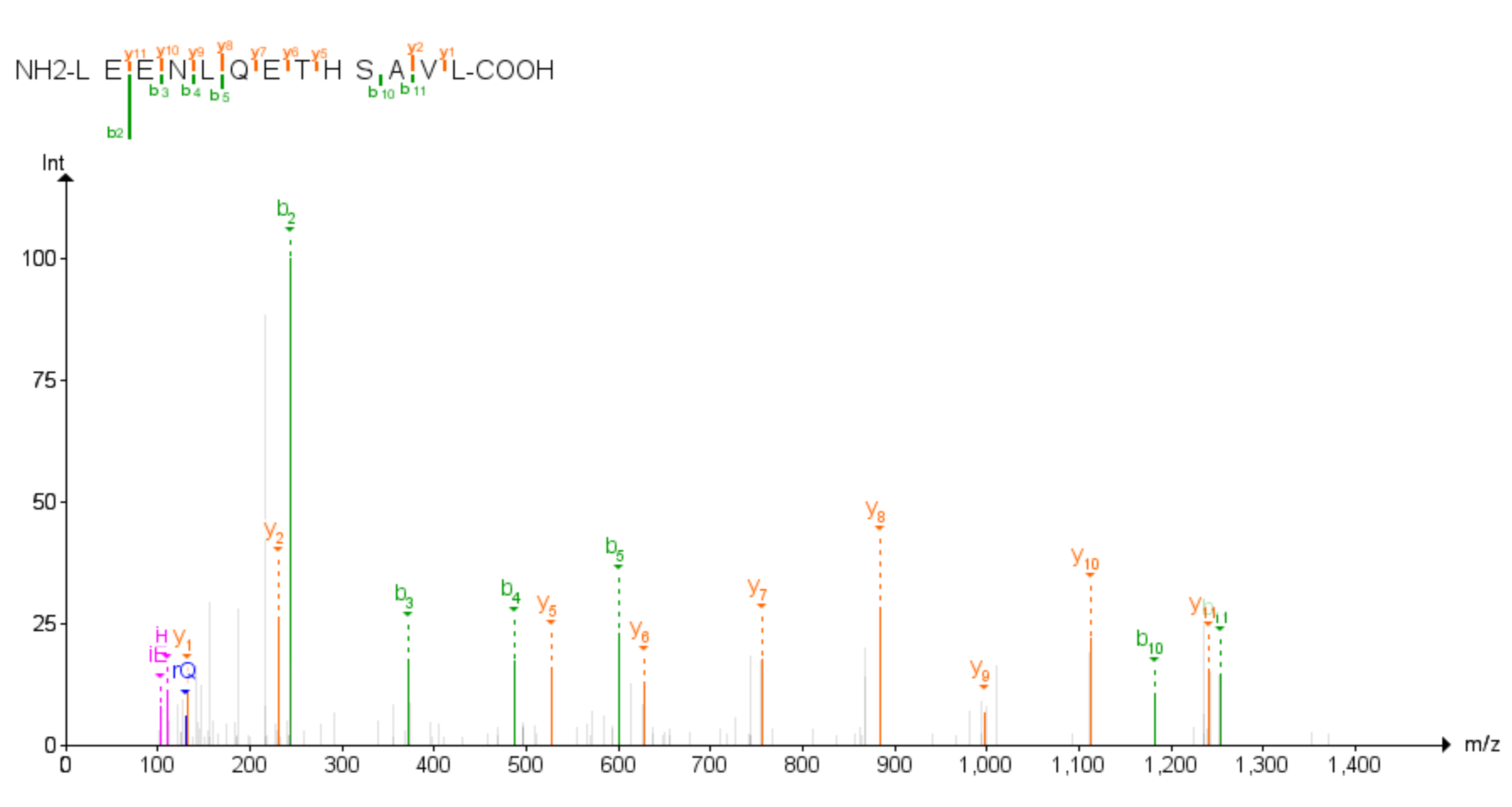
NH2-L E E N L Q E T H S A V L-COOH
Int
m/z
0
25
50
75
100
0
100
200
300
400
500
600
700
800
900
1,000
1,100
1,200
1,300
1,400
b2
b3
b4
b5
b10
b11
y1
y2
y5
y6
y7
y8
y9
y10
y11
rQ
iE
H

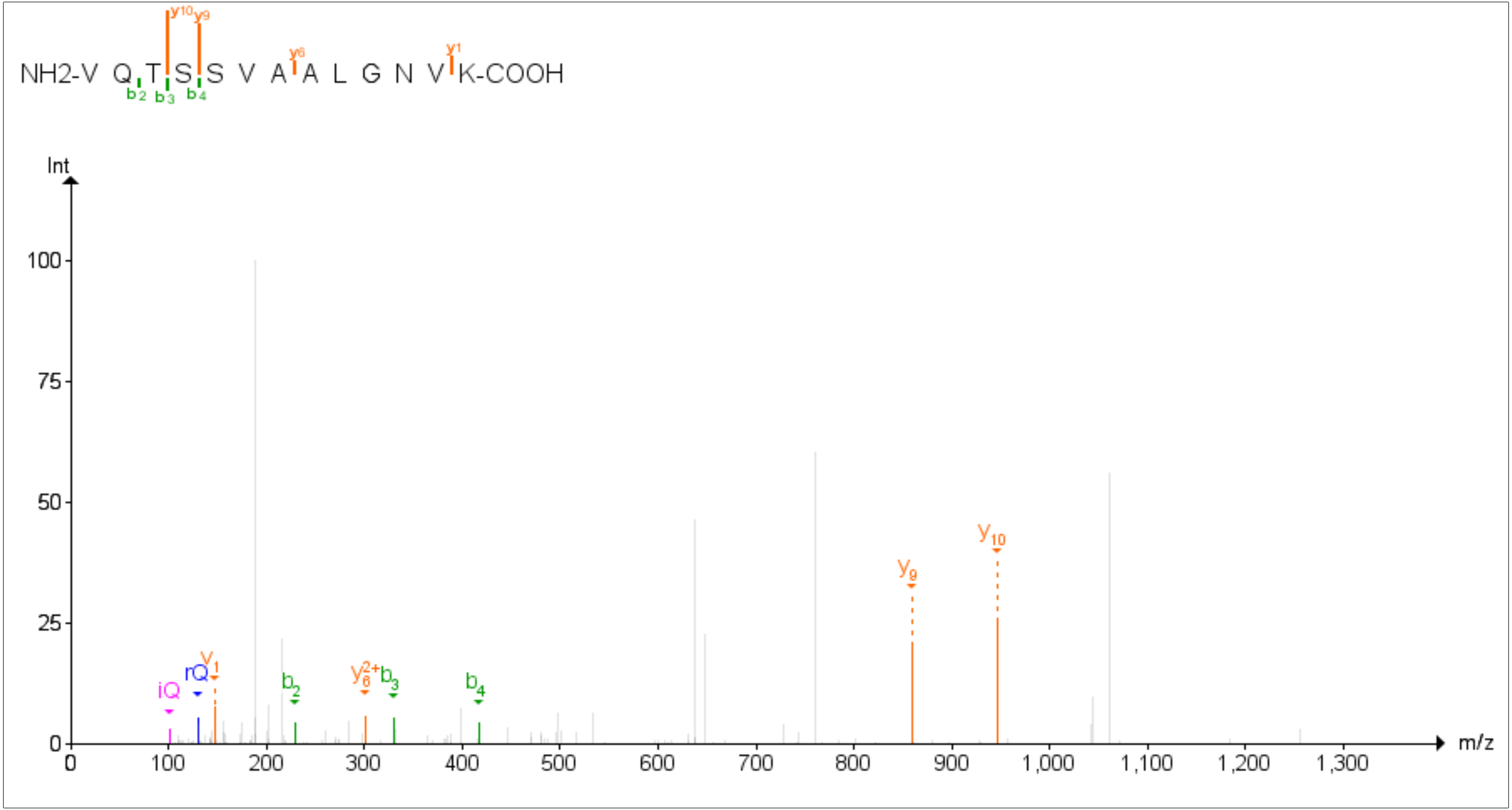

NH2-V Q T S S V A A L G N V K-COOH
y10 y9 y6 y1
b2 b3 b4
Int
100
75
50
25
0
iQ
rQ
y1
b2
y6 2+
b3
b4
y9
y10
0 100 200 300 400 500 600 700 800 900 1,000 1,100 1,200 1,300
m/z

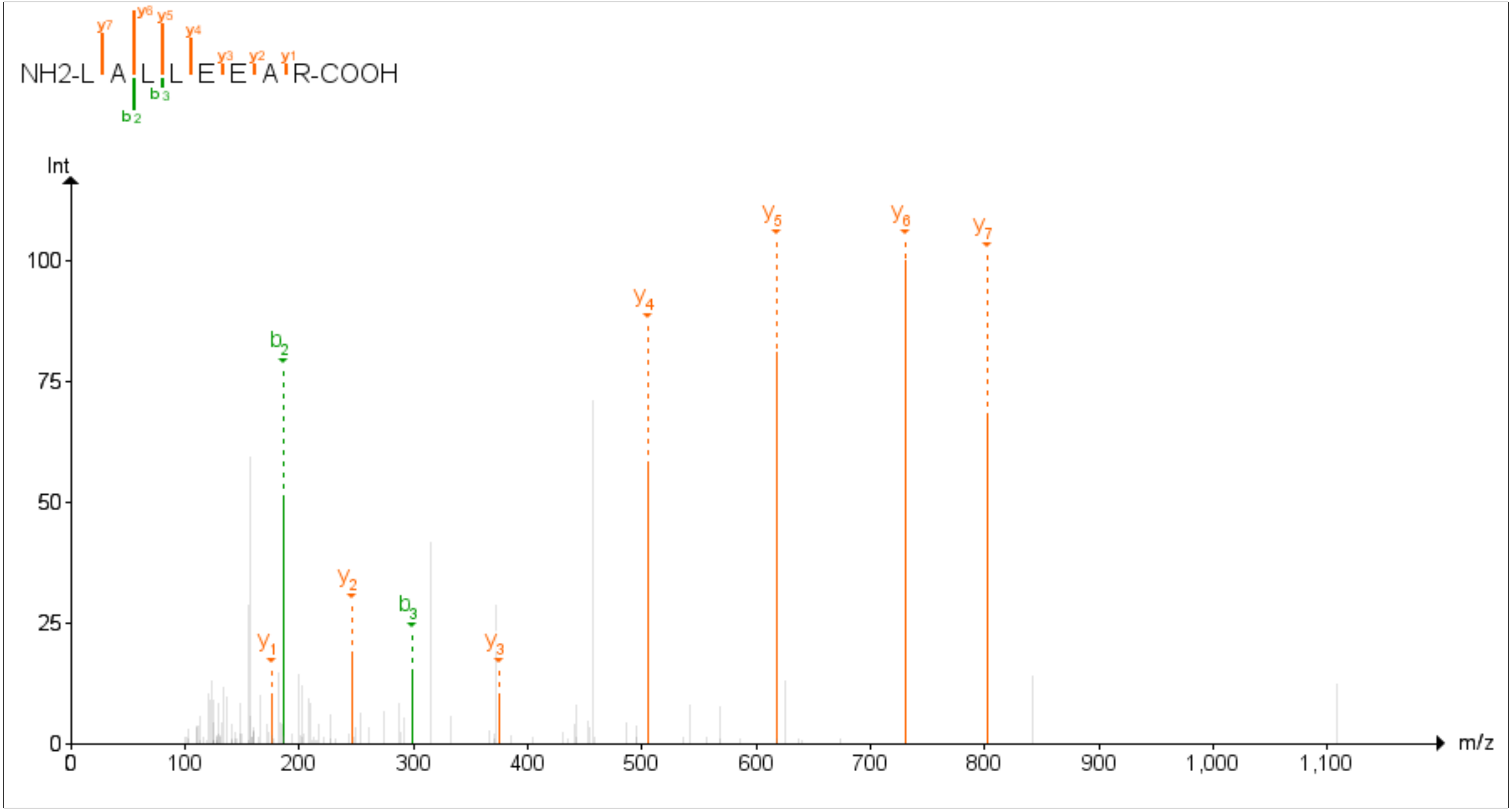

NH2-L A L L E E A R-COOH
y7 y6 y5 y4 y3 y2 y1
b2 b3
Int
100
75
50
25
0
b_2
b_3
y_1
y_2
y_3
y_4
y_5
y_6
y_7
0 100 200 300 400 500 600 700 800 900 1,000 1,100
m/z

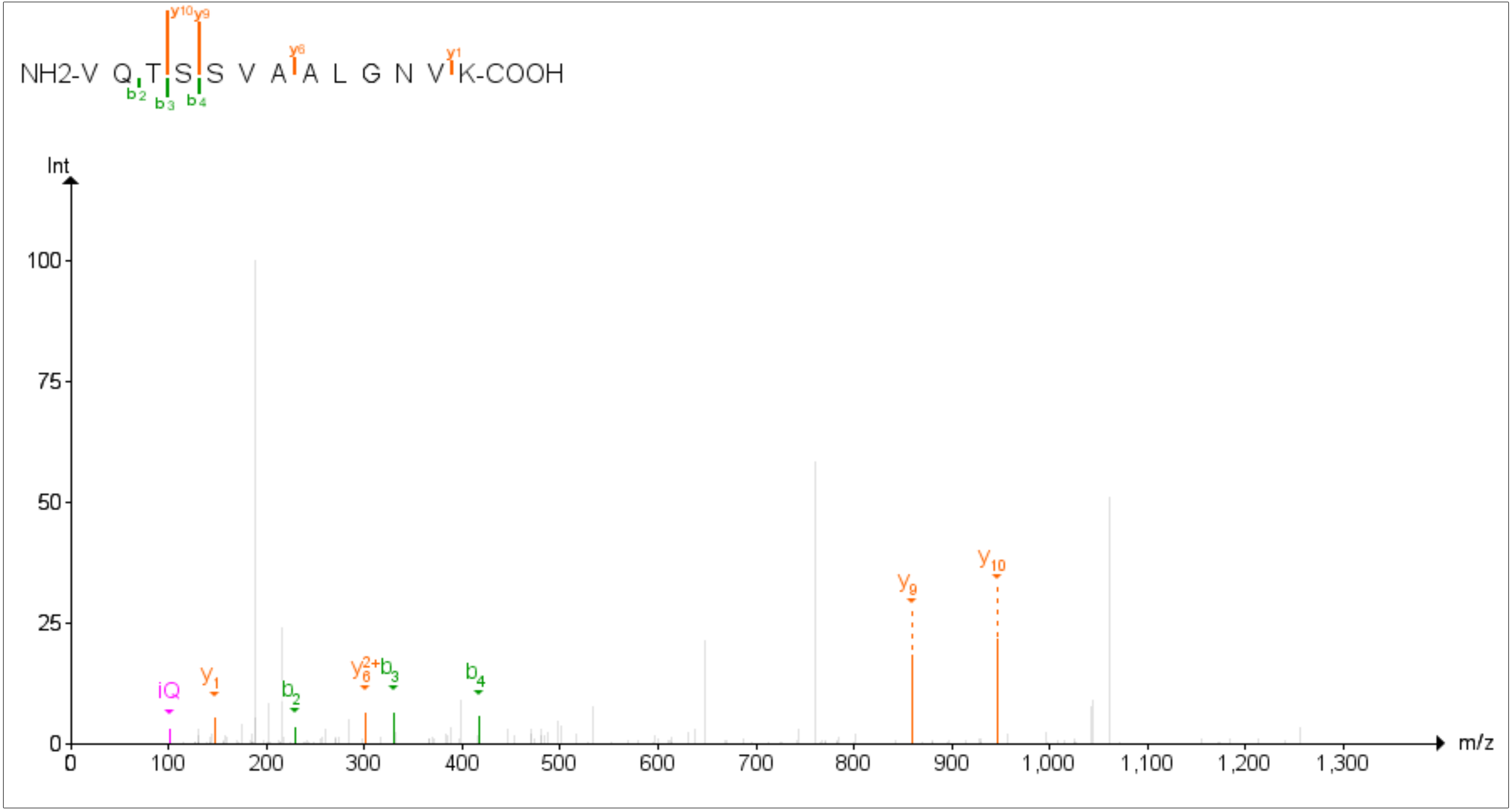

NH2-V Q T S S V A A L G N V K-COOH
y10 y9 y6 y1
b2 b3 b4
Int
100
75
50
25
0
iQ
y1
b2
y6 2+ +b3
b4
y9
y10
0 100 200 300 400 500 600 700 800 900 1,000 1,100 1,200 1,300
m/z

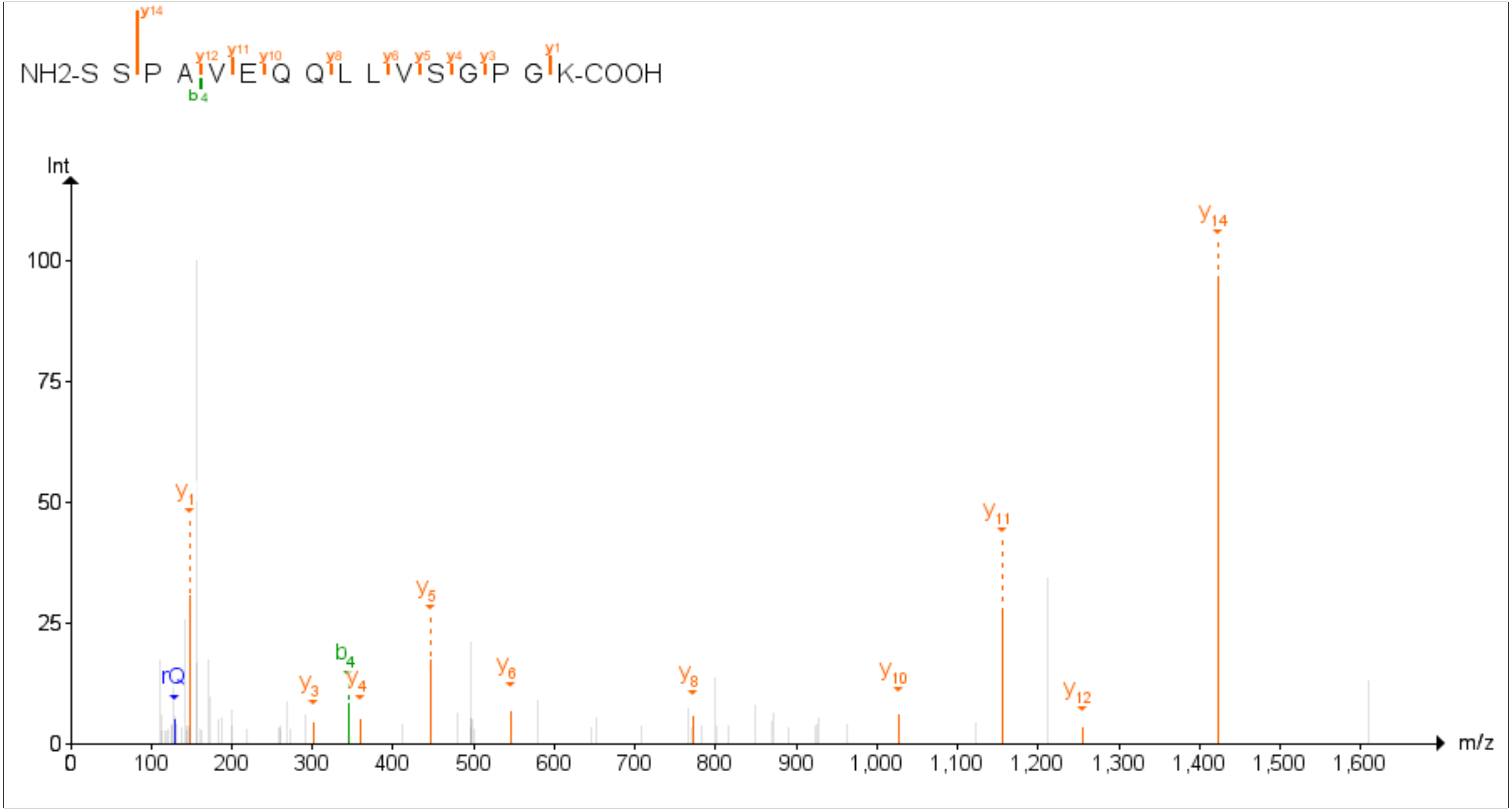

NH2-S S P A V E Q Q L L V S G P G K-COOH
y14 y12 y11 y10 y8 y6 y5 y4 y3 y1
b4
Int
100
75
50
25
0
m/z
0 100 200 300 400 500 600 700 800 900 1,000 1,100 1,200 1,300 1,400 1,500 1,600
rQ
y1
y3
b4
y4
y5
y6
y8
y10
y11
y12
y14

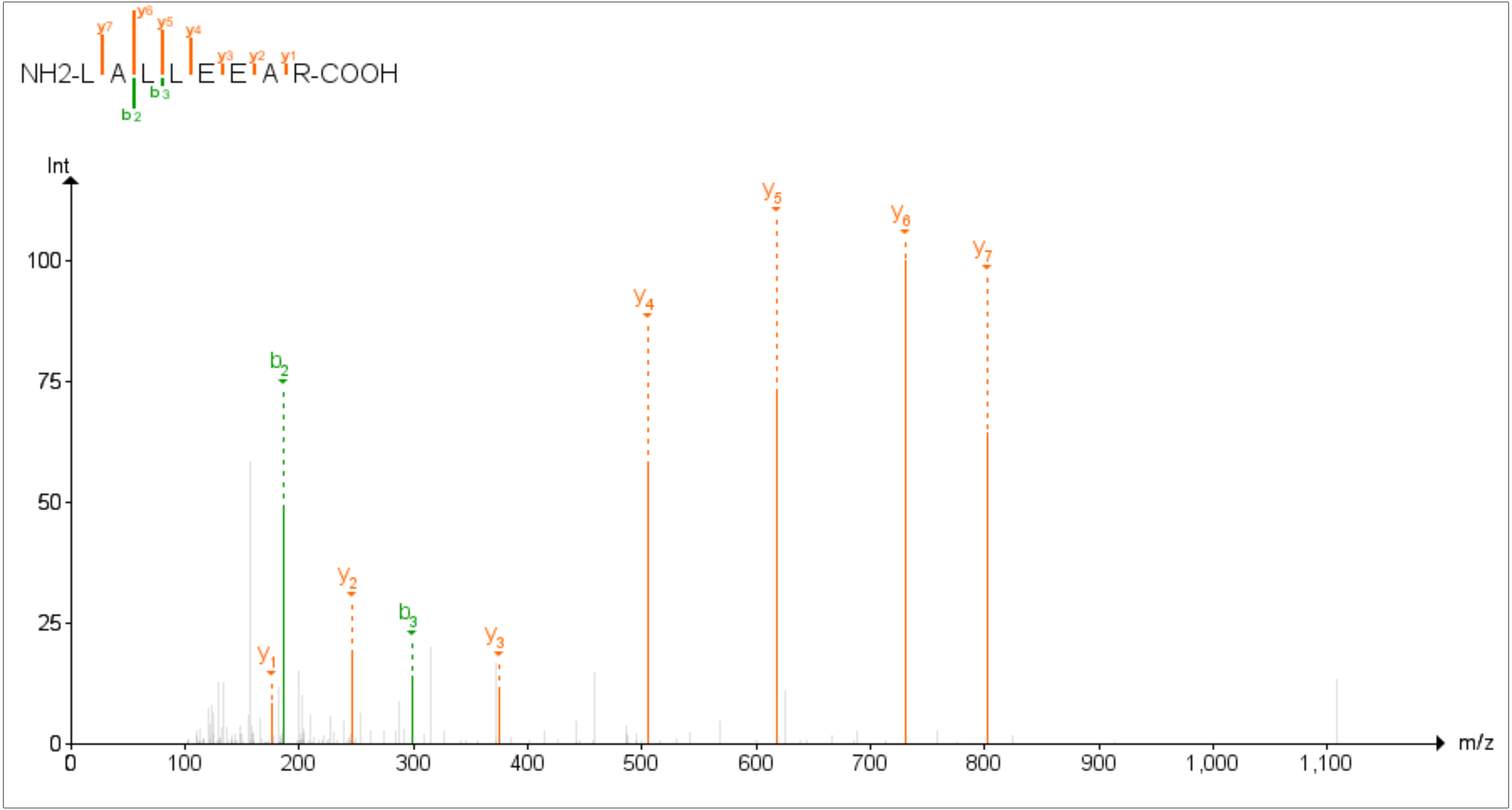

NH2-L A L L E E A R-COOH
y7
y6
y5
y4
y3
y2
y1
b2
b3
Int
m/z
0
25
50
75
100
0
100
200
300
400
500
600
700
800
900
1,000
1,100
y1
b2
y2
b3
y3
y4
y5
y6
y7

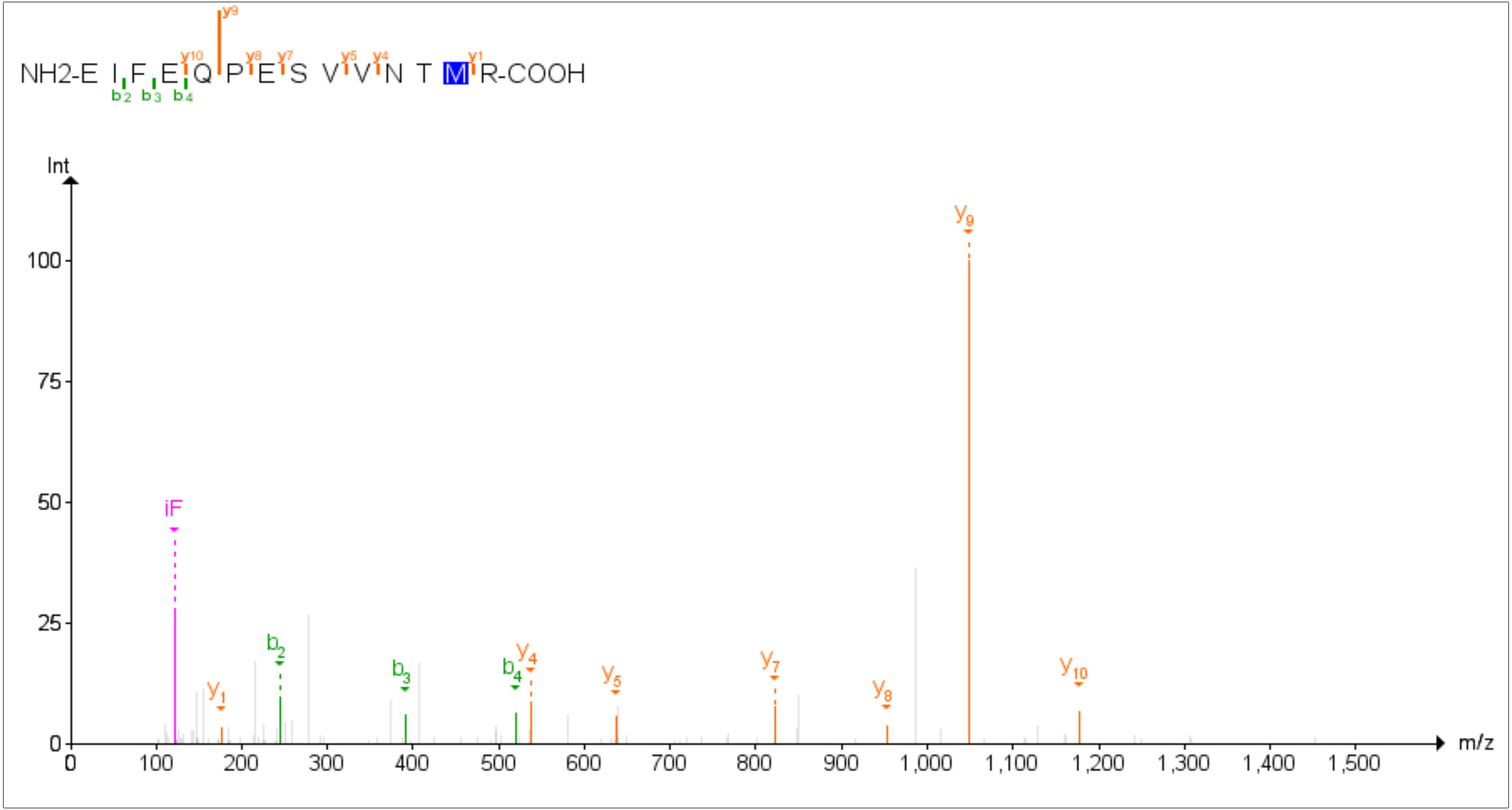

NH2-E I F E Q P E S V V N T M R-COOH
y9
y10
y8
y7
y5
y4
y1
b2
b3
b4
Int
100
75
50
25
0
iF
y1
b2
b3
b4
y4
y5
y7
y8
y9
y10
0
100
200
300
400
500
600
700
800
900
1,000
1,100
1,200
1,300
1,400
1,500
m/z

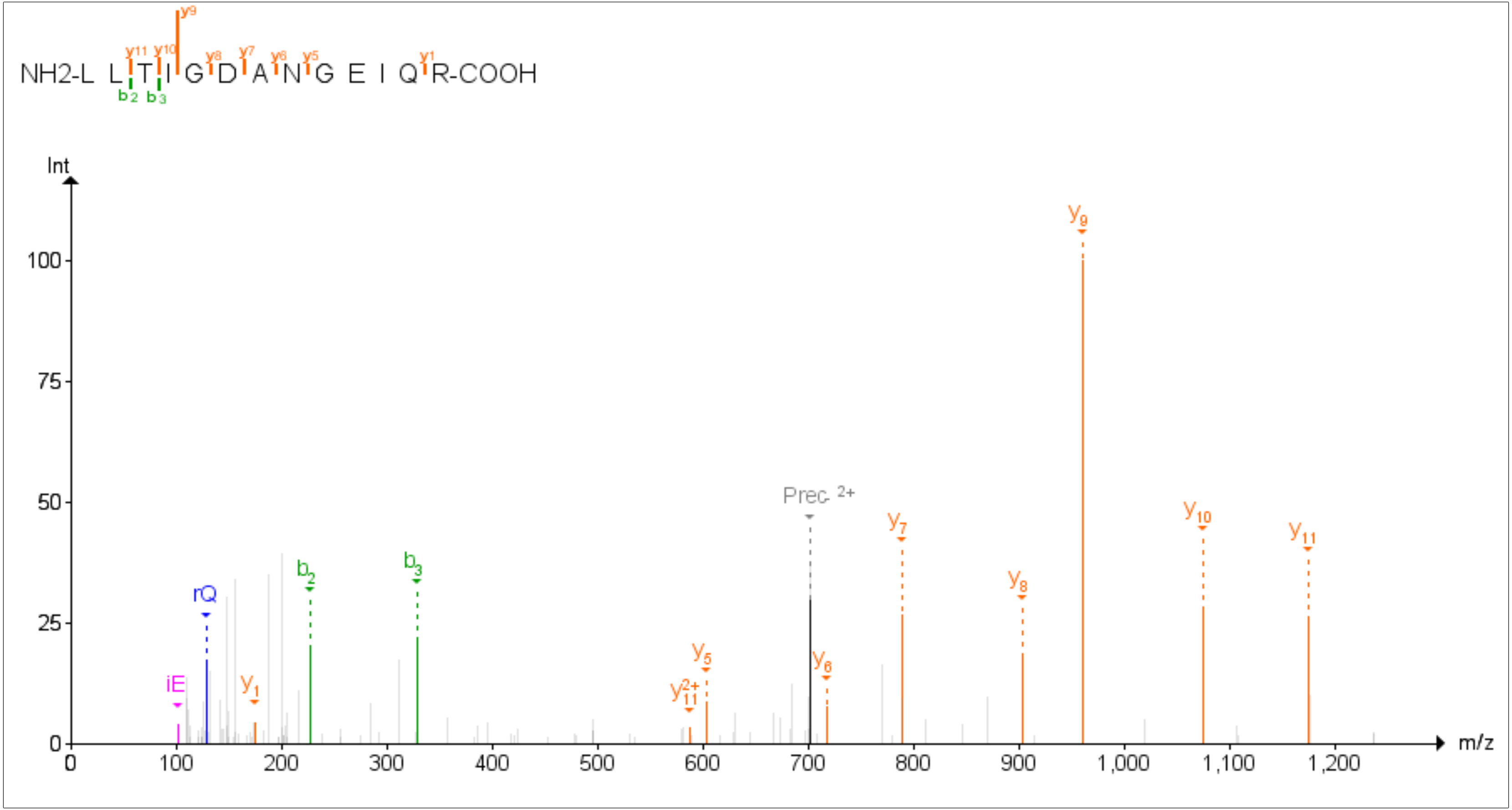

NH2-L L T I G D A N G E I Q R-COOH
y9
y11
y10
y8
y7
y6
y5
y1
b2
b3
Int
100
75
50
25
0
Prec 2+
iE
rQ
b2
b3
y1
y11 2+
y5
y6
y7
y8
y9
y10
y11
0
100
200
300
400
500
600
700
800
900
1,000
1,100
1,200
m/z

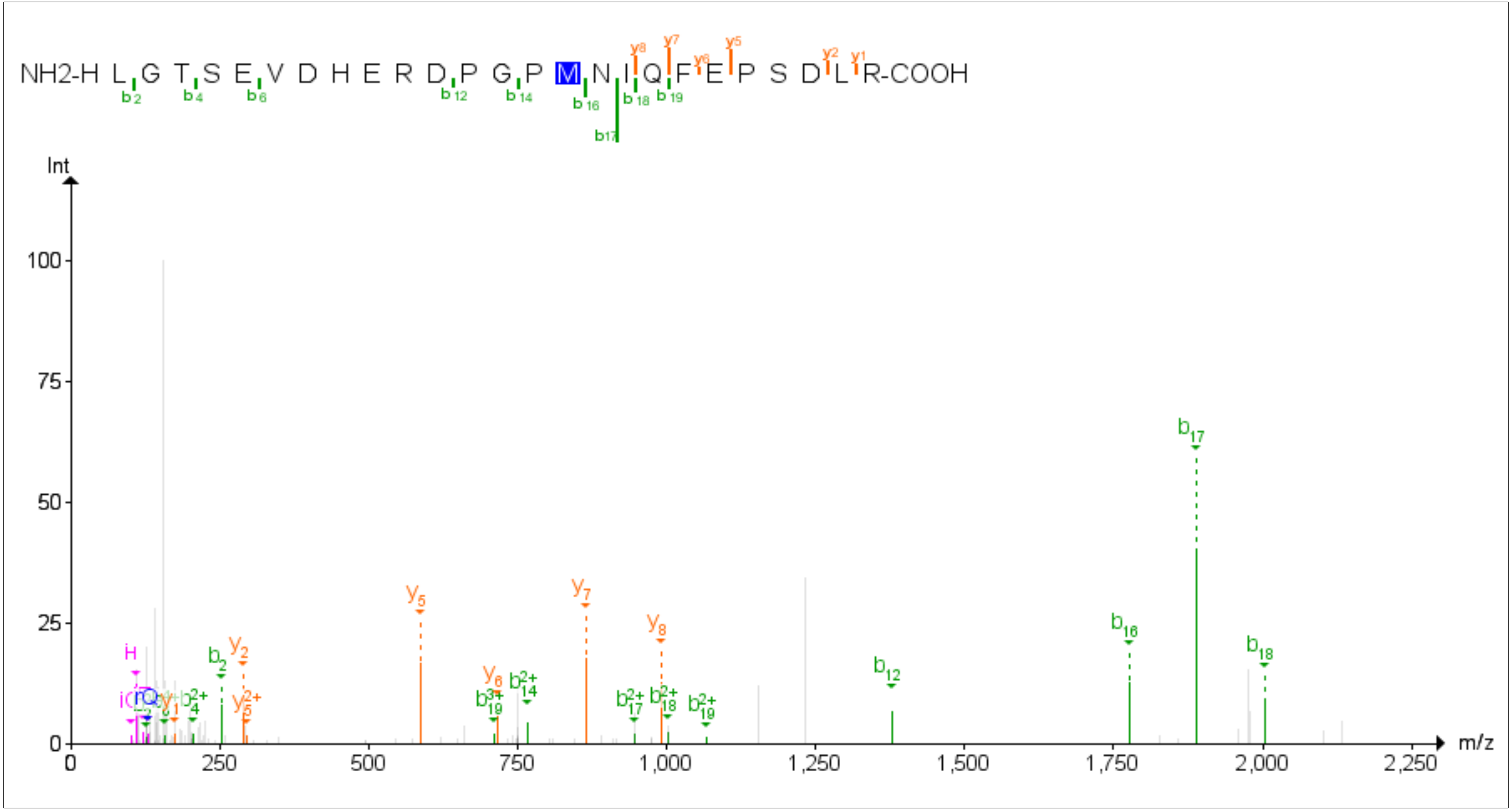
NH2-H L G T S E V D H E R D P G P M N I Q F E P S D L R-COOH
b2 b4 b6 b12 b14 b16 b17 b18 b19
y8 y7 y6 y5 y2 y1
Int
100
75
50
25
0
0 250 500 750 1,000 1,250 1,500 1,750 2,000 2,250
m/z
b17
y5
y7
y8
b16
b18
b12
y2
b2
H
y6
b2+14
b3+19
b2+17
b2+18
b2+19
y2+5
b2+4
y1

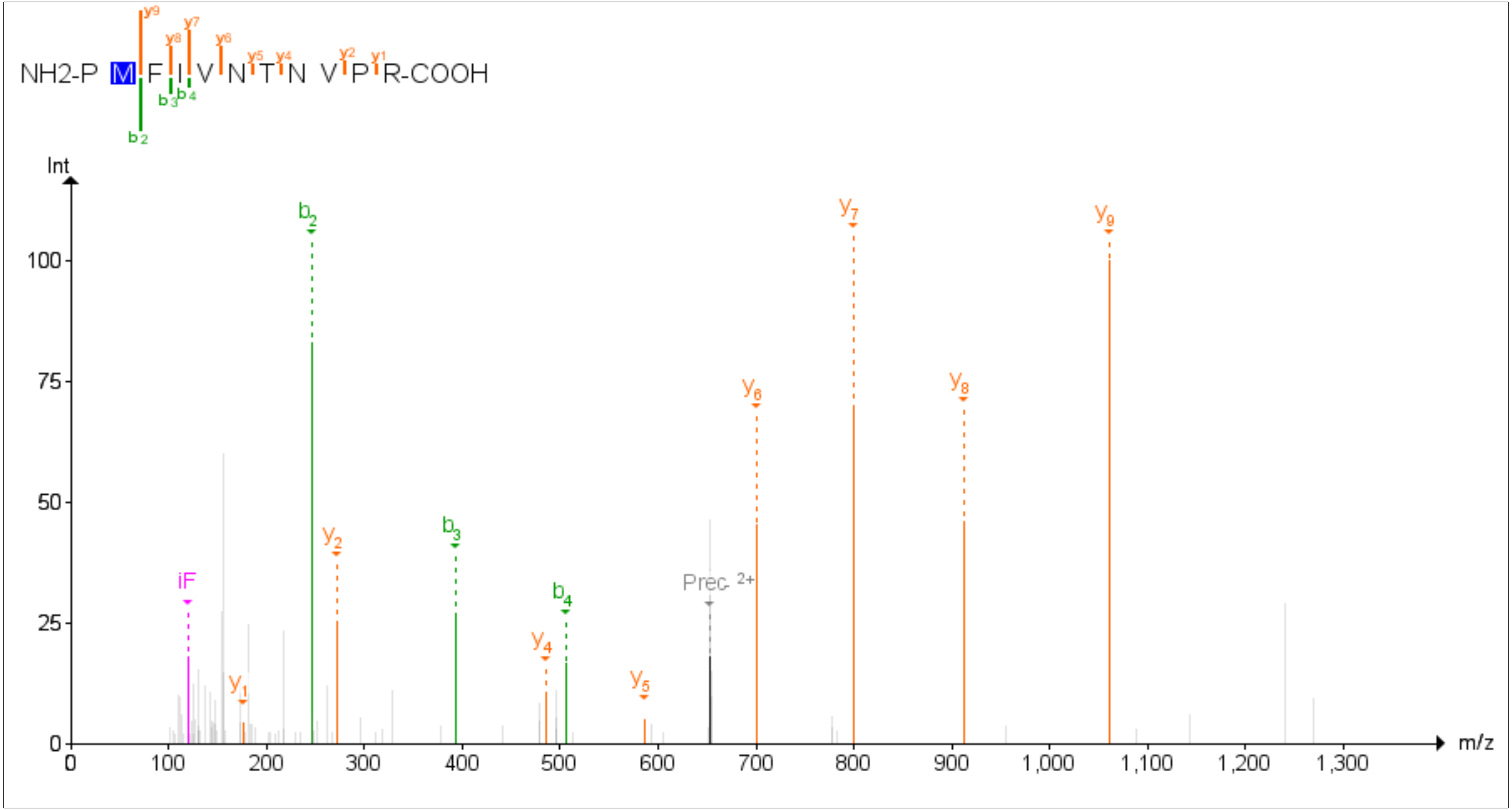

NH2-P M F I V N T N V P R-COOH
y9 y8 y7 y6 y5 y4 y2 y1
b2 b3 b4
Int
100
75
50
25
0
b2
y7
y9
y6
y8
y2
b3
iF
b4
Prec 2+
y4
y1
y5
0 100 200 300 400 500 600 700 800 900 1,000 1,100 1,200 1,300
m/z

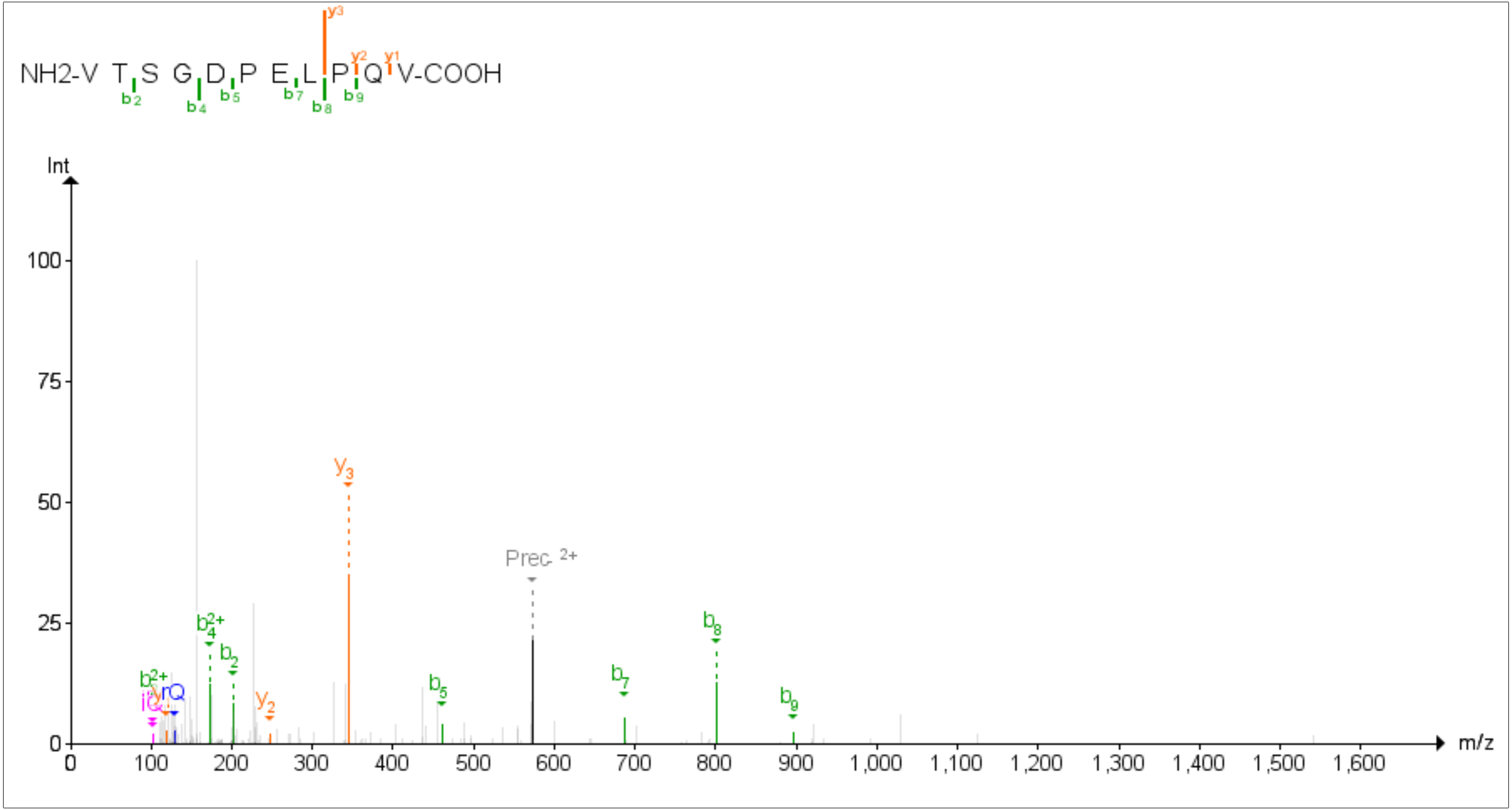

NH2-V T S G D P E L P Q V-COOH
b2
b4
b5
b7
b8
b9
y3
y2
y1
Int
100
75
50
25
0
y3
Prec 2+
b4 2+
b2
y2
rQ
b5
b7
b8
b9
0
100
200
300
400
500
600
700
800
900
1,000
1,100
1,200
1,300
1,400
1,500
1,600
m/z

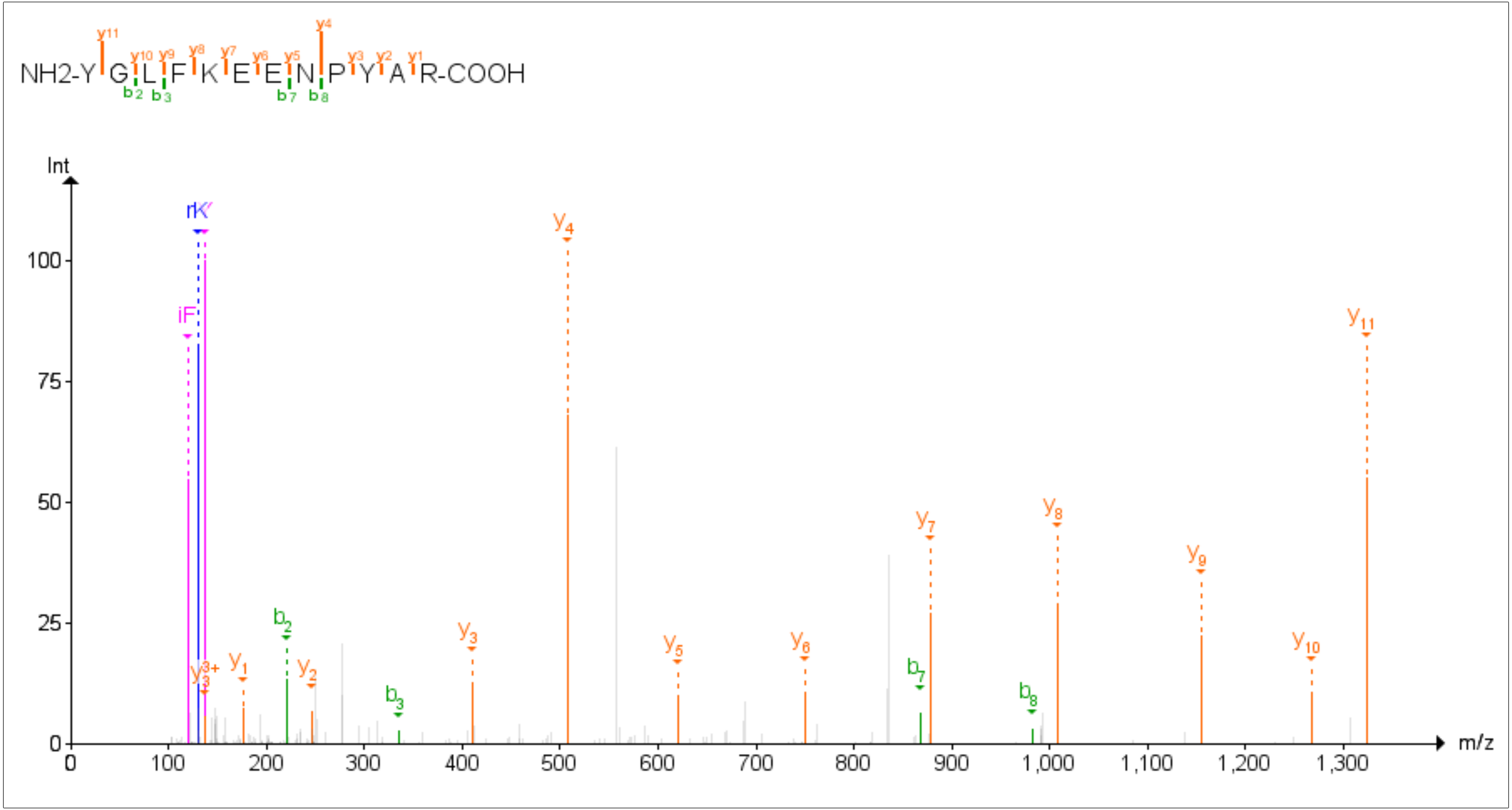

NH2-Y G L F K E E N P Y A R-COOH
y11 y10 y9 y8 y7 y6 y5 y4 y3 y2 y1
b2 b3 b7 b8
Int
m/z
rK
iF
y4
y11
y7
y8
y9
y10
y3
y5
y6
b2
b3
b7
b8
y1
y2
y3 3+
0
25
50
75
100
0
100
200
300
400
500
600
700
800
900
1,000
1,100
1,200
1,300

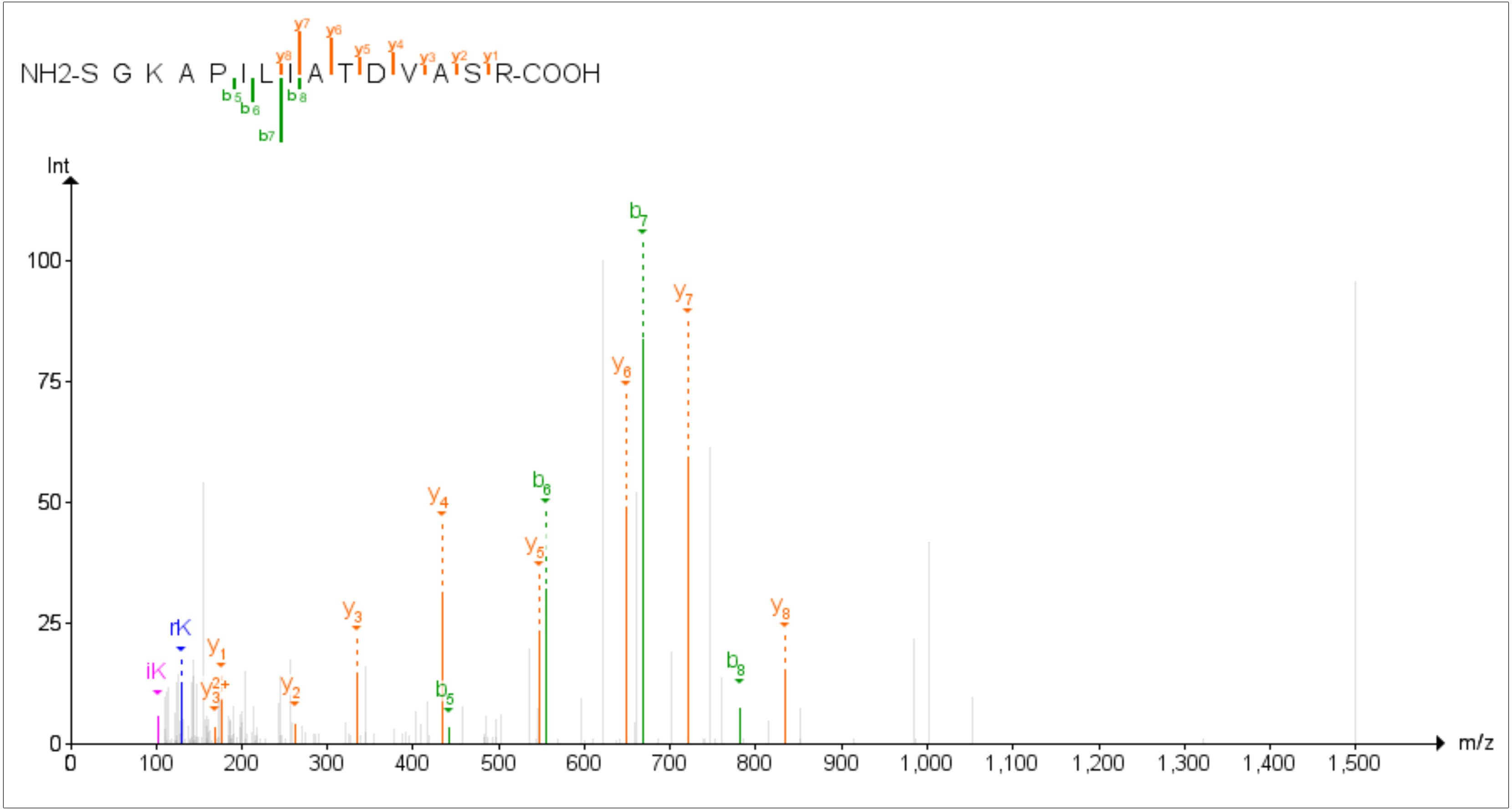

NH2-S G K A P I L I A T D V A S R-COOH
y8 y7 y6 y5 y4 y3 y2 y1
b5 b6 b7 b8
Int
m/z
0
25
50
75
100
0
100
200
300
400
500
600
700
800
900
1,000
1,100
1,200
1,300
1,400
1,500
iK
rK
y1
y2
y3
y4
y5
y6
y7
y8
b5
b6
b7
b8

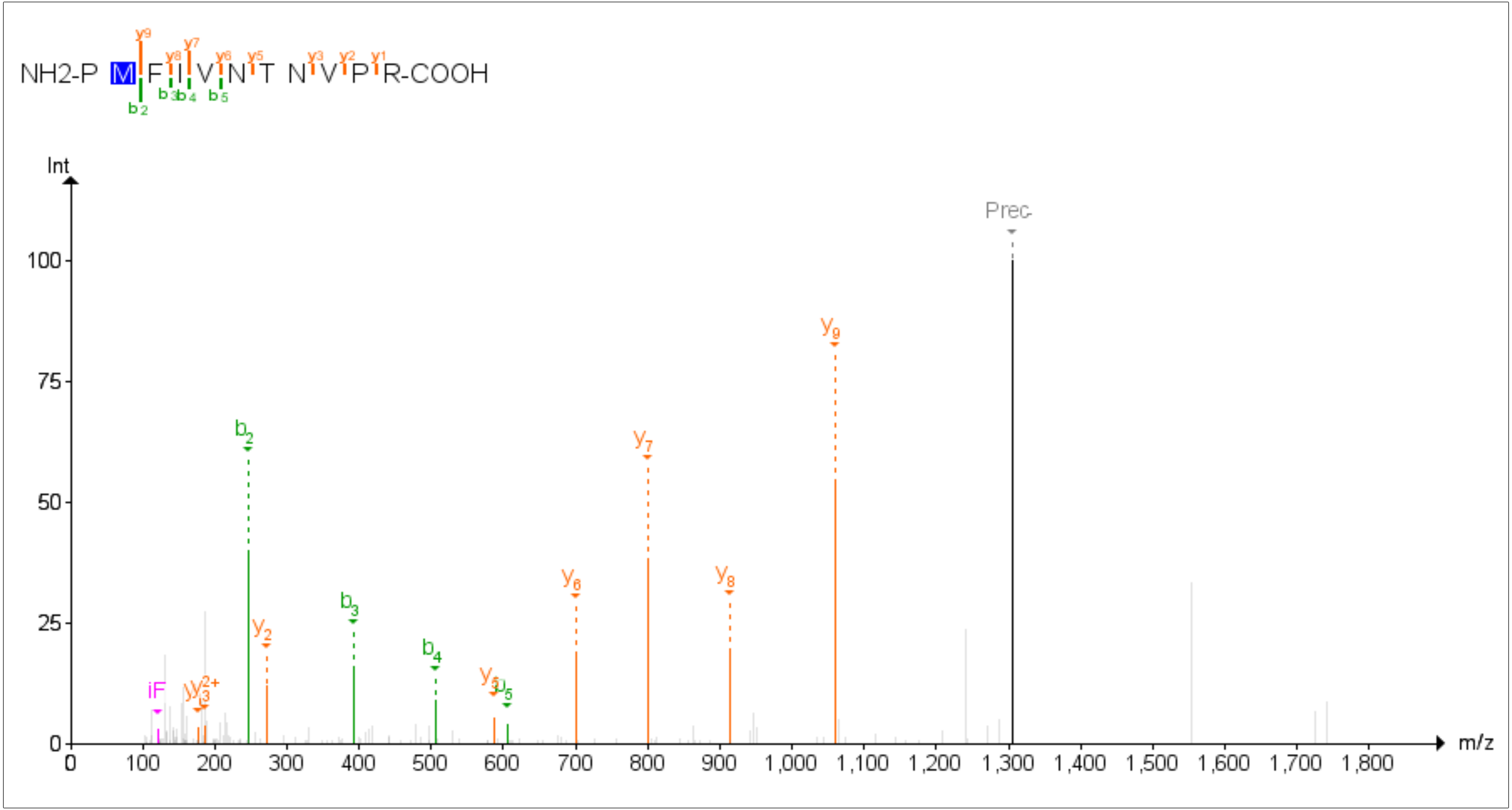
NH2-P M F I I V N T N V P R-COOH
Int
Prec
m/z
b2
b3
b4
b5
y2
y5
y6
y7
y8
y9
iF
0
25
50
75
100
0
100
200
300
400
500
600
700
800
900
1,000
1,100
1,200
1,300
1,400
1,500
1,600
1,700
1,800

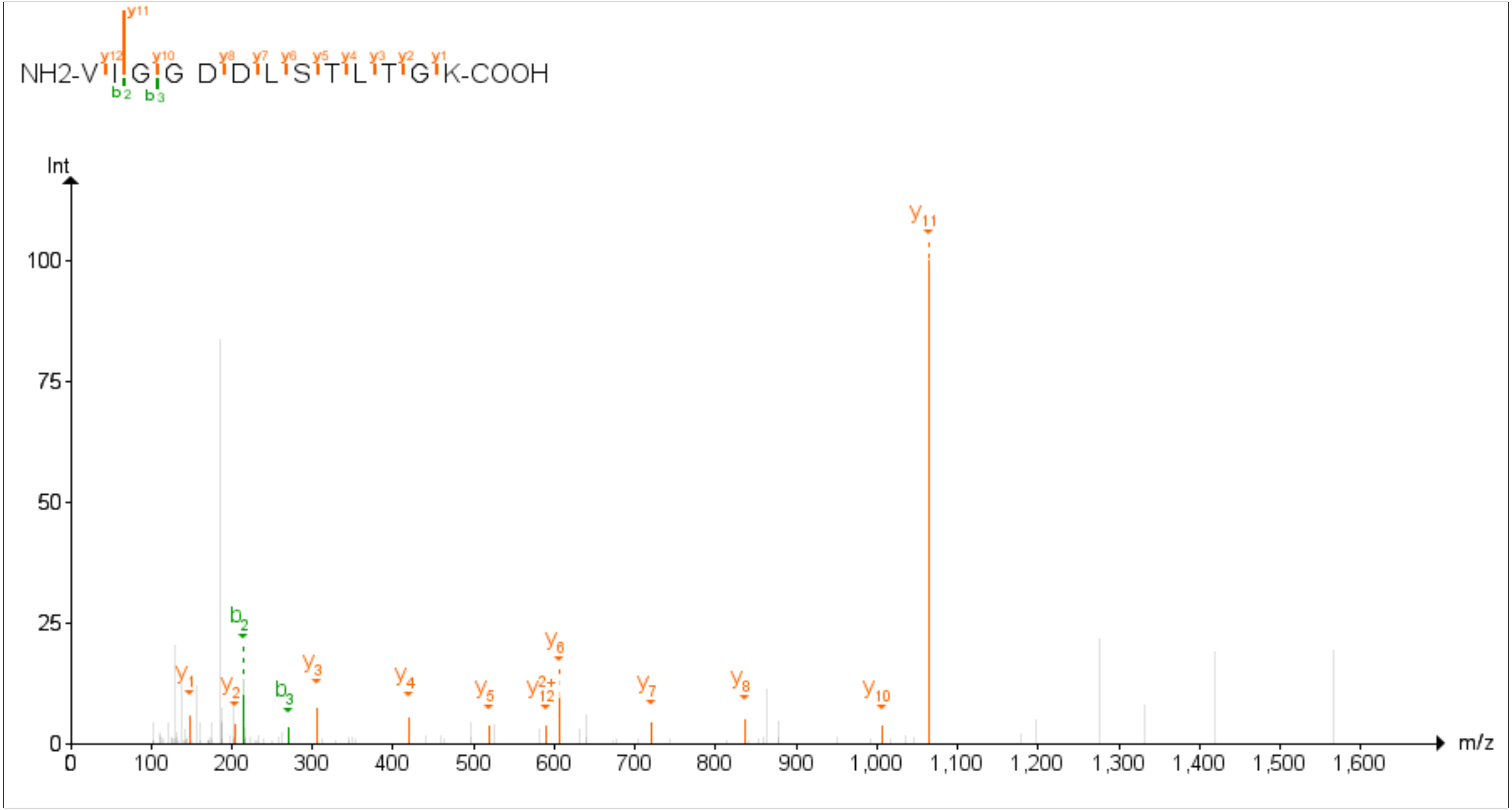

NH2-VIGGDDLSTLTGK-COOH
y12 y11 y10 y8 y7 y6 y5 y4 y3 y2 y1
b2 b3
Int
100
75
50
25
0
m/z
0 100 200 300 400 500 600 700 800 900 1,000 1,100 1,200 1,300 1,400 1,500 1,600
y11
b2
y6
y3
y1
y4
y2
b3
y5
y12 2+
y7
y8
y10

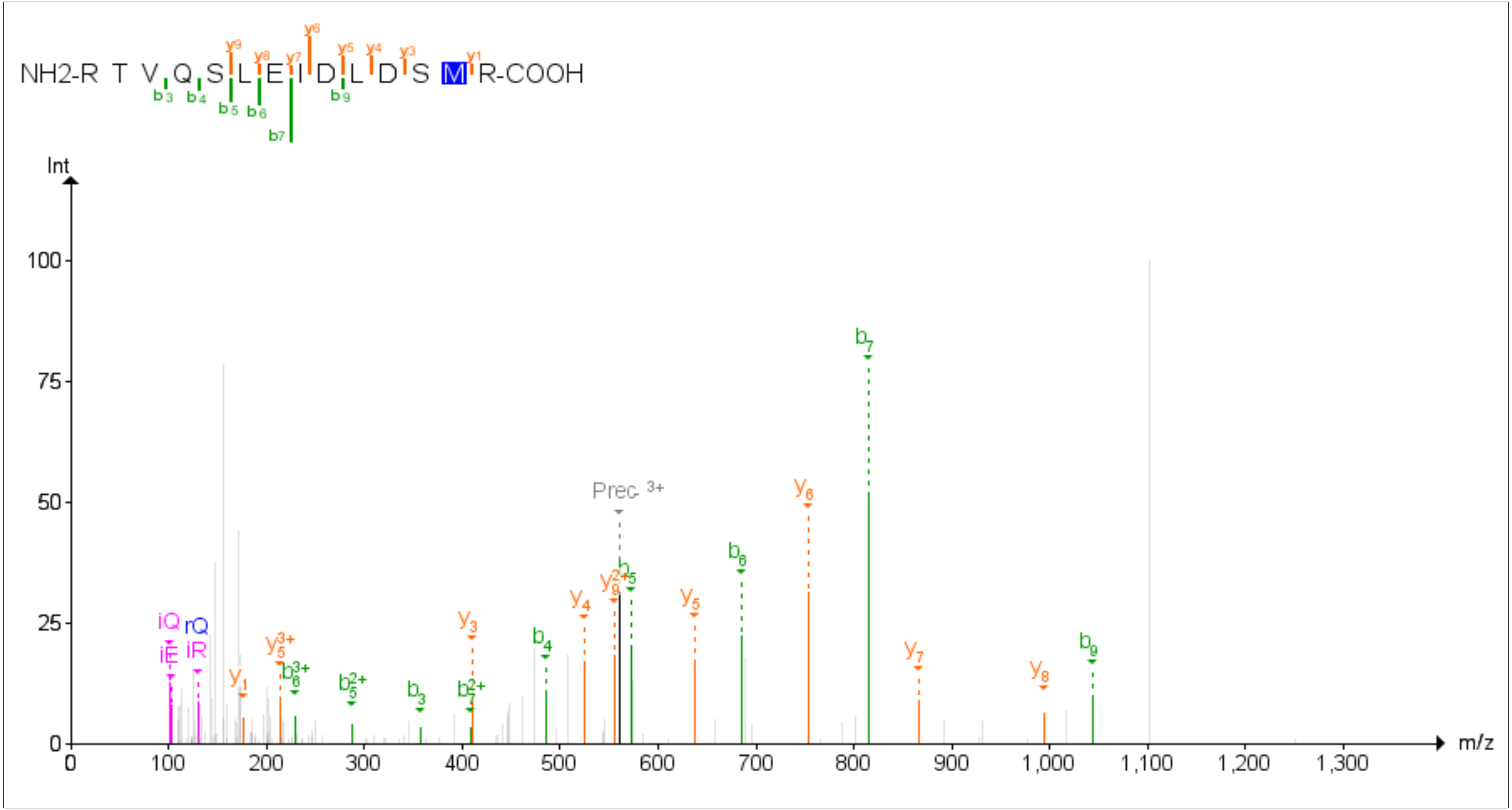

NH2-R T V Q S L E I D L D S M R-COOH
y9 y8 y7 y6 y5 y4 y3 y1
b3 b4 b5 b6 b7 b9
Int
m/z
Prec 3+
iQ rQ iE iR

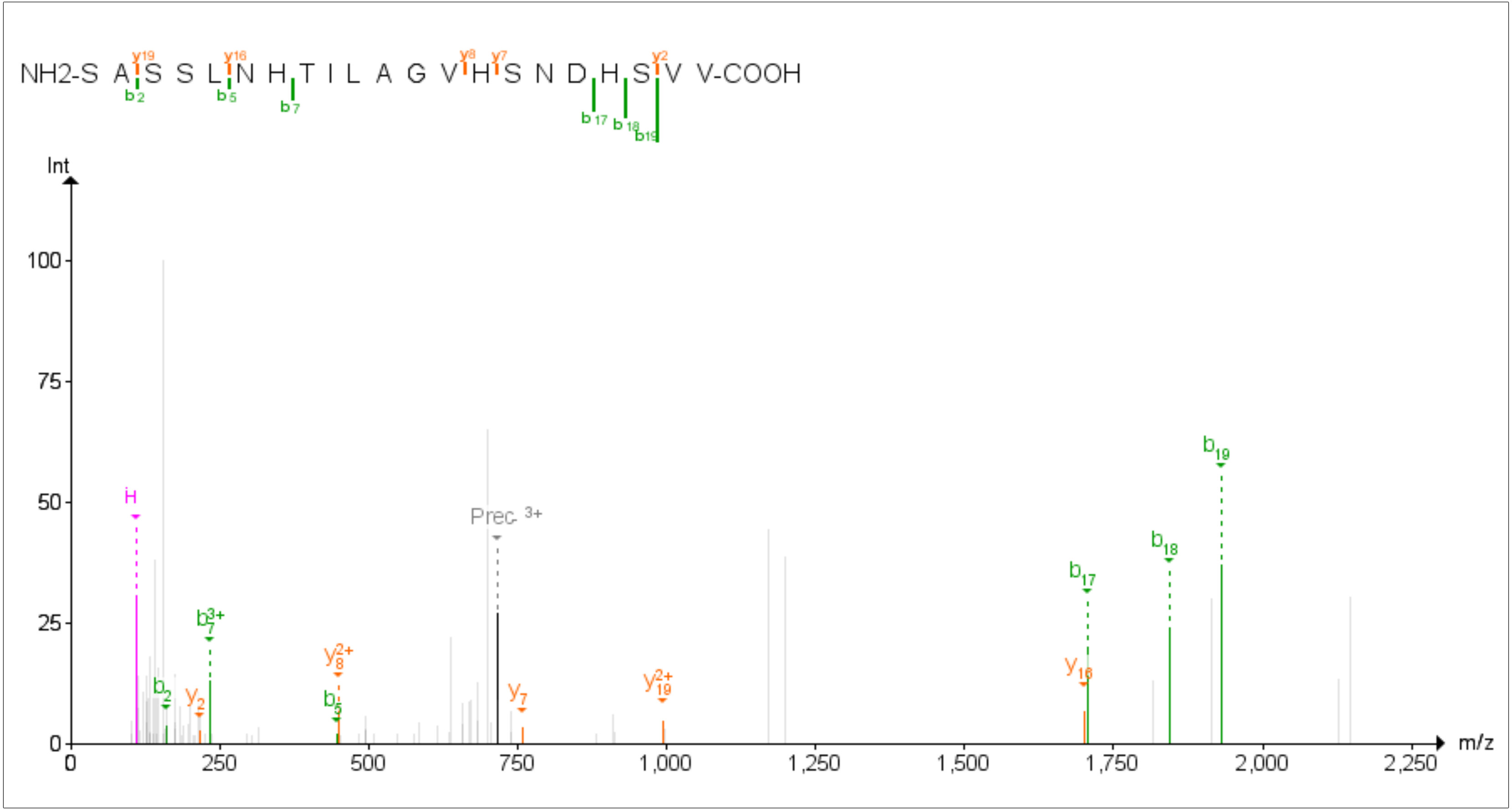

NH2-S A S S L N H T I L A G V H S N D H S V V-COOH
y19
y16
y8
y7
y2
b2
b5
b7
b17
b18
b19
Int
m/z
100
75
50
25
0
250
500
750
1,000
1,250
1,500
1,750
2,000
2,250
Prec 3+
b19
b18
b17
y16
b7 3+
y8 2+
y19 2+
y7
b2
y2
b5

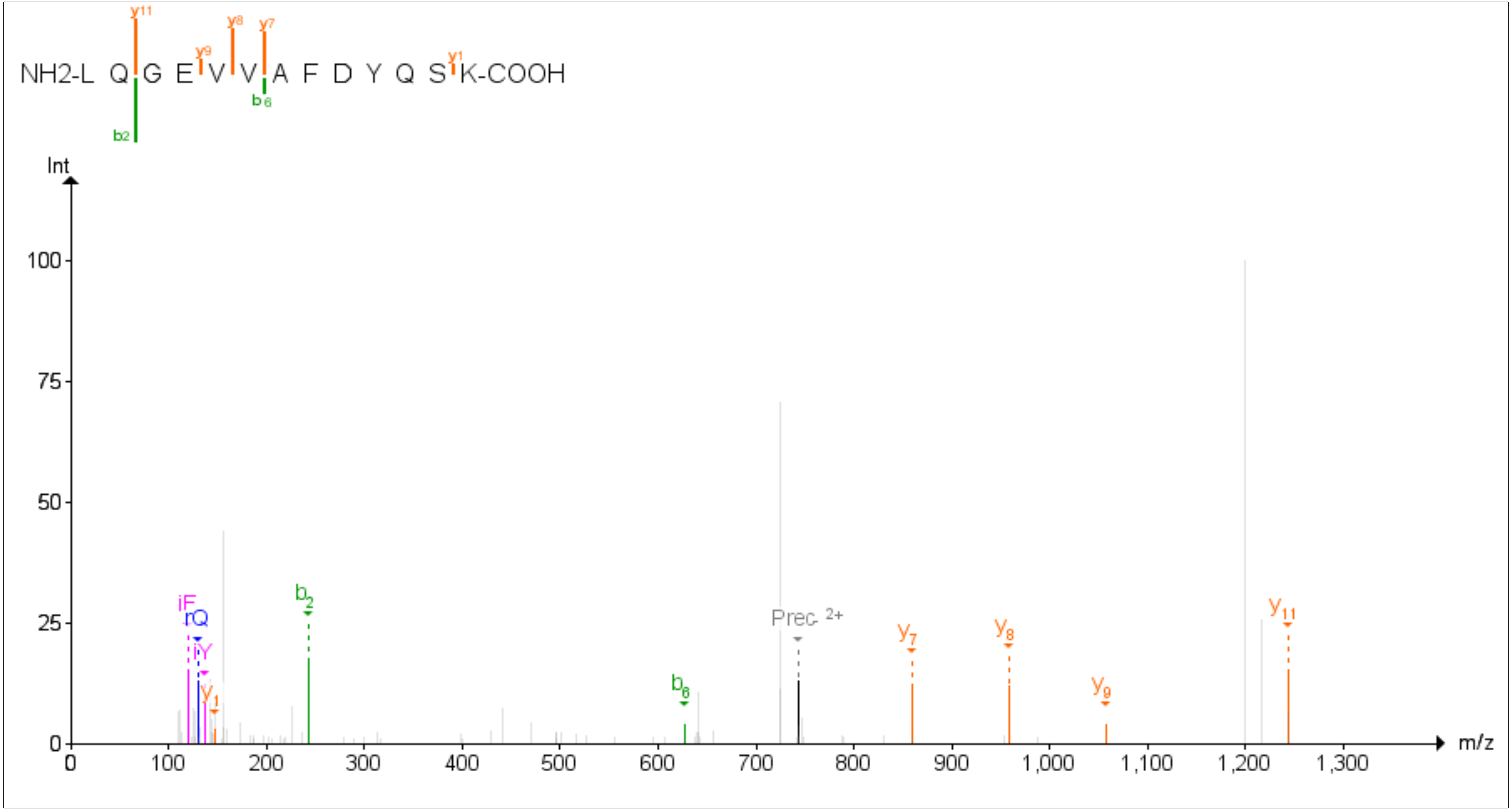

NH2-L Q G E V V A F D Y Q S K-COOH
y11
y9
y8
y7
y1
b2
b 6
Int
100
75
50
25
0
iF
rQ
iY
y1
b2
b6
Prec 2+
y7
y8
y9
y11
0
100
200
300
400
500
600
700
800
900
1,000
1,100
1,200
1,300
m/z

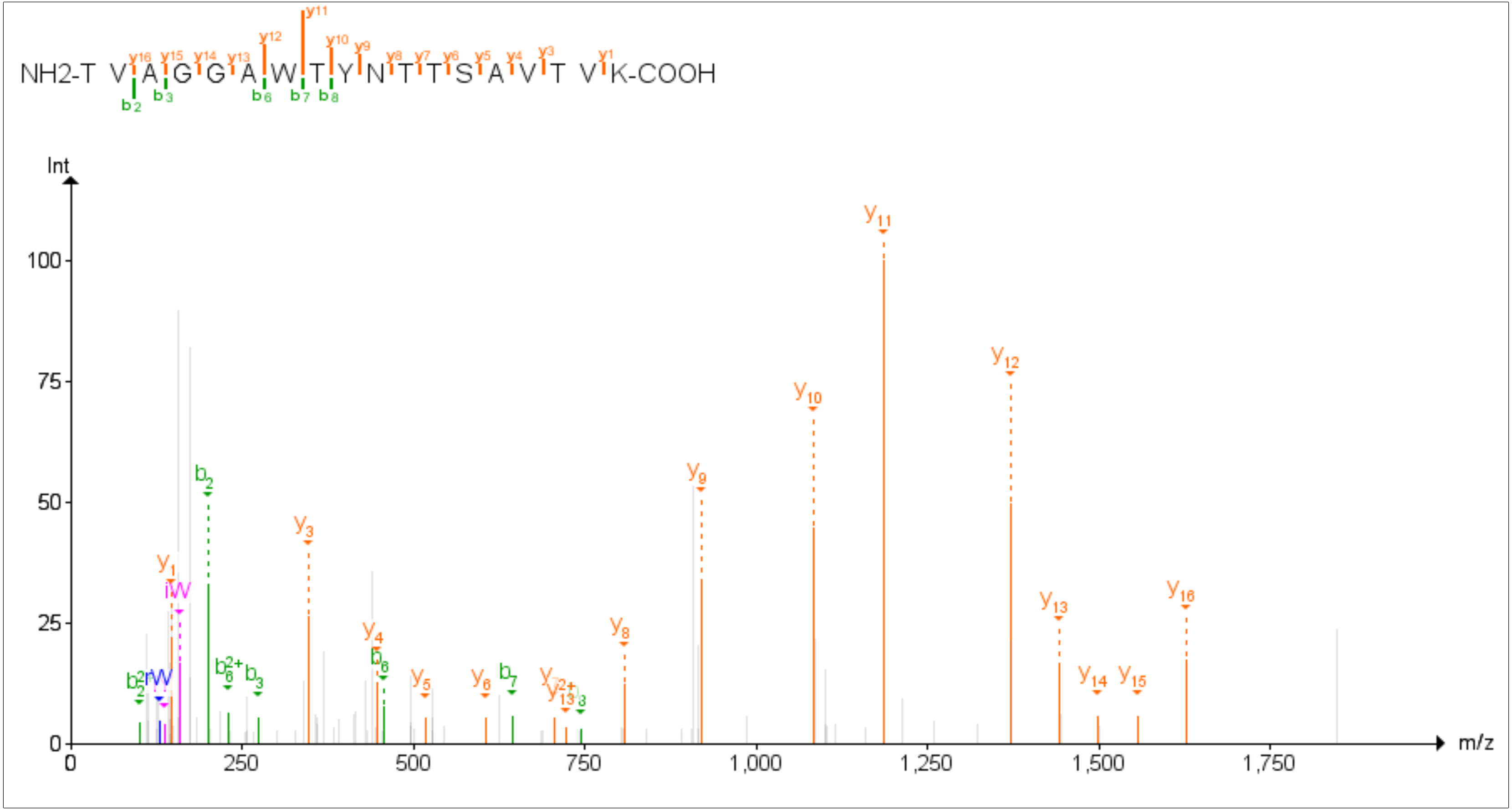

NH2-T V A G G A W T Y N T T S A V T V K-COOH
y16 y15 y14 y13 y12 y11 y10 y9 y8 y7 y6 y5 y4 y3 y1
b2 b3 b6 b7 b8
Int
100
75
50
25
0
m/z
0
250
500
750
1,000
1,250
1,500
1,750

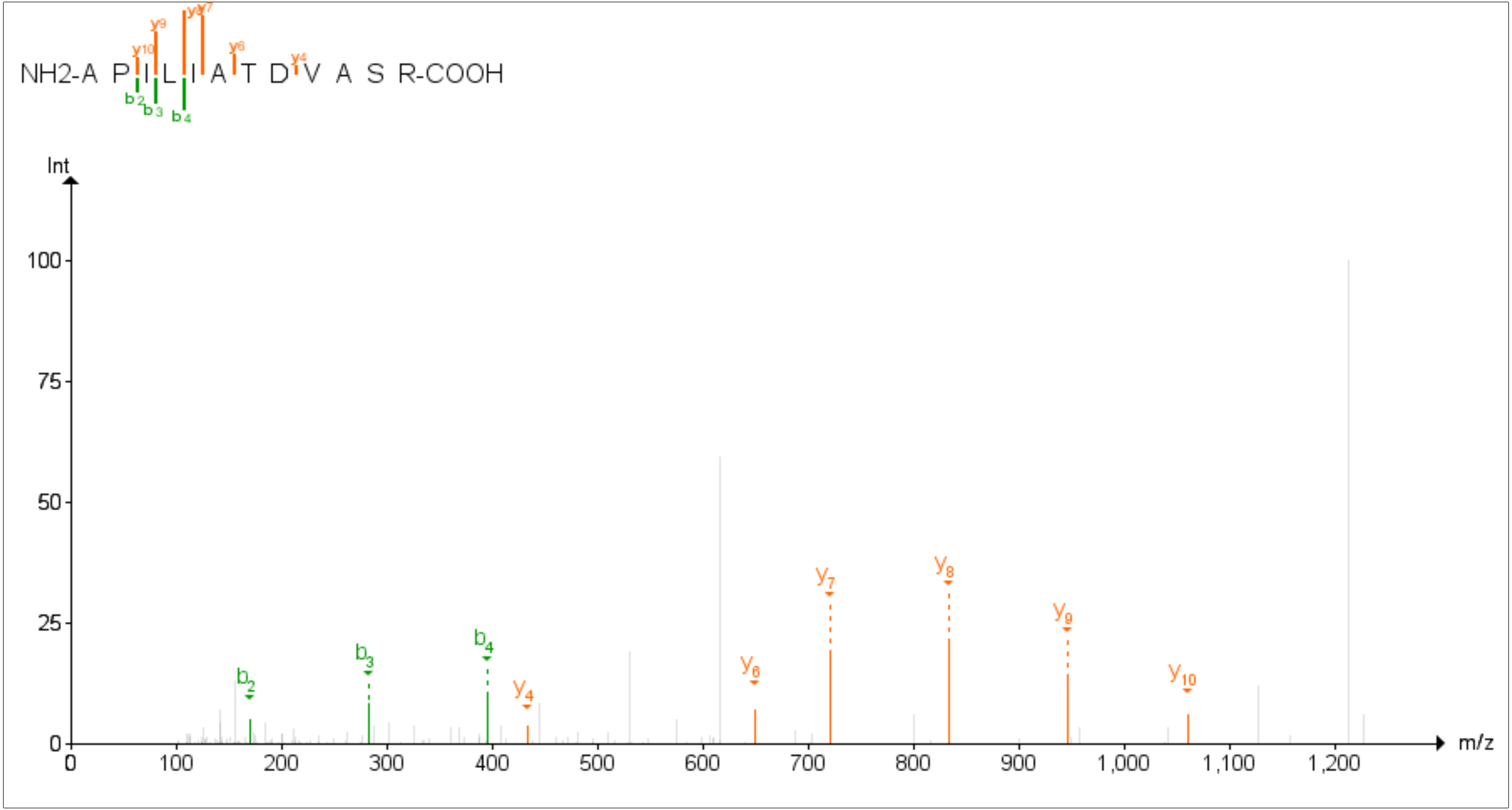
NH2-A P I L I A T D V A S R-COOH
y10
y9
y8
y7
y6
y4
b2
b3
b4
Int
100
75
50
25
0
m/z
0
100
200
300
400
500
600
700
800
900
1,000
1,100
1,200
b2
b3
b4
y4
y6
y7
y8
y9
y10

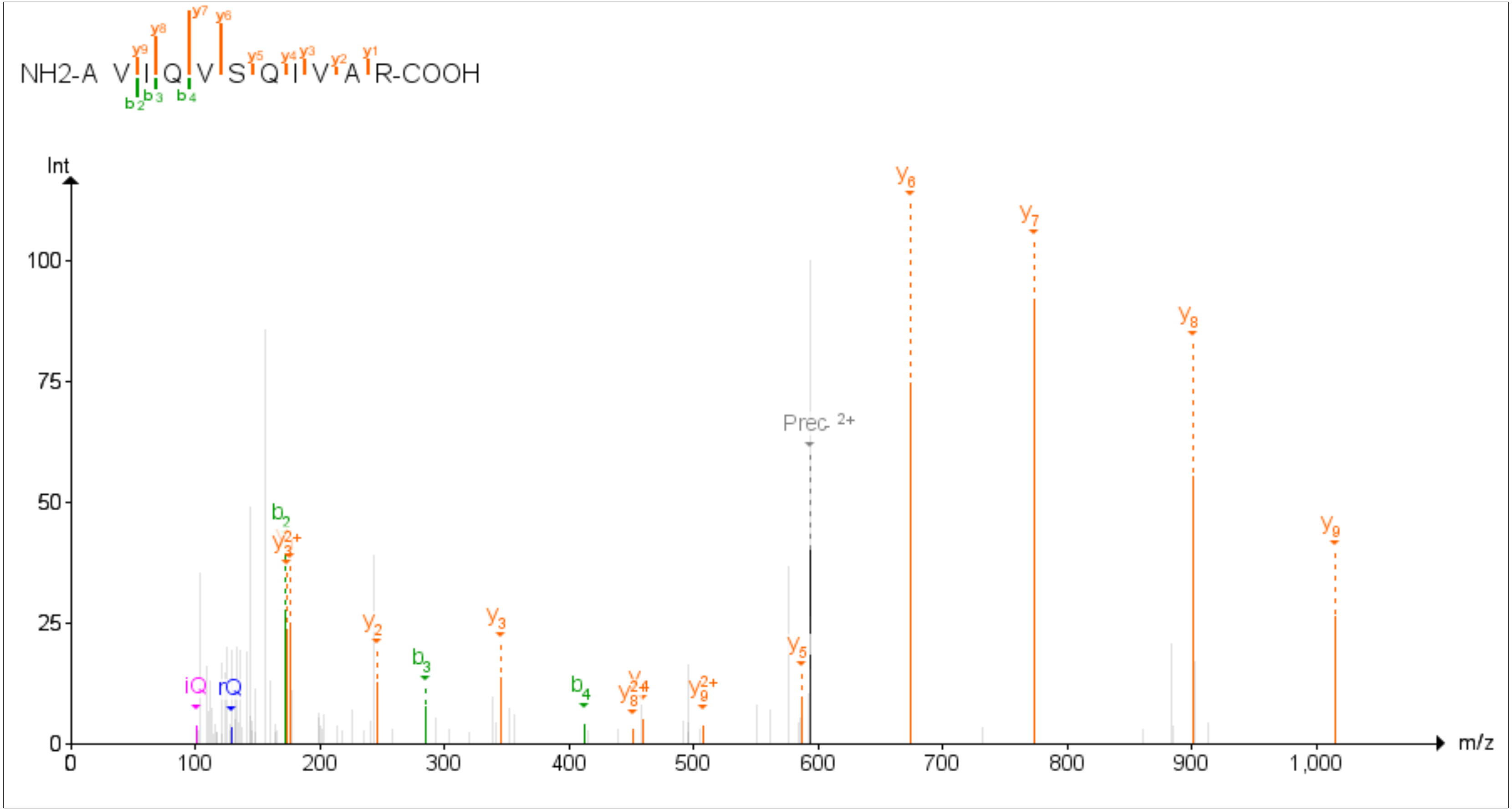

NH2-A V I Q V S Q I V A R-COOH
y9 y8 y7 y6 y5 y4 y3 y2 y1
b2 b3 b4
Int
m/z
0
25
50
75
100
0
100
200
300
400
500
600
700
800
900
1,000
Prec. 2+
y6
y7
y8
y9
y5
y3
y2
b2
b3
b4
iQ
rQ

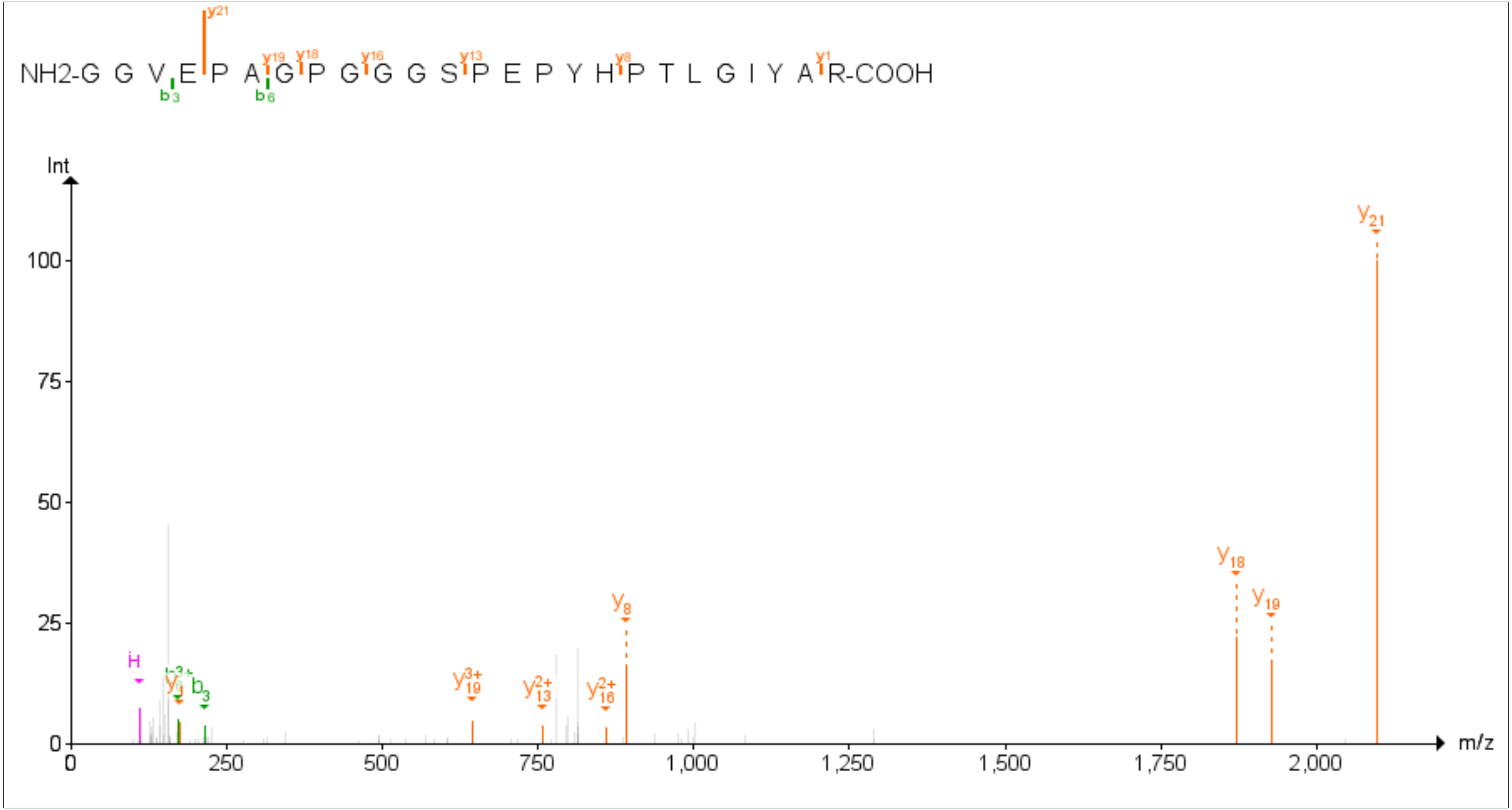

NH2-G G V E P A G P G G G S P E P Y H P T L G I Y A R-COOH
y21
y19
y18
y16
y13
y8
y1
b3
b6
Int
100
75
50
25
0
m/z
0
250
500
750
1,000
1,250
1,500
1,750
2,000
H
b3
y3+ 19
y2+ 13
y2+ 16
y8
y18
y19
y21

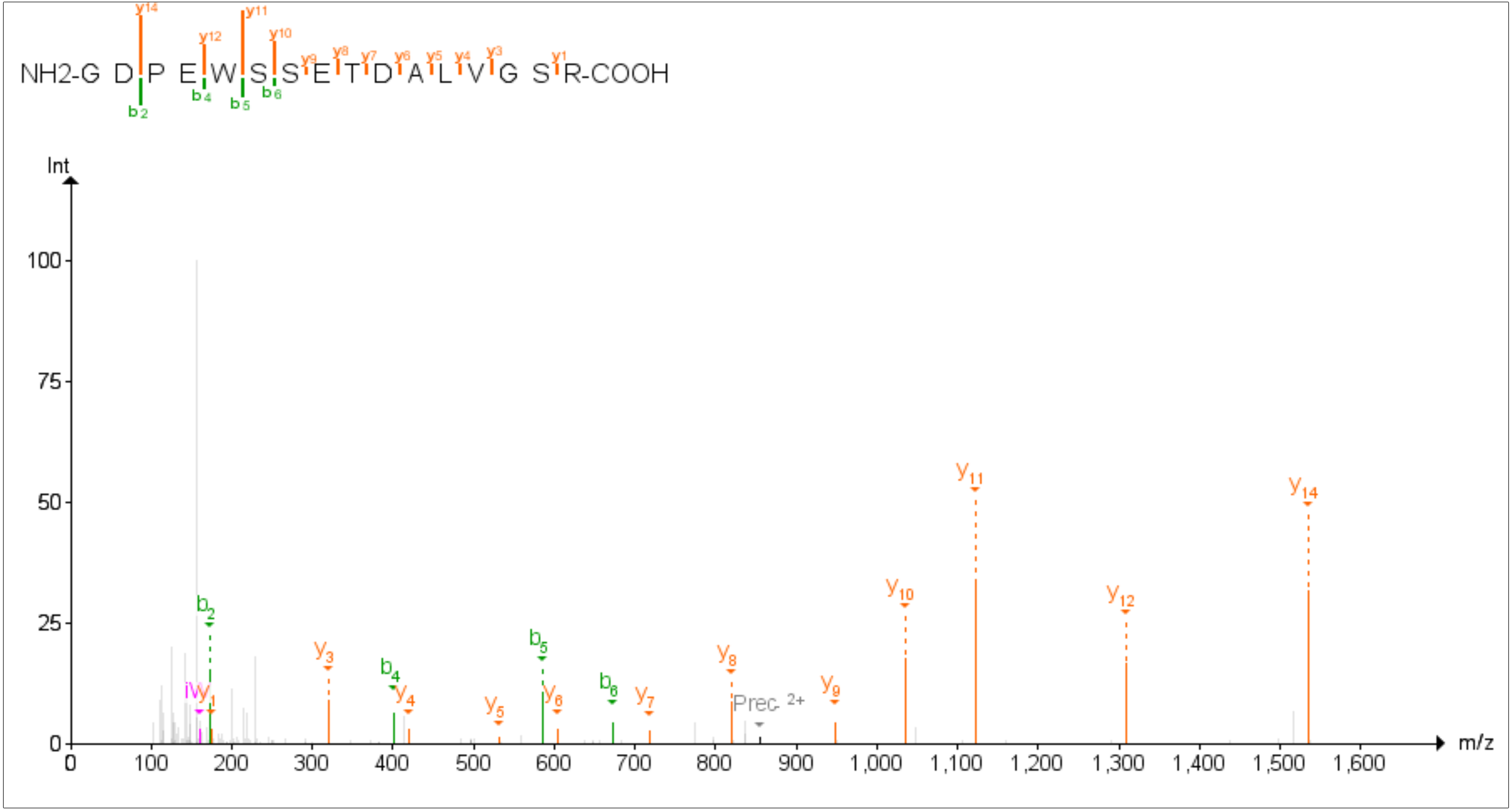

NH2-G D P E W S S E T D A L V G S R-COOH
Int
m/z
Prec 2+

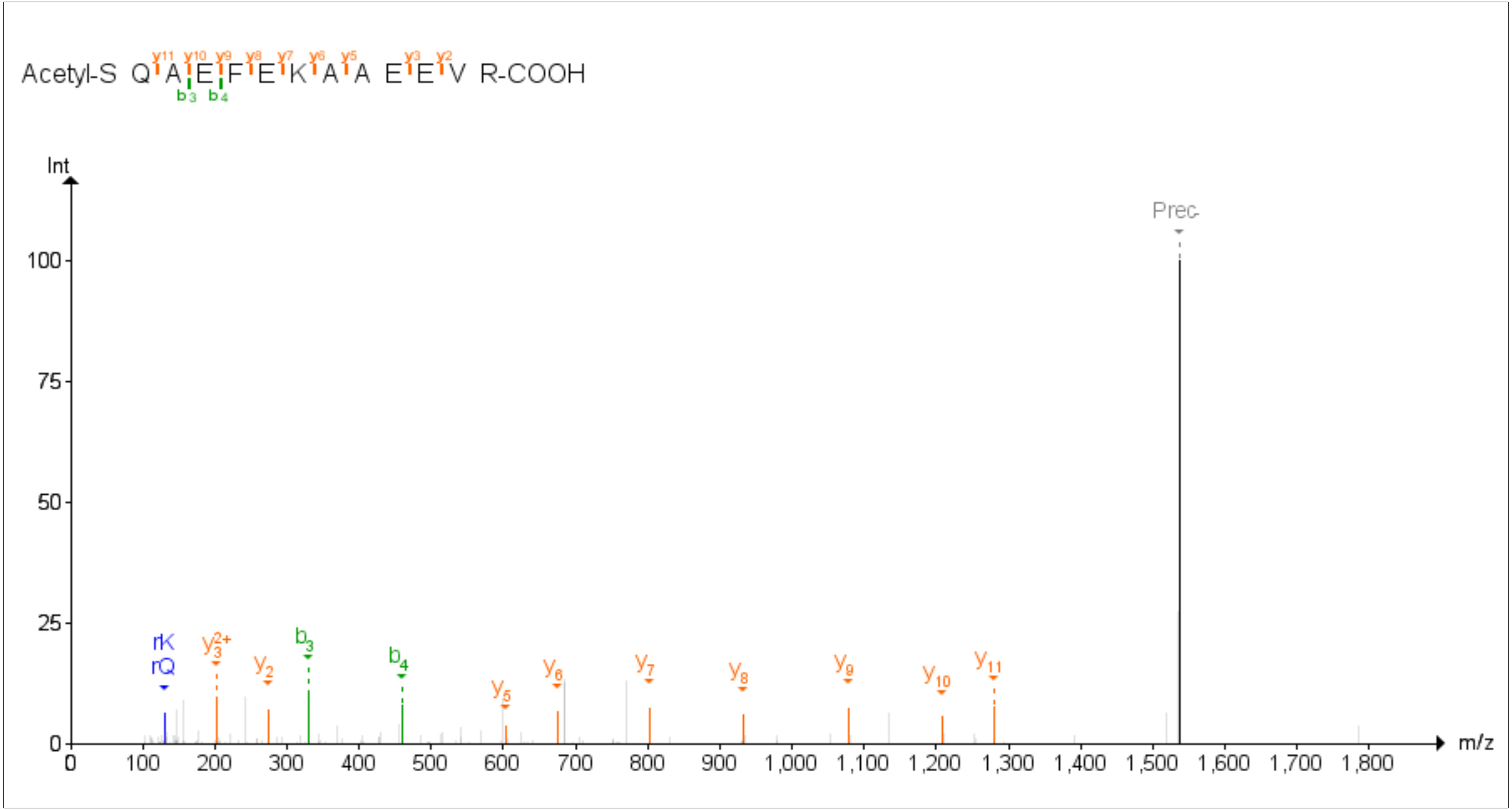
Acetyl-S Q A E F E K A A E E V R-COOH
y11 y10 y9 y8 y7 y6 y5 y3 y2
b3 b4
Int
100
75
50
25
0
Prec
rK
rQ
y3 2+
y2
b3
b4
y5
y6
y7
y8
y9
y10
y11
0 100 200 300 400 500 600 700 800 900 1,000 1,100 1,200 1,300 1,400 1,500 1,600 1,700 1,800
m/z

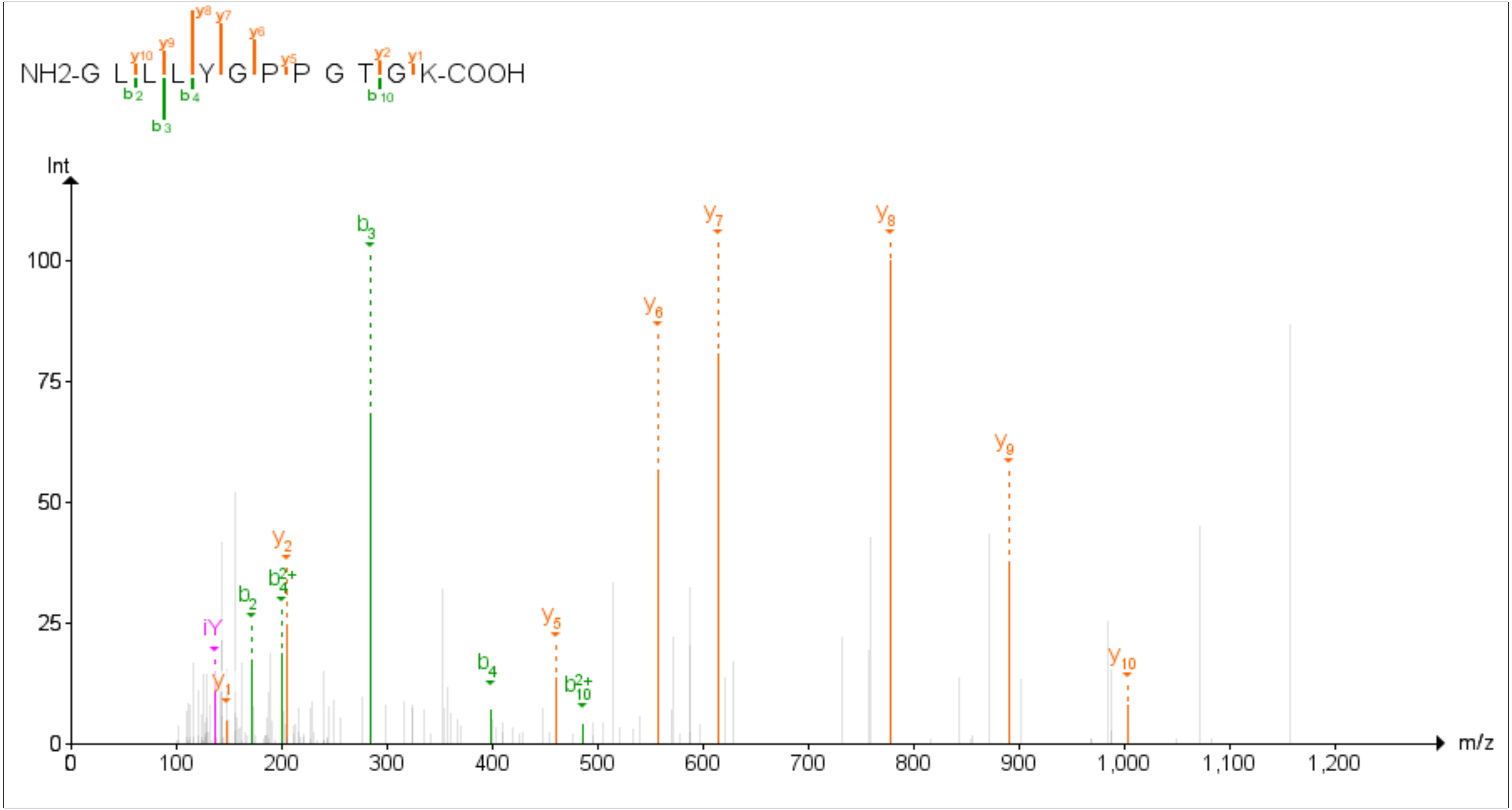

NH2-G L L L Y G P P G T G K-COOH
y10 y9 y8 y7 y6 y5 y2 y1
b2 b3 b4 b10
Int
m/z
iY
y1 y2 y5 y6 y7 y8 y9 y10
b2 b3 b4 b4 2+ b10 2+
0
25
50
75
100
0 100 200 300 400 500 600 700 800 900 1,000 1,100 1,200

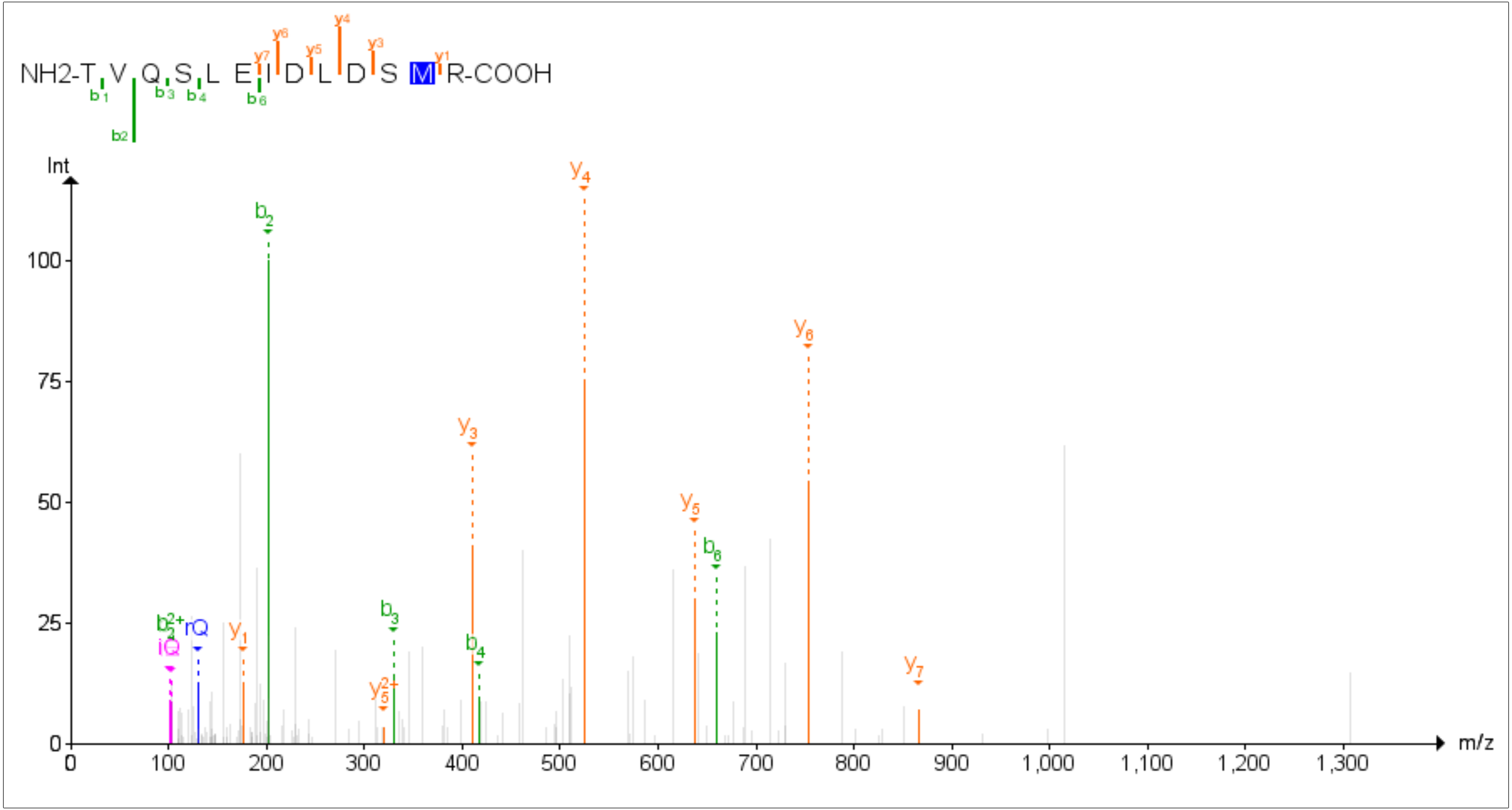

NH2-T V Q S L E I D L D S M R-COOH
Int
m/z
b2
y4
y6
y3
y5
b6
b3
b4
y7
y1
rQ
iQ

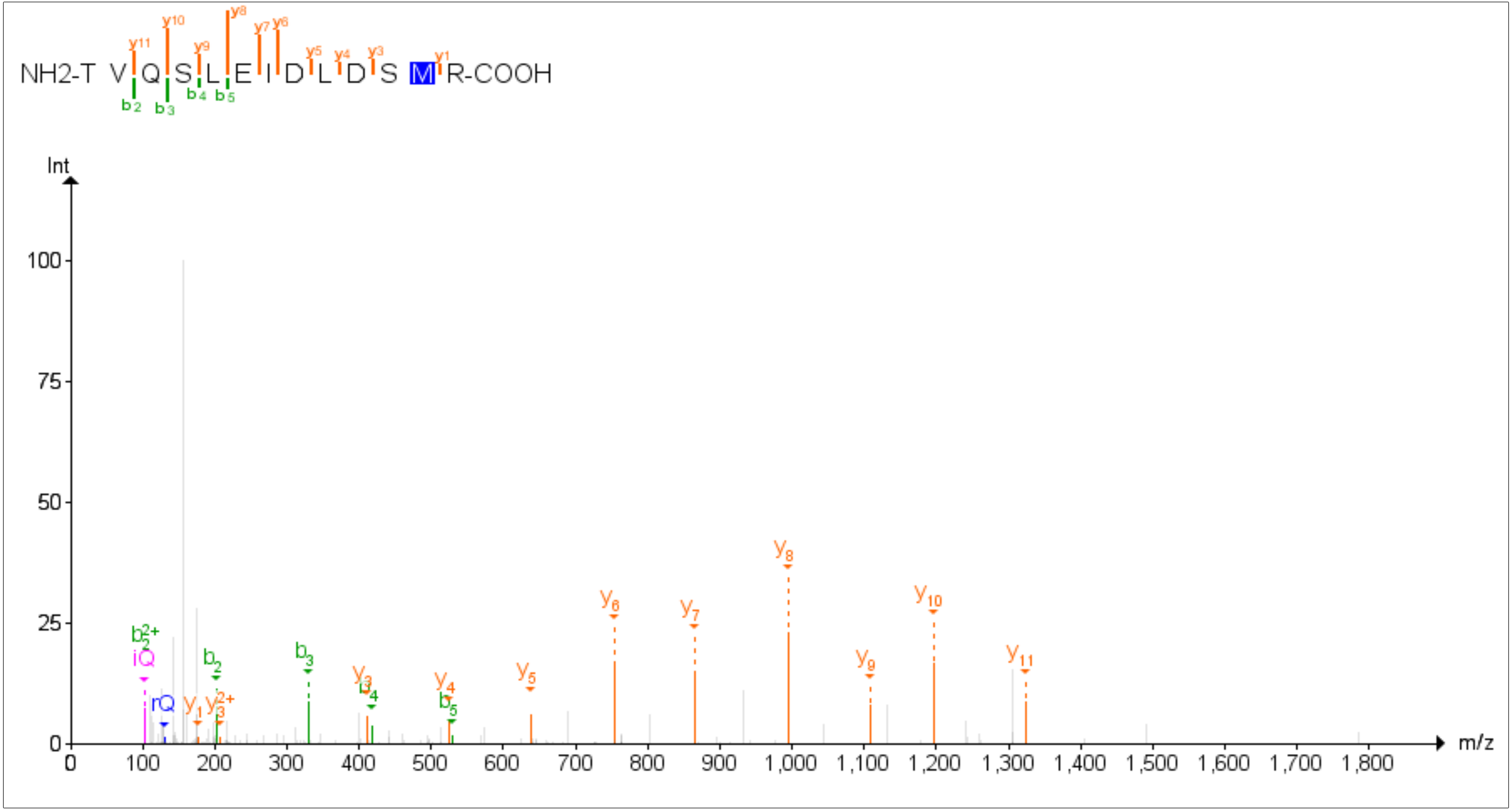

NH2-T V Q S L E I D L D S M R-COOH
Int
m/z
100
75
50
25
0
0 100 200 300 400 500 600 700 800 900 1,000 1,100 1,200 1,300 1,400 1,500 1,600 1,700 1,800

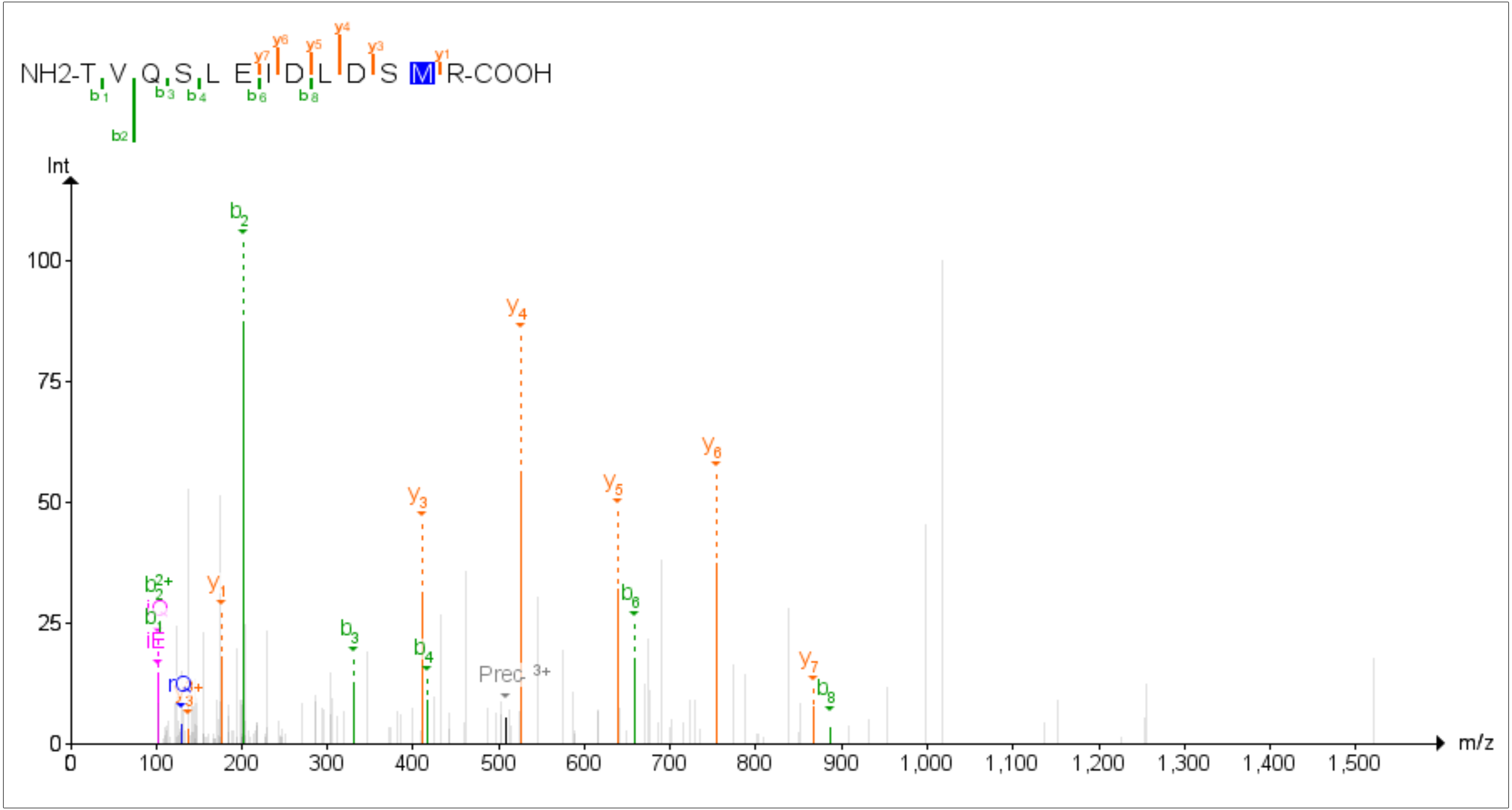

NH2-T V Q S L E I D L D S M R-COOH
Int
m/z
Prec 3+

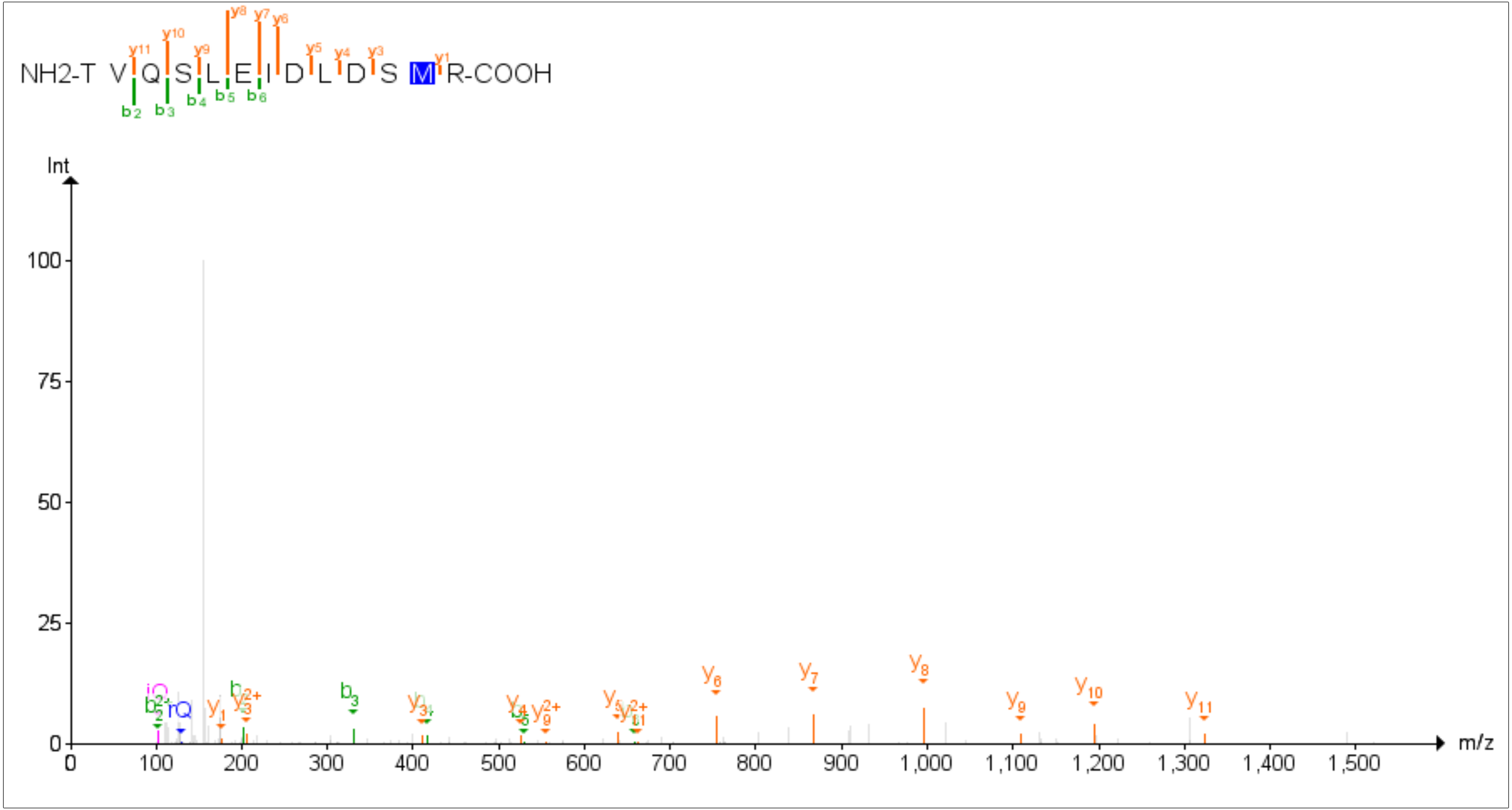
NH2-T V Q S L E I D L D S M R-COOH
Int
m/z
100
75
50
25
0
0
100
200
300
400
500
600
700
800
900
1,000
1,100
1,200
1,300
1,400
1,500

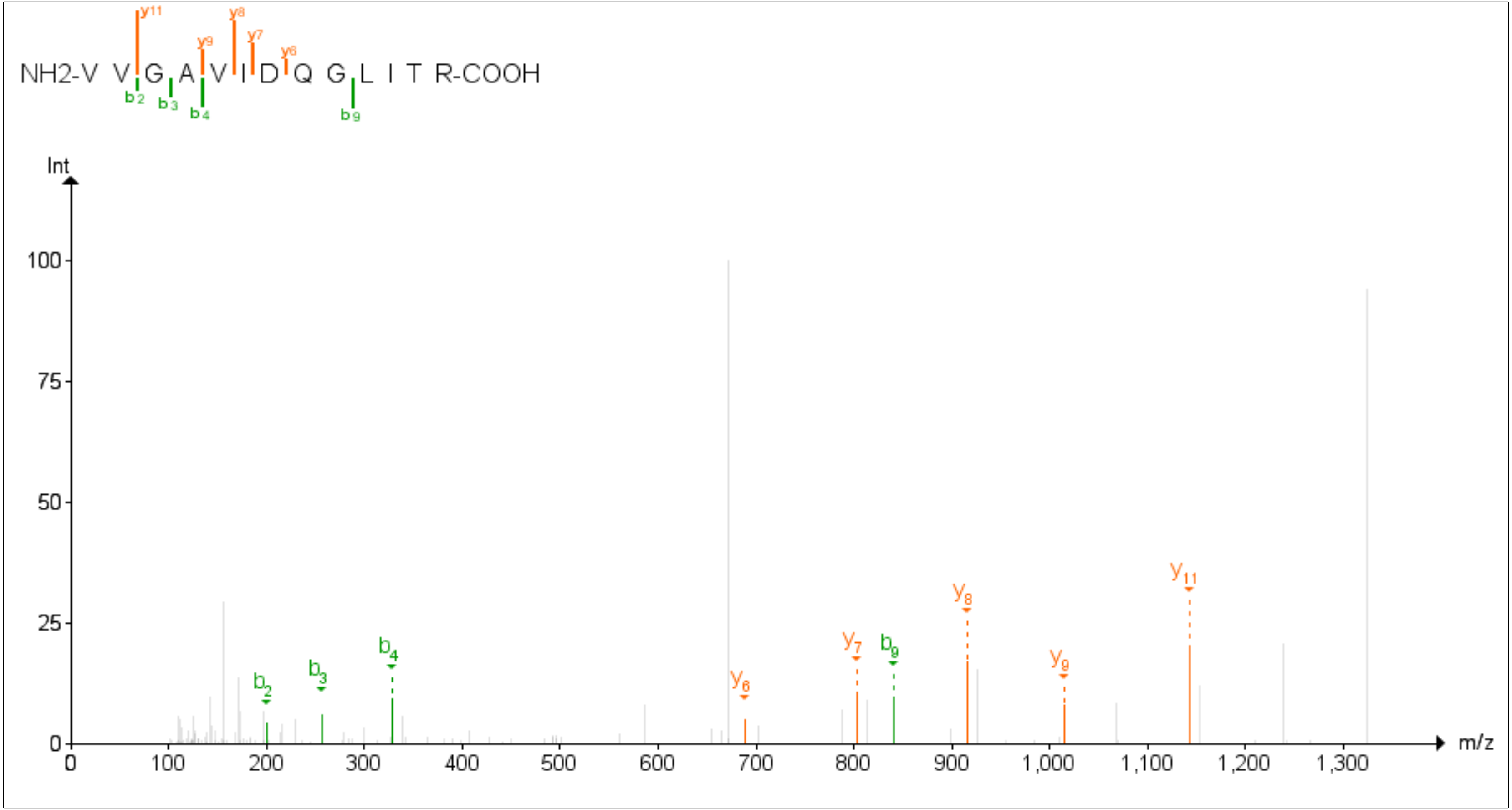

NH2-V V G A V I D Q G L I T R-COOH
y11
y9
y8
y7
y6
b2
b3
b4
b9
Int
100
75
50
25
0
m/z
0
100
200
300
400
500
600
700
800
900
1,000
1,100
1,200
1,300
b2
b3
b4
y6
y7
b9
y8
y9
y11

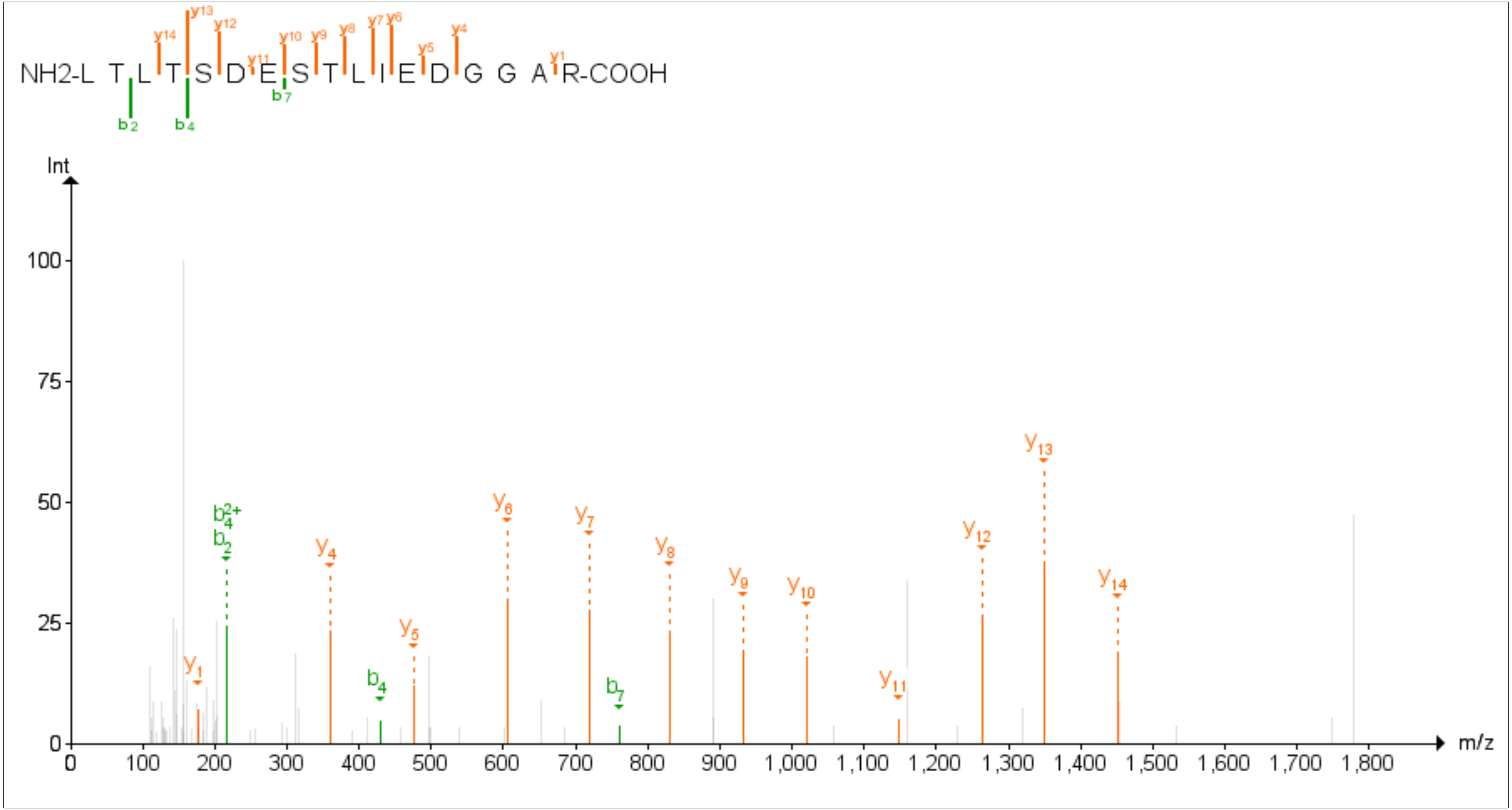

NH2-L T L T S D E S T L I E D G G A R-COOH
y14 y13 y12 y11 y10 y9 y8 y7 y6 y5 y4 y1
b2 b4 b7
Int
100
75
50
25
0
m/z
0 100 200 300 400 500 600 700 800 900 1,000 1,100 1,200 1,300 1,400 1,500 1,600 1,700 1,800
b4 2+
b2
y1 y4 b4 y5 y6 y7 b7 y8 y9 y10 y11 y12 y13 y14

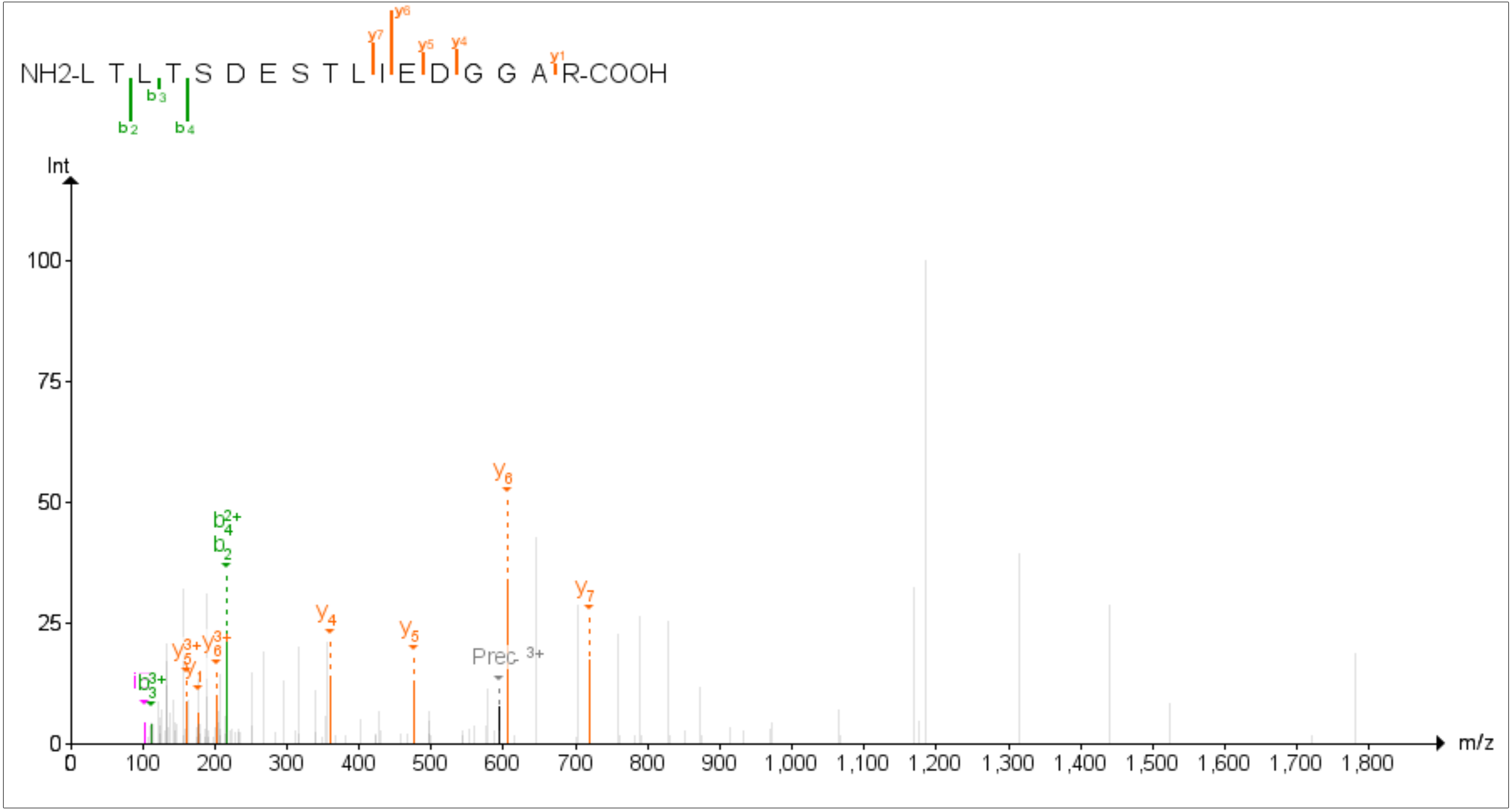

NH2-L T L T S D E S T L I E D G G A R-COOH
b2
b3
b4
y7
y6
y5
y4
y1
Int
m/z
100
75
50
25
0
0
100
200
300
400
500
600
700
800
900
1,000
1,100
1,200
1,300
1,400
1,500
1,600
1,700
1,800
Prec 3+

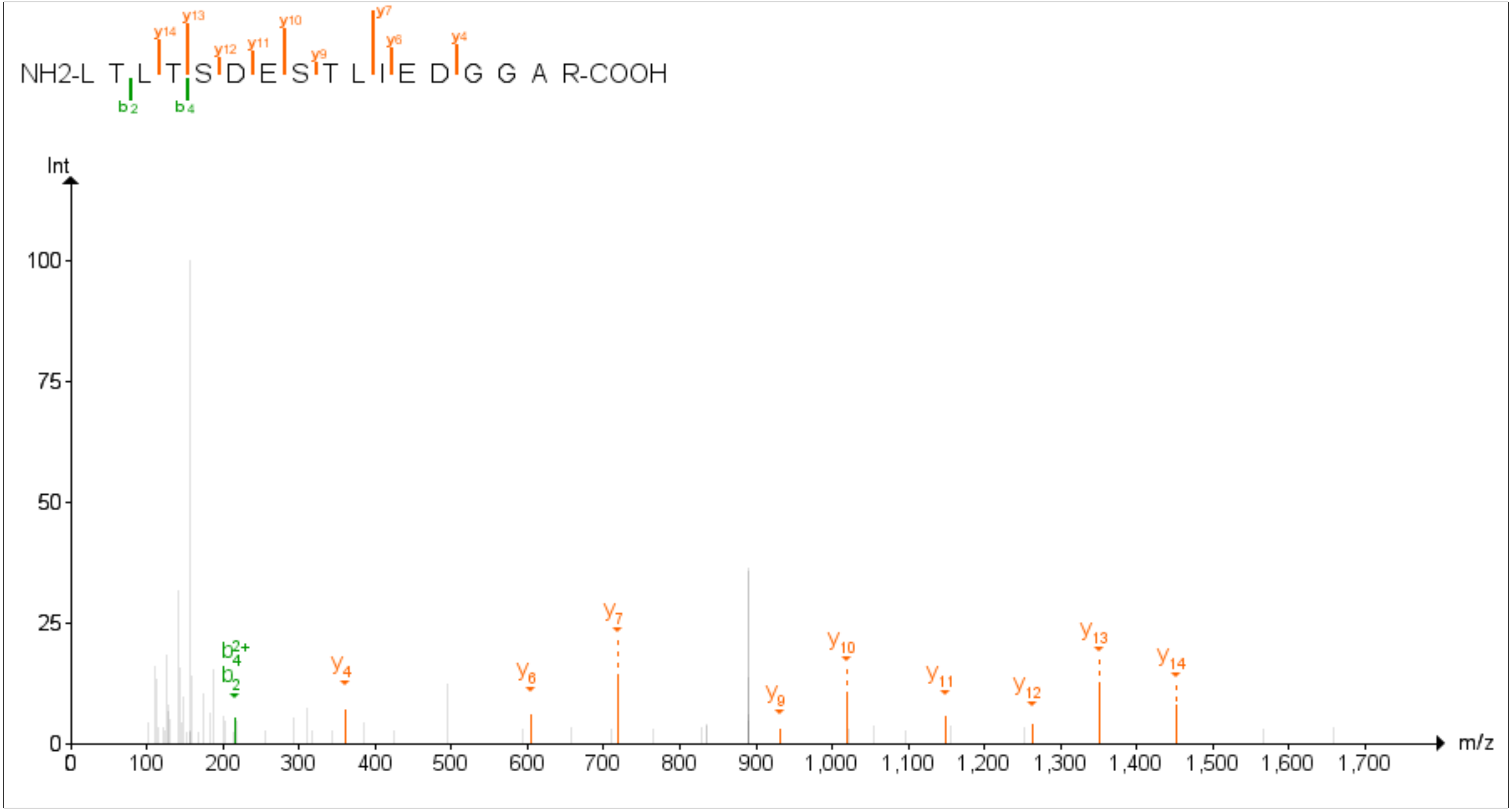

NH2-L T L T S D E S T L I E D G G A R-COOH
y14 y13 y12 y11 y10 y9 y7 y6 y4
b2 b4
Int
100
75
50
25
0
b4 2+
b2
y4
y6
y7
y9
y10
y11
y12
y13
y14
0 100 200 300 400 500 600 700 800 900 1,000 1,100 1,200 1,300 1,400 1,500 1,600 1,700
m/z

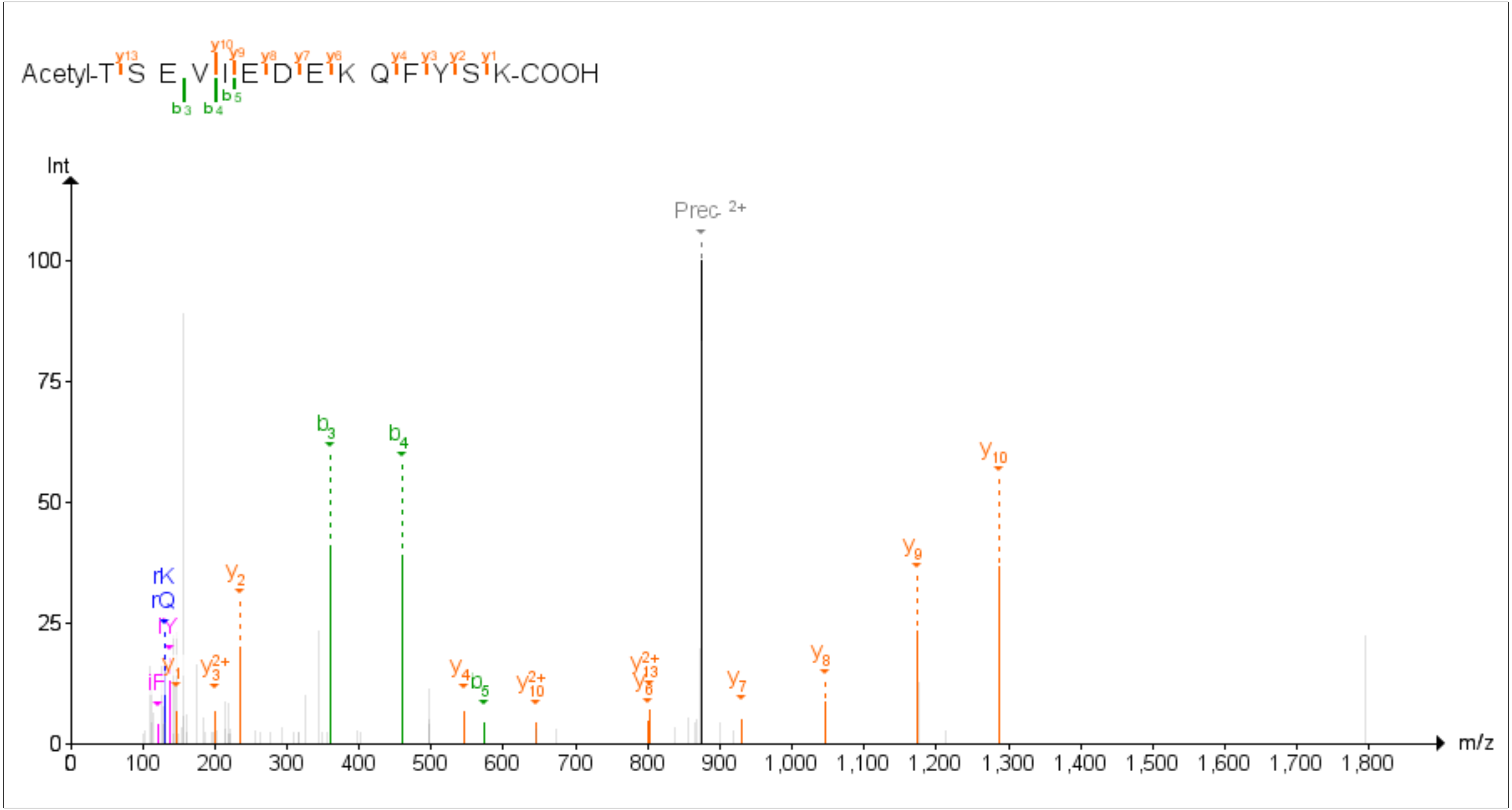

Acetyl-T S E V I I E D E K Q F Y S K-COOH
y13 y10 y9 y8 y7 y6 y4 y3 y2 y1
b3 b4 b5
Int
Prec 2+
b3
b4
y10
y9
y8
y7
y2
rK
rQ
iY
iF
y1
y4
b5
0
25
50
75
100
0 100 200 300 400 500 600 700 800 900 1,000 1,100 1,200 1,300 1,400 1,500 1,600 1,700 1,800
m/z

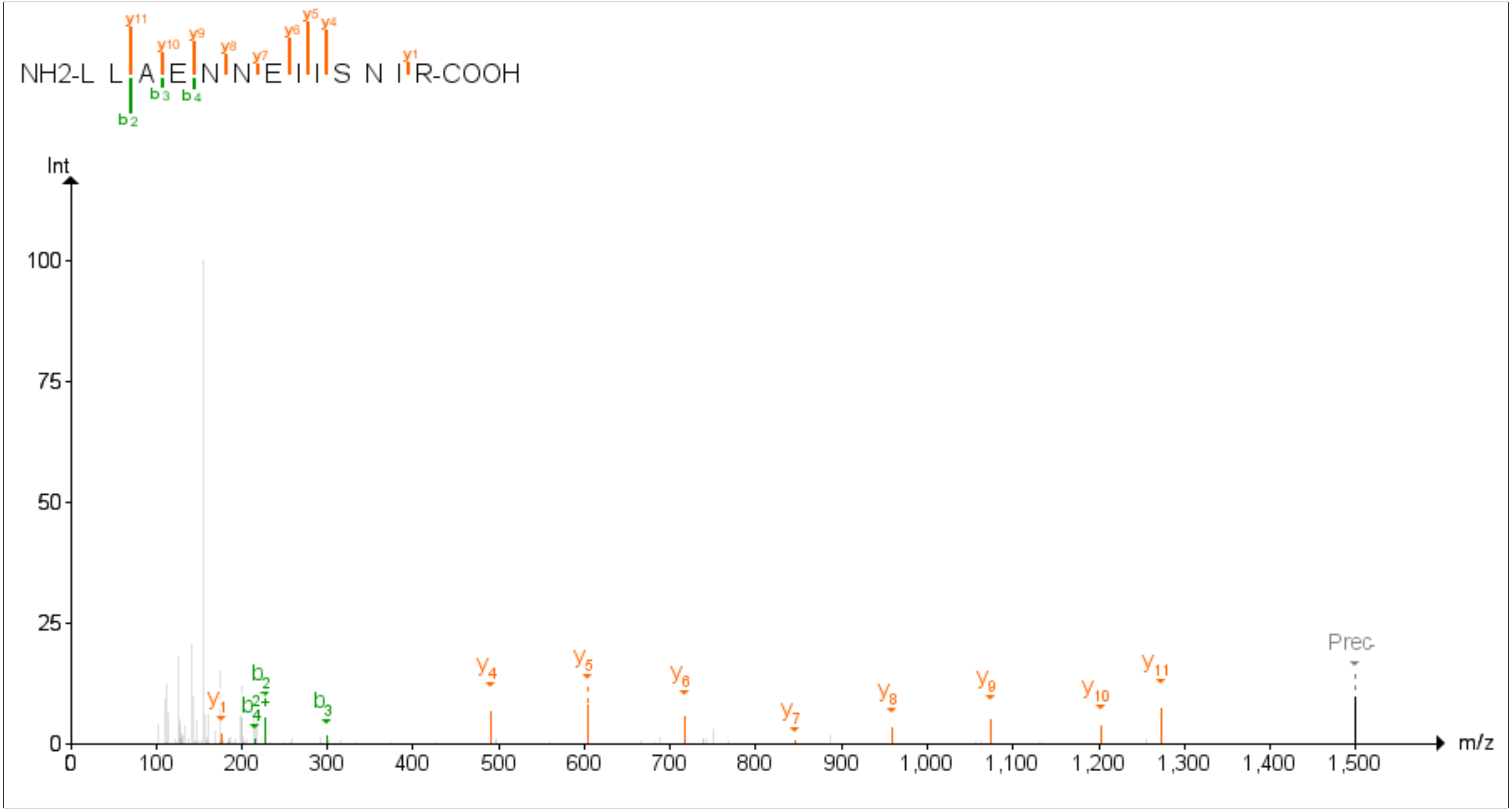

NH2-L L A E N N E I I S N I R-COOH
y11 y10 y9 y8 y7 y6 y5 y4 y1
b2 b3 b4
Int
100
75
50
25
0
m/z
0 100 200 300 400 500 600 700 800 900 1,000 1,100 1,200 1,300 1,400 1,500
y1 b2 b4 b3 y4 y5 y6 y7 y8 y9 y10 y11
Prec

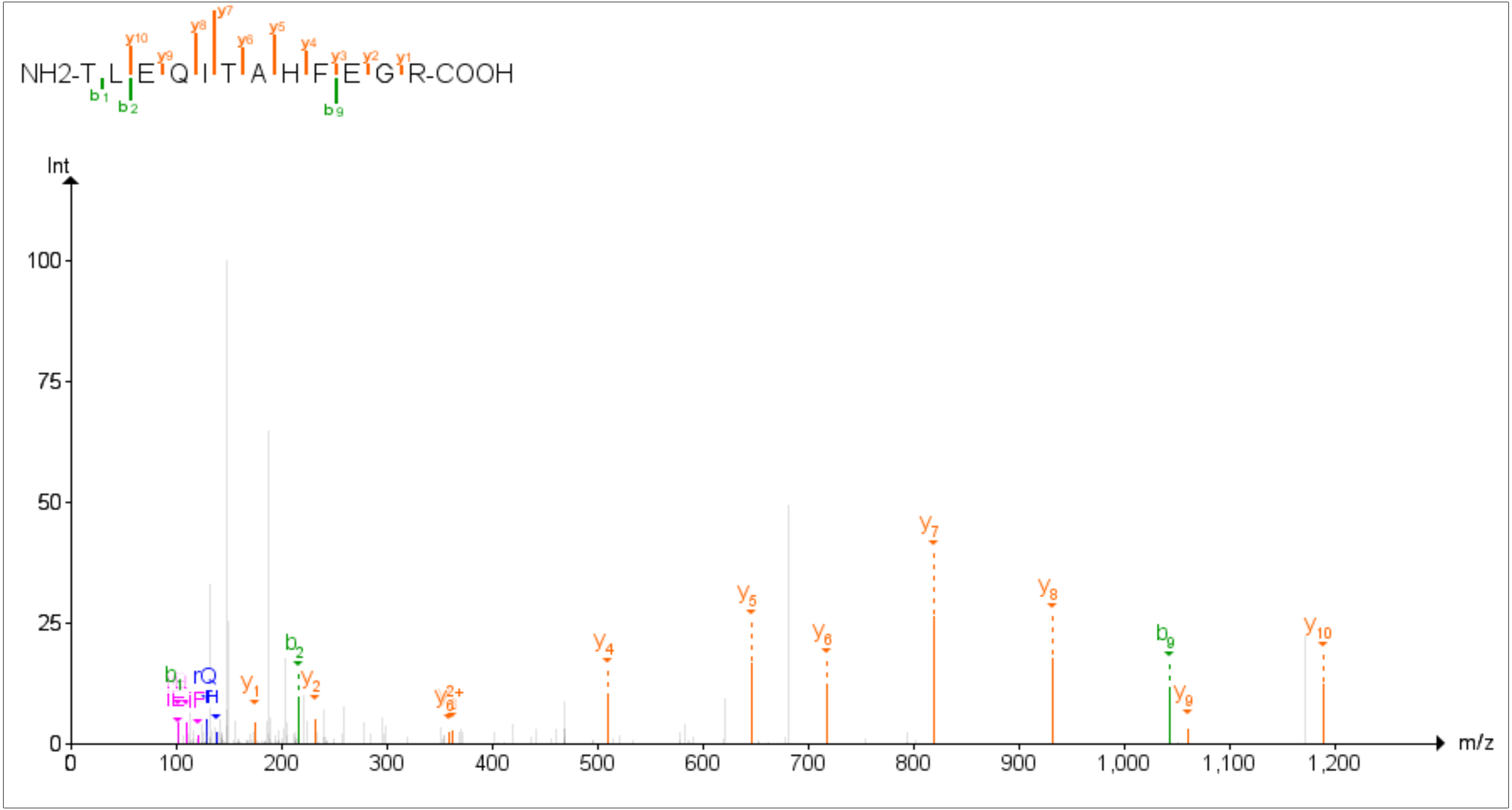

NH2-T L E Q I T A H F E G R-COOH
Int
m/z

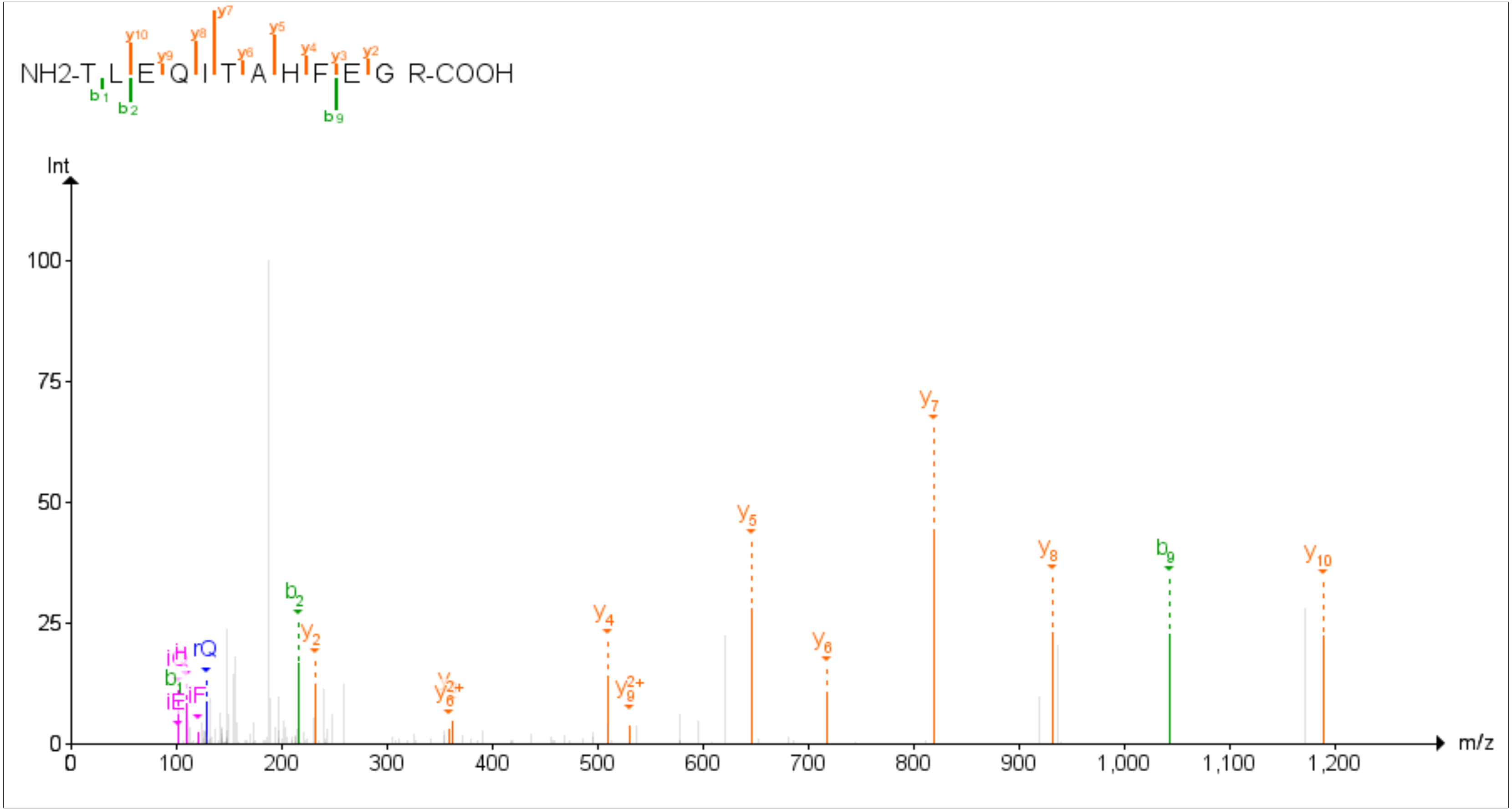

NH2-T L E Q I T A H F E G R-COOH
y10
y9
y8
y7
y6
y5
y4
y3
y2
b1
b2
b9
Int
100
75
50
25
0
rQ
m/z
0
100
200
300
400
500
600
700
800
900
1,000
1,100
1,200

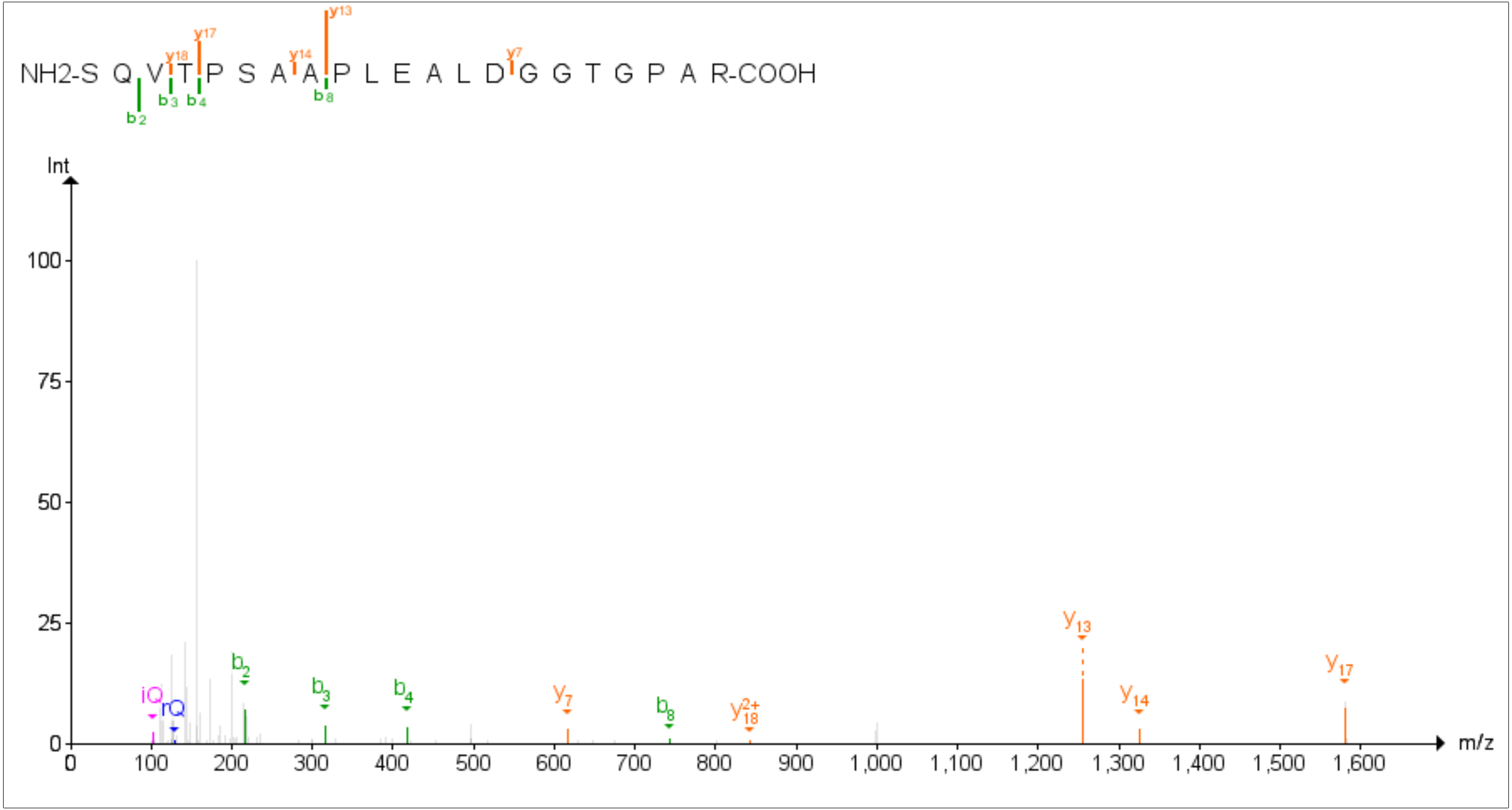

NH2-S Q V T P S A A P L E A L D G G T G P A R-COOH
y13
y17
y18
y14
y7
b2
b3
b4
b8
Int
100
75
50
25
0
iQ
rQ
b2
b3
b4
y7
b8
y18 2+
y13
y14
y17
0
100
200
300
400
500
600
700
800
900
1,000
1,100
1,200
1,300
1,400
1,500
1,600
m/z

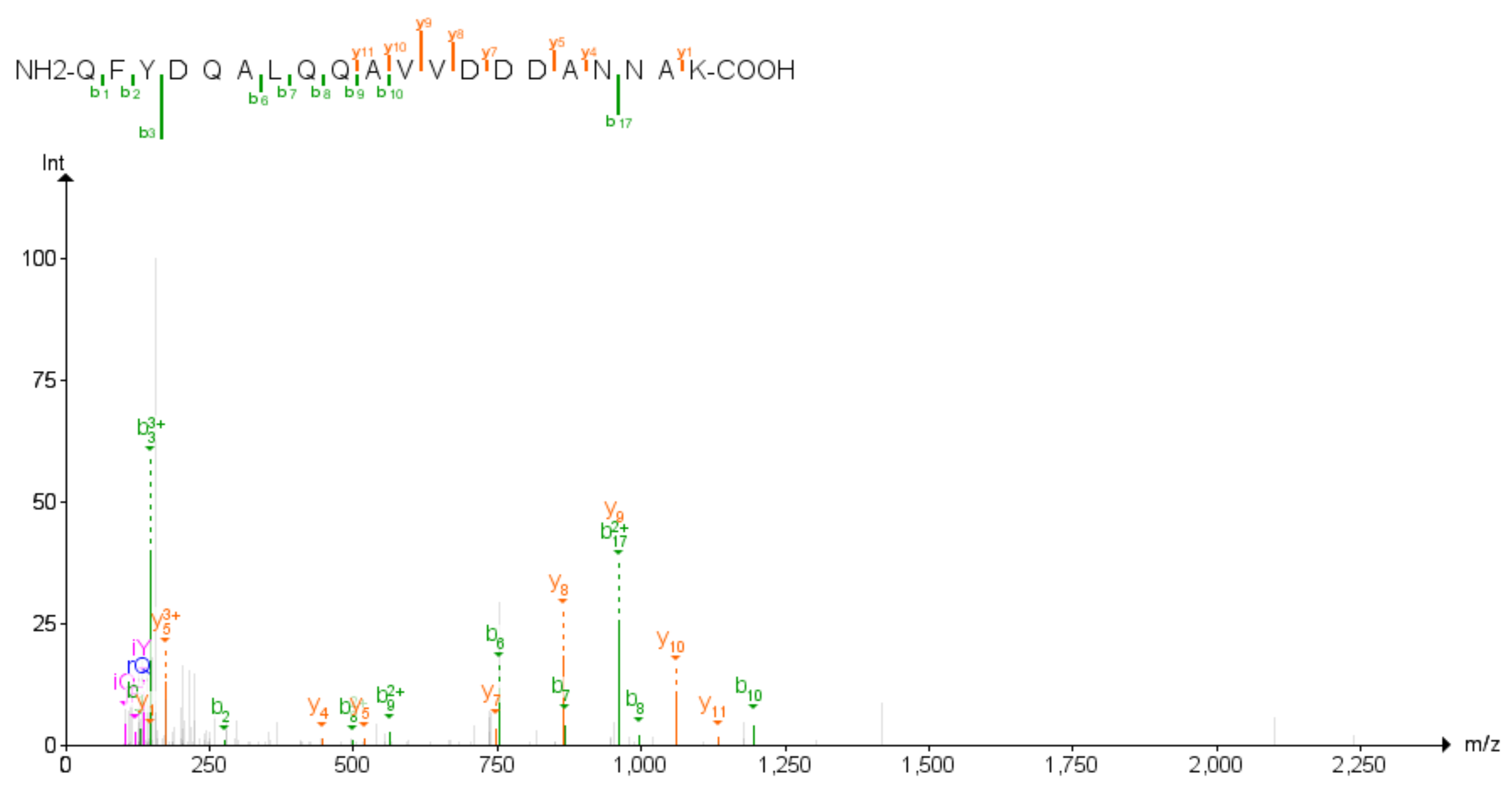
NH2-Q F Y D Q A L Q Q A V V D D D A N N A K-COOH
Int
m/z
0
25
50
75
100
250
500
750
1,000
1,250
1,500
1,750
2,000
2,250

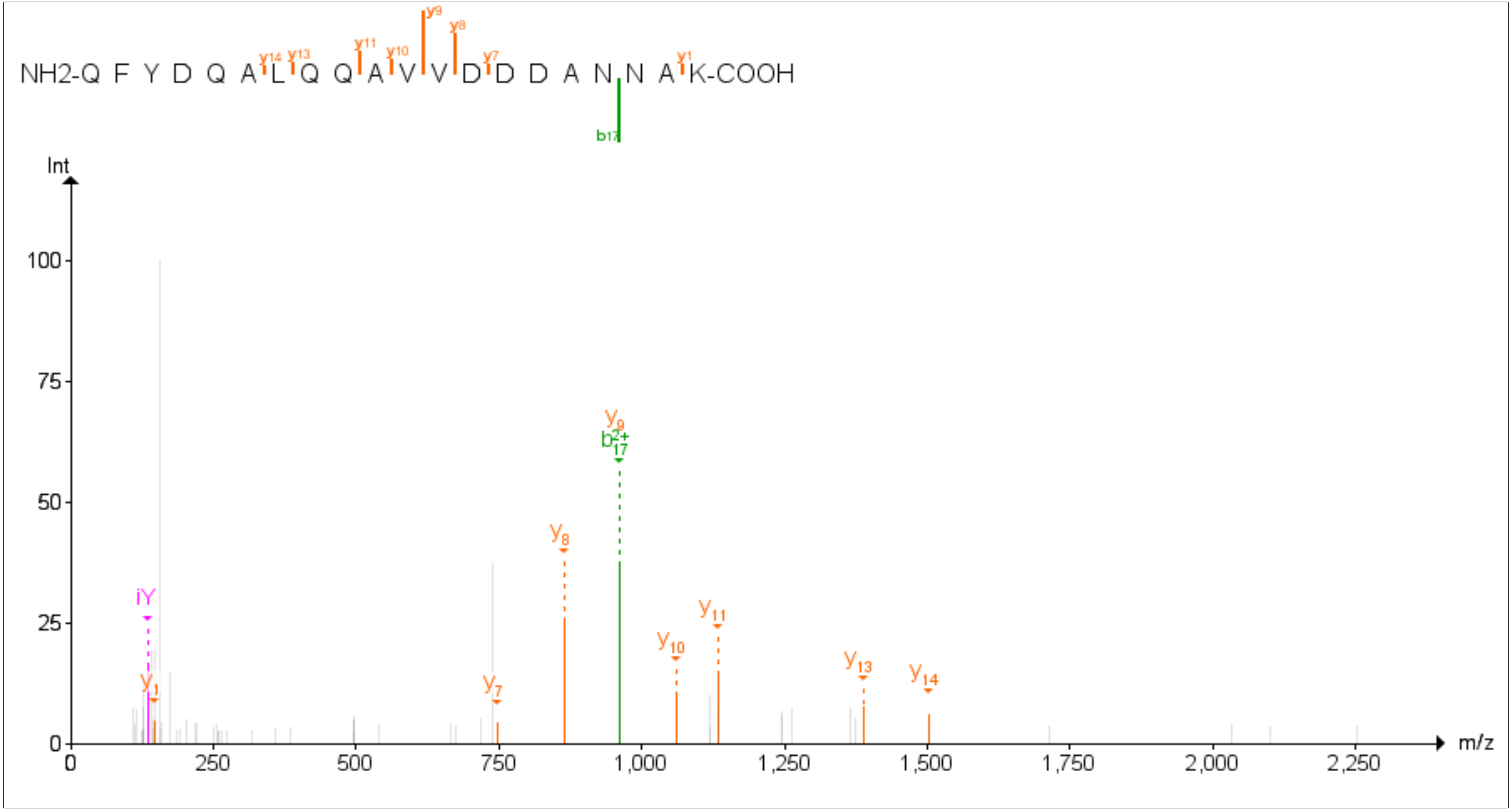

NH2-Q F Y D Q A L Q Q A V V D D D A N N A K-COOH
y14
y13
y11
y10
y9
y8
y7
y1
b17
Int
m/z
iY
y_1
y_7
y_8
y_9
b_{17}^{2+}
y_{10}
y_{11}
y_{13}
y_{14}

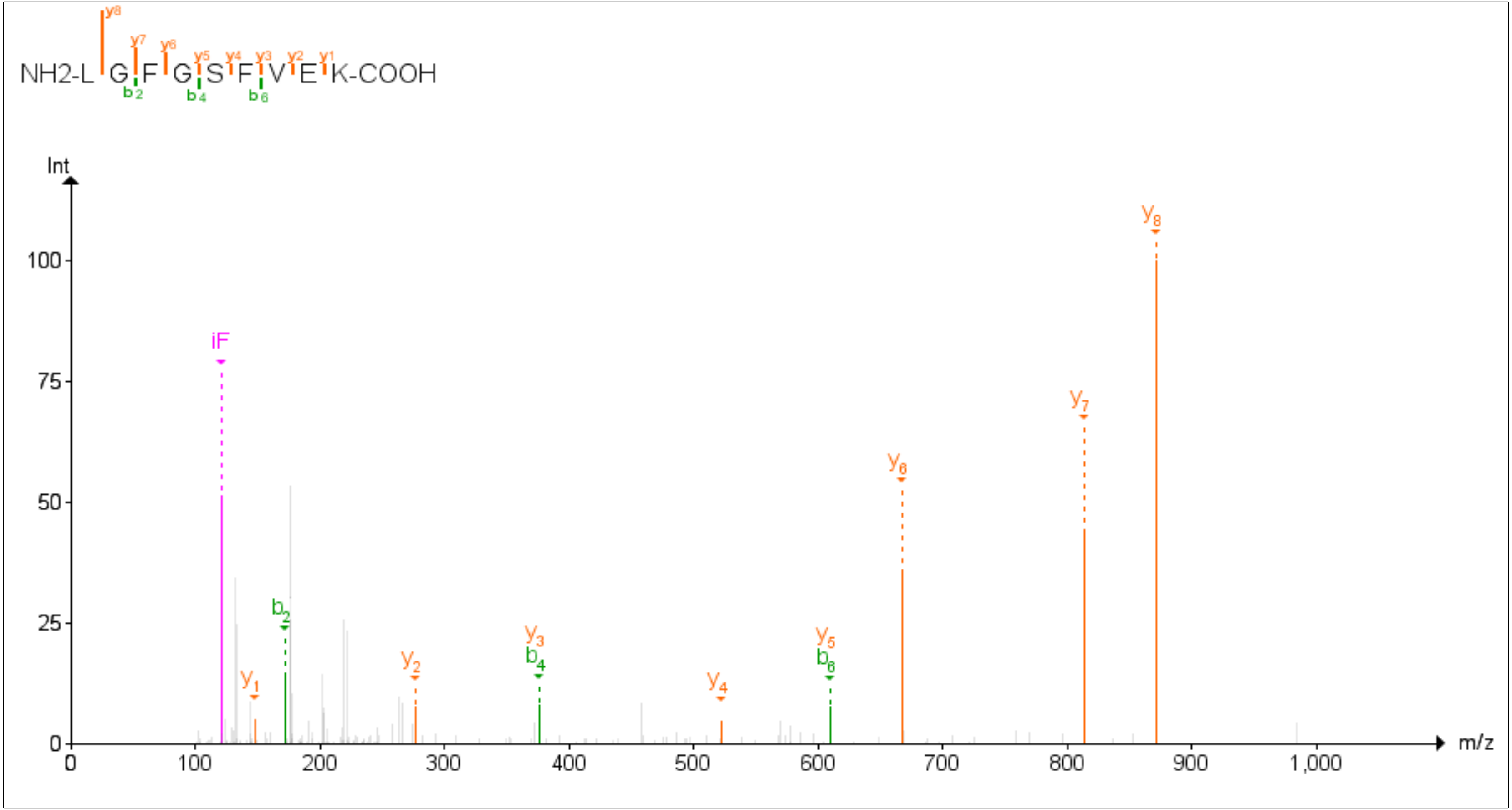

NH2-L G F G S F V E K-COOH
y8 y7 y6 y5 y4 y3 y2 y1
b2 b4 b6
Int
100
75
50
25
0
iF
y1
b2
y2
y3
b4
y4
y5
b6
y6
y7
y8
0 100 200 300 400 500 600 700 800 900 1,000
m/z

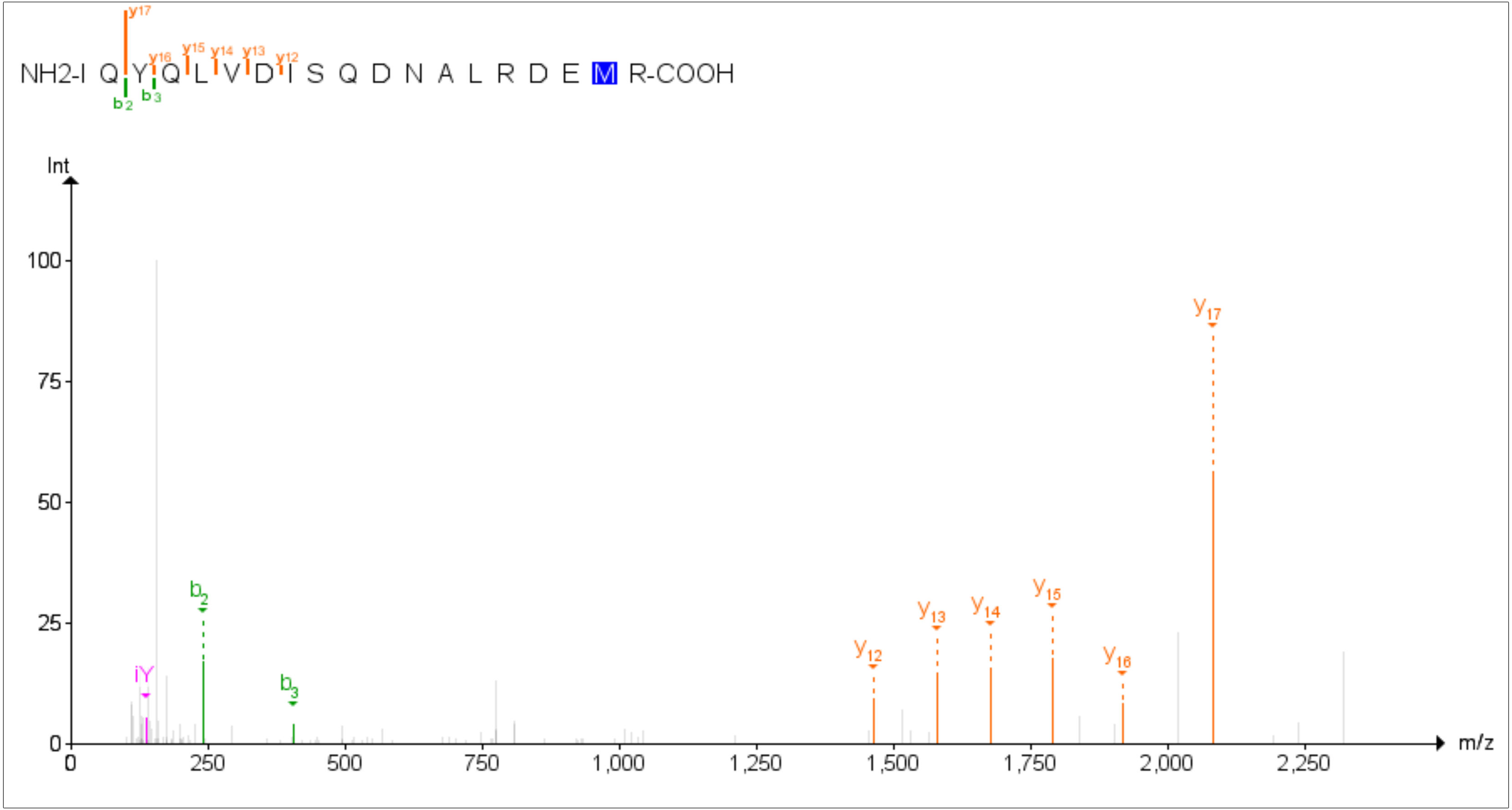
NH2-I Q Y Q L V D I S Q D N A L R D E M R-COOH
y17
y16
y15
y14
y13
y12
b2
b3
Int
100
75
50
25
0
iY
m/z
0
250
500
750
1,000
1,250
1,500
1,750
2,000
2,250

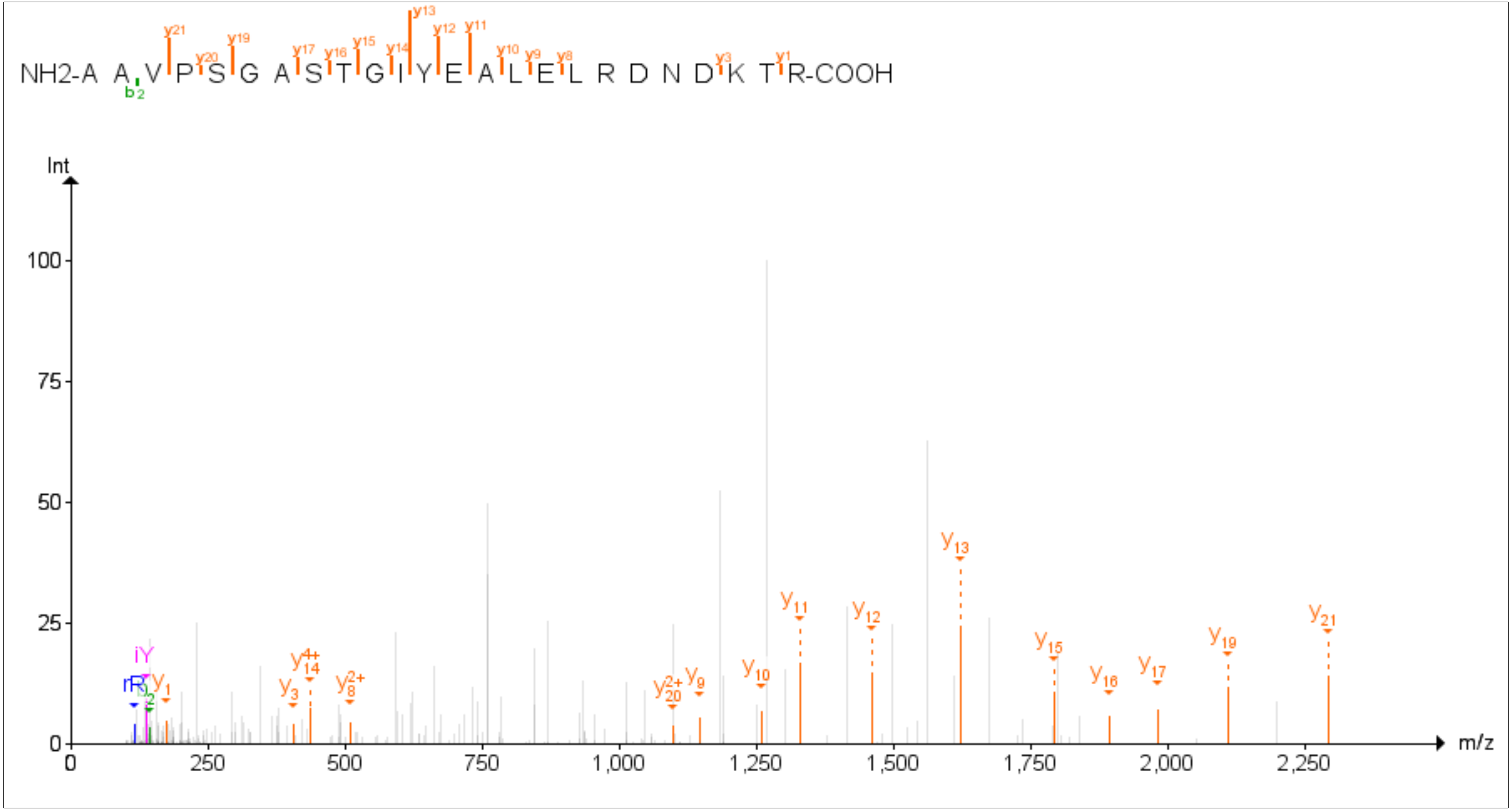

NH2-A A V P S G A S T G I Y E A L E L R D N D K T R-COOH
b2
y21 y20 y19 y17 y16 y15 y14 y13 y12 y11 y10 y9 y8 y3 y1
Int
m/z
0
25
50
75
100
0
250
500
750
1,000
1,250
1,500
1,750
2,000
2,250
iY
rR
y1
y3
y14 4+
y8 2+
y20 2+
y9
y10
y11
y12
y13
y15
y16
y17
y19
y21

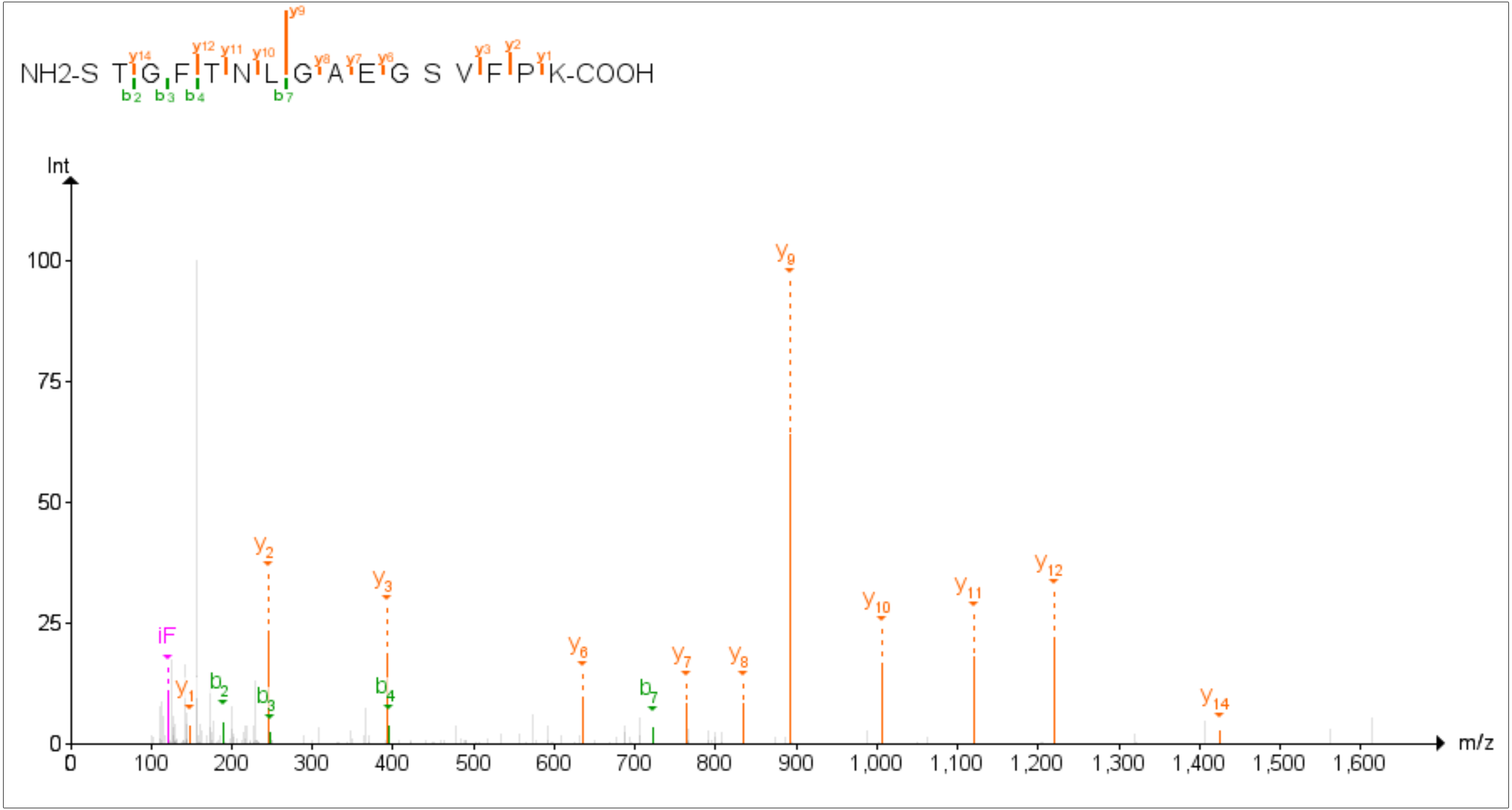

NH2-S T G F T N L G A E G S V F P K-COOH
y14 y12 y11 y10 y9 y8 y7 y6 y3 y2 y1
b2 b3 b4 b7
Int
100
75
50
25
0
iF
y1
b2
b3
y2
y3
b4
y6
b7
y7
y8
y9
y10
y11
y12
y14
0 100 200 300 400 500 600 700 800 900 1,000 1,100 1,200 1,300 1,400 1,500 1,600
m/z

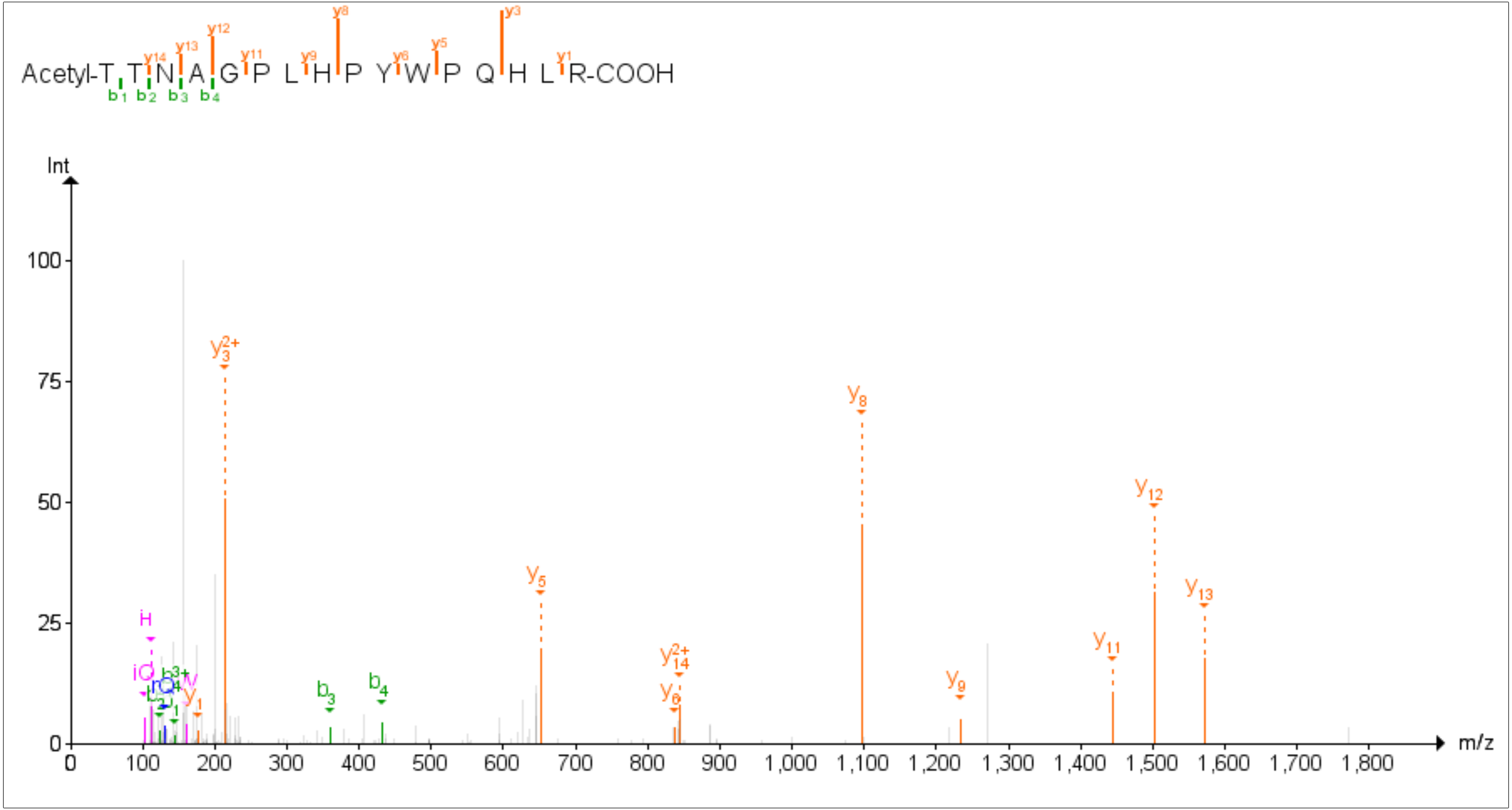

Acetyl-T T N A G P L H P Y W P Q H L R-COOH
Int
m/z

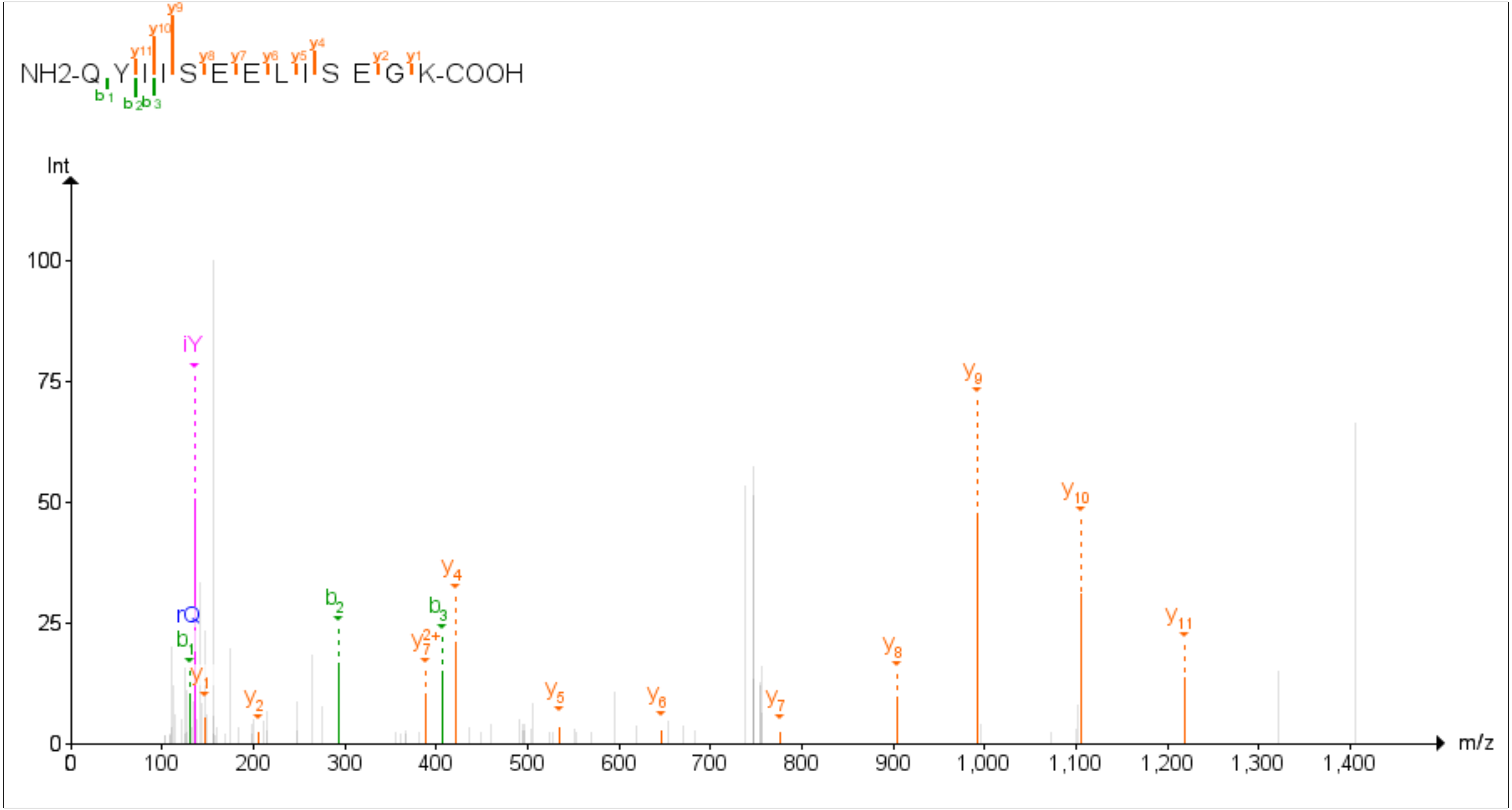

NH2-Q Y I I S E E L I S E G K-COOH
b1 b2 b3
y11 y10 y9 y8 y7 y6 y5 y4 y2 y1
Int
100
75
50
25
0
iY
rQ
b1
y1
y2
b2
y7 2+
b3
y4
y5
y6
y7
y8
y9
y10
y11
0 100 200 300 400 500 600 700 800 900 1,000 1,100 1,200 1,300 1,400
m/z

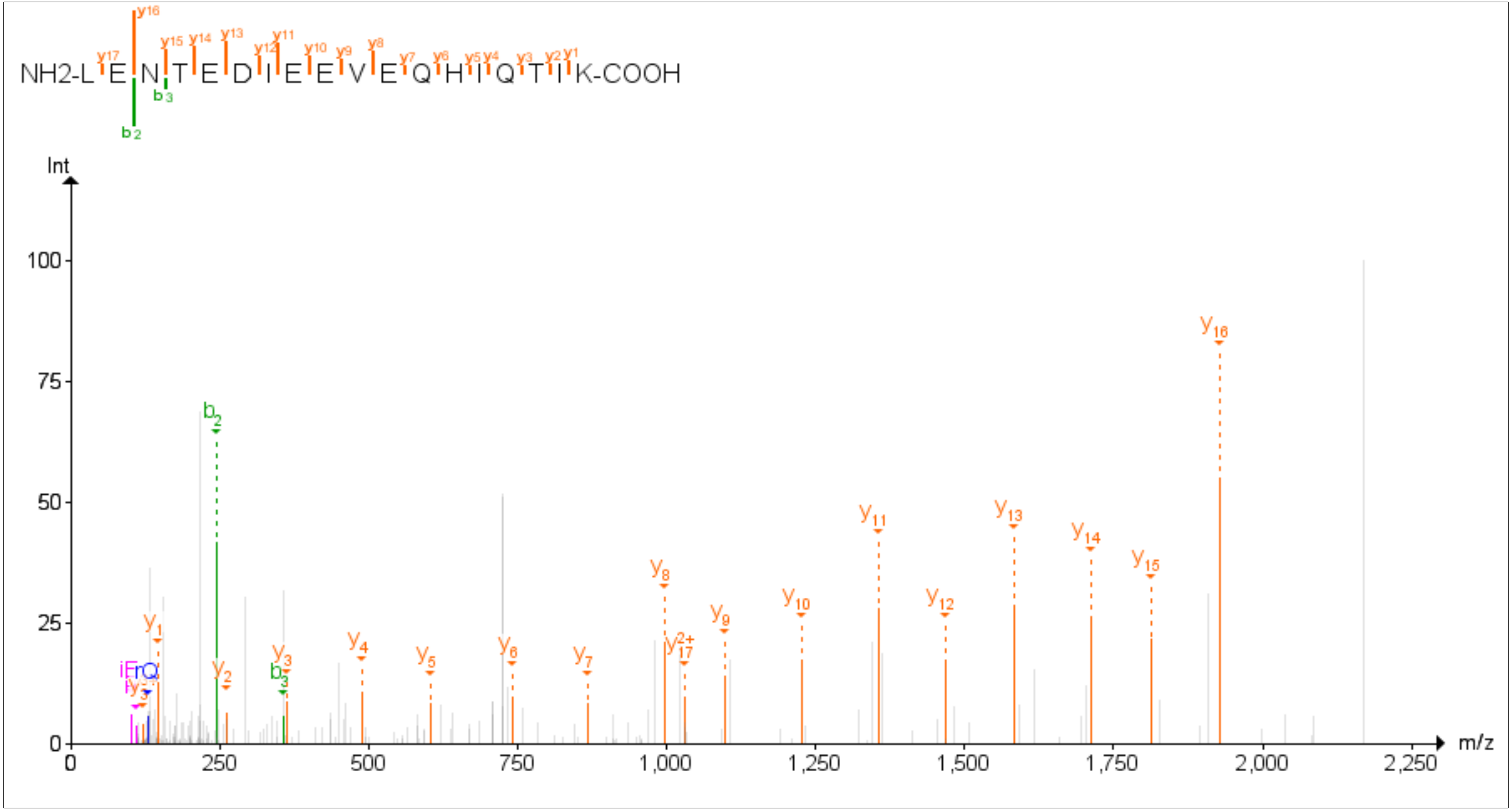

NH2-L E N T E D I E E V E Q H I Q T I K-COOH
y17 y16 y15 y14 y13 y12 y11 y10 y9 y8 y7 y6 y5 y4 y3 y2 y1
b2 b3
Int
100
75
50
25
0
m/z
0 250 500 750 1,000 1,250 1,500 1,750 2,000 2,250
y16
b2
y11 y13 y14 y15
y8 y10 y12
y9
y1 y4 y5 y6 y7 y_{17}^{2+}
y3 y2 b3
iFrQ

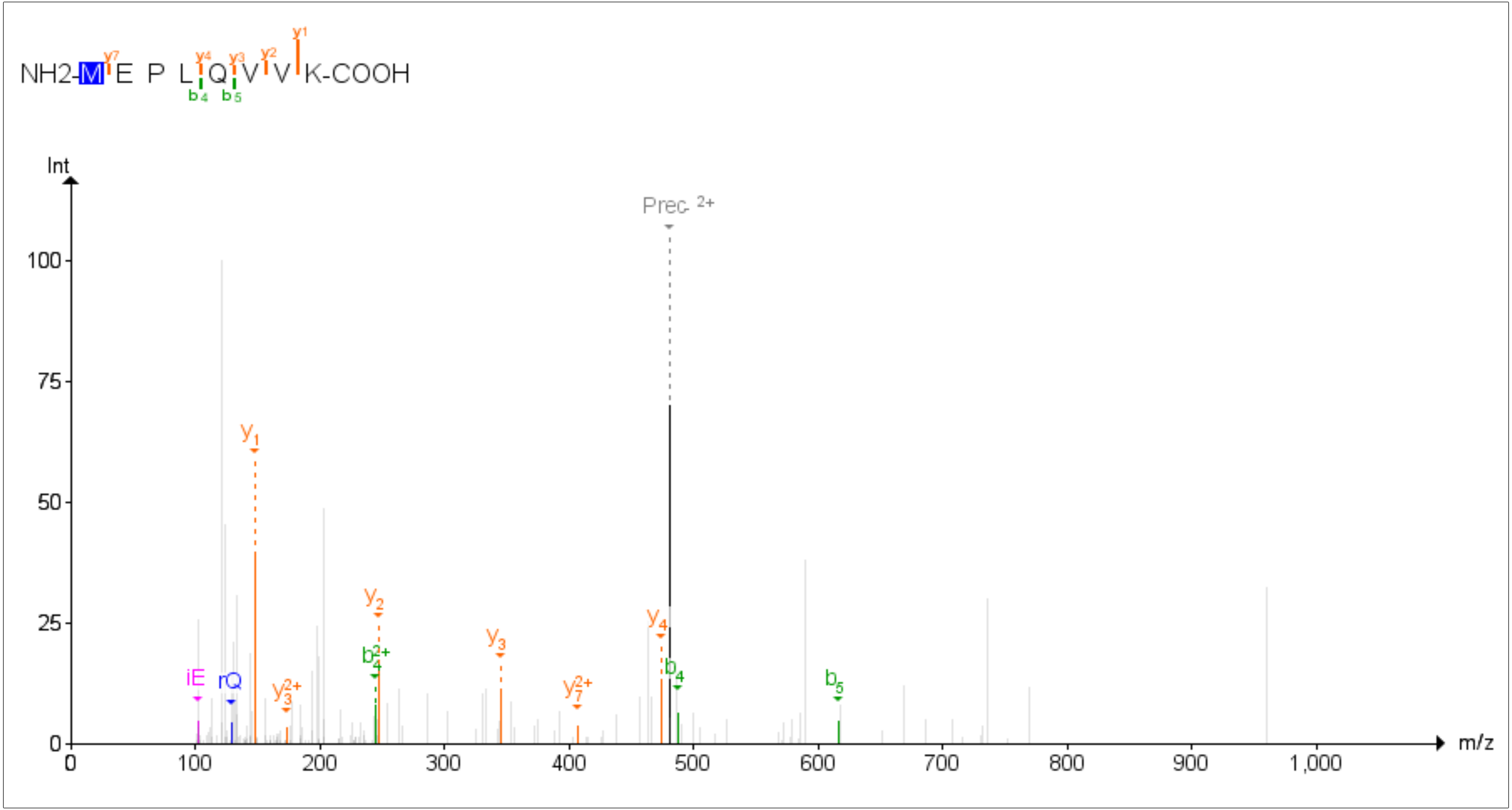

NH2-M E P L Q V V K-COOH
y7 y4 y3 y2 y1
b4 b5
Int
Prec 2+
y1
y2
y3
y4
b4 2+
b4
b5
y3 2+
y7 2+
iE
rQ
0
25
50
75
100
0
100
200
300
400
500
600
700
800
900
1,000
m/z

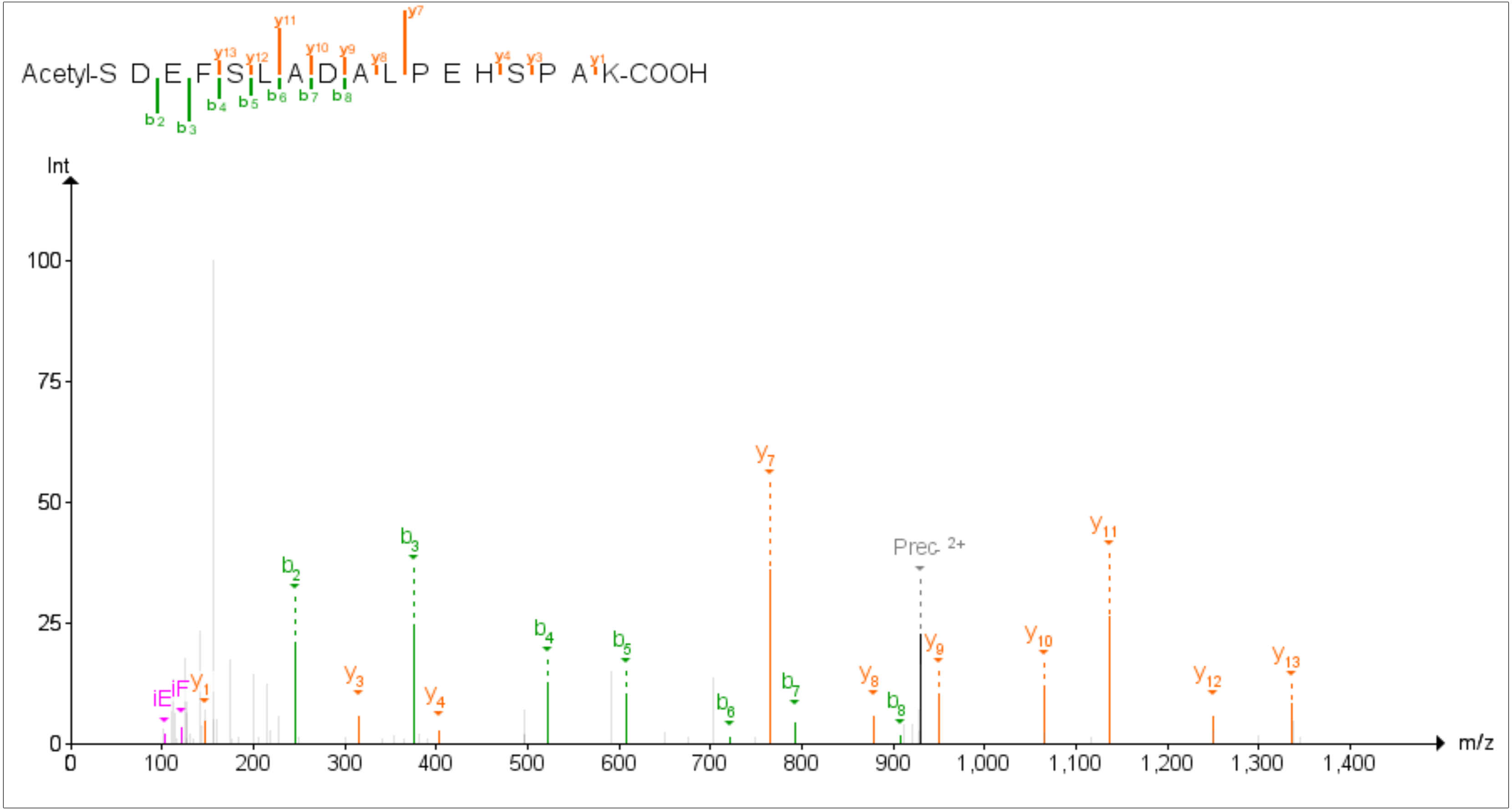

Acetyl-S D E F S L A D A L P E H S P A K-COOH
y13 y12 y11 y10 y9 y8 y7 y4 y3 y1
b2 b3 b4 b5 b6 b7 b8
Int
100
75
50
25
0
m/z
0 100 200 300 400 500 600 700 800 900 1,000 1,100 1,200 1,300 1,400
iE iF y1 y3 y4 y7 y8 y9 y10 y11 y12 y13
b2 b3 b4 b5 b6 b7 b8
Prec 2+

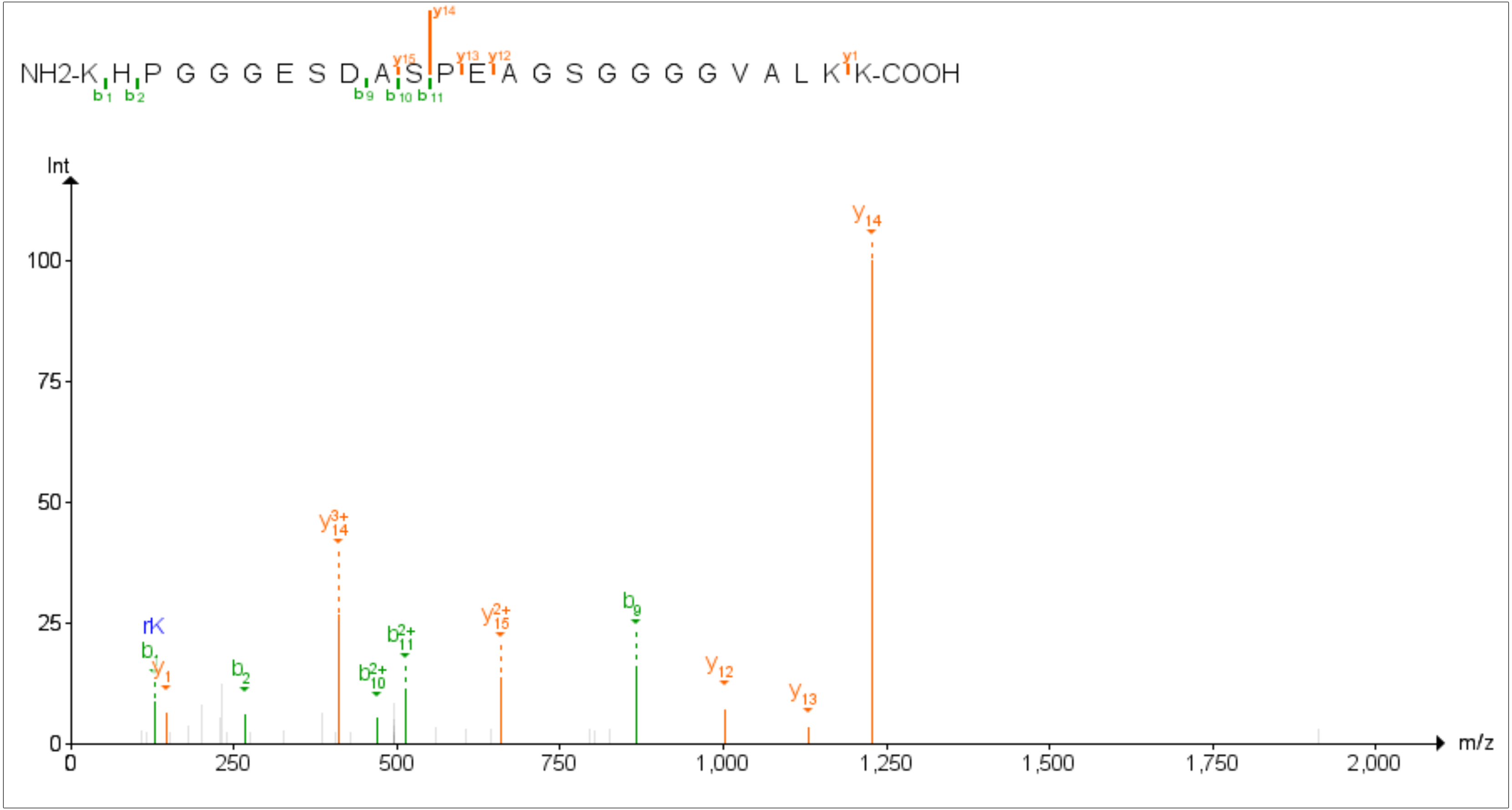
NH2-K H P G G G E S D A S P E A G S G G G G V A L K K-COOH
b1 b2 b9 b10 b11
y15 y14 y13 y12 y1
Int
100
75
50
25
0
m/z
0
250
500
750
1,000
1,250
1,500
1,750
2,000
y14
y14 3+
y15 2+
b9
b11 2+
b10 2+
b2
y1
b1
rK
y12
y13

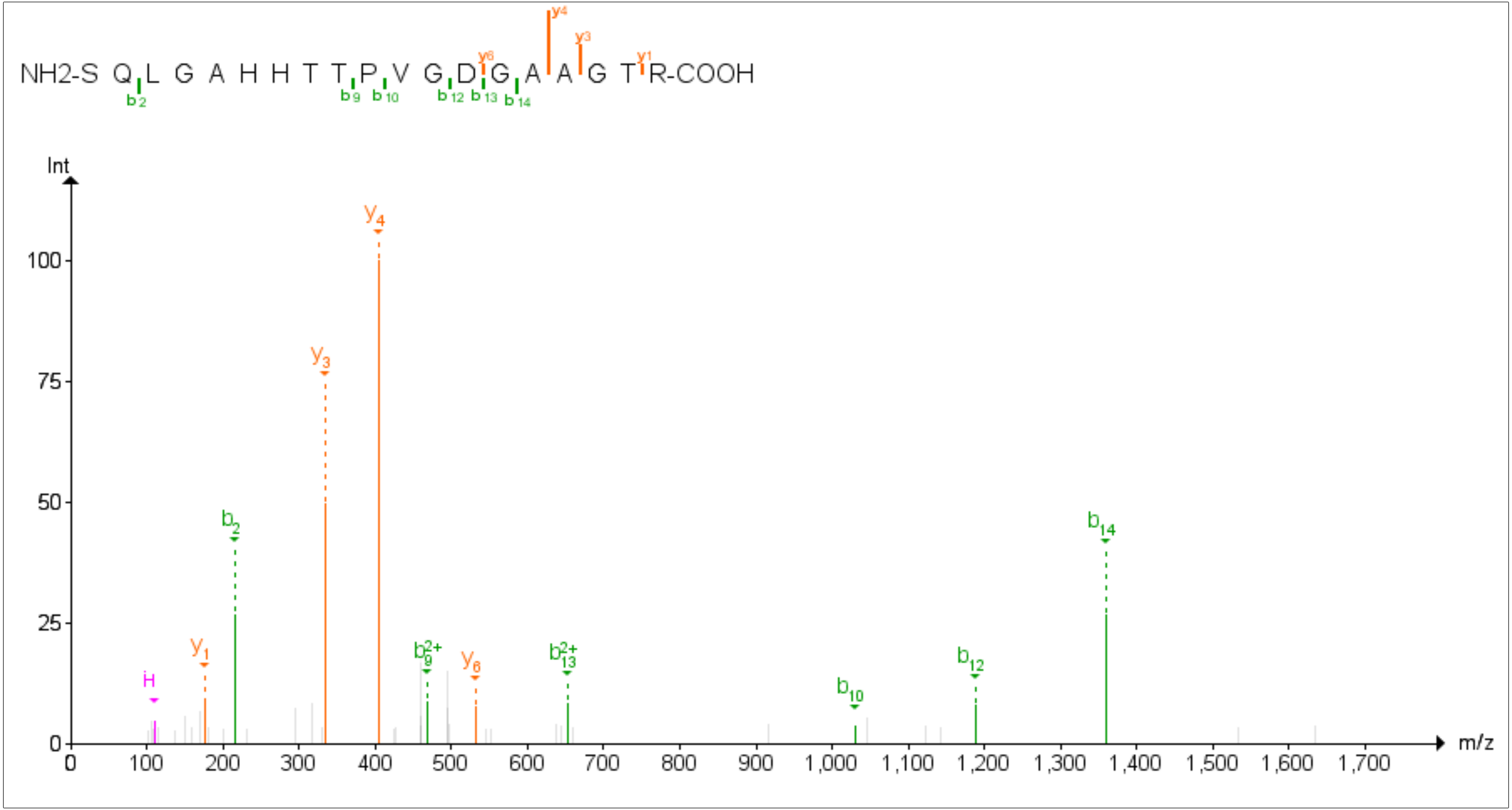

NH2-S Q L G A H H T T P V G D G A A G T R-COOH
b2 b9 b10 b12 b13 b14
y6 y4 y3 y1
Int
m/z
y4
y3
b2
b14
y1
H
b9 2+
y6
b13 2+
b10
b12

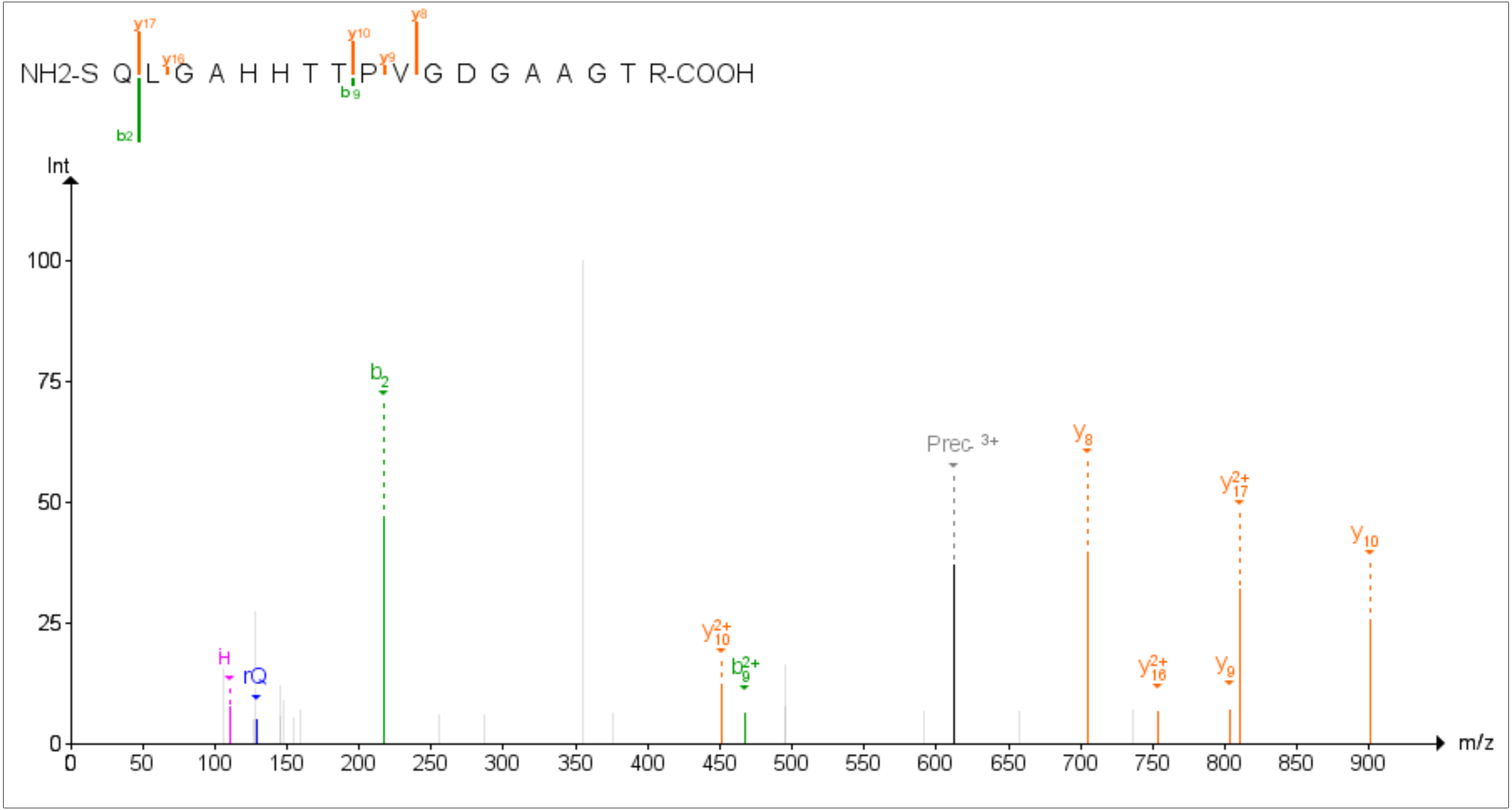
NH2-S Q L G A H H T T P V G D G A A G T R-COOH
y17 y16 y10 y9 y8
b2 b9
Int
100
75
50
25
0
b2
Prec 3+
y8
y17 2+
y10
y10 2+
b9 2+
y16 2+
y9
iH
rQ
0 50 100 150 200 250 300 350 400 450 500 550 600 650 700 750 800 850 900
m/z

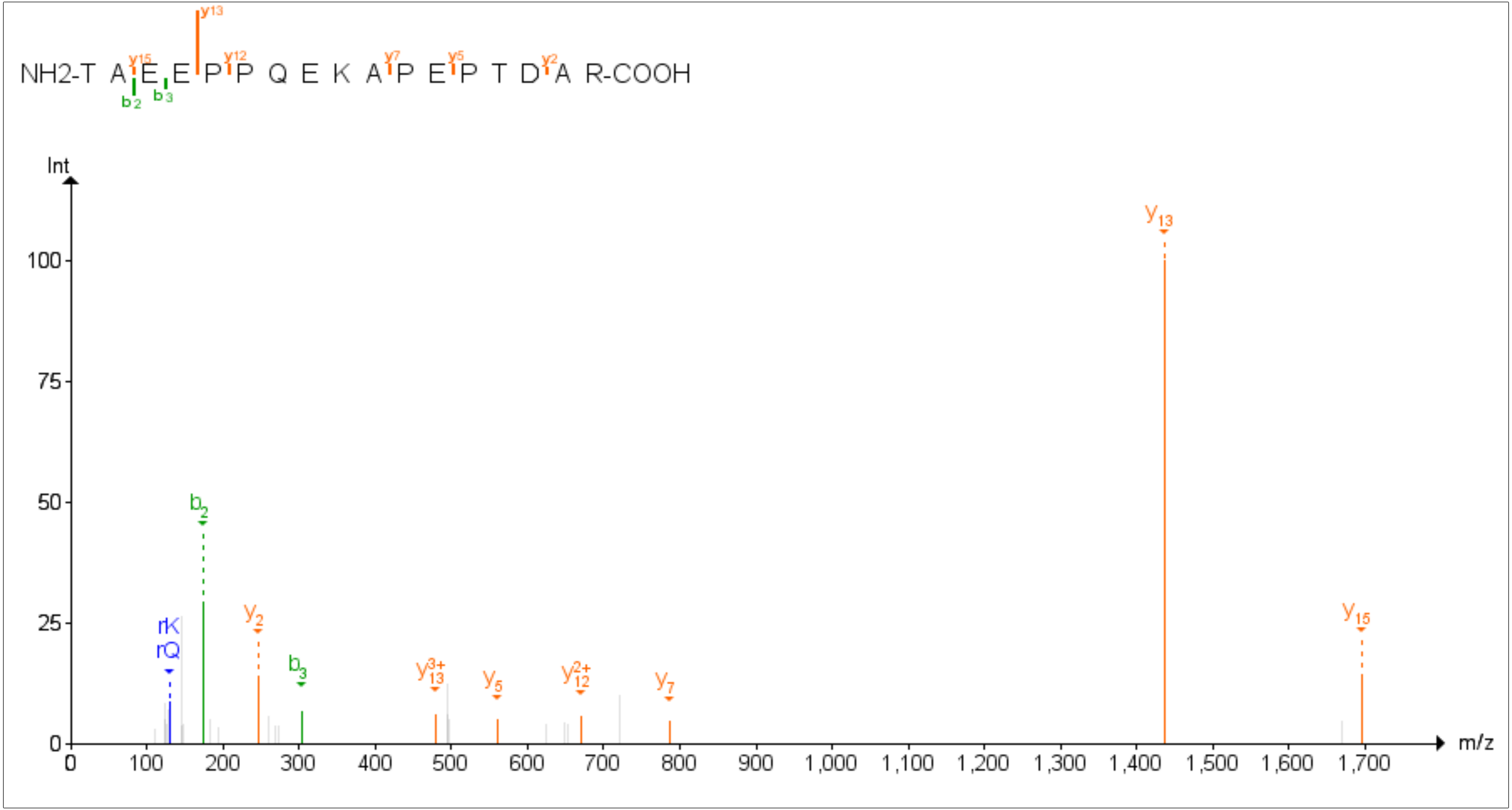

NH2-T A E E P P Q E K A P E P T D A R-COOH
y13
y15
y12
y7
y5
y2
b2
b3
Int
100
75
50
25
0
y13
b2
y2
rK
rQ
b3
y13 3+
y5
y12 2+
y7
y15
m/z
0
100
200
300
400
500
600
700
800
900
1,000
1,100
1,200
1,300
1,400
1,500
1,600
1,700

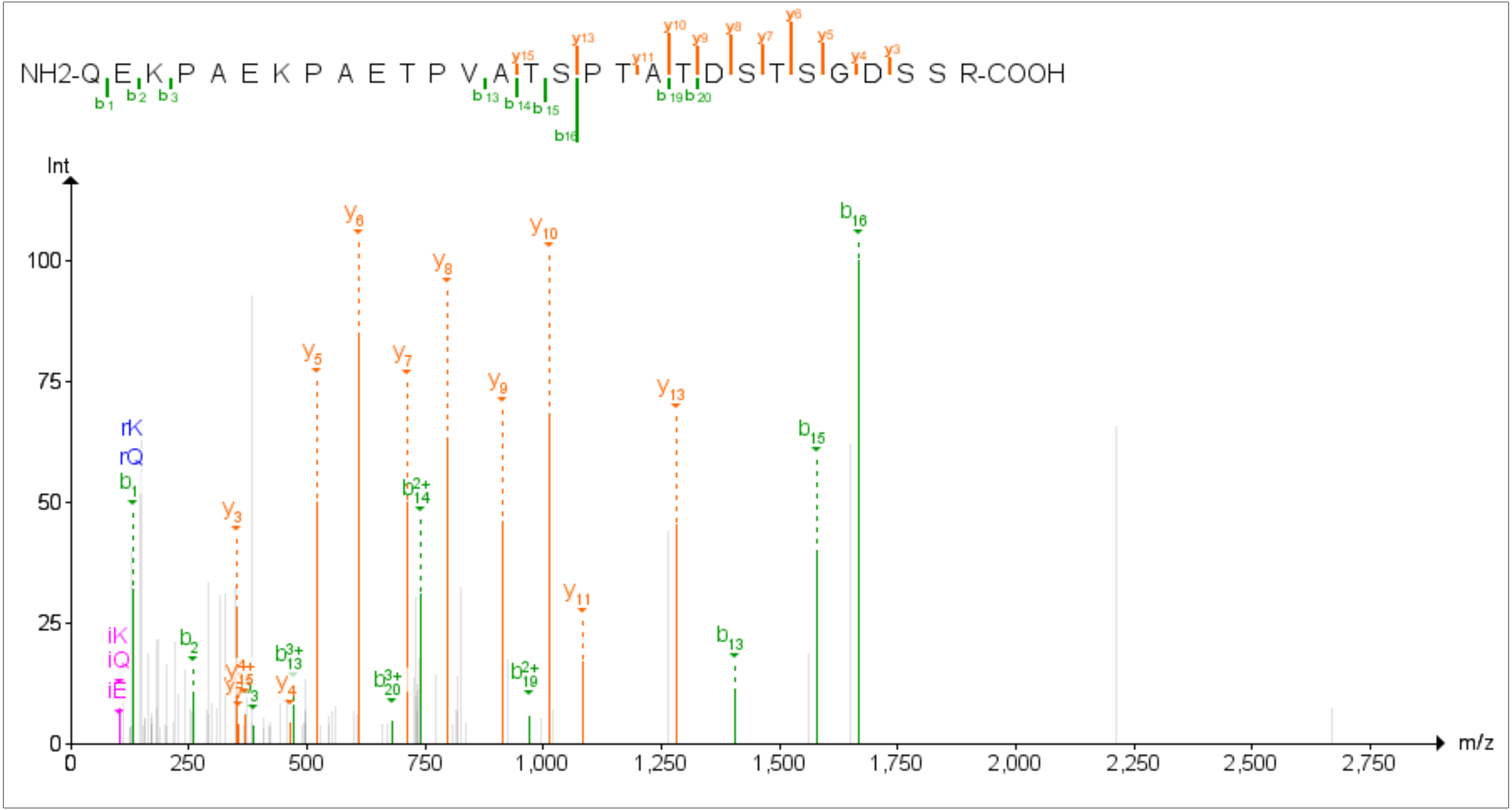
NH2-Q E K P A E K P A E T P V A T S P T A T D S T S G D S S R-COOH
Int
m/z
y6
y10
y8
y5
y7
y9
y13
b16
b15
b13
y11
y3
b1
rQ
rK
b2
iK
iQ
iE
y4

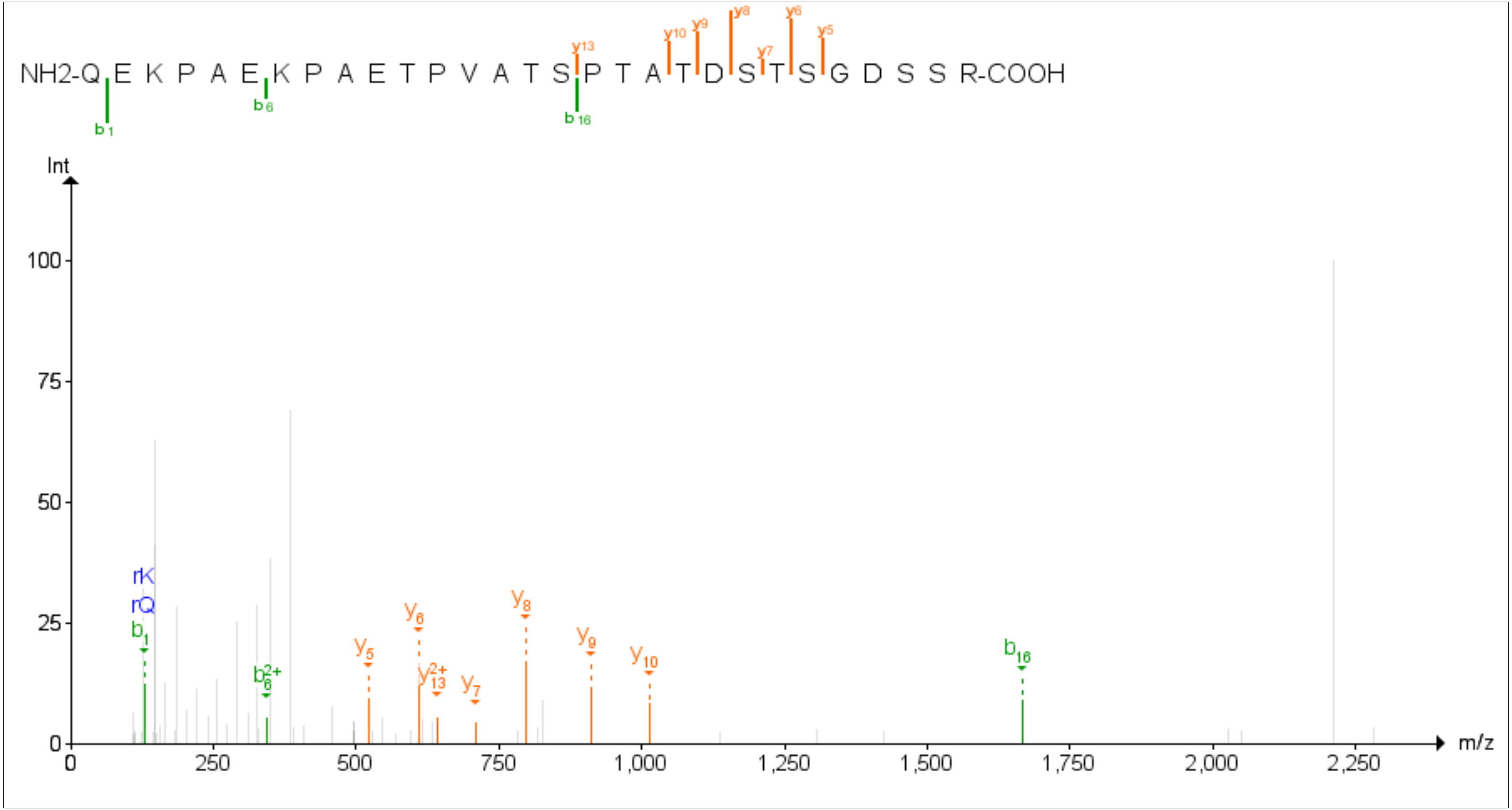
NH2-Q E K P A E K P A E T P V A T S P T A T D S T S G D S S R-COOH
b1
b6
b16
y13
y10
y9
y8
y7
y6
y5
Int
100
75
50
25
0
rK
rQ
b1
b6 2+
y5
y6
y13 2+
y7
y8
y9
y10
b16
0
250
500
750
1,000
1,250
1,500
1,750
2,000
2,250
m/z
